# Supplementary material for: A young child formula with Limosilactobacillus reuteri and GOS modulates gut microbiome and enhances bone and muscle development: a randomized trial
Source: Nat Commun. 2025 Dec 12;17:237. doi: 10.1038/s41467-025-66930-2 (PMC12783733; doi:10.1038/s41467-025-66930-2)
Supplement: Supplementary file 1 — Supplementary Information [file 41467_2025_66930_MOESM1_ESM.pdf]

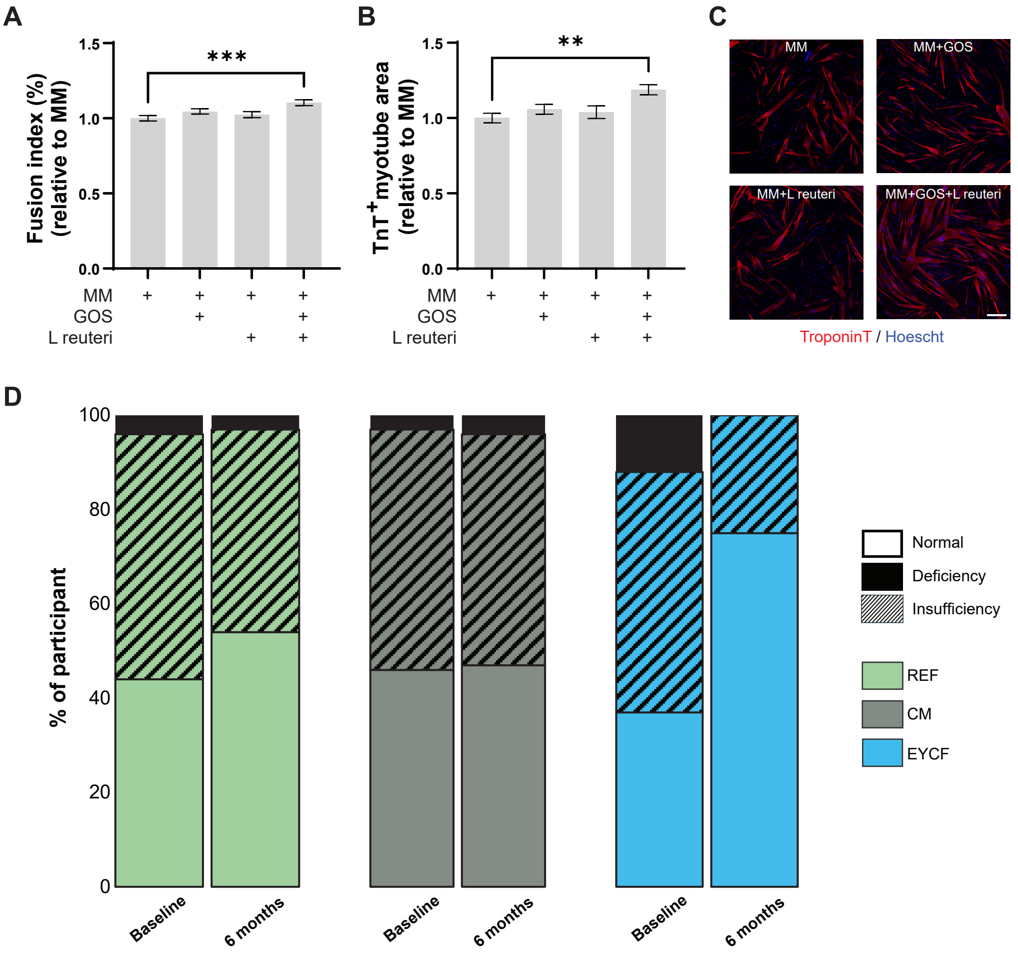

Supplemental Figure S1. EYCF impacts muscle development in vitro. (A) Fusion index of myotube after 6 days of treatment with final product of ex vivo colonic incubation (1% final concentration). (B) Troponin T positive area of myotube after 6 days of treatment with final product of ex vivo colonic incubation (1% final concentration). (C) Representative images, scale bar: 200µm. MM=Milk matrix, GOS=Galacto-oligosaccharide. Statistical analysis: One-way ANOVA Dunnett's test. **p<0.01, ***p<0.001.D) Vitamin D insufficiency (%) and deficiency (%) among study participants (at baseline n=91 per group, at 6 months n= 87 (REF), 69 (CM), 69 (EYCF).

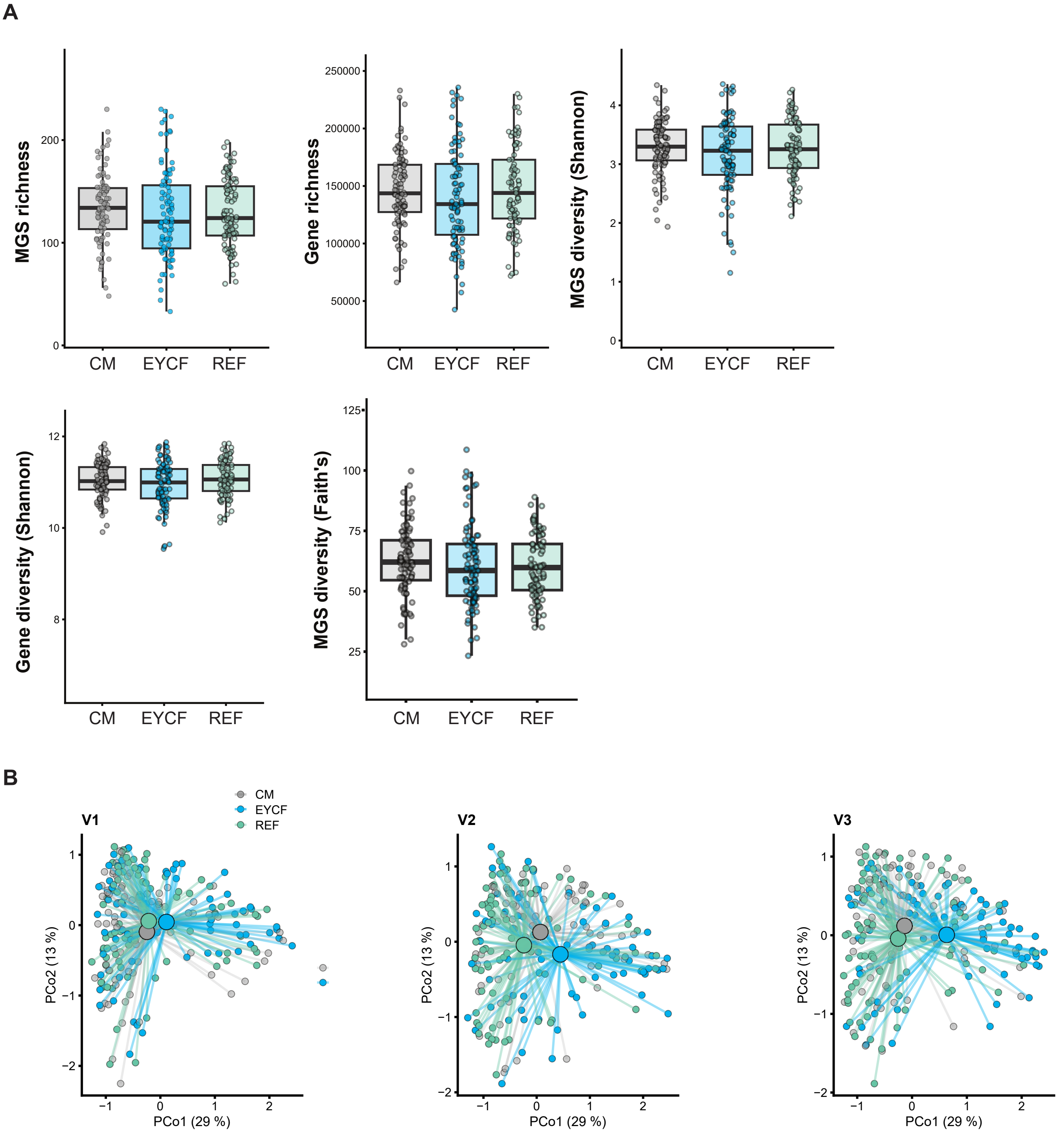

Supplemental Figure S2. Alpha and beta diversity of subjects grouped by intervention at baseline. (A) Alpha-diversity is represented by boxplots showing differences in species (MGS) richness, species (MGS) diversity (Shannon and Faith's) as well as gene richness and diversity (Shannon). Intervention groups were compared by Kruskal-Wallis and Dunn's test. No significant differences were observed for any alpha-diversity metric at baseline between intervention groups. (B) Beta diversity is represented in a Principal Coordinates Analysis (PCoA) based on weighted UniFrac distance between samples (color-coded by intervention group).

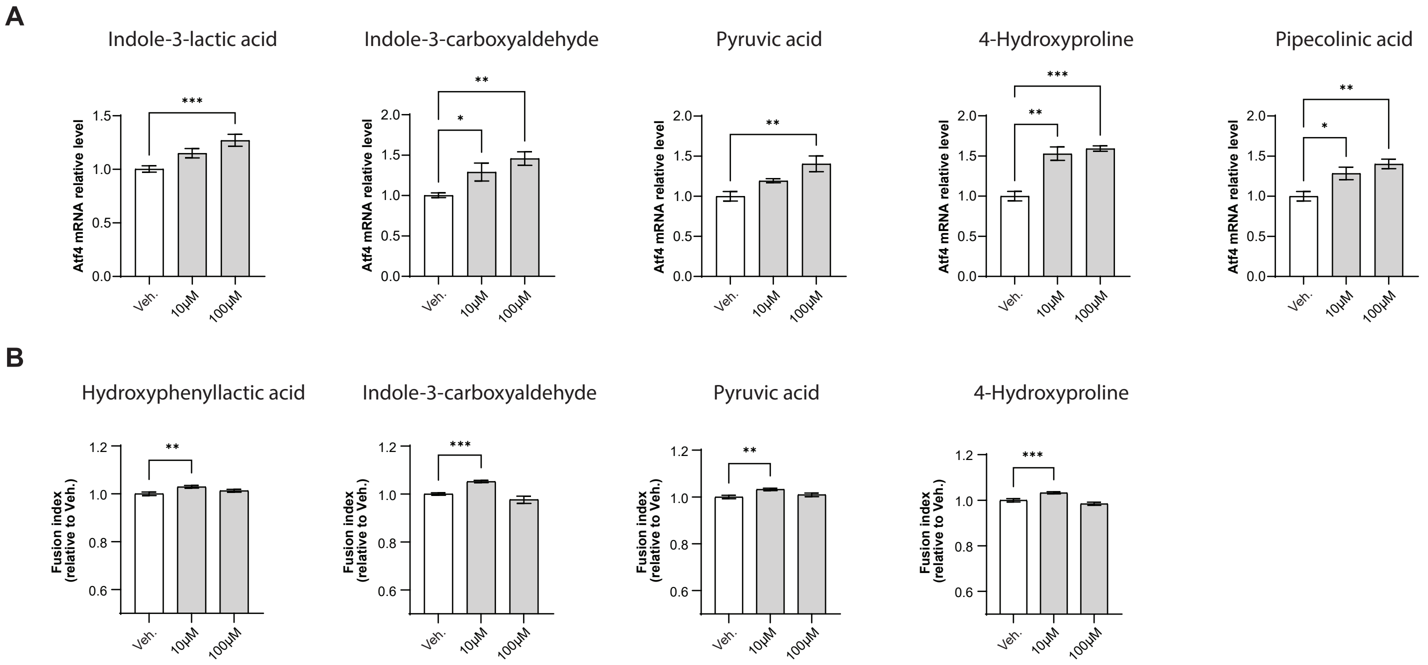

Supplemental Figure S3. Effect of metabolites on osteoblasts and human muscle progenitor *in vitro*. (A) Impact of indole-3-lactic acid, indole-3-carboxyaldehyde, pyruvic acid, 4-hydroxyproline and pipecolinic acid on Activating transcription factor 4 (*Atf4*) gene expression in osteoblasts. (B) Impact of hydroxyphenyl lactic acid, indole-3- carboxaldehyde, pyruvic acid and 4-hydroxyproline on fusion of muscle progenitors. Statistical analysis: One-way ANOVA Dunnett's test. *p<0.05, **p<0.01, ***p<0.001.
