# Supplementary material for: A young child formula with Limosilactobacillus reuteri and GOS modulates gut microbiome and enhances bone and muscle development: a randomized trial
Source: Nat Commun. 2025 Dec 12;17:237. doi: 10.1038/s41467-025-66930-2 (PMC12783733; doi:10.1038/s41467-025-66930-2)
Supplement: Supplementary file 2 — Description of Additional Supplementary Files [file 41467_2025_66930_MOESM2_ESM.pdf]

Description of Additional Supplementary Files

Supplementary Data 1. Demographics and baseline characteristics of subjects. Characteristics of the subjects in the ITT population.

Supplementary Data 2. Descriptive statistics of subjects at all visits. Characteristics of the subjects in the Reference group (REF), Control milk group (CM) and Experimental young child formula group (EYCF) at baseline (V1), 3 months, if recorded, (V2) and 6 months (V3).

Supplementary Data 3. Clinical outcomes comparisons between the experimental and control blends versus the habitual diet arm. Comparison between Experimental young child formula group and Reference group (EYCF – REF) or Control milk group and Reference group (CM – REF) at 3 months, if recorded, (V2) and 6 months (V3) with ANCOVA models.

Supplementary Data 4. Comparisons of the blood vitamin D level between Experimental group and Habitual diet group. Comparison between Experimental group (EYCF) and Habitual diet group (REF) at 6 months (V3) with ANCOVA models. Three models are proposed to evaluate the differences between the Experimental group and the Reference group, by taking into account that the Reference group was not randomized in this trial. The first model, Model 1 is a model computed without propensity scores but includes as covariates: Arm (Experimental or Reference), Visit (Visit 2 or Visit 3), Arm in interaction with Visit, baseline Vitamin D level, measurement, sex, gestational age, delivery method, duration of breastfeeding, age at baseline (measured in months), BMI (kg/m2) at enrolment and vitamin D (ng/mL) at enrolment. The other two models have additional corrections: the propensity scores included either as covariate in Model 2 or as weights in Model 3.

Supplementary Data 5. Correlation of the vitamin B3/B6 ratio and mineral excretion (adjusted to intake) and the clinical outcome. Spearman correlation of ratio of vitamin B3/B6, the calcium, magnesium and phosphorus excretion adjusted to intake and the clinical outcomes tibia SOS, Radius SOS, Tibia length, Radius length and Handgrip at 6 months (V3) after the beginning of the trial. n=222 for the ratio B3/B6 (participants of the 3 groups were measured), n=137 for the calcium, magnesium and phosphorus (only participants from the CM and EYCF were measured)

Supplementary Data 6. PERMANOVA analysis on microbiome beta diversity. P-values for tests comparing beta-diversity between intervention groups, overall and pairwise

Supplementary Data 7. Comparison of microbiome taxonomic composition between EYCF and CM. Results of the linear model at each visit and taxonomic level.

Supplementary Data 8. Comparison of metabolome intensities between EYCF and CM. Results of the linear model at each visit comparing metabolites intensity between EYCF and CM.

Supplementary Data 9. Procrustes analysis of microbiome-metabolome associations. Results from a Procrustes analysis stratified by intervention group and visit. P-values are derived from 10 000 permutations (thus the smallest possible P-value being 0.0001)

Supplementary Data 10. Associations between microbiome taxonomic composition and clinical outcomes. Results of the linear model at species (MGS) level considering EYCF and CM samples.

Supplementary Data 11. Associations between metabolome intensities and clinical outcomes. Results of the linear model for metabolites considering EYCF and CM samples.

Supplementary Data 12. Effect of metabolites on muscle progenitors and osteoblasts in vitro. (A) Impact of metabolites on osteoblasts proliferation, and Runt-related transcription factor 2 (*Runx2*), Activating transcription factor 4 (*Atf4*) and Catenin beta 1 (*Ctnnb1*) gene expression in osteoblasts. (B) Impact of metabolites on fusion of muscle progenitors and myotube area. Statistical analysis: One-way ANOVA Dunnett's test. *$p<0.05$, **$p<0.01$, ***$p<0.001$.

Supplementary Data 13. Associations between *L. reuteri* "Increased" and "Non-increased" groups and clinical outcomes. The comparisons were run considering EYCF+CM samples, and EYCF+CM+REF samples.

Supplementary Data 14. Stool consistency and Descriptive statistics for the TCGQ questionnaire. Average consistency of stools averaged over 3 days and Descriptive statistics of the questionnaire "Toddler Gut Comfort Questionnaire" in the ITT population.

Supplementary Data 15. Comparison of stool consistency and Gastrointestinal total score. Stool consistency at baseline is extracted from "24h Recall GI Symptom and Behavior Recall", from question 3: "If yes, thinking about the past 24 hours, mark the one picture that looks the most like your child's stool". The stool consistency was transformed using a 5-point stool scale: 1=watery, 2=runny, 3=mushy soft, 4=formed, and 5=hard. The effect of treatment group on stool consistency at V2 and V3 is evaluated based on a linear mixed model with a log transformation of the response and the following explanatory variables: stool consistency at baseline, treatment group, mode of delivery, sex, visit and interaction between treatment and visit as covariates. A subject specific random effect has been included to consider the repeated measurements. Effect of treatment group on the log-transformed scores of GI TOTAL at V2 and V3 is evaluated based on a linear mixed model, including GI TOTAL at baseline with log transformation, treatment group, mode of delivery, sex, visit and interaction between treatment and visit as covariates. A subject specific random effect has been included to consider the repeated measurements.

Supplementary Data 16. Complete formulation of the control milk (CM) and the experimental blend (EYCF).

Supplementary Data 17. Relative species abundance for each sample.
