# Supplementary material for: A young child formula with Limosilactobacillus reuteri and GOS modulates gut microbiome and enhances bone and muscle development: a randomized trial
Source: Nat Commun. 2025 Dec 12;17:237. doi: 10.1038/s41467-025-66930-2 (PMC12783733; doi:10.1038/s41467-025-66930-2)
Supplement: Supplementary file 3 — Supplementary data 1 [file 41467_2025_66930_MOESM3_ESM.pdf]

Demographic and baseline characteristics of subjects.			
	**CM**	**EYCF**	**REF**
	**(n=91)**	**(n=91)**	**(n=91)**
	**N (%) or**	**N (%) or**	**N (%) or**
	**mean (SD)**	**mean (SD)**	**mean (SD)**
	**range**	**range**	**range**
Sex, n (%)			
Male	43 (47%)	43 (47%)	42 (47%)
Female	48 (53%)	48 (53%)	49 (53%)
Age at enrollment in months, mean (SD); range	29.7 (3.8)	29.5 (3.4)	29.7 (3.7)
	(24, 36)	(25, 36)	(25, 36)
Consumed Growing Up Milk in the past day	9 (9.9%)	9 (9.9%)	0 (0.0%)
Consumed fortified cow milk in the past day	80 (87.9%)	81 (89.0%)	91 (100.0%)
Consumed cow milk in the past day	6 (6.6%)	4 (4.4%)	0 (0.0%)
Weight (kg)	12.17 (1.7)	12.13 (1.7)	11.93 (1.66)
Height (cm)	86.95 (4.16)	86.94 (3.96)	86.22 (4.1)
