# Supplementary material for: A young child formula with Limosilactobacillus reuteri and GOS modulates gut microbiome and enhances bone and muscle development: a randomized trial
Source: Nat Commun. 2025 Dec 12;17:237. doi: 10.1038/s41467-025-66930-2 (PMC12783733; doi:10.1038/s41467-025-66930-2)
Supplement: Supplementary file 4 — Supplementary data 2 [file 41467_2025_66930_MOESM4_ESM.pdf]

Descriptive statistics of subjects at all visits

	Visit	Arm	n	min	Q1	Median	Q3	max	mean	sd
Height (cm)	V1	REF	91	76.1	83.85	86.7	88.85	97.1	86.22	4.1
	V1	CM	91	77.9	83.5	86.5	90.25	98.3	86.95	4.16
	V1	EYCF	91	79.4	84.4	87	89.5	97.5	86.94	3.96
	V3	REF	87	80	87.35	90.2	93	98.6	90.19	4.07
	V3	CM	69	81.9	88.1	91.4	93.7	104.1	91.49	4.34
	V3	EYCF	69	84	88.9	92	94.1	101.5	91.63	4.1
Weight (kg)	V1	REF	91	9.32	10.62	11.72	12.93	18.86	11.93	1.66
	V1	CM	91	9.13	10.94	11.81	12.88	18	12.17	1.7
	V1	EYCF	91	9.31	11	11.69	13.04	18.74	12.13	1.7
	V3	REF	87	10.14	11.57	12.52	13.98	20.5	12.94	1.97
	V3	CM	69	10.58	12.29	12.97	14.43	20.26	13.73	2.13
	V3	EYCF	69	10.7	12.15	13.16	14.58	20.52	13.78	2.25
Head circ (cm)	V1	REF	91	45	46.8	47.4	48.2	50.2	47.55	1.14
	V1	CM	91	43.6	46.8	47.7	48.5	50.8	47.68	1.35
	V1	EYCF	91	40.2	46.8	47.7	48.5	51.2	47.53	1.62
	V3	REF	87	46	47.7	48.2	49.1	51.2	48.42	1.07
	V3	CM	69	44.9	47.6	48.5	49.4	51.6	48.54	1.3
	V3	EYCF	69	45.2	47.6	48.5	49.5	51.4	48.54	1.36
BMI (kg/m2)	V1	REF	91	13.89	15.01	15.76	16.55	22.53	15.99	1.37
	V1	CM	91	13.1	15.11	15.69	16.66	22.54	16.05	1.49
	V1	EYCF	91	13.46	15.13	15.75	16.66	22	16	1.35
	V3	REF	87	13.85	14.83	15.52	16.2	23.25	15.84	1.53
	V3	CM	69	13.17	15.35	15.92	16.91	23.37	16.36	1.86
	V3	EYCF	69	13.66	15.23	16.18	16.89	21.45	16.33	1.67
Tibia SOS (m/s)	V1	REF	91	3103	3306	3372	3469	3709	3384	125.5
	V1	CM	91	3128	3321	3414	3486	3721	3408	131
	V1	EYCF	91	3114	3308	3396	3493	3724	3399	140.9
	V2	REF	84	3068	3310	3412	3495	3897	3406	144.6
	V2	CM	45	2988	3304	3391	3427	3712	3370	135.8
	V2	EYCF	43	3213	3366	3421	3456	3651	3417	87.36
	V3	REF	87	3133	3310	3397	3482	3699	3399	127.9
	V3	CM	69	3032	3341	3400	3469	3760	3406	131.3
	V3	EYCF	69	2889	3374	3466	3535	3719	3458	133.9
Radius SOS (m/s)	V1	REF	91	2916	3193	3284	3372	3668	3287	144.2
	V1	CM	91	2861	3142	3298	3410	3719	3285	169.1
	V1	EYCF	91	2890	3154	3272	3343	3521	3249	140.7
	V2	REF	87	2877	3248	3361	3448	3721	3345	149.6
	V2	CM	71	2964	3244	3328	3424	3683	3333	141.9
	V2	EYCF	70	3090	3253	3336	3445	3726	3354	131.1
	V3	REF	87	2950	3284	3356	3472	3693	3357	159.7
	V3	CM	69	3015	3276	3384	3478	3683	3377	134.1
	V3	EYCF	69	3130	3293	3372	3469	3616	3375	123.5
Tibia length (cm)	V1	REF	91	13	23.6	24.4	25.75	28	24.21	2.37
	V1	CM	91	15	22.7	24.1	25.4	30	23.29	3.53
	V1	EYCF	91	13.4	23	24.4	25.6	29	23.52	3.57
	V2	REF	87	16	24.55	25.4	26.55	28.7	25.46	1.7
	V2	CM	72	23	24.87	25.55	26.63	30	25.71	1.46
	V2	EYCF	70	17	24.9	25.8	26.6	30	25.72	1.8
	V3	REF	87	23.3	25.35	26.2	27.35	29.4	26.33	1.36
	V3	CM	69	23.3	25.8	26.6	27.5	31.5	26.72	1.47
	V3	EYCF	69	23.6	25.6	26.7	27.6	30.7	26.77	1.47
Radius length (cm)	V1	REF	91	11.5	13.15	13.7	14.2	16	13.71	0.91
	V1	CM	91	11	13	13.9	14.15	16	13.7	1.04
	V1	EYCF	91	11	13	13.7	14.05	18	13.65	1.26
	V2	REF	87	12.1	13.5	14.2	14.6	16.4	14.14	0.85
	V2	CM	72	11.5	13.6	14.2	15	17	14.29	1.11
	V2	EYCF	70	11.2	13.9	14.3	14.9	19	14.35	1.22
	V3	REF	87	12.8	14	14.5	15	16.7	14.6	0.87
	V3	CM	69	11.9	14.1	14.8	15.5	17.4	14.78	1.11
	V3	EYCF	69	11.5	14.3	14.9	15.7	19.1	14.95	1.17
Handgrip (right hand) (kg)	V1	REF	91	1.07	1.58	1.9	2.15	4.2	1.98	0.61
	V1	CM	91	1.1	1.63	1.9	2.25	3.5	1.97	0.49
	V1	EYCF	91	1.07	1.6	1.8	2.1	3.9	1.92	0.52
	V3	REF	87	1.1	2.1	2.5	3.1	5.47	2.59	0.79
	V3	CM	69	1.1	1.97	2.27	3.03	5.03	2.52	0.77
	V3	EYCF	69	1.27	2.2	2.67	3.43	6.4	2.82	0.89
Bone turnover ([CTx – P1NP]/P1NP)	V1	REF	91	-4026	-1506	-755.2	-346.1	-61.54	-1011	790.8
	V1	CM	91	-2846	-870.6	-424.3	-256.5	-39.68	-691.4	628.2
	V1	EYCF	91	-3928	-1121	-531.2	-299.2	-39.99	-779.9	725.6
	V3	REF	87	-3621	-1160	-715.1	-359.4	-59.59	-926	785.5
	V3	CM	68	-3275	-1387	-782.7	-418.1	-107.8	-1038	766.2
	V3	EYCF	68	-3327	-1002	-568.1	-382.4	-120.7	-791.6	607.7
