# Supplementary material for: A young child formula with Limosilactobacillus reuteri and GOS modulates gut microbiome and enhances bone and muscle development: a randomized trial
Source: Nat Commun. 2025 Dec 12;17:237. doi: 10.1038/s41467-025-66930-2 (PMC12783733; doi:10.1038/s41467-025-66930-2)
Supplement: Supplementary file 5 — Supplementary data 3 [file 41467_2025_66930_MOESM5_ESM.pdf]

Clinical outcomes comparisons between of the experimental and control blends versus the habitual

	Model	Treatment	Visit	Estimate	95% CI	p-value
Height	Model 1	EYCF - REF	V3	0,52	[0.24; 0.8]	< 0.001
	Model 2	EYCF - REF	V3	0,51	[0.23; 0.79]	< 0.001
	Model 3	EYCF - REF	V3	0,52	[0.23; 0.81]	< 0.001
	Model 1	CM - REF	V3	0,72	[0.42; 1.02]	< 0.001
	Model 2	CM - REF	V3	0,72	[0.42; 1.02]	< 0.001
	Model 3	CM - REF	V3	0,74	[0.43; 1.05]	< 0.001
Weight	Model 1	EYCF - REF	V3	0,54	[0.31; 0.77]	< 0.001
	Model 2	EYCF - REF	V3	0,52	[0.28; 0.76]	< 0.001
	Model 3	EYCF - REF	V3	0,54	[0.3; 0.78]	< 0.001
	Model 1	CM - REF	V3	0,73	[0.48; 0.98]	< 0.001
	Model 2	CM - REF	V3	0,73	[0.48; 0.98]	< 0.001
	Model 3	CM - REF	V3	0,75	[0.49; 1.01]	< 0.001
Head circumference	Model 1	EYCF - REF	V3	0,03	[-0.11; 0.17]	0,671
	Model 2	EYCF - REF	V3	0,03	[-0.11; 0.17]	0,676
	Model 3	EYCF - REF	V3	0,04	[-0.1; 0.18]	0,582
	Model 1	CM - REF	V3	0,03	[-0.11; 0.17]	0,665
	Model 2	CM - REF	V3	0,03	[-0.11; 0.17]	0,665
	Model 3	CM - REF	V3	0,03	[-0.11; 0.17]	0,675
BMI	Model 1	EYCF - REF	V3	0,51	[0.26; 0.76]	< 0.001
	Model 2	EYCF - REF	V3	0,48	[0.22; 0.74]	< 0.001
	Model 3	EYCF - REF	V3	0,52	[0.26; 0.78]	< 0.001
	Model 1	CM - REF	V3	0,63	[0.36; 0.9]	< 0.001
	Model 2	CM - REF	V3	0,63	[0.36; 0.9]	< 0.001
	Model 3	CM - REF	V3	0,64	[0.36; 0.92]	< 0.001
Tibia length	Model 1	EYCF - REF	V2	0,24	[-0.14; 0.62]	0,211
	Model 1	EYCF - REF	V3	0,42	[0.04; 0.8]	0,03
	Model 2	EYCF - REF	V2	0,26	[-0.12; 0.64]	0,181
	Model 2	EYCF - REF	V3	0,44	[0.06; 0.82]	0,025
	Model 3	EYCF - REF	V2	0,23	[-0.15; 0.61]	0,23
	Model 3	EYCF - REF	V3	0,44	[0.06; 0.82]	0,023
	Model 1	CM - REF	V2	0,3	[-0.07; 0.67]	0,109
	Model 1	CM - REF	V3	0,42	[0.05; 0.79]	0,026
	Model 2	CM - REF	V2	0,3	[-0.07; 0.67]	0,111
	Model 2	CM - REF	V3	0,42	[0.05; 0.79]	0,027
	Model 3	CM - REF	V2	0,23	[-0.15; 0.61]	0,23
	Model 3	CM - REF	V3	0,44	[0.06; 0.82]	0,023
Tibia SoS	Model 1	EYCF - REF	V2	10,14	[-34.51; 54.79]	0,655
	Model 1	EYCF - REF	V3	53,38	[13.82; 92.94]	0,008
	Model 2	EYCF - REF	V2	4,11	[-40.54; 48.76]	0,856
	Model 2	EYCF - REF	V3	47,35	[7.78; 86.92]	0,019
	Model 3	EYCF - REF	V2	9,76	[-34.54; 54.06]	0,663
	Model 3	EYCF - REF	V3	55,01	[15.65; 94.37]	0,007
	Model 1	CM - REF	V2	-42,03	[-84.1; 0.04]	0,05

	Model 1	CM - REF	V3	-5,83	[-43.85; 32.19]	0,763
	Model 2	CM - REF	V2	-41,81	[-83.33; -0.29]	0,048
	Model 2	CM - REF	V3	-6,89	[-44.34; 30.56]	0,717
	Model 3	CM - REF	V2	-40,62	[-82.72; 1.48]	0,058
	Model 3	CM - REF	V3	-5,06	[-43.29; 33.17]	0,794
Radius length	Model 1	EYCF - REF	V2	0,13	[-0.02; 0.28]	0,096
	Model 1	EYCF - REF	V3	0,25	[0.1; 0.4]	0,002
	Model 2	EYCF - REF	V2	0,13	[-0.03; 0.29]	0,101
	Model 2	EYCF - REF	V3	0,25	[0.09; 0.41]	0,002
	Model 3	EYCF - REF	V2	0,13	[-0.02; 0.28]	0,097
	Model 3	EYCF - REF	V3	0,25	[0.1; 0.4]	0,002
	Model 1	CM - REF	V2	0,11	[-0.05; 0.27]	0,173
	Model 1	CM - REF	V3	0,16	[0; 0.32]	0,049
	Model 2	CM - REF	V2	0,11	[-0.05; 0.27]	0,173
	Model 2	CM - REF	V3	0,16	[0; 0.32]	0,049
	Model 3	CM - REF	V2	0,11	[-0.05; 0.27]	0,175
	Model 3	CM - REF	V3	0,16	[0; 0.32]	0,05
Radius SoS	Model 1	EYCF - REF	V2	15,01	[-23.49; 53.51]	0,444
	Model 1	EYCF - REF	V3	24,55	[-14.1; 63.2]	0,212
	Model 2	EYCF - REF	V2	16,31	[-22.64; 55.26]	0,41
	Model 2	EYCF - REF	V3	25,87	[-13.23; 64.97]	0,194
	Model 3	EYCF - REF	V2	14,11	[-24.64; 52.86]	0,472
	Model 3	EYCF - REF	V3	26,23	[-12.66; 65.12]	0,184
	Model 1	CM - REF	V2	-20,14	[-57.54; 17.26]	0,29
	Model 1	CM - REF	V3	14,14	[-23.51; 51.79]	0,46
	Model 2	CM - REF	V2	-20	[-57.46; 17.46]	0,294
	Model 2	CM - REF	V3	14,27	[-23.44; 51.98]	0,457
	Model 3	CM - REF	V2	-19,4	[-57.03; 18.23]	0,309
	Model 3	CM - REF	V3	13,88	[-24; 51.76]	0,469
Handgrip (right hand)	Model 1	(EYCF/REF-1)%	V3	7%	[-2%; 16%]	0,12
	Model 2	(EYCF/REF-1)%	V3	8%	[-1%; 17%]	0,079
	Model 3	(EYCF/REF-1)%	V3	8%	[-1%; 17%]	0,079
	Model 1	(CM/REF-1)%	V3	-3%	[-12%; 6%]	0,52
	Model 2	(CM/REF-1)%	V3	-3%	[-12%; 6%]	0,521
	Model 3	(CM/REF-1)%	V3	-3%	[-12%; 6%]	0,532
Bone turnover index	Model 1	(EYCF- REF)	V3	121,7	[-112.15; 355.47]	0,305
	Model 2	(EYCF- REF)	V3	119,8	[-117.24; 356.84]	0,32
	Model 3	(EYCF- REF)	V3	115,9	[-124.48; 356.26]	0,342
	Model 1	(CM- REF)	V3	-194,5	[-448.56; 59.52]	0,132
	Model 2	(CM- REF)	V3	-194,8	[-449.72; 60.04]	0,133
	Model 3	(CM- REF)	V3	-186,6	[-448.62; 75.5]	0,162
