# Supplementary material for: A young child formula with Limosilactobacillus reuteri and GOS modulates gut microbiome and enhances bone and muscle development: a randomized trial
Source: Nat Commun. 2025 Dec 12;17:237. doi: 10.1038/s41467-025-66930-2 (PMC12783733; doi:10.1038/s41467-025-66930-2)
Supplement: Supplementary file 6 — Supplementary data 4 [file 41467_2025_66930_MOESM6_ESM.pdf]

Comparisons of the blood vitamin level between Control milk group and Habitual diet group

	Model	Treatment	Visit	Estimate	95% CI	p-value
Vitamin D	Model 1	(EYCF/REF-1)%	V3	10%	[5%; 15%]	< 0.001
	Model 2	(EYCF/REF-1)%	V3	10%	[5%; 15%]	< 0.001
	Model 3	(EYCF/REF-1)%	V3	10%	[5%; 15%]	< 0.001
