# Supplementary material for: A young child formula with Limosilactobacillus reuteri and GOS modulates gut microbiome and enhances bone and muscle development: a randomized trial
Source: Nat Commun. 2025 Dec 12;17:237. doi: 10.1038/s41467-025-66930-2 (PMC12783733; doi:10.1038/s41467-025-66930-2)
Supplement: Supplementary file 7 — Supplementary data 5 [file 41467_2025_66930_MOESM7_ESM.pdf]

Correlation of ratio B3/B6 and mineral excretion (adjusted to intake) in the overall population and the clinical outcomes

	Ratio B3/B6	Calcium	Magnesium	Phosphorus
(p-value)	n=222	n=137	n=137	n=137
**TIBIA SOS V3**	-0.11 (0.11)	-0.05 (0.58)	-0.07 (0.42)	-0.05 (0.53)
**RADIUS SOS V3**	-0.07 (0.29)	-0.14 (0.1)	-0.03 (0.69)	-0.13 (0.15)
**TIBIA LENGTH V3**	-0.13 (0.05)	-0.02 (0.81)	-0.1 (0.25)	-0.09 (0.28)
**RADIUS LENGTH V3**	-0.11 (0.09)	-0.06 (0.46)	-0.11 (0.2)	-0.13 (0.13)
**HANDGRIP V3**	-0.05 (0.45)	-0.16 (0.06)	-0.16 (0.06)	-0.18 (0.03)
