# Supplementary material for: A young child formula with Limosilactobacillus reuteri and GOS modulates gut microbiome and enhances bone and muscle development: a randomized trial
Source: Nat Commun. 2025 Dec 12;17:237. doi: 10.1038/s41467-025-66930-2 (PMC12783733; doi:10.1038/s41467-025-66930-2)
Supplement: Supplementary file 8 — Supplementary data 6 [file 41467_2025_66930_MOESM8_ESM.pdf]

Comparisons	Baseline	3 months	6 months
All	0,088911	0,000999	0,000999
CM vs. REF	0,334665	0,038961	0,120879
CM vs. EYCF	0,05994	0,012987	0,000999
REF vs. EYCF	0,120879	0,001998	0,000999
