# Supplementary material for: A young child formula with Limosilactobacillus reuteri and GOS modulates gut microbiome and enhances bone and muscle development: a randomized trial
Source: Nat Commun. 2025 Dec 12;17:237. doi: 10.1038/s41467-025-66930-2 (PMC12783733; doi:10.1038/s41467-025-66930-2)
Supplement: Supplementary file 9 — Supplementary data 7 [file 41467_2025_66930_MOESM9_ESM.pdf]

Visit	Category	Item
baseline	MGS	Hgn3C.0001 - Phocaeicola vulgatus
baseline	MGS	Hgn3C.0002 - Bacteroides uniformis
baseline	MGS	Hgn3C.0003 - Lachnospiraceae sp.
baseline	MGS	Hgn3C.0004 - Blautia wexlerae
baseline	MGS	Hgn3C.0005 - Fusicatenibacter saccharivorans
baseline	MGS	Hgn3C.0006 - Bacteroides ovatus
baseline	MGS	Hgn3C.0007 - Parabacteroides distasonis
baseline	MGS	Hgn3C.0008 - Alistipes putredinis
baseline	MGS	Hgn3C.0009 - Faecalibacterium longum CLA-AA-H243
baseline	MGS	Hgn3C.0010 - Anaerostipes hadrus
baseline	MGS	Hgn3C.0011 - Roseburia faecis
baseline	MGS	Hgn3C.0012 - Lachnospira sp.
baseline	MGS	Hgn3C.0013 - Ruminococcus bromii
baseline	MGS	Hgn3C.0014 - Lacrimispora saccharolytica
baseline	MGS	Hgn3C.0015 - Faecalibacterium sp. Marseille-Q4896
baseline	MGS	Hgn3C.0016 - Parabacteroides merdae
baseline	MGS	Hgn3C.0017 - Subdoligranulum sp. APC924/74
baseline	MGS	Hgn3C.0018 - Faecalibacterium prausnitzii
baseline	MGS	Hgn3C.0019 - Phocaeicola dorei
baseline	MGS	Hgn3C.0020 - Collinsella aerofaciens
baseline	MGS	Hgn3C.0021 - Bifidobacterium longum subsp. longum
baseline	MGS	Hgn3C.0022 - Alistipes shahii
baseline	MGS	Hgn3C.0023 - Bacteroides xylanisolvens
baseline	MGS	Hgn3C.0024 - Bacteroides caccae
baseline	MGS	Hgn3C.0025 - Ruminococcus bicirculans
baseline	MGS	Hgn3C.0026 - Dorea longicatena
baseline	MGS	Hgn3C.0027 - Barnesiella intestinihominis
baseline	MGS	Hgn3C.0028 - Oscillibacter sp. ER4
baseline	MGS	Hgn3C.0029 - Anaerobutyricum hallii
baseline	MGS	Hgn3C.0030 - Faecalibacterium prausnitzii
baseline	MGS	Hgn3C.0031 - Blautia massiliensis
baseline	MGS	Hgn3C.0032 - Alistipes onderdonkii subsp. vulgaris
baseline	MGS	Hgn3C.0033 - Gemmiger formicilis
baseline	MGS	Hgn3C.0034 - Roseburia inulinivorans
baseline	MGS	Hgn3C.0035 - Odoribacter splanchnicus
baseline	MGS	Hgn3C.0036 - Bacteroides thetaiotaomicron
baseline	MGS	Hgn3C.0037 - Roseburia intestinalis
baseline	MGS	Hgn3C.0038 - Bifidobacterium adolescentis
baseline	MGS	Hgn3C.0039 - Coprococcus comes
baseline	MGS	Hgn3C.0040 - Eubacteriales sp.
baseline	MGS	Hgn3C.0041 - Ruminococcus sp.
baseline	MGS	Hgn3C.0042 - Blautia faecis
baseline	MGS	Hgn3C.0043 - Lachnoclostridium sp. 210928-DFI.6.3
baseline	MGS	Hgn3C.0044 - Lachnospira pectinoschiza
baseline	MGS	Hgn3C.0045 - Faecalibacterium sp.
baseline	MGS	Hgn3C.0046 - Clostridium sp. AF37-5
baseline	MGS	Hgn3C.0047 - Dysosmobacter sp. BX15

baseline	MGS	Hgn3C.0048 - Akkermansia muciniphila
baseline	MGS	Hgn3C.0050 - Escherichia coli
baseline	MGS	Hgn3C.0051 - Alistipes communis
baseline	MGS	Hgn3C.0052 - Phocaeicola massiliensis
baseline	MGS	Hgn3C.0053 - Eubacteriales sp.
baseline	MGS	Hgn3C.0054 - Prevotella copri
baseline	MGS	Hgn3C.0055 - Alistipes finegoldii
baseline	MGS	Hgn3C.0056 - Bacteroides fragilis
baseline	MGS	Hgn3C.0057 - Oscillibacter sp. MSJ-31
baseline	MGS	Hgn3C.0058 - Lachnospiraceae sp.
baseline	MGS	Hgn3C.0059 - Bacteroides cellulosilyticus
baseline	MGS	Hgn3C.0060 - Eubacteriales sp.
baseline	MGS	Hgn3C.0061 - Faecalibacillus sp. TM498
baseline	MGS	Hgn3C.0062 - Waltera intestinalis
baseline	MGS	Hgn3C.0063 - Eubacteriales sp.
baseline	MGS	Hgn3C.0064 - Blautia sp. DFI.9.9
baseline	MGS	Hgn3C.0065 - Blautia obeum
baseline	MGS	Hgn3C.0067 - Roseburia hominis
baseline	MGS	Hgn3C.0068 - [Ruminococcus] lactaris
baseline	MGS	Hgn3C.0069 - Oscillibacter sp. KLE 1728
baseline	MGS	Hgn3C.0070 - Faecalibacterium sp. OF04-11AC
baseline	MGS	Hgn3C.0071 - Faecalicatena fissicatena
baseline	MGS	Hgn3C.0072 - Clostridium fessum
baseline	MGS	Hgn3C.0073 - Blautia sp.
baseline	MGS	Hgn3C.0074 - Clostridium sp. D43t1_170807_D5
baseline	MGS	Hgn3C.0075 - Agathobaculum butyriciproducens
baseline	MGS	Hgn3C.0076 - Lachnospira pectinoschiza
baseline	MGS	Hgn3C.0077 - Sutterella wadsworthensis
baseline	MGS	Hgn3C.0078 - Clostridium sp. MCC328
baseline	MGS	Hgn3C.0079 - [Ruminococcus] torques
baseline	MGS	Hgn3C.0080 - Phascolarctobacterium faecium
baseline	MGS	Hgn3C.0082 - Roseburia sp. CLA-AA-H204
baseline	MGS	Hgn3C.0083 - Eubacteriales sp.
baseline	MGS	Hgn3C.0084 - Flavonifractor plautii
baseline	MGS	Hgn3C.0085 - Faecalibacterium sp.
baseline	MGS	Hgn3C.0086 - Dialister invisus
baseline	MGS	Hgn3C.0087 - Eubacteriales sp.
baseline	MGS	Hgn3C.0088 - [Ruminococcus] gnavus
baseline	MGS	Hgn3C.0089 - Paraprevotella clara
baseline	MGS	Hgn3C.0092 - Lachnospiraceae sp.
baseline	MGS	Hgn3C.0093 - Bilophila wadsworthia
baseline	MGS	Hgn3C.0094 - Eubacteriales sp.
baseline	MGS	Hgn3C.0095 - Eubacteriales sp.
baseline	MGS	Hgn3C.0096 - Eubacteriales sp.
baseline	MGS	Hgn3C.0097 - Eubacterium ventriosum
baseline	MGS	Hgn3C.0098 - Coprococcus catus
baseline	MGS	Hgn3C.0099 - Dorea longicatena
baseline	MGS	Hgn3C.0100 - Bifidobacterium bifidum

baseline	MGS	Hgn3C.0101 - Bifidobacterium pseudocatenulatum
baseline	MGS	Hgn3C.0102 - Eubacteriales sp.
baseline	MGS	Hgn3C.0103 - Holdemanella porci
baseline	MGS	Hgn3C.0104 - Clostridium sp. AM32-2
baseline	MGS	Hgn3C.0105 - Vescimonas coprocola
baseline	MGS	Hgn3C.0106 - Faecalibacterium sp. CLA-AA-H233
baseline	MGS	Hgn3C.0111 - Eubacteriales sp.
baseline	MGS	Hgn3C.0113 - Clostridium sp. MCC345
baseline	MGS	Hgn3C.0114 - Eubacteriales sp.
baseline	MGS	Hgn3C.0115 - Eubacteriales sp.
baseline	MGS	Hgn3C.0120 - Flavonifractor plautii
baseline	MGS	Hgn3C.0121 - Oscillospiraceae sp.
baseline	MGS	Hgn3C.0122 - Bacteroides finegoldii
baseline	MGS	Hgn3C.0123 - Eubacteriales sp.
baseline	MGS	Hgn3C.0124 - Ruthenibacterium lactatiformans
baseline	MGS	Hgn3C.0125 - Sutterella sp. KLE1602
baseline	MGS	Hgn3C.0126 - Ruminococcus sp. BSD2780120874_150323_B10
baseline	MGS	Hgn3C.0127 - Faecalibacterium sp.
baseline	MGS	Hgn3C.0128 - Eubacteriales sp.
baseline	MGS	Hgn3C.0129 - Haemophilus parainfluenzae
baseline	MGS	Hgn3C.0131 - Streptococcus thermophilus
baseline	MGS	Hgn3C.0132 - Eubacteriales sp.
baseline	MGS	Hgn3C.0136 - Alistipes ihumii
baseline	MGS	Hgn3C.0137 - Clostridium sp. OF03-18AA
baseline	MGS	Hgn3C.0138 - Anaerobutyricum soehngenii
baseline	MGS	Hgn3C.0139 - Ruminococcus callidus
baseline	MGS	Hgn3C.0140 - Veillonella parvula
baseline	MGS	Hgn3C.0141 - Adlercreutzia equolifaciens subsp. celatus
baseline	MGS	Hgn3C.0142 - Eubacterium ramulus
baseline	MGS	Hgn3C.0143 - Streptococcus salivarius
baseline	MGS	Hgn3C.0144 - Eubacteriales sp.
baseline	MGS	Hgn3C.0146 - Phascolarctobacterium succinatutens
baseline	MGS	Hgn3C.0147 - Oscillospiraceae sp.
baseline	MGS	Hgn3C.0153 - Lachnospiraceae sp.
baseline	MGS	Hgn3C.0154 - Prevotella copri
baseline	MGS	Hgn3C.0155 - Blautia sp.
baseline	MGS	Hgn3C.0157 - Eubacteriales sp.
baseline	MGS	Hgn3C.0161 - Blautia sp.
baseline	MGS	Hgn3C.0162 - Prevotella sp.
baseline	MGS	Hgn3C.0168 - Evtepia gabavorous
baseline	MGS	Hgn3C.0169 - Bacteroides intestinalis
baseline	MGS	Hgn3C.0170 - Oscillospiraceae sp.
baseline	MGS	Hgn3C.0171 - Catenibacterium mitsuokai
baseline	MGS	Hgn3C.0174 - Lachnospiraceae sp.
baseline	MGS	Hgn3C.0175 - Oscillospiraceae sp.
baseline	MGS	Hgn3C.0176 - Alistipes indistinctus
baseline	MGS	Hgn3C.0179 - Bacteroidales sp.
baseline	MGS	Hgn3C.0181 - Alistipes onderdonkii

baseline	MGS	Hgn3C.0182 - Butyricimonas faecihominis
baseline	MGS	Hgn3C.0183 - Intestinibacter bartlettii
baseline	MGS	Hgn3C.0184 - Eubacteriales sp.
baseline	MGS	Hgn3C.0185 - Bifidobacterium catenulatum subsp. kashiwanohense
baseline	MGS	Hgn3C.0186 - Butyricimonas virosa
baseline	MGS	Hgn3C.0191 - Prevotella sp.
baseline	MGS	Hgn3C.0192 - Erysipelatoclostridium ramosum
baseline	MGS	Hgn3C.0193 - Eubacteriales sp.
baseline	MGS	Hgn3C.0195 - Eubacteriales sp.
baseline	MGS	Hgn3C.0201 - Veillonella dispar
baseline	MGS	Hgn3C.0202 - Eggerthella lenta
baseline	MGS	Hgn3C.0203 - Faecalibacterium sp. Marseille-Q3530
baseline	MGS	Hgn3C.0206 - Gemmiger formicilis
baseline	MGS	Hgn3C.0207 - Eubacteriales sp.
baseline	MGS	Hgn3C.0208 - Romboutsia timonensis
baseline	MGS	Hgn3C.0209 - Bifidobacterium breve
baseline	MGS	Hgn3C.0212 - Holdemanella biformis
baseline	MGS	Hgn3C.0213 - Akkermansia sp. GGCC_0220
baseline	MGS	Hgn3C.0214 - Duodenibacillus massiliensis
baseline	MGS	Hgn3C.0215 - Alistipes sp. cv1
baseline	MGS	Hgn3C.0217 - Eubacteriales sp.
baseline	MGS	Hgn3C.0218 - Eubacteriales sp.
baseline	MGS	Hgn3C.0219 - Tyzzerella nexilis
baseline	MGS	Hgn3C.0220 - Clostridium sp. MCC334
baseline	MGS	Hgn3C.0221 - Clostridium sp.
baseline	MGS	Hgn3C.0222 - Parabacteroides merdae
baseline	MGS	Hgn3C.0224 - Veillonellales sp.
baseline	MGS	Hgn3C.0226 - Eubacteriales sp.
baseline	MGS	Hgn3C.0232 - Megamonas funiformis
baseline	MGS	Hgn3C.0236 - Oscillospiraceae sp.
baseline	MGS	Hgn3C.0238 - Oscillospiraceae sp.
baseline	MGS	Hgn3C.0239 - Blautia sp. BIOML-A1
baseline	MGS	Hgn3C.0242 - Enterocloster bolteae
baseline	MGS	Hgn3C.0244 - Desulfovibrio piger
baseline	MGS	Hgn3C.0245 - Blautia stercoris
baseline	MGS	Hgn3C.0248 - Coprococcus catus
baseline	MGS	Hgn3C.0249 - Clostridium sp. AM33-3
baseline	MGS	Hgn3C.0250 - Eubacteriales sp.
baseline	MGS	Hgn3C.0251 - Alistipes senegalensis
baseline	MGS	Hgn3C.0253 - Waltera sp.
baseline	MGS	Hgn3C.0260 - Hydrogenoanaerobacterium saccharovorans
baseline	MGS	Hgn3C.0262 - Oscillospiraceae sp.
baseline	MGS	Hgn3C.0264 - Clostridium sp. AF27-2AA
baseline	MGS	Hgn3C.0266 - Dorea sp. AF36-15AT
baseline	MGS	Hgn3C.0267 - Oscillospiraceae sp.
baseline	MGS	Hgn3C.0269 - Flintibacter sp. NSJ-23
baseline	MGS	Hgn3C.0272 - Blautia faecicola
baseline	MGS	Hgn3C.0273 - Slackia isoflavoniconvertens

baseline	MGS	Hgn3C.0275 - Lachnospiraceae sp.
baseline	MGS	Hgn3C.0281 - Eubacteriales sp.
baseline	MGS	Hgn3C.0284 - Erysipelotrichaceae sp.
baseline	MGS	Hgn3C.0285 - Colidextribacter sp.
baseline	MGS	Hgn3C.0286 - Clostridia sp.
baseline	MGS	Hgn3C.0290 - Sellimonas intestinalis
baseline	MGS	Hgn3C.0295 - Eubacteriales sp.
baseline	MGS	Hgn3C.0299 - Escherichia coli
baseline	MGS	Hgn3C.0300 - Clostridia sp.
baseline	MGS	Hgn3C.0301 - Prevotella hominis
baseline	MGS	Hgn3C.0304 - Eubacteriales sp.
baseline	MGS	Hgn3C.0306 - Bacteroides nordii
baseline	MGS	Hgn3C.0307 - Veillonella atypica
baseline	MGS	Hgn3C.0308 - [Clostridium] symbiosum
baseline	MGS	Hgn3C.0309 - Eubacteriales sp.
baseline	MGS	Hgn3C.0310 - Ligilactobacillus ruminis
baseline	MGS	Hgn3C.0313 - Ruminococcus bromii
baseline	MGS	Hgn3C.0314 - Ruminococcus sp.
baseline	MGS	Hgn3C.0317 - Intestinimonas butyriciproducens
baseline	MGS	Hgn3C.0319 - Clostridiaceae sp.
baseline	MGS	Hgn3C.0323 - Bacteroides sp.
baseline	MGS	Hgn3C.0324 - Prevotella stercorea
baseline	MGS	Hgn3C.0330 - Faecalibacillus intestinalis
baseline	MGS	Hgn3C.0332 - Klebsiella pneumoniae subsp. pneumoniae
baseline	MGS	Hgn3C.0334 - Bacteroides cellulosilyticus
baseline	MGS	Hgn3C.0336 - Parabacteroides goldsteinii
baseline	MGS	Hgn3C.0337 - Eubacteriales sp.
baseline	MGS	Hgn3C.0338 - Eubacteriales sp.
baseline	MGS	Hgn3C.0345 - Oscillospiraceae sp.
baseline	MGS	Hgn3C.0348 - Streptococcus sp.
baseline	MGS	Hgn3C.0350 - [Clostridium] symbiosum
baseline	MGS	Hgn3C.0351 - Prevotella sp.
baseline	MGS	Hgn3C.0354 - Sutterella seckii
baseline	MGS	Hgn3C.0356 - Blautia sp. M29
baseline	MGS	Hgn3C.0357 - Eubacteriales sp.
baseline	MGS	Hgn3C.0361 - Dorea sp. AF24-7LB
baseline	MGS	Hgn3C.0362 - Dysosmobacter sp. NSJ-60
baseline	MGS	Hgn3C.0365 - Eubacteriales sp.
baseline	MGS	Hgn3C.0367 - Lachnospiraceae sp.
baseline	MGS	Hgn3C.0369 - Prevotella sp.
baseline	MGS	Hgn3C.0371 - Lachnospiraceae sp.
baseline	MGS	Hgn3C.0373 - Enterocloster clostridioformis
baseline	MGS	Hgn3C.0375 - Ellagibacter isourolithinifaciens
baseline	MGS	Hgn3C.0381 - Clostridium sp. AM30-24
baseline	MGS	Hgn3C.0385 - Veillonella rogosae
baseline	MGS	Hgn3C.0386 - Clostridium sp. AT4
baseline	MGS	Hgn3C.0388 - Clostridium sp.
baseline	MGS	Hgn3C.0392 - Clostridia sp.

baseline	MGS	Hgn3C.0394 - Hungatella hathewayi
baseline	MGS	Hgn3C.0397 - Parabacteroides sp.
baseline	MGS	Hgn3C.0400 - Prevotellamassilia timonensis
baseline	MGS	Hgn3C.0401 - Eubacteriales sp.
baseline	MGS	Hgn3C.0405 - Bacteroides ovatus
baseline	MGS	Hgn3C.0407 - Eubacteriales sp.
baseline	MGS	Hgn3C.0408 - Eubacteriales sp.
baseline	MGS	Hgn3C.0413 - Blautia caecimuris
baseline	MGS	Hgn3C.0415 - Clostridium sp. C5-48
baseline	MGS	Hgn3C.0417 - Eubacteriales sp.
baseline	MGS	Hgn3C.0418 - Blautia sp.
baseline	MGS	Hgn3C.0419 - Prevotella sp. 885
baseline	MGS	Hgn3C.0420 - Clostridium sp. 1001270J_160509_D11
baseline	MGS	Hgn3C.0430 - Enterocloster aldenensis
baseline	MGS	Hgn3C.0435 - Eubacteriales sp.
baseline	MGS	Hgn3C.0436 - Eubacteriales sp.
baseline	MGS	Hgn3C.0437 - Prevotella sp.
baseline	MGS	Hgn3C.0438 - Coprobacillus cateniformis
baseline	MGS	Hgn3C.0439 - Enterococcus faecalis
baseline	MGS	Hgn3C.0442 - Holdemanella sp.
baseline	MGS	Hgn3C.0443 - Prevotella stercorea
baseline	MGS	Hgn3C.0445 - Aeromonadales sp.
baseline	MGS	Hgn3C.0452 - Oscillospiraceae sp.
baseline	MGS	Hgn3C.0453 - Blautia sp. Marseille-P3087
baseline	MGS	Hgn3C.0455 - Peptostreptococcaceae sp.
baseline	MGS	Hgn3C.0458 - Eubacteriales sp.
baseline	MGS	Hgn3C.0459 - Eubacteriales sp.
baseline	MGS	Hgn3C.0464 - Bifidobacterium longum subsp. infantis
baseline	MGS	Hgn3C.0465 - Eisenbergiella massiliensis
baseline	MGS	Hgn3C.0468 - Clostridium sp. NSJ-42
baseline	MGS	Hgn3C.0470 - Eubacteriales sp.
baseline	MGS	Hgn3C.0472 - Parabacteroides goldsteinii
baseline	MGS	Hgn3C.0476 - Streptococcus sp.
baseline	MGS	Hgn3C.0482 - Lachnospiraceae sp.
baseline	MGS	Hgn3C.0493 - Eisenbergiella tayi
baseline	MGS	Hgn3C.0494 - Prevotellaceae sp.
baseline	MGS	Hgn3C.0500 - Anaerotignum sp.
baseline	MGS	Hgn3C.0501 - Oscillospiraceae sp.
baseline	MGS	Hgn3C.0504 - Megasphaera sp. BL7
baseline	MGS	Hgn3C.0507 - [Clostridium] spiroforme
baseline	MGS	Hgn3C.0514 - Blautia glucerasea
baseline	MGS	Hgn3C.0523 - Phascolarctobacterium succinatutens
baseline	MGS	Hgn3C.0527 - Sutterella sp.
baseline	MGS	Hgn3C.0528 - Coprococcus sp. AM27-12LB
baseline	MGS	Hgn3C.0533 - Flintibacter sp. KGMB00164
baseline	MGS	Hgn3C.0537 - Bifidobacterium dentium
baseline	MGS	Hgn3C.0542 - [Clostridium] innocuum
baseline	MGS	Hgn3C.0543 - Oscillospiraceae sp.

baseline	MGS	Hgn3C.0549 - Longicatena caecimuris
baseline	MGS	Hgn3C.0557 - Enterocloster bolteae
baseline	MGS	Hgn3C.0558 - Olsenella sp.
baseline	MGS	Hgn3C.0560 - Oscillospiraceae sp.
baseline	MGS	Hgn3C.0573 - Prevotella sp. Marseille-P4119
baseline	MGS	Hgn3C.0582 - Bacteroides sp.
baseline	MGS	Hgn3C.0584 - Bacteroides fragilis
baseline	MGS	Hgn3C.0598 - Eubacteriales sp.
baseline	MGS	Hgn3C.0604 - Megasphaera elsdenii
baseline	MGS	Hgn3C.0607 - Eubacteriales sp.
baseline	MGS	Hgn3C.0608 - Lachnospiraceae sp.
baseline	MGS	Hgn3C.0610 - Eubacteriales sp.
baseline	MGS	Hgn3C.0613 - Clostridiaceae sp.
baseline	MGS	Hgn3C.0616 - Lachnospiraceae sp.
baseline	MGS	Hgn3C.0617 - Prevotella sp.
baseline	MGS	Hgn3C.0629 - Collinsella intestinalis
baseline	MGS	Hgn3C.0630 - Allisonella histaminiformans
baseline	MGS	Hgn3C.0635 - Eubacteriales sp.
baseline	MGS	Hgn3C.0636 - Anaerostipes caccae
baseline	MGS	Hgn3C.0640 - Eubacterium sp. AF22-8LB
baseline	MGS	Hgn3C.0643 - Eubacteriales sp.
baseline	MGS	Hgn3C.0644 - Bacteroides cellulosilyticus
baseline	MGS	Hgn3C.0649 - Lachnospiraceae sp.
baseline	MGS	Hgn3C.0657 - Parolsenella catena
baseline	MGS	Hgn3C.0664 - Blautia hansenii
baseline	MGS	Hgn3C.0675 - Eubacteriales sp.
baseline	MGS	Hgn3C.0676 - Bacteroides sp.
baseline	MGS	Hgn3C.0677 - Bacteroides sp.
baseline	MGS	Hgn3C.0679 - Holdemania filiformis
baseline	MGS	Hgn3C.0680 - Eubacteriales sp.
baseline	MGS	Hgn3C.0682 - [Clostridium] scindens
baseline	MGS	Hgn3C.0683 - Clostridium disporicum
baseline	MGS	Hgn3C.0693 - Hungatella hathewayi
baseline	MGS	Hgn3C.0694 - Anaeromassilibacillus sp. An250
baseline	MGS	Hgn3C.0701 - Bacteroides sp.
baseline	MGS	Hgn3C.0704 - [Clostridium] scindens
baseline	MGS	Hgn3C.0711 - Enterocloster asparagiformis
baseline	MGS	Hgn3C.0712 - Megasphaera micronuciformis
baseline	MGS	Hgn3C.0713 - Veillonellales sp.
baseline	MGS	Hgn3C.0714 - Intestinimonas sp. MSJ-38
baseline	MGS	Hgn3C.0715 - Desulfovibrio fairfieldensis
baseline	MGS	Hgn3C.0718 - Eubacteriales sp.
baseline	MGS	Hgn3C.0729 - Eubacteriales sp.
baseline	MGS	Hgn3C.0731 - Eubacteriales sp.
baseline	MGS	Hgn3C.0732 - Eubacterium ramulus
baseline	MGS	Hgn3C.0736 - Coprococcus sp.
baseline	MGS	Hgn3C.0737 - Alistipes timonensis
baseline	MGS	Hgn3C.0740 - Anaerostipes sp. NSJ-7

baseline	MGS	Hgn3C.0741 - Blautia sp. OF01-4LB
baseline	MGS	Hgn3C.0743 - Enterobacter sp.
baseline	MGS	Hgn3C.0749 - Eubacteriales sp.
baseline	MGS	Hgn3C.0754 - Collinsella bouchesdurhonensis
baseline	MGS	Hgn3C.0761 - Bacteroides sp.
baseline	MGS	Hgn3C.0762 - Enterocloster lavalensis
baseline	MGS	Hgn3C.0767 - Citrobacter freundii
baseline	MGS	Hgn3C.0777 - Holdemanella sp.
baseline	MGS	Hgn3C.0781 - Collinsella sp.
baseline	MGS	Hgn3C.0782 - Streptococcus sp.
baseline	MGS	Hgn3C.0790 - Oscillospiraceae sp.
baseline	MGS	Hgn3C.0793 - Prevotellaceae sp.
baseline	MGS	Hgn3C.0794 - Bacteroides sp.
baseline	MGS	Hgn3C.0797 - Senegalimassilia sp.
baseline	MGS	Hgn3C.0798 - Streptococcus vestibularis
baseline	MGS	Hgn3C.0814 - Massilimicrobiota timonensis
baseline	MGS	Hgn3C.0817 - Prevotella sp.
baseline	MGS	Hgn3C.0818 - Rothia sp.
baseline	MGS	Hgn3C.0823 - Anaerotignum lactatifermentans
baseline	MGS	Hgn3C.0829 - Dorea phocaeensis
baseline	MGS	Hgn3C.0832 - Veillonellales sp.
baseline	MGS	Hgn3C.0833 - Eisenbergiella tayi
baseline	MGS	Hgn3C.0837 - Anaerotruncus colihominis
baseline	MGS	Hgn3C.0851 - Oscillospiraceae sp.
baseline	MGS	Hgn3C.0855 - Eubacteriales sp.
baseline	MGS	Hgn3C.0858 - Turicibacter sanguinis
baseline	MGS	Hgn3C.0859 - Hydrogeniiclostidium mannosilyticum
baseline	MGS	Hgn3C.0873 - Clostridium perfringens
baseline	MGS	Hgn3C.0875 - Lachnospiraceae sp.
baseline	MGS	Hgn3C.0886 - Bacteroides sp.
baseline	MGS	Hgn3C.0889 - Clostridium paraputrificum
baseline	MGS	Hgn3C.0890 - Enterobacter roggenkampii
baseline	MGS	Hgn3C.0891 - Eubacteriales sp.
baseline	MGS	Hgn3C.0893 - Phocea massiliensis
baseline	MGS	Hgn3C.0908 - Eubacteriales sp.
baseline	MGS	Hgn3C.0913 - Erysipelotrichaceae sp.
baseline	MGS	Hgn3C.0921 - Bacteroides sp.
baseline	MGS	Hgn3C.0922 - Eubacteriales sp.
baseline	MGS	Hgn3C.0940 - Eubacteriales sp.
baseline	MGS	Hgn3C.0948 - Enterobacter sp.
baseline	MGS	Hgn3C.0978 - Veillonella sp.
baseline	MGS	Hgn3C.0987 - Fusobacterium mortiferum
baseline	MGS	Hgn3C.0996 - Oscillospiraceae sp.
baseline	MGS	Hgn3C.0998 - Eubacteriales sp.
baseline	MGS	Hgn3C.1012 - Erysipelotrichaceae sp.
baseline	MGS	Hgn3C.1035 - Klebsiella sp.
baseline	MGS	Hgn3C.1036 - Escherichia coli
baseline	MGS	Hgn3C.1037 - Rothia sp.

baseline	MGS	Hgn3C.1038 - Veillonella sp.
baseline	MGS	Hgn3C.1040 - Lachnospiraceae sp.
baseline	MGS	Hgn3C.1041 - Bacteroides stercoris
baseline	MGS	Hgn3C.1042 - Dorea formicigenerans
baseline	MGS	Hgn3C.1043 - Clostridium phoceensis
baseline	MGS	Hgn3C.1044 - Eubacteriales sp.
baseline	MGS	Hgn3C.1045 - Lactococcus lactis subsp. lactis
baseline	MGS	Hgn3C.1049 - Enterococcus faecium
baseline	MGS	Hgn3C.1050 - Staphylococcus epidermidis
baseline	MGS	Hgn3C.1051 - Holdemania massiliensis
baseline	MGS	Hgn3C.1052 - Enterococcus gallinarum
baseline	MGS	Hgn3C.1054 - Streptococcus lutetiensis
baseline	MGS	Hgn3C.1058 - Mediterraneibacter glycyrrhizinilyticus
baseline	MGS	Hgn3C.1059 - Faecalicatena sp.
baseline	MGS	Hgn3C.1063 - Enterococcus avium
baseline	MGS	Hgn3C.1065 - Klebsiella variicola subsp. variicola
baseline	MGS	Hgn3C.1067 - Lachnospiraceae sp.
baseline	MGS	Hgn3C.1073 - Streptococcus gallolyticus subsp. gallolyticus
baseline	MGS	Hgn3C.1081 - Lacticaseibacillus rhamnosus
baseline	MGS	Hgn3C.1082 - Lacticaseibacillus paracasei subsp. paracasei
baseline	MGS	Hgn3C.1084 - Massilimicrobiota timonensis
baseline	MGS	Hgn3C.1087 - Ligilactobacillus salivarius
baseline	MGS	Hgn3C.1090 - Turicibacter sanguinis
baseline	MGS	Hgn3C.1093 - Enterobacter kobei
baseline	MGS	Hgn3C.1107 - Eubacteriales sp.
baseline	MGS	Hgn3C.1110 - Porphyromonas sp.
baseline	MGS	Hgn3C.1117 - Atopobiaceae sp.
baseline	MGS	Hgn3C.1120 - Actinomycetaceae sp.
baseline	MGS	Hgn3C.1146 - Bacteria sp.
baseline	MGS	Hgn3C.1148 - Eggerthellales sp.
baseline	MGS	Hgn3C.1154 - Lactococcus garvieae
baseline	MGS	Hgn3C.1180 - Enterocloster citroniae
baseline	MGS	Hgn3C.1188 - Actinomyces oris
baseline	MGS	Hgn3C.1190 - Fenollaria massiliensis
baseline	MGS	Hgn3C.1193 - Atopobium sp.
baseline	MGS	Hgn3C.1196 - Blautia marasmi
baseline	MGS	Hgn3C.1197 - Blautia hansenii
baseline	MGS	Hgn3C.1210 - Dielma sp.
baseline	MGS	Hgn3C.1211 - Lachnoclostridium pacaense
baseline	MGS	Hgn3C.1214 - Corynebacterium tuberculostearicum
baseline	MGS	Hgn3C.1218 - Enterobacter bugandensis
baseline	MGS	Hgn3C.1219 - Enterobacter sp. NFIX58
baseline	MGS	Hgn3C.1225 - Enterococcus faecium
baseline	MGS	Hgn3C.1229 - Escherichia sp. 93.0750
baseline	MGS	Hgn3C.1230 - Eubacterium limosum
baseline	MGS	Hgn3C.1234 - Granulicatella adiacens
baseline	MGS	Hgn3C.1238 - Klebsiella quasivariicola
baseline	MGS	Hgn3C.1242 - Limosilactobacillus vaginalis

baseline	MGS	Hgn3C.1248 - Neisseria sp. HMSC061E12
baseline	MGS	Hgn3C.1258 - Rothia sp. HMSC072E10
baseline	MGS	Hgn3C.1264 - Staphylococcus hominis subsp. hominis
baseline	MGS	Hgn3C.1268 - Streptococcus sp.
baseline	MGS	Hgn3C.1269 - Streptococcus constellatus subsp. constellatus
baseline	MGS	Hgn3C.1270 - Streptococcus sp.
baseline	MGS	Hgn3C.1271 - Streptococcus agalactiae
baseline	MGS	Hgn3C.1275 - Lachnospiraceae sp.
baseline	MGS	Hgn3C.1277 - Lachnospiraceae sp.
baseline	MGS	Hgn3C.1278 - Clostridium sp. NSJ-6
baseline	MGS	Hgn3C.1282 - Terrisporobacter othiniensis
baseline	MGS	Hgn3C.1286 - Corynebacterium variabile
baseline	MGS	Hgn3C.1288 - Enterobacter hormaechei
baseline	MGS	Hgn3C.1292 - Limosilactobacillus portuensis
baseline	MGS	Hgn3C.1295 - Butyricicoccus pullicaecorum
baseline	MGS	Hgn3C.1299 - Erysipelotrichaceae sp.
baseline	MGS	Hgn3C.1300 - Rothia sp.
baseline	MGS	Hgn3C.1302 - Schaalia sp.
baseline	MGS	Hgn3C.1303 - Clostridium paraputrificum
baseline	MGS	Hgn3C.1305 - Isoptericola variabilis
baseline	MGS	Hgn3C.1307 - Bifidobacterium longum subsp. spp
baseline	MGS	Hgn3C.1308 - Eisenbergiella sp.
baseline	MGS	Hgn3C.1309 - Enterobacter cloacae subsp. cloacae
baseline	MGS	Hgn3C.1310 - Erysipelotrichaceae sp.
baseline	MGS	Hgn3C.1311 - Lachnospiraceae sp.
baseline	MGS	Hgn3C.1312 - Candida tropicalis
baseline	MGS	Hgn3C.1315 - Eubacteriales sp.
baseline	MGS	Hgn3C.1316 - Eubacteriales sp.
baseline	MGS	Hgn3C.1319 - Atlantibacter hermannii
baseline	MGS	Hgn3C.1321 - Veillonellaceae sp.
baseline	MGS	Hgn3C.1322 - Enterococcus hirae
baseline	MGS	Hgn3C.1323 - Lachnospiraceae sp.
baseline	MGS	Hgn3C.1324 - Eubacterium sp. c-25
baseline	MGS	Hgn3C.1325 - Luxibacter massiliensis
baseline	MGS	Hgn3C.1326 - Eggerthella guodeyinii
baseline	MGS	Hgn3C.1329 - Eubacteriales sp.
baseline	MGS	Hgn3C.1330 - Lachnospiraceae sp.
baseline	MGS	Hgn3C.1331 - Lachnospiraceae sp.
baseline	MGS	Hgn3C.1334 - Eubacteriales sp.
baseline	MGS	Hgn3C.1338 - Erysipelatoclostridium sp.
baseline	MGS	Hgn3C.1340 - Clostridiaceae sp.
baseline	MGS	Hgn3C.1343 - Enterococcus italicus
baseline	MGS	Hgn3C.1345 - Limosilactobacillus mucosae
baseline	MGS	Hgn3C.1346 - Erysipelotrichales sp.
baseline	MGS	Hgn3C.1347 - [Clostridium] hylemonae
baseline	MGS	Hgn3C.1348 - Eubacteriales sp.
baseline	MGS	Hgn3C.1351 - Actinomyces sp. ICM47
baseline	MGS	Hgn3C.1352 - Akkermansia sp.

baseline	MGS	Hgn3C.1354 - Actinomyces sp.
baseline	MGS	Hgn3C.1355 - Collinsella stercoris
baseline	MGS	Hgn3C.1358 - Lachnospiraceae sp.
baseline	MGS	Hgn3C.1359 - Extibacter muris
baseline	MGS	Hgn3C.1360 - Bifidobacterium crudilactis
baseline	MGS	Hgn3C.1361 - Senegalimassilia anaerobia
baseline	MGS	Hgn3C.1362 - Christensenella intestinihominis
baseline	MGS	Hgn3C.1366 - Lachnospiraceae sp.
baseline	MGS	Hgn3C.1369 - Lachnospiraceae sp.
baseline	MGS	Hgn3C.1370 - Kluyvera genomosp. 3
baseline	MGS	Hgn3C.1372 - Eubacteriales sp.
baseline	MGS	Hgn3C.1373 - Eubacteriales sp.
baseline	MGS	Hgn3C.1375 - Atopobiaceae sp.
baseline	MGS	Hgn3C.1376 - Kluyvera sp. CRP
baseline	MGS	Hgn3C.1377 - Collinsella tanakaei
baseline	MGS	Hgn3C.1378 - Collinsella sp. AF08-23
baseline	MGS	Hgn3C.1379 - Gordonibacter urolithinfaciens
baseline	MGS	Hgn3C.1380 - Kluyvera georgiana
baseline	MGS	Hgn3C.1382 - Oscillospiraceae sp.
baseline	MGS	Hgn3C.1384 - Anaerococcus sp.
baseline	MGS	Hgn3C.1386 - Veillonella sp.
baseline	MGS	Hgn3C.1387 - Alistipes sp.
baseline	MGS	Hgn3C.1388 - Streptococcus infantarius subsp. infantarius
baseline	MGS	Hgn3C.1390 - Erysipelatoclostridium sp.
baseline	MGS	Hgn3C.1392 - Enterobacter sp. JBIWA005
baseline	MGS	Hgn3C.1393 - Anaerofustis stercorihominis
baseline	MGS	Hgn3C.1394 - Eubacteriales sp.
baseline	MGS	Hgn3C.1398 - Actinomyces sp.
baseline	MGS	Hgn3C.1400 - Eubacterium sp.
baseline	MGS	Hgn3C.1401 - Eubacteriales sp.
baseline	MGS	Hgn3C.1403 - Eubacteriaceae sp.
baseline	MGS	Hgn3C.1404 - Clostridium sp.
baseline	MGS	Hgn3C.1406 - Slackia piriformis
baseline	MGS	Hgn3C.1407 - Christensenella minuta
baseline	MGS	Hgn3C.1408 - Eubacterium callanderi
baseline	MGS	Hgn3C.1409 - Eggerthella timonensis
baseline	MGS	Hgn3C.1412 - Christensenella hongkongensis
baseline	MGS	Hgn3C.1413 - Streptococcus sp.
baseline	MGS	Hgn3C.1414 - Collinsella sp.
baseline	MGS	Hgn3C.1415 - Klebsiella quasipneumoniae subsp. quasipneumoniae
baseline	MGS	Hgn3C.1416 - Limosilactobacillus fermentum
baseline	MGS	Hgn3C.1418 - Carnobacteriaceae sp.
baseline	MGS	Hgn3C.1419 - Agathobaculum sp. NSJ-28
baseline	MGS	Hgn3C.1420 - Eubacteriales sp.
baseline	MGS	Hgn3C.1423 - Lachnospiraceae sp.
baseline	MGS	Hgn3C.1424 - Mogibacterium sp. NSJ-24
baseline	MGS	Hgn3C.1425 - Eubacteriales sp.
baseline	MGS	Hgn3C.1426 - Limosilactobacillus oris

baseline	MGS	Hgn3C.1429 - Lachnospiraceae sp.
baseline	MGS	Hgn3C.1431 - Bacteroidales sp.
baseline	MGS	Hgn3C.1434 - Blautia sp.
baseline	MGS	Hgn3C.1435 - Acinetobacter baumannii
baseline	MGS	Hgn3C.1436 - Bacteria sp.
baseline	MGS	Hgn3C.1437 - Candidatus Stoquefichus sp. SB1
baseline	MGS	Hgn3C.1438 - Dielma fastidiosa
baseline	MGS	Hgn3C.1439 - Streptococcus sp. IMAU 99161
baseline	MGS	Hgn3C.1441 - Lancefieldella sp.
baseline	MGS	Hgn3C.1443 - Streptococcus sp.
baseline	MGS	Hgn3C.1444 - Ruminococcus sp. 1001175B_160314_E1
baseline	MGS	Hgn3C.1446 - Eubacteriales sp.
baseline	MGS	Hgn3C.1447 - Bacteria sp.
baseline	MGS	Hgn3C.1448 - Leuconostoc falkenbergense
baseline	MGS	Hgn3C.1450 - Lactococcus sp. NH2-7C
baseline	MGS	Hgn3C.1452 - Raoultibacter timonensis
baseline	MGS	Hgn3C.1453 - Collinsella sp.
baseline	MGS	Hgn3C.1455 - Enterocloster sp.
baseline	MGS	Hgn3C.1456 - Eubacteriales sp.
baseline	MGS	Hgn3C.1460 - Blautia sp.
baseline	MGS	Hgn3C.1463 - Enterobacter sp.
baseline	MGS	Hgn3C.1465 - Clostridia sp.
baseline	MGS	Hgn3C.1467 - Eubacteriales sp.
baseline	MGS	Hgn3C.1468 - Staphylococcus aureus
baseline	MGS	Hgn3C.1469 - Lachnospiraceae sp.
baseline	MGS	Hgn3C.1471 - Eggerthella sp.
baseline	MGS	Hgn3C.1472 - Amedibacillus dolichus
baseline	genus	Acinetobacter
baseline	genus	Actinomyces
baseline	genus	Adlercreutzia
baseline	genus	Agathobaculum
baseline	genus	Akkermansia
baseline	genus	Alistipes
baseline	genus	Allisonella
baseline	genus	Amedibacillus
baseline	genus	Anaerobutyricum
baseline	genus	Anaerococcus
baseline	genus	Anaerofustis
baseline	genus	Anaeromassilibacillus
baseline	genus	Anaerostipes
baseline	genus	Anaerotignum
baseline	genus	Anaerotruncus
baseline	genus	Atlantibacter
baseline	genus	Atopobium
baseline	genus	Bacteroides
baseline	genus	Barnesiella
baseline	genus	Bifidobacterium
baseline	genus	Bilophila

baseline	genus	Blautia
baseline	genus	Butyricicoccus
baseline	genus	Butyricimonas
baseline	genus	Candida
baseline	genus	Candidatus Stoquefichus
baseline	genus	Catenibacterium
baseline	genus	Christensenella
baseline	genus	Citrobacter
baseline	genus	Clostridium
baseline	genus	Colidextribacter
baseline	genus	Collinsella
baseline	genus	Coprobacillus
baseline	genus	Coprobacter
baseline	genus	Coprococcus
baseline	genus	Corynebacterium
baseline	genus	Desulfovibrio
baseline	genus	Dialister
baseline	genus	Dielma
baseline	genus	Dorea
baseline	genus	Duodenibacillus
baseline	genus	Dysosmobacter
baseline	genus	Eggerthella
baseline	genus	Eisenbergiella
baseline	genus	Ellagibacter
baseline	genus	Enterobacter
baseline	genus	Enterocloster
baseline	genus	Enterococcus
baseline	genus	Erysipelatoclostridium
baseline	genus	Escherichia
baseline	genus	Eubacterium
baseline	genus	Evtepia
baseline	genus	Extibacter
baseline	genus	Faecalibacillus
baseline	genus	Faecalibacterium
baseline	genus	Faecalicatena
baseline	genus	Fenollaria
baseline	genus	Flavonifractor
baseline	genus	Flintibacter
baseline	genus	Fusicatenibacter
baseline	genus	Fusobacterium
baseline	genus	Gemmiger
baseline	genus	Gordonibacter
baseline	genus	Granulicatella
baseline	genus	Haemophilus
baseline	genus	Holdemanella
baseline	genus	Holdemania
baseline	genus	Hungatella
baseline	genus	Hydrogeniiclostidium

baseline	genus	Hydrogenoanaerobacterium
baseline	genus	Intestinibacter
baseline	genus	Intestinimonas
baseline	genus	Isoptericola
baseline	genus	Klebsiella
baseline	genus	Kluyvera
baseline	genus	Lachnoclostridium
baseline	genus	Lachnospira
baseline	genus	Lacrimispora
baseline	genus	Lacticaseibacillus
baseline	genus	Lactobacillus
baseline	genus	Lactococcus
baseline	genus	Lancefieldella
baseline	genus	Leuconostoc
baseline	genus	Ligilactobacillus
baseline	genus	Limosilactobacillus
baseline	genus	Longicatena
baseline	genus	Luxibacter
baseline	genus	Massilimicrobiota
baseline	genus	Mediterraneibacter
baseline	genus	Megamonas
baseline	genus	Megasphaera
baseline	genus	Mogibacterium
baseline	genus	Neisseria
baseline	genus	Odoribacter
baseline	genus	Olsenella
baseline	genus	Oscillibacter
baseline	genus	Parabacteroides
baseline	genus	Paraprevotella
baseline	genus	Parasutterella
baseline	genus	Parolsenella
baseline	genus	Phascolarctobacterium
baseline	genus	Phocaeicola
baseline	genus	Phocea
baseline	genus	Porphyromonas
baseline	genus	Prevotella
baseline	genus	Prevotellamassilia
baseline	genus	Raoultibacter
baseline	genus	Romboutsia
baseline	genus	Roseburia
baseline	genus	Rothia
baseline	genus	Ruminococcus
baseline	genus	Ruthenibacterium
baseline	genus	Schaalia
baseline	genus	Sellimonas
baseline	genus	Senegalimassilia
baseline	genus	Slackia
baseline	genus	Staphylococcus

baseline	genus	Streptococcus
baseline	genus	Subdoligranulum
baseline	genus	Sutterella
baseline	genus	Terrisporobacter
baseline	genus	Turicibacter
baseline	genus	Tyzzerella
baseline	genus	Veillonella
baseline	genus	Vescimonas
baseline	genus	Waltera
baseline	family	Acidaminococcaceae
baseline	family	Actinomycetaceae
baseline	family	Akkermansiaceae
baseline	family	Atopobiaceae
baseline	family	Bacteroidaceae
baseline	family	Barnesiellaceae
baseline	family	Bifidobacteriaceae
baseline	family	Carnobacteriaceae
baseline	family	Christensenellaceae
baseline	family	Clostridiaceae
baseline	family	Coprobacillaceae
baseline	family	Coriobacteriaceae
baseline	family	Corynebacteriaceae
baseline	family	Debaryomycetaceae
baseline	family	Desulfovibrionaceae
baseline	family	Eggerthellaceae
baseline	family	Enterobacteriaceae
baseline	family	Enterococcaceae
baseline	family	Erysipelotrichaceae
baseline	family	Eubacteriaceae
baseline	family	Eubacteriales Family XIII. Incertae Sedis
baseline	family	Fusobacteriaceae
baseline	family	Lachnospiraceae
baseline	family	Lactobacillaceae
baseline	family	Micrococcaceae
baseline	family	Moraxellaceae
baseline	family	Neisseriaceae
baseline	family	Odoribacteraceae
baseline	family	Oscillospiraceae
baseline	family	Pasteurellaceae
baseline	family	Peptoniphilaceae
baseline	family	Peptostreptococcaceae
baseline	family	Porphyromonadaceae
baseline	family	Prevotellaceae
baseline	family	Promicromonosporaceae
baseline	family	Rikenellaceae
baseline	family	Selenomonadaceae
baseline	family	Staphylococcaceae
baseline	family	Streptococcaceae

baseline	family	Sutterellaceae
baseline	family	Tannerellaceae
baseline	family	Turicibacteraceae
baseline	family	Veillonellaceae
baseline	phylum	Actinobacteria
baseline	phylum	Ascomycota
baseline	phylum	Bacteroidetes
baseline	phylum	Firmicutes
baseline	phylum	Fusobacteria
baseline	phylum	Proteobacteria
baseline	phylum	Verrucomicrobia
3 months	MGS	Hgn3C.0001 - Phocaeicola vulgatus
3 months	MGS	Hgn3C.0002 - Bacteroides uniformis
3 months	MGS	Hgn3C.0003 - Lachnospiraceae sp.
3 months	MGS	Hgn3C.0004 - Blautia wexlerae
3 months	MGS	Hgn3C.0005 - Fusicatenibacter saccharivorans
3 months	MGS	Hgn3C.0006 - Bacteroides ovatus
3 months	MGS	Hgn3C.0007 - Parabacteroides distasonis
3 months	MGS	Hgn3C.0008 - Alistipes putredinis
3 months	MGS	Hgn3C.0009 - Faecalibacterium longum CLA-AA-H243
3 months	MGS	Hgn3C.0010 - Anaerostipes hadrus
3 months	MGS	Hgn3C.0011 - Roseburia faecis
3 months	MGS	Hgn3C.0012 - Lachnospira sp.
3 months	MGS	Hgn3C.0013 - Ruminococcus bromii
3 months	MGS	Hgn3C.0014 - Lacrimispora saccharolytica
3 months	MGS	Hgn3C.0015 - Faecalibacterium sp. Marseille-Q4896
3 months	MGS	Hgn3C.0016 - Parabacteroides merdae
3 months	MGS	Hgn3C.0017 - Subdoligranulum sp. APC924/74
3 months	MGS	Hgn3C.0018 - Faecalibacterium prausnitzii
3 months	MGS	Hgn3C.0019 - Phocaeicola dorei
3 months	MGS	Hgn3C.0020 - Collinsella aerofaciens
3 months	MGS	Hgn3C.0021 - Bifidobacterium longum subsp. longum
3 months	MGS	Hgn3C.0022 - Alistipes shahii
3 months	MGS	Hgn3C.0023 - Bacteroides xylanisolvens
3 months	MGS	Hgn3C.0024 - Bacteroides caccae
3 months	MGS	Hgn3C.0025 - Ruminococcus bicirculans
3 months	MGS	Hgn3C.0026 - Dorea longicatena
3 months	MGS	Hgn3C.0027 - Barnesiella intestinihominis
3 months	MGS	Hgn3C.0028 - Oscillibacter sp. ER4
3 months	MGS	Hgn3C.0029 - Anaerobutyricum hallii
3 months	MGS	Hgn3C.0030 - Faecalibacterium prausnitzii
3 months	MGS	Hgn3C.0031 - Blautia massiliensis
3 months	MGS	Hgn3C.0032 - Alistipes onderdonkii subsp. vulgaris
3 months	MGS	Hgn3C.0033 - Gemmiger formicilis
3 months	MGS	Hgn3C.0034 - Roseburia inulinivorans
3 months	MGS	Hgn3C.0035 - Odoribacter splanchnicus
3 months	MGS	Hgn3C.0036 - Bacteroides thetaiotaomicron
3 months	MGS	Hgn3C.0037 - Roseburia intestinalis

3 months	MGS	Hgn3C.0038 - Bifidobacterium adolescentis
3 months	MGS	Hgn3C.0039 - Coprococcus comes
3 months	MGS	Hgn3C.0040 - Eubacteriales sp.
3 months	MGS	Hgn3C.0041 - Ruminococcus sp.
3 months	MGS	Hgn3C.0042 - Blautia faecis
3 months	MGS	Hgn3C.0043 - Lachnoclostridium sp. 210928-DFI.6.3
3 months	MGS	Hgn3C.0045 - Faecalibacterium sp.
3 months	MGS	Hgn3C.0046 - Clostridium sp. AF37-5
3 months	MGS	Hgn3C.0047 - Dysosmobacter sp. BX15
3 months	MGS	Hgn3C.0048 - Akkermansia muciniphila
3 months	MGS	Hgn3C.0049 - Eubacteriales sp.
3 months	MGS	Hgn3C.0050 - Escherichia coli
3 months	MGS	Hgn3C.0051 - Alistipes communis
3 months	MGS	Hgn3C.0053 - Eubacteriales sp.
3 months	MGS	Hgn3C.0054 - Prevotella copri
3 months	MGS	Hgn3C.0055 - Alistipes finegoldii
3 months	MGS	Hgn3C.0056 - Bacteroides fragilis
3 months	MGS	Hgn3C.0057 - Oscillibacter sp. MSJ-31
3 months	MGS	Hgn3C.0058 - Lachnospiraceae sp.
3 months	MGS	Hgn3C.0059 - Bacteroides cellulosilyticus
3 months	MGS	Hgn3C.0060 - Eubacteriales sp.
3 months	MGS	Hgn3C.0061 - Faecalibacillus sp. TM498
3 months	MGS	Hgn3C.0062 - Waltera intestinalis
3 months	MGS	Hgn3C.0063 - Eubacteriales sp.
3 months	MGS	Hgn3C.0064 - Blautia sp. DFI.9.9
3 months	MGS	Hgn3C.0065 - Blautia obeum
3 months	MGS	Hgn3C.0067 - Roseburia hominis
3 months	MGS	Hgn3C.0068 - [Ruminococcus] lactaris
3 months	MGS	Hgn3C.0069 - Oscillibacter sp. KLE 1728
3 months	MGS	Hgn3C.0070 - Faecalibacterium sp. OF04-11AC
3 months	MGS	Hgn3C.0071 - Faecalicatena fissicatena
3 months	MGS	Hgn3C.0072 - Clostridium fessum
3 months	MGS	Hgn3C.0073 - Blautia sp.
3 months	MGS	Hgn3C.0075 - Agathobaculum butyriciproducens
3 months	MGS	Hgn3C.0077 - Sutterella wadsworthensis
3 months	MGS	Hgn3C.0078 - Clostridium sp. MCC328
3 months	MGS	Hgn3C.0079 - [Ruminococcus] torques
3 months	MGS	Hgn3C.0080 - Phascolarctobacterium faecium
3 months	MGS	Hgn3C.0082 - Roseburia sp. CLA-AA-H204
3 months	MGS	Hgn3C.0083 - Eubacteriales sp.
3 months	MGS	Hgn3C.0084 - Flavonifractor plautii
3 months	MGS	Hgn3C.0085 - Faecalibacterium sp.
3 months	MGS	Hgn3C.0086 - Dialister invisus
3 months	MGS	Hgn3C.0087 - Eubacteriales sp.
3 months	MGS	Hgn3C.0088 - [Ruminococcus] gnavus
3 months	MGS	Hgn3C.0089 - Paraprevotella clara
3 months	MGS	Hgn3C.0092 - Lachnospiraceae sp.
3 months	MGS	Hgn3C.0093 - Bilophila wadsworthia

3 months	MGS	Hgn3C.0094 - Eubacteriales sp.
3 months	MGS	Hgn3C.0095 - Eubacteriales sp.
3 months	MGS	Hgn3C.0096 - Eubacteriales sp.
3 months	MGS	Hgn3C.0097 - Eubacterium ventriosum
3 months	MGS	Hgn3C.0098 - Coprococcus catus
3 months	MGS	Hgn3C.0099 - Dorea longicatena
3 months	MGS	Hgn3C.0100 - Bifidobacterium bifidum
3 months	MGS	Hgn3C.0101 - Bifidobacterium pseudocatenulatum
3 months	MGS	Hgn3C.0102 - Eubacteriales sp.
3 months	MGS	Hgn3C.0103 - Holdemanella porci
3 months	MGS	Hgn3C.0104 - Clostridium sp. AM32-2
3 months	MGS	Hgn3C.0105 - Vescimonas coprocola
3 months	MGS	Hgn3C.0106 - Faecalibacterium sp. CLA-AA-H233
3 months	MGS	Hgn3C.0111 - Eubacteriales sp.
3 months	MGS	Hgn3C.0113 - Clostridium sp. MCC345
3 months	MGS	Hgn3C.0114 - Eubacteriales sp.
3 months	MGS	Hgn3C.0120 - Flavonifractor plautii
3 months	MGS	Hgn3C.0121 - Oscillospiraceae sp.
3 months	MGS	Hgn3C.0122 - Bacteroides finegoldii
3 months	MGS	Hgn3C.0123 - Eubacteriales sp.
3 months	MGS	Hgn3C.0124 - Ruthenibacterium lactatiformans
3 months	MGS	Hgn3C.0125 - Sutterella sp. KLE1602
3 months	MGS	Hgn3C.0126 - Ruminococcus sp. BSD2780120874_150323_B10
3 months	MGS	Hgn3C.0127 - Faecalibacterium sp.
3 months	MGS	Hgn3C.0128 - Eubacteriales sp.
3 months	MGS	Hgn3C.0129 - Haemophilus parainfluenzae
3 months	MGS	Hgn3C.0131 - Streptococcus thermophilus
3 months	MGS	Hgn3C.0135 - Firmicutes sp.
3 months	MGS	Hgn3C.0136 - Alistipes ihumii
3 months	MGS	Hgn3C.0137 - Clostridium sp. OF03-18AA
3 months	MGS	Hgn3C.0138 - Anaerobutyricum soehngenii
3 months	MGS	Hgn3C.0139 - Ruminococcus callidus
3 months	MGS	Hgn3C.0140 - Veillonella parvula
3 months	MGS	Hgn3C.0141 - Adlercreutzia equolifaciens subsp. celatus
3 months	MGS	Hgn3C.0142 - Eubacterium ramulus
3 months	MGS	Hgn3C.0143 - Streptococcus salivarius
3 months	MGS	Hgn3C.0144 - Eubacteriales sp.
3 months	MGS	Hgn3C.0146 - Phascolarctobacterium succinatutens
3 months	MGS	Hgn3C.0147 - Oscillospiraceae sp.
3 months	MGS	Hgn3C.0153 - Lachnospiraceae sp.
3 months	MGS	Hgn3C.0154 - Prevotella copri
3 months	MGS	Hgn3C.0155 - Blautia sp.
3 months	MGS	Hgn3C.0157 - Eubacteriales sp.
3 months	MGS	Hgn3C.0161 - Blautia sp.
3 months	MGS	Hgn3C.0162 - Prevotella sp.
3 months	MGS	Hgn3C.0168 - Evtepia gabavorous
3 months	MGS	Hgn3C.0169 - Bacteroides intestinalis
3 months	MGS	Hgn3C.0170 - Oscillospiraceae sp.

3 months	MGS	Hgn3C.0171 - Catenibacterium mitsuokai
3 months	MGS	Hgn3C.0174 - Lachnospiraceae sp.
3 months	MGS	Hgn3C.0175 - Oscillospiraceae sp.
3 months	MGS	Hgn3C.0176 - Alistipes indistinctus
3 months	MGS	Hgn3C.0179 - Bacteroidales sp.
3 months	MGS	Hgn3C.0181 - Alistipes onderdonkii
3 months	MGS	Hgn3C.0182 - Butyricimonas faecihominis
3 months	MGS	Hgn3C.0183 - Intestinibacter bartlettii
3 months	MGS	Hgn3C.0185 - Bifidobacterium catenulatum subsp. kashiwanohense
3 months	MGS	Hgn3C.0186 - Butyricimonas virosa
3 months	MGS	Hgn3C.0191 - Prevotella sp.
3 months	MGS	Hgn3C.0192 - Erysipelatoclostridium ramosum
3 months	MGS	Hgn3C.0195 - Eubacteriales sp.
3 months	MGS	Hgn3C.0201 - Veillonella dispar
3 months	MGS	Hgn3C.0202 - Eggerthella lenta
3 months	MGS	Hgn3C.0203 - Faecalibacterium sp. Marseille-Q3530
3 months	MGS	Hgn3C.0207 - Eubacteriales sp.
3 months	MGS	Hgn3C.0208 - Romboutsia timonensis
3 months	MGS	Hgn3C.0209 - Bifidobacterium breve
3 months	MGS	Hgn3C.0212 - Holdemanella biformis
3 months	MGS	Hgn3C.0213 - Akkermansia sp. GGCC_0220
3 months	MGS	Hgn3C.0214 - Duodenibacillus massiliensis
3 months	MGS	Hgn3C.0215 - Alistipes sp. cv1
3 months	MGS	Hgn3C.0217 - Eubacteriales sp.
3 months	MGS	Hgn3C.0218 - Eubacteriales sp.
3 months	MGS	Hgn3C.0219 - Tyzzerella nexilis
3 months	MGS	Hgn3C.0220 - Clostridium sp. MCC334
3 months	MGS	Hgn3C.0221 - Clostridium sp.
3 months	MGS	Hgn3C.0222 - Parabacteroides merdae
3 months	MGS	Hgn3C.0224 - Veillonellales sp.
3 months	MGS	Hgn3C.0226 - Eubacteriales sp.
3 months	MGS	Hgn3C.0232 - Megamonas funiformis
3 months	MGS	Hgn3C.0236 - Oscillospiraceae sp.
3 months	MGS	Hgn3C.0238 - Oscillospiraceae sp.
3 months	MGS	Hgn3C.0239 - Blautia sp. BIOML-A1
3 months	MGS	Hgn3C.0242 - Enterocloster bolteae
3 months	MGS	Hgn3C.0244 - Desulfovibrio piger
3 months	MGS	Hgn3C.0245 - Blautia stercoris
3 months	MGS	Hgn3C.0248 - Coprococcus catus
3 months	MGS	Hgn3C.0249 - Clostridium sp. AM33-3
3 months	MGS	Hgn3C.0250 - Eubacteriales sp.
3 months	MGS	Hgn3C.0251 - Alistipes senegalensis
3 months	MGS	Hgn3C.0253 - Waltera sp.
3 months	MGS	Hgn3C.0260 - Hydrogenoanaerobacterium saccharovorans
3 months	MGS	Hgn3C.0264 - Clostridium sp. AF27-2AA
3 months	MGS	Hgn3C.0266 - Dorea sp. AF36-15AT
3 months	MGS	Hgn3C.0267 - Oscillospiraceae sp.
3 months	MGS	Hgn3C.0269 - Flintibacter sp. NSJ-23

3 months	MGS	Hgn3C.0272 - Blautia faecicola
3 months	MGS	Hgn3C.0273 - Slackia isoflavoniconvertens
3 months	MGS	Hgn3C.0275 - Lachnospiraceae sp.
3 months	MGS	Hgn3C.0281 - Eubacteriales sp.
3 months	MGS	Hgn3C.0284 - Erysipelotrichaceae sp.
3 months	MGS	Hgn3C.0290 - Sellimonas intestinalis
3 months	MGS	Hgn3C.0295 - Eubacteriales sp.
3 months	MGS	Hgn3C.0299 - Escherichia coli
3 months	MGS	Hgn3C.0301 - Prevotella hominis
3 months	MGS	Hgn3C.0304 - Eubacteriales sp.
3 months	MGS	Hgn3C.0306 - Bacteroides nordii
3 months	MGS	Hgn3C.0307 - Veillonella atypica
3 months	MGS	Hgn3C.0308 - [Clostridium] symbiosum
3 months	MGS	Hgn3C.0309 - Eubacteriales sp.
3 months	MGS	Hgn3C.0310 - Ligilactobacillus ruminis
3 months	MGS	Hgn3C.0313 - Ruminococcus bromii
3 months	MGS	Hgn3C.0314 - Ruminococcus sp.
3 months	MGS	Hgn3C.0317 - Intestinimonas butyriciproducens
3 months	MGS	Hgn3C.0319 - Clostridiaceae sp.
3 months	MGS	Hgn3C.0323 - Bacteroides sp.
3 months	MGS	Hgn3C.0324 - Prevotella stercorea
3 months	MGS	Hgn3C.0330 - Faecalibacillus intestinalis
3 months	MGS	Hgn3C.0332 - Klebsiella pneumoniae subsp. pneumoniae
3 months	MGS	Hgn3C.0334 - Bacteroides cellulosilyticus
3 months	MGS	Hgn3C.0336 - Parabacteroides goldsteinii
3 months	MGS	Hgn3C.0338 - Eubacteriales sp.
3 months	MGS	Hgn3C.0348 - Streptococcus sp.
3 months	MGS	Hgn3C.0350 - [Clostridium] symbiosum
3 months	MGS	Hgn3C.0351 - Prevotella sp.
3 months	MGS	Hgn3C.0354 - Sutterella seckii
3 months	MGS	Hgn3C.0356 - Blautia sp. M29
3 months	MGS	Hgn3C.0361 - Dorea sp. AF24-7LB
3 months	MGS	Hgn3C.0362 - Dysosmobacter sp. NSJ-60
3 months	MGS	Hgn3C.0365 - Eubacteriales sp.
3 months	MGS	Hgn3C.0367 - Lachnospiraceae sp.
3 months	MGS	Hgn3C.0369 - Prevotella sp.
3 months	MGS	Hgn3C.0371 - Lachnospiraceae sp.
3 months	MGS	Hgn3C.0373 - Enterocloster clostridioformis
3 months	MGS	Hgn3C.0375 - Ellagibacter isourolithinifaciens
3 months	MGS	Hgn3C.0381 - Clostridium sp. AM30-24
3 months	MGS	Hgn3C.0385 - Veillonella rogosae
3 months	MGS	Hgn3C.0386 - Clostridium sp. AT4
3 months	MGS	Hgn3C.0388 - Clostridium sp.
3 months	MGS	Hgn3C.0392 - Clostridia sp.
3 months	MGS	Hgn3C.0394 - Hungatella hathewayi
3 months	MGS	Hgn3C.0397 - Parabacteroides sp.
3 months	MGS	Hgn3C.0400 - Prevotellamassilia timonensis
3 months	MGS	Hgn3C.0405 - Bacteroides ovatus

3 months	MGS	Hgn3C.0407 - Eubacteriales sp.
3 months	MGS	Hgn3C.0408 - Eubacteriales sp.
3 months	MGS	Hgn3C.0413 - Blautia caecimuris
3 months	MGS	Hgn3C.0415 - Clostridium sp. C5-48
3 months	MGS	Hgn3C.0417 - Eubacteriales sp.
3 months	MGS	Hgn3C.0418 - Blautia sp.
3 months	MGS	Hgn3C.0419 - Prevotella sp. 885
3 months	MGS	Hgn3C.0420 - Clostridium sp. 1001270J_160509_D11
3 months	MGS	Hgn3C.0430 - Enterocloster aldenensis
3 months	MGS	Hgn3C.0436 - Eubacteriales sp.
3 months	MGS	Hgn3C.0437 - Prevotella sp.
3 months	MGS	Hgn3C.0438 - Coprobacillus cateniformis
3 months	MGS	Hgn3C.0439 - Enterococcus faecalis
3 months	MGS	Hgn3C.0442 - Holdemanella sp.
3 months	MGS	Hgn3C.0453 - Blautia sp. Marseille-P3087
3 months	MGS	Hgn3C.0455 - Peptostreptococcaceae sp.
3 months	MGS	Hgn3C.0458 - Eubacteriales sp.
3 months	MGS	Hgn3C.0459 - Eubacteriales sp.
3 months	MGS	Hgn3C.0464 - Bifidobacterium longum subsp. infantis
3 months	MGS	Hgn3C.0468 - Clostridium sp. NSJ-42
3 months	MGS	Hgn3C.0470 - Eubacteriales sp.
3 months	MGS	Hgn3C.0472 - Parabacteroides goldsteinii
3 months	MGS	Hgn3C.0476 - Streptococcus sp.
3 months	MGS	Hgn3C.0482 - Lachnospiraceae sp.
3 months	MGS	Hgn3C.0493 - Eisenbergiella tayi
3 months	MGS	Hgn3C.0494 - Prevotellaceae sp.
3 months	MGS	Hgn3C.0499 - Bacteroides sp.
3 months	MGS	Hgn3C.0500 - Anaerotignum sp.
3 months	MGS	Hgn3C.0504 - Megasphaera sp. BL7
3 months	MGS	Hgn3C.0507 - [Clostridium] spiroforme
3 months	MGS	Hgn3C.0514 - Blautia glucerasea
3 months	MGS	Hgn3C.0523 - Phascolarctobacterium succinatutens
3 months	MGS	Hgn3C.0527 - Sutterella sp.
3 months	MGS	Hgn3C.0528 - Coprococcus sp. AM27-12LB
3 months	MGS	Hgn3C.0533 - Flintibacter sp. KGMB00164
3 months	MGS	Hgn3C.0537 - Bifidobacterium dentium
3 months	MGS	Hgn3C.0542 - [Clostridium] innocuum
3 months	MGS	Hgn3C.0543 - Oscillospiraceae sp.
3 months	MGS	Hgn3C.0549 - Longicatena caecimuris
3 months	MGS	Hgn3C.0557 - Enterocloster bolteae
3 months	MGS	Hgn3C.0558 - Olsenella sp.
3 months	MGS	Hgn3C.0560 - Oscillospiraceae sp.
3 months	MGS	Hgn3C.0573 - Prevotella sp. Marseille-P4119
3 months	MGS	Hgn3C.0584 - Bacteroides fragilis
3 months	MGS	Hgn3C.0598 - Eubacteriales sp.
3 months	MGS	Hgn3C.0604 - Megasphaera elsdenii
3 months	MGS	Hgn3C.0608 - Lachnospiraceae sp.
3 months	MGS	Hgn3C.0610 - Eubacteriales sp.

3 months	MGS	Hgn3C.0613 - Clostridiaceae sp.
3 months	MGS	Hgn3C.0617 - Prevotella sp.
3 months	MGS	Hgn3C.0629 - Collinsella intestinalis
3 months	MGS	Hgn3C.0630 - Allisonella histaminiformans
3 months	MGS	Hgn3C.0634 - Blautia schinkii
3 months	MGS	Hgn3C.0636 - Anaerostipes caccae
3 months	MGS	Hgn3C.0640 - Eubacterium sp. AF22-8LB
3 months	MGS	Hgn3C.0649 - Lachnospiraceae sp.
3 months	MGS	Hgn3C.0657 - Parolsenella catena
3 months	MGS	Hgn3C.0664 - Blautia hansenii
3 months	MGS	Hgn3C.0675 - Eubacteriales sp.
3 months	MGS	Hgn3C.0676 - Bacteroides sp.
3 months	MGS	Hgn3C.0677 - Bacteroides sp.
3 months	MGS	Hgn3C.0679 - Holdemania filiformis
3 months	MGS	Hgn3C.0680 - Eubacteriales sp.
3 months	MGS	Hgn3C.0682 - [Clostridium] scindens
3 months	MGS	Hgn3C.0683 - Clostridium disporicum
3 months	MGS	Hgn3C.0693 - Hungatella hathewayi
3 months	MGS	Hgn3C.0701 - Bacteroides sp.
3 months	MGS	Hgn3C.0704 - [Clostridium] scindens
3 months	MGS	Hgn3C.0711 - Enterocloster asparagiformis
3 months	MGS	Hgn3C.0712 - Megasphaera micronuciformis
3 months	MGS	Hgn3C.0713 - Veillonellales sp.
3 months	MGS	Hgn3C.0714 - Intestinimonas sp. MSJ-38
3 months	MGS	Hgn3C.0715 - Desulfovibrio fairfieldensis
3 months	MGS	Hgn3C.0718 - Eubacteriales sp.
3 months	MGS	Hgn3C.0729 - Eubacteriales sp.
3 months	MGS	Hgn3C.0731 - Eubacteriales sp.
3 months	MGS	Hgn3C.0732 - Eubacterium ramulus
3 months	MGS	Hgn3C.0736 - Coprococcus sp.
3 months	MGS	Hgn3C.0737 - Alistipes timonensis
3 months	MGS	Hgn3C.0740 - Anaerostipes sp. NSJ-7
3 months	MGS	Hgn3C.0741 - Blautia sp. OF01-4LB
3 months	MGS	Hgn3C.0743 - Enterobacter sp.
3 months	MGS	Hgn3C.0754 - Collinsella bouchesdurhonensis
3 months	MGS	Hgn3C.0761 - Bacteroides sp.
3 months	MGS	Hgn3C.0762 - Enterocloster lavalensis
3 months	MGS	Hgn3C.0777 - Holdemanella sp.
3 months	MGS	Hgn3C.0781 - Collinsella sp.
3 months	MGS	Hgn3C.0782 - Streptococcus sp.
3 months	MGS	Hgn3C.0790 - Oscillospiraceae sp.
3 months	MGS	Hgn3C.0793 - Prevotellaceae sp.
3 months	MGS	Hgn3C.0797 - Senegalimassilia sp.
3 months	MGS	Hgn3C.0798 - Streptococcus vestibularis
3 months	MGS	Hgn3C.0807 - Enorma massiliensis
3 months	MGS	Hgn3C.0817 - Prevotella sp.
3 months	MGS	Hgn3C.0818 - Rothia sp.
3 months	MGS	Hgn3C.0829 - Dorea phocaeensis

3 months	MGS	Hgn3C.0832 - Veillonellales sp.
3 months	MGS	Hgn3C.0833 - Eisenbergiella tayi
3 months	MGS	Hgn3C.0837 - Anaerotruncus colihominis
3 months	MGS	Hgn3C.0851 - Oscillospiraceae sp.
3 months	MGS	Hgn3C.0855 - Eubacteriales sp.
3 months	MGS	Hgn3C.0858 - Turicibacter sanguinis
3 months	MGS	Hgn3C.0859 - Hydrogeniiclostidium mannosilyticum
3 months	MGS	Hgn3C.0873 - Clostridium perfringens
3 months	MGS	Hgn3C.0875 - Lachnospiraceae sp.
3 months	MGS	Hgn3C.0886 - Bacteroides sp.
3 months	MGS	Hgn3C.0889 - Clostridium paraputrificum
3 months	MGS	Hgn3C.0890 - Enterobacter roggenkampii
3 months	MGS	Hgn3C.0891 - Eubacteriales sp.
3 months	MGS	Hgn3C.0893 - Phocea massiliensis
3 months	MGS	Hgn3C.0908 - Eubacteriales sp.
3 months	MGS	Hgn3C.0913 - Erysipelotrichaceae sp.
3 months	MGS	Hgn3C.0921 - Bacteroides sp.
3 months	MGS	Hgn3C.0948 - Enterobacter sp.
3 months	MGS	Hgn3C.0978 - Veillonella sp.
3 months	MGS	Hgn3C.0987 - Fusobacterium mortiferum
3 months	MGS	Hgn3C.0996 - Oscillospiraceae sp.
3 months	MGS	Hgn3C.1035 - Klebsiella sp.
3 months	MGS	Hgn3C.1036 - Escherichia coli
3 months	MGS	Hgn3C.1037 - Rothia sp.
3 months	MGS	Hgn3C.1038 - Veillonella sp.
3 months	MGS	Hgn3C.1040 - Lachnospiraceae sp.
3 months	MGS	Hgn3C.1041 - Bacteroides stercoris
3 months	MGS	Hgn3C.1042 - Dorea formicigenerans
3 months	MGS	Hgn3C.1043 - Clostridium phoceensis
3 months	MGS	Hgn3C.1045 - Lactococcus lactis subsp. lactis
3 months	MGS	Hgn3C.1049 - Enterococcus faecium
3 months	MGS	Hgn3C.1050 - Staphylococcus epidermidis
3 months	MGS	Hgn3C.1051 - Holdemania massiliensis
3 months	MGS	Hgn3C.1054 - Streptococcus lutetiensis
3 months	MGS	Hgn3C.1058 - Mediterraneibacter glycyrrhizinilyticus
3 months	MGS	Hgn3C.1059 - Faecalicatena sp.
3 months	MGS	Hgn3C.1063 - Enterococcus avium
3 months	MGS	Hgn3C.1065 - Klebsiella variicola subsp. variicola
3 months	MGS	Hgn3C.1067 - Lachnospiraceae sp.
3 months	MGS	Hgn3C.1073 - Streptococcus gallolyticus subsp. gallolyticus
3 months	MGS	Hgn3C.1081 - Lacticaseibacillus rhamnosus
3 months	MGS	Hgn3C.1082 - Lacticaseibacillus paracasei subsp. paracasei
3 months	MGS	Hgn3C.1084 - Massilimicrobiota timonensis
3 months	MGS	Hgn3C.1087 - Ligilactobacillus salivarius
3 months	MGS	Hgn3C.1090 - Turicibacter sanguinis
3 months	MGS	Hgn3C.1093 - Enterobacter kobei
3 months	MGS	Hgn3C.1110 - Porphyromonas sp.
3 months	MGS	Hgn3C.1117 - Atopobiaceae sp.

3 months	MGS	Hgn3C.1120 - Actinomycetaceae sp.
3 months	MGS	Hgn3C.1148 - Eggerthellales sp.
3 months	MGS	Hgn3C.1153 - Eubacterium sp. OM08-24
3 months	MGS	Hgn3C.1154 - Lactococcus garvieae
3 months	MGS	Hgn3C.1180 - Enterocloster citroniae
3 months	MGS	Hgn3C.1188 - Actinomyces oris
3 months	MGS	Hgn3C.1190 - Fenollaria massiliensis
3 months	MGS	Hgn3C.1193 - Atopobium sp.
3 months	MGS	Hgn3C.1196 - Blautia marasmi
3 months	MGS	Hgn3C.1197 - Blautia hansenii
3 months	MGS	Hgn3C.1210 - Dielma sp.
3 months	MGS	Hgn3C.1211 - Lachnoclostridium pacaense
3 months	MGS	Hgn3C.1214 - Corynebacterium tuberculostearicum
3 months	MGS	Hgn3C.1219 - Enterobacter sp. NFIX58
3 months	MGS	Hgn3C.1229 - Escherichia sp. 93.0750
3 months	MGS	Hgn3C.1230 - Eubacterium limosum
3 months	MGS	Hgn3C.1234 - Granulicatella adiacens
3 months	MGS	Hgn3C.1238 - Klebsiella quasivariicola
3 months	MGS	Hgn3C.1241 - Limosilactobacillus reuteri subsp. reuteri
3 months	MGS	Hgn3C.1242 - Limosilactobacillus vaginalis
3 months	MGS	Hgn3C.1248 - Neisseria sp. HMSC061E12
3 months	MGS	Hgn3C.1258 - Rothia sp. HMSC072E10
3 months	MGS	Hgn3C.1264 - Staphylococcus hominis subsp. hominis
3 months	MGS	Hgn3C.1268 - Streptococcus sp.
3 months	MGS	Hgn3C.1269 - Streptococcus constellatus subsp. constellatus
3 months	MGS	Hgn3C.1270 - Streptococcus sp.
3 months	MGS	Hgn3C.1275 - Lachnospiraceae sp.
3 months	MGS	Hgn3C.1277 - Lachnospiraceae sp.
3 months	MGS	Hgn3C.1282 - Terrisporobacter othiniensis
3 months	MGS	Hgn3C.1286 - Corynebacterium variabile
3 months	MGS	Hgn3C.1288 - Enterobacter hormaechei
3 months	MGS	Hgn3C.1299 - Erysipelotrichaceae sp.
3 months	MGS	Hgn3C.1300 - Rothia sp.
3 months	MGS	Hgn3C.1302 - Schaalia sp.
3 months	MGS	Hgn3C.1303 - Clostridium paraputrificum
3 months	MGS	Hgn3C.1305 - Isoptericola variabilis
3 months	MGS	Hgn3C.1307 - Bifidobacterium longum subsp. spp
3 months	MGS	Hgn3C.1309 - Enterobacter cloacae subsp. cloacae
3 months	MGS	Hgn3C.1310 - Erysipelotrichaceae sp.
3 months	MGS	Hgn3C.1311 - Lachnospiraceae sp.
3 months	MGS	Hgn3C.1312 - Candida tropicalis
3 months	MGS	Hgn3C.1315 - Eubacteriales sp.
3 months	MGS	Hgn3C.1316 - Eubacteriales sp.
3 months	MGS	Hgn3C.1321 - Veillonellaceae sp.
3 months	MGS	Hgn3C.1322 - Enterococcus hirae
3 months	MGS	Hgn3C.1323 - Lachnospiraceae sp.
3 months	MGS	Hgn3C.1324 - Eubacterium sp. c-25
3 months	MGS	Hgn3C.1325 - Luxibacter massiliensis

3 months	MGS	Hgn3C.1326 - Eggerthella guodeyinii
3 months	MGS	Hgn3C.1329 - Eubacteriales sp.
3 months	MGS	Hgn3C.1330 - Lachnospiraceae sp.
3 months	MGS	Hgn3C.1331 - Lachnospiraceae sp.
3 months	MGS	Hgn3C.1334 - Eubacteriales sp.
3 months	MGS	Hgn3C.1336 - Eubacteriales sp.
3 months	MGS	Hgn3C.1338 - Erysipelatoclostridium sp.
3 months	MGS	Hgn3C.1340 - Clostridiaceae sp.
3 months	MGS	Hgn3C.1343 - Enterococcus italicus
3 months	MGS	Hgn3C.1347 - [Clostridium] hylemonae
3 months	MGS	Hgn3C.1349 - Corynebacterium falsenii
3 months	MGS	Hgn3C.1351 - Actinomyces sp. ICM47
3 months	MGS	Hgn3C.1352 - Akkermansia sp.
3 months	MGS	Hgn3C.1354 - Actinomyces sp.
3 months	MGS	Hgn3C.1355 - Collinsella stercoris
3 months	MGS	Hgn3C.1356 - Eubacteriales sp.
3 months	MGS	Hgn3C.1358 - Lachnospiraceae sp.
3 months	MGS	Hgn3C.1359 - Extibacter muris
3 months	MGS	Hgn3C.1360 - Bifidobacterium crudilactis
3 months	MGS	Hgn3C.1361 - Senegalimassilia anaerobia
3 months	MGS	Hgn3C.1362 - Christensenella intestinihominis
3 months	MGS	Hgn3C.1366 - Lachnospiraceae sp.
3 months	MGS	Hgn3C.1369 - Lachnospiraceae sp.
3 months	MGS	Hgn3C.1372 - Eubacteriales sp.
3 months	MGS	Hgn3C.1373 - Eubacteriales sp.
3 months	MGS	Hgn3C.1375 - Atopobiaceae sp.
3 months	MGS	Hgn3C.1376 - Kluyvera sp. CRP
3 months	MGS	Hgn3C.1377 - Collinsella tanakaei
3 months	MGS	Hgn3C.1378 - Collinsella sp. AF08-23
3 months	MGS	Hgn3C.1379 - Gordonibacter urolithinfaciens
3 months	MGS	Hgn3C.1380 - Kluyvera georgiana
3 months	MGS	Hgn3C.1382 - Oscillospiraceae sp.
3 months	MGS	Hgn3C.1384 - Anaerococcus sp.
3 months	MGS	Hgn3C.1386 - Veillonella sp.
3 months	MGS	Hgn3C.1387 - Alistipes sp.
3 months	MGS	Hgn3C.1388 - Streptococcus infantarius subsp. infantarius
3 months	MGS	Hgn3C.1390 - Erysipelatoclostridium sp.
3 months	MGS	Hgn3C.1393 - Anaerofustis stercorihominis
3 months	MGS	Hgn3C.1394 - Eubacteriales sp.
3 months	MGS	Hgn3C.1398 - Actinomyces sp.
3 months	MGS	Hgn3C.1400 - Eubacterium sp.
3 months	MGS	Hgn3C.1401 - Eubacteriales sp.
3 months	MGS	Hgn3C.1402 - Collinsella phocaeensis
3 months	MGS	Hgn3C.1403 - Eubacteriaceae sp.
3 months	MGS	Hgn3C.1404 - Clostridium sp.
3 months	MGS	Hgn3C.1406 - Slackia piriformis
3 months	MGS	Hgn3C.1407 - Christensenella minuta
3 months	MGS	Hgn3C.1408 - Eubacterium callanderi

3 months	MGS	Hgn3C.1409 - Eggerthella timonensis
3 months	MGS	Hgn3C.1412 - Christensenella hongkongensis
3 months	MGS	Hgn3C.1413 - Streptococcus sp.
3 months	MGS	Hgn3C.1414 - Collinsella sp.
3 months	MGS	Hgn3C.1415 - Klebsiella quasipneumoniae subsp. quasipneumoniae
3 months	MGS	Hgn3C.1416 - Limosilactobacillus fermentum
3 months	MGS	Hgn3C.1418 - Carnobacteriaceae sp.
3 months	MGS	Hgn3C.1419 - Agathobaculum sp. NSJ-28
3 months	MGS	Hgn3C.1420 - Eubacteriales sp.
3 months	MGS	Hgn3C.1423 - Lachnospiraceae sp.
3 months	MGS	Hgn3C.1424 - Mogibacterium sp. NSJ-24
3 months	MGS	Hgn3C.1425 - Eubacteriales sp.
3 months	MGS	Hgn3C.1426 - Limosilactobacillus oris
3 months	MGS	Hgn3C.1429 - Lachnospiraceae sp.
3 months	MGS	Hgn3C.1431 - Bacteroidales sp.
3 months	MGS	Hgn3C.1434 - Blautia sp.
3 months	MGS	Hgn3C.1435 - Acinetobacter baumannii
3 months	MGS	Hgn3C.1436 - Bacteria sp.
3 months	MGS	Hgn3C.1437 - Candidatus Stoquefichus sp. SB1
3 months	MGS	Hgn3C.1438 - Dielma fastidiosa
3 months	MGS	Hgn3C.1439 - Streptococcus sp. IMAU 99161
3 months	MGS	Hgn3C.1441 - Lancefieldella sp.
3 months	MGS	Hgn3C.1443 - Streptococcus sp.
3 months	MGS	Hgn3C.1444 - Ruminococcus sp. 1001175B_160314_E1
3 months	MGS	Hgn3C.1446 - Eubacteriales sp.
3 months	MGS	Hgn3C.1447 - Bacteria sp.
3 months	MGS	Hgn3C.1448 - Leuconostoc falkenbergense
3 months	MGS	Hgn3C.1450 - Lactococcus sp. NH2-7C
3 months	MGS	Hgn3C.1452 - Raoultibacter timonensis
3 months	MGS	Hgn3C.1453 - Collinsella sp.
3 months	MGS	Hgn3C.1455 - Enterocloster sp.
3 months	MGS	Hgn3C.1456 - Eubacteriales sp.
3 months	MGS	Hgn3C.1460 - Blautia sp.
3 months	MGS	Hgn3C.1463 - Enterobacter sp.
3 months	MGS	Hgn3C.1465 - Clostridia sp.
3 months	MGS	Hgn3C.1467 - Eubacteriales sp.
3 months	MGS	Hgn3C.1468 - Staphylococcus aureus
3 months	MGS	Hgn3C.1469 - Lachnospiraceae sp.
3 months	MGS	Hgn3C.1471 - Eggerthella sp.
3 months	MGS	Hgn3C.1472 - Amedibacillus dolichus
3 months	genus	Acinetobacter
3 months	genus	Actinomyces
3 months	genus	Adlercreutzia
3 months	genus	Agathobaculum
3 months	genus	Akkermansia
3 months	genus	Alistipes
3 months	genus	Allisonella
3 months	genus	Amedibacillus

3 months	genus	Anaerobutyricum
3 months	genus	Anaerococcus
3 months	genus	Anaerofustis
3 months	genus	Anaerostipes
3 months	genus	Anaerotignum
3 months	genus	Anaerotruncus
3 months	genus	Atopobium
3 months	genus	Bacteroides
3 months	genus	Barnesiella
3 months	genus	Bifidobacterium
3 months	genus	Bilophila
3 months	genus	Blautia
3 months	genus	Butyricicoccus
3 months	genus	Butyricimonas
3 months	genus	Candida
3 months	genus	Candidatus Stoquefichus
3 months	genus	Catenibacterium
3 months	genus	Christensenella
3 months	genus	Citrobacter
3 months	genus	Clostridium
3 months	genus	Collinsella
3 months	genus	Coprobacillus
3 months	genus	Coprococcus
3 months	genus	Corynebacterium
3 months	genus	Desulfovibrio
3 months	genus	Dialister
3 months	genus	Dielma
3 months	genus	Dorea
3 months	genus	Duodenibacillus
3 months	genus	Dysosmobacter
3 months	genus	Eggerthella
3 months	genus	Eisenbergiella
3 months	genus	Ellagibacter
3 months	genus	Enorma
3 months	genus	Enterobacter
3 months	genus	Enterocloster
3 months	genus	Enterococcus
3 months	genus	Erysipelatoclostridium
3 months	genus	Escherichia
3 months	genus	Eubacterium
3 months	genus	Evtepia
3 months	genus	Extibacter
3 months	genus	Faecalibacillus
3 months	genus	Faecalibacterium
3 months	genus	Faecalicatena
3 months	genus	Fenollaria
3 months	genus	Flavonifractor
3 months	genus	Flintibacter

3 months	genus	Fusicatenibacter
3 months	genus	Fusobacterium
3 months	genus	Gemmiger
3 months	genus	Gordonibacter
3 months	genus	Granulicatella
3 months	genus	Haemophilus
3 months	genus	Holdemanella
3 months	genus	Holdemania
3 months	genus	Hungatella
3 months	genus	Hydrogeniiclostidium
3 months	genus	Hydrogenoanaerobacterium
3 months	genus	Intestinibacter
3 months	genus	Intestinimonas
3 months	genus	Isoptericola
3 months	genus	Klebsiella
3 months	genus	Kluyvera
3 months	genus	Lachnoclostridium
3 months	genus	Lachnospira
3 months	genus	Lacrimispora
3 months	genus	Lacticaseibacillus
3 months	genus	Lactobacillus
3 months	genus	Lactococcus
3 months	genus	Lancefieldella
3 months	genus	Leuconostoc
3 months	genus	Ligilactobacillus
3 months	genus	Limosilactobacillus
3 months	genus	Longicatena
3 months	genus	Luxibacter
3 months	genus	Massilimicrobiota
3 months	genus	Mediterraneibacter
3 months	genus	Megamonas
3 months	genus	Megasphaera
3 months	genus	Mogibacterium
3 months	genus	Neisseria
3 months	genus	Odoribacter
3 months	genus	Olsenella
3 months	genus	Oscillibacter
3 months	genus	Parabacteroides
3 months	genus	Paraprevotella
3 months	genus	Parolsenella
3 months	genus	Phascolarctobacterium
3 months	genus	Phocaeicola
3 months	genus	Phocea
3 months	genus	Porphyromonas
3 months	genus	Prevotella
3 months	genus	Prevotellamassilia
3 months	genus	Raoultibacter
3 months	genus	Romboutsia

3 months	genus	Roseburia
3 months	genus	Rothia
3 months	genus	Ruminococcus
3 months	genus	Ruthenibacterium
3 months	genus	Schaalia
3 months	genus	Sellimonas
3 months	genus	Senegalimassilia
3 months	genus	Slackia
3 months	genus	Staphylococcus
3 months	genus	Streptococcus
3 months	genus	Subdoligranulum
3 months	genus	Sutterella
3 months	genus	Terrisporobacter
3 months	genus	Turicibacter
3 months	genus	Tyzzerella
3 months	genus	Veillonella
3 months	genus	Vescimonas
3 months	genus	Waltera
3 months	family	Acidaminococcaceae
3 months	family	Actinomycetaceae
3 months	family	Akkermansiaceae
3 months	family	Atopobiaceae
3 months	family	Bacteroidaceae
3 months	family	Barnesiellaceae
3 months	family	Bifidobacteriaceae
3 months	family	Carnobacteriaceae
3 months	family	Christensenellaceae
3 months	family	Clostridiaceae
3 months	family	Coprobacillaceae
3 months	family	Coriobacteriaceae
3 months	family	Corynebacteriaceae
3 months	family	Debaryomycetaceae
3 months	family	Desulfovibrionaceae
3 months	family	Eggerthellaceae
3 months	family	Enterobacteriaceae
3 months	family	Enterococcaceae
3 months	family	Erysipelotrichaceae
3 months	family	Eubacteriaceae
3 months	family	Eubacteriales Family XIII. Incertae Sedis
3 months	family	Fusobacteriaceae
3 months	family	Lachnospiraceae
3 months	family	Lactobacillaceae
3 months	family	Micrococcaceae
3 months	family	Moraxellaceae
3 months	family	Neisseriaceae
3 months	family	Odoribacteraceae
3 months	family	Oscillospiraceae
3 months	family	Pasteurellaceae

3 months	family	Peptoniphilaceae
3 months	family	Peptostreptococcaceae
3 months	family	Porphyromonadaceae
3 months	family	Prevotellaceae
3 months	family	Promicromonosporaceae
3 months	family	Rikenellaceae
3 months	family	Selenomonadaceae
3 months	family	Staphylococcaceae
3 months	family	Streptococcaceae
3 months	family	Sutterellaceae
3 months	family	Tannerellaceae
3 months	family	Turicibacteraceae
3 months	family	Veillonellaceae
3 months	phylum	Actinobacteria
3 months	phylum	Ascomycota
3 months	phylum	Bacteroidetes
3 months	phylum	Firmicutes
3 months	phylum	Fusobacteria
3 months	phylum	Proteobacteria
3 months	phylum	Verrucomicrobia
6 months	MGS	Hgn3C.0001 - Phocaeicola vulgatus
6 months	MGS	Hgn3C.0002 - Bacteroides uniformis
6 months	MGS	Hgn3C.0003 - Lachnospiraceae sp.
6 months	MGS	Hgn3C.0004 - Blautia wexlerae
6 months	MGS	Hgn3C.0005 - Fusicatenibacter saccharivorans
6 months	MGS	Hgn3C.0006 - Bacteroides ovatus
6 months	MGS	Hgn3C.0007 - Parabacteroides distasonis
6 months	MGS	Hgn3C.0008 - Alistipes putredinis
6 months	MGS	Hgn3C.0009 - Faecalibacterium longum CLA-AA-H243
6 months	MGS	Hgn3C.0010 - Anaerostipes hadrus
6 months	MGS	Hgn3C.0011 - Roseburia faecis
6 months	MGS	Hgn3C.0012 - Lachnospira sp.
6 months	MGS	Hgn3C.0013 - Ruminococcus bromii
6 months	MGS	Hgn3C.0014 - Lacrimispora saccharolytica
6 months	MGS	Hgn3C.0015 - Faecalibacterium sp. Marseille-Q4896
6 months	MGS	Hgn3C.0016 - Parabacteroides merdae
6 months	MGS	Hgn3C.0017 - Subdoligranulum sp. APC924/74
6 months	MGS	Hgn3C.0018 - Faecalibacterium prausnitzii
6 months	MGS	Hgn3C.0019 - Phocaeicola dorei
6 months	MGS	Hgn3C.0020 - Collinsella aerofaciens
6 months	MGS	Hgn3C.0021 - Bifidobacterium longum subsp. longum
6 months	MGS	Hgn3C.0022 - Alistipes shahii
6 months	MGS	Hgn3C.0023 - Bacteroides xylanisolvens
6 months	MGS	Hgn3C.0024 - Bacteroides caccae
6 months	MGS	Hgn3C.0025 - Ruminococcus bicirculans
6 months	MGS	Hgn3C.0026 - Dorea longicatena
6 months	MGS	Hgn3C.0027 - Barnesiella intestinihominis
6 months	MGS	Hgn3C.0028 - Oscillibacter sp. ER4

6 months	MGS	Hgn3C.0029 - Anaerobutyricum hallii
6 months	MGS	Hgn3C.0030 - Faecalibacterium prausnitzii
6 months	MGS	Hgn3C.0031 - Blautia massiliensis
6 months	MGS	Hgn3C.0032 - Alistipes onderdonkii subsp. vulgaris
6 months	MGS	Hgn3C.0033 - Gemmiger formicilis
6 months	MGS	Hgn3C.0034 - Roseburia inulinivorans
6 months	MGS	Hgn3C.0035 - Odoribacter splanchnicus
6 months	MGS	Hgn3C.0036 - Bacteroides thetaiotaomicron
6 months	MGS	Hgn3C.0037 - Roseburia intestinalis
6 months	MGS	Hgn3C.0038 - Bifidobacterium adolescentis
6 months	MGS	Hgn3C.0039 - Coprococcus comes
6 months	MGS	Hgn3C.0040 - Eubacteriales sp.
6 months	MGS	Hgn3C.0041 - Ruminococcus sp.
6 months	MGS	Hgn3C.0042 - Blautia faecis
6 months	MGS	Hgn3C.0043 - Lachnoclostridium sp. 210928-DFI.6.3
6 months	MGS	Hgn3C.0044 - Lachnospira pectinoschiza
6 months	MGS	Hgn3C.0045 - Faecalibacterium sp.
6 months	MGS	Hgn3C.0046 - Clostridium sp. AF37-5
6 months	MGS	Hgn3C.0047 - Dysosmobacter sp. BX15
6 months	MGS	Hgn3C.0048 - Akkermansia muciniphila
6 months	MGS	Hgn3C.0049 - Eubacteriales sp.
6 months	MGS	Hgn3C.0050 - Escherichia coli
6 months	MGS	Hgn3C.0051 - Alistipes communis
6 months	MGS	Hgn3C.0052 - Phocaeicola massiliensis
6 months	MGS	Hgn3C.0053 - Eubacteriales sp.
6 months	MGS	Hgn3C.0054 - Prevotella copri
6 months	MGS	Hgn3C.0055 - Alistipes finegoldii
6 months	MGS	Hgn3C.0056 - Bacteroides fragilis
6 months	MGS	Hgn3C.0057 - Oscillibacter sp. MSJ-31
6 months	MGS	Hgn3C.0058 - Lachnospiraceae sp.
6 months	MGS	Hgn3C.0059 - Bacteroides cellulosilyticus
6 months	MGS	Hgn3C.0060 - Eubacteriales sp.
6 months	MGS	Hgn3C.0061 - Faecalibacillus sp. TM498
6 months	MGS	Hgn3C.0062 - Waltera intestinalis
6 months	MGS	Hgn3C.0063 - Eubacteriales sp.
6 months	MGS	Hgn3C.0064 - Blautia sp. DFI.9.9
6 months	MGS	Hgn3C.0065 - Blautia obeum
6 months	MGS	Hgn3C.0066 - Oscillospiraceae sp.
6 months	MGS	Hgn3C.0067 - Roseburia hominis
6 months	MGS	Hgn3C.0068 - [Ruminococcus] lactaris
6 months	MGS	Hgn3C.0069 - Oscillibacter sp. KLE 1728
6 months	MGS	Hgn3C.0070 - Faecalibacterium sp. OF04-11AC
6 months	MGS	Hgn3C.0071 - Faecalicatena fissicatena
6 months	MGS	Hgn3C.0072 - Clostridium fessum
6 months	MGS	Hgn3C.0073 - Blautia sp.
6 months	MGS	Hgn3C.0074 - Clostridium sp. D43t1_170807_D5
6 months	MGS	Hgn3C.0075 - Agathobaculum butyriciproducens
6 months	MGS	Hgn3C.0077 - Sutterella wadsworthensis

6 months	MGS	Hgn3C.0078 - Clostridium sp. MCC328
6 months	MGS	Hgn3C.0079 - [Ruminococcus] torques
6 months	MGS	Hgn3C.0080 - Phascolarctobacterium faecium
6 months	MGS	Hgn3C.0081 - Coprococcus eutactus
6 months	MGS	Hgn3C.0082 - Roseburia sp. CLA-AA-H204
6 months	MGS	Hgn3C.0083 - Eubacteriales sp.
6 months	MGS	Hgn3C.0084 - Flavonifractor plautii
6 months	MGS	Hgn3C.0085 - Faecalibacterium sp.
6 months	MGS	Hgn3C.0087 - Eubacteriales sp.
6 months	MGS	Hgn3C.0088 - [Ruminococcus] gnavus
6 months	MGS	Hgn3C.0089 - Paraprevotella clara
6 months	MGS	Hgn3C.0092 - Lachnospiraceae sp.
6 months	MGS	Hgn3C.0093 - Bilophila wadsworthia
6 months	MGS	Hgn3C.0094 - Eubacteriales sp.
6 months	MGS	Hgn3C.0095 - Eubacteriales sp.
6 months	MGS	Hgn3C.0096 - Eubacteriales sp.
6 months	MGS	Hgn3C.0097 - Eubacterium ventriosum
6 months	MGS	Hgn3C.0098 - Coprococcus catus
6 months	MGS	Hgn3C.0099 - Dorea longicatena
6 months	MGS	Hgn3C.0100 - Bifidobacterium bifidum
6 months	MGS	Hgn3C.0101 - Bifidobacterium pseudocatenulatum
6 months	MGS	Hgn3C.0102 - Eubacteriales sp.
6 months	MGS	Hgn3C.0103 - Holdemanella porci
6 months	MGS	Hgn3C.0104 - Clostridium sp. AM32-2
6 months	MGS	Hgn3C.0105 - Vescimonas coprocola
6 months	MGS	Hgn3C.0106 - Faecalibacterium sp. CLA-AA-H233
6 months	MGS	Hgn3C.0108 - Eubacteriales sp.
6 months	MGS	Hgn3C.0111 - Eubacteriales sp.
6 months	MGS	Hgn3C.0113 - Clostridium sp. MCC345
6 months	MGS	Hgn3C.0114 - Eubacteriales sp.
6 months	MGS	Hgn3C.0115 - Eubacteriales sp.
6 months	MGS	Hgn3C.0120 - Flavonifractor plautii
6 months	MGS	Hgn3C.0121 - Oscillospiraceae sp.
6 months	MGS	Hgn3C.0123 - Eubacteriales sp.
6 months	MGS	Hgn3C.0124 - Ruthenibacterium lactatiformans
6 months	MGS	Hgn3C.0125 - Sutterella sp. KLE1602
6 months	MGS	Hgn3C.0126 - Ruminococcus sp. BSD2780120874_150323_B10
6 months	MGS	Hgn3C.0127 - Faecalibacterium sp.
6 months	MGS	Hgn3C.0129 - Haemophilus parainfluenzae
6 months	MGS	Hgn3C.0130 - Phocaeicola plebeius
6 months	MGS	Hgn3C.0131 - Streptococcus thermophilus
6 months	MGS	Hgn3C.0132 - Eubacteriales sp.
6 months	MGS	Hgn3C.0134 - Eubacteriales sp.
6 months	MGS	Hgn3C.0135 - Firmicutes sp.
6 months	MGS	Hgn3C.0136 - Alistipes ihumii
6 months	MGS	Hgn3C.0137 - Clostridium sp. OF03-18AA
6 months	MGS	Hgn3C.0138 - Anaerobutyricum soehngenii
6 months	MGS	Hgn3C.0139 - Ruminococcus callidus

6 months	MGS	Hgn3C.0140 - Veillonella parvula
6 months	MGS	Hgn3C.0141 - Adlercreutzia equolifaciens subsp. celatus
6 months	MGS	Hgn3C.0142 - Eubacterium ramulus
6 months	MGS	Hgn3C.0143 - Streptococcus salivarius
6 months	MGS	Hgn3C.0144 - Eubacteriales sp.
6 months	MGS	Hgn3C.0146 - Phascolarctobacterium succinatutens
6 months	MGS	Hgn3C.0147 - Oscillospiraceae sp.
6 months	MGS	Hgn3C.0149 - Eggerthellales sp.
6 months	MGS	Hgn3C.0153 - Lachnospiraceae sp.
6 months	MGS	Hgn3C.0154 - Prevotella copri
6 months	MGS	Hgn3C.0155 - Blautia sp.
6 months	MGS	Hgn3C.0157 - Eubacteriales sp.
6 months	MGS	Hgn3C.0159 - Firmicutes sp.
6 months	MGS	Hgn3C.0161 - Blautia sp.
6 months	MGS	Hgn3C.0162 - Prevotella sp.
6 months	MGS	Hgn3C.0168 - Evtepia gabavorous
6 months	MGS	Hgn3C.0169 - Bacteroides intestinalis
6 months	MGS	Hgn3C.0170 - Oscillospiraceae sp.
6 months	MGS	Hgn3C.0171 - Catenibacterium mitsuokai
6 months	MGS	Hgn3C.0174 - Lachnospiraceae sp.
6 months	MGS	Hgn3C.0175 - Oscillospiraceae sp.
6 months	MGS	Hgn3C.0176 - Alistipes indistinctus
6 months	MGS	Hgn3C.0179 - Bacteroidales sp.
6 months	MGS	Hgn3C.0181 - Alistipes onderdonkii
6 months	MGS	Hgn3C.0182 - Butyricimonas faecihominis
6 months	MGS	Hgn3C.0183 - Intestinibacter bartlettii
6 months	MGS	Hgn3C.0184 - Eubacteriales sp.
6 months	MGS	Hgn3C.0185 - Bifidobacterium catenulatum subsp. kashiwanohense
6 months	MGS	Hgn3C.0186 - Butyricimonas virosa
6 months	MGS	Hgn3C.0191 - Prevotella sp.
6 months	MGS	Hgn3C.0192 - Erysipelatoclostridium ramosum
6 months	MGS	Hgn3C.0193 - Eubacteriales sp.
6 months	MGS	Hgn3C.0195 - Eubacteriales sp.
6 months	MGS	Hgn3C.0201 - Veillonella dispar
6 months	MGS	Hgn3C.0202 - Eggerthella lenta
6 months	MGS	Hgn3C.0203 - Faecalibacterium sp. Marseille-Q3530
6 months	MGS	Hgn3C.0207 - Eubacteriales sp.
6 months	MGS	Hgn3C.0208 - Romboutsia timonensis
6 months	MGS	Hgn3C.0209 - Bifidobacterium breve
6 months	MGS	Hgn3C.0211 - Eubacteriales sp.
6 months	MGS	Hgn3C.0212 - Holdemanella biformis
6 months	MGS	Hgn3C.0213 - Akkermansia sp. GGCC_0220
6 months	MGS	Hgn3C.0214 - Duodenibacillus massiliensis
6 months	MGS	Hgn3C.0215 - Alistipes sp. cv1
6 months	MGS	Hgn3C.0217 - Eubacteriales sp.
6 months	MGS	Hgn3C.0218 - Eubacteriales sp.
6 months	MGS	Hgn3C.0219 - Tyzzerella nexilis
6 months	MGS	Hgn3C.0220 - Clostridium sp. MCC334

6 months	MGS	Hgn3C.0221 - Clostridium sp.
6 months	MGS	Hgn3C.0222 - Parabacteroides merdae
6 months	MGS	Hgn3C.0224 - Veillonellales sp.
6 months	MGS	Hgn3C.0226 - Eubacteriales sp.
6 months	MGS	Hgn3C.0230 - Gemmiger sp.
6 months	MGS	Hgn3C.0232 - Megamonas funiformis
6 months	MGS	Hgn3C.0236 - Oscillospiraceae sp.
6 months	MGS	Hgn3C.0238 - Oscillospiraceae sp.
6 months	MGS	Hgn3C.0239 - Blautia sp. BIOML-A1
6 months	MGS	Hgn3C.0242 - Enterocloster bolteae
6 months	MGS	Hgn3C.0244 - Desulfovibrio piger
6 months	MGS	Hgn3C.0245 - Blautia stercoris
6 months	MGS	Hgn3C.0248 - Coprococcus catus
6 months	MGS	Hgn3C.0249 - Clostridium sp. AM33-3
6 months	MGS	Hgn3C.0250 - Eubacteriales sp.
6 months	MGS	Hgn3C.0251 - Alistipes senegalensis
6 months	MGS	Hgn3C.0253 - Waltera sp.
6 months	MGS	Hgn3C.0260 - Hydrogenoanaerobacterium saccharovorans
6 months	MGS	Hgn3C.0262 - Oscillospiraceae sp.
6 months	MGS	Hgn3C.0264 - Clostridium sp. AF27-2AA
6 months	MGS	Hgn3C.0266 - Dorea sp. AF36-15AT
6 months	MGS	Hgn3C.0267 - Oscillospiraceae sp.
6 months	MGS	Hgn3C.0269 - Flintibacter sp. NSJ-23
6 months	MGS	Hgn3C.0272 - Blautia faecicola
6 months	MGS	Hgn3C.0273 - Slackia isoflavoniconvertens
6 months	MGS	Hgn3C.0275 - Lachnospiraceae sp.
6 months	MGS	Hgn3C.0281 - Eubacteriales sp.
6 months	MGS	Hgn3C.0284 - Erysipelotrichaceae sp.
6 months	MGS	Hgn3C.0286 - Clostridia sp.
6 months	MGS	Hgn3C.0288 - Butyricimonas paravirosa
6 months	MGS	Hgn3C.0290 - Sellimonas intestinalis
6 months	MGS	Hgn3C.0295 - Eubacteriales sp.
6 months	MGS	Hgn3C.0299 - Escherichia coli
6 months	MGS	Hgn3C.0300 - Clostridia sp.
6 months	MGS	Hgn3C.0301 - Prevotella hominis
6 months	MGS	Hgn3C.0304 - Eubacteriales sp.
6 months	MGS	Hgn3C.0306 - Bacteroides nordii
6 months	MGS	Hgn3C.0307 - Veillonella atypica
6 months	MGS	Hgn3C.0308 - [Clostridium] symbiosum
6 months	MGS	Hgn3C.0309 - Eubacteriales sp.
6 months	MGS	Hgn3C.0310 - Ligilactobacillus ruminis
6 months	MGS	Hgn3C.0313 - Ruminococcus bromii
6 months	MGS	Hgn3C.0314 - Ruminococcus sp.
6 months	MGS	Hgn3C.0315 - Bacteroidia sp.
6 months	MGS	Hgn3C.0317 - Intestinimonas butyriciproducens
6 months	MGS	Hgn3C.0319 - Clostridiaceae sp.
6 months	MGS	Hgn3C.0323 - Bacteroides sp.
6 months	MGS	Hgn3C.0324 - Prevotella stercorea

6 months	MGS	Hgn3C.0330 - Faecalibacillus intestinalis
6 months	MGS	Hgn3C.0332 - Klebsiella pneumoniae subsp. pneumoniae
6 months	MGS	Hgn3C.0334 - Bacteroides cellulosilyticus
6 months	MGS	Hgn3C.0336 - Parabacteroides goldsteinii
6 months	MGS	Hgn3C.0337 - Eubacteriales sp.
6 months	MGS	Hgn3C.0338 - Eubacteriales sp.
6 months	MGS	Hgn3C.0345 - Oscillospiraceae sp.
6 months	MGS	Hgn3C.0348 - Streptococcus sp.
6 months	MGS	Hgn3C.0350 - [Clostridium] symbiosum
6 months	MGS	Hgn3C.0351 - Prevotella sp.
6 months	MGS	Hgn3C.0353 - Prevotella sp. P4-67
6 months	MGS	Hgn3C.0354 - Sutterella seckii
6 months	MGS	Hgn3C.0356 - Blautia sp. M29
6 months	MGS	Hgn3C.0359 - Eubacterium sp. BX4
6 months	MGS	Hgn3C.0361 - Dorea sp. AF24-7LB
6 months	MGS	Hgn3C.0362 - Dysosmobacter sp. NSJ-60
6 months	MGS	Hgn3C.0365 - Eubacteriales sp.
6 months	MGS	Hgn3C.0367 - Lachnospiraceae sp.
6 months	MGS	Hgn3C.0369 - Prevotella sp.
6 months	MGS	Hgn3C.0371 - Lachnospiraceae sp.
6 months	MGS	Hgn3C.0373 - Enterocloster clostridioformis
6 months	MGS	Hgn3C.0375 - Ellagibacter isourolithinifaciens
6 months	MGS	Hgn3C.0385 - Veillonella rogosae
6 months	MGS	Hgn3C.0386 - Clostridium sp. AT4
6 months	MGS	Hgn3C.0388 - Clostridium sp.
6 months	MGS	Hgn3C.0392 - Clostridia sp.
6 months	MGS	Hgn3C.0394 - Hungatella hathewayi
6 months	MGS	Hgn3C.0397 - Parabacteroides sp.
6 months	MGS	Hgn3C.0400 - Prevotellamassilia timonensis
6 months	MGS	Hgn3C.0405 - Bacteroides ovatus
6 months	MGS	Hgn3C.0407 - Eubacteriales sp.
6 months	MGS	Hgn3C.0413 - Blautia caecimuris
6 months	MGS	Hgn3C.0417 - Eubacteriales sp.
6 months	MGS	Hgn3C.0418 - Blautia sp.
6 months	MGS	Hgn3C.0419 - Prevotella sp. 885
6 months	MGS	Hgn3C.0420 - Clostridium sp. 1001270J_160509_D11
6 months	MGS	Hgn3C.0430 - Enterocloster aldenensis
6 months	MGS	Hgn3C.0435 - Eubacteriales sp.
6 months	MGS	Hgn3C.0436 - Eubacteriales sp.
6 months	MGS	Hgn3C.0437 - Prevotella sp.
6 months	MGS	Hgn3C.0438 - Coprobacillus cateniformis
6 months	MGS	Hgn3C.0439 - Enterococcus faecalis
6 months	MGS	Hgn3C.0442 - Holdemanella sp.
6 months	MGS	Hgn3C.0443 - Prevotella stercorea
6 months	MGS	Hgn3C.0445 - Aeromonadales sp.
6 months	MGS	Hgn3C.0453 - Blautia sp. Marseille-P3087
6 months	MGS	Hgn3C.0455 - Peptostreptococcaceae sp.
6 months	MGS	Hgn3C.0458 - Eubacteriales sp.

6 months	MGS	Hgn3C.0459 - Eubacteriales sp.
6 months	MGS	Hgn3C.0468 - Clostridium sp. NSJ-42
6 months	MGS	Hgn3C.0470 - Eubacteriales sp.
6 months	MGS	Hgn3C.0472 - Parabacteroides goldsteinii
6 months	MGS	Hgn3C.0476 - Streptococcus sp.
6 months	MGS	Hgn3C.0482 - Lachnospiraceae sp.
6 months	MGS	Hgn3C.0489 - Eubacteriales sp.
6 months	MGS	Hgn3C.0493 - Eisenbergiella tayi
6 months	MGS	Hgn3C.0494 - Prevotellaceae sp.
6 months	MGS	Hgn3C.0495 - Ruminococcus sp.
6 months	MGS	Hgn3C.0500 - Anaerotignum sp.
6 months	MGS	Hgn3C.0501 - Oscillospiraceae sp.
6 months	MGS	Hgn3C.0503 - Eubacteriales sp.
6 months	MGS	Hgn3C.0504 - Megasphaera sp. BL7
6 months	MGS	Hgn3C.0507 - [Clostridium] spiroforme
6 months	MGS	Hgn3C.0514 - Blautia glucerasea
6 months	MGS	Hgn3C.0523 - Phascolarctobacterium succinatutens
6 months	MGS	Hgn3C.0526 - Anaerostipes sp.
6 months	MGS	Hgn3C.0527 - Sutterella sp.
6 months	MGS	Hgn3C.0528 - Coprococcus sp. AM27-12LB
6 months	MGS	Hgn3C.0533 - Flintibacter sp. KGMB00164
6 months	MGS	Hgn3C.0537 - Bifidobacterium dentium
6 months	MGS	Hgn3C.0542 - [Clostridium] innocuum
6 months	MGS	Hgn3C.0543 - Oscillospiraceae sp.
6 months	MGS	Hgn3C.0549 - Longicatena caecimuris
6 months	MGS	Hgn3C.0557 - Enterocloster bolteae
6 months	MGS	Hgn3C.0558 - Olsenella sp.
6 months	MGS	Hgn3C.0560 - Oscillospiraceae sp.
6 months	MGS	Hgn3C.0573 - Prevotella sp. Marseille-P4119
6 months	MGS	Hgn3C.0584 - Bacteroides fragilis
6 months	MGS	Hgn3C.0596 - Butyricimonas sp.
6 months	MGS	Hgn3C.0598 - Eubacteriales sp.
6 months	MGS	Hgn3C.0607 - Eubacteriales sp.
6 months	MGS	Hgn3C.0608 - Lachnospiraceae sp.
6 months	MGS	Hgn3C.0610 - Eubacteriales sp.
6 months	MGS	Hgn3C.0613 - Clostridiaceae sp.
6 months	MGS	Hgn3C.0616 - Lachnospiraceae sp.
6 months	MGS	Hgn3C.0617 - Prevotella sp.
6 months	MGS	Hgn3C.0629 - Collinsella intestinalis
6 months	MGS	Hgn3C.0630 - Allisonella histaminiformans
6 months	MGS	Hgn3C.0634 - Blautia schinkii
6 months	MGS	Hgn3C.0635 - Eubacteriales sp.
6 months	MGS	Hgn3C.0636 - Anaerostipes caccae
6 months	MGS	Hgn3C.0640 - Eubacterium sp. AF22-8LB
6 months	MGS	Hgn3C.0649 - Lachnospiraceae sp.
6 months	MGS	Hgn3C.0657 - Parolsenella catena
6 months	MGS	Hgn3C.0660 - Eubacteriales sp.
6 months	MGS	Hgn3C.0661 - Alistipes sp.

6 months	MGS	Hgn3C.0664 - Blautia hansenii
6 months	MGS	Hgn3C.0675 - Eubacteriales sp.
6 months	MGS	Hgn3C.0676 - Bacteroides sp.
6 months	MGS	Hgn3C.0677 - Bacteroides sp.
6 months	MGS	Hgn3C.0679 - Holdemania filiformis
6 months	MGS	Hgn3C.0680 - Eubacteriales sp.
6 months	MGS	Hgn3C.0682 - [Clostridium] scindens
6 months	MGS	Hgn3C.0683 - Clostridium disporicum
6 months	MGS	Hgn3C.0693 - Hungatella hathewayi
6 months	MGS	Hgn3C.0694 - Anaeromassilibacillus sp. An250
6 months	MGS	Hgn3C.0700 - Eubacteriales sp.
6 months	MGS	Hgn3C.0701 - Bacteroides sp.
6 months	MGS	Hgn3C.0704 - [Clostridium] scindens
6 months	MGS	Hgn3C.0711 - Enterocloster asparagiformis
6 months	MGS	Hgn3C.0712 - Megasphaera micronuciformis
6 months	MGS	Hgn3C.0713 - Veillonellales sp.
6 months	MGS	Hgn3C.0714 - Intestinimonas sp. MSJ-38
6 months	MGS	Hgn3C.0715 - Desulfovibrio fairfieldensis
6 months	MGS	Hgn3C.0716 - Eubacteriales sp.
6 months	MGS	Hgn3C.0718 - Eubacteriales sp.
6 months	MGS	Hgn3C.0729 - Eubacteriales sp.
6 months	MGS	Hgn3C.0731 - Eubacteriales sp.
6 months	MGS	Hgn3C.0736 - Coprococcus sp.
6 months	MGS	Hgn3C.0737 - Alistipes timonensis
6 months	MGS	Hgn3C.0740 - Anaerostipes sp. NSJ-7
6 months	MGS	Hgn3C.0741 - Blautia sp. OF01-4LB
6 months	MGS	Hgn3C.0743 - Enterobacter sp.
6 months	MGS	Hgn3C.0749 - Eubacteriales sp.
6 months	MGS	Hgn3C.0754 - Collinsella bouchesdurhonensis
6 months	MGS	Hgn3C.0755 - Oscillibacter sp.
6 months	MGS	Hgn3C.0761 - Bacteroides sp.
6 months	MGS	Hgn3C.0762 - Enterocloster lavalensis
6 months	MGS	Hgn3C.0777 - Holdemanella sp.
6 months	MGS	Hgn3C.0781 - Collinsella sp.
6 months	MGS	Hgn3C.0782 - Streptococcus sp.
6 months	MGS	Hgn3C.0790 - Oscillospiraceae sp.
6 months	MGS	Hgn3C.0793 - Prevotellaceae sp.
6 months	MGS	Hgn3C.0794 - Bacteroides sp.
6 months	MGS	Hgn3C.0797 - Senegalimassilia sp.
6 months	MGS	Hgn3C.0798 - Streptococcus vestibularis
6 months	MGS	Hgn3C.0814 - Massilimicrobiota timonensis
6 months	MGS	Hgn3C.0817 - Prevotella sp.
6 months	MGS	Hgn3C.0818 - Rothia sp.
6 months	MGS	Hgn3C.0823 - Anaerotignum lactatifermentans
6 months	MGS	Hgn3C.0829 - Dorea phocaeensis
6 months	MGS	Hgn3C.0832 - Veillonellales sp.
6 months	MGS	Hgn3C.0833 - Eisenbergiella tayi
6 months	MGS	Hgn3C.0837 - Anaerotruncus colihominis

6 months	MGS	Hgn3C.0851 - Oscillospiraceae sp.
6 months	MGS	Hgn3C.0855 - Eubacteriales sp.
6 months	MGS	Hgn3C.0858 - Turicibacter sanguinis
6 months	MGS	Hgn3C.0859 - Hydrogeniiclostidium mannosilyticum
6 months	MGS	Hgn3C.0873 - Clostridium perfringens
6 months	MGS	Hgn3C.0875 - Lachnospiraceae sp.
6 months	MGS	Hgn3C.0886 - Bacteroides sp.
6 months	MGS	Hgn3C.0889 - Clostridium paraputrificum
6 months	MGS	Hgn3C.0890 - Enterobacter roggenkampii
6 months	MGS	Hgn3C.0891 - Eubacteriales sp.
6 months	MGS	Hgn3C.0893 - Phocea massiliensis
6 months	MGS	Hgn3C.0908 - Eubacteriales sp.
6 months	MGS	Hgn3C.0913 - Erysipelotrichaceae sp.
6 months	MGS	Hgn3C.0921 - Bacteroides sp.
6 months	MGS	Hgn3C.0922 - Eubacteriales sp.
6 months	MGS	Hgn3C.0948 - Enterobacter sp.
6 months	MGS	Hgn3C.0978 - Veillonella sp.
6 months	MGS	Hgn3C.0987 - Fusobacterium mortiferum
6 months	MGS	Hgn3C.0998 - Eubacteriales sp.
6 months	MGS	Hgn3C.1035 - Klebsiella sp.
6 months	MGS	Hgn3C.1036 - Escherichia coli
6 months	MGS	Hgn3C.1037 - Rothia sp.
6 months	MGS	Hgn3C.1038 - Veillonella sp.
6 months	MGS	Hgn3C.1040 - Lachnospiraceae sp.
6 months	MGS	Hgn3C.1041 - Bacteroides stercoris
6 months	MGS	Hgn3C.1042 - Dorea formicigenerans
6 months	MGS	Hgn3C.1043 - Clostridium phoceensis
6 months	MGS	Hgn3C.1044 - Eubacteriales sp.
6 months	MGS	Hgn3C.1045 - Lactococcus lactis subsp. lactis
6 months	MGS	Hgn3C.1049 - Enterococcus faecium
6 months	MGS	Hgn3C.1050 - Staphylococcus epidermidis
6 months	MGS	Hgn3C.1051 - Holdemania massiliensis
6 months	MGS	Hgn3C.1052 - Enterococcus gallinarum
6 months	MGS	Hgn3C.1054 - Streptococcus lutetiensis
6 months	MGS	Hgn3C.1058 - Mediterraneibacter glycyrrhizinilyticus
6 months	MGS	Hgn3C.1059 - Faecalicatena sp.
6 months	MGS	Hgn3C.1063 - Enterococcus avium
6 months	MGS	Hgn3C.1065 - Klebsiella variicola subsp. variicola
6 months	MGS	Hgn3C.1067 - Lachnospiraceae sp.
6 months	MGS	Hgn3C.1073 - Streptococcus gallolyticus subsp. gallolyticus
6 months	MGS	Hgn3C.1081 - Lacticaseibacillus rhamnosus
6 months	MGS	Hgn3C.1082 - Lacticaseibacillus paracasei subsp. paracasei
6 months	MGS	Hgn3C.1087 - Ligilactobacillus salivarius
6 months	MGS	Hgn3C.1090 - Turicibacter sanguinis
6 months	MGS	Hgn3C.1093 - Enterobacter kobei
6 months	MGS	Hgn3C.1107 - Eubacteriales sp.
6 months	MGS	Hgn3C.1110 - Porphyromonas sp.
6 months	MGS	Hgn3C.1117 - Atopobiaceae sp.

6 months	MGS	Hgn3C.1120 - Actinomycetaceae sp.
6 months	MGS	Hgn3C.1148 - Eggerthellales sp.
6 months	MGS	Hgn3C.1154 - Lactococcus garvieae
6 months	MGS	Hgn3C.1180 - Enterocloster citroniae
6 months	MGS	Hgn3C.1188 - Actinomyces oris
6 months	MGS	Hgn3C.1190 - Fenollaria massiliensis
6 months	MGS	Hgn3C.1193 - Atopobium sp.
6 months	MGS	Hgn3C.1196 - Blautia marasmi
6 months	MGS	Hgn3C.1197 - Blautia hansenii
6 months	MGS	Hgn3C.1203 - Citrobacter sp. CRE-46
6 months	MGS	Hgn3C.1210 - Dielma sp.
6 months	MGS	Hgn3C.1211 - Lachnoclostridium pacaense
6 months	MGS	Hgn3C.1214 - Corynebacterium tuberculostearicum
6 months	MGS	Hgn3C.1218 - Enterobacter bugandensis
6 months	MGS	Hgn3C.1219 - Enterobacter sp. NFIX58
6 months	MGS	Hgn3C.1225 - Enterococcus faecium
6 months	MGS	Hgn3C.1229 - Escherichia sp. 93.0750
6 months	MGS	Hgn3C.1230 - Eubacterium limosum
6 months	MGS	Hgn3C.1234 - Granulicatella adiacens
6 months	MGS	Hgn3C.1238 - Klebsiella quasivariicola
6 months	MGS	Hgn3C.1241 - Limosilactobacillus reuteri subsp. reuteri
6 months	MGS	Hgn3C.1242 - Limosilactobacillus vaginalis
6 months	MGS	Hgn3C.1248 - Neisseria sp. HMSC061E12
6 months	MGS	Hgn3C.1258 - Rothia sp. HMSC072E10
6 months	MGS	Hgn3C.1264 - Staphylococcus hominis subsp. hominis
6 months	MGS	Hgn3C.1268 - Streptococcus sp.
6 months	MGS	Hgn3C.1269 - Streptococcus constellatus subsp. constellatus
6 months	MGS	Hgn3C.1270 - Streptococcus sp.
6 months	MGS	Hgn3C.1275 - Lachnospiraceae sp.
6 months	MGS	Hgn3C.1282 - Terrisporobacter othiniensis
6 months	MGS	Hgn3C.1283 - Clostridium baratii
6 months	MGS	Hgn3C.1286 - Corynebacterium variabile
6 months	MGS	Hgn3C.1288 - Enterobacter hormaechei
6 months	MGS	Hgn3C.1299 - Erysipelotrichaceae sp.
6 months	MGS	Hgn3C.1300 - Rothia sp.
6 months	MGS	Hgn3C.1302 - Schaalia sp.
6 months	MGS	Hgn3C.1303 - Clostridium paraputrificum
6 months	MGS	Hgn3C.1305 - Isoptericola variabilis
6 months	MGS	Hgn3C.1307 - Bifidobacterium longum subsp. spp
6 months	MGS	Hgn3C.1309 - Enterobacter cloacae subsp. cloacae
6 months	MGS	Hgn3C.1310 - Erysipelotrichaceae sp.
6 months	MGS	Hgn3C.1311 - Lachnospiraceae sp.
6 months	MGS	Hgn3C.1312 - Candida tropicalis
6 months	MGS	Hgn3C.1315 - Eubacteriales sp.
6 months	MGS	Hgn3C.1316 - Eubacteriales sp.
6 months	MGS	Hgn3C.1321 - Veillonellaceae sp.
6 months	MGS	Hgn3C.1322 - Enterococcus hirae
6 months	MGS	Hgn3C.1323 - Lachnospiraceae sp.

6 months	MGS	Hgn3C.1324 - Eubacterium sp. c-25
6 months	MGS	Hgn3C.1325 - Luxibacter massiliensis
6 months	MGS	Hgn3C.1326 - Eggerthella guodeyinii
6 months	MGS	Hgn3C.1329 - Eubacteriales sp.
6 months	MGS	Hgn3C.1330 - Lachnospiraceae sp.
6 months	MGS	Hgn3C.1331 - Lachnospiraceae sp.
6 months	MGS	Hgn3C.1334 - Eubacteriales sp.
6 months	MGS	Hgn3C.1336 - Eubacteriales sp.
6 months	MGS	Hgn3C.1338 - Erysipelatoclostridium sp.
6 months	MGS	Hgn3C.1340 - Clostridiaceae sp.
6 months	MGS	Hgn3C.1343 - Enterococcus italicus
6 months	MGS	Hgn3C.1347 - [Clostridium] hylemonae
6 months	MGS	Hgn3C.1349 - Corynebacterium falsenii
6 months	MGS	Hgn3C.1351 - Actinomyces sp. ICM47
6 months	MGS	Hgn3C.1352 - Akkermansia sp.
6 months	MGS	Hgn3C.1354 - Actinomyces sp.
6 months	MGS	Hgn3C.1355 - Collinsella stercoris
6 months	MGS	Hgn3C.1358 - Lachnospiraceae sp.
6 months	MGS	Hgn3C.1361 - Senegalimassilia anaerobia
6 months	MGS	Hgn3C.1362 - Christensenella intestinihominis
6 months	MGS	Hgn3C.1366 - Lachnospiraceae sp.
6 months	MGS	Hgn3C.1369 - Lachnospiraceae sp.
6 months	MGS	Hgn3C.1370 - Kluyvera genomosp. 3
6 months	MGS	Hgn3C.1372 - Eubacteriales sp.
6 months	MGS	Hgn3C.1373 - Eubacteriales sp.
6 months	MGS	Hgn3C.1375 - Atopobiaceae sp.
6 months	MGS	Hgn3C.1376 - Kluyvera sp. CRP
6 months	MGS	Hgn3C.1377 - Collinsella tanakaei
6 months	MGS	Hgn3C.1378 - Collinsella sp. AF08-23
6 months	MGS	Hgn3C.1379 - Gordonibacter urolithinfaciens
6 months	MGS	Hgn3C.1380 - Kluyvera georgiana
6 months	MGS	Hgn3C.1381 - Eubacteriales sp.
6 months	MGS	Hgn3C.1382 - Oscillospiraceae sp.
6 months	MGS	Hgn3C.1384 - Anaerococcus sp.
6 months	MGS	Hgn3C.1386 - Veillonella sp.
6 months	MGS	Hgn3C.1388 - Streptococcus infantarius subsp. infantarius
6 months	MGS	Hgn3C.1390 - Erysipelatoclostridium sp.
6 months	MGS	Hgn3C.1392 - Enterobacter sp. JBIWA005
6 months	MGS	Hgn3C.1393 - Anaerofustis stercorihominis
6 months	MGS	Hgn3C.1398 - Actinomyces sp.
6 months	MGS	Hgn3C.1400 - Eubacterium sp.
6 months	MGS	Hgn3C.1401 - Eubacteriales sp.
6 months	MGS	Hgn3C.1402 - Collinsella phocaeensis
6 months	MGS	Hgn3C.1403 - Eubacteriaceae sp.
6 months	MGS	Hgn3C.1404 - Clostridium sp.
6 months	MGS	Hgn3C.1406 - Slackia piriformis
6 months	MGS	Hgn3C.1407 - Christensenella minuta
6 months	MGS	Hgn3C.1408 - Eubacterium callanderi

6 months	MGS	Hgn3C.1409 - Eggerthella timonensis
6 months	MGS	Hgn3C.1412 - Christensenella hongkongensis
6 months	MGS	Hgn3C.1413 - Streptococcus sp.
6 months	MGS	Hgn3C.1414 - Collinsella sp.
6 months	MGS	Hgn3C.1415 - Klebsiella quasipneumoniae subsp. quasipneumoniae
6 months	MGS	Hgn3C.1416 - Limosilactobacillus fermentum
6 months	MGS	Hgn3C.1418 - Carnobacteriaceae sp.
6 months	MGS	Hgn3C.1419 - Agathobaculum sp. NSJ-28
6 months	MGS	Hgn3C.1420 - Eubacteriales sp.
6 months	MGS	Hgn3C.1423 - Lachnospiraceae sp.
6 months	MGS	Hgn3C.1424 - Mogibacterium sp. NSJ-24
6 months	MGS	Hgn3C.1425 - Eubacteriales sp.
6 months	MGS	Hgn3C.1426 - Limosilactobacillus oris
6 months	MGS	Hgn3C.1434 - Blautia sp.
6 months	MGS	Hgn3C.1435 - Acinetobacter baumannii
6 months	MGS	Hgn3C.1436 - Bacteria sp.
6 months	MGS	Hgn3C.1437 - Candidatus Stoquefichus sp. SB1
6 months	MGS	Hgn3C.1438 - Dielma fastidiosa
6 months	MGS	Hgn3C.1439 - Streptococcus sp. IMAU 99161
6 months	MGS	Hgn3C.1441 - Lancefieldella sp.
6 months	MGS	Hgn3C.1443 - Streptococcus sp.
6 months	MGS	Hgn3C.1444 - Ruminococcus sp. 1001175B_160314_E1
6 months	MGS	Hgn3C.1446 - Eubacteriales sp.
6 months	MGS	Hgn3C.1447 - Bacteria sp.
6 months	MGS	Hgn3C.1450 - Lactococcus sp. NH2-7C
6 months	MGS	Hgn3C.1452 - Raoultibacter timonensis
6 months	MGS	Hgn3C.1453 - Collinsella sp.
6 months	MGS	Hgn3C.1455 - Enterocloster sp.
6 months	MGS	Hgn3C.1456 - Eubacteriales sp.
6 months	MGS	Hgn3C.1460 - Blautia sp.
6 months	MGS	Hgn3C.1463 - Enterobacter sp.
6 months	MGS	Hgn3C.1465 - Clostridia sp.
6 months	MGS	Hgn3C.1467 - Eubacteriales sp.
6 months	MGS	Hgn3C.1468 - Staphylococcus aureus
6 months	MGS	Hgn3C.1469 - Lachnospiraceae sp.
6 months	MGS	Hgn3C.1471 - Eggerthella sp.
6 months	MGS	Hgn3C.1472 - Amedibacillus dolichus
6 months	genus	Acinetobacter
6 months	genus	Actinomyces
6 months	genus	Adlercreutzia
6 months	genus	Agathobaculum
6 months	genus	Akkermansia
6 months	genus	Alistipes
6 months	genus	Allisonella
6 months	genus	Amedibacillus
6 months	genus	Anaerobutyricum
6 months	genus	Anaerococcus
6 months	genus	Anaerofustis

6 months	genus	Anaeromassilibacillus
6 months	genus	Anaerostipes
6 months	genus	Anaerotignum
6 months	genus	Anaerotruncus
6 months	genus	Atopobium
6 months	genus	Bacteroides
6 months	genus	Barnesiella
6 months	genus	Bifidobacterium
6 months	genus	Bilophila
6 months	genus	Blautia
6 months	genus	Butyricicoccus
6 months	genus	Butyricimonas
6 months	genus	Candida
6 months	genus	Candidatus Stoquefichus
6 months	genus	Catenibacterium
6 months	genus	Christensenella
6 months	genus	Citrobacter
6 months	genus	Clostridium
6 months	genus	Collinsella
6 months	genus	Coprobacillus
6 months	genus	Coprococcus
6 months	genus	Corynebacterium
6 months	genus	Desulfovibrio
6 months	genus	Dielma
6 months	genus	Dorea
6 months	genus	Duodenibacillus
6 months	genus	Dysosmobacter
6 months	genus	Eggerthella
6 months	genus	Eisenbergiella
6 months	genus	Ellagibacter
6 months	genus	Enterobacter
6 months	genus	Enterocloster
6 months	genus	Enterococcus
6 months	genus	Erysipelatoclostridium
6 months	genus	Escherichia
6 months	genus	Eubacterium
6 months	genus	Evtepia
6 months	genus	Faecalibacillus
6 months	genus	Faecalibacterium
6 months	genus	Faecalicatena
6 months	genus	Fenollaria
6 months	genus	Flavonifractor
6 months	genus	Flintibacter
6 months	genus	Fusicatenibacter
6 months	genus	Fusobacterium
6 months	genus	Gemmiger
6 months	genus	Gordonibacter
6 months	genus	Granulicatella

6 months	genus	Haemophilus
6 months	genus	Holdemanella
6 months	genus	Holdemania
6 months	genus	Hungatella
6 months	genus	Hydrogeniiclostidium
6 months	genus	Hydrogenoanaerobacterium
6 months	genus	Intestinibacter
6 months	genus	Intestinimonas
6 months	genus	Isoptericola
6 months	genus	Klebsiella
6 months	genus	Kluyvera
6 months	genus	Lachnoclostridium
6 months	genus	Lachnospira
6 months	genus	Lacrimispora
6 months	genus	Lacticaseibacillus
6 months	genus	Lactobacillus
6 months	genus	Lactococcus
6 months	genus	Lancefieldella
6 months	genus	Ligilactobacillus
6 months	genus	Limosilactobacillus
6 months	genus	Longicatena
6 months	genus	Luxibacter
6 months	genus	Massilimicrobiota
6 months	genus	Mediterraneibacter
6 months	genus	Megamonas
6 months	genus	Megasphaera
6 months	genus	Mogibacterium
6 months	genus	Neisseria
6 months	genus	Odoribacter
6 months	genus	Olsenella
6 months	genus	Oscillibacter
6 months	genus	Parabacteroides
6 months	genus	Paraprevotella
6 months	genus	Parolsenella
6 months	genus	Phascolarctobacterium
6 months	genus	Phocaeicola
6 months	genus	Phocea
6 months	genus	Porphyromonas
6 months	genus	Prevotella
6 months	genus	Prevotellamassilia
6 months	genus	Raoultibacter
6 months	genus	Romboutsia
6 months	genus	Roseburia
6 months	genus	Rothia
6 months	genus	Ruminococcus
6 months	genus	Ruthenibacterium
6 months	genus	Schaalia
6 months	genus	Sellimonas

6 months	genus	Senegalimassilia
6 months	genus	Slackia
6 months	genus	Staphylococcus
6 months	genus	Streptococcus
6 months	genus	Subdoligranulum
6 months	genus	Sutterella
6 months	genus	Terrisporobacter
6 months	genus	Turicibacter
6 months	genus	Tyzzerella
6 months	genus	Veillonella
6 months	genus	Vescimonas
6 months	genus	Waltera
6 months	family	Acidaminococcaceae
6 months	family	Actinomycetaceae
6 months	family	Akkermansiaceae
6 months	family	Atopobiaceae
6 months	family	Bacteroidaceae
6 months	family	Barnesiellaceae
6 months	family	Bifidobacteriaceae
6 months	family	Carnobacteriaceae
6 months	family	Christensenellaceae
6 months	family	Clostridiaceae
6 months	family	Coprobacillaceae
6 months	family	Coriobacteriaceae
6 months	family	Corynebacteriaceae
6 months	family	Debaryomycetaceae
6 months	family	Desulfovibrionaceae
6 months	family	Eggerthellaceae
6 months	family	Enterobacteriaceae
6 months	family	Enterococcaceae
6 months	family	Erysipelotrichaceae
6 months	family	Eubacteriaceae
6 months	family	Eubacteriales Family XIII. Incertae Sedis
6 months	family	Fusobacteriaceae
6 months	family	Lachnospiraceae
6 months	family	Lactobacillaceae
6 months	family	Micrococcaceae
6 months	family	Moraxellaceae
6 months	family	Neisseriaceae
6 months	family	Odoribacteraceae
6 months	family	Oscillospiraceae
6 months	family	Pasteurellaceae
6 months	family	Peptoniphilaceae
6 months	family	Peptostreptococcaceae
6 months	family	Porphyromonadaceae
6 months	family	Prevotellaceae
6 months	family	Promicromonosporaceae
6 months	family	Rikenellaceae

6 months	family	Selenomonadaceae
6 months	family	Staphylococcaceae
6 months	family	Streptococcaceae
6 months	family	Sutterellaceae
6 months	family	Tannerellaceae
6 months	family	Turicibacteraceae
6 months	family	Veillonellaceae
6 months	phylum	Actinobacteria
6 months	phylum	Ascomycota
6 months	phylum	Bacteroidetes
6 months	phylum	Firmicutes
6 months	phylum	Fusobacteria
6 months	phylum	Proteobacteria
6 months	phylum	Verrucomicrobia

Feature	Contrast
Intervention	Experimental -
Intervention	Experimental -
Intervention	Experimental -
Intervention	Experimental -
Intervention	Experimental -
Intervention	Experimental -
Intervention	Experimental -
Intervention	Experimental -
Intervention	Experimental -
Intervention	Experimental -
Intervention	Experimental -
Intervention	Experimental -
Intervention	Experimental -
Intervention	Experimental -
Intervention	Experimental -
Intervention	Experimental -
Intervention	Experimental -
Intervention	Experimental -
Intervention	Experimental -
Intervention	Experimental -
Intervention	Experimental -
Intervention	Experimental -
Intervention	Experimental -
Intervention	Experimental -
Intervention	Experimental -
Intervention	Experimental -
Intervention	Experimental -
Intervention	Experimental -
Intervention	Experimental -
Intervention	Experimental -
Intervention	Experimental -
Intervention	Experimental -
Intervention	Experimental -
Intervention	Experimental -
Intervention	Experimental -
Intervention	Experimental -
Intervention	Experimental -
Intervention	Experimental -
Intervention	Experimental -
Intervention	Experimental -
Intervention	Experimental -
Intervention	Experimental -
Intervention	Experimental -
Intervention	Experimental -
Intervention	Experimental -
Intervention	Experimental -
Intervention	Experimental -

Intervention	Experimental -
Intervention	Experimental -
Intervention	Experimental -
Intervention	Experimental -
Intervention	Experimental -
Intervention	Experimental -
Intervention	Experimental -
Intervention	Experimental -
Intervention	Experimental -
Intervention	Experimental -
Intervention	Experimental -
Intervention	Experimental -
Intervention	Experimental -
Intervention	Experimental -
Intervention	Experimental -
Intervention	Experimental -
Intervention	Experimental -
Intervention	Experimental -
Intervention	Experimental -
Intervention	Experimental -
Intervention	Experimental -
Intervention	Experimental -
Intervention	Experimental -
Intervention	Experimental -
Intervention	Experimental -
Intervention	Experimental -
Intervention	Experimental -
Intervention	Experimental -
Intervention	Experimental -
Intervention	Experimental -
Intervention	Experimental -
Intervention	Experimental -
Intervention	Experimental -
Intervention	Experimental -
Intervention	Experimental -
Intervention	Experimental -
Intervention	Experimental -
Intervention	Experimental -
Intervention	Experimental -
Intervention	Experimental -
Intervention	Experimental -
Intervention	Experimental -
Intervention	Experimental -
Intervention	Experimental -
Intervention	Experimental -
Intervention	Experimental -
Intervention	Experimental -
Intervention	Experimental -

Intervention	Experimental -
Intervention	Experimental -
Intervention	Experimental -
Intervention	Experimental -
Intervention	Experimental -
Intervention	Experimental -
Intervention	Experimental -
Intervention	Experimental -
Intervention	Experimental -
Intervention	Experimental -
Intervention	Experimental -
Intervention	Experimental -
Intervention	Experimental -
Intervention	Experimental -
Intervention	Experimental -
Intervention	Experimental -
Intervention	Experimental -
Intervention	Experimental -
Intervention	Experimental -
Intervention	Experimental -
Intervention	Experimental -
Intervention	Experimental -
Intervention	Experimental -
Intervention	Experimental -
Intervention	Experimental -
Intervention	Experimental -
Intervention	Experimental -
Intervention	Experimental -
Intervention	Experimental -
Intervention	Experimental -
Intervention	Experimental -
Intervention	Experimental -
Intervention	Experimental -
Intervention	Experimental -
Intervention	Experimental -
Intervention	Experimental -
Intervention	Experimental -
Intervention	Experimental -
Intervention	Experimental -
Intervention	Experimental -
Intervention	Experimental -
Intervention	Experimental -
Intervention	Experimental -
Intervention	Experimental -
Intervention	Experimental -
Intervention	Experimental -
Intervention	Experimental -
Intervention	Experimental -

Intervention	Experimental -
Intervention	Experimental -
Intervention	Experimental -
Intervention	Experimental -
Intervention	Experimental -
Intervention	Experimental -
Intervention	Experimental -
Intervention	Experimental -
Intervention	Experimental -
Intervention	Experimental -
Intervention	Experimental -
Intervention	Experimental -
Intervention	Experimental -
Intervention	Experimental -
Intervention	Experimental -
Intervention	Experimental -
Intervention	Experimental -
Intervention	Experimental -
Intervention	Experimental -
Intervention	Experimental -
Intervention	Experimental -
Intervention	Experimental -
Intervention	Experimental -
Intervention	Experimental -
Intervention	Experimental -
Intervention	Experimental -
Intervention	Experimental -
Intervention	Experimental -
Intervention	Experimental -
Intervention	Experimental -
Intervention	Experimental -
Intervention	Experimental -
Intervention	Experimental -
Intervention	Experimental -
Intervention	Experimental -
Intervention	Experimental -
Intervention	Experimental -
Intervention	Experimental -
Intervention	Experimental -
Intervention	Experimental -
Intervention	Experimental -
Intervention	Experimental -
Intervention	Experimental -
Intervention	Experimental -
Intervention	Experimental -
Intervention	Experimental -
Intervention	Experimental -
Intervention	Experimental -

Intervention	Experimental -
Intervention	Experimental -
Intervention	Experimental -
Intervention	Experimental -
Intervention	Experimental -
Intervention	Experimental -
Intervention	Experimental -
Intervention	Experimental -
Intervention	Experimental -
Intervention	Experimental -
Intervention	Experimental -
Intervention	Experimental -
Intervention	Experimental -
Intervention	Experimental -
Intervention	Experimental -
Intervention	Experimental -
Intervention	Experimental -
Intervention	Experimental -
Intervention	Experimental -
Intervention	Experimental -
Intervention	Experimental -
Intervention	Experimental -
Intervention	Experimental -
Intervention	Experimental -
Intervention	Experimental -
Intervention	Experimental -
Intervention	Experimental -
Intervention	Experimental -
Intervention	Experimental -
Intervention	Experimental -
Intervention	Experimental -
Intervention	Experimental -
Intervention	Experimental -
Intervention	Experimental -
Intervention	Experimental -
Intervention	Experimental -
Intervention	Experimental -
Intervention	Experimental -
Intervention	Experimental -
Intervention	Experimental -
Intervention	Experimental -
Intervention	Experimental -
Intervention	Experimental -
Intervention	Experimental -
Intervention	Experimental -
Intervention	Experimental -
Intervention	Experimental -
Intervention	Experimental -

Intervention	Experimental -
Intervention	Experimental -
Intervention	Experimental -
Intervention	Experimental -
Intervention	Experimental -
Intervention	Experimental -
Intervention	Experimental -
Intervention	Experimental -
Intervention	Experimental -
Intervention	Experimental -
Intervention	Experimental -
Intervention	Experimental -
Intervention	Experimental -
Intervention	Experimental -
Intervention	Experimental -
Intervention	Experimental -
Intervention	Experimental -
Intervention	Experimental -
Intervention	Experimental -
Intervention	Experimental -
Intervention	Experimental -
Intervention	Experimental -
Intervention	Experimental -
Intervention	Experimental -
Intervention	Experimental -
Intervention	Experimental -
Intervention	Experimental -
Intervention	Experimental -
Intervention	Experimental -
Intervention	Experimental -
Intervention	Experimental -
Intervention	Experimental -
Intervention	Experimental -
Intervention	Experimental -
Intervention	Experimental -
Intervention	Experimental -
Intervention	Experimental -
Intervention	Experimental -
Intervention	Experimental -
Intervention	Experimental -
Intervention	Experimental -
Intervention	Experimental -
Intervention	Experimental -
Intervention	Experimental -
Intervention	Experimental -
Intervention	Experimental -
Intervention	Experimental -
Intervention	Experimental -

Intervention	Experimental -
Intervention	Experimental -
Intervention	Experimental -
Intervention	Experimental -
Intervention	Experimental -
Intervention	Experimental -
Intervention	Experimental -
Intervention	Experimental -
Intervention	Experimental -
Intervention	Experimental -
Intervention	Experimental -
Intervention	Experimental -
Intervention	Experimental -
Intervention	Experimental -
Intervention	Experimental -
Intervention	Experimental -
Intervention	Experimental -
Intervention	Experimental -
Intervention	Experimental -
Intervention	Experimental -
Intervention	Experimental -
Intervention	Experimental -
Intervention	Experimental -
Intervention	Experimental -
Intervention	Experimental -
Intervention	Experimental -
Intervention	Experimental -
Intervention	Experimental -
Intervention	Experimental -
Intervention	Experimental -
Intervention	Experimental -
Intervention	Experimental -
Intervention	Experimental -
Intervention	Experimental -
Intervention	Experimental -
Intervention	Experimental -
Intervention	Experimental -
Intervention	Experimental -
Intervention	Experimental -
Intervention	Experimental -
Intervention	Experimental -
Intervention	Experimental -
Intervention	Experimental -
Intervention	Experimental -
Intervention	Experimental -
Intervention	Experimental -
Intervention	Experimental -
Intervention	Experimental -

Intervention	Experimental -
Intervention	Experimental -
Intervention	Experimental -
Intervention	Experimental -
Intervention	Experimental -
Intervention	Experimental -
Intervention	Experimental -
Intervention	Experimental -
Intervention	Experimental -
Intervention	Experimental -
Intervention	Experimental -
Intervention	Experimental -
Intervention	Experimental -
Intervention	Experimental -
Intervention	Experimental -
Intervention	Experimental -
Intervention	Experimental -
Intervention	Experimental -
Intervention	Experimental -
Intervention	Experimental -
Intervention	Experimental -
Intervention	Experimental -
Intervention	Experimental -
Intervention	Experimental -
Intervention	Experimental -
Intervention	Experimental -
Intervention	Experimental -
Intervention	Experimental -
Intervention	Experimental -
Intervention	Experimental -
Intervention	Experimental -
Intervention	Experimental -
Intervention	Experimental -
Intervention	Experimental -
Intervention	Experimental -
Intervention	Experimental -
Intervention	Experimental -
Intervention	Experimental -
Intervention	Experimental -
Intervention	Experimental -
Intervention	Experimental -
Intervention	Experimental -
Intervention	Experimental -
Intervention	Experimental -
Intervention	Experimental -
Intervention	Experimental -
Intervention	Experimental -
Intervention	Experimental -

Intervention	Experimental -
Intervention	Experimental -
Intervention	Experimental -
Intervention	Experimental -
Intervention	Experimental -
Intervention	Experimental -
Intervention	Experimental -
Intervention	Experimental -
Intervention	Experimental -
Intervention	Experimental -
Intervention	Experimental -
Intervention	Experimental -
Intervention	Experimental -
Intervention	Experimental -
Intervention	Experimental -
Intervention	Experimental -
Intervention	Experimental -
Intervention	Experimental -
Intervention	Experimental -
Intervention	Experimental -
Intervention	Experimental -
Intervention	Experimental -
Intervention	Experimental -
Intervention	Experimental -
Intervention	Experimental -
Intervention	Experimental -
Intervention	Experimental -
Intervention	Experimental -
Intervention	Experimental -
Intervention	Experimental -
Intervention	Experimental -
Intervention	Experimental -
Intervention	Experimental -
Intervention	Experimental -
Intervention	Experimental -
Intervention	Experimental -
Intervention	Experimental -
Intervention	Experimental -
Intervention	Experimental -
Intervention	Experimental -
Intervention	Experimental -
Intervention	Experimental -
Intervention	Experimental -
Intervention	Experimental -
Intervention	Experimental -
Intervention	Experimental -
Intervention	Experimental -
Intervention	Experimental -

Intervention	Experimental -
Intervention	Experimental -
Intervention	Experimental -
Intervention	Experimental -
Intervention	Experimental -
Intervention	Experimental -
Intervention	Experimental -
Intervention	Experimental -
Intervention	Experimental -
Intervention	Experimental -
Intervention	Experimental -
Intervention	Experimental -
Intervention	Experimental -
Intervention	Experimental -
Intervention	Experimental -
Intervention	Experimental -
Intervention	Experimental -
Intervention	Experimental -
Intervention	Experimental -
Intervention	Experimental -
Intervention	Experimental -
Intervention	Experimental -
Intervention	Experimental -
Intervention	Experimental -
Intervention	Experimental -
Intervention	Experimental -
Intervention	Experimental -
Intervention	Experimental -
Intervention	Experimental -
Intervention	Experimental -
Intervention	Experimental -
Intervention	Experimental -
Intervention	Experimental -
Intervention	Experimental -
Intervention	Experimental -
Intervention	Experimental -
Intervention	Experimental -
Intervention	Experimental -
Intervention	Experimental -
Intervention	Experimental -
Intervention	Experimental -
Intervention	Experimental -
Intervention	Experimental -
Intervention	Experimental -
Intervention	Experimental -
Intervention	Experimental -
Intervention	Experimental -
Intervention	Experimental -

Intervention	Experimental -
Intervention	Experimental -
Intervention	Experimental -
Intervention	Experimental -
Intervention	Experimental -
Intervention	Experimental -
Intervention	Experimental -
Intervention	Experimental -
Intervention	Experimental -
Intervention	Experimental -
Intervention	Experimental -
Intervention	Experimental -
Intervention	Experimental -
Intervention	Experimental -
Intervention	Experimental -
Intervention	Experimental -
Intervention	Experimental -
Intervention	Experimental -
Intervention	Experimental -
Intervention	Experimental -
Intervention	Experimental -
Intervention	Experimental -
Intervention	Experimental -
Intervention	Experimental -
Intervention	Experimental -
Intervention	Experimental -
Intervention	Experimental -
Intervention	Experimental -
Intervention	Experimental -
Intervention	Experimental -
Intervention	Experimental -
Intervention	Experimental -
Intervention	Experimental -
Intervention	Experimental -
Intervention	Experimental -
Intervention	Experimental -
Intervention	Experimental -
Intervention	Experimental -
Intervention	Experimental -
Intervention	Experimental -
Intervention	Experimental -
Intervention	Experimental -
Intervention	Experimental -
Intervention	Experimental -
Intervention	Experimental -
Intervention	Experimental -
Intervention	Experimental -
Intervention	Experimental -

Intervention	Experimental -
Intervention	Experimental -
Intervention	Experimental -
Intervention	Experimental -
Intervention	Experimental -
Intervention	Experimental -
Intervention	Experimental -
Intervention	Experimental -
Intervention	Experimental -
Intervention	Experimental -
Intervention	Experimental -
Intervention	Experimental -
Intervention	Experimental -
Intervention	Experimental -
Intervention	Experimental -
Intervention	Experimental -
Intervention	Experimental -
Intervention	Experimental -
Intervention	Experimental -
Intervention	Experimental -
Intervention	Experimental -
Intervention	Experimental -
Intervention	Experimental -
Intervention	Experimental -
Intervention	Experimental -
Intervention	Experimental -
Intervention	Experimental -
Intervention	Experimental -
Intervention	Experimental -
Intervention	Experimental -
Intervention	Experimental -
Intervention	Experimental -
Intervention	Experimental -
Intervention	Experimental -
Intervention	Experimental -
Intervention	Experimental -
Intervention	Experimental -
Intervention	Experimental -
Intervention	Experimental -
Intervention	Experimental -
Intervention	Experimental -
Intervention	Experimental -
Intervention	Experimental -
Intervention	Experimental -
Intervention	Experimental -
Intervention	Experimental -
Intervention	Experimental -
Intervention	Experimental -

Intervention	Experimental -
Intervention	Experimental -
Intervention	Experimental -
Intervention	Experimental -
Intervention	Experimental -
Intervention	Experimental -
Intervention	Experimental -
Intervention	Experimental -
Intervention	Experimental -
Intervention	Experimental -
Intervention	Experimental -
Intervention	Experimental -
Intervention	Experimental -
Intervention	Experimental -
Intervention	Experimental -
Intervention	Experimental -
Intervention	Experimental -
Intervention	Experimental -
Intervention	Experimental -
Intervention	Experimental -
Intervention	Experimental -
Intervention	Experimental -
Intervention	Experimental -
Intervention	Experimental -
Intervention	Experimental -
Intervention	Experimental -
Intervention	Experimental -
Intervention	Experimental -
Intervention	Experimental -
Intervention	Experimental -
Intervention	Experimental -
Intervention	Experimental -
Intervention	Experimental -
Intervention	Experimental -
Intervention	Experimental -
Intervention	Experimental -
Intervention	Experimental -
Intervention	Experimental -
Intervention	Experimental -
Intervention	Experimental -
Intervention	Experimental -
Intervention	Experimental -
Intervention	Experimental -
Intervention	Experimental -
Intervention	Experimental -
Intervention	Experimental -
Intervention	Experimental -
Intervention	Experimental -

Intervention	Experimental -
Intervention	Experimental -
Intervention	Experimental -
Intervention	Experimental -
Intervention	Experimental -
Intervention	Experimental -
Intervention	Experimental -
Intervention	Experimental -
Intervention	Experimental -
Intervention	Experimental -
Intervention	Experimental -
Intervention	Experimental -
Intervention	Experimental -
Intervention	Experimental -
Intervention	Experimental -
Intervention	Experimental -
Intervention	Experimental -
Intervention	Experimental -
Intervention	Experimental -
Intervention	Experimental -
Intervention	Experimental -
Intervention	Experimental -
Intervention	Experimental -
Intervention	Experimental -
Intervention	Experimental -
Intervention	Experimental -
Intervention	Experimental -
Intervention	Experimental -
Intervention	Experimental -
Intervention	Experimental -
Intervention	Experimental -
Intervention	Experimental -
Intervention	Experimental -
Intervention	Experimental -
Intervention	Experimental -
Intervention	Experimental -
Intervention	Experimental -
Intervention	Experimental -
Intervention	Experimental -
Intervention	Experimental -
Intervention	Experimental -
Intervention	Experimental -
Intervention	Experimental -
Intervention	Experimental -
Intervention	Experimental -
Intervention	Experimental -
Intervention	Experimental -
Intervention	Experimental -

Intervention	Experimental -
Intervention	Experimental -
Intervention	Experimental -
Intervention	Experimental -
Intervention	Experimental -
Intervention	Experimental -
Intervention	Experimental -
Intervention	Experimental -
Intervention	Experimental -
Intervention	Experimental -
Intervention	Experimental -
Intervention	Experimental -
Intervention	Experimental -
Intervention	Experimental -
Intervention	Experimental -
Intervention	Experimental -
Intervention	Experimental -
Intervention	Experimental -
Intervention	Experimental -
Intervention	Experimental -
Intervention	Experimental -
Intervention	Experimental -
Intervention	Experimental -
Intervention	Experimental -
Intervention	Experimental -
Intervention	Experimental -
Intervention	Experimental -
Intervention	Experimental -
Intervention	Experimental -
Intervention	Experimental -
Intervention	Experimental -
Intervention	Experimental -
Intervention	Experimental -
Intervention	Experimental -
Intervention	Experimental -
Intervention	Experimental -
Intervention	Experimental -
Intervention	Experimental -
Intervention	Experimental -
Intervention	Experimental -
Intervention	Experimental -
Intervention	Experimental -
Intervention	Experimental -
Intervention	Experimental -
Intervention	Experimental -
Intervention	Experimental -
Intervention	Experimental -
Intervention	Experimental -

Intervention	Experimental -
Intervention	Experimental -
Intervention	Experimental -
Intervention	Experimental -
Intervention	Experimental -
Intervention	Experimental -
Intervention	Experimental -
Intervention	Experimental -
Intervention	Experimental -
Intervention	Experimental -
Intervention	Experimental -
Intervention	Experimental -
Intervention	Experimental -
Intervention	Experimental -
Intervention	Experimental -
Intervention	Experimental -
Intervention	Experimental -
Intervention	Experimental -
Intervention	Experimental -
Intervention	Experimental -
Intervention	Experimental -
Intervention	Experimental -
Intervention	Experimental -
Intervention	Experimental -
Intervention	Experimental -
Intervention	Experimental -
Intervention	Experimental -
Intervention	Experimental -
Intervention	Experimental -
Intervention	Experimental -
Intervention	Experimental -
Intervention	Experimental -
Intervention	Experimental -
Intervention	Experimental -
Intervention	Experimental -
Intervention	Experimental -
Intervention	Experimental -
Intervention	Experimental -
Intervention	Experimental -
Intervention	Experimental -
Intervention	Experimental -
Intervention	Experimental -
Intervention	Experimental -
Intervention	Experimental -
Intervention	Experimental -
Intervention	Experimental -
Intervention	Experimental -
Intervention	Experimental -

Intervention	Experimental -
Intervention	Experimental -
Intervention	Experimental -
Intervention	Experimental -
Intervention	Experimental -
Intervention	Experimental -
Intervention	Experimental -
Intervention	Experimental -
Intervention	Experimental -
Intervention	Experimental -
Intervention	Experimental -
Intervention	Experimental -
Intervention	Experimental -
Intervention	Experimental -
Intervention	Experimental -
Intervention	Experimental -
Intervention	Experimental -
Intervention	Experimental -
Intervention	Experimental -
Intervention	Experimental -
Intervention	Experimental -
Intervention	Experimental -
Intervention	Experimental -
Intervention	Experimental -
Intervention	Experimental -
Intervention	Experimental -
Intervention	Experimental -
Intervention	Experimental -
Intervention	Experimental -
Intervention	Experimental -
Intervention	Experimental -
Intervention	Experimental -
Intervention	Experimental -
Intervention	Experimental -
Intervention	Experimental -
Intervention	Experimental -
Intervention	Experimental -
Intervention	Experimental -
Intervention	Experimental -
Intervention	Experimental -
Intervention	Experimental -
Intervention	Experimental -
Intervention	Experimental -
Intervention	Experimental -
Intervention	Experimental -
Intervention	Experimental -
Intervention	Experimental -
Intervention	Experimental -

Intervention	Experimental -
Intervention	Experimental -
Intervention	Experimental -
Intervention	Experimental -
Intervention	Experimental -
Intervention	Experimental -
Intervention	Experimental -
Intervention	Experimental -
Intervention	Experimental -
Intervention	Experimental -
Intervention	Experimental -
Intervention	Experimental -
Intervention	Experimental -
Intervention	Experimental -
Intervention	Experimental -
Intervention	Experimental -
Intervention	Experimental -
Intervention	Experimental -
Intervention	Experimental -
Intervention	Experimental -
Intervention	Experimental -
Intervention	Experimental -
Intervention	Experimental -
Intervention	Experimental -
Intervention	Experimental -
Intervention	Experimental -
Intervention	Experimental -
Intervention	Experimental -
Intervention	Experimental -
Intervention	Experimental -
Intervention	Experimental -
Intervention	Experimental -
Intervention	Experimental -
Intervention	Experimental -
Intervention	Experimental -
Intervention	Experimental -
Intervention	Experimental -
Intervention	Experimental -
Intervention	Experimental -
Intervention	Experimental -
Intervention	Experimental -
Intervention	Experimental -
Intervention	Experimental -
Intervention	Experimental -
Intervention	Experimental -
Intervention	Experimental -
Intervention	Experimental -
Intervention	Experimental -

Intervention	Experimental -
Intervention	Experimental -
Intervention	Experimental -
Intervention	Experimental -
Intervention	Experimental -
Intervention	Experimental -
Intervention	Experimental -
Intervention	Experimental -
Intervention	Experimental -
Intervention	Experimental -
Intervention	Experimental -
Intervention	Experimental -
Intervention	Experimental -
Intervention	Experimental -
Intervention	Experimental -
Intervention	Experimental -
Intervention	Experimental -
Intervention	Experimental -
Intervention	Experimental -
Intervention	Experimental -
Intervention	Experimental -
Intervention	Experimental -
Intervention	Experimental -
Intervention	Experimental -
Intervention	Experimental -
Intervention	Experimental -
Intervention	Experimental -
Intervention	Experimental -
Intervention	Experimental -
Intervention	Experimental -
Intervention	Experimental -
Intervention	Experimental -
Intervention	Experimental -
Intervention	Experimental -
Intervention	Experimental -
Intervention	Experimental -
Intervention	Experimental -
Intervention	Experimental -
Intervention	Experimental -
Intervention	Experimental -
Intervention	Experimental -
Intervention	Experimental -
Intervention	Experimental -
Intervention	Experimental -
Intervention	Experimental -
Intervention	Experimental -
Intervention	Experimental -
Intervention	Experimental -

Intervention	Experimental -
Intervention	Experimental -
Intervention	Experimental -
Intervention	Experimental -
Intervention	Experimental -
Intervention	Experimental -
Intervention	Experimental -
Intervention	Experimental -
Intervention	Experimental -
Intervention	Experimental -
Intervention	Experimental -
Intervention	Experimental -
Intervention	Experimental -
Intervention	Experimental -
Intervention	Experimental -
Intervention	Experimental -
Intervention	Experimental -
Intervention	Experimental -
Intervention	Experimental -
Intervention	Experimental -
Intervention	Experimental -
Intervention	Experimental -
Intervention	Experimental -
Intervention	Experimental -
Intervention	Experimental -
Intervention	Experimental -
Intervention	Experimental -
Intervention	Experimental -
Intervention	Experimental -
Intervention	Experimental -
Intervention	Experimental -
Intervention	Experimental -
Intervention	Experimental -
Intervention	Experimental -
Intervention	Experimental -
Intervention	Experimental -
Intervention	Experimental -
Intervention	Experimental -
Intervention	Experimental -
Intervention	Experimental -
Intervention	Experimental -
Intervention	Experimental -
Intervention	Experimental -
Intervention	Experimental -
Intervention	Experimental -
Intervention	Experimental -
Intervention	Experimental -
Intervention	Experimental -

Intervention	Experimental -
Intervention	Experimental -
Intervention	Experimental -
Intervention	Experimental -
Intervention	Experimental -
Intervention	Experimental -
Intervention	Experimental -
Intervention	Experimental -
Intervention	Experimental -
Intervention	Experimental -
Intervention	Experimental -
Intervention	Experimental -
Intervention	Experimental -
Intervention	Experimental -
Intervention	Experimental -
Intervention	Experimental -
Intervention	Experimental -
Intervention	Experimental -
Intervention	Experimental -
Intervention	Experimental -
Intervention	Experimental -
Intervention	Experimental -
Intervention	Experimental -
Intervention	Experimental -
Intervention	Experimental -
Intervention	Experimental -
Intervention	Experimental -
Intervention	Experimental -
Intervention	Experimental -
Intervention	Experimental -
Intervention	Experimental -
Intervention	Experimental -
Intervention	Experimental -
Intervention	Experimental -
Intervention	Experimental -
Intervention	Experimental -
Intervention	Experimental -
Intervention	Experimental -
Intervention	Experimental -
Intervention	Experimental -
Intervention	Experimental -
Intervention	Experimental -
Intervention	Experimental -
Intervention	Experimental -
Intervention	Experimental -
Intervention	Experimental -
Intervention	Experimental -
Intervention	Experimental -

Intervention	Experimental -
Intervention	Experimental -
Intervention	Experimental -
Intervention	Experimental -
Intervention	Experimental -
Intervention	Experimental -
Intervention	Experimental -
Intervention	Experimental -
Intervention	Experimental -
Intervention	Experimental -
Intervention	Experimental -
Intervention	Experimental -
Intervention	Experimental -
Intervention	Experimental -
Intervention	Experimental -
Intervention	Experimental -
Intervention	Experimental -
Intervention	Experimental -
Intervention	Experimental -
Intervention	Experimental -
Intervention	Experimental -
Intervention	Experimental -
Intervention	Experimental -
Intervention	Experimental -
Intervention	Experimental -
Intervention	Experimental -
Intervention	Experimental -
Intervention	Experimental -
Intervention	Experimental -
Intervention	Experimental -
Intervention	Experimental -
Intervention	Experimental -
Intervention	Experimental -
Intervention	Experimental -
Intervention	Experimental -
Intervention	Experimental -
Intervention	Experimental -
Intervention	Experimental -
Intervention	Experimental -
Intervention	Experimental -
Intervention	Experimental -
Intervention	Experimental -
Intervention	Experimental -
Intervention	Experimental -
Intervention	Experimental -
Intervention	Experimental -
Intervention	Experimental -
Intervention	Experimental -

Intervention	Experimental -
Intervention	Experimental -
Intervention	Experimental -
Intervention	Experimental -
Intervention	Experimental -
Intervention	Experimental -
Intervention	Experimental -
Intervention	Experimental -
Intervention	Experimental -
Intervention	Experimental -
Intervention	Experimental -
Intervention	Experimental -
Intervention	Experimental -
Intervention	Experimental -
Intervention	Experimental -
Intervention	Experimental -
Intervention	Experimental -
Intervention	Experimental -
Intervention	Experimental -
Intervention	Experimental -
Intervention	Experimental -
Intervention	Experimental -
Intervention	Experimental -
Intervention	Experimental -
Intervention	Experimental -
Intervention	Experimental -
Intervention	Experimental -
Intervention	Experimental -
Intervention	Experimental -
Intervention	Experimental -
Intervention	Experimental -
Intervention	Experimental -
Intervention	Experimental -
Intervention	Experimental -
Intervention	Experimental -
Intervention	Experimental -
Intervention	Experimental -
Intervention	Experimental -
Intervention	Experimental -
Intervention	Experimental -
Intervention	Experimental -
Intervention	Experimental -
Intervention	Experimental -
Intervention	Experimental -
Intervention	Experimental -
Intervention	Experimental -
Intervention	Experimental -
Intervention	Experimental -

Intervention	Experimental -
Intervention	Experimental -
Intervention	Experimental -
Intervention	Experimental -
Intervention	Experimental -
Intervention	Experimental -
Intervention	Experimental -
Intervention	Experimental -
Intervention	Experimental -
Intervention	Experimental -
Intervention	Experimental -
Intervention	Experimental -
Intervention	Experimental -
Intervention	Experimental -
Intervention	Experimental -
Intervention	Experimental -
Intervention	Experimental -
Intervention	Experimental -
Intervention	Experimental -
Intervention	Experimental -
Intervention	Experimental -
Intervention	Experimental -
Intervention	Experimental -
Intervention	Experimental -
Intervention	Experimental -
Intervention	Experimental -
Intervention	Experimental -
Intervention	Experimental -
Intervention	Experimental -
Intervention	Experimental -
Intervention	Experimental -
Intervention	Experimental -
Intervention	Experimental -
Intervention	Experimental -
Intervention	Experimental -
Intervention	Experimental -
Intervention	Experimental -
Intervention	Experimental -
Intervention	Experimental -
Intervention	Experimental -
Intervention	Experimental -
Intervention	Experimental -
Intervention	Experimental -
Intervention	Experimental -
Intervention	Experimental -
Intervention	Experimental -
Intervention	Experimental -
Intervention	Experimental -

Intervention	Experimental -
Intervention	Experimental -
Intervention	Experimental -
Intervention	Experimental -
Intervention	Experimental -
Intervention	Experimental -
Intervention	Experimental -
Intervention	Experimental -
Intervention	Experimental -
Intervention	Experimental -
Intervention	Experimental -
Intervention	Experimental -
Intervention	Experimental -
Intervention	Experimental -
Intervention	Experimental -
Intervention	Experimental -
Intervention	Experimental -
Intervention	Experimental -
Intervention	Experimental -
Intervention	Experimental -
Intervention	Experimental -
Intervention	Experimental -
Intervention	Experimental -
Intervention	Experimental -
Intervention	Experimental -
Intervention	Experimental -
Intervention	Experimental -
Intervention	Experimental -
Intervention	Experimental -
Intervention	Experimental -
Intervention	Experimental -
Intervention	Experimental -
Intervention	Experimental -
Intervention	Experimental -
Intervention	Experimental -
Intervention	Experimental -
Intervention	Experimental -
Intervention	Experimental -
Intervention	Experimental -
Intervention	Experimental -
Intervention	Experimental -
Intervention	Experimental -
Intervention	Experimental -
Intervention	Experimental -
Intervention	Experimental -
Intervention	Experimental -
Intervention	Experimental -
Intervention	Experimental -

Intervention	Experimental -
Intervention	Experimental -
Intervention	Experimental -
Intervention	Experimental -
Intervention	Experimental -
Intervention	Experimental -
Intervention	Experimental -
Intervention	Experimental -
Intervention	Experimental -
Intervention	Experimental -
Intervention	Experimental -
Intervention	Experimental -
Intervention	Experimental -
Intervention	Experimental -
Intervention	Experimental -
Intervention	Experimental -
Intervention	Experimental -
Intervention	Experimental -
Intervention	Experimental -
Intervention	Experimental -
Intervention	Experimental -
Intervention	Experimental -
Intervention	Experimental -
Intervention	Experimental -
Intervention	Experimental -
Intervention	Experimental -
Intervention	Experimental -
Intervention	Experimental -
Intervention	Experimental -
Intervention	Experimental -
Intervention	Experimental -
Intervention	Experimental -
Intervention	Experimental -
Intervention	Experimental -
Intervention	Experimental -
Intervention	Experimental -
Intervention	Experimental -
Intervention	Experimental -
Intervention	Experimental -
Intervention	Experimental -
Intervention	Experimental -
Intervention	Experimental -
Intervention	Experimental -
Intervention	Experimental -
Intervention	Experimental -
Intervention	Experimental -
Intervention	Experimental -
Intervention	Experimental -

Intervention	Experimental -
Intervention	Experimental -
Intervention	Experimental -
Intervention	Experimental -
Intervention	Experimental -
Intervention	Experimental -
Intervention	Experimental -
Intervention	Experimental -
Intervention	Experimental -
Intervention	Experimental -
Intervention	Experimental -
Intervention	Experimental -
Intervention	Experimental -
Intervention	Experimental -
Intervention	Experimental -
Intervention	Experimental -
Intervention	Experimental -
Intervention	Experimental -
Intervention	Experimental -
Intervention	Experimental -
Intervention	Experimental -
Intervention	Experimental -
Intervention	Experimental -
Intervention	Experimental -
Intervention	Experimental -
Intervention	Experimental -
Intervention	Experimental -
Intervention	Experimental -
Intervention	Experimental -
Intervention	Experimental -
Intervention	Experimental -
Intervention	Experimental -
Intervention	Experimental -
Intervention	Experimental -
Intervention	Experimental -
Intervention	Experimental -
Intervention	Experimental -
Intervention	Experimental -
Intervention	Experimental -
Intervention	Experimental -
Intervention	Experimental -
Intervention	Experimental -
Intervention	Experimental -
Intervention	Experimental -
Intervention	Experimental -
Intervention	Experimental -
Intervention	Experimental -
Intervention	Experimental -

Intervention	Experimental -
Intervention	Experimental -
Intervention	Experimental -
Intervention	Experimental -
Intervention	Experimental -
Intervention	Experimental -
Intervention	Experimental -
Intervention	Experimental -
Intervention	Experimental -
Intervention	Experimental -
Intervention	Experimental -
Intervention	Experimental -
Intervention	Experimental -
Intervention	Experimental -
Intervention	Experimental -
Intervention	Experimental -
Intervention	Experimental -
Intervention	Experimental -
Intervention	Experimental -
Intervention	Experimental -
Intervention	Experimental -
Intervention	Experimental -
Intervention	Experimental -
Intervention	Experimental -
Intervention	Experimental -
Intervention	Experimental -
Intervention	Experimental -
Intervention	Experimental -
Intervention	Experimental -
Intervention	Experimental -
Intervention	Experimental -
Intervention	Experimental -
Intervention	Experimental -
Intervention	Experimental -
Intervention	Experimental -
Intervention	Experimental -
Intervention	Experimental -
Intervention	Experimental -
Intervention	Experimental -
Intervention	Experimental -
Intervention	Experimental -
Intervention	Experimental -
Intervention	Experimental -
Intervention	Experimental -
Intervention	Experimental -
Intervention	Experimental -
Intervention	Experimental -
Intervention	Experimental -

Intervention	Experimental -
Intervention	Experimental -
Intervention	Experimental -
Intervention	Experimental -
Intervention	Experimental -
Intervention	Experimental -
Intervention	Experimental -
Intervention	Experimental -
Intervention	Experimental -
Intervention	Experimental -
Intervention	Experimental -
Intervention	Experimental -
Intervention	Experimental -
Intervention	Experimental -
Intervention	Experimental -
Intervention	Experimental -
Intervention	Experimental -
Intervention	Experimental -
Intervention	Experimental -
Intervention	Experimental -
Intervention	Experimental -
Intervention	Experimental -
Intervention	Experimental -
Intervention	Experimental -
Intervention	Experimental -
Intervention	Experimental -
Intervention	Experimental -
Intervention	Experimental -
Intervention	Experimental -
Intervention	Experimental -
Intervention	Experimental -
Intervention	Experimental -
Intervention	Experimental -
Intervention	Experimental -
Intervention	Experimental -
Intervention	Experimental -
Intervention	Experimental -
Intervention	Experimental -
Intervention	Experimental -
Intervention	Experimental -
Intervention	Experimental -
Intervention	Experimental -
Intervention	Experimental -
Intervention	Experimental -
Intervention	Experimental -
Intervention	Experimental -
Intervention	Experimental -
Intervention	Experimental -

Intervention	Experimental -
Intervention	Experimental -
Intervention	Experimental -
Intervention	Experimental -
Intervention	Experimental -
Intervention	Experimental -
Intervention	Experimental -
Intervention	Experimental -
Intervention	Experimental -
Intervention	Experimental -
Intervention	Experimental -
Intervention	Experimental -
Intervention	Experimental -
Intervention	Experimental -
Intervention	Experimental -
Intervention	Experimental -
Intervention	Experimental -
Intervention	Experimental -
Intervention	Experimental -
Intervention	Experimental -
Intervention	Experimental -
Intervention	Experimental -
Intervention	Experimental -
Intervention	Experimental -
Intervention	Experimental -
Intervention	Experimental -
Intervention	Experimental -
Intervention	Experimental -
Intervention	Experimental -
Intervention	Experimental -
Intervention	Experimental -
Intervention	Experimental -
Intervention	Experimental -
Intervention	Experimental -
Intervention	Experimental -
Intervention	Experimental -
Intervention	Experimental -
Intervention	Experimental -
Intervention	Experimental -
Intervention	Experimental -
Intervention	Experimental -
Intervention	Experimental -
Intervention	Experimental -
Intervention	Experimental -
Intervention	Experimental -
Intervention	Experimental -
Intervention	Experimental -
Intervention	Experimental -

Intervention	Experimental -
Intervention	Experimental -
Intervention	Experimental -
Intervention	Experimental -
Intervention	Experimental -
Intervention	Experimental -
Intervention	Experimental -
Intervention	Experimental -
Intervention	Experimental -
Intervention	Experimental -
Intervention	Experimental -
Intervention	Experimental -
Intervention	Experimental -
Intervention	Experimental -
Intervention	Experimental -
Intervention	Experimental -
Intervention	Experimental -
Intervention	Experimental -
Intervention	Experimental -
Intervention	Experimental -
Intervention	Experimental -
Intervention	Experimental -
Intervention	Experimental -
Intervention	Experimental -
Intervention	Experimental -
Intervention	Experimental -
Intervention	Experimental -
Intervention	Experimental -
Intervention	Experimental -
Intervention	Experimental -
Intervention	Experimental -
Intervention	Experimental -
Intervention	Experimental -
Intervention	Experimental -
Intervention	Experimental -
Intervention	Experimental -
Intervention	Experimental -
Intervention	Experimental -
Intervention	Experimental -
Intervention	Experimental -
Intervention	Experimental -
Intervention	Experimental -
Intervention	Experimental -
Intervention	Experimental -
Intervention	Experimental -
Intervention	Experimental -
Intervention	Experimental -
Intervention	Experimental -

Intervention	Experimental -
Intervention	Experimental -
Intervention	Experimental -
Intervention	Experimental -
Intervention	Experimental -
Intervention	Experimental -
Intervention	Experimental -
Intervention	Experimental -
Intervention	Experimental -
Intervention	Experimental -
Intervention	Experimental -
Intervention	Experimental -
Intervention	Experimental -
Intervention	Experimental -
Intervention	Experimental -
Intervention	Experimental -
Intervention	Experimental -
Intervention	Experimental -
Intervention	Experimental -
Intervention	Experimental -
Intervention	Experimental -
Intervention	Experimental -
Intervention	Experimental -
Intervention	Experimental -
Intervention	Experimental -
Intervention	Experimental -
Intervention	Experimental -
Intervention	Experimental -
Intervention	Experimental -
Intervention	Experimental -
Intervention	Experimental -
Intervention	Experimental -
Intervention	Experimental -
Intervention	Experimental -
Intervention	Experimental -
Intervention	Experimental -
Intervention	Experimental -
Intervention	Experimental -
Intervention	Experimental -
Intervention	Experimental -
Intervention	Experimental -
Intervention	Experimental -
Intervention	Experimental -
Intervention	Experimental -
Intervention	Experimental -
Intervention	Experimental -
Intervention	Experimental -
Intervention	Experimental -

Intervention	Experimental -
Intervention	Experimental -
Intervention	Experimental -
Intervention	Experimental -
Intervention	Experimental -
Intervention	Experimental -
Intervention	Experimental -
Intervention	Experimental -
Intervention	Experimental -
Intervention	Experimental -
Intervention	Experimental -
Intervention	Experimental -
Intervention	Experimental -
Intervention	Experimental -
Intervention	Experimental -
Intervention	Experimental -
Intervention	Experimental -
Intervention	Experimental -
Intervention	Experimental -
Intervention	Experimental -
Intervention	Experimental -
Intervention	Experimental -
Intervention	Experimental -
Intervention	Experimental -
Intervention	Experimental -
Intervention	Experimental -
Intervention	Experimental -
Intervention	Experimental -
Intervention	Experimental -
Intervention	Experimental -
Intervention	Experimental -
Intervention	Experimental -
Intervention	Experimental -
Intervention	Experimental -
Intervention	Experimental -
Intervention	Experimental -
Intervention	Experimental -
Intervention	Experimental -
Intervention	Experimental -
Intervention	Experimental -
Intervention	Experimental -
Intervention	Experimental -
Intervention	Experimental -
Intervention	Experimental -
Intervention	Experimental -
Intervention	Experimental -
Intervention	Experimental -
Intervention	Experimental -

Intervention	Experimental -
Intervention	Experimental -
Intervention	Experimental -
Intervention	Experimental -
Intervention	Experimental -
Intervention	Experimental -
Intervention	Experimental -
Intervention	Experimental -
Intervention	Experimental -
Intervention	Experimental -
Intervention	Experimental -
Intervention	Experimental -
Intervention	Experimental -
Intervention	Experimental -
Intervention	Experimental -
Intervention	Experimental -
Intervention	Experimental -
Intervention	Experimental -
Intervention	Experimental -
Intervention	Experimental -
Intervention	Experimental -
Intervention	Experimental -
Intervention	Experimental -
Intervention	Experimental -
Intervention	Experimental -
Intervention	Experimental -
Intervention	Experimental -
Intervention	Experimental -
Intervention	Experimental -
Intervention	Experimental -
Intervention	Experimental -
Intervention	Experimental -
Intervention	Experimental -
Intervention	Experimental -
Intervention	Experimental -
Intervention	Experimental -
Intervention	Experimental -
Intervention	Experimental -
Intervention	Experimental -
Intervention	Experimental -
Intervention	Experimental -
Intervention	Experimental -
Intervention	Experimental -
Intervention	Experimental -
Intervention	Experimental -
Intervention	Experimental -
Intervention	Experimental -
Intervention	Experimental -

Intervention	Experimental -
Intervention	Experimental -
Intervention	Experimental -
Intervention	Experimental -
Intervention	Experimental -
Intervention	Experimental -
Intervention	Experimental -
Intervention	Experimental -
Intervention	Experimental -
Intervention	Experimental -
Intervention	Experimental -
Intervention	Experimental -
Intervention	Experimental -
Intervention	Experimental -
Intervention	Experimental -
Intervention	Experimental -
Intervention	Experimental -
Intervention	Experimental -
Intervention	Experimental -
Intervention	Experimental -
Intervention	Experimental -
Intervention	Experimental -
Intervention	Experimental -
Intervention	Experimental -
Intervention	Experimental -
Intervention	Experimental -
Intervention	Experimental -
Intervention	Experimental -
Intervention	Experimental -
Intervention	Experimental -
Intervention	Experimental -
Intervention	Experimental -
Intervention	Experimental -
Intervention	Experimental -
Intervention	Experimental -
Intervention	Experimental -
Intervention	Experimental -
Intervention	Experimental -
Intervention	Experimental -
Intervention	Experimental -
Intervention	Experimental -
Intervention	Experimental -
Intervention	Experimental -
Intervention	Experimental -
Intervention	Experimental -
Intervention	Experimental -
Intervention	Experimental -
Intervention	Experimental -

Intervention	Experimental -
Intervention	Experimental -
Intervention	Experimental -
Intervention	Experimental -
Intervention	Experimental -
Intervention	Experimental -
Intervention	Experimental -
Intervention	Experimental -
Intervention	Experimental -
Intervention	Experimental -
Intervention	Experimental -
Intervention	Experimental -
Intervention	Experimental -
Intervention	Experimental -
Intervention	Experimental -
Intervention	Experimental -
Intervention	Experimental -
Intervention	Experimental -
Intervention	Experimental -
Intervention	Experimental -
Intervention	Experimental -
Intervention	Experimental -
Intervention	Experimental -
Intervention	Experimental -
Intervention	Experimental -
Intervention	Experimental -
Intervention	Experimental -
Intervention	Experimental -
Intervention	Experimental -
Intervention	Experimental -
Intervention	Experimental -
Intervention	Experimental -
Intervention	Experimental -
Intervention	Experimental -
Intervention	Experimental -
Intervention	Experimental -
Intervention	Experimental -
Intervention	Experimental -
Intervention	Experimental -
Intervention	Experimental -
Intervention	Experimental -
Intervention	Experimental -
Intervention	Experimental -
Intervention	Experimental -
Intervention	Experimental -
Intervention	Experimental -
Intervention	Experimental -
Intervention	Experimental -

Intervention	Experimental -
Intervention	Experimental -
Intervention	Experimental -
Intervention	Experimental -
Intervention	Experimental -
Intervention	Experimental -
Intervention	Experimental -
Intervention	Experimental -
Intervention	Experimental -
Intervention	Experimental -
Intervention	Experimental -
Intervention	Experimental -
Intervention	Experimental -
Intervention	Experimental -
Intervention	Experimental -
Intervention	Experimental -
Intervention	Experimental -
Intervention	Experimental -
Intervention	Experimental -
Intervention	Experimental -
Intervention	Experimental -
Intervention	Experimental -
Intervention	Experimental -
Intervention	Experimental -
Intervention	Experimental -
Intervention	Experimental -
Intervention	Experimental -
Intervention	Experimental -
Intervention	Experimental -
Intervention	Experimental -
Intervention	Experimental -
Intervention	Experimental -
Intervention	Experimental -
Intervention	Experimental -
Intervention	Experimental -
Intervention	Experimental -
Intervention	Experimental -
Intervention	Experimental -
Intervention	Experimental -
Intervention	Experimental -
Intervention	Experimental -
Intervention	Experimental -
Intervention	Experimental -
Intervention	Experimental -
Intervention	Experimental -
Intervention	Experimental -
Intervention	Experimental -
Intervention	Experimental -

Intervention	Experimental -
Intervention	Experimental -
Intervention	Experimental -
Intervention	Experimental -
Intervention	Experimental -
Intervention	Experimental -
Intervention	Experimental -
Intervention	Experimental -
Intervention	Experimental -
Intervention	Experimental -
Intervention	Experimental -
Intervention	Experimental -
Intervention	Experimental -
Intervention	Experimental -
Intervention	Experimental -
Intervention	Experimental -
Intervention	Experimental -
Intervention	Experimental -
Intervention	Experimental -
Intervention	Experimental -
Intervention	Experimental -
Intervention	Experimental -
Intervention	Experimental -
Intervention	Experimental -
Intervention	Experimental -
Intervention	Experimental -
Intervention	Experimental -
Intervention	Experimental -
Intervention	Experimental -
Intervention	Experimental -
Intervention	Experimental -
Intervention	Experimental -
Intervention	Experimental -
Intervention	Experimental -
Intervention	Experimental -
Intervention	Experimental -
Intervention	Experimental -
Intervention	Experimental -
Intervention	Experimental -
Intervention	Experimental -
Intervention	Experimental -
Intervention	Experimental -
Intervention	Experimental -
Intervention	Experimental -
Intervention	Experimental -
Intervention	Experimental -
Intervention	Experimental -
Intervention	Experimental -

Intervention	Experimental -
Intervention	Experimental -
Intervention	Experimental -
Intervention	Experimental -
Intervention	Experimental -
Intervention	Experimental -
Intervention	Experimental -
Intervention	Experimental -
Intervention	Experimental -
Intervention	Experimental -
Intervention	Experimental -
Intervention	Experimental -
Intervention	Experimental -
Intervention	Experimental -
Intervention	Experimental -
Intervention	Experimental -
Intervention	Experimental -
Intervention	Experimental -
Intervention	Experimental -
Intervention	Experimental -
Intervention	Experimental -
Intervention	Experimental -
Intervention	Experimental -
Intervention	Experimental -
Intervention	Experimental -
Intervention	Experimental -
Intervention	Experimental -
Intervention	Experimental -
Intervention	Experimental -
Intervention	Experimental -
Intervention	Experimental -
Intervention	Experimental -
Intervention	Experimental -
Intervention	Experimental -
Intervention	Experimental -
Intervention	Experimental -
Intervention	Experimental -
Intervention	Experimental -
Intervention	Experimental -
Intervention	Experimental -
Intervention	Experimental -
Intervention	Experimental -
Intervention	Experimental -
Intervention	Experimental -
Intervention	Experimental -
Intervention	Experimental -
Intervention	Experimental -
Intervention	Experimental -

Intervention	Experimental -
Intervention	Experimental -
Intervention	Experimental -
Intervention	Experimental -
Intervention	Experimental -
Intervention	Experimental -
Intervention	Experimental -
Intervention	Experimental -
Intervention	Experimental -
Intervention	Experimental -
Intervention	Experimental -
Intervention	Experimental -
Intervention	Experimental -
Intervention	Experimental -
Intervention	Experimental -
Intervention	Experimental -
Intervention	Experimental -
Intervention	Experimental -
Intervention	Experimental -
Intervention	Experimental -
Intervention	Experimental -
Intervention	Experimental -
Intervention	Experimental -
Intervention	Experimental -
Intervention	Experimental -
Intervention	Experimental -
Intervention	Experimental -
Intervention	Experimental -
Intervention	Experimental -
Intervention	Experimental -
Intervention	Experimental -
Intervention	Experimental -
Intervention	Experimental -
Intervention	Experimental -
Intervention	Experimental -
Intervention	Experimental -
Intervention	Experimental -
Intervention	Experimental -
Intervention	Experimental -
Intervention	Experimental -
Intervention	Experimental -
Intervention	Experimental -
Intervention	Experimental -
Intervention	Experimental -
Intervention	Experimental -
Intervention	Experimental -
Intervention	Experimental -
Intervention	Experimental -

Intervention	Experimental -
Intervention	Experimental -
Intervention	Experimental -
Intervention	Experimental -
Intervention	Experimental -
Intervention	Experimental -
Intervention	Experimental -
Intervention	Experimental -
Intervention	Experimental -
Intervention	Experimental -
Intervention	Experimental -
Intervention	Experimental -
Intervention	Experimental -
Intervention	Experimental -
Intervention	Experimental -
Intervention	Experimental -
Intervention	Experimental -
Intervention	Experimental -
Intervention	Experimental -
Intervention	Experimental -
Intervention	Experimental -
Intervention	Experimental -
Intervention	Experimental -
Intervention	Experimental -
Intervention	Experimental -
Intervention	Experimental -
Intervention	Experimental -
Intervention	Experimental -
Intervention	Experimental -
Intervention	Experimental -
Intervention	Experimental -
Intervention	Experimental -
Intervention	Experimental -
Intervention	Experimental -
Intervention	Experimental -
Intervention	Experimental -
Intervention	Experimental -
Intervention	Experimental -
Intervention	Experimental -
Intervention	Experimental -
Intervention	Experimental -
Intervention	Experimental -
Intervention	Experimental -
Intervention	Experimental -
Intervention	Experimental -
Intervention	Experimental -
Intervention	Experimental -
Intervention	Experimental -

Intervention	Experimental -
Intervention	Experimental -
Intervention	Experimental -
Intervention	Experimental -
Intervention	Experimental -
Intervention	Experimental -
Intervention	Experimental -
Intervention	Experimental -
Intervention	Experimental -
Intervention	Experimental -
Intervention	Experimental -
Intervention	Experimental -
Intervention	Experimental -
Intervention	Experimental -
Intervention	Experimental -
Intervention	Experimental -
Intervention	Experimental -
Intervention	Experimental -
Intervention	Experimental -
Intervention	Experimental -
Intervention	Experimental -
Intervention	Experimental -
Intervention	Experimental -
Intervention	Experimental -
Intervention	Experimental -
Intervention	Experimental -
Intervention	Experimental -
Intervention	Experimental -
Intervention	Experimental -
Intervention	Experimental -
Intervention	Experimental -
Intervention	Experimental -
Intervention	Experimental -
Intervention	Experimental -
Intervention	Experimental -
Intervention	Experimental -
Intervention	Experimental -
Intervention	Experimental -
Intervention	Experimental -
Intervention	Experimental -
Intervention	Experimental -
Intervention	Experimental -
Intervention	Experimental -
Intervention	Experimental -
Intervention	Experimental -
Intervention	Experimental -
Intervention	Experimental -
Intervention	Experimental -

Intervention	Experimental -
Intervention	Experimental -
Intervention	Experimental -
Intervention	Experimental -
Intervention	Experimental -
Intervention	Experimental -
Intervention	Experimental -
Intervention	Experimental -
Intervention	Experimental -
Intervention	Experimental -
Intervention	Experimental -
Intervention	Experimental -
Intervention	Experimental -
Intervention	Experimental -
Intervention	Experimental -
Intervention	Experimental -
Intervention	Experimental -
Intervention	Experimental -
Intervention	Experimental -
Intervention	Experimental -
Intervention	Experimental -
Intervention	Experimental -
Intervention	Experimental -
Intervention	Experimental -
Intervention	Experimental -
Intervention	Experimental -
Intervention	Experimental -
Intervention	Experimental -
Intervention	Experimental -
Intervention	Experimental -
Intervention	Experimental -
Intervention	Experimental -
Intervention	Experimental -
Intervention	Experimental -
Intervention	Experimental -
Intervention	Experimental -
Intervention	Experimental -
Intervention	Experimental -
Intervention	Experimental -
Intervention	Experimental -
Intervention	Experimental -
Intervention	Experimental -
Intervention	Experimental -
Intervention	Experimental -
Intervention	Experimental -
Intervention	Experimental -
Intervention	Experimental -
Intervention	Experimental -

Intervention	Experimental -
Intervention	Experimental -
Intervention	Experimental -
Intervention	Experimental -
Intervention	Experimental -
Intervention	Experimental -
Intervention	Experimental -
Intervention	Experimental -
Intervention	Experimental -
Intervention	Experimental -
Intervention	Experimental -
Intervention	Experimental -
Intervention	Experimental -
Intervention	Experimental -
Intervention	Experimental -
Intervention	Experimental -
Intervention	Experimental -
Intervention	Experimental -
Intervention	Experimental -
Intervention	Experimental -
Intervention	Experimental -
Intervention	Experimental -
Intervention	Experimental -
Intervention	Experimental -
Intervention	Experimental -
Intervention	Experimental -
Intervention	Experimental -
Intervention	Experimental -
Intervention	Experimental -
Intervention	Experimental -
Intervention	Experimental -
Intervention	Experimental -
Intervention	Experimental -
Intervention	Experimental -
Intervention	Experimental -
Intervention	Experimental -
Intervention	Experimental -
Intervention	Experimental -
Intervention	Experimental -
Intervention	Experimental -
Intervention	Experimental -
Intervention	Experimental -
Intervention	Experimental -
Intervention	Experimental -
Intervention	Experimental -
Intervention	Experimental -
Intervention	Experimental -
Intervention	Experimental -

Intervention	Experimental -
Intervention	Experimental -
Intervention	Experimental -
Intervention	Experimental -
Intervention	Experimental -
Intervention	Experimental -
Intervention	Experimental -
Intervention	Experimental -
Intervention	Experimental -
Intervention	Experimental -
Intervention	Experimental -
Intervention	Experimental -
Intervention	Experimental -
Intervention	Experimental -

Alternative group	Reference group	FDR	P-value	Effect size
Experimental	Control	0,970062739	0,650759425	-0,21189443
Experimental	Control	0,970062739	0,534073748	-0,306524892
Experimental	Control	0,970062739	0,276437863	-0,99281447
Experimental	Control	0,970062739	0,575902513	-0,180460269
Experimental	Control	0,99640621	0,988121404	-0,006772247
Experimental	Control	0,970062739	0,34355193	-0,472971223
Experimental	Control	0,87952483	0,111131296	-0,697111087
Experimental	Control	0,78357626	0,044978235	-1,347641877
Experimental	Control	0,78357626	0,030410529	-0,96636063
Experimental	Control	0,988586169	0,961916922	-0,026610055
Experimental	Control	0,970062739	0,253816224	-0,78605949
Experimental	Control	0,870685429	0,07464048	-0,748855096
Experimental	Control	0,970062739	0,481102054	-0,528283637
Experimental	Control	0,970062739	0,222440006	-0,657904262
Experimental	Control	0,694796407	0,012122597	-1,826717914
Experimental	Control	0,970062739	0,555539844	-0,336364912
Experimental	Control	0,970062739	0,400557698	0,386744088
Experimental	Control	0,970062739	0,497580853	-0,553270509
Experimental	Control	0,98331311	0,938203422	-0,042008688
Experimental	Control	0,970062739	0,435144181	0,264269499
Experimental	Control	0,970062739	0,435100331	0,409757933
Experimental	Control	0,970062739	0,18944268	0,709529109
Experimental	Control	0,98331311	0,923229872	0,044301136
Experimental	Control	0,970062739	0,727676874	-0,220911337
Experimental	Control	0,970062739	0,500369741	0,210869963
Experimental	Control	0,899730559	0,122821576	1,02439221
Experimental	Control	0,970062739	0,331056629	-0,478116209
Experimental	Control	0,87952483	0,09316543	-1,146680364
Experimental	Control	0,78357626	0,030568796	-1,719346343
Experimental	Control	0,970062739	0,69751051	-0,233777301
Experimental	Control	0,970062739	0,699190003	0,221093584
Experimental	Control	0,78357626	0,044022783	-0,872886816
Experimental	Control	0,87952483	0,10486208	-1,58367612
Experimental	Control	0,870685429	0,07672779	-1,207287048
Experimental	Control	0,970062739	0,187646107	-0,723762982
Experimental	Control	0,970062739	0,701589708	-0,180049764
Experimental	Control	0,012359826	2,23102E-05	-2,106345056
Experimental	Control	0,970062739	0,653878028	-0,403758709
Experimental	Control	0,98331311	0,914038379	0,052748297
Experimental	Control	0,98331311	0,878825237	0,104004172
Experimental	Control	0,970062739	0,332035898	-0,728177334
Experimental	Control	0,970062739	0,528293441	-0,386212096
Experimental	Control	0,970062739	0,542802211	-0,320499787
Experimental	Control	0,970062739	0,425111436	-0,308916984
Experimental	Control	0,970062739	0,190676924	-0,845080399
Experimental	Control	0,970062739	0,256847058	-0,3653499
Experimental	Control	0,970062739	0,721549482	-0,16694102

Experimental	Control	0,868668495	0,059680137	-0,867010148
Experimental	Control	0,78357626	0,042116188	-1,179658157
Experimental	Control	0,899730559	0,124969413	-0,687439475
Experimental	Control	0,96190808	0,177024859	0,355522695
Experimental	Control	0,98331311	0,937623388	0,039467392
Experimental	Control	0,899730559	0,133224922	-1,079930265
Experimental	Control	0,694796407	0,004635114	-1,494725287
Experimental	Control	0,78357626	0,047891032	-1,394480252
Experimental	Control	0,885468273	0,113480591	-0,71193324
Experimental	Control	0,991002471	0,967747901	-0,020859062
Experimental	Control	0,970062739	0,64514097	0,123245791
Experimental	Control	0,98331311	0,905345751	-0,070308762
Experimental	Control	0,970062739	0,610233203	0,336204672
Experimental	Control	0,870685429	0,077764411	-0,859386195
Experimental	Control	0,899730559	0,149413739	-0,66408938
Experimental	Control	0,973116013	0,790270458	-0,172442958
Experimental	Control	0,970062739	0,203825972	-0,774664506
Experimental	Control	0,87952483	0,107139099	-1,051742395
Experimental	Control	0,970062739	0,233685495	-0,779545538
Experimental	Control	0,970062739	0,626244742	0,207052713
Experimental	Control	0,98331311	0,916072869	-0,056338555
Experimental	Control	0,973116013	0,764301455	0,137289429
Experimental	Control	0,87952483	0,109774845	-0,979394965
Experimental	Control	0,970062739	0,341663554	0,343466593
Experimental	Control	0,970062739	0,582953175	0,189718322
Experimental	Control	0,970062739	0,552662958	-0,382062012
Experimental	Control	0,868668495	0,061151753	0,395298829
Experimental	Control	0,981816738	0,850482586	-0,056843318
Experimental	Control	0,694796407	0,01486486	-1,007305099
Experimental	Control	0,970062739	0,723601049	0,276067665
Experimental	Control	0,973116013	0,772444835	0,162985945
Experimental	Control	0,970062739	0,242804369	-0,596977076
Experimental	Control	0,970062739	0,665009309	-0,336984912
Experimental	Control	0,970062739	0,505456658	-0,248006812
Experimental	Control	0,927436375	0,165349054	-0,874115275
Experimental	Control	0,87952483	0,110796941	-0,548704728
Experimental	Control	0,870685429	0,068747236	-1,864977817
Experimental	Control	0,970062739	0,504232443	-0,44448198
Experimental	Control	0,970062739	0,486984094	0,264306925
Experimental	Control	0,973116013	0,769108995	-0,101914543
Experimental	Control	0,970062739	0,285910984	-0,553721381
Experimental	Control	0,927436375	0,160379658	-0,560713755
Experimental	Control	0,988586169	0,958437185	-0,025596259
Experimental	Control	0,899730559	0,146305762	-1,004226586
Experimental	Control	0,970062739	0,697958191	0,198170134
Experimental	Control	0,970062739	0,216521768	-0,829240459
Experimental	Control	0,899730559	0,142622986	-1,070175769
Experimental	Control	0,999707234	0,99817574	-0,002522927

Experimental	Control	0,970062739	0,629112373	-0,365372005
Experimental	Control	0,970062739	0,302358093	-0,482599553
Experimental	Control	0,970062739	0,326100559	-0,999950187
Experimental	Control	0,694796407	0,005463171	-1,211942473
Experimental	Control	0,87952483	0,102710494	-1,250611368
Experimental	Control	0,981816738	0,829285624	0,119814711
Experimental	Control	0,98331311	0,891473711	-0,08113974
Experimental	Control	0,970062739	0,332565734	-0,508061903
Experimental	Control	0,970062739	0,419076159	0,346216361
Experimental	Control	0,970062739	0,400357668	-0,263175865
Experimental	Control	0,981816738	0,859526936	-0,074162372
Experimental	Control	0,970062739	0,229927096	-0,416866041
Experimental	Control	0,981816738	0,834354347	0,05882421
Experimental	Control	0,970062739	0,527302198	0,243376092
Experimental	Control	0,970062739	0,437526315	0,339059207
Experimental	Control	0,986356047	0,951265488	-0,026684967
Experimental	Control	0,970062739	0,356527044	-0,49231504
Experimental	Control	0,970062739	0,412574491	-0,307017947
Experimental	Control	0,970062739	0,598154161	0,276979874
Experimental	Control	0,694796407	0,009426922	-1,399437904
Experimental	Control	0,970062739	0,635821852	0,197782343
Experimental	Control	0,970062739	0,267486882	0,393610093
Experimental	Control	0,970062739	0,230175971	-0,430798163
Experimental	Control	0,970062739	0,676439189	-0,160080124
Experimental	Control	0,98331311	0,871925533	-0,124198165
Experimental	Control	0,972536115	0,756351388	-0,140402215
Experimental	Control	0,970062739	0,41320437	-0,394581111
Experimental	Control	0,970062739	0,254994776	0,328148376
Experimental	Control	0,981816738	0,842945414	-0,147208788
Experimental	Control	0,99640621	0,983955894	-0,010267509
Experimental	Control	0,970062739	0,449834829	0,386745037
Experimental	Control	0,970062739	0,712580932	0,26724192
Experimental	Control	0,981816738	0,829844364	0,142541396
Experimental	Control	0,99640621	0,988022128	-0,007344202
Experimental	Control	0,870685429	0,080153352	-1,259856595
Experimental	Control	0,970062739	0,651544126	0,264117133
Experimental	Control	0,970062739	0,660383043	-0,201193301
Experimental	Control	0,970062739	0,341053772	-0,530555826
Experimental	Control	0,970062739	0,554363074	-0,277852125
Experimental	Control	0,735000532	0,018574021	1,570420003
Experimental	Control	0,970062739	0,63720742	-0,206342924
Experimental	Control	0,970062739	0,539748039	-0,208439752
Experimental	Control	0,970062739	0,341617363	-0,841030219
Experimental	Control	0,870685429	0,07650319	-0,856263566
Experimental	Control	0,970062739	0,494107315	-0,188752225
Experimental	Control	0,970062739	0,386676726	-0,436640043
Experimental	Control	0,970062739	0,737917996	-0,168733761
Experimental	Control	0,78357626	0,044020493	-0,756782651

Experimental	Control	0,970062739	0,655758741	0,154835618
Experimental	Control	0,970062739	0,436261443	0,454385194
Experimental	Control	0,970062739	0,568500283	0,237836162
Experimental	Control	0,973959896	0,79639681	-0,18188184
Experimental	Control	0,973116013	0,777626737	0,097135012
Experimental	Control	0,970062739	0,715837793	-0,183791297
Experimental	Control	0,981816738	0,820459992	-0,14649311
Experimental	Control	0,970062739	0,72126241	-0,072151035
Experimental	Control	0,87952483	0,105434381	-0,806937604
Experimental	Control	0,970062739	0,491446827	-0,374441306
Experimental	Control	0,970062739	0,315405799	0,526050906
Experimental	Control	0,970062739	0,374502775	0,604915906
Experimental	Control	0,78357626	0,042619729	0,702382571
Experimental	Control	0,970062739	0,553576255	0,327898374
Experimental	Control	0,970062739	0,592894029	0,327260551
Experimental	Control	0,899730559	0,14680433	1,443481912
Experimental	Control	0,973116013	0,790437195	-0,216613414
Experimental	Control	0,98331311	0,868483423	0,112149004
Experimental	Control	0,970062739	0,364292095	-0,138817412
Experimental	Control	0,970062739	0,276539835	-0,409995632
Experimental	Control	0,970062739	0,410381043	0,206245949
Experimental	Control	0,98331311	0,871769253	-0,079174738
Experimental	Control	0,970062739	0,258914605	-0,792255883
Experimental	Control	0,970062739	0,435854797	0,431265877
Experimental	Control	0,870685429	0,075362443	-0,849313126
Experimental	Control	0,970062739	0,444726523	-0,323539678
Experimental	Control	0,970062739	0,514417255	-0,409490027
Experimental	Control	0,970062739	0,34387338	-0,276584076
Experimental	Control	0,78357626	0,040520613	-1,41185282
Experimental	Control	0,98331311	0,907791096	0,040267991
Experimental	Control	0,972536115	0,751739086	-0,077624066
Experimental	Control	0,927436375	0,164435758	0,737371524
Experimental	Control	0,970062739	0,298464306	-0,456839161
Experimental	Control	0,970062739	0,363913179	0,459617007
Experimental	Control	0,98331311	0,934357992	0,041327997
Experimental	Control	0,98331311	0,939628161	-0,033602758
Experimental	Control	0,970062739	0,683320785	-0,174948495
Experimental	Control	0,970062739	0,40028721	-0,285615969
Experimental	Control	0,981816738	0,856246029	-0,055281321
Experimental	Control	0,970062739	0,366835579	-0,385503137
Experimental	Control	0,970062739	0,449807398	-0,262455673
Experimental	Control	0,970062739	0,328641132	-0,284366833
Experimental	Control	0,694796407	0,002727909	-0,940298606
Experimental	Control	0,98331311	0,885562413	-0,069451549
Experimental	Control	0,970062739	0,564589213	0,302965
Experimental	Control	0,970062739	0,321754823	-0,20806914
Experimental	Control	0,98331311	0,925366727	-0,053340081
Experimental	Control	0,972536115	0,754601096	0,100342022

Experimental	Control	0,981816738	0,850982853	0,043529249
Experimental	Control	0,970062739	0,673995303	-0,121430102
Experimental	Control	0,87952483	0,085033409	1,024183533
Experimental	Control	0,970062739	0,70337637	0,066068875
Experimental	Control	0,970062739	0,240208389	0,214543341
Experimental	Control	0,970062739	0,436177848	-0,601790286
Experimental	Control	0,986356047	0,952527951	-0,023988885
Experimental	Control	0,870685429	0,066088219	-1,068958325
Experimental	Control	0,973116013	0,780023747	-0,038867107
Experimental	Control	0,868668495	0,057155333	-0,938651603
Experimental	Control	0,970062739	0,492988572	0,231896197
Experimental	Control	0,970062739	0,188566573	-0,595894716
Experimental	Control	0,981816738	0,859532704	0,086450283
Experimental	Control	0,970062739	0,520138615	-0,315794832
Experimental	Control	0,970062739	0,671384997	-0,204835468
Experimental	Control	0,981816738	0,856634255	0,141756756
Experimental	Control	0,970062739	0,379800212	-0,257455104
Experimental	Control	0,970062739	0,250814477	-0,827321026
Experimental	Control	0,970062739	0,396945041	-0,319774346
Experimental	Control	0,970062739	0,311962399	-0,746860298
Experimental	Control	0,99640621	0,989108206	0,006247958
Experimental	Control	0,970062739	0,719907828	-0,155387972
Experimental	Control	0,970062739	0,739849459	0,244170613
Experimental	Control	0,970062739	0,706922443	0,213562901
Experimental	Control	0,997096582	0,993496955	-0,002294959
Experimental	Control	0,970062739	0,29556379	-0,35270128
Experimental	Control	0,98331311	0,927147988	-0,021196247
Experimental	Control	0,970062739	0,575251302	0,258777405
Experimental	Control	0,970062739	0,393361804	0,116152096
Experimental	Control	0,970062739	0,732877364	0,101893001
Experimental	Control	0,970062739	0,539639455	-0,320323503
Experimental	Control	0,78357626	0,041941598	-1,035736386
Experimental	Control	0,970062739	0,411961029	0,185059495
Experimental	Control	0,972536115	0,758511599	-0,139855653
Experimental	Control	0,98331311	0,912305191	-0,031772257
Experimental	Control	0,970062739	0,686715364	0,265197474
Experimental	Control	0,98331311	0,886459339	-0,036116886
Experimental	Control	0,78357626	0,03230963	0,942641345
Experimental	Control	0,970062739	0,416978425	0,264604472
Experimental	Control	0,970062739	0,520940117	0,349808904
Experimental	Control	0,970062739	0,233903982	0,238125767
Experimental	Control	0,981816738	0,822179402	0,054099412
Experimental	Control	0,970062739	0,301127033	0,372768694
Experimental	Control	0,981816738	0,814527675	0,041280242
Experimental	Control	0,970062739	0,382273808	-0,209242752
Experimental	Control	0,970062739	0,333017594	-0,590808949
Experimental	Control	0,927436375	0,165669147	-0,991068234
Experimental	Control	0,970062739	0,656465424	0,119913674

Experimental	Control	0,970062739	0,485656002	-0,286499863
Experimental	Control	0,899730559	0,124424835	-0,614929226
Experimental	Control	0,99640621	0,989211941	-0,007554744
Experimental	Control	0,993132522	0,975205942	0,008685328
Experimental	Control	0,970062739	0,33831839	-0,431001131
Experimental	Control	0,970062739	0,23287107	-0,488661671
Experimental	Control	0,970062739	0,557050182	-0,112398044
Experimental	Control	0,970062739	0,452205903	0,612466323
Experimental	Control	0,78357626	0,035307865	0,745090156
Experimental	Control	0,970062739	0,713140057	-0,205617799
Experimental	Control	0,970062739	0,333009193	-0,376705623
Experimental	Control	0,970062739	0,543072737	-0,190756959
Experimental	Control	0,981816738	0,851359086	0,092812734
Experimental	Control	0,981816738	0,836297828	-0,075928185
Experimental	Control	0,970062739	0,255202334	-0,392680075
Experimental	Control	0,970062739	0,73562113	0,127607777
Experimental	Control	0,868668495	0,05934936	-0,952442575
Experimental	Control	0,98331311	0,930695808	0,04772251
Experimental	Control	0,970062739	0,523252587	0,169609961
Experimental	Control	0,970062739	0,21967439	-1,118758265
Experimental	Control	0,972536115	0,761331715	0,118069119
Experimental	Control	0,970062739	0,601170404	0,192545652
Experimental	Control	0,98331311	0,93763168	-0,015050488
Experimental	Control	0,970062739	0,287626988	0,407649117
Experimental	Control	0,970062739	0,3842793	0,533221913
Experimental	Control	0,970062739	0,507061189	0,325523513
Experimental	Control	0,970062739	0,468844472	0,206018595
Experimental	Control	0,98331311	0,933510972	-0,050040564
Experimental	Control	0,87952483	0,101313729	-0,454962571
Experimental	Control	0,970062739	0,417492476	-0,546110201
Experimental	Control	0,970062739	0,398052614	-0,302204145
Experimental	Control	0,970062739	0,369554774	-0,245841925
Experimental	Control	0,970062739	0,44161322	0,60639475
Experimental	Control	0,970062739	0,655359706	-0,251854556
Experimental	Control	0,972536115	0,756261318	0,171740815
Experimental	Control	0,927436375	0,165733215	-0,419779205
Experimental	Control	0,973116013	0,789587281	0,188508081
Experimental	Control	0,970062739	0,600465947	0,136797714
Experimental	Control	0,970062739	0,532094128	-0,408503998
Experimental	Control	0,981816738	0,838160263	-0,114647507
Experimental	Control	0,970062739	0,466491741	0,254601561
Experimental	Control	0,970062739	0,309182017	0,333823483
Experimental	Control	0,970062739	0,298099435	0,226558381
Experimental	Control	0,981816738	0,849047548	0,121992807
Experimental	Control	0,970062739	0,349599321	-0,326728293
Experimental	Control	0,970062739	0,588069376	-0,27514946
Experimental	Control	0,970062739	0,211154586	0,544900792
Experimental	Control	0,970062739	0,67396112	0,129695901

Experimental	Control	0,970062739	0,338007157	-0,313903697
Experimental	Control	0,970062739	0,45554879	-0,339665016
Experimental	Control	0,98331311	0,941788555	-0,045462864
Experimental	Control	0,981816738	0,833585721	-0,034816488
Experimental	Control	0,87952483	0,108179387	-0,582093847
Experimental	Control	0,870685429	0,063843747	-0,310180804
Experimental	Control	0,970062739	0,257156655	0,668946027
Experimental	Control	0,970062739	0,430417435	-0,538789328
Experimental	Control	0,970062739	0,654029975	-0,112382741
Experimental	Control	0,970062739	0,555618908	0,188377295
Experimental	Control	0,981816738	0,857563769	-0,11207261
Experimental	Control	0,970062739	0,598044611	-0,296888656
Experimental	Control	0,970062739	0,426424346	-0,478807054
Experimental	Control	0,970062739	0,611906228	0,145948901
Experimental	Control	0,970062739	0,196586356	-0,826791299
Experimental	Control	0,98331311	0,928826155	0,054360779
Experimental	Control	0,970062739	0,63629629	-0,236064499
Experimental	Control	0,899730559	0,124872605	0,356504384
Experimental	Control	0,87952483	0,110133268	1,026218232
Experimental	Control	0,970062739	0,394051994	-0,325193703
Experimental	Control	0,970062739	0,244059405	-0,239481409
Experimental	Control	0,890654702	0,115752958	0,215572695
Experimental	Control	0,970062739	0,402321577	-0,438749888
Experimental	Control	0,899730559	0,140927985	0,287149863
Experimental	Control	0,942205427	0,170073182	1,179047638
Experimental	Control	0,970062739	0,421473436	0,258944107
Experimental	Control	0,970062739	0,381707996	-0,374196204
Experimental	Control	0,970062739	0,678516461	-0,174290303
Experimental	Control	0,870685429	0,067784555	-0,796222022
Experimental	Control	0,970062739	0,533291318	-0,269454596
Experimental	Control	0,970062739	0,514432342	-0,397729622
Experimental	Control	0,970062739	0,490819515	-0,392614815
Experimental	Control	0,870685429	0,072501533	-0,654172364
Experimental	Control	0,970062739	0,73998404	0,093633265
Experimental	Control	0,98331311	0,87998176	-0,083060314
Experimental	Control	0,98331311	0,919405913	0,044197035
Experimental	Control	0,98331311	0,94372116	-0,022921671
Experimental	Control	0,855140552	0,054025125	0,817579736
Experimental	Control	0,970062739	0,245150688	-0,365733594
Experimental	Control	0,970062739	0,33362099	-0,266115942
Experimental	Control	0,78357626	0,048089518	-0,404766263
Experimental	Control	0,970062739	0,408610482	0,241775131
Experimental	Control	0,970062739	0,730158671	0,169998691
Experimental	Control	0,970062739	0,705409266	0,058927783
Experimental	Control	0,981816738	0,812532015	0,049282941
Experimental	Control	0,999707234	0,999707234	0,000153082
Experimental	Control	0,970062739	0,242493762	0,266315299
Experimental	Control	0,981816738	0,829381631	0,053752174

Experimental	Control	0,970062739	0,486407184	-0,211511573
Experimental	Control	0,981816738	0,85485549	-0,095975419
Experimental	Control	0,970062739	0,47850376	-0,380107984
Experimental	Control	0,970062739	0,508929559	0,269112605
Experimental	Control	0,970062739	0,642420724	0,157212381
Experimental	Control	0,98331311	0,896463501	-0,031576581
Experimental	Control	0,970062739	0,631465782	-0,072129451
Experimental	Control	0,970062739	0,552968044	-0,141553326
Experimental	Control	0,973116013	0,776691011	0,124991589
Experimental	Control	0,970062739	0,625677089	-0,174033671
Experimental	Control	0,78357626	0,047254501	-0,851837031
Experimental	Control	0,78357626	0,024760057	-0,89421457
Experimental	Control	0,970062739	0,303371218	0,301736847
Experimental	Control	0,970062739	0,337398752	-0,382339081
Experimental	Control	0,985213025	0,947867405	-0,026038716
Experimental	Control	0,970062739	0,717487136	0,091739021
Experimental	Control	0,970062739	0,28573923	-0,429556358
Experimental	Control	0,973959896	0,795361342	-0,130829273
Experimental	Control	0,694796407	0,008893436	0,581402325
Experimental	Control	0,970062739	0,44778176	0,384093909
Experimental	Control	0,694796407	0,012590433	-0,904098187
Experimental	Control	0,98331311	0,942311623	-0,035896498
Experimental	Control	0,970062739	0,596548259	-0,199509639
Experimental	Control	0,970062739	0,679150762	-0,183431768
Experimental	Control	0,973116013	0,787259007	0,054926728
Experimental	Control	0,973116013	0,777984336	-0,139409212
Experimental	Control	0,970062739	0,349357128	0,329801056
Experimental	Control	0,981816738	0,811884724	0,13501512
Experimental	Control	0,970062739	0,685287659	-0,17900167
Experimental	Control	0,970062739	0,362222342	-0,222153552
Experimental	Control	0,970062739	0,721710496	0,076077164
Experimental	Control	0,970062739	0,288320329	-0,611843366
Experimental	Control	0,98331311	0,944264575	0,019483667
Experimental	Control	0,970062739	0,64871399	0,12670613
Experimental	Control	0,981816738	0,846558723	-0,079745656
Experimental	Control	0,970062739	0,33451972	0,166782607
Experimental	Control	0,970062739	0,710954323	0,150217049
Experimental	Control	0,970062739	0,693525105	0,080810367
Experimental	Control	0,970062739	0,722747449	0,093615925
Experimental	Control	0,981816738	0,81861247	0,059857089
Experimental	Control	0,970062739	0,459934079	-0,281464209
Experimental	Control	0,970062739	0,194835746	-0,618610401
Experimental	Control	0,970062739	0,303010467	0,245217797
Experimental	Control	0,981816738	0,842070732	0,042596103
Experimental	Control	0,981816738	0,820377318	0,043203821
Experimental	Control	0,981816738	0,845751663	-0,109985684
Experimental	Control	0,78357626	0,03968095	-1,163859747
Experimental	Control	0,98331311	0,920919496	-0,048530005

Experimental	Control	0,970062739	0,420260501	-0,321127914
Experimental	Control	0,970062739	0,566013634	0,328121888
Experimental	Control	0,87952483	0,09449919	0,851697502
Experimental	Control	0,970062739	0,593050527	-0,267230568
Experimental	Control	0,970062739	0,431371615	0,38641063
Experimental	Control	0,970062739	0,59244661	-0,146989384
Experimental	Control	0,970062739	0,4277195	-0,563076191
Experimental	Control	0,970062739	0,329326049	0,273507945
Experimental	Control	0,972536115	0,761721807	0,046279533
Experimental	Control	0,87952483	0,094026069	0,706351249
Experimental	Control	0,78357626	0,045546627	0,416222615
Experimental	Control	0,970062739	0,292634172	-0,883403175
Experimental	Control	0,98331311	0,873045722	0,102288475
Experimental	Control	0,970062739	0,21632939	0,494143599
Experimental	Control	0,970062739	0,418338181	-0,228953275
Experimental	Control	0,970062739	0,538036884	0,269653549
Experimental	Control	0,96190808	0,178838506	0,865710648
Experimental	Control	0,694796407	0,015049742	-1,791430705
Experimental	Control	0,970062739	0,725854999	-0,129776951
Experimental	Control	0,970062739	0,466068507	0,374781522
Experimental	Control	0,981816738	0,82396388	-0,069502117
Experimental	Control	0,970062739	0,430835859	0,516034812
Experimental	Control	0,972536115	0,748764858	-0,121413135
Experimental	Control	0,973116013	0,784420637	-0,113335019
Experimental	Control	0,970062739	0,73761458	0,051562926
Experimental	Control	0,98331311	0,937505802	0,034572789
Experimental	Control	0,970062739	0,469741424	0,257561758
Experimental	Control	0,970062739	0,632648579	-0,124602573
Experimental	Control	0,981816738	0,852484511	-0,023259788
Experimental	Control	0,970062739	0,56987082	-0,155194947
Experimental	Control	0,970062739	0,571590594	0,301744773
Experimental	Control	0,970062739	0,287128539	-0,30126831
Experimental	Control	0,970062739	0,217312519	0,295838896
Experimental	Control	0,899730559	0,125118253	-0,390369457
Experimental	Control	0,694796407	0,011916826	1,029180014
Experimental	Control	0,970062739	0,578025401	0,217151156
Experimental	Control	0,78357626	0,038151972	1,225313303
Experimental	Control	0,970062739	0,203089521	-0,295179759
Experimental	Control	0,98331311	0,887183336	-0,057344025
Experimental	Control	0,98331311	0,916750508	0,026092472
Experimental	Control	0,899730559	0,137019573	-0,400525224
Experimental	Control	0,899730559	0,148625118	-0,54949257
Experimental	Control	0,899730559	0,136299905	0,363009254
Experimental	Control	0,87952483	0,08892748	-0,725752885
Experimental	Control	0,87952483	0,0884033	1,040680333
Experimental	Control	0,98331311	0,89945553	0,043438554
Experimental	Control	0,98331311	0,942054464	0,033688526
Experimental	Control	0,970062739	0,311085016	0,325000827

Experimental	Control	0,98331311	0,891447281	-0,028598727
Experimental	Control	0,98331311	0,930436999	-0,049622519
Experimental	Control	0,970062739	0,429887158	-0,157074633
Experimental	Control	0,970062739	0,562234382	-0,218504398
Experimental	Control	0,970062739	0,738444879	-0,09694129
Experimental	Control	0,988586169	0,962577675	-0,018072918
Experimental	Control	0,992708935	0,971206214	-0,011672181
Experimental	Control	0,970062739	0,653055181	-0,180782373
Experimental	Control	0,78357626	0,032641491	0,601258382
Experimental	Control	0,970062739	0,480521953	0,135624695
Experimental	Control	0,970062739	0,419272786	0,199537736
Experimental	Control	0,87952483	0,099414643	0,303417569
Experimental	Control	0,970062739	0,622365193	-0,211817311
Experimental	Control	0,970062739	0,546900995	0,119822839
Experimental	Control	0,987814822	0,955719756	-0,016879399
Experimental	Control	0,970062739	0,197255063	0,494413847
Experimental	Control	0,970062739	0,199094591	-0,387703047
Experimental	Control	0,98331311	0,921413101	0,040854451
Experimental	Control	0,970062739	0,285996343	0,206147023
Experimental	Control	0,970062739	0,244823189	0,595801261
Experimental	Control	0,899730559	0,147142593	0,77896506
Experimental	Control	0,970062739	0,317223473	0,154816752
Experimental	Control	0,970062739	0,685590024	-0,229894067
Experimental	Control	0,970062739	0,577317996	-0,23437144
Experimental	Control	0,970062739	0,30754081	0,231243513
Experimental	Control	0,899730559	0,130062469	0,415985825
Experimental	Control	0,970062739	0,312138827	0,655495143
Experimental	Control	0,87952483	0,103048087	0,682134933
Experimental	Control	0,981816738	0,823422754	0,069154338
Experimental	Control	0,970062739	0,438906392	0,393780225
Experimental	Control	0,970062739	0,568181092	-0,32159584
Experimental	Control	0,973116013	0,777234635	-0,150621082
Experimental	Control	0,970062739	0,451553747	0,312280388
Experimental	Control	0,970062739	0,577057209	-0,175625247
Experimental	Control	0,993132522	0,974583465	0,011147511
Experimental	Control	0,96190808	0,177718137	-0,602110917
Experimental	Control	0,970062739	0,453012347	0,291566368
Experimental	Control	0,970062739	0,359524817	0,612246007
Experimental	Control	0,899730559	0,128439897	0,583209017
Experimental	Control	0,970062739	0,594496552	-0,248394132
Experimental	Control	0,970062739	0,669993382	0,146000216
Experimental	Control	0,970062739	0,63442013	-0,215075285
Experimental	Control	0,99640621	0,986257652	-0,005605439
Experimental	Control	0,970062739	0,594891155	0,118326315
Experimental	Control	0,970062739	0,542422378	-0,331098928
Experimental	Control	0,98331311	0,923643761	-0,021744813
Experimental	Control	0,870685429	0,078847319	0,788718278
Experimental	Control	0,970062739	0,622925408	-0,192700266

Experimental	Control	0,970062739	0,474193929	-0,297274395
Experimental	Control	0,970062739	0,305662237	0,479498226
Experimental	Control	0,970062739	0,261673906	0,430849005
Experimental	Control	0,98331311	0,89931009	0,041435144
Experimental	Control	0,983153927	0,862477994	0,042268262
Experimental	Control	0,970062739	0,471607735	-0,344997122
Experimental	Control	0,970062739	0,381418448	0,225738382
Experimental	Control	0,899730559	0,140349349	0,800701553
Experimental	Control	0,904627753	0,151859893	-0,484131976
Experimental	Control	0,970062739	0,660172653	0,103829177
Experimental	Control	0,98331311	0,892363347	0,033502741
Experimental	Control	0,970062739	0,553569912	0,221117235
Experimental	Control	0,99681224	0,99141434	-0,005206995
Experimental	Control	0,970062739	0,60829948	-0,220395648
Experimental	Control	0,970062739	0,467959419	0,435919589
Experimental	Control	0,970062739	0,535265978	0,264718526
Experimental	Control	0,899730559	0,148507133	-0,717191566
Experimental	Control	0,972536115	0,744798348	-0,10683126
Experimental	Control	0,970062739	0,390979369	0,275992113
Experimental	Control	0,970062739	0,505738693	0,291637183
Experimental	Control	0,970062739	0,340674893	-0,383973856
Experimental	Control	0,98331311	0,930711245	0,024865673
Experimental	Control	0,973116013	0,774826486	-0,213828612
Experimental	Control	0,970062739	0,284469053	0,515529506
Experimental	Control	0,970062739	0,311694913	-0,279370906
Experimental	Control	0,970062739	0,397921301	0,401329141
Experimental	Control	0,973116013	0,785654074	-0,109477747
Experimental	Control	0,87952483	0,084977792	0,824260679
Experimental	Control	0,970062739	0,196945714	0,632656563
Experimental	Control	0,899730559	0,139462618	0,644158771
Experimental	Control	0,970062739	0,289469502	0,58389163
Experimental	Control	0,970062739	0,717682983	-0,117936981
Experimental	Control	0,970062739	0,624920268	-0,290240405
Experimental	Control	0,970062739	0,707443487	-0,156042328
Experimental	Control	0,970062739	0,587950258	0,253481993
Experimental	Control	0,970062739	0,582275735	-0,183028707
Experimental	Control	0,98331311	0,869985979	0,083866982
Experimental	Control	0,972536115	0,755261655	0,120505322
Experimental	Control	0,970062739	0,637628373	0,268766915
Experimental	Control	0,970062739	0,634410657	-0,210585494
Experimental	Control	0,970062739	0,715072235	0,14968039
Experimental	Control	0,970062739	0,388592499	-0,335955463
Experimental	Control	0,970062739	0,655420373	0,202437345
Experimental	Control	0,78357626	0,046712087	-0,623369571
Experimental	Control	0,970062739	0,525485075	-0,263460194
Experimental	Control	0,970062739	0,725375626	0,236501109
Experimental	Control	0,970062739	0,670334205	0,241520928
Experimental	Control	0,694796407	0,014184723	0,828971555

Experimental	Control	0,970062739	0,740679672	0,083711835
Experimental	Control	0,970062739	0,631280219	0,101496797
Experimental	Control	0,735000532	0,018262501	1,200223098
Experimental	Control	0,970062739	0,679482379	0,108848986
Experimental	Control	0,988586169	0,963603847	-0,014141703
Experimental	Control	0,970062739	0,30071299	0,303600754
Experimental	Control	0,970062739	0,327449997	-0,379666203
Experimental	Control	0,970062739	0,509686026	0,328270173
Experimental	Control	0,970062739	0,36835808	0,435343173
Experimental	Control	0,970062739	0,665765394	0,158081297
Experimental	Control	0,970062739	0,284177693	0,748898592
Experimental	Control	0,970062739	0,667710818	0,213944568
Experimental	Control	0,972536115	0,761878473	-0,129060741
Experimental	Control	0,970062739	0,614412715	-0,100898625
Experimental	Control	0,970062739	0,568430971	-0,223516811
Experimental	Control	0,927436375	0,163369338	-0,728596343
Experimental	Control	0,970062739	0,496653748	0,271616212
Experimental	Control	0,970062739	0,512395264	0,16960232
Experimental	Control	0,970062739	0,387353509	0,276592974
Experimental	Control	0,973116013	0,781468048	0,107445089
Experimental	Control	0,970062739	0,398966914	-0,317793027
Experimental	Control	0,970062739	0,62245394	0,272134378
Experimental	Control	0,973959896	0,795832408	-0,142398942
Experimental	Control	0,981816738	0,852515284	-0,078282034
Experimental	Control	0,970062739	0,447509896	0,373325555
Experimental	Control	0,970062739	0,52772037	-0,189173635
Experimental	Control	0,98331311	0,934186691	0,024487068
Experimental	Control	0,995080182	0,909544426	0,032914098
Experimental	Control	0,714386358	0,141743325	0,360948896
Experimental	Control	0,795738716	0,326827618	0,282590331
Experimental	Control	0,994641719	0,8405716	-0,073874006
Experimental	Control	0,77418217	0,198133343	-1,102436784
Experimental	Control	0,159397495	0,002530119	-1,745831312
Experimental	Control	0,884317616	0,572569687	-0,283225157
Experimental	Control	0,995080182	0,912257255	-0,033141388
Experimental	Control	0,491272929	0,066282856	-1,506424363
Experimental	Control	0,880511036	0,538090077	0,268036215
Experimental	Control	0,829721397	0,455319928	0,3492079
Experimental	Control	0,994995072	0,860749705	0,050667518
Experimental	Control	0,795738716	0,371965721	0,364389449
Experimental	Control	0,905060951	0,617740014	0,333851842
Experimental	Control	0,795738716	0,401399771	-0,304586244
Experimental	Control	0,995080182	0,992388111	0,002976695
Experimental	Control	0,275596245	0,015743348	0,983909396
Experimental	Control	0,795738716	0,245152096	-0,466583568
Experimental	Control	0,795738716	0,353569769	-0,469880315
Experimental	Control	0,795738716	0,393518703	0,343211494
Experimental	Control	0,275596245	0,014487276	-1,156594731

Experimental	Control	0,934383027	0,702293087	0,102391991
Experimental	Control	0,995080182	0,890590445	0,05290861
Experimental	Control	0,994995072	0,860502336	-0,081559063
Experimental	Control	0,77418217	0,186185304	0,369199852
Experimental	Control	0,795738716	0,400938772	0,246228189
Experimental	Control	0,795738716	0,328736626	-0,868582136
Experimental	Control	0,995080182	0,964922073	-0,02339825
Experimental	Control	0,930626776	0,686891192	-0,088065449
Experimental	Control	0,491272929	0,064825599	-0,549432348
Experimental	Control	0,995080182	0,874526358	0,030215514
Experimental	Control	0,795738716	0,358059258	0,268776883
Experimental	Control	0,995080182	0,981282885	-0,012712082
Experimental	Control	0,795738716	0,361768694	-0,180530538
Experimental	Control	0,930626776	0,681086844	0,274693846
Experimental	Control	0,96948655	0,76846744	0,100881985
Experimental	Control	0,930626776	0,676580535	-0,22391642
Experimental	Control	0,415491504	0,042868171	-0,771220716
Experimental	Control	0,795738716	0,26480878	-0,428799561
Experimental	Control	0,930626776	0,668338921	-0,155168012
Experimental	Control	0,795738716	0,3230444	-0,176977307
Experimental	Control	0,829721397	0,49946856	-0,30251442
Experimental	Control	0,829721397	0,484646362	0,361888816
Experimental	Control	0,995080182	0,877251878	0,085539477
Experimental	Control	0,795738716	0,373444604	0,336546241
Experimental	Control	0,795738716	0,376331793	-0,502587101
Experimental	Control	0,795738716	0,221559666	-0,616936145
Experimental	Control	0,980766794	0,810799084	-0,170425505
Experimental	Control	0,829721397	0,476842145	0,36992483
Experimental	Control	0,415491504	0,037959652	-1,186988966
Experimental	Control	0,795738716	0,358854228	0,447948045
Experimental	Control	0,275596245	0,01919754	1,525153693
Experimental	Control	0,995080182	0,983046833	-0,006948524
Experimental	Control	0,920122287	0,649927647	0,349395188
Experimental	Control	0,159397495	0,002335977	-1,280301771
Experimental	Control	0,733100473	0,162911216	0,575139755
Experimental	Control	0,632698353	0,095406895	-0,431927016
Experimental	Control	0,795738716	0,404184745	-0,318451183
Experimental	Control	0,795738716	0,21817927	-0,433472821
Experimental	Control	0,995080182	0,929016968	-0,039583536
Experimental	Control	0,720110796	0,154309456	-0,675510248
Experimental	Control	0,415491504	0,042748919	-1,95645076
Experimental	Control	0,713226318	0,120005831	-0,752279226
Experimental	Control	0,995080182	0,995080182	-0,002216143
Experimental	Control	0,275596245	0,008750763	-1,434710524
Experimental	Control	0,795738716	0,385660497	-0,919613674
Experimental	Control	0,77418217	0,188668471	0,645605004
Experimental	Control	0,77418217	0,202761997	-0,556369691
Experimental	Control	0,795738716	0,392599428	0,297993664

Experimental	Control	0,795738716	0,396977831	-0,305548029
Experimental	Control	0,829721397	0,495054933	0,389898268
Experimental	Control	0,795738716	0,256595318	-0,489203033
Experimental	Control	0,795738716	0,270261209	0,564037505
Experimental	Control	0,96948655	0,774514402	0,162663818
Experimental	Control	0,884317616	0,575092164	-0,240621873
Experimental	Control	0,713226318	0,122418237	-0,53248115
Experimental	Control	0,795738716	0,350996749	-0,501883724
Experimental	Control	0,491272929	0,058723176	-0,963836936
Experimental	Control	0,895182615	0,596782103	0,302218943
Experimental	Control	0,96948655	0,792008663	0,089542377
Experimental	Control	0,795738716	0,351627937	-0,711470391
Experimental	Control	0,807413917	0,422931099	0,388613005
Experimental	Control	0,829721397	0,448736153	-0,161521673
Experimental	Control	0,995080182	0,973238428	-0,02965955
Experimental	Control	0,77418217	0,194749535	0,735121419
Experimental	Control	0,795738716	0,293651185	-0,358734747
Experimental	Control	0,829721397	0,481400345	-0,21712037
Experimental	Control	0,980766794	0,817305662	-0,096656137
Experimental	Control	0,96948655	0,773585523	-0,145602481
Experimental	Control	0,415491504	0,033539064	-1,469154677
Experimental	Control	0,895182615	0,603893034	0,392060502
Experimental	Control	0,906259401	0,625750539	0,324001608
Experimental	Control	0,96948655	0,792516783	-0,061401722
Experimental	Control	0,714386358	0,132268612	-0,805301949
Experimental	Control	0,994641719	0,844656063	-0,137510942
Experimental	Control	0,275596245	0,019685446	-1,427276337
Experimental	Control	0,720110796	0,153617199	-0,532919546
Experimental	Control	0,884317616	0,57550829	0,215096359
Experimental	Control	0,713226318	0,124531579	0,474707484
Experimental	Control	0,795738716	0,248133767	0,239596775
Experimental	Control	0,950058396	0,723854016	0,249997247
Experimental	Control	0,934383027	0,704495139	-0,161069879
Experimental	Control	0,96948655	0,785576437	0,07373105
Experimental	Control	0,995080182	0,990689153	-0,005076574
Experimental	Control	0,275596245	0,019633043	-1,509638103
Experimental	Control	0,995080182	0,937470703	-0,044308156
Experimental	Control	0,714386358	0,141243982	-0,759059884
Experimental	Control	0,915171283	0,639167245	0,281715915
Experimental	Control	0,275596245	0,012066572	-1,530435843
Experimental	Control	0,96948655	0,769339228	-0,149868628
Experimental	Control	0,829721397	0,469335403	-0,35518597
Experimental	Control	0,829721397	0,500466874	0,278183643
Experimental	Control	0,995080182	0,994312481	-0,002924171
Experimental	Control	0,795738716	0,352526242	-0,724946295
Experimental	Control	0,795738716	0,372087123	-0,452641595
Experimental	Control	0,884317616	0,555796061	-0,381492891
Experimental	Control	0,895182615	0,597395994	-0,224494498

Experimental	Control	0,807413917	0,418164788	-0,315826569
Experimental	Control	0,829721397	0,453214657	0,35503001
Experimental	Control	0,995080182	0,924695831	-0,038336247
Experimental	Control	0,882163331	0,54610111	0,150662636
Experimental	Control	0,995080182	0,918662057	0,056604391
Experimental	Control	0,795738716	0,234419903	-0,825543626
Experimental	Control	0,795738716	0,32438001	-0,495894212
Experimental	Control	0,564787144	0,080683878	-1,333680512
Experimental	Control	0,491272929	0,058271863	-1,061305723
Experimental	Control	0,882414063	0,714006675	0,261815686
Experimental	Control	0,767190285	0,413546284	0,19030758
Experimental	Control	0,435518578	0,140823427	-1,252853989
Experimental	Control	0,982189463	0,982189463	0,013190388
Experimental	Control	0,435518578	0,13742448	-0,567836218
Experimental	Control	0,592615022	0,23428966	-0,611289948
Experimental	Control	0,882414063	0,633187101	0,188004478
Experimental	Control	0,725868593	0,320732634	-0,398049213
Experimental	Control	0,882414063	0,710267517	-0,201637888
Experimental	Control	0,107641242	0,017522993	-0,688126979
Experimental	Control	0,435518578	0,11370904	-1,418696069
Experimental	Control	0,904982648	0,797562197	0,073476795
Experimental	Control	0,945044287	0,91992781	-0,034156897
Experimental	Control	0,767190285	0,463882498	0,206095732
Experimental	Control	0,103128262	0,011991658	-1,217938595
Experimental	Control	0,767190285	0,406400449	0,265728522
Experimental	Control	0,182301359	0,033916532	-0,948438375
Experimental	Control	0,882414063	0,644435894	-0,320846441
Experimental	Control	0,767190285	0,45640675	-0,225175193
Experimental	Control	0,767190285	0,463068724	0,316949611
Experimental	Control	0,945044287	0,923066513	0,063595458
Experimental	Control	0,309455441	0,07078204	-0,839177232
Experimental	Control	0,839436686	0,566131718	-0,121801766
Experimental	Control	0,904982648	0,820798215	0,191069508
Experimental	Control	0,839436686	0,543935653	-0,303241455
Experimental	Control	0,882414063	0,67342171	-0,119891593
Experimental	Control	0,738724983	0,343593015	-0,216594566
Experimental	Control	0,532423493	0,198111067	-0,699198177
Experimental	Control	0,070709936	0,006577668	-0,557078509
Experimental	Control	0,062463066	0,002905259	-1,604769396
Experimental	Control	0,904982648	0,781653967	0,119194762
Experimental	Control	0,839436686	0,563568623	0,299065987
Experimental	Control	0,882414063	0,718244005	-0,154175407
Experimental	Control	0,070709936	0,00653399	-1,693885769
Experimental	Control	0,767190285	0,404840074	0,416387108
Experimental	Control	0,052489085	0,001220676	-1,915132044
Experimental	Control	0,107641242	0,01607936	-1,521883672
Experimental	Control	0,597791958	0,250238494	-0,494515841
Experimental	Control	0,435518578	0,151608457	-0,518925138

Experimental	Control	0,904982648	0,8074438	0,088385198
Experimental	Control	0,309455441	0,071966382	-0,680053677
Experimental	Control	0,915182732	0,851332774	-0,10395336
Experimental	Control	0,435518578	0,151925085	-0,703863159
Experimental	Control	0,064287302	0,0183678	0,755843292
Experimental	Control	0,026609416	0,003801345	0,773823932
Experimental	Control	0,247388387	0,106023595	-0,619396985
Experimental	Control	0,289345287	0,165340164	0,242697719
Experimental	Control	0,534121185	0,534121185	-0,262898224
Experimental	Control	0,452121961	0,356059881	-0,361807175
Experimental	Control	0,452121961	0,38753311	-0,650989951
Experimental	Control	0,624192061	0,016348642	1,058685604
Experimental	Control	0,704891642	0,072012506	0,875288911
Experimental	Control	0,790420277	0,217700715	-1,127208754
Experimental	Control	0,772193668	0,160537896	-0,651079864
Experimental	Control	0,858120076	0,3123778	-0,826346418
Experimental	Control	0,915172456	0,449900366	-0,376356802
Experimental	Control	0,983491561	0,957148622	-0,021734766
Experimental	Control	0,885436121	0,406929077	0,502835618
Experimental	Control	0,983491561	0,932354773	0,040212878
Experimental	Control	0,637944107	0,03539273	-1,169489815
Experimental	Control	0,870817871	0,362081185	-0,603240996
Experimental	Control	0,772193668	0,154733844	-0,654305596
Experimental	Control	0,983491561	0,846685214	-0,133686513
Experimental	Control	0,983491561	0,889993121	-0,076831204
Experimental	Control	0,772193668	0,122610713	-1,163195578
Experimental	Control	0,942203073	0,615893087	0,29952553
Experimental	Control	0,772193668	0,186268349	-0,638142521
Experimental	Control	0,814348357	0,25530163	-0,839788111
Experimental	Control	0,621747057	0,008074685	1,35767187
Experimental	Control	0,885436121	0,401941417	-0,339891053
Experimental	Control	0,783663238	0,197222228	0,745324408
Experimental	Control	0,790420277	0,233910526	0,569899645
Experimental	Control	0,858120076	0,304693374	0,499434205
Experimental	Control	0,885436121	0,401812809	0,49717511
Experimental	Control	0,772193668	0,171773355	-0,523869293
Experimental	Control	0,692638815	0,066678393	-1,252403406
Experimental	Control	0,983491561	0,811311758	-0,103621343
Experimental	Control	0,885436121	0,394977169	-0,506266484
Experimental	Control	0,624192061	0,022370334	-1,893469818
Experimental	Control	0,858120076	0,304348228	-0,486980784
Experimental	Control	0,98145184	0,722336944	-0,267904374
Experimental	Control	0,783663238	0,2005763	0,448072623
Experimental	Control	0,908003536	0,428025954	-0,520620495
Experimental	Control	0,790420277	0,230393432	-0,805597592
Experimental	Control	0,983491561	0,828668761	0,095568752
Experimental	Control	0,772193668	0,142749167	0,676178771
Experimental	Control	0,983491561	0,967412026	-0,020038111

Experimental	Control	0,942203073	0,621965094	-0,437346032
Experimental	Control	0,772193668	0,149271601	-1,036512848
Experimental	Control	0,772193668	0,151098587	-1,15728352
Experimental	Control	0,818172512	0,270045151	-0,971891143
Experimental	Control	0,621747057	0,006483081	-1,682559409
Experimental	Control	0,919104202	0,463444218	0,317493314
Experimental	Control	0,754607597	0,095579528	-0,777487051
Experimental	Control	0,98145184	0,725510866	-0,101987283
Experimental	Control	0,840958856	0,27962138	0,391655602
Experimental	Control	0,989996372	0,978326474	-0,02023123
Experimental	Control	0,92316567	0,531442631	-0,172018156
Experimental	Control	0,815638754	0,261803018	0,790616212
Experimental	Control	0,772193668	0,14334935	0,487674256
Experimental	Control	0,92316567	0,530965087	0,458629541
Experimental	Control	0,933825177	0,558700894	0,353753719
Experimental	Control	0,885436121	0,408441945	-0,390925824
Experimental	Control	0,790420277	0,229321097	-0,815378179
Experimental	Control	0,919104202	0,475340443	0,261041853
Experimental	Control	0,816993398	0,268050879	-0,732965478
Experimental	Control	0,98145184	0,745989921	-0,058445157
Experimental	Control	0,917990807	0,459897163	-0,322082726
Experimental	Control	0,98145184	0,720261294	-0,242673951
Experimental	Control	0,983491561	0,946200026	-0,031235978
Experimental	Control	0,790420277	0,228714408	0,508747014
Experimental	Control	0,68521937	0,049809659	-1,334790534
Experimental	Control	0,624192061	0,020054334	-1,369505097
Experimental	Control	0,885436121	0,397885331	-0,488081538
Experimental	Control	0,983491561	0,817610499	-0,171423368
Experimental	Control	0,814348357	0,255983766	0,46499518
Experimental	Control	0,885436121	0,410536197	-0,306479628
Experimental	Control	0,948299071	0,643125122	-0,245115833
Experimental	Control	0,692638815	0,054544813	-1,00767304
Experimental	Control	0,948260112	0,633415399	-0,214777283
Experimental	Control	0,983491561	0,878062814	-0,082548054
Experimental	Control	0,692638815	0,058009445	0,570723943
Experimental	Control	0,983491561	0,830927383	-0,082056355
Experimental	Control	0,878603114	0,376297601	-0,595082485
Experimental	Control	0,790420277	0,233439987	-0,748654127
Experimental	Control	0,98145184	0,736278543	0,188838343
Experimental	Control	0,870817871	0,346063501	-0,744684588
Experimental	Control	0,983491561	0,949965945	-0,023629083
Experimental	Control	0,964525247	0,691685464	-0,220647994
Experimental	Control	0,772193668	0,159720134	-0,475429585
Experimental	Control	0,772193668	0,166306056	-1,445042588
Experimental	Control	0,919104202	0,47734995	-0,43686723
Experimental	Control	0,772193668	0,147616407	0,292637769
Experimental	Control	0,885436121	0,403799038	-0,193367027
Experimental	Control	0,98145184	0,752311032	0,129695415

Experimental	Control	0,983491561	0,792766776	0,063798178
Experimental	Control	0,999642631	0,999642631	0,00011395
Experimental	Control	0,783663238	0,195063602	-0,829316339
Experimental	Control	0,919104202	0,468960085	-0,344997541
Experimental	Control	0,858120076	0,323892619	-0,734225895
Experimental	Control	0,772193668	0,145593503	-1,142650772
Experimental	Control	0,959244846	0,669120087	-0,395311504
Experimental	Control	0,983491561	0,792150172	0,144948955
Experimental	Control	0,983491561	0,968033933	0,012824579
Experimental	Control	0,624192061	0,028027581	-1,955635743
Experimental	Control	0,942203073	0,578110478	-0,191514411
Experimental	Control	0,942203073	0,618752693	-0,359965088
Experimental	Control	0,915172456	0,445641024	-0,372219535
Experimental	Control	0,98145184	0,719913042	0,205708117
Experimental	Control	0,983491561	0,919888557	0,050464368
Experimental	Control	0,772193668	0,178977137	0,802711034
Experimental	Control	0,983491561	0,887177613	-0,055399524
Experimental	Control	0,98145184	0,767422067	0,109992355
Experimental	Control	0,637944107	0,036346521	0,435333794
Experimental	Control	0,772193668	0,11931435	-0,627902458
Experimental	Control	0,983491561	0,929321832	0,043932601
Experimental	Control	0,920859684	0,495639423	-0,312749309
Experimental	Control	0,959244846	0,676824843	0,207924576
Experimental	Control	0,858120076	0,322602496	-0,250601393
Experimental	Control	0,933825177	0,559481713	0,373577311
Experimental	Control	0,736049836	0,079805917	0,923004518
Experimental	Control	0,983491561	0,894580057	-0,054010213
Experimental	Control	0,983491561	0,840300106	-0,072213566
Experimental	Control	0,920859684	0,499326666	0,234916098
Experimental	Control	0,878603114	0,375365515	0,312149444
Experimental	Control	0,942203073	0,604653141	-0,452200819
Experimental	Control	0,983491561	0,794658375	0,105700885
Experimental	Control	0,797872703	0,23876264	0,605607343
Experimental	Control	0,893662802	0,416106256	0,243606192
Experimental	Control	0,754607597	0,090963582	-1,014690087
Experimental	Control	0,983491561	0,819024535	-0,12832342
Experimental	Control	0,925818599	0,544807915	0,176942106
Experimental	Control	0,624192061	0,028205142	1,064831287
Experimental	Control	0,692638815	0,064180158	-1,04282722
Experimental	Control	0,922188811	0,502775289	-0,212127696
Experimental	Control	0,983491561	0,873313074	0,138914336
Experimental	Control	0,772193668	0,178134855	-0,609450842
Experimental	Control	0,983491561	0,88125794	-0,047973224
Experimental	Control	0,624192061	0,016823282	-1,035278091
Experimental	Control	0,772193668	0,161069644	0,867839532
Experimental	Control	0,959244846	0,669174406	0,284917592
Experimental	Control	0,624192061	0,013261292	0,995424437
Experimental	Control	0,772193668	0,164104376	0,316614727

Experimental	Control	0,771535076	0,105375239	-1,267988834
Experimental	Control	0,692638815	0,055393973	-0,838029448
Experimental	Control	0,942203073	0,609045563	-0,172613382
Experimental	Control	0,98145184	0,751246018	0,162029503
Experimental	Control	0,983491561	0,940178522	-0,044152572
Experimental	Control	0,797872703	0,24296713	0,346218609
Experimental	Control	0,621747057	0,002974348	0,928170466
Experimental	Control	0,624192061	0,01665875	-1,341998486
Experimental	Control	0,885436121	0,407388989	0,606266479
Experimental	Control	0,983491561	0,956474312	0,019526798
Experimental	Control	0,870817871	0,345026141	0,557033714
Experimental	Control	0,983491561	0,837907713	0,13722634
Experimental	Control	0,858120076	0,322704443	0,393929142
Experimental	Control	0,844224726	0,286936891	0,576855178
Experimental	Control	0,983491561	0,788738184	0,162450398
Experimental	Control	0,922188811	0,505257604	-0,393583229
Experimental	Control	0,983491561	0,873080876	0,082571853
Experimental	Control	0,789234341	0,215527649	-0,724731109
Experimental	Control	0,624192061	0,027690139	2,150789641
Experimental	Control	0,772193668	0,15974216	-1,163150227
Experimental	Control	0,843858323	0,283496607	0,693049548
Experimental	Control	0,98145184	0,753661426	-0,056307084
Experimental	Control	0,990728512	0,981201648	0,00950379
Experimental	Control	0,983491561	0,781245555	-0,061793604
Experimental	Control	0,983491561	0,873890212	0,088446238
Experimental	Control	0,98145184	0,74319096	-0,251246602
Experimental	Control	0,950766093	0,650032614	0,248086487
Experimental	Control	0,942203073	0,60394764	-0,254660189
Experimental	Control	0,959244846	0,672358564	0,15190845
Experimental	Control	0,933692218	0,553978487	-0,351379846
Experimental	Control	0,983491561	0,821104872	-0,060476888
Experimental	Control	0,915172456	0,451781956	-0,552762718
Experimental	Control	0,98145184	0,731718657	-0,083317534
Experimental	Control	0,637944107	0,034371645	0,933114743
Experimental	Control	0,92316567	0,535669522	0,272059989
Experimental	Control	0,925818599	0,545669116	0,250706945
Experimental	Control	0,736049836	0,080979943	0,636506663
Experimental	Control	0,772193668	0,189200981	-0,444555234
Experimental	Control	0,990728512	0,985306837	0,005475975
Experimental	Control	0,942203073	0,58851632	0,219231796
Experimental	Control	0,942203073	0,616634943	0,102077822
Experimental	Control	0,68521937	0,047750541	0,455595519
Experimental	Control	0,98145184	0,762881437	-0,132587177
Experimental	Control	0,676638733	0,042397727	0,662685889
Experimental	Control	0,772193668	0,141928249	-0,375397769
Experimental	Control	0,772193668	0,189634987	-0,629612632
Experimental	Control	0,816993398	0,267555772	-0,52942343
Experimental	Control	0,92316567	0,536244405	0,134784489

Experimental	Control	0,626448651	0,031999342	-1,271023014
Experimental	Control	0,983491561	0,814865144	0,105762148
Experimental	Control	0,624192061	0,010653513	0,67682121
Experimental	Control	0,983491561	0,827425286	-0,144638547
Experimental	Control	0,915172456	0,442212543	0,476057881
Experimental	Control	0,983491561	0,908145909	0,079998175
Experimental	Control	0,983491561	0,919558308	-0,036051065
Experimental	Control	0,783663238	0,200210503	0,829444152
Experimental	Control	0,870817871	0,365391141	-0,462591039
Experimental	Control	0,919104202	0,477848424	0,236739635
Experimental	Control	0,885436121	0,39878601	-0,325538149
Experimental	Control	0,772193668	0,18428159	0,640267278
Experimental	Control	0,769746662	0,101322252	0,572441233
Experimental	Control	0,990728512	0,987358296	0,010811463
Experimental	Control	0,920859684	0,498440416	0,401988385
Experimental	Control	0,983491561	0,951755796	0,018387654
Experimental	Control	0,866291532	0,33792363	-0,523057122
Experimental	Control	0,983491561	0,845151096	-0,068213344
Experimental	Control	0,919104202	0,484953509	-0,49366141
Experimental	Control	0,754607597	0,09570564	0,65685969
Experimental	Control	0,98145184	0,736606048	0,121814214
Experimental	Control	0,933825177	0,563230509	-0,37644608
Experimental	Control	0,98145184	0,718616132	0,22997942
Experimental	Control	0,919104202	0,486561202	0,110801825
Experimental	Control	0,942203073	0,597270774	-0,158609137
Experimental	Control	0,790420277	0,227349326	-0,491147343
Experimental	Control	0,772193668	0,177803912	-0,562334334
Experimental	Control	0,692638815	0,063315241	0,710780857
Experimental	Control	0,919104202	0,489849092	0,362800951
Experimental	Control	0,92316567	0,534872517	-0,148641633
Experimental	Control	0,951184868	0,65218766	-0,109799235
Experimental	Control	0,790420277	0,234486173	0,828798408
Experimental	Control	0,815638754	0,258059594	0,251068889
Experimental	Control	0,624192061	0,023349422	0,735046966
Experimental	Control	0,983491561	0,861020604	-0,037406523
Experimental	Control	0,98145184	0,724524495	0,155451881
Experimental	Control	0,692638815	0,065804624	0,326089775
Experimental	Control	0,983491561	0,854552289	0,044235236
Experimental	Control	0,692638815	0,056882621	0,749701555
Experimental	Control	0,870817871	0,366132702	-0,2050025
Experimental	Control	0,772193668	0,1341956	0,340185571
Experimental	Control	0,919104202	0,468863468	0,345922051
Experimental	Control	0,704891642	0,070247487	-1,159940538
Experimental	Control	0,858120076	0,311787904	-0,181256939
Experimental	Control	0,915172456	0,451093022	0,343374869
Experimental	Control	0,942203073	0,581991683	0,154298219
Experimental	Control	0,885436121	0,395383933	0,263919493
Experimental	Control	0,983491561	0,9573986	0,022501918

Experimental	Control	0,92316567	0,515832079	-0,25403641
Experimental	Control	0,626448651	0,031594205	0,402970914
Experimental	Control	0,942203073	0,604114036	-0,410063944
Experimental	Control	0,754607597	0,093779051	0,58855212
Experimental	Control	0,790420277	0,23286364	-0,600117049
Experimental	Control	0,937006646	0,568831147	0,214740413
Experimental	Control	0,772193668	0,130553048	0,898752679
Experimental	Control	0,964525247	0,688330326	-0,182611377
Experimental	Control	0,98145184	0,721050075	0,12031103
Experimental	Control	0,983491561	0,943910394	-0,021619049
Experimental	Control	0,858120076	0,299030654	0,538127426
Experimental	Control	0,866291532	0,339848962	0,365641311
Experimental	Control	0,92316567	0,525571759	0,244226602
Experimental	Control	0,624192061	0,020469953	-1,758645288
Experimental	Control	0,870817871	0,349691838	-0,397333929
Experimental	Control	0,917990807	0,457192737	-0,431319046
Experimental	Control	0,92316567	0,526060411	0,311658646
Experimental	Control	0,983491561	0,935645558	0,022861956
Experimental	Control	0,959244846	0,681545245	0,244545225
Experimental	Control	0,772193668	0,115642282	-0,927165136
Experimental	Control	0,772193668	0,135199828	0,472598331
Experimental	Control	0,98145184	0,759472812	-0,069035188
Experimental	Control	0,908003536	0,432526352	-0,613647562
Experimental	Control	0,772193668	0,186881078	-0,756517679
Experimental	Control	0,866291532	0,337485642	0,450524664
Experimental	Control	0,983491561	0,788875886	0,075456389
Experimental	Control	0,772193668	0,124756756	0,232869849
Experimental	Control	0,942203073	0,609040401	-0,327138644
Experimental	Control	0,624192061	0,020059391	1,151767008
Experimental	Control	0,942203073	0,618072722	-0,228342567
Experimental	Control	0,797872703	0,241302767	-0,375568126
Experimental	Control	0,621747057	0,005987037	0,700034009
Experimental	Control	0,983491561	0,780223066	-0,074375026
Experimental	Control	0,92316567	0,521242974	-0,371522194
Experimental	Control	0,983491561	0,947288149	-0,017485935
Experimental	Control	0,937006646	0,568084145	0,293424364
Experimental	Control	0,92316567	0,538862597	-0,308441406
Experimental	Control	0,942203073	0,585685871	0,134069696
Experimental	Control	0,989996372	0,977775524	-0,009175624
Experimental	Control	0,772193668	0,189140119	0,502638039
Experimental	Control	0,858120076	0,316768906	0,402739091
Experimental	Control	0,870817871	0,367429992	0,128768103
Experimental	Control	0,983491561	0,952669674	-0,033770677
Experimental	Control	0,983491561	0,875292158	-0,075052127
Experimental	Control	0,92316567	0,535406146	-0,327646046
Experimental	Control	0,983491561	0,942647934	0,019226494
Experimental	Control	0,866291532	0,338949486	0,593316662
Experimental	Control	0,68521937	0,048898102	-0,928857998

Experimental	Control	0,92316567	0,538039936	-0,290797007
Experimental	Control	0,959244846	0,67552195	0,267129431
Experimental	Control	0,983491561	0,837881229	-0,120687485
Experimental	Control	0,983491561	0,953114283	0,023973229
Experimental	Control	0,941268639	0,575116987	0,18734564
Experimental	Control	0,990728512	0,988782091	-0,008661516
Experimental	Control	0,983491561	0,951876423	-0,021018883
Experimental	Control	0,626448651	0,030955739	-1,033314615
Experimental	Control	0,948299071	0,641874252	0,071516285
Experimental	Control	0,858120076	0,315684179	0,860837183
Experimental	Control	0,983491561	0,916769685	0,024938663
Experimental	Control	0,908003536	0,433486953	0,316402338
Experimental	Control	0,624192061	0,02580597	0,713426735
Experimental	Control	0,919104202	0,483116305	-0,208668745
Experimental	Control	0,754607597	0,092841861	0,633857029
Experimental	Control	0,871454353	0,36981167	-0,554710698
Experimental	Control	0,772193668	0,134326283	-0,834060144
Experimental	Control	0,919104202	0,477899201	0,181019651
Experimental	Control	0,983491561	0,866022618	-0,051909435
Experimental	Control	0,866291532	0,334150078	0,514770001
Experimental	Control	0,919104202	0,489263361	0,242695498
Experimental	Control	0,858120076	0,314437287	0,352972166
Experimental	Control	0,942203073	0,62183084	0,073755296
Experimental	Control	0,885436121	0,407686088	-0,231735724
Experimental	Control	0,919104202	0,479179186	0,194903117
Experimental	Control	0,947375571	0,629102049	-0,093155659
Experimental	Control	0,983491561	0,833973846	-0,102021646
Experimental	Control	0,983491561	0,868079876	0,035856675
Experimental	Control	0,983491561	0,788419119	-0,074487604
Experimental	Control	0,772193668	0,131140577	-0,621669277
Experimental	Control	0,92316567	0,525739397	-0,127992601
Experimental	Control	0,948299071	0,638585026	-0,131796136
Experimental	Control	0,959244846	0,669225259	-0,132659914
Experimental	Control	0,920859684	0,496348792	0,270940399
Experimental	Control	0,98145184	0,767072906	0,089931991
Experimental	Control	0,813614216	0,252104492	0,379141354
Experimental	Control	0,772193668	0,135092081	0,341739539
Experimental	Control	0,915172456	0,453091275	0,216860924
Experimental	Control	0,98145184	0,736655817	-0,137836944
Experimental	Control	0,885436121	0,404722112	-0,370664316
Experimental	Control	0,858120076	0,327063447	-0,527963145
Experimental	Control	0,915172456	0,443929679	0,426896543
Experimental	Control	0,919104202	0,486642784	-0,221382058
Experimental	Control	0,983491561	0,967444709	0,016187073
Experimental	Control	0,942203073	0,581032361	0,175053316
Experimental	Control	0,933825177	0,561276759	0,216995969
Experimental	Control	0,983491561	0,804586581	-0,111594519
Experimental	Control	0,98145184	0,767048279	0,10403229

Experimental	Control	0,983491561	0,87828715	0,054788217
Experimental	Control	0,790420277	0,230327743	0,503124539
Experimental	Control	0,754607597	0,097499462	0,666244418
Experimental	Control	0,870817871	0,358635231	0,295714432
Experimental	Control	0,813614216	0,252370308	0,183163649
Experimental	Control	0,858120076	0,324534173	-0,425763518
Experimental	Control	0,707023515	0,073619344	0,80424213
Experimental	Control	0,98145184	0,748944771	0,159711263
Experimental	Control	0,783663238	0,204768587	-0,47287906
Experimental	Control	0,621747057	0,00804813	0,566205778
Experimental	Control	0,816993398	0,265640687	-0,260007169
Experimental	Control	0,983491561	0,956056598	0,026833081
Experimental	Control	0,942203073	0,611261837	-0,128608954
Experimental	Control	0,942203073	0,590518708	-0,144760213
Experimental	Control	0,948260112	0,631947743	0,162953477
Experimental	Control	0,983491561	0,870427036	-0,023959726
Experimental	Control	0,621747057	0,008550549	0,96685568
Experimental	Control	0,983491561	0,816977097	0,048046572
Experimental	Control	0,772193668	0,155224466	0,557934245
Experimental	Control	0,772193668	0,170466052	-0,590533949
Experimental	Control	0,858120076	0,316019991	0,152784485
Experimental	Control	0,858120076	0,323275145	0,535234424
Experimental	Control	0,772193668	0,179161288	0,914644
Experimental	Control	0,983491561	0,8709438	0,087084365
Experimental	Control	0,870817871	0,354293474	0,281707716
Experimental	Control	0,983491561	0,964436938	-0,025617492
Experimental	Control	0,789234341	0,215107327	0,669550866
Experimental	Control	0,771535076	0,109136592	-0,836409714
Experimental	Control	0,919104202	0,491151951	-0,286715979
Experimental	Control	0,692638815	0,056985507	-1,25272902
Experimental	Control	0,772193668	0,178077656	0,35083638
Experimental	Control	0,92316567	0,540478133	-0,115698674
Experimental	Control	0,983491561	0,926538898	0,046531601
Experimental	Control	0,772193668	0,181351942	1,037996113
Experimental	Control	0,870817871	0,359587516	-0,54104632
Experimental	Control	0,770427777	0,102925518	-0,628094594
Experimental	Control	0,789234341	0,21091824	0,451014671
Experimental	Control	0,953177433	0,656558563	0,159567325
Experimental	Control	0,983491561	0,807882046	-0,174097685
Experimental	Control	0,790420277	0,229753574	0,885187047
Experimental	Control	0,772193668	0,17380504	0,323313226
Experimental	Control	0,983491561	0,900036991	-0,054299421
Experimental	Control	0,942203073	0,609483829	0,130559565
Experimental	Control	0,941268639	0,574247624	-0,303747643
Experimental	Control	0,790420277	0,233086805	-0,378085209
Experimental	Control	0,959244846	0,682213427	0,132371824
Experimental	Control	0,983491561	0,828853879	0,080770363
Experimental	Control	0,933825177	0,562363235	0,214867352

Experimental	Control	0,771535076	0,10697383	-0,440520961
Experimental	Control	0,797872703	0,241818211	0,235276021
Experimental	Control	0,858120076	0,313998655	0,199172339
Experimental	Control	0,676638733	0,040850994	0,938668025
Experimental	Control	0,858120076	0,321659594	0,280426793
Experimental	Control	0,692638815	0,065826966	-0,55574665
Experimental	Control	0,983491561	0,871756079	0,034953594
Experimental	Control	0,983491561	0,963850061	-0,016572217
Experimental	Control	0,98145184	0,757811617	-0,106482726
Experimental	Control	0,704891642	0,071103044	-1,005456143
Experimental	Control	0,92316567	0,508992315	0,119545795
Experimental	Control	0,772193668	0,148074194	0,417564684
Experimental	Control	0,983491561	0,832053331	0,039972353
Experimental	Control	0,92316567	0,53067002	0,169688894
Experimental	Control	0,772193668	0,152474767	0,657983216
Experimental	Control	0,919104202	0,471378447	0,410484477
Experimental	Control	0,983491561	0,776772169	-0,091854386
Experimental	Control	0,885436121	0,388606844	-0,331452676
Experimental	Control	3,83704E-08	7,53838E-11	3,337521835
Experimental	Control	0,98145184	0,738326725	-0,105537658
Experimental	Control	0,772193668	0,116956074	0,413883021
Experimental	Control	0,983491561	0,883964756	0,078042771
Experimental	Control	0,98145184	0,766756707	-0,052655483
Experimental	Control	0,983491561	0,956165485	-0,02281581
Experimental	Control	0,772193668	0,137852727	-0,515530229
Experimental	Control	0,783663238	0,204686881	-0,445568025
Experimental	Control	0,783701121	0,207857861	0,341324462
Experimental	Control	0,983491561	0,782939097	-0,077395544
Experimental	Control	0,885436121	0,388356532	-0,207975915
Experimental	Control	0,942203073	0,617215868	-0,095869388
Experimental	Control	0,983491561	0,939982767	0,019702698
Experimental	Control	0,772193668	0,165719113	-0,498276076
Experimental	Control	0,692638815	0,059485433	0,487264847
Experimental	Control	0,953177433	0,657299173	0,190411031
Experimental	Control	0,983491561	0,894685799	-0,024710224
Experimental	Control	0,870817871	0,348495469	-0,373847482
Experimental	Control	0,624192061	0,021587708	1,294117467
Experimental	Control	0,983491561	0,961107133	0,021743332
Experimental	Control	0,983491561	0,773702822	0,094522918
Experimental	Control	0,983491561	0,946389385	-0,01376267
Experimental	Control	0,959244846	0,680149667	0,07023106
Experimental	Control	0,983491561	0,952855058	-0,042678287
Experimental	Control	0,983491561	0,965336556	0,009602362
Experimental	Control	0,68521937	0,049744862	0,700718444
Experimental	Control	0,754607597	0,096723236	1,097032569
Experimental	Control	0,870817871	0,366956354	-0,444496089
Experimental	Control	0,98145184	0,761758649	0,144540405
Experimental	Control	0,908003536	0,426145434	0,217688256

Experimental	Control	0,983491561	0,798207646	-0,077282201
Experimental	Control	0,942203073	0,608010719	-0,197564385
Experimental	Control	0,858120076	0,297826816	0,296364018
Experimental	Control	0,948299071	0,636043469	-0,260484472
Experimental	Control	0,983491561	0,909284056	-0,061787953
Experimental	Control	0,772193668	0,126221277	-0,590574744
Experimental	Control	0,950766093	0,648942381	-0,174305144
Experimental	Control	0,754607597	0,097846958	0,4650607
Experimental	Control	0,983491561	0,843959769	0,084290376
Experimental	Control	0,947375571	0,628044852	-0,273777781
Experimental	Control	0,915172456	0,440925434	0,123314474
Experimental	Control	0,870817871	0,351063413	0,470746495
Experimental	Control	0,783663238	0,196632408	0,293619921
Experimental	Control	0,964525247	0,693548606	-0,1554113
Experimental	Control	0,948299071	0,644619801	0,247861545
Experimental	Control	0,922188811	0,505482668	0,136751523
Experimental	Control	0,870817871	0,36400146	-0,339807428
Experimental	Control	0,815638754	0,259747165	-0,390352642
Experimental	Control	0,948299071	0,640494412	-0,146732764
Experimental	Control	0,983491561	0,808668934	-0,124688778
Experimental	Control	0,959244846	0,681496982	-0,110651678
Experimental	Control	0,92316567	0,512637859	-0,362727291
Experimental	Control	0,840958856	0,280870345	0,38530731
Experimental	Control	0,919104202	0,478709662	0,118649859
Experimental	Control	0,908003536	0,430389911	0,210189944
Experimental	Control	0,858120076	0,302958472	-0,626755501
Experimental	Control	0,983491561	0,913620992	-0,031468906
Experimental	Control	0,676638733	0,042539174	1,011532219
Experimental	Control	0,917990807	0,459598775	0,343998134
Experimental	Control	0,692638815	0,061949257	-0,942740345
Experimental	Control	0,983491561	0,906691574	0,028549218
Experimental	Control	0,844057035	0,285221631	0,289860998
Experimental	Control	0,927175792	0,548290596	-0,21969721
Experimental	Control	0,772193668	0,167317331	0,5171583
Experimental	Control	0,870817871	0,367830731	-0,223254085
Experimental	Control	0,983491561	0,817246419	0,168826693
Experimental	Control	0,92316567	0,529921355	0,275357118
Experimental	Control	0,98145184	0,720619465	0,152559952
Experimental	Control	0,983491561	0,960886317	0,016153264
Experimental	Control	0,68521937	0,047010338	0,823548007
Experimental	Control	0,964525247	0,690201999	0,199714431
Experimental	Control	0,884905186	0,382473754	-0,276545843
Experimental	Control	0,959244846	0,667419321	-0,178499957
Experimental	Control	0,92316567	0,519890478	0,280066241
Experimental	Control	0,772193668	0,137059911	0,477285148
Experimental	Control	0,858120076	0,325432331	-0,583704881
Experimental	Control	0,98145184	0,734414687	0,119479137
Experimental	Control	0,908003536	0,430250775	0,36106462

Experimental	Control	0,725293596	0,076946668	-0,662984911
Experimental	Control	0,990728512	0,985128955	-0,009811587
Experimental	Control	0,915172456	0,446787843	-0,2779679
Experimental	Control	0,983491561	0,890463097	-0,07297762
Experimental	Control	0,983491561	0,939716835	0,027536257
Experimental	Control	0,789234341	0,212922218	-0,41832082
Experimental	Control	0,772193668	0,161844574	-0,43918077
Experimental	Control	0,983491561	0,894490958	-0,06342979
Experimental	Control	0,983491561	0,79098973	0,07211279
Experimental	Control	0,885436121	0,409116596	0,291515834
Experimental	Control	0,942203073	0,600372267	-0,325295698
Experimental	Control	0,772193668	0,171205498	0,994891629
Experimental	Control	0,983491561	0,806719255	-0,068290329
Experimental	Control	0,783663238	0,204476438	-0,347240281
Experimental	Control	0,942203073	0,608413501	-0,086730715
Experimental	Control	0,754607597	0,097288159	-0,697375011
Experimental	Control	0,772193668	0,147805739	0,368538523
Experimental	Control	0,983491561	0,846739893	-0,033090572
Experimental	Control	0,98145184	0,757269108	-0,089994504
Experimental	Control	0,884905186	0,380754524	0,282085571
Experimental	Control	0,858120076	0,304515989	-0,523398841
Experimental	Control	0,983491561	0,955380092	-0,019002419
Experimental	Control	0,98145184	0,766224152	-0,136993459
Experimental	Control	0,983491561	0,937089758	-0,053019512
Experimental	Control	0,772193668	0,171397245	-0,714508476
Experimental	Control	0,783701121	0,207350818	-0,539722295
Experimental	Control	0,98145184	0,714839395	-0,07017043
Experimental	Control	0,771535076	0,108770612	-0,530178417
Experimental	Control	0,983491561	0,944821038	0,033446669
Experimental	Control	0,942203073	0,584433479	-0,257641026
Experimental	Control	0,866291532	0,3403896	0,143499423
Experimental	Control	0,983491561	0,900440434	-0,037058363
Experimental	Control	0,983491561	0,862326924	-0,078832489
Experimental	Control	0,752251491	0,084240344	-0,63264253
Experimental	Control	0,772193668	0,158708389	0,965212187
Experimental	Control	0,813614216	0,252556083	-0,536220566
Experimental	Control	0,624192061	0,016850549	-0,948461586
Experimental	Control	0,98145184	0,71658101	-0,136766795
Experimental	Control	0,815638754	0,262799127	0,274087825
Experimental	Control	0,983491561	0,932129172	0,019802523
Experimental	Control	0,837851424	0,212896673	0,349489512
Experimental	Control	0,96532044	0,49659351	-0,200418096
Experimental	Control	0,96532044	0,40807026	0,257715905
Experimental	Control	0,96532044	0,807958935	-0,108055193
Experimental	Control	0,96532044	0,609152363	0,448128825
Experimental	Control	0,96532044	0,886903144	-0,076878538
Experimental	Control	0,96532044	0,92470743	0,038953573
Experimental	Control	0,96532044	0,912402162	0,026731792

Experimental	Control	0,71152862	0,12868639	-1,078055492
Experimental	Control	0,96532044	0,601567121	-0,19463906
Experimental	Control	0,96532044	0,685566444	0,170949464
Experimental	Control	0,422845864	0,043500191	-0,834458968
Experimental	Control	0,96532044	0,488525362	-0,427765615
Experimental	Control	0,515135012	0,076003526	0,683526537
Experimental	Control	0,987064451	0,987064451	-0,005999908
Experimental	Control	0,96532044	0,744193058	0,131153536
Experimental	Control	0,96532044	0,816071808	-0,101495062
Experimental	Control	0,96532044	0,355732549	0,371404656
Experimental	Control	0,96532044	0,733596181	0,129663073
Experimental	Control	0,422845864	0,045057346	-0,57431432
Experimental	Control	0,96532044	0,949495515	-0,018373937
Experimental	Control	0,71152862	0,145805045	0,629475845
Experimental	Control	0,96532044	0,74160759	0,061488407
Experimental	Control	0,96532044	0,803856608	-0,075644838
Experimental	Control	0,646772158	0,111329634	-1,248219001
Experimental	Control	0,96532044	0,838512153	0,107014876
Experimental	Control	0,979963996	0,971931504	0,008854499
Experimental	Control	0,96532044	0,600201154	-0,16777924
Experimental	Control	0,96532044	0,903661674	-0,03585748
Experimental	Control	0,96532044	0,305084534	0,387899843
Experimental	Control	0,452915377	0,051973896	-1,220295086
Experimental	Control	0,96532044	0,616844756	-0,151465853
Experimental	Control	0,575644074	0,089649487	0,689011415
Experimental	Control	0,71152862	0,145004106	-0,503145235
Experimental	Control	0,96532044	0,330354166	0,317598327
Experimental	Control	0,302511593	0,017357223	-1,043893598
Experimental	Control	0,96532044	0,833295865	-0,043150531
Experimental	Control	0,96532044	0,310182478	0,360972928
Experimental	Control	0,96532044	0,916094333	-0,060155393
Experimental	Control	0,96532044	0,399948201	0,379472381
Experimental	Control	0,474333398	0,062207659	0,766840254
Experimental	Control	0,96532044	0,535561468	0,205523525
Experimental	Control	0,96532044	0,398425425	-0,418288995
Experimental	Control	0,96532044	0,445789488	0,318208716
Experimental	Control	0,422845864	0,033963617	1,63152558
Experimental	Control	0,96532044	0,811330208	-0,131279629
Experimental	Control	0,837851424	0,207863028	0,878514162
Experimental	Control	0,96532044	0,679203618	-0,192032262
Experimental	Control	0,96532044	0,627699808	0,314299733
Experimental	Control	0,96532044	0,290122495	-0,349279037
Experimental	Control	0,96532044	0,762485353	-0,230892455
Experimental	Control	0,96532044	0,872541462	-0,059811074
Experimental	Control	0,422845864	0,044216415	-0,881109571
Experimental	Control	0,96532044	0,874672434	0,037550589
Experimental	Control	0,96532044	0,697589257	-0,134485931
Experimental	Control	0,96532044	0,594573014	0,176515068

Experimental	Control	0,96532044	0,303209909	-0,830048729
Experimental	Control	0,74932928	0,165835169	-0,604950697
Experimental	Control	0,96532044	0,286345517	-0,690613069
Experimental	Control	0,474333398	0,058331481	-0,925361424
Experimental	Control	0,96532044	0,810134696	-0,08061033
Experimental	Control	0,589019868	0,096560634	0,901072814
Experimental	Control	0,422845864	0,037479849	-1,959956326
Experimental	Control	0,96532044	0,841049399	0,091360625
Experimental	Control	0,96532044	0,430606127	0,338367024
Experimental	Control	0,493819748	0,068810948	0,802082025
Experimental	Control	0,422845864	0,040141587	0,670652526
Experimental	Control	0,265523961	0,012975283	-1,340904821
Experimental	Control	0,96532044	0,722869318	0,144749392
Experimental	Control	0,96532044	0,370294165	-0,352685355
Experimental	Control	0,96532044	0,525260099	0,398453582
Experimental	Control	0,96532044	0,85879498	-0,061987937
Experimental	Control	0,96532044	0,667928329	-0,179425429
Experimental	Control	0,96532044	0,457860953	-0,413949365
Experimental	Control	0,96532044	0,866198889	0,084224682
Experimental	Control	0,96532044	0,729902973	0,183471783
Experimental	Control	0,96532044	0,623317073	0,102926624
Experimental	Control	0,96532044	0,358489688	-0,631664569
Experimental	Control	0,96532044	0,94307462	-0,024579711
Experimental	Control	0,96532044	0,819015459	-0,048572
Experimental	Control	0,96532044	0,874173231	-0,110200997
Experimental	Control	6,06056E-05	4,96767E-07	2,715309904
Experimental	Control	0,96532044	0,942166052	0,023781871
Experimental	Control	0,96532044	0,377617882	0,236137569
Experimental	Control	0,96532044	0,472259829	0,236314157
Experimental	Control	0,265523961	0,011687004	-1,13906127
Experimental	Control	0,96532044	0,46068962	-0,542113819
Experimental	Control	0,265523961	0,013058555	1,665368297
Experimental	Control	0,96532044	0,934225521	0,050845745
Experimental	Control	0,71152862	0,139090955	0,417547947
Experimental	Control	0,96532044	0,718563706	0,153659354
Experimental	Control	0,96532044	0,448759852	0,314973406
Experimental	Control	0,96532044	0,681945039	0,22496009
Experimental	Control	0,96532044	0,885695976	0,058163009
Experimental	Control	0,737236159	0,157115903	0,310178211
Experimental	Control	0,96532044	0,683027818	0,07724492
Experimental	Control	0,96532044	0,569679197	0,376870148
Experimental	Control	0,265523961	0,012020837	1,072137509
Experimental	Control	0,96532044	0,625744047	-0,131802037
Experimental	Control	0,96532044	0,807446897	0,09102732
Experimental	Control	0,96532044	0,623870246	0,340389564
Experimental	Control	0,96532044	0,431350715	0,263445494
Experimental	Control	0,96532044	0,923607826	0,044725339
Experimental	Control	0,832111546	0,19097642	-0,742213858

Experimental	Control	0,96532044	0,505478142	-0,402345627
Experimental	Control	0,96532044	0,878356363	0,08925902
Experimental	Control	0,96532044	0,302010025	-0,45618472
Experimental	Control	0,96532044	0,925890614	0,043427039
Experimental	Control	0,96532044	0,623334075	0,216481469
Experimental	Control	0,96532044	0,936363171	0,05460961
Experimental	Control	0,96532044	0,723409252	-0,189238881
Experimental	Control	0,96532044	0,253979837	-0,803094564
Experimental	Control	0,265523961	0,007434625	-1,092568019
Experimental	Control	0,96532044	0,775669499	-0,123650919
Experimental	Control	0,837851424	0,207093794	-0,625740606
Experimental	Control	0,96532044	0,736681184	-0,152818202
Experimental	Control	0,96532044	0,448661997	-0,19024051
Experimental	Control	0,96532044	0,269360243	-0,533725437
Experimental	Control	0,96532044	0,765981059	-0,226339627
Experimental	Control	0,96532044	0,392916132	0,470878879
Experimental	Control	0,96532044	0,698223762	-0,272615028
Experimental	Control	0,96532044	0,671257296	-0,222247103
Experimental	Control	0,952834963	0,4210201	0,517978923
Experimental	Control	0,97102509	0,640908398	-0,135611802
Experimental	Control	0,97102509	0,588886409	0,475327076
Experimental	Control	0,97102509	0,925911039	0,057493572
Experimental	Control	0,663258616	0,222247585	0,455514093
Experimental	Control	0,97102509	0,800138595	-0,127823363
Experimental	Control	0,785606885	0,293819104	0,431031155
Experimental	Control	0,663258616	0,231369285	-0,490919127
Experimental	Control	0,97102509	0,782997846	0,149043118
Experimental	Control	0,97102509	0,925593179	-0,02851582
Experimental	Control	0,346490135	0,056405371	-1,577481282
Experimental	Control	0,97102509	0,922484117	-0,028084453
Experimental	Control	0,97102509	0,7102276	-0,111598688
Experimental	Control	0,97102509	0,556287471	0,111029994
Experimental	Control	0,663258616	0,229819891	0,491924108
Experimental	Control	0,97102509	0,630383127	-0,167263689
Experimental	Control	0,97102509	0,485443536	0,354901361
Experimental	Control	0,236400034	0,027488376	1,679720798
Experimental	Control	0,236400034	0,023644753	-0,655064015
Experimental	Control	0,97102509	0,903892314	-0,049491316
Experimental	Control	0,97102509	0,915954601	0,064265506
Experimental	Control	0,663258616	0,197353239	-0,546964545
Experimental	Control	0,236400034	0,023344044	-0,537981681
Experimental	Control	0,006098733	0,000141831	2,468454515
Experimental	Control	0,97102509	0,848265592	0,106674612
Experimental	Control	0,637127317	0,162986058	0,391992782
Experimental	Control	0,356058957	0,071741506	0,488061027
Experimental	Control	0,97102509	0,889628942	0,061516549
Experimental	Control	0,97102509	0,714555663	-0,094357283
Experimental	Control	0,356058957	0,074523968	0,91993359

Experimental	Control	0,97102509	0,620738091	-0,18651103
Experimental	Control	0,423151993	0,09840744	-0,949630331
Experimental	Control	0,97102509	0,707973211	0,138248508
Experimental	Control	0,97102509	0,635889376	0,321784763
Experimental	Control	0,97102509	0,458940138	-0,291486224
Experimental	Control	0,97102509	0,948443111	-0,035529956
Experimental	Control	0,904032312	0,378432131	-0,61033553
Experimental	Control	0,173411762	0,008065663	-1,076310493
Experimental	Control	0,97102509	0,507810434	-0,273465932
Experimental	Control	0,997364336	0,997364336	-0,001250055
Experimental	Control	0,97102509	0,724213636	0,144907495
Experimental	Control	0,785606885	0,310588769	-0,494373927
Experimental	Control	0,265397478	0,037032206	1,15331987
Experimental	Control	0,778339522	0,497824217	0,214454185
Experimental	Control	0,849753109	0,849753109	0,037329965
Experimental	Control	0,778339522	0,639454394	0,182360259
Experimental	Control	0,511665502	0,146190144	-0,291837586
Experimental	Control	0,511665502	0,07456208	-0,698071582
Experimental	Control	0,778339522	0,546969156	0,260615961
Experimental	Control	0,778339522	0,667148161	0,329560518
Experimental	Control	0,057956521	0,00277627	1,409240892
Experimental	Control	0,057956521	0,001723478	1,360625359
Experimental	Control	0,174985748	0,016787442	-2,164062143
Experimental	Control	0,572808567	0,196480329	-0,692041181
Experimental	Control	0,298114484	0,040705471	-1,43464469
Experimental	Control	0,121663398	0,009170409	1,309127913
Experimental	Control	0,993927207	0,993541535	0,004157195
Experimental	Control	0,837515875	0,507122219	0,393118921
Experimental	Control	0,63768051	0,239861476	0,518818034
Experimental	Control	0,838863437	0,514092455	-0,353792888
Experimental	Control	0,785579079	0,423780274	-0,462001282
Experimental	Control	0,993927207	0,981400445	0,008741022
Experimental	Control	0,90534009	0,70046368	0,293183748
Experimental	Control	0,863880358	0,579132729	-0,297055894
Experimental	Control	0,451865161	0,114417233	-1,11763514
Experimental	Control	0,477641458	0,122965507	1,07303249
Experimental	Control	0,529467057	0,157382868	-0,924699446
Experimental	Control	0,554948252	0,180010257	-1,063529539
Experimental	Control	0,20593209	0,021177752	1,123220272
Experimental	Control	0,837079944	0,499176113	0,359489587
Experimental	Control	0,041440413	0,000716497	1,879034643
Experimental	Control	0,648729218	0,252620998	-0,612573936
Experimental	Control	0,121663398	0,010045602	1,189902925
Experimental	Control	0,522163094	0,148017151	0,949331418
Experimental	Control	0,763168505	0,393086236	-0,318167496
Experimental	Control	0,732971373	0,326811089	-0,711739074
Experimental	Control	0,958500764	0,810447485	0,145467416
Experimental	Control	0,863880358	0,597159975	-0,335391771

Experimental	Control	0,897159618	0,662499826	-0,364549265
Experimental	Control	0,993927207	0,978559633	0,015088858
Experimental	Control	0,814252818	0,459805857	-0,546207015
Experimental	Control	0,733532311	0,328909194	0,473044016
Experimental	Control	0,712792386	0,305577245	-0,779006957
Experimental	Control	0,329988443	0,053057768	-1,286204091
Experimental	Control	0,572808567	0,196541655	0,681412228
Experimental	Control	0,298114484	0,042118927	0,856972949
Experimental	Control	0,218578096	0,024865765	-1,154828828
Experimental	Control	0,988656375	0,932364052	-0,065900788
Experimental	Control	0,057956521	0,003206616	-2,008626889
Experimental	Control	0,395419923	0,085985857	-1,363184708
Experimental	Control	0,742113995	0,340460929	-0,719824576
Experimental	Control	0,218578096	0,02463803	-1,451013654
Experimental	Control	0,703604491	0,290870921	-0,602076089
Experimental	Control	0,930764102	0,751979761	-0,086070276
Experimental	Control	0,517912006	0,1438521	-0,750878376
Experimental	Control	0,395419923	0,085684664	-0,604498297
Experimental	Control	0,851026926	0,529522269	-0,367242853
Experimental	Control	0,969496774	0,852734738	0,117835716
Experimental	Control	0,90221013	0,683154272	-0,117736888
Experimental	Control	0,686739223	0,280996049	-0,718603923
Experimental	Control	0,969496774	0,857461312	0,088651359
Experimental	Control	0,766140345	0,405655059	0,282598344
Experimental	Control	0,513480967	0,13783206	0,953033805
Experimental	Control	0,318433538	0,046742538	-1,144257294
Experimental	Control	0,863880358	0,594408589	0,25798567
Experimental	Control	0,993927207	0,976662425	-0,019199163
Experimental	Control	0,712792386	0,304404178	-0,567854575
Experimental	Control	0,90221013	0,688659475	0,288427163
Experimental	Control	0,81933177	0,470552007	-0,122827841
Experimental	Control	0,90221013	0,68384018	-0,214443549
Experimental	Control	0,766015405	0,400576863	-0,678681983
Experimental	Control	0,657888881	0,263155552	0,585448134
Experimental	Control	0,416183377	0,101245842	-0,693158789
Experimental	Control	0,508976901	0,135274311	-1,191915417
Experimental	Control	0,72873926	0,323586974	-0,71169923
Experimental	Control	0,758652695	0,365656176	0,283076334
Experimental	Control	0,869288498	0,61248951	0,288741164
Experimental	Control	0,135705582	0,012201052	-1,320566313
Experimental	Control	0,057956521	0,00274956	1,404289594
Experimental	Control	0,758652695	0,369243997	-0,480442833
Experimental	Control	0,536258408	0,165305344	-0,751915256
Experimental	Control	0,394821831	0,084035472	-0,795104834
Experimental	Control	0,875638058	0,622960301	-0,230672119
Experimental	Control	0,778142834	0,418242502	-0,293091487
Experimental	Control	0,532043431	0,161737742	-0,748542966
Experimental	Control	0,559060106	0,185021148	-0,475092993

Experimental	Control	0,298114484	0,041634908	-0,693460221
Experimental	Control	0,387976049	0,073679369	1,209814363
Experimental	Control	0,942023842	0,786319763	0,129055632
Experimental	Control	0,63768051	0,238736573	-0,444718483
Experimental	Control	0,942023842	0,770935791	0,18051778
Experimental	Control	0,442655649	0,111273071	-1,435741082
Experimental	Control	0,885956934	0,643741185	0,164788385
Experimental	Control	0,576705782	0,201053392	-0,741117139
Experimental	Control	0,919846846	0,739253062	0,336252977
Experimental	Control	0,993927207	0,958461995	0,034094925
Experimental	Control	0,416183377	0,09359265	0,477974185
Experimental	Control	0,789179454	0,428618566	0,264759867
Experimental	Control	0,930764102	0,753150402	0,149865708
Experimental	Control	0,863880358	0,593455776	-0,232222901
Experimental	Control	0,960150973	0,828678941	0,116148763
Experimental	Control	0,298114484	0,039251984	1,604383353
Experimental	Control	0,887212907	0,646281696	0,194838396
Experimental	Control	0,416183377	0,097412475	-1,050995479
Experimental	Control	0,121663398	0,009952367	-2,061357576
Experimental	Control	0,041440413	0,000369879	3,405684521
Experimental	Control	0,645797995	0,247654644	0,841003041
Experimental	Control	0,958734741	0,819762182	-0,120805051
Experimental	Control	0,90221013	0,685639185	-0,295480684
Experimental	Control	0,057956521	0,003138734	-1,05558398
Experimental	Control	0,988656375	0,932420875	-0,055559733
Experimental	Control	0,969496774	0,891058195	-0,058250788
Experimental	Control	0,837186636	0,502836169	-0,284904043
Experimental	Control	0,330471664	0,058211522	1,234481732
Experimental	Control	0,554948252	0,175793699	-0,684361893
Experimental	Control	0,90221013	0,687110126	0,272013561
Experimental	Control	0,837186636	0,503952366	-0,172250026
Experimental	Control	0,969496774	0,901898833	-0,054218652
Experimental	Control	0,899664766	0,66690746	-0,239711233
Experimental	Control	0,758652695	0,365734961	-0,417775755
Experimental	Control	0,841882069	0,517487143	0,301976959
Experimental	Control	0,703604491	0,292117388	-0,527990644
Experimental	Control	0,958734741	0,813456514	0,135466774
Experimental	Control	0,969496774	0,852867887	-0,049783986
Experimental	Control	0,330471664	0,056581936	-0,842701625
Experimental	Control	0,942023842	0,769781837	0,094527701
Experimental	Control	0,057956521	0,002231549	-1,518243565
Experimental	Control	0,969496774	0,892302333	-0,035763466
Experimental	Control	0,734874952	0,331705024	-0,206529161
Experimental	Control	0,758652695	0,381991762	0,267322948
Experimental	Control	0,552296958	0,172556182	0,494153094
Experimental	Control	0,942023842	0,782817466	-0,143344928
Experimental	Control	0,416183377	0,100969006	-1,705925126
Experimental	Control	0,879402206	0,631941216	-0,178567779

Experimental	Control	0,298114484	0,041827054	-1,318262172
Experimental	Control	0,90884232	0,708116177	-0,097574813
Experimental	Control	0,411214661	0,091021432	-1,118201065
Experimental	Control	0,057956521	0,001816162	-1,505524996
Experimental	Control	0,993927207	0,993927207	0,003129775
Experimental	Control	0,814457542	0,46177501	0,356684294
Experimental	Control	0,993927207	0,975535197	-0,018033668
Experimental	Control	0,973992691	0,911442701	-0,021101069
Experimental	Control	0,176805734	0,017528928	-0,677257476
Experimental	Control	0,857987221	0,54473702	-0,454952818
Experimental	Control	0,532043431	0,162499087	-0,765848978
Experimental	Control	0,62370955	0,226595396	-0,47203351
Experimental	Control	0,969496774	0,895015082	-0,0590556
Experimental	Control	0,121663398	0,00983903	-1,159721717
Experimental	Control	0,543317864	0,168478383	-0,696422702
Experimental	Control	0,961029126	0,83406748	0,138757103
Experimental	Control	0,969496774	0,867627061	0,066098255
Experimental	Control	0,820490176	0,475746704	0,27202335
Experimental	Control	0,060227846	0,003646824	-2,933455668
Experimental	Control	0,069458163	0,00433317	-1,201832392
Experimental	Control	0,712792386	0,308335928	-0,404429126
Experimental	Control	0,911914311	0,716317382	0,199665922
Experimental	Control	0,960150973	0,822783141	-0,112735358
Experimental	Control	0,525664202	0,15046535	0,550783272
Experimental	Control	0,041440413	0,000356456	1,126372037
Experimental	Control	0,837186636	0,505384226	-0,369482146
Experimental	Control	0,969496774	0,88309258	-0,074283957
Experimental	Control	0,712792386	0,309646185	0,838784551
Experimental	Control	0,969496774	0,862612666	0,051788868
Experimental	Control	0,709413773	0,299153906	-0,507219307
Experimental	Control	0,529545898	0,159349591	1,121396015
Experimental	Control	0,837186636	0,505366923	-0,132958238
Experimental	Control	0,814252818	0,460001849	-0,298376126
Experimental	Control	0,041440413	0,000865905	-2,315818212
Experimental	Control	0,969496774	0,871594469	-0,103780063
Experimental	Control	0,712792386	0,305808234	-0,515441529
Experimental	Control	0,529467057	0,156781726	0,662774745
Experimental	Control	0,742113995	0,343978927	-0,562370373
Experimental	Control	0,057956521	0,001687061	3,574125838
Experimental	Control	0,576326596	0,199863719	-0,381093287
Experimental	Control	0,792267412	0,436110502	-0,587856775
Experimental	Control	0,636069717	0,236501305	0,660184897
Experimental	Control	0,814252818	0,460164896	-0,171815241
Experimental	Control	0,825110389	0,482954521	0,298472143
Experimental	Control	0,969496774	0,873476595	0,043048306
Experimental	Control	0,942023842	0,774684595	0,100184384
Experimental	Control	0,969496774	0,856414555	-0,129119311
Experimental	Control	0,927785783	0,747335704	0,142388885

Experimental	Control	0,742113995	0,339273539	0,474473193
Experimental	Control	0,057956521	0,003296609	1,154401908
Experimental	Control	0,440573542	0,109941288	-1,082423146
Experimental	Control	0,899774764	0,670290925	-0,142889054
Experimental	Control	0,477641458	0,124449701	-0,513532578
Experimental	Control	0,526624713	0,153379965	-0,956426571
Experimental	Control	0,559060106	0,185669503	-0,39429847
Experimental	Control	0,554948252	0,178027283	-0,512100041
Experimental	Control	0,712792386	0,309966597	-0,601932217
Experimental	Control	0,471613712	0,12028313	0,603229971
Experimental	Control	0,91467373	0,729621834	0,152637526
Experimental	Control	0,11915024	0,008744972	-1,497790685
Experimental	Control	0,581552158	0,203810022	-0,736938121
Experimental	Control	0,877481963	0,626312814	-0,162365024
Experimental	Control	0,421631689	0,103667241	-0,428519565
Experimental	Control	0,416183377	0,101151286	-0,417141069
Experimental	Control	0,829986385	0,489525781	-0,307896269
Experimental	Control	0,958734741	0,818626248	-0,065894547
Experimental	Control	0,829986385	0,491900188	-0,175706826
Experimental	Control	0,969496774	0,884095109	-0,03657998
Experimental	Control	0,650951978	0,255603162	-0,52777699
Experimental	Control	0,877687664	0,628070072	0,238348609
Experimental	Control	0,81933177	0,466215576	0,193478338
Experimental	Control	0,328429023	0,048812387	-1,07494274
Experimental	Control	0,969496774	0,867783125	0,105536586
Experimental	Control	0,961029126	0,833891418	0,045239429
Experimental	Control	0,911914311	0,717338129	-0,090522747
Experimental	Control	0,969496774	0,846170383	0,123059708
Experimental	Control	0,863880358	0,560941231	-0,128945595
Experimental	Control	0,758652695	0,360986924	0,176602093
Experimental	Control	0,757619695	0,353092482	0,676922084
Experimental	Control	0,477641458	0,123608035	-0,715297798
Experimental	Control	0,851026926	0,530915881	-0,469983899
Experimental	Control	0,941568699	0,765348502	-0,059421083
Experimental	Control	0,559060106	0,184579389	-0,468394176
Experimental	Control	0,993927207	0,993585443	-0,002938766
Experimental	Control	0,703604491	0,290653101	0,485098345
Experimental	Control	0,057956521	0,002573829	-1,440167136
Experimental	Control	0,863880358	0,585662634	0,248238525
Experimental	Control	0,993927207	0,955047654	-0,036212213
Experimental	Control	0,969496774	0,898763613	0,07521152
Experimental	Control	0,90221013	0,681729536	-0,159381885
Experimental	Control	0,857260587	0,541096591	-0,397705867
Experimental	Control	0,969496774	0,87310114	0,06956082
Experimental	Control	0,090375866	0,006301437	0,862490613
Experimental	Control	0,71998115	0,314633675	-0,707978792
Experimental	Control	0,057956521	0,002899642	1,090829871
Experimental	Control	0,71998115	0,316618535	-0,402464111

Experimental	Control	0,59276098	0,208825886	-0,893150764
Experimental	Control	0,863880358	0,596155137	-0,360594627
Experimental	Control	0,81933177	0,468930074	-0,134873137
Experimental	Control	0,958734741	0,819136597	0,099527739
Experimental	Control	0,833767336	0,495670857	-0,147917622
Experimental	Control	0,90884232	0,70873025	0,227731002
Experimental	Control	0,90534009	0,702676804	-0,06476201
Experimental	Control	0,041440413	0,000457632	-1,404465071
Experimental	Control	0,758652695	0,356420389	0,387350991
Experimental	Control	0,394135539	0,082198577	-0,750164693
Experimental	Control	0,919665864	0,735732691	0,089028784
Experimental	Control	0,942023842	0,786043969	0,052169756
Experimental	Control	0,742113995	0,344504295	-0,394503716
Experimental	Control	0,758652695	0,375226667	-0,271639583
Experimental	Control	0,703604491	0,29435197	0,746670703
Experimental	Control	0,863880358	0,596573814	0,180247219
Experimental	Control	0,041440413	0,000912449	1,140773624
Experimental	Control	0,763168505	0,393157459	0,234139191
Experimental	Control	0,508976901	0,135415873	-0,89186557
Experimental	Control	0,993927207	0,991490511	0,002838145
Experimental	Control	0,209215047	0,022265088	0,806629622
Experimental	Control	0,329988443	0,051729413	0,501796919
Experimental	Control	0,758652695	0,369189661	-0,389780016
Experimental	Control	0,672382709	0,271420543	0,707339265
Experimental	Control	0,526624713	0,153639136	-0,905101363
Experimental	Control	0,742113995	0,343890613	-0,164497343
Experimental	Control	0,90221013	0,675969498	-0,241719996
Experimental	Control	0,95347105	0,803015068	0,117227024
Experimental	Control	0,238560017	0,028014387	-1,054027487
Experimental	Control	0,041440413	0,000433613	1,738116156
Experimental	Control	0,517912006	0,14539548	-0,538425583
Experimental	Control	0,960150973	0,825702998	0,164858273
Experimental	Control	0,394135539	0,080004738	-1,028511823
Experimental	Control	0,829986385	0,490179556	-0,296529692
Experimental	Control	0,863880358	0,583221251	-0,315996237
Experimental	Control	0,627709367	0,230352061	-0,618483194
Experimental	Control	0,394135539	0,08209431	0,560516798
Experimental	Control	0,763168505	0,39177822	-0,326267568
Experimental	Control	0,919846846	0,738727056	-0,095577097
Experimental	Control	0,758652695	0,378871259	-0,540568442
Experimental	Control	0,993927207	0,974245585	-0,016759047
Experimental	Control	0,646503812	0,250297806	-0,527940717
Experimental	Control	0,95347105	0,800069718	-0,213627919
Experimental	Control	0,517912006	0,142413701	-0,595668071
Experimental	Control	0,942023842	0,78595891	-0,082463227
Experimental	Control	0,394135539	0,083166215	-0,73534555
Experimental	Control	0,174985748	0,017016963	-1,545261543
Experimental	Control	0,969496774	0,881436834	-0,074942855

Experimental	Control	0,993927207	0,957946006	0,013770779
Experimental	Control	0,554948252	0,1802309	-0,955533523
Experimental	Control	0,863880358	0,589419442	0,174743609
Experimental	Control	0,897159618	0,663404268	0,159257169
Experimental	Control	0,394135539	0,079889467	-1,367501389
Experimental	Control	0,758652695	0,368756883	0,48307547
Experimental	Control	0,330471664	0,05744981	0,358542776
Experimental	Control	0,330471664	0,057612678	0,953254382
Experimental	Control	0,057956521	0,003283607	-1,268231233
Experimental	Control	0,851026926	0,530827213	0,20440384
Experimental	Control	0,71998115	0,318376985	0,636916149
Experimental	Control	0,90221013	0,68472078	-0,131867158
Experimental	Control	0,99096641	0,9364178	-0,018536227
Experimental	Control	0,562434821	0,190873014	0,607051416
Experimental	Control	0,660164871	0,26527726	0,430515833
Experimental	Control	0,657777016	0,259601516	-0,487330421
Experimental	Control	0,709413773	0,299385629	0,369780816
Experimental	Control	0,913007919	0,725380603	-0,091903101
Experimental	Control	0,993927207	0,947818715	0,015912061
Experimental	Control	0,712792386	0,308656696	-0,541097636
Experimental	Control	0,758652695	0,380616726	0,216315146
Experimental	Control	0,758652695	0,377683283	-0,487034347
Experimental	Control	0,673660499	0,273172422	-0,538978872
Experimental	Control	0,359410474	0,065287407	-0,589905285
Experimental	Control	0,973937655	0,907817117	0,030906974
Experimental	Control	0,387976049	0,074035797	0,895554987
Experimental	Control	0,416183377	0,100418336	1,094192809
Experimental	Control	0,871121383	0,615379325	0,070300578
Experimental	Control	0,394135539	0,077914969	-0,869677546
Experimental	Control	0,758652695	0,378085091	0,567443844
Experimental	Control	0,802180208	0,445982758	0,129503084
Experimental	Control	0,766140345	0,402690221	-0,478446491
Experimental	Control	0,911914311	0,717047834	0,081884168
Experimental	Control	0,869288498	0,611402106	-0,323673072
Experimental	Control	0,763168505	0,39142591	-0,387257636
Experimental	Control	0,764864297	0,397168066	-0,39301373
Experimental	Control	0,992179212	0,939384355	-0,027547608
Experimental	Control	0,882164852	0,636129884	-0,270254578
Experimental	Control	0,960150973	0,82808111	0,122243108
Experimental	Control	0,057956521	0,00226993	-1,097169867
Experimental	Control	0,858224921	0,54800417	0,287645291
Experimental	Control	0,626836806	0,228881696	0,275908446
Experimental	Control	0,856198414	0,537284143	0,36179934
Experimental	Control	0,911914311	0,719492025	-0,152319373
Experimental	Control	0,863880358	0,575380325	-0,27154978
Experimental	Control	0,489434128	0,128420331	0,422618222
Experimental	Control	0,863880358	0,567930771	-0,13899169
Experimental	Control	0,641870357	0,244970705	0,226792864

Experimental	Control	0,758652695	0,369128414	0,802649015
Experimental	Control	0,394135539	0,080874128	-0,520013721
Experimental	Control	0,329988443	0,053139078	0,972336743
Experimental	Control	0,992904123	0,941892535	0,026188336
Experimental	Control	0,904608978	0,692150355	-0,12228657
Experimental	Control	0,969496774	0,865388006	-0,061366219
Experimental	Control	0,529545898	0,159344986	0,895163567
Experimental	Control	0,394135539	0,082645227	-0,937741015
Experimental	Control	0,291305327	0,035906109	0,604479271
Experimental	Control	0,648729218	0,253540043	0,306423034
Experimental	Control	0,988656375	0,929320655	0,014384052
Experimental	Control	0,646335809	0,249046825	0,509242515
Experimental	Control	0,371551164	0,069538016	0,80201795
Experimental	Control	0,802180208	0,44541743	0,277207922
Experimental	Control	0,301277473	0,043118611	-0,796693802
Experimental	Control	0,073387073	0,004982242	0,986401335
Experimental	Control	0,993927207	0,967737571	0,011909951
Experimental	Control	0,416183377	0,101350899	0,403377226
Experimental	Control	0,993927207	0,979844605	0,006580072
Experimental	Control	0,135705582	0,012003224	0,670151422
Experimental	Control	0,329988443	0,055686583	0,90833285
Experimental	Control	0,758652695	0,359797636	-0,231016268
Experimental	Control	0,041440413	0,000805418	-1,47880337
Experimental	Control	0,993927207	0,94553179	0,01342205
Experimental	Control	0,875638058	0,621459957	0,093749006
Experimental	Control	0,758652695	0,372395031	0,278032355
Experimental	Control	0,20593209	0,021537852	-1,10803067
Experimental	Control	0,857260587	0,54096619	0,326644515
Experimental	Control	0,993927207	0,988685188	-0,004083179
Experimental	Control	0,792267412	0,434214889	-0,170351625
Experimental	Control	0,073387073	0,004972265	0,763146138
Experimental	Control	0,806123861	0,450507483	0,143755424
Experimental	Control	0,522163094	0,1485051	-0,475872807
Experimental	Control	0,291305327	0,036346353	0,962709906
Experimental	Control	0,942023842	0,783763549	-0,121550745
Experimental	Control	0,357558091	0,06363878	-0,730367934
Experimental	Control	0,758652695	0,359140841	-0,409553291
Experimental	Control	0,942023842	0,78167014	-0,06215276
Experimental	Control	0,517912006	0,141827093	-0,558414866
Experimental	Control	0,057956521	0,003059205	-1,151908667
Experimental	Control	0,562434821	0,190918242	0,279570784
Experimental	Control	0,763168505	0,39488719	-0,320491592
Experimental	Control	0,395419923	0,086339396	-0,749690035
Experimental	Control	0,517912006	0,143629835	0,478459796
Experimental	Control	0,863880358	0,561833856	0,224430571
Experimental	Control	0,883362092	0,638614063	-0,194967173
Experimental	Control	0,394135539	0,076754066	0,800577844
Experimental	Control	0,217858521	0,023584684	0,742855992

Experimental	Control	0,91467373	0,730060683	-0,129910254
Experimental	Control	0,703604491	0,293132733	0,195221699
Experimental	Control	0,96779014	0,841711057	-0,095500728
Experimental	Control	0,993927207	0,956905756	0,024948507
Experimental	Control	0,865761837	0,605239009	-0,284172341
Experimental	Control	0,942023842	0,781039785	-0,131379147
Experimental	Control	0,951958206	0,796500811	0,044567692
Experimental	Control	0,517912006	0,144879453	-0,394082777
Experimental	Control	0,616834884	0,22070239	-0,696293858
Experimental	Control	0,62370955	0,225833029	-0,346181772
Experimental	Control	0,778142834	0,417346932	-0,301184582
Experimental	Control	0,857987221	0,545646945	0,253441892
Experimental	Control	0,618092173	0,22228636	-0,318588253
Experimental	Control	0,251610475	0,030008589	0,983555979
Experimental	Control	0,969496774	0,901812611	0,021045389
Experimental	Control	0,59713352	0,211461962	-0,303237561
Experimental	Control	0,361430551	0,066980708	-0,780676142
Experimental	Control	0,863880358	0,599168395	-0,305540329
Experimental	Control	0,806123861	0,451133537	0,11606966
Experimental	Control	0,969496774	0,876084473	-0,094606285
Experimental	Control	0,640631523	0,242146961	-0,777629962
Experimental	Control	0,057956521	0,002630449	-1,804349138
Experimental	Control	0,149288225	0,013696167	-0,844124068
Experimental	Control	0,969496774	0,889582616	-0,077326963
Experimental	Control	0,958500764	0,810768536	0,15982695
Experimental	Control	0,863880358	0,599066565	-0,21182927
Experimental	Control	0,993927207	0,960004036	-0,020706423
Experimental	Control	0,969496774	0,886005608	0,050853188
Experimental	Control	0,42651097	0,105649506	-1,07691684
Experimental	Control	0,969496774	0,895327018	-0,043902795
Experimental	Control	0,329988443	0,0534157	-0,495035586
Experimental	Control	0,90534009	0,701438061	0,161290946
Experimental	Control	0,329988443	0,053200537	0,542895408
Experimental	Control	0,091936922	0,006578972	-2,178288457
Experimental	Control	0,763168505	0,390665849	-0,515792817
Experimental	Control	0,605930629	0,215689068	-0,527566798
Experimental	Control	0,685118193	0,279075668	0,411839713
Experimental	Control	0,969496774	0,866318028	0,072415199
Experimental	Control	0,765222281	0,398758033	0,617102461
Experimental	Control	0,071676165	0,004603056	-2,280856213
Experimental	Control	0,993927207	0,972915566	-0,005242106
Experimental	Control	0,416183377	0,098021765	0,838356828
Experimental	Control	0,900027447	0,672130589	-0,210508784
Experimental	Control	0,90534009	0,698814311	0,152125406
Experimental	Control	0,985587547	0,924101351	0,040665106
Experimental	Control	0,298114484	0,040990387	0,321487547
Experimental	Control	0,913007919	0,724452271	-0,149668087
Experimental	Control	0,416183377	0,09821208	0,768983715

Experimental	Control	0,885956934	0,643578419	0,218032318
Experimental	Control	0,562434821	0,190266728	-0,488445988
Experimental	Control	0,937032606	0,75994204	-0,129136576
Experimental	Control	0,863880358	0,593073071	0,146759673
Experimental	Control	0,837515875	0,508656431	-0,187903289
Experimental	Control	0,778142834	0,417016881	0,197766067
Experimental	Control	0,060227846	0,003613154	-1,079112424
Experimental	Control	0,857987221	0,546278102	-0,160313898
Experimental	Control	0,847421357	0,52400183	-0,314040574
Experimental	Control	0,758652695	0,366976037	0,184681062
Experimental	Control	0,820490176	0,478520728	0,184609266
Experimental	Control	0,802180208	0,443347837	0,259998826
Experimental	Control	0,742113995	0,339238433	-0,254717621
Experimental	Control	0,829986385	0,49085432	-0,142298483
Experimental	Control	0,552296958	0,173289504	-0,399591369
Experimental	Control	0,758652695	0,377886045	0,201612291
Experimental	Control	0,942023842	0,781237829	-0,109386463
Experimental	Control	0,763168505	0,393829776	0,487761841
Experimental	Control	0,411214661	0,0912972	-0,581213554
Experimental	Control	0,969496774	0,899942511	0,049570985
Experimental	Control	3,59396E-12	6,59443E-15	4,227754641
Experimental	Control	0,856198414	0,536614446	0,137070415
Experimental	Control	0,329988443	0,05565878	-0,459743525
Experimental	Control	0,057956521	0,001907853	-1,707414196
Experimental	Control	0,27381969	0,033159816	-0,515309243
Experimental	Control	0,863131153	0,552720683	0,210807986
Experimental	Control	0,121663398	0,009915013	-0,833040339
Experimental	Control	0,298114484	0,041079409	-1,05948377
Experimental	Control	0,863880358	0,56492289	0,165507909
Experimental	Control	0,766140345	0,403865934	-0,194736449
Experimental	Control	0,960150973	0,82978185	-0,035998382
Experimental	Control	0,863880358	0,564924599	0,109670613
Experimental	Control	0,329988443	0,054467466	-0,662037942
Experimental	Control	0,993927207	0,984517415	-0,006417194
Experimental	Control	0,993927207	0,97611221	0,008719391
Experimental	Control	0,636069717	0,236921381	-0,492472402
Experimental	Control	0,71998115	0,31798719	-0,190774666
Experimental	Control	0,416183377	0,101564017	-0,811468127
Experimental	Control	0,041440413	0,000231231	2,555015507
Experimental	Control	0,628864508	0,231929846	-0,624555616
Experimental	Control	0,758652695	0,378245781	0,35409256
Experimental	Control	0,847421357	0,523342166	0,09520371
Experimental	Control	0,863880358	0,577031976	-0,114874004
Experimental	Control	0,973992691	0,910516407	0,076627966
Experimental	Control	0,792267412	0,434804893	0,297631341
Experimental	Control	0,298114484	0,040070811	-0,79413301
Experimental	Control	0,176805734	0,01784278	-1,327440907
Experimental	Control	0,969496774	0,879947261	0,061728945

Experimental	Control	0,041440413	0,000839544	1,633410469
Experimental	Control	0,394135539	0,082634205	0,358271674
Experimental	Control	0,899774764	0,669152361	-0,16746037
Experimental	Control	0,969496774	0,861453044	0,067051875
Experimental	Control	0,913007919	0,723336388	-0,080811681
Experimental	Control	0,742113995	0,33672254	-0,641359327
Experimental	Control	0,81933177	0,469131742	0,232748279
Experimental	Control	0,863880358	0,572080644	0,218956543
Experimental	Control	0,865761837	0,602986688	-0,193007295
Experimental	Control	0,358800439	0,064518244	0,557129863
Experimental	Control	0,993927207	0,964916751	0,019002793
Experimental	Control	0,837835519	0,511849615	0,382752935
Experimental	Control	0,911914311	0,718465743	0,081940272
Experimental	Control	0,820490176	0,478744727	-0,347256821
Experimental	Control	0,969496774	0,870730983	0,050813755
Experimental	Control	0,050038837	0,001193587	-1,360435856
Experimental	Control	0,657777016	0,261685064	0,567855644
Experimental	Control	0,879402206	0,632524156	-0,168985813
Experimental	Control	0,329988443	0,055704471	-1,56073299
Experimental	Control	0,298114484	0,038419037	0,575299461
Experimental	Control	0,863880358	0,579284779	0,324593239
Experimental	Control	0,863880358	0,597265492	-0,221715272
Experimental	Control	0,863880358	0,589425872	0,141572412
Experimental	Control	0,416183377	0,096521354	0,312840349
Experimental	Control	0,758652695	0,382806406	-0,286955518
Experimental	Control	0,865761837	0,60435316	-0,343219936
Experimental	Control	0,90534009	0,697932142	0,122041237
Experimental	Control	0,969496774	0,898107026	0,063504444
Experimental	Control	0,789179454	0,428303824	0,388525736
Experimental	Control	0,863880358	0,559572263	-0,276937326
Experimental	Control	0,993927207	0,993471741	0,002267596
Experimental	Control	0,875638058	0,62339003	0,206490338
Experimental	Control	0,863880358	0,560674558	-0,151762122
Experimental	Control	0,318433538	0,046358347	0,836708865
Experimental	Control	0,129384111	0,010920494	-0,934782771
Experimental	Control	0,057956521	0,002109983	-2,640146094
Experimental	Control	0,891576681	0,652732286	0,201269057
Experimental	Control	0,576326596	0,199637299	-0,287258122
Experimental	Control	0,733532311	0,329753057	0,449694536
Experimental	Control	0,993927207	0,950588416	0,036082435
Experimental	Control	0,837835519	0,511925189	0,298752887
Experimental	Control	0,554948252	0,180066008	0,370616037
Experimental	Control	0,820490176	0,478740282	0,349249704
Experimental	Control	0,993927207	0,972508165	0,015554453
Experimental	Control	0,360356586	0,066120474	-0,618618216
Experimental	Control	0,863880358	0,59118755	-0,287474971
Experimental	Control	0,657777016	0,261903876	0,377297662
Experimental	Control	0,562434821	0,188866061	0,71242243

Experimental	Control	0,869288498	0,611172158	-0,174369167
Experimental	Control	0,218578096	0,024636061	1,447317213
Experimental	Control	0,041440413	0,000898039	-1,694568424
Experimental	Control	0,416183377	0,10141254	0,963475065
Experimental	Control	0,863880358	0,583079365	-0,24487346
Experimental	Control	0,893774272	0,655981117	-0,163901836
Experimental	Control	0,329988443	0,054987807	-0,686245417
Experimental	Control	0,057956521	0,003155281	1,305724749
Experimental	Control	0,64075595	0,243369691	0,361333897
Experimental	Control	0,556427534	0,181732295	0,609385675
Experimental	Control	0,942023842	0,786460272	-0,167700522
Experimental	Control	0,792267412	0,432610568	-0,514897134
Experimental	Control	0,863880358	0,567944065	0,155392668
Experimental	Control	0,958734741	0,817730823	0,08766029
Experimental	Control	0,969496774	0,870400608	-0,042096533
Experimental	Control	0,526624713	0,152131141	0,417263123
Experimental	Control	0,90221013	0,680990584	-0,108774493
Experimental	Control	0,863880358	0,598414746	0,244165821
Experimental	Control	0,130507735	0,011254796	-1,281330531
Experimental	Control	0,820490176	0,474609147	-0,269072286
Experimental	Control	0,220412032	0,025478822	-0,974716618
Experimental	Control	0,554948252	0,179687231	0,884541104
Experimental	Control	0,993927207	0,958011702	-0,03051296
Experimental	Control	0,157501496	0,014738672	-1,045996845
Experimental	Control	0,329988443	0,052419388	-0,673192608
Experimental	Control	0,993927207	0,988905515	0,006175341
Experimental	Control	0,969496774	0,861884879	0,064274896
Experimental	Control	0,887720145	0,648280033	0,076618457
Experimental	Control	0,513480967	0,138498536	-0,474139748
Experimental	Control	0,766140345	0,406265247	-0,388049079
Experimental	Control	0,394135539	0,082994376	-0,790709573
Experimental	Control	0,778142834	0,41834101	-0,609737043
Experimental	Control	0,90534009	0,696656462	0,207523297
Experimental	Control	0,897159618	0,661450823	-0,171606852
Experimental	Control	0,529467057	0,155582831	0,594481596
Experimental	Control	0,95347105	0,801813353	-0,083812625
Experimental	Control	0,532043431	0,163029822	-0,258690152
Experimental	Control	0,9597389	0,879088572	0,054210444
Experimental	Control	0,614328094	0,314907678	-0,28387826
Experimental	Control	0,936720063	0,826517703	-0,059300073
Experimental	Control	0,935024688	0,800779925	-0,075227273
Experimental	Control	0,709691399	0,43481533	0,602445742
Experimental	Control	0,455706215	0,179984807	0,76490625
Experimental	Control	0,058168303	0,003799158	-1,084259567
Experimental	Control	0,576843266	0,269389241	-0,223416704
Experimental	Control	0,496231003	0,216580025	-1,120612197
Experimental	Control	0,255131401	0,040735266	0,859578412
Experimental	Control	0,602303154	0,298620892	0,488378607

Experimental	Control	0,496231003	0,220491515	0,342902385
Experimental	Control	0,940655603	0,85082932	-0,080851935
Experimental	Control	0,790012353	0,544301518	0,450312563
Experimental	Control	0,162071329	0,017705271	0,760579139
Experimental	Control	0,067181439	0,006210049	-1,043842558
Experimental	Control	0,131981702	0,013309079	1,039253064
Experimental	Control	0,935024688	0,755411491	0,186276327
Experimental	Control	0,058168303	0,004399283	1,32136302
Experimental	Control	0,964945899	0,901501205	-0,055225139
Experimental	Control	0,421673968	0,131108713	-0,426410347
Experimental	Control	0,806518916	0,586016904	0,154417277
Experimental	Control	0,214321828	0,025214333	1,154439938
Experimental	Control	0,935024688	0,789244042	-0,059003431
Experimental	Control	0,935024688	0,76437656	-0,082950984
Experimental	Control	0,058168303	0,004034873	-2,885316466
Experimental	Control	0,062602198	0,005260689	1,796618084
Experimental	Control	0,535966654	0,243211759	0,475946133
Experimental	Control	0,451974304	0,15968099	-0,439435331
Experimental	Control	0,455706215	0,175451246	0,539504536
Experimental	Control	0,975869894	0,958711902	0,026891495
Experimental	Control	0,022618616	0,000570217	-2,587146473
Experimental	Control	0,685444734	0,403202785	-0,297839326
Experimental	Control	0,496231003	0,221010447	0,580140834
Experimental	Control	0,790012353	0,535620207	0,28651851
Experimental	Control	0,576843266	0,280632971	-0,413752837
Experimental	Control	0,806518916	0,58963988	-0,13298099
Experimental	Control	0,790012353	0,564294538	-0,250886616
Experimental	Control	0,996742863	0,996742863	-0,002540864
Experimental	Control	0,301035244	0,060747701	0,977463541
Experimental	Control	0,269622363	0,047580417	0,542975363
Experimental	Control	0,255131401	0,040042045	-1,1776085
Experimental	Control	0,449601171	0,147348283	0,599589774
Experimental	Control	0,451974304	0,163318446	-1,005906051
Experimental	Control	0,646895742	0,348661133	0,558168784
Experimental	Control	0,576843266	0,274763187	-0,766702712
Experimental	Control	0,935024688	0,786373217	0,121542196
Experimental	Control	0,935024688	0,800616653	0,162514938
Experimental	Control	0,646895742	0,36525893	-0,782249667
Experimental	Control	0,964945899	0,924401954	-0,041835481
Experimental	Control	0,255131401	0,039600785	-0,807976653
Experimental	Control	0,646895742	0,344608684	0,24050954
Experimental	Control	0,790012353	0,54091339	0,201426458
Experimental	Control	0,463899761	0,187119231	0,453760038
Experimental	Control	0,258501644	0,043445654	-1,387210991
Experimental	Control	0,845432624	0,6465073	-0,263358181
Experimental	Control	0,613636191	0,309396399	-0,75927612
Experimental	Control	0,811961081	0,600441808	-0,241832672
Experimental	Control	0,421673968	0,123304619	-0,550930322

Experimental	Control	0,326198004	0,076752471	-0,804098922
Experimental	Control	0,835257961	0,624688727	-0,530133448
Experimental	Control	0,841681174	0,636565594	0,215463407
Experimental	Control	0,767365703	0,487066099	0,393769995
Experimental	Control	0,940655603	0,853704244	0,083387985
Experimental	Control	0,964945899	0,898074919	-0,039196552
Experimental	Control	0,790012353	0,552134219	-0,323836727
Experimental	Control	0,255131401	0,033645867	0,792401604
Experimental	Control	0,391397583	0,10524977	-0,788062669
Experimental	Control	0,935024688	0,809307083	-0,166617817
Experimental	Control	0,905600813	0,707738451	0,144893255
Experimental	Control	0,614355622	0,320084441	0,395343405
Experimental	Control	0,996742863	0,995981383	-0,002181289
Experimental	Control	0,767365703	0,496530749	0,355351153
Experimental	Control	0,326198004	0,075664712	0,912179085
Experimental	Control	0,646895742	0,36965471	-0,260755641
Experimental	Control	0,301035244	0,063198904	-1,249162074
Experimental	Control	0,774049739	0,507360333	-0,25552278
Experimental	Control	0,935024688	0,805798221	0,173971368
Experimental	Control	1,23994E-08	1,04197E-10	3,468783118
Experimental	Control	0,936720063	0,825093446	0,06054355
Experimental	Control	0,301120976	0,065791138	0,392982631
Experimental	Control	0,32829181	0,082762641	0,830664751
Experimental	Control	0,470886195	0,193894316	-0,623259667
Experimental	Control	0,451974304	0,15628875	-0,930099419
Experimental	Control	0,935024688	0,761628767	0,215757979
Experimental	Control	0,935024688	0,783281853	0,169438328
Experimental	Control	0,359854499	0,093743609	-0,444971488
Experimental	Control	0,451974304	0,161521133	0,72173189
Experimental	Control	0,421673968	0,130364187	1,017707499
Experimental	Control	0,671568526	0,389396876	0,493778902
Experimental	Control	0,455706215	0,17679361	0,631361489
Experimental	Control	0,32829181	0,082476648	0,515512206
Experimental	Control	0,418818992	0,116143082	0,463984928
Experimental	Control	0,646895742	0,360680143	0,514374271
Experimental	Control	0,027765826	0,000933305	1,490986969
Experimental	Control	0,767365703	0,486399713	-0,25489357
Experimental	Control	0,936720168	0,834389393	-0,08774261
Experimental	Control	0,301035244	0,063242698	-0,995171386
Experimental	Control	0,255131401	0,034406868	-1,014411935
Experimental	Control	0,964945899	0,912513167	0,047627269
Experimental	Control	0,646895742	0,367463927	-0,529857068
Experimental	Control	0,767365703	0,490545847	-0,460544037
Experimental	Control	0,058168303	0,003069601	-1,980566092
Experimental	Control	0,873958874	0,675665684	-0,221568553
Experimental	Control	0,709691399	0,435356908	0,343468856
Experimental	Control	0,576843266	0,281150499	-0,461713139
Experimental	Control	0,485330744	0,203920481	0,919252915

Experimental	Control	0,301035244	0,061104244	-1,2132611
Experimental	Control	0,790012353	0,552396679	-0,409749127
Experimental	Control	0,42500133	0,13571471	-0,801877213
Experimental	Control	8,72317E-05	1,46608E-06	-2,271885172
Experimental	Control	0,455706215	0,176942637	-0,889588394
Experimental	Control	0,421673968	0,127730681	-0,627825975
Experimental	Control	0,790012353	0,561147805	-0,143835515
Experimental	Control	0,975869894	0,959468719	0,025252619
Experimental	Control	0,964945899	0,91970892	-0,071708476
Experimental	Control	0,058168303	0,00409947	-1,774535673
Experimental	Control	0,975869894	0,949425271	-0,04099464
Experimental	Control	0,703607686	0,419799544	0,439177869
Experimental	Control	0,509529902	0,248840185	0,674975165
Experimental	Control	0,759620943	0,662668517	-0,116422108
Experimental	Control	0,544978752	0,304174187	0,792557364
Experimental	Control	0,810464525	0,79131204	0,177019722
Experimental	Control	0,0123664	0,000948574	1,288095584
Experimental	Control	0,56621753	0,381867637	0,522373364
Experimental	Control	0,0123664	0,001150363	1,480319736
Experimental	Control	0,283914554	0,092437297	-0,757900133
Experimental	Control	0,023252391	0,002703766	1,971331687
Experimental	Control	0,661239618	0,552792511	-0,18317185
Experimental	Control	0,154900604	0,032421057	-1,991482062
Experimental	Control	0,241893146	0,062291347	0,720079448
Experimental	Control	0,775876751	0,71072682	-0,130169829
Experimental	Control	0,661239618	0,553595959	0,122931246
Experimental	Control	0,509529902	0,248159868	0,543001013
Experimental	Control	0,661239618	0,493981594	0,193647286
Experimental	Control	0,561186449	0,328855049	-0,4767009
Experimental	Control	0,509529902	0,234967891	-0,842395473
Experimental	Control	0,661239618	0,531644936	-0,206965297
Experimental	Control	0,661239618	0,549128881	0,235845173
Experimental	Control	0,561186449	0,339322039	0,584993139
Experimental	Control	0,922337234	0,922337234	-0,054112911
Experimental	Control	0,775876751	0,721745815	-0,080545734
Experimental	Control	8,86826E-06	2,06239E-07	3,435820515
Experimental	Control	0,043275009	0,006038373	-1,768411835
Experimental	Control	0,648261476	0,467351297	0,252176438
Experimental	Control	0,543790166	0,290864507	-0,27311871
Experimental	Control	0,068057752	0,012661907	1,31371884
Experimental	Control	0,582912633	0,406683233	0,210786067
Experimental	Control	0,416555	0,164684535	-0,603651678
Experimental	Control	0,060183506	0,009797315	1,031861002
Experimental	Control	0,56621753	0,380367591	-0,443138962
Experimental	Control	0,810464525	0,791616513	0,10942684
Experimental	Control	0,241893146	0,067505064	-0,952495429
Experimental	Control	0,489307476	0,204826385	-0,603281597
Experimental	Control	0,321631169	0,112196919	0,954368128

Experimental	Control	0,375099133	0,13957177	-0,977673295
Experimental	Control	0,529853819	0,271088	-0,575791502
Experimental	Control	0,000452955	2,10677E-05	-1,811285044
Experimental	Control	0,56621753	0,368305451	-0,340796255
Experimental	Control	0,267042863	0,080733889	0,84844189
Experimental	Control	0,759620943	0,671292926	0,213277336
Experimental	Control	0,165549574	0,038499901	-1,094729479
Experimental	Control	0,010680887	0,001525841	0,989648936
Experimental	Control	0,554084369	0,465381647	-0,15357193
Experimental	Control	0,73757631	0,73757631	0,105731962
Experimental	Control	0,07952014	0,02272004	-0,49981832
Experimental	Control	0,554084369	0,474929459	-0,340584798
Experimental	Control	0,158812762	0,068062612	-0,711485921
Experimental	Control	0,554084369	0,43767182	0,524525191

Standard error	Sample size	N alternative grou	N reference group	Degrees of freedom
0,467046619	142	70	72	139
0,491735245	142	70	72	139
0,908630377	142	70	72	139
0,321846281	142	70	72	139
0,454057063	142	70	72	139
0,497647978	142	70	72	139
0,434788985	142	70	72	139
0,66612106	142	70	72	139
0,44185375	142	70	72	139
0,556295958	142	70	72	139
0,685991241	142	70	72	139
0,416919232	142	70	72	139
0,747819636	142	70	72	139
0,536823064	142	70	72	139
0,718650886	142	70	72	139
0,569231322	142	70	72	139
0,458654967	142	70	72	139
0,813529669	142	70	72	139
0,540871451	142	70	72	139
0,33764527	142	70	72	139
0,52347921	142	70	72	139
0,538057688	142	70	72	139
0,458883595	142	70	72	139
0,633127759	142	70	72	139
0,312087981	142	70	72	139
0,659842891	142	70	72	139
0,490174794	142	70	72	139
0,678291259	142	70	72	139
0,786906877	142	70	72	139
0,600215352	142	70	72	139
0,570985495	142	70	72	139
0,4294963	142	70	72	139
0,97016173	142	70	72	139
0,677014648	142	70	72	139
0,546611272	142	70	72	139
0,468920033	142	70	72	139
0,479844225	142	70	72	139
0,898536607	142	70	72	139
0,487765302	142	70	72	139
0,680925361	142	70	72	139
0,748061344	142	70	72	139
0,610899907	142	70	72	139
0,525337056	142	70	72	139
0,386175937	142	70	72	139
0,642649202	142	70	72	139
0,320888939	142	70	72	139
0,467475128	142	70	72	139

0,45664017	142	70	72	139
0,575079882	142	70	72	139
0,445360718	142	70	72	139
0,262018543	142	70	72	139
0,503416803	142	70	72	139
0,715021438	142	70	72	139
0,519367861	142	70	72	139
0,698647608	142	70	72	139
0,446974634	142	70	72	139
0,514963139	142	70	72	139
0,267038348	142	70	72	139
0,59019389	142	70	72	139
0,658066552	142	70	72	139
0,483631128	142	70	72	139
0,458109016	142	70	72	139
0,647133019	142	70	72	139
0,606756145	142	70	72	139
0,648553123	142	70	72	139
0,651727684	142	70	72	139
0,424197315	142	70	72	139
0,533642425	142	70	72	139
0,456983239	142	70	72	139
0,608507108	142	70	72	139
0,359970874	142	70	72	139
0,34471433	142	70	72	139
0,641881637	142	70	72	139
0,20940842	142	70	72	139
0,300990552	142	70	72	139
0,408413328	142	70	72	139
0,779045	142	70	72	139
0,562519574	142	70	72	139
0,508934416	142	70	72	139
0,77658145	142	70	72	139
0,371455883	142	70	72	139
0,626767353	142	70	72	139
0,341904512	142	70	72	139
1,016722114	142	70	72	139
0,663816065	142	70	72	139
0,379221525	142	70	72	139
0,346512911	142	70	72	139
0,516890015	142	70	72	139
0,397292571	142	70	72	139
0,490266063	142	70	72	139
0,687413143	142	70	72	139
0,509588825	142	70	72	139
0,667948563	142	70	72	139
0,725815759	142	70	72	139
1,101479736	142	70	72	139

0,754823355	142	70	72	139
0,466175104	142	70	72	139
1,014683236	142	70	72	139
0,429372756	142	70	72	139
0,761326023	142	70	72	139
0,554635758	142	70	72	139
0,593604592	142	70	72	139
0,522509549	142	70	72	139
0,427198722	142	70	72	139
0,311977739	142	70	72	139
0,418275712	142	70	72	139
0,345705428	142	70	72	139
0,280766054	142	70	72	139
0,384040973	142	70	72	139
0,435461013	142	70	72	139
0,435828654	142	70	72	139
0,532185895	142	70	72	139
0,373573117	142	70	72	139
0,524314712	142	70	72	139
0,531541794	142	70	72	139
0,416740945	142	70	72	139
0,353542005	142	70	72	139
0,357451127	142	70	72	139
0,382774761	142	70	72	139
0,768976886	142	70	72	139
0,451616514	142	70	72	139
0,48076742	142	70	72	139
0,287088688	142	70	72	139
0,741613143	142	70	72	139
0,509658147	142	70	72	139
0,510331268	142	70	72	139
0,723947237	142	70	72	139
0,662041055	142	70	72	139
0,488323293	142	70	72	139
0,714741818	142	70	72	139
0,583558396	142	70	72	139
0,456916179	142	70	72	139
0,555345962	142	70	72	139
0,468811956	142	70	72	139
0,659292	142	70	72	139
0,436570195	142	70	72	139
0,339083666	142	70	72	139
0,881358852	142	70	72	139
0,479799897	142	70	72	139
0,275309594	142	70	72	139
0,502814549	142	70	72	139
0,503259525	142	70	72	139
0,372364173	142	70	72	139

0,346590741	142	70	72	139
0,581965529	142	70	72	139
0,416071375	142	70	72	139
0,703581407	142	70	72	139
0,343279054	142	70	72	139
0,503855881	142	70	72	139
0,644256911	142	70	72	139
0,20182308	142	70	72	139
0,495152551	142	70	72	139
0,542796814	142	70	72	139
0,522092244	142	70	72	139
0,67896633	142	70	72	139
0,343258409	142	70	72	139
0,552154958	142	70	72	139
0,610690973	142	70	72	139
0,989322259	142	70	72	139
0,813555514	142	70	72	139
0,67603848	142	70	72	139
0,152512897	142	70	72	139
0,375310733	142	70	72	139
0,249778779	142	70	72	139
0,489610196	142	70	72	139
0,698886123	142	70	72	139
0,551864507	142	70	72	139
0,47403595	142	70	72	139
0,422144495	142	70	72	139
0,626466906	142	70	72	139
0,291209099	142	70	72	139
0,682806308	142	70	72	139
0,347028688	142	70	72	139
0,244891132	142	70	72	139
0,527568087	142	70	72	139
0,437736958	142	70	72	139
0,504562117	142	70	72	139
0,500870383	142	70	72	139
0,442873766	142	70	72	139
0,427963636	142	70	72	139
0,338528254	142	70	72	139
0,304594196	142	70	72	139
0,425795826	142	70	72	139
0,346303637	142	70	72	139
0,290081398	142	70	72	139
0,30813892	142	70	72	139
0,481680164	142	70	72	139
0,524686506	142	70	72	139
0,209240342	142	70	72	139
0,568379591	142	70	72	139
0,320380759	142	70	72	139

0,231274448	142	70	72	139
0,288047413	142	70	72	139
0,590453153	142	70	72	139
0,173158277	142	70	72	139
0,18189129	142	70	72	139
0,770617661	142	70	72	139
0,402227548	142	70	72	139
0,577064051	142	70	72	139
0,138895314	142	70	72	139
0,489378021	142	70	72	139
0,337362407	142	70	72	139
0,450985475	142	70	72	139
0,48759975	142	70	72	139
0,489778641	142	70	72	139
0,481796107	142	70	72	139
0,783203233	142	70	72	139
0,292206924	142	70	72	139
0,717423941	142	70	72	139
0,376331549	142	70	72	139
0,735965027	142	70	72	139
0,456860924	142	70	72	139
0,432461143	142	70	72	139
0,73386957	142	70	72	139
0,566832471	142	70	72	139
0,281068536	142	70	72	139
0,335922425	142	70	72	139
0,231400901	142	70	72	139
0,460735701	142	70	72	139
0,135660307	142	70	72	139
0,29794428	142	70	72	139
0,520953136	142	70	72	139
0,504482848	142	70	72	139
0,224880544	142	70	72	139
0,454015084	142	70	72	139
0,28796946	142	70	72	139
0,656167603	142	70	72	139
0,252480684	142	70	72	139
0,435951918	142	70	72	139
0,325025791	142	70	72	139
0,543577645	142	70	72	139
0,199175094	142	70	72	139
0,240262601	142	70	72	139
0,359162838	142	70	72	139
0,175634653	142	70	72	139
0,238726974	142	70	72	139
0,608179744	142	70	72	139
0,71116789	142	70	72	139
0,269010566	142	70	72	139

0,409810828	142	70	72	139
0,397804576	142	70	72	139
0,557727465	142	70	72	139
0,278950331	142	70	72	139
0,448583164	142	70	72	139
0,407823572	142	70	72	139
0,190941457	142	70	72	139
0,812435886	142	70	72	139
0,350537744	142	70	72	139
0,558146712	142	70	72	139
0,387774653	142	70	72	139
0,312883249	142	70	72	139
0,494384708	142	70	72	139
0,366768814	142	70	72	139
0,343696628	142	70	72	139
0,377163811	142	70	72	139
0,500976651	142	70	72	139
0,547733892	142	70	72	139
0,265032479	142	70	72	139
0,907381022	142	70	72	139
0,387964256	142	70	72	139
0,3675115	142	70	72	139
0,191998447	142	70	72	139
0,381896861	142	70	72	139
0,610935056	142	70	72	139
0,489403845	142	70	72	139
0,283634716	142	70	72	139
0,598717776	142	70	72	139
0,275826933	142	70	72	139
0,67155467	142	70	72	139
0,356491545	142	70	72	139
0,273087846	142	70	72	139
0,785809971	142	70	72	139
0,563063851	142	70	72	139
0,552209084	142	70	72	139
0,301269878	142	70	72	139
0,70506869	142	70	72	139
0,260600283	142	70	72	139
0,652168148	142	70	72	139
0,560266356	142	70	72	139
0,348669754	142	70	72	139
0,327061765	142	70	72	139
0,216920591	142	70	72	139
0,639747741	142	70	72	139
0,348127158	142	70	72	139
0,506815943	142	70	72	139
0,433772862	142	70	72	139
0,307620673	142	70	72	139

0,326497796	142	70	72	139
0,453920922	142	70	72	139
0,621465999	142	70	72	139
0,165399	142	70	72	139
0,360018733	142	70	72	139
0,166034703	142	70	72	139
0,587923436	142	70	72	139
0,681335965	142	70	72	139
0,250217476	142	70	72	139
0,318855294	142	70	72	139
0,623284285	142	70	72	139
0,561833104	142	70	72	139
0,600259351	142	70	72	139
0,28701637	142	70	72	139
0,637187106	142	70	72	139
0,607492282	142	70	72	139
0,498104189	142	70	72	139
0,230903181	142	70	72	139
0,63824795	142	70	72	139
0,380367343	142	70	72	139
0,204709501	142	70	72	139
0,13620204	142	70	72	139
0,522288677	142	70	72	139
0,193917782	142	70	72	139
0,854928317	142	70	72	139
0,321169236	142	70	72	139
0,426415303	142	70	72	139
0,41960933	142	70	72	139
0,432543535	142	70	72	139
0,431439119	142	70	72	139
0,608496865	142	70	72	139
0,568316901	142	70	72	139
0,361479407	142	70	72	139
0,281571643	142	70	72	139
0,549085149	142	70	72	139
0,43601393	142	70	72	139
0,324112364	142	70	72	139
0,420760521	142	70	72	139
0,31335782	142	70	72	139
0,274283502	142	70	72	139
0,202974548	142	70	72	139
0,291699614	142	70	72	139
0,491880778	142	70	72	139
0,155561545	142	70	72	139
0,207409417	142	70	72	139
0,41644819	142	70	72	139
0,226888516	142	70	72	139
0,248967082	142	70	72	139

0,303069733	142	70	72	139
0,52369144	142	70	72	139
0,534888206	142	70	72	139
0,406382213	142	70	72	139
0,337848689	142	70	72	139
0,242210513	142	70	72	139
0,150042201	142	70	72	139
0,237999073	142	70	72	139
0,439823709	142	70	72	139
0,35596377	142	70	72	139
0,425539567	142	70	72	139
0,393966213	142	70	72	139
0,292081476	142	70	72	139
0,397176702	142	70	72	139
0,397517699	142	70	72	139
0,253033958	142	70	72	139
0,400840454	142	70	72	139
0,503473359	142	70	72	139
0,219105557	142	70	72	139
0,504539535	142	70	72	139
0,357636857	142	70	72	139
0,49515334	142	70	72	139
0,376013318	142	70	72	139
0,442543018	142	70	72	139
0,203136669	142	70	72	139
0,493492989	142	70	72	139
0,351224047	142	70	72	139
0,566224312	142	70	72	139
0,440773411	142	70	72	139
0,243017186	142	70	72	139
0,213163082	142	70	72	139
0,574019956	142	70	72	139
0,278189578	142	70	72	139
0,277535398	142	70	72	139
0,411330338	142	70	72	139
0,172222445	142	70	72	139
0,404533747	142	70	72	139
0,204637197	142	70	72	139
0,263329492	142	70	72	139
0,260514859	142	70	72	139
0,379835808	142	70	72	139
0,474872631	142	70	72	139
0,237193285	142	70	72	139
0,213387615	142	70	72	139
0,189915581	142	70	72	139
0,564303132	142	70	72	139
0,560467097	142	70	72	139
0,487954404	142	70	72	139

0,397254931	142	70	72	139
0,570341983	142	70	72	139
0,505872058	142	70	72	139
0,49888219	142	70	72	139
0,489657721	142	70	72	139
0,273960537	142	70	72	139
0,707888856	142	70	72	139
0,279401032	142	70	72	139
0,152327123	142	70	72	139
0,418934001	142	70	72	139
0,206285082	142	70	72	139
0,836267255	142	70	72	139
0,638959472	142	70	72	139
0,397862354	142	70	72	139
0,282058211	142	70	72	139
0,436816746	142	70	72	139
0,640715393	142	70	72	139
0,727703947	142	70	72	139
0,369362661	142	70	72	139
0,512764892	142	70	72	139
0,311850524	142	70	72	139
0,653155279	142	70	72	139
0,378346819	142	70	72	139
0,41349225	142	70	72	139
0,153605109	142	70	72	139
0,440153417	142	70	72	139
0,355314238	142	70	72	139
0,260097938	142	70	72	139
0,124854149	142	70	72	139
0,272464742	142	70	72	139
0,532124973	142	70	72	139
0,281943503	142	70	72	139
0,238709123	142	70	72	139
0,253003196	142	70	72	139
0,403895516	142	70	72	139
0,389450187	142	70	72	139
0,58538946	142	70	72	139
0,230821021	142	70	72	139
0,403462554	142	70	72	139
0,249168852	142	70	72	139
0,267798709	142	70	72	139
0,378316445	142	70	72	139
0,242266422	142	70	72	139
0,423653978	142	70	72	139
0,606482793	142	70	72	139
0,343170016	142	70	72	139
0,462630804	142	70	72	139
0,319677351	142	70	72	139

0,209172179	142	70	72	139
0,567416792	142	70	72	139
0,198402822	142	70	72	139
0,376134411	142	70	72	139
0,289738655	142	70	72	139
0,384499196	142	70	72	139
0,322787641	142	70	72	139
0,401296854	142	70	72	139
0,278611048	142	70	72	139
0,191731389	142	70	72	139
0,246315323	142	70	72	139
0,182915933	142	70	72	139
0,429124341	142	70	72	139
0,19841828	142	70	72	139
0,30344529	142	70	72	139
0,381605073	142	70	72	139
0,300478995	142	70	72	139
0,413367713	142	70	72	139
0,192469171	142	70	72	139
0,510122217	142	70	72	139
0,534331253	142	70	72	139
0,154232021	142	70	72	139
0,566666299	142	70	72	139
0,419552255	142	70	72	139
0,225789416	142	70	72	139
0,273158703	142	70	72	139
0,64616914	142	70	72	139
0,415670056	142	70	72	139
0,309323136	142	70	72	139
0,507269942	142	70	72	139
0,562136252	142	70	72	139
0,531338437	142	70	72	139
0,413641523	142	70	72	139
0,314174231	142	70	72	139
0,349257919	142	70	72	139
0,444468101	142	70	72	139
0,387454549	142	70	72	139
0,665982601	142	70	72	139
0,381333944	142	70	72	139
0,465539115	142	70	72	139
0,34186952	142	70	72	139
0,451303034	142	70	72	139
0,324852307	142	70	72	139
0,222004477	142	70	72	139
0,542199122	142	70	72	139
0,2264636	142	70	72	139
0,445494793	142	70	72	139
0,391024551	142	70	72	139

0,414248562	142	70	72	139
0,466367161	142	70	72	139
0,382288909	142	70	72	139
0,32686745	142	70	72	139
0,243562331	142	70	72	139
0,47794379	142	70	72	139
0,257083355	142	70	72	139
0,539935413	142	70	72	139
0,335991192	142	70	72	139
0,235643392	142	70	72	139
0,247138879	142	70	72	139
0,372337992	142	70	72	139
0,483018705	142	70	72	139
0,429062194	142	70	72	139
0,598954124	142	70	72	139
0,425911189	142	70	72	139
0,493629845	142	70	72	139
0,32754849	142	70	72	139
0,320723779	142	70	72	139
0,437093944	142	70	72	139
0,401598679	142	70	72	139
0,285458877	142	70	72	139
0,746022981	142	70	72	139
0,479794221	142	70	72	139
0,275142714	142	70	72	139
0,473291094	142	70	72	139
0,40177644	142	70	72	139
0,47510975	142	70	72	139
0,487966259	142	70	72	139
0,433395495	142	70	72	139
0,549108527	142	70	72	139
0,325528874	142	70	72	139
0,592351111	142	70	72	139
0,414936777	142	70	72	139
0,466756062	142	70	72	139
0,331961789	142	70	72	139
0,51144995	142	70	72	139
0,385833206	142	70	72	139
0,569356133	142	70	72	139
0,441869522	142	70	72	139
0,409189138	142	70	72	139
0,388439799	142	70	72	139
0,452668491	142	70	72	139
0,310630991	142	70	72	139
0,413907892	142	70	72	139
0,671889062	142	70	72	139
0,566159596	142	70	72	139
0,333742116	142	70	72	139

0,252437039	142	70	72	139
0,211016661	142	70	72	139
0,502508496	142	70	72	139
0,262894319	142	70	72	139
0,309351946	142	70	72	139
0,292268067	142	70	72	139
0,386340044	142	70	72	139
0,496602642	142	70	72	139
0,482380256	142	70	72	139
0,365176323	142	70	72	139
0,696563418	142	70	72	139
0,497305295	142	70	72	139
0,425086263	142	70	72	139
0,199829844	142	70	72	139
0,390950899	142	70	72	139
0,519963262	142	70	72	139
0,398523701	142	70	72	139
0,258225081	142	70	72	139
0,318967307	142	70	72	139
0,386570459	142	70	72	139
0,375610036	142	70	72	139
0,551462447	142	70	72	139
0,549290566	142	70	72	139
0,420291887	142	70	72	139
0,490100298	142	70	72	139
0,298813854	142	70	72	139
0,295994275	142	70	72	139
0,289175818	142	70	72	139
0,24425963	142	70	72	139
0,287186744	142	70	72	139
0,366549021	142	70	72	139
0,852576028	142	70	72	139
0,567638707	142	70	72	139
0,500741363	142	70	72	139
0,30021387	142	70	72	139
0,813815873	142	70	72	139
0,434253702	142	70	72	139
0,466436459	142	70	72	139
0,28830061	142	70	72	139
0,406824446	142	70	72	139
0,667464766	142	70	72	139
0,36186885	142	70	72	139
0,311454218	142	70	72	139
0,402444716	142	70	72	139
0,399766524	142	70	72	139
0,5048123	142	70	72	139
0,400988558	142	70	72	139
0,467121971	142	70	72	139

0,267330921	142	70	72	139
0,383925841	142	70	72	139
0,463243455	142	70	72	139
0,277904346	142	70	72	139
0,292248602	142	70	72	139
0,886212499	142	70	72	139
0,531088532	142	70	72	139
0,21802614	142	70	72	139
0,295200384	142	70	72	139
0,190991907	142	70	72	139
0,291474381	142	70	72	139
0,540874438	142	70	72	139
0,197298037	142	70	72	139
0,666982269	142	70	72	139
0,342023631	142	70	72	139
0,535664808	142	70	72	139
0,377358156	142	70	72	139
0,382988398	142	70	72	139
0,361408669	142	70	72	139
0,178450115	142	70	72	139
0,446780284	142	70	72	139
0,516449587	142	70	72	139
0,552799951	142	70	72	139
0,376906285	142	70	72	139
0,566282179	142	70	72	139
0,50243182	142	70	72	139
0,710547303	142	70	72	139
0,518594112	142	70	72	139
0,566505153	142	70	72	139
0,486582672	142	70	72	139
0,643718805	142	70	72	139
0,326413142	142	70	72	139
0,768156869	142	70	72	139
0,412870594	142	70	72	139
0,409999103	142	70	72	139
0,257259018	142	70	72	139
0,380593531	142	70	72	139
0,350427541	142	70	72	139
0,443546032	142	70	72	139
0,471633696	142	70	72	139
0,956733489	142	70	72	139
0,480884875	142	70	72	139
0,358760723	142	70	72	139
0,539514564	142	70	72	139
1,056715321	142	70	72	139
0,488720605	142	70	72	139
0,43474588	142	70	72	139
0,34748116	142	70	72	139

0,35961412	142	70	72	139
0,569948639	142	70	72	139
0,429441465	142	70	72	139
0,509577975	142	70	72	139
0,566707503	142	70	72	139
0,428232449	142	70	72	139
0,34261488	142	70	72	139
0,53631249	142	70	72	139
0,505703039	142	70	72	139
0,5699516	142	70	72	139
0,338904793	142	70	72	139
0,761276695	142	70	72	139
0,483517968	142	70	72	139
0,212619812	142	70	72	139
0,882530175	142	70	72	139
0,564201988	142	70	72	139
0,340312791	142	70	72	139
0,307557434	142	70	72	139
0,417611478	142	70	72	139
0,505127462	142	70	72	139
0,684323921	142	70	72	139
0,753967269	142	70	72	139
0,662845144	142	70	72	139
0,232979169	142	70	72	139
0,531865036	142	70	72	139
0,700486805	142	70	72	139
0,604879555	142	70	72	139
0,371448789	142	70	72	139
0,383222536	142	70	72	139
0,307181357	142	70	72	139
0,206591237	142	70	72	139
0,706150156	142	70	72	139
0,423822507	142	70	72	139
0,270487862	142	70	72	139
0,434240699	142	70	72	139
0,63950542	142	70	72	139
0,563779125	142	70	72	139
0,513018407	142	70	72	139
0,599531018	142	70	72	139
0,601688856	142	70	72	139
0,510082063	142	70	72	139
0,489541334	142	70	72	139
0,41180587	142	70	72	139
0,409482257	142	70	72	139
0,777148857	142	70	72	139
0,505482686	142	70	72	139
0,646020709	142	70	72	139
0,424081819	142	70	72	139

0,388936207	142	70	72	139
0,472001396	142	70	72	139
0,404851967	142	70	72	139
0,248989207	142	70	72	139
0,553290767	142	70	72	139
0,691273837	142	70	72	139
0,501408056	142	70	72	139
0,757966055	142	70	72	139
0,555833788	142	70	72	139
0,712949293	142	70	72	139
0,232045691	142	70	72	139
0,845851841	142	70	72	139
0,589797675	142	70	72	139
0,380060503	142	70	72	139
0,511724046	142	70	72	139
0,393066937	142	70	72	139
0,399442134	142	70	72	139
0,541661286	142	70	72	139
0,286212005	142	70	72	139
0,891271957	142	70	72	139
0,285906827	142	70	72	139
0,33916951	142	70	72	139
0,280593964	142	70	72	139
0,478402411	142	70	72	139
0,31908817	142	70	72	139
0,442731429	142	70	72	139
0,693700892	142	70	72	139
0,301494	142	70	72	139
0,430732418	142	70	72	139
0,657336443	142	70	72	139
0,46087388	142	70	72	139
0,211780483	142	70	72	139
0,841911641	142	70	72	139
0,498449937	142	70	72	139
0,283867464	142	70	72	139
0,227914616	142	70	72	139
0,540702048	142	70	72	139
0,201908277	142	70	72	139
0,529399905	142	70	72	139
0,429218693	142	70	72	139
0,51657773	142	70	72	139
0,426439567	142	70	72	139
0,613423302	142	70	72	139
0,498336856	142	70	72	139
0,580004975	142	70	72	139
0,624524309	142	70	72	139
0,428303387	142	70	72	139
0,359915143	142	70	72	139

0,361952684	142	70	72	139
0,37506772	142	70	72	139
0,553628205	142	70	72	139
0,488564605	142	70	72	139
0,316747986	142	70	72	139
0,262868271	142	70	72	139
0,38072267	142	70	72	139
0,174017994	142	70	72	139
0,421797202	142	70	72	139
0,390727789	142	70	72	139
0,751007165	142	70	72	139
0,435527909	141	69	72	138
0,482797478	141	69	72	138
0,910274336	141	69	72	138
0,461479156	141	69	72	138
0,81497198	141	69	72	138
0,496685204	141	69	72	138
0,403768342	141	69	72	138
0,604476567	141	69	72	138
0,472883695	141	69	72	138
0,550421714	141	69	72	138
0,659684208	141	69	72	138
0,457284638	141	69	72	138
0,69012561	141	69	72	138
0,554463626	141	69	72	138
0,748791529	141	69	72	138
0,595692609	141	69	72	138
0,48041694	141	69	72	138
0,73516404	141	69	72	138
0,505099197	141	69	72	138
0,404270406	141	69	72	138
0,575203724	141	69	72	138
0,476671724	141	69	72	138
0,484768336	141	69	72	138
0,591183944	141	69	72	138
0,381366376	141	69	72	138
0,677544504	141	69	72	138
0,433214504	141	69	72	138
0,593311198	141	69	72	138
0,819675436	141	69	72	138
0,472342081	141	69	72	138
0,752406686	141	69	72	138
0,348407676	141	69	72	138
0,654935713	141	69	72	138
0,66872998	141	69	72	138
0,440774428	141	69	72	138
0,458725783	141	69	72	138
0,489587832	141	69	72	138

0,884994198	141	69	72	138
0,714737538	141	69	72	138
0,801626941	141	69	72	138
0,877628967	141	69	72	138
0,608659636	141	69	72	138
0,431825771	141	69	72	138
0,463297929	141	69	72	138
0,289885895	141	69	72	138
0,360828129	141	69	72	138
0,743347746	141	69	72	138
0,274182126	141	69	72	138
0,701677517	141	69	72	138
0,331343488	141	69	72	138
0,730165214	141	69	72	138
0,603472692	141	69	72	138
0,471468264	141	69	72	138
0,675284729	141	69	72	138
0,364698834	141	69	72	138
0,659112929	141	69	72	138
0,180063557	141	69	72	138
0,434605872	141	69	72	138
0,676268608	141	69	72	138
0,462054563	141	69	72	138
0,420785511	141	69	72	138
0,674486804	141	69	72	138
0,58211848	141	69	72	138
0,575542093	141	69	72	138
0,741900161	141	69	72	138
0,407651596	141	69	72	138
0,37128425	141	69	72	138
0,527862962	141	69	72	138
0,519693009	141	69	72	138
0,449336616	141	69	72	138
0,537037034	141	69	72	138
0,298568985	141	69	72	138
0,383589362	141	69	72	138
0,670436117	141	69	72	138
0,625551274	141	69	72	138
0,559577723	141	69	72	138
0,787621222	141	69	72	138
0,375877597	141	69	72	138
0,555226984	141	69	72	138
0,336316422	141	69	72	138
1,03846151	141	69	72	138
0,613135548	141	69	72	138
0,200963723	141	69	72	138
0,230905387	141	69	72	138
0,410139067	141	69	72	138

0,242367685	141	69	72	138
0,25395184	141	69	72	138
0,6369244	141	69	72	138
0,475087359	141	69	72	138
0,741620532	141	69	72	138
0,780744035	141	69	72	138
0,923036341	141	69	72	138
0,548985304	141	69	72	138
0,319440321	141	69	72	138
0,880787407	141	69	72	138
0,343542997	141	69	72	138
0,721740927	141	69	72	138
0,486629231	141	69	72	138
0,57251013	141	69	72	138
0,500845688	141	69	72	138
0,5942581	141	69	72	138
0,389756299	141	69	72	138
0,371179509	141	69	72	138
0,205965304	141	69	72	138
0,400604498	141	69	72	138
0,494401259	141	69	72	138
0,457785633	141	69	72	138
0,497799492	141	69	72	138
0,252448894	141	69	72	138
0,638559021	141	69	72	138
0,523001577	141	69	72	138
0,406839457	141	69	72	138
0,357687859	141	69	72	138
0,346823532	141	69	72	138
0,350988135	141	69	72	138
0,871439997	141	69	72	138
0,405340958	141	69	72	138
0,511836809	141	69	72	138
0,298665321	141	69	72	138
0,596094083	141	69	72	138
0,559786273	141	69	72	138
0,291473334	141	69	72	138
0,480134476	141	69	72	138
0,558891281	141	69	72	138
0,315716623	141	69	72	138
0,86958355	141	69	72	138
0,45030495	141	69	72	138
0,320565931	141	69	72	138
0,427806745	141	69	72	138
0,615903899	141	69	72	138
0,66538724	141	69	72	138
0,396729232	141	69	72	138
0,226340886	141	69	72	138

0,777892183	141	69	72	138
0,433736238	141	69	72	138
0,336736105	141	69	72	138
0,510120924	141	69	72	138
0,58727327	141	69	72	138
0,295251356	141	69	72	138
0,306923139	141	69	72	138
0,553699545	141	69	72	138
0,729531188	141	69	72	138
0,357125419	141	69	72	138
0,587881106	141	69	72	138
0,669537198	141	69	72	138
0,396917207	141	69	72	138
0,539622328	141	69	72	138
0,605096157	141	69	72	138
0,589208005	141	69	72	138
0,515934001	141	69	72	138
0,582478147	141	69	72	138
0,966555488	141	69	72	138
0,822850159	141	69	72	138
0,643684229	141	69	72	138
0,17907074	141	69	72	138
0,402614798	141	69	72	138
0,222088427	141	69	72	138
0,556216171	141	69	72	138
0,765322053	141	69	72	138
0,545593186	141	69	72	138
0,489800106	141	69	72	138
0,358428665	141	69	72	138
0,592286819	141	69	72	138
0,266941074	141	69	72	138
0,73253571	141	69	72	138
0,242529798	141	69	72	138
0,436672181	141	69	72	138
0,438148802	141	69	72	138
0,413871707	141	69	72	138
0,362081439	141	69	72	138
0,336922629	141	69	72	138
0,296807363	141	69	72	138
0,404295304	141	69	72	138
0,203438967	141	69	72	138
0,228096944	141	69	72	138
0,438600258	141	69	72	138
0,32348401	141	69	72	138
0,254145735	141	69	72	138
0,477646296	141	69	72	138
0,47558494	141	69	72	138
0,217375212	141	69	72	138

0,586702664	141	69	72	138
0,450814613	141	69	72	138
0,26141009	141	69	72	138
0,662206609	141	69	72	138
0,61770941	141	69	72	138
0,692088569	141	69	72	138
0,356323466	141	69	72	138
0,644423506	141	69	72	138
0,509386907	141	69	72	138
0,332636985	141	69	72	138
0,38460769	141	69	72	138
0,479828854	141	69	72	138
0,347041856	141	69	72	138
0,681104533	141	69	72	138
0,59225835	141	69	72	138
0,303365859	141	69	72	138
0,543934629	141	69	72	138
0,34860238	141	69	72	138
0,70498439	141	69	72	138
0,391567461	141	69	72	138
0,361433955	141	69	72	138
0,649661364	141	69	72	138
0,636978464	141	69	72	138
0,158818346	141	69	72	138
0,299513503	141	69	72	138
0,405031971	141	69	72	138
0,415173037	141	69	72	138
0,379679261	141	69	72	138
0,523975312	141	69	72	138
0,238917651	141	69	72	138
0,243075079	141	69	72	138
0,694076121	141	69	72	138
0,221073885	141	69	72	138
0,320536979	141	69	72	138
0,213261249	141	69	72	138
0,440202609	141	69	72	138
0,175838765	141	69	72	138
0,240858164	141	69	72	138
0,390408002	141	69	72	138
0,226091127	141	69	72	138
0,225794944	141	69	72	138
0,476256764	141	69	72	138
0,635776287	141	69	72	138
0,178543383	141	69	72	138
0,4543557	141	69	72	138
0,279637334	141	69	72	138
0,309563396	141	69	72	138
0,420475026	141	69	72	138

0,3899485	141	69	72	138
0,185563131	141	69	72	138
0,789058815	141	69	72	138
0,348785411	141	69	72	138
0,500817267	141	69	72	138
0,375983108	141	69	72	138
0,590903966	141	69	72	138
0,454294667	141	69	72	138
0,336265295	141	69	72	138
0,306722354	141	69	72	138
0,516219265	141	69	72	138
0,381759423	141	69	72	138
0,383764463	141	69	72	138
0,750061087	141	69	72	138
0,423428234	141	69	72	138
0,578505899	141	69	72	138
0,490302864	141	69	72	138
0,282625481	141	69	72	138
0,594674559	141	69	72	138
0,585590467	141	69	72	138
0,314498226	141	69	72	138
0,225031165	141	69	72	138
0,779551769	141	69	72	138
0,57033184	141	69	72	138
0,468081102	141	69	72	138
0,281248196	141	69	72	138
0,150773365	141	69	72	138
0,638176495	141	69	72	138
0,489587578	141	69	72	138
0,456947058	141	69	72	138
0,31914464	141	69	72	138
0,250754993	141	69	72	138
0,266029985	141	69	72	138
0,577728227	141	69	72	138
0,264006217	141	69	72	138
0,51275617	141	69	72	138
0,500658969	141	69	72	138
0,245377348	141	69	72	138
0,328776353	141	69	72	138
0,380890102	141	69	72	138
0,400829427	141	69	72	138
0,142400612	141	69	72	138
0,567931373	141	69	72	138
0,477334955	141	69	72	138
0,527328389	141	69	72	138
0,266763101	141	69	72	138
0,618314643	141	69	72	138
0,467458493	141	69	72	138

0,471062482	141	69	72	138
0,636819835	141	69	72	138
0,58874537	141	69	72	138
0,406992472	141	69	72	138
0,333433206	141	69	72	138
0,614923092	141	69	72	138
0,347647097	141	69	72	138
0,474004502	141	69	72	138
0,153433404	141	69	72	138
0,854830127	141	69	72	138
0,238202543	141	69	72	138
0,402786122	141	69	72	138
0,31658936	141	69	72	138
0,29674203	141	69	72	138
0,374549454	141	69	72	138
0,616504835	141	69	72	138
0,553786863	141	69	72	138
0,254375613	141	69	72	138
0,307106686	141	69	72	138
0,531138771	141	69	72	138
0,350039715	141	69	72	138
0,349603807	141	69	72	138
0,149190428	141	69	72	138
0,279028856	141	69	72	138
0,274684783	141	69	72	138
0,19244172	141	69	72	138
0,485806811	141	69	72	138
0,215475175	141	69	72	138
0,277023169	141	69	72	138
0,409358954	141	69	72	138
0,201202217	141	69	72	138
0,279989352	141	69	72	138
0,309860301	141	69	72	138
0,397242611	141	69	72	138
0,303015345	141	69	72	138
0,329668316	141	69	72	138
0,227352861	141	69	72	138
0,2882251	141	69	72	138
0,409055169	141	69	72	138
0,443493826	141	69	72	138
0,536799393	141	69	72	138
0,556014205	141	69	72	138
0,317378585	141	69	72	138
0,395893318	141	69	72	138
0,316446715	141	69	72	138
0,37261699	141	69	72	138
0,450174271	141	69	72	138
0,350486487	141	69	72	138

0,35710067	141	69	72	138
0,417586656	141	69	72	138
0,399326202	141	69	72	138
0,321064691	141	69	72	138
0,159352869	141	69	72	138
0,430624151	141	69	72	138
0,446134774	141	69	72	138
0,498053277	141	69	72	138
0,37114679	141	69	72	138
0,210556673	141	69	72	138
0,232628692	141	69	72	138
0,486080329	141	69	72	138
0,25245468	141	69	72	138
0,268400576	141	69	72	138
0,33944694	141	69	72	138
0,146614631	141	69	72	138
0,362425865	141	69	72	138
0,207207216	141	69	72	138
0,390399288	141	69	72	138
0,428577	141	69	72	138
0,151824033	141	69	72	138
0,539933035	141	69	72	138
0,677412419	141	69	72	138
0,535040611	141	69	72	138
0,303099987	141	69	72	138
0,573515729	141	69	72	138
0,537633396	141	69	72	138
0,518701932	141	69	72	138
0,415337401	141	69	72	138
0,65263441	141	69	72	138
0,259188086	141	69	72	138
0,188551733	141	69	72	138
0,503758265	141	69	72	138
0,772671491	141	69	72	138
0,588596631	141	69	72	138
0,382583786	141	69	72	138
0,358835074	141	69	72	138
0,358066539	141	69	72	138
0,714609541	141	69	72	138
0,733784029	141	69	72	138
0,23648919	141	69	72	138
0,431475168	141	69	72	138
0,255009445	141	69	72	138
0,539373997	141	69	72	138
0,315675959	141	69	72	138
0,322612621	141	69	72	138
0,37293185	141	69	72	138
0,369989232	141	69	72	138

0,27150373	141	69	72	138
0,200149482	141	69	72	138
0,197092281	141	69	72	138
0,454689525	141	69	72	138
0,281942291	141	69	72	138
0,299702692	141	69	72	138
0,21612492	141	69	72	138
0,364985883	141	69	72	138
0,344639658	141	69	72	138
0,552798914	141	69	72	138
0,180547399	141	69	72	138
0,287081652	141	69	72	138
0,188131108	141	69	72	138
0,2699605	141	69	72	138
0,457317663	141	69	72	138
0,568361216	141	69	72	138
0,323335835	141	69	72	138
0,383236456	141	69	72	138
0,472925679	141	69	72	138
0,315279055	141	69	72	138
0,26235626	141	69	72	138
0,533752227	141	69	72	138
0,177168744	141	69	72	138
0,41433533	141	69	72	138
0,345415625	141	69	72	138
0,34964781	141	69	72	138
0,26973493	141	69	72	138
0,280389668	141	69	72	138
0,240341384	141	69	72	138
0,191381404	141	69	72	138
0,261209151	141	69	72	138
0,357580337	141	69	72	138
0,256420665	141	69	72	138
0,428266684	141	69	72	138
0,186321206	141	69	72	138
0,397408445	141	69	72	138
0,556857407	141	69	72	138
0,445077316	141	69	72	138
0,328091233	141	69	72	138
0,204302863	141	69	72	138
0,169995838	141	69	72	138
0,720558888	141	69	72	138
0,220557342	141	69	72	138
0,353980209	141	69	72	138
0,655986938	141	69	72	138
0,491067182	141	69	72	138
0,475817735	141	69	72	138
0,272735496	141	69	72	138

0,301693347	141	69	72	138
0,384298582	141	69	72	138
0,283588274	141	69	72	138
0,549210481	141	69	72	138
0,541282935	141	69	72	138
0,383869182	141	69	72	138
0,382056115	141	69	72	138
0,279034879	141	69	72	138
0,427432063	141	69	72	138
0,563832134	141	69	72	138
0,159555496	141	69	72	138
0,503096817	141	69	72	138
0,226300951	141	69	72	138
0,39357604	141	69	72	138
0,536190137	141	69	72	138
0,204830298	141	69	72	138
0,373096539	141	69	72	138
0,344943254	141	69	72	138
0,31350482	141	69	72	138
0,513950665	141	69	72	138
0,269034823	141	69	72	138
0,552572155	141	69	72	138
0,355906473	141	69	72	138
0,167039205	141	69	72	138
0,265778029	141	69	72	138
0,606163757	141	69	72	138
0,289578585	141	69	72	138
0,494113231	141	69	72	138
0,463868556	141	69	72	138
0,50093908	141	69	72	138
0,243120894	141	69	72	138
0,270184524	141	69	72	138
0,365063077	141	69	72	138
0,372542497	141	69	72	138
0,247096928	141	69	72	138
0,729180041	141	69	72	138
0,437271633	141	69	72	138
0,425713745	141	69	72	138
0,328782674	141	69	72	138
0,410919773	141	69	72	138
0,500012393	141	69	72	138
0,315639366	141	69	72	138
0,414522242	141	69	72	138
0,434099182	141	69	72	138
0,319141443	141	69	72	138
0,591469457	141	69	72	138
0,351465914	141	69	72	138
0,456415855	141	69	72	138

0,372043232	141	69	72	138
0,525443071	141	69	72	138
0,364326939	141	69	72	138
0,528927906	141	69	72	138
0,363449306	141	69	72	138
0,334294356	141	69	72	138
0,312266532	141	69	72	138
0,477387863	141	69	72	138
0,271570293	141	69	72	138
0,352084663	141	69	72	138
0,61952005	141	69	72	138
0,723295209	141	69	72	138
0,278587233	141	69	72	138
0,27236031	141	69	72	138
0,168896731	141	69	72	138
0,417718539	141	69	72	138
0,253206427	141	69	72	138
0,17088412	141	69	72	138
0,290601948	141	69	72	138
0,320798419	141	69	72	138
0,507842226	141	69	72	138
0,339003738	141	69	72	138
0,4598571	141	69	72	138
0,67052045	141	69	72	138
0,519688372	141	69	72	138
0,426042404	141	69	72	138
0,191661892	141	69	72	138
0,328448712	141	69	72	138
0,482372181	141	69	72	138
0,469967813	141	69	72	138
0,149993519	141	69	72	138
0,295673565	141	69	72	138
0,453746691	141	69	72	138
0,363767804	141	69	72	138
0,681119583	141	69	72	138
0,46669694	141	69	72	138
0,392031231	141	69	72	138
0,375962942	141	69	72	138
0,243765557	141	69	72	138
0,232092011	141	69	72	138
0,279273241	141	69	72	138
0,294012798	141	69	72	138
0,310566132	141	69	72	138
0,443709585	141	69	72	138
0,87447578	141	69	72	138
0,539547612	141	69	72	138
0,411429416	141	69	72	138
0,242551056	141	69	72	138

0,705319718	141	69	72	138
0,371906899	141	69	72	138
0,42133384	141	69	72	138
0,409528752	141	69	72	138
0,615917719	141	69	72	138
0,382329241	141	69	72	138
0,369397378	141	69	72	138
0,401129429	141	69	72	138
0,435517107	141	69	72	138
0,400809033	141	69	72	138
0,380205002	141	69	72	138
0,28396327	141	69	72	138
0,28955568	141	69	72	138
0,430332399	141	69	72	138
0,186109485	141	69	72	138
0,303993193	141	69	72	138
0,778915532	141	69	72	138
0,524115345	141	69	72	138
0,251193011	141	69	72	138
0,319382533	141	69	72	138
0,295707983	141	69	72	138
0,376814996	141	69	72	138
0,622500949	141	69	72	138
0,302048269	141	69	72	138
0,403103664	141	69	72	138
0,343279193	141	69	72	138
0,325125942	141	69	72	138
0,433494697	141	69	72	138
0,204626091	141	69	72	138
0,354386681	141	69	72	138
0,569934565	141	69	72	138
0,449437867	141	69	72	138
0,407880449	141	69	72	138
0,330904981	141	69	72	138
0,493809709	141	69	72	138
0,416153096	141	69	72	138
0,761745877	141	69	72	138
0,548901484	141	69	72	138
0,694262178	141	69	72	138
0,463366171	141	69	72	138
0,646635445	141	69	72	138
0,328909773	141	69	72	138
0,762481963	141	69	72	138
0,372122442	141	69	72	138
0,433915823	141	69	72	138
0,237632645	141	69	72	138
0,345377547	141	69	72	138
0,330886871	141	69	72	138

0,803197068	141	69	72	138
0,434253666	141	69	72	138
0,645242202	141	69	72	138
0,484723323	141	69	72	138
0,334881298	141	69	72	138
0,53854458	141	69	72	138
0,932969718	141	69	72	138
0,45468954	141	69	72	138
0,428054753	141	69	72	138
0,43734464	141	69	72	138
0,323698028	141	69	72	138
0,532711815	141	69	72	138
0,407342183	141	69	72	138
0,392370635	141	69	72	138
0,62563672	141	69	72	138
0,347775672	141	69	72	138
0,417351326	141	69	72	138
0,5560359	141	69	72	138
0,498952983	141	69	72	138
0,530333391	141	69	72	138
0,209089545	141	69	72	138
0,685606022	141	69	72	138
0,343598431	141	69	72	138
0,211875207	141	69	72	138
0,694598479	141	69	72	138
0,514546322	141	69	72	138
0,327213713	141	69	72	138
0,266778269	141	69	72	138
0,32785575	141	69	72	138
0,445730511	141	69	72	138
0,732803274	141	69	72	138
0,662235051	141	69	72	138
0,614967755	141	69	72	138
0,280652082	141	69	72	138
0,425510456	141	69	72	138
0,414629902	141	69	72	138
0,547776253	141	69	72	138
0,40385795	141	69	72	138
0,218039974	141	69	72	138
0,188770911	141	69	72	138
0,661303837	141	69	72	138
0,421238724	141	69	72	138
0,269632262	141	69	72	138
0,372773597	141	69	72	138
0,692585626	141	69	72	138
0,333814641	141	69	72	138
0,465570741	141	69	72	138
0,564786884	141	69	72	138

0,602638238	141	69	72	138
0,582109385	141	69	72	138
0,440328998	141	69	72	138
0,466023252	141	69	72	138
0,439791256	141	69	72	138
0,682727823	141	69	72	138
0,533625669	141	69	72	138
0,701079248	141	69	72	138
0,402142404	141	69	72	138
0,433063999	141	69	72	138
0,49366196	141	69	72	138
0,453560061	141	69	72	138
0,250377825	141	69	72	138
0,481268449	141	69	72	138
0,758959478	141	69	72	138
0,549433337	141	69	72	138
0,701660388	141	69	72	138
0,522526349	141	69	72	138
0,641808839	141	69	72	138
0,290103789	141	69	72	138
0,877443895	141	69	72	138
0,617144295	141	69	72	138
0,371513362	141	69	72	138
0,503923414	141	69	72	138
0,409028164	141	69	72	138
0,408372106	141	69	72	138
0,540105489	141	69	72	138
0,304781493	141	69	72	138
0,819870345	141	69	72	138
0,288095314	141	69	72	138
0,299740626	141	69	72	138
0,188249423	141	69	72	138
0,407843417	141	69	72	138
0,34683082	141	69	72	138
0,507395246	141	69	72	138
0,753861448	141	69	72	138
0,286277579	141	69	72	138
0,409127244	141	69	72	138
0,607859199	141	69	72	138
0,422243797	141	69	72	138
0,234592073	141	69	72	138
0,630614827	141	69	72	138
0,556490944	141	69	72	138
0,279478653	141	69	72	138
0,268948275	141	69	72	138
0,442468852	141	69	72	138
0,25745728	141	69	72	138
0,511929815	141	69	72	138

0,376089182	141	69	72	138
0,570735604	141	69	72	138
0,368314603	141	69	72	138
0,678147388	141	69	72	138
0,392480958	141	69	72	138
0,548473203	141	69	72	138
0,690712959	141	69	72	138
0,400364819	141	69	72	138
0,411858203	141	69	72	138
0,377738903	141	69	72	138
0,409861035	141	69	72	138
0,485762928	141	69	72	138
0,547685022	141	69	72	138
0,315506542	141	69	72	138
0,196691608	141	69	72	138
0,388415066	141	69	72	138
0,19970283	141	69	72	138
0,38851842	141	69	72	138
0,431627689	141	69	72	138
0,764659468	141	69	72	138
0,462418598	138	69	69	135
0,42544243	138	69	69	135
0,893709457	138	69	69	135
0,533135305	138	69	69	135
0,694283908	138	69	69	135
0,495181133	138	69	69	135
0,512627715	138	69	69	135
0,591068843	138	69	69	135
0,439475305	138	69	69	135
0,540797937	138	69	69	135
0,575834517	138	69	69	135
0,374244719	138	69	69	135
0,760501607	138	69	69	135
0,534275348	138	69	69	135
0,703392004	138	69	69	135
0,691316322	138	69	69	135
0,650360371	138	69	69	135
0,789140557	138	69	69	135
0,481645881	138	69	69	135
0,530522659	138	69	69	135
0,542631948	138	69	69	135
0,53317425	138	69	69	135
0,455709081	138	69	69	135
0,652504593	138	69	69	135
0,371353747	138	69	69	135
0,723215045	138	69	69	135
0,605309583	138	69	69	135
0,633121181	138	69	69	135

0,833392258	138	69	69	135
0,560411615	138	69	69	135
0,73683423	138	69	69	135
0,482768196	138	69	69	135
0,757457151	138	69	69	135
0,659055793	138	69	69	135
0,525019349	138	69	69	135
0,417662211	138	69	69	135
0,508999965	138	69	69	135
0,775036054	138	69	69	135
0,669329478	138	69	69	135
0,788146358	138	69	69	135
0,752453887	138	69	69	135
0,638516504	138	69	69	135
0,567799541	138	69	69	135
0,271794153	138	69	69	135
0,510754855	138	69	69	135
0,34916068	138	69	69	135
0,582588866	138	69	69	135
0,633572294	138	69	69	135
0,28783304	138	69	69	135
0,663887098	138	69	69	135
0,492643208	138	69	69	135
0,338775168	138	69	69	135
0,638435235	138	69	69	135
0,570122637	138	69	69	135
0,483366948	138	69	69	135
0,655088176	138	69	69	135
0,550803031	138	69	69	135
0,718307212	138	69	69	135
0,169741168	138	69	69	135
0,525456531	138	69	69	135
0,804834974	138	69	69	135
0,521023419	138	69	69	135
0,420069032	138	69	69	135
0,793228147	138	69	69	135
0,718354127	138	69	69	135
0,311862406	138	69	69	135
0,568719648	138	69	69	135
0,519804558	138	69	69	135
0,460324609	138	69	69	135
0,533290005	138	69	69	135
0,539005597	138	69	69	135
0,45680415	138	69	69	135
0,46809249	138	69	69	135
0,360966739	138	69	69	135
0,532029195	138	69	69	135
0,35660828	138	69	69	135

0,337161185	138	69	69	135
0,671143714	138	69	69	135
0,475111632	138	69	69	135
0,375802265	138	69	69	135
0,618769612	138	69	69	135
0,89566198	138	69	69	135
0,355518436	138	69	69	135
0,576823173	138	69	69	135
1,008169477	138	69	69	135
0,653403438	138	69	69	135
0,283048935	138	69	69	135
0,333472187	138	69	69	135
0,475569623	138	69	69	135
0,4339742	138	69	69	135
0,535701636	138	69	69	135
0,770682571	138	69	69	135
0,423594497	138	69	69	135
0,629673133	138	69	69	135
0,788446735	138	69	69	135
0,932303857	138	69	69	135
0,724322642	138	69	69	135
0,529161558	138	69	69	135
0,728406052	138	69	69	135
0,35093668	138	69	69	135
0,653969287	138	69	69	135
0,424494203	138	69	69	135
0,424067806	138	69	69	135
0,64621418	138	69	69	135
0,502852091	138	69	69	135
0,673887811	138	69	69	135
0,257059491	138	69	69	135
0,439035319	138	69	69	135
0,555733095	138	69	69	135
0,460335307	138	69	69	135
0,465347188	138	69	69	135
0,499225123	138	69	69	135
0,572965103	138	69	69	135
0,267920738	138	69	69	135
0,438215587	138	69	69	135
0,322345772	138	69	69	135
0,487055388	138	69	69	135
0,263650817	138	69	69	135
0,212001105	138	69	69	135
0,304781282	138	69	69	135
0,360353432	138	69	69	135
0,518991144	138	69	69	135
1,032980112	138	69	69	135
0,371947393	138	69	69	135

0,641552818	138	69	69	135
0,260075397	138	69	69	135
0,656919155	138	69	69	135
0,473160365	138	69	69	135
0,410446217	138	69	69	135
0,483290026	138	69	69	135
0,586961106	138	69	69	135
0,189370063	138	69	69	135
0,281605383	138	69	69	135
0,74926149	138	69	69	135
0,545324742	138	69	69	135
0,388596268	138	69	69	135
0,446680462	138	69	69	135
0,442884194	138	69	69	135
0,50300058	138	69	69	135
0,661084387	138	69	69	135
0,395819449	138	69	69	135
0,380368449	138	69	69	135
0,991395057	138	69	69	135
0,414156943	138	69	69	135
0,395499196	138	69	69	135
0,548310164	138	69	69	135
0,502379377	138	69	69	135
0,380873583	138	69	69	135
0,307466328	138	69	69	135
0,553260411	138	69	69	135
0,504206461	138	69	69	135
0,822497002	138	69	69	135
0,298702002	138	69	69	135
0,486653208	138	69	69	135
0,792464983	138	69	69	135
0,199082786	138	69	69	135
0,402686117	138	69	69	135
0,679731668	138	69	69	135
0,64085122	138	69	69	135
0,50142313	138	69	69	135
0,465463332	138	69	69	135
0,592176931	138	69	69	135
1,115252696	138	69	69	135
0,29582323	138	69	69	135
0,75259989	138	69	69	135
0,555213387	138	69	69	135
0,231964977	138	69	69	135
0,424266235	138	69	69	135
0,26981627	138	69	69	135
0,349285144	138	69	69	135
0,712238762	138	69	69	135
0,441086744	138	69	69	135

0,494758717	138	69	69	135
0,385836555	138	69	69	135
0,672709154	138	69	69	135
0,334884524	138	69	69	135
0,332172212	138	69	69	135
0,66614048	138	69	69	135
0,296403007	138	69	69	135
0,378237868	138	69	69	135
0,590636395	138	69	69	135
0,385827545	138	69	69	135
0,440708067	138	69	69	135
0,562946544	138	69	69	135
0,577105647	138	69	69	135
0,332687546	138	69	69	135
0,261547855	138	69	69	135
0,252725674	138	69	69	135
0,444321153	138	69	69	135
0,286797654	138	69	69	135
0,254954904	138	69	69	135
0,250451878	138	69	69	135
0,462276222	138	69	69	135
0,490879078	138	69	69	135
0,264777132	138	69	69	135
0,540661935	138	69	69	135
0,632742956	138	69	69	135
0,215303629	138	69	69	135
0,249526605	138	69	69	135
0,633087621	138	69	69	135
0,221218559	138	69	69	135
0,192670896	138	69	69	135
0,726450758	138	69	69	135
0,46163797	138	69	69	135
0,748110258	138	69	69	135
0,198687916	138	69	69	135
0,351224324	138	69	69	135
0,364862639	138	69	69	135
0,457274043	138	69	69	135
0,468832494	138	69	69	135
0,454279737	138	69	69	135
0,641218469	138	69	69	135
0,590065448	138	69	69	135
0,387797352	138	69	69	135
0,649089502	138	69	69	135
0,434689175	138	69	69	135
0,310795285	138	69	69	135
0,701451334	138	69	69	135
0,359589539	138	69	69	135
0,40039845	138	69	69	135

0,707259309	138	69	69	135
0,678834033	138	69	69	135
0,185706388	138	69	69	135
0,434426042	138	69	69	135
0,216516894	138	69	69	135
0,608334852	138	69	69	135
0,169303323	138	69	69	135
0,390940004	138	69	69	135
0,418586631	138	69	69	135
0,428394273	138	69	69	135
0,263237658	138	69	69	135
0,191806459	138	69	69	135
0,415867142	138	69	69	135
0,305326819	138	69	69	135
0,709280583	138	69	69	135
0,339710138	138	69	69	135
0,336372241	138	69	69	135
0,273320156	138	69	69	135
0,593759734	138	69	69	135
0,265618656	138	69	69	135
0,348818987	138	69	69	135
0,255657989	138	69	69	135
0,432605319	138	69	69	135
0,640522581	138	69	69	135
0,630793463	138	69	69	135
0,173184168	138	69	69	135
0,577062576	138	69	69	135
0,469024026	138	69	69	135
0,474564239	138	69	69	135
0,481751757	138	69	69	135
0,367663671	138	69	69	135
0,747168692	138	69	69	135
0,583077698	138	69	69	135
0,428564295	138	69	69	135
0,57452802	138	69	69	135
0,513309092	138	69	69	135
0,319982387	138	69	69	135
0,379754199	138	69	69	135
0,285964768	138	69	69	135
0,612279416	138	69	69	135
0,518151993	138	69	69	135
0,457253596	138	69	69	135
0,841861751	138	69	69	135
0,403713053	138	69	69	135
0,303059411	138	69	69	135
0,421271301	138	69	69	135
0,639521348	138	69	69	135
0,501522646	138	69	69	135

0,260665537	138	69	69	135
0,709369016	138	69	69	135
0,323018743	138	69	69	135
0,365119621	138	69	69	135
0,774955995	138	69	69	135
0,535665325	138	69	69	135
0,18710862	138	69	69	135
0,497793053	138	69	69	135
0,423699489	138	69	69	135
0,325295325	138	69	69	135
0,635962357	138	69	69	135
0,324072063	138	69	69	135
0,231929537	138	69	69	135
0,461776184	138	69	69	135
0,384854011	138	69	69	135
0,430467941	138	69	69	135
0,354958249	138	69	69	135
0,261081888	138	69	69	135
0,242680052	138	69	69	135
0,529502343	138	69	69	135
0,245912873	138	69	69	135
0,550267017	138	69	69	135
0,489860406	138	69	69	135
0,317423271	138	69	69	135
0,266416786	138	69	69	135
0,497429699	138	69	69	135
0,661479463	138	69	69	135
0,139602417	138	69	69	135
0,489565516	138	69	69	135
0,641658158	138	69	69	135
0,169425346	138	69	69	135
0,569939342	138	69	69	135
0,225472217	138	69	69	135
0,635576567	138	69	69	135
0,45040656	138	69	69	135
0,462701154	138	69	69	135
0,361586254	138	69	69	135
0,569927859	138	69	69	135
0,561818099	138	69	69	135
0,352584617	138	69	69	135
0,477601287	138	69	69	135
0,228264207	138	69	69	135
0,584957063	138	69	69	135
0,423239074	138	69	69	135
0,483605616	138	69	69	135
0,276267566	138	69	69	135
0,242777278	138	69	69	135
0,194215534	138	69	69	135

0,890722118	138	69	69	135
0,295660832	138	69	69	135
0,498401792	138	69	69	135
0,358610551	138	69	69	135
0,308191207	138	69	69	135
0,361311834	138	69	69	135
0,632584637	138	69	69	135
0,536302222	138	69	69	135
0,285241958	138	69	69	135
0,267225062	138	69	69	135
0,161863541	138	69	69	135
0,439889473	138	69	69	135
0,438391861	138	69	69	135
0,362212197	138	69	69	135
0,390191199	138	69	69	135
0,345487019	138	69	69	135
0,29391991	138	69	69	135
0,244531185	138	69	69	135
0,259973124	138	69	69	135
0,263166049	138	69	69	135
0,470604751	138	69	69	135
0,251410933	138	69	69	135
0,431340221	138	69	69	135
0,196096601	138	69	69	135
0,189421911	138	69	69	135
0,310660438	138	69	69	135
0,476477431	138	69	69	135
0,532939357	138	69	69	135
0,287390328	138	69	69	135
0,217191118	138	69	69	135
0,267228664	138	69	69	135
0,189959669	138	69	69	135
0,3274787	138	69	69	135
0,455377716	138	69	69	135
0,442059083	138	69	69	135
0,390555088	138	69	69	135
0,445098864	138	69	69	135
0,223815396	138	69	69	135
0,377903971	138	69	69	135
0,381903964	138	69	69	135
0,212687709	138	69	69	135
0,375497364	138	69	69	135
0,433938452	138	69	69	135
0,325271116	138	69	69	135
0,385912243	138	69	69	135
0,414206179	138	69	69	135
0,448883737	138	69	69	135
0,324421328	138	69	69	135

0,375722875	138	69	69	135
0,184976136	138	69	69	135
0,477288141	138	69	69	135
0,460837106	138	69	69	135
0,548493089	138	69	69	135
0,47170544	138	69	69	135
0,172483441	138	69	69	135
0,268751497	138	69	69	135
0,565927538	138	69	69	135
0,284520106	138	69	69	135
0,370219511	138	69	69	135
0,418340454	138	69	69	135
0,259833571	138	69	69	135
0,448466408	138	69	69	135
0,170264535	138	69	69	135
0,241525149	138	69	69	135
0,422741792	138	69	69	135
0,579947532	138	69	69	135
0,153588133	138	69	69	135
0,605554572	138	69	69	135
0,661931349	138	69	69	135
0,588722619	138	69	69	135
0,337940717	138	69	69	135
0,555930414	138	69	69	135
0,666212005	138	69	69	135
0,401961951	138	69	69	135
0,412136139	138	69	69	135
0,354053068	138	69	69	135
0,661100618	138	69	69	135
0,333064197	138	69	69	135
0,25404498	138	69	69	135
0,419814811	138	69	69	135
0,278351013	138	69	69	135
0,789163954	138	69	69	135
0,598937239	138	69	69	135
0,424120515	138	69	69	135
0,378958031	138	69	69	135
0,429360992	138	69	69	135
0,72898317	138	69	69	135
0,791497449	138	69	69	135
0,154112374	138	69	69	135
0,50319967	138	69	69	135
0,496305876	138	69	69	135
0,392332099	138	69	69	135
0,426049257	138	69	69	135
0,155804605	138	69	69	135
0,423689241	138	69	69	135
0,461824587	138	69	69	135

0,470157192	138	69	69	135
0,371043665	138	69	69	135
0,421771863	138	69	69	135
0,273977767	138	69	69	135
0,2835446	138	69	69	135
0,242923685	138	69	69	135
0,364323458	138	69	69	135
0,265036865	138	69	69	135
0,491570395	138	69	69	135
0,204024072	138	69	69	135
0,259771476	138	69	69	135
0,338182579	138	69	69	135
0,265588392	138	69	69	135
0,205979917	138	69	69	135
0,291897053	138	69	69	135
0,227885166	138	69	69	135
0,393107559	138	69	69	135
0,570196098	138	69	69	135
0,341744739	138	69	69	135
0,393511951	138	69	69	135
0,481770985	138	69	69	135
0,221250662	138	69	69	135
0,238164424	138	69	69	135
0,539216195	138	69	69	135
0,239433067	138	69	69	135
0,354193021	138	69	69	135
0,318464287	138	69	69	135
0,51369418	138	69	69	135
0,286858734	138	69	69	135
0,232557901	138	69	69	135
0,167124702	138	69	69	135
0,190082224	138	69	69	135
0,341260761	138	69	69	135
0,330072988	138	69	69	135
0,290656716	138	69	69	135
0,414540933	138	69	69	135
0,19033504	138	69	69	135
0,492229771	138	69	69	135
0,675230883	138	69	69	135
0,520114453	138	69	69	135
0,400538621	138	69	69	135
0,148786242	138	69	69	135
0,205468939	138	69	69	135
0,68054372	138	69	69	135
0,379956079	138	69	69	135
0,383077872	138	69	69	135
0,553513241	138	69	69	135
0,407929892	138	69	69	135

0,478147637	138	69	69	135
0,204891243	138	69	69	135
0,391035123	138	69	69	135
0,383464329	138	69	69	135
0,227804052	138	69	69	135
0,665245557	138	69	69	135
0,320616069	138	69	69	135
0,386594296	138	69	69	135
0,370216478	138	69	69	135
0,298917215	138	69	69	135
0,431232889	138	69	69	135
0,581955077	138	69	69	135
0,226814558	138	69	69	135
0,488889093	138	69	69	135
0,311664914	138	69	69	135
0,410911481	138	69	69	135
0,503804722	138	69	69	135
0,352592228	138	69	69	135
0,808671376	138	69	69	135
0,275155136	138	69	69	135
0,584037431	138	69	69	135
0,418653913	138	69	69	135
0,261705327	138	69	69	135
0,186924661	138	69	69	135
0,327726953	138	69	69	135
0,660838878	138	69	69	135
0,313777812	138	69	69	135
0,494990507	138	69	69	135
0,489024422	138	69	69	135
0,473455139	138	69	69	135
0,27662998	138	69	69	135
0,419540511	138	69	69	135
0,260185158	138	69	69	135
0,416146404	138	69	69	135
0,362186174	138	69	69	135
0,84218697	138	69	69	135
0,446299227	138	69	69	135
0,222870851	138	69	69	135
0,45974302	138	69	69	135
0,581195054	138	69	69	135
0,454319035	138	69	69	135
0,275033114	138	69	69	135
0,491689815	138	69	69	135
0,450505317	138	69	69	135
0,333915202	138	69	69	135
0,533943125	138	69	69	135
0,334894905	138	69	69	135
0,53946493	138	69	69	135

0,342176761	138	69	69	135
0,63688099	138	69	69	135
0,498969268	138	69	69	135
0,584174374	138	69	69	135
0,445048396	138	69	69	135
0,367115169	138	69	69	135
0,354510363	138	69	69	135
0,434344304	138	69	69	135
0,308376698	138	69	69	135
0,453965889	138	69	69	135
0,617797239	138	69	69	135
0,654180735	138	69	69	135
0,271434257	138	69	69	135
0,379622304	138	69	69	135
0,257533933	138	69	69	135
0,289729557	138	69	69	135
0,264013757	138	69	69	135
0,462496763	138	69	69	135
0,498573423	138	69	69	135
0,375272063	138	69	69	135
0,431459014	138	69	69	135
0,65584077	138	69	69	135
0,578480938	138	69	69	135
0,423394745	138	69	69	135
0,344004579	138	69	69	135
0,443277239	138	69	69	135
0,368744154	138	69	69	135
0,167591056	138	69	69	135
0,318167144	138	69	69	135
0,465794713	138	69	69	135
0,452733239	138	69	69	135
0,751101636	138	69	69	135
0,531197488	138	69	69	135
0,391011775	138	69	69	135
0,416285039	138	69	69	135
0,333256753	138	69	69	135
0,184435355	138	69	69	135
0,355680678	138	69	69	135
0,281420841	138	69	69	135
0,270042008	138	69	69	135
0,29753447	138	69	69	135
0,769099564	138	69	69	135
0,567528224	138	69	69	135
0,36811298	138	69	69	135
0,201452459	138	69	69	135
0,902639816	138	69	69	135
0,416048122	138	69	69	135
0,468057791	138	69	69	135

0,278573143	138	69	69	135
0,429101804	138	69	69	135
0,740816184	138	69	69	135
0,316754041	138	69	69	135
0,375465091	138	69	69	135
0,414296675	138	69	69	135
0,596761066	138	69	69	135
0,456141167	138	69	69	135
0,445370818	138	69	69	135
0,280723474	138	69	69	135
0,282853178	138	69	69	135
0,510074367	138	69	69	135
0,220307247	138	69	69	135
0,276186314	138	69	69	135
0,98624175	138	69	69	135
0,633341318	138	69	69	135
0,406054555	138	69	69	135
0,310787456	138	69	69	135
0,396098159	138	69	69	135
0,518477323	138	69	69	135
0,733044786	138	69	69	135
0,355182685	138	69	69	135
0,471838192	138	69	69	135
0,461352445	138	69	69	135
0,38195871	138	69	69	135
0,245965105	138	69	69	135
0,434135241	138	69	69	135
0,621269382	138	69	69	135
0,516863556	138	69	69	135
0,271579063	138	69	69	135
0,567977331	138	69	69	135
0,411431131	138	69	69	135
0,717664931	138	69	69	135
0,593505803	138	69	69	135
0,699155646	138	69	69	135
0,447566081	138	69	69	135
0,64223169	138	69	69	135
0,861080829	138	69	69	135
0,440059502	138	69	69	135
0,388818785	138	69	69	135
0,253588816	138	69	69	135
0,328595941	138	69	69	135
0,342233666	138	69	69	135
0,680483795	138	69	69	135
0,57295456	138	69	69	135
0,744146702	138	69	69	135
0,460630491	138	69	69	135
0,355268775	138	69	69	135

0,450855538	138	69	69	135
1,081152945	138	69	69	135
0,454968921	138	69	69	135
0,565033986	138	69	69	135
0,451361617	138	69	69	135
0,305423906	138	69	69	135
0,543296869	138	69	69	135
0,369212672	138	69	69	135
0,483217797	138	69	69	135
0,689091407	138	69	69	135
0,385674316	138	69	69	135
0,396151786	138	69	69	135
0,432285514	138	69	69	135
0,521192306	138	69	69	135
0,509543147	138	69	69	135
0,289687108	138	69	69	135
0,666849379	138	69	69	135
0,384403781	138	69	69	135
0,70624318	138	69	69	135
0,494936952	138	69	69	135
0,273422672	138	69	69	135
0,211861275	138	69	69	135
0,475248003	138	69	69	135
0,477358094	138	69	69	135
0,652421577	138	69	69	135
0,709830977	138	69	69	135
0,61481296	138	69	69	135
0,263627762	138	69	69	135
0,512706308	138	69	69	135
0,668692983	138	69	69	135
0,571837571	138	69	69	135
0,464989955	138	69	69	135
0,294662208	138	69	69	135
0,293414677	138	69	69	135
0,560817062	138	69	69	135
0,440511561	138	69	69	135
0,365195506	138	69	69	135
0,418859285	138	69	69	135
0,53134894	138	69	69	135
0,474705783	138	69	69	135
0,432679205	138	69	69	135
0,585951076	138	69	69	135
0,666171881	138	69	69	135
0,656876514	138	69	69	135
0,528428933	138	69	69	135
0,439000804	138	69	69	135
0,426694367	138	69	69	135
0,720055592	138	69	69	135

0,642446531	138	69	69	135
0,687885738	138	69	69	135
0,534261555	138	69	69	135
0,45074988	138	69	69	135
0,655397994	138	69	69	135
0,409665124	138	69	69	135
0,246893982	138	69	69	135
0,495978594	138	69	69	135
0,710061318	138	69	69	135
0,6076545	138	69	69	135
0,645111757	138	69	69	135
0,542702313	138	69	69	135
0,58279544	138	69	69	135
0,266293339	138	69	69	135
0,768391476	138	69	69	135
0,667665904	138	69	69	135
0,38111223	138	69	69	135
0,595414635	138	69	69	135
0,445620136	138	69	69	135
0,447217043	138	69	69	135
0,6450602	138	69	69	135
0,307815387	138	69	69	135
0,921345705	138	69	69	135
0,383063024	138	69	69	135
0,350236621	138	69	69	135
0,207001876	138	69	69	135
0,468167651	138	69	69	135
0,282344935	138	69	69	135
0,486445601	138	69	69	135
0,706124375	138	69	69	135
0,330029203	138	69	69	135
0,392697831	138	69	69	135
0,610065276	138	69	69	135
0,554024137	138	69	69	135
0,225700052	138	69	69	135
0,627527369	138	69	69	135
0,633895238	138	69	69	135
0,345988615	138	69	69	135
0,257566462	138	69	69	135
0,519897707	138	69	69	135
0,253243089	138	69	69	135
0,43208392	138	69	69	135
0,393826402	138	69	69	135
0,503508122	138	69	69	135
0,413343059	138	69	69	135
0,516784907	138	69	69	135
0,473506171	138	69	69	135
0,596917704	138	69	69	135

0,657857537	138	69	69	135
0,521037797	138	69	69	135
0,410868809	138	69	69	135
0,377539787	138	69	69	135
0,482167636	138	69	69	135
0,501471411	138	69	69	135
0,523810879	138	69	69	135
0,305843949	138	69	69	135
0,209771442	138	69	69	135
0,314907386	138	69	69	135
0,216886788	138	69	69	135
0,475354975	138	69	69	135
0,386816545	138	69	69	135
0,673815672	138	69	69	135

Test statistic	Model formula fit
-0,453690106	y ~ Intervention + Sex
-0,623353512	y ~ Intervention + Sex
-1,092649437	y ~ Intervention + Sex
-0,560703291	y ~ Intervention + Sex
-0,01491497	y ~ Intervention + Sex
-0,950413232	y ~ Intervention + Sex
-1,603331987	y ~ Intervention + Sex
-2,023118555	y ~ Intervention + Sex
-2,187059929	y ~ Intervention + Sex
-0,047834348	y ~ Intervention + Sex
-1,145873945	y ~ Intervention + Sex
-1,796163474	y ~ Intervention + Sex
-0,706431887	y ~ Intervention + Sex
-1,22555141	y ~ Intervention + Sex
-2,541871094	y ~ Intervention + Sex
-0,590910759	y ~ Intervention + Sex
0,843213562	y ~ Intervention + Sex
-0,680086455	y ~ Intervention + Sex
-0,077668525	y ~ Intervention + Sex
0,782683846	y ~ Intervention + Sex
0,782758751	y ~ Intervention + Sex
1,31868594	y ~ Intervention + Sex
0,09654112	y ~ Intervention + Sex
-0,348920631	y ~ Intervention + Sex
0,675674731	y ~ Intervention + Sex
1,552478966	y ~ Intervention + Sex
-0,975399418	y ~ Intervention + Sex
-1,690542741	y ~ Intervention + Sex
-2,18494258	y ~ Intervention + Sex
-0,389489039	y ~ Intervention + Sex
0,387214012	y ~ Intervention + Sex
-2,032350022	y ~ Intervention + Sex
-1,632383623	y ~ Intervention + Sex
-1,783250999	y ~ Intervention + Sex
-1,324090846	y ~ Intervention + Sex
-0,383966884	y ~ Intervention + Sex
-4,389643445	y ~ Intervention + Sex
-0,44935143	y ~ Intervention + Sex
0,108142782	y ~ Intervention + Sex
0,15273946	y ~ Intervention + Sex
-0,973419279	y ~ Intervention + Sex
-0,632201923	y ~ Intervention + Sex
-0,610084103	y ~ Intervention + Sex
-0,799938459	y ~ Intervention + Sex
-1,314994863	y ~ Intervention + Sex
-1,138555607	y ~ Intervention + Sex
-0,35711209	y ~ Intervention + Sex

-1,898672535	y ~ Intervention + Sex
-2,051294429	y ~ Intervention + Sex
-1,543556596	y ~ Intervention + Sex
1,35686082	y ~ Intervention + Sex
0,078399036	y ~ Intervention + Sex
-1,510346693	y ~ Intervention + Sex
-2,877970318	y ~ Intervention + Sex
-1,995970839	y ~ Intervention + Sex
-1,592782198	y ~ Intervention + Sex
-0,040505932	y ~ Intervention + Sex
0,461528435	y ~ Intervention + Sex
-0,119128245	y ~ Intervention + Sex
0,510897675	y ~ Intervention + Sex
-1,776945581	y ~ Intervention + Sex
-1,44963176	y ~ Intervention + Sex
-0,266472198	y ~ Intervention + Sex
-1,276731207	y ~ Intervention + Sex
-1,621675014	y ~ Intervention + Sex
-1,196121566	y ~ Intervention + Sex
0,488104724	y ~ Intervention + Sex
-0,105573605	y ~ Intervention + Sex
0,300425525	y ~ Intervention + Sex
-1,60950456	y ~ Intervention + Sex
0,954151067	y ~ Intervention + Sex
0,550363898	y ~ Intervention + Sex
-0,595221906	y ~ Intervention + Sex
1,887693102	y ~ Intervention + Sex
-0,188854159	y ~ Intervention + Sex
-2,46638645	y ~ Intervention + Sex
0,354366776	y ~ Intervention + Sex
0,289742709	y ~ Intervention + Sex
-1,172994118	y ~ Intervention + Sex
-0,433933764	y ~ Intervention + Sex
-0,667661554	y ~ Intervention + Sex
-1,394640724	y ~ Intervention + Sex
-1,604847872	y ~ Intervention + Sex
-1,83430437	y ~ Intervention + Sex
-0,669586054	y ~ Intervention + Sex
0,69697237	y ~ Intervention + Sex
-0,294114705	y ~ Intervention + Sex
-1,071255711	y ~ Intervention + Sex
-1,411337125	y ~ Intervention + Sex
-0,052208915	y ~ Intervention + Sex
-1,46087778	y ~ Intervention + Sex
0,388882417	y ~ Intervention + Sex
-1,241473528	y ~ Intervention + Sex
-1,474445485	y ~ Intervention + Sex
-0,002290489	y ~ Intervention + Sex

-0,484049682	y ~ Intervention + Sex
-1,035232361	y ~ Intervention + Sex
-0,985480149	y ~ Intervention + Sex
-2,822588195	y ~ Intervention + Sex
-1,642675189	y ~ Intervention + Sex
0,216024136	y ~ Intervention + Sex
-0,136689879	y ~ Intervention + Sex
-0,972349509	y ~ Intervention + Sex
0,810433982	y ~ Intervention + Sex
-0,843572575	y ~ Intervention + Sex
-0,177304993	y ~ Intervention + Sex
-1,205841757	y ~ Intervention + Sex
0,209513254	y ~ Intervention + Sex
0,633724289	y ~ Intervention + Sex
0,77862127	y ~ Intervention + Sex
-0,061228116	y ~ Intervention + Sex
-0,925080963	y ~ Intervention + Sex
-0,821841649	y ~ Intervention + Sex
0,52827027	y ~ Intervention + Sex
-2,632789969	y ~ Intervention + Sex
0,47459302	y ~ Intervention + Sex
1,113333314	y ~ Intervention + Sex
-1,205194587	y ~ Intervention + Sex
-0,418209717	y ~ Intervention + Sex
-0,161510921	y ~ Intervention + Sex
-0,310888133	y ~ Intervention + Sex
-0,820731802	y ~ Intervention + Sex
1,14302092	y ~ Intervention + Sex
-0,198498084	y ~ Intervention + Sex
-0,020145875	y ~ Intervention + Sex
0,757831357	y ~ Intervention + Sex
0,369145577	y ~ Intervention + Sex
0,215305976	y ~ Intervention + Sex
-0,015039631	y ~ Intervention + Sex
-1,762673685	y ~ Intervention + Sex
0,452597605	y ~ Intervention + Sex
-0,440328687	y ~ Intervention + Sex
-0,955360915	y ~ Intervention + Sex
-0,592672864	y ~ Intervention + Sex
2,381979462	y ~ Intervention + Sex
-0,472645467	y ~ Intervention + Sex
-0,614714813	y ~ Intervention + Sex
-0,954242664	y ~ Intervention + Sex
-1,784626407	y ~ Intervention + Sex
-0,68559988	y ~ Intervention + Sex
-0,868391823	y ~ Intervention + Sex
-0,335281803	y ~ Intervention + Sex
-2,032372352	y ~ Intervention + Sex

0,446739048	y ~ Intervention + Sex
0,780776818	y ~ Intervention + Sex
0,571623468	y ~ Intervention + Sex
-0,258508593	y ~ Intervention + Sex
0,282962246	y ~ Intervention + Sex
-0,364769577	y ~ Intervention + Sex
-0,227383064	y ~ Intervention + Sex
-0,357496453	y ~ Intervention + Sex
-1,629674737	y ~ Intervention + Sex
-0,689836964	y ~ Intervention + Sex
1,007582305	y ~ Intervention + Sex
0,890936529	y ~ Intervention + Sex
2,046221021	y ~ Intervention + Sex
0,593852087	y ~ Intervention + Sex
0,535885685	y ~ Intervention + Sex
1,459061391	y ~ Intervention + Sex
-0,266255234	y ~ Intervention + Sex
0,165891451	y ~ Intervention + Sex
-0,910201134	y ~ Intervention + Sex
-1,092416486	y ~ Intervention + Sex
0,825714456	y ~ Intervention + Sex
-0,16170974	y ~ Intervention + Sex
-1,13359796	y ~ Intervention + Sex
0,781470582	y ~ Intervention + Sex
-1,791663958	y ~ Intervention + Sex
-0,766419275	y ~ Intervention + Sex
-0,653649893	y ~ Intervention + Sex
-0,949778279	y ~ Intervention + Sex
-2,067720822	y ~ Intervention + Sex
0,11603649	y ~ Intervention + Sex
-0,316973771	y ~ Intervention + Sex
1,397680303	y ~ Intervention + Sex
-1,043638544	y ~ Intervention + Sex
0,910922544	y ~ Intervention + Sex
0,082512359	y ~ Intervention + Sex
-0,075874349	y ~ Intervention + Sex
-0,408792897	y ~ Intervention + Sex
-0,843699059	y ~ Intervention + Sex
-0,181491708	y ~ Intervention + Sex
-0,905370869	y ~ Intervention + Sex
-0,757877322	y ~ Intervention + Sex
-0,980300133	y ~ Intervention + Sex
-3,051541193	y ~ Intervention + Sex
-0,144186027	y ~ Intervention + Sex
0,577420987	y ~ Intervention + Sex
-0,9944026	y ~ Intervention + Sex
-0,093845876	y ~ Intervention + Sex
0,313196155	y ~ Intervention + Sex

0,188214694	y ~ Intervention + Sex
-0,421562898	y ~ Intervention + Sex
1,734572045	y ~ Intervention + Sex
0,381551932	y ~ Intervention + Sex
1,179514103	y ~ Intervention + Sex
-0,780919406	y ~ Intervention + Sex
-0,059640086	y ~ Intervention + Sex
-1,852408452	y ~ Intervention + Sex
-0,279830228	y ~ Intervention + Sex
-1,918050183	y ~ Intervention + Sex
0,687380076	y ~ Intervention + Sex
-1,321316869	y ~ Intervention + Sex
0,177297635	y ~ Intervention + Sex
-0,644770527	y ~ Intervention + Sex
-0,425149695	y ~ Intervention + Sex
0,180996133	y ~ Intervention + Sex
-0,881071196	y ~ Intervention + Sex
-1,153182907	y ~ Intervention + Sex
-0,849714427	y ~ Intervention + Sex
-1,014804061	y ~ Intervention + Sex
0,013675843	y ~ Intervention + Sex
-0,359310829	y ~ Intervention + Sex
0,332716633	y ~ Intervention + Sex
0,376765468	y ~ Intervention + Sex
-0,008165121	y ~ Intervention + Sex
-1,049948602	y ~ Intervention + Sex
-0,091599674	y ~ Intervention + Sex
0,561661284	y ~ Intervention + Sex
0,856198089	y ~ Intervention + Sex
0,341986768	y ~ Intervention + Sex
-0,61487969	y ~ Intervention + Sex
-2,053065611	y ~ Intervention + Sex
0,822923548	y ~ Intervention + Sex
-0,308041865	y ~ Intervention + Sex
-0,110332038	y ~ Intervention + Sex
0,404161182	y ~ Intervention + Sex
-0,143048115	y ~ Intervention + Sex
2,162259888	y ~ Intervention + Sex
0,814103004	y ~ Intervention + Sex
0,643530704	y ~ Intervention + Sex
1,195559961	y ~ Intervention + Sex
0,225167845	y ~ Intervention + Sex
1,037882137	y ~ Intervention + Sex
0,235034725	y ~ Intervention + Sex
-0,876493968	y ~ Intervention + Sex
-0,971438058	y ~ Intervention + Sex
-1,393578434	y ~ Intervention + Sex
0,445758231	y ~ Intervention + Sex

-0,699102717	y ~ Intervention + Sex
-1,545807322	y ~ Intervention + Sex
-0,013545584	y ~ Intervention + Sex
0,031135749	y ~ Intervention + Sex
-0,960805412	y ~ Intervention + Sex
-1,198218308	y ~ Intervention + Sex
-0,58865186	y ~ Intervention + Sex
0,753864193	y ~ Intervention + Sex
2,125563278	y ~ Intervention + Sex
-0,368393819	y ~ Intervention + Sex
-0,971454994	y ~ Intervention + Sex
-0,609674568	y ~ Intervention + Sex
0,187733827	y ~ Intervention + Sex
-0,207019197	y ~ Intervention + Sex
-1,142519426	y ~ Intervention + Sex
0,338335157	y ~ Intervention + Sex
-1,901171588	y ~ Intervention + Sex
0,087127182	y ~ Intervention + Sex
0,639959154	y ~ Intervention + Sex
-1,232953124	y ~ Intervention + Sex
0,304329888	y ~ Intervention + Sex
0,523917351	y ~ Intervention + Sex
-0,078388592	y ~ Intervention + Sex
1,067432487	y ~ Intervention + Sex
0,872796393	y ~ Intervention + Sex
0,665142942	y ~ Intervention + Sex
0,726351828	y ~ Intervention + Sex
-0,083579553	y ~ Intervention + Sex
-1,649449405	y ~ Intervention + Sex
-0,813202894	y ~ Intervention + Sex
-0,847717567	y ~ Intervention + Sex
-0,900230198	y ~ Intervention + Sex
0,77168116	y ~ Intervention + Sex
-0,447293066	y ~ Intervention + Sex
0,311006863	y ~ Intervention + Sex
-1,393365999	y ~ Intervention + Sex
0,2673613	y ~ Intervention + Sex
0,524933098	y ~ Intervention + Sex
-0,626378334	y ~ Intervention + Sex
-0,204630362	y ~ Intervention + Sex
0,730208337	y ~ Intervention + Sex
1,020674131	y ~ Intervention + Sex
1,044430035	y ~ Intervention + Sex
0,190688921	y ~ Intervention + Sex
-0,938531467	y ~ Intervention + Sex
-0,542898195	y ~ Intervention + Sex
1,256189216	y ~ Intervention + Sex
0,421609834	y ~ Intervention + Sex

-0,961426695	y ~ Intervention + Sex
-0,748291166	y ~ Intervention + Sex
-0,073154226	y ~ Intervention + Sex
-0,210499988	y ~ Intervention + Sex
-1,616843219	y ~ Intervention + Sex
-1,868168513	y ~ Intervention + Sex
1,137811467	y ~ Intervention + Sex
-0,79078363	y ~ Intervention + Sex
-0,449140255	y ~ Intervention + Sex
0,590792433	y ~ Intervention + Sex
-0,17980978	y ~ Intervention + Sex
-0,528428557	y ~ Intervention + Sex
-0,797666964	y ~ Intervention + Sex
0,508503752	y ~ Intervention + Sex
-1,29756439	y ~ Intervention + Sex
0,0894839	y ~ Intervention + Sex
-0,473925947	y ~ Intervention + Sex
1,543956136	y ~ Intervention + Sex
1,60786765	y ~ Intervention + Sex
-0,85494643	y ~ Intervention + Sex
-1,169859768	y ~ Intervention + Sex
1,582742043	y ~ Intervention + Sex
-0,84005246	y ~ Intervention + Sex
1,480781498	y ~ Intervention + Sex
1,37911871	y ~ Intervention + Sex
0,806254392	y ~ Intervention + Sex
-0,877539341	y ~ Intervention + Sex
-0,415363269	y ~ Intervention + Sex
-1,840790482	y ~ Intervention + Sex
-0,624548365	y ~ Intervention + Sex
-0,653626411	y ~ Intervention + Sex
-0,69083783	y ~ Intervention + Sex
-1,809708523	y ~ Intervention + Sex
0,33253798	y ~ Intervention + Sex
-0,15127037	y ~ Intervention + Sex
0,101366107	y ~ Intervention + Sex
-0,070721371	y ~ Intervention + Sex
1,943099921	y ~ Intervention + Sex
-1,167143665	y ~ Intervention + Sex
-0,970222196	y ~ Intervention + Sex
-1,994172503	y ~ Intervention + Sex
0,828849679	y ~ Intervention + Sex
0,345609542	y ~ Intervention + Sex
0,378806875	y ~ Intervention + Sex
0,237611877	y ~ Intervention + Sex
0,000367589	y ~ Intervention + Sex
1,17377161	y ~ Intervention + Sex
0,215900728	y ~ Intervention + Sex

-0,697897381	y ~ Intervention + Sex
-0,183267115	y ~ Intervention + Sex
-0,710630708	y ~ Intervention + Sex
0,66221551	y ~ Intervention + Sex
0,465333702	y ~ Intervention + Sex
-0,130368332	y ~ Intervention + Sex
-0,480727759	y ~ Intervention + Sex
-0,594764194	y ~ Intervention + Sex
0,284185655	y ~ Intervention + Sex
-0,488908383	y ~ Intervention + Sex
-2,001781024	y ~ Intervention + Sex
-2,26977477	y ~ Intervention + Sex
1,033057113	y ~ Intervention + Sex
-0,962642267	y ~ Intervention + Sex
-0,065503287	y ~ Intervention + Sex
0,362556163	y ~ Intervention + Sex
-1,071639236	y ~ Intervention + Sex
-0,259853418	y ~ Intervention + Sex
2,653526146	y ~ Intervention + Sex
0,761276139	y ~ Intervention + Sex
-2,527978223	y ~ Intervention + Sex
-0,072495721	y ~ Intervention + Sex
-0,530591949	y ~ Intervention + Sex
-0,414494774	y ~ Intervention + Sex
0,270392976	y ~ Intervention + Sex
-0,282494817	y ~ Intervention + Sex
0,939004771	y ~ Intervention + Sex
0,238448115	y ~ Intervention + Sex
-0,406108138	y ~ Intervention + Sex
-0,914147496	y ~ Intervention + Sex
0,356896531	y ~ Intervention + Sex
-1,065892151	y ~ Intervention + Sex
0,070037372	y ~ Intervention + Sex
0,456540432	y ~ Intervention + Sex
-0,193872537	y ~ Intervention + Sex
0,968413884	y ~ Intervention + Sex
0,371333789	y ~ Intervention + Sex
0,394895787	y ~ Intervention + Sex
0,355508697	y ~ Intervention + Sex
0,229764588	y ~ Intervention + Sex
-0,741015467	y ~ Intervention + Sex
-1,302686995	y ~ Intervention + Sex
1,033831109	y ~ Intervention + Sex
0,19961844	y ~ Intervention + Sex
0,227489605	y ~ Intervention + Sex
-0,194905322	y ~ Intervention + Sex
-2,076588891	y ~ Intervention + Sex
-0,099456024	y ~ Intervention + Sex

-0,808367344	y ~ Intervention + Sex
0,575307268	y ~ Intervention + Sex
1,683622347	y ~ Intervention + Sex
-0,535658665	y ~ Intervention + Sex
0,789144362	y ~ Intervention + Sex
-0,53653488	y ~ Intervention + Sex
-0,795430224	y ~ Intervention + Sex
0,978908141	y ~ Intervention + Sex
0,303816763	y ~ Intervention + Sex
1,686068087	y ~ Intervention + Sex
2,017705839	y ~ Intervention + Sex
-1,05636466	y ~ Intervention + Sex
0,160086013	y ~ Intervention + Sex
1,241996367	y ~ Intervention + Sex
-0,811723487	y ~ Intervention + Sex
0,617315044	y ~ Intervention + Sex
1,351162557	y ~ Intervention + Sex
-2,461757575	y ~ Intervention + Sex
-0,351353736	y ~ Intervention + Sex
0,730903243	y ~ Intervention + Sex
-0,222869971	y ~ Intervention + Sex
0,790064521	y ~ Intervention + Sex
-0,320904336	y ~ Intervention + Sex
-0,274092245	y ~ Intervention + Sex
0,335684971	y ~ Intervention + Sex
0,078547132	y ~ Intervention + Sex
0,724884429	y ~ Intervention + Sex
-0,479060212	y ~ Intervention + Sex
-0,186295672	y ~ Intervention + Sex
-0,569596442	y ~ Intervention + Sex
0,567056215	y ~ Intervention + Sex
-1,068541414	y ~ Intervention + Sex
1,239327985	y ~ Intervention + Sex
-1,542942792	y ~ Intervention + Sex
2,54813429	y ~ Intervention + Sex
0,557583905	y ~ Intervention + Sex
2,093159149	y ~ Intervention + Sex
-1,278825291	y ~ Intervention + Sex
-0,142129732	y ~ Intervention + Sex
0,104718031	y ~ Intervention + Sex
-1,495620447	y ~ Intervention + Sex
-1,452468104	y ~ Intervention + Sex
1,49838864	y ~ Intervention + Sex
-1,713079361	y ~ Intervention + Sex
1,715927222	y ~ Intervention + Sex
0,126580272	y ~ Intervention + Sex
0,072819461	y ~ Intervention + Sex
1,016652651	y ~ Intervention + Sex

-0,136723378	y ~ Intervention + Sex
-0,087453385	y ~ Intervention + Sex
-0,79169556	y ~ Intervention + Sex
-0,580921053	y ~ Intervention + Sex
-0,334581832	y ~ Intervention + Sex
-0,047003787	y ~ Intervention + Sex
-0,036160558	y ~ Intervention + Sex
-0,450495365	y ~ Intervention + Sex
2,158056491	y ~ Intervention + Sex
0,707368238	y ~ Intervention + Sex
0,810090635	y ~ Intervention + Sex
1,658781519	y ~ Intervention + Sex
-0,49360358	y ~ Intervention + Sex
0,603890124	y ~ Intervention + Sex
-0,055625839	y ~ Intervention + Sex
1,295616549	y ~ Intervention + Sex
-1,29028336	y ~ Intervention + Sex
0,098833193	y ~ Intervention + Sex
1,071065162	y ~ Intervention + Sex
1,167957876	y ~ Intervention + Sex
1,457831741	y ~ Intervention + Sex
1,003791248	y ~ Intervention + Sex
-0,405695676	y ~ Intervention + Sex
-0,558622764	y ~ Intervention + Sex
1,024155684	y ~ Intervention + Sex
1,522872312	y ~ Intervention + Sex
1,014432758	y ~ Intervention + Sex
1,641049009	y ~ Intervention + Sex
0,223566653	y ~ Intervention + Sex
0,776273523	y ~ Intervention + Sex
-0,572095891	y ~ Intervention + Sex
-0,283474846	y ~ Intervention + Sex
0,754954159	y ~ Intervention + Sex
-0,559005894	y ~ Intervention + Sex
0,031917704	y ~ Intervention + Sex
-1,354677457	y ~ Intervention + Sex
0,7525176	y ~ Intervention + Sex
0,919312315	y ~ Intervention + Sex
1,529391825	y ~ Intervention + Sex
-0,533562325	y ~ Intervention + Sex
0,427064149	y ~ Intervention + Sex
-0,476565122	y ~ Intervention + Sex
-0,017255346	y ~ Intervention + Sex
0,532990668	y ~ Intervention + Sex
-0,610659285	y ~ Intervention + Sex
-0,096019023	y ~ Intervention + Sex
1,770432089	y ~ Intervention + Sex
-0,492808611	y ~ Intervention + Sex

-0,71762324	y ~ Intervention + Sex
1,028156067	y ~ Intervention + Sex
1,127024599	y ~ Intervention + Sex
0,126764363	y ~ Intervention + Sex
0,173541867	y ~ Intervention + Sex
-0,721836184	y ~ Intervention + Sex
0,87807467	y ~ Intervention + Sex
1,482958025	y ~ Intervention + Sex
-1,440906752	y ~ Intervention + Sex
0,44061994	y ~ Intervention + Sex
0,135562406	y ~ Intervention + Sex
0,593861596	y ~ Intervention + Sex
-0,010780111	y ~ Intervention + Sex
-0,513668302	y ~ Intervention + Sex
0,727801298	y ~ Intervention + Sex
0,621534567	y ~ Intervention + Sex
-1,452893446	y ~ Intervention + Sex
-0,32615403	y ~ Intervention + Sex
0,860528999	y ~ Intervention + Sex
0,66721854	y ~ Intervention + Sex
-0,956113343	y ~ Intervention + Sex
0,087107725	y ~ Intervention + Sex
-0,286624699	y ~ Intervention + Sex
1,074480442	y ~ Intervention + Sex
-1,015367271	y ~ Intervention + Sex
0,847954137	y ~ Intervention + Sex
-0,272484237	y ~ Intervention + Sex
1,73488479	y ~ Intervention + Sex
1,296517027	y ~ Intervention + Sex
1,486307029	y ~ Intervention + Sex
1,063344679	y ~ Intervention + Sex
-0,362293455	y ~ Intervention + Sex
-0,489980351	y ~ Intervention + Sex
-0,376062901	y ~ Intervention + Sex
0,543071667	y ~ Intervention + Sex
-0,551354744	y ~ Intervention + Sex
0,163978865	y ~ Intervention + Sex
0,312324912	y ~ Intervention + Sex
0,472054132	y ~ Intervention + Sex
-0,476578456	y ~ Intervention + Sex
0,365797564	y ~ Intervention + Sex
-0,864884248	y ~ Intervention + Sex
0,447208828	y ~ Intervention + Sex
-2,006784861	y ~ Intervention + Sex
-0,636518895	y ~ Intervention + Sex
0,351994284	y ~ Intervention + Sex
0,426595131	y ~ Intervention + Sex
2,483868575	y ~ Intervention + Sex

0,331614708	y ~ Intervention + Sex
0,480989493	y ~ Intervention + Sex
2,388463295	y ~ Intervention + Sex
0,414040845	y ~ Intervention + Sex
-0,04571396	y ~ Intervention + Sex
1,038774975	y ~ Intervention + Sex
-0,982725475	y ~ Intervention + Sex
0,66103187	y ~ Intervention + Sex
0,902489619	y ~ Intervention + Sex
0,432890324	y ~ Intervention + Sex
1,075133395	y ~ Intervention + Sex
0,430207702	y ~ Intervention + Sex
-0,303610708	y ~ Intervention + Sex
-0,504922704	y ~ Intervention + Sex
-0,571726043	y ~ Intervention + Sex
-1,401245811	y ~ Intervention + Sex
0,681555981	y ~ Intervention + Sex
0,656800338	y ~ Intervention + Sex
0,867151485	y ~ Intervention + Sex
0,277944387	y ~ Intervention + Sex
-0,846071714	y ~ Intervention + Sex
0,493477624	y ~ Intervention + Sex
-0,259241557	y ~ Intervention + Sex
-0,186256352	y ~ Intervention + Sex
0,76173297	y ~ Intervention + Sex
-0,633081876	y ~ Intervention + Sex
0,082728181	y ~ Intervention + Sex
0,113820369	y ~ Intervention + Sex
1,477726365	y ~ Intervention + Sex
0,983995039	y ~ Intervention + Sex
-0,201539225	y ~ Intervention + Sex
-1,293065659	y ~ Intervention + Sex
-3,075603003	y ~ Intervention + Sex
-0,565611667	y ~ Intervention + Sex
-0,110392594	y ~ Intervention + Sex
-1,851062892	y ~ Intervention + Sex
0,61723415	y ~ Intervention + Sex
0,748671963	y ~ Intervention + Sex
0,17574544	y ~ Intervention + Sex
0,895692117	y ~ Intervention + Sex
0,500178975	y ~ Intervention + Sex
-0,841703406	y ~ Intervention + Sex
0,009557408	y ~ Intervention + Sex
2,444831196	y ~ Intervention + Sex
-1,167140167	y ~ Intervention + Sex
-0,930802031	y ~ Intervention + Sex
0,855913436	y ~ Intervention + Sex
-2,476001563	y ~ Intervention + Sex

0,38301589	y ~ Intervention + Sex
0,137809452	y ~ Intervention + Sex
-0,176060907	y ~ Intervention + Sex
1,328514138	y ~ Intervention + Sex
0,842529914	y ~ Intervention + Sex
-0,980105941	y ~ Intervention + Sex
-0,044057154	y ~ Intervention + Sex
-0,403921514	y ~ Intervention + Sex
-1,861218271	y ~ Intervention + Sex
0,158203109	y ~ Intervention + Sex
0,922128669	y ~ Intervention + Sex
-0,023502833	y ~ Intervention + Sex
-0,915014365	y ~ Intervention + Sex
0,4118458	y ~ Intervention + Sex
0,294956183	y ~ Intervention + Sex
-0,418015924	y ~ Intervention + Sex
-2,04373671	y ~ Intervention + Sex
-1,119615016	y ~ Intervention + Sex
-0,429342252	y ~ Intervention + Sex
-0,991746664	y ~ Intervention + Sex
-0,677098856	y ~ Intervention + Sex
0,700724379	y ~ Intervention + Sex
0,154738576	y ~ Intervention + Sex
0,892917563	y ~ Intervention + Sex
-0,887520603	y ~ Intervention + Sex
-1,227900225	y ~ Intervention + Sex
-0,239851034	y ~ Intervention + Sex
0,713322465	y ~ Intervention + Sex
-2,095283619	y ~ Intervention + Sex
0,920600075	y ~ Intervention + Sex
2,369285595	y ~ Intervention + Sex
-0,021287512	y ~ Intervention + Sex
0,454848744	y ~ Intervention + Sex
-3,100975922	y ~ Intervention + Sex
1,402782958	y ~ Intervention + Sex
-1,678957727	y ~ Intervention + Sex
-0,83672253	y ~ Intervention + Sex
-1,236982743	y ~ Intervention + Sex
-0,089243355	y ~ Intervention + Sex
-1,432277325	y ~ Intervention + Sex
-2,044927644	y ~ Intervention + Sex
-1,564364497	y ~ Intervention + Sex
-0,006177217	y ~ Intervention + Sex
-2,6592619	y ~ Intervention + Sex
-0,870256781	y ~ Intervention + Sex
1,321010406	y ~ Intervention + Sex
-1,279758397	y ~ Intervention + Sex
0,85758222	y ~ Intervention + Sex

-0,849655259	y ~ Intervention + Sex
0,68409369	y ~ Intervention + Sex
-1,139161148	y ~ Intervention + Sex
1,106871827	y ~ Intervention + Sex
0,287033112	y ~ Intervention + Sex
-0,56189547	y ~ Intervention + Sex
-1,554168196	y ~ Intervention + Sex
-0,935804654	y ~ Intervention + Sex
-1,905934635	y ~ Intervention + Sex
0,530253698	y ~ Intervention + Sex
0,264211009	y ~ Intervention + Sex
-0,934575294	y ~ Intervention + Sex
0,803719884	y ~ Intervention + Sex
-0,759673668	y ~ Intervention + Sex
-0,033607406	y ~ Intervention + Sex
1,302940144	y ~ Intervention + Sex
-1,054132421	y ~ Intervention + Sex
-0,70595065	y ~ Intervention + Sex
-0,231449906	y ~ Intervention + Sex
-0,288248991	y ~ Intervention + Sex
-2,146870263	y ~ Intervention + Sex
0,519996714	y ~ Intervention + Sex
0,488804377	y ~ Intervention + Sex
-0,263550266	y ~ Intervention + Sex
-1,514109586	y ~ Intervention + Sex
-0,196307684	y ~ Intervention + Sex
-2,359604197	y ~ Intervention + Sex
-1,434705301	y ~ Intervention + Sex
0,561283168	y ~ Intervention + Sex
1,545365541	y ~ Intervention + Sex
1,159762525	y ~ Intervention + Sex
0,354028453	y ~ Intervention + Sex
-0,38004088	y ~ Intervention + Sex
0,27258543	y ~ Intervention + Sex
-0,011690691	y ~ Intervention + Sex
-2,360633789	y ~ Intervention + Sex
-0,078591338	y ~ Intervention + Sex
-1,479595809	y ~ Intervention + Sex
0,469893812	y ~ Intervention + Sex
-2,543566873	y ~ Intervention + Sex
-0,293812779	y ~ Intervention + Sex
-0,725548478	y ~ Intervention + Sex
0,675521315	y ~ Intervention + Sex
-0,007141141	y ~ Intervention + Sex
-0,932828105	y ~ Intervention + Sex
-0,895464093	y ~ Intervention + Sex
-0,590527341	y ~ Intervention + Sex
-0,529366006	y ~ Intervention + Sex

-0,812026661	y ~ Intervention + Sex
0,752180001	y ~ Intervention + Sex
-0,094692012	y ~ Intervention + Sex
0,605097056	y ~ Intervention + Sex
0,102304962	y ~ Intervention + Sex
-1,194235313	y ~ Intervention + Sex
-0,989003279	y ~ Intervention + Sex
-1,759551766	y ~ Intervention + Sex
-1,909394042	y ~ Intervention + Sex
0,367229042	y ~ Intervention + Sex
0,820129773	y ~ Intervention + Sex
-1,481174276	y ~ Intervention + Sex
0,022364258	y ~ Intervention + Sex
-1,494067956	y ~ Intervention + Sex
-1,19456952	y ~ Intervention + Sex
0,478301429	y ~ Intervention + Sex
-0,996512833	y ~ Intervention + Sex
-0,37225826	y ~ Intervention + Sex
-2,404256167	y ~ Intervention + Sex
-1,59176563	y ~ Intervention + Sex
0,256995596	y ~ Intervention + Sex
-0,100707452	y ~ Intervention + Sex
0,734498096	y ~ Intervention + Sex
-2,545845435	y ~ Intervention + Sex
0,832774595	y ~ Intervention + Sex
-2,142243157	y ~ Intervention + Sex
-0,462514096	y ~ Intervention + Sex
-0,746864592	y ~ Intervention + Sex
0,735838766	y ~ Intervention + Sex
0,096747196	y ~ Intervention + Sex
-1,820839213	y ~ Intervention + Sex
-0,575132158	y ~ Intervention + Sex
0,226947221	y ~ Intervention + Sex
-0,608368931	y ~ Intervention + Sex
-0,422350599	y ~ Intervention + Sex
-0,950332056	y ~ Intervention + Sex
-1,293130254	y ~ Intervention + Sex
-2,759067227	y ~ Intervention + Sex
-3,031298987	y ~ Intervention + Sex
0,277701703	y ~ Intervention + Sex
0,57893705	y ~ Intervention + Sex
-0,361541044	y ~ Intervention + Sex
-2,761365217	y ~ Intervention + Sex
0,835553507	y ~ Intervention + Sex
-3,301923476	y ~ Intervention + Sex
-2,436868588	y ~ Intervention + Sex
-1,154592413	y ~ Intervention + Sex
-1,441798569	y ~ Intervention + Sex

0,244189924	y ~ Intervention + Sex
-1,813149039	y ~ Intervention + Sex
-0,187767456	y ~ Intervention + Sex
-1,440675709	y ~ Intervention + Sex
2,386260768	y ~ Intervention + Sex
2,943770769	y ~ Intervention + Sex
-1,626898092	y ~ Intervention + Sex
1,394670251	y ~ Intervention + Sex
-0,623281099	y ~ Intervention + Sex
-0,925982706	y ~ Intervention + Sex
-0,866822556	y ~ Intervention + Sex
2,43081002	y ~ Intervention + Sex
1,812952532	y ~ Intervention + Sex
-1,238317626	y ~ Intervention + Sex
-1,410854328	y ~ Intervention + Sex
-1,013956847	y ~ Intervention + Sex
-0,757737093	y ~ Intervention + Sex
-0,053829793	y ~ Intervention + Sex
0,831852955	y ~ Intervention + Sex
0,085037564	y ~ Intervention + Sex
-2,124715987	y ~ Intervention + Sex
-0,914439044	y ~ Intervention + Sex
-1,430849719	y ~ Intervention + Sex
-0,193713306	y ~ Intervention + Sex
-0,138568521	y ~ Intervention + Sex
-1,553430472	y ~ Intervention + Sex
0,502818947	y ~ Intervention + Sex
-1,328309782	y ~ Intervention + Sex
-1,142313913	y ~ Intervention + Sex
2,687931159	y ~ Intervention + Sex
-0,840751752	y ~ Intervention + Sex
1,295757271	y ~ Intervention + Sex
1,195580976	y ~ Intervention + Sex
1,030253357	y ~ Intervention + Sex
0,840982092	y ~ Intervention + Sex
-1,373664081	y ~ Intervention + Sex
-1,848444493	y ~ Intervention + Sex
-0,239191768	y ~ Intervention + Sex
-0,853289952	y ~ Intervention + Sex
-2,310023865	y ~ Intervention + Sex
-1,03099174	y ~ Intervention + Sex
-0,356063255	y ~ Intervention + Sex
1,286058415	y ~ Intervention + Sex
-0,794918471	y ~ Intervention + Sex
-1,204667977	y ~ Intervention + Sex
0,216820092	y ~ Intervention + Sex
1,474036987	y ~ Intervention + Sex
-0,040928532	y ~ Intervention + Sex

-0,494179548	y ~ Intervention + Sex
-1,450200658	y ~ Intervention + Sex
-1,443668445	y ~ Intervention + Sex
-1,107405497	y ~ Intervention + Sex
-2,764368311	y ~ Intervention + Sex
0,735234753	y ~ Intervention + Sex
-1,678157839	y ~ Intervention + Sex
-0,351818713	y ~ Intervention + Sex
1,085435338	y ~ Intervention + Sex
-0,027216374	y ~ Intervention + Sex
-0,627386468	y ~ Intervention + Sex
1,126751525	y ~ Intervention + Sex
1,471808785	y ~ Intervention + Sex
0,628117489	y ~ Intervention + Sex
0,58619673	y ~ Intervention + Sex
-0,829166784	y ~ Intervention + Sex
-1,207458342	y ~ Intervention + Sex
0,715773753	y ~ Intervention + Sex
-1,112048399	y ~ Intervention + Sex
-0,32458071	y ~ Intervention + Sex
-0,741091519	y ~ Intervention + Sex
-0,358842549	y ~ Intervention + Sex
-0,067602358	y ~ Intervention + Sex
1,209041189	y ~ Intervention + Sex
-1,978972053	y ~ Intervention + Sex
-2,35262261	y ~ Intervention + Sex
-0,848037953	y ~ Intervention + Sex
-0,231059888	y ~ Intervention + Sex
1,140668122	y ~ Intervention + Sex
-0,825458198	y ~ Intervention + Sex
-0,464355052	y ~ Intervention + Sex
-1,938977479	y ~ Intervention + Sex
-0,477987495	y ~ Intervention + Sex
-0,153710171	y ~ Intervention + Sex
1,911531242	y ~ Intervention + Sex
-0,213917182	y ~ Intervention + Sex
-0,887605052	y ~ Intervention + Sex
-1,196790988	y ~ Intervention + Sex
0,337465798	y ~ Intervention + Sex
-0,945485682	y ~ Intervention + Sex
-0,062863772	y ~ Intervention + Sex
-0,397401424	y ~ Intervention + Sex
-1,413637733	y ~ Intervention + Sex
-1,39152253	y ~ Intervention + Sex
-0,712513296	y ~ Intervention + Sex
1,456172116	y ~ Intervention + Sex
-0,83742969	y ~ Intervention + Sex
0,316223021	y ~ Intervention + Sex

0,263228893	y ~ Intervention + Sex
0,000448708	y ~ Intervention + Sex
-1,30206401	y ~ Intervention + Sex
-0,726177058	y ~ Intervention + Sex
-0,990029083	y ~ Intervention + Sex
-1,463540828	y ~ Intervention + Sex
-0,428272958	y ~ Intervention + Sex
0,264030665	y ~ Intervention + Sex
0,040147028	y ~ Intervention + Sex
-2,220326639	y ~ Intervention + Sex
-0,557468533	y ~ Intervention + Sex
-0,498745567	y ~ Intervention + Sex
-0,764893498	y ~ Intervention + Sex
0,359309131	y ~ Intervention + Sex
0,100758316	y ~ Intervention + Sex
1,350778447	y ~ Intervention + Sex
-0,14213888	y ~ Intervention + Sex
0,296331969	y ~ Intervention + Sex
2,113626831	y ~ Intervention + Sex
-1,567387436	y ~ Intervention + Sex
0,088860213	y ~ Intervention + Sex
-0,683178515	y ~ Intervention + Sex
0,4176874	y ~ Intervention + Sex
-0,992681681	y ~ Intervention + Sex
0,585031765	y ~ Intervention + Sex
1,764821672	y ~ Intervention + Sex
-0,132755591	y ~ Intervention + Sex
-0,2018899	y ~ Intervention + Sex
0,677336098	y ~ Intervention + Sex
0,889344718	y ~ Intervention + Sex
-0,518912168	y ~ Intervention + Sex
0,260770304	y ~ Intervention + Sex
1,183203969	y ~ Intervention + Sex
0,815649407	y ~ Intervention + Sex
-1,702231437	y ~ Intervention + Sex
-0,229236453	y ~ Intervention + Sex
0,607061042	y ~ Intervention + Sex
2,217777186	y ~ Intervention + Sex
-1,865885648	y ~ Intervention + Sex
-0,671892704	y ~ Intervention + Sex
0,159748119	y ~ Intervention + Sex
-1,35341804	y ~ Intervention + Sex
-0,149651661	y ~ Intervention + Sex
-2,419966734	y ~ Intervention + Sex
1,409050232	y ~ Intervention + Sex
0,428198161	y ~ Intervention + Sex
2,50907762	y ~ Intervention + Sex
1,398840186	y ~ Intervention + Sex

-1,630031592	y ~ Intervention + Sex
-1,932117666	y ~ Intervention + Sex
-0,512607289	y ~ Intervention + Sex
0,317629596	y ~ Intervention + Sex
-0,075182328	y ~ Intervention + Sex
1,172623265	y ~ Intervention + Sex
3,024113689	y ~ Intervention + Sex
-2,4236944	y ~ Intervention + Sex
0,831035723	y ~ Intervention + Sex
0,054677703	y ~ Intervention + Sex
0,947527839	y ~ Intervention + Sex
0,204957007	y ~ Intervention + Sex
0,992471817	y ~ Intervention + Sex
1,068997979	y ~ Intervention + Sex
0,268470384	y ~ Intervention + Sex
-0,6679869	y ~ Intervention + Sex
0,160043442	y ~ Intervention + Sex
-1,244220256	y ~ Intervention + Sex
2,225210727	y ~ Intervention + Sex
-1,41356262	y ~ Intervention + Sex
1,076691826	y ~ Intervention + Sex
-0,314440447	y ~ Intervention + Sex
0,023605168	y ~ Intervention + Sex
-0,278238742	y ~ Intervention + Sex
0,159014143	y ~ Intervention + Sex
-0,328288726	y ~ Intervention + Sex
0,45470965	y ~ Intervention + Sex
-0,519926773	y ~ Intervention + Sex
0,423817803	y ~ Intervention + Sex
-0,593259607	y ~ Intervention + Sex
-0,226555199	y ~ Intervention + Sex
-0,754588084	y ~ Intervention + Sex
-0,343535246	y ~ Intervention + Sex
2,136877005	y ~ Intervention + Sex
0,620930579	y ~ Intervention + Sex
0,605760049	y ~ Intervention + Sex
1,757910222	y ~ Intervention + Sex
-1,319457929	y ~ Intervention + Sex
0,018449594	y ~ Intervention + Sex
0,542256598	y ~ Intervention + Sex
0,501761403	y ~ Intervention + Sex
1,997376691	y ~ Intervention + Sex
-0,302296167	y ~ Intervention + Sex
2,048589325	y ~ Intervention + Sex
-1,477096472	y ~ Intervention + Sex
-1,318156632	y ~ Intervention + Sex
-1,113204782	y ~ Intervention + Sex
0,62005455	y ~ Intervention + Sex

-2,166383572	y ~ Intervention + Sex
0,234602307	y ~ Intervention + Sex
2,589116625	y ~ Intervention + Sex
-0,218419062	y ~ Intervention + Sex
0,77068258	y ~ Intervention + Sex
0,115589505	y ~ Intervention + Sex
-0,101175107	y ~ Intervention + Sex
1,287110331	y ~ Intervention + Sex
-0,90813296	y ~ Intervention + Sex
0,71170569	y ~ Intervention + Sex
-0,846416121	y ~ Intervention + Sex
1,334365935	y ~ Intervention + Sex
1,649487585	y ~ Intervention + Sex
0,015873426	y ~ Intervention + Sex
0,678738231	y ~ Intervention + Sex
0,060612141	y ~ Intervention + Sex
-0,961617616	y ~ Intervention + Sex
-0,195676644	y ~ Intervention + Sex
-0,700244456	y ~ Intervention + Sex
1,677513469	y ~ Intervention + Sex
0,337030355	y ~ Intervention + Sex
-0,579449696	y ~ Intervention + Sex
0,361047402	y ~ Intervention + Sex
0,697663894	y ~ Intervention + Sex
-0,529555882	y ~ Intervention + Sex
-1,212613762	y ~ Intervention + Sex
-1,354457742	y ~ Intervention + Sex
1,872056048	y ~ Intervention + Sex
0,692400848	y ~ Intervention + Sex
-0,622145881	y ~ Intervention + Sex
-0,45170914	y ~ Intervention + Sex
1,194103043	y ~ Intervention + Sex
1,135678639	y ~ Intervention + Sex
2,293173688	y ~ Intervention + Sex
-0,175402343	y ~ Intervention + Sex
0,353137118	y ~ Intervention + Sex
1,854481721	y ~ Intervention + Sex
0,183656787	y ~ Intervention + Sex
1,920302738	y ~ Intervention + Sex
-0,906725103	y ~ Intervention + Sex
1,506612882	y ~ Intervention + Sex
0,726335199	y ~ Intervention + Sex
-1,824447628	y ~ Intervention + Sex
-1,015198301	y ~ Intervention + Sex
0,755740204	y ~ Intervention + Sex
0,551779754	y ~ Intervention + Sex
0,85255394	y ~ Intervention + Sex
0,053515467	y ~ Intervention + Sex

-0,651461436	y ~ Intervention + Sex
2,171610881	y ~ Intervention + Sex
-0,519687426	y ~ Intervention + Sex
1,687433309	y ~ Intervention + Sex
-1,198275477	y ~ Intervention + Sex
0,57114378	y ~ Intervention + Sex
1,520979264	y ~ Intervention + Sex
-0,401966807	y ~ Intervention + Sex
0,357786044	y ~ Intervention + Sex
-0,0704841	y ~ Intervention + Sex
1,042439641	y ~ Intervention + Sex
0,9577794	y ~ Intervention + Sex
0,636397127	y ~ Intervention + Sex
-2,344669412	y ~ Intervention + Sex
-0,938373723	y ~ Intervention + Sex
-0,745574155	y ~ Intervention + Sex
0,635645167	y ~ Intervention + Sex
0,080891348	y ~ Intervention + Sex
0,411225302	y ~ Intervention + Sex
-1,583299573	y ~ Intervention + Sex
1,502705871	y ~ Intervention + Sex
-0,306780566	y ~ Intervention + Sex
-0,78718	y ~ Intervention + Sex
-1,326451771	y ~ Intervention + Sex
0,962492743	y ~ Intervention + Sex
0,268291102	y ~ Intervention + Sex
1,544502565	y ~ Intervention + Sex
-0,512614687	y ~ Intervention + Sex
2,352524983	y ~ Intervention + Sex
-0,499713399	y ~ Intervention + Sex
-1,17679597	y ~ Intervention + Sex
2,791705168	y ~ Intervention + Sex
-0,279573844	y ~ Intervention + Sex
-0,643074333	y ~ Intervention + Sex
-0,066233041	y ~ Intervention + Sex
0,572249309	y ~ Intervention + Sex
-0,616070869	y ~ Intervention + Sex
0,546381713	y ~ Intervention + Sex
-0,027908405	y ~ Intervention + Sex
1,319640591	y ~ Intervention + Sex
1,004764282	y ~ Intervention + Sex
0,904266505	y ~ Intervention + Sex
-0,059462602	y ~ Intervention + Sex
-0,157231575	y ~ Intervention + Sex
-0,621332082	y ~ Intervention + Sex
0,07207329	y ~ Intervention + Sex
0,959570776	y ~ Intervention + Sex
-1,987038446	y ~ Intervention + Sex

-0,617321518	y ~ Intervention + Sex
0,419474106	y ~ Intervention + Sex
-0,204990971	y ~ Intervention + Sex
0,058903372	y ~ Intervention + Sex
0,561868575	y ~ Intervention + Sex
-0,014085527	y ~ Intervention + Sex
-0,060460403	y ~ Intervention + Sex
-2,179967934	y ~ Intervention + Sex
0,466106357	y ~ Intervention + Sex
1,007027192	y ~ Intervention + Sex
0,104695199	y ~ Intervention + Sex
0,785534358	y ~ Intervention + Sex
2,253476663	y ~ Intervention + Sex
-0,703199158	y ~ Intervention + Sex
1,692318656	y ~ Intervention + Sex
-0,899766987	y ~ Intervention + Sex
-1,50610316	y ~ Intervention + Sex
0,711623449	y ~ Intervention + Sex
-0,169027369	y ~ Intervention + Sex
0,969181746	y ~ Intervention + Sex
0,693337036	y ~ Intervention + Sex
1,009634789	y ~ Intervention + Sex
0,494370164	y ~ Intervention + Sex
-0,830508095	y ~ Intervention + Sex
0,709551927	y ~ Intervention + Sex
-0,484072055	y ~ Intervention + Sex
-0,210004561	y ~ Intervention + Sex
0,166407453	y ~ Intervention + Sex
-0,268885827	y ~ Intervention + Sex
-1,518640963	y ~ Intervention + Sex
-0,636139117	y ~ Intervention + Sex
-0,470718387	y ~ Intervention + Sex
-0,428128139	y ~ Intervention + Sex
0,68205271	y ~ Intervention + Sex
0,296790219	y ~ Intervention + Sex
1,150069131	y ~ Intervention + Sex
1,503123983	y ~ Intervention + Sex
0,752401244	y ~ Intervention + Sex
-0,336964189	y ~ Intervention + Sex
-0,835782359	y ~ Intervention + Sex
-0,983539013	y ~ Intervention + Sex
0,767779923	y ~ Intervention + Sex
-0,697533069	y ~ Intervention + Sex
0,040887462	y ~ Intervention + Sex
0,553184179	y ~ Intervention + Sex
0,582356614	y ~ Intervention + Sex
-0,247891819	y ~ Intervention + Sex
0,296822543	y ~ Intervention + Sex

0,153425132	y ~ Intervention + Sex
1,204838641	y ~ Intervention + Sex
1,668421492	y ~ Intervention + Sex
0,921043144	y ~ Intervention + Sex
1,149421718	y ~ Intervention + Sex
-0,988712585	y ~ Intervention + Sex
1,802688733	y ~ Intervention + Sex
0,320671042	y ~ Intervention + Sex
-1,274102521	y ~ Intervention + Sex
2,68908968	y ~ Intervention + Sex
-1,117691749	y ~ Intervention + Sex
0,055202977	y ~ Intervention + Sex
-0,509433826	y ~ Intervention + Sex
-0,539343899	y ~ Intervention + Sex
0,480055814	y ~ Intervention + Sex
-0,16341975	y ~ Intervention + Sex
2,66773366	y ~ Intervention + Sex
0,231876926	y ~ Intervention + Sex
1,429137456	y ~ Intervention + Sex
-1,377894636	y ~ Intervention + Sex
1,006326085	y ~ Intervention + Sex
0,99129779	y ~ Intervention + Sex
1,350202587	y ~ Intervention + Sex
0,16276216	y ~ Intervention + Sex
0,929421736	y ~ Intervention + Sex
-0,044667462	y ~ Intervention + Sex
1,245366957	y ~ Intervention + Sex
-1,612505492	y ~ Intervention + Sex
-0,690320636	y ~ Intervention + Sex
-1,919495818	y ~ Intervention + Sex
1,353597636	y ~ Intervention + Sex
-0,613617662	y ~ Intervention + Sex
0,092368908	y ~ Intervention + Sex
1,343386064	y ~ Intervention + Sex
-0,919214096	y ~ Intervention + Sex
-1,641717752	y ~ Intervention + Sex
1,256885694	y ~ Intervention + Sex
0,445635958	y ~ Intervention + Sex
-0,243626309	y ~ Intervention + Sex
1,206331851	y ~ Intervention + Sex
1,367137441	y ~ Intervention + Sex
-0,125845993	y ~ Intervention + Sex
0,51197933	y ~ Intervention + Sex
-0,563148473	y ~ Intervention + Sex
-1,19770036	y ~ Intervention + Sex
0,410311981	y ~ Intervention + Sex
0,2165821	y ~ Intervention + Sex
0,580739476	y ~ Intervention + Sex

-1,622522685	y ~ Intervention + Sex
1,175501522	y ~ Intervention + Sex
1,010553724	y ~ Intervention + Sex
2,064415327	y ~ Intervention + Sex
0,994624793	y ~ Intervention + Sex
-1,854326519	y ~ Intervention + Sex
0,161728663	y ~ Intervention + Sex
-0,045405091	y ~ Intervention + Sex
-0,308968289	y ~ Intervention + Sex
-1,818846089	y ~ Intervention + Sex
0,662129699	y ~ Intervention + Sex
1,454515403	y ~ Intervention + Sex
0,212470727	y ~ Intervention + Sex
0,628569343	y ~ Intervention + Sex
1,438788108	y ~ Intervention + Sex
0,722224644	y ~ Intervention + Sex
-0,284083531	y ~ Intervention + Sex
-0,864877731	y ~ Intervention + Sex
7,057180401	y ~ Intervention + Sex
-0,334743639	y ~ Intervention + Sex
1,577561064	y ~ Intervention + Sex
0,146215354	y ~ Intervention + Sex
-0,297205263	y ~ Intervention + Sex
-0,05506605	y ~ Intervention + Sex
-1,492492499	y ~ Intervention + Sex
-1,274333806	y ~ Intervention + Sex
1,265406977	y ~ Intervention + Sex
-0,276028516	y ~ Intervention + Sex
-0,865335428	y ~ Intervention + Sex
-0,500933667	y ~ Intervention + Sex
0,075428818	y ~ Intervention + Sex
-1,393466097	y ~ Intervention + Sex
1,900255763	y ~ Intervention + Sex
0,444608554	y ~ Intervention + Sex
-0,13262164	y ~ Intervention + Sex
-0,940713481	y ~ Intervention + Sex
2,323965615	y ~ Intervention + Sex
0,048852932	y ~ Intervention + Sex
0,288099493	y ~ Intervention + Sex
-0,067364056	y ~ Intervention + Sex
0,413134	y ~ Intervention + Sex
-0,059229423	y ~ Intervention + Sex
0,043536807	y ~ Intervention + Sex
1,979541299	y ~ Intervention + Sex
1,672339044	y ~ Intervention + Sex
-0,9051635	y ~ Intervention + Sex
0,30377263	y ~ Intervention + Sex
0,798166205	y ~ Intervention + Sex

-0,256161437	y ~ Intervention + Sex
-0,514090851	y ~ Intervention + Sex
1,045050326	y ~ Intervention + Sex
-0,474288967	y ~ Intervention + Sex
-0,114150934	y ~ Intervention + Sex
-1,53847918	y ~ Intervention + Sex
-0,456229168	y ~ Intervention + Sex
1,666675869	y ~ Intervention + Sex
0,197201809	y ~ Intervention + Sex
-0,485566119	y ~ Intervention + Sex
0,772862591	y ~ Intervention + Sex
0,935697621	y ~ Intervention + Sex
1,297475418	y ~ Intervention + Sex
-0,394869821	y ~ Intervention + Sex
0,462264274	y ~ Intervention + Sex
0,667633277	y ~ Intervention + Sex
-0,910776145	y ~ Intervention + Sex
-1,131643069	y ~ Intervention + Sex
-0,468039898	y ~ Intervention + Sex
-0,242608457	y ~ Intervention + Sex
-0,411291286	y ~ Intervention + Sex
-0,656434254	y ~ Intervention + Sex
1,082608323	y ~ Intervention + Sex
0,710311448	y ~ Intervention + Sex
0,790847706	y ~ Intervention + Sex
-1,033970595	y ~ Intervention + Sex
-0,108671384	y ~ Intervention + Sex
2,047166837	y ~ Intervention + Sex
0,741585367	y ~ Intervention + Sex
-1,881946095	y ~ Intervention + Sex
0,117428074	y ~ Intervention + Sex
1,072826056	y ~ Intervention + Sex
-0,601806164	y ~ Intervention + Sex
1,388186056	y ~ Intervention + Sex
-0,903508139	y ~ Intervention + Sex
0,231529504	y ~ Intervention + Sex
0,629716398	y ~ Intervention + Sex
0,358362759	y ~ Intervention + Sex
0,04913052	y ~ Intervention + Sex
2,004157649	y ~ Intervention + Sex
0,399418962	y ~ Intervention + Sex
-0,876144971	y ~ Intervention + Sex
-0,430616114	y ~ Intervention + Sex
0,645166479	y ~ Intervention + Sex
1,495528574	y ~ Intervention + Sex
-0,986872398	y ~ Intervention + Sex
0,339945163	y ~ Intervention + Sex
0,791086936	y ~ Intervention + Sex

-1,782010404	y ~ Intervention + Sex
-0,018672979	y ~ Intervention + Sex
-0,762962797	y ~ Intervention + Sex
-0,137972716	y ~ Intervention + Sex
0,075763681	y ~ Intervention + Sex
-1,251354721	y ~ Intervention + Sex
-1,406429202	y ~ Intervention + Sex
-0,132868461	y ~ Intervention + Sex
0,265540054	y ~ Intervention + Sex
0,827970839	y ~ Intervention + Sex
-0,525076949	y ~ Intervention + Sex
1,375498713	y ~ Intervention + Sex
-0,245130862	y ~ Intervention + Sex
-1,274929822	y ~ Intervention + Sex
-0,513513283	y ~ Intervention + Sex
-1,66948542	y ~ Intervention + Sex
1,45548645	y ~ Intervention + Sex
-0,193643342	y ~ Intervention + Sex
-0,309683071	y ~ Intervention + Sex
0,879323444	y ~ Intervention + Sex
-1,030632772	y ~ Intervention + Sex
-0,056053714	y ~ Intervention + Sex
-0,297904413	y ~ Intervention + Sex
-0,079072177	y ~ Intervention + Sex
-1,374878704	y ~ Intervention + Sex
-1,266827643	y ~ Intervention + Sex
-0,366115711	y ~ Intervention + Sex
-1,61418936	y ~ Intervention + Sex
0,06933789	y ~ Intervention + Sex
-0,54820994	y ~ Intervention + Sex
0,956704156	y ~ Intervention + Sex
-0,125335396	y ~ Intervention + Sex
-0,17373678	y ~ Intervention + Sex
-1,739138326	y ~ Intervention + Sex
1,417096515	y ~ Intervention + Sex
-1,148969535	y ~ Intervention + Sex
-2,419352109	y ~ Intervention + Sex
-0,363777329	y ~ Intervention + Sex
1,124391106	y ~ Intervention + Sex
0,085321862	y ~ Intervention + Sex
1,251424985	y ~ Intervention + Sex
-0,68166453	y ~ Intervention + Sex
0,829826173	y ~ Intervention + Sex
-0,24352684	y ~ Intervention + Sex
0,512454245	y ~ Intervention + Sex
-0,142487033	y ~ Intervention + Sex
0,094678628	y ~ Intervention + Sex
0,11021099	y ~ Intervention + Sex

-1,528463567	y ~ Intervention + Sex
-0,523354259	y ~ Intervention + Sex
0,405733999	y ~ Intervention + Sex
-2,03760777	y ~ Intervention + Sex
-0,694517468	y ~ Intervention + Sex
1,787795607	y ~ Intervention + Sex
-0,016242423	y ~ Intervention + Sex
0,326960644	y ~ Intervention + Sex
-0,233044948	y ~ Intervention + Sex
0,926637438	y ~ Intervention + Sex
0,341034633	y ~ Intervention + Sex
-2,022495093	y ~ Intervention + Sex
-0,063455626	y ~ Intervention + Sex
1,462766563	y ~ Intervention + Sex
0,330388358	y ~ Intervention + Sex
-0,248837277	y ~ Intervention + Sex
-1,602508808	y ~ Intervention + Sex
0,204181917	y ~ Intervention + Sex
0,035249781	y ~ Intervention + Sex
-0,52532378	y ~ Intervention + Sex
-0,121259765	y ~ Intervention + Sex
1,029417213	y ~ Intervention + Sex
-1,960310405	y ~ Intervention + Sex
-0,501462409	y ~ Intervention + Sex
1,709266067	y ~ Intervention + Sex
-1,465702687	y ~ Intervention + Sex
0,976847081	y ~ Intervention + Sex
-2,408088506	y ~ Intervention + Sex
-0,21087502	y ~ Intervention + Sex
1,018584916	y ~ Intervention + Sex
-0,105547894	y ~ Intervention + Sex
0,844326677	y ~ Intervention + Sex
1,880061315	y ~ Intervention + Sex
0,621095289	y ~ Intervention + Sex
-0,84706515	y ~ Intervention + Sex
0,764643394	y ~ Intervention + Sex
2,141823973	y ~ Intervention + Sex
-0,239167925	y ~ Intervention + Sex
1,265392513	y ~ Intervention + Sex
-0,414428748	y ~ Intervention + Sex
0,486053982	y ~ Intervention + Sex
-1,061929641	y ~ Intervention + Sex
-0,30281694	y ~ Intervention + Sex
-0,160729553	y ~ Intervention + Sex
-2,030600229	y ~ Intervention + Sex
0,158019488	y ~ Intervention + Sex
-0,389388169	y ~ Intervention + Sex
0,533460476	y ~ Intervention + Sex

-1,033430975	y ~ Intervention + Sex
-1,393081382	y ~ Intervention + Sex
-1,070316025	y ~ Intervention + Sex
-1,90905075	y ~ Intervention + Sex
-0,240713145	y ~ Intervention + Sex
1,673162904	y ~ Intervention + Sex
-2,10077164	y ~ Intervention + Sex
0,200929683	y ~ Intervention + Sex
0,790476035	y ~ Intervention + Sex
1,833981607	y ~ Intervention + Sex
2,071846192	y ~ Intervention + Sex
-2,517129869	y ~ Intervention + Sex
0,355350854	y ~ Intervention + Sex
-0,898857669	y ~ Intervention + Sex
0,636876911	y ~ Intervention + Sex
-0,178241154	y ~ Intervention + Sex
-0,429914602	y ~ Intervention + Sex
-0,744465178	y ~ Intervention + Sex
0,168802843	y ~ Intervention + Sex
0,345955555	y ~ Intervention + Sex
0,492260979	y ~ Intervention + Sex
-0,921322958	y ~ Intervention + Sex
-0,071536155	y ~ Intervention + Sex
-0,229248154	y ~ Intervention + Sex
-0,158654244	y ~ Intervention + Sex
5,277095157	y ~ Intervention + Sex
0,072679933	y ~ Intervention + Sex
0,885145442	y ~ Intervention + Sex
0,720786983	y ~ Intervention + Sex
-2,555493153	y ~ Intervention + Sex
-0,739780836	y ~ Intervention + Sex
2,514769181	y ~ Intervention + Sex
0,082680344	y ~ Intervention + Sex
1,487777834	y ~ Intervention + Sex
0,361117692	y ~ Intervention + Sex
0,75964952	y ~ Intervention + Sex
0,410678792	y ~ Intervention + Sex
0,144018483	y ~ Intervention + Sex
1,42257498	y ~ Intervention + Sex
0,409199275	y ~ Intervention + Sex
0,569889554	y ~ Intervention + Sex
2,545201681	y ~ Intervention + Sex
-0,488821465	y ~ Intervention + Sex
0,24418929	y ~ Intervention + Sex
0,491476507	y ~ Intervention + Sex
0,789196943	y ~ Intervention + Sex
0,096065614	y ~ Intervention + Sex
-1,314148538	y ~ Intervention + Sex

-0,667640388	y ~ Intervention + Sex
0,153337194	y ~ Intervention + Sex
-1,036008807	y ~ Intervention + Sex
0,093186421	y ~ Intervention + Sex
0,492236864	y ~ Intervention + Sex
0,079987381	y ~ Intervention + Sex
-0,35462852	y ~ Intervention + Sex
-1,145511818	y ~ Intervention + Sex
-2,716868475	y ~ Intervention + Sex
-0,285525741	y ~ Intervention + Sex
-1,26754876	y ~ Intervention + Sex
-0,336930465	y ~ Intervention + Sex
-0,759813733	y ~ Intervention + Sex
-1,108997355	y ~ Intervention + Sex
-0,298223599	y ~ Intervention + Sex
0,857026407	y ~ Intervention + Sex
-0,388528457	y ~ Intervention + Sex
-0,425331858	y ~ Intervention + Sex
0,807061062	y ~ Intervention + Sex
-0,467459603	y ~ Intervention + Sex
0,541717913	y ~ Intervention + Sex
0,093160664	y ~ Intervention + Sex
1,2261042	y ~ Intervention + Sex
-0,253656329	y ~ Intervention + Sex
1,053793339	y ~ Intervention + Sex
-1,202136776	y ~ Intervention + Sex
0,275951867	y ~ Intervention + Sex
-0,09356152	y ~ Intervention + Sex
-1,924061884	y ~ Intervention + Sex
-0,097483198	y ~ Intervention + Sex
-0,372317526	y ~ Intervention + Sex
0,589802576	y ~ Intervention + Sex
1,206159248	y ~ Intervention + Sex
-0,482263051	y ~ Intervention + Sex
0,699457403	y ~ Intervention + Sex
2,228155853	y ~ Intervention + Sex
-2,288212779	y ~ Intervention + Sex
-0,120968028	y ~ Intervention + Sex
0,105724329	y ~ Intervention + Sex
-1,295376151	y ~ Intervention + Sex
-2,293264529	y ~ Intervention + Sex
3,914361685	y ~ Intervention + Sex
0,19169155	y ~ Intervention + Sex
1,40258577	y ~ Intervention + Sex
1,814702205	y ~ Intervention + Sex
0,139030237	y ~ Intervention + Sex
-0,366496855	y ~ Intervention + Sex
1,796991625	y ~ Intervention + Sex

-0,495922348	y ~ Intervention + Sex
-1,663870843	y ~ Intervention + Sex
0,375354402	y ~ Intervention + Sex
0,474505644	y ~ Intervention + Sex
-0,742676093	y ~ Intervention + Sex
-0,064779749	y ~ Intervention + Sex
-0,883631213	y ~ Intervention + Sex
-2,688324354	y ~ Intervention + Sex
-0,663980784	y ~ Intervention + Sex
-0,003309311	y ~ Intervention + Sex
0,353552747	y ~ Intervention + Sex
-1,017726752	y ~ Intervention + Sex
2,105808672	y ~ Intervention + Sex
0,679713908	y ~ Intervention + Sex
0,189789311	y ~ Intervention + Sex
0,469498416	y ~ Intervention + Sex
-1,461359292	y ~ Intervention + Sex
-1,796752859	y ~ Intervention + Sex
0,603798059	y ~ Intervention + Sex
0,430989913	y ~ Intervention + Sex
3,047543711	y ~ Intervention + Sex
3,198142129	y ~ Intervention + Sex
-2,421438114	y ~ Intervention + Sex
-1,298059186	y ~ Intervention + Sex
-2,066366041	y ~ Intervention + Sex
2,643735447	y ~ Intervention + Sex
0,008109579	y ~ Intervention + Sex
0,665098365	y ~ Intervention + Sex
1,180539677	y ~ Intervention + Sex
-0,654205322	y ~ Intervention + Sex
-0,802316063	y ~ Intervention + Sex
0,023356433	y ~ Intervention + Sex
0,385513647	y ~ Intervention + Sex
-0,555997754	y ~ Intervention + Sex
-1,588922157	y ~ Intervention + Sex
1,552158478	y ~ Intervention + Sex
-1,421826248	y ~ Intervention + Sex
-1,347706095	y ~ Intervention + Sex
2,332045839	y ~ Intervention + Sex
0,677614011	y ~ Intervention + Sex
3,462816094	y ~ Intervention + Sex
-1,148918831	y ~ Intervention + Sex
2,611102072	y ~ Intervention + Sex
1,45490381	y ~ Intervention + Sex
-0,856777394	y ~ Intervention + Sex
-0,984132008	y ~ Intervention + Sex
0,240319038	y ~ Intervention + Sex
-0,529743406	y ~ Intervention + Sex

-0,437428188	y ~ Intervention + Sex
0,026924599	y ~ Intervention + Sex
-0,741288871	y ~ Intervention + Sex
0,979857455	y ~ Intervention + Sex
-1,028450197	y ~ Intervention + Sex
-1,951586049	y ~ Intervention + Sex
1,29788022	y ~ Intervention + Sex
2,051832621	y ~ Intervention + Sex
-2,268819071	y ~ Intervention + Sex
-0,085029319	y ~ Intervention + Sex
-3,000953873	y ~ Intervention + Sex
-1,729608585	y ~ Intervention + Sex
-0,956636132	y ~ Intervention + Sex
-2,272476348	y ~ Intervention + Sex
-1,060367341	y ~ Intervention + Sex
-0,316674496	y ~ Intervention + Sex
-1,470134581	y ~ Intervention + Sex
-1,731289725	y ~ Intervention + Sex
-0,630363666	y ~ Intervention + Sex
0,18598622	y ~ Intervention + Sex
-0,409045772	y ~ Intervention + Sex
-1,08241887	y ~ Intervention + Sex
0,179950435	y ~ Intervention + Sex
0,834176676	y ~ Intervention + Sex
1,492765049	y ~ Intervention + Sex
-2,007037118	y ~ Intervention + Sex
0,533726337	y ~ Intervention + Sex
-0,029307753	y ~ Intervention + Sex
-1,030957608	y ~ Intervention + Sex
0,401537334	y ~ Intervention + Sex
-0,723618447	y ~ Intervention + Sex
-0,408109018	y ~ Intervention + Sex
-0,843256077	y ~ Intervention + Sex
1,123650325	y ~ Intervention + Sex
-1,650106856	y ~ Intervention + Sex
-1,502613619	y ~ Intervention + Sex
-0,990735911	y ~ Intervention + Sex
0,90769624	y ~ Intervention + Sex
0,507703866	y ~ Intervention + Sex
-2,540505449	y ~ Intervention + Sex
3,05065071	y ~ Intervention + Sex
-0,900903502	y ~ Intervention + Sex
-1,395004544	y ~ Intervention + Sex
-1,74058146	y ~ Intervention + Sex
-0,492791754	y ~ Intervention + Sex
-0,811962585	y ~ Intervention + Sex
-1,40695844	y ~ Intervention + Sex
-1,33225452	y ~ Intervention + Sex

-2,05676173	y ~ Intervention + Sex
1,80261595	y ~ Intervention + Sex
0,27163223	y ~ Intervention + Sex
-1,183384255	y ~ Intervention + Sex
0,29173666	y ~ Intervention + Sex
-1,602994338	y ~ Intervention + Sex
0,463515723	y ~ Intervention + Sex
-1,28482553	y ~ Intervention + Sex
0,333528226	y ~ Intervention + Sex
0,052180511	y ~ Intervention + Sex
1,688662725	y ~ Intervention + Sex
0,793948871	y ~ Intervention + Sex
0,315128849	y ~ Intervention + Sex
-0,53510762	y ~ Intervention + Sex
0,216816144	y ~ Intervention + Sex
2,081769347	y ~ Intervention + Sex
0,459964417	y ~ Intervention + Sex
-1,669112789	y ~ Intervention + Sex
-2,614453817	y ~ Intervention + Sex
3,652976972	y ~ Intervention + Sex
1,161088985	y ~ Intervention + Sex
-0,228295214	y ~ Intervention + Sex
-0,405653801	y ~ Intervention + Sex
-3,007904387	y ~ Intervention + Sex
-0,084957709	y ~ Intervention + Sex
-0,137223989	y ~ Intervention + Sex
-0,671836057	y ~ Intervention + Sex
1,910329066	y ~ Intervention + Sex
-1,360960618	y ~ Intervention + Sex
0,40364814	y ~ Intervention + Sex
-0,670078452	y ~ Intervention + Sex
-0,123494967	y ~ Intervention + Sex
-0,431342376	y ~ Intervention + Sex
-0,907546627	y ~ Intervention + Sex
0,648928298	y ~ Intervention + Sex
-1,057620339	y ~ Intervention + Sex
0,236431107	y ~ Intervention + Sex
-0,185816097	y ~ Intervention + Sex
-1,923029783	y ~ Intervention + Sex
0,29324939	y ~ Intervention + Sex
-3,11718873	y ~ Intervention + Sex
-0,135647089	y ~ Intervention + Sex
-0,974189077	y ~ Intervention + Sex
0,877097656	y ~ Intervention + Sex
1,371301201	y ~ Intervention + Sex
-0,276199179	y ~ Intervention + Sex
-1,651459796	y ~ Intervention + Sex
-0,480088805	y ~ Intervention + Sex

-2,05479913	y ~ Intervention + Sex
-0,375178945	y ~ Intervention + Sex
-1,702189769	y ~ Intervention + Sex
-3,181849344	y ~ Intervention + Sex
0,0076253	y ~ Intervention + Sex
0,738033633	y ~ Intervention + Sex
-0,030723786	y ~ Intervention + Sex
-0,11142769	y ~ Intervention + Sex
-2,404987673	y ~ Intervention + Sex
-0,607201656	y ~ Intervention + Sex
-1,404390665	y ~ Intervention + Sex
-1,214714469	y ~ Intervention + Sex
-0,132209945	y ~ Intervention + Sex
-2,618566509	y ~ Intervention + Sex
-1,384536579	y ~ Intervention + Sex
0,209893178	y ~ Intervention + Sex
0,166990924	y ~ Intervention + Sex
0,715157503	y ~ Intervention + Sex
-2,95891698	y ~ Intervention + Sex
-2,901876724	y ~ Intervention + Sex
-1,022578881	y ~ Intervention + Sex
0,364147768	y ~ Intervention + Sex
-0,224402838	y ~ Intervention + Sex
1,446105207	y ~ Intervention + Sex
3,663399648	y ~ Intervention + Sex
-0,667826829	y ~ Intervention + Sex
-0,14732845	y ~ Intervention + Sex
1,019802562	y ~ Intervention + Sex
0,173379713	y ~ Intervention + Sex
-1,042260276	y ~ Intervention + Sex
1,415073269	y ~ Intervention + Sex
-0,667854018	y ~ Intervention + Sex
-0,740964522	y ~ Intervention + Sex
-3,406959425	y ~ Intervention + Sex
-0,161940962	y ~ Intervention + Sex
-1,027957224	y ~ Intervention + Sex
1,423903237	y ~ Intervention + Sex
-0,949666129	y ~ Intervention + Sex
3,204767719	y ~ Intervention + Sex
-1,288246657	y ~ Intervention + Sex
-0,781101329	y ~ Intervention + Sex
1,189065165	y ~ Intervention + Sex
-0,740694753	y ~ Intervention + Sex
0,703501995	y ~ Intervention + Sex
0,159546739	y ~ Intervention + Sex
0,286826925	y ~ Intervention + Sex
-0,181286555	y ~ Intervention + Sex
0,322813793	y ~ Intervention + Sex

0,958999158	y ~ Intervention + Sex
2,991945407	y ~ Intervention + Sex
-1,609050715	y ~ Intervention + Sex
-0,426681569	y ~ Intervention + Sex
-1,545982955	y ~ Intervention + Sex
-1,435773083	y ~ Intervention + Sex
-1,330278241	y ~ Intervention + Sex
-1,353910026	y ~ Intervention + Sex
-1,019124832	y ~ Intervention + Sex
1,563470466	y ~ Intervention + Sex
0,346346114	y ~ Intervention + Sex
-2,660626839	y ~ Intervention + Sex
-1,276955312	y ~ Intervention + Sex
-0,488040583	y ~ Intervention + Sex
-1,638398311	y ~ Intervention + Sex
-1,650568631	y ~ Intervention + Sex
-0,692958836	y ~ Intervention + Sex
-0,229759715	y ~ Intervention + Sex
-0,689168252	y ~ Intervention + Sex
-0,14605592	y ~ Intervention + Sex
-1,14169184	y ~ Intervention + Sex
0,485554629	y ~ Intervention + Sex
0,730721481	y ~ Intervention + Sex
-1,988197561	y ~ Intervention + Sex
0,166792195	y ~ Intervention + Sex
0,210119214	y ~ Intervention + Sex
-0,362777936	y ~ Intervention + Sex
0,194380215	y ~ Intervention + Sex
-0,582887781	y ~ Intervention + Sex
0,916599743	y ~ Intervention + Sex
0,931821017	y ~ Intervention + Sex
-1,549477827	y ~ Intervention + Sex
-0,628228117	y ~ Intervention + Sex
-0,299067422	y ~ Intervention + Sex
-1,333604037	y ~ Intervention + Sex
-0,008054444	y ~ Intervention + Sex
1,060848199	y ~ Intervention + Sex
-3,071815954	y ~ Intervention + Sex
0,546444194	y ~ Intervention + Sex
-0,056474065	y ~ Intervention + Sex
0,127463013	y ~ Intervention + Sex
-0,410992711	y ~ Intervention + Sex
-0,612713449	y ~ Intervention + Sex
0,160024275	y ~ Intervention + Sex
2,775108425	y ~ Intervention + Sex
-1,009305645	y ~ Intervention + Sex
3,033541723	y ~ Intervention + Sex
-1,005159013	y ~ Intervention + Sex

-1,262833522	y ~ Intervention + Sex
-0,531197036	y ~ Intervention + Sex
-0,726270853	y ~ Intervention + Sex
0,229101688	y ~ Intervention + Sex
-0,683168962	y ~ Intervention + Sex
0,374351398	y ~ Intervention + Sex
-0,38252061	y ~ Intervention + Sex
-3,592533529	y ~ Intervention + Sex
0,925378314	y ~ Intervention + Sex
-1,751108127	y ~ Intervention + Sex
0,338206867	y ~ Intervention + Sex
0,271991653	y ~ Intervention + Sex
-0,948629206	y ~ Intervention + Sex
-0,889668272	y ~ Intervention + Sex
1,052715555	y ~ Intervention + Sex
0,530591228	y ~ Intervention + Sex
3,391402398	y ~ Intervention + Sex
0,856648093	y ~ Intervention + Sex
-1,502064756	y ~ Intervention + Sex
0,010685036	y ~ Intervention + Sex
2,312459049	y ~ Intervention + Sex
1,962766431	y ~ Intervention + Sex
-0,901006064	y ~ Intervention + Sex
1,104315891	y ~ Intervention + Sex
-1,434861672	y ~ Intervention + Sex
-0,949840534	y ~ Intervention + Sex
-0,418880042	y ~ Intervention + Sex
0,249938207	y ~ Intervention + Sex
-2,221042802	y ~ Intervention + Sex
3,607908288	y ~ Intervention + Sex
-1,464451413	y ~ Intervention + Sex
0,220643979	y ~ Intervention + Sex
-1,76393614	y ~ Intervention + Sex
-0,691914131	y ~ Intervention + Sex
-0,550010141	y ~ Intervention + Sex
-1,204894291	y ~ Intervention + Sex
1,751711407	y ~ Intervention + Sex
-0,859154602	y ~ Intervention + Sex
-0,334226828	y ~ Intervention + Sex
-0,88287868	y ~ Intervention + Sex
-0,032343882	y ~ Intervention + Sex
-1,154590627	y ~ Intervention + Sex
-0,253756532	y ~ Intervention + Sex
-1,475473894	y ~ Intervention + Sex
-0,272102512	y ~ Intervention + Sex
-1,745539155	y ~ Intervention + Sex
-2,416278279	y ~ Intervention + Sex
-0,14943065	y ~ Intervention + Sex

0,052829306	y ~ Intervention + Sex
-1,347018973	y ~ Intervention + Sex
0,540970495	y ~ Intervention + Sex
0,436178063	y ~ Intervention + Sex
-1,764618117	y ~ Intervention + Sex
0,901823299	y ~ Intervention + Sex
1,916228001	y ~ Intervention + Sex
1,914961199	y ~ Intervention + Sex
-2,993232859	y ~ Intervention + Sex
0,628363904	y ~ Intervention + Sex
1,001499762	y ~ Intervention + Sex
-0,406906899	y ~ Intervention + Sex
-0,079921806	y ~ Intervention + Sex
1,314600961	y ~ Intervention + Sex
1,118647126	y ~ Intervention + Sex
-1,132094576	y ~ Intervention + Sex
1,04175862	y ~ Intervention + Sex
-0,352008719	y ~ Intervention + Sex
0,065568062	y ~ Intervention + Sex
-1,021898474	y ~ Intervention + Sex
0,879641409	y ~ Intervention + Sex
-0,885087297	y ~ Intervention + Sex
-1,100270332	y ~ Intervention + Sex
-1,858418518	y ~ Intervention + Sex
0,116009861	y ~ Intervention + Sex
1,800364933	y ~ Intervention + Sex
1,654159908	y ~ Intervention + Sex
0,503577086	y ~ Intervention + Sex
-1,776427299	y ~ Intervention + Sex
0,884339795	y ~ Intervention + Sex
0,764366646	y ~ Intervention + Sex
-0,839469142	y ~ Intervention + Sex
0,363167439	y ~ Intervention + Sex
-0,509258976	y ~ Intervention + Sex
-0,859795729	y ~ Intervention + Sex
-0,849389993	y ~ Intervention + Sex
-0,076185439	y ~ Intervention + Sex
-0,474190854	y ~ Intervention + Sex
0,217584852	y ~ Intervention + Sex
-3,111791651	y ~ Intervention + Sex
0,602270763	y ~ Intervention + Sex
1,208724093	y ~ Intervention + Sex
0,618505806	y ~ Intervention + Sex
-0,359889676	y ~ Intervention + Sex
-0,561510808	y ~ Intervention + Sex
1,529742446	y ~ Intervention + Sex
-0,572506995	y ~ Intervention + Sex
1,167738024	y ~ Intervention + Sex

0,901121684	y ~ Intervention + Sex
-1,758818436	y ~ Intervention + Sex
1,950909404	y ~ Intervention + Sex
0,073027231	y ~ Intervention + Sex
-0,396787992	y ~ Intervention + Sex
-0,169842816	y ~ Intervention + Sex
1,415089008	y ~ Intervention + Sex
-1,748530915	y ~ Intervention + Sex
2,119180762	y ~ Intervention + Sex
1,146685242	y ~ Intervention + Sex
0,088865302	y ~ Intervention + Sex
1,157660153	y ~ Intervention + Sex
1,829454471	y ~ Intervention + Sex
0,765319128	y ~ Intervention + Sex
-2,04180362	y ~ Intervention + Sex
2,855103903	y ~ Intervention + Sex
0,040521075	y ~ Intervention + Sex
1,649594207	y ~ Intervention + Sex
0,025310586	y ~ Intervention + Sex
2,546496497	y ~ Intervention + Sex
1,930139566	y ~ Intervention + Sex
-0,918879166	y ~ Intervention + Sex
-3,428392019	y ~ Intervention + Sex
0,068446112	y ~ Intervention + Sex
0,49492166	y ~ Intervention + Sex
0,894971877	y ~ Intervention + Sex
-2,325463072	y ~ Intervention + Sex
0,612911227	y ~ Intervention + Sex
-0,014207781	y ~ Intervention + Sex
-0,784339738	y ~ Intervention + Sex
2,855779494	y ~ Intervention + Sex
0,756768134	y ~ Intervention + Sex
-1,453141249	y ~ Intervention + Sex
2,114090945	y ~ Intervention + Sex
-0,274964931	y ~ Intervention + Sex
-1,870076606	y ~ Intervention + Sex
-0,92014005	y ~ Intervention + Sex
-0,277696535	y ~ Intervention + Sex
-1,477663397	y ~ Intervention + Sex
-3,016226005	y ~ Intervention + Sex
1,314466102	y ~ Intervention + Sex
-0,853512229	y ~ Intervention + Sex
-1,727641398	y ~ Intervention + Sex
1,470956911	y ~ Intervention + Sex
0,581558567	y ~ Intervention + Sex
-0,470700783	y ~ Intervention + Sex
1,783485964	y ~ Intervention + Sex
2,28978778	y ~ Intervention + Sex

-0,345760832	y ~ Intervention + Sex
1,055388567	y ~ Intervention + Sex
-0,200090301	y ~ Intervention + Sex
0,054137366	y ~ Intervention + Sex
-0,518096485	y ~ Intervention + Sex
-0,278519467	y ~ Intervention + Sex
0,258388237	y ~ Intervention + Sex
-1,466346348	y ~ Intervention + Sex
-1,230358679	y ~ Intervention + Sex
-1,216721647	y ~ Intervention + Sex
-0,813529738	y ~ Intervention + Sex
0,60582688	y ~ Intervention + Sex
-1,226124291	y ~ Intervention + Sex
2,193154183	y ~ Intervention + Sex
0,123604067	y ~ Intervention + Sex
-1,25551133	y ~ Intervention + Sex
-1,846697338	y ~ Intervention + Sex
-0,526841329	y ~ Intervention + Sex
0,755720236	y ~ Intervention + Sex
-0,156230816	y ~ Intervention + Sex
-1,174789444	y ~ Intervention + Sex
-3,064854449	y ~ Intervention + Sex
-2,497846588	y ~ Intervention + Sex
-0,139094681	y ~ Intervention + Sex
0,239904038	y ~ Intervention + Sex
-0,526988362	y ~ Intervention + Sex
-0,050241707	y ~ Intervention + Sex
0,143631543	y ~ Intervention + Sex
-1,628975695	y ~ Intervention + Sex
-0,131814814	y ~ Intervention + Sex
-1,948613928	y ~ Intervention + Sex
0,384195464	y ~ Intervention + Sex
1,950398534	y ~ Intervention + Sex
-2,760248295	y ~ Intervention + Sex
-0,861180076	y ~ Intervention + Sex
-1,243907755	y ~ Intervention + Sex
1,086768665	y ~ Intervention + Sex
0,168658077	y ~ Intervention + Sex
0,846524977	y ~ Intervention + Sex
-2,881697491	y ~ Intervention + Sex
-0,03401483	y ~ Intervention + Sex
1,666052021	y ~ Intervention + Sex
-0,424151302	y ~ Intervention + Sex
0,387746521	y ~ Intervention + Sex
0,09544696	y ~ Intervention + Sex
2,063402084	y ~ Intervention + Sex
-0,353249676	y ~ Intervention + Sex
1,665099123	y ~ Intervention + Sex

0,463743449	y ~ Intervention + Sex
-1,316411071	y ~ Intervention + Sex
-0,306176365	y ~ Intervention + Sex
0,535662712	y ~ Intervention + Sex
-0,662693943	y ~ Intervention + Sex
0,814107799	y ~ Intervention + Sex
-2,961962511	y ~ Intervention + Sex
-0,604873962	y ~ Intervention + Sex
-0,638851682	y ~ Intervention + Sex
0,905192514	y ~ Intervention + Sex
0,710660263	y ~ Intervention + Sex
0,768812004	y ~ Intervention + Sex
-0,959069104	y ~ Intervention + Sex
-0,690836683	y ~ Intervention + Sex
-1,368946225	y ~ Intervention + Sex
0,884710027	y ~ Intervention + Sex
-0,278260898	y ~ Intervention + Sex
0,855428234	y ~ Intervention + Sex
-1,700724218	y ~ Intervention + Sex
0,125970723	y ~ Intervention + Sex
8,775444702	y ~ Intervention + Sex
0,619525445	y ~ Intervention + Sex
-1,930361877	y ~ Intervention + Sex
-3,166474246	y ~ Intervention + Sex
-2,152205825	y ~ Intervention + Sex
0,59517826	y ~ Intervention + Sex
-2,615804579	y ~ Intervention + Sex
-2,062479607	y ~ Intervention + Sex
0,576966602	y ~ Intervention + Sex
-0,83736759	y ~ Intervention + Sex
-0,215398333	y ~ Intervention + Sex
0,576964066	y ~ Intervention + Sex
-1,93997675	y ~ Intervention + Sex
-0,019441744	y ~ Intervention + Sex
0,02999893	y ~ Intervention + Sex
-1,18799463	y ~ Intervention + Sex
-1,00230975	y ~ Intervention + Sex
-1,648555564	y ~ Intervention + Sex
3,783913873	y ~ Intervention + Sex
-1,200804193	y ~ Intervention + Sex
0,884040994	y ~ Intervention + Sex
0,639869043	y ~ Intervention + Sex
-0,559082089	y ~ Intervention + Sex
0,112598153	y ~ Intervention + Sex
0,78333091	y ~ Intervention + Sex
-2,073032844	y ~ Intervention + Sex
-2,398209851	y ~ Intervention + Sex
0,151322437	y ~ Intervention + Sex

3,416121597	y ~ Intervention + Sex
1,748594374	y ~ Intervention + Sex
-0,428248924	y ~ Intervention + Sex
0,174858181	y ~ Intervention + Sex
-0,354742069	y ~ Intervention + Sex
-0,964094116	y ~ Intervention + Sex
0,725940779	y ~ Intervention + Sex
0,566372926	y ~ Intervention + Sex
-0,521336317	y ~ Intervention + Sex
1,863826621	y ~ Intervention + Sex
0,044066196	y ~ Intervention + Sex
0,657701858	y ~ Intervention + Sex
0,361265488	y ~ Intervention + Sex
-0,710297747	y ~ Intervention + Sex
0,163039702	y ~ Intervention + Sex
-3,310775967	y ~ Intervention + Sex
1,127134421	y ~ Intervention + Sex
-0,479266981	y ~ Intervention + Sex
-1,929996581	y ~ Intervention + Sex
2,090818547	y ~ Intervention + Sex
0,55577472	y ~ Intervention + Sex
-0,529590827	y ~ Intervention + Sex
0,54096114	y ~ Intervention + Sex
1,673617314	y ~ Intervention + Sex
-0,87559328	y ~ Intervention + Sex
-0,519370073	y ~ Intervention + Sex
0,388941577	y ~ Intervention + Sex
0,128294266	y ~ Intervention + Sex
0,794491478	y ~ Intervention + Sex
-0,584928334	y ~ Intervention + Sex
0,008197218	y ~ Intervention + Sex
0,49218212	y ~ Intervention + Sex
-0,583285087	y ~ Intervention + Sex
2,010611785	y ~ Intervention + Sex
-2,580945489	y ~ Intervention + Sex
-3,13486932	y ~ Intervention + Sex
0,450973349	y ~ Intervention + Sex
-1,28889947	y ~ Intervention + Sex
0,978143261	y ~ Intervention + Sex
0,062083177	y ~ Intervention + Sex
0,657583909	y ~ Intervention + Sex
1,347532418	y ~ Intervention + Sex
0,71030494	y ~ Intervention + Sex
0,03452668	y ~ Intervention + Sex
-1,8526207	y ~ Intervention + Sex
-0,538399985	y ~ Intervention + Sex
1,126615115	y ~ Intervention + Sex
1,320609349	y ~ Intervention + Sex

-0,509587987	y ~ Intervention + Sex
2,272508108	y ~ Intervention + Sex
-3,396137861	y ~ Intervention + Sex
1,649293614	y ~ Intervention + Sex
-0,550217599	y ~ Intervention + Sex
-0,446458904	y ~ Intervention + Sex
-1,935755588	y ~ Intervention + Sex
3,006197474	y ~ Intervention + Sex
1,171728923	y ~ Intervention + Sex
1,342360054	y ~ Intervention + Sex
-0,271449128	y ~ Intervention + Sex
-0,787086971	y ~ Intervention + Sex
0,572487309	y ~ Intervention + Sex
0,230914489	y ~ Intervention + Sex
-0,163460139	y ~ Intervention + Sex
1,440181414	y ~ Intervention + Sex
-0,412003124	y ~ Intervention + Sex
0,527929795	y ~ Intervention + Sex
-2,569993648	y ~ Intervention + Sex
-0,717005908	y ~ Intervention + Sex
-2,259117522	y ~ Intervention + Sex
1,3487132	y ~ Intervention + Sex
-0,052746699	y ~ Intervention + Sex
-2,470500301	y ~ Intervention + Sex
-1,956929206	y ~ Intervention + Sex
0,013931102	y ~ Intervention + Sex
0,174307566	y ~ Intervention + Sex
0,457175096	y ~ Intervention + Sex
-1,490222223	y ~ Intervention + Sex
-0,833090347	y ~ Intervention + Sex
-1,746524232	y ~ Intervention + Sex
-0,811790327	y ~ Intervention + Sex
0,390670705	y ~ Intervention + Sex
-0,438878988	y ~ Intervention + Sex
1,428063804	y ~ Intervention + Sex
-0,251495653	y ~ Intervention + Sex
-1,402606087	y ~ Intervention + Sex
0,152413237	y ~ Intervention + Sex
-1,008732186	y ~ Intervention + Sex
-0,219595735	y ~ Intervention + Sex
-0,252835487	y ~ Intervention + Sex
0,783313072	y ~ Intervention + Sex
1,347785391	y ~ Intervention + Sex
-2,945453232	y ~ Intervention + Sex
-1,109029419	y ~ Intervention + Sex
-1,241483234	y ~ Intervention + Sex
2,066055261	y ~ Intervention + Sex
1,043415188	y ~ Intervention + Sex

1,230924065	y ~ Intervention + Sex
-0,188421336	y ~ Intervention + Sex
0,60786005	y ~ Intervention + Sex
2,401166328	y ~ Intervention + Sex
-2,780132113	y ~ Intervention + Sex
2,50847551	y ~ Intervention + Sex
0,312145577	y ~ Intervention + Sex
2,89682913	y ~ Intervention + Sex
-0,123998109	y ~ Intervention + Sex
-1,518969327	y ~ Intervention + Sex
0,545927319	y ~ Intervention + Sex
2,263277695	y ~ Intervention + Sex
-0,267823378	y ~ Intervention + Sex
-0,300344295	y ~ Intervention + Sex
-2,92556715	y ~ Intervention + Sex
2,836729634	y ~ Intervention + Sex
1,172123615	y ~ Intervention + Sex
-1,413941662	y ~ Intervention + Sex
1,362047574	y ~ Intervention + Sex
0,05186629	y ~ Intervention + Sex
-3,529315699	y ~ Intervention + Sex
-0,838552493	y ~ Intervention + Sex
1,229533436	y ~ Intervention + Sex
0,621040407	y ~ Intervention + Sex
-1,083239698	y ~ Intervention + Sex
-0,540649822	y ~ Intervention + Sex
-0,577899678	y ~ Intervention + Sex
-0,004089794	y ~ Intervention + Sex
1,891144249	y ~ Intervention + Sex
1,999327036	y ~ Intervention + Sex
-2,07333715	y ~ Intervention + Sex
1,45732719	y ~ Intervention + Sex
-1,401637459	y ~ Intervention + Sex
0,940460533	y ~ Intervention + Sex
-1,096612344	y ~ Intervention + Sex
0,271562571	y ~ Intervention + Sex
0,253047211	y ~ Intervention + Sex
-0,908450915	y ~ Intervention + Sex
-0,095067783	y ~ Intervention + Sex
-2,078028853	y ~ Intervention + Sex
0,948423294	y ~ Intervention + Sex
0,612991316	y ~ Intervention + Sex
1,325877851	y ~ Intervention + Sex
-2,038565798	y ~ Intervention + Sex
-0,459649333	y ~ Intervention + Sex
-1,020331231	y ~ Intervention + Sex
-0,525003614	y ~ Intervention + Sex
-1,550742315	y ~ Intervention + Sex

-1,783495718	y ~ Intervention + Sex
-0,490340844	y ~ Intervention + Sex
0,473578298	y ~ Intervention + Sex
0,696896124	y ~ Intervention + Sex
0,184747621	y ~ Intervention + Sex
-0,128334917	y ~ Intervention + Sex
-0,596058518	y ~ Intervention + Sex
2,14619287	y ~ Intervention + Sex
-1,630864331	y ~ Intervention + Sex
-0,241793491	y ~ Intervention + Sex
0,37568811	y ~ Intervention + Sex
0,997959415	y ~ Intervention + Sex
-0,005045946	y ~ Intervention + Sex
0,681804295	y ~ Intervention + Sex
1,790190076	y ~ Intervention + Sex
-0,900128564	y ~ Intervention + Sex
-1,873229719	y ~ Intervention + Sex
-0,66472494	y ~ Intervention + Sex
0,24633352	y ~ Intervention + Sex
7,008535332	y ~ Intervention + Sex
0,22142842	y ~ Intervention + Sex
1,854905437	y ~ Intervention + Sex
1,747855321	y ~ Intervention + Sex
-1,305643867	y ~ Intervention + Sex
-1,425611065	y ~ Intervention + Sex
0,303956838	y ~ Intervention + Sex
0,275593293	y ~ Intervention + Sex
-1,687877955	y ~ Intervention + Sex
1,407690678	y ~ Intervention + Sex
1,521935364	y ~ Intervention + Sex
0,863495032	y ~ Intervention + Sex
1,357795976	y ~ Intervention + Sex
1,749502286	y ~ Intervention + Sex
1,581328285	y ~ Intervention + Sex
0,917187272	y ~ Intervention + Sex
3,384671599	y ~ Intervention + Sex
-0,697964696	y ~ Intervention + Sex
-0,209479922	y ~ Intervention + Sex
-1,872914974	y ~ Intervention + Sex
-2,136927695	y ~ Intervention + Sex
0,110075244	y ~ Intervention + Sex
-0,904268444	y ~ Intervention + Sex
-0,691329146	y ~ Intervention + Sex
-3,015126969	y ~ Intervention + Sex
-0,419296785	y ~ Intervention + Sex
0,78238776	y ~ Intervention + Sex
-1,082069918	y ~ Intervention + Sex
1,27664159	y ~ Intervention + Sex

-1,888501286	y ~ Intervention + Sex
-0,595664519	y ~ Intervention + Sex
-1,50090757	y ~ Intervention + Sex
-5,040234672	y ~ Intervention + Sex
-1,357325475	y ~ Intervention + Sex
-1,532534593	y ~ Intervention + Sex
-0,582580079	y ~ Intervention + Sex
0,050914736	y ~ Intervention + Sex
-0,100989132	y ~ Intervention + Sex
-2,920303681	y ~ Intervention + Sex
-0,063546571	y ~ Intervention + Sex
0,80924267	y ~ Intervention + Sex
1,158168234	y ~ Intervention + Sex
-0,43719497	y ~ Intervention + Sex
1,031449969	y ~ Intervention + Sex
0,265132188	y ~ Intervention + Sex
3,379832717	y ~ Intervention + Sex
0,877327048	y ~ Intervention + Sex
3,321931882	y ~ Intervention + Sex
-1,694703155	y ~ Intervention + Sex
3,056042965	y ~ Intervention + Sex
-0,595070482	y ~ Intervention + Sex
-2,16149275	y ~ Intervention + Sex
1,879793671	y ~ Intervention + Sex
-0,37166253	y ~ Intervention + Sex
0,593865369	y ~ Intervention + Sex
1,159843085	y ~ Intervention + Sex
0,685853584	y ~ Intervention + Sex
-0,979967541	y ~ Intervention + Sex
-1,192984554	y ~ Intervention + Sex
-0,62711207	y ~ Intervention + Sex
0,600576715	y ~ Intervention + Sex
0,958902534	y ~ Intervention + Sex
-0,097672479	y ~ Intervention + Sex
-0,356870694	y ~ Intervention + Sex
5,475172377	y ~ Intervention + Sex
-2,789754091	y ~ Intervention + Sex
0,728857617	y ~ Intervention + Sex
-1,060381496	y ~ Intervention + Sex
2,526879466	y ~ Intervention + Sex
0,832346769	y ~ Intervention + Sex
-1,397070455	y ~ Intervention + Sex
2,620090974	y ~ Intervention + Sex
-0,880102907	y ~ Intervention + Sex
0,264736126	y ~ Intervention + Sex
-1,84311774	y ~ Intervention + Sex
-1,274073357	y ~ Intervention + Sex
1,598826977	y ~ Intervention + Sex

-1,486147441	y ~ Intervention + Sex
-1,10508586	y ~ Intervention + Sex
-4,408426743	y ~ Intervention + Sex
-0,902676399	y ~ Intervention + Sex
1,759640893	y ~ Intervention + Sex
0,42530308	y ~ Intervention + Sex
-2,089932689	y ~ Intervention + Sex
3,235797015	y ~ Intervention + Sex
-0,73209169	y ~ Intervention + Sex
0,335755738	y ~ Intervention + Sex
-2,304512527	y ~ Intervention + Sex
-0,716485187	y ~ Intervention + Sex
-1,839336841	y ~ Intervention + Sex
0,778440177	y ~ Intervention + Sex
