# Supplementary material for: A young child formula with Limosilactobacillus reuteri and GOS modulates gut microbiome and enhances bone and muscle development: a randomized trial
Source: Nat Commun. 2025 Dec 12;17:237. doi: 10.1038/s41467-025-66930-2 (PMC12783733; doi:10.1038/s41467-025-66930-2)
Supplement: Supplementary file 10 — Supplementary data 8 [file 41467_2025_66930_MOESM10_ESM.pdf]

Visit	Test type	Item	name	ation_level	Feature	Contrast	Alternative	Reference	FDR
baseline	abundance	2-Methylpr	2-Methylpr	CFA_panel	Interventio	Experimen	Experimen	Control	0,97445
baseline	abundance	3-Methylbu	3-Methylbu	CFA_panel	Interventio	Experimen	Experimen	Control	0,957875
baseline	abundance	Acetic acid	Acetic acid	CFA_panel	Interventio	Experimen	Experimen	Control	0,936399
baseline	abundance	Butanoic a	Butanoic a	CFA_panel	Interventio	Experimen	Experimen	Control	0,936399
baseline	abundance	Hexanoic a	Hexanoic a	CFA_panel	Interventio	Experimen	Experimen	Control	0,948031
baseline	abundance	M1	M1	NA_module	Interventio	Experimen	Experimen	Control	0,97445
baseline	abundance	M10	M10	NA_module	Interventio	Experimen	Experimen	Control	0,936399
baseline	abundance	M11	M11	NA_module	Interventio	Experimen	Experimen	Control	0,945105
baseline	abundance	M12	M12	NA_module	Interventio	Experimen	Experimen	Control	0,936399
baseline	abundance	M13	M13	NA_module	Interventio	Experimen	Experimen	Control	0,936399
baseline	abundance	M14	M14	NA_module	Interventio	Experimen	Experimen	Control	0,986763
baseline	abundance	M15	M15	NA_module	Interventio	Experimen	Experimen	Control	0,936399
baseline	abundance	M16	M16	NA_module	Interventio	Experimen	Experimen	Control	0,957875
baseline	abundance	M17	M17	NA_module	Interventio	Experimen	Experimen	Control	0,97445
baseline	abundance	M18	M18	NA_module	Interventio	Experimen	Experimen	Control	0,97445
baseline	abundance	M19	M19	NA_module	Interventio	Experimen	Experimen	Control	0,974375
baseline	abundance	M2	M2	NA_module	Interventio	Experimen	Experimen	Control	0,936399
baseline	abundance	M20	M20	NA_module	Interventio	Experimen	Experimen	Control	0,936399
baseline	abundance	M21	M21	NA_module	Interventio	Experimen	Experimen	Control	0,957875
baseline	abundance	M22	M22	NA_module	Interventio	Experimen	Experimen	Control	0,97445
baseline	abundance	M23	M23	NA_module	Interventio	Experimen	Experimen	Control	0,936399
baseline	abundance	M24	M24	NA_module	Interventio	Experimen	Experimen	Control	0,97445
baseline	abundance	M25	M25	NA_module	Interventio	Experimen	Experimen	Control	0,983461
baseline	abundance	M26	M26	NA_module	Interventio	Experimen	Experimen	Control	0,97445
baseline	abundance	M27	M27	NA_module	Interventio	Experimen	Experimen	Control	0,936399
baseline	abundance	M28	M28	NA_module	Interventio	Experimen	Experimen	Control	0,936399
baseline	abundance	M29	M29	NA_module	Interventio	Experimen	Experimen	Control	0,936399
baseline	abundance	M3	M3	NA_module	Interventio	Experimen	Experimen	Control	0,945105
baseline	abundance	M30	M30	NA_module	Interventio	Experimen	Experimen	Control	0,936399
baseline	abundance	M31	M31	NA_module	Interventio	Experimen	Experimen	Control	0,988948
baseline	abundance	M32	M32	NA_module	Interventio	Experimen	Experimen	Control	0,97445
baseline	abundance	M33	M33	NA_module	Interventio	Experimen	Experimen	Control	0,936399
baseline	abundance	M34	M34	NA_module	Interventio	Experimen	Experimen	Control	0,936399
baseline	abundance	M4	M4	NA_module	Interventio	Experimen	Experimen	Control	0,945105
baseline	abundance	M5	M5	NA_module	Interventio	Experimen	Experimen	Control	0,988948
baseline	abundance	M6	M6	NA_module	Interventio	Experimen	Experimen	Control	0,936399
baseline	abundance	M7	M7	NA_module	Interventio	Experimen	Experimen	Control	0,957875
baseline	abundance	M8	M8	NA_module	Interventio	Experimen	Experimen	Control	0,936399
baseline	abundance	M9	M9	NA_module	Interventio	Experimen	Experimen	Control	0,936399
baseline	abundance	Pentanoic	Pentanoic	CFA_panel	Interventio	Experimen	Experimen	Control	0,945105
baseline	abundance	Propanoic	Propanoic	CFA_panel	Interventio	Experimen	Experimen	Control	0,936399
baseline	abundance	SL00001	Deoxycytid	2a	Interventio	Experimen	Experimen	Control	0,948031
baseline	abundance	SL00009	N-Acetylhis	2a	Interventio	Experimen	Experimen	Control	0,97445
baseline	abundance	SL00017	Aspartic ac	2a	Interventio	Experimen	Experimen	Control	0,936399
baseline	abundance	SL00020	Carnitine	2a	Interventio	Experimen	Experimen	Control	0,936399
baseline	abundance	SL00024	Creatine	2a	Interventio	Experimen	Experimen	Control	0,955424
baseline	abundance	SL00035	Glycylglyci	2a	Interventio	Experimen	Experimen	Control	0,936399

baseline	abundance	SL00040	Kynurenine	2a	Interventio	Experimen	Experimen	Control	0,945105
baseline	abundance	SL00049	N-Acetylar	2a	Interventio	Experimen	Experimen	Control	0,948031
baseline	abundance	SL00052	N-Isovalery	2a	Interventio	Experimen	Experimen	Control	0,936399
baseline	abundance	SL00054	Ornithine	1	Interventio	Experimen	Experimen	Control	0,945105
baseline	abundance	SL00061	Proline	1	Interventio	Experimen	Experimen	Control	0,936399
baseline	abundance	SL00063	Pyridoxal	1	Interventio	Experimen	Experimen	Control	0,97445
baseline	abundance	SL00074	Valine/ 5-A	2a	Interventio	Experimen	Experimen	Control	0,998355
baseline	abundance	SL00081	5-Methylcy	2a	Interventio	Experimen	Experimen	Control	0,97445
baseline	abundance	SL00082	Allopurinol	1	Interventio	Experimen	Experimen	Control	0,962445
baseline	abundance	SL00089	Dissaccari	2a	Interventio	Experimen	Experimen	Control	0,936399
baseline	abundance	SL00096	Hexoses III	1	Interventio	Experimen	Experimen	Control	0,945105
baseline	abundance	SL00097	Hexoses II	1	Interventio	Experimen	Experimen	Control	0,989213
baseline	abundance	SL00103	Gulonic ac	2a	Interventio	Experimen	Experimen	Control	0,936399
baseline	abundance	SL00104	Hexoses I	1	Interventio	Experimen	Experimen	Control	0,97445
baseline	abundance	SL00108	Histidinol	2a	Interventio	Experimen	Experimen	Control	0,945105
baseline	abundance	SL00124	Dissaccari	2a	Interventio	Experimen	Experimen	Control	0,97445
baseline	abundance	SL00128	N-Acetylala	2a	Interventio	Experimen	Experimen	Control	0,948031
baseline	abundance	SL00133	N-Acetylglu	2a	Interventio	Experimen	Experimen	Control	0,936399
baseline	abundance	SL00137	Phenylacet	2a	Interventio	Experimen	Experimen	Control	0,945105
baseline	abundance	SL00140	Pyridoxami	1	Interventio	Experimen	Experimen	Control	0,945105
baseline	abundance	SL00149	Dissaccari	2a	Interventio	Experimen	Experimen	Control	0,945105
baseline	abundance	SL00154	Trigonelline	1	Interventio	Experimen	Experimen	Control	0,948031
baseline	abundance	SL00159	Uracil	1	Interventio	Experimen	Experimen	Control	0,936399
baseline	abundance	SL00161	Uridine	2a	Interventio	Experimen	Experimen	Control	0,936399
baseline	abundance	SL00185	2-Oxo-3-ph	1	Interventio	Experimen	Experimen	Control	0,945105
baseline	abundance	SL00189	3-Hydroxyb	2a	Interventio	Experimen	Experimen	Control	0,936399
baseline	abundance	SL00197	4-Methyl-2	2a	Interventio	Experimen	Experimen	Control	0,936399
baseline	abundance	SL00199	5-Hydroxyi	2a	Interventio	Experimen	Experimen	Control	0,936399
baseline	abundance	SL00200	8-Aminooc	2a	Interventio	Experimen	Experimen	Control	0,936399
baseline	abundance	SL00201	Pentose II	2a	Interventio	Experimen	Experimen	Control	0,937501
baseline	abundance	SL00205	Citraconic	2a	Interventio	Experimen	Experimen	Control	0,945105
baseline	abundance	SL00210	Galactonic	2a	Interventio	Experimen	Experimen	Control	0,948031
baseline	abundance	SL00212	Galactosar	2a	Interventio	Experimen	Experimen	Control	0,936399
baseline	abundance	SL00215	Glycolic ac	2a	Interventio	Experimen	Experimen	Control	0,97445
baseline	abundance	SL00216	Glyoxylic a	2a	Interventio	Experimen	Experimen	Control	0,97445
baseline	abundance	SL00222	Lactic acid	1	Interventio	Experimen	Experimen	Control	0,97445
baseline	abundance	SL00226	N-Acetylgly	2a	Interventio	Experimen	Experimen	Control	0,945105
baseline	abundance	SL00239	Pinitol	2a	Interventio	Experimen	Experimen	Control	0,97445
baseline	abundance	SL00240	Pyruvic aci	1	Interventio	Experimen	Experimen	Control	0,983461
baseline	abundance	SL00243	Deoxysuga	2a	Interventio	Experimen	Experimen	Control	0,957875
baseline	abundance	SL00245	sugar alcol	2a	Interventio	Experimen	Experimen	Control	0,936399
baseline	abundance	SL00246	Pentose III	2a	Interventio	Experimen	Experimen	Control	0,937501
baseline	abundance	SL00247	Shikimic ac	2a	Interventio	Experimen	Experimen	Control	0,936399
baseline	abundance	SL00248	Gluconic a	2a	Interventio	Experimen	Experimen	Control	0,936399
baseline	abundance	SL00260	Pentose I	2a	Interventio	Experimen	Experimen	Control	0,945786
baseline	abundance	SL00262	cis-Aconiti	2a	Interventio	Experimen	Experimen	Control	0,99606
baseline	abundance	SL00264	Methyl ace	2a	Interventio	Experimen	Experimen	Control	0,936399
baseline	abundance	SL00265	Purine	2a	Interventio	Experimen	Experimen	Control	0,936399

baseline	abundance	SL00268	Thymine	1	Interventio	Experimen	Experimen	Control	0,936399
baseline	abundance	SL00270	3-hydroxy-4	2a	Interventio	Experimen	Experimen	Control	0,936399
baseline	abundance	SL00275	Dopamine	2a	Interventio	Experimen	Experimen	Control	0,936399
baseline	abundance	SL00282	3-Hydroxyh	2a	Interventio	Experimen	Experimen	Control	0,936399
baseline	abundance	SL00285	Dihydrofer	2a	Interventio	Experimen	Experimen	Control	0,936399
baseline	abundance	SL00287	DOPA	2a	Interventio	Experimen	Experimen	Control	0,957875
baseline	abundance	SL00288	Propionylc	2a	Interventio	Experimen	Experimen	Control	0,9737
baseline	abundance	SL00290	N-Acetylm	2a	Interventio	Experimen	Experimen	Control	0,97445
baseline	abundance	SL00293	Butyrylcarn	2a	Interventio	Experimen	Experimen	Control	0,97445
baseline	abundance	SL00295	5-Methylur	2a	Interventio	Experimen	Experimen	Control	0,936399
baseline	abundance	SL00297	Acetylmura	2a	Interventio	Experimen	Experimen	Control	0,936399
baseline	abundance	SL00299	β-Murichol	2a	Interventio	Experimen	Experimen	Control	0,945105
baseline	abundance	SL00309	1-Aminocy	2a	Interventio	Experimen	Experimen	Control	0,936399
baseline	abundance	SL00311	N,N-Dimet	2a	Interventio	Experimen	Experimen	Control	0,936399
baseline	abundance	SL00313	2-Aminoiso	2a	Interventio	Experimen	Experimen	Control	0,936399
baseline	abundance	SL00315	Malonic ac	2a	Interventio	Experimen	Experimen	Control	0,936399
baseline	abundance	SL00318	4-Aminoph	1	Interventio	Experimen	Experimen	Control	0,936399
baseline	abundance	SL00320	γ-Caprolac	2a	Interventio	Experimen	Experimen	Control	0,936399
baseline	abundance	SL00325	2-Methylm	2a	Interventio	Experimen	Experimen	Control	0,97445
baseline	abundance	SL00328	2-(hydroxy	2a	Interventio	Experimen	Experimen	Control	0,978464
baseline	abundance	SL00329	2-Hydroxy-	2a	Interventio	Experimen	Experimen	Control	0,945105
baseline	abundance	SL00334	Imidazolea	2a	Interventio	Experimen	Experimen	Control	0,936399
baseline	abundance	SL00346	N-Methylni	2a	Interventio	Experimen	Experimen	Control	0,97445
baseline	abundance	SL00349	1-Aminocy	2a	Interventio	Experimen	Experimen	Control	0,948031
baseline	abundance	SL00350	Stachydrin	1	Interventio	Experimen	Experimen	Control	0,936399
baseline	abundance	SL00353	Adipic acid	1	Interventio	Experimen	Experimen	Control	0,97445
baseline	abundance	SL00356	N-(5-Amin	2a	Interventio	Experimen	Experimen	Control	0,945105
baseline	abundance	SL00368	N-Methylty	1	Interventio	Experimen	Experimen	Control	0,936399
baseline	abundance	SL00373	N1-Methyl-	2a	Interventio	Experimen	Experimen	Control	0,97445
baseline	abundance	SL00376	N-AcetylPr	2a	Interventio	Experimen	Experimen	Control	0,936399
baseline	abundance	SL00380	2-Aminoad	2a	Interventio	Experimen	Experimen	Control	0,936399
baseline	abundance	SL00383	3-(2-Hydro	2a	Interventio	Experimen	Experimen	Control	0,945105
baseline	abundance	SL00384	p-Coumari	1	Interventio	Experimen	Experimen	Control	0,936399
baseline	abundance	SL00390	Gallic acid	2a	Interventio	Experimen	Experimen	Control	0,936399
baseline	abundance	SL00391	N-Acetylle	1	Interventio	Experimen	Experimen	Control	0,936399
baseline	abundance	SL00401	Theophylli	1	Interventio	Experimen	Experimen	Control	0,936399
baseline	abundance	SL00407	Homovanil	2a	Interventio	Experimen	Experimen	Control	0,936399
baseline	abundance	SL00420	4-Hydroxy-	2a	Interventio	Experimen	Experimen	Control	0,957875
baseline	abundance	SL00421	Asymmetri	2a	Interventio	Experimen	Experimen	Control	0,978464
baseline	abundance	SL00430	Homocarn	2a	Interventio	Experimen	Experimen	Control	0,936399
baseline	abundance	SL00431	Tiglylcarnit	2a	Interventio	Experimen	Experimen	Control	0,936399
baseline	abundance	SL00433	Isovalerylc	2a	Interventio	Experimen	Experimen	Control	0,948031
baseline	abundance	SL00436	1-Carboxye	2a	Interventio	Experimen	Experimen	Control	0,936399
baseline	abundance	SL00438	Daidzein	2a	Interventio	Experimen	Experimen	Control	0,957875
baseline	abundance	SL00440	5-Methylcy	2a	Interventio	Experimen	Experimen	Control	0,936399
baseline	abundance	SL00445	Glucosami	2a	Interventio	Experimen	Experimen	Control	0,945105
baseline	abundance	SL00447	Apigenin	2a	Interventio	Experimen	Experimen	Control	0,945105
baseline	abundance	SL00455	12,13-DHO	2a	Interventio	Experimen	Experimen	Control	0,936399

baseline	abundance	SL00467	Sucralose	2a	Interventio	Experimen	Experimen	Control	0,936399
baseline	abundance	SL00502	2-Ketobuty	1	Interventio	Experimen	Experimen	Control	0,945105
baseline	abundance	X00011	Glutamine	2a	Interventio	Experimen	Experimen	Control	0,936399
baseline	abundance	X00018	Succinic a	1	Interventio	Experimen	Experimen	Control	0,955424
baseline	abundance	X00020	sugar alcol	1	Interventio	Experimen	Experimen	Control	0,936399
baseline	abundance	X00022	N-Methyla	1	Interventio	Experimen	Experimen	Control	0,936399
baseline	abundance	X00023	N-Formylm	1	Interventio	Experimen	Experimen	Control	0,945105
baseline	abundance	X00025	Acesulfam	1	Interventio	Experimen	Experimen	Control	0,936399
baseline	abundance	X00026	Inosine	1	Interventio	Experimen	Experimen	Control	0,936399
baseline	abundance	X00027	N-Acetylglu	1	Interventio	Experimen	Experimen	Control	0,936399
baseline	abundance	X00029	Urocanic a	1	Interventio	Experimen	Experimen	Control	0,936399
baseline	abundance	X00030	3,4-Dihydr	1	Interventio	Experimen	Experimen	Control	0,936399
baseline	abundance	X00032	Indole-3-pr	1	Interventio	Experimen	Experimen	Control	0,936399
baseline	abundance	X00033	Deoxycholi	1	Interventio	Experimen	Experimen	Control	0,957875
baseline	abundance	X00034	Pyroglutam	1	Interventio	Experimen	Experimen	Control	0,945105
baseline	abundance	X00035	Choline	1	Interventio	Experimen	Experimen	Control	0,97445
baseline	abundance	X00036	Serine	1	Interventio	Experimen	Experimen	Control	0,936399
baseline	abundance	X00038	Histamine	1	Interventio	Experimen	Experimen	Control	0,936399
baseline	abundance	X00040	N-Acetylty	1	Interventio	Experimen	Experimen	Control	0,987751
baseline	abundance	X00042	Glucuronic	1	Interventio	Experimen	Experimen	Control	0,936399
baseline	abundance	X00045	Threonine	1	Interventio	Experimen	Experimen	Control	0,97445
baseline	abundance	X00050	β-Hydroxyi	1	Interventio	Experimen	Experimen	Control	0,955424
baseline	abundance	X00051	β-D-Gluco	2b	Interventio	Experimen	Experimen	Control	0,936399
baseline	abundance	X00056	3-(4-hydro	1	Interventio	Experimen	Experimen	Control	0,936399
baseline	abundance	X00057	N-Acetylor	1	Interventio	Experimen	Experimen	Control	0,948031
baseline	abundance	X00059	3,4-Dihydr	1	Interventio	Experimen	Experimen	Control	0,936399
baseline	abundance	X00060	Ethylmalor	1	Interventio	Experimen	Experimen	Control	0,98802
baseline	abundance	X00061	Thiamine	1	Interventio	Experimen	Experimen	Control	0,945105
baseline	abundance	X00063	2-Hydroxy	1	Interventio	Experimen	Experimen	Control	0,97445
baseline	abundance	X00064	Traumatic	1	Interventio	Experimen	Experimen	Control	0,957875
baseline	abundance	X00066	Tryptamine	1	Interventio	Experimen	Experimen	Control	0,945105
baseline	abundance	X00067	Hypoxanth	1	Interventio	Experimen	Experimen	Control	0,936399
baseline	abundance	X00068	N-Acetylm	1	Interventio	Experimen	Experimen	Control	0,936399
baseline	abundance	X00070	α-aminobu	1	Interventio	Experimen	Experimen	Control	0,936399
baseline	abundance	X00071	Tyrosine	1	Interventio	Experimen	Experimen	Control	0,936399
baseline	abundance	X00072	3,5-Dihydr	1	Interventio	Experimen	Experimen	Control	0,936399
baseline	abundance	X00073	Indole-3-la	1	Interventio	Experimen	Experimen	Control	0,97445
baseline	abundance	X00074	Methylsuc	1	Interventio	Experimen	Experimen	Control	0,985326
baseline	abundance	X00076	Isoleucine	1	Interventio	Experimen	Experimen	Control	0,945105
baseline	abundance	X00078	Acetylagm	1	Interventio	Experimen	Experimen	Control	0,97445
baseline	abundance	X00082	Cholic acid	1	Interventio	Experimen	Experimen	Control	0,945105
baseline	abundance	X00083	1,7-Dimet	1	Interventio	Experimen	Experimen	Control	0,936399
baseline	abundance	X00084	Tricarbally	1	Interventio	Experimen	Experimen	Control	0,936399
baseline	abundance	X00088	Pantotheni	1	Interventio	Experimen	Experimen	Control	0,948031
baseline	abundance	X00089	4-Hydroxyl	1	Interventio	Experimen	Experimen	Control	0,946333
baseline	abundance	X00090	Malic acid	1	Interventio	Experimen	Experimen	Control	0,992737
baseline	abundance	X00092	3-Methylhi	1	Interventio	Experimen	Experimen	Control	0,936399
baseline	abundance	X00093	Deoxyinosi	1	Interventio	Experimen	Experimen	Control	0,97445

baseline	abundance	X00094	Methionine	1	Interventio	Experimen	Experimen	Control	0,945105
baseline	abundance	X00097	2,6-Dihydr	1	Interventio	Experimen	Experimen	Control	0,936399
baseline	abundance	X00099	7-Methylgu	1	Interventio	Experimen	Experimen	Control	0,936399
baseline	abundance	X00101	Threonic a	1	Interventio	Experimen	Experimen	Control	0,986763
baseline	abundance	X00102	Tryptophar	1	Interventio	Experimen	Experimen	Control	0,936399
baseline	abundance	X00106	N-Acetyltry	1	Interventio	Experimen	Experimen	Control	0,97445
baseline	abundance	X00107	N6-Acetylly	1	Interventio	Experimen	Experimen	Control	0,992737
baseline	abundance	X00112	Xanthosine	1	Interventio	Experimen	Experimen	Control	0,945105
baseline	abundance	X00113	N-Acetylph	1	Interventio	Experimen	Experimen	Control	0,982558
baseline	abundance	X00114	Indole-3-m	1	Interventio	Experimen	Experimen	Control	0,936399
baseline	abundance	X00115	Histidine	1	Interventio	Experimen	Experimen	Control	0,936399
baseline	abundance	X00116	Cytidine	1	Interventio	Experimen	Experimen	Control	0,945105
baseline	abundance	X00120	Leucylalan	1	Interventio	Experimen	Experimen	Control	0,936399
baseline	abundance	X00123	Phenylalar	1	Interventio	Experimen	Experimen	Control	0,945105
baseline	abundance	X00124	Leucine	1	Interventio	Experimen	Experimen	Control	0,936399
baseline	abundance	X00125	Taurine	1	Interventio	Experimen	Experimen	Control	0,936399
baseline	abundance	X00127	Nicotinic a	1	Interventio	Experimen	Experimen	Control	0,936399
baseline	abundance	X00129	Quinic acic	1	Interventio	Experimen	Experimen	Control	0,936399
baseline	abundance	X00130	Deoxyuridi	1	Interventio	Experimen	Experimen	Control	0,936399
baseline	abundance	X00132	Glyceric ac	1	Interventio	Experimen	Experimen	Control	0,936399
baseline	abundance	X00135	N-Acetylglu	1	Interventio	Experimen	Experimen	Control	0,936399
baseline	abundance	X00136	leu-gln_a	3	Interventio	Experimen	Experimen	Control	0,936399
baseline	abundance	X00163	primidone_	3	Interventio	Experimen	Experimen	Control	0,936399
baseline	abundance	X00180	1-(2-Carbo	3	Interventio	Experimen	Experimen	Control	0,945105
baseline	abundance	X00182	3-(Butylsul	3	Interventio	Experimen	Experimen	Control	0,945105
baseline	abundance	X00199	tert-Butyl 3	3	Interventio	Experimen	Experimen	Control	0,936399
baseline	abundance	X00204	4-O-{3-O-[(	3	Interventio	Experimen	Experimen	Control	0,945105
baseline	abundance	X00221	Astemizole	3	Interventio	Experimen	Experimen	Control	0,948031
baseline	abundance	X00224	Nifedipine	3	Interventio	Experimen	Experimen	Control	0,945105
baseline	abundance	X00233	g-Aminobu	3	Interventio	Experimen	Experimen	Control	0,945105
baseline	abundance	X00237	OI1700000	3	Interventio	Experimen	Experimen	Control	0,936399
baseline	abundance	X00242	IN00258	3	Interventio	Experimen	Experimen	Control	0,989213
baseline	abundance	X00253	TDP-2_a	3	Interventio	Experimen	Experimen	Control	0,991989
baseline	abundance	X00261	Mexiletine	3	Interventio	Experimen	Experimen	Control	0,945105
baseline	abundance	X00264	PEG n12	2b	Interventio	Experimen	Experimen	Control	0,957875
baseline	abundance	X00266	2-Oxo-3-(p	3	Interventio	Experimen	Experimen	Control	0,945105
baseline	abundance	X00305	Formylkynu	3	Interventio	Experimen	Experimen	Control	0,936399
baseline	abundance	X00320	Panthenol_	3	Interventio	Experimen	Experimen	Control	0,945105
baseline	abundance	X00327	3-Hydroxy-	3	Interventio	Experimen	Experimen	Control	0,936399
baseline	abundance	X00331	4-(9H-beta	3	Interventio	Experimen	Experimen	Control	0,936399
baseline	abundance	X00344	SECONAL_	3	Interventio	Experimen	Experimen	Control	0,936399
baseline	abundance	X00361	nitecapone	3	Interventio	Experimen	Experimen	Control	0,97445
baseline	abundance	X00367	hexobarbit	3	Interventio	Experimen	Experimen	Control	0,959387
baseline	abundance	X00375	N-[(2S)-2-I	3	Interventio	Experimen	Experimen	Control	0,945105
baseline	abundance	X00383	pretazettin	3	Interventio	Experimen	Experimen	Control	0,957875
baseline	abundance	X00384	3-Formyl-2	3	Interventio	Experimen	Experimen	Control	0,97445
baseline	abundance	X00403	7alpha-Hy	3	Interventio	Experimen	Experimen	Control	0,945105
baseline	abundance	X00404	piscidic ac	3	Interventio	Experimen	Experimen	Control	0,936399

baseline	abundance	X00432	8-(Methyls	3	Interventio	Experimen	Experimen	Control	0,945105
baseline	abundance	X00450	Gly-Ser	3	Interventio	Experimen	Experimen	Control	0,957875
baseline	abundance	X00477	Sparfloxac	2b	Interventio	Experimen	Experimen	Control	0,936399
baseline	abundance	X00518	Nisinic aci	3	Interventio	Experimen	Experimen	Control	0,936399
baseline	abundance	X00528	R-(+)-Etira	3	Interventio	Experimen	Experimen	Control	0,936399
baseline	abundance	X00545	3-hydroxyc	3	Interventio	Experimen	Experimen	Control	0,936399
baseline	abundance	X00549	3-[(2Z)-1-C	3	Interventio	Experimen	Experimen	Control	0,945105
baseline	abundance	X00574	miglustat	3	Interventio	Experimen	Experimen	Control	0,936399
baseline	abundance	X00594	Nicotinami	3	Interventio	Experimen	Experimen	Control	0,936399
baseline	abundance	X00635	4-(5,6-Dihy	3	Interventio	Experimen	Experimen	Control	0,945105
baseline	abundance	X00637	N~6~,N~6	3	Interventio	Experimen	Experimen	Control	0,936399
baseline	abundance	X00646	Deacetyldi	3	Interventio	Experimen	Experimen	Control	0,936399
baseline	abundance	X00670	7-Chloro-5	3	Interventio	Experimen	Experimen	Control	0,97445
baseline	abundance	X00683	Zinecard_a	3	Interventio	Experimen	Experimen	Control	0,936399
baseline	abundance	X00693	n-Ribosylh	3	Interventio	Experimen	Experimen	Control	0,936399
baseline	abundance	X00698	valgancicl	3	Interventio	Experimen	Experimen	Control	0,936399
baseline	abundance	X00702	N-[(2S)-2-I	3	Interventio	Experimen	Experimen	Control	0,936399
baseline	abundance	X00722	(3S,5R,6E)	3	Interventio	Experimen	Experimen	Control	0,945105
baseline	abundance	X00723	Aspartyl-L-	3	Interventio	Experimen	Experimen	Control	0,97445
baseline	abundance	X00742	MFCD1869	3	Interventio	Experimen	Experimen	Control	0,989769
baseline	abundance	X00743	11beta,13-	3	Interventio	Experimen	Experimen	Control	0,945105
baseline	abundance	X00744	(1R,2S)-1-(	3	Interventio	Experimen	Experimen	Control	0,936399
baseline	abundance	X00748	3-[3-Metho	3	Interventio	Experimen	Experimen	Control	0,945786
baseline	abundance	X00764	5-Allyl-5-se	3	Interventio	Experimen	Experimen	Control	0,97445
baseline	abundance	X00807	Midodrine_	3	Interventio	Experimen	Experimen	Control	0,945105
baseline	abundance	X00828	Fenoterol	3	Interventio	Experimen	Experimen	Control	0,957875
baseline	abundance	X00836	3-Mercapt	3	Interventio	Experimen	Experimen	Control	0,987642
baseline	abundance	X00853	4-O-beta-D	3	Interventio	Experimen	Experimen	Control	0,936399
baseline	abundance	X00889	LW800000	3	Interventio	Experimen	Experimen	Control	0,945105
baseline	abundance	X00899	N-(2,3,4-Tr	3	Interventio	Experimen	Experimen	Control	0,936399
baseline	abundance	X00907	1,2,3,4-Tet	3	Interventio	Experimen	Experimen	Control	0,936399
baseline	abundance	X00928	(7R)-7-(5-c	3	Interventio	Experimen	Experimen	Control	0,945105
baseline	abundance	X00948	Linamarin	3	Interventio	Experimen	Experimen	Control	0,97445
baseline	abundance	X00949	primidone_	3	Interventio	Experimen	Experimen	Control	0,992737
baseline	abundance	X00950	2-Phenylet	3	Interventio	Experimen	Experimen	Control	0,936399
baseline	abundance	X00963	(4S)-4-[(2E	3	Interventio	Experimen	Experimen	Control	0,936399
baseline	abundance	X00985	1-(4-Aminc	3	Interventio	Experimen	Experimen	Control	0,97445
baseline	abundance	X00998	epsilon-(ga	3	Interventio	Experimen	Experimen	Control	0,959387
baseline	abundance	X01007	pentobarbi	3	Interventio	Experimen	Experimen	Control	0,97445
baseline	abundance	X01011	DIBEHENII	3	Interventio	Experimen	Experimen	Control	0,97445
baseline	abundance	X01017	hexobarbit	3	Interventio	Experimen	Experimen	Control	0,936399
baseline	abundance	X01031	3-(14-Ethy	3	Interventio	Experimen	Experimen	Control	0,945105
baseline	abundance	X01045	Ethyl maltc	3	Interventio	Experimen	Experimen	Control	0,936399
baseline	abundance	X01059	coronatine	3	Interventio	Experimen	Experimen	Control	0,945105
baseline	abundance	X01067	g-Aminobu	3	Interventio	Experimen	Experimen	Control	0,936399
baseline	abundance	X01078	Dihydroura	3	Interventio	Experimen	Experimen	Control	0,936399
baseline	abundance	X01081	Methyl alpl	3	Interventio	Experimen	Experimen	Control	0,945105
baseline	abundance	X01092	17,21-Dihy	3	Interventio	Experimen	Experimen	Control	0,936399

baseline	abundance	X01098	(-)-Physost	3	Interventio	Experimen	Experimen	Control	0,945105
baseline	abundance	X01100	(betaS)-be	3	Interventio	Experimen	Experimen	Control	0,97445
baseline	abundance	X01154	N-(2,3,4-Tr	3	Interventio	Experimen	Experimen	Control	0,957875
baseline	abundance	X01163	Prephenic	3	Interventio	Experimen	Experimen	Control	0,948031
baseline	abundance	X01164	DL-Carboc	3	Interventio	Experimen	Experimen	Control	0,957875
baseline	abundance	X01181	2-(3,4-Dim	3	Interventio	Experimen	Experimen	Control	0,936399
baseline	abundance	X01186	asn-val_b	3	Interventio	Experimen	Experimen	Control	0,936399
baseline	abundance	X01208	Lys-Pro_c	3	Interventio	Experimen	Experimen	Control	0,945105
baseline	abundance	X01223	DL-Mevalo	3	Interventio	Experimen	Experimen	Control	0,936399
baseline	abundance	X01235	3-[(3-Hydr	3	Interventio	Experimen	Experimen	Control	0,936399
baseline	abundance	X01236	bis(4-isoth	3	Interventio	Experimen	Experimen	Control	0,936399
baseline	abundance	X01242	3-Morpholi	2b	Interventio	Experimen	Experimen	Control	0,97445
baseline	abundance	X01246	(2R)-1-{[(2-	3	Interventio	Experimen	Experimen	Control	0,973066
baseline	abundance	X01252	(−)-nabilor	3	Interventio	Experimen	Experimen	Control	0,97445
baseline	abundance	X01285	FB950000(	3	Interventio	Experimen	Experimen	Control	0,97445
baseline	abundance	X01286	(2,4-Dihyd	3	Interventio	Experimen	Experimen	Control	0,945105
baseline	abundance	X01288	L-gamma-(	3	Interventio	Experimen	Experimen	Control	0,936399
baseline	abundance	X01316	5-Hydanto	3	Interventio	Experimen	Experimen	Control	0,936399
baseline	abundance	X01327	threonylph	3	Interventio	Experimen	Experimen	Control	0,97445
baseline	abundance	X01340	Cadralazin	3	Interventio	Experimen	Experimen	Control	0,962445
baseline	abundance	X01341	metixene	3	Interventio	Experimen	Experimen	Control	0,936399
baseline	abundance	X01346	IN00260_a	3	Interventio	Experimen	Experimen	Control	0,936399
baseline	abundance	X01363	Seryltyrosi	3	Interventio	Experimen	Experimen	Control	0,959788
baseline	abundance	X01364	Oleuropeir	3	Interventio	Experimen	Experimen	Control	0,936399
baseline	abundance	X01367	MFCD1869	3	Interventio	Experimen	Experimen	Control	0,945105
baseline	abundance	X01380	Spermic ac	3	Interventio	Experimen	Experimen	Control	0,936399
baseline	abundance	X01387	Methyl 4-(4	3	Interventio	Experimen	Experimen	Control	0,945105
baseline	abundance	X01441	1-{3-Carbo	3	Interventio	Experimen	Experimen	Control	0,989213
baseline	abundance	X01463	4-Thiapent	3	Interventio	Experimen	Experimen	Control	0,948031
baseline	abundance	X01474	Glyceropho	3	Interventio	Experimen	Experimen	Control	0,945105
baseline	abundance	X01518	L-gamma-(	3	Interventio	Experimen	Experimen	Control	0,97445
baseline	abundance	X01519	Leucyltrypt	3	Interventio	Experimen	Experimen	Control	0,985326
baseline	abundance	X01528	butalbital_	3	Interventio	Experimen	Experimen	Control	0,945105
baseline	abundance	X01530	INK (Peptic	2b	Interventio	Experimen	Experimen	Control	0,957875
baseline	abundance	X01549	3-(Sulfooxy	3	Interventio	Experimen	Experimen	Control	0,962445
baseline	abundance	X01553	uridine 5'-(	3	Interventio	Experimen	Experimen	Control	0,936399
baseline	abundance	X01558	2-Furoylgly	2b	Interventio	Experimen	Experimen	Control	0,936399
baseline	abundance	X01561	2-Ammoni(	3	Interventio	Experimen	Experimen	Control	0,97445
baseline	abundance	X01570	carglumic a	3	Interventio	Experimen	Experimen	Control	0,936399
baseline	abundance	X01574	N-Acetylpr	3	Interventio	Experimen	Experimen	Control	0,945786
baseline	abundance	X01577	Prunasin	3	Interventio	Experimen	Experimen	Control	0,945105
baseline	abundance	X01621	2-(2,4-Dihy	3	Interventio	Experimen	Experimen	Control	0,945105
baseline	abundance	X01640	Ro 20-1724	3	Interventio	Experimen	Experimen	Control	0,989209
baseline	abundance	X01656	Guanadrel	3	Interventio	Experimen	Experimen	Control	0,945105
baseline	abundance	X01664	5-methylth	3	Interventio	Experimen	Experimen	Control	0,936399
baseline	abundance	X01671	N-(3,5-Dim	3	Interventio	Experimen	Experimen	Control	0,936399
baseline	abundance	X01672	Triethyl citr	3	Interventio	Experimen	Experimen	Control	0,936399
baseline	abundance	X01679	meprobam	3	Interventio	Experimen	Experimen	Control	0,945105

baseline	abundance	X01689	Ethyl mala	3	Interventio	Experimen	Experimen	Control	0,936399
baseline	abundance	X01732	2-Hydroxy-	3	Interventio	Experimen	Experimen	Control	0,936399
baseline	abundance	X01738	2-Acetami	3	Interventio	Experimen	Experimen	Control	0,945105
baseline	abundance	X01746	Astemizole	3	Interventio	Experimen	Experimen	Control	0,936399
baseline	abundance	X01768	Butabarbit	3	Interventio	Experimen	Experimen	Control	0,936399
baseline	abundance	X01776	N-(Carboxy	3	Interventio	Experimen	Experimen	Control	0,957875
baseline	abundance	X01793	Lys-phe_b	3	Interventio	Experimen	Experimen	Control	0,936399
baseline	abundance	X01813	Erythorbic	3	Interventio	Experimen	Experimen	Control	0,972498
baseline	abundance	X01833	2,3,4,9-Tet	2b	Interventio	Experimen	Experimen	Control	0,945105
baseline	abundance	X01873	Methyl 2,3-	3	Interventio	Experimen	Experimen	Control	0,97445
baseline	abundance	X01879	Tetramethy	2b	Interventio	Experimen	Experimen	Control	0,945105
baseline	abundance	X01881	(2S)-3-Met	3	Interventio	Experimen	Experimen	Control	0,97445
baseline	abundance	X01884	Roxane	3	Interventio	Experimen	Experimen	Control	0,936399
baseline	abundance	X01887	lys-tyr_b	3	Interventio	Experimen	Experimen	Control	0,936399
baseline	abundance	X01893	4-(METHYL	3	Interventio	Experimen	Experimen	Control	0,955719
baseline	abundance	X01911	3-(2,3-Dihy	3	Interventio	Experimen	Experimen	Control	0,97477
baseline	abundance	X01920	N-(Carboxy	3	Interventio	Experimen	Experimen	Control	0,936399
baseline	abundance	X01932	asn-val_c	3	Interventio	Experimen	Experimen	Control	0,945105
baseline	abundance	X01943	Tocainide	3	Interventio	Experimen	Experimen	Control	0,936399
baseline	abundance	X01950	Histidylgly	3	Interventio	Experimen	Experimen	Control	0,936399
baseline	abundance	X01968	7-Chloro-5	3	Interventio	Experimen	Experimen	Control	0,936399
baseline	abundance	X01996	3-Hydroxy-	3	Interventio	Experimen	Experimen	Control	0,936399
baseline	abundance	X02000	11-(4-Hydr	3	Interventio	Experimen	Experimen	Control	0,957232
baseline	abundance	X02009	Arctiopicri	3	Interventio	Experimen	Experimen	Control	0,945105
baseline	abundance	X02013	Ile-cys	3	Interventio	Experimen	Experimen	Control	0,936399
baseline	abundance	X02020	Val-Ser_b	3	Interventio	Experimen	Experimen	Control	0,982558
baseline	abundance	X02023	beta-D-Eth	3	Interventio	Experimen	Experimen	Control	0,957875
baseline	abundance	X02073	N-acetyl-9-	3	Interventio	Experimen	Experimen	Control	0,936399
baseline	abundance	X02080	(-)-Aspidos	3	Interventio	Experimen	Experimen	Control	0,986763
baseline	abundance	X02082	6-Hydroxyr	3	Interventio	Experimen	Experimen	Control	0,97499
baseline	abundance	X02101	Succinic ar	3	Interventio	Experimen	Experimen	Control	0,957875
baseline	abundance	X02108	Homovanil	3	Interventio	Experimen	Experimen	Control	0,936399
baseline	abundance	X02121	2-Acetami	3	Interventio	Experimen	Experimen	Control	0,97445
baseline	abundance	X02139	SECONAL_	3	Interventio	Experimen	Experimen	Control	0,945105
baseline	abundance	X02145	3'-Hydroxy	3	Interventio	Experimen	Experimen	Control	0,936399
baseline	abundance	X02153	Asarone	3	Interventio	Experimen	Experimen	Control	0,945105
baseline	abundance	X02181	YWA1	3	Interventio	Experimen	Experimen	Control	0,941185
baseline	abundance	X02184	Piperonylo	2b	Interventio	Experimen	Experimen	Control	0,936399
baseline	abundance	X02195	alliin	3	Interventio	Experimen	Experimen	Control	0,97445
baseline	abundance	X02202	D-2-Amino	3	Interventio	Experimen	Experimen	Control	0,97445
baseline	abundance	X02208	SECONAL_	3	Interventio	Experimen	Experimen	Control	0,945105
baseline	abundance	X02214	(E)-4-Meth	3	Interventio	Experimen	Experimen	Control	0,945105
baseline	abundance	X02219	Homoanse	3	Interventio	Experimen	Experimen	Control	0,97445
baseline	abundance	X02231	Ala-Tyr	3	Interventio	Experimen	Experimen	Control	0,945105
baseline	abundance	X02238	6-Myoporo	3	Interventio	Experimen	Experimen	Control	0,936399
baseline	abundance	X02251	Hostmania	3	Interventio	Experimen	Experimen	Control	0,936399
baseline	abundance	X02256	hexobarbit	3	Interventio	Experimen	Experimen	Control	0,936399
baseline	abundance	X02265	ophthalmi	3	Interventio	Experimen	Experimen	Control	0,945105

baseline	abundance	X02268	5-Hydroxy-	3	Interventio	Experimen	Experimen	Control	0,97445
baseline	abundance	X02277	tert-Butyl 3	3	Interventio	Experimen	Experimen	Control	0,97445
baseline	abundance	X02281	tert-Butyl 3	3	Interventio	Experimen	Experimen	Control	0,97445
baseline	abundance	X02288	9-Methylur	2b	Interventio	Experimen	Experimen	Control	0,97445
baseline	abundance	X02289	Kynurenic a	2a	Interventio	Experimen	Experimen	Control	0,982558
baseline	abundance	X02317	2-Aminooc	2b	Interventio	Experimen	Experimen	Control	0,955424
baseline	abundance	X02327	1H-Pyrazol	3	Interventio	Experimen	Experimen	Control	0,945105
baseline	abundance	X02333	N-Propiony	3	Interventio	Experimen	Experimen	Control	0,986763
baseline	abundance	X02337	Methyl 1-hy	3	Interventio	Experimen	Experimen	Control	0,992433
baseline	abundance	X02348	N-Benzoyla	3	Interventio	Experimen	Experimen	Control	0,936399
baseline	abundance	X02352	Indole-3-ca	3	Interventio	Experimen	Experimen	Control	0,992433
baseline	abundance	X02380	Propafenor	3	Interventio	Experimen	Experimen	Control	0,936399
baseline	abundance	X02419	Glu-Glu	3	Interventio	Experimen	Experimen	Control	0,936399
baseline	abundance	X02426	Raltitrexed	3	Interventio	Experimen	Experimen	Control	0,936399
baseline	abundance	X02427	2-(3,5-dim	2b	Interventio	Experimen	Experimen	Control	0,958229
baseline	abundance	X02442	3-(Sulfooxy	3	Interventio	Experimen	Experimen	Control	0,936399
baseline	abundance	X02466	Validamyci	3	Interventio	Experimen	Experimen	Control	0,945105
baseline	abundance	X02468	N,N-Diethy	3	Interventio	Experimen	Experimen	Control	0,936399
baseline	abundance	X02471	n-Propyl Ga	3	Interventio	Experimen	Experimen	Control	0,936399
baseline	abundance	X02492	N-(3,5-Dim	3	Interventio	Experimen	Experimen	Control	0,948031
baseline	abundance	X02494	Butylphtha	3	Interventio	Experimen	Experimen	Control	0,945105
baseline	abundance	X02504	DLK (Pepti	2b	Interventio	Experimen	Experimen	Control	0,936399
baseline	abundance	X02513	7-Methylac	2b	Interventio	Experimen	Experimen	Control	0,936399
baseline	abundance	X02514	3-Ureidopr	1	Interventio	Experimen	Experimen	Control	0,936399
baseline	abundance	X02521	6-(1-Hydro	3	Interventio	Experimen	Experimen	Control	0,945105
baseline	abundance	X02529	tenivastati	3	Interventio	Experimen	Experimen	Control	0,936399
baseline	abundance	X02537	Leu-pro_a	3	Interventio	Experimen	Experimen	Control	0,936399
baseline	abundance	X02565	Dopamine	3	Interventio	Experimen	Experimen	Control	0,97445
baseline	abundance	X02583	Ethylvanilli	3	Interventio	Experimen	Experimen	Control	0,957875
baseline	abundance	X02599	Dihydrothy	3	Interventio	Experimen	Experimen	Control	0,936399
baseline	abundance	X02601	(7E,7'E)-5,	3	Interventio	Experimen	Experimen	Control	0,97445
baseline	abundance	X02606	4-Amino-1	3	Interventio	Experimen	Experimen	Control	0,936399
baseline	abundance	X02615	6-APA_a	3	Interventio	Experimen	Experimen	Control	0,936399
baseline	abundance	X02640	thyronine	3	Interventio	Experimen	Experimen	Control	0,945105
baseline	abundance	X02651	L-gamma-(	3	Interventio	Experimen	Experimen	Control	0,936399
baseline	abundance	X02652	Leucyltyros	3	Interventio	Experimen	Experimen	Control	0,936399
baseline	abundance	X02661	4-(3-Oxope	3	Interventio	Experimen	Experimen	Control	0,945105
baseline	abundance	X02676	Val-Trp_a	3	Interventio	Experimen	Experimen	Control	0,936399
baseline	abundance	X02687	S-Propylcy	3	Interventio	Experimen	Experimen	Control	0,936399
baseline	abundance	X02689	piscidic ac	3	Interventio	Experimen	Experimen	Control	0,97445
baseline	abundance	X02702	R-(+)-Etira	3	Interventio	Experimen	Experimen	Control	0,945105
baseline	abundance	X02720	ala-ser_b	3	Interventio	Experimen	Experimen	Control	0,936399
baseline	abundance	X02730	N-Phenyla	3	Interventio	Experimen	Experimen	Control	0,936399
baseline	abundance	X02733	Calcitriol	3	Interventio	Experimen	Experimen	Control	0,957875
baseline	abundance	X02746	N,N-Diethy	3	Interventio	Experimen	Experimen	Control	0,970482
baseline	abundance	X02760	6-hydroxyp	3	Interventio	Experimen	Experimen	Control	0,957875
baseline	abundance	X02765	L-Pyrrolysi	3	Interventio	Experimen	Experimen	Control	0,936399
baseline	abundance	X02767	6-APA_b	3	Interventio	Experimen	Experimen	Control	0,972498

baseline	abundance	X02769	Tetraacety	3	Interventio	Experimen	Experimen	Control	0,945105
baseline	abundance	X02774	9-ribosylze	3	Interventio	Experimen	Experimen	Control	0,936399
baseline	abundance	X02793	3-Methoxy-	3	Interventio	Experimen	Experimen	Control	0,936399
baseline	abundance	X02820	FC250500	3	Interventio	Experimen	Experimen	Control	0,945105
baseline	abundance	X02823	6,8-Dimeth	3	Interventio	Experimen	Experimen	Control	0,945105
baseline	abundance	X02824	Eslicarbaz	3	Interventio	Experimen	Experimen	Control	0,936399
baseline	abundance	X02832	(+/-)-2-Hyc	3	Interventio	Experimen	Experimen	Control	0,936399
baseline	abundance	X02841	Kyotorphin	3	Interventio	Experimen	Experimen	Control	0,945105
baseline	abundance	X02843	4-(1-Hydro	3	Interventio	Experimen	Experimen	Control	0,936399
baseline	abundance	X02853	7alpha-Hy	3	Interventio	Experimen	Experimen	Control	0,945105
baseline	abundance	X02854	Marimasta	3	Interventio	Experimen	Experimen	Control	0,936399
baseline	abundance	X02868	2-(3-Hydro	3	Interventio	Experimen	Experimen	Control	0,957875
baseline	abundance	X02872	folinic acid	3	Interventio	Experimen	Experimen	Control	0,945105
baseline	abundance	X02878	N~6~-[5-(1	3	Interventio	Experimen	Experimen	Control	0,97445
baseline	abundance	X02881	N-(2,3,4-Tr	3	Interventio	Experimen	Experimen	Control	0,97445
baseline	abundance	X02894	Toluene_a	3	Interventio	Experimen	Experimen	Control	0,945105
baseline	abundance	X02905	MFCD0005	3	Interventio	Experimen	Experimen	Control	0,945105
baseline	abundance	X02920	L-gamma-(	3	Interventio	Experimen	Experimen	Control	0,936399
baseline	abundance	X02922	(2E)-3-Met	3	Interventio	Experimen	Experimen	Control	0,97445
baseline	abundance	X02928	N-Acetylpr	3	Interventio	Experimen	Experimen	Control	0,945105
baseline	abundance	X02943	3-Methoxy-	3	Interventio	Experimen	Experimen	Control	0,957875
baseline	abundance	X02944	Valylvaline	3	Interventio	Experimen	Experimen	Control	0,936399
baseline	abundance	X02948	N-(2-Cyan	3	Interventio	Experimen	Experimen	Control	0,962445
baseline	abundance	X02952	Vorinostat_	3	Interventio	Experimen	Experimen	Control	0,948031
baseline	abundance	X02968	Ro 20-172	3	Interventio	Experimen	Experimen	Control	0,936399
baseline	abundance	X02972	3-(Sulfoox	3	Interventio	Experimen	Experimen	Control	0,936399
baseline	abundance	X02986	Diacetin_b	3	Interventio	Experimen	Experimen	Control	0,936399
baseline	abundance	X02990	Pentoxifylli	3	Interventio	Experimen	Experimen	Control	0,945105
baseline	abundance	X03001	Pseudouric	2b	Interventio	Experimen	Experimen	Control	0,945105
baseline	abundance	X03004	Bicine_b	3	Interventio	Experimen	Experimen	Control	0,936399
baseline	abundance	X03017	Pro-tyr	3	Interventio	Experimen	Experimen	Control	0,936399
baseline	abundance	X03023	NPK (Pepti	2b	Interventio	Experimen	Experimen	Control	0,936399
baseline	abundance	X03064	Formimino	3	Interventio	Experimen	Experimen	Control	0,936399
baseline	abundance	X03069	N-COUMAI	3	Interventio	Experimen	Experimen	Control	0,936399
baseline	abundance	X03070	2-Methoxy-	3	Interventio	Experimen	Experimen	Control	0,97445
baseline	abundance	X03077	nicotianam	3	Interventio	Experimen	Experimen	Control	0,936399
baseline	abundance	X03097	6-hydroxyn	3	Interventio	Experimen	Experimen	Control	0,984935
baseline	abundance	X03109	Guanidino	3	Interventio	Experimen	Experimen	Control	0,936399
baseline	abundance	X03132	(3S,4S)-7,1	3	Interventio	Experimen	Experimen	Control	0,936399
baseline	abundance	X03134	Tetrahydro	3	Interventio	Experimen	Experimen	Control	0,97445
baseline	abundance	X03158	5-Hydroxy-	3	Interventio	Experimen	Experimen	Control	0,97445
baseline	abundance	X03223	1-(2-Carbo	3	Interventio	Experimen	Experimen	Control	0,958749
baseline	abundance	X03254	Gly-Trp_b	3	Interventio	Experimen	Experimen	Control	0,945105
baseline	abundance	X03268	Dihydrouri	3	Interventio	Experimen	Experimen	Control	0,957232
baseline	abundance	X03271	1Î²-Hydrox	3	Interventio	Experimen	Experimen	Control	0,957875
baseline	abundance	X03275	Methdilazi	3	Interventio	Experimen	Experimen	Control	0,957875
baseline	abundance	X03276	2'-Deoxyac	3	Interventio	Experimen	Experimen	Control	0,97445
baseline	abundance	X03278	Hydroxyph	3	Interventio	Experimen	Experimen	Control	0,988867

baseline	abundance	X03294	4,4'-Thiobi	3	Interventio	Experimen	Experimen	Control	0,97445
baseline	abundance	X03340	His-pro_b	3	Interventio	Experimen	Experimen	Control	0,936399
baseline	abundance	X03343	L-alpha-As	3	Interventio	Experimen	Experimen	Control	0,97445
baseline	abundance	X03357	2-Hydroxyh	3	Interventio	Experimen	Experimen	Control	0,936399
baseline	abundance	X03363	Choline Alf	3	Interventio	Experimen	Experimen	Control	0,945105
baseline	abundance	X03376	4-(9H-beta	3	Interventio	Experimen	Experimen	Control	0,97445
baseline	abundance	X03413	Val-Trp_b	3	Interventio	Experimen	Experimen	Control	0,936399
baseline	abundance	X03416	9-(alpha-D	3	Interventio	Experimen	Experimen	Control	0,97445
baseline	abundance	X03434	SECONAL_	3	Interventio	Experimen	Experimen	Control	0,983487
baseline	abundance	X03480	epsilon-(ga	3	Interventio	Experimen	Experimen	Control	0,945105
baseline	abundance	X03488	3,4-Dihydr	3	Interventio	Experimen	Experimen	Control	0,957232
baseline	abundance	X03535	(1S,3R,4s)-	3	Interventio	Experimen	Experimen	Control	0,97445
baseline	abundance	X03552	4-Hydroxya	3	Interventio	Experimen	Experimen	Control	0,957875
baseline	abundance	X03563	6-imino-5-	3	Interventio	Experimen	Experimen	Control	0,948031
baseline	abundance	X03568	3-[(2Z)-1-C	3	Interventio	Experimen	Experimen	Control	0,936399
baseline	abundance	X03592	2-glyceryl 1	3	Interventio	Experimen	Experimen	Control	0,945105
baseline	abundance	X03595	Methylol D	3	Interventio	Experimen	Experimen	Control	0,948031
baseline	abundance	X03602	o-Succinyl	3	Interventio	Experimen	Experimen	Control	0,955424
baseline	abundance	X03604	Gly-Trp_a	3	Interventio	Experimen	Experimen	Control	0,962445
baseline	abundance	X03629	Glycylglycy	3	Interventio	Experimen	Experimen	Control	0,936399
baseline	abundance	X03660	trp-ser	3	Interventio	Experimen	Experimen	Control	0,945786
baseline	abundance	X03675	asp-gln_a	3	Interventio	Experimen	Experimen	Control	0,945105
baseline	abundance	X03704	N,N-Dimet	3	Interventio	Experimen	Experimen	Control	0,988867
baseline	abundance	X03707	9-[(5R)-5-E	3	Interventio	Experimen	Experimen	Control	0,945105
baseline	abundance	X03709	Guanfacine	3	Interventio	Experimen	Experimen	Control	0,945105
baseline	abundance	X03714	Sinapinic a	3	Interventio	Experimen	Experimen	Control	0,97445
baseline	abundance	X03718	Propanthel	3	Interventio	Experimen	Experimen	Control	0,945105
baseline	abundance	X03719	Losalen	3	Interventio	Experimen	Experimen	Control	0,936399
baseline	abundance	X03760	2-Methoxy-	3	Interventio	Experimen	Experimen	Control	0,97445
baseline	abundance	X03793	Desonide	3	Interventio	Experimen	Experimen	Control	0,945105
baseline	abundance	X03837	2-Methoxy-	3	Interventio	Experimen	Experimen	Control	0,936399
baseline	abundance	X03855	2-{(2S,4S)-	3	Interventio	Experimen	Experimen	Control	0,955424
baseline	abundance	X03860	2-BUTYL PI	3	Interventio	Experimen	Experimen	Control	0,945105
baseline	abundance	X03892	shinorine	3	Interventio	Experimen	Experimen	Control	0,945105
baseline	abundance	X03901	ferrileghen	3	Interventio	Experimen	Experimen	Control	0,936399
baseline	abundance	X03916	2-(1-Ethoxy	3	Interventio	Experimen	Experimen	Control	0,936399
baseline	abundance	X03917	Uramustine	3	Interventio	Experimen	Experimen	Control	0,936399
baseline	abundance	X03934	2-Aminomu	3	Interventio	Experimen	Experimen	Control	0,936399
baseline	abundance	X03936	(3R)-2-(3,4	3	Interventio	Experimen	Experimen	Control	0,936399
baseline	abundance	X03961	Artesunate	3	Interventio	Experimen	Experimen	Control	0,959387
baseline	abundance	X04021	Bentazone	2b	Interventio	Experimen	Experimen	Control	0,936399
baseline	abundance	X04040	3-(3,4-dihy	3	Interventio	Experimen	Experimen	Control	0,987642
baseline	abundance	X04117	2,3,4,5-tet	3	Interventio	Experimen	Experimen	Control	0,945105
baseline	abundance	X04119	Tetraacety	3	Interventio	Experimen	Experimen	Control	0,97445
baseline	abundance	X04146	L-fucopyra	3	Interventio	Experimen	Experimen	Control	0,957875
baseline	abundance	X04150	4-(METHYL	3	Interventio	Experimen	Experimen	Control	0,957875
baseline	abundance	X04157	3-Methoxy-	3	Interventio	Experimen	Experimen	Control	0,945105
baseline	abundance	X04181	3-Methoxy-	3	Interventio	Experimen	Experimen	Control	0,936399

baseline	abundance	X04183	Spaglumic	3	Interventio	Experimen	Experimen	Control	0,992185
baseline	abundance	X04229	gamma-L-g	3	Interventio	Experimen	Experimen	Control	0,957875
baseline	abundance	X04259	3-Hydroxyt	3	Interventio	Experimen	Experimen	Control	0,936399
baseline	abundance	X04274	6-[(Z)-2-(3,	3	Interventio	Experimen	Experimen	Control	0,936399
baseline	abundance	X04286	Zinecard_b	3	Interventio	Experimen	Experimen	Control	0,936399
baseline	abundance	X04310	Aminohipp	3	Interventio	Experimen	Experimen	Control	0,945105
baseline	abundance	X04315	Benzamide	3	Interventio	Experimen	Experimen	Control	0,989213
baseline	abundance	X04334	3-Benzyl-6	3	Interventio	Experimen	Experimen	Control	0,936399
baseline	abundance	X04339	L-Glutamic	3	Interventio	Experimen	Experimen	Control	0,936399
baseline	abundance	X04351	Pyrimidine	3	Interventio	Experimen	Experimen	Control	0,986763
baseline	abundance	X04377	Dihydroura	3	Interventio	Experimen	Experimen	Control	0,97445
baseline	abundance	X04378	9,11-Dihyc	3	Interventio	Experimen	Experimen	Control	0,936399
baseline	abundance	X04379	2-Amino-6	3	Interventio	Experimen	Experimen	Control	0,945105
baseline	abundance	X04440	Mono(3-ca	3	Interventio	Experimen	Experimen	Control	0,945105
baseline	abundance	X04450	3-Methylac	3	Interventio	Experimen	Experimen	Control	0,936399
baseline	abundance	X04483	(2E)-5-Hyd	3	Interventio	Experimen	Experimen	Control	0,936399
baseline	abundance	X04493	Tyrosol	3	Interventio	Experimen	Experimen	Control	0,936399
baseline	abundance	X04526	N-Nonanoy	3	Interventio	Experimen	Experimen	Control	0,945105
baseline	abundance	X04538	Valylvaline	3	Interventio	Experimen	Experimen	Control	0,955424
baseline	abundance	X04543	N-Acetyl-S	3	Interventio	Experimen	Experimen	Control	0,936399
baseline	abundance	X04544	L-gamma-(	3	Interventio	Experimen	Experimen	Control	0,945105
baseline	abundance	X04553	N-(4-Amin	3	Interventio	Experimen	Experimen	Control	0,936399
baseline	abundance	X04557	1,4-Naphth	3	Interventio	Experimen	Experimen	Control	0,936399
baseline	abundance	X04562	Lisdexamfe	3	Interventio	Experimen	Experimen	Control	0,945105
baseline	abundance	X04564	asp-gln_b	3	Interventio	Experimen	Experimen	Control	0,945105
baseline	abundance	X04566	pterin	3	Interventio	Experimen	Experimen	Control	0,936399
baseline	abundance	X04571	13a-Hydro:	3	Interventio	Experimen	Experimen	Control	0,945105
baseline	abundance	X04579	6-(alpha-D	3	Interventio	Experimen	Experimen	Control	0,936399
baseline	abundance	X04582	Dinoseb	3	Interventio	Experimen	Experimen	Control	0,945105
baseline	abundance	X04593	Methional	3	Interventio	Experimen	Experimen	Control	0,945105
baseline	abundance	X04636	epsilon-(ga	3	Interventio	Experimen	Experimen	Control	0,936399
baseline	abundance	X04639	hydroxyhe:	3	Interventio	Experimen	Experimen	Control	0,957875
baseline	abundance	X04682	1-Methylin	3	Interventio	Experimen	Experimen	Control	0,945105
baseline	abundance	X04684	AAMU_b	3	Interventio	Experimen	Experimen	Control	0,936399
baseline	abundance	X04688	Minoxidil	3	Interventio	Experimen	Experimen	Control	0,936399
baseline	abundance	X04695	N-[(4-Meth	3	Interventio	Experimen	Experimen	Control	0,936399
baseline	abundance	X04697	Ethosuxim	3	Interventio	Experimen	Experimen	Control	0,945105
baseline	abundance	X04743	5-Methoxy-	3	Interventio	Experimen	Experimen	Control	0,936399
baseline	abundance	X04744	16alpha-hy	3	Interventio	Experimen	Experimen	Control	0,936399
baseline	abundance	X04759	7-Aminom	3	Interventio	Experimen	Experimen	Control	0,945786
baseline	abundance	X04788	2-(4-Isopro	3	Interventio	Experimen	Experimen	Control	0,970482
baseline	abundance	X04792	his-asn	3	Interventio	Experimen	Experimen	Control	0,957875
baseline	abundance	X04814	meprobam	3	Interventio	Experimen	Experimen	Control	0,987642
baseline	abundance	X04822	6-Sulfatoxy	3	Interventio	Experimen	Experimen	Control	0,98802
baseline	abundance	X04852	Toluene_b	3	Interventio	Experimen	Experimen	Control	0,936399
baseline	abundance	X04877	Trifluorome	3	Interventio	Experimen	Experimen	Control	0,94488
baseline	abundance	X04897	Taxifolin	3	Interventio	Experimen	Experimen	Control	0,958229
baseline	abundance	X04898	17-Hydroxy	3	Interventio	Experimen	Experimen	Control	0,936399

baseline	abundance	X04919	5-O-alpha-	3	Interventio	Experimen	Experimen	Control	0,957875
baseline	abundance	X04922	Sinapinic a	3	Interventio	Experimen	Experimen	Control	0,936399
baseline	abundance	X04939	N-Desalkyl	3	Interventio	Experimen	Experimen	Control	0,97445
baseline	abundance	X04975	MFCD0995	3	Interventio	Experimen	Experimen	Control	0,936399
baseline	abundance	X04986	L-Proline, 4	3	Interventio	Experimen	Experimen	Control	0,936399
baseline	abundance	X04990	Cilazapril	3	Interventio	Experimen	Experimen	Control	0,936399
baseline	abundance	X04996	MFCD0272	3	Interventio	Experimen	Experimen	Control	0,936399
baseline	abundance	X04999	2-Hydroxyp	1	Interventio	Experimen	Experimen	Control	0,936399
baseline	abundance	X05017	N-(1-{[Metl	3	Interventio	Experimen	Experimen	Control	0,991989
baseline	abundance	X05029	Hypericin	3	Interventio	Experimen	Experimen	Control	0,936399
baseline	abundance	X05033	(2E)-N-3,7-	3	Interventio	Experimen	Experimen	Control	0,936399
baseline	abundance	X05046	Casimiroin	3	Interventio	Experimen	Experimen	Control	0,945105
baseline	abundance	X05068	pro-gln_c	3	Interventio	Experimen	Experimen	Control	0,936399
baseline	abundance	X05071	Histidylphe	3	Interventio	Experimen	Experimen	Control	0,97445
baseline	abundance	X05072	L-gamma-(	3	Interventio	Experimen	Experimen	Control	0,945105
baseline	abundance	X05081	ELK (Peptic	2b	Interventio	Experimen	Experimen	Control	0,936399
baseline	abundance	X05126	(3aS,5S,6F	3	Interventio	Experimen	Experimen	Control	0,945786
baseline	abundance	X05146	UQ367500	3	Interventio	Experimen	Experimen	Control	0,936399
baseline	abundance	X05149	S-Sulfocys	3	Interventio	Experimen	Experimen	Control	0,936399
baseline	abundance	X05177	cis-3-Hexe	3	Interventio	Experimen	Experimen	Control	0,988867
baseline	abundance	X05183	3,6-Dichlo	3	Interventio	Experimen	Experimen	Control	0,936399
baseline	abundance	X05195	Lys-phe_a	3	Interventio	Experimen	Experimen	Control	0,936399
baseline	abundance	X05203	felbamate	3	Interventio	Experimen	Experimen	Control	0,936399
baseline	abundance	X05204	Urothion	3	Interventio	Experimen	Experimen	Control	0,957875
baseline	abundance	X05214	[7-Hydroxy	3	Interventio	Experimen	Experimen	Control	0,97445
baseline	abundance	X05225	4-(METHYL	3	Interventio	Experimen	Experimen	Control	0,992737
baseline	abundance	X05234	3,4-dihydr	3	Interventio	Experimen	Experimen	Control	0,936399
baseline	abundance	X05237	imazameth	3	Interventio	Experimen	Experimen	Control	0,936399
baseline	abundance	X05240	Dimeric m	3	Interventio	Experimen	Experimen	Control	0,937501
baseline	abundance	X05243	N-(4-Hydro	3	Interventio	Experimen	Experimen	Control	0,936399
baseline	abundance	X05275	H-DL-MET-	3	Interventio	Experimen	Experimen	Control	0,97445
baseline	abundance	X05295	3-(4,7-Dim	3	Interventio	Experimen	Experimen	Control	0,936399
baseline	abundance	X05306	2-methoxy	3	Interventio	Experimen	Experimen	Control	0,936399
baseline	abundance	X05357	NPYR	3	Interventio	Experimen	Experimen	Control	0,936399
baseline	abundance	X05391	2-Acetami	3	Interventio	Experimen	Experimen	Control	0,936399
baseline	abundance	X05398	DIBOA	3	Interventio	Experimen	Experimen	Control	0,936399
baseline	abundance	X05411	3-(Sulfoox	3	Interventio	Experimen	Experimen	Control	0,97445
baseline	abundance	X05415	Redul	3	Interventio	Experimen	Experimen	Control	0,936399
baseline	abundance	X05417	(S)-2-hydra	3	Interventio	Experimen	Experimen	Control	0,936399
baseline	abundance	X05421	S-Allylcyst	3	Interventio	Experimen	Experimen	Control	0,945105
baseline	abundance	X05459	Scopoletin	3	Interventio	Experimen	Experimen	Control	0,97445
baseline	abundance	X05467	Azulfidine	3	Interventio	Experimen	Experimen	Control	0,936399
baseline	abundance	X05478	S(8)-aminc	3	Interventio	Experimen	Experimen	Control	0,957875
baseline	abundance	X05499	GLY-MET	3	Interventio	Experimen	Experimen	Control	0,945105
baseline	abundance	X05510	3-Benzyl-6	3	Interventio	Experimen	Experimen	Control	0,945105
baseline	abundance	X05514	(3aS,5S,6F	3	Interventio	Experimen	Experimen	Control	0,97445
baseline	abundance	X05551	Trolox	3	Interventio	Experimen	Experimen	Control	0,936399
baseline	abundance	X05561	1Î²-Hydroxy	3	Interventio	Experimen	Experimen	Control	0,948031

baseline	abundance	X05580	Xanthureni	3	Interventio	Experimen	Experimen	Control	0,945105
baseline	abundance	X05581	N-[(10Z)-7-	3	Interventio	Experimen	Experimen	Control	0,945105
baseline	abundance	X05584	Serotonin	1	Interventio	Experimen	Experimen	Control	0,987751
baseline	abundance	X05656	mesifurane	3	Interventio	Experimen	Experimen	Control	0,936399
baseline	abundance	X05709	2,3-Dihydr	3	Interventio	Experimen	Experimen	Control	0,945105
baseline	abundance	X05726	methocarb	3	Interventio	Experimen	Experimen	Control	0,936399
baseline	abundance	X05734	N-[(2S)-2-I	3	Interventio	Experimen	Experimen	Control	0,97445
baseline	abundance	X05741	g-Aminobu	3	Interventio	Experimen	Experimen	Control	0,936399
baseline	abundance	X05758	quinol sulf	3	Interventio	Experimen	Experimen	Control	0,97499
baseline	abundance	X05786	Nitrendipir	3	Interventio	Experimen	Experimen	Control	0,936399
baseline	abundance	X05812	(2S)-3-(1H	3	Interventio	Experimen	Experimen	Control	0,957875
baseline	abundance	X05857	N-D-Gluco	3	Interventio	Experimen	Experimen	Control	0,97445
baseline	abundance	X05859	3,7,12,17-	3	Interventio	Experimen	Experimen	Control	0,985326
baseline	abundance	X05878	1,2-dihydr	3	Interventio	Experimen	Experimen	Control	0,97445
baseline	abundance	X05892	(5Z)-2-Ami	3	Interventio	Experimen	Experimen	Control	0,97445
baseline	abundance	X05919	2-(2-Aminc	3	Interventio	Experimen	Experimen	Control	0,945105
baseline	abundance	X05968	Tetraacety	3	Interventio	Experimen	Experimen	Control	0,945105
baseline	abundance	X05969	4-(METHYL	3	Interventio	Experimen	Experimen	Control	0,936399
baseline	abundance	X05971	L-gamma-(	3	Interventio	Experimen	Experimen	Control	0,936399
baseline	abundance	X05982	(19R,25S)-	3	Interventio	Experimen	Experimen	Control	0,97445
baseline	abundance	X05984	1-(4-Methy	2b	Interventio	Experimen	Experimen	Control	0,936399
baseline	abundance	X05988	Selsun	3	Interventio	Experimen	Experimen	Control	0,957875
baseline	abundance	X05995	Diacetin_a	3	Interventio	Experimen	Experimen	Control	0,936399
baseline	abundance	X05997	FB950000(	3	Interventio	Experimen	Experimen	Control	0,945105
baseline	abundance	X06011	Coprine_a	3	Interventio	Experimen	Experimen	Control	0,992737
baseline	abundance	X06016	N,N-Dimet	3	Interventio	Experimen	Experimen	Control	0,955424
baseline	abundance	X06034	N-[(2S)-2-I	3	Interventio	Experimen	Experimen	Control	0,97445
baseline	abundance	X06039	gamma-Gl	3	Interventio	Experimen	Experimen	Control	0,948031
baseline	abundance	X06041	(DL)-3-O-M	3	Interventio	Experimen	Experimen	Control	0,936399
baseline	abundance	X06043	vinyl sulfid	3	Interventio	Experimen	Experimen	Control	0,945105
baseline	abundance	X06046	Valylvaline	3	Interventio	Experimen	Experimen	Control	0,987751
baseline	abundance	X06062	1-(2,3-Dihy	3	Interventio	Experimen	Experimen	Control	0,948031
baseline	abundance	X06079	Glycylleuci	1	Interventio	Experimen	Experimen	Control	0,945105
baseline	abundance	X06082	O-heptano	3	Interventio	Experimen	Experimen	Control	0,936399
baseline	abundance	X06085	Tetraacety	3	Interventio	Experimen	Experimen	Control	0,945105
baseline	abundance	X06089	riboprine	3	Interventio	Experimen	Experimen	Control	0,97445
baseline	abundance	X06100	Midodrine_	3	Interventio	Experimen	Experimen	Control	0,989769
baseline	abundance	X06107	Hydroxycar	3	Interventio	Experimen	Experimen	Control	0,97445
baseline	abundance	X06112	Val-Ser_a	3	Interventio	Experimen	Experimen	Control	0,936399
baseline	abundance	X06124	MFCD0995	3	Interventio	Experimen	Experimen	Control	0,936399
baseline	abundance	X06127	2-Methoxy-	3	Interventio	Experimen	Experimen	Control	0,987642
baseline	abundance	X06143	2-Methylbu	2a	Interventio	Experimen	Experimen	Control	0,945105
baseline	abundance	X06146	S-Methyl-1	3	Interventio	Experimen	Experimen	Control	0,945105
baseline	abundance	X06149	Yangonin	3	Interventio	Experimen	Experimen	Control	0,945786
baseline	abundance	X06150	Zalcitabine	3	Interventio	Experimen	Experimen	Control	0,936399
baseline	abundance	X06152	alpha-keto	3	Interventio	Experimen	Experimen	Control	0,993031
baseline	abundance	X06167	N-Acetylas	2a	Interventio	Experimen	Experimen	Control	0,936399
baseline	abundance	X06168	leu-gln_b	3	Interventio	Experimen	Experimen	Control	0,945105

baseline	abundance	X06183	Nisinic aci	3	Interventio	Experimen	Experimen	Control	0,936399
baseline	abundance	X06189	delta-Guar	3	Interventio	Experimen	Experimen	Control	0,994656
baseline	abundance	X06197	4-(Nitrosoa	3	Interventio	Experimen	Experimen	Control	0,936399
baseline	abundance	X06220	Tetrahydro	3	Interventio	Experimen	Experimen	Control	0,936399
baseline	abundance	X06222	Leucylaspa	3	Interventio	Experimen	Experimen	Control	0,936399
baseline	abundance	X06227	lys-tyr_a	3	Interventio	Experimen	Experimen	Control	0,936399
baseline	abundance	X06230	Glu-Gly	3	Interventio	Experimen	Experimen	Control	0,936399
baseline	abundance	X06240	His-pro_a	3	Interventio	Experimen	Experimen	Control	0,945105
baseline	abundance	X06250	[3-({3-[(Cy	2b	Interventio	Experimen	Experimen	Control	0,945105
baseline	abundance	X06253	N-(4-Amin	3	Interventio	Experimen	Experimen	Control	0,955719
baseline	abundance	X06254	D-Alanyl-D	3	Interventio	Experimen	Experimen	Control	0,936399
baseline	abundance	X06259	(S)-?-glyce	3	Interventio	Experimen	Experimen	Control	0,945105
baseline	abundance	X06268	mesifurane	3	Interventio	Experimen	Experimen	Control	0,936399
baseline	abundance	X06270	9-Methylur	2b	Interventio	Experimen	Experimen	Control	0,936399
baseline	abundance	X06276	asn-pro_b	3	Interventio	Experimen	Experimen	Control	0,936399
baseline	abundance	X06278	3-Hydroxy-	3	Interventio	Experimen	Experimen	Control	0,936399
baseline	abundance	X06289	Nicotinatel	3	Interventio	Experimen	Experimen	Control	0,988948
baseline	abundance	X06291	Corticoster	2b	Interventio	Experimen	Experimen	Control	0,945105
baseline	abundance	X06292	mesifurane	3	Interventio	Experimen	Experimen	Control	0,936399
baseline	abundance	X06293	(4R)-4-{[3-	3	Interventio	Experimen	Experimen	Control	0,945105
baseline	abundance	X06313	Isoquinolir	2b	Interventio	Experimen	Experimen	Control	0,95582
baseline	abundance	X06318	Propamoca	2b	Interventio	Experimen	Experimen	Control	0,948031
baseline	abundance	X06320	7alpha-Hy	3	Interventio	Experimen	Experimen	Control	0,97445
baseline	abundance	X06329	Leu-Leu_d	3	Interventio	Experimen	Experimen	Control	0,936399
baseline	abundance	X06334	Bis-D-fruct	3	Interventio	Experimen	Experimen	Control	0,97445
baseline	abundance	X06337	Zalcitabine	3	Interventio	Experimen	Experimen	Control	0,945105
baseline	abundance	X06354	trans-Zeati	2b	Interventio	Experimen	Experimen	Control	0,936399
baseline	abundance	X06361	Oxprenolol	3	Interventio	Experimen	Experimen	Control	0,936399
baseline	abundance	X06368	TDP-2_b	3	Interventio	Experimen	Experimen	Control	0,936399
baseline	abundance	X06371	L-gamma-(	3	Interventio	Experimen	Experimen	Control	0,936399
baseline	abundance	X06372	N,N-dimetl	2b	Interventio	Experimen	Experimen	Control	0,945105
baseline	abundance	X06381	MFCD1869	3	Interventio	Experimen	Experimen	Control	0,936399
baseline	abundance	X06388	Caffeic aci	1	Interventio	Experimen	Experimen	Control	0,936399
baseline	abundance	X06400	Phloionolic	3	Interventio	Experimen	Experimen	Control	0,998245
baseline	abundance	X06401	Isophthalic	2b	Interventio	Experimen	Experimen	Control	0,936399
baseline	abundance	X06404	Flemichap	3	Interventio	Experimen	Experimen	Control	0,962445
baseline	abundance	X06405	Asparaginy	3	Interventio	Experimen	Experimen	Control	0,98802
baseline	abundance	X06409	Glycylproli	2b	Interventio	Experimen	Experimen	Control	0,945105
baseline	abundance	X06412	Butenylcar	3	Interventio	Experimen	Experimen	Control	0,936399
baseline	abundance	X06416	KYNURAMI	3	Interventio	Experimen	Experimen	Control	0,936399
baseline	abundance	X06419	Spermic ac	3	Interventio	Experimen	Experimen	Control	0,945105
baseline	abundance	X06433	4-(2,5-Diflu	2b	Interventio	Experimen	Experimen	Control	0,97445
baseline	abundance	X06434	butyrin	3	Interventio	Experimen	Experimen	Control	0,97445
baseline	abundance	X06437	2-Acetami	3	Interventio	Experimen	Experimen	Control	0,957875
baseline	abundance	X06448	3,8,9-trihy	2b	Interventio	Experimen	Experimen	Control	0,936399
baseline	abundance	X06454	8-Amino-7-	3	Interventio	Experimen	Experimen	Control	0,936399
baseline	abundance	X06455	4-Hydroxyp	3	Interventio	Experimen	Experimen	Control	0,982558
baseline	abundance	X06462	DNOP_d	3	Interventio	Experimen	Experimen	Control	0,97445

baseline	abundance	X06463	8-Amino-7-	3	Interventio	Experimen	Experimen	Control	0,936399
baseline	abundance	X06472	N(alpha)-B	3	Interventio	Experimen	Experimen	Control	0,936399
baseline	abundance	X06473	MFCD0002	3	Interventio	Experimen	Experimen	Control	0,945105
baseline	abundance	X06477	APM_b	3	Interventio	Experimen	Experimen	Control	0,936399
baseline	abundance	X06478	Caffeic aci	3	Interventio	Experimen	Experimen	Control	0,936399
baseline	abundance	X06482	glu-pro	3	Interventio	Experimen	Experimen	Control	0,957875
baseline	abundance	X06486	Leu-Val_b	3	Interventio	Experimen	Experimen	Control	0,97445
baseline	abundance	X06495	2-Acetami	3	Interventio	Experimen	Experimen	Control	0,945786
baseline	abundance	X06502	MFCD2836	3	Interventio	Experimen	Experimen	Control	0,945105
baseline	abundance	X06506	L-Homocys	3	Interventio	Experimen	Experimen	Control	0,945105
baseline	abundance	X06507	Gly-Lys	3	Interventio	Experimen	Experimen	Control	0,945105
baseline	abundance	X06508	Asparaginy	3	Interventio	Experimen	Experimen	Control	0,987751
baseline	abundance	X06510	N-{4-[(2R,3	2b	Interventio	Experimen	Experimen	Control	0,936399
baseline	abundance	X06529	Methanesu	2a	Interventio	Experimen	Experimen	Control	0,985326
baseline	abundance	X06530	3-(1-hydro:	2b	Interventio	Experimen	Experimen	Control	0,97445
baseline	abundance	X06532	Leupeptin	3	Interventio	Experimen	Experimen	Control	0,936399
baseline	abundance	X06546	4-Acetami	2b	Interventio	Experimen	Experimen	Control	0,936399
baseline	abundance	X06549	Panthenol_	3	Interventio	Experimen	Experimen	Control	0,936399
baseline	abundance	X06551	Methyl 1-hy	3	Interventio	Experimen	Experimen	Control	0,97445
baseline	abundance	X06560	2,6-Dimeth	2b	Interventio	Experimen	Experimen	Control	0,936399
baseline	abundance	X06590	3-hydroxy-3	3	Interventio	Experimen	Experimen	Control	0,948031
baseline	abundance	X06600	MFCD0014	3	Interventio	Experimen	Experimen	Control	0,988948
baseline	abundance	X06606	Leu-Val_d	3	Interventio	Experimen	Experimen	Control	0,97445
baseline	abundance	X06612	Spermic ac	3	Interventio	Experimen	Experimen	Control	0,936399
baseline	abundance	X06622	Lys-Pro_b	3	Interventio	Experimen	Experimen	Control	0,97445
baseline	abundance	X06623	quinol sulf	3	Interventio	Experimen	Experimen	Control	0,945105
baseline	abundance	X06626	N-Butyryl-L	3	Interventio	Experimen	Experimen	Control	0,936399
baseline	abundance	X06630	4-(METHYL	3	Interventio	Experimen	Experimen	Control	0,936399
baseline	abundance	X06631	2-Isopropy	2b	Interventio	Experimen	Experimen	Control	0,936399
baseline	abundance	X06634	Glycitein	3	Interventio	Experimen	Experimen	Control	0,945105
baseline	abundance	X06642	Homocitru	2a	Interventio	Experimen	Experimen	Control	0,936399
baseline	abundance	X06646	(S)-3-sulfo	3	Interventio	Experimen	Experimen	Control	0,936399
baseline	abundance	X06655	Hydroxycar	3	Interventio	Experimen	Experimen	Control	0,936399
baseline	abundance	X06656	Valylvaline	3	Interventio	Experimen	Experimen	Control	0,945105
baseline	abundance	X06663	Leucyltyros	3	Interventio	Experimen	Experimen	Control	0,945105
baseline	abundance	X06675	L-gamma-(	3	Interventio	Experimen	Experimen	Control	0,957875
baseline	abundance	X06680	7-Hydroxy-	2b	Interventio	Experimen	Experimen	Control	0,936399
baseline	abundance	X06681	Scymnol	3	Interventio	Experimen	Experimen	Control	0,936399
baseline	abundance	X06684	Bromazine	3	Interventio	Experimen	Experimen	Control	0,945105
baseline	abundance	X06690	4-Methylca	2b	Interventio	Experimen	Experimen	Control	0,936399
baseline	abundance	X06698	entecavir	3	Interventio	Experimen	Experimen	Control	0,957875
baseline	abundance	X06703	N6-METHY	3	Interventio	Experimen	Experimen	Control	0,97445
baseline	abundance	X06704	feruloylagr	3	Interventio	Experimen	Experimen	Control	0,945105
baseline	abundance	X06709	Tetraacety	3	Interventio	Experimen	Experimen	Control	0,955424
baseline	abundance	X06722	LW800000	3	Interventio	Experimen	Experimen	Control	0,945105
baseline	abundance	X06723	3-Hydroxy-	3	Interventio	Experimen	Experimen	Control	0,945105
baseline	abundance	X06727	MFCD0087	3	Interventio	Experimen	Experimen	Control	0,948031
baseline	abundance	X06735	7-Methylxa	2b	Interventio	Experimen	Experimen	Control	0,945105

baseline	abundance	X06744	Diethylpyro	3	Interventio	Experimen	Experimen	Control	0,978464
baseline	abundance	X06750	1-PYRENYL	3	Interventio	Experimen	Experimen	Control	0,97445
baseline	abundance	X06764	N-(4-Hydro	3	Interventio	Experimen	Experimen	Control	0,936399
baseline	abundance	X06767	2-Hydroxyb	1	Interventio	Experimen	Experimen	Control	0,945105
baseline	abundance	X06768	Sinapyl alc	3	Interventio	Experimen	Experimen	Control	0,936399
baseline	abundance	X06771	MFCD0015	3	Interventio	Experimen	Experimen	Control	0,937501
baseline	abundance	X06783	N~6~-Octa	3	Interventio	Experimen	Experimen	Control	0,936399
baseline	abundance	X06797	pro-gln_a	3	Interventio	Experimen	Experimen	Control	0,957232
baseline	abundance	X06805	(9cis)-O~1	3	Interventio	Experimen	Experimen	Control	0,957875
baseline	abundance	X06807	1,1'-[1,12-I	3	Interventio	Experimen	Experimen	Control	0,97445
baseline	abundance	X06812	2-Hydroxy-	3	Interventio	Experimen	Experimen	Control	0,936399
baseline	abundance	X06814	N-Benzoyla	3	Interventio	Experimen	Experimen	Control	0,936399
baseline	abundance	X06818	Homoanse	3	Interventio	Experimen	Experimen	Control	0,936399
baseline	abundance	X06819	Primaquine	3	Interventio	Experimen	Experimen	Control	0,945105
baseline	abundance	X06820	1-(3,4-dim	2b	Interventio	Experimen	Experimen	Control	0,945105
baseline	abundance	X06836	asn-lys	3	Interventio	Experimen	Experimen	Control	0,982558
baseline	abundance	X06850	Leu-Val_c	3	Interventio	Experimen	Experimen	Control	0,97445
baseline	abundance	X06856	Tiglic acid_	2b	Interventio	Experimen	Experimen	Control	0,936399
baseline	abundance	X06858	tert-Butyl 3	3	Interventio	Experimen	Experimen	Control	0,945105
baseline	abundance	X06859	Methylol D	3	Interventio	Experimen	Experimen	Control	0,936399
baseline	abundance	X06861	4-(4-Deoxy	3	Interventio	Experimen	Experimen	Control	0,936399
baseline	abundance	X06866	tert-Butyl 3	3	Interventio	Experimen	Experimen	Control	0,957875
baseline	abundance	X06870	Menadiol	3	Interventio	Experimen	Experimen	Control	0,945105
baseline	abundance	X06874	N-Acetyl-5	3	Interventio	Experimen	Experimen	Control	0,936399
baseline	abundance	X06875	Nicotine gl	3	Interventio	Experimen	Experimen	Control	0,936399
baseline	abundance	X06880	pentobarbi	3	Interventio	Experimen	Experimen	Control	0,945105
baseline	abundance	X06883	Agomelatin	3	Interventio	Experimen	Experimen	Control	0,962445
baseline	abundance	X06889	3,4-Dimeth	2a	Interventio	Experimen	Experimen	Control	0,97445
baseline	abundance	X06896	Pirbuterol	3	Interventio	Experimen	Experimen	Control	0,97445
baseline	abundance	X06902	Homocyste	3	Interventio	Experimen	Experimen	Control	0,936399
baseline	abundance	X06903	Esculin	2b	Interventio	Experimen	Experimen	Control	0,936399
baseline	abundance	X06905	Ectoine	3	Interventio	Experimen	Experimen	Control	0,955424
baseline	abundance	X06906	Arg-pro	3	Interventio	Experimen	Experimen	Control	0,945105
baseline	abundance	X06917	Alanyltrypt	3	Interventio	Experimen	Experimen	Control	0,936399
baseline	abundance	X06933	3-Methoxy-	3	Interventio	Experimen	Experimen	Control	0,968601
baseline	abundance	X06943	NSC 92778	3	Interventio	Experimen	Experimen	Control	0,945105
baseline	abundance	X06946	SECONAL_	3	Interventio	Experimen	Experimen	Control	0,97445
baseline	abundance	X06948	(1R,3R,5R)	3	Interventio	Experimen	Experimen	Control	0,945105
baseline	abundance	X06958	2-[(carboxy	2b	Interventio	Experimen	Experimen	Control	0,936399
baseline	abundance	X06961	Zalcitabine	3	Interventio	Experimen	Experimen	Control	0,948031
baseline	abundance	X06977	4-Hydroxyp	3	Interventio	Experimen	Experimen	Control	0,945786
baseline	abundance	X06985	N-Ethylpro	3	Interventio	Experimen	Experimen	Control	0,948031
baseline	abundance	X07002	Paraldehyd	3	Interventio	Experimen	Experimen	Control	0,936399
baseline	abundance	X07013	pimethixen	3	Interventio	Experimen	Experimen	Control	0,936399
baseline	abundance	X07014	Octyl benz	3	Interventio	Experimen	Experimen	Control	0,936399
baseline	abundance	X07026	7α-Hydroxy	2b	Interventio	Experimen	Experimen	Control	0,978198
baseline	abundance	X07027	Allyl merca	3	Interventio	Experimen	Experimen	Control	0,936399
baseline	abundance	X07028	N-Pentano	3	Interventio	Experimen	Experimen	Control	0,936399

baseline	abundance	X07038	Piperidine_	3	Interventio	Experimen	Experimen	Control	0,945105
baseline	abundance	X07040	6-Hydroxyp	3	Interventio	Experimen	Experimen	Control	0,97445
baseline	abundance	X07050	2-Methylth	3	Interventio	Experimen	Experimen	Control	0,945105
baseline	abundance	X07051	O-succinyl	3	Interventio	Experimen	Experimen	Control	0,97445
baseline	abundance	X07054	Valylprolin	2b	Interventio	Experimen	Experimen	Control	0,987276
baseline	abundance	X07057	Leu-Leu_c	3	Interventio	Experimen	Experimen	Control	0,968601
baseline	abundance	X07073	asn-val_a	3	Interventio	Experimen	Experimen	Control	0,936399
baseline	abundance	X07077	Hyodeoxyc	1	Interventio	Experimen	Experimen	Control	0,945105
baseline	abundance	X07079	N-Pentano	3	Interventio	Experimen	Experimen	Control	0,936399
baseline	abundance	X07081	N-LACTOYI	3	Interventio	Experimen	Experimen	Control	0,97445
baseline	abundance	X07084	heptabarbi	3	Interventio	Experimen	Experimen	Control	0,945105
baseline	abundance	X07089	Pregabalin	3	Interventio	Experimen	Experimen	Control	0,936399
baseline	abundance	X07092	Dibutyl ma	3	Interventio	Experimen	Experimen	Control	0,945105
baseline	abundance	X07107	Lysylvaline	3	Interventio	Experimen	Experimen	Control	0,957875
baseline	abundance	X07112	MFCD0272	3	Interventio	Experimen	Experimen	Control	0,954102
baseline	abundance	X07113	Sulfoaceta	3	Interventio	Experimen	Experimen	Control	0,936399
baseline	abundance	X07125	Zalcitabine	3	Interventio	Experimen	Experimen	Control	0,936399
baseline	abundance	X07126	Leu-Val_f	3	Interventio	Experimen	Experimen	Control	0,936399
baseline	abundance	X07127	N~5~-[P-Ai	3	Interventio	Experimen	Experimen	Control	0,936399
baseline	abundance	X07134	4-Phenolsu	2b	Interventio	Experimen	Experimen	Control	0,97445
baseline	abundance	X07136	hexobarbit	3	Interventio	Experimen	Experimen	Control	0,97445
baseline	abundance	X07139	N,N-Diethy	3	Interventio	Experimen	Experimen	Control	0,936399
baseline	abundance	X07146	meprobam	3	Interventio	Experimen	Experimen	Control	0,991989
baseline	abundance	X07155	Naphthale	3	Interventio	Experimen	Experimen	Control	0,945105
baseline	abundance	X07164	N-Acetyl-5	3	Interventio	Experimen	Experimen	Control	0,97445
baseline	abundance	X07165	pro-gln_b	3	Interventio	Experimen	Experimen	Control	0,991989
baseline	abundance	X07168	L-(+)-Eryth	3	Interventio	Experimen	Experimen	Control	0,948031
baseline	abundance	X07193	N-(1-Methy	3	Interventio	Experimen	Experimen	Control	0,97445
baseline	abundance	X07201	2,4-Quinol	2b	Interventio	Experimen	Experimen	Control	0,97445
baseline	abundance	X07216	pentobarbi	3	Interventio	Experimen	Experimen	Control	0,936399
baseline	abundance	X07219	3,3-Dimetl	2b	Interventio	Experimen	Experimen	Control	0,97445
baseline	abundance	X07220	Glycylproly	3	Interventio	Experimen	Experimen	Control	0,936399
baseline	abundance	X07226	threonylph	3	Interventio	Experimen	Experimen	Control	0,97445
baseline	abundance	X07236	2-methylci	3	Interventio	Experimen	Experimen	Control	0,945105
baseline	abundance	X07250	Leucylaspa	3	Interventio	Experimen	Experimen	Control	0,997375
baseline	abundance	X07260	(7E,7'E)-5,	3	Interventio	Experimen	Experimen	Control	0,936399
baseline	abundance	X07263	3,3-Dimetl	2b	Interventio	Experimen	Experimen	Control	0,945105
baseline	abundance	X07269	(3aR,4R,5F	3	Interventio	Experimen	Experimen	Control	0,962445
baseline	abundance	X07270	5-(2-Carbo	3	Interventio	Experimen	Experimen	Control	0,936399
baseline	abundance	X07278	5-Phospho	3	Interventio	Experimen	Experimen	Control	0,936399
baseline	abundance	X07283	8-Hydroxyl	3	Interventio	Experimen	Experimen	Control	0,957875
baseline	abundance	X07285	2-(Carboxy	3	Interventio	Experimen	Experimen	Control	0,936399
baseline	abundance	X07294	6-Acetami	3	Interventio	Experimen	Experimen	Control	0,957875
baseline	abundance	X07303	Triacetin	3	Interventio	Experimen	Experimen	Control	0,945786
baseline	abundance	X07308	Nicotinic a	2a	Interventio	Experimen	Experimen	Control	0,936399
baseline	abundance	X07309	3,4,15-Trih	3	Interventio	Experimen	Experimen	Control	0,988867
baseline	abundance	X07315	Rivastigmi	3	Interventio	Experimen	Experimen	Control	0,945105
baseline	abundance	X07320	asn-pro_d	3	Interventio	Experimen	Experimen	Control	0,97445

baseline	abundance	X07327	Aspartyl-L-	3	Interventio	Experimen	Experimen	Control	0,936399
baseline	abundance	X07334	2,2-Bis(hyd	3	Interventio	Experimen	Experimen	Control	0,972498
baseline	abundance	X07336	Threonylse	3	Interventio	Experimen	Experimen	Control	0,945105
baseline	abundance	X07344	(−)-nabilor	3	Interventio	Experimen	Experimen	Control	0,983487
baseline	abundance	X07345	Phenyl D-g	3	Interventio	Experimen	Experimen	Control	0,936399
baseline	abundance	X07375	porphobilir	3	Interventio	Experimen	Experimen	Control	0,97445
baseline	abundance	X07381	Bis-D-fruct	3	Interventio	Experimen	Experimen	Control	0,941185
baseline	abundance	X07394	Hydroxypro	3	Interventio	Experimen	Experimen	Control	0,936399
baseline	abundance	X07399	Homoanse	3	Interventio	Experimen	Experimen	Control	0,968601
baseline	abundance	X07400	Tranexami	2b	Interventio	Experimen	Experimen	Control	0,936399
baseline	abundance	X07413	Chenodeo:	1	Interventio	Experimen	Experimen	Control	0,974375
baseline	abundance	X07414	2,2-Bis(hyd	3	Interventio	Experimen	Experimen	Control	0,936399
baseline	abundance	X07417	δ-Valerolac	2b	Interventio	Experimen	Experimen	Control	0,986763
baseline	abundance	X07420	DNOP_h	3	Interventio	Experimen	Experimen	Control	0,97445
baseline	abundance	X07424	Rutinose (b	3	Interventio	Experimen	Experimen	Control	0,945105
baseline	abundance	X07425	N-Acetylas	2a	Interventio	Experimen	Experimen	Control	0,936399
baseline	abundance	X07428	N6-METHY	3	Interventio	Experimen	Experimen	Control	0,936399
baseline	abundance	X07429	DNOP_c	3	Interventio	Experimen	Experimen	Control	0,97445
baseline	abundance	X07434	N'-Hydroxy	2b	Interventio	Experimen	Experimen	Control	0,97445
baseline	abundance	X07439	4-pyridoxic	1	Interventio	Experimen	Experimen	Control	0,936399
baseline	abundance	X07442	coenzyme	3	Interventio	Experimen	Experimen	Control	0,987642
baseline	abundance	X07443	Butylparab	3	Interventio	Experimen	Experimen	Control	0,97445
baseline	abundance	X07445	3-(Sulfooxy	3	Interventio	Experimen	Experimen	Control	0,948031
baseline	abundance	X07451	Valylvaline	3	Interventio	Experimen	Experimen	Control	0,936399
baseline	abundance	X07452	4-Amino-1-	3	Interventio	Experimen	Experimen	Control	0,936399
baseline	abundance	X07454	2,4-Quinol	2b	Interventio	Experimen	Experimen	Control	0,945105
baseline	abundance	X07457	Glucosami	1	Interventio	Experimen	Experimen	Control	0,97445
baseline	abundance	X07460	1-pyrroline	3	Interventio	Experimen	Experimen	Control	0,957875
baseline	abundance	X07465	TO0127900	3	Interventio	Experimen	Experimen	Control	0,957875
baseline	abundance	X07469	1,1'-[1,12-I	3	Interventio	Experimen	Experimen	Control	0,945105
baseline	abundance	X07475	2'-Deoxy-5	3	Interventio	Experimen	Experimen	Control	0,936399
baseline	abundance	X07476	4-Hydroxyb	1	Interventio	Experimen	Experimen	Control	0,936399
baseline	abundance	X07480	Naringenin	2a	Interventio	Experimen	Experimen	Control	0,945105
baseline	abundance	X07484	MFCD0002	3	Interventio	Experimen	Experimen	Control	0,983461
baseline	abundance	X07487	Oxypeucec	3	Interventio	Experimen	Experimen	Control	0,989213
baseline	abundance	X07491	1-Vinylimic	2b	Interventio	Experimen	Experimen	Control	0,936399
baseline	abundance	X07504	4-Acetami	2b	Interventio	Experimen	Experimen	Control	0,959701
baseline	abundance	X07508	Procaine_a	2b	Interventio	Experimen	Experimen	Control	0,97445
baseline	abundance	X07511	Piperidine_	3	Interventio	Experimen	Experimen	Control	0,936399
baseline	abundance	X07512	4-{[(3-Hydr	3	Interventio	Experimen	Experimen	Control	0,936399
baseline	abundance	X07513	(−)-nabilor	3	Interventio	Experimen	Experimen	Control	0,957875
baseline	abundance	X07514	L-(+)-Eryth	3	Interventio	Experimen	Experimen	Control	0,97445
baseline	abundance	X07518	(+/-)-2-Hyc	3	Interventio	Experimen	Experimen	Control	0,945105
baseline	abundance	X07519	Pyrrolidine	3	Interventio	Experimen	Experimen	Control	0,936399
baseline	abundance	X07523	Ursodeoxy	1	Interventio	Experimen	Experimen	Control	0,945105
baseline	abundance	X07524	Prolinamid	2b	Interventio	Experimen	Experimen	Control	0,936399
baseline	abundance	X07527	1,7-Dimeth	2b	Interventio	Experimen	Experimen	Control	0,936399
baseline	abundance	X07530	Alanine	1	Interventio	Experimen	Experimen	Control	0,936399

baseline	abundance	X07537	alpha-Cha	3	Interventio	Experimen	Experimen	Control	0,957875
baseline	abundance	X07538	Homoanse	3	Interventio	Experimen	Experimen	Control	0,936399
baseline	abundance	X07541	Saccharin	1	Interventio	Experimen	Experimen	Control	0,97445
baseline	abundance	X07544	Ethyl sulfat	3	Interventio	Experimen	Experimen	Control	0,957875
baseline	abundance	X07558	Leucylproli	2b	Interventio	Experimen	Experimen	Control	0,97445
baseline	abundance	X07563	Xanthine	1	Interventio	Experimen	Experimen	Control	0,948031
baseline	abundance	X07565	5-Hydroxyi	2b	Interventio	Experimen	Experimen	Control	0,945105
baseline	abundance	X07566	5-Allyl-5-se	3	Interventio	Experimen	Experimen	Control	0,97445
baseline	abundance	X07572	8-(3-Furyl)-	3	Interventio	Experimen	Experimen	Control	0,936399
baseline	abundance	X07574	7-ketodeox	3	Interventio	Experimen	Experimen	Control	0,957875
baseline	abundance	X07577	2-[(Sulfoox	3	Interventio	Experimen	Experimen	Control	0,936399
baseline	abundance	X07578	5-Allyl-5-se	3	Interventio	Experimen	Experimen	Control	0,936399
baseline	abundance	X07580	N-Pentano	3	Interventio	Experimen	Experimen	Control	0,945105
baseline	abundance	X07581	N-Acetylva	3	Interventio	Experimen	Experimen	Control	0,945105
baseline	abundance	X07582	4-methylpy	2b	Interventio	Experimen	Experimen	Control	0,97445
baseline	abundance	X07596	5-amino-2-	2b	Interventio	Experimen	Experimen	Control	0,936399
baseline	abundance	X07597	p-Cresylsu	3	Interventio	Experimen	Experimen	Control	0,936399
baseline	abundance	X07601	Asp-lys	3	Interventio	Experimen	Experimen	Control	0,992737
baseline	abundance	X07613	1-(4-Aminc	3	Interventio	Experimen	Experimen	Control	0,988948
baseline	abundance	X07618	N-(3-aceta	3	Interventio	Experimen	Experimen	Control	0,945105
baseline	abundance	X07630	Methionine	1	Interventio	Experimen	Experimen	Control	0,948059
baseline	abundance	X07634	3,8,9-trihy	2b	Interventio	Experimen	Experimen	Control	0,945105
baseline	abundance	X07638	7-ketodeox	3	Interventio	Experimen	Experimen	Control	0,955424
baseline	abundance	X07645	O-propeno	3	Interventio	Experimen	Experimen	Control	0,962445
baseline	abundance	X07646	(2Z)-2-({[(6	3	Interventio	Experimen	Experimen	Control	0,936399
baseline	abundance	X07647	Genistein	1	Interventio	Experimen	Experimen	Control	0,936399
baseline	abundance	X07648	NL851300	3	Interventio	Experimen	Experimen	Control	0,936399
baseline	abundance	X07657	Docosahex	2b	Interventio	Experimen	Experimen	Control	0,936399
baseline	abundance	X07658	δ-Glucono	1	Interventio	Experimen	Experimen	Control	0,945105
baseline	abundance	X07674	Lysine	1	Interventio	Experimen	Experimen	Control	0,936399
baseline	abundance	X07678	(2E)-3-(3,4	2b	Interventio	Experimen	Experimen	Control	0,936399
baseline	abundance	X07680	Indole-3-ac	1	Interventio	Experimen	Experimen	Control	0,978464
baseline	abundance	X07694	DNOP_a	3	Interventio	Experimen	Experimen	Control	0,97445
baseline	abundance	X07699	N-(1-Methy	3	Interventio	Experimen	Experimen	Control	0,945105
baseline	abundance	X07701	3-Benzyl-6	3	Interventio	Experimen	Experimen	Control	0,97445
baseline	abundance	X07710	3-O-beta-D	3	Interventio	Experimen	Experimen	Control	0,970763
baseline	abundance	X07712	paracetam	3	Interventio	Experimen	Experimen	Control	0,936399
baseline	abundance	X07731	3-[4-methy	2b	Interventio	Experimen	Experimen	Control	0,936399
baseline	abundance	X07732	N-Acetylva	2b	Interventio	Experimen	Experimen	Control	0,97499
baseline	abundance	X07743	D-Alanine r	2b	Interventio	Experimen	Experimen	Control	0,936399
baseline	abundance	X07745	APM_c	3	Interventio	Experimen	Experimen	Control	0,936399
baseline	abundance	X07746	3-(2-Oxo-2	2b	Interventio	Experimen	Experimen	Control	0,936399
baseline	abundance	X07747	Diethylpyrc	3	Interventio	Experimen	Experimen	Control	0,958749
baseline	abundance	X07749	(DL)-3-O-M	3	Interventio	Experimen	Experimen	Control	0,936399
baseline	abundance	X07753	Lovastatin_	3	Interventio	Experimen	Experimen	Control	0,957875
baseline	abundance	X07754	hexobarbit	3	Interventio	Experimen	Experimen	Control	0,945105
baseline	abundance	X07764	(S)-2-meth	3	Interventio	Experimen	Experimen	Control	0,957875
baseline	abundance	X07770	Piperine	1	Interventio	Experimen	Experimen	Control	0,981877

baseline	abundance	X07771	Caprolacta	2b	Interventio	Experimen	Experimen	Control	0,945105
baseline	abundance	X07774	7alpha-Hy	3	Interventio	Experimen	Experimen	Control	0,988948
baseline	abundance	X07777	Arginine	1	Interventio	Experimen	Experimen	Control	0,97445
baseline	abundance	X07782	Cytosine	1	Interventio	Experimen	Experimen	Control	0,962445
baseline	abundance	X07783	1,3-dimeth	3	Interventio	Experimen	Experimen	Control	0,945105
baseline	abundance	X07792	N-Acetylhi	2b	Interventio	Experimen	Experimen	Control	0,936399
baseline	abundance	X07794	Prolylleuci	2b	Interventio	Experimen	Experimen	Control	0,978464
baseline	abundance	X07798	N-lauroylgl	3	Interventio	Experimen	Experimen	Control	0,936399
baseline	abundance	X07799	Capryloylg	2b	Interventio	Experimen	Experimen	Control	0,97445
baseline	abundance	X07811	Metirosine	3	Interventio	Experimen	Experimen	Control	0,936399
baseline	abundance	X07812	Triethyl citi	3	Interventio	Experimen	Experimen	Control	0,97445
baseline	abundance	X07815	3-[2-[(Z)-[3	3	Interventio	Experimen	Experimen	Control	0,936399
baseline	abundance	X07820	Creatinine	1	Interventio	Experimen	Experimen	Control	0,945105
baseline	abundance	X07825	Maltotriose	1	Interventio	Experimen	Experimen	Control	0,936399
baseline	abundance	X07830	1,5-Isoquir	2b	Interventio	Experimen	Experimen	Control	0,945105
baseline	abundance	X07834	Choline su	3	Interventio	Experimen	Experimen	Control	0,945105
baseline	abundance	X07835	7-ketodeo	3	Interventio	Experimen	Experimen	Control	0,945105
baseline	abundance	X07841	2-(5-Benzy	3	Interventio	Experimen	Experimen	Control	0,97445
baseline	abundance	X07843	Piperidine_	3	Interventio	Experimen	Experimen	Control	0,936399
baseline	abundance	X07846	Carbofurar	3	Interventio	Experimen	Experimen	Control	0,945105
baseline	abundance	X07848	(−)-nabilor	3	Interventio	Experimen	Experimen	Control	0,97445
baseline	abundance	X07849	Crotamitor	3	Interventio	Experimen	Experimen	Control	0,936399
baseline	abundance	X07852	N6,N6,N6-	3	Interventio	Experimen	Experimen	Control	0,980969
baseline	abundance	X07854	TO012790	3	Interventio	Experimen	Experimen	Control	0,945105
baseline	abundance	X07856	(3R,4S,5S,	3	Interventio	Experimen	Experimen	Control	0,97445
baseline	abundance	X07866	3,3,5,5-Tet	2b	Interventio	Experimen	Experimen	Control	0,936399
baseline	abundance	X07867	N-Acetylva	3	Interventio	Experimen	Experimen	Control	0,992737
baseline	abundance	X07868	Biotin	2b	Interventio	Experimen	Experimen	Control	0,94488
baseline	abundance	X07869	Hexamethy	2a	Interventio	Experimen	Experimen	Control	0,945105
baseline	abundance	X07873	(3Z,6Z,9Z,	3	Interventio	Experimen	Experimen	Control	0,98802
baseline	abundance	X07874	δ-Valerola	2b	Interventio	Experimen	Experimen	Control	0,94488
baseline	abundance	X07886	L-Urobilin	3	Interventio	Experimen	Experimen	Control	0,97445
baseline	abundance	X07889	UROBILIN,	3	Interventio	Experimen	Experimen	Control	0,936399
baseline	abundance	X07890	N-(5-aceta	2b	Interventio	Experimen	Experimen	Control	0,97445
baseline	abundance	X07891	Cyclamic a	2b	Interventio	Experimen	Experimen	Control	0,936399
baseline	abundance	X07892	urobilinoge	3	Interventio	Experimen	Experimen	Control	0,936399
baseline	abundance	X07899	N-Acetylpu	2b	Interventio	Experimen	Experimen	Control	0,957875
baseline	abundance	X07900	presqualer	3	Interventio	Experimen	Experimen	Control	0,945105
baseline	abundance	X07903	Acetophen	2b	Interventio	Experimen	Experimen	Control	0,948031
baseline	abundance	X07905	Prilocaine	2b	Interventio	Experimen	Experimen	Control	0,945105
baseline	abundance	X07907	4-Acetami	1	Interventio	Experimen	Experimen	Control	0,988948
baseline	abundance	X07909	(±)-Albuter	2b	Interventio	Experimen	Experimen	Control	0,957875
baseline	abundance	X07914	(-)-Erythror	3	Interventio	Experimen	Experimen	Control	0,945105
baseline	abundance	X07916	N6,N6,N6-	3	Interventio	Experimen	Experimen	Control	0,968601
baseline	abundance	X07921	Pipecolinic	1	Interventio	Experimen	Experimen	Control	0,936399
baseline	abundance	X07925	DNOP_f	3	Interventio	Experimen	Experimen	Control	0,97445
baseline	abundance	X07930	N-Methylca	2b	Interventio	Experimen	Experimen	Control	0,945105
baseline	abundance	X07935	Limonin	3	Interventio	Experimen	Experimen	Control	0,936399

baseline	abundance	X07937	Atenolol	1	Interventio	Experimen	Experimen	Control	0,97445
baseline	abundance	X07944	Styrene	3	Interventio	Experimen	Experimen	Control	0,97445
baseline	abundance	X07955	Valylvaline	3	Interventio	Experimen	Experimen	Control	0,982558
baseline	abundance	X07961	2-(Hydroxy	3	Interventio	Experimen	Experimen	Control	0,936399
baseline	abundance	X07963	Theobromi	1	Interventio	Experimen	Experimen	Control	0,936399
baseline	abundance	X07969	6-hydroxyp	3	Interventio	Experimen	Experimen	Control	0,936399
baseline	abundance	X07974	Isoprene	3	Interventio	Experimen	Experimen	Control	0,945105
baseline	abundance	X07977	Tyramine	2b	Interventio	Experimen	Experimen	Control	0,945105
baseline	abundance	X07978	1-[(4E)-4-(	3	Interventio	Experimen	Experimen	Control	0,957875
baseline	abundance	X07981	Methylimic	2b	Interventio	Experimen	Experimen	Control	0,946624
baseline	abundance	X07983	butalbital_	3	Interventio	Experimen	Experimen	Control	0,936399
baseline	abundance	X07989	Piperidine_	3	Interventio	Experimen	Experimen	Control	0,936399
baseline	abundance	X07993	UROBILIN,	3	Interventio	Experimen	Experimen	Control	0,936399
baseline	abundance	X08002	(2S)-6-Ami	3	Interventio	Experimen	Experimen	Control	0,97445
baseline	abundance	X08004	δ-Valerola	2b	Interventio	Experimen	Experimen	Control	0,945105
baseline	abundance	X08007	MFCD0002	3	Interventio	Experimen	Experimen	Control	0,97445
baseline	abundance	X08008	N-Acetylne	1	Interventio	Experimen	Experimen	Control	0,936399
baseline	abundance	X08012	Methylol D	3	Interventio	Experimen	Experimen	Control	0,936399
baseline	abundance	X08013	Deoxysuga	1	Interventio	Experimen	Experimen	Control	0,97477
baseline	abundance	X08014	DNOP_e	3	Interventio	Experimen	Experimen	Control	0,970763
baseline	abundance	X08017	Tropinone	2b	Interventio	Experimen	Experimen	Control	0,936399
baseline	abundance	X08019	Thymidine	1	Interventio	Experimen	Experimen	Control	0,936399
baseline	abundance	X08024	8-Methyl-8	3	Interventio	Experimen	Experimen	Control	0,983461
baseline	abundance	X08028	Solanidine	3	Interventio	Experimen	Experimen	Control	0,945105
baseline	abundance	X08035	Tetrahydro	3	Interventio	Experimen	Experimen	Control	0,945105
baseline	abundance	X08037	Crotonic a	2b	Interventio	Experimen	Experimen	Control	0,936399
baseline	abundance	X08038	N-{6-[(7-C	2b	Interventio	Experimen	Experimen	Control	0,945105
baseline	abundance	X08040	Prolylleuci	2b	Interventio	Experimen	Experimen	Control	0,97445
baseline	abundance	X08044	3-[2-[(Z)-[3	3	Interventio	Experimen	Experimen	Control	0,945105
baseline	abundance	X08046	3-Hydroxy-	3	Interventio	Experimen	Experimen	Control	0,945105
baseline	abundance	X08054	Docosahe	2b	Interventio	Experimen	Experimen	Control	0,97445
baseline	abundance	X08056	3-(2-Oxo-2	2b	Interventio	Experimen	Experimen	Control	0,936399
baseline	abundance	X08057	N-{3-Carb	3	Interventio	Experimen	Experimen	Control	0,936399
baseline	abundance	X08058	2-Hydroxy-	3	Interventio	Experimen	Experimen	Control	0,972498
baseline	abundance	X08059	Cadaverin	3	Interventio	Experimen	Experimen	Control	0,936399
baseline	abundance	X08062	Indole-3-c	1	Interventio	Experimen	Experimen	Control	0,936399
baseline	abundance	X08072	4-Hydroxy	3	Interventio	Experimen	Experimen	Control	0,936399
baseline	abundance	X08076	3',5,7-Trih	2b	Interventio	Experimen	Experimen	Control	0,945105
baseline	abundance	X08077	6-(alpha-D	3	Interventio	Experimen	Experimen	Control	0,936399
baseline	abundance	X08078	Ethyl mala	3	Interventio	Experimen	Experimen	Control	0,945105
baseline	abundance	X08092	Safrole	3	Interventio	Experimen	Experimen	Control	0,945105
baseline	abundance	X08094	1,1'-[1,12-	3	Interventio	Experimen	Experimen	Control	0,945105
baseline	abundance	X08095	DNOP_g	3	Interventio	Experimen	Experimen	Control	0,983461
baseline	abundance	X08096	(2E)-3-Met	3	Interventio	Experimen	Experimen	Control	0,936399
baseline	abundance	X08098	Citrulline	1	Interventio	Experimen	Experimen	Control	0,936399
baseline	abundance	X08099	g-Butyrobe	3	Interventio	Experimen	Experimen	Control	0,936399
baseline	abundance	X08100	Piperidine_	3	Interventio	Experimen	Experimen	Control	0,936399
baseline	abundance	X08103	Isopelletie	3	Interventio	Experimen	Experimen	Control	0,957875

baseline	abundance	X08105	2-Acetami	3	Interventio	Experimen	Experimen	Control	0,97445
baseline	abundance	X08106	4-Vinylphe	1	Interventio	Experimen	Experimen	Control	0,936399
baseline	abundance	X08111	2-Hydroxy	2b	Interventio	Experimen	Experimen	Control	0,945105
baseline	abundance	X08116	Methyl (2Z,	3	Interventio	Experimen	Experimen	Control	0,936399
baseline	abundance	X08123	Tiglic acid_	2b	Interventio	Experimen	Experimen	Control	0,936399
baseline	abundance	X08146	O-Ethyl (4-	3	Interventio	Experimen	Experimen	Control	0,987642
baseline	abundance	X08147	porphobilir	3	Interventio	Experimen	Experimen	Control	0,945105
baseline	abundance	X08150	DL-4-Hydro	2b	Interventio	Experimen	Experimen	Control	0,945105
baseline	abundance	X08154	4-(METHYL	3	Interventio	Experimen	Experimen	Control	0,945105
baseline	abundance	X08157	Leu-Val_e	3	Interventio	Experimen	Experimen	Control	0,945105
baseline	abundance	X08162	Valylvaline	3	Interventio	Experimen	Experimen	Control	0,986763
baseline	abundance	X08163	Phenyl D-g	3	Interventio	Experimen	Experimen	Control	0,945105
baseline	abundance	X08167	Lanthionin	3	Interventio	Experimen	Experimen	Control	0,97445
baseline	abundance	X08173	9-Methylur	2b	Interventio	Experimen	Experimen	Control	0,948031
baseline	abundance	X08188	2-Hydroxy-	2b	Interventio	Experimen	Experimen	Control	0,945105
baseline	abundance	X08193	Seryltyrosi	3	Interventio	Experimen	Experimen	Control	0,945105
baseline	abundance	X08204	2-Amino-4	2b	Interventio	Experimen	Experimen	Control	0,936399
baseline	abundance	X08212	LU345300	3	Interventio	Experimen	Experimen	Control	0,993446
baseline	abundance	X08221	Brilliant blu	2b	Interventio	Experimen	Experimen	Control	0,97499
baseline	abundance	X08230	Coumaron	3	Interventio	Experimen	Experimen	Control	0,936399
baseline	abundance	X08235	1-Methylhi	2b	Interventio	Experimen	Experimen	Control	0,936399
baseline	abundance	X08244	Setoclavin	3	Interventio	Experimen	Experimen	Control	0,945105
baseline	abundance	X08270	Butabarbit	3	Interventio	Experimen	Experimen	Control	0,936399
baseline	abundance	X08274	Mevalonic	2b	Interventio	Experimen	Experimen	Control	0,936399
baseline	abundance	X08277	adrenaline	3	Interventio	Experimen	Experimen	Control	0,936399
baseline	abundance	X08279	DNOP_b	3	Interventio	Experimen	Experimen	Control	0,945105
baseline	abundance	X08288	Capryloylg	3	Interventio	Experimen	Experimen	Control	0,936399
baseline	abundance	X08292	Lidocaine	2b	Interventio	Experimen	Experimen	Control	0,957875
baseline	abundance	X08298	N6,N6,N6-	2b	Interventio	Experimen	Experimen	Control	0,936399
baseline	abundance	X08300	Indole-3-a	3	Interventio	Experimen	Experimen	Control	0,987276
baseline	abundance	X08305	Prolylhydro	2a	Interventio	Experimen	Experimen	Control	0,957875
baseline	abundance	X08306	1-Methylxa	2b	Interventio	Experimen	Experimen	Control	0,945105
baseline	abundance	X08311	Methyl 3-fo	3	Interventio	Experimen	Experimen	Control	0,985903
baseline	abundance	X08317	8-Hydroxy	2b	Interventio	Experimen	Experimen	Control	0,945105
baseline	abundance	X08322	Glycylproly	3	Interventio	Experimen	Experimen	Control	0,936399
baseline	abundance	X08323	(15Z)-9,12	2b	Interventio	Experimen	Experimen	Control	0,936399
baseline	abundance	X08326	2-(1,3-Ben	3	Interventio	Experimen	Experimen	Control	0,945105
baseline	abundance	X08338	Methyl [9-(	3	Interventio	Experimen	Experimen	Control	0,936399
baseline	abundance	X08341	Midodrine_	3	Interventio	Experimen	Experimen	Control	0,97445
baseline	abundance	X08352	7-[4-(tert-b	2b	Interventio	Experimen	Experimen	Control	0,936399
baseline	abundance	X08365	Lysylvaline	3	Interventio	Experimen	Experimen	Control	0,936399
baseline	abundance	X08367	11-dehydro	2b	Interventio	Experimen	Experimen	Control	0,945105
baseline	abundance	X08373	2-Hydroxy-	3	Interventio	Experimen	Experimen	Control	0,936399
baseline	abundance	X08386	3b-Hydroxy	3	Interventio	Experimen	Experimen	Control	0,936399
baseline	abundance	X08387	4-[(2E,4Z)-	3	Interventio	Experimen	Experimen	Control	0,936399
baseline	abundance	X08412	2-Hydroxyp	2b	Interventio	Experimen	Experimen	Control	0,957875
baseline	abundance	X08417	asn-pro_c	3	Interventio	Experimen	Experimen	Control	0,97445
baseline	abundance	X08420	Dodecane	1	Interventio	Experimen	Experimen	Control	0,945105

baseline	abundance	X08421	Cys-tyr	3	Interventio	Experimen	Experimen	Control	0,955424
baseline	abundance	X08422	N-(3-aceta	3	Interventio	Experimen	Experimen	Control	0,936399
baseline	abundance	X08425	MFCD1297	3	Interventio	Experimen	Experimen	Control	0,936399
baseline	abundance	X08436	N,N-Dimet	3	Interventio	Experimen	Experimen	Control	0,988948
baseline	abundance	X08439	Leucyltyro	3	Interventio	Experimen	Experimen	Control	0,992737
baseline	abundance	X08451	L-gamma-(	3	Interventio	Experimen	Experimen	Control	0,936399
baseline	abundance	X08460	α-Murichol	1	Interventio	Experimen	Experimen	Control	0,936399
baseline	abundance	X08461	Dihydrouri	3	Interventio	Experimen	Experimen	Control	0,945105
baseline	abundance	X08475	1-(4-Amino	3	Interventio	Experimen	Experimen	Control	0,936399
baseline	abundance	X08476	Coprine_c	3	Interventio	Experimen	Experimen	Control	0,945105
baseline	abundance	X08478	1-(beta-D-I	3	Interventio	Experimen	Experimen	Control	0,936399
baseline	abundance	X08485	Isoprenalir	3	Interventio	Experimen	Experimen	Control	0,945105
baseline	abundance	X08486	(2E,6E)-9-[	3	Interventio	Experimen	Experimen	Control	0,936399
baseline	abundance	X08504	Indole-3-ca	2a	Interventio	Experimen	Experimen	Control	0,948031
baseline	abundance	X08509	Piceid	3	Interventio	Experimen	Experimen	Control	0,936399
baseline	abundance	X08512	Bile acid I (	2b	Interventio	Experimen	Experimen	Control	0,945105
baseline	abundance	X08514	N-Ethylpro	3	Interventio	Experimen	Experimen	Control	0,936399
baseline	abundance	X08525	MFCD1869	3	Interventio	Experimen	Experimen	Control	0,97445
baseline	abundance	X08529	7,8-Diamir	3	Interventio	Experimen	Experimen	Control	0,945105
baseline	abundance	X08533	tyramine su	3	Interventio	Experimen	Experimen	Control	0,936399
baseline	abundance	X08535	Nitrosohep	2b	Interventio	Experimen	Experimen	Control	0,936399
baseline	abundance	X08546	1,3,7-Trime	2b	Interventio	Experimen	Experimen	Control	0,957875
baseline	abundance	X08548	N2-Acetyll	1	Interventio	Experimen	Experimen	Control	0,936399
baseline	abundance	X08549	3-Methylsu	3	Interventio	Experimen	Experimen	Control	0,945105
baseline	abundance	X08561	N-(Carboxy	3	Interventio	Experimen	Experimen	Control	0,97445
baseline	abundance	X08578	tert-Butyl 3	3	Interventio	Experimen	Experimen	Control	0,991763
baseline	abundance	X08584	meticillin	3	Interventio	Experimen	Experimen	Control	0,936399
baseline	abundance	X08588	2-Acetami	3	Interventio	Experimen	Experimen	Control	0,948031
baseline	abundance	X08593	Uric acid	1	Interventio	Experimen	Experimen	Control	0,936399
baseline	abundance	X08594	7-ketodeox	3	Interventio	Experimen	Experimen	Control	0,97445
baseline	abundance	X08600	N-Propiony	3	Interventio	Experimen	Experimen	Control	0,945105
baseline	abundance	X08606	Butabarbit	3	Interventio	Experimen	Experimen	Control	0,936399
baseline	abundance	X08610	Leucyltyro	3	Interventio	Experimen	Experimen	Control	0,936399
baseline	abundance	X08616	MFCD0002	3	Interventio	Experimen	Experimen	Control	0,936399
baseline	abundance	X08625	Hept-2-ulo	3	Interventio	Experimen	Experimen	Control	0,936399
baseline	abundance	X08634	1,5-Isoquir	2b	Interventio	Experimen	Experimen	Control	0,936399
baseline	abundance	X08639	hypaphorir	3	Interventio	Experimen	Experimen	Control	0,936399
baseline	abundance	X08641	meprobam	3	Interventio	Experimen	Experimen	Control	0,936399
baseline	abundance	X08644	2,5-Dimetl	2b	Interventio	Experimen	Experimen	Control	0,936399
baseline	abundance	X08646	N-Stearoyl	3	Interventio	Experimen	Experimen	Control	0,945105
baseline	abundance	X08657	1-(4-Amino	3	Interventio	Experimen	Experimen	Control	0,97445
baseline	abundance	X08659	3,3-Dimetl	2b	Interventio	Experimen	Experimen	Control	0,945105
baseline	abundance	X08663	Pilocarpine	2b	Interventio	Experimen	Experimen	Control	0,936399
baseline	abundance	X08670	Hydroxypro	3	Interventio	Experimen	Experimen	Control	0,945105
baseline	abundance	X08673	NPC	3	Interventio	Experimen	Experimen	Control	0,97445
baseline	abundance	X08677	1-methylhy	3	Interventio	Experimen	Experimen	Control	0,936399
baseline	abundance	X08690	Ethanoic a	3	Interventio	Experimen	Experimen	Control	0,982558
baseline	abundance	X08693	7-Sulfocho	3	Interventio	Experimen	Experimen	Control	0,97445

baseline	abundance	X08695	3b-Hydroxy	3	Interventio	Experimen	Experimen	Control	0,979456
baseline	abundance	X08699	Aurorix	3	Interventio	Experimen	Experimen	Control	0,936399
baseline	abundance	X08707	2-(1-Ethoxy	3	Interventio	Experimen	Experimen	Control	0,948031
baseline	abundance	X08711	2-Acetami	3	Interventio	Experimen	Experimen	Control	0,97445
baseline	abundance	X08712	4-Iodoanis	3	Interventio	Experimen	Experimen	Control	0,948031
baseline	abundance	X08713	Phenethyla	2b	Interventio	Experimen	Experimen	Control	0,97445
baseline	abundance	X08714	Arabic acic	3	Interventio	Experimen	Experimen	Control	0,992737
baseline	abundance	X08719	6-Methylqu	2b	Interventio	Experimen	Experimen	Control	0,945105
baseline	abundance	X08723	S-Allylcyst	3	Interventio	Experimen	Experimen	Control	0,957875
baseline	abundance	X08726	YV8195000	3	Interventio	Experimen	Experimen	Control	0,936399
baseline	abundance	X08733	N-Methyl-1	3	Interventio	Experimen	Experimen	Control	0,936399
baseline	abundance	X08739	1,1'-[1,12-I	3	Interventio	Experimen	Experimen	Control	0,936399
baseline	abundance	X08758	Methohexit	3	Interventio	Experimen	Experimen	Control	0,936399
baseline	abundance	X08777	3-Aminosa	2b	Interventio	Experimen	Experimen	Control	0,936399
baseline	abundance	X08779	3-(1-hydro:	2b	Interventio	Experimen	Experimen	Control	0,945105
baseline	abundance	X08781	Procaine_b	3	Interventio	Experimen	Experimen	Control	0,936399
baseline	abundance	X08789	Acrylic acic	2b	Interventio	Experimen	Experimen	Control	0,985903
baseline	abundance	X08792	3,4-Methyl	3	Interventio	Experimen	Experimen	Control	0,945105
baseline	abundance	X08795	N-Acetylva	3	Interventio	Experimen	Experimen	Control	0,936399
baseline	abundance	X08801	3,7-Dimeth	2b	Interventio	Experimen	Experimen	Control	0,936399
baseline	abundance	X08810	N~6~,N~6~	3	Interventio	Experimen	Experimen	Control	0,936399
baseline	abundance	X08822	CYS-ASP	3	Interventio	Experimen	Experimen	Control	0,945105
baseline	abundance	X08825	Chenodeo:	3	Interventio	Experimen	Experimen	Control	0,936399
baseline	abundance	X08842	Spermic ac	3	Interventio	Experimen	Experimen	Control	0,936399
baseline	abundance	X08843	(2R,3S)-3-I	3	Interventio	Experimen	Experimen	Control	0,936399
baseline	abundance	X08844	4-Hydroxy-	3	Interventio	Experimen	Experimen	Control	0,97445
baseline	abundance	X08845	14-Hydroxy	3	Interventio	Experimen	Experimen	Control	0,945105
baseline	abundance	X08847	1-Vinylimic	2b	Interventio	Experimen	Experimen	Control	0,936399
baseline	abundance	X08869	Lanthionin	3	Interventio	Experimen	Experimen	Control	0,945105
baseline	abundance	X08870	Tetraacety	3	Interventio	Experimen	Experimen	Control	0,945105
baseline	abundance	X08883	3-Phenylpr	3	Interventio	Experimen	Experimen	Control	0,957875
baseline	abundance	X08893	5beta-Cho	3	Interventio	Experimen	Experimen	Control	0,936399
baseline	abundance	X08895	pentoxyl	3	Interventio	Experimen	Experimen	Control	0,936399
baseline	abundance	X08908	Vorinostat_	3	Interventio	Experimen	Experimen	Control	0,986763
baseline	abundance	X08909	GLK (Pepti	2b	Interventio	Experimen	Experimen	Control	0,936399
baseline	abundance	X08923	Semilicois	3	Interventio	Experimen	Experimen	Control	0,945105
baseline	abundance	X08938	Nonivamid	3	Interventio	Experimen	Experimen	Control	0,936399
baseline	abundance	X08944	Dipivefrin	3	Interventio	Experimen	Experimen	Control	0,936399
baseline	abundance	X08965	1,9-Nonan	3	Interventio	Experimen	Experimen	Control	0,936399
baseline	abundance	X08973	Aminohipp	3	Interventio	Experimen	Experimen	Control	0,936399
baseline	abundance	X08983	IN00150	3	Interventio	Experimen	Experimen	Control	0,957875
baseline	abundance	X08987	feruloylser	3	Interventio	Experimen	Experimen	Control	0,945105
baseline	abundance	X08990	Procaine_c	3	Interventio	Experimen	Experimen	Control	0,986763
baseline	abundance	X08992	N(2)-succi	3	Interventio	Experimen	Experimen	Control	0,936399
baseline	abundance	X09001	Varanic ac	3	Interventio	Experimen	Experimen	Control	0,957875
baseline	abundance	X09004	Arenaine	3	Interventio	Experimen	Experimen	Control	0,97445
baseline	abundance	X09008	5-guanidin	3	Interventio	Experimen	Experimen	Control	0,97445
baseline	abundance	X09010	4-Hydroxyp	1	Interventio	Experimen	Experimen	Control	0,936399

baseline	abundance	X09055	N-[(1R,2S,(	3	Interventio	Experimen	Experimen	Control	0,936399
baseline	abundance	X09064	Tetrahydro	3	Interventio	Experimen	Experimen	Control	0,945105
baseline	abundance	X09068	N,N-Diethy	3	Interventio	Experimen	Experimen	Control	0,945105
baseline	abundance	X09082	1_2-Dihydr	3	Interventio	Experimen	Experimen	Control	0,97445
baseline	abundance	X09091	N-(3-aceta	3	Interventio	Experimen	Experimen	Control	0,936399
baseline	abundance	X09092	LysoSM(d1	3	Interventio	Experimen	Experimen	Control	0,945105
baseline	abundance	X09099	1-(2-Hydro	3	Interventio	Experimen	Experimen	Control	0,936399
baseline	abundance	X09100	Ethyl aceta	3	Interventio	Experimen	Experimen	Control	0,98802
baseline	abundance	X09110	Valylvaline	3	Interventio	Experimen	Experimen	Control	0,94488
baseline	abundance	X09122	Valyl-4-hyc	3	Interventio	Experimen	Experimen	Control	0,945105
baseline	abundance	X09129	Coprine_d	3	Interventio	Experimen	Experimen	Control	0,945105
baseline	abundance	X09152	Ferulic aci	1	Interventio	Experimen	Experimen	Control	0,936399
baseline	abundance	X09163	Prenisteine	3	Interventio	Experimen	Experimen	Control	0,97445
baseline	abundance	X09165	N-[1-Carbc	3	Interventio	Experimen	Experimen	Control	0,936399
baseline	abundance	X09187	Salicylic ac	1	Interventio	Experimen	Experimen	Control	0,945105
baseline	abundance	X09189	4-[(E)-2-(3,	3	Interventio	Experimen	Experimen	Control	0,936399
baseline	abundance	X09207	AAMU_a	3	Interventio	Experimen	Experimen	Control	0,936399
baseline	abundance	X09208	IN00260_b	3	Interventio	Experimen	Experimen	Control	0,957875
baseline	abundance	X09222	L-gamma-(	3	Interventio	Experimen	Experimen	Control	0,945105
baseline	abundance	X09231	2-Keto-glut	3	Interventio	Experimen	Experimen	Control	0,936399
baseline	abundance	X09244	gamma-Gl	3	Interventio	Experimen	Experimen	Control	0,945105
baseline	abundance	X09245	L-gamma-(	3	Interventio	Experimen	Experimen	Control	0,936399
baseline	abundance	X09256	Aspartyl-L-	3	Interventio	Experimen	Experimen	Control	0,945105
baseline	abundance	X09260	(4S)-4-[(2E	3	Interventio	Experimen	Experimen	Control	0,936399
baseline	abundance	X09284	4-(Nitrosoa	3	Interventio	Experimen	Experimen	Control	0,957875
baseline	abundance	X09285	MFCD0272	3	Interventio	Experimen	Experimen	Control	0,957875
baseline	abundance	X09302	Sular	3	Interventio	Experimen	Experimen	Control	0,945105
baseline	abundance	X09318	butalbital_	3	Interventio	Experimen	Experimen	Control	0,945105
baseline	abundance	X09336	1,1'-[1,12-I	3	Interventio	Experimen	Experimen	Control	0,97445
baseline	abundance	X09357	5-(5-Methy	3	Interventio	Experimen	Experimen	Control	0,936399
baseline	abundance	X09374	3-Deoxy-D-	3	Interventio	Experimen	Experimen	Control	0,936399
baseline	abundance	X09385	2-(1-Napht	3	Interventio	Experimen	Experimen	Control	0,936399
baseline	abundance	X09390	asn-pro_a	3	Interventio	Experimen	Experimen	Control	0,936399
baseline	abundance	X09391	ophthalmic	3	Interventio	Experimen	Experimen	Control	0,957875
baseline	abundance	X09393	2-Isopropy	3	Interventio	Experimen	Experimen	Control	0,957875
baseline	abundance	X09399	L-gamma-(	3	Interventio	Experimen	Experimen	Control	0,936399
baseline	abundance	X09454	2-(2-Hydro	3	Interventio	Experimen	Experimen	Control	0,936399
baseline	abundance	X09476	N(1),N(8)-I	3	Interventio	Experimen	Experimen	Control	0,936399
baseline	abundance	X09480	Spermic ac	3	Interventio	Experimen	Experimen	Control	0,982558
baseline	abundance	X09490	2-Hydroxy-	3	Interventio	Experimen	Experimen	Control	0,936399
baseline	abundance	X09503	4-(METHYL	3	Interventio	Experimen	Experimen	Control	0,936399
baseline	abundance	X09505	(-)-Physost	3	Interventio	Experimen	Experimen	Control	0,936399
baseline	abundance	X09523	N-(3,5-Dim	3	Interventio	Experimen	Experimen	Control	0,97445
baseline	abundance	X09525	Homoanse	3	Interventio	Experimen	Experimen	Control	0,985326
baseline	abundance	X09533	S(6)-acetyl	3	Interventio	Experimen	Experimen	Control	0,97445
baseline	abundance	X09545	4-Methyler	3	Interventio	Experimen	Experimen	Control	0,945105
baseline	abundance	X09546	asn-phe	3	Interventio	Experimen	Experimen	Control	0,97445
baseline	abundance	X09548	2-O-beta-D	3	Interventio	Experimen	Experimen	Control	0,97445

baseline	abundance	X09554	N-Propiony	3	Interventio	Experimen	Experimen	Control	0,992737
baseline	abundance	X09555	N4-(beta-N	3	Interventio	Experimen	Experimen	Control	0,936399
baseline	abundance	X09565	3-Methoxy-	3	Interventio	Experimen	Experimen	Control	0,957875
baseline	abundance	X09577	Spermic ac	3	Interventio	Experimen	Experimen	Control	0,936399
baseline	abundance	X09581	L-gamma-(	3	Interventio	Experimen	Experimen	Control	0,936399
baseline	abundance	X09607	N-(1H-Pyrr	3	Interventio	Experimen	Experimen	Control	0,948031
baseline	abundance	X09608	N-(3,5-Dim	3	Interventio	Experimen	Experimen	Control	0,945105
baseline	abundance	X09609	N-{3-[(4-Ac	3	Interventio	Experimen	Experimen	Control	0,936399
baseline	abundance	X09612	3-hydroxy-:	3	Interventio	Experimen	Experimen	Control	0,936399
baseline	abundance	X09615	tyramine su	3	Interventio	Experimen	Experimen	Control	0,945105
baseline	abundance	X09622	N-Hydroxy-	3	Interventio	Experimen	Experimen	Control	0,936399
baseline	abundance	X09623	Endothal	2b	Interventio	Experimen	Experimen	Control	0,936399
baseline	abundance	X09635	2,4,6-Triisc	3	Interventio	Experimen	Experimen	Control	0,994656
baseline	abundance	X09643	N-(3,5-Dim	3	Interventio	Experimen	Experimen	Control	0,97445
baseline	abundance	X09645	TOLMETIN	3	Interventio	Experimen	Experimen	Control	0,97445
baseline	abundance	X09654	linatine	3	Interventio	Experimen	Experimen	Control	0,936399
baseline	abundance	X09657	2,3,4,5,6-F	3	Interventio	Experimen	Experimen	Control	0,936399
baseline	abundance	X09675	UK387000	3	Interventio	Experimen	Experimen	Control	0,97445
baseline	abundance	X09684	(+)-Etomid	3	Interventio	Experimen	Experimen	Control	0,936399
baseline	abundance	X09703	7,8-Didehy	3	Interventio	Experimen	Experimen	Control	0,936399
baseline	abundance	X09710	LW800000	3	Interventio	Experimen	Experimen	Control	0,945105
baseline	abundance	X09713	N(2)-succi	3	Interventio	Experimen	Experimen	Control	0,97445
baseline	abundance	X09721	3-(2,3-Dihy	3	Interventio	Experimen	Experimen	Control	0,955424
baseline	abundance	X09724	Formylkynu	3	Interventio	Experimen	Experimen	Control	0,936399
baseline	abundance	X09726	indoline-2-	2b	Interventio	Experimen	Experimen	Control	0,936399
baseline	abundance	X09728	{[(15-Hydro	3	Interventio	Experimen	Experimen	Control	0,936399
baseline	abundance	X09738	(7E,7'E)-5,:	3	Interventio	Experimen	Experimen	Control	0,936399
baseline	abundance	X09740	MFCD0166	3	Interventio	Experimen	Experimen	Control	0,945105
baseline	abundance	X09741	Estrone glu	3	Interventio	Experimen	Experimen	Control	0,982558
baseline	abundance	X09746	ELK (Peptic	2b	Interventio	Experimen	Experimen	Control	0,936399
baseline	abundance	X09765	Tetrahydro	3	Interventio	Experimen	Experimen	Control	0,945105
baseline	abundance	X09771	2-(3-CARB	3	Interventio	Experimen	Experimen	Control	0,936399
baseline	abundance	X09787	Queuosine	3	Interventio	Experimen	Experimen	Control	0,945105
baseline	abundance	X09805	Methyl alpl	3	Interventio	Experimen	Experimen	Control	0,945105
baseline	abundance	X09810	Zalcitabine	3	Interventio	Experimen	Experimen	Control	0,936399
baseline	abundance	X09825	(2S)-6-Ami	3	Interventio	Experimen	Experimen	Control	0,945105
baseline	abundance	X09849	3-Methylac	3	Interventio	Experimen	Experimen	Control	0,988948
baseline	abundance	X09854	N-Benzyl-3	2b	Interventio	Experimen	Experimen	Control	0,97445
baseline	abundance	X09875	4-[(E)-2-(3,	3	Interventio	Experimen	Experimen	Control	0,945786
baseline	abundance	X09882	Harmane	3	Interventio	Experimen	Experimen	Control	0,936399
baseline	abundance	X09895	1-(beta-D-r	3	Interventio	Experimen	Experimen	Control	0,936399
baseline	abundance	X09898	N(6),N(6)-l	3	Interventio	Experimen	Experimen	Control	0,936399
baseline	abundance	X09912	MC055530	3	Interventio	Experimen	Experimen	Control	0,945786
baseline	abundance	X09919	4-ethylphe	3	Interventio	Experimen	Experimen	Control	0,936399
baseline	abundance	X09921	5-Hydroxy-	3	Interventio	Experimen	Experimen	Control	0,936399
baseline	abundance	X09932	g-Aminobu	3	Interventio	Experimen	Experimen	Control	0,936399
baseline	abundance	X09940	pentobarbi	3	Interventio	Experimen	Experimen	Control	0,936399
baseline	abundance	X09942	Spermic ac	3	Interventio	Experimen	Experimen	Control	0,982558

baseline	abundance	X09950	3-Hydroxyl	3	Interventio	Experimen	Experimen	Control	0,945105
baseline	abundance	X09951	5beta-Cypr	3	Interventio	Experimen	Experimen	Control	0,936399
baseline	abundance	X09963	Iminoglycir	3	Interventio	Experimen	Experimen	Control	0,936399
baseline	abundance	X09965	Biocytin	2b	Interventio	Experimen	Experimen	Control	0,97445
baseline	abundance	X09969	3-Methylac	3	Interventio	Experimen	Experimen	Control	0,945105
baseline	abundance	X09977	TLK (Peptic	2b	Interventio	Experimen	Experimen	Control	0,945105
baseline	abundance	X09980	N-(4-Hepta	3	Interventio	Experimen	Experimen	Control	0,936399
baseline	abundance	X09981	N-Acetylva	3	Interventio	Experimen	Experimen	Control	0,936399
baseline	abundance	X09998	N-[(10Z)-7-	3	Interventio	Experimen	Experimen	Control	0,945105
baseline	abundance	X10011	Midodrine_	3	Interventio	Experimen	Experimen	Control	0,936399
baseline	abundance	X10025	2-[4-(3-Hyd	3	Interventio	Experimen	Experimen	Control	0,945105
baseline	abundance	X10032	4,9a-Dimet	3	Interventio	Experimen	Experimen	Control	0,945105
baseline	abundance	X10033	epsilon-(ga	3	Interventio	Experimen	Experimen	Control	0,962143
baseline	abundance	X10036	Nimodipine	3	Interventio	Experimen	Experimen	Control	0,978464
baseline	abundance	X10037	his-gln_a	3	Interventio	Experimen	Experimen	Control	0,936399
baseline	abundance	X10046	Dehydroac	2b	Interventio	Experimen	Experimen	Control	0,936399
baseline	abundance	X10076	trimethadi	3	Interventio	Experimen	Experimen	Control	0,936399
baseline	abundance	X10091	(2E,6E)-9-[	3	Interventio	Experimen	Experimen	Control	0,957875
baseline	abundance	X10097	Coixol	3	Interventio	Experimen	Experimen	Control	0,97445
baseline	abundance	X10098	Methyl 2,3-	3	Interventio	Experimen	Experimen	Control	0,945105
baseline	abundance	X10099	ala-met	3	Interventio	Experimen	Experimen	Control	0,98802
baseline	abundance	X10121	Leu-pro_b	3	Interventio	Experimen	Experimen	Control	0,945105
baseline	abundance	X10129	SECONAL_	3	Interventio	Experimen	Experimen	Control	0,945105
baseline	abundance	X10130	N-(4-Hydro	3	Interventio	Experimen	Experimen	Control	0,936399
baseline	abundance	X10137	1-Methyl-1	3	Interventio	Experimen	Experimen	Control	0,963031
baseline	abundance	X10144	imazameth	3	Interventio	Experimen	Experimen	Control	0,986763
baseline	abundance	X10148	cys-met	3	Interventio	Experimen	Experimen	Control	0,936399
baseline	abundance	X10151	2-(3-CARB	3	Interventio	Experimen	Experimen	Control	0,936399
baseline	abundance	X10162	Quinaldic a	2a	Interventio	Experimen	Experimen	Control	0,960702
baseline	abundance	X10177	Gly-DL-Phe	3	Interventio	Experimen	Experimen	Control	0,957875
baseline	abundance	X10180	4-Formyl-2	3	Interventio	Experimen	Experimen	Control	0,936399
baseline	abundance	X10203	L-gamma-G	3	Interventio	Experimen	Experimen	Control	0,936399
baseline	abundance	X10224	Imidazolela	2b	Interventio	Experimen	Experimen	Control	0,936399
baseline	abundance	X10246	Ethynodiol	3	Interventio	Experimen	Experimen	Control	0,957875
baseline	abundance	X10248	(+)-Etomid	3	Interventio	Experimen	Experimen	Control	0,97445
baseline	abundance	X10265	3-methyl-4	3	Interventio	Experimen	Experimen	Control	0,945786
baseline	abundance	X10276	Maleamate	3	Interventio	Experimen	Experimen	Control	0,936399
baseline	abundance	X10279	Lys-Pro_d	3	Interventio	Experimen	Experimen	Control	0,97029
baseline	abundance	X10304	2,3,8,9-Tet	3	Interventio	Experimen	Experimen	Control	0,945105
baseline	abundance	X10314	4-morpholi	2b	Interventio	Experimen	Experimen	Control	0,97445
baseline	abundance	X10319	1-Methyl-1	3	Interventio	Experimen	Experimen	Control	0,970482
baseline	abundance	X10325	Lovastatin_	3	Interventio	Experimen	Experimen	Control	0,97499
baseline	abundance	X10330	Sulfurous a	3	Interventio	Experimen	Experimen	Control	0,936399
baseline	abundance	X10339	4H-1-Benz	2b	Interventio	Experimen	Experimen	Control	0,936399
baseline	abundance	X10345	S-Allylcyste	3	Interventio	Experimen	Experimen	Control	0,936399
baseline	abundance	X10353	L-gamma-G	3	Interventio	Experimen	Experimen	Control	0,945105
baseline	abundance	X10367	2-(1-Napht	3	Interventio	Experimen	Experimen	Control	0,941349
baseline	abundance	X10370	L-Arogenat	3	Interventio	Experimen	Experimen	Control	0,936399

baseline	abundance	X10375	4-Indoleca	2b	Interventio	Experimen	Experimen	Control	0,936399
baseline	abundance	X10379	Phenyl D-g	3	Interventio	Experimen	Experimen	Control	0,945786
baseline	abundance	X10382	Zalcitabine	3	Interventio	Experimen	Experimen	Control	0,936399
baseline	abundance	X10384	L-N2-(2-Ca	3	Interventio	Experimen	Experimen	Control	0,957875
baseline	abundance	X10387	Coenzyme	3	Interventio	Experimen	Experimen	Control	0,936399
baseline	abundance	X10389	Leucylaspa	3	Interventio	Experimen	Experimen	Control	0,936399
baseline	abundance	X10393	Ro 20-1724	3	Interventio	Experimen	Experimen	Control	0,936399
baseline	abundance	X10401	3-Methylxa	1	Interventio	Experimen	Experimen	Control	0,936399
baseline	abundance	X10415	beta-D-Glc	3	Interventio	Experimen	Experimen	Control	0,97445
baseline	abundance	X10417	L-Sacchar(	2b	Interventio	Experimen	Experimen	Control	0,936399
baseline	abundance	X10429	1,5-Isoquir	2b	Interventio	Experimen	Experimen	Control	0,936399
baseline	abundance	X10442	(6alpha,11	3	Interventio	Experimen	Experimen	Control	0,97445
baseline	abundance	X10445	2-(Carboxy	3	Interventio	Experimen	Experimen	Control	0,97445
baseline	abundance	X10451	Ro 20-1724	3	Interventio	Experimen	Experimen	Control	0,945105
baseline	abundance	X10454	8-Amino-7-	3	Interventio	Experimen	Experimen	Control	0,936399
baseline	abundance	X10457	glu-ser	3	Interventio	Experimen	Experimen	Control	0,970772
baseline	abundance	X10476	GAMMA-HY	3	Interventio	Experimen	Experimen	Control	0,957875
baseline	abundance	X10486	Coumarin	2b	Interventio	Experimen	Experimen	Control	0,94488
baseline	abundance	X10488	4-Guanidir	1	Interventio	Experimen	Experimen	Control	0,982558
baseline	abundance	X10494	26Q0EO75	3	Interventio	Experimen	Experimen	Control	0,97445
baseline	abundance	X10497	4-Guanidir	3	Interventio	Experimen	Experimen	Control	0,97445
baseline	abundance	X10503	APM_a	3	Interventio	Experimen	Experimen	Control	0,936399
baseline	abundance	X10511	dopaquino	3	Interventio	Experimen	Experimen	Control	0,936399
baseline	abundance	X10513	ophthalmic	3	Interventio	Experimen	Experimen	Control	0,957875
baseline	abundance	X10518	MFCD0002	3	Interventio	Experimen	Experimen	Control	0,936399
baseline	abundance	X10534	L-N2-(2-Ca	3	Interventio	Experimen	Experimen	Control	0,936399
baseline	abundance	X10536	CMPF	3	Interventio	Experimen	Experimen	Control	0,97445
baseline	abundance	X10549	Glaucine	2b	Interventio	Experimen	Experimen	Control	0,97445
baseline	abundance	X10550	pentobarbi	3	Interventio	Experimen	Experimen	Control	0,945105
baseline	abundance	X10554	Glycine	1	Interventio	Experimen	Experimen	Control	0,945105
baseline	abundance	X10564	2-(1-Ethoxy	3	Interventio	Experimen	Experimen	Control	0,936399
baseline	abundance	X10580	Leu-Leu_a	3	Interventio	Experimen	Experimen	Control	0,97445
baseline	abundance	X10588	(8)-Ginger(	3	Interventio	Experimen	Experimen	Control	0,97445
baseline	abundance	X10593	Vorinostat_	3	Interventio	Experimen	Experimen	Control	0,936399
baseline	abundance	X10600	Glycocyam	3	Interventio	Experimen	Experimen	Control	0,936399
baseline	abundance	X10611	2-Acrylami	3	Interventio	Experimen	Experimen	Control	0,97445
baseline	abundance	X10614	Monometh	2a	Interventio	Experimen	Experimen	Control	0,957875
baseline	abundance	X10617	2-methyl-1	2b	Interventio	Experimen	Experimen	Control	0,936399
baseline	abundance	X10623	N-Nonanoy	3	Interventio	Experimen	Experimen	Control	0,936399
baseline	abundance	X10636	8-hydroxy-	2b	Interventio	Experimen	Experimen	Control	0,945105
baseline	abundance	X10647	4-Hydroxyp	3	Interventio	Experimen	Experimen	Control	0,955424
baseline	abundance	X10651	Methyl 1-hy	3	Interventio	Experimen	Experimen	Control	0,936399
baseline	abundance	X10660	2-Methylth	3	Interventio	Experimen	Experimen	Control	0,936399
baseline	abundance	X10664	N1-(5-meth	2b	Interventio	Experimen	Experimen	Control	0,985903
baseline	abundance	X10665	Aprobarbit	3	Interventio	Experimen	Experimen	Control	0,936399
baseline	abundance	X10667	N-Phenyla(	3	Interventio	Experimen	Experimen	Control	0,936399
baseline	abundance	X10676	6-(1-Hydro	3	Interventio	Experimen	Experimen	Control	0,945105
baseline	abundance	X10680	8-hydroxy-7	3	Interventio	Experimen	Experimen	Control	0,945105

baseline	abundance	X10685	Glycylvalin	2a	Interventio	Experimen	Experimen	Control	0,986763
baseline	abundance	X10687	Gln-Gln	3	Interventio	Experimen	Experimen	Control	0,945105
baseline	abundance	X10691	(−)-nabilor	3	Interventio	Experimen	Experimen	Control	0,97445
baseline	abundance	X10706	Furaneol	3	Interventio	Experimen	Experimen	Control	0,936399
baseline	abundance	X10713	norhaman	3	Interventio	Experimen	Experimen	Control	0,97445
baseline	abundance	X10722	Acetanilide	2b	Interventio	Experimen	Experimen	Control	0,936399
baseline	abundance	X10725	Ethyl malat	3	Interventio	Experimen	Experimen	Control	0,97445
baseline	abundance	X10736	Toxopyrimi	3	Interventio	Experimen	Experimen	Control	0,936399
baseline	abundance	X10741	5-Nitro-2-p	3	Interventio	Experimen	Experimen	Control	0,988948
baseline	abundance	X10757	Glycyrin	3	Interventio	Experimen	Experimen	Control	0,945105
baseline	abundance	X10769	Cystine	1	Interventio	Experimen	Experimen	Control	0,936399
baseline	abundance	X10770	2-(4-Isoprc	3	Interventio	Experimen	Experimen	Control	0,945105
baseline	abundance	X10772	Bicine_a	3	Interventio	Experimen	Experimen	Control	0,936399
baseline	abundance	X10776	Histidylgly	3	Interventio	Experimen	Experimen	Control	0,97445
baseline	abundance	X10793	Zalcitabine	3	Interventio	Experimen	Experimen	Control	0,945105
baseline	abundance	X10818	butalbital_	3	Interventio	Experimen	Experimen	Control	0,97445
baseline	abundance	X10819	Hyocholic	2a	Interventio	Experimen	Experimen	Control	0,936399
baseline	abundance	X10864	5-amino-2-	2b	Interventio	Experimen	Experimen	Control	0,945105
baseline	abundance	X10865	Melatonin	3	Interventio	Experimen	Experimen	Control	0,945105
baseline	abundance	X10881	L-gamma-(	3	Interventio	Experimen	Experimen	Control	0,936399
baseline	abundance	X10886	L-gamma-(	3	Interventio	Experimen	Experimen	Control	0,936399
baseline	abundance	X10889	N-acetyl-b	3	Interventio	Experimen	Experimen	Control	0,957875
baseline	abundance	X10896	6-Hydroxyr	2b	Interventio	Experimen	Experimen	Control	0,945105
baseline	abundance	X10901	bis-noryan	3	Interventio	Experimen	Experimen	Control	0,945105
baseline	abundance	X10902	Isoquinolir	2b	Interventio	Experimen	Experimen	Control	0,936399
baseline	abundance	X10918	meprobam	3	Interventio	Experimen	Experimen	Control	0,936399
baseline	abundance	X10929	Temozolon	3	Interventio	Experimen	Experimen	Control	0,936399
baseline	abundance	X10930	MFCD0995	3	Interventio	Experimen	Experimen	Control	0,948031
baseline	abundance	X10936	clavulanic	3	Interventio	Experimen	Experimen	Control	0,936399
baseline	abundance	X10947	1,1'-[1,12-	3	Interventio	Experimen	Experimen	Control	0,945105
baseline	abundance	X10951	8-Methyl-8	3	Interventio	Experimen	Experimen	Control	0,936399
baseline	abundance	X10953	Vorinostat_	3	Interventio	Experimen	Experimen	Control	0,936399
baseline	abundance	X10974	Threonylglu	3	Interventio	Experimen	Experimen	Control	0,941185
baseline	abundance	X10977	pentobarbi	3	Interventio	Experimen	Experimen	Control	0,97445
baseline	abundance	X10979	acetyltauri	3	Interventio	Experimen	Experimen	Control	0,936399
baseline	abundance	X10982	8-Methyl-8	3	Interventio	Experimen	Experimen	Control	0,936399
baseline	abundance	X10987	2-BUTYL PI	3	Interventio	Experimen	Experimen	Control	0,945105
baseline	abundance	X11001	2-[(carbox	2b	Interventio	Experimen	Experimen	Control	0,936399
baseline	abundance	X11012	Furan	3	Interventio	Experimen	Experimen	Control	0,936399
baseline	abundance	X11028	butalbital_	3	Interventio	Experimen	Experimen	Control	0,945105
baseline	abundance	X11032	2,4-Bis(3-r	3	Interventio	Experimen	Experimen	Control	0,978464
baseline	abundance	X11033	N-Acetyl-5	3	Interventio	Experimen	Experimen	Control	0,983461
baseline	abundance	X11034	1-(3-Aminc	3	Interventio	Experimen	Experimen	Control	0,945105
baseline	abundance	X11036	3-Hydroxyp	2b	Interventio	Experimen	Experimen	Control	0,936399
baseline	abundance	X11038	(2E)-3-(3,4	3	Interventio	Experimen	Experimen	Control	0,936399
baseline	abundance	X11042	Leu-arg	3	Interventio	Experimen	Experimen	Control	0,957875
baseline	abundance	X11059	Dihydrocor	3	Interventio	Experimen	Experimen	Control	0,945105
baseline	abundance	X11067	Resveratro	3	Interventio	Experimen	Experimen	Control	0,936399

baseline	abundance	X11068	N,N'-Bis[4-	3	Interventio	Experimen	Experimen	Control	0,945786
baseline	abundance	X11110	Tauropine	3	Interventio	Experimen	Experimen	Control	0,936399
baseline	abundance	X11115	Hydroxyca	3	Interventio	Experimen	Experimen	Control	0,936399
baseline	abundance	X11117	Coprine_b	3	Interventio	Experimen	Experimen	Control	0,936399
baseline	abundance	X11125	5,8,12-Trih	3	Interventio	Experimen	Experimen	Control	0,936399
baseline	abundance	X11140	Leu-Leu_e	3	Interventio	Experimen	Experimen	Control	0,936399
baseline	abundance	X11163	Lys-Pro_a	3	Interventio	Experimen	Experimen	Control	0,936399
baseline	abundance	X11170	QJ972000(	3	Interventio	Experimen	Experimen	Control	0,936399
baseline	abundance	X11186	Fluocinolo	2b	Interventio	Experimen	Experimen	Control	0,988867
baseline	abundance	X11215	Leu-Leu_b	3	Interventio	Experimen	Experimen	Control	0,936399
baseline	abundance	X11223	Ethyl mala	3	Interventio	Experimen	Experimen	Control	0,936399
baseline	abundance	X11245	MFCD0003	3	Interventio	Experimen	Experimen	Control	0,936399
baseline	abundance	X11251	N-Nonanoy	3	Interventio	Experimen	Experimen	Control	0,958229
baseline	abundance	X11254	(4R,5S,9S,	3	Interventio	Experimen	Experimen	Control	0,945105
baseline	abundance	X11257	1-Methylgu	2b	Interventio	Experimen	Experimen	Control	0,955424
baseline	abundance	X11258	Valyl-4-hyc	3	Interventio	Experimen	Experimen	Control	0,945105
baseline	abundance	X11265	Sulfurol	3	Interventio	Experimen	Experimen	Control	0,97445
baseline	abundance	X11273	trp-pro	3	Interventio	Experimen	Experimen	Control	0,936399
baseline	abundance	X11277	lys-leu	3	Interventio	Experimen	Experimen	Control	0,957875
baseline	abundance	X11281	Penbutolol	3	Interventio	Experimen	Experimen	Control	0,960702
baseline	abundance	X11301	(6S)-2-Ami	3	Interventio	Experimen	Experimen	Control	0,97445
baseline	abundance	X11310	Leu-Val_a	3	Interventio	Experimen	Experimen	Control	0,936399
baseline	abundance	X11319	Methohexi	3	Interventio	Experimen	Experimen	Control	0,987751
baseline	abundance	X11323	dihydroxyb	3	Interventio	Experimen	Experimen	Control	0,936399
baseline	abundance	X11331	N~6~-Octa	3	Interventio	Experimen	Experimen	Control	0,955424
baseline	abundance	X11339	L-gamma-(	3	Interventio	Experimen	Experimen	Control	0,97445
baseline	abundance	X11347	Voglibose	3	Interventio	Experimen	Experimen	Control	0,945105
baseline	abundance	X11349	Butabarbit	3	Interventio	Experimen	Experimen	Control	0,962445
baseline	abundance	X11354	3-Oxo-4,6-	3	Interventio	Experimen	Experimen	Control	0,987751
baseline	abundance	X11361	1-Methylhi	3	Interventio	Experimen	Experimen	Control	0,936399
baseline	abundance	X11366	3-Sulfino-L	3	Interventio	Experimen	Experimen	Control	0,936399
baseline	abundance	X11373	3-Succinoy	2b	Interventio	Experimen	Experimen	Control	0,945105
baseline	abundance	X11375	L-gamma-(	3	Interventio	Experimen	Experimen	Control	0,948031
baseline	abundance	X11383	N-{3-[(4-Ac	3	Interventio	Experimen	Experimen	Control	0,957875
baseline	abundance	X11408	5-Amino-6	3	Interventio	Experimen	Experimen	Control	0,97445
baseline	abundance	X11421	8-Amino-7	3	Interventio	Experimen	Experimen	Control	0,97445
baseline	abundance	X11438	pro-met	3	Interventio	Experimen	Experimen	Control	0,945105
baseline	abundance	X11441	beta-D-Eth	3	Interventio	Experimen	Experimen	Control	0,988948
baseline	abundance	X11477	gamma-Gl	3	Interventio	Experimen	Experimen	Control	0,945105
baseline	abundance	X11481	his-gln_b	3	Interventio	Experimen	Experimen	Control	0,955424
baseline	abundance	X11494	3-Hydroxys	3	Interventio	Experimen	Experimen	Control	0,962445
baseline	abundance	X11498	Valylvaline	3	Interventio	Experimen	Experimen	Control	0,957875
baseline	abundance	X11505	FB950000(	3	Interventio	Experimen	Experimen	Control	0,945105
baseline	abundance	X11515	13(S)-HOT	2b	Interventio	Experimen	Experimen	Control	0,936399
baseline	abundance	X11522	thr-trp	3	Interventio	Experimen	Experimen	Control	0,988867
baseline	abundance	X11524	6-hydroxyp	3	Interventio	Experimen	Experimen	Control	0,936399
baseline	abundance	X11529	2-Aminooc	2b	Interventio	Experimen	Experimen	Control	0,988948
baseline	abundance	X11549	1,1'-[1,12-	3	Interventio	Experimen	Experimen	Control	0,945105

baseline	abundance	X11556	N-[(2E)-3-(	3	Interventio	Experimen	Experimen	Control	0,936399
baseline	abundance	X11562	ALA-PRO	3	Interventio	Experimen	Experimen	Control	0,957875
baseline	abundance	X11563	threonylph	3	Interventio	Experimen	Experimen	Control	0,936399
baseline	abundance	X11576	Phenyl D-g	3	Interventio	Experimen	Experimen	Control	0,945105
baseline	abundance	X11605	3-Methyl-2	3	Interventio	Experimen	Experimen	Control	0,957875
baseline	abundance	X11614	2-Methylth	3	Interventio	Experimen	Experimen	Control	0,936399
baseline	abundance	X11627	N-(3-aceta	3	Interventio	Experimen	Experimen	Control	0,936399
baseline	abundance	X11639	Midodrine_	3	Interventio	Experimen	Experimen	Control	0,957875
baseline	abundance	X11645	glu-thr	3	Interventio	Experimen	Experimen	Control	0,945105
baseline	abundance	X11666	N-(4-Hydro	3	Interventio	Experimen	Experimen	Control	0,987541
baseline	abundance	X11667	Tetraacety	3	Interventio	Experimen	Experimen	Control	0,962445
baseline	abundance	X11692	2_7-Anhyd	3	Interventio	Experimen	Experimen	Control	0,936399
baseline	abundance	X11698	1-(beta-D-r	3	Interventio	Experimen	Experimen	Control	0,948225
baseline	abundance	X11699	{2-[2-(Isob	3	Interventio	Experimen	Experimen	Control	0,936399
baseline	abundance	X11716	ala-ser_a	3	Interventio	Experimen	Experimen	Control	0,945105
baseline	abundance	X11717	nicotianam	3	Interventio	Experimen	Experimen	Control	0,988948
baseline	abundance	X11733	1,3-Dihydr	3	Interventio	Experimen	Experimen	Control	0,97445
baseline	abundance	X11745	2-Despiper	3	Interventio	Experimen	Experimen	Control	0,936399
baseline	abundance	X11755	Furfuranol	3	Interventio	Experimen	Experimen	Control	0,945105
baseline	abundance	p_cresol	p-Cresol	1	Interventio	Experimen	Experimen	Control	0,97445
3 months	abundance	2-Methylpr	2-Methylpr	CFA_panel	Interventio	Experimen	Experimen	Control	0,106066
3 months	abundance	3-Methylbu	3-Methylbu	CFA_panel	Interventio	Experimen	Experimen	Control	0,151734
3 months	abundance	Acetic acid	Acetic acid	CFA_panel	Interventio	Experimen	Experimen	Control	0,918443
3 months	abundance	Butanoic a	Butanoic a	CFA_panel	Interventio	Experimen	Experimen	Control	0,626645
3 months	abundance	Hexanoic a	Hexanoic a	CFA_panel	Interventio	Experimen	Experimen	Control	0,108736
3 months	abundance	M1	M1	IA_module	Interventio	Experimen	Experimen	Control	0,141328
3 months	abundance	M10	M10	IA_module	Interventio	Experimen	Experimen	Control	0,967096
3 months	abundance	M11	M11	IA_module	Interventio	Experimen	Experimen	Control	0,787558
3 months	abundance	M12	M12	IA_module	Interventio	Experimen	Experimen	Control	0,017773
3 months	abundance	M13	M13	IA_module	Interventio	Experimen	Experimen	Control	0,718544
3 months	abundance	M14	M14	IA_module	Interventio	Experimen	Experimen	Control	0,581247
3 months	abundance	M15	M15	IA_module	Interventio	Experimen	Experimen	Control	0,819081
3 months	abundance	M16	M16	IA_module	Interventio	Experimen	Experimen	Control	0,648353
3 months	abundance	M17	M17	IA_module	Interventio	Experimen	Experimen	Control	0,953271
3 months	abundance	M18	M18	IA_module	Interventio	Experimen	Experimen	Control	0,927867
3 months	abundance	M19	M19	IA_module	Interventio	Experimen	Experimen	Control	0,922757
3 months	abundance	M2	M2	IA_module	Interventio	Experimen	Experimen	Control	0,136956
3 months	abundance	M20	M20	IA_module	Interventio	Experimen	Experimen	Control	0,100587
3 months	abundance	M21	M21	IA_module	Interventio	Experimen	Experimen	Control	0,639019
3 months	abundance	M22	M22	IA_module	Interventio	Experimen	Experimen	Control	0,55784
3 months	abundance	M23	M23	IA_module	Interventio	Experimen	Experimen	Control	0,960119
3 months	abundance	M24	M24	IA_module	Interventio	Experimen	Experimen	Control	0,980029
3 months	abundance	M25	M25	IA_module	Interventio	Experimen	Experimen	Control	0,174141
3 months	abundance	M26	M26	IA_module	Interventio	Experimen	Experimen	Control	0,862952
3 months	abundance	M27	M27	IA_module	Interventio	Experimen	Experimen	Control	0,854716
3 months	abundance	M28	M28	IA_module	Interventio	Experimen	Experimen	Control	0,633187
3 months	abundance	M29	M29	IA_module	Interventio	Experimen	Experimen	Control	0,232653
3 months	abundance	M3	M3	IA_module	Interventio	Experimen	Experimen	Control	0,867514

3 months	abundance	M30	M30	\A_module	Interventio	Experimen	Experimen	Control	0,123709
3 months	abundance	M31	M31	\A_module	Interventio	Experimen	Experimen	Control	0,834977
3 months	abundance	M32	M32	\A_module	Interventio	Experimen	Experimen	Control	0,59163
3 months	abundance	M33	M33	\A_module	Interventio	Experimen	Experimen	Control	0,217561
3 months	abundance	M34	M34	\A_module	Interventio	Experimen	Experimen	Control	0,985356
3 months	abundance	M4	M4	\A_module	Interventio	Experimen	Experimen	Control	0,541151
3 months	abundance	M5	M5	\A_module	Interventio	Experimen	Experimen	Control	0,004656
3 months	abundance	M6	M6	\A_module	Interventio	Experimen	Experimen	Control	0,960119
3 months	abundance	M7	M7	\A_module	Interventio	Experimen	Experimen	Control	0,651767
3 months	abundance	M8	M8	\A_module	Interventio	Experimen	Experimen	Control	0,876495
3 months	abundance	M9	M9	\A_module	Interventio	Experimen	Experimen	Control	0,098744
3 months	abundance	Pentanoic	Pentanoic	CFA_panel	Interventio	Experimen	Experimen	Control	0,740178
3 months	abundance	Propanoic	Propanoic	CFA_panel	Interventio	Experimen	Experimen	Control	0,400419
3 months	abundance	SL00001	Deoxycytid	2a	Interventio	Experimen	Experimen	Control	0,997328
3 months	abundance	SL00009	N-Acetylhis	2a	Interventio	Experimen	Experimen	Control	0,515877
3 months	abundance	SL00017	Aspartic ac	2a	Interventio	Experimen	Experimen	Control	0,73207
3 months	abundance	SL00020	Carnitine	2a	Interventio	Experimen	Experimen	Control	0,916188
3 months	abundance	SL00024	Creatine	2a	Interventio	Experimen	Experimen	Control	0,808744
3 months	abundance	SL00035	Glycylglyci	2a	Interventio	Experimen	Experimen	Control	0,808747
3 months	abundance	SL00040	Kynurenine	2a	Interventio	Experimen	Experimen	Control	0,747665
3 months	abundance	SL00049	N-Acetylar	2a	Interventio	Experimen	Experimen	Control	0,277347
3 months	abundance	SL00052	N-Isovalery	2a	Interventio	Experimen	Experimen	Control	0,343109
3 months	abundance	SL00054	Ornithine	1	Interventio	Experimen	Experimen	Control	0,513969
3 months	abundance	SL00061	Proline	1	Interventio	Experimen	Experimen	Control	0,400419
3 months	abundance	SL00063	Pyridoxal	1	Interventio	Experimen	Experimen	Control	0,448346
3 months	abundance	SL00074	Valine/ 5-A	2a	Interventio	Experimen	Experimen	Control	0,220826
3 months	abundance	SL00081	5-Methylcy	2a	Interventio	Experimen	Experimen	Control	0,30026
3 months	abundance	SL00082	Allopurinol	1	Interventio	Experimen	Experimen	Control	0,853666
3 months	abundance	SL00089	Dissaccari	2a	Interventio	Experimen	Experimen	Control	0,235652
3 months	abundance	SL00096	Hexoses III	1	Interventio	Experimen	Experimen	Control	0,096259
3 months	abundance	SL00097	Hexoses II	1	Interventio	Experimen	Experimen	Control	0,115645
3 months	abundance	SL00103	Gulonic ac	2a	Interventio	Experimen	Experimen	Control	0,100093
3 months	abundance	SL00104	Hexoses I	1	Interventio	Experimen	Experimen	Control	0,099053
3 months	abundance	SL00108	Histidinol	2a	Interventio	Experimen	Experimen	Control	0,030613
3 months	abundance	SL00124	Dissaccari	2a	Interventio	Experimen	Experimen	Control	0,503169
3 months	abundance	SL00128	N-Acetylala	2a	Interventio	Experimen	Experimen	Control	0,778801
3 months	abundance	SL00133	N-Acetylglu	2a	Interventio	Experimen	Experimen	Control	0,909515
3 months	abundance	SL00137	Phenylacet	2a	Interventio	Experimen	Experimen	Control	0,613297
3 months	abundance	SL00140	Pyridoxami	1	Interventio	Experimen	Experimen	Control	0,385109
3 months	abundance	SL00149	Dissaccari	2a	Interventio	Experimen	Experimen	Control	0,017773
3 months	abundance	SL00154	Trigonelline	1	Interventio	Experimen	Experimen	Control	0,449163
3 months	abundance	SL00159	Uracil	1	Interventio	Experimen	Experimen	Control	0,98068
3 months	abundance	SL00161	Uridine	2a	Interventio	Experimen	Experimen	Control	0,993459
3 months	abundance	SL00185	2-Oxo-3-ph	1	Interventio	Experimen	Experimen	Control	0,056989
3 months	abundance	SL00189	3-Hydroxyb	2a	Interventio	Experimen	Experimen	Control	0,105225
3 months	abundance	SL00197	4-Methyl-2	2a	Interventio	Experimen	Experimen	Control	0,323676
3 months	abundance	SL00199	5-Hydroxyi	2a	Interventio	Experimen	Experimen	Control	0,798192
3 months	abundance	SL00200	8-Aminooc	2a	Interventio	Experimen	Experimen	Control	0,143909

3 months	abundance	SL00201	Pentose II	2a	Interventio	Experimen	Experimen	Control	0,497383
3 months	abundance	SL00205	Citraconic	2a	Interventio	Experimen	Experimen	Control	0,985356
3 months	abundance	SL00210	Galactonic	2a	Interventio	Experimen	Experimen	Control	0,243875
3 months	abundance	SL00212	Galactosar	2a	Interventio	Experimen	Experimen	Control	0,71076
3 months	abundance	SL00215	Glycolic ac	2a	Interventio	Experimen	Experimen	Control	0,867667
3 months	abundance	SL00216	Glyoxylic a	2a	Interventio	Experimen	Experimen	Control	0,584092
3 months	abundance	SL00222	Lactic acid	1	Interventio	Experimen	Experimen	Control	0,250053
3 months	abundance	SL00226	N-Acetylgly	2a	Interventio	Experimen	Experimen	Control	0,198023
3 months	abundance	SL00239	Pinitol	2a	Interventio	Experimen	Experimen	Control	0,876028
3 months	abundance	SL00240	Pyruvic aci	1	Interventio	Experimen	Experimen	Control	0,798192
3 months	abundance	SL00243	Deoxysuga	2a	Interventio	Experimen	Experimen	Control	0,150298
3 months	abundance	SL00245	sugar alcol	2a	Interventio	Experimen	Experimen	Control	0,68636
3 months	abundance	SL00246	Pentose III	2a	Interventio	Experimen	Experimen	Control	0,139689
3 months	abundance	SL00247	Shikimic ac	2a	Interventio	Experimen	Experimen	Control	0,163182
3 months	abundance	SL00248	Gluconic a	2a	Interventio	Experimen	Experimen	Control	0,747665
3 months	abundance	SL00260	Pentose I	2a	Interventio	Experimen	Experimen	Control	0,29731
3 months	abundance	SL00262	cis-Aconiti	2a	Interventio	Experimen	Experimen	Control	0,365312
3 months	abundance	SL00264	Methyl ace	2a	Interventio	Experimen	Experimen	Control	0,560103
3 months	abundance	SL00265	Purine	2a	Interventio	Experimen	Experimen	Control	0,222442
3 months	abundance	SL00268	Thymine	1	Interventio	Experimen	Experimen	Control	0,915997
3 months	abundance	SL00270	3-hydroxy-	2a	Interventio	Experimen	Experimen	Control	0,98692
3 months	abundance	SL00275	Dopamine	2a	Interventio	Experimen	Experimen	Control	0,985504
3 months	abundance	SL00282	3-Hydroxyh	2a	Interventio	Experimen	Experimen	Control	0,886266
3 months	abundance	SL00285	Dihydrofer	2a	Interventio	Experimen	Experimen	Control	0,988551
3 months	abundance	SL00287	DOPA	2a	Interventio	Experimen	Experimen	Control	0,776369
3 months	abundance	SL00288	Propionylc	2a	Interventio	Experimen	Experimen	Control	0,419167
3 months	abundance	SL00290	N-Acetylma	2a	Interventio	Experimen	Experimen	Control	0,264089
3 months	abundance	SL00293	Butyrylcarr	2a	Interventio	Experimen	Experimen	Control	0,960119
3 months	abundance	SL00295	5-Methylur	2a	Interventio	Experimen	Experimen	Control	0,254073
3 months	abundance	SL00297	Acetylmura	2a	Interventio	Experimen	Experimen	Control	0,765006
3 months	abundance	SL00299	β-Murichol	2a	Interventio	Experimen	Experimen	Control	0,499773
3 months	abundance	SL00309	1-Aminocy	2a	Interventio	Experimen	Experimen	Control	0,087526
3 months	abundance	SL00311	N,N-Dimet	2a	Interventio	Experimen	Experimen	Control	0,836327
3 months	abundance	SL00313	2-Aminoiso	2a	Interventio	Experimen	Experimen	Control	0,957479
3 months	abundance	SL00315	Malonic ac	2a	Interventio	Experimen	Experimen	Control	0,404129
3 months	abundance	SL00318	4-Aminoph	1	Interventio	Experimen	Experimen	Control	0,944871
3 months	abundance	SL00320	γ-Caprolac	2a	Interventio	Experimen	Experimen	Control	0,437457
3 months	abundance	SL00325	2-Methylm	2a	Interventio	Experimen	Experimen	Control	0,375891
3 months	abundance	SL00328	2-(hydroxy	2a	Interventio	Experimen	Experimen	Control	0,143745
3 months	abundance	SL00329	2-Hydroxy-	2a	Interventio	Experimen	Experimen	Control	0,718544
3 months	abundance	SL00334	Imidazolea	2a	Interventio	Experimen	Experimen	Control	0,73403
3 months	abundance	SL00346	N-Methylni	2a	Interventio	Experimen	Experimen	Control	0,9752
3 months	abundance	SL00349	1-Aminocy	2a	Interventio	Experimen	Experimen	Control	0,845389
3 months	abundance	SL00350	Stachydrin	1	Interventio	Experimen	Experimen	Control	0,561912
3 months	abundance	SL00353	Adipic acid	1	Interventio	Experimen	Experimen	Control	0,985504
3 months	abundance	SL00356	N-(5-Amin	2a	Interventio	Experimen	Experimen	Control	0,066304
3 months	abundance	SL00368	N-Methylty	1	Interventio	Experimen	Experimen	Control	0,36547
3 months	abundance	SL00373	N1-Methyl-	2a	Interventio	Experimen	Experimen	Control	0,004228

3 months	abundance	SL00376	N-AcetylPr	2a	Interventio	Experimen	Experimen	Control	0,776369
3 months	abundance	SL00380	2-Aminoad	2a	Interventio	Experimen	Experimen	Control	0,325689
3 months	abundance	SL00383	3-(2-Hydro	2a	Interventio	Experimen	Experimen	Control	0,655638
3 months	abundance	SL00384	p-Coumari	1	Interventio	Experimen	Experimen	Control	0,685901
3 months	abundance	SL00390	Gallic acid	2a	Interventio	Experimen	Experimen	Control	0,511679
3 months	abundance	SL00391	N-Acetyllе	1	Interventio	Experimen	Experimen	Control	0,704502
3 months	abundance	SL00401	Theophyllі	1	Interventio	Experimen	Experimen	Control	0,820887
3 months	abundance	SL00407	Homovanil	2a	Interventio	Experimen	Experimen	Control	0,691158
3 months	abundance	SL00420	4-Hydroxy-	2a	Interventio	Experimen	Experimen	Control	0,84521
3 months	abundance	SL00421	Asymmetri	2a	Interventio	Experimen	Experimen	Control	0,988551
3 months	abundance	SL00430	Homocarn	2a	Interventio	Experimen	Experimen	Control	0,419167
3 months	abundance	SL00431	Tiglylcarnit	2a	Interventio	Experimen	Experimen	Control	0,268012
3 months	abundance	SL00433	Isovalerylc	2a	Interventio	Experimen	Experimen	Control	0,788303
3 months	abundance	SL00436	1-Carboxye	2a	Interventio	Experimen	Experimen	Control	0,666592
3 months	abundance	SL00438	Daidzein	2a	Interventio	Experimen	Experimen	Control	0,532452
3 months	abundance	SL00440	5-Methylcy	2a	Interventio	Experimen	Experimen	Control	0,988198
3 months	abundance	SL00445	Glucosami	2a	Interventio	Experimen	Experimen	Control	0,860527
3 months	abundance	SL00447	Apigenin	2a	Interventio	Experimen	Experimen	Control	0,798192
3 months	abundance	SL00455	12,13-DHC	2a	Interventio	Experimen	Experimen	Control	0,867514
3 months	abundance	SL00467	Sucralose	2a	Interventio	Experimen	Experimen	Control	0,997328
3 months	abundance	SL00502	2-Ketobuty	1	Interventio	Experimen	Experimen	Control	0,53654
3 months	abundance	X00011	Glutamine	2a	Interventio	Experimen	Experimen	Control	0,651289
3 months	abundance	X00018	Succinic ac	1	Interventio	Experimen	Experimen	Control	0,511237
3 months	abundance	X00020	sugar alcol	1	Interventio	Experimen	Experimen	Control	0,423696
3 months	abundance	X00022	N-Methylas	1	Interventio	Experimen	Experimen	Control	0,014777
3 months	abundance	X00023	N-Formylm	1	Interventio	Experimen	Experimen	Control	0,142786
3 months	abundance	X00025	Acesulfam	1	Interventio	Experimen	Experimen	Control	0,998514
3 months	abundance	X00026	Inosine	1	Interventio	Experimen	Experimen	Control	0,364717
3 months	abundance	X00027	N-Acetylglu	1	Interventio	Experimen	Experimen	Control	0,104321
3 months	abundance	X00029	Urocanic a	1	Interventio	Experimen	Experimen	Control	0,971921
3 months	abundance	X00030	3,4-Dihydro	1	Interventio	Experimen	Experimen	Control	0,587315
3 months	abundance	X00032	Indole-3-pı	1	Interventio	Experimen	Experimen	Control	0,002538
3 months	abundance	X00033	Deoxycholi	1	Interventio	Experimen	Experimen	Control	0,718544
3 months	abundance	X00034	Pyroglutam	1	Interventio	Experimen	Experimen	Control	0,960119
3 months	abundance	X00035	Choline	1	Interventio	Experimen	Experimen	Control	0,244993
3 months	abundance	X00036	Serine	1	Interventio	Experimen	Experimen	Control	0,261424
3 months	abundance	X00038	Histamine	1	Interventio	Experimen	Experimen	Control	0,605819
3 months	abundance	X00040	N-Acetyltyı	1	Interventio	Experimen	Experimen	Control	0,99116
3 months	abundance	X00042	Glucuronic	1	Interventio	Experimen	Experimen	Control	0,98336
3 months	abundance	X00045	Threonine	1	Interventio	Experimen	Experimen	Control	0,181353
3 months	abundance	X00050	β-Hydroxyi	1	Interventio	Experimen	Experimen	Control	0,135093
3 months	abundance	X00051	β-D-Glucoן	2b	Interventio	Experimen	Experimen	Control	0,755448
3 months	abundance	X00056	3-(4-hydro	1	Interventio	Experimen	Experimen	Control	0,823326
3 months	abundance	X00057	N-Acetylor	1	Interventio	Experimen	Experimen	Control	0,341701
3 months	abundance	X00059	3,4-Dihydro	1	Interventio	Experimen	Experimen	Control	0,385799
3 months	abundance	X00060	Ethylmalor	1	Interventio	Experimen	Experimen	Control	0,468958
3 months	abundance	X00061	Thiamine	1	Interventio	Experimen	Experimen	Control	0,94039
3 months	abundance	X00063	2-Hydroxyo	1	Interventio	Experimen	Experimen	Control	0,907733

3 months	abundance	X00064	Traumatic	1	Interventio	Experimen	Experimen	Control	0,21397
3 months	abundance	X00066	Tryptamine	1	Interventio	Experimen	Experimen	Control	0,98336
3 months	abundance	X00067	Hypoxanth	1	Interventio	Experimen	Experimen	Control	0,143745
3 months	abundance	X00068	N-Acetylm	1	Interventio	Experimen	Experimen	Control	0,560103
3 months	abundance	X00070	α-aminobu	1	Interventio	Experimen	Experimen	Control	0,923057
3 months	abundance	X00071	Tyrosine	1	Interventio	Experimen	Experimen	Control	0,833128
3 months	abundance	X00072	3,5-Dihydr	1	Interventio	Experimen	Experimen	Control	0,736809
3 months	abundance	X00073	Indole-3-la	1	Interventio	Experimen	Experimen	Control	0,025224
3 months	abundance	X00074	Methylsuc	1	Interventio	Experimen	Experimen	Control	0,613297
3 months	abundance	X00076	Isoleucine	1	Interventio	Experimen	Experimen	Control	0,560103
3 months	abundance	X00078	Acetylagm	1	Interventio	Experimen	Experimen	Control	0,718544
3 months	abundance	X00082	Cholic acic	1	Interventio	Experimen	Experimen	Control	0,270355
3 months	abundance	X00083	1,7-Dimetl	1	Interventio	Experimen	Experimen	Control	0,798192
3 months	abundance	X00084	Tricarballyl	1	Interventio	Experimen	Experimen	Control	0,761041
3 months	abundance	X00088	Pantotheni	1	Interventio	Experimen	Experimen	Control	0,122267
3 months	abundance	X00089	4-Hydroxyl	1	Interventio	Experimen	Experimen	Control	0,840004
3 months	abundance	X00090	Malic acid	1	Interventio	Experimen	Experimen	Control	0,387203
3 months	abundance	X00092	3-Methylhi	1	Interventio	Experimen	Experimen	Control	0,339934
3 months	abundance	X00093	Deoxyinosi	1	Interventio	Experimen	Experimen	Control	0,000237
3 months	abundance	X00094	Methionine	1	Interventio	Experimen	Experimen	Control	0,230723
3 months	abundance	X00097	2,6-Dihydr	1	Interventio	Experimen	Experimen	Control	0,932813
3 months	abundance	X00099	7-Methylgu	1	Interventio	Experimen	Experimen	Control	0,988551
3 months	abundance	X00101	Threonic ac	1	Interventio	Experimen	Experimen	Control	0,221894
3 months	abundance	X00102	Tryptophar	1	Interventio	Experimen	Experimen	Control	0,141328
3 months	abundance	X00106	N-Acetyltry	1	Interventio	Experimen	Experimen	Control	0,161346
3 months	abundance	X00107	N6-Acetylly	1	Interventio	Experimen	Experimen	Control	0,017773
3 months	abundance	X00112	Xanthosine	1	Interventio	Experimen	Experimen	Control	0,091781
3 months	abundance	X00113	N-Acetylph	1	Interventio	Experimen	Experimen	Control	0,361332
3 months	abundance	X00114	Indole-3-m	1	Interventio	Experimen	Experimen	Control	0,325689
3 months	abundance	X00115	Histidine	1	Interventio	Experimen	Experimen	Control	0,042091
3 months	abundance	X00116	Cytidine	1	Interventio	Experimen	Experimen	Control	0,988551
3 months	abundance	X00120	Leucylalan	1	Interventio	Experimen	Experimen	Control	0,850869
3 months	abundance	X00123	Phenylalar	1	Interventio	Experimen	Experimen	Control	0,836327
3 months	abundance	X00124	Leucine	1	Interventio	Experimen	Experimen	Control	0,449163
3 months	abundance	X00125	Taurine	1	Interventio	Experimen	Experimen	Control	0,740265
3 months	abundance	X00127	Nicotinic a	1	Interventio	Experimen	Experimen	Control	0,04166
3 months	abundance	X00129	Quinic acic	1	Interventio	Experimen	Experimen	Control	0,888652
3 months	abundance	X00130	Deoxyuridi	1	Interventio	Experimen	Experimen	Control	0,082572
3 months	abundance	X00132	Glyceric ac	1	Interventio	Experimen	Experimen	Control	0,957479
3 months	abundance	X00135	N-Acetylglu	1	Interventio	Experimen	Experimen	Control	0,235652
3 months	abundance	X00136	leu-gln_a	3	Interventio	Experimen	Experimen	Control	0,960119
3 months	abundance	X00163	primidone_	3	Interventio	Experimen	Experimen	Control	0,790121
3 months	abundance	X00180	1-(2-Carbo	3	Interventio	Experimen	Experimen	Control	0,696041
3 months	abundance	X00182	3-(Butylsul	3	Interventio	Experimen	Experimen	Control	0,968857
3 months	abundance	X00199	tert-Butyl 3	3	Interventio	Experimen	Experimen	Control	0,151919
3 months	abundance	X00204	4-O-{3-O-[(	3	Interventio	Experimen	Experimen	Control	0,333178
3 months	abundance	X00221	Astemizole	3	Interventio	Experimen	Experimen	Control	0,003817
3 months	abundance	X00224	Nifedipine	3	Interventio	Experimen	Experimen	Control	0,765006

3 months	abundance	X00233	g-Aminobu	3	Interventio	Experimen	Experimen	Control	0,212863
3 months	abundance	X00237	OI1700000	3	Interventio	Experimen	Experimen	Control	0,625098
3 months	abundance	X00242	IN00258	3	Interventio	Experimen	Experimen	Control	0,099053
3 months	abundance	X00253	TDP-2_a	3	Interventio	Experimen	Experimen	Control	0,684644
3 months	abundance	X00261	Mexiletine	3	Interventio	Experimen	Experimen	Control	0,986565
3 months	abundance	X00264	PEG n12	2b	Interventio	Experimen	Experimen	Control	0,460268
3 months	abundance	X00266	2-Oxo-3-(p	3	Interventio	Experimen	Experimen	Control	0,423696
3 months	abundance	X00305	Formylkynu	3	Interventio	Experimen	Experimen	Control	0,486342
3 months	abundance	X00320	Panthenol_	3	Interventio	Experimen	Experimen	Control	0,277347
3 months	abundance	X00327	3-Hydroxy-	3	Interventio	Experimen	Experimen	Control	0,992999
3 months	abundance	X00331	4-(9H-beta	3	Interventio	Experimen	Experimen	Control	0,520085
3 months	abundance	X00344	SECONAL_	3	Interventio	Experimen	Experimen	Control	0,475034
3 months	abundance	X00361	nitecapone	3	Interventio	Experimen	Experimen	Control	0,143745
3 months	abundance	X00367	hexobarbit	3	Interventio	Experimen	Experimen	Control	0,174141
3 months	abundance	X00375	N-[(2S)-2-H	3	Interventio	Experimen	Experimen	Control	0,021942
3 months	abundance	X00383	pretazettin	3	Interventio	Experimen	Experimen	Control	0,405763
3 months	abundance	X00384	3-Formyl-2	3	Interventio	Experimen	Experimen	Control	0,136644
3 months	abundance	X00403	7alpha-Hyd	3	Interventio	Experimen	Experimen	Control	0,613297
3 months	abundance	X00404	piscidic ac	3	Interventio	Experimen	Experimen	Control	0,449163
3 months	abundance	X00432	8-(Methyls	3	Interventio	Experimen	Experimen	Control	0,98336
3 months	abundance	X00450	Gly-Ser	3	Interventio	Experimen	Experimen	Control	0,876028
3 months	abundance	X00477	Sparfloxac	2b	Interventio	Experimen	Experimen	Control	0,63113
3 months	abundance	X00518	Nisinic aci	3	Interventio	Experimen	Experimen	Control	0,505325
3 months	abundance	X00528	R-(+)-Etira	3	Interventio	Experimen	Experimen	Control	0,787558
3 months	abundance	X00545	3-hydroxyc	3	Interventio	Experimen	Experimen	Control	0,530275
3 months	abundance	X00549	3-[(2Z)-1-C	3	Interventio	Experimen	Experimen	Control	0,798192
3 months	abundance	X00574	miglustat	3	Interventio	Experimen	Experimen	Control	0,22934
3 months	abundance	X00594	Nicotinami	3	Interventio	Experimen	Experimen	Control	0,988551
3 months	abundance	X00635	4-(5,6-Dihy	3	Interventio	Experimen	Experimen	Control	0,860527
3 months	abundance	X00637	N~6~,N~6	3	Interventio	Experimen	Experimen	Control	0,128284
3 months	abundance	X00646	Deacetyldi	3	Interventio	Experimen	Experimen	Control	0,986931
3 months	abundance	X00670	7-Chloro-5	3	Interventio	Experimen	Experimen	Control	0,894218
3 months	abundance	X00683	Zinecard_a	3	Interventio	Experimen	Experimen	Control	0,109824
3 months	abundance	X00693	n-Ribosylh	3	Interventio	Experimen	Experimen	Control	0,225072
3 months	abundance	X00698	valganciclo	3	Interventio	Experimen	Experimen	Control	0,106496
3 months	abundance	X00702	N-[(2S)-2-H	3	Interventio	Experimen	Experimen	Control	0,096259
3 months	abundance	X00722	(3S,5R,6E)	3	Interventio	Experimen	Experimen	Control	0,301015
3 months	abundance	X00723	Aspartyl-L-	3	Interventio	Experimen	Experimen	Control	0,817686
3 months	abundance	X00742	MFCD1869	3	Interventio	Experimen	Experimen	Control	0,84915
3 months	abundance	X00743	11beta,13-	3	Interventio	Experimen	Experimen	Control	0,835202
3 months	abundance	X00744	(1R,2S)-1-(	3	Interventio	Experimen	Experimen	Control	0,713464
3 months	abundance	X00748	3-[3-Metho	3	Interventio	Experimen	Experimen	Control	0,867667
3 months	abundance	X00764	5-Allyl-5-se	3	Interventio	Experimen	Experimen	Control	0,26602
3 months	abundance	X00807	Midodrine_	3	Interventio	Experimen	Experimen	Control	0,667762
3 months	abundance	X00828	Fenoterol	3	Interventio	Experimen	Experimen	Control	0,613297
3 months	abundance	X00836	3-Mercapto	3	Interventio	Experimen	Experimen	Control	0,354732
3 months	abundance	X00853	4-O-beta-D	3	Interventio	Experimen	Experimen	Control	0,725009
3 months	abundance	X00889	LW800000	3	Interventio	Experimen	Experimen	Control	0,176658

3 months	abundance	X00899	N-(2,3,4-Tr	3	Interventio	Experimen	Experimen	Control	0,446333
3 months	abundance	X00907	1,2,3,4-Tet	3	Interventio	Experimen	Experimen	Control	0,935319
3 months	abundance	X00928	(7R)-7-(5-c	3	Interventio	Experimen	Experimen	Control	0,932813
3 months	abundance	X00948	Linamarin	3	Interventio	Experimen	Experimen	Control	0,647023
3 months	abundance	X00949	primidone_	3	Interventio	Experimen	Experimen	Control	0,404129
3 months	abundance	X00950	2-Phenylet	3	Interventio	Experimen	Experimen	Control	0,268483
3 months	abundance	X00963	(4S)-4-[(2E	3	Interventio	Experimen	Experimen	Control	0,099053
3 months	abundance	X00985	1-(4-Aminc	3	Interventio	Experimen	Experimen	Control	0,324406
3 months	abundance	X00998	epsilon-(ga	3	Interventio	Experimen	Experimen	Control	0,106066
3 months	abundance	X01007	pentobarbi	3	Interventio	Experimen	Experimen	Control	0,992999
3 months	abundance	X01011	DIBEHENIN	3	Interventio	Experimen	Experimen	Control	0,724969
3 months	abundance	X01017	hexobarbit	3	Interventio	Experimen	Experimen	Control	0,424219
3 months	abundance	X01031	3-(14-Ethy	3	Interventio	Experimen	Experimen	Control	0,181353
3 months	abundance	X01045	Ethyl maltc	3	Interventio	Experimen	Experimen	Control	0,19078
3 months	abundance	X01059	coronatine	3	Interventio	Experimen	Experimen	Control	0,992999
3 months	abundance	X01067	g-Aminobu	3	Interventio	Experimen	Experimen	Control	0,918473
3 months	abundance	X01078	Dihydroura	3	Interventio	Experimen	Experimen	Control	0,860697
3 months	abundance	X01081	Methyl alpl	3	Interventio	Experimen	Experimen	Control	0,927509
3 months	abundance	X01092	17,21-Dihy	3	Interventio	Experimen	Experimen	Control	0,908976
3 months	abundance	X01098	(-)-Physost	3	Interventio	Experimen	Experimen	Control	0,758265
3 months	abundance	X01100	(betaS)-be	3	Interventio	Experimen	Experimen	Control	0,907733
3 months	abundance	X01154	N-(2,3,4-Tr	3	Interventio	Experimen	Experimen	Control	0,262176
3 months	abundance	X01163	Prephenic	3	Interventio	Experimen	Experimen	Control	0,944776
3 months	abundance	X01164	DL-Carboc	3	Interventio	Experimen	Experimen	Control	0,98692
3 months	abundance	X01181	2-(3,4-Dim	3	Interventio	Experimen	Experimen	Control	0,63113
3 months	abundance	X01186	asn-val_b	3	Interventio	Experimen	Experimen	Control	0,698621
3 months	abundance	X01208	Lys-Pro_c	3	Interventio	Experimen	Experimen	Control	0,002122
3 months	abundance	X01223	DL-Mevalo	3	Interventio	Experimen	Experimen	Control	0,514326
3 months	abundance	X01235	3-[(3-Hydro	3	Interventio	Experimen	Experimen	Control	0,006023
3 months	abundance	X01236	bis(4-isoth	3	Interventio	Experimen	Experimen	Control	0,915997
3 months	abundance	X01242	3-Morpholi	2b	Interventio	Experimen	Experimen	Control	0,967096
3 months	abundance	X01246	(2R)-1-{[(2-	3	Interventio	Experimen	Experimen	Control	0,002122
3 months	abundance	X01252	(−)-nabilor	3	Interventio	Experimen	Experimen	Control	0,808744
3 months	abundance	X01285	FB9500000	3	Interventio	Experimen	Experimen	Control	0,780575
3 months	abundance	X01286	(2,4-Dihyd	3	Interventio	Experimen	Experimen	Control	0,76164
3 months	abundance	X01288	L-gamma-(	3	Interventio	Experimen	Experimen	Control	0,925953
3 months	abundance	X01316	5-Hydanto	3	Interventio	Experimen	Experimen	Control	0,143745
3 months	abundance	X01327	threonylph	3	Interventio	Experimen	Experimen	Control	0,94858
3 months	abundance	X01340	Cadralazin	3	Interventio	Experimen	Experimen	Control	0,250053
3 months	abundance	X01341	metixene	3	Interventio	Experimen	Experimen	Control	0,36583
3 months	abundance	X01346	IN00260_a	3	Interventio	Experimen	Experimen	Control	0,997328
3 months	abundance	X01363	Seryltyrosi	3	Interventio	Experimen	Experimen	Control	0,98692
3 months	abundance	X01364	Oleuropeir	3	Interventio	Experimen	Experimen	Control	0,862952
3 months	abundance	X01367	MFCD1869	3	Interventio	Experimen	Experimen	Control	0,423696
3 months	abundance	X01380	Spermic ac	3	Interventio	Experimen	Experimen	Control	0,96467
3 months	abundance	X01387	Methyl 4-(4	3	Interventio	Experimen	Experimen	Control	0,830617
3 months	abundance	X01441	1-{3-Carbo	3	Interventio	Experimen	Experimen	Control	0,510076
3 months	abundance	X01463	4-Thiapent	3	Interventio	Experimen	Experimen	Control	0,997328

3 months	abundance	X01474	Glyceropho	3	Interventio	Experimen	Experimen	Control	0,004656
3 months	abundance	X01518	L-gamma-(	3	Interventio	Experimen	Experimen	Control	0,979909
3 months	abundance	X01519	Leucyltrypt	3	Interventio	Experimen	Experimen	Control	0,884928
3 months	abundance	X01528	butalbital_	3	Interventio	Experimen	Experimen	Control	0,588242
3 months	abundance	X01530	INK (Peptic	2b	Interventio	Experimen	Experimen	Control	0,977246
3 months	abundance	X01549	3-(Sulfooxy	3	Interventio	Experimen	Experimen	Control	0,198011
3 months	abundance	X01553	uridine 5'-c	3	Interventio	Experimen	Experimen	Control	0,854716
3 months	abundance	X01558	2-Furoylgly	2b	Interventio	Experimen	Experimen	Control	0,778801
3 months	abundance	X01561	2-Ammoni	3	Interventio	Experimen	Experimen	Control	0,680872
3 months	abundance	X01570	carglumic a	3	Interventio	Experimen	Experimen	Control	0,949849
3 months	abundance	X01574	N-Acetylpr	3	Interventio	Experimen	Experimen	Control	0,960119
3 months	abundance	X01577	Prunasin	3	Interventio	Experimen	Experimen	Control	0,883461
3 months	abundance	X01621	2-(2,4-Dihy	3	Interventio	Experimen	Experimen	Control	0,042091
3 months	abundance	X01640	Ro 20-172	3	Interventio	Experimen	Experimen	Control	0,405692
3 months	abundance	X01656	Guanadrel	3	Interventio	Experimen	Experimen	Control	0,015478
3 months	abundance	X01664	5-methylth	3	Interventio	Experimen	Experimen	Control	0,565249
3 months	abundance	X01671	N-(3,5-Dim	3	Interventio	Experimen	Experimen	Control	0,9752
3 months	abundance	X01672	Triethyl citı	3	Interventio	Experimen	Experimen	Control	0,867667
3 months	abundance	X01679	meprobam	3	Interventio	Experimen	Experimen	Control	0,988551
3 months	abundance	X01689	Ethyl mala	3	Interventio	Experimen	Experimen	Control	0,003588
3 months	abundance	X01732	2-Hydroxy-	3	Interventio	Experimen	Experimen	Control	0,985356
3 months	abundance	X01738	2-Acetami	3	Interventio	Experimen	Experimen	Control	0,765006
3 months	abundance	X01746	Astemizole	3	Interventio	Experimen	Experimen	Control	0,949849
3 months	abundance	X01768	Butabarbit	3	Interventio	Experimen	Experimen	Control	2,67E-13
3 months	abundance	X01776	N-(Carboxy	3	Interventio	Experimen	Experimen	Control	0,483353
3 months	abundance	X01793	Lys-phe_b	3	Interventio	Experimen	Experimen	Control	0,701873
3 months	abundance	X01813	Erythorbic	3	Interventio	Experimen	Experimen	Control	0,94878
3 months	abundance	X01833	2,3,4,9-Tet	2b	Interventio	Experimen	Experimen	Control	0,808744
3 months	abundance	X01873	Methyl 2,3	3	Interventio	Experimen	Experimen	Control	0,817974
3 months	abundance	X01879	Tetramethy	2b	Interventio	Experimen	Experimen	Control	0,572678
3 months	abundance	X01881	(2S)-3-Met	3	Interventio	Experimen	Experimen	Control	0,867514
3 months	abundance	X01884	Roxane	3	Interventio	Experimen	Experimen	Control	5,30E-05
3 months	abundance	X01887	lys-tyr_b	3	Interventio	Experimen	Experimen	Control	0,124404
3 months	abundance	X01893	4-(METHYL	3	Interventio	Experimen	Experimen	Control	0,960119
3 months	abundance	X01911	3-(2,3-Dihy	3	Interventio	Experimen	Experimen	Control	0,872597
3 months	abundance	X01920	N-(Carboxy	3	Interventio	Experimen	Experimen	Control	0,725856
3 months	abundance	X01932	asn-val_c	3	Interventio	Experimen	Experimen	Control	0,26602
3 months	abundance	X01943	Tocainide	3	Interventio	Experimen	Experimen	Control	0,859603
3 months	abundance	X01950	Histidylgly	3	Interventio	Experimen	Experimen	Control	0,835202
3 months	abundance	X01968	7-Chloro-5	3	Interventio	Experimen	Experimen	Control	0,835202
3 months	abundance	X01996	3-Hydroxy-	3	Interventio	Experimen	Experimen	Control	0,687176
3 months	abundance	X02000	11-(4-Hydı	3	Interventio	Experimen	Experimen	Control	0,877325
3 months	abundance	X02009	Arctiopicri	3	Interventio	Experimen	Experimen	Control	0,416668
3 months	abundance	X02013	Ile-cys	3	Interventio	Experimen	Experimen	Control	0,778801
3 months	abundance	X02020	Val-Ser_b	3	Interventio	Experimen	Experimen	Control	0,884928
3 months	abundance	X02023	beta-D-Eth	3	Interventio	Experimen	Experimen	Control	0,397203
3 months	abundance	X02073	N-acetyl-9	3	Interventio	Experimen	Experimen	Control	0,096437
3 months	abundance	X02080	(-)-Aspidos	3	Interventio	Experimen	Experimen	Control	0,210987

3 months	abundance	X02082	6-Hydroxyr	3	Interventio	Experimen	Experimen	Control	0,647508
3 months	abundance	X02101	Succinic ar	3	Interventio	Experimen	Experimen	Control	0,59787
3 months	abundance	X02108	Homovanil	3	Interventio	Experimen	Experimen	Control	0,456954
3 months	abundance	X02121	2-Acetamio	3	Interventio	Experimen	Experimen	Control	0,77242
3 months	abundance	X02139	SECONAL_	3	Interventio	Experimen	Experimen	Control	0,150157
3 months	abundance	X02145	3'-Hydroxy	3	Interventio	Experimen	Experimen	Control	0,992999
3 months	abundance	X02153	Asarone	3	Interventio	Experimen	Experimen	Control	0,56915
3 months	abundance	X02181	YWA1	3	Interventio	Experimen	Experimen	Control	0,174141
3 months	abundance	X02184	Piperonylo	2b	Interventio	Experimen	Experimen	Control	0,971921
3 months	abundance	X02195	alliin	3	Interventio	Experimen	Experimen	Control	0,988551
3 months	abundance	X02202	D-2-Amino	3	Interventio	Experimen	Experimen	Control	0,950648
3 months	abundance	X02208	SECONAL_	3	Interventio	Experimen	Experimen	Control	0,021942
3 months	abundance	X02214	(E)-4-Meth	3	Interventio	Experimen	Experimen	Control	0,46054
3 months	abundance	X02219	Homoanse	3	Interventio	Experimen	Experimen	Control	0,36547
3 months	abundance	X02231	Ala-Tyr	3	Interventio	Experimen	Experimen	Control	0,725339
3 months	abundance	X02238	6-Myoporo	3	Interventio	Experimen	Experimen	Control	0,830535
3 months	abundance	X02251	Hostmania	3	Interventio	Experimen	Experimen	Control	0,998514
3 months	abundance	X02256	hexobarbit	3	Interventio	Experimen	Experimen	Control	0,052565
3 months	abundance	X02265	ophthalmio	3	Interventio	Experimen	Experimen	Control	0,21873
3 months	abundance	X02268	5-Hydroxy-	3	Interventio	Experimen	Experimen	Control	0,640356
3 months	abundance	X02277	tert-Butyl 3	3	Interventio	Experimen	Experimen	Control	0,235652
3 months	abundance	X02281	tert-Butyl 3	3	Interventio	Experimen	Experimen	Control	0,63113
3 months	abundance	X02288	9-Methylur	2b	Interventio	Experimen	Experimen	Control	0,392246
3 months	abundance	X02289	Kynurenic a	2a	Interventio	Experimen	Experimen	Control	0,824813
3 months	abundance	X02317	2-Aminooc	2b	Interventio	Experimen	Experimen	Control	0,876083
3 months	abundance	X02327	1H-Pyrazol	3	Interventio	Experimen	Experimen	Control	0,924699
3 months	abundance	X02333	N-Propiony	3	Interventio	Experimen	Experimen	Control	0,350171
3 months	abundance	X02337	Methyl 1-hy	3	Interventio	Experimen	Experimen	Control	0,698621
3 months	abundance	X02348	N-Benzoyla	3	Interventio	Experimen	Experimen	Control	0,63213
3 months	abundance	X02352	Indole-3-ca	3	Interventio	Experimen	Experimen	Control	0,251118
3 months	abundance	X02380	Propafenor	3	Interventio	Experimen	Experimen	Control	0,142786
3 months	abundance	X02419	Glu-Glu	3	Interventio	Experimen	Experimen	Control	0,980029
3 months	abundance	X02426	Raltitrexed	3	Interventio	Experimen	Experimen	Control	0,927159
3 months	abundance	X02427	2-(3,5-dim	2b	Interventio	Experimen	Experimen	Control	0,690566
3 months	abundance	X02442	3-(Sulfooxy	3	Interventio	Experimen	Experimen	Control	0,655638
3 months	abundance	X02466	Validamyci	3	Interventio	Experimen	Experimen	Control	0,59163
3 months	abundance	X02468	N,N-Diethy	3	Interventio	Experimen	Experimen	Control	0,819081
3 months	abundance	X02471	n-Propyl Ga	3	Interventio	Experimen	Experimen	Control	0,60023
3 months	abundance	X02492	N-(3,5-Dim	3	Interventio	Experimen	Experimen	Control	0,88099
3 months	abundance	X02494	Butylphtha	3	Interventio	Experimen	Experimen	Control	0,419167
3 months	abundance	X02504	DLK (Peptio	2b	Interventio	Experimen	Experimen	Control	0,854716
3 months	abundance	X02513	7-Methylac	2b	Interventio	Experimen	Experimen	Control	0,012524
3 months	abundance	X02514	3-Ureidopr	1	Interventio	Experimen	Experimen	Control	0,625098
3 months	abundance	X02521	6-(1-Hydro	3	Interventio	Experimen	Experimen	Control	0,837165
3 months	abundance	X02529	tenivastati	3	Interventio	Experimen	Experimen	Control	0,920024
3 months	abundance	X02537	Leu-pro_a	3	Interventio	Experimen	Experimen	Control	0,968857
3 months	abundance	X02565	Dopamine	3	Interventio	Experimen	Experimen	Control	0,680872
3 months	abundance	X02583	Ethylvanilli	3	Interventio	Experimen	Experimen	Control	0,276947

3 months	abundance	X02599	Dihydrothy	3	Interventio	Experimen	Experimen	Control		0,141328
3 months	abundance	X02601	(7E,7'E)-5,	3	Interventio	Experimen	Experimen	Control		0,15445
3 months	abundance	X02606	4-Amino-1	3	Interventio	Experimen	Experimen	Control		0,808747
3 months	abundance	X02615	6-APA_a	3	Interventio	Experimen	Experimen	Control		0,860527
3 months	abundance	X02640	thyronine	3	Interventio	Experimen	Experimen	Control		0,325689
3 months	abundance	X02651	L-gamma-(	3	Interventio	Experimen	Experimen	Control		0,466427
3 months	abundance	X02652	Leucyltyros	3	Interventio	Experimen	Experimen	Control		0,244161
3 months	abundance	X02661	4-(3-Oxope	3	Interventio	Experimen	Experimen	Control		0,988551
3 months	abundance	X02676	Val-Trp_a	3	Interventio	Experimen	Experimen	Control		0,949849
3 months	abundance	X02687	S-Propylcy	3	Interventio	Experimen	Experimen	Control		0,927509
3 months	abundance	X02689	piscidic ac	3	Interventio	Experimen	Experimen	Control		0,988551
3 months	abundance	X02702	R-(+)-Etira	3	Interventio	Experimen	Experimen	Control		0,639019
3 months	abundance	X02720	ala-ser_b	3	Interventio	Experimen	Experimen	Control		0,150157
3 months	abundance	X02730	N-Phenyla	3	Interventio	Experimen	Experimen	Control		0,048889
3 months	abundance	X02733	Calcitriol	3	Interventio	Experimen	Experimen	Control		0,860527
3 months	abundance	X02746	N,N-Diethy	3	Interventio	Experimen	Experimen	Control		0,177953
3 months	abundance	X02760	6-hydroxyp	3	Interventio	Experimen	Experimen	Control		4,83E-14
3 months	abundance	X02765	L-Pyrrolysi	3	Interventio	Experimen	Experimen	Control		0,997328
3 months	abundance	X02767	6-APA_b	3	Interventio	Experimen	Experimen	Control		0,181353
3 months	abundance	X02769	Tetraacety	3	Interventio	Experimen	Experimen	Control		0,517729
3 months	abundance	X02774	9-ribosylze	3	Interventio	Experimen	Experimen	Control		0,947336
3 months	abundance	X02793	3-Methoxy-	3	Interventio	Experimen	Experimen	Control		0,9752
3 months	abundance	X02820	FC250500	3	Interventio	Experimen	Experimen	Control		0,116825
3 months	abundance	X02823	6,8-Dimetl	3	Interventio	Experimen	Experimen	Control		0,960119
3 months	abundance	X02824	Eslicarbaz	3	Interventio	Experimen	Experimen	Control		0,834977
3 months	abundance	X02832	(+/-)-2-Hyc	3	Interventio	Experimen	Experimen	Control		0,765006
3 months	abundance	X02841	Kyotorphin	3	Interventio	Experimen	Experimen	Control		0,021942
3 months	abundance	X02843	4-(1-Hydro	3	Interventio	Experimen	Experimen	Control		0,631492
3 months	abundance	X02853	7alpha-Hy	3	Interventio	Experimen	Experimen	Control		0,350171
3 months	abundance	X02854	Marimasta	3	Interventio	Experimen	Experimen	Control		0,511237
3 months	abundance	X02868	2-(3-Hydro	3	Interventio	Experimen	Experimen	Control		0,729281
3 months	abundance	X02872	folinic acid	3	Interventio	Experimen	Experimen	Control		0,059894
3 months	abundance	X02878	N~6~-[5-(1	3	Interventio	Experimen	Experimen	Control		0,924699
3 months	abundance	X02881	N-(2,3,4-Tr	3	Interventio	Experimen	Experimen	Control		0,856619
3 months	abundance	X02894	Toluene_a	3	Interventio	Experimen	Experimen	Control		0,244993
3 months	abundance	X02905	MFCD0005	3	Interventio	Experimen	Experimen	Control		0,442109
3 months	abundance	X02920	L-gamma-(	3	Interventio	Experimen	Experimen	Control		0,552202
3 months	abundance	X02922	(2E)-3-Met	3	Interventio	Experimen	Experimen	Control		0,913384
3 months	abundance	X02928	N-Acetylpr	3	Interventio	Experimen	Experimen	Control		0,021942
3 months	abundance	X02943	3-Methoxy-	3	Interventio	Experimen	Experimen	Control		0,758687
3 months	abundance	X02944	Valylvaline	3	Interventio	Experimen	Experimen	Control		0,255035
3 months	abundance	X02948	N-(2-Cyan	3	Interventio	Experimen	Experimen	Control		0,570659
3 months	abundance	X02952	Vorinostat_	3	Interventio	Experimen	Experimen	Control		0,915997
3 months	abundance	X02968	Ro 20-172	3	Interventio	Experimen	Experimen	Control		0,052565
3 months	abundance	X02972	3-(Sulfoox	3	Interventio	Experimen	Experimen	Control		0,445572
3 months	abundance	X02986	Diacetin_b	3	Interventio	Experimen	Experimen	Control		0,505325
3 months	abundance	X02990	Pentoxifylli	3	Interventio	Experimen	Experimen	Control		0,002026
3 months	abundance	X03001	Pseudouric	2b	Interventio	Experimen	Experimen	Control		0,988551

3 months	abundance	X03004	Bicine_b	3	Interventio	Experimen	Experimen	Control	0,934681
3 months	abundance	X03017	Pro-tyr	3	Interventio	Experimen	Experimen	Control	0,94878
3 months	abundance	X03023	NPK (Pepti	2b	Interventio	Experimen	Experimen	Control	0,015774
3 months	abundance	X03064	Formimino	3	Interventio	Experimen	Experimen	Control	0,465566
3 months	abundance	X03069	N-COUMAI	3	Interventio	Experimen	Experimen	Control	0,033766
3 months	abundance	X03070	2-Methoxy-	3	Interventio	Experimen	Experimen	Control	0,801038
3 months	abundance	X03077	nicotianam	3	Interventio	Experimen	Experimen	Control	0,593214
3 months	abundance	X03097	6-hydroxyn	3	Interventio	Experimen	Experimen	Control	0,879562
3 months	abundance	X03109	Guanidino	3	Interventio	Experimen	Experimen	Control	0,988551
3 months	abundance	X03132	(3S,4S)-7,1	3	Interventio	Experimen	Experimen	Control	0,010133
3 months	abundance	X03134	Tetrahydro	3	Interventio	Experimen	Experimen	Control	0,949849
3 months	abundance	X03158	5-Hydroxy-	3	Interventio	Experimen	Experimen	Control	0,004863
3 months	abundance	X03223	1-(2-Carbo	3	Interventio	Experimen	Experimen	Control	0,449163
3 months	abundance	X03254	Gly-Trp_b	3	Interventio	Experimen	Experimen	Control	0,971877
3 months	abundance	X03268	Dihydrouri	3	Interventio	Experimen	Experimen	Control	0,953271
3 months	abundance	X03271	1Î²-Hydroxy	3	Interventio	Experimen	Experimen	Control	0,71541
3 months	abundance	X03275	Methdilazi	3	Interventio	Experimen	Experimen	Control	0,860527
3 months	abundance	X03276	2'-Deoxyac	3	Interventio	Experimen	Experimen	Control	0,577829
3 months	abundance	X03278	Hydroxyph	3	Interventio	Experimen	Experimen	Control	0,385347
3 months	abundance	X03294	4,4'-Thiobi	3	Interventio	Experimen	Experimen	Control	0,647023
3 months	abundance	X03340	His-pro_b	3	Interventio	Experimen	Experimen	Control	0,232653
3 months	abundance	X03343	L-alpha-As	3	Interventio	Experimen	Experimen	Control	0,380548
3 months	abundance	X03357	2-Hydroxyh	3	Interventio	Experimen	Experimen	Control	0,718544
3 months	abundance	X03363	Choline Alf	3	Interventio	Experimen	Experimen	Control	0,61536
3 months	abundance	X03376	4-(9H-beta	3	Interventio	Experimen	Experimen	Control	0,817974
3 months	abundance	X03413	Val-Trp_b	3	Interventio	Experimen	Experimen	Control	0,657878
3 months	abundance	X03416	9-(alpha-D	3	Interventio	Experimen	Experimen	Control	0,991184
3 months	abundance	X03434	SECONAL_	3	Interventio	Experimen	Experimen	Control	0,907192
3 months	abundance	X03480	epsilon-(ga	3	Interventio	Experimen	Experimen	Control	0,836327
3 months	abundance	X03488	3,4-Dihydr	3	Interventio	Experimen	Experimen	Control	0,997328
3 months	abundance	X03535	(1S,3R,4s)-	3	Interventio	Experimen	Experimen	Control	0,876028
3 months	abundance	X03552	4-Hydroxya	3	Interventio	Experimen	Experimen	Control	0,88099
3 months	abundance	X03563	6-imino-5-	3	Interventio	Experimen	Experimen	Control	0,244993
3 months	abundance	X03568	3-[(2Z)-1-C	3	Interventio	Experimen	Experimen	Control	0,808744
3 months	abundance	X03592	2-glyceryl 1	3	Interventio	Experimen	Experimen	Control	0,181353
3 months	abundance	X03595	Methylol D	3	Interventio	Experimen	Experimen	Control	0,758265
3 months	abundance	X03602	o-Succinyl	3	Interventio	Experimen	Experimen	Control	0,217561
3 months	abundance	X03604	Gly-Trp_a	3	Interventio	Experimen	Experimen	Control	1,36E-07
3 months	abundance	X03629	Glycylglycy	3	Interventio	Experimen	Experimen	Control	0,931973
3 months	abundance	X03660	trp-ser	3	Interventio	Experimen	Experimen	Control	0,949849
3 months	abundance	X03675	asp-gln_a	3	Interventio	Experimen	Experimen	Control	0,748275
3 months	abundance	X03704	N,N-Dimet	3	Interventio	Experimen	Experimen	Control	0,88099
3 months	abundance	X03707	9-[(5R)-5-E	3	Interventio	Experimen	Experimen	Control	0,019284
3 months	abundance	X03709	Guanfacine	3	Interventio	Experimen	Experimen	Control	0,907733
3 months	abundance	X03714	Sinapinic a	3	Interventio	Experimen	Experimen	Control	0,718544
3 months	abundance	X03718	Propanthel	3	Interventio	Experimen	Experimen	Control	0,808115
3 months	abundance	X03719	Losalen	3	Interventio	Experimen	Experimen	Control	0,970457
3 months	abundance	X03760	2-Methoxy-	3	Interventio	Experimen	Experimen	Control	0,834104

3 months	abundance	X03793	Desonide	3	Interventio	Experimen	Experimen	Control	6,33E-05
3 months	abundance	X03837	2-Methoxy-	3	Interventio	Experimen	Experimen	Control	0,448346
3 months	abundance	X03855	2-{(2S,4S)-	3	Interventio	Experimen	Experimen	Control	0,988551
3 months	abundance	X03860	2-BUTYL PI	3	Interventio	Experimen	Experimen	Control	0,968857
3 months	abundance	X03892	shinorine	3	Interventio	Experimen	Experimen	Control	0,988551
3 months	abundance	X03901	ferrileghem	3	Interventio	Experimen	Experimen	Control	0,899996
3 months	abundance	X03916	2-(1-Ethoxy	3	Interventio	Experimen	Experimen	Control	0,960119
3 months	abundance	X03917	Uramustine	3	Interventio	Experimen	Experimen	Control	0,232653
3 months	abundance	X03934	2-Aminom	3	Interventio	Experimen	Experimen	Control	0,845389
3 months	abundance	X03936	(3R)-2-(3,4	3	Interventio	Experimen	Experimen	Control	0,73207
3 months	abundance	X03961	Artesunate	3	Interventio	Experimen	Experimen	Control	0,001034
3 months	abundance	X04021	Bentazone	2b	Interventio	Experimen	Experimen	Control	1,31E-21
3 months	abundance	X04040	3-(3,4-dihy	3	Interventio	Experimen	Experimen	Control	0,98336
3 months	abundance	X04117	2,3,4,5-tet	3	Interventio	Experimen	Experimen	Control	0,604864
3 months	abundance	X04119	Tetraacety	3	Interventio	Experimen	Experimen	Control	0,349017
3 months	abundance	X04146	L-fucopyra	3	Interventio	Experimen	Experimen	Control	0,571812
3 months	abundance	X04150	4-(METHYL	3	Interventio	Experimen	Experimen	Control	0,61536
3 months	abundance	X04157	3-Methoxy-	3	Interventio	Experimen	Experimen	Control	0,741986
3 months	abundance	X04181	3-Methoxy-	3	Interventio	Experimen	Experimen	Control	0,83895
3 months	abundance	X04183	Spaglumic	3	Interventio	Experimen	Experimen	Control	0,99116
3 months	abundance	X04229	gamma-L-g	3	Interventio	Experimen	Experimen	Control	0,59787
3 months	abundance	X04259	3-Hydroxyt	3	Interventio	Experimen	Experimen	Control	0,646655
3 months	abundance	X04274	6-[(Z)-2-(3,	3	Interventio	Experimen	Experimen	Control	0,012053
3 months	abundance	X04286	Zinecard_b	3	Interventio	Experimen	Experimen	Control	0,761041
3 months	abundance	X04310	Aminohipp	3	Interventio	Experimen	Experimen	Control	0,141328
3 months	abundance	X04315	Benzamide	3	Interventio	Experimen	Experimen	Control	0,446773
3 months	abundance	X04334	3-Benzyl-6	3	Interventio	Experimen	Experimen	Control	0,73207
3 months	abundance	X04339	L-Glutamic	3	Interventio	Experimen	Experimen	Control	0,385109
3 months	abundance	X04351	Pyrimidine	3	Interventio	Experimen	Experimen	Control	0,949849
3 months	abundance	X04377	Dihydroura	3	Interventio	Experimen	Experimen	Control	0,582285
3 months	abundance	X04378	9,11-Dihyd	3	Interventio	Experimen	Experimen	Control	0,856135
3 months	abundance	X04379	2-Amino-6-	3	Interventio	Experimen	Experimen	Control	0,820266
3 months	abundance	X04440	Mono(3-ca	3	Interventio	Experimen	Experimen	Control	0,867373
3 months	abundance	X04450	3-Methylac	3	Interventio	Experimen	Experimen	Control	0,250532
3 months	abundance	X04483	(2E)-5-Hyd	3	Interventio	Experimen	Experimen	Control	0,582285
3 months	abundance	X04493	Tyrosol	3	Interventio	Experimen	Experimen	Control	0,722718
3 months	abundance	X04526	N-Nonanoy	3	Interventio	Experimen	Experimen	Control	0,198023
3 months	abundance	X04538	Valylvaline	3	Interventio	Experimen	Experimen	Control	0,833128
3 months	abundance	X04543	N-Acetyl-S	3	Interventio	Experimen	Experimen	Control	0,728208
3 months	abundance	X04544	L-gamma-G	3	Interventio	Experimen	Experimen	Control	0,995163
3 months	abundance	X04553	N-(4-Amino	3	Interventio	Experimen	Experimen	Control	0,960119
3 months	abundance	X04557	1,4-Naphth	3	Interventio	Experimen	Experimen	Control	0,431024
3 months	abundance	X04562	Lisdexamfe	3	Interventio	Experimen	Experimen	Control	0,958777
3 months	abundance	X04564	asp-gln_b	3	Interventio	Experimen	Experimen	Control	0,625098
3 months	abundance	X04566	pterin	3	Interventio	Experimen	Experimen	Control	0,840004
3 months	abundance	X04571	13a-Hydrox	3	Interventio	Experimen	Experimen	Control	0,639019
3 months	abundance	X04579	6-(alpha-D	3	Interventio	Experimen	Experimen	Control	0,861311
3 months	abundance	X04582	Dinoseb	3	Interventio	Experimen	Experimen	Control	0,46054

3 months	abundance	X04593	Methional	3	Interventio	Experimen	Experimen	Control	0,619252
3 months	abundance	X04636	epsilon-(ga	3	Interventio	Experimen	Experimen	Control	0,725339
3 months	abundance	X04639	hydroxyhex	3	Interventio	Experimen	Experimen	Control	0,124404
3 months	abundance	X04682	1-Methylin	3	Interventio	Experimen	Experimen	Control	0,282328
3 months	abundance	X04684	AAMU_b	3	Interventio	Experimen	Experimen	Control	0,862952
3 months	abundance	X04688	Minoxidil	3	Interventio	Experimen	Experimen	Control	0,393572
3 months	abundance	X04695	N-[(4-Meth	3	Interventio	Experimen	Experimen	Control	0,718199
3 months	abundance	X04697	Ethosuxim	3	Interventio	Experimen	Experimen	Control	0,820266
3 months	abundance	X04743	5-Methoxy-	3	Interventio	Experimen	Experimen	Control	0,798841
3 months	abundance	X04744	16alpha-hy	3	Interventio	Experimen	Experimen	Control	0,948674
3 months	abundance	X04759	7-Aminome	3	Interventio	Experimen	Experimen	Control	0,960119
3 months	abundance	X04788	2-(4-Isopro	3	Interventio	Experimen	Experimen	Control	0,446309
3 months	abundance	X04792	his-asn	3	Interventio	Experimen	Experimen	Control	0,203782
3 months	abundance	X04814	meprobam	3	Interventio	Experimen	Experimen	Control	0,927159
3 months	abundance	X04822	6-Sulfatoxy	3	Interventio	Experimen	Experimen	Control	0,141328
3 months	abundance	X04852	Toluene_b	3	Interventio	Experimen	Experimen	Control	0,058295
3 months	abundance	X04877	Trifluorome	3	Interventio	Experimen	Experimen	Control	0,216079
3 months	abundance	X04897	Taxifolin	3	Interventio	Experimen	Experimen	Control	0,082572
3 months	abundance	X04898	17-Hydroxy	3	Interventio	Experimen	Experimen	Control	0,491513
3 months	abundance	X04919	5-O-alpha-	3	Interventio	Experimen	Experimen	Control	0,646655
3 months	abundance	X04922	Sinapinic a	3	Interventio	Experimen	Experimen	Control	0,830535
3 months	abundance	X04939	N-Desalkyl	3	Interventio	Experimen	Experimen	Control	0,522871
3 months	abundance	X04975	MFCD0995	3	Interventio	Experimen	Experimen	Control	0,639019
3 months	abundance	X04986	L-Proline, 4	3	Interventio	Experimen	Experimen	Control	0,642236
3 months	abundance	X04990	Cilazapril	3	Interventio	Experimen	Experimen	Control	0,282368
3 months	abundance	X04996	MFCD0272	3	Interventio	Experimen	Experimen	Control	0,807551
3 months	abundance	X04999	2-Hydroxyp	1	Interventio	Experimen	Experimen	Control	0,819081
3 months	abundance	X05017	N-(1-{[Metl	3	Interventio	Experimen	Experimen	Control	0,078386
3 months	abundance	X05029	Hypericin	3	Interventio	Experimen	Experimen	Control	0,083526
3 months	abundance	X05033	(2E)-N-3,7-	3	Interventio	Experimen	Experimen	Control	1,92E-05
3 months	abundance	X05046	Casimiroin	3	Interventio	Experimen	Experimen	Control	0,960119
3 months	abundance	X05068	pro-gln_c	3	Interventio	Experimen	Experimen	Control	0,141328
3 months	abundance	X05071	Histidylphe	3	Interventio	Experimen	Experimen	Control	0,613297
3 months	abundance	X05072	L-gamma-(	3	Interventio	Experimen	Experimen	Control	0,113776
3 months	abundance	X05081	ELK (Peptic	2b	Interventio	Experimen	Experimen	Control	0,322462
3 months	abundance	X05126	(3aS,5S,6F	3	Interventio	Experimen	Experimen	Control	0,008795
3 months	abundance	X05146	UQ367500	3	Interventio	Experimen	Experimen	Control	0,949849
3 months	abundance	X05149	S-Sulfocys	3	Interventio	Experimen	Experimen	Control	0,907192
3 months	abundance	X05177	cis-3-Hexe	3	Interventio	Experimen	Experimen	Control	0,833128
3 months	abundance	X05183	3,6-Dichlo	3	Interventio	Experimen	Experimen	Control	0,778801
3 months	abundance	X05195	Lys-phe_a	3	Interventio	Experimen	Experimen	Control	0,651289
3 months	abundance	X05203	felbamate	3	Interventio	Experimen	Experimen	Control	0,820266
3 months	abundance	X05204	Urothion	3	Interventio	Experimen	Experimen	Control	0,082572
3 months	abundance	X05214	[7-Hydroxy	3	Interventio	Experimen	Experimen	Control	0,423696
3 months	abundance	X05225	4-(METHYL	3	Interventio	Experimen	Experimen	Control	0,503169
3 months	abundance	X05234	3,4-dihydro	3	Interventio	Experimen	Experimen	Control	0,988198
3 months	abundance	X05237	imazameth	3	Interventio	Experimen	Experimen	Control	0,985356
3 months	abundance	X05240	Dimeric mo	3	Interventio	Experimen	Experimen	Control	0,854716

3 months	abundance	X05243	N-(4-Hydro	3	Interventio	Experimen	Experimen	Control	0,971921
3 months	abundance	X05275	H-DL-MET-	3	Interventio	Experimen	Experimen	Control	0,042091
3 months	abundance	X05295	3-(4,7-Dim	3	Interventio	Experimen	Experimen	Control	0,78879
3 months	abundance	X05306	2-methoxy	3	Interventio	Experimen	Experimen	Control	0,63213
3 months	abundance	X05357	NPYR	3	Interventio	Experimen	Experimen	Control	0,193817
3 months	abundance	X05391	2-Acetami	3	Interventio	Experimen	Experimen	Control	0,206211
3 months	abundance	X05398	DIBOA	3	Interventio	Experimen	Experimen	Control	0,424219
3 months	abundance	X05411	3-(Sulfoox	3	Interventio	Experimen	Experimen	Control	0,88266
3 months	abundance	X05415	Redul	3	Interventio	Experimen	Experimen	Control	0,923057
3 months	abundance	X05417	(S)-2-hydra	3	Interventio	Experimen	Experimen	Control	0,143745
3 months	abundance	X05421	S-Allylcyst	3	Interventio	Experimen	Experimen	Control	0,953271
3 months	abundance	X05459	Scopoletin	3	Interventio	Experimen	Experimen	Control	0,142786
3 months	abundance	X05467	Azulfidine	3	Interventio	Experimen	Experimen	Control	0,333178
3 months	abundance	X05478	S(8)-amino	3	Interventio	Experimen	Experimen	Control	0,385347
3 months	abundance	X05499	GLY-MET	3	Interventio	Experimen	Experimen	Control	0,000124
3 months	abundance	X05510	3-Benzyl-6	3	Interventio	Experimen	Experimen	Control	0,988551
3 months	abundance	X05514	(3aS,5S,6F	3	Interventio	Experimen	Experimen	Control	0,936365
3 months	abundance	X05551	Trolox	3	Interventio	Experimen	Experimen	Control	0,474449
3 months	abundance	X05561	1Î²-Hydrox	3	Interventio	Experimen	Experimen	Control	0,956306
3 months	abundance	X05580	Xanthureni	3	Interventio	Experimen	Experimen	Control	0,948674
3 months	abundance	X05581	N-[(10Z)-7-	3	Interventio	Experimen	Experimen	Control	0,656167
3 months	abundance	X05584	Serotonin	1	Interventio	Experimen	Experimen	Control	0,815986
3 months	abundance	X05656	mesifurane	3	Interventio	Experimen	Experimen	Control	0,947758
3 months	abundance	X05709	2,3-Dihydr	3	Interventio	Experimen	Experimen	Control	0,915552
3 months	abundance	X05726	methocarb	3	Interventio	Experimen	Experimen	Control	0,835202
3 months	abundance	X05734	N-[(2S)-2-I	3	Interventio	Experimen	Experimen	Control	0,773264
3 months	abundance	X05741	g-Aminobu	3	Interventio	Experimen	Experimen	Control	0,001817
3 months	abundance	X05758	quinol sulf	3	Interventio	Experimen	Experimen	Control	0,60023
3 months	abundance	X05786	Nitrendipir	3	Interventio	Experimen	Experimen	Control	0,988551
3 months	abundance	X05812	(2S)-3-(1H	3	Interventio	Experimen	Experimen	Control	0,864576
3 months	abundance	X05857	N-D-Gluco	3	Interventio	Experimen	Experimen	Control	0,217561
3 months	abundance	X05859	3,7,12,17-	3	Interventio	Experimen	Experimen	Control	0,960119
3 months	abundance	X05878	1,2-dihydr	3	Interventio	Experimen	Experimen	Control	0,034991
3 months	abundance	X05892	(5Z)-2-Ami	3	Interventio	Experimen	Experimen	Control	0,821708
3 months	abundance	X05919	2-(2-Amino	3	Interventio	Experimen	Experimen	Control	0,651767
3 months	abundance	X05968	Tetraacety	3	Interventio	Experimen	Experimen	Control	0,631492
3 months	abundance	X05969	4-(METHYL	3	Interventio	Experimen	Experimen	Control	0,820266
3 months	abundance	X05971	L-gamma-(	3	Interventio	Experimen	Experimen	Control	0,681178
3 months	abundance	X05982	(19R,25S)-	3	Interventio	Experimen	Experimen	Control	0,10419
3 months	abundance	X05984	1-(4-Methy	2b	Interventio	Experimen	Experimen	Control	0,820408
3 months	abundance	X05988	Selsun	3	Interventio	Experimen	Experimen	Control	0,179615
3 months	abundance	X05995	Diacetin_a	3	Interventio	Experimen	Experimen	Control	0,234997
3 months	abundance	X05997	FB950000(	3	Interventio	Experimen	Experimen	Control	0,287585
3 months	abundance	X06011	Coprine_a	3	Interventio	Experimen	Experimen	Control	0,392246
3 months	abundance	X06016	N,N-Dimet	3	Interventio	Experimen	Experimen	Control	0,161121
3 months	abundance	X06034	N-[(2S)-2-I	3	Interventio	Experimen	Experimen	Control	0,59787
3 months	abundance	X06039	gamma-Gl	3	Interventio	Experimen	Experimen	Control	1,83E-11
3 months	abundance	X06041	(DL)-3-O-M	3	Interventio	Experimen	Experimen	Control	0,798841

3 months	abundance	X06043	vinyl sulfid	3	Interventio	Experimen	Experimen	Control	0,22934
3 months	abundance	X06046	Valylvaline	3	Interventio	Experimen	Experimen	Control	0,000496
3 months	abundance	X06062	1-(2,3-Dihy	3	Interventio	Experimen	Experimen	Control	0,957479
3 months	abundance	X06079	Glycylleuci	1	Interventio	Experimen	Experimen	Control	0,627129
3 months	abundance	X06082	O-heptano	3	Interventio	Experimen	Experimen	Control	0,830535
3 months	abundance	X06085	Tetraacety	3	Interventio	Experimen	Experimen	Control	0,992999
3 months	abundance	X06089	riboprine	3	Interventio	Experimen	Experimen	Control	0,820408
3 months	abundance	X06100	Midodrine_	3	Interventio	Experimen	Experimen	Control	0,927509
3 months	abundance	X06107	Hydroxycai	3	Interventio	Experimen	Experimen	Control	0,787558
3 months	abundance	X06112	Val-Ser_a	3	Interventio	Experimen	Experimen	Control	0,724599
3 months	abundance	X06124	MFCD0995	3	Interventio	Experimen	Experimen	Control	0,860527
3 months	abundance	X06127	2-Methoxy-	3	Interventio	Experimen	Experimen	Control	0,625098
3 months	abundance	X06143	2-Methylbu	2a	Interventio	Experimen	Experimen	Control	0,938964
3 months	abundance	X06146	S-Methyl-1	3	Interventio	Experimen	Experimen	Control	0,823267
3 months	abundance	X06149	Yangonin	3	Interventio	Experimen	Experimen	Control	0,034991
3 months	abundance	X06150	Zalcitabine	3	Interventio	Experimen	Experimen	Control	0,707386
3 months	abundance	X06152	alpha-keto	3	Interventio	Experimen	Experimen	Control	0,605613
3 months	abundance	X06167	N-Acetylas	2a	Interventio	Experimen	Experimen	Control	0,198023
3 months	abundance	X06168	leu-gln_b	3	Interventio	Experimen	Experimen	Control	0,988551
3 months	abundance	X06183	Nisinic aci	3	Interventio	Experimen	Experimen	Control	0,718544
3 months	abundance	X06189	delta-Guar	3	Interventio	Experimen	Experimen	Control	0,008037
3 months	abundance	X06197	4-(Nitrosoa	3	Interventio	Experimen	Experimen	Control	0,853633
3 months	abundance	X06220	Tetrahydro	3	Interventio	Experimen	Experimen	Control	0,907192
3 months	abundance	X06222	Leucylaspa	3	Interventio	Experimen	Experimen	Control	0,739004
3 months	abundance	X06227	lys-tyr_a	3	Interventio	Experimen	Experimen	Control	0,698621
3 months	abundance	X06230	Glu-Gly	3	Interventio	Experimen	Experimen	Control	0,315213
3 months	abundance	X06240	His-pro_a	3	Interventio	Experimen	Experimen	Control	0,105225
3 months	abundance	X06250	[3-({3-[(Cy	2b	Interventio	Experimen	Experimen	Control	0,10419
3 months	abundance	X06253	N-(4-Amin	3	Interventio	Experimen	Experimen	Control	0,718544
3 months	abundance	X06254	D-Alanyl-D	3	Interventio	Experimen	Experimen	Control	0,113776
3 months	abundance	X06259	(S)-?-glyce	3	Interventio	Experimen	Experimen	Control	0,836327
3 months	abundance	X06268	mesifurane	3	Interventio	Experimen	Experimen	Control	0,985356
3 months	abundance	X06270	9-Methylur	2b	Interventio	Experimen	Experimen	Control	0,625098
3 months	abundance	X06276	asn-pro_b	3	Interventio	Experimen	Experimen	Control	0,431744
3 months	abundance	X06278	3-Hydroxy-	3	Interventio	Experimen	Experimen	Control	0,718544
3 months	abundance	X06289	Nicotinatel	3	Interventio	Experimen	Experimen	Control	0,08584
3 months	abundance	X06291	Corticoste	2b	Interventio	Experimen	Experimen	Control	0,803437
3 months	abundance	X06292	mesifurane	3	Interventio	Experimen	Experimen	Control	0,990649
3 months	abundance	X06293	(4R)-4-{[3-	3	Interventio	Experimen	Experimen	Control	0,250575
3 months	abundance	X06313	Isoquinolir	2b	Interventio	Experimen	Experimen	Control	0,881276
3 months	abundance	X06318	Propamoca	2b	Interventio	Experimen	Experimen	Control	0,819701
3 months	abundance	X06320	7alpha-Hy	3	Interventio	Experimen	Experimen	Control	0,876495
3 months	abundance	X06329	Leu-Leu_d	3	Interventio	Experimen	Experimen	Control	0,394274
3 months	abundance	X06334	Bis-D-fruct	3	Interventio	Experimen	Experimen	Control	0,980029
3 months	abundance	X06337	Zalcitabine	3	Interventio	Experimen	Experimen	Control	0,387209
3 months	abundance	X06354	trans-Zeati	2b	Interventio	Experimen	Experimen	Control	0,449163
3 months	abundance	X06361	Oxprenolol	3	Interventio	Experimen	Experimen	Control	0,124383
3 months	abundance	X06368	TDP-2_b	3	Interventio	Experimen	Experimen	Control	0,562487

3 months	abundance	X06371	L-gamma-(	3	Interventio	Experimen	Experimen	Control	0,909315
3 months	abundance	X06372	N,N-dimetl	2b	Interventio	Experimen	Experimen	Control	0,089222
3 months	abundance	X06381	MFCD1869	3	Interventio	Experimen	Experimen	Control	0,99116
3 months	abundance	X06388	Caffeic aci	1	Interventio	Experimen	Experimen	Control	0,71076
3 months	abundance	X06400	Phloionolic	3	Interventio	Experimen	Experimen	Control	0,819701
3 months	abundance	X06401	Isophthalic	2b	Interventio	Experimen	Experimen	Control	0,817715
3 months	abundance	X06404	Flemichap	3	Interventio	Experimen	Experimen	Control	0,210209
3 months	abundance	X06405	Asparaginy	3	Interventio	Experimen	Experimen	Control	0,322462
3 months	abundance	X06409	Glycylproli	2b	Interventio	Experimen	Experimen	Control	0,490686
3 months	abundance	X06412	Butenylcar	3	Interventio	Experimen	Experimen	Control	0,991184
3 months	abundance	X06416	KYNURAMI	3	Interventio	Experimen	Experimen	Control	0,960119
3 months	abundance	X06419	Spermic ac	3	Interventio	Experimen	Experimen	Control	0,988551
3 months	abundance	X06433	4-(2,5-Diflu	2b	Interventio	Experimen	Experimen	Control	0,935128
3 months	abundance	X06434	butyrin	3	Interventio	Experimen	Experimen	Control	0,860304
3 months	abundance	X06437	2-Acetami	3	Interventio	Experimen	Experimen	Control	0,336689
3 months	abundance	X06448	3,8,9-trihy	2b	Interventio	Experimen	Experimen	Control	0,761478
3 months	abundance	X06454	8-Amino-7	3	Interventio	Experimen	Experimen	Control	0,284646
3 months	abundance	X06455	4-Hydroxyp	3	Interventio	Experimen	Experimen	Control	0,63213
3 months	abundance	X06462	DNOP_d	3	Interventio	Experimen	Experimen	Control	0,605613
3 months	abundance	X06463	8-Amino-7	3	Interventio	Experimen	Experimen	Control	0,696041
3 months	abundance	X06472	N(alpha)-B	3	Interventio	Experimen	Experimen	Control	0,927509
3 months	abundance	X06473	MFCD0002	3	Interventio	Experimen	Experimen	Control	0,967096
3 months	abundance	X06477	APM_b	3	Interventio	Experimen	Experimen	Control	0,303703
3 months	abundance	X06478	Caffeic aci	3	Interventio	Experimen	Experimen	Control	0,860527
3 months	abundance	X06482	glu-pro	3	Interventio	Experimen	Experimen	Control	0,967096
3 months	abundance	X06486	Leu-Val_b	3	Interventio	Experimen	Experimen	Control	0,096259
3 months	abundance	X06495	2-Acetami	3	Interventio	Experimen	Experimen	Control	0,633187
3 months	abundance	X06502	MFCD2836	3	Interventio	Experimen	Experimen	Control	0,665073
3 months	abundance	X06506	L-Homocys	3	Interventio	Experimen	Experimen	Control	0,758687
3 months	abundance	X06507	Gly-Lys	3	Interventio	Experimen	Experimen	Control	0,226642
3 months	abundance	X06508	Asparaginy	3	Interventio	Experimen	Experimen	Control	0,475833
3 months	abundance	X06510	N-{4-[(2R,3	2b	Interventio	Experimen	Experimen	Control	0,235092
3 months	abundance	X06529	Methanesu	2a	Interventio	Experimen	Experimen	Control	0,862952
3 months	abundance	X06530	3-(1-hydro:	2b	Interventio	Experimen	Experimen	Control	0,998514
3 months	abundance	X06532	Leupeptin	3	Interventio	Experimen	Experimen	Control	0,000266
3 months	abundance	X06546	4-Acetami	2b	Interventio	Experimen	Experimen	Control	0,106066
3 months	abundance	X06549	Panthenol_	3	Interventio	Experimen	Experimen	Control	0,853605
3 months	abundance	X06551	Methyl 1-hy	3	Interventio	Experimen	Experimen	Control	0,988551
3 months	abundance	X06560	2,6-Dimeth	2b	Interventio	Experimen	Experimen	Control	0,150647
3 months	abundance	X06590	3-hydroxy-:	3	Interventio	Experimen	Experimen	Control	0,303703
3 months	abundance	X06600	MFCD0014	3	Interventio	Experimen	Experimen	Control	0,08584
3 months	abundance	X06606	Leu-Val_d	3	Interventio	Experimen	Experimen	Control	0,503169
3 months	abundance	X06612	Spermic ac	3	Interventio	Experimen	Experimen	Control	0,77242
3 months	abundance	X06622	Lys-Pro_b	3	Interventio	Experimen	Experimen	Control	0,152323
3 months	abundance	X06623	quinol sulf	3	Interventio	Experimen	Experimen	Control	0,329628
3 months	abundance	X06626	N-Butyryl-L	3	Interventio	Experimen	Experimen	Control	0,953271
3 months	abundance	X06630	4-(METHYL	3	Interventio	Experimen	Experimen	Control	0,187996
3 months	abundance	X06631	2-Isopropy	2b	Interventio	Experimen	Experimen	Control	0,136002

3 months	abundance	X06634	Glycitein	3	Interventio	Experimen	Experimen	Control	0,143819
3 months	abundance	X06642	Homocitru	2a	Interventio	Experimen	Experimen	Control	0,798192
3 months	abundance	X06646	(S)-3-sulfo	3	Interventio	Experimen	Experimen	Control	0,385799
3 months	abundance	X06655	Hydroxyca	3	Interventio	Experimen	Experimen	Control	0,654003
3 months	abundance	X06656	Valylvaline	3	Interventio	Experimen	Experimen	Control	0,84521
3 months	abundance	X06663	Leucyltyro	3	Interventio	Experimen	Experimen	Control	0,898308
3 months	abundance	X06675	L-gamma-	3	Interventio	Experimen	Experimen	Control	0,430971
3 months	abundance	X06680	7-Hydroxy-	2b	Interventio	Experimen	Experimen	Control	0,270117
3 months	abundance	X06681	Scymnol	3	Interventio	Experimen	Experimen	Control	0,249675
3 months	abundance	X06684	Bromazine	3	Interventio	Experimen	Experimen	Control	0,854716
3 months	abundance	X06690	4-Methylca	2b	Interventio	Experimen	Experimen	Control	0,860527
3 months	abundance	X06698	entecavir	3	Interventio	Experimen	Experimen	Control	0,997937
3 months	abundance	X06703	N6-METHY	3	Interventio	Experimen	Experimen	Control	0,60023
3 months	abundance	X06704	feruloylagr	3	Interventio	Experimen	Experimen	Control	0,552202
3 months	abundance	X06709	Tetraacety	3	Interventio	Experimen	Experimen	Control	0,798192
3 months	abundance	X06722	LW800000	3	Interventio	Experimen	Experimen	Control	0,146246
3 months	abundance	X06723	3-Hydroxy-	3	Interventio	Experimen	Experimen	Control	0,836327
3 months	abundance	X06727	MFCD0087	3	Interventio	Experimen	Experimen	Control	0,854716
3 months	abundance	X06735	7-Methylxa	2b	Interventio	Experimen	Experimen	Control	0,198011
3 months	abundance	X06744	Diethylpyr	3	Interventio	Experimen	Experimen	Control	0,150298
3 months	abundance	X06750	1-PYRENYL	3	Interventio	Experimen	Experimen	Control	0,835202
3 months	abundance	X06764	N-(4-Hydro	3	Interventio	Experimen	Experimen	Control	0,71541
3 months	abundance	X06767	2-Hydroxyb	1	Interventio	Experimen	Experimen	Control	0,750158
3 months	abundance	X06768	Sinapyl alc	3	Interventio	Experimen	Experimen	Control	0,413227
3 months	abundance	X06771	MFCD0015	3	Interventio	Experimen	Experimen	Control	0,198023
3 months	abundance	X06783	N~6~-Octa	3	Interventio	Experimen	Experimen	Control	0,385109
3 months	abundance	X06797	pro-gln_a	3	Interventio	Experimen	Experimen	Control	0,718544
3 months	abundance	X06805	(9cis)-O~1	3	Interventio	Experimen	Experimen	Control	0,698241
3 months	abundance	X06807	1,1'-[1,12-	3	Interventio	Experimen	Experimen	Control	0,413564
3 months	abundance	X06812	2-Hydroxy-	3	Interventio	Experimen	Experimen	Control	0,182946
3 months	abundance	X06814	N-Benzoyla	3	Interventio	Experimen	Experimen	Control	0,748275
3 months	abundance	X06818	Homoanse	3	Interventio	Experimen	Experimen	Control	0,025414
3 months	abundance	X06819	Primaquine	3	Interventio	Experimen	Experimen	Control	0,004762
3 months	abundance	X06820	1-(3,4-dim	2b	Interventio	Experimen	Experimen	Control	0,993797
3 months	abundance	X06836	asn-lys	3	Interventio	Experimen	Experimen	Control	0,194947
3 months	abundance	X06850	Leu-Val_c	3	Interventio	Experimen	Experimen	Control	0,01685
3 months	abundance	X06856	Tiglic acid_	2b	Interventio	Experimen	Experimen	Control	0,440554
3 months	abundance	X06858	tert-Butyl 3	3	Interventio	Experimen	Experimen	Control	0,056989
3 months	abundance	X06859	Methylol D	3	Interventio	Experimen	Experimen	Control	0,697678
3 months	abundance	X06861	4-(4-Deoxy	3	Interventio	Experimen	Experimen	Control	0,894218
3 months	abundance	X06866	tert-Butyl 3	3	Interventio	Experimen	Experimen	Control	0,043633
3 months	abundance	X06870	Menadiol	3	Interventio	Experimen	Experimen	Control	0,206211
3 months	abundance	X06874	N-Acetyl-5	3	Interventio	Experimen	Experimen	Control	0,119178
3 months	abundance	X06875	Nicotine gl	3	Interventio	Experimen	Experimen	Control	0,909234
3 months	abundance	X06880	pentobarbi	3	Interventio	Experimen	Experimen	Control	0,029867
3 months	abundance	X06883	Agomelatir	3	Interventio	Experimen	Experimen	Control	0,948674
3 months	abundance	X06889	3,4-Dimetl	2a	Interventio	Experimen	Experimen	Control	0,968857
3 months	abundance	X06896	Pirbuterol	3	Interventio	Experimen	Experimen	Control	0,642549

3 months	abundance	X06902	Homocyste	3	Interventio	Experimen	Experimen	Control	0,867667
3 months	abundance	X06903	Esculin	2b	Interventio	Experimen	Experimen	Control	0,515877
3 months	abundance	X06905	Ectoine	3	Interventio	Experimen	Experimen	Control	0,997328
3 months	abundance	X06906	Arg-pro	3	Interventio	Experimen	Experimen	Control	0,503169
3 months	abundance	X06917	Alanyltrypt	3	Interventio	Experimen	Experimen	Control	0,131627
3 months	abundance	X06933	3-Methoxy-	3	Interventio	Experimen	Experimen	Control	0,968857
3 months	abundance	X06943	NSC 9277	3	Interventio	Experimen	Experimen	Control	0,404129
3 months	abundance	X06946	SECONAL_	3	Interventio	Experimen	Experimen	Control	0,58814
3 months	abundance	X06948	(1R,3R,5R)	3	Interventio	Experimen	Experimen	Control	0,86519
3 months	abundance	X06958	2-[(carboxy	2b	Interventio	Experimen	Experimen	Control	0,000193
3 months	abundance	X06961	Zalcitabine	3	Interventio	Experimen	Experimen	Control	0,892548
3 months	abundance	X06977	4-Hydroxyp	3	Interventio	Experimen	Experimen	Control	0,988551
3 months	abundance	X06985	N-Ethylpro	3	Interventio	Experimen	Experimen	Control	0,116825
3 months	abundance	X07002	Paraldehyc	3	Interventio	Experimen	Experimen	Control	0,980029
3 months	abundance	X07013	pimethixer	3	Interventio	Experimen	Experimen	Control	0,21959
3 months	abundance	X07014	Octyl benz	3	Interventio	Experimen	Experimen	Control	0,821708
3 months	abundance	X07026	7α-Hydroxy	2b	Interventio	Experimen	Experimen	Control	0,820408
3 months	abundance	X07027	Allyl merca	3	Interventio	Experimen	Experimen	Control	0,886266
3 months	abundance	X07028	N-Pentano	3	Interventio	Experimen	Experimen	Control	0,947336
3 months	abundance	X07038	Piperidine_	3	Interventio	Experimen	Experimen	Control	0,207459
3 months	abundance	X07040	6-Hydroxyp	3	Interventio	Experimen	Experimen	Control	0,988551
3 months	abundance	X07050	2-Methylth	3	Interventio	Experimen	Experimen	Control	0,22934
3 months	abundance	X07051	O-succinyl	3	Interventio	Experimen	Experimen	Control	0,655638
3 months	abundance	X07054	Valylprolin	2b	Interventio	Experimen	Experimen	Control	0,223785
3 months	abundance	X07057	Leu-Leu_c	3	Interventio	Experimen	Experimen	Control	0,000943
3 months	abundance	X07073	asn-val_a	3	Interventio	Experimen	Experimen	Control	0,468958
3 months	abundance	X07077	Hyodeoxyc	1	Interventio	Experimen	Experimen	Control	0,314021
3 months	abundance	X07079	N-Pentano	3	Interventio	Experimen	Experimen	Control	0,854716
3 months	abundance	X07081	N-LACTOYI	3	Interventio	Experimen	Experimen	Control	0,274678
3 months	abundance	X07084	heptabarbi	3	Interventio	Experimen	Experimen	Control	0,867667
3 months	abundance	X07089	Pregabalin	3	Interventio	Experimen	Experimen	Control	0,992999
3 months	abundance	X07092	Dibutyl ma	3	Interventio	Experimen	Experimen	Control	0,541151
3 months	abundance	X07107	Lysylvaline	3	Interventio	Experimen	Experimen	Control	0,004656
3 months	abundance	X07112	MFCD0272	3	Interventio	Experimen	Experimen	Control	0,270117
3 months	abundance	X07113	Sulfoaceta	3	Interventio	Experimen	Experimen	Control	0,685901
3 months	abundance	X07125	Zalcitabine	3	Interventio	Experimen	Experimen	Control	0,823267
3 months	abundance	X07126	Leu-Val_f	3	Interventio	Experimen	Experimen	Control	0,124404
3 months	abundance	X07127	N~5~-[P-Ar	3	Interventio	Experimen	Experimen	Control	0,997328
3 months	abundance	X07134	4-Phenolsu	2b	Interventio	Experimen	Experimen	Control	0,44396
3 months	abundance	X07136	hexobarbit	3	Interventio	Experimen	Experimen	Control	0,972998
3 months	abundance	X07139	N,N-Diethy	3	Interventio	Experimen	Experimen	Control	0,232653
3 months	abundance	X07146	meprobam	3	Interventio	Experimen	Experimen	Control	0,988551
3 months	abundance	X07155	Naphthale	3	Interventio	Experimen	Experimen	Control	0,952532
3 months	abundance	X07164	N-Acetyl-5	3	Interventio	Experimen	Experimen	Control	0,024021
3 months	abundance	X07165	pro-gln_b	3	Interventio	Experimen	Experimen	Control	0,613297
3 months	abundance	X07168	L-(+)-Eryth	3	Interventio	Experimen	Experimen	Control	0,172075
3 months	abundance	X07193	N-(1-Methy	3	Interventio	Experimen	Experimen	Control	0,651767
3 months	abundance	X07201	2,4-Quinol	2b	Interventio	Experimen	Experimen	Control	0,530275

3 months	abundance	X07216	pentobarbi	3	Interventio	Experimen	Experimen	Control	0,988551
3 months	abundance	X07219	3,3-Dimeth	2b	Interventio	Experimen	Experimen	Control	0,808747
3 months	abundance	X07220	Glycylproly	3	Interventio	Experimen	Experimen	Control	0,836327
3 months	abundance	X07226	threonylph	3	Interventio	Experimen	Experimen	Control	0,824533
3 months	abundance	X07236	2-methylci	3	Interventio	Experimen	Experimen	Control	0,988551
3 months	abundance	X07250	Leucylaspa	3	Interventio	Experimen	Experimen	Control	0,988551
3 months	abundance	X07260	(7E,7'E)-5,	3	Interventio	Experimen	Experimen	Control	0,748275
3 months	abundance	X07263	3,3-Dimeth	2b	Interventio	Experimen	Experimen	Control	0,798192
3 months	abundance	X07269	(3aR,4R,5R	3	Interventio	Experimen	Experimen	Control	0,433847
3 months	abundance	X07270	5-(2-Carbo	3	Interventio	Experimen	Experimen	Control	0,149754
3 months	abundance	X07278	5-Phospho	3	Interventio	Experimen	Experimen	Control	0,631492
3 months	abundance	X07283	8-Hydroxyh	3	Interventio	Experimen	Experimen	Control	0,102756
3 months	abundance	X07285	2-(Carboxy	3	Interventio	Experimen	Experimen	Control	0,141328
3 months	abundance	X07294	6-Acetami	3	Interventio	Experimen	Experimen	Control	0,325689
3 months	abundance	X07303	Triacetin	3	Interventio	Experimen	Experimen	Control	0,317237
3 months	abundance	X07308	Nicotinic a	2a	Interventio	Experimen	Experimen	Control	0,409881
3 months	abundance	X07309	3,4,15-Trih	3	Interventio	Experimen	Experimen	Control	0,633187
3 months	abundance	X07315	Rivastigmi	3	Interventio	Experimen	Experimen	Control	0,384971
3 months	abundance	X07320	asn-pro_d	3	Interventio	Experimen	Experimen	Control	0,456954
3 months	abundance	X07327	Aspartyl-L-	3	Interventio	Experimen	Experimen	Control	0,854716
3 months	abundance	X07334	2,2-Bis(hyd	3	Interventio	Experimen	Experimen	Control	0,324406
3 months	abundance	X07336	Threonylse	3	Interventio	Experimen	Experimen	Control	0,830535
3 months	abundance	X07344	(−)-nabilor	3	Interventio	Experimen	Experimen	Control	0,755448
3 months	abundance	X07345	Phenyl D-g	3	Interventio	Experimen	Experimen	Control	0,661083
3 months	abundance	X07375	porphobili	3	Interventio	Experimen	Experimen	Control	0,898308
3 months	abundance	X07381	Bis-D-fruct	3	Interventio	Experimen	Experimen	Control	0,014081
3 months	abundance	X07394	Hydroxypro	3	Interventio	Experimen	Experimen	Control	0,000235
3 months	abundance	X07399	Homoanse	3	Interventio	Experimen	Experimen	Control	0,273699
3 months	abundance	X07400	Tranexami	2b	Interventio	Experimen	Experimen	Control	0,440554
3 months	abundance	X07413	Chenodeo	1	Interventio	Experimen	Experimen	Control	0,15162
3 months	abundance	X07414	2,2-Bis(hyd	3	Interventio	Experimen	Experimen	Control	0,115261
3 months	abundance	X07417	δ-Valerolac	2b	Interventio	Experimen	Experimen	Control	0,836327
3 months	abundance	X07420	DNOP_h	3	Interventio	Experimen	Experimen	Control	0,819081
3 months	abundance	X07424	Rutinose (b	3	Interventio	Experimen	Experimen	Control	0,860527
3 months	abundance	X07425	N-Acetylas	2a	Interventio	Experimen	Experimen	Control	0,056989
3 months	abundance	X07428	N6-METHY	3	Interventio	Experimen	Experimen	Control	0,216079
3 months	abundance	X07429	DNOP_c	3	Interventio	Experimen	Experimen	Control	0,579428
3 months	abundance	X07434	N'-Hydroxy	2b	Interventio	Experimen	Experimen	Control	0,945538
3 months	abundance	X07439	4-pyridoxic	1	Interventio	Experimen	Experimen	Control	0,052565
3 months	abundance	X07442	coenzyme	3	Interventio	Experimen	Experimen	Control	0,817974
3 months	abundance	X07443	Butylparab	3	Interventio	Experimen	Experimen	Control	0,514326
3 months	abundance	X07445	3-(Sulfooxy	3	Interventio	Experimen	Experimen	Control	0,988551
3 months	abundance	X07451	Valylvaline	3	Interventio	Experimen	Experimen	Control	0,949849
3 months	abundance	X07452	4-Amino-1-	3	Interventio	Experimen	Experimen	Control	0,99116
3 months	abundance	X07454	2,4-Quinol	2b	Interventio	Experimen	Experimen	Control	0,988551
3 months	abundance	X07457	Glucosami	1	Interventio	Experimen	Experimen	Control	0,14094
3 months	abundance	X07460	1-pyrroline	3	Interventio	Experimen	Experimen	Control	0,404129
3 months	abundance	X07465	TO0127900	3	Interventio	Experimen	Experimen	Control	0,988551

3 months	abundance	X07469	1,1'-[1,12-	3	Interventio	Experimen	Experimen	Control	0,260224
3 months	abundance	X07475	2'-Deoxy-5	3	Interventio	Experimen	Experimen	Control	0,935609
3 months	abundance	X07476	4-Hydroxyb	1	Interventio	Experimen	Experimen	Control	0,821708
3 months	abundance	X07480	Naringenin	2a	Interventio	Experimen	Experimen	Control	0,532452
3 months	abundance	X07484	MFCD0002	3	Interventio	Experimen	Experimen	Control	0,377363
3 months	abundance	X07487	Oxypeucec	3	Interventio	Experimen	Experimen	Control	0,762066
3 months	abundance	X07491	1-Vinylimic	2b	Interventio	Experimen	Experimen	Control	0,908976
3 months	abundance	X07504	4-Acetami	2b	Interventio	Experimen	Experimen	Control	0,88099
3 months	abundance	X07508	Procaine_a	2b	Interventio	Experimen	Experimen	Control	0,250053
3 months	abundance	X07511	Piperidine_	3	Interventio	Experimen	Experimen	Control	0,744844
3 months	abundance	X07512	4-{[(3-Hydr	3	Interventio	Experimen	Experimen	Control	0,314021
3 months	abundance	X07513	(−)-nabilor	3	Interventio	Experimen	Experimen	Control	0,698315
3 months	abundance	X07514	L-(+)-Eryth	3	Interventio	Experimen	Experimen	Control	0,10419
3 months	abundance	X07518	(+/-)-2-Hyc	3	Interventio	Experimen	Experimen	Control	0,106066
3 months	abundance	X07519	Pyrrolidine	3	Interventio	Experimen	Experimen	Control	0,817974
3 months	abundance	X07523	Ursodeoxy	1	Interventio	Experimen	Experimen	Control	0,429858
3 months	abundance	X07524	Prolinamid	2b	Interventio	Experimen	Experimen	Control	0,593214
3 months	abundance	X07527	1,7-Dimetl	2b	Interventio	Experimen	Experimen	Control	0,960119
3 months	abundance	X07530	Alanine	1	Interventio	Experimen	Experimen	Control	0,854716
3 months	abundance	X07537	alpha-Cha	3	Interventio	Experimen	Experimen	Control	0,135093
3 months	abundance	X07538	Homoanse	3	Interventio	Experimen	Experimen	Control	0,052565
3 months	abundance	X07541	Saccharin	1	Interventio	Experimen	Experimen	Control	0,503169
3 months	abundance	X07544	Ethyl sulfa	3	Interventio	Experimen	Experimen	Control	0,439076
3 months	abundance	X07558	Leucylproli	2b	Interventio	Experimen	Experimen	Control	0,082572
3 months	abundance	X07563	Xanthine	1	Interventio	Experimen	Experimen	Control	0,884928
3 months	abundance	X07565	5-Hydroxyi	2b	Interventio	Experimen	Experimen	Control	0,042091
3 months	abundance	X07566	5-Allyl-5-se	3	Interventio	Experimen	Experimen	Control	0,817974
3 months	abundance	X07572	8-(3-Furyl)-	3	Interventio	Experimen	Experimen	Control	0,744844
3 months	abundance	X07574	7-ketodeo	3	Interventio	Experimen	Experimen	Control	0,988551
3 months	abundance	X07577	2-[(Sulfoox	3	Interventio	Experimen	Experimen	Control	0,681513
3 months	abundance	X07578	5-Allyl-5-se	3	Interventio	Experimen	Experimen	Control	0,956306
3 months	abundance	X07580	N-Pentano	3	Interventio	Experimen	Experimen	Control	0,333178
3 months	abundance	X07581	N-Acetylva	3	Interventio	Experimen	Experimen	Control	0,513969
3 months	abundance	X07582	4-methylpy	2b	Interventio	Experimen	Experimen	Control	0,96692
3 months	abundance	X07596	5-amino-2-	2b	Interventio	Experimen	Experimen	Control	0,835202
3 months	abundance	X07597	p-Cresylsu	3	Interventio	Experimen	Experimen	Control	0,990949
3 months	abundance	X07601	Asp-lys	3	Interventio	Experimen	Experimen	Control	0,656167
3 months	abundance	X07613	1-(4-Aminc	3	Interventio	Experimen	Experimen	Control	0,483229
3 months	abundance	X07618	N-(3-aceta	3	Interventio	Experimen	Experimen	Control	0,833088
3 months	abundance	X07630	Methionine	1	Interventio	Experimen	Experimen	Control	0,617664
3 months	abundance	X07634	3,8,9-trihy	2b	Interventio	Experimen	Experimen	Control	0,141328
3 months	abundance	X07638	7-ketodeo	3	Interventio	Experimen	Experimen	Control	0,651767
3 months	abundance	X07645	O-propeno	3	Interventio	Experimen	Experimen	Control	0,968857
3 months	abundance	X07646	(2Z)-2-({[(6	3	Interventio	Experimen	Experimen	Control	0,834104
3 months	abundance	X07647	Genistein	1	Interventio	Experimen	Experimen	Control	0,023171
3 months	abundance	X07648	NL851300	3	Interventio	Experimen	Experimen	Control	0,886266
3 months	abundance	X07657	Docosahex	2b	Interventio	Experimen	Experimen	Control	0,323676
3 months	abundance	X07658	δ-Glucono	1	Interventio	Experimen	Experimen	Control	0,469877

3 months	abundance	X07674	Lysine	1	Interventio	Experimen	Experimen	Control	0,26602
3 months	abundance	X07678	(2E)-3-(3,4	2b	Interventio	Experimen	Experimen	Control	0,322462
3 months	abundance	X07680	Indole-3-ac	1	Interventio	Experimen	Experimen	Control	0,84521
3 months	abundance	X07694	DNOP_a	3	Interventio	Experimen	Experimen	Control	0,985356
3 months	abundance	X07699	N-(1-Methy	3	Interventio	Experimen	Experimen	Control	0,915997
3 months	abundance	X07701	3-Benzyl-6	3	Interventio	Experimen	Experimen	Control	0,505748
3 months	abundance	X07710	3-O-beta-D	3	Interventio	Experimen	Experimen	Control	0,755239
3 months	abundance	X07712	paracetam	3	Interventio	Experimen	Experimen	Control	0,635505
3 months	abundance	X07731	3-[4-methy	2b	Interventio	Experimen	Experimen	Control	0,124383
3 months	abundance	X07732	N-Acetylva	2b	Interventio	Experimen	Experimen	Control	0,923057
3 months	abundance	X07743	D-Alanine r	2b	Interventio	Experimen	Experimen	Control	0,694902
3 months	abundance	X07745	APM_c	3	Interventio	Experimen	Experimen	Control	0,691158
3 months	abundance	X07746	3-(2-Oxo-2	2b	Interventio	Experimen	Experimen	Control	0,718544
3 months	abundance	X07747	Diethylpyrc	3	Interventio	Experimen	Experimen	Control	0,108736
3 months	abundance	X07749	(DL)-3-O-M	3	Interventio	Experimen	Experimen	Control	0,632225
3 months	abundance	X07753	Lovastatin_	3	Interventio	Experimen	Experimen	Control	0,755448
3 months	abundance	X07754	hexobarbit	3	Interventio	Experimen	Experimen	Control	0,787558
3 months	abundance	X07764	(S)-2-meth	3	Interventio	Experimen	Experimen	Control	0,833128
3 months	abundance	X07770	Piperine	1	Interventio	Experimen	Experimen	Control	0,798841
3 months	abundance	X07771	Caprolacta	2b	Interventio	Experimen	Experimen	Control	0,60023
3 months	abundance	X07774	7alpha-Hyd	3	Interventio	Experimen	Experimen	Control	0,593214
3 months	abundance	X07777	Arginine	1	Interventio	Experimen	Experimen	Control	0,980029
3 months	abundance	X07782	Cytosine	1	Interventio	Experimen	Experimen	Control	0,172283
3 months	abundance	X07783	1,3-dimeth	3	Interventio	Experimen	Experimen	Control	0,886266
3 months	abundance	X07792	N-Acetylhis	2b	Interventio	Experimen	Experimen	Control	0,867667
3 months	abundance	X07794	Prolylleuci	2b	Interventio	Experimen	Experimen	Control	0,867667
3 months	abundance	X07798	N-lauroylgl	3	Interventio	Experimen	Experimen	Control	0,713464
3 months	abundance	X07799	Capryloylg	2b	Interventio	Experimen	Experimen	Control	0,329628
3 months	abundance	X07811	Metirosine	3	Interventio	Experimen	Experimen	Control	0,835202
3 months	abundance	X07812	Triethyl citr	3	Interventio	Experimen	Experimen	Control	0,718544
3 months	abundance	X07815	3-[2-[(Z)-[3	3	Interventio	Experimen	Experimen	Control	0,260022
3 months	abundance	X07820	Creatinine	1	Interventio	Experimen	Experimen	Control	0,238218
3 months	abundance	X07825	Maltotriose	1	Interventio	Experimen	Experimen	Control	0,009306
3 months	abundance	X07830	1,5-Isoquir	2b	Interventio	Experimen	Experimen	Control	0,458416
3 months	abundance	X07834	Choline su	3	Interventio	Experimen	Experimen	Control	0,907733
3 months	abundance	X07835	7-ketodeox	3	Interventio	Experimen	Experimen	Control	0,659543
3 months	abundance	X07841	2-(5-Benzy	3	Interventio	Experimen	Experimen	Control	0,718544
3 months	abundance	X07843	Piperidine_	3	Interventio	Experimen	Experimen	Control	0,115261
3 months	abundance	X07846	Carbofurar	3	Interventio	Experimen	Experimen	Control	0,161121
3 months	abundance	X07848	(−)-nabilor	3	Interventio	Experimen	Experimen	Control	0,633187
3 months	abundance	X07849	Crotamitor	3	Interventio	Experimen	Experimen	Control	0,142786
3 months	abundance	X07852	N6,N6,N6-	3	Interventio	Experimen	Experimen	Control	0,503169
3 months	abundance	X07854	TO0127900	3	Interventio	Experimen	Experimen	Control	4,85E-05

3 months	abundance	X07856	,6R,7R,9R ,11R,12R, 13S,14R)- 6- {[(2S,3R,4 S,6R)-4- (Dimethyl amino)-3- hydroxy-6- methyltetr ahydro- 2H-pyran- 2-yl]oxy}- 12,13- dihydroxy- 14-[(1S)-1- hydroxyet hyl]-4- {[(2R,4R,5 S,6S)-5- hydroxy-4- methoxy- 4 ,6- dimethylt etrahydro- 2H-pyran- 2-yl]oxy}-7-	3	Interventio	Experimen	Experimen	Control	0,222442
3 months	abundance	X07866	3,3,5,5-Tet	2b	Interventio	Experimen	Experimen	Control	0,001034
3 months	abundance	X07867	N-Acetylva	3	Interventio	Experimen	Experimen	Control	0,083448
3 months	abundance	X07868	Biotin	2b	Interventio	Experimen	Experimen	Control	0,997328
3 months	abundance	X07869	Hexamethy	2a	Interventio	Experimen	Experimen	Control	0,081509
3 months	abundance	X07873	(3Z,6Z,9Z,1	3	Interventio	Experimen	Experimen	Control	0,392246
3 months	abundance	X07874	δ-Valerolac	2b	Interventio	Experimen	Experimen	Control	0,985504
3 months	abundance	X07886	L-Urobilin	3	Interventio	Experimen	Experimen	Control	0,900417
3 months	abundance	X07889	UROBILIN,	3	Interventio	Experimen	Experimen	Control	0,996617
3 months	abundance	X07890	N-(5-aceta	2b	Interventio	Experimen	Experimen	Control	0,680872
3 months	abundance	X07891	Cyclamic a	2b	Interventio	Experimen	Experimen	Control	0,867667
3 months	abundance	X07892	urobilinoge	3	Interventio	Experimen	Experimen	Control	0,909722
3 months	abundance	X07899	N-Acetylpu	2b	Interventio	Experimen	Experimen	Control	0,755448
3 months	abundance	X07900	presqualer	3	Interventio	Experimen	Experimen	Control	0,021235
3 months	abundance	X07903	Acetophen	2b	Interventio	Experimen	Experimen	Control	0,985504
3 months	abundance	X07905	Prilocaine	2b	Interventio	Experimen	Experimen	Control	0,113035
3 months	abundance	X07907	4-Acetamid	1	Interventio	Experimen	Experimen	Control	0,821708
3 months	abundance	X07909	(±)-Albuter	2b	Interventio	Experimen	Experimen	Control	0,620467
3 months	abundance	X07914	(-)-Erythror	3	Interventio	Experimen	Experimen	Control	0,231572
3 months	abundance	X07916	N6,N6,N6-	3	Interventio	Experimen	Experimen	Control	5,47E-05

3 months	abundance	X07921	Pipecolinic	1	Interventio	Experimen	Experimen	Control	0,000266
3 months	abundance	X07925	DNOP_f	3	Interventio	Experimen	Experimen	Control	0,656554
3 months	abundance	X07930	N-Methylca	2b	Interventio	Experimen	Experimen	Control	0,064289
3 months	abundance	X07935	Limonin	3	Interventio	Experimen	Experimen	Control	0,997328
3 months	abundance	X07937	Atenolol	1	Interventio	Experimen	Experimen	Control	0,712596
3 months	abundance	X07944	Styrene	3	Interventio	Experimen	Experimen	Control	0,725856
3 months	abundance	X07955	Valylvaline	3	Interventio	Experimen	Experimen	Control	0,860527
3 months	abundance	X07961	2-(Hydroxy	3	Interventio	Experimen	Experimen	Control	0,560103
3 months	abundance	X07963	Theobromi	1	Interventio	Experimen	Experimen	Control	0,817715
3 months	abundance	X07969	6-hydroxyp	3	Interventio	Experimen	Experimen	Control	0,265534
3 months	abundance	X07974	Isoprene	3	Interventio	Experimen	Experimen	Control	0,276947
3 months	abundance	X07977	Tyramine	2b	Interventio	Experimen	Experimen	Control	0,94858
3 months	abundance	X07978	1-[(4E)-4-(4	3	Interventio	Experimen	Experimen	Control	0,325689
3 months	abundance	X07981	Methylimic	2b	Interventio	Experimen	Experimen	Control	0,817974
3 months	abundance	X07983	butalbital_	3	Interventio	Experimen	Experimen	Control	0,297127
3 months	abundance	X07989	Piperidine_	3	Interventio	Experimen	Experimen	Control	0,448277
3 months	abundance	X07993	UROBILIN,	3	Interventio	Experimen	Experimen	Control	0,99116
3 months	abundance	X08002	(2S)-6-Ami	3	Interventio	Experimen	Experimen	Control	0,506018
3 months	abundance	X08004	δ-Valerolac	2b	Interventio	Experimen	Experimen	Control	0,988551
3 months	abundance	X08007	MFCD0002	3	Interventio	Experimen	Experimen	Control	0,000518
3 months	abundance	X08008	N-Acetylne	1	Interventio	Experimen	Experimen	Control	0,086617
3 months	abundance	X08012	Methylol D	3	Interventio	Experimen	Experimen	Control	0,242449
3 months	abundance	X08013	Deoxysuga	1	Interventio	Experimen	Experimen	Control	0,049638
3 months	abundance	X08014	DNOP_e	3	Interventio	Experimen	Experimen	Control	0,703544
3 months	abundance	X08017	Tropinone	2b	Interventio	Experimen	Experimen	Control	0,750825
3 months	abundance	X08019	Thymidine	1	Interventio	Experimen	Experimen	Control	0,082877
3 months	abundance	X08024	8-Methyl-8	3	Interventio	Experimen	Experimen	Control	0,124404
3 months	abundance	X08028	Solanidine	3	Interventio	Experimen	Experimen	Control	0,080062
3 months	abundance	X08035	Tetrahydro	3	Interventio	Experimen	Experimen	Control	0,75812
3 months	abundance	X08037	Crotonic ac	2b	Interventio	Experimen	Experimen	Control	0,892974
3 months	abundance	X08038	N-{6-[(7-Cl	2b	Interventio	Experimen	Experimen	Control	0,85207
3 months	abundance	X08040	Prolylleuci	2b	Interventio	Experimen	Experimen	Control	0,98336
3 months	abundance	X08044	3-[2-[(Z)-[3	3	Interventio	Experimen	Experimen	Control	0,633187
3 months	abundance	X08046	3-Hydroxy-	3	Interventio	Experimen	Experimen	Control	0,614079
3 months	abundance	X08054	Docosahex	2b	Interventio	Experimen	Experimen	Control	0,694902
3 months	abundance	X08056	3-(2-Oxo-2	2b	Interventio	Experimen	Experimen	Control	0,333178
3 months	abundance	X08057	N-{3-Carbo	3	Interventio	Experimen	Experimen	Control	0,9752
3 months	abundance	X08058	2-Hydroxy-	3	Interventio	Experimen	Experimen	Control	0,879562
3 months	abundance	X08059	Cadaverine	3	Interventio	Experimen	Experimen	Control	0,082877
3 months	abundance	X08062	Indole-3-ca	1	Interventio	Experimen	Experimen	Control	0,436028
3 months	abundance	X08072	4-Hydroxyp	3	Interventio	Experimen	Experimen	Control	0,021942
3 months	abundance	X08076	3',5,7-Trihy	2b	Interventio	Experimen	Experimen	Control	0,613297
3 months	abundance	X08077	6-(alpha-D	3	Interventio	Experimen	Experimen	Control	0,581247
3 months	abundance	X08078	Ethyl malat	3	Interventio	Experimen	Experimen	Control	0,915997
3 months	abundance	X08092	Safrole	3	Interventio	Experimen	Experimen	Control	0,94878
3 months	abundance	X08094	1,1'-[1,12-I	3	Interventio	Experimen	Experimen	Control	0,503169
3 months	abundance	X08095	DNOP_g	3	Interventio	Experimen	Experimen	Control	0,821708
3 months	abundance	X08096	(2E)-3-Met	3	Interventio	Experimen	Experimen	Control	0,625098

3 months	abundance	X08098	Citrulline	1	Interventio	Experimen	Experimen	Control	0,30026
3 months	abundance	X08099	g-Butyrobe	3	Interventio	Experimen	Experimen	Control	0,702384
3 months	abundance	X08100	Piperidine_	3	Interventio	Experimen	Experimen	Control	0,001936
3 months	abundance	X08103	Isopelletie	3	Interventio	Experimen	Experimen	Control	0,399618
3 months	abundance	X08105	2-Acetami	3	Interventio	Experimen	Experimen	Control	0,915366
3 months	abundance	X08106	4-Vinylphe	1	Interventio	Experimen	Experimen	Control	0,581247
3 months	abundance	X08111	2-Hydroxy	2b	Interventio	Experimen	Experimen	Control	0,552202
3 months	abundance	X08116	Methyl (2Z,	3	Interventio	Experimen	Experimen	Control	0,003352
3 months	abundance	X08123	Tiglic acid_	2b	Interventio	Experimen	Experimen	Control	0,765006
3 months	abundance	X08146	O-Ethyl (4-	3	Interventio	Experimen	Experimen	Control	0,98719
3 months	abundance	X08147	porphobili	3	Interventio	Experimen	Experimen	Control	0,935319
3 months	abundance	X08150	DL-4-Hydr	2b	Interventio	Experimen	Experimen	Control	0,404129
3 months	abundance	X08154	4-(METHYL	3	Interventio	Experimen	Experimen	Control	0,752586
3 months	abundance	X08157	Leu-Val_e	3	Interventio	Experimen	Experimen	Control	0,787558
3 months	abundance	X08162	Valylvaline	3	Interventio	Experimen	Experimen	Control	0,244161
3 months	abundance	X08163	Phenyl D-g	3	Interventio	Experimen	Experimen	Control	0,613297
3 months	abundance	X08167	Lanthionin	3	Interventio	Experimen	Experimen	Control	0,993803
3 months	abundance	X08173	9-Methylur	2b	Interventio	Experimen	Experimen	Control	0,718544
3 months	abundance	X08188	2-Hydroxy-	2b	Interventio	Experimen	Experimen	Control	0,681178
3 months	abundance	X08193	Seryltyrosi	3	Interventio	Experimen	Experimen	Control	0,761041
3 months	abundance	X08204	2-Amino-4	2b	Interventio	Experimen	Experimen	Control	0,038414
3 months	abundance	X08212	LU345300	3	Interventio	Experimen	Experimen	Control	0,070241
3 months	abundance	X08221	Brilliant blu	2b	Interventio	Experimen	Experimen	Control	0,238218
3 months	abundance	X08230	Coumaron	3	Interventio	Experimen	Experimen	Control	0,46992
3 months	abundance	X08235	1-Methylhi	2b	Interventio	Experimen	Experimen	Control	0,821708
3 months	abundance	X08244	Setoclavin	3	Interventio	Experimen	Experimen	Control	0,800324
3 months	abundance	X08270	Butabarbit	3	Interventio	Experimen	Experimen	Control	0,787558
3 months	abundance	X08274	Mevalonic	2b	Interventio	Experimen	Experimen	Control	0,244993
3 months	abundance	X08277	adrenaline	3	Interventio	Experimen	Experimen	Control	0,703692
3 months	abundance	X08279	DNOP_b	3	Interventio	Experimen	Experimen	Control	0,90642
3 months	abundance	X08288	Capryloylg	3	Interventio	Experimen	Experimen	Control	0,907733
3 months	abundance	X08292	Lidocaine	2b	Interventio	Experimen	Experimen	Control	0,778801
3 months	abundance	X08298	N6,N6,N6-	2b	Interventio	Experimen	Experimen	Control	0,333178
3 months	abundance	X08300	Indole-3-a	3	Interventio	Experimen	Experimen	Control	0,808903
3 months	abundance	X08305	Prolylhydr	2a	Interventio	Experimen	Experimen	Control	0,808744
3 months	abundance	X08306	1-Methylxa	2b	Interventio	Experimen	Experimen	Control	0,522871
3 months	abundance	X08311	Methyl 3-f	3	Interventio	Experimen	Experimen	Control	0,755448
3 months	abundance	X08317	8-Hydroxy	2b	Interventio	Experimen	Experimen	Control	0,529387
3 months	abundance	X08322	Glycylproly	3	Interventio	Experimen	Experimen	Control	0,860527
3 months	abundance	X08323	(15Z)-9,12	2b	Interventio	Experimen	Experimen	Control	0,854716
3 months	abundance	X08326	2-(1,3-Ben	3	Interventio	Experimen	Experimen	Control	0,188874
3 months	abundance	X08338	Methyl [9-(	3	Interventio	Experimen	Experimen	Control	0,322462
3 months	abundance	X08341	Midodrine_	3	Interventio	Experimen	Experimen	Control	0,892548
3 months	abundance	X08352	7-[4-(tert-b	2b	Interventio	Experimen	Experimen	Control	0,953271
3 months	abundance	X08365	Lysylvaline	3	Interventio	Experimen	Experimen	Control	0,003817
3 months	abundance	X08367	11-dehydr	2b	Interventio	Experimen	Experimen	Control	0,59787
3 months	abundance	X08373	2-Hydroxy-	3	Interventio	Experimen	Experimen	Control	0,854716
3 months	abundance	X08386	3b-Hydroxy	3	Interventio	Experimen	Experimen	Control	0,509355

3 months	abundance	X08387	4-[(2E,4Z)-	3	Interventio	Experimen	Experimen	Control	0,807551
3 months	abundance	X08412	2-Hydroxyp	2b	Interventio	Experimen	Experimen	Control	0,830535
3 months	abundance	X08417	asn-pro_c	3	Interventio	Experimen	Experimen	Control	0,798192
3 months	abundance	X08420	Dodecane	1	Interventio	Experimen	Experimen	Control	0,047927
3 months	abundance	X08421	Cys-tyr	3	Interventio	Experimen	Experimen	Control	0,109563
3 months	abundance	X08422	N-(3-aceta	3	Interventio	Experimen	Experimen	Control	0,765006
3 months	abundance	X08425	MFCD1297	3	Interventio	Experimen	Experimen	Control	0,008475
3 months	abundance	X08436	N,N-Dimet	3	Interventio	Experimen	Experimen	Control	0,521176
3 months	abundance	X08439	Leucyltyro	3	Interventio	Experimen	Experimen	Control	0,070241
3 months	abundance	X08451	L-gamma-	3	Interventio	Experimen	Experimen	Control	0,985356
3 months	abundance	X08460	α-Murichol	1	Interventio	Experimen	Experimen	Control	0,985356
3 months	abundance	X08461	Dihydrouri	3	Interventio	Experimen	Experimen	Control	0,208353
3 months	abundance	X08475	1-(4-Aminc	3	Interventio	Experimen	Experimen	Control	0,657878
3 months	abundance	X08476	Coprine_c	3	Interventio	Experimen	Experimen	Control	0,679693
3 months	abundance	X08478	1-(beta-D-	3	Interventio	Experimen	Experimen	Control	0,99116
3 months	abundance	X08485	Isoprenalir	3	Interventio	Experimen	Experimen	Control	0,980029
3 months	abundance	X08486	(2E,6E)-9-[	3	Interventio	Experimen	Experimen	Control	0,216079
3 months	abundance	X08504	Indole-3-c	2a	Interventio	Experimen	Experimen	Control	0,988551
3 months	abundance	X08509	Piceid	3	Interventio	Experimen	Experimen	Control	0,412774
3 months	abundance	X08512	Bile acid I (	2b	Interventio	Experimen	Experimen	Control	0,645886
3 months	abundance	X08514	N-Ethylpro	3	Interventio	Experimen	Experimen	Control	0,242305
3 months	abundance	X08525	MFCD186	3	Interventio	Experimen	Experimen	Control	0,023171
3 months	abundance	X08529	7,8-Diamir	3	Interventio	Experimen	Experimen	Control	0,014515
3 months	abundance	X08533	tyramine s	3	Interventio	Experimen	Experimen	Control	0,598888
3 months	abundance	X08535	Nitrosohep	2b	Interventio	Experimen	Experimen	Control	0,385109
3 months	abundance	X08546	1,3,7-Trim	2b	Interventio	Experimen	Experimen	Control	0,819701
3 months	abundance	X08548	N2-Acetyll	1	Interventio	Experimen	Experimen	Control	0,405692
3 months	abundance	X08549	3-Methylsu	3	Interventio	Experimen	Experimen	Control	0,243875
3 months	abundance	X08561	N-(Carbox	3	Interventio	Experimen	Experimen	Control	0,00049
3 months	abundance	X08578	tert-Butyl 3	3	Interventio	Experimen	Experimen	Control	0,625098
3 months	abundance	X08584	meticillin	3	Interventio	Experimen	Experimen	Control	0,467786
3 months	abundance	X08588	2-Acetami	3	Interventio	Experimen	Experimen	Control	0,486342
3 months	abundance	X08593	Uric acid	1	Interventio	Experimen	Experimen	Control	0,084515
3 months	abundance	X08594	7-ketodeo	3	Interventio	Experimen	Experimen	Control	0,997328
3 months	abundance	X08600	N-Propiony	3	Interventio	Experimen	Experimen	Control	0,60023
3 months	abundance	X08606	Butabarbit	3	Interventio	Experimen	Experimen	Control	0,217561
3 months	abundance	X08610	Leucyltyro	3	Interventio	Experimen	Experimen	Control	0,833128
3 months	abundance	X08616	MFCD0002	3	Interventio	Experimen	Experimen	Control	0,718544
3 months	abundance	X08625	Hept-2-ulo	3	Interventio	Experimen	Experimen	Control	0,322462
3 months	abundance	X08634	1,5-Isoquir	2b	Interventio	Experimen	Experimen	Control	0,798571
3 months	abundance	X08639	hypaphorir	3	Interventio	Experimen	Experimen	Control	0,99116
3 months	abundance	X08641	meprobam	3	Interventio	Experimen	Experimen	Control	0,76643
3 months	abundance	X08644	2,5-Dimetl	2b	Interventio	Experimen	Experimen	Control	0,466427
3 months	abundance	X08646	N-Stearoyl	3	Interventio	Experimen	Experimen	Control	0,84915
3 months	abundance	X08657	1-(4-Aminc	3	Interventio	Experimen	Experimen	Control	0,99587
3 months	abundance	X08659	3,3-Dimetl	2b	Interventio	Experimen	Experimen	Control	0,96467
3 months	abundance	X08663	Pilocarpine	2b	Interventio	Experimen	Experimen	Control	0,59787
3 months	abundance	X08670	Hydroxypro	3	Interventio	Experimen	Experimen	Control	0,004228

3 months	abundance	X08673	NPC	3	Interventio	Experimen	Experimen	Control	0,854716
3 months	abundance	X08677	1-methylhy	3	Interventio	Experimen	Experimen	Control	0,126143
3 months	abundance	X08690	Ethanoic a	3	Interventio	Experimen	Experimen	Control	0,146137
3 months	abundance	X08693	7-Sulfocho	3	Interventio	Experimen	Experimen	Control	0,96467
3 months	abundance	X08695	3b-Hydroxy	3	Interventio	Experimen	Experimen	Control	0,231572
3 months	abundance	X08699	Aurorix	3	Interventio	Experimen	Experimen	Control	0,124383
3 months	abundance	X08707	2-(1-Ethoxy	3	Interventio	Experimen	Experimen	Control	0,58465
3 months	abundance	X08711	2-Acetami	3	Interventio	Experimen	Experimen	Control	0,871745
3 months	abundance	X08712	4-Iodoanis	3	Interventio	Experimen	Experimen	Control	0,038414
3 months	abundance	X08713	Phenethyla	2b	Interventio	Experimen	Experimen	Control	0,838687
3 months	abundance	X08714	Arabic acic	3	Interventio	Experimen	Experimen	Control	0,256516
3 months	abundance	X08719	6-Methylqu	2b	Interventio	Experimen	Experimen	Control	0,993459
3 months	abundance	X08723	S-Allylcyste	3	Interventio	Experimen	Experimen	Control	0,142786
3 months	abundance	X08726	YV8195000	3	Interventio	Experimen	Experimen	Control	0,000211
3 months	abundance	X08733	N-Methyl-1	3	Interventio	Experimen	Experimen	Control	0,977246
3 months	abundance	X08739	1,1'-[1,12-	3	Interventio	Experimen	Experimen	Control	0,758687
3 months	abundance	X08758	Methohexit	3	Interventio	Experimen	Experimen	Control	0,217561
3 months	abundance	X08777	3-Aminosa	2b	Interventio	Experimen	Experimen	Control	0,385799
3 months	abundance	X08779	3-(1-hydro:	2b	Interventio	Experimen	Experimen	Control	0,665073
3 months	abundance	X08781	Procaine_b	3	Interventio	Experimen	Experimen	Control	0,174141
3 months	abundance	X08789	Acrylic acic	2b	Interventio	Experimen	Experimen	Control	0,124404
3 months	abundance	X08792	3,4-Methyl	3	Interventio	Experimen	Experimen	Control	0,967096
3 months	abundance	X08795	N-Acetylva	3	Interventio	Experimen	Experimen	Control	0,614079
3 months	abundance	X08801	3,7-Dimeth	2b	Interventio	Experimen	Experimen	Control	0,787558
3 months	abundance	X08810	N~6~,N~6	3	Interventio	Experimen	Experimen	Control	0,011582
3 months	abundance	X08822	CYS-ASP	3	Interventio	Experimen	Experimen	Control	0,267725
3 months	abundance	X08825	Chenodeo:	3	Interventio	Experimen	Experimen	Control	0,584092
3 months	abundance	X08842	Spermic ac	3	Interventio	Experimen	Experimen	Control	0,992999
3 months	abundance	X08843	(2R,3S)-3-I	3	Interventio	Experimen	Experimen	Control	0,002229
3 months	abundance	X08844	4-Hydroxy-	3	Interventio	Experimen	Experimen	Control	0,321752
3 months	abundance	X08845	14-Hydroxy	3	Interventio	Experimen	Experimen	Control	0,351458
3 months	abundance	X08847	1-Vinylimic	2b	Interventio	Experimen	Experimen	Control	0,960119
3 months	abundance	X08869	Lanthionin	3	Interventio	Experimen	Experimen	Control	0,9765
3 months	abundance	X08870	Tetraacety	3	Interventio	Experimen	Experimen	Control	0,144456
3 months	abundance	X08883	3-Phenylpr	3	Interventio	Experimen	Experimen	Control	0,981401
3 months	abundance	X08893	5beta-Cho	3	Interventio	Experimen	Experimen	Control	0,115645
3 months	abundance	X08895	pentoxyl	3	Interventio	Experimen	Experimen	Control	0,565249
3 months	abundance	X08908	Vorinostat_	3	Interventio	Experimen	Experimen	Control	0,191691
3 months	abundance	X08909	GLK (Pepti	2b	Interventio	Experimen	Experimen	Control	0,442109
3 months	abundance	X08923	Semilicois	3	Interventio	Experimen	Experimen	Control	0,28244
3 months	abundance	X08938	Nonivamid	3	Interventio	Experimen	Experimen	Control	8,90E-05
3 months	abundance	X08944	Dipivefrin	3	Interventio	Experimen	Experimen	Control	0,239969
3 months	abundance	X08965	1,9-Nonan	3	Interventio	Experimen	Experimen	Control	0,820266
3 months	abundance	X08973	Aminohipp	3	Interventio	Experimen	Experimen	Control	0,419167
3 months	abundance	X08983	IN00150	3	Interventio	Experimen	Experimen	Control	0,830535
3 months	abundance	X08987	feruloylser	3	Interventio	Experimen	Experimen	Control	0,436347
3 months	abundance	X08990	Procaine_c	3	Interventio	Experimen	Experimen	Control	0,656554
3 months	abundance	X08992	N(2)-succi	3	Interventio	Experimen	Experimen	Control	0,71541

3 months	abundance	X09001	Varanic ac	3	Interventio	Experimen	Experimen	Control	0,957479
3 months	abundance	X09004	Arenaine	3	Interventio	Experimen	Experimen	Control	0,070143
3 months	abundance	X09008	5-guanidin	3	Interventio	Experimen	Experimen	Control	0,778801
3 months	abundance	X09010	4-Hydroxyp	1	Interventio	Experimen	Experimen	Control	0,532452
3 months	abundance	X09055	N-[(1R,2S,(	3	Interventio	Experimen	Experimen	Control	0,605819
3 months	abundance	X09064	Tetrahydro	3	Interventio	Experimen	Experimen	Control	0,518924
3 months	abundance	X09068	N,N-Diethy	3	Interventio	Experimen	Experimen	Control	0,000404
3 months	abundance	X09082	1_2-Dihydr	3	Interventio	Experimen	Experimen	Control	0,99116
3 months	abundance	X09091	N-(3-aceta	3	Interventio	Experimen	Experimen	Control	0,155891
3 months	abundance	X09092	LysoSM(d1	3	Interventio	Experimen	Experimen	Control	0,787162
3 months	abundance	X09099	1-(2-Hydro	3	Interventio	Experimen	Experimen	Control	0,149795
3 months	abundance	X09100	Ethyl aceta	3	Interventio	Experimen	Experimen	Control	0,613297
3 months	abundance	X09110	Valylvaline	3	Interventio	Experimen	Experimen	Control	0,100587
3 months	abundance	X09122	Valyl-4-hyc	3	Interventio	Experimen	Experimen	Control	0,413564
3 months	abundance	X09129	Coprine_d	3	Interventio	Experimen	Experimen	Control	0,655532
3 months	abundance	X09152	Ferulic aci	1	Interventio	Experimen	Experimen	Control	0,268018
3 months	abundance	X09163	Prenisteine	3	Interventio	Experimen	Experimen	Control	0,938964
3 months	abundance	X09165	N-[1-Carbo	3	Interventio	Experimen	Experimen	Control	0,012368
3 months	abundance	X09187	Salicylic ac	1	Interventio	Experimen	Experimen	Control	0,76643
3 months	abundance	X09189	4-[(E)-2-(3,	3	Interventio	Experimen	Experimen	Control	0,333863
3 months	abundance	X09207	AAMU_a	3	Interventio	Experimen	Experimen	Control	0,333178
3 months	abundance	X09208	IN00260_b	3	Interventio	Experimen	Experimen	Control	0,798192
3 months	abundance	X09222	L-gamma-(	3	Interventio	Experimen	Experimen	Control	0,619252
3 months	abundance	X09231	2-Keto-glut	3	Interventio	Experimen	Experimen	Control	0,988551
3 months	abundance	X09244	gamma-Gl	3	Interventio	Experimen	Experimen	Control	0,409881
3 months	abundance	X09245	L-gamma-(	3	Interventio	Experimen	Experimen	Control	0,390151
3 months	abundance	X09256	Aspartyl-L-	3	Interventio	Experimen	Experimen	Control	0,451712
3 months	abundance	X09260	(4S)-4-[(2E	3	Interventio	Experimen	Experimen	Control	0,23349
3 months	abundance	X09284	4-(Nitrosoa	3	Interventio	Experimen	Experimen	Control	0,913373
3 months	abundance	X09285	MFCD0272	3	Interventio	Experimen	Experimen	Control	0,991184
3 months	abundance	X09302	Sular	3	Interventio	Experimen	Experimen	Control	0,836327
3 months	abundance	X09318	butalbital_	3	Interventio	Experimen	Experimen	Control	0,971877
3 months	abundance	X09336	1,1'-[1,12-l	3	Interventio	Experimen	Experimen	Control	0,627129
3 months	abundance	X09357	5-(5-Methy	3	Interventio	Experimen	Experimen	Control	0,469773
3 months	abundance	X09374	3-Deoxy-D-	3	Interventio	Experimen	Experimen	Control	0,46054
3 months	abundance	X09385	2-(1-Napht	3	Interventio	Experimen	Experimen	Control	0,503169
3 months	abundance	X09390	asn-pro_a	3	Interventio	Experimen	Experimen	Control	0,142786
3 months	abundance	X09391	ophthalmic	3	Interventio	Experimen	Experimen	Control	0,096259
3 months	abundance	X09393	2-Isopropy	3	Interventio	Experimen	Experimen	Control	0,943743
3 months	abundance	X09399	L-gamma-(	3	Interventio	Experimen	Experimen	Control	0,718544
3 months	abundance	X09454	2-(2-Hydro	3	Interventio	Experimen	Experimen	Control	0,80026
3 months	abundance	X09476	N(1),N(8)-l	3	Interventio	Experimen	Experimen	Control	0,927159
3 months	abundance	X09480	Spermic ac	3	Interventio	Experimen	Experimen	Control	0,739091
3 months	abundance	X09490	2-Hydroxy-	3	Interventio	Experimen	Experimen	Control	0,226184
3 months	abundance	X09503	4-(METHYL	3	Interventio	Experimen	Experimen	Control	0,60023
3 months	abundance	X09505	(-)-Physost	3	Interventio	Experimen	Experimen	Control	0,991184
3 months	abundance	X09523	N-(3,5-Dim	3	Interventio	Experimen	Experimen	Control	0,45097
3 months	abundance	X09525	Homoanse	3	Interventio	Experimen	Experimen	Control	0,268483

3 months	abundance	X09533	S(6)-acetyl	3	Interventio	Experimen	Experimen	Control	0,873316
3 months	abundance	X09545	4-Methyler	3	Interventio	Experimen	Experimen	Control	0,691471
3 months	abundance	X09546	asn-phe	3	Interventio	Experimen	Experimen	Control	0,625098
3 months	abundance	X09548	2-O-beta-D	3	Interventio	Experimen	Experimen	Control	0,021942
3 months	abundance	X09554	N-Propiony	3	Interventio	Experimen	Experimen	Control	0,953482
3 months	abundance	X09555	N4-(beta-N	3	Interventio	Experimen	Experimen	Control	0,449163
3 months	abundance	X09565	3-Methoxy-	3	Interventio	Experimen	Experimen	Control	0,30859
3 months	abundance	X09577	Spermic ac	3	Interventio	Experimen	Experimen	Control	0,291778
3 months	abundance	X09581	L-gamma-(	3	Interventio	Experimen	Experimen	Control	0,76164
3 months	abundance	X09607	N-(1H-Pyrr	3	Interventio	Experimen	Experimen	Control	0,244993
3 months	abundance	X09608	N-(3,5-Dim	3	Interventio	Experimen	Experimen	Control	0,802409
3 months	abundance	X09609	N-{3-[(4-Ac	3	Interventio	Experimen	Experimen	Control	0,886266
3 months	abundance	X09612	3-hydroxy-3	3	Interventio	Experimen	Experimen	Control	0,60023
3 months	abundance	X09615	tyramine su	3	Interventio	Experimen	Experimen	Control	0,691158
3 months	abundance	X09622	N-Hydroxy-	3	Interventio	Experimen	Experimen	Control	0,243875
3 months	abundance	X09623	Endothal	2b	Interventio	Experimen	Experimen	Control	0,161121
3 months	abundance	X09635	2,4,6-Triiso	3	Interventio	Experimen	Experimen	Control	0,712279
3 months	abundance	X09643	N-(3,5-Dim	3	Interventio	Experimen	Experimen	Control	0,854716
3 months	abundance	X09645	TOLMETIN	3	Interventio	Experimen	Experimen	Control	0,76643
3 months	abundance	X09654	linatine	3	Interventio	Experimen	Experimen	Control	0,860527
3 months	abundance	X09657	2,3,4,5,6-P	3	Interventio	Experimen	Experimen	Control	0,771075
3 months	abundance	X09675	UK387000	3	Interventio	Experimen	Experimen	Control	0,821708
3 months	abundance	X09684	(+)-Etomid	3	Interventio	Experimen	Experimen	Control	0,598888
3 months	abundance	X09703	7,8-Didehy	3	Interventio	Experimen	Experimen	Control	0,468958
3 months	abundance	X09710	LW800000	3	Interventio	Experimen	Experimen	Control	0,093704
3 months	abundance	X09713	N(2)-succi	3	Interventio	Experimen	Experimen	Control	0,581247
3 months	abundance	X09721	3-(2,3-Dihy	3	Interventio	Experimen	Experimen	Control	0,124404
3 months	abundance	X09724	Formylkynu	3	Interventio	Experimen	Experimen	Control	0,151919
3 months	abundance	X09726	indoline-2-	2b	Interventio	Experimen	Experimen	Control	0,143745
3 months	abundance	X09728	{[(15-Hydro	3	Interventio	Experimen	Experimen	Control	0,915997
3 months	abundance	X09738	(7E,7'E)-5,5	3	Interventio	Experimen	Experimen	Control	0,694902
3 months	abundance	X09740	MFCD0166	3	Interventio	Experimen	Experimen	Control	0,365259
3 months	abundance	X09741	Estrone glu	3	Interventio	Experimen	Experimen	Control	0,415989
3 months	abundance	X09746	ELK (Peptic	2b	Interventio	Experimen	Experimen	Control	0,98336
3 months	abundance	X09765	Tetrahydro	3	Interventio	Experimen	Experimen	Control	0,657878
3 months	abundance	X09771	2-(3-CARB	3	Interventio	Experimen	Experimen	Control	0,953271
3 months	abundance	X09787	Queuosine	3	Interventio	Experimen	Experimen	Control	0,713464
3 months	abundance	X09805	Methyl alpl	3	Interventio	Experimen	Experimen	Control	0,787558
3 months	abundance	X09810	Zalcitabine	3	Interventio	Experimen	Experimen	Control	0,798192
3 months	abundance	X09825	(2S)-6-Ami	3	Interventio	Experimen	Experimen	Control	0,927159
3 months	abundance	X09849	3-Methylac	3	Interventio	Experimen	Experimen	Control	0,002308
3 months	abundance	X09854	N-Benzyl-3	2b	Interventio	Experimen	Experimen	Control	0,755448
3 months	abundance	X09875	4-[(E)-2-(3,	3	Interventio	Experimen	Experimen	Control	0,778801
3 months	abundance	X09882	Harmane	3	Interventio	Experimen	Experimen	Control	0,986565
3 months	abundance	X09895	1-(beta-D-r	3	Interventio	Experimen	Experimen	Control	0,886266
3 months	abundance	X09898	N(6),N(6)-I	3	Interventio	Experimen	Experimen	Control	0,099053
3 months	abundance	X09912	MC055530	3	Interventio	Experimen	Experimen	Control	0,988551
3 months	abundance	X09919	4-ethylphe	3	Interventio	Experimen	Experimen	Control	0,627129

3 months	abundance	X09921	5-Hydroxy-	3	Interventio	Experimen	Experimen	Control	0,082425
3 months	abundance	X09932	g-Aminobu	3	Interventio	Experimen	Experimen	Control	0,001482
3 months	abundance	X09940	pentobarbi	3	Interventio	Experimen	Experimen	Control	0,598888
3 months	abundance	X09942	Spermic ac	3	Interventio	Experimen	Experimen	Control	0,96467
3 months	abundance	X09950	3-Hydroxyl	3	Interventio	Experimen	Experimen	Control	0,230103
3 months	abundance	X09951	5beta-Cypr	3	Interventio	Experimen	Experimen	Control	0,821759
3 months	abundance	X09963	Iminoglycir	3	Interventio	Experimen	Experimen	Control	0,691471
3 months	abundance	X09965	Biocytin	2b	Interventio	Experimen	Experimen	Control	0,798341
3 months	abundance	X09969	3-Methylac	3	Interventio	Experimen	Experimen	Control	0,005269
3 months	abundance	X09977	TLK (Peptic	2b	Interventio	Experimen	Experimen	Control	0,898308
3 months	abundance	X09980	N-(4-Hepta	3	Interventio	Experimen	Experimen	Control	0,052265
3 months	abundance	X09981	N-Acetylva	3	Interventio	Experimen	Experimen	Control	0,503169
3 months	abundance	X09998	N-[(10Z)-7-	3	Interventio	Experimen	Experimen	Control	0,766128
3 months	abundance	X10011	Midodrine_	3	Interventio	Experimen	Experimen	Control	0,718544
3 months	abundance	X10025	2-[4-(3-Hy	3	Interventio	Experimen	Experimen	Control	0,46992
3 months	abundance	X10032	4,9a-Dimet	3	Interventio	Experimen	Experimen	Control	0,680872
3 months	abundance	X10033	epsilon-(ga	3	Interventio	Experimen	Experimen	Control	0,916051
3 months	abundance	X10036	Nimodipine	3	Interventio	Experimen	Experimen	Control	0,9752
3 months	abundance	X10037	his-gln_a	3	Interventio	Experimen	Experimen	Control	0,88099
3 months	abundance	X10046	Dehydroac	2b	Interventio	Experimen	Experimen	Control	0,986931
3 months	abundance	X10076	trimethadi	3	Interventio	Experimen	Experimen	Control	0,924699
3 months	abundance	X10091	(2E,6E)-9-[	3	Interventio	Experimen	Experimen	Control	0,977246
3 months	abundance	X10097	Coixol	3	Interventio	Experimen	Experimen	Control	0,298368
3 months	abundance	X10098	Methyl 2,3-	3	Interventio	Experimen	Experimen	Control	0,583521
3 months	abundance	X10099	ala-met	3	Interventio	Experimen	Experimen	Control	0,419167
3 months	abundance	X10121	Leu-pro_b	3	Interventio	Experimen	Experimen	Control	0,262176
3 months	abundance	X10129	SECONAL_	3	Interventio	Experimen	Experimen	Control	0,664257
3 months	abundance	X10130	N-(4-Hydro	3	Interventio	Experimen	Experimen	Control	0,833128
3 months	abundance	X10137	1-Methyl-1	3	Interventio	Experimen	Experimen	Control	0,61536
3 months	abundance	X10144	imazameth	3	Interventio	Experimen	Experimen	Control	0,907192
3 months	abundance	X10148	cys-met	3	Interventio	Experimen	Experimen	Control	0,144997
3 months	abundance	X10151	2-(3-CARB	3	Interventio	Experimen	Experimen	Control	0,741794
3 months	abundance	X10162	Quinaldic a	2a	Interventio	Experimen	Experimen	Control	0,144456
3 months	abundance	X10177	Gly-DL-Phe	3	Interventio	Experimen	Experimen	Control	0,706727
3 months	abundance	X10180	4-Formyl-2	3	Interventio	Experimen	Experimen	Control	0,328214
3 months	abundance	X10203	L-gamma-C	3	Interventio	Experimen	Experimen	Control	0,282689
3 months	abundance	X10224	Imidazolela	2b	Interventio	Experimen	Experimen	Control	0,433847
3 months	abundance	X10246	Ethynodiol	3	Interventio	Experimen	Experimen	Control	0,23701
3 months	abundance	X10248	(+)-Etomid	3	Interventio	Experimen	Experimen	Control	0,448962
3 months	abundance	X10265	3-methyl-4	3	Interventio	Experimen	Experimen	Control	0,099053
3 months	abundance	X10276	Maleamate	3	Interventio	Experimen	Experimen	Control	0,718544
3 months	abundance	X10279	Lys-Pro_d	3	Interventio	Experimen	Experimen	Control	0,143745
3 months	abundance	X10304	2,3,8,9-Tet	3	Interventio	Experimen	Experimen	Control	0,480445
3 months	abundance	X10314	4-morpholi	2b	Interventio	Experimen	Experimen	Control	0,517388
3 months	abundance	X10319	1-Methyl-1	3	Interventio	Experimen	Experimen	Control	0,232653
3 months	abundance	X10325	Lovastatin_	3	Interventio	Experimen	Experimen	Control	0,633187
3 months	abundance	X10330	Sulfurous a	3	Interventio	Experimen	Experimen	Control	0,71076
3 months	abundance	X10339	4H-1-Benz	2b	Interventio	Experimen	Experimen	Control	0,965837

3 months	abundance	X10345	S-Allylcyst	3	Interventio	Experimen	Experimen	Control	0,631492
3 months	abundance	X10353	L-gamma-(	3	Interventio	Experimen	Experimen	Control	0,468958
3 months	abundance	X10367	2-(1-Napht	3	Interventio	Experimen	Experimen	Control	0,468958
3 months	abundance	X10370	L-Arogenat	3	Interventio	Experimen	Experimen	Control	0,613297
3 months	abundance	X10375	4-Indoleca	2b	Interventio	Experimen	Experimen	Control	0,143745
3 months	abundance	X10379	Phenyl D-g	3	Interventio	Experimen	Experimen	Control	0,625098
3 months	abundance	X10382	Zalcitabine	3	Interventio	Experimen	Experimen	Control	0,724259
3 months	abundance	X10384	L-N2-(2-Ca	3	Interventio	Experimen	Experimen	Control	0,239969
3 months	abundance	X10387	Coenzyme	3	Interventio	Experimen	Experimen	Control	0,886266
3 months	abundance	X10389	Leucylaspa	3	Interventio	Experimen	Experimen	Control	0,63213
3 months	abundance	X10393	Ro 20-1724	3	Interventio	Experimen	Experimen	Control	0,124404
3 months	abundance	X10401	3-Methylxa	1	Interventio	Experimen	Experimen	Control	0,99116
3 months	abundance	X10415	beta-D-Glc	3	Interventio	Experimen	Experimen	Control	0,143745
3 months	abundance	X10417	L-Sacchar(	2b	Interventio	Experimen	Experimen	Control	0,198011
3 months	abundance	X10429	1,5-Isoquir	2b	Interventio	Experimen	Experimen	Control	0,246675
3 months	abundance	X10442	(6alpha,11	3	Interventio	Experimen	Experimen	Control	0,055322
3 months	abundance	X10445	2-(Carboxy	3	Interventio	Experimen	Experimen	Control	0,675486
3 months	abundance	X10451	Ro 20-1724	3	Interventio	Experimen	Experimen	Control	0,629552
3 months	abundance	X10454	8-Amino-7-	3	Interventio	Experimen	Experimen	Control	0,61536
3 months	abundance	X10457	glu-ser	3	Interventio	Experimen	Experimen	Control	0,98719
3 months	abundance	X10476	GAMMA-HY	3	Interventio	Experimen	Experimen	Control	0,03075
3 months	abundance	X10486	Coumarin	2b	Interventio	Experimen	Experimen	Control	0,468958
3 months	abundance	X10488	4-Guanidir	1	Interventio	Experimen	Experimen	Control	0,46992
3 months	abundance	X10494	26Q0EO75	3	Interventio	Experimen	Experimen	Control	0,835202
3 months	abundance	X10497	4-Guanidir	3	Interventio	Experimen	Experimen	Control	0,490686
3 months	abundance	X10503	APM_a	3	Interventio	Experimen	Experimen	Control	0,980029
3 months	abundance	X10511	dopaquino	3	Interventio	Experimen	Experimen	Control	0,833128
3 months	abundance	X10513	ophthalmic	3	Interventio	Experimen	Experimen	Control	0,297155
3 months	abundance	X10518	MFCD0002	3	Interventio	Experimen	Experimen	Control	0,907733
3 months	abundance	X10534	L-N2-(2-Ca	3	Interventio	Experimen	Experimen	Control	0,718544
3 months	abundance	X10536	CMPF	3	Interventio	Experimen	Experimen	Control	0,988551
3 months	abundance	X10549	Glaucine	2b	Interventio	Experimen	Experimen	Control	0,901344
3 months	abundance	X10550	pentobarbi	3	Interventio	Experimen	Experimen	Control	0,423696
3 months	abundance	X10554	Glycine	1	Interventio	Experimen	Experimen	Control	0,440598
3 months	abundance	X10564	2-(1-Ethox	3	Interventio	Experimen	Experimen	Control	0,206211
3 months	abundance	X10580	Leu-Leu_a	3	Interventio	Experimen	Experimen	Control	0,773109
3 months	abundance	X10588	(8)-Ginger(	3	Interventio	Experimen	Experimen	Control	0,765006
3 months	abundance	X10593	Vorinostat_	3	Interventio	Experimen	Experimen	Control	0,701955
3 months	abundance	X10600	Glycocyam	3	Interventio	Experimen	Experimen	Control	0,830535
3 months	abundance	X10611	2-Acrylami	3	Interventio	Experimen	Experimen	Control	0,143745
3 months	abundance	X10614	Monometh	2a	Interventio	Experimen	Experimen	Control	0,552894
3 months	abundance	X10617	2-methyl-1	2b	Interventio	Experimen	Experimen	Control	0,000106
3 months	abundance	X10623	N-Nonano	3	Interventio	Experimen	Experimen	Control	0,960119
3 months	abundance	X10636	8-hydroxy-	2b	Interventio	Experimen	Experimen	Control	0,882862
3 months	abundance	X10647	4-Hydroxy	3	Interventio	Experimen	Experimen	Control	0,949849
3 months	abundance	X10651	Methyl 1-hy	3	Interventio	Experimen	Experimen	Control	0,949849
3 months	abundance	X10660	2-Methylth	3	Interventio	Experimen	Experimen	Control	0,98719
3 months	abundance	X10664	N1-(5-metl	2b	Interventio	Experimen	Experimen	Control	0,584092

3 months	abundance	X10665	Aprobarbit	3	Interventio	Experimen	Experimen	Control	0,011491
3 months	abundance	X10667	N-Phenyla	3	Interventio	Experimen	Experimen	Control	0,997328
3 months	abundance	X10676	6-(1-Hydro	3	Interventio	Experimen	Experimen	Control	0,932175
3 months	abundance	X10680	8-hydroxy-	3	Interventio	Experimen	Experimen	Control	0,714434
3 months	abundance	X10685	Glycylvalin	2a	Interventio	Experimen	Experimen	Control	0,960119
3 months	abundance	X10687	Gln-Gln	3	Interventio	Experimen	Experimen	Control	0,960135
3 months	abundance	X10691	(−)-nabilor	3	Interventio	Experimen	Experimen	Control	0,468958
3 months	abundance	X10706	Furaneol	3	Interventio	Experimen	Experimen	Control	0,242305
3 months	abundance	X10713	norhaman	3	Interventio	Experimen	Experimen	Control	0,748275
3 months	abundance	X10722	Acetanilide	2b	Interventio	Experimen	Experimen	Control	0,495802
3 months	abundance	X10725	Ethyl mala	3	Interventio	Experimen	Experimen	Control	0,354443
3 months	abundance	X10736	Toxopyrimi	3	Interventio	Experimen	Experimen	Control	0,217561
3 months	abundance	X10741	5-Nitro-2-p	3	Interventio	Experimen	Experimen	Control	0,915997
3 months	abundance	X10757	Glycyrin	3	Interventio	Experimen	Experimen	Control	0,082425
3 months	abundance	X10769	Cystine	1	Interventio	Experimen	Experimen	Control	0,63213
3 months	abundance	X10770	2-(4-Isopro	3	Interventio	Experimen	Experimen	Control	0,698621
3 months	abundance	X10772	Bicine_a	3	Interventio	Experimen	Experimen	Control	0,042091
3 months	abundance	X10776	Histidylgly	3	Interventio	Experimen	Experimen	Control	0,821708
3 months	abundance	X10793	Zalcitabine	3	Interventio	Experimen	Experimen	Control	0,122346
3 months	abundance	X10818	butalbital_	3	Interventio	Experimen	Experimen	Control	0,614079
3 months	abundance	X10819	Hyocholic	2a	Interventio	Experimen	Experimen	Control	0,892772
3 months	abundance	X10864	5-amino-2-	2b	Interventio	Experimen	Experimen	Control	0,884928
3 months	abundance	X10865	Melatonin	3	Interventio	Experimen	Experimen	Control	0,664511
3 months	abundance	X10881	L-gamma-(	3	Interventio	Experimen	Experimen	Control	0,94878
3 months	abundance	X10886	L-gamma-(	3	Interventio	Experimen	Experimen	Control	0,838286
3 months	abundance	X10889	N-acetyl-b	3	Interventio	Experimen	Experimen	Control	0,988551
3 months	abundance	X10896	6-Hydroxyr	2b	Interventio	Experimen	Experimen	Control	0,960119
3 months	abundance	X10901	bis-noryan	3	Interventio	Experimen	Experimen	Control	0,143745
3 months	abundance	X10902	Isoquinolir	2b	Interventio	Experimen	Experimen	Control	0,392246
3 months	abundance	X10918	meprobam	3	Interventio	Experimen	Experimen	Control	0,625098
3 months	abundance	X10929	Temozolon	3	Interventio	Experimen	Experimen	Control	0,021942
3 months	abundance	X10930	MFCD0995	3	Interventio	Experimen	Experimen	Control	0,76643
3 months	abundance	X10936	clavulanic	3	Interventio	Experimen	Experimen	Control	0,1745
3 months	abundance	X10947	1,1'-[1,12-	3	Interventio	Experimen	Experimen	Control	0,815986
3 months	abundance	X10951	8-Methyl-8	3	Interventio	Experimen	Experimen	Control	0,946232
3 months	abundance	X10953	Vorinostat_	3	Interventio	Experimen	Experimen	Control	0,078386
3 months	abundance	X10974	Threonylglu	3	Interventio	Experimen	Experimen	Control	0,902223
3 months	abundance	X10977	pentobarbi	3	Interventio	Experimen	Experimen	Control	0,952305
3 months	abundance	X10979	acetyltauri	3	Interventio	Experimen	Experimen	Control	0,986565
3 months	abundance	X10982	8-Methyl-8	3	Interventio	Experimen	Experimen	Control	0,330615
3 months	abundance	X10987	2-BUTYL PI	3	Interventio	Experimen	Experimen	Control	0,560103
3 months	abundance	X11001	2-[(carboxy	2b	Interventio	Experimen	Experimen	Control	0,820266
3 months	abundance	X11012	Furan	3	Interventio	Experimen	Experimen	Control	0,765006
3 months	abundance	X11028	butalbital_	3	Interventio	Experimen	Experimen	Control	0,604864
3 months	abundance	X11032	2,4-Bis(3-r	3	Interventio	Experimen	Experimen	Control	0,960119
3 months	abundance	X11033	N-Acetyl-5	3	Interventio	Experimen	Experimen	Control	0,967096
3 months	abundance	X11034	1-(3-Aminc	3	Interventio	Experimen	Experimen	Control	0,565249
3 months	abundance	X11036	3-Hydroxyp	2b	Interventio	Experimen	Experimen	Control	0,259613

3 months	abundance	X11038	(2E)-3-(3,4	3	Interventio	Experimen	Experimen	Control	0,722718
3 months	abundance	X11042	Leu-arg	3	Interventio	Experimen	Experimen	Control	0,396367
3 months	abundance	X11059	Dihydrocor	3	Interventio	Experimen	Experimen	Control	0,521176
3 months	abundance	X11067	Resveratro	3	Interventio	Experimen	Experimen	Control	0,755448
3 months	abundance	X11068	N,N'-Bis[4-	3	Interventio	Experimen	Experimen	Control	0,567851
3 months	abundance	X11110	Tauropine	3	Interventio	Experimen	Experimen	Control	0,248637
3 months	abundance	X11115	Hydroxycar	3	Interventio	Experimen	Experimen	Control	0,80026
3 months	abundance	X11117	Coprine_b	3	Interventio	Experimen	Experimen	Control	0,067442
3 months	abundance	X11125	5,8,12-Trih	3	Interventio	Experimen	Experimen	Control	0,029867
3 months	abundance	X11140	Leu-Leu_e	3	Interventio	Experimen	Experimen	Control	0,251604
3 months	abundance	X11163	Lys-Pro_a	3	Interventio	Experimen	Experimen	Control	0,176658
3 months	abundance	X11170	QJ972000(	3	Interventio	Experimen	Experimen	Control	0,510076
3 months	abundance	X11186	Fluocinolo	2b	Interventio	Experimen	Experimen	Control	0,99116
3 months	abundance	X11215	Leu-Leu_b	3	Interventio	Experimen	Experimen	Control	0,099053
3 months	abundance	X11223	Ethyl malat	3	Interventio	Experimen	Experimen	Control	0,988551
3 months	abundance	X11245	MFCD0003	3	Interventio	Experimen	Experimen	Control	0,718452
3 months	abundance	X11251	N-Nonanoy	3	Interventio	Experimen	Experimen	Control	0,685901
3 months	abundance	X11254	(4R,5S,9S,	3	Interventio	Experimen	Experimen	Control	0,614079
3 months	abundance	X11257	1-Methylgu	2b	Interventio	Experimen	Experimen	Control	0,191691
3 months	abundance	X11258	Valyl-4-hyc	3	Interventio	Experimen	Experimen	Control	0,972998
3 months	abundance	X11265	Sulfurol	3	Interventio	Experimen	Experimen	Control	0,342788
3 months	abundance	X11273	trp-pro	3	Interventio	Experimen	Experimen	Control	0,191691
3 months	abundance	X11277	lys-leu	3	Interventio	Experimen	Experimen	Control	0,000943
3 months	abundance	X11281	Penbutolol	3	Interventio	Experimen	Experimen	Control	0,798192
3 months	abundance	X11301	(6S)-2-Ami	3	Interventio	Experimen	Experimen	Control	0,552894
3 months	abundance	X11310	Leu-Val_a	3	Interventio	Experimen	Experimen	Control	0,633187
3 months	abundance	X11319	Methohexit	3	Interventio	Experimen	Experimen	Control	0,248187
3 months	abundance	X11323	dihydroxyb	3	Interventio	Experimen	Experimen	Control	0,003846
3 months	abundance	X11331	N~6~-Octa	3	Interventio	Experimen	Experimen	Control	0,058295
3 months	abundance	X11339	L-gamma-(	3	Interventio	Experimen	Experimen	Control	0,981401
3 months	abundance	X11347	Voglibose	3	Interventio	Experimen	Experimen	Control	0,953271
3 months	abundance	X11349	Butabarbit	3	Interventio	Experimen	Experimen	Control	0,198469
3 months	abundance	X11354	3-Oxo-4,6-	3	Interventio	Experimen	Experimen	Control	0,78879
3 months	abundance	X11361	1-Methylhi	3	Interventio	Experimen	Experimen	Control	0,150507
3 months	abundance	X11366	3-Sulfino-L	3	Interventio	Experimen	Experimen	Control	0,990745
3 months	abundance	X11373	3-Succinoy	2b	Interventio	Experimen	Experimen	Control	0,698621
3 months	abundance	X11375	L-gamma-(	3	Interventio	Experimen	Experimen	Control	0,698621
3 months	abundance	X11383	N-{3-[(4-Ac	3	Interventio	Experimen	Experimen	Control	0,835202
3 months	abundance	X11408	5-Amino-6	3	Interventio	Experimen	Experimen	Control	0,767238
3 months	abundance	X11421	8-Amino-7	3	Interventio	Experimen	Experimen	Control	0,338283
3 months	abundance	X11438	pro-met	3	Interventio	Experimen	Experimen	Control	0,748275
3 months	abundance	X11441	beta-D-Eth	3	Interventio	Experimen	Experimen	Control	0,958101
3 months	abundance	X11477	gamma-Gl	3	Interventio	Experimen	Experimen	Control	0,988198
3 months	abundance	X11481	his-gln_b	3	Interventio	Experimen	Experimen	Control	0,000404
3 months	abundance	X11494	3-Hydroxys	3	Interventio	Experimen	Experimen	Control	0,988551
3 months	abundance	X11498	Valylvaline	3	Interventio	Experimen	Experimen	Control	0,104321
3 months	abundance	X11505	FB950000(	3	Interventio	Experimen	Experimen	Control	0,60023
3 months	abundance	X11515	13(S)-HOT	2b	Interventio	Experimen	Experimen	Control	0,992999

3 months	abundance	X11522	thr-trp	3	Interventio	Experimen	Experimen	Control	0,61996
3 months	abundance	X11524	6-hydroxyp	3	Interventio	Experimen	Experimen	Control	0,142786
3 months	abundance	X11529	2-Aminooc	2b	Interventio	Experimen	Experimen	Control	0,301015
3 months	abundance	X11549	1,1'-[1,12-	3	Interventio	Experimen	Experimen	Control	0,797019
3 months	abundance	X11556	N-[(2E)-3-(	3	Interventio	Experimen	Experimen	Control	0,960119
3 months	abundance	X11562	ALA-PRO	3	Interventio	Experimen	Experimen	Control	0,876028
3 months	abundance	X11563	threonylph	3	Interventio	Experimen	Experimen	Control	0,76643
3 months	abundance	X11576	Phenyl D-g	3	Interventio	Experimen	Experimen	Control	0,988551
3 months	abundance	X11605	3-Methyl-2	3	Interventio	Experimen	Experimen	Control	0,985356
3 months	abundance	X11614	2-Methylth	3	Interventio	Experimen	Experimen	Control	0,46054
3 months	abundance	X11627	N-(3-aceta	3	Interventio	Experimen	Experimen	Control	0,821708
3 months	abundance	X11639	Midodrine_	3	Interventio	Experimen	Experimen	Control	0,821708
3 months	abundance	X11645	glu-thr	3	Interventio	Experimen	Experimen	Control	0,985356
3 months	abundance	X11666	N-(4-Hydrc	3	Interventio	Experimen	Experimen	Control	0,801038
3 months	abundance	X11667	Tetraacety	3	Interventio	Experimen	Experimen	Control	0,613297
3 months	abundance	X11692	2_7-Anhyd	3	Interventio	Experimen	Experimen	Control	0,05742
3 months	abundance	X11698	1-(beta-D-	3	Interventio	Experimen	Experimen	Control	1,75E-11
3 months	abundance	X11699	{2-[2-(Isob	3	Interventio	Experimen	Experimen	Control	0,511679
3 months	abundance	X11716	ala-ser_a	3	Interventio	Experimen	Experimen	Control	0,76643
3 months	abundance	X11717	nicotianam	3	Interventio	Experimen	Experimen	Control	0,430971
3 months	abundance	X11733	1,3-Dihydr	3	Interventio	Experimen	Experimen	Control	0,816795
3 months	abundance	X11745	2-Despipe	3	Interventio	Experimen	Experimen	Control	0,834104
3 months	abundance	X11755	Furfuranol	3	Interventio	Experimen	Experimen	Control	0,765006
3 months	abundance	p_cresol	p-Cresol	1	Interventio	Experimen	Experimen	Control	0,575427
6 months	abundance	X11698	1-(beta-D-	3	Interventio	Experimen	Experimen	Control	1,72E-21
6 months	abundance	X06039	gamma-Gl	3	Interventio	Experimen	Experimen	Control	1,72E-21
6 months	abundance	X08843	(2R,3S)-3-	3	Interventio	Experimen	Experimen	Control	6,48E-07
6 months	abundance	X07921	Pipecolinic	1	Interventio	Experimen	Experimen	Control	2,83E-06
6 months	abundance	X03132	(3S,4S)-7,	3	Interventio	Experimen	Experimen	Control	2,83E-06
6 months	abundance	X10617	2-methyl-1	2b	Interventio	Experimen	Experimen	Control	5,94E-06
6 months	abundance	X08726	YV819500	3	Interventio	Experimen	Experimen	Control	3,82E-05
6 months	abundance	X02928	N-Acetylpr	3	Interventio	Experimen	Experimen	Control	5,71E-06
6 months	abundance	X00375	N-[(2S)-2-	3	Interventio	Experimen	Experimen	Control	0,000814
6 months	abundance	X00107	N6-Acetyll	1	Interventio	Experimen	Experimen	Control	5,65E-05
6 months	abundance	X08561	N-(Carbox	3	Interventio	Experimen	Experimen	Control	8,18E-05
6 months	abundance	X07782	Cytosine	1	Interventio	Experimen	Experimen	Control	0,000821
6 months	abundance	X01768	Butabarbit	3	Interventio	Experimen	Experimen	Control	0,000235
6 months	abundance	X00115	Histidine	1	Interventio	Experimen	Experimen	Control	0,002657
6 months	abundance	X03023	NPK (Pepti	2b	Interventio	Experimen	Experimen	Control	0,001953
6 months	abundance	X01689	Ethyl mala	3	Interventio	Experimen	Experimen	Control	0,002657
6 months	abundance	X10611	2-Acrylami	3	Interventio	Experimen	Experimen	Control	0,003073
6 months	abundance	X08204	2-Amino-4	2b	Interventio	Experimen	Experimen	Control	0,002868
6 months	abundance	SL00309	1-Aminocy	2a	Interventio	Experimen	Experimen	Control	0,000833
6 months	abundance	X07674	Lysine	1	Interventio	Experimen	Experimen	Control	0,00107
6 months	abundance	X06046	Valylvaline	3	Interventio	Experimen	Experimen	Control	0,003235
6 months	abundance	X00022	N-Methyla	1	Interventio	Experimen	Experimen	Control	0,003235
6 months	abundance	X06477	APM_b	3	Interventio	Experimen	Experimen	Control	0,002657
6 months	abundance	X05499	GLY-MET	3	Interventio	Experimen	Experimen	Control	0,003235

6 months	abundance	X07394	Hydroxypro	3	Interventio	Experimen	Experimen	Control	0,002808
6 months	abundance	X03961	Artesunate	3	Interventio	Experimen	Experimen	Control	0,007941
6 months	abundance	X00032	Indole-3-pr	1	Interventio	Experimen	Experimen	Control	0,007096
6 months	abundance	X09256	Aspartyl-L-	3	Interventio	Experimen	Experimen	Control	0,003479
6 months	abundance	M12	M12	IA_module	Interventio	Experimen	Experimen	Control	0,002657
6 months	abundance	X09969	3-Methylac	3	Interventio	Experimen	Experimen	Control	0,003399
6 months	abundance	X00702	N-[(2S)-2-H	3	Interventio	Experimen	Experimen	Control	0,008517
6 months	abundance	X05195	Lys-phe_a	3	Interventio	Experimen	Experimen	Control	0,008517
6 months	abundance	X06630	4-(METHYL	3	Interventio	Experimen	Experimen	Control	0,007523
6 months	abundance	X09980	N-(4-Hepta	3	Interventio	Experimen	Experimen	Control	0,002868
6 months	abundance	M2	M2	IA_module	Interventio	Experimen	Experimen	Control	0,012138
6 months	abundance	X09165	N-[1-Carbo	3	Interventio	Experimen	Experimen	Control	0,006049
6 months	abundance	X00027	N-Acetylglu	1	Interventio	Experimen	Experimen	Control	0,004511
6 months	abundance	X06675	L-gamma-(	3	Interventio	Experimen	Experimen	Control	0,006888
6 months	abundance	X08944	Dipivefrin	3	Interventio	Experimen	Experimen	Control	0,004096
6 months	abundance	X06958	2-[(carboxy	2b	Interventio	Experimen	Experimen	Control	0,012263
6 months	abundance	X07825	Maltotriose	1	Interventio	Experimen	Experimen	Control	0,002657
6 months	abundance	X05149	S-Sulfocys	3	Interventio	Experimen	Experimen	Control	0,007941
6 months	abundance	X07381	Bis-D-fruct	3	Interventio	Experimen	Experimen	Control	0,002868
6 months	abundance	X03604	Gly-Trp_a	3	Interventio	Experimen	Experimen	Control	0,012184
6 months	abundance	X04526	N-Nonanoy	3	Interventio	Experimen	Experimen	Control	0,019271
6 months	abundance	X02843	4-(1-Hydro	3	Interventio	Experimen	Experimen	Control	0,009848
6 months	abundance	X08938	Nonivamid	3	Interventio	Experimen	Experimen	Control	0,002516
6 months	abundance	X10901	bis-noryan	3	Interventio	Experimen	Experimen	Control	0,012138
6 months	abundance	SL00049	N-Acetylar	2a	Interventio	Experimen	Experimen	Control	0,012184
6 months	abundance	X01549	3-(Sulfooxy	3	Interventio	Experimen	Experimen	Control	0,012184
6 months	abundance	Hexanoic a	Hexanoic a	CFA_panel	Interventio	Experimen	Experimen	Control	0,06731
6 months	abundance	X10929	Temozolon	3	Interventio	Experimen	Experimen	Control	0,014596
6 months	abundance	X06866	tert-Butyl 3	3	Interventio	Experimen	Experimen	Control	0,013902
6 months	abundance	X02181	YWA1	3	Interventio	Experimen	Experimen	Control	0,012466
6 months	abundance	X10564	2-(1-Ethoxy	3	Interventio	Experimen	Experimen	Control	0,031241
6 months	abundance	X00093	Deoxyinosi	1	Interventio	Experimen	Experimen	Control	0,022391
6 months	abundance	X08777	3-Aminosa	2b	Interventio	Experimen	Experimen	Control	0,012184
6 months	abundance	X06361	Oxprenolol	3	Interventio	Experimen	Experimen	Control	0,003235
6 months	abundance	X00045	Threonine	1	Interventio	Experimen	Experimen	Control	0,025332
6 months	abundance	X01884	Roxane	3	Interventio	Experimen	Experimen	Control	0,006888
6 months	abundance	X11421	8-Amino-7-	3	Interventio	Experimen	Experimen	Control	0,005507
6 months	abundance	X11163	Lys-Pro_a	3	Interventio	Experimen	Experimen	Control	0,022035
6 months	abundance	X00114	Indole-3-m	1	Interventio	Experimen	Experimen	Control	0,017055
6 months	abundance	X08062	Indole-3-ca	1	Interventio	Experimen	Experimen	Control	0,013207
6 months	abundance	X10099	ala-met	3	Interventio	Experimen	Experimen	Control	0,021862
6 months	abundance	X00237	OI1700000	3	Interventio	Experimen	Experimen	Control	0,025324
6 months	abundance	SL00054	Ornithine	1	Interventio	Experimen	Experimen	Control	0,009848
6 months	abundance	X08670	Hydroxypro	3	Interventio	Experimen	Experimen	Control	0,015345
6 months	abundance	X10902	Isoquinolir	2b	Interventio	Experimen	Experimen	Control	0,019271
6 months	abundance	X02513	7-Methylac	2b	Interventio	Experimen	Experimen	Control	0,024983
6 months	abundance	X05275	H-DL-MET-	3	Interventio	Experimen	Experimen	Control	0,012138
6 months	abundance	X02720	ala-ser_b	3	Interventio	Experimen	Experimen	Control	0,024328

6 months	abundance	X07961	2-(Hydroxy	3	Interventio	Experimen	Experimen	Control	0,022718
6 months	abundance	SL00373	N1-Methyl-	2a	Interventio	Experimen	Experimen	Control	0,027979
6 months	abundance	X04897	Taxifolin	3	Interventio	Experimen	Experimen	Control	0,019271
6 months	abundance	X10736	Toxopyrimi	3	Interventio	Experimen	Experimen	Control	0,012184
6 months	abundance	X09622	N-Hydroxy-	3	Interventio	Experimen	Experimen	Control	0,031688
6 months	abundance	SL00017	Aspartic ac	2a	Interventio	Experimen	Experimen	Control	0,032825
6 months	abundance	X11717	nicotianam	3	Interventio	Experimen	Experimen	Control	0,024334
6 months	abundance	SL00189	3-Hydroxyb	2a	Interventio	Experimen	Experimen	Control	0,009848
6 months	abundance	SL00328	2-(hydroxy	2a	Interventio	Experimen	Experimen	Control	0,01944
6 months	abundance	X00320	Panthenol_	3	Interventio	Experimen	Experimen	Control	0,034024
6 months	abundance	X07558	Leucylproli	2b	Interventio	Experimen	Experimen	Control	0,012263
6 months	abundance	SL00140	Pyridoxami	1	Interventio	Experimen	Experimen	Control	0,021043
6 months	abundance	X10554	Glycine	1	Interventio	Experimen	Experimen	Control	0,030586
6 months	abundance	X08548	N2-Acetylly	1	Interventio	Experimen	Experimen	Control	0,021401
6 months	abundance	X10265	3-methyl-4	3	Interventio	Experimen	Experimen	Control	0,012184
6 months	abundance	X09963	Iminoglycir	3	Interventio	Experimen	Experimen	Control	0,040501
6 months	abundance	X11170	QJ972000(	3	Interventio	Experimen	Experimen	Control	0,030712
6 months	abundance	X10687	Gln-Gln	3	Interventio	Experimen	Experimen	Control	0,030712
6 months	abundance	X01246	(2R)-1-{[(2-	3	Interventio	Experimen	Experimen	Control	0,04742
6 months	abundance	X02380	Propafenoı	3	Interventio	Experimen	Experimen	Control	0,027049
6 months	abundance	X07630	Methionine	1	Interventio	Experimen	Experimen	Control	0,020533
6 months	abundance	X00036	Serine	1	Interventio	Experimen	Experimen	Control	0,04742
6 months	abundance	X00127	Nicotinic a	1	Interventio	Experimen	Experimen	Control	0,035097
6 months	abundance	X09849	3-Methylac	3	Interventio	Experimen	Experimen	Control	0,035097
6 months	abundance	X05033	(2E)-N-3,7-	3	Interventio	Experimen	Experimen	Control	0,020208
6 months	abundance	X01235	3-[(3-Hydro	3	Interventio	Experimen	Experimen	Control	0,012184
6 months	abundance	SL00149	Dissaccari	2a	Interventio	Experimen	Experimen	Control	0,017511
6 months	abundance	X08712	4-Iodoanis	3	Interventio	Experimen	Experimen	Control	0,041133
6 months	abundance	X10706	Furaneol	3	Interventio	Experimen	Experimen	Control	0,012552
6 months	abundance	X11614	2-Methylth	3	Interventio	Experimen	Experimen	Control	0,056436
6 months	abundance	SL00009	N-Acetylhi:	2a	Interventio	Experimen	Experimen	Control	0,055668
6 months	abundance	X08012	Methylol D	3	Interventio	Experimen	Experimen	Control	0,032515
6 months	abundance	X10276	Maleamate	3	Interventio	Experimen	Experimen	Control	0,053894
6 months	abundance	SL00081	5-Methylcy	2a	Interventio	Experimen	Experimen	Control	0,031688
6 months	abundance	X00998	epsilon-(ga	3	Interventio	Experimen	Experimen	Control	0,046863
6 months	abundance	X06240	His-pro_a	3	Interventio	Experimen	Experimen	Control	0,032515
6 months	abundance	X02760	6-hydroxyp	3	Interventio	Experimen	Experimen	Control	0,03552
6 months	abundance	X08895	pentoxyl	3	Interventio	Experimen	Experimen	Control	0,04788
6 months	abundance	X11036	3-Hydroxyp	2b	Interventio	Experimen	Experimen	Control	0,035388
6 months	abundance	X09981	N-Acetylva	3	Interventio	Experimen	Experimen	Control	0,025324
6 months	abundance	X07125	Zalcitabine	3	Interventio	Experimen	Experimen	Control	0,035357
6 months	abundance	X07856	(3R,4S,5S,	3	Interventio	Experimen	Experimen	Control	0,032515
6 months	abundance	X01340	Cadralazin	3	Interventio	Experimen	Experimen	Control	0,045594
6 months	abundance	X08116	Methyl (2Z,	3	Interventio	Experimen	Experimen	Control	0,056436
6 months	abundance	X10415	beta-D-Glc	3	Interventio	Experimen	Experimen	Control	0,025324
6 months	abundance	X06409	Glycylproli	2b	Interventio	Experimen	Experimen	Control	0,035097
6 months	abundance	X06771	MFCD0015	3	Interventio	Experimen	Experimen	Control	0,056436
6 months	abundance	X08525	MFCD1869	3	Interventio	Experimen	Experimen	Control	0,072606

6 months	abundance	X08230	Coumaron	3	Interventio	Experimen	Experimen	Control	0,066068
6 months	abundance	X00683	Zinecard_a	3	Interventio	Experimen	Experimen	Control	0,068028
6 months	abundance	X00057	N-Acetylor	1	Interventio	Experimen	Experimen	Control	0,068026
6 months	abundance	X00094	Methionine	1	Interventio	Experimen	Experimen	Control	0,080527
6 months	abundance	X06089	riboprine	3	Interventio	Experimen	Experimen	Control	0,074642
6 months	abundance	X07460	1-pyrroline	3	Interventio	Experimen	Experimen	Control	0,050313
6 months	abundance	X04339	L-Glutamic	3	Interventio	Experimen	Experimen	Control	0,055563
6 months	abundance	X00124	Leucine	1	Interventio	Experimen	Experimen	Control	0,056436
6 months	abundance	X10486	Coumarin	2b	Interventio	Experimen	Experimen	Control	0,077439
6 months	abundance	X10722	Acetanilide	2b	Interventio	Experimen	Experimen	Control	0,06731
6 months	abundance	X07512	4-{[(3-Hydr	3	Interventio	Experimen	Experimen	Control	0,046863
6 months	abundance	X09724	Formylkynu	3	Interventio	Experimen	Experimen	Control	0,099094
6 months	abundance	X04822	6-Sulfatoxy	3	Interventio	Experimen	Experimen	Control	0,055312
6 months	abundance	SL00074	Valine/ 5-A	2a	Interventio	Experimen	Experimen	Control	0,059922
6 months	abundance	X10713	norhaman	3	Interventio	Experimen	Experimen	Control	0,065238
6 months	abundance	X04571	13a-Hydro:	3	Interventio	Experimen	Experimen	Control	0,06731
6 months	abundance	X05029	Hypericin	3	Interventio	Experimen	Experimen	Control	0,068026
6 months	abundance	X05459	Scopoletin	3	Interventio	Experimen	Experimen	Control	0,077865
6 months	abundance	SL00061	Proline	1	Interventio	Experimen	Experimen	Control	0,065283
6 months	abundance	X00076	Isoleucine	1	Interventio	Experimen	Experimen	Control	0,065793
6 months	abundance	X06858	tert-Butyl 3	3	Interventio	Experimen	Experimen	Control	0,060957
6 months	abundance	X00073	Indole-3-la	1	Interventio	Experimen	Experimen	Control	0,047694
6 months	abundance	X07647	Genistein	1	Interventio	Experimen	Experimen	Control	0,100431
6 months	abundance	X07334	2,2-Bis(hyd	3	Interventio	Experimen	Experimen	Control	0,080527
6 months	abundance	X09385	2-(1-Napht	3	Interventio	Experimen	Experimen	Control	0,103356
6 months	abundance	X07989	Piperidine_	3	Interventio	Experimen	Experimen	Control	0,071535
6 months	abundance	X06043	vinyl sulfid	3	Interventio	Experimen	Experimen	Control	0,103571
6 months	abundance	X07050	2-Methylth	3	Interventio	Experimen	Experimen	Control	0,103571
6 months	abundance	X09391	ophthalmic	3	Interventio	Experimen	Experimen	Control	0,110798
6 months	abundance	X08549	3-Methylsu	3	Interventio	Experimen	Experimen	Control	0,106358
6 months	abundance	X08002	(2S)-6-Ami	3	Interventio	Experimen	Experimen	Control	0,102658
6 months	abundance	X06454	8-Amino-7-	3	Interventio	Experimen	Experimen	Control	0,103571
6 months	abundance	X11117	Coprine_b	3	Interventio	Experimen	Experimen	Control	0,059922
6 months	abundance	X07974	Isoprene	3	Interventio	Experimen	Experimen	Control	0,080527
6 months	abundance	X02442	3-(Sulfooxy	3	Interventio	Experimen	Experimen	Control	0,099711
6 months	abundance	X05995	Diacetin_a	3	Interventio	Experimen	Experimen	Control	0,094328
6 months	abundance	X05478	S(8)-amino	3	Interventio	Experimen	Experimen	Control	0,074642
6 months	abundance	X10442	(6alpha,11	3	Interventio	Experimen	Experimen	Control	0,113797
6 months	abundance	X07054	Valylprolin	2b	Interventio	Experimen	Experimen	Control	0,062463
6 months	abundance	X08699	Aurorix	3	Interventio	Experimen	Experimen	Control	0,095545
6 months	abundance	X06812	2-Hydroxy-	3	Interventio	Experimen	Experimen	Control	0,127044
6 months	abundance	X06404	Flemichap	3	Interventio	Experimen	Experimen	Control	0,12884
6 months	abundance	X09554	N-Propiony	3	Interventio	Experimen	Experimen	Control	0,075736
6 months	abundance	X09726	indoline-2-	2b	Interventio	Experimen	Experimen	Control	0,126909
6 months	abundance	X10375	4-Indoleca	2b	Interventio	Experimen	Experimen	Control	0,126909
6 months	abundance	X00050	β-Hydroxyi	1	Interventio	Experimen	Experimen	Control	0,143926
6 months	abundance	X00068	N-Acetylm	1	Interventio	Experimen	Experimen	Control	0,126909
6 months	abundance	X08111	2-Hydroxy	2b	Interventio	Experimen	Experimen	Control	0,137332

6 months	abundance	X07914	(-)-Erythrom	3	Interventio	Experimen	Experimen	Control	0,063697
6 months	abundance	X05357	NPYR	3	Interventio	Experimen	Experimen	Control	0,113797
6 months	abundance	X01621	2-(2,4-Dihy	3	Interventio	Experimen	Experimen	Control	0,148418
6 months	abundance	M9	M9	IA_module	Interventio	Experimen	Experimen	Control	0,118447
6 months	abundance	X03363	Choline Alf	3	Interventio	Experimen	Experimen	Control	0,130162
6 months	abundance	X08421	Cys-tyr	3	Interventio	Experimen	Experimen	Control	0,133886
6 months	abundance	X00106	N-Acetyltry	1	Interventio	Experimen	Experimen	Control	0,097673
6 months	abundance	X08288	Capryloylg	3	Interventio	Experimen	Experimen	Control	0,071535
6 months	abundance	X04877	Trifluorome	3	Interventio	Experimen	Experimen	Control	0,174109
6 months	abundance	X06622	Lys-Pro_b	3	Interventio	Experimen	Experimen	Control	0,105786
6 months	abundance	SL00247	Shikimic ac	2a	Interventio	Experimen	Experimen	Control	0,068028
6 months	abundance	X01208	Lys-Pro_c	3	Interventio	Experimen	Experimen	Control	0,089671
6 months	abundance	X08677	1-methylhy	3	Interventio	Experimen	Experimen	Control	0,126909
6 months	abundance	X09122	Valyl-4-hyc	3	Interventio	Experimen	Experimen	Control	0,115704
6 months	abundance	SL00103	Gulonic ac	2a	Interventio	Experimen	Experimen	Control	0,125436
6 months	abundance	X08098	Citrulline	1	Interventio	Experimen	Experimen	Control	0,109405
6 months	abundance	X05240	Dimeric me	3	Interventio	Experimen	Experimen	Control	0,157793
6 months	abundance	X10769	Cystine	1	Interventio	Experimen	Experimen	Control	0,161409
6 months	abundance	SL00210	Galactonic	2a	Interventio	Experimen	Experimen	Control	0,146968
6 months	abundance	SL00334	Imidazolea	2a	Interventio	Experimen	Experimen	Control	0,067529
6 months	abundance	SL00108	Histidinol	2a	Interventio	Experimen	Experimen	Control	0,110798
6 months	abundance	X05214	[7-Hydroxy	3	Interventio	Experimen	Experimen	Control	0,0902
6 months	abundance	X00130	Deoxyuridi	1	Interventio	Experimen	Experimen	Control	0,161409
6 months	abundance	X07476	4-Hydroxyb	1	Interventio	Experimen	Experimen	Control	0,145494
6 months	abundance	SL00215	Glycolic ac	2a	Interventio	Experimen	Experimen	Control	0,142606
6 months	abundance	X08019	Thymidine	1	Interventio	Experimen	Experimen	Control	0,149045
6 months	abundance	X01031	3-(14-Ethy	3	Interventio	Experimen	Experimen	Control	0,126909
6 months	abundance	X07424	Rutinose (b	3	Interventio	Experimen	Experimen	Control	0,160244
6 months	abundance	X08822	CYS-ASP	3	Interventio	Experimen	Experimen	Control	0,170002
6 months	abundance	X06034	N-[(2S)-2-H	3	Interventio	Experimen	Experimen	Control	0,170002
6 months	abundance	X00071	Tyrosine	1	Interventio	Experimen	Experimen	Control	0,165608
6 months	abundance	X08150	DL-4-Hydro	2b	Interventio	Experimen	Experimen	Control	0,160244
6 months	abundance	X08616	MFCD0002	3	Interventio	Experimen	Experimen	Control	0,187472
6 months	abundance	X02080	(-)-Aspidos	3	Interventio	Experimen	Experimen	Control	0,248374
6 months	abundance	X10476	GAMMA-HY	3	Interventio	Experimen	Experimen	Control	0,196868
6 months	abundance	X00011	Glutamine	2a	Interventio	Experimen	Experimen	Control	0,114299
6 months	abundance	X09721	3-(2,3-Dihy	3	Interventio	Experimen	Experimen	Control	0,22431
6 months	abundance	X07216	pentobarbi	3	Interventio	Experimen	Experimen	Control	0,153848
6 months	abundance	X04788	2-(4-Isopro	3	Interventio	Experimen	Experimen	Control	0,172247
6 months	abundance	X00102	Tryptophan	1	Interventio	Experimen	Experimen	Control	0,171599
6 months	abundance	X06870	Menadiol	3	Interventio	Experimen	Experimen	Control	0,160244
6 months	abundance	X02101	Succinic an	3	Interventio	Experimen	Experimen	Control	0,172965
6 months	abundance	X09741	Estrone glu	3	Interventio	Experimen	Experimen	Control	0,10858
6 months	abundance	X02468	N,N-Diethy	3	Interventio	Experimen	Experimen	Control	0,169648
6 months	abundance	X07537	alpha-Cha	3	Interventio	Experimen	Experimen	Control	0,171874
6 months	abundance	X06507	Gly-Lys	3	Interventio	Experimen	Experimen	Control	0,201493
6 months	abundance	X10793	Zalcitabine	3	Interventio	Experimen	Experimen	Control	0,103211
6 months	abundance	SL00440	5-Methylcy	2a	Interventio	Experimen	Experimen	Control	0,162202

6 months	abundance	SL00246	Pentose III	2a	Interventio	Experimen	Experimen	Control	0,171874
6 months	abundance	X00090	Malic acid	1	Interventio	Experimen	Experimen	Control	0,133886
6 months	abundance	X06656	Valylvaline	3	Interventio	Experimen	Experimen	Control	0,20627
6 months	abundance	X11556	N-[(2E)-3-(	3	Interventio	Experimen	Experimen	Control	0,148764
6 months	abundance	X10248	(+)-Etomid	3	Interventio	Experimen	Experimen	Control	0,167056
6 months	abundance	X02277	tert-Butyl 3	3	Interventio	Experimen	Experimen	Control	0,187192
6 months	abundance	X01367	MFCD1869	3	Interventio	Experimen	Experimen	Control	0,225907
6 months	abundance	X00060	Ethylmalor	1	Interventio	Experimen	Experimen	Control	0,186012
6 months	abundance	SL00436	1-Carboxye	2a	Interventio	Experimen	Experimen	Control	0,143926
6 months	abundance	X09654	linatine	3	Interventio	Experimen	Experimen	Control	0,11721
6 months	abundance	X08436	N,N-Dimet	3	Interventio	Experimen	Experimen	Control	0,287584
6 months	abundance	X10148	cys-met	3	Interventio	Experimen	Experimen	Control	0,215732
6 months	abundance	X00088	Pantotheni	1	Interventio	Experimen	Experimen	Control	0,254807
6 months	abundance	X00367	hexobarbit	3	Interventio	Experimen	Experimen	Control	0,179358
6 months	abundance	X09390	asn-pro_a	3	Interventio	Experimen	Experimen	Control	0,138188
6 months	abundance	X06634	Glycitein	3	Interventio	Experimen	Experimen	Control	0,186692
6 months	abundance	X11067	Resveratro	3	Interventio	Experimen	Experimen	Control	0,174109
6 months	abundance	X02599	Dihydrothy	3	Interventio	Experimen	Experimen	Control	0,201493
6 months	abundance	X00204	4-O-{3-O-[(	3	Interventio	Experimen	Experimen	Control	0,23628
6 months	abundance	X11375	L-gamma-(	3	Interventio	Experimen	Experimen	Control	0,180061
6 months	abundance	X06631	2-Isopropy	2b	Interventio	Experimen	Experimen	Control	0,174109
6 months	abundance	X05878	1,2-dihydro	3	Interventio	Experimen	Experimen	Control	0,23628
6 months	abundance	X09533	S(6)-acetyl	3	Interventio	Experimen	Experimen	Control	0,234335
6 months	abundance	X06546	4-Acetami	2b	Interventio	Experimen	Experimen	Control	0,186094
6 months	abundance	M25	M25	NA_module	Interventio	Experimen	Experimen	Control	0,246099
6 months	abundance	X06167	N-Acetylas	2a	Interventio	Experimen	Experimen	Control	0,169034
6 months	abundance	X07530	Alanine	1	Interventio	Experimen	Experimen	Control	0,189396
6 months	abundance	X07873	(3Z,6Z,9Z,1	3	Interventio	Experimen	Experimen	Control	0,261581
6 months	abundance	X02746	N,N-Diethy	3	Interventio	Experimen	Experimen	Control	0,247541
6 months	abundance	X02514	3-Ureidopr	1	Interventio	Experimen	Experimen	Control	0,220238
6 months	abundance	X05982	(19R,25S)-	3	Interventio	Experimen	Experimen	Control	0,25478
6 months	abundance	X00574	miglustat	3	Interventio	Experimen	Experimen	Control	0,217996
6 months	abundance	X00018	Succinic ac	1	Interventio	Experimen	Experimen	Control	0,196145
6 months	abundance	X02466	Validamyci	3	Interventio	Experimen	Experimen	Control	0,186094
6 months	abundance	X07451	Valylvaline	3	Interventio	Experimen	Experimen	Control	0,179439
6 months	abundance	X01045	Ethyl malto	3	Interventio	Experimen	Experimen	Control	0,134826
6 months	abundance	X11257	1-Methylgu	2b	Interventio	Experimen	Experimen	Control	0,287584
6 months	abundance	X10647	4-Hydroxyp	3	Interventio	Experimen	Experimen	Control	0,278551
6 months	abundance	SL00089	Dissaccari	2a	Interventio	Experimen	Experimen	Control	0,162222
6 months	abundance	X06254	D-Alanyl-D	3	Interventio	Experimen	Experimen	Control	0,274433
6 months	abundance	X05177	cis-3-Hexe	3	Interventio	Experimen	Experimen	Control	0,284006
6 months	abundance	X09635	2,4,6-Triiso	3	Interventio	Experimen	Experimen	Control	0,301875
6 months	abundance	X03340	His-pro_b	3	Interventio	Experimen	Experimen	Control	0,20627
6 months	abundance	X07270	5-(2-Carbo	3	Interventio	Experimen	Experimen	Control	0,250568
6 months	abundance	X07504	4-Acetami	2b	Interventio	Experimen	Experimen	Control	0,254963
6 months	abundance	X10664	N1-(5-metl	2b	Interventio	Experimen	Experimen	Control	0,248374
6 months	abundance	X08013	Deoxysuga	1	Interventio	Experimen	Experimen	Control	0,262216
6 months	abundance	X09609	N-{3-[(4-Ac	3	Interventio	Experimen	Experimen	Control	0,270217

6 months	abundance	X02854	Marimasta	3	Interventio	Experimen	Experimen	Control	0,301288
6 months	abundance	X09657	2,3,4,5,6-P	3	Interventio	Experimen	Experimen	Control	0,303634
6 months	abundance	X08028	Solanidine	3	Interventio	Experimen	Experimen	Control	0,273643
6 months	abundance	X09555	N4-(beta-N	3	Interventio	Experimen	Experimen	Control	0,287584
6 months	abundance	X06977	4-Hydroxyp	3	Interventio	Experimen	Experimen	Control	0,287584
6 months	abundance	X06943	NSC 92778	3	Interventio	Experimen	Experimen	Control	0,303982
6 months	abundance	X04310	Aminohipp	3	Interventio	Experimen	Experimen	Control	0,22431
6 months	abundance	X00123	Phenylalar	1	Interventio	Experimen	Experimen	Control	0,275763
6 months	abundance	X07524	Prolinamid	2b	Interventio	Experimen	Experimen	Control	0,301288
6 months	abundance	X09951	5beta-Cypr	3	Interventio	Experimen	Experimen	Control	0,310912
6 months	abundance	X00101	Threonic ac	1	Interventio	Experimen	Experimen	Control	0,275484
6 months	abundance	X04682	1-Methylin	3	Interventio	Experimen	Experimen	Control	0,334391
6 months	abundance	X10036	Nimodipine	3	Interventio	Experimen	Experimen	Control	0,316696
6 months	abundance	X02606	4-Amino-1-	3	Interventio	Experimen	Experimen	Control	0,33088
6 months	abundance	X07164	N-Acetyl-5	3	Interventio	Experimen	Experimen	Control	0,391188
6 months	abundance	X00344	SECONAL_	3	Interventio	Experimen	Experimen	Control	0,324575
6 months	abundance	X06510	N-{4-[(2R,3	2b	Interventio	Experimen	Experimen	Control	0,213812
6 months	abundance	X08870	Tetraacety	3	Interventio	Experimen	Experimen	Control	0,310448
6 months	abundance	X07084	heptabarbi	3	Interventio	Experimen	Experimen	Control	0,33088
6 months	abundance	X09336	1,1'-[1,12-I	3	Interventio	Experimen	Experimen	Control	0,312951
6 months	abundance	X00182	3-(Butylsul	3	Interventio	Experimen	Experimen	Control	0,278551
6 months	abundance	X00264	PEG n12	2b	Interventio	Experimen	Experimen	Control	0,373224
6 months	abundance	X02073	N-acetyl-9-	3	Interventio	Experimen	Experimen	Control	0,25478
6 months	abundance	X04553	N-(4-Amino	3	Interventio	Experimen	Experimen	Control	0,346785
6 months	abundance	X08714	Arabic acid	3	Interventio	Experimen	Experimen	Control	0,288871
6 months	abundance	X01285	FB9500000	3	Interventio	Experimen	Experimen	Control	0,365232
6 months	abundance	X04759	7-Aminome	3	Interventio	Experimen	Experimen	Control	0,378791
6 months	abundance	X09476	N(1),N(8)-I	3	Interventio	Experimen	Experimen	Control	0,324575
6 months	abundance	X03097	6-hydroxym	3	Interventio	Experimen	Experimen	Control	0,375103
6 months	abundance	X06906	Arg-pro	3	Interventio	Experimen	Experimen	Control	0,310912
6 months	abundance	X06735	7-Methylxa	2b	Interventio	Experimen	Experimen	Control	0,355054
6 months	abundance	X01996	3-Hydroxy-	3	Interventio	Experimen	Experimen	Control	0,28123
6 months	abundance	X07420	DNOP_h	3	Interventio	Experimen	Experimen	Control	0,310912
6 months	abundance	X07002	Paraldehyd	3	Interventio	Experimen	Experimen	Control	0,291867
6 months	abundance	X08412	2-Hydroxyp	2b	Interventio	Experimen	Experimen	Control	0,320284
6 months	abundance	X06836	asn-lys	3	Interventio	Experimen	Experimen	Control	0,358168
6 months	abundance	X02000	11-(4-Hydr	3	Interventio	Experimen	Experimen	Control	0,355054
6 months	abundance	X09010	4-Hydroxyp	1	Interventio	Experimen	Experimen	Control	0,260834
6 months	abundance	X10896	6-Hydroxyn	2b	Interventio	Experimen	Experimen	Control	0,310448
6 months	abundance	X09091	N-(3-aceta	3	Interventio	Experimen	Experimen	Control	0,299663
6 months	abundance	X00040	N-Acetyltyı	1	Interventio	Experimen	Experimen	Control	0,346785
6 months	abundance	X02504	DLK (Pepti	2b	Interventio	Experimen	Experimen	Control	0,30493
6 months	abundance	X05561	1Î²-Hydroxy	3	Interventio	Experimen	Experimen	Control	0,326255
6 months	abundance	SL00325	2-Methylm	2a	Interventio	Experimen	Experimen	Control	0,34199
6 months	abundance	X07907	4-Acetamic	1	Interventio	Experimen	Experimen	Control	0,380677
6 months	abundance	X10224	Imidazolela	2b	Interventio	Experimen	Experimen	Control	0,307119
6 months	abundance	X11310	Leu-Val_a	3	Interventio	Experimen	Experimen	Control	0,34021
6 months	abundance	X00020	sugar alcol	1	Interventio	Experimen	Experimen	Control	0,381461

6 months	abundance	X08096	(2E)-3-Met	3	Interventio	Experimen	Experimen	Control	0,320854
6 months	abundance	X06016	N,N-Dimet	3	Interventio	Experimen	Experimen	Control	0,402458
6 months	abundance	X07400	Tranexami	2b	Interventio	Experimen	Experimen	Control	0,34199
6 months	abundance	Acetic acid	Acetic acid	CFA_panel	Interventio	Experimen	Experimen	Control	0,310448
6 months	abundance	X00067	Hypoxanth	1	Interventio	Experimen	Experimen	Control	0,299663
6 months	abundance	X00403	7alpha-Hy	3	Interventio	Experimen	Experimen	Control	0,370881
6 months	abundance	X06529	Methanesu	2a	Interventio	Experimen	Experimen	Control	0,375103
6 months	abundance	X06230	Glu-Gly	3	Interventio	Experimen	Experimen	Control	0,286639
6 months	abundance	X11258	Valyl-4-hyc	3	Interventio	Experimen	Experimen	Control	0,378866
6 months	abundance	X02214	(E)-4-Meth	3	Interventio	Experimen	Experimen	Control	0,402458
6 months	abundance	X07073	asn-val_a	3	Interventio	Experimen	Experimen	Control	0,410744
6 months	abundance	X11281	Penbutolol	3	Interventio	Experimen	Experimen	Control	0,294471
6 months	abundance	SL00265	Purine	2a	Interventio	Experimen	Experimen	Control	0,417706
6 months	abundance	X10680	8-hydroxy-	3	Interventio	Experimen	Experimen	Control	0,452861
6 months	abundance	X09260	(4S)-4-[(2E	3	Interventio	Experimen	Experimen	Control	0,303733
6 months	abundance	SL00315	Malonic ac	2a	Interventio	Experimen	Experimen	Control	0,428209
6 months	abundance	X04975	MFCD0995	3	Interventio	Experimen	Experimen	Control	0,425528
6 months	abundance	X11319	Methohexi	3	Interventio	Experimen	Experimen	Control	0,328207
6 months	abundance	X00242	IN00258	3	Interventio	Experimen	Experimen	Control	0,296902
6 months	abundance	X10367	2-(1-Napht	3	Interventio	Experimen	Experimen	Control	0,39359
6 months	abundance	X08476	Coprine_c	3	Interventio	Experimen	Experimen	Control	0,401596
6 months	abundance	X07518	(+/-)-2-Hyc	3	Interventio	Experimen	Experimen	Control	0,375606
6 months	abundance	X11186	Fluocinolo	2b	Interventio	Experimen	Experimen	Control	0,433028
6 months	abundance	X00670	7-Chloro-5	3	Interventio	Experimen	Experimen	Control	0,392683
6 months	abundance	SL00097	Hexoses II	1	Interventio	Experimen	Experimen	Control	0,275484
6 months	abundance	X09895	1-(beta-D-	3	Interventio	Experimen	Experimen	Control	0,414019
6 months	abundance	X09713	N(2)-succi	3	Interventio	Experimen	Experimen	Control	0,375103
6 months	abundance	X02317	2-Aminooc	2b	Interventio	Experimen	Experimen	Control	0,402458
6 months	abundance	X06372	N,N-dimet	2b	Interventio	Experimen	Experimen	Control	0,42661
6 months	abundance	M16	M16	IA_module	Interventio	Experimen	Experimen	Control	0,387982
6 months	abundance	SL00264	Methyl ace	2a	Interventio	Experimen	Experimen	Control	0,411713
6 months	abundance	X01316	5-Hydanto	3	Interventio	Experimen	Experimen	Control	0,371899
6 months	abundance	X08758	Methohexi	3	Interventio	Experimen	Experimen	Control	0,375103
6 months	abundance	X02687	S-Propylcy	3	Interventio	Experimen	Experimen	Control	0,336351
6 months	abundance	X10454	8-Amino-7	3	Interventio	Experimen	Experimen	Control	0,428209
6 months	abundance	X01776	N-(Carboxy	3	Interventio	Experimen	Experimen	Control	0,425873
6 months	abundance	X03070	2-Methoxy	3	Interventio	Experimen	Experimen	Control	0,429158
6 months	abundance	X09810	Zalcitabine	3	Interventio	Experimen	Experimen	Control	0,511339
6 months	abundance	X02265	ophthalmic	3	Interventio	Experimen	Experimen	Control	0,452966
6 months	abundance	X10417	L-Sacchard	2b	Interventio	Experimen	Experimen	Control	0,428209
6 months	abundance	X01873	Methyl 2,3	3	Interventio	Experimen	Experimen	Control	0,472806
6 months	abundance	X08244	Setoclavin	3	Interventio	Experimen	Experimen	Control	0,391188
6 months	abundance	X04593	Methional	3	Interventio	Experimen	Experimen	Control	0,405943
6 months	abundance	X00384	3-Formyl-2	3	Interventio	Experimen	Experimen	Control	0,452191
6 months	abundance	X00807	Midodrine_	3	Interventio	Experimen	Experimen	Control	0,279022
6 months	abundance	X03855	2-{(2S,4S)-	3	Interventio	Experimen	Experimen	Control	0,401596
6 months	abundance	X08323	(15Z)-9,12	2b	Interventio	Experimen	Experimen	Control	0,441241
6 months	abundance	X09950	3-Hydroxyl	3	Interventio	Experimen	Experimen	Control	0,478267

6 months	abundance	X06722	LW800000	3	Interventio	Experimen	Experimen	Control	0,320854
6 months	abundance	X10660	2-Methylth	3	Interventio	Experimen	Experimen	Control	0,447107
6 months	abundance	M13	M13	IA_module	Interventio	Experimen	Experimen	Control	0,433028
6 months	abundance	X02702	R-(+)-Etira	3	Interventio	Experimen	Experimen	Control	0,459973
6 months	abundance	X07900	presqualer	3	Interventio	Experimen	Experimen	Control	0,476616
6 months	abundance	SL00293	Butyrylcarn	2a	Interventio	Experimen	Experimen	Control	0,416238
6 months	abundance	SL00240	Pyruvic aci	1	Interventio	Experimen	Experimen	Control	0,33088
6 months	abundance	X10772	Bicine_a	3	Interventio	Experimen	Experimen	Control	0,455427
6 months	abundance	X00889	LW800000	3	Interventio	Experimen	Experimen	Control	0,428209
6 months	abundance	X08625	Hept-2-ulo	3	Interventio	Experimen	Experimen	Control	0,463949
6 months	abundance	X11733	1,3-Dihydr	3	Interventio	Experimen	Experimen	Control	0,455427
6 months	abundance	X02238	6-Myoporo	3	Interventio	Experimen	Experimen	Control	0,485832
6 months	abundance	X08663	Pilocarpine	2b	Interventio	Experimen	Experimen	Control	0,451974
6 months	abundance	SL00104	Hexoses I	1	Interventio	Experimen	Experimen	Control	0,403055
6 months	abundance	X08306	1-Methylxa	2b	Interventio	Experimen	Experimen	Control	0,474044
6 months	abundance	X06146	S-Methyl-1	3	Interventio	Experimen	Experimen	Control	0,402458
6 months	abundance	M31	M31	IA_module	Interventio	Experimen	Experimen	Control	0,373224
6 months	abundance	X07414	2,2-Bis(hyc	3	Interventio	Experimen	Experimen	Control	0,472806
6 months	abundance	X08212	LU345300	3	Interventio	Experimen	Experimen	Control	0,365141
6 months	abundance	X04229	gamma-L-g	3	Interventio	Experimen	Experimen	Control	0,401596
6 months	abundance	X11273	trp-pro	3	Interventio	Experimen	Experimen	Control	0,476616
6 months	abundance	X06642	Homocitru	2a	Interventio	Experimen	Experimen	Control	0,472806
6 months	abundance	X09222	L-gamma-(	3	Interventio	Experimen	Experimen	Control	0,462525
6 months	abundance	X05306	2-methoxy	3	Interventio	Experimen	Experimen	Control	0,472806
6 months	abundance	X01341	metixene	3	Interventio	Experimen	Experimen	Control	0,474371
6 months	abundance	X00305	Formylkynu	3	Interventio	Experimen	Experimen	Control	0,462525
6 months	abundance	X09008	5-guanidin	3	Interventio	Experimen	Experimen	Control	0,452966
6 months	abundance	X08058	2-Hydroxy-	3	Interventio	Experimen	Experimen	Control	0,472806
6 months	abundance	X10137	1-Methyl-1	3	Interventio	Experimen	Experimen	Control	0,484284
6 months	abundance	X00163	primidone_	3	Interventio	Experimen	Experimen	Control	0,462525
6 months	abundance	X02601	(7E,7'E)-5,	3	Interventio	Experimen	Experimen	Control	0,474913
6 months	abundance	X02184	Piperonylo	2b	Interventio	Experimen	Experimen	Control	0,455918
6 months	abundance	X06549	Panthenol_	3	Interventio	Experimen	Experimen	Control	0,491198
6 months	abundance	X08154	4-(METHYL	3	Interventio	Experimen	Experimen	Control	0,511339
6 months	abundance	X02767	6-APA_b	3	Interventio	Experimen	Experimen	Control	0,487202
6 months	abundance	X03592	2-glyceryl 1	3	Interventio	Experimen	Experimen	Control	0,547533
6 months	abundance	X10246	Ethynodiol	3	Interventio	Experimen	Experimen	Control	0,477197
6 months	abundance	X08461	Dihydrouri	3	Interventio	Experimen	Experimen	Control	0,446583
6 months	abundance	X06107	Hydroxycar	3	Interventio	Experimen	Experimen	Control	0,495451
6 months	abundance	SL00243	Deoxysuga	2a	Interventio	Experimen	Experimen	Control	0,444616
6 months	abundance	X04562	Lisdexamfe	3	Interventio	Experimen	Experimen	Control	0,539856
6 months	abundance	SL00063	Pyridoxal	1	Interventio	Experimen	Experimen	Control	0,452966
6 months	abundance	X06875	Nicotine gl	3	Interventio	Experimen	Experimen	Control	0,539856
6 months	abundance	X02952	Vorinostat_	3	Interventio	Experimen	Experimen	Control	0,494948
6 months	abundance	X10384	L-N2-(2-Ca	3	Interventio	Experimen	Experimen	Control	0,536933
6 months	abundance	X06902	Homocyste	3	Interventio	Experimen	Experimen	Control	0,513677
6 months	abundance	X07026	7α-Hydroxy	2b	Interventio	Experimen	Experimen	Control	0,566482
6 months	abundance	X10345	S-Allylcyste	3	Interventio	Experimen	Experimen	Control	0,497615

6 months	abundance	X05415	Redul	3	Interventio	Experimen	Experimen	Control	0,462222
6 months	abundance	X04792	his-asn	3	Interventio	Experimen	Experimen	Control	0,449538
6 months	abundance	SL00222	Lactic acid	1	Interventio	Experimen	Experimen	Control	0,462222
6 months	abundance	X07457	Glucosami	1	Interventio	Experimen	Experimen	Control	0,472806
6 months	abundance	X06727	MFCD0087	3	Interventio	Experimen	Experimen	Control	0,516851
6 months	abundance	X03837	2-Methoxy-	3	Interventio	Experimen	Experimen	Control	0,522714
6 months	abundance	X06478	Caffeic aci	3	Interventio	Experimen	Experimen	Control	0,536933
6 months	abundance	X08274	Mevalonic	2b	Interventio	Experimen	Experimen	Control	0,536933
6 months	abundance	X05581	N-[(10Z)-7-	3	Interventio	Experimen	Experimen	Control	0,536933
6 months	abundance	X09244	gamma-Gl	3	Interventio	Experimen	Experimen	Control	0,452966
6 months	abundance	X06626	N-Butyryl-L	3	Interventio	Experimen	Experimen	Control	0,512311
6 months	abundance	SL00262	cis-Aconiti	2a	Interventio	Experimen	Experimen	Control	0,477197
6 months	abundance	SL00380	2-Aminoad	2a	Interventio	Experimen	Experimen	Control	0,535443
6 months	abundance	SL00020	Carnitine	2a	Interventio	Experimen	Experimen	Control	0,534558
6 months	abundance	X10304	2,3,8,9-Tet	3	Interventio	Experimen	Experimen	Control	0,481144
6 months	abundance	X01474	Glyceroph	3	Interventio	Experimen	Experimen	Control	0,547533
6 months	abundance	SL00128	N-Acetylal	2a	Interventio	Experimen	Experimen	Control	0,507098
6 months	abundance	M1	M1	IA_module	Interventio	Experimen	Experimen	Control	0,526151
6 months	abundance	X02121	2-Acetami	3	Interventio	Experimen	Experimen	Control	0,550871
6 months	abundance	X07260	(7E,7'E)-5,	3	Interventio	Experimen	Experimen	Control	0,530382
6 months	abundance	X00089	4-Hydroxyb	1	Interventio	Experimen	Experimen	Control	0,574199
6 months	abundance	X07014	Octyl benz	3	Interventio	Experimen	Experimen	Control	0,574621
6 months	abundance	X07484	MFCD0002	3	Interventio	Experimen	Experimen	Control	0,532547
6 months	abundance	X08008	N-Acetylne	1	Interventio	Experimen	Experimen	Control	0,547533
6 months	abundance	X07815	3-[2-[(Z)-[3	3	Interventio	Experimen	Experimen	Control	0,519242
6 months	abundance	X07563	Xanthine	1	Interventio	Experimen	Experimen	Control	0,558374
6 months	abundance	X04315	Benzamide	3	Interventio	Experimen	Experimen	Control	0,574621
6 months	abundance	X07732	N-Acetylva	2b	Interventio	Experimen	Experimen	Control	0,52723
6 months	abundance	X10097	Coixol	3	Interventio	Experimen	Experimen	Control	0,584085
6 months	abundance	X11692	2_7-Anhyd	3	Interventio	Experimen	Experimen	Control	0,566785
6 months	abundance	X00594	Nicotinami	3	Interventio	Experimen	Experimen	Control	0,596698
6 months	abundance	X04119	Tetraacety	3	Interventio	Experimen	Experimen	Control	0,544099
6 months	abundance	X01968	7-Chloro-5	3	Interventio	Experimen	Experimen	Control	0,539856
6 months	abundance	SL00124	Dissaccari	2a	Interventio	Experimen	Experimen	Control	0,497615
6 months	abundance	X11563	threonylph	3	Interventio	Experimen	Experimen	Control	0,547533
6 months	abundance	X08659	3,3-Dimeth	2b	Interventio	Experimen	Experimen	Control	0,622746
6 months	abundance	X07925	DNOP_f	3	Interventio	Experimen	Experimen	Control	0,533701
6 months	abundance	X00635	4-(5,6-Dihy	3	Interventio	Experimen	Experimen	Control	0,587711
6 months	abundance	X07514	L-(+)-Eryth	3	Interventio	Experimen	Experimen	Control	0,545162
6 months	abundance	X07155	Naphthale	3	Interventio	Experimen	Experimen	Control	0,604328
6 months	abundance	X00035	Choline	1	Interventio	Experimen	Experimen	Control	0,530382
6 months	abundance	X08046	3-Hydroxy-	3	Interventio	Experimen	Experimen	Control	0,596842
6 months	abundance	X04990	Cilazapril	3	Interventio	Experimen	Experimen	Control	0,612948
6 months	abundance	X08789	Acrylic aci	2b	Interventio	Experimen	Experimen	Control	0,542307
6 months	abundance	X09152	Ferulic aci	1	Interventio	Experimen	Experimen	Control	0,573484
6 months	abundance	X10091	(2E,6E)-9-[	3	Interventio	Experimen	Experimen	Control	0,579165
6 months	abundance	X06473	MFCD0002	3	Interventio	Experimen	Experimen	Control	0,588894
6 months	abundance	X08606	Butabarbit	3	Interventio	Experimen	Experimen	Control	0,607249

6 months	abundance	X02583	Ethylvanilli	3	Interventio	Experimen	Experimen	Control	0,613206
6 months	abundance	X09525	Homoanse	3	Interventio	Experimen	Experimen	Control	0,519176
6 months	abundance	X02872	folinic acid	3	Interventio	Experimen	Experimen	Control	0,57055
6 months	abundance	X06150	Zalcitabine	3	Interventio	Experimen	Experimen	Control	0,624583
6 months	abundance	X07596	5-amino-2-	2b	Interventio	Experimen	Experimen	Control	0,587711
6 months	abundance	X05786	Nitrendipir	3	Interventio	Experimen	Experimen	Control	0,615628
6 months	abundance	X11001	2-[(carboxy	2b	Interventio	Experimen	Experimen	Control	0,578228
6 months	abundance	X03294	4,4'-Thiobi	3	Interventio	Experimen	Experimen	Control	0,566482
6 months	abundance	X06183	Nisinic aci	3	Interventio	Experimen	Experimen	Control	0,614024
6 months	abundance	X08965	1,9-Nonan	3	Interventio	Experimen	Experimen	Control	0,644196
6 months	abundance	SL00159	Uracil	1	Interventio	Experimen	Experimen	Control	0,622672
6 months	abundance	X05017	N-(1-{[Metl	3	Interventio	Experimen	Experimen	Control	0,574621
6 months	abundance	X08723	S-Allylcyst	3	Interventio	Experimen	Experimen	Control	0,622672
6 months	abundance	X06293	(4R)-4-{[3-	3	Interventio	Experimen	Experimen	Control	0,608921
6 months	abundance	X06416	KYNURAMI	3	Interventio	Experimen	Experimen	Control	0,662984
6 months	abundance	SL00245	sugar alcol	2a	Interventio	Experimen	Experimen	Control	0,559923
6 months	abundance	X08035	Tetrahydro	3	Interventio	Experimen	Experimen	Control	0,587711
6 months	abundance	X02139	SECONAL_	3	Interventio	Experimen	Experimen	Control	0,662457
6 months	abundance	X10518	MFCD0002	3	Interventio	Experimen	Experimen	Control	0,538141
6 months	abundance	SL00288	Propionylc	2a	Interventio	Experimen	Experimen	Control	0,596842
6 months	abundance	M23	M23	IA_module	Interventio	Experimen	Experimen	Control	0,66921
6 months	abundance	X07777	Arginine	1	Interventio	Experimen	Experimen	Control	0,57257
6 months	abundance	X06337	Zalcitabine	3	Interventio	Experimen	Experimen	Control	0,622746
6 months	abundance	X07811	Metirosine	3	Interventio	Experimen	Experimen	Control	0,66921
6 months	abundance	X02905	MFCD0005	3	Interventio	Experimen	Experimen	Control	0,639371
6 months	abundance	X01879	Tetramethy	2b	Interventio	Experimen	Experimen	Control	0,67553
6 months	abundance	X01154	N-(2,3,4-Tr	3	Interventio	Experimen	Experimen	Control	0,679153
6 months	abundance	X09882	Harmane	3	Interventio	Experimen	Experimen	Control	0,67553
6 months	abundance	X08014	DNOP_e	3	Interventio	Experimen	Experimen	Control	0,621405
6 months	abundance	X08099	g-Butyrobe	3	Interventio	Experimen	Experimen	Control	0,67553
6 months	abundance	X08279	DNOP_b	3	Interventio	Experimen	Experimen	Control	0,668366
6 months	abundance	X07783	1,3-dimeth	3	Interventio	Experimen	Experimen	Control	0,660061
6 months	abundance	X00029	Urocanic a	1	Interventio	Experimen	Experimen	Control	0,622672
6 months	abundance	X03278	Hydroxyph	3	Interventio	Experimen	Experimen	Control	0,660061
6 months	abundance	X06253	N-(4-Amin	3	Interventio	Experimen	Experimen	Control	0,660061
6 months	abundance	X09231	2-Keto-glut	3	Interventio	Experimen	Experimen	Control	0,632396
6 months	abundance	X08425	MFCD1297	3	Interventio	Experimen	Experimen	Control	0,621405
6 months	abundance	X04939	N-Desalkyl	3	Interventio	Experimen	Experimen	Control	0,613206
6 months	abundance	X03934	2-Aminom	3	Interventio	Experimen	Experimen	Control	0,622746
6 months	abundance	X10667	N-Phenyla	3	Interventio	Experimen	Experimen	Control	0,669161
6 months	abundance	X00072	3,5-Dihydr	1	Interventio	Experimen	Experimen	Control	0,62929
6 months	abundance	X06318	Propamoca	2b	Interventio	Experimen	Experimen	Control	0,660061
6 months	abundance	M19	M19	IA_module	Interventio	Experimen	Experimen	Control	0,687737
6 months	abundance	X09612	3-hydroxy-	3	Interventio	Experimen	Experimen	Control	0,667479
6 months	abundance	X08157	Leu-Val_e	3	Interventio	Experimen	Experimen	Control	0,660061
6 months	abundance	X06278	3-Hydroxy-	3	Interventio	Experimen	Experimen	Control	0,694759
6 months	abundance	SL00329	2-Hydroxy-	2a	Interventio	Experimen	Experimen	Control	0,644196
6 months	abundance	X09805	Methyl alpl	3	Interventio	Experimen	Experimen	Control	0,641729

6 months	abundance	X10691	(−)-nabilor	3	Interventio	Experimen	Experimen	Control	0,626113
6 months	abundance	X06463	8-Amino-7-	3	Interventio	Experimen	Experimen	Control	0,660061
6 months	abundance	X11549	1,1'-[1,12-I	3	Interventio	Experimen	Experimen	Control	0,672927
6 months	abundance	X08588	2-Acetami	3	Interventio	Experimen	Experimen	Control	0,660061
6 months	abundance	X04744	16alpha-hy	3	Interventio	Experimen	Experimen	Control	0,682743
6 months	abundance	X07648	NL851300	3	Interventio	Experimen	Experimen	Control	0,679833
6 months	abundance	X08123	Tiglic acid_	2b	Interventio	Experimen	Experimen	Control	0,660061
6 months	abundance	X08146	O-Ethyl (4-	3	Interventio	Experimen	Experimen	Control	0,696038
6 months	abundance	X07601	Asp-lys	3	Interventio	Experimen	Experimen	Control	0,662346
6 months	abundance	X09921	5-Hydroxy-	3	Interventio	Experimen	Experimen	Control	0,663456
6 months	abundance	X03860	2-BUTYL PI	3	Interventio	Experimen	Experimen	Control	0,660061
6 months	abundance	X07294	6-Acetami	3	Interventio	Experimen	Experimen	Control	0,662984
6 months	abundance	X08188	2-Hydroxy-	2b	Interventio	Experimen	Experimen	Control	0,659507
6 months	abundance	X11605	3-Methyl-2	3	Interventio	Experimen	Experimen	Control	0,662984
6 months	abundance	X06698	entecavir	3	Interventio	Experimen	Experimen	Control	0,660061
6 months	abundance	X08987	feruloylser	3	Interventio	Experimen	Experimen	Control	0,709598
6 months	abundance	X04040	3-(3,4-dihy	3	Interventio	Experimen	Experimen	Control	0,662984
6 months	abundance	X08707	2-(1-Ethoxy	3	Interventio	Experimen	Experimen	Control	0,696038
6 months	abundance	X08578	tert-Butyl 3	3	Interventio	Experimen	Experimen	Control	0,709124
6 months	abundance	X03276	2'-Deoxyad	3	Interventio	Experimen	Experimen	Control	0,679833
6 months	abundance	X07434	N'-Hydroxy	2b	Interventio	Experimen	Experimen	Control	0,696038
6 months	abundance	SL00201	Pentose II	2a	Interventio	Experimen	Experimen	Control	0,660061
6 months	abundance	X00518	Nisinic aci	3	Interventio	Experimen	Experimen	Control	0,723284
6 months	abundance	X01943	Tocainide	3	Interventio	Experimen	Experimen	Control	0,723552
6 months	abundance	X01223	DL-Mevalo	3	Interventio	Experimen	Experimen	Control	0,694971
6 months	abundance	X11562	ALA-PRO	3	Interventio	Experimen	Experimen	Control	0,696038
6 months	abundance	SL00154	Trigonelline	1	Interventio	Experimen	Experimen	Control	0,682898
6 months	abundance	X07747	Diethylpyrc	3	Interventio	Experimen	Experimen	Control	0,65482
6 months	abundance	X08739	1,1'-[1,12-I	3	Interventio	Experimen	Experimen	Control	0,732912
6 months	abundance	X05510	3-Benzyl-6	3	Interventio	Experimen	Experimen	Control	0,75248
6 months	abundance	X08733	N-Methyl-1	3	Interventio	Experimen	Experimen	Control	0,744735
6 months	abundance	X11110	Tauropine	3	Interventio	Experimen	Experimen	Control	0,746475
6 months	abundance	X01950	Histidylgly	3	Interventio	Experimen	Experimen	Control	0,741224
6 months	abundance	SL00216	Glyoxylic a	2a	Interventio	Experimen	Experimen	Control	0,66921
6 months	abundance	X07442	coenzyme	3	Interventio	Experimen	Experimen	Control	0,766405
6 months	abundance	X11524	6-hydroxyp	3	Interventio	Experimen	Experimen	Control	0,728006
6 months	abundance	X08092	Safrole	3	Interventio	Experimen	Experimen	Control	0,696038
6 months	abundance	X09854	N-Benzyl-3	2b	Interventio	Experimen	Experimen	Control	0,71955
6 months	abundance	X08270	Butabarbit	3	Interventio	Experimen	Experimen	Control	0,739135
6 months	abundance	X08095	DNOP_g	3	Interventio	Experimen	Experimen	Control	0,740789
6 months	abundance	SL00137	Phenylacet	2a	Interventio	Experimen	Experimen	Control	0,750878
6 months	abundance	X09675	UK387000	3	Interventio	Experimen	Experimen	Control	0,734771
6 months	abundance	X02730	N-Phenylac	3	Interventio	Experimen	Experimen	Control	0,67553
6 months	abundance	X07079	N-Pentano	3	Interventio	Experimen	Experimen	Control	0,739135
6 months	abundance	X04538	Valylvaline	3	Interventio	Experimen	Experimen	Control	0,770082
6 months	abundance	X10588	(8)-Ginger	3	Interventio	Experimen	Experimen	Control	0,766405
6 months	abundance	X06684	Bromazine	3	Interventio	Experimen	Experimen	Control	0,731466
6 months	abundance	X08825	Chenodeox	3	Interventio	Experimen	Experimen	Control	0,746475

6 months	abundance	X10534	L-N2-(2-Ca	3	Interventio	Experimen	Experimen	Control	0,751594
6 months	abundance	X00828	Fenoterol	3	Interventio	Experimen	Experimen	Control	0,752189
6 months	abundance	X09998	N-[(10Z)-7-	3	Interventio	Experimen	Experimen	Control	0,729309
6 months	abundance	SL00311	N,N-Dimet	2a	Interventio	Experimen	Experimen	Control	0,741993
6 months	abundance	X09503	4-(METHYL	3	Interventio	Experimen	Experimen	Control	0,686987
6 months	abundance	X05857	N-D-Gluco	3	Interventio	Experimen	Experimen	Control	0,749793
6 months	abundance	X09548	2-O-beta-D	3	Interventio	Experimen	Experimen	Control	0,756336
6 months	abundance	X02195	alliin	3	Interventio	Experimen	Experimen	Control	0,762795
6 months	abundance	X06334	Bis-D-fruct	3	Interventio	Experimen	Experimen	Control	0,756336
6 months	abundance	X03004	Bicine_b	3	Interventio	Experimen	Experimen	Control	0,723106
6 months	abundance	SL00430	Homocarn	2a	Interventio	Experimen	Experimen	Control	0,767195
6 months	abundance	X05146	UQ367500	3	Interventio	Experimen	Experimen	Control	0,726086
6 months	abundance	X03413	Val-Trp_b	3	Interventio	Experimen	Experimen	Control	0,733178
6 months	abundance	X07168	L-(+)-Eryth	3	Interventio	Experimen	Experimen	Control	0,734771
6 months	abundance	X05859	3,7,12,17-T	3	Interventio	Experimen	Experimen	Control	0,755323
6 months	abundance	SL00383	3-(2-Hydro	2a	Interventio	Experimen	Experimen	Control	0,682743
6 months	abundance	X08486	(2E,6E)-9-[	3	Interventio	Experimen	Experimen	Control	0,722158
6 months	abundance	X09684	(+)-Etomid	3	Interventio	Experimen	Experimen	Control	0,755986
6 months	abundance	X01463	4-Thiapent	3	Interventio	Experimen	Experimen	Control	0,75033
6 months	abundance	SL00268	Thymine	1	Interventio	Experimen	Experimen	Control	0,741993
6 months	abundance	X09393	2-Isopropy	3	Interventio	Experimen	Experimen	Control	0,761405
6 months	abundance	X07812	Triethyl citr	3	Interventio	Experimen	Experimen	Control	0,709124
6 months	abundance	SL00096	Hexoses III	1	Interventio	Experimen	Experimen	Control	0,742624
6 months	abundance	X10600	Glycocyam	3	Interventio	Experimen	Experimen	Control	0,770082
6 months	abundance	X00722	(3S,5R,6E)	3	Interventio	Experimen	Experimen	Control	0,729456
6 months	abundance	X11699	{2-[2-(Isob	3	Interventio	Experimen	Experimen	Control	0,767935
6 months	abundance	X06329	Leu-Leu_d	3	Interventio	Experimen	Experimen	Control	0,740789
6 months	abundance	X02333	N-Propiony	3	Interventio	Experimen	Experimen	Control	0,769575
6 months	abundance	M14	M14	NA_module	Interventio	Experimen	Experimen	Control	0,761405
6 months	abundance	X06124	MFCD0995	3	Interventio	Experimen	Experimen	Control	0,770082
6 months	abundance	X03001	Pseudouric	2b	Interventio	Experimen	Experimen	Control	0,756336
6 months	abundance	X03936	(3R)-2-(3,4	3	Interventio	Experimen	Experimen	Control	0,761405
6 months	abundance	X08594	7-ketodeox	3	Interventio	Experimen	Experimen	Control	0,760607
6 months	abundance	X07544	Ethyl sulfat	3	Interventio	Experimen	Experimen	Control	0,772129
6 months	abundance	SL00260	Pentose I	2a	Interventio	Experimen	Experimen	Control	0,767935
6 months	abundance	SL00433	Isovalerylc	2a	Interventio	Experimen	Experimen	Control	0,662984
6 months	abundance	X04259	3-Hydroxyt	3	Interventio	Experimen	Experimen	Control	0,740789
6 months	abundance	X02832	(+/-)-2-Hyd	3	Interventio	Experimen	Experimen	Control	0,766405
6 months	abundance	X00199	tert-Butyl 3	3	Interventio	Experimen	Experimen	Control	0,761405
6 months	abundance	X07909	(±)-Albuter	2b	Interventio	Experimen	Experimen	Control	0,79043
6 months	abundance	X00331	4-(9H-beta	3	Interventio	Experimen	Experimen	Control	0,772129
6 months	abundance	X08792	3,4-Methyl	3	Interventio	Experimen	Experimen	Control	0,762795
6 months	abundance	X03760	2-Methoxy-	3	Interventio	Experimen	Experimen	Control	0,749793
6 months	abundance	X07699	N-(1-Methy	3	Interventio	Experimen	Experimen	Control	0,726086
6 months	abundance	X08387	4-[(2E,4Z)-	3	Interventio	Experimen	Experimen	Control	0,79043
6 months	abundance	X09374	3-Deoxy-D-	3	Interventio	Experimen	Experimen	Control	0,769575
6 months	abundance	X02023	beta-D-Eth	3	Interventio	Experimen	Experimen	Control	0,766405
6 months	abundance	X09001	Varanic ac	3	Interventio	Experimen	Experimen	Control	0,783629

6 months	abundance	X07618	N-(3-aceta	3	Interventio	Experimen	Experimen	Control	0,79043
6 months	abundance	X01932	asn-val_c	3	Interventio	Experimen	Experimen	Control	0,79043
6 months	abundance	X08634	1,5-Isoquir	2b	Interventio	Experimen	Experimen	Control	0,780431
6 months	abundance	X10947	1,1'-[1,12-	3	Interventio	Experimen	Experimen	Control	0,798106
6 months	abundance	SL00082	Allopurinol	1	Interventio	Experimen	Experimen	Control	0,77966
6 months	abundance	X02922	(2E)-3-Met	3	Interventio	Experimen	Experimen	Control	0,79043
6 months	abundance	X08893	5beta-Cho	3	Interventio	Experimen	Experimen	Control	0,79043
6 months	abundance	X02009	Arctiopicri	3	Interventio	Experimen	Experimen	Control	0,790718
6 months	abundance	X07678	(2E)-3-(3,4	2b	Interventio	Experimen	Experimen	Control	0,772129
6 months	abundance	X07774	7alpha-Hy	3	Interventio	Experimen	Experimen	Control	0,79043
6 months	abundance	SL00197	4-Methyl-2	2a	Interventio	Experimen	Experimen	Control	0,772129
6 months	abundance	X01092	17,21-Dihy	3	Interventio	Experimen	Experimen	Control	0,79043
6 months	abundance	X00963	(4S)-4-[(2E	3	Interventio	Experimen	Experimen	Control	0,782484
6 months	abundance	X10325	Lovastatin_	3	Interventio	Experimen	Experimen	Control	0,792475
6 months	abundance	X10370	L-Arogenat	3	Interventio	Experimen	Experimen	Control	0,782484
6 months	abundance	X08711	2-Acetami	3	Interventio	Experimen	Experimen	Control	0,79043
6 months	abundance	X10623	N-Nonanoy	3	Interventio	Experimen	Experimen	Control	0,79043
6 months	abundance	X00034	Pyroglutam	1	Interventio	Experimen	Experimen	Control	0,785613
6 months	abundance	X04564	asp-gln_b	3	Interventio	Experimen	Experimen	Control	0,79043
6 months	abundance	X07308	Nicotinic a	2a	Interventio	Experimen	Experimen	Control	0,770082
6 months	abundance	X01364	Oleuropeir	3	Interventio	Experimen	Experimen	Control	0,792475
6 months	abundance	X06905	Ectoine	3	Interventio	Experimen	Experimen	Control	0,79043
6 months	abundance	SL00248	Gluconic a	2a	Interventio	Experimen	Experimen	Control	0,783629
6 months	abundance	X03109	Guanidino	3	Interventio	Experimen	Experimen	Control	0,828594
6 months	abundance	X03595	Methylol D	3	Interventio	Experimen	Experimen	Control	0,821549
6 months	abundance	X06455	4-Hydroxyp	3	Interventio	Experimen	Experimen	Control	0,811341
6 months	abundance	X10918	meprobam	3	Interventio	Experimen	Experimen	Control	0,79043
6 months	abundance	X06368	TDP-2_b	3	Interventio	Experimen	Experimen	Control	0,825116
6 months	abundance	X03718	Propanthel	3	Interventio	Experimen	Experimen	Control	0,823738
6 months	abundance	X00743	11beta,13-	3	Interventio	Experimen	Experimen	Control	0,836775
6 months	abundance	X06917	Alanyltrypt	3	Interventio	Experimen	Experimen	Control	0,838691
6 months	abundance	X06472	N(alpha)-B	3	Interventio	Experimen	Experimen	Control	0,821549
6 months	abundance	X10770	2-(4-Isopro	3	Interventio	Experimen	Experimen	Control	0,801
6 months	abundance	X07165	pro-gln_b	3	Interventio	Experimen	Experimen	Control	0,838691
6 months	abundance	SL00455	12,13-DHC	2a	Interventio	Experimen	Experimen	Control	0,801727
6 months	abundance	X09581	L-gamma-(	3	Interventio	Experimen	Experimen	Control	0,825133
6 months	abundance	X00136	leu-gln_a	3	Interventio	Experimen	Experimen	Control	0,838691
6 months	abundance	X01181	2-(3,4-Dim	3	Interventio	Experimen	Experimen	Control	0,826734
6 months	abundance	X10162	Quinaldic a	2a	Interventio	Experimen	Experimen	Control	0,843934
6 months	abundance	X10379	Phenyl D-g	3	Interventio	Experimen	Experimen	Control	0,857307
6 months	abundance	X09919	4-ethylphe	3	Interventio	Experimen	Experimen	Control	0,856311
6 months	abundance	X04150	4-(METHYL	3	Interventio	Experimen	Experimen	Control	0,836775
6 months	abundance	X11494	3-Hydroxys	3	Interventio	Experimen	Experimen	Control	0,836775
6 months	abundance	X07746	3-(2-Oxo-2	2b	Interventio	Experimen	Experimen	Control	0,840699
6 months	abundance	X04440	Mono(3-ca	3	Interventio	Experimen	Experimen	Control	0,852491
6 months	abundance	X07899	N-Acetylpu	2b	Interventio	Experimen	Experimen	Control	0,856311
6 months	abundance	X02268	5-Hydroxy-	3	Interventio	Experimen	Experimen	Control	0,856311
6 months	abundance	X02327	1H-Pyrazol	3	Interventio	Experimen	Experimen	Control	0,836775

6 months	abundance	X00928	(7R)-7-(5-c	3	Interventio	Experimen	Experimen	Control		0,847789
6 months	abundance	X08422	N-(3-aceta	3	Interventio	Experimen	Experimen	Control		0,856311
6 months	abundance	X09082	1_2-Dihydr	3	Interventio	Experimen	Experimen	Control		0,856186
6 months	abundance	X07527	1,7-Dimetl	2b	Interventio	Experimen	Experimen	Control		0,864593
6 months	abundance	X10076	trimethadi	3	Interventio	Experimen	Experimen	Control		0,838691
6 months	abundance	X08844	4-Hydroxy-	3	Interventio	Experimen	Experimen	Control		0,852491
6 months	abundance	X04919	5-O-alpha-	3	Interventio	Experimen	Experimen	Control		0,856186
6 months	abundance	X08690	Ethanoic a	3	Interventio	Experimen	Experimen	Control		0,848721
6 months	abundance	M21	M21	IA_module	Interventio	Experimen	Experimen	Control		0,856311
6 months	abundance	X09898	N(6),N(6)-I	3	Interventio	Experimen	Experimen	Control		0,856311
6 months	abundance	X05225	4-(METHYL	3	Interventio	Experimen	Experimen	Control		0,864593
6 months	abundance	SL00239	Pinitol	2a	Interventio	Experimen	Experimen	Control		0,85403
6 months	abundance	X10725	Ethyl mala	3	Interventio	Experimen	Experimen	Control		0,863282
6 months	abundance	X07040	6-Hydroxyp	3	Interventio	Experimen	Experimen	Control		0,864593
6 months	abundance	X00646	Deacetyldi	3	Interventio	Experimen	Experimen	Control		0,864593
6 months	abundance	M7	M7	IA_module	Interventio	Experimen	Experimen	Control		0,856311
6 months	abundance	M27	M27	IA_module	Interventio	Experimen	Experimen	Control		0,864593
6 months	abundance	X08292	Lidocaine	2b	Interventio	Experimen	Experimen	Control		0,864593
6 months	abundance	X01346	IN00260_a	3	Interventio	Experimen	Experimen	Control		0,874528
6 months	abundance	X09825	(2S)-6-Ami	3	Interventio	Experimen	Experimen	Control		0,864593
6 months	abundance	X07375	porphobilir	3	Interventio	Experimen	Experimen	Control		0,864593
6 months	abundance	X06388	Caffeic aci	1	Interventio	Experimen	Experimen	Control		0,864593
6 months	abundance	X05984	1-(4-Methy	2b	Interventio	Experimen	Experimen	Control		0,864593
6 months	abundance	X06268	mesifurane	3	Interventio	Experimen	Experimen	Control		0,880429
6 months	abundance	X00135	N-Acetylglu	1	Interventio	Experimen	Experimen	Control		0,864593
6 months	abundance	SL00285	Dihydrofer	2a	Interventio	Experimen	Experimen	Control		0,864593
6 months	abundance	X00061	Thiamine	1	Interventio	Experimen	Experimen	Control		0,861845
6 months	abundance	X02820	FC250500	3	Interventio	Experimen	Experimen	Control		0,880429
6 months	abundance	X06681	Scymnol	3	Interventio	Experimen	Experimen	Control		0,880429
6 months	abundance	X05126	(3aS,5S,6F	3	Interventio	Experimen	Experimen	Control		0,864593
6 months	abundance	X07028	N-Pentano	3	Interventio	Experimen	Experimen	Control		0,873138
6 months	abundance	SL00290	N-Acetylm	2a	Interventio	Experimen	Experimen	Control		0,872367
6 months	abundance	X06502	MFCD2836	3	Interventio	Experimen	Experimen	Control		0,872258
6 months	abundance	X08352	7-[4-(tert-b	2b	Interventio	Experimen	Experimen	Control		0,885146
6 months	abundance	X10032	4,9a-Dime	3	Interventio	Experimen	Experimen	Control		0,897356
6 months	abundance	X09710	LW800000	3	Interventio	Experimen	Experimen	Control		0,901301
6 months	abundance	X07770	Piperine	1	Interventio	Experimen	Experimen	Control		0,885778
6 months	abundance	SL00384	p-Coumari	1	Interventio	Experimen	Experimen	Control		0,888946
6 months	abundance	X01380	Spermic ac	3	Interventio	Experimen	Experimen	Control		0,880429
6 months	abundance	X04898	17-Hydroxy	3	Interventio	Experimen	Experimen	Control		0,907254
6 months	abundance	X08460	α-Murichol	1	Interventio	Experimen	Experimen	Control		0,905579
6 months	abundance	X07754	hexobarbit	3	Interventio	Experimen	Experimen	Control		0,907254
6 months	abundance	X05892	(5Z)-2-Ami	3	Interventio	Experimen	Experimen	Control		0,905579
6 months	abundance	X07886	L-Urobilin	3	Interventio	Experimen	Experimen	Control		0,922666
6 months	abundance	X03675	asp-gln_a	3	Interventio	Experimen	Experimen	Control		0,916184
6 months	abundance	X10445	2-(Carboxy	3	Interventio	Experimen	Experimen	Control		0,921647
6 months	abundance	X06434	butyrin	3	Interventio	Experimen	Experimen	Control		0,923492
6 months	abundance	X00907	1,2,3,4-Tet	3	Interventio	Experimen	Experimen	Control		0,921679

6 months	abundance	X09208	IN00260_b	3	Interventio	Experimen	Experimen	Control	0,919926
6 months	abundance	X09546	asn-phe	3	Interventio	Experimen	Experimen	Control	0,922666
6 months	abundance	X09055	N-[(1R,2S,(	3	Interventio	Experimen	Experimen	Control	0,922779
6 months	abundance	X10451	Ro 20-172	3	Interventio	Experimen	Experimen	Control	0,921679
6 months	abundance	X07541	Saccharin	1	Interventio	Experimen	Experimen	Control	0,922779
6 months	abundance	SL00287	DOPA	2a	Interventio	Experimen	Experimen	Control	0,922674
6 months	abundance	X11265	Sulfurol	3	Interventio	Experimen	Experimen	Control	0,916193
6 months	abundance	X07841	2-(5-Benzy	3	Interventio	Experimen	Experimen	Control	0,916184
6 months	abundance	X07764	(S)-2-meth	3	Interventio	Experimen	Experimen	Control	0,922666
6 months	abundance	X00063	2-Hydroxy	1	Interventio	Experimen	Experimen	Control	0,922666
6 months	abundance	X11438	pro-met	3	Interventio	Experimen	Experimen	Control	0,922674
6 months	abundance	X02202	D-2-Amino	3	Interventio	Experimen	Experimen	Control	0,922779
6 months	abundance	X08485	Isoprenalir	3	Interventio	Experimen	Experimen	Control	0,938072
6 months	abundance	X00078	Acetylagm	1	Interventio	Experimen	Experimen	Control	0,93201
6 months	abundance	X00116	Cytidine	1	Interventio	Experimen	Experimen	Control	0,929246
6 months	abundance	X06127	2-Methoxy-	3	Interventio	Experimen	Experimen	Control	0,938072
6 months	abundance	X07278	5-Phospho	3	Interventio	Experimen	Experimen	Control	0,934752
6 months	abundance	X11515	13(S)-HOT	2b	Interventio	Experimen	Experimen	Control	0,939435
6 months	abundance	X02661	4-(3-Oxope	3	Interventio	Experimen	Experimen	Control	0,932328
6 months	abundance	X07638	7-ketodeo	3	Interventio	Experimen	Experimen	Control	0,937075
6 months	abundance	X11059	Dihydrocor	3	Interventio	Experimen	Experimen	Control	0,938375
6 months	abundance	X04286	Zinecard_b	3	Interventio	Experimen	Experimen	Control	0,939203
6 months	abundance	X10580	Leu-Leu_a	3	Interventio	Experimen	Experimen	Control	0,938375
6 months	abundance	X04183	Spaglumic	3	Interventio	Experimen	Experimen	Control	0,939525
6 months	abundance	X03064	Formimino	3	Interventio	Experimen	Experimen	Control	0,939525
6 months	abundance	SL00346	N-Methylni	2a	Interventio	Experimen	Experimen	Control	0,940033
6 months	abundance	X00528	R-(+)-Etira	3	Interventio	Experimen	Experimen	Control	0,946138
6 months	abundance	X07753	Lovastatin_	3	Interventio	Experimen	Experimen	Control	0,946963
6 months	abundance	X10488	4-Guanidir	1	Interventio	Experimen	Experimen	Control	0,950361
6 months	abundance	X01252	(−)-nabilor	3	Interventio	Experimen	Experimen	Control	0,950361
6 months	abundance	X08078	Ethyl mala	3	Interventio	Experimen	Experimen	Control	0,951718
6 months	abundance	X02537	Leu-pro_a	3	Interventio	Experimen	Experimen	Control	0,949352
6 months	abundance	X08317	8-Hydroxy	2b	Interventio	Experimen	Experimen	Control	0,962036
6 months	abundance	X02765	L-Pyrrolysi	3	Interventio	Experimen	Experimen	Control	0,962302
6 months	abundance	X09875	4-[(E)-2-(3,	3	Interventio	Experimen	Experimen	Control	0,962036
6 months	abundance	X08024	8-Methyl-8	3	Interventio	Experimen	Experimen	Control	0,954086
6 months	abundance	X07219	3,3-Dimet	2b	Interventio	Experimen	Experimen	Control	0,966723
6 months	abundance	X05969	4-(METHYL	3	Interventio	Experimen	Experimen	Control	0,966723
6 months	abundance	X10818	butalbital_	3	Interventio	Experimen	Experimen	Control	0,968054
6 months	abundance	X07835	7-ketodeo	3	Interventio	Experimen	Experimen	Control	0,969893
6 months	abundance	X10536	CMPF	3	Interventio	Experimen	Experimen	Control	0,962036
6 months	abundance	M4	M4	IA_module	Interventio	Experimen	Experimen	Control	0,9714
6 months	abundance	SL00133	N-Acetylgl	2a	Interventio	Experimen	Experimen	Control	0,9714
6 months	abundance	X03552	4-Hydroxy	3	Interventio	Experimen	Experimen	Control	0,97202
6 months	abundance	X08693	7-Sulfocho	3	Interventio	Experimen	Experimen	Control	0,9714
6 months	abundance	X00083	1,7-Dimet	1	Interventio	Experimen	Experimen	Control	0,973253
6 months	abundance	X06291	Corticoste	2b	Interventio	Experimen	Experimen	Control	0,9714
6 months	abundance	X11245	MFCD000	3	Interventio	Experimen	Experimen	Control	0,9714

6 months	abundance	X05988	Selsun	3	Interventio	Experimen	Experimen	Control	0,977372
6 months	abundance	X06462	DNOP_d	3	Interventio	Experimen	Experimen	Control	0,983736
6 months	abundance	X02013	Ile-cys	3	Interventio	Experimen	Experimen	Control	0,98383
6 months	abundance	X10685	Glycylvalin	2a	Interventio	Experimen	Experimen	Control	0,98383
6 months	abundance	X04334	3-Benzyl-6	3	Interventio	Experimen	Experimen	Control	0,986192
6 months	abundance	X04639	hydroxyhe	3	Interventio	Experimen	Experimen	Control	0,986192
6 months	abundance	X03343	L-alpha-As	3	Interventio	Experimen	Experimen	Control	0,989662
6 months	abundance	X09480	Spermic ac	3	Interventio	Experimen	Experimen	Control	0,990034
6 months	abundance	X01679	meprobam	3	Interventio	Experimen	Experimen	Control	0,990034
6 months	abundance	X06082	O-heptano	3	Interventio	Experimen	Experimen	Control	0,991611
6 months	abundance	X02427	2-(3,5-dim	2b	Interventio	Experimen	Experimen	Control	0,991713
6 months	abundance	M22	M22	IA_module	Interventio	Experimen	Experimen	Control	0,991713
6 months	abundance	X07613	1-(4-Aminc	3	Interventio	Experimen	Experimen	Control	0,992985
6 months	abundance	X02426	Raltitrexed	3	Interventio	Experimen	Experimen	Control	0,992985
6 months	abundance	X08167	Lanthionin	3	Interventio	Experimen	Experimen	Control	0,996256
6 months	abundance	X08105	2-Acetami	3	Interventio	Experimen	Experimen	Control	0,999683
6 months	abundance	X02769	Tetraacety	3	Interventio	Experimen	Experimen	Control	0,999683
6 months	abundance	X09302	Sular	3	Interventio	Experimen	Experimen	Control	0,999683
6 months	abundance	X07469	1,1'-[1,12-	3	Interventio	Experimen	Experimen	Control	0,999683
6 months	abundance	X03535	(1S,3R,4s)-	3	Interventio	Experimen	Experimen	Control	0,999683
6 months	abundance	X09245	L-gamma-(	3	Interventio	Experimen	Experimen	Control	0,996256
6 months	abundance	X01017	hexobarbit	3	Interventio	Experimen	Experimen	Control	0,996256
6 months	abundance	X08646	N-Stearoyl	3	Interventio	Experimen	Experimen	Control	0,992514
6 months	abundance	X02853	7alpha-Hy	3	Interventio	Experimen	Experimen	Control	0,992514
6 months	abundance	X02948	N-(2-Cyan	3	Interventio	Experimen	Experimen	Control	0,989662
6 months	abundance	X06412	Butenylcar	3	Interventio	Experimen	Experimen	Control	0,990034
6 months	abundance	SL00502	2-Ketobuty	1	Interventio	Experimen	Experimen	Control	0,989662
6 months	abundance	X07890	N-(5-aceta	2b	Interventio	Experimen	Experimen	Control	0,989662
6 months	abundance	X00477	Sparfloxac	2b	Interventio	Experimen	Experimen	Control	0,989662
6 months	abundance	X07771	Caprolacta	2b	Interventio	Experimen	Experimen	Control	0,989662
6 months	abundance	X10819	Hyocholic	2a	Interventio	Experimen	Experimen	Control	0,989662
6 months	abundance	X00253	TDP-2_a	3	Interventio	Experimen	Experimen	Control	0,989662
6 months	abundance	X08584	meticillin	3	Interventio	Experimen	Experimen	Control	0,986192
6 months	abundance	X09765	Tetrahydro	3	Interventio	Experimen	Experimen	Control	0,986192
6 months	abundance	X01664	5-methylth	3	Interventio	Experimen	Experimen	Control	0,983955
6 months	abundance	X08057	N-{3-Carbo	3	Interventio	Experimen	Experimen	Control	0,986192
6 months	abundance	X11716	ala-ser_a	3	Interventio	Experimen	Experimen	Control	0,98383
6 months	abundance	X08235	1-Methylhi	2b	Interventio	Experimen	Experimen	Control	0,98383
6 months	abundance	X11755	Furfuranol	3	Interventio	Experimen	Experimen	Control	0,977927
6 months	abundance	X08017	Tropinone	2b	Interventio	Experimen	Experimen	Control	0,973414
6 months	abundance	X03660	trp-ser	3	Interventio	Experimen	Experimen	Control	0,975253
6 months	abundance	X02920	L-gamma-(	3	Interventio	Experimen	Experimen	Control	0,972448
6 months	abundance	X01577	Prunasin	3	Interventio	Experimen	Experimen	Control	0,973253
6 months	abundance	X10741	5-Nitro-2-p	3	Interventio	Experimen	Experimen	Control	0,9714
6 months	abundance	X09187	Salicylic ac	1	Interventio	Experimen	Experimen	Control	0,9714
6 months	abundance	X00099	7-Methylgu	1	Interventio	Experimen	Experimen	Control	0,972448
6 months	abundance	X07345	Phenyl D-g	3	Interventio	Experimen	Experimen	Control	0,9714
6 months	abundance	X06011	Coprine_a	3	Interventio	Experimen	Experimen	Control	0,9714

6 months	abundance	X00224	Nifedipine	3	Interventio	Experimen	Experimen	Control	0,9714
6 months	abundance	X06805	(9cis)-O~1	3	Interventio	Experimen	Experimen	Control	0,96818
6 months	abundance	X07429	DNOP_c	3	Interventio	Experimen	Experimen	Control	0,962036
6 months	abundance	SL00270	3-hydroxy-	2a	Interventio	Experimen	Experimen	Control	0,95757
6 months	abundance	X07566	5-Allyl-5-se	3	Interventio	Experimen	Experimen	Control	0,952514
6 months	abundance	SL00313	2-Aminoisc	2a	Interventio	Experimen	Experimen	Control	0,947377
6 months	abundance	X08695	3b-Hydroxy	3	Interventio	Experimen	Experimen	Control	0,948936
6 months	abundance	X11529	2-Aminooc	2b	Interventio	Experimen	Experimen	Control	0,948936
6 months	abundance	M10	M10	NA_module	Interventio	Experimen	Experimen	Control	0,949352
6 months	abundance	X03017	Pro-tyr	3	Interventio	Experimen	Experimen	Control	0,938375
6 months	abundance	X00836	3-Mercapto	3	Interventio	Experimen	Experimen	Control	0,944459
6 months	abundance	X08094	1,1'-[1,12-	3	Interventio	Experimen	Experimen	Control	0,946138
6 months	abundance	X02878	N~6~-[5-(1	3	Interventio	Experimen	Experimen	Control	0,939435
6 months	abundance	X04351	Pyrimidine	3	Interventio	Experimen	Experimen	Control	0,946875
6 months	abundance	X07634	3,8,9-trihy	2b	Interventio	Experimen	Experimen	Control	0,939525
6 months	abundance	X10046	Dehydroac	2b	Interventio	Experimen	Experimen	Control	0,939203
6 months	abundance	X09545	4-Methyler	3	Interventio	Experimen	Experimen	Control	0,937636
6 months	abundance	X07092	Dibutyl ma	3	Interventio	Experimen	Experimen	Control	0,938375
6 months	abundance	X08533	tyramine s	3	Interventio	Experimen	Experimen	Control	0,93201
6 months	abundance	X09728	{[(15-Hydro	3	Interventio	Experimen	Experimen	Control	0,924496
6 months	abundance	X07226	threonylph	3	Interventio	Experimen	Experimen	Control	0,922674
6 months	abundance	X01078	Dihydroura	3	Interventio	Experimen	Experimen	Control	0,922779
6 months	abundance	SL00297	Acetylmura	2a	Interventio	Experimen	Experimen	Control	0,922779
6 months	abundance	X11349	Butabarbit	3	Interventio	Experimen	Experimen	Control	0,93201
6 months	abundance	X06606	Leu-Val_d	3	Interventio	Experimen	Experimen	Control	0,929246
6 months	abundance	X07743	D-Alanine	2b	Interventio	Experimen	Experimen	Control	0,923004
6 months	abundance	X10614	Monometh	2a	Interventio	Experimen	Experimen	Control	0,922779
6 months	abundance	X07892	urobilinoge	3	Interventio	Experimen	Experimen	Control	0,929246
6 months	abundance	X07452	4-Amino-1	3	Interventio	Experimen	Experimen	Control	0,923492
6 months	abundance	X06313	Isoquinolir	2b	Interventio	Experimen	Experimen	Control	0,922674
6 months	abundance	X05421	S-Allylcyst	3	Interventio	Experimen	Experimen	Control	0,919926
6 months	abundance	X02208	SECONAL_	3	Interventio	Experimen	Experimen	Control	0,922666
6 months	abundance	X08037	Crotonic a	2b	Interventio	Experimen	Experimen	Control	0,919926
6 months	abundance	X10339	4H-1-Benz	2b	Interventio	Experimen	Experimen	Control	0,922666
6 months	abundance	X09284	4-(Nitrosoa	3	Interventio	Experimen	Experimen	Control	0,922779
6 months	abundance	X07874	δ-Valerola	2b	Interventio	Experimen	Experimen	Control	0,922666
6 months	abundance	X11032	2,4-Bis(3-r	3	Interventio	Experimen	Experimen	Control	0,922666
6 months	abundance	X02944	Valylvaline	3	Interventio	Experimen	Experimen	Control	0,907254
6 months	abundance	X02256	hexobarbit	3	Interventio	Experimen	Experimen	Control	0,922779
6 months	abundance	X10776	Histidylgly	3	Interventio	Experimen	Experimen	Control	0,905579
6 months	abundance	X06197	4-(Nitrosoa	3	Interventio	Experimen	Experimen	Control	0,922666
6 months	abundance	X09490	2-Hydroxy-	3	Interventio	Experimen	Experimen	Control	0,913548
6 months	abundance	X03480	epsilon-(ga	3	Interventio	Experimen	Experimen	Control	0,910919
6 months	abundance	X07868	Biotin	2b	Interventio	Experimen	Experimen	Control	0,922666
6 months	abundance	X03416	9-(alpha-D	3	Interventio	Experimen	Experimen	Control	0,907254
6 months	abundance	X05580	Xanthureni	3	Interventio	Experimen	Experimen	Control	0,906599
6 months	abundance	X00074	Methylsuc	1	Interventio	Experimen	Experimen	Control	0,919926
6 months	abundance	X08923	Semilicois	3	Interventio	Experimen	Experimen	Control	0,911969

6 months	abundance	X04814	meprobam	3	Interventio	Experimen	Experimen	Control	0,913038
6 months	abundance	X01911	3-(2,3-Dihy	3	Interventio	Experimen	Experimen	Control	0,916412
6 months	abundance	X09100	Ethyl aceta	3	Interventio	Experimen	Experimen	Control	0,917684
6 months	abundance	X02492	N-(3,5-Dim	3	Interventio	Experimen	Experimen	Control	0,8995
6 months	abundance	X09623	Endothal	2b	Interventio	Experimen	Experimen	Control	0,910969
6 months	abundance	X07193	N-(1-Methy	3	Interventio	Experimen	Experimen	Control	0,8995
6 months	abundance	X10144	imazameth	3	Interventio	Experimen	Experimen	Control	0,905717
6 months	abundance	X06767	2-Hydroxyb	1	Interventio	Experimen	Experimen	Control	0,902955
6 months	abundance	M20	M20	IA_module	Interventio	Experimen	Experimen	Control	0,901376
6 months	abundance	X03077	nicotianam	3	Interventio	Experimen	Experimen	Control	0,897356
6 months	abundance	X11115	Hydroxyca	3	Interventio	Experimen	Experimen	Control	0,902955
6 months	abundance	Butanoic a	Butanoic a	CFA_panel	Interventio	Experimen	Experimen	Control	0,878814
6 months	abundance	X01011	DIBEHENIN	3	Interventio	Experimen	Experimen	Control	0,897356
6 months	abundance	SL00438	Daidzein	2a	Interventio	Experimen	Experimen	Control	0,8995
6 months	abundance	X08847	1-Vinylimic	2b	Interventio	Experimen	Experimen	Control	0,864906
6 months	abundance	X11627	N-(3-aceta	3	Interventio	Experimen	Experimen	Control	0,885146
6 months	abundance	SL00299	β-Murichol	2a	Interventio	Experimen	Experimen	Control	0,896401
6 months	abundance	SL00205	Citraconic	2a	Interventio	Experimen	Experimen	Control	0,865759
6 months	abundance	X07051	O-succinyl	3	Interventio	Experimen	Experimen	Control	0,885146
6 months	abundance	X02972	3-(Sulfoox	3	Interventio	Experimen	Experimen	Control	0,885146
6 months	abundance	X03901	ferrileghen	3	Interventio	Experimen	Experimen	Control	0,887588
6 months	abundance	X02881	N-(2,3,4-Tr	3	Interventio	Experimen	Experimen	Control	0,887588
6 months	abundance	X04579	6-(alpha-D	3	Interventio	Experimen	Experimen	Control	0,889563
6 months	abundance	X08719	6-Methylqu	2b	Interventio	Experimen	Experimen	Control	0,880429
6 months	abundance	X01059	coronatine	3	Interventio	Experimen	Experimen	Control	0,889563
6 months	abundance	X05237	imazameth	3	Interventio	Experimen	Experimen	Control	0,877813
6 months	abundance	X02529	tenivastati	3	Interventio	Experimen	Experimen	Control	0,872258
6 months	abundance	X00033	Deoxycholi	1	Interventio	Experimen	Experimen	Control	0,877813
6 months	abundance	X10330	Sulfurous a	3	Interventio	Experimen	Experimen	Control	0,864593
6 months	abundance	X09942	Spermic ac	3	Interventio	Experimen	Experimen	Control	0,864593
6 months	abundance	X02615	6-APA_a	3	Interventio	Experimen	Experimen	Control	0,883824
6 months	abundance	SL00035	Glycylglyci	2a	Interventio	Experimen	Experimen	Control	0,864593
6 months	abundance	X06530	3-(1-hydro:	2b	Interventio	Experimen	Experimen	Control	0,875244
6 months	abundance	X11223	Ethyl mala	3	Interventio	Experimen	Experimen	Control	0,864593
6 months	abundance	X06903	Esculin	2b	Interventio	Experimen	Experimen	Control	0,873138
6 months	abundance	X06292	mesifurane	3	Interventio	Experimen	Experimen	Control	0,864593
6 months	abundance	X08054	Docosahex	2b	Interventio	Experimen	Experimen	Control	0,864906
6 months	abundance	X07201	2,4-Quinol	2b	Interventio	Experimen	Experimen	Control	0,867822
6 months	abundance	X05656	mesifurane	3	Interventio	Experimen	Experimen	Control	0,864906
6 months	abundance	X06320	7alpha-Hy	3	Interventio	Experimen	Experimen	Control	0,864593
6 months	abundance	X09746	ELK (Peptic	2b	Interventio	Experimen	Experimen	Control	0,851221
6 months	abundance	X10497	4-Guanidir	3	Interventio	Experimen	Experimen	Control	0,864593
6 months	abundance	X07694	DNOP_a	3	Interventio	Experimen	Experimen	Control	0,864593
6 months	abundance	X00125	Taurine	1	Interventio	Experimen	Experimen	Control	0,857307
6 months	abundance	X09740	MFCD0166	3	Interventio	Experimen	Experimen	Control	0,864593
6 months	abundance	X02281	tert-Butyl 3	3	Interventio	Experimen	Experimen	Control	0,864593
6 months	abundance	X00084	Tricarballyl	1	Interventio	Experimen	Experimen	Control	0,864593
6 months	abundance	X08600	N-Propiony	3	Interventio	Experimen	Experimen	Control	0,861845

6 months	abundance	X01746	Astemizole	3	Interventio	Experimen	Experimen	Control	0,860024
6 months	abundance	X00082	Cholic acid	1	Interventio	Experimen	Experimen	Control	0,864593
6 months	abundance	X01833	2,3,4,9-Tet	2b	Interventio	Experimen	Experimen	Control	0,864593
6 months	abundance	X04157	3-Methoxy-	3	Interventio	Experimen	Experimen	Control	0,864593
6 months	abundance	X09189	4-[(E)-2-(3,	3	Interventio	Experimen	Experimen	Control	0,857746
6 months	abundance	X06948	(1R,3R,5R)	3	Interventio	Experimen	Experimen	Control	0,864593
6 months	abundance	X08004	δ-Valerolac	2b	Interventio	Experimen	Experimen	Control	0,856311
6 months	abundance	X02145	3'-Hydroxy	3	Interventio	Experimen	Experimen	Control	0,850568
6 months	abundance	X00723	Aspartyl-L-	3	Interventio	Experimen	Experimen	Control	0,856311
6 months	abundance	X07523	Ursodeoxy	1	Interventio	Experimen	Experimen	Control	0,856311
6 months	abundance	X00764	5-Allyl-5-se	3	Interventio	Experimen	Experimen	Control	0,856311
6 months	abundance	X07799	Capryloylg	2b	Interventio	Experimen	Experimen	Control	0,815129
6 months	abundance	X06818	Homoanse	3	Interventio	Experimen	Experimen	Control	0,814559
6 months	abundance	X07710	3-O-beta-D	3	Interventio	Experimen	Experimen	Control	0,860361
6 months	abundance	X01067	g-Aminobu	3	Interventio	Experimen	Experimen	Control	0,852621
6 months	abundance	X07344	(−)-nabilor	3	Interventio	Experimen	Experimen	Control	0,856311
6 months	abundance	SL00391	N-Acetylle	1	Interventio	Experimen	Experimen	Control	0,82397
6 months	abundance	X06551	Methyl 1-h	3	Interventio	Experimen	Experimen	Control	0,838691
6 months	abundance	X10974	Threonylgl	3	Interventio	Experimen	Experimen	Control	0,811341
6 months	abundance	X00066	Tryptamine	1	Interventio	Experimen	Experimen	Control	0,842213
6 months	abundance	X07645	O-propeno	3	Interventio	Experimen	Experimen	Control	0,838204
6 months	abundance	X02108	Homovanil	3	Interventio	Experimen	Experimen	Control	0,825116
6 months	abundance	SL00052	N-Isovaler	2a	Interventio	Experimen	Experimen	Control	0,81676
6 months	abundance	X06448	3,8,9-trihy	2b	Interventio	Experimen	Experimen	Control	0,825116
6 months	abundance	X00113	N-Acetylph	1	Interventio	Experimen	Experimen	Control	0,79043
6 months	abundance	X05204	Urothion	3	Interventio	Experimen	Experimen	Control	0,836775
6 months	abundance	SL00199	5-Hydroxyi	2a	Interventio	Experimen	Experimen	Control	0,851221
6 months	abundance	SL00226	N-Acetylgly	2a	Interventio	Experimen	Experimen	Control	0,798663
6 months	abundance	X09645	TOLMETIN	3	Interventio	Experimen	Experimen	Control	0,836775
6 months	abundance	X00432	8-(Methyls	3	Interventio	Experimen	Experimen	Control	0,838691
6 months	abundance	X07027	Allyl merca	3	Interventio	Experimen	Experimen	Control	0,829112
6 months	abundance	X07978	1-[(4E)-4-(	3	Interventio	Experimen	Experimen	Control	0,79043
6 months	abundance	X10353	L-gamma-(	3	Interventio	Experimen	Experimen	Control	0,79043
6 months	abundance	M24	M24	\A_module	Interventio	Experimen	Experimen	Control	0,801727
6 months	abundance	X07578	5-Allyl-5-se	3	Interventio	Experimen	Experimen	Control	0,825133
6 months	abundance	X03709	Guanfacine	3	Interventio	Experimen	Experimen	Control	0,792475
6 months	abundance	X11354	3-Oxo-4,6-	3	Interventio	Experimen	Experimen	Control	0,814559
6 months	abundance	X10881	L-gamma-(	3	Interventio	Experimen	Experimen	Control	0,79043
6 months	abundance	X08341	Midodrine_	3	Interventio	Experimen	Experimen	Control	0,82397
6 months	abundance	X07658	δ-Glucono	1	Interventio	Experimen	Experimen	Control	0,822447
6 months	abundance	X08173	9-Methylur	2b	Interventio	Experimen	Experimen	Control	0,815129
6 months	abundance	X08221	Brilliant blu	2b	Interventio	Experimen	Experimen	Control	0,815764
6 months	abundance	X10098	Methyl 2,3-	3	Interventio	Experimen	Experimen	Control	0,79043
6 months	abundance	X00853	4-O-beta-D	3	Interventio	Experimen	Experimen	Control	0,767935
6 months	abundance	X08277	adrenaline	3	Interventio	Experimen	Experimen	Control	0,81084
6 months	abundance	X00327	3-Hydroxy-	3	Interventio	Experimen	Experimen	Control	0,806712
6 months	abundance	X06797	pro-gln_a	3	Interventio	Experimen	Experimen	Control	0,790718
6 months	abundance	X02733	Calcitriol	3	Interventio	Experimen	Experimen	Control	0,811341

6 months	abundance	X07399	Homoanse	3	Interventio	Experimen	Experimen	Control	0,79043
6 months	abundance	X11441	beta-D-Eth	3	Interventio	Experimen	Experimen	Control	0,772129
6 months	abundance	X07935	Limonin	3	Interventio	Experimen	Experimen	Control	0,811341
6 months	abundance	X03134	Tetrahydro	3	Interventio	Experimen	Experimen	Control	0,770919
6 months	abundance	Pentanoic	Pentanoic	CFA_panel	Interventio	Experimen	Experimen	Control	0,77651
6 months	abundance	X03916	2-(1-Ethox	3	Interventio	Experimen	Experimen	Control	0,79043
6 months	abundance	X10593	Vorinostat_	3	Interventio	Experimen	Experimen	Control	0,79043
6 months	abundance	X08386	3b-Hydrox	3	Interventio	Experimen	Experimen	Control	0,806712
6 months	abundance	X10930	MFCD0995	3	Interventio	Experimen	Experimen	Control	0,79043
6 months	abundance	X10177	Gly-DL-Phe	3	Interventio	Experimen	Experimen	Control	0,801
6 months	abundance	X09940	pentobarbi	3	Interventio	Experimen	Experimen	Control	0,79043
6 months	abundance	X08326	2-(1,3-Ben	3	Interventio	Experimen	Experimen	Control	0,803444
6 months	abundance	X07680	Indole-3-a	1	Interventio	Experimen	Experimen	Control	0,786166
6 months	abundance	X06623	quinol sulf	3	Interventio	Experimen	Experimen	Control	0,798346
6 months	abundance	X07519	Pyrrolidine	3	Interventio	Experimen	Experimen	Control	0,769775
6 months	abundance	X06100	Midodrine_	3	Interventio	Experimen	Experimen	Control	0,777511
6 months	abundance	X06723	3-Hydroxy-	3	Interventio	Experimen	Experimen	Control	0,790718
6 months	abundance	SL00349	1-Aminocy	2a	Interventio	Experimen	Experimen	Control	0,79043
6 months	abundance	X07320	asn-pro_d	3	Interventio	Experimen	Experimen	Control	0,790718
6 months	abundance	X11639	Midodrine_	3	Interventio	Experimen	Experimen	Control	0,772129
6 months	abundance	X11505	FB950000(	3	Interventio	Experimen	Experimen	Control	0,801
6 months	abundance	X01738	2-Acetami	3	Interventio	Experimen	Experimen	Control	0,796588
6 months	abundance	X06807	1,1'-[1,12-I	3	Interventio	Experimen	Experimen	Control	0,780414
6 months	abundance	X08795	N-Acetylva	3	Interventio	Experimen	Experimen	Control	0,780178
6 months	abundance	X04557	1,4-Naphtl	3	Interventio	Experimen	Experimen	Control	0,79043
6 months	abundance	X06508	Asparaginy	3	Interventio	Experimen	Experimen	Control	0,714671
6 months	abundance	X01920	N-(Carbox	3	Interventio	Experimen	Experimen	Control	0,770082
6 months	abundance	X01518	L-gamma-(	3	Interventio	Experimen	Experimen	Control	0,782484
6 months	abundance	X03271	1Î²-Hydrox	3	Interventio	Experimen	Experimen	Control	0,767953
6 months	abundance	X06861	4-(4-Deox	3	Interventio	Experimen	Experimen	Control	0,79043
6 months	abundance	X11576	Phenyl D-g	3	Interventio	Experimen	Experimen	Control	0,79043
6 months	abundance	X10987	2-BUTYL PI	3	Interventio	Experimen	Experimen	Control	0,766405
6 months	abundance	X03434	SECONAL_	3	Interventio	Experimen	Experimen	Control	0,79043
6 months	abundance	X04274	6-[(Z)-2-(3,	3	Interventio	Experimen	Experimen	Control	0,79043
6 months	abundance	X01236	bis(4-isoth	3	Interventio	Experimen	Experimen	Control	0,767935
6 months	abundance	X02419	Glu-Glu	3	Interventio	Experimen	Experimen	Control	0,755323
6 months	abundance	X08077	6-(alpha-D	3	Interventio	Experimen	Experimen	Control	0,769575
6 months	abundance	X09577	Spermic ac	3	Interventio	Experimen	Experimen	Control	0,769775
6 months	abundance	X02288	9-Methylur	2b	Interventio	Experimen	Experimen	Control	0,770082
6 months	abundance	X07480	Naringenin	2a	Interventio	Experimen	Experimen	Control	0,772129
6 months	abundance	X07513	(−)-nabilor	3	Interventio	Experimen	Experimen	Control	0,769575
6 months	abundance	X07580	N-Pentano	3	Interventio	Experimen	Experimen	Control	0,758222
6 months	abundance	X07220	Glycylprol	3	Interventio	Experimen	Experimen	Control	0,779289
6 months	abundance	X11033	N-Acetyl-5	3	Interventio	Experimen	Experimen	Control	0,788346
6 months	abundance	X06985	N-Ethylpro	3	Interventio	Experimen	Experimen	Control	0,772129
6 months	abundance	X08992	N(2)-succi	3	Interventio	Experimen	Experimen	Control	0,758222
6 months	abundance	X10033	epsilon-(ga	3	Interventio	Experimen	Experimen	Control	0,767646
6 months	abundance	X08417	asn-pro_c	3	Interventio	Experimen	Experimen	Control	0,762795

6 months	abundance	X04636	epsilon-(ga	3	Interventio	Experimen	Experimen	Control	0,756336
6 months	abundance	X08040	Prolylleuci	2b	Interventio	Experimen	Experimen	Control	0,769775
6 months	abundance	X10865	Melatonin	3	Interventio	Experimen	Experimen	Control	0,769775
6 months	abundance	X05411	3-(Sulfooxy	3	Interventio	Experimen	Experimen	Control	0,768316
6 months	abundance	X09523	N-(3,5-Dim	3	Interventio	Experimen	Experimen	Control	0,766405
6 months	abundance	X07413	Chenodeox	1	Interventio	Experimen	Experimen	Control	0,767935
6 months	abundance	SL00421	Asymmetri	2a	Interventio	Experimen	Experimen	Control	0,76776
6 months	abundance	X00899	N-(2,3,4-Tr	3	Interventio	Experimen	Experimen	Control	0,767646
6 months	abundance	X04379	2-Amino-6	3	Interventio	Experimen	Experimen	Control	0,729456
6 months	abundance	SL00390	Gallic acid	2a	Interventio	Experimen	Experimen	Control	0,728006
6 months	abundance	X06646	(S)-3-sulfo	3	Interventio	Experimen	Experimen	Control	0,762795
6 months	abundance	X01288	L-gamma-(	3	Interventio	Experimen	Experimen	Control	0,751594
6 months	abundance	X10387	Coenzyme	3	Interventio	Experimen	Experimen	Control	0,762795
6 months	abundance	X09771	2-(3-CARB	3	Interventio	Experimen	Experimen	Control	0,770082
6 months	abundance	X07889	UROBILIN,	3	Interventio	Experimen	Experimen	Control	0,766405
6 months	abundance	X07969	6-hydroxyp	3	Interventio	Experimen	Experimen	Control	0,769575
6 months	abundance	X02868	2-(3-Hydro	3	Interventio	Experimen	Experimen	Control	0,767935
6 months	abundance	X07491	1-Vinylimic	2b	Interventio	Experimen	Experimen	Control	0,735877
6 months	abundance	X10951	8-Methyl-8	3	Interventio	Experimen	Experimen	Control	0,741993
6 months	abundance	X09505	(-)-Physost	3	Interventio	Experimen	Experimen	Control	0,749793
6 months	abundance	X06437	2-Acetamid	3	Interventio	Experimen	Experimen	Control	0,731466
6 months	abundance	X08305	Prolylhydro	2a	Interventio	Experimen	Experimen	Control	0,71955
6 months	abundance	X07597	p-Cresylsu	3	Interventio	Experimen	Experimen	Control	0,735906
6 months	abundance	X07136	hexobarbit	3	Interventio	Experimen	Experimen	Control	0,71955
6 months	abundance	X07089	Pregabalin	3	Interventio	Experimen	Experimen	Control	0,766405
6 months	abundance	X07993	UROBILIN,	3	Interventio	Experimen	Experimen	Control	0,752328
6 months	abundance	X10401	3-Methylxa	1	Interventio	Experimen	Experimen	Control	0,741993
6 months	abundance	X00059	3,4-Dihydr	1	Interventio	Experimen	Experimen	Control	0,752328
6 months	abundance	X05971	L-gamma-(	3	Interventio	Experimen	Experimen	Control	0,733425
6 months	abundance	X04688	Minoxidil	3	Interventio	Experimen	Experimen	Control	0,750878
6 months	abundance	X02352	Indole-3-ca	3	Interventio	Experimen	Experimen	Control	0,756336
6 months	abundance	X06371	L-gamma-(	3	Interventio	Experimen	Experimen	Control	0,697971
6 months	abundance	X07327	Aspartyl-L-	3	Interventio	Experimen	Experimen	Control	0,713864
6 months	abundance	X11645	glu-thr	3	Interventio	Experimen	Experimen	Control	0,643904
6 months	abundance	X08883	3-Phenylpr	3	Interventio	Experimen	Experimen	Control	0,71955
6 months	abundance	X06079	Glycylleuci	1	Interventio	Experimen	Experimen	Control	0,686259
6 months	abundance	X07127	N~5~-[P-Ar	3	Interventio	Experimen	Experimen	Control	0,741993
6 months	abundance	X08162	Valylvaline	3	Interventio	Experimen	Experimen	Control	0,728006
6 months	abundance	X07445	3-(Sulfooxy	3	Interventio	Experimen	Experimen	Control	0,695875
6 months	abundance	SL00353	Adipic acid	1	Interventio	Experimen	Experimen	Control	0,701195
6 months	abundance	X06085	Tetraacety	3	Interventio	Experimen	Experimen	Control	0,707314
6 months	abundance	X07443	Butylparab	3	Interventio	Experimen	Experimen	Control	0,698931
6 months	abundance	X05812	(2S)-3-(1H	3	Interventio	Experimen	Experimen	Control	0,662346
6 months	abundance	X05758	quinol sulfa	3	Interventio	Experimen	Experimen	Control	0,679833
6 months	abundance	X06143	2-Methylbu	2a	Interventio	Experimen	Experimen	Control	0,696038
6 months	abundance	X06783	N~6~-Octa	3	Interventio	Experimen	Experimen	Control	0,71955
6 months	abundance	X00092	3-Methylhi	1	Interventio	Experimen	Experimen	Control	0,718213
6 months	abundance	SL00420	4-Hydroxy-	2a	Interventio	Experimen	Experimen	Control	0,70518

6 months	abundance	X10511	dopaquino	3	Interventio	Experimen	Experimen	Control	0,694971
6 months	abundance	X05514	(3aS,5S,6F	3	Interventio	Experimen	Experimen	Control	0,718952
6 months	abundance	X11667	Tetraacety	3	Interventio	Experimen	Experimen	Control	0,660061
6 months	abundance	X08801	3,7-Dimetl	2b	Interventio	Experimen	Experimen	Control	0,701195
6 months	abundance	X11301	(6S)-2-Ami	3	Interventio	Experimen	Experimen	Control	0,679833
6 months	abundance	X06690	4-Methylca	2b	Interventio	Experimen	Experimen	Control	0,714671
6 months	abundance	X06961	Zalcitabine	3	Interventio	Experimen	Experimen	Control	0,669742
6 months	abundance	SL00001	Deoxycytid	2a	Interventio	Experimen	Experimen	Control	0,64451
6 months	abundance	M32	M32	IA_module	Interventio	Experimen	Experimen	Control	0,679015
6 months	abundance	X09129	Coprine_d	3	Interventio	Experimen	Experimen	Control	0,644196
6 months	abundance	X01671	N-(3,5-Dim	3	Interventio	Experimen	Experimen	Control	0,707314
6 months	abundance	X05068	pro-gln_c	3	Interventio	Experimen	Experimen	Control	0,686987
6 months	abundance	X08657	1-(4-Aminc	3	Interventio	Experimen	Experimen	Control	0,713054
6 months	abundance	X07963	Theobromi	1	Interventio	Experimen	Experimen	Control	0,679833
6 months	abundance	SL00024	Creatine	2a	Interventio	Experimen	Experimen	Control	0,652884
6 months	abundance	X00132	Glyceric ac	1	Interventio	Experimen	Experimen	Control	0,679833
6 months	abundance	X03719	Losalen	3	Interventio	Experimen	Experimen	Control	0,679494
6 months	abundance	X07538	Homoanse	3	Interventio	Experimen	Experimen	Control	0,696038
6 months	abundance	M26	M26	IA_module	Interventio	Experimen	Experimen	Control	0,696038
6 months	abundance	X03704	N,N-Dimet	3	Interventio	Experimen	Experimen	Control	0,716787
6 months	abundance	X08163	Phenyl D-g	3	Interventio	Experimen	Experimen	Control	0,709598
6 months	abundance	X08512	Bile acid I (	2b	Interventio	Experimen	Experimen	Control	0,659423
6 months	abundance	X07794	Prolylleuci	2b	Interventio	Experimen	Experimen	Control	0,67553
6 months	abundance	X02565	Dopamine	3	Interventio	Experimen	Experimen	Control	0,67778
6 months	abundance	X09285	MFCD0272	3	Interventio	Experimen	Experimen	Control	0,67553
6 months	abundance	X08983	IN00150	3	Interventio	Experimen	Experimen	Control	0,66921
6 months	abundance	X11012	Furan	3	Interventio	Experimen	Experimen	Control	0,622746
6 months	abundance	X06889	3,4-Dimetl	2a	Interventio	Experimen	Experimen	Control	0,696038
6 months	abundance	X06112	Val-Ser_a	3	Interventio	Experimen	Experimen	Control	0,660061
6 months	abundance	X09092	LysoSM(d1	3	Interventio	Experimen	Experimen	Control	0,658755
6 months	abundance	X06764	N-(4-Hydrc	3	Interventio	Experimen	Experimen	Control	0,652283
6 months	abundance	X01793	Lys-phe_b	3	Interventio	Experimen	Experimen	Control	0,609036
6 months	abundance	X11347	Voglibose	3	Interventio	Experimen	Experimen	Control	0,665468
6 months	abundance	X08367	11-dehydrc	2b	Interventio	Experimen	Experimen	Control	0,66921
6 months	abundance	X07134	4-Phenolsu	2b	Interventio	Experimen	Experimen	Control	0,660061
6 months	abundance	X03892	shinorine	3	Interventio	Experimen	Experimen	Control	0,660061
6 months	abundance	X07792	N-Acetylhis	2b	Interventio	Experimen	Experimen	Control	0,579512
6 months	abundance	X07263	3,3-Dimetl	2b	Interventio	Experimen	Experimen	Control	0,662457
6 months	abundance	SL00401	Theophyllin	1	Interventio	Experimen	Experimen	Control	0,643904
6 months	abundance	X05072	L-gamma-(	3	Interventio	Experimen	Experimen	Control	0,66921
6 months	abundance	X07336	Threonylse	3	Interventio	Experimen	Experimen	Control	0,574621
6 months	abundance	X07646	(2Z)-2-({[(6	3	Interventio	Experimen	Experimen	Control	0,640371
6 months	abundance	X06405	Asparaginy	3	Interventio	Experimen	Experimen	Control	0,535443
6 months	abundance	X09207	AAMU_a	3	Interventio	Experimen	Experimen	Control	0,587711
6 months	abundance	X07712	paracetam	3	Interventio	Experimen	Experimen	Control	0,644196
6 months	abundance	X07981	Methylimic	2b	Interventio	Experimen	Experimen	Control	0,66245
6 months	abundance	X08869	Lanthionin	3	Interventio	Experimen	Experimen	Control	0,610394
6 months	abundance	X03917	Uramustin	3	Interventio	Experimen	Experimen	Control	0,574621

6 months	abundance	X07848	(−)-nabilor	3	Interventio	Experimen	Experimen	Control	0,645111
6 months	abundance	X06222	Leucylaspa	3	Interventio	Experimen	Experimen	Control	0,588937
6 months	abundance	X07798	N-lauroylgl	3	Interventio	Experimen	Experimen	Control	0,662984
6 months	abundance	X01164	DL-Carboc	3	Interventio	Experimen	Experimen	Control	0,607861
6 months	abundance	X10203	L-gamma-(	3	Interventio	Experimen	Experimen	Control	0,643904
6 months	abundance	X10389	Leucylaspa	3	Interventio	Experimen	Experimen	Control	0,567954
6 months	abundance	X08300	Indole-3-ac	3	Interventio	Experimen	Experimen	Control	0,646446
6 months	abundance	X00064	Traumatic	1	Interventio	Experimen	Experimen	Control	0,608921
6 months	abundance	X06149	Yangonin	3	Interventio	Experimen	Experimen	Control	0,644181
6 months	abundance	X02774	9-ribosylze	3	Interventio	Experimen	Experimen	Control	0,622672
6 months	abundance	X04544	L-gamma-(	3	Interventio	Experimen	Experimen	Control	0,574621
6 months	abundance	SL00376	N-AcetylPr	2a	Interventio	Experimen	Experimen	Control	0,547533
6 months	abundance	X11068	N,N'-Bis[4-	3	Interventio	Experimen	Experimen	Control	0,622672
6 months	abundance	X05584	Serotonin	1	Interventio	Experimen	Experimen	Control	0,628857
6 months	abundance	X08845	14-Hydroxy	3	Interventio	Experimen	Experimen	Control	0,621405
6 months	abundance	X01553	uridine 5'-c	3	Interventio	Experimen	Experimen	Control	0,628365
6 months	abundance	X02494	Butylphtha	3	Interventio	Experimen	Experimen	Control	0,639371
6 months	abundance	X10429	1,5-Isoquir	2b	Interventio	Experimen	Experimen	Control	0,613206
6 months	abundance	X06883	Agomelatir	3	Interventio	Experimen	Experimen	Control	0,614024
6 months	abundance	X07657	Docosahex	2b	Interventio	Experimen	Experimen	Control	0,622672
6 months	abundance	X05046	Casimiroin	3	Interventio	Experimen	Experimen	Control	0,622746
6 months	abundance	X10279	Lys-Pro_d	3	Interventio	Experimen	Experimen	Control	0,602457
6 months	abundance	X08673	NPC	3	Interventio	Experimen	Experimen	Control	0,613827
6 months	abundance	X05391	2-Acetamic	3	Interventio	Experimen	Experimen	Control	0,497615
6 months	abundance	X02153	Asarone	3	Interventio	Experimen	Experimen	Control	0,607249
6 months	abundance	M18	M18	NA_module	Interventio	Experimen	Experimen	Control	0,628777
6 months	abundance	X08909	GLK (Pepti	2b	Interventio	Experimen	Experimen	Control	0,495451
6 months	abundance	X03254	Gly-Trp_b	3	Interventio	Experimen	Experimen	Control	0,547533
6 months	abundance	X06259	(S)-?-glyce	3	Interventio	Experimen	Experimen	Control	0,612837
6 months	abundance	X00070	α-aminobu	1	Interventio	Experimen	Experimen	Control	0,549392
6 months	abundance	X00744	(1R,2S)-1-(	3	Interventio	Experimen	Experimen	Control	0,614024
6 months	abundance	X08044	3-[2-[(Z)-[3	3	Interventio	Experimen	Experimen	Control	0,574621
6 months	abundance	X08908	Vorinostat_	3	Interventio	Experimen	Experimen	Control	0,610394
6 months	abundance	X03629	Glycylglycy	3	Interventio	Experimen	Experimen	Control	0,547533
6 months	abundance	X08593	Uric acid	1	Interventio	Experimen	Experimen	Control	0,52723
6 months	abundance	X01186	asn-val_b	3	Interventio	Experimen	Experimen	Control	0,530545
6 months	abundance	X06270	9-Methylur	2b	Interventio	Experimen	Experimen	Control	0,584085
6 months	abundance	X08973	Aminohipp	3	Interventio	Experimen	Experimen	Control	0,541353
6 months	abundance	X06946	SECONAL_	3	Interventio	Experimen	Experimen	Control	0,611916
6 months	abundance	X07574	7-ketodeox	3	Interventio	Experimen	Experimen	Control	0,541353
6 months	abundance	X11408	5-Amino-6	3	Interventio	Experimen	Experimen	Control	0,587711
6 months	abundance	X07417	δ-Valerolac	2b	Interventio	Experimen	Experimen	Control	0,567461
6 months	abundance	X11498	Valylvaline	3	Interventio	Experimen	Experimen	Control	0,547533
6 months	abundance	X00042	Glucuronic	1	Interventio	Experimen	Experimen	Control	0,587711
6 months	abundance	X09965	Biocytin	2b	Interventio	Experimen	Experimen	Control	0,536933
6 months	abundance	X08546	1,3,7-Trime	2b	Interventio	Experimen	Experimen	Control	0,60451
6 months	abundance	X01441	1-{3-Carbo	3	Interventio	Experimen	Experimen	Control	0,566785
6 months	abundance	X10382	Zalcitabine	3	Interventio	Experimen	Experimen	Control	0,536933

6 months	abundance	X09318	butalbital_	3	Interventio	Experimen	Experimen	Control	0,536933
6 months	abundance	X04986	L-Proline, 4	3	Interventio	Experimen	Experimen	Control	0,526151
6 months	abundance	X10977	pentobarbi	3	Interventio	Experimen	Experimen	Control	0,60451
6 months	abundance	X00030	3,4-Dihydr	1	Interventio	Experimen	Experimen	Control	0,547533
6 months	abundance	X05071	Histidylphe	3	Interventio	Experimen	Experimen	Control	0,519242
6 months	abundance	X06704	feruloylagr	3	Interventio	Experimen	Experimen	Control	0,621405
6 months	abundance	X00949	primidone_	3	Interventio	Experimen	Experimen	Control	0,547533
6 months	abundance	X07891	Cyclamic a	2b	Interventio	Experimen	Experimen	Control	0,547533
6 months	abundance	X10037	his-gln_a	3	Interventio	Experimen	Experimen	Control	0,536149
6 months	abundance	SL00350	Stachydrin	1	Interventio	Experimen	Experimen	Control	0,559923
6 months	abundance	X00056	3-(4-hydro:	1	Interventio	Experimen	Experimen	Control	0,482527
6 months	abundance	X07511	Piperidine_	3	Interventio	Experimen	Experimen	Control	0,496369
6 months	abundance	X08373	2-Hydroxy-	3	Interventio	Experimen	Experimen	Control	0,579512
6 months	abundance	X02943	3-Methoxy-	3	Interventio	Experimen	Experimen	Control	0,552443
6 months	abundance	X04378	9,11-Dihyd	3	Interventio	Experimen	Experimen	Control	0,573484
6 months	abundance	X08475	1-(4-Amino	3	Interventio	Experimen	Experimen	Control	0,547533
6 months	abundance	X00742	MFCD1869	3	Interventio	Experimen	Experimen	Control	0,581344
6 months	abundance	X06896	Pirbuterol	3	Interventio	Experimen	Experimen	Control	0,552645
6 months	abundance	X11373	3-Succinoy	2b	Interventio	Experimen	Experimen	Control	0,507626
6 months	abundance	SL00431	Tiglylcarnit	2a	Interventio	Experimen	Experimen	Control	0,519243
6 months	abundance	X08779	3-(1-hydro:	2b	Interventio	Experimen	Experimen	Control	0,448848
6 months	abundance	M15	M15	NA_module	Interventio	Experimen	Experimen	Control	0,433769
6 months	abundance	p_cresol	p-Cresol	1	Interventio	Experimen	Experimen	Control	0,475754
6 months	abundance	X01574	N-Acetylpr	3	Interventio	Experimen	Experimen	Control	0,458898
6 months	abundance	X10180	4-Formyl-2	3	Interventio	Experimen	Experimen	Control	0,530382
6 months	abundance	X02348	N-Benzoyla	3	Interventio	Experimen	Experimen	Control	0,494948
6 months	abundance	X07077	Hyodeoxyc	1	Interventio	Experimen	Experimen	Control	0,536933
6 months	abundance	X01813	Erythorbic	3	Interventio	Experimen	Experimen	Control	0,536933
6 months	abundance	SL00447	Apigenin	2a	Interventio	Experimen	Experimen	Control	0,495451
6 months	abundance	X07572	8-(3-Furyl)-	3	Interventio	Experimen	Experimen	Control	0,514474
6 months	abundance	X06062	1-(2,3-Dihy	3	Interventio	Experimen	Experimen	Control	0,461611
6 months	abundance	X02841	Kyotorphin	3	Interventio	Experimen	Experimen	Control	0,402458
6 months	abundance	X01561	2-Ammonio	3	Interventio	Experimen	Experimen	Control	0,547533
6 months	abundance	X06859	Methylol D	3	Interventio	Experimen	Experimen	Control	0,522714
6 months	abundance	X11366	3-Sulfino-L	3	Interventio	Experimen	Experimen	Control	0,526151
6 months	abundance	X01007	pentobarbi	3	Interventio	Experimen	Experimen	Control	0,487464
6 months	abundance	X02082	6-Hydroxyn	3	Interventio	Experimen	Experimen	Control	0,535443
6 months	abundance	X02231	Ala-Tyr	3	Interventio	Experimen	Experimen	Control	0,530382
6 months	abundance	X07508	Procaine_a	2b	Interventio	Experimen	Experimen	Control	0,481265
6 months	abundance	X06482	glu-pro	3	Interventio	Experimen	Experimen	Control	0,458898
6 months	abundance	X01732	2-Hydroxy-	3	Interventio	Experimen	Experimen	Control	0,530382
6 months	abundance	X03275	Methdilazi	3	Interventio	Experimen	Experimen	Control	0,474418
6 months	abundance	X07834	Choline su	3	Interventio	Experimen	Experimen	Control	0,497529
6 months	abundance	X06381	MFCD1869	3	Interventio	Experimen	Experimen	Control	0,493814
6 months	abundance	X08509	Piceid	3	Interventio	Experimen	Experimen	Control	0,495451
6 months	abundance	X06703	N6-METHY	3	Interventio	Experimen	Experimen	Control	0,413749
6 months	abundance	X03568	3-[(2Z)-1-C	3	Interventio	Experimen	Experimen	Control	0,494948
6 months	abundance	X01640	Ro 20-1724	3	Interventio	Experimen	Experimen	Control	0,424411

6 months	abundance	X08420	Dodecane	1	Interventio	Experimen	Experimen	Control	0,474418
6 months	abundance	X10886	L-gamma-(	3	Interventio	Experimen	Experimen	Control	0,471375
6 months	abundance	X06856	Tiglic acid_	2b	Interventio	Experimen	Experimen	Control	0,445492
6 months	abundance	X09064	Tetrahydro	3	Interventio	Experimen	Experimen	Control	0,477197
6 months	abundance	X07081	N-LACTOYI	3	Interventio	Experimen	Experimen	Control	0,487464
6 months	abundance	X09787	Queuosine	3	Interventio	Experimen	Experimen	Control	0,497615
6 months	abundance	SL00040	Kynurenine	2a	Interventio	Experimen	Experimen	Control	0,474418
6 months	abundance	X10151	2-(3-CARB	3	Interventio	Experimen	Experimen	Control	0,477197
6 months	abundance	X10549	Glaucine	2b	Interventio	Experimen	Experimen	Control	0,455427
6 months	abundance	X09912	MC055530	3	Interventio	Experimen	Experimen	Control	0,485449
6 months	abundance	X04543	N-Acetyl-S	3	Interventio	Experimen	Experimen	Control	0,477197
6 months	abundance	X10129	SECONAL_	3	Interventio	Experimen	Experimen	Control	0,45459
6 months	abundance	X03069	N-COUMAI	3	Interventio	Experimen	Experimen	Control	0,475519
6 months	abundance	X06041	(DL)-3-O-M	3	Interventio	Experimen	Experimen	Control	0,428209
6 months	abundance	X07236	2-methylci	3	Interventio	Experimen	Experimen	Control	0,495451
6 months	abundance	X08147	porphobilir	3	Interventio	Experimen	Experimen	Control	0,472806
6 months	abundance	X04852	Toluene_b	3	Interventio	Experimen	Experimen	Control	0,464749
6 months	abundance	X07112	MFCD0272	3	Interventio	Experimen	Experimen	Control	0,462525
6 months	abundance	X07425	N-Acetylas	2a	Interventio	Experimen	Experimen	Control	0,425825
6 months	abundance	X02289	Kynurenic a	2a	Interventio	Experimen	Experimen	Control	0,451534
6 months	abundance	X06709	Tetraacety	3	Interventio	Experimen	Experimen	Control	0,402821
6 months	abundance	Propanoic	Propanoic	CFA_panel	Interventio	Experimen	Experimen	Control	0,446583
6 months	abundance	X01570	carglumic a	3	Interventio	Experimen	Experimen	Control	0,472806
6 months	abundance	X11383	N-{3-[(4-Ac	3	Interventio	Experimen	Experimen	Control	0,444616
6 months	abundance	X00450	Gly-Ser	3	Interventio	Experimen	Experimen	Control	0,421892
6 months	abundance	X05243	N-(4-Hydro	3	Interventio	Experimen	Experimen	Control	0,471112
6 months	abundance	X06506	L-Homocys	3	Interventio	Experimen	Experimen	Control	0,474951
6 months	abundance	X07830	1,5-Isoquir	2b	Interventio	Experimen	Experimen	Control	0,472806
6 months	abundance	X01519	Leucyltrypt	3	Interventio	Experimen	Experimen	Control	0,472806
6 months	abundance	SL00368	N-Methylty	1	Interventio	Experimen	Experimen	Control	0,472806
6 months	abundance	X10550	pentobarbi	3	Interventio	Experimen	Experimen	Control	0,485832
6 months	abundance	X07701	3-Benzyl-6	3	Interventio	Experimen	Experimen	Control	0,483186
6 months	abundance	M8	M8	NA_module	Interventio	Experimen	Experimen	Control	0,465501
6 months	abundance	X07745	APM_c	3	Interventio	Experimen	Experimen	Control	0,462155
6 months	abundance	X07565	5-Hydroxyi	2b	Interventio	Experimen	Experimen	Control	0,444616
6 months	abundance	X10979	acetyltauri	3	Interventio	Experimen	Experimen	Control	0,463049
6 months	abundance	X00051	β-D-Glucop	2b	Interventio	Experimen	Experimen	Control	0,429158
6 months	abundance	SL00161	Uridine	2a	Interventio	Experimen	Experimen	Control	0,411713
6 months	abundance	X07315	Rivastigmir	3	Interventio	Experimen	Experimen	Control	0,437868
6 months	abundance	X07846	Carbofurar	3	Interventio	Experimen	Experimen	Control	0,373897
6 months	abundance	X09615	tyramine su	3	Interventio	Experimen	Experimen	Control	0,476616
6 months	abundance	X07749	(DL)-3-O-M	3	Interventio	Experimen	Experimen	Control	0,425825
6 months	abundance	X02521	6-(1-Hydro	3	Interventio	Experimen	Experimen	Control	0,464749
6 months	abundance	X11034	1-(3-Aminc	3	Interventio	Experimen	Experimen	Control	0,416238
6 months	abundance	X11477	gamma-Gl	3	Interventio	Experimen	Experimen	Control	0,363159
6 months	abundance	X06612	Spermic ac	3	Interventio	Experimen	Experimen	Control	0,315603
6 months	abundance	X00545	3-hydroxyc	3	Interventio	Experimen	Experimen	Control	0,434372
6 months	abundance	X04684	AAMU_b	3	Interventio	Experimen	Experimen	Control	0,473588

6 months	abundance	X04181	3-Methoxy-	3	Interventio	Experimen	Experimen	Control	0,413749
6 months	abundance	3-Methylbu	3-Methylbu	CFA_panel	Interventio	Experimen	Experimen	Control	0,366113
6 months	abundance	SL00445	Glucosami	2a	Interventio	Experimen	Experimen	Control	0,462525
6 months	abundance	SL00185	2-Oxo-3-pl	1	Interventio	Experimen	Experimen	Control	0,375103
6 months	abundance	X04996	MFCD0272	3	Interventio	Experimen	Experimen	Control	0,497529
6 months	abundance	X05551	Trolox	3	Interventio	Experimen	Experimen	Control	0,435244
6 months	abundance	X04922	Sinapinic a	3	Interventio	Experimen	Experimen	Control	0,433769
6 months	abundance	X08504	Indole-3-ca	2a	Interventio	Experimen	Experimen	Control	0,374505
6 months	abundance	X09454	2-(2-Hydro	3	Interventio	Experimen	Experimen	Control	0,410744
6 months	abundance	X10457	glu-ser	3	Interventio	Experimen	Experimen	Control	0,310448
6 months	abundance	X09004	Arenaine	3	Interventio	Experimen	Experimen	Control	0,455294
6 months	abundance	SL00212	Galactosai	2a	Interventio	Experimen	Experimen	Control	0,346393
6 months	abundance	X08322	Glycylproly	3	Interventio	Experimen	Experimen	Control	0,402458
6 months	abundance	X10503	APM_a	3	Interventio	Experimen	Experimen	Control	0,408947
6 months	abundance	X06820	1-(3,4-dim	2b	Interventio	Experimen	Experimen	Control	0,472806
6 months	abundance	X01528	butalbital_	3	Interventio	Experimen	Experimen	Control	0,346785
6 months	abundance	X04566	pterin	3	Interventio	Experimen	Experimen	Control	0,464749
6 months	abundance	X02689	piscidic ac	3	Interventio	Experimen	Experimen	Control	0,402458
6 months	abundance	X01881	(2S)-3-Met	3	Interventio	Experimen	Experimen	Control	0,408947
6 months	abundance	X07439	4-pyridoxic	1	Interventio	Experimen	Experimen	Control	0,328207
6 months	abundance	X10025	2-[4-(3-Hy	3	Interventio	Experimen	Experimen	Control	0,416238
6 months	abundance	X11038	(2E)-3-(3,4	3	Interventio	Experimen	Experimen	Control	0,346785
6 months	abundance	X00266	2-Oxo-3-(p	3	Interventio	Experimen	Experimen	Control	0,411713
6 months	abundance	2-Methylpr	2-Methylpr	CFA_panel	Interventio	Experimen	Experimen	Control	0,299663
6 months	abundance	X08076	3',5,7-Trihy	2b	Interventio	Experimen	Experimen	Control	0,400134
6 months	abundance	X11028	butalbital_	3	Interventio	Experimen	Experimen	Control	0,395346
6 months	abundance	X08311	Methyl 3-fo	3	Interventio	Experimen	Experimen	Control	0,421892
6 months	abundance	X05398	DIBOA	3	Interventio	Experimen	Experimen	Control	0,373224
6 months	abundance	X06590	3-hydroxy-	3	Interventio	Experimen	Experimen	Control	0,392683
6 months	abundance	X00383	pretazettin	3	Interventio	Experimen	Experimen	Control	0,362327
6 months	abundance	X01100	(betaS)-be	3	Interventio	Experimen	Experimen	Control	0,408413
6 months	abundance	X09977	TLK (Peptic	2b	Interventio	Experimen	Experimen	Control	0,297276
6 months	abundance	X04377	Dihydroura	3	Interventio	Experimen	Experimen	Control	0,327144
6 months	abundance	X05417	(S)-2-hydra	3	Interventio	Experimen	Experimen	Control	0,346785
6 months	abundance	X08535	Nitrosohep	2b	Interventio	Experimen	Experimen	Control	0,324575
6 months	abundance	X05734	N-[(2S)-2-H	3	Interventio	Experimen	Experimen	Control	0,310912
6 months	abundance	X06220	Tetrahydro	3	Interventio	Experimen	Experimen	Control	0,40852
6 months	abundance	X02968	Ro 20-172	3	Interventio	Experimen	Experimen	Control	0,387234
6 months	abundance	X08990	Procaine_c	3	Interventio	Experimen	Experimen	Control	0,357085
6 months	abundance	X05968	Tetraacety	3	Interventio	Experimen	Experimen	Control	0,303733
6 months	abundance	X10011	Midodrine_	3	Interventio	Experimen	Experimen	Control	0,33088
6 months	abundance	X02823	6,8-Dimetl	3	Interventio	Experimen	Experimen	Control	0,319626
6 months	abundance	X06486	Leu-Val_b	3	Interventio	Experimen	Experimen	Control	0,301288
6 months	abundance	X09357	5-(5-Methy	3	Interventio	Experimen	Experimen	Control	0,346785
6 months	abundance	X06600	MFCD0014	3	Interventio	Experimen	Experimen	Control	0,189922
6 months	abundance	X07849	Crotamitor	3	Interventio	Experimen	Experimen	Control	0,36404
6 months	abundance	X06289	Nicotinatel	3	Interventio	Experimen	Experimen	Control	0,189396
6 months	abundance	X10936	clavulanic	3	Interventio	Experimen	Experimen	Control	0,248374

6 months	abundance	X04697	Ethosuxim	3	Interventio	Experimen	Experimen	Control	0,283406
6 months	abundance	X07250	Leucylaspa	3	Interventio	Experimen	Experimen	Control	0,250568
6 months	abundance	X07869	Hexamethy	2a	Interventio	Experimen	Experimen	Control	0,30878
6 months	abundance	X10864	5-amino-2-	2b	Interventio	Experimen	Experimen	Control	0,25478
6 months	abundance	X01530	INK (Peptic	2b	Interventio	Experimen	Experimen	Control	0,20049
6 months	abundance	X06655	Hydroxycar	3	Interventio	Experimen	Experimen	Control	0,313913
6 months	abundance	X06933	3-Methoxy-	3	Interventio	Experimen	Experimen	Control	0,316818
6 months	abundance	X04999	2-Hydroxyp	1	Interventio	Experimen	Experimen	Control	0,316818
6 months	abundance	X07577	2-[(Sulfoox	3	Interventio	Experimen	Experimen	Control	0,22808
6 months	abundance	X06152	alpha-keto	3	Interventio	Experimen	Experimen	Control	0,330185
6 months	abundance	M11	M11	NA_module	Interventio	Experimen	Experimen	Control	0,301288
6 months	abundance	X03714	Sinapinic a	3	Interventio	Experimen	Experimen	Control	0,375103
6 months	abundance	SL00407	Homovanil	2a	Interventio	Experimen	Experimen	Control	0,250568
6 months	abundance	X00120	Leucylalan	1	Interventio	Experimen	Experimen	Control	0,275681
6 months	abundance	X04146	L-fucopyra	3	Interventio	Experimen	Experimen	Control	0,326255
6 months	abundance	X08439	Leucyltyros	3	Interventio	Experimen	Experimen	Control	0,292527
6 months	abundance	X06400	Phloionolic	3	Interventio	Experimen	Experimen	Control	0,37321
6 months	abundance	X05726	methocarb	3	Interventio	Experimen	Experimen	Control	0,27927
6 months	abundance	X00404	piscidic ac	3	Interventio	Experimen	Experimen	Control	0,301288
6 months	abundance	X06495	2-Acetamid	3	Interventio	Experimen	Experimen	Control	0,248374
6 months	abundance	X10757	Glycyrin	3	Interventio	Experimen	Experimen	Control	0,272268
6 months	abundance	X06880	pentobarbi	3	Interventio	Experimen	Experimen	Control	0,327464
6 months	abundance	X07038	Piperidine_	3	Interventio	Experimen	Experimen	Control	0,303488
6 months	abundance	X00549	3-[(2Z)-1-C	3	Interventio	Experimen	Experimen	Control	0,283861
6 months	abundance	X05081	ELK (Peptic	2b	Interventio	Experimen	Experimen	Control	0,251302
6 months	abundance	M29	M29	NA_module	Interventio	Experimen	Experimen	Control	0,301288
6 months	abundance	X00233	g-Aminobu	3	Interventio	Experimen	Experimen	Control	0,317066
6 months	abundance	X10636	8-hydroxy-	2b	Interventio	Experimen	Experimen	Control	0,315603
6 months	abundance	X07465	TO0127900	3	Interventio	Experimen	Experimen	Control	0,246789
6 months	abundance	X07454	2,4-Quinol	2b	Interventio	Experimen	Experimen	Control	0,179358
6 months	abundance	X01672	Triethyl citr	3	Interventio	Experimen	Experimen	Control	0,254807
6 months	abundance	X04450	3-Methylac	3	Interventio	Experimen	Experimen	Control	0,305574
6 months	abundance	X07487	Oxypeucec	3	Interventio	Experimen	Experimen	Control	0,316818
6 months	abundance	X06768	Sinapyl alc	3	Interventio	Experimen	Experimen	Control	0,279022
6 months	abundance	X07820	Creatinine	1	Interventio	Experimen	Experimen	Control	0,287584
6 months	abundance	X08072	4-Hydroxyp	3	Interventio	Experimen	Experimen	Control	0,303733
6 months	abundance	X10130	N-(4-Hydro	3	Interventio	Experimen	Experimen	Control	0,2532
6 months	abundance	X07013	pimethixer	3	Interventio	Experimen	Experimen	Control	0,310448
6 months	abundance	X07937	Atenolol	1	Interventio	Experimen	Experimen	Control	0,274405
6 months	abundance	X10314	4-morpholi	2b	Interventio	Experimen	Experimen	Control	0,278551
6 months	abundance	X10665	Aprobarbit	3	Interventio	Experimen	Experimen	Control	0,309332
6 months	abundance	X08038	N-{6-[(7-Cl	2b	Interventio	Experimen	Experimen	Control	0,327144
6 months	abundance	M3	M3	NA_module	Interventio	Experimen	Experimen	Control	0,27927
6 months	abundance	X05997	FB9500000	3	Interventio	Experimen	Experimen	Control	0,118447
6 months	abundance	M17	M17	NA_module	Interventio	Experimen	Experimen	Control	0,170002
6 months	abundance	X08451	L-gamma-(	3	Interventio	Experimen	Experimen	Control	0,162035
6 months	abundance	X11251	N-Nonanoy	3	Interventio	Experimen	Experimen	Control	0,246099
6 months	abundance	X10651	Methyl 1-hy	3	Interventio	Experimen	Experimen	Control	0,246099

6 months	abundance	X08056	3-(2-Oxo-2	2b	Interventio	Experimen	Experimen	Control	0,278551
6 months	abundance	M6	M6	NA_module	Interventio	Experimen	Experimen	Control	0,287584
6 months	abundance	X10982	8-Methyl-8	3	Interventio	Experimen	Experimen	Control	0,157063
6 months	abundance	X06354	trans-Zeati	2b	Interventio	Experimen	Experimen	Control	0,245841
6 months	abundance	X06680	7-Hydroxy-	2b	Interventio	Experimen	Experimen	Control	0,215732
6 months	abundance	X07113	Sulfoaceta	3	Interventio	Experimen	Experimen	Control	0,243384
6 months	abundance	X06168	leu-gln_b	3	Interventio	Experimen	Experimen	Control	0,167056
6 months	abundance	X07428	N6-METHY	3	Interventio	Experimen	Experimen	Control	0,213235
6 months	abundance	X02640	thyronine	3	Interventio	Experimen	Experimen	Control	0,25111
6 months	abundance	X07303	Triacetin	3	Interventio	Experimen	Experimen	Control	0,217996
6 months	abundance	SL00320	γ-Caprolac	2a	Interventio	Experimen	Experimen	Control	0,160244
6 months	abundance	X02020	Val-Ser_b	3	Interventio	Experimen	Experimen	Control	0,153848
6 months	abundance	X07057	Leu-Leu_c	3	Interventio	Experimen	Experimen	Control	0,276451
6 months	abundance	X10676	6-(1-Hydro	3	Interventio	Experimen	Experimen	Control	0,225907
6 months	abundance	X06250	[3-({3-[(Cy	2b	Interventio	Experimen	Experimen	Control	0,187192
6 months	abundance	X00026	Inosine	1	Interventio	Experimen	Experimen	Control	0,195944
6 months	abundance	SL00282	3-Hydroxyl	2a	Interventio	Experimen	Experimen	Control	0,196579
6 months	abundance	X09163	Prenisteine	3	Interventio	Experimen	Experimen	Control	0,224766
6 months	abundance	X07139	N,N-Diethy	3	Interventio	Experimen	Experimen	Control	0,179358
6 months	abundance	SL00200	8-Aminooc	2a	Interventio	Experimen	Experimen	Control	0,246099
6 months	abundance	X04582	Dinoseb	3	Interventio	Experimen	Experimen	Control	0,218277
6 months	abundance	X11666	N-(4-Hydro	3	Interventio	Experimen	Experimen	Control	0,213812
6 months	abundance	X06850	Leu-Val_c	3	Interventio	Experimen	Experimen	Control	0,251476
6 months	abundance	X01363	Seryltyrosi	3	Interventio	Experimen	Experimen	Control	0,276451
6 months	abundance	X00097	2,6-Dihydr	1	Interventio	Experimen	Experimen	Control	0,199156
6 months	abundance	X05183	3,6-Dichlo	3	Interventio	Experimen	Experimen	Control	0,248374
6 months	abundance	X09643	N-(3,5-Dim	3	Interventio	Experimen	Experimen	Control	0,187115
6 months	abundance	X05295	3-(4,7-Dim	3	Interventio	Experimen	Experimen	Control	0,270217
6 months	abundance	X10953	Vorinostat_	3	Interventio	Experimen	Experimen	Control	0,196579
6 months	abundance	X01893	4-(METHYL	3	Interventio	Experimen	Experimen	Control	0,232202
6 months	abundance	X10889	N-acetyl-b	3	Interventio	Experimen	Experimen	Control	0,141367
6 months	abundance	X11254	(4R,5S,9S,	3	Interventio	Experimen	Experimen	Control	0,222774
6 months	abundance	X01163	Prephenic	3	Interventio	Experimen	Experimen	Control	0,251601
6 months	abundance	X00748	3-[3-Metho	3	Interventio	Experimen	Experimen	Control	0,209548
6 months	abundance	X09099	1-(2-Hydro	3	Interventio	Experimen	Experimen	Control	0,200132
6 months	abundance	X08106	4-Vinylphe	1	Interventio	Experimen	Experimen	Control	0,107899
6 months	abundance	X11042	Leu-arg	3	Interventio	Experimen	Experimen	Control	0,08278
6 months	abundance	X07852	N6,N6,N6-	3	Interventio	Experimen	Experimen	Control	0,139315
6 months	abundance	X11125	5,8,12-Trih	3	Interventio	Experimen	Experimen	Control	0,143983
6 months	abundance	X00261	Mexiletine	3	Interventio	Experimen	Experimen	Control	0,18655
6 months	abundance	X00948	Linamarin	3	Interventio	Experimen	Experimen	Control	0,126909
6 months	abundance	SL00275	Dopamine	2a	Interventio	Experimen	Experimen	Control	0,246789
6 months	abundance	X04117	2,3,4,5-tet	3	Interventio	Experimen	Experimen	Control	0,196471
6 months	abundance	X00038	Histamine	1	Interventio	Experimen	Experimen	Control	0,221015
6 months	abundance	X03357	2-Hydroxyl	3	Interventio	Experimen	Experimen	Control	0,12884
6 months	abundance	X11481	his-gln_b	3	Interventio	Experimen	Experimen	Control	0,216874
6 months	abundance	X06189	delta-Guar	3	Interventio	Experimen	Experimen	Control	0,165482
6 months	abundance	M28	M28	NA_module	Interventio	Experimen	Experimen	Control	0,136337

6 months	abundance	X00221	Astemizole	3	Interventio	Experimen	Experimen	Control	0,159111
6 months	abundance	X07475	2'-Deoxy-5	3	Interventio	Experimen	Experimen	Control	0,213235
6 months	abundance	X06814	N-Benzoyla	3	Interventio	Experimen	Experimen	Control	0,171599
6 months	abundance	X06276	asn-pro_b	3	Interventio	Experimen	Experimen	Control	0,094335
6 months	abundance	X02824	Eslicarbaz	3	Interventio	Experimen	Experimen	Control	0,187823
6 months	abundance	X03268	Dihydrouri	3	Interventio	Experimen	Experimen	Control	0,171599
6 months	abundance	X08781	Procaine_b	3	Interventio	Experimen	Experimen	Control	0,179439
6 months	abundance	X11215	Leu-Leu_b	3	Interventio	Experimen	Experimen	Control	0,117512
6 months	abundance	M34	M34	IA_module	Interventio	Experimen	Experimen	Control	0,196868
6 months	abundance	X02986	Diacetin_b	3	Interventio	Experimen	Experimen	Control	0,173912
6 months	abundance	X08338	Methyl [9-(	3	Interventio	Experimen	Experimen	Control	0,164527
6 months	abundance	X05919	2-(2-Aminc	3	Interventio	Experimen	Experimen	Control	0,175218
6 months	abundance	X01327	threonylph	3	Interventio	Experimen	Experimen	Control	0,222739
6 months	abundance	SL00467	Sucralose	2a	Interventio	Experimen	Experimen	Control	0,20049
6 months	abundance	X01242	3-Morpholi	2b	Interventio	Experimen	Experimen	Control	0,080975
6 months	abundance	X11522	thr-trp	3	Interventio	Experimen	Experimen	Control	0,098395
6 months	abundance	X11339	L-gamma-(	3	Interventio	Experimen	Experimen	Control	0,136337
6 months	abundance	X01558	2-Furoylgly	2b	Interventio	Experimen	Experimen	Control	0,145494
6 months	abundance	X07731	3-[4-methy	2b	Interventio	Experimen	Experimen	Control	0,113797
6 months	abundance	X09608	N-(3,5-Dim	3	Interventio	Experimen	Experimen	Control	0,12884
6 months	abundance	X06419	Spermic ac	3	Interventio	Experimen	Experimen	Control	0,068876
6 months	abundance	X07283	8-Hydroxyh	3	Interventio	Experimen	Experimen	Control	0,107899
6 months	abundance	X01387	Methyl 4-(4	3	Interventio	Experimen	Experimen	Control	0,139315
6 months	abundance	X06227	lys-tyr_a	3	Interventio	Experimen	Experimen	Control	0,103211
6 months	abundance	X04695	N-[(4-Meth	3	Interventio	Experimen	Experimen	Control	0,124409
6 months	abundance	X00129	Quinic acic	1	Interventio	Experimen	Experimen	Control	0,115704
6 months	abundance	X09399	L-gamma-(	3	Interventio	Experimen	Experimen	Control	0,062463
6 months	abundance	X05203	felbamate	3	Interventio	Experimen	Experimen	Control	0,107855
6 months	abundance	X03158	5-Hydroxy-	3	Interventio	Experimen	Experimen	Control	0,103211
6 months	abundance	M33	M33	IA_module	Interventio	Experimen	Experimen	Control	0,092456
6 months	abundance	X08713	Phenethyla	2b	Interventio	Experimen	Experimen	Control	0,056436
6 months	abundance	X07977	Tyramine	2b	Interventio	Experimen	Experimen	Control	0,095967
6 months	abundance	X08298	N6,N6,N6-	2b	Interventio	Experimen	Experimen	Control	0,095031
6 months	abundance	X07905	Prilocaine	2b	Interventio	Experimen	Experimen	Control	0,125216
6 months	abundance	X03563	6-imino-5-	3	Interventio	Experimen	Experimen	Control	0,077552
6 months	abundance	X01887	lys-tyr_b	3	Interventio	Experimen	Experimen	Control	0,0902
6 months	abundance	X07146	meprobam	3	Interventio	Experimen	Experimen	Control	0,055405
6 months	abundance	X07930	N-Methylca	2b	Interventio	Experimen	Experimen	Control	0,130126
6 months	abundance	X09607	N-(1H-Pyrr	3	Interventio	Experimen	Experimen	Control	0,06731
6 months	abundance	X08639	hypaphorir	3	Interventio	Experimen	Experimen	Control	0,080527
6 months	abundance	SL00356	N-(5-Amin	2a	Interventio	Experimen	Experimen	Control	0,126909
6 months	abundance	X11140	Leu-Leu_e	3	Interventio	Experimen	Experimen	Control	0,095967
6 months	abundance	X04483	(2E)-5-Hyd	3	Interventio	Experimen	Experimen	Control	0,134826
6 months	abundance	X06401	Isophthalic	2b	Interventio	Experimen	Experimen	Control	0,118447
6 months	abundance	X00693	n-Ribosylh	3	Interventio	Experimen	Experimen	Control	0,045594
6 months	abundance	X00985	1-(4-Aminc	3	Interventio	Experimen	Experimen	Control	0,068028
6 months	abundance	M30	M30	IA_module	Interventio	Experimen	Experimen	Control	0,080527
6 months	abundance	X10513	ophthalmic	3	Interventio	Experimen	Experimen	Control	0,06242

6 months	abundance	X02219	Homoanse	3	Interventio	Experimen	Experimen	Control	0,110798
6 months	abundance	X05234	3,4-dihydro	3	Interventio	Experimen	Experimen	Control	0,114862
6 months	abundance	X06433	4-(2,5-Diflu	2b	Interventio	Experimen	Experimen	Control	0,092456
6 months	abundance	X07285	2-(Carboxy	3	Interventio	Experimen	Experimen	Control	0,113467
6 months	abundance	X10393	Ro 20-1724	3	Interventio	Experimen	Experimen	Control	0,081606
6 months	abundance	X08478	1-(beta-D-l	3	Interventio	Experimen	Experimen	Control	0,074642
6 months	abundance	X03223	1-(2-Carbo	3	Interventio	Experimen	Experimen	Control	0,095967
6 months	abundance	X00025	Acesulfam	1	Interventio	Experimen	Experimen	Control	0,077865
6 months	abundance	X07309	3,4,15-Trih	3	Interventio	Experimen	Experimen	Control	0,086596
6 months	abundance	SL00318	4-Aminoph	1	Interventio	Experimen	Experimen	Control	0,097102
6 months	abundance	X01081	Methyl alpl	3	Interventio	Experimen	Experimen	Control	0,048201
6 months	abundance	X08103	Isopelletier	3	Interventio	Experimen	Experimen	Control	0,053894
6 months	abundance	X09738	(7E,7'E)-5,	3	Interventio	Experimen	Experimen	Control	0,060452
6 months	abundance	X07944	Styrene	3	Interventio	Experimen	Experimen	Control	0,032515
6 months	abundance	X03376	4-(9H-beta	3	Interventio	Experimen	Experimen	Control	0,080527
6 months	abundance	X00180	1-(2-Carbo	3	Interventio	Experimen	Experimen	Control	0,072309
6 months	abundance	X07903	Acetophen	2b	Interventio	Experimen	Experimen	Control	0,068026
6 months	abundance	X02990	Pentoxifylli	3	Interventio	Experimen	Experimen	Control	0,080527
6 months	abundance	X03602	o-Succinyl	3	Interventio	Experimen	Experimen	Control	0,06595
6 months	abundance	X02337	Methyl 1-hy	3	Interventio	Experimen	Experimen	Control	0,080527
6 months	abundance	X07107	Lysylvaline	3	Interventio	Experimen	Experimen	Control	0,109347
6 months	abundance	X10121	Leu-pro_b	3	Interventio	Experimen	Experimen	Control	0,053894
6 months	abundance	X02793	3-Methoxy-	3	Interventio	Experimen	Experimen	Control	0,065283
6 months	abundance	X07581	N-Acetylva	3	Interventio	Experimen	Experimen	Control	0,06595
6 months	abundance	X06663	Leucyltyros	3	Interventio	Experimen	Experimen	Control	0,095545
6 months	abundance	X00361	nitecapone	3	Interventio	Experimen	Experimen	Control	0,085643
6 months	abundance	X05709	2,3-Dihydro	3	Interventio	Experimen	Experimen	Control	0,053894
6 months	abundance	X03793	Desonide	3	Interventio	Experimen	Experimen	Control	0,07652
6 months	abundance	X08193	Seryltyrosi	3	Interventio	Experimen	Experimen	Control	0,065283
6 months	abundance	X01656	Guanadrel	3	Interventio	Experimen	Experimen	Control	0,055563
6 months	abundance	X04493	Tyrosol	3	Interventio	Experimen	Experimen	Control	0,080975
6 months	abundance	X11323	dihydroxyb	3	Interventio	Experimen	Experimen	Control	0,039418
6 months	abundance	X06750	1-PYRENYL	3	Interventio	Experimen	Experimen	Control	0,080975
6 months	abundance	X10494	26Q0EO75	3	Interventio	Experimen	Experimen	Control	0,080943
6 months	abundance	M5	M5	IA_module	Interventio	Experimen	Experimen	Control	0,056436
6 months	abundance	X06560	2,6-Dimeth	2b	Interventio	Experimen	Experimen	Control	0,053803
6 months	abundance	X07582	4-methylpy	2b	Interventio	Experimen	Experimen	Control	0,074642
6 months	abundance	X06874	N-Acetyl-5	3	Interventio	Experimen	Experimen	Control	0,046544
6 months	abundance	X11361	1-Methylhi	3	Interventio	Experimen	Experimen	Control	0,052526
6 months	abundance	X02651	L-gamma-(	3	Interventio	Experimen	Experimen	Control	0,046272
6 months	abundance	X06744	Diethylpyro	3	Interventio	Experimen	Experimen	Control	0,053894
6 months	abundance	X09565	3-Methoxy-	3	Interventio	Experimen	Experimen	Control	0,061587
6 months	abundance	X04743	5-Methoxy-	3	Interventio	Experimen	Experimen	Control	0,074608
6 months	abundance	X01098	(-)-Physost	3	Interventio	Experimen	Experimen	Control	0,042505
6 months	abundance	X08610	Leucyltyros	3	Interventio	Experimen	Experimen	Control	0,060452
6 months	abundance	X09932	g-Aminobu	3	Interventio	Experimen	Experimen	Control	0,03552
6 months	abundance	X00637	N~6~,N~6~	3	Interventio	Experimen	Experimen	Control	0,023843
6 months	abundance	X00698	valganciclo	3	Interventio	Experimen	Experimen	Control	0,046272

6 months	abundance	X03488	3,4-Dihydr	3	Interventio	Experimen	Experimen	Control	0,046125
6 months	abundance	X09703	7,8-Didehy	3	Interventio	Experimen	Experimen	Control	0,050313
6 months	abundance	X08644	2,5-Dimeth	2b	Interventio	Experimen	Experimen	Control	0,040318
6 months	abundance	X01286	(2,4-Dihyd	3	Interventio	Experimen	Experimen	Control	0,022499
6 months	abundance	X07955	Valylvaline	3	Interventio	Experimen	Experimen	Control	0,012138
6 months	abundance	X08842	Spermic ac	3	Interventio	Experimen	Experimen	Control	0,016813
6 months	abundance	X02652	Leucyltyros	3	Interventio	Experimen	Experimen	Control	0,023682
6 months	abundance	X05467	Azulfidine	3	Interventio	Experimen	Experimen	Control	0,025893
6 months	abundance	X08641	meprobam	3	Interventio	Experimen	Experimen	Control	0,017053
6 months	abundance	X02676	Val-Trp_a	3	Interventio	Experimen	Experimen	Control	0,060957
6 months	abundance	X08529	7,8-Diamir	3	Interventio	Experimen	Experimen	Control	0,014596
6 months	abundance	X08514	N-Ethylpro	3	Interventio	Experimen	Experimen	Control	0,025324
6 months	abundance	X05741	g-Aminobu	3	Interventio	Experimen	Experimen	Control	0,023022
6 months	abundance	X07867	N-Acetylva	3	Interventio	Experimen	Experimen	Control	0,038212
6 months	abundance	X11331	N~6~-Octa	3	Interventio	Experimen	Experimen	Control	0,017053
6 months	abundance	X00950	2-Phenylet	3	Interventio	Experimen	Experimen	Control	0,005134
6 months	abundance	X11277	lys-leu	3	Interventio	Experimen	Experimen	Control	0,035097
6 months	abundance	X10319	1-Methyl-1	3	Interventio	Experimen	Experimen	Control	0,014596
6 months	abundance	X03707	9-[(5R)-5-E	3	Interventio	Experimen	Experimen	Control	0,032515
6 months	abundance	X02471	n-Propyl G	3	Interventio	Experimen	Experimen	Control	0,013146
6 months	abundance	X07126	Leu-Val_f	3	Interventio	Experimen	Experimen	Control	0,012184
6 months	abundance	X02894	Toluene_a	3	Interventio	Experimen	Experimen	Control	0,009848
6 months	abundance	X06532	Leupeptin	3	Interventio	Experimen	Experimen	Control	0,00969
6 months	abundance	X07843	Piperidine_	3	Interventio	Experimen	Experimen	Control	0,006888
6 months	abundance	X02251	Hostmania	3	Interventio	Experimen	Experimen	Control	0,003046
6 months	abundance	X06819	Primaquin	3	Interventio	Experimen	Experimen	Control	0,009224
6 months	abundance	X00023	N-Formylm	1	Interventio	Experimen	Experimen	Control	0,006049
6 months	abundance	X08007	MFCD0002	3	Interventio	Experimen	Experimen	Control	0,008517
6 months	abundance	X08100	Piperidine_	3	Interventio	Experimen	Experimen	Control	0,007323
6 months	abundance	X08059	Cadaverin	3	Interventio	Experimen	Experimen	Control	0,005507
6 months	abundance	X08810	N~6~,N~6	3	Interventio	Experimen	Experimen	Control	0,004096
6 months	abundance	X07269	(3aR,4R,5	3	Interventio	Experimen	Experimen	Control	0,012184
6 months	abundance	X07866	3,3,5,5-Tet	2b	Interventio	Experimen	Experimen	Control	0,006888
6 months	abundance	SL00295	5-Methylur	2a	Interventio	Experimen	Experimen	Control	0,002657
6 months	abundance	X09110	Valylvaline	3	Interventio	Experimen	Experimen	Control	0,002657
6 months	abundance	X08365	Lysylvaline	3	Interventio	Experimen	Experimen	Control	0,003479
6 months	abundance	X11745	2-Despipe	3	Interventio	Experimen	Experimen	Control	0,003399
6 months	abundance	X07854	TO012790	3	Interventio	Experimen	Experimen	Control	0,00194
6 months	abundance	X09068	N,N-Diethy	3	Interventio	Experimen	Experimen	Control	0,003525
6 months	abundance	X07983	butalbital_	3	Interventio	Experimen	Experimen	Control	0,002657
6 months	abundance	X07916	N6,N6,N6-	3	Interventio	Experimen	Experimen	Control	0,000536
6 months	abundance	X00112	Xanthosin	1	Interventio	Experimen	Experimen	Control	5,65E-05
6 months	abundance	X04021	Bentazone	2b	Interventio	Experimen	Experimen	Control	1,23E-18

P-value	Effect size	Standard e	Sample siz	N alternati	N referenc	Degrees of	Test statist	Model formula fit
0,887212	-0,0202	0,142142	140	69	71	136	-0,1421	y ~ Intervention + Sex +
0,680706	-0,06074	0,147281	137	68	69	133	-0,41241	y ~ Intervention + Sex +
0,074152	-0,27675	0,153806	142	70	72	138	-1,79933	y ~ Intervention + Sex +
0,002816	-0,44677	0,146879	142	70	72	138	-3,04176	y ~ Intervention + Sex +
0,627082	-0,11769	0,241146	72	31	41	68	-0,48805	y ~ Intervention + Sex +
0,792364	-0,04171	0,158143	142	70	72	138	-0,26375	y ~ Intervention + Sex +
0,273916	-0,16803	0,152968	142	70	72	138	-1,09846	y ~ Intervention + Sex +
0,587785	-0,09077	0,167061	142	70	72	138	-0,54332	y ~ Intervention + Sex +
0,081652	0,220251	0,125571	142	70	72	138	1,753992	y ~ Intervention + Sex +
0,107509	0,256055	0,158056	142	70	72	138	1,620029	y ~ Intervention + Sex +
0,935522	-0,01382	0,170514	142	70	72	138	-0,08105	y ~ Intervention + Sex +
0,326071	-0,15097	0,153183	142	70	72	138	-0,98557	y ~ Intervention + Sex +
0,669916	0,068003	0,159191	142	70	72	138	0,427177	y ~ Intervention + Sex +
0,806273	-0,03563	0,145013	142	70	72	138	-0,24571	y ~ Intervention + Sex +
0,817678	0,036569	0,158327	142	70	72	138	0,230973	y ~ Intervention + Sex +
0,759715	0,046793	0,152688	142	70	72	138	0,306461	y ~ Intervention + Sex +
0,250178	0,180989	0,156731	142	70	72	138	1,154776	y ~ Intervention + Sex +
0,156925	0,227993	0,160194	142	70	72	138	1,423234	y ~ Intervention + Sex +
0,715584	-0,05696	0,156002	142	70	72	138	-0,36512	y ~ Intervention + Sex +
0,781694	-0,04056	0,14607	142	70	72	138	-0,27765	y ~ Intervention + Sex +
0,141139	0,231593	0,156476	142	70	72	138	1,480053	y ~ Intervention + Sex +
0,817358	-0,03787	0,163668	142	70	72	138	-0,23139	y ~ Intervention + Sex +
0,919247	0,017117	0,168527	142	70	72	138	0,101569	y ~ Intervention + Sex +
0,849855	-0,03079	0,162366	142	70	72	138	-0,18966	y ~ Intervention + Sex +
0,258621	-0,1727	0,152244	142	70	72	138	-1,13433	y ~ Intervention + Sex +
0,210827	-0,20755	0,1651	142	70	72	138	-1,25714	y ~ Intervention + Sex +
0,358	0,145924	0,158223	142	70	72	138	0,922266	y ~ Intervention + Sex +
0,539028	-0,09796	0,159066	142	70	72	138	-0,61582	y ~ Intervention + Sex +
0,038895	-0,29874	0,143269	142	70	72	138	-2,08518	y ~ Intervention + Sex +
0,964279	0,006996	0,15592	142	70	72	138	0,044866	y ~ Intervention + Sex +
0,876181	-0,02323	0,148797	142	70	72	138	-0,1561	y ~ Intervention + Sex +
0,109782	-0,2649	0,164581	142	70	72	138	-1,60955	y ~ Intervention + Sex +
0,304308	-0,14241	0,138117	142	70	72	138	-1,03108	y ~ Intervention + Sex +
0,485933	0,113639	0,16265	142	70	72	138	0,698672	y ~ Intervention + Sex +
0,966471	-0,00657	0,156001	142	70	72	138	-0,04211	y ~ Intervention + Sex +
0,304995	-0,16968	0,164804	142	70	72	138	-1,02961	y ~ Intervention + Sex +
0,705916	0,057331	0,151619	142	70	72	138	0,378128	y ~ Intervention + Sex +
0,09434	-0,25929	0,153922	142	70	72	138	-1,68453	y ~ Intervention + Sex +
0,321498	0,150025	0,150785	142	70	72	138	0,994959	y ~ Intervention + Sex +
0,567671	-0,08807	0,153726	136	67	69	132	-0,57292	y ~ Intervention + Sex +
0,128262	-0,21001	0,137247	142	70	72	138	-1,53018	y ~ Intervention + Sex +
0,637742	-0,07313	0,154965	142	70	72	138	-0,4719	y ~ Intervention + Sex +
0,783485	-0,03737	0,135727	142	70	72	138	-0,27532	y ~ Intervention + Sex +
0,169367	0,209832	0,15189	142	70	72	138	1,38147	y ~ Intervention + Sex +
0,371913	0,122736	0,137011	142	70	72	138	0,895813	y ~ Intervention + Sex +
0,654915	0,061171	0,136567	142	70	72	138	0,447918	y ~ Intervention + Sex +
0,373772	-0,12406	0,139031	142	70	72	138	-0,89232	y ~ Intervention + Sex +

0,571832	-0,09384	0,165596	142	70	72	138	-0,56671	y ~ Intervention + Sex +
0,63443	-0,0781	0,163874	142	70	72	138	-0,47656	y ~ Intervention + Sex +
0,322011	0,155308	0,156261	142	70	72	138	0,993901	y ~ Intervention + Sex +
0,593192	0,057069	0,10658	142	70	72	138	0,535463	y ~ Intervention + Sex +
0,334213	0,14214	0,146679	142	70	72	138	0,969055	y ~ Intervention + Sex +
0,837692	-0,02798	0,136342	142	70	72	138	-0,20523	y ~ Intervention + Sex +
0,998355	0,000345	0,166939	142	70	72	138	0,002066	y ~ Intervention + Sex +
0,863253	-0,02496	0,144666	142	70	72	138	-0,17256	y ~ Intervention + Sex +
0,736323	-0,04822	0,142911	142	70	72	138	-0,33741	y ~ Intervention + Sex +
0,083789	0,250961	0,14409	142	70	72	138	1,741698	y ~ Intervention + Sex +
0,5456	-0,1004	0,165709	142	70	72	138	-0,60587	y ~ Intervention + Sex +
0,968465	0,005249	0,13253	142	70	72	138	0,039605	y ~ Intervention + Sex +
0,176385	-0,18705	0,137644	142	70	72	138	-1,35893	y ~ Intervention + Sex +
0,794404	-0,03541	0,135609	142	70	72	138	-0,2611	y ~ Intervention + Sex +
0,478465	-0,11008	0,154894	142	70	72	138	-0,71071	y ~ Intervention + Sex +
0,841699	-0,03168	0,158308	142	70	72	138	-0,2001	y ~ Intervention + Sex +
0,615292	0,077871	0,154605	142	70	72	138	0,503676	y ~ Intervention + Sex +
0,389469	0,109753	0,127131	142	70	72	138	0,863303	y ~ Intervention + Sex +
0,535012	-0,0867	0,139404	142	70	72	138	-0,62193	y ~ Intervention + Sex +
0,562818	0,078146	0,13472	142	70	72	138	0,580063	y ~ Intervention + Sex +
0,466935	0,10384	0,142345	142	70	72	138	0,729495	y ~ Intervention + Sex +
0,617871	-0,06748	0,134968	142	70	72	138	-0,5	y ~ Intervention + Sex +
0,292869	-0,15024	0,142285	142	70	72	138	-1,05588	y ~ Intervention + Sex +
0,075765	0,253878	0,141889	142	70	72	138	1,789269	y ~ Intervention + Sex +
0,580376	0,085555	0,15439	142	70	72	138	0,554146	y ~ Intervention + Sex +
0,185058	-0,19028	0,142855	142	70	72	138	-1,33199	y ~ Intervention + Sex +
0,141436	0,219571	0,148465	142	70	72	138	1,478937	y ~ Intervention + Sex +
0,164654	-0,20203	0,144616	142	70	72	138	-1,39701	y ~ Intervention + Sex +
0,366908	0,142449	0,157358	142	70	72	138	0,905255	y ~ Intervention + Sex +
0,398222	-0,13685	0,161489	142	70	72	138	-0,84743	y ~ Intervention + Sex +
0,509683	0,094366	0,142752	142	70	72	138	0,66105	y ~ Intervention + Sex +
0,636458	-0,07001	0,147794	142	70	72	138	-0,47371	y ~ Intervention + Sex +
0,354242	-0,11421	0,122869	142	70	72	138	-0,92952	y ~ Intervention + Sex +
0,788053	-0,04214	0,156439	142	70	72	138	-0,26936	y ~ Intervention + Sex +
0,76252	0,041493	0,137045	142	70	72	138	0,302772	y ~ Intervention + Sex +
0,882378	-0,0236	0,159234	142	70	72	138	-0,14823	y ~ Intervention + Sex +
0,43195	0,126642	0,160678	142	70	72	138	0,788169	y ~ Intervention + Sex +
0,881597	0,020755	0,139089	142	70	72	138	0,149221	y ~ Intervention + Sex +
0,920418	-0,01397	0,139579	142	70	72	138	-0,10009	y ~ Intervention + Sex +
0,716162	-0,05847	0,160489	142	70	72	138	-0,36434	y ~ Intervention + Sex +
0,196967	-0,21693	0,167317	142	70	72	138	-1,2965	y ~ Intervention + Sex +
0,398764	-0,14461	0,170847	142	70	72	138	-0,84646	y ~ Intervention + Sex +
0,169617	-0,18022	0,130534	142	70	72	138	-1,38066	y ~ Intervention + Sex +
0,30978	-0,15509	0,152133	142	70	72	138	-1,01944	y ~ Intervention + Sex +
0,607869	0,077295	0,150293	142	70	72	138	0,514294	y ~ Intervention + Sex +
0,994077	0,001019	0,137044	142	70	72	138	0,007437	y ~ Intervention + Sex +
0,188842	0,214119	0,162146	142	70	72	138	1,320536	y ~ Intervention + Sex +
0,32045	-0,13265	0,133031	142	70	72	138	-0,99712	y ~ Intervention + Sex +

0,198147	-0,18109	0,140045	142	70	72	138	-1,29307	y ~ Intervention + Sex +
0,161822	-0,19428	0,138131	142	70	72	138	-1,40651	y ~ Intervention + Sex +
0,179349	-0,18457	0,136756	142	70	72	138	-1,34962	y ~ Intervention + Sex +
0,241709	-0,17646	0,150078	142	70	72	138	-1,17578	y ~ Intervention + Sex +
0,03068	-0,22814	0,104478	142	70	72	138	-2,18362	y ~ Intervention + Sex +
0,711331	-0,05965	0,160863	142	70	72	138	-0,37083	y ~ Intervention + Sex +
0,757897	-0,04098	0,132683	142	70	72	138	-0,30886	y ~ Intervention + Sex +
0,78058	0,039614	0,141932	142	70	72	138	0,279107	y ~ Intervention + Sex +
0,831609	-0,0299	0,140327	142	70	72	138	-0,21304	y ~ Intervention + Sex +
0,328039	0,13358	0,136091	142	70	72	138	0,98155	y ~ Intervention + Sex +
0,021146	-0,33744	0,144696	142	70	72	138	-2,33204	y ~ Intervention + Sex +
0,510951	-0,09828	0,149115	142	70	72	138	-0,65907	y ~ Intervention + Sex +
0,323798	0,144666	0,146094	142	70	72	138	0,990222	y ~ Intervention + Sex +
0,226684	-0,1696	0,139662	142	70	72	138	-1,21436	y ~ Intervention + Sex +
0,056757	-0,27494	0,143103	142	70	72	138	-1,92129	y ~ Intervention + Sex +
0,275787	0,17477	0,159729	142	70	72	138	1,09417	y ~ Intervention + Sex +
0,09714	-0,26129	0,156441	142	70	72	138	-1,67023	y ~ Intervention + Sex +
0,07515	-0,29319	0,163511	142	70	72	138	-1,79308	y ~ Intervention + Sex +
0,886217	0,021843	0,152368	142	70	72	138	0,143358	y ~ Intervention + Sex +
0,902623	-0,01922	0,15679	142	70	72	138	-0,12257	y ~ Intervention + Sex +
0,580827	0,092019	0,166255	142	70	72	138	0,553485	y ~ Intervention + Sex +
0,213623	-0,19309	0,154543	142	70	72	138	-1,24943	y ~ Intervention + Sex +
0,835261	0,03152	0,151283	142	70	72	138	0,208352	y ~ Intervention + Sex +
0,628421	-0,07629	0,157295	142	70	72	138	-0,48503	y ~ Intervention + Sex +
0,046047	-0,3054	0,151704	142	70	72	138	-2,01312	y ~ Intervention + Sex +
0,816208	0,03776	0,16215	142	70	72	138	0,23287	y ~ Intervention + Sex +
0,489599	-0,10131	0,146238	142	70	72	138	-0,6928	y ~ Intervention + Sex +
0,181533	-0,14709	0,109539	142	70	72	138	-1,34283	y ~ Intervention + Sex +
0,833612	-0,0313	0,148697	142	70	72	138	-0,21047	y ~ Intervention + Sex +
0,265183	0,168567	0,150672	142	70	72	138	1,118768	y ~ Intervention + Sex +
0,115322	0,234092	0,14772	142	70	72	138	1,584707	y ~ Intervention + Sex +
0,477125	0,093609	0,131311	142	70	72	138	0,712877	y ~ Intervention + Sex +
0,258888	0,173055	0,152647	142	70	72	138	1,133696	y ~ Intervention + Sex +
0,244353	-0,17583	0,150391	142	70	72	138	-1,16916	y ~ Intervention + Sex +
0,312783	0,146902	0,145002	142	70	72	138	1,013105	y ~ Intervention + Sex +
0,008416	-0,39018	0,145953	142	70	72	138	-2,67335	y ~ Intervention + Sex +
0,004353	-0,39653	0,136769	142	70	72	138	-2,89926	y ~ Intervention + Sex +
0,717612	0,050522	0,139412	142	70	72	138	0,362395	y ~ Intervention + Sex +
0,901499	-0,01984	0,160022	142	70	72	138	-0,124	y ~ Intervention + Sex +
0,373845	-0,13358	0,149722	142	70	72	138	-0,89219	y ~ Intervention + Sex +
0,393145	0,131696	0,153741	142	70	72	138	0,856611	y ~ Intervention + Sex +
0,637892	0,071619	0,151833	142	70	72	138	0,471691	y ~ Intervention + Sex +
0,34715	-0,11879	0,125923	142	70	72	138	-0,94335	y ~ Intervention + Sex +
0,702346	0,056336	0,14711	142	70	72	138	0,38295	y ~ Intervention + Sex +
0,311338	0,152978	0,150547	142	70	72	138	1,016145	y ~ Intervention + Sex +
0,421056	0,123516	0,153056	142	70	72	138	0,806999	y ~ Intervention + Sex +
0,550993	-0,0879	0,14706	142	70	72	138	-0,59774	y ~ Intervention + Sex +
0,329656	0,147392	0,150667	142	70	72	138	0,978263	y ~ Intervention + Sex +

0,251758	-0,15097	0,131172	142	70	72	138	-1,15091	y ~ Intervention + Sex +
0,571736	-0,08931	0,157547	142	70	72	138	-0,56685	y ~ Intervention + Sex +
0,134653	0,217591	0,144595	142	70	72	138	1,504829	y ~ Intervention + Sex +
0,655012	0,080706	0,180234	142	70	72	138	0,447783	y ~ Intervention + Sex +
0,092862	-0,26934	0,159162	142	70	72	138	-1,69221	y ~ Intervention + Sex +
0,132505	0,206607	0,136533	142	70	72	138	1,51324	y ~ Intervention + Sex +
0,521923	-0,09471	0,14751	142	70	72	138	-0,64202	y ~ Intervention + Sex +
0,300052	-0,15382	0,147875	142	70	72	138	-1,04023	y ~ Intervention + Sex +
0,304463	0,149174	0,144724	142	70	72	138	1,030747	y ~ Intervention + Sex +
0,130855	0,209335	0,13774	142	70	72	138	1,519777	y ~ Intervention + Sex +
0,28711	-0,16469	0,154115	142	70	72	138	-1,06861	y ~ Intervention + Sex +
0,240322	-0,17247	0,146251	142	70	72	138	-1,17927	y ~ Intervention + Sex +
0,243963	-0,15593	0,133259	142	70	72	138	-1,17014	y ~ Intervention + Sex +
0,693994	-0,06306	0,159937	142	70	72	138	-0,39426	y ~ Intervention + Sex +
0,480116	-0,11776	0,166319	142	70	72	138	-0,70804	y ~ Intervention + Sex +
0,857627	-0,02993	0,166506	142	70	72	138	-0,17973	y ~ Intervention + Sex +
0,284731	0,157875	0,147007	142	70	72	138	1,073924	y ~ Intervention + Sex +
0,200564	-0,20558	0,159848	142	70	72	138	-1,28609	y ~ Intervention + Sex +
0,949077	-0,00876	0,13686	142	70	72	138	-0,06398	y ~ Intervention + Sex +
0,342507	0,128585	0,134996	142	70	72	138	0,952504	y ~ Intervention + Sex +
0,765057	0,047638	0,159092	142	70	72	138	0,299437	y ~ Intervention + Sex +
0,64975	-0,06892	0,151447	142	70	72	138	-0,4551	y ~ Intervention + Sex +
0,140238	0,201693	0,135963	142	70	72	138	1,483438	y ~ Intervention + Sex +
0,303667	-0,14853	0,143857	142	70	72	138	-1,03245	y ~ Intervention + Sex +
0,634306	-0,0655	0,137401	142	70	72	138	-0,47673	y ~ Intervention + Sex +
0,163261	-0,21752	0,155185	142	70	72	138	-1,40166	y ~ Intervention + Sex +
0,953112	0,009134	0,155068	142	70	72	138	0,058906	y ~ Intervention + Sex +
0,564647	-0,08588	0,148752	142	70	72	138	-0,57735	y ~ Intervention + Sex +
0,772019	0,048054	0,165529	142	70	72	138	0,290304	y ~ Intervention + Sex +
0,702274	0,053739	0,140292	142	70	72	138	0,383047	y ~ Intervention + Sex +
0,43832	0,115543	0,148649	142	70	72	138	0,777286	y ~ Intervention + Sex +
0,08805	-0,24043	0,139951	142	70	72	138	-1,71794	y ~ Intervention + Sex +
0,203435	0,182995	0,143202	142	70	72	138	1,277885	y ~ Intervention + Sex +
0,039928	-0,32227	0,155377	142	70	72	138	-2,07411	y ~ Intervention + Sex +
0,324558	0,150811	0,15254	142	70	72	138	0,988663	y ~ Intervention + Sex +
0,078365	-0,27871	0,157157	142	70	72	138	-1,77342	y ~ Intervention + Sex +
0,805595	-0,03865	0,156742	142	70	72	138	-0,24659	y ~ Intervention + Sex +
0,926003	-0,01525	0,163865	142	70	72	138	-0,09304	y ~ Intervention + Sex +
0,438986	0,12511	0,161192	142	70	72	138	0,776154	y ~ Intervention + Sex +
0,846628	-0,03157	0,162894	142	70	72	138	-0,19379	y ~ Intervention + Sex +
0,602025	0,081432	0,155792	142	70	72	138	0,522695	y ~ Intervention + Sex +
0,015583	-0,35813	0,146239	142	70	72	138	-2,44891	y ~ Intervention + Sex +
0,362828	0,15801	0,173065	142	70	72	138	0,913012	y ~ Intervention + Sex +
0,629293	-0,06141	0,126931	142	70	72	138	-0,4838	y ~ Intervention + Sex +
0,612887	-0,07318	0,144301	142	70	72	138	-0,50711	y ~ Intervention + Sex +
0,986963	0,002677	0,163518	142	70	72	138	0,01637	y ~ Intervention + Sex +
0,078602	-0,28066	0,158386	142	70	72	138	-1,772	y ~ Intervention + Sex +
0,830113	0,031929	0,148532	142	70	72	138	0,214964	y ~ Intervention + Sex +

0,576056	0,083855	0,149611	142	70	72	138	0,560487	y ~ Intervention + Sex +	
0,040598	-0,2974	0,143877	142	70	72	138	-2,06706	y ~ Intervention + Sex +	
0,359576	0,133048	0,144737	142	70	72	138	0,919237	y ~ Intervention + Sex +	
0,938309	0,011695	0,150834	142	70	72	138	0,077537	y ~ Intervention + Sex +	
0,324051	0,138855	0,1403	142	70	72	138	0,989703	y ~ Intervention + Sex +	
0,805009	-0,03886	0,157094	142	70	72	138	-0,24734	y ~ Intervention + Sex +	
0,984249	0,003055	0,154455	142	70	72	138	0,019778	y ~ Intervention + Sex +	
0,571068	0,086795	0,152852	142	70	72	138	0,567838	y ~ Intervention + Sex +	
0,915528	0,016589	0,156115	142	70	72	138	0,106263	y ~ Intervention + Sex +	
0,167018	0,208431	0,15004	142	70	72	138	1,389174	y ~ Intervention + Sex +	
0,081128	0,251709	0,143257	142	70	72	138	1,757047	y ~ Intervention + Sex +	
0,593585	0,073822	0,138012	142	70	72	138	0,534892	y ~ Intervention + Sex +	
0,097094	-0,27211	0,162896	142	70	72	138	-1,67046	y ~ Intervention + Sex +	
0,527553	0,100961	0,159407	142	70	72	138	0,633351	y ~ Intervention + Sex +	
0,386437	0,137869	0,158679	142	70	72	138	0,868851	y ~ Intervention + Sex +	
0,161777	-0,21271	0,15122	142	70	72	138	-1,40666	y ~ Intervention + Sex +	
0,188945	-0,17892	0,135526	142	70	72	138	-1,32023	y ~ Intervention + Sex +	
0,051485	-0,31541	0,160557	142	70	72	138	-1,96447	y ~ Intervention + Sex +	
0,130345	0,242223	0,159167	142	70	72	138	1,521809	y ~ Intervention + Sex +	
0,243694	-0,16798	0,143477	142	70	72	138	-1,17081	y ~ Intervention + Sex +	
0,228943	0,160414	0,132744	142	70	72	138	1,208443	y ~ Intervention + Sex +	
0,385739	0,143897	0,165374	142	70	72	138	0,870133	y ~ Intervention + Sex +	
0,235235	0,186572	0,156496	142	70	72	138	1,192184	y ~ Intervention + Sex +	
0,486787	-0,10935	0,156821	142	70	72	138	-0,6973	y ~ Intervention + Sex +	
0,58978	0,100217	0,185444	142	70	72	138	0,540418	y ~ Intervention + Sex +	
0,321628	0,137428	0,138162	142	70	72	138	0,994689	y ~ Intervention + Sex +	
0,421202	0,133621	0,16563	142	70	72	138	0,806745	y ~ Intervention + Sex +	
0,622984	0,075713	0,153658	142	70	72	138	0,492733	y ~ Intervention + Sex +	
0,542139	0,087769	0,143625	142	70	72	138	0,6111	y ~ Intervention + Sex +	
0,476272	0,114222	0,159917	142	70	72	138	0,714261	y ~ Intervention + Sex +	
0,132106	-0,2316	0,152889	142	70	72	138	-1,51482	y ~ Intervention + Sex +	
0,970734	0,005704	0,155194	142	70	72	138	0,036754	y ~ Intervention + Sex +	
0,976448	0,004281	0,144748	142	70	72	138	0,029576	y ~ Intervention + Sex +	
0,571291	0,08943	0,157583	142	70	72	138	0,567508	y ~ Intervention + Sex +	
0,689893	-0,06736	0,168458	142	70	72	138	-0,39984	y ~ Intervention + Sex +	
0,557718	-0,09938	0,169102	142	70	72	138	-0,58766	y ~ Intervention + Sex +	
0,223849	0,182961	0,149741	142	70	72	138	1,221847	y ~ Intervention + Sex +	
0,436935	-0,11632	0,149196	142	70	72	138	-0,77964	y ~ Intervention + Sex +	
0,390065	-0,12711	0,147421	142	70	72	138	-0,86221	y ~ Intervention + Sex +	
0,381469	-0,1422	0,161953	142	70	72	138	-0,878	y ~ Intervention + Sex +	
0,305665	-0,18245	0,177445	142	70	72	138	-1,02818	y ~ Intervention + Sex +	
0,765338	-0,04385	0,146636	142	70	72	138	-0,29907	y ~ Intervention + Sex +	
0,723837	0,059477	0,167988	142	70	72	138	0,354056	y ~ Intervention + Sex +	
0,55282	-0,08354	0,140397	142	70	72	138	-0,595	y ~ Intervention + Sex +	
0,698698	0,053949	0,139085	142	70	72	138	0,387887	y ~ Intervention + Sex +	
0,85075	-0,02568	0,136247	142	70	72	138	-0,18851	y ~ Intervention + Sex +	
0,449255	0,128755	0,169678	142	70	72	138	0,758819	y ~ Intervention + Sex +	
0,281372	-0,18656	0,172507	142	70	72	138	-1,08147	y ~ Intervention + Sex +	

0,600942	-0,08554	0,163168	142	70	72	138	-0,52425	y ~ Intervention + Sex +
0,698842	-0,06028	0,155491	142	70	72	138	-0,38769	y ~ Intervention + Sex +
0,125555	0,241452	0,156664	142	70	72	138	1,541211	y ~ Intervention + Sex +
0,043056	0,329088	0,161159	142	70	72	138	2,042003	y ~ Intervention + Sex +
0,136177	0,234101	0,156179	142	70	72	138	1,498928	y ~ Intervention + Sex +
0,287512	0,16065	0,150461	142	70	72	138	1,067719	y ~ Intervention + Sex +
0,411982	-0,13959	0,169632	142	70	72	138	-0,8229	y ~ Intervention + Sex +
0,172636	-0,20884	0,15234	142	70	72	138	-1,37089	y ~ Intervention + Sex +
0,068187	0,300085	0,163252	142	70	72	138	1,838174	y ~ Intervention + Sex +
0,558012	0,094499	0,160925	142	70	72	138	0,587226	y ~ Intervention + Sex +
0,047456	-0,30462	0,152303	142	70	72	138	-2,00006	y ~ Intervention + Sex +
0,257839	-0,19485	0,171491	142	70	72	138	-1,13621	y ~ Intervention + Sex +
0,814132	-0,03637	0,1544	142	70	72	138	-0,23555	y ~ Intervention + Sex +
0,237802	-0,20474	0,172684	142	70	72	138	-1,18564	y ~ Intervention + Sex +
0,050925	0,315112	0,160014	142	70	72	138	1,969275	y ~ Intervention + Sex +
0,166985	0,192238	0,138372	142	70	72	138	1,389281	y ~ Intervention + Sex +
0,280948	0,161435	0,149141	142	70	72	138	1,082432	y ~ Intervention + Sex +
0,523428	-0,10282	0,160731	142	70	72	138	-0,6397	y ~ Intervention + Sex +
0,864097	0,024806	0,144659	142	70	72	138	0,171481	y ~ Intervention + Sex +
0,972693	0,005462	0,159264	142	70	72	138	0,034294	y ~ Intervention + Sex +
0,527767	-0,10946	0,172915	142	70	72	138	-0,63302	y ~ Intervention + Sex +
0,33075	-0,16206	0,16604	142	70	72	138	-0,97604	y ~ Intervention + Sex +
0,606525	-0,08629	0,167162	142	70	72	138	-0,51622	y ~ Intervention + Sex +
0,859786	0,023903	0,135062	142	70	72	138	0,176977	y ~ Intervention + Sex +
0,536909	0,087747	0,141747	142	70	72	138	0,619042	y ~ Intervention + Sex +
0,690542	0,064982	0,162879	142	70	72	138	0,398956	y ~ Intervention + Sex +
0,944915	-0,01135	0,163958	142	70	72	138	-0,06922	y ~ Intervention + Sex +
0,211813	0,166262	0,132542	142	70	72	138	1,254411	y ~ Intervention + Sex +
0,534592	0,102739	0,165024	142	70	72	138	0,622573	y ~ Intervention + Sex +
0,375485	-0,14785	0,166292	142	70	72	138	-0,88912	y ~ Intervention + Sex +
0,250064	-0,17995	0,155792	142	70	72	138	-1,15505	y ~ Intervention + Sex +
0,485925	0,104037	0,148904	142	70	72	138	0,698685	y ~ Intervention + Sex +
0,79365	-0,04406	0,168101	142	70	72	138	-0,26208	y ~ Intervention + Sex +
0,987467	0,002595	0,164929	142	70	72	138	0,015737	y ~ Intervention + Sex +
0,221688	0,185704	0,151274	142	70	72	138	1,227599	y ~ Intervention + Sex +
0,023463	0,285638	0,124664	142	70	72	138	2,291266	y ~ Intervention + Sex +
0,794333	-0,03983	0,152477	142	70	72	138	-0,26119	y ~ Intervention + Sex +
0,722595	0,059418	0,167036	142	70	72	138	0,355718	y ~ Intervention + Sex +
0,822419	-0,03412	0,15174	142	70	72	138	-0,22486	y ~ Intervention + Sex +
0,864582	-0,02872	0,168105	142	70	72	138	-0,17086	y ~ Intervention + Sex +
0,195005	-0,18241	0,140077	142	70	72	138	-1,30223	y ~ Intervention + Sex +
0,452153	-0,12461	0,165278	142	70	72	138	-0,75397	y ~ Intervention + Sex +
0,326748	0,189061	0,192099	142	70	72	138	0,984183	y ~ Intervention + Sex +
0,446448	-0,12572	0,164661	142	70	72	138	-0,76353	y ~ Intervention + Sex +
0,315771	-0,13628	0,135356	142	70	72	138	-1,00685	y ~ Intervention + Sex +
0,317076	-0,15084	0,150224	142	70	72	138	-1,00412	y ~ Intervention + Sex +
0,558527	0,089331	0,152324	142	70	72	138	0,586456	y ~ Intervention + Sex +
0,125178	0,257099	0,166648	142	70	72	138	1,542764	y ~ Intervention + Sex +

0,58737	-0,08269	0,152021	142	70	72	138	-0,54393	y ~ Intervention + Sex
0,811257	-0,03558	0,148727	142	70	72	138	-0,23926	y ~ Intervention + Sex
0,689486	0,05652	0,14116	142	70	72	138	0,400393	y ~ Intervention + Sex
0,62992	-0,07113	0,14729	142	70	72	138	-0,48292	y ~ Intervention + Sex
0,66601	-0,07074	0,163546	142	70	72	138	-0,43256	y ~ Intervention + Sex
0,05122	-0,35036	0,178143	142	70	72	138	-1,96673	y ~ Intervention + Sex
0,352915	-0,14257	0,152962	142	70	72	138	-0,93209	y ~ Intervention + Sex
0,410871	0,118562	0,143735	142	70	72	138	0,824867	y ~ Intervention + Sex
0,182611	-0,20634	0,154045	142	70	72	138	-1,3395	y ~ Intervention + Sex
0,257071	0,177465	0,155938	142	70	72	138	1,138051	y ~ Intervention + Sex
0,162256	0,193495	0,137715	142	70	72	138	1,405041	y ~ Intervention + Sex
0,769799	0,041872	0,142802	142	70	72	138	0,293214	y ~ Intervention + Sex
0,756757	0,043721	0,140872	142	70	72	138	0,310358	y ~ Intervention + Sex
0,815977	-0,04	0,171547	142	70	72	138	-0,23317	y ~ Intervention + Sex
0,870546	-0,02558	0,156646	142	70	72	138	-0,16327	y ~ Intervention + Sex
0,431191	-0,1267	0,160486	142	70	72	138	-0,78947	y ~ Intervention + Sex
0,228925	-0,18851	0,15599	142	70	72	138	-1,20849	y ~ Intervention + Sex
0,384851	0,142929	0,163953	142	70	72	138	0,871764	y ~ Intervention + Sex
0,793454	-0,04076	0,155368	142	70	72	138	-0,26234	y ~ Intervention + Sex
0,733179	0,053732	0,157299	142	70	72	138	0,34159	y ~ Intervention + Sex
0,324606	0,154728	0,156518	142	70	72	138	0,988565	y ~ Intervention + Sex
0,180716	-0,21744	0,161623	142	70	72	138	-1,34536	y ~ Intervention + Sex
0,725413	0,056594	0,160803	142	70	72	138	0,351949	y ~ Intervention + Sex
0,045177	0,322769	0,15968	142	70	72	138	2,021354	y ~ Intervention + Sex
0,589452	0,08149	0,150657	142	70	72	138	0,540895	y ~ Intervention + Sex
0,071017	0,253497	0,139329	142	70	72	138	1,819408	y ~ Intervention + Sex
0,559205	0,102933	0,175821	142	70	72	138	0,585444	y ~ Intervention + Sex
0,970834	0,005379	0,146858	142	70	72	138	0,036629	y ~ Intervention + Sex
0,630582	0,073832	0,153183	142	70	72	138	0,481982	y ~ Intervention + Sex
0,518083	-0,10754	0,165971	142	70	72	138	-0,64797	y ~ Intervention + Sex
0,794966	-0,04256	0,163468	142	70	72	138	-0,26037	y ~ Intervention + Sex
0,926698	-0,01325	0,143773	142	70	72	138	-0,09217	y ~ Intervention + Sex
0,574765	-0,09004	0,160101	142	70	72	138	-0,56239	y ~ Intervention + Sex
0,710713	-0,05757	0,154887	142	70	72	138	-0,37166	y ~ Intervention + Sex
0,7333	0,051697	0,151413	142	70	72	138	0,341429	y ~ Intervention + Sex
0,029863	0,326815	0,148916	142	70	72	138	2,194623	y ~ Intervention + Sex
0,349261	-0,14402	0,153344	142	70	72	138	-0,93922	y ~ Intervention + Sex
0,796407	-0,04065	0,157262	142	70	72	138	-0,2585	y ~ Intervention + Sex
0,117581	-0,24478	0,155431	142	70	72	138	-1,57485	y ~ Intervention + Sex
0,610065	0,083611	0,163576	142	70	72	138	0,511147	y ~ Intervention + Sex
0,45894	0,111953	0,150742	142	70	72	138	0,742677	y ~ Intervention + Sex
0,449327	0,12279	0,161843	142	70	72	138	0,758698	y ~ Intervention + Sex
0,967547	0,006633	0,162731	142	70	72	138	0,040759	y ~ Intervention + Sex
0,543558	-0,1014	0,166516	142	70	72	138	-0,60895	y ~ Intervention + Sex
0,232149	-0,1915	0,15957	142	70	72	138	-1,20012	y ~ Intervention + Sex
0,361983	-0,13184	0,144143	142	70	72	138	-0,91463	y ~ Intervention + Sex
0,162837	-0,24669	0,175822	142	70	72	138	-1,40308	y ~ Intervention + Sex
0,558317	0,103587	0,176538	142	70	72	138	0,58677	y ~ Intervention + Sex

0,213135	0,19047	0,152282	142	70	72	138	1,250768	y ~ Intervention + Sex +
0,231793	-0,2136	0,177846	142	70	72	138	-1,20104	y ~ Intervention + Sex +
0,434654	0,124782	0,159254	142	70	72	138	0,783538	y ~ Intervention + Sex +
0,156194	0,22944	0,160924	142	70	72	138	1,425767	y ~ Intervention + Sex +
0,176242	0,184895	0,136014	142	70	72	138	1,359385	y ~ Intervention + Sex +
0,691304	-0,06667	0,167551	142	70	72	138	-0,39792	y ~ Intervention + Sex +
0,356159	-0,13928	0,150441	142	70	72	138	-0,92581	y ~ Intervention + Sex +
0,754913	-0,04086	0,130638	142	70	72	138	-0,31279	y ~ Intervention + Sex +
0,587623	-0,08248	0,151741	142	70	72	138	-0,54356	y ~ Intervention + Sex +
0,838462	0,031806	0,155724	142	70	72	138	0,204246	y ~ Intervention + Sex +
0,601525	-0,08463	0,161696	142	70	72	138	-0,52342	y ~ Intervention + Sex +
0,814169	0,037338	0,158549	142	70	72	138	0,235501	y ~ Intervention + Sex +
0,135838	0,216841	0,144538	142	70	72	138	1,500235	y ~ Intervention + Sex +
0,117433	-0,2384	0,151319	142	70	72	138	-1,57549	y ~ Intervention + Sex +
0,657017	-0,06684	0,150204	142	70	72	138	-0,445	y ~ Intervention + Sex +
0,891329	-0,02301	0,168104	142	70	72	138	-0,13687	y ~ Intervention + Sex +
0,241438	-0,17603	0,14963	142	70	72	138	-1,17645	y ~ Intervention + Sex +
0,514203	0,101947	0,155884	142	70	72	138	0,653995	y ~ Intervention + Sex +
0,119362	0,257763	0,164475	142	70	72	138	1,567185	y ~ Intervention + Sex +
0,388406	-0,13337	0,154136	142	70	72	138	-0,86525	y ~ Intervention + Sex +
0,322163	-0,172	0,173111	142	70	72	138	-0,99359	y ~ Intervention + Sex +
0,131649	0,251453	0,165798	142	70	72	138	1,516623	y ~ Intervention + Sex +
0,659475	-0,07309	0,165509	142	70	72	138	-0,44159	y ~ Intervention + Sex +
0,552981	0,101012	0,169838	142	70	72	138	0,594755	y ~ Intervention + Sex +
0,223374	-0,18271	0,14938	142	70	72	138	-1,22311	y ~ Intervention + Sex +
0,915529	-0,01609	0,151462	142	70	72	138	-0,10626	y ~ Intervention + Sex +
0,676618	-0,06814	0,163034	142	70	72	138	-0,41797	y ~ Intervention + Sex +
0,119797	-0,24511	0,15659	142	70	72	138	-1,56533	y ~ Intervention + Sex +
0,937786	0,012418	0,158811	142	70	72	138	0,078196	y ~ Intervention + Sex +
0,894234	0,022916	0,172051	142	70	72	138	0,133194	y ~ Intervention + Sex +
0,71745	0,067688	0,186668	142	70	72	138	0,362611	y ~ Intervention + Sex +
0,063124	-0,32252	0,172156	142	70	72	138	-1,87343	y ~ Intervention + Sex +
0,812157	-0,03969	0,166707	142	70	72	138	-0,2381	y ~ Intervention + Sex +
0,550997	0,081301	0,136015	142	70	72	138	0,597734	y ~ Intervention + Sex +
0,154606	0,220853	0,154303	142	70	72	138	1,431297	y ~ Intervention + Sex +
0,438134	0,126149	0,162228	142	70	72	138	0,777603	y ~ Intervention + Sex +
0,401662	0,128846	0,153159	142	70	72	138	0,841253	y ~ Intervention + Sex +
0,388289	0,123407	0,142591	142	70	72	138	0,865459	y ~ Intervention + Sex +
0,772503	-0,04564	0,157568	142	70	72	138	-0,28967	y ~ Intervention + Sex +
0,87958	0,024045	0,158414	142	70	72	138	0,151783	y ~ Intervention + Sex +
0,585922	-0,08201	0,150191	142	70	72	138	-0,54604	y ~ Intervention + Sex +
0,517586	0,105883	0,163214	142	70	72	138	0,648738	y ~ Intervention + Sex +
0,763996	0,049043	0,163027	142	70	72	138	0,300831	y ~ Intervention + Sex +
0,594493	-0,07307	0,136947	142	70	72	138	-0,53358	y ~ Intervention + Sex +
0,253443	-0,18005	0,156996	142	70	72	138	-1,14681	y ~ Intervention + Sex +
0,26986	-0,17629	0,15913	142	70	72	138	-1,10784	y ~ Intervention + Sex +
0,145566	0,21152	0,144516	142	70	72	138	1,46364	y ~ Intervention + Sex +
0,566205	0,097819	0,170109	142	70	72	138	0,575034	y ~ Intervention + Sex +

0,77662	-0,04611	0,162212	142	70	72	138	-0,28428	y ~ Intervention + Sex +
0,805934	0,040346	0,16391	142	70	72	138	0,246148	y ~ Intervention + Sex +
0,816467	-0,03326	0,143039	142	70	72	138	-0,23253	y ~ Intervention + Sex +
0,846829	-0,02921	0,150921	142	70	72	138	-0,19353	y ~ Intervention + Sex +
0,91355	0,018505	0,170144	142	70	72	138	0,108761	y ~ Intervention + Sex +
0,647505	0,073021	0,159352	142	70	72	138	0,458234	y ~ Intervention + Sex +
0,547002	0,090638	0,150125	142	70	72	138	0,603749	y ~ Intervention + Sex +
0,932498	0,014255	0,167989	142	70	72	138	0,084857	y ~ Intervention + Sex +
0,980579	-0,00377	0,15447	142	70	72	138	-0,02439	y ~ Intervention + Sex +
0,147331	0,209731	0,143926	142	70	72	138	1,457207	y ~ Intervention + Sex +
0,980505	-0,00362	0,147782	142	70	72	138	-0,02448	y ~ Intervention + Sex +
0,324851	0,163146	0,165117	142	70	72	138	0,988062	y ~ Intervention + Sex +
0,20693	0,167932	0,132438	142	70	72	138	1,268007	y ~ Intervention + Sex +
0,294151	-0,17777	0,168816	142	70	72	138	-1,05307	y ~ Intervention + Sex +
0,719216	0,049769	0,138153	142	70	72	138	0,360243	y ~ Intervention + Sex +
0,222949	0,190373	0,155503	142	70	72	138	1,224238	y ~ Intervention + Sex +
0,515133	0,11295	0,17309	142	70	72	138	0,652549	y ~ Intervention + Sex +
0,169948	-0,20788	0,150687	142	70	72	138	-1,37958	y ~ Intervention + Sex +
0,165507	-0,2094	0,150197	142	70	72	138	-1,39417	y ~ Intervention + Sex +
0,618097	-0,07545	0,150988	142	70	72	138	-0,49968	y ~ Intervention + Sex +
0,496969	0,107863	0,158373	142	70	72	138	0,68107	y ~ Intervention + Sex +
0,061969	-0,29063	0,154443	142	70	72	138	-1,8818	y ~ Intervention + Sex +
0,368601	-0,14177	0,157164	142	70	72	138	-0,90205	y ~ Intervention + Sex +
0,182933	-0,20636	0,154169	142	70	72	138	-1,3385	y ~ Intervention + Sex +
0,433355	-0,10617	0,135119	142	70	72	138	-0,78576	y ~ Intervention + Sex +
0,263397	0,174533	0,15542	142	70	72	138	1,122978	y ~ Intervention + Sex +
0,358664	-0,15335	0,166503	142	70	72	138	-0,92099	y ~ Intervention + Sex +
0,802805	0,040809	0,163107	142	70	72	138	0,2502	y ~ Intervention + Sex +
0,694216	0,063887	0,162164	142	70	72	138	0,393963	y ~ Intervention + Sex +
0,321283	0,157697	0,158426	142	70	72	138	0,995401	y ~ Intervention + Sex +
0,819536	-0,03689	0,161411	142	70	72	138	-0,22858	y ~ Intervention + Sex +
0,211914	0,202317	0,161321	142	70	72	138	1,254132	y ~ Intervention + Sex +
0,143485	-0,24234	0,164712	142	70	72	138	-1,47131	y ~ Intervention + Sex +
0,503284	-0,11459	0,170758	142	70	72	138	-0,67109	y ~ Intervention + Sex +
0,369676	-0,14528	0,161414	142	70	72	138	-0,90002	y ~ Intervention + Sex +
0,157946	-0,21295	0,149997	142	70	72	138	-1,41971	y ~ Intervention + Sex +
0,409216	-0,14178	0,171279	142	70	72	138	-0,82779	y ~ Intervention + Sex +
0,11958	-0,25622	0,163586	142	70	72	138	-1,56625	y ~ Intervention + Sex +
0,329403	0,148943	0,152173	142	70	72	138	0,978777	y ~ Intervention + Sex +
0,871118	0,027121	0,166855	142	70	72	138	0,16254	y ~ Intervention + Sex +
0,506637	0,096135	0,144386	142	70	72	138	0,66582	y ~ Intervention + Sex +
0,216302	0,202699	0,163189	142	70	72	138	1,242113	y ~ Intervention + Sex +
0,220218	0,214	0,173767	142	70	72	138	1,231532	y ~ Intervention + Sex +
0,693037	-0,05668	0,1433	142	70	72	138	-0,39556	y ~ Intervention + Sex +
0,749574	-0,05713	0,178611	142	70	72	138	-0,31984	y ~ Intervention + Sex +
0,684912	0,060344	0,148402	142	70	72	138	0,406627	y ~ Intervention + Sex +
0,060196	0,293157	0,154708	142	70	72	138	1,894908	y ~ Intervention + Sex +
0,754441	-0,04622	0,147459	142	70	72	138	-0,31341	y ~ Intervention + Sex +

0,476277	-0,11219	0,157073	142	70	72	138	-0,71425	y ~ Intervention + Sex +
0,240823	-0,20187	0,171368	142	70	72	138	-1,178	y ~ Intervention + Sex +
0,128786	-0,23162	0,151575	142	70	72	138	-1,52806	y ~ Intervention + Sex +
0,499616	-0,1112	0,164286	142	70	72	138	-0,67688	y ~ Intervention + Sex +
0,505104	-0,10869	0,162655	142	70	72	138	-0,66823	y ~ Intervention + Sex +
0,175238	-0,2232	0,163808	142	70	72	138	-1,36257	y ~ Intervention + Sex +
0,387759	0,127602	0,147274	142	70	72	138	0,866429	y ~ Intervention + Sex +
0,481497	0,107605	0,152456	142	70	72	138	0,705808	y ~ Intervention + Sex +
0,365971	0,144948	0,159805	142	70	72	138	0,907033	y ~ Intervention + Sex +
0,416364	0,127592	0,156517	142	70	72	138	0,815198	y ~ Intervention + Sex +
0,318807	-0,1639	0,163811	142	70	72	138	-1,00053	y ~ Intervention + Sex +
0,690138	-0,06182	0,154736	142	70	72	138	-0,39951	y ~ Intervention + Sex +
0,563706	0,084394	0,145824	142	70	72	138	0,578743	y ~ Intervention + Sex +
0,840179	0,02975	0,147242	142	70	72	138	0,202045	y ~ Intervention + Sex +
0,838666	-0,0293	0,143636	142	70	72	138	-0,20398	y ~ Intervention + Sex +
0,534496	-0,09732	0,156279	142	70	72	138	-0,62272	y ~ Intervention + Sex +
0,56315	-0,09825	0,169529	142	70	72	138	-0,57957	y ~ Intervention + Sex +
0,113114	0,262376	0,164551	142	70	72	138	1,594492	y ~ Intervention + Sex +
0,845073	-0,02989	0,152649	142	70	72	138	-0,19578	y ~ Intervention + Sex +
0,599551	0,086713	0,164772	142	70	72	138	0,526263	y ~ Intervention + Sex +
0,713732	0,058218	0,158372	142	70	72	138	0,367603	y ~ Intervention + Sex +
0,365186	0,151226	0,166452	142	70	72	138	0,908524	y ~ Intervention + Sex +
0,732282	0,047068	0,137311	142	70	72	138	0,342785	y ~ Intervention + Sex +
0,634862	-0,07253	0,152398	142	70	72	138	-0,47595	y ~ Intervention + Sex +
0,318334	-0,15505	0,154812	142	70	72	138	-1,00151	y ~ Intervention + Sex +
0,382521	-0,1424	0,162549	142	70	72	138	-0,87606	y ~ Intervention + Sex +
0,259091	-0,17699	0,156183	142	70	72	138	-1,13321	y ~ Intervention + Sex +
0,489148	0,111347	0,160553	142	70	72	138	0,693521	y ~ Intervention + Sex +
0,448423	-0,10987	0,144522	142	70	72	138	-0,76021	y ~ Intervention + Sex +
0,093502	0,2787	0,165022	142	70	72	138	1,688871	y ~ Intervention + Sex +
0,256975	0,152599	0,134061	142	70	72	138	1,138283	y ~ Intervention + Sex +
0,375443	0,140486	0,157991	142	70	72	138	0,8892	y ~ Intervention + Sex +
0,103914	0,243832	0,148953	142	70	72	138	1,636975	y ~ Intervention + Sex +
0,395769	0,14814	0,173902	142	70	72	138	0,851858	y ~ Intervention + Sex +
0,856243	-0,02638	0,145333	142	70	72	138	-0,1815	y ~ Intervention + Sex +
0,323065	-0,1722	0,173641	142	70	72	138	-0,99173	y ~ Intervention + Sex +
0,924152	-0,01546	0,162089	142	70	72	138	-0,09538	y ~ Intervention + Sex +
0,11886	-0,25779	0,164265	142	70	72	138	-1,56933	y ~ Intervention + Sex +
0,189817	0,204793	0,155427	142	70	72	138	1,317611	y ~ Intervention + Sex +
0,889648	-0,02254	0,162144	142	70	72	138	-0,13901	y ~ Intervention + Sex +
0,793735	-0,03638	0,138867	142	70	72	138	-0,26197	y ~ Intervention + Sex +
0,721268	-0,05606	0,156812	142	70	72	138	-0,35749	y ~ Intervention + Sex +
0,544032	-0,09416	0,154812	142	70	72	138	-0,60823	y ~ Intervention + Sex +
0,661162	0,067055	0,152654	142	70	72	138	0,439258	y ~ Intervention + Sex +
0,699837	-0,05823	0,150719	142	70	72	138	-0,38634	y ~ Intervention + Sex +
0,68202	-0,06961	0,169552	142	70	72	138	-0,41058	y ~ Intervention + Sex +
0,871516	0,027206	0,167901	142	70	72	138	0,162034	y ~ Intervention + Sex +
0,957692	-0,00863	0,162422	142	70	72	138	-0,05315	y ~ Intervention + Sex +

0,822427	-0,03305	0,146979	142	70	72	138	-0,22485	y ~ Intervention + Sex +
0,39096	0,13238	0,153825	142	70	72	138	0,860584	y ~ Intervention + Sex +
0,813798	0,034862	0,147733	142	70	72	138	0,235981	y ~ Intervention + Sex +
0,298262	-0,17336	0,166039	142	70	72	138	-1,04411	y ~ Intervention + Sex +
0,587086	-0,08553	0,157123	142	70	72	138	-0,54434	y ~ Intervention + Sex +
0,855791	-0,03009	0,165287	142	70	72	138	-0,18207	y ~ Intervention + Sex +
0,278812	0,165455	0,152174	142	70	72	138	1,087272	y ~ Intervention + Sex +
0,787801	0,046051	0,170753	142	70	72	138	0,269691	y ~ Intervention + Sex +
0,922142	-0,01458	0,148933	142	70	72	138	-0,09792	y ~ Intervention + Sex +
0,516417	0,104898	0,161245	142	70	72	138	0,650553	y ~ Intervention + Sex +
0,65999	-0,07606	0,172524	142	70	72	138	-0,44088	y ~ Intervention + Sex +
0,885094	0,025568	0,176596	142	70	72	138	0,144782	y ~ Intervention + Sex +
0,668071	0,073018	0,169921	142	70	72	138	0,429718	y ~ Intervention + Sex +
0,624284	0,072352	0,147389	142	70	72	138	0,49089	y ~ Intervention + Sex +
0,2925	-0,15778	0,149317	142	70	72	138	-1,05669	y ~ Intervention + Sex +
0,505169	-0,10986	0,164436	142	70	72	138	-0,66813	y ~ Intervention + Sex +
0,621674	0,086206	0,174297	142	70	72	138	0,494592	y ~ Intervention + Sex +
0,64936	-0,07872	0,172763	142	70	72	138	-0,45565	y ~ Intervention + Sex +
0,730997	0,050355	0,14617	142	70	72	138	0,344496	y ~ Intervention + Sex +
0,281539	0,175208	0,162065	142	70	72	138	1,081098	y ~ Intervention + Sex +
0,607087	-0,08655	0,167918	142	70	72	138	-0,51542	y ~ Intervention + Sex +
0,489185	-0,10732	0,154763	142	70	72	138	-0,69346	y ~ Intervention + Sex +
0,956906	-0,00805	0,148671	142	70	72	138	-0,05413	y ~ Intervention + Sex +
0,572694	0,087669	0,155046	142	70	72	138	0,565438	y ~ Intervention + Sex +
0,569934	-0,0841	0,147672	142	70	72	138	-0,56951	y ~ Intervention + Sex +
0,828081	-0,03622	0,166473	142	70	72	138	-0,21758	y ~ Intervention + Sex +
0,544371	-0,09345	0,153765	142	70	72	138	-0,60772	y ~ Intervention + Sex +
0,071404	-0,29266	0,161078	142	70	72	138	-1,81689	y ~ Intervention + Sex +
0,869992	0,021975	0,134015	142	70	72	138	0,163973	y ~ Intervention + Sex +
0,471848	0,116605	0,161623	142	70	72	138	0,721459	y ~ Intervention + Sex +
0,286334	0,170977	0,15974	142	70	72	138	1,070342	y ~ Intervention + Sex +
0,653302	0,071358	0,158517	142	70	72	138	0,450159	y ~ Intervention + Sex +
0,554915	-0,09267	0,156574	142	70	72	138	-0,59186	y ~ Intervention + Sex +
0,584881	0,076096	0,138974	142	70	72	138	0,547557	y ~ Intervention + Sex +
0,014485	-0,3775	0,152444	142	70	72	138	-2,4763	y ~ Intervention + Sex +
0,338867	-0,1492	0,155456	142	70	72	138	-0,95974	y ~ Intervention + Sex +
0,361755	0,158559	0,173276	142	70	72	138	0,915063	y ~ Intervention + Sex +
0,024846	0,368606	0,162483	142	70	72	138	2,268584	y ~ Intervention + Sex +
0,346259	-0,15008	0,158799	142	70	72	138	-0,9451	y ~ Intervention + Sex +
0,72381	-0,05083	0,143557	142	70	72	138	-0,35409	y ~ Intervention + Sex +
0,187794	0,198966	0,150311	142	70	72	138	1,323692	y ~ Intervention + Sex +
0,944463	0,010823	0,155082	142	70	72	138	0,069788	y ~ Intervention + Sex +
0,54482	0,078988	0,13012	142	70	72	138	0,607043	y ~ Intervention + Sex +
0,799039	0,039175	0,153576	142	70	72	138	0,255082	y ~ Intervention + Sex +
0,680472	0,072683	0,176119	142	70	72	138	0,412693	y ~ Intervention + Sex +
0,704822	-0,0594	0,156475	142	70	72	138	-0,3796	y ~ Intervention + Sex +
0,553015	-0,10029	0,168637	142	70	72	138	-0,5947	y ~ Intervention + Sex +
0,069046	-0,28985	0,15818	142	70	72	138	-1,83241	y ~ Intervention + Sex +

0,979018	0,004221	0,16019	142	70	72	138	0,026348	y ~ Intervention + Sex +
0,692679	0,060974	0,153955	142	70	72	138	0,396051	y ~ Intervention + Sex +
0,358064	0,143188	0,155278	142	70	72	138	0,922141	y ~ Intervention + Sex +
0,190953	0,186355	0,141799	142	70	72	138	1,314218	y ~ Intervention + Sex +
0,339634	0,175556	0,183213	142	70	72	138	0,958207	y ~ Intervention + Sex +
0,510616	0,097594	0,147961	142	70	72	138	0,659591	y ~ Intervention + Sex +
0,969115	-0,00556	0,143233	142	70	72	138	-0,03879	y ~ Intervention + Sex +
0,367087	0,143976	0,159104	142	70	72	138	0,904917	y ~ Intervention + Sex +
0,120373	-0,25916	0,165821	142	70	72	138	-1,56287	y ~ Intervention + Sex +
0,935885	0,013062	0,162074	142	70	72	138	0,08059	y ~ Intervention + Sex +
0,804824	-0,03676	0,148481	142	70	72	138	-0,24758	y ~ Intervention + Sex +
0,096432	-0,26414	0,157809	142	70	72	138	-1,67381	y ~ Intervention + Sex +
0,462303	0,121213	0,164442	142	70	72	138	0,737117	y ~ Intervention + Sex +
0,558246	0,095753	0,163157	142	70	72	138	0,586875	y ~ Intervention + Sex +
0,143446	-0,22853	0,155307	142	70	72	138	-1,47145	y ~ Intervention + Sex +
0,149914	-0,22633	0,156317	142	70	72	138	-1,4479	y ~ Intervention + Sex +
0,08648	-0,26313	0,152401	142	70	72	138	-1,72659	y ~ Intervention + Sex +
0,53246	-0,09516	0,15205	142	70	72	138	-0,62583	y ~ Intervention + Sex +
0,653719	0,068596	0,152579	142	70	72	138	0,449579	y ~ Intervention + Sex +
0,342513	-0,15656	0,164373	142	70	72	138	-0,95249	y ~ Intervention + Sex +
0,559885	-0,09837	0,168318	142	70	72	138	-0,58443	y ~ Intervention + Sex +
0,188374	-0,22056	0,166842	142	70	72	138	-1,32194	y ~ Intervention + Sex +
0,354679	0,150468	0,162025	142	70	72	138	0,928675	y ~ Intervention + Sex +
0,57704	-0,096	0,171722	142	70	72	138	-0,55904	y ~ Intervention + Sex +
0,593008	0,078021	0,145635	142	70	72	138	0,535729	y ~ Intervention + Sex +
0,353061	-0,14259	0,153024	142	70	72	138	-0,93181	y ~ Intervention + Sex +
0,430179	-0,13215	0,167019	142	70	72	138	-0,79121	y ~ Intervention + Sex +
0,275747	-0,16903	0,154468	142	70	72	138	-1,09426	y ~ Intervention + Sex +
0,591873	0,082389	0,153317	142	70	72	138	0,537377	y ~ Intervention + Sex +
0,50827	0,095818	0,144466	142	70	72	138	0,66326	y ~ Intervention + Sex +
0,293855	-0,17272	0,163919	142	70	72	138	-1,05371	y ~ Intervention + Sex +
0,699135	-0,0634	0,163711	142	70	72	138	-0,38729	y ~ Intervention + Sex +
0,574383	0,08984	0,159588	142	70	72	138	0,562949	y ~ Intervention + Sex +
0,368347	-0,13128	0,145453	142	70	72	138	-0,90253	y ~ Intervention + Sex +
0,263246	-0,1923	0,171191	142	70	72	138	-1,12333	y ~ Intervention + Sex +
0,24696	0,190794	0,164096	142	70	72	138	1,162695	y ~ Intervention + Sex +
0,543196	-0,10585	0,173668	142	70	72	138	-0,6095	y ~ Intervention + Sex +
0,335293	-0,15755	0,162948	142	70	72	138	-0,96689	y ~ Intervention + Sex +
0,00667	-0,42865	0,155614	142	70	72	138	-2,75455	y ~ Intervention + Sex +
0,611905	0,082658	0,162548	142	70	72	138	0,508514	y ~ Intervention + Sex +
0,748609	0,052996	0,165037	142	70	72	138	0,321115	y ~ Intervention + Sex +
0,698498	0,053421	0,137626	142	70	72	138	0,388157	y ~ Intervention + Sex +
0,944578	0,010048	0,144277	142	70	72	138	0,069644	y ~ Intervention + Sex +
0,951957	0,0098	0,16237	142	70	72	138	0,060359	y ~ Intervention + Sex +
0,304002	0,16977	0,164549	142	70	72	138	1,031734	y ~ Intervention + Sex +
0,406665	-0,12502	0,150212	142	70	72	138	-0,83232	y ~ Intervention + Sex +
0,719784	0,053256	0,148147	142	70	72	138	0,359481	y ~ Intervention + Sex +
0,287217	0,171374	0,160406	142	70	72	138	1,068374	y ~ Intervention + Sex +

0,702344	0,055377	0,144605	142	70	72	138	0,382953	y ~ Intervention + Sex +
0,002099	-0,47833	0,15257	142	70	72	138	-3,13515	y ~ Intervention + Sex +
0,810361	0,042335	0,176086	142	70	72	138	0,240421	y ~ Intervention + Sex +
0,379312	0,140155	0,158907	142	70	72	138	0,881998	y ~ Intervention + Sex +
0,1346	0,273509	0,181729	142	70	72	138	1,505037	y ~ Intervention + Sex +
0,251322	0,185889	0,161365	142	70	72	138	1,151978	y ~ Intervention + Sex +
0,274311	-0,16864	0,153653	142	70	72	138	-1,09755	y ~ Intervention + Sex +
0,073837	-0,24968	0,138613	142	70	72	138	-1,80131	y ~ Intervention + Sex +
0,978166	-0,00445	0,162166	142	70	72	138	-0,02742	y ~ Intervention + Sex +
0,392417	0,14073	0,164034	142	70	72	138	0,857932	y ~ Intervention + Sex +
0,125809	0,224388	0,14569	142	70	72	138	1,54017	y ~ Intervention + Sex +
0,584469	-0,09573	0,174636	142	70	72	138	-0,54816	y ~ Intervention + Sex +
0,031411	-0,36511	0,167943	142	70	72	138	-2,17399	y ~ Intervention + Sex +
0,797615	0,042585	0,165747	142	70	72	138	0,256931	y ~ Intervention + Sex +
0,576503	-0,09014	0,161009	142	70	72	138	-0,55983	y ~ Intervention + Sex +
0,108111	-0,21467	0,132739	142	70	72	138	-1,61723	y ~ Intervention + Sex +
0,607977	-0,08153	0,158575	142	70	72	138	-0,51414	y ~ Intervention + Sex +
0,197924	-0,19891	0,153752	142	70	72	138	-1,29372	y ~ Intervention + Sex +
0,264058	0,175176	0,15621	142	70	72	138	1,121416	y ~ Intervention + Sex +
0,957656	-0,00801	0,150589	142	70	72	138	-0,05319	y ~ Intervention + Sex +
0,354243	-0,14178	0,152529	142	70	72	138	-0,92952	y ~ Intervention + Sex +
0,166677	0,201474	0,144915	142	70	72	138	1,390296	y ~ Intervention + Sex +
0,309457	-0,14084	0,138066	142	70	72	138	-1,02012	y ~ Intervention + Sex +
0,684395	0,069395	0,170363	142	70	72	138	0,407333	y ~ Intervention + Sex +
0,862431	0,024998	0,143995	142	70	72	138	0,173605	y ~ Intervention + Sex +
0,985942	0,002848	0,161341	142	70	72	138	0,017653	y ~ Intervention + Sex +
0,159317	-0,20133	0,142282	142	70	72	138	-1,41502	y ~ Intervention + Sex +
0,008819	-0,47268	0,177915	142	70	72	138	-2,65677	y ~ Intervention + Sex +
0,397666	0,138441	0,163172	142	70	72	138	0,848434	y ~ Intervention + Sex +
0,301316	-0,16561	0,159623	142	70	72	138	-1,0375	y ~ Intervention + Sex +
0,814781	-0,03155	0,134417	142	70	72	138	-0,23471	y ~ Intervention + Sex +
0,295224	-0,17636	0,167847	142	70	72	138	-1,05072	y ~ Intervention + Sex +
0,007075	0,437121	0,159875	142	70	72	138	2,734149	y ~ Intervention + Sex +
0,284542	0,169908	0,15815	142	70	72	138	1,074348	y ~ Intervention + Sex +
0,18803	0,208733	0,157775	142	70	72	138	1,322979	y ~ Intervention + Sex +
0,313447	0,159696	0,157847	142	70	72	138	1,01171	y ~ Intervention + Sex +
0,864448	0,027403	0,160222	142	70	72	138	0,171033	y ~ Intervention + Sex +
0,206767	0,183108	0,144354	142	70	72	138	1,268467	y ~ Intervention + Sex +
0,310155	0,117593	0,115441	142	70	72	138	1,018643	y ~ Intervention + Sex +
0,523989	-0,10414	0,163008	142	70	72	138	-0,63884	y ~ Intervention + Sex +
0,847724	0,029717	0,154468	142	70	72	138	0,192384	y ~ Intervention + Sex +
0,251126	-0,18154	0,157529	142	70	72	138	-1,15246	y ~ Intervention + Sex +
0,67959	0,0578	0,139648	142	70	72	138	0,4139	y ~ Intervention + Sex +
0,431578	-0,11824	0,149899	142	70	72	138	-0,78881	y ~ Intervention + Sex +
0,557274	0,092129	0,156594	142	70	72	138	0,588328	y ~ Intervention + Sex +
0,829162	0,036301	0,167915	142	70	72	138	0,216186	y ~ Intervention + Sex +
0,084594	-0,27463	0,158096	142	70	72	138	-1,73714	y ~ Intervention + Sex +
0,634693	-0,07368	0,154738	142	70	72	138	-0,47619	y ~ Intervention + Sex +

0,474345	0,121223	0,168977	142	70	72	138	0,717391	y ~ Intervention + Sex
0,573396	0,090966	0,161172	142	70	72	138	0,564403	y ~ Intervention + Sex
0,946732	0,010682	0,1596	142	70	72	138	0,066932	y ~ Intervention + Sex
0,174268	0,222552	0,162963	142	70	72	138	1,365657	y ~ Intervention + Sex
0,520571	-0,10636	0,165121	142	70	72	138	-0,64411	y ~ Intervention + Sex
0,125087	-0,24247	0,157129	142	70	72	138	-1,54314	y ~ Intervention + Sex
0,888881	-0,02187	0,156223	142	70	72	138	-0,13998	y ~ Intervention + Sex
0,274567	0,183367	0,167159	142	70	72	138	1,096966	y ~ Intervention + Sex
0,893063	0,023197	0,172239	142	70	72	138	0,134678	y ~ Intervention + Sex
0,104881	0,258789	0,158535	142	70	72	138	1,632374	y ~ Intervention + Sex
0,704684	-0,06654	0,175197	142	70	72	138	-0,37979	y ~ Intervention + Sex
0,768121	-0,04707	0,159346	142	70	72	138	-0,29541	y ~ Intervention + Sex
0,927343	-0,01473	0,161286	142	70	72	138	-0,09136	y ~ Intervention + Sex
0,860516	0,027459	0,155974	142	70	72	138	0,176046	y ~ Intervention + Sex
0,866353	0,027481	0,162991	142	70	72	138	0,168607	y ~ Intervention + Sex
0,45116	-0,12786	0,169217	142	70	72	138	-0,75563	y ~ Intervention + Sex
0,468945	-0,11792	0,162376	142	70	72	138	-0,7262	y ~ Intervention + Sex
0,010091	-0,43608	0,167167	142	70	72	138	-2,60867	y ~ Intervention + Sex
0,286644	-0,15901	0,14866	142	70	72	138	-1,06965	y ~ Intervention + Sex
0,792816	-0,04585	0,174231	142	70	72	138	-0,26317	y ~ Intervention + Sex
0,143802	0,220305	0,149854	142	70	72	138	1,470135	y ~ Intervention + Sex
0,691091	0,059714	0,149957	142	70	72	138	0,39821	y ~ Intervention + Sex
0,158912	-0,23921	0,168887	142	70	72	138	-1,4164	y ~ Intervention + Sex
0,552088	-0,0873	0,146457	142	70	72	138	-0,5961	y ~ Intervention + Sex
0,987232	-0,00245	0,152895	142	70	72	138	-0,01603	y ~ Intervention + Sex
0,649655	0,068693	0,150895	142	70	72	138	0,455236	y ~ Intervention + Sex
0,769455	0,040675	0,138509	142	70	72	138	0,293665	y ~ Intervention + Sex
0,628774	0,070924	0,146375	142	70	72	138	0,484536	y ~ Intervention + Sex
0,316498	-0,14372	0,142955	142	70	72	138	-1,00533	y ~ Intervention + Sex
0,474664	0,105825	0,14762	142	70	72	138	0,716874	y ~ Intervention + Sex
0,945959	0,010358	0,152537	142	70	72	138	0,067906	y ~ Intervention + Sex
0,620363	0,087576	0,176403	142	70	72	138	0,496455	y ~ Intervention + Sex
0,464356	-0,10613	0,144646	142	70	72	138	-0,73373	y ~ Intervention + Sex
0,09982	-0,25802	0,155727	142	70	72	138	-1,65686	y ~ Intervention + Sex
0,586928	-0,08821	0,161979	142	70	72	138	-0,54457	y ~ Intervention + Sex
0,869768	0,025641	0,156099	142	70	72	138	0,164259	y ~ Intervention + Sex
0,972508	0,00627	0,181593	142	70	72	138	0,034526	y ~ Intervention + Sex
0,878994	0,025089	0,164486	142	70	72	138	0,152528	y ~ Intervention + Sex
0,176945	0,230392	0,16976	142	70	72	138	1,357164	y ~ Intervention + Sex
0,132068	0,194866	0,128627	142	70	72	138	1,514967	y ~ Intervention + Sex
0,942808	0,012053	0,167706	142	70	72	138	0,071872	y ~ Intervention + Sex
0,581757	-0,08482	0,153628	142	70	72	138	-0,55212	y ~ Intervention + Sex
0,487222	0,109681	0,157451	142	70	72	138	0,696604	y ~ Intervention + Sex
0,61134	-0,0865	0,16983	142	70	72	138	-0,50932	y ~ Intervention + Sex
0,3933	-0,13836	0,16157	142	70	72	138	-0,85633	y ~ Intervention + Sex
0,988418	0,00201	0,138192	142	70	72	138	0,014542	y ~ Intervention + Sex
0,102739	-0,26089	0,158827	142	70	72	138	-1,64262	y ~ Intervention + Sex
0,491712	-0,11031	0,160009	142	70	72	138	-0,68943	y ~ Intervention + Sex

0,16935	0,233365	0,168918	142	70	72	138	1,381525	y ~ Intervention + Sex +
0,992016	0,001223	0,121952	142	70	72	138	0,010025	y ~ Intervention + Sex +
0,122777	-0,22163	0,142738	142	70	72	138	-1,55273	y ~ Intervention + Sex +
0,348546	-0,15812	0,168101	142	70	72	138	-0,94061	y ~ Intervention + Sex +
0,241544	-0,1778	0,151164	142	70	72	138	-1,17619	y ~ Intervention + Sex +
0,184099	-0,18626	0,139525	142	70	72	138	-1,33492	y ~ Intervention + Sex +
0,394886	0,1214	0,142245	142	70	72	138	0,853455	y ~ Intervention + Sex +
0,410562	0,125554	0,152111	142	70	72	138	0,825412	y ~ Intervention + Sex +
0,467241	0,098021	0,134461	142	70	72	138	0,728994	y ~ Intervention + Sex +
0,656473	-0,06832	0,153276	142	70	72	138	-0,44575	y ~ Intervention + Sex +
0,030814	-0,31072	0,142413	142	70	72	138	-2,18185	y ~ Intervention + Sex +
0,565407	-0,08817	0,153023	142	70	72	138	-0,57622	y ~ Intervention + Sex +
0,296287	0,163037	0,15551	142	70	72	138	1,0484	y ~ Intervention + Sex +
0,349334	0,151176	0,160984	142	70	72	138	0,939072	y ~ Intervention + Sex +
0,291091	-0,15511	0,146362	142	70	72	138	-1,05979	y ~ Intervention + Sex +
0,250294	0,195062	0,168959	142	70	72	138	1,154493	y ~ Intervention + Sex +
0,963162	-0,00686	0,148225	142	70	72	138	-0,04627	y ~ Intervention + Sex +
0,418794	0,142103	0,175231	142	70	72	138	0,810944	y ~ Intervention + Sex +
0,146851	0,231715	0,158823	142	70	72	138	1,458949	y ~ Intervention + Sex +
0,49254	-0,11419	0,165953	142	70	72	138	-0,68811	y ~ Intervention + Sex +
0,657721	-0,07568	0,17045	142	70	72	138	-0,44402	y ~ Intervention + Sex +
0,618617	-0,0769	0,154129	142	70	72	138	-0,49894	y ~ Intervention + Sex +
0,805714	0,037194	0,150929	142	70	72	138	0,246433	y ~ Intervention + Sex +
0,324734	-0,17592	0,178006	142	70	72	138	-0,9883	y ~ Intervention + Sex +
0,792878	-0,03681	0,139899	142	70	72	138	-0,26308	y ~ Intervention + Sex +
0,463053	0,110298	0,149886	142	70	72	138	0,73588	y ~ Intervention + Sex +
0,201486	-0,22022	0,171585	142	70	72	138	-1,28345	y ~ Intervention + Sex +
0,019723	0,283957	0,120369	142	70	72	138	2,359061	y ~ Intervention + Sex +
0,182944	0,207276	0,154861	142	70	72	138	1,33847	y ~ Intervention + Sex +
0,070957	-0,27461	0,150901	142	70	72	138	-1,8198	y ~ Intervention + Sex +
0,466235	0,112565	0,154063	142	70	72	138	0,730644	y ~ Intervention + Sex +
0,36544	-0,12578	0,138514	142	70	72	138	-0,90804	y ~ Intervention + Sex +
0,309828	-0,14436	0,141621	142	70	72	138	-1,01934	y ~ Intervention + Sex +
0,997583	0,000495	0,163141	142	70	72	138	0,003035	y ~ Intervention + Sex +
0,066391	-0,30019	0,162226	142	70	72	138	-1,85042	y ~ Intervention + Sex +
0,735514	0,053638	0,158466	142	70	72	138	0,338482	y ~ Intervention + Sex +
0,950299	-0,01003	0,160566	142	70	72	138	-0,06245	y ~ Intervention + Sex +
0,443566	0,113263	0,147402	142	70	72	138	0,768394	y ~ Intervention + Sex +
0,295169	0,157961	0,150319	142	70	72	138	1,050839	y ~ Intervention + Sex +
0,183994	0,201285	0,150748	142	70	72	138	1,335246	y ~ Intervention + Sex +
0,584302	-0,08112	0,147928	142	70	72	138	-0,5484	y ~ Intervention + Sex +
0,857532	0,027906	0,155158	142	70	72	138	0,179853	y ~ Intervention + Sex +
0,807946	-0,04222	0,173357	142	70	72	138	-0,24354	y ~ Intervention + Sex +
0,666219	0,066965	0,154914	142	70	72	138	0,432272	y ~ Intervention + Sex +
0,240322	0,188743	0,160051	142	70	72	138	1,179265	y ~ Intervention + Sex +
0,102211	0,232557	0,141357	142	70	72	138	1,645166	y ~ Intervention + Sex +
0,915794	0,014409	0,136026	142	70	72	138	0,105927	y ~ Intervention + Sex +
0,855349	-0,02741	0,150081	142	70	72	138	-0,18264	y ~ Intervention + Sex +

0,252453	-0,15867	0,138066	142	70	72	138	-1,14922	y ~ Intervention + Sex +
0,073342	0,277726	0,153912	142	70	72	138	1,80445	y ~ Intervention + Sex +
0,41008	0,102836	0,124459	142	70	72	138	0,826266	y ~ Intervention + Sex +
0,062533	0,292216	0,155625	142	70	72	138	1,877697	y ~ Intervention + Sex +
0,274489	0,180976	0,164952	142	70	72	138	1,097145	y ~ Intervention + Sex +
0,70537	-0,06241	0,164719	142	70	72	138	-0,37887	y ~ Intervention + Sex +
0,883842	0,02457	0,167862	142	70	72	138	0,146371	y ~ Intervention + Sex +
0,606957	-0,08407	0,163057	142	70	72	138	-0,5156	y ~ Intervention + Sex +
0,557301	0,092993	0,158074	142	70	72	138	0,588287	y ~ Intervention + Sex +
0,601167	-0,08665	0,165376	142	70	72	138	-0,52393	y ~ Intervention + Sex +
0,573853	0,076448	0,135612	142	70	72	138	0,56373	y ~ Intervention + Sex +
0,948428	0,010655	0,164427	142	70	72	138	0,064798	y ~ Intervention + Sex +
0,287858	0,179482	0,16822	142	70	72	138	1,066949	y ~ Intervention + Sex +
0,927789	0,014288	0,157373	142	70	72	138	0,090793	y ~ Intervention + Sex +
0,787256	0,045391	0,167867	142	70	72	138	0,2704	y ~ Intervention + Sex +
0,280339	0,173443	0,160031	142	70	72	138	1,083811	y ~ Intervention + Sex +
0,13589	-0,23986	0,1599	142	70	72	138	-1,50003	y ~ Intervention + Sex +
0,18259	0,207864	0,155174	142	70	72	138	1,33956	y ~ Intervention + Sex +
0,846614	-0,03505	0,180854	142	70	72	138	-0,1938	y ~ Intervention + Sex +
0,086512	-0,27392	0,158663	142	70	72	138	-1,72641	y ~ Intervention + Sex +
0,621849	0,074865	0,151443	142	70	72	138	0,494344	y ~ Intervention + Sex +
0,964252	-0,00666	0,148342	142	70	72	138	-0,0449	y ~ Intervention + Sex +
0,877838	-0,02185	0,141894	142	70	72	138	-0,154	y ~ Intervention + Sex +
0,154545	-0,21402	0,149508	142	70	72	138	-1,43151	y ~ Intervention + Sex +
0,829832	0,033179	0,154087	142	70	72	138	0,215324	y ~ Intervention + Sex +
0,56778	-0,09533	0,166455	142	70	72	138	-0,5727	y ~ Intervention + Sex +
0,395358	-0,1247	0,146257	142	70	72	138	-0,8526	y ~ Intervention + Sex +
0,044076	-0,29147	0,143442	142	70	72	138	-2,03196	y ~ Intervention + Sex +
0,347693	0,151791	0,161088	142	70	72	138	0,942286	y ~ Intervention + Sex +
0,456191	0,121265	0,162284	142	70	72	138	0,747238	y ~ Intervention + Sex +
0,366857	0,147695	0,163135	142	70	72	138	0,905351	y ~ Intervention + Sex +
0,251151	0,183591	0,159313	142	70	72	138	1,152396	y ~ Intervention + Sex +
0,333128	0,153888	0,158445	142	70	72	138	0,97124	y ~ Intervention + Sex +
0,566902	0,091927	0,160151	142	70	72	138	0,574	y ~ Intervention + Sex +
0,56509	-0,08833	0,153161	142	70	72	138	-0,57669	y ~ Intervention + Sex +
0,716474	-0,06317	0,173588	142	70	72	138	-0,36392	y ~ Intervention + Sex +
0,122262	0,215809	0,138794	142	70	72	138	1,554894	y ~ Intervention + Sex +
0,258277	-0,1665	0,146675	142	70	72	138	-1,13516	y ~ Intervention + Sex +
0,462336	0,107444	0,145774	142	70	72	138	0,737062	y ~ Intervention + Sex +
0,04903	-0,28248	0,142244	142	70	72	138	-1,98586	y ~ Intervention + Sex +
0,664951	-0,06546	0,150818	142	70	72	138	-0,43402	y ~ Intervention + Sex +
0,886838	0,021368	0,14988	142	70	72	138	0,14257	y ~ Intervention + Sex +
0,532023	-0,10654	0,17005	142	70	72	138	-0,6265	y ~ Intervention + Sex +
0,652089	-0,07038	0,15577	142	70	72	138	-0,45185	y ~ Intervention + Sex +
0,517198	0,11415	0,175793	142	70	72	138	0,649341	y ~ Intervention + Sex +
0,445056	0,135425	0,176823	142	70	72	138	0,765879	y ~ Intervention + Sex +
0,634819	0,067957	0,142763	142	70	72	138	0,476011	y ~ Intervention + Sex +
0,425244	-0,12275	0,153493	142	70	72	138	-0,79973	y ~ Intervention + Sex +

0,900983	-0,01863	0,149461	142	70	72	138	-0,12465	y ~ Intervention + Sex +
0,80624	0,03537	0,143926	142	70	72	138	0,245751	y ~ Intervention + Sex +
0,296155	-0,16063	0,153172	142	70	72	138	-1,04869	y ~ Intervention + Sex +
0,459589	-0,11876	0,160144	142	70	72	138	-0,7416	y ~ Intervention + Sex +
0,385949	-0,1295	0,14889	142	70	72	138	-0,86975	y ~ Intervention + Sex +
0,397912	0,120215	0,141765	142	70	72	138	0,84799	y ~ Intervention + Sex +
0,373469	0,157548	0,176447	142	70	72	138	0,892892	y ~ Intervention + Sex +
0,661233	0,07721	0,175814	142	70	72	138	0,439159	y ~ Intervention + Sex +
0,698837	0,054757	0,141236	142	70	72	138	0,387698	y ~ Intervention + Sex +
0,794818	0,041966	0,161058	142	70	72	138	0,260563	y ~ Intervention + Sex +
0,371248	0,139831	0,155877	142	70	72	138	0,897063	y ~ Intervention + Sex +
0,140356	-0,24931	0,168113	142	70	72	138	-1,483	y ~ Intervention + Sex +
0,197408	0,177321	0,136905	142	70	72	138	1,295216	y ~ Intervention + Sex +
0,491003	0,114661	0,166041	142	70	72	138	0,690558	y ~ Intervention + Sex +
0,513837	-0,09873	0,150832	142	70	72	138	-0,65456	y ~ Intervention + Sex +
0,912865	-0,01771	0,161515	142	70	72	138	-0,10963	y ~ Intervention + Sex +
0,872644	-0,02569	0,159979	142	70	72	138	-0,1606	y ~ Intervention + Sex +
0,093056	0,267546	0,158199	142	70	72	138	1,691198	y ~ Intervention + Sex +
0,420032	0,124257	0,153634	142	70	72	138	0,808782	y ~ Intervention + Sex +
0,392357	0,150173	0,175018	142	70	72	138	0,858041	y ~ Intervention + Sex +
0,010648	0,374002	0,14444	142	70	72	138	2,589315	y ~ Intervention + Sex +
0,705059	0,054144	0,142752	142	70	72	138	0,379286	y ~ Intervention + Sex +
0,572615	-0,08838	0,156262	142	70	72	138	-0,56556	y ~ Intervention + Sex +
0,168115	0,203893	0,147155	142	70	72	138	1,385564	y ~ Intervention + Sex +
0,268728	0,16588	0,149378	142	70	72	138	1,110469	y ~ Intervention + Sex +
0,467277	0,108251	0,148506	142	70	72	138	0,728935	y ~ Intervention + Sex +
0,734398	-0,05683	0,167173	142	70	72	138	-0,33997	y ~ Intervention + Sex +
0,836381	-0,02861	0,138252	142	70	72	138	-0,20692	y ~ Intervention + Sex +
0,865405	-0,02747	0,161754	142	70	72	138	-0,16981	y ~ Intervention + Sex +
0,023598	-0,30818	0,134637	142	70	72	138	-2,289	y ~ Intervention + Sex +
0,259268	0,196695	0,173638	142	70	72	138	1,132786	y ~ Intervention + Sex +
0,654263	0,077747	0,173225	142	70	72	138	0,448824	y ~ Intervention + Sex +
0,493806	0,09975	0,145388	142	70	72	138	0,686092	y ~ Intervention + Sex +
0,051106	0,319545	0,162394	142	70	72	138	1,967711	y ~ Intervention + Sex +
0,745471	-0,056	0,172167	142	70	72	138	-0,32527	y ~ Intervention + Sex +
0,426152	-0,11372	0,142481	142	70	72	138	-0,79815	y ~ Intervention + Sex +
0,839282	0,032029	0,157629	142	70	72	138	0,203195	y ~ Intervention + Sex +
0,563072	-0,09992	0,172373	142	70	72	138	-0,57969	y ~ Intervention + Sex +
0,027293	0,32877	0,147362	142	70	72	138	2,23103	y ~ Intervention + Sex +
0,625062	0,076497	0,156185	142	70	72	138	0,489787	y ~ Intervention + Sex +
0,611192	0,077625	0,152346	142	70	72	138	0,509534	y ~ Intervention + Sex +
0,625368	-0,07572	0,154725	142	70	72	138	-0,48935	y ~ Intervention + Sex +
0,241488	0,191344	0,162662	142	70	72	138	1,176331	y ~ Intervention + Sex +
0,282945	0,179317	0,166353	142	70	72	138	1,077932	y ~ Intervention + Sex +
0,210048	-0,1993	0,158266	142	70	72	138	-1,2593	y ~ Intervention + Sex +
0,898359	0,019987	0,156186	142	70	72	138	0,127971	y ~ Intervention + Sex +
0,290921	-0,17513	0,165195	142	70	72	138	-1,06017	y ~ Intervention + Sex +
0,223948	0,194943	0,159582	142	70	72	138	1,221583	y ~ Intervention + Sex +

0,578818	-0,0861	0,154734	142	70	72	138	-0,55643	y ~ Intervention + Sex +
0,887711	-0,02429	0,171691	142	70	72	138	-0,14146	y ~ Intervention + Sex +
0,474664	0,105825	0,14762	142	70	72	138	0,716874	y ~ Intervention + Sex +
0,815256	0,040982	0,175066	142	70	72	138	0,234097	y ~ Intervention + Sex +
0,940107	-0,01169	0,155338	142	70	72	138	-0,07527	y ~ Intervention + Sex +
0,745572	0,05312	0,163379	142	70	72	138	0,325134	y ~ Intervention + Sex +
0,378732	0,168996	0,191373	142	70	72	138	0,883074	y ~ Intervention + Sex +
0,428452	-0,12658	0,159379	142	70	72	138	-0,79418	y ~ Intervention + Sex +
0,296489	0,173341	0,165408	142	70	72	138	1,04796	y ~ Intervention + Sex +
0,865989	0,024154	0,142865	142	70	72	138	0,16907	y ~ Intervention + Sex +
0,516781	0,10386	0,159788	142	70	72	138	0,649988	y ~ Intervention + Sex +
0,050949	-0,29228	0,148435	142	70	72	138	-1,96906	y ~ Intervention + Sex +
0,572971	0,08929	0,158027	142	70	72	138	0,56503	y ~ Intervention + Sex +
0,705183	0,058091	0,153227	142	70	72	138	0,379118	y ~ Intervention + Sex +
0,643876	0,070709	0,152619	142	70	72	138	0,463304	y ~ Intervention + Sex +
0,174021	-0,19304	0,141271	142	70	72	138	-1,36645	y ~ Intervention + Sex +
0,237112	-0,17803	0,14993	142	70	72	138	-1,1874	y ~ Intervention + Sex +
0,252304	-0,17446	0,15176	142	70	72	138	-1,14958	y ~ Intervention + Sex +
0,232375	0,164283	0,136955	142	70	72	138	1,199537	y ~ Intervention + Sex +
0,841353	-0,03327	0,165911	142	70	72	138	-0,20054	y ~ Intervention + Sex +
0,834304	-0,03621	0,172783	142	70	72	138	-0,20958	y ~ Intervention + Sex +
0,377003	-0,13822	0,15595	142	70	72	138	-0,88629	y ~ Intervention + Sex +
0,97793	-0,00389	0,140488	142	70	72	138	-0,02771	y ~ Intervention + Sex +
0,507767	0,105069	0,158225	142	70	72	138	0,664049	y ~ Intervention + Sex +
0,852994	0,027419	0,147695	142	70	72	138	0,185647	y ~ Intervention + Sex +
0,97749	0,004574	0,161801	142	70	72	138	0,028267	y ~ Intervention + Sex +
0,625591	0,06356	0,129969	142	70	72	138	0,489039	y ~ Intervention + Sex +
0,869218	-0,02451	0,148576	142	70	72	138	-0,16496	y ~ Intervention + Sex +
0,832773	0,033753	0,159553	142	70	72	138	0,211546	y ~ Intervention + Sex +
0,333252	0,170181	0,175266	142	70	72	138	0,97099	y ~ Intervention + Sex +
0,844783	-0,02983	0,152097	142	70	72	138	-0,19615	y ~ Intervention + Sex +
0,239425	-0,16896	0,143004	142	70	72	138	-1,18153	y ~ Intervention + Sex +
0,819329	0,035265	0,154101	142	70	72	138	0,228844	y ~ Intervention + Sex +
0,547436	-0,09335	0,154791	142	70	72	138	-0,60309	y ~ Intervention + Sex +
0,996052	0,000696	0,140305	142	70	72	138	0,004957	y ~ Intervention + Sex +
0,18828	-0,21101	0,159583	142	70	72	138	-1,32223	y ~ Intervention + Sex +
0,455448	-0,1095	0,146297	142	70	72	138	-0,74847	y ~ Intervention + Sex +
0,737162	-0,05052	0,150218	142	70	72	138	-0,33629	y ~ Intervention + Sex +
0,338127	0,163102	0,169684	142	70	72	138	0,961211	y ~ Intervention + Sex +
0,395379	-0,12818	0,150344	142	70	72	138	-0,85256	y ~ Intervention + Sex +
0,693455	0,061656	0,156092	142	70	72	138	0,394996	y ~ Intervention + Sex +
0,144639	0,240335	0,163823	142	70	72	138	1,467044	y ~ Intervention + Sex +
0,68973	-0,05591	0,139749	142	70	72	138	-0,40006	y ~ Intervention + Sex +
0,611644	-0,07819	0,153642	142	70	72	138	-0,50889	y ~ Intervention + Sex +
0,284512	0,178139	0,165801	142	70	72	138	1,074414	y ~ Intervention + Sex +
0,958027	0,007312	0,13868	142	70	72	138	0,052726	y ~ Intervention + Sex +
0,423493	-0,13213	0,164591	142	70	72	138	-0,80276	y ~ Intervention + Sex +
0,877451	-0,0233	0,150839	142	70	72	138	-0,15449	y ~ Intervention + Sex +

0,171394	-0,18153	0,13203	142	70	72	138	-1,37489	y ~ Intervention + Sex +
0,75567	-0,05323	0,170735	142	70	72	138	-0,31179	y ~ Intervention + Sex +
0,598502	-0,08187	0,155115	142	70	72	138	-0,52778	y ~ Intervention + Sex +
0,921664	-0,01623	0,164709	142	70	72	138	-0,09852	y ~ Intervention + Sex +
0,35306	0,149027	0,159933	142	70	72	138	0,931814	y ~ Intervention + Sex +
0,800037	-0,04291	0,169068	142	70	72	138	-0,25379	y ~ Intervention + Sex +
0,401823	-0,13702	0,162927	142	70	72	138	-0,84096	y ~ Intervention + Sex +
0,378608	-0,12653	0,143248	142	70	72	138	-0,88331	y ~ Intervention + Sex +
0,74401	-0,05021	0,153457	142	70	72	138	-0,3272	y ~ Intervention + Sex +
0,341937	0,151647	0,159021	142	70	72	138	0,953633	y ~ Intervention + Sex +
0,759573	0,049489	0,161386	142	70	72	138	0,306649	y ~ Intervention + Sex +
0,127047	-0,2312	0,150605	142	70	72	138	-1,53511	y ~ Intervention + Sex +
0,934966	0,013574	0,166051	142	70	72	138	0,081748	y ~ Intervention + Sex +
0,811721	0,039831	0,166893	142	70	72	138	0,238663	y ~ Intervention + Sex +
0,555649	-0,09537	0,161443	142	70	72	138	-0,59076	y ~ Intervention + Sex +
0,106801	0,236678	0,145798	142	70	72	138	1,623331	y ~ Intervention + Sex +
0,089962	-0,2839	0,166256	142	70	72	138	-1,70759	y ~ Intervention + Sex +
0,797003	0,044089	0,171069	142	70	72	138	0,257726	y ~ Intervention + Sex +
0,76214	0,049633	0,163659	142	70	72	138	0,303271	y ~ Intervention + Sex +
0,371707	0,151352	0,168882	142	70	72	138	0,896199	y ~ Intervention + Sex +
0,942861	0,012365	0,1722	142	70	72	138	0,071805	y ~ Intervention + Sex +
0,788602	0,041981	0,156266	142	70	72	138	0,268647	y ~ Intervention + Sex +
0,635344	0,068078	0,143239	142	70	72	138	0,475273	y ~ Intervention + Sex +
0,375449	-0,12654	0,142309	142	70	72	138	-0,88919	y ~ Intervention + Sex +
0,108415	-0,27212	0,168407	142	70	72	138	-1,61583	y ~ Intervention + Sex +
0,519596	0,106495	0,164949	142	70	72	138	0,645623	y ~ Intervention + Sex +
0,767618	-0,04	0,135095	142	70	72	138	-0,29607	y ~ Intervention + Sex +
0,701118	0,056966	0,148115	142	70	72	138	0,38461	y ~ Intervention + Sex +
0,679965	0,072178	0,174601	142	70	72	138	0,413386	y ~ Intervention + Sex +
0,482375	0,113512	0,161149	142	70	72	138	0,704393	y ~ Intervention + Sex +
0,258676	-0,16152	0,142406	142	70	72	138	-1,1342	y ~ Intervention + Sex +
0,03013	0,341657	0,155936	142	70	72	138	2,191005	y ~ Intervention + Sex +
0,555883	0,088379	0,149691	142	70	72	138	0,590407	y ~ Intervention + Sex +
0,919166	0,016659	0,163852	142	70	72	138	0,10167	y ~ Intervention + Sex +
0,96963	-0,00529	0,138756	142	70	72	138	-0,03814	y ~ Intervention + Sex +
0,371491	-0,14776	0,164798	142	70	72	138	-0,89661	y ~ Intervention + Sex +
0,724712	0,049466	0,140176	142	70	72	138	0,352887	y ~ Intervention + Sex +
0,886675	-0,02308	0,161623	142	70	72	138	-0,14278	y ~ Intervention + Sex +
0,317348	-0,15942	0,158852	142	70	72	138	-1,00356	y ~ Intervention + Sex +
0,359539	0,149656	0,162792	142	70	72	138	0,919307	y ~ Intervention + Sex +
0,70637	0,057866	0,153282	142	70	72	138	0,377516	y ~ Intervention + Sex +
0,887755	-0,02023	0,143053	142	70	72	138	-0,14141	y ~ Intervention + Sex +
0,508122	0,110046	0,165858	142	70	72	138	0,663493	y ~ Intervention + Sex +
0,050005	-0,30766	0,155601	142	70	72	138	-1,97726	y ~ Intervention + Sex +
0,573326	0,093441	0,165527	142	70	72	138	0,564506	y ~ Intervention + Sex +
0,117506	-0,22982	0,145903	142	70	72	138	-1,57518	y ~ Intervention + Sex +
0,166759	-0,21981	0,158135	142	70	72	138	-1,39003	y ~ Intervention + Sex +
0,11127	0,247167	0,154211	142	70	72	138	1,602779	y ~ Intervention + Sex +

0,704945	0,064977	0,171245	142	70	72	138	0,379439	y ~ Intervention + Sex +
0,112679	0,221416	0,138694	142	70	72	138	1,596436	y ~ Intervention + Sex +
0,761755	0,047553	0,156539	142	70	72	138	0,303777	y ~ Intervention + Sex +
0,666146	-0,05712	0,132107	142	70	72	138	-0,43237	y ~ Intervention + Sex +
0,826082	0,034956	0,158784	142	70	72	138	0,220148	y ~ Intervention + Sex +
0,637652	-0,0655	0,138773	142	70	72	138	-0,47203	y ~ Intervention + Sex +
0,510429	-0,10541	0,159745	142	70	72	138	-0,65988	y ~ Intervention + Sex +
0,780549	0,03521	0,126133	142	70	72	138	0,279148	y ~ Intervention + Sex +
0,085565	-0,25444	0,146933	142	70	72	138	-1,73168	y ~ Intervention + Sex +
0,670223	-0,06322	0,148133	142	70	72	138	-0,42675	y ~ Intervention + Sex +
0,166141	-0,21019	0,150992	142	70	72	138	-1,39207	y ~ Intervention + Sex +
0,261523	-0,15585	0,13824	142	70	72	138	-1,12742	y ~ Intervention + Sex +
0,423241	0,121973	0,151859	142	70	72	138	0,803199	y ~ Intervention + Sex +
0,580436	0,066473	0,119976	142	70	72	138	0,554058	y ~ Intervention + Sex +
0,855506	-0,02437	0,133602	142	70	72	138	-0,18244	y ~ Intervention + Sex +
0,230437	0,186268	0,154637	142	70	72	138	1,204555	y ~ Intervention + Sex +
0,158673	0,207703	0,146557	142	70	72	138	1,417216	y ~ Intervention + Sex +
0,986173	0,002676	0,154126	142	70	72	138	0,017362	y ~ Intervention + Sex +
0,963328	0,007081	0,153728	142	70	72	138	0,046061	y ~ Intervention + Sex +
0,602057	0,079483	0,152077	142	70	72	138	0,522648	y ~ Intervention + Sex +
0,63854	0,068949	0,146458	142	70	72	138	0,470781	y ~ Intervention + Sex +
0,57721	-0,09223	0,165053	142	70	72	138	-0,55879	y ~ Intervention + Sex +
0,646452	0,06723	0,146246	142	70	72	138	0,459705	y ~ Intervention + Sex +
0,735402	-0,05333	0,157482	142	70	72	138	-0,33863	y ~ Intervention + Sex +
0,323361	-0,16242	0,163871	142	70	72	138	-0,99112	y ~ Intervention + Sex +
0,178888	0,218621	0,161815	142	70	72	138	1,351056	y ~ Intervention + Sex +
0,186556	0,206176	0,155319	142	70	72	138	1,327438	y ~ Intervention + Sex +
0,261368	-0,17522	0,155363	142	70	72	138	-1,12779	y ~ Intervention + Sex +
0,511595	0,089361	0,135795	142	70	72	138	0,658061	y ~ Intervention + Sex +
0,329992	0,161309	0,165009	142	70	72	138	0,977581	y ~ Intervention + Sex +
0,05723	-0,32147	0,167646	142	70	72	138	-1,91759	y ~ Intervention + Sex +
0,902449	-0,02042	0,166292	142	70	72	138	-0,12279	y ~ Intervention + Sex +
0,799368	0,042713	0,167728	142	70	72	138	0,254655	y ~ Intervention + Sex +
0,598867	-0,08074	0,153139	142	70	72	138	-0,52725	y ~ Intervention + Sex +
0,826262	0,036548	0,166191	142	70	72	138	0,219915	y ~ Intervention + Sex +
0,751101	-0,04689	0,147536	142	70	72	138	-0,31782	y ~ Intervention + Sex +
0,134644	-0,24012	0,15956	142	70	72	138	-1,50487	y ~ Intervention + Sex +
0,2282	0,159122	0,131464	142	70	72	138	1,210385	y ~ Intervention + Sex +
0,894765	0,02142	0,161636	142	70	72	138	0,132521	y ~ Intervention + Sex +
0,057021	-0,30363	0,158208	142	70	72	138	-1,91922	y ~ Intervention + Sex +
0,082684	-0,24476	0,140021	142	70	72	138	-1,74802	y ~ Intervention + Sex +
0,310565	-0,15529	0,152574	142	70	72	138	-1,01778	y ~ Intervention + Sex +
0,721447	-0,04617	0,129238	142	70	72	138	-0,35725	y ~ Intervention + Sex +
0,261022	-0,1406	0,124582	142	70	72	138	-1,12861	y ~ Intervention + Sex +
0,675152	0,059983	0,142822	142	70	72	138	0,419981	y ~ Intervention + Sex +
0,581883	-0,08237	0,149229	142	70	72	138	-0,55194	y ~ Intervention + Sex +
0,68146	0,064666	0,157209	142	70	72	138	0,411342	y ~ Intervention + Sex +
0,908253	0,018802	0,162853	142	70	72	138	0,115455	y ~ Intervention + Sex +

0,524702	0,100399	0,157431	142	70	72	138	0,637737	y ~ Intervention + Sex +
0,965564	0,007263	0,167928	142	70	72	138	0,043251	y ~ Intervention + Sex +
0,763148	0,042648	0,141244	142	70	72	138	0,301946	y ~ Intervention + Sex +
0,73764	-0,0463	0,137947	142	70	72	138	-0,33566	y ~ Intervention + Sex +
0,480976	0,113518	0,160643	142	70	72	138	0,706649	y ~ Intervention + Sex +
0,07575	-0,26596	0,148631	142	70	72	138	-1,78936	y ~ Intervention + Sex +
0,903147	0,019605	0,160812	142	70	72	138	0,12191	y ~ Intervention + Sex +
0,062099	-0,25098	0,13344	142	70	72	138	-1,88085	y ~ Intervention + Sex +
0,863444	-0,02675	0,155245	142	70	72	138	-0,17231	y ~ Intervention + Sex +
0,231205	0,171187	0,142351	142	70	72	138	1,202563	y ~ Intervention + Sex +
0,793049	0,042155	0,160369	142	70	72	138	0,262862	y ~ Intervention + Sex +
0,341628	0,137	0,143569	142	70	72	138	0,954245	y ~ Intervention + Sex +
0,444819	-0,11313	0,147629	142	70	72	138	-0,76628	y ~ Intervention + Sex +
0,240005	-0,17954	0,152147	142	70	72	138	-1,18007	y ~ Intervention + Sex +
0,474322	0,108987	0,151913	142	70	72	138	0,71743	y ~ Intervention + Sex +
0,576962	-0,08956	0,160163	142	70	72	138	-0,55916	y ~ Intervention + Sex +
0,585049	0,077195	0,141043	142	70	72	138	0,547311	y ~ Intervention + Sex +
0,880632	-0,0194	0,128967	142	70	72	138	-0,15045	y ~ Intervention + Sex +
0,353328	-0,14453	0,155195	142	70	72	138	-0,93129	y ~ Intervention + Sex +
0,493063	0,104798	0,152483	142	70	72	138	0,687275	y ~ Intervention + Sex +
0,889743	0,022887	0,164794	142	70	72	138	0,138886	y ~ Intervention + Sex +
0,161458	0,233985	0,166214	142	70	72	138	1,407736	y ~ Intervention + Sex +
0,906762	0,020366	0,173564	142	70	72	138	0,117339	y ~ Intervention + Sex +
0,594533	-0,08378	0,15703	142	70	72	138	-0,53352	y ~ Intervention + Sex +
0,810718	0,038989	0,162482	142	70	72	138	0,239959	y ~ Intervention + Sex +
0,223179	-0,17983	0,146965	142	70	72	138	-1,22362	y ~ Intervention + Sex +
0,982215	-0,00277	0,124211	142	70	72	138	-0,02233	y ~ Intervention + Sex +
0,406662	0,132706	0,15944	142	70	72	138	0,832328	y ~ Intervention + Sex +
0,446078	-0,11049	0,144586	142	70	72	138	-0,76416	y ~ Intervention + Sex +
0,952697	0,010443	0,175729	142	70	72	138	0,059428	y ~ Intervention + Sex +
0,40752	-0,13611	0,163824	142	70	72	138	-0,8308	y ~ Intervention + Sex +
0,869445	-0,02511	0,152483	142	70	72	138	-0,16467	y ~ Intervention + Sex +
0,232674	0,198583	0,165656	142	70	72	138	1,198763	y ~ Intervention + Sex +
0,84853	-0,02788	0,145718	142	70	72	138	-0,19135	y ~ Intervention + Sex +
0,068022	-0,29381	0,159743	142	70	72	138	-1,83929	y ~ Intervention + Sex +
0,095084	0,245718	0,1462	142	70	72	138	1,680697	y ~ Intervention + Sex +
0,711834	0,059942	0,161938	142	70	72	138	0,370156	y ~ Intervention + Sex +
0,582771	-0,0826	0,149999	142	70	72	138	-0,55064	y ~ Intervention + Sex +
0,62624	0,077953	0,1597	142	70	72	138	0,488119	y ~ Intervention + Sex +
0,44603	0,117874	0,154238	142	70	72	138	0,764239	y ~ Intervention + Sex +
0,960362	-0,00759	0,152502	142	70	72	138	-0,04979	y ~ Intervention + Sex +
0,670626	0,065002	0,152515	142	70	72	138	0,426201	y ~ Intervention + Sex +
0,595445	0,076534	0,143808	142	70	72	138	0,532198	y ~ Intervention + Sex +
0,744907	-0,04889	0,149958	142	70	72	138	-0,32601	y ~ Intervention + Sex +
0,384974	0,123129	0,141277	142	70	72	138	0,871538	y ~ Intervention + Sex +
0,770365	0,046136	0,157743	142	70	72	138	0,292472	y ~ Intervention + Sex +
0,499879	-0,09881	0,146075	142	70	72	138	-0,67646	y ~ Intervention + Sex +
0,318646	-0,15322	0,15309	142	70	72	138	-1,00086	y ~ Intervention + Sex +

0,851331	0,028393	0,151211	142	70	72	138	0,187772	y ~ Intervention + Sex +
0,849043	0,028628	0,150122	142	70	72	138	0,190697	y ~ Intervention + Sex +
0,913379	0,015356	0,140912	142	70	72	138	0,108977	y ~ Intervention + Sex +
0,353442	0,164288	0,17645	142	70	72	138	0,931073	y ~ Intervention + Sex +
0,036217	-0,31051	0,146804	142	70	72	138	-2,11511	y ~ Intervention + Sex +
0,329106	0,149526	0,152674	142	70	72	138	0,979381	y ~ Intervention + Sex +
0,460087	0,119067	0,160732	142	70	72	138	0,740778	y ~ Intervention + Sex +
0,570276	0,090191	0,158506	142	70	72	138	0,569008	y ~ Intervention + Sex +
0,696896	-0,0632	0,161904	142	70	72	138	-0,39033	y ~ Intervention + Sex +
0,613704	-0,07077	0,139884	142	70	72	138	-0,50594	y ~ Intervention + Sex +
0,173567	-0,20731	0,151551	142	70	72	138	-1,3679	y ~ Intervention + Sex +
0,336337	0,150119	0,155597	142	70	72	138	0,964791	y ~ Intervention + Sex +
0,337563	0,124434	0,129304	142	70	72	138	0,962339	y ~ Intervention + Sex +
0,82802	0,033215	0,152606	142	70	72	138	0,217655	y ~ Intervention + Sex +
0,467765	-0,12101	0,166193	142	70	72	138	-0,72814	y ~ Intervention + Sex +
0,866777	0,025719	0,15303	142	70	72	138	0,168066	y ~ Intervention + Sex +
0,186162	0,191053	0,143797	142	70	72	138	1,328631	y ~ Intervention + Sex +
0,206147	0,197155	0,155215	142	70	72	138	1,27021	y ~ Intervention + Sex +
0,890976	0,021744	0,158344	142	70	72	138	0,137323	y ~ Intervention + Sex +
0,750464	0,054668	0,171554	142	70	72	138	0,318663	y ~ Intervention + Sex +
0,252059	0,168736	0,146704	142	70	72	138	1,150179	y ~ Intervention + Sex +
0,319596	0,152548	0,152717	142	70	72	138	0,998891	y ~ Intervention + Sex +
0,919242	0,01478	0,145508	142	70	72	138	0,101575	y ~ Intervention + Sex +
0,444519	0,123125	0,160573	142	70	72	138	0,766785	y ~ Intervention + Sex +
0,546727	0,100447	0,166258	142	70	72	138	0,604164	y ~ Intervention + Sex +
0,030911	-0,35673	0,163595	142	70	72	138	-2,18056	y ~ Intervention + Sex +
0,454936	-0,10152	0,135479	142	70	72	138	-0,74933	y ~ Intervention + Sex +
0,844114	-0,03187	0,161789	142	70	72	138	-0,197	y ~ Intervention + Sex +
0,412083	0,140241	0,170459	142	70	72	138	0,822726	y ~ Intervention + Sex +
0,580091	0,082312	0,148426	142	70	72	138	0,554563	y ~ Intervention + Sex +
0,792129	-0,04053	0,153498	142	70	72	138	-0,26406	y ~ Intervention + Sex +
0,391086	0,127596	0,148306	142	70	72	138	0,860354	y ~ Intervention + Sex +
0,393657	-0,13275	0,155139	142	70	72	138	-0,85568	y ~ Intervention + Sex +
0,755472	-0,04433	0,142057	142	70	72	138	-0,31205	y ~ Intervention + Sex +
0,363072	-0,14049	0,153956	142	70	72	138	-0,91255	y ~ Intervention + Sex +
0,013172	0,371708	0,147998	142	70	72	138	2,511576	y ~ Intervention + Sex +
0,34822	0,140221	0,148972	142	70	72	138	0,941254	y ~ Intervention + Sex +
0,494726	-0,1053	0,153801	142	70	72	138	-0,68463	y ~ Intervention + Sex +
0,374436	-0,14484	0,162543	142	70	72	138	-0,89108	y ~ Intervention + Sex +
0,428034	0,129185	0,162516	142	70	72	138	0,794904	y ~ Intervention + Sex +
0,500308	-0,09594	0,141962	142	70	72	138	-0,67578	y ~ Intervention + Sex +
0,503212	0,113611	0,169265	142	70	72	138	0,671205	y ~ Intervention + Sex +
0,920811	-0,01537	0,154293	142	70	72	138	-0,09959	y ~ Intervention + Sex +
0,203692	-0,17555	0,137453	142	70	72	138	-1,27715	y ~ Intervention + Sex +
0,225191	0,191498	0,157185	142	70	72	138	1,218295	y ~ Intervention + Sex +
0,07671	0,292192	0,163835	142	70	72	138	1,783455	y ~ Intervention + Sex +
0,240607	-0,17907	0,151942	142	70	72	138	-1,17855	y ~ Intervention + Sex +
0,681598	-0,06772	0,164705	142	70	72	138	-0,41115	y ~ Intervention + Sex +

0,871722	-0,02333	0,144231	142	70	72	138	-0,16177	y ~ Intervention + Sex +
0,163911	-0,22624	0,161659	142	70	72	138	-1,39949	y ~ Intervention + Sex +
0,489624	0,098202	0,141754	142	70	72	138	0,69276	y ~ Intervention + Sex +
0,012141	-0,38176	0,150206	142	70	72	138	-2,54155	y ~ Intervention + Sex +
0,351152	-0,14891	0,159172	142	70	72	138	-0,93552	y ~ Intervention + Sex +
0,945043	0,011139	0,161306	142	70	72	138	0,069058	y ~ Intervention + Sex +
0,562697	-0,0888	0,153031	142	70	72	138	-0,58024	y ~ Intervention + Sex +
0,486753	0,10073	0,144446	142	70	72	138	0,697356	y ~ Intervention + Sex +
0,475615	-0,11046	0,154421	142	70	72	138	-0,71533	y ~ Intervention + Sex +
0,560658	0,083292	0,1428	142	70	72	138	0,583278	y ~ Intervention + Sex +
0,937646	-0,01164	0,148529	142	70	72	138	-0,07837	y ~ Intervention + Sex +
0,545675	-0,08719	0,143934	142	70	72	138	-0,60575	y ~ Intervention + Sex +
0,886611	0,020488	0,143416	142	70	72	138	0,142858	y ~ Intervention + Sex +
0,635723	0,072977	0,153719	142	70	72	138	0,47474	y ~ Intervention + Sex +
0,523107	0,097097	0,151667	142	70	72	138	0,640196	y ~ Intervention + Sex +
0,58187	-0,08907	0,16138	142	70	72	138	-0,55196	y ~ Intervention + Sex +
0,07939	0,252477	0,142861	142	70	72	138	1,767292	y ~ Intervention + Sex +
0,989491	0,001906	0,144412	142	70	72	138	0,013195	y ~ Intervention + Sex +
0,894494	0,022564	0,169829	142	70	72	138	0,132865	y ~ Intervention + Sex +
0,307736	0,152706	0,149161	142	70	72	138	1,023769	y ~ Intervention + Sex +
0,29392	-0,15568	0,147763	142	70	72	138	-1,05357	y ~ Intervention + Sex +
0,424796	-0,12997	0,162364	142	70	72	138	-0,8005	y ~ Intervention + Sex +
0,333306	-0,15319	0,157785	142	70	72	138	-0,97088	y ~ Intervention + Sex +
0,099623	-0,25183	0,151906	142	70	72	138	-1,65783	y ~ Intervention + Sex +
0,090901	0,276965	0,162675	142	70	72	138	1,702566	y ~ Intervention + Sex +
0,413845	0,136892	0,167018	142	70	72	138	0,819622	y ~ Intervention + Sex +
0,12452	-0,25319	0,163823	142	70	72	138	-1,54548	y ~ Intervention + Sex +
0,713692	0,064012	0,174107	142	70	72	138	0,367658	y ~ Intervention + Sex +
0,216933	-0,20358	0,164122	142	70	72	138	-1,2404	y ~ Intervention + Sex +
0,940095	0,012016	0,159598	142	70	72	138	0,075288	y ~ Intervention + Sex +
0,698465	-0,06309	0,162518	142	70	72	138	-0,3882	y ~ Intervention + Sex +
0,529823	-0,09314	0,147877	142	70	72	138	-0,62987	y ~ Intervention + Sex +
0,929653	-0,01421	0,160688	142	70	72	138	-0,08844	y ~ Intervention + Sex +
0,54778	0,088074	0,146163	142	70	72	138	0,602575	y ~ Intervention + Sex +
0,389859	-0,13969	0,161942	142	70	72	138	-0,86259	y ~ Intervention + Sex +
0,144724	0,191715	0,130709	142	70	72	138	1,466733	y ~ Intervention + Sex +
0,590194	-0,08826	0,163502	142	70	72	138	-0,53982	y ~ Intervention + Sex +
0,08267	0,238767	0,136586	142	70	72	138	1,748106	y ~ Intervention + Sex +
0,789749	-0,03906	0,146199	142	70	72	138	-0,26716	y ~ Intervention + Sex +
0,283289	0,180179	0,167272	142	70	72	138	1,077159	y ~ Intervention + Sex +
0,332235	-0,13692	0,140714	142	70	72	138	-0,97304	y ~ Intervention + Sex +
0,480741	0,115938	0,163979	142	70	72	138	0,707029	y ~ Intervention + Sex +
0,354325	-0,13962	0,150236	142	70	72	138	-0,92936	y ~ Intervention + Sex +
0,325365	-0,15899	0,161084	142	70	72	138	-0,98701	y ~ Intervention + Sex +
0,38918	-0,14929	0,172821	142	70	72	138	-0,86383	y ~ Intervention + Sex +
0,711164	0,052334	0,14104	142	70	72	138	0,371057	y ~ Intervention + Sex +
0,820772	0,038265	0,168579	142	70	72	138	0,226984	y ~ Intervention + Sex +
0,564141	0,082678	0,143018	142	70	72	138	0,578097	y ~ Intervention + Sex +

0,655397	-0,06435	0,143878	142	70	72	138	-0,44725	y ~ Intervention + Sex +
0,195011	-0,20135	0,154624	142	70	72	138	-1,30222	y ~ Intervention + Sex +
0,249882	0,195337	0,16905	142	70	72	138	1,1555	y ~ Intervention + Sex +
0,966636	-0,00658	0,157017	142	70	72	138	-0,0419	y ~ Intervention + Sex +
0,985745	0,00262	0,146364	142	70	72	138	0,017899	y ~ Intervention + Sex +
0,373709	-0,12199	0,136693	142	70	72	138	-0,89244	y ~ Intervention + Sex +
0,299181	-0,1737	0,166683	142	70	72	138	-1,04211	y ~ Intervention + Sex +
0,568461	-0,08256	0,144411	142	70	72	138	-0,57169	y ~ Intervention + Sex +
0,162884	-0,21587	0,153869	142	70	72	138	-1,40293	y ~ Intervention + Sex +
0,502606	0,086269	0,128346	142	70	72	138	0,672159	y ~ Intervention + Sex +
0,178024	-0,21921	0,161927	142	70	72	138	-1,35377	y ~ Intervention + Sex +
0,585885	0,085485	0,15654	142	70	72	138	0,546092	y ~ Intervention + Sex +
0,043132	0,314072	0,153863	142	70	72	138	2,041247	y ~ Intervention + Sex +
0,620213	0,082859	0,16683	142	70	72	138	0,496669	y ~ Intervention + Sex +
0,049477	-0,26979	0,136129	142	70	72	138	-1,9819	y ~ Intervention + Sex +
0,490163	-0,11227	0,162263	142	70	72	138	-0,6919	y ~ Intervention + Sex +
0,048513	-0,32712	0,164342	142	70	72	138	-1,99048	y ~ Intervention + Sex +
0,828934	0,032653	0,150838	142	70	72	138	0,216479	y ~ Intervention + Sex +
0,44811	-0,12909	0,169691	142	70	72	138	-0,76074	y ~ Intervention + Sex +
0,226412	-0,18846	0,155105	142	70	72	138	-1,21508	y ~ Intervention + Sex +
0,118985	-0,25978	0,165594	142	70	72	138	-1,5688	y ~ Intervention + Sex +
0,707536	-0,05133	0,136531	142	70	72	138	-0,37594	y ~ Intervention + Sex +
0,269745	0,176657	0,159423	142	70	72	138	1,108102	y ~ Intervention + Sex +
0,493306	0,101798	0,148202	142	70	72	138	0,686888	y ~ Intervention + Sex +
0,839979	-0,02936	0,145115	142	70	72	138	-0,2023	y ~ Intervention + Sex +
0,97531	0,005685	0,183347	142	70	72	138	0,031005	y ~ Intervention + Sex +
0,034929	-0,36092	0,169432	142	70	72	138	-2,13021	y ~ Intervention + Sex +
0,628684	0,064885	0,133876	142	70	72	138	0,484662	y ~ Intervention + Sex +
0,049726	0,311584	0,157389	142	70	72	138	1,979711	y ~ Intervention + Sex +
0,836799	0,030838	0,149425	142	70	72	138	0,206379	y ~ Intervention + Sex +
0,55422	0,074672	0,125943	142	70	72	138	0,592898	y ~ Intervention + Sex +
0,146657	0,208684	0,142968	142	70	72	138	1,459656	y ~ Intervention + Sex +
0,17727	-0,1796	0,132433	142	70	72	138	-1,35614	y ~ Intervention + Sex +
0,013025	-0,35898	0,142693	142	70	72	138	-2,51573	y ~ Intervention + Sex +
0,306232	-0,11842	0,115313	142	70	72	138	-1,02697	y ~ Intervention + Sex +
0,361895	-0,13973	0,152744	142	70	72	138	-0,9148	y ~ Intervention + Sex +
0,33803	-0,17182	0,178714	142	70	72	138	-0,96141	y ~ Intervention + Sex +
0,102497	0,22842	0,13896	142	70	72	138	1,643786	y ~ Intervention + Sex +
0,134638	-0,21983	0,146078	142	70	72	138	-1,50489	y ~ Intervention + Sex +
0,559798	-0,09559	0,163521	142	70	72	138	-0,58456	y ~ Intervention + Sex +
0,866747	0,026874	0,159863	142	70	72	138	0,168104	y ~ Intervention + Sex +
0,582034	0,094768	0,171769	142	70	72	138	0,551718	y ~ Intervention + Sex +
0,325905	0,15357	0,155765	142	70	72	138	0,985905	y ~ Intervention + Sex +
0,537267	-0,09979	0,161344	142	70	72	138	-0,6185	y ~ Intervention + Sex +
0,862233	-0,02409	0,13855	142	70	72	138	-0,17386	y ~ Intervention + Sex +
0,364919	-0,13948	0,153442	142	70	72	138	-0,90903	y ~ Intervention + Sex +
0,913652	0,016342	0,150434	142	70	72	138	0,108632	y ~ Intervention + Sex +
0,819759	-0,03571	0,156436	142	70	72	138	-0,22829	y ~ Intervention + Sex +

0,904713	0,020645	0,172144	142	70	72	138	0,11993	y ~ Intervention + Sex +
0,049185	0,331347	0,166968	142	70	72	138	1,98449	y ~ Intervention + Sex +
0,629984	-0,07829	0,162153	142	70	72	138	-0,48283	y ~ Intervention + Sex +
0,871001	-0,02541	0,156186	142	70	72	138	-0,16269	y ~ Intervention + Sex +
0,630753	0,071189	0,147774	142	70	72	138	0,481741	y ~ Intervention + Sex +
0,888158	0,021912	0,155517	142	70	72	138	0,140896	y ~ Intervention + Sex +
0,986536	0,002541	0,150274	142	70	72	138	0,016906	y ~ Intervention + Sex +
0,591462	0,077865	0,144738	142	70	72	138	0,537974	y ~ Intervention + Sex +
0,692098	0,061266	0,154384	142	70	72	138	0,39684	y ~ Intervention + Sex +
0,385295	0,12617	0,144865	142	70	72	138	0,870948	y ~ Intervention + Sex +
0,174087	0,224725	0,164485	142	70	72	138	1,366238	y ~ Intervention + Sex +
0,237847	0,190686	0,160845	142	70	72	138	1,185525	y ~ Intervention + Sex +
0,39581	0,133762	0,157038	142	70	72	138	0,851783	y ~ Intervention + Sex +
0,231543	0,182159	0,151586	142	70	72	138	1,201688	y ~ Intervention + Sex +
0,426152	-0,13417	0,168099	142	70	72	138	-0,79815	y ~ Intervention + Sex +
0,133583	0,240304	0,159246	142	70	72	138	1,509009	y ~ Intervention + Sex +
0,929312	-0,01251	0,140819	142	70	72	138	-0,08887	y ~ Intervention + Sex +
0,464758	0,102147	0,139341	142	70	72	138	0,733072	y ~ Intervention + Sex +
0,111733	-0,23682	0,147952	142	70	72	138	-1,60069	y ~ Intervention + Sex +
0,395446	-0,13096	0,153626	142	70	72	138	-0,85244	y ~ Intervention + Sex +
0,101866	-0,25437	0,154457	142	70	72	138	-1,64684	y ~ Intervention + Sex +
0,540435	-0,08329	0,135717	142	70	72	138	-0,61368	y ~ Intervention + Sex +
0,134426	0,238697	0,158527	142	70	72	138	1,505715	y ~ Intervention + Sex +
0,074487	0,238048	0,132453	142	70	72	138	1,797222	y ~ Intervention + Sex +
0,010529	0,378585	0,145983	142	70	72	138	2,593358	y ~ Intervention + Sex +
0,874613	0,021979	0,139021	142	70	72	138	0,158096	y ~ Intervention + Sex +
0,523329	-0,10916	0,170607	142	70	72	138	-0,63985	y ~ Intervention + Sex +
0,088022	-0,26197	0,152478	142	70	72	138	-1,7181	y ~ Intervention + Sex +
0,591769	-0,0905	0,168362	142	70	72	138	-0,53753	y ~ Intervention + Sex +
0,600304	0,078761	0,149971	142	70	72	138	0,525176	y ~ Intervention + Sex +
0,685217	0,064536	0,158874	142	70	72	138	0,406211	y ~ Intervention + Sex +
0,275358	-0,17508	0,15987	142	70	72	138	-1,09515	y ~ Intervention + Sex +
0,207608	-0,21249	0,167827	142	70	72	138	-1,26611	y ~ Intervention + Sex +
0,936306	-0,01357	0,169465	142	70	72	138	-0,08006	y ~ Intervention + Sex +
0,155973	-0,24181	0,169512	142	70	72	138	-1,42653	y ~ Intervention + Sex +
0,59421	0,08059	0,150922	142	70	72	138	0,533987	y ~ Intervention + Sex +
0,170298	0,195336	0,141708	142	70	72	138	1,378442	y ~ Intervention + Sex +
0,112345	0,201529	0,126119	142	70	72	138	1,597935	y ~ Intervention + Sex +
0,085724	0,277284	0,160207	142	70	72	138	1,730792	y ~ Intervention + Sex +
0,097837	-0,22651	0,135904	142	70	72	138	-1,66672	y ~ Intervention + Sex +
0,66708	-0,07691	0,17842	142	70	72	138	-0,43108	y ~ Intervention + Sex +
0,496029	-0,12285	0,179982	142	70	72	138	-0,68256	y ~ Intervention + Sex +
0,935128	0,012281	0,150608	142	70	72	138	0,081544	y ~ Intervention + Sex +
0,041344	-0,31624	0,153564	142	70	72	138	-2,05931	y ~ Intervention + Sex +
0,67764	-0,06308	0,151426	142	70	72	138	-0,41657	y ~ Intervention + Sex +
0,838849	0,034834	0,170967	142	70	72	138	0,20375	y ~ Intervention + Sex +
0,785859	-0,03627	0,133234	142	70	72	138	-0,27222	y ~ Intervention + Sex +
0,262661	-0,17329	0,15407	142	70	72	138	-1,12472	y ~ Intervention + Sex +

0,355711	0,139373	0,1504	142	70	72	138	0,926678	y ~ Intervention + Sex +
0,419912	-0,13574	0,167786	142	70	72	138	-0,80899	y ~ Intervention + Sex +
0,426657	-0,12709	0,15941	142	70	72	138	-0,79728	y ~ Intervention + Sex +
0,874812	0,023354	0,14796	142	70	72	138	0,157843	y ~ Intervention + Sex +
0,057219	0,264924	0,138149	142	70	72	138	1,917671	y ~ Intervention + Sex +
0,490993	-0,1088	0,157547	142	70	72	138	-0,69057	y ~ Intervention + Sex +
0,177222	-0,22404	0,165185	142	70	72	138	-1,35629	y ~ Intervention + Sex +
0,953272	0,009604	0,163606	142	70	72	138	0,058705	y ~ Intervention + Sex +
0,405924	-0,12648	0,151722	142	70	72	138	-0,83364	y ~ Intervention + Sex +
0,506341	0,098776	0,148249	142	70	72	138	0,666286	y ~ Intervention + Sex +
0,460391	-0,11578	0,156396	142	70	72	138	-0,74027	y ~ Intervention + Sex +
0,086934	0,267189	0,154976	142	70	72	138	1,724071	y ~ Intervention + Sex +
0,839394	-0,03321	0,163536	142	70	72	138	-0,20305	y ~ Intervention + Sex +
0,24937	0,183188	0,158363	142	70	72	138	1,156756	y ~ Intervention + Sex +
0,461974	-0,11075	0,150142	142	70	72	138	-0,73766	y ~ Intervention + Sex +
0,244854	0,181391	0,155311	142	70	72	138	1,167917	y ~ Intervention + Sex +
0,314764	-0,15274	0,15139	142	70	72	138	-1,00895	y ~ Intervention + Sex +
0,680885	-0,06985	0,169475	142	70	72	138	-0,41213	y ~ Intervention + Sex +
0,52672	0,09957	0,156895	142	70	72	138	0,634631	y ~ Intervention + Sex +
0,003764	0,483493	0,164042	142	70	72	138	2,947368	y ~ Intervention + Sex +
0,589925	-0,07867	0,14563	142	70	72	138	-0,54021	y ~ Intervention + Sex +
0,014515	-0,35306	0,14262	142	70	72	138	-2,47553	y ~ Intervention + Sex +
0,419046	0,123992	0,152982	142	70	72	138	0,810504	y ~ Intervention + Sex +
0,080258	0,215189	0,122117	142	70	72	138	1,762151	y ~ Intervention + Sex +
0,688426	0,059409	0,147844	142	70	72	138	0,401836	y ~ Intervention + Sex +
0,694781	0,06252	0,159003	142	70	72	138	0,393197	y ~ Intervention + Sex +
0,499482	0,121679	0,179708	142	70	72	138	0,67709	y ~ Intervention + Sex +
0,576278	-0,09418	0,168123	142	70	72	138	-0,56016	y ~ Intervention + Sex +
0,855844	-0,03036	0,166796	142	70	72	138	-0,18201	y ~ Intervention + Sex +
0,197936	-0,21733	0,167993	142	70	72	138	-1,29368	y ~ Intervention + Sex +
0,362276	-0,14473	0,158332	142	70	72	138	-0,91407	y ~ Intervention + Sex +
0,150836	-0,24755	0,17136	142	70	72	138	-1,4446	y ~ Intervention + Sex +
0,338182	0,150101	0,156176	142	70	72	138	0,961102	y ~ Intervention + Sex +
0,680531	-0,06606	0,1601	142	70	72	138	-0,41261	y ~ Intervention + Sex +
0,680805	0,068245	0,165548	142	70	72	138	0,412238	y ~ Intervention + Sex +
0,208598	-0,17932	0,141939	142	70	72	138	-1,26334	y ~ Intervention + Sex +
0,243679	-0,20222	0,172711	142	70	72	138	-1,17085	y ~ Intervention + Sex +
0,204123	-0,20358	0,159555	142	70	72	138	-1,27593	y ~ Intervention + Sex +
0,916055	0,015352	0,145378	142	70	72	138	0,105598	y ~ Intervention + Sex +
0,321366	-0,16856	0,169368	142	70	72	138	-0,99523	y ~ Intervention + Sex +
0,219634	-0,20054	0,162633	142	70	72	138	-1,2331	y ~ Intervention + Sex +
0,266819	-0,16616	0,149034	142	70	72	138	-1,11493	y ~ Intervention + Sex +
0,867546	-0,02544	0,152238	142	70	72	138	-0,16709	y ~ Intervention + Sex +
0,9263	0,014189	0,153107	142	70	72	138	0,092671	y ~ Intervention + Sex +
0,824034	0,034411	0,154461	142	70	72	138	0,222783	y ~ Intervention + Sex +
0,586444	0,093403	0,171295	142	70	72	138	0,545276	y ~ Intervention + Sex +
0,824958	-0,03003	0,135506	142	70	72	138	-0,22159	y ~ Intervention + Sex +
0,830702	0,03267	0,152515	142	70	72	138	0,214207	y ~ Intervention + Sex +

0,98684	0,00254	0,153737	142	70	72	138	0,016524	y ~ Intervention + Sex +
0,118052	0,24102	0,153241	142	70	72	138	1,572813	y ~ Intervention + Sex +
0,715537	-0,05513	0,150975	142	70	72	138	-0,36518	y ~ Intervention + Sex +
0,0864	-0,24659	0,142781	142	70	72	138	-1,72703	y ~ Intervention + Sex +
0,137672	0,212797	0,142513	142	70	72	138	1,493182	y ~ Intervention + Sex +
0,620653	0,073104	0,147374	142	70	72	138	0,496043	y ~ Intervention + Sex +
0,510167	-0,1024	0,155078	142	70	72	138	-0,66029	y ~ Intervention + Sex +
0,13027	-0,2267	0,148937	142	70	72	138	-1,52211	y ~ Intervention + Sex +
0,326188	-0,15397	0,156265	142	70	72	138	-0,98533	y ~ Intervention + Sex +
0,438712	-0,11676	0,150343	142	70	72	138	-0,77662	y ~ Intervention + Sex +
0,051698	0,296196	0,150916	142	70	72	138	1,962651	y ~ Intervention + Sex +
0,280236	-0,17462	0,161079	142	70	72	138	-1,08404	y ~ Intervention + Sex +
0,991842	0,001727	0,168563	142	70	72	138	0,010243	y ~ Intervention + Sex +
0,836659	0,029762	0,144084	142	70	72	138	0,206558	y ~ Intervention + Sex +
0,803111	-0,04266	0,170761	142	70	72	138	-0,2498	y ~ Intervention + Sex +
0,38769	0,142579	0,164535	142	70	72	138	0,866555	y ~ Intervention + Sex +
0,13511	0,194252	0,129239	142	70	72	138	1,503053	y ~ Intervention + Sex +
0,765761	0,041852	0,140202	142	70	72	138	0,298513	y ~ Intervention + Sex +
0,218011	0,174668	0,141148	142	70	72	138	1,237478	y ~ Intervention + Sex +
0,227029	-0,1997	0,164568	142	70	72	138	-1,21346	y ~ Intervention + Sex +
0,563777	0,095093	0,164339	142	70	72	138	0,578638	y ~ Intervention + Sex +
0,882499	-0,02391	0,161446	142	70	72	138	-0,14808	y ~ Intervention + Sex +
0,655546	0,070509	0,157724	142	70	72	138	0,447041	y ~ Intervention + Sex +
0,068703	0,297717	0,162269	142	70	72	138	1,834706	y ~ Intervention + Sex +
0,310835	0,15181	0,149242	142	70	72	138	1,017207	y ~ Intervention + Sex +
0,034935	-0,29732	0,13958	142	70	72	138	-2,13013	y ~ Intervention + Sex +
0,207975	-0,20593	0,162778	142	70	72	138	-1,26508	y ~ Intervention + Sex +
0,494803	0,089487	0,130732	142	70	72	138	0,684507	y ~ Intervention + Sex +
0,915901	0,01533	0,144905	142	70	72	138	0,105792	y ~ Intervention + Sex +
0,174892	-0,23137	0,169669	142	70	72	138	-1,36367	y ~ Intervention + Sex +
0,596125	0,089244	0,168001	142	70	72	138	0,531214	y ~ Intervention + Sex +
0,315881	-0,15661	0,155584	142	70	72	138	-1,00662	y ~ Intervention + Sex +
0,481662	-0,10891	0,154364	142	70	72	138	-0,70554	y ~ Intervention + Sex +
0,421301	0,124748	0,154665	142	70	72	138	0,806572	y ~ Intervention + Sex +
0,285741	0,157249	0,146733	142	70	72	138	1,071666	y ~ Intervention + Sex +
0,427354	-0,1299	0,163178	142	70	72	138	-0,79608	y ~ Intervention + Sex +
0,965107	-0,00632	0,14421	142	70	72	138	-0,04382	y ~ Intervention + Sex +
0,761642	-0,04961	0,163227	142	70	72	138	-0,30393	y ~ Intervention + Sex +
0,609855	0,085461	0,167096	142	70	72	138	0,511448	y ~ Intervention + Sex +
0,103915	0,246988	0,150881	142	70	72	138	1,636971	y ~ Intervention + Sex +
0,337709	0,122114	0,126931	142	70	72	138	0,962047	y ~ Intervention + Sex +
0,312159	-0,144	0,141954	142	70	72	138	-1,01442	y ~ Intervention + Sex +
0,607521	-0,08169	0,158684	142	70	72	138	-0,51479	y ~ Intervention + Sex +
0,142658	-0,23867	0,161877	142	70	72	138	-1,47438	y ~ Intervention + Sex +
0,023507	0,345904	0,151015	142	70	72	138	2,29052	y ~ Intervention + Sex +
0,281419	0,179431	0,16593	142	70	72	138	1,081368	y ~ Intervention + Sex +
0,0772	0,262548	0,14746	142	70	72	138	1,780467	y ~ Intervention + Sex +
0,9114	-0,01701	0,152609	142	70	72	138	-0,11148	y ~ Intervention + Sex +

0,595323	-0,08801	0,165315	142	70	72	138	-0,53237	y ~ Intervention + Sex +
0,277459	-0,16842	0,154463	142	70	72	138	-1,09035	y ~ Intervention + Sex +
0,183146	0,202268	0,151189	142	70	72	138	1,337851	y ~ Intervention + Sex +
0,783668	0,040721	0,148035	142	70	72	138	0,275077	y ~ Intervention + Sex +
0,490493	0,097444	0,140943	142	70	72	138	0,691373	y ~ Intervention + Sex +
0,599549	-0,08294	0,157598	142	70	72	138	-0,52626	y ~ Intervention + Sex +
0,05386	0,252357	0,129775	142	70	72	138	1,944573	y ~ Intervention + Sex +
0,125673	0,27505	0,178519	142	70	72	138	1,54073	y ~ Intervention + Sex +
0,594742	-0,08382	0,157197	142	70	72	138	-0,53322	y ~ Intervention + Sex +
0,249428	-0,17734	0,153326	142	70	72	138	-1,15661	y ~ Intervention + Sex +
0,574017	-0,09505	0,168683	142	70	72	138	-0,56349	y ~ Intervention + Sex +
0,422295	0,139651	0,173513	142	70	72	138	0,804842	y ~ Intervention + Sex +
0,729109	-0,05571	0,160535	142	70	72	138	-0,34701	y ~ Intervention + Sex +
0,899559	-0,01972	0,155975	142	70	72	138	-0,12645	y ~ Intervention + Sex +
0,230776	-0,18981	0,157693	142	70	72	138	-1,20368	y ~ Intervention + Sex +
0,113087	-0,24335	0,15261	142	70	72	138	-1,59461	y ~ Intervention + Sex +
0,007435	-0,40137	0,147733	142	70	72	138	-2,71686	y ~ Intervention + Sex +
0,716752	0,059776	0,164424	142	70	72	138	0,363547	y ~ Intervention + Sex +
0,873364	-0,02312	0,144812	142	70	72	138	-0,15968	y ~ Intervention + Sex +
0,524351	-0,10705	0,167714	142	70	72	138	-0,63828	y ~ Intervention + Sex +
0,95135	-0,0093	0,152079	142	70	72	138	-0,06112	y ~ Intervention + Sex +
0,536425	-0,09926	0,160157	142	70	72	138	-0,61978	y ~ Intervention + Sex +
0,55163	0,100551	0,168489	142	70	72	138	0,596784	y ~ Intervention + Sex +
0,272762	-0,1811	0,164466	142	70	72	138	-1,10112	y ~ Intervention + Sex +
0,738728	0,057375	0,171674	142	70	72	138	0,33421	y ~ Intervention + Sex +
0,936488	0,012804	0,16039	142	70	72	138	0,07983	y ~ Intervention + Sex +
0,099294	-0,24405	0,147064	142	70	72	138	-1,65946	y ~ Intervention + Sex +
0,380836	-0,14431	0,164145	142	70	72	138	-0,87917	y ~ Intervention + Sex +
0,7272	-0,05616	0,160661	142	70	72	138	-0,34956	y ~ Intervention + Sex +
0,709274	0,057917	0,155023	142	70	72	138	0,373601	y ~ Intervention + Sex +
0,286404	0,186782	0,174532	142	70	72	138	1,070186	y ~ Intervention + Sex +
0,29182	-0,15357	0,14513	142	70	72	138	-1,05818	y ~ Intervention + Sex +
0,332585	0,141731	0,145764	142	70	72	138	0,972335	y ~ Intervention + Sex +
0,665745	0,074205	0,171403	142	70	72	138	0,432926	y ~ Intervention + Sex +
0,788008	0,041854	0,155346	142	70	72	138	0,269421	y ~ Intervention + Sex +
0,603356	0,085288	0,16377	142	70	72	138	0,520779	y ~ Intervention + Sex +
0,146534	0,220161	0,150784	142	70	72	138	1,460106	y ~ Intervention + Sex +
0,747516	0,055842	0,173121	142	70	72	138	0,322561	y ~ Intervention + Sex +
0,542353	0,087517	0,143288	142	70	72	138	0,610776	y ~ Intervention + Sex +
0,843679	-0,02931	0,148349	142	70	72	138	-0,19756	y ~ Intervention + Sex +
0,749596	0,04453	0,139239	142	70	72	138	0,319811	y ~ Intervention + Sex +
0,892314	0,022013	0,162305	142	70	72	138	0,135627	y ~ Intervention + Sex +
0,125588	-0,23841	0,154701	142	70	72	138	-1,54108	y ~ Intervention + Sex +
0,295269	0,180784	0,172073	142	70	72	138	1,05062	y ~ Intervention + Sex +
0,0961	0,27699	0,165317	142	70	72	138	1,675503	y ~ Intervention + Sex +
0,416207	-0,12865	0,157756	142	70	72	138	-0,81547	y ~ Intervention + Sex +
0,4029	0,141219	0,168311	142	70	72	138	0,839036	y ~ Intervention + Sex +
0,190329	0,194714	0,14795	142	70	72	138	1,316081	y ~ Intervention + Sex +

0,310835	0,15181	0,149242	142	70	72	138	1,017207	y ~ Intervention + Sex +
0,609215	0,08246	0,160941	142	70	72	138	0,512364	y ~ Intervention + Sex +
0,264103	-0,18667	0,166474	142	70	72	138	-1,12131	y ~ Intervention + Sex +
0,688152	0,06225	0,154771	142	70	72	138	0,402209	y ~ Intervention + Sex +
0,043493	0,346757	0,170173	142	70	72	138	2,037674	y ~ Intervention + Sex +
0,244618	-0,16296	0,139456	142	70	72	138	-1,1685	y ~ Intervention + Sex +
0,000735	-0,50377	0,145865	142	70	72	138	-3,45368	y ~ Intervention + Sex +
0,392739	-0,1285	0,149877	142	70	72	138	-0,85735	y ~ Intervention + Sex +
0,767909	0,036439	0,123232	142	70	72	138	0,295693	y ~ Intervention + Sex +
0,189273	0,20277	0,153702	142	70	72	138	1,319242	y ~ Intervention + Sex +
0,357955	0,15454	0,16755	142	70	72	138	0,922352	y ~ Intervention + Sex +
0,845557	0,030113	0,154302	142	70	72	138	0,195157	y ~ Intervention + Sex +
0,782473	0,042347	0,153078	142	70	72	138	0,276637	y ~ Intervention + Sex +
0,57196	0,089031	0,157155	142	70	72	138	0,566521	y ~ Intervention + Sex +
0,311504	-0,16289	0,160354	142	70	72	138	-1,0158	y ~ Intervention + Sex +
0,751752	-0,04451	0,14044	142	70	72	138	-0,31696	y ~ Intervention + Sex +
0,717084	-0,05584	0,153776	142	70	72	138	-0,3631	y ~ Intervention + Sex +
0,407546	0,121333	0,146051	142	70	72	138	0,830757	y ~ Intervention + Sex +
0,910581	-0,01763	0,156682	142	70	72	138	-0,11251	y ~ Intervention + Sex +
0,803675	0,035946	0,14432	142	70	72	138	0,249073	y ~ Intervention + Sex +
0,772229	0,036634	0,126312	142	70	72	138	0,29003	y ~ Intervention + Sex +
0,298812	-0,14902	0,142889	142	70	72	138	-1,04291	y ~ Intervention + Sex +
0,267035	-0,19323	0,173392	142	70	72	138	-1,11442	y ~ Intervention + Sex +
0,686458	-0,05614	0,138776	142	70	72	138	-0,40452	y ~ Intervention + Sex +
0,299998	-0,14947	0,143671	142	70	72	138	-1,04035	y ~ Intervention + Sex +
0,150959	0,232016	0,160658	142	70	72	138	1,444166	y ~ Intervention + Sex +
0,851289	0,029824	0,158785	142	70	72	138	0,187826	y ~ Intervention + Sex +
0,845699	-0,03127	0,160378	142	70	72	138	-0,19497	y ~ Intervention + Sex +
0,553471	-0,09339	0,15721	142	70	72	138	-0,59402	y ~ Intervention + Sex +
0,56886	0,092577	0,162102	142	70	72	138	0,571102	y ~ Intervention + Sex +
0,344433	-0,15043	0,158569	142	70	72	138	-0,9487	y ~ Intervention + Sex +
0,785431	0,046794	0,171544	142	70	72	138	0,272779	y ~ Intervention + Sex +
0,858372	0,029	0,162209	142	70	72	138	0,178781	y ~ Intervention + Sex +
0,239699	-0,19078	0,16156	142	70	72	138	-1,18084	y ~ Intervention + Sex +
0,278648	-0,16415	0,150924	142	70	72	138	-1,08765	y ~ Intervention + Sex +
0,871304	0,02323	0,143127	142	70	72	138	0,162304	y ~ Intervention + Sex +
0,709867	0,062955	0,16887	142	70	72	138	0,372803	y ~ Intervention + Sex +
0,360737	0,131817	0,143747	142	70	72	138	0,91701	y ~ Intervention + Sex +
0,132721	0,224997	0,148769	142	70	72	138	1,512392	y ~ Intervention + Sex +
0,589062	-0,07546	0,139354	142	70	72	138	-0,54146	y ~ Intervention + Sex +
0,650326	-0,07125	0,156831	142	70	72	138	-0,4543	y ~ Intervention + Sex +
0,023276	-0,35608	0,155193	142	70	72	138	-2,29442	y ~ Intervention + Sex +
0,247904	0,173562	0,149575	142	70	72	138	1,160364	y ~ Intervention + Sex +
0,930295	0,015553	0,177473	142	70	72	138	0,087634	y ~ Intervention + Sex +
0,209632	-0,16161	0,128218	142	70	72	138	-1,26046	y ~ Intervention + Sex +
0,111346	-0,22644	0,141311	142	70	72	138	-1,60244	y ~ Intervention + Sex +
0,513191	-0,09857	0,150362	142	70	72	138	-0,65557	y ~ Intervention + Sex +
0,439755	0,129305	0,166878	142	70	72	138	0,774847	y ~ Intervention + Sex +

0,936147	0,012757	0,15895	142	70	72	138	0,080259	y ~ Intervention + Sex +
0,534347	0,11054	0,177447	142	70	72	138	0,622948	y ~ Intervention + Sex +
0,843941	0,034381	0,174321	142	70	72	138	0,197225	y ~ Intervention + Sex +
0,329753	0,133048	0,136031	142	70	72	138	0,978065	y ~ Intervention + Sex +
0,877793	0,022997	0,149277	142	70	72	138	0,154053	y ~ Intervention + Sex +
0,217621	0,185587	0,149844	142	70	72	138	1,238534	y ~ Intervention + Sex +
0,815168	-0,04274	0,182475	142	70	72	138	-0,23421	y ~ Intervention + Sex +
0,259488	-0,14523	0,128268	142	70	72	138	-1,13226	y ~ Intervention + Sex +
0,965455	0,006495	0,149688	142	70	72	138	0,043388	y ~ Intervention + Sex +
0,482873	-0,09849	0,139979	142	70	72	138	-0,70359	y ~ Intervention + Sex +
0,352282	0,149007	0,159652	142	70	72	138	0,933326	y ~ Intervention + Sex +
0,587614	-0,08557	0,15742	142	70	72	138	-0,54357	y ~ Intervention + Sex +
0,07375	0,283186	0,157163	142	70	72	138	1,80186	y ~ Intervention + Sex +
0,82599	-0,03235	0,146882	142	70	72	138	-0,22027	y ~ Intervention + Sex +
0,555558	0,093955	0,159004	142	70	72	138	0,590894	y ~ Intervention + Sex +
0,879943	-0,02047	0,135291	142	70	72	138	-0,15132	y ~ Intervention + Sex +
0,363162	0,15594	0,170916	142	70	72	138	0,912377	y ~ Intervention + Sex +
0,46597	-0,11484	0,157089	142	70	72	138	-0,73108	y ~ Intervention + Sex +
0,598266	0,085806	0,162475	142	70	72	138	0,528118	y ~ Intervention + Sex +
0,350694	-0,14316	0,152876	142	70	72	138	-0,93642	y ~ Intervention + Sex +
0,064138	-0,25432	0,136278	142	70	72	138	-1,86619	y ~ Intervention + Sex +
0,682195	0,059513	0,145033	142	70	72	138	0,410338	y ~ Intervention + Sex +
0,53374	-0,10076	0,161504	142	70	72	138	-0,62387	y ~ Intervention + Sex +
0,457036	0,118452	0,158819	142	70	72	138	0,745834	y ~ Intervention + Sex +
0,289246	0,162511	0,152755	142	70	72	138	1,063869	y ~ Intervention + Sex +
0,025528	0,354285	0,156916	142	70	72	138	2,2578	y ~ Intervention + Sex +
0,013769	-0,34468	0,138138	142	70	72	138	-2,49517	y ~ Intervention + Sex +
0,630886	-0,07104	0,147523	142	70	72	138	-0,48155	y ~ Intervention + Sex +
0,38956	-0,1332	0,154321	142	70	72	138	-0,86314	y ~ Intervention + Sex +
0,422436	0,126426	0,157129	142	70	72	138	0,804598	y ~ Intervention + Sex +
0,086527	0,305149	0,176762	142	70	72	138	1,726327	y ~ Intervention + Sex +
0,002415	-0,45299	0,146556	142	70	72	138	-3,09087	y ~ Intervention + Sex +
0,402205	-0,13023	0,154978	142	70	72	138	-0,84028	y ~ Intervention + Sex +
0,867802	0,026006	0,155949	142	70	72	138	0,166762	y ~ Intervention + Sex +
0,050565	-0,30872	0,156523	142	70	72	138	-1,97238	y ~ Intervention + Sex +
0,332938	0,164541	0,169347	142	70	72	138	0,971624	y ~ Intervention + Sex +
0,498055	-0,09806	0,14434	142	70	72	138	-0,67935	y ~ Intervention + Sex +
0,130165	0,209748	0,137763	142	70	72	138	1,522527	y ~ Intervention + Sex +
0,234208	-0,16857	0,141085	142	70	72	138	-1,19482	y ~ Intervention + Sex +
0,504933	-0,09536	0,142652	142	70	72	138	-0,6685	y ~ Intervention + Sex +
0,899942	0,021931	0,174106	142	70	72	138	0,125966	y ~ Intervention + Sex +
0,920409	0,01635	0,163331	142	70	72	138	0,100101	y ~ Intervention + Sex +
0,509453	-0,1044	0,157846	142	70	72	138	-0,66141	y ~ Intervention + Sex +
0,323935	0,151557	0,153096	142	70	72	138	0,989942	y ~ Intervention + Sex +
0,098698	-0,24548	0,147666	142	70	72	138	-1,66242	y ~ Intervention + Sex +
0,706962	-0,05443	0,144477	142	70	72	138	-0,37672	y ~ Intervention + Sex +
0,444404	0,135951	0,177255	142	70	72	138	0,766979	y ~ Intervention + Sex +
0,372005	0,141294	0,157757	142	70	72	138	0,895639	y ~ Intervention + Sex +

0,603865	0,084998	0,163443	142	70	72	138	0,520046	y ~ Intervention + Sex +
0,004877	-0,43451	0,151861	142	70	72	138	-2,86124	y ~ Intervention + Sex +
0,216448	-0,18826	0,151616	142	70	72	138	-1,24171	y ~ Intervention + Sex +
0,133675	0,233074	0,154492	142	70	72	138	1,508646	y ~ Intervention + Sex +
0,030916	-0,35469	0,162667	142	70	72	138	-2,18049	y ~ Intervention + Sex +
0,343333	-0,14581	0,153341	142	70	72	138	-0,95087	y ~ Intervention + Sex +
0,063671	0,291169	0,155746	142	70	72	138	1,869512	y ~ Intervention + Sex +
0,015069	0,355418	0,144391	142	70	72	138	2,461507	y ~ Intervention + Sex +
0,956689	0,009238	0,169787	142	70	72	138	0,054408	y ~ Intervention + Sex +
0,247806	-0,18991	0,163633	142	70	72	138	-1,16061	y ~ Intervention + Sex +
0,197902	-0,20716	0,16012	142	70	72	138	-1,29378	y ~ Intervention + Sex +
0,044419	-0,30303	0,149376	142	70	72	138	-2,02863	y ~ Intervention + Sex +
0,718658	0,053154	0,147244	142	70	72	138	0,360991	y ~ Intervention + Sex +
0,579072	-0,08455	0,152058	142	70	72	138	-0,55606	y ~ Intervention + Sex +
0,650806	-0,06644	0,146454	142	70	72	138	-0,45363	y ~ Intervention + Sex +
0,453708	0,120095	0,159834	142	70	72	138	0,751373	y ~ Intervention + Sex +
0,843378	0,037117	0,187512	142	70	72	138	0,197946	y ~ Intervention + Sex +
0,227919	0,18079	0,149275	142	70	72	138	1,21112	y ~ Intervention + Sex +
0,684763	0,067798	0,16665	142	70	72	138	0,40683	y ~ Intervention + Sex +
0,727379	-0,05416	0,155044	142	70	72	138	-0,34932	y ~ Intervention + Sex +
0,797706	-0,03794	0,147721	142	70	72	138	-0,25681	y ~ Intervention + Sex +
0,081052	0,284335	0,161785	142	70	72	138	1,757489	y ~ Intervention + Sex +
0,94805	-0,01089	0,166776	142	70	72	138	-0,06527	y ~ Intervention + Sex +
0,268122	0,180299	0,162156	142	70	72	138	1,111883	y ~ Intervention + Sex +
0,647433	-0,07428	0,162062	142	70	72	138	-0,45833	y ~ Intervention + Sex +
0,82016	0,037849	0,166169	142	70	72	138	0,227772	y ~ Intervention + Sex +
0,558081	-0,08832	0,15043	142	70	72	138	-0,58712	y ~ Intervention + Sex +
0,736567	0,054516	0,161728	142	70	72	138	0,337082	y ~ Intervention + Sex +
0,94908	-0,01045	0,163286	142	70	72	138	-0,06398	y ~ Intervention + Sex +
0,085327	-0,27457	0,158433	142	70	72	138	-1,73302	y ~ Intervention + Sex +
0,067919	-0,26918	0,146294	142	70	72	138	-1,83999	y ~ Intervention + Sex +
0,444605	0,134304	0,175186	142	70	72	138	0,76664	y ~ Intervention + Sex +
0,635636	0,070673	0,148828	142	70	72	138	0,474861	y ~ Intervention + Sex +
0,71191	0,05873	0,158708	142	70	72	138	0,370053	y ~ Intervention + Sex +
0,875099	-0,02631	0,16708	142	70	72	138	-0,15748	y ~ Intervention + Sex +
0,793662	-0,03733	0,142429	142	70	72	138	-0,26207	y ~ Intervention + Sex +
0,577198	0,092446	0,165434	142	70	72	138	0,558809	y ~ Intervention + Sex +
0,963676	0,006607	0,144808	142	70	72	138	0,045624	y ~ Intervention + Sex +
0,426808	-0,13292	0,166766	142	70	72	138	-0,79702	y ~ Intervention + Sex +
0,647766	-0,07693	0,168019	142	70	72	138	-0,45787	y ~ Intervention + Sex +
0,732767	-0,05822	0,17017	142	70	72	138	-0,34214	y ~ Intervention + Sex +
0,704274	0,058339	0,153383	142	70	72	138	0,380345	y ~ Intervention + Sex +
0,551511	-0,08707	0,14585	142	70	72	138	-0,59696	y ~ Intervention + Sex +
0,163976	0,180664	0,129113	142	70	72	138	1,39927	y ~ Intervention + Sex +
0,954897	-0,01014	0,178988	142	70	72	138	-0,05666	y ~ Intervention + Sex +
0,291036	-0,15346	0,144786	142	70	72	138	-1,05991	y ~ Intervention + Sex +
0,959971	0,008193	0,162936	142	70	72	138	0,050281	y ~ Intervention + Sex +
0,491651	-0,10478	0,151962	142	70	72	138	-0,68952	y ~ Intervention + Sex +

0,220888	0,196693	0,159947	142	70	72	138	1,229738	y ~ Intervention + Sex +
0,671497	0,064959	0,152845	142	70	72	138	0,425002	y ~ Intervention + Sex +
0,33643	0,147163	0,152563	142	70	72	138	0,964606	y ~ Intervention + Sex +
0,524189	0,102536	0,160582	142	70	72	138	0,638527	y ~ Intervention + Sex +
0,705931	0,058598	0,154977	142	70	72	138	0,378108	y ~ Intervention + Sex +
0,210878	0,185869	0,147867	142	70	72	138	1,256996	y ~ Intervention + Sex +
0,217303	-0,20808	0,167891	142	70	72	138	-1,2394	y ~ Intervention + Sex +
0,702675	-0,06165	0,161178	142	70	72	138	-0,38251	y ~ Intervention + Sex +
0,508622	-0,09512	0,14353	142	70	72	138	-0,66271	y ~ Intervention + Sex +
0,941014	-0,01162	0,156801	142	70	72	138	-0,07413	y ~ Intervention + Sex +
0,733827	0,050569	0,148414	142	70	72	138	0,340727	y ~ Intervention + Sex +
0,128288	0,231009	0,150979	142	70	72	138	1,530072	y ~ Intervention + Sex +
0,639281	0,066554	0,141682	142	70	72	138	0,469742	y ~ Intervention + Sex +
0,278288	0,18778	0,172518	142	70	72	138	1,088464	y ~ Intervention + Sex +
0,426978	-0,11497	0,144304	142	70	72	138	-0,79673	y ~ Intervention + Sex +
0,964134	0,007482	0,166101	142	70	72	138	0,045048	y ~ Intervention + Sex +
0,879081	-0,02611	0,171335	142	70	72	138	-0,15242	y ~ Intervention + Sex +
0,301221	0,164853	0,158863	142	70	72	138	1,037708	y ~ Intervention + Sex +
0,448868	-0,11255	0,148194	142	70	72	138	-0,75947	y ~ Intervention + Sex +
0,814407	0,036123	0,15359	142	70	72	138	0,235193	y ~ Intervention + Sex +
0,01208	-0,4175	0,1641	139	68	71	135	-2,54417	y ~ Intervention + Sex +
0,025474	-0,36409	0,161106	135	65	70	131	-2,25997	y ~ Intervention + Sex +
0,756939	0,049375	0,159211	141	69	72	137	0,310123	y ~ Intervention + Sex +
0,30563	-0,17339	0,168618	140	69	71	136	-1,02831	y ~ Intervention + Sex +
0,012561	0,49606	0,194369	86	42	44	82	2,552152	y ~ Intervention + Sex +
0,020243	0,386427	0,164494	141	69	72	137	2,34919	y ~ Intervention + Sex +
0,868813	0,027618	0,166903	141	69	72	137	0,165476	y ~ Intervention + Sex +
0,50849	-0,09815	0,148057	141	69	72	137	-0,66293	y ~ Intervention + Sex +
0,000837	0,559172	0,163704	141	69	72	137	3,415753	y ~ Intervention + Sex +
0,416379	-0,11699	0,143517	141	69	72	137	-0,81519	y ~ Intervention + Sex +
0,255718	-0,18456	0,161708	141	69	72	137	-1,14134	y ~ Intervention + Sex +
0,55906	0,089399	0,152644	141	69	72	137	0,585672	y ~ Intervention + Sex +
0,332996	0,132341	0,136219	141	69	72	137	0,971531	y ~ Intervention + Sex +
0,826894	0,033363	0,152268	141	69	72	137	0,219106	y ~ Intervention + Sex +
0,776404	-0,04159	0,14615	141	69	72	137	-0,28457	y ~ Intervention + Sex +
0,762331	-0,05151	0,169976	141	69	72	137	-0,30302	y ~ Intervention + Sex +
0,018903	0,32949	0,138695	141	69	72	137	2,375646	y ~ Intervention + Sex +
0,010679	-0,40953	0,158213	141	69	72	137	-2,5885	y ~ Intervention + Sex +
0,323268	0,141605	0,142843	141	69	72	137	0,991339	y ~ Intervention + Sex +
0,237276	0,191743	0,161534	141	69	72	137	1,187014	y ~ Intervention + Sex +
0,85373	-0,02835	0,153464	141	69	72	137	-0,18471	y ~ Intervention + Sex +
0,898675	0,022818	0,178867	141	69	72	137	0,127572	y ~ Intervention + Sex +
0,031084	0,320875	0,147296	141	69	72	137	2,178438	y ~ Intervention + Sex +
0,657651	-0,06306	0,141997	141	69	72	137	-0,44413	y ~ Intervention + Sex +
0,636839	-0,07797	0,164769	141	69	72	137	-0,47318	y ~ Intervention + Sex +
0,317284	-0,1627	0,162098	141	69	72	137	-1,00372	y ~ Intervention + Sex +
0,051634	-0,31325	0,159552	141	69	72	137	-1,96332	y ~ Intervention + Sex +
0,665352	-0,06138	0,14161	141	69	72	137	-0,43347	y ~ Intervention + Sex +

0,015597	-0,37292	0,152287	141	69	72	137	-2,44879	y ~ Intervention + Sex +
0,598391	0,079465	0,150517	141	69	72	137	0,527946	y ~ Intervention + Sex +
0,264878	0,177959	0,15896	141	69	72	137	1,119518	y ~ Intervention + Sex +
0,044837	-0,31165	0,153919	141	69	72	137	-2,02474	y ~ Intervention + Sex +
0,915518	0,019698	0,185348	141	69	72	137	0,106276	y ~ Intervention + Sex +
0,227749	0,195294	0,161186	141	69	72	137	1,211606	y ~ Intervention + Sex +
0,000148	-0,61155	0,156648	141	69	72	137	-3,90394	y ~ Intervention + Sex +
0,850683	-0,02704	0,143374	141	69	72	137	-0,1886	y ~ Intervention + Sex +
0,337334	-0,16067	0,166878	141	69	72	137	-0,96282	y ~ Intervention + Sex +
0,682619	0,062415	0,152318	141	69	72	137	0,409764	y ~ Intervention + Sex +
0,009894	0,400988	0,153282	141	69	72	137	2,616016	y ~ Intervention + Sex +
0,440079	-0,1246	0,160891	135	66	69	131	-0,77442	y ~ Intervention + Sex +
0,130993	-0,22751	0,149744	141	69	72	137	-1,51929	y ~ Intervention + Sex +
0,99468	0,000834	0,124866	141	69	72	137	0,006679	y ~ Intervention + Sex +
0,211554	0,183107	0,145882	141	69	72	137	1,25517	y ~ Intervention + Sex +
0,432799	0,126723	0,161076	141	69	72	137	0,78673	y ~ Intervention + Sex +
0,754472	0,050638	0,161588	141	69	72	137	0,313376	y ~ Intervention + Sex +
0,541904	0,087136	0,142503	141	69	72	137	0,611466	y ~ Intervention + Sex +
0,543637	0,087212	0,143242	141	69	72	137	0,608843	y ~ Intervention + Sex +
0,448004	-0,10797	0,141896	141	69	72	137	-0,76093	y ~ Intervention + Sex +
0,073432	0,260768	0,144551	141	69	72	137	1,80398	y ~ Intervention + Sex +
0,103138	-0,25869	0,157664	141	69	72	137	-1,64078	y ~ Intervention + Sex +
0,209245	0,175637	0,13922	141	69	72	137	1,261577	y ~ Intervention + Sex +
0,130914	0,197306	0,12984	141	69	72	137	1,519607	y ~ Intervention + Sex +
0,162142	0,258361	0,183824	141	69	72	137	1,405479	y ~ Intervention + Sex +
0,046451	0,269768	0,134248	141	69	72	137	2,009474	y ~ Intervention + Sex +
0,08191	0,276567	0,157805	141	69	72	137	1,752587	y ~ Intervention + Sex +
0,631611	-0,06645	0,138288	141	69	72	137	-0,48054	y ~ Intervention + Sex +
0,053479	0,291585	0,149697	141	69	72	137	1,947839	y ~ Intervention + Sex +
0,009517	0,459596	0,174757	141	69	72	137	2,629922	y ~ Intervention + Sex +
0,01412	0,33533	0,134885	141	69	72	137	2,486036	y ~ Intervention + Sex +
0,010494	0,404146	0,155751	141	69	72	137	2,594821	y ~ Intervention + Sex +
0,010303	0,396095	0,15226	141	69	72	137	2,601439	y ~ Intervention + Sex +
0,001788	0,587052	0,184269	141	69	72	137	3,185845	y ~ Intervention + Sex +
0,198676	0,190323	0,147356	141	69	72	137	1,291584	y ~ Intervention + Sex +
0,497204	0,095327	0,140041	141	69	72	137	0,680709	y ~ Intervention + Sex +
0,740527	-0,05232	0,157685	141	69	72	137	-0,33183	y ~ Intervention + Sex +
0,287773	0,157712	0,147785	141	69	72	137	1,067167	y ~ Intervention + Sex +
0,121035	0,255683	0,163886	141	69	72	137	1,560134	y ~ Intervention + Sex +
0,000827	0,498791	0,145869	141	69	72	137	3,41945	y ~ Intervention + Sex +
0,163444	0,220932	0,157684	141	69	72	137	1,401104	y ~ Intervention + Sex +
0,901939	-0,02106	0,17058	141	69	72	137	-0,12344	y ~ Intervention + Sex +
0,979615	0,003867	0,151053	141	69	72	137	0,025599	y ~ Intervention + Sex +
0,004271	-0,39305	0,135257	141	69	72	137	-2,90595	y ~ Intervention + Sex +
0,01173	0,474093	0,185599	141	69	72	137	2,554391	y ~ Intervention + Sex +
0,092141	-0,24862	0,146582	141	69	72	137	-1,69608	y ~ Intervention + Sex +
0,519661	-0,10547	0,163391	141	69	72	137	-0,64553	y ~ Intervention + Sex +
0,022728	-0,37026	0,160704	141	69	72	137	-2,30399	y ~ Intervention + Sex +

0,195058	0,186567	0,143279	141	69	72	137	1,302125	y ~ Intervention + Sex +
0,912129	-0,01947	0,176099	141	69	72	137	-0,11056	y ~ Intervention + Sex +
0,057059	0,276151	0,143901	141	69	72	137	1,919036	y ~ Intervention + Sex +
0,399007	-0,13414	0,158546	141	69	72	137	-0,84604	y ~ Intervention + Sex +
0,669939	0,060526	0,141696	141	69	72	137	0,427153	y ~ Intervention + Sex +
0,259295	0,186079	0,164271	141	69	72	137	1,132757	y ~ Intervention + Sex +
0,060797	0,327172	0,173057	141	69	72	137	1,890543	y ~ Intervention + Sex +
0,038616	-0,30672	0,14687	141	69	72	137	-2,08836	y ~ Intervention + Sex +
0,680337	-0,07042	0,170562	141	69	72	137	-0,41288	y ~ Intervention + Sex +
0,522951	0,085588	0,133638	141	69	72	137	0,640448	y ~ Intervention + Sex +
0,024769	0,351944	0,155041	141	69	72	137	2,270002	y ~ Intervention + Sex +
0,370734	0,119243	0,132779	141	69	72	137	0,89805	y ~ Intervention + Sex +
0,019373	0,373794	0,157973	141	69	72	137	2,366186	y ~ Intervention + Sex +
0,028478	0,392283	0,177179	141	69	72	137	2,214053	y ~ Intervention + Sex +
0,447537	0,100964	0,132548	141	69	72	137	0,761719	y ~ Intervention + Sex +
0,08069	0,267202	0,151845	141	69	72	137	1,759705	y ~ Intervention + Sex +
0,112236	0,264604	0,165533	141	69	72	137	1,598499	y ~ Intervention + Sex +
0,23974	-0,16778	0,14209	141	69	72	137	-1,18077	y ~ Intervention + Sex +
0,047234	0,341857	0,170738	141	69	72	137	2,002232	y ~ Intervention + Sex +
0,752382	-0,05814	0,183921	141	69	72	137	-0,31613	y ~ Intervention + Sex +
0,925504	0,016182	0,172744	141	69	72	137	0,093675	y ~ Intervention + Sex +
0,919873	-0,01342	0,133128	141	69	72	137	-0,10078	y ~ Intervention + Sex +
0,702601	0,060054	0,156957	141	69	72	137	0,382611	y ~ Intervention + Sex +
0,951388	0,00789	0,129193	141	69	72	137	0,061075	y ~ Intervention + Sex +
0,492508	-0,10456	0,15194	141	69	72	137	-0,68817	y ~ Intervention + Sex +
0,142496	0,22094	0,149786	141	69	72	137	1,475042	y ~ Intervention + Sex +
0,066767	0,280522	0,151802	141	69	72	137	1,847943	y ~ Intervention + Sex +
0,844852	0,034113	0,173989	141	69	72	137	0,196062	y ~ Intervention + Sex +
0,062717	-0,31991	0,170484	141	69	72	137	-1,87647	y ~ Intervention + Sex +
0,474293	-0,11525	0,160633	141	69	72	137	-0,71749	y ~ Intervention + Sex +
0,196328	0,191415	0,147423	141	69	72	137	1,298411	y ~ Intervention + Sex +
0,008247	0,383554	0,143076	141	69	72	137	2,680769	y ~ Intervention + Sex +
0,607725	-0,08042	0,156308	141	69	72	137	-0,51451	y ~ Intervention + Sex +
0,836757	-0,03353	0,162412	141	69	72	137	-0,20644	y ~ Intervention + Sex +
0,132907	0,226353	0,149731	141	69	72	137	1,511727	y ~ Intervention + Sex +
0,800664	-0,0404	0,159699	141	69	72	137	-0,25298	y ~ Intervention + Sex +
0,15414	-0,21392	0,149281	141	69	72	137	-1,43298	y ~ Intervention + Sex +
0,116484	0,229239	0,145117	141	69	72	137	1,57969	y ~ Intervention + Sex +
0,021617	0,362457	0,155987	141	69	72	137	2,323629	y ~ Intervention + Sex +
0,415871	0,11509	0,141027	141	69	72	137	0,81608	y ~ Intervention + Sex +
0,434476	0,108772	0,138764	141	69	72	137	0,783859	y ~ Intervention + Sex +
0,888486	-0,01817	0,129308	141	69	72	137	-0,14048	y ~ Intervention + Sex +
0,621392	0,081534	0,164714	141	69	72	137	0,495002	y ~ Intervention + Sex +
0,241245	-0,18782	0,159576	141	69	72	137	-1,17698	y ~ Intervention + Sex +
0,919396	-0,01684	0,166152	141	69	72	137	-0,10138	y ~ Intervention + Sex +
0,005236	-0,52256	0,184152	141	69	72	137	-2,83766	y ~ Intervention + Sex +
0,112769	-0,20766	0,130103	141	69	72	137	-1,59611	y ~ Intervention + Sex +
0,000124	0,657292	0,166379	141	69	72	137	3,950563	y ~ Intervention + Sex +

0,49241	-0,10677	0,155108	141	69	72	137	-0,68833	y ~ Intervention + Sex +
0,094009	0,282217	0,167356	141	69	72	137	1,686328	y ~ Intervention + Sex +
0,341285	-0,12737	0,133378	141	69	72	137	-0,95495	y ~ Intervention + Sex +
0,369777	0,139787	0,155344	141	69	72	137	0,899854	y ~ Intervention + Sex +
0,207795	0,172746	0,136491	141	69	72	137	1,265626	y ~ Intervention + Sex +
0,393156	-0,12889	0,150462	141	69	72	137	-0,85661	y ~ Intervention + Sex +
0,568139	0,106855	0,186752	141	69	72	137	0,572179	y ~ Intervention + Sex +
0,37535	-0,13118	0,147492	141	69	72	137	-0,8894	y ~ Intervention + Sex +
0,620044	0,076363	0,153674	141	69	72	137	0,496918	y ~ Intervention + Sex +
0,936552	-0,0137	0,171733	141	69	72	137	-0,07975	y ~ Intervention + Sex +
0,142073	0,213534	0,14461	141	69	72	137	1,476618	y ~ Intervention + Sex +
0,068826	0,286125	0,156013	141	69	72	137	1,833987	y ~ Intervention + Sex +
0,509494	0,121283	0,183385	141	69	72	137	0,661358	y ~ Intervention + Sex +
0,353422	0,157192	0,168818	141	69	72	137	0,931134	y ~ Intervention + Sex +
0,222994	0,217918	0,178014	141	69	72	137	1,224158	y ~ Intervention + Sex +
0,93131	-0,01334	0,154444	141	69	72	137	-0,08636	y ~ Intervention + Sex +
0,647652	-0,0805	0,175741	141	69	72	137	-0,45804	y ~ Intervention + Sex +
0,522526	0,097789	0,152532	141	69	72	137	0,641104	y ~ Intervention + Sex +
0,665209	0,062406	0,143901	141	69	72	137	0,433671	y ~ Intervention + Sex +
0,993393	-0,00155	0,186902	141	69	72	137	-0,0083	y ~ Intervention + Sex +
0,225368	-0,16817	0,138089	141	69	72	137	-1,21787	y ~ Intervention + Sex +
0,335311	0,150307	0,155457	141	69	72	137	0,966873	y ~ Intervention + Sex +
0,206937	0,196139	0,15468	141	69	72	137	1,268032	y ~ Intervention + Sex +
0,145347	0,242675	0,165705	141	69	72	137	1,464504	y ~ Intervention + Sex +
0,000637	0,489705	0,140084	141	69	72	137	3,495797	y ~ Intervention + Sex +
0,021267	-0,36879	0,15828	141	69	72	137	-2,33	y ~ Intervention + Sex +
0,998311	0,000378	0,178304	141	69	72	137	0,002121	y ~ Intervention + Sex +
0,111569	-0,25382	0,158487	141	69	72	137	-1,6015	y ~ Intervention + Sex +
0,011491	0,378028	0,147556	141	69	72	137	2,561918	y ~ Intervention + Sex +
0,880983	0,024143	0,160949	141	69	72	137	0,150003	y ~ Intervention + Sex +
0,261505	-0,18207	0,161485	141	69	72	137	-1,12749	y ~ Intervention + Sex +
6,40E-05	0,68682	0,166506	141	69	72	137	4,124887	y ~ Intervention + Sex +
0,41033	-0,13175	0,15954	141	69	72	137	-0,82584	y ~ Intervention + Sex +
0,845161	0,025619	0,130933	141	69	72	137	0,195667	y ~ Intervention + Sex +
0,058267	0,326874	0,171169	141	69	72	137	1,909659	y ~ Intervention + Sex +
0,065573	0,272638	0,146879	141	69	72	137	1,856204	y ~ Intervention + Sex +
0,281308	-0,17113	0,158215	141	69	72	137	-1,08165	y ~ Intervention + Sex +
0,962358	-0,00714	0,151023	141	69	72	137	-0,04728	y ~ Intervention + Sex +
0,908549	-0,01612	0,140047	141	69	72	137	-0,11508	y ~ Intervention + Sex +
0,033292	0,300085	0,139559	141	69	72	137	2,150239	y ~ Intervention + Sex +
0,018377	0,390103	0,163463	141	69	72	137	2,386486	y ~ Intervention + Sex +
0,460421	-0,11427	0,154363	141	69	72	137	-0,74024	y ~ Intervention + Sex +
0,577476	-0,07977	0,142851	141	69	72	137	-0,55841	y ~ Intervention + Sex +
0,102261	0,279446	0,169875	141	69	72	137	1,645008	y ~ Intervention + Sex +
0,122435	0,22759	0,146432	141	69	72	137	1,554237	y ~ Intervention + Sex +
0,178188	0,235619	0,174107	141	69	72	137	1,353302	y ~ Intervention + Sex +
0,794995	0,036845	0,141529	141	69	72	137	0,260337	y ~ Intervention + Sex +
0,734421	0,043158	0,126957	141	69	72	137	0,339942	y ~ Intervention + Sex +

0,043305	-0,33002	0,161801	141	69	72	137	-2,03967	y ~ Intervention + Sex +
0,908408	-0,01795	0,155757	141	69	72	137	-0,11526	y ~ Intervention + Sex +
0,022402	0,38377	0,166158	141	69	72	137	2,309663	y ~ Intervention + Sex +
0,240097	0,157816	0,133757	141	69	72	137	1,179869	y ~ Intervention + Sex +
0,763895	0,051123	0,169863	141	69	72	137	0,300968	y ~ Intervention + Sex +
0,593866	0,075109	0,140523	141	69	72	137	0,534494	y ~ Intervention + Sex +
0,43661	0,125853	0,161306	141	69	72	137	0,780216	y ~ Intervention + Sex +
0,001406	0,452345	0,13876	141	69	72	137	3,259909	y ~ Intervention + Sex +
0,289232	0,177891	0,167202	141	69	72	137	1,063929	y ~ Intervention + Sex +
0,238726	0,149282	0,126154	141	69	72	137	1,183335	y ~ Intervention + Sex +
0,41712	0,139933	0,171931	141	69	72	137	0,813891	y ~ Intervention + Sex +
0,070504	0,304071	0,166809	141	69	72	137	1,822866	y ~ Intervention + Sex +
0,519356	-0,09789	0,151532	141	69	72	137	-0,64601	y ~ Intervention + Sex +
0,468509	-0,11742	0,161533	141	69	72	137	-0,72693	y ~ Intervention + Sex +
0,015253	0,434845	0,176969	141	69	72	137	2,457176	y ~ Intervention + Sex +
0,614665	-0,0754	0,149432	141	69	72	137	-0,50458	y ~ Intervention + Sex +
0,123329	0,266667	0,171988	141	69	72	137	1,550497	y ~ Intervention + Sex +
0,101507	-0,24699	0,14981	141	69	72	137	-1,64867	y ~ Intervention + Sex +
2,83E-06	0,715066	0,146355	141	69	72	137	4,885824	y ~ Intervention + Sex +
0,05037	0,272813	0,138189	141	69	72	137	1,9742	y ~ Intervention + Sex +
0,783018	-0,0436	0,157997	141	69	72	137	-0,27593	y ~ Intervention + Sex +
0,955914	0,010389	0,187577	141	69	72	137	0,055383	y ~ Intervention + Sex +
0,046823	0,30274	0,150916	141	69	72	137	2,006021	y ~ Intervention + Sex +
0,020444	0,341364	0,145549	141	69	72	137	2,345357	y ~ Intervention + Sex +
0,028051	0,330169	0,148714	141	69	72	137	2,220163	y ~ Intervention + Sex +
0,000823	0,487846	0,142606	141	69	72	137	3,420935	y ~ Intervention + Sex +
0,00877	-0,44705	0,168123	141	69	72	137	-2,65904	y ~ Intervention + Sex +
0,110294	-0,2291	0,142541	141	69	72	137	-1,60729	y ~ Intervention + Sex +
0,093524	0,257005	0,152178	141	69	72	137	1,688843	y ~ Intervention + Sex +
0,002728	0,495162	0,162226	141	69	72	137	3,052291	y ~ Intervention + Sex +
0,948917	-0,00959	0,149459	141	69	72	137	-0,06418	y ~ Intervention + Sex +
0,627283	0,077232	0,158701	141	69	72	137	0,486651	y ~ Intervention + Sex +
0,606813	0,066902	0,129701	141	69	72	137	0,515818	y ~ Intervention + Sex +
0,163651	0,183548	0,131067	141	69	72	137	1,400412	y ~ Intervention + Sex +
0,440622	0,131822	0,170447	141	69	72	137	0,773393	y ~ Intervention + Sex +
0,002626	0,509838	0,166368	141	69	72	137	3,064526	y ~ Intervention + Sex +
0,70644	-0,0629	0,166645	141	69	72	137	-0,37743	y ~ Intervention + Sex +
0,007191	0,422905	0,154978	141	69	72	137	2,728798	y ~ Intervention + Sex +
0,837397	-0,03532	0,171767	141	69	72	137	-0,20561	y ~ Intervention + Sex +
0,053277	0,291257	0,1494	141	69	72	137	1,949512	y ~ Intervention + Sex +
0,845192	-0,03226	0,164883	141	69	72	137	-0,19563	y ~ Intervention + Sex +
0,512242	0,097196	0,147925	141	69	72	137	0,657065	y ~ Intervention + Sex +
0,381362	-0,13016	0,148204	141	69	72	137	-0,87822	y ~ Intervention + Sex +
0,873825	-0,02607	0,163859	141	69	72	137	-0,1591	y ~ Intervention + Sex +
0,025622	0,342971	0,151991	141	69	72	137	2,256516	y ~ Intervention + Sex +
0,098037	0,278798	0,167365	141	69	72	137	1,665806	y ~ Intervention + Sex +
0,000106	-0,61649	0,154436	141	69	72	137	-3,99185	y ~ Intervention + Sex +
0,476112	-0,12266	0,171671	141	69	72	137	-0,71454	y ~ Intervention + Sex +

0,04294	-0,3329	0,162924	141	69	72	137	-2,0433	y ~ Intervention + Sex +
0,304299	0,165575	0,160577	141	69	72	137	1,031125	y ~ Intervention + Sex +
0,010319	0,356151	0,136935	141	69	72	137	2,60088	y ~ Intervention + Sex +
0,367991	-0,14872	0,164649	141	69	72	137	-0,90323	y ~ Intervention + Sex +
0,923437	-0,01573	0,163425	141	69	72	137	-0,09628	y ~ Intervention + Sex +
0,170424	0,242477	0,175952	141	69	72	137	1,378083	y ~ Intervention + Sex +
0,144815	-0,24332	0,165925	141	69	72	137	-1,46646	y ~ Intervention + Sex +
0,188822	0,213869	0,161943	141	69	72	137	1,320645	y ~ Intervention + Sex +
0,073364	0,293293	0,162542	141	69	72	137	1,804407	y ~ Intervention + Sex +
0,974453	0,005228	0,162963	141	69	72	137	0,032082	y ~ Intervention + Sex +
0,21466	0,212744	0,170655	141	69	72	137	1,24663	y ~ Intervention + Sex +
0,182826	0,213947	0,159795	141	69	72	137	1,338882	y ~ Intervention + Sex +
0,022415	-0,39923	0,172868	141	69	72	137	-2,30944	y ~ Intervention + Sex +
0,030854	0,360311	0,165168	141	69	72	137	2,181474	y ~ Intervention + Sex +
0,001118	0,557623	0,167474	141	69	72	137	3,329601	y ~ Intervention + Sex +
0,134895	-0,2474	0,164499	141	69	72	137	-1,50395	y ~ Intervention + Sex +
0,018769	0,420459	0,176784	141	69	72	137	2,378375	y ~ Intervention + Sex +
0,288222	0,163984	0,153807	141	69	72	137	1,066171	y ~ Intervention + Sex +
0,164345	0,213878	0,152979	141	69	72	137	1,398091	y ~ Intervention + Sex +
0,908147	-0,01827	0,158037	141	69	72	137	-0,11559	y ~ Intervention + Sex +
0,68071	0,065152	0,157992	141	69	72	137	0,412374	y ~ Intervention + Sex +
0,310352	-0,15437	0,151607	141	69	72	137	-1,01825	y ~ Intervention + Sex +
0,201962	0,205539	0,16031	141	69	72	137	1,282131	y ~ Intervention + Sex +
0,506056	-0,0966	0,144878	141	69	72	137	-0,66675	y ~ Intervention + Sex +
0,221329	-0,20079	0,163433	141	69	72	137	-1,2286	y ~ Intervention + Sex +
0,522257	-0,10706	0,166886	141	69	72	137	-0,64152	y ~ Intervention + Sex +
0,049626	0,30737	0,155181	141	69	72	137	1,980717	y ~ Intervention + Sex +
0,952425	0,009242	0,154615	141	69	72	137	0,059772	y ~ Intervention + Sex +
0,653247	-0,07082	0,157295	141	69	72	137	-0,45024	y ~ Intervention + Sex +
0,017195	-0,36658	0,151989	141	69	72	137	-2,41187	y ~ Intervention + Sex +
0,927336	0,012802	0,140116	141	69	72	137	0,091365	y ~ Intervention + Sex +
0,714425	-0,05997	0,163546	141	69	72	137	-0,36668	y ~ Intervention + Sex +
0,012899	0,393761	0,156282	141	69	72	137	2,519547	y ~ Intervention + Sex +
0,048091	-0,35575	0,178371	141	69	72	137	-1,99442	y ~ Intervention + Sex +
0,012225	-0,39604	0,155965	141	69	72	137	-2,53927	y ~ Intervention + Sex +
0,00946	0,450451	0,171138	141	69	72	137	2,632087	y ~ Intervention + Sex +
0,082694	0,267272	0,152897	141	69	72	137	1,748058	y ~ Intervention + Sex +
0,552359	-0,10145	0,170308	141	69	72	137	-0,5957	y ~ Intervention + Sex +
0,625452	0,069013	0,14106	141	69	72	137	0,489243	y ~ Intervention + Sex +
0,601788	0,080881	0,154634	141	69	72	137	0,523045	y ~ Intervention + Sex +
0,402567	-0,13024	0,155107	141	69	72	137	-0,83965	y ~ Intervention + Sex +
0,669356	-0,06633	0,155	141	69	72	137	-0,42796	y ~ Intervention + Sex +
0,067727	-0,35365	0,192056	141	69	72	137	-1,84139	y ~ Intervention + Sex +
0,354486	0,137179	0,147652	141	69	72	137	0,929073	y ~ Intervention + Sex +
0,289352	-0,16237	0,152656	141	69	72	137	-1,06366	y ~ Intervention + Sex +
0,108044	-0,25107	0,155211	141	69	72	137	-1,61762	y ~ Intervention + Sex +
0,424325	0,121261	0,151324	141	69	72	137	0,801335	y ~ Intervention + Sex +
0,031885	-0,32621	0,150466	141	69	72	137	-2,16801	y ~ Intervention + Sex +

0,16023	0,203396	0,144052	141	69	72	137	1,411958	y ~ Intervention + Sex
0,787605	-0,04636	0,171733	141	69	72	137	-0,26995	y ~ Intervention + Sex
0,78256	0,044714	0,161696	141	69	72	137	0,276527	y ~ Intervention + Sex
0,331455	-0,1454	0,149178	141	69	72	137	-0,97464	y ~ Intervention + Sex
0,132639	-0,2484	0,164201	141	69	72	137	-1,51278	y ~ Intervention + Sex
0,069465	-0,24418	0,133449	141	69	72	137	-1,82973	y ~ Intervention + Sex
0,010198	-0,36422	0,139809	141	69	72	137	-2,60516	y ~ Intervention + Sex
0,09278	-0,25961	0,15337	141	69	72	137	-1,69273	y ~ Intervention + Sex
0,012106	0,40571	0,159548	141	69	72	137	2,542874	y ~ Intervention + Sex
0,97752	-0,00495	0,175354	141	69	72	137	-0,02823	y ~ Intervention + Sex
0,423821	-0,13288	0,165638	141	69	72	137	-0,80221	y ~ Intervention + Sex
0,146661	0,24833	0,170124	141	69	72	137	1,459703	y ~ Intervention + Sex
0,033422	0,327341	0,152349	141	69	72	137	2,148629	y ~ Intervention + Sex
0,0357	0,3087	0,145526	141	69	72	137	2,121268	y ~ Intervention + Sex
0,977053	0,004376	0,151844	141	69	72	137	0,028816	y ~ Intervention + Sex
0,757573	0,052185	0,168725	141	69	72	137	0,309287	y ~ Intervention + Sex
0,653947	-0,07201	0,160292	141	69	72	137	-0,44927	y ~ Intervention + Sex
0,775488	0,043148	0,150989	141	69	72	137	0,285767	y ~ Intervention + Sex
0,737757	-0,05678	0,16923	141	69	72	137	-0,33551	y ~ Intervention + Sex
0,463543	-0,12398	0,168661	141	69	72	137	-0,73509	y ~ Intervention + Sex
0,735664	0,053089	0,156934	141	69	72	137	0,338288	y ~ Intervention + Sex
0,066109	0,310179	0,16744	141	69	72	137	1,852476	y ~ Intervention + Sex
0,799956	-0,03928	0,154717	141	69	72	137	-0,2539	y ~ Intervention + Sex
0,926015	0,014069	0,151232	141	69	72	137	0,093031	y ~ Intervention + Sex
0,310749	0,135081	0,132769	141	69	72	137	1,017416	y ~ Intervention + Sex
0,38709	0,133244	0,153565	141	69	72	137	0,867675	y ~ Intervention + Sex
4,93E-05	0,661843	0,157877	141	69	72	137	4,192139	y ~ Intervention + Sex
0,21023	0,222461	0,176719	141	69	72	137	1,258837	y ~ Intervention + Sex
0,000208	0,519234	0,136222	141	69	72	137	3,811675	y ~ Intervention + Sex
0,751713	-0,04945	0,155972	141	69	72	137	-0,31702	y ~ Intervention + Sex
0,866341	0,024355	0,144436	141	69	72	137	0,168624	y ~ Intervention + Sex
4,90E-05	0,663808	0,158279	141	69	72	137	4,193897	y ~ Intervention + Sex
0,541133	0,091606	0,149528	141	69	72	137	0,612635	y ~ Intervention + Sex
0,49932	0,105509	0,155765	141	69	72	137	0,67736	y ~ Intervention + Sex
0,470529	-0,10336	0,14284	141	69	72	137	-0,72363	y ~ Intervention + Sex
0,769272	0,047975	0,163232	141	69	72	137	0,29391	y ~ Intervention + Sex
0,022418	-0,33413	0,144683	141	69	72	137	-2,30938	y ~ Intervention + Sex
0,808212	-0,03428	0,140934	141	69	72	137	-0,2432	y ~ Intervention + Sex
0,060895	0,301796	0,159696	141	69	72	137	1,889815	y ~ Intervention + Sex
0,113123	0,246824	0,154795	141	69	72	137	1,594523	y ~ Intervention + Sex
0,991965	-0,00163	0,161239	141	69	72	137	-0,01009	y ~ Intervention + Sex
0,925497	-0,01426	0,152169	141	69	72	137	-0,09368	y ~ Intervention + Sex
0,658324	-0,06588	0,148638	141	69	72	137	-0,4432	y ~ Intervention + Sex
0,145918	0,250458	0,171263	141	69	72	137	1,462415	y ~ Intervention + Sex
0,861532	-0,02813	0,160997	141	69	72	137	-0,17475	y ~ Intervention + Sex
0,588101	-0,08362	0,15404	141	69	72	137	-0,54287	y ~ Intervention + Sex
0,205672	-0,20379	0,160264	141	69	72	137	-1,27159	y ~ Intervention + Sex
0,988693	-0,00202	0,142334	141	69	72	137	-0,0142	y ~ Intervention + Sex

0,000146	0,607536	0,155505	141	69	72	137	3,906859	y ~ Intervention + Sex +
0,896028	-0,02281	0,174216	141	69	72	137	-0,13092	y ~ Intervention + Sex +
0,698782	0,063102	0,162728	141	69	72	137	0,387778	y ~ Intervention + Sex +
0,262699	0,165316	0,146992	141	69	72	137	1,124663	y ~ Intervention + Sex +
0,892521	-0,02056	0,151908	141	69	72	137	-0,13537	y ~ Intervention + Sex +
0,038104	0,324283	0,154864	141	69	72	137	2,093979	y ~ Intervention + Sex +
0,633012	-0,07374	0,154094	141	69	72	137	-0,47856	y ~ Intervention + Sex +
0,497668	-0,11802	0,173567	141	69	72	137	-0,67997	y ~ Intervention + Sex +
0,363247	-0,16347	0,179194	141	69	72	137	-0,91224	y ~ Intervention + Sex +
0,817203	-0,03629	0,156704	141	69	72	137	-0,23159	y ~ Intervention + Sex +
0,851716	-0,02883	0,153928	141	69	72	137	-0,18728	y ~ Intervention + Sex +
0,695279	0,062997	0,160491	141	69	72	137	0,392527	y ~ Intervention + Sex +
0,002793	0,518418	0,170265	141	69	72	137	3,044778	y ~ Intervention + Sex +
0,134603	-0,22743	0,151104	141	69	72	137	-1,50509	y ~ Intervention + Sex +
0,000678	-0,5266	0,151417	141	69	72	137	-3,47785	y ~ Intervention + Sex +
0,24406	0,167765	0,143397	141	69	72	137	1,16993	y ~ Intervention + Sex +
0,887962	0,0224	0,158704	141	69	72	137	0,141146	y ~ Intervention + Sex +
0,67	0,06611	0,154799	141	69	72	137	0,427068	y ~ Intervention + Sex +
0,955072	-0,00857	0,151896	141	69	72	137	-0,05644	y ~ Intervention + Sex +
9,52E-05	0,584732	0,145416	141	69	72	137	4,021092	y ~ Intervention + Sex +
0,916634	-0,01604	0,152918	141	69	72	137	-0,10487	y ~ Intervention + Sex +
0,477095	0,115517	0,162029	141	69	72	137	0,712941	y ~ Intervention + Sex +
0,819483	-0,03497	0,152929	141	69	72	137	-0,22865	y ~ Intervention + Sex +
5,31E-16	1,408143	0,153056	141	69	72	137	9,200162	y ~ Intervention + Sex +
0,187311	0,221273	0,166974	141	69	72	137	1,325198	y ~ Intervention + Sex +
0,38936	-0,13832	0,16018	141	69	72	137	-0,86352	y ~ Intervention + Sex +
0,812716	0,036884	0,155379	141	69	72	137	0,237381	y ~ Intervention + Sex +
0,541687	0,090118	0,147301	141	69	72	137	0,611795	y ~ Intervention + Sex +
0,556172	-0,10226	0,173318	141	69	72	137	-0,58999	y ~ Intervention + Sex +
0,249288	0,182016	0,157318	141	69	72	137	1,156995	y ~ Intervention + Sex +
0,665459	0,060703	0,140085	141	69	72	137	0,433327	y ~ Intervention + Sex +
3,17E-07	0,858172	0,159578	141	69	72	137	5,377746	y ~ Intervention + Sex +
0,016356	-0,3719	0,152993	141	69	72	137	-2,43085	y ~ Intervention + Sex +
0,849387	0,032832	0,172563	141	69	72	137	0,19026	y ~ Intervention + Sex +
0,675148	0,060271	0,143505	141	69	72	137	0,419993	y ~ Intervention + Sex +
0,426674	-0,12873	0,161462	141	69	72	137	-0,79727	y ~ Intervention + Sex +
0,06794	-0,27259	0,148152	141	69	72	137	-1,83995	y ~ Intervention + Sex +
0,64513	0,075047	0,162595	141	69	72	137	0,461558	y ~ Intervention + Sex +
0,602934	0,08262	0,158459	141	69	72	137	0,521394	y ~ Intervention + Sex +
0,600739	0,090356	0,172251	141	69	72	137	0,524557	y ~ Intervention + Sex +
0,371631	0,12967	0,144663	141	69	72	137	0,896363	y ~ Intervention + Sex +
0,684046	0,066918	0,164088	141	69	72	137	0,407816	y ~ Intervention + Sex +
0,140732	0,247009	0,166713	141	69	72	137	1,48164	y ~ Intervention + Sex +
0,496885	-0,10472	0,153727	141	69	72	137	-0,68122	y ~ Intervention + Sex +
0,697767	0,057936	0,148878	141	69	72	137	0,389153	y ~ Intervention + Sex +
0,12915	0,248421	0,162721	141	69	72	137	1,526662	y ~ Intervention + Sex +
0,009599	0,403701	0,153681	141	69	72	137	2,626867	y ~ Intervention + Sex +
0,042421	0,323137	0,157744	141	69	72	137	2,048491	y ~ Intervention + Sex +

0,332133	0,159852	0,164241	141	69	72	137	0,973274	y ~ Intervention + Sex +
0,270923	0,164247	0,148586	141	69	72	137	1,105401	y ~ Intervention + Sex +
0,168411	0,220246	0,159063	141	69	72	137	1,384648	y ~ Intervention + Sex +
0,487952	0,112077	0,161158	141	69	72	137	0,695448	y ~ Intervention + Sex +
0,024611	0,380349	0,167368	141	69	72	137	2,272537	y ~ Intervention + Sex +
0,974694	0,004928	0,155083	141	69	72	137	0,031779	y ~ Intervention + Sex +
0,246619	0,188199	0,161742	141	69	72	137	1,163574	y ~ Intervention + Sex +
0,031051	0,324137	0,148763	141	69	72	137	2,178877	y ~ Intervention + Sex +
0,879796	-0,02386	0,157453	141	69	72	137	-0,15151	y ~ Intervention + Sex +
0,943467	0,010693	0,150522	141	69	72	137	0,071043	y ~ Intervention + Sex +
0,821329	0,035434	0,156603	141	69	72	137	0,226269	y ~ Intervention + Sex +
0,001135	0,54718	0,164564	141	69	72	137	3,325033	y ~ Intervention + Sex +
0,171748	0,211235	0,153759	141	69	72	137	1,3738	y ~ Intervention + Sex +
0,112543	-0,26065	0,163199	141	69	72	137	-1,59712	y ~ Intervention + Sex +
0,42514	-0,13842	0,173046	141	69	72	137	-0,79992	y ~ Intervention + Sex +
0,585827	0,093732	0,171612	141	69	72	137	0,546186	y ~ Intervention + Sex +
0,998514	0,000292	0,1563	141	69	72	137	0,001866	y ~ Intervention + Sex +
0,003761	-0,49383	0,167512	141	69	72	137	-2,94804	y ~ Intervention + Sex +
0,04572	0,314309	0,155881	141	69	72	137	2,016334	y ~ Intervention + Sex +
0,325065	-0,1469	0,148736	141	69	72	137	-0,98765	y ~ Intervention + Sex +
0,053419	0,294617	0,151215	141	69	72	137	1,948336	y ~ Intervention + Sex +
0,310512	-0,16188	0,159028	141	69	72	137	-1,01792	y ~ Intervention + Sex +
0,126332	-0,25037	0,162777	141	69	72	137	-1,53809	y ~ Intervention + Sex +
0,579613	0,086603	0,155964	141	69	72	137	0,555274	y ~ Intervention + Sex +
0,681333	0,069065	0,167829	141	69	72	137	0,411522	y ~ Intervention + Sex +
0,767617	0,047769	0,161339	141	69	72	137	0,296081	y ~ Intervention + Sex +
0,105794	0,253813	0,155892	141	69	72	137	1,628136	y ~ Intervention + Sex +
0,386572	-0,13672	0,157395	141	69	72	137	-0,86862	y ~ Intervention + Sex +
0,315016	-0,15365	0,152363	141	69	72	137	-1,00845	y ~ Intervention + Sex +
0,061655	-0,31467	0,167006	141	69	72	137	-1,88421	y ~ Intervention + Sex +
0,021313	0,34854	0,149642	141	69	72	137	2,329162	y ~ Intervention + Sex +
0,90043	0,020013	0,159653	141	69	72	137	0,125351	y ~ Intervention + Sex +
0,772324	0,043875	0,15134	141	69	72	137	0,28991	y ~ Intervention + Sex +
0,373923	-0,13667	0,153203	141	69	72	137	-0,89206	y ~ Intervention + Sex +
0,340844	0,144705	0,151393	141	69	72	137	0,955825	y ~ Intervention + Sex +
0,264997	-0,16664	0,148885	141	69	72	137	-1,11924	y ~ Intervention + Sex +
0,559823	0,10126	0,173233	141	69	72	137	0,584533	y ~ Intervention + Sex +
0,274812	-0,18048	0,164608	141	69	72	137	-1,09644	y ~ Intervention + Sex +
0,689864	-0,06015	0,150424	141	69	72	137	-0,39989	y ~ Intervention + Sex +
0,142967	0,217939	0,147927	141	69	72	137	1,473287	y ~ Intervention + Sex +
0,639761	-0,0704	0,150082	141	69	72	137	-0,46908	y ~ Intervention + Sex +
0,000515	0,530051	0,149005	141	69	72	137	3,557267	y ~ Intervention + Sex +
0,30124	0,183092	0,176441	141	69	72	137	1,037695	y ~ Intervention + Sex +
0,609958	-0,09441	0,184653	141	69	72	137	-0,51131	y ~ Intervention + Sex +
0,759463	0,04659	0,15186	141	69	72	137	0,306798	y ~ Intervention + Sex +
0,873424	0,025447	0,159436	141	69	72	137	0,159609	y ~ Intervention + Sex +
0,364156	0,145046	0,159303	141	69	72	137	0,910504	y ~ Intervention + Sex +
0,072958	-0,30893	0,170962	141	69	72	137	-1,80699	y ~ Intervention + Sex +

0,020378	-0,33863	0,144306	141	69	72	137	-2,34662	y ~ Intervention + Sex +
0,02634	0,334376	0,148911	141	69	72	137	2,245472	y ~ Intervention + Sex +
0,543634	0,103066	0,169281	141	69	72	137	0,608848	y ~ Intervention + Sex +
0,653094	0,079857	0,177281	141	69	72	137	0,450456	y ~ Intervention + Sex +
0,094227	-0,24102	0,143022	141	69	72	137	-1,6852	y ~ Intervention + Sex +
0,174721	-0,20563	0,150723	141	69	72	137	-1,36427	y ~ Intervention + Sex +
0,057356	-0,30469	0,158964	141	69	72	137	-1,91672	y ~ Intervention + Sex +
0,935662	0,0114	0,140958	141	69	72	137	0,080872	y ~ Intervention + Sex +
0,817564	-0,03551	0,153656	141	69	72	137	-0,23112	y ~ Intervention + Sex +
0,773881	-0,04102	0,142484	141	69	72	137	-0,28787	y ~ Intervention + Sex +
0,951153	-0,00998	0,162617	141	69	72	137	-0,06137	y ~ Intervention + Sex +
0,322876	-0,14981	0,150994	141	69	72	137	-0,99215	y ~ Intervention + Sex +
0,024564	0,374758	0,164852	141	69	72	137	2,273293	y ~ Intervention + Sex +
0,003341	0,457755	0,153262	141	69	72	137	2,986755	y ~ Intervention + Sex +
0,653197	-0,07658	0,170067	141	69	72	137	-0,45031	y ~ Intervention + Sex +
0,032237	0,303102	0,140098	141	69	72	137	2,163506	y ~ Intervention + Sex +
6,41E-17	1,28004	0,133806	141	69	72	137	9,566385	y ~ Intervention + Sex +
0,992655	-0,00152	0,164605	141	69	72	137	-0,00922	y ~ Intervention + Sex +
0,033455	0,34682	0,161445	141	69	72	137	2,148226	y ~ Intervention + Sex +
0,213001	-0,18923	0,151243	141	69	72	137	-1,25118	y ~ Intervention + Sex +
0,804939	-0,03844	0,15534	141	69	72	137	-0,24744	y ~ Intervention + Sex +
0,88634	-0,02359	0,164757	141	69	72	137	-0,1432	y ~ Intervention + Sex +
0,014419	0,397349	0,160336	141	69	72	137	2,478223	y ~ Intervention + Sex +
0,854359	-0,02879	0,156531	141	69	72	137	-0,18391	y ~ Intervention + Sex +
0,598057	-0,08242	0,155981	141	69	72	137	-0,52843	y ~ Intervention + Sex +
0,47549	0,1203	0,168124	141	69	72	137	0,715544	y ~ Intervention + Sex +
0,001148	-0,5163	0,15544	141	69	72	137	-3,32152	y ~ Intervention + Sex +
0,312095	0,157477	0,155214	141	69	72	137	1,014579	y ~ Intervention + Sex +
0,105957	-0,24574	0,151005	141	69	72	137	-1,62736	y ~ Intervention + Sex +
0,206721	0,192339	0,151611	141	69	72	137	1,268639	y ~ Intervention + Sex +
0,429729	0,12728	0,160707	141	69	72	137	0,792001	y ~ Intervention + Sex +
0,00465	0,457438	0,158966	141	69	72	137	2,877586	y ~ Intervention + Sex +
0,767257	0,051817	0,174733	141	69	72	137	0,296553	y ~ Intervention + Sex +
0,642322	0,071537	0,153682	141	69	72	137	0,465486	y ~ Intervention + Sex +
0,058374	-0,29854	0,156396	141	69	72	137	-1,90884	y ~ Intervention + Sex +
0,15754	0,225154	0,158428	141	69	72	137	1,421169	y ~ Intervention + Sex +
0,233779	-0,1745	0,14591	141	69	72	137	-1,19596	y ~ Intervention + Sex +
0,745496	0,048761	0,149924	141	69	72	137	0,325239	y ~ Intervention + Sex +
0,001143	0,52366	0,157596	141	69	72	137	3,322807	y ~ Intervention + Sex +
0,465202	-0,1102	0,150474	141	69	72	137	-0,73236	y ~ Intervention + Sex +
0,063124	-0,25912	0,138307	141	69	72	137	-1,87354	y ~ Intervention + Sex +
0,247652	0,204087	0,175782	141	69	72	137	1,161023	y ~ Intervention + Sex +
0,752768	0,052695	0,166956	141	69	72	137	0,315624	y ~ Intervention + Sex +
0,003802	-0,46117	0,156624	141	69	72	137	-2,94445	y ~ Intervention + Sex +
0,159365	0,242161	0,17115	141	69	72	137	1,414906	y ~ Intervention + Sex +
0,202197	-0,21094	0,164606	141	69	72	137	-1,28146	y ~ Intervention + Sex +
4,44E-05	-0,66796	0,158321	141	69	72	137	-4,21902	y ~ Intervention + Sex +
0,938098	0,013093	0,16828	141	69	72	137	0,077803	y ~ Intervention + Sex +

0,785207	0,041663	0,152569	141	69	72	137	0,273075	y ~ Intervention + Sex +
0,811673	-0,038	0,159166	141	69	72	137	-0,23873	y ~ Intervention + Sex +
0,000701	0,565246	0,162993	141	69	72	137	3,467912	y ~ Intervention + Sex +
0,173931	-0,2034	0,148815	141	69	72	137	-1,36679	y ~ Intervention + Sex +
0,002017	-0,44492	0,141326	141	69	72	137	-3,1482	y ~ Intervention + Sex +
0,531545	0,093764	0,149487	141	69	72	137	0,627242	y ~ Intervention + Sex +
0,266887	0,182822	0,163995	141	69	72	137	1,114802	y ~ Intervention + Sex +
0,686747	0,065502	0,162081	141	69	72	137	0,404131	y ~ Intervention + Sex +
0,940013	-0,01236	0,163969	141	69	72	137	-0,07539	y ~ Intervention + Sex +
0,000383	0,58317	0,160143	141	69	72	137	3,641566	y ~ Intervention + Sex +
0,815392	-0,03342	0,14288	141	69	72	137	-0,23393	y ~ Intervention + Sex +
0,000161	-0,64103	0,165194	141	69	72	137	-3,88045	y ~ Intervention + Sex +
0,164383	-0,20976	0,15005	141	69	72	137	-1,39796	y ~ Intervention + Sex +
0,878832	-0,02508	0,164224	141	69	72	137	-0,15274	y ~ Intervention + Sex +
0,828982	0,037342	0,172545	141	69	72	137	0,216421	y ~ Intervention + Sex +
0,405669	-0,13868	0,166256	141	69	72	137	-0,83411	y ~ Intervention + Sex +
0,651768	0,071263	0,157557	141	69	72	137	0,4523	y ~ Intervention + Sex +
0,252297	0,184032	0,160079	141	69	72	137	1,149636	y ~ Intervention + Sex +
0,121715	0,251756	0,161666	141	69	72	137	1,557262	y ~ Intervention + Sex +
0,331196	0,151965	0,155835	141	69	72	137	0,975168	y ~ Intervention + Sex +
0,051284	0,309078	0,157187	141	69	72	137	1,966312	y ~ Intervention + Sex +
0,118432	0,241643	0,15379	141	69	72	137	1,571247	y ~ Intervention + Sex +
0,411025	-0,12972	0,157314	141	69	72	137	-0,82461	y ~ Intervention + Sex +
0,293592	0,17207	0,163205	141	69	72	137	1,05432	y ~ Intervention + Sex +
0,555605	-0,09827	0,166328	141	69	72	137	-0,59083	y ~ Intervention + Sex +
0,345285	0,159016	0,167907	141	69	72	137	0,947043	y ~ Intervention + Sex +
0,970795	-0,00567	0,154665	141	69	72	137	-0,03668	y ~ Intervention + Sex +
0,732014	-0,05255	0,153152	141	69	72	137	-0,34315	y ~ Intervention + Sex +
0,606961	0,080854	0,156813	141	69	72	137	0,515606	y ~ Intervention + Sex +
0,994637	-0,00102	0,152172	141	69	72	137	-0,00673	y ~ Intervention + Sex +
0,680403	0,07263	0,175948	141	69	72	137	0,412794	y ~ Intervention + Sex +
0,690943	-0,05851	0,146848	141	69	72	137	-0,39842	y ~ Intervention + Sex +
0,05838	-0,30335	0,158921	141	69	72	137	-1,90879	y ~ Intervention + Sex +
0,542025	-0,10016	0,163859	141	69	72	137	-0,61128	y ~ Intervention + Sex +
0,033202	0,353832	0,164469	141	69	72	137	2,151355	y ~ Intervention + Sex +
0,463915	0,11585	0,157732	141	69	72	137	0,734474	y ~ Intervention + Sex +
0,044916	-0,36429	0,179989	141	69	72	137	-2,02399	y ~ Intervention + Sex +
5,40E-10	1,098728	0,164388	141	69	72	137	6,683756	y ~ Intervention + Sex +
0,780458	0,041213	0,147575	141	69	72	137	0,279271	y ~ Intervention + Sex +
0,819011	-0,0384	0,167508	141	69	72	137	-0,22926	y ~ Intervention + Sex +
0,450288	0,100293	0,13247	141	69	72	137	0,757103	y ~ Intervention + Sex +
0,690995	-0,06448	0,161868	141	69	72	137	-0,39835	y ~ Intervention + Sex +
0,000921	-0,56579	0,167027	141	69	72	137	-3,3874	y ~ Intervention + Sex +
0,735664	-0,05475	0,16184	141	69	72	137	-0,33829	y ~ Intervention + Sex +
0,415807	-0,12656	0,155062	141	69	72	137	-0,81619	y ~ Intervention + Sex +
0,538922	-0,09681	0,157164	141	69	72	137	-0,61599	y ~ Intervention + Sex +
0,876438	0,02389	0,15336	141	69	72	137	0,155778	y ~ Intervention + Sex +
0,596658	0,082889	0,156262	141	69	72	137	0,53045	y ~ Intervention + Sex +

4,62E-07	-0,73646	0,139091	141	69	72	137	-5,29482	y ~ Intervention + Sex +
0,161995	-0,21337	0,151757	141	69	72	137	-1,40598	y ~ Intervention + Sex +
0,934186	0,011867	0,143441	141	69	72	137	0,082731	y ~ Intervention + Sex +
0,874247	0,024709	0,155831	141	69	72	137	0,158562	y ~ Intervention + Sex +
0,945057	-0,01123	0,162608	141	69	72	137	-0,06904	y ~ Intervention + Sex +
0,72143	-0,05641	0,157885	141	69	72	137	-0,35728	y ~ Intervention + Sex +
0,846471	0,030511	0,15728	141	69	72	137	0,19399	y ~ Intervention + Sex +
0,051856	-0,29123	0,148476	141	69	72	137	-1,96143	y ~ Intervention + Sex +
0,62156	-0,07523	0,152059	141	69	72	137	-0,49476	y ~ Intervention + Sex +
0,43283	-0,12984	0,165047	141	69	72	137	-0,78668	y ~ Intervention + Sex +
1,99E-05	0,655359	0,148268	141	69	72	137	4,420107	y ~ Intervention + Sex +
8,72E-25	-1,45305	0,114855	141	69	72	137	-12,6512	y ~ Intervention + Sex +
0,907012	-0,019	0,162321	141	69	72	137	-0,11702	y ~ Intervention + Sex +
0,279246	-0,16881	0,155398	141	69	72	137	-1,08632	y ~ Intervention + Sex +
0,105145	0,254844	0,156231	141	69	72	137	1,631199	y ~ Intervention + Sex +
0,248531	-0,1925	0,166112	141	69	72	137	-1,15885	y ~ Intervention + Sex +
0,292627	-0,17388	0,164593	141	69	72	137	-1,05644	y ~ Intervention + Sex +
0,442632	0,103664	0,13463	141	69	72	137	0,76999	y ~ Intervention + Sex +
0,612929	0,07646	0,150792	141	69	72	137	0,507058	y ~ Intervention + Sex +
0,966419	0,006515	0,154476	141	69	72	137	0,042177	y ~ Intervention + Sex +
0,270966	0,18109	0,163837	141	69	72	137	1,105303	y ~ Intervention + Sex +
0,330184	-0,15572	0,159353	141	69	72	137	-0,97722	y ~ Intervention + Sex +
0,00048	-0,63306	0,176949	141	69	72	137	-3,57765	y ~ Intervention + Sex +
0,468394	0,117814	0,162028	141	69	72	137	0,727119	y ~ Intervention + Sex +
0,020397	0,371761	0,158448	141	69	72	137	2,34626	y ~ Intervention + Sex +
0,160684	-0,20953	0,148562	141	69	72	137	-1,41041	y ~ Intervention + Sex +
0,432801	0,122223	0,155356	141	69	72	137	0,786727	y ~ Intervention + Sex +
0,120765	0,240614	0,154113	141	69	72	137	1,561281	y ~ Intervention + Sex +
0,820009	0,0369	0,161861	141	69	72	137	0,22797	y ~ Intervention + Sex +
0,256902	0,196552	0,172642	141	69	72	137	1,138492	y ~ Intervention + Sex +
0,641391	0,076043	0,162907	141	69	72	137	0,466791	y ~ Intervention + Sex +
0,565376	0,090907	0,15775	141	69	72	137	0,576273	y ~ Intervention + Sex +
0,663624	-0,06819	0,156457	141	69	72	137	-0,43586	y ~ Intervention + Sex +
0,061178	-0,3408	0,180536	141	69	72	137	-1,88772	y ~ Intervention + Sex +
0,256947	-0,18992	0,166837	141	69	72	137	-1,13838	y ~ Intervention + Sex +
0,421066	-0,12129	0,150295	141	69	72	137	-0,807	y ~ Intervention + Sex +
0,038544	0,366998	0,175668	141	69	72	137	2,089154	y ~ Intervention + Sex +
0,594095	-0,07396	0,138467	141	69	72	137	-0,53416	y ~ Intervention + Sex +
0,428614	-0,12175	0,153352	141	69	72	137	-0,79392	y ~ Intervention + Sex +
0,983276	0,003451	0,164355	141	69	72	137	0,021	y ~ Intervention + Sex +
0,854213	-0,02835	0,154017	141	69	72	137	-0,18409	y ~ Intervention + Sex +
0,150158	-0,24559	0,169714	141	69	72	137	-1,44708	y ~ Intervention + Sex +
0,839804	-0,03397	0,167718	141	69	72	137	-0,20253	y ~ Intervention + Sex +
0,30446	0,154317	0,149709	141	69	72	137	1,03078	y ~ Intervention + Sex +
0,614814	0,088375	0,17522	141	69	72	137	0,504367	y ~ Intervention + Sex +
0,323962	0,158336	0,15995	141	69	72	137	0,989913	y ~ Intervention + Sex +
0,654985	-0,07336	0,163821	141	69	72	137	-0,44783	y ~ Intervention + Sex +
0,171352	-0,22086	0,160617	141	69	72	137	-1,37508	y ~ Intervention + Sex +

0,296682	0,150522	0,143687	141	69	72	137	1,047569	y ~ Intervention + Sex +
0,425481	0,134795	0,168635	141	69	72	137	0,799334	y ~ Intervention + Sex +
0,016272	-0,375	0,154141	141	69	72	137	-2,4328	y ~ Intervention + Sex +
0,074938	0,28953	0,161342	141	69	72	137	1,794505	y ~ Intervention + Sex +
0,657153	-0,06947	0,156169	141	69	72	137	-0,44482	y ~ Intervention + Sex +
0,127186	-0,22803	0,148588	141	69	72	137	-1,53461	y ~ Intervention + Sex +
0,407949	-0,1371	0,165173	141	69	72	137	-0,83006	y ~ Intervention + Sex +
0,56495	-0,08785	0,152286	141	69	72	137	-0,57691	y ~ Intervention + Sex +
0,527436	-0,10071	0,15896	141	69	72	137	-0,63354	y ~ Intervention + Sex +
0,809944	0,040173	0,166718	141	69	72	137	0,240963	y ~ Intervention + Sex +
0,852993	-0,03005	0,161874	141	69	72	137	-0,18565	y ~ Intervention + Sex +
0,159925	0,208459	0,14753	141	69	72	137	1,412996	y ~ Intervention + Sex +
0,040026	0,343052	0,165469	141	69	72	137	2,073211	y ~ Intervention + Sex +
0,772729	0,051055	0,17643	141	69	72	137	0,289379	y ~ Intervention + Sex +
0,020361	0,312251	0,133046	141	69	72	137	2,34694	y ~ Intervention + Sex +
0,004477	-0,38397	0,132847	141	69	72	137	-2,8903	y ~ Intervention + Sex +
0,044109	0,339979	0,16733	141	69	72	137	2,031782	y ~ Intervention + Sex +
0,007204	0,440925	0,16162	141	69	72	137	2,728166	y ~ Intervention + Sex +
0,192104	0,22148	0,16896	141	69	72	137	1,310841	y ~ Intervention + Sex +
0,330408	0,17454	0,178692	141	69	72	137	0,976764	y ~ Intervention + Sex +
0,586787	-0,09201	0,168884	141	69	72	137	-0,54478	y ~ Intervention + Sex +
0,217198	0,164374	0,132589	141	69	72	137	1,239721	y ~ Intervention + Sex +
0,323876	0,148255	0,149739	141	69	72	137	0,99009	y ~ Intervention + Sex +
0,326445	0,155228	0,157619	141	69	72	137	0,984827	y ~ Intervention + Sex +
0,075136	0,27661	0,154249	141	69	72	137	1,79327	y ~ Intervention + Sex +
0,53801	0,096639	0,156531	141	69	72	137	0,617378	y ~ Intervention + Sex +
0,559498	-0,08622	0,147376	141	69	72	137	-0,58502	y ~ Intervention + Sex +
0,006502	0,460055	0,166464	141	69	72	137	2,763685	y ~ Intervention + Sex +
0,007593	0,366144	0,135119	141	69	72	137	2,709788	y ~ Intervention + Sex +
8,90E-08	0,877761	0,155336	141	69	72	137	5,650729	y ~ Intervention + Sex +
0,852202	-0,0268	0,143557	141	69	72	137	-0,18666	y ~ Intervention + Sex +
0,020397	0,374318	0,159538	141	69	72	137	2,346259	y ~ Intervention + Sex +
0,287056	0,154985	0,145013	141	69	72	137	1,068763	y ~ Intervention + Sex +
0,013581	-0,40059	0,160206	141	69	72	137	-2,50048	y ~ Intervention + Sex +
0,091368	-0,26185	0,154014	141	69	72	137	-1,70017	y ~ Intervention + Sex +
0,000321	0,498503	0,135043	141	69	72	137	3,691423	y ~ Intervention + Sex +
0,817018	-0,03748	0,161664	141	69	72	137	-0,23183	y ~ Intervention + Sex +
0,731232	0,056401	0,163867	141	69	72	137	0,344189	y ~ Intervention + Sex +
0,592586	-0,08426	0,157091	141	69	72	137	-0,53635	y ~ Intervention + Sex +
0,496839	0,116781	0,171412	141	69	72	137	0,681288	y ~ Intervention + Sex +
0,335368	0,170198	0,17605	141	69	72	137	0,966759	y ~ Intervention + Sex +
0,565532	0,085653	0,148693	141	69	72	137	0,576042	y ~ Intervention + Sex +
0,007263	0,440403	0,161596	141	69	72	137	2,72533	y ~ Intervention + Sex +
0,145522	0,234323	0,160072	141	69	72	137	1,463864	y ~ Intervention + Sex +
0,200088	0,194412	0,150998	141	69	72	137	1,287509	y ~ Intervention + Sex +
0,932453	-0,01291	0,151979	141	69	72	137	-0,08492	y ~ Intervention + Sex +
0,916625	-0,0157	0,149694	141	69	72	137	-0,10488	y ~ Intervention + Sex +
0,638784	0,074813	0,159025	141	69	72	137	0,470447	y ~ Intervention + Sex +

0,880985	-0,02037	0,1358	141	69	72	137	-0,15	y ~ Intervention + Sex +
0,002775	0,480931	0,157847	141	69	72	137	3,04682	y ~ Intervention + Sex +
0,510856	-0,10367	0,157266	141	69	72	137	-0,65923	y ~ Intervention + Sex +
0,313978	0,158647	0,156979	141	69	72	137	1,010624	y ~ Intervention + Sex +
0,036783	-0,29796	0,141297	141	69	72	137	-2,10879	y ~ Intervention + Sex +
0,040914	0,328937	0,159376	141	69	72	137	2,063906	y ~ Intervention + Sex +
0,146466	-0,22586	0,154656	141	69	72	137	-1,46042	y ~ Intervention + Sex +
0,693477	-0,05918	0,149833	141	69	72	137	-0,39497	y ~ Intervention + Sex +
0,764416	-0,05226	0,174033	141	69	72	137	-0,30028	y ~ Intervention + Sex +
0,022511	-0,37746	0,163563	141	69	72	137	-2,30776	y ~ Intervention + Sex +
0,828589	-0,03547	0,163511	141	69	72	137	-0,21693	y ~ Intervention + Sex +
0,020983	0,33371	0,142902	141	69	72	137	2,335237	y ~ Intervention + Sex +
0,098605	-0,25424	0,152881	141	69	72	137	-1,66297	y ~ Intervention + Sex +
0,121586	0,242955	0,155959	141	69	72	137	1,557809	y ~ Intervention + Sex +
1,15E-06	0,799692	0,157051	141	69	72	137	5,091932	y ~ Intervention + Sex +
0,936269	-0,0131	0,163522	141	69	72	137	-0,08011	y ~ Intervention + Sex +
0,789728	0,035938	0,134505	141	69	72	137	0,267186	y ~ Intervention + Sex +
0,182287	-0,20947	0,156259	141	69	72	137	-1,34055	y ~ Intervention + Sex +
0,833833	-0,03454	0,164329	141	69	72	137	-0,21019	y ~ Intervention + Sex +
0,808967	-0,03853	0,159086	141	69	72	137	-0,24223	y ~ Intervention + Sex +
0,34267	0,173834	0,18256	141	69	72	137	0,952206	y ~ Intervention + Sex +
0,550127	-0,08921	0,148922	141	69	72	137	-0,59905	y ~ Intervention + Sex +
0,806254	-0,03639	0,148099	141	69	72	137	-0,24574	y ~ Intervention + Sex +
0,74848	0,051582	0,160547	141	69	72	137	0,32129	y ~ Intervention + Sex +
0,603367	-0,08447	0,162194	141	69	72	137	-0,52077	y ~ Intervention + Sex +
0,489511	-0,10431	0,150535	141	69	72	137	-0,69295	y ~ Intervention + Sex +
3,74E-05	-0,65221	0,153006	141	69	72	137	-4,26264	y ~ Intervention + Sex +
0,27626	0,160512	0,146839	141	69	72	137	1,093118	y ~ Intervention + Sex +
0,951111	-0,01025	0,166886	141	69	72	137	-0,06142	y ~ Intervention + Sex +
0,660336	0,060821	0,138102	141	69	72	137	0,440407	y ~ Intervention + Sex +
0,045331	0,349097	0,172819	141	69	72	137	2,020019	y ~ Intervention + Sex +
0,847866	-0,02922	0,152008	141	69	72	137	-0,19221	y ~ Intervention + Sex +
0,002121	0,531586	0,169711	141	69	72	137	3,132304	y ~ Intervention + Sex +
0,573457	-0,09594	0,170002	141	69	72	137	-0,56432	y ~ Intervention + Sex +
0,337345	-0,14599	0,151627	141	69	72	137	-0,9628	y ~ Intervention + Sex +
0,312603	-0,16808	0,165842	141	69	72	137	-1,01351	y ~ Intervention + Sex +
0,563224	0,087335	0,150715	141	69	72	137	0,57947	y ~ Intervention + Sex +
0,365058	-0,14589	0,160532	141	69	72	137	-0,90879	y ~ Intervention + Sex +
0,011339	0,394792	0,153808	141	69	72	137	2,566775	y ~ Intervention + Sex +
0,566759	-0,0892	0,155345	141	69	72	137	-0,57422	y ~ Intervention + Sex +
0,032657	0,325662	0,150897	141	69	72	137	2,158176	y ~ Intervention + Sex +
0,052707	0,299012	0,153005	141	69	72	137	1,954264	y ~ Intervention + Sex +
0,077287	-0,28011	0,157363	141	69	72	137	-1,78003	y ~ Intervention + Sex +
0,126087	0,248702	0,161589	141	69	72	137	1,539098	y ~ Intervention + Sex +
0,027846	0,326625	0,146922	141	69	72	137	2,22312	y ~ Intervention + Sex +
0,270788	0,149248	0,134979	141	69	72	137	1,105716	y ~ Intervention + Sex +
6,08E-14	1,146761	0,137066	141	69	72	137	8,366469	y ~ Intervention + Sex +
0,527003	0,096907	0,1528	141	69	72	137	0,634207	y ~ Intervention + Sex +

0,049764	0,292762	0,147897	141	69	72	137	1,979502	y ~ Intervention + Sex +
7,90E-06	0,644442	0,138751	141	69	72	137	4,644582	y ~ Intervention + Sex +
0,836115	0,029039	0,140112	141	69	72	137	0,207259	y ~ Intervention + Sex +
0,306452	0,165763	0,161479	141	69	72	137	1,026527	y ~ Intervention + Sex +
0,58578	-0,0789	0,144432	141	69	72	137	-0,54625	y ~ Intervention + Sex +
0,977122	-0,00442	0,153953	141	69	72	137	-0,02873	y ~ Intervention + Sex +
0,567263	-0,09294	0,162059	141	69	72	137	-0,57348	y ~ Intervention + Sex +
0,774694	0,045929	0,160141	141	69	72	137	0,286807	y ~ Intervention + Sex +
0,507543	0,118446	0,178272	141	69	72	137	0,664413	y ~ Intervention + Sex +
0,423124	-0,13325	0,165848	141	69	72	137	-0,80342	y ~ Intervention + Sex +
0,649323	-0,0689	0,151189	141	69	72	137	-0,45571	y ~ Intervention + Sex +
0,303752	0,157576	0,152646	141	69	72	137	1,032297	y ~ Intervention + Sex +
0,793166	-0,04192	0,159561	141	69	72	137	-0,26271	y ~ Intervention + Sex +
0,576888	0,095729	0,171166	141	69	72	137	0,559274	y ~ Intervention + Sex +
0,002136	-0,46869	0,149739	141	69	72	137	-3,13009	y ~ Intervention + Sex +
0,395705	-0,12943	0,151918	141	69	72	137	-0,85199	y ~ Intervention + Sex +
0,280503	0,191057	0,176338	141	69	72	137	1,08347	y ~ Intervention + Sex +
0,038443	0,346443	0,165742	141	69	72	137	2,090253	y ~ Intervention + Sex +
0,940106	0,011364	0,150967	141	69	72	137	0,075274	y ~ Intervention + Sex +
0,409438	0,135044	0,16321	141	69	72	137	0,82742	y ~ Intervention + Sex +
0,000283	-0,54951	0,147446	141	69	72	137	-3,72687	y ~ Intervention + Sex +
0,63102	-0,0824	0,171186	141	69	72	137	-0,48137	y ~ Intervention + Sex +
0,731615	-0,05364	0,156074	141	69	72	137	-0,34368	y ~ Intervention + Sex +
0,438401	0,120999	0,155693	141	69	72	137	0,777166	y ~ Intervention + Sex +
0,385356	0,151987	0,174526	141	69	72	137	0,870856	y ~ Intervention + Sex +
0,08785	0,220605	0,128324	141	69	72	137	1,719128	y ~ Intervention + Sex +
0,011703	0,4007	0,156814	141	69	72	137	2,555253	y ~ Intervention + Sex +
0,011305	-0,38462	0,149783	141	69	72	137	-2,56787	y ~ Intervention + Sex +
0,41516	0,130224	0,159329	141	69	72	137	0,817329	y ~ Intervention + Sex +
0,01359	0,419588	0,167818	141	69	72	137	2,500259	y ~ Intervention + Sex +
0,608792	0,084213	0,164164	141	69	72	137	0,512979	y ~ Intervention + Sex +
0,914686	-0,01692	0,157629	141	69	72	137	-0,10733	y ~ Intervention + Sex +
0,303006	-0,14499	0,140233	141	69	72	137	-1,0339	y ~ Intervention + Sex +
0,150695	-0,20221	0,139921	141	69	72	137	-1,44516	y ~ Intervention + Sex +
0,417121	0,137303	0,1687	141	69	72	137	0,813889	y ~ Intervention + Sex +
0,007974	-0,3813	0,141608	141	69	72	137	-2,69261	y ~ Intervention + Sex +
0,534203	-0,08494	0,136299	141	69	72	137	-0,62318	y ~ Intervention + Sex +
0,959753	0,007864	0,15555	141	69	72	137	0,050556	y ~ Intervention + Sex +
0,061355	0,28976	0,153603	141	69	72	137	1,886418	y ~ Intervention + Sex +
0,691805	-0,05763	0,145084	141	69	72	137	-0,39725	y ~ Intervention + Sex +
0,561598	-0,10187	0,175061	141	69	72	137	-0,58189	y ~ Intervention + Sex +
0,682817	0,069874	0,170635	141	69	72	137	0,409494	y ~ Intervention + Sex +
0,127675	-0,20819	0,135836	141	69	72	137	-1,53262	y ~ Intervention + Sex +
0,897032	0,021209	0,163583	141	69	72	137	0,129652	y ~ Intervention + Sex +
0,123588	-0,25983	0,167693	141	69	72	137	-1,54942	y ~ Intervention + Sex +
0,164524	-0,21442	0,153434	141	69	72	137	-1,39749	y ~ Intervention + Sex +
0,01593	0,387488	0,158752	141	69	72	137	2,440841	y ~ Intervention + Sex +
0,241866	-0,18993	0,161583	141	69	72	137	-1,17542	y ~ Intervention + Sex +

0,739761	0,049654	0,149181	141	69	72	137	0,332844	y ~ Intervention + Sex +
0,008466	0,397691	0,148864	141	69	72	137	2,671517	y ~ Intervention + Sex +
0,965474	0,006295	0,145174	141	69	72	137	0,043364	y ~ Intervention + Sex +
0,398699	0,135887	0,16051	141	69	72	137	0,846591	y ~ Intervention + Sex +
0,561104	-0,10077	0,172964	141	69	72	137	-0,58262	y ~ Intervention + Sex +
0,553343	-0,08746	0,147189	141	69	72	137	-0,59422	y ~ Intervention + Sex +
0,042126	0,337281	0,164409	141	69	72	137	2,051479	y ~ Intervention + Sex +
0,091325	-0,24205	0,142349	141	69	72	137	-1,7004	y ~ Intervention + Sex +
0,191267	0,203009	0,154576	141	69	72	137	1,313329	y ~ Intervention + Sex +
0,970319	0,005722	0,153492	141	69	72	137	0,037276	y ~ Intervention + Sex +
0,847046	-0,03111	0,16096	141	69	72	137	-0,19325	y ~ Intervention + Sex +
0,942873	0,011409	0,158915	141	69	72	137	0,071791	y ~ Intervention + Sex +
0,786203	-0,04358	0,160339	141	69	72	137	-0,27178	y ~ Intervention + Sex +
0,646227	-0,08159	0,177354	141	69	72	137	-0,46003	y ~ Intervention + Sex +
0,100091	0,238041	0,143779	141	69	72	137	1,655604	y ~ Intervention + Sex +
0,469418	-0,10401	0,14337	141	69	72	137	-0,72544	y ~ Intervention + Sex +
0,076308	0,310706	0,173966	141	69	72	137	1,786017	y ~ Intervention + Sex +
0,313577	0,166993	0,1651	141	69	72	137	1,011465	y ~ Intervention + Sex +
0,280437	0,16324	0,150643	141	69	72	137	1,083619	y ~ Intervention + Sex +
0,381506	0,160218	0,182491	141	69	72	137	0,877954	y ~ Intervention + Sex +
0,775334	0,044998	0,157351	141	69	72	137	0,285968	y ~ Intervention + Sex +
0,867766	0,023426	0,140436	141	69	72	137	0,166809	y ~ Intervention + Sex +
0,083836	0,299095	0,171743	141	69	72	137	1,741527	y ~ Intervention + Sex +
0,649253	-0,07496	0,164448	141	69	72	137	-0,4558	y ~ Intervention + Sex +
0,866958	-0,02444	0,145614	141	69	72	137	-0,16784	y ~ Intervention + Sex +
0,009432	-0,44101	0,167483	141	69	72	137	-2,63313	y ~ Intervention + Sex +
0,318724	-0,1582	0,158085	141	69	72	137	-1,00073	y ~ Intervention + Sex +
0,351804	0,149621	0,160146	141	69	72	137	0,934278	y ~ Intervention + Sex +
0,465684	-0,12587	0,172051	141	69	72	137	-0,73157	y ~ Intervention + Sex +
0,048727	0,349005	0,175495	141	69	72	137	1,988694	y ~ Intervention + Sex +
0,18345	-0,19209	0,143673	141	69	72	137	-1,33697	y ~ Intervention + Sex +
0,052884	0,315836	0,161736	141	69	72	137	1,952782	y ~ Intervention + Sex +
0,658523	0,06236	0,140794	141	69	72	137	0,442919	y ~ Intervention + Sex +
0,998316	-0,00031	0,148882	141	69	72	137	-0,00211	y ~ Intervention + Sex +
3,36E-06	-0,7956	0,164175	141	69	72	137	-4,84604	y ~ Intervention + Sex +
0,011935	0,411737	0,161588	141	69	72	137	2,548066	y ~ Intervention + Sex +
0,630433	0,08072	0,1674	141	69	72	137	0,4822	y ~ Intervention + Sex +
0,952939	0,008963	0,151595	141	69	72	137	0,059125	y ~ Intervention + Sex +
0,025091	-0,37421	0,165226	141	69	72	137	-2,26486	y ~ Intervention + Sex +
0,083806	-0,28384	0,162966	141	69	72	137	-1,7417	y ~ Intervention + Sex +
0,00797	-0,38157	0,141701	141	69	72	137	-2,69281	y ~ Intervention + Sex +
0,200042	-0,2098	0,162937	141	69	72	137	-1,28764	y ~ Intervention + Sex +
0,48777	0,10469	0,150472	141	69	72	137	0,695741	y ~ Intervention + Sex +
0,025876	0,357372	0,15865	141	69	72	137	2,252577	y ~ Intervention + Sex +
0,096023	0,246945	0,147344	141	69	72	137	1,675978	y ~ Intervention + Sex +
0,828249	-0,03081	0,141754	141	69	72	137	-0,21736	y ~ Intervention + Sex +
0,03493	0,358518	0,168291	141	69	72	137	2,130348	y ~ Intervention + Sex +
0,018591	0,379049	0,159128	141	69	72	137	2,382045	y ~ Intervention + Sex +

0,022618	0,364262	0,15797	141	69	72	137	2,305894	y ~ Intervention + Sex +
0,519963	0,094012	0,14574	141	69	72	137	0,645066	y ~ Intervention + Sex +
0,122609	-0,24226	0,155947	141	69	72	137	-1,55351	y ~ Intervention + Sex +
0,338936	-0,1502	0,156521	141	69	72	137	-0,95962	y ~ Intervention + Sex +
0,620307	-0,07538	0,151816	141	69	72	137	-0,49654	y ~ Intervention + Sex +
0,719394	0,05484	0,152328	141	69	72	137	0,360011	y ~ Intervention + Sex +
0,149791	0,223666	0,154423	141	69	72	137	1,448396	y ~ Intervention + Sex +
0,070263	-0,28985	0,158871	141	69	72	137	-1,82445	y ~ Intervention + Sex +
0,060306	0,301955	0,15941	141	69	72	137	1,8942	y ~ Intervention + Sex +
0,639695	0,062259	0,132701	141	69	72	137	0,469168	y ~ Intervention + Sex +
0,649913	0,069106	0,151919	141	69	72	137	0,454883	y ~ Intervention + Sex +
0,99595	0,000851	0,167457	141	69	72	137	0,005085	y ~ Intervention + Sex +
0,276204	-0,17867	0,163433	141	69	72	137	-1,09325	y ~ Intervention + Sex +
0,233582	-0,19681	0,164497	141	69	72	137	-1,19646	y ~ Intervention + Sex +
0,524185	0,090892	0,142343	141	69	72	137	0,638545	y ~ Intervention + Sex +
0,023582	0,3536	0,154447	141	69	72	137	2,289453	y ~ Intervention + Sex +
0,608763	-0,07711	0,150305	141	69	72	137	-0,51302	y ~ Intervention + Sex +
0,638672	-0,07323	0,155618	141	69	72	137	-0,4706	y ~ Intervention + Sex +
0,037976	0,333273	0,15905	141	69	72	137	2,095397	y ~ Intervention + Sex +
0,024834	-0,36709	0,161789	141	69	72	137	-2,26896	y ~ Intervention + Sex +
0,60072	0,084507	0,161092	141	69	72	137	0,524584	y ~ Intervention + Sex +
0,405509	0,109766	0,131551	141	69	72	137	0,834398	y ~ Intervention + Sex +
0,452484	0,120314	0,159688	141	69	72	137	0,753431	y ~ Intervention + Sex +
0,138474	-0,24841	0,166698	141	69	72	137	-1,49019	y ~ Intervention + Sex +
0,038632	0,274906	0,131648	141	69	72	137	2,088186	y ~ Intervention + Sex +
0,121129	-0,24718	0,158474	141	69	72	137	-1,55974	y ~ Intervention + Sex +
0,415398	-0,12031	0,14727	141	69	72	137	-0,81691	y ~ Intervention + Sex +
0,383639	0,148364	0,169749	141	69	72	137	0,874017	y ~ Intervention + Sex +
0,139135	0,234861	0,157871	141	69	72	137	1,487673	y ~ Intervention + Sex +
0,03387	0,375048	0,175001	141	69	72	137	2,143124	y ~ Intervention + Sex +
0,450852	-0,11508	0,152195	141	69	72	137	-0,75616	y ~ Intervention + Sex +
0,001433	-0,57732	0,177418	141	69	72	137	-3,25399	y ~ Intervention + Sex +
0,000155	-0,60518	0,15551	141	69	72	137	-3,89157	y ~ Intervention + Sex +
0,980608	-0,00377	0,154718	141	69	72	137	-0,02435	y ~ Intervention + Sex +
0,037127	0,330069	0,15681	141	69	72	137	2,104893	y ~ Intervention + Sex +
0,00076	-0,54669	0,158728	141	69	72	137	-3,44421	y ~ Intervention + Sex +
0,156089	-0,22879	0,160422	141	69	72	137	-1,42619	y ~ Intervention + Sex +
0,004246	0,42785	0,147132	141	69	72	137	2,907938	y ~ Intervention + Sex +
0,382866	-0,14024	0,160198	141	69	72	137	-0,87544	y ~ Intervention + Sex +
0,714205	-0,05724	0,15597	141	69	72	137	-0,36697	y ~ Intervention + Sex +
0,002924	0,468586	0,154649	141	69	72	137	3,02999	y ~ Intervention + Sex +
0,040886	0,333473	0,161551	141	69	72	137	2,064194	y ~ Intervention + Sex +
0,014789	-0,31936	0,12936	141	69	72	137	-2,46877	y ~ Intervention + Sex +
0,739092	-0,05286	0,158389	141	69	72	137	-0,33373	y ~ Intervention + Sex +
0,001724	-0,51939	0,162459	141	69	72	137	-3,19706	y ~ Intervention + Sex +
0,810182	-0,03492	0,145111	141	69	72	137	-0,24066	y ~ Intervention + Sex +
0,872683	0,024038	0,14972	141	69	72	137	0,160552	y ~ Intervention + Sex +
0,32703	-0,16796	0,170754	141	69	72	137	-0,98363	y ~ Intervention + Sex +

0,668411	0,0706	0,164471	141	69	72	137	0,429256	y ~ Intervention + Sex +
0,211503	0,188404	0,150086	141	69	72	137	1,25531	y ~ Intervention + Sex +
0,991309	0,001599	0,146483	141	69	72	137	0,010913	y ~ Intervention + Sex +
0,199526	0,193757	0,150301	141	69	72	137	1,289127	y ~ Intervention + Sex +
0,017731	0,350518	0,146038	141	69	72	137	2,400181	y ~ Intervention + Sex +
0,872084	-0,02554	0,158345	141	69	72	137	-0,16131	y ~ Intervention + Sex +
0,133092	0,279145	0,184742	141	69	72	137	1,510997	y ~ Intervention + Sex +
0,262263	-0,18307	0,162631	141	69	72	137	-1,1257	y ~ Intervention + Sex +
0,66138	-0,06864	0,156359	141	69	72	137	-0,43896	y ~ Intervention + Sex +
1,92E-06	0,728475	0,146427	141	69	72	137	4,97501	y ~ Intervention + Sex +
0,710721	0,05217	0,140371	141	69	72	137	0,371659	y ~ Intervention + Sex +
0,946344	-0,01028	0,152456	141	69	72	137	-0,06742	y ~ Intervention + Sex +
0,014351	-0,38734	0,156186	141	69	72	137	-2,47998	y ~ Intervention + Sex +
0,898951	0,019333	0,151965	141	69	72	137	0,127223	y ~ Intervention + Sex +
0,046046	-0,32947	0,163648	141	69	72	137	-2,01327	y ~ Intervention + Sex +
0,57416	0,09824	0,174404	141	69	72	137	0,563288	y ~ Intervention + Sex +
0,566204	-0,0947	0,164675	141	69	72	137	-0,57505	y ~ Intervention + Sex +
0,70113	0,05914	0,153771	141	69	72	137	0,3846	y ~ Intervention + Sex +
0,805267	-0,039	0,157896	141	69	72	137	-0,24701	y ~ Intervention + Sex +
0,041299	-0,36779	0,178546	141	69	72	137	-2,05992	y ~ Intervention + Sex +
0,951321	0,009548	0,156122	141	69	72	137	0,06116	y ~ Intervention + Sex +
0,049764	0,292762	0,147897	141	69	72	137	1,979502	y ~ Intervention + Sex +
0,341523	0,162957	0,170729	141	69	72	137	0,954477	y ~ Intervention + Sex +
0,047668	0,320456	0,160367	141	69	72	137	1,998263	y ~ Intervention + Sex +
1,63E-05	-0,64575	0,14449	141	69	72	137	-4,46917	y ~ Intervention + Sex +
0,178229	0,206621	0,152694	141	69	72	137	1,353173	y ~ Intervention + Sex +
0,087309	-0,26964	0,156574	141	69	72	137	-1,7221	y ~ Intervention + Sex +
0,63923	0,073432	0,156298	141	69	72	137	0,46982	y ~ Intervention + Sex +
0,071996	-0,28624	0,157867	141	69	72	137	-1,81316	y ~ Intervention + Sex +
0,669739	0,073048	0,170901	141	69	72	137	0,427427	y ~ Intervention + Sex +
0,97533	-0,00456	0,147154	141	69	72	137	-0,03098	y ~ Intervention + Sex +
0,228023	-0,18812	0,155358	141	69	72	137	-1,21089	y ~ Intervention + Sex +
0,000148	-0,6537	0,167477	141	69	72	137	-3,90322	y ~ Intervention + Sex +
0,070096	-0,28337	0,155225	141	69	72	137	-1,82555	y ~ Intervention + Sex +
0,369125	-0,12191	0,135291	141	69	72	137	-0,90108	y ~ Intervention + Sex +
0,576373	0,075591	0,134976	141	69	72	137	0,560031	y ~ Intervention + Sex +
0,016399	-0,37636	0,15489	141	69	72	137	-2,42987	y ~ Intervention + Sex +
0,989553	-0,00213	0,162602	141	69	72	137	-0,01312	y ~ Intervention + Sex +
0,158494	-0,23322	0,164482	141	69	72	137	-1,41789	y ~ Intervention + Sex +
0,883252	0,021789	0,148103	141	69	72	137	0,147122	y ~ Intervention + Sex +
0,051872	-0,32033	0,163327	141	69	72	137	-1,9613	y ~ Intervention + Sex +
0,952586	-0,00864	0,145086	141	69	72	137	-0,05957	y ~ Intervention + Sex +
0,824222	0,034263	0,153959	141	69	72	137	0,222544	y ~ Intervention + Sex +
0,001323	0,53961	0,164591	141	69	72	137	3,278482	y ~ Intervention + Sex +
0,287594	-0,18494	0,173232	141	69	72	137	-1,06757	y ~ Intervention + Sex +
0,030145	0,33357	0,152248	141	69	72	137	2,190971	y ~ Intervention + Sex +
0,336881	-0,16234	0,168455	141	69	72	137	-0,96373	y ~ Intervention + Sex +
0,221113	0,209377	0,170339	141	69	72	137	1,229176	y ~ Intervention + Sex +

0,95633	0,008608	0,156911	141	69	72	137	0,054859	y ~ Intervention + Sex +
0,543413	-0,0903	0,148226	141	69	72	137	-0,60918	y ~ Intervention + Sex +
0,606383	-0,07987	0,15466	141	69	72	137	-0,51644	y ~ Intervention + Sex +
0,57887	0,082022	0,147425	141	69	72	137	0,556364	y ~ Intervention + Sex +
0,957065	0,008136	0,150854	141	69	72	137	0,053936	y ~ Intervention + Sex +
0,956256	-0,00864	0,157134	141	69	72	137	-0,05495	y ~ Intervention + Sex +
0,450202	0,128114	0,169183	141	69	72	137	0,757247	y ~ Intervention + Sex +
0,520935	-0,09813	0,152479	141	69	72	137	-0,64356	y ~ Intervention + Sex +
0,151897	-0,23527	0,163278	141	69	72	137	-1,44089	y ~ Intervention + Sex +
0,024247	-0,33109	0,145314	141	69	72	137	-2,27845	y ~ Intervention + Sex +
0,312544	-0,16917	0,166894	141	69	72	137	-1,01363	y ~ Intervention + Sex +
0,010978	-0,35927	0,139331	141	69	72	137	-2,57851	y ~ Intervention + Sex +
0,02025	-0,37539	0,159805	141	69	72	137	-2,34906	y ~ Intervention + Sex +
0,094119	0,260264	0,15439	141	69	72	137	1,685758	y ~ Intervention + Sex +
0,088624	-0,28462	0,165972	141	69	72	137	-1,7149	y ~ Intervention + Sex +
0,136661	0,247441	0,165277	141	69	72	137	1,497127	y ~ Intervention + Sex +
0,318904	0,168704	0,168645	141	69	72	137	1,000351	y ~ Intervention + Sex +
0,120064	-0,25131	0,160656	141	69	72	137	-1,56426	y ~ Intervention + Sex +
0,168591	0,219424	0,158537	141	69	72	137	1,384059	y ~ Intervention + Sex +
0,636489	0,070038	0,147862	141	69	72	137	0,473671	y ~ Intervention + Sex +
0,092737	0,292334	0,172677	141	69	72	137	1,692952	y ~ Intervention + Sex +
0,586906	0,083395	0,153127	141	69	72	137	0,544612	y ~ Intervention + Sex +
0,459865	-0,11661	0,157338	141	69	72	137	-0,74116	y ~ Intervention + Sex +
0,348308	-0,15614	0,165913	141	69	72	137	-0,9411	y ~ Intervention + Sex +
0,719481	0,055893	0,155303	141	69	72	137	0,359894	y ~ Intervention + Sex +
0,000589	0,540284	0,15354	141	69	72	137	3,518857	y ~ Intervention + Sex +
2,65E-06	0,734066	0,149768	141	69	72	137	4,901361	y ~ Intervention + Sex +
0,071558	0,307357	0,16925	141	69	72	137	1,815996	y ~ Intervention + Sex +
0,156109	0,233824	0,163958	141	69	72	137	1,426118	y ~ Intervention + Sex +
0,025354	0,367598	0,162603	141	69	72	137	2,260707	y ~ Intervention + Sex +
0,013884	0,4642	0,186252	141	69	72	137	2,492316	y ~ Intervention + Sex +
0,605138	-0,08223	0,15867	141	69	72	137	-0,51822	y ~ Intervention + Sex +
0,559707	-0,10089	0,172549	141	69	72	137	-0,58471	y ~ Intervention + Sex +
0,651169	0,076566	0,168969	141	69	72	137	0,453135	y ~ Intervention + Sex +
0,004273	-0,43648	0,15021	141	69	72	137	-2,90579	y ~ Intervention + Sex +
0,044092	-0,32332	0,159116	141	69	72	137	-2,03195	y ~ Intervention + Sex +
0,253379	0,187935	0,163849	141	69	72	137	1,147003	y ~ Intervention + Sex +
0,801857	-0,04001	0,159134	141	69	72	137	-0,25143	y ~ Intervention + Sex +
0,003708	-0,41176	0,139453	141	69	72	137	-2,95269	y ~ Intervention + Sex +
0,556201	0,098806	0,167484	141	69	72	137	0,589943	y ~ Intervention + Sex +
0,210236	-0,22988	0,182616	141	69	72	137	-1,25882	y ~ Intervention + Sex +
0,95093	-0,01053	0,170718	141	69	72	137	-0,06165	y ~ Intervention + Sex +
0,818431	-0,037	0,160859	141	69	72	137	-0,23001	y ~ Intervention + Sex +
0,967395	0,006181	0,150936	141	69	72	137	0,040951	y ~ Intervention + Sex +
0,953991	0,007475	0,129321	141	69	72	137	0,057802	y ~ Intervention + Sex +
0,01964	0,368849	0,156232	141	69	72	137	2,360901	y ~ Intervention + Sex +
0,133492	0,230259	0,152547	141	69	72	137	1,509431	y ~ Intervention + Sex +
0,952654	-0,00961	0,161518	141	69	72	137	-0,05948	y ~ Intervention + Sex +

0,065099	0,302315	0,162577	141	69	72	137	1,859513	y ~ Intervention + Sex +
0,78847	0,048216	0,17936	141	69	72	137	0,268824	y ~ Intervention + Sex +
0,569453	-0,07886	0,138286	141	69	72	137	-0,57023	y ~ Intervention + Sex +
0,223298	0,200196	0,163646	141	69	72	137	1,223349	y ~ Intervention + Sex +
0,11719	-0,20818	0,13204	141	69	72	137	-1,57662	y ~ Intervention + Sex +
0,471298	0,116489	0,161259	141	69	72	137	0,722371	y ~ Intervention + Sex +
0,738279	-0,05103	0,152418	141	69	72	137	-0,33481	y ~ Intervention + Sex +
0,688997	0,073017	0,182058	141	69	72	137	0,401065	y ~ Intervention + Sex +
0,06058	-0,29932	0,158191	141	69	72	137	-1,89216	y ~ Intervention + Sex +
0,445306	-0,11493	0,15014	141	69	72	137	-0,76547	y ~ Intervention + Sex +
0,087265	0,232045	0,134727	141	69	72	137	1,722338	y ~ Intervention + Sex +
0,384143	0,14625	0,167509	141	69	72	137	0,873088	y ~ Intervention + Sex +
0,011237	0,396719	0,154363	141	69	72	137	2,570038	y ~ Intervention + Sex +
0,011973	0,418179	0,16419	141	69	72	137	2,546921	y ~ Intervention + Sex +
0,556896	0,088204	0,149777	141	69	72	137	0,588904	y ~ Intervention + Sex +
0,148896	0,2395	0,164989	141	69	72	137	1,451611	y ~ Intervention + Sex +
0,266589	0,175556	0,157379	141	69	72	137	1,1155	y ~ Intervention + Sex +
0,848626	-0,03064	0,160211	141	69	72	137	-0,19123	y ~ Intervention + Sex +
0,63599	0,062515	0,131784	141	69	72	137	0,474372	y ~ Intervention + Sex +
0,018353	0,377547	0,158169	141	69	72	137	2,386978	y ~ Intervention + Sex +
0,003776	-0,50745	0,17221	141	69	72	137	-2,94668	y ~ Intervention + Sex +
0,198836	0,21703	0,168094	141	69	72	137	1,291121	y ~ Intervention + Sex +
0,155002	0,232436	0,162546	141	69	72	137	1,42997	y ~ Intervention + Sex +
0,007287	0,426983	0,15674	141	69	72	137	2,724143	y ~ Intervention + Sex +
0,697423	0,058878	0,151116	141	69	72	137	0,389619	y ~ Intervention + Sex +
0,002744	-0,48562	0,159195	141	69	72	137	-3,05049	y ~ Intervention + Sex +
0,55648	-0,09593	0,162724	141	69	72	137	-0,58952	y ~ Intervention + Sex +
0,445325	-0,11737	0,153331	141	69	72	137	-0,76544	y ~ Intervention + Sex +
0,940406	-0,01155	0,154253	141	69	72	137	-0,0749	y ~ Intervention + Sex +
0,365855	-0,15704	0,173088	141	69	72	137	-0,90727	y ~ Intervention + Sex +
0,833819	0,031966	0,152068	141	69	72	137	0,210206	y ~ Intervention + Sex +
0,097867	-0,26066	0,156397	141	69	72	137	-1,66666	y ~ Intervention + Sex +
0,209407	-0,21248	0,168487	141	69	72	137	-1,26112	y ~ Intervention + Sex +
0,864903	0,026503	0,155485	141	69	72	137	0,170456	y ~ Intervention + Sex +
0,601261	0,0854	0,163038	141	69	72	137	0,523805	y ~ Intervention + Sex +
0,961359	0,007368	0,15181	141	69	72	137	0,048537	y ~ Intervention + Sex +
0,342628	0,15156	0,159153	141	69	72	137	0,952289	y ~ Intervention + Sex +
0,186943	0,194082	0,146332	141	69	72	137	1,326314	y ~ Intervention + Sex +
0,590403	0,095698	0,177376	141	69	72	137	0,539521	y ~ Intervention + Sex +
0,295101	0,155588	0,148036	141	69	72	137	1,051016	y ~ Intervention + Sex +
0,019956	-0,40522	0,172087	141	69	72	137	-2,35473	y ~ Intervention + Sex +
0,336176	0,146976	0,152284	141	69	72	137	0,965138	y ~ Intervention + Sex +
0,87435	0,024658	0,155637	141	69	72	137	0,158431	y ~ Intervention + Sex +
0,596074	0,081496	0,153392	141	69	72	137	0,531297	y ~ Intervention + Sex +
0,001261	0,540867	0,164241	141	69	72	137	3,293126	y ~ Intervention + Sex +
0,703016	-0,05892	0,154208	141	69	72	137	-0,38205	y ~ Intervention + Sex +
0,092113	-0,26173	0,1543	141	69	72	137	-1,69623	y ~ Intervention + Sex +
0,179283	0,242418	0,179586	141	69	72	137	1,349873	y ~ Intervention + Sex +

0,067961	0,226704	0,123222	141	69	72	137	1,839805	y ~ Intervention + Sex +
0,090662	0,23709	0,139144	141	69	72	137	1,703925	y ~ Intervention + Sex +
0,620071	-0,07524	0,15143	141	69	72	137	-0,49688	y ~ Intervention + Sex +
0,916343	-0,01756	0,166834	141	69	72	137	-0,10524	y ~ Intervention + Sex +
0,751842	-0,04389	0,13852	141	69	72	137	-0,31685	y ~ Intervention + Sex +
0,202702	-0,18972	0,14822	141	69	72	137	-1,28002	y ~ Intervention + Sex +
0,457052	-0,12682	0,170041	141	69	72	137	-0,74582	y ~ Intervention + Sex +
0,320494	0,149814	0,150256	141	69	72	137	0,997059	y ~ Intervention + Sex +
0,015794	-0,38102	0,155896	141	69	72	137	-2,44407	y ~ Intervention + Sex +
0,764407	-0,04005	0,133364	141	69	72	137	-0,3003	y ~ Intervention + Sex +
0,379778	-0,1537	0,174427	141	69	72	137	-0,88115	y ~ Intervention + Sex +
0,375619	-0,13002	0,146274	141	69	72	137	-0,88889	y ~ Intervention + Sex +
0,412856	-0,13646	0,166136	141	69	72	137	-0,82138	y ~ Intervention + Sex +
0,012627	0,402733	0,159347	141	69	72	137	2,527398	y ~ Intervention + Sex +
0,315483	-0,14831	0,14721	141	69	72	137	-1,00747	y ~ Intervention + Sex +
0,459656	0,12152	0,163883	141	69	72	137	0,741506	y ~ Intervention + Sex +
0,507146	0,10732	0,161375	141	69	72	137	0,665035	y ~ Intervention + Sex +
0,594208	0,068376	0,128045	141	69	72	137	0,533999	y ~ Intervention + Sex +
0,52675	0,105903	0,166882	141	69	72	137	0,634597	y ~ Intervention + Sex +
0,275528	0,192674	0,175991	141	69	72	137	1,094794	y ~ Intervention + Sex +
0,266762	-0,18872	0,169241	141	69	72	137	-1,1151	y ~ Intervention + Sex +
0,899991	-0,01964	0,155956	141	69	72	137	-0,12591	y ~ Intervention + Sex +
0,030295	0,353662	0,161568	141	69	72	137	2,188937	y ~ Intervention + Sex +
0,70352	0,062238	0,163197	141	69	72	137	0,381369	y ~ Intervention + Sex +
0,670182	-0,06398	0,149904	141	69	72	137	-0,42682	y ~ Intervention + Sex +
0,667223	-0,07519	0,174495	141	69	72	137	-0,43089	y ~ Intervention + Sex +
0,402892	-0,1278	0,15231	141	69	72	137	-0,83907	y ~ Intervention + Sex +
0,095853	-0,2731	0,162868	141	69	72	137	-1,67685	y ~ Intervention + Sex +
0,602481	0,086432	0,165563	141	69	72	137	0,522047	y ~ Intervention + Sex +
0,410903	0,121577	0,147396	141	69	72	137	0,824828	y ~ Intervention + Sex +
0,064876	0,303006	0,162812	141	69	72	137	1,86108	y ~ Intervention + Sex +
0,054402	-0,30265	0,155982	141	69	72	137	-1,94026	y ~ Intervention + Sex +
0,000346	0,567916	0,154722	141	69	72	137	3,670549	y ~ Intervention + Sex +
0,169434	-0,22801	0,165068	141	69	72	137	-1,3813	y ~ Intervention + Sex +
0,733778	0,053642	0,157402	141	69	72	137	0,340798	y ~ Intervention + Sex +
0,347059	0,147004	0,155799	141	69	72	137	0,943553	y ~ Intervention + Sex +
0,417599	-0,1383	0,170106	141	69	72	137	-0,81305	y ~ Intervention + Sex +
0,01392	-0,42965	0,172458	141	69	72	137	-2,49134	y ~ Intervention + Sex +
0,027846	-0,34435	0,154894	141	69	72	137	-2,22312	y ~ Intervention + Sex +
0,318566	0,165709	0,165534	141	69	72	137	1,001054	y ~ Intervention + Sex +
0,02086	-0,37761	0,161541	141	69	72	137	-2,33752	y ~ Intervention + Sex +
0,200544	-0,21028	0,163492	141	69	72	137	-1,2862	y ~ Intervention + Sex +
2,57E-07	-0,75959	0,140071	141	69	72	137	-5,42292	y ~ Intervention + Sex +

0,0472	0,329172	0,164377	141	69	72	137	2,002539	y ~ Intervention + Sex +
1,94E-05	-0,75113	0,169688	141	69	72	137	-4,42652	y ~ Intervention + Sex +
0,007531	-0,40172	0,148089	141	69	72	137	-2,71268	y ~ Intervention + Sex +
0,991267	0,001956	0,178405	141	69	72	137	0,010966	y ~ Intervention + Sex +
0,006869	-0,44721	0,162938	141	69	72	137	-2,74469	y ~ Intervention + Sex +
0,126497	0,245538	0,159708	141	69	72	137	1,537418	y ~ Intervention + Sex +
0,920763	0,017333	0,173931	141	69	72	137	0,099656	y ~ Intervention + Sex +
0,722365	-0,05898	0,165658	141	69	72	137	-0,35603	y ~ Intervention + Sex +
0,986036	-0,00282	0,160553	141	69	72	137	-0,01753	y ~ Intervention + Sex +
0,363779	-0,15253	0,167394	141	69	72	137	-0,91122	y ~ Intervention + Sex +
0,666588	0,078079	0,180834	141	69	72	137	0,431768	y ~ Intervention + Sex +
0,7413	-0,0555	0,167778	141	69	72	137	-0,3308	y ~ Intervention + Sex +
0,458143	0,135342	0,181908	141	69	72	137	0,744013	y ~ Intervention + Sex +
0,001029	0,459583	0,137005	141	69	72	137	3,354509	y ~ Intervention + Sex +
0,920142	-0,01575	0,156824	141	69	72	137	-0,10044	y ~ Intervention + Sex +
0,013351	-0,39365	0,15703	141	69	72	137	-2,50682	y ~ Intervention + Sex +
0,572353	0,097455	0,172196	141	69	72	137	0,565951	y ~ Intervention + Sex +
0,298088	0,174014	0,166599	141	69	72	137	1,044513	y ~ Intervention + Sex +
0,050863	0,318982	0,161925	141	69	72	137	1,969934	y ~ Intervention + Sex +
3,63E-07	-0,7986	0,14933	141	69	72	137	-5,34787	y ~ Intervention + Sex +

3,52E-06	0,763247	0,157853	141	69	72	137	4,835165	y ~ Intervention + Sex +
0,343688	-0,15906	0,167396	141	69	72	137	-0,95019	y ~ Intervention + Sex +
0,005034	-0,52466	0,18403	141	69	72	137	-2,85093	y ~ Intervention + Sex +
0,9928	-0,00149	0,164558	141	69	72	137	-0,00904	y ~ Intervention + Sex +
0,400983	-0,13887	0,164832	141	69	72	137	-0,84249	y ~ Intervention + Sex +
0,426747	-0,1222	0,153295	141	69	72	137	-0,79714	y ~ Intervention + Sex +
0,649681	0,068499	0,150478	141	69	72	137	0,455207	y ~ Intervention + Sex +
0,239432	0,209044	0,176924	141	69	72	137	1,181548	y ~ Intervention + Sex +
0,553463	0,102046	0,171782	141	69	72	137	0,594042	y ~ Intervention + Sex +
0,067308	-0,33663	0,182529	141	69	72	137	-1,84424	y ~ Intervention + Sex +
0,072928	0,263113	0,145593	141	69	72	137	1,80718	y ~ Intervention + Sex +
0,808158	0,038837	0,159643	141	69	72	137	0,243273	y ~ Intervention + Sex +
0,094113	-0,25416	0,150769	141	69	72	137	-1,68579	y ~ Intervention + Sex +
0,55593	-0,09027	0,152906	141	69	72	137	-0,59035	y ~ Intervention + Sex +
0,080246	-0,25335	0,143759	141	69	72	137	-1,76232	y ~ Intervention + Sex +
0,161523	0,197329	0,140191	141	69	72	137	1,407572	y ~ Intervention + Sex +
0,967657	0,007021	0,172828	141	69	72	137	0,040622	y ~ Intervention + Sex +
0,203146	0,213555	0,167002	141	69	72	137	1,278753	y ~ Intervention + Sex +
0,943425	-0,01163	0,163614	141	69	72	137	-0,0711	y ~ Intervention + Sex +
8,60E-06	-0,66624	0,144073	141	69	72	137	-4,62433	y ~ Intervention + Sex +
0,008104	0,429649	0,159903	141	69	72	137	2,686938	y ~ Intervention + Sex +
0,056309	0,282351	0,14668	141	69	72	137	1,924946	y ~ Intervention + Sex +
0,003426	0,489194	0,164234	141	69	72	137	2,978642	y ~ Intervention + Sex +
0,391688	-0,14434	0,167979	141	69	72	137	-0,85928	y ~ Intervention + Sex +
0,453385	-0,11841	0,157471	141	69	72	137	-0,75193	y ~ Intervention + Sex +
0,007424	0,425631	0,156617	141	69	72	137	2,717653	y ~ Intervention + Sex +
0,01651	0,418907	0,172581	141	69	72	137	2,427308	y ~ Intervention + Sex +
0,006694	0,44183	0,160454	141	69	72	137	2,753625	y ~ Intervention + Sex +
0,462821	0,102108	0,138682	141	69	72	137	0,736277	y ~ Intervention + Sex +
0,712246	0,062111	0,168046	141	69	72	137	0,369607	y ~ Intervention + Sex +
0,628734	0,088756	0,183153	141	69	72	137	0,484601	y ~ Intervention + Sex +
0,909159	-0,01964	0,171855	141	69	72	137	-0,11431	y ~ Intervention + Sex +
0,31802	-0,16527	0,164905	141	69	72	137	-1,00219	y ~ Intervention + Sex +
0,291352	0,173184	0,163497	141	69	72	137	1,059246	y ~ Intervention + Sex +
0,379124	-0,14232	0,16129	141	69	72	137	-0,88237	y ~ Intervention + Sex +
0,098213	-0,28102	0,168787	141	69	72	137	-1,66493	y ~ Intervention + Sex +
0,888464	-0,02369	0,16859	141	69	72	137	-0,14051	y ~ Intervention + Sex +
0,686957	0,065835	0,16302	141	69	72	137	0,403844	y ~ Intervention + Sex +
0,007384	-0,46337	0,170383	141	69	72	137	-2,71955	y ~ Intervention + Sex +
0,153058	0,209836	0,146045	141	69	72	137	1,436787	y ~ Intervention + Sex +
0,001152	-0,53019	0,159676	141	69	72	137	-3,32038	y ~ Intervention + Sex +
0,286869	0,166113	0,155365	141	69	72	137	1,06918	y ~ Intervention + Sex +
0,255535	0,178112	0,155994	141	69	72	137	1,141786	y ~ Intervention + Sex +
0,753099	0,049525	0,157129	141	69	72	137	0,315187	y ~ Intervention + Sex +
0,812791	-0,03505	0,147723	141	69	72	137	-0,23728	y ~ Intervention + Sex +
0,198207	0,216669	0,167578	141	69	72	137	1,292944	y ~ Intervention + Sex +
0,571653	0,095149	0,167815	141	69	72	137	0,566984	y ~ Intervention + Sex +
0,303729	0,160554	0,155524	141	69	72	137	1,032345	y ~ Intervention + Sex +

0,082088	0,252854	0,14436	141	69	72	137	1,751555	y ~ Intervention + Sex +
0,390576	0,141566	0,164362	141	69	72	137	0,861304	y ~ Intervention + Sex +
4,11E-05	-0,70423	0,166154	141	69	72	137	-4,2384	y ~ Intervention + Sex +
0,130201	-0,24664	0,162001	141	69	72	137	-1,52245	y ~ Intervention + Sex +
0,747721	0,048706	0,151123	141	69	72	137	0,322294	y ~ Intervention + Sex +
0,255277	-0,17744	0,155324	141	69	72	137	-1,14241	y ~ Intervention + Sex +
0,233618	0,183145	0,153084	141	69	72	137	1,196371	y ~ Intervention + Sex +
8,67E-05	0,594265	0,146889	141	69	72	137	4,045675	y ~ Intervention + Sex +
0,477324	0,116699	0,163772	141	69	72	137	0,71257	y ~ Intervention + Sex +
0,92919	0,014103	0,158414	141	69	72	137	0,089028	y ~ Intervention + Sex +
0,787102	0,040844	0,150937	141	69	72	137	0,270605	y ~ Intervention + Sex +
0,133548	0,211777	0,140323	141	69	72	137	1,509212	y ~ Intervention + Sex +
0,454947	0,12203	0,162853	141	69	72	137	0,749323	y ~ Intervention + Sex +
0,507169	-0,10837	0,162964	141	69	72	137	-0,665	y ~ Intervention + Sex +
0,057516	-0,3217	0,167948	141	69	72	137	-1,91547	y ~ Intervention + Sex +
0,288998	-0,16058	0,150856	141	69	72	137	-1,06445	y ~ Intervention + Sex +
0,981273	-0,00365	0,155129	141	69	72	137	-0,02352	y ~ Intervention + Sex +
0,415428	0,139836	0,171188	141	69	72	137	0,816857	y ~ Intervention + Sex +
0,365224	0,117843	0,129716	141	69	72	137	0,908473	y ~ Intervention + Sex +
0,468644	-0,11174	0,153755	141	69	72	137	-0,72671	y ~ Intervention + Sex +
0,002392	0,503059	0,162576	141	69	72	137	3,094289	y ~ Intervention + Sex +
0,005733	0,420177	0,149698	141	69	72	137	2,806836	y ~ Intervention + Sex +
0,054536	0,316307	0,163114	141	69	72	137	1,939174	y ~ Intervention + Sex +
0,17991	0,194016	0,143938	141	69	72	137	1,347915	y ~ Intervention + Sex +
0,574038	-0,09508	0,168741	141	69	72	137	-0,56347	y ~ Intervention + Sex +
0,530009	0,099019	0,157275	141	69	72	137	0,629594	y ~ Intervention + Sex +
0,505851	-0,10596	0,158848	141	69	72	137	-0,66707	y ~ Intervention + Sex +
0,058525	0,338143	0,177253	141	69	72	137	1,907681	y ~ Intervention + Sex +
0,392237	-0,13287	0,154813	141	69	72	137	-0,85828	y ~ Intervention + Sex +
0,728985	-0,05527	0,159198	141	69	72	137	-0,34718	y ~ Intervention + Sex +
0,734117	0,053321	0,156666	141	69	72	137	0,340347	y ~ Intervention + Sex +
0,496006	-0,10975	0,160778	141	69	72	137	-0,68261	y ~ Intervention + Sex +
0,097538	-0,2592	0,155368	141	69	72	137	-1,66831	y ~ Intervention + Sex +
0,544278	-0,09026	0,148489	141	69	72	137	-0,60787	y ~ Intervention + Sex +
0,540041	-0,10281	0,167363	141	69	72	137	-0,61429	y ~ Intervention + Sex +
0,217151	0,183277	0,147822	141	69	72	137	1,239847	y ~ Intervention + Sex +
0,460086	-0,12291	0,165917	141	69	72	137	-0,74079	y ~ Intervention + Sex +
0,220256	-0,19672	0,15974	141	69	72	137	-1,23147	y ~ Intervention + Sex +
0,648175	-0,06977	0,152578	141	69	72	137	-0,45731	y ~ Intervention + Sex +
0,639247	0,086349	0,1838	141	69	72	137	0,469797	y ~ Intervention + Sex +
0,035218	-0,33332	0,156714	141	69	72	137	-2,12693	y ~ Intervention + Sex +
0,090825	0,260797	0,153135	141	69	72	137	1,703056	y ~ Intervention + Sex +
0,710671	0,063551	0,170962	141	69	72	137	0,371726	y ~ Intervention + Sex +
0,829124	0,032012	0,148041	141	69	72	137	0,216237	y ~ Intervention + Sex +
0,000106	-0,64514	0,161608	141	69	72	137	-3,99203	y ~ Intervention + Sex +
0,270961	-0,15451	0,139789	141	69	72	137	-1,10531	y ~ Intervention + Sex +
0,63545	-0,07369	0,155092	141	69	72	137	-0,47513	y ~ Intervention + Sex +
0,204823	-0,19803	0,155443	141	69	72	137	-1,27399	y ~ Intervention + Sex +

0,537958	-0,09963	0,161358	141	69	72	137	-0,61746	y ~ Intervention + Sex +
0,587341	0,099327	0,182594	141	69	72	137	0,543978	y ~ Intervention + Sex +
0,52436	-0,09244	0,144825	141	69	72	137	-0,63828	y ~ Intervention + Sex +
0,003244	-0,44093	0,147152	141	69	72	137	-2,9964	y ~ Intervention + Sex +
0,012796	0,407968	0,161731	141	69	72	137	2,522509	y ~ Intervention + Sex +
0,477685	0,11395	0,160046	141	69	72	137	0,711985	y ~ Intervention + Sex +
0,000304	0,506405	0,136612	141	69	72	137	3,706885	y ~ Intervention + Sex +
0,215775	0,236859	0,190464	141	69	72	137	1,243588	y ~ Intervention + Sex +
0,005724	-0,43515	0,155003	141	69	72	137	-2,80739	y ~ Intervention + Sex +
0,918009	-0,01588	0,154021	141	69	72	137	-0,10313	y ~ Intervention + Sex +
0,917483	0,016925	0,163058	141	69	72	137	0,103796	y ~ Intervention + Sex +
0,041615	0,356248	0,173216	141	69	72	137	2,056673	y ~ Intervention + Sex +
0,345373	-0,15407	0,162713	141	69	72	137	-0,94687	y ~ Intervention + Sex +
0,361721	0,118143	0,129097	141	69	72	137	0,91515	y ~ Intervention + Sex +
0,965505	-0,00637	0,147121	141	69	72	137	-0,04333	y ~ Intervention + Sex +
0,900173	0,019691	0,156681	141	69	72	137	0,125675	y ~ Intervention + Sex +
0,044162	-0,28525	0,140429	141	69	72	137	-2,03127	y ~ Intervention + Sex +
0,943978	0,010776	0,153067	141	69	72	137	0,070399	y ~ Intervention + Sex +
0,138048	-0,23659	0,158594	141	69	72	137	-1,49181	y ~ Intervention + Sex +
0,329158	-0,15685	0,160161	141	69	72	137	-0,9793	y ~ Intervention + Sex +
0,056086	-0,29836	0,154853	141	69	72	137	-1,92671	y ~ Intervention + Sex +
0,001248	0,493685	0,149774	141	69	72	137	3,296201	y ~ Intervention + Sex +
0,000616	-0,48791	0,139185	141	69	72	137	-3,5055	y ~ Intervention + Sex +
0,272619	-0,16989	0,154237	141	69	72	137	-1,10148	y ~ Intervention + Sex +
0,120428	-0,24428	0,156316	141	69	72	137	-1,56271	y ~ Intervention + Sex +
0,561878	-0,09381	0,161337	141	69	72	137	-0,58147	y ~ Intervention + Sex +
0,134365	0,207992	0,138107	141	69	72	137	1,506019	y ~ Intervention + Sex +
0,057125	0,28235	0,147171	141	69	72	137	1,918519	y ~ Intervention + Sex +
7,48E-06	0,7952	0,170733	141	69	72	137	4,657568	y ~ Intervention + Sex +
0,301559	0,172937	0,166765	141	69	72	137	1,037009	y ~ Intervention + Sex +
0,175691	0,239049	0,175619	141	69	72	137	1,36118	y ~ Intervention + Sex +
0,189115	0,189554	0,143627	141	69	72	137	1,319763	y ~ Intervention + Sex +
0,007739	-0,37893	0,140181	141	69	72	137	-2,70312	y ~ Intervention + Sex +
0,989779	0,002195	0,171013	141	69	72	137	0,012834	y ~ Intervention + Sex +
0,273839	-0,17667	0,160806	141	69	72	137	-1,09867	y ~ Intervention + Sex +
0,044699	0,319362	0,157627	141	69	72	137	2,026066	y ~ Intervention + Sex +
0,592234	-0,0811	0,151067	141	69	72	137	-0,53686	y ~ Intervention + Sex +
0,411303	0,145284	0,176289	141	69	72	137	0,824122	y ~ Intervention + Sex +
0,091337	0,263518	0,15498	141	69	72	137	1,700336	y ~ Intervention + Sex +
0,525669	-0,10688	0,167979	141	69	72	137	-0,63626	y ~ Intervention + Sex +
0,968141	-0,00703	0,175667	141	69	72	137	-0,04001	y ~ Intervention + Sex +
0,481555	-0,10833	0,153501	141	69	72	137	-0,70573	y ~ Intervention + Sex +
0,174871	-0,223	0,163515	141	69	72	137	-1,36379	y ~ Intervention + Sex +
0,625323	0,078626	0,16065	141	69	72	137	0,489426	y ~ Intervention + Sex +
0,984636	0,003184	0,16502	141	69	72	137	0,019292	y ~ Intervention + Sex +
0,860649	0,029404	0,167185	141	69	72	137	0,175879	y ~ Intervention + Sex +
0,269698	0,162787	0,146887	141	69	72	137	1,108244	y ~ Intervention + Sex +
0,000126	0,575987	0,145956	141	69	72	137	3,946314	y ~ Intervention + Sex +

0,637089	0,073262	0,154945	141	69	72	137	0,472828	y ~ Intervention + Sex +
0,016825	0,386124	0,159545	141	69	72	137	2,420153	y ~ Intervention + Sex +
0,023467	0,357405	0,155978	141	69	72	137	2,291375	y ~ Intervention + Sex +
0,860345	-0,02872	0,162935	141	69	72	137	-0,17627	y ~ Intervention + Sex +
0,050822	0,305112	0,154857	141	69	72	137	1,970287	y ~ Intervention + Sex +
0,015856	0,316303	0,129495	141	69	72	137	2,442583	y ~ Intervention + Sex +
0,259931	0,190177	0,168114	141	69	72	137	1,131239	y ~ Intervention + Sex +
0,673911	-0,0731	0,173357	141	69	72	137	-0,42169	y ~ Intervention + Sex +
0,002396	0,468512	0,151438	141	69	72	137	3,093758	y ~ Intervention + Sex +
0,612181	-0,07947	0,1564	141	69	72	137	-0,50813	y ~ Intervention + Sex +
0,063661	0,301375	0,16119	141	69	72	137	1,869692	y ~ Intervention + Sex +
0,979467	-0,00401	0,155628	141	69	72	137	-0,02578	y ~ Intervention + Sex +
0,021318	0,389878	0,167397	141	69	72	137	2,329061	y ~ Intervention + Sex +
2,24E-06	0,788783	0,159681	141	69	72	137	4,939732	y ~ Intervention + Sex +
0,892945	-0,0219	0,16243	141	69	72	137	-0,13483	y ~ Intervention + Sex +
0,465043	0,130932	0,178718	141	69	72	137	0,732617	y ~ Intervention + Sex +
0,045088	0,325818	0,16111	141	69	72	137	2,022333	y ~ Intervention + Sex +
0,122626	0,253357	0,163095	141	69	72	137	1,553435	y ~ Intervention + Sex +
0,352175	-0,15022	0,160913	141	69	72	137	-0,93356	y ~ Intervention + Sex +
0,030923	-0,36094	0,165526	141	69	72	137	-2,18057	y ~ Intervention + Sex +
0,016032	0,376918	0,154575	141	69	72	137	2,438414	y ~ Intervention + Sex +
0,86891	-0,02584	0,156245	141	69	72	137	-0,16535	y ~ Intervention + Sex +
0,291177	-0,16845	0,158966	141	69	72	137	-1,05963	y ~ Intervention + Sex +
0,505588	-0,10731	0,16077	141	69	72	137	-0,66748	y ~ Intervention + Sex +
0,000453	-0,53378	0,148527	141	69	72	137	-3,59385	y ~ Intervention + Sex +
0,068574	0,308781	0,168211	141	69	72	137	1,835672	y ~ Intervention + Sex +
0,259153	0,181837	0,160478	141	69	72	137	1,133096	y ~ Intervention + Sex +
0,977844	-0,00454	0,163163	141	69	72	137	-0,02782	y ~ Intervention + Sex +
5,33E-05	0,699655	0,16769	141	69	72	137	4,172319	y ~ Intervention + Sex +
0,090099	0,264133	0,154741	141	69	72	137	1,706937	y ~ Intervention + Sex +
0,10658	-0,25134	0,154724	141	69	72	137	-1,62444	y ~ Intervention + Sex +
0,843998	0,030357	0,153973	141	69	72	137	0,197156	y ~ Intervention + Sex +
0,890319	0,019122	0,13841	141	69	72	137	0,138158	y ~ Intervention + Sex +
0,022997	0,36201	0,157439	141	69	72	137	2,299359	y ~ Intervention + Sex +
0,903905	-0,01766	0,146013	141	69	72	137	-0,12095	y ~ Intervention + Sex +
0,014055	0,399286	0,160502	141	69	72	137	2,48774	y ~ Intervention + Sex +
0,244179	0,20649	0,176542	141	69	72	137	1,169635	y ~ Intervention + Sex +
0,036017	-0,34009	0,160603	141	69	72	137	-2,11758	y ~ Intervention + Sex +
0,157524	0,215906	0,151915	141	69	72	137	1,421224	y ~ Intervention + Sex +
0,075342	0,274978	0,153449	141	69	72	137	1,791987	y ~ Intervention + Sex +
7,08E-07	0,744818	0,143225	141	69	72	137	5,200321	y ~ Intervention + Sex +
0,055255	0,298196	0,154238	141	69	72	137	1,933355	y ~ Intervention + Sex +
0,563425	0,082075	0,141712	141	69	72	137	0,579171	y ~ Intervention + Sex +
0,142392	-0,27966	0,189544	141	69	72	137	-1,47543	y ~ Intervention + Sex +
0,58531	-0,07839	0,143323	141	69	72	137	-0,54694	y ~ Intervention + Sex +
0,15346	0,206245	0,143687	141	69	72	137	1,435374	y ~ Intervention + Sex +
0,343743	-0,15641	0,164624	141	69	72	137	-0,95008	y ~ Intervention + Sex +
0,40589	0,131257	0,157435	141	69	72	137	0,833721	y ~ Intervention + Sex +

0,836954	0,035001	0,169755	141	69	72	137	0,206183	y ~ Intervention + Sex +
0,005632	-0,45575	0,16202	141	69	72	137	-2,8129	y ~ Intervention + Sex +
0,496225	-0,11768	0,17249	141	69	72	137	-0,68226	y ~ Intervention + Sex +
0,222882	0,184086	0,150341	141	69	72	137	1,224454	y ~ Intervention + Sex +
0,281402	0,157246	0,145405	141	69	72	137	1,081438	y ~ Intervention + Sex +
0,213837	-0,18896	0,151304	141	69	72	137	-1,24889	y ~ Intervention + Sex +
5,79E-06	-0,72664	0,153991	141	69	72	137	-4,71874	y ~ Intervention + Sex +
0,963664	0,006819	0,149413	141	69	72	137	0,04564	y ~ Intervention + Sex +
0,026689	0,325061	0,145104	141	69	72	137	2,240194	y ~ Intervention + Sex +
0,504056	-0,11557	0,172515	141	69	72	137	-0,66989	y ~ Intervention + Sex +
0,024353	-0,36299	0,159434	141	69	72	137	-2,27672	y ~ Intervention + Sex +
0,288462	0,178856	0,16784	141	69	72	137	1,065639	y ~ Intervention + Sex +
0,010673	-0,40533	0,156576	141	69	72	137	-2,58871	y ~ Intervention + Sex +
0,138959	0,2354	0,158163	141	69	72	137	1,488343	y ~ Intervention + Sex +
0,340163	-0,14213	0,148489	141	69	72	137	-0,95718	y ~ Intervention + Sex +
0,069005	0,251536	0,137242	141	69	72	137	1,832787	y ~ Intervention + Sex +
0,793136	0,043449	0,165362	141	69	72	137	0,262753	y ~ Intervention + Sex +
0,000501	0,594436	0,166717	141	69	72	137	3,565533	y ~ Intervention + Sex +
0,479978	0,115302	0,162793	141	69	72	137	0,708275	y ~ Intervention + Sex +
0,099029	-0,26243	0,158006	141	69	72	137	-1,66086	y ~ Intervention + Sex +
0,098543	-0,25937	0,155937	141	69	72	137	-1,66327	y ~ Intervention + Sex +
0,523434	0,100621	0,157294	141	69	72	137	0,639703	y ~ Intervention + Sex +
0,296508	0,160571	0,153224	141	69	72	137	1,047948	y ~ Intervention + Sex +
0,954378	0,009366	0,16341	141	69	72	137	0,057315	y ~ Intervention + Sex +
0,136808	0,218222	0,145816	141	69	72	137	1,496559	y ~ Intervention + Sex +
0,124786	0,219936	0,142404	141	69	72	137	1,54445	y ~ Intervention + Sex +
0,166057	0,201382	0,144629	141	69	72	137	1,392399	y ~ Intervention + Sex +
0,052214	-0,24985	0,127577	141	69	72	137	-1,95841	y ~ Intervention + Sex +
0,744881	-0,04828	0,14807	141	69	72	137	-0,32605	y ~ Intervention + Sex +
0,970435	-0,00634	0,170835	141	69	72	137	-0,03713	y ~ Intervention + Sex +
0,607973	-0,07145	0,138965	141	69	72	137	-0,51415	y ~ Intervention + Sex +
0,87901	-0,02339	0,153384	141	69	72	137	-0,15251	y ~ Intervention + Sex +
0,307114	-0,15544	0,151627	141	69	72	137	-1,02512	y ~ Intervention + Sex +
0,178931	-0,20326	0,150451	141	69	72	137	-1,35097	y ~ Intervention + Sex +
0,171406	0,208391	0,151568	141	69	72	137	1,374901	y ~ Intervention + Sex +
0,200667	0,198344	0,154252	141	69	72	137	1,285844	y ~ Intervention + Sex +
0,021079	0,356215	0,152655	141	69	72	137	2,333465	y ~ Intervention + Sex +
0,009506	0,406396	0,154502	141	69	72	137	2,63036	y ~ Intervention + Sex +
0,798455	-0,04045	0,158089	141	69	72	137	-0,25584	y ~ Intervention + Sex +
0,413788	-0,1246	0,152001	141	69	72	137	-0,81974	y ~ Intervention + Sex +
0,529436	0,092363	0,146498	141	69	72	137	0,630473	y ~ Intervention + Sex +
0,772735	0,044632	0,154239	141	69	72	137	0,289371	y ~ Intervention + Sex +
0,438942	-0,13194	0,169972	141	69	72	137	-0,77625	y ~ Intervention + Sex +
0,048479	0,267877	0,134549	141	69	72	137	1,990922	y ~ Intervention + Sex +
0,276416	0,177913	0,16281	141	69	72	137	1,092762	y ~ Intervention + Sex +
0,969886	0,00619	0,163663	141	69	72	137	0,03782	y ~ Intervention + Sex +
0,165485	-0,20503	0,14705	141	69	72	137	-1,3943	y ~ Intervention + Sex +
0,069481	0,305966	0,16723	141	69	72	137	1,829616	y ~ Intervention + Sex +

0,676284	0,07125	0,170277	141	69	72	137	0,418436	y ~ Intervention + Sex +
0,376609	-0,13664	0,154035	141	69	72	137	-0,88705	y ~ Intervention + Sex +
0,303064	0,162429	0,157122	141	69	72	137	1,033773	y ~ Intervention + Sex +
0,001088	0,502365	0,150515	141	69	72	137	3,337647	y ~ Intervention + Sex +
0,830105	0,030792	0,143234	141	69	72	137	0,214977	y ~ Intervention + Sex +
0,163896	0,206244	0,14736	141	69	72	137	1,399593	y ~ Intervention + Sex +
0,085389	-0,26183	0,151103	141	69	72	137	-1,73276	y ~ Intervention + Sex +
0,078608	-0,28526	0,160979	141	69	72	137	-1,77206	y ~ Intervention + Sex +
0,47017	0,10893	0,150411	141	69	72	137	0,724213	y ~ Intervention + Sex +
0,057982	-0,30494	0,159498	141	69	72	137	-1,91186	y ~ Intervention + Sex +
0,532987	-0,09362	0,149783	141	69	72	137	-0,62504	y ~ Intervention + Sex +
0,703955	-0,06859	0,180125	141	69	72	137	-0,38078	y ~ Intervention + Sex +
0,276209	-0,1833	0,167666	141	69	72	137	-1,09324	y ~ Intervention + Sex +
0,375056	-0,14892	0,167336	141	69	72	137	-0,88994	y ~ Intervention + Sex +
0,057052	0,296228	0,154358	141	69	72	137	1,919095	y ~ Intervention + Sex +
0,027905	0,377515	0,169878	141	69	72	137	2,222271	y ~ Intervention + Sex +
0,400332	0,120402	0,142715	141	69	72	137	0,843657	y ~ Intervention + Sex +
0,636356	-0,06646	0,140255	141	69	72	137	-0,47386	y ~ Intervention + Sex +
0,480334	0,107252	0,151551	141	69	72	137	0,707699	y ~ Intervention + Sex +
0,651856	-0,07194	0,159097	141	69	72	137	-0,45218	y ~ Intervention + Sex +
0,486079	-0,11515	0,164871	141	69	72	137	-0,69845	y ~ Intervention + Sex +
0,571056	-0,08624	0,151865	141	69	72	137	-0,56787	y ~ Intervention + Sex +
0,27261	0,171602	0,155789	141	69	72	137	1,101501	y ~ Intervention + Sex +
0,177034	-0,2118	0,156086	141	69	72	137	-1,35693	y ~ Intervention + Sex +
0,009016	0,424467	0,160224	141	69	72	137	2,649216	y ~ Intervention + Sex +
0,255287	0,160574	0,14056	141	69	72	137	1,142385	y ~ Intervention + Sex +
0,016432	0,396343	0,163165	141	69	72	137	2,4291	y ~ Intervention + Sex +
0,025706	0,303834	0,134726	141	69	72	137	2,255201	y ~ Intervention + Sex +
0,022116	0,360714	0,155836	141	69	72	137	2,314699	y ~ Intervention + Sex +
0,751113	-0,05074	0,159658	141	69	72	137	-0,31781	y ~ Intervention + Sex +
0,379959	-0,1298	0,147359	141	69	72	137	-0,88082	y ~ Intervention + Sex +
0,111977	0,294319	0,183988	141	69	72	137	1,599662	y ~ Intervention + Sex +
0,140227	0,237974	0,160409	141	69	72	137	1,483543	y ~ Intervention + Sex +
0,909625	0,01734	0,152479	141	69	72	137	0,113722	y ~ Intervention + Sex +
0,345746	-0,16146	0,170649	141	69	72	137	-0,94613	y ~ Intervention + Sex +
0,826884	-0,03286	0,149951	141	69	72	137	-0,21912	y ~ Intervention + Sex +
0,402424	-0,12857	0,153079	141	69	72	137	-0,83991	y ~ Intervention + Sex +
0,50837	0,104777	0,158007	141	69	72	137	0,663116	y ~ Intervention + Sex +
0,521602	0,112177	0,174586	141	69	72	137	0,642531	y ~ Intervention + Sex +
0,772154	0,044848	0,154578	141	69	72	137	0,290132	y ~ Intervention + Sex +
5,67E-05	0,592487	0,14255	141	69	72	137	4,156334	y ~ Intervention + Sex +
0,459704	0,123056	0,165972	141	69	72	137	0,741426	y ~ Intervention + Sex +
0,495139	-0,10906	0,15945	141	69	72	137	-0,68399	y ~ Intervention + Sex +
0,923718	0,015663	0,163278	141	69	72	137	0,095928	y ~ Intervention + Sex +
0,703616	-0,06265	0,164324	141	69	72	137	-0,38124	y ~ Intervention + Sex +
0,010257	0,423721	0,162778	141	69	72	137	2,603057	y ~ Intervention + Sex +
0,933702	0,013147	0,157751	141	69	72	137	0,083342	y ~ Intervention + Sex +
0,307052	-0,15841	0,154509	141	69	72	137	-1,02525	y ~ Intervention + Sex +

0,007043	0,397702	0,145359	141	69	72	137	2,735996	y ~ Intervention + Sex +
2,95E-05	-0,66178	0,153113	141	69	72	137	-4,32215	y ~ Intervention + Sex +
0,27223	0,162913	0,147783	141	69	72	137	1,102379	y ~ Intervention + Sex +
0,861609	0,030561	0,174979	141	69	72	137	0,174654	y ~ Intervention + Sex +
0,050082	0,336537	0,170251	141	69	72	137	1,976712	y ~ Intervention + Sex +
0,574741	0,09175	0,16313	141	69	72	137	0,562432	y ~ Intervention + Sex +
0,376707	0,146407	0,165084	141	69	72	137	0,886862	y ~ Intervention + Sex +
0,524987	-0,0984	0,154399	141	69	72	137	-0,63731	y ~ Intervention + Sex +
0,000178	0,512178	0,132917	141	69	72	137	3,853371	y ~ Intervention + Sex +
0,718486	-0,0536	0,148385	141	69	72	137	-0,36123	y ~ Intervention + Sex +
0,003642	0,459622	0,15535	141	69	72	137	2,958627	y ~ Intervention + Sex +
0,200204	0,203648	0,158213	141	69	72	137	1,287175	y ~ Intervention + Sex +
0,478893	0,125334	0,176519	141	69	72	137	0,710028	y ~ Intervention + Sex +
0,417681	0,116649	0,143495	141	69	72	137	0,812909	y ~ Intervention + Sex +
0,179986	-0,20707	0,153647	141	69	72	137	-1,34768	y ~ Intervention + Sex +
0,363325	-0,13705	0,150261	141	69	72	137	-0,91209	y ~ Intervention + Sex +
0,753751	-0,05302	0,168679	141	69	72	137	-0,31433	y ~ Intervention + Sex +
0,887705	0,021141	0,149436	141	69	72	137	0,141472	y ~ Intervention + Sex +
0,689982	-0,06424	0,1607	141	69	72	137	-0,39972	y ~ Intervention + Sex +
0,926997	-0,01303	0,141994	141	69	72	137	-0,09179	y ~ Intervention + Sex +
0,767017	0,046675	0,157225	141	69	72	137	0,296868	y ~ Intervention + Sex +
0,892422	-0,02087	0,154067	141	69	72	137	-0,13549	y ~ Intervention + Sex +
0,081175	-0,27017	0,153778	141	69	72	137	-1,75686	y ~ Intervention + Sex +
0,25788	0,178529	0,157136	141	69	72	137	1,136144	y ~ Intervention + Sex +
0,142908	0,21641	0,146867	141	69	72	137	1,473509	y ~ Intervention + Sex +
0,06607	-0,2642	0,142598	141	69	72	137	-1,85275	y ~ Intervention + Sex +
0,350421	-0,15013	0,16023	141	69	72	137	-0,93697	y ~ Intervention + Sex +
0,591395	0,079945	0,148574	141	69	72	137	0,53808	y ~ Intervention + Sex +
0,293571	0,159375	0,151157	141	69	72	137	1,054367	y ~ Intervention + Sex +
0,730726	0,055531	0,161024	141	69	72	137	0,344864	y ~ Intervention + Sex +
0,023188	0,368448	0,160467	141	69	72	137	2,296097	y ~ Intervention + Sex +
0,442025	-0,11852	0,153714	141	69	72	137	-0,77102	y ~ Intervention + Sex +
0,023006	-0,37038	0,161089	141	69	72	137	-2,29921	y ~ Intervention + Sex +
0,394867	0,134428	0,1575	141	69	72	137	0,853509	y ~ Intervention + Sex +
0,095175	0,244485	0,1455	141	69	72	137	1,680312	y ~ Intervention + Sex +
0,075596	-0,28648	0,160008	141	69	72	137	-1,79041	y ~ Intervention + Sex +
0,152005	0,238936	0,165869	141	69	72	137	1,440509	y ~ Intervention + Sex +
0,053944	-0,27683	0,142403	141	69	72	137	-1,944	y ~ Intervention + Sex +
0,162663	0,211536	0,150696	141	69	72	137	1,403726	y ~ Intervention + Sex +
0,010244	0,405577	0,155781	141	69	72	137	2,603519	y ~ Intervention + Sex +
0,412223	0,135053	0,164199	141	69	72	137	0,822498	y ~ Intervention + Sex +
0,022201	-0,37036	0,160108	141	69	72	137	-2,3132	y ~ Intervention + Sex +
0,185547	0,210868	0,158482	141	69	72	137	1,330552	y ~ Intervention + Sex +
0,212517	-0,18867	0,150635	141	69	72	137	-1,25251	y ~ Intervention + Sex +
0,051549	-0,33744	0,17181	141	69	72	137	-1,96405	y ~ Intervention + Sex +
0,318174	-0,17165	0,17133	141	69	72	137	-1,00187	y ~ Intervention + Sex +
0,39831	0,127354	0,150307	141	69	72	137	0,847293	y ~ Intervention + Sex +
0,863293	-0,03014	0,174707	141	69	72	137	-0,17251	y ~ Intervention + Sex +

0,311572	0,15608	0,15367	141	69	72	137	1,015681	y ~ Intervention + Sex
0,177638	0,193191	0,142573	141	69	72	137	1,355031	y ~ Intervention + Sex
0,176676	0,220364	0,162263	141	69	72	137	1,358064	y ~ Intervention + Sex
0,285984	-0,16324	0,152398	141	69	72	137	-1,07115	y ~ Intervention + Sex
0,022116	0,360714	0,155836	141	69	72	137	2,314699	y ~ Intervention + Sex
0,302208	-0,16248	0,156895	141	69	72	137	-1,03561	y ~ Intervention + Sex
0,422444	-0,12998	0,16154	141	69	72	137	-0,8046	y ~ Intervention + Sex
0,05524	0,289675	0,149821	141	69	72	137	1,933478	y ~ Intervention + Sex
0,701644	-0,05992	0,156087	141	69	72	137	-0,38391	y ~ Intervention + Sex
0,314832	0,168951	0,167471	141	69	72	137	1,008835	y ~ Intervention + Sex
0,01642	-0,3887	0,159998	141	69	72	137	-2,42939	y ~ Intervention + Sex
0,968088	0,0069	0,172163	141	69	72	137	0,040079	y ~ Intervention + Sex
0,021906	0,367507	0,158515	141	69	72	137	2,318444	y ~ Intervention + Sex
0,038045	0,32044	0,152981	141	69	72	137	2,094639	y ~ Intervention + Sex
0,059091	-0,31348	0,164698	141	69	72	137	-1,90336	y ~ Intervention + Sex
0,004038	0,43629	0,149181	141	69	72	137	2,924559	y ~ Intervention + Sex
0,359034	0,152544	0,165754	141	69	72	137	0,920299	y ~ Intervention + Sex
0,308718	0,153658	0,150392	141	69	72	137	1,021711	y ~ Intervention + Sex
0,292895	0,163783	0,15512	141	69	72	137	1,05585	y ~ Intervention + Sex
0,928365	0,013503	0,149916	141	69	72	137	0,090068	y ~ Intervention + Sex
0,001816	0,457605	0,143859	141	69	72	137	3,180934	y ~ Intervention + Sex
0,17831	0,196532	0,145265	141	69	72	137	1,352919	y ~ Intervention + Sex
0,180235	-0,20465	0,151939	141	69	72	137	-1,3469	y ~ Intervention + Sex
0,600447	0,084781	0,161493	141	69	72	137	0,524979	y ~ Intervention + Sex
0,191456	-0,20256	0,154297	141	69	72	137	-1,31277	y ~ Intervention + Sex
0,90069	-0,02097	0,167703	141	69	72	137	-0,12502	y ~ Intervention + Sex
0,594302	0,089439	0,167532	141	69	72	137	0,533863	y ~ Intervention + Sex
0,080451	-0,27886	0,158342	141	69	72	137	-1,76111	y ~ Intervention + Sex
0,736065	-0,05681	0,168196	141	69	72	137	-0,33776	y ~ Intervention + Sex
0,415234	-0,12027	0,14717	141	69	72	137	-0,8172	y ~ Intervention + Sex
0,947049	0,01063	0,159768	141	69	72	137	0,066535	y ~ Intervention + Sex
0,723707	0,058806	0,166007	141	69	72	137	0,354236	y ~ Intervention + Sex
0,145683	-0,24382	0,166624	141	69	72	137	-1,46327	y ~ Intervention + Sex
0,156417	0,197525	0,138609	141	69	72	137	1,425051	y ~ Intervention + Sex
0,040789	0,355381	0,172081	141	69	72	137	2,065201	y ~ Intervention + Sex
0,488901	-0,11106	0,16004	141	69	72	137	-0,69393	y ~ Intervention + Sex
0,475952	-0,12038	0,168412	141	69	72	137	-0,7148	y ~ Intervention + Sex
0,389872	0,136101	0,157782	141	69	72	137	0,862588	y ~ Intervention + Sex
0,587492	-0,08635	0,158795	141	69	72	137	-0,54376	y ~ Intervention + Sex
0,022111	0,328326	0,141838	141	69	72	137	2,31479	y ~ Intervention + Sex
0,234671	-0,16037	0,134347	141	69	72	137	-1,19367	y ~ Intervention + Sex
9,11E-07	0,74622	0,145063	141	69	72	137	5,144123	y ~ Intervention + Sex
0,844353	0,029079	0,147832	141	69	72	137	0,196701	y ~ Intervention + Sex
0,694221	0,061979	0,157323	141	69	72	137	0,393962	y ~ Intervention + Sex
0,816004	0,039954	0,171376	141	69	72	137	0,233136	y ~ Intervention + Sex
0,819105	0,034272	0,149572	141	69	72	137	0,229136	y ~ Intervention + Sex
0,929544	-0,01259	0,142133	141	69	72	137	-0,08858	y ~ Intervention + Sex
0,258787	0,170083	0,149989	141	69	72	137	1,13397	y ~ Intervention + Sex

0,000442	-0,53605	0,148864	141	69	72	137	-3,60096	y ~ Intervention + Sex +
0,991354	0,001722	0,158606	141	69	72	137	0,010856	y ~ Intervention + Sex +
0,781245	-0,04527	0,162689	141	69	72	137	-0,27824	y ~ Intervention + Sex +
0,403914	-0,12819	0,153104	141	69	72	137	-0,83724	y ~ Intervention + Sex +
0,846219	0,029819	0,153461	141	69	72	137	0,194312	y ~ Intervention + Sex +
0,855011	0,029937	0,163524	141	69	72	137	0,183074	y ~ Intervention + Sex +
0,177295	-0,22072	0,162759	141	69	72	137	-1,35611	y ~ Intervention + Sex +
0,056114	0,275396	0,142952	141	69	72	137	1,926486	y ~ Intervention + Sex +
0,449133	0,117927	0,155363	141	69	72	137	0,759039	y ~ Intervention + Sex +
0,19411	0,188914	0,144772	141	69	72	137	1,304914	y ~ Intervention + Sex +
0,107721	0,238921	0,147562	141	69	72	137	1,619123	y ~ Intervention + Sex +
0,045195	0,325938	0,161251	141	69	72	137	2,021311	y ~ Intervention + Sex +
0,752909	0,046471	0,147322	141	69	72	137	0,315438	y ~ Intervention + Sex +
0,007056	-0,4244	0,15515	141	69	72	137	-2,73539	y ~ Intervention + Sex +
0,314493	0,168655	0,16706	141	69	72	137	1,009546	y ~ Intervention + Sex +
0,387093	-0,14483	0,166914	141	69	72	137	-0,86767	y ~ Intervention + Sex +
0,002732	0,459647	0,150614	141	69	72	137	3,051814	y ~ Intervention + Sex +
0,571585	0,085839	0,151369	141	69	72	137	0,567084	y ~ Intervention + Sex +
0,015344	0,357617	0,145672	141	69	72	137	2,454943	y ~ Intervention + Sex +
0,290708	-0,17471	0,16472	141	69	72	137	-1,06067	y ~ Intervention + Sex +
0,711493	-0,0593	0,160007	141	69	72	137	-0,37062	y ~ Intervention + Sex +
0,698455	-0,06287	0,161932	141	69	72	137	-0,38822	y ~ Intervention + Sex +
0,350996	-0,14567	0,155656	141	69	72	137	-0,93585	y ~ Intervention + Sex +
0,81206	0,036568	0,1535	141	69	72	137	0,238228	y ~ Intervention + Sex +
0,611332	-0,08275	0,162463	141	69	72	137	-0,50934	y ~ Intervention + Sex +
0,942701	-0,01158	0,160781	141	69	72	137	-0,07201	y ~ Intervention + Sex +
0,844043	0,031086	0,157721	141	69	72	137	0,197098	y ~ Intervention + Sex +
0,022056	0,332754	0,143691	141	69	72	137	2,315768	y ~ Intervention + Sex +
0,125796	0,231143	0,150065	141	69	72	137	1,540289	y ~ Intervention + Sex +
0,301563	0,175099	0,168852	141	69	72	137	1,036999	y ~ Intervention + Sex +
0,001165	0,540283	0,162877	141	69	72	137	3,317114	y ~ Intervention + Sex +
0,482134	-0,11636	0,165092	141	69	72	137	-0,7048	y ~ Intervention + Sex +
0,031264	-0,38847	0,17852	141	69	72	137	-2,17608	y ~ Intervention + Sex +
0,549601	0,094446	0,157451	141	69	72	137	0,599844	y ~ Intervention + Sex +
0,803073	0,037034	0,148222	141	69	72	137	0,249856	y ~ Intervention + Sex +
0,006476	-0,42079	0,152181	141	69	72	137	-2,76504	y ~ Intervention + Sex +
0,725011	0,057268	0,162465	141	69	72	137	0,352492	y ~ Intervention + Sex +
0,823393	0,03364	0,15044	141	69	72	137	0,223611	y ~ Intervention + Sex +
0,922491	-0,01568	0,160811	141	69	72	137	-0,09748	y ~ Intervention + Sex +
0,09653	-0,25408	0,151835	141	69	72	137	-1,6734	y ~ Intervention + Sex +
0,239329	-0,19073	0,161388	141	69	72	137	-1,18181	y ~ Intervention + Sex +
0,565071	0,080291	0,139219	141	69	72	137	0,576726	y ~ Intervention + Sex +
0,477389	-0,12355	0,173412	141	69	72	137	-0,71246	y ~ Intervention + Sex +
0,279353	-0,16875	0,155373	141	69	72	137	-1,08607	y ~ Intervention + Sex +
0,854028	0,029691	0,161075	141	69	72	137	0,184328	y ~ Intervention + Sex +
0,868419	0,026054	0,156975	141	69	72	137	0,165978	y ~ Intervention + Sex +
0,244085	-0,17376	0,148532	141	69	72	137	-1,16987	y ~ Intervention + Sex +
0,064602	0,278315	0,14939	141	69	72	137	1,863013	y ~ Intervention + Sex +

0,420765	-0,148	0,183277	141	69	72	137	-0,80752	y ~ Intervention + Sex +
0,128615	-0,22971	0,150252	141	69	72	137	-1,52882	y ~ Intervention + Sex +
0,215802	-0,17613	0,141638	141	69	72	137	-1,24351	y ~ Intervention + Sex +
0,460688	0,121968	0,164866	141	69	72	137	0,739799	y ~ Intervention + Sex +
0,245679	-0,17734	0,152108	141	69	72	137	-1,1659	y ~ Intervention + Sex +
0,059891	0,306877	0,161743	141	69	72	137	1,897317	y ~ Intervention + Sex +
0,529014	0,103347	0,163752	141	69	72	137	0,631119	y ~ Intervention + Sex +
0,00537	0,442877	0,156546	141	69	72	137	2,829061	y ~ Intervention + Sex +
0,001712	-0,47974	0,149951	141	69	72	137	-3,19931	y ~ Intervention + Sex +
0,061941	-0,29951	0,159132	141	69	72	137	-1,88212	y ~ Intervention + Sex +
0,031842	0,332981	0,153549	141	69	72	137	2,168568	y ~ Intervention + Sex +
0,20579	0,184397	0,145051	141	69	72	137	1,27126	y ~ Intervention + Sex +
0,963947	-0,00726	0,160394	141	69	72	137	-0,04528	y ~ Intervention + Sex +
0,010168	-0,41751	0,160198	141	69	72	137	-2,60621	y ~ Intervention + Sex +
0,953164	0,008473	0,143999	141	69	72	137	0,058842	y ~ Intervention + Sex +
0,408569	0,116641	0,140707	141	69	72	137	0,828961	y ~ Intervention + Sex +
0,370032	-0,1512	0,168116	141	69	72	137	-0,89937	y ~ Intervention + Sex +
0,290545	-0,1636	0,154189	141	69	72	137	-1,06103	y ~ Intervention + Sex +
0,036252	0,341858	0,161645	141	69	72	137	2,114866	y ~ Intervention + Sex +
0,883034	0,024877	0,168772	141	69	72	137	0,147399	y ~ Intervention + Sex +
0,102814	0,266705	0,162393	141	69	72	137	1,642337	y ~ Intervention + Sex +
0,036161	0,320413	0,15143	141	69	72	137	2,115911	y ~ Intervention + Sex +
1,69E-05	-0,68308	0,153137	141	69	72	137	-4,46059	y ~ Intervention + Sex +
0,523775	0,100001	0,156452	141	69	72	137	0,639177	y ~ Intervention + Sex +
0,234806	-0,1753	0,146902	141	69	72	137	-1,19332	y ~ Intervention + Sex +
0,317809	0,151909	0,151511	141	69	72	137	1,002626	y ~ Intervention + Sex +
0,059618	0,309424	0,162908	141	69	72	137	1,899373	y ~ Intervention + Sex +
0,00011	-0,50927	0,127842	141	69	72	137	-3,98359	y ~ Intervention + Sex +
0,004487	-0,44759	0,154903	141	69	72	137	-2,8895	y ~ Intervention + Sex +
0,903614	-0,02062	0,169938	141	69	72	137	-0,12132	y ~ Intervention + Sex +
0,829289	-0,03016	0,139625	141	69	72	137	-0,21603	y ~ Intervention + Sex +
0,038851	0,31011	0,148676	141	69	72	137	2,085806	y ~ Intervention + Sex +
0,510344	0,103897	0,157413	141	69	72	137	0,660028	y ~ Intervention + Sex +
0,024968	-0,37683	0,166237	141	69	72	137	-2,26681	y ~ Intervention + Sex +
0,960504	0,007849	0,158213	141	69	72	137	0,049612	y ~ Intervention + Sex +
0,387077	-0,12502	0,144084	141	69	72	137	-0,8677	y ~ Intervention + Sex +
0,385333	0,135715	0,155833	141	69	72	137	0,870899	y ~ Intervention + Sex +
0,60354	-0,08951	0,171965	141	69	72	137	-0,52052	y ~ Intervention + Sex +
0,483151	0,102257	0,145425	141	69	72	137	0,703156	y ~ Intervention + Sex +
0,100789	0,236286	0,143015	141	69	72	137	1,652176	y ~ Intervention + Sex +
0,450731	0,119754	0,158329	141	69	72	137	0,756362	y ~ Intervention + Sex +
0,838577	-0,03659	0,179268	141	69	72	137	-0,2041	y ~ Intervention + Sex +
0,93246	0,013673	0,161031	141	69	72	137	0,084906	y ~ Intervention + Sex +
5,89E-06	-0,67738	0,143682	141	69	72	137	-4,71444	y ~ Intervention + Sex +
0,939073	0,011129	0,145336	141	69	72	137	0,076575	y ~ Intervention + Sex +
0,011491	-0,41426	0,1617	141	69	72	137	-2,56191	y ~ Intervention + Sex +
0,275496	-0,17698	0,161641	141	69	72	137	-1,09487	y ~ Intervention + Sex +
0,975032	0,004891	0,155999	141	69	72	137	0,031355	y ~ Intervention + Sex +

0,297433	-0,15902	0,15204	141	69	72	137	-1,04594	y ~ Intervention + Sex +
0,021177	0,378139	0,162176	141	69	72	137	2,331656	y ~ Intervention + Sex +
0,082593	-0,25896	0,148091	141	69	72	137	-1,74864	y ~ Intervention + Sex +
0,517243	0,100589	0,154923	141	69	72	137	0,649282	y ~ Intervention + Sex +
0,848479	0,025002	0,13061	141	69	72	137	0,191421	y ~ Intervention + Sex +
0,680279	0,066262	0,160455	141	69	72	137	0,412963	y ~ Intervention + Sex +
0,480769	0,11933	0,168784	141	69	72	137	0,706997	y ~ Intervention + Sex +
0,956958	0,008691	0,160732	141	69	72	137	0,05407	y ~ Intervention + Sex +
0,917647	-0,01471	0,141992	141	69	72	137	-0,10359	y ~ Intervention + Sex +
0,171522	0,190408	0,138526	141	69	72	137	1,374527	y ~ Intervention + Sex +
0,572651	-0,08522	0,150694	141	69	72	137	-0,56551	y ~ Intervention + Sex +
0,570747	-0,09108	0,160257	141	69	72	137	-0,56832	y ~ Intervention + Sex +
0,917874	-0,01428	0,1382	141	69	72	137	-0,1033	y ~ Intervention + Sex +
0,531165	-0,09575	0,15251	141	69	72	137	-0,62782	y ~ Intervention + Sex +
0,286019	0,161109	0,150418	141	69	72	137	1,071075	y ~ Intervention + Sex +
0,004344	0,471267	0,162486	141	69	72	137	2,900347	y ~ Intervention + Sex +
4,65E-14	1,181914	0,140466	141	69	72	137	8,414223	y ~ Intervention + Sex +
0,207667	-0,20071	0,15854	141	69	72	137	-1,26599	y ~ Intervention + Sex +
0,481675	0,107642	0,152568	141	69	72	137	0,705535	y ~ Intervention + Sex +
0,149853	0,232236	0,160365	141	69	72	137	1,448173	y ~ Intervention + Sex +
0,551214	-0,09522	0,159393	141	69	72	137	-0,59742	y ~ Intervention + Sex +
0,596259	-0,08635	0,162612	141	69	72	137	-0,53103	y ~ Intervention + Sex +
0,474527	-0,12685	0,176892	141	69	72	137	-0,71711	y ~ Intervention + Sex +
0,250867	-0,20104	0,174344	141	69	72	137	-1,15313	y ~ Intervention + Sex +
1,32E-24	1,320778	0,104473	138	69	69	134	12,64228	y ~ Intervention + Sex +
2,28E-24	1,286363	0,102514	138	69	69	134	12,54822	y ~ Intervention + Sex +
1,72E-09	0,998532	0,154404	138	69	69	134	6,466987	y ~ Intervention + Sex +
1,02E-08	0,986572	0,161514	138	69	69	134	6,108277	y ~ Intervention + Sex +
1,13E-08	0,980408	0,16103	138	69	69	134	6,088337	y ~ Intervention + Sex +
3,15E-08	0,850782	0,14478	138	69	69	134	5,876397	y ~ Intervention + Sex +
2,28E-07	0,838897	0,153767	138	69	69	134	5,455629	y ~ Intervention + Sex +
2,65E-08	0,836341	0,141463	138	69	69	134	5,912097	y ~ Intervention + Sex +
8,11E-06	0,816675	0,17591	138	69	69	134	4,642567	y ~ Intervention + Sex +
4,09E-07	0,78665	0,14765	138	69	69	134	5,327819	y ~ Intervention + Sex +
6,51E-07	0,776208	0,148561	138	69	69	134	5,224834	y ~ Intervention + Sex +
8,71E-06	0,712439	0,154034	138	69	69	134	4,625217	y ~ Intervention + Sex +
2,03E-06	0,705348	0,141992	138	69	69	134	4,967532	y ~ Intervention + Sex +
4,63E-05	0,689158	0,163647	138	69	69	134	4,211244	y ~ Intervention + Sex +
2,59E-05	0,686156	0,157449	138	69	69	134	4,357959	y ~ Intervention + Sex +
4,3E-05	0,68525	0,161993	138	69	69	134	4,230125	y ~ Intervention + Sex +
7,14E-05	0,679982	0,16587	138	69	69	134	4,099501	y ~ Intervention + Sex +
6,18E-05	0,678074	0,163907	138	69	69	134	4,136947	y ~ Intervention + Sex +
9,4E-06	0,6667	0,144715	138	69	69	134	4,607004	y ~ Intervention + Sex +
1,28E-05	0,659396	0,145478	138	69	69	134	4,532616	y ~ Intervention + Sex +
8,07E-05	0,652237	0,16036	138	69	69	134	4,067339	y ~ Intervention + Sex +
8,37E-05	0,650966	0,160426	138	69	69	134	4,057733	y ~ Intervention + Sex +
5,07E-05	0,633257	0,151206	138	69	69	134	4,188038	y ~ Intervention + Sex +
8,21E-05	0,621806	0,153048	138	69	69	134	4,062822	y ~ Intervention + Sex +

5,59E-05	0,618088	0,148476	138	69	69	134	4,162884	y ~ Intervention + Sex +
0,000321	0,616342	0,166886	138	69	69	134	3,69319	y ~ Intervention + Sex +
0,000268	0,613909	0,163995	138	69	69	134	3,743462	y ~ Intervention + Sex +
9,93E-05	0,611897	0,152485	138	69	69	134	4,012843	y ~ Intervention + Sex +
4,9E-05	0,611564	0,145717	138	69	69	134	4,196932	y ~ Intervention + Sex +
9,25E-05	0,609762	0,151246	138	69	69	134	4,031598	y ~ Intervention + Sex +
0,000358	0,602637	0,164508	138	69	69	134	3,663274	y ~ Intervention + Sex +
0,000362	0,601645	0,164384	138	69	69	134	3,659996	y ~ Intervention + Sex +
0,000295	0,59819	0,160907	138	69	69	134	3,717616	y ~ Intervention + Sex +
6,09E-05	0,596912	0,144152	138	69	69	134	4,140846	y ~ Intervention + Sex +
0,000586	0,59136	0,167913	138	69	69	134	3,521833	y ~ Intervention + Sex +
0,000209	0,58924	0,154542	138	69	69	134	3,812807	y ~ Intervention + Sex +
0,000141	0,574872	0,146654	138	69	69	134	3,919917	y ~ Intervention + Sex +
0,000249	0,573138	0,152271	138	69	69	134	3,763921	y ~ Intervention + Sex +
0,000123	0,571918	0,144546	138	69	69	134	3,956652	y ~ Intervention + Sex +
0,000692	0,568272	0,163591	138	69	69	134	3,473744	y ~ Intervention + Sex +
4,38E-05	0,567857	0,134398	138	69	69	134	4,225195	y ~ Intervention + Sex +
0,000316	0,562406	0,152097	138	69	69	134	3,697691	y ~ Intervention + Sex +
6,28E-05	0,561675	0,13591	138	69	69	134	4,132713	y ~ Intervention + Sex +
0,000668	0,5608	0,160966	138	69	69	134	3,483973	y ~ Intervention + Sex +
0,001292	0,555576	0,169012	138	69	69	134	3,287191	y ~ Intervention + Sex +
0,000457	0,555545	0,154604	138	69	69	134	3,593336	y ~ Intervention + Sex +
3,51E-05	0,553618	0,12929	138	69	69	134	4,281984	y ~ Intervention + Sex +
0,000596	0,552842	0,157186	138	69	69	134	3,517127	y ~ Intervention + Sex +
0,000669	0,543593	0,156041	138	69	69	134	3,483657	y ~ Intervention + Sex +
0,000671	0,543199	0,155975	138	69	69	134	3,482613	y ~ Intervention + Sex +
0,009287	0,542055	0,20333	84	45	39	80	2,665892	y ~ Intervention + Sex +
0,000895	0,541746	0,159446	138	69	69	134	3,397689	y ~ Intervention + Sex +
0,00083	0,536595	0,156899	138	69	69	134	3,420007	y ~ Intervention + Sex +
0,000711	0,535588	0,154547	138	69	69	134	3,465529	y ~ Intervention + Sex +
0,002695	0,535073	0,175003	138	69	69	134	3,057515	y ~ Intervention + Sex +
0,001634	0,531991	0,165468	138	69	69	134	3,215078	y ~ Intervention + Sex +
0,000655	0,528321	0,151388	138	69	69	134	3,489856	y ~ Intervention + Sex +
8,22E-05	0,525817	0,129431	138	69	69	134	4,06251	y ~ Intervention + Sex +
0,002068	0,525618	0,167303	138	69	69	134	3,141719	y ~ Intervention + Sex +
0,000248	0,524307	0,139244	138	69	69	134	3,765386	y ~ Intervention + Sex +
0,000179	0,524028	0,135956	138	69	69	134	3,854388	y ~ Intervention + Sex +
0,001594	0,522863	0,162235	138	69	69	134	3,222868	y ~ Intervention + Sex +
0,001109	0,521401	0,156412	138	69	69	134	3,333507	y ~ Intervention + Sex +
0,00078	0,520916	0,151497	138	69	69	134	3,438462	y ~ Intervention + Sex +
0,001567	0,517865	0,160422	138	69	69	134	3,228148	y ~ Intervention + Sex +
0,002021	0,516995	0,16418	138	69	69	134	3,148958	y ~ Intervention + Sex +
0,000455	0,516442	0,143662	138	69	69	134	3,594838	y ~ Intervention + Sex +
0,000957	0,515287	0,152555	138	69	69	134	3,377715	y ~ Intervention + Sex +
0,001302	0,515152	0,156825	138	69	69	134	3,284875	y ~ Intervention + Sex +
0,001956	0,513813	0,162645	138	69	69	134	3,159111	y ~ Intervention + Sex +
0,000595	0,512742	0,145759	138	69	69	134	3,517744	y ~ Intervention + Sex +
0,001873	0,504537	0,159021	138	69	69	134	3,17277	y ~ Intervention + Sex +

0,001688	0,501848	0,156582	138	69	69	134	3,20501	y ~ Intervention + Sex +
0,002339	0,500192	0,161212	138	69	69	134	3,102691	y ~ Intervention + Sex +
0,001304	0,499581	0,15211	138	69	69	134	3,284349	y ~ Intervention + Sex +
0,000629	0,499339	0,142616	138	69	69	134	3,501291	y ~ Intervention + Sex +
0,002776	0,497149	0,163104	138	69	69	134	3,048046	y ~ Intervention + Sex +
0,003006	0,495809	0,164048	138	69	69	134	3,022333	y ~ Intervention + Sex +
0,001889	0,495579	0,156333	138	69	69	134	3,170016	y ~ Intervention + Sex +
0,000439	0,495172	0,137359	138	69	69	134	3,604961	y ~ Intervention + Sex +
0,001329	0,494718	0,150888	138	69	69	134	3,278713	y ~ Intervention + Sex +
0,003138	0,491877	0,163503	138	69	69	134	3,008363	y ~ Intervention + Sex +
0,000692	0,491788	0,141572	138	69	69	134	3,473762	y ~ Intervention + Sex +
0,00148	0,488881	0,150626	138	69	69	134	3,245662	y ~ Intervention + Sex +
0,002578	0,488021	0,158872	138	69	69	134	3,07178	y ~ Intervention + Sex +
0,00152	0,486826	0,150367	138	69	69	134	3,237575	y ~ Intervention + Sex +
0,000665	0,48032	0,13782	138	69	69	134	3,485114	y ~ Intervention + Sex +
0,004058	0,479015	0,16382	138	69	69	134	2,924036	y ~ Intervention + Sex +
0,002629	0,47761	0,155803	138	69	69	134	3,065468	y ~ Intervention + Sex +
0,002616	0,476502	0,155359	138	69	69	134	3,067106	y ~ Intervention + Sex +
0,005129	0,469055	0,164832	138	69	69	134	2,845654	y ~ Intervention + Sex +
0,002244	0,46773	0,150109	138	69	69	134	3,115934	y ~ Intervention + Sex +
0,001431	0,466817	0,143367	138	69	69	134	3,256095	y ~ Intervention + Sex +
0,005114	0,466328	0,163817	138	69	69	134	2,846646	y ~ Intervention + Sex +
0,003316	0,465357	0,155615	138	69	69	134	2,990434	y ~ Intervention + Sex +
0,003294	0,46469	0,15528	138	69	69	134	2,992591	y ~ Intervention + Sex +
0,001395	0,464134	0,142201	138	69	69	134	3,263926	y ~ Intervention + Sex +
0,000646	0,459641	0,131561	138	69	69	134	3,493734	y ~ Intervention + Sex +
0,00115	0,45694	0,13753	138	69	69	134	3,322479	y ~ Intervention + Sex +
0,004149	0,456556	0,156531	138	69	69	134	2,916713	y ~ Intervention + Sex +
0,000725	0,455517	0,131648	138	69	69	134	3,460117	y ~ Intervention + Sex +
0,006844	0,45396	0,165261	138	69	69	134	2,746924	y ~ Intervention + Sex +
0,006649	0,453083	0,164345	138	69	69	134	2,756907	y ~ Intervention + Sex +
0,002912	0,451897	0,149012	138	69	69	134	3,032613	y ~ Intervention + Sex +
0,006142	0,451758	0,162254	138	69	69	134	2,784257	y ~ Intervention + Sex +
0,002769	0,451672	0,148148	138	69	69	134	3,048795	y ~ Intervention + Sex +
0,004993	0,451311	0,158092	138	69	69	134	2,854735	y ~ Intervention + Sex +
0,002926	0,44951	0,1483	138	69	69	134	3,031082	y ~ Intervention + Sex +
0,003443	0,448708	0,150666	138	69	69	134	2,978172	y ~ Intervention + Sex +
0,005242	0,447422	0,157639	138	69	69	134	2,838265	y ~ Intervention + Sex +
0,003405	0,444463	0,149059	138	69	69	134	2,981801	y ~ Intervention + Sex +
0,002008	0,444308	0,14101	138	69	69	134	3,150891	y ~ Intervention + Sex +
0,003379	0,440465	0,147592	138	69	69	134	2,984341	y ~ Intervention + Sex +
0,002956	0,438022	0,144669	138	69	69	134	3,027753	y ~ Intervention + Sex +
0,00466	0,436104	0,151535	138	69	69	134	2,877913	y ~ Intervention + Sex +
0,006864	0,435907	0,158748	138	69	69	134	2,745901	y ~ Intervention + Sex +
0,00205	0,43473	0,138256	138	69	69	134	3,144387	y ~ Intervention + Sex +
0,003303	0,434299	0,145167	138	69	69	134	2,991728	y ~ Intervention + Sex +
0,006885	0,433023	0,15776	138	69	69	134	2,744818	y ~ Intervention + Sex +
0,010648	0,427039	0,164856	138	69	69	134	2,590384	y ~ Intervention + Sex +

0,008987	0,426194	0,160755	138	69	69	134	2,651207	y ~ Intervention + Sex +
0,009727	0,425942	0,16239	138	69	69	134	2,622956	y ~ Intervention + Sex +
0,009615	0,421414	0,16041	138	69	69	134	2,627114	y ~ Intervention + Sex +
0,012763	0,420247	0,166486	138	69	69	134	2,524218	y ~ Intervention + Sex +
0,011154	0,418085	0,162456	138	69	69	134	2,573526	y ~ Intervention + Sex +
0,005607	0,414554	0,147245	138	69	69	134	2,815401	y ~ Intervention + Sex +
0,006574	0,413706	0,149848	138	69	69	134	2,760832	y ~ Intervention + Sex +
0,006891	0,413681	0,15073	138	69	69	134	2,744508	y ~ Intervention + Sex +
0,011767	0,413532	0,161915	138	69	69	134	2,554015	y ~ Intervention + Sex +
0,009335	0,413294	0,156689	138	69	69	134	2,63768	y ~ Intervention + Sex +
0,005007	0,413216	0,144794	138	69	69	134	2,853812	y ~ Intervention + Sex +
0,017425	0,412538	0,171357	138	69	69	134	2,407474	y ~ Intervention + Sex +
0,00646	0,412009	0,148907	138	69	69	134	2,766882	y ~ Intervention + Sex +
0,007454	0,411957	0,151612	138	69	69	134	2,717176	y ~ Intervention + Sex +
0,008571	0,409321	0,153417	138	69	69	134	2,668027	y ~ Intervention + Sex +
0,009307	0,408965	0,154985	138	69	69	134	2,638739	y ~ Intervention + Sex +
0,00958	0,405284	0,154194	138	69	69	134	2,628399	y ~ Intervention + Sex +
0,011987	0,404341	0,158737	138	69	69	134	2,547246	y ~ Intervention + Sex +
0,008707	0,403979	0,151732	138	69	69	134	2,66245	y ~ Intervention + Sex +
0,008819	0,403437	0,151786	138	69	69	134	2,657931	y ~ Intervention + Sex +
0,007736	0,395046	0,146088	138	69	69	134	2,704167	y ~ Intervention + Sex +
0,00519	0,394845	0,13895	138	69	69	134	2,84164	y ~ Intervention + Sex +
0,017794	0,393484	0,163987	138	69	69	134	2,399484	y ~ Intervention + Sex +
0,012518	0,393266	0,155358	138	69	69	134	2,531348	y ~ Intervention + Sex +
0,018655	0,393059	0,165056	138	69	69	134	2,381368	y ~ Intervention + Sex +
0,010374	0,392444	0,150952	138	69	69	134	2,599799	y ~ Intervention + Sex +
0,0189	0,390413	0,164291	138	69	69	134	2,376352	y ~ Intervention + Sex +
0,0189	0,390413	0,164291	138	69	69	134	2,376352	y ~ Intervention + Sex +
0,021027	0,387182	0,165815	138	69	69	134	2,335031	y ~ Intervention + Sex +
0,01955	0,386841	0,163686	138	69	69	134	2,363314	y ~ Intervention + Sex +
0,018256	0,385741	0,161421	138	69	69	134	2,389658	y ~ Intervention + Sex +
0,018785	0,384472	0,161632	138	69	69	134	2,378694	y ~ Intervention + Sex +
0,007475	0,382101	0,140676	138	69	69	134	2,716173	y ~ Intervention + Sex +
0,012661	0,381538	0,150975	138	69	69	134	2,527164	y ~ Intervention + Sex +
0,0176	0,381009	0,158512	138	69	69	134	2,403668	y ~ Intervention + Sex +
0,015899	0,377544	0,154588	138	69	69	134	2,442261	y ~ Intervention + Sex +
0,011074	0,377369	0,146485	138	69	69	134	2,576154	y ~ Intervention + Sex +
0,021898	0,375772	0,162028	138	69	69	134	2,319176	y ~ Intervention + Sex +
0,008092	0,374304	0,139232	138	69	69	134	2,688347	y ~ Intervention + Sex +
0,016357	0,373134	0,153458	138	69	69	134	2,431506	y ~ Intervention + Sex +
0,026134	0,371931	0,165365	138	69	69	134	2,249161	y ~ Intervention + Sex +
0,026683	0,370526	0,165352	138	69	69	134	2,240834	y ~ Intervention + Sex +
0,011408	0,368561	0,14367	138	69	69	134	2,565333	y ~ Intervention + Sex +
0,026022	0,367856	0,163428	138	69	69	134	2,25088	y ~ Intervention + Sex +
0,026022	0,367856	0,163428	138	69	69	134	2,25088	y ~ Intervention + Sex +
0,031421	0,367809	0,169144	138	69	69	134	2,17453	y ~ Intervention + Sex +
0,025556	0,364358	0,161356	138	69	69	134	2,258095	y ~ Intervention + Sex +
0,029344	0,363412	0,165004	138	69	69	134	2,202447	y ~ Intervention + Sex +

0,008327	0,362312	0,135278	138	69	69	134	2,678271	y ~ Intervention + Sex +
0,021866	0,360641	0,155465	138	69	69	134	2,319756	y ~ Intervention + Sex +
0,032894	0,359546	0,166789	138	69	69	134	2,155691	y ~ Intervention + Sex +
0,0234	0,35854	0,156357	138	69	69	134	2,293089	y ~ Intervention + Sex +
0,027207	0,357231	0,159977	138	69	69	134	2,23301	y ~ Intervention + Sex +
0,028151	0,356018	0,160422	138	69	69	134	2,219262	y ~ Intervention + Sex +
0,017046	0,355463	0,147137	138	69	69	134	2,415862	y ~ Intervention + Sex +
0,010396	0,353631	0,136062	138	69	69	134	2,599039	y ~ Intervention + Sex +
0,042766	0,353269	0,172708	138	69	69	134	2,045467	y ~ Intervention + Sex +
0,019374	0,352524	0,148946	138	69	69	134	2,366792	y ~ Intervention + Sex +
0,009695	0,35223	0,134227	138	69	69	134	2,624146	y ~ Intervention + Sex +
0,014816	0,350511	0,141978	138	69	69	134	2,468774	y ~ Intervention + Sex +
0,026005	0,349895	0,15543	138	69	69	134	2,251145	y ~ Intervention + Sex +
0,022511	0,34931	0,151325	138	69	69	134	2,308338	y ~ Intervention + Sex +
0,025137	0,34621	0,152873	138	69	69	134	2,264689	y ~ Intervention + Sex +
0,020545	0,345356	0,147333	138	69	69	134	2,34406	y ~ Intervention + Sex +
0,0356	0,343089	0,161613	138	69	69	134	2,122908	y ~ Intervention + Sex +
0,037166	0,342738	0,162828	138	69	69	134	2,104914	y ~ Intervention + Sex +
0,032475	0,342289	0,158396	138	69	69	134	2,160971	y ~ Intervention + Sex +
0,00941	0,341847	0,129742	138	69	69	134	2,634811	y ~ Intervention + Sex +
0,020975	0,341548	0,146211	138	69	69	134	2,335997	y ~ Intervention + Sex +
0,015023	0,341291	0,138534	138	69	69	134	2,463586	y ~ Intervention + Sex +
0,037102	0,341147	0,162017	138	69	69	134	2,105632	y ~ Intervention + Sex +
0,032053	0,340779	0,157305	138	69	69	134	2,166357	y ~ Intervention + Sex +
0,030944	0,340509	0,15614	138	69	69	134	2,180801	y ~ Intervention + Sex +
0,033231	0,340472	0,15825	138	69	69	134	2,151487	y ~ Intervention + Sex +
0,025996	0,339023	0,150592	138	69	69	134	2,25127	y ~ Intervention + Sex +
0,036624	0,338566	0,160377	138	69	69	134	2,111064	y ~ Intervention + Sex +
0,040551	0,337035	0,162966	138	69	69	134	2,068128	y ~ Intervention + Sex +
0,040404	0,335566	0,162135	138	69	69	134	2,069668	y ~ Intervention + Sex +
0,038792	0,335386	0,16071	138	69	69	134	2,086898	y ~ Intervention + Sex +
0,036685	0,332246	0,157435	138	69	69	134	2,110371	y ~ Intervention + Sex +
0,048143	0,332213	0,166577	138	69	69	134	1,994349	y ~ Intervention + Sex +
0,07275	0,329091	0,181956	138	69	69	134	1,808634	y ~ Intervention + Sex +
0,051988	0,327792	0,167181	138	69	69	134	1,960703	y ~ Intervention + Sex +
0,022071	0,326241	0,140858	138	69	69	134	2,316093	y ~ Intervention + Sex +
0,062855	0,325987	0,173783	138	69	69	134	1,875827	y ~ Intervention + Sex +
0,034506	0,325331	0,152316	138	69	69	134	2,135896	y ~ Intervention + Sex +
0,041833	0,325163	0,158239	138	69	69	134	2,054885	y ~ Intervention + Sex +
0,041334	0,32489	0,157714	138	69	69	134	2,06	y ~ Intervention + Sex +
0,036659	0,324669	0,153822	138	69	69	134	2,110672	y ~ Intervention + Sex +
0,042122	0,324264	0,158027	138	69	69	134	2,051946	y ~ Intervention + Sex +
0,020246	0,323945	0,137863	138	69	69	134	2,349756	y ~ Intervention + Sex +
0,040189	0,322636	0,155717	138	69	69	134	2,071939	y ~ Intervention + Sex +
0,041601	0,318247	0,154694	138	69	69	134	2,057261	y ~ Intervention + Sex +
0,054017	0,317164	0,163168	138	69	69	134	1,943789	y ~ Intervention + Sex +
0,01856	0,316465	0,132783	138	69	69	134	2,383323	y ~ Intervention + Sex +
0,037564	0,316151	0,150516	138	69	69	134	2,100444	y ~ Intervention + Sex +

0,041629	0,313418	0,152368	138	69	69	134	2,056975	y ~ Intervention + Sex +
0,028163	0,313263	0,141168	138	69	69	134	2,219083	y ~ Intervention + Sex +
0,055504	0,312447	0,161745	138	69	69	134	1,931727	y ~ Intervention + Sex +
0,03307	0,311622	0,144705	138	69	69	134	2,153496	y ~ Intervention + Sex +
0,039308	0,309507	0,148707	138	69	69	134	2,08132	y ~ Intervention + Sex +
0,047947	0,309402	0,155001	138	69	69	134	1,996127	y ~ Intervention + Sex +
0,06386	0,309184	0,165461	138	69	69	134	1,868616	y ~ Intervention + Sex +
0,046781	0,308619	0,153785	138	69	69	134	2,006822	y ~ Intervention + Sex +
0,031367	0,307556	0,14139	138	69	69	134	2,17524	y ~ Intervention + Sex +
0,022944	0,307352	0,133582	138	69	69	134	2,300846	y ~ Intervention + Sex +
0,094033	0,307296	0,182213	138	69	69	134	1,686467	y ~ Intervention + Sex +
0,059122	0,307163	0,161369	138	69	69	134	1,903481	y ~ Intervention + Sex +
0,07744	0,307093	0,172581	138	69	69	134	1,779408	y ~ Intervention + Sex +
0,044517	0,306566	0,151149	138	69	69	134	2,028242	y ~ Intervention + Sex +
0,029618	0,306271	0,139299	138	69	69	134	2,198659	y ~ Intervention + Sex +
0,047447	0,305082	0,152489	138	69	69	134	2,000683	y ~ Intervention + Sex +
0,04283	0,303144	0,14825	138	69	69	134	2,04482	y ~ Intervention + Sex +
0,053975	0,303091	0,155901	138	69	69	134	1,944128	y ~ Intervention + Sex +
0,067576	0,302041	0,163908	138	69	69	134	1,842749	y ~ Intervention + Sex +
0,045165	0,30175	0,149232	138	69	69	134	2,022023	y ~ Intervention + Sex +
0,042863	0,301113	0,14728	138	69	69	134	2,044494	y ~ Intervention + Sex +
0,067508	0,30077	0,163177	138	69	69	134	1,843212	y ~ Intervention + Sex +
0,066708	0,299863	0,162204	138	69	69	134	1,848677	y ~ Intervention + Sex +
0,046962	0,298419	0,148826	138	69	69	134	2,005147	y ~ Intervention + Sex +
0,071146	0,297838	0,163738	138	69	69	134	1,81899	y ~ Intervention + Sex +
0,039931	0,297111	0,143209	138	69	69	134	2,074665	y ~ Intervention + Sex +
0,048914	0,294094	0,147977	138	69	69	134	1,987427	y ~ Intervention + Sex +
0,080019	0,294087	0,16672	138	69	69	134	1,763951	y ~ Intervention + Sex +
0,072275	0,293648	0,162086	138	69	69	134	1,811682	y ~ Intervention + Sex +
0,061088	0,29336	0,15532	138	69	69	134	1,888742	y ~ Intervention + Sex +
0,077075	0,293147	0,164539	138	69	69	134	1,781628	y ~ Intervention + Sex +
0,060177	0,292684	0,154408	138	69	69	134	1,895523	y ~ Intervention + Sex +
0,051151	0,292312	0,148545	138	69	69	134	1,967841	y ~ Intervention + Sex +
0,047048	0,288915	0,144144	138	69	69	134	2,004349	y ~ Intervention + Sex +
0,04477	0,286764	0,141556	138	69	69	134	2,025801	y ~ Intervention + Sex +
0,028472	0,286163	0,129212	138	69	69	134	2,214677	y ~ Intervention + Sex +
0,093878	0,285266	0,16907	138	69	69	134	1,687269	y ~ Intervention + Sex +
0,08809	0,283991	0,165295	138	69	69	134	1,718087	y ~ Intervention + Sex +
0,037676	0,283779	0,135185	138	69	69	134	2,099189	y ~ Intervention + Sex +
0,085225	0,283514	0,163507	138	69	69	134	1,733959	y ~ Intervention + Sex +
0,09159	0,283435	0,166799	138	69	69	134	1,699261	y ~ Intervention + Sex +
0,10196	0,278499	0,169124	138	69	69	134	1,646712	y ~ Intervention + Sex +
0,055571	0,277221	0,14355	138	69	69	134	1,931189	y ~ Intervention + Sex +
0,074356	0,275057	0,152941	138	69	69	134	1,798456	y ~ Intervention + Sex +
0,077656	0,274125	0,154168	138	69	69	134	1,778095	y ~ Intervention + Sex +
0,073342	0,274029	0,151828	138	69	69	134	1,80486	y ~ Intervention + Sex +
0,080387	0,273578	0,155285	138	69	69	134	1,761778	y ~ Intervention + Sex +
0,083199	0,272721	0,156247	138	69	69	134	1,74545	y ~ Intervention + Sex +

0,100838	0,272099	0,16469	138	69	69	134	1,652188	y ~ Intervention + Sex +
0,102957	0,271681	0,165468	138	69	69	134	1,641891	y ~ Intervention + Sex +
0,084617	0,2708	0,155867	138	69	69	134	1,737384	y ~ Intervention + Sex +
0,093709	0,270126	0,160013	138	69	69	134	1,688149	y ~ Intervention + Sex +
0,093485	0,269033	0,159256	138	69	69	134	1,689311	y ~ Intervention + Sex +
0,103882	0,268112	0,163737	138	69	69	134	1,637452	y ~ Intervention + Sex +
0,062962	0,26785	0,142849	138	69	69	134	1,875054	y ~ Intervention + Sex +
0,086371	0,267397	0,154783	138	69	69	134	1,727563	y ~ Intervention + Sex +
0,101562	0,26586	0,161259	138	69	69	134	1,648649	y ~ Intervention + Sex +
0,109002	0,265678	0,164666	138	69	69	134	1,613437	y ~ Intervention + Sex +
0,085815	0,265306	0,153298	138	69	69	134	1,730656	y ~ Intervention + Sex +
0,123816	0,263902	0,170404	138	69	69	134	1,548687	y ~ Intervention + Sex +
0,11222	0,261491	0,163554	138	69	69	134	1,598801	y ~ Intervention + Sex +
0,122296	0,260887	0,167769	138	69	69	134	1,555037	y ~ Intervention + Sex +
0,156085	0,260819	0,182854	138	69	69	134	1,426378	y ~ Intervention + Sex +
0,117096	0,260331	0,165054	138	69	69	134	1,577249	y ~ Intervention + Sex +
0,058312	0,260326	0,13632	138	69	69	134	1,909675	y ~ Intervention + Sex +
0,108118	0,259927	0,160695	138	69	69	134	1,617518	y ~ Intervention + Sex +
0,122103	0,259224	0,166613	138	69	69	134	1,555846	y ~ Intervention + Sex +
0,110062	0,259199	0,161136	138	69	69	134	1,608578	y ~ Intervention + Sex +
0,088352	0,259017	0,150884	138	69	69	134	1,716657	y ~ Intervention + Sex +
0,144513	0,257306	0,175305	138	69	69	134	1,467759	y ~ Intervention + Sex +
0,076891	0,256611	0,143941	138	69	69	134	1,782751	y ~ Intervention + Sex +
0,130889	0,254389	0,167371	138	69	69	134	1,519912	y ~ Intervention + Sex +
0,094693	0,25423	0,151053	138	69	69	134	1,683056	y ~ Intervention + Sex +
0,140082	0,254157	0,171232	138	69	69	134	1,484283	y ~ Intervention + Sex +
0,149556	0,253902	0,175175	138	69	69	134	1,449423	y ~ Intervention + Sex +
0,117381	0,253094	0,160592	138	69	69	134	1,576011	y ~ Intervention + Sex +
0,146898	0,251845	0,172612	138	69	69	134	1,459026	y ~ Intervention + Sex +
0,108942	0,251643	0,15594	138	69	69	134	1,613713	y ~ Intervention + Sex +
0,134351	0,250187	0,166097	138	69	69	134	1,506271	y ~ Intervention + Sex +
0,090135	0,249914	0,146404	138	69	69	134	1,707015	y ~ Intervention + Sex +
0,108634	0,249108	0,154234	138	69	69	134	1,615132	y ~ Intervention + Sex +
0,095869	0,248592	0,148234	138	69	69	134	1,677025	y ~ Intervention + Sex +
0,114767	0,24851	0,156546	138	69	69	134	1,587455	y ~ Intervention + Sex +
0,136185	0,248452	0,165728	138	69	69	134	1,499159	y ~ Intervention + Sex +
0,13453	0,248334	0,164943	138	69	69	134	1,505577	y ~ Intervention + Sex +
0,079618	0,248096	0,140458	138	69	69	134	1,76633	y ~ Intervention + Sex +
0,108152	0,248026	0,153352	138	69	69	134	1,61736	y ~ Intervention + Sex +
0,099781	0,247874	0,149557	138	69	69	134	1,657383	y ~ Intervention + Sex +
0,130233	0,24513	0,161002	138	69	69	134	1,52253	y ~ Intervention + Sex +
0,104409	0,244285	0,149415	138	69	69	134	1,63494	y ~ Intervention + Sex +
0,118269	0,244258	0,155364	138	69	69	134	1,57217	y ~ Intervention + Sex +
0,127537	0,242623	0,158226	138	69	69	134	1,533391	y ~ Intervention + Sex +
0,150806	0,242288	0,167679	138	69	69	134	1,444952	y ~ Intervention + Sex +
0,105566	0,240394	0,14753	138	69	69	134	1,629453	y ~ Intervention + Sex +
0,126422	0,240197	0,156181	138	69	69	134	1,537939	y ~ Intervention + Sex +
0,151369	0,240059	0,166367	138	69	69	134	1,442946	y ~ Intervention + Sex +

0,115397	0,238762	0,150669	138	69	69	134	1,584678	y ~ Intervention + Sex +
0,164775	0,238718	0,1709	138	69	69	134	1,396825	y ~ Intervention + Sex +
0,127505	0,237183	0,154666	138	69	69	134	1,533522	y ~ Intervention + Sex +
0,107419	0,237038	0,146251	138	69	69	134	1,620764	y ~ Intervention + Sex +
0,099821	0,236352	0,142623	138	69	69	134	1,657186	y ~ Intervention + Sex +
0,142741	0,23547	0,159714	138	69	69	134	1,47432	y ~ Intervention + Sex +
0,146865	0,234153	0,160473	138	69	69	134	1,459146	y ~ Intervention + Sex +
0,09263	0,232725	0,1374	138	69	69	134	1,693783	y ~ Intervention + Sex +
0,149837	0,23174	0,159995	138	69	69	134	1,448415	y ~ Intervention + Sex +
0,164196	0,231256	0,16533	138	69	69	134	1,398756	y ~ Intervention + Sex +
0,170419	0,23124	0,167776	138	69	69	134	1,378264	y ~ Intervention + Sex +
0,097115	0,231174	0,138369	138	69	69	134	1,670701	y ~ Intervention + Sex +
0,176285	0,231113	0,170004	138	69	69	134	1,359459	y ~ Intervention + Sex +
0,201939	0,23075	0,179945	138	69	69	134	1,282334	y ~ Intervention + Sex +
0,103596	0,230038	0,140368	138	69	69	134	1,638823	y ~ Intervention + Sex +
0,184411	0,228734	0,171443	138	69	69	134	1,334169	y ~ Intervention + Sex +
0,180715	0,228691	0,169959	138	69	69	134	1,345565	y ~ Intervention + Sex +
0,120219	0,227895	0,14573	138	69	69	134	1,563817	y ~ Intervention + Sex +
0,098114	0,227724	0,136715	138	69	69	134	1,665679	y ~ Intervention + Sex +
0,158011	0,227564	0,160287	138	69	69	134	1,419722	y ~ Intervention + Sex +
0,162557	0,227237	0,16182	138	69	69	134	1,404253	y ~ Intervention + Sex +
0,148049	0,226415	0,155628	138	69	69	134	1,454851	y ~ Intervention + Sex +
0,187571	0,225964	0,170596	138	69	69	134	1,324561	y ~ Intervention + Sex +
0,157386	0,225738	0,158761	138	69	69	134	1,421876	y ~ Intervention + Sex +
0,085917	0,225413	0,13029	138	69	69	134	1,730085	y ~ Intervention + Sex +
0,17363	0,224034	0,163778	138	69	69	134	1,36791	y ~ Intervention + Sex +
0,147602	0,221684	0,152207	138	69	69	134	1,456471	y ~ Intervention + Sex +
0,163237	0,221332	0,157872	138	69	69	134	1,401969	y ~ Intervention + Sex +
0,182307	0,221256	0,165039	138	69	69	134	1,340635	y ~ Intervention + Sex +
0,154472	0,221246	0,154501	138	69	69	134	1,432001	y ~ Intervention + Sex +
0,171573	0,221184	0,160916	138	69	69	134	1,374527	y ~ Intervention + Sex +
0,14338	0,220534	0,149824	138	69	69	134	1,471948	y ~ Intervention + Sex +
0,147376	0,220501	0,151309	138	69	69	134	1,457288	y ~ Intervention + Sex +
0,124764	0,2205	0,142742	138	69	69	134	1,544753	y ~ Intervention + Sex +
0,184048	0,220429	0,165081	138	69	69	134	1,335282	y ~ Intervention + Sex +
0,181709	0,220395	0,16417	138	69	69	134	1,342483	y ~ Intervention + Sex +
0,185389	0,220098	0,165341	138	69	69	134	1,331183	y ~ Intervention + Sex +
0,259251	0,219351	0,193608	138	69	69	134	1,132968	y ~ Intervention + Sex +
0,203189	0,218947	0,171217	138	69	69	134	1,278769	y ~ Intervention + Sex +
0,184231	0,217128	0,162676	138	69	69	134	1,33472	y ~ Intervention + Sex +
0,222286	0,216741	0,176763	138	69	69	134	1,226168	y ~ Intervention + Sex +
0,156267	0,216626	0,151939	138	69	69	134	1,425745	y ~ Intervention + Sex +
0,16701	0,216377	0,155732	138	69	69	134	1,389417	y ~ Intervention + Sex +
0,20134	0,215941	0,168172	138	69	69	134	1,284049	y ~ Intervention + Sex +
0,088872	0,215766	0,125897	138	69	69	134	1,71383	y ~ Intervention + Sex +
0,162361	0,215674	0,153514	138	69	69	134	1,404913	y ~ Intervention + Sex +
0,192952	0,214664	0,164056	138	69	69	134	1,308476	y ~ Intervention + Sex +
0,232628	0,212822	0,177494	138	69	69	134	1,199042	y ~ Intervention + Sex +

0,115368	0,211455	0,133427	138	69	69	134	1,584806	y ~ Intervention + Sex +
0,197594	0,211123	0,163047	138	69	69	134	1,294865	y ~ Intervention + Sex +
0,187636	0,206704	0,156078	138	69	69	134	1,324366	y ~ Intervention + Sex +
0,209078	0,205307	0,162661	138	69	69	134	1,262174	y ~ Intervention + Sex +
0,229394	0,204289	0,169193	138	69	69	134	1,207429	y ~ Intervention + Sex +
0,174866	0,203718	0,149357	138	69	69	134	1,363961	y ~ Intervention + Sex +
0,122022	0,20339	0,130698	138	69	69	134	1,55619	y ~ Intervention + Sex +
0,205781	0,203049	0,159702	138	69	69	134	1,271423	y ~ Intervention + Sex +
0,183486	0,202571	0,151511	138	69	69	134	1,337006	y ~ Intervention + Sex +
0,214272	0,201326	0,161342	138	69	69	134	1,247823	y ~ Intervention + Sex +
0,205338	0,201018	0,15795	138	69	69	134	1,272671	y ~ Intervention + Sex +
0,238886	0,200832	0,169759	138	69	69	134	1,183045	y ~ Intervention + Sex +
0,200944	0,200197	0,155773	138	69	69	134	1,285187	y ~ Intervention + Sex +
0,165555	0,199958	0,143418	138	69	69	134	1,394233	y ~ Intervention + Sex +
0,225226	0,199002	0,163336	138	69	69	134	1,218365	y ~ Intervention + Sex +
0,164436	0,198728	0,142156	138	69	69	134	1,397957	y ~ Intervention + Sex +
0,144881	0,198305	0,135232	138	69	69	134	1,466404	y ~ Intervention + Sex +
0,223417	0,198015	0,161889	138	69	69	134	1,223158	y ~ Intervention + Sex +
0,139805	0,197861	0,13321	138	69	69	134	1,48533	y ~ Intervention + Sex +
0,16228	0,197535	0,140575	138	69	69	134	1,405186	y ~ Intervention + Sex +
0,229797	0,195744	0,162257	138	69	69	134	1,206378	y ~ Intervention + Sex +
0,22401	0,195021	0,159646	138	69	69	134	1,221583	y ~ Intervention + Sex +
0,212582	0,194058	0,154941	138	69	69	134	1,252465	y ~ Intervention + Sex +
0,221332	0,193864	0,157778	138	69	69	134	1,228716	y ~ Intervention + Sex +
0,225696	0,193817	0,159242	138	69	69	134	1,217124	y ~ Intervention + Sex +
0,213001	0,192526	0,153859	138	69	69	134	1,251312	y ~ Intervention + Sex +
0,202602	0,19092	0,149105	138	69	69	134	1,280441	y ~ Intervention + Sex +
0,222304	0,190693	0,155526	138	69	69	134	1,22612	y ~ Intervention + Sex +
0,237161	0,189559	0,159639	138	69	69	134	1,187424	y ~ Intervention + Sex +
0,212938	0,18806	0,150269	138	69	69	134	1,251484	y ~ Intervention + Sex +
0,227215	0,187703	0,154726	138	69	69	134	1,213129	y ~ Intervention + Sex +
0,206328	0,186569	0,146918	138	69	69	134	1,269881	y ~ Intervention + Sex +
0,242828	0,186077	0,158617	138	69	69	134	1,173121	y ~ Intervention + Sex +
0,259572	0,186	0,164281	138	69	69	134	1,132202	y ~ Intervention + Sex +
0,239883	0,185594	0,157214	138	69	69	134	1,180524	y ~ Intervention + Sex +
0,295457	0,184891	0,176032	138	69	69	134	1,05033	y ~ Intervention + Sex +
0,23179	0,184645	0,153716	138	69	69	134	1,201206	y ~ Intervention + Sex +
0,197066	0,184346	0,142198	138	69	69	134	1,296401	y ~ Intervention + Sex +
0,247136	0,18429	0,158541	138	69	69	134	1,162408	y ~ Intervention + Sex +
0,194805	0,182646	0,140172	138	69	69	134	1,303013	y ~ Intervention + Sex +
0,28805	0,182139	0,170759	138	69	69	134	1,066646	y ~ Intervention + Sex +
0,202961	0,181927	0,142195	138	69	69	134	1,279417	y ~ Intervention + Sex +
0,288735	0,181867	0,170747	138	69	69	134	1,065125	y ~ Intervention + Sex +
0,24569	0,18186	0,155971	138	69	69	134	1,165989	y ~ Intervention + Sex +
0,28549	0,181512	0,169266	138	69	69	134	1,07235	y ~ Intervention + Sex +
0,26144	0,18055	0,160097	138	69	69	134	1,127752	y ~ Intervention + Sex +
0,312845	0,179944	0,17762	138	69	69	134	1,013086	y ~ Intervention + Sex +
0,251002	0,179539	0,155727	138	69	69	134	1,152904	y ~ Intervention + Sex +

0,211189	0,179224	0,142659	138	69	69	134	1,256312	y ~ Intervention + Sex +
0,199264	0,178909	0,138687	138	69	69	134	1,290024	y ~ Intervention + Sex +
0,211328	0,178724	0,142305	138	69	69	134	1,255927	y ~ Intervention + Sex +
0,221869	0,17796	0,145004	138	69	69	134	1,22728	y ~ Intervention + Sex +
0,263742	0,177506	0,158163	138	69	69	134	1,1223	y ~ Intervention + Sex +
0,268814	0,177504	0,159856	138	69	69	134	1,110402	y ~ Intervention + Sex +
0,283034	0,177081	0,16429	138	69	69	134	1,077857	y ~ Intervention + Sex +
0,283924	0,176261	0,163833	138	69	69	134	1,075857	y ~ Intervention + Sex +
0,284161	0,176151	0,163812	138	69	69	134	1,075326	y ~ Intervention + Sex +
0,203076	0,174108	0,136119	138	69	69	134	1,27909	y ~ Intervention + Sex +
0,260405	0,17376	0,153741	138	69	69	134	1,130214	y ~ Intervention + Sex +
0,231764	0,172676	0,143744	138	69	69	134	1,201275	y ~ Intervention + Sex +
0,281388	0,172297	0,159304	138	69	69	134	1,081566	y ~ Intervention + Sex +
0,279871	0,172168	0,158681	138	69	69	134	1,084996	y ~ Intervention + Sex +
0,234346	0,172125	0,144084	138	69	69	134	1,194619	y ~ Intervention + Sex +
0,298823	0,171864	0,164777	138	69	69	134	1,043007	y ~ Intervention + Sex +
0,256409	0,170892	0,149933	138	69	69	134	1,139785	y ~ Intervention + Sex +
0,271629	0,170864	0,154786	138	69	69	134	1,103866	y ~ Intervention + Sex +
0,301937	0,169574	0,163637	138	69	69	134	1,036281	y ~ Intervention + Sex +
0,275658	0,168561	0,153994	138	69	69	134	1,094593	y ~ Intervention + Sex +
0,32082	0,167462	0,168056	138	69	69	134	0,996466	y ~ Intervention + Sex +
0,323359	0,167443	0,168924	138	69	69	134	0,991233	y ~ Intervention + Sex +
0,278112	0,167218	0,153553	138	69	69	134	1,088993	y ~ Intervention + Sex +
0,297219	0,166345	0,158955	138	69	69	134	1,046489	y ~ Intervention + Sex +
0,265652	0,166327	0,148799	138	69	69	134	1,117802	y ~ Intervention + Sex +
0,307161	0,166069	0,162002	138	69	69	134	1,025104	y ~ Intervention + Sex +
0,322972	0,165634	0,166964	138	69	69	134	0,992028	y ~ Intervention + Sex +
0,272886	0,165046	0,14991	138	69	69	134	1,100964	y ~ Intervention + Sex +
0,331547	0,164642	0,168945	138	69	69	134	0,974534	y ~ Intervention + Sex +
0,314045	0,164047	0,162332	138	69	69	134	1,010568	y ~ Intervention + Sex +
0,342498	0,163203	0,17132	138	69	69	134	0,952621	y ~ Intervention + Sex +
0,292449	0,163152	0,154365	138	69	69	134	1,056922	y ~ Intervention + Sex +
0,288535	0,163133	0,153095	138	69	69	134	1,065568	y ~ Intervention + Sex +
0,251284	0,162727	0,141229	138	69	69	134	1,152215	y ~ Intervention + Sex +
0,29777	0,161981	0,154962	138	69	69	134	1,045293	y ~ Intervention + Sex +
0,373746	0,159977	0,179254	138	69	69	134	0,892461	y ~ Intervention + Sex +
0,279069	0,159844	0,147075	138	69	69	134	1,086817	y ~ Intervention + Sex +
0,335041	0,158679	0,164011	138	69	69	134	0,967493	y ~ Intervention + Sex +
0,293382	0,158551	0,150303	138	69	69	134	1,054872	y ~ Intervention + Sex +
0,348481	0,15855	0,16852	138	69	69	134	0,940837	y ~ Intervention + Sex +
0,276277	0,158499	0,14499	138	69	69	134	1,093178	y ~ Intervention + Sex +
0,343372	0,158355	0,166534	138	69	69	134	0,950889	y ~ Intervention + Sex +
0,358739	0,158248	0,171835	138	69	69	134	0,920935	y ~ Intervention + Sex +
0,291126	0,157568	0,148672	138	69	69	134	1,059836	y ~ Intervention + Sex +
0,32004	0,1572	0,157503	138	69	69	134	0,998079	y ~ Intervention + Sex +
0,327053	0,155436	0,158018	138	69	69	134	0,983665	y ~ Intervention + Sex +
0,337236	0,154509	0,16043	138	69	69	134	0,963091	y ~ Intervention + Sex +
0,351448	0,153158	0,163798	138	69	69	134	0,935044	y ~ Intervention + Sex +

0,359365	0,152628	0,165948	138	69	69	134	0,919734	y ~ Intervention + Sex +
0,265272	0,150565	0,13459	138	69	69	134	1,118694	y ~ Intervention + Sex +
0,317267	0,150346	0,149771	138	69	69	134	1,003836	y ~ Intervention + Sex +
0,375496	0,150217	0,168937	138	69	69	134	0,889188	y ~ Intervention + Sex +
0,335091	0,150206	0,155269	138	69	69	134	0,967391	y ~ Intervention + Sex +
0,363576	0,149238	0,163696	138	69	69	134	0,911677	y ~ Intervention + Sex +
0,32614	0,148783	0,150968	138	69	69	134	0,985529	y ~ Intervention + Sex +
0,313125	0,148775	0,146939	138	69	69	134	1,012498	y ~ Intervention + Sex +
0,362216	0,145929	0,159612	138	69	69	134	0,914273	y ~ Intervention + Sex +
0,393977	0,143471	0,167767	138	69	69	134	0,855183	y ~ Intervention + Sex +
0,371309	0,14311	0,159537	138	69	69	134	0,897035	y ~ Intervention + Sex +
0,323725	0,142501	0,143871	138	69	69	134	0,99048	y ~ Intervention + Sex +
0,370812	0,141463	0,157536	138	69	69	134	0,89797	y ~ Intervention + Sex +
0,353732	0,13985	0,150278	138	69	69	134	0,930604	y ~ Intervention + Sex +
0,420176	0,138782	0,171631	138	69	69	134	0,808605	y ~ Intervention + Sex +
0,308691	0,138677	0,135711	138	69	69	134	1,021854	y ~ Intervention + Sex +
0,335916	0,137675	0,14256	138	69	69	134	0,965735	y ~ Intervention + Sex +
0,418774	0,137462	0,169486	138	69	69	134	0,811052	y ~ Intervention + Sex +
0,286747	0,137271	0,128345	138	69	69	134	1,069546	y ~ Intervention + Sex +
0,343049	0,137231	0,144222	138	69	69	134	0,951529	y ~ Intervention + Sex +
0,428555	0,137188	0,172764	138	69	69	134	0,794077	y ~ Intervention + Sex +
0,31877	0,13665	0,136553	138	69	69	134	1,000712	y ~ Intervention + Sex +
0,373978	0,13602	0,152484	138	69	69	134	0,892026	y ~ Intervention + Sex +
0,430746	0,135619	0,171603	138	69	69	134	0,790306	y ~ Intervention + Sex +
0,38778	0,135508	0,156391	138	69	69	134	0,866472	y ~ Intervention + Sex +
0,437701	0,13546	0,174021	138	69	69	134	0,778408	y ~ Intervention + Sex +
0,442103	0,134665	0,174677	138	69	69	134	0,770935	y ~ Intervention + Sex +
0,437673	0,134262	0,172473	138	69	69	134	0,778455	y ~ Intervention + Sex +
0,368375	0,133838	0,148286	138	69	69	134	0,902567	y ~ Intervention + Sex +
0,437634	0,131953	0,169492	138	69	69	134	0,778522	y ~ Intervention + Sex +
0,427098	0,13161	0,165216	138	69	69	134	0,796591	y ~ Intervention + Sex +
0,410945	0,13136	0,15926	138	69	69	134	0,824811	y ~ Intervention + Sex +
0,371103	0,131162	0,146154	138	69	69	134	0,897422	y ~ Intervention + Sex +
0,414607	0,130953	0,160019	138	69	69	134	0,818356	y ~ Intervention + Sex +
0,412046	0,130163	0,158182	138	69	69	134	0,822866	y ~ Intervention + Sex +
0,382711	0,130119	0,148573	138	69	69	134	0,875791	y ~ Intervention + Sex +
0,367592	0,129547	0,143297	138	69	69	134	0,904048	y ~ Intervention + Sex +
0,360111	0,129347	0,140855	138	69	69	134	0,918302	y ~ Intervention + Sex +
0,373187	0,129224	0,144626	138	69	69	134	0,893508	y ~ Intervention + Sex +
0,42805	0,129222	0,162555	138	69	69	134	0,794947	y ~ Intervention + Sex +
0,380413	0,128133	0,145599	138	69	69	134	0,88004	y ~ Intervention + Sex +
0,412765	0,127651	0,155369	138	69	69	134	0,821598	y ~ Intervention + Sex +
0,45408	0,127384	0,169661	138	69	69	134	0,750816	y ~ Intervention + Sex +
0,426088	0,127277	0,159427	138	69	69	134	0,798336	y ~ Intervention + Sex +
0,411999	0,126932	0,15424	138	69	69	134	0,822949	y ~ Intervention + Sex +
0,459177	0,126639	0,170593	138	69	69	134	0,742346	y ~ Intervention + Sex +
0,394927	0,126305	0,147992	138	69	69	134	0,853461	y ~ Intervention + Sex +
0,390062	0,125921	0,14603	138	69	69	134	0,862301	y ~ Intervention + Sex +

0,376831	0,125896	0,141983	138	69	69	134	0,886696	y ~ Intervention + Sex +
0,410284	0,125662	0,152137	138	69	69	134	0,825979	y ~ Intervention + Sex +
0,434031	0,125328	0,159721	138	69	69	134	0,784671	y ~ Intervention + Sex +
0,410334	0,125082	0,151452	138	69	69	134	0,82589	y ~ Intervention + Sex +
0,448289	0,125066	0,164452	138	69	69	134	0,760504	y ~ Intervention + Sex +
0,444816	0,124462	0,162408	138	69	69	134	0,76635	y ~ Intervention + Sex +
0,415221	0,124281	0,152068	138	69	69	134	0,817277	y ~ Intervention + Sex +
0,464128	0,124248	0,169236	138	69	69	134	0,734169	y ~ Intervention + Sex +
0,417261	0,123821	0,15217	138	69	69	134	0,8137	y ~ Intervention + Sex +
0,422639	0,123647	0,15373	138	69	69	134	0,804316	y ~ Intervention + Sex +
0,409945	0,12308	0,148903	138	69	69	134	0,82658	y ~ Intervention + Sex +
0,421539	0,121863	0,151152	138	69	69	134	0,80623	y ~ Intervention + Sex +
0,408308	0,12172	0,146742	138	69	69	134	0,829481	y ~ Intervention + Sex +
0,420255	0,121716	0,150552	138	69	69	134	0,808466	y ~ Intervention + Sex +
0,414151	0,121379	0,148175	138	69	69	134	0,819158	y ~ Intervention + Sex +
0,479723	0,119224	0,168223	138	69	69	134	0,70873	y ~ Intervention + Sex +
0,421899	0,119066	0,147798	138	69	69	134	0,805603	y ~ Intervention + Sex +
0,462629	0,118734	0,161183	138	69	69	134	0,736641	y ~ Intervention + Sex +
0,478381	0,117871	0,165806	138	69	69	134	0,710901	y ~ Intervention + Sex +
0,445153	0,117515	0,153457	138	69	69	134	0,765782	y ~ Intervention + Sex +
0,463561	0,117265	0,159522	138	69	69	134	0,735104	y ~ Intervention + Sex +
0,413692	0,116589	0,142188	138	69	69	134	0,819966	y ~ Intervention + Sex +
0,496268	0,114316	0,167561	138	69	69	134	0,682236	y ~ Intervention + Sex +
0,496932	0,114112	0,167521	138	69	69	134	0,681182	y ~ Intervention + Sex +
0,460118	0,113744	0,153544	138	69	69	134	0,740789	y ~ Intervention + Sex +
0,4628	0,113266	0,153819	138	69	69	134	0,736358	y ~ Intervention + Sex +
0,449073	0,112551	0,148251	138	69	69	134	0,75919	y ~ Intervention + Sex +
0,404103	0,112484	0,134395	138	69	69	134	0,836965	y ~ Intervention + Sex +
0,50871	0,111208	0,16783	138	69	69	134	0,662623	y ~ Intervention + Sex +
0,53877	0,108976	0,176834	138	69	69	134	0,616258	y ~ Intervention + Sex +
0,526306	0,108299	0,170465	138	69	69	134	0,635316	y ~ Intervention + Sex +
0,5285	0,107513	0,17013	138	69	69	134	0,631945	y ~ Intervention + Sex +
0,520382	0,107403	0,166657	138	69	69	134	0,644456	y ~ Intervention + Sex +
0,430305	0,107366	0,135724	138	69	69	134	0,791063	y ~ Intervention + Sex +
0,562591	0,106657	0,183751	138	69	69	134	0,580443	y ~ Intervention + Sex +
0,502123	0,106473	0,158213	138	69	69	134	0,672973	y ~ Intervention + Sex +
0,465484	0,106037	0,144871	138	69	69	134	0,731939	y ~ Intervention + Sex +
0,491524	0,10592	0,153557	138	69	69	134	0,689781	y ~ Intervention + Sex +
0,516499	0,105549	0,162264	138	69	69	134	0,650475	y ~ Intervention + Sex +
0,519261	0,105438	0,163168	138	69	69	134	0,646191	y ~ Intervention + Sex +
0,534633	0,105033	0,168713	138	69	69	134	0,622558	y ~ Intervention + Sex +
0,51195	0,10461	0,159088	138	69	69	134	0,657558	y ~ Intervention + Sex +
0,43821	0,104263	0,134093	138	69	69	134	0,777542	y ~ Intervention + Sex +
0,516953	0,103814	0,159771	138	69	69	134	0,64977	y ~ Intervention + Sex +
0,576953	0,103308	0,18474	138	69	69	134	0,559208	y ~ Intervention + Sex +
0,560938	0,102693	0,176175	138	69	69	134	0,582904	y ~ Intervention + Sex +
0,507172	0,101384	0,152449	138	69	69	134	0,665034	y ~ Intervention + Sex +
0,528526	0,101101	0,159994	138	69	69	134	0,631905	y ~ Intervention + Sex +

0,53614	0,100731	0,162401	138	69	69	134	0,62026	y ~ Intervention + Sex +
0,537064	0,100525	0,162437	138	69	69	134	0,618853	y ~ Intervention + Sex +
0,503789	0,100402	0,149776	138	69	69	134	0,670349	y ~ Intervention + Sex +
0,5232	0,100184	0,156512	138	69	69	134	0,640101	y ~ Intervention + Sex +
0,452868	0,09978	0,132539	138	69	69	134	0,752838	y ~ Intervention + Sex +
0,531519	0,099766	0,159036	138	69	69	134	0,627316	y ~ Intervention + Sex +
0,544732	0,099382	0,163667	138	69	69	134	0,60722	y ~ Intervention + Sex +
0,555783	0,099309	0,168149	138	69	69	134	0,590601	y ~ Intervention + Sex +
0,545545	0,098864	0,163143	138	69	69	134	0,605992	y ~ Intervention + Sex +
0,495666	0,098657	0,144406	138	69	69	134	0,683191	y ~ Intervention + Sex +
0,564069	0,09855	0,17043	138	69	69	134	0,578245	y ~ Intervention + Sex +
0,499636	0,098412	0,145386	138	69	69	134	0,6769	y ~ Intervention + Sex +
0,509381	0,097629	0,147571	138	69	69	134	0,661572	y ~ Intervention + Sex +
0,511788	0,097337	0,147972	138	69	69	134	0,657811	y ~ Intervention + Sex +
0,541579	0,097053	0,158586	138	69	69	134	0,611994	y ~ Intervention + Sex +
0,448517	0,096787	0,127331	138	69	69	134	0,760121	y ~ Intervention + Sex +
0,494537	0,096786	0,141296	138	69	69	134	0,684985	y ~ Intervention + Sex +
0,542785	0,096569	0,158266	138	69	69	134	0,610167	y ~ Intervention + Sex +
0,533247	0,09654	0,154544	138	69	69	134	0,624674	y ~ Intervention + Sex +
0,523383	0,096426	0,150709	138	69	69	134	0,639818	y ~ Intervention + Sex +
0,552684	0,09618	0,16158	138	69	69	134	0,595245	y ~ Intervention + Sex +
0,478553	0,096107	0,135243	138	69	69	134	0,710623	y ~ Intervention + Sex +
0,524321	0,095474	0,149559	138	69	69	134	0,638372	y ~ Intervention + Sex +
0,578438	0,094895	0,17036	138	69	69	134	0,557027	y ~ Intervention + Sex +
0,504859	0,09469	0,14161	138	69	69	134	0,668666	y ~ Intervention + Sex +
0,569095	0,09465	0,165821	138	69	69	134	0,570795	y ~ Intervention + Sex +
0,519585	0,093867	0,145374	138	69	69	134	0,645689	y ~ Intervention + Sex +
0,573761	0,093653	0,166079	138	69	69	134	0,563905	y ~ Intervention + Sex +
0,5517	0,093549	0,156771	138	69	69	134	0,596722	y ~ Intervention + Sex +
0,578061	0,093439	0,167579	138	69	69	134	0,557581	y ~ Intervention + Sex +
0,54515	0,093366	0,153919	138	69	69	134	0,606589	y ~ Intervention + Sex +
0,551835	0,092959	0,155836	138	69	69	134	0,596519	y ~ Intervention + Sex +
0,550141	0,091007	0,151916	138	69	69	134	0,599066	y ~ Intervention + Sex +
0,584463	0,09072	0,165485	138	69	69	134	0,548206	y ~ Intervention + Sex +
0,567965	0,090708	0,158452	138	69	69	134	0,572467	y ~ Intervention + Sex +
0,421877	0,090502	0,112336	138	69	69	134	0,805641	y ~ Intervention + Sex +
0,519095	0,08984	0,138974	138	69	69	134	0,646448	y ~ Intervention + Sex +
0,559955	0,089257	0,152742	138	69	69	134	0,584368	y ~ Intervention + Sex +
0,552738	0,089136	0,149767	138	69	69	134	0,595163	y ~ Intervention + Sex +
0,614942	0,088779	0,176075	138	69	69	134	0,50421	y ~ Intervention + Sex +
0,586142	0,08755	0,160421	138	69	69	134	0,545755	y ~ Intervention + Sex +
0,554597	0,08642	0,145888	138	69	69	134	0,592376	y ~ Intervention + Sex +
0,532368	0,085584	0,136712	138	69	69	134	0,626018	y ~ Intervention + Sex +
0,499599	0,084974	0,125523	138	69	69	134	0,676959	y ~ Intervention + Sex +
0,622492	0,084936	0,172123	138	69	69	134	0,493465	y ~ Intervention + Sex +
0,57399	0,084426	0,149806	138	69	69	134	0,563568	y ~ Intervention + Sex +
0,56298	0,084348	0,145462	138	69	69	134	0,579864	y ~ Intervention + Sex +
0,601112	0,083937	0,160171	138	69	69	134	0,524047	y ~ Intervention + Sex +

0,61031	0,083719	0,163888	138	69	69	134	0,510832	y ~ Intervention + Sex +
0,610144	0,082732	0,16188	138	69	69	134	0,511069	y ~ Intervention + Sex +
0,596069	0,082642	0,155538	138	69	69	134	0,531332	y ~ Intervention + Sex +
0,633931	0,081569	0,170899	138	69	69	134	0,477294	y ~ Intervention + Sex +
0,593928	0,081547	0,152586	138	69	69	134	0,534432	y ~ Intervention + Sex +
0,613715	0,08101	0,160111	138	69	69	134	0,505961	y ~ Intervention + Sex +
0,615717	0,081002	0,161005	138	69	69	134	0,503104	y ~ Intervention + Sex +
0,624627	0,080326	0,163785	138	69	69	134	0,490437	y ~ Intervention + Sex +
0,582893	0,079831	0,145015	138	69	69	134	0,550501	y ~ Intervention + Sex +
0,618981	0,079763	0,160021	138	69	69	134	0,498455	y ~ Intervention + Sex +
0,583153	0,079593	0,144683	138	69	69	134	0,550119	y ~ Intervention + Sex +
0,622097	0,078271	0,158435	138	69	69	134	0,494025	y ~ Intervention + Sex +
0,598666	0,077554	0,147001	138	69	69	134	0,527576	y ~ Intervention + Sex +
0,628406	0,077072	0,158882	138	69	69	134	0,485088	y ~ Intervention + Sex +
0,599054	0,076534	0,145222	138	69	69	134	0,527016	y ~ Intervention + Sex +
0,620693	0,076529	0,154286	138	69	69	134	0,49602	y ~ Intervention + Sex +
0,623113	0,076367	0,155035	138	69	69	134	0,492583	y ~ Intervention + Sex +
0,603155	0,075566	0,145011	138	69	69	134	0,521103	y ~ Intervention + Sex +
0,611327	0,074964	0,147168	138	69	69	134	0,509375	y ~ Intervention + Sex +
0,579989	0,07432	0,13397	138	69	69	134	0,554752	y ~ Intervention + Sex +
0,627914	0,074012	0,152356	138	69	69	134	0,485783	y ~ Intervention + Sex +
0,611702	0,074006	0,145441	138	69	69	134	0,508839	y ~ Intervention + Sex +
0,60078	0,072987	0,139148	138	69	69	134	0,524524	y ~ Intervention + Sex +
0,676841	0,072604	0,173823	138	69	69	134	0,417692	y ~ Intervention + Sex +
0,665089	0,072182	0,166373	138	69	69	134	0,433858	y ~ Intervention + Sex +
0,652264	0,071206	0,157664	138	69	69	134	0,451633	y ~ Intervention + Sex +
0,62113	0,07073	0,142773	138	69	69	134	0,495399	y ~ Intervention + Sex +
0,671393	0,07033	0,165414	138	69	69	134	0,425172	y ~ Intervention + Sex +
0,667955	0,069271	0,16113	138	69	69	134	0,429906	y ~ Intervention + Sex +
0,686907	0,069148	0,171187	138	69	69	134	0,403931	y ~ Intervention + Sex +
0,692585	0,068759	0,173545	138	69	69	134	0,396204	y ~ Intervention + Sex +
0,664899	0,067661	0,155857	138	69	69	134	0,434121	y ~ Intervention + Sex +
0,638886	0,067423	0,143353	138	69	69	134	0,470327	y ~ Intervention + Sex +
0,692201	0,066449	0,167495	138	69	69	134	0,396725	y ~ Intervention + Sex +
0,640531	0,066349	0,141765	138	69	69	134	0,46802	y ~ Intervention + Sex +
0,672919	0,065417	0,154622	138	69	69	134	0,423075	y ~ Intervention + Sex +
0,690686	0,064241	0,161092	138	69	69	134	0,398785	y ~ Intervention + Sex +
0,674773	0,064113	0,152459	138	69	69	134	0,420528	y ~ Intervention + Sex +
0,698892	0,064026	0,165166	138	69	69	134	0,387648	y ~ Intervention + Sex +
0,723944	0,063451	0,179272	138	69	69	134	0,353935	y ~ Intervention + Sex +
0,719393	0,063022	0,175046	138	69	69	134	0,360028	y ~ Intervention + Sex +
0,687411	0,062867	0,155904	138	69	69	134	0,403245	y ~ Intervention + Sex +
0,685926	0,062432	0,154051	138	69	69	134	0,40527	y ~ Intervention + Sex +
0,695097	0,06159	0,156802	138	69	69	134	0,392792	y ~ Intervention + Sex +
0,709938	0,061109	0,163948	138	69	69	134	0,372731	y ~ Intervention + Sex +
0,720929	0,059121	0,165156	138	69	69	134	0,357971	y ~ Intervention + Sex +
0,716909	0,059085	0,162609	138	69	69	134	0,363359	y ~ Intervention + Sex +
0,686114	0,058794	0,145166	138	69	69	134	0,405013	y ~ Intervention + Sex +

0,702647	0,05852	0,152966	138	69	69	134	0,382567	y ~ Intervention + Sex +
0,721	0,058505	0,163479	138	69	69	134	0,357875	y ~ Intervention + Sex +
0,715286	0,058276	0,159425	138	69	69	134	0,365538	y ~ Intervention + Sex +
0,738257	0,058126	0,173584	138	69	69	134	0,334856	y ~ Intervention + Sex +
0,69288	0,058075	0,146728	138	69	69	134	0,395803	y ~ Intervention + Sex +
0,709614	0,057499	0,154084	138	69	69	134	0,373166	y ~ Intervention + Sex +
0,715287	0,057322	0,156816	138	69	69	134	0,365537	y ~ Intervention + Sex +
0,703982	0,05698	0,149648	138	69	69	134	0,380763	y ~ Intervention + Sex +
0,722211	0,056486	0,158555	138	69	69	134	0,356254	y ~ Intervention + Sex +
0,716204	0,05631	0,154567	138	69	69	134	0,364306	y ~ Intervention + Sex +
0,735779	0,055994	0,165587	138	69	69	134	0,338151	y ~ Intervention + Sex +
0,712353	0,054963	0,148758	138	69	69	134	0,36948	y ~ Intervention + Sex +
0,732673	0,054419	0,158989	138	69	69	134	0,342285	y ~ Intervention + Sex +
0,749374	0,054419	0,169994	138	69	69	134	0,320122	y ~ Intervention + Sex +
0,746451	0,053951	0,166521	138	69	69	134	0,323989	y ~ Intervention + Sex +
0,719959	0,053697	0,149462	138	69	69	134	0,35927	y ~ Intervention + Sex +
0,73574	0,052788	0,156083	138	69	69	134	0,338202	y ~ Intervention + Sex +
0,749367	0,052445	0,163822	138	69	69	134	0,320132	y ~ Intervention + Sex +
0,764849	0,052375	0,174742	138	69	69	134	0,299728	y ~ Intervention + Sex +
0,747297	0,052281	0,161926	138	69	69	134	0,32287	y ~ Intervention + Sex +
0,741586	0,051881	0,157006	138	69	69	134	0,330436	y ~ Intervention + Sex +
0,744314	0,051803	0,158506	138	69	69	134	0,326821	y ~ Intervention + Sex +
0,749213	0,05129	0,160115	138	69	69	134	0,320336	y ~ Intervention + Sex +
0,7739	0,051106	0,177538	138	69	69	134	0,287859	y ~ Intervention + Sex +
0,741648	0,050939	0,154194	138	69	69	134	0,330354	y ~ Intervention + Sex +
0,745898	0,050608	0,15585	138	69	69	134	0,324722	y ~ Intervention + Sex +
0,730407	0,050445	0,146088	138	69	69	134	0,345305	y ~ Intervention + Sex +
0,775268	0,048576	0,169808	138	69	69	134	0,286067	y ~ Intervention + Sex +
0,774481	0,048484	0,168875	138	69	69	134	0,287098	y ~ Intervention + Sex +
0,736877	0,048112	0,142896	138	69	69	134	0,33669	y ~ Intervention + Sex +
0,763054	0,046879	0,155184	138	69	69	134	0,302087	y ~ Intervention + Sex +
0,761223	0,046798	0,153691	138	69	69	134	0,304496	y ~ Intervention + Sex +
0,760538	0,046441	0,152069	138	69	69	134	0,305397	y ~ Intervention + Sex +
0,782092	0,042998	0,155143	138	69	69	134	0,27715	y ~ Intervention + Sex +
0,79892	0,041535	0,16272	138	69	69	134	0,255252	y ~ Intervention + Sex +
0,805011	0,03979	0,16086	138	69	69	134	0,247356	y ~ Intervention + Sex +
0,783505	0,039584	0,14378	138	69	69	134	0,275306	y ~ Intervention + Sex +
0,788077	0,039527	0,14675	138	69	69	134	0,269347	y ~ Intervention + Sex +
0,774798	0,038854	0,13553	138	69	69	134	0,286682	y ~ Intervention + Sex +
0,815856	0,03869	0,165811	138	69	69	134	0,233337	y ~ Intervention + Sex +
0,811811	0,038385	0,160902	138	69	69	134	0,23856	y ~ Intervention + Sex +
0,817552	0,037604	0,162684	138	69	69	134	0,231148	y ~ Intervention + Sex +
0,811877	0,036281	0,152135	138	69	69	134	0,238475	y ~ Intervention + Sex +
0,844394	0,034332	0,17458	138	69	69	134	0,196656	y ~ Intervention + Sex +
0,829855	0,032898	0,152798	138	69	69	134	0,215307	y ~ Intervention + Sex +
0,839696	0,032533	0,16052	138	69	69	134	0,202676	y ~ Intervention + Sex +
0,857508	0,031781	0,176666	138	69	69	134	0,179894	y ~ Intervention + Sex +
0,840948	0,031728	0,157793	138	69	69	134	0,201071	y ~ Intervention + Sex +

0,836852	0,031606	0,153186	138	69	69	134	0,206322	y ~ Intervention + Sex +
0,847664	0,030953	0,16082	138	69	69	134	0,192472	y ~ Intervention + Sex +
0,851215	0,030527	0,162436	138	69	69	134	0,187931	y ~ Intervention + Sex +
0,840582	0,029399	0,145872	138	69	69	134	0,201539	y ~ Intervention + Sex +
0,854994	0,029009	0,15843	138	69	69	134	0,183104	y ~ Intervention + Sex +
0,849701	0,029002	0,152751	138	69	69	134	0,189867	y ~ Intervention + Sex +
0,830471	0,028657	0,13359	138	69	69	134	0,214516	y ~ Intervention + Sex +
0,829374	0,028372	0,131398	138	69	69	134	0,215926	y ~ Intervention + Sex +
0,843989	0,02816	0,142816	138	69	69	134	0,197176	y ~ Intervention + Sex +
0,847971	0,026945	0,140283	138	69	69	134	0,192079	y ~ Intervention + Sex +
0,850428	0,02694	0,142586	138	69	69	134	0,188938	y ~ Intervention + Sex +
0,854343	0,026709	0,14521	138	69	69	134	0,183935	y ~ Intervention + Sex +
0,879062	0,026301	0,172523	138	69	69	134	0,152449	y ~ Intervention + Sex +
0,869871	0,026232	0,159817	138	69	69	134	0,164137	y ~ Intervention + Sex +
0,865734	0,026078	0,153937	138	69	69	134	0,169405	y ~ Intervention + Sex +
0,879559	0,025628	0,168808	138	69	69	134	0,151818	y ~ Intervention + Sex +
0,873966	0,024847	0,156345	138	69	69	134	0,158927	y ~ Intervention + Sex +
0,885523	0,024517	0,169965	138	69	69	134	0,144246	y ~ Intervention + Sex +
0,87108	0,024285	0,149356	138	69	69	134	0,162598	y ~ Intervention + Sex +
0,876759	0,02423	0,155943	138	69	69	134	0,155376	y ~ Intervention + Sex +
0,882152	0,024011	0,161663	138	69	69	134	0,148525	y ~ Intervention + Sex +
0,88403	0,023374	0,159944	138	69	69	134	0,146141	y ~ Intervention + Sex +
0,881387	0,023302	0,155874	138	69	69	134	0,149496	y ~ Intervention + Sex +
0,887006	0,023102	0,162277	138	69	69	134	0,142365	y ~ Intervention + Sex +
0,88778	0,02218	0,156878	138	69	69	134	0,141383	y ~ Intervention + Sex +
0,888883	0,02087	0,149087	138	69	69	134	0,139984	y ~ Intervention + Sex +
0,896539	0,019708	0,151273	138	69	69	134	0,130281	y ~ Intervention + Sex +
0,898578	0,019114	0,149677	138	69	69	134	0,1277	y ~ Intervention + Sex +
0,905732	0,018753	0,158054	138	69	69	134	0,118647	y ~ Intervention + Sex +
0,906217	0,018663	0,158117	138	69	69	134	0,118035	y ~ Intervention + Sex +
0,908142	0,018397	0,159142	138	69	69	134	0,1156	y ~ Intervention + Sex +
0,903994	0,018358	0,151912	138	69	69	134	0,120846	y ~ Intervention + Sex +
0,922457	0,016967	0,173982	138	69	69	134	0,097523	y ~ Intervention + Sex +
0,92335	0,016092	0,166932	138	69	69	134	0,096396	y ~ Intervention + Sex +
0,921697	0,016015	0,162623	138	69	69	134	0,098482	y ~ Intervention + Sex +
0,911668	0,01545	0,139006	138	69	69	134	0,111145	y ~ Intervention + Sex +
0,928835	0,014611	0,163286	138	69	69	134	0,089479	y ~ Intervention + Sex +
0,928875	0,013991	0,156452	138	69	69	134	0,089428	y ~ Intervention + Sex +
0,930797	0,01351	0,155278	138	69	69	134	0,087006	y ~ Intervention + Sex +
0,933852	0,013447	0,161712	138	69	69	134	0,083155	y ~ Intervention + Sex +
0,922139	0,013208	0,13488	138	69	69	134	0,097923	y ~ Intervention + Sex +
0,938933	0,012522	0,163145	138	69	69	134	0,076755	y ~ Intervention + Sex +
0,937225	0,012513	0,15858	138	69	69	134	0,078907	y ~ Intervention + Sex +
0,942995	0,012295	0,171625	138	69	69	134	0,07164	y ~ Intervention + Sex +
0,941297	0,011527	0,156245	138	69	69	134	0,073777	y ~ Intervention + Sex +
0,946775	0,011473	0,171539	138	69	69	134	0,066883	y ~ Intervention + Sex +
0,941749	0,01129	0,154214	138	69	69	134	0,073209	y ~ Intervention + Sex +
0,939334	0,009796	0,128476	138	69	69	134	0,07625	y ~ Intervention + Sex +

0,952727	0,008954	0,150753	138	69	69	134	0,059394	y ~ Intervention + Sex +
0,960236	0,007963	0,159411	138	69	69	134	0,049951	y ~ Intervention + Sex +
0,96246	0,007778	0,164952	138	69	69	134	0,047155	y ~ Intervention + Sex +
0,962939	0,006879	0,147777	138	69	69	134	0,046553	y ~ Intervention + Sex +
0,96848	0,006509	0,164413	138	69	69	134	0,039589	y ~ Intervention + Sex +
0,969178	0,006503	0,167989	138	69	69	134	0,038712	y ~ Intervention + Sex +
0,976695	0,004694	0,160381	138	69	69	134	0,029268	y ~ Intervention + Sex +
0,979468	0,004337	0,168219	138	69	69	134	0,025784	y ~ Intervention + Sex +
0,98018	0,003861	0,155139	138	69	69	134	0,02489	y ~ Intervention + Sex +
0,982399	0,003487	0,157787	138	69	69	134	0,022102	y ~ Intervention + Sex +
0,983816	0,003281	0,161425	138	69	69	134	0,020323	y ~ Intervention + Sex +
0,983741	0,003021	0,147945	138	69	69	134	0,020417	y ~ Intervention + Sex +
0,987681	0,002519	0,162841	138	69	69	134	0,01547	y ~ Intervention + Sex +
0,987713	0,002464	0,15969	138	69	69	134	0,015428	y ~ Intervention + Sex +
0,991787	0,001666	0,161561	138	69	69	134	0,010312	y ~ Intervention + Sex +
0,998627	0,000269	0,155849	138	69	69	134	0,001724	y ~ Intervention + Sex +
0,998967	0,000205	0,158257	138	69	69	134	0,001297	y ~ Intervention + Sex +
0,999683	-6,7E-05	0,167181	138	69	69	134	-0,0004	y ~ Intervention + Sex +
0,99929	-0,00015	0,166237	138	69	69	134	-0,00089	y ~ Intervention + Sex +
0,997527	-0,00041	0,133334	138	69	69	134	-0,00311	y ~ Intervention + Sex +
0,992951	-0,00123	0,139019	138	69	69	134	-0,00885	y ~ Intervention + Sex +
0,99259	-0,00137	0,147667	138	69	69	134	-0,00931	y ~ Intervention + Sex +
0,985928	-0,00264	0,149566	138	69	69	134	-0,01767	y ~ Intervention + Sex +
0,985741	-0,00276	0,154403	138	69	69	134	-0,0179	y ~ Intervention + Sex +
0,976508	-0,00424	0,143792	138	69	69	134	-0,0295	y ~ Intervention + Sex +
0,97953	-0,00426	0,165542	138	69	69	134	-0,02571	y ~ Intervention + Sex +
0,977841	-0,00432	0,155176	138	69	69	134	-0,02783	y ~ Intervention + Sex +
0,977324	-0,00456	0,160085	138	69	69	134	-0,02848	y ~ Intervention + Sex +
0,977017	-0,00465	0,161004	138	69	69	134	-0,02886	y ~ Intervention + Sex +
0,976619	-0,00486	0,165613	138	69	69	134	-0,02936	y ~ Intervention + Sex +
0,975193	-0,00516	0,165489	138	69	69	134	-0,03115	y ~ Intervention + Sex +
0,974408	-0,00565	0,175947	138	69	69	134	-0,03214	y ~ Intervention + Sex +
0,968917	-0,00614	0,157365	138	69	69	134	-0,03904	y ~ Intervention + Sex +
0,96905	-0,00656	0,168637	138	69	69	134	-0,03887	y ~ Intervention + Sex +
0,963714	-0,00664	0,145764	138	69	69	134	-0,04558	y ~ Intervention + Sex +
0,968752	-0,00682	0,173738	138	69	69	134	-0,03925	y ~ Intervention + Sex +
0,962645	-0,00717	0,152717	138	69	69	134	-0,04692	y ~ Intervention + Sex +
0,9626	-0,00814	0,173242	138	69	69	134	-0,04698	y ~ Intervention + Sex +
0,953917	-0,00841	0,1453	138	69	69	134	-0,0579	y ~ Intervention + Sex +
0,947577	-0,0103	0,156302	138	69	69	134	-0,06587	y ~ Intervention + Sex +
0,950014	-0,01065	0,169509	138	69	69	134	-0,06281	y ~ Intervention + Sex +
0,944701	-0,01098	0,157957	138	69	69	134	-0,06949	y ~ Intervention + Sex +
0,946603	-0,01117	0,166495	138	69	69	134	-0,0671	y ~ Intervention + Sex +
0,940566	-0,01154	0,154543	138	69	69	134	-0,0747	y ~ Intervention + Sex +
0,940462	-0,01231	0,164531	138	69	69	134	-0,07483	y ~ Intervention + Sex +
0,944092	-0,01245	0,177165	138	69	69	134	-0,07026	y ~ Intervention + Sex +
0,939477	-0,01283	0,168662	138	69	69	134	-0,07607	y ~ Intervention + Sex +
0,937362	-0,0131	0,166424	138	69	69	134	-0,07873	y ~ Intervention + Sex +

0,938943	-0,01312	0,17099	138	69	69	134	-0,07674	y ~ Intervention + Sex +
0,93156	-0,01382	0,160637	138	69	69	134	-0,08604	y ~ Intervention + Sex +
0,920663	-0,01612	0,16154	138	69	69	134	-0,09979	y ~ Intervention + Sex +
0,915633	-0,01668	0,15714	138	69	69	134	-0,10614	y ~ Intervention + Sex +
0,909534	-0,01697	0,149029	138	69	69	134	-0,11384	y ~ Intervention + Sex +
0,899599	-0,01825	0,144375	138	69	69	134	-0,12641	y ~ Intervention + Sex +
0,90234	-0,01904	0,154872	138	69	69	134	-0,12294	y ~ Intervention + Sex +
0,902202	-0,01913	0,155353	138	69	69	134	-0,12311	y ~ Intervention + Sex +
0,903448	-0,0193	0,158803	138	69	69	134	-0,12154	y ~ Intervention + Sex +
0,882334	-0,02022	0,136327	138	69	69	134	-0,14829	y ~ Intervention + Sex +
0,893695	-0,02114	0,157911	138	69	69	134	-0,13388	y ~ Intervention + Sex +
0,895985	-0,02136	0,163111	138	69	69	134	-0,13098	y ~ Intervention + Sex +
0,885824	-0,022	0,152919	138	69	69	134	-0,14386	y ~ Intervention + Sex +
0,897866	-0,02215	0,172208	138	69	69	134	-0,1286	y ~ Intervention + Sex +
0,887586	-0,02339	0,16513	138	69	69	134	-0,14163	y ~ Intervention + Sex +
0,884359	-0,02354	0,161531	138	69	69	134	-0,14572	y ~ Intervention + Sex +
0,877906	-0,0249	0,161749	138	69	69	134	-0,15392	y ~ Intervention + Sex +
0,880963	-0,02549	0,169899	138	69	69	134	-0,15003	y ~ Intervention + Sex +
0,870164	-0,02558	0,156226	138	69	69	134	-0,16376	y ~ Intervention + Sex +
0,859469	-0,02588	0,145906	138	69	69	134	-0,17739	y ~ Intervention + Sex +
0,850206	-0,02597	0,137234	138	69	69	134	-0,18922	y ~ Intervention + Sex +
0,854624	-0,02652	0,144444	138	69	69	134	-0,18357	y ~ Intervention + Sex +
0,855423	-0,02685	0,147093	138	69	69	134	-0,18256	y ~ Intervention + Sex +
0,868978	-0,027	0,163374	138	69	69	134	-0,16527	y ~ Intervention + Sex +
0,865441	-0,02719	0,160158	138	69	69	134	-0,16978	y ~ Intervention + Sex +
0,856244	-0,02762	0,152174	138	69	69	134	-0,18151	y ~ Intervention + Sex +
0,853908	-0,02821	0,152927	138	69	69	134	-0,18449	y ~ Intervention + Sex +
0,864815	-0,02828	0,16582	138	69	69	134	-0,17058	y ~ Intervention + Sex +
0,857922	-0,02943	0,164052	138	69	69	134	-0,17936	y ~ Intervention + Sex +
0,84943	-0,02959	0,155573	138	69	69	134	-0,19021	y ~ Intervention + Sex +
0,83724	-0,0304	0,147714	138	69	69	134	-0,20583	y ~ Intervention + Sex +
0,847786	-0,03044	0,158294	138	69	69	134	-0,19232	y ~ Intervention + Sex +
0,837517	-0,03083	0,150062	138	69	69	134	-0,20547	y ~ Intervention + Sex +
0,846604	-0,03091	0,159482	138	69	69	134	-0,19383	y ~ Intervention + Sex +
0,852373	-0,03098	0,166147	138	69	69	134	-0,18645	y ~ Intervention + Sex +
0,846661	-0,03099	0,15996	138	69	69	134	-0,19376	y ~ Intervention + Sex +
0,843143	-0,03137	0,158232	138	69	69	134	-0,19826	y ~ Intervention + Sex +
0,817134	-0,03191	0,13773	138	69	69	134	-0,23169	y ~ Intervention + Sex +
0,852946	-0,03203	0,172489	138	69	69	134	-0,18572	y ~ Intervention + Sex +
0,812437	-0,03214	0,135185	138	69	69	134	-0,23775	y ~ Intervention + Sex +
0,84637	-0,03217	0,165704	138	69	69	134	-0,19413	y ~ Intervention + Sex +
0,826255	-0,03217	0,146259	138	69	69	134	-0,21994	y ~ Intervention + Sex +
0,821459	-0,03317	0,146682	138	69	69	134	-0,22611	y ~ Intervention + Sex +
0,844311	-0,03423	0,173971	138	69	69	134	-0,19676	y ~ Intervention + Sex +
0,816474	-0,03441	0,147981	138	69	69	134	-0,23254	y ~ Intervention + Sex +
0,814555	-0,0364	0,154863	138	69	69	134	-0,23502	y ~ Intervention + Sex +
0,835721	-0,03734	0,179691	138	69	69	134	-0,20777	y ~ Intervention + Sex +
0,823617	-0,03741	0,167523	138	69	69	134	-0,22333	y ~ Intervention + Sex +

0,825187	-0,03755	0,169662	138	69	69	134	-0,22131	y ~ Intervention + Sex +
0,831277	-0,03777	0,176942	138	69	69	134	-0,21348	y ~ Intervention + Sex +
0,83304	-0,03819	0,180822	138	69	69	134	-0,21122	y ~ Intervention + Sex +
0,802806	-0,03877	0,154947	138	69	69	134	-0,25021	y ~ Intervention + Sex +
0,822109	-0,03881	0,172272	138	69	69	134	-0,22527	y ~ Intervention + Sex +
0,802431	-0,03891	0,155219	138	69	69	134	-0,2507	y ~ Intervention + Sex +
0,813162	-0,03892	0,164361	138	69	69	134	-0,23681	y ~ Intervention + Sex +
0,80795	-0,03917	0,160845	138	69	69	134	-0,24355	y ~ Intervention + Sex +
0,805676	-0,03975	0,161249	138	69	69	134	-0,2465	y ~ Intervention + Sex +
0,799105	-0,03976	0,155896	138	69	69	134	-0,25501	y ~ Intervention + Sex +
0,808286	-0,03989	0,164064	138	69	69	134	-0,24312	y ~ Intervention + Sex +
0,77093	-0,04039	0,138432	138	69	69	134	-0,29175	y ~ Intervention + Sex +
0,798191	-0,04049	0,158056	138	69	69	134	-0,2562	y ~ Intervention + Sex +
0,801719	-0,04091	0,16258	138	69	69	134	-0,25162	y ~ Intervention + Sex +
0,751842	-0,04155	0,131129	138	69	69	134	-0,31686	y ~ Intervention + Sex +
0,781513	-0,04155	0,14952	138	69	69	134	-0,27791	y ~ Intervention + Sex +
0,79647	-0,04159	0,160951	138	69	69	134	-0,25843	y ~ Intervention + Sex +
0,753159	-0,04163	0,132102	138	69	69	134	-0,31512	y ~ Intervention + Sex +
0,781969	-0,04206	0,151674	138	69	69	134	-0,27731	y ~ Intervention + Sex +
0,782358	-0,04366	0,157716	138	69	69	134	-0,2768	y ~ Intervention + Sex +
0,786256	-0,04375	0,161026	138	69	69	134	-0,27172	y ~ Intervention + Sex +
0,786284	-0,04387	0,161482	138	69	69	134	-0,27168	y ~ Intervention + Sex +
0,789805	-0,0451	0,168852	138	69	69	134	-0,2671	y ~ Intervention + Sex +
0,773528	-0,04555	0,157977	138	69	69	134	-0,28835	y ~ Intervention + Sex +
0,789415	-0,04558	0,17034	138	69	69	134	-0,26761	y ~ Intervention + Sex +
0,76947	-0,04584	0,156114	138	69	69	134	-0,29366	y ~ Intervention + Sex +
0,760549	-0,04648	0,152206	138	69	69	134	-0,30538	y ~ Intervention + Sex +
0,768986	-0,0468	0,159025	138	69	69	134	-0,2943	y ~ Intervention + Sex +
0,744498	-0,04698	0,143853	138	69	69	134	-0,32658	y ~ Intervention + Sex +
0,743828	-0,04702	0,143591	138	69	69	134	-0,32746	y ~ Intervention + Sex +
0,778844	-0,04722	0,167793	138	69	69	134	-0,28139	y ~ Intervention + Sex +
0,742442	-0,04756	0,144441	138	69	69	134	-0,3293	y ~ Intervention + Sex +
0,766057	-0,04808	0,161266	138	69	69	134	-0,29814	y ~ Intervention + Sex +
0,746333	-0,04829	0,148965	138	69	69	134	-0,32415	y ~ Intervention + Sex +
0,762791	-0,04843	0,160121	138	69	69	134	-0,30243	y ~ Intervention + Sex +
0,748015	-0,04868	0,151222	138	69	69	134	-0,32192	y ~ Intervention + Sex +
0,75143	-0,04991	0,157236	138	69	69	134	-0,3174	y ~ Intervention + Sex +
0,755529	-0,05117	0,164001	138	69	69	134	-0,312	y ~ Intervention + Sex +
0,751199	-0,05128	0,161413	138	69	69	134	-0,31771	y ~ Intervention + Sex +
0,743507	-0,05143	0,156855	138	69	69	134	-0,32789	y ~ Intervention + Sex +
0,70775	-0,05155	0,13721	138	69	69	134	-0,37568	y ~ Intervention + Sex +
0,735299	-0,05159	0,15227	138	69	69	134	-0,33879	y ~ Intervention + Sex +
0,74847	-0,05174	0,161016	138	69	69	134	-0,32132	y ~ Intervention + Sex +
0,724189	-0,05212	0,147393	138	69	69	134	-0,35361	y ~ Intervention + Sex +
0,738266	-0,0527	0,157382	138	69	69	134	-0,33484	y ~ Intervention + Sex +
0,737097	-0,05302	0,157602	138	69	69	134	-0,3364	y ~ Intervention + Sex +
0,749849	-0,05348	0,167387	138	69	69	134	-0,31949	y ~ Intervention + Sex +
0,730881	-0,05348	0,155172	138	69	69	134	-0,34467	y ~ Intervention + Sex +

0,727625	-0,0537	0,153872	138	69	69	134	-0,34902	y ~ Intervention + Sex +
0,745146	-0,05437	0,166916	138	69	69	134	-0,32572	y ~ Intervention + Sex +
0,744477	-0,05502	0,168468	138	69	69	134	-0,3266	y ~ Intervention + Sex +
0,74107	-0,05549	0,167591	138	69	69	134	-0,33112	y ~ Intervention + Sex +
0,725128	-0,05553	0,15759	138	69	69	134	-0,35235	y ~ Intervention + Sex +
0,745936	-0,05573	0,171638	138	69	69	134	-0,32467	y ~ Intervention + Sex +
0,721297	-0,05642	0,157828	138	69	69	134	-0,35748	y ~ Intervention + Sex +
0,706079	-0,05715	0,151222	138	69	69	134	-0,37793	y ~ Intervention + Sex +
0,721852	-0,05743	0,160987	138	69	69	134	-0,35673	y ~ Intervention + Sex +
0,720158	-0,05851	0,162989	138	69	69	134	-0,359	y ~ Intervention + Sex +
0,717841	-0,05874	0,162214	138	69	69	134	-0,36211	y ~ Intervention + Sex +
0,657729	-0,05914	0,133176	138	69	69	134	-0,44404	y ~ Intervention + Sex +
0,656188	-0,06039	0,135352	138	69	69	134	-0,44618	y ~ Intervention + Sex +
0,728481	-0,06039	0,173611	138	69	69	134	-0,34787	y ~ Intervention + Sex +
0,710611	-0,06041	0,162457	138	69	69	134	-0,37182	y ~ Intervention + Sex +
0,719517	-0,06044	0,167961	138	69	69	134	-0,35986	y ~ Intervention + Sex +
0,669237	-0,06082	0,142052	138	69	69	134	-0,42814	y ~ Intervention + Sex +
0,692853	-0,06143	0,155187	138	69	69	134	-0,39584	y ~ Intervention + Sex +
0,651253	-0,06237	0,137675	138	69	69	134	-0,45304	y ~ Intervention + Sex +
0,696908	-0,06285	0,161022	138	69	69	134	-0,39034	y ~ Intervention + Sex +
0,68914	-0,06288	0,156861	138	69	69	134	-0,40089	y ~ Intervention + Sex +
0,67181	-0,06395	0,150619	138	69	69	134	-0,4246	y ~ Intervention + Sex +
0,660128	-0,06446	0,146256	138	69	69	134	-0,44072	y ~ Intervention + Sex +
0,671732	-0,06453	0,151931	138	69	69	134	-0,42471	y ~ Intervention + Sex +
0,610384	-0,06586	0,128961	138	69	69	134	-0,51072	y ~ Intervention + Sex +
0,684686	-0,06667	0,163835	138	69	69	134	-0,40696	y ~ Intervention + Sex +
0,707246	-0,06737	0,179004	138	69	69	134	-0,37636	y ~ Intervention + Sex +
0,635432	-0,06778	0,142643	138	69	69	134	-0,47518	y ~ Intervention + Sex +
0,687006	-0,06787	0,16809	138	69	69	134	-0,4038	y ~ Intervention + Sex +
0,691995	-0,06788	0,170968	138	69	69	134	-0,39701	y ~ Intervention + Sex +
0,677814	-0,06906	0,165876	138	69	69	134	-0,41636	y ~ Intervention + Sex +
0,612339	-0,06927	0,13638	138	69	69	134	-0,50793	y ~ Intervention + Sex +
0,613857	-0,07006	0,138523	138	69	69	134	-0,50576	y ~ Intervention + Sex +
0,640311	-0,07016	0,14982	138	69	69	134	-0,46833	y ~ Intervention + Sex +
0,672692	-0,07035	0,166153	138	69	69	134	-0,42339	y ~ Intervention + Sex +
0,627694	-0,07079	0,145623	138	69	69	134	-0,48609	y ~ Intervention + Sex +
0,656072	-0,07106	0,159206	138	69	69	134	-0,44634	y ~ Intervention + Sex +
0,620944	-0,07113	0,143501	138	69	69	134	-0,49566	y ~ Intervention + Sex +
0,668708	-0,07114	0,165886	138	69	69	134	-0,42887	y ~ Intervention + Sex +
0,666362	-0,07149	0,165456	138	69	69	134	-0,4321	y ~ Intervention + Sex +
0,657454	-0,07184	0,161655	138	69	69	134	-0,44442	y ~ Intervention + Sex +
0,658782	-0,07187	0,162398	138	69	69	134	-0,44258	y ~ Intervention + Sex +
0,616443	-0,07211	0,143618	138	69	69	134	-0,50207	y ~ Intervention + Sex +
0,568896	-0,07321	0,128198	138	69	69	134	-0,57109	y ~ Intervention + Sex +
0,649963	-0,07409	0,162884	138	69	69	134	-0,45484	y ~ Intervention + Sex +
0,646119	-0,07449	0,161864	138	69	69	134	-0,4602	y ~ Intervention + Sex +
0,624142	-0,07473	0,152167	138	69	69	134	-0,49112	y ~ Intervention + Sex +
0,652519	-0,07476	0,165658	138	69	69	134	-0,45128	y ~ Intervention + Sex +

0,61865	-0,07549	0,151298	138	69	69	134	-0,49893	y ~ Intervention + Sex +
0,585151	-0,07653	0,13985	138	69	69	134	-0,5472	y ~ Intervention + Sex +
0,652203	-0,07699	0,170429	138	69	69	134	-0,45172	y ~ Intervention + Sex +
0,581131	-0,07701	0,139236	138	69	69	134	-0,55308	y ~ Intervention + Sex +
0,589982	-0,07707	0,142676	134	67	67	130	-0,5402	y ~ Intervention + Sex +
0,61145	-0,07716	0,15154	138	69	69	134	-0,5092	y ~ Intervention + Sex +
0,617896	-0,0773	0,15459	138	69	69	134	-0,5	y ~ Intervention + Sex +
0,645814	-0,07758	0,168432	138	69	69	134	-0,46063	y ~ Intervention + Sex +
0,610577	-0,07762	0,152066	138	69	69	134	-0,51045	y ~ Intervention + Sex +
0,638613	-0,07854	0,166857	138	69	69	134	-0,47071	y ~ Intervention + Sex +
0,615767	-0,07889	0,156824	138	69	69	134	-0,50303	y ~ Intervention + Sex +
0,642435	-0,07909	0,169967	138	69	69	134	-0,46535	y ~ Intervention + Sex +
0,604101	-0,07914	0,152276	138	69	69	134	-0,51974	y ~ Intervention + Sex +
0,634651	-0,07921	0,166315	138	69	69	134	-0,47628	y ~ Intervention + Sex +
0,575791	-0,07929	0,141352	138	69	69	134	-0,56092	y ~ Intervention + Sex +
0,591259	-0,07999	0,148601	138	69	69	134	-0,53831	y ~ Intervention + Sex +
0,625278	-0,08044	0,164318	138	69	69	134	-0,48951	y ~ Intervention + Sex +
0,623112	-0,08063	0,163679	138	69	69	134	-0,49258	y ~ Intervention + Sex +
0,625438	-0,08091	0,165363	138	69	69	134	-0,48929	y ~ Intervention + Sex +
0,585938	-0,08099	0,148311	138	69	69	134	-0,54605	y ~ Intervention + Sex +
0,63866	-0,08109	0,172288	138	69	69	134	-0,47064	y ~ Intervention + Sex +
0,632196	-0,08138	0,169644	138	69	69	134	-0,47974	y ~ Intervention + Sex +
0,595538	-0,08162	0,153389	138	69	69	134	-0,5321	y ~ Intervention + Sex +
0,59484	-0,08308	0,155836	138	69	69	134	-0,53311	y ~ Intervention + Sex +
0,621609	-0,08309	0,167963	138	69	69	134	-0,49472	y ~ Intervention + Sex +
0,485142	-0,0832	0,118856	138	69	69	134	-0,7	y ~ Intervention + Sex +
0,579707	-0,08338	0,150184	138	69	69	134	-0,55517	y ~ Intervention + Sex +
0,599195	-0,08371	0,158898	138	69	69	134	-0,52681	y ~ Intervention + Sex +
0,569722	-0,08399	0,147378	138	69	69	134	-0,56987	y ~ Intervention + Sex +
0,620494	-0,08477	0,170794	138	69	69	134	-0,4963	y ~ Intervention + Sex +
0,617193	-0,08522	0,170092	138	69	69	134	-0,501	y ~ Intervention + Sex +
0,559663	-0,0856	0,146367	138	69	69	134	-0,5848	y ~ Intervention + Sex +
0,618645	-0,08609	0,172555	138	69	69	134	-0,49893	y ~ Intervention + Sex +
0,616936	-0,08657	0,172658	138	69	69	134	-0,50137	y ~ Intervention + Sex +
0,569199	-0,08661	0,15178	138	69	69	134	-0,57064	y ~ Intervention + Sex +
0,541808	-0,08682	0,141946	138	69	69	134	-0,61165	y ~ Intervention + Sex +
0,572208	-0,08687	0,153423	138	69	69	134	-0,5662	y ~ Intervention + Sex +
0,574688	-0,08698	0,154622	138	69	69	134	-0,56254	y ~ Intervention + Sex +
0,57931	-0,08727	0,157038	138	69	69	134	-0,55575	y ~ Intervention + Sex +
0,585851	-0,08741	0,160034	138	69	69	134	-0,54618	y ~ Intervention + Sex +
0,572872	-0,08741	0,154655	138	69	69	134	-0,56522	y ~ Intervention + Sex +
0,547757	-0,08782	0,145718	138	69	69	134	-0,60265	y ~ Intervention + Sex +
0,593128	-0,08789	0,164102	138	69	69	134	-0,53559	y ~ Intervention + Sex +
0,606299	-0,08793	0,170216	138	69	69	134	-0,51658	y ~ Intervention + Sex +
0,583649	-0,08858	0,161231	138	69	69	134	-0,54939	y ~ Intervention + Sex +
0,547912	-0,08897	0,147686	138	69	69	134	-0,60242	y ~ Intervention + Sex +
0,565293	-0,08915	0,154668	138	69	69	134	-0,57643	y ~ Intervention + Sex +
0,555786	-0,09028	0,152866	138	69	69	134	-0,5906	y ~ Intervention + Sex +

0,545224	-0,09067	0,149496	138	69	69	134	-0,60648	y ~ Intervention + Sex +
0,576182	-0,0912	0,162763	138	69	69	134	-0,56034	y ~ Intervention + Sex +
0,575221	-0,09239	0,164468	138	69	69	134	-0,56176	y ~ Intervention + Sex +
0,570501	-0,09242	0,162506	138	69	69	134	-0,56872	y ~ Intervention + Sex +
0,562292	-0,09271	0,159604	138	69	69	134	-0,58089	y ~ Intervention + Sex +
0,568052	-0,09302	0,162532	138	69	69	134	-0,57234	y ~ Intervention + Sex +
0,566013	-0,09345	0,162427	138	69	69	134	-0,57536	y ~ Intervention + Sex +
0,565419	-0,09388	0,162915	138	69	69	134	-0,57624	y ~ Intervention + Sex +
0,504697	-0,09539	0,142606	138	69	69	134	-0,66892	y ~ Intervention + Sex +
0,501644	-0,09568	0,142013	138	69	69	134	-0,67373	y ~ Intervention + Sex +
0,556279	-0,09602	0,162793	138	69	69	134	-0,58986	y ~ Intervention + Sex +
0,536038	-0,09607	0,154841	138	69	69	134	-0,62042	y ~ Intervention + Sex +
0,555462	-0,09632	0,162961	138	69	69	134	-0,59108	y ~ Intervention + Sex +
0,579698	-0,09651	0,173831	138	69	69	134	-0,55518	y ~ Intervention + Sex +
0,562333	-0,0966	0,166319	138	69	69	134	-0,58083	y ~ Intervention + Sex +
0,573556	-0,09674	0,17147	138	69	69	134	-0,56421	y ~ Intervention + Sex +
0,567261	-0,09698	0,169092	138	69	69	134	-0,57351	y ~ Intervention + Sex +
0,51321	-0,09707	0,148069	138	69	69	134	-0,65559	y ~ Intervention + Sex +
0,52303	-0,09715	0,151711	138	69	69	134	-0,64036	y ~ Intervention + Sex +
0,532219	-0,09725	0,155283	138	69	69	134	-0,62625	y ~ Intervention + Sex +
0,507221	-0,09917	0,149142	138	69	69	134	-0,66496	y ~ Intervention + Sex +
0,491886	-0,10025	0,14546	138	69	69	134	-0,68921	y ~ Intervention + Sex +
0,513718	-0,10061	0,153655	138	69	69	134	-0,6548	y ~ Intervention + Sex +
0,49164	-0,10109	0,14659	138	69	69	134	-0,6896	y ~ Intervention + Sex +
0,561279	-0,10165	0,174542	138	69	69	134	-0,5824	y ~ Intervention + Sex +
0,538162	-0,1019	0,165103	138	69	69	134	-0,61718	y ~ Intervention + Sex +
0,523367	-0,10256	0,16029	138	69	69	134	-0,63984	y ~ Intervention + Sex +
0,537948	-0,1035	0,167614	138	69	69	134	-0,61751	y ~ Intervention + Sex +
0,51004	-0,10388	0,15726	138	69	69	134	-0,66054	y ~ Intervention + Sex +
0,534435	-0,10505	0,168655	138	69	69	134	-0,62286	y ~ Intervention + Sex +
0,544887	-0,10554	0,173879	138	69	69	134	-0,60699	y ~ Intervention + Sex +
0,467321	-0,10627	0,145797	138	69	69	134	-0,72892	y ~ Intervention + Sex +
0,483646	-0,10672	0,151931	138	69	69	134	-0,7024	y ~ Intervention + Sex +
0,39264	-0,10778	0,12568	138	69	69	134	-0,85761	y ~ Intervention + Sex +
0,491334	-0,10795	0,156431	138	69	69	134	-0,69008	y ~ Intervention + Sex +
0,451738	-0,10798	0,143069	138	69	69	134	-0,75473	y ~ Intervention + Sex +
0,523298	-0,10802	0,168791	138	69	69	134	-0,63995	y ~ Intervention + Sex +
0,502406	-0,10803	0,160636	138	69	69	134	-0,67253	y ~ Intervention + Sex +
0,4613	-0,11007	0,148984	138	69	69	134	-0,73883	y ~ Intervention + Sex +
0,470876	-0,11093	0,153408	138	69	69	134	-0,7231	y ~ Intervention + Sex +
0,476136	-0,11102	0,15537	138	69	69	134	-0,71454	y ~ Intervention + Sex +
0,468427	-0,11159	0,153477	138	69	69	134	-0,72711	y ~ Intervention + Sex +
0,417537	-0,11187	0,137567	138	69	69	134	-0,81321	y ~ Intervention + Sex +
0,445649	-0,11205	0,146483	138	69	69	134	-0,76495	y ~ Intervention + Sex +
0,465565	-0,11232	0,153482	138	69	69	134	-0,73181	y ~ Intervention + Sex +
0,492273	-0,11286	0,163898	138	69	69	134	-0,68859	y ~ Intervention + Sex +
0,488499	-0,11294	0,162594	138	69	69	134	-0,69461	y ~ Intervention + Sex +
0,474019	-0,11295	0,157311	138	69	69	134	-0,71798	y ~ Intervention + Sex +

0,460239	-0,113	0,152578	138	69	69	134	-0,74059	y ~ Intervention + Sex +
0,489479	-0,11366	0,164	138	69	69	134	-0,69305	y ~ Intervention + Sex +
0,413999	-0,11443	0,139649	138	69	69	134	-0,81942	y ~ Intervention + Sex +
0,470773	-0,11542	0,159587	138	69	69	134	-0,72327	y ~ Intervention + Sex +
0,444021	-0,11704	0,152458	138	69	69	134	-0,76769	y ~ Intervention + Sex +
0,485085	-0,11768	0,168093	138	69	69	134	-0,70009	y ~ Intervention + Sex +
0,431532	-0,11811	0,149699	138	69	69	134	-0,78895	y ~ Intervention + Sex +
0,395602	-0,11819	0,13868	138	69	69	134	-0,85224	y ~ Intervention + Sex +
0,441563	-0,11846	0,153479	138	69	69	134	-0,77185	y ~ Intervention + Sex +
0,394878	-0,11859	0,138934	138	69	69	134	-0,85355	y ~ Intervention + Sex +
0,476393	-0,11861	0,166088	138	69	69	134	-0,71412	y ~ Intervention + Sex +
0,453129	-0,11877	0,157848	138	69	69	134	-0,7524	y ~ Intervention + Sex +
0,482624	-0,11982	0,170193	138	69	69	134	-0,70405	y ~ Intervention + Sex +
0,444244	-0,12024	0,156697	138	69	69	134	-0,76732	y ~ Intervention + Sex +
0,402475	-0,12047	0,143442	138	69	69	134	-0,83987	y ~ Intervention + Sex +
0,445704	-0,12115	0,158395	138	69	69	134	-0,76485	y ~ Intervention + Sex +
0,442776	-0,12145	0,157769	138	69	69	134	-0,7698	y ~ Intervention + Sex +
0,465558	-0,12206	0,166793	138	69	69	134	-0,73182	y ~ Intervention + Sex +
0,465469	-0,12348	0,168702	138	69	69	134	-0,73196	y ~ Intervention + Sex +
0,487053	-0,12392	0,17781	138	69	69	134	-0,69693	y ~ Intervention + Sex +
0,479815	-0,12525	0,176767	138	69	69	134	-0,70858	y ~ Intervention + Sex +
0,407818	-0,12544	0,151069	138	69	69	134	-0,83035	y ~ Intervention + Sex +
0,437172	-0,12651	0,162342	138	69	69	134	-0,77931	y ~ Intervention + Sex +
0,44031	-0,12656	0,163517	138	69	69	134	-0,77397	y ~ Intervention + Sex +
0,438399	-0,12667	0,162972	138	69	69	134	-0,77722	y ~ Intervention + Sex +
0,429946	-0,12733	0,160838	138	69	69	134	-0,79168	y ~ Intervention + Sex +
0,372694	-0,12756	0,142611	138	69	69	134	-0,89443	y ~ Intervention + Sex +
0,463551	-0,12846	0,17475	138	69	69	134	-0,73512	y ~ Intervention + Sex +
0,413777	-0,12942	0,157863	138	69	69	134	-0,81982	y ~ Intervention + Sex +
0,406968	-0,12996	0,156229	138	69	69	134	-0,83186	y ~ Intervention + Sex +
0,401671	-0,13032	0,154895	138	69	69	134	-0,84131	y ~ Intervention + Sex +
0,354429	-0,13109	0,141068	138	69	69	134	-0,92925	y ~ Intervention + Sex +
0,424363	-0,13128	0,163824	138	69	69	134	-0,80132	y ~ Intervention + Sex +
0,429443	-0,13144	0,165839	138	69	69	134	-0,79255	y ~ Intervention + Sex +
0,415071	-0,13195	0,161398	138	69	69	134	-0,81754	y ~ Intervention + Sex +
0,414369	-0,13291	0,162331	138	69	69	134	-0,81877	y ~ Intervention + Sex +
0,328018	-0,13295	0,135425	138	69	69	134	-0,9817	y ~ Intervention + Sex +
0,418926	-0,13297	0,164001	138	69	69	134	-0,81079	y ~ Intervention + Sex +
0,391841	-0,13308	0,154913	138	69	69	134	-0,85906	y ~ Intervention + Sex +
0,429432	-0,13347	0,168405	138	69	69	134	-0,79257	y ~ Intervention + Sex +
0,322524	-0,13358	0,134531	138	69	69	134	-0,99295	y ~ Intervention + Sex +
0,388812	-0,13363	0,154558	138	69	69	134	-0,86458	y ~ Intervention + Sex +
0,281401	-0,13372	0,12364	138	69	69	134	-1,08154	y ~ Intervention + Sex +
0,335891	-0,13531	0,1401	138	69	69	134	-0,96579	y ~ Intervention + Sex +
0,394982	-0,13561	0,158917	138	69	69	134	-0,85336	y ~ Intervention + Sex +
0,418043	-0,13574	0,167103	138	69	69	134	-0,81233	y ~ Intervention + Sex +
0,355978	-0,13669	0,147573	138	69	69	134	-0,92626	y ~ Intervention + Sex +
0,322147	-0,13717	0,138036	138	69	69	134	-0,99373	y ~ Intervention + Sex +

0,396399	-0,13898	0,163348	138	69	69	134	-0,8508	y ~ Intervention + Sex +
0,337652	-0,139	0,144454	138	69	69	134	-0,96226	y ~ Intervention + Sex +
0,421038	-0,13952	0,172866	138	69	69	134	-0,8071	y ~ Intervention + Sex +
0,352535	-0,14067	0,150781	138	69	69	134	-0,93293	y ~ Intervention + Sex +
0,392666	-0,14103	0,164456	138	69	69	134	-0,85756	y ~ Intervention + Sex +
0,315446	-0,14115	0,140085	138	69	69	134	-1,00763	y ~ Intervention + Sex +
0,397648	-0,14143	0,166676	138	69	69	134	-0,84854	y ~ Intervention + Sex +
0,353958	-0,1422	0,152875	138	69	69	134	-0,93017	y ~ Intervention + Sex +
0,393262	-0,14311	0,167096	138	69	69	134	-0,85648	y ~ Intervention + Sex +
0,371868	-0,14329	0,159929	138	69	69	134	-0,89598	y ~ Intervention + Sex +
0,323622	-0,14439	0,145751	138	69	69	134	-0,99069	y ~ Intervention + Sex +
0,29837	-0,14571	0,139571	138	69	69	134	-1,04399	y ~ Intervention + Sex +
0,371831	-0,14619	0,163149	138	69	69	134	-0,89605	y ~ Intervention + Sex +
0,379735	-0,1466	0,166348	138	69	69	134	-0,8813	y ~ Intervention + Sex +
0,368637	-0,14716	0,163134	138	69	69	134	-0,90207	y ~ Intervention + Sex +
0,378603	-0,14742	0,166882	138	69	69	134	-0,8834	y ~ Intervention + Sex +
0,387438	-0,14891	0,171736	138	69	69	134	-0,8671	y ~ Intervention + Sex +
0,359915	-0,14897	0,16216	138	69	69	134	-0,91868	y ~ Intervention + Sex +
0,362221	-0,14912	0,163099	138	69	69	134	-0,91426	y ~ Intervention + Sex +
0,370434	-0,15202	0,169164	138	69	69	134	-0,89868	y ~ Intervention + Sex +
0,373566	-0,15228	0,17056	138	69	69	134	-0,8928	y ~ Intervention + Sex +
0,347002	-0,15254	0,161631	138	69	69	134	-0,94374	y ~ Intervention + Sex +
0,360883	-0,15287	0,166734	138	69	69	134	-0,91682	y ~ Intervention + Sex +
0,251083	-0,15335	0,133033	138	69	69	134	-1,15271	y ~ Intervention + Sex +
0,351778	-0,15411	0,164933	138	69	69	134	-0,9344	y ~ Intervention + Sex +
0,379269	-0,15412	0,174711	138	69	69	134	-0,88216	y ~ Intervention + Sex +
0,2473	-0,15462	0,133065	138	69	69	134	-1,162	y ~ Intervention + Sex +
0,298327	-0,15465	0,148119	138	69	69	134	-1,04408	y ~ Intervention + Sex +
0,358268	-0,15519	0,168352	138	69	69	134	-0,92184	y ~ Intervention + Sex +
0,300762	-0,15523	0,149428	138	69	69	134	-1,03881	y ~ Intervention + Sex +
0,361654	-0,15542	0,169798	138	69	69	134	-0,91535	y ~ Intervention + Sex +
0,323567	-0,15587	0,157314	138	69	69	134	-0,9908	y ~ Intervention + Sex +
0,356029	-0,15607	0,168511	138	69	69	134	-0,92616	y ~ Intervention + Sex +
0,297789	-0,15635	0,149585	138	69	69	134	-1,04525	y ~ Intervention + Sex +
0,272766	-0,15678	0,142368	138	69	69	134	-1,10124	y ~ Intervention + Sex +
0,276714	-0,15688	0,143636	138	69	69	134	-1,09218	y ~ Intervention + Sex +
0,33177	-0,1578	0,162002	138	69	69	134	-0,97408	y ~ Intervention + Sex +
0,289956	-0,15838	0,149075	138	69	69	134	-1,06242	y ~ Intervention + Sex +
0,357323	-0,15876	0,171884	138	69	69	134	-0,92366	y ~ Intervention + Sex +
0,290254	-0,15938	0,150109	138	69	69	134	-1,06176	y ~ Intervention + Sex +
0,336169	-0,15939	0,165133	138	69	69	134	-0,96523	y ~ Intervention + Sex +
0,314796	-0,15951	0,158083	138	69	69	134	-1,00899	y ~ Intervention + Sex +
0,298258	-0,1596	0,152837	138	69	69	134	-1,04423	y ~ Intervention + Sex +
0,335225	-0,16019	0,165631	138	69	69	134	-0,96712	y ~ Intervention + Sex +
0,28369	-0,16223	0,150716	138	69	69	134	-1,07638	y ~ Intervention + Sex +
0,349351	-0,16295	0,173515	138	69	69	134	-0,93914	y ~ Intervention + Sex +
0,313712	-0,16309	0,161276	138	69	69	134	-1,01127	y ~ Intervention + Sex +
0,285656	-0,16342	0,152446	138	69	69	134	-1,07198	y ~ Intervention + Sex +

0,285747	-0,16351	0,152559	138	69	69	134	-1,07178	y ~ Intervention + Sex +
0,271592	-0,16352	0,148122	138	69	69	134	-1,10395	y ~ Intervention + Sex +
0,349388	-0,16368	0,174299	138	69	69	134	-0,93906	y ~ Intervention + Sex +
0,296011	-0,16376	0,15609	138	69	69	134	-1,04912	y ~ Intervention + Sex +
0,265995	-0,16379	0,146632	138	69	69	134	-1,117	y ~ Intervention + Sex +
0,368375	-0,16418	0,181901	138	69	69	134	-0,90257	y ~ Intervention + Sex +
0,299381	-0,16477	0,158163	138	69	69	134	-1,0418	y ~ Intervention + Sex +
0,299028	-0,16551	0,158755	138	69	69	134	-1,04256	y ~ Intervention + Sex +
0,282127	-0,16569	0,153432	138	69	69	134	-1,0799	y ~ Intervention + Sex +
0,308757	-0,16665	0,163107	138	69	69	134	-1,02172	y ~ Intervention + Sex +
0,23566	-0,1677	0,140777	138	69	69	134	-1,19125	y ~ Intervention + Sex +
0,248679	-0,16807	0,145063	138	69	69	134	-1,1586	y ~ Intervention + Sex +
0,327912	-0,1699	0,17303	138	69	69	134	-0,98191	y ~ Intervention + Sex +
0,303165	-0,17052	0,164966	138	69	69	134	-1,03364	y ~ Intervention + Sex +
0,319665	-0,17104	0,171236	138	69	69	134	-0,99886	y ~ Intervention + Sex +
0,297531	-0,17228	0,164731	138	69	69	134	-1,04581	y ~ Intervention + Sex +
0,329441	-0,1727	0,176444	138	69	69	134	-0,9788	y ~ Intervention + Sex +
0,303643	-0,1729	0,167441	138	69	69	134	-1,03262	y ~ Intervention + Sex +
0,257013	-0,174	0,152856	138	69	69	134	-1,13833	y ~ Intervention + Sex +
0,26634	-0,17527	0,157022	138	69	69	134	-1,11619	y ~ Intervention + Sex +
0,198661	-0,17713	0,137122	138	69	69	134	-1,29177	y ~ Intervention + Sex +
0,188533	-0,17724	0,134108	138	69	69	134	-1,32166	y ~ Intervention + Sex +
0,228564	-0,17795	0,147111	138	69	69	134	-1,20959	y ~ Intervention + Sex +
0,208286	-0,17932	0,141822	138	69	69	134	-1,26439	y ~ Intervention + Sex +
0,275925	-0,17969	0,164256	138	69	69	134	-1,09398	y ~ Intervention + Sex +
0,245996	-0,18029	0,154727	138	69	69	134	-1,16523	y ~ Intervention + Sex +
0,284708	-0,18071	0,168247	138	69	69	134	-1,0741	y ~ Intervention + Sex +
0,285572	-0,18142	0,169204	138	69	69	134	-1,07217	y ~ Intervention + Sex +
0,24789	-0,18158	0,156464	138	69	69	134	-1,16055	y ~ Intervention + Sex +
0,262187	-0,18243	0,162023	138	69	69	134	-1,12598	y ~ Intervention + Sex +
0,210129	-0,18271	0,145092	138	69	69	134	-1,25925	y ~ Intervention + Sex +
0,164532	-0,18336	0,13119	138	69	69	134	-1,39764	y ~ Intervention + Sex +
0,29525	-0,18471	0,175787	138	69	69	134	-1,05078	y ~ Intervention + Sex +
0,268578	-0,18602	0,167442	138	69	69	134	-1,11095	y ~ Intervention + Sex +
0,271369	-0,18675	0,169087	138	69	69	134	-1,10447	y ~ Intervention + Sex +
0,240556	-0,18678	0,158446	138	69	69	134	-1,17883	y ~ Intervention + Sex +
0,280981	-0,18682	0,172589	138	69	69	134	-1,08249	y ~ Intervention + Sex +
0,275275	-0,18684	0,170557	138	69	69	134	-1,09547	y ~ Intervention + Sex +
0,234724	-0,187	0,156658	138	69	69	134	-1,19365	y ~ Intervention + Sex +
0,20807	-0,18743	0,148166	138	69	69	134	-1,26499	y ~ Intervention + Sex +
0,275693	-0,1896	0,173224	138	69	69	134	-1,09451	y ~ Intervention + Sex +
0,226663	-0,18976	0,156234	138	69	69	134	-1,21458	y ~ Intervention + Sex +
0,24992	-0,18983	0,164272	138	69	69	134	-1,15555	y ~ Intervention + Sex +
0,244449	-0,18995	0,162481	138	69	69	134	-1,16907	y ~ Intervention + Sex +
0,247864	-0,19031	0,163974	138	69	69	134	-1,16061	y ~ Intervention + Sex +
0,173012	-0,19087	0,139334	138	69	69	134	-1,36989	y ~ Intervention + Sex +
0,245629	-0,19213	0,164761	138	69	69	134	-1,16614	y ~ Intervention + Sex +
0,179959	-0,19236	0,142709	138	69	69	134	-1,34792	y ~ Intervention + Sex +

0,226473	-0,19288	0,158735	138	69	69	134	-1,21508	y ~ Intervention + Sex +
0,219579	-0,19315	0,156599	138	69	69	134	-1,23342	y ~ Intervention + Sex +
0,195993	-0,19344	0,148852	138	69	69	134	-1,29953	y ~ Intervention + Sex +
0,231588	-0,19425	0,161645	138	69	69	134	-1,20173	y ~ Intervention + Sex +
0,240659	-0,19429	0,164854	138	69	69	134	-1,17857	y ~ Intervention + Sex +
0,250713	-0,19438	0,168494	138	69	69	134	-1,15361	y ~ Intervention + Sex +
0,226307	-0,19441	0,15994	138	69	69	134	-1,21551	y ~ Intervention + Sex +
0,231464	-0,19658	0,163539	138	69	69	134	-1,20205	y ~ Intervention + Sex +
0,205803	-0,19665	0,154679	138	69	69	134	-1,27136	y ~ Intervention + Sex +
0,238054	-0,19676	0,166017	138	69	69	134	-1,18516	y ~ Intervention + Sex +
0,230827	-0,19731	0,16392	138	69	69	134	-1,2037	y ~ Intervention + Sex +
0,204219	-0,19802	0,155205	138	69	69	134	-1,27584	y ~ Intervention + Sex +
0,228135	-0,19837	0,163842	138	69	69	134	-1,21072	y ~ Intervention + Sex +
0,183978	-0,19861	0,148716	138	69	69	134	-1,3355	y ~ Intervention + Sex +
0,246586	-0,19887	0,170883	138	69	69	134	-1,16377	y ~ Intervention + Sex +
0,223111	-0,19925	0,162787	138	69	69	134	-1,22397	y ~ Intervention + Sex +
0,215321	-0,20019	0,1608	138	69	69	134	-1,24496	y ~ Intervention + Sex +
0,212953	-0,20048	0,160202	138	69	69	134	-1,25144	y ~ Intervention + Sex +
0,181406	-0,20066	0,149362	138	69	69	134	-1,34342	y ~ Intervention + Sex +
0,200449	-0,20085	0,15611	138	69	69	134	-1,28661	y ~ Intervention + Sex +
0,165191	-0,20094	0,144	138	69	69	134	-1,39544	y ~ Intervention + Sex +
0,196942	-0,201	0,155003	138	69	69	134	-1,29676	y ~ Intervention + Sex +
0,223872	-0,20104	0,164526	138	69	69	134	-1,22195	y ~ Intervention + Sex +
0,195232	-0,20123	0,154586	138	69	69	134	-1,30176	y ~ Intervention + Sex +
0,178611	-0,20133	0,1489	138	69	69	134	-1,35213	y ~ Intervention + Sex +
0,219144	-0,20136	0,163102	138	69	69	134	-1,23459	y ~ Intervention + Sex +
0,227548	-0,20185	0,166512	138	69	69	134	-1,21226	y ~ Intervention + Sex +
0,222911	-0,20216	0,165096	138	69	69	134	-1,2245	y ~ Intervention + Sex +
0,222132	-0,2031	0,165579	138	69	69	134	-1,22658	y ~ Intervention + Sex +
0,223891	-0,20333	0,166407	138	69	69	134	-1,2219	y ~ Intervention + Sex +
0,238813	-0,20347	0,171963	138	69	69	134	-1,18323	y ~ Intervention + Sex +
0,236303	-0,20356	0,171118	138	69	69	134	-1,18961	y ~ Intervention + Sex +
0,216225	-0,20405	0,164228	138	69	69	134	-1,24249	y ~ Intervention + Sex +
0,210684	-0,20457	0,162651	138	69	69	134	-1,25771	y ~ Intervention + Sex +
0,195313	-0,20576	0,158091	138	69	69	134	-1,30152	y ~ Intervention + Sex +
0,213549	-0,20676	0,165436	138	69	69	134	-1,2498	y ~ Intervention + Sex +
0,185154	-0,20709	0,155486	138	69	69	134	-1,3319	y ~ Intervention + Sex +
0,171795	-0,20762	0,151126	138	69	69	134	-1,37381	y ~ Intervention + Sex +
0,191186	-0,20867	0,15884	138	69	69	134	-1,31372	y ~ Intervention + Sex +
0,14539	-0,20874	0,142532	138	69	69	134	-1,46453	y ~ Intervention + Sex +
0,229927	-0,20879	0,173124	138	69	69	134	-1,20604	y ~ Intervention + Sex +
0,181356	-0,20894	0,155509	138	69	69	134	-1,34358	y ~ Intervention + Sex +
0,215567	-0,20936	0,168256	138	69	69	134	-1,24428	y ~ Intervention + Sex +
0,175389	-0,2098	0,154004	138	69	69	134	-1,3623	y ~ Intervention + Sex +
0,138564	-0,21018	0,141056	138	69	69	134	-1,49004	y ~ Intervention + Sex +
0,111487	-0,21018	0,131191	138	69	69	134	-1,6021	y ~ Intervention + Sex +
0,189083	-0,21113	0,159949	138	69	69	134	-1,32001	y ~ Intervention + Sex +
0,224695	-0,21179	0,173631	138	69	69	134	-1,21977	y ~ Intervention + Sex +

0,173242	-0,21274	0,15538	138	69	69	134	-1,36915	y ~ Intervention + Sex +
0,140663	-0,2137	0,144183	137	68	69	133	-1,48216	y ~ Intervention + Sex +
0,211834	-0,21374	0,170371	138	69	69	134	-1,25453	y ~ Intervention + Sex +
0,146785	-0,21455	0,147006	138	69	69	134	-1,45944	y ~ Intervention + Sex +
0,249906	-0,21548	0,186472	138	69	69	134	-1,15559	y ~ Intervention + Sex +
0,189751	-0,21616	0,164004	138	69	69	134	-1,318	y ~ Intervention + Sex +
0,188493	-0,21672	0,163961	138	69	69	134	-1,32178	y ~ Intervention + Sex +
0,145876	-0,21828	0,149222	138	69	69	134	-1,46276	y ~ Intervention + Sex +
0,170621	-0,21864	0,158713	138	69	69	134	-1,37761	y ~ Intervention + Sex +
0,108086	-0,21905	0,135409	138	69	69	134	-1,61767	y ~ Intervention + Sex +
0,204837	-0,2191	0,171966	138	69	69	134	-1,27409	y ~ Intervention + Sex +
0,129409	-0,21944	0,143816	138	69	69	134	-1,52583	y ~ Intervention + Sex +
0,164771	-0,22008	0,157559	138	69	69	134	-1,39684	y ~ Intervention + Sex +
0,169177	-0,22105	0,159914	138	69	69	134	-1,38231	y ~ Intervention + Sex +
0,223234	-0,22125	0,180814	138	69	69	134	-1,22364	y ~ Intervention + Sex +
0,129786	-0,22139	0,145239	138	69	69	134	-1,52432	y ~ Intervention + Sex +
0,215047	-0,22233	0,178475	138	69	69	134	-1,2457	y ~ Intervention + Sex +
0,164541	-0,22354	0,159942	138	69	69	134	-1,39761	y ~ Intervention + Sex +
0,169332	-0,22475	0,162648	138	69	69	134	-1,3818	y ~ Intervention + Sex +
0,120209	-0,22748	0,145458	138	69	69	134	-1,56386	y ~ Intervention + Sex +
0,175336	-0,2275	0,166975	138	69	69	134	-1,36247	y ~ Intervention + Sex +
0,130325	-0,22885	0,150343	138	69	69	134	-1,52216	y ~ Intervention + Sex +
0,171843	-0,23111	0,168243	138	69	69	134	-1,37365	y ~ Intervention + Sex +
0,09968	-0,2316	0,139696	138	69	69	134	-1,65788	y ~ Intervention + Sex +
0,161169	-0,23211	0,16474	138	69	69	134	-1,40894	y ~ Intervention + Sex +
0,158978	-0,23276	0,164328	138	69	69	134	-1,4164	y ~ Intervention + Sex +
0,178564	-0,23368	0,172802	138	69	69	134	-1,35228	y ~ Intervention + Sex +
0,144753	-0,23651	0,161231	138	69	69	134	-1,46687	y ~ Intervention + Sex +
0,157338	-0,23655	0,166344	138	69	69	134	-1,42204	y ~ Intervention + Sex +
0,138006	-0,23832	0,159716	138	69	69	134	-1,49217	y ~ Intervention + Sex +
0,168298	-0,23911	0,172622	138	69	69	134	-1,38518	y ~ Intervention + Sex +
0,098434	-0,24061	0,144589	138	69	69	134	-1,66407	y ~ Intervention + Sex +
0,11909	-0,24062	0,153395	138	69	69	134	-1,56864	y ~ Intervention + Sex +
0,130886	-0,24077	0,158412	138	69	69	134	-1,51992	y ~ Intervention + Sex +
0,117301	-0,24134	0,153099	138	69	69	134	-1,57636	y ~ Intervention + Sex +
0,109139	-0,2414	0,149677	138	69	69	134	-1,61281	y ~ Intervention + Sex +
0,168613	-0,2448	0,17686	138	69	69	134	-1,38415	y ~ Intervention + Sex +
0,153917	-0,24496	0,170826	138	69	69	134	-1,43395	y ~ Intervention + Sex +
0,135536	-0,24519	0,16328	138	69	69	134	-1,50167	y ~ Intervention + Sex +
0,103365	-0,24554	0,149723	138	69	69	134	-1,63993	y ~ Intervention + Sex +
0,121706	-0,24649	0,15826	138	69	69	134	-1,55752	y ~ Intervention + Sex +
0,114319	-0,24662	0,155161	138	69	69	134	-1,58943	y ~ Intervention + Sex +
0,101275	-0,2467	0,149511	138	69	69	134	-1,65005	y ~ Intervention + Sex +
0,130936	-0,24694	0,162491	138	69	69	134	-1,51973	y ~ Intervention + Sex +
0,049276	-0,24742	0,124695	138	69	69	134	-1,9842	y ~ Intervention + Sex +
0,139142	-0,24766	0,166457	138	69	69	134	-1,48784	y ~ Intervention + Sex +
0,049014	-0,24786	0,124771	138	69	69	134	-1,98653	y ~ Intervention + Sex +
0,073187	-0,24848	0,137596	138	69	69	134	-1,80585	y ~ Intervention + Sex +

0,091021	-0,25201	0,14804	138	69	69	134	-1,70228	y ~ Intervention + Sex +
0,074245	-0,25217	0,140159	138	69	69	134	-1,79915	y ~ Intervention + Sex +
0,106342	-0,25221	0,15513	138	69	69	134	-1,6258	y ~ Intervention + Sex +
0,077093	-0,25367	0,14239	138	69	69	134	-1,78152	y ~ Intervention + Sex +
0,053452	-0,25371	0,130214	138	69	69	134	-1,94844	y ~ Intervention + Sex +
0,110609	-0,25464	0,158547	138	69	69	134	-1,60609	y ~ Intervention + Sex +
0,112511	-0,25532	0,159827	138	69	69	134	-1,5975	y ~ Intervention + Sex +
0,112894	-0,25546	0,160082	138	69	69	134	-1,59578	y ~ Intervention + Sex +
0,064625	-0,25649	0,13766	138	69	69	134	-1,86319	y ~ Intervention + Sex +
0,121163	-0,25649	0,164438	138	69	69	134	-1,55981	y ~ Intervention + Sex +
0,101398	-0,25672	0,155641	138	69	69	134	-1,64945	y ~ Intervention + Sex +
0,147541	-0,25784	0,177001	138	69	69	134	-1,45669	y ~ Intervention + Sex +
0,074489	-0,25885	0,143993	138	69	69	134	-1,79762	y ~ Intervention + Sex +
0,086162	-0,26207	0,151596	138	69	69	134	-1,72872	y ~ Intervention + Sex +
0,118422	-0,26216	0,166817	138	69	69	134	-1,57151	y ~ Intervention + Sex +
0,09628	-0,26397	0,157598	138	69	69	134	-1,67493	y ~ Intervention + Sex +
0,144133	-0,26403	0,179713	138	69	69	134	-1,46916	y ~ Intervention + Sex +
0,08927	-0,26472	0,154653	138	69	69	134	-1,71168	y ~ Intervention + Sex +
0,101414	-0,26582	0,161162	138	69	69	134	-1,64937	y ~ Intervention + Sex +
0,073226	-0,26607	0,147359	138	69	69	134	-1,8056	y ~ Intervention + Sex +
0,084011	-0,26699	0,153368	138	69	69	134	-1,74082	y ~ Intervention + Sex +
0,119512	-0,2688	0,171558	138	69	69	134	-1,56683	y ~ Intervention + Sex +
0,102707	-0,26886	0,16363	138	69	69	134	-1,6431	y ~ Intervention + Sex +
0,091355	-0,26988	0,158705	138	69	69	134	-1,70051	y ~ Intervention + Sex +
0,075041	-0,27014	0,150565	138	69	69	134	-1,79417	y ~ Intervention + Sex +
0,100799	-0,27136	0,164226	138	69	69	134	-1,65238	y ~ Intervention + Sex +
0,113193	-0,27146	0,170254	138	69	69	134	-1,59444	y ~ Intervention + Sex +
0,111623	-0,2721	0,169907	138	69	69	134	-1,60149	y ~ Intervention + Sex +
0,071891	-0,27237	0,150136	138	69	69	134	-1,81415	y ~ Intervention + Sex +
0,0446	-0,27311	0,134708	138	69	69	134	-2,02745	y ~ Intervention + Sex +
0,077293	-0,27368	0,153729	138	69	69	134	-1,7803	y ~ Intervention + Sex +
0,104832	-0,27371	0,167622	138	69	69	134	-1,63293	y ~ Intervention + Sex +
0,112829	-0,2741	0,171734	138	69	69	134	-1,59607	y ~ Intervention + Sex +
0,088744	-0,27411	0,159876	138	69	69	134	-1,71453	y ~ Intervention + Sex +
0,09408	-0,27612	0,163748	138	69	69	134	-1,68622	y ~ Intervention + Sex +
0,10353	-0,27716	0,169086	138	69	69	134	-1,63914	y ~ Intervention + Sex +
0,076111	-0,27779	0,155403	138	69	69	134	-1,78754	y ~ Intervention + Sex +
0,107825	-0,27836	0,171945	138	69	69	134	-1,61888	y ~ Intervention + Sex +
0,085035	-0,28108	0,162002	138	69	69	134	-1,73503	y ~ Intervention + Sex +
0,088164	-0,282	0,164177	138	69	69	134	-1,71769	y ~ Intervention + Sex +
0,106737	-0,28324	0,174413	138	69	69	134	-1,62395	y ~ Intervention + Sex +
0,119179	-0,28404	0,18112	138	69	69	134	-1,56826	y ~ Intervention + Sex +
0,089322	-0,28531	0,166711	138	69	69	134	-1,7114	y ~ Intervention + Sex +
0,023501	-0,28706	0,125279	138	69	69	134	-2,29139	y ~ Intervention + Sex +
0,040611	-0,28748	0,139045	138	69	69	134	-2,0675	y ~ Intervention + Sex +
0,037418	-0,28891	0,13744	138	69	69	134	-2,10208	y ~ Intervention + Sex +
0,071054	-0,29069	0,159755	138	69	69	134	-1,81959	y ~ Intervention + Sex +
0,071352	-0,29156	0,160408	138	69	69	134	-1,81764	y ~ Intervention + Sex +

0,088196	-0,29327	0,170753	138	69	69	134	-1,71751	y ~ Intervention + Sex +
0,093148	-0,29383	0,173755	138	69	69	134	-1,69107	y ~ Intervention + Sex +
0,035331	-0,29437	0,138456	138	69	69	134	-2,12607	y ~ Intervention + Sex +
0,070637	-0,29465	0,161689	138	69	69	134	-1,82232	y ~ Intervention + Sex +
0,059086	-0,29488	0,154891	138	69	69	134	-1,90376	y ~ Intervention + Sex +
0,069769	-0,29653	0,162211	138	69	69	134	-1,82803	y ~ Intervention + Sex +
0,039353	-0,29748	0,14296	138	69	69	134	-2,08084	y ~ Intervention + Sex +
0,057872	-0,2979	0,155719	138	69	69	134	-1,91307	y ~ Intervention + Sex +
0,074816	-0,29848	0,166229	138	69	69	134	-1,79557	y ~ Intervention + Sex +
0,060096	-0,29916	0,157776	138	69	69	134	-1,89613	y ~ Intervention + Sex +
0,036398	-0,29943	0,141663	138	69	69	134	-2,11365	y ~ Intervention + Sex +
0,034404	-0,30038	0,140555	138	69	69	134	-2,13712	y ~ Intervention + Sex +
0,086926	-0,30064	0,174338	138	69	69	134	-1,72448	y ~ Intervention + Sex +
0,063732	-0,30189	0,16148	138	69	69	134	-1,86953	y ~ Intervention + Sex +
0,047937	-0,30257	0,151573	138	69	69	134	-1,99621	y ~ Intervention + Sex +
0,050969	-0,30356	0,15414	138	69	69	134	-1,96941	y ~ Intervention + Sex +
0,051642	-0,30365	0,154636	138	69	69	134	-1,96364	y ~ Intervention + Sex +
0,063239	-0,30399	0,162295	138	69	69	134	-1,87306	y ~ Intervention + Sex +
0,044631	-0,30399	0,149959	138	69	69	134	-2,02714	y ~ Intervention + Sex +
0,071364	-0,30444	0,167498	138	69	69	134	-1,81757	y ~ Intervention + Sex +
0,060399	-0,30449	0,160776	138	69	69	134	-1,89386	y ~ Intervention + Sex +
0,058306	-0,30675	0,160625	138	69	69	134	-1,90972	y ~ Intervention + Sex +
0,075259	-0,30702	0,171249	138	69	69	134	-1,79281	y ~ Intervention + Sex +
0,086953	-0,30747	0,178312	138	69	69	134	-1,72434	y ~ Intervention + Sex +
0,052729	-0,30908	0,158142	138	69	69	134	-1,95446	y ~ Intervention + Sex +
0,073224	-0,30911	0,171192	138	69	69	134	-1,80561	y ~ Intervention + Sex +
0,047679	-0,3092	0,154713	138	69	69	134	-1,99857	y ~ Intervention + Sex +
0,083144	-0,30954	0,177311	138	69	69	134	-1,74576	y ~ Intervention + Sex +
0,051656	-0,31053	0,158149	138	69	69	134	-1,96352	y ~ Intervention + Sex +
0,065947	-0,31095	0,167724	138	69	69	134	-1,85394	y ~ Intervention + Sex +
0,030581	-0,31107	0,142328	138	69	69	134	-2,18562	y ~ Intervention + Sex +
0,062235	-0,31966	0,170001	138	69	69	134	-1,88032	y ~ Intervention + Sex +
0,075464	-0,32074	0,179029	138	69	69	134	-1,79154	y ~ Intervention + Sex +
0,056593	-0,32249	0,167698	138	69	69	134	-1,92306	y ~ Intervention + Sex +
0,053121	-0,32333	0,165711	138	69	69	134	-1,95119	y ~ Intervention + Sex +
0,02003	-0,32387	0,137587	138	69	69	134	-2,35392	y ~ Intervention + Sex +
0,013513	-0,32488	0,129792	138	69	69	134	-2,50309	y ~ Intervention + Sex +
0,030045	-0,32532	0,148356	138	69	69	134	-2,19284	y ~ Intervention + Sex +
0,031529	-0,32588	0,149961	138	69	69	134	-2,17312	y ~ Intervention + Sex +
0,047287	-0,32631	0,16298	138	69	69	134	-2,00215	y ~ Intervention + Sex +
0,025875	-0,32668	0,14499	138	69	69	134	-2,25314	y ~ Intervention + Sex +
0,071856	-0,32716	0,180317	138	69	69	134	-1,81438	y ~ Intervention + Sex +
0,051367	-0,32782	0,166746	138	69	69	134	-1,96599	y ~ Intervention + Sex +
0,06145	-0,32799	0,173904	138	69	69	134	-1,88607	y ~ Intervention + Sex +
0,026606	-0,32818	0,14638	138	69	69	134	-2,24198	y ~ Intervention + Sex +
0,059579	-0,32921	0,173268	138	69	69	134	-1,90002	y ~ Intervention + Sex +
0,038653	-0,33035	0,158183	138	69	69	134	-2,08842	y ~ Intervention + Sex +
0,029041	-0,33544	0,152011	138	69	69	134	-2,20666	y ~ Intervention + Sex +

0,036003	-0,33545	0,158364	138	69	69	134	-2,11821	y ~ Intervention + Sex +
0,057775	-0,33663	0,175894	138	69	69	134	-1,91382	y ~ Intervention + Sex +
0,041281	-0,33843	0,164241	138	69	69	134	-2,06055	y ~ Intervention + Sex +
0,015963	-0,34171	0,140004	138	69	69	134	-2,44075	y ~ Intervention + Sex +
0,048358	-0,3422	0,171752	138	69	69	134	-1,99241	y ~ Intervention + Sex +
0,041332	-0,34278	0,166395	138	69	69	134	-2,06001	y ~ Intervention + Sex +
0,04489	-0,34409	0,169951	138	69	69	134	-2,02466	y ~ Intervention + Sex +
0,023081	-0,34436	0,149822	138	69	69	134	-2,2985	y ~ Intervention + Sex +
0,051993	-0,34665	0,176804	138	69	69	134	-1,96066	y ~ Intervention + Sex +
0,042468	-0,34692	0,169358	138	69	69	134	-2,04845	y ~ Intervention + Sex +
0,038321	-0,34847	0,166569	138	69	69	134	-2,09205	y ~ Intervention + Sex +
0,043252	-0,34871	0,170886	138	69	69	134	-2,04062	y ~ Intervention + Sex +
0,062077	-0,34945	0,185732	138	69	69	134	-1,88147	y ~ Intervention + Sex +
0,053482	-0,35032	0,179817	138	69	69	134	-1,9482	y ~ Intervention + Sex +
0,013111	-0,35322	0,140485	138	69	69	134	-2,51428	y ~ Intervention + Sex +
0,017237	-0,35521	0,147291	138	69	69	134	-2,41161	y ~ Intervention + Sex +
0,028959	-0,35597	0,161235	138	69	69	134	-2,2078	y ~ Intervention + Sex +
0,032016	-0,35908	0,165715	138	69	69	134	-2,16684	y ~ Intervention + Sex +
0,021899	-0,35947	0,154998	138	69	69	134	-2,31917	y ~ Intervention + Sex +
0,02676	-0,35969	0,160601	138	69	69	134	-2,23967	y ~ Intervention + Sex +
0,009918	-0,3628	0,138686	138	69	69	134	-2,61598	y ~ Intervention + Sex +
0,020048	-0,36462	0,154922	138	69	69	134	-2,35358	y ~ Intervention + Sex +
0,030029	-0,36487	0,166378	138	69	69	134	-2,19305	y ~ Intervention + Sex +
0,018495	-0,36512	0,153111	138	69	69	134	-2,38467	y ~ Intervention + Sex +
0,024766	-0,3664	0,161367	138	69	69	134	-2,27061	y ~ Intervention + Sex +
0,022573	-0,36943	0,160114	138	69	69	134	-2,30727	y ~ Intervention + Sex +
0,008124	-0,37003	0,137711	138	69	69	134	-2,68697	y ~ Intervention + Sex +
0,019896	-0,37115	0,1575	138	69	69	134	-2,35651	y ~ Intervention + Sex +
0,018531	-0,37254	0,15627	138	69	69	134	-2,38393	y ~ Intervention + Sex +
0,015522	-0,37505	0,153	138	69	69	134	-2,4513	y ~ Intervention + Sex +
0,006966	-0,37549	0,137003	138	69	69	134	-2,74079	y ~ Intervention + Sex +
0,016577	-0,37735	0,155517	138	69	69	134	-2,42645	y ~ Intervention + Sex +
0,016143	-0,37812	0,155189	138	69	69	134	-2,43649	y ~ Intervention + Sex +
0,02501	-0,37819	0,166845	138	69	69	134	-2,26671	y ~ Intervention + Sex +
0,011836	-0,37831	0,148249	138	69	69	134	-2,55189	y ~ Intervention + Sex +
0,015023	-0,37872	0,153729	138	69	69	134	-2,46357	y ~ Intervention + Sex +
0,006507	-0,37962	0,137327	138	69	69	134	-2,76435	y ~ Intervention + Sex +
0,027113	-0,38003	0,170082	138	69	69	134	-2,2344	y ~ Intervention + Sex +
0,009318	-0,38362	0,145402	138	69	69	134	-2,63833	y ~ Intervention + Sex +
0,012824	-0,38439	0,152387	138	69	69	134	-2,52243	y ~ Intervention + Sex +
0,025824	-0,38469	0,170675	138	69	69	134	-2,25393	y ~ Intervention + Sex +
0,016508	-0,38525	0,158667	138	69	69	134	-2,42803	y ~ Intervention + Sex +
0,02854	-0,38569	0,174226	138	69	69	134	-2,21371	y ~ Intervention + Sex +
0,023461	-0,38693	0,168813	138	69	69	134	-2,29206	y ~ Intervention + Sex +
0,004689	-0,38803	0,134928	138	69	69	134	-2,87583	y ~ Intervention + Sex +
0,009751	-0,38819	0,148046	138	69	69	134	-2,62209	y ~ Intervention + Sex +
0,012807	-0,38828	0,153901	138	69	69	134	-2,52294	y ~ Intervention + Sex +
0,008036	-0,39208	0,14571	138	69	69	134	-2,69082	y ~ Intervention + Sex +

0,020987	-0,39277	0,168155	138	69	69	134	-2,33579	y ~ Intervention + Sex +
0,022256	-0,39371	0,170228	138	69	69	134	-2,31282	y ~ Intervention + Sex +
0,015477	-0,39394	0,160635	138	69	69	134	-2,4524	y ~ Intervention + Sex +
0,021609	-0,39502	0,169948	138	69	69	134	-2,32437	y ~ Intervention + Sex +
0,013267	-0,39541	0,15754	138	69	69	134	-2,5099	y ~ Intervention + Sex +
0,011194	-0,39832	0,154855	138	69	69	134	-2,57224	y ~ Intervention + Sex +
0,016621	-0,39834	0,164231	138	69	69	134	-2,42545	y ~ Intervention + Sex +
0,01195	-0,39964	0,15682	138	69	69	134	-2,54838	y ~ Intervention + Sex +
0,014251	-0,4005	0,161278	138	69	69	134	-2,48332	y ~ Intervention + Sex +
0,016882	-0,40179	0,166059	138	69	69	134	-2,41954	y ~ Intervention + Sex +
0,005309	-0,40353	0,14239	138	69	69	134	-2,83395	y ~ Intervention + Sex +
0,006166	-0,40384	0,145117	138	69	69	134	-2,78288	y ~ Intervention + Sex +
0,0076	-0,40751	0,150349	138	69	69	134	-2,71041	y ~ Intervention + Sex +
0,002951	-0,40809	0,134758	138	69	69	134	-3,02833	y ~ Intervention + Sex +
0,012469	-0,40814	0,161142	138	69	69	134	-2,53279	y ~ Intervention + Sex +
0,010556	-0,40833	0,157444	138	69	69	134	-2,5935	y ~ Intervention + Sex +
0,009576	-0,4103	0,156094	138	69	69	134	-2,62856	y ~ Intervention + Sex +
0,01275	-0,41119	0,162874	138	69	69	134	-2,52458	y ~ Intervention + Sex +
0,008886	-0,41281	0,155467	138	69	69	134	-2,65526	y ~ Intervention + Sex +
0,012671	-0,4146	0,164075	138	69	69	134	-2,52687	y ~ Intervention + Sex +
0,020462	-0,41524	0,177025	138	69	69	134	-2,34564	y ~ Intervention + Sex +
0,006258	-0,41771	0,150375	138	69	69	134	-2,77779	y ~ Intervention + Sex +
0,008641	-0,418	0,156837	138	69	69	134	-2,66517	y ~ Intervention + Sex +
0,008927	-0,41892	0,157868	138	69	69	134	-2,65358	y ~ Intervention + Sex +
0,016314	-0,42141	0,173242	138	69	69	134	-2,4325	y ~ Intervention + Sex +
0,014037	-0,42282	0,169879	138	69	69	134	-2,48895	y ~ Intervention + Sex +
0,006254	-0,43152	0,155331	138	69	69	134	-2,77805	y ~ Intervention + Sex +
0,011577	-0,4319	0,168711	138	69	69	134	-2,55998	y ~ Intervention + Sex +
0,008674	-0,43442	0,163084	138	69	69	134	-2,66379	y ~ Intervention + Sex +
0,0066	-0,43494	0,157616	138	69	69	134	-2,75949	y ~ Intervention + Sex +
0,013075	-0,43802	0,174144	138	69	69	134	-2,51528	y ~ Intervention + Sex +
0,003897	-0,43884	0,149396	138	69	69	134	-2,93742	y ~ Intervention + Sex +
0,01305	-0,44195	0,175655	138	69	69	134	-2,51599	y ~ Intervention + Sex +
0,012945	-0,44222	0,175555	138	69	69	134	-2,51899	y ~ Intervention + Sex +
0,006957	-0,44364	0,16184	138	69	69	134	-2,74123	y ~ Intervention + Sex +
0,006069	-0,44417	0,159297	138	69	69	134	-2,78833	y ~ Intervention + Sex +
0,011178	-0,44437	0,172721	138	69	69	134	-2,57276	y ~ Intervention + Sex +
0,004911	-0,4448	0,155506	138	69	69	134	-2,86033	y ~ Intervention + Sex +
0,00589	-0,4469	0,159687	138	69	69	134	-2,79858	y ~ Intervention + Sex +
0,004851	-0,4471	0,156087	138	69	69	134	-2,86442	y ~ Intervention + Sex +
0,006218	-0,45037	0,162002	138	69	69	134	-2,78001	y ~ Intervention + Sex +
0,007887	-0,45417	0,168375	138	69	69	134	-2,69737	y ~ Intervention + Sex +
0,010991	-0,45461	0,176283	138	69	69	134	-2,57889	y ~ Intervention + Sex +
0,004315	-0,45644	0,157196	138	69	69	134	-2,90362	y ~ Intervention + Sex +
0,007622	-0,45803	0,169053	138	69	69	134	-2,70939	y ~ Intervention + Sex +
0,003465	-0,46058	0,154762	138	69	69	134	-2,9761	y ~ Intervention + Sex +
0,001819	-0,46225	0,145283	138	69	69	134	-3,18176	y ~ Intervention + Sex +
0,004832	-0,46565	0,16249	138	69	69	134	-2,86574	y ~ Intervention + Sex +

0,004775	-0,47096	0,164109	138	69	69	134	-2,86978	y ~ Intervention + Sex +
0,005609	-0,47237	0,167787	138	69	69	134	-2,81531	y ~ Intervention + Sex +
0,004013	-0,47409	0,161932	138	69	69	134	-2,92774	y ~ Intervention + Sex +
0,001657	-0,47854	0,14904	138	69	69	134	-3,21079	y ~ Intervention + Sex +
0,000584	-0,48361	0,137267	138	69	69	134	-3,52315	y ~ Intervention + Sex +
0,00106	-0,48374	0,144523	138	69	69	134	-3,34716	y ~ Intervention + Sex +
0,001792	-0,4856	0,15239	138	69	69	134	-3,18659	y ~ Intervention + Sex +
0,002131	-0,48603	0,155168	138	69	69	134	-3,13227	y ~ Intervention + Sex +
0,001088	-0,48844	0,146269	138	69	69	134	-3,33931	y ~ Intervention + Sex +
0,007766	-0,48848	0,180731	138	69	69	134	-2,70281	y ~ Intervention + Sex +
0,000901	-0,49244	0,145013	138	69	69	134	-3,39582	y ~ Intervention + Sex +
0,002049	-0,49384	0,157044	138	69	69	134	-3,14463	y ~ Intervention + Sex +
0,001726	-0,50071	0,156565	138	69	69	134	-3,19813	y ~ Intervention + Sex +
0,003753	-0,50204	0,170189	138	69	69	134	-2,94989	y ~ Intervention + Sex +
0,001098	-0,50241	0,150574	138	69	69	134	-3,33663	y ~ Intervention + Sex +
0,000164	-0,51024	0,131529	138	69	69	134	-3,87932	y ~ Intervention + Sex +
0,00333	-0,51259	0,17149	138	69	69	134	-2,98903	y ~ Intervention + Sex +
0,000884	-0,51649	0,151847	138	69	69	134	-3,40136	y ~ Intervention + Sex +
0,002892	-0,5172	0,170422	138	69	69	134	-3,03484	y ~ Intervention + Sex +
0,000768	-0,52119	0,151371	138	69	69	134	-3,44314	y ~ Intervention + Sex +
0,000637	-0,5271	0,150699	138	69	69	134	-3,49771	y ~ Intervention + Sex +
0,000457	-0,53866	0,149897	138	69	69	134	-3,59354	y ~ Intervention + Sex +
0,000424	-0,56575	0,156513	138	69	69	134	-3,61472	y ~ Intervention + Sex +
0,000255	-0,56882	0,151355	138	69	69	134	-3,7582	y ~ Intervention + Sex +
6,87E-05	-0,56971	0,138637	138	69	69	134	-4,10934	y ~ Intervention + Sex +
0,000398	-0,57032	0,156981	138	69	69	134	-3,63304	y ~ Intervention + Sex +
0,000208	-0,5787	0,151739	138	69	69	134	-3,81376	y ~ Intervention + Sex +
0,000355	-0,58054	0,158406	138	69	69	134	-3,66488	y ~ Intervention + Sex +
0,000282	-0,58118	0,155818	138	69	69	134	-3,72989	y ~ Intervention + Sex +
0,000183	-0,58908	0,153039	138	69	69	134	-3,84919	y ~ Intervention + Sex +
0,000125	-0,59101	0,149565	138	69	69	134	-3,9515	y ~ Intervention + Sex +
0,000636	-0,59103	0,168939	138	69	69	134	-3,49849	y ~ Intervention + Sex +
0,000256	-0,61888	0,164744	138	69	69	134	-3,75665	y ~ Intervention + Sex +
4,54E-05	-0,63768	0,151249	138	69	69	134	-4,21609	y ~ Intervention + Sex +
4,98E-05	-0,64057	0,152784	138	69	69	134	-4,19265	y ~ Intervention + Sex +
9,89E-05	-0,6433	0,160266	138	69	69	134	-4,01397	y ~ Intervention + Sex +
9,05E-05	-0,64481	0,159713	138	69	69	134	-4,03732	y ~ Intervention + Sex +
2,45E-05	-0,64498	0,147509	138	69	69	134	-4,37248	y ~ Intervention + Sex +
0,000103	-0,6739	0,168336	138	69	69	134	-4,00329	y ~ Intervention + Sex +
5,11E-05	-0,69057	0,164978	138	69	69	134	-4,18581	y ~ Intervention + Sex +
4,98E-06	-0,72042	0,151392	138	69	69	134	-4,75862	y ~ Intervention + Sex +
4,12E-07	-0,76718	0,144041	138	69	69	134	-5,32613	y ~ Intervention + Sex +
2,45E-21	-1,47592	0,130046	138	69	69	134	-11,3492	y ~ Intervention + Sex +

├ storage_time
├ storage_time
├ storage_time
├ storage_time
├ storage_time
├ storage_time
├ storage_time
├ storage_time
├ storage_time
├ storage_time
├ storage_time
├ storage_time
├ storage_time
├ storage_time
├ storage_time
├ storage_time
├ storage_time
├ storage_time
├ storage_time
├ storage_time
├ storage_time
├ storage_time
├ storage_time
├ storage_time
├ storage_time
├ storage_time
├ storage_time
├ storage_time
├ storage_time
├ storage_time
├ storage_time
├ storage_time
├ storage_time
├ storage_time
├ storage_time
├ storage_time
├ storage_time
├ storage_time
├ storage_time
├ storage_time
├ storage_time
├ storage_time
├ storage_time
├ storage_time
├ storage_time
├ storage_time
├ storage_time

⊦ storage_time
⊦ storage_time
⊦ storage_time
⊦ storage_time
⊦ storage_time
⊦ storage_time
⊦ storage_time
⊦ storage_time
⊦ storage_time
⊦ storage_time
⊦ storage_time
⊦ storage_time
⊦ storage_time
⊦ storage_time
⊦ storage_time
⊦ storage_time
⊦ storage_time
⊦ storage_time
⊦ storage_time
⊦ storage_time
⊦ storage_time
⊦ storage_time
⊦ storage_time
⊦ storage_time
⊦ storage_time
⊦ storage_time
⊦ storage_time
⊦ storage_time
⊦ storage_time
⊦ storage_time
⊦ storage_time
⊦ storage_time
⊦ storage_time
⊦ storage_time
⊦ storage_time
⊦ storage_time
⊦ storage_time
⊦ storage_time
⊦ storage_time
⊦ storage_time
⊦ storage_time
⊦ storage_time
⊦ storage_time
⊦ storage_time
⊦ storage_time
⊦ storage_time
⊦ storage_time
⊦ storage_time

⊦ storage_time
⊦ storage_time
⊦ storage_time
⊦ storage_time
⊦ storage_time
⊦ storage_time
⊦ storage_time
⊦ storage_time
⊦ storage_time
⊦ storage_time
⊦ storage_time
⊦ storage_time
⊦ storage_time
⊦ storage_time
⊦ storage_time
⊦ storage_time
⊦ storage_time
⊦ storage_time
⊦ storage_time
⊦ storage_time
⊦ storage_time
⊦ storage_time
⊦ storage_time
⊦ storage_time
⊦ storage_time
⊦ storage_time
⊦ storage_time
⊦ storage_time
⊦ storage_time
⊦ storage_time
⊦ storage_time
⊦ storage_time
⊦ storage_time
⊦ storage_time
⊦ storage_time
⊦ storage_time
⊦ storage_time
⊦ storage_time
⊦ storage_time
⊦ storage_time
⊦ storage_time
⊦ storage_time
⊦ storage_time
⊦ storage_time
⊦ storage_time
⊦ storage_time
⊦ storage_time
⊦ storage_time

├ storage_time
├ storage_time
├ storage_time
├ storage_time
├ storage_time
├ storage_time
├ storage_time
├ storage_time
├ storage_time
├ storage_time
├ storage_time
├ storage_time
├ storage_time
├ storage_time
├ storage_time
├ storage_time
├ storage_time
├ storage_time
├ storage_time
├ storage_time
├ storage_time
├ storage_time
├ storage_time
├ storage_time
├ storage_time
├ storage_time
├ storage_time
├ storage_time
├ storage_time
├ storage_time
├ storage_time
├ storage_time
├ storage_time
├ storage_time
├ storage_time
├ storage_time
├ storage_time
├ storage_time
├ storage_time
├ storage_time
├ storage_time
├ storage_time
├ storage_time
├ storage_time
├ storage_time
├ storage_time
├ storage_time
├ storage_time

⊦ storage_time
⊦ storage_time
⊦ storage_time
⊦ storage_time
⊦ storage_time
⊦ storage_time
⊦ storage_time
⊦ storage_time
⊦ storage_time
⊦ storage_time
⊦ storage_time
⊦ storage_time
⊦ storage_time
⊦ storage_time
⊦ storage_time
⊦ storage_time
⊦ storage_time
⊦ storage_time
⊦ storage_time
⊦ storage_time
⊦ storage_time
⊦ storage_time
⊦ storage_time
⊦ storage_time
⊦ storage_time
⊦ storage_time
⊦ storage_time
⊦ storage_time
⊦ storage_time
⊦ storage_time
⊦ storage_time
⊦ storage_time
⊦ storage_time
⊦ storage_time
⊦ storage_time
⊦ storage_time
⊦ storage_time
⊦ storage_time
⊦ storage_time
⊦ storage_time
⊦ storage_time
⊦ storage_time
⊦ storage_time
⊦ storage_time
⊦ storage_time
⊦ storage_time
⊦ storage_time
⊦ storage_time

├ storage_time
├ storage_time
├ storage_time
├ storage_time
├ storage_time
├ storage_time
├ storage_time
├ storage_time
├ storage_time
├ storage_time
├ storage_time
├ storage_time
├ storage_time
├ storage_time
├ storage_time
├ storage_time
├ storage_time
├ storage_time
├ storage_time
├ storage_time
├ storage_time
├ storage_time
├ storage_time
├ storage_time
├ storage_time
├ storage_time
├ storage_time
├ storage_time
├ storage_time
├ storage_time
├ storage_time
├ storage_time
├ storage_time
├ storage_time
├ storage_time
├ storage_time
├ storage_time
├ storage_time
├ storage_time
├ storage_time
├ storage_time
├ storage_time
├ storage_time
├ storage_time
├ storage_time
├ storage_time
├ storage_time
├ storage_time

├ storage_time
├ storage_time
├ storage_time
├ storage_time
├ storage_time
├ storage_time
├ storage_time
├ storage_time
├ storage_time
├ storage_time
├ storage_time
├ storage_time
├ storage_time
├ storage_time
├ storage_time
├ storage_time
├ storage_time
├ storage_time
├ storage_time
├ storage_time
├ storage_time
├ storage_time
├ storage_time
├ storage_time
├ storage_time
├ storage_time
├ storage_time
├ storage_time
├ storage_time
├ storage_time
├ storage_time
├ storage_time
├ storage_time
├ storage_time
├ storage_time
├ storage_time
├ storage_time
├ storage_time
├ storage_time
├ storage_time
├ storage_time
├ storage_time
├ storage_time
├ storage_time
├ storage_time
├ storage_time
├ storage_time
├ storage_time

├ storage_time
├ storage_time
├ storage_time
├ storage_time
├ storage_time
├ storage_time
├ storage_time
├ storage_time
├ storage_time
├ storage_time
├ storage_time
├ storage_time
├ storage_time
├ storage_time
├ storage_time
├ storage_time
├ storage_time
├ storage_time
├ storage_time
├ storage_time
├ storage_time
├ storage_time
├ storage_time
├ storage_time
├ storage_time
├ storage_time
├ storage_time
├ storage_time
├ storage_time
├ storage_time
├ storage_time
├ storage_time
├ storage_time
├ storage_time
├ storage_time
├ storage_time
├ storage_time
├ storage_time
├ storage_time
├ storage_time
├ storage_time
├ storage_time
├ storage_time
├ storage_time
├ storage_time
├ storage_time
├ storage_time
├ storage_time

├ storage_time
├ storage_time
├ storage_time
├ storage_time
├ storage_time
├ storage_time
├ storage_time
├ storage_time
├ storage_time
├ storage_time
├ storage_time
├ storage_time
├ storage_time
├ storage_time
├ storage_time
├ storage_time
├ storage_time
├ storage_time
├ storage_time
├ storage_time
├ storage_time
├ storage_time
├ storage_time
├ storage_time
├ storage_time
├ storage_time
├ storage_time
├ storage_time
├ storage_time
├ storage_time
├ storage_time
├ storage_time
├ storage_time
├ storage_time
├ storage_time
├ storage_time
├ storage_time
├ storage_time
├ storage_time
├ storage_time
├ storage_time
├ storage_time
├ storage_time
├ storage_time
├ storage_time
├ storage_time
├ storage_time
├ storage_time

├ storage_time
├ storage_time
├ storage_time
├ storage_time
├ storage_time
├ storage_time
├ storage_time
├ storage_time
├ storage_time
├ storage_time
├ storage_time
├ storage_time
├ storage_time
├ storage_time
├ storage_time
├ storage_time
├ storage_time
├ storage_time
├ storage_time
├ storage_time
├ storage_time
├ storage_time
├ storage_time
├ storage_time
├ storage_time
├ storage_time
├ storage_time
├ storage_time
├ storage_time
├ storage_time
├ storage_time
├ storage_time
├ storage_time
├ storage_time
├ storage_time
├ storage_time
├ storage_time
├ storage_time
├ storage_time
├ storage_time
├ storage_time
├ storage_time
├ storage_time
├ storage_time
├ storage_time
├ storage_time
├ storage_time
├ storage_time

├ storage_time
├ storage_time
├ storage_time
├ storage_time
├ storage_time
├ storage_time
├ storage_time
├ storage_time
├ storage_time
├ storage_time
├ storage_time
├ storage_time
├ storage_time
├ storage_time
├ storage_time
├ storage_time
├ storage_time
├ storage_time
├ storage_time
├ storage_time
├ storage_time
├ storage_time
├ storage_time
├ storage_time
├ storage_time
├ storage_time
├ storage_time
├ storage_time
├ storage_time
├ storage_time
├ storage_time
├ storage_time
├ storage_time
├ storage_time
├ storage_time
├ storage_time
├ storage_time
├ storage_time
├ storage_time
├ storage_time
├ storage_time
├ storage_time
├ storage_time
├ storage_time
├ storage_time
├ storage_time
├ storage_time
├ storage_time

⊦ storage_time
⊦ storage_time
⊦ storage_time
⊦ storage_time
⊦ storage_time
⊦ storage_time
⊦ storage_time
⊦ storage_time
⊦ storage_time
⊦ storage_time
⊦ storage_time
⊦ storage_time
⊦ storage_time
⊦ storage_time
⊦ storage_time
⊦ storage_time
⊦ storage_time
⊦ storage_time
⊦ storage_time
⊦ storage_time
⊦ storage_time
⊦ storage_time
⊦ storage_time
⊦ storage_time
⊦ storage_time
⊦ storage_time
⊦ storage_time
⊦ storage_time
⊦ storage_time
⊦ storage_time
⊦ storage_time
⊦ storage_time
⊦ storage_time
⊦ storage_time
⊦ storage_time
⊦ storage_time
⊦ storage_time
⊦ storage_time
⊦ storage_time
⊦ storage_time
⊦ storage_time
⊦ storage_time
⊦ storage_time
⊦ storage_time
⊦ storage_time
⊦ storage_time
⊦ storage_time
⊦ storage_time

├ storage_time
├ storage_time
├ storage_time
├ storage_time
├ storage_time
├ storage_time
├ storage_time
├ storage_time
├ storage_time
├ storage_time
├ storage_time
├ storage_time
├ storage_time
├ storage_time
├ storage_time
├ storage_time
├ storage_time
├ storage_time
├ storage_time
├ storage_time
├ storage_time
├ storage_time
├ storage_time
├ storage_time
├ storage_time
├ storage_time
├ storage_time
├ storage_time
├ storage_time
├ storage_time
├ storage_time
├ storage_time
├ storage_time
├ storage_time
├ storage_time
├ storage_time
├ storage_time
├ storage_time
├ storage_time
├ storage_time
├ storage_time
├ storage_time
├ storage_time
├ storage_time
├ storage_time
├ storage_time
├ storage_time
├ storage_time

├ storage_time
├ storage_time
├ storage_time
├ storage_time
├ storage_time
├ storage_time
├ storage_time
├ storage_time
├ storage_time
├ storage_time
├ storage_time
├ storage_time
├ storage_time
├ storage_time
├ storage_time
├ storage_time
├ storage_time
├ storage_time
├ storage_time
├ storage_time
├ storage_time
├ storage_time
├ storage_time
├ storage_time
├ storage_time
├ storage_time
├ storage_time
├ storage_time
├ storage_time
├ storage_time
├ storage_time
├ storage_time
├ storage_time
├ storage_time
├ storage_time
├ storage_time
├ storage_time
├ storage_time
├ storage_time
├ storage_time
├ storage_time
├ storage_time
├ storage_time
├ storage_time
├ storage_time
├ storage_time
├ storage_time
├ storage_time

├ storage_time
├ storage_time
├ storage_time
├ storage_time
├ storage_time
├ storage_time
├ storage_time
├ storage_time
├ storage_time
├ storage_time
├ storage_time
├ storage_time
├ storage_time
├ storage_time
├ storage_time
├ storage_time
├ storage_time
├ storage_time
├ storage_time
├ storage_time
├ storage_time
├ storage_time
├ storage_time
├ storage_time
├ storage_time
├ storage_time
├ storage_time
├ storage_time
├ storage_time
├ storage_time
├ storage_time
├ storage_time
├ storage_time
├ storage_time
├ storage_time
├ storage_time
├ storage_time
├ storage_time
├ storage_time
├ storage_time
├ storage_time
├ storage_time
├ storage_time
├ storage_time
├ storage_time
├ storage_time
├ storage_time
├ storage_time

├ storage_time
├ storage_time
├ storage_time
├ storage_time
├ storage_time
├ storage_time
├ storage_time
├ storage_time
├ storage_time
├ storage_time
├ storage_time
├ storage_time
├ storage_time
├ storage_time
├ storage_time
├ storage_time
├ storage_time
├ storage_time
├ storage_time
├ storage_time
├ storage_time
├ storage_time
├ storage_time
├ storage_time
├ storage_time
├ storage_time
├ storage_time
├ storage_time
├ storage_time
├ storage_time
├ storage_time
├ storage_time
├ storage_time
├ storage_time
├ storage_time
├ storage_time
├ storage_time
├ storage_time
├ storage_time
├ storage_time
├ storage_time
├ storage_time
├ storage_time
├ storage_time
├ storage_time
├ storage_time
├ storage_time
├ storage_time

⊦ storage_time
⊦ storage_time
⊦ storage_time
⊦ storage_time
⊦ storage_time
⊦ storage_time
⊦ storage_time
⊦ storage_time
⊦ storage_time
⊦ storage_time
⊦ storage_time
⊦ storage_time
⊦ storage_time
⊦ storage_time
⊦ storage_time
⊦ storage_time
⊦ storage_time
⊦ storage_time
⊦ storage_time
⊦ storage_time
⊦ storage_time
⊦ storage_time
⊦ storage_time
⊦ storage_time
⊦ storage_time
⊦ storage_time
⊦ storage_time
⊦ storage_time
⊦ storage_time
⊦ storage_time
⊦ storage_time
⊦ storage_time
⊦ storage_time
⊦ storage_time
⊦ storage_time
⊦ storage_time
⊦ storage_time
⊦ storage_time
⊦ storage_time
⊦ storage_time
⊦ storage_time
⊦ storage_time
⊦ storage_time
⊦ storage_time
⊦ storage_time
⊦ storage_time
⊦ storage_time
⊦ storage_time

├ storage_time
├ storage_time
├ storage_time
├ storage_time
├ storage_time
├ storage_time
├ storage_time
├ storage_time
├ storage_time
├ storage_time
├ storage_time
├ storage_time
├ storage_time
├ storage_time
├ storage_time
├ storage_time
├ storage_time
├ storage_time
├ storage_time
├ storage_time
├ storage_time
├ storage_time
├ storage_time
├ storage_time
├ storage_time
├ storage_time
├ storage_time
├ storage_time
├ storage_time
├ storage_time
├ storage_time
├ storage_time
├ storage_time
├ storage_time
├ storage_time
├ storage_time
├ storage_time
├ storage_time
├ storage_time
├ storage_time
├ storage_time
├ storage_time
├ storage_time
├ storage_time
├ storage_time
├ storage_time
├ storage_time
├ storage_time

⊦ storage_time
⊦ storage_time
⊦ storage_time
⊦ storage_time
⊦ storage_time
⊦ storage_time
⊦ storage_time
⊦ storage_time
⊦ storage_time
⊦ storage_time
⊦ storage_time
⊦ storage_time
⊦ storage_time
⊦ storage_time
⊦ storage_time
⊦ storage_time
⊦ storage_time
⊦ storage_time
⊦ storage_time
⊦ storage_time
⊦ storage_time
⊦ storage_time
⊦ storage_time
⊦ storage_time
⊦ storage_time
⊦ storage_time
⊦ storage_time
⊦ storage_time
⊦ storage_time
⊦ storage_time
⊦ storage_time
⊦ storage_time
⊦ storage_time
⊦ storage_time
⊦ storage_time
⊦ storage_time
⊦ storage_time
⊦ storage_time
⊦ storage_time
⊦ storage_time
⊦ storage_time
⊦ storage_time
⊦ storage_time
⊦ storage_time
⊦ storage_time
⊦ storage_time
⊦ storage_time
⊦ storage_time

├ storage_time
├ storage_time
├ storage_time
├ storage_time
├ storage_time
├ storage_time
├ storage_time
├ storage_time
├ storage_time
├ storage_time
├ storage_time
├ storage_time
├ storage_time
├ storage_time
├ storage_time
├ storage_time
├ storage_time
├ storage_time
├ storage_time
├ storage_time
├ storage_time
├ storage_time
├ storage_time
├ storage_time
├ storage_time
├ storage_time
├ storage_time
├ storage_time
├ storage_time
├ storage_time
├ storage_time
├ storage_time
├ storage_time
├ storage_time
├ storage_time
├ storage_time
├ storage_time
├ storage_time
├ storage_time
├ storage_time
├ storage_time
├ storage_time
├ storage_time
├ storage_time
├ storage_time
├ storage_time
├ storage_time
├ storage_time

⊦ storage_time
⊦ storage_time
⊦ storage_time
⊦ storage_time
⊦ storage_time
⊦ storage_time
⊦ storage_time
⊦ storage_time
⊦ storage_time
⊦ storage_time
⊦ storage_time
⊦ storage_time
⊦ storage_time
⊦ storage_time
⊦ storage_time
⊦ storage_time
⊦ storage_time
⊦ storage_time
⊦ storage_time
⊦ storage_time
⊦ storage_time
⊦ storage_time
⊦ storage_time
⊦ storage_time
⊦ storage_time
⊦ storage_time
⊦ storage_time
⊦ storage_time
⊦ storage_time
⊦ storage_time
⊦ storage_time
⊦ storage_time
⊦ storage_time
⊦ storage_time
⊦ storage_time
⊦ storage_time
⊦ storage_time
⊦ storage_time
⊦ storage_time
⊦ storage_time
⊦ storage_time
⊦ storage_time
⊦ storage_time
⊦ storage_time
⊦ storage_time
⊦ storage_time
⊦ storage_time
⊦ storage_time

⊦ storage_time
⊦ storage_time
⊦ storage_time
⊦ storage_time
⊦ storage_time
⊦ storage_time
⊦ storage_time
⊦ storage_time
⊦ storage_time
⊦ storage_time
⊦ storage_time
⊦ storage_time
⊦ storage_time
⊦ storage_time
⊦ storage_time
⊦ storage_time
⊦ storage_time
⊦ storage_time
⊦ storage_time
⊦ storage_time
⊦ storage_time
⊦ storage_time
⊦ storage_time
⊦ storage_time
⊦ storage_time
⊦ storage_time
⊦ storage_time
⊦ storage_time
⊦ storage_time
⊦ storage_time
⊦ storage_time
⊦ storage_time
⊦ storage_time
⊦ storage_time
⊦ storage_time
⊦ storage_time
⊦ storage_time
⊦ storage_time
⊦ storage_time
⊦ storage_time
⊦ storage_time
⊦ storage_time
⊦ storage_time
⊦ storage_time
⊦ storage_time
⊦ storage_time
⊦ storage_time
⊦ storage_time

⊦ storage_time
⊦ storage_time
⊦ storage_time
⊦ storage_time
⊦ storage_time
⊦ storage_time
⊦ storage_time
⊦ storage_time
⊦ storage_time
⊦ storage_time
⊦ storage_time
⊦ storage_time
⊦ storage_time
⊦ storage_time
⊦ storage_time
⊦ storage_time
⊦ storage_time
⊦ storage_time
⊦ storage_time
⊦ storage_time
⊦ storage_time
⊦ storage_time
⊦ storage_time
⊦ storage_time
⊦ storage_time
⊦ storage_time
⊦ storage_time
⊦ storage_time
⊦ storage_time
⊦ storage_time
⊦ storage_time
⊦ storage_time
⊦ storage_time
⊦ storage_time
⊦ storage_time
⊦ storage_time
⊦ storage_time
⊦ storage_time
⊦ storage_time
⊦ storage_time
⊦ storage_time
⊦ storage_time
⊦ storage_time
⊦ storage_time
⊦ storage_time
⊦ storage_time
⊦ storage_time
⊦ storage_time

├ storage_time
├ storage_time
├ storage_time
├ storage_time
├ storage_time
├ storage_time
├ storage_time
├ storage_time
├ storage_time
├ storage_time
├ storage_time
├ storage_time
├ storage_time
├ storage_time
├ storage_time
├ storage_time
├ storage_time
├ storage_time
├ storage_time
├ storage_time
├ storage_time
├ storage_time
├ storage_time
├ storage_time
├ storage_time
├ storage_time
├ storage_time
├ storage_time
├ storage_time
├ storage_time
├ storage_time
├ storage_time
├ storage_time
├ storage_time
├ storage_time
├ storage_time
├ storage_time
├ storage_time
├ storage_time
├ storage_time
├ storage_time
├ storage_time
├ storage_time
├ storage_time
├ storage_time
├ storage_time
├ storage_time
├ storage_time

├ storage_time
├ storage_time
├ storage_time
├ storage_time
├ storage_time
├ storage_time
├ storage_time
├ storage_time
├ storage_time
├ storage_time
├ storage_time
├ storage_time
├ storage_time
├ storage_time
├ storage_time
├ storage_time
├ storage_time
├ storage_time
├ storage_time
├ storage_time
├ storage_time
├ storage_time
├ storage_time
├ storage_time
├ storage_time
├ storage_time
├ storage_time
├ storage_time
├ storage_time
├ storage_time
├ storage_time
├ storage_time
├ storage_time
├ storage_time
├ storage_time
├ storage_time
├ storage_time
├ storage_time
├ storage_time
├ storage_time
├ storage_time
├ storage_time
├ storage_time
├ storage_time
├ storage_time
├ storage_time
├ storage_time
├ storage_time

├ storage_time
├ storage_time
├ storage_time
├ storage_time
├ storage_time
├ storage_time
├ storage_time
├ storage_time
├ storage_time
├ storage_time
├ storage_time
├ storage_time
├ storage_time
├ storage_time
├ storage_time
├ storage_time
├ storage_time
├ storage_time
├ storage_time
├ storage_time
├ storage_time
├ storage_time
├ storage_time
├ storage_time
├ storage_time
├ storage_time
├ storage_time
├ storage_time
├ storage_time
├ storage_time
├ storage_time
├ storage_time
├ storage_time
├ storage_time
├ storage_time
├ storage_time
├ storage_time
├ storage_time
├ storage_time
├ storage_time
├ storage_time
├ storage_time
├ storage_time
├ storage_time
├ storage_time
├ storage_time
├ storage_time
├ storage_time

⊦ storage_time
⊦ storage_time
⊦ storage_time
⊦ storage_time
⊦ storage_time
⊦ storage_time
⊦ storage_time
⊦ storage_time
⊦ storage_time
⊦ storage_time
⊦ storage_time
⊦ storage_time
⊦ storage_time
⊦ storage_time
⊦ storage_time
⊦ storage_time
⊦ storage_time
⊦ storage_time
⊦ storage_time
⊦ storage_time
⊦ storage_time
⊦ storage_time
⊦ storage_time
⊦ storage_time
⊦ storage_time
⊦ storage_time
⊦ storage_time
⊦ storage_time
⊦ storage_time
⊦ storage_time
⊦ storage_time
⊦ storage_time
⊦ storage_time
⊦ storage_time
⊦ storage_time
⊦ storage_time
⊦ storage_time
⊦ storage_time
⊦ storage_time
⊦ storage_time
⊦ storage_time
⊦ storage_time
⊦ storage_time
⊦ storage_time
⊦ storage_time
⊦ storage_time
⊦ storage_time
⊦ storage_time

├ storage_time
├ storage_time
├ storage_time
├ storage_time
├ storage_time
├ storage_time
├ storage_time
├ storage_time
├ storage_time
├ storage_time
├ storage_time
├ storage_time
├ storage_time
├ storage_time
├ storage_time
├ storage_time
├ storage_time
├ storage_time
├ storage_time
├ storage_time
├ storage_time
├ storage_time
├ storage_time
├ storage_time
├ storage_time
├ storage_time
├ storage_time
├ storage_time
├ storage_time
├ storage_time
├ storage_time
├ storage_time
├ storage_time
├ storage_time
├ storage_time
├ storage_time
├ storage_time
├ storage_time
├ storage_time
├ storage_time
├ storage_time
├ storage_time
├ storage_time
├ storage_time
├ storage_time
├ storage_time
├ storage_time
├ storage_time

⊦ storage_time
⊦ storage_time
⊦ storage_time
⊦ storage_time
⊦ storage_time
⊦ storage_time
⊦ storage_time
⊦ storage_time
⊦ storage_time
⊦ storage_time
⊦ storage_time
⊦ storage_time
⊦ storage_time
⊦ storage_time
⊦ storage_time
⊦ storage_time
⊦ storage_time
⊦ storage_time
⊦ storage_time
⊦ storage_time
⊦ storage_time
⊦ storage_time
⊦ storage_time
⊦ storage_time
⊦ storage_time
⊦ storage_time
⊦ storage_time
⊦ storage_time
⊦ storage_time
⊦ storage_time
⊦ storage_time
⊦ storage_time
⊦ storage_time
⊦ storage_time
⊦ storage_time
⊦ storage_time
⊦ storage_time
⊦ storage_time
⊦ storage_time
⊦ storage_time
⊦ storage_time
⊦ storage_time
⊦ storage_time
⊦ storage_time
⊦ storage_time
⊦ storage_time
⊦ storage_time
⊦ storage_time

⊦ storage_time
⊦ storage_time
⊦ storage_time
⊦ storage_time
⊦ storage_time
⊦ storage_time
⊦ storage_time
⊦ storage_time
⊦ storage_time
⊦ storage_time
⊦ storage_time
⊦ storage_time
⊦ storage_time
⊦ storage_time
⊦ storage_time
⊦ storage_time
⊦ storage_time
⊦ storage_time
⊦ storage_time
⊦ storage_time
⊦ storage_time
⊦ storage_time
⊦ storage_time
⊦ storage_time
⊦ storage_time
⊦ storage_time
⊦ storage_time
⊦ storage_time
⊦ storage_time
⊦ storage_time
⊦ storage_time
⊦ storage_time
⊦ storage_time
⊦ storage_time
⊦ storage_time
⊦ storage_time
⊦ storage_time
⊦ storage_time
⊦ storage_time
⊦ storage_time
⊦ storage_time
⊦ storage_time
⊦ storage_time
⊦ storage_time
⊦ storage_time
⊦ storage_time
⊦ storage_time
⊦ storage_time

├ storage_time
├ storage_time
├ storage_time
├ storage_time
├ storage_time
├ storage_time
├ storage_time
├ storage_time
├ storage_time
├ storage_time
├ storage_time
├ storage_time
├ storage_time
├ storage_time
├ storage_time
├ storage_time
├ storage_time
├ storage_time
├ storage_time
├ storage_time
├ storage_time
├ storage_time
├ storage_time
├ storage_time
├ storage_time
├ storage_time
├ storage_time
├ storage_time
├ storage_time
├ storage_time
├ storage_time
├ storage_time
├ storage_time
├ storage_time
├ storage_time
├ storage_time
├ storage_time
├ storage_time
├ storage_time
├ storage_time
├ storage_time
├ storage_time
├ storage_time
├ storage_time
├ storage_time
├ storage_time
├ storage_time
├ storage_time

⊦ storage_time
⊦ storage_time
⊦ storage_time
⊦ storage_time
⊦ storage_time
⊦ storage_time
⊦ storage_time
⊦ storage_time
⊦ storage_time
⊦ storage_time
⊦ storage_time
⊦ storage_time
⊦ storage_time
⊦ storage_time
⊦ storage_time
⊦ storage_time
⊦ storage_time
⊦ storage_time
⊦ storage_time
⊦ storage_time
⊦ storage_time
⊦ storage_time
⊦ storage_time
⊦ storage_time
⊦ storage_time
⊦ storage_time
⊦ storage_time
⊦ storage_time
⊦ storage_time
⊦ storage_time
⊦ storage_time
⊦ storage_time
⊦ storage_time
⊦ storage_time
⊦ storage_time
⊦ storage_time
⊦ storage_time
⊦ storage_time
⊦ storage_time
⊦ storage_time
⊦ storage_time
⊦ storage_time
⊦ storage_time
⊦ storage_time
⊦ storage_time
⊦ storage_time
⊦ storage_time
⊦ storage_time

⊦ storage_time
⊦ storage_time
⊦ storage_time
⊦ storage_time
⊦ storage_time
⊦ storage_time
⊦ storage_time
⊦ storage_time
⊦ storage_time
⊦ storage_time
⊦ storage_time
⊦ storage_time
⊦ storage_time
⊦ storage_time
⊦ storage_time
⊦ storage_time
⊦ storage_time
⊦ storage_time
⊦ storage_time
⊦ storage_time
⊦ storage_time
⊦ storage_time
⊦ storage_time
⊦ storage_time
⊦ storage_time
⊦ storage_time
⊦ storage_time
⊦ storage_time
⊦ storage_time
⊦ storage_time
⊦ storage_time
⊦ storage_time
⊦ storage_time
⊦ storage_time
⊦ storage_time
⊦ storage_time
⊦ storage_time
⊦ storage_time
⊦ storage_time
⊦ storage_time
⊦ storage_time
⊦ storage_time
⊦ storage_time
⊦ storage_time
⊦ storage_time
⊦ storage_time
⊦ storage_time
⊦ storage_time

⊦ storage_time
⊦ storage_time
⊦ storage_time
⊦ storage_time
⊦ storage_time
⊦ storage_time
⊦ storage_time
⊦ storage_time
⊦ storage_time
⊦ storage_time
⊦ storage_time
⊦ storage_time
⊦ storage_time
⊦ storage_time
⊦ storage_time
⊦ storage_time
⊦ storage_time
⊦ storage_time
⊦ storage_time
⊦ storage_time
⊦ storage_time
⊦ storage_time
⊦ storage_time
⊦ storage_time
⊦ storage_time
⊦ storage_time
⊦ storage_time
⊦ storage_time
⊦ storage_time
⊦ storage_time
⊦ storage_time
⊦ storage_time
⊦ storage_time
⊦ storage_time
⊦ storage_time
⊦ storage_time
⊦ storage_time
⊦ storage_time
⊦ storage_time
⊦ storage_time
⊦ storage_time
⊦ storage_time
⊦ storage_time
⊦ storage_time
⊦ storage_time
⊦ storage_time
⊦ storage_time
⊦ storage_time

├ storage_time
├ storage_time
├ storage_time
├ storage_time
├ storage_time
├ storage_time
├ storage_time
├ storage_time
├ storage_time
├ storage_time
├ storage_time
├ storage_time
├ storage_time
├ storage_time
├ storage_time
├ storage_time
├ storage_time
├ storage_time
├ storage_time
├ storage_time
├ storage_time
├ storage_time
├ storage_time
├ storage_time
├ storage_time
├ storage_time
├ storage_time
├ storage_time
├ storage_time
├ storage_time
├ storage_time
├ storage_time
├ storage_time
├ storage_time
├ storage_time
├ storage_time
├ storage_time
├ storage_time
├ storage_time
├ storage_time
├ storage_time
├ storage_time
├ storage_time
├ storage_time
├ storage_time
├ storage_time
├ storage_time
├ storage_time

⊦ storage_time
⊦ storage_time
⊦ storage_time
⊦ storage_time
⊦ storage_time
⊦ storage_time
⊦ storage_time
⊦ storage_time
⊦ storage_time
⊦ storage_time
⊦ storage_time
⊦ storage_time
⊦ storage_time
⊦ storage_time
⊦ storage_time
⊦ storage_time
⊦ storage_time
⊦ storage_time
⊦ storage_time
⊦ storage_time
⊦ storage_time
⊦ storage_time
⊦ storage_time
⊦ storage_time
⊦ storage_time
⊦ storage_time
⊦ storage_time
⊦ storage_time
⊦ storage_time
⊦ storage_time
⊦ storage_time
⊦ storage_time
⊦ storage_time
⊦ storage_time
⊦ storage_time
⊦ storage_time
⊦ storage_time
⊦ storage_time
⊦ storage_time
⊦ storage_time
⊦ storage_time
⊦ storage_time
⊦ storage_time
⊦ storage_time
⊦ storage_time
⊦ storage_time
⊦ storage_time
⊦ storage_time

├ storage_time
├ storage_time
├ storage_time
├ storage_time
├ storage_time
├ storage_time
├ storage_time
├ storage_time
├ storage_time
├ storage_time
├ storage_time
├ storage_time
├ storage_time
├ storage_time
├ storage_time
├ storage_time
├ storage_time
├ storage_time
├ storage_time
├ storage_time
├ storage_time
├ storage_time
├ storage_time
├ storage_time
├ storage_time
├ storage_time
├ storage_time
├ storage_time
├ storage_time
├ storage_time
├ storage_time
├ storage_time
├ storage_time
├ storage_time
├ storage_time
├ storage_time
├ storage_time
├ storage_time
├ storage_time
├ storage_time
├ storage_time
├ storage_time
├ storage_time
├ storage_time
├ storage_time
├ storage_time
├ storage_time
├ storage_time

├ storage_time
├ storage_time
├ storage_time
├ storage_time
├ storage_time
├ storage_time
├ storage_time
├ storage_time
├ storage_time
├ storage_time
├ storage_time
├ storage_time
├ storage_time
├ storage_time
├ storage_time
├ storage_time
├ storage_time
├ storage_time
├ storage_time
├ storage_time
├ storage_time
├ storage_time
├ storage_time
├ storage_time
├ storage_time
├ storage_time
├ storage_time
├ storage_time
├ storage_time
├ storage_time
├ storage_time
├ storage_time
├ storage_time
├ storage_time
├ storage_time
├ storage_time
├ storage_time
├ storage_time
├ storage_time
├ storage_time
├ storage_time
├ storage_time
├ storage_time
├ storage_time
├ storage_time
├ storage_time
├ storage_time
├ storage_time

├ storage_time
├ storage_time
├ storage_time
├ storage_time
├ storage_time
├ storage_time
├ storage_time
├ storage_time
├ storage_time
├ storage_time
├ storage_time
├ storage_time
├ storage_time
├ storage_time
├ storage_time
├ storage_time
├ storage_time
├ storage_time
├ storage_time
├ storage_time
├ storage_time
├ storage_time
├ storage_time
├ storage_time
├ storage_time
├ storage_time
├ storage_time
├ storage_time
├ storage_time
├ storage_time
├ storage_time
├ storage_time
├ storage_time
├ storage_time
├ storage_time
├ storage_time
├ storage_time
├ storage_time
├ storage_time
├ storage_time
├ storage_time
├ storage_time
├ storage_time
├ storage_time
├ storage_time
├ storage_time
├ storage_time
├ storage_time

⊦ storage_time
⊦ storage_time
⊦ storage_time
⊦ storage_time
⊦ storage_time
⊦ storage_time
⊦ storage_time
⊦ storage_time
⊦ storage_time
⊦ storage_time
⊦ storage_time
⊦ storage_time
⊦ storage_time
⊦ storage_time
⊦ storage_time
⊦ storage_time
⊦ storage_time
⊦ storage_time
⊦ storage_time
⊦ storage_time
⊦ storage_time
⊦ storage_time
⊦ storage_time
⊦ storage_time
⊦ storage_time
⊦ storage_time
⊦ storage_time
⊦ storage_time
⊦ storage_time
⊦ storage_time
⊦ storage_time
⊦ storage_time
⊦ storage_time
⊦ storage_time
⊦ storage_time
⊦ storage_time
⊦ storage_time
⊦ storage_time
⊦ storage_time
⊦ storage_time
⊦ storage_time
⊦ storage_time
⊦ storage_time
⊦ storage_time
⊦ storage_time
⊦ storage_time
⊦ storage_time
⊦ storage_time

├ storage_time
├ storage_time
├ storage_time
├ storage_time
├ storage_time
├ storage_time
├ storage_time
├ storage_time
├ storage_time
├ storage_time
├ storage_time
├ storage_time
├ storage_time
├ storage_time
├ storage_time
├ storage_time
├ storage_time
├ storage_time
├ storage_time
├ storage_time
├ storage_time
├ storage_time
├ storage_time
├ storage_time
├ storage_time
├ storage_time
├ storage_time
├ storage_time
├ storage_time
├ storage_time
├ storage_time
├ storage_time
├ storage_time
├ storage_time
├ storage_time
├ storage_time
├ storage_time
├ storage_time
├ storage_time
├ storage_time
├ storage_time
├ storage_time
├ storage_time
├ storage_time
├ storage_time
├ storage_time
├ storage_time
├ storage_time

├ storage_time
├ storage_time
├ storage_time
├ storage_time
├ storage_time
├ storage_time
├ storage_time
├ storage_time
├ storage_time
├ storage_time
├ storage_time
├ storage_time
├ storage_time
├ storage_time
├ storage_time
├ storage_time
├ storage_time
├ storage_time
├ storage_time
├ storage_time
├ storage_time
├ storage_time
├ storage_time
├ storage_time
├ storage_time
├ storage_time
├ storage_time
├ storage_time
├ storage_time
├ storage_time
├ storage_time
├ storage_time
├ storage_time
├ storage_time
├ storage_time
├ storage_time
├ storage_time
├ storage_time
├ storage_time
├ storage_time
├ storage_time
├ storage_time
├ storage_time
├ storage_time
├ storage_time
├ storage_time
├ storage_time
├ storage_time

⊦ storage_time
⊦ storage_time
⊦ storage_time
⊦ storage_time
⊦ storage_time
⊦ storage_time
⊦ storage_time
⊦ storage_time
⊦ storage_time
⊦ storage_time
⊦ storage_time
⊦ storage_time
⊦ storage_time
⊦ storage_time
⊦ storage_time
⊦ storage_time
⊦ storage_time
⊦ storage_time
⊦ storage_time
⊦ storage_time
⊦ storage_time
⊦ storage_time
⊦ storage_time
⊦ storage_time
⊦ storage_time
⊦ storage_time
⊦ storage_time
⊦ storage_time
⊦ storage_time
⊦ storage_time
⊦ storage_time
⊦ storage_time
⊦ storage_time
⊦ storage_time
⊦ storage_time
⊦ storage_time
⊦ storage_time
⊦ storage_time
⊦ storage_time
⊦ storage_time
⊦ storage_time
⊦ storage_time
⊦ storage_time
⊦ storage_time
⊦ storage_time
⊦ storage_time
⊦ storage_time
⊦ storage_time

⊦ storage_time
⊦ storage_time
⊦ storage_time
⊦ storage_time
⊦ storage_time
⊦ storage_time
⊦ storage_time
⊦ storage_time
⊦ storage_time
⊦ storage_time
⊦ storage_time
⊦ storage_time
⊦ storage_time
⊦ storage_time
⊦ storage_time
⊦ storage_time
⊦ storage_time
⊦ storage_time
⊦ storage_time
⊦ storage_time
⊦ storage_time
⊦ storage_time
⊦ storage_time
⊦ storage_time
⊦ storage_time
⊦ storage_time
⊦ storage_time
⊦ storage_time
⊦ storage_time
⊦ storage_time
⊦ storage_time
⊦ storage_time
⊦ storage_time
⊦ storage_time
⊦ storage_time
⊦ storage_time
⊦ storage_time
⊦ storage_time
⊦ storage_time
⊦ storage_time
⊦ storage_time
⊦ storage_time
⊦ storage_time
⊦ storage_time
⊦ storage_time
⊦ storage_time
⊦ storage_time
⊦ storage_time

⊦ storage_time
⊦ storage_time
⊦ storage_time
⊦ storage_time
⊦ storage_time
⊦ storage_time
⊦ storage_time
⊦ storage_time
⊦ storage_time
⊦ storage_time
⊦ storage_time
⊦ storage_time
⊦ storage_time
⊦ storage_time
⊦ storage_time
⊦ storage_time
⊦ storage_time
⊦ storage_time
⊦ storage_time
⊦ storage_time
⊦ storage_time
⊦ storage_time
⊦ storage_time
⊦ storage_time
⊦ storage_time
⊦ storage_time
⊦ storage_time
⊦ storage_time
⊦ storage_time
⊦ storage_time
⊦ storage_time
⊦ storage_time
⊦ storage_time
⊦ storage_time
⊦ storage_time
⊦ storage_time
⊦ storage_time
⊦ storage_time
⊦ storage_time
⊦ storage_time
⊦ storage_time
⊦ storage_time
⊦ storage_time
⊦ storage_time
⊦ storage_time
⊦ storage_time
⊦ storage_time
⊦ storage_time

⊦ storage_time
⊦ storage_time
⊦ storage_time
⊦ storage_time
⊦ storage_time
⊦ storage_time
⊦ storage_time
⊦ storage_time
⊦ storage_time
⊦ storage_time
⊦ storage_time
⊦ storage_time
⊦ storage_time
⊦ storage_time
⊦ storage_time
⊦ storage_time
⊦ storage_time
⊦ storage_time
⊦ storage_time
⊦ storage_time
⊦ storage_time
⊦ storage_time
⊦ storage_time
⊦ storage_time
⊦ storage_time
⊦ storage_time
⊦ storage_time
⊦ storage_time
⊦ storage_time
⊦ storage_time
⊦ storage_time
⊦ storage_time
⊦ storage_time
⊦ storage_time
⊦ storage_time
⊦ storage_time
⊦ storage_time
⊦ storage_time
⊦ storage_time
⊦ storage_time
⊦ storage_time
⊦ storage_time
⊦ storage_time
⊦ storage_time
⊦ storage_time
⊦ storage_time
⊦ storage_time
⊦ storage_time

⊦ storage_time
⊦ storage_time
⊦ storage_time
⊦ storage_time
⊦ storage_time
⊦ storage_time
⊦ storage_time
⊦ storage_time
⊦ storage_time
⊦ storage_time
⊦ storage_time
⊦ storage_time
⊦ storage_time
⊦ storage_time
⊦ storage_time
⊦ storage_time
⊦ storage_time
⊦ storage_time
⊦ storage_time
⊦ storage_time
⊦ storage_time
⊦ storage_time
⊦ storage_time
⊦ storage_time
⊦ storage_time
⊦ storage_time
⊦ storage_time
⊦ storage_time
⊦ storage_time
⊦ storage_time
⊦ storage_time
⊦ storage_time
⊦ storage_time
⊦ storage_time
⊦ storage_time
⊦ storage_time
⊦ storage_time
⊦ storage_time
⊦ storage_time
⊦ storage_time
⊦ storage_time
⊦ storage_time
⊦ storage_time
⊦ storage_time
⊦ storage_time
⊦ storage_time
⊦ storage_time
⊦ storage_time

⊦ storage_time
⊦ storage_time
⊦ storage_time
⊦ storage_time
⊦ storage_time
⊦ storage_time
⊦ storage_time
⊦ storage_time
⊦ storage_time
⊦ storage_time
⊦ storage_time
⊦ storage_time
⊦ storage_time
⊦ storage_time
⊦ storage_time
⊦ storage_time
⊦ storage_time
⊦ storage_time
⊦ storage_time
⊦ storage_time
⊦ storage_time
⊦ storage_time
⊦ storage_time
⊦ storage_time
⊦ storage_time
⊦ storage_time
⊦ storage_time
⊦ storage_time
⊦ storage_time
⊦ storage_time
⊦ storage_time
⊦ storage_time
⊦ storage_time
⊦ storage_time
⊦ storage_time
⊦ storage_time
⊦ storage_time
⊦ storage_time
⊦ storage_time
⊦ storage_time
⊦ storage_time
⊦ storage_time
⊦ storage_time
⊦ storage_time
⊦ storage_time
⊦ storage_time
⊦ storage_time
⊦ storage_time

⊦ storage_time
⊦ storage_time
⊦ storage_time
⊦ storage_time
⊦ storage_time
⊦ storage_time
⊦ storage_time
⊦ storage_time
⊦ storage_time
⊦ storage_time
⊦ storage_time
⊦ storage_time
⊦ storage_time
⊦ storage_time
⊦ storage_time
⊦ storage_time
⊦ storage_time
⊦ storage_time
⊦ storage_time
⊦ storage_time
⊦ storage_time
⊦ storage_time
⊦ storage_time
⊦ storage_time
⊦ storage_time
⊦ storage_time
⊦ storage_time
⊦ storage_time
⊦ storage_time
⊦ storage_time
⊦ storage_time
⊦ storage_time
⊦ storage_time
⊦ storage_time
⊦ storage_time
⊦ storage_time
⊦ storage_time
⊦ storage_time
⊦ storage_time
⊦ storage_time
⊦ storage_time
⊦ storage_time
⊦ storage_time
⊦ storage_time
⊦ storage_time
⊦ storage_time
⊦ storage_time
⊦ storage_time

├ storage_time
├ storage_time
├ storage_time
├ storage_time
├ storage_time
├ storage_time
├ storage_time
├ storage_time
├ storage_time
├ storage_time
├ storage_time
├ storage_time
├ storage_time
├ storage_time
├ storage_time
├ storage_time
├ storage_time
├ storage_time
├ storage_time
├ storage_time
├ storage_time
├ storage_time
├ storage_time
├ storage_time
├ storage_time
├ storage_time
├ storage_time
├ storage_time
├ storage_time
├ storage_time
├ storage_time
├ storage_time
├ storage_time
├ storage_time
├ storage_time
├ storage_time
├ storage_time
├ storage_time
├ storage_time
├ storage_time
├ storage_time
├ storage_time
├ storage_time
├ storage_time
├ storage_time
├ storage_time
├ storage_time
├ storage_time

⊦ storage_time
⊦ storage_time
⊦ storage_time
⊦ storage_time
⊦ storage_time
⊦ storage_time
⊦ storage_time
⊦ storage_time
⊦ storage_time
⊦ storage_time
⊦ storage_time
⊦ storage_time
⊦ storage_time
⊦ storage_time
⊦ storage_time
⊦ storage_time
⊦ storage_time
⊦ storage_time
⊦ storage_time
⊦ storage_time
⊦ storage_time
⊦ storage_time
⊦ storage_time
⊦ storage_time
⊦ storage_time
⊦ storage_time
⊦ storage_time
⊦ storage_time
⊦ storage_time
⊦ storage_time
⊦ storage_time
⊦ storage_time
⊦ storage_time
⊦ storage_time
⊦ storage_time
⊦ storage_time
⊦ storage_time
⊦ storage_time
⊦ storage_time
⊦ storage_time
⊦ storage_time
⊦ storage_time
⊦ storage_time
⊦ storage_time
⊦ storage_time
⊦ storage_time
⊦ storage_time
⊦ storage_time

⊦ storage_time
⊦ storage_time
⊦ storage_time
⊦ storage_time
⊦ storage_time
⊦ storage_time
⊦ storage_time
⊦ storage_time
⊦ storage_time
⊦ storage_time
⊦ storage_time
⊦ storage_time
⊦ storage_time
⊦ storage_time
⊦ storage_time
⊦ storage_time
⊦ storage_time
⊦ storage_time
⊦ storage_time
⊦ storage_time
⊦ storage_time
⊦ storage_time
⊦ storage_time
⊦ storage_time
⊦ storage_time
⊦ storage_time
⊦ storage_time
⊦ storage_time
⊦ storage_time
⊦ storage_time
⊦ storage_time
⊦ storage_time
⊦ storage_time
⊦ storage_time
⊦ storage_time
⊦ storage_time
⊦ storage_time
⊦ storage_time
⊦ storage_time
⊦ storage_time
⊦ storage_time
⊦ storage_time
⊦ storage_time

⊦ storage_time
⊦ storage_time
⊦ storage_time
⊦ storage_time
⊦ storage_time
⊦ storage_time
⊦ storage_time
⊦ storage_time
⊦ storage_time
⊦ storage_time
⊦ storage_time
⊦ storage_time
⊦ storage_time
⊦ storage_time
⊦ storage_time
⊦ storage_time
⊦ storage_time
⊦ storage_time
⊦ storage_time
⊦ storage_time

⊦ storage_time
⊦ storage_time
⊦ storage_time
⊦ storage_time
⊦ storage_time
⊦ storage_time
⊦ storage_time
⊦ storage_time
⊦ storage_time
⊦ storage_time
⊦ storage_time
⊦ storage_time
⊦ storage_time
⊦ storage_time
⊦ storage_time
⊦ storage_time
⊦ storage_time
⊦ storage_time
⊦ storage_time
⊦ storage_time
⊦ storage_time
⊦ storage_time
⊦ storage_time
⊦ storage_time
⊦ storage_time
⊦ storage_time
⊦ storage_time
⊦ storage_time
⊦ storage_time
⊦ storage_time
⊦ storage_time
⊦ storage_time
⊦ storage_time
⊦ storage_time
⊦ storage_time
⊦ storage_time
⊦ storage_time
⊦ storage_time
⊦ storage_time
⊦ storage_time
⊦ storage_time
⊦ storage_time
⊦ storage_time
⊦ storage_time
⊦ storage_time
⊦ storage_time
⊦ storage_time
⊦ storage_time

├ storage_time
├ storage_time
├ storage_time
├ storage_time
├ storage_time
├ storage_time
├ storage_time
├ storage_time
├ storage_time
├ storage_time
├ storage_time
├ storage_time
├ storage_time
├ storage_time
├ storage_time
├ storage_time
├ storage_time
├ storage_time
├ storage_time
├ storage_time
├ storage_time
├ storage_time
├ storage_time
├ storage_time
├ storage_time
├ storage_time
├ storage_time
├ storage_time
├ storage_time
├ storage_time
├ storage_time
├ storage_time
├ storage_time
├ storage_time
├ storage_time
├ storage_time
├ storage_time
├ storage_time
├ storage_time
├ storage_time
├ storage_time
├ storage_time
├ storage_time
├ storage_time
├ storage_time
├ storage_time
├ storage_time
├ storage_time

├ storage_time
├ storage_time
├ storage_time
├ storage_time
├ storage_time
├ storage_time
├ storage_time
├ storage_time
├ storage_time
├ storage_time
├ storage_time
├ storage_time
├ storage_time
├ storage_time
├ storage_time
├ storage_time
├ storage_time
├ storage_time
├ storage_time
├ storage_time
├ storage_time
├ storage_time
├ storage_time
├ storage_time
├ storage_time
├ storage_time
├ storage_time
├ storage_time
├ storage_time
├ storage_time
├ storage_time
├ storage_time
├ storage_time
├ storage_time
├ storage_time
├ storage_time
├ storage_time
├ storage_time
├ storage_time
├ storage_time
├ storage_time
├ storage_time
├ storage_time
├ storage_time
├ storage_time
├ storage_time
├ storage_time
├ storage_time

⊦ storage_time
⊦ storage_time
⊦ storage_time
⊦ storage_time
⊦ storage_time
⊦ storage_time
⊦ storage_time
⊦ storage_time
⊦ storage_time
⊦ storage_time
⊦ storage_time
⊦ storage_time
⊦ storage_time
⊦ storage_time
⊦ storage_time
⊦ storage_time
⊦ storage_time
⊦ storage_time
⊦ storage_time
⊦ storage_time
⊦ storage_time
⊦ storage_time
⊦ storage_time
⊦ storage_time
⊦ storage_time
⊦ storage_time
⊦ storage_time
⊦ storage_time
⊦ storage_time
⊦ storage_time
⊦ storage_time
⊦ storage_time
⊦ storage_time
⊦ storage_time
⊦ storage_time
⊦ storage_time
⊦ storage_time
⊦ storage_time
⊦ storage_time
⊦ storage_time
⊦ storage_time
⊦ storage_time
⊦ storage_time
⊦ storage_time
⊦ storage_time
⊦ storage_time
⊦ storage_time
⊦ storage_time

├ storage_time
├ storage_time
├ storage_time
├ storage_time
├ storage_time
├ storage_time
├ storage_time
├ storage_time
├ storage_time
├ storage_time
├ storage_time
├ storage_time
├ storage_time
├ storage_time
├ storage_time
├ storage_time
├ storage_time
├ storage_time
├ storage_time
├ storage_time
├ storage_time
├ storage_time
├ storage_time
├ storage_time
├ storage_time
├ storage_time
├ storage_time
├ storage_time
├ storage_time
├ storage_time
├ storage_time
├ storage_time
├ storage_time
├ storage_time
├ storage_time
├ storage_time
├ storage_time
├ storage_time
├ storage_time
├ storage_time
├ storage_time
├ storage_time
├ storage_time
├ storage_time
├ storage_time
├ storage_time
├ storage_time
├ storage_time

⊦ storage_time
⊦ storage_time
⊦ storage_time
⊦ storage_time
⊦ storage_time
⊦ storage_time
⊦ storage_time
⊦ storage_time
⊦ storage_time
⊦ storage_time
⊦ storage_time
⊦ storage_time
⊦ storage_time
⊦ storage_time
⊦ storage_time
⊦ storage_time
⊦ storage_time
⊦ storage_time
⊦ storage_time
⊦ storage_time
⊦ storage_time
⊦ storage_time
⊦ storage_time
⊦ storage_time
⊦ storage_time
⊦ storage_time
⊦ storage_time
⊦ storage_time
⊦ storage_time
⊦ storage_time
⊦ storage_time
⊦ storage_time
⊦ storage_time
⊦ storage_time
⊦ storage_time
⊦ storage_time
⊦ storage_time
⊦ storage_time
⊦ storage_time
⊦ storage_time
⊦ storage_time
⊦ storage_time
⊦ storage_time
⊦ storage_time
⊦ storage_time
⊦ storage_time
⊦ storage_time
⊦ storage_time

├ storage_time
├ storage_time
├ storage_time
├ storage_time
├ storage_time
├ storage_time
├ storage_time
├ storage_time
├ storage_time
├ storage_time
├ storage_time
├ storage_time
├ storage_time
├ storage_time
├ storage_time
├ storage_time
├ storage_time
├ storage_time
├ storage_time
├ storage_time
├ storage_time
├ storage_time
├ storage_time
├ storage_time
├ storage_time
├ storage_time
├ storage_time
├ storage_time
├ storage_time
├ storage_time
├ storage_time
├ storage_time
├ storage_time
├ storage_time
├ storage_time
├ storage_time
├ storage_time
├ storage_time
├ storage_time
├ storage_time
├ storage_time
├ storage_time
├ storage_time
├ storage_time
├ storage_time
├ storage_time
├ storage_time
├ storage_time

⊦ storage_time
⊦ storage_time
⊦ storage_time
⊦ storage_time
⊦ storage_time
⊦ storage_time
⊦ storage_time
⊦ storage_time
⊦ storage_time
⊦ storage_time
⊦ storage_time
⊦ storage_time
⊦ storage_time
⊦ storage_time
⊦ storage_time
⊦ storage_time
⊦ storage_time
⊦ storage_time
⊦ storage_time
⊦ storage_time
⊦ storage_time
⊦ storage_time
⊦ storage_time
⊦ storage_time
⊦ storage_time
⊦ storage_time
⊦ storage_time
⊦ storage_time
⊦ storage_time
⊦ storage_time
⊦ storage_time
⊦ storage_time
⊦ storage_time
⊦ storage_time
⊦ storage_time
⊦ storage_time
⊦ storage_time
⊦ storage_time
⊦ storage_time
⊦ storage_time
⊦ storage_time
⊦ storage_time
⊦ storage_time
⊦ storage_time
⊦ storage_time
⊦ storage_time
⊦ storage_time
⊦ storage_time

⊦ storage_time
⊦ storage_time
⊦ storage_time
⊦ storage_time
⊦ storage_time
⊦ storage_time
⊦ storage_time
⊦ storage_time
⊦ storage_time
⊦ storage_time
⊦ storage_time
⊦ storage_time
⊦ storage_time
⊦ storage_time
⊦ storage_time
⊦ storage_time
⊦ storage_time
⊦ storage_time
⊦ storage_time
⊦ storage_time
⊦ storage_time
⊦ storage_time
⊦ storage_time
⊦ storage_time
⊦ storage_time
⊦ storage_time
⊦ storage_time
⊦ storage_time
⊦ storage_time
⊦ storage_time
⊦ storage_time
⊦ storage_time
⊦ storage_time
⊦ storage_time
⊦ storage_time
⊦ storage_time
⊦ storage_time
⊦ storage_time
⊦ storage_time
⊦ storage_time
⊦ storage_time
⊦ storage_time
⊦ storage_time
⊦ storage_time
⊦ storage_time
⊦ storage_time
⊦ storage_time
⊦ storage_time

⊦ storage_time
⊦ storage_time
⊦ storage_time
⊦ storage_time
⊦ storage_time
⊦ storage_time
⊦ storage_time
⊦ storage_time
⊦ storage_time
⊦ storage_time
⊦ storage_time
⊦ storage_time
⊦ storage_time
⊦ storage_time
⊦ storage_time
⊦ storage_time
⊦ storage_time
⊦ storage_time
⊦ storage_time
⊦ storage_time
⊦ storage_time
⊦ storage_time
⊦ storage_time
⊦ storage_time
⊦ storage_time
⊦ storage_time
⊦ storage_time
⊦ storage_time
⊦ storage_time
⊦ storage_time
⊦ storage_time
⊦ storage_time
⊦ storage_time
⊦ storage_time
⊦ storage_time
⊦ storage_time
⊦ storage_time
⊦ storage_time
⊦ storage_time
⊦ storage_time
⊦ storage_time
⊦ storage_time
⊦ storage_time
⊦ storage_time
⊦ storage_time
⊦ storage_time
⊦ storage_time
⊦ storage_time

├ storage_time
├ storage_time
├ storage_time
├ storage_time
├ storage_time
├ storage_time
├ storage_time
├ storage_time
├ storage_time
├ storage_time
├ storage_time
├ storage_time
├ storage_time
├ storage_time
├ storage_time
├ storage_time
├ storage_time
├ storage_time
├ storage_time
├ storage_time
├ storage_time
├ storage_time
├ storage_time
├ storage_time
├ storage_time
├ storage_time
├ storage_time
├ storage_time
├ storage_time
├ storage_time
├ storage_time
├ storage_time
├ storage_time
├ storage_time
├ storage_time
├ storage_time
├ storage_time
├ storage_time
├ storage_time
├ storage_time
├ storage_time
├ storage_time
├ storage_time
├ storage_time
├ storage_time
├ storage_time
├ storage_time
├ storage_time

⊦ storage_time
⊦ storage_time
⊦ storage_time
⊦ storage_time
⊦ storage_time
⊦ storage_time
⊦ storage_time
⊦ storage_time
⊦ storage_time
⊦ storage_time
⊦ storage_time
⊦ storage_time
⊦ storage_time
⊦ storage_time
⊦ storage_time
⊦ storage_time
⊦ storage_time
⊦ storage_time
⊦ storage_time
⊦ storage_time
⊦ storage_time
⊦ storage_time
⊦ storage_time
⊦ storage_time
⊦ storage_time
⊦ storage_time
⊦ storage_time
⊦ storage_time
⊦ storage_time
⊦ storage_time
⊦ storage_time
⊦ storage_time
⊦ storage_time
⊦ storage_time
⊦ storage_time
⊦ storage_time
⊦ storage_time
⊦ storage_time
⊦ storage_time
⊦ storage_time
⊦ storage_time
⊦ storage_time
⊦ storage_time
⊦ storage_time
⊦ storage_time
⊦ storage_time
⊦ storage_time
⊦ storage_time

├ storage_time
├ storage_time
├ storage_time
├ storage_time
├ storage_time
├ storage_time
├ storage_time
├ storage_time
├ storage_time
├ storage_time
├ storage_time
├ storage_time
├ storage_time
├ storage_time
├ storage_time
├ storage_time
├ storage_time
├ storage_time
├ storage_time
├ storage_time
├ storage_time
├ storage_time
├ storage_time
├ storage_time
├ storage_time
├ storage_time
├ storage_time
├ storage_time
├ storage_time
├ storage_time
├ storage_time
├ storage_time
├ storage_time
├ storage_time
├ storage_time
├ storage_time
├ storage_time
├ storage_time
├ storage_time
├ storage_time
├ storage_time
├ storage_time
├ storage_time
├ storage_time
├ storage_time
├ storage_time
├ storage_time
├ storage_time

⊦ storage_time
⊦ storage_time
⊦ storage_time
⊦ storage_time
⊦ storage_time
⊦ storage_time
⊦ storage_time
⊦ storage_time
⊦ storage_time
⊦ storage_time
⊦ storage_time
⊦ storage_time
⊦ storage_time
⊦ storage_time
⊦ storage_time
⊦ storage_time
⊦ storage_time
⊦ storage_time
⊦ storage_time
⊦ storage_time
⊦ storage_time
⊦ storage_time
⊦ storage_time
⊦ storage_time
⊦ storage_time
⊦ storage_time
⊦ storage_time
⊦ storage_time
⊦ storage_time
⊦ storage_time
⊦ storage_time
⊦ storage_time
⊦ storage_time
⊦ storage_time
⊦ storage_time
⊦ storage_time
⊦ storage_time
⊦ storage_time
⊦ storage_time
⊦ storage_time
⊦ storage_time
⊦ storage_time
⊦ storage_time
⊦ storage_time
⊦ storage_time
⊦ storage_time
⊦ storage_time
⊦ storage_time

⊦ storage_time
⊦ storage_time
⊦ storage_time
⊦ storage_time
⊦ storage_time
⊦ storage_time
⊦ storage_time
⊦ storage_time
⊦ storage_time
⊦ storage_time
⊦ storage_time
⊦ storage_time
⊦ storage_time
⊦ storage_time
⊦ storage_time
⊦ storage_time
⊦ storage_time
⊦ storage_time
⊦ storage_time
⊦ storage_time
⊦ storage_time
⊦ storage_time
⊦ storage_time
⊦ storage_time
⊦ storage_time
⊦ storage_time
⊦ storage_time
⊦ storage_time
⊦ storage_time
⊦ storage_time
⊦ storage_time
⊦ storage_time
⊦ storage_time
⊦ storage_time
⊦ storage_time
⊦ storage_time
⊦ storage_time
⊦ storage_time
⊦ storage_time
⊦ storage_time
⊦ storage_time
⊦ storage_time
⊦ storage_time
⊦ storage_time
⊦ storage_time
⊦ storage_time
⊦ storage_time
⊦ storage_time

├ storage_time
├ storage_time
├ storage_time
├ storage_time
├ storage_time
├ storage_time
├ storage_time
├ storage_time
├ storage_time
├ storage_time
├ storage_time
├ storage_time
├ storage_time
├ storage_time
├ storage_time
├ storage_time
├ storage_time
├ storage_time
├ storage_time
├ storage_time
├ storage_time
├ storage_time
├ storage_time
├ storage_time
├ storage_time
├ storage_time
├ storage_time
├ storage_time
├ storage_time
├ storage_time
├ storage_time
├ storage_time
├ storage_time
├ storage_time
├ storage_time
├ storage_time
├ storage_time
├ storage_time
├ storage_time
├ storage_time
├ storage_time
├ storage_time
├ storage_time
├ storage_time
├ storage_time
├ storage_time
├ storage_time
├ storage_time

├ storage_time
├ storage_time
├ storage_time
├ storage_time
├ storage_time
├ storage_time
├ storage_time
├ storage_time
├ storage_time
├ storage_time
├ storage_time
├ storage_time
├ storage_time
├ storage_time
├ storage_time
├ storage_time
├ storage_time
├ storage_time
├ storage_time
├ storage_time
├ storage_time
├ storage_time
├ storage_time
├ storage_time
├ storage_time
├ storage_time
├ storage_time
├ storage_time
├ storage_time
├ storage_time
├ storage_time
├ storage_time
├ storage_time
├ storage_time
├ storage_time
├ storage_time
├ storage_time
├ storage_time
├ storage_time
├ storage_time
├ storage_time
├ storage_time
├ storage_time
├ storage_time
├ storage_time
├ storage_time
├ storage_time
├ storage_time

├ storage_time
├ storage_time
├ storage_time
├ storage_time
├ storage_time
├ storage_time
├ storage_time
├ storage_time
├ storage_time
├ storage_time
├ storage_time
├ storage_time
├ storage_time
├ storage_time
├ storage_time
├ storage_time
├ storage_time
├ storage_time
├ storage_time
├ storage_time
├ storage_time
├ storage_time
├ storage_time
├ storage_time
├ storage_time
├ storage_time
├ storage_time
├ storage_time
├ storage_time
├ storage_time
├ storage_time
├ storage_time
├ storage_time
├ storage_time
├ storage_time
├ storage_time
├ storage_time
├ storage_time
├ storage_time
├ storage_time
├ storage_time
├ storage_time
├ storage_time
├ storage_time
├ storage_time
├ storage_time
├ storage_time
├ storage_time

⊦ storage_time
⊦ storage_time
⊦ storage_time
⊦ storage_time
⊦ storage_time
⊦ storage_time
⊦ storage_time
⊦ storage_time
⊦ storage_time
⊦ storage_time
⊦ storage_time
⊦ storage_time
⊦ storage_time
⊦ storage_time
⊦ storage_time
⊦ storage_time
⊦ storage_time
⊦ storage_time
⊦ storage_time
⊦ storage_time
⊦ storage_time
⊦ storage_time
⊦ storage_time
⊦ storage_time
⊦ storage_time
⊦ storage_time
⊦ storage_time
⊦ storage_time
⊦ storage_time
⊦ storage_time
⊦ storage_time
⊦ storage_time
⊦ storage_time
⊦ storage_time
⊦ storage_time
⊦ storage_time
⊦ storage_time
⊦ storage_time
⊦ storage_time
⊦ storage_time
⊦ storage_time
⊦ storage_time
⊦ storage_time
⊦ storage_time
⊦ storage_time
⊦ storage_time
⊦ storage_time
⊦ storage_time

⊦ storage_time
⊦ storage_time
⊦ storage_time
⊦ storage_time
⊦ storage_time
⊦ storage_time
⊦ storage_time
⊦ storage_time
⊦ storage_time
⊦ storage_time
⊦ storage_time
⊦ storage_time
⊦ storage_time
⊦ storage_time
⊦ storage_time
⊦ storage_time
⊦ storage_time
⊦ storage_time
⊦ storage_time
⊦ storage_time
⊦ storage_time
⊦ storage_time
⊦ storage_time
⊦ storage_time
⊦ storage_time
⊦ storage_time
⊦ storage_time
⊦ storage_time
⊦ storage_time
⊦ storage_time
⊦ storage_time
⊦ storage_time
⊦ storage_time
⊦ storage_time
⊦ storage_time
⊦ storage_time
⊦ storage_time
⊦ storage_time
⊦ storage_time
⊦ storage_time
⊦ storage_time
⊦ storage_time
⊦ storage_time
⊦ storage_time
⊦ storage_time
⊦ storage_time
⊦ storage_time
⊦ storage_time

⊦ storage_time
⊦ storage_time
⊦ storage_time
⊦ storage_time
⊦ storage_time
⊦ storage_time
⊦ storage_time
⊦ storage_time
⊦ storage_time
⊦ storage_time
⊦ storage_time
⊦ storage_time
⊦ storage_time
⊦ storage_time
⊦ storage_time
⊦ storage_time
⊦ storage_time
⊦ storage_time
⊦ storage_time
⊦ storage_time
⊦ storage_time
⊦ storage_time
⊦ storage_time
⊦ storage_time
⊦ storage_time
⊦ storage_time
⊦ storage_time
⊦ storage_time
⊦ storage_time
⊦ storage_time
⊦ storage_time
⊦ storage_time
⊦ storage_time
⊦ storage_time
⊦ storage_time
⊦ storage_time
⊦ storage_time
⊦ storage_time
⊦ storage_time
⊦ storage_time
⊦ storage_time
⊦ storage_time
⊦ storage_time
⊦ storage_time
⊦ storage_time
⊦ storage_time
⊦ storage_time
⊦ storage_time

├ storage_time
├ storage_time
├ storage_time
├ storage_time
├ storage_time
├ storage_time
├ storage_time
├ storage_time
├ storage_time
├ storage_time
├ storage_time
├ storage_time
├ storage_time
├ storage_time
├ storage_time
├ storage_time
├ storage_time
├ storage_time
├ storage_time
├ storage_time
├ storage_time
├ storage_time
├ storage_time
├ storage_time
├ storage_time
├ storage_time
├ storage_time
├ storage_time
├ storage_time
├ storage_time
├ storage_time
├ storage_time
├ storage_time
├ storage_time
├ storage_time
├ storage_time
├ storage_time
├ storage_time
├ storage_time
├ storage_time
├ storage_time
├ storage_time
├ storage_time
├ storage_time
├ storage_time
├ storage_time
├ storage_time
├ storage_time

⊦ storage_time
⊦ storage_time
⊦ storage_time
⊦ storage_time
⊦ storage_time
⊦ storage_time
⊦ storage_time
⊦ storage_time
⊦ storage_time
⊦ storage_time
⊦ storage_time
⊦ storage_time
⊦ storage_time
⊦ storage_time
⊦ storage_time
⊦ storage_time
⊦ storage_time
⊦ storage_time
⊦ storage_time
⊦ storage_time
⊦ storage_time
⊦ storage_time
⊦ storage_time
⊦ storage_time
⊦ storage_time
⊦ storage_time
⊦ storage_time
⊦ storage_time
⊦ storage_time
⊦ storage_time
⊦ storage_time
⊦ storage_time
⊦ storage_time
⊦ storage_time
⊦ storage_time
⊦ storage_time
⊦ storage_time
⊦ storage_time
⊦ storage_time
⊦ storage_time
⊦ storage_time
⊦ storage_time
⊦ storage_time
⊦ storage_time
⊦ storage_time
⊦ storage_time
⊦ storage_time
⊦ storage_time

├ storage_time
├ storage_time
├ storage_time
├ storage_time
├ storage_time
├ storage_time
├ storage_time
├ storage_time
├ storage_time
├ storage_time
├ storage_time
├ storage_time
├ storage_time
├ storage_time
├ storage_time
├ storage_time
├ storage_time
├ storage_time
├ storage_time
├ storage_time
├ storage_time
├ storage_time
├ storage_time
├ storage_time
├ storage_time
├ storage_time
├ storage_time
├ storage_time
├ storage_time
├ storage_time
├ storage_time
├ storage_time
├ storage_time
├ storage_time
├ storage_time
├ storage_time
├ storage_time
├ storage_time
├ storage_time
├ storage_time
├ storage_time
├ storage_time
├ storage_time
├ storage_time
├ storage_time
├ storage_time
├ storage_time
├ storage_time

├ storage_time
├ storage_time
├ storage_time
├ storage_time
├ storage_time
├ storage_time
├ storage_time
├ storage_time
├ storage_time
├ storage_time
├ storage_time
├ storage_time
├ storage_time
├ storage_time
├ storage_time
├ storage_time
├ storage_time
├ storage_time
├ storage_time
├ storage_time
├ storage_time
├ storage_time
├ storage_time
├ storage_time
├ storage_time
├ storage_time
├ storage_time
├ storage_time
├ storage_time
├ storage_time
├ storage_time
├ storage_time
├ storage_time
├ storage_time
├ storage_time
├ storage_time
├ storage_time
├ storage_time
├ storage_time
├ storage_time
├ storage_time
├ storage_time
├ storage_time
├ storage_time
├ storage_time
├ storage_time
├ storage_time
├ storage_time

⊦ storage_time
⊦ storage_time
⊦ storage_time
⊦ storage_time
⊦ storage_time
⊦ storage_time
⊦ storage_time
⊦ storage_time
⊦ storage_time
⊦ storage_time
⊦ storage_time
⊦ storage_time
⊦ storage_time
⊦ storage_time
⊦ storage_time
⊦ storage_time
⊦ storage_time
⊦ storage_time
⊦ storage_time
⊦ storage_time
⊦ storage_time
⊦ storage_time
⊦ storage_time
⊦ storage_time
⊦ storage_time
⊦ storage_time
⊦ storage_time
⊦ storage_time
⊦ storage_time
⊦ storage_time
⊦ storage_time
⊦ storage_time
⊦ storage_time
⊦ storage_time
⊦ storage_time
⊦ storage_time
⊦ storage_time
⊦ storage_time
⊦ storage_time
⊦ storage_time
⊦ storage_time
⊦ storage_time
⊦ storage_time
⊦ storage_time
⊦ storage_time
⊦ storage_time
⊦ storage_time
⊦ storage_time

├ storage_time
├ storage_time
├ storage_time
├ storage_time
├ storage_time
├ storage_time
├ storage_time
├ storage_time
├ storage_time
├ storage_time
├ storage_time
├ storage_time
├ storage_time
├ storage_time
├ storage_time
├ storage_time
├ storage_time
├ storage_time
├ storage_time
├ storage_time
├ storage_time
├ storage_time
├ storage_time
├ storage_time
├ storage_time
├ storage_time
├ storage_time
├ storage_time
├ storage_time
├ storage_time
├ storage_time
├ storage_time
├ storage_time
├ storage_time
├ storage_time
├ storage_time
├ storage_time
├ storage_time
├ storage_time
├ storage_time
├ storage_time
├ storage_time
├ storage_time
├ storage_time
├ storage_time
├ storage_time
├ storage_time
├ storage_time

├ storage_time
├ storage_time
├ storage_time
├ storage_time
├ storage_time
├ storage_time
├ storage_time
├ storage_time
├ storage_time
├ storage_time
├ storage_time
├ storage_time
├ storage_time
├ storage_time
├ storage_time
├ storage_time
├ storage_time
├ storage_time
├ storage_time
├ storage_time
├ storage_time
├ storage_time
├ storage_time
├ storage_time
├ storage_time
├ storage_time
├ storage_time
├ storage_time
├ storage_time
├ storage_time
├ storage_time
├ storage_time
├ storage_time
├ storage_time
├ storage_time
├ storage_time
├ storage_time
├ storage_time
├ storage_time
├ storage_time
├ storage_time
├ storage_time
├ storage_time
├ storage_time
├ storage_time
├ storage_time
├ storage_time
├ storage_time

⊦ storage_time
⊦ storage_time
⊦ storage_time
⊦ storage_time
⊦ storage_time
⊦ storage_time
⊦ storage_time
⊦ storage_time
⊦ storage_time
⊦ storage_time
⊦ storage_time
⊦ storage_time
⊦ storage_time
⊦ storage_time
⊦ storage_time
⊦ storage_time
⊦ storage_time
⊦ storage_time
⊦ storage_time
⊦ storage_time
⊦ storage_time
⊦ storage_time
⊦ storage_time
⊦ storage_time
⊦ storage_time
⊦ storage_time
⊦ storage_time
⊦ storage_time
⊦ storage_time
⊦ storage_time
⊦ storage_time
⊦ storage_time
⊦ storage_time
⊦ storage_time
⊦ storage_time
⊦ storage_time
⊦ storage_time
⊦ storage_time
⊦ storage_time
⊦ storage_time
⊦ storage_time
⊦ storage_time
⊦ storage_time
⊦ storage_time
⊦ storage_time
⊦ storage_time
⊦ storage_time
⊦ storage_time

├ storage_time
├ storage_time
├ storage_time
├ storage_time
├ storage_time
├ storage_time
├ storage_time
├ storage_time
├ storage_time
├ storage_time
├ storage_time
├ storage_time
├ storage_time
├ storage_time
├ storage_time
├ storage_time
├ storage_time
├ storage_time
├ storage_time
├ storage_time
├ storage_time
├ storage_time
├ storage_time
├ storage_time
├ storage_time
├ storage_time
├ storage_time
├ storage_time
├ storage_time
├ storage_time
├ storage_time
├ storage_time
├ storage_time
├ storage_time
├ storage_time
├ storage_time
├ storage_time
├ storage_time
├ storage_time
├ storage_time
├ storage_time
├ storage_time
├ storage_time
├ storage_time
├ storage_time
├ storage_time
├ storage_time
├ storage_time

⊦ storage_time
⊦ storage_time
⊦ storage_time
⊦ storage_time
⊦ storage_time
⊦ storage_time
⊦ storage_time
⊦ storage_time
⊦ storage_time
⊦ storage_time
⊦ storage_time
⊦ storage_time
⊦ storage_time
⊦ storage_time
⊦ storage_time
⊦ storage_time
⊦ storage_time
⊦ storage_time
⊦ storage_time
⊦ storage_time
⊦ storage_time
⊦ storage_time
⊦ storage_time
⊦ storage_time
⊦ storage_time
⊦ storage_time
⊦ storage_time
⊦ storage_time
⊦ storage_time
⊦ storage_time
⊦ storage_time
⊦ storage_time
⊦ storage_time
⊦ storage_time
⊦ storage_time
⊦ storage_time
⊦ storage_time
⊦ storage_time
⊦ storage_time
⊦ storage_time
⊦ storage_time
⊦ storage_time
⊦ storage_time
⊦ storage_time
⊦ storage_time
⊦ storage_time
⊦ storage_time
⊦ storage_time

⊦ storage_time
⊦ storage_time
⊦ storage_time
⊦ storage_time
⊦ storage_time
⊦ storage_time
⊦ storage_time
⊦ storage_time
⊦ storage_time
⊦ storage_time
⊦ storage_time
⊦ storage_time
⊦ storage_time
⊦ storage_time
⊦ storage_time
⊦ storage_time
⊦ storage_time
⊦ storage_time
⊦ storage_time
⊦ storage_time
⊦ storage_time
⊦ storage_time
⊦ storage_time
⊦ storage_time
⊦ storage_time
⊦ storage_time
⊦ storage_time
⊦ storage_time
⊦ storage_time
⊦ storage_time
⊦ storage_time
⊦ storage_time
⊦ storage_time
⊦ storage_time
⊦ storage_time
⊦ storage_time
⊦ storage_time
⊦ storage_time
⊦ storage_time
⊦ storage_time
⊦ storage_time
⊦ storage_time
⊦ storage_time
⊦ storage_time
⊦ storage_time
⊦ storage_time
⊦ storage_time
⊦ storage_time

⊦ storage_time
⊦ storage_time
⊦ storage_time
⊦ storage_time
⊦ storage_time
⊦ storage_time
⊦ storage_time
⊦ storage_time
⊦ storage_time
⊦ storage_time
⊦ storage_time
⊦ storage_time
⊦ storage_time
⊦ storage_time
⊦ storage_time
⊦ storage_time
⊦ storage_time
⊦ storage_time
⊦ storage_time
⊦ storage_time
⊦ storage_time
⊦ storage_time
⊦ storage_time
⊦ storage_time
⊦ storage_time
⊦ storage_time
⊦ storage_time
⊦ storage_time
⊦ storage_time
⊦ storage_time
⊦ storage_time
⊦ storage_time
⊦ storage_time
⊦ storage_time
⊦ storage_time
⊦ storage_time
⊦ storage_time
⊦ storage_time
⊦ storage_time
⊦ storage_time
⊦ storage_time
⊦ storage_time
⊦ storage_time
⊦ storage_time
⊦ storage_time
⊦ storage_time
⊦ storage_time
⊦ storage_time

⊦ storage_time
⊦ storage_time
⊦ storage_time
⊦ storage_time
⊦ storage_time
⊦ storage_time
⊦ storage_time
⊦ storage_time
⊦ storage_time
⊦ storage_time
⊦ storage_time
⊦ storage_time
⊦ storage_time
⊦ storage_time
⊦ storage_time
⊦ storage_time
⊦ storage_time
⊦ storage_time
⊦ storage_time
⊦ storage_time
⊦ storage_time
⊦ storage_time
⊦ storage_time
⊦ storage_time
⊦ storage_time
⊦ storage_time
⊦ storage_time
⊦ storage_time
⊦ storage_time
⊦ storage_time
⊦ storage_time
⊦ storage_time
⊦ storage_time
⊦ storage_time
⊦ storage_time
⊦ storage_time
⊦ storage_time
⊦ storage_time
⊦ storage_time
⊦ storage_time
⊦ storage_time
⊦ storage_time
⊦ storage_time
⊦ storage_time
⊦ storage_time
⊦ storage_time
⊦ storage_time
⊦ storage_time

├ storage_time
├ storage_time
├ storage_time
├ storage_time
├ storage_time
├ storage_time
├ storage_time
├ storage_time
├ storage_time
├ storage_time
├ storage_time
├ storage_time
├ storage_time
├ storage_time
├ storage_time
├ storage_time
├ storage_time
├ storage_time
├ storage_time
├ storage_time
├ storage_time
├ storage_time
├ storage_time
├ storage_time
├ storage_time
├ storage_time
├ storage_time
├ storage_time
├ storage_time
├ storage_time
├ storage_time
├ storage_time
├ storage_time
├ storage_time
├ storage_time
├ storage_time
├ storage_time
├ storage_time
├ storage_time
├ storage_time
├ storage_time
├ storage_time
├ storage_time
├ storage_time
├ storage_time
├ storage_time
├ storage_time
├ storage_time

├ storage_time
├ storage_time
├ storage_time
├ storage_time
├ storage_time
├ storage_time
├ storage_time
├ storage_time
├ storage_time
├ storage_time
├ storage_time
├ storage_time
├ storage_time
├ storage_time
├ storage_time
├ storage_time
├ storage_time
├ storage_time
├ storage_time
├ storage_time
├ storage_time
├ storage_time
├ storage_time
├ storage_time
├ storage_time
├ storage_time
├ storage_time
├ storage_time
├ storage_time
├ storage_time
├ storage_time
├ storage_time
├ storage_time
├ storage_time
├ storage_time
├ storage_time
├ storage_time
├ storage_time
├ storage_time
├ storage_time
├ storage_time
├ storage_time
├ storage_time
├ storage_time
├ storage_time
├ storage_time
├ storage_time
├ storage_time

├ storage_time
├ storage_time
├ storage_time
├ storage_time
├ storage_time
├ storage_time
├ storage_time
├ storage_time
├ storage_time
├ storage_time
├ storage_time
├ storage_time
├ storage_time
├ storage_time
├ storage_time
├ storage_time
├ storage_time
├ storage_time
├ storage_time
├ storage_time
├ storage_time
├ storage_time
├ storage_time
├ storage_time
├ storage_time
├ storage_time
├ storage_time
├ storage_time
├ storage_time
├ storage_time
├ storage_time
├ storage_time
├ storage_time
├ storage_time
├ storage_time
├ storage_time
├ storage_time
├ storage_time
├ storage_time
├ storage_time
├ storage_time
├ storage_time
├ storage_time
├ storage_time
├ storage_time
├ storage_time
├ storage_time
├ storage_time

├ storage_time
├ storage_time
├ storage_time
├ storage_time
├ storage_time
├ storage_time
├ storage_time
├ storage_time
├ storage_time
├ storage_time
├ storage_time
├ storage_time
├ storage_time
├ storage_time
├ storage_time
├ storage_time
├ storage_time
├ storage_time
├ storage_time
├ storage_time
├ storage_time
├ storage_time
├ storage_time
├ storage_time
├ storage_time
├ storage_time
├ storage_time
├ storage_time
├ storage_time
├ storage_time
├ storage_time
├ storage_time
├ storage_time
├ storage_time
├ storage_time
├ storage_time
├ storage_time
├ storage_time
├ storage_time
├ storage_time
├ storage_time
├ storage_time
├ storage_time
├ storage_time
├ storage_time
├ storage_time
├ storage_time
├ storage_time

⊦ storage_time
⊦ storage_time
⊦ storage_time
⊦ storage_time
⊦ storage_time
⊦ storage_time
⊦ storage_time
⊦ storage_time
⊦ storage_time
⊦ storage_time
⊦ storage_time
⊦ storage_time
⊦ storage_time
⊦ storage_time
⊦ storage_time
⊦ storage_time
⊦ storage_time
⊦ storage_time
⊦ storage_time
⊦ storage_time
⊦ storage_time
⊦ storage_time
⊦ storage_time
⊦ storage_time
⊦ storage_time
⊦ storage_time
⊦ storage_time
⊦ storage_time
⊦ storage_time
⊦ storage_time
⊦ storage_time
⊦ storage_time
⊦ storage_time
⊦ storage_time
⊦ storage_time
⊦ storage_time
⊦ storage_time
⊦ storage_time
⊦ storage_time
⊦ storage_time
⊦ storage_time
⊦ storage_time
⊦ storage_time
⊦ storage_time
⊦ storage_time
⊦ storage_time
⊦ storage_time
⊦ storage_time

⊦ storage_time
⊦ storage_time
⊦ storage_time
⊦ storage_time
⊦ storage_time
⊦ storage_time
⊦ storage_time
⊦ storage_time
⊦ storage_time
⊦ storage_time
⊦ storage_time
⊦ storage_time
⊦ storage_time
⊦ storage_time
⊦ storage_time
⊦ storage_time
⊦ storage_time
⊦ storage_time
⊦ storage_time
⊦ storage_time
⊦ storage_time
⊦ storage_time
⊦ storage_time
⊦ storage_time
⊦ storage_time
⊦ storage_time
⊦ storage_time
⊦ storage_time
⊦ storage_time
⊦ storage_time
⊦ storage_time
⊦ storage_time
⊦ storage_time
⊦ storage_time
⊦ storage_time
⊦ storage_time
⊦ storage_time
⊦ storage_time
⊦ storage_time
⊦ storage_time
⊦ storage_time
⊦ storage_time
⊦ storage_time
⊦ storage_time
⊦ storage_time
⊦ storage_time
⊦ storage_time
⊦ storage_time

├ storage_time
├ storage_time
├ storage_time
├ storage_time
├ storage_time
├ storage_time
├ storage_time
├ storage_time
├ storage_time
├ storage_time
├ storage_time
├ storage_time
├ storage_time
├ storage_time
├ storage_time
├ storage_time
├ storage_time
├ storage_time
├ storage_time
├ storage_time
├ storage_time
├ storage_time
├ storage_time
├ storage_time
├ storage_time
├ storage_time
├ storage_time
├ storage_time
├ storage_time
├ storage_time
├ storage_time
├ storage_time
├ storage_time
├ storage_time
├ storage_time
├ storage_time
├ storage_time
├ storage_time
├ storage_time
├ storage_time
├ storage_time
├ storage_time
├ storage_time
├ storage_time
├ storage_time
├ storage_time
├ storage_time
├ storage_time

⊦ storage_time
⊦ storage_time
⊦ storage_time
⊦ storage_time
⊦ storage_time
⊦ storage_time
⊦ storage_time
⊦ storage_time
⊦ storage_time
⊦ storage_time
⊦ storage_time
⊦ storage_time
⊦ storage_time
⊦ storage_time
⊦ storage_time
⊦ storage_time
⊦ storage_time
⊦ storage_time
⊦ storage_time
⊦ storage_time
⊦ storage_time
⊦ storage_time
⊦ storage_time
⊦ storage_time
⊦ storage_time
⊦ storage_time
⊦ storage_time
⊦ storage_time
⊦ storage_time
⊦ storage_time
⊦ storage_time
⊦ storage_time
⊦ storage_time
⊦ storage_time
⊦ storage_time
⊦ storage_time
⊦ storage_time
⊦ storage_time
⊦ storage_time
⊦ storage_time
⊦ storage_time
⊦ storage_time
⊦ storage_time
