# Supplementary material for: A young child formula with Limosilactobacillus reuteri and GOS modulates gut microbiome and enhances bone and muscle development: a randomized trial
Source: Nat Commun. 2025 Dec 12;17:237. doi: 10.1038/s41467-025-66930-2 (PMC12783733; doi:10.1038/s41467-025-66930-2)
Supplement: Supplementary file 11 — Supplementary data 9 [file 41467_2025_66930_MOESM11_ESM.pdf]

Intervention	Visit	P-value	Correlation coefficient
Pooled	All	0,0001	0,342
Pooled	V1	0,0001	0,405
Pooled	V2	0,0001	0,296
Pooled	V3	0,0001	0,322
Experimental	All	0,0001	0,42
Experimental	V1	0,0001	0,462
Experimental	V2	0,0004	0,339
Experimental	V3	0,0001	0,469
Control	All	0,0001	0,323
Control	V1	0,0001	0,425
Control	V2	0,012	0,265
Control	V3	0,0007	0,335
Reference	All	0,0001	0,27
Reference	V1	0,0001	0,363
Reference	V2	0,0001	0,276
Reference	V3	0,0947	0,191
