# Supplementary material for: A young child formula with Limosilactobacillus reuteri and GOS modulates gut microbiome and enhances bone and muscle development: a randomized trial
Source: Nat Commun. 2025 Dec 12;17:237. doi: 10.1038/s41467-025-66930-2 (PMC12783733; doi:10.1038/s41467-025-66930-2)
Supplement: Supplementary file 12 — Supplementary data 10 [file 41467_2025_66930_MOESM12_ESM.pdf]

Category	Test type	Item	Feature
MGS	abundance	Hgn3C.0001 - Phocaeicola vulgatus	Tibia_length
MGS	abundance	Hgn3C.0001 - Phocaeicola vulgatus	Radius_length
MGS	abundance	Hgn3C.0001 - Phocaeicola vulgatus	Radius_SOS
MGS	abundance	Hgn3C.0001 - Phocaeicola vulgatus	Tibia_SOS
MGS	abundance	Hgn3C.0001 - Phocaeicola vulgatus	Handgrip
MGS	abundance	Hgn3C.0002 - Bacteroides uniformis	Tibia_length
MGS	abundance	Hgn3C.0002 - Bacteroides uniformis	Radius_length
MGS	abundance	Hgn3C.0002 - Bacteroides uniformis	Radius_SOS
MGS	abundance	Hgn3C.0002 - Bacteroides uniformis	Tibia_SOS
MGS	abundance	Hgn3C.0002 - Bacteroides uniformis	Handgrip
MGS	abundance	Hgn3C.0003 - Lachnospiraceae sp.	Tibia_length
MGS	abundance	Hgn3C.0003 - Lachnospiraceae sp.	Radius_length
MGS	abundance	Hgn3C.0003 - Lachnospiraceae sp.	Radius_SOS
MGS	abundance	Hgn3C.0003 - Lachnospiraceae sp.	Tibia_SOS
MGS	abundance	Hgn3C.0003 - Lachnospiraceae sp.	Handgrip
MGS	abundance	Hgn3C.0004 - Blautia wexlerae	Tibia_length
MGS	abundance	Hgn3C.0004 - Blautia wexlerae	Radius_length
MGS	abundance	Hgn3C.0004 - Blautia wexlerae	Radius_SOS
MGS	abundance	Hgn3C.0004 - Blautia wexlerae	Tibia_SOS
MGS	abundance	Hgn3C.0004 - Blautia wexlerae	Handgrip
MGS	abundance	Hgn3C.0005 - Fusicatenibacter saccharivorans	Tibia_length
MGS	abundance	Hgn3C.0005 - Fusicatenibacter saccharivorans	Radius_length
MGS	abundance	Hgn3C.0005 - Fusicatenibacter saccharivorans	Radius_SOS
MGS	abundance	Hgn3C.0005 - Fusicatenibacter saccharivorans	Tibia_SOS
MGS	abundance	Hgn3C.0005 - Fusicatenibacter saccharivorans	Handgrip
MGS	abundance	Hgn3C.0006 - Bacteroides ovatus	Tibia_length
MGS	abundance	Hgn3C.0006 - Bacteroides ovatus	Radius_length
MGS	abundance	Hgn3C.0006 - Bacteroides ovatus	Radius_SOS
MGS	abundance	Hgn3C.0006 - Bacteroides ovatus	Tibia_SOS
MGS	abundance	Hgn3C.0006 - Bacteroides ovatus	Handgrip
MGS	abundance	Hgn3C.0007 - Parabacteroides distasonis	Tibia_length
MGS	abundance	Hgn3C.0007 - Parabacteroides distasonis	Radius_length
MGS	abundance	Hgn3C.0007 - Parabacteroides distasonis	Radius_SOS
MGS	abundance	Hgn3C.0007 - Parabacteroides distasonis	Tibia_SOS
MGS	abundance	Hgn3C.0007 - Parabacteroides distasonis	Handgrip
MGS	abundance	Hgn3C.0008 - Alistipes putredinis	Tibia_length
MGS	abundance	Hgn3C.0008 - Alistipes putredinis	Radius_length
MGS	abundance	Hgn3C.0008 - Alistipes putredinis	Radius_SOS
MGS	abundance	Hgn3C.0008 - Alistipes putredinis	Tibia_SOS
MGS	abundance	Hgn3C.0008 - Alistipes putredinis	Handgrip
MGS	abundance	Hgn3C.0009 - Faecalibacterium longum CLA-AA-H243	Tibia_length
MGS	abundance	Hgn3C.0009 - Faecalibacterium longum CLA-AA-H243	Radius_length
MGS	abundance	Hgn3C.0009 - Faecalibacterium longum CLA-AA-H243	Radius_SOS
MGS	abundance	Hgn3C.0009 - Faecalibacterium longum CLA-AA-H243	Tibia_SOS
MGS	abundance	Hgn3C.0009 - Faecalibacterium longum CLA-AA-H243	Handgrip
MGS	abundance	Hgn3C.0010 - Anaerostipes hadrus	Tibia_length
MGS	abundance	Hgn3C.0010 - Anaerostipes hadrus	Radius_length

MGS	abundance	Hgn3C.0010 - Anaerostipes hadrus	Radius_SOS
MGS	abundance	Hgn3C.0010 - Anaerostipes hadrus	Tibia_SOS
MGS	abundance	Hgn3C.0010 - Anaerostipes hadrus	Handgrip
MGS	abundance	Hgn3C.0011 - Roseburia faecis	Tibia_length
MGS	abundance	Hgn3C.0011 - Roseburia faecis	Radius_length
MGS	abundance	Hgn3C.0011 - Roseburia faecis	Radius_SOS
MGS	abundance	Hgn3C.0011 - Roseburia faecis	Tibia_SOS
MGS	abundance	Hgn3C.0011 - Roseburia faecis	Handgrip
MGS	abundance	Hgn3C.0012 - Lachnospira sp.	Tibia_length
MGS	abundance	Hgn3C.0012 - Lachnospira sp.	Radius_length
MGS	abundance	Hgn3C.0012 - Lachnospira sp.	Radius_SOS
MGS	abundance	Hgn3C.0012 - Lachnospira sp.	Tibia_SOS
MGS	abundance	Hgn3C.0012 - Lachnospira sp.	Handgrip
MGS	abundance	Hgn3C.0013 - Ruminococcus bromii	Tibia_length
MGS	abundance	Hgn3C.0013 - Ruminococcus bromii	Radius_length
MGS	abundance	Hgn3C.0013 - Ruminococcus bromii	Radius_SOS
MGS	abundance	Hgn3C.0013 - Ruminococcus bromii	Tibia_SOS
MGS	abundance	Hgn3C.0013 - Ruminococcus bromii	Handgrip
MGS	abundance	Hgn3C.0014 - Lacrimispora saccharolytica	Tibia_length
MGS	abundance	Hgn3C.0014 - Lacrimispora saccharolytica	Radius_length
MGS	abundance	Hgn3C.0014 - Lacrimispora saccharolytica	Radius_SOS
MGS	abundance	Hgn3C.0014 - Lacrimispora saccharolytica	Tibia_SOS
MGS	abundance	Hgn3C.0014 - Lacrimispora saccharolytica	Handgrip
MGS	abundance	Hgn3C.0015 - Faecalibacterium sp. Marseille-Q4896	Tibia_length
MGS	abundance	Hgn3C.0015 - Faecalibacterium sp. Marseille-Q4896	Radius_length
MGS	abundance	Hgn3C.0015 - Faecalibacterium sp. Marseille-Q4896	Radius_SOS
MGS	abundance	Hgn3C.0015 - Faecalibacterium sp. Marseille-Q4896	Tibia_SOS
MGS	abundance	Hgn3C.0015 - Faecalibacterium sp. Marseille-Q4896	Handgrip
MGS	abundance	Hgn3C.0016 - Parabacteroides merdae	Tibia_length
MGS	abundance	Hgn3C.0016 - Parabacteroides merdae	Radius_length
MGS	abundance	Hgn3C.0016 - Parabacteroides merdae	Radius_SOS
MGS	abundance	Hgn3C.0016 - Parabacteroides merdae	Tibia_SOS
MGS	abundance	Hgn3C.0016 - Parabacteroides merdae	Handgrip
MGS	abundance	Hgn3C.0017 - Subdoligranulum sp. APC924/74	Tibia_length
MGS	abundance	Hgn3C.0017 - Subdoligranulum sp. APC924/74	Radius_length
MGS	abundance	Hgn3C.0017 - Subdoligranulum sp. APC924/74	Radius_SOS
MGS	abundance	Hgn3C.0017 - Subdoligranulum sp. APC924/74	Tibia_SOS
MGS	abundance	Hgn3C.0017 - Subdoligranulum sp. APC924/74	Handgrip
MGS	abundance	Hgn3C.0018 - Faecalibacterium prausnitzii	Tibia_length
MGS	abundance	Hgn3C.0018 - Faecalibacterium prausnitzii	Radius_length
MGS	abundance	Hgn3C.0018 - Faecalibacterium prausnitzii	Radius_SOS
MGS	abundance	Hgn3C.0018 - Faecalibacterium prausnitzii	Tibia_SOS
MGS	abundance	Hgn3C.0018 - Faecalibacterium prausnitzii	Handgrip
MGS	abundance	Hgn3C.0019 - Phocaeicola dorei	Tibia_length
MGS	abundance	Hgn3C.0019 - Phocaeicola dorei	Radius_length
MGS	abundance	Hgn3C.0019 - Phocaeicola dorei	Radius_SOS
MGS	abundance	Hgn3C.0019 - Phocaeicola dorei	Tibia_SOS
MGS	abundance	Hgn3C.0019 - Phocaeicola dorei	Handgrip

MGS	abundance	Hgn3C.0020 - Collinsella aerofaciens	Tibia_length
MGS	abundance	Hgn3C.0020 - Collinsella aerofaciens	Radius_length
MGS	abundance	Hgn3C.0020 - Collinsella aerofaciens	Radius_SOS
MGS	abundance	Hgn3C.0020 - Collinsella aerofaciens	Tibia_SOS
MGS	abundance	Hgn3C.0020 - Collinsella aerofaciens	Handgrip
MGS	abundance	Hgn3C.0021 - Bifidobacterium longum subsp. longum	Tibia_length
MGS	abundance	Hgn3C.0021 - Bifidobacterium longum subsp. longum	Radius_length
MGS	abundance	Hgn3C.0021 - Bifidobacterium longum subsp. longum	Radius_SOS
MGS	abundance	Hgn3C.0021 - Bifidobacterium longum subsp. longum	Tibia_SOS
MGS	abundance	Hgn3C.0021 - Bifidobacterium longum subsp. longum	Handgrip
MGS	abundance	Hgn3C.0022 - Alistipes shahii	Tibia_length
MGS	abundance	Hgn3C.0022 - Alistipes shahii	Radius_length
MGS	abundance	Hgn3C.0022 - Alistipes shahii	Radius_SOS
MGS	abundance	Hgn3C.0022 - Alistipes shahii	Tibia_SOS
MGS	abundance	Hgn3C.0022 - Alistipes shahii	Handgrip
MGS	abundance	Hgn3C.0023 - Bacteroides xylanisolvens	Tibia_length
MGS	abundance	Hgn3C.0023 - Bacteroides xylanisolvens	Radius_length
MGS	abundance	Hgn3C.0023 - Bacteroides xylanisolvens	Radius_SOS
MGS	abundance	Hgn3C.0023 - Bacteroides xylanisolvens	Tibia_SOS
MGS	abundance	Hgn3C.0023 - Bacteroides xylanisolvens	Handgrip
MGS	abundance	Hgn3C.0024 - Bacteroides caccae	Tibia_length
MGS	abundance	Hgn3C.0024 - Bacteroides caccae	Radius_length
MGS	abundance	Hgn3C.0024 - Bacteroides caccae	Radius_SOS
MGS	abundance	Hgn3C.0024 - Bacteroides caccae	Tibia_SOS
MGS	abundance	Hgn3C.0024 - Bacteroides caccae	Handgrip
MGS	abundance	Hgn3C.0025 - Ruminococcus bicirculans	Tibia_length
MGS	abundance	Hgn3C.0025 - Ruminococcus bicirculans	Radius_length
MGS	abundance	Hgn3C.0025 - Ruminococcus bicirculans	Radius_SOS
MGS	abundance	Hgn3C.0025 - Ruminococcus bicirculans	Tibia_SOS
MGS	abundance	Hgn3C.0025 - Ruminococcus bicirculans	Handgrip
MGS	abundance	Hgn3C.0026 - Dorea longicatena	Tibia_length
MGS	abundance	Hgn3C.0026 - Dorea longicatena	Radius_length
MGS	abundance	Hgn3C.0026 - Dorea longicatena	Radius_SOS
MGS	abundance	Hgn3C.0026 - Dorea longicatena	Tibia_SOS
MGS	abundance	Hgn3C.0026 - Dorea longicatena	Handgrip
MGS	abundance	Hgn3C.0027 - Barnesiella intestinihominis	Tibia_length
MGS	abundance	Hgn3C.0027 - Barnesiella intestinihominis	Radius_length
MGS	abundance	Hgn3C.0027 - Barnesiella intestinihominis	Radius_SOS
MGS	abundance	Hgn3C.0027 - Barnesiella intestinihominis	Tibia_SOS
MGS	abundance	Hgn3C.0027 - Barnesiella intestinihominis	Handgrip
MGS	abundance	Hgn3C.0028 - Oscillibacter sp. ER4	Tibia_length
MGS	abundance	Hgn3C.0028 - Oscillibacter sp. ER4	Radius_length
MGS	abundance	Hgn3C.0028 - Oscillibacter sp. ER4	Radius_SOS
MGS	abundance	Hgn3C.0028 - Oscillibacter sp. ER4	Tibia_SOS
MGS	abundance	Hgn3C.0028 - Oscillibacter sp. ER4	Handgrip
MGS	abundance	Hgn3C.0029 - Anaerobutyricum hallii	Tibia_length
MGS	abundance	Hgn3C.0029 - Anaerobutyricum hallii	Radius_length
MGS	abundance	Hgn3C.0029 - Anaerobutyricum hallii	Radius_SOS

MGS	abundance	Hgn3C.0029 - Anaerobutyricum hallii	Tibia_SOS
MGS	abundance	Hgn3C.0029 - Anaerobutyricum hallii	Handgrip
MGS	abundance	Hgn3C.0030 - Faecalibacterium prausnitzii	Tibia_length
MGS	abundance	Hgn3C.0030 - Faecalibacterium prausnitzii	Radius_length
MGS	abundance	Hgn3C.0030 - Faecalibacterium prausnitzii	Radius_SOS
MGS	abundance	Hgn3C.0030 - Faecalibacterium prausnitzii	Tibia_SOS
MGS	abundance	Hgn3C.0030 - Faecalibacterium prausnitzii	Handgrip
MGS	abundance	Hgn3C.0031 - Blautia massiliensis	Tibia_length
MGS	abundance	Hgn3C.0031 - Blautia massiliensis	Radius_length
MGS	abundance	Hgn3C.0031 - Blautia massiliensis	Radius_SOS
MGS	abundance	Hgn3C.0031 - Blautia massiliensis	Tibia_SOS
MGS	abundance	Hgn3C.0031 - Blautia massiliensis	Handgrip
MGS	abundance	Hgn3C.0032 - Alistipes onderdonkii subsp. vulgaris	Tibia_length
MGS	abundance	Hgn3C.0032 - Alistipes onderdonkii subsp. vulgaris	Radius_length
MGS	abundance	Hgn3C.0032 - Alistipes onderdonkii subsp. vulgaris	Radius_SOS
MGS	abundance	Hgn3C.0032 - Alistipes onderdonkii subsp. vulgaris	Tibia_SOS
MGS	abundance	Hgn3C.0032 - Alistipes onderdonkii subsp. vulgaris	Handgrip
MGS	abundance	Hgn3C.0033 - Gemmiger formicilis	Tibia_length
MGS	abundance	Hgn3C.0033 - Gemmiger formicilis	Radius_length
MGS	abundance	Hgn3C.0033 - Gemmiger formicilis	Radius_SOS
MGS	abundance	Hgn3C.0033 - Gemmiger formicilis	Tibia_SOS
MGS	abundance	Hgn3C.0033 - Gemmiger formicilis	Handgrip
MGS	abundance	Hgn3C.0034 - Roseburia inulinivorans	Tibia_length
MGS	abundance	Hgn3C.0034 - Roseburia inulinivorans	Radius_length
MGS	abundance	Hgn3C.0034 - Roseburia inulinivorans	Radius_SOS
MGS	abundance	Hgn3C.0034 - Roseburia inulinivorans	Tibia_SOS
MGS	abundance	Hgn3C.0034 - Roseburia inulinivorans	Handgrip
MGS	abundance	Hgn3C.0035 - Odoribacter splanchnicus	Tibia_length
MGS	abundance	Hgn3C.0035 - Odoribacter splanchnicus	Radius_length
MGS	abundance	Hgn3C.0035 - Odoribacter splanchnicus	Radius_SOS
MGS	abundance	Hgn3C.0035 - Odoribacter splanchnicus	Tibia_SOS
MGS	abundance	Hgn3C.0035 - Odoribacter splanchnicus	Handgrip
MGS	abundance	Hgn3C.0036 - Bacteroides thetaiotaomicron	Tibia_length
MGS	abundance	Hgn3C.0036 - Bacteroides thetaiotaomicron	Radius_length
MGS	abundance	Hgn3C.0036 - Bacteroides thetaiotaomicron	Radius_SOS
MGS	abundance	Hgn3C.0036 - Bacteroides thetaiotaomicron	Tibia_SOS
MGS	abundance	Hgn3C.0036 - Bacteroides thetaiotaomicron	Handgrip
MGS	abundance	Hgn3C.0037 - Roseburia intestinalis	Tibia_length
MGS	abundance	Hgn3C.0037 - Roseburia intestinalis	Radius_length
MGS	abundance	Hgn3C.0037 - Roseburia intestinalis	Radius_SOS
MGS	abundance	Hgn3C.0037 - Roseburia intestinalis	Tibia_SOS
MGS	abundance	Hgn3C.0037 - Roseburia intestinalis	Handgrip
MGS	abundance	Hgn3C.0038 - Bifidobacterium adolescentis	Tibia_length
MGS	abundance	Hgn3C.0038 - Bifidobacterium adolescentis	Radius_length
MGS	abundance	Hgn3C.0038 - Bifidobacterium adolescentis	Radius_SOS
MGS	abundance	Hgn3C.0038 - Bifidobacterium adolescentis	Tibia_SOS
MGS	abundance	Hgn3C.0038 - Bifidobacterium adolescentis	Handgrip
MGS	abundance	Hgn3C.0039 - Coprococcus comes	Tibia_length

MGS	abundance	Hgn3C.0039 - Coprococcus comes	Radius_length
MGS	abundance	Hgn3C.0039 - Coprococcus comes	Radius_SOS
MGS	abundance	Hgn3C.0039 - Coprococcus comes	Tibia_SOS
MGS	abundance	Hgn3C.0039 - Coprococcus comes	Handgrip
MGS	abundance	Hgn3C.0040 - Eubacteriales sp.	Tibia_length
MGS	abundance	Hgn3C.0040 - Eubacteriales sp.	Radius_length
MGS	abundance	Hgn3C.0040 - Eubacteriales sp.	Radius_SOS
MGS	abundance	Hgn3C.0040 - Eubacteriales sp.	Tibia_SOS
MGS	abundance	Hgn3C.0040 - Eubacteriales sp.	Handgrip
MGS	abundance	Hgn3C.0041 - Ruminococcus sp.	Tibia_length
MGS	abundance	Hgn3C.0041 - Ruminococcus sp.	Radius_length
MGS	abundance	Hgn3C.0041 - Ruminococcus sp.	Radius_SOS
MGS	abundance	Hgn3C.0041 - Ruminococcus sp.	Tibia_SOS
MGS	abundance	Hgn3C.0041 - Ruminococcus sp.	Handgrip
MGS	abundance	Hgn3C.0042 - Blautia faecis	Tibia_length
MGS	abundance	Hgn3C.0042 - Blautia faecis	Radius_length
MGS	abundance	Hgn3C.0042 - Blautia faecis	Radius_SOS
MGS	abundance	Hgn3C.0042 - Blautia faecis	Tibia_SOS
MGS	abundance	Hgn3C.0042 - Blautia faecis	Handgrip
MGS	abundance	Hgn3C.0043 - Lachnoclostridium sp. 210928-DFI.6.3	Tibia_length
MGS	abundance	Hgn3C.0043 - Lachnoclostridium sp. 210928-DFI.6.3	Radius_length
MGS	abundance	Hgn3C.0043 - Lachnoclostridium sp. 210928-DFI.6.3	Radius_SOS
MGS	abundance	Hgn3C.0043 - Lachnoclostridium sp. 210928-DFI.6.3	Tibia_SOS
MGS	abundance	Hgn3C.0043 - Lachnoclostridium sp. 210928-DFI.6.3	Handgrip
MGS	abundance	Hgn3C.0044 - Lachnospira pectinoschiza	Tibia_length
MGS	abundance	Hgn3C.0044 - Lachnospira pectinoschiza	Radius_length
MGS	abundance	Hgn3C.0044 - Lachnospira pectinoschiza	Radius_SOS
MGS	abundance	Hgn3C.0044 - Lachnospira pectinoschiza	Tibia_SOS
MGS	abundance	Hgn3C.0044 - Lachnospira pectinoschiza	Handgrip
MGS	abundance	Hgn3C.0045 - Faecalibacterium sp.	Tibia_length
MGS	abundance	Hgn3C.0045 - Faecalibacterium sp.	Radius_length
MGS	abundance	Hgn3C.0045 - Faecalibacterium sp.	Radius_SOS
MGS	abundance	Hgn3C.0045 - Faecalibacterium sp.	Tibia_SOS
MGS	abundance	Hgn3C.0045 - Faecalibacterium sp.	Handgrip
MGS	abundance	Hgn3C.0046 - Clostridium sp. AF37-5	Tibia_length
MGS	abundance	Hgn3C.0046 - Clostridium sp. AF37-5	Radius_length
MGS	abundance	Hgn3C.0046 - Clostridium sp. AF37-5	Radius_SOS
MGS	abundance	Hgn3C.0046 - Clostridium sp. AF37-5	Tibia_SOS
MGS	abundance	Hgn3C.0046 - Clostridium sp. AF37-5	Handgrip
MGS	abundance	Hgn3C.0047 - Dysosmobacter sp. BX15	Tibia_length
MGS	abundance	Hgn3C.0047 - Dysosmobacter sp. BX15	Radius_length
MGS	abundance	Hgn3C.0047 - Dysosmobacter sp. BX15	Radius_SOS
MGS	abundance	Hgn3C.0047 - Dysosmobacter sp. BX15	Tibia_SOS
MGS	abundance	Hgn3C.0047 - Dysosmobacter sp. BX15	Handgrip
MGS	abundance	Hgn3C.0048 - Akkermansia muciniphila	Tibia_length
MGS	abundance	Hgn3C.0048 - Akkermansia muciniphila	Radius_length
MGS	abundance	Hgn3C.0048 - Akkermansia muciniphila	Radius_SOS
MGS	abundance	Hgn3C.0048 - Akkermansia muciniphila	Tibia_SOS

MGS	abundance	Hgn3C.0048 - Akkermansia muciniphila	Handgrip
MGS	abundance	Hgn3C.0049 - Eubacteriales sp.	Tibia_length
MGS	abundance	Hgn3C.0049 - Eubacteriales sp.	Radius_length
MGS	abundance	Hgn3C.0049 - Eubacteriales sp.	Radius_SOS
MGS	abundance	Hgn3C.0049 - Eubacteriales sp.	Tibia_SOS
MGS	abundance	Hgn3C.0049 - Eubacteriales sp.	Handgrip
MGS	abundance	Hgn3C.0050 - Escherichia coli	Tibia_length
MGS	abundance	Hgn3C.0050 - Escherichia coli	Radius_length
MGS	abundance	Hgn3C.0050 - Escherichia coli	Radius_SOS
MGS	abundance	Hgn3C.0050 - Escherichia coli	Tibia_SOS
MGS	abundance	Hgn3C.0050 - Escherichia coli	Handgrip
MGS	abundance	Hgn3C.0051 - Alistipes communis	Tibia_length
MGS	abundance	Hgn3C.0051 - Alistipes communis	Radius_length
MGS	abundance	Hgn3C.0051 - Alistipes communis	Radius_SOS
MGS	abundance	Hgn3C.0051 - Alistipes communis	Tibia_SOS
MGS	abundance	Hgn3C.0051 - Alistipes communis	Handgrip
MGS	abundance	Hgn3C.0052 - Phocaeicola massiliensis	Tibia_length
MGS	abundance	Hgn3C.0052 - Phocaeicola massiliensis	Radius_length
MGS	abundance	Hgn3C.0052 - Phocaeicola massiliensis	Radius_SOS
MGS	abundance	Hgn3C.0052 - Phocaeicola massiliensis	Tibia_SOS
MGS	abundance	Hgn3C.0052 - Phocaeicola massiliensis	Handgrip
MGS	abundance	Hgn3C.0053 - Eubacteriales sp.	Tibia_length
MGS	abundance	Hgn3C.0053 - Eubacteriales sp.	Radius_length
MGS	abundance	Hgn3C.0053 - Eubacteriales sp.	Radius_SOS
MGS	abundance	Hgn3C.0053 - Eubacteriales sp.	Tibia_SOS
MGS	abundance	Hgn3C.0053 - Eubacteriales sp.	Handgrip
MGS	abundance	Hgn3C.0054 - Prevotella copri	Tibia_length
MGS	abundance	Hgn3C.0054 - Prevotella copri	Radius_length
MGS	abundance	Hgn3C.0054 - Prevotella copri	Radius_SOS
MGS	abundance	Hgn3C.0054 - Prevotella copri	Tibia_SOS
MGS	abundance	Hgn3C.0054 - Prevotella copri	Handgrip
MGS	abundance	Hgn3C.0055 - Alistipes finegoldii	Tibia_length
MGS	abundance	Hgn3C.0055 - Alistipes finegoldii	Radius_length
MGS	abundance	Hgn3C.0055 - Alistipes finegoldii	Radius_SOS
MGS	abundance	Hgn3C.0055 - Alistipes finegoldii	Tibia_SOS
MGS	abundance	Hgn3C.0055 - Alistipes finegoldii	Handgrip
MGS	abundance	Hgn3C.0056 - Bacteroides fragilis	Tibia_length
MGS	abundance	Hgn3C.0056 - Bacteroides fragilis	Radius_length
MGS	abundance	Hgn3C.0056 - Bacteroides fragilis	Radius_SOS
MGS	abundance	Hgn3C.0056 - Bacteroides fragilis	Tibia_SOS
MGS	abundance	Hgn3C.0056 - Bacteroides fragilis	Handgrip
MGS	abundance	Hgn3C.0057 - Oscillibacter sp. MSJ-31	Tibia_length
MGS	abundance	Hgn3C.0057 - Oscillibacter sp. MSJ-31	Radius_length
MGS	abundance	Hgn3C.0057 - Oscillibacter sp. MSJ-31	Radius_SOS
MGS	abundance	Hgn3C.0057 - Oscillibacter sp. MSJ-31	Tibia_SOS
MGS	abundance	Hgn3C.0057 - Oscillibacter sp. MSJ-31	Handgrip
MGS	abundance	Hgn3C.0058 - Lachnospiraceae sp.	Tibia_length
MGS	abundance	Hgn3C.0058 - Lachnospiraceae sp.	Radius_length

MGS	abundance	Hgn3C.0058 - Lachnospiraceae sp.	Radius_SOS
MGS	abundance	Hgn3C.0058 - Lachnospiraceae sp.	Tibia_SOS
MGS	abundance	Hgn3C.0058 - Lachnospiraceae sp.	Handgrip
MGS	abundance	Hgn3C.0059 - Bacteroides cellulosilyticus	Tibia_length
MGS	abundance	Hgn3C.0059 - Bacteroides cellulosilyticus	Radius_length
MGS	abundance	Hgn3C.0059 - Bacteroides cellulosilyticus	Radius_SOS
MGS	abundance	Hgn3C.0059 - Bacteroides cellulosilyticus	Tibia_SOS
MGS	abundance	Hgn3C.0059 - Bacteroides cellulosilyticus	Handgrip
MGS	abundance	Hgn3C.0060 - Eubacteriales sp.	Tibia_length
MGS	abundance	Hgn3C.0060 - Eubacteriales sp.	Radius_length
MGS	abundance	Hgn3C.0060 - Eubacteriales sp.	Radius_SOS
MGS	abundance	Hgn3C.0060 - Eubacteriales sp.	Tibia_SOS
MGS	abundance	Hgn3C.0060 - Eubacteriales sp.	Handgrip
MGS	abundance	Hgn3C.0061 - Faecalibacillus sp. TM498	Tibia_length
MGS	abundance	Hgn3C.0061 - Faecalibacillus sp. TM498	Radius_length
MGS	abundance	Hgn3C.0061 - Faecalibacillus sp. TM498	Radius_SOS
MGS	abundance	Hgn3C.0061 - Faecalibacillus sp. TM498	Tibia_SOS
MGS	abundance	Hgn3C.0061 - Faecalibacillus sp. TM498	Handgrip
MGS	abundance	Hgn3C.0062 - Waltera intestinalis	Tibia_length
MGS	abundance	Hgn3C.0062 - Waltera intestinalis	Radius_length
MGS	abundance	Hgn3C.0062 - Waltera intestinalis	Radius_SOS
MGS	abundance	Hgn3C.0062 - Waltera intestinalis	Tibia_SOS
MGS	abundance	Hgn3C.0062 - Waltera intestinalis	Handgrip
MGS	abundance	Hgn3C.0063 - Eubacteriales sp.	Tibia_length
MGS	abundance	Hgn3C.0063 - Eubacteriales sp.	Radius_length
MGS	abundance	Hgn3C.0063 - Eubacteriales sp.	Radius_SOS
MGS	abundance	Hgn3C.0063 - Eubacteriales sp.	Tibia_SOS
MGS	abundance	Hgn3C.0063 - Eubacteriales sp.	Handgrip
MGS	abundance	Hgn3C.0064 - Blautia sp. DFI.9.9	Tibia_length
MGS	abundance	Hgn3C.0064 - Blautia sp. DFI.9.9	Radius_length
MGS	abundance	Hgn3C.0064 - Blautia sp. DFI.9.9	Radius_SOS
MGS	abundance	Hgn3C.0064 - Blautia sp. DFI.9.9	Tibia_SOS
MGS	abundance	Hgn3C.0064 - Blautia sp. DFI.9.9	Handgrip
MGS	abundance	Hgn3C.0065 - Blautia obeum	Tibia_length
MGS	abundance	Hgn3C.0065 - Blautia obeum	Radius_length
MGS	abundance	Hgn3C.0065 - Blautia obeum	Radius_SOS
MGS	abundance	Hgn3C.0065 - Blautia obeum	Tibia_SOS
MGS	abundance	Hgn3C.0065 - Blautia obeum	Handgrip
MGS	abundance	Hgn3C.0066 - Oscillospiraceae sp.	Tibia_length
MGS	abundance	Hgn3C.0066 - Oscillospiraceae sp.	Radius_length
MGS	abundance	Hgn3C.0066 - Oscillospiraceae sp.	Radius_SOS
MGS	abundance	Hgn3C.0066 - Oscillospiraceae sp.	Tibia_SOS
MGS	abundance	Hgn3C.0066 - Oscillospiraceae sp.	Handgrip
MGS	abundance	Hgn3C.0067 - Roseburia hominis	Tibia_length
MGS	abundance	Hgn3C.0067 - Roseburia hominis	Radius_length
MGS	abundance	Hgn3C.0067 - Roseburia hominis	Radius_SOS
MGS	abundance	Hgn3C.0067 - Roseburia hominis	Tibia_SOS
MGS	abundance	Hgn3C.0067 - Roseburia hominis	Handgrip

MGS	abundance	Hgn3C.0068 - [Ruminococcus] lactaris	Tibia_length
MGS	abundance	Hgn3C.0068 - [Ruminococcus] lactaris	Radius_length
MGS	abundance	Hgn3C.0068 - [Ruminococcus] lactaris	Radius_SOS
MGS	abundance	Hgn3C.0068 - [Ruminococcus] lactaris	Tibia_SOS
MGS	abundance	Hgn3C.0068 - [Ruminococcus] lactaris	Handgrip
MGS	abundance	Hgn3C.0069 - Oscillibacter sp. KLE 1728	Tibia_length
MGS	abundance	Hgn3C.0069 - Oscillibacter sp. KLE 1728	Radius_length
MGS	abundance	Hgn3C.0069 - Oscillibacter sp. KLE 1728	Radius_SOS
MGS	abundance	Hgn3C.0069 - Oscillibacter sp. KLE 1728	Tibia_SOS
MGS	abundance	Hgn3C.0069 - Oscillibacter sp. KLE 1728	Handgrip
MGS	abundance	Hgn3C.0070 - Faecalibacterium sp. OF04-11AC	Tibia_length
MGS	abundance	Hgn3C.0070 - Faecalibacterium sp. OF04-11AC	Radius_length
MGS	abundance	Hgn3C.0070 - Faecalibacterium sp. OF04-11AC	Radius_SOS
MGS	abundance	Hgn3C.0070 - Faecalibacterium sp. OF04-11AC	Tibia_SOS
MGS	abundance	Hgn3C.0070 - Faecalibacterium sp. OF04-11AC	Handgrip
MGS	abundance	Hgn3C.0071 - Faecalicatena fissicatena	Tibia_length
MGS	abundance	Hgn3C.0071 - Faecalicatena fissicatena	Radius_length
MGS	abundance	Hgn3C.0071 - Faecalicatena fissicatena	Radius_SOS
MGS	abundance	Hgn3C.0071 - Faecalicatena fissicatena	Tibia_SOS
MGS	abundance	Hgn3C.0071 - Faecalicatena fissicatena	Handgrip
MGS	abundance	Hgn3C.0072 - Clostridium fessum	Tibia_length
MGS	abundance	Hgn3C.0072 - Clostridium fessum	Radius_length
MGS	abundance	Hgn3C.0072 - Clostridium fessum	Radius_SOS
MGS	abundance	Hgn3C.0072 - Clostridium fessum	Tibia_SOS
MGS	abundance	Hgn3C.0072 - Clostridium fessum	Handgrip
MGS	abundance	Hgn3C.0073 - Blautia sp.	Tibia_length
MGS	abundance	Hgn3C.0073 - Blautia sp.	Radius_length
MGS	abundance	Hgn3C.0073 - Blautia sp.	Radius_SOS
MGS	abundance	Hgn3C.0073 - Blautia sp.	Tibia_SOS
MGS	abundance	Hgn3C.0073 - Blautia sp.	Handgrip
MGS	abundance	Hgn3C.0074 - Clostridium sp. D43t1_170807_D5	Tibia_length
MGS	abundance	Hgn3C.0074 - Clostridium sp. D43t1_170807_D5	Radius_length
MGS	abundance	Hgn3C.0074 - Clostridium sp. D43t1_170807_D5	Radius_SOS
MGS	abundance	Hgn3C.0074 - Clostridium sp. D43t1_170807_D5	Tibia_SOS
MGS	abundance	Hgn3C.0074 - Clostridium sp. D43t1_170807_D5	Handgrip
MGS	abundance	Hgn3C.0075 - Agathobaculum butyriciproducens	Tibia_length
MGS	abundance	Hgn3C.0075 - Agathobaculum butyriciproducens	Radius_length
MGS	abundance	Hgn3C.0075 - Agathobaculum butyriciproducens	Radius_SOS
MGS	abundance	Hgn3C.0075 - Agathobaculum butyriciproducens	Tibia_SOS
MGS	abundance	Hgn3C.0075 - Agathobaculum butyriciproducens	Handgrip
MGS	abundance	Hgn3C.0076 - Lachnospira pectinoschiza	Tibia_length
MGS	abundance	Hgn3C.0076 - Lachnospira pectinoschiza	Radius_length
MGS	abundance	Hgn3C.0076 - Lachnospira pectinoschiza	Radius_SOS
MGS	abundance	Hgn3C.0076 - Lachnospira pectinoschiza	Tibia_SOS
MGS	abundance	Hgn3C.0076 - Lachnospira pectinoschiza	Handgrip
MGS	abundance	Hgn3C.0077 - Sutterella wadsworthensis	Tibia_length
MGS	abundance	Hgn3C.0077 - Sutterella wadsworthensis	Radius_length
MGS	abundance	Hgn3C.0077 - Sutterella wadsworthensis	Radius_SOS

MGS	abundance	Hgn3C.0077 - Sutterella wadsworthensis	Tibia_SOS
MGS	abundance	Hgn3C.0077 - Sutterella wadsworthensis	Handgrip
MGS	abundance	Hgn3C.0078 - Clostridium sp. MCC328	Tibia_length
MGS	abundance	Hgn3C.0078 - Clostridium sp. MCC328	Radius_length
MGS	abundance	Hgn3C.0078 - Clostridium sp. MCC328	Radius_SOS
MGS	abundance	Hgn3C.0078 - Clostridium sp. MCC328	Tibia_SOS
MGS	abundance	Hgn3C.0078 - Clostridium sp. MCC328	Handgrip
MGS	abundance	Hgn3C.0079 - [Ruminococcus] torques	Tibia_length
MGS	abundance	Hgn3C.0079 - [Ruminococcus] torques	Radius_length
MGS	abundance	Hgn3C.0079 - [Ruminococcus] torques	Radius_SOS
MGS	abundance	Hgn3C.0079 - [Ruminococcus] torques	Tibia_SOS
MGS	abundance	Hgn3C.0079 - [Ruminococcus] torques	Handgrip
MGS	abundance	Hgn3C.0080 - Phascolarctobacterium faecium	Tibia_length
MGS	abundance	Hgn3C.0080 - Phascolarctobacterium faecium	Radius_length
MGS	abundance	Hgn3C.0080 - Phascolarctobacterium faecium	Radius_SOS
MGS	abundance	Hgn3C.0080 - Phascolarctobacterium faecium	Tibia_SOS
MGS	abundance	Hgn3C.0080 - Phascolarctobacterium faecium	Handgrip
MGS	abundance	Hgn3C.0081 - Coprococcus eutactus	Tibia_length
MGS	abundance	Hgn3C.0081 - Coprococcus eutactus	Radius_length
MGS	abundance	Hgn3C.0081 - Coprococcus eutactus	Radius_SOS
MGS	abundance	Hgn3C.0081 - Coprococcus eutactus	Tibia_SOS
MGS	abundance	Hgn3C.0081 - Coprococcus eutactus	Handgrip
MGS	abundance	Hgn3C.0082 - Roseburia sp. CLA-AA-H204	Tibia_length
MGS	abundance	Hgn3C.0082 - Roseburia sp. CLA-AA-H204	Radius_length
MGS	abundance	Hgn3C.0082 - Roseburia sp. CLA-AA-H204	Radius_SOS
MGS	abundance	Hgn3C.0082 - Roseburia sp. CLA-AA-H204	Tibia_SOS
MGS	abundance	Hgn3C.0082 - Roseburia sp. CLA-AA-H204	Handgrip
MGS	abundance	Hgn3C.0083 - Eubacteriales sp.	Tibia_length
MGS	abundance	Hgn3C.0083 - Eubacteriales sp.	Radius_length
MGS	abundance	Hgn3C.0083 - Eubacteriales sp.	Radius_SOS
MGS	abundance	Hgn3C.0083 - Eubacteriales sp.	Tibia_SOS
MGS	abundance	Hgn3C.0083 - Eubacteriales sp.	Handgrip
MGS	abundance	Hgn3C.0084 - Flavonifractor plautii	Tibia_length
MGS	abundance	Hgn3C.0084 - Flavonifractor plautii	Radius_length
MGS	abundance	Hgn3C.0084 - Flavonifractor plautii	Radius_SOS
MGS	abundance	Hgn3C.0084 - Flavonifractor plautii	Tibia_SOS
MGS	abundance	Hgn3C.0084 - Flavonifractor plautii	Handgrip
MGS	abundance	Hgn3C.0085 - Faecalibacterium sp.	Tibia_length
MGS	abundance	Hgn3C.0085 - Faecalibacterium sp.	Radius_length
MGS	abundance	Hgn3C.0085 - Faecalibacterium sp.	Radius_SOS
MGS	abundance	Hgn3C.0085 - Faecalibacterium sp.	Tibia_SOS
MGS	abundance	Hgn3C.0085 - Faecalibacterium sp.	Handgrip
MGS	abundance	Hgn3C.0086 - Dialister invisus	Tibia_length
MGS	abundance	Hgn3C.0086 - Dialister invisus	Radius_length
MGS	abundance	Hgn3C.0086 - Dialister invisus	Radius_SOS
MGS	abundance	Hgn3C.0086 - Dialister invisus	Tibia_SOS
MGS	abundance	Hgn3C.0086 - Dialister invisus	Handgrip
MGS	abundance	Hgn3C.0087 - Eubacteriales sp.	Tibia_length

MGS	abundance	Hgn3C.0087 - Eubacteriales sp.	Radius_length
MGS	abundance	Hgn3C.0087 - Eubacteriales sp.	Radius_SOS
MGS	abundance	Hgn3C.0087 - Eubacteriales sp.	Tibia_SOS
MGS	abundance	Hgn3C.0087 - Eubacteriales sp.	Handgrip
MGS	abundance	Hgn3C.0088 - [Ruminococcus] gnavus	Tibia_length
MGS	abundance	Hgn3C.0088 - [Ruminococcus] gnavus	Radius_length
MGS	abundance	Hgn3C.0088 - [Ruminococcus] gnavus	Radius_SOS
MGS	abundance	Hgn3C.0088 - [Ruminococcus] gnavus	Tibia_SOS
MGS	abundance	Hgn3C.0088 - [Ruminococcus] gnavus	Handgrip
MGS	abundance	Hgn3C.0089 - Paraprevotella clara	Tibia_length
MGS	abundance	Hgn3C.0089 - Paraprevotella clara	Radius_length
MGS	abundance	Hgn3C.0089 - Paraprevotella clara	Radius_SOS
MGS	abundance	Hgn3C.0089 - Paraprevotella clara	Tibia_SOS
MGS	abundance	Hgn3C.0089 - Paraprevotella clara	Handgrip
MGS	abundance	Hgn3C.0091 - Lachnospira sp. NSJ-43	Tibia_length
MGS	abundance	Hgn3C.0091 - Lachnospira sp. NSJ-43	Radius_length
MGS	abundance	Hgn3C.0091 - Lachnospira sp. NSJ-43	Radius_SOS
MGS	abundance	Hgn3C.0091 - Lachnospira sp. NSJ-43	Tibia_SOS
MGS	abundance	Hgn3C.0091 - Lachnospira sp. NSJ-43	Handgrip
MGS	abundance	Hgn3C.0092 - Lachnospiraceae sp.	Tibia_length
MGS	abundance	Hgn3C.0092 - Lachnospiraceae sp.	Radius_length
MGS	abundance	Hgn3C.0092 - Lachnospiraceae sp.	Radius_SOS
MGS	abundance	Hgn3C.0092 - Lachnospiraceae sp.	Tibia_SOS
MGS	abundance	Hgn3C.0092 - Lachnospiraceae sp.	Handgrip
MGS	abundance	Hgn3C.0093 - Bilophila wadsworthia	Tibia_length
MGS	abundance	Hgn3C.0093 - Bilophila wadsworthia	Radius_length
MGS	abundance	Hgn3C.0093 - Bilophila wadsworthia	Radius_SOS
MGS	abundance	Hgn3C.0093 - Bilophila wadsworthia	Tibia_SOS
MGS	abundance	Hgn3C.0093 - Bilophila wadsworthia	Handgrip
MGS	abundance	Hgn3C.0094 - Eubacteriales sp.	Tibia_length
MGS	abundance	Hgn3C.0094 - Eubacteriales sp.	Radius_length
MGS	abundance	Hgn3C.0094 - Eubacteriales sp.	Radius_SOS
MGS	abundance	Hgn3C.0094 - Eubacteriales sp.	Tibia_SOS
MGS	abundance	Hgn3C.0094 - Eubacteriales sp.	Handgrip
MGS	abundance	Hgn3C.0095 - Eubacteriales sp.	Tibia_length
MGS	abundance	Hgn3C.0095 - Eubacteriales sp.	Radius_length
MGS	abundance	Hgn3C.0095 - Eubacteriales sp.	Radius_SOS
MGS	abundance	Hgn3C.0095 - Eubacteriales sp.	Tibia_SOS
MGS	abundance	Hgn3C.0095 - Eubacteriales sp.	Handgrip
MGS	abundance	Hgn3C.0096 - Eubacteriales sp.	Tibia_length
MGS	abundance	Hgn3C.0096 - Eubacteriales sp.	Radius_length
MGS	abundance	Hgn3C.0096 - Eubacteriales sp.	Radius_SOS
MGS	abundance	Hgn3C.0096 - Eubacteriales sp.	Tibia_SOS
MGS	abundance	Hgn3C.0096 - Eubacteriales sp.	Handgrip
MGS	abundance	Hgn3C.0097 - Eubacterium ventriosum	Tibia_length
MGS	abundance	Hgn3C.0097 - Eubacterium ventriosum	Radius_length
MGS	abundance	Hgn3C.0097 - Eubacterium ventriosum	Radius_SOS
MGS	abundance	Hgn3C.0097 - Eubacterium ventriosum	Tibia_SOS

MGS	abundance	Hgn3C.0097 - Eubacterium ventriosum	Handgrip
MGS	abundance	Hgn3C.0098 - Coprococcus catus	Tibia_length
MGS	abundance	Hgn3C.0098 - Coprococcus catus	Radius_length
MGS	abundance	Hgn3C.0098 - Coprococcus catus	Radius_SOS
MGS	abundance	Hgn3C.0098 - Coprococcus catus	Tibia_SOS
MGS	abundance	Hgn3C.0098 - Coprococcus catus	Handgrip
MGS	abundance	Hgn3C.0099 - Dorea longicatena	Tibia_length
MGS	abundance	Hgn3C.0099 - Dorea longicatena	Radius_length
MGS	abundance	Hgn3C.0099 - Dorea longicatena	Radius_SOS
MGS	abundance	Hgn3C.0099 - Dorea longicatena	Tibia_SOS
MGS	abundance	Hgn3C.0099 - Dorea longicatena	Handgrip
MGS	abundance	Hgn3C.0100 - Bifidobacterium bifidum	Tibia_length
MGS	abundance	Hgn3C.0100 - Bifidobacterium bifidum	Radius_length
MGS	abundance	Hgn3C.0100 - Bifidobacterium bifidum	Radius_SOS
MGS	abundance	Hgn3C.0100 - Bifidobacterium bifidum	Tibia_SOS
MGS	abundance	Hgn3C.0100 - Bifidobacterium bifidum	Handgrip
MGS	abundance	Hgn3C.0101 - Bifidobacterium pseudocatenulatum	Tibia_length
MGS	abundance	Hgn3C.0101 - Bifidobacterium pseudocatenulatum	Radius_length
MGS	abundance	Hgn3C.0101 - Bifidobacterium pseudocatenulatum	Radius_SOS
MGS	abundance	Hgn3C.0101 - Bifidobacterium pseudocatenulatum	Tibia_SOS
MGS	abundance	Hgn3C.0101 - Bifidobacterium pseudocatenulatum	Handgrip
MGS	abundance	Hgn3C.0102 - Eubacteriales sp.	Tibia_length
MGS	abundance	Hgn3C.0102 - Eubacteriales sp.	Radius_length
MGS	abundance	Hgn3C.0102 - Eubacteriales sp.	Radius_SOS
MGS	abundance	Hgn3C.0102 - Eubacteriales sp.	Tibia_SOS
MGS	abundance	Hgn3C.0102 - Eubacteriales sp.	Handgrip
MGS	abundance	Hgn3C.0103 - Holdemanella porci	Tibia_length
MGS	abundance	Hgn3C.0103 - Holdemanella porci	Radius_length
MGS	abundance	Hgn3C.0103 - Holdemanella porci	Radius_SOS
MGS	abundance	Hgn3C.0103 - Holdemanella porci	Tibia_SOS
MGS	abundance	Hgn3C.0103 - Holdemanella porci	Handgrip
MGS	abundance	Hgn3C.0104 - Clostridium sp. AM32-2	Tibia_length
MGS	abundance	Hgn3C.0104 - Clostridium sp. AM32-2	Radius_length
MGS	abundance	Hgn3C.0104 - Clostridium sp. AM32-2	Radius_SOS
MGS	abundance	Hgn3C.0104 - Clostridium sp. AM32-2	Tibia_SOS
MGS	abundance	Hgn3C.0104 - Clostridium sp. AM32-2	Handgrip
MGS	abundance	Hgn3C.0105 - Vescimonas coprocola	Tibia_length
MGS	abundance	Hgn3C.0105 - Vescimonas coprocola	Radius_length
MGS	abundance	Hgn3C.0105 - Vescimonas coprocola	Radius_SOS
MGS	abundance	Hgn3C.0105 - Vescimonas coprocola	Tibia_SOS
MGS	abundance	Hgn3C.0105 - Vescimonas coprocola	Handgrip
MGS	abundance	Hgn3C.0106 - Faecalibacterium sp. CLA-AA-H233	Tibia_length
MGS	abundance	Hgn3C.0106 - Faecalibacterium sp. CLA-AA-H233	Radius_length
MGS	abundance	Hgn3C.0106 - Faecalibacterium sp. CLA-AA-H233	Radius_SOS
MGS	abundance	Hgn3C.0106 - Faecalibacterium sp. CLA-AA-H233	Tibia_SOS
MGS	abundance	Hgn3C.0106 - Faecalibacterium sp. CLA-AA-H233	Handgrip
MGS	abundance	Hgn3C.0107 - Parasutterella excrementihominis	Tibia_length
MGS	abundance	Hgn3C.0107 - Parasutterella excrementihominis	Radius_length

MGS	abundance	Hgn3C.0107 - Parasutterella excrementihominis	Radius_SOS
MGS	abundance	Hgn3C.0107 - Parasutterella excrementihominis	Tibia_SOS
MGS	abundance	Hgn3C.0107 - Parasutterella excrementihominis	Handgrip
MGS	abundance	Hgn3C.0108 - Eubacteriales sp.	Tibia_length
MGS	abundance	Hgn3C.0108 - Eubacteriales sp.	Radius_length
MGS	abundance	Hgn3C.0108 - Eubacteriales sp.	Radius_SOS
MGS	abundance	Hgn3C.0108 - Eubacteriales sp.	Tibia_SOS
MGS	abundance	Hgn3C.0108 - Eubacteriales sp.	Handgrip
MGS	abundance	Hgn3C.0109 - Bacteroides eggerthii	Tibia_length
MGS	abundance	Hgn3C.0109 - Bacteroides eggerthii	Radius_length
MGS	abundance	Hgn3C.0109 - Bacteroides eggerthii	Radius_SOS
MGS	abundance	Hgn3C.0109 - Bacteroides eggerthii	Tibia_SOS
MGS	abundance	Hgn3C.0109 - Bacteroides eggerthii	Handgrip
MGS	abundance	Hgn3C.0110 - Eubacteriales sp.	Tibia_length
MGS	abundance	Hgn3C.0110 - Eubacteriales sp.	Radius_length
MGS	abundance	Hgn3C.0110 - Eubacteriales sp.	Radius_SOS
MGS	abundance	Hgn3C.0110 - Eubacteriales sp.	Tibia_SOS
MGS	abundance	Hgn3C.0110 - Eubacteriales sp.	Handgrip
MGS	abundance	Hgn3C.0111 - Eubacteriales sp.	Tibia_length
MGS	abundance	Hgn3C.0111 - Eubacteriales sp.	Radius_length
MGS	abundance	Hgn3C.0111 - Eubacteriales sp.	Radius_SOS
MGS	abundance	Hgn3C.0111 - Eubacteriales sp.	Tibia_SOS
MGS	abundance	Hgn3C.0111 - Eubacteriales sp.	Handgrip
MGS	abundance	Hgn3C.0113 - Clostridium sp. MCC345	Tibia_length
MGS	abundance	Hgn3C.0113 - Clostridium sp. MCC345	Radius_length
MGS	abundance	Hgn3C.0113 - Clostridium sp. MCC345	Radius_SOS
MGS	abundance	Hgn3C.0113 - Clostridium sp. MCC345	Tibia_SOS
MGS	abundance	Hgn3C.0113 - Clostridium sp. MCC345	Handgrip
MGS	abundance	Hgn3C.0114 - Eubacteriales sp.	Tibia_length
MGS	abundance	Hgn3C.0114 - Eubacteriales sp.	Radius_length
MGS	abundance	Hgn3C.0114 - Eubacteriales sp.	Radius_SOS
MGS	abundance	Hgn3C.0114 - Eubacteriales sp.	Tibia_SOS
MGS	abundance	Hgn3C.0114 - Eubacteriales sp.	Handgrip
MGS	abundance	Hgn3C.0115 - Eubacteriales sp.	Tibia_length
MGS	abundance	Hgn3C.0115 - Eubacteriales sp.	Radius_length
MGS	abundance	Hgn3C.0115 - Eubacteriales sp.	Radius_SOS
MGS	abundance	Hgn3C.0115 - Eubacteriales sp.	Tibia_SOS
MGS	abundance	Hgn3C.0115 - Eubacteriales sp.	Handgrip
MGS	abundance	Hgn3C.0116 - Anaerostipes hadrus	Tibia_length
MGS	abundance	Hgn3C.0116 - Anaerostipes hadrus	Radius_length
MGS	abundance	Hgn3C.0116 - Anaerostipes hadrus	Radius_SOS
MGS	abundance	Hgn3C.0116 - Anaerostipes hadrus	Tibia_SOS
MGS	abundance	Hgn3C.0116 - Anaerostipes hadrus	Handgrip
MGS	abundance	Hgn3C.0117 - Methanobrevibacter smithii	Tibia_length
MGS	abundance	Hgn3C.0117 - Methanobrevibacter smithii	Radius_length
MGS	abundance	Hgn3C.0117 - Methanobrevibacter smithii	Radius_SOS
MGS	abundance	Hgn3C.0117 - Methanobrevibacter smithii	Tibia_SOS
MGS	abundance	Hgn3C.0117 - Methanobrevibacter smithii	Handgrip

MGS	abundance	Hgn3C.0118 - Eubacteriales sp.	Tibia_length
MGS	abundance	Hgn3C.0118 - Eubacteriales sp.	Radius_length
MGS	abundance	Hgn3C.0118 - Eubacteriales sp.	Radius_SOS
MGS	abundance	Hgn3C.0118 - Eubacteriales sp.	Tibia_SOS
MGS	abundance	Hgn3C.0118 - Eubacteriales sp.	Handgrip
MGS	abundance	Hgn3C.0119 - Bacteroides faecis	Tibia_length
MGS	abundance	Hgn3C.0119 - Bacteroides faecis	Radius_length
MGS	abundance	Hgn3C.0119 - Bacteroides faecis	Radius_SOS
MGS	abundance	Hgn3C.0119 - Bacteroides faecis	Tibia_SOS
MGS	abundance	Hgn3C.0119 - Bacteroides faecis	Handgrip
MGS	abundance	Hgn3C.0120 - Flavonifractor plautii	Tibia_length
MGS	abundance	Hgn3C.0120 - Flavonifractor plautii	Radius_length
MGS	abundance	Hgn3C.0120 - Flavonifractor plautii	Radius_SOS
MGS	abundance	Hgn3C.0120 - Flavonifractor plautii	Tibia_SOS
MGS	abundance	Hgn3C.0120 - Flavonifractor plautii	Handgrip
MGS	abundance	Hgn3C.0121 - Oscillospiraceae sp.	Tibia_length
MGS	abundance	Hgn3C.0121 - Oscillospiraceae sp.	Radius_length
MGS	abundance	Hgn3C.0121 - Oscillospiraceae sp.	Radius_SOS
MGS	abundance	Hgn3C.0121 - Oscillospiraceae sp.	Tibia_SOS
MGS	abundance	Hgn3C.0121 - Oscillospiraceae sp.	Handgrip
MGS	abundance	Hgn3C.0122 - Bacteroides finegoldii	Tibia_length
MGS	abundance	Hgn3C.0122 - Bacteroides finegoldii	Radius_length
MGS	abundance	Hgn3C.0122 - Bacteroides finegoldii	Radius_SOS
MGS	abundance	Hgn3C.0122 - Bacteroides finegoldii	Tibia_SOS
MGS	abundance	Hgn3C.0122 - Bacteroides finegoldii	Handgrip
MGS	abundance	Hgn3C.0123 - Eubacteriales sp.	Tibia_length
MGS	abundance	Hgn3C.0123 - Eubacteriales sp.	Radius_length
MGS	abundance	Hgn3C.0123 - Eubacteriales sp.	Radius_SOS
MGS	abundance	Hgn3C.0123 - Eubacteriales sp.	Tibia_SOS
MGS	abundance	Hgn3C.0123 - Eubacteriales sp.	Handgrip
MGS	abundance	Hgn3C.0124 - Ruthenibacterium lactatiformans	Tibia_length
MGS	abundance	Hgn3C.0124 - Ruthenibacterium lactatiformans	Radius_length
MGS	abundance	Hgn3C.0124 - Ruthenibacterium lactatiformans	Radius_SOS
MGS	abundance	Hgn3C.0124 - Ruthenibacterium lactatiformans	Tibia_SOS
MGS	abundance	Hgn3C.0124 - Ruthenibacterium lactatiformans	Handgrip
MGS	abundance	Hgn3C.0125 - Sutterella sp. KLE1602	Tibia_length
MGS	abundance	Hgn3C.0125 - Sutterella sp. KLE1602	Radius_length
MGS	abundance	Hgn3C.0125 - Sutterella sp. KLE1602	Radius_SOS
MGS	abundance	Hgn3C.0125 - Sutterella sp. KLE1602	Tibia_SOS
MGS	abundance	Hgn3C.0125 - Sutterella sp. KLE1602	Handgrip
MGS	abundance	Hgn3C.0126 - Ruminococcus sp. BSD2780120874_150323_B10	Tibia_length
MGS	abundance	Hgn3C.0126 - Ruminococcus sp. BSD2780120874_150323_B10	Radius_length
MGS	abundance	Hgn3C.0126 - Ruminococcus sp. BSD2780120874_150323_B10	Radius_SOS
MGS	abundance	Hgn3C.0126 - Ruminococcus sp. BSD2780120874_150323_B10	Tibia_SOS
MGS	abundance	Hgn3C.0126 - Ruminococcus sp. BSD2780120874_150323_B10	Handgrip
MGS	abundance	Hgn3C.0127 - Faecalibacterium sp.	Tibia_length
MGS	abundance	Hgn3C.0127 - Faecalibacterium sp.	Radius_length
MGS	abundance	Hgn3C.0127 - Faecalibacterium sp.	Radius_SOS

MGS	abundance	Hgn3C.0127 - Faecalibacterium sp.	Tibia_SOS
MGS	abundance	Hgn3C.0127 - Faecalibacterium sp.	Handgrip
MGS	abundance	Hgn3C.0128 - Eubacteriales sp.	Tibia_length
MGS	abundance	Hgn3C.0128 - Eubacteriales sp.	Radius_length
MGS	abundance	Hgn3C.0128 - Eubacteriales sp.	Radius_SOS
MGS	abundance	Hgn3C.0128 - Eubacteriales sp.	Tibia_SOS
MGS	abundance	Hgn3C.0128 - Eubacteriales sp.	Handgrip
MGS	abundance	Hgn3C.0129 - Haemophilus parainfluenzae	Tibia_length
MGS	abundance	Hgn3C.0129 - Haemophilus parainfluenzae	Radius_length
MGS	abundance	Hgn3C.0129 - Haemophilus parainfluenzae	Radius_SOS
MGS	abundance	Hgn3C.0129 - Haemophilus parainfluenzae	Tibia_SOS
MGS	abundance	Hgn3C.0129 - Haemophilus parainfluenzae	Handgrip
MGS	abundance	Hgn3C.0130 - Phocaeicola plebeius	Tibia_length
MGS	abundance	Hgn3C.0130 - Phocaeicola plebeius	Radius_length
MGS	abundance	Hgn3C.0130 - Phocaeicola plebeius	Radius_SOS
MGS	abundance	Hgn3C.0130 - Phocaeicola plebeius	Tibia_SOS
MGS	abundance	Hgn3C.0130 - Phocaeicola plebeius	Handgrip
MGS	abundance	Hgn3C.0131 - Streptococcus thermophilus	Tibia_length
MGS	abundance	Hgn3C.0131 - Streptococcus thermophilus	Radius_length
MGS	abundance	Hgn3C.0131 - Streptococcus thermophilus	Radius_SOS
MGS	abundance	Hgn3C.0131 - Streptococcus thermophilus	Tibia_SOS
MGS	abundance	Hgn3C.0131 - Streptococcus thermophilus	Handgrip
MGS	abundance	Hgn3C.0132 - Eubacteriales sp.	Tibia_length
MGS	abundance	Hgn3C.0132 - Eubacteriales sp.	Radius_length
MGS	abundance	Hgn3C.0132 - Eubacteriales sp.	Radius_SOS
MGS	abundance	Hgn3C.0132 - Eubacteriales sp.	Tibia_SOS
MGS	abundance	Hgn3C.0132 - Eubacteriales sp.	Handgrip
MGS	abundance	Hgn3C.0134 - Eubacteriales sp.	Tibia_length
MGS	abundance	Hgn3C.0134 - Eubacteriales sp.	Radius_length
MGS	abundance	Hgn3C.0134 - Eubacteriales sp.	Radius_SOS
MGS	abundance	Hgn3C.0134 - Eubacteriales sp.	Tibia_SOS
MGS	abundance	Hgn3C.0134 - Eubacteriales sp.	Handgrip
MGS	abundance	Hgn3C.0135 - Firmicutes sp.	Tibia_length
MGS	abundance	Hgn3C.0135 - Firmicutes sp.	Radius_length
MGS	abundance	Hgn3C.0135 - Firmicutes sp.	Radius_SOS
MGS	abundance	Hgn3C.0135 - Firmicutes sp.	Tibia_SOS
MGS	abundance	Hgn3C.0135 - Firmicutes sp.	Handgrip
MGS	abundance	Hgn3C.0136 - Alistipes ihumii	Tibia_length
MGS	abundance	Hgn3C.0136 - Alistipes ihumii	Radius_length
MGS	abundance	Hgn3C.0136 - Alistipes ihumii	Radius_SOS
MGS	abundance	Hgn3C.0136 - Alistipes ihumii	Tibia_SOS
MGS	abundance	Hgn3C.0136 - Alistipes ihumii	Handgrip
MGS	abundance	Hgn3C.0137 - Clostridium sp. OF03-18AA	Tibia_length
MGS	abundance	Hgn3C.0137 - Clostridium sp. OF03-18AA	Radius_length
MGS	abundance	Hgn3C.0137 - Clostridium sp. OF03-18AA	Radius_SOS
MGS	abundance	Hgn3C.0137 - Clostridium sp. OF03-18AA	Tibia_SOS
MGS	abundance	Hgn3C.0137 - Clostridium sp. OF03-18AA	Handgrip
MGS	abundance	Hgn3C.0138 - Anaerobutyricum soehngenii	Tibia_length

MGS	abundance	Hgn3C.0138 - Anaerobutyricum soehngenii	Radius_length
MGS	abundance	Hgn3C.0138 - Anaerobutyricum soehngenii	Radius_SOS
MGS	abundance	Hgn3C.0138 - Anaerobutyricum soehngenii	Tibia_SOS
MGS	abundance	Hgn3C.0138 - Anaerobutyricum soehngenii	Handgrip
MGS	abundance	Hgn3C.0139 - Ruminococcus callidus	Tibia_length
MGS	abundance	Hgn3C.0139 - Ruminococcus callidus	Radius_length
MGS	abundance	Hgn3C.0139 - Ruminococcus callidus	Radius_SOS
MGS	abundance	Hgn3C.0139 - Ruminococcus callidus	Tibia_SOS
MGS	abundance	Hgn3C.0139 - Ruminococcus callidus	Handgrip
MGS	abundance	Hgn3C.0140 - Veillonella parvula	Tibia_length
MGS	abundance	Hgn3C.0140 - Veillonella parvula	Radius_length
MGS	abundance	Hgn3C.0140 - Veillonella parvula	Radius_SOS
MGS	abundance	Hgn3C.0140 - Veillonella parvula	Tibia_SOS
MGS	abundance	Hgn3C.0140 - Veillonella parvula	Handgrip
MGS	abundance	Hgn3C.0141 - Adlercreutzia equolifaciens subsp. celatus	Tibia_length
MGS	abundance	Hgn3C.0141 - Adlercreutzia equolifaciens subsp. celatus	Radius_length
MGS	abundance	Hgn3C.0141 - Adlercreutzia equolifaciens subsp. celatus	Radius_SOS
MGS	abundance	Hgn3C.0141 - Adlercreutzia equolifaciens subsp. celatus	Tibia_SOS
MGS	abundance	Hgn3C.0141 - Adlercreutzia equolifaciens subsp. celatus	Handgrip
MGS	abundance	Hgn3C.0142 - Eubacterium ramulus	Tibia_length
MGS	abundance	Hgn3C.0142 - Eubacterium ramulus	Radius_length
MGS	abundance	Hgn3C.0142 - Eubacterium ramulus	Radius_SOS
MGS	abundance	Hgn3C.0142 - Eubacterium ramulus	Tibia_SOS
MGS	abundance	Hgn3C.0142 - Eubacterium ramulus	Handgrip
MGS	abundance	Hgn3C.0143 - Streptococcus salivarius	Tibia_length
MGS	abundance	Hgn3C.0143 - Streptococcus salivarius	Radius_length
MGS	abundance	Hgn3C.0143 - Streptococcus salivarius	Radius_SOS
MGS	abundance	Hgn3C.0143 - Streptococcus salivarius	Tibia_SOS
MGS	abundance	Hgn3C.0143 - Streptococcus salivarius	Handgrip
MGS	abundance	Hgn3C.0144 - Eubacteriales sp.	Tibia_length
MGS	abundance	Hgn3C.0144 - Eubacteriales sp.	Radius_length
MGS	abundance	Hgn3C.0144 - Eubacteriales sp.	Radius_SOS
MGS	abundance	Hgn3C.0144 - Eubacteriales sp.	Tibia_SOS
MGS	abundance	Hgn3C.0144 - Eubacteriales sp.	Handgrip
MGS	abundance	Hgn3C.0146 - Phascolarctobacterium succinatutens	Tibia_length
MGS	abundance	Hgn3C.0146 - Phascolarctobacterium succinatutens	Radius_length
MGS	abundance	Hgn3C.0146 - Phascolarctobacterium succinatutens	Radius_SOS
MGS	abundance	Hgn3C.0146 - Phascolarctobacterium succinatutens	Tibia_SOS
MGS	abundance	Hgn3C.0146 - Phascolarctobacterium succinatutens	Handgrip
MGS	abundance	Hgn3C.0147 - Oscillospiraceae sp.	Tibia_length
MGS	abundance	Hgn3C.0147 - Oscillospiraceae sp.	Radius_length
MGS	abundance	Hgn3C.0147 - Oscillospiraceae sp.	Radius_SOS
MGS	abundance	Hgn3C.0147 - Oscillospiraceae sp.	Tibia_SOS
MGS	abundance	Hgn3C.0147 - Oscillospiraceae sp.	Handgrip
MGS	abundance	Hgn3C.0148 - Agathobaculum sp. M2	Tibia_length
MGS	abundance	Hgn3C.0148 - Agathobaculum sp. M2	Radius_length
MGS	abundance	Hgn3C.0148 - Agathobaculum sp. M2	Radius_SOS
MGS	abundance	Hgn3C.0148 - Agathobaculum sp. M2	Tibia_SOS

MGS	abundance	Hgn3C.0148 - Agathobaculum sp. M2	Handgrip
MGS	abundance	Hgn3C.0149 - Eggerthellales sp.	Tibia_length
MGS	abundance	Hgn3C.0149 - Eggerthellales sp.	Radius_length
MGS	abundance	Hgn3C.0149 - Eggerthellales sp.	Radius_SOS
MGS	abundance	Hgn3C.0149 - Eggerthellales sp.	Tibia_SOS
MGS	abundance	Hgn3C.0149 - Eggerthellales sp.	Handgrip
MGS	abundance	Hgn3C.0151 - Clostridium sp. AF34-13	Tibia_length
MGS	abundance	Hgn3C.0151 - Clostridium sp. AF34-13	Radius_length
MGS	abundance	Hgn3C.0151 - Clostridium sp. AF34-13	Radius_SOS
MGS	abundance	Hgn3C.0151 - Clostridium sp. AF34-13	Tibia_SOS
MGS	abundance	Hgn3C.0151 - Clostridium sp. AF34-13	Handgrip
MGS	abundance	Hgn3C.0152 - Bacteroides salyersiae	Tibia_length
MGS	abundance	Hgn3C.0152 - Bacteroides salyersiae	Radius_length
MGS	abundance	Hgn3C.0152 - Bacteroides salyersiae	Radius_SOS
MGS	abundance	Hgn3C.0152 - Bacteroides salyersiae	Tibia_SOS
MGS	abundance	Hgn3C.0152 - Bacteroides salyersiae	Handgrip
MGS	abundance	Hgn3C.0153 - Lachnospiraceae sp.	Tibia_length
MGS	abundance	Hgn3C.0153 - Lachnospiraceae sp.	Radius_length
MGS	abundance	Hgn3C.0153 - Lachnospiraceae sp.	Radius_SOS
MGS	abundance	Hgn3C.0153 - Lachnospiraceae sp.	Tibia_SOS
MGS	abundance	Hgn3C.0153 - Lachnospiraceae sp.	Handgrip
MGS	abundance	Hgn3C.0154 - Prevotella copri	Tibia_length
MGS	abundance	Hgn3C.0154 - Prevotella copri	Radius_length
MGS	abundance	Hgn3C.0154 - Prevotella copri	Radius_SOS
MGS	abundance	Hgn3C.0154 - Prevotella copri	Tibia_SOS
MGS	abundance	Hgn3C.0154 - Prevotella copri	Handgrip
MGS	abundance	Hgn3C.0155 - Blautia sp.	Tibia_length
MGS	abundance	Hgn3C.0155 - Blautia sp.	Radius_length
MGS	abundance	Hgn3C.0155 - Blautia sp.	Radius_SOS
MGS	abundance	Hgn3C.0155 - Blautia sp.	Tibia_SOS
MGS	abundance	Hgn3C.0155 - Blautia sp.	Handgrip
MGS	abundance	Hgn3C.0157 - Eubacteriales sp.	Tibia_length
MGS	abundance	Hgn3C.0157 - Eubacteriales sp.	Radius_length
MGS	abundance	Hgn3C.0157 - Eubacteriales sp.	Radius_SOS
MGS	abundance	Hgn3C.0157 - Eubacteriales sp.	Tibia_SOS
MGS	abundance	Hgn3C.0157 - Eubacteriales sp.	Handgrip
MGS	abundance	Hgn3C.0159 - Firmicutes sp.	Tibia_length
MGS	abundance	Hgn3C.0159 - Firmicutes sp.	Radius_length
MGS	abundance	Hgn3C.0159 - Firmicutes sp.	Radius_SOS
MGS	abundance	Hgn3C.0159 - Firmicutes sp.	Tibia_SOS
MGS	abundance	Hgn3C.0159 - Firmicutes sp.	Handgrip
MGS	abundance	Hgn3C.0161 - Blautia sp.	Tibia_length
MGS	abundance	Hgn3C.0161 - Blautia sp.	Radius_length
MGS	abundance	Hgn3C.0161 - Blautia sp.	Radius_SOS
MGS	abundance	Hgn3C.0161 - Blautia sp.	Tibia_SOS
MGS	abundance	Hgn3C.0161 - Blautia sp.	Handgrip
MGS	abundance	Hgn3C.0162 - Prevotella sp.	Tibia_length
MGS	abundance	Hgn3C.0162 - Prevotella sp.	Radius_length

MGS	abundance	Hgn3C.0162 - Prevotella sp.	Radius_SOS
MGS	abundance	Hgn3C.0162 - Prevotella sp.	Tibia_SOS
MGS	abundance	Hgn3C.0162 - Prevotella sp.	Handgrip
MGS	abundance	Hgn3C.0164 - Kineothrix sp.	Tibia_length
MGS	abundance	Hgn3C.0164 - Kineothrix sp.	Radius_length
MGS	abundance	Hgn3C.0164 - Kineothrix sp.	Radius_SOS
MGS	abundance	Hgn3C.0164 - Kineothrix sp.	Tibia_SOS
MGS	abundance	Hgn3C.0164 - Kineothrix sp.	Handgrip
MGS	abundance	Hgn3C.0166 - Eubacteriales sp.	Tibia_length
MGS	abundance	Hgn3C.0166 - Eubacteriales sp.	Radius_length
MGS	abundance	Hgn3C.0166 - Eubacteriales sp.	Radius_SOS
MGS	abundance	Hgn3C.0166 - Eubacteriales sp.	Tibia_SOS
MGS	abundance	Hgn3C.0166 - Eubacteriales sp.	Handgrip
MGS	abundance	Hgn3C.0167 - Lachnospiraceae sp.	Tibia_length
MGS	abundance	Hgn3C.0167 - Lachnospiraceae sp.	Radius_length
MGS	abundance	Hgn3C.0167 - Lachnospiraceae sp.	Radius_SOS
MGS	abundance	Hgn3C.0167 - Lachnospiraceae sp.	Tibia_SOS
MGS	abundance	Hgn3C.0167 - Lachnospiraceae sp.	Handgrip
MGS	abundance	Hgn3C.0168 - Evtepia gabavorous	Tibia_length
MGS	abundance	Hgn3C.0168 - Evtepia gabavorous	Radius_length
MGS	abundance	Hgn3C.0168 - Evtepia gabavorous	Radius_SOS
MGS	abundance	Hgn3C.0168 - Evtepia gabavorous	Tibia_SOS
MGS	abundance	Hgn3C.0168 - Evtepia gabavorous	Handgrip
MGS	abundance	Hgn3C.0169 - Bacteroides intestinalis	Tibia_length
MGS	abundance	Hgn3C.0169 - Bacteroides intestinalis	Radius_length
MGS	abundance	Hgn3C.0169 - Bacteroides intestinalis	Radius_SOS
MGS	abundance	Hgn3C.0169 - Bacteroides intestinalis	Tibia_SOS
MGS	abundance	Hgn3C.0169 - Bacteroides intestinalis	Handgrip
MGS	abundance	Hgn3C.0170 - Oscillospiraceae sp.	Tibia_length
MGS	abundance	Hgn3C.0170 - Oscillospiraceae sp.	Radius_length
MGS	abundance	Hgn3C.0170 - Oscillospiraceae sp.	Radius_SOS
MGS	abundance	Hgn3C.0170 - Oscillospiraceae sp.	Tibia_SOS
MGS	abundance	Hgn3C.0170 - Oscillospiraceae sp.	Handgrip
MGS	abundance	Hgn3C.0171 - Catenibacterium mitsuokai	Tibia_length
MGS	abundance	Hgn3C.0171 - Catenibacterium mitsuokai	Radius_length
MGS	abundance	Hgn3C.0171 - Catenibacterium mitsuokai	Radius_SOS
MGS	abundance	Hgn3C.0171 - Catenibacterium mitsuokai	Tibia_SOS
MGS	abundance	Hgn3C.0171 - Catenibacterium mitsuokai	Handgrip
MGS	abundance	Hgn3C.0174 - Lachnospiraceae sp.	Tibia_length
MGS	abundance	Hgn3C.0174 - Lachnospiraceae sp.	Radius_length
MGS	abundance	Hgn3C.0174 - Lachnospiraceae sp.	Radius_SOS
MGS	abundance	Hgn3C.0174 - Lachnospiraceae sp.	Tibia_SOS
MGS	abundance	Hgn3C.0174 - Lachnospiraceae sp.	Handgrip
MGS	abundance	Hgn3C.0175 - Oscillospiraceae sp.	Tibia_length
MGS	abundance	Hgn3C.0175 - Oscillospiraceae sp.	Radius_length
MGS	abundance	Hgn3C.0175 - Oscillospiraceae sp.	Radius_SOS
MGS	abundance	Hgn3C.0175 - Oscillospiraceae sp.	Tibia_SOS
MGS	abundance	Hgn3C.0175 - Oscillospiraceae sp.	Handgrip

MGS	abundance	Hgn3C.0176 - Alistipes indistinctus	Tibia_length
MGS	abundance	Hgn3C.0176 - Alistipes indistinctus	Radius_length
MGS	abundance	Hgn3C.0176 - Alistipes indistinctus	Radius_SOS
MGS	abundance	Hgn3C.0176 - Alistipes indistinctus	Tibia_SOS
MGS	abundance	Hgn3C.0176 - Alistipes indistinctus	Handgrip
MGS	abundance	Hgn3C.0178 - Clostridia sp.	Tibia_length
MGS	abundance	Hgn3C.0178 - Clostridia sp.	Radius_length
MGS	abundance	Hgn3C.0178 - Clostridia sp.	Radius_SOS
MGS	abundance	Hgn3C.0178 - Clostridia sp.	Tibia_SOS
MGS	abundance	Hgn3C.0178 - Clostridia sp.	Handgrip
MGS	abundance	Hgn3C.0179 - Bacteroidales sp.	Tibia_length
MGS	abundance	Hgn3C.0179 - Bacteroidales sp.	Radius_length
MGS	abundance	Hgn3C.0179 - Bacteroidales sp.	Radius_SOS
MGS	abundance	Hgn3C.0179 - Bacteroidales sp.	Tibia_SOS
MGS	abundance	Hgn3C.0179 - Bacteroidales sp.	Handgrip
MGS	abundance	Hgn3C.0180 - Bacteria sp.	Tibia_length
MGS	abundance	Hgn3C.0180 - Bacteria sp.	Radius_length
MGS	abundance	Hgn3C.0180 - Bacteria sp.	Radius_SOS
MGS	abundance	Hgn3C.0180 - Bacteria sp.	Tibia_SOS
MGS	abundance	Hgn3C.0180 - Bacteria sp.	Handgrip
MGS	abundance	Hgn3C.0181 - Alistipes onderdonkii	Tibia_length
MGS	abundance	Hgn3C.0181 - Alistipes onderdonkii	Radius_length
MGS	abundance	Hgn3C.0181 - Alistipes onderdonkii	Radius_SOS
MGS	abundance	Hgn3C.0181 - Alistipes onderdonkii	Tibia_SOS
MGS	abundance	Hgn3C.0181 - Alistipes onderdonkii	Handgrip
MGS	abundance	Hgn3C.0182 - Butyricimonas faecihominis	Tibia_length
MGS	abundance	Hgn3C.0182 - Butyricimonas faecihominis	Radius_length
MGS	abundance	Hgn3C.0182 - Butyricimonas faecihominis	Radius_SOS
MGS	abundance	Hgn3C.0182 - Butyricimonas faecihominis	Tibia_SOS
MGS	abundance	Hgn3C.0182 - Butyricimonas faecihominis	Handgrip
MGS	abundance	Hgn3C.0183 - Intestinibacter bartlettii	Tibia_length
MGS	abundance	Hgn3C.0183 - Intestinibacter bartlettii	Radius_length
MGS	abundance	Hgn3C.0183 - Intestinibacter bartlettii	Radius_SOS
MGS	abundance	Hgn3C.0183 - Intestinibacter bartlettii	Tibia_SOS
MGS	abundance	Hgn3C.0183 - Intestinibacter bartlettii	Handgrip
MGS	abundance	Hgn3C.0184 - Eubacteriales sp.	Tibia_length
MGS	abundance	Hgn3C.0184 - Eubacteriales sp.	Radius_length
MGS	abundance	Hgn3C.0184 - Eubacteriales sp.	Radius_SOS
MGS	abundance	Hgn3C.0184 - Eubacteriales sp.	Tibia_SOS
MGS	abundance	Hgn3C.0184 - Eubacteriales sp.	Handgrip
MGS	abundance	Hgn3C.0185 - Bifidobacterium catenulatum subsp. kashiwanohense	Tibia_length
MGS	abundance	Hgn3C.0185 - Bifidobacterium catenulatum subsp. kashiwanohense	Radius_length
MGS	abundance	Hgn3C.0185 - Bifidobacterium catenulatum subsp. kashiwanohense	Radius_SOS
MGS	abundance	Hgn3C.0185 - Bifidobacterium catenulatum subsp. kashiwanohense	Tibia_SOS
MGS	abundance	Hgn3C.0185 - Bifidobacterium catenulatum subsp. kashiwanohense	Handgrip
MGS	abundance	Hgn3C.0186 - Butyricimonas virosa	Tibia_length
MGS	abundance	Hgn3C.0186 - Butyricimonas virosa	Radius_length
MGS	abundance	Hgn3C.0186 - Butyricimonas virosa	Radius_SOS

MGS	abundance	Hgn3C.0186 - Butyricimonas virosa	Tibia_SOS
MGS	abundance	Hgn3C.0186 - Butyricimonas virosa	Handgrip
MGS	abundance	Hgn3C.0191 - Prevotella sp.	Tibia_length
MGS	abundance	Hgn3C.0191 - Prevotella sp.	Radius_length
MGS	abundance	Hgn3C.0191 - Prevotella sp.	Radius_SOS
MGS	abundance	Hgn3C.0191 - Prevotella sp.	Tibia_SOS
MGS	abundance	Hgn3C.0191 - Prevotella sp.	Handgrip
MGS	abundance	Hgn3C.0192 - Erysipelatoclostridium ramosum	Tibia_length
MGS	abundance	Hgn3C.0192 - Erysipelatoclostridium ramosum	Radius_length
MGS	abundance	Hgn3C.0192 - Erysipelatoclostridium ramosum	Radius_SOS
MGS	abundance	Hgn3C.0192 - Erysipelatoclostridium ramosum	Tibia_SOS
MGS	abundance	Hgn3C.0192 - Erysipelatoclostridium ramosum	Handgrip
MGS	abundance	Hgn3C.0193 - Eubacteriales sp.	Tibia_length
MGS	abundance	Hgn3C.0193 - Eubacteriales sp.	Radius_length
MGS	abundance	Hgn3C.0193 - Eubacteriales sp.	Radius_SOS
MGS	abundance	Hgn3C.0193 - Eubacteriales sp.	Tibia_SOS
MGS	abundance	Hgn3C.0193 - Eubacteriales sp.	Handgrip
MGS	abundance	Hgn3C.0194 - Faecalibacillus faecis	Tibia_length
MGS	abundance	Hgn3C.0194 - Faecalibacillus faecis	Radius_length
MGS	abundance	Hgn3C.0194 - Faecalibacillus faecis	Radius_SOS
MGS	abundance	Hgn3C.0194 - Faecalibacillus faecis	Tibia_SOS
MGS	abundance	Hgn3C.0194 - Faecalibacillus faecis	Handgrip
MGS	abundance	Hgn3C.0195 - Eubacteriales sp.	Tibia_length
MGS	abundance	Hgn3C.0195 - Eubacteriales sp.	Radius_length
MGS	abundance	Hgn3C.0195 - Eubacteriales sp.	Radius_SOS
MGS	abundance	Hgn3C.0195 - Eubacteriales sp.	Tibia_SOS
MGS	abundance	Hgn3C.0195 - Eubacteriales sp.	Handgrip
MGS	abundance	Hgn3C.0196 - Phocaeicola coprocola	Tibia_length
MGS	abundance	Hgn3C.0196 - Phocaeicola coprocola	Radius_length
MGS	abundance	Hgn3C.0196 - Phocaeicola coprocola	Radius_SOS
MGS	abundance	Hgn3C.0196 - Phocaeicola coprocola	Tibia_SOS
MGS	abundance	Hgn3C.0196 - Phocaeicola coprocola	Handgrip
MGS	abundance	Hgn3C.0197 - Bacteroides clarus	Tibia_length
MGS	abundance	Hgn3C.0197 - Bacteroides clarus	Radius_length
MGS	abundance	Hgn3C.0197 - Bacteroides clarus	Radius_SOS
MGS	abundance	Hgn3C.0197 - Bacteroides clarus	Tibia_SOS
MGS	abundance	Hgn3C.0197 - Bacteroides clarus	Handgrip
MGS	abundance	Hgn3C.0199 - Bacteroidales sp.	Tibia_length
MGS	abundance	Hgn3C.0199 - Bacteroidales sp.	Radius_length
MGS	abundance	Hgn3C.0199 - Bacteroidales sp.	Radius_SOS
MGS	abundance	Hgn3C.0199 - Bacteroidales sp.	Tibia_SOS
MGS	abundance	Hgn3C.0199 - Bacteroidales sp.	Handgrip
MGS	abundance	Hgn3C.0201 - Veillonella dispar	Tibia_length
MGS	abundance	Hgn3C.0201 - Veillonella dispar	Radius_length
MGS	abundance	Hgn3C.0201 - Veillonella dispar	Radius_SOS
MGS	abundance	Hgn3C.0201 - Veillonella dispar	Tibia_SOS
MGS	abundance	Hgn3C.0201 - Veillonella dispar	Handgrip
MGS	abundance	Hgn3C.0202 - Eggerthella lenta	Tibia_length

MGS	abundance	Hgn3C.0202 - Eggerthella lenta	Radius_length
MGS	abundance	Hgn3C.0202 - Eggerthella lenta	Radius_SOS
MGS	abundance	Hgn3C.0202 - Eggerthella lenta	Tibia_SOS
MGS	abundance	Hgn3C.0202 - Eggerthella lenta	Handgrip
MGS	abundance	Hgn3C.0203 - Faecalibacterium sp. Marseille-Q3530	Tibia_length
MGS	abundance	Hgn3C.0203 - Faecalibacterium sp. Marseille-Q3530	Radius_length
MGS	abundance	Hgn3C.0203 - Faecalibacterium sp. Marseille-Q3530	Radius_SOS
MGS	abundance	Hgn3C.0203 - Faecalibacterium sp. Marseille-Q3530	Tibia_SOS
MGS	abundance	Hgn3C.0203 - Faecalibacterium sp. Marseille-Q3530	Handgrip
MGS	abundance	Hgn3C.0204 - Coprobacter fastidiosus	Tibia_length
MGS	abundance	Hgn3C.0204 - Coprobacter fastidiosus	Radius_length
MGS	abundance	Hgn3C.0204 - Coprobacter fastidiosus	Radius_SOS
MGS	abundance	Hgn3C.0204 - Coprobacter fastidiosus	Tibia_SOS
MGS	abundance	Hgn3C.0204 - Coprobacter fastidiosus	Handgrip
MGS	abundance	Hgn3C.0206 - Gemmiger formicilis	Tibia_length
MGS	abundance	Hgn3C.0206 - Gemmiger formicilis	Radius_length
MGS	abundance	Hgn3C.0206 - Gemmiger formicilis	Radius_SOS
MGS	abundance	Hgn3C.0206 - Gemmiger formicilis	Tibia_SOS
MGS	abundance	Hgn3C.0206 - Gemmiger formicilis	Handgrip
MGS	abundance	Hgn3C.0207 - Eubacteriales sp.	Tibia_length
MGS	abundance	Hgn3C.0207 - Eubacteriales sp.	Radius_length
MGS	abundance	Hgn3C.0207 - Eubacteriales sp.	Radius_SOS
MGS	abundance	Hgn3C.0207 - Eubacteriales sp.	Tibia_SOS
MGS	abundance	Hgn3C.0207 - Eubacteriales sp.	Handgrip
MGS	abundance	Hgn3C.0208 - Romboutsia timonensis	Tibia_length
MGS	abundance	Hgn3C.0208 - Romboutsia timonensis	Radius_length
MGS	abundance	Hgn3C.0208 - Romboutsia timonensis	Radius_SOS
MGS	abundance	Hgn3C.0208 - Romboutsia timonensis	Tibia_SOS
MGS	abundance	Hgn3C.0208 - Romboutsia timonensis	Handgrip
MGS	abundance	Hgn3C.0209 - Bifidobacterium breve	Tibia_length
MGS	abundance	Hgn3C.0209 - Bifidobacterium breve	Radius_length
MGS	abundance	Hgn3C.0209 - Bifidobacterium breve	Radius_SOS
MGS	abundance	Hgn3C.0209 - Bifidobacterium breve	Tibia_SOS
MGS	abundance	Hgn3C.0209 - Bifidobacterium breve	Handgrip
MGS	abundance	Hgn3C.0211 - Eubacteriales sp.	Tibia_length
MGS	abundance	Hgn3C.0211 - Eubacteriales sp.	Radius_length
MGS	abundance	Hgn3C.0211 - Eubacteriales sp.	Radius_SOS
MGS	abundance	Hgn3C.0211 - Eubacteriales sp.	Tibia_SOS
MGS	abundance	Hgn3C.0211 - Eubacteriales sp.	Handgrip
MGS	abundance	Hgn3C.0212 - Holdemanella biformis	Tibia_length
MGS	abundance	Hgn3C.0212 - Holdemanella biformis	Radius_length
MGS	abundance	Hgn3C.0212 - Holdemanella biformis	Radius_SOS
MGS	abundance	Hgn3C.0212 - Holdemanella biformis	Tibia_SOS
MGS	abundance	Hgn3C.0212 - Holdemanella biformis	Handgrip
MGS	abundance	Hgn3C.0213 - Akkermansia sp. GGCC_0220	Tibia_length
MGS	abundance	Hgn3C.0213 - Akkermansia sp. GGCC_0220	Radius_length
MGS	abundance	Hgn3C.0213 - Akkermansia sp. GGCC_0220	Radius_SOS
MGS	abundance	Hgn3C.0213 - Akkermansia sp. GGCC_0220	Tibia_SOS

MGS	abundance	Hgn3C.0213 - Akkermansia sp. GGCC_0220	Handgrip
MGS	abundance	Hgn3C.0214 - Duodenibacillus massiliensis	Tibia_length
MGS	abundance	Hgn3C.0214 - Duodenibacillus massiliensis	Radius_length
MGS	abundance	Hgn3C.0214 - Duodenibacillus massiliensis	Radius_SOS
MGS	abundance	Hgn3C.0214 - Duodenibacillus massiliensis	Tibia_SOS
MGS	abundance	Hgn3C.0214 - Duodenibacillus massiliensis	Handgrip
MGS	abundance	Hgn3C.0215 - Alistipes sp. cv1	Tibia_length
MGS	abundance	Hgn3C.0215 - Alistipes sp. cv1	Radius_length
MGS	abundance	Hgn3C.0215 - Alistipes sp. cv1	Radius_SOS
MGS	abundance	Hgn3C.0215 - Alistipes sp. cv1	Tibia_SOS
MGS	abundance	Hgn3C.0215 - Alistipes sp. cv1	Handgrip
MGS	abundance	Hgn3C.0217 - Eubacteriales sp.	Tibia_length
MGS	abundance	Hgn3C.0217 - Eubacteriales sp.	Radius_length
MGS	abundance	Hgn3C.0217 - Eubacteriales sp.	Radius_SOS
MGS	abundance	Hgn3C.0217 - Eubacteriales sp.	Tibia_SOS
MGS	abundance	Hgn3C.0217 - Eubacteriales sp.	Handgrip
MGS	abundance	Hgn3C.0218 - Eubacteriales sp.	Tibia_length
MGS	abundance	Hgn3C.0218 - Eubacteriales sp.	Radius_length
MGS	abundance	Hgn3C.0218 - Eubacteriales sp.	Radius_SOS
MGS	abundance	Hgn3C.0218 - Eubacteriales sp.	Tibia_SOS
MGS	abundance	Hgn3C.0218 - Eubacteriales sp.	Handgrip
MGS	abundance	Hgn3C.0219 - Tyzzerella nexilis	Tibia_length
MGS	abundance	Hgn3C.0219 - Tyzzerella nexilis	Radius_length
MGS	abundance	Hgn3C.0219 - Tyzzerella nexilis	Radius_SOS
MGS	abundance	Hgn3C.0219 - Tyzzerella nexilis	Tibia_SOS
MGS	abundance	Hgn3C.0219 - Tyzzerella nexilis	Handgrip
MGS	abundance	Hgn3C.0220 - Clostridium sp. MCC334	Tibia_length
MGS	abundance	Hgn3C.0220 - Clostridium sp. MCC334	Radius_length
MGS	abundance	Hgn3C.0220 - Clostridium sp. MCC334	Radius_SOS
MGS	abundance	Hgn3C.0220 - Clostridium sp. MCC334	Tibia_SOS
MGS	abundance	Hgn3C.0220 - Clostridium sp. MCC334	Handgrip
MGS	abundance	Hgn3C.0221 - Clostridium sp.	Tibia_length
MGS	abundance	Hgn3C.0221 - Clostridium sp.	Radius_length
MGS	abundance	Hgn3C.0221 - Clostridium sp.	Radius_SOS
MGS	abundance	Hgn3C.0221 - Clostridium sp.	Tibia_SOS
MGS	abundance	Hgn3C.0221 - Clostridium sp.	Handgrip
MGS	abundance	Hgn3C.0222 - Parabacteroides merdae	Tibia_length
MGS	abundance	Hgn3C.0222 - Parabacteroides merdae	Radius_length
MGS	abundance	Hgn3C.0222 - Parabacteroides merdae	Radius_SOS
MGS	abundance	Hgn3C.0222 - Parabacteroides merdae	Tibia_SOS
MGS	abundance	Hgn3C.0222 - Parabacteroides merdae	Handgrip
MGS	abundance	Hgn3C.0224 - Veillonellales sp.	Tibia_length
MGS	abundance	Hgn3C.0224 - Veillonellales sp.	Radius_length
MGS	abundance	Hgn3C.0224 - Veillonellales sp.	Radius_SOS
MGS	abundance	Hgn3C.0224 - Veillonellales sp.	Tibia_SOS
MGS	abundance	Hgn3C.0224 - Veillonellales sp.	Handgrip
MGS	abundance	Hgn3C.0226 - Eubacteriales sp.	Tibia_length
MGS	abundance	Hgn3C.0226 - Eubacteriales sp.	Radius_length

MGS	abundance	Hgn3C.0226 - Eubacteriales sp.	Radius_SOS
MGS	abundance	Hgn3C.0226 - Eubacteriales sp.	Tibia_SOS
MGS	abundance	Hgn3C.0226 - Eubacteriales sp.	Handgrip
MGS	abundance	Hgn3C.0228 - Coprococcus eutactus	Tibia_length
MGS	abundance	Hgn3C.0228 - Coprococcus eutactus	Radius_length
MGS	abundance	Hgn3C.0228 - Coprococcus eutactus	Radius_SOS
MGS	abundance	Hgn3C.0228 - Coprococcus eutactus	Tibia_SOS
MGS	abundance	Hgn3C.0228 - Coprococcus eutactus	Handgrip
MGS	abundance	Hgn3C.0230 - Gemmiger sp.	Tibia_length
MGS	abundance	Hgn3C.0230 - Gemmiger sp.	Radius_length
MGS	abundance	Hgn3C.0230 - Gemmiger sp.	Radius_SOS
MGS	abundance	Hgn3C.0230 - Gemmiger sp.	Tibia_SOS
MGS	abundance	Hgn3C.0230 - Gemmiger sp.	Handgrip
MGS	abundance	Hgn3C.0232 - Megamonas funiformis	Tibia_length
MGS	abundance	Hgn3C.0232 - Megamonas funiformis	Radius_length
MGS	abundance	Hgn3C.0232 - Megamonas funiformis	Radius_SOS
MGS	abundance	Hgn3C.0232 - Megamonas funiformis	Tibia_SOS
MGS	abundance	Hgn3C.0232 - Megamonas funiformis	Handgrip
MGS	abundance	Hgn3C.0236 - Oscillospiraceae sp.	Tibia_length
MGS	abundance	Hgn3C.0236 - Oscillospiraceae sp.	Radius_length
MGS	abundance	Hgn3C.0236 - Oscillospiraceae sp.	Radius_SOS
MGS	abundance	Hgn3C.0236 - Oscillospiraceae sp.	Tibia_SOS
MGS	abundance	Hgn3C.0236 - Oscillospiraceae sp.	Handgrip
MGS	abundance	Hgn3C.0238 - Oscillospiraceae sp.	Tibia_length
MGS	abundance	Hgn3C.0238 - Oscillospiraceae sp.	Radius_length
MGS	abundance	Hgn3C.0238 - Oscillospiraceae sp.	Radius_SOS
MGS	abundance	Hgn3C.0238 - Oscillospiraceae sp.	Tibia_SOS
MGS	abundance	Hgn3C.0238 - Oscillospiraceae sp.	Handgrip
MGS	abundance	Hgn3C.0239 - Blautia sp. BIOML-A1	Tibia_length
MGS	abundance	Hgn3C.0239 - Blautia sp. BIOML-A1	Radius_length
MGS	abundance	Hgn3C.0239 - Blautia sp. BIOML-A1	Radius_SOS
MGS	abundance	Hgn3C.0239 - Blautia sp. BIOML-A1	Tibia_SOS
MGS	abundance	Hgn3C.0239 - Blautia sp. BIOML-A1	Handgrip
MGS	abundance	Hgn3C.0242 - Enterocloster bolteae	Tibia_length
MGS	abundance	Hgn3C.0242 - Enterocloster bolteae	Radius_length
MGS	abundance	Hgn3C.0242 - Enterocloster bolteae	Radius_SOS
MGS	abundance	Hgn3C.0242 - Enterocloster bolteae	Tibia_SOS
MGS	abundance	Hgn3C.0242 - Enterocloster bolteae	Handgrip
MGS	abundance	Hgn3C.0244 - Desulfovibrio piger	Tibia_length
MGS	abundance	Hgn3C.0244 - Desulfovibrio piger	Radius_length
MGS	abundance	Hgn3C.0244 - Desulfovibrio piger	Radius_SOS
MGS	abundance	Hgn3C.0244 - Desulfovibrio piger	Tibia_SOS
MGS	abundance	Hgn3C.0244 - Desulfovibrio piger	Handgrip
MGS	abundance	Hgn3C.0245 - Blautia stercoris	Tibia_length
MGS	abundance	Hgn3C.0245 - Blautia stercoris	Radius_length
MGS	abundance	Hgn3C.0245 - Blautia stercoris	Radius_SOS
MGS	abundance	Hgn3C.0245 - Blautia stercoris	Tibia_SOS
MGS	abundance	Hgn3C.0245 - Blautia stercoris	Handgrip

MGS	abundance	Hgn3C.0248 - Coprococcus catus	Tibia_length
MGS	abundance	Hgn3C.0248 - Coprococcus catus	Radius_length
MGS	abundance	Hgn3C.0248 - Coprococcus catus	Radius_SOS
MGS	abundance	Hgn3C.0248 - Coprococcus catus	Tibia_SOS
MGS	abundance	Hgn3C.0248 - Coprococcus catus	Handgrip
MGS	abundance	Hgn3C.0249 - Clostridium sp. AM33-3	Tibia_length
MGS	abundance	Hgn3C.0249 - Clostridium sp. AM33-3	Radius_length
MGS	abundance	Hgn3C.0249 - Clostridium sp. AM33-3	Radius_SOS
MGS	abundance	Hgn3C.0249 - Clostridium sp. AM33-3	Tibia_SOS
MGS	abundance	Hgn3C.0249 - Clostridium sp. AM33-3	Handgrip
MGS	abundance	Hgn3C.0250 - Eubacteriales sp.	Tibia_length
MGS	abundance	Hgn3C.0250 - Eubacteriales sp.	Radius_length
MGS	abundance	Hgn3C.0250 - Eubacteriales sp.	Radius_SOS
MGS	abundance	Hgn3C.0250 - Eubacteriales sp.	Tibia_SOS
MGS	abundance	Hgn3C.0250 - Eubacteriales sp.	Handgrip
MGS	abundance	Hgn3C.0251 - Alistipes senegalensis	Tibia_length
MGS	abundance	Hgn3C.0251 - Alistipes senegalensis	Radius_length
MGS	abundance	Hgn3C.0251 - Alistipes senegalensis	Radius_SOS
MGS	abundance	Hgn3C.0251 - Alistipes senegalensis	Tibia_SOS
MGS	abundance	Hgn3C.0251 - Alistipes senegalensis	Handgrip
MGS	abundance	Hgn3C.0253 - Waltera sp.	Tibia_length
MGS	abundance	Hgn3C.0253 - Waltera sp.	Radius_length
MGS	abundance	Hgn3C.0253 - Waltera sp.	Radius_SOS
MGS	abundance	Hgn3C.0253 - Waltera sp.	Tibia_SOS
MGS	abundance	Hgn3C.0253 - Waltera sp.	Handgrip
MGS	abundance	Hgn3C.0260 - Hydrogenoanaerobacterium saccharovorans	Tibia_length
MGS	abundance	Hgn3C.0260 - Hydrogenoanaerobacterium saccharovorans	Radius_length
MGS	abundance	Hgn3C.0260 - Hydrogenoanaerobacterium saccharovorans	Radius_SOS
MGS	abundance	Hgn3C.0260 - Hydrogenoanaerobacterium saccharovorans	Tibia_SOS
MGS	abundance	Hgn3C.0260 - Hydrogenoanaerobacterium saccharovorans	Handgrip
MGS	abundance	Hgn3C.0262 - Oscillospiraceae sp.	Tibia_length
MGS	abundance	Hgn3C.0262 - Oscillospiraceae sp.	Radius_length
MGS	abundance	Hgn3C.0262 - Oscillospiraceae sp.	Radius_SOS
MGS	abundance	Hgn3C.0262 - Oscillospiraceae sp.	Tibia_SOS
MGS	abundance	Hgn3C.0262 - Oscillospiraceae sp.	Handgrip
MGS	abundance	Hgn3C.0264 - Clostridium sp. AF27-2AA	Tibia_length
MGS	abundance	Hgn3C.0264 - Clostridium sp. AF27-2AA	Radius_length
MGS	abundance	Hgn3C.0264 - Clostridium sp. AF27-2AA	Radius_SOS
MGS	abundance	Hgn3C.0264 - Clostridium sp. AF27-2AA	Tibia_SOS
MGS	abundance	Hgn3C.0264 - Clostridium sp. AF27-2AA	Handgrip
MGS	abundance	Hgn3C.0266 - Dorea sp. AF36-15AT	Tibia_length
MGS	abundance	Hgn3C.0266 - Dorea sp. AF36-15AT	Radius_length
MGS	abundance	Hgn3C.0266 - Dorea sp. AF36-15AT	Radius_SOS
MGS	abundance	Hgn3C.0266 - Dorea sp. AF36-15AT	Tibia_SOS
MGS	abundance	Hgn3C.0266 - Dorea sp. AF36-15AT	Handgrip
MGS	abundance	Hgn3C.0267 - Oscillospiraceae sp.	Tibia_length
MGS	abundance	Hgn3C.0267 - Oscillospiraceae sp.	Radius_length
MGS	abundance	Hgn3C.0267 - Oscillospiraceae sp.	Radius_SOS

MGS	abundance	Hgn3C.0267 - Oscillospiraceae sp.	Tibia_SOS
MGS	abundance	Hgn3C.0267 - Oscillospiraceae sp.	Handgrip
MGS	abundance	Hgn3C.0269 - Flintibacter sp. NSJ-23	Tibia_length
MGS	abundance	Hgn3C.0269 - Flintibacter sp. NSJ-23	Radius_length
MGS	abundance	Hgn3C.0269 - Flintibacter sp. NSJ-23	Radius_SOS
MGS	abundance	Hgn3C.0269 - Flintibacter sp. NSJ-23	Tibia_SOS
MGS	abundance	Hgn3C.0269 - Flintibacter sp. NSJ-23	Handgrip
MGS	abundance	Hgn3C.0271 - Oscillibacter sp.	Tibia_length
MGS	abundance	Hgn3C.0271 - Oscillibacter sp.	Radius_length
MGS	abundance	Hgn3C.0271 - Oscillibacter sp.	Radius_SOS
MGS	abundance	Hgn3C.0271 - Oscillibacter sp.	Tibia_SOS
MGS	abundance	Hgn3C.0271 - Oscillibacter sp.	Handgrip
MGS	abundance	Hgn3C.0272 - Blautia faecicola	Tibia_length
MGS	abundance	Hgn3C.0272 - Blautia faecicola	Radius_length
MGS	abundance	Hgn3C.0272 - Blautia faecicola	Radius_SOS
MGS	abundance	Hgn3C.0272 - Blautia faecicola	Tibia_SOS
MGS	abundance	Hgn3C.0272 - Blautia faecicola	Handgrip
MGS	abundance	Hgn3C.0273 - Slackia isoflavoniconvertens	Tibia_length
MGS	abundance	Hgn3C.0273 - Slackia isoflavoniconvertens	Radius_length
MGS	abundance	Hgn3C.0273 - Slackia isoflavoniconvertens	Radius_SOS
MGS	abundance	Hgn3C.0273 - Slackia isoflavoniconvertens	Tibia_SOS
MGS	abundance	Hgn3C.0273 - Slackia isoflavoniconvertens	Handgrip
MGS	abundance	Hgn3C.0275 - Lachnospiraceae sp.	Tibia_length
MGS	abundance	Hgn3C.0275 - Lachnospiraceae sp.	Radius_length
MGS	abundance	Hgn3C.0275 - Lachnospiraceae sp.	Radius_SOS
MGS	abundance	Hgn3C.0275 - Lachnospiraceae sp.	Tibia_SOS
MGS	abundance	Hgn3C.0275 - Lachnospiraceae sp.	Handgrip
MGS	abundance	Hgn3C.0279 - Oscillospiraceae sp.	Tibia_length
MGS	abundance	Hgn3C.0279 - Oscillospiraceae sp.	Radius_length
MGS	abundance	Hgn3C.0279 - Oscillospiraceae sp.	Radius_SOS
MGS	abundance	Hgn3C.0279 - Oscillospiraceae sp.	Tibia_SOS
MGS	abundance	Hgn3C.0279 - Oscillospiraceae sp.	Handgrip
MGS	abundance	Hgn3C.0281 - Eubacteriales sp.	Tibia_length
MGS	abundance	Hgn3C.0281 - Eubacteriales sp.	Radius_length
MGS	abundance	Hgn3C.0281 - Eubacteriales sp.	Radius_SOS
MGS	abundance	Hgn3C.0281 - Eubacteriales sp.	Tibia_SOS
MGS	abundance	Hgn3C.0281 - Eubacteriales sp.	Handgrip
MGS	abundance	Hgn3C.0284 - Erysipelotrichaceae sp.	Tibia_length
MGS	abundance	Hgn3C.0284 - Erysipelotrichaceae sp.	Radius_length
MGS	abundance	Hgn3C.0284 - Erysipelotrichaceae sp.	Radius_SOS
MGS	abundance	Hgn3C.0284 - Erysipelotrichaceae sp.	Tibia_SOS
MGS	abundance	Hgn3C.0284 - Erysipelotrichaceae sp.	Handgrip
MGS	abundance	Hgn3C.0285 - Colidextribacter sp.	Tibia_length
MGS	abundance	Hgn3C.0285 - Colidextribacter sp.	Radius_length
MGS	abundance	Hgn3C.0285 - Colidextribacter sp.	Radius_SOS
MGS	abundance	Hgn3C.0285 - Colidextribacter sp.	Tibia_SOS
MGS	abundance	Hgn3C.0285 - Colidextribacter sp.	Handgrip
MGS	abundance	Hgn3C.0286 - Clostridia sp.	Tibia_length

MGS	abundance	Hgn3C.0286 - Clostridia sp.	Radius_length
MGS	abundance	Hgn3C.0286 - Clostridia sp.	Radius_SOS
MGS	abundance	Hgn3C.0286 - Clostridia sp.	Tibia_SOS
MGS	abundance	Hgn3C.0286 - Clostridia sp.	Handgrip
MGS	abundance	Hgn3C.0288 - Butyricimonas paravirosa	Tibia_length
MGS	abundance	Hgn3C.0288 - Butyricimonas paravirosa	Radius_length
MGS	abundance	Hgn3C.0288 - Butyricimonas paravirosa	Radius_SOS
MGS	abundance	Hgn3C.0288 - Butyricimonas paravirosa	Tibia_SOS
MGS	abundance	Hgn3C.0288 - Butyricimonas paravirosa	Handgrip
MGS	abundance	Hgn3C.0289 - Bacteroides cellulosilyticus	Tibia_length
MGS	abundance	Hgn3C.0289 - Bacteroides cellulosilyticus	Radius_length
MGS	abundance	Hgn3C.0289 - Bacteroides cellulosilyticus	Radius_SOS
MGS	abundance	Hgn3C.0289 - Bacteroides cellulosilyticus	Tibia_SOS
MGS	abundance	Hgn3C.0289 - Bacteroides cellulosilyticus	Handgrip
MGS	abundance	Hgn3C.0290 - Sellimonas intestinalis	Tibia_length
MGS	abundance	Hgn3C.0290 - Sellimonas intestinalis	Radius_length
MGS	abundance	Hgn3C.0290 - Sellimonas intestinalis	Radius_SOS
MGS	abundance	Hgn3C.0290 - Sellimonas intestinalis	Tibia_SOS
MGS	abundance	Hgn3C.0290 - Sellimonas intestinalis	Handgrip
MGS	abundance	Hgn3C.0291 - Parabacteroides johnsonii	Tibia_length
MGS	abundance	Hgn3C.0291 - Parabacteroides johnsonii	Radius_length
MGS	abundance	Hgn3C.0291 - Parabacteroides johnsonii	Radius_SOS
MGS	abundance	Hgn3C.0291 - Parabacteroides johnsonii	Tibia_SOS
MGS	abundance	Hgn3C.0291 - Parabacteroides johnsonii	Handgrip
MGS	abundance	Hgn3C.0295 - Eubacteriales sp.	Tibia_length
MGS	abundance	Hgn3C.0295 - Eubacteriales sp.	Radius_length
MGS	abundance	Hgn3C.0295 - Eubacteriales sp.	Radius_SOS
MGS	abundance	Hgn3C.0295 - Eubacteriales sp.	Tibia_SOS
MGS	abundance	Hgn3C.0295 - Eubacteriales sp.	Handgrip
MGS	abundance	Hgn3C.0298 - Monoglobus pectinilyticus	Tibia_length
MGS	abundance	Hgn3C.0298 - Monoglobus pectinilyticus	Radius_length
MGS	abundance	Hgn3C.0298 - Monoglobus pectinilyticus	Radius_SOS
MGS	abundance	Hgn3C.0298 - Monoglobus pectinilyticus	Tibia_SOS
MGS	abundance	Hgn3C.0298 - Monoglobus pectinilyticus	Handgrip
MGS	abundance	Hgn3C.0299 - Escherichia coli	Tibia_length
MGS	abundance	Hgn3C.0299 - Escherichia coli	Radius_length
MGS	abundance	Hgn3C.0299 - Escherichia coli	Radius_SOS
MGS	abundance	Hgn3C.0299 - Escherichia coli	Tibia_SOS
MGS	abundance	Hgn3C.0299 - Escherichia coli	Handgrip
MGS	abundance	Hgn3C.0300 - Clostridia sp.	Tibia_length
MGS	abundance	Hgn3C.0300 - Clostridia sp.	Radius_length
MGS	abundance	Hgn3C.0300 - Clostridia sp.	Radius_SOS
MGS	abundance	Hgn3C.0300 - Clostridia sp.	Tibia_SOS
MGS	abundance	Hgn3C.0300 - Clostridia sp.	Handgrip
MGS	abundance	Hgn3C.0301 - Prevotella hominis	Tibia_length
MGS	abundance	Hgn3C.0301 - Prevotella hominis	Radius_length
MGS	abundance	Hgn3C.0301 - Prevotella hominis	Radius_SOS
MGS	abundance	Hgn3C.0301 - Prevotella hominis	Tibia_SOS

MGS	abundance	Hgn3C.0301 - Prevotella hominis	Handgrip
MGS	abundance	Hgn3C.0302 - Lachnospiraceae sp.	Tibia_length
MGS	abundance	Hgn3C.0302 - Lachnospiraceae sp.	Radius_length
MGS	abundance	Hgn3C.0302 - Lachnospiraceae sp.	Radius_SOS
MGS	abundance	Hgn3C.0302 - Lachnospiraceae sp.	Tibia_SOS
MGS	abundance	Hgn3C.0302 - Lachnospiraceae sp.	Handgrip
MGS	abundance	Hgn3C.0304 - Eubacteriales sp.	Tibia_length
MGS	abundance	Hgn3C.0304 - Eubacteriales sp.	Radius_length
MGS	abundance	Hgn3C.0304 - Eubacteriales sp.	Radius_SOS
MGS	abundance	Hgn3C.0304 - Eubacteriales sp.	Tibia_SOS
MGS	abundance	Hgn3C.0304 - Eubacteriales sp.	Handgrip
MGS	abundance	Hgn3C.0305 - Candidatus Borkfalkia ceftriaxoniphila	Tibia_length
MGS	abundance	Hgn3C.0305 - Candidatus Borkfalkia ceftriaxoniphila	Radius_length
MGS	abundance	Hgn3C.0305 - Candidatus Borkfalkia ceftriaxoniphila	Radius_SOS
MGS	abundance	Hgn3C.0305 - Candidatus Borkfalkia ceftriaxoniphila	Tibia_SOS
MGS	abundance	Hgn3C.0305 - Candidatus Borkfalkia ceftriaxoniphila	Handgrip
MGS	abundance	Hgn3C.0306 - Bacteroides nordii	Tibia_length
MGS	abundance	Hgn3C.0306 - Bacteroides nordii	Radius_length
MGS	abundance	Hgn3C.0306 - Bacteroides nordii	Radius_SOS
MGS	abundance	Hgn3C.0306 - Bacteroides nordii	Tibia_SOS
MGS	abundance	Hgn3C.0306 - Bacteroides nordii	Handgrip
MGS	abundance	Hgn3C.0307 - Veillonella atypica	Tibia_length
MGS	abundance	Hgn3C.0307 - Veillonella atypica	Radius_length
MGS	abundance	Hgn3C.0307 - Veillonella atypica	Radius_SOS
MGS	abundance	Hgn3C.0307 - Veillonella atypica	Tibia_SOS
MGS	abundance	Hgn3C.0307 - Veillonella atypica	Handgrip
MGS	abundance	Hgn3C.0308 - [Clostridium] symbiosum	Tibia_length
MGS	abundance	Hgn3C.0308 - [Clostridium] symbiosum	Radius_length
MGS	abundance	Hgn3C.0308 - [Clostridium] symbiosum	Radius_SOS
MGS	abundance	Hgn3C.0308 - [Clostridium] symbiosum	Tibia_SOS
MGS	abundance	Hgn3C.0308 - [Clostridium] symbiosum	Handgrip
MGS	abundance	Hgn3C.0309 - Eubacteriales sp.	Tibia_length
MGS	abundance	Hgn3C.0309 - Eubacteriales sp.	Radius_length
MGS	abundance	Hgn3C.0309 - Eubacteriales sp.	Radius_SOS
MGS	abundance	Hgn3C.0309 - Eubacteriales sp.	Tibia_SOS
MGS	abundance	Hgn3C.0309 - Eubacteriales sp.	Handgrip
MGS	abundance	Hgn3C.0310 - Ligilactobacillus ruminis	Tibia_length
MGS	abundance	Hgn3C.0310 - Ligilactobacillus ruminis	Radius_length
MGS	abundance	Hgn3C.0310 - Ligilactobacillus ruminis	Radius_SOS
MGS	abundance	Hgn3C.0310 - Ligilactobacillus ruminis	Tibia_SOS
MGS	abundance	Hgn3C.0310 - Ligilactobacillus ruminis	Handgrip
MGS	abundance	Hgn3C.0313 - Ruminococcus bromii	Tibia_length
MGS	abundance	Hgn3C.0313 - Ruminococcus bromii	Radius_length
MGS	abundance	Hgn3C.0313 - Ruminococcus bromii	Radius_SOS
MGS	abundance	Hgn3C.0313 - Ruminococcus bromii	Tibia_SOS
MGS	abundance	Hgn3C.0313 - Ruminococcus bromii	Handgrip
MGS	abundance	Hgn3C.0314 - Ruminococcus sp.	Tibia_length
MGS	abundance	Hgn3C.0314 - Ruminococcus sp.	Radius_length

MGS	abundance	Hgn3C.0314 - Ruminococcus sp.	Radius_SOS
MGS	abundance	Hgn3C.0314 - Ruminococcus sp.	Tibia_SOS
MGS	abundance	Hgn3C.0314 - Ruminococcus sp.	Handgrip
MGS	abundance	Hgn3C.0315 - Bacteroidia sp.	Tibia_length
MGS	abundance	Hgn3C.0315 - Bacteroidia sp.	Radius_length
MGS	abundance	Hgn3C.0315 - Bacteroidia sp.	Radius_SOS
MGS	abundance	Hgn3C.0315 - Bacteroidia sp.	Tibia_SOS
MGS	abundance	Hgn3C.0315 - Bacteroidia sp.	Handgrip
MGS	abundance	Hgn3C.0317 - Intestinimonas butyriciproducens	Tibia_length
MGS	abundance	Hgn3C.0317 - Intestinimonas butyriciproducens	Radius_length
MGS	abundance	Hgn3C.0317 - Intestinimonas butyriciproducens	Radius_SOS
MGS	abundance	Hgn3C.0317 - Intestinimonas butyriciproducens	Tibia_SOS
MGS	abundance	Hgn3C.0317 - Intestinimonas butyriciproducens	Handgrip
MGS	abundance	Hgn3C.0319 - Clostridiaceae sp.	Tibia_length
MGS	abundance	Hgn3C.0319 - Clostridiaceae sp.	Radius_length
MGS	abundance	Hgn3C.0319 - Clostridiaceae sp.	Radius_SOS
MGS	abundance	Hgn3C.0319 - Clostridiaceae sp.	Tibia_SOS
MGS	abundance	Hgn3C.0319 - Clostridiaceae sp.	Handgrip
MGS	abundance	Hgn3C.0323 - Bacteroides sp.	Tibia_length
MGS	abundance	Hgn3C.0323 - Bacteroides sp.	Radius_length
MGS	abundance	Hgn3C.0323 - Bacteroides sp.	Radius_SOS
MGS	abundance	Hgn3C.0323 - Bacteroides sp.	Tibia_SOS
MGS	abundance	Hgn3C.0323 - Bacteroides sp.	Handgrip
MGS	abundance	Hgn3C.0324 - Prevotella stercorea	Tibia_length
MGS	abundance	Hgn3C.0324 - Prevotella stercorea	Radius_length
MGS	abundance	Hgn3C.0324 - Prevotella stercorea	Radius_SOS
MGS	abundance	Hgn3C.0324 - Prevotella stercorea	Tibia_SOS
MGS	abundance	Hgn3C.0324 - Prevotella stercorea	Handgrip
MGS	abundance	Hgn3C.0325 - Roseburia sp. BX0805	Tibia_length
MGS	abundance	Hgn3C.0325 - Roseburia sp. BX0805	Radius_length
MGS	abundance	Hgn3C.0325 - Roseburia sp. BX0805	Radius_SOS
MGS	abundance	Hgn3C.0325 - Roseburia sp. BX0805	Tibia_SOS
MGS	abundance	Hgn3C.0325 - Roseburia sp. BX0805	Handgrip
MGS	abundance	Hgn3C.0328 - Eubacteriales sp.	Tibia_length
MGS	abundance	Hgn3C.0328 - Eubacteriales sp.	Radius_length
MGS	abundance	Hgn3C.0328 - Eubacteriales sp.	Radius_SOS
MGS	abundance	Hgn3C.0328 - Eubacteriales sp.	Tibia_SOS
MGS	abundance	Hgn3C.0328 - Eubacteriales sp.	Handgrip
MGS	abundance	Hgn3C.0329 - Eubacteriales sp.	Tibia_length
MGS	abundance	Hgn3C.0329 - Eubacteriales sp.	Radius_length
MGS	abundance	Hgn3C.0329 - Eubacteriales sp.	Radius_SOS
MGS	abundance	Hgn3C.0329 - Eubacteriales sp.	Tibia_SOS
MGS	abundance	Hgn3C.0329 - Eubacteriales sp.	Handgrip
MGS	abundance	Hgn3C.0330 - Faecalibacillus intestinalis	Tibia_length
MGS	abundance	Hgn3C.0330 - Faecalibacillus intestinalis	Radius_length
MGS	abundance	Hgn3C.0330 - Faecalibacillus intestinalis	Radius_SOS
MGS	abundance	Hgn3C.0330 - Faecalibacillus intestinalis	Tibia_SOS
MGS	abundance	Hgn3C.0330 - Faecalibacillus intestinalis	Handgrip

MGS	abundance	Hgn3C.0332 - Klebsiella pneumoniae subsp. pneumoniae	Tibia_length
MGS	abundance	Hgn3C.0332 - Klebsiella pneumoniae subsp. pneumoniae	Radius_length
MGS	abundance	Hgn3C.0332 - Klebsiella pneumoniae subsp. pneumoniae	Radius_SOS
MGS	abundance	Hgn3C.0332 - Klebsiella pneumoniae subsp. pneumoniae	Tibia_SOS
MGS	abundance	Hgn3C.0332 - Klebsiella pneumoniae subsp. pneumoniae	Handgrip
MGS	abundance	Hgn3C.0334 - Bacteroides cellulosilyticus	Tibia_length
MGS	abundance	Hgn3C.0334 - Bacteroides cellulosilyticus	Radius_length
MGS	abundance	Hgn3C.0334 - Bacteroides cellulosilyticus	Radius_SOS
MGS	abundance	Hgn3C.0334 - Bacteroides cellulosilyticus	Tibia_SOS
MGS	abundance	Hgn3C.0334 - Bacteroides cellulosilyticus	Handgrip
MGS	abundance	Hgn3C.0335 - Alistipes sp. Marseille-P2263	Tibia_length
MGS	abundance	Hgn3C.0335 - Alistipes sp. Marseille-P2263	Radius_length
MGS	abundance	Hgn3C.0335 - Alistipes sp. Marseille-P2263	Radius_SOS
MGS	abundance	Hgn3C.0335 - Alistipes sp. Marseille-P2263	Tibia_SOS
MGS	abundance	Hgn3C.0335 - Alistipes sp. Marseille-P2263	Handgrip
MGS	abundance	Hgn3C.0336 - Parabacteroides goldsteinii	Tibia_length
MGS	abundance	Hgn3C.0336 - Parabacteroides goldsteinii	Radius_length
MGS	abundance	Hgn3C.0336 - Parabacteroides goldsteinii	Radius_SOS
MGS	abundance	Hgn3C.0336 - Parabacteroides goldsteinii	Tibia_SOS
MGS	abundance	Hgn3C.0336 - Parabacteroides goldsteinii	Handgrip
MGS	abundance	Hgn3C.0337 - Eubacteriales sp.	Tibia_length
MGS	abundance	Hgn3C.0337 - Eubacteriales sp.	Radius_length
MGS	abundance	Hgn3C.0337 - Eubacteriales sp.	Radius_SOS
MGS	abundance	Hgn3C.0337 - Eubacteriales sp.	Tibia_SOS
MGS	abundance	Hgn3C.0337 - Eubacteriales sp.	Handgrip
MGS	abundance	Hgn3C.0338 - Eubacteriales sp.	Tibia_length
MGS	abundance	Hgn3C.0338 - Eubacteriales sp.	Radius_length
MGS	abundance	Hgn3C.0338 - Eubacteriales sp.	Radius_SOS
MGS	abundance	Hgn3C.0338 - Eubacteriales sp.	Tibia_SOS
MGS	abundance	Hgn3C.0338 - Eubacteriales sp.	Handgrip
MGS	abundance	Hgn3C.0345 - Oscillospiraceae sp.	Tibia_length
MGS	abundance	Hgn3C.0345 - Oscillospiraceae sp.	Radius_length
MGS	abundance	Hgn3C.0345 - Oscillospiraceae sp.	Radius_SOS
MGS	abundance	Hgn3C.0345 - Oscillospiraceae sp.	Tibia_SOS
MGS	abundance	Hgn3C.0345 - Oscillospiraceae sp.	Handgrip
MGS	abundance	Hgn3C.0348 - Streptococcus sp.	Tibia_length
MGS	abundance	Hgn3C.0348 - Streptococcus sp.	Radius_length
MGS	abundance	Hgn3C.0348 - Streptococcus sp.	Radius_SOS
MGS	abundance	Hgn3C.0348 - Streptococcus sp.	Tibia_SOS
MGS	abundance	Hgn3C.0348 - Streptococcus sp.	Handgrip
MGS	abundance	Hgn3C.0349 - Eubacteriales sp.	Tibia_length
MGS	abundance	Hgn3C.0349 - Eubacteriales sp.	Radius_length
MGS	abundance	Hgn3C.0349 - Eubacteriales sp.	Radius_SOS
MGS	abundance	Hgn3C.0349 - Eubacteriales sp.	Tibia_SOS
MGS	abundance	Hgn3C.0349 - Eubacteriales sp.	Handgrip
MGS	abundance	Hgn3C.0350 - [Clostridium] symbiosum	Tibia_length
MGS	abundance	Hgn3C.0350 - [Clostridium] symbiosum	Radius_length
MGS	abundance	Hgn3C.0350 - [Clostridium] symbiosum	Radius_SOS

MGS	abundance	Hgn3C.0350 - [Clostridium] symbiosum	Tibia_SOS
MGS	abundance	Hgn3C.0350 - [Clostridium] symbiosum	Handgrip
MGS	abundance	Hgn3C.0351 - Prevotella sp.	Tibia_length
MGS	abundance	Hgn3C.0351 - Prevotella sp.	Radius_length
MGS	abundance	Hgn3C.0351 - Prevotella sp.	Radius_SOS
MGS	abundance	Hgn3C.0351 - Prevotella sp.	Tibia_SOS
MGS	abundance	Hgn3C.0351 - Prevotella sp.	Handgrip
MGS	abundance	Hgn3C.0352 - Eubacteriales sp.	Tibia_length
MGS	abundance	Hgn3C.0352 - Eubacteriales sp.	Radius_length
MGS	abundance	Hgn3C.0352 - Eubacteriales sp.	Radius_SOS
MGS	abundance	Hgn3C.0352 - Eubacteriales sp.	Tibia_SOS
MGS	abundance	Hgn3C.0352 - Eubacteriales sp.	Handgrip
MGS	abundance	Hgn3C.0353 - Prevotella sp. P4-67	Tibia_length
MGS	abundance	Hgn3C.0353 - Prevotella sp. P4-67	Radius_length
MGS	abundance	Hgn3C.0353 - Prevotella sp. P4-67	Radius_SOS
MGS	abundance	Hgn3C.0353 - Prevotella sp. P4-67	Tibia_SOS
MGS	abundance	Hgn3C.0353 - Prevotella sp. P4-67	Handgrip
MGS	abundance	Hgn3C.0354 - Sutterella seckii	Tibia_length
MGS	abundance	Hgn3C.0354 - Sutterella seckii	Radius_length
MGS	abundance	Hgn3C.0354 - Sutterella seckii	Radius_SOS
MGS	abundance	Hgn3C.0354 - Sutterella seckii	Tibia_SOS
MGS	abundance	Hgn3C.0354 - Sutterella seckii	Handgrip
MGS	abundance	Hgn3C.0356 - Blautia sp. M29	Tibia_length
MGS	abundance	Hgn3C.0356 - Blautia sp. M29	Radius_length
MGS	abundance	Hgn3C.0356 - Blautia sp. M29	Radius_SOS
MGS	abundance	Hgn3C.0356 - Blautia sp. M29	Tibia_SOS
MGS	abundance	Hgn3C.0356 - Blautia sp. M29	Handgrip
MGS	abundance	Hgn3C.0357 - Eubacteriales sp.	Tibia_length
MGS	abundance	Hgn3C.0357 - Eubacteriales sp.	Radius_length
MGS	abundance	Hgn3C.0357 - Eubacteriales sp.	Radius_SOS
MGS	abundance	Hgn3C.0357 - Eubacteriales sp.	Tibia_SOS
MGS	abundance	Hgn3C.0357 - Eubacteriales sp.	Handgrip
MGS	abundance	Hgn3C.0359 - Eubacterium sp. BX4	Tibia_length
MGS	abundance	Hgn3C.0359 - Eubacterium sp. BX4	Radius_length
MGS	abundance	Hgn3C.0359 - Eubacterium sp. BX4	Radius_SOS
MGS	abundance	Hgn3C.0359 - Eubacterium sp. BX4	Tibia_SOS
MGS	abundance	Hgn3C.0359 - Eubacterium sp. BX4	Handgrip
MGS	abundance	Hgn3C.0361 - Dorea sp. AF24-7LB	Tibia_length
MGS	abundance	Hgn3C.0361 - Dorea sp. AF24-7LB	Radius_length
MGS	abundance	Hgn3C.0361 - Dorea sp. AF24-7LB	Radius_SOS
MGS	abundance	Hgn3C.0361 - Dorea sp. AF24-7LB	Tibia_SOS
MGS	abundance	Hgn3C.0361 - Dorea sp. AF24-7LB	Handgrip
MGS	abundance	Hgn3C.0362 - Dysosmobacter sp. NSJ-60	Tibia_length
MGS	abundance	Hgn3C.0362 - Dysosmobacter sp. NSJ-60	Radius_length
MGS	abundance	Hgn3C.0362 - Dysosmobacter sp. NSJ-60	Radius_SOS
MGS	abundance	Hgn3C.0362 - Dysosmobacter sp. NSJ-60	Tibia_SOS
MGS	abundance	Hgn3C.0362 - Dysosmobacter sp. NSJ-60	Handgrip
MGS	abundance	Hgn3C.0364 - Eubacteriales sp.	Tibia_length

MGS	abundance	Hgn3C.0364 - Eubacteriales sp.	Radius_length
MGS	abundance	Hgn3C.0364 - Eubacteriales sp.	Radius_SOS
MGS	abundance	Hgn3C.0364 - Eubacteriales sp.	Tibia_SOS
MGS	abundance	Hgn3C.0364 - Eubacteriales sp.	Handgrip
MGS	abundance	Hgn3C.0365 - Eubacteriales sp.	Tibia_length
MGS	abundance	Hgn3C.0365 - Eubacteriales sp.	Radius_length
MGS	abundance	Hgn3C.0365 - Eubacteriales sp.	Radius_SOS
MGS	abundance	Hgn3C.0365 - Eubacteriales sp.	Tibia_SOS
MGS	abundance	Hgn3C.0365 - Eubacteriales sp.	Handgrip
MGS	abundance	Hgn3C.0367 - Lachnospiraceae sp.	Tibia_length
MGS	abundance	Hgn3C.0367 - Lachnospiraceae sp.	Radius_length
MGS	abundance	Hgn3C.0367 - Lachnospiraceae sp.	Radius_SOS
MGS	abundance	Hgn3C.0367 - Lachnospiraceae sp.	Tibia_SOS
MGS	abundance	Hgn3C.0367 - Lachnospiraceae sp.	Handgrip
MGS	abundance	Hgn3C.0368 - Eubacteriales sp.	Tibia_length
MGS	abundance	Hgn3C.0368 - Eubacteriales sp.	Radius_length
MGS	abundance	Hgn3C.0368 - Eubacteriales sp.	Radius_SOS
MGS	abundance	Hgn3C.0368 - Eubacteriales sp.	Tibia_SOS
MGS	abundance	Hgn3C.0368 - Eubacteriales sp.	Handgrip
MGS	abundance	Hgn3C.0369 - Prevotella sp.	Tibia_length
MGS	abundance	Hgn3C.0369 - Prevotella sp.	Radius_length
MGS	abundance	Hgn3C.0369 - Prevotella sp.	Radius_SOS
MGS	abundance	Hgn3C.0369 - Prevotella sp.	Tibia_SOS
MGS	abundance	Hgn3C.0369 - Prevotella sp.	Handgrip
MGS	abundance	Hgn3C.0371 - Lachnospiraceae sp.	Tibia_length
MGS	abundance	Hgn3C.0371 - Lachnospiraceae sp.	Radius_length
MGS	abundance	Hgn3C.0371 - Lachnospiraceae sp.	Radius_SOS
MGS	abundance	Hgn3C.0371 - Lachnospiraceae sp.	Tibia_SOS
MGS	abundance	Hgn3C.0371 - Lachnospiraceae sp.	Handgrip
MGS	abundance	Hgn3C.0373 - Enterocloster clostridioformis	Tibia_length
MGS	abundance	Hgn3C.0373 - Enterocloster clostridioformis	Radius_length
MGS	abundance	Hgn3C.0373 - Enterocloster clostridioformis	Radius_SOS
MGS	abundance	Hgn3C.0373 - Enterocloster clostridioformis	Tibia_SOS
MGS	abundance	Hgn3C.0373 - Enterocloster clostridioformis	Handgrip
MGS	abundance	Hgn3C.0375 - Ellagibacter isourolithinifaciens	Tibia_length
MGS	abundance	Hgn3C.0375 - Ellagibacter isourolithinifaciens	Radius_length
MGS	abundance	Hgn3C.0375 - Ellagibacter isourolithinifaciens	Radius_SOS
MGS	abundance	Hgn3C.0375 - Ellagibacter isourolithinifaciens	Tibia_SOS
MGS	abundance	Hgn3C.0375 - Ellagibacter isourolithinifaciens	Handgrip
MGS	abundance	Hgn3C.0378 - Firmicutes sp.	Tibia_length
MGS	abundance	Hgn3C.0378 - Firmicutes sp.	Radius_length
MGS	abundance	Hgn3C.0378 - Firmicutes sp.	Radius_SOS
MGS	abundance	Hgn3C.0378 - Firmicutes sp.	Tibia_SOS
MGS	abundance	Hgn3C.0378 - Firmicutes sp.	Handgrip
MGS	abundance	Hgn3C.0379 - Eubacteriales sp.	Tibia_length
MGS	abundance	Hgn3C.0379 - Eubacteriales sp.	Radius_length
MGS	abundance	Hgn3C.0379 - Eubacteriales sp.	Radius_SOS
MGS	abundance	Hgn3C.0379 - Eubacteriales sp.	Tibia_SOS

MGS	abundance	Hgn3C.0379 - Eubacteriales sp.	Handgrip
MGS	abundance	Hgn3C.0381 - Clostridium sp. AM30-24	Tibia_length
MGS	abundance	Hgn3C.0381 - Clostridium sp. AM30-24	Radius_length
MGS	abundance	Hgn3C.0381 - Clostridium sp. AM30-24	Radius_SOS
MGS	abundance	Hgn3C.0381 - Clostridium sp. AM30-24	Tibia_SOS
MGS	abundance	Hgn3C.0381 - Clostridium sp. AM30-24	Handgrip
MGS	abundance	Hgn3C.0385 - Veillonella rogosae	Tibia_length
MGS	abundance	Hgn3C.0385 - Veillonella rogosae	Radius_length
MGS	abundance	Hgn3C.0385 - Veillonella rogosae	Radius_SOS
MGS	abundance	Hgn3C.0385 - Veillonella rogosae	Tibia_SOS
MGS	abundance	Hgn3C.0385 - Veillonella rogosae	Handgrip
MGS	abundance	Hgn3C.0386 - Clostridium sp. AT4	Tibia_length
MGS	abundance	Hgn3C.0386 - Clostridium sp. AT4	Radius_length
MGS	abundance	Hgn3C.0386 - Clostridium sp. AT4	Radius_SOS
MGS	abundance	Hgn3C.0386 - Clostridium sp. AT4	Tibia_SOS
MGS	abundance	Hgn3C.0386 - Clostridium sp. AT4	Handgrip
MGS	abundance	Hgn3C.0388 - Clostridium sp.	Tibia_length
MGS	abundance	Hgn3C.0388 - Clostridium sp.	Radius_length
MGS	abundance	Hgn3C.0388 - Clostridium sp.	Radius_SOS
MGS	abundance	Hgn3C.0388 - Clostridium sp.	Tibia_SOS
MGS	abundance	Hgn3C.0388 - Clostridium sp.	Handgrip
MGS	abundance	Hgn3C.0389 - Vescimonas fastidiosa	Tibia_length
MGS	abundance	Hgn3C.0389 - Vescimonas fastidiosa	Radius_length
MGS	abundance	Hgn3C.0389 - Vescimonas fastidiosa	Radius_SOS
MGS	abundance	Hgn3C.0389 - Vescimonas fastidiosa	Tibia_SOS
MGS	abundance	Hgn3C.0389 - Vescimonas fastidiosa	Handgrip
MGS	abundance	Hgn3C.0392 - Clostridia sp.	Tibia_length
MGS	abundance	Hgn3C.0392 - Clostridia sp.	Radius_length
MGS	abundance	Hgn3C.0392 - Clostridia sp.	Radius_SOS
MGS	abundance	Hgn3C.0392 - Clostridia sp.	Tibia_SOS
MGS	abundance	Hgn3C.0392 - Clostridia sp.	Handgrip
MGS	abundance	Hgn3C.0394 - Hungatella hathewayi	Tibia_length
MGS	abundance	Hgn3C.0394 - Hungatella hathewayi	Radius_length
MGS	abundance	Hgn3C.0394 - Hungatella hathewayi	Radius_SOS
MGS	abundance	Hgn3C.0394 - Hungatella hathewayi	Tibia_SOS
MGS	abundance	Hgn3C.0394 - Hungatella hathewayi	Handgrip
MGS	abundance	Hgn3C.0395 - Bacteria sp.	Tibia_length
MGS	abundance	Hgn3C.0395 - Bacteria sp.	Radius_length
MGS	abundance	Hgn3C.0395 - Bacteria sp.	Radius_SOS
MGS	abundance	Hgn3C.0395 - Bacteria sp.	Tibia_SOS
MGS	abundance	Hgn3C.0395 - Bacteria sp.	Handgrip
MGS	abundance	Hgn3C.0396 - Bacteroidales sp.	Tibia_length
MGS	abundance	Hgn3C.0396 - Bacteroidales sp.	Radius_length
MGS	abundance	Hgn3C.0396 - Bacteroidales sp.	Radius_SOS
MGS	abundance	Hgn3C.0396 - Bacteroidales sp.	Tibia_SOS
MGS	abundance	Hgn3C.0396 - Bacteroidales sp.	Handgrip
MGS	abundance	Hgn3C.0397 - Parabacteroides sp.	Tibia_length
MGS	abundance	Hgn3C.0397 - Parabacteroides sp.	Radius_length

MGS	abundance	Hgn3C.0397 - Parabacteroides sp.	Radius_SOS
MGS	abundance	Hgn3C.0397 - Parabacteroides sp.	Tibia_SOS
MGS	abundance	Hgn3C.0397 - Parabacteroides sp.	Handgrip
MGS	abundance	Hgn3C.0400 - Prevotellamassilia timonensis	Tibia_length
MGS	abundance	Hgn3C.0400 - Prevotellamassilia timonensis	Radius_length
MGS	abundance	Hgn3C.0400 - Prevotellamassilia timonensis	Radius_SOS
MGS	abundance	Hgn3C.0400 - Prevotellamassilia timonensis	Tibia_SOS
MGS	abundance	Hgn3C.0400 - Prevotellamassilia timonensis	Handgrip
MGS	abundance	Hgn3C.0401 - Eubacteriales sp.	Tibia_length
MGS	abundance	Hgn3C.0401 - Eubacteriales sp.	Radius_length
MGS	abundance	Hgn3C.0401 - Eubacteriales sp.	Radius_SOS
MGS	abundance	Hgn3C.0401 - Eubacteriales sp.	Tibia_SOS
MGS	abundance	Hgn3C.0401 - Eubacteriales sp.	Handgrip
MGS	abundance	Hgn3C.0404 - Eubacteriales sp.	Tibia_length
MGS	abundance	Hgn3C.0404 - Eubacteriales sp.	Radius_length
MGS	abundance	Hgn3C.0404 - Eubacteriales sp.	Radius_SOS
MGS	abundance	Hgn3C.0404 - Eubacteriales sp.	Tibia_SOS
MGS	abundance	Hgn3C.0404 - Eubacteriales sp.	Handgrip
MGS	abundance	Hgn3C.0405 - Bacteroides ovatus	Tibia_length
MGS	abundance	Hgn3C.0405 - Bacteroides ovatus	Radius_length
MGS	abundance	Hgn3C.0405 - Bacteroides ovatus	Radius_SOS
MGS	abundance	Hgn3C.0405 - Bacteroides ovatus	Tibia_SOS
MGS	abundance	Hgn3C.0405 - Bacteroides ovatus	Handgrip
MGS	abundance	Hgn3C.0407 - Eubacteriales sp.	Tibia_length
MGS	abundance	Hgn3C.0407 - Eubacteriales sp.	Radius_length
MGS	abundance	Hgn3C.0407 - Eubacteriales sp.	Radius_SOS
MGS	abundance	Hgn3C.0407 - Eubacteriales sp.	Tibia_SOS
MGS	abundance	Hgn3C.0407 - Eubacteriales sp.	Handgrip
MGS	abundance	Hgn3C.0408 - Eubacteriales sp.	Tibia_length
MGS	abundance	Hgn3C.0408 - Eubacteriales sp.	Radius_length
MGS	abundance	Hgn3C.0408 - Eubacteriales sp.	Radius_SOS
MGS	abundance	Hgn3C.0408 - Eubacteriales sp.	Tibia_SOS
MGS	abundance	Hgn3C.0408 - Eubacteriales sp.	Handgrip
MGS	abundance	Hgn3C.0412 - Eubacteriales sp.	Tibia_length
MGS	abundance	Hgn3C.0412 - Eubacteriales sp.	Radius_length
MGS	abundance	Hgn3C.0412 - Eubacteriales sp.	Radius_SOS
MGS	abundance	Hgn3C.0412 - Eubacteriales sp.	Tibia_SOS
MGS	abundance	Hgn3C.0412 - Eubacteriales sp.	Handgrip
MGS	abundance	Hgn3C.0413 - Blautia caecimuris	Tibia_length
MGS	abundance	Hgn3C.0413 - Blautia caecimuris	Radius_length
MGS	abundance	Hgn3C.0413 - Blautia caecimuris	Radius_SOS
MGS	abundance	Hgn3C.0413 - Blautia caecimuris	Tibia_SOS
MGS	abundance	Hgn3C.0413 - Blautia caecimuris	Handgrip
MGS	abundance	Hgn3C.0415 - Clostridium sp. C5-48	Tibia_length
MGS	abundance	Hgn3C.0415 - Clostridium sp. C5-48	Radius_length
MGS	abundance	Hgn3C.0415 - Clostridium sp. C5-48	Radius_SOS
MGS	abundance	Hgn3C.0415 - Clostridium sp. C5-48	Tibia_SOS
MGS	abundance	Hgn3C.0415 - Clostridium sp. C5-48	Handgrip

MGS	abundance	Hgn3C.0417 - Eubacteriales sp.	Tibia_length
MGS	abundance	Hgn3C.0417 - Eubacteriales sp.	Radius_length
MGS	abundance	Hgn3C.0417 - Eubacteriales sp.	Radius_SOS
MGS	abundance	Hgn3C.0417 - Eubacteriales sp.	Tibia_SOS
MGS	abundance	Hgn3C.0417 - Eubacteriales sp.	Handgrip
MGS	abundance	Hgn3C.0418 - Blautia sp.	Tibia_length
MGS	abundance	Hgn3C.0418 - Blautia sp.	Radius_length
MGS	abundance	Hgn3C.0418 - Blautia sp.	Radius_SOS
MGS	abundance	Hgn3C.0418 - Blautia sp.	Tibia_SOS
MGS	abundance	Hgn3C.0418 - Blautia sp.	Handgrip
MGS	abundance	Hgn3C.0419 - Prevotella sp. 885	Tibia_length
MGS	abundance	Hgn3C.0419 - Prevotella sp. 885	Radius_length
MGS	abundance	Hgn3C.0419 - Prevotella sp. 885	Radius_SOS
MGS	abundance	Hgn3C.0419 - Prevotella sp. 885	Tibia_SOS
MGS	abundance	Hgn3C.0419 - Prevotella sp. 885	Handgrip
MGS	abundance	Hgn3C.0420 - Clostridium sp. 1001270J_160509_D11	Tibia_length
MGS	abundance	Hgn3C.0420 - Clostridium sp. 1001270J_160509_D11	Radius_length
MGS	abundance	Hgn3C.0420 - Clostridium sp. 1001270J_160509_D11	Radius_SOS
MGS	abundance	Hgn3C.0420 - Clostridium sp. 1001270J_160509_D11	Tibia_SOS
MGS	abundance	Hgn3C.0420 - Clostridium sp. 1001270J_160509_D11	Handgrip
MGS	abundance	Hgn3C.0428 - Eubacteriales sp.	Tibia_length
MGS	abundance	Hgn3C.0428 - Eubacteriales sp.	Radius_length
MGS	abundance	Hgn3C.0428 - Eubacteriales sp.	Radius_SOS
MGS	abundance	Hgn3C.0428 - Eubacteriales sp.	Tibia_SOS
MGS	abundance	Hgn3C.0428 - Eubacteriales sp.	Handgrip
MGS	abundance	Hgn3C.0430 - Enterocloster aldenensis	Tibia_length
MGS	abundance	Hgn3C.0430 - Enterocloster aldenensis	Radius_length
MGS	abundance	Hgn3C.0430 - Enterocloster aldenensis	Radius_SOS
MGS	abundance	Hgn3C.0430 - Enterocloster aldenensis	Tibia_SOS
MGS	abundance	Hgn3C.0430 - Enterocloster aldenensis	Handgrip
MGS	abundance	Hgn3C.0434 - Firmicutes sp.	Tibia_length
MGS	abundance	Hgn3C.0434 - Firmicutes sp.	Radius_length
MGS	abundance	Hgn3C.0434 - Firmicutes sp.	Radius_SOS
MGS	abundance	Hgn3C.0434 - Firmicutes sp.	Tibia_SOS
MGS	abundance	Hgn3C.0434 - Firmicutes sp.	Handgrip
MGS	abundance	Hgn3C.0435 - Eubacteriales sp.	Tibia_length
MGS	abundance	Hgn3C.0435 - Eubacteriales sp.	Radius_length
MGS	abundance	Hgn3C.0435 - Eubacteriales sp.	Radius_SOS
MGS	abundance	Hgn3C.0435 - Eubacteriales sp.	Tibia_SOS
MGS	abundance	Hgn3C.0435 - Eubacteriales sp.	Handgrip
MGS	abundance	Hgn3C.0436 - Eubacteriales sp.	Tibia_length
MGS	abundance	Hgn3C.0436 - Eubacteriales sp.	Radius_length
MGS	abundance	Hgn3C.0436 - Eubacteriales sp.	Radius_SOS
MGS	abundance	Hgn3C.0436 - Eubacteriales sp.	Tibia_SOS
MGS	abundance	Hgn3C.0436 - Eubacteriales sp.	Handgrip
MGS	abundance	Hgn3C.0437 - Prevotella sp.	Tibia_length
MGS	abundance	Hgn3C.0437 - Prevotella sp.	Radius_length
MGS	abundance	Hgn3C.0437 - Prevotella sp.	Radius_SOS

MGS	abundance	Hgn3C.0437 - Prevotella sp.	Tibia_SOS
MGS	abundance	Hgn3C.0437 - Prevotella sp.	Handgrip
MGS	abundance	Hgn3C.0438 - Coprobacillus cateniformis	Tibia_length
MGS	abundance	Hgn3C.0438 - Coprobacillus cateniformis	Radius_length
MGS	abundance	Hgn3C.0438 - Coprobacillus cateniformis	Radius_SOS
MGS	abundance	Hgn3C.0438 - Coprobacillus cateniformis	Tibia_SOS
MGS	abundance	Hgn3C.0438 - Coprobacillus cateniformis	Handgrip
MGS	abundance	Hgn3C.0439 - Enterococcus faecalis	Tibia_length
MGS	abundance	Hgn3C.0439 - Enterococcus faecalis	Radius_length
MGS	abundance	Hgn3C.0439 - Enterococcus faecalis	Radius_SOS
MGS	abundance	Hgn3C.0439 - Enterococcus faecalis	Tibia_SOS
MGS	abundance	Hgn3C.0439 - Enterococcus faecalis	Handgrip
MGS	abundance	Hgn3C.0442 - Holdemanella sp.	Tibia_length
MGS	abundance	Hgn3C.0442 - Holdemanella sp.	Radius_length
MGS	abundance	Hgn3C.0442 - Holdemanella sp.	Radius_SOS
MGS	abundance	Hgn3C.0442 - Holdemanella sp.	Tibia_SOS
MGS	abundance	Hgn3C.0442 - Holdemanella sp.	Handgrip
MGS	abundance	Hgn3C.0443 - Prevotella stercorea	Tibia_length
MGS	abundance	Hgn3C.0443 - Prevotella stercorea	Radius_length
MGS	abundance	Hgn3C.0443 - Prevotella stercorea	Radius_SOS
MGS	abundance	Hgn3C.0443 - Prevotella stercorea	Tibia_SOS
MGS	abundance	Hgn3C.0443 - Prevotella stercorea	Handgrip
MGS	abundance	Hgn3C.0444 - Eubacteriales sp.	Tibia_length
MGS	abundance	Hgn3C.0444 - Eubacteriales sp.	Radius_length
MGS	abundance	Hgn3C.0444 - Eubacteriales sp.	Radius_SOS
MGS	abundance	Hgn3C.0444 - Eubacteriales sp.	Tibia_SOS
MGS	abundance	Hgn3C.0444 - Eubacteriales sp.	Handgrip
MGS	abundance	Hgn3C.0445 - Aeromonadales sp.	Tibia_length
MGS	abundance	Hgn3C.0445 - Aeromonadales sp.	Radius_length
MGS	abundance	Hgn3C.0445 - Aeromonadales sp.	Radius_SOS
MGS	abundance	Hgn3C.0445 - Aeromonadales sp.	Tibia_SOS
MGS	abundance	Hgn3C.0445 - Aeromonadales sp.	Handgrip
MGS	abundance	Hgn3C.0448 - Firmicutes sp.	Tibia_length
MGS	abundance	Hgn3C.0448 - Firmicutes sp.	Radius_length
MGS	abundance	Hgn3C.0448 - Firmicutes sp.	Radius_SOS
MGS	abundance	Hgn3C.0448 - Firmicutes sp.	Tibia_SOS
MGS	abundance	Hgn3C.0448 - Firmicutes sp.	Handgrip
MGS	abundance	Hgn3C.0451 - Roseburia sp. AM16-25	Tibia_length
MGS	abundance	Hgn3C.0451 - Roseburia sp. AM16-25	Radius_length
MGS	abundance	Hgn3C.0451 - Roseburia sp. AM16-25	Radius_SOS
MGS	abundance	Hgn3C.0451 - Roseburia sp. AM16-25	Tibia_SOS
MGS	abundance	Hgn3C.0451 - Roseburia sp. AM16-25	Handgrip
MGS	abundance	Hgn3C.0452 - Oscillospiraceae sp.	Tibia_length
MGS	abundance	Hgn3C.0452 - Oscillospiraceae sp.	Radius_length
MGS	abundance	Hgn3C.0452 - Oscillospiraceae sp.	Radius_SOS
MGS	abundance	Hgn3C.0452 - Oscillospiraceae sp.	Tibia_SOS
MGS	abundance	Hgn3C.0452 - Oscillospiraceae sp.	Handgrip
MGS	abundance	Hgn3C.0453 - Blautia sp. Marseille-P3087	Tibia_length

MGS	abundance	Hgn3C.0453 - Blautia sp. Marseille-P3087	Radius_length
MGS	abundance	Hgn3C.0453 - Blautia sp. Marseille-P3087	Radius_SOS
MGS	abundance	Hgn3C.0453 - Blautia sp. Marseille-P3087	Tibia_SOS
MGS	abundance	Hgn3C.0453 - Blautia sp. Marseille-P3087	Handgrip
MGS	abundance	Hgn3C.0455 - Peptostreptococcaceae sp.	Tibia_length
MGS	abundance	Hgn3C.0455 - Peptostreptococcaceae sp.	Radius_length
MGS	abundance	Hgn3C.0455 - Peptostreptococcaceae sp.	Radius_SOS
MGS	abundance	Hgn3C.0455 - Peptostreptococcaceae sp.	Tibia_SOS
MGS	abundance	Hgn3C.0455 - Peptostreptococcaceae sp.	Handgrip
MGS	abundance	Hgn3C.0456 - Alistipes sp.	Tibia_length
MGS	abundance	Hgn3C.0456 - Alistipes sp.	Radius_length
MGS	abundance	Hgn3C.0456 - Alistipes sp.	Radius_SOS
MGS	abundance	Hgn3C.0456 - Alistipes sp.	Tibia_SOS
MGS	abundance	Hgn3C.0456 - Alistipes sp.	Handgrip
MGS	abundance	Hgn3C.0457 - Coprobacter secundus subsp. similis	Tibia_length
MGS	abundance	Hgn3C.0457 - Coprobacter secundus subsp. similis	Radius_length
MGS	abundance	Hgn3C.0457 - Coprobacter secundus subsp. similis	Radius_SOS
MGS	abundance	Hgn3C.0457 - Coprobacter secundus subsp. similis	Tibia_SOS
MGS	abundance	Hgn3C.0457 - Coprobacter secundus subsp. similis	Handgrip
MGS	abundance	Hgn3C.0458 - Eubacteriales sp.	Tibia_length
MGS	abundance	Hgn3C.0458 - Eubacteriales sp.	Radius_length
MGS	abundance	Hgn3C.0458 - Eubacteriales sp.	Radius_SOS
MGS	abundance	Hgn3C.0458 - Eubacteriales sp.	Tibia_SOS
MGS	abundance	Hgn3C.0458 - Eubacteriales sp.	Handgrip
MGS	abundance	Hgn3C.0459 - Eubacteriales sp.	Tibia_length
MGS	abundance	Hgn3C.0459 - Eubacteriales sp.	Radius_length
MGS	abundance	Hgn3C.0459 - Eubacteriales sp.	Radius_SOS
MGS	abundance	Hgn3C.0459 - Eubacteriales sp.	Tibia_SOS
MGS	abundance	Hgn3C.0459 - Eubacteriales sp.	Handgrip
MGS	abundance	Hgn3C.0464 - Bifidobacterium longum subsp. infantis	Tibia_length
MGS	abundance	Hgn3C.0464 - Bifidobacterium longum subsp. infantis	Radius_length
MGS	abundance	Hgn3C.0464 - Bifidobacterium longum subsp. infantis	Radius_SOS
MGS	abundance	Hgn3C.0464 - Bifidobacterium longum subsp. infantis	Tibia_SOS
MGS	abundance	Hgn3C.0464 - Bifidobacterium longum subsp. infantis	Handgrip
MGS	abundance	Hgn3C.0465 - Eisenbergiella massiliensis	Tibia_length
MGS	abundance	Hgn3C.0465 - Eisenbergiella massiliensis	Radius_length
MGS	abundance	Hgn3C.0465 - Eisenbergiella massiliensis	Radius_SOS
MGS	abundance	Hgn3C.0465 - Eisenbergiella massiliensis	Tibia_SOS
MGS	abundance	Hgn3C.0465 - Eisenbergiella massiliensis	Handgrip
MGS	abundance	Hgn3C.0468 - Clostridium sp. NSJ-42	Tibia_length
MGS	abundance	Hgn3C.0468 - Clostridium sp. NSJ-42	Radius_length
MGS	abundance	Hgn3C.0468 - Clostridium sp. NSJ-42	Radius_SOS
MGS	abundance	Hgn3C.0468 - Clostridium sp. NSJ-42	Tibia_SOS
MGS	abundance	Hgn3C.0468 - Clostridium sp. NSJ-42	Handgrip
MGS	abundance	Hgn3C.0470 - Eubacteriales sp.	Tibia_length
MGS	abundance	Hgn3C.0470 - Eubacteriales sp.	Radius_length
MGS	abundance	Hgn3C.0470 - Eubacteriales sp.	Radius_SOS
MGS	abundance	Hgn3C.0470 - Eubacteriales sp.	Tibia_SOS

MGS	abundance	Hgn3C.0470 - Eubacteriales sp.	Handgrip
MGS	abundance	Hgn3C.0472 - Parabacteroides goldsteinii	Tibia_length
MGS	abundance	Hgn3C.0472 - Parabacteroides goldsteinii	Radius_length
MGS	abundance	Hgn3C.0472 - Parabacteroides goldsteinii	Radius_SOS
MGS	abundance	Hgn3C.0472 - Parabacteroides goldsteinii	Tibia_SOS
MGS	abundance	Hgn3C.0472 - Parabacteroides goldsteinii	Handgrip
MGS	abundance	Hgn3C.0475 - Coriobacteriia sp.	Tibia_length
MGS	abundance	Hgn3C.0475 - Coriobacteriia sp.	Radius_length
MGS	abundance	Hgn3C.0475 - Coriobacteriia sp.	Radius_SOS
MGS	abundance	Hgn3C.0475 - Coriobacteriia sp.	Tibia_SOS
MGS	abundance	Hgn3C.0475 - Coriobacteriia sp.	Handgrip
MGS	abundance	Hgn3C.0476 - Streptococcus sp.	Tibia_length
MGS	abundance	Hgn3C.0476 - Streptococcus sp.	Radius_length
MGS	abundance	Hgn3C.0476 - Streptococcus sp.	Radius_SOS
MGS	abundance	Hgn3C.0476 - Streptococcus sp.	Tibia_SOS
MGS	abundance	Hgn3C.0476 - Streptococcus sp.	Handgrip
MGS	abundance	Hgn3C.0477 - Eubacteriales sp.	Tibia_length
MGS	abundance	Hgn3C.0477 - Eubacteriales sp.	Radius_length
MGS	abundance	Hgn3C.0477 - Eubacteriales sp.	Radius_SOS
MGS	abundance	Hgn3C.0477 - Eubacteriales sp.	Tibia_SOS
MGS	abundance	Hgn3C.0477 - Eubacteriales sp.	Handgrip
MGS	abundance	Hgn3C.0482 - Lachnospiraceae sp.	Tibia_length
MGS	abundance	Hgn3C.0482 - Lachnospiraceae sp.	Radius_length
MGS	abundance	Hgn3C.0482 - Lachnospiraceae sp.	Radius_SOS
MGS	abundance	Hgn3C.0482 - Lachnospiraceae sp.	Tibia_SOS
MGS	abundance	Hgn3C.0482 - Lachnospiraceae sp.	Handgrip
MGS	abundance	Hgn3C.0489 - Eubacteriales sp.	Tibia_length
MGS	abundance	Hgn3C.0489 - Eubacteriales sp.	Radius_length
MGS	abundance	Hgn3C.0489 - Eubacteriales sp.	Radius_SOS
MGS	abundance	Hgn3C.0489 - Eubacteriales sp.	Tibia_SOS
MGS	abundance	Hgn3C.0489 - Eubacteriales sp.	Handgrip
MGS	abundance	Hgn3C.0492 - Prevotellaceae sp.	Tibia_length
MGS	abundance	Hgn3C.0492 - Prevotellaceae sp.	Radius_length
MGS	abundance	Hgn3C.0492 - Prevotellaceae sp.	Radius_SOS
MGS	abundance	Hgn3C.0492 - Prevotellaceae sp.	Tibia_SOS
MGS	abundance	Hgn3C.0492 - Prevotellaceae sp.	Handgrip
MGS	abundance	Hgn3C.0493 - Eisenbergiella tayi	Tibia_length
MGS	abundance	Hgn3C.0493 - Eisenbergiella tayi	Radius_length
MGS	abundance	Hgn3C.0493 - Eisenbergiella tayi	Radius_SOS
MGS	abundance	Hgn3C.0493 - Eisenbergiella tayi	Tibia_SOS
MGS	abundance	Hgn3C.0493 - Eisenbergiella tayi	Handgrip
MGS	abundance	Hgn3C.0494 - Prevotellaceae sp.	Tibia_length
MGS	abundance	Hgn3C.0494 - Prevotellaceae sp.	Radius_length
MGS	abundance	Hgn3C.0494 - Prevotellaceae sp.	Radius_SOS
MGS	abundance	Hgn3C.0494 - Prevotellaceae sp.	Tibia_SOS
MGS	abundance	Hgn3C.0494 - Prevotellaceae sp.	Handgrip
MGS	abundance	Hgn3C.0495 - Ruminococcus sp.	Tibia_length
MGS	abundance	Hgn3C.0495 - Ruminococcus sp.	Radius_length

MGS	abundance	Hgn3C.0495 - Ruminococcus sp.	Radius_SOS
MGS	abundance	Hgn3C.0495 - Ruminococcus sp.	Tibia_SOS
MGS	abundance	Hgn3C.0495 - Ruminococcus sp.	Handgrip
MGS	abundance	Hgn3C.0498 - Prevotella sp.	Tibia_length
MGS	abundance	Hgn3C.0498 - Prevotella sp.	Radius_length
MGS	abundance	Hgn3C.0498 - Prevotella sp.	Radius_SOS
MGS	abundance	Hgn3C.0498 - Prevotella sp.	Tibia_SOS
MGS	abundance	Hgn3C.0498 - Prevotella sp.	Handgrip
MGS	abundance	Hgn3C.0499 - Bacteroides sp.	Tibia_length
MGS	abundance	Hgn3C.0499 - Bacteroides sp.	Radius_length
MGS	abundance	Hgn3C.0499 - Bacteroides sp.	Radius_SOS
MGS	abundance	Hgn3C.0499 - Bacteroides sp.	Tibia_SOS
MGS	abundance	Hgn3C.0499 - Bacteroides sp.	Handgrip
MGS	abundance	Hgn3C.0500 - Anaerotignum sp.	Tibia_length
MGS	abundance	Hgn3C.0500 - Anaerotignum sp.	Radius_length
MGS	abundance	Hgn3C.0500 - Anaerotignum sp.	Radius_SOS
MGS	abundance	Hgn3C.0500 - Anaerotignum sp.	Tibia_SOS
MGS	abundance	Hgn3C.0500 - Anaerotignum sp.	Handgrip
MGS	abundance	Hgn3C.0501 - Oscillospiraceae sp.	Tibia_length
MGS	abundance	Hgn3C.0501 - Oscillospiraceae sp.	Radius_length
MGS	abundance	Hgn3C.0501 - Oscillospiraceae sp.	Radius_SOS
MGS	abundance	Hgn3C.0501 - Oscillospiraceae sp.	Tibia_SOS
MGS	abundance	Hgn3C.0501 - Oscillospiraceae sp.	Handgrip
MGS	abundance	Hgn3C.0503 - Eubacteriales sp.	Tibia_length
MGS	abundance	Hgn3C.0503 - Eubacteriales sp.	Radius_length
MGS	abundance	Hgn3C.0503 - Eubacteriales sp.	Radius_SOS
MGS	abundance	Hgn3C.0503 - Eubacteriales sp.	Tibia_SOS
MGS	abundance	Hgn3C.0503 - Eubacteriales sp.	Handgrip
MGS	abundance	Hgn3C.0504 - Megasphaera sp. BL7	Tibia_length
MGS	abundance	Hgn3C.0504 - Megasphaera sp. BL7	Radius_length
MGS	abundance	Hgn3C.0504 - Megasphaera sp. BL7	Radius_SOS
MGS	abundance	Hgn3C.0504 - Megasphaera sp. BL7	Tibia_SOS
MGS	abundance	Hgn3C.0504 - Megasphaera sp. BL7	Handgrip
MGS	abundance	Hgn3C.0507 - [Clostridium] spiroforme	Tibia_length
MGS	abundance	Hgn3C.0507 - [Clostridium] spiroforme	Radius_length
MGS	abundance	Hgn3C.0507 - [Clostridium] spiroforme	Radius_SOS
MGS	abundance	Hgn3C.0507 - [Clostridium] spiroforme	Tibia_SOS
MGS	abundance	Hgn3C.0507 - [Clostridium] spiroforme	Handgrip
MGS	abundance	Hgn3C.0514 - Blautia glucerasea	Tibia_length
MGS	abundance	Hgn3C.0514 - Blautia glucerasea	Radius_length
MGS	abundance	Hgn3C.0514 - Blautia glucerasea	Radius_SOS
MGS	abundance	Hgn3C.0514 - Blautia glucerasea	Tibia_SOS
MGS	abundance	Hgn3C.0514 - Blautia glucerasea	Handgrip
MGS	abundance	Hgn3C.0518 - Eubacteriales sp.	Tibia_length
MGS	abundance	Hgn3C.0518 - Eubacteriales sp.	Radius_length
MGS	abundance	Hgn3C.0518 - Eubacteriales sp.	Radius_SOS
MGS	abundance	Hgn3C.0518 - Eubacteriales sp.	Tibia_SOS
MGS	abundance	Hgn3C.0518 - Eubacteriales sp.	Handgrip

MGS	abundance	Hgn3C.0522 - Roseburia sp. BX1005	Tibia_length
MGS	abundance	Hgn3C.0522 - Roseburia sp. BX1005	Radius_length
MGS	abundance	Hgn3C.0522 - Roseburia sp. BX1005	Radius_SOS
MGS	abundance	Hgn3C.0522 - Roseburia sp. BX1005	Tibia_SOS
MGS	abundance	Hgn3C.0522 - Roseburia sp. BX1005	Handgrip
MGS	abundance	Hgn3C.0523 - Phascolarctobacterium succinatutens	Tibia_length
MGS	abundance	Hgn3C.0523 - Phascolarctobacterium succinatutens	Radius_length
MGS	abundance	Hgn3C.0523 - Phascolarctobacterium succinatutens	Radius_SOS
MGS	abundance	Hgn3C.0523 - Phascolarctobacterium succinatutens	Tibia_SOS
MGS	abundance	Hgn3C.0523 - Phascolarctobacterium succinatutens	Handgrip
MGS	abundance	Hgn3C.0524 - Butyricicoccus sp.	Tibia_length
MGS	abundance	Hgn3C.0524 - Butyricicoccus sp.	Radius_length
MGS	abundance	Hgn3C.0524 - Butyricicoccus sp.	Radius_SOS
MGS	abundance	Hgn3C.0524 - Butyricicoccus sp.	Tibia_SOS
MGS	abundance	Hgn3C.0524 - Butyricicoccus sp.	Handgrip
MGS	abundance	Hgn3C.0526 - Anaerostipes sp.	Tibia_length
MGS	abundance	Hgn3C.0526 - Anaerostipes sp.	Radius_length
MGS	abundance	Hgn3C.0526 - Anaerostipes sp.	Radius_SOS
MGS	abundance	Hgn3C.0526 - Anaerostipes sp.	Tibia_SOS
MGS	abundance	Hgn3C.0526 - Anaerostipes sp.	Handgrip
MGS	abundance	Hgn3C.0527 - Sutterella sp.	Tibia_length
MGS	abundance	Hgn3C.0527 - Sutterella sp.	Radius_length
MGS	abundance	Hgn3C.0527 - Sutterella sp.	Radius_SOS
MGS	abundance	Hgn3C.0527 - Sutterella sp.	Tibia_SOS
MGS	abundance	Hgn3C.0527 - Sutterella sp.	Handgrip
MGS	abundance	Hgn3C.0528 - Coprococcus sp. AM27-12LB	Tibia_length
MGS	abundance	Hgn3C.0528 - Coprococcus sp. AM27-12LB	Radius_length
MGS	abundance	Hgn3C.0528 - Coprococcus sp. AM27-12LB	Radius_SOS
MGS	abundance	Hgn3C.0528 - Coprococcus sp. AM27-12LB	Tibia_SOS
MGS	abundance	Hgn3C.0528 - Coprococcus sp. AM27-12LB	Handgrip
MGS	abundance	Hgn3C.0532 - Paraprevotella xylaniphila	Tibia_length
MGS	abundance	Hgn3C.0532 - Paraprevotella xylaniphila	Radius_length
MGS	abundance	Hgn3C.0532 - Paraprevotella xylaniphila	Radius_SOS
MGS	abundance	Hgn3C.0532 - Paraprevotella xylaniphila	Tibia_SOS
MGS	abundance	Hgn3C.0532 - Paraprevotella xylaniphila	Handgrip
MGS	abundance	Hgn3C.0533 - Flintibacter sp. KGMB00164	Tibia_length
MGS	abundance	Hgn3C.0533 - Flintibacter sp. KGMB00164	Radius_length
MGS	abundance	Hgn3C.0533 - Flintibacter sp. KGMB00164	Radius_SOS
MGS	abundance	Hgn3C.0533 - Flintibacter sp. KGMB00164	Tibia_SOS
MGS	abundance	Hgn3C.0533 - Flintibacter sp. KGMB00164	Handgrip
MGS	abundance	Hgn3C.0535 - Victivallis vadensis	Tibia_length
MGS	abundance	Hgn3C.0535 - Victivallis vadensis	Radius_length
MGS	abundance	Hgn3C.0535 - Victivallis vadensis	Radius_SOS
MGS	abundance	Hgn3C.0535 - Victivallis vadensis	Tibia_SOS
MGS	abundance	Hgn3C.0535 - Victivallis vadensis	Handgrip
MGS	abundance	Hgn3C.0537 - Bifidobacterium dentium	Tibia_length
MGS	abundance	Hgn3C.0537 - Bifidobacterium dentium	Radius_length
MGS	abundance	Hgn3C.0537 - Bifidobacterium dentium	Radius_SOS

MGS	abundance	Hgn3C.0537 - Bifidobacterium dentium	Tibia_SOS
MGS	abundance	Hgn3C.0537 - Bifidobacterium dentium	Handgrip
MGS	abundance	Hgn3C.0540 - Firmicutes sp.	Tibia_length
MGS	abundance	Hgn3C.0540 - Firmicutes sp.	Radius_length
MGS	abundance	Hgn3C.0540 - Firmicutes sp.	Radius_SOS
MGS	abundance	Hgn3C.0540 - Firmicutes sp.	Tibia_SOS
MGS	abundance	Hgn3C.0540 - Firmicutes sp.	Handgrip
MGS	abundance	Hgn3C.0541 - Phocaeicola plebeius	Tibia_length
MGS	abundance	Hgn3C.0541 - Phocaeicola plebeius	Radius_length
MGS	abundance	Hgn3C.0541 - Phocaeicola plebeius	Radius_SOS
MGS	abundance	Hgn3C.0541 - Phocaeicola plebeius	Tibia_SOS
MGS	abundance	Hgn3C.0541 - Phocaeicola plebeius	Handgrip
MGS	abundance	Hgn3C.0542 - [Clostridium] innocuum	Tibia_length
MGS	abundance	Hgn3C.0542 - [Clostridium] innocuum	Radius_length
MGS	abundance	Hgn3C.0542 - [Clostridium] innocuum	Radius_SOS
MGS	abundance	Hgn3C.0542 - [Clostridium] innocuum	Tibia_SOS
MGS	abundance	Hgn3C.0542 - [Clostridium] innocuum	Handgrip
MGS	abundance	Hgn3C.0543 - Oscillospiraceae sp.	Tibia_length
MGS	abundance	Hgn3C.0543 - Oscillospiraceae sp.	Radius_length
MGS	abundance	Hgn3C.0543 - Oscillospiraceae sp.	Radius_SOS
MGS	abundance	Hgn3C.0543 - Oscillospiraceae sp.	Tibia_SOS
MGS	abundance	Hgn3C.0543 - Oscillospiraceae sp.	Handgrip
MGS	abundance	Hgn3C.0544 - Eubacteriales sp.	Tibia_length
MGS	abundance	Hgn3C.0544 - Eubacteriales sp.	Radius_length
MGS	abundance	Hgn3C.0544 - Eubacteriales sp.	Radius_SOS
MGS	abundance	Hgn3C.0544 - Eubacteriales sp.	Tibia_SOS
MGS	abundance	Hgn3C.0544 - Eubacteriales sp.	Handgrip
MGS	abundance	Hgn3C.0546 - Bacteroidia sp.	Tibia_length
MGS	abundance	Hgn3C.0546 - Bacteroidia sp.	Radius_length
MGS	abundance	Hgn3C.0546 - Bacteroidia sp.	Radius_SOS
MGS	abundance	Hgn3C.0546 - Bacteroidia sp.	Tibia_SOS
MGS	abundance	Hgn3C.0546 - Bacteroidia sp.	Handgrip
MGS	abundance	Hgn3C.0549 - Longicatena caecimuris	Tibia_length
MGS	abundance	Hgn3C.0549 - Longicatena caecimuris	Radius_length
MGS	abundance	Hgn3C.0549 - Longicatena caecimuris	Radius_SOS
MGS	abundance	Hgn3C.0549 - Longicatena caecimuris	Tibia_SOS
MGS	abundance	Hgn3C.0549 - Longicatena caecimuris	Handgrip
MGS	abundance	Hgn3C.0557 - Enterocloster bolteae	Tibia_length
MGS	abundance	Hgn3C.0557 - Enterocloster bolteae	Radius_length
MGS	abundance	Hgn3C.0557 - Enterocloster bolteae	Radius_SOS
MGS	abundance	Hgn3C.0557 - Enterocloster bolteae	Tibia_SOS
MGS	abundance	Hgn3C.0557 - Enterocloster bolteae	Handgrip
MGS	abundance	Hgn3C.0558 - Olsenella sp.	Tibia_length
MGS	abundance	Hgn3C.0558 - Olsenella sp.	Radius_length
MGS	abundance	Hgn3C.0558 - Olsenella sp.	Radius_SOS
MGS	abundance	Hgn3C.0558 - Olsenella sp.	Tibia_SOS
MGS	abundance	Hgn3C.0558 - Olsenella sp.	Handgrip
MGS	abundance	Hgn3C.0560 - Oscillospiraceae sp.	Tibia_length

MGS	abundance	Hgn3C.0560 - Oscillospiraceae sp.	Radius_length
MGS	abundance	Hgn3C.0560 - Oscillospiraceae sp.	Radius_SOS
MGS	abundance	Hgn3C.0560 - Oscillospiraceae sp.	Tibia_SOS
MGS	abundance	Hgn3C.0560 - Oscillospiraceae sp.	Handgrip
MGS	abundance	Hgn3C.0561 - Enterocloster clostridioformis	Tibia_length
MGS	abundance	Hgn3C.0561 - Enterocloster clostridioformis	Radius_length
MGS	abundance	Hgn3C.0561 - Enterocloster clostridioformis	Radius_SOS
MGS	abundance	Hgn3C.0561 - Enterocloster clostridioformis	Tibia_SOS
MGS	abundance	Hgn3C.0561 - Enterocloster clostridioformis	Handgrip
MGS	abundance	Hgn3C.0570 - Eubacteriales sp.	Tibia_length
MGS	abundance	Hgn3C.0570 - Eubacteriales sp.	Radius_length
MGS	abundance	Hgn3C.0570 - Eubacteriales sp.	Radius_SOS
MGS	abundance	Hgn3C.0570 - Eubacteriales sp.	Tibia_SOS
MGS	abundance	Hgn3C.0570 - Eubacteriales sp.	Handgrip
MGS	abundance	Hgn3C.0573 - Prevotella sp. Marseille-P4119	Tibia_length
MGS	abundance	Hgn3C.0573 - Prevotella sp. Marseille-P4119	Radius_length
MGS	abundance	Hgn3C.0573 - Prevotella sp. Marseille-P4119	Radius_SOS
MGS	abundance	Hgn3C.0573 - Prevotella sp. Marseille-P4119	Tibia_SOS
MGS	abundance	Hgn3C.0573 - Prevotella sp. Marseille-P4119	Handgrip
MGS	abundance	Hgn3C.0575 - Bacteria sp.	Tibia_length
MGS	abundance	Hgn3C.0575 - Bacteria sp.	Radius_length
MGS	abundance	Hgn3C.0575 - Bacteria sp.	Radius_SOS
MGS	abundance	Hgn3C.0575 - Bacteria sp.	Tibia_SOS
MGS	abundance	Hgn3C.0575 - Bacteria sp.	Handgrip
MGS	abundance	Hgn3C.0579 - Eubacteriales sp.	Tibia_length
MGS	abundance	Hgn3C.0579 - Eubacteriales sp.	Radius_length
MGS	abundance	Hgn3C.0579 - Eubacteriales sp.	Radius_SOS
MGS	abundance	Hgn3C.0579 - Eubacteriales sp.	Tibia_SOS
MGS	abundance	Hgn3C.0579 - Eubacteriales sp.	Handgrip
MGS	abundance	Hgn3C.0581 - Negativibacillus massiliensis	Tibia_length
MGS	abundance	Hgn3C.0581 - Negativibacillus massiliensis	Radius_length
MGS	abundance	Hgn3C.0581 - Negativibacillus massiliensis	Radius_SOS
MGS	abundance	Hgn3C.0581 - Negativibacillus massiliensis	Tibia_SOS
MGS	abundance	Hgn3C.0581 - Negativibacillus massiliensis	Handgrip
MGS	abundance	Hgn3C.0582 - Bacteroides sp.	Tibia_length
MGS	abundance	Hgn3C.0582 - Bacteroides sp.	Radius_length
MGS	abundance	Hgn3C.0582 - Bacteroides sp.	Radius_SOS
MGS	abundance	Hgn3C.0582 - Bacteroides sp.	Tibia_SOS
MGS	abundance	Hgn3C.0582 - Bacteroides sp.	Handgrip
MGS	abundance	Hgn3C.0584 - Bacteroides fragilis	Tibia_length
MGS	abundance	Hgn3C.0584 - Bacteroides fragilis	Radius_length
MGS	abundance	Hgn3C.0584 - Bacteroides fragilis	Radius_SOS
MGS	abundance	Hgn3C.0584 - Bacteroides fragilis	Tibia_SOS
MGS	abundance	Hgn3C.0584 - Bacteroides fragilis	Handgrip
MGS	abundance	Hgn3C.0590 - Clostridia sp.	Tibia_length
MGS	abundance	Hgn3C.0590 - Clostridia sp.	Radius_length
MGS	abundance	Hgn3C.0590 - Clostridia sp.	Radius_SOS
MGS	abundance	Hgn3C.0590 - Clostridia sp.	Tibia_SOS

MGS	abundance	Hgn3C.0590 - Clostridia sp.	Handgrip
MGS	abundance	Hgn3C.0595 - Eubacteriales sp.	Tibia_length
MGS	abundance	Hgn3C.0595 - Eubacteriales sp.	Radius_length
MGS	abundance	Hgn3C.0595 - Eubacteriales sp.	Radius_SOS
MGS	abundance	Hgn3C.0595 - Eubacteriales sp.	Tibia_SOS
MGS	abundance	Hgn3C.0595 - Eubacteriales sp.	Handgrip
MGS	abundance	Hgn3C.0596 - Butyricimonas sp.	Tibia_length
MGS	abundance	Hgn3C.0596 - Butyricimonas sp.	Radius_length
MGS	abundance	Hgn3C.0596 - Butyricimonas sp.	Radius_SOS
MGS	abundance	Hgn3C.0596 - Butyricimonas sp.	Tibia_SOS
MGS	abundance	Hgn3C.0596 - Butyricimonas sp.	Handgrip
MGS	abundance	Hgn3C.0598 - Eubacteriales sp.	Tibia_length
MGS	abundance	Hgn3C.0598 - Eubacteriales sp.	Radius_length
MGS	abundance	Hgn3C.0598 - Eubacteriales sp.	Radius_SOS
MGS	abundance	Hgn3C.0598 - Eubacteriales sp.	Tibia_SOS
MGS	abundance	Hgn3C.0598 - Eubacteriales sp.	Handgrip
MGS	abundance	Hgn3C.0601 - Bacteroidales sp.	Tibia_length
MGS	abundance	Hgn3C.0601 - Bacteroidales sp.	Radius_length
MGS	abundance	Hgn3C.0601 - Bacteroidales sp.	Radius_SOS
MGS	abundance	Hgn3C.0601 - Bacteroidales sp.	Tibia_SOS
MGS	abundance	Hgn3C.0601 - Bacteroidales sp.	Handgrip
MGS	abundance	Hgn3C.0604 - Megasphaera elsdenii	Tibia_length
MGS	abundance	Hgn3C.0604 - Megasphaera elsdenii	Radius_length
MGS	abundance	Hgn3C.0604 - Megasphaera elsdenii	Radius_SOS
MGS	abundance	Hgn3C.0604 - Megasphaera elsdenii	Tibia_SOS
MGS	abundance	Hgn3C.0604 - Megasphaera elsdenii	Handgrip
MGS	abundance	Hgn3C.0607 - Eubacteriales sp.	Tibia_length
MGS	abundance	Hgn3C.0607 - Eubacteriales sp.	Radius_length
MGS	abundance	Hgn3C.0607 - Eubacteriales sp.	Radius_SOS
MGS	abundance	Hgn3C.0607 - Eubacteriales sp.	Tibia_SOS
MGS	abundance	Hgn3C.0607 - Eubacteriales sp.	Handgrip
MGS	abundance	Hgn3C.0608 - Lachnospiraceae sp.	Tibia_length
MGS	abundance	Hgn3C.0608 - Lachnospiraceae sp.	Radius_length
MGS	abundance	Hgn3C.0608 - Lachnospiraceae sp.	Radius_SOS
MGS	abundance	Hgn3C.0608 - Lachnospiraceae sp.	Tibia_SOS
MGS	abundance	Hgn3C.0608 - Lachnospiraceae sp.	Handgrip
MGS	abundance	Hgn3C.0610 - Eubacteriales sp.	Tibia_length
MGS	abundance	Hgn3C.0610 - Eubacteriales sp.	Radius_length
MGS	abundance	Hgn3C.0610 - Eubacteriales sp.	Radius_SOS
MGS	abundance	Hgn3C.0610 - Eubacteriales sp.	Tibia_SOS
MGS	abundance	Hgn3C.0610 - Eubacteriales sp.	Handgrip
MGS	abundance	Hgn3C.0612 - Bacteroidales sp.	Tibia_length
MGS	abundance	Hgn3C.0612 - Bacteroidales sp.	Radius_length
MGS	abundance	Hgn3C.0612 - Bacteroidales sp.	Radius_SOS
MGS	abundance	Hgn3C.0612 - Bacteroidales sp.	Tibia_SOS
MGS	abundance	Hgn3C.0612 - Bacteroidales sp.	Handgrip
MGS	abundance	Hgn3C.0613 - Clostridiaceae sp.	Tibia_length
MGS	abundance	Hgn3C.0613 - Clostridiaceae sp.	Radius_length

MGS	abundance	Hgn3C.0613 - Clostridiaceae sp.	Radius_SOS
MGS	abundance	Hgn3C.0613 - Clostridiaceae sp.	Tibia_SOS
MGS	abundance	Hgn3C.0613 - Clostridiaceae sp.	Handgrip
MGS	abundance	Hgn3C.0616 - Lachnospiraceae sp.	Tibia_length
MGS	abundance	Hgn3C.0616 - Lachnospiraceae sp.	Radius_length
MGS	abundance	Hgn3C.0616 - Lachnospiraceae sp.	Radius_SOS
MGS	abundance	Hgn3C.0616 - Lachnospiraceae sp.	Tibia_SOS
MGS	abundance	Hgn3C.0616 - Lachnospiraceae sp.	Handgrip
MGS	abundance	Hgn3C.0617 - Prevotella sp.	Tibia_length
MGS	abundance	Hgn3C.0617 - Prevotella sp.	Radius_length
MGS	abundance	Hgn3C.0617 - Prevotella sp.	Radius_SOS
MGS	abundance	Hgn3C.0617 - Prevotella sp.	Tibia_SOS
MGS	abundance	Hgn3C.0617 - Prevotella sp.	Handgrip
MGS	abundance	Hgn3C.0629 - Collinsella intestinalis	Tibia_length
MGS	abundance	Hgn3C.0629 - Collinsella intestinalis	Radius_length
MGS	abundance	Hgn3C.0629 - Collinsella intestinalis	Radius_SOS
MGS	abundance	Hgn3C.0629 - Collinsella intestinalis	Tibia_SOS
MGS	abundance	Hgn3C.0629 - Collinsella intestinalis	Handgrip
MGS	abundance	Hgn3C.0630 - Allisonella histaminiformans	Tibia_length
MGS	abundance	Hgn3C.0630 - Allisonella histaminiformans	Radius_length
MGS	abundance	Hgn3C.0630 - Allisonella histaminiformans	Radius_SOS
MGS	abundance	Hgn3C.0630 - Allisonella histaminiformans	Tibia_SOS
MGS	abundance	Hgn3C.0630 - Allisonella histaminiformans	Handgrip
MGS	abundance	Hgn3C.0634 - Blautia schinkii	Tibia_length
MGS	abundance	Hgn3C.0634 - Blautia schinkii	Radius_length
MGS	abundance	Hgn3C.0634 - Blautia schinkii	Radius_SOS
MGS	abundance	Hgn3C.0634 - Blautia schinkii	Tibia_SOS
MGS	abundance	Hgn3C.0634 - Blautia schinkii	Handgrip
MGS	abundance	Hgn3C.0635 - Eubacteriales sp.	Tibia_length
MGS	abundance	Hgn3C.0635 - Eubacteriales sp.	Radius_length
MGS	abundance	Hgn3C.0635 - Eubacteriales sp.	Radius_SOS
MGS	abundance	Hgn3C.0635 - Eubacteriales sp.	Tibia_SOS
MGS	abundance	Hgn3C.0635 - Eubacteriales sp.	Handgrip
MGS	abundance	Hgn3C.0636 - Anaerostipes caccae	Tibia_length
MGS	abundance	Hgn3C.0636 - Anaerostipes caccae	Radius_length
MGS	abundance	Hgn3C.0636 - Anaerostipes caccae	Radius_SOS
MGS	abundance	Hgn3C.0636 - Anaerostipes caccae	Tibia_SOS
MGS	abundance	Hgn3C.0636 - Anaerostipes caccae	Handgrip
MGS	abundance	Hgn3C.0640 - Eubacterium sp. AF22-8LB	Tibia_length
MGS	abundance	Hgn3C.0640 - Eubacterium sp. AF22-8LB	Radius_length
MGS	abundance	Hgn3C.0640 - Eubacterium sp. AF22-8LB	Radius_SOS
MGS	abundance	Hgn3C.0640 - Eubacterium sp. AF22-8LB	Tibia_SOS
MGS	abundance	Hgn3C.0640 - Eubacterium sp. AF22-8LB	Handgrip
MGS	abundance	Hgn3C.0643 - Eubacteriales sp.	Tibia_length
MGS	abundance	Hgn3C.0643 - Eubacteriales sp.	Radius_length
MGS	abundance	Hgn3C.0643 - Eubacteriales sp.	Radius_SOS
MGS	abundance	Hgn3C.0643 - Eubacteriales sp.	Tibia_SOS
MGS	abundance	Hgn3C.0643 - Eubacteriales sp.	Handgrip

MGS	abundance	Hgn3C.0644 - Bacteroides cellulosilyticus	Tibia_length
MGS	abundance	Hgn3C.0644 - Bacteroides cellulosilyticus	Radius_length
MGS	abundance	Hgn3C.0644 - Bacteroides cellulosilyticus	Radius_SOS
MGS	abundance	Hgn3C.0644 - Bacteroides cellulosilyticus	Tibia_SOS
MGS	abundance	Hgn3C.0644 - Bacteroides cellulosilyticus	Handgrip
MGS	abundance	Hgn3C.0645 - Eubacteriales sp.	Tibia_length
MGS	abundance	Hgn3C.0645 - Eubacteriales sp.	Radius_length
MGS	abundance	Hgn3C.0645 - Eubacteriales sp.	Radius_SOS
MGS	abundance	Hgn3C.0645 - Eubacteriales sp.	Tibia_SOS
MGS	abundance	Hgn3C.0645 - Eubacteriales sp.	Handgrip
MGS	abundance	Hgn3C.0649 - Lachnospiraceae sp.	Tibia_length
MGS	abundance	Hgn3C.0649 - Lachnospiraceae sp.	Radius_length
MGS	abundance	Hgn3C.0649 - Lachnospiraceae sp.	Radius_SOS
MGS	abundance	Hgn3C.0649 - Lachnospiraceae sp.	Tibia_SOS
MGS	abundance	Hgn3C.0649 - Lachnospiraceae sp.	Handgrip
MGS	abundance	Hgn3C.0651 - Eubacteriales sp.	Tibia_length
MGS	abundance	Hgn3C.0651 - Eubacteriales sp.	Radius_length
MGS	abundance	Hgn3C.0651 - Eubacteriales sp.	Radius_SOS
MGS	abundance	Hgn3C.0651 - Eubacteriales sp.	Tibia_SOS
MGS	abundance	Hgn3C.0651 - Eubacteriales sp.	Handgrip
MGS	abundance	Hgn3C.0657 - Parolsenella catena	Tibia_length
MGS	abundance	Hgn3C.0657 - Parolsenella catena	Radius_length
MGS	abundance	Hgn3C.0657 - Parolsenella catena	Radius_SOS
MGS	abundance	Hgn3C.0657 - Parolsenella catena	Tibia_SOS
MGS	abundance	Hgn3C.0657 - Parolsenella catena	Handgrip
MGS	abundance	Hgn3C.0659 - Oscillospiraceae sp.	Tibia_length
MGS	abundance	Hgn3C.0659 - Oscillospiraceae sp.	Radius_length
MGS	abundance	Hgn3C.0659 - Oscillospiraceae sp.	Radius_SOS
MGS	abundance	Hgn3C.0659 - Oscillospiraceae sp.	Tibia_SOS
MGS	abundance	Hgn3C.0659 - Oscillospiraceae sp.	Handgrip
MGS	abundance	Hgn3C.0660 - Eubacteriales sp.	Tibia_length
MGS	abundance	Hgn3C.0660 - Eubacteriales sp.	Radius_length
MGS	abundance	Hgn3C.0660 - Eubacteriales sp.	Radius_SOS
MGS	abundance	Hgn3C.0660 - Eubacteriales sp.	Tibia_SOS
MGS	abundance	Hgn3C.0660 - Eubacteriales sp.	Handgrip
MGS	abundance	Hgn3C.0661 - Alistipes sp.	Tibia_length
MGS	abundance	Hgn3C.0661 - Alistipes sp.	Radius_length
MGS	abundance	Hgn3C.0661 - Alistipes sp.	Radius_SOS
MGS	abundance	Hgn3C.0661 - Alistipes sp.	Tibia_SOS
MGS	abundance	Hgn3C.0661 - Alistipes sp.	Handgrip
MGS	abundance	Hgn3C.0664 - Blautia hansenii	Tibia_length
MGS	abundance	Hgn3C.0664 - Blautia hansenii	Radius_length
MGS	abundance	Hgn3C.0664 - Blautia hansenii	Radius_SOS
MGS	abundance	Hgn3C.0664 - Blautia hansenii	Tibia_SOS
MGS	abundance	Hgn3C.0664 - Blautia hansenii	Handgrip
MGS	abundance	Hgn3C.0665 - Desulfovibrionales sp.	Tibia_length
MGS	abundance	Hgn3C.0665 - Desulfovibrionales sp.	Radius_length
MGS	abundance	Hgn3C.0665 - Desulfovibrionales sp.	Radius_SOS

MGS	abundance	Hgn3C.0665 - Desulfovibrionales sp.	Tibia_SOS
MGS	abundance	Hgn3C.0665 - Desulfovibrionales sp.	Handgrip
MGS	abundance	Hgn3C.0669 - Burkholderiales sp.	Tibia_length
MGS	abundance	Hgn3C.0669 - Burkholderiales sp.	Radius_length
MGS	abundance	Hgn3C.0669 - Burkholderiales sp.	Radius_SOS
MGS	abundance	Hgn3C.0669 - Burkholderiales sp.	Tibia_SOS
MGS	abundance	Hgn3C.0669 - Burkholderiales sp.	Handgrip
MGS	abundance	Hgn3C.0675 - Eubacteriales sp.	Tibia_length
MGS	abundance	Hgn3C.0675 - Eubacteriales sp.	Radius_length
MGS	abundance	Hgn3C.0675 - Eubacteriales sp.	Radius_SOS
MGS	abundance	Hgn3C.0675 - Eubacteriales sp.	Tibia_SOS
MGS	abundance	Hgn3C.0675 - Eubacteriales sp.	Handgrip
MGS	abundance	Hgn3C.0676 - Bacteroides sp.	Tibia_length
MGS	abundance	Hgn3C.0676 - Bacteroides sp.	Radius_length
MGS	abundance	Hgn3C.0676 - Bacteroides sp.	Radius_SOS
MGS	abundance	Hgn3C.0676 - Bacteroides sp.	Tibia_SOS
MGS	abundance	Hgn3C.0676 - Bacteroides sp.	Handgrip
MGS	abundance	Hgn3C.0677 - Bacteroides sp.	Tibia_length
MGS	abundance	Hgn3C.0677 - Bacteroides sp.	Radius_length
MGS	abundance	Hgn3C.0677 - Bacteroides sp.	Radius_SOS
MGS	abundance	Hgn3C.0677 - Bacteroides sp.	Tibia_SOS
MGS	abundance	Hgn3C.0677 - Bacteroides sp.	Handgrip
MGS	abundance	Hgn3C.0679 - Holdemania filiformis	Tibia_length
MGS	abundance	Hgn3C.0679 - Holdemania filiformis	Radius_length
MGS	abundance	Hgn3C.0679 - Holdemania filiformis	Radius_SOS
MGS	abundance	Hgn3C.0679 - Holdemania filiformis	Tibia_SOS
MGS	abundance	Hgn3C.0679 - Holdemania filiformis	Handgrip
MGS	abundance	Hgn3C.0680 - Eubacteriales sp.	Tibia_length
MGS	abundance	Hgn3C.0680 - Eubacteriales sp.	Radius_length
MGS	abundance	Hgn3C.0680 - Eubacteriales sp.	Radius_SOS
MGS	abundance	Hgn3C.0680 - Eubacteriales sp.	Tibia_SOS
MGS	abundance	Hgn3C.0680 - Eubacteriales sp.	Handgrip
MGS	abundance	Hgn3C.0681 - Pseudoflavonifractor sp.	Tibia_length
MGS	abundance	Hgn3C.0681 - Pseudoflavonifractor sp.	Radius_length
MGS	abundance	Hgn3C.0681 - Pseudoflavonifractor sp.	Radius_SOS
MGS	abundance	Hgn3C.0681 - Pseudoflavonifractor sp.	Tibia_SOS
MGS	abundance	Hgn3C.0681 - Pseudoflavonifractor sp.	Handgrip
MGS	abundance	Hgn3C.0682 - [Clostridium] scindens	Tibia_length
MGS	abundance	Hgn3C.0682 - [Clostridium] scindens	Radius_length
MGS	abundance	Hgn3C.0682 - [Clostridium] scindens	Radius_SOS
MGS	abundance	Hgn3C.0682 - [Clostridium] scindens	Tibia_SOS
MGS	abundance	Hgn3C.0682 - [Clostridium] scindens	Handgrip
MGS	abundance	Hgn3C.0683 - Clostridium disporicum	Tibia_length
MGS	abundance	Hgn3C.0683 - Clostridium disporicum	Radius_length
MGS	abundance	Hgn3C.0683 - Clostridium disporicum	Radius_SOS
MGS	abundance	Hgn3C.0683 - Clostridium disporicum	Tibia_SOS
MGS	abundance	Hgn3C.0683 - Clostridium disporicum	Handgrip
MGS	abundance	Hgn3C.0685 - Lachnospiraceae sp.	Tibia_length

MGS	abundance	Hgn3C.0685 - Lachnospiraceae sp.	Radius_length
MGS	abundance	Hgn3C.0685 - Lachnospiraceae sp.	Radius_SOS
MGS	abundance	Hgn3C.0685 - Lachnospiraceae sp.	Tibia_SOS
MGS	abundance	Hgn3C.0685 - Lachnospiraceae sp.	Handgrip
MGS	abundance	Hgn3C.0690 - Eubacteriales sp.	Tibia_length
MGS	abundance	Hgn3C.0690 - Eubacteriales sp.	Radius_length
MGS	abundance	Hgn3C.0690 - Eubacteriales sp.	Radius_SOS
MGS	abundance	Hgn3C.0690 - Eubacteriales sp.	Tibia_SOS
MGS	abundance	Hgn3C.0690 - Eubacteriales sp.	Handgrip
MGS	abundance	Hgn3C.0693 - Hungatella hathewayi	Tibia_length
MGS	abundance	Hgn3C.0693 - Hungatella hathewayi	Radius_length
MGS	abundance	Hgn3C.0693 - Hungatella hathewayi	Radius_SOS
MGS	abundance	Hgn3C.0693 - Hungatella hathewayi	Tibia_SOS
MGS	abundance	Hgn3C.0693 - Hungatella hathewayi	Handgrip
MGS	abundance	Hgn3C.0694 - Anaeromassilibacillus sp. An250	Tibia_length
MGS	abundance	Hgn3C.0694 - Anaeromassilibacillus sp. An250	Radius_length
MGS	abundance	Hgn3C.0694 - Anaeromassilibacillus sp. An250	Radius_SOS
MGS	abundance	Hgn3C.0694 - Anaeromassilibacillus sp. An250	Tibia_SOS
MGS	abundance	Hgn3C.0694 - Anaeromassilibacillus sp. An250	Handgrip
MGS	abundance	Hgn3C.0700 - Eubacteriales sp.	Tibia_length
MGS	abundance	Hgn3C.0700 - Eubacteriales sp.	Radius_length
MGS	abundance	Hgn3C.0700 - Eubacteriales sp.	Radius_SOS
MGS	abundance	Hgn3C.0700 - Eubacteriales sp.	Tibia_SOS
MGS	abundance	Hgn3C.0700 - Eubacteriales sp.	Handgrip
MGS	abundance	Hgn3C.0701 - Bacteroides sp.	Tibia_length
MGS	abundance	Hgn3C.0701 - Bacteroides sp.	Radius_length
MGS	abundance	Hgn3C.0701 - Bacteroides sp.	Radius_SOS
MGS	abundance	Hgn3C.0701 - Bacteroides sp.	Tibia_SOS
MGS	abundance	Hgn3C.0701 - Bacteroides sp.	Handgrip
MGS	abundance	Hgn3C.0704 - [Clostridium] scindens	Tibia_length
MGS	abundance	Hgn3C.0704 - [Clostridium] scindens	Radius_length
MGS	abundance	Hgn3C.0704 - [Clostridium] scindens	Radius_SOS
MGS	abundance	Hgn3C.0704 - [Clostridium] scindens	Tibia_SOS
MGS	abundance	Hgn3C.0704 - [Clostridium] scindens	Handgrip
MGS	abundance	Hgn3C.0706 - Eubacteriales sp.	Tibia_length
MGS	abundance	Hgn3C.0706 - Eubacteriales sp.	Radius_length
MGS	abundance	Hgn3C.0706 - Eubacteriales sp.	Radius_SOS
MGS	abundance	Hgn3C.0706 - Eubacteriales sp.	Tibia_SOS
MGS	abundance	Hgn3C.0706 - Eubacteriales sp.	Handgrip
MGS	abundance	Hgn3C.0711 - Enterocloster asparagiformis	Tibia_length
MGS	abundance	Hgn3C.0711 - Enterocloster asparagiformis	Radius_length
MGS	abundance	Hgn3C.0711 - Enterocloster asparagiformis	Radius_SOS
MGS	abundance	Hgn3C.0711 - Enterocloster asparagiformis	Tibia_SOS
MGS	abundance	Hgn3C.0711 - Enterocloster asparagiformis	Handgrip
MGS	abundance	Hgn3C.0712 - Megasphaera micronuciformis	Tibia_length
MGS	abundance	Hgn3C.0712 - Megasphaera micronuciformis	Radius_length
MGS	abundance	Hgn3C.0712 - Megasphaera micronuciformis	Radius_SOS
MGS	abundance	Hgn3C.0712 - Megasphaera micronuciformis	Tibia_SOS

MGS	abundance Hgn3C.0712 - Megasphaera micronuciformis	Handgrip
MGS	abundance Hgn3C.0713 - Veillonellales sp.	Tibia_length
MGS	abundance Hgn3C.0713 - Veillonellales sp.	Radius_length
MGS	abundance Hgn3C.0713 - Veillonellales sp.	Radius_SOS
MGS	abundance Hgn3C.0713 - Veillonellales sp.	Tibia_SOS
MGS	abundance Hgn3C.0713 - Veillonellales sp.	Handgrip
MGS	abundance Hgn3C.0714 - Intestinimonas sp. MSJ-38	Tibia_length
MGS	abundance Hgn3C.0714 - Intestinimonas sp. MSJ-38	Radius_length
MGS	abundance Hgn3C.0714 - Intestinimonas sp. MSJ-38	Radius_SOS
MGS	abundance Hgn3C.0714 - Intestinimonas sp. MSJ-38	Tibia_SOS
MGS	abundance Hgn3C.0714 - Intestinimonas sp. MSJ-38	Handgrip
MGS	abundance Hgn3C.0715 - Desulfovibrio fairfieldensis	Tibia_length
MGS	abundance Hgn3C.0715 - Desulfovibrio fairfieldensis	Radius_length
MGS	abundance Hgn3C.0715 - Desulfovibrio fairfieldensis	Radius_SOS
MGS	abundance Hgn3C.0715 - Desulfovibrio fairfieldensis	Tibia_SOS
MGS	abundance Hgn3C.0715 - Desulfovibrio fairfieldensis	Handgrip
MGS	abundance Hgn3C.0716 - Eubacteriales sp.	Tibia_length
MGS	abundance Hgn3C.0716 - Eubacteriales sp.	Radius_length
MGS	abundance Hgn3C.0716 - Eubacteriales sp.	Radius_SOS
MGS	abundance Hgn3C.0716 - Eubacteriales sp.	Tibia_SOS
MGS	abundance Hgn3C.0716 - Eubacteriales sp.	Handgrip
MGS	abundance Hgn3C.0717 - Eubacteriales sp.	Tibia_length
MGS	abundance Hgn3C.0717 - Eubacteriales sp.	Radius_length
MGS	abundance Hgn3C.0717 - Eubacteriales sp.	Radius_SOS
MGS	abundance Hgn3C.0717 - Eubacteriales sp.	Tibia_SOS
MGS	abundance Hgn3C.0717 - Eubacteriales sp.	Handgrip
MGS	abundance Hgn3C.0718 - Eubacteriales sp.	Tibia_length
MGS	abundance Hgn3C.0718 - Eubacteriales sp.	Radius_length
MGS	abundance Hgn3C.0718 - Eubacteriales sp.	Radius_SOS
MGS	abundance Hgn3C.0718 - Eubacteriales sp.	Tibia_SOS
MGS	abundance Hgn3C.0718 - Eubacteriales sp.	Handgrip
MGS	abundance Hgn3C.0729 - Eubacteriales sp.	Tibia_length
MGS	abundance Hgn3C.0729 - Eubacteriales sp.	Radius_length
MGS	abundance Hgn3C.0729 - Eubacteriales sp.	Radius_SOS
MGS	abundance Hgn3C.0729 - Eubacteriales sp.	Tibia_SOS
MGS	abundance Hgn3C.0729 - Eubacteriales sp.	Handgrip
MGS	abundance Hgn3C.0731 - Eubacteriales sp.	Tibia_length
MGS	abundance Hgn3C.0731 - Eubacteriales sp.	Radius_length
MGS	abundance Hgn3C.0731 - Eubacteriales sp.	Radius_SOS
MGS	abundance Hgn3C.0731 - Eubacteriales sp.	Tibia_SOS
MGS	abundance Hgn3C.0731 - Eubacteriales sp.	Handgrip
MGS	abundance Hgn3C.0732 - Eubacterium ramulus	Tibia_length
MGS	abundance Hgn3C.0732 - Eubacterium ramulus	Radius_length
MGS	abundance Hgn3C.0732 - Eubacterium ramulus	Radius_SOS
MGS	abundance Hgn3C.0732 - Eubacterium ramulus	Tibia_SOS
MGS	abundance Hgn3C.0732 - Eubacterium ramulus	Handgrip
MGS	abundance Hgn3C.0736 - Coprococcus sp.	Tibia_length
MGS	abundance Hgn3C.0736 - Coprococcus sp.	Radius_length

MGS	abundance	Hgn3C.0736 - Coprococcus sp.	Radius_SOS
MGS	abundance	Hgn3C.0736 - Coprococcus sp.	Tibia_SOS
MGS	abundance	Hgn3C.0736 - Coprococcus sp.	Handgrip
MGS	abundance	Hgn3C.0737 - Alistipes timonensis	Tibia_length
MGS	abundance	Hgn3C.0737 - Alistipes timonensis	Radius_length
MGS	abundance	Hgn3C.0737 - Alistipes timonensis	Radius_SOS
MGS	abundance	Hgn3C.0737 - Alistipes timonensis	Tibia_SOS
MGS	abundance	Hgn3C.0737 - Alistipes timonensis	Handgrip
MGS	abundance	Hgn3C.0740 - Anaerostipes sp. NSJ-7	Tibia_length
MGS	abundance	Hgn3C.0740 - Anaerostipes sp. NSJ-7	Radius_length
MGS	abundance	Hgn3C.0740 - Anaerostipes sp. NSJ-7	Radius_SOS
MGS	abundance	Hgn3C.0740 - Anaerostipes sp. NSJ-7	Tibia_SOS
MGS	abundance	Hgn3C.0740 - Anaerostipes sp. NSJ-7	Handgrip
MGS	abundance	Hgn3C.0741 - Blautia sp. OF01-4LB	Tibia_length
MGS	abundance	Hgn3C.0741 - Blautia sp. OF01-4LB	Radius_length
MGS	abundance	Hgn3C.0741 - Blautia sp. OF01-4LB	Radius_SOS
MGS	abundance	Hgn3C.0741 - Blautia sp. OF01-4LB	Tibia_SOS
MGS	abundance	Hgn3C.0741 - Blautia sp. OF01-4LB	Handgrip
MGS	abundance	Hgn3C.0742 - Eubacteriales sp.	Tibia_length
MGS	abundance	Hgn3C.0742 - Eubacteriales sp.	Radius_length
MGS	abundance	Hgn3C.0742 - Eubacteriales sp.	Radius_SOS
MGS	abundance	Hgn3C.0742 - Eubacteriales sp.	Tibia_SOS
MGS	abundance	Hgn3C.0742 - Eubacteriales sp.	Handgrip
MGS	abundance	Hgn3C.0743 - Enterobacter sp.	Tibia_length
MGS	abundance	Hgn3C.0743 - Enterobacter sp.	Radius_length
MGS	abundance	Hgn3C.0743 - Enterobacter sp.	Radius_SOS
MGS	abundance	Hgn3C.0743 - Enterobacter sp.	Tibia_SOS
MGS	abundance	Hgn3C.0743 - Enterobacter sp.	Handgrip
MGS	abundance	Hgn3C.0749 - Eubacteriales sp.	Tibia_length
MGS	abundance	Hgn3C.0749 - Eubacteriales sp.	Radius_length
MGS	abundance	Hgn3C.0749 - Eubacteriales sp.	Radius_SOS
MGS	abundance	Hgn3C.0749 - Eubacteriales sp.	Tibia_SOS
MGS	abundance	Hgn3C.0749 - Eubacteriales sp.	Handgrip
MGS	abundance	Hgn3C.0750 - Merdimonas faecis	Tibia_length
MGS	abundance	Hgn3C.0750 - Merdimonas faecis	Radius_length
MGS	abundance	Hgn3C.0750 - Merdimonas faecis	Radius_SOS
MGS	abundance	Hgn3C.0750 - Merdimonas faecis	Tibia_SOS
MGS	abundance	Hgn3C.0750 - Merdimonas faecis	Handgrip
MGS	abundance	Hgn3C.0754 - Collinsella bouchesdurhonensis	Tibia_length
MGS	abundance	Hgn3C.0754 - Collinsella bouchesdurhonensis	Radius_length
MGS	abundance	Hgn3C.0754 - Collinsella bouchesdurhonensis	Radius_SOS
MGS	abundance	Hgn3C.0754 - Collinsella bouchesdurhonensis	Tibia_SOS
MGS	abundance	Hgn3C.0754 - Collinsella bouchesdurhonensis	Handgrip
MGS	abundance	Hgn3C.0755 - Oscillibacter sp.	Tibia_length
MGS	abundance	Hgn3C.0755 - Oscillibacter sp.	Radius_length
MGS	abundance	Hgn3C.0755 - Oscillibacter sp.	Radius_SOS
MGS	abundance	Hgn3C.0755 - Oscillibacter sp.	Tibia_SOS
MGS	abundance	Hgn3C.0755 - Oscillibacter sp.	Handgrip

MGS	abundance	Hgn3C.0756 - Oscillibacter valericigenes	Tibia_length
MGS	abundance	Hgn3C.0756 - Oscillibacter valericigenes	Radius_length
MGS	abundance	Hgn3C.0756 - Oscillibacter valericigenes	Radius_SOS
MGS	abundance	Hgn3C.0756 - Oscillibacter valericigenes	Tibia_SOS
MGS	abundance	Hgn3C.0756 - Oscillibacter valericigenes	Handgrip
MGS	abundance	Hgn3C.0758 - Bacteroides sp.	Tibia_length
MGS	abundance	Hgn3C.0758 - Bacteroides sp.	Radius_length
MGS	abundance	Hgn3C.0758 - Bacteroides sp.	Radius_SOS
MGS	abundance	Hgn3C.0758 - Bacteroides sp.	Tibia_SOS
MGS	abundance	Hgn3C.0758 - Bacteroides sp.	Handgrip
MGS	abundance	Hgn3C.0760 - Klebsiella oxytoca	Tibia_length
MGS	abundance	Hgn3C.0760 - Klebsiella oxytoca	Radius_length
MGS	abundance	Hgn3C.0760 - Klebsiella oxytoca	Radius_SOS
MGS	abundance	Hgn3C.0760 - Klebsiella oxytoca	Tibia_SOS
MGS	abundance	Hgn3C.0760 - Klebsiella oxytoca	Handgrip
MGS	abundance	Hgn3C.0761 - Bacteroides sp.	Tibia_length
MGS	abundance	Hgn3C.0761 - Bacteroides sp.	Radius_length
MGS	abundance	Hgn3C.0761 - Bacteroides sp.	Radius_SOS
MGS	abundance	Hgn3C.0761 - Bacteroides sp.	Tibia_SOS
MGS	abundance	Hgn3C.0761 - Bacteroides sp.	Handgrip
MGS	abundance	Hgn3C.0762 - Enterocloster lavalensis	Tibia_length
MGS	abundance	Hgn3C.0762 - Enterocloster lavalensis	Radius_length
MGS	abundance	Hgn3C.0762 - Enterocloster lavalensis	Radius_SOS
MGS	abundance	Hgn3C.0762 - Enterocloster lavalensis	Tibia_SOS
MGS	abundance	Hgn3C.0762 - Enterocloster lavalensis	Handgrip
MGS	abundance	Hgn3C.0767 - Citrobacter freundii	Tibia_length
MGS	abundance	Hgn3C.0767 - Citrobacter freundii	Radius_length
MGS	abundance	Hgn3C.0767 - Citrobacter freundii	Radius_SOS
MGS	abundance	Hgn3C.0767 - Citrobacter freundii	Tibia_SOS
MGS	abundance	Hgn3C.0767 - Citrobacter freundii	Handgrip
MGS	abundance	Hgn3C.0777 - Holdemanella sp.	Tibia_length
MGS	abundance	Hgn3C.0777 - Holdemanella sp.	Radius_length
MGS	abundance	Hgn3C.0777 - Holdemanella sp.	Radius_SOS
MGS	abundance	Hgn3C.0777 - Holdemanella sp.	Tibia_SOS
MGS	abundance	Hgn3C.0777 - Holdemanella sp.	Handgrip
MGS	abundance	Hgn3C.0781 - Collinsella sp.	Tibia_length
MGS	abundance	Hgn3C.0781 - Collinsella sp.	Radius_length
MGS	abundance	Hgn3C.0781 - Collinsella sp.	Radius_SOS
MGS	abundance	Hgn3C.0781 - Collinsella sp.	Tibia_SOS
MGS	abundance	Hgn3C.0781 - Collinsella sp.	Handgrip
MGS	abundance	Hgn3C.0782 - Streptococcus sp.	Tibia_length
MGS	abundance	Hgn3C.0782 - Streptococcus sp.	Radius_length
MGS	abundance	Hgn3C.0782 - Streptococcus sp.	Radius_SOS
MGS	abundance	Hgn3C.0782 - Streptococcus sp.	Tibia_SOS
MGS	abundance	Hgn3C.0782 - Streptococcus sp.	Handgrip
MGS	abundance	Hgn3C.0790 - Oscillospiraceae sp.	Tibia_length
MGS	abundance	Hgn3C.0790 - Oscillospiraceae sp.	Radius_length
MGS	abundance	Hgn3C.0790 - Oscillospiraceae sp.	Radius_SOS

MGS	abundance	Hgn3C.0790 - Oscillospiraceae sp.	Tibia_SOS
MGS	abundance	Hgn3C.0790 - Oscillospiraceae sp.	Handgrip
MGS	abundance	Hgn3C.0793 - Prevotellaceae sp.	Tibia_length
MGS	abundance	Hgn3C.0793 - Prevotellaceae sp.	Radius_length
MGS	abundance	Hgn3C.0793 - Prevotellaceae sp.	Radius_SOS
MGS	abundance	Hgn3C.0793 - Prevotellaceae sp.	Tibia_SOS
MGS	abundance	Hgn3C.0793 - Prevotellaceae sp.	Handgrip
MGS	abundance	Hgn3C.0794 - Bacteroides sp.	Tibia_length
MGS	abundance	Hgn3C.0794 - Bacteroides sp.	Radius_length
MGS	abundance	Hgn3C.0794 - Bacteroides sp.	Radius_SOS
MGS	abundance	Hgn3C.0794 - Bacteroides sp.	Tibia_SOS
MGS	abundance	Hgn3C.0794 - Bacteroides sp.	Handgrip
MGS	abundance	Hgn3C.0797 - Senegalimassilia sp.	Tibia_length
MGS	abundance	Hgn3C.0797 - Senegalimassilia sp.	Radius_length
MGS	abundance	Hgn3C.0797 - Senegalimassilia sp.	Radius_SOS
MGS	abundance	Hgn3C.0797 - Senegalimassilia sp.	Tibia_SOS
MGS	abundance	Hgn3C.0797 - Senegalimassilia sp.	Handgrip
MGS	abundance	Hgn3C.0798 - Streptococcus vestibularis	Tibia_length
MGS	abundance	Hgn3C.0798 - Streptococcus vestibularis	Radius_length
MGS	abundance	Hgn3C.0798 - Streptococcus vestibularis	Radius_SOS
MGS	abundance	Hgn3C.0798 - Streptococcus vestibularis	Tibia_SOS
MGS	abundance	Hgn3C.0798 - Streptococcus vestibularis	Handgrip
MGS	abundance	Hgn3C.0807 - Enorma massiliensis	Tibia_length
MGS	abundance	Hgn3C.0807 - Enorma massiliensis	Radius_length
MGS	abundance	Hgn3C.0807 - Enorma massiliensis	Radius_SOS
MGS	abundance	Hgn3C.0807 - Enorma massiliensis	Tibia_SOS
MGS	abundance	Hgn3C.0807 - Enorma massiliensis	Handgrip
MGS	abundance	Hgn3C.0814 - Massilimicrobiota timonensis	Tibia_length
MGS	abundance	Hgn3C.0814 - Massilimicrobiota timonensis	Radius_length
MGS	abundance	Hgn3C.0814 - Massilimicrobiota timonensis	Radius_SOS
MGS	abundance	Hgn3C.0814 - Massilimicrobiota timonensis	Tibia_SOS
MGS	abundance	Hgn3C.0814 - Massilimicrobiota timonensis	Handgrip
MGS	abundance	Hgn3C.0815 - Eubacteriales sp.	Tibia_length
MGS	abundance	Hgn3C.0815 - Eubacteriales sp.	Radius_length
MGS	abundance	Hgn3C.0815 - Eubacteriales sp.	Radius_SOS
MGS	abundance	Hgn3C.0815 - Eubacteriales sp.	Tibia_SOS
MGS	abundance	Hgn3C.0815 - Eubacteriales sp.	Handgrip
MGS	abundance	Hgn3C.0817 - Prevotella sp.	Tibia_length
MGS	abundance	Hgn3C.0817 - Prevotella sp.	Radius_length
MGS	abundance	Hgn3C.0817 - Prevotella sp.	Radius_SOS
MGS	abundance	Hgn3C.0817 - Prevotella sp.	Tibia_SOS
MGS	abundance	Hgn3C.0817 - Prevotella sp.	Handgrip
MGS	abundance	Hgn3C.0818 - Rothia sp.	Tibia_length
MGS	abundance	Hgn3C.0818 - Rothia sp.	Radius_length
MGS	abundance	Hgn3C.0818 - Rothia sp.	Radius_SOS
MGS	abundance	Hgn3C.0818 - Rothia sp.	Tibia_SOS
MGS	abundance	Hgn3C.0818 - Rothia sp.	Handgrip
MGS	abundance	Hgn3C.0823 - Anaerotignum lactatifermentans	Tibia_length

MGS	abundance	Hgn3C.0823 - Anaerotignum lactatifermentans	Radius_length
MGS	abundance	Hgn3C.0823 - Anaerotignum lactatifermentans	Radius_SOS
MGS	abundance	Hgn3C.0823 - Anaerotignum lactatifermentans	Tibia_SOS
MGS	abundance	Hgn3C.0823 - Anaerotignum lactatifermentans	Handgrip
MGS	abundance	Hgn3C.0828 - Bacteroides congonensis	Tibia_length
MGS	abundance	Hgn3C.0828 - Bacteroides congonensis	Radius_length
MGS	abundance	Hgn3C.0828 - Bacteroides congonensis	Radius_SOS
MGS	abundance	Hgn3C.0828 - Bacteroides congonensis	Tibia_SOS
MGS	abundance	Hgn3C.0828 - Bacteroides congonensis	Handgrip
MGS	abundance	Hgn3C.0829 - Dorea phocaeensis	Tibia_length
MGS	abundance	Hgn3C.0829 - Dorea phocaeensis	Radius_length
MGS	abundance	Hgn3C.0829 - Dorea phocaeensis	Radius_SOS
MGS	abundance	Hgn3C.0829 - Dorea phocaeensis	Tibia_SOS
MGS	abundance	Hgn3C.0829 - Dorea phocaeensis	Handgrip
MGS	abundance	Hgn3C.0832 - Veillonellales sp.	Tibia_length
MGS	abundance	Hgn3C.0832 - Veillonellales sp.	Radius_length
MGS	abundance	Hgn3C.0832 - Veillonellales sp.	Radius_SOS
MGS	abundance	Hgn3C.0832 - Veillonellales sp.	Tibia_SOS
MGS	abundance	Hgn3C.0832 - Veillonellales sp.	Handgrip
MGS	abundance	Hgn3C.0833 - Eisenbergiella tayi	Tibia_length
MGS	abundance	Hgn3C.0833 - Eisenbergiella tayi	Radius_length
MGS	abundance	Hgn3C.0833 - Eisenbergiella tayi	Radius_SOS
MGS	abundance	Hgn3C.0833 - Eisenbergiella tayi	Tibia_SOS
MGS	abundance	Hgn3C.0833 - Eisenbergiella tayi	Handgrip
MGS	abundance	Hgn3C.0836 - Lachnospiraceae sp.	Tibia_length
MGS	abundance	Hgn3C.0836 - Lachnospiraceae sp.	Radius_length
MGS	abundance	Hgn3C.0836 - Lachnospiraceae sp.	Radius_SOS
MGS	abundance	Hgn3C.0836 - Lachnospiraceae sp.	Tibia_SOS
MGS	abundance	Hgn3C.0836 - Lachnospiraceae sp.	Handgrip
MGS	abundance	Hgn3C.0837 - Anaerotruncus colihominis	Tibia_length
MGS	abundance	Hgn3C.0837 - Anaerotruncus colihominis	Radius_length
MGS	abundance	Hgn3C.0837 - Anaerotruncus colihominis	Radius_SOS
MGS	abundance	Hgn3C.0837 - Anaerotruncus colihominis	Tibia_SOS
MGS	abundance	Hgn3C.0837 - Anaerotruncus colihominis	Handgrip
MGS	abundance	Hgn3C.0840 - Eubacteriales sp.	Tibia_length
MGS	abundance	Hgn3C.0840 - Eubacteriales sp.	Radius_length
MGS	abundance	Hgn3C.0840 - Eubacteriales sp.	Radius_SOS
MGS	abundance	Hgn3C.0840 - Eubacteriales sp.	Tibia_SOS
MGS	abundance	Hgn3C.0840 - Eubacteriales sp.	Handgrip
MGS	abundance	Hgn3C.0851 - Oscillospiraceae sp.	Tibia_length
MGS	abundance	Hgn3C.0851 - Oscillospiraceae sp.	Radius_length
MGS	abundance	Hgn3C.0851 - Oscillospiraceae sp.	Radius_SOS
MGS	abundance	Hgn3C.0851 - Oscillospiraceae sp.	Tibia_SOS
MGS	abundance	Hgn3C.0851 - Oscillospiraceae sp.	Handgrip
MGS	abundance	Hgn3C.0855 - Eubacteriales sp.	Tibia_length
MGS	abundance	Hgn3C.0855 - Eubacteriales sp.	Radius_length
MGS	abundance	Hgn3C.0855 - Eubacteriales sp.	Radius_SOS
MGS	abundance	Hgn3C.0855 - Eubacteriales sp.	Tibia_SOS

MGS	abundance	Hgn3C.0855 - Eubacteriales sp.	Handgrip
MGS	abundance	Hgn3C.0858 - Turicibacter sanguinis	Tibia_length
MGS	abundance	Hgn3C.0858 - Turicibacter sanguinis	Radius_length
MGS	abundance	Hgn3C.0858 - Turicibacter sanguinis	Radius_SOS
MGS	abundance	Hgn3C.0858 - Turicibacter sanguinis	Tibia_SOS
MGS	abundance	Hgn3C.0858 - Turicibacter sanguinis	Handgrip
MGS	abundance	Hgn3C.0859 - Hydrogeniiclostidium mannosilyticum	Tibia_length
MGS	abundance	Hgn3C.0859 - Hydrogeniiclostidium mannosilyticum	Radius_length
MGS	abundance	Hgn3C.0859 - Hydrogeniiclostidium mannosilyticum	Radius_SOS
MGS	abundance	Hgn3C.0859 - Hydrogeniiclostidium mannosilyticum	Tibia_SOS
MGS	abundance	Hgn3C.0859 - Hydrogeniiclostidium mannosilyticum	Handgrip
MGS	abundance	Hgn3C.0873 - Clostridium perfringens	Tibia_length
MGS	abundance	Hgn3C.0873 - Clostridium perfringens	Radius_length
MGS	abundance	Hgn3C.0873 - Clostridium perfringens	Radius_SOS
MGS	abundance	Hgn3C.0873 - Clostridium perfringens	Tibia_SOS
MGS	abundance	Hgn3C.0873 - Clostridium perfringens	Handgrip
MGS	abundance	Hgn3C.0875 - Lachnospiraceae sp.	Tibia_length
MGS	abundance	Hgn3C.0875 - Lachnospiraceae sp.	Radius_length
MGS	abundance	Hgn3C.0875 - Lachnospiraceae sp.	Radius_SOS
MGS	abundance	Hgn3C.0875 - Lachnospiraceae sp.	Tibia_SOS
MGS	abundance	Hgn3C.0875 - Lachnospiraceae sp.	Handgrip
MGS	abundance	Hgn3C.0882 - Eubacteriales sp.	Tibia_length
MGS	abundance	Hgn3C.0882 - Eubacteriales sp.	Radius_length
MGS	abundance	Hgn3C.0882 - Eubacteriales sp.	Radius_SOS
MGS	abundance	Hgn3C.0882 - Eubacteriales sp.	Tibia_SOS
MGS	abundance	Hgn3C.0882 - Eubacteriales sp.	Handgrip
MGS	abundance	Hgn3C.0885 - Eubacteriales sp.	Tibia_length
MGS	abundance	Hgn3C.0885 - Eubacteriales sp.	Radius_length
MGS	abundance	Hgn3C.0885 - Eubacteriales sp.	Radius_SOS
MGS	abundance	Hgn3C.0885 - Eubacteriales sp.	Tibia_SOS
MGS	abundance	Hgn3C.0885 - Eubacteriales sp.	Handgrip
MGS	abundance	Hgn3C.0886 - Bacteroides sp.	Tibia_length
MGS	abundance	Hgn3C.0886 - Bacteroides sp.	Radius_length
MGS	abundance	Hgn3C.0886 - Bacteroides sp.	Radius_SOS
MGS	abundance	Hgn3C.0886 - Bacteroides sp.	Tibia_SOS
MGS	abundance	Hgn3C.0886 - Bacteroides sp.	Handgrip
MGS	abundance	Hgn3C.0889 - Clostridium paraputrificum	Tibia_length
MGS	abundance	Hgn3C.0889 - Clostridium paraputrificum	Radius_length
MGS	abundance	Hgn3C.0889 - Clostridium paraputrificum	Radius_SOS
MGS	abundance	Hgn3C.0889 - Clostridium paraputrificum	Tibia_SOS
MGS	abundance	Hgn3C.0889 - Clostridium paraputrificum	Handgrip
MGS	abundance	Hgn3C.0890 - Enterobacter roggenkampii	Tibia_length
MGS	abundance	Hgn3C.0890 - Enterobacter roggenkampii	Radius_length
MGS	abundance	Hgn3C.0890 - Enterobacter roggenkampii	Radius_SOS
MGS	abundance	Hgn3C.0890 - Enterobacter roggenkampii	Tibia_SOS
MGS	abundance	Hgn3C.0890 - Enterobacter roggenkampii	Handgrip
MGS	abundance	Hgn3C.0891 - Eubacteriales sp.	Tibia_length
MGS	abundance	Hgn3C.0891 - Eubacteriales sp.	Radius_length

MGS	abundance	Hgn3C.0891 - Eubacteriales sp.	Radius_SOS
MGS	abundance	Hgn3C.0891 - Eubacteriales sp.	Tibia_SOS
MGS	abundance	Hgn3C.0891 - Eubacteriales sp.	Handgrip
MGS	abundance	Hgn3C.0893 - Phocea massiliensis	Tibia_length
MGS	abundance	Hgn3C.0893 - Phocea massiliensis	Radius_length
MGS	abundance	Hgn3C.0893 - Phocea massiliensis	Radius_SOS
MGS	abundance	Hgn3C.0893 - Phocea massiliensis	Tibia_SOS
MGS	abundance	Hgn3C.0893 - Phocea massiliensis	Handgrip
MGS	abundance	Hgn3C.0904 - Bacteroidia sp.	Tibia_length
MGS	abundance	Hgn3C.0904 - Bacteroidia sp.	Radius_length
MGS	abundance	Hgn3C.0904 - Bacteroidia sp.	Radius_SOS
MGS	abundance	Hgn3C.0904 - Bacteroidia sp.	Tibia_SOS
MGS	abundance	Hgn3C.0904 - Bacteroidia sp.	Handgrip
MGS	abundance	Hgn3C.0908 - Eubacteriales sp.	Tibia_length
MGS	abundance	Hgn3C.0908 - Eubacteriales sp.	Radius_length
MGS	abundance	Hgn3C.0908 - Eubacteriales sp.	Radius_SOS
MGS	abundance	Hgn3C.0908 - Eubacteriales sp.	Tibia_SOS
MGS	abundance	Hgn3C.0908 - Eubacteriales sp.	Handgrip
MGS	abundance	Hgn3C.0913 - Erysipelotrichaceae sp.	Tibia_length
MGS	abundance	Hgn3C.0913 - Erysipelotrichaceae sp.	Radius_length
MGS	abundance	Hgn3C.0913 - Erysipelotrichaceae sp.	Radius_SOS
MGS	abundance	Hgn3C.0913 - Erysipelotrichaceae sp.	Tibia_SOS
MGS	abundance	Hgn3C.0913 - Erysipelotrichaceae sp.	Handgrip
MGS	abundance	Hgn3C.0921 - Bacteroides sp.	Tibia_length
MGS	abundance	Hgn3C.0921 - Bacteroides sp.	Radius_length
MGS	abundance	Hgn3C.0921 - Bacteroides sp.	Radius_SOS
MGS	abundance	Hgn3C.0921 - Bacteroides sp.	Tibia_SOS
MGS	abundance	Hgn3C.0921 - Bacteroides sp.	Handgrip
MGS	abundance	Hgn3C.0922 - Eubacteriales sp.	Tibia_length
MGS	abundance	Hgn3C.0922 - Eubacteriales sp.	Radius_length
MGS	abundance	Hgn3C.0922 - Eubacteriales sp.	Radius_SOS
MGS	abundance	Hgn3C.0922 - Eubacteriales sp.	Tibia_SOS
MGS	abundance	Hgn3C.0922 - Eubacteriales sp.	Handgrip
MGS	abundance	Hgn3C.0924 - Clostridioides difficile	Tibia_length
MGS	abundance	Hgn3C.0924 - Clostridioides difficile	Radius_length
MGS	abundance	Hgn3C.0924 - Clostridioides difficile	Radius_SOS
MGS	abundance	Hgn3C.0924 - Clostridioides difficile	Tibia_SOS
MGS	abundance	Hgn3C.0924 - Clostridioides difficile	Handgrip
MGS	abundance	Hgn3C.0940 - Eubacteriales sp.	Tibia_length
MGS	abundance	Hgn3C.0940 - Eubacteriales sp.	Radius_length
MGS	abundance	Hgn3C.0940 - Eubacteriales sp.	Radius_SOS
MGS	abundance	Hgn3C.0940 - Eubacteriales sp.	Tibia_SOS
MGS	abundance	Hgn3C.0940 - Eubacteriales sp.	Handgrip
MGS	abundance	Hgn3C.0943 - Lactiplantibacillus plantarum subsp. plantarum	Tibia_length
MGS	abundance	Hgn3C.0943 - Lactiplantibacillus plantarum subsp. plantarum	Radius_length
MGS	abundance	Hgn3C.0943 - Lactiplantibacillus plantarum subsp. plantarum	Radius_SOS
MGS	abundance	Hgn3C.0943 - Lactiplantibacillus plantarum subsp. plantarum	Tibia_SOS
MGS	abundance	Hgn3C.0943 - Lactiplantibacillus plantarum subsp. plantarum	Handgrip

MGS	abundanc	Hgn3C.0946 - Eubacteriales sp.	Tibia_length
MGS	abundanc	Hgn3C.0946 - Eubacteriales sp.	Radius_length
MGS	abundanc	Hgn3C.0946 - Eubacteriales sp.	Radius_SOS
MGS	abundanc	Hgn3C.0946 - Eubacteriales sp.	Tibia_SOS
MGS	abundanc	Hgn3C.0946 - Eubacteriales sp.	Handgrip
MGS	abundanc	Hgn3C.0948 - Enterobacter sp.	Tibia_length
MGS	abundanc	Hgn3C.0948 - Enterobacter sp.	Radius_length
MGS	abundanc	Hgn3C.0948 - Enterobacter sp.	Radius_SOS
MGS	abundanc	Hgn3C.0948 - Enterobacter sp.	Tibia_SOS
MGS	abundanc	Hgn3C.0948 - Enterobacter sp.	Handgrip
MGS	abundanc	Hgn3C.0951 - Eubacteriales sp.	Tibia_length
MGS	abundanc	Hgn3C.0951 - Eubacteriales sp.	Radius_length
MGS	abundanc	Hgn3C.0951 - Eubacteriales sp.	Radius_SOS
MGS	abundanc	Hgn3C.0951 - Eubacteriales sp.	Tibia_SOS
MGS	abundanc	Hgn3C.0951 - Eubacteriales sp.	Handgrip
MGS	abundanc	Hgn3C.0973 - Desulfovibrio piger	Tibia_length
MGS	abundanc	Hgn3C.0973 - Desulfovibrio piger	Radius_length
MGS	abundanc	Hgn3C.0973 - Desulfovibrio piger	Radius_SOS
MGS	abundanc	Hgn3C.0973 - Desulfovibrio piger	Tibia_SOS
MGS	abundanc	Hgn3C.0973 - Desulfovibrio piger	Handgrip
MGS	abundanc	Hgn3C.0978 - Veillonella sp.	Tibia_length
MGS	abundanc	Hgn3C.0978 - Veillonella sp.	Radius_length
MGS	abundanc	Hgn3C.0978 - Veillonella sp.	Radius_SOS
MGS	abundanc	Hgn3C.0978 - Veillonella sp.	Tibia_SOS
MGS	abundanc	Hgn3C.0978 - Veillonella sp.	Handgrip
MGS	abundanc	Hgn3C.0986 - Desulfovibrio sp.	Tibia_length
MGS	abundanc	Hgn3C.0986 - Desulfovibrio sp.	Radius_length
MGS	abundanc	Hgn3C.0986 - Desulfovibrio sp.	Radius_SOS
MGS	abundanc	Hgn3C.0986 - Desulfovibrio sp.	Tibia_SOS
MGS	abundanc	Hgn3C.0986 - Desulfovibrio sp.	Handgrip
MGS	abundanc	Hgn3C.0987 - Fusobacterium mortiferum	Tibia_length
MGS	abundanc	Hgn3C.0987 - Fusobacterium mortiferum	Radius_length
MGS	abundanc	Hgn3C.0987 - Fusobacterium mortiferum	Radius_SOS
MGS	abundanc	Hgn3C.0987 - Fusobacterium mortiferum	Tibia_SOS
MGS	abundanc	Hgn3C.0987 - Fusobacterium mortiferum	Handgrip
MGS	abundanc	Hgn3C.0988 - Eubacteriales sp.	Tibia_length
MGS	abundanc	Hgn3C.0988 - Eubacteriales sp.	Radius_length
MGS	abundanc	Hgn3C.0988 - Eubacteriales sp.	Radius_SOS
MGS	abundanc	Hgn3C.0988 - Eubacteriales sp.	Tibia_SOS
MGS	abundanc	Hgn3C.0988 - Eubacteriales sp.	Handgrip
MGS	abundanc	Hgn3C.0993 - Faecalitalea cylindroides	Tibia_length
MGS	abundanc	Hgn3C.0993 - Faecalitalea cylindroides	Radius_length
MGS	abundanc	Hgn3C.0993 - Faecalitalea cylindroides	Radius_SOS
MGS	abundanc	Hgn3C.0993 - Faecalitalea cylindroides	Tibia_SOS
MGS	abundanc	Hgn3C.0993 - Faecalitalea cylindroides	Handgrip
MGS	abundanc	Hgn3C.0996 - Oscillospiraceae sp.	Tibia_length
MGS	abundanc	Hgn3C.0996 - Oscillospiraceae sp.	Radius_length
MGS	abundanc	Hgn3C.0996 - Oscillospiraceae sp.	Radius_SOS

MGS	abundance	Hgn3C.0996 - Oscillospiraceae sp.	Tibia_SOS
MGS	abundance	Hgn3C.0996 - Oscillospiraceae sp.	Handgrip
MGS	abundance	Hgn3C.0998 - Eubacteriales sp.	Tibia_length
MGS	abundance	Hgn3C.0998 - Eubacteriales sp.	Radius_length
MGS	abundance	Hgn3C.0998 - Eubacteriales sp.	Radius_SOS
MGS	abundance	Hgn3C.0998 - Eubacteriales sp.	Tibia_SOS
MGS	abundance	Hgn3C.0998 - Eubacteriales sp.	Handgrip
MGS	abundance	Hgn3C.1012 - Erysipelotrichaceae sp.	Tibia_length
MGS	abundance	Hgn3C.1012 - Erysipelotrichaceae sp.	Radius_length
MGS	abundance	Hgn3C.1012 - Erysipelotrichaceae sp.	Radius_SOS
MGS	abundance	Hgn3C.1012 - Erysipelotrichaceae sp.	Tibia_SOS
MGS	abundance	Hgn3C.1012 - Erysipelotrichaceae sp.	Handgrip
MGS	abundance	Hgn3C.1017 - Emergencia timonensis	Tibia_length
MGS	abundance	Hgn3C.1017 - Emergencia timonensis	Radius_length
MGS	abundance	Hgn3C.1017 - Emergencia timonensis	Radius_SOS
MGS	abundance	Hgn3C.1017 - Emergencia timonensis	Tibia_SOS
MGS	abundance	Hgn3C.1017 - Emergencia timonensis	Handgrip
MGS	abundance	Hgn3C.1035 - Klebsiella sp.	Tibia_length
MGS	abundance	Hgn3C.1035 - Klebsiella sp.	Radius_length
MGS	abundance	Hgn3C.1035 - Klebsiella sp.	Radius_SOS
MGS	abundance	Hgn3C.1035 - Klebsiella sp.	Tibia_SOS
MGS	abundance	Hgn3C.1035 - Klebsiella sp.	Handgrip
MGS	abundance	Hgn3C.1036 - Escherichia coli	Tibia_length
MGS	abundance	Hgn3C.1036 - Escherichia coli	Radius_length
MGS	abundance	Hgn3C.1036 - Escherichia coli	Radius_SOS
MGS	abundance	Hgn3C.1036 - Escherichia coli	Tibia_SOS
MGS	abundance	Hgn3C.1036 - Escherichia coli	Handgrip
MGS	abundance	Hgn3C.1037 - Rothia sp.	Tibia_length
MGS	abundance	Hgn3C.1037 - Rothia sp.	Radius_length
MGS	abundance	Hgn3C.1037 - Rothia sp.	Radius_SOS
MGS	abundance	Hgn3C.1037 - Rothia sp.	Tibia_SOS
MGS	abundance	Hgn3C.1037 - Rothia sp.	Handgrip
MGS	abundance	Hgn3C.1038 - Veillonella sp.	Tibia_length
MGS	abundance	Hgn3C.1038 - Veillonella sp.	Radius_length
MGS	abundance	Hgn3C.1038 - Veillonella sp.	Radius_SOS
MGS	abundance	Hgn3C.1038 - Veillonella sp.	Tibia_SOS
MGS	abundance	Hgn3C.1038 - Veillonella sp.	Handgrip
MGS	abundance	Hgn3C.1040 - Lachnospiraceae sp.	Tibia_length
MGS	abundance	Hgn3C.1040 - Lachnospiraceae sp.	Radius_length
MGS	abundance	Hgn3C.1040 - Lachnospiraceae sp.	Radius_SOS
MGS	abundance	Hgn3C.1040 - Lachnospiraceae sp.	Tibia_SOS
MGS	abundance	Hgn3C.1040 - Lachnospiraceae sp.	Handgrip
MGS	abundance	Hgn3C.1041 - Bacteroides stercoris	Tibia_length
MGS	abundance	Hgn3C.1041 - Bacteroides stercoris	Radius_length
MGS	abundance	Hgn3C.1041 - Bacteroides stercoris	Radius_SOS
MGS	abundance	Hgn3C.1041 - Bacteroides stercoris	Tibia_SOS
MGS	abundance	Hgn3C.1041 - Bacteroides stercoris	Handgrip
MGS	abundance	Hgn3C.1042 - Dorea formicigenerans	Tibia_length

MGS	abundance	Hgn3C.1042 - Dorea formicigenerans	Radius_length
MGS	abundance	Hgn3C.1042 - Dorea formicigenerans	Radius_SOS
MGS	abundance	Hgn3C.1042 - Dorea formicigenerans	Tibia_SOS
MGS	abundance	Hgn3C.1042 - Dorea formicigenerans	Handgrip
MGS	abundance	Hgn3C.1043 - Clostridium phoceensis	Tibia_length
MGS	abundance	Hgn3C.1043 - Clostridium phoceensis	Radius_length
MGS	abundance	Hgn3C.1043 - Clostridium phoceensis	Radius_SOS
MGS	abundance	Hgn3C.1043 - Clostridium phoceensis	Tibia_SOS
MGS	abundance	Hgn3C.1043 - Clostridium phoceensis	Handgrip
MGS	abundance	Hgn3C.1044 - Eubacteriales sp.	Tibia_length
MGS	abundance	Hgn3C.1044 - Eubacteriales sp.	Radius_length
MGS	abundance	Hgn3C.1044 - Eubacteriales sp.	Radius_SOS
MGS	abundance	Hgn3C.1044 - Eubacteriales sp.	Tibia_SOS
MGS	abundance	Hgn3C.1044 - Eubacteriales sp.	Handgrip
MGS	abundance	Hgn3C.1045 - Lactococcus lactis subsp. lactis	Tibia_length
MGS	abundance	Hgn3C.1045 - Lactococcus lactis subsp. lactis	Radius_length
MGS	abundance	Hgn3C.1045 - Lactococcus lactis subsp. lactis	Radius_SOS
MGS	abundance	Hgn3C.1045 - Lactococcus lactis subsp. lactis	Tibia_SOS
MGS	abundance	Hgn3C.1045 - Lactococcus lactis subsp. lactis	Handgrip
MGS	abundance	Hgn3C.1046 - Bacteroidales sp.	Tibia_length
MGS	abundance	Hgn3C.1046 - Bacteroidales sp.	Radius_length
MGS	abundance	Hgn3C.1046 - Bacteroidales sp.	Radius_SOS
MGS	abundance	Hgn3C.1046 - Bacteroidales sp.	Tibia_SOS
MGS	abundance	Hgn3C.1046 - Bacteroidales sp.	Handgrip
MGS	abundance	Hgn3C.1047 - Eubacteriales sp.	Tibia_length
MGS	abundance	Hgn3C.1047 - Eubacteriales sp.	Radius_length
MGS	abundance	Hgn3C.1047 - Eubacteriales sp.	Radius_SOS
MGS	abundance	Hgn3C.1047 - Eubacteriales sp.	Tibia_SOS
MGS	abundance	Hgn3C.1047 - Eubacteriales sp.	Handgrip
MGS	abundance	Hgn3C.1049 - Enterococcus faecium	Tibia_length
MGS	abundance	Hgn3C.1049 - Enterococcus faecium	Radius_length
MGS	abundance	Hgn3C.1049 - Enterococcus faecium	Radius_SOS
MGS	abundance	Hgn3C.1049 - Enterococcus faecium	Tibia_SOS
MGS	abundance	Hgn3C.1049 - Enterococcus faecium	Handgrip
MGS	abundance	Hgn3C.1050 - Staphylococcus epidermidis	Tibia_length
MGS	abundance	Hgn3C.1050 - Staphylococcus epidermidis	Radius_length
MGS	abundance	Hgn3C.1050 - Staphylococcus epidermidis	Radius_SOS
MGS	abundance	Hgn3C.1050 - Staphylococcus epidermidis	Tibia_SOS
MGS	abundance	Hgn3C.1050 - Staphylococcus epidermidis	Handgrip
MGS	abundance	Hgn3C.1051 - Holdemania massiliensis	Tibia_length
MGS	abundance	Hgn3C.1051 - Holdemania massiliensis	Radius_length
MGS	abundance	Hgn3C.1051 - Holdemania massiliensis	Radius_SOS
MGS	abundance	Hgn3C.1051 - Holdemania massiliensis	Tibia_SOS
MGS	abundance	Hgn3C.1051 - Holdemania massiliensis	Handgrip
MGS	abundance	Hgn3C.1052 - Enterococcus gallinarum	Tibia_length
MGS	abundance	Hgn3C.1052 - Enterococcus gallinarum	Radius_length
MGS	abundance	Hgn3C.1052 - Enterococcus gallinarum	Radius_SOS
MGS	abundance	Hgn3C.1052 - Enterococcus gallinarum	Tibia_SOS

MGS	abundance	Hgn3C.1052 - Enterococcus gallinarum	Handgrip
MGS	abundance	Hgn3C.1054 - Streptococcus lutetiensis	Tibia_length
MGS	abundance	Hgn3C.1054 - Streptococcus lutetiensis	Radius_length
MGS	abundance	Hgn3C.1054 - Streptococcus lutetiensis	Radius_SOS
MGS	abundance	Hgn3C.1054 - Streptococcus lutetiensis	Tibia_SOS
MGS	abundance	Hgn3C.1054 - Streptococcus lutetiensis	Handgrip
MGS	abundance	Hgn3C.1058 - Mediterraneibacter glycyrrhizinilyticus	Tibia_length
MGS	abundance	Hgn3C.1058 - Mediterraneibacter glycyrrhizinilyticus	Radius_length
MGS	abundance	Hgn3C.1058 - Mediterraneibacter glycyrrhizinilyticus	Radius_SOS
MGS	abundance	Hgn3C.1058 - Mediterraneibacter glycyrrhizinilyticus	Tibia_SOS
MGS	abundance	Hgn3C.1058 - Mediterraneibacter glycyrrhizinilyticus	Handgrip
MGS	abundance	Hgn3C.1059 - Faecalicatena sp.	Tibia_length
MGS	abundance	Hgn3C.1059 - Faecalicatena sp.	Radius_length
MGS	abundance	Hgn3C.1059 - Faecalicatena sp.	Radius_SOS
MGS	abundance	Hgn3C.1059 - Faecalicatena sp.	Tibia_SOS
MGS	abundance	Hgn3C.1059 - Faecalicatena sp.	Handgrip
MGS	abundance	Hgn3C.1063 - Enterococcus avium	Tibia_length
MGS	abundance	Hgn3C.1063 - Enterococcus avium	Radius_length
MGS	abundance	Hgn3C.1063 - Enterococcus avium	Radius_SOS
MGS	abundance	Hgn3C.1063 - Enterococcus avium	Tibia_SOS
MGS	abundance	Hgn3C.1063 - Enterococcus avium	Handgrip
MGS	abundance	Hgn3C.1065 - Klebsiella variicola subsp. variicola	Tibia_length
MGS	abundance	Hgn3C.1065 - Klebsiella variicola subsp. variicola	Radius_length
MGS	abundance	Hgn3C.1065 - Klebsiella variicola subsp. variicola	Radius_SOS
MGS	abundance	Hgn3C.1065 - Klebsiella variicola subsp. variicola	Tibia_SOS
MGS	abundance	Hgn3C.1065 - Klebsiella variicola subsp. variicola	Handgrip
MGS	abundance	Hgn3C.1067 - Lachnospiraceae sp.	Tibia_length
MGS	abundance	Hgn3C.1067 - Lachnospiraceae sp.	Radius_length
MGS	abundance	Hgn3C.1067 - Lachnospiraceae sp.	Radius_SOS
MGS	abundance	Hgn3C.1067 - Lachnospiraceae sp.	Tibia_SOS
MGS	abundance	Hgn3C.1067 - Lachnospiraceae sp.	Handgrip
MGS	abundance	Hgn3C.1071 - Lactobacillus gasseri	Tibia_length
MGS	abundance	Hgn3C.1071 - Lactobacillus gasseri	Radius_length
MGS	abundance	Hgn3C.1071 - Lactobacillus gasseri	Radius_SOS
MGS	abundance	Hgn3C.1071 - Lactobacillus gasseri	Tibia_SOS
MGS	abundance	Hgn3C.1071 - Lactobacillus gasseri	Handgrip
MGS	abundance	Hgn3C.1073 - Streptococcus gallolyticus subsp. gallolyticus	Tibia_length
MGS	abundance	Hgn3C.1073 - Streptococcus gallolyticus subsp. gallolyticus	Radius_length
MGS	abundance	Hgn3C.1073 - Streptococcus gallolyticus subsp. gallolyticus	Radius_SOS
MGS	abundance	Hgn3C.1073 - Streptococcus gallolyticus subsp. gallolyticus	Tibia_SOS
MGS	abundance	Hgn3C.1073 - Streptococcus gallolyticus subsp. gallolyticus	Handgrip
MGS	abundance	Hgn3C.1081 - Lacticaseibacillus rhamnosus	Tibia_length
MGS	abundance	Hgn3C.1081 - Lacticaseibacillus rhamnosus	Radius_length
MGS	abundance	Hgn3C.1081 - Lacticaseibacillus rhamnosus	Radius_SOS
MGS	abundance	Hgn3C.1081 - Lacticaseibacillus rhamnosus	Tibia_SOS
MGS	abundance	Hgn3C.1081 - Lacticaseibacillus rhamnosus	Handgrip
MGS	abundance	Hgn3C.1082 - Lacticaseibacillus paracasei subsp. paracasei	Tibia_length
MGS	abundance	Hgn3C.1082 - Lacticaseibacillus paracasei subsp. paracasei	Radius_length

MGS	abundance	Hgn3C.1082 - Lacticaseibacillus paracasei subsp. paracasei	Radius_SOS
MGS	abundance	Hgn3C.1082 - Lacticaseibacillus paracasei subsp. paracasei	Tibia_SOS
MGS	abundance	Hgn3C.1082 - Lacticaseibacillus paracasei subsp. paracasei	Handgrip
MGS	abundance	Hgn3C.1084 - Massilimicrobiota timonensis	Tibia_length
MGS	abundance	Hgn3C.1084 - Massilimicrobiota timonensis	Radius_length
MGS	abundance	Hgn3C.1084 - Massilimicrobiota timonensis	Radius_SOS
MGS	abundance	Hgn3C.1084 - Massilimicrobiota timonensis	Tibia_SOS
MGS	abundance	Hgn3C.1084 - Massilimicrobiota timonensis	Handgrip
MGS	abundance	Hgn3C.1087 - Ligilactobacillus salivarius	Tibia_length
MGS	abundance	Hgn3C.1087 - Ligilactobacillus salivarius	Radius_length
MGS	abundance	Hgn3C.1087 - Ligilactobacillus salivarius	Radius_SOS
MGS	abundance	Hgn3C.1087 - Ligilactobacillus salivarius	Tibia_SOS
MGS	abundance	Hgn3C.1087 - Ligilactobacillus salivarius	Handgrip
MGS	abundance	Hgn3C.1088 - Enterococcus casseliflavus	Tibia_length
MGS	abundance	Hgn3C.1088 - Enterococcus casseliflavus	Radius_length
MGS	abundance	Hgn3C.1088 - Enterococcus casseliflavus	Radius_SOS
MGS	abundance	Hgn3C.1088 - Enterococcus casseliflavus	Tibia_SOS
MGS	abundance	Hgn3C.1088 - Enterococcus casseliflavus	Handgrip
MGS	abundance	Hgn3C.1090 - Turicibacter sanguinis	Tibia_length
MGS	abundance	Hgn3C.1090 - Turicibacter sanguinis	Radius_length
MGS	abundance	Hgn3C.1090 - Turicibacter sanguinis	Radius_SOS
MGS	abundance	Hgn3C.1090 - Turicibacter sanguinis	Tibia_SOS
MGS	abundance	Hgn3C.1090 - Turicibacter sanguinis	Handgrip
MGS	abundance	Hgn3C.1093 - Enterobacter kobei	Tibia_length
MGS	abundance	Hgn3C.1093 - Enterobacter kobei	Radius_length
MGS	abundance	Hgn3C.1093 - Enterobacter kobei	Radius_SOS
MGS	abundance	Hgn3C.1093 - Enterobacter kobei	Tibia_SOS
MGS	abundance	Hgn3C.1093 - Enterobacter kobei	Handgrip
MGS	abundance	Hgn3C.1097 - Alistipes provencensis	Tibia_length
MGS	abundance	Hgn3C.1097 - Alistipes provencensis	Radius_length
MGS	abundance	Hgn3C.1097 - Alistipes provencensis	Radius_SOS
MGS	abundance	Hgn3C.1097 - Alistipes provencensis	Tibia_SOS
MGS	abundance	Hgn3C.1097 - Alistipes provencensis	Handgrip
MGS	abundance	Hgn3C.1102 - Eubacteriales sp.	Tibia_length
MGS	abundance	Hgn3C.1102 - Eubacteriales sp.	Radius_length
MGS	abundance	Hgn3C.1102 - Eubacteriales sp.	Radius_SOS
MGS	abundance	Hgn3C.1102 - Eubacteriales sp.	Tibia_SOS
MGS	abundance	Hgn3C.1102 - Eubacteriales sp.	Handgrip
MGS	abundance	Hgn3C.1106 - Clostridium butyricum	Tibia_length
MGS	abundance	Hgn3C.1106 - Clostridium butyricum	Radius_length
MGS	abundance	Hgn3C.1106 - Clostridium butyricum	Radius_SOS
MGS	abundance	Hgn3C.1106 - Clostridium butyricum	Tibia_SOS
MGS	abundance	Hgn3C.1106 - Clostridium butyricum	Handgrip
MGS	abundance	Hgn3C.1107 - Eubacteriales sp.	Tibia_length
MGS	abundance	Hgn3C.1107 - Eubacteriales sp.	Radius_length
MGS	abundance	Hgn3C.1107 - Eubacteriales sp.	Radius_SOS
MGS	abundance	Hgn3C.1107 - Eubacteriales sp.	Tibia_SOS
MGS	abundance	Hgn3C.1107 - Eubacteriales sp.	Handgrip

MGS	abundance	Hgn3C.1110 - Porphyromonas sp.	Tibia_length
MGS	abundance	Hgn3C.1110 - Porphyromonas sp.	Radius_length
MGS	abundance	Hgn3C.1110 - Porphyromonas sp.	Radius_SOS
MGS	abundance	Hgn3C.1110 - Porphyromonas sp.	Tibia_SOS
MGS	abundance	Hgn3C.1110 - Porphyromonas sp.	Handgrip
MGS	abundance	Hgn3C.1117 - Atopobiaceae sp.	Tibia_length
MGS	abundance	Hgn3C.1117 - Atopobiaceae sp.	Radius_length
MGS	abundance	Hgn3C.1117 - Atopobiaceae sp.	Radius_SOS
MGS	abundance	Hgn3C.1117 - Atopobiaceae sp.	Tibia_SOS
MGS	abundance	Hgn3C.1117 - Atopobiaceae sp.	Handgrip
MGS	abundance	Hgn3C.1120 - Actinomycetaceae sp.	Tibia_length
MGS	abundance	Hgn3C.1120 - Actinomycetaceae sp.	Radius_length
MGS	abundance	Hgn3C.1120 - Actinomycetaceae sp.	Radius_SOS
MGS	abundance	Hgn3C.1120 - Actinomycetaceae sp.	Tibia_SOS
MGS	abundance	Hgn3C.1120 - Actinomycetaceae sp.	Handgrip
MGS	abundance	Hgn3C.1122 - Ruminococcus sp.	Tibia_length
MGS	abundance	Hgn3C.1122 - Ruminococcus sp.	Radius_length
MGS	abundance	Hgn3C.1122 - Ruminococcus sp.	Radius_SOS
MGS	abundance	Hgn3C.1122 - Ruminococcus sp.	Tibia_SOS
MGS	abundance	Hgn3C.1122 - Ruminococcus sp.	Handgrip
MGS	abundance	Hgn3C.1124 - Clostridium porci	Tibia_length
MGS	abundance	Hgn3C.1124 - Clostridium porci	Radius_length
MGS	abundance	Hgn3C.1124 - Clostridium porci	Radius_SOS
MGS	abundance	Hgn3C.1124 - Clostridium porci	Tibia_SOS
MGS	abundance	Hgn3C.1124 - Clostridium porci	Handgrip
MGS	abundance	Hgn3C.1126 - Phocaeicola sartorii	Tibia_length
MGS	abundance	Hgn3C.1126 - Phocaeicola sartorii	Radius_length
MGS	abundance	Hgn3C.1126 - Phocaeicola sartorii	Radius_SOS
MGS	abundance	Hgn3C.1126 - Phocaeicola sartorii	Tibia_SOS
MGS	abundance	Hgn3C.1126 - Phocaeicola sartorii	Handgrip
MGS	abundance	Hgn3C.1146 - Bacteria sp.	Tibia_length
MGS	abundance	Hgn3C.1146 - Bacteria sp.	Radius_length
MGS	abundance	Hgn3C.1146 - Bacteria sp.	Radius_SOS
MGS	abundance	Hgn3C.1146 - Bacteria sp.	Tibia_SOS
MGS	abundance	Hgn3C.1146 - Bacteria sp.	Handgrip
MGS	abundance	Hgn3C.1148 - Eggerthellales sp.	Tibia_length
MGS	abundance	Hgn3C.1148 - Eggerthellales sp.	Radius_length
MGS	abundance	Hgn3C.1148 - Eggerthellales sp.	Radius_SOS
MGS	abundance	Hgn3C.1148 - Eggerthellales sp.	Tibia_SOS
MGS	abundance	Hgn3C.1148 - Eggerthellales sp.	Handgrip
MGS	abundance	Hgn3C.1153 - Eubacterium sp. OM08-24	Tibia_length
MGS	abundance	Hgn3C.1153 - Eubacterium sp. OM08-24	Radius_length
MGS	abundance	Hgn3C.1153 - Eubacterium sp. OM08-24	Radius_SOS
MGS	abundance	Hgn3C.1153 - Eubacterium sp. OM08-24	Tibia_SOS
MGS	abundance	Hgn3C.1153 - Eubacterium sp. OM08-24	Handgrip
MGS	abundance	Hgn3C.1154 - Lactococcus garvieae	Tibia_length
MGS	abundance	Hgn3C.1154 - Lactococcus garvieae	Radius_length
MGS	abundance	Hgn3C.1154 - Lactococcus garvieae	Radius_SOS

MGS	abundance	Hgn3C.1154 - Lactococcus garvieae	Tibia_SOS
MGS	abundance	Hgn3C.1154 - Lactococcus garvieae	Handgrip
MGS	abundance	Hgn3C.1169 - Oscillospiraceae sp.	Tibia_length
MGS	abundance	Hgn3C.1169 - Oscillospiraceae sp.	Radius_length
MGS	abundance	Hgn3C.1169 - Oscillospiraceae sp.	Radius_SOS
MGS	abundance	Hgn3C.1169 - Oscillospiraceae sp.	Tibia_SOS
MGS	abundance	Hgn3C.1169 - Oscillospiraceae sp.	Handgrip
MGS	abundance	Hgn3C.1180 - Enterocloster citroniae	Tibia_length
MGS	abundance	Hgn3C.1180 - Enterocloster citroniae	Radius_length
MGS	abundance	Hgn3C.1180 - Enterocloster citroniae	Radius_SOS
MGS	abundance	Hgn3C.1180 - Enterocloster citroniae	Tibia_SOS
MGS	abundance	Hgn3C.1180 - Enterocloster citroniae	Handgrip
MGS	abundance	Hgn3C.1182 - Comamonas kerstersii	Tibia_length
MGS	abundance	Hgn3C.1182 - Comamonas kerstersii	Radius_length
MGS	abundance	Hgn3C.1182 - Comamonas kerstersii	Radius_SOS
MGS	abundance	Hgn3C.1182 - Comamonas kerstersii	Tibia_SOS
MGS	abundance	Hgn3C.1182 - Comamonas kerstersii	Handgrip
MGS	abundance	Hgn3C.1185 - Acinetobacter ursingii	Tibia_length
MGS	abundance	Hgn3C.1185 - Acinetobacter ursingii	Radius_length
MGS	abundance	Hgn3C.1185 - Acinetobacter ursingii	Radius_SOS
MGS	abundance	Hgn3C.1185 - Acinetobacter ursingii	Tibia_SOS
MGS	abundance	Hgn3C.1185 - Acinetobacter ursingii	Handgrip
MGS	abundance	Hgn3C.1188 - Actinomyces oris	Tibia_length
MGS	abundance	Hgn3C.1188 - Actinomyces oris	Radius_length
MGS	abundance	Hgn3C.1188 - Actinomyces oris	Radius_SOS
MGS	abundance	Hgn3C.1188 - Actinomyces oris	Tibia_SOS
MGS	abundance	Hgn3C.1188 - Actinomyces oris	Handgrip
MGS	abundance	Hgn3C.1190 - Fenollaria massiliensis	Tibia_length
MGS	abundance	Hgn3C.1190 - Fenollaria massiliensis	Radius_length
MGS	abundance	Hgn3C.1190 - Fenollaria massiliensis	Radius_SOS
MGS	abundance	Hgn3C.1190 - Fenollaria massiliensis	Tibia_SOS
MGS	abundance	Hgn3C.1190 - Fenollaria massiliensis	Handgrip
MGS	abundance	Hgn3C.1193 - Atopobium sp.	Tibia_length
MGS	abundance	Hgn3C.1193 - Atopobium sp.	Radius_length
MGS	abundance	Hgn3C.1193 - Atopobium sp.	Radius_SOS
MGS	abundance	Hgn3C.1193 - Atopobium sp.	Tibia_SOS
MGS	abundance	Hgn3C.1193 - Atopobium sp.	Handgrip
MGS	abundance	Hgn3C.1196 - Blautia marasmi	Tibia_length
MGS	abundance	Hgn3C.1196 - Blautia marasmi	Radius_length
MGS	abundance	Hgn3C.1196 - Blautia marasmi	Radius_SOS
MGS	abundance	Hgn3C.1196 - Blautia marasmi	Tibia_SOS
MGS	abundance	Hgn3C.1196 - Blautia marasmi	Handgrip
MGS	abundance	Hgn3C.1197 - Blautia hansenii	Tibia_length
MGS	abundance	Hgn3C.1197 - Blautia hansenii	Radius_length
MGS	abundance	Hgn3C.1197 - Blautia hansenii	Radius_SOS
MGS	abundance	Hgn3C.1197 - Blautia hansenii	Tibia_SOS
MGS	abundance	Hgn3C.1197 - Blautia hansenii	Handgrip
MGS	abundance	Hgn3C.1203 - Citrobacter sp. CRE-46	Tibia_length

MGS	abundance	Hgn3C.1203 - Citrobacter sp. CRE-46	Radius_length
MGS	abundance	Hgn3C.1203 - Citrobacter sp. CRE-46	Radius_SOS
MGS	abundance	Hgn3C.1203 - Citrobacter sp. CRE-46	Tibia_SOS
MGS	abundance	Hgn3C.1203 - Citrobacter sp. CRE-46	Handgrip
MGS	abundance	Hgn3C.1206 - Citrobacter pasteurii	Tibia_length
MGS	abundance	Hgn3C.1206 - Citrobacter pasteurii	Radius_length
MGS	abundance	Hgn3C.1206 - Citrobacter pasteurii	Radius_SOS
MGS	abundance	Hgn3C.1206 - Citrobacter pasteurii	Tibia_SOS
MGS	abundance	Hgn3C.1206 - Citrobacter pasteurii	Handgrip
MGS	abundance	Hgn3C.1207 - Citrobacter sp.	Tibia_length
MGS	abundance	Hgn3C.1207 - Citrobacter sp.	Radius_length
MGS	abundance	Hgn3C.1207 - Citrobacter sp.	Radius_SOS
MGS	abundance	Hgn3C.1207 - Citrobacter sp.	Tibia_SOS
MGS	abundance	Hgn3C.1207 - Citrobacter sp.	Handgrip
MGS	abundance	Hgn3C.1210 - Dielma sp.	Tibia_length
MGS	abundance	Hgn3C.1210 - Dielma sp.	Radius_length
MGS	abundance	Hgn3C.1210 - Dielma sp.	Radius_SOS
MGS	abundance	Hgn3C.1210 - Dielma sp.	Tibia_SOS
MGS	abundance	Hgn3C.1210 - Dielma sp.	Handgrip
MGS	abundance	Hgn3C.1211 - Lachnoclostridium pacaense	Tibia_length
MGS	abundance	Hgn3C.1211 - Lachnoclostridium pacaense	Radius_length
MGS	abundance	Hgn3C.1211 - Lachnoclostridium pacaense	Radius_SOS
MGS	abundance	Hgn3C.1211 - Lachnoclostridium pacaense	Tibia_SOS
MGS	abundance	Hgn3C.1211 - Lachnoclostridium pacaense	Handgrip
MGS	abundance	Hgn3C.1214 - Corynebacterium tuberculostearicum	Tibia_length
MGS	abundance	Hgn3C.1214 - Corynebacterium tuberculostearicum	Radius_length
MGS	abundance	Hgn3C.1214 - Corynebacterium tuberculostearicum	Radius_SOS
MGS	abundance	Hgn3C.1214 - Corynebacterium tuberculostearicum	Tibia_SOS
MGS	abundance	Hgn3C.1214 - Corynebacterium tuberculostearicum	Handgrip
MGS	abundance	Hgn3C.1215 - Cutibacterium acnes subsp. acnes	Tibia_length
MGS	abundance	Hgn3C.1215 - Cutibacterium acnes subsp. acnes	Radius_length
MGS	abundance	Hgn3C.1215 - Cutibacterium acnes subsp. acnes	Radius_SOS
MGS	abundance	Hgn3C.1215 - Cutibacterium acnes subsp. acnes	Tibia_SOS
MGS	abundance	Hgn3C.1215 - Cutibacterium acnes subsp. acnes	Handgrip
MGS	abundance	Hgn3C.1218 - Enterobacter bugandensis	Tibia_length
MGS	abundance	Hgn3C.1218 - Enterobacter bugandensis	Radius_length
MGS	abundance	Hgn3C.1218 - Enterobacter bugandensis	Radius_SOS
MGS	abundance	Hgn3C.1218 - Enterobacter bugandensis	Tibia_SOS
MGS	abundance	Hgn3C.1218 - Enterobacter bugandensis	Handgrip
MGS	abundance	Hgn3C.1219 - Enterobacter sp. NFIX58	Tibia_length
MGS	abundance	Hgn3C.1219 - Enterobacter sp. NFIX58	Radius_length
MGS	abundance	Hgn3C.1219 - Enterobacter sp. NFIX58	Radius_SOS
MGS	abundance	Hgn3C.1219 - Enterobacter sp. NFIX58	Tibia_SOS
MGS	abundance	Hgn3C.1219 - Enterobacter sp. NFIX58	Handgrip
MGS	abundance	Hgn3C.1221 - Phytobacter diazotrophicus	Tibia_length
MGS	abundance	Hgn3C.1221 - Phytobacter diazotrophicus	Radius_length
MGS	abundance	Hgn3C.1221 - Phytobacter diazotrophicus	Radius_SOS
MGS	abundance	Hgn3C.1221 - Phytobacter diazotrophicus	Tibia_SOS

MGS	abundance	Hgn3C.1221 - Phytobacter diazotrophicus	Handgrip
MGS	abundance	Hgn3C.1224 - Enterococcus raffinosus	Tibia_length
MGS	abundance	Hgn3C.1224 - Enterococcus raffinosus	Radius_length
MGS	abundance	Hgn3C.1224 - Enterococcus raffinosus	Radius_SOS
MGS	abundance	Hgn3C.1224 - Enterococcus raffinosus	Tibia_SOS
MGS	abundance	Hgn3C.1224 - Enterococcus raffinosus	Handgrip
MGS	abundance	Hgn3C.1225 - Enterococcus faecium	Tibia_length
MGS	abundance	Hgn3C.1225 - Enterococcus faecium	Radius_length
MGS	abundance	Hgn3C.1225 - Enterococcus faecium	Radius_SOS
MGS	abundance	Hgn3C.1225 - Enterococcus faecium	Tibia_SOS
MGS	abundance	Hgn3C.1225 - Enterococcus faecium	Handgrip
MGS	abundance	Hgn3C.1228 - Escherichia marmotae	Tibia_length
MGS	abundance	Hgn3C.1228 - Escherichia marmotae	Radius_length
MGS	abundance	Hgn3C.1228 - Escherichia marmotae	Radius_SOS
MGS	abundance	Hgn3C.1228 - Escherichia marmotae	Tibia_SOS
MGS	abundance	Hgn3C.1228 - Escherichia marmotae	Handgrip
MGS	abundance	Hgn3C.1229 - Escherichia sp. 93.0750	Tibia_length
MGS	abundance	Hgn3C.1229 - Escherichia sp. 93.0750	Radius_length
MGS	abundance	Hgn3C.1229 - Escherichia sp. 93.0750	Radius_SOS
MGS	abundance	Hgn3C.1229 - Escherichia sp. 93.0750	Tibia_SOS
MGS	abundance	Hgn3C.1229 - Escherichia sp. 93.0750	Handgrip
MGS	abundance	Hgn3C.1230 - Eubacterium limosum	Tibia_length
MGS	abundance	Hgn3C.1230 - Eubacterium limosum	Radius_length
MGS	abundance	Hgn3C.1230 - Eubacterium limosum	Radius_SOS
MGS	abundance	Hgn3C.1230 - Eubacterium limosum	Tibia_SOS
MGS	abundance	Hgn3C.1230 - Eubacterium limosum	Handgrip
MGS	abundance	Hgn3C.1234 - Granulicatella adiacens	Tibia_length
MGS	abundance	Hgn3C.1234 - Granulicatella adiacens	Radius_length
MGS	abundance	Hgn3C.1234 - Granulicatella adiacens	Radius_SOS
MGS	abundance	Hgn3C.1234 - Granulicatella adiacens	Tibia_SOS
MGS	abundance	Hgn3C.1234 - Granulicatella adiacens	Handgrip
MGS	abundance	Hgn3C.1238 - Klebsiella quasivariicola	Tibia_length
MGS	abundance	Hgn3C.1238 - Klebsiella quasivariicola	Radius_length
MGS	abundance	Hgn3C.1238 - Klebsiella quasivariicola	Radius_SOS
MGS	abundance	Hgn3C.1238 - Klebsiella quasivariicola	Tibia_SOS
MGS	abundance	Hgn3C.1238 - Klebsiella quasivariicola	Handgrip
MGS	abundance	Hgn3C.1241 - Limosilactobacillus reuteri subsp. reuteri	Tibia_length
MGS	abundance	Hgn3C.1241 - Limosilactobacillus reuteri subsp. reuteri	Radius_length
MGS	abundance	Hgn3C.1241 - Limosilactobacillus reuteri subsp. reuteri	Radius_SOS
MGS	abundance	Hgn3C.1241 - Limosilactobacillus reuteri subsp. reuteri	Tibia_SOS
MGS	abundance	Hgn3C.1241 - Limosilactobacillus reuteri subsp. reuteri	Handgrip
MGS	abundance	Hgn3C.1242 - Limosilactobacillus vaginalis	Tibia_length
MGS	abundance	Hgn3C.1242 - Limosilactobacillus vaginalis	Radius_length
MGS	abundance	Hgn3C.1242 - Limosilactobacillus vaginalis	Radius_SOS
MGS	abundance	Hgn3C.1242 - Limosilactobacillus vaginalis	Tibia_SOS
MGS	abundance	Hgn3C.1242 - Limosilactobacillus vaginalis	Handgrip
MGS	abundance	Hgn3C.1248 - Neisseria sp. HMSC061E12	Tibia_length
MGS	abundance	Hgn3C.1248 - Neisseria sp. HMSC061E12	Radius_length

MGS	abundanc	Hgn3C.1248 - Neisseria sp. HMSC061E12	Radius_SOS
MGS	abundanc	Hgn3C.1248 - Neisseria sp. HMSC061E12	Tibia_SOS
MGS	abundanc	Hgn3C.1248 - Neisseria sp. HMSC061E12	Handgrip
MGS	abundanc	Hgn3C.1250 - Parabacteroides gordonii	Tibia_length
MGS	abundanc	Hgn3C.1250 - Parabacteroides gordonii	Radius_length
MGS	abundanc	Hgn3C.1250 - Parabacteroides gordonii	Radius_SOS
MGS	abundanc	Hgn3C.1250 - Parabacteroides gordonii	Tibia_SOS
MGS	abundanc	Hgn3C.1250 - Parabacteroides gordonii	Handgrip
MGS	abundanc	Hgn3C.1258 - Rothia sp. HMSC072E10	Tibia_length
MGS	abundanc	Hgn3C.1258 - Rothia sp. HMSC072E10	Radius_length
MGS	abundanc	Hgn3C.1258 - Rothia sp. HMSC072E10	Radius_SOS
MGS	abundanc	Hgn3C.1258 - Rothia sp. HMSC072E10	Tibia_SOS
MGS	abundanc	Hgn3C.1258 - Rothia sp. HMSC072E10	Handgrip
MGS	abundanc	Hgn3C.1264 - Staphylococcus hominis subsp. hominis	Tibia_length
MGS	abundanc	Hgn3C.1264 - Staphylococcus hominis subsp. hominis	Radius_length
MGS	abundanc	Hgn3C.1264 - Staphylococcus hominis subsp. hominis	Radius_SOS
MGS	abundanc	Hgn3C.1264 - Staphylococcus hominis subsp. hominis	Tibia_SOS
MGS	abundanc	Hgn3C.1264 - Staphylococcus hominis subsp. hominis	Handgrip
MGS	abundanc	Hgn3C.1268 - Streptococcus sp.	Tibia_length
MGS	abundanc	Hgn3C.1268 - Streptococcus sp.	Radius_length
MGS	abundanc	Hgn3C.1268 - Streptococcus sp.	Radius_SOS
MGS	abundanc	Hgn3C.1268 - Streptococcus sp.	Tibia_SOS
MGS	abundanc	Hgn3C.1268 - Streptococcus sp.	Handgrip
MGS	abundanc	Hgn3C.1269 - Streptococcus constellatus subsp. constellatus	Tibia_length
MGS	abundanc	Hgn3C.1269 - Streptococcus constellatus subsp. constellatus	Radius_length
MGS	abundanc	Hgn3C.1269 - Streptococcus constellatus subsp. constellatus	Radius_SOS
MGS	abundanc	Hgn3C.1269 - Streptococcus constellatus subsp. constellatus	Tibia_SOS
MGS	abundanc	Hgn3C.1269 - Streptococcus constellatus subsp. constellatus	Handgrip
MGS	abundanc	Hgn3C.1270 - Streptococcus sp.	Tibia_length
MGS	abundanc	Hgn3C.1270 - Streptococcus sp.	Radius_length
MGS	abundanc	Hgn3C.1270 - Streptococcus sp.	Radius_SOS
MGS	abundanc	Hgn3C.1270 - Streptococcus sp.	Tibia_SOS
MGS	abundanc	Hgn3C.1270 - Streptococcus sp.	Handgrip
MGS	abundanc	Hgn3C.1271 - Streptococcus agalactiae	Tibia_length
MGS	abundanc	Hgn3C.1271 - Streptococcus agalactiae	Radius_length
MGS	abundanc	Hgn3C.1271 - Streptococcus agalactiae	Radius_SOS
MGS	abundanc	Hgn3C.1271 - Streptococcus agalactiae	Tibia_SOS
MGS	abundanc	Hgn3C.1271 - Streptococcus agalactiae	Handgrip
MGS	abundanc	Hgn3C.1275 - Lachnospiraceae sp.	Tibia_length
MGS	abundanc	Hgn3C.1275 - Lachnospiraceae sp.	Radius_length
MGS	abundanc	Hgn3C.1275 - Lachnospiraceae sp.	Radius_SOS
MGS	abundanc	Hgn3C.1275 - Lachnospiraceae sp.	Tibia_SOS
MGS	abundanc	Hgn3C.1275 - Lachnospiraceae sp.	Handgrip
MGS	abundanc	Hgn3C.1277 - Lachnospiraceae sp.	Tibia_length
MGS	abundanc	Hgn3C.1277 - Lachnospiraceae sp.	Radius_length
MGS	abundanc	Hgn3C.1277 - Lachnospiraceae sp.	Radius_SOS
MGS	abundanc	Hgn3C.1277 - Lachnospiraceae sp.	Tibia_SOS
MGS	abundanc	Hgn3C.1277 - Lachnospiraceae sp.	Handgrip

MGS	abundance	Hgn3C.1278 - Clostridium sp. NSJ-6	Tibia_length
MGS	abundance	Hgn3C.1278 - Clostridium sp. NSJ-6	Radius_length
MGS	abundance	Hgn3C.1278 - Clostridium sp. NSJ-6	Radius_SOS
MGS	abundance	Hgn3C.1278 - Clostridium sp. NSJ-6	Tibia_SOS
MGS	abundance	Hgn3C.1278 - Clostridium sp. NSJ-6	Handgrip
MGS	abundance	Hgn3C.1282 - Terrisporobacter othiniensis	Tibia_length
MGS	abundance	Hgn3C.1282 - Terrisporobacter othiniensis	Radius_length
MGS	abundance	Hgn3C.1282 - Terrisporobacter othiniensis	Radius_SOS
MGS	abundance	Hgn3C.1282 - Terrisporobacter othiniensis	Tibia_SOS
MGS	abundance	Hgn3C.1282 - Terrisporobacter othiniensis	Handgrip
MGS	abundance	Hgn3C.1283 - Clostridium baratii	Tibia_length
MGS	abundance	Hgn3C.1283 - Clostridium baratii	Radius_length
MGS	abundance	Hgn3C.1283 - Clostridium baratii	Radius_SOS
MGS	abundance	Hgn3C.1283 - Clostridium baratii	Tibia_SOS
MGS	abundance	Hgn3C.1283 - Clostridium baratii	Handgrip
MGS	abundance	Hgn3C.1285 - Enterobacteriaceae sp.	Tibia_length
MGS	abundance	Hgn3C.1285 - Enterobacteriaceae sp.	Radius_length
MGS	abundance	Hgn3C.1285 - Enterobacteriaceae sp.	Radius_SOS
MGS	abundance	Hgn3C.1285 - Enterobacteriaceae sp.	Tibia_SOS
MGS	abundance	Hgn3C.1285 - Enterobacteriaceae sp.	Handgrip
MGS	abundance	Hgn3C.1286 - Corynebacterium variabile	Tibia_length
MGS	abundance	Hgn3C.1286 - Corynebacterium variabile	Radius_length
MGS	abundance	Hgn3C.1286 - Corynebacterium variabile	Radius_SOS
MGS	abundance	Hgn3C.1286 - Corynebacterium variabile	Tibia_SOS
MGS	abundance	Hgn3C.1286 - Corynebacterium variabile	Handgrip
MGS	abundance	Hgn3C.1288 - Enterobacter hormaechei	Tibia_length
MGS	abundance	Hgn3C.1288 - Enterobacter hormaechei	Radius_length
MGS	abundance	Hgn3C.1288 - Enterobacter hormaechei	Radius_SOS
MGS	abundance	Hgn3C.1288 - Enterobacter hormaechei	Tibia_SOS
MGS	abundance	Hgn3C.1288 - Enterobacter hormaechei	Handgrip
MGS	abundance	Hgn3C.1292 - Limosilactobacillus portuensis	Tibia_length
MGS	abundance	Hgn3C.1292 - Limosilactobacillus portuensis	Radius_length
MGS	abundance	Hgn3C.1292 - Limosilactobacillus portuensis	Radius_SOS
MGS	abundance	Hgn3C.1292 - Limosilactobacillus portuensis	Tibia_SOS
MGS	abundance	Hgn3C.1292 - Limosilactobacillus portuensis	Handgrip
MGS	abundance	Hgn3C.1295 - Butyricicoccus pullicaecorum	Tibia_length
MGS	abundance	Hgn3C.1295 - Butyricicoccus pullicaecorum	Radius_length
MGS	abundance	Hgn3C.1295 - Butyricicoccus pullicaecorum	Radius_SOS
MGS	abundance	Hgn3C.1295 - Butyricicoccus pullicaecorum	Tibia_SOS
MGS	abundance	Hgn3C.1295 - Butyricicoccus pullicaecorum	Handgrip
MGS	abundance	Hgn3C.1297 - Clostridium sp.	Tibia_length
MGS	abundance	Hgn3C.1297 - Clostridium sp.	Radius_length
MGS	abundance	Hgn3C.1297 - Clostridium sp.	Radius_SOS
MGS	abundance	Hgn3C.1297 - Clostridium sp.	Tibia_SOS
MGS	abundance	Hgn3C.1297 - Clostridium sp.	Handgrip
MGS	abundance	Hgn3C.1298 - Firmicutes sp.	Tibia_length
MGS	abundance	Hgn3C.1298 - Firmicutes sp.	Radius_length
MGS	abundance	Hgn3C.1298 - Firmicutes sp.	Radius_SOS

MGS	abundance	Hgn3C.1298 - Firmicutes sp.	Tibia_SOS
MGS	abundance	Hgn3C.1298 - Firmicutes sp.	Handgrip
MGS	abundance	Hgn3C.1299 - Erysipelotrichaceae sp.	Tibia_length
MGS	abundance	Hgn3C.1299 - Erysipelotrichaceae sp.	Radius_length
MGS	abundance	Hgn3C.1299 - Erysipelotrichaceae sp.	Radius_SOS
MGS	abundance	Hgn3C.1299 - Erysipelotrichaceae sp.	Tibia_SOS
MGS	abundance	Hgn3C.1299 - Erysipelotrichaceae sp.	Handgrip
MGS	abundance	Hgn3C.1300 - Rothia sp.	Tibia_length
MGS	abundance	Hgn3C.1300 - Rothia sp.	Radius_length
MGS	abundance	Hgn3C.1300 - Rothia sp.	Radius_SOS
MGS	abundance	Hgn3C.1300 - Rothia sp.	Tibia_SOS
MGS	abundance	Hgn3C.1300 - Rothia sp.	Handgrip
MGS	abundance	Hgn3C.1302 - Schaalia sp.	Tibia_length
MGS	abundance	Hgn3C.1302 - Schaalia sp.	Radius_length
MGS	abundance	Hgn3C.1302 - Schaalia sp.	Radius_SOS
MGS	abundance	Hgn3C.1302 - Schaalia sp.	Tibia_SOS
MGS	abundance	Hgn3C.1302 - Schaalia sp.	Handgrip
MGS	abundance	Hgn3C.1303 - Clostridium paraputrificum	Tibia_length
MGS	abundance	Hgn3C.1303 - Clostridium paraputrificum	Radius_length
MGS	abundance	Hgn3C.1303 - Clostridium paraputrificum	Radius_SOS
MGS	abundance	Hgn3C.1303 - Clostridium paraputrificum	Tibia_SOS
MGS	abundance	Hgn3C.1303 - Clostridium paraputrificum	Handgrip
MGS	abundance	Hgn3C.1305 - Isoptericola variabilis	Tibia_length
MGS	abundance	Hgn3C.1305 - Isoptericola variabilis	Radius_length
MGS	abundance	Hgn3C.1305 - Isoptericola variabilis	Radius_SOS
MGS	abundance	Hgn3C.1305 - Isoptericola variabilis	Tibia_SOS
MGS	abundance	Hgn3C.1305 - Isoptericola variabilis	Handgrip
MGS	abundance	Hgn3C.1308 - Eisenbergiella sp.	Tibia_length
MGS	abundance	Hgn3C.1308 - Eisenbergiella sp.	Radius_length
MGS	abundance	Hgn3C.1308 - Eisenbergiella sp.	Radius_SOS
MGS	abundance	Hgn3C.1308 - Eisenbergiella sp.	Tibia_SOS
MGS	abundance	Hgn3C.1308 - Eisenbergiella sp.	Handgrip
MGS	abundance	Hgn3C.1309 - Enterobacter cloacae subsp. cloacae	Tibia_length
MGS	abundance	Hgn3C.1309 - Enterobacter cloacae subsp. cloacae	Radius_length
MGS	abundance	Hgn3C.1309 - Enterobacter cloacae subsp. cloacae	Radius_SOS
MGS	abundance	Hgn3C.1309 - Enterobacter cloacae subsp. cloacae	Tibia_SOS
MGS	abundance	Hgn3C.1309 - Enterobacter cloacae subsp. cloacae	Handgrip
MGS	abundance	Hgn3C.1310 - Erysipelotrichaceae sp.	Tibia_length
MGS	abundance	Hgn3C.1310 - Erysipelotrichaceae sp.	Radius_length
MGS	abundance	Hgn3C.1310 - Erysipelotrichaceae sp.	Radius_SOS
MGS	abundance	Hgn3C.1310 - Erysipelotrichaceae sp.	Tibia_SOS
MGS	abundance	Hgn3C.1310 - Erysipelotrichaceae sp.	Handgrip
MGS	abundance	Hgn3C.1311 - Lachnospiraceae sp.	Tibia_length
MGS	abundance	Hgn3C.1311 - Lachnospiraceae sp.	Radius_length
MGS	abundance	Hgn3C.1311 - Lachnospiraceae sp.	Radius_SOS
MGS	abundance	Hgn3C.1311 - Lachnospiraceae sp.	Tibia_SOS
MGS	abundance	Hgn3C.1311 - Lachnospiraceae sp.	Handgrip
MGS	abundance	Hgn3C.1312 - Candida tropicalis	Tibia_length

MGS	abundance	Hgn3C.1312 - Candida tropicalis	Radius_length
MGS	abundance	Hgn3C.1312 - Candida tropicalis	Radius_SOS
MGS	abundance	Hgn3C.1312 - Candida tropicalis	Tibia_SOS
MGS	abundance	Hgn3C.1312 - Candida tropicalis	Handgrip
MGS	abundance	Hgn3C.1313 - Klebsiella aerogenes	Tibia_length
MGS	abundance	Hgn3C.1313 - Klebsiella aerogenes	Radius_length
MGS	abundance	Hgn3C.1313 - Klebsiella aerogenes	Radius_SOS
MGS	abundance	Hgn3C.1313 - Klebsiella aerogenes	Tibia_SOS
MGS	abundance	Hgn3C.1313 - Klebsiella aerogenes	Handgrip
MGS	abundance	Hgn3C.1314 - Eubacteriales sp.	Tibia_length
MGS	abundance	Hgn3C.1314 - Eubacteriales sp.	Radius_length
MGS	abundance	Hgn3C.1314 - Eubacteriales sp.	Radius_SOS
MGS	abundance	Hgn3C.1314 - Eubacteriales sp.	Tibia_SOS
MGS	abundance	Hgn3C.1314 - Eubacteriales sp.	Handgrip
MGS	abundance	Hgn3C.1315 - Eubacteriales sp.	Tibia_length
MGS	abundance	Hgn3C.1315 - Eubacteriales sp.	Radius_length
MGS	abundance	Hgn3C.1315 - Eubacteriales sp.	Radius_SOS
MGS	abundance	Hgn3C.1315 - Eubacteriales sp.	Tibia_SOS
MGS	abundance	Hgn3C.1315 - Eubacteriales sp.	Handgrip
MGS	abundance	Hgn3C.1316 - Eubacteriales sp.	Tibia_length
MGS	abundance	Hgn3C.1316 - Eubacteriales sp.	Radius_length
MGS	abundance	Hgn3C.1316 - Eubacteriales sp.	Radius_SOS
MGS	abundance	Hgn3C.1316 - Eubacteriales sp.	Tibia_SOS
MGS	abundance	Hgn3C.1316 - Eubacteriales sp.	Handgrip
MGS	abundance	Hgn3C.1319 - Atlantibacter hermannii	Tibia_length
MGS	abundance	Hgn3C.1319 - Atlantibacter hermannii	Radius_length
MGS	abundance	Hgn3C.1319 - Atlantibacter hermannii	Radius_SOS
MGS	abundance	Hgn3C.1319 - Atlantibacter hermannii	Tibia_SOS
MGS	abundance	Hgn3C.1319 - Atlantibacter hermannii	Handgrip
MGS	abundance	Hgn3C.1321 - Veillonellaceae sp.	Tibia_length
MGS	abundance	Hgn3C.1321 - Veillonellaceae sp.	Radius_length
MGS	abundance	Hgn3C.1321 - Veillonellaceae sp.	Radius_SOS
MGS	abundance	Hgn3C.1321 - Veillonellaceae sp.	Tibia_SOS
MGS	abundance	Hgn3C.1321 - Veillonellaceae sp.	Handgrip
MGS	abundance	Hgn3C.1322 - Enterococcus hirae	Tibia_length
MGS	abundance	Hgn3C.1322 - Enterococcus hirae	Radius_length
MGS	abundance	Hgn3C.1322 - Enterococcus hirae	Radius_SOS
MGS	abundance	Hgn3C.1322 - Enterococcus hirae	Tibia_SOS
MGS	abundance	Hgn3C.1322 - Enterococcus hirae	Handgrip
MGS	abundance	Hgn3C.1323 - Lachnospiraceae sp.	Tibia_length
MGS	abundance	Hgn3C.1323 - Lachnospiraceae sp.	Radius_length
MGS	abundance	Hgn3C.1323 - Lachnospiraceae sp.	Radius_SOS
MGS	abundance	Hgn3C.1323 - Lachnospiraceae sp.	Tibia_SOS
MGS	abundance	Hgn3C.1323 - Lachnospiraceae sp.	Handgrip
MGS	abundance	Hgn3C.1324 - Eubacterium sp. c-25	Tibia_length
MGS	abundance	Hgn3C.1324 - Eubacterium sp. c-25	Radius_length
MGS	abundance	Hgn3C.1324 - Eubacterium sp. c-25	Radius_SOS
MGS	abundance	Hgn3C.1324 - Eubacterium sp. c-25	Tibia_SOS

MGS	abundance	Hgn3C.1324 - Eubacterium sp. c-25	Handgrip
MGS	abundance	Hgn3C.1325 - Luxibacter massiliensis	Tibia_length
MGS	abundance	Hgn3C.1325 - Luxibacter massiliensis	Radius_length
MGS	abundance	Hgn3C.1325 - Luxibacter massiliensis	Radius_SOS
MGS	abundance	Hgn3C.1325 - Luxibacter massiliensis	Tibia_SOS
MGS	abundance	Hgn3C.1325 - Luxibacter massiliensis	Handgrip
MGS	abundance	Hgn3C.1326 - Eggerthella guodeyinii	Tibia_length
MGS	abundance	Hgn3C.1326 - Eggerthella guodeyinii	Radius_length
MGS	abundance	Hgn3C.1326 - Eggerthella guodeyinii	Radius_SOS
MGS	abundance	Hgn3C.1326 - Eggerthella guodeyinii	Tibia_SOS
MGS	abundance	Hgn3C.1326 - Eggerthella guodeyinii	Handgrip
MGS	abundance	Hgn3C.1328 - Lacrimispora saccharolytica	Tibia_length
MGS	abundance	Hgn3C.1328 - Lacrimispora saccharolytica	Radius_length
MGS	abundance	Hgn3C.1328 - Lacrimispora saccharolytica	Radius_SOS
MGS	abundance	Hgn3C.1328 - Lacrimispora saccharolytica	Tibia_SOS
MGS	abundance	Hgn3C.1328 - Lacrimispora saccharolytica	Handgrip
MGS	abundance	Hgn3C.1329 - Eubacteriales sp.	Tibia_length
MGS	abundance	Hgn3C.1329 - Eubacteriales sp.	Radius_length
MGS	abundance	Hgn3C.1329 - Eubacteriales sp.	Radius_SOS
MGS	abundance	Hgn3C.1329 - Eubacteriales sp.	Tibia_SOS
MGS	abundance	Hgn3C.1329 - Eubacteriales sp.	Handgrip
MGS	abundance	Hgn3C.1330 - Lachnospiraceae sp.	Tibia_length
MGS	abundance	Hgn3C.1330 - Lachnospiraceae sp.	Radius_length
MGS	abundance	Hgn3C.1330 - Lachnospiraceae sp.	Radius_SOS
MGS	abundance	Hgn3C.1330 - Lachnospiraceae sp.	Tibia_SOS
MGS	abundance	Hgn3C.1330 - Lachnospiraceae sp.	Handgrip
MGS	abundance	Hgn3C.1331 - Lachnospiraceae sp.	Tibia_length
MGS	abundance	Hgn3C.1331 - Lachnospiraceae sp.	Radius_length
MGS	abundance	Hgn3C.1331 - Lachnospiraceae sp.	Radius_SOS
MGS	abundance	Hgn3C.1331 - Lachnospiraceae sp.	Tibia_SOS
MGS	abundance	Hgn3C.1331 - Lachnospiraceae sp.	Handgrip
MGS	abundance	Hgn3C.1334 - Eubacteriales sp.	Tibia_length
MGS	abundance	Hgn3C.1334 - Eubacteriales sp.	Radius_length
MGS	abundance	Hgn3C.1334 - Eubacteriales sp.	Radius_SOS
MGS	abundance	Hgn3C.1334 - Eubacteriales sp.	Tibia_SOS
MGS	abundance	Hgn3C.1334 - Eubacteriales sp.	Handgrip
MGS	abundance	Hgn3C.1335 - Eubacteriales sp.	Tibia_length
MGS	abundance	Hgn3C.1335 - Eubacteriales sp.	Radius_length
MGS	abundance	Hgn3C.1335 - Eubacteriales sp.	Radius_SOS
MGS	abundance	Hgn3C.1335 - Eubacteriales sp.	Tibia_SOS
MGS	abundance	Hgn3C.1335 - Eubacteriales sp.	Handgrip
MGS	abundance	Hgn3C.1336 - Eubacteriales sp.	Tibia_length
MGS	abundance	Hgn3C.1336 - Eubacteriales sp.	Radius_length
MGS	abundance	Hgn3C.1336 - Eubacteriales sp.	Radius_SOS
MGS	abundance	Hgn3C.1336 - Eubacteriales sp.	Tibia_SOS
MGS	abundance	Hgn3C.1336 - Eubacteriales sp.	Handgrip
MGS	abundance	Hgn3C.1338 - Erysipelatoclostridium sp.	Tibia_length
MGS	abundance	Hgn3C.1338 - Erysipelatoclostridium sp.	Radius_length

MGS	abundance	Hgn3C.1338 - Erysipelatoclostridium sp.	Radius_SOS
MGS	abundance	Hgn3C.1338 - Erysipelatoclostridium sp.	Tibia_SOS
MGS	abundance	Hgn3C.1338 - Erysipelatoclostridium sp.	Handgrip
MGS	abundance	Hgn3C.1340 - Clostridiaceae sp.	Tibia_length
MGS	abundance	Hgn3C.1340 - Clostridiaceae sp.	Radius_length
MGS	abundance	Hgn3C.1340 - Clostridiaceae sp.	Radius_SOS
MGS	abundance	Hgn3C.1340 - Clostridiaceae sp.	Tibia_SOS
MGS	abundance	Hgn3C.1340 - Clostridiaceae sp.	Handgrip
MGS	abundance	Hgn3C.1341 - Lachnospiraceae sp.	Tibia_length
MGS	abundance	Hgn3C.1341 - Lachnospiraceae sp.	Radius_length
MGS	abundance	Hgn3C.1341 - Lachnospiraceae sp.	Radius_SOS
MGS	abundance	Hgn3C.1341 - Lachnospiraceae sp.	Tibia_SOS
MGS	abundance	Hgn3C.1341 - Lachnospiraceae sp.	Handgrip
MGS	abundance	Hgn3C.1342 - Eubacteriales sp.	Tibia_length
MGS	abundance	Hgn3C.1342 - Eubacteriales sp.	Radius_length
MGS	abundance	Hgn3C.1342 - Eubacteriales sp.	Radius_SOS
MGS	abundance	Hgn3C.1342 - Eubacteriales sp.	Tibia_SOS
MGS	abundance	Hgn3C.1342 - Eubacteriales sp.	Handgrip
MGS	abundance	Hgn3C.1343 - Enterococcus italicus	Tibia_length
MGS	abundance	Hgn3C.1343 - Enterococcus italicus	Radius_length
MGS	abundance	Hgn3C.1343 - Enterococcus italicus	Radius_SOS
MGS	abundance	Hgn3C.1343 - Enterococcus italicus	Tibia_SOS
MGS	abundance	Hgn3C.1343 - Enterococcus italicus	Handgrip
MGS	abundance	Hgn3C.1345 - Limosilactobacillus mucosae	Tibia_length
MGS	abundance	Hgn3C.1345 - Limosilactobacillus mucosae	Radius_length
MGS	abundance	Hgn3C.1345 - Limosilactobacillus mucosae	Radius_SOS
MGS	abundance	Hgn3C.1345 - Limosilactobacillus mucosae	Tibia_SOS
MGS	abundance	Hgn3C.1345 - Limosilactobacillus mucosae	Handgrip
MGS	abundance	Hgn3C.1346 - Erysipelotrichales sp.	Tibia_length
MGS	abundance	Hgn3C.1346 - Erysipelotrichales sp.	Radius_length
MGS	abundance	Hgn3C.1346 - Erysipelotrichales sp.	Radius_SOS
MGS	abundance	Hgn3C.1346 - Erysipelotrichales sp.	Tibia_SOS
MGS	abundance	Hgn3C.1346 - Erysipelotrichales sp.	Handgrip
MGS	abundance	Hgn3C.1347 - [Clostridium] hylemonae	Tibia_length
MGS	abundance	Hgn3C.1347 - [Clostridium] hylemonae	Radius_length
MGS	abundance	Hgn3C.1347 - [Clostridium] hylemonae	Radius_SOS
MGS	abundance	Hgn3C.1347 - [Clostridium] hylemonae	Tibia_SOS
MGS	abundance	Hgn3C.1347 - [Clostridium] hylemonae	Handgrip
MGS	abundance	Hgn3C.1348 - Eubacteriales sp.	Tibia_length
MGS	abundance	Hgn3C.1348 - Eubacteriales sp.	Radius_length
MGS	abundance	Hgn3C.1348 - Eubacteriales sp.	Radius_SOS
MGS	abundance	Hgn3C.1348 - Eubacteriales sp.	Tibia_SOS
MGS	abundance	Hgn3C.1348 - Eubacteriales sp.	Handgrip
MGS	abundance	Hgn3C.1349 - Corynebacterium falsenii	Tibia_length
MGS	abundance	Hgn3C.1349 - Corynebacterium falsenii	Radius_length
MGS	abundance	Hgn3C.1349 - Corynebacterium falsenii	Radius_SOS
MGS	abundance	Hgn3C.1349 - Corynebacterium falsenii	Tibia_SOS
MGS	abundance	Hgn3C.1349 - Corynebacterium falsenii	Handgrip

MGS	abundance	Hgn3C.1351 - Actinomyces sp. ICM47	Tibia_length
MGS	abundance	Hgn3C.1351 - Actinomyces sp. ICM47	Radius_length
MGS	abundance	Hgn3C.1351 - Actinomyces sp. ICM47	Radius_SOS
MGS	abundance	Hgn3C.1351 - Actinomyces sp. ICM47	Tibia_SOS
MGS	abundance	Hgn3C.1351 - Actinomyces sp. ICM47	Handgrip
MGS	abundance	Hgn3C.1352 - Akkermansia sp.	Tibia_length
MGS	abundance	Hgn3C.1352 - Akkermansia sp.	Radius_length
MGS	abundance	Hgn3C.1352 - Akkermansia sp.	Radius_SOS
MGS	abundance	Hgn3C.1352 - Akkermansia sp.	Tibia_SOS
MGS	abundance	Hgn3C.1352 - Akkermansia sp.	Handgrip
MGS	abundance	Hgn3C.1353 - Beduini sp.	Tibia_length
MGS	abundance	Hgn3C.1353 - Beduini sp.	Radius_length
MGS	abundance	Hgn3C.1353 - Beduini sp.	Radius_SOS
MGS	abundance	Hgn3C.1353 - Beduini sp.	Tibia_SOS
MGS	abundance	Hgn3C.1353 - Beduini sp.	Handgrip
MGS	abundance	Hgn3C.1354 - Actinomyces sp.	Tibia_length
MGS	abundance	Hgn3C.1354 - Actinomyces sp.	Radius_length
MGS	abundance	Hgn3C.1354 - Actinomyces sp.	Radius_SOS
MGS	abundance	Hgn3C.1354 - Actinomyces sp.	Tibia_SOS
MGS	abundance	Hgn3C.1354 - Actinomyces sp.	Handgrip
MGS	abundance	Hgn3C.1355 - Collinsella stercoris	Tibia_length
MGS	abundance	Hgn3C.1355 - Collinsella stercoris	Radius_length
MGS	abundance	Hgn3C.1355 - Collinsella stercoris	Radius_SOS
MGS	abundance	Hgn3C.1355 - Collinsella stercoris	Tibia_SOS
MGS	abundance	Hgn3C.1355 - Collinsella stercoris	Handgrip
MGS	abundance	Hgn3C.1356 - Eubacteriales sp.	Tibia_length
MGS	abundance	Hgn3C.1356 - Eubacteriales sp.	Radius_length
MGS	abundance	Hgn3C.1356 - Eubacteriales sp.	Radius_SOS
MGS	abundance	Hgn3C.1356 - Eubacteriales sp.	Tibia_SOS
MGS	abundance	Hgn3C.1356 - Eubacteriales sp.	Handgrip
MGS	abundance	Hgn3C.1357 - Bacteroidales sp.	Tibia_length
MGS	abundance	Hgn3C.1357 - Bacteroidales sp.	Radius_length
MGS	abundance	Hgn3C.1357 - Bacteroidales sp.	Radius_SOS
MGS	abundance	Hgn3C.1357 - Bacteroidales sp.	Tibia_SOS
MGS	abundance	Hgn3C.1357 - Bacteroidales sp.	Handgrip
MGS	abundance	Hgn3C.1358 - Lachnospiraceae sp.	Tibia_length
MGS	abundance	Hgn3C.1358 - Lachnospiraceae sp.	Radius_length
MGS	abundance	Hgn3C.1358 - Lachnospiraceae sp.	Radius_SOS
MGS	abundance	Hgn3C.1358 - Lachnospiraceae sp.	Tibia_SOS
MGS	abundance	Hgn3C.1358 - Lachnospiraceae sp.	Handgrip
MGS	abundance	Hgn3C.1359 - Extibacter muris	Tibia_length
MGS	abundance	Hgn3C.1359 - Extibacter muris	Radius_length
MGS	abundance	Hgn3C.1359 - Extibacter muris	Radius_SOS
MGS	abundance	Hgn3C.1359 - Extibacter muris	Tibia_SOS
MGS	abundance	Hgn3C.1359 - Extibacter muris	Handgrip
MGS	abundance	Hgn3C.1360 - Bifidobacterium crudilactis	Tibia_length
MGS	abundance	Hgn3C.1360 - Bifidobacterium crudilactis	Radius_length
MGS	abundance	Hgn3C.1360 - Bifidobacterium crudilactis	Radius_SOS

MGS	abundance	Hgn3C.1360 - Bifidobacterium crudilactis	Tibia_SOS
MGS	abundance	Hgn3C.1360 - Bifidobacterium crudilactis	Handgrip
MGS	abundance	Hgn3C.1361 - Senegalimassilia anaerobia	Tibia_length
MGS	abundance	Hgn3C.1361 - Senegalimassilia anaerobia	Radius_length
MGS	abundance	Hgn3C.1361 - Senegalimassilia anaerobia	Radius_SOS
MGS	abundance	Hgn3C.1361 - Senegalimassilia anaerobia	Tibia_SOS
MGS	abundance	Hgn3C.1361 - Senegalimassilia anaerobia	Handgrip
MGS	abundance	Hgn3C.1362 - Christensenella intestinihominis	Tibia_length
MGS	abundance	Hgn3C.1362 - Christensenella intestinihominis	Radius_length
MGS	abundance	Hgn3C.1362 - Christensenella intestinihominis	Radius_SOS
MGS	abundance	Hgn3C.1362 - Christensenella intestinihominis	Tibia_SOS
MGS	abundance	Hgn3C.1362 - Christensenella intestinihominis	Handgrip
MGS	abundance	Hgn3C.1366 - Lachnospiraceae sp.	Tibia_length
MGS	abundance	Hgn3C.1366 - Lachnospiraceae sp.	Radius_length
MGS	abundance	Hgn3C.1366 - Lachnospiraceae sp.	Radius_SOS
MGS	abundance	Hgn3C.1366 - Lachnospiraceae sp.	Tibia_SOS
MGS	abundance	Hgn3C.1366 - Lachnospiraceae sp.	Handgrip
MGS	abundance	Hgn3C.1369 - Lachnospiraceae sp.	Tibia_length
MGS	abundance	Hgn3C.1369 - Lachnospiraceae sp.	Radius_length
MGS	abundance	Hgn3C.1369 - Lachnospiraceae sp.	Radius_SOS
MGS	abundance	Hgn3C.1369 - Lachnospiraceae sp.	Tibia_SOS
MGS	abundance	Hgn3C.1369 - Lachnospiraceae sp.	Handgrip
MGS	abundance	Hgn3C.1370 - Kluyvera genomosp. 3	Tibia_length
MGS	abundance	Hgn3C.1370 - Kluyvera genomosp. 3	Radius_length
MGS	abundance	Hgn3C.1370 - Kluyvera genomosp. 3	Radius_SOS
MGS	abundance	Hgn3C.1370 - Kluyvera genomosp. 3	Tibia_SOS
MGS	abundance	Hgn3C.1370 - Kluyvera genomosp. 3	Handgrip
MGS	abundance	Hgn3C.1371 - Lachnospiraceae sp.	Tibia_length
MGS	abundance	Hgn3C.1371 - Lachnospiraceae sp.	Radius_length
MGS	abundance	Hgn3C.1371 - Lachnospiraceae sp.	Radius_SOS
MGS	abundance	Hgn3C.1371 - Lachnospiraceae sp.	Tibia_SOS
MGS	abundance	Hgn3C.1371 - Lachnospiraceae sp.	Handgrip
MGS	abundance	Hgn3C.1372 - Eubacteriales sp.	Tibia_length
MGS	abundance	Hgn3C.1372 - Eubacteriales sp.	Radius_length
MGS	abundance	Hgn3C.1372 - Eubacteriales sp.	Radius_SOS
MGS	abundance	Hgn3C.1372 - Eubacteriales sp.	Tibia_SOS
MGS	abundance	Hgn3C.1372 - Eubacteriales sp.	Handgrip
MGS	abundance	Hgn3C.1373 - Eubacteriales sp.	Tibia_length
MGS	abundance	Hgn3C.1373 - Eubacteriales sp.	Radius_length
MGS	abundance	Hgn3C.1373 - Eubacteriales sp.	Radius_SOS
MGS	abundance	Hgn3C.1373 - Eubacteriales sp.	Tibia_SOS
MGS	abundance	Hgn3C.1373 - Eubacteriales sp.	Handgrip
MGS	abundance	Hgn3C.1375 - Atopobiaceae sp.	Tibia_length
MGS	abundance	Hgn3C.1375 - Atopobiaceae sp.	Radius_length
MGS	abundance	Hgn3C.1375 - Atopobiaceae sp.	Radius_SOS
MGS	abundance	Hgn3C.1375 - Atopobiaceae sp.	Tibia_SOS
MGS	abundance	Hgn3C.1375 - Atopobiaceae sp.	Handgrip
MGS	abundance	Hgn3C.1376 - Kluyvera sp. CRP	Tibia_length

MGS	abundance	Hgn3C.1376 - Kluyvera sp. CRP	Radius_length
MGS	abundance	Hgn3C.1376 - Kluyvera sp. CRP	Radius_SOS
MGS	abundance	Hgn3C.1376 - Kluyvera sp. CRP	Tibia_SOS
MGS	abundance	Hgn3C.1376 - Kluyvera sp. CRP	Handgrip
MGS	abundance	Hgn3C.1377 - Collinsella tanakaei	Tibia_length
MGS	abundance	Hgn3C.1377 - Collinsella tanakaei	Radius_length
MGS	abundance	Hgn3C.1377 - Collinsella tanakaei	Radius_SOS
MGS	abundance	Hgn3C.1377 - Collinsella tanakaei	Tibia_SOS
MGS	abundance	Hgn3C.1377 - Collinsella tanakaei	Handgrip
MGS	abundance	Hgn3C.1378 - Collinsella sp. AF08-23	Tibia_length
MGS	abundance	Hgn3C.1378 - Collinsella sp. AF08-23	Radius_length
MGS	abundance	Hgn3C.1378 - Collinsella sp. AF08-23	Radius_SOS
MGS	abundance	Hgn3C.1378 - Collinsella sp. AF08-23	Tibia_SOS
MGS	abundance	Hgn3C.1378 - Collinsella sp. AF08-23	Handgrip
MGS	abundance	Hgn3C.1379 - Gordonibacter urolithinfaciens	Tibia_length
MGS	abundance	Hgn3C.1379 - Gordonibacter urolithinfaciens	Radius_length
MGS	abundance	Hgn3C.1379 - Gordonibacter urolithinfaciens	Radius_SOS
MGS	abundance	Hgn3C.1379 - Gordonibacter urolithinfaciens	Tibia_SOS
MGS	abundance	Hgn3C.1379 - Gordonibacter urolithinfaciens	Handgrip
MGS	abundance	Hgn3C.1380 - Kluyvera georgiana	Tibia_length
MGS	abundance	Hgn3C.1380 - Kluyvera georgiana	Radius_length
MGS	abundance	Hgn3C.1380 - Kluyvera georgiana	Radius_SOS
MGS	abundance	Hgn3C.1380 - Kluyvera georgiana	Tibia_SOS
MGS	abundance	Hgn3C.1380 - Kluyvera georgiana	Handgrip
MGS	abundance	Hgn3C.1381 - Eubacteriales sp.	Tibia_length
MGS	abundance	Hgn3C.1381 - Eubacteriales sp.	Radius_length
MGS	abundance	Hgn3C.1381 - Eubacteriales sp.	Radius_SOS
MGS	abundance	Hgn3C.1381 - Eubacteriales sp.	Tibia_SOS
MGS	abundance	Hgn3C.1381 - Eubacteriales sp.	Handgrip
MGS	abundance	Hgn3C.1382 - Oscillospiraceae sp.	Tibia_length
MGS	abundance	Hgn3C.1382 - Oscillospiraceae sp.	Radius_length
MGS	abundance	Hgn3C.1382 - Oscillospiraceae sp.	Radius_SOS
MGS	abundance	Hgn3C.1382 - Oscillospiraceae sp.	Tibia_SOS
MGS	abundance	Hgn3C.1382 - Oscillospiraceae sp.	Handgrip
MGS	abundance	Hgn3C.1383 - Sellimonas monacensis	Tibia_length
MGS	abundance	Hgn3C.1383 - Sellimonas monacensis	Radius_length
MGS	abundance	Hgn3C.1383 - Sellimonas monacensis	Radius_SOS
MGS	abundance	Hgn3C.1383 - Sellimonas monacensis	Tibia_SOS
MGS	abundance	Hgn3C.1383 - Sellimonas monacensis	Handgrip
MGS	abundance	Hgn3C.1384 - Anaerococcus sp.	Tibia_length
MGS	abundance	Hgn3C.1384 - Anaerococcus sp.	Radius_length
MGS	abundance	Hgn3C.1384 - Anaerococcus sp.	Radius_SOS
MGS	abundance	Hgn3C.1384 - Anaerococcus sp.	Tibia_SOS
MGS	abundance	Hgn3C.1384 - Anaerococcus sp.	Handgrip
MGS	abundance	Hgn3C.1385 - Eubacteriales sp.	Tibia_length
MGS	abundance	Hgn3C.1385 - Eubacteriales sp.	Radius_length
MGS	abundance	Hgn3C.1385 - Eubacteriales sp.	Radius_SOS
MGS	abundance	Hgn3C.1385 - Eubacteriales sp.	Tibia_SOS

MGS	abundance	Hgn3C.1385 - Eubacteriales sp.	Handgrip
MGS	abundance	Hgn3C.1386 - Veillonella sp.	Tibia_length
MGS	abundance	Hgn3C.1386 - Veillonella sp.	Radius_length
MGS	abundance	Hgn3C.1386 - Veillonella sp.	Radius_SOS
MGS	abundance	Hgn3C.1386 - Veillonella sp.	Tibia_SOS
MGS	abundance	Hgn3C.1386 - Veillonella sp.	Handgrip
MGS	abundance	Hgn3C.1387 - Alistipes sp.	Tibia_length
MGS	abundance	Hgn3C.1387 - Alistipes sp.	Radius_length
MGS	abundance	Hgn3C.1387 - Alistipes sp.	Radius_SOS
MGS	abundance	Hgn3C.1387 - Alistipes sp.	Tibia_SOS
MGS	abundance	Hgn3C.1387 - Alistipes sp.	Handgrip
MGS	abundance	Hgn3C.1388 - Streptococcus infantarius subsp. infantarius	Tibia_length
MGS	abundance	Hgn3C.1388 - Streptococcus infantarius subsp. infantarius	Radius_length
MGS	abundance	Hgn3C.1388 - Streptococcus infantarius subsp. infantarius	Radius_SOS
MGS	abundance	Hgn3C.1388 - Streptococcus infantarius subsp. infantarius	Tibia_SOS
MGS	abundance	Hgn3C.1388 - Streptococcus infantarius subsp. infantarius	Handgrip
MGS	abundance	Hgn3C.1390 - Erysipelatoclostridium sp.	Tibia_length
MGS	abundance	Hgn3C.1390 - Erysipelatoclostridium sp.	Radius_length
MGS	abundance	Hgn3C.1390 - Erysipelatoclostridium sp.	Radius_SOS
MGS	abundance	Hgn3C.1390 - Erysipelatoclostridium sp.	Tibia_SOS
MGS	abundance	Hgn3C.1390 - Erysipelatoclostridium sp.	Handgrip
MGS	abundance	Hgn3C.1392 - Enterobacter sp. JBIWA005	Tibia_length
MGS	abundance	Hgn3C.1392 - Enterobacter sp. JBIWA005	Radius_length
MGS	abundance	Hgn3C.1392 - Enterobacter sp. JBIWA005	Radius_SOS
MGS	abundance	Hgn3C.1392 - Enterobacter sp. JBIWA005	Tibia_SOS
MGS	abundance	Hgn3C.1392 - Enterobacter sp. JBIWA005	Handgrip
MGS	abundance	Hgn3C.1393 - Anaerofustis stercorihominis	Tibia_length
MGS	abundance	Hgn3C.1393 - Anaerofustis stercorihominis	Radius_length
MGS	abundance	Hgn3C.1393 - Anaerofustis stercorihominis	Radius_SOS
MGS	abundance	Hgn3C.1393 - Anaerofustis stercorihominis	Tibia_SOS
MGS	abundance	Hgn3C.1393 - Anaerofustis stercorihominis	Handgrip
MGS	abundance	Hgn3C.1394 - Eubacteriales sp.	Tibia_length
MGS	abundance	Hgn3C.1394 - Eubacteriales sp.	Radius_length
MGS	abundance	Hgn3C.1394 - Eubacteriales sp.	Radius_SOS
MGS	abundance	Hgn3C.1394 - Eubacteriales sp.	Tibia_SOS
MGS	abundance	Hgn3C.1394 - Eubacteriales sp.	Handgrip
MGS	abundance	Hgn3C.1397 - Erysipelotrichales sp.	Tibia_length
MGS	abundance	Hgn3C.1397 - Erysipelotrichales sp.	Radius_length
MGS	abundance	Hgn3C.1397 - Erysipelotrichales sp.	Radius_SOS
MGS	abundance	Hgn3C.1397 - Erysipelotrichales sp.	Tibia_SOS
MGS	abundance	Hgn3C.1397 - Erysipelotrichales sp.	Handgrip
MGS	abundance	Hgn3C.1398 - Actinomyces sp.	Tibia_length
MGS	abundance	Hgn3C.1398 - Actinomyces sp.	Radius_length
MGS	abundance	Hgn3C.1398 - Actinomyces sp.	Radius_SOS
MGS	abundance	Hgn3C.1398 - Actinomyces sp.	Tibia_SOS
MGS	abundance	Hgn3C.1398 - Actinomyces sp.	Handgrip
MGS	abundance	Hgn3C.1400 - Eubacterium sp.	Tibia_length
MGS	abundance	Hgn3C.1400 - Eubacterium sp.	Radius_length

MGS	abundance	Hgn3C.1400 - Eubacterium sp.	Radius_SOS
MGS	abundance	Hgn3C.1400 - Eubacterium sp.	Tibia_SOS
MGS	abundance	Hgn3C.1400 - Eubacterium sp.	Handgrip
MGS	abundance	Hgn3C.1401 - Eubacteriales sp.	Tibia_length
MGS	abundance	Hgn3C.1401 - Eubacteriales sp.	Radius_length
MGS	abundance	Hgn3C.1401 - Eubacteriales sp.	Radius_SOS
MGS	abundance	Hgn3C.1401 - Eubacteriales sp.	Tibia_SOS
MGS	abundance	Hgn3C.1401 - Eubacteriales sp.	Handgrip
MGS	abundance	Hgn3C.1402 - Collinsella phocaeensis	Tibia_length
MGS	abundance	Hgn3C.1402 - Collinsella phocaeensis	Radius_length
MGS	abundance	Hgn3C.1402 - Collinsella phocaeensis	Radius_SOS
MGS	abundance	Hgn3C.1402 - Collinsella phocaeensis	Tibia_SOS
MGS	abundance	Hgn3C.1402 - Collinsella phocaeensis	Handgrip
MGS	abundance	Hgn3C.1403 - Eubacteriaceae sp.	Tibia_length
MGS	abundance	Hgn3C.1403 - Eubacteriaceae sp.	Radius_length
MGS	abundance	Hgn3C.1403 - Eubacteriaceae sp.	Radius_SOS
MGS	abundance	Hgn3C.1403 - Eubacteriaceae sp.	Tibia_SOS
MGS	abundance	Hgn3C.1403 - Eubacteriaceae sp.	Handgrip
MGS	abundance	Hgn3C.1404 - Clostridium sp.	Tibia_length
MGS	abundance	Hgn3C.1404 - Clostridium sp.	Radius_length
MGS	abundance	Hgn3C.1404 - Clostridium sp.	Radius_SOS
MGS	abundance	Hgn3C.1404 - Clostridium sp.	Tibia_SOS
MGS	abundance	Hgn3C.1404 - Clostridium sp.	Handgrip
MGS	abundance	Hgn3C.1405 - Eubacteriales sp.	Tibia_length
MGS	abundance	Hgn3C.1405 - Eubacteriales sp.	Radius_length
MGS	abundance	Hgn3C.1405 - Eubacteriales sp.	Radius_SOS
MGS	abundance	Hgn3C.1405 - Eubacteriales sp.	Tibia_SOS
MGS	abundance	Hgn3C.1405 - Eubacteriales sp.	Handgrip
MGS	abundance	Hgn3C.1406 - Slackia piriformis	Tibia_length
MGS	abundance	Hgn3C.1406 - Slackia piriformis	Radius_length
MGS	abundance	Hgn3C.1406 - Slackia piriformis	Radius_SOS
MGS	abundance	Hgn3C.1406 - Slackia piriformis	Tibia_SOS
MGS	abundance	Hgn3C.1406 - Slackia piriformis	Handgrip
MGS	abundance	Hgn3C.1407 - Christensenella minuta	Tibia_length
MGS	abundance	Hgn3C.1407 - Christensenella minuta	Radius_length
MGS	abundance	Hgn3C.1407 - Christensenella minuta	Radius_SOS
MGS	abundance	Hgn3C.1407 - Christensenella minuta	Tibia_SOS
MGS	abundance	Hgn3C.1407 - Christensenella minuta	Handgrip
MGS	abundance	Hgn3C.1408 - Eubacterium callanderi	Tibia_length
MGS	abundance	Hgn3C.1408 - Eubacterium callanderi	Radius_length
MGS	abundance	Hgn3C.1408 - Eubacterium callanderi	Radius_SOS
MGS	abundance	Hgn3C.1408 - Eubacterium callanderi	Tibia_SOS
MGS	abundance	Hgn3C.1408 - Eubacterium callanderi	Handgrip
MGS	abundance	Hgn3C.1409 - Eggerthella timonensis	Tibia_length
MGS	abundance	Hgn3C.1409 - Eggerthella timonensis	Radius_length
MGS	abundance	Hgn3C.1409 - Eggerthella timonensis	Radius_SOS
MGS	abundance	Hgn3C.1409 - Eggerthella timonensis	Tibia_SOS
MGS	abundance	Hgn3C.1409 - Eggerthella timonensis	Handgrip

MGS	abundance	Hgn3C.1411 - Eubacteriales sp.	Tibia_length
MGS	abundance	Hgn3C.1411 - Eubacteriales sp.	Radius_length
MGS	abundance	Hgn3C.1411 - Eubacteriales sp.	Radius_SOS
MGS	abundance	Hgn3C.1411 - Eubacteriales sp.	Tibia_SOS
MGS	abundance	Hgn3C.1411 - Eubacteriales sp.	Handgrip
MGS	abundance	Hgn3C.1412 - Christensenella hongkongensis	Tibia_length
MGS	abundance	Hgn3C.1412 - Christensenella hongkongensis	Radius_length
MGS	abundance	Hgn3C.1412 - Christensenella hongkongensis	Radius_SOS
MGS	abundance	Hgn3C.1412 - Christensenella hongkongensis	Tibia_SOS
MGS	abundance	Hgn3C.1412 - Christensenella hongkongensis	Handgrip
MGS	abundance	Hgn3C.1413 - Streptococcus sp.	Tibia_length
MGS	abundance	Hgn3C.1413 - Streptococcus sp.	Radius_length
MGS	abundance	Hgn3C.1413 - Streptococcus sp.	Radius_SOS
MGS	abundance	Hgn3C.1413 - Streptococcus sp.	Tibia_SOS
MGS	abundance	Hgn3C.1413 - Streptococcus sp.	Handgrip
MGS	abundance	Hgn3C.1414 - Collinsella sp.	Tibia_length
MGS	abundance	Hgn3C.1414 - Collinsella sp.	Radius_length
MGS	abundance	Hgn3C.1414 - Collinsella sp.	Radius_SOS
MGS	abundance	Hgn3C.1414 - Collinsella sp.	Tibia_SOS
MGS	abundance	Hgn3C.1414 - Collinsella sp.	Handgrip
MGS	abundance	Hgn3C.1415 - Klebsiella quasipneumoniae subsp. quasipneumoniae	Tibia_length
MGS	abundance	Hgn3C.1415 - Klebsiella quasipneumoniae subsp. quasipneumoniae	Radius_length
MGS	abundance	Hgn3C.1415 - Klebsiella quasipneumoniae subsp. quasipneumoniae	Radius_SOS
MGS	abundance	Hgn3C.1415 - Klebsiella quasipneumoniae subsp. quasipneumoniae	Tibia_SOS
MGS	abundance	Hgn3C.1415 - Klebsiella quasipneumoniae subsp. quasipneumoniae	Handgrip
MGS	abundance	Hgn3C.1416 - Limosilactobacillus fermentum	Tibia_length
MGS	abundance	Hgn3C.1416 - Limosilactobacillus fermentum	Radius_length
MGS	abundance	Hgn3C.1416 - Limosilactobacillus fermentum	Radius_SOS
MGS	abundance	Hgn3C.1416 - Limosilactobacillus fermentum	Tibia_SOS
MGS	abundance	Hgn3C.1416 - Limosilactobacillus fermentum	Handgrip
MGS	abundance	Hgn3C.1418 - Carnobacteriaceae sp.	Tibia_length
MGS	abundance	Hgn3C.1418 - Carnobacteriaceae sp.	Radius_length
MGS	abundance	Hgn3C.1418 - Carnobacteriaceae sp.	Radius_SOS
MGS	abundance	Hgn3C.1418 - Carnobacteriaceae sp.	Tibia_SOS
MGS	abundance	Hgn3C.1418 - Carnobacteriaceae sp.	Handgrip
MGS	abundance	Hgn3C.1419 - Agathobaculum sp. NSJ-28	Tibia_length
MGS	abundance	Hgn3C.1419 - Agathobaculum sp. NSJ-28	Radius_length
MGS	abundance	Hgn3C.1419 - Agathobaculum sp. NSJ-28	Radius_SOS
MGS	abundance	Hgn3C.1419 - Agathobaculum sp. NSJ-28	Tibia_SOS
MGS	abundance	Hgn3C.1419 - Agathobaculum sp. NSJ-28	Handgrip
MGS	abundance	Hgn3C.1420 - Eubacteriales sp.	Tibia_length
MGS	abundance	Hgn3C.1420 - Eubacteriales sp.	Radius_length
MGS	abundance	Hgn3C.1420 - Eubacteriales sp.	Radius_SOS
MGS	abundance	Hgn3C.1420 - Eubacteriales sp.	Tibia_SOS
MGS	abundance	Hgn3C.1420 - Eubacteriales sp.	Handgrip
MGS	abundance	Hgn3C.1423 - Lachnospiraceae sp.	Tibia_length
MGS	abundance	Hgn3C.1423 - Lachnospiraceae sp.	Radius_length
MGS	abundance	Hgn3C.1423 - Lachnospiraceae sp.	Radius_SOS

MGS	abundance	Hgn3C.1423 - Lachnospiraceae sp.	Tibia_SOS
MGS	abundance	Hgn3C.1423 - Lachnospiraceae sp.	Handgrip
MGS	abundance	Hgn3C.1424 - Mogibacterium sp. NSJ-24	Tibia_length
MGS	abundance	Hgn3C.1424 - Mogibacterium sp. NSJ-24	Radius_length
MGS	abundance	Hgn3C.1424 - Mogibacterium sp. NSJ-24	Radius_SOS
MGS	abundance	Hgn3C.1424 - Mogibacterium sp. NSJ-24	Tibia_SOS
MGS	abundance	Hgn3C.1424 - Mogibacterium sp. NSJ-24	Handgrip
MGS	abundance	Hgn3C.1425 - Eubacteriales sp.	Tibia_length
MGS	abundance	Hgn3C.1425 - Eubacteriales sp.	Radius_length
MGS	abundance	Hgn3C.1425 - Eubacteriales sp.	Radius_SOS
MGS	abundance	Hgn3C.1425 - Eubacteriales sp.	Tibia_SOS
MGS	abundance	Hgn3C.1425 - Eubacteriales sp.	Handgrip
MGS	abundance	Hgn3C.1426 - Limosilactobacillus oris	Tibia_length
MGS	abundance	Hgn3C.1426 - Limosilactobacillus oris	Radius_length
MGS	abundance	Hgn3C.1426 - Limosilactobacillus oris	Radius_SOS
MGS	abundance	Hgn3C.1426 - Limosilactobacillus oris	Tibia_SOS
MGS	abundance	Hgn3C.1426 - Limosilactobacillus oris	Handgrip
MGS	abundance	Hgn3C.1427 - Erysipelotrichales sp.	Tibia_length
MGS	abundance	Hgn3C.1427 - Erysipelotrichales sp.	Radius_length
MGS	abundance	Hgn3C.1427 - Erysipelotrichales sp.	Radius_SOS
MGS	abundance	Hgn3C.1427 - Erysipelotrichales sp.	Tibia_SOS
MGS	abundance	Hgn3C.1427 - Erysipelotrichales sp.	Handgrip
MGS	abundance	Hgn3C.1429 - Lachnospiraceae sp.	Tibia_length
MGS	abundance	Hgn3C.1429 - Lachnospiraceae sp.	Radius_length
MGS	abundance	Hgn3C.1429 - Lachnospiraceae sp.	Radius_SOS
MGS	abundance	Hgn3C.1429 - Lachnospiraceae sp.	Tibia_SOS
MGS	abundance	Hgn3C.1429 - Lachnospiraceae sp.	Handgrip
MGS	abundance	Hgn3C.1430 - Corynebacterium provencense	Tibia_length
MGS	abundance	Hgn3C.1430 - Corynebacterium provencense	Radius_length
MGS	abundance	Hgn3C.1430 - Corynebacterium provencense	Radius_SOS
MGS	abundance	Hgn3C.1430 - Corynebacterium provencense	Tibia_SOS
MGS	abundance	Hgn3C.1430 - Corynebacterium provencense	Handgrip
MGS	abundance	Hgn3C.1431 - Bacteroidales sp.	Tibia_length
MGS	abundance	Hgn3C.1431 - Bacteroidales sp.	Radius_length
MGS	abundance	Hgn3C.1431 - Bacteroidales sp.	Radius_SOS
MGS	abundance	Hgn3C.1431 - Bacteroidales sp.	Tibia_SOS
MGS	abundance	Hgn3C.1431 - Bacteroidales sp.	Handgrip
MGS	abundance	Hgn3C.1432 - Eggerthella timonensis	Tibia_length
MGS	abundance	Hgn3C.1432 - Eggerthella timonensis	Radius_length
MGS	abundance	Hgn3C.1432 - Eggerthella timonensis	Radius_SOS
MGS	abundance	Hgn3C.1432 - Eggerthella timonensis	Tibia_SOS
MGS	abundance	Hgn3C.1432 - Eggerthella timonensis	Handgrip
MGS	abundance	Hgn3C.1434 - Blautia sp.	Tibia_length
MGS	abundance	Hgn3C.1434 - Blautia sp.	Radius_length
MGS	abundance	Hgn3C.1434 - Blautia sp.	Radius_SOS
MGS	abundance	Hgn3C.1434 - Blautia sp.	Tibia_SOS
MGS	abundance	Hgn3C.1434 - Blautia sp.	Handgrip
MGS	abundance	Hgn3C.1435 - Acinetobacter baumannii	Tibia_length

MGS	abundance	Hgn3C.1435 - Acinetobacter baumannii	Radius_length
MGS	abundance	Hgn3C.1435 - Acinetobacter baumannii	Radius_SOS
MGS	abundance	Hgn3C.1435 - Acinetobacter baumannii	Tibia_SOS
MGS	abundance	Hgn3C.1435 - Acinetobacter baumannii	Handgrip
MGS	abundance	Hgn3C.1436 - Bacteria sp.	Tibia_length
MGS	abundance	Hgn3C.1436 - Bacteria sp.	Radius_length
MGS	abundance	Hgn3C.1436 - Bacteria sp.	Radius_SOS
MGS	abundance	Hgn3C.1436 - Bacteria sp.	Tibia_SOS
MGS	abundance	Hgn3C.1436 - Bacteria sp.	Handgrip
MGS	abundance	Hgn3C.1437 - Candidatus Stoquefichus sp. SB1	Tibia_length
MGS	abundance	Hgn3C.1437 - Candidatus Stoquefichus sp. SB1	Radius_length
MGS	abundance	Hgn3C.1437 - Candidatus Stoquefichus sp. SB1	Radius_SOS
MGS	abundance	Hgn3C.1437 - Candidatus Stoquefichus sp. SB1	Tibia_SOS
MGS	abundance	Hgn3C.1437 - Candidatus Stoquefichus sp. SB1	Handgrip
MGS	abundance	Hgn3C.1438 - Dielma fastidiosa	Tibia_length
MGS	abundance	Hgn3C.1438 - Dielma fastidiosa	Radius_length
MGS	abundance	Hgn3C.1438 - Dielma fastidiosa	Radius_SOS
MGS	abundance	Hgn3C.1438 - Dielma fastidiosa	Tibia_SOS
MGS	abundance	Hgn3C.1438 - Dielma fastidiosa	Handgrip
MGS	abundance	Hgn3C.1439 - Streptococcus sp. IMAU 99161	Tibia_length
MGS	abundance	Hgn3C.1439 - Streptococcus sp. IMAU 99161	Radius_length
MGS	abundance	Hgn3C.1439 - Streptococcus sp. IMAU 99161	Radius_SOS
MGS	abundance	Hgn3C.1439 - Streptococcus sp. IMAU 99161	Tibia_SOS
MGS	abundance	Hgn3C.1439 - Streptococcus sp. IMAU 99161	Handgrip
MGS	abundance	Hgn3C.1440 - Eubacteriales sp.	Tibia_length
MGS	abundance	Hgn3C.1440 - Eubacteriales sp.	Radius_length
MGS	abundance	Hgn3C.1440 - Eubacteriales sp.	Radius_SOS
MGS	abundance	Hgn3C.1440 - Eubacteriales sp.	Tibia_SOS
MGS	abundance	Hgn3C.1440 - Eubacteriales sp.	Handgrip
MGS	abundance	Hgn3C.1441 - Lancefieldella sp.	Tibia_length
MGS	abundance	Hgn3C.1441 - Lancefieldella sp.	Radius_length
MGS	abundance	Hgn3C.1441 - Lancefieldella sp.	Radius_SOS
MGS	abundance	Hgn3C.1441 - Lancefieldella sp.	Tibia_SOS
MGS	abundance	Hgn3C.1441 - Lancefieldella sp.	Handgrip
MGS	abundance	Hgn3C.1443 - Streptococcus sp.	Tibia_length
MGS	abundance	Hgn3C.1443 - Streptococcus sp.	Radius_length
MGS	abundance	Hgn3C.1443 - Streptococcus sp.	Radius_SOS
MGS	abundance	Hgn3C.1443 - Streptococcus sp.	Tibia_SOS
MGS	abundance	Hgn3C.1443 - Streptococcus sp.	Handgrip
MGS	abundance	Hgn3C.1444 - Ruminococcus sp. 1001175B_160314_E1	Tibia_length
MGS	abundance	Hgn3C.1444 - Ruminococcus sp. 1001175B_160314_E1	Radius_length
MGS	abundance	Hgn3C.1444 - Ruminococcus sp. 1001175B_160314_E1	Radius_SOS
MGS	abundance	Hgn3C.1444 - Ruminococcus sp. 1001175B_160314_E1	Tibia_SOS
MGS	abundance	Hgn3C.1444 - Ruminococcus sp. 1001175B_160314_E1	Handgrip
MGS	abundance	Hgn3C.1445 - Oscillospiraceae sp.	Tibia_length
MGS	abundance	Hgn3C.1445 - Oscillospiraceae sp.	Radius_length
MGS	abundance	Hgn3C.1445 - Oscillospiraceae sp.	Radius_SOS
MGS	abundance	Hgn3C.1445 - Oscillospiraceae sp.	Tibia_SOS

MGS	abundance	Hgn3C.1445 - Oscillospiraceae sp.	Handgrip
MGS	abundance	Hgn3C.1446 - Eubacteriales sp.	Tibia_length
MGS	abundance	Hgn3C.1446 - Eubacteriales sp.	Radius_length
MGS	abundance	Hgn3C.1446 - Eubacteriales sp.	Radius_SOS
MGS	abundance	Hgn3C.1446 - Eubacteriales sp.	Tibia_SOS
MGS	abundance	Hgn3C.1446 - Eubacteriales sp.	Handgrip
MGS	abundance	Hgn3C.1447 - Bacteria sp.	Tibia_length
MGS	abundance	Hgn3C.1447 - Bacteria sp.	Radius_length
MGS	abundance	Hgn3C.1447 - Bacteria sp.	Radius_SOS
MGS	abundance	Hgn3C.1447 - Bacteria sp.	Tibia_SOS
MGS	abundance	Hgn3C.1447 - Bacteria sp.	Handgrip
MGS	abundance	Hgn3C.1448 - Leuconostoc falkenbergense	Tibia_length
MGS	abundance	Hgn3C.1448 - Leuconostoc falkenbergense	Radius_length
MGS	abundance	Hgn3C.1448 - Leuconostoc falkenbergense	Radius_SOS
MGS	abundance	Hgn3C.1448 - Leuconostoc falkenbergense	Tibia_SOS
MGS	abundance	Hgn3C.1448 - Leuconostoc falkenbergense	Handgrip
MGS	abundance	Hgn3C.1450 - Lactococcus sp. NH2-7C	Tibia_length
MGS	abundance	Hgn3C.1450 - Lactococcus sp. NH2-7C	Radius_length
MGS	abundance	Hgn3C.1450 - Lactococcus sp. NH2-7C	Radius_SOS
MGS	abundance	Hgn3C.1450 - Lactococcus sp. NH2-7C	Tibia_SOS
MGS	abundance	Hgn3C.1450 - Lactococcus sp. NH2-7C	Handgrip
MGS	abundance	Hgn3C.1451 - Faecalicatena orotica	Tibia_length
MGS	abundance	Hgn3C.1451 - Faecalicatena orotica	Radius_length
MGS	abundance	Hgn3C.1451 - Faecalicatena orotica	Radius_SOS
MGS	abundance	Hgn3C.1451 - Faecalicatena orotica	Tibia_SOS
MGS	abundance	Hgn3C.1451 - Faecalicatena orotica	Handgrip
MGS	abundance	Hgn3C.1452 - Raoultibacter timonensis	Tibia_length
MGS	abundance	Hgn3C.1452 - Raoultibacter timonensis	Radius_length
MGS	abundance	Hgn3C.1452 - Raoultibacter timonensis	Radius_SOS
MGS	abundance	Hgn3C.1452 - Raoultibacter timonensis	Tibia_SOS
MGS	abundance	Hgn3C.1452 - Raoultibacter timonensis	Handgrip
MGS	abundance	Hgn3C.1453 - Collinsella sp.	Tibia_length
MGS	abundance	Hgn3C.1453 - Collinsella sp.	Radius_length
MGS	abundance	Hgn3C.1453 - Collinsella sp.	Radius_SOS
MGS	abundance	Hgn3C.1453 - Collinsella sp.	Tibia_SOS
MGS	abundance	Hgn3C.1453 - Collinsella sp.	Handgrip
MGS	abundance	Hgn3C.1454 - Escherichia fergusonii	Tibia_length
MGS	abundance	Hgn3C.1454 - Escherichia fergusonii	Radius_length
MGS	abundance	Hgn3C.1454 - Escherichia fergusonii	Radius_SOS
MGS	abundance	Hgn3C.1454 - Escherichia fergusonii	Tibia_SOS
MGS	abundance	Hgn3C.1454 - Escherichia fergusonii	Handgrip
MGS	abundance	Hgn3C.1455 - Enterocloster sp.	Tibia_length
MGS	abundance	Hgn3C.1455 - Enterocloster sp.	Radius_length
MGS	abundance	Hgn3C.1455 - Enterocloster sp.	Radius_SOS
MGS	abundance	Hgn3C.1455 - Enterocloster sp.	Tibia_SOS
MGS	abundance	Hgn3C.1455 - Enterocloster sp.	Handgrip
MGS	abundance	Hgn3C.1456 - Eubacteriales sp.	Tibia_length
MGS	abundance	Hgn3C.1456 - Eubacteriales sp.	Radius_length

MGS	abundance	Hgn3C.1456 - Eubacteriales sp.	Radius_SOS
MGS	abundance	Hgn3C.1456 - Eubacteriales sp.	Tibia_SOS
MGS	abundance	Hgn3C.1456 - Eubacteriales sp.	Handgrip
MGS	abundance	Hgn3C.1458 - Intestinimonas timonensis	Tibia_length
MGS	abundance	Hgn3C.1458 - Intestinimonas timonensis	Radius_length
MGS	abundance	Hgn3C.1458 - Intestinimonas timonensis	Radius_SOS
MGS	abundance	Hgn3C.1458 - Intestinimonas timonensis	Tibia_SOS
MGS	abundance	Hgn3C.1458 - Intestinimonas timonensis	Handgrip
MGS	abundance	Hgn3C.1460 - Blautia sp.	Tibia_length
MGS	abundance	Hgn3C.1460 - Blautia sp.	Radius_length
MGS	abundance	Hgn3C.1460 - Blautia sp.	Radius_SOS
MGS	abundance	Hgn3C.1460 - Blautia sp.	Tibia_SOS
MGS	abundance	Hgn3C.1460 - Blautia sp.	Handgrip
MGS	abundance	Hgn3C.1461 - Eubacteriales sp.	Tibia_length
MGS	abundance	Hgn3C.1461 - Eubacteriales sp.	Radius_length
MGS	abundance	Hgn3C.1461 - Eubacteriales sp.	Radius_SOS
MGS	abundance	Hgn3C.1461 - Eubacteriales sp.	Tibia_SOS
MGS	abundance	Hgn3C.1461 - Eubacteriales sp.	Handgrip
MGS	abundance	Hgn3C.1463 - Enterobacter sp.	Tibia_length
MGS	abundance	Hgn3C.1463 - Enterobacter sp.	Radius_length
MGS	abundance	Hgn3C.1463 - Enterobacter sp.	Radius_SOS
MGS	abundance	Hgn3C.1463 - Enterobacter sp.	Tibia_SOS
MGS	abundance	Hgn3C.1463 - Enterobacter sp.	Handgrip
MGS	abundance	Hgn3C.1465 - Clostridia sp.	Tibia_length
MGS	abundance	Hgn3C.1465 - Clostridia sp.	Radius_length
MGS	abundance	Hgn3C.1465 - Clostridia sp.	Radius_SOS
MGS	abundance	Hgn3C.1465 - Clostridia sp.	Tibia_SOS
MGS	abundance	Hgn3C.1465 - Clostridia sp.	Handgrip
MGS	abundance	Hgn3C.1467 - Eubacteriales sp.	Tibia_length
MGS	abundance	Hgn3C.1467 - Eubacteriales sp.	Radius_length
MGS	abundance	Hgn3C.1467 - Eubacteriales sp.	Radius_SOS
MGS	abundance	Hgn3C.1467 - Eubacteriales sp.	Tibia_SOS
MGS	abundance	Hgn3C.1467 - Eubacteriales sp.	Handgrip
MGS	abundance	Hgn3C.1468 - Staphylococcus aureus	Tibia_length
MGS	abundance	Hgn3C.1468 - Staphylococcus aureus	Radius_length
MGS	abundance	Hgn3C.1468 - Staphylococcus aureus	Radius_SOS
MGS	abundance	Hgn3C.1468 - Staphylococcus aureus	Tibia_SOS
MGS	abundance	Hgn3C.1468 - Staphylococcus aureus	Handgrip
MGS	abundance	Hgn3C.1469 - Lachnospiraceae sp.	Tibia_length
MGS	abundance	Hgn3C.1469 - Lachnospiraceae sp.	Radius_length
MGS	abundance	Hgn3C.1469 - Lachnospiraceae sp.	Radius_SOS
MGS	abundance	Hgn3C.1469 - Lachnospiraceae sp.	Tibia_SOS
MGS	abundance	Hgn3C.1469 - Lachnospiraceae sp.	Handgrip
MGS	abundance	Hgn3C.1471 - Eggerthella sp.	Tibia_length
MGS	abundance	Hgn3C.1471 - Eggerthella sp.	Radius_length
MGS	abundance	Hgn3C.1471 - Eggerthella sp.	Radius_SOS
MGS	abundance	Hgn3C.1471 - Eggerthella sp.	Tibia_SOS
MGS	abundance	Hgn3C.1471 - Eggerthella sp.	Handgrip

MGS	abundance	Hgn3C.1472 - Amedibacillus dolichus	Tibia_length
MGS	abundance	Hgn3C.1472 - Amedibacillus dolichus	Radius_length
MGS	abundance	Hgn3C.1472 - Amedibacillus dolichus	Radius_SOS
MGS	abundance	Hgn3C.1472 - Amedibacillus dolichus	Tibia_SOS
MGS	abundance	Hgn3C.1472 - Amedibacillus dolichus	Handgrip

Effect size	Standard e	Bias mode	Test statist	Sample siz	Degrees of	P-value	FDR	Model formula fit
0,00423	0,049202	-0,07874	0,085979	421	413,0724	0,931525	0,991839	y ~ current_feature + S
0,024423	0,061765	0,034007	0,395421	421	261,9694	0,692854	0,916569	y ~ current_feature + S
0,101956	0,049514	-0,04751	2,059146	420	403,6533	0,040121	0,612508	y ~ current_feature + S
-0,01113	0,054585	0,061498	-0,20396	367	335,5832	0,838509	0,996179	y ~ current_feature + S
0,009443	0,060435	-0,02429	0,156243	280	273,7698	0,875956	0,984468	y ~ current_feature + S
0,01462	0,050486	-0,07874	0,289595	415	392,254	0,772279	0,965962	y ~ current_feature + S
0,027272	0,065364	0,034007	0,417237	415	233,8815	0,676887	0,912427	y ~ current_feature + S
0,043118	0,051355	-0,04751	0,839599	414	378,4587	0,401663	0,803665	y ~ current_feature + S
0,045009	0,054776	0,061498	0,821707	361	332,6186	0,411832	0,934833	y ~ current_feature + S
0,032157	0,06081	-0,02429	0,528806	276	270,1439	0,597374	0,924682	y ~ current_feature + S
-0,04648	0,05257	-0,07874	-0,8841	388	361,0588	0,377229	0,789252	y ~ current_feature + S
-0,14828	0,06754	0,034007	-2,19542	388	214,3972	0,029206	0,347616	y ~ current_feature + S
-0,04404	0,053134	-0,04751	-0,82886	387	353,5153	0,407742	0,803665	y ~ current_feature + S
0,016179	0,058231	0,061498	0,27784	335	294,8309	0,78133	0,978149	y ~ current_feature + S
-0,10497	0,062276	-0,02429	-1,68562	258	255	0,093092	0,605474	y ~ current_feature + S
0,056146	0,051914	-0,07874	1,081504	421	369,8727	0,280178	0,71741	y ~ current_feature + S
0,071604	0,068417	0,034007	1,046583	421	212,5409	0,296481	0,718742	y ~ current_feature + S
0,090739	0,053434	-0,04751	1,698145	420	347,3555	0,090376	0,640171	y ~ current_feature + S
0,015664	0,056697	0,061498	0,276277	367	311,0055	0,782519	0,978149	y ~ current_feature + S
0,155065	0,059357	-0,02429	2,612392	280	277	0,009482	0,295269	y ~ current_feature + S
0,045947	0,05055	-0,07874	0,908944	403	390,5224	0,36394	0,773896	y ~ current_feature + S
-0,04476	0,063625	0,034007	-0,70353	403	246,5305	0,482391	0,831402	y ~ current_feature + S
-0,03818	0,051093	-0,04751	-0,74724	402	382,5131	0,455375	0,832044	y ~ current_feature + S
-0,01836	0,054228	0,061498	-0,33853	349	339,9453	0,735175	0,972573	y ~ current_feature + S
0,013528	0,062863	-0,02429	0,215196	268	253,0027	0,829788	0,976969	y ~ current_feature + S
-0,02123	0,051389	-0,07874	-0,41306	395	378,4938	0,679797	0,934653	y ~ current_feature + S
0,005939	0,065397	0,034007	0,090814	395	233,8104	0,927718	0,9732	y ~ current_feature + S
-0,02164	0,052277	-0,04751	-0,414	394	365,7453	0,679114	0,92829	y ~ current_feature + S
-0,01992	0,056462	0,061498	-0,35273	343	313,5582	0,724529	0,972573	y ~ current_feature + S
-0,01512	0,063085	-0,02429	-0,23964	262	251,2185	0,810808	0,974251	y ~ current_feature + S
0,029819	0,05051	-0,07874	0,590367	418	391,6176	0,555285	0,891348	y ~ current_feature + S
-0,04913	0,065719	0,034007	-0,74762	418	230,9758	0,455451	0,821488	y ~ current_feature + S
-0,0321	0,05145	-0,04751	-0,62393	417	377,3792	0,533051	0,883994	y ~ current_feature + S
-0,0579	0,055981	0,061498	-1,03436	365	318,0232	0,301756	0,893818	y ~ current_feature + S
0,057939	0,060218	-0,02429	0,96216	278	274,849	0,336815	0,785829	y ~ current_feature + S
-0,01021	0,056058	-0,07874	-0,18215	338	318,1874	0,855584	0,983548	y ~ current_feature + S
0,011115	0,073101	0,034007	0,152045	338	187,1093	0,879315	0,96591	y ~ current_feature + S
0,096416	0,057229	-0,04751	1,684759	337	302,4954	0,093066	0,640171	y ~ current_feature + S
0,009498	0,059531	0,061498	0,15954	293	282,1464	0,873358	0,996179	y ~ current_feature + S
0,066181	0,068457	-0,02429	0,966753	225	212,4478	0,334767	0,785829	y ~ current_feature + S
0,008659	0,049947	-0,07874	0,173369	421	400,8257	0,862449	0,985453	y ~ current_feature + S
0,023878	0,064311	0,034007	0,371283	421	241,6481	0,710752	0,920814	y ~ current_feature + S
0,047071	0,050744	-0,04751	0,927614	420	387,4983	0,354185	0,795089	y ~ current_feature + S
-0,00941	0,055588	0,061498	-0,16936	367	323,5944	0,86562	0,996179	y ~ current_feature + S
0,04392	0,060214	-0,02429	0,729394	280	275,2754	0,466381	0,876224	y ~ current_feature + S
0,064893	0,051208	-0,07874	1,267243	415	379,7486	0,205845	0,622938	y ~ current_feature + S
0,086323	0,067115	0,034007	1,286197	415	220,352	0,199724	0,647595	y ~ current_feature + S

0,088952	0,052428	-0,04751	1,69666		414	360,9328	0,090623	0,640171	y ~ current_feature + S
0,06274	0,055771	0,061498	1,124967		361	320,2409	0,261445	0,893818	y ~ current_feature + S
0,149647	0,061257	-0,02429	2,442932		276	260,5251	0,015233	0,417502	y ~ current_feature + S
-0,05607	0,054469	-0,07874	-1,02932		339	336	0,304069	0,718687	y ~ current_feature + S
-0,10861	0,054232	0,034007	-2,00276		339	336	0,046006	0,400524	y ~ current_feature + S
-0,05682	0,054548	-0,04751	-1,04164		338	335	0,29833	0,753461	y ~ current_feature + S
-0,13668	0,058373	0,061498	-2,34144		291	288	0,019891	0,609646	y ~ current_feature + S
-0,0973	0,066647	-0,02429	-1,45989		226	223	0,145728	0,619762	y ~ current_feature + S
-0,081	0,05904	-0,07874	-1,37195		288	285	0,171158	0,550681	y ~ current_feature + S
-0,11002	0,058875	0,034007	-1,8687		288	285	0,062691	0,437643	y ~ current_feature + S
0,001515	0,059339	-0,04751	0,025537		287	284	0,979644	0,992311	y ~ current_feature + S
-0,03373	0,063721	0,061498	-0,52926		249	246	0,597102	0,945257	y ~ current_feature + S
-0,01922	0,072726	-0,02429	-0,2643		192	189	0,791835	0,967756	y ~ current_feature + S
-0,02246	0,063872	-0,07874	-0,35165		248	245	0,725402	0,955155	y ~ current_feature + S
0,133254	0,063318	0,034007	2,104525		248	245	0,036352	0,384289	y ~ current_feature + S
0,041277	0,063833	-0,04751	0,646644		248	245	0,518467	0,873954	y ~ current_feature + S
-0,02168	0,067558	0,061498	-0,32097		222	219	0,748542	0,972573	y ~ current_feature + S
0,110687	0,078085	-0,02429	1,41753		165	162	0,158249	0,623497	y ~ current_feature + S
0,031857	0,052006	-0,07874	0,612564		389	369,3679	0,540542	0,882344	y ~ current_feature + S
-0,02603	0,066591	0,034007	-0,39095		389	225,3586	0,696204	0,918206	y ~ current_feature + S
0,046829	0,052746	-0,04751	0,887822		389	358,6482	0,375232	0,803665	y ~ current_feature + S
0,118537	0,057733	0,061498	2,053191		336	295,8037	0,040933	0,658491	y ~ current_feature + S
0,095521	0,062882	-0,02429	1,519038		259	250,5888	0,130013	0,617591	y ~ current_feature + S
0,003856	0,055888	-0,07874	0,068988		347	320,1479	0,945042	0,994607	y ~ current_feature + S
-0,05808	0,072054	0,034007	-0,80605		347	191,9622	0,421212	0,791109	y ~ current_feature + S
0,046247	0,056075	-0,04751	0,824745		346	317,3493	0,410136	0,803665	y ~ current_feature + S
-0,06022	0,06074	0,061498	-0,99137		298	270,0692	0,322392	0,893818	y ~ current_feature + S
-0,04808	0,067013	-0,02429	-0,7175		231	222,1673	0,473817	0,876561	y ~ current_feature + S
-0,04313	0,05343	-0,07874	-0,80723		377	349,6365	0,420081	0,811887	y ~ current_feature + S
-0,03943	0,069184	0,034007	-0,56994		377	208,5995	0,569333	0,863333	y ~ current_feature + S
0,124461	0,055114	-0,04751	2,258246		376	324,1139	0,024595	0,568759	y ~ current_feature + S
0,006877	0,057329	0,061498	0,119959		328	304,2476	0,904595	0,996179	y ~ current_feature + S
0,079123	0,063766	-0,02429	1,240844		251	244,3973	0,215853	0,682612	y ~ current_feature + S
0,008179	0,071426	-0,07874	0,114508		199	196	0,908953	0,987929	y ~ current_feature + S
-0,12397	0,070878	0,034007	-1,74905		199	196	0,081847	0,473527	y ~ current_feature + S
-0,17694	0,070482	-0,04751	-2,51046		198	195	0,01287	0,526293	y ~ current_feature + S
-0,06962	0,082972	0,061498	-0,83903		164	144,5521	0,402836	0,93433	y ~ current_feature + S
-0,17429	0,086697	-0,02429	-2,01035		132	129	0,046479	0,525585	y ~ current_feature + S
-0,01745	0,056069	-0,07874	-0,31123		321	318	0,75583	0,964335	y ~ current_feature + S
-0,03924	0,056034	0,034007	-0,70033		321	318	0,484235	0,831402	y ~ current_feature + S
0,095482	0,055821	-0,04751	1,710509		321	318	0,088147	0,640171	y ~ current_feature + S
-0,01578	0,060738	0,061498	-0,2598		274	271	0,795212	0,984075	y ~ current_feature + S
-0,10518	0,068461	-0,02429	-1,53639		214	211	0,125942	0,617591	y ~ current_feature + S
-0,00379	0,05194	-0,07874	-0,07289		374	370,6763	0,941933	0,994607	y ~ current_feature + S
-0,06038	0,062688	0,034007	-0,96317		374	253,5397	0,33638	0,73474	y ~ current_feature + S
0,043731	0,052184	-0,04751	0,838023		374	366,5236	0,402564	0,803665	y ~ current_feature + S
0,022577	0,05659	0,061498	0,398954		322	312,1068	0,6902	0,971004	y ~ current_feature + S
-0,0787	0,06547	-0,02429	-1,20208		249	231,8541	0,23056	0,695919	y ~ current_feature + S

-0,00645	0,049744	-0,07874	-0,12964	418	404,116	0,896916	0,987929	y ~ current_feature + S
0,049664	0,063271	0,034007	0,784935	418	249,1829	0,433237	0,796709	y ~ current_feature + S
0,028905	0,050399	-0,04751	0,573525	417	393,3659	0,566617	0,903656	y ~ current_feature + S
0,089076	0,055059	0,061498	1,617827	364	327,2508	0,106663	0,8197	y ~ current_feature + S
0,024455	0,062381	-0,02429	0,392033	278	256,8262	0,695359	0,928819	y ~ current_feature + S
0,146297	0,050073	-0,07874	2,921669	421	390,2971	0,003684	0,069632	y ~ current_feature + S
0,229413	0,06443	0,034007	3,560683	421	228,2173	4,50E-04	0,067708	y ~ current_feature + S
0,094593	0,051466	-0,04751	1,837992	420	374,1632	0,066856	0,640171	y ~ current_feature + S
-0,00812	0,056506	0,061498	-0,14377	367	313,1697	0,885774	0,996179	y ~ current_feature + S
0,144817	0,060025	-0,02429	2,412625	280	271,7285	0,016501	0,428958	y ~ current_feature + S
0,133796	0,059053	-0,07874	2,265715	321	281,6286	0,024228	0,197019	y ~ current_feature + S
0,054279	0,078542	0,034007	0,691086	321	161,6276	0,490503	0,837983	y ~ current_feature + S
0,157421	0,059105	-0,04751	2,663414	320	279,1598	0,008185	0,432614	y ~ current_feature + S
0,037046	0,063	0,061498	0,588036	277	251,6101	0,557035	0,937665	y ~ current_feature + S
0,090102	0,068563	-0,02429	1,31415	214	211	0,190223	0,65347	y ~ current_feature + S
0,046425	0,050061	-0,07874	0,927366	418	398,1673	0,354298	0,765762	y ~ current_feature + S
-0,0301	0,064391	0,034007	-0,46747	418	240,9649	0,640584	0,899624	y ~ current_feature + S
0,004264	0,051018	-0,04751	0,083569	417	384,1917	0,933443	0,988194	y ~ current_feature + S
0,04176	0,055191	0,061498	0,756645	365	327,7222	0,449806	0,937665	y ~ current_feature + S
-0,0233	0,061203	-0,02429	-0,38069	278	266,8181	0,703736	0,93118	y ~ current_feature + S
0,076012	0,053767	-0,07874	1,413726	367	343,9168	0,158347	0,527823	y ~ current_feature + S
0,024445	0,068828	0,034007	0,355167	367	210,965	0,722819	0,920814	y ~ current_feature + S
0,019455	0,054754	-0,04751	0,355312	367	333,4332	0,722581	0,93314	y ~ current_feature + S
-0,03516	0,057169	0,061498	-0,61505	321	305,5936	0,53898	0,937665	y ~ current_feature + S
0,074341	0,064752	-0,02429	1,148089	245	237,1848	0,252089	0,717483	y ~ current_feature + S
0,040621	0,084146	-0,07874	0,482743	144	141	0,630027	0,92504	y ~ current_feature + S
-0,04034	0,084147	0,034007	-0,47944	144	141	0,632369	0,899624	y ~ current_feature + S
-0,05826	0,084072	-0,04751	-0,69302	144	141	0,489436	0,860292	y ~ current_feature + S
-0,07301	0,090294	0,061498	-0,80853	125	122	0,420358	0,937665	y ~ current_feature + S
-0,12516	0,10288	-0,02429	-1,21661	96	93	0,226833	0,695919	y ~ current_feature + S
0,004643	0,055265	-0,07874	0,084016	346	327,4043	0,933095	0,992084	y ~ current_feature + S
-0,00704	0,070757	0,034007	-0,09949	346	199,7282	0,920852	0,9732	y ~ current_feature + S
-0,07437	0,056063	-0,04751	-1,3266	346	316,3962	0,185597	0,704811	y ~ current_feature + S
-0,06865	0,059217	0,061498	-1,15938	300	283,8319	0,247274	0,893818	y ~ current_feature + S
-0,03681	0,067033	-0,02429	-0,54914	230	222,2451	0,583459	0,924682	y ~ current_feature + S
0,139526	0,063654	-0,07874	2,191951	245	242	0,029336	0,219281	y ~ current_feature + S
0,065354	0,064145	0,034007	1,018845	245	242	0,309294	0,718742	y ~ current_feature + S
0,120015	0,06395	-0,04751	1,876691	244	241	0,061769	0,640171	y ~ current_feature + S
0,120692	0,069164	0,061498	1,745008	209	206	0,082475	0,753473	y ~ current_feature + S
0,126236	0,078425	-0,02429	1,609648	163	160	0,109446	0,605474	y ~ current_feature + S
0,051181	0,065133	-0,07874	0,785799	272	235,1069	0,432777	0,814177	y ~ current_feature + S
-0,07469	0,087642	0,034007	-0,85224	272	129,4642	0,395656	0,775482	y ~ current_feature + S
0,040947	0,06593	-0,04751	0,621062	271	229,6687	0,535175	0,883994	y ~ current_feature + S
0,066306	0,06873	0,061498	0,96473	232	210,7647	0,335786	0,893818	y ~ current_feature + S
-0,0826	0,075427	-0,02429	-1,09504	181	174,5719	0,275006	0,734108	y ~ current_feature + S
0,033861	0,055541	-0,07874	0,609648	376	323,7965	0,542522	0,882344	y ~ current_feature + S
0,010155	0,072483	0,034007	0,140108	376	190,3204	0,888723	0,96591	y ~ current_feature + S
0,021319	0,057398	-0,04751	0,371427	375	303,3978	0,710579	0,93314	y ~ current_feature + S

0,036049	0,061519	0,061498	0,585976	326	263,883	0,558393	0,937665	y ~ current_feature + S
0,062229	0,063505	-0,02429	0,979902	250	247	0,328093	0,775683	y ~ current_feature + S
0,067826	0,069012	-0,07874	0,982807	212	209	0,326839	0,726391	y ~ current_feature + S
-0,03828	0,069121	0,034007	-0,5538	212	209	0,58031	0,869291	y ~ current_feature + S
-0,06848	0,069009	-0,04751	-0,99228	212	209	0,322208	0,774585	y ~ current_feature + S
-0,07339	0,074751	0,061498	-0,98177	181	178	0,327546	0,893818	y ~ current_feature + S
-0,03692	0,085068	-0,02429	-0,43398	141	138	0,664982	0,926342	y ~ current_feature + S
0,101502	0,053945	-0,07874	1,881563	377	340,0885	0,060749	0,325854	y ~ current_feature + S
-0,04308	0,069857	0,034007	-0,61671	377	204,5375	0,538113	0,850862	y ~ current_feature + S
0,10383	0,053697	-0,04751	1,93362	376	343,0754	0,053982	0,640171	y ~ current_feature + S
0,057469	0,057363	0,061498	1,001842	326	302,8957	0,317219	0,893818	y ~ current_feature + S
0,051372	0,064895	-0,02429	0,791621	251	236,8271	0,429374	0,849564	y ~ current_feature + S
0,171304	0,071206	-0,07874	2,405741	211	191,4376	0,017091	0,176938	y ~ current_feature + S
0,137604	0,094045	0,034007	1,463168	211	110,9233	0,146249	0,594724	y ~ current_feature + S
0,077269	0,077633	-0,04751	0,995304	210	164,9309	0,321047	0,774585	y ~ current_feature + S
-0,00979	0,077544	0,061498	-0,12627	180	166,2876	0,899671	0,996179	y ~ current_feature + S
0,052733	0,085317	-0,02429	0,618086	140	137	0,537545	0,906112	y ~ current_feature + S
0,032766	0,053611	-0,07874	0,611184	380	347,5507	0,541477	0,882344	y ~ current_feature + S
-3,07E-04	0,069086	0,034007	-0,00445	380	209,5202	0,996453	0,997989	y ~ current_feature + S
0,096914	0,054331	-0,04751	1,783775	380	335,593	0,075364	0,640171	y ~ current_feature + S
0,028918	0,057752	0,061498	0,500729	328	299,5712	0,61693	0,948092	y ~ current_feature + S
9,79E-04	0,06355	-0,02429	0,015406	253	247,6082	0,98772	0,99444	y ~ current_feature + S
-0,01147	0,050273	-0,07874	-0,22815	404	395,6195	0,819649	0,979871	y ~ current_feature + S
-0,16999	0,062758	0,034007	-2,70873	404	246,5636	0,007227	0,205704	y ~ current_feature + S
-0,05735	0,050667	-0,04751	-1,13194	403	388,261	0,258358	0,742928	y ~ current_feature + S
-0,02922	0,055772	0,061498	-0,52385	350	321,2142	0,600742	0,945257	y ~ current_feature + S
-0,11705	0,063684	-0,02429	-1,83801	269	243,195	0,06728	0,592706	y ~ current_feature + S
-0,00132	0,05517	-0,07874	-0,02395	368	328,5474	0,980904	0,996318	y ~ current_feature + S
-0,01533	0,069757	0,034007	-0,21982	368	205,4557	0,826233	0,953842	y ~ current_feature + S
0,090133	0,056288	-0,04751	1,601303	367	313,0642	0,110318	0,640171	y ~ current_feature + S
-0,0173	0,057864	0,061498	-0,299	319	298,5787	0,765151	0,972573	y ~ current_feature + S
0,120285	0,064424	-0,02429	1,867069	245	237,449	0,063124	0,569659	y ~ current_feature + S
0,029436	0,049508	-0,07874	0,59458	415	407,6361	0,552454	0,889995	y ~ current_feature + S
-0,03534	0,061958	0,034007	-0,57035	415	260,1755	0,568932	0,863333	y ~ current_feature + S
0,009662	0,049982	-0,04751	0,193316	414	400,2445	0,84681	0,980218	y ~ current_feature + S
0,024787	0,053472	0,061498	0,463553	362	349,5264	0,643256	0,956496	y ~ current_feature + S
0,042812	0,061574	-0,02429	0,695301	276	263,2748	0,48748	0,888665	y ~ current_feature + S
-0,01679	0,055208	-0,07874	-0,30418	331	328	0,761186	0,965008	y ~ current_feature + S
-0,05867	0,055121	0,034007	-1,06448	331	328	0,287894	0,718742	y ~ current_feature + S
0,087046	0,05509	-0,04751	1,580069	330	327	0,115058	0,640171	y ~ current_feature + S
-0,05849	0,059237	0,061498	-0,98736	287	284	0,324304	0,893818	y ~ current_feature + S
-0,01709	0,067875	-0,02429	-0,25185	220	217	0,801392	0,968297	y ~ current_feature + S
-0,11221	0,056898	-0,07874	-1,97221	308	305	0,049489	0,29534	y ~ current_feature + S
-0,07935	0,057079	0,034007	-1,39013	308	305	0,165504	0,612363	y ~ current_feature + S
-0,00315	0,05726	-0,04751	-0,05507	308	305	0,956122	0,990156	y ~ current_feature + S
-0,09867	0,060787	0,061498	-1,62315	271	268	0,105733	0,8197	y ~ current_feature + S
-0,15758	0,069481	-0,02429	-2,268	205	202	0,024387	0,485321	y ~ current_feature + S
0,059304	0,055554	-0,07874	1,067495	359	322,8767	0,286546	0,718687	y ~ current_feature + S

-0,07735	0,073023	0,034007	-1,05918	359	186,4109	0,290887	0,718742	y ~ current_feature + S
-0,02665	0,056983	-0,04751	-0,46771	358	307,7521	0,640324	0,925756	y ~ current_feature + S
-0,035	0,059862	0,061498	-0,58474	310	278,7221	0,559198	0,937665	y ~ current_feature + S
-0,04748	0,065525	-0,02429	-0,7246	239	232,3822	0,469424	0,876224	y ~ current_feature + S
-0,02505	0,050326	-0,07874	-0,49778	410	394,5809	0,618918	0,919937	y ~ current_feature + S
-0,00943	0,065108	0,034007	-0,14477	410	235,883	0,88502	0,96591	y ~ current_feature + S
0,082642	0,051073	-0,04751	1,618134	409	380,7567	0,106462	0,640171	y ~ current_feature + S
-0,02437	0,054733	0,061498	-0,44527	356	333,612	0,656415	0,957207	y ~ current_feature + S
-0,04011	0,062516	-0,02429	-0,64162	273	255,4593	0,521692	0,899808	y ~ current_feature + S
0,090898	0,052198	-0,07874	1,741416	401	363,9958	0,082456	0,388948	y ~ current_feature + S
-0,01541	0,067466	0,034007	-0,22848	401	219,6476	0,819488	0,952501	y ~ current_feature + S
0,116319	0,052528	-0,04751	2,214391	400	357,515	0,027432	0,588545	y ~ current_feature + S
0,016219	0,05697	0,061498	0,284691	348	308,0302	0,776072	0,975031	y ~ current_feature + S
-0,00317	0,062637	-0,02429	-0,05065	266	254,8826	0,959642	0,99444	y ~ current_feature + S
0,067043	0,05429	-0,07874	1,234898	374	337,7541	0,217727	0,639357	y ~ current_feature + S
-0,03816	0,07161	0,034007	-0,53284	374	194,7217	0,594753	0,878477	y ~ current_feature + S
-0,00768	0,055041	-0,04751	-0,13945	373	330,0627	0,889179	0,985217	y ~ current_feature + S
0,002409	0,060359	0,061498	0,039918	324	274,4847	0,968188	0,996179	y ~ current_feature + S
-0,03917	0,063709	-0,02429	-0,6149	249	246	0,539192	0,906823	y ~ current_feature + S
-0,0085	0,054634	-0,07874	-0,15552	338	335	0,876505	0,987929	y ~ current_feature + S
-0,05888	0,054541	0,034007	-1,07953	338	335	0,281127	0,715049	y ~ current_feature + S
0,067268	0,054594	-0,04751	1,232162	337	334	0,218755	0,742928	y ~ current_feature + S
-0,04374	0,058565	0,061498	-0,7469	294	291	0,455729	0,937665	y ~ current_feature + S
0,030025	0,067085	-0,02429	0,447559	225	222	0,654908	0,924682	y ~ current_feature + S
-0,01867	0,078554	-0,07874	-0,23763	165	162	0,81247	0,979871	y ~ current_feature + S
-0,2389	0,076293	0,034007	-3,13131	165	162	0,002065	0,133248	y ~ current_feature + S
-0,01791	0,078555	-0,04751	-0,228	165	162	0,819932	0,972405	y ~ current_feature + S
-0,01994	0,084198	0,061498	-0,23683	144	141	0,813136	0,995348	y ~ current_feature + S
-0,14576	0,095641	-0,02429	-1,52408	110	107	0,130438	0,617591	y ~ current_feature + S
-0,14206	0,063111	-0,07874	-2,251	249	246	0,025269	0,201065	y ~ current_feature + S
-0,22018	0,062193	0,034007	-3,54023	249	246	4,78E-04	0,067708	y ~ current_feature + S
-0,01765	0,063748	-0,04751	-0,27684	249	246	0,782136	0,94882	y ~ current_feature + S
-0,04988	0,068757	0,061498	-0,72551	214	211	0,468943	0,937665	y ~ current_feature + S
-0,20313	0,076693	-0,02429	-2,64857	166	163	0,008878	0,295269	y ~ current_feature + S
-0,03726	0,083275	-0,07874	-0,44743	147	144	0,655235	0,932255	y ~ current_feature + S
-0,04993	0,083229	0,034007	-0,5999	147	144	0,549519	0,854294	y ~ current_feature + S
-0,05952	0,083186	-0,04751	-0,71548	147	144	0,47547	0,842057	y ~ current_feature + S
-0,13212	0,089015	0,061498	-1,48429	127	124	0,140269	0,8197	y ~ current_feature + S
0,04913	0,102474	-0,02429	0,479443	98	95	0,632726	0,924682	y ~ current_feature + S
0,060997	0,061845	-0,07874	0,98629	298	260,4785	0,324906	0,726376	y ~ current_feature + S
0,008941	0,080804	0,034007	0,110647	298	153,1437	0,912041	0,9732	y ~ current_feature + S
0,029545	0,064445	-0,04751	0,458452	298	240,571	0,647041	0,925756	y ~ current_feature + S
0,034691	0,067542	0,061498	0,513627	258	218,9438	0,60803	0,945257	y ~ current_feature + S
0,02427	0,071408	-0,02429	0,339876	199	196	0,734314	0,944216	y ~ current_feature + S
0,071884	0,071795	-0,07874	1,001235	196	193	0,317967	0,723052	y ~ current_feature + S
0,065876	0,071825	0,034007	0,917177	196	193	0,360195	0,7498	y ~ current_feature + S
0,008696	0,072166	-0,04751	0,120502	195	192	0,904212	0,985217	y ~ current_feature + S
-0,04881	0,07729	0,061498	-0,63148	170	167	0,52859	0,937665	y ~ current_feature + S

0,136693	0,087903	-0,02429	1,555044	130	127	0,122424	0,617591	y ~ current_feature + S
-0,03933	0,097982	-0,07874	-0,4014	107	104	0,688949	0,93638	y ~ current_feature + S
0,109092	0,097473	0,034007	1,1192	107	104	0,265633	0,71095	y ~ current_feature + S
0,073796	0,097791	-0,04751	0,754631	107	104	0,452176	0,832044	y ~ current_feature + S
-0,1143	0,107754	0,061498	-1,06078	88	85	0,291794	0,893818	y ~ current_feature + S
0,018881	0,121246	-0,02429	0,155728	71	68	0,876709	0,984468	y ~ current_feature + S
-0,02793	0,049934	-0,07874	-0,55933	421	400,7485	0,57625	0,899631	y ~ current_feature + S
-0,07971	0,063929	0,034007	-1,24682	421	243,1296	0,213663	0,664036	y ~ current_feature + S
-0,0219	0,050827	-0,04751	-0,43086	420	386,8972	0,66681	0,92822	y ~ current_feature + S
-0,04421	0,055146	0,061498	-0,80177	367	328,1921	0,423268	0,937665	y ~ current_feature + S
0,008246	0,061593	-0,02429	0,133872	280	263,5805	0,893606	0,99444	y ~ current_feature + S
-0,0915	0,068392	-0,07874	-1,33788	215	212	0,18237	0,581699	y ~ current_feature + S
-0,0666	0,068528	0,034007	-0,97184	215	212	0,33224	0,734392	y ~ current_feature + S
0,132781	0,068072	-0,04751	1,950598	215	212	0,052423	0,640171	y ~ current_feature + S
0,078983	0,07493	0,061498	1,054089	180	177	0,293279	0,893818	y ~ current_feature + S
0,016858	0,084503	-0,02429	0,199491	143	140	0,842168	0,977734	y ~ current_feature + S
-0,00157	0,095346	-0,07874	-0,01647	113	110	0,986893	0,996318	y ~ current_feature + S
-0,09946	0,094873	0,034007	-1,04834	113	110	0,296781	0,718742	y ~ current_feature + S
0,125936	0,09502	-0,04751	1,325363	112	109	0,187822	0,709124	y ~ current_feature + S
0,163396	0,104221	0,061498	1,567792	95	89,60662	0,120454	0,8197	y ~ current_feature + S
-0,0414	0,11775	-0,02429	-0,35157	75	72	0,726191	0,942774	y ~ current_feature + S
0,017915	0,056784	-0,07874	0,315498	337	310,0368	0,752596	0,963531	y ~ current_feature + S
0,104123	0,075444	0,034007	1,380132	337	173,7862	0,169319	0,61875	y ~ current_feature + S
0,0639	0,059309	-0,04751	1,077414	337	283,1313	0,282213	0,747915	y ~ current_feature + S
0,153869	0,061209	0,061498	2,51381	292	260,5907	0,012547	0,55988	y ~ current_feature + S
0,121318	0,066674	-0,02429	1,819569	225	221,6377	0,070174	0,605474	y ~ current_feature + S
0,017291	0,05117	-0,07874	0,337909	394	381,8067	0,735618	0,959225	y ~ current_feature + S
-0,04136	0,065687	0,034007	-0,62968	394	231,3622	0,529523	0,848387	y ~ current_feature + S
0,090154	0,052192	-0,04751	1,727344	393	364,1217	0,084954	0,640171	y ~ current_feature + S
0,029325	0,055869	0,061498	0,524881	341	320,1009	0,600029	0,945257	y ~ current_feature + S
0,086709	0,062425	-0,02429	1,38901	262	254,6835	0,166043	0,624945	y ~ current_feature + S
0,031012	0,059733	-0,07874	0,519173	283	280	0,604051	0,908531	y ~ current_feature + S
-0,04349	0,059705	0,034007	-0,72842	283	280	0,466968	0,831402	y ~ current_feature + S
9,34E-04	0,059868	-0,04751	0,015606	282	279	0,98756	0,994138	y ~ current_feature + S
0,024168	0,069591	0,061498	0,347286	243	206,3672	0,72873	0,972573	y ~ current_feature + S
-0,07809	0,073366	-0,02429	-1,0644	188	184,6503	0,288536	0,734108	y ~ current_feature + S
-0,03758	0,052133	-0,07874	-0,72084	379	367,4114	0,471468	0,840689	y ~ current_feature + S
-0,12924	0,067201	0,034007	-1,92323	379	217,7347	0,055756	0,420946	y ~ current_feature + S
-0,12438	0,05277	-0,04751	-2,35698	378	353,549	0,018969	0,54566	y ~ current_feature + S
-0,16462	0,055592	0,061498	-2,96116	328	314,8076	0,003298	0,305054	y ~ current_feature + S
-0,00713	0,065427	-0,02429	-0,10896	252	233,5987	0,913329	0,99444	y ~ current_feature + S
0,139968	0,064454	-0,07874	2,171612	239	236	0,030882	0,224043	y ~ current_feature + S
0,039684	0,065043	0,034007	0,610112	239	236	0,542375	0,852506	y ~ current_feature + S
0,11231	0,064683	-0,04751	1,736321	239	236	0,083812	0,640171	y ~ current_feature + S
0,002896	0,075514	0,061498	0,038352	201	175,3643	0,969451	0,996179	y ~ current_feature + S
0,046186	0,080846	-0,02429	0,571285	159	152,6719	0,568647	0,924682	y ~ current_feature + S
-0,09255	0,054979	-0,07874	-1,6834	331	328	0,093248	0,42595	y ~ current_feature + S
-0,10533	0,054909	0,034007	-1,91836	331	328	0,055934	0,420946	y ~ current_feature + S

-0,01728	0,055208	-0,04751	-0,31296	331	328	0,754508	0,9394	y ~ current_feature + S
-0,00441	0,063015	0,061498	-0,07005	287	251,8253	0,944211	0,996179	y ~ current_feature + S
-0,10637	0,067344	-0,02429	-1,57948	221	218	0,115675	0,614731	y ~ current_feature + S
-0,03877	0,081862	-0,07874	-0,47359	152	149	0,636489	0,927169	y ~ current_feature + S
-0,18839	0,080456	0,034007	-2,34154	152	149	0,020529	0,31947	y ~ current_feature + S
-0,01191	0,081917	-0,04751	-0,14537	152	149	0,884616	0,985217	y ~ current_feature + S
-0,16225	0,088258	0,061498	-1,83833	128	125	0,068387	0,715249	y ~ current_feature + S
-0,00629	0,101013	-0,02429	-0,06225	101	98	0,950492	0,99444	y ~ current_feature + S
0,007869	0,072355	-0,07874	0,108756	194	191	0,91351	0,987929	y ~ current_feature + S
-0,12442	0,071795	0,034007	-1,73294	194	191	0,08472	0,478574	y ~ current_feature + S
-0,02802	0,072329	-0,04751	-0,38735	194	191	0,698926	0,931902	y ~ current_feature + S
-0,06637	0,077915	0,061498	-0,85185	167	164	0,395537	0,93433	y ~ current_feature + S
-0,08744	0,088746	-0,02429	-0,98532	129	126	0,326353	0,774043	y ~ current_feature + S
-0,05919	0,057992	-0,07874	-1,02062	337	296,3059	0,308269	0,718687	y ~ current_feature + S
-0,08226	0,07583	0,034007	-1,08475	337	172,7329	0,279545	0,715049	y ~ current_feature + S
-0,0475	0,059983	-0,04751	-0,79197	337	277,3074	0,429057	0,819126	y ~ current_feature + S
-0,04093	0,064435	0,061498	-0,63519	294	240,4521	0,525911	0,937665	y ~ current_feature + S
-0,01107	0,067263	-0,02429	-0,16458	224	221	0,869421	0,984468	y ~ current_feature + S
-0,04207	0,061169	-0,07874	-0,68769	319	266,7861	0,492244	0,853069	y ~ current_feature + S
-0,0364	0,078986	0,034007	-0,46081	319	160,0757	0,645563	0,900251	y ~ current_feature + S
0,10738	0,061647	-0,04751	1,741855	318	260,0981	0,082716	0,640171	y ~ current_feature + S
0,099486	0,063268	0,061498	1,572459	275	247,3516	0,117122	0,8197	y ~ current_feature + S
0,030517	0,069286	-0,02429	0,440454	212	208,1159	0,660065	0,924682	y ~ current_feature + S
-0,03538	0,066923	-0,07874	-0,52865	226	223	0,597575	0,90386	y ~ current_feature + S
-0,04286	0,066903	0,034007	-0,64068	226	223	0,52239	0,845883	y ~ current_feature + S
-0,01286	0,066959	-0,04751	-0,19202	226	223	0,847898	0,980218	y ~ current_feature + S
0,029665	0,072137	0,061498	0,411229	195	192	0,681363	0,962245	y ~ current_feature + S
-0,0837	0,082189	-0,02429	-1,01834	150	147	0,310189	0,762591	y ~ current_feature + S
-0,00829	0,056886	-0,07874	-0,14574	312	309	0,884219	0,987929	y ~ current_feature + S
-0,02009	0,056877	0,034007	-0,35316	312	309	0,724208	0,920814	y ~ current_feature + S
-0,05634	0,05689	-0,04751	-0,99029	311	308	0,322807	0,774585	y ~ current_feature + S
0,068751	0,061054	0,061498	1,126058	270	267	0,261152	0,893818	y ~ current_feature + S
-0,00645	0,070013	-0,02429	-0,09213	207	204	0,926685	0,99444	y ~ current_feature + S
0,051311	0,05697	-0,07874	0,90066	346	307,2964	0,368474	0,77906	y ~ current_feature + S
-0,04151	0,075681	0,034007	-0,54851	346	174,2911	0,584045	0,871357	y ~ current_feature + S
-3,43E-04	0,057981	-0,04751	-0,00592	345	297,4593	0,995282	0,995481	y ~ current_feature + S
-0,01589	0,060174	0,061498	-0,26404	299	276,1036	0,791943	0,983887	y ~ current_feature + S
0,023253	0,067488	-0,02429	0,344554	230	219,4367	0,73076	0,944216	y ~ current_feature + S
-0,01515	0,103683	-0,07874	-0,14612	96	93	0,88414	0,987929	y ~ current_feature + S
0,296826	0,099022	0,034007	2,997581	96	93	0,00349	0,151911	y ~ current_feature + S
0,007356	0,103692	-0,04751	0,070937	96	93	0,9436	0,990156	y ~ current_feature + S
-0,03136	0,111748	0,061498	-0,28067	83	80	0,779684	0,977909	y ~ current_feature + S
0,197741	0,125509	-0,02429	1,575517	64	61	0,12031	0,615361	y ~ current_feature + S
-0,06331	0,051062	-0,07874	-1,23996	385	382	0,215752	0,639357	y ~ current_feature + S
-0,07288	0,051028	0,034007	-1,42826	385	382	0,154035	0,60427	y ~ current_feature + S
0,043637	0,051183	-0,04751	0,852582	384	381	0,394427	0,803665	y ~ current_feature + S
0,063467	0,060122	0,061498	1,05564	334	275,5365	0,292058	0,893818	y ~ current_feature + S
0,049434	0,063001	-0,02429	0,784655	256	251,3319	0,433395	0,852958	y ~ current_feature + S

-0,0888	0,062498	-0,07874	-1,42084	257	254	0,15659	0,524327	y ~ current_feature + S
-0,19031	0,061599	0,034007	-3,08951	257	254	0,002228	0,133248	y ~ current_feature + S
-0,11584	0,062323	-0,04751	-1,85877	257	254	0,064216	0,640171	y ~ current_feature + S
-0,07368	0,066783	0,061498	-1,10321	226	223	0,271125	0,893818	y ~ current_feature + S
-0,09595	0,076796	-0,02429	-1,24948	171	168	0,213229	0,679441	y ~ current_feature + S
0,083019	0,053055	-0,07874	1,56476	390	352,8107	0,118536	0,474142	y ~ current_feature + S
0,235206	0,068611	0,034007	3,428092	390	200,6748	7,37E-04	0,068218	y ~ current_feature + S
0,092753	0,055455	-0,04751	1,672583	389	322,3821	0,09538	0,640171	y ~ current_feature + S
0,034432	0,059176	0,061498	0,581856	339	285,2321	0,561123	0,937665	y ~ current_feature + S
0,183571	0,061633	-0,02429	2,978434	260	254,3791	0,003177	0,18344	y ~ current_feature + S
0,016105	0,069162	-0,07874	0,232863	212	209	0,816096	0,979871	y ~ current_feature + S
-0,06376	0,069031	0,034007	-0,92371	212	209	0,356705	0,7498	y ~ current_feature + S
-0,06351	0,069032	-0,04751	-0,91994	212	209	0,358664	0,798092	y ~ current_feature + S
0,022232	0,074517	0,061498	0,298343	183	180	0,765785	0,972573	y ~ current_feature + S
-0,06588	0,084941	-0,02429	-0,77561	141	138	0,439307	0,858965	y ~ current_feature + S
-0,01299	0,069499	-0,07874	-0,18696	210	207	0,851873	0,983548	y ~ current_feature + S
-0,14959	0,068723	0,034007	-2,17672	210	207	0,030633	0,347616	y ~ current_feature + S
-0,02513	0,069483	-0,04751	-0,36165	210	207	0,71798	0,93314	y ~ current_feature + S
-0,04879	0,07572	0,061498	-0,6444	177	174	0,520165	0,937665	y ~ current_feature + S
-0,10136	0,084996	-0,02429	-1,19255	140	137	0,235108	0,695919	y ~ current_feature + S
-0,01599	0,052681	-0,07874	-0,3036	381	360,2311	0,761604	0,965008	y ~ current_feature + S
-0,21262	0,069351	0,034007	-3,06584	381	198,5221	0,002473	0,133248	y ~ current_feature + S
-0,0241	0,05435	-0,04751	-0,44339	381	338,3301	0,657767	0,925756	y ~ current_feature + S
-0,09213	0,0571	0,061498	-1,61344	330	304,1096	0,107687	0,8197	y ~ current_feature + S
-0,04882	0,063492	-0,02429	-0,76891	254	247,4684	0,442679	0,858965	y ~ current_feature + S
0,115448	0,056903	-0,07874	2,02886	332	304,7228	0,043342	0,277028	y ~ current_feature + S
-0,05323	0,0753	0,034007	-0,70693	332	175,8621	0,480543	0,831402	y ~ current_feature + S
-0,00729	0,058428	-0,04751	-0,12476	331	292,9125	0,900797	0,985217	y ~ current_feature + S
0,035172	0,062727	0,061498	0,560719	284	253,8332	0,575484	0,937665	y ~ current_feature + S
0,036818	0,068685	-0,02429	0,536042	221	211,6828	0,592492	0,924682	y ~ current_feature + S
0,082775	0,084834	-0,07874	0,975734	141	138	0,330903	0,730951	y ~ current_feature + S
0,020668	0,085107	0,034007	0,242845	141	138	0,808486	0,952501	y ~ current_feature + S
-0,23493	0,082743	-0,04751	-2,83922	141	138	0,005206	0,422981	y ~ current_feature + S
-0,05582	0,090767	0,061498	-0,615	124	121	0,539713	0,937665	y ~ current_feature + S
-0,00409	0,104828	-0,02429	-0,03904	94	91	0,968947	0,99444	y ~ current_feature + S
-0,02792	0,050947	-0,07874	-0,54807	401	384,9627	0,583963	0,902886	y ~ current_feature + S
-0,04719	0,065591	0,034007	-0,71946	401	231,9232	0,472579	0,831402	y ~ current_feature + S
0,006715	0,052037	-0,04751	0,129047	400	369,286	0,897391	0,985217	y ~ current_feature + S
0,038952	0,055845	0,061498	0,697497	347	320,1641	0,485998	0,937665	y ~ current_feature + S
-0,01765	0,062905	-0,02429	-0,28066	267	252,6377	0,779203	0,961068	y ~ current_feature + S
-0,00999	0,092843	-0,07874	-0,10764	119	116	0,914466	0,987929	y ~ current_feature + S
-0,10016	0,092381	0,034007	-1,08426	119	116	0,280499	0,715049	y ~ current_feature + S
-0,03387	0,092794	-0,04751	-0,36502	119	116	0,71576	0,93314	y ~ current_feature + S
-0,21191	0,096766	0,061498	-2,18987	105	102	0,030812	0,658491	y ~ current_feature + S
0,058333	0,114513	-0,02429	0,509405	79	76	0,611944	0,924682	y ~ current_feature + S
0,067998	0,061993	-0,07874	1,096856	262	259	0,273723	0,705864	y ~ current_feature + S
0,050939	0,062056	0,034007	0,820855	262	259	0,412484	0,785427	y ~ current_feature + S
0,009004	0,062134	-0,04751	0,144913	262	259	0,884892	0,985217	y ~ current_feature + S

-0,0507	0,067029	0,061498	-0,75633	225	222	0,45025	0,937665	y ~ current_feature + S
0,017741	0,076237	-0,02429	0,232709	175	172	0,816264	0,974251	y ~ current_feature + S
-0,00932	0,052292	-0,07874	-0,17827	385	365,6684	0,858611	0,983548	y ~ current_feature + S
-0,09005	0,069025	0,034007	-1,30455	385	208,1883	0,193485	0,6438	y ~ current_feature + S
-0,03037	0,053501	-0,04751	-0,56771	384	349,0444	0,570599	0,903741	y ~ current_feature + S
-0,08719	0,05782	0,061498	-1,50787	333	296,84	0,132652	0,8197	y ~ current_feature + S
-0,01327	0,063065	-0,02429	-0,21043	256	251,3861	0,833503	0,977484	y ~ current_feature + S
0,082032	0,053783	-0,07874	1,525228	402	343,3762	0,128123	0,481525	y ~ current_feature + S
-0,00428	0,069743	0,034007	-0,06141	402	205,5871	0,951095	0,988497	y ~ current_feature + S
0,032036	0,054545	-0,04751	0,587327	401	335,7694	0,557379	0,897386	y ~ current_feature + S
-0,07062	0,056647	0,061498	-1,24664	348	310,0843	0,213469	0,893818	y ~ current_feature + S
0,016106	0,063	-0,02429	0,255644	267	251,8847	0,798434	0,968297	y ~ current_feature + S
-0,08	0,064655	-0,07874	-1,23737	286	237,6887	0,21717	0,639357	y ~ current_feature + S
-0,18558	0,084445	0,034007	-2,19766	286	135,4033	0,029673	0,347616	y ~ current_feature + S
-0,02859	0,062676	-0,04751	-0,45618	285	254,3601	0,648652	0,925756	y ~ current_feature + S
-0,14426	0,066674	0,061498	-2,16364	242	220,2706	0,031568	0,658491	y ~ current_feature + S
-0,21321	0,071446	-0,02429	-2,98423	190	186,9975	0,003223	0,18344	y ~ current_feature + S
-0,02919	0,115421	-0,07874	-0,25292	78	75	0,801023	0,979102	y ~ current_feature + S
-0,03959	0,11538	0,034007	-0,3431	78	75	0,732483	0,922572	y ~ current_feature + S
0,257753	0,111568	-0,04751	2,310273	78	75	0,023625	0,563956	y ~ current_feature + S
-0,12319	0,12309	0,061498	-1,00079	68	65	0,320639	0,893818	y ~ current_feature + S
-0,09876	0,142159	-0,02429	-0,69472	52	49	0,490512	0,889654	y ~ current_feature + S
0,107707	0,065707	-0,07874	1,639191	281	228,9327	0,102547	0,449022	y ~ current_feature + S
0,020591	0,059963	0,034007	0,343386	281	278	0,731567	0,922572	y ~ current_feature + S
-0,01142	0,06008	-0,04751	-0,19016	280	277	0,849322	0,980218	y ~ current_feature + S
0,076092	0,071138	0,061498	1,06964	243	196,463	0,286094	0,893818	y ~ current_feature + S
-0,06063	0,073786	-0,02429	-0,82173	186	183	0,4123	0,837702	y ~ current_feature + S
-0,03554	0,056578	-0,07874	-0,62813	315	312	0,530378	0,880735	y ~ current_feature + S
-0,00152	0,056614	0,034007	-0,02687	315	312	0,978579	0,989859	y ~ current_feature + S
-0,00754	0,056612	-0,04751	-0,13317	315	312	0,894142	0,985217	y ~ current_feature + S
0,022423	0,06073	0,061498	0,369223	274	271	0,71225	0,972444	y ~ current_feature + S
-0,00976	0,06967	-0,02429	-0,14007	209	206	0,888738	0,992711	y ~ current_feature + S
0,115658	0,048583	-0,07874	2,380604	421	418	0,017732	0,177316	y ~ current_feature + S
0,049021	0,048853	0,034007	1,003446	421	418	0,316226	0,718742	y ~ current_feature + S
0,022926	0,048957	-0,04751	0,46828	420	417	0,639829	0,925756	y ~ current_feature + S
-0,02771	0,052394	0,061498	-0,52887	367	364	0,597216	0,945257	y ~ current_feature + S
0,083094	0,059876	-0,02429	1,387751	280	277	0,166328	0,624945	y ~ current_feature + S
-0,01076	0,059192	-0,07874	-0,18185	309	285,3801	0,855828	0,983548	y ~ current_feature + S
-0,10433	0,078369	0,034007	-1,33121	309	161,0496	0,185002	0,638805	y ~ current_feature + S
-0,02447	0,0607	-0,04751	-0,4032	309	271,2456	0,687117	0,929555	y ~ current_feature + S
0,030297	0,064048	0,061498	0,473044	267	243,5517	0,636605	0,955689	y ~ current_feature + S
-0,07068	0,070011	-0,02429	-1,00953	206	203	0,313922	0,765236	y ~ current_feature + S
-0,18151	0,089399	-0,07874	-2,03039	124	121	0,044509	0,279123	y ~ current_feature + S
-0,10369	0,090419	0,034007	-1,14677	124	121	0,25374	0,704827	y ~ current_feature + S
-0,07618	0,091022	-0,04751	-0,83692	123	120	0,404299	0,803665	y ~ current_feature + S
-0,10572	0,09846	0,061498	-1,07374	105	102	0,285473	0,893818	y ~ current_feature + S
-0,20971	0,110007	-0,02429	-1,90632	82	79	0,060246	0,558147	y ~ current_feature + S
-0,00908	0,056794	-0,07874	-0,15988	313	310	0,873084	0,987929	y ~ current_feature + S

-0,05763	0,056702	0,034007	-1,01641	313	310	0,310226	0,718742	y ~ current_feature + S
-0,01742	0,056879	-0,04751	-0,30631	312	309	0,759579	0,9394	y ~ current_feature + S
0,003558	0,060745	0,061498	0,058568	274	271	0,95334	0,996179	y ~ current_feature + S
-0,06291	0,069705	-0,02429	-0,90246	208	205	0,36787	0,807787	y ~ current_feature + S
0,051411	0,050357	-0,07874	1,020942	421	393,3087	0,307909	0,718687	y ~ current_feature + S
0,052183	0,065351	0,034007	0,798502	421	233,5108	0,425391	0,791701	y ~ current_feature + S
0,046661	0,05134	-0,04751	0,908858	420	378,5697	0,364003	0,801674	y ~ current_feature + S
-0,06747	0,055153	0,061498	-1,22335	367	327,2501	0,222078	0,893818	y ~ current_feature + S
-0,02062	0,060472	-0,02429	-0,34095	280	273,3434	0,7334	0,944216	y ~ current_feature + S
-0,03394	0,093605	-0,07874	-0,36258	117	114	0,717593	0,948276	y ~ current_feature + S
-0,12556	0,092917	0,034007	-1,35134	117	114	0,179262	0,630217	y ~ current_feature + S
-0,01547	0,093647	-0,04751	-0,16524	117	114	0,86905	0,985217	y ~ current_feature + S
0,03106	0,103092	0,061498	0,301282	97	94	0,763865	0,972573	y ~ current_feature + S
0,057235	0,115281	-0,02429	0,496483	78	75	0,621006	0,924682	y ~ current_feature + S
-0,15625	0,14724	-0,07874	-1,06118	48	45	0,294273	0,718687	y ~ current_feature + S
-0,13345	0,147738	0,034007	-0,9033	48	45	0,371175	0,750317	y ~ current_feature + S
0,03499	0,14898	-0,04751	0,234861	48	45	0,815382	0,972405	y ~ current_feature + S
0,091001	0,161548	0,061498	0,563304	41	38	0,576537	0,937665	y ~ current_feature + S
0,117869	0,184401	-0,02429	0,639201	32	29	0,527709	0,899808	y ~ current_feature + S
0,013799	0,074118	-0,07874	0,186171	185	182	0,852518	0,983548	y ~ current_feature + S
0,050882	0,074029	0,034007	0,687323	185	182	0,492754	0,838248	y ~ current_feature + S
0,084952	0,073857	-0,04751	1,150219	185	182	0,251563	0,742928	y ~ current_feature + S
-0,00972	0,080578	0,061498	-0,1206	157	154	0,904162	0,996179	y ~ current_feature + S
-0,00361	0,091286	-0,02429	-0,03953	123	120	0,968533	0,99444	y ~ current_feature + S
0,028698	0,05017	-0,07874	0,572009	410	396,9642	0,56764	0,898449	y ~ current_feature + S
0,049649	0,063637	0,034007	0,780195	410	246,3282	0,436025	0,79866	y ~ current_feature + S
0,017572	0,050825	-0,04751	0,345736	409	386,9961	0,729729	0,93314	y ~ current_feature + S
-0,03447	0,054498	0,061498	-0,63244	359	336,3011	0,527526	0,937665	y ~ current_feature + S
0,070604	0,062041	-0,02429	1,138022	272	258,505	0,256165	0,718089	y ~ current_feature + S
0,126944	0,081811	-0,07874	1,55167	150	147	0,122891	0,477837	y ~ current_feature + S
0,050055	0,082375	0,034007	0,607648	150	147	0,544358	0,852506	y ~ current_feature + S
0,025225	0,082452	-0,04751	0,30594	150	147	0,760083	0,9394	y ~ current_feature + S
-0,00834	0,090533	0,061498	-0,09209	125	122	0,926778	0,996179	y ~ current_feature + S
0,044564	0,101434	-0,02429	0,439342	100	97	0,661391	0,924682	y ~ current_feature + S
-0,01311	0,084208	-0,07874	-0,15565	144	141	0,876532	0,987929	y ~ current_feature + S
0,044408	0,084132	0,034007	0,527833	144	141	0,598445	0,878669	y ~ current_feature + S
-0,05255	0,084099	-0,04751	-0,62487	144	141	0,533068	0,883994	y ~ current_feature + S
0,030292	0,091628	0,061498	0,330595	122	119	0,741531	0,972573	y ~ current_feature + S
0,070923	0,103434	-0,02429	0,685687	96	93	0,494616	0,892722	y ~ current_feature + S
0,132422	0,063068	-0,07874	2,099668	250	247	0,036773	0,251962	y ~ current_feature + S
0,023378	0,063611	0,034007	0,367516	250	247	0,713549	0,920814	y ~ current_feature + S
-0,00359	0,063757	-0,04751	-0,05625	249	246	0,955192	0,990156	y ~ current_feature + S
0,026151	0,068657	0,061498	0,380887	215	212	0,703668	0,972444	y ~ current_feature + S
0,24718	0,075896	-0,02429	3,256848	166	163	0,001371	0,144895	y ~ current_feature + S
-0,0546	0,06315	-0,07874	-0,86468	277	250,0103	0,388044	0,789252	y ~ current_feature + S
-0,08319	0,08151	0,034007	-1,02063	277	149,4721	0,309081	0,718742	y ~ current_feature + S
0,019379	0,067021	-0,04751	0,289147	276	222,5468	0,772738	0,945414	y ~ current_feature + S
0,070647	0,072432	0,061498	0,975349	239	189,6544	0,330629	0,893818	y ~ current_feature + S

-0,08102	0,075009	-0,02429	-1,08021	184	176,5706	0,281523	0,734108	y ~ current_feature + S
0,047485	0,061593	-0,07874	0,770946	266	263	0,441431	0,818694	y ~ current_feature + S
3,47E-04	0,061663	0,034007	0,00562	266	263	0,99552	0,997989	y ~ current_feature + S
-0,05768	0,06156	-0,04751	-0,93696	266	263	0,349641	0,795089	y ~ current_feature + S
-0,0455	0,066746	0,061498	-0,68165	227	224	0,496162	0,937665	y ~ current_feature + S
-0,08026	0,075565	-0,02429	-1,06207	177	174	0,289675	0,734108	y ~ current_feature + S
0,009713	0,052431	-0,07874	0,18525	386	363,7374	0,853136	0,983548	y ~ current_feature + S
-0,0374	0,068102	0,034007	-0,54915	386	215,3119	0,583469	0,871357	y ~ current_feature + S
0,06301	0,053652	-0,04751	1,174411	386	346,0166	0,241038	0,742928	y ~ current_feature + S
0,021198	0,058117	0,061498	0,364743	334	295,9392	0,715564	0,972573	y ~ current_feature + S
-0,06079	0,063223	-0,02429	-0,96146	257	249,2541	0,337253	0,785829	y ~ current_feature + S
-0,05518	0,055586	-0,07874	-0,99262	370	322,656	0,321639	0,725761	y ~ current_feature + S
0,101761	0,071603	0,034007	1,421184	370	193,0279	0,156877	0,60427	y ~ current_feature + S
0,050098	0,056609	-0,04751	0,884985	369	311,2752	0,376848	0,803665	y ~ current_feature + S
0,135231	0,062633	0,061498	2,159093	320	250,2506	0,031794	0,658491	y ~ current_feature + S
0,036176	0,064108	-0,02429	0,564305	246	243	0,573067	0,924682	y ~ current_feature + S
0,12623	0,051791	-0,07874	2,43732	415	366,8788	0,015271	0,174676	y ~ current_feature + S
0,191026	0,067212	0,034007	2,842162	415	213,289	0,004916	0,167881	y ~ current_feature + S
0,056813	0,053431	-0,04751	1,063303	414	349,1476	0,28838	0,747915	y ~ current_feature + S
0,060721	0,057824	0,061498	1,050087	361	297,9722	0,294529	0,893818	y ~ current_feature + S
0,100496	0,060482	-0,02429	1,661589	276	270,6107	0,097753	0,605474	y ~ current_feature + S
0,175653	0,076639	-0,07874	2,291934	168	165	0,023173	0,194866	y ~ current_feature + S
0,070901	0,077654	0,034007	0,913033	168	165	0,362557	0,7498	y ~ current_feature + S
0,253689	0,075303	-0,04751	3,368903	168	165	9,39E-04	0,132163	y ~ current_feature + S
-0,00494	0,084214	0,061498	-0,0586	144	141	0,953352	0,996179	y ~ current_feature + S
0,131466	0,094951	-0,02429	1,384559	112	109	0,169015	0,625356	y ~ current_feature + S
-0,02955	0,054209	-0,07874	-0,54505	343	340	0,586074	0,902886	y ~ current_feature + S
-0,0863	0,05403	0,034007	-1,59732	343	340	0,111124	0,540997	y ~ current_feature + S
0,011222	0,054309	-0,04751	0,20663	342	339	0,836423	0,979827	y ~ current_feature + S
0,024693	0,058106	0,061498	0,424972	299	296	0,671166	0,958606	y ~ current_feature + S
0,076723	0,06647	-0,02429	1,154247	228	225	0,249623	0,717451	y ~ current_feature + S
-0,1943	0,06204	-0,07874	-3,13189	253	250	0,001944	0,055318	y ~ current_feature + S
-0,17902	0,062224	0,034007	-2,877	253	250	0,004361	0,167881	y ~ current_feature + S
-0,10033	0,063053	-0,04751	-1,59121	252	249	0,112831	0,640171	y ~ current_feature + S
-0,03844	0,067991	0,061498	-0,56542	219	216	0,572375	0,937665	y ~ current_feature + S
-0,13205	0,076935	-0,02429	-1,7164	169	166	0,087954	0,605474	y ~ current_feature + S
-0,02399	0,053743	-0,07874	-0,44646	379	346,0236	0,655542	0,932255	y ~ current_feature + S
-0,05608	0,069506	0,034007	-0,80677	379	206,3397	0,420729	0,791109	y ~ current_feature + S
0,069484	0,054137	-0,04751	1,283491	378	339,5592	0,200195	0,723562	y ~ current_feature + S
0,077126	0,057274	0,061498	1,346615	328	303,0392	0,179111	0,855109	y ~ current_feature + S
-0,01308	0,064859	-0,02429	-0,20168	252	237,6755	0,840338	0,977734	y ~ current_feature + S
-0,0705	0,067252	-0,07874	-1,04831	223	220	0,295645	0,718687	y ~ current_feature + S
-0,11431	0,066978	0,034007	-1,70661	223	220	0,089305	0,487845	y ~ current_feature + S
-0,13509	0,066802	-0,04751	-2,02231	223	220	0,044355	0,63563	y ~ current_feature + S
0,026042	0,071587	0,061498	0,363785	198	195	0,716413	0,972573	y ~ current_feature + S
-0,05972	0,082897	-0,02429	-0,72038	148	145	0,472451	0,876224	y ~ current_feature + S
-0,36916	0,131432	-0,07874	-2,80875	53	50	0,007077	0,109106	y ~ current_feature + S
-0,0374	0,141322	0,034007	-0,26465	53	50	0,792369	0,945731	y ~ current_feature + S

0,008527	0,141416	-0,04751	0,060296	53	50	0,95216	0,990156	y ~ current_feature + S
0,12745	0,149526	0,061498	0,852357	47	44	0,398632	0,93433	y ~ current_feature + S
-0,37203	0,161583	-0,02429	-2,30238	36	33	0,02776	0,485321	y ~ current_feature + S
4,44E-04	0,105409	-0,07874	0,004215	93	90	0,996647	0,996647	y ~ current_feature + S
0,118842	0,104662	0,034007	1,135483	93	90	0,259188	0,71095	y ~ current_feature + S
0,102735	0,104852	-0,04751	0,979818	93	90	0,329803	0,779476	y ~ current_feature + S
-0,0367	0,111728	0,061498	-0,32849	83	80	0,743401	0,972573	y ~ current_feature + S
-0,04019	0,130084	-0,02429	-0,30894	62	59	0,758457	0,951727	y ~ current_feature + S
-0,2172	0,111903	-0,07874	-1,94094	81	76,09054	0,055969	0,312071	y ~ current_feature + S
0,019392	0,159132	0,034007	0,121859	81	39,4748	0,903629	0,970516	y ~ current_feature + S
-0,13108	0,117893	-0,04751	-1,11188	81	70,71222	0,269957	0,747915	y ~ current_feature + S
0,217818	0,121853	0,061498	1,787552	69	64,15347	0,078572	0,738262	y ~ current_feature + S
-0,18191	0,137692	-0,02429	-1,32115	54	51	0,192349	0,65347	y ~ current_feature + S
-0,0515	0,148873	-0,07874	-0,34591	48	45	0,731025	0,957449	y ~ current_feature + S
0,130298	0,1478	0,034007	0,88158	48	45	0,382689	0,759222	y ~ current_feature + S
0,333845	0,140519	-0,04751	2,375806	48	45	0,021829	0,557019	y ~ current_feature + S
0,013991	0,162206	0,061498	0,086255	41	38	0,931717	0,996179	y ~ current_feature + S
0,013795	0,185678	-0,02429	0,074294	32	29	0,941286	0,99444	y ~ current_feature + S
0,092975	0,053918	-0,07874	1,724395	367	341,0103	0,085543	0,400643	y ~ current_feature + S
0,016593	0,071188	0,034007	0,233083	367	197,274	0,815939	0,952501	y ~ current_feature + S
0,00621	0,055476	-0,04751	0,111949	366	324,9168	0,910933	0,985217	y ~ current_feature + S
-0,03716	0,058491	0,061498	-0,6353	318	291,8962	0,525728	0,937665	y ~ current_feature + S
0,100228	0,064335	-0,02429	1,557907	244	239,177	0,120578	0,615361	y ~ current_feature + S
0,012201	0,058617	-0,07874	0,208142	294	291	0,835264	0,982764	y ~ current_feature + S
-0,0248	0,058603	0,034007	-0,42316	294	291	0,672494	0,910826	y ~ current_feature + S
0,032136	0,058591	-0,04751	0,548479	294	291	0,583784	0,907563	y ~ current_feature + S
5,20E-04	0,065703	0,061498	0,007918	254	231,6502	0,99369	0,999005	y ~ current_feature + S
-0,11382	0,071514	-0,02429	-1,59156	196	193	0,113121	0,614731	y ~ current_feature + S
0,189294	0,078617	-0,07874	2,407811	159	156	0,017216	0,176938	y ~ current_feature + S
-0,0425	0,079992	0,034007	-0,53126	159	156	0,595994	0,878557	y ~ current_feature + S
0,108737	0,079589	-0,04751	1,366223	159	156	0,173835	0,700235	y ~ current_feature + S
0,089811	0,086361	0,061498	1,039955	136	133	0,300247	0,893818	y ~ current_feature + S
0,184857	0,096835	-0,02429	1,908996	106	103	0,059046	0,558147	y ~ current_feature + S
0,069031	0,089952	-0,07874	0,767418	126	123	0,444303	0,819912	y ~ current_feature + S
0,002776	0,090167	0,034007	0,030793	126	123	0,975485	0,989859	y ~ current_feature + S
0,048438	0,090061	-0,04751	0,537835	126	123	0,591663	0,91049	y ~ current_feature + S
-0,1441	0,097505	0,061498	-1,47788	106	103	0,14249	0,8197	y ~ current_feature + S
-0,04808	0,110983	-0,02429	-0,43321	84	81	0,666013	0,926342	y ~ current_feature + S
-0,06292	0,153998	-0,07874	-0,40856	45	42	0,684941	0,93638	y ~ current_feature + S
-0,17359	0,151961	0,034007	-1,14237	45	42	0,259773	0,71095	y ~ current_feature + S
0,055046	0,154069	-0,04751	0,357282	45	42	0,72267	0,93314	y ~ current_feature + S
0,225229	0,160175	0,061498	1,406145	40	37	0,168024	0,834482	y ~ current_feature + S
-0,07838	0,191858	-0,02429	-0,40854	30	27	0,686098	0,926342	y ~ current_feature + S
0,134596	0,143024	-0,07874	0,941073	51	48	0,35138	0,765193	y ~ current_feature + S
0,305217	0,13745	0,034007	2,220565	51	48	0,031135	0,347616	y ~ current_feature + S
-0,18548	0,141833	-0,04751	-1,30771	51	48	0,1972	0,723562	y ~ current_feature + S
-0,07104	0,157714	0,061498	-0,45045	43	40	0,654816	0,957207	y ~ current_feature + S
-0,10149	0,178678	-0,02429	-0,56799	34	31	0,574131	0,924682	y ~ current_feature + S

0,162828	0,127377	-0,07874	1,278324	63	60	0,206058	0,622938	y ~ current_feature + S
0,24295	0,125231	0,034007	1,940005	63	60	0,057083	0,420946	y ~ current_feature + S
-0,03368	0,129026	-0,04751	-0,26103	63	60	0,794962	0,959661	y ~ current_feature + S
0,104642	0,142073	0,061498	0,736535	52	49	0,464917	0,937665	y ~ current_feature + S
0,21167	0,1565	-0,02429	1,352525	42	39	0,183998	0,649807	y ~ current_feature + S
-0,11277	0,107145	-0,07874	-1,05245	89	86	0,29554	0,718687	y ~ current_feature + S
-0,14767	0,10665	0,034007	-1,38466	89	86	0,169738	0,61875	y ~ current_feature + S
-0,11855	0,107072	-0,04751	-1,10723	89	86	0,271281	0,747915	y ~ current_feature + S
-0,08928	0,113505	0,061498	-0,7866	80	77	0,433933	0,937665	y ~ current_feature + S
-0,136	0,132389	-0,02429	-1,02731	59	56	0,308691	0,761472	y ~ current_feature + S
0,090937	0,04997	-0,07874	1,819837	421	397,1683	0,069537	0,352445	y ~ current_feature + S
0,042221	0,064683	0,034007	0,652743	421	238,5886	0,51455	0,841996	y ~ current_feature + S
-0,01789	0,051026	-0,04751	-0,35053	420	383,9504	0,726135	0,93314	y ~ current_feature + S
0,019735	0,056546	0,061498	0,34901	367	312,6242	0,727317	0,972573	y ~ current_feature + S
0,080155	0,059891	-0,02429	1,338355	280	277	0,181878	0,647067	y ~ current_feature + S
0,191158	0,078841	-0,07874	2,424611	158	155	0,016475	0,174676	y ~ current_feature + S
0,089046	0,080003	0,034007	1,113033	158	155	0,267417	0,71095	y ~ current_feature + S
0,018462	0,080308	-0,04751	0,22989	158	155	0,81848	0,972405	y ~ current_feature + S
0,22963	0,084078	0,061498	2,731139	137	134	0,007162	0,417322	y ~ current_feature + S
-0,03595	0,098469	-0,02429	-0,36507	106	103	0,71581	0,936058	y ~ current_feature + S
-0,12957	0,116705	-0,07874	-1,11023	83	72,18831	0,270586	0,702573	y ~ current_feature + S
-0,10042	0,126714	0,034007	-0,79252	83	61,65206	0,431094	0,795535	y ~ current_feature + S
-0,00981	0,113556	-0,04751	-0,08641	82	77,54157	0,931361	0,987403	y ~ current_feature + S
-0,2176	0,116257	0,061498	-1,87174	74	70,48528	0,065392	0,715249	y ~ current_feature + S
-0,19129	0,150529	-0,02429	-1,27081	55	42,5177	0,210704	0,675518	y ~ current_feature + S
0,004167	0,084214	-0,07874	0,049483	144	141	0,960605	0,994607	y ~ current_feature + S
-0,08831	0,083886	0,034007	-1,05279	144	141	0,29424	0,718742	y ~ current_feature + S
-0,05528	0,084086	-0,04751	-0,65747	144	141	0,511952	0,868909	y ~ current_feature + S
-0,06585	0,090712	0,061498	-0,72597	124	121	0,469258	0,937665	y ~ current_feature + S
-0,15519	0,102439	-0,02429	-1,51497	96	93	0,133171	0,617591	y ~ current_feature + S
0,114045	0,049461	-0,07874	2,305741	421	403,4468	0,021632	0,19062	y ~ current_feature + S
0,15349	0,064961	0,034007	2,362819	421	231,3906	0,018965	0,311868	y ~ current_feature + S
0,033361	0,050582	-0,04751	0,659533	420	390,4124	0,509942	0,868909	y ~ current_feature + S
0,064569	0,05559	0,061498	1,161533	367	322,252	0,246285	0,893818	y ~ current_feature + S
0,078567	0,060092	-0,02429	1,30745	280	275,2184	0,192151	0,65347	y ~ current_feature + S
0,119861	0,052882	-0,07874	2,266567	374	352,4479	0,024022	0,197019	y ~ current_feature + S
0,040341	0,069744	0,034007	0,578416	374	205,2487	0,563617	0,861729	y ~ current_feature + S
0,094032	0,055777	-0,04751	1,685862	373	318,5915	0,092801	0,640171	y ~ current_feature + S
0,011687	0,059344	0,061498	0,196936	326	283,9133	0,844019	0,996179	y ~ current_feature + S
0,115044	0,064604	-0,02429	1,780754	248	236,425	0,076237	0,605474	y ~ current_feature + S
0,16884	0,072271	-0,07874	2,336211	189	186	0,020546	0,189455	y ~ current_feature + S
0,016349	0,073314	0,034007	0,222995	189	186	0,823784	0,952501	y ~ current_feature + S
-0,00169	0,073323	-0,04751	-0,02311	189	186	0,981584	0,992311	y ~ current_feature + S
-0,00233	0,078811	0,061498	-0,02961	164	161	0,976417	0,996179	y ~ current_feature + S
0,057628	0,090017	-0,02429	0,64019	126	123	0,52324	0,899808	y ~ current_feature + S
0,093405	0,075696	-0,07874	1,233945	176	173	0,218897	0,639583	y ~ current_feature + S
-0,02143	0,076011	0,034007	-0,28187	176	173	0,77838	0,942718	y ~ current_feature + S
-0,12981	0,075385	-0,04751	-1,7219	176	173	0,086875	0,640171	y ~ current_feature + S

-0,06619	0,082298	0,061498	-0,80424	150	147	0,42256	0,937665	y ~ current_feature + S
-0,08406	0,093327	-0,02429	-0,90066	117	114	0,36967	0,809337	y ~ current_feature + S
0,019203	0,082463	-0,07874	0,232867	150	147	0,816188	0,979871	y ~ current_feature + S
-0,04988	0,082376	0,034007	-0,60551	150	147	0,545773	0,852506	y ~ current_feature + S
0,007666	0,082476	-0,04751	0,092948	150	147	0,926071	0,985217	y ~ current_feature + S
0,031507	0,090122	0,061498	0,349604	126	123	0,727234	0,972573	y ~ current_feature + S
-0,11549	0,100855	-0,02429	-1,14511	100	97	0,254981	0,718089	y ~ current_feature + S
-0,10709	0,050475	-0,07874	-2,12153	391	388	0,034511	0,238671	y ~ current_feature + S
-0,12694	0,050357	0,034007	-2,52091	391	388	0,012105	0,235731	y ~ current_feature + S
0,061805	0,050736	-0,04751	1,218176	390	387	0,223899	0,742928	y ~ current_feature + S
-0,00877	0,054471	0,061498	-0,16107	340	337	0,872139	0,996179	y ~ current_feature + S
-0,10912	0,062006	-0,02429	-1,75987	260	257	0,079619	0,605474	y ~ current_feature + S
0,252212	0,122894	-0,07874	2,052263	65	62	0,044372	0,279123	y ~ current_feature + S
0,10806	0,162355	0,034007	0,665581	65	37,49448	0,509753	0,841996	y ~ current_feature + S
0,104911	0,126299	-0,04751	0,83065	65	62	0,409359	0,803665	y ~ current_feature + S
0,204638	0,169877	0,061498	1,204625	56	33,20111	0,236865	0,893818	y ~ current_feature + S
0,332484	0,149119	-0,02429	2,22966	43	40	0,03145	0,485321	y ~ current_feature + S
-0,16136	0,050478	-0,07874	-3,19664	418	382,2453	0,001506	0,048726	y ~ current_feature + S
-0,08783	0,066389	0,034007	-1,3229	418	225,1389	0,18721	0,638805	y ~ current_feature + S
-0,02916	0,052544	-0,04751	-0,55496	417	361,8939	0,579266	0,904733	y ~ current_feature + S
-0,0819	0,057138	0,061498	-1,43331	364	304,2436	0,152796	0,8197	y ~ current_feature + S
-0,14182	0,060008	-0,02429	-2,36338	278	272,1195	0,018812	0,428958	y ~ current_feature + S
-0,07043	0,101809	-0,07874	-0,69174	99	96	0,490768	0,852508	y ~ current_feature + S
-0,05659	0,101899	0,034007	-0,55532	99	96	0,579966	0,869291	y ~ current_feature + S
0,00318	0,102062	-0,04751	0,031161	99	96	0,975206	0,992311	y ~ current_feature + S
-0,03937	0,111025	0,061498	-0,35461	84	81	0,723802	0,972573	y ~ current_feature + S
-0,07664	0,125618	-0,02429	-0,6101	66	63	0,543988	0,911914	y ~ current_feature + S
0,287093	0,104516	-0,07874	2,746885	87	84	0,00736	0,110197	y ~ current_feature + S
0,173329	0,107457	0,034007	1,613005	87	84	0,110494	0,540997	y ~ current_feature + S
-0,04558	0,108996	-0,04751	-0,4182	87	84	0,676866	0,928258	y ~ current_feature + S
-0,23275	0,114615	0,061498	-2,0307	75	72	0,045979	0,700056	y ~ current_feature + S
0,078904	0,13442	-0,02429	0,587	58	55	0,559606	0,92024	y ~ current_feature + S
0,075613	0,099219	-0,07874	0,762083	104	101	0,447786	0,822238	y ~ current_feature + S
0,064418	0,099297	0,034007	0,648739	104	101	0,517979	0,841996	y ~ current_feature + S
-0,18345	0,098303	-0,04751	-1,86614	103	100	0,064952	0,640171	y ~ current_feature + S
-0,04969	0,1077	0,061498	-0,4614	89	86	0,645679	0,956496	y ~ current_feature + S
0,269332	0,118543	-0,02429	2,272022	69	66	0,02635	0,485321	y ~ current_feature + S
0,009053	0,077379	-0,07874	0,117	170	167	0,907001	0,987929	y ~ current_feature + S
0,089855	0,077069	0,034007	1,165894	170	167	0,24532	0,702074	y ~ current_feature + S
0,073856	0,077403	-0,04751	0,95418	169	166	0,34138	0,789051	y ~ current_feature + S
0,130583	0,08764	0,061498	1,489983	144	127,9742	0,138689	0,8197	y ~ current_feature + S
0,165805	0,095433	-0,02429	1,737405	113	106,7821	0,085199	0,605474	y ~ current_feature + S
-0,03783	0,057125	-0,07874	-0,66214	309	306	0,508378	0,863164	y ~ current_feature + S
-0,01847	0,057156	0,034007	-0,32314	309	306	0,746807	0,928802	y ~ current_feature + S
0,040502	0,057119	-0,04751	0,709077	309	306	0,478817	0,845643	y ~ current_feature + S
-0,02603	0,061877	0,061498	-0,42073	264	261	0,674302	0,958606	y ~ current_feature + S
0,058763	0,070238	-0,02429	0,836619	205	202	0,403795	0,830024	y ~ current_feature + S
0,011515	0,055358	-0,07874	0,208012	387	326,2689	0,835349	0,982764	y ~ current_feature + S

0,073273	0,072975	0,034007	1,004088	387	186,7743	0,316635	0,718742	y ~ current_feature + S
0,038196	0,056778	-0,04751	0,672727	386	309,7411	0,501623	0,868909	y ~ current_feature + S
0,00391	0,058865	0,061498	0,066421	336	288,5871	0,947088	0,996179	y ~ current_feature + S
0,034126	0,062668	-0,02429	0,544547	258	254,3337	0,586542	0,924682	y ~ current_feature + S
0,009534	0,080579	-0,07874	0,11832	157	154	0,905969	0,987929	y ~ current_feature + S
-0,04106	0,080514	0,034007	-0,51002	157	154	0,61077	0,889068	y ~ current_feature + S
0,067982	0,080658	-0,04751	0,842838	156	153	0,400635	0,803665	y ~ current_feature + S
-0,10671	0,085894	0,061498	-1,24241	137	134	0,216257	0,893818	y ~ current_feature + S
0,015855	0,099491	-0,02429	0,159356	104	101	0,873706	0,984468	y ~ current_feature + S
-0,15828	0,05231	-0,07874	-3,02582	415	356,2919	0,00266	0,065908	y ~ current_feature + S
-0,16757	0,068464	0,034007	-2,44755	415	207,3512	0,015215	0,268069	y ~ current_feature + S
-0,057	0,054653	-0,04751	-1,04293	414	333,7028	0,297734	0,753461	y ~ current_feature + S
-0,06859	0,059046	0,061498	-1,16166	361	285,4785	0,246343	0,893818	y ~ current_feature + S
-0,02547	0,060533	-0,02429	-0,42074	276	272,734	0,674275	0,926342	y ~ current_feature + S
-0,02114	0,088025	-0,07874	-0,24014	132	129	0,810604	0,979871	y ~ current_feature + S
-0,06687	0,087848	0,034007	-0,76116	132	129	0,44795	0,814299	y ~ current_feature + S
0,117611	0,087434	-0,04751	1,345145	132	129	0,180938	0,704707	y ~ current_feature + S
0,051075	0,099016	0,061498	0,515821	111	101,7313	0,607099	0,945257	y ~ current_feature + S
0,042188	0,110831	-0,02429	0,380656	88	81,26514	0,704452	0,93118	y ~ current_feature + S
0,033818	0,05879	-0,07874	0,57523	292	289	0,565583	0,898449	y ~ current_feature + S
0,031601	0,058794	0,034007	0,537488	292	289	0,591344	0,876338	y ~ current_feature + S
0,007155	0,058822	-0,04751	0,121637	292	289	0,903271	0,985217	y ~ current_feature + S
0,019947	0,063233	0,061498	0,315455	253	250	0,752679	0,972573	y ~ current_feature + S
-0,0346	0,072126	-0,02429	-0,47973	195	192	0,631967	0,924682	y ~ current_feature + S
0,02466	0,049437	-0,07874	0,498823	421	408,9081	0,618172	0,919937	y ~ current_feature + S
-0,01606	0,0628	0,034007	-0,25571	421	253,4937	0,798383	0,94832	y ~ current_feature + S
0,024673	0,050117	-0,04751	0,492304	420	397,8867	0,622776	0,925756	y ~ current_feature + S
-0,02692	0,054771	0,061498	-0,49143	367	333,1073	0,623446	0,950526	y ~ current_feature + S
0,007388	0,062081	-0,02429	0,119007	280	259,4523	0,905362	0,99444	y ~ current_feature + S
0,039015	0,077093	-0,07874	0,506082	171	168	0,613462	0,918951	y ~ current_feature + S
0,011574	0,077147	0,034007	0,150026	171	168	0,880924	0,96591	y ~ current_feature + S
-0,05979	0,077014	-0,04751	-0,77635	171	168	0,438637	0,823749	y ~ current_feature + S
0,199861	0,083108	0,061498	2,404848	142	139	0,017496	0,562908	y ~ current_feature + S
-0,10715	0,094369	-0,02429	-1,13541	114	111	0,25865	0,719554	y ~ current_feature + S
-0,04367	0,064895	-0,07874	-0,67287	240	237	0,501688	0,863164	y ~ current_feature + S
-0,02608	0,064935	0,034007	-0,40162	240	237	0,688322	0,916569	y ~ current_feature + S
0,113204	0,064539	-0,04751	1,754022	240	237	0,080719	0,640171	y ~ current_feature + S
0,107978	0,071937	0,061498	1,50101	209	190,9857	0,135004	0,8197	y ~ current_feature + S
-0,00413	0,080525	-0,02429	-0,05129	160	154,2164	0,959159	0,99444	y ~ current_feature + S
-0,00805	0,068197	-0,07874	-0,11806	218	215	0,906133	0,987929	y ~ current_feature + S
-0,08108	0,067975	0,034007	-1,19284	218	215	0,234246	0,685147	y ~ current_feature + S
-0,10416	0,067828	-0,04751	-1,53567	218	215	0,126091	0,654671	y ~ current_feature + S
-0,13307	0,080589	0,061498	-1,65116	187	151,2481	0,100781	0,8197	y ~ current_feature + S
-0,04432	0,084229	-0,02429	-0,52617	145	140,6773	0,5996	0,924682	y ~ current_feature + S
0,197028	0,146149	-0,07874	1,348127	48	45	0,184365	0,585536	y ~ current_feature + S
0,135418	0,147698	0,034007	0,916855	48	45	0,364106	0,7498	y ~ current_feature + S
0,290058	0,142662	-0,04751	2,033177	48	45	0,047958	0,640171	y ~ current_feature + S
0,058161	0,159857	0,061498	0,363831	42	39	0,71795	0,972573	y ~ current_feature + S

0,241747	0,180187	-0,02429	1,341642	32	29	0,190124	0,65347	y ~ current_feature + S
0,219543	0,111899	-0,07874	1,961984	81	76,01447	0,053425	0,30647	y ~ current_feature + S
0,176781	0,157753	0,034007	1,120619	81	38,92753	0,269316	0,71095	y ~ current_feature + S
-0,21869	0,131083	-0,04751	-1,66834	81	55,41461	0,10089	0,640171	y ~ current_feature + S
-0,13274	0,130623	0,061498	-1,01617	72	57,57574	0,3138	0,893818	y ~ current_feature + S
0,12483	0,144592	-0,02429	0,863327	54	47,08584	0,392334	0,821036	y ~ current_feature + S
0,272486	0,148464	-0,07874	1,835358	45	42	0,07354	0,362799	y ~ current_feature + S
-0,04257	0,154163	0,034007	-0,27611	45	42	0,783819	0,945731	y ~ current_feature + S
0,321282	0,146123	-0,04751	2,198714	45	42	0,033459	0,603897	y ~ current_feature + S
0,174825	0,157662	0,061498	1,108862	42	39	0,274281	0,893818	y ~ current_feature + S
0,09882	0,194004	-0,02429	0,50937	30	26,30968	0,614739	0,924682	y ~ current_feature + S
-0,44557	0,121827	-0,07874	-3,65742	57	54	5,79E-04	0,031617	y ~ current_feature + S
-0,25756	0,131492	0,034007	-1,95877	57	54	0,055312	0,420946	y ~ current_feature + S
-0,21628	0,132862	-0,04751	-1,62786	57	54	0,109376	0,640171	y ~ current_feature + S
-0,03069	0,145796	0,061498	-0,21048	50	47	0,8342	0,996179	y ~ current_feature + S
-0,21082	0,165232	-0,02429	-1,27588	38	35	0,2104	0,675518	y ~ current_feature + S
-0,06756	0,067574	-0,07874	-0,99976	221	218	0,318534	0,723052	y ~ current_feature + S
-0,10405	0,067361	0,034007	-1,54467	221	218	0,123876	0,572145	y ~ current_feature + S
0,0358	0,067685	-0,04751	0,528914	221	218	0,597403	0,911502	y ~ current_feature + S
0,082185	0,072686	0,061498	1,130685	191	188	0,259628	0,893818	y ~ current_feature + S
-0,06333	0,083166	-0,02429	-0,76148	147	144	0,447616	0,858965	y ~ current_feature + S
-0,03374	0,056835	-0,07874	-0,59356	343	309,2236	0,55324	0,889995	y ~ current_feature + S
0,048127	0,075463	0,034007	0,637756	343	175,1952	0,524465	0,84739	y ~ current_feature + S
-0,00108	0,057637	-0,04751	-0,01875	342	301,0169	0,985055	0,993835	y ~ current_feature + S
-0,05668	0,059922	0,061498	-0,94593	295	277,6028	0,345008	0,907332	y ~ current_feature + S
0,068342	0,066781	-0,02429	1,023368	228	223,1819	0,307242	0,761472	y ~ current_feature + S
0,013646	0,062864	-0,07874	0,21708	256	253	0,828321	0,981192	y ~ current_feature + S
-0,09313	0,062596	0,034007	-1,48787	256	253	0,138031	0,58036	y ~ current_feature + S
-0,07493	0,062693	-0,04751	-1,19526	256	253	0,233104	0,742928	y ~ current_feature + S
-0,09084	0,067917	0,061498	-1,33756	218	215	0,182453	0,857147	y ~ current_feature + S
-0,12929	0,076733	-0,02429	-1,68488	170	167	0,09388	0,605474	y ~ current_feature + S
0,177053	0,058437	-0,07874	3,029797	325	283,6538	0,002673	0,065908	y ~ current_feature + S
0,183088	0,078358	0,034007	2,336559	325	157,4071	0,020722	0,31947	y ~ current_feature + S
0,167799	0,060227	-0,04751	2,786089	325	267,9223	0,005716	0,422981	y ~ current_feature + S
-0,00723	0,06451	0,061498	-0,11201	277	240,2799	0,91091	0,996179	y ~ current_feature + S
0,265591	0,066118	-0,02429	4,016928	216	212,6149	8,18E-05	0,020175	y ~ current_feature + S
0,36762	0,093942	-0,07874	3,91328	101	98	1,68E-04	0,015552	y ~ current_feature + S
0,152483	0,099834	0,034007	1,527361	101	98	0,129892	0,572145	y ~ current_feature + S
0,123538	0,100757	-0,04751	1,226097	100	97	0,22313	0,742928	y ~ current_feature + S
-0,01074	0,112502	0,061498	-0,09542	82	79	0,924221	0,996179	y ~ current_feature + S
0,263132	0,120595	-0,02429	2,181946	67	64	0,032792	0,485321	y ~ current_feature + S
-0,07605	0,067225	-0,07874	-1,13128	223	220	0,259168	0,689873	y ~ current_feature + S
-0,10114	0,067074	0,034007	-1,50789	223	220	0,133017	0,572281	y ~ current_feature + S
-0,03591	0,067377	-0,04751	-0,53293	223	220	0,594617	0,91049	y ~ current_feature + S
-0,00215	0,072739	0,061498	-0,02955	192	189	0,976458	0,996179	y ~ current_feature + S
-0,13815	0,082249	-0,02429	-1,67961	148	145	0,095187	0,605474	y ~ current_feature + S
-0,11777	0,079002	-0,07874	-1,49066	161	158	0,138045	0,498495	y ~ current_feature + S
-0,04267	0,079483	0,034007	-0,53686	161	158	0,59212	0,876338	y ~ current_feature + S

-0,07367	0,07934	-0,04751	-0,9285	161	158	0,354567	0,795089	y ~ current_feature + S
0,133543	0,085613	0,061498	1,559847	137	134	0,121155	0,8197	y ~ current_feature + S
-0,17249	0,096588	-0,02429	-1,78579	107	104	0,077047	0,605474	y ~ current_feature + S
0,084763	0,119953	-0,07874	0,706639	72	69	0,48217	0,845512	y ~ current_feature + S
0,003392	0,120385	0,034007	0,028179	72	69	0,977601	0,989859	y ~ current_feature + S
-0,02843	0,120337	-0,04751	-0,23627	72	69	0,813924	0,972405	y ~ current_feature + S
-0,03563	0,134754	0,061498	-0,26444	58	55	0,792428	0,983887	y ~ current_feature + S
-0,01052	0,149063	-0,02429	-0,0706	48	45	0,944032	0,99444	y ~ current_feature + S
0,311853	0,116953	-0,07874	2,666485	69	66	0,00963	0,129567	y ~ current_feature + S
0,043585	0,122975	0,034007	0,35442	69	66	0,724154	0,920814	y ~ current_feature + S
0,059376	0,122874	-0,04751	0,483224	69	66	0,630536	0,925756	y ~ current_feature + S
0,138987	0,134762	0,061498	1,031351	57	54	0,306974	0,893818	y ~ current_feature + S
0,051358	0,152297	-0,02429	0,337223	46	43	0,737589	0,944216	y ~ current_feature + S
0,032277	0,139955	-0,07874	0,230621	54	51	0,818532	0,979871	y ~ current_feature + S
0,047314	0,139871	0,034007	0,338267	54	51	0,73655	0,923809	y ~ current_feature + S
0,008649	0,140023	-0,04751	0,06177	54	51	0,950987	0,990156	y ~ current_feature + S
-0,04699	0,15233	0,061498	-0,3085	46	43	0,759195	0,972573	y ~ current_feature + S
0,03367	0,173979	-0,02429	0,193532	36	33	0,847729	0,977734	y ~ current_feature + S
0,081727	0,054814	-0,07874	1,490969	385	330,5978	0,136923	0,498495	y ~ current_feature + S
0,043574	0,072484	0,034007	0,60115	385	189,9723	0,548456	0,854294	y ~ current_feature + S
0,00766	0,056547	-0,04751	0,135456	384	312,7196	0,892338	0,985217	y ~ current_feature + S
-0,00228	0,059418	0,061498	-0,03836	333	283,2437	0,969429	0,996179	y ~ current_feature + S
0,107321	0,062796	-0,02429	1,709027	256	250,668	0,088684	0,605474	y ~ current_feature + S
-0,09194	0,073015	-0,07874	-1,25917	222	185,9907	0,209547	0,630344	y ~ current_feature + S
-0,03785	0,095317	0,034007	-0,39713	222	109,9107	0,692044	0,916569	y ~ current_feature + S
-0,00232	0,07646	-0,04751	-0,0304	222	171,0533	0,97578	0,992311	y ~ current_feature + S
0,069906	0,081661	0,061498	0,856051	192	149,2266	0,393342	0,93433	y ~ current_feature + S
0,010025	0,083045	-0,02429	0,120719	148	144,9878	0,904081	0,99444	y ~ current_feature + S
0,083334	0,082192	-0,07874	1,013898	150	147	0,312298	0,718687	y ~ current_feature + S
0,106753	0,082007	0,034007	1,301745	150	147	0,19504	0,6438	y ~ current_feature + S
-0,08121	0,082206	-0,04751	-0,98789	150	147	0,32483	0,775402	y ~ current_feature + S
-2,20E-04	0,088045	0,061498	-0,0025	132	129	0,998006	0,999005	y ~ current_feature + S
-0,06662	0,101309	-0,02429	-0,65755	100	97	0,512383	0,899808	y ~ current_feature + S
0,052293	0,056446	-0,07874	0,926421	316	313	0,354941	0,765762	y ~ current_feature + S
-0,09937	0,056244	0,034007	-1,76673	316	313	0,078249	0,470764	y ~ current_feature + S
0,038136	0,056573	-0,04751	0,674099	315	312	0,500748	0,868909	y ~ current_feature + S
0,006272	0,061084	0,061498	0,102683	271	268	0,918291	0,996179	y ~ current_feature + S
-0,04123	0,069446	-0,02429	-0,5937	210	207	0,553364	0,918138	y ~ current_feature + S
-0,1316	0,061478	-0,07874	-2,14063	263	260	0,033234	0,236472	y ~ current_feature + S
-0,19211	0,060862	0,034007	-3,15652	263	260	0,001785	0,132069	y ~ current_feature + S
0,149978	0,061316	-0,04751	2,445991	263	260	0,015109	0,526293	y ~ current_feature + S
-0,00495	0,066518	0,061498	-0,07439	229	226	0,940765	0,996179	y ~ current_feature + S
-0,17574	0,075063	-0,02429	-2,34124	175	172	0,020366	0,443263	y ~ current_feature + S
0,280645	0,084836	-0,07874	3,308083	131	128	0,00122	0,047511	y ~ current_feature + S
0,142703	0,087484	0,034007	1,631199	131	128	0,105306	0,526532	y ~ current_feature + S
0,046045	0,088295	-0,04751	0,521497	131	128	0,602922	0,91263	y ~ current_feature + S
-0,08718	0,094983	0,061498	-0,91786	113	110	0,3607	0,920619	y ~ current_feature + S
0,21893	0,106462	-0,02429	2,056419	87	84	0,042846	0,525585	y ~ current_feature + S

-0,06038	0,06147	-0,07874	-0,98225	295	263,6904	0,326876	0,726391	y ~ current_feature + S
-0,09855	0,07973	0,034007	-1,23608	295	155,7829	0,218288	0,664036	y ~ current_feature + S
-0,0015	0,063512	-0,04751	-0,02367	294	247,906	0,981133	0,992311	y ~ current_feature + S
0,028544	0,065398	0,061498	0,43646	249	233,6215	0,662906	0,957207	y ~ current_feature + S
0,044493	0,07191	-0,02429	0,618728	196	193	0,536825	0,906112	y ~ current_feature + S
0,395577	0,115712	-0,07874	3,418648	66	63	0,001108	0,045561	y ~ current_feature + S
0,189	0,123717	0,034007	1,527675	66	63	0,131599	0,572281	y ~ current_feature + S
0,117528	0,125115	-0,04751	0,939359	66	63	0,351134	0,795089	y ~ current_feature + S
-0,0082	0,13867	0,061498	-0,0591	55	52	0,953098	0,996179	y ~ current_feature + S
0,101071	0,155374	-0,02429	0,650501	44	41	0,518998	0,899808	y ~ current_feature + S
0,019597	0,077946	-0,07874	0,251417	203	164,5307	0,801805	0,979102	y ~ current_feature + S
0,030972	0,070677	0,034007	0,438215	203	200	0,661704	0,900251	y ~ current_feature + S
0,072344	0,079039	-0,04751	0,915296	203	159,2341	0,36142	0,798361	y ~ current_feature + S
-0,05461	0,081111	0,061498	-0,6733	176	151,5449	0,501781	0,937665	y ~ current_feature + S
-0,04985	0,087842	-0,02429	-0,56745	135	129,2762	0,571394	0,924682	y ~ current_feature + S
0,076726	0,133237	-0,07874	0,575863	59	56	0,567015	0,898449	y ~ current_feature + S
-0,00575	0,133628	0,034007	-0,04302	59	56	0,965839	0,989859	y ~ current_feature + S
0,102832	0,132922	-0,04751	0,773623	59	56	0,442408	0,824639	y ~ current_feature + S
-0,06802	0,145527	0,061498	-0,46737	50	47	0,642393	0,956496	y ~ current_feature + S
0,009684	0,166659	-0,02429	0,058105	39	36	0,953986	0,99444	y ~ current_feature + S
0,155135	0,0754	-0,07874	2,057502	181	171,6655	0,04115	0,274334	y ~ current_feature + S
0,055772	0,096122	0,034007	0,580215	181	107,8946	0,562979	0,861729	y ~ current_feature + S
0,07073	0,078678	-0,04751	0,898984	180	160,7366	0,370006	0,802018	y ~ current_feature + S
-0,07074	0,081056	0,061498	-0,8727	155	151,4424	0,384209	0,93433	y ~ current_feature + S
0,121529	0,094178	-0,02429	1,290415	120	111,0808	0,199586	0,660578	y ~ current_feature + S
0,155631	0,07554	-0,07874	2,060244	174	171	0,04089	0,274334	y ~ current_feature + S
0,124411	0,075878	0,034007	1,639619	174	171	0,102923	0,525261	y ~ current_feature + S
0,075692	0,076253	-0,04751	0,992654	174	171	0,322281	0,774585	y ~ current_feature + S
-0,04427	0,089839	0,061498	-0,4928	149	123,6574	0,623026	0,950526	y ~ current_feature + S
0,225315	0,093501	-0,02429	2,409761	116	108,5781	0,017644	0,428958	y ~ current_feature + S
0,157043	0,050551	-0,07874	3,106641	418	381,683	0,002034	0,055741	y ~ current_feature + S
-0,03777	0,06744	0,034007	-0,55999	418	219,5579	0,576058	0,869291	y ~ current_feature + S
0,04131	0,05242	-0,04751	0,788062	417	363,2968	0,431175	0,820106	y ~ current_feature + S
-0,04804	0,055718	0,061498	-0,86219	364	321,3747	0,389226	0,93433	y ~ current_feature + S
-0,03963	0,061235	-0,02429	-0,64715	278	266,2707	0,518094	0,899808	y ~ current_feature + S
-0,03079	0,08244	-0,07874	-0,37343	150	147	0,709368	0,9454	y ~ current_feature + S
-0,03287	0,082434	0,034007	-0,39872	150	147	0,690675	0,916569	y ~ current_feature + S
-0,04395	0,082399	-0,04751	-0,53339	150	147	0,594569	0,91049	y ~ current_feature + S
0,0309	0,090493	0,061498	0,341465	125	122	0,733341	0,972573	y ~ current_feature + S
-0,06595	0,101314	-0,02429	-0,65093	100	97	0,516632	0,899808	y ~ current_feature + S
0,06849	0,049151	-0,07874	1,393464	415	412	0,164231	0,535378	y ~ current_feature + S
0,077241	0,049119	0,034007	1,572512	415	412	0,1166	0,552558	y ~ current_feature + S
0,045135	0,049276	-0,04751	0,91597	414	411	0,36022	0,798092	y ~ current_feature + S
0,087904	0,058238	0,061498	1,509396	361	292,5666	0,132277	0,8197	y ~ current_feature + S
0,115977	0,060567	-0,02429	1,914858	276	268,9353	0,056571	0,558147	y ~ current_feature + S
0,148459	0,077754	-0,07874	1,909358	187	161,7635	0,057986	0,318142	y ~ current_feature + S
-0,06472	0,099942	0,034007	-0,64757	187	99,69689	0,518753	0,841996	y ~ current_feature + S
0,089204	0,083924	-0,04751	1,06291	187	140,8494	0,289642	0,747915	y ~ current_feature + S

0,175935	0,084547	0,061498	2,080925	162	135,566	0,039323	0,658491	y ~ current_feature + S
0,035443	0,090852	-0,02429	0,390116	124	121	0,697137	0,929516	y ~ current_feature + S
-0,10318	0,06706	-0,07874	-1,53864	223	220	0,12533	0,478063	y ~ current_feature + S
-0,14328	0,09654	0,034007	-1,48412	223	105,0938	0,140771	0,588533	y ~ current_feature + S
-0,11242	0,074023	-0,04751	-1,51871	222	180,1928	0,130588	0,654671	y ~ current_feature + S
0,019002	0,073151	0,061498	0,259767	190	186,8113	0,79533	0,984075	y ~ current_feature + S
-0,20629	0,081456	-0,02429	-2,53255	148	144,2993	0,012393	0,366843	y ~ current_feature + S
0,101476	0,051011	-0,07874	1,989314	421	380,3491	0,047382	0,289576	y ~ current_feature + S
0,158052	0,066468	0,034007	2,377848	421	220,69	0,018267	0,307222	y ~ current_feature + S
0,067772	0,051901	-0,04751	1,305786	420	369,5291	0,192438	0,717449	y ~ current_feature + S
-0,00608	0,056377	0,061498	-0,10789	367	314,6106	0,914154	0,996179	y ~ current_feature + S
0,036417	0,060044	-0,02429	0,606494	280	277	0,544684	0,911914	y ~ current_feature + S
0,200336	0,097487	-0,07874	2,055012	104	101	0,042459	0,275611	y ~ current_feature + S
-0,0977	0,099028	0,034007	-0,98657	104	101	0,326212	0,728793	y ~ current_feature + S
0,132288	0,098629	-0,04751	1,341261	104	101	0,182844	0,704811	y ~ current_feature + S
-0,01319	0,107823	0,061498	-0,12231	89	86	0,902942	0,996179	y ~ current_feature + S
-0,03394	0,123021	-0,02429	-0,27592	69	66	0,783468	0,964545	y ~ current_feature + S
-0,33314	0,128309	-0,07874	-2,59638	57	54	0,012111	0,144555	y ~ current_feature + S
-0,18446	0,133748	0,034007	-1,37914	57	54	0,173538	0,619418	y ~ current_feature + S
-0,27806	0,130716	-0,04751	-2,12719	57	54	0,037988	0,611115	y ~ current_feature + S
0,007713	0,154299	0,061498	0,049987	45	42	0,96037	0,996179	y ~ current_feature + S
0,036159	0,16892	-0,02429	0,214058	38	35	0,831744	0,976969	y ~ current_feature + S
-0,08811	0,056214	-0,07874	-1,56742	317	314	0,118024	0,474142	y ~ current_feature + S
-0,07458	0,056276	0,034007	-1,32532	317	314	0,186027	0,638805	y ~ current_feature + S
-0,07103	0,056291	-0,04751	-1,2619	317	314	0,207923	0,725768	y ~ current_feature + S
-0,05725	0,066339	0,061498	-0,86292	272	226,4809	0,389093	0,93433	y ~ current_feature + S
-0,07725	0,069257	-0,02429	-1,11537	211	207,2387	0,265984	0,723659	y ~ current_feature + S
0,005866	0,15108	-0,07874	0,038825	47	43,80964	0,969206	0,994607	y ~ current_feature + S
0,249239	0,197012	0,034007	1,265097	47	24,16372	0,217901	0,664036	y ~ current_feature + S
-0,03514	0,162965	-0,04751	-0,21563	46	37,60743	0,830441	0,975438	y ~ current_feature + S
0,273003	0,177892	0,061498	1,534657	44	29,24494	0,135617	0,8197	y ~ current_feature + S
0,20889	0,184813	-0,02429	1,130276	31	28	0,267949	0,723659	y ~ current_feature + S
-0,11198	0,128287	-0,07874	-0,87288	63	60	0,386207	0,789252	y ~ current_feature + S
-0,20779	0,126282	0,034007	-1,64546	63	60	0,105104	0,526532	y ~ current_feature + S
-0,1856	0,126857	-0,04751	-1,46303	63	60	0,148678	0,661823	y ~ current_feature + S
-0,11478	0,136453	0,061498	-0,84117	56	53	0,404035	0,93433	y ~ current_feature + S
0,064267	0,159797	-0,02429	0,402177	42	39	0,689749	0,926342	y ~ current_feature + S
-0,01405	0,137347	-0,07874	-0,10229	56	53	0,918912	0,989804	y ~ current_feature + S
0,367897	0,127727	0,034007	2,880343	56	53	0,005721	0,170187	y ~ current_feature + S
0,488785	0,120981	-0,04751	4,040184	55	52	1,77E-04	0,067562	y ~ current_feature + S
-0,1175	0,146421	0,061498	-0,80252	49	46	0,426382	0,937665	y ~ current_feature + S
0,033408	0,172467	-0,02429	0,193708	37	33,58171	0,847572	0,977734	y ~ current_feature + S
-0,07129	0,049503	-0,07874	-1,44009	409	406	0,150612	0,513608	y ~ current_feature + S
-0,10439	0,049358	0,034007	-2,11492	409	406	0,035045	0,375847	y ~ current_feature + S
0,002571	0,04969	-0,04751	0,051735	408	405	0,958766	0,990156	y ~ current_feature + S
-0,0013	0,0606	0,061498	-0,02138	356	272,3059	0,982959	0,997791	y ~ current_feature + S
-0,02609	0,06095	-0,02429	-0,4281	272	269	0,668917	0,926342	y ~ current_feature + S
0,143452	0,049683	-0,07874	2,887364	421	396,7904	0,004097	0,073642	y ~ current_feature + S

0,168382	0,063963	0,034007	2,632507	421	237,4967	0,009032	0,215592	y ~ current_feature + S
0,060533	0,050679	-0,04751	1,194454	420	387,9304	0,23303	0,742928	y ~ current_feature + S
-0,05354	0,054864	0,061498	-0,97578	367	331,2657	0,329886	0,893818	y ~ current_feature + S
0,078447	0,060049	-0,02429	1,306391	280	275,618	0,192509	0,65347	y ~ current_feature + S
-0,10387	0,079377	-0,07874	-1,30856	160	157	0,192595	0,598826	y ~ current_feature + S
-0,07292	0,079596	0,034007	-0,91612	160	157	0,361007	0,7498	y ~ current_feature + S
-0,15183	0,078883	-0,04751	-1,9247	160	157	0,056075	0,640171	y ~ current_feature + S
-0,07143	0,086166	0,061498	-0,82895	137	134	0,408604	0,934833	y ~ current_feature + S
-0,09913	0,098048	-0,02429	-1,01104	106	103	0,314367	0,765236	y ~ current_feature + S
-0,00744	0,140024	-0,07874	-0,0531	54	51	0,957858	0,994607	y ~ current_feature + S
0,269583	0,134844	0,034007	1,999223	54	51	0,050926	0,420946	y ~ current_feature + S
0,050601	0,139849	-0,04751	0,361829	54	51	0,718975	0,93314	y ~ current_feature + S
0,112513	0,148125	0,061498	0,759585	48	45	0,451464	0,937665	y ~ current_feature + S
0,493781	0,151376	-0,02429	3,261956	36	33	0,002574	0,18344	y ~ current_feature + S
-0,20124	0,092974	-0,07874	-2,16451	114	111	0,032568	0,233981	y ~ current_feature + S
-0,17362	0,093474	0,034007	-1,85744	114	111	0,065899	0,439525	y ~ current_feature + S
-0,11002	0,09434	-0,04751	-1,16626	114	111	0,246009	0,742928	y ~ current_feature + S
-0,13174	0,103348	0,061498	-1,27475	95	92	0,205609	0,893818	y ~ current_feature + S
-0,1875	0,114965	-0,02429	-1,63095	76	73	0,107208	0,605474	y ~ current_feature + S
0,003574	0,055222	-0,07874	0,064727	351	327,9198	0,948431	0,994607	y ~ current_feature + S
0,052104	0,072355	0,034007	0,720114	351	190,4954	0,472337	0,831402	y ~ current_feature + S
-0,00148	0,056188	-0,04751	-0,0263	350	316,7468	0,979036	0,992311	y ~ current_feature + S
0,009462	0,059467	0,061498	0,159107	310	282,7584	0,873699	0,996179	y ~ current_feature + S
-0,0046	0,06616	-0,02429	-0,06949	233	228,4541	0,944664	0,99444	y ~ current_feature + S
0,029225	0,051764	-0,07874	0,564583	416	372,8847	0,572697	0,899533	y ~ current_feature + S
-0,00712	0,066982	0,034007	-0,1063	416	222,8756	0,915444	0,9732	y ~ current_feature + S
0,065474	0,052287	-0,04751	1,252189	415	364,2024	0,211305	0,733915	y ~ current_feature + S
-0,02168	0,05605	0,061498	-0,38674	362	318,1562	0,699204	0,972444	y ~ current_feature + S
0,011865	0,061228	-0,02429	0,193787	276	266,706	0,84649	0,977734	y ~ current_feature + S
0,120085	0,051954	-0,07874	2,311357	397	365,1299	0,021369	0,19062	y ~ current_feature + S
0,146844	0,06631	0,034007	2,214514	397	222,5257	0,027808	0,347616	y ~ current_feature + S
0,046161	0,053003	-0,04751	0,870914	396	355,195	0,384389	0,803665	y ~ current_feature + S
-0,03259	0,056895	0,061498	-0,57287	345	308,598	0,567151	0,937665	y ~ current_feature + S
0,088377	0,062381	-0,02429	1,416733	264	254,9731	0,157782	0,623497	y ~ current_feature + S
0,390503	0,104238	-0,07874	3,746278	81	78	3,42E-04	0,023018	y ~ current_feature + S
0,240091	0,109916	0,034007	2,184314	81	78	0,031943	0,347616	y ~ current_feature + S
0,046298	0,113106	-0,04751	0,409334	81	78	0,683417	0,928814	y ~ current_feature + S
0,001741	0,120386	0,061498	0,01446	72	69	0,988505	0,997945	y ~ current_feature + S
-0,07448	0,139639	-0,02429	-0,53339	54	51	0,596078	0,924682	y ~ current_feature + S
-0,01256	0,05717	-0,07874	-0,21972	323	305,9105	0,82624	0,981192	y ~ current_feature + S
0,050766	0,071041	0,034007	0,714593	323	197,6321	0,475704	0,831402	y ~ current_feature + S
0,016917	0,056741	-0,04751	0,298146	322	310,5128	0,765791	0,941338	y ~ current_feature + S
-0,09207	0,060592	0,061498	-1,51942	283	270,0634	0,129826	0,8197	y ~ current_feature + S
-0,04658	0,072026	-0,02429	-0,64677	215	192,3416	0,518549	0,899808	y ~ current_feature + S
0,024251	0,065214	-0,07874	0,371866	238	235	0,710327	0,9454	y ~ current_feature + S
-0,00351	0,065232	0,034007	-0,05376	238	235	0,957175	0,989859	y ~ current_feature + S
-0,02574	0,065211	-0,04751	-0,39474	238	235	0,693395	0,930326	y ~ current_feature + S
-0,06203	0,069709	0,061498	-0,88986	208	205	0,374586	0,932536	y ~ current_feature + S

-0,02683	0,080035	-0,02429	-0,33517	159	156	0,737949	0,944216	y ~ current_feature + S
0,468594	0,07493	-0,07874	6,253737	142	139	4,63E-09	3,42E-06	y ~ current_feature + S
0,081515	0,084537	0,034007	0,964259	142	139	0,33659	0,73474	y ~ current_feature + S
0,03018	0,08478	-0,04751	0,355978	142	139	0,722397	0,93314	y ~ current_feature + S
-0,12687	0,089077	0,061498	-1,42428	127	124	0,156877	0,8197	y ~ current_feature + S
0,211048	0,101909	-0,02429	2,070946	95	92	0,041165	0,525585	y ~ current_feature + S
0,001741	0,09759	-0,07874	0,017839	108	105	0,985801	0,996318	y ~ current_feature + S
0,04326	0,145022	0,034007	0,298297	108	47,45907	0,766778	0,93943	y ~ current_feature + S
0,042298	0,113624	-0,04751	0,372266	108	77,3185	0,710713	0,93314	y ~ current_feature + S
-0,10928	0,117467	0,061498	-0,93026	93	71,60574	0,35536	0,920619	y ~ current_feature + S
-0,04341	0,120272	-0,02429	-0,36095	72	69	0,719243	0,937042	y ~ current_feature + S
0,309994	0,072283	-0,07874	4,288594	176	173	2,98E-05	0,005519	y ~ current_feature + S
0,131337	0,07537	0,034007	1,742562	176	173	0,083187	0,473527	y ~ current_feature + S
0,149925	0,075169	-0,04751	1,994498	176	173	0,047668	0,640171	y ~ current_feature + S
0,01441	0,083616	0,061498	0,172336	146	143	0,863417	0,996179	y ~ current_feature + S
0,082416	0,093341	-0,02429	0,882951	117	113,9963	0,379121	0,815551	y ~ current_feature + S
0,173603	0,05809	-0,07874	2,988518	331	287,4152	0,003045	0,066821	y ~ current_feature + S
0,126773	0,076706	0,034007	1,652712	331	167,2271	0,100266	0,518857	y ~ current_feature + S
0,026613	0,060212	-0,04751	0,441987	331	275,631	0,658845	0,925756	y ~ current_feature + S
0,082772	0,06216	0,061498	1,331602	282	257,0365	0,184171	0,857147	y ~ current_feature + S
0,097767	0,067559	-0,02429	1,447133	220	217	0,149303	0,622449	y ~ current_feature + S
0,058938	0,055822	-0,07874	1,055817	390	319,7989	0,291849	0,718687	y ~ current_feature + S
0,036575	0,072425	0,034007	0,505006	390	190,3897	0,614138	0,889359	y ~ current_feature + S
-0,01712	0,056398	-0,04751	-0,30354	389	314,3013	0,761675	0,9394	y ~ current_feature + S
-0,014	0,060508	0,061498	-0,23145	338	273,0816	0,817138	0,996179	y ~ current_feature + S
-0,05544	0,062718	-0,02429	-0,88395	259	253,4416	0,37756	0,815551	y ~ current_feature + S
0,083133	0,051393	-0,07874	1,617599	379	376	0,106588	0,449749	y ~ current_feature + S
0,095999	0,051333	0,034007	1,870124	379	376	0,062243	0,437643	y ~ current_feature + S
0,043974	0,05159	-0,04751	0,852368	378	375	0,394554	0,803665	y ~ current_feature + S
-0,03731	0,062375	0,061498	-0,59821	328	256,6674	0,550227	0,937665	y ~ current_feature + S
0,107655	0,063049	-0,02429	1,707473	252	248,6449	0,088982	0,605474	y ~ current_feature + S
-0,07765	0,050702	-0,07874	-1,53159	409	386,6566	0,12644	0,479825	y ~ current_feature + S
-0,05758	0,065713	0,034007	-0,87624	409	230,8137	0,381811	0,759222	y ~ current_feature + S
-0,09418	0,051696	-0,04751	-1,82174	408	370,868	0,069299	0,640171	y ~ current_feature + S
-0,06317	0,055957	0,061498	-1,1289	356	318,0943	0,259791	0,893818	y ~ current_feature + S
-0,01871	0,061272	-0,02429	-0,30536	272	266,2748	0,760332	0,951727	y ~ current_feature + S
0,01743	0,055969	-0,07874	0,311416	341	319,13	0,755687	0,964335	y ~ current_feature + S
0,032122	0,073318	0,034007	0,438123	341	185,837	0,661806	0,900251	y ~ current_feature + S
0,096228	0,057902	-0,04751	1,661917	340	295,5132	0,09759	0,640171	y ~ current_feature + S
0,034778	0,060469	0,061498	0,575134	295	273,1586	0,565674	0,937665	y ~ current_feature + S
0,119735	0,067413	-0,02429	1,776137	227	216,8905	0,077112	0,605474	y ~ current_feature + S
-0,05295	0,060401	-0,07874	-0,87657	326	273,3363	0,38149	0,789252	y ~ current_feature + S
-0,03494	0,079312	0,034007	-0,44056	326	158,778	0,660133	0,900251	y ~ current_feature + S
-0,05142	0,061471	-0,04751	-0,83642	326	263,9444	0,403673	0,803665	y ~ current_feature + S
-0,08731	0,062007	0,061498	-1,40813	279	258,1057	0,160295	0,8197	y ~ current_feature + S
0,005887	0,068834	-0,02429	0,085521	217	211,0439	0,931928	0,99444	y ~ current_feature + S
-5,82E-04	0,077152	-0,07874	-0,00754	171	168	0,993995	0,996647	y ~ current_feature + S
-0,0094	0,077148	0,034007	-0,1219	171	168	0,903124	0,970516	y ~ current_feature + S

0,021939	0,077133	-0,04751	0,284429	171	168	0,776432	0,948119	y ~ current_feature + S
-0,07122	0,083705	0,061498	-0,85082	145	142	0,396303	0,93433	y ~ current_feature + S
-0,03523	0,094857	-0,02429	-0,3714	114	111	0,71105	0,934596	y ~ current_feature + S
0,291249	0,123503	-0,07874	2,358243	63	60	0,021638	0,19062	y ~ current_feature + S
0,103642	0,128404	0,034007	0,807154	63	60	0,422766	0,791701	y ~ current_feature + S
-0,04325	0,128979	-0,04751	-0,3353	63	60	0,738568	0,933594	y ~ current_feature + S
0,127835	0,140261	0,061498	0,911406	53	50	0,366455	0,925517	y ~ current_feature + S
0,071923	0,159713	-0,02429	0,450327	42	39	0,654968	0,924682	y ~ current_feature + S
0,17984	0,0974	-0,07874	1,846403	105	102	0,067733	0,34663	y ~ current_feature + S
0,103737	0,098481	0,034007	1,053375	105	102	0,294658	0,718742	y ~ current_feature + S
0,019259	0,098996	-0,04751	0,194538	105	102	0,846142	0,980218	y ~ current_feature + S
0,050461	0,106465	0,061498	0,47397	91	88	0,636695	0,955689	y ~ current_feature + S
-0,00255	0,122169	-0,02429	-0,02088	70	67	0,983404	0,99444	y ~ current_feature + S
-0,04156	0,052805	-0,07874	-0,78709	388	358,0103	0,431752	0,814177	y ~ current_feature + S
-0,05815	0,069632	0,034007	-0,83516	388	205,5493	0,4046	0,779245	y ~ current_feature + S
0,02045	0,054981	-0,04751	0,37195	387	330,6664	0,710168	0,93314	y ~ current_feature + S
-0,07265	0,058363	0,061498	-1,24474	335	292,0254	0,214224	0,893818	y ~ current_feature + S
-0,03965	0,062791	-0,02429	-0,63144	258	253,2322	0,528324	0,899808	y ~ current_feature + S
0,007546	0,064474	-0,07874	0,117045	269	240,5504	0,906922	0,987929	y ~ current_feature + S
-0,03968	0,084822	0,034007	-0,46778	269	138,7724	0,640678	0,899624	y ~ current_feature + S
0,081323	0,066209	-0,04751	1,228268	269	226,6106	0,220621	0,742928	y ~ current_feature + S
0,007594	0,069065	0,061498	0,109951	229	209,6347	0,912554	0,996179	y ~ current_feature + S
-0,1034	0,075657	-0,02429	-1,36665	179	172,8359	0,173511	0,626332	y ~ current_feature + S
0,119582	0,076598	-0,07874	1,561157	171	168	0,120368	0,477837	y ~ current_feature + S
-0,02005	0,077136	0,034007	-0,25987	171	168	0,795279	0,946875	y ~ current_feature + S
-0,03074	0,077115	-0,04751	-0,39867	171	168	0,690644	0,930326	y ~ current_feature + S
-0,02535	0,085098	0,061498	-0,2979	141	138	0,766229	0,972573	y ~ current_feature + S
-0,19184	0,093153	-0,02429	-2,05944	114	110,9997	0,041792	0,525585	y ~ current_feature + S
0,007824	0,074951	-0,07874	0,104387	181	178	0,91698	0,989162	y ~ current_feature + S
0,023861	0,074932	0,034007	0,318434	181	178	0,750529	0,931865	y ~ current_feature + S
-0,03122	0,074917	-0,04751	-0,41673	181	178	0,677378	0,928258	y ~ current_feature + S
0,062458	0,080165	0,061498	0,779111	158	155	0,437102	0,937665	y ~ current_feature + S
0,078187	0,091776	-0,02429	0,851936	121	118	0,395975	0,823699	y ~ current_feature + S
-3,48E-04	0,054404	-0,07874	-0,0064	385	337,8655	0,994898	0,996647	y ~ current_feature + S
-0,04711	0,072882	0,034007	-0,64634	385	187,8441	0,518846	0,841996	y ~ current_feature + S
-0,06699	0,056837	-0,04751	-1,17862	384	308,1699	0,239457	0,742928	y ~ current_feature + S
-0,11766	0,061155	0,061498	-1,92403	335	263,6863	0,055427	0,715249	y ~ current_feature + S
-0,08093	0,062991	-0,02429	-1,2848	256	250,3734	0,200049	0,660578	y ~ current_feature + S
0,077584	0,070006	-0,07874	1,10824	222	202,8177	0,269071	0,702573	y ~ current_feature + S
0,059067	0,097529	0,034007	0,605639	222	104,7643	0,546065	0,852506	y ~ current_feature + S
-0,0789	0,07164	-0,04751	-1,10127	222	193,6297	0,272146	0,747915	y ~ current_feature + S
-0,0994	0,074466	0,061498	-1,3348	191	178,5526	0,183641	0,857147	y ~ current_feature + S
-0,06911	0,083064	-0,02429	-0,832	148	144,2416	0,406786	0,831551	y ~ current_feature + S
0,193169	0,079323	-0,07874	2,435231	156	153	0,016032	0,174676	y ~ current_feature + S
-0,07552	0,080614	0,034007	-0,93686	156	153	0,350308	0,749214	y ~ current_feature + S
0,114345	0,080315	-0,04751	1,423708	156	153	0,156567	0,673603	y ~ current_feature + S
0,075059	0,088485	0,061498	0,84826	130	127	0,397889	0,93433	y ~ current_feature + S
0,062292	0,09931	-0,02429	0,627247	104	101	0,531912	0,901895	y ~ current_feature + S

0,053898	0,071507	-0,07874	0,753743	198	195	0,451913	0,82458	y ~ current_feature + S
0,029638	0,07158	0,034007	0,414047	198	195	0,679294	0,912427	y ~ current_feature + S
0,067408	0,071449	-0,04751	0,943443	198	195	0,346622	0,795089	y ~ current_feature + S
0,382207	0,07692	0,061498	4,968861	171	144,3222	1,88E-06	0,001391	y ~ current_feature + S
0,042735	0,087965	-0,02429	0,485823	132	129	0,627917	0,924682	y ~ current_feature + S
0,043725	0,056024	-0,07874	0,780468	321	318	0,435696	0,81418	y ~ current_feature + S
-0,05944	0,055978	0,034007	-1,06187	321	318	0,289099	0,718742	y ~ current_feature + S
-0,03647	0,05604	-0,04751	-0,65073	321	318	0,515688	0,871254	y ~ current_feature + S
-0,13923	0,06513	0,061498	-2,13775	277	231,1735	0,033587	0,658491	y ~ current_feature + S
-0,08254	0,068608	-0,02429	-1,20314	214	211	0,23027	0,695919	y ~ current_feature + S
-0,01757	0,089163	-0,07874	-0,19701	168	125,7468	0,844139	0,983548	y ~ current_feature + S
0,038266	0,100262	0,034007	0,381664	168	99,33279	0,703526	0,920814	y ~ current_feature + S
0,22618	0,085559	-0,04751	2,643556	168	129,6171	0,009216	0,454673	y ~ current_feature + S
-0,05664	0,090274	0,061498	-0,62742	142	122,3158	0,531551	0,937665	y ~ current_feature + S
0,131857	0,096091	-0,02429	1,372213	112	106,4192	0,172883	0,626332	y ~ current_feature + S
0,013531	0,086703	-0,07874	0,156059	136	133	0,876224	0,987929	y ~ current_feature + S
-0,0893	0,086365	0,034007	-1,03403	136	133	0,302999	0,718742	y ~ current_feature + S
0,06869	0,086506	-0,04751	0,794046	136	133	0,428583	0,819126	y ~ current_feature + S
-0,0542	0,095206	0,061498	-0,56926	113	110	0,57034	0,937665	y ~ current_feature + S
-0,14199	0,106125	-0,02429	-1,33793	90	87	0,184405	0,649807	y ~ current_feature + S
0,075575	0,063004	-0,07874	1,199528	275	250,4814	0,231456	0,657483	y ~ current_feature + S
0,057648	0,08524	0,034007	0,676305	275	137,1719	0,499986	0,841996	y ~ current_feature + S
0,067403	0,068105	-0,04751	0,989687	275	214,6174	0,323442	0,774585	y ~ current_feature + S
0,041666	0,067352	0,061498	0,618621	239	220,0598	0,536806	0,937665	y ~ current_feature + S
0,02973	0,074687	-0,02429	0,398063	183	179,1106	0,691058	0,926418	y ~ current_feature + S
-0,00293	0,078811	-0,07874	-0,03715	164	161	0,970414	0,994607	y ~ current_feature + S
-0,05264	0,078702	0,034007	-0,66886	164	161	0,504545	0,841996	y ~ current_feature + S
0,065529	0,078887	-0,04751	0,83067	163	160	0,407398	0,803665	y ~ current_feature + S
-0,04831	0,085336	0,061498	-0,56607	140	137	0,572269	0,937665	y ~ current_feature + S
-0,06998	0,09689	-0,02429	-0,72223	109	106	0,471745	0,876224	y ~ current_feature + S
0,110151	0,106746	-0,07874	1,031905	93	86,69588	0,304987	0,718687	y ~ current_feature + S
0,020691	0,152218	0,034007	0,135929	93	43,14031	0,89251	0,96591	y ~ current_feature + S
-0,07409	0,115072	-0,04751	-0,64387	93	75,10476	0,521619	0,875279	y ~ current_feature + S
0,010661	0,128176	0,061498	0,083173	82	60,86082	0,933987	0,996179	y ~ current_feature + S
0,024585	0,131098	-0,02429	0,18753	62	58,1495	0,851898	0,980411	y ~ current_feature + S
-0,01083	0,076692	-0,07874	-0,14115	173	170	0,887915	0,987929	y ~ current_feature + S
-0,08028	0,076449	0,034007	-1,0501	173	170	0,295162	0,718742	y ~ current_feature + S
-0,08295	0,076432	-0,04751	-1,08531	173	170	0,279322	0,747915	y ~ current_feature + S
-0,04096	0,081854	0,061498	-0,50039	152	149	0,617541	0,948092	y ~ current_feature + S
-0,11341	0,093882	-0,02429	-1,208	115	112	0,229593	0,695919	y ~ current_feature + S
0,072048	0,062955	-0,07874	1,144422	254	251	0,253539	0,679778	y ~ current_feature + S
0,02102	0,063105	0,034007	0,333098	254	251	0,739339	0,925737	y ~ current_feature + S
0,046005	0,063053	-0,04751	0,729636	254	251	0,466293	0,842057	y ~ current_feature + S
0,031524	0,068166	0,061498	0,462456	218	215	0,644222	0,956496	y ~ current_feature + S
0,039445	0,077555	-0,02429	0,508611	169	166	0,6117	0,924682	y ~ current_feature + S
0,076641	0,050359	-0,07874	1,5219	395	392	0,12884	0,481525	y ~ current_feature + S
0,07696	0,050358	0,034007	1,528268	395	392	0,127253	0,572145	y ~ current_feature + S
0,060703	0,050479	-0,04751	1,202552	394	391	0,229878	0,742928	y ~ current_feature + S

-0,08399	0,054041	0,061498	-1,55419	343	340	0,121069	0,8197	y ~ current_feature + S
0,085421	0,061791	-0,02429	1,382419	263	260	0,168029	0,624945	y ~ current_feature + S
0,066079	0,076529	-0,07874	0,863447	173	170	0,389108	0,789252	y ~ current_feature + S
0,08021	0,076449	0,034007	1,049196	173	170	0,295577	0,718742	y ~ current_feature + S
0,085634	0,076641	-0,04751	1,117347	172	169	0,265432	0,747915	y ~ current_feature + S
-0,01027	0,083041	0,061498	-0,12366	148	145	0,901759	0,996179	y ~ current_feature + S
0,157079	0,093318	-0,02429	1,683262	115	112	0,09511	0,605474	y ~ current_feature + S
0,255912	0,144107	-0,07874	1,775849	48	45	0,08252	0,388948	y ~ current_feature + S
0,040821	0,148947	0,034007	0,274065	48	45	0,785289	0,945731	y ~ current_feature + S
-0,07966	0,148598	-0,04751	-0,53606	48	45	0,59456	0,91049	y ~ current_feature + S
0,048268	0,164207	0,061498	0,293948	40	37	0,770441	0,973246	y ~ current_feature + S
-0,05247	0,18544	-0,02429	-0,28292	32	29	0,779245	0,961068	y ~ current_feature + S
0,028468	0,063534	-0,07874	0,448077	259	247,5361	0,65449	0,932255	y ~ current_feature + S
-0,11177	0,081457	0,034007	-1,3721	259	148,8271	0,172097	0,618964	y ~ current_feature + S
-0,02742	0,065105	-0,04751	-0,42118	258	235,7475	0,674007	0,928258	y ~ current_feature + S
-0,00532	0,06825	0,061498	-0,07789	226	214,677	0,937986	0,996179	y ~ current_feature + S
-0,12924	0,077412	-0,02429	-1,66949	172	164,0867	0,096927	0,605474	y ~ current_feature + S
-0,13715	0,062774	-0,07874	-2,18483	252	249	0,029834	0,220772	y ~ current_feature + S
-0,11514	0,062951	0,034007	-1,82909	252	249	0,068582	0,441313	y ~ current_feature + S
0,05914	0,063389	-0,04751	0,932966	251	248	0,351745	0,795089	y ~ current_feature + S
-0,07095	0,06787	0,061498	-1,04537	219	216	0,297019	0,893818	y ~ current_feature + S
-0,07815	0,077612	-0,02429	-1,00697	168	165	0,315425	0,765294	y ~ current_feature + S
0,050505	0,079454	-0,07874	0,635648	161	158	0,525925	0,876542	y ~ current_feature + S
0,087927	0,079248	0,034007	1,109521	161	158	0,268892	0,71095	y ~ current_feature + S
0,168283	0,078421	-0,04751	2,145891	161	158	0,033409	0,603897	y ~ current_feature + S
0,007719	0,092185	0,061498	0,083737	133	117,6675	0,933408	0,996179	y ~ current_feature + S
0,023004	0,098032	-0,02429	0,234654	107	104	0,814939	0,974251	y ~ current_feature + S
0,264963	0,109887	-0,07874	2,411222	80	77	0,018282	0,180385	y ~ current_feature + S
0,152498	0,112628	0,034007	1,354	80	77	0,179697	0,630217	y ~ current_feature + S
-0,0429	0,113856	-0,04751	-0,3768	80	77	0,707357	0,93314	y ~ current_feature + S
-0,06117	0,168162	0,061498	-0,36376	67	35,23012	0,718213	0,972573	y ~ current_feature + S
0,110724	0,145781	-0,02429	0,75952	53	46,4774	0,451377	0,861794	y ~ current_feature + S
0,0321	0,085082	-0,07874	0,37728	141	138	0,706545	0,94376	y ~ current_feature + S
0,038742	0,085062	0,034007	0,455454	141	138	0,649499	0,900251	y ~ current_feature + S
0,066904	0,084935	-0,04751	0,787709	141	138	0,432218	0,820106	y ~ current_feature + S
0,125064	0,09095	0,061498	1,375089	122	119	0,171687	0,840578	y ~ current_feature + S
-0,17577	0,103196	-0,02429	-1,70326	94	91	0,091932	0,605474	y ~ current_feature + S
0,068127	0,051997	-0,07874	1,310223	418	368,1549	0,190937	0,596175	y ~ current_feature + S
0,115343	0,068216	0,034007	1,690866	418	212,0394	0,092332	0,495112	y ~ current_feature + S
0,034646	0,053201	-0,04751	0,651234	417	352,8872	0,515319	0,871254	y ~ current_feature + S
-0,03735	0,057985	0,061498	-0,64416	364	297,0007	0,519967	0,937665	y ~ current_feature + S
-0,07036	0,060153	-0,02429	-1,16975	278	275	0,243115	0,711086	y ~ current_feature + S
0,107556	0,091914	-0,07874	1,170186	120	117	0,244304	0,67447	y ~ current_feature + S
0,00501	0,092449	0,034007	0,054188	120	117	0,956878	0,989859	y ~ current_feature + S
-0,0453	0,092355	-0,04751	-0,49051	120	117	0,62469	0,925756	y ~ current_feature + S
0,108763	0,098913	0,061498	1,099579	104	101	0,274129	0,893818	y ~ current_feature + S
-0,14387	0,112775	-0,02429	-1,27571	80	77	0,205893	0,674164	y ~ current_feature + S
-0,10973	0,100921	-0,07874	-1,08733	100	97	0,279586	0,71741	y ~ current_feature + S

0,063869	0,101327	0,034007	0,630322	100	97	0,529966	0,848387	y ~ current_feature + S
-0,01105	0,101528	-0,04751	-0,10882	100	97	0,913574	0,985217	y ~ current_feature + S
-0,05253	0,108315	0,061498	-0,48496	88	85	0,628952	0,952306	y ~ current_feature + S
-0,05955	0,125765	-0,02429	-0,47352	66	63	0,637479	0,924682	y ~ current_feature + S
-0,06095	0,113749	-0,07874	-0,53581	80	77	0,593637	0,903286	y ~ current_feature + S
0,108981	0,113282	0,034007	0,962032	80	77	0,339045	0,737921	y ~ current_feature + S
0,119148	0,113149	-0,04751	1,053019	80	77	0,295625	0,752909	y ~ current_feature + S
0,017806	0,12215	0,061498	0,145773	70	67	0,884538	0,996179	y ~ current_feature + S
0,326983	0,133647	-0,02429	2,446609	53	50	0,01798	0,428958	y ~ current_feature + S
-0,04971	0,154113	-0,07874	-0,32255	45	42	0,748637	0,962676	y ~ current_feature + S
-0,25271	0,149295	0,034007	-1,69272	45	42	0,097916	0,517555	y ~ current_feature + S
0,099687	0,153535	-0,04751	0,649277	45	42	0,519695	0,874032	y ~ current_feature + S
-0,13582	0,158644	0,061498	-0,85616	42	39	0,397141	0,93433	y ~ current_feature + S
-0,22887	0,18736	-0,02429	-1,22152	30	26,99481	0,232448	0,695919	y ~ current_feature + S
0,03911	0,061499	-0,07874	0,635943	267	264	0,525365	0,876542	y ~ current_feature + S
0,06015	0,061434	0,034007	0,979092	267	264	0,328431	0,729847	y ~ current_feature + S
0,050985	0,061582	-0,04751	0,827911	266	263	0,408471	0,803665	y ~ current_feature + S
0,042138	0,074284	0,061498	0,567257	230	180,9015	0,571243	0,937665	y ~ current_feature + S
0,082945	0,075332	-0,02429	1,101047	178	175	0,272388	0,732972	y ~ current_feature + S
0,119235	0,12926	-0,07874	0,922445	62	59	0,360053	0,770057	y ~ current_feature + S
-0,31152	0,12371	0,034007	-2,51818	62	59	0,014529	0,262237	y ~ current_feature + S
-0,03771	0,130096	-0,04751	-0,28986	62	59	0,77294	0,945414	y ~ current_feature + S
0,014722	0,141406	0,061498	0,104109	53	50	0,917499	0,996179	y ~ current_feature + S
-0,13906	0,160645	-0,02429	-0,86563	41	38	0,392124	0,821036	y ~ current_feature + S
0,129906	0,069937	-0,07874	1,857481	204	201	0,064706	0,339589	y ~ current_feature + S
0,064233	0,070389	0,034007	0,912548	204	201	0,362574	0,7498	y ~ current_feature + S
0,154407	0,069689	-0,04751	2,215667	204	201	0,027837	0,588545	y ~ current_feature + S
0,080627	0,075781	0,061498	1,063953	176	173	0,288833	0,893818	y ~ current_feature + S
-0,01208	0,086705	-0,02429	-0,13931	136	133	0,889416	0,992711	y ~ current_feature + S
-0,11489	0,135182	-0,07874	-0,8499	57	54	0,399132	0,791912	y ~ current_feature + S
-0,19462	0,133481	0,034007	-1,45805	57	54	0,150619	0,604094	y ~ current_feature + S
-0,19294	0,133526	-0,04751	-1,445	57	54	0,154236	0,672434	y ~ current_feature + S
-0,03417	0,144253	0,061498	-0,23687	51	48	0,813764	0,995348	y ~ current_feature + S
-0,07832	0,168512	-0,02429	-0,46475	38	35	0,644992	0,924682	y ~ current_feature + S
-8,67E-04	0,051109	-0,07874	-0,01697	412	382,8314	0,986473	0,996318	y ~ current_feature + S
-0,07414	0,06611	0,034007	-1,12141	412	227,5464	0,263295	0,71095	y ~ current_feature + S
-0,02782	0,052352	-0,04751	-0,53134	411	364,5853	0,59551	0,91049	y ~ current_feature + S
-0,06434	0,056721	0,061498	-1,13427	359	309,5388	0,257558	0,893818	y ~ current_feature + S
0,019596	0,06132	-0,02429	0,319566	274	265,8431	0,749548	0,94977	y ~ current_feature + S
0,040697	0,091594	-0,07874	0,44432	122	119	0,657618	0,932255	y ~ current_feature + S
0,025864	0,091639	0,034007	0,282234	122	119	0,778255	0,942718	y ~ current_feature + S
0,174215	0,090268	-0,04751	1,92998	122	119	0,055989	0,640171	y ~ current_feature + S
-0,19537	0,094371	0,061498	-2,07027	111	108	0,040812	0,658491	y ~ current_feature + S
0,022312	0,112481	-0,02429	0,198359	82	79	0,843274	0,977734	y ~ current_feature + S
-0,2084	0,06594	-0,07874	-3,16049	223	220	0,001797	0,053181	y ~ current_feature + S
-0,13699	0,066784	0,034007	-2,05119	223	220	0,041434	0,398193	y ~ current_feature + S
-0,04572	0,067349	-0,04751	-0,67882	223	220	0,497965	0,868909	y ~ current_feature + S
0,008601	0,07789	0,061498	0,110419	192	164,8168	0,912212	0,996179	y ~ current_feature + S

-0,13765	0,081983	-0,02429	-1,67897	149	145,9644	0,095297	0,605474	y ~ current_feature + S
-0,00141	0,11547	-0,07874	-0,01223	78	75	0,990278	0,996647	y ~ current_feature + S
-0,23219	0,112314	0,034007	-2,06729	78	75	0,042158	0,399959	y ~ current_feature + S
-0,09543	0,114943	-0,04751	-0,83027	78	75	0,409023	0,803665	y ~ current_feature + S
-0,0469	0,122956	0,061498	-0,38141	69	66	0,704126	0,972444	y ~ current_feature + S
0,136241	0,141525	-0,02429	0,962662	52	49	0,340444	0,785829	y ~ current_feature + S
0,120188	0,07526	-0,07874	1,596962	177	174	0,112089	0,465986	y ~ current_feature + S
0,117595	0,075284	0,034007	1,562023	177	174	0,1201	0,558954	y ~ current_feature + S
0,138944	0,075074	-0,04751	1,850749	177	174	0,065901	0,640171	y ~ current_feature + S
0,127698	0,080447	0,061498	1,587366	155	152	0,114508	0,8197	y ~ current_feature + S
0,163192	0,092	-0,02429	1,773819	118	115	0,07874	0,605474	y ~ current_feature + S
0,129742	0,147811	-0,07874	0,877757	48	45	0,384738	0,789252	y ~ current_feature + S
0,130748	0,147792	0,034007	0,88468	48	45	0,381032	0,759222	y ~ current_feature + S
0,015205	0,149054	-0,04751	0,102009	48	45	0,919203	0,985217	y ~ current_feature + S
0,29635	0,157014	0,061498	1,887413	40	37	0,066968	0,715249	y ~ current_feature + S
0,346119	0,174218	-0,02429	1,986707	32	29	0,056476	0,558147	y ~ current_feature + S
-0,00852	0,060488	-0,07874	-0,14078	318	273,2951	0,88815	0,987929	y ~ current_feature + S
-0,10617	0,077451	0,034007	-1,37078	318	164,8232	0,172306	0,618964	y ~ current_feature + S
-0,01218	0,061057	-0,04751	-0,19942	317	268,2046	0,842084	0,980218	y ~ current_feature + S
-0,01323	0,06641	0,061498	-0,19919	272	226,7035	0,84229	0,996179	y ~ current_feature + S
-0,00346	0,069337	-0,02429	-0,0499	211	208	0,96025	0,99444	y ~ current_feature + S
-0,09595	0,049957	-0,07874	-1,92067	400	397	0,05549	0,312071	y ~ current_feature + S
-0,09651	0,049954	0,034007	-1,93198	400	397	0,054073	0,420946	y ~ current_feature + S
-0,04195	0,050208	-0,04751	-0,83552	399	396	0,403928	0,803665	y ~ current_feature + S
-0,02418	0,053901	0,061498	-0,44862	347	344	0,653988	0,957207	y ~ current_feature + S
-0,06059	0,061549	-0,02429	-0,98445	266	263	0,325801	0,774043	y ~ current_feature + S
0,023324	0,054287	-0,07874	0,429637	386	339,131	0,667732	0,934653	y ~ current_feature + S
-0,0321	0,071234	0,034007	-0,45065	386	196,8688	0,652741	0,900251	y ~ current_feature + S
-0,06439	0,056155	-0,04751	-1,14671	385	315,8066	0,252368	0,742928	y ~ current_feature + S
-0,02904	0,063563	0,061498	-0,45693	337	247,2979	0,648126	0,957207	y ~ current_feature + S
0,014668	0,062739	-0,02429	0,233795	257	254	0,815332	0,974251	y ~ current_feature + S
0,169527	0,066444	-0,07874	2,551427	223	220	0,011407	0,140685	y ~ current_feature + S
0,119393	0,066938	0,034007	1,783646	223	220	0,07586	0,464477	y ~ current_feature + S
0,140167	0,066907	-0,04751	2,09497	222	219	0,037323	0,611115	y ~ current_feature + S
0,083969	0,072102	0,061498	1,164592	194	191	0,245637	0,893818	y ~ current_feature + S
0,203443	0,081309	-0,02429	2,502103	148	145	0,013456	0,382986	y ~ current_feature + S
-0,05063	0,079203	-0,07874	-0,63923	162	159	0,523595	0,876542	y ~ current_feature + S
-0,10731	0,078847	0,034007	-1,361	162	159	0,17544	0,621173	y ~ current_feature + S
-0,09575	0,07919	-0,04751	-1,20911	161	158	0,228429	0,742928	y ~ current_feature + S
-0,11703	0,083637	0,061498	-1,39924	144	141	0,163937	0,8197	y ~ current_feature + S
-0,1959	0,095699	-0,02429	-2,04707	108	105	0,043148	0,525585	y ~ current_feature + S
-0,07884	0,074511	-0,07874	-1,05813	182	179	0,291421	0,718687	y ~ current_feature + S
0,127883	0,07413	0,034007	1,725128	182	179	0,08623	0,479777	y ~ current_feature + S
0,079038	0,07451	-0,04751	1,060769	182	179	0,290224	0,747915	y ~ current_feature + S
0,041889	0,078742	0,061498	0,531983	164	161	0,595471	0,945257	y ~ current_feature + S
0,087695	0,091703	-0,02429	0,956292	121	118	0,34088	0,785829	y ~ current_feature + S
0,043069	0,05287	-0,07874	0,814627	400	357,0904	0,415829	0,811887	y ~ current_feature + S
-0,08606	0,068778	0,034007	-1,25123	400	209,8294	0,212245	0,664036	y ~ current_feature + S

0,130506	0,053154	-0,04751	2,455242	400	347,9118	0,014568	0,526293	y ~ current_feature + S
-0,00587	0,057817	0,061498	-0,10151	348	299,1399	0,919211	0,996179	y ~ current_feature + S
-0,02621	0,062613	-0,02429	-0,41868	266	254,9002	0,675806	0,926342	y ~ current_feature + S
0,004356	0,109763	-0,07874	0,039682	86	83	0,968442	0,994607	y ~ current_feature + S
0,116433	0,109018	0,034007	1,068015	86	83	0,28861	0,718742	y ~ current_feature + S
0,043034	0,109663	-0,04751	0,392424	86	83	0,695751	0,931023	y ~ current_feature + S
0,081162	0,119129	0,061498	0,681294	73	70	0,497933	0,937665	y ~ current_feature + S
0,187228	0,133676	-0,02429	1,400606	57	54	0,167052	0,624945	y ~ current_feature + S
0,010751	0,060189	-0,07874	0,178612	279	276	0,858374	0,983548	y ~ current_feature + S
-0,07304	0,060032	0,034007	-1,21669	279	276	0,224762	0,678874	y ~ current_feature + S
-0,0255	0,060283	-0,04751	-0,42303	278	275	0,672604	0,928258	y ~ current_feature + S
0,063916	0,064961	0,061498	0,983906	239	236	0,326169	0,893818	y ~ current_feature + S
0,147469	0,073114	-0,02429	2,01697	186	183	0,04516	0,525585	y ~ current_feature + S
-0,01794	0,051907	-0,07874	-0,34554	401	371,0319	0,729883	0,957449	y ~ current_feature + S
-0,07842	0,06767	0,034007	-1,15889	401	217,0338	0,247774	0,702074	y ~ current_feature + S
0,074778	0,052008	-0,04751	1,437832	400	367,6464	0,151332	0,666583	y ~ current_feature + S
0,112476	0,0581	0,061498	1,935897	350	292,4904	0,053844	0,715249	y ~ current_feature + S
0,003511	0,062009	-0,02429	0,056628	267	260,0631	0,954885	0,99444	y ~ current_feature + S
0,041109	0,058361	-0,07874	0,704396	327	293,1028	0,481746	0,845512	y ~ current_feature + S
0,048024	0,078774	0,034007	0,609645	327	160,7812	0,542958	0,852506	y ~ current_feature + S
-0,06923	0,061207	-0,04751	-1,13112	326	265,649	0,259023	0,742928	y ~ current_feature + S
-7,89E-05	0,063151	0,061498	-0,00125	282	250,7455	0,999005	0,999005	y ~ current_feature + S
-8,73E-04	0,068199	-0,02429	-0,0128	218	215	0,989803	0,995182	y ~ current_feature + S
-0,08763	0,086377	-0,07874	-1,01445	136	133	0,31221	0,718687	y ~ current_feature + S
-0,15979	0,085597	0,034007	-1,86678	136	133	0,064134	0,439434	y ~ current_feature + S
-0,06217	0,086543	-0,04751	-0,71831	136	133	0,473825	0,842057	y ~ current_feature + S
0,110847	0,097667	0,061498	1,134948	118	103,5472	0,259017	0,893818	y ~ current_feature + S
-0,17524	0,104951	-0,02429	-1,66972	91	88	0,098529	0,605474	y ~ current_feature + S
0,519514	0,124636	-0,07874	4,168252	50	47	1,31E-04	0,013812	y ~ current_feature + S
0,279166	0,140066	0,034007	1,993104	50	47	0,052073	0,420946	y ~ current_feature + S
0,260339	0,140835	-0,04751	1,848537	50	47	0,070821	0,640171	y ~ current_feature + S
0,064448	0,155849	0,061498	0,41353	44	41	0,681374	0,962245	y ~ current_feature + S
0,367222	0,169818	-0,02429	2,162437	33	30	0,038684	0,520476	y ~ current_feature + S
0,050486	0,128935	-0,07874	0,391562	63	60	0,696769	0,939646	y ~ current_feature + S
0,179064	0,127013	0,034007	1,40981	63	60	0,163759	0,61196	y ~ current_feature + S
0,057653	0,128885	-0,04751	0,44732	63	60	0,656255	0,925756	y ~ current_feature + S
0,216782	0,13946	0,061498	1,554439	52	49	0,126516	0,8197	y ~ current_feature + S
0,277424	0,153843	-0,02429	1,8033	42	39	0,079071	0,605474	y ~ current_feature + S
0,104383	0,114839	-0,07874	0,908945	78	75	0,366291	0,776663	y ~ current_feature + S
0,218034	0,112692	0,034007	1,934777	78	75	0,056789	0,420946	y ~ current_feature + S
-0,1711	0,113767	-0,04751	-1,50394	78	75	0,136798	0,657012	y ~ current_feature + S
-0,12772	0,123976	0,061498	-1,03016	67	64	0,306813	0,893818	y ~ current_feature + S
0,048049	0,142692	-0,02429	0,336731	52	49	0,737758	0,944216	y ~ current_feature + S
-0,08931	0,055248	-0,07874	-1,61645	328	325	0,106967	0,449749	y ~ current_feature + S
-0,06842	0,05534	0,034007	-1,2363	328	325	0,21724	0,664036	y ~ current_feature + S
-0,02899	0,055447	-0,04751	-0,52282	328	325	0,601454	0,91263	y ~ current_feature + S
-0,05483	0,066257	0,061498	-0,82759	285	227,1069	0,408771	0,934833	y ~ current_feature + S
-0,00425	0,068199	-0,02429	-0,06235	218	215	0,950344	0,99444	y ~ current_feature + S

-0,03414	0,051202	-0,07874	-0,66681	421	381,0016	0,505296	0,863164	y ~ current_feature + S
-0,06037	0,067236	0,034007	-0,89785	421	220,4002	0,370245	0,750317	y ~ current_feature + S
-0,02639	0,052602	-0,04751	-0,50164	420	361,1513	0,616223	0,924009	y ~ current_feature + S
-0,01667	0,05716	0,061498	-0,29171	367	305,9762	0,770706	0,973246	y ~ current_feature + S
-0,05762	0,06029	-0,02429	-0,95579	280	274,1998	0,340023	0,785829	y ~ current_feature + S
-0,10724	0,083142	-0,07874	-1,28987	146	143	0,199179	0,611596	y ~ current_feature + S
-0,21035	0,081753	0,034007	-2,57295	146	143	0,011102	0,233774	y ~ current_feature + S
-0,0263	0,083595	-0,04751	-0,31457	146	143	0,753549	0,9394	y ~ current_feature + S
0,01391	0,090527	0,061498	0,153652	125	122	0,878138	0,996179	y ~ current_feature + S
-0,04749	0,103026	-0,02429	-0,46094	97	94	0,645904	0,924682	y ~ current_feature + S
0,075677	0,133247	-0,07874	0,567947	59	56	0,572341	0,899533	y ~ current_feature + S
0,207718	0,130716	0,034007	1,589076	59	56	0,117674	0,552558	y ~ current_feature + S
-0,11681	0,133917	-0,04751	-0,87225	58	55	0,386865	0,803665	y ~ current_feature + S
0,113204	0,157097	0,061498	0,7206	43	40	0,475346	0,937665	y ~ current_feature + S
0,095127	0,165911	-0,02429	0,57336	39	36	0,569965	0,924682	y ~ current_feature + S
-0,12887	0,064827	-0,07874	-1,98792	237	234	0,047986	0,289576	y ~ current_feature + S
-0,1517	0,064615	0,034007	-2,34771	237	234	0,019723	0,317291	y ~ current_feature + S
-0,08756	0,065121	-0,04751	-1,34461	237	234	0,180053	0,704707	y ~ current_feature + S
0,046475	0,069598	0,061498	0,667758	209	206	0,505036	0,937665	y ~ current_feature + S
-0,12179	0,079982	-0,02429	-1,5227	157	154	0,129884	0,617591	y ~ current_feature + S
0,089004	0,088038	-0,07874	1,010982	131	128	0,313932	0,718687	y ~ current_feature + S
0,021339	0,088368	0,034007	0,24148	131	128	0,809569	0,952501	y ~ current_feature + S
0,080992	0,088098	-0,04751	0,919345	131	128	0,359645	0,798092	y ~ current_feature + S
-0,04294	0,095694	0,061498	-0,44873	112	109	0,65452	0,957207	y ~ current_feature + S
-0,00883	0,109105	-0,02429	-0,08093	87	84	0,935688	0,99444	y ~ current_feature + S
0,125282	0,067979	-0,07874	1,842956	216	213	0,066725	0,346137	y ~ current_feature + S
0,089465	0,068244	0,034007	1,31096	216	213	0,191283	0,643406	y ~ current_feature + S
0,111824	0,068089	-0,04751	1,642325	216	213	0,101998	0,640171	y ~ current_feature + S
0,097626	0,073771	0,061498	1,323365	185	182	0,187374	0,866606	y ~ current_feature + S
0,13494	0,083445	-0,02429	1,617112	144	141	0,108089	0,605474	y ~ current_feature + S
-0,02227	0,111084	-0,07874	-0,20046	84	81	0,841623	0,983548	y ~ current_feature + S
0,084691	0,110712	0,034007	0,764966	84	81	0,446515	0,813845	y ~ current_feature + S
0,225317	0,108254	-0,04751	2,081375	84	81	0,040558	0,612508	y ~ current_feature + S
0,031617	0,123973	0,061498	0,255032	68	65	0,799503	0,984413	y ~ current_feature + S
-0,02945	0,137301	-0,02429	-0,21451	56	53	0,83097	0,976969	y ~ current_feature + S
-0,01636	0,049602	-0,07874	-0,32978	418	406,3361	0,741736	0,959588	y ~ current_feature + S
-0,028	0,063231	0,034007	-0,44283	418	249,9155	0,658269	0,900251	y ~ current_feature + S
-0,00897	0,050414	-0,04751	-0,17796	417	393,4194	0,858849	0,983821	y ~ current_feature + S
-0,08201	0,055131	0,061498	-1,4876	364	326,8001	0,137821	0,8197	y ~ current_feature + S
-0,05789	0,061141	-0,02429	-0,94687	278	266,6141	0,344564	0,789404	y ~ current_feature + S
0,09416	0,135478	-0,07874	0,695022	57	54	0,490022	0,852508	y ~ current_feature + S
0,618827	0,106897	0,034007	5,789018	57	54	3,71E-07	2,74E-04	y ~ current_feature + S
0,146674	0,134611	-0,04751	1,089617	57	54	0,280721	0,747915	y ~ current_feature + S
0,393827	0,134077	0,061498	2,937322	50	47	0,005114	0,378455	y ~ current_feature + S
0,3367	0,17174	-0,02429	1,960515	38	30,06068	0,05926	0,558147	y ~ current_feature + S
-0,02464	0,051419	-0,07874	-0,47922	381	378	0,632062	0,92619	y ~ current_feature + S
-0,06155	0,051337	0,034007	-1,1989	381	378	0,23132	0,681978	y ~ current_feature + S
-0,07969	0,051339	-0,04751	-1,55222	380	377	0,121448	0,651244	y ~ current_feature + S

-0,00979	0,055046	0,061498	-0,17782	333	330	0,858974	0,996179	y ~ current_feature + S
0,028258	0,06322	-0,02429	0,44697	253	250	0,655284	0,924682	y ~ current_feature + S
-0,05431	0,052595	-0,07874	-1,03254	379	360,4322	0,302512	0,718687	y ~ current_feature + S
-0,11854	0,06755	0,034007	-1,75484	379	216,0769	0,080704	0,473527	y ~ current_feature + S
0,024029	0,053503	-0,04751	0,449114	378	349,1395	0,653628	0,925756	y ~ current_feature + S
0,025974	0,057282	0,061498	0,453452	327	304,5624	0,650546	0,957207	y ~ current_feature + S
-0,01979	0,065403	-0,02429	-0,30264	252	233,6869	0,76243	0,951727	y ~ current_feature + S
0,00662	0,113225	-0,07874	0,058464	81	78	0,953529	0,994607	y ~ current_feature + S
-0,07318	0,112924	0,034007	-0,64806	81	78	0,518851	0,841996	y ~ current_feature + S
0,063144	0,113002	-0,04751	0,558792	81	78	0,577904	0,904733	y ~ current_feature + S
-0,26089	0,116217	0,061498	-2,24489	72	69	0,027983	0,658491	y ~ current_feature + S
0,064163	0,144216	-0,02429	0,444906	54	47,88314	0,658392	0,924682	y ~ current_feature + S
0,072525	0,108822	-0,07874	0,666458	87	84	0,506945	0,863164	y ~ current_feature + S
-0,04836	0,108981	0,034007	-0,44375	87	84	0,658363	0,900251	y ~ current_feature + S
-0,01375	0,109099	-0,04751	-0,12601	87	84	0,900028	0,985217	y ~ current_feature + S
-0,08781	0,117396	0,061498	-0,74802	75	72	0,456887	0,937665	y ~ current_feature + S
0,054817	0,134637	-0,02429	0,407146	58	55	0,685481	0,926342	y ~ current_feature + S
-0,14765	0,08242	-0,07874	-1,79141	147	144	0,075329	0,366731	y ~ current_feature + S
-5,31E-04	0,083333	0,034007	-0,00638	147	144	0,994921	0,997989	y ~ current_feature + S
0,070627	0,083125	-0,04751	0,849647	147	144	0,396932	0,803665	y ~ current_feature + S
-0,10977	0,091245	0,061498	-1,20298	124	118,6646	0,231379	0,893818	y ~ current_feature + S
0,061051	0,102435	-0,02429	0,595996	98	94,94784	0,552596	0,918138	y ~ current_feature + S
0,053723	0,074222	-0,07874	0,723818	184	181	0,470112	0,840689	y ~ current_feature + S
-0,05165	0,07423	0,034007	-0,69587	184	181	0,487401	0,834899	y ~ current_feature + S
-0,05908	0,0742	-0,04751	-0,79619	184	181	0,426964	0,819126	y ~ current_feature + S
-0,09617	0,080209	0,061498	-1,199	157	154	0,232368	0,893818	y ~ current_feature + S
-0,11721	0,090658	-0,02429	-1,29285	123	120	0,198547	0,660578	y ~ current_feature + S
0,091557	0,114985	-0,07874	0,796254	78	75	0,428398	0,814177	y ~ current_feature + S
0,016119	0,115455	0,034007	0,13961	78	75	0,889342	0,96591	y ~ current_feature + S
-0,11242	0,114738	-0,04751	-0,97981	78	75	0,330328	0,779476	y ~ current_feature + S
-0,45487	0,11132	0,061498	-4,08615	67	64	1,25E-04	0,023038	y ~ current_feature + S
-0,196	0,140086	-0,02429	-1,39917	52	49	0,168059	0,624945	y ~ current_feature + S
0,138223	0,093169	-0,07874	1,483576	116	113	0,140705	0,498495	y ~ current_feature + S
-0,02196	0,094049	0,034007	-0,23346	116	113	0,815824	0,952501	y ~ current_feature + S
0,136408	0,093608	-0,04751	1,45723	115	112	0,147851	0,661823	y ~ current_feature + S
-0,01837	0,101517	0,061498	-0,18094	100	97	0,856794	0,996179	y ~ current_feature + S
0,189914	0,114132	-0,02429	1,663983	77	74	0,100345	0,605474	y ~ current_feature + S
-0,09388	0,064399	-0,07874	-1,45772	242	239	0,146229	0,506121	y ~ current_feature + S
-0,04549	0,064618	0,034007	-0,70402	242	239	0,482103	0,831402	y ~ current_feature + S
-0,03925	0,064635	-0,04751	-0,6073	242	239	0,544227	0,892966	y ~ current_feature + S
0,127212	0,069616	0,061498	1,827345	206	203	0,069116	0,715249	y ~ current_feature + S
-0,09002	0,079233	-0,02429	-1,13621	161	158	0,257591	0,71931	y ~ current_feature + S
0,214205	0,058796	-0,07874	3,643196	279	276	3,22E-04	0,023018	y ~ current_feature + S
0,12057	0,059754	0,034007	2,017774	279	276	0,044582	0,400524	y ~ current_feature + S
0,136288	0,05974	-0,04751	2,281364	278	275	0,023291	0,563956	y ~ current_feature + S
-0,05708	0,064988	0,061498	-0,87833	239	236	0,380657	0,93433	y ~ current_feature + S
0,203664	0,072373	-0,02429	2,814096	186	183	0,005427	0,236215	y ~ current_feature + S
0,206472	0,137011	-0,07874	1,506974	54	51	0,137987	0,498495	y ~ current_feature + S

-0,09834	0,139349	0,034007	-0,70571	54	51	0,483582	0,831402	y ~ current_feature + S
-0,05551	0,139812	-0,04751	-0,39705	54	51	0,692991	0,930326	y ~ current_feature + S
0,136034	0,151081	0,061498	0,900406	46	43	0,372919	0,932536	y ~ current_feature + S
0,085878	0,173435	-0,02429	0,49516	36	33	0,623768	0,924682	y ~ current_feature + S
0,078342	0,062925	-0,07874	1,244992	254	251	0,214296	0,639357	y ~ current_feature + S
0,088418	0,062872	0,034007	1,406318	254	251	0,160867	0,60427	y ~ current_feature + S
0,064695	0,06781	-0,04751	0,95407	253	216,5665	0,341112	0,789051	y ~ current_feature + S
-0,00676	0,07023	0,061498	-0,09628	222	202,7387	0,923393	0,996179	y ~ current_feature + S
0,052374	0,077509	-0,02429	0,675722	169	166	0,500157	0,89399	y ~ current_feature + S
-0,04019	0,093996	-0,07874	-0,42757	116	113	0,669774	0,934653	y ~ current_feature + S
-0,14846	0,09303	0,034007	-1,5958	116	113	0,113327	0,548117	y ~ current_feature + S
0,063784	0,094299	-0,04751	0,676402	115	112	0,50018	0,868909	y ~ current_feature + S
0,118148	0,101347	0,061498	1,16577	99	96	0,246596	0,893818	y ~ current_feature + S
0,09244	0,11575	-0,02429	0,79862	77	74	0,427067	0,847264	y ~ current_feature + S
-0,02338	0,121235	-0,07874	-0,19287	71	68	0,847638	0,983548	y ~ current_feature + S
0,04115	0,121165	0,034007	0,339617	71	68	0,735192	0,923671	y ~ current_feature + S
-0,05056	0,122013	-0,04751	-0,41439	70	67	0,67991	0,92829	y ~ current_feature + S
-0,08034	0,129768	0,061498	-0,6191	62	59	0,538236	0,937665	y ~ current_feature + S
-0,01229	0,150744	-0,02429	-0,08151	47	44	0,935408	0,99444	y ~ current_feature + S
-0,04043	0,075316	-0,07874	-0,53683	179	176	0,59206	0,903286	y ~ current_feature + S
-0,07801	0,075148	0,034007	-1,03804	179	176	0,300675	0,718742	y ~ current_feature + S
-0,09828	0,075013	-0,04751	-1,31024	179	176	0,191822	0,717449	y ~ current_feature + S
-0,03933	0,081316	0,061498	-0,48371	154	151	0,629294	0,952306	y ~ current_feature + S
0,02057	0,092828	-0,02429	0,221589	119	116	0,825024	0,976828	y ~ current_feature + S
-0,01532	0,104919	-0,07874	-0,14597	123	90,82179	0,884266	0,987929	y ~ current_feature + S
0,061794	0,135483	0,034007	0,456102	123	54,271	0,650136	0,900251	y ~ current_feature + S
0,097676	0,09563	-0,04751	1,021398	123	108,3051	0,309342	0,76529	y ~ current_feature + S
0,066483	0,100912	0,061498	0,658826	104	97,76654	0,511556	0,937665	y ~ current_feature + S
0,053705	0,113644	-0,02429	0,472574	82	77,20606	0,63785	0,924682	y ~ current_feature + S
0,20698	0,086138	-0,07874	2,40287	132	129	0,017691	0,177316	y ~ current_feature + S
0,041264	0,08797	0,034007	0,469064	132	129	0,639816	0,899624	y ~ current_feature + S
0,074291	0,087802	-0,04751	0,84612	132	129	0,399053	0,803665	y ~ current_feature + S
-0,0496	0,095229	0,061498	-0,52083	113	110	0,603529	0,945257	y ~ current_feature + S
0,064924	0,108236	-0,02429	0,599835	88	85	0,550212	0,91702	y ~ current_feature + S
-0,13072	0,088675	-0,07874	-1,47419	128	125	0,142944	0,501016	y ~ current_feature + S
-0,11925	0,088804	0,034007	-1,34289	128	125	0,181741	0,634381	y ~ current_feature + S
0,049628	0,089333	-0,04751	0,55554	128	125	0,579518	0,904733	y ~ current_feature + S
-0,04845	0,096112	0,061498	-0,50407	111	108	0,615236	0,948092	y ~ current_feature + S
0,108634	0,109778	-0,02429	0,98958	85	82	0,32529	0,774043	y ~ current_feature + S
-0,02785	0,109067	-0,07874	-0,25537	87	84	0,79906	0,979102	y ~ current_feature + S
-0,08321	0,108731	0,034007	-0,76528	87	84	0,446249	0,813845	y ~ current_feature + S
0,06431	0,108883	-0,04751	0,590631	87	84	0,556353	0,897386	y ~ current_feature + S
0,119451	0,117007	0,061498	1,020883	75	72	0,310728	0,893818	y ~ current_feature + S
-0,0812	0,134395	-0,02429	-0,60421	58	55	0,54819	0,915712	y ~ current_feature + S
0,109287	0,153379	-0,07874	0,712529	45	42	0,480077	0,845512	y ~ current_feature + S
0,15766	0,152374	0,034007	1,034697	45	42	0,306732	0,718742	y ~ current_feature + S
-0,1592	0,152336	-0,04751	-1,04503	45	42	0,301987	0,754967	y ~ current_feature + S
-0,1031	0,175835	0,061498	-0,58636	35	32	0,561744	0,937665	y ~ current_feature + S

0,016513	0,192424	-0,02429	0,085815	30	27	0,932247	0,99444	y ~ current_feature + S
0,244613	0,090027	-0,07874	2,71711	119	116	0,007595	0,110197	y ~ current_feature + S
0,054882	0,092708	0,034007	0,591988	119	116	0,55501	0,857986	y ~ current_feature + S
0,039212	0,092776	-0,04751	0,422653	119	116	0,673331	0,928258	y ~ current_feature + S
0,060987	0,099814	0,061498	0,611007	103	100	0,542581	0,937665	y ~ current_feature + S
-0,00362	0,114707	-0,02429	-0,0316	79	76	0,974875	0,99444	y ~ current_feature + S
0,05842	0,067925	-0,07874	0,860061	219	216	0,390709	0,789252	y ~ current_feature + S
-0,00389	0,068041	0,034007	-0,05718	219	216	0,954457	0,989859	y ~ current_feature + S
-0,019	0,068029	-0,04751	-0,27933	219	216	0,780262	0,94882	y ~ current_feature + S
-0,03103	0,073288	0,061498	-0,42346	189	186	0,672446	0,958606	y ~ current_feature + S
-0,01375	0,083616	-0,02429	-0,1645	146	143	0,869573	0,984468	y ~ current_feature + S
0,095614	0,049585	-0,07874	1,928273	406	403	0,054523	0,310364	y ~ current_feature + S
0,110379	0,049509	0,034007	2,229459	406	403	0,026334	0,347616	y ~ current_feature + S
0,066927	0,049764	-0,04751	1,344892	405	402	0,179418	0,704707	y ~ current_feature + S
-0,05756	0,060216	0,061498	-0,95598	352	274,8787	0,339924	0,901591	y ~ current_feature + S
0,11684	0,060796	-0,02429	1,921839	270	266,8605	0,055691	0,558147	y ~ current_feature + S
-0,05922	0,053656	-0,07874	-1,10368	402	346,1297	0,2705	0,702573	y ~ current_feature + S
-0,11908	0,069412	0,034007	-1,71551	402	204,6139	0,087766	0,484679	y ~ current_feature + S
0,096863	0,053442	-0,04751	1,812476	401	346,8447	0,070777	0,640171	y ~ current_feature + S
0,034889	0,058038	0,061498	0,601136	350	296,5128	0,548209	0,937665	y ~ current_feature + S
-0,03347	0,062024	-0,02429	-0,53956	267	259,6492	0,589963	0,924682	y ~ current_feature + S
0,054871	0,122906	-0,07874	0,446443	69	66	0,656738	0,932255	y ~ current_feature + S
0,03606	0,123011	0,034007	0,29314	69	66	0,770335	0,940673	y ~ current_feature + S
-0,0426	0,12298	-0,04751	-0,34643	69	66	0,730119	0,93314	y ~ current_feature + S
-0,09894	0,135415	0,061498	-0,73064	57	54	0,468157	0,937665	y ~ current_feature + S
-0,2923	0,145838	-0,02429	-2,00431	46	43	0,051361	0,558147	y ~ current_feature + S
-0,07901	0,092161	-0,07874	-0,85731	120	117	0,393026	0,789252	y ~ current_feature + S
-0,20305	0,090524	0,034007	-2,24304	120	117	0,026777	0,347616	y ~ current_feature + S
-0,1366	0,091583	-0,04751	-1,49156	120	117	0,138505	0,657012	y ~ current_feature + S
-0,09082	0,099092	0,061498	-0,91654	104	101	0,361568	0,920619	y ~ current_feature + S
-0,32253	0,10787	-0,02429	-2,99002	80	77	0,003744	0,184706	y ~ current_feature + S
-0,04627	0,052721	-0,07874	-0,87758	362	359	0,380761	0,789252	y ~ current_feature + S
-0,17502	0,072679	0,034007	-2,40808	362	183,5137	0,017027	0,293019	y ~ current_feature + S
-0,09138	0,059154	-0,04751	-1,54484	361	283,393	0,1235	0,652786	y ~ current_feature + S
-0,08255	0,064873	0,061498	-1,27249	315	235,9981	0,20445	0,893818	y ~ current_feature + S
-0,09534	0,064974	-0,02429	-1,46739	240	234,726	0,143607	0,618941	y ~ current_feature + S
0,064757	0,11299	-0,07874	0,573125	81	78	0,568208	0,898449	y ~ current_feature + S
0,079586	0,112869	0,034007	0,705125	81	78	0,482833	0,831402	y ~ current_feature + S
-0,04467	0,113115	-0,04751	-0,39493	81	78	0,693973	0,930326	y ~ current_feature + S
-0,21361	0,115939	0,061498	-1,8424	74	71	0,069592	0,715249	y ~ current_feature + S
-0,11795	0,139051	-0,02429	-0,84827	54	51	0,40025	0,825029	y ~ current_feature + S
-0,26039	0,135198	-0,07874	-1,92597	54	51	0,059691	0,324792	y ~ current_feature + S
0,006435	0,140025	0,034007	0,045956	54	51	0,963525	0,989859	y ~ current_feature + S
-0,17692	0,137819	-0,04751	-1,28369	54	51	0,205051	0,723562	y ~ current_feature + S
0,152985	0,156253	0,061498	0,979087	43	40	0,333422	0,893818	y ~ current_feature + S
0,038841	0,173946	-0,02429	0,223292	36	33	0,824685	0,976828	y ~ current_feature + S
0,030565	0,056407	-0,07874	0,541866	317	314	0,588295	0,902886	y ~ current_feature + S
-0,05675	0,056342	0,034007	-1,00727	317	314	0,314583	0,718742	y ~ current_feature + S

0,032394	0,056404	-0,04751	0,574318	317	314	0,566164	0,903656	y ~ current_feature + S
-0,02601	0,060502	0,061498	-0,4299	276	273	0,667604	0,958606	y ~ current_feature + S
0,032278	0,069301	-0,02429	0,46576	211	208	0,641875	0,924682	y ~ current_feature + S
-0,04485	0,078732	-0,07874	-0,56965	164	161	0,56971	0,898903	y ~ current_feature + S
-0,01761	0,078799	0,034007	-0,22345	164	161	0,823467	0,952501	y ~ current_feature + S
-0,00714	0,078809	-0,04751	-0,09054	164	161	0,927968	0,985217	y ~ current_feature + S
0,064944	0,093031	0,061498	0,69809	141	115,0553	0,486529	0,937665	y ~ current_feature + S
-0,03672	0,097063	-0,02429	-0,37827	109	106	0,705987	0,93118	y ~ current_feature + S
-0,05889	0,109463	-0,07874	-0,538	87	83,16845	0,592011	0,903286	y ~ current_feature + S
0,021913	0,165309	0,034007	0,132559	87	36,57635	0,895269	0,96591	y ~ current_feature + S
-0,17593	0,113686	-0,04751	-1,54748	87	74,97713	0,125958	0,654671	y ~ current_feature + S
0,078066	0,141085	0,061498	0,55333	70	49,93257	0,582507	0,945257	y ~ current_feature + S
-0,14433	0,133428	-0,02429	-1,08168	58	55	0,284115	0,734108	y ~ current_feature + S
0,258526	0,140977	-0,07874	1,833821	50	46,95283	0,073023	0,362665	y ~ current_feature + S
-0,09174	0,209613	0,034007	-0,43766	50	22,56807	0,665792	0,904011	y ~ current_feature + S
-0,06131	0,145894	-0,04751	-0,42021	50	46,80485	0,676258	0,928258	y ~ current_feature + S
-0,17537	0,194549	0,061498	-0,9014	42	25,60797	0,375776	0,932536	y ~ current_feature + S
0,008241	0,184317	-0,02429	0,04471	33	29,43348	0,96464	0,99444	y ~ current_feature + S
-0,03917	0,05328	-0,07874	-0,73513	383	351,7216	0,462748	0,835203	y ~ current_feature + S
-0,08764	0,067909	0,034007	-1,29062	383	215,1796	0,198222	0,646185	y ~ current_feature + S
-0,04155	0,053803	-0,04751	-0,77233	382	344,8515	0,440449	0,823749	y ~ current_feature + S
0,030076	0,058412	0,061498	0,514895	333	292,8222	0,607015	0,945257	y ~ current_feature + S
-0,01493	0,063349	-0,02429	-0,23575	254	249,1272	0,813821	0,974251	y ~ current_feature + S
0,108459	0,057882	-0,07874	1,873794	332	294,9678	0,061947	0,329792	y ~ current_feature + S
0,036457	0,0727	0,034007	0,501471	332	188,9524	0,616623	0,889725	y ~ current_feature + S
0,069154	0,05986	-0,04751	1,155264	331	277,7472	0,248975	0,742928	y ~ current_feature + S
-0,00671	0,063341	0,061498	-0,10588	287	249,235	0,915762	0,996179	y ~ current_feature + S
0,128848	0,06753	-0,02429	1,908015	221	215,6435	0,057718	0,558147	y ~ current_feature + S
-0,04945	0,093957	-0,07874	-0,52628	116	113	0,599723	0,90386	y ~ current_feature + S
-0,08012	0,09377	0,034007	-0,85446	116	113	0,394659	0,775482	y ~ current_feature + S
0,014848	0,094062	-0,04751	0,157854	116	113	0,874854	0,985217	y ~ current_feature + S
0,06463	0,099791	0,061498	0,647654	103	100	0,518693	0,937665	y ~ current_feature + S
-0,07759	0,115897	-0,02429	-0,6695	77	74	0,505263	0,894767	y ~ current_feature + S
0,173382	0,130447	-0,07874	1,329136	60	57	0,189099	0,592937	y ~ current_feature + S
0,235348	0,128733	0,034007	1,828189	60	57	0,072756	0,460165	y ~ current_feature + S
0,00916	0,132448	-0,04751	0,069159	60	57	0,945105	0,990156	y ~ current_feature + S
-0,12268	0,144763	0,061498	-0,84748	50	47	0,401025	0,93433	y ~ current_feature + S
-0,02699	0,164339	-0,02429	-0,16426	40	37	0,870421	0,984468	y ~ current_feature + S
0,153748	0,051209	-0,07874	3,002377	412	372,3271	0,00286	0,066128	y ~ current_feature + S
0,197335	0,066801	0,034007	2,954063	412	215,3687	0,003485	0,151911	y ~ current_feature + S
0,125197	0,052705	-0,04751	2,375456	411	354,3583	0,018059	0,54566	y ~ current_feature + S
0,029884	0,055784	0,061498	0,535709	359	321,0656	0,592531	0,945257	y ~ current_feature + S
0,091	0,061245	-0,02429	1,485823	274	264,3886	0,138517	0,618941	y ~ current_feature + S
-0,03819	0,091603	-0,07874	-0,41693	122	119	0,677481	0,934653	y ~ current_feature + S
0,017796	0,091655	0,034007	0,194168	122	119	0,846376	0,959445	y ~ current_feature + S
0,152458	0,090598	-0,04751	1,682796	122	119	0,095037	0,640171	y ~ current_feature + S
0,036754	0,098466	0,061498	0,373261	106	103	0,709721	0,972444	y ~ current_feature + S
-0,00473	0,113226	-0,02429	-0,04173	81	78	0,96682	0,99444	y ~ current_feature + S

0,033041	0,057042	-0,07874	0,579246	310	307	0,562848	0,897646	y ~ current_feature + S
0,033577	0,057041	0,034007	0,588646	310	307	0,556531	0,857986	y ~ current_feature + S
0,092037	0,056831	-0,04751	1,619498	310	307	0,106367	0,640171	y ~ current_feature + S
0,040373	0,068104	0,061498	0,592809	267	215,252	0,553931	0,937665	y ~ current_feature + S
-0,06051	0,069886	-0,02429	-0,86585	207	204	0,387591	0,819479	y ~ current_feature + S
-0,03533	0,052291	-0,07874	-0,67572	421	365,2638	0,499649	0,861866	y ~ current_feature + S
-0,0164	0,068501	0,034007	-0,23946	421	213,0547	0,810976	0,952501	y ~ current_feature + S
0,091643	0,05355	-0,04751	1,711359	420	345,7982	0,087912	0,640171	y ~ current_feature + S
0,019087	0,058311	0,061498	0,327327	367	293,9961	0,743654	0,972573	y ~ current_feature + S
0,12715	0,059597	-0,02429	2,133513	280	277	0,033761	0,489869	y ~ current_feature + S
0,054078	0,070084	-0,07874	0,771626	206	203	0,441233	0,818694	y ~ current_feature + S
-0,00794	0,070184	0,034007	-0,11309	206	203	0,910069	0,9732	y ~ current_feature + S
0,006373	0,070185	-0,04751	0,090805	206	203	0,927737	0,985217	y ~ current_feature + S
0,045408	0,079784	0,061498	0,569146	173	156,7752	0,570071	0,937665	y ~ current_feature + S
-0,01058	0,087664	-0,02429	-0,12069	138	130,1093	0,904126	0,99444	y ~ current_feature + S
0,19113	0,055199	-0,07874	3,462577	337	316,2139	6,09E-04	0,031617	y ~ current_feature + S
-0,0025	0,077616	0,034007	-0,0322	337	165,9947	0,974348	0,989859	y ~ current_feature + S
-0,00739	0,062104	-0,04751	-0,11893	336	259,2602	0,905426	0,985217	y ~ current_feature + S
0,011281	0,065028	0,061498	0,173472	296	236,4516	0,862429	0,996179	y ~ current_feature + S
0,063873	0,067818	-0,02429	0,94183	224	216,5366	0,347329	0,790842	y ~ current_feature + S
-0,01373	0,149057	-0,07874	-0,09214	48	45	0,926997	0,990519	y ~ current_feature + S
0,169198	0,146922	0,034007	1,15162	48	45	0,255559	0,705649	y ~ current_feature + S
0,138527	0,147634	-0,04751	0,938313	48	45	0,353093	0,795089	y ~ current_feature + S
0,174415	0,159735	0,061498	1,091905	41	38	0,281749	0,893818	y ~ current_feature + S
-0,109	0,184589	-0,02429	-0,59049	32	29	0,559437	0,92024	y ~ current_feature + S
-0,00653	0,056432	-0,07874	-0,11577	317	314	0,907909	0,987929	y ~ current_feature + S
-9,48E-04	0,056433	0,034007	-0,01681	317	314	0,986602	0,994667	y ~ current_feature + S
-0,02031	0,056512	-0,04751	-0,35938	316	313	0,719556	0,93314	y ~ current_feature + S
-0,06326	0,060291	0,061498	-1,04929	277	274	0,294968	0,893818	y ~ current_feature + S
-0,03106	0,069471	-0,02429	-0,44705	210	207	0,655308	0,924682	y ~ current_feature + S
0,113859	0,108399	-0,07874	1,050366	87	84	0,296562	0,718687	y ~ current_feature + S
0,234632	0,106063	0,034007	2,212198	87	84	0,029667	0,347616	y ~ current_feature + S
0,112826	0,108412	-0,04751	1,040709	87	84	0,300997	0,754967	y ~ current_feature + S
0,130454	0,115254	0,061498	1,131877	77	74	0,261341	0,893818	y ~ current_feature + S
0,220061	0,131535	-0,02429	1,673031	58	55	0,100001	0,605474	y ~ current_feature + S
-0,04078	0,093994	-0,07874	-0,43388	116	113	0,6652	0,934653	y ~ current_feature + S
0,054722	0,093931	0,034007	0,582577	116	113	0,561339	0,861729	y ~ current_feature + S
0,022457	0,094048	-0,04751	0,238779	116	113	0,811709	0,972405	y ~ current_feature + S
0,070536	0,100764	0,061498	0,700014	101	98	0,485577	0,937665	y ~ current_feature + S
0,005649	0,116246	-0,02429	0,048591	77	74	0,961376	0,99444	y ~ current_feature + S
0,138945	0,068997	-0,07874	2,013772	209	206	0,045334	0,281911	y ~ current_feature + S
-0,00132	0,069673	0,034007	-0,01891	209	206	0,984932	0,994338	y ~ current_feature + S
0,019385	0,06966	-0,04751	0,278281	209	206	0,781076	0,94882	y ~ current_feature + S
-0,03101	0,079069	0,061498	-0,39217	181	159,7991	0,695454	0,972444	y ~ current_feature + S
0,013778	0,085741	-0,02429	0,160697	139	136	0,872571	0,984468	y ~ current_feature + S
-0,02636	0,066204	-0,07874	-0,39814	231	228	0,690897	0,93638	y ~ current_feature + S
-0,05389	0,06613	0,034007	-0,81487	231	228	0,415997	0,787309	y ~ current_feature + S
-0,02249	0,066356	-0,04751	-0,33886	230	227	0,735027	0,933491	y ~ current_feature + S

-0,04509	0,080109	0,061498	-0,56288	201	155,5065	0,574324	0,937665	y ~ current_feature + S
-0,08641	0,081147	-0,02429	-1,06486	154	150,7284	0,28864	0,734108	y ~ current_feature + S
-0,10835	0,059839	-0,07874	-1,81062	279	276	0,071287	0,356433	y ~ current_feature + S
-0,05274	0,060109	0,034007	-0,87742	279	276	0,381019	0,759222	y ~ current_feature + S
-0,06648	0,060169	-0,04751	-1,10495	278	275	0,270146	0,747915	y ~ current_feature + S
4,94E-04	0,06455	0,061498	0,007655	243	240	0,993899	0,999005	y ~ current_feature + S
-0,01613	0,074115	-0,02429	-0,21768	185	182	0,827924	0,976969	y ~ current_feature + S
0,179352	0,069219	-0,07874	2,591082	205	202	0,010265	0,135649	y ~ current_feature + S
-0,01008	0,070356	0,034007	-0,14329	205	202	0,886204	0,96591	y ~ current_feature + S
0,076876	0,070326	-0,04751	1,093145	204	201	0,275639	0,747915	y ~ current_feature + S
-0,1817	0,073913	0,061498	-2,4583	180	177	0,014922	0,55988	y ~ current_feature + S
0,126819	0,086011	-0,02429	1,474455	136	133	0,142722	0,618941	y ~ current_feature + S
-0,01824	0,054224	-0,07874	-0,33634	343	340	0,736821	0,959225	y ~ current_feature + S
-0,0809	0,054055	0,034007	-1,49669	343	340	0,135401	0,576909	y ~ current_feature + S
0,036081	0,054277	-0,04751	0,664748	342	339	0,506664	0,868909	y ~ current_feature + S
0,056404	0,058031	0,061498	0,971961	299	296	0,331864	0,893818	y ~ current_feature + S
0,095833	0,06636	-0,02429	1,444147	228	225	0,150089	0,622449	y ~ current_feature + S
0,184005	0,0993	-0,07874	1,853022	101	97,98083	0,066889	0,346137	y ~ current_feature + S
0,018132	0,135434	0,034007	0,133883	101	54,50041	0,893988	0,96591	y ~ current_feature + S
0,296172	0,113503	-0,04751	2,609384	101	70,81371	0,01106	0,481414	y ~ current_feature + S
0,043558	0,109687	0,061498	0,397111	86	82,95917	0,692306	0,972118	y ~ current_feature + S
0,06997	0,125398	-0,02429	0,557986	67	63,28333	0,578823	0,924682	y ~ current_feature + S
0,037942	0,125897	-0,07874	0,301369	66	63	0,764126	0,965008	y ~ current_feature + S
-0,08545	0,125527	0,034007	-0,68076	66	63	0,498519	0,841996	y ~ current_feature + S
-0,04819	0,125842	-0,04751	-0,38298	66	63	0,703027	0,93314	y ~ current_feature + S
0,103937	0,137924	0,061498	0,753583	55	52	0,454499	0,937665	y ~ current_feature + S
0,01273	0,156161	-0,02429	0,081519	44	41	0,935426	0,99444	y ~ current_feature + S
0,039299	0,095273	-0,07874	0,412493	113	110	0,680781	0,934653	y ~ current_feature + S
0,139486	0,094414	0,034007	1,477387	113	110	0,142429	0,588814	y ~ current_feature + S
0,101379	0,094855	-0,04751	1,068773	113	110	0,287512	0,747915	y ~ current_feature + S
-0,04267	0,100412	0,061498	-0,42492	102	99	0,671814	0,958606	y ~ current_feature + S
-0,0019	0,117851	-0,02429	-0,01611	75	72	0,987193	0,99444	y ~ current_feature + S
0,054122	0,154077	-0,07874	0,351264	45	42	0,727146	0,955752	y ~ current_feature + S
-0,03868	0,154188	0,034007	-0,25087	45	42	0,803138	0,952439	y ~ current_feature + S
0,137493	0,152838	-0,04751	0,899597	45	42	0,373464	0,803381	y ~ current_feature + S
-0,10148	0,16355	0,061498	-0,62046	40	37	0,538756	0,937665	y ~ current_feature + S
-0,26649	0,185491	-0,02429	-1,43665	30	27	0,162304	0,623497	y ~ current_feature + S
-0,00849	0,129095	-0,07874	-0,06573	63	60	0,947808	0,994607	y ~ current_feature + S
-0,10256	0,128419	0,034007	-0,79861	63	60	0,427664	0,791701	y ~ current_feature + S
-0,10871	0,128334	-0,04751	-0,84712	63	60	0,400297	0,803665	y ~ current_feature + S
0,048143	0,15157	0,061498	0,317629	50	43,42769	0,752288	0,972573	y ~ current_feature + S
0,177738	0,181554	-0,02429	0,978987	42	29,37985	0,33558	0,785829	y ~ current_feature + S
0,121595	0,102926	-0,07874	1,181382	96	93	0,240463	0,668958	y ~ current_feature + S
0,039834	0,103613	0,034007	0,384454	96	93	0,70152	0,920814	y ~ current_feature + S
-0,09988	0,103177	-0,04751	-0,96803	96	93	0,335541	0,785761	y ~ current_feature + S
-0,12652	0,121993	0,061498	-1,03712	79	66,11877	0,303458	0,893818	y ~ current_feature + S
0,110743	0,127249	-0,02429	0,870283	64	61	0,387558	0,819479	y ~ current_feature + S
0,026024	0,062848	-0,07874	0,414073	256	253	0,679171	0,934653	y ~ current_feature + S

-0,11496	0,062453	0,034007	-1,84079	256	253	0,066823	0,439525	y ~ current_feature + S
-0,03036	0,06284	-0,04751	-0,48309	256	253	0,62945	0,925756	y ~ current_feature + S
0,020502	0,068027	0,061498	0,301386	219	216	0,76341	0,972573	y ~ current_feature + S
-0,05046	0,077284	-0,02429	-0,65288	170	167	0,514731	0,899808	y ~ current_feature + S
0,083328	0,053943	-0,07874	1,54475	390	341,2762	0,123334	0,477837	y ~ current_feature + S
-0,08071	0,070287	0,034007	-1,14833	390	201,0973	0,252198	0,704252	y ~ current_feature + S
0,032372	0,05518	-0,04751	0,586662	389	328,0834	0,557834	0,897386	y ~ current_feature + S
0,048185	0,060749	0,061498	0,793178	340	270,3439	0,42837	0,937665	y ~ current_feature + S
0,04269	0,062712	-0,02429	0,680729	259	253,807	0,496664	0,89399	y ~ current_feature + S
0,23883	0,150962	-0,07874	1,582049	45	41,37678	0,121253	0,477837	y ~ current_feature + S
0,007452	0,190293	0,034007	0,039159	45	27,61393	0,969046	0,989859	y ~ current_feature + S
0,206761	0,159002	-0,04751	1,300372	45	37,86367	0,201333	0,723562	y ~ current_feature + S
-0,13147	0,161258	0,061498	-0,81529	41	37,79088	0,420014	0,937665	y ~ current_feature + S
-0,08487	0,191756	-0,02429	-0,44257	30	27	0,661601	0,924682	y ~ current_feature + S
-0,36171	0,121374	-0,07874	-2,98013	62	59	0,00418	0,073642	y ~ current_feature + S
-0,04622	0,13005	0,034007	-0,35537	62	59	0,723579	0,920814	y ~ current_feature + S
-0,01422	0,130176	-0,04751	-0,1092	62	59	0,913414	0,985217	y ~ current_feature + S
0,089854	0,147036	0,061498	0,6111	49	45,88062	0,544149	0,937665	y ~ current_feature + S
0,140042	0,16378	-0,02429	0,85506	41	36,54916	0,398088	0,823699	y ~ current_feature + S
-0,03266	0,070149	-0,07874	-0,46554	206	203	0,642044	0,928694	y ~ current_feature + S
-0,00643	0,070185	0,034007	-0,09167	206	203	0,927054	0,9732	y ~ current_feature + S
-0,06955	0,070189	-0,04751	-0,99092	205	202	0,322909	0,774585	y ~ current_feature + S
-0,14391	0,078666	0,061498	-1,82938	173	158,2489	0,069225	0,715249	y ~ current_feature + S
-0,06022	0,08623	-0,02429	-0,69839	137	134	0,486141	0,888665	y ~ current_feature + S
0,122987	0,072767	-0,07874	1,690155	189	186	0,092674	0,42595	y ~ current_feature + S
0,067677	0,073155	0,034007	0,925116	189	186	0,356104	0,7498	y ~ current_feature + S
0,007099	0,073322	-0,04751	0,096823	189	186	0,922971	0,985217	y ~ current_feature + S
-0,08969	0,078986	0,061498	-1,13555	162	159	0,257853	0,893818	y ~ current_feature + S
-0,01944	0,090247	-0,02429	-0,21539	126	122,7343	0,829824	0,976969	y ~ current_feature + S
0,036447	0,085692	-0,07874	0,425328	139	136	0,67127	0,934653	y ~ current_feature + S
0,03069	0,085709	0,034007	0,358075	139	136	0,720842	0,920814	y ~ current_feature + S
0,026744	0,085719	-0,04751	0,312003	139	136	0,755516	0,9394	y ~ current_feature + S
-0,04893	0,092736	0,061498	-0,52765	119	116	0,59875	0,945257	y ~ current_feature + S
-0,07849	0,105084	-0,02429	-0,74695	93	90	0,457038	0,866856	y ~ current_feature + S
-0,07083	0,100762	-0,07874	-0,70294	101	98	0,483758	0,846291	y ~ current_feature + S
0,062896	0,100815	0,034007	0,623877	101	98	0,534158	0,849319	y ~ current_feature + S
0,096448	0,101061	-0,04751	0,954352	100	97	0,342278	0,789051	y ~ current_feature + S
0,008121	0,111107	0,061498	0,073094	84	81	0,941911	0,996179	y ~ current_feature + S
-0,02071	0,124973	-0,02429	-0,16575	67	64	0,868875	0,984468	y ~ current_feature + S
-0,04281	0,054243	-0,07874	-0,78922	395	339,2445	0,430537	0,814177	y ~ current_feature + S
-0,12903	0,050085	0,034007	-2,57613	395	392	0,010357	0,232238	y ~ current_feature + S
0,031198	0,055095	-0,04751	0,56627	394	329,1236	0,571596	0,903741	y ~ current_feature + S
-3,13E-04	0,059316	0,061498	-0,00529	343	284,2256	0,995787	0,999005	y ~ current_feature + S
-0,00988	0,062014	-0,02429	-0,15929	263	260	0,87356	0,984468	y ~ current_feature + S
0,065751	0,053557	-0,07874	1,227686	408	347,1218	0,220397	0,639583	y ~ current_feature + S
-0,00907	0,069769	0,034007	-0,12995	408	205,4197	0,89673	0,96591	y ~ current_feature + S
0,008906	0,05547	-0,04751	0,160553	407	324,9797	0,872546	0,985217	y ~ current_feature + S
-0,04101	0,059045	0,061498	-0,69453	354	286,3581	0,487915	0,937665	y ~ current_feature + S

0,085336	0,060862	-0,02429	1,402128	271	268	0,162034	0,623497	y ~ current_feature + S
-0,13958	0,070194	-0,07874	-1,98845	202	199	0,048132	0,289576	y ~ current_feature + S
-0,15215	0,070063	0,034007	-2,17157	202	199	0,031071	0,347616	y ~ current_feature + S
-0,06149	0,070754	-0,04751	-0,8691	202	199	0,385837	0,803665	y ~ current_feature + S
0,102689	0,075193	0,061498	1,36567	178	175	0,173795	0,840578	y ~ current_feature + S
-0,15293	0,086343	-0,02429	-1,77124	134	131	0,078847	0,605474	y ~ current_feature + S
0,072757	0,120946	-0,07874	0,601565	71	68	0,549464	0,888464	y ~ current_feature + S
0,122588	0,120353	0,034007	1,018571	71	68	0,312017	0,718742	y ~ current_feature + S
0,171956	0,119461	-0,04751	1,439423	71	68	0,154618	0,672434	y ~ current_feature + S
-0,18767	0,128973	0,061498	-1,45513	61	58	0,151023	0,8197	y ~ current_feature + S
0,125634	0,149561	-0,02429	0,840014	47	44	0,405442	0,8311	y ~ current_feature + S
-0,0879	0,052424	-0,07874	-1,67663	385	361,0536	0,09448	0,42893	y ~ current_feature + S
-0,06883	0,06995	0,034007	-0,98403	385	203,4078	0,326272	0,728793	y ~ current_feature + S
-0,07667	0,054735	-0,04751	-1,40069	384	331,8299	0,162242	0,693983	y ~ current_feature + S
-0,10151	0,057559	0,061498	-1,76364	335	298,7285	0,078814	0,738262	y ~ current_feature + S
-0,00144	0,063209	-0,02429	-0,02274	256	250,2879	0,981875	0,99444	y ~ current_feature + S
0,096039	0,11571	-0,07874	0,829991	77	74	0,409213	0,806796	y ~ current_feature + S
0,084567	0,115831	0,034007	0,730092	77	74	0,467639	0,831402	y ~ current_feature + S
-0,0499	0,116103	-0,04751	-0,42983	77	74	0,668569	0,92822	y ~ current_feature + S
-0,02598	0,126957	0,061498	-0,20462	65	62	0,838539	0,996179	y ~ current_feature + S
-0,03938	0,144226	-0,02429	-0,27307	51	48	0,785974	0,964545	y ~ current_feature + S
0,050082	0,052785	-0,07874	0,948784	361	358	0,343371	0,751758	y ~ current_feature + S
5,97E-04	0,052852	0,034007	0,011296	361	358	0,990993	0,997735	y ~ current_feature + S
0,003218	0,052851	-0,04751	0,060885	361	358	0,951485	0,990156	y ~ current_feature + S
-0,06504	0,056585	0,061498	-1,14949	314	311	0,251238	0,893818	y ~ current_feature + S
-0,04389	0,064894	-0,02429	-0,67628	240	237	0,499525	0,89399	y ~ current_feature + S
-0,0878	0,106797	-0,07874	-0,82207	90	87	0,413283	0,811218	y ~ current_feature + S
0,118599	0,106455	0,034007	1,114083	90	87	0,268312	0,71095	y ~ current_feature + S
-0,08479	0,106825	-0,04751	-0,79377	90	87	0,429488	0,819126	y ~ current_feature + S
0,028108	0,113916	0,061498	0,246746	80	77	0,805761	0,988828	y ~ current_feature + S
0,085569	0,131967	-0,02429	0,648407	60	57	0,519325	0,899808	y ~ current_feature + S
0,236903	0,125424	-0,07874	1,888811	63	60	0,063753	0,336982	y ~ current_feature + S
-0,07038	0,128779	0,034007	-0,54651	63	60	0,586745	0,87187	y ~ current_feature + S
0,164969	0,127331	-0,04751	1,295593	63	60	0,200078	0,723562	y ~ current_feature + S
-0,29734	0,132403	0,061498	-2,24569	55	52	0,029	0,658491	y ~ current_feature + S
0,017789	0,160103	-0,02429	0,111107	42	39	0,912101	0,99444	y ~ current_feature + S
0,024282	0,053911	-0,07874	0,450412	392	343,8666	0,652697	0,932255	y ~ current_feature + S
0,084742	0,0714	0,034007	1,186853	392	194,7466	0,236731	0,68969	y ~ current_feature + S
0,04902	0,054643	-0,04751	0,897101	391	334,1121	0,370311	0,802018	y ~ current_feature + S
-0,01066	0,058758	0,061498	-0,1814	342	289,6107	0,85618	0,996179	y ~ current_feature + S
0,088451	0,062134	-0,02429	1,423552	260	257	0,155789	0,623156	y ~ current_feature + S
0,015225	0,102586	-0,07874	0,148411	98	95	0,882333	0,987929	y ~ current_feature + S
-0,01983	0,102578	0,034007	-0,19327	98	95	0,847159	0,959445	y ~ current_feature + S
-0,07189	0,102332	-0,04751	-0,70253	98	95	0,484065	0,852876	y ~ current_feature + S
0,017129	0,109093	0,061498	0,157015	87	84	0,87561	0,996179	y ~ current_feature + S
-0,10474	0,126302	-0,02429	-0,8293	65	62	0,410115	0,836046	y ~ current_feature + S
0,078661	0,099195	-0,07874	0,792987	104	101	0,429644	0,814177	y ~ current_feature + S
-0,05175	0,09937	0,034007	-0,52073	104	101	0,603691	0,883662	y ~ current_feature + S

-0,173	0,098003	-0,04751	-1,7652	104	101	0,080553	0,640171	y ~ current_feature + S
0,135138	0,105027	0,061498	1,28669	92	89	0,201539	0,893818	y ~ current_feature + S
-0,12306	0,122156	-0,02429	-1,00743	69	66	0,317405	0,766329	y ~ current_feature + S
0,09461	0,153611	-0,07874	0,615906	45	42	0,54128	0,882344	y ~ current_feature + S
-0,03496	0,154209	0,034007	-0,22673	45	42	0,821734	0,952501	y ~ current_feature + S
-0,10372	0,153471	-0,04751	-0,67583	45	42	0,502854	0,868909	y ~ current_feature + S
0,186742	0,166057	0,061498	1,124564	38	35	0,268427	0,893818	y ~ current_feature + S
0,086969	0,191721	-0,02429	0,453622	30	27	0,653726	0,924682	y ~ current_feature + S
0,079551	0,105664	-0,07874	0,752864	92	89	0,453518	0,82458	y ~ current_feature + S
0,051104	0,105861	0,034007	0,482748	92	89	0,63046	0,899624	y ~ current_feature + S
0,166702	0,104517	-0,04751	1,594978	92	89	0,114262	0,640171	y ~ current_feature + S
-0,15591	0,113305	0,061498	-1,37601	79	76	0,172859	0,840578	y ~ current_feature + S
0,031323	0,131242	-0,02429	0,238666	61	58	0,812206	0,974251	y ~ current_feature + S
0,038985	0,050729	-0,07874	0,768508	391	388	0,442653	0,818908	y ~ current_feature + S
0,096854	0,050529	0,034007	1,916809	391	388	0,055996	0,420946	y ~ current_feature + S
0,011149	0,05083	-0,04751	0,219333	390	387	0,826507	0,975438	y ~ current_feature + S
0,010204	0,063445	0,061498	0,160832	339	248,4026	0,872356	0,996179	y ~ current_feature + S
0,078084	0,062188	-0,02429	1,255615	260	257	0,210396	0,675518	y ~ current_feature + S
0,13848	0,094001	-0,07874	1,473174	114	111	0,143534	0,501016	y ~ current_feature + S
-0,05781	0,094757	0,034007	-0,61011	114	111	0,543034	0,852506	y ~ current_feature + S
5,39E-04	0,094916	-0,04751	0,005677	114	111	0,995481	0,995481	y ~ current_feature + S
-0,13701	0,107375	0,061498	-1,276	99	85,10716	0,205426	0,893818	y ~ current_feature + S
-0,13906	0,115904	-0,02429	-1,19974	76	73	0,234118	0,695919	y ~ current_feature + S
0,197909	0,100043	-0,07874	1,978234	99	96	0,050769	0,30055	y ~ current_feature + S
0,086286	0,101681	0,034007	0,84859	99	96	0,398221	0,775482	y ~ current_feature + S
0,06303	0,101859	-0,04751	0,618795	99	96	0,537517	0,885885	y ~ current_feature + S
-0,02566	0,112472	0,061498	-0,2281	82	79	0,820154	0,996179	y ~ current_feature + S
-0,02857	0,125937	-0,02429	-0,2269	66	63	0,821237	0,976828	y ~ current_feature + S
0,003667	0,071795	-0,07874	0,051079	197	194	0,959316	0,994607	y ~ current_feature + S
-0,04099	0,071735	0,034007	-0,57143	197	194	0,568372	0,863333	y ~ current_feature + S
-7,51E-04	0,071796	-0,04751	-0,01046	197	194	0,991663	0,99446	y ~ current_feature + S
-0,08641	0,076863	0,061498	-1,12418	171	168	0,26254	0,893818	y ~ current_feature + S
-0,00712	0,088386	-0,02429	-0,08056	131	128	0,935915	0,99444	y ~ current_feature + S
0,058917	0,069384	-0,07874	0,849147	210	207	0,39678	0,791422	y ~ current_feature + S
-0,0308	0,069472	0,034007	-0,44337	210	207	0,657962	0,900251	y ~ current_feature + S
0,134491	0,068873	-0,04751	1,952722	210	207	0,052201	0,640171	y ~ current_feature + S
-0,04847	0,074448	0,061498	-0,65109	183	180	0,515819	0,937665	y ~ current_feature + S
-0,13308	0,084676	-0,02429	-1,57158	140	137	0,118354	0,615361	y ~ current_feature + S
0,062904	0,070047	-0,07874	0,898016	206	203	0,370241	0,780565	y ~ current_feature + S
-0,11534	0,069718	0,034007	-1,6544	206	203	0,099592	0,518857	y ~ current_feature + S
-0,02625	0,070162	-0,04751	-0,37416	206	203	0,708677	0,93314	y ~ current_feature + S
0,024559	0,076006	0,061498	0,323116	176	173	0,746997	0,972573	y ~ current_feature + S
-0,10375	0,085921	-0,02429	-1,20748	137	134	0,229376	0,695919	y ~ current_feature + S
-0,1509	0,144195	-0,07874	-1,04653	50	47	0,300667	0,718687	y ~ current_feature + S
-0,17552	0,143601	0,034007	-1,22226	50	47	0,227703	0,681978	y ~ current_feature + S
-0,30134	0,140589	-0,04751	-2,1434	49	46	0,037402	0,611115	y ~ current_feature + S
0,178848	0,155565	0,061498	1,149673	43	40	0,257106	0,893818	y ~ current_feature + S
-0,12215	0,181207	-0,02429	-0,67408	33	30	0,505423	0,894767	y ~ current_feature + S

-0,03805	0,135984	-0,07874	-0,27978	57	54	0,780717	0,97261	y ~ current_feature + S
-0,09637	0,135449	0,034007	-0,71149	57	54	0,479841	0,831402	y ~ current_feature + S
0,004147	0,136082	-0,04751	0,030472	57	54	0,975803	0,992311	y ~ current_feature + S
0,045626	0,147288	0,061498	0,309775	49	46	0,758131	0,972573	y ~ current_feature + S
-0,04386	0,168868	-0,02429	-0,25971	38	35	0,796608	0,968297	y ~ current_feature + S
0,028555	0,073892	-0,07874	0,386449	186	183	0,699613	0,939646	y ~ current_feature + S
-0,02766	0,073894	0,034007	-0,37429	186	183	0,708623	0,920814	y ~ current_feature + S
0,111003	0,073465	-0,04751	1,510958	186	183	0,132524	0,657012	y ~ current_feature + S
0,045379	0,084405	0,061498	0,53763	161	140,0773	0,591685	0,945257	y ~ current_feature + S
-0,00609	0,09092	-0,02429	-0,06703	124	120,9674	0,946665	0,99444	y ~ current_feature + S
0,319973	0,132666	-0,07874	2,411861	54	51	0,019509	0,187487	y ~ current_feature + S
-0,1632	0,138151	0,034007	-1,18134	54	51	0,242948	0,702074	y ~ current_feature + S
0,018245	0,140005	-0,04751	0,130314	54	51	0,89683	0,985217	y ~ current_feature + S
-0,07138	0,15211	0,061498	-0,46928	46	43	0,641244	0,956496	y ~ current_feature + S
-0,64636	0,132828	-0,02429	-4,86612	36	33	2,74E-05	0,010121	y ~ current_feature + S
0,049261	0,100893	-0,07874	0,488256	101	98	0,626459	0,92163	y ~ current_feature + S
0,277217	0,097056	0,034007	2,856256	101	98	0,005234	0,168399	y ~ current_feature + S
0,13952	0,100542	-0,04751	1,387683	100	97	0,168413	0,700235	y ~ current_feature + S
-0,00958	0,110426	0,061498	-0,08678	85	82	0,931056	0,996179	y ~ current_feature + S
0,249193	0,121057	-0,02429	2,058481	67	64	0,04362	0,525585	y ~ current_feature + S
-0,01016	0,078322	-0,07874	-0,12977	166	163	0,896912	0,987929	y ~ current_feature + S
-0,08299	0,078056	0,034007	-1,06322	166	163	0,289254	0,718742	y ~ current_feature + S
0,06562	0,078157	-0,04751	0,839589	166	163	0,402369	0,803665	y ~ current_feature + S
-0,12664	0,084136	0,061498	-1,50517	142	139	0,134549	0,8197	y ~ current_feature + S
-0,04693	0,096567	-0,02429	-0,48597	110	107	0,62798	0,924682	y ~ current_feature + S
0,014584	0,07254	-0,07874	0,201047	193	190	0,840877	0,983548	y ~ current_feature + S
-0,14831	0,071745	0,034007	-2,06712	193	190	0,040078	0,396441	y ~ current_feature + S
0,001867	0,072739	-0,04751	0,025664	192	189	0,979552	0,992311	y ~ current_feature + S
-0,01664	0,078557	0,061498	-0,21187	165	162	0,832474	0,996179	y ~ current_feature + S
-0,1041	0,089219	-0,02429	-1,16673	128	124,2658	0,245552	0,715387	y ~ current_feature + S
-0,05056	0,139849	-0,07874	-0,36155	54	51	0,71918	0,948651	y ~ current_feature + S
-0,26581	0,134991	0,034007	-1,96909	54	51	0,054387	0,420946	y ~ current_feature + S
0,026891	0,139977	-0,04751	0,192109	54	51	0,84842	0,980218	y ~ current_feature + S
0,035065	0,156078	0,061498	0,224663	44	41	0,823357	0,996179	y ~ current_feature + S
0,089164	0,173384	-0,02429	0,514256	36	33	0,6105	0,924682	y ~ current_feature + S
-0,00424	0,090908	-0,07874	-0,04664	124	121	0,962876	0,994607	y ~ current_feature + S
0,077061	0,090639	0,034007	0,850203	124	121	0,396891	0,775482	y ~ current_feature + S
0,044085	0,091198	-0,04751	0,483401	123	120	0,629692	0,925756	y ~ current_feature + S
0,062134	0,097401	0,061498	0,637913	108	105	0,524919	0,937665	y ~ current_feature + S
0,008074	0,1118	-0,02429	0,072215	83	80	0,942611	0,99444	y ~ current_feature + S
0,444551	0,121897	-0,07874	3,646952	57	54	5,98E-04	0,031617	y ~ current_feature + S
0,269277	0,131056	0,034007	2,054667	57	54	0,044763	0,400524	y ~ current_feature + S
0,062489	0,135817	-0,04751	0,460096	57	54	0,647295	0,925756	y ~ current_feature + S
-0,09611	0,14519	0,061498	-0,66195	50	47	0,511235	0,937665	y ~ current_feature + S
0,239171	0,164125	-0,02429	1,45725	38	35	0,153962	0,623156	y ~ current_feature + S
0,139478	0,060971	-0,07874	2,287613	304	263,7675	0,022952	0,194866	y ~ current_feature + S
0,097962	0,081044	0,034007	1,20875	304	150,7879	0,228651	0,681978	y ~ current_feature + S
0,073159	0,062661	-0,04751	1,167536	304	253,324	0,244092	0,742928	y ~ current_feature + S

-0,13171	0,066869	0,061498	-1,96961	259	219,7618	0,050139	0,700056	y ~ current_feature + S
0,107165	0,070692	-0,02429	1,515934	203	197,8049	0,131132	0,617591	y ~ current_feature + S
0,165013	0,121404	-0,07874	1,359202	69	66	0,178709	0,572488	y ~ current_feature + S
0,152106	0,121659	0,034007	1,25026	69	66	0,215619	0,664036	y ~ current_feature + S
0,020367	0,123066	-0,04751	0,165493	69	66	0,869062	0,985217	y ~ current_feature + S
-0,16056	0,129603	0,061498	-1,23889	61	58	0,220377	0,893818	y ~ current_feature + S
-0,22073	0,148737	-0,02429	-1,48405	46	43	0,145089	0,619762	y ~ current_feature + S
0,165064	0,14227	-0,07874	1,160215	59	48,05913	0,251693	0,679754	y ~ current_feature + S
0,160062	0,15743	0,034007	1,016714	59	39,31432	0,315506	0,718742	y ~ current_feature + S
0,208548	0,140683	-0,04751	1,482398	59	48,32859	0,144726	0,661823	y ~ current_feature + S
-0,18802	0,184285	0,061498	-1,02025	51	28,40453	0,316223	0,893818	y ~ current_feature + S
0,58531	0,135162	-0,02429	4,330449	39	35,98578	1,14E-04	0,021049	y ~ current_feature + S
0,058516	0,051919	-0,07874	1,127058	421	369,7019	0,260449	0,690796	y ~ current_feature + S
0,043417	0,068324	0,034007	0,63546	421	213,8108	0,525808	0,847708	y ~ current_feature + S
0,01224	0,053045	-0,04751	0,230743	420	355,3474	0,817647	0,972405	y ~ current_feature + S
-0,09259	0,057747	0,061498	-1,60331	367	297,3071	0,109927	0,8197	y ~ current_feature + S
-0,10265	0,059767	-0,02429	-1,71756	280	277	0,086994	0,605474	y ~ current_feature + S
-0,0905	0,073821	-0,07874	-1,22596	185	182	0,221799	0,641138	y ~ current_feature + S
-0,06937	0,073946	0,034007	-0,93807	185	182	0,34945	0,749214	y ~ current_feature + S
-0,03453	0,074081	-0,04751	-0,46605	185	182	0,641734	0,925756	y ~ current_feature + S
0,168354	0,080217	0,061498	2,098721	154	151	0,037506	0,658491	y ~ current_feature + S
-0,07422	0,091035	-0,02429	-0,81524	123	120	0,416553	0,839968	y ~ current_feature + S
-0,01497	0,129085	-0,07874	-0,11598	63	60	0,908056	0,987929	y ~ current_feature + S
0,164779	0,127335	0,034007	1,294062	63	60	0,200603	0,647595	y ~ current_feature + S
-0,17076	0,127203	-0,04751	-1,34243	63	60	0,184515	0,704811	y ~ current_feature + S
0,107409	0,143503	0,061498	0,748482	51	48	0,457822	0,937665	y ~ current_feature + S
-0,0846	0,159554	-0,02429	-0,53023	42	39	0,598961	0,924682	y ~ current_feature + S
-0,02711	0,123046	-0,07874	-0,22029	69	66	0,826328	0,981192	y ~ current_feature + S
0,03042	0,123035	0,034007	0,24725	69	66	0,805482	0,952501	y ~ current_feature + S
0,009313	0,123086	-0,04751	0,075666	69	66	0,939913	0,990156	y ~ current_feature + S
0,085394	0,133143	0,061498	0,641371	59	56	0,523898	0,937665	y ~ current_feature + S
0,132109	0,151162	-0,02429	0,873958	46	43	0,386996	0,819479	y ~ current_feature + S
-0,01005	0,082474	-0,07874	-0,12188	150	147	0,903161	0,987929	y ~ current_feature + S
0,015548	0,082469	0,034007	0,188538	150	147	0,850715	0,959445	y ~ current_feature + S
0,073682	0,082254	-0,04751	0,895779	150	147	0,371835	0,80221	y ~ current_feature + S
0,022947	0,089064	0,061498	0,257651	129	126	0,797097	0,984075	y ~ current_feature + S
0,153038	0,100339	-0,02429	1,525212	100	97	0,13046	0,617591	y ~ current_feature + S
-0,07597	0,052191	-0,07874	-1,45559	368	365	0,146365	0,506121	y ~ current_feature + S
-0,00492	0,052342	0,034007	-0,09404	368	365	0,925128	0,9732	y ~ current_feature + S
-0,05084	0,052346	-0,04751	-0,97113	367	364	0,332126	0,780234	y ~ current_feature + S
-0,00811	0,056164	0,061498	-0,14442	320	317	0,885261	0,996179	y ~ current_feature + S
-0,05938	0,064169	-0,02429	-0,92539	245	242	0,355687	0,800026	y ~ current_feature + S
0,025774	0,067093	-0,07874	0,384153	225	222	0,701233	0,940058	y ~ current_feature + S
0,012098	0,067111	0,034007	0,180266	225	222	0,857108	0,961	y ~ current_feature + S
-0,00826	0,067113	-0,04751	-0,12312	225	222	0,902126	0,985217	y ~ current_feature + S
-0,04788	0,072656	0,061498	-0,65905	192	189	0,510665	0,937665	y ~ current_feature + S
-0,00289	0,082478	-0,02429	-0,03501	150	147	0,972118	0,99444	y ~ current_feature + S
0,11119	0,086173	-0,07874	1,290309	136	133	0,199182	0,611596	y ~ current_feature + S

0,02696	0,086679	0,034007	0,311026	136	133	0,756267	0,93416	y ~ current_feature + S
0,077949	0,086774	-0,04751	0,898298	135	132	0,370662	0,802018	y ~ current_feature + S
0,056657	0,093101	0,061498	0,608557	118	115	0,544018	0,937665	y ~ current_feature + S
0,159964	0,105228	-0,02429	1,520173	91	88	0,132053	0,617591	y ~ current_feature + S
0,113397	0,115498	-0,07874	0,981814	77	74	0,329391	0,729788	y ~ current_feature + S
-0,07585	0,115913	0,034007	-0,6544	77	74	0,514886	0,841996	y ~ current_feature + S
-0,02503	0,116211	-0,04751	-0,21538	77	74	0,830067	0,975438	y ~ current_feature + S
-0,19051	0,124674	0,061498	-1,52807	65	62	0,131581	0,8197	y ~ current_feature + S
-0,02934	0,144275	-0,02429	-0,20339	51	48	0,839694	0,977734	y ~ current_feature + S
0,052255	0,113073	-0,07874	0,462131	81	78	0,645274	0,930804	y ~ current_feature + S
-0,02135	0,113202	0,034007	-0,18862	81	78	0,850878	0,959445	y ~ current_feature + S
-0,04064	0,113134	-0,04751	-0,35923	81	78	0,720391	0,93314	y ~ current_feature + S
0,063972	0,12674	0,061498	0,504749	65	62	0,615524	0,948092	y ~ current_feature + S
-0,06661	0,139717	-0,02429	-0,47676	54	51	0,635569	0,924682	y ~ current_feature + S
0,021176	0,086367	-0,07874	0,24518	137	134	0,806692	0,979871	y ~ current_feature + S
-0,06126	0,086225	0,034007	-0,71051	137	134	0,478626	0,831402	y ~ current_feature + S
-0,03751	0,086326	-0,04751	-0,43451	137	134	0,664615	0,927953	y ~ current_feature + S
-0,11609	0,099361	0,061498	-1,16835	116	99,9245	0,245446	0,893818	y ~ current_feature + S
-0,12832	0,110236	-0,02429	-1,16406	91	80,93679	0,24782	0,717451	y ~ current_feature + S
0,01146	0,138666	-0,07874	0,082645	55	52	0,934451	0,9921	y ~ current_feature + S
-0,12571	0,137575	0,034007	-0,91375	55	52	0,365064	0,7498	y ~ current_feature + S
-0,18967	0,137486	-0,04751	-1,37954	54	51	0,173747	0,700235	y ~ current_feature + S
0,067548	0,153951	0,061498	0,438764	45	42	0,663081	0,957207	y ~ current_feature + S
-0,05762	0,173788	-0,02429	-0,33154	36	33	0,74233	0,945481	y ~ current_feature + S
0,015786	0,136066	-0,07874	0,116019	57	54	0,908067	0,987929	y ~ current_feature + S
-0,06118	0,135828	0,034007	-0,4504	57	54	0,654224	0,900251	y ~ current_feature + S
0,109079	0,135271	-0,04751	0,806375	57	54	0,423564	0,819126	y ~ current_feature + S
0,030195	0,150687	0,061498	0,200383	47	44	0,842104	0,996179	y ~ current_feature + S
-0,25657	0,163373	-0,02429	-1,57046	38	35	0,125305	0,617591	y ~ current_feature + S
0,019257	0,140002	-0,07874	0,137547	54	51	0,89114	0,987929	y ~ current_feature + S
0,144539	0,138558	0,034007	1,043168	54	51	0,30179	0,718742	y ~ current_feature + S
0,229773	0,136281	-0,04751	1,686021	54	51	0,097901	0,640171	y ~ current_feature + S
-0,00654	0,147439	0,061498	-0,04437	49	46	0,964802	0,996179	y ~ current_feature + S
-0,0912	0,173352	-0,02429	-0,52609	36	33	0,602347	0,924682	y ~ current_feature + S
-0,11603	0,109023	-0,07874	-1,06431	86	83	0,290274	0,718687	y ~ current_feature + S
-0,11633	0,109019	0,034007	-1,06702	86	83	0,289057	0,718742	y ~ current_feature + S
-0,15244	0,108481	-0,04751	-1,40521	86	83	0,163691	0,694976	y ~ current_feature + S
-0,23956	0,126327	0,061498	-1,89632	77	59,06604	0,062812	0,715249	y ~ current_feature + S
-0,01474	0,136068	-0,02429	-0,10835	57	54	0,914122	0,99444	y ~ current_feature + S
-0,01667	0,063491	-0,07874	-0,26255	251	248	0,793117	0,979102	y ~ current_feature + S
-0,11808	0,063056	0,034007	-1,87264	251	248	0,062295	0,437643	y ~ current_feature + S
0,001709	0,0635	-0,04751	0,026913	251	248	0,97855	0,992311	y ~ current_feature + S
0,051121	0,067952	0,061498	0,75231	219	216	0,452684	0,937665	y ~ current_feature + S
-0,02639	0,07806	-0,02429	-0,33804	167	164	0,735769	0,944216	y ~ current_feature + S
0,117288	0,143341	-0,07874	0,818242	51	48	0,417262	0,811887	y ~ current_feature + S
0,047887	0,144172	0,034007	0,332148	51	48	0,741223	0,926529	y ~ current_feature + S
0,226466	0,140588	-0,04751	1,610854	51	48	0,113769	0,640171	y ~ current_feature + S
0,045278	0,156014	0,061498	0,290219	44	41	0,773112	0,974621	y ~ current_feature + S

-0,02351	0,179556	-0,02429	-0,13094	34	31	0,896665	0,99444	y ~ current_feature + S
-0,04876	0,116109	-0,07874	-0,41997	77	74	0,675723	0,934653	y ~ current_feature + S
0,107018	0,11558	0,034007	0,925918	77	74	0,357498	0,7498	y ~ current_feature + S
0,139271	0,1159	-0,04751	1,201645	76	73	0,233385	0,742928	y ~ current_feature + S
0,020037	0,129074	0,061498	0,155239	63	60	0,877154	0,996179	y ~ current_feature + S
0,161588	0,142441	-0,02429	1,134421	51	48	0,262251	0,723659	y ~ current_feature + S
-0,00405	0,11785	-0,07874	-0,03435	75	72	0,972694	0,995565	y ~ current_feature + S
0,131923	0,116821	0,034007	1,129272	75	72	0,262532	0,71095	y ~ current_feature + S
0,190536	0,115692	-0,04751	1,64692	75	72	0,103933	0,640171	y ~ current_feature + S
0,051407	0,135258	0,061498	0,380068	63	54,51612	0,705371	0,972444	y ~ current_feature + S
0,310279	0,138666	-0,02429	2,237602	50	47	0,030026	0,485321	y ~ current_feature + S
0,037862	0,052888	-0,07874	0,715891	360	357	0,474526	0,84411	y ~ current_feature + S
0,043249	0,052876	0,034007	0,817933	360	357	0,413941	0,785427	y ~ current_feature + S
0,091335	0,052778	-0,04751	1,730545	359	356	0,084399	0,640171	y ~ current_feature + S
0,035893	0,061989	0,061498	0,579011	314	259,8996	0,563083	0,937665	y ~ current_feature + S
0,194216	0,063813	-0,02429	3,043526	240	236,3125	0,002603	0,18344	y ~ current_feature + S
0,044221	0,121149	-0,07874	0,365015	71	68	0,716233	0,948276	y ~ current_feature + S
0,087292	0,120805	0,034007	0,722586	71	68	0,472412	0,831402	y ~ current_feature + S
-0,21311	0,118482	-0,04751	-1,79864	71	68	0,076514	0,640171	y ~ current_feature + S
0,073286	0,134477	0,061498	0,544967	58	55	0,587979	0,945257	y ~ current_feature + S
-0,13715	0,149331	-0,02429	-0,91841	47	44	0,363412	0,805981	y ~ current_feature + S
-0,00255	0,102062	-0,07874	-0,02501	99	96	0,980101	0,996318	y ~ current_feature + S
0,023061	0,102035	0,034007	0,226009	99	96	0,821675	0,952501	y ~ current_feature + S
0,088602	0,102194	-0,04751	0,866995	98	95	0,388129	0,803665	y ~ current_feature + S
-0,13819	0,112681	0,061498	-1,22639	88	77,2552	0,223776	0,893818	y ~ current_feature + S
-0,00819	0,126996	-0,02429	-0,06451	65	62	0,948769	0,99444	y ~ current_feature + S
-0,1553	0,097813	-0,07874	-1,58776	105	102	0,115436	0,469356	y ~ current_feature + S
-0,01317	0,099006	0,034007	-0,13307	105	102	0,894403	0,96591	y ~ current_feature + S
0,106009	0,098457	-0,04751	1,076703	105	102	0,284153	0,747915	y ~ current_feature + S
-0,09794	0,107314	0,061498	-0,9126	89	86	0,364002	0,92247	y ~ current_feature + S
0,012787	0,122159	-0,02429	0,104674	70	67	0,916947	0,99444	y ~ current_feature + S
-0,00741	0,052264	-0,07874	-0,14169	403	366,0763	0,887405	0,987929	y ~ current_feature + S
-0,03897	0,068341	0,034007	-0,57023	403	213,7846	0,569118	0,863333	y ~ current_feature + S
-0,05965	0,05273	-0,04751	-1,13132	402	358,3706	0,258678	0,742928	y ~ current_feature + S
-0,11004	0,05575	0,061498	-1,97387	353	317,8444	0,049263	0,700056	y ~ current_feature + S
-0,03836	0,061384	-0,02429	-0,62485	268	265	0,532606	0,901895	y ~ current_feature + S
-0,03625	0,059152	-0,07874	-0,61275	296	285,4264	0,540528	0,882344	y ~ current_feature + S
0,094568	0,078152	0,034007	1,210064	296	162,2644	0,228014	0,681978	y ~ current_feature + S
-0,03223	0,060103	-0,04751	-0,5363	295	276,5356	0,592181	0,91049	y ~ current_feature + S
-0,12898	0,065171	0,061498	-1,97906	255	231,5265	0,048995	0,700056	y ~ current_feature + S
0,020961	0,072261	-0,02429	0,290073	197	191,4259	0,772074	0,957298	y ~ current_feature + S
-0,00682	0,140025	-0,07874	-0,04868	54	51	0,961366	0,994607	y ~ current_feature + S
-0,10076	0,139315	0,034007	-0,72326	54	51	0,472825	0,831402	y ~ current_feature + S
-0,10377	0,139272	-0,04751	-0,74508	54	51	0,459642	0,837772	y ~ current_feature + S
-0,14249	0,15458	0,061498	-0,9218	44	41	0,362027	0,920619	y ~ current_feature + S
-0,28119	0,167054	-0,02429	-1,68325	36	33	0,101766	0,605474	y ~ current_feature + S
0,071515	0,056052	-0,07874	1,275863	370	316,6576	0,202939	0,620557	y ~ current_feature + S
-0,00961	0,07175	0,034007	-0,13399	370	194,2287	0,893552	0,96591	y ~ current_feature + S

0,035778	0,05694	-0,04751	0,628338	369	308,0358	0,530248	0,883747	y ~ current_feature + S
-0,11825	0,059769	0,061498	-1,9784	322	276,0135	0,048877	0,700056	y ~ current_feature + S
0,026727	0,064403	-0,02429	0,414991	246	240,9248	0,678518	0,926342	y ~ current_feature + S
-0,07911	0,105079	-0,07874	-0,75283	93	90	0,453519	0,82458	y ~ current_feature + S
0,036752	0,105338	0,034007	0,3489	93	90	0,727979	0,922439	y ~ current_feature + S
-0,11425	0,104719	-0,04751	-1,09102	93	90	0,278177	0,747915	y ~ current_feature + S
-0,04598	0,123811	0,061498	-0,3714	78	65,09683	0,711542	0,972444	y ~ current_feature + S
0,195117	0,127687	-0,02429	1,528092	62	59	0,131834	0,617591	y ~ current_feature + S
-0,08191	0,056362	-0,07874	-1,45329	352	312,6795	0,147145	0,506453	y ~ current_feature + S
-0,03686	0,075361	0,034007	-0,48906	352	175,8396	0,625409	0,896904	y ~ current_feature + S
-0,02111	0,057267	-0,04751	-0,36857	351	304,788	0,712701	0,93314	y ~ current_feature + S
-0,02943	0,06002	0,061498	-0,49036	305	277,3538	0,624264	0,950526	y ~ current_feature + S
-0,02934	0,066934	-0,02429	-0,43842	234	223,0132	0,661509	0,924682	y ~ current_feature + S
-0,03117	0,072897	-0,07874	-0,42756	191	188	0,66946	0,934653	y ~ current_feature + S
0,028893	0,072902	0,034007	0,396333	191	188	0,692309	0,916569	y ~ current_feature + S
0,032795	0,072893	-0,04751	0,449901	191	188	0,6533	0,925756	y ~ current_feature + S
0,139275	0,077091	0,061498	1,806629	168	165	0,072642	0,721272	y ~ current_feature + S
0,078171	0,089528	-0,02429	0,87315	127	124	0,384269	0,819479	y ~ current_feature + S
-0,06258	0,059432	-0,07874	-1,053	285	282	0,293241	0,718687	y ~ current_feature + S
-0,10835	0,059199	0,034007	-1,83031	285	282	0,068259	0,441313	y ~ current_feature + S
0,001064	0,059655	-0,04751	0,017843	284	281	0,985776	0,993835	y ~ current_feature + S
-0,18466	0,07286	0,061498	-2,53446	246	181,9515	0,012105	0,55988	y ~ current_feature + S
-0,19584	0,073545	-0,02429	-2,66281	189	177,7904	0,00846	0,295269	y ~ current_feature + S
0,121798	0,092156	-0,07874	1,321641	119	116	0,188888	0,592937	y ~ current_feature + S
0,047145	0,092744	0,034007	0,508336	119	116	0,612183	0,889068	y ~ current_feature + S
-0,0404	0,092772	-0,04751	-0,43548	119	116	0,664026	0,927953	y ~ current_feature + S
0,113763	0,098858	0,061498	1,150772	104	101	0,252543	0,893818	y ~ current_feature + S
-0,10096	0,114122	-0,02429	-0,88468	79	76	0,379118	0,815551	y ~ current_feature + S
0,116276	0,093434	-0,07874	1,244472	116	113	0,215901	0,639357	y ~ current_feature + S
0,012334	0,094065	0,034007	0,131119	116	113	0,895915	0,96591	y ~ current_feature + S
-0,10011	0,094016	-0,04751	-1,06479	115	112	0,289261	0,747915	y ~ current_feature + S
-0,12841	0,101217	0,061498	-1,26865	99	96	0,207633	0,893818	y ~ current_feature + S
0,123737	0,115354	-0,02429	1,072669	77	74	0,286905	0,734108	y ~ current_feature + S
0,069949	0,060097	-0,07874	1,163934	308	275,525	0,245458	0,67447	y ~ current_feature + S
0,052392	0,075991	0,034007	0,68945	308	172,697	0,491466	0,837983	y ~ current_feature + S
0,016795	0,060476	-0,04751	0,277713	307	273,3446	0,781443	0,94882	y ~ current_feature + S
-0,06113	0,06276	0,061498	-0,974	273	252,9319	0,330987	0,893818	y ~ current_feature + S
0,001231	0,070892	-0,02429	0,017369	205	198,9781	0,98616	0,99444	y ~ current_feature + S
0,142303	0,062762	-0,07874	2,267368	280	248,7301	0,024226	0,197019	y ~ current_feature + S
0,143961	0,079359	0,034007	1,814034	280	155,4919	0,0716	0,456761	y ~ current_feature + S
0,046846	0,065432	-0,04751	0,71595	279	233,0565	0,474739	0,842057	y ~ current_feature + S
0,051249	0,06921	0,061498	0,74049	241	208,2196	0,459837	0,937665	y ~ current_feature + S
0,086329	0,074822	-0,02429	1,153793	186	177,2937	0,250138	0,717451	y ~ current_feature + S
0,197088	0,10006	-0,07874	1,969689	99	96	0,051756	0,303964	y ~ current_feature + S
0,217402	0,099621	0,034007	2,18229	99	96	0,031528	0,347616	y ~ current_feature + S
0,156947	0,100797	-0,04751	1,557053	99	96	0,122749	0,652786	y ~ current_feature + S
0,202158	0,106856	0,061498	1,891867	87	84	0,061954	0,715249	y ~ current_feature + S
-0,06598	0,125714	-0,02429	-0,52481	66	63	0,601556	0,924682	y ~ current_feature + S

-0,00108	0,120386	-0,07874	-0,00895	72	69	0,992884	0,996647	y ~ current_feature + S
-0,27076	0,115889	0,034007	-2,33638	72	69	0,022382	0,331255	y ~ current_feature + S
-0,03672	0,120305	-0,04751	-0,30521	72	69	0,761126	0,9394	y ~ current_feature + S
0,023182	0,130154	0,061498	0,178111	62	59	0,859246	0,996179	y ~ current_feature + S
-0,12096	0,148031	-0,02429	-0,81714	48	44,967	0,418154	0,840853	y ~ current_feature + S
0,160451	0,148802	-0,07874	1,078281	47	44	0,286784	0,718687	y ~ current_feature + S
-0,01143	0,150746	0,034007	-0,07582	47	44	0,939906	0,981002	y ~ current_feature + S
-0,02348	0,152457	-0,04751	-0,15398	46	43	0,878346	0,985217	y ~ current_feature + S
-0,18064	0,16625	0,061498	-1,08656	38	35	0,284659	0,893818	y ~ current_feature + S
-0,27973	0,181438	-0,02429	-1,54172	31	28	0,134368	0,617591	y ~ current_feature + S
0,077282	0,066467	-0,07874	1,162704	228	225	0,246181	0,67447	y ~ current_feature + S
-0,07716	0,066468	0,034007	-1,16086	228	225	0,24693	0,702074	y ~ current_feature + S
-0,04391	0,066602	-0,04751	-0,65925	228	225	0,51041	0,868909	y ~ current_feature + S
0,003654	0,077671	0,061498	0,047043	194	165,7597	0,962536	0,996179	y ~ current_feature + S
-0,06309	0,08176	-0,02429	-0,7717	152	149	0,441515	0,858965	y ~ current_feature + S
0,263176	0,145419	-0,07874	1,809785	48	44,01372	0,077161	0,370448	y ~ current_feature + S
0,432439	0,161435	0,034007	2,678719	48	31,19554	0,011689	0,233774	y ~ current_feature + S
-0,07975	0,171167	-0,04751	-0,4659	48	33,91496	0,644263	0,925756	y ~ current_feature + S
0,090709	0,166733	0,061498	0,544033	41	35,67523	0,589803	0,945257	y ~ current_feature + S
0,644876	0,177531	-0,02429	3,632464	32	18,53378	0,001832	0,169424	y ~ current_feature + S
0,012348	0,09016	-0,07874	0,136957	126	123	0,891288	0,987929	y ~ current_feature + S
-0,02721	0,090134	0,034007	-0,30186	126	123	0,763268	0,93668	y ~ current_feature + S
0,090687	0,089795	-0,04751	1,009928	126	123	0,314512	0,77066	y ~ current_feature + S
-0,44101	0,091018	0,061498	-4,84528	111	97,23336	4,78E-06	0,001769	y ~ current_feature + S
-0,05208	0,11096	-0,02429	-0,46934	84	81	0,640089	0,924682	y ~ current_feature + S
-0,12336	0,153125	-0,07874	-0,80562	45	42	0,425002	0,814177	y ~ current_feature + S
-0,08604	0,153731	0,034007	-0,55966	45	42	0,578682	0,869291	y ~ current_feature + S
0,080044	0,153808	-0,04751	0,520413	45	42	0,605509	0,914441	y ~ current_feature + S
-0,18695	0,159361	0,061498	-1,17315	41	38	0,248037	0,893818	y ~ current_feature + S
-0,10161	0,191454	-0,02429	-0,53074	30	27	0,599935	0,924682	y ~ current_feature + S
-0,07713	0,105095	-0,07874	-0,73394	93	90	0,464893	0,837033	y ~ current_feature + S
-0,15267	0,104174	0,034007	-1,4655	93	90	0,14627	0,594724	y ~ current_feature + S
-0,08065	0,105066	-0,04751	-0,76758	93	90	0,444749	0,824847	y ~ current_feature + S
0,004811	0,113226	0,061498	0,042486	81	78	0,96622	0,996179	y ~ current_feature + S
-0,24828	0,126112	-0,02429	-1,96872	62	59	0,053688	0,558147	y ~ current_feature + S
0,16642	0,110244	-0,07874	1,509556	83	80	0,135096	0,49737	y ~ current_feature + S
0,018679	0,111784	0,034007	0,167098	83	80	0,867714	0,96591	y ~ current_feature + S
-0,14103	0,110686	-0,04751	-1,27411	83	80	0,206313	0,723562	y ~ current_feature + S
-0,09407	0,119852	0,061498	-0,78492	72	69	0,43519	0,937665	y ~ current_feature + S
0,207742	0,139086	-0,02429	1,493626	55	49,46225	0,141626	0,618941	y ~ current_feature + S
0,101056	0,052146	-0,07874	1,937953	367	364	0,053402	0,30647	y ~ current_feature + S
0,093099	0,052187	0,034007	1,78397	367	364	0,075261	0,464477	y ~ current_feature + S
0,054839	0,052407	-0,04751	1,046392	366	363	0,296076	0,752909	y ~ current_feature + S
-0,04498	0,062726	0,061498	-0,71711	319	253,6479	0,473967	0,937665	y ~ current_feature + S
-0,00954	0,064413	-0,02429	-0,14818	244	241	0,882325	0,987777	y ~ current_feature + S
0,031243	0,117794	-0,07874	0,265231	75	72	0,791589	0,979102	y ~ current_feature + S
0,187955	0,115751	0,034007	1,623794	75	72	0,10879	0,536698	y ~ current_feature + S
-0,05425	0,117678	-0,04751	-0,46096	75	72	0,646213	0,925756	y ~ current_feature + S

0,069838	0,150148	0,061498	0,465132	64	44,14075	0,644124	0,956496	y ~ current_feature + S
0,234878	0,141784	-0,02429	1,656584	50	47	0,104265	0,605474	y ~ current_feature + S
-0,0432	0,128979	-0,07874	-0,33491	63	60	0,738863	0,959225	y ~ current_feature + S
-0,19295	0,126673	0,034007	-1,52322	63	60	0,132957	0,572281	y ~ current_feature + S
0,139182	0,127843	-0,04751	1,088696	63	60	0,280642	0,747915	y ~ current_feature + S
0,065826	0,142547	0,061498	0,461783	52	49	0,646281	0,956496	y ~ current_feature + S
0,025613	0,160076	-0,02429	0,160008	42	39	0,873701	0,984468	y ~ current_feature + S
-0,15147	0,080175	-0,07874	-1,88922	155	152	0,060767	0,325854	y ~ current_feature + S
-0,12264	0,080498	0,034007	-1,52348	155	152	0,129716	0,572145	y ~ current_feature + S
-0,10247	0,080684	-0,04751	-1,26998	155	152	0,206031	0,723562	y ~ current_feature + S
0,006001	0,087369	0,061498	0,068687	134	131	0,945344	0,996179	y ~ current_feature + S
-0,23056	0,096823	-0,02429	-2,38123	104	101	0,019129	0,428958	y ~ current_feature + S
-0,02826	0,050371	-0,07874	-0,56107	406	393,8134	0,575072	0,899631	y ~ current_feature + S
-0,08983	0,063289	0,034007	-1,41937	406	247,6449	0,157048	0,60427	y ~ current_feature + S
-0,01504	0,050963	-0,04751	-0,29516	405	384,9326	0,76803	0,942525	y ~ current_feature + S
-0,05808	0,054322	0,061498	-1,06917	353	337,7405	0,285757	0,893818	y ~ current_feature + S
-0,01912	0,062116	-0,02429	-0,30789	270	259,0823	0,758414	0,951727	y ~ current_feature + S
0,064893	0,062368	-0,07874	1,040482	259	256	0,299098	0,718687	y ~ current_feature + S
-0,0313	0,062469	0,034007	-0,50101	259	256	0,616796	0,889725	y ~ current_feature + S
0,0721	0,062337	-0,04751	1,156616	259	256	0,248507	0,742928	y ~ current_feature + S
-0,20714	0,072329	0,061498	-2,8638	227	182,9491	0,004675	0,378455	y ~ current_feature + S
0,113378	0,076241	-0,02429	1,487093	173	169,8256	0,138845	0,618941	y ~ current_feature + S
-0,02204	0,06665	-0,07874	-0,33063	228	225	0,741231	0,959588	y ~ current_feature + S
-0,01769	0,066656	0,034007	-0,26541	228	225	0,790935	0,945731	y ~ current_feature + S
0,043955	0,066602	-0,04751	0,659964	228	225	0,509952	0,868909	y ~ current_feature + S
0,022622	0,072339	0,061498	0,312725	194	191	0,754831	0,972573	y ~ current_feature + S
0,059035	0,08178	-0,02429	0,721873	152	149	0,471504	0,876224	y ~ current_feature + S
-0,06964	0,062606	-0,07874	-1,11243	278	253,8973	0,267007	0,702573	y ~ current_feature + S
0,038917	0,083126	0,034007	0,468172	278	144,5008	0,640367	0,899624	y ~ current_feature + S
0,048474	0,067542	-0,04751	0,717678	277	218,6889	0,473722	0,842057	y ~ current_feature + S
-0,06376	0,065966	0,061498	-0,96653	243	228,87	0,334801	0,893818	y ~ current_feature + S
0,131095	0,073485	-0,02429	1,78396	185	182	0,076096	0,605474	y ~ current_feature + S
-0,07059	0,15038	-0,07874	-0,46941	47	44	0,641097	0,928694	y ~ current_feature + S
0,003961	0,150754	0,034007	0,026274	47	44	0,979158	0,989859	y ~ current_feature + S
-0,25058	0,147633	-0,04751	-1,69728	46	43	0,096871	0,640171	y ~ current_feature + S
-0,2328	0,15377	0,061498	-1,51397	43	40	0,137897	0,8197	y ~ current_feature + S
0,205111	0,184964	-0,02429	1,108924	31	28	0,276897	0,734108	y ~ current_feature + S
0,083069	0,051325	-0,07874	1,61851	380	377	0,106389	0,449749	y ~ current_feature + S
0,097029	0,05126	0,034007	1,892898	380	377	0,059137	0,424869	y ~ current_feature + S
0,056918	0,051487	-0,04751	1,105465	379	376	0,269665	0,747915	y ~ current_feature + S
-0,009	0,060721	0,061498	-0,14827	330	271,2016	0,882239	0,996179	y ~ current_feature + S
0,139146	0,062786	-0,02429	2,216191	253	248,7608	0,027584	0,485321	y ~ current_feature + S
0,047194	0,055901	-0,07874	0,844242	381	319,2924	0,399166	0,791912	y ~ current_feature + S
-0,05146	0,072378	0,034007	-0,71103	381	190,385	0,477936	0,831402	y ~ current_feature + S
0,038637	0,056755	-0,04751	0,680773	380	309,9889	0,496523	0,868909	y ~ current_feature + S
-0,01068	0,060986	0,061498	-0,17507	332	268,8377	0,861153	0,996179	y ~ current_feature + S
0,057754	0,063475	-0,02429	0,909863	253	247,3655	0,363781	0,805981	y ~ current_feature + S
-0,12738	0,153046	-0,07874	-0,83231	45	42	0,40994	0,806796	y ~ current_feature + S

-0,18988	0,151496	0,034007	-1,25339	45	42	0,216998	0,664036	y ~ current_feature + S
-0,13878	0,15281	-0,04751	-0,90816	45	42	0,368977	0,802018	y ~ current_feature + S
0,129778	0,172622	0,061498	0,751806	36	32,99385	0,457497	0,937665	y ~ current_feature + S
0,001943	0,19245	-0,02429	0,010096	30	27	0,992019	0,996057	y ~ current_feature + S
-0,01479	0,136068	-0,07874	-0,10867	57	54	0,913868	0,987929	y ~ current_feature + S
0,056459	0,135866	0,034007	0,415547	57	54	0,679388	0,912427	y ~ current_feature + S
-0,16125	0,134302	-0,04751	-1,20067	57	54	0,235118	0,742928	y ~ current_feature + S
-0,06305	0,14405	0,061498	-0,43769	51	48	0,663577	0,957207	y ~ current_feature + S
0,124896	0,167707	-0,02429	0,744727	38	35	0,461408	0,871026	y ~ current_feature + S
0,028773	0,056773	-0,07874	0,506819	313	310	0,612642	0,918951	y ~ current_feature + S
-0,00158	0,056796	0,034007	-0,02785	313	310	0,977798	0,989859	y ~ current_feature + S
-0,01348	0,056883	-0,04751	-0,23705	312	309	0,812774	0,972405	y ~ current_feature + S
-0,07551	0,060572	0,061498	-1,24669	274	271	0,213589	0,893818	y ~ current_feature + S
0,053884	0,069742	-0,02429	0,772624	208	205	0,440635	0,858965	y ~ current_feature + S
-0,0567	0,092698	-0,07874	-0,61164	119	116	0,541973	0,882344	y ~ current_feature + S
-0,18414	0,09126	0,034007	-2,01772	119	116	0,045928	0,400524	y ~ current_feature + S
0,0093	0,093246	-0,04751	0,099736	118	115	0,920727	0,985217	y ~ current_feature + S
-0,26402	0,096452	0,061498	-2,73738	103	100	0,007331	0,417322	y ~ current_feature + S
0,055766	0,114529	-0,02429	0,486918	79	76	0,627718	0,924682	y ~ current_feature + S
0,16901	0,102757	-0,07874	1,644751	95	92	0,103434	0,449749	y ~ current_feature + S
0,098526	0,10375	0,034007	0,949648	95	92	0,344779	0,746014	y ~ current_feature + S
0,277679	0,100706	-0,04751	2,757321	94	91	0,007042	0,432614	y ~ current_feature + S
-0,05538	0,108942	0,061498	-0,50833	87	84	0,612555	0,948092	y ~ current_feature + S
0,274799	0,124129	-0,02429	2,213809	63	60	0,030654	0,485321	y ~ current_feature + S
-0,0153	0,058715	-0,07874	-0,26055	293	290	0,794627	0,979102	y ~ current_feature + S
-0,08262	0,058521	0,034007	-1,41183	293	290	0,159072	0,60427	y ~ current_feature + S
-0,02869	0,058698	-0,04751	-0,48876	293	290	0,625378	0,925756	y ~ current_feature + S
-0,04412	0,062933	0,061498	-0,70114	255	252	0,483863	0,937665	y ~ current_feature + S
-0,03155	0,072133	-0,02429	-0,43746	195	192	0,662272	0,924682	y ~ current_feature + S
0,138368	0,062066	-0,07874	2,229356	315	254,6211	0,026663	0,204832	y ~ current_feature + S
0,077378	0,056444	0,034007	1,370878	315	312	0,171398	0,618964	y ~ current_feature + S
0,041209	0,056657	-0,04751	0,727354	314	311	0,467556	0,842057	y ~ current_feature + S
-0,06716	0,066227	0,061498	-1,01416	274	226,9713	0,311585	0,893818	y ~ current_feature + S
0,07075	0,069332	-0,02429	1,020453	210	206,9904	0,308705	0,761472	y ~ current_feature + S
-0,02658	0,132406	-0,07874	-0,20073	60	57	0,841622	0,983548	y ~ current_feature + S
-0,12806	0,131363	0,034007	-0,97487	60	57	0,333746	0,734392	y ~ current_feature + S
-0,00818	0,132449	-0,04751	-0,06176	60	57	0,950967	0,990156	y ~ current_feature + S
-0,08141	0,140952	0,061498	-0,57758	53	50	0,566141	0,937665	y ~ current_feature + S
-0,12535	0,163102	-0,02429	-0,76854	40	37	0,447046	0,858965	y ~ current_feature + S
0,007134	0,051707	-0,07874	0,137963	377	374	0,890344	0,987929	y ~ current_feature + S
-1,31E-04	0,051709	0,034007	-0,00253	377	374	0,997984	0,997989	y ~ current_feature + S
-0,01121	0,051775	-0,04751	-0,2166	376	373	0,828635	0,975438	y ~ current_feature + S
-0,08442	0,055357	0,061498	-1,52504	327	324	0,128224	0,8197	y ~ current_feature + S
-0,03655	0,063458	-0,02429	-0,57597	251	248	0,56516	0,924682	y ~ current_feature + S
0,036249	0,056577	-0,07874	0,640712	315	312	0,52218	0,876542	y ~ current_feature + S
-0,02599	0,056595	0,034007	-0,45927	315	312	0,646357	0,900251	y ~ current_feature + S
-0,09571	0,056354	-0,04751	-1,6983	315	312	0,090448	0,640171	y ~ current_feature + S
-0,15464	0,059905	0,061498	-2,58139	275	272	0,010364	0,547829	y ~ current_feature + S

-0,06919	0,069338	-0,02429	-0,99791	210	207	0,31949	0,767607	y ~ current_feature + S
0,011045	0,064546	-0,07874	0,171126	243	240	0,864269	0,985453	y ~ current_feature + S
0,022679	0,064533	0,034007	0,351439	243	240	0,725567	0,92096	y ~ current_feature + S
0,065228	0,064412	-0,04751	1,012666	243	240	0,312239	0,767632	y ~ current_feature + S
0,020174	0,068993	0,061498	0,292405	213	210	0,770265	0,973246	y ~ current_feature + S
0,05639	0,079179	-0,02429	0,712187	162	159	0,477392	0,878782	y ~ current_feature + S
0,075837	0,075161	-0,07874	1,008991	179	176	0,314364	0,718687	y ~ current_feature + S
-0,07444	0,075169	0,034007	-0,9903	179	176	0,323387	0,728793	y ~ current_feature + S
0,063595	0,07544	-0,04751	0,842985	178	175	0,400387	0,803665	y ~ current_feature + S
-0,01726	0,081911	0,061498	-0,21069	152	149	0,83342	0,996179	y ~ current_feature + S
-0,07056	0,092616	-0,02429	-0,76191	119	116	0,447663	0,858965	y ~ current_feature + S
-0,00855	0,089084	-0,07874	-0,09597	129	126	0,923695	0,990519	y ~ current_feature + S
-0,01348	0,089079	0,034007	-0,15131	129	126	0,87997	0,96591	y ~ current_feature + S
-0,01271	0,08908	-0,04751	-0,14272	129	126	0,886736	0,985217	y ~ current_feature + S
0,209506	0,094528	0,061498	2,216333	110	107	0,028785	0,658491	y ~ current_feature + S
0,035773	0,109694	-0,02429	0,326119	86	83	0,745155	0,947448	y ~ current_feature + S
0,249916	0,107585	-0,07874	2,322958	84	81	0,022691	0,194866	y ~ current_feature + S
0,114964	0,110374	0,034007	1,041586	84	81	0,300703	0,718742	y ~ current_feature + S
0,25148	0,10754	-0,04751	2,33847	84	81	0,021829	0,557019	y ~ current_feature + S
0,167031	0,118695	0,061498	1,407233	72	69	0,163847	0,8197	y ~ current_feature + S
0,177194	0,135187	-0,02429	1,310732	56	53	0,1956	0,657926	y ~ current_feature + S
0,064361	0,117607	-0,07874	0,547255	75	72	0,585896	0,902886	y ~ current_feature + S
0,122796	0,116959	0,034007	1,049903	75	72	0,297274	0,718742	y ~ current_feature + S
-0,07856	0,117487	-0,04751	-0,66865	75	72	0,505855	0,868909	y ~ current_feature + S
-0,12905	0,12802	0,061498	-1,00803	63	60	0,31749	0,893818	y ~ current_feature + S
-0,00972	0,145858	-0,02429	-0,06663	50	47	0,94716	0,99444	y ~ current_feature + S
0,13278	0,08347	-0,07874	1,590764	144	141	0,113902	0,467692	y ~ current_feature + S
0,11972	0,083609	0,034007	1,431901	144	141	0,154385	0,60427	y ~ current_feature + S
0,002623	0,084215	-0,04751	0,031148	144	141	0,975195	0,992311	y ~ current_feature + S
0,027981	0,092414	0,061498	0,302777	120	117	0,762597	0,972573	y ~ current_feature + S
0,198716	0,101627	-0,02429	1,955342	96	93	0,053545	0,558147	y ~ current_feature + S
0,079029	0,054828	-0,07874	1,441382	370	330,5736	0,150423	0,513608	y ~ current_feature + S
0,138545	0,07032	0,034007	1,970209	370	198,346	0,050206	0,420946	y ~ current_feature + S
0,081481	0,055151	-0,04751	1,477405	369	326,5831	0,140531	0,660782	y ~ current_feature + S
-0,00579	0,059111	0,061498	-0,09796	318	286,1873	0,922036	0,996179	y ~ current_feature + S
0,087737	0,06461	-0,02429	1,357951	246	237,7075	0,175767	0,631395	y ~ current_feature + S
0,003596	0,090535	-0,07874	0,039719	125	122	0,968382	0,994607	y ~ current_feature + S
0,02966	0,090496	0,034007	0,327751	125	122	0,743661	0,927328	y ~ current_feature + S
-0,01287	0,090528	-0,04751	-0,14217	125	122	0,887183	0,985217	y ~ current_feature + S
-0,1411	0,097077	0,061498	-1,45351	107	104	0,149092	0,8197	y ~ current_feature + S
-0,1341	0,110794	-0,02429	-1,21037	83	80	0,2297	0,695919	y ~ current_feature + S
-0,13861	0,098543	-0,07874	-1,40663	104	101	0,162605	0,532425	y ~ current_feature + S
-0,20228	0,097447	0,034007	-2,0758	104	101	0,040452	0,396441	y ~ current_feature + S
-0,15634	0,09828	-0,04751	-1,59079	104	101	0,114783	0,640171	y ~ current_feature + S
0,078643	0,113606	0,061498	0,692248	84	77,00274	0,490864	0,937665	y ~ current_feature + S
0,062077	0,132073	-0,02429	0,470017	69	57,1079	0,640133	0,924682	y ~ current_feature + S
0,058169	0,057498	-0,07874	1,01167	344	301,4535	0,312507	0,718687	y ~ current_feature + S
-0,0993	0,07645	0,034007	-1,29888	344	169,4119	0,19575	0,6438	y ~ current_feature + S

-0,08204	0,059939	-0,04751	-1,36873	343	276,4727	0,172193	0,700235	y ~ current_feature + S
-0,06964	0,061008	0,061498	-1,14155	298	267,3674	0,254661	0,893818	y ~ current_feature + S
-0,07992	0,067063	-0,02429	-1,19176	229	220,925	0,234634	0,695919	y ~ current_feature + S
0,021505	0,099481	-0,07874	0,216169	104	101	0,829292	0,981192	y ~ current_feature + S
0,087428	0,099123	0,034007	0,882021	104	101	0,37986	0,759222	y ~ current_feature + S
-0,15506	0,09879	-0,04751	-1,56963	103	100	0,119661	0,651097	y ~ current_feature + S
-0,19629	0,105126	0,061498	-1,86722	90	87	0,065239	0,715249	y ~ current_feature + S
-0,05912	0,122876	-0,02429	-0,48114	69	66	0,632008	0,924682	y ~ current_feature + S
0,044916	0,098915	-0,07874	0,454091	105	102	0,650728	0,932255	y ~ current_feature + S
0,00486	0,099014	0,034007	0,049085	105	102	0,960948	0,989859	y ~ current_feature + S
0,112774	0,098383	-0,04751	1,146274	105	102	0,254364	0,742928	y ~ current_feature + S
0,156645	0,124387	0,061498	1,259333	89	63,04657	0,212552	0,893818	y ~ current_feature + S
-0,05139	0,124962	-0,02429	-0,41122	70	63,86965	0,682284	0,926342	y ~ current_feature + S
0,015121	0,095335	-0,07874	0,158605	113	110	0,874271	0,987929	y ~ current_feature + S
-0,00387	0,095346	0,034007	-0,04056	113	110	0,96772	0,989859	y ~ current_feature + S
0,292855	0,091583	-0,04751	3,197696	112	109	0,001814	0,191719	y ~ current_feature + S
-0,11804	0,100825	0,061498	-1,17078	100	97	0,244555	0,893818	y ~ current_feature + S
0,074509	0,117524	-0,02429	0,633991	75	72	0,528096	0,899808	y ~ current_feature + S
-0,00737	0,141418	-0,07874	-0,05211	53	50	0,958649	0,994607	y ~ current_feature + S
-0,10387	0,140656	0,034007	-0,73846	53	50	0,463688	0,830821	y ~ current_feature + S
0,008216	0,142852	-0,04751	0,057514	52	49	0,95437	0,990156	y ~ current_feature + S
-0,05709	0,15225	0,061498	-0,37495	46	43	0,70954	0,972444	y ~ current_feature + S
0,215543	0,172621	-0,02429	1,248647	35	32	0,220852	0,69545	y ~ current_feature + S
-0,01703	0,052844	-0,07874	-0,32221	361	358	0,74748	0,962676	y ~ current_feature + S
-0,04834	0,05279	0,034007	-0,91569	361	358	0,360446	0,7498	y ~ current_feature + S
-0,01685	0,052918	-0,04751	-0,31847	360	357	0,750315	0,9394	y ~ current_feature + S
-0,00218	0,05698	0,061498	-0,03819	311	308	0,969562	0,996179	y ~ current_feature + S
-0,12869	0,064417	-0,02429	-1,99784	240	237	0,046876	0,525585	y ~ current_feature + S
0,080835	0,105653	-0,07874	0,765104	92	89	0,446234	0,821425	y ~ current_feature + S
0,010523	0,105994	0,034007	0,099276	92	89	0,921142	0,9732	y ~ current_feature + S
-0,05989	0,105809	-0,04751	-0,56606	92	89	0,572776	0,903741	y ~ current_feature + S
0,083463	0,112833	0,061498	0,739702	81	78	0,461701	0,937665	y ~ current_feature + S
0,068987	0,130994	-0,02429	0,526644	61	58	0,600449	0,924682	y ~ current_feature + S
-0,01133	0,113953	-0,07874	-0,0994	80	77	0,921082	0,990519	y ~ current_feature + S
2,88E-04	0,113961	0,034007	0,002528	80	77	0,997989	0,997989	y ~ current_feature + S
-0,02015	0,114685	-0,04751	-0,17569	79	76	0,861005	0,984767	y ~ current_feature + S
-0,13043	0,12204	0,061498	-1,06876	69	66	0,289072	0,893818	y ~ current_feature + S
0,15167	0,139785	-0,02429	1,08502	53	50	0,283117	0,734108	y ~ current_feature + S
0,019825	0,076681	-0,07874	0,258539	173	170	0,796304	0,979102	y ~ current_feature + S
0,021628	0,076679	0,034007	0,282058	173	170	0,778242	0,942718	y ~ current_feature + S
-0,01906	0,076683	-0,04751	-0,24861	173	170	0,80396	0,968942	y ~ current_feature + S
-0,10689	0,084516	0,061498	-1,26469	148	138,3972	0,208108	0,893818	y ~ current_feature + S
-0,06819	0,094293	-0,02429	-0,72319	115	111,9488	0,471074	0,876224	y ~ current_feature + S
-0,13825	0,108524	-0,07874	-1,2739	90	83,28586	0,206243	0,622938	y ~ current_feature + S
-0,16926	0,151737	0,034007	-1,1155	90	42,18825	0,27095	0,71095	y ~ current_feature + S
0,109013	0,106805	-0,04751	1,02067	90	86,6212	0,310253	0,76529	y ~ current_feature + S
0,125346	0,11618	0,061498	1,078901	76	72,92249	0,284188	0,893818	y ~ current_feature + S
-0,06949	0,133499	-0,02429	-0,52053	60	55,83949	0,604749	0,924682	y ~ current_feature + S

0,00749	0,150751	-0,07874	0,049683	47	44	0,960599	0,994607	y ~ current_feature + S
-0,1264	0,149546	0,034007	-0,84524	47	44	0,402552	0,778997	y ~ current_feature + S
0,148357	0,150811	-0,04751	0,983731	46	43	0,330751	0,779476	y ~ current_feature + S
-0,263	0,152548	0,061498	-1,72404	43	40	0,092423	0,795265	y ~ current_feature + S
0,14908	0,18687	-0,02429	0,797772	31	28	0,431717	0,851922	y ~ current_feature + S
0,030371	0,12593	-0,07874	0,241175	66	63	0,810202	0,979871	y ~ current_feature + S
0,180503	0,123919	0,034007	1,456626	66	63	0,150186	0,604094	y ~ current_feature + S
0,07454	0,125638	-0,04751	0,593297	66	63	0,555106	0,897386	y ~ current_feature + S
-0,07908	0,133212	0,061498	-0,59366	59	56	0,555128	0,937665	y ~ current_feature + S
0,316638	0,148138	-0,02429	2,137449	44	41	0,038573	0,520476	y ~ current_feature + S
0,427882	0,139465	-0,07874	3,068031	45	42	0,003764	0,069632	y ~ current_feature + S
0,026127	0,154251	0,034007	0,169379	45	42	0,866311	0,96591	y ~ current_feature + S
0,058483	0,154039	-0,04751	0,379666	45	42	0,706107	0,93314	y ~ current_feature + S
0,07356	0,166215	0,061498	0,44256	39	36	0,660732	0,957207	y ~ current_feature + S
-0,08077	0,191821	-0,02429	-0,42109	30	27	0,677025	0,926342	y ~ current_feature + S
-0,03496	0,062063	-0,07874	-0,56324	297	259,2979	0,573756	0,899533	y ~ current_feature + S
-0,0731	0,082583	0,034007	-0,88515	297	145,8459	0,377534	0,759171	y ~ current_feature + S
-0,13233	0,064233	-0,04751	-2,0601	297	238,1319	0,040476	0,612508	y ~ current_feature + S
-0,16813	0,069191	0,061498	-2,43002	261	202,9781	0,015968	0,55988	y ~ current_feature + S
-0,1028	0,071232	-0,02429	-1,44321	198	195	0,150565	0,622449	y ~ current_feature + S
-0,01241	0,065648	-0,07874	-0,18906	235	232	0,850209	0,983548	y ~ current_feature + S
-0,05552	0,065552	0,034007	-0,84694	235	232	0,397901	0,775482	y ~ current_feature + S
-0,01242	0,06579	-0,04751	-0,18881	234	231	0,850405	0,980218	y ~ current_feature + S
-0,21177	0,069107	0,061498	-3,06431	203	200	0,002483	0,305054	y ~ current_feature + S
-0,1337	0,080119	-0,02429	-1,66874	156	153	0,097214	0,605474	y ~ current_feature + S
0,011791	0,09534	-0,07874	0,123677	113	110	0,901797	0,987929	y ~ current_feature + S
-0,07587	0,095071	0,034007	-0,79806	113	110	0,426554	0,791701	y ~ current_feature + S
-0,02296	0,095757	-0,04751	-0,23974	112	109	0,810984	0,972405	y ~ current_feature + S
0,075124	0,103402	0,061498	0,72652	96	93	0,469345	0,937665	y ~ current_feature + S
0,088794	0,117386	-0,02429	0,756429	75	72	0,45186	0,861794	y ~ current_feature + S
-0,02864	0,071399	-0,07874	-0,40117	199	196	0,68873	0,93638	y ~ current_feature + S
-0,14255	0,070699	0,034007	-2,01624	199	196	0,04514	0,400524	y ~ current_feature + S
0,007073	0,071427	-0,04751	0,099021	199	196	0,921223	0,985217	y ~ current_feature + S
-0,09265	0,076592	0,061498	-1,2097	172	169	0,228084	0,893818	y ~ current_feature + S
-0,18642	0,086172	-0,02429	-2,16338	133	129,9891	0,032341	0,485321	y ~ current_feature + S
-0,01913	0,05024	-0,07874	-0,38086	418	396,036	0,703512	0,941409	y ~ current_feature + S
0,058014	0,064627	0,034007	0,897685	418	238,6234	0,370258	0,750317	y ~ current_feature + S
0,037431	0,051094	-0,04751	0,732592	417	382,5106	0,464256	0,842057	y ~ current_feature + S
0,052518	0,056117	0,061498	0,935867	364	316,6787	0,350055	0,915337	y ~ current_feature + S
0,032605	0,061064	-0,02429	0,53395	278	267,9008	0,593819	0,924682	y ~ current_feature + S
0,037848	0,05429	-0,07874	0,697144	372	338,7953	0,486191	0,84854	y ~ current_feature + S
-0,02412	0,070634	0,034007	-0,34152	372	200,3192	0,733071	0,922572	y ~ current_feature + S
-0,01137	0,05585	-0,04751	-0,20364	372	320,5535	0,838765	0,980218	y ~ current_feature + S
-0,03937	0,059972	0,061498	-0,65653	325	277,6094	0,512029	0,937665	y ~ current_feature + S
-0,12872	0,063801	-0,02429	-2,0175	248	241,5994	0,044749	0,525585	y ~ current_feature + S
0,029091	0,053603	-0,07874	0,542703	377	347,7346	0,587682	0,902886	y ~ current_feature + S
0,003106	0,069048	0,034007	0,044983	377	209,7488	0,964164	0,989859	y ~ current_feature + S
0,087361	0,053745	-0,04751	1,625494	377	343,5614	0,104974	0,640171	y ~ current_feature + S

0,035545	0,058396	0,061498	0,608687	325	292,8723	0,543204	0,937665	y ~ current_feature + S
-0,04023	0,063811	-0,02429	-0,63052	251	245,1905	0,528941	0,899808	y ~ current_feature + S
-0,06566	0,05539	-0,07874	-1,18537	358	324,5374	0,236739	0,663586	y ~ current_feature + S
-0,10567	0,07204	0,034007	-1,46688	358	190,5351	0,144058	0,592239	y ~ current_feature + S
0,009247	0,056288	-0,04751	0,164272	357	315,5954	0,869622	0,985217	y ~ current_feature + S
0,005694	0,061827	0,061498	0,092104	309	261,5993	0,926686	0,996179	y ~ current_feature + S
-0,02747	0,065435	-0,02429	-0,4198	238	233,3743	0,675017	0,926342	y ~ current_feature + S
0,032309	0,083581	-0,07874	0,386562	146	143	0,699655	0,939646	y ~ current_feature + S
-0,22829	0,081416	0,034007	-2,80398	146	143	0,00575	0,170187	y ~ current_feature + S
0,090114	0,083284	-0,04751	1,082011	146	143	0,281069	0,747915	y ~ current_feature + S
0,011633	0,090161	0,061498	0,129021	126	123	0,897552	0,996179	y ~ current_feature + S
-0,0231	0,103115	-0,02429	-0,22398	97	94	0,823256	0,976828	y ~ current_feature + S
0,150897	0,061952	-0,07874	2,435718	281	254,6187	0,015549	0,174676	y ~ current_feature + S
-0,03568	0,088731	0,034007	-0,4021	281	126,8511	0,68829	0,916569	y ~ current_feature + S
0,075817	0,06714	-0,04751	1,129236	281	220,5655	0,260025	0,742928	y ~ current_feature + S
-0,04523	0,067905	0,061498	-0,66602	244	216,4244	0,506108	0,937665	y ~ current_feature + S
0,029495	0,073689	-0,02429	0,400264	187	184	0,689426	0,926342	y ~ current_feature + S
-0,04	0,051139	-0,07874	-0,78218	407	381,7646	0,434595	0,814177	y ~ current_feature + S
-0,07159	0,065912	0,034007	-1,08617	407	229,0004	0,278545	0,715049	y ~ current_feature + S
-0,03034	0,052129	-0,04751	-0,58205	406	367,6531	0,56089	0,900344	y ~ current_feature + S
-0,0846	0,057097	0,061498	-1,48168	353	304,5423	0,13946	0,8197	y ~ current_feature + S
-0,00162	0,061541	-0,02429	-0,02626	271	264,0364	0,979067	0,99444	y ~ current_feature + S
0,268666	0,103868	-0,07874	2,586611	89	86	0,011374	0,140685	y ~ current_feature + S
0,020675	0,10781	0,034007	0,191776	89	86	0,848369	0,959445	y ~ current_feature + S
0,156733	0,107125	-0,04751	1,463089	88	85	0,147131	0,661823	y ~ current_feature + S
-0,04022	0,116946	0,061498	-0,34393	76	73	0,731887	0,972573	y ~ current_feature + S
0,005816	0,133628	-0,02429	0,043521	59	56	0,965441	0,99444	y ~ current_feature + S
-0,12082	0,101846	-0,07874	-1,1863	98	95	0,238464	0,6659	y ~ current_feature + S
-0,03897	0,10252	0,034007	-0,38011	98	95	0,70471	0,920814	y ~ current_feature + S
-0,0213	0,103119	-0,04751	-0,20653	97	94	0,836825	0,979827	y ~ current_feature + S
0,040588	0,107744	0,061498	0,37671	89	86	0,707317	0,972444	y ~ current_feature + S
0,116551	0,126135	-0,02429	0,924023	65	62	0,359057	0,800662	y ~ current_feature + S
0,089604	0,148472	-0,07874	0,60351	48	45	0,5492	0,888464	y ~ current_feature + S
-0,26018	0,143937	0,034007	-1,80757	48	45	0,077361	0,469239	y ~ current_feature + S
0,34053	0,140162	-0,04751	2,429551	48	45	0,019172	0,54566	y ~ current_feature + S
0,184996	0,155385	0,061498	1,190565	43	40	0,240842	0,893818	y ~ current_feature + S
0,087055	0,18499	-0,02429	0,470593	32	29	0,641452	0,924682	y ~ current_feature + S
-0,03964	0,062267	-0,07874	-0,63668	276	257,5156	0,5249	0,876542	y ~ current_feature + S
-0,00791	0,083251	0,034007	-0,09499	276	144,2749	0,924458	0,9732	y ~ current_feature + S
-0,02169	0,062623	-0,04751	-0,34634	276	254,876	0,729375	0,93314	y ~ current_feature + S
-0,03908	0,066091	0,061498	-0,59133	241	228,5892	0,554885	0,937665	y ~ current_feature + S
-0,01906	0,076436	-0,02429	-0,2493	183	171,0993	0,803425	0,968297	y ~ current_feature + S
-0,18852	0,058698	-0,07874	-3,21175	316	279,9268	0,001473	0,048726	y ~ current_feature + S
-0,28696	0,077044	0,034007	-3,72459	316	154,597	2,74E-04	0,067708	y ~ current_feature + S
-0,07245	0,062508	-0,04751	-1,15908	315	254,5912	0,247509	0,742928	y ~ current_feature + S
-0,0596	0,064854	0,061498	-0,919	272	236,9121	0,359031	0,920619	y ~ current_feature + S
-0,15158	0,068812	-0,02429	-2,20276	210	206,3342	0,028718	0,485321	y ~ current_feature + S
-0,08147	0,080576	-0,07874	-1,0111	156	153	0,313564	0,718687	y ~ current_feature + S

-0,10456	0,080402	0,034007	-1,30045	156	153	0,195401	0,6438	y ~ current_feature + S
-0,07137	0,080639	-0,04751	-0,88507	156	153	0,377508	0,803665	y ~ current_feature + S
-0,25323	0,082954	0,061498	-3,05259	139	136	0,002729	0,305054	y ~ current_feature + S
0,008365	0,0995	-0,02429	0,084067	104	101	0,93317	0,99444	y ~ current_feature + S
0,192615	0,121712	-0,07874	1,582543	68	65	0,11838	0,474142	y ~ current_feature + S
-0,0484	0,123889	0,034007	-0,39064	68	65	0,69734	0,918206	y ~ current_feature + S
-0,05641	0,124801	-0,04751	-0,45198	67	64	0,652811	0,925756	y ~ current_feature + S
-0,05863	0,132225	0,061498	-0,44342	60	57	0,659142	0,957207	y ~ current_feature + S
0,045776	0,154142	-0,02429	0,296977	45	42	0,767948	0,955095	y ~ current_feature + S
0,079765	0,076452	-0,07874	1,043339	173	170	0,298273	0,718687	y ~ current_feature + S
0,069667	0,07651	0,034007	0,910561	173	170	0,363816	0,7498	y ~ current_feature + S
0,066285	0,076754	-0,04751	0,863608	172	169	0,389027	0,803665	y ~ current_feature + S
-0,12644	0,081817	0,061498	-1,54542	150	147	0,124395	0,8197	y ~ current_feature + S
0,13425	0,093636	-0,02429	1,433749	115	112	0,15443	0,623156	y ~ current_feature + S
-0,03094	0,066566	-0,07874	-0,46475	251	225,4672	0,642556	0,928694	y ~ current_feature + S
-0,0126	0,085384	0,034007	-0,14762	251	137,1436	0,882861	0,96591	y ~ current_feature + S
0,102745	0,070202	-0,04751	1,463549	250	200,7653	0,144881	0,661823	y ~ current_feature + S
0,05465	0,073326	0,061498	0,745296	217	185,4313	0,457036	0,937665	y ~ current_feature + S
-0,11665	0,077791	-0,02429	-1,49957	166	163	0,135661	0,618941	y ~ current_feature + S
0,016157	0,054819	-0,07874	0,29474	383	332,6814	0,768377	0,965616	y ~ current_feature + S
0,070477	0,072747	0,034007	0,968806	383	188,023	0,333887	0,734392	y ~ current_feature + S
0,033388	0,055532	-0,04751	0,601244	382	323,9126	0,548098	0,895709	y ~ current_feature + S
-0,00612	0,060051	0,061498	-0,10196	333	277,2929	0,91886	0,996179	y ~ current_feature + S
0,078404	0,062925	-0,02429	1,245983	254	251	0,213932	0,679441	y ~ current_feature + S
-0,08112	0,103353	-0,07874	-0,7849	96	93	0,434508	0,814177	y ~ current_feature + S
-0,20221	0,101553	0,034007	-1,99114	96	93	0,0494	0,420946	y ~ current_feature + S
0,02742	0,103656	-0,04751	0,264529	96	93	0,791958	0,957596	y ~ current_feature + S
-0,22809	0,109543	0,061498	-2,08223	82	79	0,040559	0,658491	y ~ current_feature + S
0,060206	0,127805	-0,02429	0,47108	64	61	0,639264	0,924682	y ~ current_feature + S
-0,01233	0,053952	-0,07874	-0,22851	381	343,4932	0,819385	0,979871	y ~ current_feature + S
-0,02533	0,067545	0,034007	-0,37497	381	219,0486	0,708046	0,920814	y ~ current_feature + S
-5,51E-04	0,053403	-0,04751	-0,01032	380	350,6493	0,991772	0,99446	y ~ current_feature + S
-0,06918	0,05868	0,061498	-1,1789	330	289,029	0,239408	0,893818	y ~ current_feature + S
-0,01166	0,063241	-0,02429	-0,18439	253	250	0,853859	0,981143	y ~ current_feature + S
-0,03324	0,113165	-0,07874	-0,29377	81	78	0,769717	0,965616	y ~ current_feature + S
-0,0582	0,113036	0,034007	-0,51492	81	78	0,608062	0,887506	y ~ current_feature + S
-0,09769	0,112686	-0,04751	-0,86693	81	78	0,388639	0,803665	y ~ current_feature + S
0,105202	0,123346	0,061498	0,852901	68	65	0,396846	0,93433	y ~ current_feature + S
0,042545	0,139901	-0,02429	0,304108	54	51	0,762283	0,951727	y ~ current_feature + S
0,084297	0,053262	-0,07874	1,582691	353	350	0,114395	0,467692	y ~ current_feature + S
0,063772	0,053343	0,034007	1,195502	353	350	0,2327	0,683327	y ~ current_feature + S
0,098021	0,053271	-0,04751	1,840052	352	349	0,06661	0,640171	y ~ current_feature + S
0,006174	0,057638	0,061498	0,107117	304	301	0,914767	0,996179	y ~ current_feature + S
0,169048	0,064708	-0,02429	2,612464	235	232	0,009576	0,295269	y ~ current_feature + S
0,00546	0,094914	-0,07874	0,057524	114	111	0,954231	0,994607	y ~ current_feature + S
0,076533	0,094637	0,034007	0,808697	114	111	0,420419	0,791109	y ~ current_feature + S
0,332915	0,089502	-0,04751	3,719657	114	111	3,15E-04	0,077615	y ~ current_feature + S
0,033862	0,100957	0,061498	0,335414	101	98	0,73803	0,972573	y ~ current_feature + S

0,063302	0,116806	-0,02429	0,541938	76	73	0,589511	0,924682	y ~ current_feature + S
0,05064	0,054404	-0,07874	0,930827	340	337	0,352609	0,765193	y ~ current_feature + S
-0,08507	0,054276	0,034007	-1,56732	340	337	0,117979	0,552558	y ~ current_feature + S
0,082663	0,054368	-0,04751	1,520444	339	336	0,12934	0,654671	y ~ current_feature + S
-0,08065	0,066019	0,061498	-1,22164	300	227,9454	0,223107	0,893818	y ~ current_feature + S
0,006485	0,066964	-0,02429	0,096847	226	223	0,922935	0,99444	y ~ current_feature + S
0,007569	0,058221	-0,07874	0,130006	298	295	0,89665	0,987929	y ~ current_feature + S
0,05996	0,058117	0,034007	1,031696	298	295	0,30306	0,718742	y ~ current_feature + S
0,116795	0,057922	-0,04751	2,016413	297	294	0,044666	0,63563	y ~ current_feature + S
-0,04582	0,062313	0,061498	-0,73532	260	257	0,462816	0,937665	y ~ current_feature + S
0,091937	0,071126	-0,02429	1,292593	199	196	0,197674	0,660578	y ~ current_feature + S
0,044453	0,050652	-0,07874	0,877627	392	389	0,380688	0,789252	y ~ current_feature + S
-0,08595	0,050514	0,034007	-1,70145	392	389	0,089658	0,487845	y ~ current_feature + S
0,070554	0,050641	-0,04751	1,393215	391	388	0,164352	0,694976	y ~ current_feature + S
0,004693	0,060532	0,061498	0,077531	340	272,9105	0,938258	0,996179	y ~ current_feature + S
-0,0098	0,062375	-0,02429	-0,15712	260	257	0,875271	0,984468	y ~ current_feature + S
0,043167	0,050232	-0,07874	0,859356	418	395,5787	0,390665	0,789252	y ~ current_feature + S
0,029163	0,06494	0,034007	0,449072	418	236,9226	0,65379	0,900251	y ~ current_feature + S
0,083265	0,05104	-0,04751	1,631389	417	381,2093	0,103634	0,640171	y ~ current_feature + S
-0,02664	0,055424	0,061498	-0,48065	365	325,3098	0,631092	0,953078	y ~ current_feature + S
0,049071	0,060313	-0,02429	0,813603	278	274,2364	0,416579	0,839968	y ~ current_feature + S
-0,05052	0,120232	-0,07874	-0,42022	72	69	0,67563	0,934653	y ~ current_feature + S
-0,10074	0,119773	0,034007	-0,84112	72	69	0,403184	0,778997	y ~ current_feature + S
0,021866	0,120357	-0,04751	0,181673	72	69	0,856372	0,983239	y ~ current_feature + S
-0,18171	0,125905	0,061498	-1,4432	64	61	0,15408	0,8197	y ~ current_feature + S
-0,16898	0,146927	-0,02429	-1,15009	48	45	0,256183	0,718089	y ~ current_feature + S
0,355841	0,123784	-0,07874	2,874697	60	57	0,005676	0,092669	y ~ current_feature + S
0,290214	0,126753	0,034007	2,289608	60	57	0,025767	0,347616	y ~ current_feature + S
-0,19393	0,129939	-0,04751	-1,49249	60	57	0,141086	0,660782	y ~ current_feature + S
0,015543	0,14284	0,061498	0,108812	52	49	0,913796	0,996179	y ~ current_feature + S
0,176519	0,161817	-0,02429	1,090855	40	37	0,28239	0,734108	y ~ current_feature + S
-0,10112	0,082056	-0,07874	-1,23233	150	147	0,219793	0,639583	y ~ current_feature + S
-0,04039	0,082411	0,034007	-0,49016	150	147	0,624752	0,896904	y ~ current_feature + S
-0,14691	0,081584	-0,04751	-1,80074	150	147	0,073794	0,640171	y ~ current_feature + S
-0,06166	0,098899	0,061498	-0,6235	134	101,8502	0,534352	0,937665	y ~ current_feature + S
-0,01329	0,101526	-0,02429	-0,1309	100	97	0,896124	0,99444	y ~ current_feature + S
0,068192	0,07375	-0,07874	0,924635	186	183	0,356373	0,766617	y ~ current_feature + S
0,019673	0,073908	0,034007	0,266179	186	183	0,790401	0,945731	y ~ current_feature + S
-0,01337	0,074118	-0,04751	-0,18043	185	182	0,857012	0,983239	y ~ current_feature + S
-0,04304	0,078984	0,061498	-0,5449	163	160	0,586581	0,945257	y ~ current_feature + S
-0,11661	0,090664	-0,02429	-1,28619	123	120	0,200852	0,660578	y ~ current_feature + S
0,020266	0,051767	-0,07874	0,391486	376	373	0,695661	0,939646	y ~ current_feature + S
-0,01584	0,051772	0,034007	-0,30595	376	373	0,759814	0,935544	y ~ current_feature + S
-0,06858	0,051726	-0,04751	-1,32578	375	372	0,185727	0,704811	y ~ current_feature + S
0,018753	0,055546	0,061498	0,337605	327	324	0,73588	0,972573	y ~ current_feature + S
-0,03044	0,063599	-0,02429	-0,47858	250	247	0,632659	0,924682	y ~ current_feature + S
-0,02293	0,079284	-0,07874	-0,28925	162	159	0,77277	0,965962	y ~ current_feature + S
0,175496	0,078074	0,034007	2,247804	162	159	0,025964	0,347616	y ~ current_feature + S

0,114148	0,078787	-0,04751	1,448819	162	159	0,149357	0,661823	y ~ current_feature + S
-0,00727	0,087037	0,061498	-0,08349	135	132	0,933586	0,996179	y ~ current_feature + S
0,108423	0,097015	-0,02429	1,117596	108	105	0,26629	0,723659	y ~ current_feature + S
0,018258	0,056156	-0,07874	0,325122	320	317	0,745303	0,96252	y ~ current_feature + S
-0,07387	0,056012	0,034007	-1,3188	320	317	0,188189	0,638805	y ~ current_feature + S
-0,01788	0,056245	-0,04751	-0,3178	319	316	0,750843	0,9394	y ~ current_feature + S
-0,04296	0,060578	0,061498	-0,70914	275	272	0,478845	0,937665	y ~ current_feature + S
-0,02627	0,068983	-0,02429	-0,3808	213	210	0,703738	0,93118	y ~ current_feature + S
0,138823	0,127849	-0,07874	1,085829	63	60	0,281898	0,718628	y ~ current_feature + S
0,023996	0,129062	0,034007	0,185928	63	60	0,853128	0,959445	y ~ current_feature + S
0,073173	0,128753	-0,04751	0,568317	63	60	0,57194	0,903741	y ~ current_feature + S
0,03567	0,138587	0,061498	0,257383	55	52	0,797899	0,984075	y ~ current_feature + S
0,074135	0,159688	-0,02429	0,464252	42	39	0,645048	0,924682	y ~ current_feature + S
0,101839	0,066767	-0,07874	1,525298	225	222	0,128608	0,481525	y ~ current_feature + S
0,085707	0,066869	0,034007	1,281715	225	222	0,20128	0,647595	y ~ current_feature + S
0,064362	0,066976	-0,04751	0,960971	225	222	0,337612	0,788117	y ~ current_feature + S
0,10642	0,081028	0,061498	1,313376	196	150,5857	0,191054	0,878136	y ~ current_feature + S
0,120581	0,081877	-0,02429	1,472709	150	147	0,142967	0,618941	y ~ current_feature + S
0,024502	0,083599	-0,07874	0,293084	146	143	0,769883	0,965616	y ~ current_feature + S
0,03682	0,083567	0,034007	0,440597	146	143	0,66017	0,900251	y ~ current_feature + S
0,130381	0,08291	-0,04751	1,572559	146	143	0,118031	0,646984	y ~ current_feature + S
-0,04756	0,091184	0,061498	-0,52162	123	120	0,602898	0,945257	y ~ current_feature + S
0,121923	0,102373	-0,02429	1,190969	97	94	0,236665	0,697311	y ~ current_feature + S
-0,03275	0,0522	-0,07874	-0,62735	383	366,6025	0,530821	0,880735	y ~ current_feature + S
-0,06065	0,066483	0,034007	-0,9122	383	225,4133	0,362639	0,7498	y ~ current_feature + S
0,087244	0,053389	-0,04751	1,634133	382	348,1625	0,103135	0,640171	y ~ current_feature + S
0,058715	0,057066	0,061498	1,028895	334	306,0117	0,304341	0,893818	y ~ current_feature + S
-0,04393	0,064016	-0,02429	-0,68622	255	243,5489	0,493225	0,892387	y ~ current_feature + S
-0,07686	0,097301	-0,07874	-0,78989	108	105	0,431372	0,814177	y ~ current_feature + S
-0,20089	0,0956	0,034007	-2,10139	108	105	0,038	0,390551	y ~ current_feature + S
-0,02632	0,097556	-0,04751	-0,26984	108	105	0,787811	0,954141	y ~ current_feature + S
-0,02891	0,105955	0,061498	-0,27285	92	89	0,785598	0,979725	y ~ current_feature + S
-0,06053	0,120165	-0,02429	-0,50375	72	69	0,616039	0,924682	y ~ current_feature + S
0,314776	0,111091	-0,07874	2,833487	76	73	0,00595	0,093673	y ~ current_feature + S
0,18982	0,114913	0,034007	1,651854	76	73	0,10286	0,525261	y ~ current_feature + S
0,186142	0,114996	-0,04751	1,618684	76	73	0,109828	0,640171	y ~ current_feature + S
-0,26718	0,123382	0,061498	-2,1655	64	61	0,034269	0,658491	y ~ current_feature + S
0,098356	0,145158	-0,02429	0,677581	50	47	0,501359	0,89399	y ~ current_feature + S
-0,18842	0,109801	-0,07874	-1,71601	83	80	0,09003	0,419008	y ~ current_feature + S
-0,16619	0,110249	0,034007	-1,50738	83	80	0,135652	0,576909	y ~ current_feature + S
-0,10085	0,111935	-0,04751	-0,901	82	79	0,370328	0,802018	y ~ current_feature + S
-0,18282	0,119224	0,061498	-1,5334	71	68	0,129816	0,8197	y ~ current_feature + S
-0,1295	0,137507	-0,02429	-0,9418	55	52	0,35065	0,795954	y ~ current_feature + S
0,118309	0,126108	-0,07874	0,938153	65	62	0,351806	0,765193	y ~ current_feature + S
-0,02865	0,126948	0,034007	-0,22572	65	62	0,822161	0,952501	y ~ current_feature + S
0,187361	0,124751	-0,04751	1,501877	65	62	0,138205	0,657012	y ~ current_feature + S
-0,04102	0,135968	0,061498	-0,30167	57	54	0,764059	0,972573	y ~ current_feature + S
0,186414	0,153436	-0,02429	1,21493	44	41	0,231343	0,695919	y ~ current_feature + S

0,091993	0,139434	-0,07874	0,659757	54	51	0,512378	0,867643	y ~ current_feature + S
-0,07114	0,139673	0,034007	-0,5093	54	51	0,612736	0,889068	y ~ current_feature + S
0,057625	0,139795	-0,04751	0,412209	54	51	0,681914	0,928814	y ~ current_feature + S
0,215222	0,150687	0,061498	1,428267	45	42	0,16061	0,8197	y ~ current_feature + S
0,017605	0,174051	-0,02429	0,101151	36	33	0,920042	0,99444	y ~ current_feature + S
-0,07198	0,072743	-0,07874	-0,98944	191	188	0,323718	0,725913	y ~ current_feature + S
-0,14499	0,072162	0,034007	-2,00921	191	188	0,045945	0,400524	y ~ current_feature + S
-0,13312	0,072283	-0,04751	-1,8417	191	188	0,067095	0,640171	y ~ current_feature + S
-0,13373	0,078593	0,061498	-1,70154	162	159	0,090797	0,795265	y ~ current_feature + S
-0,03197	0,089397	-0,02429	-0,35762	128	125	0,72123	0,937979	y ~ current_feature + S
0,100089	0,113388	-0,07874	0,882714	80	77	0,380138	0,789252	y ~ current_feature + S
-0,01692	0,113944	0,034007	-0,14849	80	77	0,882348	0,96591	y ~ current_feature + S
0,040397	0,114614	-0,04751	0,352457	79	76	0,725471	0,93314	y ~ current_feature + S
-0,29236	0,116832	0,061498	-2,50239	70	67	0,014783	0,55988	y ~ current_feature + S
-0,01312	0,141409	-0,02429	-0,09281	53	50	0,926424	0,99444	y ~ current_feature + S
0,101122	0,135385	-0,07874	0,746923	57	54	0,45835	0,829288	y ~ current_feature + S
0,042969	0,135957	0,034007	0,316047	57	54	0,753185	0,933597	y ~ current_feature + S
0,230918	0,132405	-0,04751	1,744033	57	54	0,086844	0,640171	y ~ current_feature + S
-0,02368	0,145824	0,061498	-0,1624	50	47	0,87169	0,996179	y ~ current_feature + S
0,135023	0,167483	-0,02429	0,806187	38	35	0,425579	0,847264	y ~ current_feature + S
-0,08556	0,052806	-0,07874	-1,62037	359	356	0,106039	0,449749	y ~ current_feature + S
-0,1163	0,05264	0,034007	-2,20927	359	356	0,027792	0,347616	y ~ current_feature + S
-0,03522	0,052967	-0,04751	-0,66486	359	356	0,506569	0,868909	y ~ current_feature + S
-0,02509	0,05687	0,061498	-0,44112	312	309	0,659438	0,957207	y ~ current_feature + S
-0,10576	0,064729	-0,02429	-1,63381	239	236	0,103631	0,605474	y ~ current_feature + S
0,281396	0,113089	-0,07874	2,488267	75	72	0,015151	0,174676	y ~ current_feature + S
0,017211	0,117834	0,034007	0,146058	75	72	0,884283	0,96591	y ~ current_feature + S
0,05388	0,11768	-0,04751	0,457856	75	72	0,648435	0,925756	y ~ current_feature + S
-0,36929	0,119974	0,061498	-3,07811	63	60	0,003139	0,305054	y ~ current_feature + S
0,216987	0,14239	-0,02429	1,523892	50	47	0,134237	0,617591	y ~ current_feature + S
0,198903	0,05403	-0,07874	3,681334	332	329	2,71E-04	0,022288	y ~ current_feature + S
-0,02037	0,05512	0,034007	-0,36949	332	329	0,712002	0,920814	y ~ current_feature + S
0,024844	0,055115	-0,04751	0,45076	332	329	0,65246	0,925756	y ~ current_feature + S
-0,01458	0,059229	0,061498	-0,24618	288	285	0,805723	0,988828	y ~ current_feature + S
0,007092	0,067572	-0,02429	0,10495	222	219	0,916512	0,99444	y ~ current_feature + S
0,241758	0,144649	-0,07874	1,671343	48	45	0,101595	0,447502	y ~ current_feature + S
0,030197	0,149003	0,034007	0,202663	48	45	0,840312	0,958985	y ~ current_feature + S
-0,07395	0,148663	-0,04751	-0,49742	48	45	0,621318	0,925756	y ~ current_feature + S
-0,10331	0,168126	0,061498	-0,61448	38	35	0,542872	0,937665	y ~ current_feature + S
0,06656	0,204326	-0,02429	0,325755	32	23,8464	0,747451	0,948737	y ~ current_feature + S
0,007294	0,141418	-0,07874	0,051581	53	50	0,959068	0,994607	y ~ current_feature + S
-0,0056	0,141419	0,034007	-0,03961	53	50	0,968566	0,989859	y ~ current_feature + S
0,078894	0,142412	-0,04751	0,553988	52	49	0,582107	0,906861	y ~ current_feature + S
-0,22232	0,150442	0,061498	-1,47775	45	42	0,146936	0,8197	y ~ current_feature + S
-0,27196	0,170114	-0,02429	-1,5987	35	32	0,119717	0,615361	y ~ current_feature + S
0,023656	0,096647	-0,07874	0,244764	110	107	0,807108	0,979871	y ~ current_feature + S
-0,05372	0,096534	0,034007	-0,55649	110	107	0,579035	0,869291	y ~ current_feature + S
-0,22172	0,094711	-0,04751	-2,34107	109	106	0,021099	0,557019	y ~ current_feature + S

-0,2158	0,100712	0,061498	-2,14278	97	94	0,034714	0,658491	y ~ current_feature + S
-0,09557	0,118976	-0,02429	-0,80329	73	70	0,424524	0,847264	y ~ current_feature + S
0,030358	0,090494	-0,07874	0,335471	125	122	0,737846	0,959225	y ~ current_feature + S
-0,07823	0,090258	0,034007	-0,86674	125	122	0,387784	0,767272	y ~ current_feature + S
-0,11072	0,089979	-0,04751	-1,23046	125	122	0,220892	0,742928	y ~ current_feature + S
0,00251	0,1	0,061498	0,025101	103	100	0,980025	0,996179	y ~ current_feature + S
-0,11803	0,111022	-0,02429	-1,0631	83	80	0,290933	0,734779	y ~ current_feature + S
0,068139	0,115977	-0,07874	0,587519	77	74	0,558644	0,894753	y ~ current_feature + S
-0,16106	0,11473	0,034007	-1,40378	77	74	0,164568	0,61196	y ~ current_feature + S
-0,21402	0,114329	-0,04751	-1,87193	76	73	0,065222	0,640171	y ~ current_feature + S
-0,11923	0,121298	0,061498	-0,98296	70	67	0,329161	0,893818	y ~ current_feature + S
0,036215	0,144243	-0,02429	0,251067	51	48	0,802834	0,968297	y ~ current_feature + S
6,84E-04	0,127	-0,07874	0,005388	65	62	0,995718	0,996647	y ~ current_feature + S
-0,0502	0,12684	0,034007	-0,39579	65	62	0,69362	0,916569	y ~ current_feature + S
-0,25651	0,123753	-0,04751	-2,07272	64	61	0,042431	0,627977	y ~ current_feature + S
0,003472	0,13736	0,061498	0,025277	56	53	0,979929	0,996179	y ~ current_feature + S
0,136354	0,156637	-0,02429	0,87051	43	40	0,389216	0,82057	y ~ current_feature + S
-0,04436	0,050572	-0,07874	-0,87709	418	390,2291	0,38098	0,789252	y ~ current_feature + S
-0,13179	0,065127	0,034007	-2,02363	418	231,6656	0,044157	0,400524	y ~ current_feature + S
-0,10033	0,051505	-0,04751	-1,94797	417	373,1719	0,052168	0,640171	y ~ current_feature + S
-0,02962	0,056034	0,061498	-0,52866	365	318,2085	0,597413	0,945257	y ~ current_feature + S
-0,08875	0,061903	-0,02429	-1,43376	278	258,9056	0,152848	0,623156	y ~ current_feature + S
-0,04039	0,050058	-0,07874	-0,8069	421	398,423	0,420206	0,811887	y ~ current_feature + S
-0,10459	0,064163	0,034007	-1,63013	421	240,2432	0,104384	0,526532	y ~ current_feature + S
-0,02747	0,051024	-0,04751	-0,53829	420	383,8236	0,590691	0,91049	y ~ current_feature + S
-0,05376	0,055398	0,061498	-0,97048	367	324,9004	0,33253	0,893818	y ~ current_feature + S
-0,00439	0,061726	-0,02429	-0,07107	280	262,459	0,943395	0,99444	y ~ current_feature + S
-0,04968	0,050173	-0,07874	-0,99022	412	396,2713	0,322669	0,725761	y ~ current_feature + S
-0,09014	0,063605	0,034007	-1,4172	412	245,1778	0,157693	0,60427	y ~ current_feature + S
-0,05803	0,05083	-0,04751	-1,14171	411	385,7327	0,254282	0,742928	y ~ current_feature + S
-0,01303	0,055801	0,061498	-0,23344	358	321,0981	0,815568	0,995908	y ~ current_feature + S
0,029476	0,060753	-0,02429	0,485183	274	270,6987	0,627939	0,924682	y ~ current_feature + S
-0,11505	0,056238	-0,07874	-2,04572	315	312	0,041622	0,275003	y ~ current_feature + S
-0,08669	0,056401	0,034007	-1,53703	315	312	0,125299	0,572145	y ~ current_feature + S
-0,01999	0,056603	-0,04751	-0,35324	315	312	0,724146	0,93314	y ~ current_feature + S
-1,16E-04	0,060746	0,061498	-0,00192	274	271	0,998473	0,999005	y ~ current_feature + S
-0,1242	0,068967	-0,02429	-1,80087	210	207	0,073179	0,605474	y ~ current_feature + S
0,062437	0,053842	-0,07874	1,159637	395	343,6081	0,247002	0,67447	y ~ current_feature + S
0,007009	0,070176	0,034007	0,099881	395	203,0477	0,920538	0,9732	y ~ current_feature + S
0,005018	0,0551	-0,04751	0,091075	394	329,3676	0,927489	0,985217	y ~ current_feature + S
-0,10779	0,05719	0,061498	-1,8847	343	302,1894	0,06043	0,715249	y ~ current_feature + S
-0,05378	0,062826	-0,02429	-0,85608	263	252,6157	0,392766	0,821036	y ~ current_feature + S
0,079708	0,056763	-0,07874	1,404232	345	308,391	0,161256	0,532425	y ~ current_feature + S
0,038018	0,073224	0,034007	0,519206	345	186,2386	0,604234	0,883662	y ~ current_feature + S
0,044556	0,058823	-0,04751	0,757449	344	288,4283	0,449399	0,831388	y ~ current_feature + S
-0,03317	0,062266	0,061498	-0,53274	299	257,6476	0,594672	0,945257	y ~ current_feature + S
0,02018	0,066746	-0,02429	0,302347	230	224,377	0,762667	0,951727	y ~ current_feature + S
0,08506	0,050958	-0,07874	1,669238	415	382,3224	0,095889	0,431938	y ~ current_feature + S

0,070469	0,066977	0,034007	1,05214	415	221,8114	0,29388	0,718742	y ~ current_feature + S
0,031254	0,052435	-0,04751	0,596061	414	363,3627	0,551506	0,897386	y ~ current_feature + S
0,034953	0,055933	0,061498	0,624901	361	319,2487	0,532482	0,937665	y ~ current_feature + S
0,090135	0,061486	-0,02429	1,465941	276	262,3633	0,143862	0,618941	y ~ current_feature + S
-0,03723	0,055603	-0,07874	-0,66959	326	323	0,503594	0,863164	y ~ current_feature + S
-0,00396	0,055641	0,034007	-0,07114	326	323	0,943334	0,98181	y ~ current_feature + S
0,02529	0,05571	-0,04751	0,453955	325	322	0,650167	0,925756	y ~ current_feature + S
0,001741	0,064886	0,061498	0,026839	280	237,5155	0,978611	0,996179	y ~ current_feature + S
0,068369	0,068996	-0,02429	0,990911	217	209,082	0,322874	0,773227	y ~ current_feature + S
0,068981	0,087162	-0,07874	0,791413	134	131	0,430134	0,814177	y ~ current_feature + S
0,031571	0,087327	0,034007	0,361524	134	131	0,71829	0,920814	y ~ current_feature + S
0,01026	0,087366	-0,04751	0,117434	134	131	0,906696	0,985217	y ~ current_feature + S
0,066956	0,095132	0,061498	0,703816	113	110	0,483034	0,937665	y ~ current_feature + S
0,018342	0,107815	-0,02429	0,170123	89	86	0,865313	0,984468	y ~ current_feature + S
-0,00671	0,054078	-0,07874	-0,12416	389	341,9334	0,901259	0,987929	y ~ current_feature + S
-0,04646	0,071316	0,034007	-0,65153	389	196,195	0,515464	0,841996	y ~ current_feature + S
-0,06358	0,055999	-0,04751	-1,1354	388	317,6031	0,257062	0,742928	y ~ current_feature + S
0,020866	0,059697	0,061498	0,349539	337	280,4812	0,726947	0,972573	y ~ current_feature + S
-0,0046	0,062499	-0,02429	-0,07363	259	256	0,941359	0,99444	y ~ current_feature + S
0,112207	0,140528	-0,07874	0,798466	53	50	0,428376	0,814177	y ~ current_feature + S
-0,01226	0,141411	0,034007	-0,08671	53	50	0,93125	0,974557	y ~ current_feature + S
0,042626	0,141293	-0,04751	0,301683	53	50	0,764145	0,940878	y ~ current_feature + S
-0,09716	0,150042	0,061498	-0,64753	47	44	0,520652	0,937665	y ~ current_feature + S
0,201062	0,173167	-0,02429	1,161087	35	32	0,254195	0,718089	y ~ current_feature + S
0,308431	0,129448	-0,07874	2,382658	57	54	0,020738	0,189455	y ~ current_feature + S
0,108524	0,135279	0,034007	0,802224	57	54	0,42594	0,791701	y ~ current_feature + S
0,073213	0,135718	-0,04751	0,539451	57	54	0,591793	0,91049	y ~ current_feature + S
0,110412	0,149834	0,061498	0,736898	47	44	0,465095	0,937665	y ~ current_feature + S
-0,00976	0,169023	-0,02429	-0,05777	38	35	0,954262	0,99444	y ~ current_feature + S
0,051913	0,073622	-0,07874	0,705132	187	184	0,48162	0,845512	y ~ current_feature + S
0,013534	0,073714	0,034007	0,183599	187	184	0,85453	0,959563	y ~ current_feature + S
0,085724	0,07345	-0,04751	1,167108	187	184	0,244677	0,742928	y ~ current_feature + S
0,025755	0,077589	0,061498	0,331938	169	166	0,740355	0,972573	y ~ current_feature + S
-0,03694	0,090847	-0,02429	-0,40663	124	121	0,684999	0,926342	y ~ current_feature + S
0,121077	0,081872	-0,07874	1,478859	150	147	0,141318	0,498495	y ~ current_feature + S
0,094939	0,082106	0,034007	1,156303	150	147	0,249433	0,702074	y ~ current_feature + S
0,074048	0,082252	-0,04751	0,900258	150	147	0,369455	0,802018	y ~ current_feature + S
-0,08259	0,089496	0,061498	-0,92278	127	124	0,357913	0,920619	y ~ current_feature + S
-0,02676	0,101498	-0,02429	-0,26367	100	97	0,79259	0,967756	y ~ current_feature + S
0,148652	0,050719	-0,07874	2,930921	407	380,1567	0,003584	0,069632	y ~ current_feature + S
0,144226	0,065615	0,034007	2,198067	407	227,4383	0,028953	0,347616	y ~ current_feature + S
0,050538	0,051724	-0,04751	0,977074	406	372,8218	0,329166	0,779476	y ~ current_feature + S
0,06752	0,05532	0,061498	1,220536	353	325,2761	0,223146	0,893818	y ~ current_feature + S
0,084382	0,061537	-0,02429	1,371244	271	262,1944	0,171471	0,626332	y ~ current_feature + S
0,143473	0,092286	-0,07874	1,554659	118	115	0,122775	0,477837	y ~ current_feature + S
0,126504	0,092501	0,034007	1,367592	118	115	0,174107	0,619418	y ~ current_feature + S
0,201976	0,091728	-0,04751	2,201889	117	114	0,029687	0,603897	y ~ current_feature + S
-0,05011	0,100888	0,061498	-0,49673	101	98	0,620493	0,950526	y ~ current_feature + S

0,148794	0,113431	-0,02429	1,311755	79	76	0,193552	0,65401	y ~ current_feature + S
-0,17838	0,052226	-0,07874	-3,41561	386	354,959	7,10E-04	0,031617	y ~ current_feature + S
-0,17305	0,069401	0,034007	-2,49343	386	201,4	0,013457	0,255339	y ~ current_feature + S
-0,0347	0,054448	-0,04751	-0,63736	385	336,9115	0,524323	0,876197	y ~ current_feature + S
0,101585	0,058034	0,061498	1,750419	339	293,8486	0,08109	0,750079	y ~ current_feature + S
-0,12799	0,062905	-0,02429	-2,03465	256	248,577	0,042948	0,525585	y ~ current_feature + S
0,168316	0,052074	-0,07874	3,232285	415	358,3312	0,001342	0,048726	y ~ current_feature + S
0,179864	0,069009	0,034007	2,606391	415	203,1926	0,009828	0,227273	y ~ current_feature + S
0,129207	0,053193	-0,04751	2,429011	414	347,5166	0,015647	0,526293	y ~ current_feature + S
-0,06553	0,057818	0,061498	-1,13345	361	297,8542	0,257935	0,893818	y ~ current_feature + S
0,116471	0,060111	-0,02429	1,937603	276	273	0,053703	0,558147	y ~ current_feature + S
0,102415	0,050262	-0,07874	2,037623	421	391,6883	0,042259	0,275611	y ~ current_feature + S
0,097914	0,066433	0,034007	1,473874	421	224,4139	0,141917	0,588814	y ~ current_feature + S
0,064531	0,051643	-0,04751	1,249567	420	373,398	0,21224	0,733915	y ~ current_feature + S
0,001462	0,056583	0,061498	0,025834	367	312,3406	0,979406	0,996179	y ~ current_feature + S
0,034623	0,060459	-0,02429	0,572668	280	273,2523	0,567341	0,924682	y ~ current_feature + S
3,81E-04	0,08737	-0,07874	0,004361	134	131	0,996527	0,996647	y ~ current_feature + S
-0,01886	0,087355	0,034007	-0,2159	134	131	0,829402	0,956008	y ~ current_feature + S
0,057449	0,087226	-0,04751	0,658627	134	131	0,511291	0,868909	y ~ current_feature + S
-0,07999	0,092154	0,061498	-0,868	120	117	0,38717	0,93433	y ~ current_feature + S
0,009388	0,107828	-0,02429	0,087066	89	86	0,930821	0,99444	y ~ current_feature + S
-0,05596	0,054068	-0,07874	-1,035	344	341	0,301404	0,718687	y ~ current_feature + S
-0,0599	0,054056	0,034007	-1,10806	344	341	0,268619	0,71095	y ~ current_feature + S
-0,03206	0,054125	-0,04751	-0,59236	344	341	0,554001	0,897386	y ~ current_feature + S
-0,0386	0,058179	0,061498	-0,66349	298	295	0,507533	0,937665	y ~ current_feature + S
-0,02694	0,066495	-0,02429	-0,40514	229	226	0,685759	0,926342	y ~ current_feature + S
0,038744	0,071573	-0,07874	0,541324	254	194,9147	0,588902	0,902886	y ~ current_feature + S
0,114213	0,086565	0,034007	1,319394	254	131,7088	0,189327	0,639734	y ~ current_feature + S
0,026735	0,067706	-0,04751	0,394863	253	217,9896	0,69333	0,930326	y ~ current_feature + S
-0,10793	0,076384	0,061498	-1,413	218	169,3979	0,159491	0,8197	y ~ current_feature + S
0,148684	0,076989	-0,02429	1,931235	169	164,9808	0,055168	0,558147	y ~ current_feature + S
0,052896	0,122919	-0,07874	0,430329	69	66	0,668358	0,934653	y ~ current_feature + S
0,011104	0,123084	0,034007	0,090211	69	66	0,928393	0,9732	y ~ current_feature + S
0,163028	0,121445	-0,04751	1,342405	69	66	0,184064	0,704811	y ~ current_feature + S
-0,14393	0,128833	0,061498	-1,1172	62	59	0,268437	0,893818	y ~ current_feature + S
0,156231	0,150626	-0,02429	1,037214	46	43	0,305434	0,761472	y ~ current_feature + S
-0,12614	0,057562	-0,07874	-2,1914	300	297	0,029199	0,219281	y ~ current_feature + S
-0,15303	0,057342	0,034007	-2,66867	300	297	0,008034	0,212321	y ~ current_feature + S
-0,02032	0,058112	-0,04751	-0,3497	299	296	0,72681	0,93314	y ~ current_feature + S
0,048963	0,068475	0,061498	0,715049	264	212,7608	0,475363	0,937665	y ~ current_feature + S
-0,12365	0,07088	-0,02429	-1,74456	199	196	0,082629	0,605474	y ~ current_feature + S
0,038433	0,086323	-0,07874	0,445224	137	134	0,656876	0,932255	y ~ current_feature + S
0,029735	0,086349	0,034007	0,344364	137	134	0,731113	0,922572	y ~ current_feature + S
-0,03882	0,086322	-0,04751	-0,44975	137	134	0,653619	0,925756	y ~ current_feature + S
-0,14621	0,092248	0,061498	-1,585	118	115	0,115713	0,8197	y ~ current_feature + S
-0,14732	0,104843	-0,02429	-1,40515	92	89	0,163457	0,623497	y ~ current_feature + S
0,09159	0,06938	-0,07874	1,32011	209	206	0,188263	0,592937	y ~ current_feature + S
0,183991	0,068484	0,034007	2,68663	209	206	0,007807	0,212321	y ~ current_feature + S

0,12709	0,069108	-0,04751	1,839001	209	206	0,067355	0,640171	y ~ current_feature + S
0,004972	0,075164	0,061498	0,066152	180	177	0,947332	0,996179	y ~ current_feature + S
0,059583	0,085597	-0,02429	0,696083	139	136	0,487565	0,888665	y ~ current_feature + S
0,165158	0,101189	-0,07874	1,632173	98	95	0,105954	0,449749	y ~ current_feature + S
0,02945	0,102553	0,034007	0,287165	98	95	0,774612	0,942718	y ~ current_feature + S
0,152936	0,101929	-0,04751	1,500422	97	94	0,136857	0,657012	y ~ current_feature + S
0,202857	0,105591	0,061498	1,921163	89	86	0,058024	0,715249	y ~ current_feature + S
0,1011	0,126349	-0,02429	0,800161	65	62	0,426673	0,847264	y ~ current_feature + S
-0,00865	0,062135	-0,07874	-0,13926	262	259	0,889354	0,987929	y ~ current_feature + S
0,011714	0,062133	0,034007	0,188526	262	259	0,850612	0,959445	y ~ current_feature + S
0,049264	0,062182	-0,04751	0,792263	261	258	0,428935	0,819126	y ~ current_feature + S
-0,04463	0,0672	0,061498	-0,66412	224	221	0,507308	0,937665	y ~ current_feature + S
0,015137	0,076463	-0,02429	0,197965	174	171	0,843308	0,977734	y ~ current_feature + S
-0,03945	0,139919	-0,07874	-0,28196	54	51	0,779118	0,972255	y ~ current_feature + S
0,093534	0,139414	0,034007	0,670905	54	51	0,505308	0,841996	y ~ current_feature + S
0,26073	0,135185	-0,04751	1,928698	54	51	0,059343	0,640171	y ~ current_feature + S
-0,09647	0,159381	0,061498	-0,60529	42	39	0,548492	0,937665	y ~ current_feature + S
0,163978	0,171721	-0,02429	0,954906	36	33	0,346568	0,790842	y ~ current_feature + S
0,104163	0,05881	-0,07874	1,771191	289	286	0,077594	0,370448	y ~ current_feature + S
-0,01561	0,059124	0,034007	-0,264	289	286	0,791968	0,945731	y ~ current_feature + S
0,085619	0,059017	-0,04751	1,450735	288	285	0,147953	0,661823	y ~ current_feature + S
9,26E-04	0,0635	0,061498	0,014583	251	248	0,988376	0,997945	y ~ current_feature + S
0,08172	0,072496	-0,02429	1,127237	192	189	0,261071	0,723568	y ~ current_feature + S
0,036778	0,057792	-0,07874	0,636378	302	299	0,525017	0,876542	y ~ current_feature + S
-0,02402	0,057815	0,034007	-0,41554	302	299	0,678044	0,912427	y ~ current_feature + S
-0,08762	0,057706	-0,04751	-1,51838	301	298	0,129979	0,654671	y ~ current_feature + S
-0,05088	0,061818	0,061498	-0,82312	264	261	0,41119	0,934833	y ~ current_feature + S
0,018595	0,071055	-0,02429	0,261705	201	198	0,793821	0,967756	y ~ current_feature + S
0,177942	0,127039	-0,07874	1,400689	63	60	0,166458	0,540258	y ~ current_feature + S
0,141891	0,127793	0,034007	1,110314	63	60	0,271294	0,71095	y ~ current_feature + S
-0,06479	0,128828	-0,04751	-0,50295	63	60	0,616838	0,924009	y ~ current_feature + S
0,132089	0,138801	0,061498	0,951642	54	51	0,345767	0,907332	y ~ current_feature + S
0,253588	0,154894	-0,02429	1,637171	42	39	0,10964	0,605474	y ~ current_feature + S
0,075853	0,139625	-0,07874	0,543263	54	51	0,589316	0,902886	y ~ current_feature + S
0,334977	0,131938	0,034007	2,538896	54	51	0,014214	0,262237	y ~ current_feature + S
0,17696	0,137818	-0,04751	1,284008	54	51	0,204942	0,723562	y ~ current_feature + S
-0,16808	0,145344	0,061498	-1,15646	49	46	0,253464	0,893818	y ~ current_feature + S
0,52122	0,148562	-0,02429	3,508444	36	33	0,001325	0,144895	y ~ current_feature + S
0,111263	0,121411	-0,07874	0,916415	70	67	0,362736	0,773558	y ~ current_feature + S
-0,02464	0,122132	0,034007	-0,20174	70	67	0,840733	0,958985	y ~ current_feature + S
0,091361	0,121659	-0,04751	0,750962	70	67	0,455305	0,832044	y ~ current_feature + S
-0,01623	0,130172	0,061498	-0,12472	62	59	0,90117	0,996179	y ~ current_feature + S
-0,15573	0,150638	-0,02429	-1,03381	46	43	0,307005	0,761472	y ~ current_feature + S
0,113232	0,100366	-0,07874	1,128193	101	98	0,261993	0,692409	y ~ current_feature + S
0,046468	0,100906	0,034007	0,46051	101	98	0,646171	0,900251	y ~ current_feature + S
0,117205	0,100835	-0,04751	1,162343	100	97	0,247948	0,742928	y ~ current_feature + S
-0,14557	0,103712	0,061498	-1,40356	94	91	0,163854	0,8197	y ~ current_feature + S
0,280522	0,119981	-0,02429	2,33805	67	64	0,022522	0,467656	y ~ current_feature + S

-0,0258	0,058676	-0,07874	-0,43966	338	290,2662	0,660511	0,934566	y ~ current_feature + S
-0,01611	0,077711	0,034007	-0,20731	338	165,5476	0,836022	0,958985	y ~ current_feature + S
0,115823	0,060636	-0,04751	1,910131	338	268,3332	0,057182	0,640171	y ~ current_feature + S
0,063626	0,064099	0,061498	0,99261	292	242,3988	0,32189	0,893818	y ~ current_feature + S
0,039927	0,067783	-0,02429	0,589034	225	217,3023	0,55645	0,92024	y ~ current_feature + S
0,043596	0,088304	-0,07874	0,4937	131	128	0,622364	0,919937	y ~ current_feature + S
0,041808	0,088311	0,034007	0,473418	131	128	0,636722	0,899624	y ~ current_feature + S
0,019177	0,088719	-0,04751	0,216153	130	127	0,829215	0,975438	y ~ current_feature + S
-0,1331	0,09365	0,061498	-1,42119	115	112	0,158039	0,8197	y ~ current_feature + S
0,061473	0,108903	-0,02429	0,564474	87	84	0,573936	0,924682	y ~ current_feature + S
0,169539	0,062963	-0,07874	2,692679	248	245	0,007577	0,110197	y ~ current_feature + S
0,166112	0,063	0,034007	2,636697	248	245	0,008907	0,215592	y ~ current_feature + S
0,054522	0,063793	-0,04751	0,85467	248	245	0,393569	0,803665	y ~ current_feature + S
0,045859	0,068608	0,061498	0,668418	215	212	0,504594	0,937665	y ~ current_feature + S
0,123269	0,077729	-0,02429	1,585884	166	163	0,114704	0,614731	y ~ current_feature + S
0,24162	0,149732	-0,07874	1,613686	45	42	0,114085	0,467692	y ~ current_feature + S
-0,01316	0,15429	0,034007	-0,08532	45	42	0,932414	0,974557	y ~ current_feature + S
0,171602	0,152014	-0,04751	1,128852	45	42	0,265367	0,747915	y ~ current_feature + S
-0,32114	0,151647	0,061498	-2,11767	42	39	0,040632	0,658491	y ~ current_feature + S
0,230524	0,187267	-0,02429	1,230991	30	27	0,228942	0,695919	y ~ current_feature + S
0,429982	0,119584	-0,07874	3,59566	60	57	6,77E-04	0,031617	y ~ current_feature + S
0,267713	0,127619	0,034007	2,097757	60	57	0,040372	0,396441	y ~ current_feature + S
0,003496	0,132452	-0,04751	0,026396	60	57	0,979034	0,992311	y ~ current_feature + S
0,115028	0,140483	0,061498	0,818805	53	50	0,416783	0,937665	y ~ current_feature + S
0,064929	0,164052	-0,02429	0,395782	40	37	0,694539	0,928819	y ~ current_feature + S
-0,14507	0,139925	-0,07874	-1,03676	53	50	0,304835	0,718687	y ~ current_feature + S
-0,14213	0,139986	0,034007	-1,01531	53	50	0,314847	0,718742	y ~ current_feature + S
0,020743	0,141391	-0,04751	0,146708	53	50	0,883952	0,985217	y ~ current_feature + S
-0,10324	0,148275	0,061498	-0,69627	48	45	0,489843	0,937665	y ~ current_feature + S
0,085141	0,173446	-0,02429	0,49088	36	33	0,62676	0,924682	y ~ current_feature + S
-0,00735	0,120383	-0,07874	-0,06107	72	69	0,95148	0,994607	y ~ current_feature + S
-0,06306	0,166472	0,034007	-0,37877	72	35,94068	0,707085	0,920814	y ~ current_feature + S
0,104851	0,13437	-0,04751	0,780314	72	54,7762	0,438565	0,823749	y ~ current_feature + S
-0,23367	0,16073	0,061498	-1,45382	60	36,59491	0,154519	0,8197	y ~ current_feature + S
-0,0727	0,148677	-0,02429	-0,48899	48	45	0,627225	0,924682	y ~ current_feature + S
-0,01028	0,090531	-0,07874	-0,11355	125	122	0,909778	0,987929	y ~ current_feature + S
-0,12972	0,089771	0,034007	-1,44499	125	122	0,151023	0,604094	y ~ current_feature + S
-0,04039	0,090462	-0,04751	-0,44647	125	122	0,656051	0,925756	y ~ current_feature + S
0,029225	0,097548	0,061498	0,2996	108	105	0,765075	0,972573	y ~ current_feature + S
-0,12875	0,110873	-0,02429	-1,16127	83	80	0,248987	0,717451	y ~ current_feature + S
0,103356	0,09571	-0,07874	1,079893	111	108	0,282596	0,718628	y ~ current_feature + S
0,025647	0,096193	0,034007	0,266619	111	108	0,790271	0,945731	y ~ current_feature + S
0,09944	0,095748	-0,04751	1,038563	111	108	0,301328	0,754967	y ~ current_feature + S
-0,00858	0,104253	0,061498	-0,08226	95	92	0,934619	0,996179	y ~ current_feature + S
0,034051	0,118609	-0,02429	0,287087	74	71	0,774882	0,958884	y ~ current_feature + S
0,01193	0,061658	-0,07874	0,193479	266	263	0,846734	0,983548	y ~ current_feature + S
0,013033	0,061657	0,034007	0,211372	266	263	0,832761	0,958387	y ~ current_feature + S
0,098105	0,061365	-0,04751	1,598714	266	263	0,111085	0,640171	y ~ current_feature + S

-0,08049	0,066012	0,061498	-1,21937	231	228	0,223964	0,893818	y ~ current_feature + S
0,020145	0,075578	-0,02429	0,266541	178	175	0,790136	0,967756	y ~ current_feature + S
0,17527	0,124038	-0,07874	1,413038	66	63	0,162566	0,532425	y ~ current_feature + S
0,223084	0,122813	0,034007	1,816453	66	63	0,074059	0,464437	y ~ current_feature + S
0,1358	0,124821	-0,04751	1,087959	66	63	0,280758	0,747915	y ~ current_feature + S
0,226338	0,133796	0,061498	1,691665	56	53	0,096582	0,8197	y ~ current_feature + S
0,073976	0,156674	-0,02429	0,472169	44	40,51573	0,63934	0,924682	y ~ current_feature + S
-0,01063	0,05634	-0,07874	-0,18868	318	315	0,850466	0,983548	y ~ current_feature + S
-0,15849	0,055631	0,034007	-2,849	318	315	0,004674	0,167881	y ~ current_feature + S
-0,0103	0,05643	-0,04751	-0,18256	317	314	0,85526	0,983239	y ~ current_feature + S
-0,10794	0,060059	0,061498	-1,79723	277	274	0,073401	0,721272	y ~ current_feature + S
-0,02525	0,069315	-0,02429	-0,36429	211	208	0,716014	0,936058	y ~ current_feature + S
0,115492	0,118723	-0,07874	0,972789	73	70	0,334008	0,735613	y ~ current_feature + S
-0,21864	0,116631	0,034007	-1,87461	73	70	0,065018	0,439525	y ~ current_feature + S
-0,16336	0,117917	-0,04751	-1,38536	73	70	0,170341	0,700235	y ~ current_feature + S
-0,19786	0,130989	0,061498	-1,51049	59	56	0,13654	0,8197	y ~ current_feature + S
-0,13148	0,147777	-0,02429	-0,88968	48	45	0,378367	0,815551	y ~ current_feature + S
-0,06404	0,150446	-0,07874	-0,42566	47	44	0,672434	0,934653	y ~ current_feature + S
-0,10188	0,149971	0,034007	-0,67931	47	44	0,500497	0,841996	y ~ current_feature + S
-0,20127	0,14767	-0,04751	-1,363	47	44	0,179818	0,704707	y ~ current_feature + S
-0,14073	0,160607	0,061498	-0,87621	41	38	0,386419	0,93433	y ~ current_feature + S
-0,12593	0,184217	-0,02429	-0,68359	32	29	0,499659	0,89399	y ~ current_feature + S
0,091063	0,051204	-0,07874	1,778426	421	378,2431	0,076137	0,368245	y ~ current_feature + S
0,056587	0,067106	0,034007	0,843243	421	221,3521	0,400003	0,776908	y ~ current_feature + S
0,085494	0,052587	-0,04751	1,625764	420	358,9723	0,104878	0,640171	y ~ current_feature + S
-0,02195	0,057207	0,061498	-0,38373	367	305,4143	0,701442	0,972444	y ~ current_feature + S
0,168631	0,059244	-0,02429	2,846383	280	276,8107	0,004753	0,219812	y ~ current_feature + S
-0,01908	0,068994	-0,07874	-0,27655	213	210	0,7824	0,973069	y ~ current_feature + S
0,069492	0,06884	0,034007	1,009482	213	210	0,313905	0,718742	y ~ current_feature + S
0,078842	0,068792	-0,04751	1,146094	213	210	0,253061	0,742928	y ~ current_feature + S
-0,00478	0,073323	0,061498	-0,06517	189	186	0,94811	0,996179	y ~ current_feature + S
0,053825	0,084696	-0,02429	0,635511	142	139	0,52614	0,899808	y ~ current_feature + S
0,090666	0,050667	-0,07874	1,789447	412	386,337	0,074326	0,364247	y ~ current_feature + S
0,027435	0,064984	0,034007	0,422186	412	236,6263	0,673273	0,910826	y ~ current_feature + S
0,062805	0,052169	-0,04751	1,203868	411	365,9744	0,229419	0,742928	y ~ current_feature + S
-0,01292	0,056354	0,061498	-0,22922	359	314,8288	0,818843	0,996179	y ~ current_feature + S
0,056968	0,061781	-0,02429	0,922097	274	261,1396	0,357329	0,800662	y ~ current_feature + S
-0,06556	0,076307	-0,07874	-0,85913	174	171	0,391472	0,789252	y ~ current_feature + S
-0,219	0,074616	0,034007	-2,93506	174	171	0,003793	0,15595	y ~ current_feature + S
-0,0585	0,076341	-0,04751	-0,76629	174	171	0,444557	0,824847	y ~ current_feature + S
-0,22392	0,080384	0,061498	-2,78565	150	147	0,006046	0,406759	y ~ current_feature + S
-0,1862	0,092427	-0,02429	-2,01454	116	113	0,046328	0,525585	y ~ current_feature + S
0,097674	0,063453	-0,07874	1,539319	249	246	0,125012	0,478063	y ~ current_feature + S
0,068838	0,063606	0,034007	1,082245	249	246	0,280203	0,715049	y ~ current_feature + S
0,087362	0,063643	-0,04751	1,372685	248	245	0,171105	0,700235	y ~ current_feature + S
-0,11704	0,080235	0,061498	-1,45869	212	153,2085	0,146698	0,8197	y ~ current_feature + S
0,016229	0,078316	-0,02429	0,207222	166	163	0,836095	0,977734	y ~ current_feature + S
0,173713	0,11448	-0,07874	1,517407	77	74	0,133425	0,493672	y ~ current_feature + S

0,200416	0,113889	0,034007	1,759751	77	74	0,082583	0,473527	y ~ current_feature + S
0,070411	0,116751	-0,04751	0,603089	76	73	0,548319	0,895709	y ~ current_feature + S
0,096449	0,126408	0,061498	0,762997	65	62	0,448358	0,937665	y ~ current_feature + S
0,192291	0,141644	-0,02429	1,357564	51	48	0,180951	0,646876	y ~ current_feature + S
-0,00549	0,130187	-0,07874	-0,0422	62	59	0,966485	0,994607	y ~ current_feature + S
0,047216	0,130044	0,034007	0,363076	62	59	0,717846	0,920814	y ~ current_feature + S
-0,18873	0,128947	-0,04751	-1,46366	61	58	0,148687	0,661823	y ~ current_feature + S
0,115272	0,136445	0,061498	0,844821	56	53	0,402008	0,93433	y ~ current_feature + S
0,244562	0,157295	-0,02429	1,554793	41	38	0,128286	0,617591	y ~ current_feature + S
0,193318	0,147912	-0,07874	1,30698	47	44	0,198009	0,611596	y ~ current_feature + S
0,15919	0,148833	0,034007	1,069584	47	44	0,290639	0,718742	y ~ current_feature + S
-0,19772	0,149488	-0,04751	-1,32267	46	43	0,192936	0,717449	y ~ current_feature + S
-0,20271	0,165522	0,061498	-1,22466	38	35	0,228883	0,893818	y ~ current_feature + S
0,292848	0,180697	-0,02429	1,620657	31	28	0,1163	0,614731	y ~ current_feature + S
0,172295	0,065524	-0,07874	2,629488	229	226	0,009139	0,125233	y ~ current_feature + S
0,091362	0,066241	0,034007	1,379246	229	226	0,169182	0,61875	y ~ current_feature + S
0,055617	0,066563	-0,04751	0,835551	228	225	0,404294	0,803665	y ~ current_feature + S
-0,09002	0,070958	0,061498	-1,26864	200	197	0,206066	0,893818	y ~ current_feature + S
0,169452	0,080738	-0,02429	2,098774	152	149	0,037524	0,520476	y ~ current_feature + S
-0,09123	0,054006	-0,07874	-1,68919	343	340	0,092101	0,42595	y ~ current_feature + S
-0,16124	0,053523	0,034007	-3,01259	343	340	0,002785	0,137369	y ~ current_feature + S
-0,09042	0,05409	-0,04751	-1,67163	342	339	0,09552	0,640171	y ~ current_feature + S
-0,12323	0,057487	0,061498	-2,14367	301	298	0,032868	0,658491	y ~ current_feature + S
-0,17438	0,065645	-0,02429	-2,6564	228	225	0,008464	0,295269	y ~ current_feature + S
0,017647	0,076237	-0,07874	0,231479	175	172	0,817218	0,979871	y ~ current_feature + S
-0,06807	0,076072	0,034007	-0,89485	175	172	0,372117	0,750317	y ~ current_feature + S
0,012244	0,076466	-0,04751	0,160128	174	171	0,872969	0,985217	y ~ current_feature + S
-0,15933	0,080607	0,061498	-1,97658	153	150	0,049923	0,700056	y ~ current_feature + S
0,127937	0,093299	-0,02429	1,371262	116	113	0,17301	0,626332	y ~ current_feature + S
0,215579	0,100185	-0,07874	2,151805	98	95	0,033948	0,236995	y ~ current_feature + S
0,14358	0,101535	0,034007	1,414095	98	95	0,160603	0,60427	y ~ current_feature + S
0,22003	0,100083	-0,04751	2,198464	98	95	0,030345	0,603897	y ~ current_feature + S
-0,2302	0,108801	0,061498	-2,11579	83	80	0,037473	0,658491	y ~ current_feature + S
0,206279	0,124269	-0,02429	1,659945	65	62	0,101976	0,605474	y ~ current_feature + S
-0,00224	0,084819	-0,07874	-0,02637	142	139	0,978998	0,996318	y ~ current_feature + S
-0,07061	0,084607	0,034007	-0,83452	142	139	0,405418	0,779245	y ~ current_feature + S
-0,01927	0,08511	-0,04751	-0,22644	141	138	0,821193	0,972405	y ~ current_feature + S
0,002722	0,090909	0,061498	0,029946	124	121	0,97616	0,996179	y ~ current_feature + S
-0,0933	0,104371	-0,02429	-0,89396	94	91	0,373699	0,815551	y ~ current_feature + S
-0,02675	0,066495	-0,07874	-0,4023	229	226	0,687843	0,93638	y ~ current_feature + S
-0,17331	0,065512	0,034007	-2,64549	229	226	0,00873	0,215592	y ~ current_feature + S
-0,07573	0,066328	-0,04751	-1,14182	229	226	0,254739	0,742928	y ~ current_feature + S
0,036906	0,071199	0,061498	0,518354	200	197	0,604793	0,945257	y ~ current_feature + S
-0,03633	0,081596	-0,02429	-0,44518	153	150	0,656829	0,924682	y ~ current_feature + S
0,092889	0,150104	-0,07874	0,618831	47	44	0,539218	0,882344	y ~ current_feature + S
-0,04708	0,150588	0,034007	-0,31267	47	44	0,756007	0,93416	y ~ current_feature + S
0,05134	0,150557	-0,04751	0,341002	47	44	0,734725	0,933491	y ~ current_feature + S
0,17641	0,159677	0,061498	1,104792	41	38	0,276196	0,893818	y ~ current_feature + S

-0,08017	0,188374	-0,02429	-0,42557	31	28	0,673671	0,926342	y ~ current_feature + S
0,074697	0,150334	-0,07874	0,496874	47	44	0,621752	0,919937	y ~ current_feature + S
0,136478	0,149345	0,034007	0,913841	47	44	0,365781	0,7498	y ~ current_feature + S
-0,16873	0,148594	-0,04751	-1,1355	47	44	0,262312	0,74658	y ~ current_feature + S
0,027729	0,160067	0,061498	0,173233	42	39	0,863364	0,996179	y ~ current_feature + S
-0,49158	0,164572	-0,02429	-2,98702	31	28	0,005801	0,238467	y ~ current_feature + S
0,03964	0,087637	-0,07874	0,45232	133	130	0,651793	0,932255	y ~ current_feature + S
0,041786	0,087629	0,034007	0,476855	133	130	0,634266	0,899624	y ~ current_feature + S
0,021576	0,087685	-0,04751	0,24606	133	130	0,806024	0,96985	y ~ current_feature + S
-0,01485	0,094062	0,061498	-0,15785	116	113	0,874856	0,996179	y ~ current_feature + S
0,024952	0,108431	-0,02429	0,23012	88	85	0,818552	0,975408	y ~ current_feature + S
-0,12948	0,113001	-0,07874	-1,14586	80	77	0,255404	0,682306	y ~ current_feature + S
-0,06368	0,113729	0,034007	-0,55995	80	77	0,577139	0,869291	y ~ current_feature + S
-0,01798	0,113942	-0,04751	-0,15778	80	77	0,875046	0,985217	y ~ current_feature + S
-0,04096	0,122988	0,061498	-0,33302	69	66	0,740173	0,972573	y ~ current_feature + S
-0,07072	0,141067	-0,02429	-0,50132	53	50	0,618346	0,924682	y ~ current_feature + S
-0,00175	0,055132	-0,07874	-0,03181	332	329	0,974641	0,99618	y ~ current_feature + S
-0,04372	0,055079	0,034007	-0,79369	332	329	0,427947	0,791701	y ~ current_feature + S
-0,0265	0,055112	-0,04751	-0,48079	332	329	0,630986	0,925756	y ~ current_feature + S
-0,05551	0,06976	0,061498	-0,79579	289	204,8543	0,427074	0,937665	y ~ current_feature + S
-0,00271	0,067838	-0,02429	-0,03999	221	217,2952	0,968138	0,99444	y ~ current_feature + S
0,115356	0,051295	-0,07874	2,248871	378	375	0,025101	0,201065	y ~ current_feature + S
0,032379	0,051613	0,034007	0,627345	378	375	0,530815	0,848387	y ~ current_feature + S
-0,01644	0,051702	-0,04751	-0,31803	377	374	0,750643	0,9394	y ~ current_feature + S
-0,08816	0,055169	0,061498	-1,59806	329	326	0,110998	0,8197	y ~ current_feature + S
-0,02415	0,063354	-0,02429	-0,38121	252	249	0,703369	0,93118	y ~ current_feature + S
-0,03744	0,051535	-0,07874	-0,7265	379	376	0,467984	0,840554	y ~ current_feature + S
-0,02725	0,051552	0,034007	-0,5285	379	376	0,597462	0,878669	y ~ current_feature + S
-0,01746	0,051632	-0,04751	-0,3381	378	375	0,735473	0,933491	y ~ current_feature + S
0,012483	0,061937	0,061498	0,201552	329	260,6368	0,840424	0,996179	y ~ current_feature + S
-0,07264	0,063272	-0,02429	-1,14799	252	248,4754	0,252078	0,717483	y ~ current_feature + S
-0,01874	0,061535	-0,07874	-0,30453	267	264	0,760964	0,965008	y ~ current_feature + S
-0,08153	0,061341	0,034007	-1,32919	267	264	0,184932	0,638805	y ~ current_feature + S
0,006996	0,061544	-0,04751	0,113673	267	264	0,909584	0,985217	y ~ current_feature + S
0,001041	0,066372	0,061498	0,015684	230	227	0,9875	0,997945	y ~ current_feature + S
-0,0804	0,075564	-0,02429	-1,06399	177	174	0,288807	0,734108	y ~ current_feature + S
0,270663	0,063477	-0,07874	4,263961	233	230	2,93E-05	0,005519	y ~ current_feature + S
0,220686	0,064312	0,034007	3,431474	233	230	7,12E-04	0,068218	y ~ current_feature + S
0,243284	0,063957	-0,04751	3,803866	233	230	1,83E-04	0,067562	y ~ current_feature + S
0,056542	0,070598	0,061498	0,800911	203	200	0,424133	0,937665	y ~ current_feature + S
0,33435	0,076192	-0,02429	4,388235	156	153	2,12E-05	0,010121	y ~ current_feature + S
-0,02391	0,074722	-0,07874	-0,32002	182	179	0,749326	0,962676	y ~ current_feature + S
-0,05011	0,07465	0,034007	-0,67126	182	179	0,502923	0,841996	y ~ current_feature + S
0,06154	0,074602	-0,04751	0,82491	182	179	0,410521	0,803665	y ~ current_feature + S
-0,12159	0,079726	0,061498	-1,52505	158	155	0,129284	0,8197	y ~ current_feature + S
0,002162	0,09167	-0,02429	0,023588	122	119	0,981221	0,99444	y ~ current_feature + S
0,00178	0,088388	-0,07874	0,020135	131	128	0,983967	0,996318	y ~ current_feature + S
0,042772	0,088307	0,034007	0,484359	131	128	0,628959	0,899624	y ~ current_feature + S

-0,0094	0,088384	-0,04751	-0,10637	131	128	0,915452	0,985217	y ~ current_feature + S
0,030484	0,094028	0,061498	0,324203	116	113	0,746384	0,972573	y ~ current_feature + S
0,032604	0,109051	-0,02429	0,298977	87	84	0,765696	0,953898	y ~ current_feature + S
-0,04647	0,116122	-0,07874	-0,40015	77	74	0,690195	0,93638	y ~ current_feature + S
-0,03539	0,116175	0,034007	-0,30466	77	74	0,761483	0,936042	y ~ current_feature + S
-0,04895	0,116108	-0,04751	-0,42159	77	74	0,674547	0,928258	y ~ current_feature + S
-0,12086	0,143502	0,061498	-0,84224	69	47,85124	0,403841	0,93433	y ~ current_feature + S
0,009284	0,144331	-0,02429	0,064322	51	48	0,948981	0,99444	y ~ current_feature + S
-0,03483	0,052636	-0,07874	-0,66173	394	360,5014	0,508567	0,863164	y ~ current_feature + S
-0,13251	0,069514	0,034007	-1,9063	394	203,3119	0,058022	0,420946	y ~ current_feature + S
-0,06616	0,054599	-0,04751	-1,21174	393	333,9873	0,226469	0,742928	y ~ current_feature + S
-0,00451	0,05995	0,061498	-0,07521	341	278,2356	0,940104	0,996179	y ~ current_feature + S
-0,03311	0,062103	-0,02429	-0,53307	262	259	0,594441	0,924682	y ~ current_feature + S
0,02812	0,083591	-0,07874	0,336398	146	143	0,737064	0,959225	y ~ current_feature + S
0,084317	0,083326	0,034007	1,011894	146	143	0,313298	0,718742	y ~ current_feature + S
0,137952	0,083116	-0,04751	1,659757	145	142	0,09917	0,640171	y ~ current_feature + S
-0,08755	0,09056	0,061498	-0,96675	124	121	0,335598	0,893818	y ~ current_feature + S
0,228609	0,100411	-0,02429	2,276735	97	94	0,025071	0,485321	y ~ current_feature + S
0,029285	0,055722	-0,07874	0,525562	365	321,7946	0,599554	0,90386	y ~ current_feature + S
0,048212	0,073871	0,034007	0,652643	365	182,8255	0,514806	0,841996	y ~ current_feature + S
0,022866	0,059045	-0,04751	0,387259	364	286,6822	0,698852	0,931902	y ~ current_feature + S
-0,06308	0,063229	0,061498	-0,99771	314	249,1355	0,319387	0,893818	y ~ current_feature + S
-0,00397	0,064549	-0,02429	-0,06157	243	240	0,950954	0,99444	y ~ current_feature + S
-0,01333	0,052082	-0,07874	-0,25597	412	368,5973	0,798121	0,979102	y ~ current_feature + S
-0,07751	0,067815	0,034007	-1,14298	412	216,1402	0,254309	0,704827	y ~ current_feature + S
0,01794	0,053314	-0,04751	0,336491	411	351,7055	0,736701	0,933491	y ~ current_feature + S
-0,11498	0,057164	0,061498	-2,01147	358	301,98	0,045164	0,700056	y ~ current_feature + S
-0,01551	0,061164	-0,02429	-0,25358	274	267,24	0,800017	0,968297	y ~ current_feature + S
0,03599	0,050932	-0,07874	0,706624	388	385	0,480227	0,845512	y ~ current_feature + S
-0,00503	0,050964	0,034007	-0,09873	388	385	0,921401	0,9732	y ~ current_feature + S
-0,0055	0,05103	-0,04751	-0,1077	387	384	0,914293	0,985217	y ~ current_feature + S
-0,07346	0,062085	0,061498	-1,18319	338	258,0305	0,237824	0,893818	y ~ current_feature + S
-0,06818	0,062482	-0,02429	-1,09125	258	254,9591	0,276193	0,734108	y ~ current_feature + S
0,094168	0,093242	-0,07874	1,009929	117	114	0,314668	0,718687	y ~ current_feature + S
-0,05882	0,093496	0,034007	-0,62914	117	114	0,530516	0,848387	y ~ current_feature + S
-0,10121	0,093589	-0,04751	-1,08141	116	113	0,281819	0,747915	y ~ current_feature + S
-0,16178	0,101246	0,061498	-1,59793	98	95	0,113379	0,8197	y ~ current_feature + S
0,008382	0,115466	-0,02429	0,072593	78	75	0,942323	0,99444	y ~ current_feature + S
-0,06444	0,056227	-0,07874	-1,14603	318	315	0,252651	0,679778	y ~ current_feature + S
-0,12876	0,055875	0,034007	-2,30447	318	315	0,021847	0,329927	y ~ current_feature + S
-0,06149	0,056237	-0,04751	-1,0934	318	315	0,275052	0,747915	y ~ current_feature + S
-0,12797	0,059483	0,061498	-2,15137	281	278	0,032309	0,658491	y ~ current_feature + S
-0,14857	0,068404	-0,02429	-2,17189	212	209	0,03099	0,485321	y ~ current_feature + S
0,035243	0,090853	-0,07874	0,387913	124	121	0,698762	0,939646	y ~ current_feature + S
-0,07447	0,090657	0,034007	-0,82142	124	121	0,413023	0,785427	y ~ current_feature + S
0,045811	0,090814	-0,04751	0,504453	124	121	0,61486	0,924009	y ~ current_feature + S
-0,09426	0,096696	0,061498	-0,97476	109	106	0,331896	0,893818	y ~ current_feature + S
-0,08617	0,111388	-0,02429	-0,77358	83	80	0,441457	0,858965	y ~ current_feature + S

0,004693	0,103694	-0,07874	0,045254	96	93	0,964002	0,994607	y ~ current_feature + S
-0,2858	0,09937	0,034007	-2,87616	96	93	0,004991	0,167881	y ~ current_feature + S
0,006626	0,104255	-0,04751	0,063554	95	92	0,949463	0,990156	y ~ current_feature + S
0,007722	0,1118	0,061498	0,069066	83	80	0,945109	0,996179	y ~ current_feature + S
-0,16191	0,127396	-0,02429	-1,27094	63	60	0,208657	0,675518	y ~ current_feature + S
0,123635	0,07417	-0,07874	1,666919	182	179	0,097279	0,431938	y ~ current_feature + S
0,010313	0,07474	0,034007	0,137987	182	179	0,890405	0,96591	y ~ current_feature + S
0,053502	0,074846	-0,04751	0,714833	181	178	0,475649	0,842057	y ~ current_feature + S
0,030513	0,080027	0,061498	0,38129	159	156	0,703507	0,972444	y ~ current_feature + S
0,001665	0,092057	-0,02429	0,018082	121	118	0,985604	0,99444	y ~ current_feature + S
0,396241	0,0942	-0,07874	4,206392	98	95	5,88E-05	0,008233	y ~ current_feature + S
0,122781	0,101822	0,034007	1,20584	98	95	0,230874	0,681978	y ~ current_feature + S
0,154493	0,101366	-0,04751	1,524113	98	95	0,130803	0,654671	y ~ current_feature + S
0,012748	0,1091	0,061498	0,116847	87	84	0,907261	0,996179	y ~ current_feature + S
0,024396	0,126962	-0,02429	0,192153	65	62	0,84825	0,977734	y ~ current_feature + S
0,329037	0,12619	-0,07874	2,607477	59	56	0,01167	0,141568	y ~ current_feature + S
0,099753	0,132964	0,034007	0,750227	59	56	0,456259	0,821488	y ~ current_feature + S
-0,02594	0,134795	-0,04751	-0,19247	58	55	0,848084	0,980218	y ~ current_feature + S
-0,26433	0,142198	0,061498	-1,85892	49	46	0,069442	0,715249	y ~ current_feature + S
0,27035	0,16046	-0,02429	1,68484	39	36	0,100671	0,605474	y ~ current_feature + S
0,175416	0,086016	-0,07874	2,039351	134	131	0,043426	0,277028	y ~ current_feature + S
0,096765	0,08696	0,034007	1,112747	134	131	0,267855	0,71095	y ~ current_feature + S
0,008867	0,087367	-0,04751	0,101488	134	131	0,919318	0,985217	y ~ current_feature + S
-0,05738	0,095189	0,061498	-0,60279	113	110	0,547891	0,937665	y ~ current_feature + S
0,02935	0,107165	-0,02429	0,273875	90	87	0,78483	0,964545	y ~ current_feature + S
-0,00497	0,064149	-0,07874	-0,0775	246	243	0,938287	0,994607	y ~ current_feature + S
-0,01189	0,064145	0,034007	-0,18536	246	243	0,8531	0,959445	y ~ current_feature + S
-0,00398	0,06415	-0,04751	-0,06202	246	243	0,950595	0,990156	y ~ current_feature + S
-0,0156	0,069329	0,061498	-0,22501	211	208	0,822192	0,996179	y ~ current_feature + S
-0,07119	0,078611	-0,02429	-0,90561	164	161	0,366494	0,807159	y ~ current_feature + S
-0,32263	0,104526	-0,07874	-3,08664	85	82	0,002761	0,065908	y ~ current_feature + S
-0,1377	0,10938	0,034007	-1,25894	85	82	0,211623	0,664036	y ~ current_feature + S
-0,02133	0,111086	-0,04751	-0,192	84	81	0,848221	0,980218	y ~ current_feature + S
0,008331	0,119519	0,061498	0,069708	73	70	0,944625	0,996179	y ~ current_feature + S
0,012403	0,136072	-0,02429	0,091147	57	54	0,927713	0,99444	y ~ current_feature + S
-0,00643	0,097588	-0,07874	-0,06589	108	105	0,94759	0,994607	y ~ current_feature + S
-0,0028	0,09759	0,034007	-0,02869	108	105	0,977165	0,989859	y ~ current_feature + S
0,085073	0,097236	-0,04751	0,874914	108	105	0,383617	0,803665	y ~ current_feature + S
0,112009	0,104169	0,061498	1,075264	94	91	0,285099	0,893818	y ~ current_feature + S
0,15031	0,119018	-0,02429	1,262914	72	69	0,210871	0,675518	y ~ current_feature + S
-0,08853	0,103848	-0,07874	-0,85252	95	92	0,396138	0,791422	y ~ current_feature + S
-0,05691	0,104088	0,034007	-0,5467	95	92	0,585906	0,87187	y ~ current_feature + S
0,141523	0,103773	-0,04751	1,363767	94	91	0,176005	0,700235	y ~ current_feature + S
-0,1557	0,109756	0,061498	-1,41856	84	81	0,159863	0,8197	y ~ current_feature + S
-0,15225	0,127594	-0,02429	-1,19326	63	60	0,237463	0,697311	y ~ current_feature + S
0,102816	0,128415	-0,07874	0,800654	63	60	0,426491	0,814177	y ~ current_feature + S
0,058689	0,128877	0,034007	0,455391	63	60	0,650472	0,900251	y ~ current_feature + S
0,149302	0,127652	-0,04751	1,169598	63	60	0,246788	0,742928	y ~ current_feature + S

-0,30876	0,129434	0,061498	-2,38545	57	54	0,020596	0,609646	y ~ current_feature + S
-0,11651	0,159038	-0,02429	-0,7326	42	39	0,468185	0,876224	y ~ current_feature + S
0,087949	0,06238	-0,07874	1,409893	258	255	0,15979	0,530244	y ~ current_feature + S
0,088062	0,062379	0,034007	1,411722	258	255	0,159251	0,60427	y ~ current_feature + S
0,032107	0,06259	-0,04751	0,512966	258	255	0,608419	0,916966	y ~ current_feature + S
-0,08501	0,066278	0,061498	-1,2826	229	226	0,200945	0,893818	y ~ current_feature + S
0,042001	0,076855	-0,02429	0,546494	172	169	0,585447	0,924682	y ~ current_feature + S
0,081544	0,068291	-0,07874	1,194079	216	213	0,233775	0,657771	y ~ current_feature + S
-0,03388	0,06848	0,034007	-0,49475	216	213	0,621284	0,894455	y ~ current_feature + S
0,152429	0,067718	-0,04751	2,250938	216	213	0,025411	0,569815	y ~ current_feature + S
-0,00523	0,074328	0,061498	-0,07036	184	181	0,943985	0,996179	y ~ current_feature + S
0,071277	0,084001	-0,02429	0,848524	144	141	0,397585	0,823699	y ~ current_feature + S
0,154309	0,052841	-0,07874	2,920256	396	349,6166	0,003724	0,069632	y ~ current_feature + S
0,098426	0,069095	0,034007	1,424502	396	207,4353	0,155804	0,60427	y ~ current_feature + S
0,061236	0,05404	-0,04751	1,133149	395	341,1423	0,257947	0,742928	y ~ current_feature + S
-0,02193	0,059145	0,061498	-0,3708	343	285,7334	0,711063	0,972444	y ~ current_feature + S
0,087753	0,062517	-0,02429	1,403669	263	253,8906	0,161639	0,623497	y ~ current_feature + S
0,089043	0,087023	-0,07874	1,023211	134	131	0,308094	0,718687	y ~ current_feature + S
-0,09076	0,08701	0,034007	-1,04316	134	131	0,298796	0,718742	y ~ current_feature + S
-0,11948	0,086745	-0,04751	-1,37733	134	131	0,170758	0,700235	y ~ current_feature + S
-0,04402	0,091968	0,061498	-0,4786	121	118	0,633112	0,95418	y ~ current_feature + S
-0,20105	0,105631	-0,02429	-1,90335	89	86	0,06034	0,558147	y ~ current_feature + S
0,144332	0,051963	-0,07874	2,777617	404	362,6407	0,005761	0,092669	y ~ current_feature + S
0,056516	0,068302	0,034007	0,827441	404	213,6684	0,40891	0,783921	y ~ current_feature + S
0,021849	0,053759	-0,04751	0,406424	403	345,8498	0,684682	0,928814	y ~ current_feature + S
0,010828	0,058118	0,061498	0,186318	350	296,0216	0,852323	0,996179	y ~ current_feature + S
0,102514	0,060991	-0,02429	1,680804	269	266	0,093975	0,605474	y ~ current_feature + S
0,157508	0,105873	-0,07874	1,487706	90	87	0,140444	0,498495	y ~ current_feature + S
-0,02781	0,10717	0,034007	-0,25947	90	87	0,795887	0,946875	y ~ current_feature + S
-0,04612	0,107097	-0,04751	-0,4306	90	87	0,667821	0,92822	y ~ current_feature + S
-0,05135	0,113078	0,061498	-0,45408	81	78	0,651029	0,957207	y ~ current_feature + S
-6,69E-04	0,132453	-0,02429	-0,00505	60	57	0,995989	0,998688	y ~ current_feature + S
-0,03037	0,050744	-0,07874	-0,59845	391	388	0,549887	0,888464	y ~ current_feature + S
-0,06269	0,050667	0,034007	-1,23725	391	388	0,216741	0,664036	y ~ current_feature + S
-0,06174	0,050736	-0,04751	-1,21685	390	387	0,224402	0,742928	y ~ current_feature + S
-0,03993	0,06066	0,061498	-0,65821	342	271,3292	0,510964	0,937665	y ~ current_feature + S
-0,04441	0,062317	-0,02429	-0,71273	260	257	0,47666	0,878782	y ~ current_feature + S
0,026761	0,073837	-0,07874	0,362439	200	183,2901	0,717442	0,948276	y ~ current_feature + S
0,032601	0,090078	0,034007	0,361917	200	123,1108	0,718035	0,920814	y ~ current_feature + S
0,083691	0,078081	-0,04751	1,071843	199	162,8748	0,285376	0,747915	y ~ current_feature + S
0,072157	0,078068	0,061498	0,924283	175	163,223	0,356703	0,920619	y ~ current_feature + S
0,04808	0,089476	-0,02429	0,537358	133	124,6195	0,591979	0,924682	y ~ current_feature + S
0,008448	0,078565	-0,07874	0,107529	165	162	0,914502	0,987929	y ~ current_feature + S
-0,14333	0,077756	0,034007	-1,84327	165	162	0,067117	0,439525	y ~ current_feature + S
-0,10618	0,078123	-0,04751	-1,35916	165	162	0,175984	0,700235	y ~ current_feature + S
-0,15642	0,082595	0,061498	-1,89376	146	143	0,060276	0,715249	y ~ current_feature + S
-0,13822	0,095746	-0,02429	-1,44362	110	107	0,151767	0,623156	y ~ current_feature + S
-0,06423	0,076538	-0,07874	-0,8392	173	170	0,402533	0,796456	y ~ current_feature + S

-0,01253	0,07669	0,034007	-0,16334	173	170	0,870444	0,96591	y ~ current_feature + S
0,059199	0,076562	-0,04751	0,773221	173	170	0,440466	0,823749	y ~ current_feature + S
0,002754	0,081923	0,061498	0,033621	152	149	0,973224	0,996179	y ~ current_feature + S
0,065111	0,093872	-0,02429	0,693608	116	113	0,489351	0,889654	y ~ current_feature + S
0,109131	0,105367	-0,07874	1,035725	92	89	0,303136	0,718687	y ~ current_feature + S
-0,03801	0,105923	0,034007	-0,35881	92	89	0,720588	0,920814	y ~ current_feature + S
0,017345	0,105984	-0,04751	0,163661	92	89	0,870369	0,985217	y ~ current_feature + S
-0,16253	0,114702	0,061498	-1,41699	77	74	0,160684	0,8197	y ~ current_feature + S
0,145621	0,129907	-0,02429	1,120963	61	58	0,266923	0,723659	y ~ current_feature + S
0,013919	0,102588	-0,07874	0,135682	98	95	0,89236	0,987929	y ~ current_feature + S
-0,00927	0,102593	0,034007	-0,0904	98	95	0,928159	0,9732	y ~ current_feature + S
0,096424	0,102662	-0,04751	0,939238	97	94	0,350016	0,795089	y ~ current_feature + S
0,06606	0,111559	0,061498	0,59215	83	80	0,55542	0,937665	y ~ current_feature + S
0,17344	0,125075	-0,02429	1,386687	65	62	0,170503	0,626332	y ~ current_feature + S
0,113094	0,058958	-0,07874	1,918198	287	284	0,056088	0,312071	y ~ current_feature + S
0,083639	0,059131	0,034007	1,414457	287	284	0,158323	0,60427	y ~ current_feature + S
0,054312	0,059251	-0,04751	0,916643	287	284	0,360108	0,798092	y ~ current_feature + S
-0,03101	0,063598	0,061498	-0,48753	250	247	0,626317	0,951693	y ~ current_feature + S
-0,00953	0,072736	-0,02429	-0,13103	192	189	0,895887	0,99444	y ~ current_feature + S
0,037463	0,090472	-0,07874	0,414087	125	122	0,679537	0,934653	y ~ current_feature + S
0,008142	0,090533	0,034007	0,089936	125	122	0,928486	0,9732	y ~ current_feature + S
0,001283	0,090909	-0,04751	0,014111	124	121	0,988765	0,994138	y ~ current_feature + S
-0,0117	0,099008	0,061498	-0,11818	105	102	0,906156	0,996179	y ~ current_feature + S
-0,10712	0,11116	-0,02429	-0,96369	83	80	0,338105	0,785829	y ~ current_feature + S
-0,01581	0,087695	-0,07874	-0,18034	133	130	0,857167	0,983548	y ~ current_feature + S
-0,05854	0,087555	0,034007	-0,66858	133	130	0,504949	0,841996	y ~ current_feature + S
-0,20569	0,086162	-0,04751	-2,38725	132	129	0,018426	0,54566	y ~ current_feature + S
-0,03196	0,094867	0,061498	-0,33685	114	111	0,736863	0,972573	y ~ current_feature + S
-0,05859	0,107648	-0,02429	-0,54423	89	86	0,58769	0,924682	y ~ current_feature + S
-0,08762	0,054024	-0,07874	-1,62195	343	340	0,105742	0,449749	y ~ current_feature + S
-0,09609	0,053982	0,034007	-1,78013	343	340	0,075948	0,464477	y ~ current_feature + S
-0,0694	0,054182	-0,04751	-1,28096	342	339	0,201082	0,723562	y ~ current_feature + S
-0,03178	0,058391	0,061498	-0,5443	296	293	0,586652	0,945257	y ~ current_feature + S
-0,09469	0,066367	-0,02429	-1,42669	228	225	0,155055	0,623156	y ~ current_feature + S
-0,09086	0,07873	-0,07874	-1,1541	163	160	0,25018	0,679754	y ~ current_feature + S
-0,07445	0,078838	0,034007	-0,94431	163	160	0,346436	0,747412	y ~ current_feature + S
-0,05347	0,078944	-0,04751	-0,67737	163	160	0,499148	0,868909	y ~ current_feature + S
-0,06709	0,095376	0,061498	-0,70345	144	109,437	0,483268	0,937665	y ~ current_feature + S
2,28E-05	0,09759	-0,02429	2,33E-04	108	105	0,999814	0,999814	y ~ current_feature + S
0,025455	0,063479	-0,07874	0,400999	251	248	0,688766	0,93638	y ~ current_feature + S
0,012747	0,063495	0,034007	0,200753	251	248	0,841056	0,958985	y ~ current_feature + S
0,070007	0,063472	-0,04751	1,102956	250	247	0,27112	0,747915	y ~ current_feature + S
0,07258	0,068499	0,061498	1,059572	215	212	0,290545	0,893818	y ~ current_feature + S
0,084046	0,077811	-0,02429	1,080141	167	164	0,281665	0,734108	y ~ current_feature + S
0,12232	0,085376	-0,07874	1,432719	153	135,1385	0,154248	0,518833	y ~ current_feature + S
0,025521	0,113536	0,034007	0,224786	153	77,52606	0,822737	0,952501	y ~ current_feature + S
0,149015	0,083367	-0,04751	1,787455	152	140,6878	0,076017	0,640171	y ~ current_feature + S
-0,06812	0,093844	0,061498	-0,72592	137	113,0233	0,469388	0,937665	y ~ current_feature + S

0,006673	0,100502	-0,02429	0,066396	102	99	0,947196	0,99444	y ~ current_feature + S
-0,01744	0,071785	-0,07874	-0,24301	197	194	0,808251	0,979871	y ~ current_feature + S
-0,05055	0,071704	0,034007	-0,70495	197	194	0,481686	0,831402	y ~ current_feature + S
-0,05194	0,071699	-0,04751	-0,72436	197	194	0,469719	0,842057	y ~ current_feature + S
-0,01281	0,077376	0,061498	-0,16551	170	167	0,868745	0,996179	y ~ current_feature + S
0,027719	0,088354	-0,02429	0,313721	131	128	0,754243	0,951432	y ~ current_feature + S
0,127392	0,079668	-0,07874	1,599049	158	155	0,111846	0,465986	y ~ current_feature + S
-0,00904	0,080319	0,034007	-0,11257	158	155	0,910516	0,9732	y ~ current_feature + S
0,166944	0,079451	-0,04751	2,101202	157	154	0,037252	0,611115	y ~ current_feature + S
-0,10046	0,091831	0,061498	-1,09398	135	117,3868	0,276202	0,893818	y ~ current_feature + S
0,103956	0,0991	-0,02429	1,049001	105	100,7233	0,296688	0,744235	y ~ current_feature + S
0,370654	0,135475	-0,07874	2,735955	50	47	0,008749	0,122156	y ~ current_feature + S
0,276209	0,14019	0,034007	1,970243	50	47	0,054718	0,420946	y ~ current_feature + S
0,111168	0,146528	-0,04751	0,758681	49	46	0,451914	0,832044	y ~ current_feature + S
0,17853	0,153665	0,061498	1,161814	44	41	0,25203	0,893818	y ~ current_feature + S
0,135985	0,180878	-0,02429	0,751802	33	30	0,458028	0,866856	y ~ current_feature + S
-0,10702	0,072131	-0,07874	-1,4837	193	190	0,139546	0,498495	y ~ current_feature + S
-0,0922	0,072239	0,034007	-1,2763	193	190	0,203406	0,65103	y ~ current_feature + S
-0,02421	0,072718	-0,04751	-0,33298	192	189	0,739518	0,933594	y ~ current_feature + S
0,059696	0,078186	0,061498	0,763508	166	163	0,446264	0,937665	y ~ current_feature + S
-0,14212	0,088183	-0,02429	-1,61163	129	126	0,109545	0,605474	y ~ current_feature + S
0,023829	0,094045	-0,07874	0,25338	116	113	0,800435	0,979102	y ~ current_feature + S
0,037518	0,094006	0,034007	0,399103	116	113	0,690571	0,916569	y ~ current_feature + S
0,010056	0,094486	-0,04751	0,106424	115	112	0,915436	0,985217	y ~ current_feature + S
-0,01942	0,102043	0,061498	-0,19034	99	96	0,849448	0,996179	y ~ current_feature + S
0,047445	0,116117	-0,02429	0,408601	77	74	0,684013	0,926342	y ~ current_feature + S
0,16757	0,0585	-0,07874	2,864441	287	284	0,004489	0,077255	y ~ current_feature + S
0,134077	0,058803	0,034007	2,280087	287	284	0,023344	0,338715	y ~ current_feature + S
0,067655	0,059308	-0,04751	1,140739	286	283	0,254943	0,742928	y ~ current_feature + S
0,013532	0,073447	0,061498	0,184236	248	185,3432	0,85403	0,996179	y ~ current_feature + S
0,12343	0,072377	-0,02429	1,705386	191	187,9896	0,089774	0,605474	y ~ current_feature + S
0,18306	0,078211	-0,07874	2,34058	161	158	0,020503	0,189455	y ~ current_feature + S
0,092572	0,079214	0,034007	1,168628	161	158	0,244314	0,702074	y ~ current_feature + S
0,125161	0,079181	-0,04751	1,580697	160	157	0,11596	0,640375	y ~ current_feature + S
0,008702	0,097039	0,061498	0,089673	139	106,1882	0,928716	0,996179	y ~ current_feature + S
-0,1525	0,097166	-0,02429	-1,56944	107	103,4559	0,119598	0,615361	y ~ current_feature + S
0,285021	0,124789	-0,07874	2,284025	62	59	0,025985	0,204564	y ~ current_feature + S
0,127632	0,129124	0,034007	0,988443	62	59	0,326972	0,728793	y ~ current_feature + S
0,177392	0,129224	-0,04751	1,37275	61	58	0,175115	0,700235	y ~ current_feature + S
0,204454	0,139839	0,061498	1,462065	52	49	0,150108	0,8197	y ~ current_feature + S
0,079148	0,161713	-0,02429	0,489438	41	38	0,627345	0,924682	y ~ current_feature + S
0,215477	0,088409	-0,07874	2,437275	125	122	0,016241	0,174676	y ~ current_feature + S
0,08089	0,090239	0,034007	0,896391	125	122	0,371809	0,750317	y ~ current_feature + S
0,136947	0,090053	-0,04751	1,52074	124	121	0,130934	0,654671	y ~ current_feature + S
-0,05684	0,097432	0,061498	-0,58335	108	105	0,56091	0,937665	y ~ current_feature + S
0,084933	0,111399	-0,02429	0,762414	83	80	0,448055	0,858965	y ~ current_feature + S
0,034805	0,073279	-0,07874	0,474962	189	186	0,635371	0,927169	y ~ current_feature + S
-0,02394	0,073303	0,034007	-0,32656	189	186	0,744369	0,927328	y ~ current_feature + S

-0,00534	0,073323	-0,04751	-0,0728	189	186	0,942046	0,990156	y ~ current_feature + S
-0,15872	0,076401	0,061498	-2,07745	170	167	0,03929	0,658491	y ~ current_feature + S
0,073832	0,089921	-0,02429	0,821077	126	123	0,413191	0,837702	y ~ current_feature + S
0,179255	0,056541	-0,07874	3,170346	364	302,7515	0,001679	0,051754	y ~ current_feature + S
0,027475	0,073457	0,034007	0,374031	364	185,1835	0,708809	0,920814	y ~ current_feature + S
0,070151	0,058647	-0,04751	1,196154	363	289,3098	0,232616	0,742928	y ~ current_feature + S
0,127151	0,065123	0,061498	1,952477	315	231,9832	0,052084	0,71375	y ~ current_feature + S
0,114671	0,064124	-0,02429	1,788269	243	240	0,074994	0,605474	y ~ current_feature + S
0,242295	0,101151	-0,07874	2,395387	95	92	0,018629	0,181384	y ~ current_feature + S
-0,09471	0,103789	0,034007	-0,91252	95	92	0,363882	0,7498	y ~ current_feature + S
0,178066	0,103153	-0,04751	1,726225	94	91	0,087701	0,640171	y ~ current_feature + S
-0,03433	0,112442	0,061498	-0,30531	82	79	0,760935	0,972573	y ~ current_feature + S
0,021578	0,129069	-0,02429	0,167185	63	60	0,867787	0,984468	y ~ current_feature + S
-0,1172	0,149717	-0,07874	-0,78284	47	44	0,437917	0,816268	y ~ current_feature + S
-0,11442	0,149766	0,034007	-0,76397	47	44	0,448965	0,814299	y ~ current_feature + S
0,220778	0,148736	-0,04751	1,484367	46	43	0,145006	0,661823	y ~ current_feature + S
-0,18264	0,157435	0,061498	-1,16011	42	39	0,253057	0,893818	y ~ current_feature + S
0,30016	0,180268	-0,02429	1,665073	31	28	0,10705	0,605474	y ~ current_feature + S
-0,02734	0,058802	-0,07874	-0,46502	292	289	0,642266	0,928694	y ~ current_feature + S
-0,07415	0,058662	0,034007	-1,26403	292	289	0,207237	0,655365	y ~ current_feature + S
-0,06739	0,058792	-0,04751	-1,14625	291	288	0,252644	0,742928	y ~ current_feature + S
0,064985	0,068715	0,061498	0,945715	251	210,8914	0,345376	0,907332	y ~ current_feature + S
-0,08643	0,072114	-0,02429	-1,19858	195	190,8569	0,232177	0,695919	y ~ current_feature + S
-0,00969	0,102057	-0,07874	-0,09491	99	96	0,924582	0,990519	y ~ current_feature + S
0,003855	0,102061	0,034007	0,037771	99	96	0,969949	0,989859	y ~ current_feature + S
-0,08121	0,101725	-0,04751	-0,79835	99	96	0,426639	0,819126	y ~ current_feature + S
-0,09453	0,110614	0,061498	-0,85464	84	81	0,395272	0,93433	y ~ current_feature + S
-0,20203	0,124381	-0,02429	-1,62424	65	62	0,109398	0,605474	y ~ current_feature + S
-0,02052	0,091268	-0,07874	-0,22483	123	120	0,822496	0,981192	y ~ current_feature + S
-0,15341	0,090207	0,034007	-1,70065	123	120	0,0916	0,494772	y ~ current_feature + S
-0,14468	0,090327	-0,04751	-1,60176	123	120	0,111839	0,640171	y ~ current_feature + S
0,117448	0,097851	0,061498	1,200272	106	103	0,232787	0,893818	y ~ current_feature + S
-0,10181	0,111924	-0,02429	-0,90961	82	79	0,365793	0,807159	y ~ current_feature + S
0,064222	0,053572	-0,07874	1,1988	350	347	0,231424	0,657483	y ~ current_feature + S
0,035664	0,053649	0,034007	0,664777	350	347	0,506635	0,841996	y ~ current_feature + S
0,06928	0,053631	-0,04751	1,291785	349	346	0,197294	0,723562	y ~ current_feature + S
-0,07035	0,057496	0,061498	-1,22363	304	301	0,222048	0,893818	y ~ current_feature + S
0,05569	0,065836	-0,02429	0,845892	233	230	0,398492	0,823699	y ~ current_feature + S
-0,08587	0,100133	-0,07874	-0,85756	102	99	0,393208	0,789252	y ~ current_feature + S
-0,1125	0,099866	0,034007	-1,12656	102	99	0,262653	0,71095	y ~ current_feature + S
-0,14893	0,099383	-0,04751	-1,49859	102	99	0,137161	0,657012	y ~ current_feature + S
-0,09672	0,107957	0,061498	-0,89595	88	85	0,372809	0,932536	y ~ current_feature + S
-0,02488	0,123996	-0,02429	-0,20069	68	65	0,841569	0,977734	y ~ current_feature + S
0,199876	0,093001	-0,07874	2,149189	114	111	0,033791	0,236995	y ~ current_feature + S
0,088167	0,094546	0,034007	0,932533	114	111	0,353085	0,7498	y ~ current_feature + S
-0,02149	0,094894	-0,04751	-0,22642	114	111	0,821288	0,972405	y ~ current_feature + S
0,004981	0,099999	0,061498	0,04981	103	100	0,960374	0,996179	y ~ current_feature + S
0,006164	0,117039	-0,02429	0,052668	76	73	0,95814	0,99444	y ~ current_feature + S

0,222465	0,052464	-0,07874	4,240336	393	345,3285	2,87E-05	0,005519	y ~ current_feature + S
0,142971	0,068215	0,034007	2,095907	393	210,5117	0,037285	0,388609	y ~ current_feature + S
0,139465	0,054207	-0,04751	2,572827	392	333,7012	0,010519	0,481414	y ~ current_feature + S
0,010312	0,058899	0,061498	0,175084	341	288,2289	0,861137	0,996179	y ~ current_feature + S
0,111595	0,062733	-0,02429	1,778901	261	250,9399	0,076466	0,605474	y ~ current_feature + S
0,101924	0,08233	-0,07874	1,238001	149	146	0,217703	0,639357	y ~ current_feature + S
-0,04878	0,082662	0,034007	-0,59008	149	146	0,556048	0,857986	y ~ current_feature + S
-0,01041	0,082756	-0,04751	-0,12575	149	146	0,900105	0,985217	y ~ current_feature + S
-0,15025	0,088427	0,061498	-1,69908	128	125	0,091791	0,795265	y ~ current_feature + S
-0,06567	0,101315	-0,02429	-0,64816	100	97	0,518411	0,899808	y ~ current_feature + S
-0,37481	0,12982	-0,07874	-2,88714	54	51	0,005689	0,092669	y ~ current_feature + S
-0,47529	0,123201	0,034007	-3,85782	54	51	3,23E-04	0,067708	y ~ current_feature + S
0,079911	0,13958	-0,04751	0,572507	54	51	0,569494	0,903741	y ~ current_feature + S
-0,19877	0,146097	0,061498	-1,36053	48	45	0,180437	0,85592	y ~ current_feature + S
-0,27225	0,167502	-0,02429	-1,62538	36	33	0,113596	0,614731	y ~ current_feature + S
-0,01126	0,052844	-0,07874	-0,21311	403	358,0589	0,831361	0,981192	y ~ current_feature + S
-0,05115	0,069495	0,034007	-0,736	403	206,519	0,462565	0,830821	y ~ current_feature + S
0,028474	0,054633	-0,04751	0,521191	402	334,7623	0,602578	0,91263	y ~ current_feature + S
-0,06037	0,058545	0,061498	-1,03117	351	290,6903	0,303317	0,893818	y ~ current_feature + S
0,001104	0,06145	-0,02429	0,01796	268	264,821	0,985684	0,99444	y ~ current_feature + S
0,044044	0,062439	-0,07874	0,70539	259	256	0,481209	0,845512	y ~ current_feature + S
0,01721	0,062491	0,034007	0,275399	259	256	0,783232	0,945731	y ~ current_feature + S
0,05466	0,062529	-0,04751	0,87415	258	255	0,382859	0,803665	y ~ current_feature + S
-0,00581	0,066666	0,061498	-0,08718	228	225	0,930603	0,996179	y ~ current_feature + S
0,037457	0,076869	-0,02429	0,48728	172	169	0,626691	0,924682	y ~ current_feature + S
0,214868	0,115906	-0,07874	1,853808	74	71	0,067921	0,34663	y ~ current_feature + S
0,080227	0,118296	0,034007	0,678192	74	71	0,499855	0,841996	y ~ current_feature + S
0,13876	0,118367	-0,04751	1,172291	73	70	0,245055	0,742928	y ~ current_feature + S
-0,29163	0,118643	0,061498	-2,45802	68	65	0,016645	0,55988	y ~ current_feature + S
3,09E-04	0,147442	-0,02429	0,002093	49	46	0,998339	0,99969	y ~ current_feature + S
-0,40964	0,133065	-0,07874	-3,07853	50	47	0,003467	0,069632	y ~ current_feature + S
-0,2419	0,141533	0,034007	-1,70916	50	47	0,094017	0,500522	y ~ current_feature + S
-0,23183	0,141891	-0,04751	-1,63384	50	47	0,108975	0,640171	y ~ current_feature + S
0,0081	0,162216	0,061498	0,049934	41	38	0,960437	0,996179	y ~ current_feature + S
0,314337	0,17332	-0,02429	1,813627	33	30	0,07975	0,605474	y ~ current_feature + S
0,106477	0,077407	-0,07874	1,375537	168	165	0,170829	0,550681	y ~ current_feature + S
0,048361	0,077759	0,034007	0,621938	168	165	0,534841	0,849319	y ~ current_feature + S
0,074485	0,077634	-0,04751	0,959442	168	165	0,33874	0,788263	y ~ current_feature + S
-0,13845	0,084002	0,061498	-1,6482	142	139	0,101571	0,8197	y ~ current_feature + S
0,203681	0,093775	-0,02429	2,172025	112	109	0,032021	0,485321	y ~ current_feature + S
0,022234	0,081359	-0,07874	0,273278	154	151	0,785013	0,974681	y ~ current_feature + S
-0,01067	0,081374	0,034007	-0,13107	154	151	0,895897	0,96591	y ~ current_feature + S
-0,1582	0,080621	-0,04751	-1,9623	153	150	0,051578	0,640171	y ~ current_feature + S
-0,07298	0,08781	0,061498	-0,8311	132	129	0,407451	0,934833	y ~ current_feature + S
-0,1399	0,099515	-0,02429	-1,4058	102	99	0,162913	0,623497	y ~ current_feature + S
0,008349	0,095343	-0,07874	0,087572	113	110	0,930376	0,991839	y ~ current_feature + S
-0,01481	0,095336	0,034007	-0,15535	113	110	0,87683	0,96591	y ~ current_feature + S
-0,07731	0,095496	-0,04751	-0,80956	112	109	0,419959	0,819126	y ~ current_feature + S

-0,07154	0,10399	0,061498	-0,68794	95	92	0,493222	0,937665	y ~ current_feature + S
-0,17406	0,116052	-0,02429	-1,49982	75	72	0,138032	0,618941	y ~ current_feature + S
0,080121	0,055985	-0,07874	1,431123	320	317	0,15338	0,518271	y ~ current_feature + S
0,022196	0,056152	0,034007	0,39529	320	317	0,692895	0,916569	y ~ current_feature + S
0,024779	0,056148	-0,04751	0,441312	320	317	0,659288	0,925756	y ~ current_feature + S
0,017405	0,064164	0,061498	0,271252	274	242,8208	0,786428	0,979725	y ~ current_feature + S
0,022919	0,068988	-0,02429	0,332211	213	210	0,740061	0,944216	y ~ current_feature + S
0,126275	0,085377	-0,07874	1,479019	138	135	0,141465	0,498495	y ~ current_feature + S
0,014318	0,086057	0,034007	0,16638	138	135	0,868107	0,96591	y ~ current_feature + S
0,029305	0,08635	-0,04751	0,339371	137	134	0,734862	0,933491	y ~ current_feature + S
0,071073	0,094856	0,061498	0,749272	115	110,5795	0,455284	0,937665	y ~ current_feature + S
0,218732	0,103608	-0,02429	2,111151	92	88,70006	0,037573	0,520476	y ~ current_feature + S
0,134635	0,065152	-0,07874	2,066467	272	231,3103	0,039898	0,270866	y ~ current_feature + S
0,137277	0,060394	0,034007	2,273023	272	269	0,023814	0,338887	y ~ current_feature + S
-0,02888	0,065751	-0,04751	-0,43916	271	231,1166	0,660957	0,926341	y ~ current_feature + S
-0,11286	0,072412	0,061498	-1,55864	234	188,2813	0,120761	0,8197	y ~ current_feature + S
0,035182	0,074907	-0,02429	0,469678	181	177,9998	0,63916	0,924682	y ~ current_feature + S
0,022155	0,052759	-0,07874	0,419925	421	359,08	0,674792	0,934653	y ~ current_feature + S
-0,00496	0,069097	0,034007	-0,07171	421	209,4453	0,942897	0,98181	y ~ current_feature + S
0,050789	0,054303	-0,04751	0,935283	420	338,2462	0,35031	0,795089	y ~ current_feature + S
0,018413	0,059214	0,061498	0,310962	367	285,1058	0,756057	0,972573	y ~ current_feature + S
0,090282	0,059839	-0,02429	1,508756	280	277	0,132501	0,617591	y ~ current_feature + S
0,056002	0,084383	-0,07874	0,663672	143	140	0,507992	0,863164	y ~ current_feature + S
-0,02615	0,084487	0,034007	-0,30947	143	140	0,757427	0,93416	y ~ current_feature + S
-0,03995	0,084751	-0,04751	-0,4714	142	139	0,638095	0,925756	y ~ current_feature + S
1,61E-04	0,090536	0,061498	0,001773	125	122	0,998588	0,999005	y ~ current_feature + S
0,006512	0,104255	-0,02429	0,062461	95	92	0,950331	0,99444	y ~ current_feature + S
0,090942	0,139448	-0,07874	0,652157	54	51	0,517228	0,873856	y ~ current_feature + S
0,024613	0,139986	0,034007	0,175824	54	51	0,861129	0,964047	y ~ current_feature + S
0,147744	0,138491	-0,04751	1,066813	54	51	0,291081	0,747915	y ~ current_feature + S
-0,15039	0,152548	0,061498	-0,98584	45	42	0,329856	0,893818	y ~ current_feature + S
0,139978	0,172364	-0,02429	0,812107	36	33	0,42255	0,847264	y ~ current_feature + S
0,089603	0,085092	-0,07874	1,05301	140	137	0,29419	0,718687	y ~ current_feature + S
0,110177	0,084916	0,034007	1,297489	140	137	0,196644	0,643878	y ~ current_feature + S
-0,06428	0,085572	-0,04751	-0,75119	139	136	0,453836	0,832044	y ~ current_feature + S
0,094816	0,09283	0,061498	1,021391	118	115	0,309213	0,893818	y ~ current_feature + S
0,195994	0,103365	-0,02429	1,896141	93	90	0,06115	0,558651	y ~ current_feature + S
0,056699	0,070073	-0,07874	0,809136	206	203	0,419384	0,811887	y ~ current_feature + S
0,003055	0,070186	0,034007	0,043526	206	203	0,965325	0,989859	y ~ current_feature + S
0,051216	0,070267	-0,04751	0,728866	205	202	0,466928	0,842057	y ~ current_feature + S
0,061552	0,074602	0,061498	0,825071	182	179	0,410429	0,934833	y ~ current_feature + S
0,02504	0,08636	-0,02429	0,289945	137	134	0,772307	0,957298	y ~ current_feature + S
0,05037	0,074316	-0,07874	0,677774	185	180,6036	0,498783	0,861866	y ~ current_feature + S
-0,03695	0,101348	0,034007	-0,36458	185	97,22553	0,716215	0,920814	y ~ current_feature + S
0,004707	0,077969	-0,04751	0,060369	184	164,4924	0,951935	0,990156	y ~ current_feature + S
-0,31853	0,076521	0,061498	-4,16261	159	153,4517	5,23E-05	0,012893	y ~ current_feature + S
-0,05193	0,092223	-0,02429	-0,5631	123	117,259	0,574441	0,924682	y ~ current_feature + S
-0,03874	0,063452	-0,07874	-0,61048	251	248	0,542106	0,882344	y ~ current_feature + S

-0,04203	0,063444	0,034007	-0,66253	251	248	0,508247	0,841996	y ~ current_feature + S
-0,06802	0,063481	-0,04751	-1,07149	250	247	0,284997	0,747915	y ~ current_feature + S
0,058869	0,067767	0,061498	0,8687	220	217	0,385971	0,93433	y ~ current_feature + S
-0,07799	0,077849	-0,02429	-1,00179	167	164	0,317923	0,766329	y ~ current_feature + S
0,191287	0,081232	-0,07874	2,354811	149	146	0,019863	0,188446	y ~ current_feature + S
-0,08232	0,08248	0,034007	-0,998	149	146	0,319929	0,723998	y ~ current_feature + S
0,275786	0,079825	-0,04751	3,454889	148	145	7,22E-04	0,132163	y ~ current_feature + S
-0,11289	0,088518	0,061498	-1,27537	129	126	0,204526	0,893818	y ~ current_feature + S
0,002603	0,102062	-0,02429	0,025508	99	96	0,979703	0,99444	y ~ current_feature + S
0,065093	0,080152	-0,07874	0,812118	158	155	0,417969	0,811887	y ~ current_feature + S
0,050147	0,080221	0,034007	0,62511	158	155	0,532818	0,849319	y ~ current_feature + S
0,152835	0,079636	-0,04751	1,919182	157	154	0,056811	0,640171	y ~ current_feature + S
0,075897	0,085818	0,061498	0,884396	138	135	0,378055	0,932536	y ~ current_feature + S
0,103785	0,09848	-0,02429	1,053867	105	102	0,294434	0,741092	y ~ current_feature + S
0,089637	0,053789	-0,07874	1,666463	390	342,8544	0,096534	0,431938	y ~ current_feature + S
0,21149	0,069125	0,034007	3,059519	390	199,9194	0,002521	0,133248	y ~ current_feature + S
0,114133	0,05471	-0,04751	2,086132	389	329,7367	0,037735	0,611115	y ~ current_feature + S
0,025	0,058893	0,061498	0,424503	338	288,1407	0,671516	0,958606	y ~ current_feature + S
0,107939	0,062519	-0,02429	1,726507	260	252,8664	0,085478	0,605474	y ~ current_feature + S
-0,06245	0,071472	-0,07874	-0,87379	198	195	0,383306	0,789252	y ~ current_feature + S
-0,06913	0,07144	0,034007	-0,96759	198	195	0,334446	0,734392	y ~ current_feature + S
-0,04561	0,071537	-0,04751	-0,63752	198	195	0,524534	0,876197	y ~ current_feature + S
0,058123	0,07612	0,061498	0,763565	175	172	0,446173	0,937665	y ~ current_feature + S
-0,02927	0,088007	-0,02429	-0,3326	132	129	0,739978	0,944216	y ~ current_feature + S
0,051546	0,092327	-0,07874	0,558302	120	117	0,577705	0,900004	y ~ current_feature + S
0,072408	0,092207	0,034007	0,785269	120	117	0,433883	0,796709	y ~ current_feature + S
0,142809	0,091502	-0,04751	1,560711	120	117	0,121293	0,651244	y ~ current_feature + S
0,056133	0,099347	0,061498	0,565025	104	101	0,57331	0,937665	y ~ current_feature + S
0,047542	0,113832	-0,02429	0,417648	80	77	0,677367	0,926342	y ~ current_feature + S
0,012396	0,072352	-0,07874	0,171333	194	191	0,864144	0,985453	y ~ current_feature + S
-0,01486	0,072349	0,034007	-0,20533	194	191	0,837532	0,958985	y ~ current_feature + S
0,072062	0,072169	-0,04751	0,998518	194	191	0,319291	0,774585	y ~ current_feature + S
-0,11061	0,076908	0,061498	-1,4382	170	167	0,152248	0,8197	y ~ current_feature + S
0,098272	0,088306	-0,02429	1,112855	130	127	0,267873	0,723659	y ~ current_feature + S
0,151168	0,125541	-0,07874	1,204136	65	62	0,233114	0,657771	y ~ current_feature + S
0,060255	0,126769	0,034007	0,475313	65	62	0,636234	0,899624	y ~ current_feature + S
-0,21084	0,125159	-0,04751	-1,68456	64	61	0,097184	0,640171	y ~ current_feature + S
-0,19646	0,132212	0,061498	-1,48595	58	55	0,143002	0,8197	y ~ current_feature + S
0,118092	0,157008	-0,02429	0,75214	43	40	0,45637	0,866856	y ~ current_feature + S
0,07918	0,051409	-0,07874	1,5402	379	376	0,124353	0,478063	y ~ current_feature + S
0,090311	0,05136	0,034007	1,758373	379	376	0,079498	0,473527	y ~ current_feature + S
0,039762	0,05153	-0,04751	0,771632	379	376	0,440817	0,823749	y ~ current_feature + S
-0,00274	0,060821	0,061498	-0,04505	328	270,3281	0,964097	0,996179	y ~ current_feature + S
0,067371	0,063389	-0,02429	1,062806	252	247,7376	0,288905	0,734108	y ~ current_feature + S
-0,04918	0,154117	-0,07874	-0,31912	45	42	0,751218	0,963434	y ~ current_feature + S
-0,20247	0,151108	0,034007	-1,33989	45	42	0,187485	0,638805	y ~ current_feature + S
-0,3945	0,141788	-0,04751	-2,78235	45	42	0,00805	0,432614	y ~ current_feature + S
-0,24189	0,155373	0,061498	-1,55683	42	39	0,127589	0,8197	y ~ current_feature + S

-0,26689	0,185469	-0,02429	-1,43901	30	27	0,16164	0,623497	y ~ current_feature + S
-0,14612	0,054875	-0,07874	-2,66278	328	325	0,008136	0,115775	y ~ current_feature + S
-0,14088	0,054917	0,034007	-2,56534	328	325	0,010756	0,233774	y ~ current_feature + S
-0,06406	0,055441	-0,04751	-1,15554	327	324	0,248721	0,742928	y ~ current_feature + S
0,022144	0,05922	0,061498	0,373926	288	285	0,708737	0,972444	y ~ current_feature + S
-0,10396	0,06783	-0,02429	-1,53269	218	215	0,126821	0,617591	y ~ current_feature + S
0,018132	0,096209	-0,07874	0,188469	111	108	0,850863	0,983548	y ~ current_feature + S
-0,06308	0,096033	0,034007	-0,65681	111	108	0,512703	0,841996	y ~ current_feature + S
-0,0031	0,096225	-0,04751	-0,03223	111	108	0,974348	0,992311	y ~ current_feature + S
-0,00278	0,102597	0,061498	-0,02713	98	95	0,978417	0,996179	y ~ current_feature + S
0,126514	0,117725	-0,02429	1,074663	74	71	0,286164	0,734108	y ~ current_feature + S
-0,13371	0,052273	-0,07874	-2,55797	388	359,4333	0,010938	0,140685	y ~ current_feature + S
-0,08005	0,069321	0,034007	-1,15476	388	206,7665	0,249521	0,702074	y ~ current_feature + S
-0,02404	0,054445	-0,04751	-0,44147	387	337,1572	0,659158	0,925756	y ~ current_feature + S
0,051432	0,058163	0,061498	0,884279	341	294,823	0,377266	0,932536	y ~ current_feature + S
-0,1168	0,062886	-0,02429	-1,85724	258	249,4142	0,064456	0,574667	y ~ current_feature + S
0,132606	0,069566	-0,07874	1,90618	206	203	0,058039	0,318142	y ~ current_feature + S
0,020683	0,070171	0,034007	0,294752	206	203	0,768484	0,939964	y ~ current_feature + S
0,138683	0,06968	-0,04751	1,990295	205	202	0,047907	0,640171	y ~ current_feature + S
0,08524	0,075534	0,061498	1,128504	177	174	0,260661	0,893818	y ~ current_feature + S
0,231063	0,084049	-0,02429	2,74914	137	134	0,0068	0,26484	y ~ current_feature + S
0,073326	0,084896	-0,07874	0,863707	141	138	0,389247	0,789252	y ~ current_feature + S
-0,06355	0,084954	0,034007	-0,74804	141	138	0,455707	0,821488	y ~ current_feature + S
-0,15942	0,084037	-0,04751	-1,89705	141	138	0,059911	0,640171	y ~ current_feature + S
-0,17169	0,091469	0,061498	-1,87701	119	116	0,063029	0,715249	y ~ current_feature + S
-0,01561	0,104816	-0,02429	-0,14891	94	91	0,881955	0,987777	y ~ current_feature + S
0,003047	0,068519	-0,07874	0,044468	216	213	0,964573	0,994607	y ~ current_feature + S
0,006476	0,068517	0,034007	0,094509	216	213	0,924793	0,9732	y ~ current_feature + S
0,184876	0,067338	-0,04751	2,745507	216	213	0,006558	0,432614	y ~ current_feature + S
0,008976	0,075691	0,061498	0,118587	188	174,5307	0,905739	0,996179	y ~ current_feature + S
0,093783	0,083844	-0,02429	1,118544	144	141	0,265237	0,723659	y ~ current_feature + S
-0,05993	0,102413	-0,07874	-0,58516	98	95	0,559825	0,894753	y ~ current_feature + S
-0,08454	0,102231	0,034007	-0,82695	98	95	0,41034	0,784629	y ~ current_feature + S
-0,08225	0,102793	-0,04751	-0,80015	97	94	0,425643	0,819126	y ~ current_feature + S
-0,06875	0,106958	0,061498	-0,64274	90	87	0,522081	0,937665	y ~ current_feature + S
0,010615	0,126993	-0,02429	0,083586	65	62	0,933655	0,99444	y ~ current_feature + S
0,288602	0,123606	-0,07874	2,334849	63	60	0,022915	0,194866	y ~ current_feature + S
0,222473	0,125864	0,034007	1,767565	63	60	0,082218	0,473527	y ~ current_feature + S
0,07204	0,128764	-0,04751	0,559471	63	60	0,577923	0,904733	y ~ current_feature + S
0,092847	0,135495	0,061498	0,68524	57	54	0,496124	0,937665	y ~ current_feature + S
0,138725	0,15858	-0,02429	0,874799	42	39	0,387039	0,819479	y ~ current_feature + S
0,188257	0,055161	-0,07874	3,412838	320	317	7,26E-04	0,031617	y ~ current_feature + S
0,179095	0,055257	0,034007	3,241102	320	317	0,001317	0,108307	y ~ current_feature + S
0,065591	0,056045	-0,04751	1,170339	320	317	0,242744	0,742928	y ~ current_feature + S
0,066012	0,06028	0,061498	1,095076	277	274	0,274445	0,893818	y ~ current_feature + S
0,225656	0,067227	-0,02429	3,356646	213	210	9,36E-04	0,138591	y ~ current_feature + S
0,060358	0,059759	-0,07874	1,01002	282	279	0,313361	0,718687	y ~ current_feature + S
-0,06491	0,059742	0,034007	-1,08654	282	279	0,278179	0,715049	y ~ current_feature + S

-0,06762	0,059839	-0,04751	-1,13008	281	278	0,259418	0,742928	y ~ current_feature + S
-0,06513	0,063883	0,061498	-1,01956	247	244	0,308945	0,893818	y ~ current_feature + S
-0,11923	0,072997	-0,02429	-1,63336	188	185	0,104093	0,605474	y ~ current_feature + S
-0,11588	0,07618	-0,07874	-1,52108	173	170	0,130099	0,483784	y ~ current_feature + S
-0,14193	0,07592	0,034007	-1,86944	173	170	0,063281	0,437643	y ~ current_feature + S
-0,11471	0,076415	-0,04751	-1,50116	172	169	0,135179	0,657012	y ~ current_feature + S
-0,02374	0,083022	0,061498	-0,28593	148	145	0,775341	0,975031	y ~ current_feature + S
-0,00245	0,094491	-0,02429	-0,02596	115	112	0,979336	0,99444	y ~ current_feature + S
0,212052	0,096763	-0,07874	2,191455	105	102	0,030694	0,224043	y ~ current_feature + S
0,016997	0,099	0,034007	0,171688	105	102	0,864023	0,965827	y ~ current_feature + S
0,003863	0,099014	-0,04751	0,039012	105	102	0,968957	0,992311	y ~ current_feature + S
0,14724	0,103686	0,061498	1,420058	94	91	0,159008	0,8197	y ~ current_feature + S
0,175339	0,120302	-0,02429	1,457482	70	66,97149	0,149657	0,622449	y ~ current_feature + S
0,183264	0,057236	-0,07874	3,201889	298	295	0,001514	0,048726	y ~ current_feature + S
0,089055	0,057991	0,034007	1,535671	298	295	0,125691	0,572145	y ~ current_feature + S
0,067152	0,058091	-0,04751	1,15598	298	295	0,248625	0,742928	y ~ current_feature + S
-0,11229	0,062226	0,061498	-1,80461	258	255	0,072316	0,721272	y ~ current_feature + S
0,099989	0,071071	-0,02429	1,406898	199	196	0,161041	0,623497	y ~ current_feature + S
0,042623	0,056292	-0,07874	0,757165	318	315	0,449517	0,823372	y ~ current_feature + S
-0,0332	0,056313	0,034007	-0,58949	318	315	0,555955	0,857986	y ~ current_feature + S
0,045075	0,056376	-0,04751	0,799543	317	314	0,42458	0,819126	y ~ current_feature + S
-0,00578	0,060633	0,061498	-0,09525	275	272	0,92419	0,996179	y ~ current_feature + S
0,16245	0,068253	-0,02429	2,380134	212	209	0,018205	0,428958	y ~ current_feature + S
0,054136	0,113062	-0,07874	0,47882	81	78	0,633406	0,926325	y ~ current_feature + S
0,113457	0,112497	0,034007	1,008536	81	78	0,316316	0,718742	y ~ current_feature + S
0,070439	0,112946	-0,04751	0,623648	81	78	0,534678	0,883994	y ~ current_feature + S
0,013806	0,11784	0,061498	0,11716	75	72	0,907059	0,996179	y ~ current_feature + S
0,122877	0,138967	-0,02429	0,884215	54	51	0,38073	0,816637	y ~ current_feature + S
0,065282	0,067584	-0,07874	0,965933	221	218	0,335148	0,735933	y ~ current_feature + S
-0,10589	0,067348	0,034007	-1,57225	221	218	0,117343	0,552558	y ~ current_feature + S
0,052829	0,06779	-0,04751	0,779309	220	217	0,436647	0,823749	y ~ current_feature + S
0,013102	0,073121	0,061498	0,179187	190	187	0,857985	0,996179	y ~ current_feature + S
0,002579	0,083333	-0,02429	0,030951	147	144	0,975351	0,99444	y ~ current_feature + S
-0,02042	0,083628	-0,07874	-0,24419	158	142,9257	0,807437	0,979871	y ~ current_feature + S
0,099612	0,098702	0,034007	1,009223	158	101,6297	0,315265	0,718742	y ~ current_feature + S
0,219995	0,078354	-0,04751	2,807703	158	155	0,005631	0,422981	y ~ current_feature + S
0,098623	0,091199	0,061498	1,081404	140	119,062	0,281703	0,893818	y ~ current_feature + S
0,017132	0,100612	-0,02429	0,17028	105	98,75906	0,865138	0,984468	y ~ current_feature + S
0,034669	0,063462	-0,07874	0,546294	251	248	0,585355	0,902886	y ~ current_feature + S
-0,01458	0,063493	0,034007	-0,22956	251	248	0,818623	0,952501	y ~ current_feature + S
0,003237	0,0635	-0,04751	0,050982	251	248	0,959381	0,990156	y ~ current_feature + S
-0,00285	0,068358	0,061498	-0,04162	217	214	0,966837	0,996179	y ~ current_feature + S
0,003809	0,077849	-0,02429	0,048924	168	165	0,961039	0,99444	y ~ current_feature + S
-0,00112	0,055902	-0,07874	-0,01997	323	320	0,98408	0,996318	y ~ current_feature + S
-0,00512	0,055901	0,034007	-0,09154	323	320	0,927119	0,9732	y ~ current_feature + S
0,137694	0,055456	-0,04751	2,482942	322	319	0,013544	0,526293	y ~ current_feature + S
0,001049	0,066318	0,061498	0,01581	279	227,375	0,987399	0,997945	y ~ current_feature + S
0,109309	0,068856	-0,02429	1,5875	215	208,3979	0,113915	0,614731	y ~ current_feature + S

-0,04029	0,111021	-0,07874	-0,36291	84	81	0,717614	0,948276	y ~ current_feature + S
0,023275	0,111081	0,034007	0,209531	84	81	0,83456	0,958966	y ~ current_feature + S
-0,16868	0,109519	-0,04751	-1,54021	84	81	0,127407	0,654671	y ~ current_feature + S
-0,06793	0,115203	0,061498	-0,58965	78	75	0,557195	0,937665	y ~ current_feature + S
-0,02349	0,137323	-0,02429	-0,17106	56	53	0,864831	0,984468	y ~ current_feature + S
0,015373	0,052193	-0,07874	0,294545	382	366,999	0,768508	0,965616	y ~ current_feature + S
-0,01903	0,066921	0,034007	-0,2843	382	223,2114	0,776446	0,942718	y ~ current_feature + S
0,038189	0,053084	-0,04751	0,719404	381	354,3579	0,472366	0,842057	y ~ current_feature + S
0,028183	0,055988	0,061498	0,503383	332	318,764	0,615043	0,948092	y ~ current_feature + S
0,020592	0,065082	-0,02429	0,316394	254	235,9871	0,751983	0,951227	y ~ current_feature + S
-0,06655	0,05469	-0,07874	-1,21687	370	332,8561	0,224517	0,646468	y ~ current_feature + S
-0,11561	0,072818	0,034007	-1,58758	370	186,0687	0,114079	0,548173	y ~ current_feature + S
-0,03261	0,058264	-0,04751	-0,55965	369	294,2637	0,576144	0,904733	y ~ current_feature + S
-0,1122	0,063085	0,061498	-1,77858	320	248,1103	0,076533	0,735511	y ~ current_feature + S
-0,07763	0,063956	-0,02429	-1,21383	246	243	0,225993	0,695919	y ~ current_feature + S
0,026537	0,049971	-0,07874	0,53105	412	400,1772	0,595679	0,903286	y ~ current_feature + S
0,028837	0,063552	0,034007	0,453752	412	247,3917	0,650405	0,900251	y ~ current_feature + S
-0,07539	0,050714	-0,04751	-1,48666	411	386,6077	0,137919	0,657012	y ~ current_feature + S
-0,0117	0,054192	0,061498	-0,21592	359	340,4669	0,829177	0,996179	y ~ current_feature + S
0,041203	0,063112	-0,02429	0,652855	274	250,6363	0,514448	0,899808	y ~ current_feature + S
-0,0032	0,055048	-0,07874	-0,05821	333	330	0,953617	0,994607	y ~ current_feature + S
-0,10461	0,054746	0,034007	-1,91076	333	330	0,056902	0,420946	y ~ current_feature + S
-0,01704	0,05504	-0,04751	-0,30962	333	330	0,757045	0,9394	y ~ current_feature + S
0,008116	0,059233	0,061498	0,137011	288	285	0,891119	0,996179	y ~ current_feature + S
-0,09812	0,067402	-0,02429	-1,45567	221	218	0,146921	0,621266	y ~ current_feature + S
-0,05758	0,062519	-0,07874	-0,92094	258	255	0,357952	0,767781	y ~ current_feature + S
-0,04395	0,062562	0,034007	-0,70246	258	255	0,483033	0,831402	y ~ current_feature + S
-0,06384	0,062495	-0,04751	-1,02152	258	255	0,307974	0,76529	y ~ current_feature + S
-0,04059	0,067673	0,061498	-0,59983	221	218	0,549242	0,937665	y ~ current_feature + S
-0,03088	0,076886	-0,02429	-0,40158	172	169	0,688504	0,926342	y ~ current_feature + S
-0,0785	0,054795	-0,07874	-1,43264	334	331	0,152905	0,518271	y ~ current_feature + S
-0,05143	0,054892	0,034007	-0,93692	334	331	0,349485	0,749214	y ~ current_feature + S
-0,10566	0,05474	-0,04751	-1,93015	333	330	0,054446	0,640171	y ~ current_feature + S
-0,10104	0,06908	0,061498	-1,46263	289	207,4116	0,145083	0,8197	y ~ current_feature + S
-0,02118	0,067706	-0,02429	-0,31282	222	218,0471	0,754717	0,951432	y ~ current_feature + S
0,111295	0,050087	-0,07874	2,222022	410	393,6732	0,02685	0,204832	y ~ current_feature + S
0,118201	0,064176	0,034007	1,841834	410	239,4121	0,066736	0,439525	y ~ current_feature + S
0,045083	0,051119	-0,04751	0,881911	409	381,8981	0,378379	0,803665	y ~ current_feature + S
0,087865	0,054556	0,061498	1,610565	357	333,3928	0,108221	0,8197	y ~ current_feature + S
0,101922	0,062227	-0,02429	1,637899	273	255,5664	0,102673	0,605474	y ~ current_feature + S
0,079838	0,066602	-0,07874	1,198739	227	224	0,231896	0,657483	y ~ current_feature + S
0,126474	0,066279	0,034007	1,908219	227	224	0,057641	0,420946	y ~ current_feature + S
0,070083	0,0668	-0,04751	1,049145	226	223	0,295247	0,752909	y ~ current_feature + S
0,044426	0,072286	0,061498	0,61459	194	191	0,539557	0,937665	y ~ current_feature + S
0,241856	0,080267	-0,02429	3,013144	151	146,1333	0,003048	0,18344	y ~ current_feature + S
0,070392	0,060044	-0,07874	1,172346	279	276	0,242069	0,670903	y ~ current_feature + S
0,105035	0,05986	0,034007	1,754683	279	276	0,080423	0,473527	y ~ current_feature + S
0,0269	0,06028	-0,04751	0,446243	278	275	0,655773	0,925756	y ~ current_feature + S

0,027153	0,064933	0,061498	0,418164	240	237	0,676206	0,958606	y ~ current_feature + S
0,097627	0,073771	-0,02429	1,323375	185	182	0,187371	0,65347	y ~ current_feature + S
0,091943	0,05532	-0,07874	1,66201	327	324	0,097478	0,431938	y ~ current_feature + S
0,11341	0,055197	0,034007	2,054639	327	324	0,040716	0,396441	y ~ current_feature + S
0,032637	0,055526	-0,04751	0,58777	327	324	0,557096	0,897386	y ~ current_feature + S
0,012898	0,06571	0,061498	0,196293	281	231,5606	0,844553	0,996179	y ~ current_feature + S
0,089807	0,068082	-0,02429	1,319092	217	214	0,188548	0,65347	y ~ current_feature + S
0,152467	0,063141	-0,07874	2,414714	248	245	0,016481	0,174676	y ~ current_feature + S
0,076451	0,063701	0,034007	1,200155	248	245	0,231238	0,681978	y ~ current_feature + S
0,001928	0,064018	-0,04751	0,03011	247	244	0,976004	0,992311	y ~ current_feature + S
0,043493	0,068454	0,061498	0,635364	216	213	0,525873	0,937665	y ~ current_feature + S
0,034507	0,078521	-0,02429	0,439463	165	162	0,660911	0,924682	y ~ current_feature + S
-0,00183	0,081111	-0,07874	-0,02255	155	152	0,982037	0,996318	y ~ current_feature + S
0,08841	0,080793	0,034007	1,094273	155	152	0,275566	0,715049	y ~ current_feature + S
-0,01018	0,081107	-0,04751	-0,12549	155	152	0,9003	0,985217	y ~ current_feature + S
-0,1572	0,087289	0,061498	-1,80088	131	128	0,074077	0,721272	y ~ current_feature + S
0,108092	0,098921	-0,02429	1,092713	104	101	0,277119	0,734108	y ~ current_feature + S
0,071863	0,145488	-0,07874	0,493944	50	47	0,623647	0,919937	y ~ current_feature + S
0,178814	0,143514	0,034007	1,245965	50	47	0,218952	0,664036	y ~ current_feature + S
0,202113	0,144399	-0,04751	1,39968	49	46	0,168317	0,700235	y ~ current_feature + S
-0,20934	0,150885	0,061498	-1,38739	45	42	0,172641	0,840578	y ~ current_feature + S
0,337314	0,171874	-0,02429	1,962565	33	30	0,05903	0,558147	y ~ current_feature + S
-0,06208	0,0859	-0,07874	-0,72269	138	135	0,471122	0,840689	y ~ current_feature + S
-0,05562	0,085933	0,034007	-0,64726	138	135	0,518563	0,841996	y ~ current_feature + S
-0,03821	0,086003	-0,04751	-0,44432	138	135	0,657521	0,925756	y ~ current_feature + S
-0,00339	0,09245	0,061498	-0,03663	120	117	0,970839	0,996179	y ~ current_feature + S
0,10023	0,105466	-0,02429	0,950353	92	89	0,344507	0,789404	y ~ current_feature + S
0,08032	0,150269	-0,07874	0,534509	47	44	0,59568	0,903286	y ~ current_feature + S
-0,03713	0,150652	0,034007	-0,2465	47	44	0,806445	0,952501	y ~ current_feature + S
-0,24019	0,148034	-0,04751	-1,62252	46	43	0,112003	0,640171	y ~ current_feature + S
0,065757	0,159782	0,061498	0,411541	42	39	0,682929	0,962605	y ~ current_feature + S
0,414536	0,17198	-0,02429	2,410372	31	28	0,022751	0,467656	y ~ current_feature + S
0,097387	0,093625	-0,07874	1,040181	116	113	0,300476	0,718687	y ~ current_feature + S
-0,1193	0,0934	0,034007	-1,27732	116	113	0,204107	0,65103	y ~ current_feature + S
0,003668	0,09449	-0,04751	0,038821	115	112	0,969103	0,992311	y ~ current_feature + S
0,071208	0,100759	0,061498	0,706713	101	98	0,481422	0,937665	y ~ current_feature + S
-0,0295	0,116197	-0,02429	-0,25385	77	74	0,800315	0,968297	y ~ current_feature + S
0,527493	0,12106	-0,07874	4,357278	57	49,24744	6,68E-05	0,008233	y ~ current_feature + S
0,279304	0,182817	0,034007	1,52778	57	27,58621	0,137953	0,58036	y ~ current_feature + S
0,074223	0,141827	-0,04751	0,523339	57	49,44074	0,603076	0,91263	y ~ current_feature + S
-0,0812	0,142451	0,061498	-0,57	52	48,9549	0,571286	0,937665	y ~ current_feature + S
0,467127	0,149455	-0,02429	3,125526	38	35	0,003559	0,184706	y ~ current_feature + S
0,080661	0,073481	-0,07874	1,097721	187	184	0,273761	0,705864	y ~ current_feature + S
0,063155	0,073574	0,034007	0,858387	187	184	0,391795	0,773143	y ~ current_feature + S
0,00395	0,073922	-0,04751	0,053433	186	183	0,957445	0,990156	y ~ current_feature + S
-0,0736	0,07934	0,061498	-0,9277	161	158	0,354981	0,920619	y ~ current_feature + S
0,032848	0,090487	-0,02429	0,363014	125	122	0,717223	0,936058	y ~ current_feature + S
-0,01464	0,068351	-0,07874	-0,2142	217	214	0,830593	0,981192	y ~ current_feature + S

-0,00189	0,068358	0,034007	-0,02758	217	214	0,978022	0,989859	y ~ current_feature + S
0,11563	0,0679	-0,04751	1,70295	217	214	0,090029	0,640171	y ~ current_feature + S
-0,0028	0,073127	0,061498	-0,03831	190	187	0,96948	0,996179	y ~ current_feature + S
0,030672	0,083879	-0,02429	0,365665	145	142	0,715159	0,936058	y ~ current_feature + S
0,073464	0,066193	-0,07874	1,109839	230	227	0,268243	0,702573	y ~ current_feature + S
-0,03118	0,06634	0,034007	-0,46995	230	227	0,638839	0,899624	y ~ current_feature + S
-0,03817	0,066471	-0,04751	-0,5742	229	226	0,566404	0,903656	y ~ current_feature + S
-0,13285	0,071344	0,061498	-1,86213	196	193	0,064105	0,715249	y ~ current_feature + S
-0,02797	0,081618	-0,02429	-0,34269	153	150	0,732311	0,944216	y ~ current_feature + S
0,017338	0,079293	-0,07874	0,218655	162	159	0,827199	0,981192	y ~ current_feature + S
-0,0909	0,078977	0,034007	-1,15091	162	159	0,251496	0,704252	y ~ current_feature + S
-0,01239	0,079299	-0,04751	-0,15627	162	159	0,876017	0,985217	y ~ current_feature + S
-0,11541	0,084865	0,061498	-1,35987	140	137	0,176104	0,846213	y ~ current_feature + S
-0,1177	0,096912	-0,02429	-1,21451	108	105	0,227277	0,695919	y ~ current_feature + S
0,032504	0,052243	-0,07874	0,62216	369	366	0,534224	0,882344	y ~ current_feature + S
0,060381	0,052175	0,034007	1,157277	369	366	0,247914	0,702074	y ~ current_feature + S
0,021222	0,052331	-0,04751	0,405546	368	365	0,685314	0,928814	y ~ current_feature + S
-0,0389	0,056123	0,061498	-0,69308	320	317	0,488769	0,937665	y ~ current_feature + S
0,136939	0,063546	-0,02429	2,154966	246	243	0,032147	0,485321	y ~ current_feature + S
-0,11814	0,049083	-0,07874	-2,40706	418	409,2989	0,016523	0,174676	y ~ current_feature + S
-0,10728	0,062239	0,034007	-1,72369	418	255,1828	0,085975	0,479777	y ~ current_feature + S
-0,05806	0,050245	-0,04751	-1,15564	417	394,7808	0,248528	0,742928	y ~ current_feature + S
-0,12909	0,053226	0,061498	-2,42538	364	347,1053	0,015802	0,55988	y ~ current_feature + S
-0,06311	0,062336	-0,02429	-1,01235	278	256,3273	0,312324	0,765236	y ~ current_feature + S
0,06732	0,153953	-0,07874	0,437277	45	42	0,664151	0,934653	y ~ current_feature + S
0,166113	0,15216	0,034007	1,091699	45	42	0,281188	0,715049	y ~ current_feature + S
0,0949	0,153607	-0,04751	0,617813	45	42	0,540034	0,888057	y ~ current_feature + S
0,048619	0,157927	0,061498	0,307856	43	40	0,75979	0,972573	y ~ current_feature + S
0,014621	0,19243	-0,02429	0,075982	30	27	0,939994	0,99444	y ~ current_feature + S
-0,11398	0,058746	-0,07874	-1,94016	289	286	0,053343	0,30647	y ~ current_feature + S
-0,12867	0,05864	0,034007	-2,19431	289	286	0,029018	0,347616	y ~ current_feature + S
-0,06025	0,059127	-0,04751	-1,019	288	285	0,309066	0,76529	y ~ current_feature + S
-0,10806	0,063384	0,061498	-1,70488	249	246	0,089479	0,795265	y ~ current_feature + S
-0,12619	0,072158	-0,02429	-1,74877	192	189	0,081955	0,605474	y ~ current_feature + S
0,015205	0,049795	-0,07874	0,305351	418	403,2004	0,760256	0,965008	y ~ current_feature + S
-0,00865	0,06338	0,034007	-0,13652	418	248,9187	0,891524	0,96591	y ~ current_feature + S
0,015921	0,050493	-0,04751	0,315306	417	392,1214	0,752697	0,9394	y ~ current_feature + S
-0,02347	0,054299	0,061498	-0,43215	364	338,9865	0,665908	0,958606	y ~ current_feature + S
-0,09236	0,062067	-0,02429	-1,48806	278	257,3697	0,137958	0,618941	y ~ current_feature + S
0,156191	0,052394	-0,07874	2,981093	396	355,394	0,00307	0,066821	y ~ current_feature + S
0,238673	0,067974	0,034007	3,511212	396	204,0968	5,49E-04	0,067708	y ~ current_feature + S
0,068988	0,054235	-0,04751	1,272017	395	338,35	0,20424	0,723562	y ~ current_feature + S
-0,00955	0,057842	0,061498	-0,16511	343	298,8631	0,868968	0,996179	y ~ current_feature + S
0,03276	0,062371	-0,02429	0,525239	264	256,7865	0,599871	0,924682	y ~ current_feature + S
-0,04711	0,156	-0,07874	-0,302	44	41	0,764182	0,965008	y ~ current_feature + S
-0,03034	0,156102	0,034007	-0,19439	44	41	0,846832	0,959445	y ~ current_feature + S
0,294329	0,15111	-0,04751	1,947778	43	40	0,058484	0,640171	y ~ current_feature + S
-0,09955	0,173213	0,061498	-0,57473	36	33	0,56937	0,937665	y ~ current_feature + S

0,180057	0,192911	-0,02429	0,933372	29	26	0,359216	0,800662	y ~ current_feature + S
0,082443	0,055799	-0,07874	1,477506	322	319	0,140527	0,498495	y ~ current_feature + S
0,140721	0,055432	0,034007	2,538619	322	319	0,011604	0,233774	y ~ current_feature + S
0,006373	0,056076	-0,04751	0,113645	321	318	0,909591	0,985217	y ~ current_feature + S
-0,05352	0,060107	0,061498	-0,89046	279	276	0,373994	0,932536	y ~ current_feature + S
0,074615	0,068651	-0,02429	1,086869	214	211	0,278335	0,734108	y ~ current_feature + S
-0,02426	0,049162	-0,07874	-0,49349	418	413,513	0,621925	0,919937	y ~ current_feature + S
-0,09291	0,060559	0,034007	-1,53411	418	270,3157	0,126171	0,572145	y ~ current_feature + S
-0,00476	0,049508	-0,04751	-0,09606	417	407,9831	0,923522	0,985217	y ~ current_feature + S
-0,13103	0,05393	0,061498	-2,42967	364	337,925	0,015633	0,55988	y ~ current_feature + S
-0,03604	0,061351	-0,02429	-0,58751	278	265,3297	0,557363	0,92024	y ~ current_feature + S
-0,07168	0,095536	-0,07874	-0,75028	112	109	0,454703	0,824707	y ~ current_feature + S
0,025431	0,095752	0,034007	0,265598	112	109	0,791051	0,945731	y ~ current_feature + S
-0,00739	0,096222	-0,04751	-0,07675	111	108	0,938963	0,990156	y ~ current_feature + S
0,042654	0,101442	0,061498	0,420478	100	97	0,675066	0,958606	y ~ current_feature + S
-0,05983	0,118466	-0,02429	-0,50504	74	71	0,615097	0,924682	y ~ current_feature + S
0,035069	0,070845	-0,07874	0,495018	202	199	0,621133	0,919937	y ~ current_feature + S
-0,08172	0,070651	0,034007	-1,15667	202	199	0,248795	0,702074	y ~ current_feature + S
-0,11641	0,070584	-0,04751	-1,64918	201	198	0,100697	0,640171	y ~ current_feature + S
-0,04761	0,075942	0,061498	-0,62693	176	173	0,53153	0,937665	y ~ current_feature + S
-0,03275	0,086992	-0,02429	-0,37644	135	132	0,707194	0,93118	y ~ current_feature + S
-0,10513	0,122409	-0,07874	-0,8588	69	66	0,393559	0,789252	y ~ current_feature + S
-0,18581	0,120948	0,034007	-1,53626	69	66	0,129254	0,572145	y ~ current_feature + S
-0,021	0,123064	-0,04751	-0,17063	69	66	0,86504	0,985217	y ~ current_feature + S
-0,01811	0,129078	0,061498	-0,14028	63	60	0,888907	0,996179	y ~ current_feature + S
-0,07567	0,155476	-0,02429	-0,48669	46	41,13176	0,62906	0,924682	y ~ current_feature + S
0,170014	0,057192	-0,07874	2,972668	315	296,8832	0,003194	0,067531	y ~ current_feature + S
0,126891	0,076696	0,034007	1,654452	315	167,2629	0,099911	0,518857	y ~ current_feature + S
0,197213	0,059639	-0,04751	3,30678	314	270,2169	0,001072	0,132163	y ~ current_feature + S
0,091514	0,060987	0,061498	1,500554	271	266,6084	0,134654	0,8197	y ~ current_feature + S
-0,03196	0,07198	-0,02429	-0,44397	209	192,8136	0,657561	0,924682	y ~ current_feature + S
0,049044	0,049526	-0,07874	0,990281	418	406,7206	0,322626	0,725761	y ~ current_feature + S
0,059222	0,062492	0,034007	0,947668	418	255,1641	0,344195	0,746014	y ~ current_feature + S
-0,00499	0,050223	-0,04751	-0,09941	417	396,4479	0,920866	0,985217	y ~ current_feature + S
0,036555	0,05424	0,061498	0,67396	364	339,4556	0,500795	0,937665	y ~ current_feature + S
0,043587	0,062317	-0,02429	0,699439	278	257,0169	0,48491	0,888665	y ~ current_feature + S
0,134228	0,126878	-0,07874	1,057926	64	61	0,29426	0,718687	y ~ current_feature + S
-0,12608	0,127015	0,034007	-0,99262	64	61	0,324818	0,728793	y ~ current_feature + S
-0,04528	0,127906	-0,04751	-0,35397	64	61	0,72458	0,93314	y ~ current_feature + S
0,049077	0,135919	0,061498	0,361074	57	54	0,719453	0,972573	y ~ current_feature + S
-0,20777	0,156634	-0,02429	-1,32646	42	39	0,192402	0,65347	y ~ current_feature + S
0,230983	0,075745	-0,07874	3,049498	168	165	0,002671	0,065908	y ~ current_feature + S
0,048122	0,07776	0,034007	0,618857	168	165	0,536864	0,850706	y ~ current_feature + S
0,083885	0,077576	-0,04751	1,081335	168	165	0,281126	0,747915	y ~ current_feature + S
-0,15181	0,082367	0,061498	-1,84312	147	144	0,067367	0,715249	y ~ current_feature + S
0,039425	0,095708	-0,02429	0,411931	112	109	0,681199	0,926342	y ~ current_feature + S
0,129251	0,064548	-0,07874	2,002393	239	236	0,046387	0,286052	y ~ current_feature + S
0,084241	0,064863	0,034007	1,298747	239	236	0,195298	0,6438	y ~ current_feature + S

0,019857	0,065082	-0,04751	0,305103	239	236	0,760556	0,9394	y ~ current_feature + S
-0,09758	0,069851	0,061498	-1,397	206	203	0,16394	0,8197	y ~ current_feature + S
0,060733	0,079661	-0,02429	0,762388	160	157	0,446972	0,858965	y ~ current_feature + S
0,055989	0,113782	-0,07874	0,492077	80	77	0,624065	0,919937	y ~ current_feature + S
-0,10189	0,113367	0,034007	-0,89877	80	77	0,371576	0,750317	y ~ current_feature + S
0,241056	0,1106	-0,04751	2,179527	80	77	0,032349	0,603897	y ~ current_feature + S
-0,20528	0,116977	0,061498	-1,75487	73	70	0,083658	0,754962	y ~ current_feature + S
0,12944	0,13885	-0,02429	0,932227	54	51	0,355612	0,800026	y ~ current_feature + S
0,130755	0,051229	-0,07874	2,55238	421	374,5281	0,011095	0,140685	y ~ current_feature + S
0,085689	0,067737	0,034007	1,265022	421	216,3454	0,207224	0,655365	y ~ current_feature + S
0,088359	0,052703	-0,04751	1,676549	420	357,2125	0,094506	0,640171	y ~ current_feature + S
-0,04459	0,056178	0,061498	-0,79376	367	316,2322	0,427932	0,937665	y ~ current_feature + S
0,08813	0,05985	-0,02429	1,472505	280	277	0,14202	0,618941	y ~ current_feature + S
0,01025	0,111798	-0,07874	0,09168	83	80	0,927182	0,990519	y ~ current_feature + S
0,026512	0,111764	0,034007	0,237217	83	80	0,813095	0,952501	y ~ current_feature + S
-0,08069	0,112142	-0,04751	-0,71953	82	79	0,473938	0,842057	y ~ current_feature + S
0,198098	0,128032	0,061498	1,54725	71	58,61036	0,127186	0,8197	y ~ current_feature + S
0,123003	0,137622	-0,02429	0,893777	55	52	0,375559	0,815551	y ~ current_feature + S
-0,01301	0,050375	-0,07874	-0,25821	397	394	0,796382	0,979102	y ~ current_feature + S
-0,07628	0,050232	0,034007	-1,51863	397	394	0,129659	0,572145	y ~ current_feature + S
-0,08402	0,050265	-0,04751	-1,67153	396	393	0,095412	0,640171	y ~ current_feature + S
-0,03958	0,059416	0,061498	-0,66608	346	282,8199	0,505903	0,937665	y ~ current_feature + S
-0,04144	0,061941	-0,02429	-0,66896	264	260,1902	0,504116	0,894767	y ~ current_feature + S
0,141362	0,063246	-0,07874	2,235104	248	245	0,026312	0,204832	y ~ current_feature + S
0,096003	0,063593	0,034007	1,509657	248	245	0,13242	0,572281	y ~ current_feature + S
0,029594	0,06399	-0,04751	0,462475	247	244	0,644153	0,925756	y ~ current_feature + S
0,069093	0,068516	0,061498	1,008412	215	212	0,314406	0,893818	y ~ current_feature + S
0,032967	0,078525	-0,02429	0,419831	165	162	0,675165	0,926342	y ~ current_feature + S
0,062868	0,051815	-0,07874	1,213321	374	371	0,225779	0,647583	y ~ current_feature + S
-0,03033	0,051894	0,034007	-0,58447	374	371	0,559257	0,860396	y ~ current_feature + S
-0,01722	0,05198	-0,04751	-0,33135	373	370	0,740567	0,933594	y ~ current_feature + S
-0,06956	0,064077	0,061498	-1,08561	322	242,3721	0,27873	0,893818	y ~ current_feature + S
-0,03341	0,063759	-0,02429	-0,52402	249	245,7138	0,600736	0,924682	y ~ current_feature + S
0,102589	0,056588	-0,07874	1,812914	312	309	0,070815	0,356433	y ~ current_feature + S
0,080388	0,056704	0,034007	1,417676	312	309	0,157293	0,60427	y ~ current_feature + S
0,072539	0,05683	-0,04751	1,276422	311	308	0,202768	0,723562	y ~ current_feature + S
-0,12766	0,060812	0,061498	-2,09922	269	266	0,03674	0,658491	y ~ current_feature + S
-0,02641	0,06999	-0,02429	-0,37731	207	204	0,706334	0,93118	y ~ current_feature + S
0,125084	0,080474	-0,07874	1,554352	155	152	0,12218	0,477837	y ~ current_feature + S
0,130295	0,080419	0,034007	1,620191	155	152	0,107264	0,532721	y ~ current_feature + S
0,115424	0,080835	-0,04751	1,427896	154	151	0,155387	0,672434	y ~ current_feature + S
0,033089	0,08834	0,061498	0,374559	131	128	0,708608	0,972444	y ~ current_feature + S
0,162478	0,098671	-0,02429	1,646663	103	100	0,102767	0,605474	y ~ current_feature + S
0,006689	0,07352	-0,07874	0,090979	188	185	0,927608	0,990519	y ~ current_feature + S
-0,08076	0,073281	0,034007	-1,10201	188	185	0,27189	0,71095	y ~ current_feature + S
0,157582	0,0728	-0,04751	2,164588	187	184	0,031707	0,603897	y ~ current_feature + S
-0,17059	0,07839	0,061498	-2,17618	161	158	0,031027	0,658491	y ~ current_feature + S
0,083752	0,090218	-0,02429	0,92833	125	122	0,35507	0,800026	y ~ current_feature + S

-0,10027	0,08693	-0,07874	-1,15342	134	131	0,250838	0,679754	y ~ current_feature + S
-0,16642	0,086152	0,034007	-1,93168	134	131	0,055559	0,420946	y ~ current_feature + S
-0,01292	0,087698	-0,04751	-0,14732	133	130	0,883105	0,985217	y ~ current_feature + S
0,053309	0,094357	0,061498	0,564969	115	112	0,573225	0,937665	y ~ current_feature + S
-0,06866	0,107578	-0,02429	-0,6382	89	86	0,525037	0,899808	y ~ current_feature + S

ex + (1 | Subject)

ex + (1 | Subject)

ex + (1 | Subject)

ex + (1 | Subject)

ex + (1 | Subject)

ex + (1 | Subject)

ex + (1 | Subject)

ex + (1 | Subject)

ex + (1 | Subject)

ex + (1 | Subject)

ex + (1 | Subject)

ex + (1 | Subject)

ex + (1 | Subject)

ex + (1 | Subject)

ex + (1 | Subject)

ex + (1 | Subject)

ex + (1 | Subject)

ex + (1 | Subject)

ex + (1 | Subject)

ex + (1 | Subject)

ex + (1 | Subject)

ex + (1 | Subject)

ex + (1 | Subject)

ex + (1 | Subject)

ex + (1 | Subject)

ex + (1 | Subject)

ex + (1 | Subject)

ex + (1 | Subject)

ex + (1 | Subject)

ex + (1 | Subject)

ex + (1 | Subject)

ex + (1 | Subject)

ex + (1 | Subject)

ex + (1 | Subject)

ex + (1 | Subject)

ex + (1 | Subject)

ex + (1 | Subject)

ex + (1 | Subject)

ex + (1 | Subject)

ex + (1 | Subject)

ex + (1 | Subject)

ex + (1 | Subject)

ex + (1 | Subject)

ex + (1 | Subject)

ex + (1 | Subject)

ex + (1 | Subject)

ex + (1 | Subject)

ex + (1 | Subject)
ex + (1 | Subject)
ex + (1 | Subject)
ex + (1 | Subject)
ex + (1 | Subject)
ex + (1 | Subject)
ex + (1 | Subject)
ex + (1 | Subject)
ex + (1 | Subject)
ex + (1 | Subject)
ex + (1 | Subject)
ex + (1 | Subject)
ex + (1 | Subject)
ex + (1 | Subject)
ex + (1 | Subject)
ex + (1 | Subject)
ex + (1 | Subject)
ex + (1 | Subject)
ex + (1 | Subject)
ex + (1 | Subject)
ex + (1 | Subject)
ex + (1 | Subject)
ex + (1 | Subject)
ex + (1 | Subject)
ex + (1 | Subject)
ex + (1 | Subject)
ex + (1 | Subject)
ex + (1 | Subject)
ex + (1 | Subject)
ex + (1 | Subject)
ex + (1 | Subject)
ex + (1 | Subject)
ex + (1 | Subject)
ex + (1 | Subject)
ex + (1 | Subject)
ex + (1 | Subject)
ex + (1 | Subject)
ex + (1 | Subject)
ex + (1 | Subject)
ex + (1 | Subject)
ex + (1 | Subject)
ex + (1 | Subject)
ex + (1 | Subject)
ex + (1 | Subject)
ex + (1 | Subject)
ex + (1 | Subject)
ex + (1 | Subject)
ex + (1 | Subject)

ex + (1 | Subject)
ex + (1 | Subject)
ex + (1 | Subject)
ex + (1 | Subject)
ex + (1 | Subject)
ex + (1 | Subject)
ex + (1 | Subject)
ex + (1 | Subject)
ex + (1 | Subject)
ex + (1 | Subject)
ex + (1 | Subject)
ex + (1 | Subject)
ex + (1 | Subject)
ex + (1 | Subject)
ex + (1 | Subject)
ex + (1 | Subject)
ex + (1 | Subject)
ex + (1 | Subject)
ex + (1 | Subject)
ex + (1 | Subject)
ex + (1 | Subject)
ex + (1 | Subject)
ex + (1 | Subject)
ex + (1 | Subject)
ex + (1 | Subject)
ex + (1 | Subject)
ex + (1 | Subject)
ex + (1 | Subject)
ex + (1 | Subject)
ex + (1 | Subject)
ex + (1 | Subject)
ex + (1 | Subject)
ex + (1 | Subject)
ex + (1 | Subject)
ex + (1 | Subject)
ex + (1 | Subject)
ex + (1 | Subject)
ex + (1 | Subject)
ex + (1 | Subject)
ex + (1 | Subject)
ex + (1 | Subject)
ex + (1 | Subject)
ex + (1 | Subject)
ex + (1 | Subject)
ex + (1 | Subject)
ex + (1 | Subject)
ex + (1 | Subject)
ex + (1 | Subject)

ex + (1 | Subject)
ex + (1 | Subject)
ex + (1 | Subject)
ex + (1 | Subject)
ex + (1 | Subject)
ex + (1 | Subject)
ex + (1 | Subject)
ex + (1 | Subject)
ex + (1 | Subject)
ex + (1 | Subject)
ex + (1 | Subject)
ex + (1 | Subject)
ex + (1 | Subject)
ex + (1 | Subject)
ex + (1 | Subject)
ex + (1 | Subject)
ex + (1 | Subject)
ex + (1 | Subject)
ex + (1 | Subject)
ex + (1 | Subject)
ex + (1 | Subject)
ex + (1 | Subject)
ex + (1 | Subject)
ex + (1 | Subject)
ex + (1 | Subject)
ex + (1 | Subject)
ex + (1 | Subject)
ex + (1 | Subject)
ex + (1 | Subject)
ex + (1 | Subject)
ex + (1 | Subject)
ex + (1 | Subject)
ex + (1 | Subject)
ex + (1 | Subject)
ex + (1 | Subject)
ex + (1 | Subject)
ex + (1 | Subject)
ex + (1 | Subject)
ex + (1 | Subject)
ex + (1 | Subject)
ex + (1 | Subject)
ex + (1 | Subject)
ex + (1 | Subject)
ex + (1 | Subject)
ex + (1 | Subject)
ex + (1 | Subject)
ex + (1 | Subject)
ex + (1 | Subject)

ex + (1 | Subject)
ex + (1 | Subject)
ex + (1 | Subject)
ex + (1 | Subject)
ex + (1 | Subject)
ex + (1 | Subject)
ex + (1 | Subject)
ex + (1 | Subject)
ex + (1 | Subject)
ex + (1 | Subject)
ex + (1 | Subject)
ex + (1 | Subject)
ex + (1 | Subject)
ex + (1 | Subject)
ex + (1 | Subject)
ex + (1 | Subject)
ex + (1 | Subject)
ex + (1 | Subject)
ex + (1 | Subject)
ex + (1 | Subject)
ex + (1 | Subject)
ex + (1 | Subject)
ex + (1 | Subject)
ex + (1 | Subject)
ex + (1 | Subject)
ex + (1 | Subject)
ex + (1 | Subject)
ex + (1 | Subject)
ex + (1 | Subject)
ex + (1 | Subject)
ex + (1 | Subject)
ex + (1 | Subject)
ex + (1 | Subject)
ex + (1 | Subject)
ex + (1 | Subject)
ex + (1 | Subject)
ex + (1 | Subject)
ex + (1 | Subject)
ex + (1 | Subject)
ex + (1 | Subject)
ex + (1 | Subject)
ex + (1 | Subject)
ex + (1 | Subject)
ex + (1 | Subject)
ex + (1 | Subject)
ex + (1 | Subject)
ex + (1 | Subject)
ex + (1 | Subject)

ex + (1 | Subject)
ex + (1 | Subject)
ex + (1 | Subject)
ex + (1 | Subject)
ex + (1 | Subject)
ex + (1 | Subject)
ex + (1 | Subject)
ex + (1 | Subject)
ex + (1 | Subject)
ex + (1 | Subject)
ex + (1 | Subject)
ex + (1 | Subject)
ex + (1 | Subject)
ex + (1 | Subject)
ex + (1 | Subject)
ex + (1 | Subject)
ex + (1 | Subject)
ex + (1 | Subject)
ex + (1 | Subject)
ex + (1 | Subject)
ex + (1 | Subject)
ex + (1 | Subject)
ex + (1 | Subject)
ex + (1 | Subject)
ex + (1 | Subject)
ex + (1 | Subject)
ex + (1 | Subject)
ex + (1 | Subject)
ex + (1 | Subject)
ex + (1 | Subject)
ex + (1 | Subject)
ex + (1 | Subject)
ex + (1 | Subject)
ex + (1 | Subject)
ex + (1 | Subject)
ex + (1 | Subject)
ex + (1 | Subject)
ex + (1 | Subject)
ex + (1 | Subject)
ex + (1 | Subject)
ex + (1 | Subject)
ex + (1 | Subject)
ex + (1 | Subject)
ex + (1 | Subject)
ex + (1 | Subject)
ex + (1 | Subject)
ex + (1 | Subject)
ex + (1 | Subject)

ex + (1 | Subject)
ex + (1 | Subject)
ex + (1 | Subject)
ex + (1 | Subject)
ex + (1 | Subject)
ex + (1 | Subject)
ex + (1 | Subject)
ex + (1 | Subject)
ex + (1 | Subject)
ex + (1 | Subject)
ex + (1 | Subject)
ex + (1 | Subject)
ex + (1 | Subject)
ex + (1 | Subject)
ex + (1 | Subject)
ex + (1 | Subject)
ex + (1 | Subject)
ex + (1 | Subject)
ex + (1 | Subject)
ex + (1 | Subject)
ex + (1 | Subject)
ex + (1 | Subject)
ex + (1 | Subject)
ex + (1 | Subject)
ex + (1 | Subject)
ex + (1 | Subject)
ex + (1 | Subject)
ex + (1 | Subject)
ex + (1 | Subject)
ex + (1 | Subject)
ex + (1 | Subject)
ex + (1 | Subject)
ex + (1 | Subject)
ex + (1 | Subject)
ex + (1 | Subject)
ex + (1 | Subject)
ex + (1 | Subject)
ex + (1 | Subject)
ex + (1 | Subject)
ex + (1 | Subject)
ex + (1 | Subject)
ex + (1 | Subject)
ex + (1 | Subject)
ex + (1 | Subject)
ex + (1 | Subject)
ex + (1 | Subject)
ex + (1 | Subject)
ex + (1 | Subject)

ex + (1 | Subject)
ex + (1 | Subject)
ex + (1 | Subject)
ex + (1 | Subject)
ex + (1 | Subject)
ex + (1 | Subject)
ex + (1 | Subject)
ex + (1 | Subject)
ex + (1 | Subject)
ex + (1 | Subject)
ex + (1 | Subject)
ex + (1 | Subject)
ex + (1 | Subject)
ex + (1 | Subject)
ex + (1 | Subject)
ex + (1 | Subject)
ex + (1 | Subject)
ex + (1 | Subject)
ex + (1 | Subject)
ex + (1 | Subject)
ex + (1 | Subject)
ex + (1 | Subject)
ex + (1 | Subject)
ex + (1 | Subject)
ex + (1 | Subject)
ex + (1 | Subject)
ex + (1 | Subject)
ex + (1 | Subject)
ex + (1 | Subject)
ex + (1 | Subject)
ex + (1 | Subject)
ex + (1 | Subject)
ex + (1 | Subject)
ex + (1 | Subject)
ex + (1 | Subject)
ex + (1 | Subject)
ex + (1 | Subject)
ex + (1 | Subject)
ex + (1 | Subject)
ex + (1 | Subject)
ex + (1 | Subject)
ex + (1 | Subject)
ex + (1 | Subject)
ex + (1 | Subject)
ex + (1 | Subject)
ex + (1 | Subject)
ex + (1 | Subject)
ex + (1 | Subject)

ex + (1 | Subject)
ex + (1 | Subject)
ex + (1 | Subject)
ex + (1 | Subject)
ex + (1 | Subject)
ex + (1 | Subject)
ex + (1 | Subject)
ex + (1 | Subject)
ex + (1 | Subject)
ex + (1 | Subject)
ex + (1 | Subject)
ex + (1 | Subject)
ex + (1 | Subject)
ex + (1 | Subject)
ex + (1 | Subject)
ex + (1 | Subject)
ex + (1 | Subject)
ex + (1 | Subject)
ex + (1 | Subject)
ex + (1 | Subject)
ex + (1 | Subject)
ex + (1 | Subject)
ex + (1 | Subject)
ex + (1 | Subject)
ex + (1 | Subject)
ex + (1 | Subject)
ex + (1 | Subject)
ex + (1 | Subject)
ex + (1 | Subject)
ex + (1 | Subject)
ex + (1 | Subject)
ex + (1 | Subject)
ex + (1 | Subject)
ex + (1 | Subject)
ex + (1 | Subject)
ex + (1 | Subject)
ex + (1 | Subject)
ex + (1 | Subject)
ex + (1 | Subject)
ex + (1 | Subject)
ex + (1 | Subject)
ex + (1 | Subject)
ex + (1 | Subject)
ex + (1 | Subject)
ex + (1 | Subject)
ex + (1 | Subject)
ex + (1 | Subject)
ex + (1 | Subject)

ex + (1 | Subject)
ex + (1 | Subject)
ex + (1 | Subject)
ex + (1 | Subject)
ex + (1 | Subject)
ex + (1 | Subject)
ex + (1 | Subject)
ex + (1 | Subject)
ex + (1 | Subject)
ex + (1 | Subject)
ex + (1 | Subject)
ex + (1 | Subject)
ex + (1 | Subject)
ex + (1 | Subject)
ex + (1 | Subject)
ex + (1 | Subject)
ex + (1 | Subject)
ex + (1 | Subject)
ex + (1 | Subject)
ex + (1 | Subject)
ex + (1 | Subject)
ex + (1 | Subject)
ex + (1 | Subject)
ex + (1 | Subject)
ex + (1 | Subject)
ex + (1 | Subject)
ex + (1 | Subject)
ex + (1 | Subject)
ex + (1 | Subject)
ex + (1 | Subject)
ex + (1 | Subject)
ex + (1 | Subject)
ex + (1 | Subject)
ex + (1 | Subject)
ex + (1 | Subject)
ex + (1 | Subject)
ex + (1 | Subject)
ex + (1 | Subject)
ex + (1 | Subject)
ex + (1 | Subject)
ex + (1 | Subject)
ex + (1 | Subject)
ex + (1 | Subject)
ex + (1 | Subject)
ex + (1 | Subject)
ex + (1 | Subject)
ex + (1 | Subject)
ex + (1 | Subject)

ex + (1 | Subject)
ex + (1 | Subject)
ex + (1 | Subject)
ex + (1 | Subject)
ex + (1 | Subject)
ex + (1 | Subject)
ex + (1 | Subject)
ex + (1 | Subject)
ex + (1 | Subject)
ex + (1 | Subject)
ex + (1 | Subject)
ex + (1 | Subject)
ex + (1 | Subject)
ex + (1 | Subject)
ex + (1 | Subject)
ex + (1 | Subject)
ex + (1 | Subject)
ex + (1 | Subject)
ex + (1 | Subject)
ex + (1 | Subject)
ex + (1 | Subject)
ex + (1 | Subject)
ex + (1 | Subject)
ex + (1 | Subject)
ex + (1 | Subject)
ex + (1 | Subject)
ex + (1 | Subject)
ex + (1 | Subject)
ex + (1 | Subject)
ex + (1 | Subject)
ex + (1 | Subject)
ex + (1 | Subject)
ex + (1 | Subject)
ex + (1 | Subject)
ex + (1 | Subject)
ex + (1 | Subject)
ex + (1 | Subject)
ex + (1 | Subject)
ex + (1 | Subject)
ex + (1 | Subject)
ex + (1 | Subject)
ex + (1 | Subject)
ex + (1 | Subject)
ex + (1 | Subject)
ex + (1 | Subject)
ex + (1 | Subject)
ex + (1 | Subject)
ex + (1 | Subject)

ex + (1 | Subject)
ex + (1 | Subject)
ex + (1 | Subject)
ex + (1 | Subject)
ex + (1 | Subject)
ex + (1 | Subject)
ex + (1 | Subject)
ex + (1 | Subject)
ex + (1 | Subject)
ex + (1 | Subject)
ex + (1 | Subject)
ex + (1 | Subject)
ex + (1 | Subject)
ex + (1 | Subject)
ex + (1 | Subject)
ex + (1 | Subject)
ex + (1 | Subject)
ex + (1 | Subject)
ex + (1 | Subject)
ex + (1 | Subject)
ex + (1 | Subject)
ex + (1 | Subject)
ex + (1 | Subject)
ex + (1 | Subject)
ex + (1 | Subject)
ex + (1 | Subject)
ex + (1 | Subject)
ex + (1 | Subject)
ex + (1 | Subject)
ex + (1 | Subject)
ex + (1 | Subject)
ex + (1 | Subject)
ex + (1 | Subject)
ex + (1 | Subject)
ex + (1 | Subject)
ex + (1 | Subject)
ex + (1 | Subject)
ex + (1 | Subject)
ex + (1 | Subject)
ex + (1 | Subject)
ex + (1 | Subject)
ex + (1 | Subject)
ex + (1 | Subject)
ex + (1 | Subject)
ex + (1 | Subject)
ex + (1 | Subject)
ex + (1 | Subject)
ex + (1 | Subject)

ex + (1 | Subject)
ex + (1 | Subject)
ex + (1 | Subject)
ex + (1 | Subject)
ex + (1 | Subject)
ex + (1 | Subject)
ex + (1 | Subject)
ex + (1 | Subject)
ex + (1 | Subject)
ex + (1 | Subject)
ex + (1 | Subject)
ex + (1 | Subject)
ex + (1 | Subject)
ex + (1 | Subject)
ex + (1 | Subject)
ex + (1 | Subject)
ex + (1 | Subject)
ex + (1 | Subject)
ex + (1 | Subject)
ex + (1 | Subject)
ex + (1 | Subject)
ex + (1 | Subject)
ex + (1 | Subject)
ex + (1 | Subject)
ex + (1 | Subject)
ex + (1 | Subject)
ex + (1 | Subject)
ex + (1 | Subject)
ex + (1 | Subject)
ex + (1 | Subject)
ex + (1 | Subject)
ex + (1 | Subject)
ex + (1 | Subject)
ex + (1 | Subject)
ex + (1 | Subject)
ex + (1 | Subject)
ex + (1 | Subject)
ex + (1 | Subject)
ex + (1 | Subject)
ex + (1 | Subject)
ex + (1 | Subject)
ex + (1 | Subject)
ex + (1 | Subject)
ex + (1 | Subject)
ex + (1 | Subject)
ex + (1 | Subject)
ex + (1 | Subject)
ex + (1 | Subject)

ex + (1 | Subject)
ex + (1 | Subject)
ex + (1 | Subject)
ex + (1 | Subject)
ex + (1 | Subject)
ex + (1 | Subject)
ex + (1 | Subject)
ex + (1 | Subject)
ex + (1 | Subject)
ex + (1 | Subject)
ex + (1 | Subject)
ex + (1 | Subject)
ex + (1 | Subject)
ex + (1 | Subject)
ex + (1 | Subject)
ex + (1 | Subject)
ex + (1 | Subject)
ex + (1 | Subject)
ex + (1 | Subject)
ex + (1 | Subject)
ex + (1 | Subject)
ex + (1 | Subject)
ex + (1 | Subject)
ex + (1 | Subject)
ex + (1 | Subject)
ex + (1 | Subject)
ex + (1 | Subject)
ex + (1 | Subject)
ex + (1 | Subject)
ex + (1 | Subject)
ex + (1 | Subject)
ex + (1 | Subject)
ex + (1 | Subject)
ex + (1 | Subject)
ex + (1 | Subject)
ex + (1 | Subject)
ex + (1 | Subject)
ex + (1 | Subject)
ex + (1 | Subject)
ex + (1 | Subject)
ex + (1 | Subject)
ex + (1 | Subject)
ex + (1 | Subject)
ex + (1 | Subject)
ex + (1 | Subject)
ex + (1 | Subject)
ex + (1 | Subject)
ex + (1 | Subject)

ex + (1 | Subject)
ex + (1 | Subject)
ex + (1 | Subject)
ex + (1 | Subject)
ex + (1 | Subject)
ex + (1 | Subject)
ex + (1 | Subject)
ex + (1 | Subject)
ex + (1 | Subject)
ex + (1 | Subject)
ex + (1 | Subject)
ex + (1 | Subject)
ex + (1 | Subject)
ex + (1 | Subject)
ex + (1 | Subject)
ex + (1 | Subject)
ex + (1 | Subject)
ex + (1 | Subject)
ex + (1 | Subject)
ex + (1 | Subject)
ex + (1 | Subject)
ex + (1 | Subject)
ex + (1 | Subject)
ex + (1 | Subject)
ex + (1 | Subject)
ex + (1 | Subject)
ex + (1 | Subject)
ex + (1 | Subject)
ex + (1 | Subject)
ex + (1 | Subject)
ex + (1 | Subject)
ex + (1 | Subject)
ex + (1 | Subject)
ex + (1 | Subject)
ex + (1 | Subject)
ex + (1 | Subject)
ex + (1 | Subject)
ex + (1 | Subject)
ex + (1 | Subject)
ex + (1 | Subject)
ex + (1 | Subject)
ex + (1 | Subject)
ex + (1 | Subject)
ex + (1 | Subject)
ex + (1 | Subject)
ex + (1 | Subject)
ex + (1 | Subject)
ex + (1 | Subject)

ex + (1 | Subject)
ex + (1 | Subject)
ex + (1 | Subject)
ex + (1 | Subject)
ex + (1 | Subject)
ex + (1 | Subject)
ex + (1 | Subject)
ex + (1 | Subject)
ex + (1 | Subject)
ex + (1 | Subject)
ex + (1 | Subject)
ex + (1 | Subject)
ex + (1 | Subject)
ex + (1 | Subject)
ex + (1 | Subject)
ex + (1 | Subject)
ex + (1 | Subject)
ex + (1 | Subject)
ex + (1 | Subject)
ex + (1 | Subject)
ex + (1 | Subject)
ex + (1 | Subject)
ex + (1 | Subject)
ex + (1 | Subject)
ex + (1 | Subject)
ex + (1 | Subject)
ex + (1 | Subject)
ex + (1 | Subject)
ex + (1 | Subject)
ex + (1 | Subject)
ex + (1 | Subject)
ex + (1 | Subject)
ex + (1 | Subject)
ex + (1 | Subject)
ex + (1 | Subject)
ex + (1 | Subject)
ex + (1 | Subject)
ex + (1 | Subject)
ex + (1 | Subject)
ex + (1 | Subject)
ex + (1 | Subject)
ex + (1 | Subject)
ex + (1 | Subject)
ex + (1 | Subject)
ex + (1 | Subject)
ex + (1 | Subject)
ex + (1 | Subject)
ex + (1 | Subject)

ex + (1 | Subject)
ex + (1 | Subject)
ex + (1 | Subject)
ex + (1 | Subject)
ex + (1 | Subject)
ex + (1 | Subject)
ex + (1 | Subject)
ex + (1 | Subject)
ex + (1 | Subject)
ex + (1 | Subject)
ex + (1 | Subject)
ex + (1 | Subject)
ex + (1 | Subject)
ex + (1 | Subject)
ex + (1 | Subject)
ex + (1 | Subject)
ex + (1 | Subject)
ex + (1 | Subject)
ex + (1 | Subject)
ex + (1 | Subject)
ex + (1 | Subject)
ex + (1 | Subject)
ex + (1 | Subject)
ex + (1 | Subject)
ex + (1 | Subject)
ex + (1 | Subject)
ex + (1 | Subject)
ex + (1 | Subject)
ex + (1 | Subject)
ex + (1 | Subject)
ex + (1 | Subject)
ex + (1 | Subject)
ex + (1 | Subject)
ex + (1 | Subject)
ex + (1 | Subject)
ex + (1 | Subject)
ex + (1 | Subject)
ex + (1 | Subject)
ex + (1 | Subject)
ex + (1 | Subject)
ex + (1 | Subject)
ex + (1 | Subject)
ex + (1 | Subject)
ex + (1 | Subject)
ex + (1 | Subject)
ex + (1 | Subject)
ex + (1 | Subject)
ex + (1 | Subject)

ex + (1 | Subject)
ex + (1 | Subject)
ex + (1 | Subject)
ex + (1 | Subject)
ex + (1 | Subject)
ex + (1 | Subject)
ex + (1 | Subject)
ex + (1 | Subject)
ex + (1 | Subject)
ex + (1 | Subject)
ex + (1 | Subject)
ex + (1 | Subject)
ex + (1 | Subject)
ex + (1 | Subject)
ex + (1 | Subject)
ex + (1 | Subject)
ex + (1 | Subject)
ex + (1 | Subject)
ex + (1 | Subject)
ex + (1 | Subject)
ex + (1 | Subject)
ex + (1 | Subject)
ex + (1 | Subject)
ex + (1 | Subject)
ex + (1 | Subject)
ex + (1 | Subject)
ex + (1 | Subject)
ex + (1 | Subject)
ex + (1 | Subject)
ex + (1 | Subject)
ex + (1 | Subject)
ex + (1 | Subject)
ex + (1 | Subject)
ex + (1 | Subject)
ex + (1 | Subject)
ex + (1 | Subject)
ex + (1 | Subject)
ex + (1 | Subject)
ex + (1 | Subject)
ex + (1 | Subject)
ex + (1 | Subject)
ex + (1 | Subject)
ex + (1 | Subject)
ex + (1 | Subject)
ex + (1 | Subject)
ex + (1 | Subject)
ex + (1 | Subject)
ex + (1 | Subject)

ex + (1 | Subject)
ex + (1 | Subject)
ex + (1 | Subject)
ex + (1 | Subject)
ex + (1 | Subject)
ex + (1 | Subject)
ex + (1 | Subject)
ex + (1 | Subject)
ex + (1 | Subject)
ex + (1 | Subject)
ex + (1 | Subject)
ex + (1 | Subject)
ex + (1 | Subject)
ex + (1 | Subject)
ex + (1 | Subject)
ex + (1 | Subject)
ex + (1 | Subject)
ex + (1 | Subject)
ex + (1 | Subject)
ex + (1 | Subject)
ex + (1 | Subject)
ex + (1 | Subject)
ex + (1 | Subject)
ex + (1 | Subject)
ex + (1 | Subject)
ex + (1 | Subject)
ex + (1 | Subject)
ex + (1 | Subject)
ex + (1 | Subject)
ex + (1 | Subject)
ex + (1 | Subject)
ex + (1 | Subject)
ex + (1 | Subject)
ex + (1 | Subject)
ex + (1 | Subject)
ex + (1 | Subject)
ex + (1 | Subject)
ex + (1 | Subject)
ex + (1 | Subject)
ex + (1 | Subject)
ex + (1 | Subject)
ex + (1 | Subject)
ex + (1 | Subject)
ex + (1 | Subject)
ex + (1 | Subject)
ex + (1 | Subject)
ex + (1 | Subject)
ex + (1 | Subject)

ex + (1 | Subject)
ex + (1 | Subject)
ex + (1 | Subject)
ex + (1 | Subject)
ex + (1 | Subject)
ex + (1 | Subject)
ex + (1 | Subject)
ex + (1 | Subject)
ex + (1 | Subject)
ex + (1 | Subject)
ex + (1 | Subject)
ex + (1 | Subject)
ex + (1 | Subject)
ex + (1 | Subject)
ex + (1 | Subject)
ex + (1 | Subject)
ex + (1 | Subject)
ex + (1 | Subject)
ex + (1 | Subject)
ex + (1 | Subject)
ex + (1 | Subject)
ex + (1 | Subject)
ex + (1 | Subject)
ex + (1 | Subject)
ex + (1 | Subject)
ex + (1 | Subject)
ex + (1 | Subject)
ex + (1 | Subject)
ex + (1 | Subject)
ex + (1 | Subject)
ex + (1 | Subject)
ex + (1 | Subject)
ex + (1 | Subject)
ex + (1 | Subject)
ex + (1 | Subject)
ex + (1 | Subject)
ex + (1 | Subject)
ex + (1 | Subject)
ex + (1 | Subject)
ex + (1 | Subject)
ex + (1 | Subject)
ex + (1 | Subject)
ex + (1 | Subject)
ex + (1 | Subject)
ex + (1 | Subject)
ex + (1 | Subject)
ex + (1 | Subject)
ex + (1 | Subject)

ex + (1 | Subject)
ex + (1 | Subject)
ex + (1 | Subject)
ex + (1 | Subject)
ex + (1 | Subject)
ex + (1 | Subject)
ex + (1 | Subject)
ex + (1 | Subject)
ex + (1 | Subject)
ex + (1 | Subject)
ex + (1 | Subject)
ex + (1 | Subject)
ex + (1 | Subject)
ex + (1 | Subject)
ex + (1 | Subject)
ex + (1 | Subject)
ex + (1 | Subject)
ex + (1 | Subject)
ex + (1 | Subject)
ex + (1 | Subject)
ex + (1 | Subject)
ex + (1 | Subject)
ex + (1 | Subject)
ex + (1 | Subject)
ex + (1 | Subject)
ex + (1 | Subject)
ex + (1 | Subject)
ex + (1 | Subject)
ex + (1 | Subject)
ex + (1 | Subject)
ex + (1 | Subject)
ex + (1 | Subject)
ex + (1 | Subject)
ex + (1 | Subject)
ex + (1 | Subject)
ex + (1 | Subject)
ex + (1 | Subject)
ex + (1 | Subject)
ex + (1 | Subject)
ex + (1 | Subject)
ex + (1 | Subject)
ex + (1 | Subject)
ex + (1 | Subject)
ex + (1 | Subject)
ex + (1 | Subject)
ex + (1 | Subject)
ex + (1 | Subject)
ex + (1 | Subject)

ex + (1 | Subject)
ex + (1 | Subject)
ex + (1 | Subject)
ex + (1 | Subject)
ex + (1 | Subject)
ex + (1 | Subject)
ex + (1 | Subject)
ex + (1 | Subject)
ex + (1 | Subject)
ex + (1 | Subject)
ex + (1 | Subject)
ex + (1 | Subject)
ex + (1 | Subject)
ex + (1 | Subject)
ex + (1 | Subject)
ex + (1 | Subject)
ex + (1 | Subject)
ex + (1 | Subject)
ex + (1 | Subject)
ex + (1 | Subject)
ex + (1 | Subject)
ex + (1 | Subject)
ex + (1 | Subject)
ex + (1 | Subject)
ex + (1 | Subject)
ex + (1 | Subject)
ex + (1 | Subject)
ex + (1 | Subject)
ex + (1 | Subject)
ex + (1 | Subject)
ex + (1 | Subject)
ex + (1 | Subject)
ex + (1 | Subject)
ex + (1 | Subject)
ex + (1 | Subject)
ex + (1 | Subject)
ex + (1 | Subject)
ex + (1 | Subject)
ex + (1 | Subject)
ex + (1 | Subject)
ex + (1 | Subject)
ex + (1 | Subject)
ex + (1 | Subject)
ex + (1 | Subject)
ex + (1 | Subject)
ex + (1 | Subject)
ex + (1 | Subject)
ex + (1 | Subject)

ex + (1 | Subject)
ex + (1 | Subject)
ex + (1 | Subject)
ex + (1 | Subject)
ex + (1 | Subject)
ex + (1 | Subject)
ex + (1 | Subject)
ex + (1 | Subject)
ex + (1 | Subject)
ex + (1 | Subject)
ex + (1 | Subject)
ex + (1 | Subject)
ex + (1 | Subject)
ex + (1 | Subject)
ex + (1 | Subject)
ex + (1 | Subject)
ex + (1 | Subject)
ex + (1 | Subject)
ex + (1 | Subject)
ex + (1 | Subject)
ex + (1 | Subject)
ex + (1 | Subject)
ex + (1 | Subject)
ex + (1 | Subject)
ex + (1 | Subject)
ex + (1 | Subject)
ex + (1 | Subject)
ex + (1 | Subject)
ex + (1 | Subject)
ex + (1 | Subject)
ex + (1 | Subject)
ex + (1 | Subject)
ex + (1 | Subject)
ex + (1 | Subject)
ex + (1 | Subject)
ex + (1 | Subject)
ex + (1 | Subject)
ex + (1 | Subject)
ex + (1 | Subject)
ex + (1 | Subject)
ex + (1 | Subject)
ex + (1 | Subject)
ex + (1 | Subject)
ex + (1 | Subject)
ex + (1 | Subject)
ex + (1 | Subject)
ex + (1 | Subject)
ex + (1 | Subject)

ex + (1 | Subject)
ex + (1 | Subject)
ex + (1 | Subject)
ex + (1 | Subject)
ex + (1 | Subject)
ex + (1 | Subject)
ex + (1 | Subject)
ex + (1 | Subject)
ex + (1 | Subject)
ex + (1 | Subject)
ex + (1 | Subject)
ex + (1 | Subject)
ex + (1 | Subject)
ex + (1 | Subject)
ex + (1 | Subject)
ex + (1 | Subject)
ex + (1 | Subject)
ex + (1 | Subject)
ex + (1 | Subject)
ex + (1 | Subject)
ex + (1 | Subject)
ex + (1 | Subject)
ex + (1 | Subject)
ex + (1 | Subject)
ex + (1 | Subject)
ex + (1 | Subject)
ex + (1 | Subject)
ex + (1 | Subject)
ex + (1 | Subject)
ex + (1 | Subject)
ex + (1 | Subject)
ex + (1 | Subject)
ex + (1 | Subject)
ex + (1 | Subject)
ex + (1 | Subject)
ex + (1 | Subject)
ex + (1 | Subject)
ex + (1 | Subject)
ex + (1 | Subject)
ex + (1 | Subject)
ex + (1 | Subject)
ex + (1 | Subject)
ex + (1 | Subject)
ex + (1 | Subject)
ex + (1 | Subject)
ex + (1 | Subject)
ex + (1 | Subject)
ex + (1 | Subject)

ex + (1 | Subject)
ex + (1 | Subject)
ex + (1 | Subject)
ex + (1 | Subject)
ex + (1 | Subject)
ex + (1 | Subject)
ex + (1 | Subject)
ex + (1 | Subject)
ex + (1 | Subject)
ex + (1 | Subject)
ex + (1 | Subject)
ex + (1 | Subject)
ex + (1 | Subject)
ex + (1 | Subject)
ex + (1 | Subject)
ex + (1 | Subject)
ex + (1 | Subject)
ex + (1 | Subject)
ex + (1 | Subject)
ex + (1 | Subject)
ex + (1 | Subject)
ex + (1 | Subject)
ex + (1 | Subject)
ex + (1 | Subject)
ex + (1 | Subject)
ex + (1 | Subject)
ex + (1 | Subject)
ex + (1 | Subject)
ex + (1 | Subject)
ex + (1 | Subject)
ex + (1 | Subject)
ex + (1 | Subject)
ex + (1 | Subject)
ex + (1 | Subject)
ex + (1 | Subject)
ex + (1 | Subject)
ex + (1 | Subject)
ex + (1 | Subject)
ex + (1 | Subject)
ex + (1 | Subject)
ex + (1 | Subject)
ex + (1 | Subject)
ex + (1 | Subject)
ex + (1 | Subject)
ex + (1 | Subject)
ex + (1 | Subject)
ex + (1 | Subject)
ex + (1 | Subject)

ex + (1 | Subject)
ex + (1 | Subject)
ex + (1 | Subject)
ex + (1 | Subject)
ex + (1 | Subject)
ex + (1 | Subject)
ex + (1 | Subject)
ex + (1 | Subject)
ex + (1 | Subject)
ex + (1 | Subject)
ex + (1 | Subject)
ex + (1 | Subject)
ex + (1 | Subject)
ex + (1 | Subject)
ex + (1 | Subject)
ex + (1 | Subject)
ex + (1 | Subject)
ex + (1 | Subject)
ex + (1 | Subject)
ex + (1 | Subject)
ex + (1 | Subject)
ex + (1 | Subject)
ex + (1 | Subject)
ex + (1 | Subject)
ex + (1 | Subject)
ex + (1 | Subject)
ex + (1 | Subject)
ex + (1 | Subject)
ex + (1 | Subject)
ex + (1 | Subject)
ex + (1 | Subject)
ex + (1 | Subject)
ex + (1 | Subject)
ex + (1 | Subject)
ex + (1 | Subject)
ex + (1 | Subject)
ex + (1 | Subject)
ex + (1 | Subject)
ex + (1 | Subject)
ex + (1 | Subject)
ex + (1 | Subject)
ex + (1 | Subject)
ex + (1 | Subject)
ex + (1 | Subject)
ex + (1 | Subject)
ex + (1 | Subject)
ex + (1 | Subject)
ex + (1 | Subject)

ex + (1 | Subject)
ex + (1 | Subject)
ex + (1 | Subject)
ex + (1 | Subject)
ex + (1 | Subject)
ex + (1 | Subject)
ex + (1 | Subject)
ex + (1 | Subject)
ex + (1 | Subject)
ex + (1 | Subject)
ex + (1 | Subject)
ex + (1 | Subject)
ex + (1 | Subject)
ex + (1 | Subject)
ex + (1 | Subject)
ex + (1 | Subject)
ex + (1 | Subject)
ex + (1 | Subject)
ex + (1 | Subject)
ex + (1 | Subject)
ex + (1 | Subject)
ex + (1 | Subject)
ex + (1 | Subject)
ex + (1 | Subject)
ex + (1 | Subject)
ex + (1 | Subject)
ex + (1 | Subject)
ex + (1 | Subject)
ex + (1 | Subject)
ex + (1 | Subject)
ex + (1 | Subject)
ex + (1 | Subject)
ex + (1 | Subject)
ex + (1 | Subject)
ex + (1 | Subject)
ex + (1 | Subject)
ex + (1 | Subject)
ex + (1 | Subject)
ex + (1 | Subject)
ex + (1 | Subject)
ex + (1 | Subject)
ex + (1 | Subject)
ex + (1 | Subject)
ex + (1 | Subject)
ex + (1 | Subject)
ex + (1 | Subject)
ex + (1 | Subject)
ex + (1 | Subject)

ex + (1 | Subject)
ex + (1 | Subject)
ex + (1 | Subject)
ex + (1 | Subject)
ex + (1 | Subject)
ex + (1 | Subject)
ex + (1 | Subject)
ex + (1 | Subject)
ex + (1 | Subject)
ex + (1 | Subject)
ex + (1 | Subject)
ex + (1 | Subject)
ex + (1 | Subject)
ex + (1 | Subject)
ex + (1 | Subject)
ex + (1 | Subject)
ex + (1 | Subject)
ex + (1 | Subject)
ex + (1 | Subject)
ex + (1 | Subject)
ex + (1 | Subject)
ex + (1 | Subject)
ex + (1 | Subject)
ex + (1 | Subject)
ex + (1 | Subject)
ex + (1 | Subject)
ex + (1 | Subject)
ex + (1 | Subject)
ex + (1 | Subject)
ex + (1 | Subject)
ex + (1 | Subject)
ex + (1 | Subject)
ex + (1 | Subject)
ex + (1 | Subject)
ex + (1 | Subject)
ex + (1 | Subject)
ex + (1 | Subject)
ex + (1 | Subject)
ex + (1 | Subject)
ex + (1 | Subject)
ex + (1 | Subject)
ex + (1 | Subject)
ex + (1 | Subject)
ex + (1 | Subject)
ex + (1 | Subject)
ex + (1 | Subject)
ex + (1 | Subject)
ex + (1 | Subject)

ex + (1 | Subject)
ex + (1 | Subject)
ex + (1 | Subject)
ex + (1 | Subject)
ex + (1 | Subject)
ex + (1 | Subject)
ex + (1 | Subject)
ex + (1 | Subject)
ex + (1 | Subject)
ex + (1 | Subject)
ex + (1 | Subject)
ex + (1 | Subject)
ex + (1 | Subject)
ex + (1 | Subject)
ex + (1 | Subject)
ex + (1 | Subject)
ex + (1 | Subject)
ex + (1 | Subject)
ex + (1 | Subject)
ex + (1 | Subject)
ex + (1 | Subject)
ex + (1 | Subject)
ex + (1 | Subject)
ex + (1 | Subject)
ex + (1 | Subject)
ex + (1 | Subject)
ex + (1 | Subject)
ex + (1 | Subject)
ex + (1 | Subject)
ex + (1 | Subject)
ex + (1 | Subject)
ex + (1 | Subject)
ex + (1 | Subject)
ex + (1 | Subject)
ex + (1 | Subject)
ex + (1 | Subject)
ex + (1 | Subject)
ex + (1 | Subject)
ex + (1 | Subject)
ex + (1 | Subject)
ex + (1 | Subject)
ex + (1 | Subject)
ex + (1 | Subject)
ex + (1 | Subject)
ex + (1 | Subject)
ex + (1 | Subject)
ex + (1 | Subject)
ex + (1 | Subject)

ex + (1 | Subject)
ex + (1 | Subject)
ex + (1 | Subject)
ex + (1 | Subject)
ex + (1 | Subject)
ex + (1 | Subject)
ex + (1 | Subject)
ex + (1 | Subject)
ex + (1 | Subject)
ex + (1 | Subject)
ex + (1 | Subject)
ex + (1 | Subject)
ex + (1 | Subject)
ex + (1 | Subject)
ex + (1 | Subject)
ex + (1 | Subject)
ex + (1 | Subject)
ex + (1 | Subject)
ex + (1 | Subject)
ex + (1 | Subject)
ex + (1 | Subject)
ex + (1 | Subject)
ex + (1 | Subject)
ex + (1 | Subject)
ex + (1 | Subject)
ex + (1 | Subject)
ex + (1 | Subject)
ex + (1 | Subject)
ex + (1 | Subject)
ex + (1 | Subject)
ex + (1 | Subject)
ex + (1 | Subject)
ex + (1 | Subject)
ex + (1 | Subject)
ex + (1 | Subject)
ex + (1 | Subject)
ex + (1 | Subject)
ex + (1 | Subject)
ex + (1 | Subject)
ex + (1 | Subject)
ex + (1 | Subject)
ex + (1 | Subject)
ex + (1 | Subject)
ex + (1 | Subject)
ex + (1 | Subject)
ex + (1 | Subject)
ex + (1 | Subject)
ex + (1 | Subject)

ex + (1 | Subject)
ex + (1 | Subject)
ex + (1 | Subject)
ex + (1 | Subject)
ex + (1 | Subject)
ex + (1 | Subject)
ex + (1 | Subject)
ex + (1 | Subject)
ex + (1 | Subject)
ex + (1 | Subject)
ex + (1 | Subject)
ex + (1 | Subject)
ex + (1 | Subject)
ex + (1 | Subject)
ex + (1 | Subject)
ex + (1 | Subject)
ex + (1 | Subject)
ex + (1 | Subject)
ex + (1 | Subject)
ex + (1 | Subject)
ex + (1 | Subject)
ex + (1 | Subject)
ex + (1 | Subject)
ex + (1 | Subject)
ex + (1 | Subject)
ex + (1 | Subject)
ex + (1 | Subject)
ex + (1 | Subject)
ex + (1 | Subject)
ex + (1 | Subject)
ex + (1 | Subject)
ex + (1 | Subject)
ex + (1 | Subject)
ex + (1 | Subject)
ex + (1 | Subject)
ex + (1 | Subject)
ex + (1 | Subject)
ex + (1 | Subject)
ex + (1 | Subject)
ex + (1 | Subject)
ex + (1 | Subject)
ex + (1 | Subject)
ex + (1 | Subject)
ex + (1 | Subject)
ex + (1 | Subject)
ex + (1 | Subject)
ex + (1 | Subject)
ex + (1 | Subject)

ex + (1 | Subject)
ex + (1 | Subject)
ex + (1 | Subject)
ex + (1 | Subject)
ex + (1 | Subject)
ex + (1 | Subject)
ex + (1 | Subject)
ex + (1 | Subject)
ex + (1 | Subject)
ex + (1 | Subject)
ex + (1 | Subject)
ex + (1 | Subject)
ex + (1 | Subject)
ex + (1 | Subject)
ex + (1 | Subject)
ex + (1 | Subject)
ex + (1 | Subject)
ex + (1 | Subject)
ex + (1 | Subject)
ex + (1 | Subject)
ex + (1 | Subject)
ex + (1 | Subject)
ex + (1 | Subject)
ex + (1 | Subject)
ex + (1 | Subject)
ex + (1 | Subject)
ex + (1 | Subject)
ex + (1 | Subject)
ex + (1 | Subject)
ex + (1 | Subject)
ex + (1 | Subject)
ex + (1 | Subject)
ex + (1 | Subject)
ex + (1 | Subject)
ex + (1 | Subject)
ex + (1 | Subject)
ex + (1 | Subject)
ex + (1 | Subject)
ex + (1 | Subject)
ex + (1 | Subject)
ex + (1 | Subject)
ex + (1 | Subject)
ex + (1 | Subject)
ex + (1 | Subject)
ex + (1 | Subject)
ex + (1 | Subject)
ex + (1 | Subject)
ex + (1 | Subject)

ex + (1 | Subject)
ex + (1 | Subject)
ex + (1 | Subject)
ex + (1 | Subject)
ex + (1 | Subject)
ex + (1 | Subject)
ex + (1 | Subject)
ex + (1 | Subject)
ex + (1 | Subject)
ex + (1 | Subject)
ex + (1 | Subject)
ex + (1 | Subject)
ex + (1 | Subject)
ex + (1 | Subject)
ex + (1 | Subject)
ex + (1 | Subject)
ex + (1 | Subject)
ex + (1 | Subject)
ex + (1 | Subject)
ex + (1 | Subject)
ex + (1 | Subject)
ex + (1 | Subject)
ex + (1 | Subject)
ex + (1 | Subject)
ex + (1 | Subject)
ex + (1 | Subject)
ex + (1 | Subject)
ex + (1 | Subject)
ex + (1 | Subject)
ex + (1 | Subject)
ex + (1 | Subject)
ex + (1 | Subject)
ex + (1 | Subject)
ex + (1 | Subject)
ex + (1 | Subject)
ex + (1 | Subject)
ex + (1 | Subject)
ex + (1 | Subject)
ex + (1 | Subject)
ex + (1 | Subject)
ex + (1 | Subject)
ex + (1 | Subject)
ex + (1 | Subject)
ex + (1 | Subject)
ex + (1 | Subject)
ex + (1 | Subject)
ex + (1 | Subject)
ex + (1 | Subject)

ex + (1 | Subject)
ex + (1 | Subject)
ex + (1 | Subject)
ex + (1 | Subject)
ex + (1 | Subject)
ex + (1 | Subject)
ex + (1 | Subject)
ex + (1 | Subject)
ex + (1 | Subject)
ex + (1 | Subject)
ex + (1 | Subject)
ex + (1 | Subject)
ex + (1 | Subject)
ex + (1 | Subject)
ex + (1 | Subject)
ex + (1 | Subject)
ex + (1 | Subject)
ex + (1 | Subject)
ex + (1 | Subject)
ex + (1 | Subject)
ex + (1 | Subject)
ex + (1 | Subject)
ex + (1 | Subject)
ex + (1 | Subject)
ex + (1 | Subject)
ex + (1 | Subject)
ex + (1 | Subject)
ex + (1 | Subject)
ex + (1 | Subject)
ex + (1 | Subject)
ex + (1 | Subject)
ex + (1 | Subject)
ex + (1 | Subject)
ex + (1 | Subject)
ex + (1 | Subject)
ex + (1 | Subject)
ex + (1 | Subject)
ex + (1 | Subject)
ex + (1 | Subject)
ex + (1 | Subject)
ex + (1 | Subject)
ex + (1 | Subject)
ex + (1 | Subject)
ex + (1 | Subject)
ex + (1 | Subject)
ex + (1 | Subject)
ex + (1 | Subject)
ex + (1 | Subject)

ex + (1 | Subject)
ex + (1 | Subject)
ex + (1 | Subject)
ex + (1 | Subject)
ex + (1 | Subject)
ex + (1 | Subject)
ex + (1 | Subject)
ex + (1 | Subject)
ex + (1 | Subject)
ex + (1 | Subject)
ex + (1 | Subject)
ex + (1 | Subject)
ex + (1 | Subject)
ex + (1 | Subject)
ex + (1 | Subject)
ex + (1 | Subject)
ex + (1 | Subject)
ex + (1 | Subject)
ex + (1 | Subject)
ex + (1 | Subject)
ex + (1 | Subject)
ex + (1 | Subject)
ex + (1 | Subject)
ex + (1 | Subject)
ex + (1 | Subject)
ex + (1 | Subject)
ex + (1 | Subject)
ex + (1 | Subject)
ex + (1 | Subject)
ex + (1 | Subject)
ex + (1 | Subject)
ex + (1 | Subject)
ex + (1 | Subject)
ex + (1 | Subject)
ex + (1 | Subject)
ex + (1 | Subject)
ex + (1 | Subject)
ex + (1 | Subject)
ex + (1 | Subject)
ex + (1 | Subject)
ex + (1 | Subject)
ex + (1 | Subject)
ex + (1 | Subject)
ex + (1 | Subject)
ex + (1 | Subject)
ex + (1 | Subject)
ex + (1 | Subject)
ex + (1 | Subject)

ex + (1 | Subject)
ex + (1 | Subject)
ex + (1 | Subject)
ex + (1 | Subject)
ex + (1 | Subject)
ex + (1 | Subject)
ex + (1 | Subject)
ex + (1 | Subject)
ex + (1 | Subject)
ex + (1 | Subject)
ex + (1 | Subject)
ex + (1 | Subject)
ex + (1 | Subject)
ex + (1 | Subject)
ex + (1 | Subject)
ex + (1 | Subject)
ex + (1 | Subject)
ex + (1 | Subject)
ex + (1 | Subject)
ex + (1 | Subject)
ex + (1 | Subject)
ex + (1 | Subject)
ex + (1 | Subject)
ex + (1 | Subject)
ex + (1 | Subject)
ex + (1 | Subject)
ex + (1 | Subject)
ex + (1 | Subject)
ex + (1 | Subject)
ex + (1 | Subject)
ex + (1 | Subject)
ex + (1 | Subject)
ex + (1 | Subject)
ex + (1 | Subject)
ex + (1 | Subject)
ex + (1 | Subject)
ex + (1 | Subject)
ex + (1 | Subject)
ex + (1 | Subject)
ex + (1 | Subject)
ex + (1 | Subject)
ex + (1 | Subject)
ex + (1 | Subject)
ex + (1 | Subject)
ex + (1 | Subject)
ex + (1 | Subject)
ex + (1 | Subject)
ex + (1 | Subject)

ex + (1 | Subject)
ex + (1 | Subject)
ex + (1 | Subject)
ex + (1 | Subject)
ex + (1 | Subject)
ex + (1 | Subject)
ex + (1 | Subject)
ex + (1 | Subject)
ex + (1 | Subject)
ex + (1 | Subject)
ex + (1 | Subject)
ex + (1 | Subject)
ex + (1 | Subject)
ex + (1 | Subject)
ex + (1 | Subject)
ex + (1 | Subject)
ex + (1 | Subject)
ex + (1 | Subject)
ex + (1 | Subject)
ex + (1 | Subject)
ex + (1 | Subject)
ex + (1 | Subject)
ex + (1 | Subject)
ex + (1 | Subject)
ex + (1 | Subject)
ex + (1 | Subject)
ex + (1 | Subject)
ex + (1 | Subject)
ex + (1 | Subject)
ex + (1 | Subject)
ex + (1 | Subject)
ex + (1 | Subject)
ex + (1 | Subject)
ex + (1 | Subject)
ex + (1 | Subject)
ex + (1 | Subject)
ex + (1 | Subject)
ex + (1 | Subject)
ex + (1 | Subject)
ex + (1 | Subject)
ex + (1 | Subject)
ex + (1 | Subject)
ex + (1 | Subject)
ex + (1 | Subject)
ex + (1 | Subject)
ex + (1 | Subject)
ex + (1 | Subject)
ex + (1 | Subject)

ex + (1 | Subject)
ex + (1 | Subject)
ex + (1 | Subject)
ex + (1 | Subject)
ex + (1 | Subject)
ex + (1 | Subject)
ex + (1 | Subject)
ex + (1 | Subject)
ex + (1 | Subject)
ex + (1 | Subject)
ex + (1 | Subject)
ex + (1 | Subject)
ex + (1 | Subject)
ex + (1 | Subject)
ex + (1 | Subject)
ex + (1 | Subject)
ex + (1 | Subject)
ex + (1 | Subject)
ex + (1 | Subject)
ex + (1 | Subject)
ex + (1 | Subject)
ex + (1 | Subject)
ex + (1 | Subject)
ex + (1 | Subject)
ex + (1 | Subject)
ex + (1 | Subject)
ex + (1 | Subject)
ex + (1 | Subject)
ex + (1 | Subject)
ex + (1 | Subject)
ex + (1 | Subject)
ex + (1 | Subject)
ex + (1 | Subject)
ex + (1 | Subject)
ex + (1 | Subject)
ex + (1 | Subject)
ex + (1 | Subject)
ex + (1 | Subject)
ex + (1 | Subject)
ex + (1 | Subject)
ex + (1 | Subject)
ex + (1 | Subject)
ex + (1 | Subject)
ex + (1 | Subject)
ex + (1 | Subject)
ex + (1 | Subject)
ex + (1 | Subject)
ex + (1 | Subject)

ex + (1 | Subject)
ex + (1 | Subject)
ex + (1 | Subject)
ex + (1 | Subject)
ex + (1 | Subject)
ex + (1 | Subject)
ex + (1 | Subject)
ex + (1 | Subject)
ex + (1 | Subject)
ex + (1 | Subject)
ex + (1 | Subject)
ex + (1 | Subject)
ex + (1 | Subject)
ex + (1 | Subject)
ex + (1 | Subject)
ex + (1 | Subject)
ex + (1 | Subject)
ex + (1 | Subject)
ex + (1 | Subject)
ex + (1 | Subject)
ex + (1 | Subject)
ex + (1 | Subject)
ex + (1 | Subject)
ex + (1 | Subject)
ex + (1 | Subject)
ex + (1 | Subject)
ex + (1 | Subject)
ex + (1 | Subject)
ex + (1 | Subject)
ex + (1 | Subject)
ex + (1 | Subject)
ex + (1 | Subject)
ex + (1 | Subject)
ex + (1 | Subject)
ex + (1 | Subject)
ex + (1 | Subject)
ex + (1 | Subject)
ex + (1 | Subject)
ex + (1 | Subject)
ex + (1 | Subject)
ex + (1 | Subject)
ex + (1 | Subject)
ex + (1 | Subject)
ex + (1 | Subject)
ex + (1 | Subject)
ex + (1 | Subject)
ex + (1 | Subject)
ex + (1 | Subject)

ex + (1 | Subject)
ex + (1 | Subject)
ex + (1 | Subject)
ex + (1 | Subject)
ex + (1 | Subject)
ex + (1 | Subject)
ex + (1 | Subject)
ex + (1 | Subject)
ex + (1 | Subject)
ex + (1 | Subject)
ex + (1 | Subject)
ex + (1 | Subject)
ex + (1 | Subject)
ex + (1 | Subject)
ex + (1 | Subject)
ex + (1 | Subject)
ex + (1 | Subject)
ex + (1 | Subject)
ex + (1 | Subject)
ex + (1 | Subject)
ex + (1 | Subject)
ex + (1 | Subject)
ex + (1 | Subject)
ex + (1 | Subject)
ex + (1 | Subject)
ex + (1 | Subject)
ex + (1 | Subject)
ex + (1 | Subject)
ex + (1 | Subject)
ex + (1 | Subject)
ex + (1 | Subject)
ex + (1 | Subject)
ex + (1 | Subject)
ex + (1 | Subject)
ex + (1 | Subject)
ex + (1 | Subject)
ex + (1 | Subject)
ex + (1 | Subject)
ex + (1 | Subject)
ex + (1 | Subject)
ex + (1 | Subject)
ex + (1 | Subject)
ex + (1 | Subject)
ex + (1 | Subject)
ex + (1 | Subject)
ex + (1 | Subject)
ex + (1 | Subject)
ex + (1 | Subject)

ex + (1 | Subject)
ex + (1 | Subject)
ex + (1 | Subject)
ex + (1 | Subject)
ex + (1 | Subject)
ex + (1 | Subject)
ex + (1 | Subject)
ex + (1 | Subject)
ex + (1 | Subject)
ex + (1 | Subject)
ex + (1 | Subject)
ex + (1 | Subject)
ex + (1 | Subject)
ex + (1 | Subject)
ex + (1 | Subject)
ex + (1 | Subject)
ex + (1 | Subject)
ex + (1 | Subject)
ex + (1 | Subject)
ex + (1 | Subject)
ex + (1 | Subject)
ex + (1 | Subject)
ex + (1 | Subject)
ex + (1 | Subject)
ex + (1 | Subject)
ex + (1 | Subject)
ex + (1 | Subject)
ex + (1 | Subject)
ex + (1 | Subject)
ex + (1 | Subject)
ex + (1 | Subject)
ex + (1 | Subject)
ex + (1 | Subject)
ex + (1 | Subject)
ex + (1 | Subject)
ex + (1 | Subject)
ex + (1 | Subject)
ex + (1 | Subject)
ex + (1 | Subject)
ex + (1 | Subject)
ex + (1 | Subject)
ex + (1 | Subject)
ex + (1 | Subject)
ex + (1 | Subject)
ex + (1 | Subject)
ex + (1 | Subject)
ex + (1 | Subject)
ex + (1 | Subject)

ex + (1 | Subject)
ex + (1 | Subject)
ex + (1 | Subject)
ex + (1 | Subject)
ex + (1 | Subject)
ex + (1 | Subject)
ex + (1 | Subject)
ex + (1 | Subject)
ex + (1 | Subject)
ex + (1 | Subject)
ex + (1 | Subject)
ex + (1 | Subject)
ex + (1 | Subject)
ex + (1 | Subject)
ex + (1 | Subject)
ex + (1 | Subject)
ex + (1 | Subject)
ex + (1 | Subject)
ex + (1 | Subject)
ex + (1 | Subject)
ex + (1 | Subject)
ex + (1 | Subject)
ex + (1 | Subject)
ex + (1 | Subject)
ex + (1 | Subject)
ex + (1 | Subject)
ex + (1 | Subject)
ex + (1 | Subject)
ex + (1 | Subject)
ex + (1 | Subject)
ex + (1 | Subject)
ex + (1 | Subject)
ex + (1 | Subject)
ex + (1 | Subject)
ex + (1 | Subject)
ex + (1 | Subject)
ex + (1 | Subject)
ex + (1 | Subject)
ex + (1 | Subject)
ex + (1 | Subject)
ex + (1 | Subject)
ex + (1 | Subject)
ex + (1 | Subject)
ex + (1 | Subject)
ex + (1 | Subject)
ex + (1 | Subject)
ex + (1 | Subject)
ex + (1 | Subject)

ex + (1 | Subject)
ex + (1 | Subject)
ex + (1 | Subject)
ex + (1 | Subject)
ex + (1 | Subject)
ex + (1 | Subject)
ex + (1 | Subject)
ex + (1 | Subject)
ex + (1 | Subject)
ex + (1 | Subject)
ex + (1 | Subject)
ex + (1 | Subject)
ex + (1 | Subject)
ex + (1 | Subject)
ex + (1 | Subject)
ex + (1 | Subject)
ex + (1 | Subject)
ex + (1 | Subject)
ex + (1 | Subject)
ex + (1 | Subject)
ex + (1 | Subject)
ex + (1 | Subject)
ex + (1 | Subject)
ex + (1 | Subject)
ex + (1 | Subject)
ex + (1 | Subject)
ex + (1 | Subject)
ex + (1 | Subject)
ex + (1 | Subject)
ex + (1 | Subject)
ex + (1 | Subject)
ex + (1 | Subject)
ex + (1 | Subject)
ex + (1 | Subject)
ex + (1 | Subject)
ex + (1 | Subject)
ex + (1 | Subject)
ex + (1 | Subject)
ex + (1 | Subject)
ex + (1 | Subject)
ex + (1 | Subject)
ex + (1 | Subject)
ex + (1 | Subject)
ex + (1 | Subject)
ex + (1 | Subject)
ex + (1 | Subject)
ex + (1 | Subject)
ex + (1 | Subject)

ex + (1 | Subject)
ex + (1 | Subject)
ex + (1 | Subject)
ex + (1 | Subject)
ex + (1 | Subject)
ex + (1 | Subject)
ex + (1 | Subject)
ex + (1 | Subject)
ex + (1 | Subject)
ex + (1 | Subject)
ex + (1 | Subject)
ex + (1 | Subject)
ex + (1 | Subject)
ex + (1 | Subject)
ex + (1 | Subject)
ex + (1 | Subject)
ex + (1 | Subject)
ex + (1 | Subject)
ex + (1 | Subject)
ex + (1 | Subject)
ex + (1 | Subject)
ex + (1 | Subject)
ex + (1 | Subject)
ex + (1 | Subject)
ex + (1 | Subject)
ex + (1 | Subject)
ex + (1 | Subject)
ex + (1 | Subject)
ex + (1 | Subject)
ex + (1 | Subject)
ex + (1 | Subject)
ex + (1 | Subject)
ex + (1 | Subject)
ex + (1 | Subject)
ex + (1 | Subject)
ex + (1 | Subject)
ex + (1 | Subject)
ex + (1 | Subject)
ex + (1 | Subject)
ex + (1 | Subject)
ex + (1 | Subject)
ex + (1 | Subject)
ex + (1 | Subject)
ex + (1 | Subject)
ex + (1 | Subject)
ex + (1 | Subject)
ex + (1 | Subject)
ex + (1 | Subject)

ex + (1 | Subject)
ex + (1 | Subject)
ex + (1 | Subject)
ex + (1 | Subject)
ex + (1 | Subject)
ex + (1 | Subject)
ex + (1 | Subject)
ex + (1 | Subject)
ex + (1 | Subject)
ex + (1 | Subject)
ex + (1 | Subject)
ex + (1 | Subject)
ex + (1 | Subject)
ex + (1 | Subject)
ex + (1 | Subject)
ex + (1 | Subject)
ex + (1 | Subject)
ex + (1 | Subject)
ex + (1 | Subject)
ex + (1 | Subject)
ex + (1 | Subject)
ex + (1 | Subject)
ex + (1 | Subject)
ex + (1 | Subject)
ex + (1 | Subject)
ex + (1 | Subject)
ex + (1 | Subject)
ex + (1 | Subject)
ex + (1 | Subject)
ex + (1 | Subject)
ex + (1 | Subject)
ex + (1 | Subject)
ex + (1 | Subject)
ex + (1 | Subject)
ex + (1 | Subject)
ex + (1 | Subject)
ex + (1 | Subject)
ex + (1 | Subject)
ex + (1 | Subject)
ex + (1 | Subject)
ex + (1 | Subject)
ex + (1 | Subject)
ex + (1 | Subject)
ex + (1 | Subject)
ex + (1 | Subject)
ex + (1 | Subject)
ex + (1 | Subject)
ex + (1 | Subject)

ex + (1 | Subject)
ex + (1 | Subject)
ex + (1 | Subject)
ex + (1 | Subject)
ex + (1 | Subject)
ex + (1 | Subject)
ex + (1 | Subject)
ex + (1 | Subject)
ex + (1 | Subject)
ex + (1 | Subject)
ex + (1 | Subject)
ex + (1 | Subject)
ex + (1 | Subject)
ex + (1 | Subject)
ex + (1 | Subject)
ex + (1 | Subject)
ex + (1 | Subject)
ex + (1 | Subject)
ex + (1 | Subject)
ex + (1 | Subject)
ex + (1 | Subject)
ex + (1 | Subject)
ex + (1 | Subject)
ex + (1 | Subject)
ex + (1 | Subject)
ex + (1 | Subject)
ex + (1 | Subject)
ex + (1 | Subject)
ex + (1 | Subject)
ex + (1 | Subject)
ex + (1 | Subject)
ex + (1 | Subject)
ex + (1 | Subject)
ex + (1 | Subject)
ex + (1 | Subject)
ex + (1 | Subject)
ex + (1 | Subject)
ex + (1 | Subject)
ex + (1 | Subject)
ex + (1 | Subject)
ex + (1 | Subject)
ex + (1 | Subject)
ex + (1 | Subject)
ex + (1 | Subject)
ex + (1 | Subject)
ex + (1 | Subject)
ex + (1 | Subject)
ex + (1 | Subject)

ex + (1 | Subject)
ex + (1 | Subject)
ex + (1 | Subject)
ex + (1 | Subject)
ex + (1 | Subject)
ex + (1 | Subject)
ex + (1 | Subject)
ex + (1 | Subject)
ex + (1 | Subject)
ex + (1 | Subject)
ex + (1 | Subject)
ex + (1 | Subject)
ex + (1 | Subject)
ex + (1 | Subject)
ex + (1 | Subject)
ex + (1 | Subject)
ex + (1 | Subject)
ex + (1 | Subject)
ex + (1 | Subject)
ex + (1 | Subject)
ex + (1 | Subject)
ex + (1 | Subject)
ex + (1 | Subject)
ex + (1 | Subject)
ex + (1 | Subject)
ex + (1 | Subject)
ex + (1 | Subject)
ex + (1 | Subject)
ex + (1 | Subject)
ex + (1 | Subject)
ex + (1 | Subject)
ex + (1 | Subject)
ex + (1 | Subject)
ex + (1 | Subject)
ex + (1 | Subject)
ex + (1 | Subject)
ex + (1 | Subject)
ex + (1 | Subject)
ex + (1 | Subject)
ex + (1 | Subject)
ex + (1 | Subject)
ex + (1 | Subject)
ex + (1 | Subject)
ex + (1 | Subject)
ex + (1 | Subject)
ex + (1 | Subject)
ex + (1 | Subject)
ex + (1 | Subject)

ex + (1 | Subject)
ex + (1 | Subject)
ex + (1 | Subject)
ex + (1 | Subject)
ex + (1 | Subject)
ex + (1 | Subject)
ex + (1 | Subject)
ex + (1 | Subject)
ex + (1 | Subject)
ex + (1 | Subject)
ex + (1 | Subject)
ex + (1 | Subject)
ex + (1 | Subject)
ex + (1 | Subject)
ex + (1 | Subject)
ex + (1 | Subject)
ex + (1 | Subject)
ex + (1 | Subject)
ex + (1 | Subject)
ex + (1 | Subject)
ex + (1 | Subject)
ex + (1 | Subject)
ex + (1 | Subject)
ex + (1 | Subject)
ex + (1 | Subject)
ex + (1 | Subject)
ex + (1 | Subject)
ex + (1 | Subject)
ex + (1 | Subject)
ex + (1 | Subject)
ex + (1 | Subject)
ex + (1 | Subject)
ex + (1 | Subject)
ex + (1 | Subject)
ex + (1 | Subject)
ex + (1 | Subject)
ex + (1 | Subject)
ex + (1 | Subject)
ex + (1 | Subject)
ex + (1 | Subject)
ex + (1 | Subject)
ex + (1 | Subject)
ex + (1 | Subject)
ex + (1 | Subject)
ex + (1 | Subject)
ex + (1 | Subject)
ex + (1 | Subject)
ex + (1 | Subject)

ex + (1 | Subject)
ex + (1 | Subject)
ex + (1 | Subject)
ex + (1 | Subject)
ex + (1 | Subject)
ex + (1 | Subject)
ex + (1 | Subject)
ex + (1 | Subject)
ex + (1 | Subject)
ex + (1 | Subject)
ex + (1 | Subject)
ex + (1 | Subject)
ex + (1 | Subject)
ex + (1 | Subject)
ex + (1 | Subject)
ex + (1 | Subject)
ex + (1 | Subject)
ex + (1 | Subject)
ex + (1 | Subject)
ex + (1 | Subject)
ex + (1 | Subject)
ex + (1 | Subject)
ex + (1 | Subject)
ex + (1 | Subject)
ex + (1 | Subject)
ex + (1 | Subject)
ex + (1 | Subject)
ex + (1 | Subject)
ex + (1 | Subject)
ex + (1 | Subject)
ex + (1 | Subject)
ex + (1 | Subject)
ex + (1 | Subject)
ex + (1 | Subject)
ex + (1 | Subject)
ex + (1 | Subject)
ex + (1 | Subject)
ex + (1 | Subject)
ex + (1 | Subject)
ex + (1 | Subject)
ex + (1 | Subject)
ex + (1 | Subject)
ex + (1 | Subject)
ex + (1 | Subject)
ex + (1 | Subject)
ex + (1 | Subject)
ex + (1 | Subject)
ex + (1 | Subject)

ex + (1 | Subject)
ex + (1 | Subject)
ex + (1 | Subject)
ex + (1 | Subject)
ex + (1 | Subject)
ex + (1 | Subject)
ex + (1 | Subject)
ex + (1 | Subject)
ex + (1 | Subject)
ex + (1 | Subject)
ex + (1 | Subject)
ex + (1 | Subject)
ex + (1 | Subject)
ex + (1 | Subject)
ex + (1 | Subject)
ex + (1 | Subject)
ex + (1 | Subject)
ex + (1 | Subject)
ex + (1 | Subject)
ex + (1 | Subject)
ex + (1 | Subject)
ex + (1 | Subject)
ex + (1 | Subject)
ex + (1 | Subject)
ex + (1 | Subject)
ex + (1 | Subject)
ex + (1 | Subject)
ex + (1 | Subject)
ex + (1 | Subject)
ex + (1 | Subject)
ex + (1 | Subject)
ex + (1 | Subject)
ex + (1 | Subject)
ex + (1 | Subject)
ex + (1 | Subject)
ex + (1 | Subject)
ex + (1 | Subject)
ex + (1 | Subject)
ex + (1 | Subject)
ex + (1 | Subject)
ex + (1 | Subject)
ex + (1 | Subject)
ex + (1 | Subject)
ex + (1 | Subject)
ex + (1 | Subject)
ex + (1 | Subject)
ex + (1 | Subject)
ex + (1 | Subject)

ex + (1 | Subject)
ex + (1 | Subject)
ex + (1 | Subject)
ex + (1 | Subject)
ex + (1 | Subject)
ex + (1 | Subject)
ex + (1 | Subject)
ex + (1 | Subject)
ex + (1 | Subject)
ex + (1 | Subject)
ex + (1 | Subject)
ex + (1 | Subject)
ex + (1 | Subject)
ex + (1 | Subject)
ex + (1 | Subject)
ex + (1 | Subject)
ex + (1 | Subject)
ex + (1 | Subject)
ex + (1 | Subject)
ex + (1 | Subject)
ex + (1 | Subject)
ex + (1 | Subject)
ex + (1 | Subject)
ex + (1 | Subject)
ex + (1 | Subject)
ex + (1 | Subject)
ex + (1 | Subject)
ex + (1 | Subject)
ex + (1 | Subject)
ex + (1 | Subject)
ex + (1 | Subject)
ex + (1 | Subject)
ex + (1 | Subject)
ex + (1 | Subject)
ex + (1 | Subject)
ex + (1 | Subject)
ex + (1 | Subject)
ex + (1 | Subject)
ex + (1 | Subject)
ex + (1 | Subject)
ex + (1 | Subject)
ex + (1 | Subject)
ex + (1 | Subject)
ex + (1 | Subject)
ex + (1 | Subject)
ex + (1 | Subject)
ex + (1 | Subject)
ex + (1 | Subject)

ex + (1 | Subject)
ex + (1 | Subject)
ex + (1 | Subject)
ex + (1 | Subject)
ex + (1 | Subject)
ex + (1 | Subject)
ex + (1 | Subject)
ex + (1 | Subject)
ex + (1 | Subject)
ex + (1 | Subject)
ex + (1 | Subject)
ex + (1 | Subject)
ex + (1 | Subject)
ex + (1 | Subject)
ex + (1 | Subject)
ex + (1 | Subject)
ex + (1 | Subject)
ex + (1 | Subject)
ex + (1 | Subject)
ex + (1 | Subject)
ex + (1 | Subject)
ex + (1 | Subject)
ex + (1 | Subject)
ex + (1 | Subject)
ex + (1 | Subject)
ex + (1 | Subject)
ex + (1 | Subject)
ex + (1 | Subject)
ex + (1 | Subject)
ex + (1 | Subject)
ex + (1 | Subject)
ex + (1 | Subject)
ex + (1 | Subject)
ex + (1 | Subject)
ex + (1 | Subject)
ex + (1 | Subject)
ex + (1 | Subject)
ex + (1 | Subject)
ex + (1 | Subject)
ex + (1 | Subject)
ex + (1 | Subject)
ex + (1 | Subject)
ex + (1 | Subject)
ex + (1 | Subject)
ex + (1 | Subject)
ex + (1 | Subject)
ex + (1 | Subject)
ex + (1 | Subject)

ex + (1 | Subject)
ex + (1 | Subject)
ex + (1 | Subject)
ex + (1 | Subject)
ex + (1 | Subject)
ex + (1 | Subject)
ex + (1 | Subject)
ex + (1 | Subject)
ex + (1 | Subject)
ex + (1 | Subject)
ex + (1 | Subject)
ex + (1 | Subject)
ex + (1 | Subject)
ex + (1 | Subject)
ex + (1 | Subject)
ex + (1 | Subject)
ex + (1 | Subject)
ex + (1 | Subject)
ex + (1 | Subject)
ex + (1 | Subject)
ex + (1 | Subject)
ex + (1 | Subject)
ex + (1 | Subject)
ex + (1 | Subject)
ex + (1 | Subject)
ex + (1 | Subject)
ex + (1 | Subject)
ex + (1 | Subject)
ex + (1 | Subject)
ex + (1 | Subject)
ex + (1 | Subject)
ex + (1 | Subject)
ex + (1 | Subject)
ex + (1 | Subject)
ex + (1 | Subject)
ex + (1 | Subject)
ex + (1 | Subject)
ex + (1 | Subject)
ex + (1 | Subject)
ex + (1 | Subject)
ex + (1 | Subject)
ex + (1 | Subject)
ex + (1 | Subject)
ex + (1 | Subject)
ex + (1 | Subject)
ex + (1 | Subject)
ex + (1 | Subject)
ex + (1 | Subject)

ex + (1 | Subject)
ex + (1 | Subject)
ex + (1 | Subject)
ex + (1 | Subject)
ex + (1 | Subject)
ex + (1 | Subject)
ex + (1 | Subject)
ex + (1 | Subject)
ex + (1 | Subject)
ex + (1 | Subject)
ex + (1 | Subject)
ex + (1 | Subject)
ex + (1 | Subject)
ex + (1 | Subject)
ex + (1 | Subject)
ex + (1 | Subject)
ex + (1 | Subject)
ex + (1 | Subject)
ex + (1 | Subject)
ex + (1 | Subject)
ex + (1 | Subject)
ex + (1 | Subject)
ex + (1 | Subject)
ex + (1 | Subject)
ex + (1 | Subject)
ex + (1 | Subject)
ex + (1 | Subject)
ex + (1 | Subject)
ex + (1 | Subject)
ex + (1 | Subject)
ex + (1 | Subject)
ex + (1 | Subject)
ex + (1 | Subject)
ex + (1 | Subject)
ex + (1 | Subject)
ex + (1 | Subject)
ex + (1 | Subject)
ex + (1 | Subject)
ex + (1 | Subject)
ex + (1 | Subject)
ex + (1 | Subject)
ex + (1 | Subject)
ex + (1 | Subject)
ex + (1 | Subject)
ex + (1 | Subject)
ex + (1 | Subject)
ex + (1 | Subject)
ex + (1 | Subject)

ex + (1 | Subject)
ex + (1 | Subject)
ex + (1 | Subject)
ex + (1 | Subject)
ex + (1 | Subject)
ex + (1 | Subject)
ex + (1 | Subject)
ex + (1 | Subject)
ex + (1 | Subject)
ex + (1 | Subject)
ex + (1 | Subject)
ex + (1 | Subject)
ex + (1 | Subject)
ex + (1 | Subject)
ex + (1 | Subject)
ex + (1 | Subject)
ex + (1 | Subject)
ex + (1 | Subject)
ex + (1 | Subject)
ex + (1 | Subject)
ex + (1 | Subject)
ex + (1 | Subject)
ex + (1 | Subject)
ex + (1 | Subject)
ex + (1 | Subject)
ex + (1 | Subject)
ex + (1 | Subject)
ex + (1 | Subject)
ex + (1 | Subject)
ex + (1 | Subject)
ex + (1 | Subject)
ex + (1 | Subject)
ex + (1 | Subject)
ex + (1 | Subject)
ex + (1 | Subject)
ex + (1 | Subject)
ex + (1 | Subject)
ex + (1 | Subject)
ex + (1 | Subject)
ex + (1 | Subject)
ex + (1 | Subject)
ex + (1 | Subject)
ex + (1 | Subject)
ex + (1 | Subject)
ex + (1 | Subject)
ex + (1 | Subject)
ex + (1 | Subject)
ex + (1 | Subject)

ex + (1 | Subject)
ex + (1 | Subject)
ex + (1 | Subject)
ex + (1 | Subject)
ex + (1 | Subject)
ex + (1 | Subject)
ex + (1 | Subject)
ex + (1 | Subject)
ex + (1 | Subject)
ex + (1 | Subject)
ex + (1 | Subject)
ex + (1 | Subject)
ex + (1 | Subject)
ex + (1 | Subject)
ex + (1 | Subject)
ex + (1 | Subject)
ex + (1 | Subject)
ex + (1 | Subject)
ex + (1 | Subject)
ex + (1 | Subject)
ex + (1 | Subject)
ex + (1 | Subject)
ex + (1 | Subject)
ex + (1 | Subject)
ex + (1 | Subject)
ex + (1 | Subject)
ex + (1 | Subject)
ex + (1 | Subject)
ex + (1 | Subject)
ex + (1 | Subject)
ex + (1 | Subject)
ex + (1 | Subject)
ex + (1 | Subject)
ex + (1 | Subject)
ex + (1 | Subject)
ex + (1 | Subject)
ex + (1 | Subject)
ex + (1 | Subject)
ex + (1 | Subject)
ex + (1 | Subject)
ex + (1 | Subject)
ex + (1 | Subject)
ex + (1 | Subject)
ex + (1 | Subject)
ex + (1 | Subject)
ex + (1 | Subject)
ex + (1 | Subject)
ex + (1 | Subject)

ex + (1 | Subject)
ex + (1 | Subject)
ex + (1 | Subject)
ex + (1 | Subject)
ex + (1 | Subject)
ex + (1 | Subject)
ex + (1 | Subject)
ex + (1 | Subject)
ex + (1 | Subject)
ex + (1 | Subject)
ex + (1 | Subject)
ex + (1 | Subject)
ex + (1 | Subject)
ex + (1 | Subject)
ex + (1 | Subject)
ex + (1 | Subject)
ex + (1 | Subject)
ex + (1 | Subject)
ex + (1 | Subject)
ex + (1 | Subject)
ex + (1 | Subject)
ex + (1 | Subject)
ex + (1 | Subject)
ex + (1 | Subject)
ex + (1 | Subject)
ex + (1 | Subject)
ex + (1 | Subject)
ex + (1 | Subject)
ex + (1 | Subject)
ex + (1 | Subject)
ex + (1 | Subject)
ex + (1 | Subject)
ex + (1 | Subject)
ex + (1 | Subject)
ex + (1 | Subject)
ex + (1 | Subject)
ex + (1 | Subject)
ex + (1 | Subject)
ex + (1 | Subject)
ex + (1 | Subject)
ex + (1 | Subject)
ex + (1 | Subject)
ex + (1 | Subject)
ex + (1 | Subject)
ex + (1 | Subject)
ex + (1 | Subject)
ex + (1 | Subject)
ex + (1 | Subject)

ex + (1 | Subject)
ex + (1 | Subject)
ex + (1 | Subject)
ex + (1 | Subject)
ex + (1 | Subject)
ex + (1 | Subject)
ex + (1 | Subject)
ex + (1 | Subject)
ex + (1 | Subject)
ex + (1 | Subject)
ex + (1 | Subject)
ex + (1 | Subject)
ex + (1 | Subject)
ex + (1 | Subject)
ex + (1 | Subject)
ex + (1 | Subject)
ex + (1 | Subject)
ex + (1 | Subject)
ex + (1 | Subject)
ex + (1 | Subject)
ex + (1 | Subject)
ex + (1 | Subject)
ex + (1 | Subject)
ex + (1 | Subject)
ex + (1 | Subject)
ex + (1 | Subject)
ex + (1 | Subject)
ex + (1 | Subject)
ex + (1 | Subject)
ex + (1 | Subject)
ex + (1 | Subject)
ex + (1 | Subject)
ex + (1 | Subject)
ex + (1 | Subject)
ex + (1 | Subject)
ex + (1 | Subject)
ex + (1 | Subject)
ex + (1 | Subject)
ex + (1 | Subject)
ex + (1 | Subject)
ex + (1 | Subject)
ex + (1 | Subject)
ex + (1 | Subject)
ex + (1 | Subject)
ex + (1 | Subject)
ex + (1 | Subject)
ex + (1 | Subject)
ex + (1 | Subject)

ex + (1 | Subject)
ex + (1 | Subject)
ex + (1 | Subject)
ex + (1 | Subject)
ex + (1 | Subject)
ex + (1 | Subject)
ex + (1 | Subject)
ex + (1 | Subject)
ex + (1 | Subject)
ex + (1 | Subject)
ex + (1 | Subject)
ex + (1 | Subject)
ex + (1 | Subject)
ex + (1 | Subject)
ex + (1 | Subject)
ex + (1 | Subject)
ex + (1 | Subject)
ex + (1 | Subject)
ex + (1 | Subject)
ex + (1 | Subject)
ex + (1 | Subject)
ex + (1 | Subject)
ex + (1 | Subject)
ex + (1 | Subject)
ex + (1 | Subject)
ex + (1 | Subject)
ex + (1 | Subject)
ex + (1 | Subject)
ex + (1 | Subject)
ex + (1 | Subject)
ex + (1 | Subject)
ex + (1 | Subject)
ex + (1 | Subject)
ex + (1 | Subject)
ex + (1 | Subject)
ex + (1 | Subject)
ex + (1 | Subject)
ex + (1 | Subject)
ex + (1 | Subject)
ex + (1 | Subject)
ex + (1 | Subject)
ex + (1 | Subject)
ex + (1 | Subject)
ex + (1 | Subject)
ex + (1 | Subject)
ex + (1 | Subject)
ex + (1 | Subject)
ex + (1 | Subject)

ex + (1 | Subject)
ex + (1 | Subject)
ex + (1 | Subject)
ex + (1 | Subject)
ex + (1 | Subject)
ex + (1 | Subject)
ex + (1 | Subject)
ex + (1 | Subject)
ex + (1 | Subject)
ex + (1 | Subject)
ex + (1 | Subject)
ex + (1 | Subject)
ex + (1 | Subject)
ex + (1 | Subject)
ex + (1 | Subject)
ex + (1 | Subject)
ex + (1 | Subject)
ex + (1 | Subject)
ex + (1 | Subject)
ex + (1 | Subject)
ex + (1 | Subject)
ex + (1 | Subject)
ex + (1 | Subject)
ex + (1 | Subject)
ex + (1 | Subject)
ex + (1 | Subject)
ex + (1 | Subject)
ex + (1 | Subject)
ex + (1 | Subject)
ex + (1 | Subject)
ex + (1 | Subject)
ex + (1 | Subject)
ex + (1 | Subject)
ex + (1 | Subject)
ex + (1 | Subject)
ex + (1 | Subject)
ex + (1 | Subject)
ex + (1 | Subject)
ex + (1 | Subject)
ex + (1 | Subject)
ex + (1 | Subject)
ex + (1 | Subject)
ex + (1 | Subject)
ex + (1 | Subject)
ex + (1 | Subject)
ex + (1 | Subject)
ex + (1 | Subject)
ex + (1 | Subject)

ex + (1 | Subject)
ex + (1 | Subject)
ex + (1 | Subject)
ex + (1 | Subject)
ex + (1 | Subject)
ex + (1 | Subject)
ex + (1 | Subject)
ex + (1 | Subject)
ex + (1 | Subject)
ex + (1 | Subject)
ex + (1 | Subject)
ex + (1 | Subject)
ex + (1 | Subject)
ex + (1 | Subject)
ex + (1 | Subject)
ex + (1 | Subject)
ex + (1 | Subject)
ex + (1 | Subject)
ex + (1 | Subject)
ex + (1 | Subject)
ex + (1 | Subject)
ex + (1 | Subject)
ex + (1 | Subject)
ex + (1 | Subject)
ex + (1 | Subject)
ex + (1 | Subject)
ex + (1 | Subject)
ex + (1 | Subject)
ex + (1 | Subject)
ex + (1 | Subject)
ex + (1 | Subject)
ex + (1 | Subject)
ex + (1 | Subject)
ex + (1 | Subject)
ex + (1 | Subject)
ex + (1 | Subject)
ex + (1 | Subject)
ex + (1 | Subject)
ex + (1 | Subject)
ex + (1 | Subject)
ex + (1 | Subject)
ex + (1 | Subject)
ex + (1 | Subject)
ex + (1 | Subject)
ex + (1 | Subject)
ex + (1 | Subject)
ex + (1 | Subject)
ex + (1 | Subject)

ex + (1 | Subject)
ex + (1 | Subject)
ex + (1 | Subject)
ex + (1 | Subject)
ex + (1 | Subject)
ex + (1 | Subject)
ex + (1 | Subject)
ex + (1 | Subject)
ex + (1 | Subject)
ex + (1 | Subject)
ex + (1 | Subject)
ex + (1 | Subject)
ex + (1 | Subject)
ex + (1 | Subject)
ex + (1 | Subject)
ex + (1 | Subject)
ex + (1 | Subject)
ex + (1 | Subject)
ex + (1 | Subject)
ex + (1 | Subject)
ex + (1 | Subject)
ex + (1 | Subject)
ex + (1 | Subject)
ex + (1 | Subject)
ex + (1 | Subject)
ex + (1 | Subject)
ex + (1 | Subject)
ex + (1 | Subject)
ex + (1 | Subject)
ex + (1 | Subject)
ex + (1 | Subject)
ex + (1 | Subject)
ex + (1 | Subject)
ex + (1 | Subject)
ex + (1 | Subject)
ex + (1 | Subject)
ex + (1 | Subject)
ex + (1 | Subject)
ex + (1 | Subject)
ex + (1 | Subject)
ex + (1 | Subject)
ex + (1 | Subject)
ex + (1 | Subject)
ex + (1 | Subject)
ex + (1 | Subject)
ex + (1 | Subject)
ex + (1 | Subject)
ex + (1 | Subject)

ex + (1 | Subject)
ex + (1 | Subject)
ex + (1 | Subject)
ex + (1 | Subject)
ex + (1 | Subject)
ex + (1 | Subject)
ex + (1 | Subject)
ex + (1 | Subject)
ex + (1 | Subject)
ex + (1 | Subject)
ex + (1 | Subject)
ex + (1 | Subject)
ex + (1 | Subject)
ex + (1 | Subject)
ex + (1 | Subject)
ex + (1 | Subject)
ex + (1 | Subject)
ex + (1 | Subject)
ex + (1 | Subject)
ex + (1 | Subject)
ex + (1 | Subject)
ex + (1 | Subject)
ex + (1 | Subject)
ex + (1 | Subject)
ex + (1 | Subject)
ex + (1 | Subject)
ex + (1 | Subject)
ex + (1 | Subject)
ex + (1 | Subject)
ex + (1 | Subject)
ex + (1 | Subject)
ex + (1 | Subject)
ex + (1 | Subject)
ex + (1 | Subject)
ex + (1 | Subject)
ex + (1 | Subject)
ex + (1 | Subject)
ex + (1 | Subject)
ex + (1 | Subject)
ex + (1 | Subject)
ex + (1 | Subject)
ex + (1 | Subject)
ex + (1 | Subject)
ex + (1 | Subject)
ex + (1 | Subject)
ex + (1 | Subject)
ex + (1 | Subject)
ex + (1 | Subject)

ex + (1 | Subject)
ex + (1 | Subject)
ex + (1 | Subject)
ex + (1 | Subject)
ex + (1 | Subject)
ex + (1 | Subject)
ex + (1 | Subject)
ex + (1 | Subject)
ex + (1 | Subject)
ex + (1 | Subject)
ex + (1 | Subject)
ex + (1 | Subject)
ex + (1 | Subject)
ex + (1 | Subject)
ex + (1 | Subject)
ex + (1 | Subject)
ex + (1 | Subject)
ex + (1 | Subject)
ex + (1 | Subject)
ex + (1 | Subject)
ex + (1 | Subject)
ex + (1 | Subject)
ex + (1 | Subject)
ex + (1 | Subject)
ex + (1 | Subject)
ex + (1 | Subject)
ex + (1 | Subject)
ex + (1 | Subject)
ex + (1 | Subject)
ex + (1 | Subject)
ex + (1 | Subject)
ex + (1 | Subject)
ex + (1 | Subject)
ex + (1 | Subject)
ex + (1 | Subject)
ex + (1 | Subject)
ex + (1 | Subject)
ex + (1 | Subject)
ex + (1 | Subject)
ex + (1 | Subject)
ex + (1 | Subject)
ex + (1 | Subject)
ex + (1 | Subject)
ex + (1 | Subject)
ex + (1 | Subject)
ex + (1 | Subject)
ex + (1 | Subject)
ex + (1 | Subject)

ex + (1 | Subject)
ex + (1 | Subject)
ex + (1 | Subject)
ex + (1 | Subject)
ex + (1 | Subject)
ex + (1 | Subject)
ex + (1 | Subject)
ex + (1 | Subject)
ex + (1 | Subject)
ex + (1 | Subject)
ex + (1 | Subject)
ex + (1 | Subject)
ex + (1 | Subject)
ex + (1 | Subject)
ex + (1 | Subject)
ex + (1 | Subject)
ex + (1 | Subject)
ex + (1 | Subject)
ex + (1 | Subject)
ex + (1 | Subject)
ex + (1 | Subject)
ex + (1 | Subject)
ex + (1 | Subject)
ex + (1 | Subject)
ex + (1 | Subject)
ex + (1 | Subject)
ex + (1 | Subject)
ex + (1 | Subject)
ex + (1 | Subject)
ex + (1 | Subject)
ex + (1 | Subject)
ex + (1 | Subject)
ex + (1 | Subject)
ex + (1 | Subject)
ex + (1 | Subject)
ex + (1 | Subject)
ex + (1 | Subject)
ex + (1 | Subject)
ex + (1 | Subject)
ex + (1 | Subject)
ex + (1 | Subject)
ex + (1 | Subject)
ex + (1 | Subject)
ex + (1 | Subject)
ex + (1 | Subject)
ex + (1 | Subject)
ex + (1 | Subject)
ex + (1 | Subject)

ex + (1 | Subject)
ex + (1 | Subject)
ex + (1 | Subject)
ex + (1 | Subject)
ex + (1 | Subject)
ex + (1 | Subject)
ex + (1 | Subject)
ex + (1 | Subject)
ex + (1 | Subject)
ex + (1 | Subject)
ex + (1 | Subject)
ex + (1 | Subject)
ex + (1 | Subject)
ex + (1 | Subject)
ex + (1 | Subject)
ex + (1 | Subject)
ex + (1 | Subject)
ex + (1 | Subject)
ex + (1 | Subject)
ex + (1 | Subject)
ex + (1 | Subject)
ex + (1 | Subject)
ex + (1 | Subject)
ex + (1 | Subject)
ex + (1 | Subject)
ex + (1 | Subject)
ex + (1 | Subject)
ex + (1 | Subject)
ex + (1 | Subject)
ex + (1 | Subject)
ex + (1 | Subject)
ex + (1 | Subject)
ex + (1 | Subject)
ex + (1 | Subject)
ex + (1 | Subject)
ex + (1 | Subject)
ex + (1 | Subject)
ex + (1 | Subject)
ex + (1 | Subject)
ex + (1 | Subject)
ex + (1 | Subject)
ex + (1 | Subject)
ex + (1 | Subject)
ex + (1 | Subject)
ex + (1 | Subject)
ex + (1 | Subject)
ex + (1 | Subject)
ex + (1 | Subject)

ex + (1 | Subject)
ex + (1 | Subject)
ex + (1 | Subject)
ex + (1 | Subject)
ex + (1 | Subject)
ex + (1 | Subject)
ex + (1 | Subject)
ex + (1 | Subject)
ex + (1 | Subject)
ex + (1 | Subject)
ex + (1 | Subject)
ex + (1 | Subject)
ex + (1 | Subject)
ex + (1 | Subject)
ex + (1 | Subject)
ex + (1 | Subject)
ex + (1 | Subject)
ex + (1 | Subject)
ex + (1 | Subject)
ex + (1 | Subject)
ex + (1 | Subject)
ex + (1 | Subject)
ex + (1 | Subject)
ex + (1 | Subject)
ex + (1 | Subject)
ex + (1 | Subject)
ex + (1 | Subject)
ex + (1 | Subject)
ex + (1 | Subject)
ex + (1 | Subject)
ex + (1 | Subject)
ex + (1 | Subject)
ex + (1 | Subject)
ex + (1 | Subject)
ex + (1 | Subject)
ex + (1 | Subject)
ex + (1 | Subject)
ex + (1 | Subject)
ex + (1 | Subject)
ex + (1 | Subject)
ex + (1 | Subject)
ex + (1 | Subject)
ex + (1 | Subject)
ex + (1 | Subject)
ex + (1 | Subject)
ex + (1 | Subject)
ex + (1 | Subject)
ex + (1 | Subject)

ex + (1 | Subject)
ex + (1 | Subject)
ex + (1 | Subject)
ex + (1 | Subject)
ex + (1 | Subject)
ex + (1 | Subject)
ex + (1 | Subject)
ex + (1 | Subject)
ex + (1 | Subject)
ex + (1 | Subject)
ex + (1 | Subject)
ex + (1 | Subject)
ex + (1 | Subject)
ex + (1 | Subject)
ex + (1 | Subject)
ex + (1 | Subject)
ex + (1 | Subject)
ex + (1 | Subject)
ex + (1 | Subject)
ex + (1 | Subject)
ex + (1 | Subject)
ex + (1 | Subject)
ex + (1 | Subject)
ex + (1 | Subject)
ex + (1 | Subject)
ex + (1 | Subject)
ex + (1 | Subject)
ex + (1 | Subject)
ex + (1 | Subject)
ex + (1 | Subject)
ex + (1 | Subject)
ex + (1 | Subject)
ex + (1 | Subject)
ex + (1 | Subject)
ex + (1 | Subject)
ex + (1 | Subject)
ex + (1 | Subject)
ex + (1 | Subject)
ex + (1 | Subject)
ex + (1 | Subject)
ex + (1 | Subject)
ex + (1 | Subject)
ex + (1 | Subject)
ex + (1 | Subject)
ex + (1 | Subject)
ex + (1 | Subject)
ex + (1 | Subject)
ex + (1 | Subject)

ex + (1 | Subject)
ex + (1 | Subject)
ex + (1 | Subject)
ex + (1 | Subject)
ex + (1 | Subject)
ex + (1 | Subject)
ex + (1 | Subject)
ex + (1 | Subject)
ex + (1 | Subject)
ex + (1 | Subject)
ex + (1 | Subject)
ex + (1 | Subject)
ex + (1 | Subject)
ex + (1 | Subject)
ex + (1 | Subject)
ex + (1 | Subject)
ex + (1 | Subject)
ex + (1 | Subject)
ex + (1 | Subject)
ex + (1 | Subject)
ex + (1 | Subject)
ex + (1 | Subject)
ex + (1 | Subject)
ex + (1 | Subject)
ex + (1 | Subject)
ex + (1 | Subject)
ex + (1 | Subject)
ex + (1 | Subject)
ex + (1 | Subject)
ex + (1 | Subject)
ex + (1 | Subject)
ex + (1 | Subject)
ex + (1 | Subject)
ex + (1 | Subject)
ex + (1 | Subject)
ex + (1 | Subject)
ex + (1 | Subject)
ex + (1 | Subject)
ex + (1 | Subject)
ex + (1 | Subject)
ex + (1 | Subject)
ex + (1 | Subject)
ex + (1 | Subject)
ex + (1 | Subject)
ex + (1 | Subject)
ex + (1 | Subject)
ex + (1 | Subject)
ex + (1 | Subject)

ex + (1 | Subject)
ex + (1 | Subject)
ex + (1 | Subject)
ex + (1 | Subject)
ex + (1 | Subject)
ex + (1 | Subject)
ex + (1 | Subject)
ex + (1 | Subject)
ex + (1 | Subject)
ex + (1 | Subject)
ex + (1 | Subject)
ex + (1 | Subject)
ex + (1 | Subject)
ex + (1 | Subject)
ex + (1 | Subject)
ex + (1 | Subject)
ex + (1 | Subject)
ex + (1 | Subject)
ex + (1 | Subject)
ex + (1 | Subject)
ex + (1 | Subject)
ex + (1 | Subject)
ex + (1 | Subject)
ex + (1 | Subject)
ex + (1 | Subject)
ex + (1 | Subject)
ex + (1 | Subject)
ex + (1 | Subject)
ex + (1 | Subject)
ex + (1 | Subject)
ex + (1 | Subject)
ex + (1 | Subject)
ex + (1 | Subject)
ex + (1 | Subject)
ex + (1 | Subject)
ex + (1 | Subject)
ex + (1 | Subject)
ex + (1 | Subject)
ex + (1 | Subject)
ex + (1 | Subject)
ex + (1 | Subject)
ex + (1 | Subject)
ex + (1 | Subject)
ex + (1 | Subject)
ex + (1 | Subject)
ex + (1 | Subject)
ex + (1 | Subject)
ex + (1 | Subject)

ex + (1 | Subject)
ex + (1 | Subject)
ex + (1 | Subject)
ex + (1 | Subject)
ex + (1 | Subject)
ex + (1 | Subject)
ex + (1 | Subject)
ex + (1 | Subject)
ex + (1 | Subject)
ex + (1 | Subject)
ex + (1 | Subject)
ex + (1 | Subject)
ex + (1 | Subject)
ex + (1 | Subject)
ex + (1 | Subject)
ex + (1 | Subject)
ex + (1 | Subject)
ex + (1 | Subject)
ex + (1 | Subject)
ex + (1 | Subject)
ex + (1 | Subject)
ex + (1 | Subject)
ex + (1 | Subject)
ex + (1 | Subject)
ex + (1 | Subject)
ex + (1 | Subject)
ex + (1 | Subject)
ex + (1 | Subject)
ex + (1 | Subject)
ex + (1 | Subject)
ex + (1 | Subject)
ex + (1 | Subject)
ex + (1 | Subject)
ex + (1 | Subject)
ex + (1 | Subject)
ex + (1 | Subject)
ex + (1 | Subject)
ex + (1 | Subject)
ex + (1 | Subject)
ex + (1 | Subject)
ex + (1 | Subject)
ex + (1 | Subject)
ex + (1 | Subject)
ex + (1 | Subject)
ex + (1 | Subject)
ex + (1 | Subject)
ex + (1 | Subject)
ex + (1 | Subject)

ex + (1 | Subject)
ex + (1 | Subject)
ex + (1 | Subject)
ex + (1 | Subject)
ex + (1 | Subject)
ex + (1 | Subject)
ex + (1 | Subject)
ex + (1 | Subject)
ex + (1 | Subject)
ex + (1 | Subject)
ex + (1 | Subject)
ex + (1 | Subject)
ex + (1 | Subject)
ex + (1 | Subject)
ex + (1 | Subject)
ex + (1 | Subject)
ex + (1 | Subject)
ex + (1 | Subject)
ex + (1 | Subject)
ex + (1 | Subject)
ex + (1 | Subject)
ex + (1 | Subject)
ex + (1 | Subject)
ex + (1 | Subject)
ex + (1 | Subject)
ex + (1 | Subject)
ex + (1 | Subject)
ex + (1 | Subject)
ex + (1 | Subject)
ex + (1 | Subject)
ex + (1 | Subject)
ex + (1 | Subject)
ex + (1 | Subject)
ex + (1 | Subject)
ex + (1 | Subject)
ex + (1 | Subject)
ex + (1 | Subject)
ex + (1 | Subject)
ex + (1 | Subject)
ex + (1 | Subject)
ex + (1 | Subject)
ex + (1 | Subject)
ex + (1 | Subject)
ex + (1 | Subject)
ex + (1 | Subject)
ex + (1 | Subject)
ex + (1 | Subject)
ex + (1 | Subject)

ex + (1 | Subject)
ex + (1 | Subject)
ex + (1 | Subject)
ex + (1 | Subject)
ex + (1 | Subject)
ex + (1 | Subject)
ex + (1 | Subject)
ex + (1 | Subject)
ex + (1 | Subject)
ex + (1 | Subject)
ex + (1 | Subject)
ex + (1 | Subject)
ex + (1 | Subject)
ex + (1 | Subject)
ex + (1 | Subject)
ex + (1 | Subject)
ex + (1 | Subject)
ex + (1 | Subject)
ex + (1 | Subject)
ex + (1 | Subject)
ex + (1 | Subject)
ex + (1 | Subject)
ex + (1 | Subject)
ex + (1 | Subject)
ex + (1 | Subject)
ex + (1 | Subject)
ex + (1 | Subject)
ex + (1 | Subject)
ex + (1 | Subject)
ex + (1 | Subject)
ex + (1 | Subject)
ex + (1 | Subject)
ex + (1 | Subject)
ex + (1 | Subject)
ex + (1 | Subject)
ex + (1 | Subject)
ex + (1 | Subject)
ex + (1 | Subject)
ex + (1 | Subject)
ex + (1 | Subject)
ex + (1 | Subject)
ex + (1 | Subject)
ex + (1 | Subject)
ex + (1 | Subject)
ex + (1 | Subject)
ex + (1 | Subject)
ex + (1 | Subject)
ex + (1 | Subject)

ex + (1 | Subject)
ex + (1 | Subject)
ex + (1 | Subject)
ex + (1 | Subject)
ex + (1 | Subject)
ex + (1 | Subject)
ex + (1 | Subject)
ex + (1 | Subject)
ex + (1 | Subject)
ex + (1 | Subject)
ex + (1 | Subject)
ex + (1 | Subject)
ex + (1 | Subject)
ex + (1 | Subject)
ex + (1 | Subject)
ex + (1 | Subject)
ex + (1 | Subject)
ex + (1 | Subject)
ex + (1 | Subject)
ex + (1 | Subject)
ex + (1 | Subject)
ex + (1 | Subject)
ex + (1 | Subject)
ex + (1 | Subject)
ex + (1 | Subject)
ex + (1 | Subject)
ex + (1 | Subject)
ex + (1 | Subject)
ex + (1 | Subject)
ex + (1 | Subject)
ex + (1 | Subject)
ex + (1 | Subject)
ex + (1 | Subject)
ex + (1 | Subject)
ex + (1 | Subject)
ex + (1 | Subject)
ex + (1 | Subject)
ex + (1 | Subject)
ex + (1 | Subject)
ex + (1 | Subject)
ex + (1 | Subject)
ex + (1 | Subject)
ex + (1 | Subject)
ex + (1 | Subject)
ex + (1 | Subject)
ex + (1 | Subject)
ex + (1 | Subject)
ex + (1 | Subject)

ex + (1 | Subject)
ex + (1 | Subject)
ex + (1 | Subject)
ex + (1 | Subject)
ex + (1 | Subject)
ex + (1 | Subject)
ex + (1 | Subject)
ex + (1 | Subject)
ex + (1 | Subject)
ex + (1 | Subject)
ex + (1 | Subject)
ex + (1 | Subject)
ex + (1 | Subject)
ex + (1 | Subject)
ex + (1 | Subject)
ex + (1 | Subject)
ex + (1 | Subject)
ex + (1 | Subject)
ex + (1 | Subject)
ex + (1 | Subject)
ex + (1 | Subject)
ex + (1 | Subject)
ex + (1 | Subject)
ex + (1 | Subject)
ex + (1 | Subject)
ex + (1 | Subject)
ex + (1 | Subject)
ex + (1 | Subject)
ex + (1 | Subject)
ex + (1 | Subject)
ex + (1 | Subject)
ex + (1 | Subject)
ex + (1 | Subject)
ex + (1 | Subject)
ex + (1 | Subject)
ex + (1 | Subject)
ex + (1 | Subject)
ex + (1 | Subject)
ex + (1 | Subject)
ex + (1 | Subject)
ex + (1 | Subject)
ex + (1 | Subject)
ex + (1 | Subject)
ex + (1 | Subject)
ex + (1 | Subject)
ex + (1 | Subject)
ex + (1 | Subject)
ex + (1 | Subject)

ex + (1 | Subject)
ex + (1 | Subject)
ex + (1 | Subject)
ex + (1 | Subject)
ex + (1 | Subject)
ex + (1 | Subject)
ex + (1 | Subject)
ex + (1 | Subject)
ex + (1 | Subject)
ex + (1 | Subject)
ex + (1 | Subject)
ex + (1 | Subject)
ex + (1 | Subject)
ex + (1 | Subject)
ex + (1 | Subject)
ex + (1 | Subject)
ex + (1 | Subject)
ex + (1 | Subject)
ex + (1 | Subject)
ex + (1 | Subject)
ex + (1 | Subject)
ex + (1 | Subject)
ex + (1 | Subject)
ex + (1 | Subject)
ex + (1 | Subject)
ex + (1 | Subject)
ex + (1 | Subject)
ex + (1 | Subject)
ex + (1 | Subject)
ex + (1 | Subject)
ex + (1 | Subject)
ex + (1 | Subject)
ex + (1 | Subject)
ex + (1 | Subject)
ex + (1 | Subject)
ex + (1 | Subject)
ex + (1 | Subject)
ex + (1 | Subject)
ex + (1 | Subject)
ex + (1 | Subject)
ex + (1 | Subject)
ex + (1 | Subject)
ex + (1 | Subject)
ex + (1 | Subject)
ex + (1 | Subject)
ex + (1 | Subject)
ex + (1 | Subject)
ex + (1 | Subject)

ex + (1 | Subject)
ex + (1 | Subject)
ex + (1 | Subject)
ex + (1 | Subject)
ex + (1 | Subject)
ex + (1 | Subject)
ex + (1 | Subject)
ex + (1 | Subject)
ex + (1 | Subject)
ex + (1 | Subject)
ex + (1 | Subject)
ex + (1 | Subject)
ex + (1 | Subject)
ex + (1 | Subject)
ex + (1 | Subject)
ex + (1 | Subject)
ex + (1 | Subject)
ex + (1 | Subject)
ex + (1 | Subject)
ex + (1 | Subject)
ex + (1 | Subject)
ex + (1 | Subject)
ex + (1 | Subject)
ex + (1 | Subject)
ex + (1 | Subject)
ex + (1 | Subject)
ex + (1 | Subject)
ex + (1 | Subject)
ex + (1 | Subject)
ex + (1 | Subject)
ex + (1 | Subject)
ex + (1 | Subject)
ex + (1 | Subject)
ex + (1 | Subject)
ex + (1 | Subject)
ex + (1 | Subject)
ex + (1 | Subject)
ex + (1 | Subject)
ex + (1 | Subject)
ex + (1 | Subject)
ex + (1 | Subject)
ex + (1 | Subject)
ex + (1 | Subject)
ex + (1 | Subject)
ex + (1 | Subject)
ex + (1 | Subject)
ex + (1 | Subject)
ex + (1 | Subject)

[illegible]ex + (1 | Subject)
[illegible]ex + (1 | Subject)
[illegible]ex + (1 | Subject)
[illegible]ex + (1 | Subject)
[illegible]ex + (1 | Subject)
