# Supplementary material for: A young child formula with Limosilactobacillus reuteri and GOS modulates gut microbiome and enhances bone and muscle development: a randomized trial
Source: Nat Commun. 2025 Dec 12;17:237. doi: 10.1038/s41467-025-66930-2 (PMC12783733; doi:10.1038/s41467-025-66930-2)
Supplement: Supplementary file 13 — Supplementary data 11 [file 41467_2025_66930_MOESM13_ESM.pdf]

Category	Test type	Item	Name	Feature	Effect size	Standard e	Bias mode
SCFA_panel	abundance	2-Methylpr	2-Methylpr	Tibia_length	0,05085	0,054087	NA
SCFA_panel	abundance	2-Methylpr	2-Methylpr	Radius_length	-0,03881	0,072536	NA
SCFA_panel	abundance	2-Methylpr	2-Methylpr	Radius_SOS	0,024803	0,05179	NA
SCFA_panel	abundance	2-Methylpr	2-Methylpr	Tibia_SOS	-0,03831	0,054479	NA
SCFA_panel	abundance	2-Methylpr	2-Methylpr	Handgrip	-0,12843	0,062131	NA
SCFA_panel	abundance	3-Methylbu	3-Methylbu	Tibia_length	0,032589	0,054461	NA
SCFA_panel	abundance	3-Methylbu	3-Methylbu	Radius_length	-0,03333	0,071189	NA
SCFA_panel	abundance	3-Methylbu	3-Methylbu	Radius_SOS	0,041428	0,052083	NA
SCFA_panel	abundance	3-Methylbu	3-Methylbu	Tibia_SOS	-0,03173	0,055242	NA
SCFA_panel	abundance	3-Methylbu	3-Methylbu	Handgrip	-0,16736	0,062271	NA
SCFA_panel	abundance	Acetic acid	Acetic acid	Tibia_length	-0,01186	0,055419	NA
SCFA_panel	abundance	Acetic acid	Acetic acid	Radius_length	4,79E-04	0,073843	NA
SCFA_panel	abundance	Acetic acid	Acetic acid	Radius_SOS	0,030678	0,052606	NA
SCFA_panel	abundance	Acetic acid	Acetic acid	Tibia_SOS	0,020414	0,056205	NA
SCFA_panel	abundance	Acetic acid	Acetic acid	Handgrip	0,030145	0,062326	NA
SCFA_panel	abundance	Butanoic a	Butanoic a	Tibia_length	-0,06657	0,052982	NA
SCFA_panel	abundance	Butanoic a	Butanoic a	Radius_length	-0,0443	0,071763	NA
SCFA_panel	abundance	Butanoic a	Butanoic a	Radius_SOS	0,025865	0,050837	NA
SCFA_panel	abundance	Butanoic a	Butanoic a	Tibia_SOS	0,012665	0,054702	NA
SCFA_panel	abundance	Butanoic a	Butanoic a	Handgrip	-0,00944	0,061712	NA
SCFA_panel	abundance	Hexanoic a	Hexanoic a	Tibia_length	-0,01783	0,067127	NA
SCFA_panel	abundance	Hexanoic a	Hexanoic a	Radius_length	-0,00725	0,083039	NA
SCFA_panel	abundance	Hexanoic a	Hexanoic a	Radius_SOS	0,085926	0,06696	NA
SCFA_panel	abundance	Hexanoic a	Hexanoic a	Tibia_SOS	-0,07639	0,069285	NA
SCFA_panel	abundance	Hexanoic a	Hexanoic a	Handgrip	0,058547	0,081307	NA
WGCNA_module	abundance	M1	M1	Tibia_length	-0,06523	0,053249	NA
WGCNA_module	abundance	M1	M1	Radius_length	-0,02623	0,073013	NA
WGCNA_module	abundance	M1	M1	Radius_SOS	-0,05494	0,051057	NA
WGCNA_module	abundance	M1	M1	Tibia_SOS	3,38E-04	0,054601	NA
WGCNA_module	abundance	M1	M1	Handgrip	0,077771	0,062105	NA
WGCNA_module	abundance	M10	M10	Tibia_length	-0,01209	0,051803	NA
WGCNA_module	abundance	M10	M10	Radius_length	-0,0324	0,071423	NA
WGCNA_module	abundance	M10	M10	Radius_SOS	-0,03865	0,049929	NA
WGCNA_module	abundance	M10	M10	Tibia_SOS	0,116624	0,052847	NA
WGCNA_module	abundance	M10	M10	Handgrip	0,114057	0,061076	NA
WGCNA_module	abundance	M11	M11	Tibia_length	-0,09965	0,050683	NA
WGCNA_module	abundance	M11	M11	Radius_length	-0,11481	0,069207	NA
WGCNA_module	abundance	M11	M11	Radius_SOS	0,039247	0,049322	NA
WGCNA_module	abundance	M11	M11	Tibia_SOS	-0,14646	0,052131	NA
WGCNA_module	abundance	M11	M11	Handgrip	-0,18272	0,059742	NA
WGCNA_module	abundance	M12	M12	Tibia_length	0,117163	0,049513	NA
WGCNA_module	abundance	M12	M12	Radius_length	0,187866	0,066897	NA
WGCNA_module	abundance	M12	M12	Radius_SOS	0,068866	0,048928	NA
WGCNA_module	abundance	M12	M12	Tibia_SOS	0,089343	0,052315	NA
WGCNA_module	abundance	M12	M12	Handgrip	0,086003	0,060054	NA
WGCNA_module	abundance	M13	M13	Tibia_length	0,177682	0,051891	NA
WGCNA_module	abundance	M13	M13	Radius_length	0,075706	0,07194	NA

WGCNA_module	abundance	M13	M13	Radius_SOS	0,007855	0,050487	NA
WGCNA_module	abundance	M13	M13	Tibia_SOS	0,138438	0,054394	NA
WGCNA_module	abundance	M13	M13	Handgrip	0,06133	0,060857	NA
WGCNA_module	abundance	M14	M14	Tibia_length	0,025074	0,051874	NA
WGCNA_module	abundance	M14	M14	Radius_length	0,017131	0,07124	NA
WGCNA_module	abundance	M14	M14	Radius_SOS	0,046625	0,049915	NA
WGCNA_module	abundance	M14	M14	Tibia_SOS	0,010138	0,053591	NA
WGCNA_module	abundance	M14	M14	Handgrip	0,001402	0,060907	NA
WGCNA_module	abundance	M15	M15	Tibia_length	-0,09048	0,053021	NA
WGCNA_module	abundance	M15	M15	Radius_length	0,018358	0,072291	NA
WGCNA_module	abundance	M15	M15	Radius_SOS	0,01215	0,050744	NA
WGCNA_module	abundance	M15	M15	Tibia_SOS	-0,02125	0,054363	NA
WGCNA_module	abundance	M15	M15	Handgrip	0,035758	0,060642	NA
WGCNA_module	abundance	M16	M16	Tibia_length	0,108921	0,053158	NA
WGCNA_module	abundance	M16	M16	Radius_length	0,168578	0,07193	NA
WGCNA_module	abundance	M16	M16	Radius_SOS	0,091187	0,050477	NA
WGCNA_module	abundance	M16	M16	Tibia_SOS	0,078484	0,054704	NA
WGCNA_module	abundance	M16	M16	Handgrip	0,181445	0,059805	NA
WGCNA_module	abundance	M17	M17	Tibia_length	-0,05981	0,056156	NA
WGCNA_module	abundance	M17	M17	Radius_length	0,012789	0,074785	NA
WGCNA_module	abundance	M17	M17	Radius_SOS	-0,01785	0,053496	NA
WGCNA_module	abundance	M17	M17	Tibia_SOS	0,003708	0,058081	NA
WGCNA_module	abundance	M17	M17	Handgrip	-2,03E-04	0,062841	NA
WGCNA_module	abundance	M18	M18	Tibia_length	-0,11323	0,052208	NA
WGCNA_module	abundance	M18	M18	Radius_length	-0,19213	0,070118	NA
WGCNA_module	abundance	M18	M18	Radius_SOS	-0,07564	0,050161	NA
WGCNA_module	abundance	M18	M18	Tibia_SOS	-0,09374	0,053715	NA
WGCNA_module	abundance	M18	M18	Handgrip	-0,13414	0,060931	NA
WGCNA_module	abundance	M19	M19	Tibia_length	0,013027	0,049351	NA
WGCNA_module	abundance	M19	M19	Radius_length	0,057482	0,066642	NA
WGCNA_module	abundance	M19	M19	Radius_SOS	0,039858	0,049005	NA
WGCNA_module	abundance	M19	M19	Tibia_SOS	0,065489	0,052374	NA
WGCNA_module	abundance	M19	M19	Handgrip	-0,00769	0,060356	NA
WGCNA_module	abundance	M2	M2	Tibia_length	0,123654	0,049128	NA
WGCNA_module	abundance	M2	M2	Radius_length	0,192817	0,065281	NA
WGCNA_module	abundance	M2	M2	Radius_SOS	0,022016	0,049033	NA
WGCNA_module	abundance	M2	M2	Tibia_SOS	0,152998	0,051884	NA
WGCNA_module	abundance	M2	M2	Handgrip	0,097167	0,059909	NA
WGCNA_module	abundance	M20	M20	Tibia_length	-0,02035	0,049051	NA
WGCNA_module	abundance	M20	M20	Radius_length	0,108929	0,06452	NA
WGCNA_module	abundance	M20	M20	Radius_SOS	-0,01291	0,049242	NA
WGCNA_module	abundance	M20	M20	Tibia_SOS	0,047559	0,052707	NA
WGCNA_module	abundance	M20	M20	Handgrip	0,019809	0,060182	NA
WGCNA_module	abundance	M21	M21	Tibia_length	-0,0552	0,055567	NA
WGCNA_module	abundance	M21	M21	Radius_length	-0,12045	0,073357	NA
WGCNA_module	abundance	M21	M21	Radius_SOS	-0,13912	0,052741	NA
WGCNA_module	abundance	M21	M21	Tibia_SOS	-0,0746	0,057661	NA
WGCNA_module	abundance	M21	M21	Handgrip	-0,09737	0,062208	NA

WGCNA_module	abundance	M22	M22	Tibia_length	-0,03379	0,056103	NA
WGCNA_module	abundance	M22	M22	Radius_length	0,096061	0,074195	NA
WGCNA_module	abundance	M22	M22	Radius_SOS	-0,02776	0,053351	NA
WGCNA_module	abundance	M22	M22	Tibia_SOS	0,089982	0,056122	NA
WGCNA_module	abundance	M22	M22	Handgrip	0,095737	0,062659	NA
WGCNA_module	abundance	M23	M23	Tibia_length	0,08184	0,049445	NA
WGCNA_module	abundance	M23	M23	Radius_length	0,096648	0,066935	NA
WGCNA_module	abundance	M23	M23	Radius_SOS	0,007385	0,049028	NA
WGCNA_module	abundance	M23	M23	Tibia_SOS	0,066105	0,052372	NA
WGCNA_module	abundance	M23	M23	Handgrip	-0,01463	0,060268	NA
WGCNA_module	abundance	M24	M24	Tibia_length	-0,08644	0,050717	NA
WGCNA_module	abundance	M24	M24	Radius_length	-0,10364	0,069253	NA
WGCNA_module	abundance	M24	M24	Radius_SOS	-0,08589	0,049287	NA
WGCNA_module	abundance	M24	M24	Tibia_SOS	-0,04169	0,052693	NA
WGCNA_module	abundance	M24	M24	Handgrip	0,004183	0,060837	NA
WGCNA_module	abundance	M25	M25	Tibia_length	0,057666	0,054385	NA
WGCNA_module	abundance	M25	M25	Radius_length	0,187379	0,071681	NA
WGCNA_module	abundance	M25	M25	Radius_SOS	-0,01438	0,051503	NA
WGCNA_module	abundance	M25	M25	Tibia_SOS	0,057663	0,05478	NA
WGCNA_module	abundance	M25	M25	Handgrip	0,025347	0,062768	NA
WGCNA_module	abundance	M26	M26	Tibia_length	-0,08126	0,050013	NA
WGCNA_module	abundance	M26	M26	Radius_length	-0,13346	0,067707	NA
WGCNA_module	abundance	M26	M26	Radius_SOS	-0,04655	0,04904	NA
WGCNA_module	abundance	M26	M26	Tibia_SOS	-0,09959	0,052283	NA
WGCNA_module	abundance	M26	M26	Handgrip	-0,08936	0,060036	NA
WGCNA_module	abundance	M27	M27	Tibia_length	-0,04902	0,050562	NA
WGCNA_module	abundance	M27	M27	Radius_length	-0,09568	0,069012	NA
WGCNA_module	abundance	M27	M27	Radius_SOS	0,074299	0,049113	NA
WGCNA_module	abundance	M27	M27	Tibia_SOS	-0,04491	0,052807	NA
WGCNA_module	abundance	M27	M27	Handgrip	-0,01129	0,060891	NA
WGCNA_module	abundance	M28	M28	Tibia_length	-0,13017	0,048562	NA
WGCNA_module	abundance	M28	M28	Radius_length	-0,17692	0,062616	NA
WGCNA_module	abundance	M28	M28	Radius_SOS	-0,08595	0,049331	NA
WGCNA_module	abundance	M28	M28	Tibia_SOS	-0,10031	0,052851	NA
WGCNA_module	abundance	M28	M28	Handgrip	0,001227	0,060355	NA
WGCNA_module	abundance	M29	M29	Tibia_length	-0,01999	0,057476	NA
WGCNA_module	abundance	M29	M29	Radius_length	-0,03508	0,074713	NA
WGCNA_module	abundance	M29	M29	Radius_SOS	-0,02491	0,054644	NA
WGCNA_module	abundance	M29	M29	Tibia_SOS	0,02119	0,058341	NA
WGCNA_module	abundance	M29	M29	Handgrip	-0,04709	0,060126	NA
WGCNA_module	abundance	M3	M3	Tibia_length	-0,1258	0,051409	NA
WGCNA_module	abundance	M3	M3	Radius_length	-0,14508	0,070048	NA
WGCNA_module	abundance	M3	M3	Radius_SOS	-0,02579	0,049799	NA
WGCNA_module	abundance	M3	M3	Tibia_SOS	-0,13206	0,052719	NA
WGCNA_module	abundance	M3	M3	Handgrip	-0,12915	0,060535	NA
WGCNA_module	abundance	M30	M30	Tibia_length	-0,07154	0,049495	NA
WGCNA_module	abundance	M30	M30	Radius_length	-0,11064	0,066487	NA
WGCNA_module	abundance	M30	M30	Radius_SOS	0,001665	0,04903	NA

WGCNA_module	abundance	M30	M30	Tibia_SOS	-0,04819	0,052438	NA
WGCNA_module	abundance	M30	M30	Handgrip	-0,0433	0,060206	NA
WGCNA_module	abundance	M31	M31	Tibia_length	-0,05555	0,052981	NA
WGCNA_module	abundance	M31	M31	Radius_length	0,020837	0,07219	NA
WGCNA_module	abundance	M31	M31	Radius_SOS	5,57E-04	0,050847	NA
WGCNA_module	abundance	M31	M31	Tibia_SOS	-0,0035	0,053213	NA
WGCNA_module	abundance	M31	M31	Handgrip	-0,07795	0,061121	NA
WGCNA_module	abundance	M32	M32	Tibia_length	-0,11755	0,052994	NA
WGCNA_module	abundance	M32	M32	Radius_length	-0,16893	0,071841	NA
WGCNA_module	abundance	M32	M32	Radius_SOS	0,007256	0,050777	NA
WGCNA_module	abundance	M32	M32	Tibia_SOS	-0,12512	0,053447	NA
WGCNA_module	abundance	M32	M32	Handgrip	-0,11435	0,061628	NA
WGCNA_module	abundance	M33	M33	Tibia_length	-0,02887	0,049099	NA
WGCNA_module	abundance	M33	M33	Radius_length	8,58E-04	0,064972	NA
WGCNA_module	abundance	M33	M33	Radius_SOS	0,024525	0,049176	NA
WGCNA_module	abundance	M33	M33	Tibia_SOS	-0,06878	0,052573	NA
WGCNA_module	abundance	M33	M33	Handgrip	-0,00617	0,060238	NA
WGCNA_module	abundance	M34	M34	Tibia_length	8,93E-04	0,053089	NA
WGCNA_module	abundance	M34	M34	Radius_length	-0,00203	0,072086	NA
WGCNA_module	abundance	M34	M34	Radius_SOS	0,057603	0,050758	NA
WGCNA_module	abundance	M34	M34	Tibia_SOS	-0,00637	0,054489	NA
WGCNA_module	abundance	M34	M34	Handgrip	0,035054	0,061955	NA
WGCNA_module	abundance	M4	M4	Tibia_length	0,013163	0,048986	NA
WGCNA_module	abundance	M4	M4	Radius_length	0,054728	0,062205	NA
WGCNA_module	abundance	M4	M4	Radius_SOS	0,015467	0,049758	NA
WGCNA_module	abundance	M4	M4	Tibia_SOS	-0,0355	0,052953	NA
WGCNA_module	abundance	M4	M4	Handgrip	0,012708	0,06025	NA
WGCNA_module	abundance	M5	M5	Tibia_length	0,026873	0,050822	NA
WGCNA_module	abundance	M5	M5	Radius_length	0,006341	0,069695	NA
WGCNA_module	abundance	M5	M5	Radius_SOS	-0,02389	0,049369	NA
WGCNA_module	abundance	M5	M5	Tibia_SOS	-0,022	0,053149	NA
WGCNA_module	abundance	M5	M5	Handgrip	0,041387	0,061182	NA
WGCNA_module	abundance	M6	M6	Tibia_length	-0,13703	0,051797	NA
WGCNA_module	abundance	M6	M6	Radius_length	-0,19212	0,069692	NA
WGCNA_module	abundance	M6	M6	Radius_SOS	-0,05458	0,049818	NA
WGCNA_module	abundance	M6	M6	Tibia_SOS	-0,11506	0,053189	NA
WGCNA_module	abundance	M6	M6	Handgrip	-0,08852	0,06044	NA
WGCNA_module	abundance	M7	M7	Tibia_length	0,110263	0,052358	NA
WGCNA_module	abundance	M7	M7	Radius_length	0,094861	0,071715	NA
WGCNA_module	abundance	M7	M7	Radius_SOS	0,071773	0,050505	NA
WGCNA_module	abundance	M7	M7	Tibia_SOS	0,045363	0,054039	NA
WGCNA_module	abundance	M7	M7	Handgrip	-0,05917	0,061901	NA
WGCNA_module	abundance	M8	M8	Tibia_length	-0,16236	0,050775	NA
WGCNA_module	abundance	M8	M8	Radius_length	-0,10796	0,070233	NA
WGCNA_module	abundance	M8	M8	Radius_SOS	-0,02405	0,049574	NA
WGCNA_module	abundance	M8	M8	Tibia_SOS	-0,07251	0,053315	NA
WGCNA_module	abundance	M8	M8	Handgrip	0,007986	0,060849	NA
WGCNA_module	abundance	M9	M9	Tibia_length	-0,02759	0,05053	NA

WGCNA_module	abundance	M9	M9	Radius_length	0,055507	0,069388	NA
WGCNA_module	abundance	M9	M9	Radius_SOS	-0,01354	0,049277	NA
WGCNA_module	abundance	M9	M9	Tibia_SOS	0,058617	0,052969	NA
WGCNA_module	abundance	M9	M9	Handgrip	0,044679	0,060614	NA
SCFA_panel	abundance	Pentanoic	Pentanoic	Tibia_length	-0,01685	0,053447	NA
SCFA_panel	abundance	Pentanoic	Pentanoic	Radius_length	-0,00914	0,070958	NA
SCFA_panel	abundance	Pentanoic	Pentanoic	Radius_SOS	0,026734	0,051158	NA
SCFA_panel	abundance	Pentanoic	Pentanoic	Tibia_SOS	-0,07161	0,05445	NA
SCFA_panel	abundance	Pentanoic	Pentanoic	Handgrip	-0,06883	0,062944	NA
SCFA_panel	abundance	Propanoic	Propanoic	Tibia_length	0,005412	0,051431	NA
SCFA_panel	abundance	Propanoic	Propanoic	Radius_length	-0,05697	0,070241	NA
SCFA_panel	abundance	Propanoic	Propanoic	Radius_SOS	-0,02462	0,049668	NA
SCFA_panel	abundance	Propanoic	Propanoic	Tibia_SOS	0,026991	0,053245	NA
SCFA_panel	abundance	Propanoic	Propanoic	Handgrip	-0,02213	0,061287	NA
2a	abundance	SL00001	Deoxycytid	Tibia_length	-0,11416	0,054475	NA
2a	abundance	SL00001	Deoxycytid	Radius_length	-0,07379	0,073462	NA
2a	abundance	SL00001	Deoxycytid	Radius_SOS	-0,05855	0,051853	NA
2a	abundance	SL00001	Deoxycytid	Tibia_SOS	-0,04107	0,054463	NA
2a	abundance	SL00001	Deoxycytid	Handgrip	-0,12974	0,061708	NA
2a	abundance	SL00009	N-Acetylhi:	Tibia_length	0,011843	0,053986	NA
2a	abundance	SL00009	N-Acetylhi:	Radius_length	0,045231	0,073018	NA
2a	abundance	SL00009	N-Acetylhi:	Radius_SOS	0,003155	0,051457	NA
2a	abundance	SL00009	N-Acetylhi:	Tibia_SOS	0,056383	0,055734	NA
2a	abundance	SL00009	N-Acetylhi:	Handgrip	0,077633	0,061809	NA
2a	abundance	SL00017	Aspartic ac	Tibia_length	0,108429	0,049737	NA
2a	abundance	SL00017	Aspartic ac	Radius_length	0,213994	0,066411	NA
2a	abundance	SL00017	Aspartic ac	Radius_SOS	0,014617	0,049063	NA
2a	abundance	SL00017	Aspartic ac	Tibia_SOS	0,117673	0,052312	NA
2a	abundance	SL00017	Aspartic ac	Handgrip	0,076218	0,060239	NA
2a	abundance	SL00020	Carnitine	Tibia_length	0,026401	0,052256	NA
2a	abundance	SL00020	Carnitine	Radius_length	-0,01274	0,071275	NA
2a	abundance	SL00020	Carnitine	Radius_SOS	-0,0617	0,05018	NA
2a	abundance	SL00020	Carnitine	Tibia_SOS	-0,06811	0,053621	NA
2a	abundance	SL00020	Carnitine	Handgrip	0,005252	0,061618	NA
2a	abundance	SL00024	Creatine	Tibia_length	-0,06847	0,054177	NA
2a	abundance	SL00024	Creatine	Radius_length	-0,08238	0,073133	NA
2a	abundance	SL00024	Creatine	Radius_SOS	-0,07205	0,051641	NA
2a	abundance	SL00024	Creatine	Tibia_SOS	-0,00522	0,055856	NA
2a	abundance	SL00024	Creatine	Handgrip	0,026729	0,061903	NA
2a	abundance	SL00035	Glycylglyci	Tibia_length	0,028419	0,054553	NA
2a	abundance	SL00035	Glycylglyci	Radius_length	0,045915	0,073529	NA
2a	abundance	SL00035	Glycylglyci	Radius_SOS	-0,0103	0,051964	NA
2a	abundance	SL00035	Glycylglyci	Tibia_SOS	0,055319	0,055438	NA
2a	abundance	SL00035	Glycylglyci	Handgrip	0,031751	0,062386	NA
2a	abundance	SL00040	Kynurenine	Tibia_length	-0,03947	0,050683	NA
2a	abundance	SL00040	Kynurenine	Radius_length	-0,09895	0,069193	NA
2a	abundance	SL00040	Kynurenine	Radius_SOS	-0,07539	0,049124	NA
2a	abundance	SL00040	Kynurenine	Tibia_SOS	-0,07452	0,052682	NA

2a	abundance	SL00040	Kynurenine	Handgrip	-0,09771	0,060212	NA
2a	abundance	SL00049	N-Acetylar	Tibia_length	0,068086	0,052637	NA
2a	abundance	SL00049	N-Acetylar	Radius_length	0,080969	0,0713	NA
2a	abundance	SL00049	N-Acetylar	Radius_SOS	0,001891	0,050101	NA
2a	abundance	SL00049	N-Acetylar	Tibia_SOS	0,064986	0,053687	NA
2a	abundance	SL00049	N-Acetylar	Handgrip	0,068815	0,060634	NA
2a	abundance	SL00052	N-Isovaler	Tibia_length	0,100728	0,055233	NA
2a	abundance	SL00052	N-Isovaler	Radius_length	0,20141	0,07248	NA
2a	abundance	SL00052	N-Isovaler	Radius_SOS	0,062503	0,052685	NA
2a	abundance	SL00052	N-Isovaler	Tibia_SOS	0,050761	0,056134	NA
2a	abundance	SL00052	N-Isovaler	Handgrip	-0,01302	0,063589	NA
1	abundance	SL00054	Ornithine	Tibia_length	0,10572	0,052238	NA
1	abundance	SL00054	Ornithine	Radius_length	0,102708	0,071745	NA
1	abundance	SL00054	Ornithine	Radius_SOS	0,104438	0,049804	NA
1	abundance	SL00054	Ornithine	Tibia_SOS	0,097644	0,053702	NA
1	abundance	SL00054	Ornithine	Handgrip	-0,01654	0,06137	NA
1	abundance	SL00061	Proline	Tibia_length	0,112599	0,051792	NA
1	abundance	SL00061	Proline	Radius_length	0,070543	0,071545	NA
1	abundance	SL00061	Proline	Radius_SOS	-0,00561	0,050016	NA
1	abundance	SL00061	Proline	Tibia_SOS	0,116942	0,053437	NA
1	abundance	SL00061	Proline	Handgrip	0,089723	0,060268	NA
1	abundance	SL00063	Pyridoxal	Tibia_length	0,005833	0,052027	NA
1	abundance	SL00063	Pyridoxal	Radius_length	0,086878	0,07096	NA
1	abundance	SL00063	Pyridoxal	Radius_SOS	-0,03333	0,049977	NA
1	abundance	SL00063	Pyridoxal	Tibia_SOS	0,087121	0,053268	NA
1	abundance	SL00063	Pyridoxal	Handgrip	-0,02557	0,061575	NA
2a	abundance	SL00074	Valine/ 5-A	Tibia_length	0,018432	0,053314	NA
2a	abundance	SL00074	Valine/ 5-A	Radius_length	0,049237	0,0726	NA
2a	abundance	SL00074	Valine/ 5-A	Radius_SOS	-0,07147	0,050962	NA
2a	abundance	SL00074	Valine/ 5-A	Tibia_SOS	0,11463	0,053896	NA
2a	abundance	SL00074	Valine/ 5-A	Handgrip	0,098988	0,060852	NA
2a	abundance	SL00081	5-Methylcy	Tibia_length	-0,08437	0,051669	NA
2a	abundance	SL00081	5-Methylcy	Radius_length	0,036004	0,071172	NA
2a	abundance	SL00081	5-Methylcy	Radius_SOS	-0,01615	0,04999	NA
2a	abundance	SL00081	5-Methylcy	Tibia_SOS	-0,00144	0,053266	NA
2a	abundance	SL00081	5-Methylcy	Handgrip	0,032346	0,060885	NA
1	abundance	SL00082	Allopurinol	Tibia_length	0,115899	0,056805	NA
1	abundance	SL00082	Allopurinol	Radius_length	0,172989	0,075307	NA
1	abundance	SL00082	Allopurinol	Radius_SOS	0,161732	0,053116	NA
1	abundance	SL00082	Allopurinol	Tibia_SOS	0,085539	0,057739	NA
1	abundance	SL00082	Allopurinol	Handgrip	0,140607	0,061968	NA
2a	abundance	SL00089	Dissaccari	Tibia_length	0,067076	0,051786	NA
2a	abundance	SL00089	Dissaccari	Radius_length	0,135754	0,070886	NA
2a	abundance	SL00089	Dissaccari	Radius_SOS	-0,03375	0,04986	NA
2a	abundance	SL00089	Dissaccari	Tibia_SOS	-0,01403	0,053306	NA
2a	abundance	SL00089	Dissaccari	Handgrip	0,004525	0,060976	NA
1	abundance	SL00096	Hexoses III	Tibia_length	-0,15627	0,054526	NA
1	abundance	SL00096	Hexoses III	Radius_length	-0,07404	0,074468	NA

1	abundance	SL00096	Hexoses III	Radius_SOS	-0,1168	0,052559	NA
1	abundance	SL00096	Hexoses III	Tibia_SOS	-0,07879	0,055852	NA
1	abundance	SL00096	Hexoses III	Handgrip	-0,16668	0,06151	NA
1	abundance	SL00097	Hexoses II	Tibia_length	-0,01102	0,055654	NA
1	abundance	SL00097	Hexoses II	Radius_length	0,071268	0,074328	NA
1	abundance	SL00097	Hexoses II	Radius_SOS	-0,02189	0,052864	NA
1	abundance	SL00097	Hexoses II	Tibia_SOS	0,020414	0,056451	NA
1	abundance	SL00097	Hexoses II	Handgrip	0,094793	0,06235	NA
2a	abundance	SL00103	Gulonic ac	Tibia_length	0,04138	0,053562	NA
2a	abundance	SL00103	Gulonic ac	Radius_length	0,041352	0,072925	NA
2a	abundance	SL00103	Gulonic ac	Radius_SOS	0,015481	0,051175	NA
2a	abundance	SL00103	Gulonic ac	Tibia_SOS	0,045157	0,054338	NA
2a	abundance	SL00103	Gulonic ac	Handgrip	0,104073	0,061224	NA
1	abundance	SL00104	Hexoses I	Tibia_length	-0,02603	0,056099	NA
1	abundance	SL00104	Hexoses I	Radius_length	0,015612	0,074824	NA
1	abundance	SL00104	Hexoses I	Radius_SOS	-0,04413	0,053339	NA
1	abundance	SL00104	Hexoses I	Tibia_SOS	-0,00222	0,057339	NA
1	abundance	SL00104	Hexoses I	Handgrip	0,091103	0,063361	NA
2a	abundance	SL00108	Histidinol	Tibia_length	-0,13454	0,053054	NA
2a	abundance	SL00108	Histidinol	Radius_length	-0,03618	0,072461	NA
2a	abundance	SL00108	Histidinol	Radius_SOS	-0,08422	0,051232	NA
2a	abundance	SL00108	Histidinol	Tibia_SOS	-0,01244	0,053756	NA
2a	abundance	SL00108	Histidinol	Handgrip	0,085887	0,061373	NA
2a	abundance	SL00124	Dissaccari	Tibia_length	-0,12803	0,054661	NA
2a	abundance	SL00124	Dissaccari	Radius_length	-0,02392	0,074042	NA
2a	abundance	SL00124	Dissaccari	Radius_SOS	-0,03104	0,052244	NA
2a	abundance	SL00124	Dissaccari	Tibia_SOS	0,013602	0,055792	NA
2a	abundance	SL00124	Dissaccari	Handgrip	0,038449	0,062922	NA
2a	abundance	SL00128	N-Acetylal	Tibia_length	0,001491	0,052561	NA
2a	abundance	SL00128	N-Acetylal	Radius_length	0,048435	0,071462	NA
2a	abundance	SL00128	N-Acetylal	Radius_SOS	0,012134	0,050412	NA
2a	abundance	SL00128	N-Acetylal	Tibia_SOS	0,103101	0,054187	NA
2a	abundance	SL00128	N-Acetylal	Handgrip	-0,01294	0,061673	NA
2a	abundance	SL00133	N-Acetylgl	Tibia_length	0,082277	0,052888	NA
2a	abundance	SL00133	N-Acetylgl	Radius_length	0,02883	0,071701	NA
2a	abundance	SL00133	N-Acetylgl	Radius_SOS	-0,04737	0,050496	NA
2a	abundance	SL00133	N-Acetylgl	Tibia_SOS	-0,042	0,053907	NA
2a	abundance	SL00133	N-Acetylgl	Handgrip	0,015926	0,061462	NA
2a	abundance	SL00137	Phenylace	Tibia_length	-0,00816	0,056013	NA
2a	abundance	SL00137	Phenylace	Radius_length	-0,07722	0,074054	NA
2a	abundance	SL00137	Phenylace	Radius_SOS	0,051751	0,053075	NA
2a	abundance	SL00137	Phenylace	Tibia_SOS	-0,08032	0,055937	NA
2a	abundance	SL00137	Phenylace	Handgrip	-0,00328	0,062914	NA
1	abundance	SL00140	Pyridoxam	Tibia_length	0,098317	0,052002	NA
1	abundance	SL00140	Pyridoxam	Radius_length	0,192125	0,070471	NA
1	abundance	SL00140	Pyridoxam	Radius_SOS	0,055673	0,050041	NA
1	abundance	SL00140	Pyridoxam	Tibia_SOS	0,141937	0,052933	NA
1	abundance	SL00140	Pyridoxam	Handgrip	0,065186	0,060184	NA

2a	abundance	SL00149	Dissaccari	Tibia_length	0,012652	0,053152	NA
2a	abundance	SL00149	Dissaccari	Radius_length	0,0814	0,072344	NA
2a	abundance	SL00149	Dissaccari	Radius_SOS	-0,02475	0,05076	NA
2a	abundance	SL00149	Dissaccari	Tibia_SOS	-0,01914	0,054495	NA
2a	abundance	SL00149	Dissaccari	Handgrip	0,007023	0,061698	NA
1	abundance	SL00154	Trigonellin	Tibia_length	-0,03817	0,0558	NA
1	abundance	SL00154	Trigonellin	Radius_length	0,04552	0,074413	NA
1	abundance	SL00154	Trigonellin	Radius_SOS	0,016825	0,053091	NA
1	abundance	SL00154	Trigonellin	Tibia_SOS	0,012266	0,056603	NA
1	abundance	SL00154	Trigonellin	Handgrip	0,094999	0,062669	NA
1	abundance	SL00159	Uracil	Tibia_length	0,025082	0,052136	NA
1	abundance	SL00159	Uracil	Radius_length	0,073715	0,072193	NA
1	abundance	SL00159	Uracil	Radius_SOS	0,008478	0,050098	NA
1	abundance	SL00159	Uracil	Tibia_SOS	0,133603	0,052638	NA
1	abundance	SL00159	Uracil	Handgrip	0,150821	0,060206	NA
2a	abundance	SL00161	Uridine	Tibia_length	0,008068	0,052375	NA
2a	abundance	SL00161	Uridine	Radius_length	0,079816	0,071438	NA
2a	abundance	SL00161	Uridine	Radius_SOS	-0,07158	0,050165	NA
2a	abundance	SL00161	Uridine	Tibia_SOS	-0,00335	0,053757	NA
2a	abundance	SL00161	Uridine	Handgrip	-0,00114	0,061294	NA
1	abundance	SL00185	2-Oxo-3-pł	Tibia_length	0,014134	0,057348	NA
1	abundance	SL00185	2-Oxo-3-pł	Radius_length	-0,15339	0,074252	NA
1	abundance	SL00185	2-Oxo-3-pł	Radius_SOS	-0,08692	0,054634	NA
1	abundance	SL00185	2-Oxo-3-pł	Tibia_SOS	0,025771	0,058149	NA
1	abundance	SL00185	2-Oxo-3-pł	Handgrip	-0,00393	0,063529	NA
2a	abundance	SL00189	3-Hydroxył	Tibia_length	-0,0239	0,055254	NA
2a	abundance	SL00189	3-Hydroxył	Radius_length	-0,06249	0,073562	NA
2a	abundance	SL00189	3-Hydroxył	Radius_SOS	-0,02686	0,0523	NA
2a	abundance	SL00189	3-Hydroxył	Tibia_SOS	-0,05288	0,055552	NA
2a	abundance	SL00189	3-Hydroxył	Handgrip	0,021065	0,063246	NA
2a	abundance	SL00197	4-Methyl-2	Tibia_length	0,149031	0,055656	NA
2a	abundance	SL00197	4-Methyl-2	Radius_length	0,020848	0,074836	NA
2a	abundance	SL00197	4-Methyl-2	Radius_SOS	-0,04537	0,05341	NA
2a	abundance	SL00197	4-Methyl-2	Tibia_SOS	0,117611	0,05733	NA
2a	abundance	SL00197	4-Methyl-2	Handgrip	0,069748	0,062393	NA
2a	abundance	SL00199	5-Hydroxyi	Tibia_length	-0,07121	0,052082	NA
2a	abundance	SL00199	5-Hydroxyi	Radius_length	-0,03116	0,071444	NA
2a	abundance	SL00199	5-Hydroxyi	Radius_SOS	-0,09835	0,049961	NA
2a	abundance	SL00199	5-Hydroxyi	Tibia_SOS	-0,01069	0,053853	NA
2a	abundance	SL00199	5-Hydroxyi	Handgrip	0,084362	0,061425	NA
2a	abundance	SL00200	8-Aminooc	Tibia_length	0,035841	0,052572	NA
2a	abundance	SL00200	8-Aminooc	Radius_length	0,054387	0,072008	NA
2a	abundance	SL00200	8-Aminooc	Radius_SOS	0,015453	0,050484	NA
2a	abundance	SL00200	8-Aminooc	Tibia_SOS	0,03552	0,054745	NA
2a	abundance	SL00200	8-Aminooc	Handgrip	0,047135	0,062308	NA
2a	abundance	SL00201	Pentose II	Tibia_length	0,008171	0,05211	NA
2a	abundance	SL00201	Pentose II	Radius_length	0,137927	0,071133	NA
2a	abundance	SL00201	Pentose II	Radius_SOS	0,017493	0,050016	NA

2a	abundance	SL00201	Pentose II	Tibia_SOS	0,084491	0,052916	NA
2a	abundance	SL00201	Pentose II	Handgrip	0,16256	0,060112	NA
2a	abundance	SL00205	Citraconic	Tibia_length	-0,04275	0,056946	NA
2a	abundance	SL00205	Citraconic	Radius_length	-0,02007	0,07516	NA
2a	abundance	SL00205	Citraconic	Radius_SOS	-0,07389	0,054274	NA
2a	abundance	SL00205	Citraconic	Tibia_SOS	-0,07278	0,056941	NA
2a	abundance	SL00205	Citraconic	Handgrip	0,015264	0,06284	NA
2a	abundance	SL00210	Galactonic	Tibia_length	-0,05137	0,056398	NA
2a	abundance	SL00210	Galactonic	Radius_length	-0,05215	0,074843	NA
2a	abundance	SL00210	Galactonic	Radius_SOS	-0,14495	0,054081	NA
2a	abundance	SL00210	Galactonic	Tibia_SOS	-0,02708	0,056797	NA
2a	abundance	SL00210	Galactonic	Handgrip	0,106994	0,063564	NA
2a	abundance	SL00212	Galactosar	Tibia_length	-0,03692	0,05135	NA
2a	abundance	SL00212	Galactosar	Radius_length	-0,18915	0,068997	NA
2a	abundance	SL00212	Galactosar	Radius_SOS	-0,02064	0,049596	NA
2a	abundance	SL00212	Galactosar	Tibia_SOS	-0,15177	0,052568	NA
2a	abundance	SL00212	Galactosar	Handgrip	-0,15415	0,060012	NA
2a	abundance	SL00215	Glycolic ac	Tibia_length	0,076565	0,055323	NA
2a	abundance	SL00215	Glycolic ac	Radius_length	0,185742	0,072743	NA
2a	abundance	SL00215	Glycolic ac	Radius_SOS	0,04022	0,052399	NA
2a	abundance	SL00215	Glycolic ac	Tibia_SOS	0,143253	0,056301	NA
2a	abundance	SL00215	Glycolic ac	Handgrip	0,063361	0,062167	NA
2a	abundance	SL00216	Glyoxylic a	Tibia_length	-0,0468	0,053364	NA
2a	abundance	SL00216	Glyoxylic a	Radius_length	-0,05534	0,07285	NA
2a	abundance	SL00216	Glyoxylic a	Radius_SOS	-0,03745	0,051107	NA
2a	abundance	SL00216	Glyoxylic a	Tibia_SOS	0,060596	0,054854	NA
2a	abundance	SL00216	Glyoxylic a	Handgrip	0,125939	0,061807	NA
1	abundance	SL00222	Lactic acid	Tibia_length	-0,03093	0,055746	NA
1	abundance	SL00222	Lactic acid	Radius_length	0,003612	0,074205	NA
1	abundance	SL00222	Lactic acid	Radius_SOS	-0,02295	0,053301	NA
1	abundance	SL00222	Lactic acid	Tibia_SOS	-0,01346	0,056555	NA
1	abundance	SL00222	Lactic acid	Handgrip	0,154658	0,061085	NA
2a	abundance	SL00226	N-Acetylgly	Tibia_length	0,096856	0,053905	NA
2a	abundance	SL00226	N-Acetylgly	Radius_length	0,156046	0,071969	NA
2a	abundance	SL00226	N-Acetylgly	Radius_SOS	-0,00595	0,051516	NA
2a	abundance	SL00226	N-Acetylgly	Tibia_SOS	0,098041	0,055889	NA
2a	abundance	SL00226	N-Acetylgly	Handgrip	0,058154	0,06196	NA
2a	abundance	SL00239	Pinitol	Tibia_length	-0,09634	0,055063	NA
2a	abundance	SL00239	Pinitol	Radius_length	-0,03699	0,073517	NA
2a	abundance	SL00239	Pinitol	Radius_SOS	0,02458	0,052375	NA
2a	abundance	SL00239	Pinitol	Tibia_SOS	-0,06518	0,056326	NA
2a	abundance	SL00239	Pinitol	Handgrip	-0,06071	0,063407	NA
1	abundance	SL00240	Pyruvic aci	Tibia_length	0,078354	0,055419	NA
1	abundance	SL00240	Pyruvic aci	Radius_length	0,029946	0,074345	NA
1	abundance	SL00240	Pyruvic aci	Radius_SOS	-0,06008	0,053179	NA
1	abundance	SL00240	Pyruvic aci	Tibia_SOS	0,068526	0,056074	NA
1	abundance	SL00240	Pyruvic aci	Handgrip	0,211058	0,060657	NA
2a	abundance	SL00243	Deoxysuga	Tibia_length	0,005599	0,054567	NA

2a	abundance	SL00243	Deoxysuga	Radius_length	0,08573	0,073889	NA
2a	abundance	SL00243	Deoxysuga	Radius_SOS	0,053501	0,051745	NA
2a	abundance	SL00243	Deoxysuga	Tibia_SOS	0,00575	0,054985	NA
2a	abundance	SL00243	Deoxysuga	Handgrip	0,139053	0,061127	NA
2a	abundance	SL00245	sugar alcol	Tibia_length	0,010534	0,057713	NA
2a	abundance	SL00245	sugar alcol	Radius_length	0,081711	0,07603	NA
2a	abundance	SL00245	sugar alcol	Radius_SOS	0,078648	0,054424	NA
2a	abundance	SL00245	sugar alcol	Tibia_SOS	0,009817	0,057993	NA
2a	abundance	SL00245	sugar alcol	Handgrip	0,169216	0,061121	NA
2a	abundance	SL00246	Pentose III	Tibia_length	-0,01068	0,052437	NA
2a	abundance	SL00246	Pentose III	Radius_length	0,023625	0,072241	NA
2a	abundance	SL00246	Pentose III	Radius_SOS	0,006333	0,050271	NA
2a	abundance	SL00246	Pentose III	Tibia_SOS	0,036428	0,053785	NA
2a	abundance	SL00246	Pentose III	Handgrip	0,107443	0,061282	NA
2a	abundance	SL00247	Shikimic a	Tibia_length	0,012398	0,058755	NA
2a	abundance	SL00247	Shikimic a	Radius_length	-0,06695	0,075403	NA
2a	abundance	SL00247	Shikimic a	Radius_SOS	-0,0571	0,056242	NA
2a	abundance	SL00247	Shikimic a	Tibia_SOS	0,065584	0,060221	NA
2a	abundance	SL00247	Shikimic a	Handgrip	0,010105	0,062661	NA
2a	abundance	SL00248	Gluconic a	Tibia_length	0,009536	0,05365	NA
2a	abundance	SL00248	Gluconic a	Radius_length	0,139038	0,072643	NA
2a	abundance	SL00248	Gluconic a	Radius_SOS	-3,32E-04	0,051118	NA
2a	abundance	SL00248	Gluconic a	Tibia_SOS	0,069417	0,05378	NA
2a	abundance	SL00248	Gluconic a	Handgrip	0,113344	0,061552	NA
2a	abundance	SL00260	Pentose I	Tibia_length	-0,02099	0,055023	NA
2a	abundance	SL00260	Pentose I	Radius_length	0,051073	0,073928	NA
2a	abundance	SL00260	Pentose I	Radius_SOS	-0,0408	0,052356	NA
2a	abundance	SL00260	Pentose I	Tibia_SOS	0,002856	0,055683	NA
2a	abundance	SL00260	Pentose I	Handgrip	0,115027	0,062616	NA
2a	abundance	SL00262	cis-Aconiti	Tibia_length	-0,11144	0,054552	NA
2a	abundance	SL00262	cis-Aconiti	Radius_length	-0,02852	0,073558	NA
2a	abundance	SL00262	cis-Aconiti	Radius_SOS	-0,05481	0,052164	NA
2a	abundance	SL00262	cis-Aconiti	Tibia_SOS	-0,12556	0,056242	NA
2a	abundance	SL00262	cis-Aconiti	Handgrip	-0,03078	0,062632	NA
2a	abundance	SL00264	Methyl ace	Tibia_length	0,108681	0,052703	NA
2a	abundance	SL00264	Methyl ace	Radius_length	0,129369	0,071284	NA
2a	abundance	SL00264	Methyl ace	Radius_SOS	0,027977	0,050442	NA
2a	abundance	SL00264	Methyl ace	Tibia_SOS	0,097809	0,0541	NA
2a	abundance	SL00264	Methyl ace	Handgrip	-0,03004	0,061554	NA
2a	abundance	SL00265	Purine	Tibia_length	0,01791	0,054787	NA
2a	abundance	SL00265	Purine	Radius_length	0,107272	0,073304	NA
2a	abundance	SL00265	Purine	Radius_SOS	0,033035	0,051959	NA
2a	abundance	SL00265	Purine	Tibia_SOS	0,037921	0,055948	NA
2a	abundance	SL00265	Purine	Handgrip	0,11876	0,061432	NA
1	abundance	SL00268	Thymine	Tibia_length	0,016409	0,054066	NA
1	abundance	SL00268	Thymine	Radius_length	-0,01377	0,07334	NA
1	abundance	SL00268	Thymine	Radius_SOS	-0,02366	0,051537	NA
1	abundance	SL00268	Thymine	Tibia_SOS	0,156614	0,056199	NA

	1	abundance	SL00268	Thymine	Handgrip	0,021533	0,06367	NA
2a		abundance	SL00270	3-hydroxy-:	Tibia_length	-0,07854	0,052996	NA
2a		abundance	SL00270	3-hydroxy-:	Radius_length	0,025357	0,071931	NA
2a		abundance	SL00270	3-hydroxy-:	Radius_SOS	0,037703	0,050825	NA
2a		abundance	SL00270	3-hydroxy-:	Tibia_SOS	-0,01957	0,053407	NA
2a		abundance	SL00270	3-hydroxy-:	Handgrip	0,018091	0,060948	NA
2a		abundance	SL00275	Dopamine	Tibia_length	-0,15652	0,053522	NA
2a		abundance	SL00275	Dopamine	Radius_length	-0,13046	0,071774	NA
2a		abundance	SL00275	Dopamine	Radius_SOS	-0,09189	0,051085	NA
2a		abundance	SL00275	Dopamine	Tibia_SOS	-0,0907	0,054268	NA
2a		abundance	SL00275	Dopamine	Handgrip	-0,0336	0,061516	NA
2a		abundance	SL00282	3-Hydroxyl	Tibia_length	-0,15235	0,058341	NA
2a		abundance	SL00282	3-Hydroxyl	Radius_length	-0,16759	0,07484	NA
2a		abundance	SL00282	3-Hydroxyl	Radius_SOS	-0,04951	0,055751	NA
2a		abundance	SL00282	3-Hydroxyl	Tibia_SOS	-0,1374	0,051989	NA
2a		abundance	SL00282	3-Hydroxyl	Handgrip	-0,18441	0,061177	NA
2a		abundance	SL00285	Dihydrofer	Tibia_length	-0,03433	0,052824	NA
2a		abundance	SL00285	Dihydrofer	Radius_length	0,034876	0,071722	NA
2a		abundance	SL00285	Dihydrofer	Radius_SOS	-0,03832	0,050632	NA
2a		abundance	SL00285	Dihydrofer	Tibia_SOS	-0,12032	0,054519	NA
2a		abundance	SL00285	Dihydrofer	Handgrip	0,124842	0,062391	NA
2a		abundance	SL00287	DOPA	Tibia_length	-0,04216	0,051046	NA
2a		abundance	SL00287	DOPA	Radius_length	-0,07406	0,06955	NA
2a		abundance	SL00287	DOPA	Radius_SOS	-0,00211	0,049394	NA
2a		abundance	SL00287	DOPA	Tibia_SOS	-0,09933	0,052336	NA
2a		abundance	SL00287	DOPA	Handgrip	-0,10213	0,06032	NA
2a		abundance	SL00288	Propionylc	Tibia_length	-0,01451	0,058782	NA
2a		abundance	SL00288	Propionylc	Radius_length	0,018426	0,07681	NA
2a		abundance	SL00288	Propionylc	Radius_SOS	0,084762	0,055609	NA
2a		abundance	SL00288	Propionylc	Tibia_SOS	0,008469	0,057913	NA
2a		abundance	SL00288	Propionylc	Handgrip	0,001225	0,060193	NA
2a		abundance	SL00290	N-Acetylm	Tibia_length	0,017181	0,054518	NA
2a		abundance	SL00290	N-Acetylm	Radius_length	0,061096	0,073785	NA
2a		abundance	SL00290	N-Acetylm	Radius_SOS	-0,03268	0,051996	NA
2a		abundance	SL00290	N-Acetylm	Tibia_SOS	0,022794	0,055404	NA
2a		abundance	SL00290	N-Acetylm	Handgrip	0,091184	0,062619	NA
2a		abundance	SL00293	Butyrylcarr	Tibia_length	-0,03089	0,057787	NA
2a		abundance	SL00293	Butyrylcarr	Radius_length	0,096599	0,074835	NA
2a		abundance	SL00293	Butyrylcarr	Radius_SOS	0,023373	0,055046	NA
2a		abundance	SL00293	Butyrylcarr	Tibia_SOS	0,086278	0,057419	NA
2a		abundance	SL00293	Butyrylcarr	Handgrip	-0,0112	0,062653	NA
2a		abundance	SL00295	5-Methylur	Tibia_length	0,04092	0,054029	NA
2a		abundance	SL00295	5-Methylur	Radius_length	-0,00711	0,073246	NA
2a		abundance	SL00295	5-Methylur	Radius_SOS	-0,09428	0,051184	NA
2a		abundance	SL00295	5-Methylur	Tibia_SOS	0,075648	0,054223	NA
2a		abundance	SL00295	5-Methylur	Handgrip	0,004469	0,061795	NA
2a		abundance	SL00297	Acetylmura	Tibia_length	-0,00029	0,049475	NA
2a		abundance	SL00297	Acetylmura	Radius_length	-0,07829	0,066803	NA

2a		abundance	SL00297	Acetylmura	Radius_SOS	-0,00764	0,049037	NA
2a		abundance	SL00297	Acetylmura	Tibia_SOS	0,05506	0,052517	NA
2a		abundance	SL00297	Acetylmura	Handgrip	0,033434	0,060306	NA
2a		abundance	SL00299	Î²-Murichol	Tibia_length	0,037161	0,050668	NA
2a		abundance	SL00299	Î²-Murichol	Radius_length	0,112809	0,068916	NA
2a		abundance	SL00299	Î²-Murichol	Radius_SOS	0,072249	0,049124	NA
2a		abundance	SL00299	Î²-Murichol	Tibia_SOS	-0,03557	0,053218	NA
2a		abundance	SL00299	Î²-Murichol	Handgrip	0,031215	0,061505	NA
2a		abundance	SL00309	1-Aminocy	Tibia_length	0,088526	0,050865	NA
2a		abundance	SL00309	1-Aminocy	Radius_length	0,172024	0,068701	NA
2a		abundance	SL00309	1-Aminocy	Radius_SOS	0,027035	0,049382	NA
2a		abundance	SL00309	1-Aminocy	Tibia_SOS	0,161945	0,052253	NA
2a		abundance	SL00309	1-Aminocy	Handgrip	0,102164	0,060353	NA
2a		abundance	SL00311	N,N-Dimet	Tibia_length	-0,09507	0,053519	NA
2a		abundance	SL00311	N,N-Dimet	Radius_length	-0,0583	0,072645	NA
2a		abundance	SL00311	N,N-Dimet	Radius_SOS	0,005618	0,051204	NA
2a		abundance	SL00311	N,N-Dimet	Tibia_SOS	0,012809	0,054628	NA
2a		abundance	SL00311	N,N-Dimet	Handgrip	-0,00691	0,062289	NA
2a		abundance	SL00313	2-Aminoiso	Tibia_length	-0,10501	0,054959	NA
2a		abundance	SL00313	2-Aminoiso	Radius_length	-0,05205	0,074069	NA
2a		abundance	SL00313	2-Aminoiso	Radius_SOS	-0,06592	0,052199	NA
2a		abundance	SL00313	2-Aminoiso	Tibia_SOS	-0,09447	0,056459	NA
2a		abundance	SL00313	2-Aminoiso	Handgrip	-0,06932	0,060739	NA
2a		abundance	SL00315	Malonic ac	Tibia_length	0,074932	0,049702	NA
2a		abundance	SL00315	Malonic ac	Radius_length	0,127454	0,067079	NA
2a		abundance	SL00315	Malonic ac	Radius_SOS	0,013086	0,049046	NA
2a		abundance	SL00315	Malonic ac	Tibia_SOS	0,032286	0,05246	NA
2a		abundance	SL00315	Malonic ac	Handgrip	0,128273	0,059701	NA
	1	abundance	SL00318	4-Aminoph	Tibia_length	-0,15098	0,053085	NA
	1	abundance	SL00318	4-Aminoph	Radius_length	-0,22651	0,070812	NA
	1	abundance	SL00318	4-Aminoph	Radius_SOS	-0,01327	0,050809	NA
	1	abundance	SL00318	4-Aminoph	Tibia_SOS	-0,05745	0,054488	NA
	1	abundance	SL00318	4-Aminoph	Handgrip	-0,06931	0,061647	NA
2a		abundance	SL00320	Î³-Caprolac	Tibia_length	-0,12601	0,053226	NA
2a		abundance	SL00320	Î³-Caprolac	Radius_length	-0,08914	0,072137	NA
2a		abundance	SL00320	Î³-Caprolac	Radius_SOS	0,014039	0,051008	NA
2a		abundance	SL00320	Î³-Caprolac	Tibia_SOS	-0,10028	0,053929	NA
2a		abundance	SL00320	Î³-Caprolac	Handgrip	-0,0743	0,06118	NA
2a		abundance	SL00325	2-Methylm	Tibia_length	-0,01346	0,056494	NA
2a		abundance	SL00325	2-Methylm	Radius_length	0,046891	0,074239	NA
2a		abundance	SL00325	2-Methylm	Radius_SOS	0,02923	0,053651	NA
2a		abundance	SL00325	2-Methylm	Tibia_SOS	0,052754	0,056954	NA
2a		abundance	SL00325	2-Methylm	Handgrip	-0,0536	0,06284	NA
2a		abundance	SL00328	2-(hydroxy	Tibia_length	0,07241	0,051169	NA
2a		abundance	SL00328	2-(hydroxy	Radius_length	0,001366	0,070509	NA
2a		abundance	SL00328	2-(hydroxy	Radius_SOS	0,018836	0,049602	NA
2a		abundance	SL00328	2-(hydroxy	Tibia_SOS	-0,04521	0,053191	NA
2a		abundance	SL00328	2-(hydroxy	Handgrip	-0,01753	0,061193	NA

2a	abundance	SL00329	2-Hydroxy-	Tibia_length	0,094474	0,053647	NA
2a	abundance	SL00329	2-Hydroxy-	Radius_length	0,138373	0,072522	NA
2a	abundance	SL00329	2-Hydroxy-	Radius_SOS	0,06541	0,050982	NA
2a	abundance	SL00329	2-Hydroxy-	Tibia_SOS	0,057765	0,054836	NA
2a	abundance	SL00329	2-Hydroxy-	Handgrip	0,180232	0,0601	NA
2a	abundance	SL00334	Imidazolea	Tibia_length	0,026929	0,056191	NA
2a	abundance	SL00334	Imidazolea	Radius_length	0,078178	0,073942	NA
2a	abundance	SL00334	Imidazolea	Radius_SOS	0,086138	0,053123	NA
2a	abundance	SL00334	Imidazolea	Tibia_SOS	0,026731	0,056915	NA
2a	abundance	SL00334	Imidazolea	Handgrip	0,075469	0,063575	NA
2a	abundance	SL00346	N-Methylni	Tibia_length	-0,07487	0,054864	NA
2a	abundance	SL00346	N-Methylni	Radius_length	-0,16307	0,072802	NA
2a	abundance	SL00346	N-Methylni	Radius_SOS	-0,07165	0,051805	NA
2a	abundance	SL00346	N-Methylni	Tibia_SOS	-0,05598	0,05452	NA
2a	abundance	SL00346	N-Methylni	Handgrip	-0,02755	0,060921	NA
2a	abundance	SL00349	1-Aminocy	Tibia_length	0,027664	0,05148	NA
2a	abundance	SL00349	1-Aminocy	Radius_length	-0,05581	0,070351	NA
2a	abundance	SL00349	1-Aminocy	Radius_SOS	0,00817	0,049768	NA
2a	abundance	SL00349	1-Aminocy	Tibia_SOS	0,009514	0,053005	NA
2a	abundance	SL00349	1-Aminocy	Handgrip	-0,01972	0,061861	NA
1	abundance	SL00350	Stachydrin	Tibia_length	-0,0552	0,051634	NA
1	abundance	SL00350	Stachydrin	Radius_length	-0,03224	0,070586	NA
1	abundance	SL00350	Stachydrin	Radius_SOS	-0,06207	0,049756	NA
1	abundance	SL00350	Stachydrin	Tibia_SOS	-0,05243	0,053436	NA
1	abundance	SL00350	Stachydrin	Handgrip	0,053936	0,060519	NA
1	abundance	SL00353	Adipic acid	Tibia_length	0,006779	0,051098	NA
1	abundance	SL00353	Adipic acid	Radius_length	0,095772	0,069781	NA
1	abundance	SL00353	Adipic acid	Radius_SOS	-0,00857	0,049511	NA
1	abundance	SL00353	Adipic acid	Tibia_SOS	-0,03704	0,053005	NA
1	abundance	SL00353	Adipic acid	Handgrip	-0,06198	0,061117	NA
2a	abundance	SL00356	N-(5-Amin	Tibia_length	-0,00498	0,050128	NA
2a	abundance	SL00356	N-(5-Amin	Radius_length	-0,03764	0,068219	NA
2a	abundance	SL00356	N-(5-Amin	Radius_SOS	-0,04199	0,049068	NA
2a	abundance	SL00356	N-(5-Amin	Tibia_SOS	-0,06363	0,052707	NA
2a	abundance	SL00356	N-(5-Amin	Handgrip	0,009885	0,060852	NA
1	abundance	SL00368	N-Methylty	Tibia_length	-0,03872	0,05789	NA
1	abundance	SL00368	N-Methylty	Radius_length	-0,04282	0,074852	NA
1	abundance	SL00368	N-Methylty	Radius_SOS	-0,01442	0,055139	NA
1	abundance	SL00368	N-Methylty	Tibia_SOS	0,073177	0,058224	NA
1	abundance	SL00368	N-Methylty	Handgrip	0,016485	0,060185	NA
2a	abundance	SL00373	N1-Methyl-	Tibia_length	0,037698	0,048957	NA
2a	abundance	SL00373	N1-Methyl-	Radius_length	0,153391	0,063223	NA
2a	abundance	SL00373	N1-Methyl-	Radius_SOS	0,052749	0,049351	NA
2a	abundance	SL00373	N1-Methyl-	Tibia_SOS	-4,64E-04	0,052719	NA
2a	abundance	SL00373	N1-Methyl-	Handgrip	0,131975	0,059711	NA
2a	abundance	SL00376	N-AcetylPr	Tibia_length	-0,1477	0,056404	NA
2a	abundance	SL00376	N-AcetylPr	Radius_length	-0,06527	0,074212	NA
2a	abundance	SL00376	N-AcetylPr	Radius_SOS	-0,0933	0,053314	NA

2a	abundance	SL00376	N-AcetylPr	Tibia_SOS	-0,10165	0,055891	NA
2a	abundance	SL00376	N-AcetylPr	Handgrip	-0,10658	0,062121	NA
2a	abundance	SL00380	2-Aminoad	Tibia_length	0,033022	0,051481	NA
2a	abundance	SL00380	2-Aminoad	Radius_length	0,144636	0,070157	NA
2a	abundance	SL00380	2-Aminoad	Radius_SOS	0,015641	0,049742	NA
2a	abundance	SL00380	2-Aminoad	Tibia_SOS	0,019528	0,053363	NA
2a	abundance	SL00380	2-Aminoad	Handgrip	-0,01457	0,0617	NA
2a	abundance	SL00383	3-(2-Hydro	Tibia_length	0,040579	0,053681	NA
2a	abundance	SL00383	3-(2-Hydro	Radius_length	0,026797	0,072616	NA
2a	abundance	SL00383	3-(2-Hydro	Radius_SOS	0,069628	0,051055	NA
2a	abundance	SL00383	3-(2-Hydro	Tibia_SOS	0,107294	0,055476	NA
2a	abundance	SL00383	3-(2-Hydro	Handgrip	0,001674	0,062196	NA
1	abundance	SL00384	p-Coumari	Tibia_length	0,057215	0,052527	NA
1	abundance	SL00384	p-Coumari	Radius_length	-0,05666	0,071483	NA
1	abundance	SL00384	p-Coumari	Radius_SOS	-0,0686	0,050246	NA
1	abundance	SL00384	p-Coumari	Tibia_SOS	-0,0362	0,054262	NA
1	abundance	SL00384	p-Coumari	Handgrip	-0,02449	0,062831	NA
2a	abundance	SL00390	Gallic acid	Tibia_length	-0,14184	0,052928	NA
2a	abundance	SL00390	Gallic acid	Radius_length	-0,08934	0,072237	NA
2a	abundance	SL00390	Gallic acid	Radius_SOS	-0,03733	0,050858	NA
2a	abundance	SL00390	Gallic acid	Tibia_SOS	-0,10199	0,053897	NA
2a	abundance	SL00390	Gallic acid	Handgrip	-0,056	0,061532	NA
1	abundance	SL00391	N-Acetyllei	Tibia_length	0,117539	0,052018	NA
1	abundance	SL00391	N-Acetyllei	Radius_length	0,105039	0,071058	NA
1	abundance	SL00391	N-Acetyllei	Radius_SOS	0,052039	0,050118	NA
1	abundance	SL00391	N-Acetyllei	Tibia_SOS	0,067062	0,054035	NA
1	abundance	SL00391	N-Acetyllei	Handgrip	-0,00693	0,061799	NA
1	abundance	SL00401	Theophyllii	Tibia_length	-0,0628	0,054755	NA
1	abundance	SL00401	Theophyllii	Radius_length	-0,10832	0,073023	NA
1	abundance	SL00401	Theophyllii	Radius_SOS	-0,00796	0,052294	NA
1	abundance	SL00401	Theophyllii	Tibia_SOS	-0,0203	0,054883	NA
1	abundance	SL00401	Theophyllii	Handgrip	-0,05552	0,061614	NA
2a	abundance	SL00407	Homovanil	Tibia_length	-0,05292	0,051488	NA
2a	abundance	SL00407	Homovanil	Radius_length	-0,12304	0,07015	NA
2a	abundance	SL00407	Homovanil	Radius_SOS	-0,04355	0,049687	NA
2a	abundance	SL00407	Homovanil	Tibia_SOS	-0,07294	0,053425	NA
2a	abundance	SL00407	Homovanil	Handgrip	-0,02086	0,060514	NA
2a	abundance	SL00420	4-Hydroxy-	Tibia_length	-0,05251	0,054628	NA
2a	abundance	SL00420	4-Hydroxy-	Radius_length	-0,17443	0,072572	NA
2a	abundance	SL00420	4-Hydroxy-	Radius_SOS	-0,07267	0,051859	NA
2a	abundance	SL00420	4-Hydroxy-	Tibia_SOS	-0,11234	0,054992	NA
2a	abundance	SL00420	4-Hydroxy-	Handgrip	-0,13907	0,061633	NA
2a	abundance	SL00421	Asymmetri	Tibia_length	-0,13302	0,048973	NA
2a	abundance	SL00421	Asymmetri	Radius_length	-0,1599	0,065888	NA
2a	abundance	SL00421	Asymmetri	Radius_SOS	-0,07012	0,048911	NA
2a	abundance	SL00421	Asymmetri	Tibia_SOS	-0,11276	0,052177	NA
2a	abundance	SL00421	Asymmetri	Handgrip	-0,0207	0,060374	NA
2a	abundance	SL00430	Homocarn	Tibia_length	-0,0569	0,05343	NA

2a	abundance	SL00430	Homocarn	Radius_length	-0,01705	0,072397	NA
2a	abundance	SL00430	Homocarn	Radius_SOS	0,024722	0,051051	NA
2a	abundance	SL00430	Homocarn	Tibia_SOS	0,013088	0,054475	NA
2a	abundance	SL00430	Homocarn	Handgrip	-0,01366	0,062179	NA
2a	abundance	SL00431	Tiglylcarnit	Tibia_length	-0,06788	0,053356	NA
2a	abundance	SL00431	Tiglylcarnit	Radius_length	-0,1715	0,071585	NA
2a	abundance	SL00431	Tiglylcarnit	Radius_SOS	-0,00761	0,05101	NA
2a	abundance	SL00431	Tiglylcarnit	Tibia_SOS	-0,15399	0,053619	NA
2a	abundance	SL00431	Tiglylcarnit	Handgrip	-0,1166	0,061812	NA
2a	abundance	SL00433	Isovalerylc	Tibia_length	-0,08338	0,053534	NA
2a	abundance	SL00433	Isovalerylc	Radius_length	0,122261	0,072623	NA
2a	abundance	SL00433	Isovalerylc	Radius_SOS	-0,02281	0,051298	NA
2a	abundance	SL00433	Isovalerylc	Tibia_SOS	-0,02342	0,054548	NA
2a	abundance	SL00433	Isovalerylc	Handgrip	0,01485	0,061988	NA
2a	abundance	SL00436	1-Carboxy(	Tibia_length	0,016997	0,053739	NA
2a	abundance	SL00436	1-Carboxy(	Radius_length	0,089667	0,072183	NA
2a	abundance	SL00436	1-Carboxy(	Radius_SOS	0,083109	0,051038	NA
2a	abundance	SL00436	1-Carboxy(	Tibia_SOS	0,065646	0,053994	NA
2a	abundance	SL00436	1-Carboxy(	Handgrip	0,031512	0,061049	NA
2a	abundance	SL00438	Daidzein	Tibia_length	-0,00822	0,054102	NA
2a	abundance	SL00438	Daidzein	Radius_length	-0,02087	0,073589	NA
2a	abundance	SL00438	Daidzein	Radius_SOS	0,011044	0,051641	NA
2a	abundance	SL00438	Daidzein	Tibia_SOS	0,044082	0,055274	NA
2a	abundance	SL00438	Daidzein	Handgrip	0,016786	0,063367	NA
2a	abundance	SL00440	5-Methylcy	Tibia_length	0,017369	0,052785	NA
2a	abundance	SL00440	5-Methylcy	Radius_length	0,195762	0,070411	NA
2a	abundance	SL00440	5-Methylcy	Radius_SOS	0,011286	0,05052	NA
2a	abundance	SL00440	5-Methylcy	Tibia_SOS	0,086294	0,05424	NA
2a	abundance	SL00440	5-Methylcy	Handgrip	0,121476	0,060584	NA
2a	abundance	SL00445	Glucosami	Tibia_length	-0,08718	0,055924	NA
2a	abundance	SL00445	Glucosami	Radius_length	-0,06493	0,074222	NA
2a	abundance	SL00445	Glucosami	Radius_SOS	-0,09879	0,053543	NA
2a	abundance	SL00445	Glucosami	Tibia_SOS	-0,02486	0,056434	NA
2a	abundance	SL00445	Glucosami	Handgrip	0,001549	0,062895	NA
2a	abundance	SL00447	Apigenin	Tibia_length	0,021945	0,05396	NA
2a	abundance	SL00447	Apigenin	Radius_length	-0,0033	0,073333	NA
2a	abundance	SL00447	Apigenin	Radius_SOS	-0,05346	0,051544	NA
2a	abundance	SL00447	Apigenin	Tibia_SOS	-0,02202	0,056457	NA
2a	abundance	SL00447	Apigenin	Handgrip	0,090721	0,062732	NA
2a	abundance	SL00455	12,13-DH(	Tibia_length	0,106408	0,05165	NA
2a	abundance	SL00455	12,13-DH(	Radius_length	0,175376	0,069624	NA
2a	abundance	SL00455	12,13-DH(	Radius_SOS	0,04034	0,04974	NA
2a	abundance	SL00455	12,13-DH(	Tibia_SOS	0,014666	0,053131	NA
2a	abundance	SL00455	12,13-DH(	Handgrip	0,021071	0,060772	NA
2a	abundance	SL00467	Sucralose	Tibia_length	-0,01487	0,05373	NA
2a	abundance	SL00467	Sucralose	Radius_length	-0,02797	0,072539	NA
2a	abundance	SL00467	Sucralose	Radius_SOS	0,048315	0,051291	NA
2a	abundance	SL00467	Sucralose	Tibia_SOS	-0,00145	0,054815	NA

2a		abundance	SL00467	Sucralose	Handgrip	0,040725	0,062307	NA
	1	abundance	SL00502	2-Ketobuty	Tibia_length	0,03935	0,051689	NA
	1	abundance	SL00502	2-Ketobuty	Radius_length	-0,05217	0,070893	NA
	1	abundance	SL00502	2-Ketobuty	Radius_SOS	0,020005	0,049864	NA
	1	abundance	SL00502	2-Ketobuty	Tibia_SOS	0,064163	0,054088	NA
	1	abundance	SL00502	2-Ketobuty	Handgrip	0,03979	0,061062	NA
2a		abundance	X00011	Glutamine	Tibia_length	0,083273	0,052176	NA
2a		abundance	X00011	Glutamine	Radius_length	0,110833	0,070745	NA
2a		abundance	X00011	Glutamine	Radius_SOS	-0,00756	0,050049	NA
2a		abundance	X00011	Glutamine	Tibia_SOS	0,135753	0,052907	NA
2a		abundance	X00011	Glutamine	Handgrip	0,031328	0,060691	NA
	1	abundance	X00018	Succinic a	Tibia_length	0,038165	0,055819	NA
	1	abundance	X00018	Succinic a	Radius_length	0,033086	0,074017	NA
	1	abundance	X00018	Succinic a	Radius_SOS	0,035122	0,052991	NA
	1	abundance	X00018	Succinic a	Tibia_SOS	-0,03772	0,055985	NA
	1	abundance	X00018	Succinic a	Handgrip	0,128902	0,061474	NA
	1	abundance	X00020	sugar alcol	Tibia_length	-0,06732	0,057716	NA
	1	abundance	X00020	sugar alcol	Radius_length	-0,06583	0,075423	NA
	1	abundance	X00020	sugar alcol	Radius_SOS	0,032338	0,054422	NA
	1	abundance	X00020	sugar alcol	Tibia_SOS	-0,02001	0,057789	NA
	1	abundance	X00020	sugar alcol	Handgrip	0,057451	0,062899	NA
	1	abundance	X00022	N-Methylas	Tibia_length	0,11706	0,051278	NA
	1	abundance	X00022	N-Methylas	Radius_length	0,222258	0,068789	NA
	1	abundance	X00022	N-Methylas	Radius_SOS	0,048927	0,049519	NA
	1	abundance	X00022	N-Methylas	Tibia_SOS	0,175979	0,052228	NA
	1	abundance	X00022	N-Methylas	Handgrip	0,07412	0,060424	NA
	1	abundance	X00023	N-Formylm	Tibia_length	-0,06421	0,050756	NA
	1	abundance	X00023	N-Formylm	Radius_length	-0,09939	0,069918	NA
	1	abundance	X00023	N-Formylm	Radius_SOS	-0,03467	0,049376	NA
	1	abundance	X00023	N-Formylm	Tibia_SOS	-0,04874	0,052805	NA
	1	abundance	X00023	N-Formylm	Handgrip	-0,14638	0,059744	NA
	1	abundance	X00025	Acesulfam	Tibia_length	-0,05025	0,052713	NA
	1	abundance	X00025	Acesulfam	Radius_length	-0,14053	0,071539	NA
	1	abundance	X00025	Acesulfam	Radius_SOS	0,034353	0,050389	NA
	1	abundance	X00025	Acesulfam	Tibia_SOS	-0,01141	0,054851	NA
	1	abundance	X00025	Acesulfam	Handgrip	-0,04856	0,061501	NA
	1	abundance	X00026	Inosine	Tibia_length	-0,0319	0,055859	NA
	1	abundance	X00026	Inosine	Radius_length	-0,06449	0,074004	NA
	1	abundance	X00026	Inosine	Radius_SOS	-0,11461	0,052857	NA
	1	abundance	X00026	Inosine	Tibia_SOS	0,055462	0,056122	NA
	1	abundance	X00026	Inosine	Handgrip	0,028778	0,062392	NA
	1	abundance	X00027	N-Acetylglu	Tibia_length	0,147758	0,052138	NA
	1	abundance	X00027	N-Acetylglu	Radius_length	0,194949	0,07007	NA
	1	abundance	X00027	N-Acetylglu	Radius_SOS	0,029917	0,05017	NA
	1	abundance	X00027	N-Acetylglu	Tibia_SOS	0,071167	0,052869	NA
	1	abundance	X00027	N-Acetylglu	Handgrip	0,008762	0,060524	NA
	1	abundance	X00029	Urocanic a	Tibia_length	0,006555	0,053465	NA
	1	abundance	X00029	Urocanic a	Radius_length	-0,10891	0,072467	NA

1	abundance	X00029	Urocanic a	Radius_SOS	-4,36E-04	0,051064	NA
1	abundance	X00029	Urocanic a	Tibia_SOS	0,11451	0,055037	NA
1	abundance	X00029	Urocanic a	Handgrip	0,058963	0,063068	NA
1	abundance	X00030	3,4-Dihydr	Tibia_length	-0,1607	0,053077	NA
1	abundance	X00030	3,4-Dihydr	Radius_length	-0,0642	0,072364	NA
1	abundance	X00030	3,4-Dihydr	Radius_SOS	0,006696	0,051007	NA
1	abundance	X00030	3,4-Dihydr	Tibia_SOS	-0,05225	0,054194	NA
1	abundance	X00030	3,4-Dihydr	Handgrip	0,042196	0,062511	NA
1	abundance	X00032	Indole-3-pı	Tibia_length	0,022544	0,054603	NA
1	abundance	X00032	Indole-3-pı	Radius_length	0,071272	0,073134	NA
1	abundance	X00032	Indole-3-pı	Radius_SOS	0,040054	0,051888	NA
1	abundance	X00032	Indole-3-pı	Tibia_SOS	0,061372	0,055305	NA
1	abundance	X00032	Indole-3-pı	Handgrip	0,121808	0,062633	NA
1	abundance	X00033	Deoxychol	Tibia_length	0,026075	0,052178	NA
1	abundance	X00033	Deoxychol	Radius_length	0,018066	0,071483	NA
1	abundance	X00033	Deoxychol	Radius_SOS	0,097919	0,050044	NA
1	abundance	X00033	Deoxychol	Tibia_SOS	-0,00499	0,053605	NA
1	abundance	X00033	Deoxychol	Handgrip	0,02805	0,060772	NA
1	abundance	X00034	Pyroglutan	Tibia_length	0,11027	0,053098	NA
1	abundance	X00034	Pyroglutan	Radius_length	0,117044	0,072026	NA
1	abundance	X00034	Pyroglutan	Radius_SOS	0,042281	0,050878	NA
1	abundance	X00034	Pyroglutan	Tibia_SOS	0,076614	0,054777	NA
1	abundance	X00034	Pyroglutan	Handgrip	0,083689	0,060364	NA
1	abundance	X00035	Choline	Tibia_length	-0,06916	0,054051	NA
1	abundance	X00035	Choline	Radius_length	-0,10685	0,072629	NA
1	abundance	X00035	Choline	Radius_SOS	-0,09246	0,052011	NA
1	abundance	X00035	Choline	Tibia_SOS	-0,03166	0,055196	NA
1	abundance	X00035	Choline	Handgrip	-0,00449	0,062191	NA
1	abundance	X00036	Serine	Tibia_length	0,025488	0,051632	NA
1	abundance	X00036	Serine	Radius_length	0,066225	0,070411	NA
1	abundance	X00036	Serine	Radius_SOS	-0,03455	0,049798	NA
1	abundance	X00036	Serine	Tibia_SOS	0,072152	0,053184	NA
1	abundance	X00036	Serine	Handgrip	0,113664	0,061365	NA
1	abundance	X00038	Histamine	Tibia_length	-0,1072	0,048821	NA
1	abundance	X00038	Histamine	Radius_length	-0,15892	0,064049	NA
1	abundance	X00038	Histamine	Radius_SOS	-0,0234	0,049277	NA
1	abundance	X00038	Histamine	Tibia_SOS	-0,1336	0,052202	NA
1	abundance	X00038	Histamine	Handgrip	0,026288	0,060319	NA
1	abundance	X00040	N-Acetyltyı	Tibia_length	0,091833	0,052209	NA
1	abundance	X00040	N-Acetyltyı	Radius_length	0,097689	0,071129	NA
1	abundance	X00040	N-Acetyltyı	Radius_SOS	0,08767	0,050018	NA
1	abundance	X00040	N-Acetyltyı	Tibia_SOS	0,083996	0,053246	NA
1	abundance	X00040	N-Acetyltyı	Handgrip	-0,03688	0,060931	NA
1	abundance	X00042	Glucuronic	Tibia_length	-0,0122	0,056257	NA
1	abundance	X00042	Glucuronic	Radius_length	-0,0165	0,075064	NA
1	abundance	X00042	Glucuronic	Radius_SOS	-0,04844	0,053305	NA
1	abundance	X00042	Glucuronic	Tibia_SOS	0,033226	0,056363	NA
1	abundance	X00042	Glucuronic	Handgrip	0,130063	0,06313	NA

	1	abundance	X00045	Threonine	Tibia_length	0,073615	0,051296	NA
	1	abundance	X00045	Threonine	Radius_length	0,115764	0,069903	NA
	1	abundance	X00045	Threonine	Radius_SOS	-0,02265	0,049618	NA
	1	abundance	X00045	Threonine	Tibia_SOS	0,078008	0,052968	NA
	1	abundance	X00045	Threonine	Handgrip	0,106462	0,060346	NA
	1	abundance	X00050	Î²-Hydroxyi	Tibia_length	-0,06128	0,053066	NA
	1	abundance	X00050	Î²-Hydroxyi	Radius_length	4,85E-04	0,072149	NA
	1	abundance	X00050	Î²-Hydroxyi	Radius_SOS	-0,05394	0,050806	NA
	1	abundance	X00050	Î²-Hydroxyi	Tibia_SOS	0,026945	0,054199	NA
	1	abundance	X00050	Î²-Hydroxyi	Handgrip	0,044248	0,061738	NA
2b		abundance	X00051	Î²-D-Gluco	Tibia_length	0,015004	0,057117	NA
2b		abundance	X00051	Î²-D-Gluco	Radius_length	0,014331	0,07545	NA
2b		abundance	X00051	Î²-D-Gluco	Radius_SOS	-0,00603	0,054218	NA
2b		abundance	X00051	Î²-D-Gluco	Tibia_SOS	0,058796	0,05838	NA
2b		abundance	X00051	Î²-D-Gluco	Handgrip	0,131312	0,059672	NA
	1	abundance	X00056	3-(4-hydro:	Tibia_length	0,032282	0,053616	NA
	1	abundance	X00056	3-(4-hydro:	Radius_length	-0,04725	0,072741	NA
	1	abundance	X00056	3-(4-hydro:	Radius_SOS	0,062286	0,050953	NA
	1	abundance	X00056	3-(4-hydro:	Tibia_SOS	0,007527	0,05602	NA
	1	abundance	X00056	3-(4-hydro:	Handgrip	0,052843	0,062083	NA
	1	abundance	X00057	N-Acetylor	Tibia_length	6,97E-04	0,054207	NA
	1	abundance	X00057	N-Acetylor	Radius_length	0,050254	0,073263	NA
	1	abundance	X00057	N-Acetylor	Radius_SOS	5,99E-04	0,051644	NA
	1	abundance	X00057	N-Acetylor	Tibia_SOS	0,040211	0,055159	NA
	1	abundance	X00057	N-Acetylor	Handgrip	-0,04115	0,061502	NA
	1	abundance	X00059	3,4-Dihydr	Tibia_length	-0,14414	0,054442	NA
	1	abundance	X00059	3,4-Dihydr	Radius_length	-0,04117	0,073707	NA
	1	abundance	X00059	3,4-Dihydr	Radius_SOS	-0,0563	0,052099	NA
	1	abundance	X00059	3,4-Dihydr	Tibia_SOS	-0,10612	0,055135	NA
	1	abundance	X00059	3,4-Dihydr	Handgrip	0,108751	0,060776	NA
	1	abundance	X00060	Ethylmalor	Tibia_length	-0,00341	0,05471	NA
	1	abundance	X00060	Ethylmalor	Radius_length	0,035117	0,073244	NA
	1	abundance	X00060	Ethylmalor	Radius_SOS	0,068698	0,051877	NA
	1	abundance	X00060	Ethylmalor	Tibia_SOS	-0,06361	0,054857	NA
	1	abundance	X00060	Ethylmalor	Handgrip	-0,05711	0,062465	NA
	1	abundance	X00061	Thiamine	Tibia_length	-0,00149	0,050104	NA
	1	abundance	X00061	Thiamine	Radius_length	-0,04162	0,068256	NA
	1	abundance	X00061	Thiamine	Radius_SOS	-0,0669	0,049016	NA
	1	abundance	X00061	Thiamine	Tibia_SOS	0,058668	0,052775	NA
	1	abundance	X00061	Thiamine	Handgrip	-0,05388	0,060808	NA
	1	abundance	X00063	2-Hydroxy(	Tibia_length	0,10413	0,053997	NA
	1	abundance	X00063	2-Hydroxy(	Radius_length	0,138514	0,072888	NA
	1	abundance	X00063	2-Hydroxy(	Radius_SOS	0,068549	0,05127	NA
	1	abundance	X00063	2-Hydroxy(	Tibia_SOS	0,071645	0,055668	NA
	1	abundance	X00063	2-Hydroxy(	Handgrip	0,188187	0,060442	NA
	1	abundance	X00064	Traumatic	Tibia_length	0,066096	0,053842	NA
	1	abundance	X00064	Traumatic	Radius_length	0,074945	0,072847	NA
	1	abundance	X00064	Traumatic	Radius_SOS	0,068049	0,051362	NA

1	abundance	X00064	Traumatic	Tibia_SOS	0,078011	0,054044	NA
1	abundance	X00064	Traumatic	Handgrip	-0,00349	0,061674	NA
1	abundance	X00066	Tryptamine	Tibia_length	0,037466	0,049441	NA
1	abundance	X00066	Tryptamine	Radius_length	-0,00447	0,06675	NA
1	abundance	X00066	Tryptamine	Radius_SOS	-0,04949	0,048979	NA
1	abundance	X00066	Tryptamine	Tibia_SOS	-0,02475	0,052496	NA
1	abundance	X00066	Tryptamine	Handgrip	-0,0917	0,059963	NA
1	abundance	X00067	Hypoxanth	Tibia_length	-0,09717	0,055088	NA
1	abundance	X00067	Hypoxanth	Radius_length	-0,00965	0,074436	NA
1	abundance	X00067	Hypoxanth	Radius_SOS	-0,07046	0,052641	NA
1	abundance	X00067	Hypoxanth	Tibia_SOS	0,083143	0,055658	NA
1	abundance	X00067	Hypoxanth	Handgrip	0,092361	0,062811	NA
1	abundance	X00068	N-Acetylm	Tibia_length	0,1419	0,050285	NA
1	abundance	X00068	N-Acetylm	Radius_length	0,160506	0,068082	NA
1	abundance	X00068	N-Acetylm	Radius_SOS	0,032645	0,049194	NA
1	abundance	X00068	N-Acetylm	Tibia_SOS	0,10788	0,052492	NA
1	abundance	X00068	N-Acetylm	Handgrip	0,068761	0,060121	NA
1	abundance	X00070	Î±-aminobu	Tibia_length	-0,15121	0,051843	NA
1	abundance	X00070	Î±-aminobu	Radius_length	-0,12023	0,071261	NA
1	abundance	X00070	Î±-aminobu	Radius_SOS	-0,03729	0,050003	NA
1	abundance	X00070	Î±-aminobu	Tibia_SOS	-0,06254	0,053376	NA
1	abundance	X00070	Î±-aminobu	Handgrip	0,005603	0,060405	NA
1	abundance	X00071	Tyrosine	Tibia_length	0,172024	0,048699	NA
1	abundance	X00071	Tyrosine	Radius_length	0,14535	0,065646	NA
1	abundance	X00071	Tyrosine	Radius_SOS	0,059246	0,048959	NA
1	abundance	X00071	Tyrosine	Tibia_SOS	0,103179	0,052254	NA
1	abundance	X00071	Tyrosine	Handgrip	0,035829	0,060163	NA
1	abundance	X00072	3,5-Dihydr	Tibia_length	-0,10341	0,054833	NA
1	abundance	X00072	3,5-Dihydr	Radius_length	-0,05023	0,07357	NA
1	abundance	X00072	3,5-Dihydr	Radius_SOS	-0,09287	0,052411	NA
1	abundance	X00072	3,5-Dihydr	Tibia_SOS	-0,03743	0,055137	NA
1	abundance	X00072	3,5-Dihydr	Handgrip	-0,01015	0,062739	NA
1	abundance	X00073	Indole-3-la	Tibia_length	0,098404	0,05272	NA
1	abundance	X00073	Indole-3-la	Radius_length	0,173687	0,071209	NA
1	abundance	X00073	Indole-3-la	Radius_SOS	0,064745	0,050193	NA
1	abundance	X00073	Indole-3-la	Tibia_SOS	0,095293	0,054171	NA
1	abundance	X00073	Indole-3-la	Handgrip	0,235417	0,059314	NA
1	abundance	X00074	Methylsucc	Tibia_length	0,043367	0,051023	NA
1	abundance	X00074	Methylsucc	Radius_length	0,006046	0,069757	NA
1	abundance	X00074	Methylsucc	Radius_SOS	0,052284	0,049369	NA
1	abundance	X00074	Methylsucc	Tibia_SOS	0,059673	0,052746	NA
1	abundance	X00074	Methylsucc	Handgrip	0,035955	0,060554	NA
1	abundance	X00076	Isoleucine	Tibia_length	0,119449	0,050168	NA
1	abundance	X00076	Isoleucine	Radius_length	0,147209	0,068194	NA
1	abundance	X00076	Isoleucine	Radius_SOS	0,011468	0,049183	NA
1	abundance	X00076	Isoleucine	Tibia_SOS	0,144782	0,052136	NA
1	abundance	X00076	Isoleucine	Handgrip	0,078352	0,060086	NA
1	abundance	X00078	Acetylagm	Tibia_length	8,37E-04	0,050501	NA

1	abundance	X00078	Acetylagm	Radius_length	-0,06439	0,069172	NA
1	abundance	X00078	Acetylagm	Radius_SOS	-0,08031	0,049096	NA
1	abundance	X00078	Acetylagm	Tibia_SOS	-0,05181	0,052875	NA
1	abundance	X00078	Acetylagm	Handgrip	0,011341	0,060706	NA
1	abundance	X00082	Cholic acid	Tibia_length	-4,40E-05	0,050032	NA
1	abundance	X00082	Cholic acid	Radius_length	0,060456	0,068122	NA
1	abundance	X00082	Cholic acid	Radius_SOS	-0,0068	0,049099	NA
1	abundance	X00082	Cholic acid	Tibia_SOS	-0,06909	0,052469	NA
1	abundance	X00082	Cholic acid	Handgrip	0,026596	0,060755	NA
1	abundance	X00083	1,7-Dimeth	Tibia_length	-0,06132	0,050524	NA
1	abundance	X00083	1,7-Dimeth	Radius_length	-0,07951	0,069018	NA
1	abundance	X00083	1,7-Dimeth	Radius_SOS	0,027533	0,049243	NA
1	abundance	X00083	1,7-Dimeth	Tibia_SOS	-0,05201	0,05243	NA
1	abundance	X00083	1,7-Dimeth	Handgrip	0,005264	0,060352	NA
1	abundance	X00084	Tricarballyl	Tibia_length	-0,03418	0,053194	NA
1	abundance	X00084	Tricarballyl	Radius_length	-0,08495	0,071801	NA
1	abundance	X00084	Tricarballyl	Radius_SOS	-0,02668	0,050817	NA
1	abundance	X00084	Tricarballyl	Tibia_SOS	-0,08528	0,05355	NA
1	abundance	X00084	Tricarballyl	Handgrip	-0,02332	0,061718	NA
1	abundance	X00088	Pantotheni	Tibia_length	-0,05206	0,055753	NA
1	abundance	X00088	Pantotheni	Radius_length	-0,11026	0,07357	NA
1	abundance	X00088	Pantotheni	Radius_SOS	-0,07851	0,053467	NA
1	abundance	X00088	Pantotheni	Tibia_SOS	-0,00797	0,056245	NA
1	abundance	X00088	Pantotheni	Handgrip	-0,02402	0,062171	NA
1	abundance	X00089	4-Hydroxyb	Tibia_length	-0,0299	0,052431	NA
1	abundance	X00089	4-Hydroxyb	Radius_length	-0,02723	0,071688	NA
1	abundance	X00089	4-Hydroxyb	Radius_SOS	-0,06606	0,050249	NA
1	abundance	X00089	4-Hydroxyb	Tibia_SOS	0,029211	0,053043	NA
1	abundance	X00089	4-Hydroxyb	Handgrip	0,206846	0,059743	NA
1	abundance	X00090	Malic acid	Tibia_length	-0,02968	0,053279	NA
1	abundance	X00090	Malic acid	Radius_length	-0,11538	0,07229	NA
1	abundance	X00090	Malic acid	Radius_SOS	-0,05631	0,050932	NA
1	abundance	X00090	Malic acid	Tibia_SOS	0,031112	0,054603	NA
1	abundance	X00090	Malic acid	Handgrip	0,095452	0,062647	NA
1	abundance	X00092	3-Methylhi	Tibia_length	-0,1028	0,051248	NA
1	abundance	X00092	3-Methylhi	Radius_length	-0,01893	0,070533	NA
1	abundance	X00092	3-Methylhi	Radius_SOS	4,80E-04	0,049755	NA
1	abundance	X00092	3-Methylhi	Tibia_SOS	0,035759	0,053293	NA
1	abundance	X00092	3-Methylhi	Handgrip	-0,00218	0,061297	NA
1	abundance	X00093	Deoxyinosi	Tibia_length	0,016247	0,05183	NA
1	abundance	X00093	Deoxyinosi	Radius_length	0,05206	0,070715	NA
1	abundance	X00093	Deoxyinosi	Radius_SOS	-0,06955	0,049762	NA
1	abundance	X00093	Deoxyinosi	Tibia_SOS	0,04137	0,053875	NA
1	abundance	X00093	Deoxyinosi	Handgrip	0,100349	0,061312	NA
1	abundance	X00094	Methionine	Tibia_length	0,101042	0,050688	NA
1	abundance	X00094	Methionine	Radius_length	0,179148	0,068276	NA
1	abundance	X00094	Methionine	Radius_SOS	0,023745	0,049308	NA
1	abundance	X00094	Methionine	Tibia_SOS	0,135725	0,052383	NA

1	abundance	X00094	Methionine	Handgrip	0,111918	0,060024	NA
1	abundance	X00097	2,6-Dihydr	Tibia_length	-0,11345	0,050963	NA
1	abundance	X00097	2,6-Dihydr	Radius_length	-0,09682	0,070277	NA
1	abundance	X00097	2,6-Dihydr	Radius_SOS	-0,0329	0,049594	NA
1	abundance	X00097	2,6-Dihydr	Tibia_SOS	-0,07214	0,053107	NA
1	abundance	X00097	2,6-Dihydr	Handgrip	0,003524	0,060687	NA
1	abundance	X00099	7-Methylgu	Tibia_length	-0,10301	0,051628	NA
1	abundance	X00099	7-Methylgu	Radius_length	-0,11516	0,070706	NA
1	abundance	X00099	7-Methylgu	Radius_SOS	-0,04702	0,050057	NA
1	abundance	X00099	7-Methylgu	Tibia_SOS	-0,00342	0,053679	NA
1	abundance	X00099	7-Methylgu	Handgrip	-0,02793	0,062416	NA
1	abundance	X00101	Threonic a	Tibia_length	0,019597	0,052245	NA
1	abundance	X00101	Threonic a	Radius_length	0,072193	0,0716	NA
1	abundance	X00101	Threonic a	Radius_SOS	0,051129	0,050066	NA
1	abundance	X00101	Threonic a	Tibia_SOS	0,044327	0,053714	NA
1	abundance	X00101	Threonic a	Handgrip	0,096946	0,060492	NA
1	abundance	X00102	Tryptophar	Tibia_length	0,048205	0,052881	NA
1	abundance	X00102	Tryptophar	Radius_length	0,111003	0,071996	NA
1	abundance	X00102	Tryptophar	Radius_SOS	-0,03289	0,050646	NA
1	abundance	X00102	Tryptophar	Tibia_SOS	0,097685	0,053712	NA
1	abundance	X00102	Tryptophar	Handgrip	0,029409	0,060659	NA
1	abundance	X00106	N-Acetyltry	Tibia_length	-0,02033	0,0571	NA
1	abundance	X00106	N-Acetyltry	Radius_length	-0,0523	0,075209	NA
1	abundance	X00106	N-Acetyltry	Radius_SOS	-0,0282	0,05429	NA
1	abundance	X00106	N-Acetyltry	Tibia_SOS	0,015417	0,05706	NA
1	abundance	X00106	N-Acetyltry	Handgrip	-0,01297	0,063354	NA
1	abundance	X00107	N6-Acetyll	Tibia_length	0,111332	0,051816	NA
1	abundance	X00107	N6-Acetyll	Radius_length	0,184654	0,069829	NA
1	abundance	X00107	N6-Acetyll	Radius_SOS	0,096608	0,049793	NA
1	abundance	X00107	N6-Acetyll	Tibia_SOS	0,11166	0,053278	NA
1	abundance	X00107	N6-Acetyll	Handgrip	0,059336	0,060861	NA
1	abundance	X00112	Xanthosine	Tibia_length	0,00759	0,053547	NA
1	abundance	X00112	Xanthosine	Radius_length	-0,07258	0,072597	NA
1	abundance	X00112	Xanthosine	Radius_SOS	-0,06023	0,051009	NA
1	abundance	X00112	Xanthosine	Tibia_SOS	0,093021	0,054142	NA
1	abundance	X00112	Xanthosine	Handgrip	-0,00524	0,062529	NA
1	abundance	X00113	N-Acetylph	Tibia_length	0,007381	0,055505	NA
1	abundance	X00113	N-Acetylph	Radius_length	-0,05036	0,07382	NA
1	abundance	X00113	N-Acetylph	Radius_SOS	0,03872	0,052743	NA
1	abundance	X00113	N-Acetylph	Tibia_SOS	0,066098	0,05644	NA
1	abundance	X00113	N-Acetylph	Handgrip	-0,03472	0,063526	NA
1	abundance	X00114	Indole-3-m	Tibia_length	0,102198	0,056578	NA
1	abundance	X00114	Indole-3-m	Radius_length	0,08891	0,074333	NA
1	abundance	X00114	Indole-3-m	Radius_SOS	-0,07217	0,054148	NA
1	abundance	X00114	Indole-3-m	Tibia_SOS	0,13764	0,056497	NA
1	abundance	X00114	Indole-3-m	Handgrip	0,149222	0,062358	NA
1	abundance	X00115	Histidine	Tibia_length	0,050284	0,049464	NA
1	abundance	X00115	Histidine	Radius_length	0,239689	0,064954	NA

1	abundance	X00115	Histidine	Radius_SOS	0,022313	0,049022	NA
1	abundance	X00115	Histidine	Tibia_SOS	0,107976	0,05218	NA
1	abundance	X00115	Histidine	Handgrip	0,103889	0,059867	NA
1	abundance	X00116	Cytidine	Tibia_length	-0,04349	0,054901	NA
1	abundance	X00116	Cytidine	Radius_length	0,043435	0,073282	NA
1	abundance	X00116	Cytidine	Radius_SOS	-0,08906	0,052302	NA
1	abundance	X00116	Cytidine	Tibia_SOS	0,010959	0,056053	NA
1	abundance	X00116	Cytidine	Handgrip	-0,05042	0,062611	NA
1	abundance	X00120	Leucylalan	Tibia_length	-0,03293	0,051473	NA
1	abundance	X00120	Leucylalan	Radius_length	0,026938	0,070239	NA
1	abundance	X00120	Leucylalan	Radius_SOS	0,029321	0,04962	NA
1	abundance	X00120	Leucylalan	Tibia_SOS	-0,09873	0,052795	NA
1	abundance	X00120	Leucylalan	Handgrip	0,014154	0,060649	NA
1	abundance	X00123	Phenylalar	Tibia_length	0,115299	0,050203	NA
1	abundance	X00123	Phenylalar	Radius_length	0,112802	0,068561	NA
1	abundance	X00123	Phenylalar	Radius_SOS	-0,00723	0,049226	NA
1	abundance	X00123	Phenylalar	Tibia_SOS	0,107552	0,052366	NA
1	abundance	X00123	Phenylalar	Handgrip	0,051184	0,060146	NA
1	abundance	X00124	Leucine	Tibia_length	0,1011	0,050415	NA
1	abundance	X00124	Leucine	Radius_length	0,145916	0,068538	NA
1	abundance	X00124	Leucine	Radius_SOS	0,002545	0,049262	NA
1	abundance	X00124	Leucine	Tibia_SOS	0,126055	0,052369	NA
1	abundance	X00124	Leucine	Handgrip	0,087534	0,060072	NA
1	abundance	X00125	Taurine	Tibia_length	-0,0376	0,053881	NA
1	abundance	X00125	Taurine	Radius_length	-0,10023	0,07311	NA
1	abundance	X00125	Taurine	Radius_SOS	-0,04897	0,051743	NA
1	abundance	X00125	Taurine	Tibia_SOS	-0,01511	0,055505	NA
1	abundance	X00125	Taurine	Handgrip	0,036284	0,062625	NA
1	abundance	X00127	Nicotinic a	Tibia_length	-0,01567	0,056093	NA
1	abundance	X00127	Nicotinic a	Radius_length	0,044877	0,074507	NA
1	abundance	X00127	Nicotinic a	Radius_SOS	-0,02251	0,053611	NA
1	abundance	X00127	Nicotinic a	Tibia_SOS	0,079163	0,056208	NA
1	abundance	X00127	Nicotinic a	Handgrip	0,08161	0,062995	NA
1	abundance	X00129	Quinic acic	Tibia_length	-0,03666	0,052648	NA
1	abundance	X00129	Quinic acic	Radius_length	-0,08101	0,071541	NA
1	abundance	X00129	Quinic acic	Radius_SOS	-0,10599	0,050257	NA
1	abundance	X00129	Quinic acic	Tibia_SOS	-0,17045	0,054759	NA
1	abundance	X00129	Quinic acic	Handgrip	-0,05344	0,060936	NA
1	abundance	X00130	Deoxyuridi	Tibia_length	0,027841	0,050955	NA
1	abundance	X00130	Deoxyuridi	Radius_length	0,082951	0,069456	NA
1	abundance	X00130	Deoxyuridi	Radius_SOS	-0,07317	0,049282	NA
1	abundance	X00130	Deoxyuridi	Tibia_SOS	0,061594	0,05265	NA
1	abundance	X00130	Deoxyuridi	Handgrip	0,041636	0,060709	NA
1	abundance	X00132	Glyceric ac	Tibia_length	0,020323	0,052569	NA
1	abundance	X00132	Glyceric ac	Radius_length	0,068423	0,071798	NA
1	abundance	X00132	Glyceric ac	Radius_SOS	8,13E-04	0,050349	NA
1	abundance	X00132	Glyceric ac	Tibia_SOS	0,033332	0,053681	NA
1	abundance	X00132	Glyceric ac	Handgrip	0,037799	0,061178	NA

1	abundance	X00135	N-Acetylglu	Tibia_length	0,011064	0,052674	NA
1	abundance	X00135	N-Acetylglu	Radius_length	0,049756	0,072064	NA
1	abundance	X00135	N-Acetylglu	Radius_SOS	-0,02453	0,050523	NA
1	abundance	X00135	N-Acetylglu	Tibia_SOS	0,032406	0,054054	NA
1	abundance	X00135	N-Acetylglu	Handgrip	0,073611	0,06241	NA
3	abundance	X00136	leu-gln	Tibia_length	0,223846	0,053172	NA
3	abundance	X00136	leu-gln	Radius_length	0,131445	0,072867	NA
3	abundance	X00136	leu-gln	Radius_SOS	0,086427	0,051787	NA
3	abundance	X00136	leu-gln	Tibia_SOS	0,089745	0,055596	NA
3	abundance	X00136	leu-gln	Handgrip	0,084751	0,062051	NA
3	abundance	X00163	primidone	Tibia_length	0,032576	0,053945	NA
3	abundance	X00163	primidone	Radius_length	-0,00464	0,07357	NA
3	abundance	X00163	primidone	Radius_SOS	-0,09348	0,05118	NA
3	abundance	X00163	primidone	Tibia_SOS	0,064338	0,05533	NA
3	abundance	X00163	primidone	Handgrip	0,017282	0,062033	NA
3	abundance	X00180	1-(2-Carbo	Tibia_length	-0,12078	0,055632	NA
3	abundance	X00180	1-(2-Carbo	Radius_length	-0,11496	0,074171	NA
3	abundance	X00180	1-(2-Carbo	Radius_SOS	-0,06278	0,052977	NA
3	abundance	X00180	1-(2-Carbo	Tibia_SOS	-0,0845	0,055915	NA
3	abundance	X00180	1-(2-Carbo	Handgrip	-0,13688	0,061436	NA
3	abundance	X00182	3-(Butylsul	Tibia_length	0,087168	0,051884	NA
3	abundance	X00182	3-(Butylsul	Radius_length	0,051176	0,071615	NA
3	abundance	X00182	3-(Butylsul	Radius_SOS	0,034234	0,0501	NA
3	abundance	X00182	3-(Butylsul	Tibia_SOS	-0,01955	0,053327	NA
3	abundance	X00182	3-(Butylsul	Handgrip	-0,18402	0,060219	NA
3	abundance	X00199	tert-Butyl 3	Tibia_length	-0,08159	0,054762	NA
3	abundance	X00199	tert-Butyl 3	Radius_length	-0,12476	0,072923	NA
3	abundance	X00199	tert-Butyl 3	Radius_SOS	-0,06787	0,052188	NA
3	abundance	X00199	tert-Butyl 3	Tibia_SOS	-0,07761	0,057096	NA
3	abundance	X00199	tert-Butyl 3	Handgrip	-0,09088	0,062368	NA
3	abundance	X00204	4-O-{3-O-[	Tibia_length	-0,01725	0,050659	NA
3	abundance	X00204	4-O-{3-O-[	Radius_length	0,119211	0,06891	NA
3	abundance	X00204	4-O-{3-O-[	Radius_SOS	0,045785	0,049253	NA
3	abundance	X00204	4-O-{3-O-[	Tibia_SOS	0,087712	0,052791	NA
3	abundance	X00204	4-O-{3-O-[	Handgrip	0,079784	0,060789	NA
3	abundance	X00221	Astemizole	Tibia_length	0,057138	0,049579	NA
3	abundance	X00221	Astemizole	Radius_length	0,148687	0,066342	NA
3	abundance	X00221	Astemizole	Radius_SOS	-0,03799	0,048996	NA
3	abundance	X00221	Astemizole	Tibia_SOS	0,058884	0,052407	NA
3	abundance	X00221	Astemizole	Handgrip	0,033976	0,060356	NA
3	abundance	X00224	Nifedipine	Tibia_length	0,001529	0,054104	NA
3	abundance	X00224	Nifedipine	Radius_length	-0,01471	0,07323	NA
3	abundance	X00224	Nifedipine	Radius_SOS	-0,02622	0,051651	NA
3	abundance	X00224	Nifedipine	Tibia_SOS	0,003693	0,054297	NA
3	abundance	X00224	Nifedipine	Handgrip	-0,08457	0,0621	NA
3	abundance	X00233	g-Aminobu	Tibia_length	0,042452	0,053881	NA
3	abundance	X00233	g-Aminobu	Radius_length	0,05258	0,07282	NA
3	abundance	X00233	g-Aminobu	Radius_SOS	-0,00137	0,051533	NA

	3	abundance	X00233	g-Aminobu	Tibia_SOS	-0,05241	0,054975	NA
	3	abundance	X00233	g-Aminobu	Handgrip	0,077881	0,06271	NA
	3	abundance	X00237	OI170000(	Tibia_length	0,004769	0,05137	NA
	3	abundance	X00237	OI170000(	Radius_length	0,015747	0,070239	NA
	3	abundance	X00237	OI170000(	Radius_SOS	0,047756	0,049563	NA
	3	abundance	X00237	OI170000(	Tibia_SOS	0,035211	0,053084	NA
	3	abundance	X00237	OI170000(	Handgrip	-0,0545	0,061014	NA
	3	abundance	X00242	IN00258	Tibia_length	-0,11148	0,05608	NA
	3	abundance	X00242	IN00258	Radius_length	0,028023	0,074773	NA
	3	abundance	X00242	IN00258	Radius_SOS	-0,06999	0,053748	NA
	3	abundance	X00242	IN00258	Tibia_SOS	0,063277	0,057407	NA
	3	abundance	X00242	IN00258	Handgrip	0,044024	0,063008	NA
	3	abundance	X00253	TDP-2	Tibia_length	-0,05233	0,051384	NA
	3	abundance	X00253	TDP-2	Radius_length	5,15E-04	0,070651	NA
	3	abundance	X00253	TDP-2	Radius_SOS	0,041323	0,049833	NA
	3	abundance	X00253	TDP-2	Tibia_SOS	0,017228	0,053028	NA
	3	abundance	X00253	TDP-2	Handgrip	-0,12235	0,060337	NA
	3	abundance	X00261	Mexiletine	Tibia_length	-0,06713	0,055755	NA
	3	abundance	X00261	Mexiletine	Radius_length	-0,05035	0,073981	NA
	3	abundance	X00261	Mexiletine	Radius_SOS	-0,01324	0,052874	NA
	3	abundance	X00261	Mexiletine	Tibia_SOS	-0,00345	0,056799	NA
	3	abundance	X00261	Mexiletine	Handgrip	0,084418	0,062105	NA
2b		abundance	X00264	PEG n12	Tibia_length	0,044173	0,05616	NA
2b		abundance	X00264	PEG n12	Radius_length	0,139112	0,073448	NA
2b		abundance	X00264	PEG n12	Radius_SOS	-0,06757	0,053452	NA
2b		abundance	X00264	PEG n12	Tibia_SOS	0,014748	0,056222	NA
2b		abundance	X00264	PEG n12	Handgrip	-0,00906	0,063332	NA
	3	abundance	X00266	2-Oxo-3-(p	Tibia_length	0,08667	0,054647	NA
	3	abundance	X00266	2-Oxo-3-(p	Radius_length	0,146981	0,07251	NA
	3	abundance	X00266	2-Oxo-3-(p	Radius_SOS	0,032303	0,052125	NA
	3	abundance	X00266	2-Oxo-3-(p	Tibia_SOS	0,007299	0,056268	NA
	3	abundance	X00266	2-Oxo-3-(p	Handgrip	0,021004	0,063388	NA
	3	abundance	X00305	Formylkyn	Tibia_length	0,093341	0,053145	NA
	3	abundance	X00305	Formylkyn	Radius_length	0,057459	0,072535	NA
	3	abundance	X00305	Formylkyn	Radius_SOS	-0,05581	0,051013	NA
	3	abundance	X00305	Formylkyn	Tibia_SOS	-0,02556	0,054593	NA
	3	abundance	X00305	Formylkyn	Handgrip	-0,0321	0,062632	NA
	3	abundance	X00320	Panthenol	Tibia_length	-0,04866	0,051291	NA
	3	abundance	X00320	Panthenol	Radius_length	-0,07635	0,070093	NA
	3	abundance	X00320	Panthenol	Radius_SOS	0,061502	0,049496	NA
	3	abundance	X00320	Panthenol	Tibia_SOS	-2,53E-04	0,053018	NA
	3	abundance	X00320	Panthenol	Handgrip	-0,05737	0,060985	NA
	3	abundance	X00327	3-Hydroxy-	Tibia_length	-0,09	0,051085	NA
	3	abundance	X00327	3-Hydroxy-	Radius_length	-0,16902	0,069422	NA
	3	abundance	X00327	3-Hydroxy-	Radius_SOS	-0,03207	0,049638	NA
	3	abundance	X00327	3-Hydroxy-	Tibia_SOS	-0,03714	0,052972	NA
	3	abundance	X00327	3-Hydroxy-	Handgrip	-0,0341	0,061004	NA
	3	abundance	X00331	4-(9H-beta	Tibia_length	-0,08144	0,055548	NA

3	abundance	X00331	4-(9H-beta	Radius_length	-0,03787	0,073982	NA
3	abundance	X00331	4-(9H-beta	Radius_SOS	0,054653	0,052897	NA
3	abundance	X00331	4-(9H-beta	Tibia_SOS	-0,06232	0,05583	NA
3	abundance	X00331	4-(9H-beta	Handgrip	-0,1199	0,062593	NA
3	abundance	X00344	SECONAL	Tibia_length	0,051992	0,054214	NA
3	abundance	X00344	SECONAL	Radius_length	0,154403	0,07229	NA
3	abundance	X00344	SECONAL	Radius_SOS	0,022303	0,051268	NA
3	abundance	X00344	SECONAL	Tibia_SOS	0,075401	0,054659	NA
3	abundance	X00344	SECONAL	Handgrip	0,104345	0,062688	NA
3	abundance	X00361	nitecapone	Tibia_length	-0,0489	0,051313	NA
3	abundance	X00361	nitecapone	Radius_length	-0,0033	0,07036	NA
3	abundance	X00361	nitecapone	Radius_SOS	0,0349	0,049674	NA
3	abundance	X00361	nitecapone	Tibia_SOS	-0,03044	0,053971	NA
3	abundance	X00361	nitecapone	Handgrip	-0,11201	0,06099	NA
3	abundance	X00367	hexobarbit	Tibia_length	-0,0948	0,055559	NA
3	abundance	X00367	hexobarbit	Radius_length	-0,11972	0,072966	NA
3	abundance	X00367	hexobarbit	Radius_SOS	-0,01476	0,052711	NA
3	abundance	X00367	hexobarbit	Tibia_SOS	-0,09154	0,056364	NA
3	abundance	X00367	hexobarbit	Handgrip	-0,07366	0,063311	NA
3	abundance	X00375	N-[(2S)-2-I	Tibia_length	0,032165	0,055582	NA
3	abundance	X00375	N-[(2S)-2-I	Radius_length	0,152909	0,073181	NA
3	abundance	X00375	N-[(2S)-2-I	Radius_SOS	-0,01767	0,052807	NA
3	abundance	X00375	N-[(2S)-2-I	Tibia_SOS	0,084299	0,056717	NA
3	abundance	X00375	N-[(2S)-2-I	Handgrip	0,11047	0,061769	NA
3	abundance	X00383	pretazettin	Tibia_length	-0,04474	0,052652	NA
3	abundance	X00383	pretazettin	Radius_length	-0,08874	0,071238	NA
3	abundance	X00383	pretazettin	Radius_SOS	0,017431	0,050338	NA
3	abundance	X00383	pretazettin	Tibia_SOS	-0,05677	0,053258	NA
3	abundance	X00383	pretazettin	Handgrip	-0,04261	0,062166	NA
3	abundance	X00384	3-Formyl-2	Tibia_length	-0,09333	0,055797	NA
3	abundance	X00384	3-Formyl-2	Radius_length	-0,13905	0,073387	NA
3	abundance	X00384	3-Formyl-2	Radius_SOS	-0,10139	0,053287	NA
3	abundance	X00384	3-Formyl-2	Tibia_SOS	-0,0407	0,055806	NA
3	abundance	X00384	3-Formyl-2	Handgrip	-0,08733	0,061829	NA
3	abundance	X00403	7alpha-Hy	Tibia_length	0,055278	0,054074	NA
3	abundance	X00403	7alpha-Hy	Radius_length	0,071394	0,07259	NA
3	abundance	X00403	7alpha-Hy	Radius_SOS	-0,01193	0,051494	NA
3	abundance	X00403	7alpha-Hy	Tibia_SOS	-0,02223	0,055584	NA
3	abundance	X00403	7alpha-Hy	Handgrip	0,026234	0,061224	NA
3	abundance	X00404	piscidic ac	Tibia_length	-0,13788	0,055266	NA
3	abundance	X00404	piscidic ac	Radius_length	-0,17678	0,072665	NA
3	abundance	X00404	piscidic ac	Radius_SOS	-0,04274	0,052837	NA
3	abundance	X00404	piscidic ac	Tibia_SOS	-0,104	0,055084	NA
3	abundance	X00404	piscidic ac	Handgrip	-0,11983	0,060857	NA
3	abundance	X00432	8-(Methyls	Tibia_length	-0,09103	0,055768	NA
3	abundance	X00432	8-(Methyls	Radius_length	-0,11654	0,073399	NA
3	abundance	X00432	8-(Methyls	Radius_SOS	-0,00205	0,053091	NA
3	abundance	X00432	8-(Methyls	Tibia_SOS	-0,05358	0,056416	NA

	3	abundance	X00432	8-(Methyls	Handgrip	-0,14401	0,06212	NA
	3	abundance	X00450	Gly-Ser	Tibia_length	-0,09265	0,054129	NA
	3	abundance	X00450	Gly-Ser	Radius_length	-0,01147	0,073203	NA
	3	abundance	X00450	Gly-Ser	Radius_SOS	-0,02642	0,05174	NA
	3	abundance	X00450	Gly-Ser	Tibia_SOS	-0,01809	0,055134	NA
	3	abundance	X00450	Gly-Ser	Handgrip	0,066419	0,062705	NA
2b		abundance	X00477	Sparfloxac	Tibia_length	0,017386	0,055479	NA
2b		abundance	X00477	Sparfloxac	Radius_length	0,017131	0,074173	NA
2b		abundance	X00477	Sparfloxac	Radius_SOS	-0,0548	0,052983	NA
2b		abundance	X00477	Sparfloxac	Tibia_SOS	-0,05675	0,056383	NA
2b		abundance	X00477	Sparfloxac	Handgrip	-0,05952	0,061967	NA
	3	abundance	X00518	Nisinic aci	Tibia_length	0,124295	0,052328	NA
	3	abundance	X00518	Nisinic aci	Radius_length	0,117315	0,071227	NA
	3	abundance	X00518	Nisinic aci	Radius_SOS	0,044141	0,050421	NA
	3	abundance	X00518	Nisinic aci	Tibia_SOS	-0,06242	0,053845	NA
	3	abundance	X00518	Nisinic aci	Handgrip	0,128953	0,061376	NA
	3	abundance	X00528	R-(+)-Etira	Tibia_length	0,031212	0,049821	NA
	3	abundance	X00528	R-(+)-Etira	Radius_length	-0,02202	0,067568	NA
	3	abundance	X00528	R-(+)-Etira	Radius_SOS	-0,03447	0,049018	NA
	3	abundance	X00528	R-(+)-Etira	Tibia_SOS	-0,02285	0,052485	NA
	3	abundance	X00528	R-(+)-Etira	Handgrip	-0,02518	0,060604	NA
	3	abundance	X00545	3-hydroxyc	Tibia_length	-0,05435	0,054777	NA
	3	abundance	X00545	3-hydroxyc	Radius_length	-0,10671	0,072886	NA
	3	abundance	X00545	3-hydroxyc	Radius_SOS	0,036547	0,052176	NA
	3	abundance	X00545	3-hydroxyc	Tibia_SOS	-0,05312	0,054887	NA
	3	abundance	X00545	3-hydroxyc	Handgrip	-0,02575	0,061877	NA
	3	abundance	X00549	3-[(2Z)-1-C	Tibia_length	-0,06816	0,051648	NA
	3	abundance	X00549	3-[(2Z)-1-C	Radius_length	-0,16753	0,069921	NA
	3	abundance	X00549	3-[(2Z)-1-C	Radius_SOS	-0,05345	0,049796	NA
	3	abundance	X00549	3-[(2Z)-1-C	Tibia_SOS	-0,14544	0,053014	NA
	3	abundance	X00549	3-[(2Z)-1-C	Handgrip	-0,11276	0,060612	NA
	3	abundance	X00574	miglustat	Tibia_length	-0,04392	0,052156	NA
	3	abundance	X00574	miglustat	Radius_length	-0,0954	0,071065	NA
	3	abundance	X00574	miglustat	Radius_SOS	0,020308	0,050028	NA
	3	abundance	X00574	miglustat	Tibia_SOS	-0,08934	0,05402	NA
	3	abundance	X00574	miglustat	Handgrip	-0,07351	0,061991	NA
	3	abundance	X00594	Nicotinami	Tibia_length	-0,02234	0,055547	NA
	3	abundance	X00594	Nicotinami	Radius_length	0,030035	0,073691	NA
	3	abundance	X00594	Nicotinami	Radius_SOS	-7,18E-04	0,052939	NA
	3	abundance	X00594	Nicotinami	Tibia_SOS	-0,09739	0,056931	NA
	3	abundance	X00594	Nicotinami	Handgrip	0,092581	0,062504	NA
	3	abundance	X00635	4-(5,6-Dihy	Tibia_length	0,01121	0,053008	NA
	3	abundance	X00635	4-(5,6-Dihy	Radius_length	0,087553	0,071841	NA
	3	abundance	X00635	4-(5,6-Dihy	Radius_SOS	0,023466	0,050704	NA
	3	abundance	X00635	4-(5,6-Dihy	Tibia_SOS	0,12414	0,053898	NA
	3	abundance	X00635	4-(5,6-Dihy	Handgrip	-0,02368	0,061428	NA
	3	abundance	X00637	N~6~,N~6	Tibia_length	-0,02298	0,049409	NA
	3	abundance	X00637	N~6~,N~6	Radius_length	-0,01958	0,066389	NA

3	abundance	X00637	N~6~,N~6	Radius_SOS	0,013973	0,049043	NA
3	abundance	X00637	N~6~,N~6	Tibia_SOS	-0,04685	0,052468	NA
3	abundance	X00637	N~6~,N~6	Handgrip	-0,03782	0,06015	NA
3	abundance	X00646	Deacetyldi	Tibia_length	-0,01039	0,052894	NA
3	abundance	X00646	Deacetyldi	Radius_length	-0,05388	0,071646	NA
3	abundance	X00646	Deacetyldi	Radius_SOS	0,036508	0,050592	NA
3	abundance	X00646	Deacetyldi	Tibia_SOS	-0,14126	0,053012	NA
3	abundance	X00646	Deacetyldi	Handgrip	-0,13173	0,060342	NA
3	abundance	X00670	7-Chloro-5	Tibia_length	-0,02063	0,05646	NA
3	abundance	X00670	7-Chloro-5	Radius_length	0,033057	0,074276	NA
3	abundance	X00670	7-Chloro-5	Radius_SOS	-0,02285	0,053536	NA
3	abundance	X00670	7-Chloro-5	Tibia_SOS	-0,02657	0,057164	NA
3	abundance	X00670	7-Chloro-5	Handgrip	0,126357	0,062861	NA
3	abundance	X00683	Zinecard	Tibia_length	-0,13331	0,054513	NA
3	abundance	X00683	Zinecard	Radius_length	-0,1037	0,073132	NA
3	abundance	X00683	Zinecard	Radius_SOS	-0,02173	0,052087	NA
3	abundance	X00683	Zinecard	Tibia_SOS	0,01744	0,055704	NA
3	abundance	X00683	Zinecard	Handgrip	-0,05797	0,063524	NA
3	abundance	X00693	n-Ribosylh	Tibia_length	0,040895	0,056748	NA
3	abundance	X00693	n-Ribosylh	Radius_length	0,080856	0,07451	NA
3	abundance	X00693	n-Ribosylh	Radius_SOS	-0,0177	0,053983	NA
3	abundance	X00693	n-Ribosylh	Tibia_SOS	-0,12438	0,056451	NA
3	abundance	X00693	n-Ribosylh	Handgrip	0,094676	0,063115	NA
3	abundance	X00698	valganciclo	Tibia_length	-0,03777	0,054915	NA
3	abundance	X00698	valganciclo	Radius_length	-0,12389	0,072894	NA
3	abundance	X00698	valganciclo	Radius_SOS	0,043701	0,052363	NA
3	abundance	X00698	valganciclo	Tibia_SOS	-0,06158	0,054605	NA
3	abundance	X00698	valganciclo	Handgrip	0,008073	0,061789	NA
3	abundance	X00702	N-[(2S)-2-I	Tibia_length	0,019616	0,052127	NA
3	abundance	X00702	N-[(2S)-2-I	Radius_length	0,092032	0,070769	NA
3	abundance	X00702	N-[(2S)-2-I	Radius_SOS	-0,03769	0,050058	NA
3	abundance	X00702	N-[(2S)-2-I	Tibia_SOS	0,080957	0,053372	NA
3	abundance	X00702	N-[(2S)-2-I	Handgrip	-0,01046	0,061102	NA
3	abundance	X00722	(3S,5R,6E)	Tibia_length	-0,02703	0,055521	NA
3	abundance	X00722	(3S,5R,6E)	Radius_length	0,072124	0,07412	NA
3	abundance	X00722	(3S,5R,6E)	Radius_SOS	-0,03996	0,052816	NA
3	abundance	X00722	(3S,5R,6E)	Tibia_SOS	0,042769	0,056549	NA
3	abundance	X00722	(3S,5R,6E)	Handgrip	0,071321	0,06004	NA
3	abundance	X00723	Aspartyl-L-	Tibia_length	0,032235	0,052154	NA
3	abundance	X00723	Aspartyl-L-	Radius_length	0,044171	0,071213	NA
3	abundance	X00723	Aspartyl-L-	Radius_SOS	0,032187	0,050121	NA
3	abundance	X00723	Aspartyl-L-	Tibia_SOS	0,149875	0,053005	NA
3	abundance	X00723	Aspartyl-L-	Handgrip	0,067048	0,061585	NA
3	abundance	X00742	MFCD1869	Tibia_length	-0,10032	0,054448	NA
3	abundance	X00742	MFCD1869	Radius_length	-0,16347	0,072445	NA
3	abundance	X00742	MFCD1869	Radius_SOS	-0,07481	0,051485	NA
3	abundance	X00742	MFCD1869	Tibia_SOS	-0,06463	0,056486	NA
3	abundance	X00742	MFCD1869	Handgrip	-0,09229	0,062115	NA

3	abundance	X00743	11beta,13-	Tibia_length	0,030463	0,057521	NA
3	abundance	X00743	11beta,13-	Radius_length	0,062608	0,074774	NA
3	abundance	X00743	11beta,13-	Radius_SOS	-0,10168	0,054542	NA
3	abundance	X00743	11beta,13-	Tibia_SOS	0,055698	0,056951	NA
3	abundance	X00743	11beta,13-	Handgrip	-0,0198	0,060181	NA
3	abundance	X00744	(1R,2S)-1-(	Tibia_length	-0,13375	0,055332	NA
3	abundance	X00744	(1R,2S)-1-(	Radius_length	-0,17614	0,072553	NA
3	abundance	X00744	(1R,2S)-1-(	Radius_SOS	0,052234	0,052379	NA
3	abundance	X00744	(1R,2S)-1-(	Tibia_SOS	-0,10341	0,054794	NA
3	abundance	X00744	(1R,2S)-1-(	Handgrip	-0,06975	0,061996	NA
3	abundance	X00748	3-[3-Metho	Tibia_length	-0,17031	0,054537	NA
3	abundance	X00748	3-[3-Metho	Radius_length	-0,1382	0,072745	NA
3	abundance	X00748	3-[3-Metho	Radius_SOS	-0,03839	0,052575	NA
3	abundance	X00748	3-[3-Metho	Tibia_SOS	0,017934	0,055536	NA
3	abundance	X00748	3-[3-Metho	Handgrip	-0,13125	0,061544	NA
3	abundance	X00764	5-Allyl-5-se	Tibia_length	-0,0047	0,051111	NA
3	abundance	X00764	5-Allyl-5-se	Radius_length	0,106688	0,069691	NA
3	abundance	X00764	5-Allyl-5-se	Radius_SOS	0,034191	0,049508	NA
3	abundance	X00764	5-Allyl-5-se	Tibia_SOS	-0,06466	0,052632	NA
3	abundance	X00764	5-Allyl-5-se	Handgrip	-0,01438	0,060843	NA
3	abundance	X00807	Midodrine	Tibia_length	-0,13997	0,053331	NA
3	abundance	X00807	Midodrine	Radius_length	-0,16351	0,072262	NA
3	abundance	X00807	Midodrine	Radius_SOS	-0,03994	0,051485	NA
3	abundance	X00807	Midodrine	Tibia_SOS	-0,04332	0,055591	NA
3	abundance	X00807	Midodrine	Handgrip	-0,18596	0,061356	NA
3	abundance	X00828	Fenoterol	Tibia_length	-0,00526	0,052341	NA
3	abundance	X00828	Fenoterol	Radius_length	0,058463	0,071354	NA
3	abundance	X00828	Fenoterol	Radius_SOS	0,061679	0,050331	NA
3	abundance	X00828	Fenoterol	Tibia_SOS	0,034643	0,053602	NA
3	abundance	X00828	Fenoterol	Handgrip	-5,99E-04	0,061999	NA
3	abundance	X00836	3-Mercapt	Tibia_length	0,076263	0,050727	NA
3	abundance	X00836	3-Mercapt	Radius_length	0,090852	0,069619	NA
3	abundance	X00836	3-Mercapt	Radius_SOS	0,073474	0,049272	NA
3	abundance	X00836	3-Mercapt	Tibia_SOS	0,059365	0,052873	NA
3	abundance	X00836	3-Mercapt	Handgrip	-0,03154	0,060413	NA
3	abundance	X00853	4-O-beta-D	Tibia_length	-0,07612	0,048828	NA
3	abundance	X00853	4-O-beta-D	Radius_length	-0,06024	0,048881	NA
3	abundance	X00853	4-O-beta-D	Radius_SOS	-0,03093	0,049006	NA
3	abundance	X00853	4-O-beta-D	Tibia_SOS	-0,04093	0,059374	NA
3	abundance	X00853	4-O-beta-D	Handgrip	-0,06338	0,060072	NA
3	abundance	X00889	LW800000	Tibia_length	-0,0504	0,05557	NA
3	abundance	X00889	LW800000	Radius_length	-0,02855	0,073794	NA
3	abundance	X00889	LW800000	Radius_SOS	0,107515	0,05234	NA
3	abundance	X00889	LW800000	Tibia_SOS	0,101271	0,055787	NA
3	abundance	X00889	LW800000	Handgrip	0,074882	0,062895	NA
3	abundance	X00899	N-(2,3,4-Tr	Tibia_length	-0,04371	0,054455	NA
3	abundance	X00899	N-(2,3,4-Tr	Radius_length	-0,09967	0,072699	NA
3	abundance	X00899	N-(2,3,4-Tr	Radius_SOS	-0,11822	0,051883	NA

3	abundance	X00899	N-(2,3,4-Tı	Tibia_SOS	-0,02906	0,055376	NA
3	abundance	X00899	N-(2,3,4-Tı	Handgrip	-0,04102	0,061752	NA
3	abundance	X00907	1,2,3,4-Tet	Tibia_length	-0,02053	0,053741	NA
3	abundance	X00907	1,2,3,4-Tet	Radius_length	-0,12564	0,072389	NA
3	abundance	X00907	1,2,3,4-Tet	Radius_SOS	-0,00902	0,05119	NA
3	abundance	X00907	1,2,3,4-Tet	Tibia_SOS	0,050042	0,054344	NA
3	abundance	X00907	1,2,3,4-Tet	Handgrip	0,008022	0,061678	NA
3	abundance	X00928	(7R)-7-(5-c	Tibia_length	0,089581	0,05362	NA
3	abundance	X00928	(7R)-7-(5-c	Radius_length	0,033622	0,072276	NA
3	abundance	X00928	(7R)-7-(5-c	Radius_SOS	-0,00747	0,051109	NA
3	abundance	X00928	(7R)-7-(5-c	Tibia_SOS	0,095309	0,054534	NA
3	abundance	X00928	(7R)-7-(5-c	Handgrip	0,056067	0,062002	NA
3	abundance	X00948	Linamarin	Tibia_length	-0,07275	0,051495	NA
3	abundance	X00948	Linamarin	Radius_length	-0,09689	0,070269	NA
3	abundance	X00948	Linamarin	Radius_SOS	-0,0133	0,04969	NA
3	abundance	X00948	Linamarin	Tibia_SOS	-0,15769	0,052376	NA
3	abundance	X00948	Linamarin	Handgrip	-0,17887	0,059897	NA
3	abundance	X00949	primidone	Tibia_length	0,16541	0,052738	NA
3	abundance	X00949	primidone	Radius_length	0,095181	0,072273	NA
3	abundance	X00949	primidone	Radius_SOS	0,048426	0,050862	NA
3	abundance	X00949	primidone	Tibia_SOS	-0,01145	0,054738	NA
3	abundance	X00949	primidone	Handgrip	0,02346	0,062197	NA
3	abundance	X00950	2-Phenylet	Tibia_length	-0,02198	0,055997	NA
3	abundance	X00950	2-Phenylet	Radius_length	-0,02798	0,074909	NA
3	abundance	X00950	2-Phenylet	Radius_SOS	-0,05001	0,053104	NA
3	abundance	X00950	2-Phenylet	Tibia_SOS	0,077726	0,057982	NA
3	abundance	X00950	2-Phenylet	Handgrip	-0,19653	0,06251	NA
3	abundance	X00963	(4S)-4-[(2E	Tibia_length	0,147797	0,05221	NA
3	abundance	X00963	(4S)-4-[(2E	Radius_length	0,206846	0,070845	NA
3	abundance	X00963	(4S)-4-[(2E	Radius_SOS	0,0838	0,050544	NA
3	abundance	X00963	(4S)-4-[(2E	Tibia_SOS	0,087385	0,053936	NA
3	abundance	X00963	(4S)-4-[(2E	Handgrip	0,096304	0,060618	NA
3	abundance	X00985	1-(4-Aminc	Tibia_length	-0,0991	0,052912	NA
3	abundance	X00985	1-(4-Aminc	Radius_length	-0,2064	0,070697	NA
3	abundance	X00985	1-(4-Aminc	Radius_SOS	-0,05771	0,050582	NA
3	abundance	X00985	1-(4-Aminc	Tibia_SOS	-0,20903	0,052927	NA
3	abundance	X00985	1-(4-Aminc	Handgrip	-0,23293	0,0602	NA
3	abundance	X00998	epsilon-(ga	Tibia_length	0,043734	0,053375	NA
3	abundance	X00998	epsilon-(ga	Radius_length	0,078196	0,072519	NA
3	abundance	X00998	epsilon-(ga	Radius_SOS	-0,00113	0,051021	NA
3	abundance	X00998	epsilon-(ga	Tibia_SOS	0,059686	0,054934	NA
3	abundance	X00998	epsilon-(ga	Handgrip	0,031554	0,061905	NA
3	abundance	X01007	pentobarbi	Tibia_length	-0,10395	0,053804	NA
3	abundance	X01007	pentobarbi	Radius_length	-0,20229	0,071104	NA
3	abundance	X01007	pentobarbi	Radius_SOS	-0,05482	0,051079	NA
3	abundance	X01007	pentobarbi	Tibia_SOS	-0,13688	0,053438	NA
3	abundance	X01007	pentobarbi	Handgrip	-0,01706	0,061355	NA
3	abundance	X01011	DIBEHENII	Tibia_length	-0,01065	0,054208	NA

3	abundance	X01011	DIBEHENIN	Radius_length	-0,07879	0,073147	NA
3	abundance	X01011	DIBEHENIN	Radius_SOS	-0,0922	0,051418	NA
3	abundance	X01011	DIBEHENIN	Tibia_SOS	-0,06323	0,056213	NA
3	abundance	X01011	DIBEHENIN	Handgrip	-0,1141	0,06247	NA
3	abundance	X01017	hexobarbit	Tibia_length	-0,14337	0,059203	NA
3	abundance	X01017	hexobarbit	Radius_length	-0,04494	0,076268	NA
3	abundance	X01017	hexobarbit	Radius_SOS	0,046936	0,056382	NA
3	abundance	X01017	hexobarbit	Tibia_SOS	0,050154	0,05997	NA
3	abundance	X01017	hexobarbit	Handgrip	0,11009	0,063379	NA
3	abundance	X01031	3-(14-Ethy	Tibia_length	0,066944	0,05815	NA
3	abundance	X01031	3-(14-Ethy	Radius_length	0,249939	0,073148	NA
3	abundance	X01031	3-(14-Ethy	Radius_SOS	-0,08124	0,05519	NA
3	abundance	X01031	3-(14-Ethy	Tibia_SOS	0,03813	0,058042	NA
3	abundance	X01031	3-(14-Ethy	Handgrip	0,119493	0,059762	NA
3	abundance	X01045	Ethyl malto	Tibia_length	-0,01939	0,053843	NA
3	abundance	X01045	Ethyl malto	Radius_length	0,042631	0,07257	NA
3	abundance	X01045	Ethyl malto	Radius_SOS	0,014729	0,051348	NA
3	abundance	X01045	Ethyl malto	Tibia_SOS	0,056677	0,055004	NA
3	abundance	X01045	Ethyl malto	Handgrip	0,042608	0,06286	NA
3	abundance	X01059	coronatine	Tibia_length	-0,19851	0,054915	NA
3	abundance	X01059	coronatine	Radius_length	-0,12179	0,073159	NA
3	abundance	X01059	coronatine	Radius_SOS	0,002917	0,052611	NA
3	abundance	X01059	coronatine	Tibia_SOS	-0,0543	0,056088	NA
3	abundance	X01059	coronatine	Handgrip	-0,05004	0,062056	NA
3	abundance	X01067	g-Aminobu	Tibia_length	0,040805	0,055635	NA
3	abundance	X01067	g-Aminobu	Radius_length	-0,03386	0,073998	NA
3	abundance	X01067	g-Aminobu	Radius_SOS	-0,04501	0,053016	NA
3	abundance	X01067	g-Aminobu	Tibia_SOS	0,082827	0,05544	NA
3	abundance	X01067	g-Aminobu	Handgrip	0,042921	0,062017	NA
3	abundance	X01078	Dihydroura	Tibia_length	-0,09479	0,051747	NA
3	abundance	X01078	Dihydroura	Radius_length	-0,11915	0,070596	NA
3	abundance	X01078	Dihydroura	Radius_SOS	0,063418	0,049927	NA
3	abundance	X01078	Dihydroura	Tibia_SOS	-0,08013	0,053477	NA
3	abundance	X01078	Dihydroura	Handgrip	-0,16438	0,060101	NA
3	abundance	X01081	Methyl alpl	Tibia_length	-0,07469	0,052168	NA
3	abundance	X01081	Methyl alpl	Radius_length	0,021994	0,071158	NA
3	abundance	X01081	Methyl alpl	Radius_SOS	-0,01118	0,050113	NA
3	abundance	X01081	Methyl alpl	Tibia_SOS	-0,06995	0,053723	NA
3	abundance	X01081	Methyl alpl	Handgrip	-0,05023	0,061129	NA
3	abundance	X01092	17,21-Dihy	Tibia_length	0,074914	0,050747	NA
3	abundance	X01092	17,21-Dihy	Radius_length	0,088345	0,069203	NA
3	abundance	X01092	17,21-Dihy	Radius_SOS	0,007663	0,049379	NA
3	abundance	X01092	17,21-Dihy	Tibia_SOS	0,017681	0,05266	NA
3	abundance	X01092	17,21-Dihy	Handgrip	0,001617	0,060554	NA
3	abundance	X01098	(-)-Physost	Tibia_length	-0,04128	0,057906	NA
3	abundance	X01098	(-)-Physost	Radius_length	-0,11764	0,075152	NA
3	abundance	X01098	(-)-Physost	Radius_SOS	0,029231	0,054957	NA
3	abundance	X01098	(-)-Physost	Tibia_SOS	-0,15918	0,056099	NA

3	abundance	X01098	(-)-Physost	Handgrip	-0,11407	0,062682	NA
3	abundance	X01100	(betaS)-be	Tibia_length	-0,15937	0,056142	NA
3	abundance	X01100	(betaS)-be	Radius_length	-0,13381	0,074494	NA
3	abundance	X01100	(betaS)-be	Radius_SOS	0,017202	0,054061	NA
3	abundance	X01100	(betaS)-be	Tibia_SOS	-0,13971	0,056616	NA
3	abundance	X01100	(betaS)-be	Handgrip	-0,05068	0,063319	NA
3	abundance	X01154	N-(2,3,4-Tr	Tibia_length	-0,03868	0,056177	NA
3	abundance	X01154	N-(2,3,4-Tr	Radius_length	-0,07317	0,074016	NA
3	abundance	X01154	N-(2,3,4-Tr	Radius_SOS	-0,0679	0,053285	NA
3	abundance	X01154	N-(2,3,4-Tr	Tibia_SOS	-0,09403	0,057089	NA
3	abundance	X01154	N-(2,3,4-Tr	Handgrip	-0,00732	0,062333	NA
3	abundance	X01163	Prephenic	Tibia_length	-0,11995	0,054427	NA
3	abundance	X01163	Prephenic	Radius_length	-0,25776	0,070826	NA
3	abundance	X01163	Prephenic	Radius_SOS	-0,04159	0,051816	NA
3	abundance	X01163	Prephenic	Tibia_SOS	-0,10661	0,054549	NA
3	abundance	X01163	Prephenic	Handgrip	-0,13438	0,061959	NA
3	abundance	X01164	DL-Carboc	Tibia_length	-0,05385	0,056666	NA
3	abundance	X01164	DL-Carboc	Radius_length	-0,02939	0,075091	NA
3	abundance	X01164	DL-Carboc	Radius_SOS	0,022607	0,053934	NA
3	abundance	X01164	DL-Carboc	Tibia_SOS	0,021383	0,056857	NA
3	abundance	X01164	DL-Carboc	Handgrip	-0,05853	0,063678	NA
3	abundance	X01181	2-(3,4-Dim	Tibia_length	-0,05938	0,051931	NA
3	abundance	X01181	2-(3,4-Dim	Radius_length	-0,11302	0,07067	NA
3	abundance	X01181	2-(3,4-Dim	Radius_SOS	0,001284	0,05007	NA
3	abundance	X01181	2-(3,4-Dim	Tibia_SOS	-0,12921	0,052618	NA
3	abundance	X01181	2-(3,4-Dim	Handgrip	-0,1129	0,060314	NA
3	abundance	X01186	asn-val	Tibia_length	-0,0786	0,05479	NA
3	abundance	X01186	asn-val	Radius_length	-0,0291	0,073894	NA
3	abundance	X01186	asn-val	Radius_SOS	-0,02546	0,052426	NA
3	abundance	X01186	asn-val	Tibia_SOS	-0,02454	0,057019	NA
3	abundance	X01186	asn-val	Handgrip	-0,01575	0,062022	NA
3	abundance	X01208	Lys-Pro	Tibia_length	0,094728	0,05122	NA
3	abundance	X01208	Lys-Pro	Radius_length	0,093903	0,070453	NA
3	abundance	X01208	Lys-Pro	Radius_SOS	0,084621	0,049455	NA
3	abundance	X01208	Lys-Pro	Tibia_SOS	0,00377	0,053236	NA
3	abundance	X01208	Lys-Pro	Handgrip	-0,03662	0,061132	NA
3	abundance	X01223	DL-Mevalo	Tibia_length	-0,03461	0,053506	NA
3	abundance	X01223	DL-Mevalo	Radius_length	-0,10063	0,072358	NA
3	abundance	X01223	DL-Mevalo	Radius_SOS	-0,05034	0,051079	NA
3	abundance	X01223	DL-Mevalo	Tibia_SOS	-0,00497	0,054904	NA
3	abundance	X01223	DL-Mevalo	Handgrip	0,021654	0,061531	NA
3	abundance	X01235	3-[(3-Hydro	Tibia_length	0,17846	0,056519	NA
3	abundance	X01235	3-[(3-Hydro	Radius_length	0,28961	0,072646	NA
3	abundance	X01235	3-[(3-Hydro	Radius_SOS	0,098401	0,054019	NA
3	abundance	X01235	3-[(3-Hydro	Tibia_SOS	0,088518	0,058835	NA
3	abundance	X01235	3-[(3-Hydro	Handgrip	0,143953	0,059566	NA
3	abundance	X01236	bis(4-isoth	Tibia_length	0,070204	0,056166	NA
3	abundance	X01236	bis(4-isoth	Radius_length	0,193557	0,0743	NA

3	abundance	X01236	bis(4-isoth	Radius_SOS	0,101831	0,052944	NA
3	abundance	X01236	bis(4-isoth	Tibia_SOS	0,050492	0,057475	NA
3	abundance	X01236	bis(4-isoth	Handgrip	0,161715	0,062542	NA
2b	abundance	X01242	3-Morpholi	Tibia_length	-0,0201	0,057968	NA
2b	abundance	X01242	3-Morpholi	Radius_length	0,042537	0,075308	NA
2b	abundance	X01242	3-Morpholi	Radius_SOS	0,001627	0,055146	NA
2b	abundance	X01242	3-Morpholi	Tibia_SOS	0,040567	0,058908	NA
2b	abundance	X01242	3-Morpholi	Handgrip	0,008453	0,063641	NA
3	abundance	X01246	(2R)-1-{[(2	Tibia_length	0,042204	0,054276	NA
3	abundance	X01246	(2R)-1-{[(2	Radius_length	0,107704	0,072723	NA
3	abundance	X01246	(2R)-1-{[(2	Radius_SOS	-0,03369	0,051807	NA
3	abundance	X01246	(2R)-1-{[(2	Tibia_SOS	0,019857	0,05546	NA
3	abundance	X01246	(2R)-1-{[(2	Handgrip	0,056156	0,062825	NA
3	abundance	X01252	(âˆ’)-nabil	Tibia_length	-0,00643	0,052942	NA
3	abundance	X01252	(âˆ’)-nabil	Radius_length	-0,03525	0,071809	NA
3	abundance	X01252	(âˆ’)-nabil	Radius_SOS	0,035319	0,050553	NA
3	abundance	X01252	(âˆ’)-nabil	Tibia_SOS	0,006564	0,053969	NA
3	abundance	X01252	(âˆ’)-nabil	Handgrip	-0,02432	0,062432	NA
3	abundance	X01285	FB950000	Tibia_length	-0,11127	0,054129	NA
3	abundance	X01285	FB950000	Radius_length	-0,09951	0,072561	NA
3	abundance	X01285	FB950000	Radius_SOS	0,016106	0,051573	NA
3	abundance	X01285	FB950000	Tibia_SOS	-0,06417	0,055664	NA
3	abundance	X01285	FB950000	Handgrip	-0,08813	0,062091	NA
3	abundance	X01286	(2,4-Dihyd	Tibia_length	-0,10244	0,05014	NA
3	abundance	X01286	(2,4-Dihyd	Radius_length	-0,17865	0,067827	NA
3	abundance	X01286	(2,4-Dihyd	Radius_SOS	0,011386	0,049145	NA
3	abundance	X01286	(2,4-Dihyd	Tibia_SOS	-0,19684	0,051469	NA
3	abundance	X01286	(2,4-Dihyd	Handgrip	-0,21369	0,058819	NA
3	abundance	X01288	L-gamma-(	Tibia_length	-0,07763	0,051998	NA
3	abundance	X01288	L-gamma-(	Radius_length	-0,12451	0,070531	NA
3	abundance	X01288	L-gamma-(	Radius_SOS	0,009216	0,049885	NA
3	abundance	X01288	L-gamma-(	Tibia_SOS	-0,13169	0,052651	NA
3	abundance	X01288	L-gamma-(	Handgrip	-0,0991	0,060194	NA
3	abundance	X01316	5-Hydanto	Tibia_length	0,002372	0,054771	NA
3	abundance	X01316	5-Hydanto	Radius_length	-0,08569	0,073617	NA
3	abundance	X01316	5-Hydanto	Radius_SOS	-0,03564	0,052012	NA
3	abundance	X01316	5-Hydanto	Tibia_SOS	0,10599	0,055268	NA
3	abundance	X01316	5-Hydanto	Handgrip	-0,01105	0,061043	NA
3	abundance	X01327	threonylph	Tibia_length	-0,15021	0,053071	NA
3	abundance	X01327	threonylph	Radius_length	-0,21427	0,070675	NA
3	abundance	X01327	threonylph	Radius_SOS	-0,07543	0,050756	NA
3	abundance	X01327	threonylph	Tibia_SOS	-0,14049	0,054014	NA
3	abundance	X01327	threonylph	Handgrip	-0,10511	0,060784	NA
3	abundance	X01340	Cadralazin	Tibia_length	0,004105	0,0532	NA
3	abundance	X01340	Cadralazin	Radius_length	0,188322	0,070775	NA
3	abundance	X01340	Cadralazin	Radius_SOS	-0,04548	0,050659	NA
3	abundance	X01340	Cadralazin	Tibia_SOS	-0,054	0,054856	NA
3	abundance	X01340	Cadralazin	Handgrip	-0,01938	0,061606	NA

3	abundance	X01341	metixene	Tibia_length	0,130118	0,051514	NA
3	abundance	X01341	metixene	Radius_length	0,153746	0,070172	NA
3	abundance	X01341	metixene	Radius_SOS	-0,01164	0,049805	NA
3	abundance	X01341	metixene	Tibia_SOS	0,076126	0,053566	NA
3	abundance	X01341	metixene	Handgrip	0,033096	0,061522	NA
3	abundance	X01346	IN00260	Tibia_length	-0,06848	0,050071	NA
3	abundance	X01346	IN00260	Radius_length	-0,07711	0,068376	NA
3	abundance	X01346	IN00260	Radius_SOS	-0,05841	0,049058	NA
3	abundance	X01346	IN00260	Tibia_SOS	-0,02999	0,052571	NA
3	abundance	X01346	IN00260	Handgrip	-0,00768	0,060536	NA
3	abundance	X01363	Seryltyrosi	Tibia_length	-0,11629	0,053501	NA
3	abundance	X01363	Seryltyrosi	Radius_length	-0,14981	0,071505	NA
3	abundance	X01363	Seryltyrosi	Radius_SOS	0,004969	0,050949	NA
3	abundance	X01363	Seryltyrosi	Tibia_SOS	-0,10358	0,053698	NA
3	abundance	X01363	Seryltyrosi	Handgrip	-0,06999	0,06091	NA
3	abundance	X01364	Oleuropeir	Tibia_length	-0,07947	0,056772	NA
3	abundance	X01364	Oleuropeir	Radius_length	0,041928	0,074736	NA
3	abundance	X01364	Oleuropeir	Radius_SOS	0,055862	0,053904	NA
3	abundance	X01364	Oleuropeir	Tibia_SOS	0,093015	0,058012	NA
3	abundance	X01364	Oleuropeir	Handgrip	0,053521	0,061612	NA
3	abundance	X01367	MFCD1869	Tibia_length	0,026653	0,054053	NA
3	abundance	X01367	MFCD1869	Radius_length	0,143937	0,07202	NA
3	abundance	X01367	MFCD1869	Radius_SOS	-0,01044	0,051249	NA
3	abundance	X01367	MFCD1869	Tibia_SOS	0,025865	0,054668	NA
3	abundance	X01367	MFCD1869	Handgrip	0,028587	0,062969	NA
3	abundance	X01380	Spermic ac	Tibia_length	-0,05897	0,057715	NA
3	abundance	X01380	Spermic ac	Radius_length	0,010028	0,074946	NA
3	abundance	X01380	Spermic ac	Radius_SOS	-0,03397	0,054871	NA
3	abundance	X01380	Spermic ac	Tibia_SOS	0,010718	0,05751	NA
3	abundance	X01380	Spermic ac	Handgrip	-0,05014	0,062828	NA
3	abundance	X01387	Methyl 4-(4	Tibia_length	-0,12664	0,05386	NA
3	abundance	X01387	Methyl 4-(4	Radius_length	-0,15789	0,072791	NA
3	abundance	X01387	Methyl 4-(4	Radius_SOS	-0,08734	0,051638	NA
3	abundance	X01387	Methyl 4-(4	Tibia_SOS	-0,02685	0,055908	NA
3	abundance	X01387	Methyl 4-(4	Handgrip	-0,12776	0,062504	NA
3	abundance	X01441	1-{3-Carbo	Tibia_length	0,009343	0,052441	NA
3	abundance	X01441	1-{3-Carbo	Radius_length	-0,05353	0,071267	NA
3	abundance	X01441	1-{3-Carbo	Radius_SOS	0,032015	0,050293	NA
3	abundance	X01441	1-{3-Carbo	Tibia_SOS	-0,11577	0,053544	NA
3	abundance	X01441	1-{3-Carbo	Handgrip	-0,11084	0,061543	NA
3	abundance	X01463	4-Thiapent	Tibia_length	0,124044	0,051898	NA
3	abundance	X01463	4-Thiapent	Radius_length	0,143703	0,071095	NA
3	abundance	X01463	4-Thiapent	Radius_SOS	0,051839	0,050024	NA
3	abundance	X01463	4-Thiapent	Tibia_SOS	0,103133	0,053572	NA
3	abundance	X01463	4-Thiapent	Handgrip	0,174349	0,05968	NA
3	abundance	X01474	Glyceroph	Tibia_length	-0,10666	0,05519	NA
3	abundance	X01474	Glyceroph	Radius_length	0,034048	0,073574	NA
3	abundance	X01474	Glyceroph	Radius_SOS	0,009822	0,052797	NA

3	abundance	X01474	Glycerophı	Tibia_SOS	0,023239	0,056178	NA
3	abundance	X01474	Glycerophı	Handgrip	-0,03199	0,060761	NA
3	abundance	X01518	L-gamma-(	Tibia_length	-0,04338	0,053276	NA
3	abundance	X01518	L-gamma-(	Radius_length	-0,1062	0,072147	NA
3	abundance	X01518	L-gamma-(	Radius_SOS	-0,07821	0,05076	NA
3	abundance	X01518	L-gamma-(	Tibia_SOS	-0,10354	0,053896	NA
3	abundance	X01518	L-gamma-(	Handgrip	-0,03965	0,061829	NA
3	abundance	X01519	Leucyltrypt	Tibia_length	-0,09888	0,053879	NA
3	abundance	X01519	Leucyltrypt	Radius_length	-0,11946	0,072446	NA
3	abundance	X01519	Leucyltrypt	Radius_SOS	0,016754	0,051585	NA
3	abundance	X01519	Leucyltrypt	Tibia_SOS	0,00904	0,054533	NA
3	abundance	X01519	Leucyltrypt	Handgrip	-0,16792	0,062418	NA
3	abundance	X01528	butalbital	Tibia_length	-0,07404	0,054025	NA
3	abundance	X01528	butalbital	Radius_length	0,012401	0,072938	NA
3	abundance	X01528	butalbital	Radius_SOS	-7,69E-04	0,051586	NA
3	abundance	X01528	butalbital	Tibia_SOS	-0,03468	0,055483	NA
3	abundance	X01528	butalbital	Handgrip	0,011923	0,061361	NA
2b	abundance	X01530	INK (Peptic	Tibia_length	-0,03325	0,054998	NA
2b	abundance	X01530	INK (Peptic	Radius_length	0,045609	0,073677	NA
2b	abundance	X01530	INK (Peptic	Radius_SOS	0,064445	0,052375	NA
2b	abundance	X01530	INK (Peptic	Tibia_SOS	0,051006	0,055907	NA
2b	abundance	X01530	INK (Peptic	Handgrip	0,046725	0,061577	NA
3	abundance	X01549	3-(Sulfooxy	Tibia_length	0,063169	0,050847	NA
3	abundance	X01549	3-(Sulfooxy	Radius_length	0,08732	0,069542	NA
3	abundance	X01549	3-(Sulfooxy	Radius_SOS	0,016713	0,04938	NA
3	abundance	X01549	3-(Sulfooxy	Tibia_SOS	0,132145	0,05281	NA
3	abundance	X01549	3-(Sulfooxy	Handgrip	0,014147	0,060687	NA
3	abundance	X01553	uridine 5â€	Tibia_length	0,137448	0,050815	NA
3	abundance	X01553	uridine 5â€	Radius_length	0,175188	0,068892	NA
3	abundance	X01553	uridine 5â€	Radius_SOS	-0,05553	0,049271	NA
3	abundance	X01553	uridine 5â€	Tibia_SOS	0,074429	0,052866	NA
3	abundance	X01553	uridine 5â€	Handgrip	0,040102	0,060442	NA
2b	abundance	X01558	2-Furoylgly	Tibia_length	-0,11467	0,051937	NA
2b	abundance	X01558	2-Furoylgly	Radius_length	-0,09776	0,071224	NA
2b	abundance	X01558	2-Furoylgly	Radius_SOS	-0,01483	0,050182	NA
2b	abundance	X01558	2-Furoylgly	Tibia_SOS	-0,05127	0,053666	NA
2b	abundance	X01558	2-Furoylgly	Handgrip	-0,02656	0,062111	NA
3	abundance	X01561	2-Ammoni	Tibia_length	0,026999	0,055335	NA
3	abundance	X01561	2-Ammoni	Radius_length	-0,01424	0,073665	NA
3	abundance	X01561	2-Ammoni	Radius_SOS	-0,01145	0,052631	NA
3	abundance	X01561	2-Ammoni	Tibia_SOS	-0,07799	0,055944	NA
3	abundance	X01561	2-Ammoni	Handgrip	-0,10376	0,062608	NA
3	abundance	X01570	carglumic a	Tibia_length	-0,08509	0,054904	NA
3	abundance	X01570	carglumic a	Radius_length	-0,11397	0,072683	NA
3	abundance	X01570	carglumic a	Radius_SOS	-0,091	0,051695	NA
3	abundance	X01570	carglumic a	Tibia_SOS	-0,03726	0,055726	NA
3	abundance	X01570	carglumic a	Handgrip	-0,00278	0,061298	NA
3	abundance	X01574	N-Acetylpr	Tibia_length	-0,08511	0,053093	NA

3	abundance	X01574	N-Acetylpr	Radius_length	-0,12248	0,071442	NA
3	abundance	X01574	N-Acetylpr	Radius_SOS	-0,04913	0,050765	NA
3	abundance	X01574	N-Acetylpr	Tibia_SOS	-0,11235	0,054419	NA
3	abundance	X01574	N-Acetylpr	Handgrip	-0,01369	0,062212	NA
3	abundance	X01577	Prunasin	Tibia_length	-0,04182	0,051882	NA
3	abundance	X01577	Prunasin	Radius_length	-0,04705	0,070834	NA
3	abundance	X01577	Prunasin	Radius_SOS	0,023705	0,049852	NA
3	abundance	X01577	Prunasin	Tibia_SOS	0,00679	0,053443	NA
3	abundance	X01577	Prunasin	Handgrip	-0,01824	0,061564	NA
3	abundance	X01621	2-(2,4-Dihy	Tibia_length	0,052312	0,049559	NA
3	abundance	X01621	2-(2,4-Dihy	Radius_length	0,082751	0,066965	NA
3	abundance	X01621	2-(2,4-Dihy	Radius_SOS	-0,04287	0,048984	NA
3	abundance	X01621	2-(2,4-Dihy	Tibia_SOS	0,023421	0,052478	NA
3	abundance	X01621	2-(2,4-Dihy	Handgrip	0,115619	0,060075	NA
3	abundance	X01640	Ro 20-172	Tibia_length	-0,12599	0,055069	NA
3	abundance	X01640	Ro 20-172	Radius_length	-0,11441	0,073081	NA
3	abundance	X01640	Ro 20-172	Radius_SOS	-0,02531	0,052122	NA
3	abundance	X01640	Ro 20-172	Tibia_SOS	-0,11716	0,055114	NA
3	abundance	X01640	Ro 20-172	Handgrip	-0,08174	0,06215	NA
3	abundance	X01656	Guanadrel	Tibia_length	-0,01112	0,051128	NA
3	abundance	X01656	Guanadrel	Radius_length	0,011884	0,070013	NA
3	abundance	X01656	Guanadrel	Radius_SOS	-0,02161	0,04953	NA
3	abundance	X01656	Guanadrel	Tibia_SOS	-0,03312	0,052913	NA
3	abundance	X01656	Guanadrel	Handgrip	0,053039	0,060771	NA
3	abundance	X01664	5-methylth	Tibia_length	-0,07678	0,051795	NA
3	abundance	X01664	5-methylth	Radius_length	-0,07958	0,070764	NA
3	abundance	X01664	5-methylth	Radius_SOS	-0,03399	0,049877	NA
3	abundance	X01664	5-methylth	Tibia_SOS	-0,09247	0,053705	NA
3	abundance	X01664	5-methylth	Handgrip	0,047864	0,061738	NA
3	abundance	X01671	N-(3,5-Dim	Tibia_length	-0,08812	0,054645	NA
3	abundance	X01671	N-(3,5-Dim	Radius_length	-0,08365	0,073509	NA
3	abundance	X01671	N-(3,5-Dim	Radius_SOS	-0,0489	0,052026	NA
3	abundance	X01671	N-(3,5-Dim	Tibia_SOS	-0,03375	0,055551	NA
3	abundance	X01671	N-(3,5-Dim	Handgrip	-0,06157	0,062813	NA
3	abundance	X01672	Triethyl citı	Tibia_length	-0,14076	0,054585	NA
3	abundance	X01672	Triethyl citı	Radius_length	-0,12636	0,072966	NA
3	abundance	X01672	Triethyl citı	Radius_SOS	-0,07835	0,051946	NA
3	abundance	X01672	Triethyl citı	Tibia_SOS	-0,11008	0,055898	NA
3	abundance	X01672	Triethyl citı	Handgrip	-0,07004	0,061485	NA
3	abundance	X01679	meprobam	Tibia_length	0,017934	0,057298	NA
3	abundance	X01679	meprobam	Radius_length	0,087596	0,074435	NA
3	abundance	X01679	meprobam	Radius_SOS	0,018521	0,054393	NA
3	abundance	X01679	meprobam	Tibia_SOS	-0,02577	0,057646	NA
3	abundance	X01679	meprobam	Handgrip	0,006239	0,060192	NA
3	abundance	X01689	Ethyl mala	Tibia_length	0,056396	0,052362	NA
3	abundance	X01689	Ethyl mala	Radius_length	0,021868	0,071396	NA
3	abundance	X01689	Ethyl mala	Radius_SOS	0,01668	0,050326	NA
3	abundance	X01689	Ethyl mala	Tibia_SOS	-0,02917	0,054353	NA

3	abundance	X01689	Ethyl mala	Handgrip	0,039278	0,061128	NA
3	abundance	X01732	2-Hydroxy-	Tibia_length	-0,11606	0,052571	NA
3	abundance	X01732	2-Hydroxy-	Radius_length	-0,17007	0,070815	NA
3	abundance	X01732	2-Hydroxy-	Radius_SOS	-0,07297	0,050511	NA
3	abundance	X01732	2-Hydroxy-	Tibia_SOS	-0,12418	0,053245	NA
3	abundance	X01732	2-Hydroxy-	Handgrip	-0,07387	0,06068	NA
3	abundance	X01738	2-Acetami	Tibia_length	-0,18372	0,054203	NA
3	abundance	X01738	2-Acetami	Radius_length	-0,05984	0,073398	NA
3	abundance	X01738	2-Acetami	Radius_SOS	-0,0427	0,05253	NA
3	abundance	X01738	2-Acetami	Tibia_SOS	-0,01956	0,055707	NA
3	abundance	X01738	2-Acetami	Handgrip	-0,06475	0,061576	NA
3	abundance	X01746	Astemizole	Tibia_length	-0,00959	0,057851	NA
3	abundance	X01746	Astemizole	Radius_length	-0,01186	0,074936	NA
3	abundance	X01746	Astemizole	Radius_SOS	-0,01936	0,055273	NA
3	abundance	X01746	Astemizole	Tibia_SOS	-0,00601	0,058094	NA
3	abundance	X01746	Astemizole	Handgrip	-0,04056	0,062368	NA
3	abundance	X01768	Butabarbit	Tibia_length	0,013917	0,05131	NA
3	abundance	X01768	Butabarbit	Radius_length	0,082526	0,069915	NA
3	abundance	X01768	Butabarbit	Radius_SOS	0,056203	0,049493	NA
3	abundance	X01768	Butabarbit	Tibia_SOS	0,068311	0,053731	NA
3	abundance	X01768	Butabarbit	Handgrip	-0,05468	0,060858	NA
3	abundance	X01776	N-(Carboxy	Tibia_length	-0,14683	0,055435	NA
3	abundance	X01776	N-(Carboxy	Radius_length	-0,0691	0,074407	NA
3	abundance	X01776	N-(Carboxy	Radius_SOS	-0,07437	0,05363	NA
3	abundance	X01776	N-(Carboxy	Tibia_SOS	-0,08411	0,054699	NA
3	abundance	X01776	N-(Carboxy	Handgrip	-0,01434	0,060989	NA
3	abundance	X01793	Lys-phe	Tibia_length	-0,08272	0,054968	NA
3	abundance	X01793	Lys-phe	Radius_length	0,003764	0,073708	NA
3	abundance	X01793	Lys-phe	Radius_SOS	-0,00966	0,052228	NA
3	abundance	X01793	Lys-phe	Tibia_SOS	-0,07075	0,055832	NA
3	abundance	X01793	Lys-phe	Handgrip	-0,00449	0,062955	NA
3	abundance	X01813	Erythorbic	Tibia_length	-0,15959	0,056616	NA
3	abundance	X01813	Erythorbic	Radius_length	-0,06688	0,075382	NA
3	abundance	X01813	Erythorbic	Radius_SOS	-0,07022	0,054704	NA
3	abundance	X01813	Erythorbic	Tibia_SOS	-0,0564	0,058677	NA
3	abundance	X01813	Erythorbic	Handgrip	-0,04174	0,06014	NA
2b	abundance	X01833	2,3,4,9-Tet	Tibia_length	-0,08836	0,052698	NA
2b	abundance	X01833	2,3,4,9-Tet	Radius_length	-0,08456	0,071809	NA
2b	abundance	X01833	2,3,4,9-Tet	Radius_SOS	-0,04843	0,050535	NA
2b	abundance	X01833	2,3,4,9-Tet	Tibia_SOS	-0,08154	0,053673	NA
2b	abundance	X01833	2,3,4,9-Tet	Handgrip	-0,09167	0,06083	NA
3	abundance	X01873	Methyl 2,3-	Tibia_length	0,110015	0,053316	NA
3	abundance	X01873	Methyl 2,3-	Radius_length	0,259503	0,070176	NA
3	abundance	X01873	Methyl 2,3-	Radius_SOS	0,006729	0,051117	NA
3	abundance	X01873	Methyl 2,3-	Tibia_SOS	0,004914	0,055321	NA
3	abundance	X01873	Methyl 2,3-	Handgrip	0,014346	0,062362	NA
2b	abundance	X01879	Tetramethy	Tibia_length	-0,01311	0,053256	NA
2b	abundance	X01879	Tetramethy	Radius_length	-0,00189	0,072258	NA

2b	abundance	X01879	Tetramethy	Radius_SOS	0,017281	0,050887	NA
2b	abundance	X01879	Tetramethy	Tibia_SOS	0,084343	0,053737	NA
2b	abundance	X01879	Tetramethy	Handgrip	-0,01617	0,061793	NA
3	abundance	X01881	(2S)-3-Met	Tibia_length	-0,14622	0,056426	NA
3	abundance	X01881	(2S)-3-Met	Radius_length	-0,06198	0,075507	NA
3	abundance	X01881	(2S)-3-Met	Radius_SOS	0,043216	0,054359	NA
3	abundance	X01881	(2S)-3-Met	Tibia_SOS	0,03017	0,059393	NA
3	abundance	X01881	(2S)-3-Met	Handgrip	-0,12546	0,062616	NA
3	abundance	X01884	Roxane	Tibia_length	0,018481	0,050295	NA
3	abundance	X01884	Roxane	Radius_length	0,070468	0,068699	NA
3	abundance	X01884	Roxane	Radius_SOS	0,038314	0,049111	NA
3	abundance	X01884	Roxane	Tibia_SOS	0,119016	0,052544	NA
3	abundance	X01884	Roxane	Handgrip	0,044138	0,060251	NA
3	abundance	X01887	lys-tyr	Tibia_length	-0,05453	0,054317	NA
3	abundance	X01887	lys-tyr	Radius_length	-0,08801	0,073922	NA
3	abundance	X01887	lys-tyr	Radius_SOS	0,028591	0,051542	NA
3	abundance	X01887	lys-tyr	Tibia_SOS	-0,02719	0,055034	NA
3	abundance	X01887	lys-tyr	Handgrip	0,094317	0,061248	NA
3	abundance	X01893	4-(METHYL	Tibia_length	-0,08688	0,056806	NA
3	abundance	X01893	4-(METHYL	Radius_length	-0,18771	0,073121	NA
3	abundance	X01893	4-(METHYL	Radius_SOS	-0,06295	0,053674	NA
3	abundance	X01893	4-(METHYL	Tibia_SOS	-0,12145	0,057024	NA
3	abundance	X01893	4-(METHYL	Handgrip	-0,09029	0,061926	NA
3	abundance	X01911	3-(2,3-Dihy	Tibia_length	-0,12214	0,05197	NA
3	abundance	X01911	3-(2,3-Dihy	Radius_length	-0,17433	0,070348	NA
3	abundance	X01911	3-(2,3-Dihy	Radius_SOS	-0,10667	0,049848	NA
3	abundance	X01911	3-(2,3-Dihy	Tibia_SOS	-0,07604	0,053453	NA
3	abundance	X01911	3-(2,3-Dihy	Handgrip	-0,04161	0,060671	NA
3	abundance	X01920	N-(Carboxy	Tibia_length	0,031708	0,053537	NA
3	abundance	X01920	N-(Carboxy	Radius_length	0,109102	0,072193	NA
3	abundance	X01920	N-(Carboxy	Radius_SOS	0,035366	0,051154	NA
3	abundance	X01920	N-(Carboxy	Tibia_SOS	0,012424	0,054182	NA
3	abundance	X01920	N-(Carboxy	Handgrip	-0,04827	0,062885	NA
3	abundance	X01932	asn-val	Tibia_length	0,167148	0,054909	NA
3	abundance	X01932	asn-val	Radius_length	0,163207	0,073133	NA
3	abundance	X01932	asn-val	Radius_SOS	0,081928	0,052962	NA
3	abundance	X01932	asn-val	Tibia_SOS	0,101887	0,05825	NA
3	abundance	X01932	asn-val	Handgrip	0,064987	0,062567	NA
3	abundance	X01943	Tocainide	Tibia_length	0,08615	0,049208	NA
3	abundance	X01943	Tocainide	Radius_length	0,118993	0,065955	NA
3	abundance	X01943	Tocainide	Radius_SOS	0,005509	0,049048	NA
3	abundance	X01943	Tocainide	Tibia_SOS	0,077389	0,052355	NA
3	abundance	X01943	Tocainide	Handgrip	0,010736	0,060234	NA
3	abundance	X01950	Histidylgly	Tibia_length	-0,06136	0,052937	NA
3	abundance	X01950	Histidylgly	Radius_length	-0,06685	0,071705	NA
3	abundance	X01950	Histidylgly	Radius_SOS	0,072025	0,050592	NA
3	abundance	X01950	Histidylgly	Tibia_SOS	-0,03607	0,054673	NA
3	abundance	X01950	Histidylgly	Handgrip	-0,05083	0,061816	NA

3	abundance	X01968	7-Chloro-5	Tibia_length	-0,06924	0,057515	NA
3	abundance	X01968	7-Chloro-5	Radius_length	-0,03628	0,075365	NA
3	abundance	X01968	7-Chloro-5	Radius_SOS	-0,07539	0,054927	NA
3	abundance	X01968	7-Chloro-5	Tibia_SOS	0,034944	0,058681	NA
3	abundance	X01968	7-Chloro-5	Handgrip	0,009074	0,0634	NA
3	abundance	X01996	3-Hydroxy-	Tibia_length	0,121803	0,054772	NA
3	abundance	X01996	3-Hydroxy-	Radius_length	0,14509	0,072935	NA
3	abundance	X01996	3-Hydroxy-	Radius_SOS	0,043899	0,052462	NA
3	abundance	X01996	3-Hydroxy-	Tibia_SOS	0,071474	0,055592	NA
3	abundance	X01996	3-Hydroxy-	Handgrip	0,018997	0,062948	NA
3	abundance	X02000	11-(4-Hydr	Tibia_length	0,025582	0,053667	NA
3	abundance	X02000	11-(4-Hydr	Radius_length	0,105237	0,072028	NA
3	abundance	X02000	11-(4-Hydr	Radius_SOS	0,040772	0,051164	NA
3	abundance	X02000	11-(4-Hydr	Tibia_SOS	0,0473	0,054041	NA
3	abundance	X02000	11-(4-Hydr	Handgrip	0,064741	0,062129	NA
3	abundance	X02009	Arctiopicri	Tibia_length	-0,01382	0,054493	NA
3	abundance	X02009	Arctiopicri	Radius_length	-0,06599	0,073504	NA
3	abundance	X02009	Arctiopicri	Radius_SOS	-0,0142	0,051926	NA
3	abundance	X02009	Arctiopicri	Tibia_SOS	0,068871	0,054301	NA
3	abundance	X02009	Arctiopicri	Handgrip	0,074915	0,061034	NA
3	abundance	X02013	Ile-cys	Tibia_length	0,078473	0,055803	NA
3	abundance	X02013	Ile-cys	Radius_length	0,139657	0,07361	NA
3	abundance	X02013	Ile-cys	Radius_SOS	0,0192	0,053295	NA
3	abundance	X02013	Ile-cys	Tibia_SOS	0,081101	0,056153	NA
3	abundance	X02013	Ile-cys	Handgrip	0,104265	0,061327	NA
3	abundance	X02020	Val-Ser	Tibia_length	-0,07217	0,056216	NA
3	abundance	X02020	Val-Ser	Radius_length	-0,0126	0,074629	NA
3	abundance	X02020	Val-Ser	Radius_SOS	-0,02578	0,053619	NA
3	abundance	X02020	Val-Ser	Tibia_SOS	0,004616	0,058602	NA
3	abundance	X02020	Val-Ser	Handgrip	0,01408	0,063281	NA
3	abundance	X02023	beta-D-Eth	Tibia_length	-0,15486	0,053845	NA
3	abundance	X02023	beta-D-Eth	Radius_length	-0,11068	0,073458	NA
3	abundance	X02023	beta-D-Eth	Radius_SOS	-0,06666	0,051731	NA
3	abundance	X02023	beta-D-Eth	Tibia_SOS	-0,01098	0,056356	NA
3	abundance	X02023	beta-D-Eth	Handgrip	-0,13196	0,061754	NA
3	abundance	X02073	N-acetyl-9	Tibia_length	-0,14716	0,055966	NA
3	abundance	X02073	N-acetyl-9	Radius_length	-0,02048	0,074895	NA
3	abundance	X02073	N-acetyl-9	Radius_SOS	-0,07341	0,053995	NA
3	abundance	X02073	N-acetyl-9	Tibia_SOS	-0,02239	0,056757	NA
3	abundance	X02073	N-acetyl-9	Handgrip	0,09984	0,062587	NA
3	abundance	X02080	(-)-Aspidos	Tibia_length	0,080444	0,054789	NA
3	abundance	X02080	(-)-Aspidos	Radius_length	0,143989	0,072634	NA
3	abundance	X02080	(-)-Aspidos	Radius_SOS	-0,05983	0,052182	NA
3	abundance	X02080	(-)-Aspidos	Tibia_SOS	0,014734	0,055026	NA
3	abundance	X02080	(-)-Aspidos	Handgrip	0,006147	0,061795	NA
3	abundance	X02082	6-Hydroxyr	Tibia_length	0,009107	0,052499	NA
3	abundance	X02082	6-Hydroxyr	Radius_length	-0,04453	0,071417	NA
3	abundance	X02082	6-Hydroxyr	Radius_SOS	-0,04103	0,050368	NA

	3	abundance	X02082	6-Hydroxyr	Tibia_SOS	-0,05974	0,053077	NA
	3	abundance	X02082	6-Hydroxyr	Handgrip	0,009284	0,060462	NA
	3	abundance	X02101	Succinic ai	Tibia_length	0,024177	0,056143	NA
	3	abundance	X02101	Succinic ai	Radius_length	0,038431	0,074398	NA
	3	abundance	X02101	Succinic ai	Radius_SOS	0,058141	0,053093	NA
	3	abundance	X02101	Succinic ai	Tibia_SOS	-0,01872	0,056521	NA
	3	abundance	X02101	Succinic ai	Handgrip	0,139487	0,061669	NA
	3	abundance	X02108	Homovanil	Tibia_length	-0,00819	0,053219	NA
	3	abundance	X02108	Homovanil	Radius_length	-0,05175	0,072452	NA
	3	abundance	X02108	Homovanil	Radius_SOS	-0,04183	0,051104	NA
	3	abundance	X02108	Homovanil	Tibia_SOS	-0,05205	0,054602	NA
	3	abundance	X02108	Homovanil	Handgrip	0,014549	0,061314	NA
	3	abundance	X02121	2-Acetamic	Tibia_length	-0,03059	0,053029	NA
	3	abundance	X02121	2-Acetamic	Radius_length	0,015614	0,072109	NA
	3	abundance	X02121	2-Acetamic	Radius_SOS	-0,04236	0,050664	NA
	3	abundance	X02121	2-Acetamic	Tibia_SOS	-0,06091	0,053706	NA
	3	abundance	X02121	2-Acetamic	Handgrip	0,025119	0,06113	NA
	3	abundance	X02139	SECONAL	Tibia_length	-0,03225	0,053406	NA
	3	abundance	X02139	SECONAL	Radius_length	-0,07004	0,072028	NA
	3	abundance	X02139	SECONAL	Radius_SOS	0,060401	0,050882	NA
	3	abundance	X02139	SECONAL	Tibia_SOS	0,010041	0,053995	NA
	3	abundance	X02139	SECONAL	Handgrip	-0,07488	0,061328	NA
	3	abundance	X02145	3'-Hydroxy	Tibia_length	0,062849	0,051437	NA
	3	abundance	X02145	3'-Hydroxy	Radius_length	0,055526	0,070052	NA
	3	abundance	X02145	3'-Hydroxy	Radius_SOS	0,012226	0,049665	NA
	3	abundance	X02145	3'-Hydroxy	Tibia_SOS	0,04347	0,053649	NA
	3	abundance	X02145	3'-Hydroxy	Handgrip	-0,09567	0,060241	NA
	3	abundance	X02153	Asarone	Tibia_length	-0,08039	0,053656	NA
	3	abundance	X02153	Asarone	Radius_length	-0,20268	0,071397	NA
	3	abundance	X02153	Asarone	Radius_SOS	-0,0153	0,051221	NA
	3	abundance	X02153	Asarone	Tibia_SOS	-0,15314	0,053742	NA
	3	abundance	X02153	Asarone	Handgrip	-0,09254	0,061697	NA
	3	abundance	X02181	YWA1	Tibia_length	0,112554	0,049688	NA
	3	abundance	X02181	YWA1	Radius_length	0,24793	0,066019	NA
	3	abundance	X02181	YWA1	Radius_SOS	0,04695	0,048984	NA
	3	abundance	X02181	YWA1	Tibia_SOS	0,119649	0,052111	NA
	3	abundance	X02181	YWA1	Handgrip	0,138754	0,059747	NA
2b		abundance	X02184	Piperonylo	Tibia_length	0,051869	0,053314	NA
2b		abundance	X02184	Piperonylo	Radius_length	-0,00403	0,072736	NA
2b		abundance	X02184	Piperonylo	Radius_SOS	-0,05133	0,050896	NA
2b		abundance	X02184	Piperonylo	Tibia_SOS	0,09819	0,05411	NA
2b		abundance	X02184	Piperonylo	Handgrip	-0,01987	0,061442	NA
	3	abundance	X02195	alliin	Tibia_length	-0,04466	0,053803	NA
	3	abundance	X02195	alliin	Radius_length	-0,02589	0,072677	NA
	3	abundance	X02195	alliin	Radius_SOS	0,052694	0,051269	NA
	3	abundance	X02195	alliin	Tibia_SOS	0,01807	0,054705	NA
	3	abundance	X02195	alliin	Handgrip	-0,04438	0,061102	NA
	3	abundance	X02202	D-2-Amino	Tibia_length	0,051411	0,056147	NA

3	abundance	X02202	D-2-Amino	Radius_length	-0,0446	0,074331	NA
3	abundance	X02202	D-2-Amino	Radius_SOS	0,020755	0,053372	NA
3	abundance	X02202	D-2-Amino	Tibia_SOS	0,053971	0,057329	NA
3	abundance	X02202	D-2-Amino	Handgrip	0,086933	0,06233	NA
3	abundance	X02208	SECONAL	Tibia_length	-0,04648	0,05843	NA
3	abundance	X02208	SECONAL	Radius_length	-0,08612	0,075492	NA
3	abundance	X02208	SECONAL	Radius_SOS	0,053221	0,055595	NA
3	abundance	X02208	SECONAL	Tibia_SOS	-0,04937	0,058605	NA
3	abundance	X02208	SECONAL	Handgrip	0,012048	0,060189	NA
3	abundance	X02214	(E)-4-Meth	Tibia_length	-0,06352	0,055463	NA
3	abundance	X02214	(E)-4-Meth	Radius_length	-0,0095	0,073713	NA
3	abundance	X02214	(E)-4-Meth	Radius_SOS	1,82E-04	0,052915	NA
3	abundance	X02214	(E)-4-Meth	Tibia_SOS	0,029821	0,055204	NA
3	abundance	X02214	(E)-4-Meth	Handgrip	-0,01635	0,061594	NA
3	abundance	X02219	Homoanse	Tibia_length	-0,09508	0,0538	NA
3	abundance	X02219	Homoanse	Radius_length	-0,14728	0,072133	NA
3	abundance	X02219	Homoanse	Radius_SOS	-0,04199	0,051443	NA
3	abundance	X02219	Homoanse	Tibia_SOS	-0,11015	0,053746	NA
3	abundance	X02219	Homoanse	Handgrip	-0,07574	0,061506	NA
3	abundance	X02231	Ala-Tyr	Tibia_length	-0,12612	0,052192	NA
3	abundance	X02231	Ala-Tyr	Radius_length	-0,18055	0,070678	NA
3	abundance	X02231	Ala-Tyr	Radius_SOS	-0,05059	0,050179	NA
3	abundance	X02231	Ala-Tyr	Tibia_SOS	-0,07477	0,054157	NA
3	abundance	X02231	Ala-Tyr	Handgrip	-0,01853	0,061596	NA
3	abundance	X02238	6-Myoporo	Tibia_length	0,023302	0,053292	NA
3	abundance	X02238	6-Myoporo	Radius_length	0,050495	0,071953	NA
3	abundance	X02238	6-Myoporo	Radius_SOS	-0,0367	0,050915	NA
3	abundance	X02238	6-Myoporo	Tibia_SOS	0,020407	0,053701	NA
3	abundance	X02238	6-Myoporo	Handgrip	-0,03848	0,06175	NA
3	abundance	X02251	Hostmania	Tibia_length	-0,06904	0,052451	NA
3	abundance	X02251	Hostmania	Radius_length	-0,09689	0,071472	NA
3	abundance	X02251	Hostmania	Radius_SOS	-0,00717	0,050241	NA
3	abundance	X02251	Hostmania	Tibia_SOS	-0,13804	0,053742	NA
3	abundance	X02251	Hostmania	Handgrip	-0,12963	0,060585	NA
3	abundance	X02256	hexobarbit	Tibia_length	0,015655	0,049747	NA
3	abundance	X02256	hexobarbit	Radius_length	0,15493	0,066713	NA
3	abundance	X02256	hexobarbit	Radius_SOS	0,031481	0,049021	NA
3	abundance	X02256	hexobarbit	Tibia_SOS	0,005521	0,052489	NA
3	abundance	X02256	hexobarbit	Handgrip	0,043801	0,060633	NA
3	abundance	X02265	ophthalmic	Tibia_length	-0,17397	0,053639	NA
3	abundance	X02265	ophthalmic	Radius_length	-0,08823	0,07288	NA
3	abundance	X02265	ophthalmic	Radius_SOS	-0,02568	0,051511	NA
3	abundance	X02265	ophthalmic	Tibia_SOS	-5,76E-04	0,055468	NA
3	abundance	X02265	ophthalmic	Handgrip	-0,04084	0,061901	NA
3	abundance	X02268	5-Hydroxy-	Tibia_length	-0,05708	0,051551	NA
3	abundance	X02268	5-Hydroxy-	Radius_length	-0,01356	0,070451	NA
3	abundance	X02268	5-Hydroxy-	Radius_SOS	0,079973	0,049571	NA
3	abundance	X02268	5-Hydroxy-	Tibia_SOS	-0,10435	0,052759	NA

	3	abundance	X02268	5-Hydroxy-	Handgrip	-0,10543	0,060778	NA
	3	abundance	X02277	tert-Butyl 3	Tibia_length	-0,02862	0,053253	NA
	3	abundance	X02277	tert-Butyl 3	Radius_length	0,025641	0,072765	NA
	3	abundance	X02277	tert-Butyl 3	Radius_SOS	-0,01314	0,050893	NA
	3	abundance	X02277	tert-Butyl 3	Tibia_SOS	-0,00507	0,054783	NA
	3	abundance	X02277	tert-Butyl 3	Handgrip	0,012367	0,062003	NA
	3	abundance	X02281	tert-Butyl 3	Tibia_length	0,030353	0,052089	NA
	3	abundance	X02281	tert-Butyl 3	Radius_length	0,031728	0,071053	NA
	3	abundance	X02281	tert-Butyl 3	Radius_SOS	-0,01533	0,050062	NA
	3	abundance	X02281	tert-Butyl 3	Tibia_SOS	-0,02954	0,053542	NA
	3	abundance	X02281	tert-Butyl 3	Handgrip	-0,01153	0,061071	NA
2b		abundance	X02288	9-Methylur	Tibia_length	-0,14671	0,056462	NA
2b		abundance	X02288	9-Methylur	Radius_length	-0,10514	0,074072	NA
2b		abundance	X02288	9-Methylur	Radius_SOS	0,01269	0,053995	NA
2b		abundance	X02288	9-Methylur	Tibia_SOS	0,076223	0,057148	NA
2b		abundance	X02288	9-Methylur	Handgrip	-0,10031	0,062717	NA
2a		abundance	X02289	Kynurenic a	Tibia_length	-0,10557	0,04888	NA
2a		abundance	X02289	Kynurenic a	Radius_length	-0,10922	0,065245	NA
2a		abundance	X02289	Kynurenic a	Radius_SOS	-0,0783	0,048961	NA
2a		abundance	X02289	Kynurenic a	Tibia_SOS	0,012957	0,0526	NA
2a		abundance	X02289	Kynurenic a	Handgrip	0,02109	0,060252	NA
2b		abundance	X02317	2-Aminooc	Tibia_length	-0,01079	0,055935	NA
2b		abundance	X02317	2-Aminooc	Radius_length	0,066563	0,073788	NA
2b		abundance	X02317	2-Aminooc	Radius_SOS	-0,06465	0,052994	NA
2b		abundance	X02317	2-Aminooc	Tibia_SOS	-0,06216	0,056634	NA
2b		abundance	X02317	2-Aminooc	Handgrip	-0,05407	0,063482	NA
	3	abundance	X02327	1H-Pyrazol	Tibia_length	-0,0143	0,058277	NA
	3	abundance	X02327	1H-Pyrazol	Radius_length	-0,10351	0,075531	NA
	3	abundance	X02327	1H-Pyrazol	Radius_SOS	0,047007	0,055454	NA
	3	abundance	X02327	1H-Pyrazol	Tibia_SOS	0,014322	0,060023	NA
	3	abundance	X02327	1H-Pyrazol	Handgrip	0,028476	0,062937	NA
	3	abundance	X02333	N-Propiony	Tibia_length	0,017904	0,053921	NA
	3	abundance	X02333	N-Propiony	Radius_length	0,130536	0,072014	NA
	3	abundance	X02333	N-Propiony	Radius_SOS	0,052428	0,051265	NA
	3	abundance	X02333	N-Propiony	Tibia_SOS	0,07829	0,054702	NA
	3	abundance	X02333	N-Propiony	Handgrip	-0,00515	0,061316	NA
	3	abundance	X02337	Methyl 1-hy	Tibia_length	-0,11224	0,050628	NA
	3	abundance	X02337	Methyl 1-hy	Radius_length	-0,06615	0,06984	NA
	3	abundance	X02337	Methyl 1-hy	Radius_SOS	-0,01416	0,04943	NA
	3	abundance	X02337	Methyl 1-hy	Tibia_SOS	0,067664	0,052964	NA
	3	abundance	X02337	Methyl 1-hy	Handgrip	-0,04072	0,061341	NA
	3	abundance	X02348	N-Benzoyla	Tibia_length	0,093142	0,054694	NA
	3	abundance	X02348	N-Benzoyla	Radius_length	0,059293	0,07406	NA
	3	abundance	X02348	N-Benzoyla	Radius_SOS	0,006154	0,052023	NA
	3	abundance	X02348	N-Benzoyla	Tibia_SOS	0,088237	0,057373	NA
	3	abundance	X02348	N-Benzoyla	Handgrip	0,027193	0,061823	NA
	3	abundance	X02352	Indole-3-ca	Tibia_length	0,001395	0,055199	NA
	3	abundance	X02352	Indole-3-ca	Radius_length	-0,03666	0,073275	NA

	3	abundance	X02352	Indole-3-ca	Radius_SOS	0,045118	0,052352	NA
	3	abundance	X02352	Indole-3-ca	Tibia_SOS	0,040251	0,056113	NA
	3	abundance	X02352	Indole-3-ca	Handgrip	-0,01446	0,063191	NA
	3	abundance	X02380	Propafenon	Tibia_length	0,124264	0,051538	NA
	3	abundance	X02380	Propafenon	Radius_length	0,177323	0,070085	NA
	3	abundance	X02380	Propafenon	Radius_SOS	0,039074	0,049778	NA
	3	abundance	X02380	Propafenon	Tibia_SOS	0,050736	0,053615	NA
	3	abundance	X02380	Propafenon	Handgrip	0,103024	0,060923	NA
	3	abundance	X02419	Glu-Glu	Tibia_length	-0,00791	0,053471	NA
	3	abundance	X02419	Glu-Glu	Radius_length	-0,01022	0,073002	NA
	3	abundance	X02419	Glu-Glu	Radius_SOS	-0,04692	0,050886	NA
	3	abundance	X02419	Glu-Glu	Tibia_SOS	-0,06634	0,054734	NA
	3	abundance	X02419	Glu-Glu	Handgrip	-0,12076	0,061556	NA
	3	abundance	X02426	Raltitrexed	Tibia_length	-0,15965	0,051939	NA
	3	abundance	X02426	Raltitrexed	Radius_length	-0,06655	0,071658	NA
	3	abundance	X02426	Raltitrexed	Radius_SOS	0,022428	0,050516	NA
	3	abundance	X02426	Raltitrexed	Tibia_SOS	0,027542	0,053171	NA
	3	abundance	X02426	Raltitrexed	Handgrip	-0,04309	0,060719	NA
2b		abundance	X02427	2-(3,5-dim	Tibia_length	0,092884	0,056403	NA
2b		abundance	X02427	2-(3,5-dim	Radius_length	0,007768	0,074353	NA
2b		abundance	X02427	2-(3,5-dim	Radius_SOS	-0,01103	0,053829	NA
2b		abundance	X02427	2-(3,5-dim	Tibia_SOS	0,004483	0,058162	NA
2b		abundance	X02427	2-(3,5-dim	Handgrip	-0,11532	0,059791	NA
	3	abundance	X02442	3-(Sulfooxy	Tibia_length	0,044114	0,052294	NA
	3	abundance	X02442	3-(Sulfooxy	Radius_length	0,104683	0,071019	NA
	3	abundance	X02442	3-(Sulfooxy	Radius_SOS	0,010619	0,050202	NA
	3	abundance	X02442	3-(Sulfooxy	Tibia_SOS	0,150246	0,05333	NA
	3	abundance	X02442	3-(Sulfooxy	Handgrip	0,005698	0,061036	NA
	3	abundance	X02466	Validamyci	Tibia_length	0,047329	0,056491	NA
	3	abundance	X02466	Validamyci	Radius_length	0,128316	0,074246	NA
	3	abundance	X02466	Validamyci	Radius_SOS	0,035619	0,053617	NA
	3	abundance	X02466	Validamyci	Tibia_SOS	0,022109	0,057757	NA
	3	abundance	X02466	Validamyci	Handgrip	-0,05195	0,061854	NA
	3	abundance	X02468	N,N-Diethy	Tibia_length	-0,04671	0,054255	NA
	3	abundance	X02468	N,N-Diethy	Radius_length	-0,03309	0,072858	NA
	3	abundance	X02468	N,N-Diethy	Radius_SOS	-0,0385	0,051621	NA
	3	abundance	X02468	N,N-Diethy	Tibia_SOS	0,053645	0,055113	NA
	3	abundance	X02468	N,N-Diethy	Handgrip	-0,0407	0,061805	NA
	3	abundance	X02471	n-Propyl Ga	Tibia_length	-0,0144	0,051552	NA
	3	abundance	X02471	n-Propyl Ga	Radius_length	-0,18216	0,069508	NA
	3	abundance	X02471	n-Propyl Ga	Radius_SOS	-0,00479	0,049751	NA
	3	abundance	X02471	n-Propyl Ga	Tibia_SOS	-0,07744	0,054286	NA
	3	abundance	X02471	n-Propyl Ga	Handgrip	-0,06583	0,060905	NA
	3	abundance	X02492	N-(3,5-Dim	Tibia_length	-0,14187	0,054316	NA
	3	abundance	X02492	N-(3,5-Dim	Radius_length	-0,08766	0,073227	NA
	3	abundance	X02492	N-(3,5-Dim	Radius_SOS	-0,03602	0,052032	NA
	3	abundance	X02492	N-(3,5-Dim	Tibia_SOS	-0,07963	0,055201	NA
	3	abundance	X02492	N-(3,5-Dim	Handgrip	-0,12844	0,061556	NA

3	abundance	X02494	Butylphtha	Tibia_length	-0,07335	0,052971	NA
3	abundance	X02494	Butylphtha	Radius_length	-0,18732	0,070962	NA
3	abundance	X02494	Butylphtha	Radius_SOS	-0,00811	0,050708	NA
3	abundance	X02494	Butylphtha	Tibia_SOS	-0,15785	0,053248	NA
3	abundance	X02494	Butylphtha	Handgrip	-0,09127	0,061313	NA
2b	abundance	X02504	DLK (Pepti	Tibia_length	-0,07466	0,052597	NA
2b	abundance	X02504	DLK (Pepti	Radius_length	0,046727	0,072082	NA
2b	abundance	X02504	DLK (Pepti	Radius_SOS	0,066062	0,050578	NA
2b	abundance	X02504	DLK (Pepti	Tibia_SOS	0,119891	0,053898	NA
2b	abundance	X02504	DLK (Pepti	Handgrip	0,080129	0,061929	NA
2b	abundance	X02513	7-Methylac	Tibia_length	-0,02498	0,052393	NA
2b	abundance	X02513	7-Methylac	Radius_length	0,018212	0,071481	NA
2b	abundance	X02513	7-Methylac	Radius_SOS	0,012584	0,050223	NA
2b	abundance	X02513	7-Methylac	Tibia_SOS	0,029887	0,054	NA
2b	abundance	X02513	7-Methylac	Handgrip	0,026987	0,061669	NA
1	abundance	X02514	3-Ureidopr	Tibia_length	-0,015	0,054201	NA
1	abundance	X02514	3-Ureidopr	Radius_length	-0,06923	0,072818	NA
1	abundance	X02514	3-Ureidopr	Radius_SOS	-0,06993	0,052006	NA
1	abundance	X02514	3-Ureidopr	Tibia_SOS	-0,00672	0,055436	NA
1	abundance	X02514	3-Ureidopr	Handgrip	0,049732	0,062687	NA
3	abundance	X02521	6-(1-Hydro	Tibia_length	-0,12475	0,054706	NA
3	abundance	X02521	6-(1-Hydro	Radius_length	-0,1505	0,072863	NA
3	abundance	X02521	6-(1-Hydro	Radius_SOS	-0,01699	0,052055	NA
3	abundance	X02521	6-(1-Hydro	Tibia_SOS	-0,02797	0,054746	NA
3	abundance	X02521	6-(1-Hydro	Handgrip	-0,16119	0,060542	NA
3	abundance	X02529	tenivastati	Tibia_length	0,098563	0,05171	NA
3	abundance	X02529	tenivastati	Radius_length	0,047686	0,070823	NA
3	abundance	X02529	tenivastati	Radius_SOS	-0,00633	0,049776	NA
3	abundance	X02529	tenivastati	Tibia_SOS	0,027179	0,053267	NA
3	abundance	X02529	tenivastati	Handgrip	-0,07728	0,060993	NA
3	abundance	X02537	Leu-pro	Tibia_length	-0,19564	0,053089	NA
3	abundance	X02537	Leu-pro	Radius_length	-0,2295	0,0714	NA
3	abundance	X02537	Leu-pro	Radius_SOS	-0,00644	0,051217	NA
3	abundance	X02537	Leu-pro	Tibia_SOS	-0,03867	0,054344	NA
3	abundance	X02537	Leu-pro	Handgrip	-0,19467	0,060664	NA
3	abundance	X02565	Dopamine	Tibia_length	-0,09645	0,059184	NA
3	abundance	X02565	Dopamine	Radius_length	0,019037	0,076919	NA
3	abundance	X02565	Dopamine	Radius_SOS	-0,03767	0,056866	NA
3	abundance	X02565	Dopamine	Tibia_SOS	-0,13218	0,059107	NA
3	abundance	X02565	Dopamine	Handgrip	-0,06153	0,060079	NA
3	abundance	X02583	Ethylvanilli	Tibia_length	-0,02993	0,054739	NA
3	abundance	X02583	Ethylvanilli	Radius_length	0,029025	0,073274	NA
3	abundance	X02583	Ethylvanilli	Radius_SOS	-0,05308	0,052011	NA
3	abundance	X02583	Ethylvanilli	Tibia_SOS	9,37E-04	0,054857	NA
3	abundance	X02583	Ethylvanilli	Handgrip	-0,04157	0,061747	NA
3	abundance	X02599	Dihydrothy	Tibia_length	0,100245	0,054922	NA
3	abundance	X02599	Dihydrothy	Radius_length	-0,05236	0,073925	NA
3	abundance	X02599	Dihydrothy	Radius_SOS	-0,01774	0,052514	NA

3	abundance	X02599	Dihydrothy	Tibia_SOS	0,112013	0,055555	NA
3	abundance	X02599	Dihydrothy	Handgrip	-0,02453	0,061423	NA
3	abundance	X02601	(7E,7'E)-5,	Tibia_length	0,017123	0,054236	NA
3	abundance	X02601	(7E,7'E)-5,	Radius_length	0,145194	0,07242	NA
3	abundance	X02601	(7E,7'E)-5,	Radius_SOS	0,01257	0,051632	NA
3	abundance	X02601	(7E,7'E)-5,	Tibia_SOS	-0,00816	0,054453	NA
3	abundance	X02601	(7E,7'E)-5,	Handgrip	0,067601	0,061725	NA
3	abundance	X02606	4-Amino-1	Tibia_length	0,039518	0,04979	NA
3	abundance	X02606	4-Amino-1	Radius_length	0,099891	0,067367	NA
3	abundance	X02606	4-Amino-1	Radius_SOS	0,014285	0,049047	NA
3	abundance	X02606	4-Amino-1	Tibia_SOS	0,062899	0,052433	NA
3	abundance	X02606	4-Amino-1	Handgrip	0,075191	0,060286	NA
3	abundance	X02615	6-APA	Tibia_length	-0,02579	0,053226	NA
3	abundance	X02615	6-APA	Radius_length	-0,07598	0,07188	NA
3	abundance	X02615	6-APA	Radius_SOS	-0,06532	0,050793	NA
3	abundance	X02615	6-APA	Tibia_SOS	-0,07341	0,054143	NA
3	abundance	X02615	6-APA	Handgrip	-0,01582	0,061352	NA
3	abundance	X02640	thyronine	Tibia_length	-0,07836	0,052424	NA
3	abundance	X02640	thyronine	Radius_length	-0,08933	0,07115	NA
3	abundance	X02640	thyronine	Radius_SOS	0,014475	0,050375	NA
3	abundance	X02640	thyronine	Tibia_SOS	-0,08835	0,053127	NA
3	abundance	X02640	thyronine	Handgrip	-0,10632	0,060958	NA
3	abundance	X02651	L-gamma-(	Tibia_length	-0,07453	0,053203	NA
3	abundance	X02651	L-gamma-(	Radius_length	-0,19615	0,07156	NA
3	abundance	X02651	L-gamma-(	Radius_SOS	-0,06071	0,05097	NA
3	abundance	X02651	L-gamma-(	Tibia_SOS	-0,07139	0,055236	NA
3	abundance	X02651	L-gamma-(	Handgrip	-0,01735	0,062608	NA
3	abundance	X02652	Leucyltyros	Tibia_length	-0,12852	0,052753	NA
3	abundance	X02652	Leucyltyros	Radius_length	-0,07792	0,071638	NA
3	abundance	X02652	Leucyltyros	Radius_SOS	-0,00204	0,050604	NA
3	abundance	X02652	Leucyltyros	Tibia_SOS	-0,0874	0,053749	NA
3	abundance	X02652	Leucyltyros	Handgrip	-0,04446	0,06171	NA
3	abundance	X02661	4-(3-Oxope	Tibia_length	-0,12534	0,052715	NA
3	abundance	X02661	4-(3-Oxope	Radius_length	-0,12079	0,071786	NA
3	abundance	X02661	4-(3-Oxope	Radius_SOS	-0,05813	0,051057	NA
3	abundance	X02661	4-(3-Oxope	Tibia_SOS	0,072024	0,053199	NA
3	abundance	X02661	4-(3-Oxope	Handgrip	0,043838	0,061101	NA
3	abundance	X02676	Val-Trp	Tibia_length	-0,14409	0,054296	NA
3	abundance	X02676	Val-Trp	Radius_length	-0,19325	0,07212	NA
3	abundance	X02676	Val-Trp	Radius_SOS	-0,00327	0,051706	NA
3	abundance	X02676	Val-Trp	Tibia_SOS	-0,11498	0,054863	NA
3	abundance	X02676	Val-Trp	Handgrip	-0,06815	0,061517	NA
3	abundance	X02687	S-Propylcy	Tibia_length	0,13871	0,054154	NA
3	abundance	X02687	S-Propylcy	Radius_length	0,126411	0,072937	NA
3	abundance	X02687	S-Propylcy	Radius_SOS	0,211698	0,051401	NA
3	abundance	X02687	S-Propylcy	Tibia_SOS	0,089681	0,05486	NA
3	abundance	X02687	S-Propylcy	Handgrip	0,042178	0,062169	NA
3	abundance	X02689	piscidic ac	Tibia_length	-0,14607	0,051674	NA

3	abundance	X02689	piscidic ac	Radius_length	-0,10821	0,070474	NA
3	abundance	X02689	piscidic ac	Radius_SOS	-0,00761	0,050001	NA
3	abundance	X02689	piscidic ac	Tibia_SOS	-6,89E-04	0,053043	NA
3	abundance	X02689	piscidic ac	Handgrip	-0,11083	0,06015	NA
3	abundance	X02702	R-(+)-Etira	Tibia_length	0,025673	0,054084	NA
3	abundance	X02702	R-(+)-Etira	Radius_length	-0,05132	0,072708	NA
3	abundance	X02702	R-(+)-Etira	Radius_SOS	0,032996	0,051508	NA
3	abundance	X02702	R-(+)-Etira	Tibia_SOS	-0,01726	0,055275	NA
3	abundance	X02702	R-(+)-Etira	Handgrip	-0,17876	0,060399	NA
3	abundance	X02720	ala-ser	Tibia_length	0,064492	0,051888	NA
3	abundance	X02720	ala-ser	Radius_length	0,1313	0,07052	NA
3	abundance	X02720	ala-ser	Radius_SOS	0,021854	0,049962	NA
3	abundance	X02720	ala-ser	Tibia_SOS	0,064062	0,053306	NA
3	abundance	X02720	ala-ser	Handgrip	0,068419	0,061189	NA
3	abundance	X02730	N-Phenyla	Tibia_length	-0,12157	0,05544	NA
3	abundance	X02730	N-Phenyla	Radius_length	-0,08704	0,073477	NA
3	abundance	X02730	N-Phenyla	Radius_SOS	0,079871	0,052993	NA
3	abundance	X02730	N-Phenyla	Tibia_SOS	0,001907	0,056349	NA
3	abundance	X02730	N-Phenyla	Handgrip	-0,01814	0,062023	NA
3	abundance	X02733	Calcitriol	Tibia_length	-0,01527	0,052862	NA
3	abundance	X02733	Calcitriol	Radius_length	-0,08921	0,072059	NA
3	abundance	X02733	Calcitriol	Radius_SOS	-0,01674	0,050588	NA
3	abundance	X02733	Calcitriol	Tibia_SOS	0,14098	0,053041	NA
3	abundance	X02733	Calcitriol	Handgrip	-0,14746	0,061072	NA
3	abundance	X02746	N,N-Diethy	Tibia_length	-0,07832	0,054514	NA
3	abundance	X02746	N,N-Diethy	Radius_length	0,007261	0,072869	NA
3	abundance	X02746	N,N-Diethy	Radius_SOS	0,04419	0,051532	NA
3	abundance	X02746	N,N-Diethy	Tibia_SOS	0,083217	0,054938	NA
3	abundance	X02746	N,N-Diethy	Handgrip	0,078456	0,061717	NA
3	abundance	X02760	6-hydroxyp	Tibia_length	0,007868	0,055869	NA
3	abundance	X02760	6-hydroxyp	Radius_length	0,081	0,073924	NA
3	abundance	X02760	6-hydroxyp	Radius_SOS	0,055861	0,052904	NA
3	abundance	X02760	6-hydroxyp	Tibia_SOS	0,114694	0,058325	NA
3	abundance	X02760	6-hydroxyp	Handgrip	0,058821	0,062992	NA
3	abundance	X02765	L-Pyrrolysi	Tibia_length	0,070793	0,053092	NA
3	abundance	X02765	L-Pyrrolysi	Radius_length	0,034526	0,072001	NA
3	abundance	X02765	L-Pyrrolysi	Radius_SOS	-0,06402	0,050606	NA
3	abundance	X02765	L-Pyrrolysi	Tibia_SOS	0,02575	0,054302	NA
3	abundance	X02765	L-Pyrrolysi	Handgrip	-0,09023	0,061278	NA
3	abundance	X02767	6-APA	Tibia_length	-0,04431	0,058824	NA
3	abundance	X02767	6-APA	Radius_length	-0,04373	0,075421	NA
3	abundance	X02767	6-APA	Radius_SOS	-0,03568	0,056083	NA
3	abundance	X02767	6-APA	Tibia_SOS	-0,09102	0,058507	NA
3	abundance	X02767	6-APA	Handgrip	-0,03854	0,060148	NA
3	abundance	X02769	Tetraacety	Tibia_length	-0,01263	0,052604	NA
3	abundance	X02769	Tetraacety	Radius_length	-0,04534	0,071807	NA
3	abundance	X02769	Tetraacety	Radius_SOS	-0,05994	0,050331	NA
3	abundance	X02769	Tetraacety	Tibia_SOS	0,086997	0,054033	NA

3	abundance	X02769	Tetraacety	Handgrip	-0,12967	0,061667	NA
3	abundance	X02774	9-ribosylze	Tibia_length	-0,17938	0,055749	NA
3	abundance	X02774	9-ribosylze	Radius_length	-0,18906	0,073092	NA
3	abundance	X02774	9-ribosylze	Radius_SOS	-0,10651	0,052705	NA
3	abundance	X02774	9-ribosylze	Tibia_SOS	-0,04848	0,05672	NA
3	abundance	X02774	9-ribosylze	Handgrip	-0,01334	0,062321	NA
3	abundance	X02793	3-Methoxy-	Tibia_length	-0,14847	0,04996	NA
3	abundance	X02793	3-Methoxy-	Radius_length	-0,17911	0,067804	NA
3	abundance	X02793	3-Methoxy-	Radius_SOS	-0,01192	0,049151	NA
3	abundance	X02793	3-Methoxy-	Tibia_SOS	-0,10036	0,052427	NA
3	abundance	X02793	3-Methoxy-	Handgrip	-0,12884	0,059897	NA
3	abundance	X02820	FC250500	Tibia_length	-0,13519	0,053718	NA
3	abundance	X02820	FC250500	Radius_length	-0,1809	0,072009	NA
3	abundance	X02820	FC250500	Radius_SOS	0,037194	0,051585	NA
3	abundance	X02820	FC250500	Tibia_SOS	-0,12126	0,055137	NA
3	abundance	X02820	FC250500	Handgrip	-0,05901	0,062316	NA
3	abundance	X02823	6,8-Dimetl	Tibia_length	-0,04267	0,053113	NA
3	abundance	X02823	6,8-Dimetl	Radius_length	-0,11679	0,07176	NA
3	abundance	X02823	6,8-Dimetl	Radius_SOS	-0,04745	0,051112	NA
3	abundance	X02823	6,8-Dimetl	Tibia_SOS	-0,08383	0,053857	NA
3	abundance	X02823	6,8-Dimetl	Handgrip	-0,01301	0,061554	NA
3	abundance	X02824	Eslicarbaz	Tibia_length	-0,06422	0,053719	NA
3	abundance	X02824	Eslicarbaz	Radius_length	-0,1019	0,0726	NA
3	abundance	X02824	Eslicarbaz	Radius_SOS	0,057413	0,051068	NA
3	abundance	X02824	Eslicarbaz	Tibia_SOS	-0,11185	0,054044	NA
3	abundance	X02824	Eslicarbaz	Handgrip	-0,02609	0,06142	NA
3	abundance	X02832	(+/-)-2-Hyc	Tibia_length	-0,03649	0,055465	NA
3	abundance	X02832	(+/-)-2-Hyc	Radius_length	0,068539	0,074634	NA
3	abundance	X02832	(+/-)-2-Hyc	Radius_SOS	-0,01641	0,052709	NA
3	abundance	X02832	(+/-)-2-Hyc	Tibia_SOS	0,086285	0,055887	NA
3	abundance	X02832	(+/-)-2-Hyc	Handgrip	0,093497	0,06161	NA
3	abundance	X02841	Kyotorphin	Tibia_length	-0,0656	0,053773	NA
3	abundance	X02841	Kyotorphin	Radius_length	0,043161	0,073113	NA
3	abundance	X02841	Kyotorphin	Radius_SOS	0,006374	0,051438	NA
3	abundance	X02841	Kyotorphin	Tibia_SOS	0,014966	0,056188	NA
3	abundance	X02841	Kyotorphin	Handgrip	-0,11534	0,063012	NA
3	abundance	X02843	4-(1-Hydro	Tibia_length	0,060789	0,051837	NA
3	abundance	X02843	4-(1-Hydro	Radius_length	0,083055	0,070943	NA
3	abundance	X02843	4-(1-Hydro	Radius_SOS	-0,01513	0,049802	NA
3	abundance	X02843	4-(1-Hydro	Tibia_SOS	0,139819	0,053978	NA
3	abundance	X02843	4-(1-Hydro	Handgrip	0,138506	0,06074	NA
3	abundance	X02853	7alpha-Hy	Tibia_length	0,071107	0,051711	NA
3	abundance	X02853	7alpha-Hy	Radius_length	0,112121	0,070791	NA
3	abundance	X02853	7alpha-Hy	Radius_SOS	0,021076	0,049798	NA
3	abundance	X02853	7alpha-Hy	Tibia_SOS	0,073659	0,053595	NA
3	abundance	X02853	7alpha-Hy	Handgrip	0,015418	0,061313	NA
3	abundance	X02854	Marimasta	Tibia_length	0,082213	0,055374	NA
3	abundance	X02854	Marimasta	Radius_length	0,129554	0,073222	NA

3	abundance	X02854	Marimasta	Radius_SOS	-0,05856	0,052665	NA
3	abundance	X02854	Marimasta	Tibia_SOS	0,050345	0,055883	NA
3	abundance	X02854	Marimasta	Handgrip	-0,01228	0,062287	NA
3	abundance	X02868	2-(3-Hydro	Tibia_length	-0,07597	0,056619	NA
3	abundance	X02868	2-(3-Hydro	Radius_length	-0,25473	0,072137	NA
3	abundance	X02868	2-(3-Hydro	Radius_SOS	-0,1041	0,053688	NA
3	abundance	X02868	2-(3-Hydro	Tibia_SOS	-0,15704	0,05675	NA
3	abundance	X02868	2-(3-Hydro	Handgrip	-0,14935	0,062151	NA
3	abundance	X02872	folinic acid	Tibia_length	-0,12082	0,052964	NA
3	abundance	X02872	folinic acid	Radius_length	-0,05053	0,072803	NA
3	abundance	X02872	folinic acid	Radius_SOS	-0,05349	0,050921	NA
3	abundance	X02872	folinic acid	Tibia_SOS	-0,09189	0,054496	NA
3	abundance	X02872	folinic acid	Handgrip	0,006458	0,061541	NA
3	abundance	X02878	N~6~-[5-(1	Tibia_length	-0,09259	0,050812	NA
3	abundance	X02878	N~6~-[5-(1	Radius_length	-0,06516	0,06985	NA
3	abundance	X02878	N~6~-[5-(1	Radius_SOS	-0,01211	0,049467	NA
3	abundance	X02878	N~6~-[5-(1	Tibia_SOS	-0,04293	0,053849	NA
3	abundance	X02878	N~6~-[5-(1	Handgrip	0,012858	0,062343	NA
3	abundance	X02881	N-(2,3,4-Tr	Tibia_length	-0,05739	0,054177	NA
3	abundance	X02881	N-(2,3,4-Tr	Radius_length	-0,11344	0,072362	NA
3	abundance	X02881	N-(2,3,4-Tr	Radius_SOS	-0,10311	0,05151	NA
3	abundance	X02881	N-(2,3,4-Tr	Tibia_SOS	-0,08029	0,055448	NA
3	abundance	X02881	N-(2,3,4-Tr	Handgrip	-0,04048	0,061717	NA
3	abundance	X02894	Toluene	Tibia_length	-0,08387	0,048953	NA
3	abundance	X02894	Toluene	Radius_length	-0,11858	0,064616	NA
3	abundance	X02894	Toluene	Radius_SOS	-0,12544	0,048784	NA
3	abundance	X02894	Toluene	Tibia_SOS	-0,06744	0,052444	NA
3	abundance	X02894	Toluene	Handgrip	0,034206	0,060322	NA
3	abundance	X02905	MFCD0005	Tibia_length	-0,07477	0,057209	NA
3	abundance	X02905	MFCD0005	Radius_length	-0,02891	0,075018	NA
3	abundance	X02905	MFCD0005	Radius_SOS	0,00732	0,054595	NA
3	abundance	X02905	MFCD0005	Tibia_SOS	-0,02744	0,058478	NA
3	abundance	X02905	MFCD0005	Handgrip	-0,01031	0,063122	NA
3	abundance	X02920	L-gamma-(	Tibia_length	0,01488	0,051149	NA
3	abundance	X02920	L-gamma-(	Radius_length	0,128038	0,069606	NA
3	abundance	X02920	L-gamma-(	Radius_SOS	-0,00939	0,049531	NA
3	abundance	X02920	L-gamma-(	Tibia_SOS	0,118299	0,052934	NA
3	abundance	X02920	L-gamma-(	Handgrip	-0,0983	0,060933	NA
3	abundance	X02922	(2E)-3-Met	Tibia_length	-0,0448	0,057058	NA
3	abundance	X02922	(2E)-3-Met	Radius_length	-0,12579	0,074375	NA
3	abundance	X02922	(2E)-3-Met	Radius_SOS	-0,1752	0,053835	NA
3	abundance	X02922	(2E)-3-Met	Tibia_SOS	-0,118	0,058825	NA
3	abundance	X02922	(2E)-3-Met	Handgrip	-0,07642	0,062877	NA
3	abundance	X02928	N-Acetylpr	Tibia_length	0,057297	0,055211	NA
3	abundance	X02928	N-Acetylpr	Radius_length	0,162973	0,073184	NA
3	abundance	X02928	N-Acetylpr	Radius_SOS	0,08621	0,052464	NA
3	abundance	X02928	N-Acetylpr	Tibia_SOS	0,134813	0,055826	NA
3	abundance	X02928	N-Acetylpr	Handgrip	0,100599	0,062456	NA

	3	abundance	X02943	3-Methoxy·	Tibia_length	-0,04187	0,050973	NA
	3	abundance	X02943	3-Methoxy·	Radius_length	-0,07183	0,069545	NA
	3	abundance	X02943	3-Methoxy·	Radius_SOS	-2,96E-04	0,049437	NA
	3	abundance	X02943	3-Methoxy·	Tibia_SOS	-0,08254	0,052659	NA
	3	abundance	X02943	3-Methoxy·	Handgrip	-0,066	0,060594	NA
	3	abundance	X02944	Valylvaline	Tibia_length	-0,04001	0,049797	NA
	3	abundance	X02944	Valylvaline	Radius_length	0,057807	0,067994	NA
	3	abundance	X02944	Valylvaline	Radius_SOS	-0,05188	0,048972	NA
	3	abundance	X02944	Valylvaline	Tibia_SOS	0,062885	0,05248	NA
	3	abundance	X02944	Valylvaline	Handgrip	-0,05783	0,060241	NA
	3	abundance	X02948	N-(2-Cyano	Tibia_length	-0,1379	0,0565	NA
	3	abundance	X02948	N-(2-Cyano	Radius_length	-0,11796	0,074583	NA
	3	abundance	X02948	N-(2-Cyano	Radius_SOS	-0,03001	0,054136	NA
	3	abundance	X02948	N-(2-Cyano	Tibia_SOS	-0,14302	0,056802	NA
	3	abundance	X02948	N-(2-Cyano	Handgrip	-0,13929	0,060925	NA
	3	abundance	X02952	Vorinostat	Tibia_length	-0,06217	0,053929	NA
	3	abundance	X02952	Vorinostat	Radius_length	-0,1149	0,072328	NA
	3	abundance	X02952	Vorinostat	Radius_SOS	-0,08611	0,051432	NA
	3	abundance	X02952	Vorinostat	Tibia_SOS	0,032735	0,054008	NA
	3	abundance	X02952	Vorinostat	Handgrip	0,01486	0,061064	NA
	3	abundance	X02968	Ro 20-172	Tibia_length	-0,05416	0,052502	NA
	3	abundance	X02968	Ro 20-172	Radius_length	-0,13037	0,07127	NA
	3	abundance	X02968	Ro 20-172	Radius_SOS	-0,02495	0,050361	NA
	3	abundance	X02968	Ro 20-172	Tibia_SOS	-0,0655	0,053018	NA
	3	abundance	X02968	Ro 20-172	Handgrip	-0,05611	0,061603	NA
	3	abundance	X02972	3-(Sulfoox	Tibia_length	-0,01518	0,051381	NA
	3	abundance	X02972	3-(Sulfoox	Radius_length	-0,07552	0,070386	NA
	3	abundance	X02972	3-(Sulfoox	Radius_SOS	0,02812	0,049636	NA
	3	abundance	X02972	3-(Sulfoox	Tibia_SOS	-0,01154	0,053128	NA
	3	abundance	X02972	3-(Sulfoox	Handgrip	-0,0368	0,061363	NA
	3	abundance	X02986	Diacetin	Tibia_length	-0,01675	0,049694	NA
	3	abundance	X02986	Diacetin	Radius_length	-0,04094	0,067352	NA
	3	abundance	X02986	Diacetin	Radius_SOS	-0,10748	0,048749	NA
	3	abundance	X02986	Diacetin	Tibia_SOS	-0,10738	0,052184	NA
	3	abundance	X02986	Diacetin	Handgrip	-0,06084	0,060281	NA
	3	abundance	X02990	Pentoxifylli	Tibia_length	0,044516	0,053854	NA
	3	abundance	X02990	Pentoxifylli	Radius_length	0,058584	0,072853	NA
	3	abundance	X02990	Pentoxifylli	Radius_SOS	-0,01522	0,051476	NA
	3	abundance	X02990	Pentoxifylli	Tibia_SOS	0,022525	0,055061	NA
	3	abundance	X02990	Pentoxifylli	Handgrip	0,046716	0,06256	NA
2b		abundance	X03001	Pseudouri	Tibia_length	0,039263	0,051287	NA
2b		abundance	X03001	Pseudouri	Radius_length	0,021576	0,071073	NA
2b		abundance	X03001	Pseudouri	Radius_SOS	4,49E-04	0,049684	NA
2b		abundance	X03001	Pseudouri	Tibia_SOS	0,122125	0,052666	NA
2b		abundance	X03001	Pseudouri	Handgrip	0,132103	0,060636	NA
	3	abundance	X03004	Bicine	Tibia_length	0,039915	0,054713	NA
	3	abundance	X03004	Bicine	Radius_length	0,153733	0,072481	NA
	3	abundance	X03004	Bicine	Radius_SOS	0,08817	0,051942	NA

3	abundance	X03004	Bicine	Tibia_SOS	0,07295	0,056385	NA
3	abundance	X03004	Bicine	Handgrip	-0,00636	0,06166	NA
3	abundance	X03017	Pro-tyr	Tibia_length	-0,0506	0,058091	NA
3	abundance	X03017	Pro-tyr	Radius_length	-0,07245	0,075231	NA
3	abundance	X03017	Pro-tyr	Radius_SOS	-0,00826	0,055455	NA
3	abundance	X03017	Pro-tyr	Tibia_SOS	-0,04476	0,057663	NA
3	abundance	X03017	Pro-tyr	Handgrip	-0,08283	0,063509	NA
2b	abundance	X03023	NPK (Pepti	Tibia_length	0,043231	0,053541	NA
2b	abundance	X03023	NPK (Pepti	Radius_length	0,059308	0,072177	NA
2b	abundance	X03023	NPK (Pepti	Radius_SOS	0,031447	0,051048	NA
2b	abundance	X03023	NPK (Pepti	Tibia_SOS	0,037228	0,054002	NA
2b	abundance	X03023	NPK (Pepti	Handgrip	-0,00513	0,062457	NA
3	abundance	X03064	Formimino	Tibia_length	0,116355	0,052808	NA
3	abundance	X03064	Formimino	Radius_length	-0,11559	0,071726	NA
3	abundance	X03064	Formimino	Radius_SOS	-0,03444	0,050742	NA
3	abundance	X03064	Formimino	Tibia_SOS	0,017874	0,054048	NA
3	abundance	X03064	Formimino	Handgrip	-0,15815	0,060522	NA
3	abundance	X03069	N-COUMAI	Tibia_length	-0,02129	0,052686	NA
3	abundance	X03069	N-COUMAI	Radius_length	-0,05528	0,071695	NA
3	abundance	X03069	N-COUMAI	Radius_SOS	0,011591	0,050574	NA
3	abundance	X03069	N-COUMAI	Tibia_SOS	0,015624	0,054354	NA
3	abundance	X03069	N-COUMAI	Handgrip	-0,05037	0,061392	NA
3	abundance	X03070	2-Methoxy-	Tibia_length	0,007125	0,053238	NA
3	abundance	X03070	2-Methoxy-	Radius_length	0,039055	0,072036	NA
3	abundance	X03070	2-Methoxy-	Radius_SOS	-0,00981	0,050902	NA
3	abundance	X03070	2-Methoxy-	Tibia_SOS	0,0039	0,053943	NA
3	abundance	X03070	2-Methoxy-	Handgrip	-0,08896	0,062068	NA
3	abundance	X03077	nicotianam	Tibia_length	-0,01713	0,050768	NA
3	abundance	X03077	nicotianam	Radius_length	0,037802	0,069477	NA
3	abundance	X03077	nicotianam	Radius_SOS	0,054333	0,049259	NA
3	abundance	X03077	nicotianam	Tibia_SOS	0,00484	0,052784	NA
3	abundance	X03077	nicotianam	Handgrip	0,022965	0,06036	NA
3	abundance	X03097	6-hydroxyn	Tibia_length	-0,14302	0,051777	NA
3	abundance	X03097	6-hydroxyn	Radius_length	0,006428	0,071257	NA
3	abundance	X03097	6-hydroxyn	Radius_SOS	-0,00925	0,050331	NA
3	abundance	X03097	6-hydroxyn	Tibia_SOS	0,044399	0,054206	NA
3	abundance	X03097	6-hydroxyn	Handgrip	0,031868	0,062218	NA
3	abundance	X03109	Guanidino	Tibia_length	-0,08292	0,051227	NA
3	abundance	X03109	Guanidino	Radius_length	-0,11544	0,069917	NA
3	abundance	X03109	Guanidino	Radius_SOS	0,013297	0,04968	NA
3	abundance	X03109	Guanidino	Tibia_SOS	-0,05579	0,052834	NA
3	abundance	X03109	Guanidino	Handgrip	-0,01659	0,06111	NA
3	abundance	X03132	(3S,4S)-7,:	Tibia_length	0,017459	0,052642	NA
3	abundance	X03132	(3S,4S)-7,:	Radius_length	0,112748	0,071249	NA
3	abundance	X03132	(3S,4S)-7,:	Radius_SOS	0,023906	0,050425	NA
3	abundance	X03132	(3S,4S)-7,:	Tibia_SOS	0,165976	0,05256	NA
3	abundance	X03132	(3S,4S)-7,:	Handgrip	0,133811	0,061002	NA
3	abundance	X03134	Tetrahydro	Tibia_length	-0,0644	0,055028	NA

3	abundance	X03134	Tetrahydro	Radius_length	-0,11173	0,073201	NA
3	abundance	X03134	Tetrahydro	Radius_SOS	-0,1466	0,051792	NA
3	abundance	X03134	Tetrahydro	Tibia_SOS	-0,06177	0,055705	NA
3	abundance	X03134	Tetrahydro	Handgrip	-0,1775	0,060799	NA
3	abundance	X03158	5-Hydroxy-	Tibia_length	-0,04274	0,052786	NA
3	abundance	X03158	5-Hydroxy-	Radius_length	-0,03472	0,071561	NA
3	abundance	X03158	5-Hydroxy-	Radius_SOS	0,033876	0,050479	NA
3	abundance	X03158	5-Hydroxy-	Tibia_SOS	0,010522	0,055302	NA
3	abundance	X03158	5-Hydroxy-	Handgrip	0,026982	0,061648	NA
3	abundance	X03223	1-(2-Carbo	Tibia_length	-0,11138	0,052656	NA
3	abundance	X03223	1-(2-Carbo	Radius_length	-0,14198	0,071315	NA
3	abundance	X03223	1-(2-Carbo	Radius_SOS	0,048738	0,050428	NA
3	abundance	X03223	1-(2-Carbo	Tibia_SOS	-0,10595	0,053299	NA
3	abundance	X03223	1-(2-Carbo	Handgrip	-0,20011	0,060375	NA
3	abundance	X03254	Gly-Trp	Tibia_length	-0,08887	0,054673	NA
3	abundance	X03254	Gly-Trp	Radius_length	-0,04694	0,073683	NA
3	abundance	X03254	Gly-Trp	Radius_SOS	0,044984	0,051899	NA
3	abundance	X03254	Gly-Trp	Tibia_SOS	0,001037	0,055087	NA
3	abundance	X03254	Gly-Trp	Handgrip	0,02866	0,061457	NA
3	abundance	X03268	Dihydrouri	Tibia_length	0,007021	0,053203	NA
3	abundance	X03268	Dihydrouri	Radius_length	-0,04496	0,072328	NA
3	abundance	X03268	Dihydrouri	Radius_SOS	-0,04825	0,050817	NA
3	abundance	X03268	Dihydrouri	Tibia_SOS	0,129193	0,053103	NA
3	abundance	X03268	Dihydrouri	Handgrip	0,004615	0,062327	NA
3	abundance	X03271	1ÃŽÂ²-Hyd	Tibia_length	0,0933	0,050218	NA
3	abundance	X03271	1ÃŽÂ²-Hyd	Radius_length	0,142952	0,068266	NA
3	abundance	X03271	1ÃŽÂ²-Hyd	Radius_SOS	0,038581	0,049138	NA
3	abundance	X03271	1ÃŽÂ²-Hyd	Tibia_SOS	-0,01473	0,052752	NA
3	abundance	X03271	1ÃŽÂ²-Hyd	Handgrip	0,031775	0,060434	NA
3	abundance	X03275	Methdilazi	Tibia_length	-0,11661	0,051746	NA
3	abundance	X03275	Methdilazi	Radius_length	-0,05372	0,071304	NA
3	abundance	X03275	Methdilazi	Radius_SOS	0,017544	0,050102	NA
3	abundance	X03275	Methdilazi	Tibia_SOS	-0,01067	0,053222	NA
3	abundance	X03275	Methdilazi	Handgrip	-0,06393	0,06071	NA
3	abundance	X03276	2'-Deoxyac	Tibia_length	-0,05226	0,058934	NA
3	abundance	X03276	2'-Deoxyac	Radius_length	0,012493	0,075316	NA
3	abundance	X03276	2'-Deoxyac	Radius_SOS	-0,00807	0,055904	NA
3	abundance	X03276	2'-Deoxyac	Tibia_SOS	-0,00922	0,058841	NA
3	abundance	X03276	2'-Deoxyac	Handgrip	0,170344	0,062638	NA
3	abundance	X03278	Hydroxyph	Tibia_length	-0,05165	0,055355	NA
3	abundance	X03278	Hydroxyph	Radius_length	-0,09177	0,073386	NA
3	abundance	X03278	Hydroxyph	Radius_SOS	-0,1247	0,052801	NA
3	abundance	X03278	Hydroxyph	Tibia_SOS	-0,01552	0,056977	NA
3	abundance	X03278	Hydroxyph	Handgrip	-0,04873	0,062667	NA
3	abundance	X03294	4,4'-Thiobi	Tibia_length	-0,01852	0,053217	NA
3	abundance	X03294	4,4'-Thiobi	Radius_length	-0,09553	0,071858	NA
3	abundance	X03294	4,4'-Thiobi	Radius_SOS	-0,05383	0,050919	NA
3	abundance	X03294	4,4'-Thiobi	Tibia_SOS	0,023104	0,054004	NA

3	abundance	X03294	4,4'-Thiobi	Handgrip	0,111382	0,061312	NA
3	abundance	X03340	His-pro	Tibia_length	0,007433	0,052603	NA
3	abundance	X03340	His-pro	Radius_length	0,042916	0,071844	NA
3	abundance	X03340	His-pro	Radius_SOS	-0,0051	0,050416	NA
3	abundance	X03340	His-pro	Tibia_SOS	0,020505	0,054633	NA
3	abundance	X03340	His-pro	Handgrip	0,04857	0,061184	NA
3	abundance	X03343	L-alpha-As	Tibia_length	-0,03719	0,051565	NA
3	abundance	X03343	L-alpha-As	Radius_length	-0,00794	0,070805	NA
3	abundance	X03343	L-alpha-As	Radius_SOS	-0,02192	0,049776	NA
3	abundance	X03343	L-alpha-As	Tibia_SOS	-0,0217	0,05398	NA
3	abundance	X03343	L-alpha-As	Handgrip	-0,11423	0,061007	NA
3	abundance	X03357	2-Hydroxyl	Tibia_length	-0,05717	0,050501	NA
3	abundance	X03357	2-Hydroxyl	Radius_length	-0,13059	0,068447	NA
3	abundance	X03357	2-Hydroxyl	Radius_SOS	-0,03753	0,049212	NA
3	abundance	X03357	2-Hydroxyl	Tibia_SOS	-0,13757	0,052228	NA
3	abundance	X03357	2-Hydroxyl	Handgrip	-0,13312	0,059918	NA
3	abundance	X03363	Choline Alf	Tibia_length	-0,01092	0,054602	NA
3	abundance	X03363	Choline Alf	Radius_length	0,133123	0,072507	NA
3	abundance	X03363	Choline Alf	Radius_SOS	0,016234	0,051938	NA
3	abundance	X03363	Choline Alf	Tibia_SOS	0,128949	0,055074	NA
3	abundance	X03363	Choline Alf	Handgrip	0,144786	0,061902	NA
3	abundance	X03376	4-(9H-beta	Tibia_length	-0,14392	0,05332	NA
3	abundance	X03376	4-(9H-beta	Radius_length	-0,13386	0,071772	NA
3	abundance	X03376	4-(9H-beta	Radius_SOS	0,039687	0,051054	NA
3	abundance	X03376	4-(9H-beta	Tibia_SOS	-0,12605	0,053237	NA
3	abundance	X03376	4-(9H-beta	Handgrip	-0,12313	0,06098	NA
3	abundance	X03413	Val-Trp	Tibia_length	0,043372	0,059298	NA
3	abundance	X03413	Val-Trp	Radius_length	0,086057	0,075806	NA
3	abundance	X03413	Val-Trp	Radius_SOS	0,051426	0,056871	NA
3	abundance	X03413	Val-Trp	Tibia_SOS	0,04018	0,059818	NA
3	abundance	X03413	Val-Trp	Handgrip	0,078132	0,060009	NA
3	abundance	X03416	9-(alpha-D	Tibia_length	-0,02682	0,053368	NA
3	abundance	X03416	9-(alpha-D	Radius_length	0,120262	0,072093	NA
3	abundance	X03416	9-(alpha-D	Radius_SOS	0,055874	0,050849	NA
3	abundance	X03416	9-(alpha-D	Tibia_SOS	0,041845	0,054664	NA
3	abundance	X03416	9-(alpha-D	Handgrip	0,076136	0,0611	NA
3	abundance	X03434	SECONAL	Tibia_length	-0,04593	0,056111	NA
3	abundance	X03434	SECONAL	Radius_length	0,076681	0,074052	NA
3	abundance	X03434	SECONAL	Radius_SOS	0,106691	0,053264	NA
3	abundance	X03434	SECONAL	Tibia_SOS	-0,011	0,057749	NA
3	abundance	X03434	SECONAL	Handgrip	0,003869	0,063046	NA
3	abundance	X03480	epsilon-(ga	Tibia_length	-0,00222	0,051878	NA
3	abundance	X03480	epsilon-(ga	Radius_length	0,029004	0,071015	NA
3	abundance	X03480	epsilon-(ga	Radius_SOS	0,015726	0,049972	NA
3	abundance	X03480	epsilon-(ga	Tibia_SOS	-0,02096	0,053385	NA
3	abundance	X03480	epsilon-(ga	Handgrip	0,039985	0,060663	NA
3	abundance	X03488	3,4-Dihydr	Tibia_length	-0,12168	0,054	NA
3	abundance	X03488	3,4-Dihydr	Radius_length	-0,23132	0,070827	NA

3	abundance	X03488	3,4-Dihydr	Radius_SOS	-0,02092	0,051411	NA
3	abundance	X03488	3,4-Dihydr	Tibia_SOS	-0,1845	0,054578	NA
3	abundance	X03488	3,4-Dihydr	Handgrip	-0,13385	0,060651	NA
3	abundance	X03535	(1S,3R,4s)	Tibia_length	-0,0253	0,05371	NA
3	abundance	X03535	(1S,3R,4s)	Radius_length	-0,12363	0,072433	NA
3	abundance	X03535	(1S,3R,4s)	Radius_SOS	-0,03584	0,051206	NA
3	abundance	X03535	(1S,3R,4s)	Tibia_SOS	-0,07014	0,055322	NA
3	abundance	X03535	(1S,3R,4s)	Handgrip	-0,15102	0,061801	NA
3	abundance	X03552	4-Hydroxya	Tibia_length	-0,07486	0,052728	NA
3	abundance	X03552	4-Hydroxya	Radius_length	-0,07471	0,071792	NA
3	abundance	X03552	4-Hydroxya	Radius_SOS	-0,00176	0,050521	NA
3	abundance	X03552	4-Hydroxya	Tibia_SOS	-0,0046	0,054572	NA
3	abundance	X03552	4-Hydroxya	Handgrip	-0,10986	0,062034	NA
3	abundance	X03563	6-imino-5-	Tibia_length	-0,04483	0,053051	NA
3	abundance	X03563	6-imino-5-	Radius_length	-0,02158	0,072222	NA
3	abundance	X03563	6-imino-5-	Radius_SOS	-0,00796	0,050822	NA
3	abundance	X03563	6-imino-5-	Tibia_SOS	-0,02465	0,054769	NA
3	abundance	X03563	6-imino-5-	Handgrip	-0,10538	0,061462	NA
3	abundance	X03568	3-[(2Z)-1-C	Tibia_length	-0,07234	0,052062	NA
3	abundance	X03568	3-[(2Z)-1-C	Radius_length	-0,1335	0,070662	NA
3	abundance	X03568	3-[(2Z)-1-C	Radius_SOS	-0,00225	0,050081	NA
3	abundance	X03568	3-[(2Z)-1-C	Tibia_SOS	-0,10102	0,053548	NA
3	abundance	X03568	3-[(2Z)-1-C	Handgrip	-0,08211	0,061225	NA
3	abundance	X03592	2-glyceryl :	Tibia_length	0,013164	0,05026	NA
3	abundance	X03592	2-glyceryl :	Radius_length	0,052204	0,068405	NA
3	abundance	X03592	2-glyceryl :	Radius_SOS	0,073306	0,049014	NA
3	abundance	X03592	2-glyceryl :	Tibia_SOS	-0,02034	0,05274	NA
3	abundance	X03592	2-glyceryl :	Handgrip	-0,0568	0,060504	NA
3	abundance	X03595	Methylol D	Tibia_length	-0,02016	0,056366	NA
3	abundance	X03595	Methylol D	Radius_length	-0,03923	0,073993	NA
3	abundance	X03595	Methylol D	Radius_SOS	-0,02826	0,053502	NA
3	abundance	X03595	Methylol D	Tibia_SOS	0,048507	0,056581	NA
3	abundance	X03595	Methylol D	Handgrip	0,060123	0,060084	NA
3	abundance	X03602	o-Succinyl	Tibia_length	-0,04779	0,052008	NA
3	abundance	X03602	o-Succinyl	Radius_length	-0,01044	0,071064	NA
3	abundance	X03602	o-Succinyl	Radius_SOS	0,04711	0,050031	NA
3	abundance	X03602	o-Succinyl	Tibia_SOS	0,049058	0,053924	NA
3	abundance	X03602	o-Succinyl	Handgrip	-0,01564	0,061399	NA
3	abundance	X03604	Gly-Trp	Tibia_length	0,022743	0,055543	NA
3	abundance	X03604	Gly-Trp	Radius_length	0,005689	0,074138	NA
3	abundance	X03604	Gly-Trp	Radius_SOS	0,092591	0,052536	NA
3	abundance	X03604	Gly-Trp	Tibia_SOS	0,02654	0,05743	NA
3	abundance	X03604	Gly-Trp	Handgrip	0,015348	0,063034	NA
3	abundance	X03629	Glycylglycy	Tibia_length	0,06162	0,054525	NA
3	abundance	X03629	Glycylglycy	Radius_length	0,114224	0,072635	NA
3	abundance	X03629	Glycylglycy	Radius_SOS	0,009879	0,051958	NA
3	abundance	X03629	Glycylglycy	Tibia_SOS	0,052256	0,055867	NA
3	abundance	X03629	Glycylglycy	Handgrip	0,064944	0,062434	NA

3	abundance	X03660	trp-ser	Tibia_length	0,029356	0,052204	NA
3	abundance	X03660	trp-ser	Radius_length	0,010238	0,071122	NA
3	abundance	X03660	trp-ser	Radius_SOS	0,038461	0,050147	NA
3	abundance	X03660	trp-ser	Tibia_SOS	-0,10515	0,053387	NA
3	abundance	X03660	trp-ser	Handgrip	-0,03378	0,062113	NA
3	abundance	X03675	asp-gln	Tibia_length	-0,05648	0,054118	NA
3	abundance	X03675	asp-gln	Radius_length	-0,00845	0,073224	NA
3	abundance	X03675	asp-gln	Radius_SOS	0,051211	0,05172	NA
3	abundance	X03675	asp-gln	Tibia_SOS	0,06026	0,055358	NA
3	abundance	X03675	asp-gln	Handgrip	-0,06042	0,061866	NA
3	abundance	X03704	N,N-Dimet	Tibia_length	-0,21773	0,053811	NA
3	abundance	X03704	N,N-Dimet	Radius_length	-0,18214	0,072059	NA
3	abundance	X03704	N,N-Dimet	Radius_SOS	-0,08814	0,051428	NA
3	abundance	X03704	N,N-Dimet	Tibia_SOS	-0,15215	0,053804	NA
3	abundance	X03704	N,N-Dimet	Handgrip	-0,15587	0,060367	NA
3	abundance	X03707	9-[(5R)-5-E	Tibia_length	-0,03678	0,054554	NA
3	abundance	X03707	9-[(5R)-5-E	Radius_length	-0,1303	0,072797	NA
3	abundance	X03707	9-[(5R)-5-E	Radius_SOS	-0,03587	0,05199	NA
3	abundance	X03707	9-[(5R)-5-E	Tibia_SOS	-0,12124	0,055396	NA
3	abundance	X03707	9-[(5R)-5-E	Handgrip	-0,13976	0,062401	NA
3	abundance	X03709	Guanfacin	Tibia_length	0,022809	0,055033	NA
3	abundance	X03709	Guanfacin	Radius_length	0,01744	0,073718	NA
3	abundance	X03709	Guanfacin	Radius_SOS	-0,00531	0,052346	NA
3	abundance	X03709	Guanfacin	Tibia_SOS	0,031365	0,056163	NA
3	abundance	X03709	Guanfacin	Handgrip	-0,07705	0,06111	NA
3	abundance	X03714	Sinapinic a	Tibia_length	-0,10491	0,053119	NA
3	abundance	X03714	Sinapinic a	Radius_length	-0,17783	0,071093	NA
3	abundance	X03714	Sinapinic a	Radius_SOS	-0,06201	0,050698	NA
3	abundance	X03714	Sinapinic a	Tibia_SOS	-0,09291	0,053761	NA
3	abundance	X03714	Sinapinic a	Handgrip	-0,09987	0,060561	NA
3	abundance	X03718	Propanthel	Tibia_length	-0,0386	0,056806	NA
3	abundance	X03718	Propanthel	Radius_length	-0,00999	0,074743	NA
3	abundance	X03718	Propanthel	Radius_SOS	-0,01982	0,054129	NA
3	abundance	X03718	Propanthel	Tibia_SOS	-0,04473	0,055564	NA
3	abundance	X03718	Propanthel	Handgrip	-0,02374	0,062447	NA
3	abundance	X03719	Losalen	Tibia_length	-0,09263	0,056318	NA
3	abundance	X03719	Losalen	Radius_length	-0,13902	0,073556	NA
3	abundance	X03719	Losalen	Radius_SOS	-0,03325	0,053621	NA
3	abundance	X03719	Losalen	Tibia_SOS	-0,11629	0,057717	NA
3	abundance	X03719	Losalen	Handgrip	-0,15614	0,062065	NA
3	abundance	X03760	2-Methoxy-	Tibia_length	0,031844	0,052862	NA
3	abundance	X03760	2-Methoxy-	Radius_length	-0,09028	0,071908	NA
3	abundance	X03760	2-Methoxy-	Radius_SOS	0,010177	0,050638	NA
3	abundance	X03760	2-Methoxy-	Tibia_SOS	0,050687	0,054047	NA
3	abundance	X03760	2-Methoxy-	Handgrip	0,0866	0,061171	NA
3	abundance	X03793	Desonide	Tibia_length	-0,02743	0,05337	NA
3	abundance	X03793	Desonide	Radius_length	-0,03632	0,072349	NA
3	abundance	X03793	Desonide	Radius_SOS	-0,04135	0,050998	NA

3	abundance	X03793	Desonide	Tibia_SOS	-0,00675	0,054334	NA
3	abundance	X03793	Desonide	Handgrip	-0,01389	0,061489	NA
3	abundance	X03837	2-Methoxy	Tibia_length	0,098608	0,055569	NA
3	abundance	X03837	2-Methoxy	Radius_length	0,024555	0,074131	NA
3	abundance	X03837	2-Methoxy	Radius_SOS	-0,05075	0,052967	NA
3	abundance	X03837	2-Methoxy	Tibia_SOS	0,090654	0,056937	NA
3	abundance	X03837	2-Methoxy	Handgrip	0,046847	0,062462	NA
3	abundance	X03855	2-{(2S,4S)-	Tibia_length	0,114374	0,051318	NA
3	abundance	X03855	2-{(2S,4S)-	Radius_length	0,092577	0,070369	NA
3	abundance	X03855	2-{(2S,4S)-	Radius_SOS	0,010003	0,049764	NA
3	abundance	X03855	2-{(2S,4S)-	Tibia_SOS	0,122544	0,052689	NA
3	abundance	X03855	2-{(2S,4S)-	Handgrip	0,063169	0,060566	NA
3	abundance	X03860	2-BUTYL PI	Tibia_length	0,121148	0,053354	NA
3	abundance	X03860	2-BUTYL PI	Radius_length	0,121199	0,072908	NA
3	abundance	X03860	2-BUTYL PI	Radius_SOS	0,04737	0,051304	NA
3	abundance	X03860	2-BUTYL PI	Tibia_SOS	0,135362	0,054428	NA
3	abundance	X03860	2-BUTYL PI	Handgrip	0,086317	0,061322	NA
3	abundance	X03892	shinorine	Tibia_length	-0,06935	0,054642	NA
3	abundance	X03892	shinorine	Radius_length	-0,12684	0,073083	NA
3	abundance	X03892	shinorine	Radius_SOS	-0,1415	0,052029	NA
3	abundance	X03892	shinorine	Tibia_SOS	-0,12515	0,056132	NA
3	abundance	X03892	shinorine	Handgrip	-0,0343	0,062694	NA
3	abundance	X03901	ferrileghen	Tibia_length	-0,00442	0,049493	NA
3	abundance	X03901	ferrileghen	Radius_length	-0,07509	0,066963	NA
3	abundance	X03901	ferrileghen	Radius_SOS	0,031687	0,049015	NA
3	abundance	X03901	ferrileghen	Tibia_SOS	0,053069	0,052438	NA
3	abundance	X03901	ferrileghen	Handgrip	0,081899	0,060162	NA
3	abundance	X03916	2-(1-Ethox	Tibia_length	-0,04166	0,052182	NA
3	abundance	X03916	2-(1-Ethox	Radius_length	-0,13215	0,071185	NA
3	abundance	X03916	2-(1-Ethox	Radius_SOS	-0,02492	0,049895	NA
3	abundance	X03916	2-(1-Ethox	Tibia_SOS	-0,05164	0,053574	NA
3	abundance	X03916	2-(1-Ethox	Handgrip	0,00338	0,061639	NA
3	abundance	X03917	Uramustin	Tibia_length	0,072076	0,054258	NA
3	abundance	X03917	Uramustin	Radius_length	0,119143	0,072493	NA
3	abundance	X03917	Uramustin	Radius_SOS	-0,00317	0,051844	NA
3	abundance	X03917	Uramustin	Tibia_SOS	0,055331	0,055536	NA
3	abundance	X03917	Uramustin	Handgrip	-0,00893	0,062552	NA
3	abundance	X03934	2-Aminom	Tibia_length	0,098551	0,054292	NA
3	abundance	X03934	2-Aminom	Radius_length	-0,07441	0,073142	NA
3	abundance	X03934	2-Aminom	Radius_SOS	-0,03086	0,052045	NA
3	abundance	X03934	2-Aminom	Tibia_SOS	0,054179	0,054896	NA
3	abundance	X03934	2-Aminom	Handgrip	0,038435	0,06182	NA
3	abundance	X03936	(3R)-2-(3,4	Tibia_length	-0,07006	0,05295	NA
3	abundance	X03936	(3R)-2-(3,4	Radius_length	-0,12281	0,071423	NA
3	abundance	X03936	(3R)-2-(3,4	Radius_SOS	0,079663	0,050485	NA
3	abundance	X03936	(3R)-2-(3,4	Tibia_SOS	-0,05387	0,054092	NA
3	abundance	X03936	(3R)-2-(3,4	Handgrip	-0,00793	0,061327	NA
3	abundance	X03961	Artesunate	Tibia_length	-0,04258	0,052039	NA

	3	abundance	X03961	Artesunate	Radius_length	0,033613	0,071144	NA
	3	abundance	X03961	Artesunate	Radius_SOS	-0,00389	0,050092	NA
	3	abundance	X03961	Artesunate	Tibia_SOS	0,09763	0,053903	NA
	3	abundance	X03961	Artesunate	Handgrip	0,055527	0,061692	NA
2b		abundance	X04021	Bentazone	Tibia_length	0,052097	0,054844	NA
2b		abundance	X04021	Bentazone	Radius_length	-0,02316	0,073481	NA
2b		abundance	X04021	Bentazone	Radius_SOS	0,043325	0,052323	NA
2b		abundance	X04021	Bentazone	Tibia_SOS	-0,04142	0,055901	NA
2b		abundance	X04021	Bentazone	Handgrip	-0,0215	0,060179	NA
	3	abundance	X04040	3-(3,4-dihy	Tibia_length	0,003176	0,054265	NA
	3	abundance	X04040	3-(3,4-dihy	Radius_length	0,006687	0,073346	NA
	3	abundance	X04040	3-(3,4-dihy	Radius_SOS	-0,01902	0,051622	NA
	3	abundance	X04040	3-(3,4-dihy	Tibia_SOS	0,073648	0,056976	NA
	3	abundance	X04040	3-(3,4-dihy	Handgrip	-0,06119	0,062228	NA
	3	abundance	X04117	2,3,4,5-tet	Tibia_length	0,06057	0,05403	NA
	3	abundance	X04117	2,3,4,5-tet	Radius_length	-0,01231	0,072906	NA
	3	abundance	X04117	2,3,4,5-tet	Radius_SOS	-0,08698	0,051278	NA
	3	abundance	X04117	2,3,4,5-tet	Tibia_SOS	-0,08971	0,05518	NA
	3	abundance	X04117	2,3,4,5-tet	Handgrip	0,009642	0,061967	NA
	3	abundance	X04119	Tetraacety	Tibia_length	-0,01926	0,051206	NA
	3	abundance	X04119	Tetraacety	Radius_length	0,009732	0,070471	NA
	3	abundance	X04119	Tetraacety	Radius_SOS	-0,02322	0,049577	NA
	3	abundance	X04119	Tetraacety	Tibia_SOS	0,057602	0,052793	NA
	3	abundance	X04119	Tetraacety	Handgrip	-0,06681	0,060859	NA
	3	abundance	X04146	L-fucopyra	Tibia_length	0,003521	0,052077	NA
	3	abundance	X04146	L-fucopyra	Radius_length	0,135575	0,07052	NA
	3	abundance	X04146	L-fucopyra	Radius_SOS	0,063822	0,050066	NA
	3	abundance	X04146	L-fucopyra	Tibia_SOS	0,110526	0,053886	NA
	3	abundance	X04146	L-fucopyra	Handgrip	0,028714	0,061288	NA
	3	abundance	X04150	4-(METHYL	Tibia_length	-0,11767	0,056307	NA
	3	abundance	X04150	4-(METHYL	Radius_length	-0,04685	0,074472	NA
	3	abundance	X04150	4-(METHYL	Radius_SOS	0,027588	0,053642	NA
	3	abundance	X04150	4-(METHYL	Tibia_SOS	-0,08171	0,055665	NA
	3	abundance	X04150	4-(METHYL	Handgrip	-0,03818	0,063203	NA
	3	abundance	X04157	3-Methoxy-	Tibia_length	-0,13822	0,054337	NA
	3	abundance	X04157	3-Methoxy-	Radius_length	-0,14929	0,072694	NA
	3	abundance	X04157	3-Methoxy-	Radius_SOS	-0,01693	0,051895	NA
	3	abundance	X04157	3-Methoxy-	Tibia_SOS	-0,09629	0,055164	NA
	3	abundance	X04157	3-Methoxy-	Handgrip	-0,16744	0,060508	NA
	3	abundance	X04181	3-Methoxy-	Tibia_length	-0,1309	0,052618	NA
	3	abundance	X04181	3-Methoxy-	Radius_length	-0,1617	0,071667	NA
	3	abundance	X04181	3-Methoxy-	Radius_SOS	-0,08228	0,050981	NA
	3	abundance	X04181	3-Methoxy-	Tibia_SOS	-0,2057	0,053721	NA
	3	abundance	X04181	3-Methoxy-	Handgrip	-0,13192	0,060331	NA
	3	abundance	X04183	Spaglumic	Tibia_length	-0,00423	0,053883	NA
	3	abundance	X04183	Spaglumic	Radius_length	-0,03916	0,073184	NA
	3	abundance	X04183	Spaglumic	Radius_SOS	-0,05793	0,051585	NA
	3	abundance	X04183	Spaglumic	Tibia_SOS	0,156053	0,052873	NA

3	abundance	X04183	Spaglumic	Handgrip	0,151153	0,06136	NA
3	abundance	X04229	gamma-L-g	Tibia_length	0,069597	0,053999	NA
3	abundance	X04229	gamma-L-g	Radius_length	0,024724	0,07303	NA
3	abundance	X04229	gamma-L-g	Radius_SOS	-0,07498	0,051342	NA
3	abundance	X04229	gamma-L-g	Tibia_SOS	0,090512	0,055551	NA
3	abundance	X04229	gamma-L-g	Handgrip	-0,01666	0,062514	NA
3	abundance	X04259	3-Hydroxyt	Tibia_length	0,041649	0,052266	NA
3	abundance	X04259	3-Hydroxyt	Radius_length	0,017609	0,071339	NA
3	abundance	X04259	3-Hydroxyt	Radius_SOS	0,00306	0,050295	NA
3	abundance	X04259	3-Hydroxyt	Tibia_SOS	-0,00765	0,054012	NA
3	abundance	X04259	3-Hydroxyt	Handgrip	-0,06301	0,061721	NA
3	abundance	X04274	6-[(Z)-2-(3,	Tibia_length	0,034708	0,050163	NA
3	abundance	X04274	6-[(Z)-2-(3,	Radius_length	0,145594	0,067785	NA
3	abundance	X04274	6-[(Z)-2-(3,	Radius_SOS	0,059909	0,049086	NA
3	abundance	X04274	6-[(Z)-2-(3,	Tibia_SOS	0,02155	0,052536	NA
3	abundance	X04274	6-[(Z)-2-(3,	Handgrip	0,112458	0,060421	NA
3	abundance	X04286	Zinecard	Tibia_length	-0,0355	0,054975	NA
3	abundance	X04286	Zinecard	Radius_length	-6,50E-04	0,073489	NA
3	abundance	X04286	Zinecard	Radius_SOS	-0,02027	0,052441	NA
3	abundance	X04286	Zinecard	Tibia_SOS	-0,00321	0,055851	NA
3	abundance	X04286	Zinecard	Handgrip	0,001784	0,062284	NA
3	abundance	X04310	Aminohipp	Tibia_length	-0,0314	0,053192	NA
3	abundance	X04310	Aminohipp	Radius_length	0,047388	0,072314	NA
3	abundance	X04310	Aminohipp	Radius_SOS	0,008898	0,050859	NA
3	abundance	X04310	Aminohipp	Tibia_SOS	0,059864	0,054535	NA
3	abundance	X04310	Aminohipp	Handgrip	0,096257	0,061129	NA
3	abundance	X04315	Benzamide	Tibia_length	-0,01365	0,053567	NA
3	abundance	X04315	Benzamide	Radius_length	-0,11025	0,071952	NA
3	abundance	X04315	Benzamide	Radius_SOS	-0,03729	0,051057	NA
3	abundance	X04315	Benzamide	Tibia_SOS	0,030388	0,054203	NA
3	abundance	X04315	Benzamide	Handgrip	-0,09302	0,060609	NA
3	abundance	X04334	3-Benzyl-6	Tibia_length	0,093965	0,050662	NA
3	abundance	X04334	3-Benzyl-6	Radius_length	0,139571	0,0693	NA
3	abundance	X04334	3-Benzyl-6	Radius_SOS	0,053436	0,049372	NA
3	abundance	X04334	3-Benzyl-6	Tibia_SOS	0,020399	0,052684	NA
3	abundance	X04334	3-Benzyl-6	Handgrip	-0,05554	0,060359	NA
3	abundance	X04339	L-Glutamic	Tibia_length	-0,19211	0,053343	NA
3	abundance	X04339	L-Glutamic	Radius_length	-0,05876	0,072997	NA
3	abundance	X04339	L-Glutamic	Radius_SOS	0,01528	0,05171	NA
3	abundance	X04339	L-Glutamic	Tibia_SOS	-0,08043	0,056105	NA
3	abundance	X04339	L-Glutamic	Handgrip	-0,06934	0,061972	NA
3	abundance	X04351	Pyrimidine	Tibia_length	-0,09897	0,053816	NA
3	abundance	X04351	Pyrimidine	Radius_length	-0,09654	0,072736	NA
3	abundance	X04351	Pyrimidine	Radius_SOS	-0,09014	0,051531	NA
3	abundance	X04351	Pyrimidine	Tibia_SOS	0,148846	0,052993	NA
3	abundance	X04351	Pyrimidine	Handgrip	0,089136	0,061751	NA
3	abundance	X04377	Dihydroura	Tibia_length	-0,05375	0,053322	NA
3	abundance	X04377	Dihydroura	Radius_length	-0,01681	0,072449	NA

3	abundance	X04377	Dihydroura	Radius_SOS	-0,03491	0,051066	NA
3	abundance	X04377	Dihydroura	Tibia_SOS	0,110452	0,053181	NA
3	abundance	X04377	Dihydroura	Handgrip	0,055587	0,061741	NA
3	abundance	X04378	9,11-Dihyc	Tibia_length	-0,10803	0,053348	NA
3	abundance	X04378	9,11-Dihyc	Radius_length	-0,19534	0,070959	NA
3	abundance	X04378	9,11-Dihyc	Radius_SOS	-0,0256	0,051008	NA
3	abundance	X04378	9,11-Dihyc	Tibia_SOS	0,029294	0,053794	NA
3	abundance	X04378	9,11-Dihyc	Handgrip	-0,01113	0,061212	NA
3	abundance	X04379	2-Amino-6	Tibia_length	-0,0129	0,053585	NA
3	abundance	X04379	2-Amino-6	Radius_length	0,077202	0,0725	NA
3	abundance	X04379	2-Amino-6	Radius_SOS	-5,56E-04	0,05118	NA
3	abundance	X04379	2-Amino-6	Tibia_SOS	-0,06895	0,054985	NA
3	abundance	X04379	2-Amino-6	Handgrip	0,055832	0,061812	NA
3	abundance	X04440	Mono(3-ca	Tibia_length	-0,06483	0,056577	NA
3	abundance	X04440	Mono(3-ca	Radius_length	-0,07247	0,074616	NA
3	abundance	X04440	Mono(3-ca	Radius_SOS	-0,02511	0,053635	NA
3	abundance	X04440	Mono(3-ca	Tibia_SOS	-0,06839	0,056351	NA
3	abundance	X04440	Mono(3-ca	Handgrip	-0,13461	0,06293	NA
3	abundance	X04450	3-Methylac	Tibia_length	-0,03943	0,048933	NA
3	abundance	X04450	3-Methylac	Radius_length	-0,0999	0,062796	NA
3	abundance	X04450	3-Methylac	Radius_SOS	0,006484	0,049643	NA
3	abundance	X04450	3-Methylac	Tibia_SOS	0,009815	0,052768	NA
3	abundance	X04450	3-Methylac	Handgrip	0,041087	0,060153	NA
3	abundance	X04483	(2E)-5-Hyd	Tibia_length	-0,11936	0,052373	NA
3	abundance	X04483	(2E)-5-Hyd	Radius_length	-0,21178	0,070109	NA
3	abundance	X04483	(2E)-5-Hyd	Radius_SOS	-0,00457	0,050285	NA
3	abundance	X04483	(2E)-5-Hyd	Tibia_SOS	-0,11991	0,053907	NA
3	abundance	X04483	(2E)-5-Hyd	Handgrip	-0,08083	0,061125	NA
3	abundance	X04493	Tyrosol	Tibia_length	-0,10841	0,05304	NA
3	abundance	X04493	Tyrosol	Radius_length	-0,13896	0,071565	NA
3	abundance	X04493	Tyrosol	Radius_SOS	-0,02109	0,051043	NA
3	abundance	X04493	Tyrosol	Tibia_SOS	-0,03185	0,053665	NA
3	abundance	X04493	Tyrosol	Handgrip	-0,0732	0,060843	NA
3	abundance	X04526	N-Nonano	Tibia_length	0,024383	0,05469	NA
3	abundance	X04526	N-Nonano	Radius_length	0,119188	0,072883	NA
3	abundance	X04526	N-Nonano	Radius_SOS	-0,04639	0,051968	NA
3	abundance	X04526	N-Nonano	Tibia_SOS	0,074725	0,055821	NA
3	abundance	X04526	N-Nonano	Handgrip	0,028474	0,061966	NA
3	abundance	X04538	Valylvaline	Tibia_length	0,154381	0,054328	NA
3	abundance	X04538	Valylvaline	Radius_length	0,103311	0,073546	NA
3	abundance	X04538	Valylvaline	Radius_SOS	0,072244	0,052265	NA
3	abundance	X04538	Valylvaline	Tibia_SOS	0,088308	0,056615	NA
3	abundance	X04538	Valylvaline	Handgrip	-0,00802	0,061912	NA
3	abundance	X04543	N-Acetyl-S	Tibia_length	-0,1728	0,053381	NA
3	abundance	X04543	N-Acetyl-S	Radius_length	-0,16234	0,071906	NA
3	abundance	X04543	N-Acetyl-S	Radius_SOS	-0,0364	0,05136	NA
3	abundance	X04543	N-Acetyl-S	Tibia_SOS	-0,05753	0,054388	NA
3	abundance	X04543	N-Acetyl-S	Handgrip	-0,00916	0,061954	NA

3	abundance	X04544	L-gamma-(	Tibia_length	-0,02932	0,055092	NA
3	abundance	X04544	L-gamma-(	Radius_length	-0,10594	0,073181	NA
3	abundance	X04544	L-gamma-(	Radius_SOS	0,011775	0,052396	NA
3	abundance	X04544	L-gamma-(	Tibia_SOS	-0,10166	0,055239	NA
3	abundance	X04544	L-gamma-(	Handgrip	-0,25093	0,060995	NA
3	abundance	X04553	N-(4-Amin	Tibia_length	0,027701	0,054781	NA
3	abundance	X04553	N-(4-Amin	Radius_length	-0,04549	0,074387	NA
3	abundance	X04553	N-(4-Amin	Radius_SOS	0,009745	0,052189	NA
3	abundance	X04553	N-(4-Amin	Tibia_SOS	0,042402	0,056042	NA
3	abundance	X04553	N-(4-Amin	Handgrip	0,012018	0,062992	NA
3	abundance	X04557	1,4-Naphth	Tibia_length	0,060851	0,05098	NA
3	abundance	X04557	1,4-Naphth	Radius_length	0,172622	0,068967	NA
3	abundance	X04557	1,4-Naphth	Radius_SOS	0,103933	0,049342	NA
3	abundance	X04557	1,4-Naphth	Tibia_SOS	0,174925	0,052776	NA
3	abundance	X04557	1,4-Naphth	Handgrip	0,116019	0,06041	NA
3	abundance	X04562	Lisdexamf	Tibia_length	-0,00806	0,05299	NA
3	abundance	X04562	Lisdexamf	Radius_length	-0,00715	0,072019	NA
3	abundance	X04562	Lisdexamf	Radius_SOS	-0,06261	0,050733	NA
3	abundance	X04562	Lisdexamf	Tibia_SOS	-0,05219	0,054634	NA
3	abundance	X04562	Lisdexamf	Handgrip	0,051557	0,061665	NA
3	abundance	X04564	asp-gln	Tibia_length	-0,05758	0,053542	NA
3	abundance	X04564	asp-gln	Radius_length	0,054167	0,072428	NA
3	abundance	X04564	asp-gln	Radius_SOS	0,013695	0,050935	NA
3	abundance	X04564	asp-gln	Tibia_SOS	0,082092	0,054334	NA
3	abundance	X04564	asp-gln	Handgrip	0,026374	0,061988	NA
3	abundance	X04566	pterin	Tibia_length	-0,05306	0,053836	NA
3	abundance	X04566	pterin	Radius_length	0,018692	0,07277	NA
3	abundance	X04566	pterin	Radius_SOS	0,032307	0,051401	NA
3	abundance	X04566	pterin	Tibia_SOS	0,042306	0,055947	NA
3	abundance	X04566	pterin	Handgrip	-0,02564	0,062116	NA
3	abundance	X04571	13a-Hydro	Tibia_length	-0,04686	0,058307	NA
3	abundance	X04571	13a-Hydro	Radius_length	-0,09657	0,074699	NA
3	abundance	X04571	13a-Hydro	Radius_SOS	-0,05779	0,055644	NA
3	abundance	X04571	13a-Hydro	Tibia_SOS	-0,00681	0,058309	NA
3	abundance	X04571	13a-Hydro	Handgrip	-0,12384	0,05973	NA
3	abundance	X04579	6-(alpha-D	Tibia_length	-0,12174	0,054171	NA
3	abundance	X04579	6-(alpha-D	Radius_length	-0,1076	0,072926	NA
3	abundance	X04579	6-(alpha-D	Radius_SOS	-0,0702	0,051971	NA
3	abundance	X04579	6-(alpha-D	Tibia_SOS	-0,03657	0,054871	NA
3	abundance	X04579	6-(alpha-D	Handgrip	-0,02313	0,061592	NA
3	abundance	X04582	Dinoseb	Tibia_length	-0,07468	0,053884	NA
3	abundance	X04582	Dinoseb	Radius_length	-0,07891	0,072427	NA
3	abundance	X04582	Dinoseb	Radius_SOS	-0,06597	0,050985	NA
3	abundance	X04582	Dinoseb	Tibia_SOS	-0,06061	0,054064	NA
3	abundance	X04582	Dinoseb	Handgrip	-0,0593	0,061369	NA
3	abundance	X04593	Methional	Tibia_length	0,108659	0,053878	NA
3	abundance	X04593	Methional	Radius_length	0,189573	0,072151	NA
3	abundance	X04593	Methional	Radius_SOS	0,084529	0,051055	NA

3	abundance	X04593	Methional	Tibia_SOS	0,132233	0,056204	NA
3	abundance	X04593	Methional	Handgrip	0,166006	0,060606	NA
3	abundance	X04636	epsilon-(ga	Tibia_length	-0,02348	0,050936	NA
3	abundance	X04636	epsilon-(ga	Radius_length	0,033932	0,069813	NA
3	abundance	X04636	epsilon-(ga	Radius_SOS	0,035282	0,049357	NA
3	abundance	X04636	epsilon-(ga	Tibia_SOS	-0,01753	0,052762	NA
3	abundance	X04636	epsilon-(ga	Handgrip	0,071736	0,060206	NA
3	abundance	X04639	hydroxyhe›	Tibia_length	-0,06273	0,051289	NA
3	abundance	X04639	hydroxyhe›	Radius_length	-0,02781	0,070449	NA
3	abundance	X04639	hydroxyhe›	Radius_SOS	0,020037	0,049566	NA
3	abundance	X04639	hydroxyhe›	Tibia_SOS	-0,01021	0,052779	NA
3	abundance	X04639	hydroxyhe›	Handgrip	-8,40E-04	0,061209	NA
3	abundance	X04682	1-Methylin	Tibia_length	0,045623	0,050908	NA
3	abundance	X04682	1-Methylin	Radius_length	0,015192	0,069582	NA
3	abundance	X04682	1-Methylin	Radius_SOS	-0,04858	0,049198	NA
3	abundance	X04682	1-Methylin	Tibia_SOS	-0,01289	0,052894	NA
3	abundance	X04682	1-Methylin	Handgrip	0,031538	0,060899	NA
3	abundance	X04684	AAMU	Tibia_length	-0,13334	0,05137	NA
3	abundance	X04684	AAMU	Radius_length	-0,20644	0,069402	NA
3	abundance	X04684	AAMU	Radius_SOS	-0,04256	0,049823	NA
3	abundance	X04684	AAMU	Tibia_SOS	-0,0834	0,052873	NA
3	abundance	X04684	AAMU	Handgrip	-0,11549	0,060414	NA
3	abundance	X04688	Minoxidil	Tibia_length	-0,09607	0,055939	NA
3	abundance	X04688	Minoxidil	Radius_length	-0,20095	0,072946	NA
3	abundance	X04688	Minoxidil	Radius_SOS	0,01793	0,053158	NA
3	abundance	X04688	Minoxidil	Tibia_SOS	-8,75E-04	0,05729	NA
3	abundance	X04688	Minoxidil	Handgrip	0,053966	0,06231	NA
3	abundance	X04695	N-[(4-Meth	Tibia_length	-0,04819	0,048913	NA
3	abundance	X04695	N-[(4-Meth	Radius_length	-0,04525	0,04892	NA
3	abundance	X04695	N-[(4-Meth	Radius_SOS	-0,05768	0,048947	NA
3	abundance	X04695	N-[(4-Meth	Tibia_SOS	-0,00614	0,052485	NA
3	abundance	X04695	N-[(4-Meth	Handgrip	-0,02588	0,060173	NA
3	abundance	X04697	Ethosuxim	Tibia_length	0,007677	0,056433	NA
3	abundance	X04697	Ethosuxim	Radius_length	0,025033	0,074599	NA
3	abundance	X04697	Ethosuxim	Radius_SOS	0,05822	0,053921	NA
3	abundance	X04697	Ethosuxim	Tibia_SOS	-0,01639	0,057506	NA
3	abundance	X04697	Ethosuxim	Handgrip	-0,06552	0,062946	NA
3	abundance	X04743	5-Methoxy-	Tibia_length	-0,12753	0,052456	NA
3	abundance	X04743	5-Methoxy-	Radius_length	-0,18912	0,070635	NA
3	abundance	X04743	5-Methoxy-	Radius_SOS	-0,01346	0,05015	NA
3	abundance	X04743	5-Methoxy-	Tibia_SOS	-0,03861	0,053797	NA
3	abundance	X04743	5-Methoxy-	Handgrip	0,010782	0,061153	NA
3	abundance	X04744	16alpha-hy	Tibia_length	-0,0626	0,054764	NA
3	abundance	X04744	16alpha-hy	Radius_length	0,065337	0,072942	NA
3	abundance	X04744	16alpha-hy	Radius_SOS	-0,01124	0,052173	NA
3	abundance	X04744	16alpha-hy	Tibia_SOS	0,014056	0,056905	NA
3	abundance	X04744	16alpha-hy	Handgrip	0,047513	0,06306	NA
3	abundance	X04759	7-Aminom	Tibia_length	-0,02595	0,051044	NA

3	abundance	X04759	7-Aminom	Radius_length	0,002652	0,070021	NA
3	abundance	X04759	7-Aminom	Radius_SOS	-0,01507	0,049484	NA
3	abundance	X04759	7-Aminom	Tibia_SOS	0,011796	0,053014	NA
3	abundance	X04759	7-Aminom	Handgrip	0,034785	0,060788	NA
3	abundance	X04788	2-(4-Isopro	Tibia_length	0,115183	0,0529	NA
3	abundance	X04788	2-(4-Isopro	Radius_length	0,127804	0,071963	NA
3	abundance	X04788	2-(4-Isopro	Radius_SOS	0,011732	0,050609	NA
3	abundance	X04788	2-(4-Isopro	Tibia_SOS	0,128503	0,05459	NA
3	abundance	X04788	2-(4-Isopro	Handgrip	0,100959	0,061003	NA
3	abundance	X04792	his-asn	Tibia_length	0,034947	0,054975	NA
3	abundance	X04792	his-asn	Radius_length	0,096484	0,07352	NA
3	abundance	X04792	his-asn	Radius_SOS	0,022083	0,052251	NA
3	abundance	X04792	his-asn	Tibia_SOS	0,089294	0,055822	NA
3	abundance	X04792	his-asn	Handgrip	0,140905	0,059592	NA
3	abundance	X04814	meprobam	Tibia_length	-0,07862	0,053226	NA
3	abundance	X04814	meprobam	Radius_length	-0,10195	0,071621	NA
3	abundance	X04814	meprobam	Radius_SOS	-0,02773	0,050865	NA
3	abundance	X04814	meprobam	Tibia_SOS	-0,06547	0,05408	NA
3	abundance	X04814	meprobam	Handgrip	0,044943	0,061671	NA
3	abundance	X04822	6-Sulfatoxy	Tibia_length	0,146474	0,052547	NA
3	abundance	X04822	6-Sulfatoxy	Radius_length	0,224373	0,06999	NA
3	abundance	X04822	6-Sulfatoxy	Radius_SOS	0,080234	0,050361	NA
3	abundance	X04822	6-Sulfatoxy	Tibia_SOS	0,142676	0,053398	NA
3	abundance	X04822	6-Sulfatoxy	Handgrip	0,161954	0,060426	NA
3	abundance	X04852	Toluene	Tibia_length	0,062891	0,056708	NA
3	abundance	X04852	Toluene	Radius_length	-0,06967	0,074682	NA
3	abundance	X04852	Toluene	Radius_SOS	-0,03105	0,054136	NA
3	abundance	X04852	Toluene	Tibia_SOS	0,074163	0,058401	NA
3	abundance	X04852	Toluene	Handgrip	0,006529	0,063829	NA
3	abundance	X04877	Trifluorom	Tibia_length	-0,03269	0,052843	NA
3	abundance	X04877	Trifluorom	Radius_length	-4,25E-04	0,071921	NA
3	abundance	X04877	Trifluorom	Radius_SOS	-0,04781	0,050621	NA
3	abundance	X04877	Trifluorom	Tibia_SOS	0,033659	0,053834	NA
3	abundance	X04877	Trifluorom	Handgrip	0,085664	0,061233	NA
3	abundance	X04897	Taxifolin	Tibia_length	0,091633	0,051572	NA
3	abundance	X04897	Taxifolin	Radius_length	0,104839	0,070672	NA
3	abundance	X04897	Taxifolin	Radius_SOS	-0,02522	0,049832	NA
3	abundance	X04897	Taxifolin	Tibia_SOS	0,090502	0,052892	NA
3	abundance	X04897	Taxifolin	Handgrip	0,121333	0,061152	NA
3	abundance	X04898	17-Hydroxy	Tibia_length	0,019404	0,050616	NA
3	abundance	X04898	17-Hydroxy	Radius_length	0,014218	0,069157	NA
3	abundance	X04898	17-Hydroxy	Radius_SOS	0,012545	0,049244	NA
3	abundance	X04898	17-Hydroxy	Tibia_SOS	0,024923	0,05314	NA
3	abundance	X04898	17-Hydroxy	Handgrip	-0,01257	0,061323	NA
3	abundance	X04919	5-O-alpha-	Tibia_length	-0,12821	0,057073	NA
3	abundance	X04919	5-O-alpha-	Radius_length	0,004503	0,075525	NA
3	abundance	X04919	5-O-alpha-	Radius_SOS	-0,06918	0,054482	NA
3	abundance	X04919	5-O-alpha-	Tibia_SOS	-0,01828	0,059268	NA

3	abundance	X04919	5-O-alpha-	Handgrip	-0,03088	0,062695	NA
3	abundance	X04922	Sinapinic a	Tibia_length	-0,07997	0,053735	NA
3	abundance	X04922	Sinapinic a	Radius_length	-0,17822	0,071657	NA
3	abundance	X04922	Sinapinic a	Radius_SOS	0,00221	0,051386	NA
3	abundance	X04922	Sinapinic a	Tibia_SOS	-0,06121	0,054801	NA
3	abundance	X04922	Sinapinic a	Handgrip	-0,11052	0,061186	NA
3	abundance	X04939	N-Desalkyl	Tibia_length	-0,11399	0,053833	NA
3	abundance	X04939	N-Desalkyl	Radius_length	-0,17704	0,071924	NA
3	abundance	X04939	N-Desalkyl	Radius_SOS	-0,11289	0,052083	NA
3	abundance	X04939	N-Desalkyl	Tibia_SOS	0,004675	0,05554	NA
3	abundance	X04939	N-Desalkyl	Handgrip	-0,05878	0,061925	NA
3	abundance	X04975	MFCD0995	Tibia_length	0,119025	0,054022	NA
3	abundance	X04975	MFCD0995	Radius_length	0,105006	0,073041	NA
3	abundance	X04975	MFCD0995	Radius_SOS	0,11586	0,05156	NA
3	abundance	X04975	MFCD0995	Tibia_SOS	0,110767	0,055592	NA
3	abundance	X04975	MFCD0995	Handgrip	0,06349	0,061945	NA
3	abundance	X04986	L-Proline, 4	Tibia_length	-0,08087	0,057625	NA
3	abundance	X04986	L-Proline, 4	Radius_length	-0,14865	0,074703	NA
3	abundance	X04986	L-Proline, 4	Radius_SOS	0,055089	0,054754	NA
3	abundance	X04986	L-Proline, 4	Tibia_SOS	0,006251	0,059203	NA
3	abundance	X04986	L-Proline, 4	Handgrip	-0,02857	0,062494	NA
3	abundance	X04990	Cilazapril	Tibia_length	0,090416	0,052095	NA
3	abundance	X04990	Cilazapril	Radius_length	0,105078	0,070776	NA
3	abundance	X04990	Cilazapril	Radius_SOS	0,037388	0,050002	NA
3	abundance	X04990	Cilazapril	Tibia_SOS	0,104487	0,05383	NA
3	abundance	X04990	Cilazapril	Handgrip	-0,0159	0,061843	NA
3	abundance	X04996	MFCD0272	Tibia_length	-0,11349	0,054244	NA
3	abundance	X04996	MFCD0272	Radius_length	-0,19976	0,071612	NA
3	abundance	X04996	MFCD0272	Radius_SOS	-0,00979	0,051351	NA
3	abundance	X04996	MFCD0272	Tibia_SOS	-0,10754	0,054567	NA
3	abundance	X04996	MFCD0272	Handgrip	-0,08433	0,061106	NA
1	abundance	X04999	2-Hydroxyp	Tibia_length	-0,14287	0,052941	NA
1	abundance	X04999	2-Hydroxyp	Radius_length	-0,08564	0,071949	NA
1	abundance	X04999	2-Hydroxyp	Radius_SOS	-0,04954	0,050865	NA
1	abundance	X04999	2-Hydroxyp	Tibia_SOS	-0,12464	0,053607	NA
1	abundance	X04999	2-Hydroxyp	Handgrip	-0,03095	0,061296	NA
3	abundance	X05017	N-(1-{[Metl	Tibia_length	-0,07801	0,053994	NA
3	abundance	X05017	N-(1-{[Metl	Radius_length	0,01554	0,073591	NA
3	abundance	X05017	N-(1-{[Metl	Radius_SOS	-0,10193	0,051677	NA
3	abundance	X05017	N-(1-{[Metl	Tibia_SOS	0,015959	0,054708	NA
3	abundance	X05017	N-(1-{[Metl	Handgrip	0,073357	0,061875	NA
3	abundance	X05029	Hypericin	Tibia_length	0,159747	0,05145	NA
3	abundance	X05029	Hypericin	Radius_length	0,198155	0,069424	NA
3	abundance	X05029	Hypericin	Radius_SOS	0,083323	0,049575	NA
3	abundance	X05029	Hypericin	Tibia_SOS	0,12815	0,052887	NA
3	abundance	X05029	Hypericin	Handgrip	0,131787	0,060134	NA
3	abundance	X05033	(2E)-N-3,7-	Tibia_length	0,077261	0,052782	NA
3	abundance	X05033	(2E)-N-3,7-	Radius_length	0,127394	0,07159	NA

3	abundance	X05033	(2E)-N-3,7·	Radius_SOS	0,050618	0,050591	NA
3	abundance	X05033	(2E)-N-3,7·	Tibia_SOS	0,114958	0,053919	NA
3	abundance	X05033	(2E)-N-3,7·	Handgrip	0,166641	0,060307	NA
3	abundance	X05046	Casimiroin	Tibia_length	-0,12026	0,057503	NA
3	abundance	X05046	Casimiroin	Radius_length	-0,04843	0,074893	NA
3	abundance	X05046	Casimiroin	Radius_SOS	-0,03487	0,055208	NA
3	abundance	X05046	Casimiroin	Tibia_SOS	0,015293	0,058124	NA
3	abundance	X05046	Casimiroin	Handgrip	0,012681	0,061763	NA
3	abundance	X05068	pro-gln	Tibia_length	-0,11495	0,054444	NA
3	abundance	X05068	pro-gln	Radius_length	-0,09099	0,072805	NA
3	abundance	X05068	pro-gln	Radius_SOS	-0,04142	0,051945	NA
3	abundance	X05068	pro-gln	Tibia_SOS	-0,15194	0,055248	NA
3	abundance	X05068	pro-gln	Handgrip	-0,07971	0,061406	NA
3	abundance	X05071	Histidylphe	Tibia_length	-3,36E-04	0,0583	NA
3	abundance	X05071	Histidylphe	Radius_length	0,001006	0,075843	NA
3	abundance	X05071	Histidylphe	Radius_SOS	-0,01884	0,055481	NA
3	abundance	X05071	Histidylphe	Tibia_SOS	0,016123	0,058091	NA
3	abundance	X05071	Histidylphe	Handgrip	-0,07397	0,060028	NA
3	abundance	X05072	L-gamma-(	Tibia_length	-0,07384	0,052074	NA
3	abundance	X05072	L-gamma-(	Radius_length	-0,06852	0,07097	NA
3	abundance	X05072	L-gamma-(	Radius_SOS	2,91E-04	0,04998	NA
3	abundance	X05072	L-gamma-(	Tibia_SOS	-0,08289	0,053632	NA
3	abundance	X05072	L-gamma-(	Handgrip	-0,0088	0,061256	NA
2b	abundance	X05081	ELK (Peptic	Tibia_length	-0,05935	0,055499	NA
2b	abundance	X05081	ELK (Peptic	Radius_length	0,005442	0,073838	NA
2b	abundance	X05081	ELK (Peptic	Radius_SOS	0,034576	0,052863	NA
2b	abundance	X05081	ELK (Peptic	Tibia_SOS	0,013283	0,055824	NA
2b	abundance	X05081	ELK (Peptic	Handgrip	0,031272	0,062132	NA
3	abundance	X05126	(3aS,5S,6F	Tibia_length	0,012505	0,050531	NA
3	abundance	X05126	(3aS,5S,6F	Radius_length	0,085293	0,068954	NA
3	abundance	X05126	(3aS,5S,6F	Radius_SOS	0,029314	0,049204	NA
3	abundance	X05126	(3aS,5S,6F	Tibia_SOS	-0,05533	0,052474	NA
3	abundance	X05126	(3aS,5S,6F	Handgrip	0,009086	0,060298	NA
3	abundance	X05146	UQ367500	Tibia_length	0,007026	0,053454	NA
3	abundance	X05146	UQ367500	Radius_length	-0,11075	0,072431	NA
3	abundance	X05146	UQ367500	Radius_SOS	0,002076	0,051054	NA
3	abundance	X05146	UQ367500	Tibia_SOS	0,099097	0,055224	NA
3	abundance	X05146	UQ367500	Handgrip	0,0607	0,063002	NA
3	abundance	X05149	S-Sulfocys	Tibia_length	0,107982	0,05027	NA
3	abundance	X05149	S-Sulfocys	Radius_length	0,123272	0,068527	NA
3	abundance	X05149	S-Sulfocys	Radius_SOS	0,007156	0,049188	NA
3	abundance	X05149	S-Sulfocys	Tibia_SOS	0,08751	0,052566	NA
3	abundance	X05149	S-Sulfocys	Handgrip	0,083899	0,060046	NA
3	abundance	X05177	cis-3-Hexe	Tibia_length	-0,1006	0,05563	NA
3	abundance	X05177	cis-3-Hexe	Radius_length	-0,03237	0,073876	NA
3	abundance	X05177	cis-3-Hexe	Radius_SOS	0,002912	0,053071	NA
3	abundance	X05177	cis-3-Hexe	Tibia_SOS	0,054571	0,055645	NA
3	abundance	X05177	cis-3-Hexe	Handgrip	-0,00723	0,061627	NA

3	abundance	X05183	3,6-Dichlo	Tibia_length	0,006434	0,054261	NA
3	abundance	X05183	3,6-Dichlo	Radius_length	-0,00169	0,073108	NA
3	abundance	X05183	3,6-Dichlo	Radius_SOS	0,028467	0,051726	NA
3	abundance	X05183	3,6-Dichlo	Tibia_SOS	-0,01799	0,055278	NA
3	abundance	X05183	3,6-Dichlo	Handgrip	0,024267	0,062534	NA
3	abundance	X05195	Lys-phe	Tibia_length	-0,02046	0,05644	NA
3	abundance	X05195	Lys-phe	Radius_length	-0,06424	0,074227	NA
3	abundance	X05195	Lys-phe	Radius_SOS	0,033792	0,053619	NA
3	abundance	X05195	Lys-phe	Tibia_SOS	-0,0878	0,055837	NA
3	abundance	X05195	Lys-phe	Handgrip	0,032923	0,062726	NA
3	abundance	X05203	felbamate	Tibia_length	-0,15821	0,057004	NA
3	abundance	X05203	felbamate	Radius_length	-0,02083	0,075056	NA
3	abundance	X05203	felbamate	Radius_SOS	-0,01099	0,054926	NA
3	abundance	X05203	felbamate	Tibia_SOS	-0,10441	0,057806	NA
3	abundance	X05203	felbamate	Handgrip	-0,08607	0,06337	NA
3	abundance	X05204	Urothion	Tibia_length	-0,08126	0,050622	NA
3	abundance	X05204	Urothion	Radius_length	-0,0058	0,069711	NA
3	abundance	X05204	Urothion	Radius_SOS	-0,02128	0,049466	NA
3	abundance	X05204	Urothion	Tibia_SOS	-0,0409	0,052754	NA
3	abundance	X05204	Urothion	Handgrip	0,015653	0,061032	NA
3	abundance	X05214	[7-Hydroxy	Tibia_length	0,102512	0,051164	NA
3	abundance	X05214	[7-Hydroxy	Radius_length	0,071701	0,070242	NA
3	abundance	X05214	[7-Hydroxy	Radius_SOS	-0,02571	0,049608	NA
3	abundance	X05214	[7-Hydroxy	Tibia_SOS	0,080337	0,053368	NA
3	abundance	X05214	[7-Hydroxy	Handgrip	-4,80E-04	0,061125	NA
3	abundance	X05225	4-(METHYL	Tibia_length	-0,02798	0,056395	NA
3	abundance	X05225	4-(METHYL	Radius_length	-0,00264	0,074873	NA
3	abundance	X05225	4-(METHYL	Radius_SOS	0,026211	0,053425	NA
3	abundance	X05225	4-(METHYL	Tibia_SOS	-0,04596	0,057027	NA
3	abundance	X05225	4-(METHYL	Handgrip	0,126249	0,059711	NA
3	abundance	X05234	3,4-dihydr(	Tibia_length	-0,16498	0,052238	NA
3	abundance	X05234	3,4-dihydr(	Radius_length	-0,15619	0,071045	NA
3	abundance	X05234	3,4-dihydr(	Radius_SOS	-0,02739	0,0503	NA
3	abundance	X05234	3,4-dihydr(	Tibia_SOS	-0,11151	0,053953	NA
3	abundance	X05234	3,4-dihydr(	Handgrip	-0,04684	0,060964	NA
3	abundance	X05237	imazametl	Tibia_length	-0,11546	0,057313	NA
3	abundance	X05237	imazametl	Radius_length	-0,17681	0,074551	NA
3	abundance	X05237	imazametl	Radius_SOS	-0,04992	0,054975	NA
3	abundance	X05237	imazametl	Tibia_SOS	-0,13088	0,057909	NA
3	abundance	X05237	imazametl	Handgrip	-0,01214	0,062174	NA
3	abundance	X05240	Dimeric m(	Tibia_length	-0,05444	0,053759	NA
3	abundance	X05240	Dimeric m(	Radius_length	-0,09609	0,072319	NA
3	abundance	X05240	Dimeric m(	Radius_SOS	-0,01349	0,051248	NA
3	abundance	X05240	Dimeric m(	Tibia_SOS	0,037548	0,054502	NA
3	abundance	X05240	Dimeric m(	Handgrip	-0,08016	0,061798	NA
3	abundance	X05243	N-(4-Hydrc	Tibia_length	-0,16276	0,052882	NA
3	abundance	X05243	N-(4-Hydrc	Radius_length	-0,13841	0,071725	NA
3	abundance	X05243	N-(4-Hydrc	Radius_SOS	-0,05274	0,050765	NA

3	abundance	X05243	N-(4-Hydro	Tibia_SOS	-0,10624	0,053647	NA
3	abundance	X05243	N-(4-Hydro	Handgrip	-0,06683	0,061255	NA
3	abundance	X05275	H-DL-MET-	Tibia_length	0,052213	0,056406	NA
3	abundance	X05275	H-DL-MET-	Radius_length	0,169372	0,074346	NA
3	abundance	X05275	H-DL-MET-	Radius_SOS	0,006341	0,053629	NA
3	abundance	X05275	H-DL-MET-	Tibia_SOS	0,03579	0,057555	NA
3	abundance	X05275	H-DL-MET-	Handgrip	0,085015	0,063503	NA
3	abundance	X05295	3-(4,7-Dim	Tibia_length	-0,08569	0,055151	NA
3	abundance	X05295	3-(4,7-Dim	Radius_length	-0,18008	0,07231	NA
3	abundance	X05295	3-(4,7-Dim	Radius_SOS	-0,05981	0,052201	NA
3	abundance	X05295	3-(4,7-Dim	Tibia_SOS	-0,1151	0,055593	NA
3	abundance	X05295	3-(4,7-Dim	Handgrip	-0,04404	0,061922	NA
3	abundance	X05306	2-methoxy	Tibia_length	0,062459	0,049707	NA
3	abundance	X05306	2-methoxy	Radius_length	0,183707	0,066655	NA
3	abundance	X05306	2-methoxy	Radius_SOS	-0,00957	0,049035	NA
3	abundance	X05306	2-methoxy	Tibia_SOS	0,037985	0,052472	NA
3	abundance	X05306	2-methoxy	Handgrip	0,043055	0,060442	NA
3	abundance	X05357	NPYR	Tibia_length	0,096384	0,05461	NA
3	abundance	X05357	NPYR	Radius_length	-0,06291	0,073614	NA
3	abundance	X05357	NPYR	Radius_SOS	-0,03305	0,052176	NA
3	abundance	X05357	NPYR	Tibia_SOS	0,102885	0,055284	NA
3	abundance	X05357	NPYR	Handgrip	-0,02004	0,061511	NA
3	abundance	X05391	2-Acetami	Tibia_length	-0,0144	0,056771	NA
3	abundance	X05391	2-Acetami	Radius_length	0,106734	0,074093	NA
3	abundance	X05391	2-Acetami	Radius_SOS	-0,02713	0,054029	NA
3	abundance	X05391	2-Acetami	Tibia_SOS	0,005619	0,057719	NA
3	abundance	X05391	2-Acetami	Handgrip	0,021026	0,061555	NA
3	abundance	X05398	DIBOA	Tibia_length	-0,11838	0,05495	NA
3	abundance	X05398	DIBOA	Radius_length	-0,09145	0,073934	NA
3	abundance	X05398	DIBOA	Radius_SOS	-0,02162	0,05239	NA
3	abundance	X05398	DIBOA	Tibia_SOS	-0,07671	0,055385	NA
3	abundance	X05398	DIBOA	Handgrip	-0,17129	0,062051	NA
3	abundance	X05411	3-(Sulfoox	Tibia_length	-0,07929	0,057482	NA
3	abundance	X05411	3-(Sulfoox	Radius_length	0,021068	0,075079	NA
3	abundance	X05411	3-(Sulfoox	Radius_SOS	0,103198	0,054752	NA
3	abundance	X05411	3-(Sulfoox	Tibia_SOS	0,121111	0,058274	NA
3	abundance	X05411	3-(Sulfoox	Handgrip	-0,05177	0,062641	NA
3	abundance	X05415	Redul	Tibia_length	-0,00806	0,051629	NA
3	abundance	X05415	Redul	Radius_length	0,164021	0,069676	NA
3	abundance	X05415	Redul	Radius_SOS	0,056063	0,049817	NA
3	abundance	X05415	Redul	Tibia_SOS	0,054274	0,05289	NA
3	abundance	X05415	Redul	Handgrip	0,019186	0,060487	NA
3	abundance	X05417	(S)-2-hydra	Tibia_length	-0,03656	0,053385	NA
3	abundance	X05417	(S)-2-hydra	Radius_length	-0,03855	0,072634	NA
3	abundance	X05417	(S)-2-hydra	Radius_SOS	0,036638	0,051071	NA
3	abundance	X05417	(S)-2-hydra	Tibia_SOS	-0,05801	0,054105	NA
3	abundance	X05417	(S)-2-hydra	Handgrip	-0,02425	0,061804	NA
3	abundance	X05421	S-Allylcyst	Tibia_length	-0,04054	0,054644	NA

3	abundance	X05421	S-Allylcyst	Radius_length	0,057137	0,073071	NA
3	abundance	X05421	S-Allylcyst	Radius_SOS	0,00523	0,051851	NA
3	abundance	X05421	S-Allylcyst	Tibia_SOS	-0,02598	0,055223	NA
3	abundance	X05421	S-Allylcyst	Handgrip	-0,05044	0,062544	NA
3	abundance	X05459	Scopoletin	Tibia_length	0,114587	0,05277	NA
3	abundance	X05459	Scopoletin	Radius_length	0,154163	0,071194	NA
3	abundance	X05459	Scopoletin	Radius_SOS	0,063131	0,050474	NA
3	abundance	X05459	Scopoletin	Tibia_SOS	0,131143	0,053192	NA
3	abundance	X05459	Scopoletin	Handgrip	0,146135	0,060541	NA
3	abundance	X05467	Azulfidine	Tibia_length	-0,12265	0,053254	NA
3	abundance	X05467	Azulfidine	Radius_length	-0,13575	0,071824	NA
3	abundance	X05467	Azulfidine	Radius_SOS	-0,08405	0,050985	NA
3	abundance	X05467	Azulfidine	Tibia_SOS	0,066422	0,053782	NA
3	abundance	X05467	Azulfidine	Handgrip	0,029479	0,061555	NA
3	abundance	X05478	S(8)-aminc	Tibia_length	0,042555	0,052151	NA
3	abundance	X05478	S(8)-aminc	Radius_length	0,095765	0,071392	NA
3	abundance	X05478	S(8)-aminc	Radius_SOS	0,029436	0,05006	NA
3	abundance	X05478	S(8)-aminc	Tibia_SOS	0,045698	0,053382	NA
3	abundance	X05478	S(8)-aminc	Handgrip	0,019181	0,061769	NA
3	abundance	X05499	GLY-MET	Tibia_length	-0,09102	0,055609	NA
3	abundance	X05499	GLY-MET	Radius_length	0,026006	0,074386	NA
3	abundance	X05499	GLY-MET	Radius_SOS	0,017759	0,052726	NA
3	abundance	X05499	GLY-MET	Tibia_SOS	0,012694	0,056688	NA
3	abundance	X05499	GLY-MET	Handgrip	0,056327	0,062839	NA
3	abundance	X05510	3-Benzyl-6	Tibia_length	0,072653	0,050843	NA
3	abundance	X05510	3-Benzyl-6	Radius_length	0,060784	0,069626	NA
3	abundance	X05510	3-Benzyl-6	Radius_SOS	0,053232	0,049429	NA
3	abundance	X05510	3-Benzyl-6	Tibia_SOS	0,05811	0,052943	NA
3	abundance	X05510	3-Benzyl-6	Handgrip	-0,0241	0,061016	NA
3	abundance	X05514	(3aS,5S,6F	Tibia_length	-0,02366	0,050601	NA
3	abundance	X05514	(3aS,5S,6F	Radius_length	-0,11134	0,068795	NA
3	abundance	X05514	(3aS,5S,6F	Radius_SOS	0,03792	0,049217	NA
3	abundance	X05514	(3aS,5S,6F	Tibia_SOS	-0,02015	0,052861	NA
3	abundance	X05514	(3aS,5S,6F	Handgrip	-0,04604	0,060575	NA
3	abundance	X05551	Trolox	Tibia_length	-0,07299	0,052029	NA
3	abundance	X05551	Trolox	Radius_length	-0,07521	0,070909	NA
3	abundance	X05551	Trolox	Radius_SOS	0,039625	0,050087	NA
3	abundance	X05551	Trolox	Tibia_SOS	0,002699	0,053213	NA
3	abundance	X05551	Trolox	Handgrip	-0,15207	0,059681	NA
3	abundance	X05561	1ÃŽÂ²-Hyd	Tibia_length	-0,03043	0,051651	NA
3	abundance	X05561	1ÃŽÂ²-Hyd	Radius_length	-0,10629	0,070389	NA
3	abundance	X05561	1ÃŽÂ²-Hyd	Radius_SOS	0,036089	0,04977	NA
3	abundance	X05561	1ÃŽÂ²-Hyd	Tibia_SOS	-0,05592	0,053318	NA
3	abundance	X05561	1ÃŽÂ²-Hyd	Handgrip	-0,02626	0,061065	NA
3	abundance	X05580	Xanthureni	Tibia_length	-0,06045	0,055531	NA
3	abundance	X05580	Xanthureni	Radius_length	-0,03276	0,073372	NA
3	abundance	X05580	Xanthureni	Radius_SOS	-0,09718	0,052327	NA
3	abundance	X05580	Xanthureni	Tibia_SOS	0,018266	0,055381	NA

3	abundance	X05580	Xanthureni	Handgrip	0,022769	0,062273	NA
3	abundance	X05581	N-[(10Z)-7-	Tibia_length	-0,04964	0,049312	NA
3	abundance	X05581	N-[(10Z)-7-	Radius_length	-0,03355	0,066552	NA
3	abundance	X05581	N-[(10Z)-7-	Radius_SOS	-0,02866	0,049018	NA
3	abundance	X05581	N-[(10Z)-7-	Tibia_SOS	-0,05756	0,052404	NA
3	abundance	X05581	N-[(10Z)-7-	Handgrip	-0,03494	0,060349	NA
1	abundance	X05584	Serotonin	Tibia_length	0,044454	0,050355	NA
1	abundance	X05584	Serotonin	Radius_length	-0,0383	0,068748	NA
1	abundance	X05584	Serotonin	Radius_SOS	-0,05696	0,049095	NA
1	abundance	X05584	Serotonin	Tibia_SOS	-0,00914	0,05283	NA
1	abundance	X05584	Serotonin	Handgrip	-0,09428	0,060127	NA
3	abundance	X05656	mesifurane	Tibia_length	-0,04134	0,053123	NA
3	abundance	X05656	mesifurane	Radius_length	-0,15511	0,071041	NA
3	abundance	X05656	mesifurane	Radius_SOS	-0,0311	0,050775	NA
3	abundance	X05656	mesifurane	Tibia_SOS	-0,09787	0,053624	NA
3	abundance	X05656	mesifurane	Handgrip	-0,03982	0,061055	NA
3	abundance	X05709	2,3-Dihydr	Tibia_length	-0,06542	0,052438	NA
3	abundance	X05709	2,3-Dihydr	Radius_length	-0,14541	0,070847	NA
3	abundance	X05709	2,3-Dihydr	Radius_SOS	-0,00733	0,050207	NA
3	abundance	X05709	2,3-Dihydr	Tibia_SOS	-0,1288	0,053069	NA
3	abundance	X05709	2,3-Dihydr	Handgrip	-0,22035	0,059634	NA
3	abundance	X05726	methocarb	Tibia_length	-0,11235	0,051362	NA
3	abundance	X05726	methocarb	Radius_length	-0,14246	0,069734	NA
3	abundance	X05726	methocarb	Radius_SOS	-0,03188	0,049649	NA
3	abundance	X05726	methocarb	Tibia_SOS	-0,09827	0,052726	NA
3	abundance	X05726	methocarb	Handgrip	-0,1043	0,060434	NA
3	abundance	X05734	N-[(2S)-2-I	Tibia_length	-0,08726	0,054691	NA
3	abundance	X05734	N-[(2S)-2-I	Radius_length	0,008213	0,073651	NA
3	abundance	X05734	N-[(2S)-2-I	Radius_SOS	0,018569	0,052373	NA
3	abundance	X05734	N-[(2S)-2-I	Tibia_SOS	-0,00141	0,05506	NA
3	abundance	X05734	N-[(2S)-2-I	Handgrip	-0,05445	0,062102	NA
3	abundance	X05741	g-Aminobu	Tibia_length	0,048084	0,0526	NA
3	abundance	X05741	g-Aminobu	Radius_length	0,018081	0,071537	NA
3	abundance	X05741	g-Aminobu	Radius_SOS	-0,02317	0,050448	NA
3	abundance	X05741	g-Aminobu	Tibia_SOS	-0,04953	0,054357	NA
3	abundance	X05741	g-Aminobu	Handgrip	0,029539	0,061861	NA
3	abundance	X05758	quinol sulf	Tibia_length	-0,0533	0,054778	NA
3	abundance	X05758	quinol sulf	Radius_length	-0,11793	0,073093	NA
3	abundance	X05758	quinol sulf	Radius_SOS	-0,16278	0,052645	NA
3	abundance	X05758	quinol sulf	Tibia_SOS	-0,02935	0,055855	NA
3	abundance	X05758	quinol sulf	Handgrip	-0,05739	0,062447	NA
3	abundance	X05786	Nitrendipir	Tibia_length	0,027985	0,051604	NA
3	abundance	X05786	Nitrendipir	Radius_length	0,032212	0,070944	NA
3	abundance	X05786	Nitrendipir	Radius_SOS	0,039469	0,0497	NA
3	abundance	X05786	Nitrendipir	Tibia_SOS	0,053342	0,052817	NA
3	abundance	X05786	Nitrendipir	Handgrip	-0,06186	0,06062	NA
3	abundance	X05812	(2S)-3-(1H	Tibia_length	-0,01635	0,057204	NA
3	abundance	X05812	(2S)-3-(1H	Radius_length	0,078454	0,0746	NA

3	abundance	X05812	(2S)-3-(1H	Radius_SOS	0,100206	0,054617	NA
3	abundance	X05812	(2S)-3-(1H	Tibia_SOS	-0,02215	0,059488	NA
3	abundance	X05812	(2S)-3-(1H	Handgrip	0,027733	0,063339	NA
3	abundance	X05857	N-D-Gluco	Tibia_length	-0,22338	0,053523	NA
3	abundance	X05857	N-D-Gluco	Radius_length	-0,07587	0,073167	NA
3	abundance	X05857	N-D-Gluco	Radius_SOS	-0,03423	0,05208	NA
3	abundance	X05857	N-D-Gluco	Tibia_SOS	-0,07561	0,055829	NA
3	abundance	X05857	N-D-Gluco	Handgrip	0,049048	0,062607	NA
3	abundance	X05859	3,7,12,17-1	Tibia_length	-0,03676	0,05046	NA
3	abundance	X05859	3,7,12,17-1	Radius_length	-0,11659	0,06864	NA
3	abundance	X05859	3,7,12,17-1	Radius_SOS	0,032444	0,049165	NA
3	abundance	X05859	3,7,12,17-1	Tibia_SOS	-0,00357	0,052659	NA
3	abundance	X05859	3,7,12,17-1	Handgrip	0,019612	0,060624	NA
3	abundance	X05878	1,2-dihydro	Tibia_length	0,00703	0,053187	NA
3	abundance	X05878	1,2-dihydro	Radius_length	0,044215	0,072121	NA
3	abundance	X05878	1,2-dihydro	Radius_SOS	-0,08547	0,050928	NA
3	abundance	X05878	1,2-dihydro	Tibia_SOS	0,026126	0,054789	NA
3	abundance	X05878	1,2-dihydro	Handgrip	-0,02583	0,061384	NA
3	abundance	X05892	(5Z)-2-Ami	Tibia_length	-0,04695	0,053029	NA
3	abundance	X05892	(5Z)-2-Ami	Radius_length	-0,0865	0,071731	NA
3	abundance	X05892	(5Z)-2-Ami	Radius_SOS	-0,02436	0,050693	NA
3	abundance	X05892	(5Z)-2-Ami	Tibia_SOS	-0,07948	0,054461	NA
3	abundance	X05892	(5Z)-2-Ami	Handgrip	-0,00816	0,062068	NA
3	abundance	X05919	2-(2-Amino	Tibia_length	-0,04415	0,054083	NA
3	abundance	X05919	2-(2-Amino	Radius_length	-0,16457	0,071678	NA
3	abundance	X05919	2-(2-Amino	Radius_SOS	-0,02935	0,051348	NA
3	abundance	X05919	2-(2-Amino	Tibia_SOS	-0,07899	0,055449	NA
3	abundance	X05919	2-(2-Amino	Handgrip	-0,0436	0,061478	NA
3	abundance	X05968	Tetraacety	Tibia_length	-0,10168	0,054087	NA
3	abundance	X05968	Tetraacety	Radius_length	-0,21319	0,072369	NA
3	abundance	X05968	Tetraacety	Radius_SOS	-0,08344	0,0513	NA
3	abundance	X05968	Tetraacety	Tibia_SOS	-0,10764	0,054086	NA
3	abundance	X05968	Tetraacety	Handgrip	-0,05923	0,062103	NA
3	abundance	X05969	4-(METHYL	Tibia_length	-0,17285	0,055857	NA
3	abundance	X05969	4-(METHYL	Radius_length	-0,20319	0,073371	NA
3	abundance	X05969	4-(METHYL	Radius_SOS	-0,01159	0,053434	NA
3	abundance	X05969	4-(METHYL	Tibia_SOS	0,009908	0,057084	NA
3	abundance	X05969	4-(METHYL	Handgrip	-0,06322	0,063519	NA
3	abundance	X05971	L-gamma-(	Tibia_length	0,022567	0,050688	NA
3	abundance	X05971	L-gamma-(	Radius_length	-0,05788	0,069518	NA
3	abundance	X05971	L-gamma-(	Radius_SOS	0,093733	0,049079	NA
3	abundance	X05971	L-gamma-(	Tibia_SOS	-0,00345	0,052879	NA
3	abundance	X05971	L-gamma-(	Handgrip	0,024284	0,06122	NA
3	abundance	X05982	(19R,25S)-	Tibia_length	0,044066	0,054753	NA
3	abundance	X05982	(19R,25S)-	Radius_length	0,185906	0,072199	NA
3	abundance	X05982	(19R,25S)-	Radius_SOS	-0,02122	0,051923	NA
3	abundance	X05982	(19R,25S)-	Tibia_SOS	0,037	0,054568	NA
3	abundance	X05982	(19R,25S)-	Handgrip	-0,00513	0,062475	NA

2b	abundance	X05984	1-(4-Methy	Tibia_length	0,0936	0,050629	NA
2b	abundance	X05984	1-(4-Methy	Radius_length	0,090154	0,069391	NA
2b	abundance	X05984	1-(4-Methy	Radius_SOS	0,02293	0,049395	NA
2b	abundance	X05984	1-(4-Methy	Tibia_SOS	0,087611	0,052624	NA
2b	abundance	X05984	1-(4-Methy	Handgrip	0,033179	0,060404	NA
3	abundance	X05988	Selsun	Tibia_length	-0,01441	0,053163	NA
3	abundance	X05988	Selsun	Radius_length	0,055911	0,072278	NA
3	abundance	X05988	Selsun	Radius_SOS	-0,06651	0,050901	NA
3	abundance	X05988	Selsun	Tibia_SOS	0,070994	0,054239	NA
3	abundance	X05988	Selsun	Handgrip	0,016128	0,060938	NA
3	abundance	X05995	Diacetin	Tibia_length	-0,06771	0,054764	NA
3	abundance	X05995	Diacetin	Radius_length	0,008377	0,073672	NA
3	abundance	X05995	Diacetin	Radius_SOS	0,030281	0,052036	NA
3	abundance	X05995	Diacetin	Tibia_SOS	0,008525	0,056664	NA
3	abundance	X05995	Diacetin	Handgrip	0,06998	0,060536	NA
3	abundance	X05997	FB950000(	Tibia_length	0,005926	0,054597	NA
3	abundance	X05997	FB950000(	Radius_length	-0,05752	0,073519	NA
3	abundance	X05997	FB950000(	Radius_SOS	0,024438	0,051985	NA
3	abundance	X05997	FB950000(	Tibia_SOS	0,03422	0,056314	NA
3	abundance	X05997	FB950000(	Handgrip	-0,03613	0,060154	NA
3	abundance	X06011	Coprine	Tibia_length	-0,07289	0,056247	NA
3	abundance	X06011	Coprine	Radius_length	-0,12803	0,07359	NA
3	abundance	X06011	Coprine	Radius_SOS	-0,0348	0,053584	NA
3	abundance	X06011	Coprine	Tibia_SOS	-0,05068	0,056386	NA
3	abundance	X06011	Coprine	Handgrip	-0,01839	0,061924	NA
3	abundance	X06016	N,N-Dimet	Tibia_length	0,029741	0,055103	NA
3	abundance	X06016	N,N-Dimet	Radius_length	0,059434	0,073666	NA
3	abundance	X06016	N,N-Dimet	Radius_SOS	0,161791	0,051565	NA
3	abundance	X06016	N,N-Dimet	Tibia_SOS	-0,02781	0,055191	NA
3	abundance	X06016	N,N-Dimet	Handgrip	0,064673	0,062073	NA
3	abundance	X06034	N-[(2S)-2-I	Tibia_length	0,111917	0,052607	NA
3	abundance	X06034	N-[(2S)-2-I	Radius_length	0,042594	0,071641	NA
3	abundance	X06034	N-[(2S)-2-I	Radius_SOS	-0,02349	0,050648	NA
3	abundance	X06034	N-[(2S)-2-I	Tibia_SOS	0,18852	0,053311	NA
3	abundance	X06034	N-[(2S)-2-I	Handgrip	0,010699	0,061141	NA
3	abundance	X06039	gamma-Gl	Tibia_length	-0,01647	0,053378	NA
3	abundance	X06039	gamma-Gl	Radius_length	0,189705	0,070965	NA
3	abundance	X06039	gamma-Gl	Radius_SOS	0,0897	0,050849	NA
3	abundance	X06039	gamma-Gl	Tibia_SOS	0,161061	0,053984	NA
3	abundance	X06039	gamma-Gl	Handgrip	0,095695	0,062868	NA
3	abundance	X06041	(DL)-3-O-N	Tibia_length	-0,10502	0,052675	NA
3	abundance	X06041	(DL)-3-O-N	Radius_length	-0,10188	0,071675	NA
3	abundance	X06041	(DL)-3-O-N	Radius_SOS	-0,05796	0,050476	NA
3	abundance	X06041	(DL)-3-O-N	Tibia_SOS	-0,11443	0,054396	NA
3	abundance	X06041	(DL)-3-O-N	Handgrip	-0,13361	0,061644	NA
3	abundance	X06043	vinyl sulfid	Tibia_length	0,090124	0,051078	NA
3	abundance	X06043	vinyl sulfid	Radius_length	0,167208	0,068944	NA
3	abundance	X06043	vinyl sulfid	Radius_SOS	0,02503	0,049449	NA

3	abundance	X06043	vinyl sulfid	Tibia_SOS	0,132958	0,052524	NA
3	abundance	X06043	vinyl sulfid	Handgrip	0,105814	0,060213	NA
3	abundance	X06046	Valylvaline	Tibia_length	0,078074	0,052892	NA
3	abundance	X06046	Valylvaline	Radius_length	0,191752	0,070619	NA
3	abundance	X06046	Valylvaline	Radius_SOS	0,05588	0,050393	NA
3	abundance	X06046	Valylvaline	Tibia_SOS	0,14771	0,053614	NA
3	abundance	X06046	Valylvaline	Handgrip	0,090151	0,061721	NA
3	abundance	X06062	1-(2,3-Dihy	Tibia_length	-0,05173	0,052052	NA
3	abundance	X06062	1-(2,3-Dihy	Radius_length	-0,10325	0,070673	NA
3	abundance	X06062	1-(2,3-Dihy	Radius_SOS	-0,07852	0,0497	NA
3	abundance	X06062	1-(2,3-Dihy	Tibia_SOS	0,093025	0,052933	NA
3	abundance	X06062	1-(2,3-Dihy	Handgrip	-0,11887	0,060219	NA
1	abundance	X06079	Glycylleuci	Tibia_length	-0,07355	0,054723	NA
1	abundance	X06079	Glycylleuci	Radius_length	-0,02101	0,074062	NA
1	abundance	X06079	Glycylleuci	Radius_SOS	-0,01957	0,052214	NA
1	abundance	X06079	Glycylleuci	Tibia_SOS	-0,02076	0,056536	NA
1	abundance	X06079	Glycylleuci	Handgrip	0,014957	0,061489	NA
3	abundance	X06082	O-heptano	Tibia_length	-0,04609	0,055592	NA
3	abundance	X06082	O-heptano	Radius_length	-0,10724	0,073722	NA
3	abundance	X06082	O-heptano	Radius_SOS	-0,05777	0,052667	NA
3	abundance	X06082	O-heptano	Tibia_SOS	-0,12541	0,05739	NA
3	abundance	X06082	O-heptano	Handgrip	-0,18729	0,061113	NA
3	abundance	X06085	Tetraacety	Tibia_length	-0,10727	0,051204	NA
3	abundance	X06085	Tetraacety	Radius_length	-0,09219	0,069936	NA
3	abundance	X06085	Tetraacety	Radius_SOS	-0,02889	0,049542	NA
3	abundance	X06085	Tetraacety	Tibia_SOS	-0,10632	0,052846	NA
3	abundance	X06085	Tetraacety	Handgrip	-0,12067	0,060354	NA
3	abundance	X06089	riboprine	Tibia_length	-0,06428	0,055545	NA
3	abundance	X06089	riboprine	Radius_length	0,005182	0,074244	NA
3	abundance	X06089	riboprine	Radius_SOS	-0,05389	0,053116	NA
3	abundance	X06089	riboprine	Tibia_SOS	-0,01535	0,056275	NA
3	abundance	X06089	riboprine	Handgrip	0,111784	0,062566	NA
3	abundance	X06100	Midodrine	Tibia_length	-0,18109	0,052353	NA
3	abundance	X06100	Midodrine	Radius_length	-0,10907	0,071532	NA
3	abundance	X06100	Midodrine	Radius_SOS	-0,07342	0,050503	NA
3	abundance	X06100	Midodrine	Tibia_SOS	-0,04055	0,054098	NA
3	abundance	X06100	Midodrine	Handgrip	0,063902	0,061533	NA
3	abundance	X06107	Hydroxycar	Tibia_length	0,068185	0,049585	NA
3	abundance	X06107	Hydroxycar	Radius_length	0,097444	0,067284	NA
3	abundance	X06107	Hydroxycar	Radius_SOS	0,012584	0,049026	NA
3	abundance	X06107	Hydroxycar	Tibia_SOS	0,069971	0,052382	NA
3	abundance	X06107	Hydroxycar	Handgrip	0,080614	0,060191	NA
3	abundance	X06112	Val-Ser	Tibia_length	0,071109	0,049405	NA
3	abundance	X06112	Val-Ser	Radius_length	0,074063	0,067011	NA
3	abundance	X06112	Val-Ser	Radius_SOS	-0,05864	0,048945	NA
3	abundance	X06112	Val-Ser	Tibia_SOS	-0,03476	0,052615	NA
3	abundance	X06112	Val-Ser	Handgrip	-0,11045	0,06001	NA
3	abundance	X06124	MFCD0995	Tibia_length	-0,07456	0,055394	NA

	3	abundance	X06124	MFCD0995	Radius_length	0,009873	0,073736	NA
	3	abundance	X06124	MFCD0995	Radius_SOS	0,004475	0,052676	NA
	3	abundance	X06124	MFCD0995	Tibia_SOS	0,011462	0,055978	NA
	3	abundance	X06124	MFCD0995	Handgrip	-0,08015	0,062837	NA
	3	abundance	X06127	2-Methoxy-	Tibia_length	-0,09205	0,052677	NA
	3	abundance	X06127	2-Methoxy-	Radius_length	-0,00739	0,071836	NA
	3	abundance	X06127	2-Methoxy-	Radius_SOS	-0,03529	0,050591	NA
	3	abundance	X06127	2-Methoxy-	Tibia_SOS	0,028723	0,054076	NA
	3	abundance	X06127	2-Methoxy-	Handgrip	0,003804	0,0612	NA
2a		abundance	X06143	2-Methylbu	Tibia_length	-0,00549	0,056193	NA
2a		abundance	X06143	2-Methylbu	Radius_length	0,068655	0,07411	NA
2a		abundance	X06143	2-Methylbu	Radius_SOS	0,006015	0,05344	NA
2a		abundance	X06143	2-Methylbu	Tibia_SOS	0,005753	0,056855	NA
2a		abundance	X06143	2-Methylbu	Handgrip	-0,00905	0,062658	NA
	3	abundance	X06146	S-Methyl-1	Tibia_length	0,038654	0,052256	NA
	3	abundance	X06146	S-Methyl-1	Radius_length	-0,00899	0,071276	NA
	3	abundance	X06146	S-Methyl-1	Radius_SOS	0,008988	0,050235	NA
	3	abundance	X06146	S-Methyl-1	Tibia_SOS	0,056029	0,053634	NA
	3	abundance	X06146	S-Methyl-1	Handgrip	-0,14453	0,059999	NA
	3	abundance	X06149	Yangonin	Tibia_length	-0,02286	0,052602	NA
	3	abundance	X06149	Yangonin	Radius_length	0,04252	0,071791	NA
	3	abundance	X06149	Yangonin	Radius_SOS	0,006035	0,050496	NA
	3	abundance	X06149	Yangonin	Tibia_SOS	0,014138	0,053721	NA
	3	abundance	X06149	Yangonin	Handgrip	-0,03491	0,062215	NA
	3	abundance	X06150	Zalcitabine	Tibia_length	0,012754	0,052309	NA
	3	abundance	X06150	Zalcitabine	Radius_length	-5,97E-04	0,071603	NA
	3	abundance	X06150	Zalcitabine	Radius_SOS	0,012733	0,050238	NA
	3	abundance	X06150	Zalcitabine	Tibia_SOS	0,097833	0,05358	NA
	3	abundance	X06150	Zalcitabine	Handgrip	0,03959	0,061783	NA
	3	abundance	X06152	alpha-keto	Tibia_length	-0,05837	0,055568	NA
	3	abundance	X06152	alpha-keto	Radius_length	0,021281	0,074414	NA
	3	abundance	X06152	alpha-keto	Radius_SOS	0,062542	0,052552	NA
	3	abundance	X06152	alpha-keto	Tibia_SOS	0,057128	0,057517	NA
	3	abundance	X06152	alpha-keto	Handgrip	0,067046	0,062086	NA
2a		abundance	X06167	N-Acetylas	Tibia_length	-0,07438	0,054151	NA
2a		abundance	X06167	N-Acetylas	Radius_length	0,015486	0,07353	NA
2a		abundance	X06167	N-Acetylas	Radius_SOS	-0,05619	0,051826	NA
2a		abundance	X06167	N-Acetylas	Tibia_SOS	0,021957	0,056566	NA
2a		abundance	X06167	N-Acetylas	Handgrip	-0,02495	0,061848	NA
	3	abundance	X06168	leu-gln	Tibia_length	-0,04108	0,053138	NA
	3	abundance	X06168	leu-gln	Radius_length	0,031587	0,072296	NA
	3	abundance	X06168	leu-gln	Radius_SOS	0,002424	0,050823	NA
	3	abundance	X06168	leu-gln	Tibia_SOS	-0,02657	0,054585	NA
	3	abundance	X06168	leu-gln	Handgrip	0,001045	0,061112	NA
	3	abundance	X06183	Nisinic aci	Tibia_length	0,014739	0,049657	NA
	3	abundance	X06183	Nisinic aci	Radius_length	0,082715	0,066952	NA
	3	abundance	X06183	Nisinic aci	Radius_SOS	0,029689	0,049007	NA
	3	abundance	X06183	Nisinic aci	Tibia_SOS	0,025946	0,052483	NA

3	abundance	X06183	Nisinic aci	Handgrip	0,041522	0,060616	NA
3	abundance	X06189	delta-Guar	Tibia_length	-0,06835	0,054262	NA
3	abundance	X06189	delta-Guar	Radius_length	-0,0291	0,07283	NA
3	abundance	X06189	delta-Guar	Radius_SOS	0,010987	0,051822	NA
3	abundance	X06189	delta-Guar	Tibia_SOS	0,006866	0,055908	NA
3	abundance	X06189	delta-Guar	Handgrip	-0,02197	0,062821	NA
3	abundance	X06197	4-(Nitroso	Tibia_length	-0,00241	0,05692	NA
3	abundance	X06197	4-(Nitroso	Radius_length	0,064446	0,07499	NA
3	abundance	X06197	4-(Nitroso	Radius_SOS	0,054649	0,054029	NA
3	abundance	X06197	4-(Nitroso	Tibia_SOS	-0,12386	0,056175	NA
3	abundance	X06197	4-(Nitroso	Handgrip	0,06178	0,061815	NA
3	abundance	X06220	Tetrahydro	Tibia_length	-0,08561	0,053618	NA
3	abundance	X06220	Tetrahydro	Radius_length	-0,09851	0,072373	NA
3	abundance	X06220	Tetrahydro	Radius_SOS	0,014926	0,051222	NA
3	abundance	X06220	Tetrahydro	Tibia_SOS	-0,11757	0,054559	NA
3	abundance	X06220	Tetrahydro	Handgrip	-0,02403	0,061508	NA
3	abundance	X06222	Leucylaspa	Tibia_length	-0,08619	0,05391	NA
3	abundance	X06222	Leucylaspa	Radius_length	-0,01682	0,073067	NA
3	abundance	X06222	Leucylaspa	Radius_SOS	0,009799	0,051495	NA
3	abundance	X06222	Leucylaspa	Tibia_SOS	-0,02822	0,055069	NA
3	abundance	X06222	Leucylaspa	Handgrip	0,054702	0,061129	NA
3	abundance	X06227	lys-tyr	Tibia_length	-0,13701	0,054863	NA
3	abundance	X06227	lys-tyr	Radius_length	-0,11013	0,07343	NA
3	abundance	X06227	lys-tyr	Radius_SOS	-0,04874	0,052234	NA
3	abundance	X06227	lys-tyr	Tibia_SOS	-0,0691	0,055167	NA
3	abundance	X06227	lys-tyr	Handgrip	-0,05089	0,060394	NA
3	abundance	X06230	Glu-Gly	Tibia_length	-0,10444	0,053131	NA
3	abundance	X06230	Glu-Gly	Radius_length	-0,01436	0,072573	NA
3	abundance	X06230	Glu-Gly	Radius_SOS	-0,06942	0,051276	NA
3	abundance	X06230	Glu-Gly	Tibia_SOS	-0,05161	0,055899	NA
3	abundance	X06230	Glu-Gly	Handgrip	-0,00399	0,062985	NA
3	abundance	X06240	His-pro	Tibia_length	0,00527	0,051744	NA
3	abundance	X06240	His-pro	Radius_length	0,014607	0,07088	NA
3	abundance	X06240	His-pro	Radius_SOS	-0,00381	0,049859	NA
3	abundance	X06240	His-pro	Tibia_SOS	0,060949	0,053832	NA
3	abundance	X06240	His-pro	Handgrip	0,061535	0,060792	NA
2b	abundance	X06250	[3-({3-[(Cy	Tibia_length	-0,13802	0,054848	NA
2b	abundance	X06250	[3-({3-[(Cy	Radius_length	-0,32189	0,069814	NA
2b	abundance	X06250	[3-({3-[(Cy	Radius_SOS	-0,14715	0,051927	NA
2b	abundance	X06250	[3-({3-[(Cy	Tibia_SOS	-0,18609	0,054707	NA
2b	abundance	X06250	[3-({3-[(Cy	Handgrip	-0,20076	0,061585	NA
3	abundance	X06253	N-(4-Amin	Tibia_length	-0,06751	0,054503	NA
3	abundance	X06253	N-(4-Amin	Radius_length	-0,11649	0,072572	NA
3	abundance	X06253	N-(4-Amin	Radius_SOS	-0,06298	0,051875	NA
3	abundance	X06253	N-(4-Amin	Tibia_SOS	-0,02607	0,056193	NA
3	abundance	X06253	N-(4-Amin	Handgrip	-0,07082	0,062275	NA
3	abundance	X06254	D-Alanyl-D	Tibia_length	-0,05277	0,055473	NA
3	abundance	X06254	D-Alanyl-D	Radius_length	-0,06977	0,074101	NA

3	abundance	X06254	D-Alanyl-D	Radius_SOS	-0,05579	0,053028	NA
3	abundance	X06254	D-Alanyl-D	Tibia_SOS	-0,01378	0,056824	NA
3	abundance	X06254	D-Alanyl-D	Handgrip	0,035791	0,063468	NA
3	abundance	X06259	(S)-?-glyce	Tibia_length	0,019149	0,055032	NA
3	abundance	X06259	(S)-?-glyce	Radius_length	0,026943	0,073814	NA
3	abundance	X06259	(S)-?-glyce	Radius_SOS	0,043583	0,052337	NA
3	abundance	X06259	(S)-?-glyce	Tibia_SOS	0,052428	0,055705	NA
3	abundance	X06259	(S)-?-glyce	Handgrip	0,061106	0,06206	NA
3	abundance	X06268	mesifurane	Tibia_length	0,011122	0,053762	NA
3	abundance	X06268	mesifurane	Radius_length	-0,01353	0,07281	NA
3	abundance	X06268	mesifurane	Radius_SOS	0,007958	0,051354	NA
3	abundance	X06268	mesifurane	Tibia_SOS	-0,01818	0,055859	NA
3	abundance	X06268	mesifurane	Handgrip	0,034033	0,063405	NA
2b	abundance	X06270	9-Methylur	Tibia_length	-0,02417	0,054684	NA
2b	abundance	X06270	9-Methylur	Radius_length	-0,1069	0,073237	NA
2b	abundance	X06270	9-Methylur	Radius_SOS	-0,03399	0,051879	NA
2b	abundance	X06270	9-Methylur	Tibia_SOS	-0,09685	0,055496	NA
2b	abundance	X06270	9-Methylur	Handgrip	-0,13781	0,061911	NA
3	abundance	X06276	asn-pro	Tibia_length	-0,04759	0,056281	NA
3	abundance	X06276	asn-pro	Radius_length	-0,06384	0,074691	NA
3	abundance	X06276	asn-pro	Radius_SOS	-0,06485	0,053375	NA
3	abundance	X06276	asn-pro	Tibia_SOS	-0,11471	0,054685	NA
3	abundance	X06276	asn-pro	Handgrip	-0,14474	0,061113	NA
3	abundance	X06278	3-Hydroxy-	Tibia_length	0,005425	0,049926	NA
3	abundance	X06278	3-Hydroxy-	Radius_length	0,059115	0,067989	NA
3	abundance	X06278	3-Hydroxy-	Radius_SOS	-0,0423	0,049033	NA
3	abundance	X06278	3-Hydroxy-	Tibia_SOS	0,002828	0,05259	NA
3	abundance	X06278	3-Hydroxy-	Handgrip	0,011361	0,060323	NA
3	abundance	X06289	Nicotinatel	Tibia_length	0,034658	0,053987	NA
3	abundance	X06289	Nicotinatel	Radius_length	-0,00477	0,073421	NA
3	abundance	X06289	Nicotinatel	Radius_SOS	0,052567	0,051573	NA
3	abundance	X06289	Nicotinatel	Tibia_SOS	-0,01355	0,055268	NA
3	abundance	X06289	Nicotinatel	Handgrip	0,076065	0,063531	NA
2b	abundance	X06291	Corticoste	Tibia_length	0,019422	0,050012	NA
2b	abundance	X06291	Corticoste	Radius_length	0,062755	0,068044	NA
2b	abundance	X06291	Corticoste	Radius_SOS	-0,02968	0,049042	NA
2b	abundance	X06291	Corticoste	Tibia_SOS	0,050399	0,052422	NA
2b	abundance	X06291	Corticoste	Handgrip	0,062533	0,0601	NA
3	abundance	X06292	mesifurane	Tibia_length	0,005974	0,057711	NA
3	abundance	X06292	mesifurane	Radius_length	0,020427	0,075574	NA
3	abundance	X06292	mesifurane	Radius_SOS	-0,02763	0,054885	NA
3	abundance	X06292	mesifurane	Tibia_SOS	-0,02393	0,059902	NA
3	abundance	X06292	mesifurane	Handgrip	0,026939	0,060171	NA
3	abundance	X06293	(4R)-4-{[3-	Tibia_length	-0,09859	0,053438	NA
3	abundance	X06293	(4R)-4-{[3-	Radius_length	-0,11437	0,071998	NA
3	abundance	X06293	(4R)-4-{[3-	Radius_SOS	-0,02794	0,051071	NA
3	abundance	X06293	(4R)-4-{[3-	Tibia_SOS	-0,07517	0,053638	NA
3	abundance	X06293	(4R)-4-{[3-	Handgrip	-0,02393	0,061153	NA

2b	abundance	X06313	Isoquinolir	Tibia_length	0,044929	0,054168	NA
2b	abundance	X06313	Isoquinolir	Radius_length	-0,08393	0,072726	NA
2b	abundance	X06313	Isoquinolir	Radius_SOS	-0,13437	0,051183	NA
2b	abundance	X06313	Isoquinolir	Tibia_SOS	0,057328	0,054729	NA
2b	abundance	X06313	Isoquinolir	Handgrip	-0,01431	0,06249	NA
2b	abundance	X06318	Propamoca	Tibia_length	0,060736	0,053625	NA
2b	abundance	X06318	Propamoca	Radius_length	0,072961	0,072505	NA
2b	abundance	X06318	Propamoca	Radius_SOS	0,023715	0,051363	NA
2b	abundance	X06318	Propamoca	Tibia_SOS	-0,0522	0,054825	NA
2b	abundance	X06318	Propamoca	Handgrip	0,019658	0,06241	NA
3	abundance	X06320	7alpha-Hy	Tibia_length	0,040547	0,051857	NA
3	abundance	X06320	7alpha-Hy	Radius_length	-0,00957	0,070781	NA
3	abundance	X06320	7alpha-Hy	Radius_SOS	0,020558	0,049928	NA
3	abundance	X06320	7alpha-Hy	Tibia_SOS	-0,07736	0,053788	NA
3	abundance	X06320	7alpha-Hy	Handgrip	-0,02516	0,061657	NA
3	abundance	X06329	Leu-Leu	Tibia_length	-0,07605	0,055098	NA
3	abundance	X06329	Leu-Leu	Radius_length	-0,05216	0,073348	NA
3	abundance	X06329	Leu-Leu	Radius_SOS	-0,02566	0,052379	NA
3	abundance	X06329	Leu-Leu	Tibia_SOS	-0,05281	0,056859	NA
3	abundance	X06329	Leu-Leu	Handgrip	-0,04359	0,062131	NA
3	abundance	X06334	Bis-D-fruct	Tibia_length	-0,0262	0,052361	NA
3	abundance	X06334	Bis-D-fruct	Radius_length	-0,01936	0,071783	NA
3	abundance	X06334	Bis-D-fruct	Radius_SOS	-0,03009	0,050236	NA
3	abundance	X06334	Bis-D-fruct	Tibia_SOS	0,072936	0,053501	NA
3	abundance	X06334	Bis-D-fruct	Handgrip	-0,02648	0,062344	NA
3	abundance	X06337	Zalcitabine	Tibia_length	-0,05029	0,053576	NA
3	abundance	X06337	Zalcitabine	Radius_length	-0,01442	0,072772	NA
3	abundance	X06337	Zalcitabine	Radius_SOS	0,006677	0,051127	NA
3	abundance	X06337	Zalcitabine	Tibia_SOS	-0,08015	0,053419	NA
3	abundance	X06337	Zalcitabine	Handgrip	-0,08022	0,062384	NA
2b	abundance	X06354	trans-Zeati	Tibia_length	-0,11675	0,056285	NA
2b	abundance	X06354	trans-Zeati	Radius_length	-0,12675	0,073327	NA
2b	abundance	X06354	trans-Zeati	Radius_SOS	-0,04904	0,052882	NA
2b	abundance	X06354	trans-Zeati	Tibia_SOS	-0,01896	0,056078	NA
2b	abundance	X06354	trans-Zeati	Handgrip	0,00192	0,062505	NA
3	abundance	X06361	Oxprenolol	Tibia_length	0,191295	0,049028	NA
3	abundance	X06361	Oxprenolol	Radius_length	0,200661	0,066894	NA
3	abundance	X06361	Oxprenolol	Radius_SOS	0,065064	0,048957	NA
3	abundance	X06361	Oxprenolol	Tibia_SOS	0,117601	0,052263	NA
3	abundance	X06361	Oxprenolol	Handgrip	0,107707	0,059958	NA
3	abundance	X06368	TDP-2	Tibia_length	0,053941	0,050864	NA
3	abundance	X06368	TDP-2	Radius_length	0,05697	0,069687	NA
3	abundance	X06368	TDP-2	Radius_SOS	0,019009	0,04945	NA
3	abundance	X06368	TDP-2	Tibia_SOS	0,092788	0,052489	NA
3	abundance	X06368	TDP-2	Handgrip	-0,03304	0,060332	NA
3	abundance	X06371	L-gamma-(	Tibia_length	-0,09136	0,055851	NA
3	abundance	X06371	L-gamma-(	Radius_length	-0,06769	0,074259	NA
3	abundance	X06371	L-gamma-(	Radius_SOS	-0,00788	0,053082	NA

	3	abundance	X06371	L-gamma-(	Tibia_SOS	0,007457	0,057575	NA
	3	abundance	X06371	L-gamma-(	Handgrip	-0,02258	0,062355	NA
2b		abundance	X06372	N,N-dimetl	Tibia_length	0,072157	0,051159	NA
2b		abundance	X06372	N,N-dimetl	Radius_length	0,109093	0,069705	NA
2b		abundance	X06372	N,N-dimetl	Radius_SOS	-0,01136	0,049548	NA
2b		abundance	X06372	N,N-dimetl	Tibia_SOS	0,127747	0,052424	NA
2b		abundance	X06372	N,N-dimetl	Handgrip	0,080396	0,060963	NA
	3	abundance	X06381	MFCD1869	Tibia_length	-0,18008	0,04817	NA
	3	abundance	X06381	MFCD1869	Radius_length	-0,16987	0,048259	NA
	3	abundance	X06381	MFCD1869	Radius_SOS	-0,01823	0,049021	NA
	3	abundance	X06381	MFCD1869	Tibia_SOS	-0,09552	0,052246	NA
	3	abundance	X06381	MFCD1869	Handgrip	-0,08537	0,059973	NA
	1	abundance	X06388	Caffeic aci	Tibia_length	-0,13063	0,05366	NA
	1	abundance	X06388	Caffeic aci	Radius_length	-0,10431	0,072493	NA
	1	abundance	X06388	Caffeic aci	Radius_SOS	-0,11649	0,051073	NA
	1	abundance	X06388	Caffeic aci	Tibia_SOS	-0,1475	0,054394	NA
	1	abundance	X06388	Caffeic aci	Handgrip	0,036944	0,061844	NA
	3	abundance	X06400	Phloionolic	Tibia_length	0,049637	0,049977	NA
	3	abundance	X06400	Phloionolic	Radius_length	-0,01737	0,068068	NA
	3	abundance	X06400	Phloionolic	Radius_SOS	-0,03446	0,049047	NA
	3	abundance	X06400	Phloionolic	Tibia_SOS	-0,0578	0,052557	NA
	3	abundance	X06400	Phloionolic	Handgrip	-0,04204	0,06074	NA
2b		abundance	X06401	Isophthalic	Tibia_length	-0,15365	0,050098	NA
2b		abundance	X06401	Isophthalic	Radius_length	-0,12551	0,068797	NA
2b		abundance	X06401	Isophthalic	Radius_SOS	-0,01582	0,049248	NA
2b		abundance	X06401	Isophthalic	Tibia_SOS	-0,08404	0,052416	NA
2b		abundance	X06401	Isophthalic	Handgrip	-0,11225	0,059921	NA
	3	abundance	X06404	Flemichap	Tibia_length	0,024902	0,053044	NA
	3	abundance	X06404	Flemichap	Radius_length	0,035236	0,071741	NA
	3	abundance	X06404	Flemichap	Radius_SOS	0,030225	0,050727	NA
	3	abundance	X06404	Flemichap	Tibia_SOS	0,066691	0,05493	NA
	3	abundance	X06404	Flemichap	Handgrip	-0,04765	0,060866	NA
	3	abundance	X06405	Asparaginy	Tibia_length	-0,07442	0,052787	NA
	3	abundance	X06405	Asparaginy	Radius_length	-0,07572	0,071946	NA
	3	abundance	X06405	Asparaginy	Radius_SOS	-0,04239	0,050503	NA
	3	abundance	X06405	Asparaginy	Tibia_SOS	-0,07288	0,053132	NA
	3	abundance	X06405	Asparaginy	Handgrip	-0,07977	0,061049	NA
2b		abundance	X06409	Glycylproli	Tibia_length	0,069406	0,052731	NA
2b		abundance	X06409	Glycylproli	Radius_length	0,009603	0,071587	NA
2b		abundance	X06409	Glycylproli	Radius_SOS	-0,03709	0,050466	NA
2b		abundance	X06409	Glycylproli	Tibia_SOS	0,10099	0,055239	NA
2b		abundance	X06409	Glycylproli	Handgrip	0,07444	0,061937	NA
	3	abundance	X06412	Butenylcar	Tibia_length	0,087623	0,056446	NA
	3	abundance	X06412	Butenylcar	Radius_length	0,049298	0,074404	NA
	3	abundance	X06412	Butenylcar	Radius_SOS	-0,03367	0,053716	NA
	3	abundance	X06412	Butenylcar	Tibia_SOS	0,06437	0,056819	NA
	3	abundance	X06412	Butenylcar	Handgrip	-0,07966	0,062488	NA
	3	abundance	X06416	KYNURAMI	Tibia_length	0,118456	0,049635	NA

	3	abundance	X06416	KYNURAMI	Radius_length	0,097884	0,067721	NA
	3	abundance	X06416	KYNURAMI	Radius_SOS	0,022409	0,049064	NA
	3	abundance	X06416	KYNURAMI	Tibia_SOS	0,093638	0,052329	NA
	3	abundance	X06416	KYNURAMI	Handgrip	0,039492	0,060271	NA
	3	abundance	X06419	Spermic ac	Tibia_length	-0,07428	0,054135	NA
	3	abundance	X06419	Spermic ac	Radius_length	0,067321	0,073066	NA
	3	abundance	X06419	Spermic ac	Radius_SOS	-0,01975	0,051716	NA
	3	abundance	X06419	Spermic ac	Tibia_SOS	-0,01119	0,056219	NA
	3	abundance	X06419	Spermic ac	Handgrip	0,019181	0,062404	NA
2b		abundance	X06433	4-(2,5-Diflu	Tibia_length	-0,07759	0,050959	NA
2b		abundance	X06433	4-(2,5-Diflu	Radius_length	-0,0638	0,070049	NA
2b		abundance	X06433	4-(2,5-Diflu	Radius_SOS	-0,00424	0,049496	NA
2b		abundance	X06433	4-(2,5-Diflu	Tibia_SOS	0,082756	0,052709	NA
2b		abundance	X06433	4-(2,5-Diflu	Handgrip	0,002398	0,061637	NA
	3	abundance	X06434	butyrin	Tibia_length	0,058961	0,051606	NA
	3	abundance	X06434	butyrin	Radius_length	0,058292	0,070468	NA
	3	abundance	X06434	butyrin	Radius_SOS	0,055945	0,049651	NA
	3	abundance	X06434	butyrin	Tibia_SOS	0,021276	0,053463	NA
	3	abundance	X06434	butyrin	Handgrip	-0,01428	0,061079	NA
	3	abundance	X06437	2-Acetami	Tibia_length	0,091956	0,055857	NA
	3	abundance	X06437	2-Acetami	Radius_length	0,086354	0,074512	NA
	3	abundance	X06437	2-Acetami	Radius_SOS	-0,05022	0,053428	NA
	3	abundance	X06437	2-Acetami	Tibia_SOS	0,001714	0,057231	NA
	3	abundance	X06437	2-Acetami	Handgrip	0,084135	0,063306	NA
2b		abundance	X06448	3,8,9-trihy	Tibia_length	-0,02774	0,054296	NA
2b		abundance	X06448	3,8,9-trihy	Radius_length	0,060451	0,073067	NA
2b		abundance	X06448	3,8,9-trihy	Radius_SOS	0,062404	0,051755	NA
2b		abundance	X06448	3,8,9-trihy	Tibia_SOS	-0,02379	0,055143	NA
2b		abundance	X06448	3,8,9-trihy	Handgrip	-0,09117	0,061803	NA
	3	abundance	X06454	8-Amino-7-	Tibia_length	-0,1283	0,05255	NA
	3	abundance	X06454	8-Amino-7-	Radius_length	0,010186	0,071921	NA
	3	abundance	X06454	8-Amino-7-	Radius_SOS	0,013555	0,050669	NA
	3	abundance	X06454	8-Amino-7-	Tibia_SOS	0,001984	0,054159	NA
	3	abundance	X06454	8-Amino-7-	Handgrip	-0,10008	0,061226	NA
	3	abundance	X06455	4-Hydroxyp	Tibia_length	0,041174	0,054336	NA
	3	abundance	X06455	4-Hydroxyp	Radius_length	0,077081	0,072998	NA
	3	abundance	X06455	4-Hydroxyp	Radius_SOS	0,045603	0,05182	NA
	3	abundance	X06455	4-Hydroxyp	Tibia_SOS	-0,07963	0,057052	NA
	3	abundance	X06455	4-Hydroxyp	Handgrip	0,013532	0,061714	NA
	3	abundance	X06462	DNOP	Tibia_length	0,036803	0,050273	NA
	3	abundance	X06462	DNOP	Radius_length	0,104732	0,06828	NA
	3	abundance	X06462	DNOP	Radius_SOS	0,058215	0,049047	NA
	3	abundance	X06462	DNOP	Tibia_SOS	-0,04316	0,052729	NA
	3	abundance	X06462	DNOP	Handgrip	-1,87E-04	0,060881	NA
	3	abundance	X06463	8-Amino-7-	Tibia_length	-0,1102	0,059354	NA
	3	abundance	X06463	8-Amino-7-	Radius_length	-0,11097	0,075516	NA
	3	abundance	X06463	8-Amino-7-	Radius_SOS	-0,05053	0,056734	NA
	3	abundance	X06463	8-Amino-7-	Tibia_SOS	-0,00459	0,052486	NA

3	abundance	X06463	8-Amino-7-	Handgrip	0,01064	0,06019	NA
3	abundance	X06472	N(alpha)-B	Tibia_length	0,095061	0,053743	NA
3	abundance	X06472	N(alpha)-B	Radius_length	0,097071	0,072773	NA
3	abundance	X06472	N(alpha)-B	Radius_SOS	0,095307	0,051346	NA
3	abundance	X06472	N(alpha)-B	Tibia_SOS	0,088685	0,055244	NA
3	abundance	X06472	N(alpha)-B	Handgrip	0,054292	0,061755	NA
3	abundance	X06473	MFCD0002	Tibia_length	0,112181	0,055165	NA
3	abundance	X06473	MFCD0002	Radius_length	0,125161	0,073269	NA
3	abundance	X06473	MFCD0002	Radius_SOS	0,080069	0,052333	NA
3	abundance	X06473	MFCD0002	Tibia_SOS	0,090441	0,055366	NA
3	abundance	X06473	MFCD0002	Handgrip	0,01004	0,062499	NA
3	abundance	X06477	APM	Tibia_length	-0,0017	0,056695	NA
3	abundance	X06477	APM	Radius_length	0,228024	0,07275	NA
3	abundance	X06477	APM	Radius_SOS	0,021026	0,053717	NA
3	abundance	X06477	APM	Tibia_SOS	0,025153	0,055685	NA
3	abundance	X06477	APM	Handgrip	0,003888	0,062059	NA
3	abundance	X06478	Caffeic aci	Tibia_length	-0,04645	0,056036	NA
3	abundance	X06478	Caffeic aci	Radius_length	-0,123	0,073477	NA
3	abundance	X06478	Caffeic aci	Radius_SOS	-0,06974	0,053168	NA
3	abundance	X06478	Caffeic aci	Tibia_SOS	-0,05996	0,056361	NA
3	abundance	X06478	Caffeic aci	Handgrip	-0,09454	0,062827	NA
3	abundance	X06482	glu-pro	Tibia_length	-0,06765	0,053292	NA
3	abundance	X06482	glu-pro	Radius_length	-0,13311	0,071572	NA
3	abundance	X06482	glu-pro	Radius_SOS	-0,08093	0,050744	NA
3	abundance	X06482	glu-pro	Tibia_SOS	-0,06234	0,054218	NA
3	abundance	X06482	glu-pro	Handgrip	-0,0848	0,061366	NA
3	abundance	X06486	Leu-Val	Tibia_length	-0,01324	0,053182	NA
3	abundance	X06486	Leu-Val	Radius_length	-0,07705	0,072042	NA
3	abundance	X06486	Leu-Val	Radius_SOS	0,021845	0,050773	NA
3	abundance	X06486	Leu-Val	Tibia_SOS	0,041334	0,055247	NA
3	abundance	X06486	Leu-Val	Handgrip	-0,01612	0,06248	NA
3	abundance	X06495	2-Acetami	Tibia_length	-0,0193	0,056134	NA
3	abundance	X06495	2-Acetami	Radius_length	1,89E-05	0,074378	NA
3	abundance	X06495	2-Acetami	Radius_SOS	0,035743	0,053637	NA
3	abundance	X06495	2-Acetami	Tibia_SOS	-0,04111	0,057275	NA
3	abundance	X06495	2-Acetami	Handgrip	-0,0788	0,062652	NA
3	abundance	X06502	MFCD2836	Tibia_length	-0,06256	0,051566	NA
3	abundance	X06502	MFCD2836	Radius_length	-0,07658	0,070584	NA
3	abundance	X06502	MFCD2836	Radius_SOS	-0,00964	0,049629	NA
3	abundance	X06502	MFCD2836	Tibia_SOS	-0,01539	0,053262	NA
3	abundance	X06502	MFCD2836	Handgrip	0,026111	0,061344	NA
3	abundance	X06506	L-Homocys	Tibia_length	0,055354	0,054099	NA
3	abundance	X06506	L-Homocys	Radius_length	0,072092	0,072551	NA
3	abundance	X06506	L-Homocys	Radius_SOS	0,063445	0,051474	NA
3	abundance	X06506	L-Homocys	Tibia_SOS	1,10E-04	0,055116	NA
3	abundance	X06506	L-Homocys	Handgrip	0,012217	0,063125	NA
3	abundance	X06507	Gly-Lys	Tibia_length	-0,00309	0,055844	NA
3	abundance	X06507	Gly-Lys	Radius_length	-0,02209	0,074097	NA

3	abundance	X06507	Gly-Lys	Radius_SOS	0,069145	0,052917	NA
3	abundance	X06507	Gly-Lys	Tibia_SOS	0,04169	0,057081	NA
3	abundance	X06507	Gly-Lys	Handgrip	0,039007	0,06356	NA
3	abundance	X06508	Asparaginy	Tibia_length	-0,08607	0,05509	NA
3	abundance	X06508	Asparaginy	Radius_length	-0,12188	0,073385	NA
3	abundance	X06508	Asparaginy	Radius_SOS	-0,03701	0,052438	NA
3	abundance	X06508	Asparaginy	Tibia_SOS	-0,08406	0,054066	NA
3	abundance	X06508	Asparaginy	Handgrip	-0,07456	0,061585	NA
2b	abundance	X06510	N-{4-[(2R,3	Tibia_length	-0,04655	0,053173	NA
2b	abundance	X06510	N-{4-[(2R,3	Radius_length	0,027708	0,072668	NA
2b	abundance	X06510	N-{4-[(2R,3	Radius_SOS	0,003778	0,050859	NA
2b	abundance	X06510	N-{4-[(2R,3	Tibia_SOS	0,015784	0,054478	NA
2b	abundance	X06510	N-{4-[(2R,3	Handgrip	0,095043	0,061003	NA
2a	abundance	X06529	Methanesu	Tibia_length	-0,00534	0,055554	NA
2a	abundance	X06529	Methanesu	Radius_length	0,070077	0,073366	NA
2a	abundance	X06529	Methanesu	Radius_SOS	0,03024	0,052799	NA
2a	abundance	X06529	Methanesu	Tibia_SOS	0,116933	0,055832	NA
2a	abundance	X06529	Methanesu	Handgrip	0,055134	0,06173	NA
2b	abundance	X06530	3-(1-hydro:	Tibia_length	-0,04252	0,051674	NA
2b	abundance	X06530	3-(1-hydro:	Radius_length	-0,06248	0,070604	NA
2b	abundance	X06530	3-(1-hydro:	Radius_SOS	-0,03073	0,049769	NA
2b	abundance	X06530	3-(1-hydro:	Tibia_SOS	-0,0177	0,053124	NA
2b	abundance	X06530	3-(1-hydro:	Handgrip	-0,0808	0,060237	NA
3	abundance	X06532	Leupeptin	Tibia_length	-6,36E-04	0,052485	NA
3	abundance	X06532	Leupeptin	Radius_length	0,030462	0,071392	NA
3	abundance	X06532	Leupeptin	Radius_SOS	-0,01795	0,050318	NA
3	abundance	X06532	Leupeptin	Tibia_SOS	-0,02827	0,054194	NA
3	abundance	X06532	Leupeptin	Handgrip	0,041097	0,063223	NA
2b	abundance	X06546	4-Acetami	Tibia_length	-0,07817	0,052361	NA
2b	abundance	X06546	4-Acetami	Radius_length	0,001242	0,071485	NA
2b	abundance	X06546	4-Acetami	Radius_SOS	-0,00304	0,050334	NA
2b	abundance	X06546	4-Acetami	Tibia_SOS	-0,00929	0,05352	NA
2b	abundance	X06546	4-Acetami	Handgrip	0,060077	0,061484	NA
3	abundance	X06549	Panthenol	Tibia_length	0,025992	0,056796	NA
3	abundance	X06549	Panthenol	Radius_length	0,058043	0,074702	NA
3	abundance	X06549	Panthenol	Radius_SOS	-0,00359	0,053964	NA
3	abundance	X06549	Panthenol	Tibia_SOS	-0,0039	0,056695	NA
3	abundance	X06549	Panthenol	Handgrip	0,01808	0,062824	NA
3	abundance	X06551	Methyl 1-hy	Tibia_length	-0,17448	0,053912	NA
3	abundance	X06551	Methyl 1-hy	Radius_length	-0,1325	0,072452	NA
3	abundance	X06551	Methyl 1-hy	Radius_SOS	-0,09407	0,051672	NA
3	abundance	X06551	Methyl 1-hy	Tibia_SOS	-0,14404	0,054813	NA
3	abundance	X06551	Methyl 1-hy	Handgrip	-0,12509	0,061957	NA
2b	abundance	X06560	2,6-Dimeth	Tibia_length	-0,13895	0,048762	NA
2b	abundance	X06560	2,6-Dimeth	Radius_length	-0,19891	0,064282	NA
2b	abundance	X06560	2,6-Dimeth	Radius_SOS	-0,04648	0,049121	NA
2b	abundance	X06560	2,6-Dimeth	Tibia_SOS	-0,07618	0,052666	NA
2b	abundance	X06560	2,6-Dimeth	Handgrip	-0,06395	0,060081	NA

3	abundance	X06590	3-hydroxy-	Tibia_length	-0,07176	0,056861	NA
3	abundance	X06590	3-hydroxy-	Radius_length	-0,09218	0,074156	NA
3	abundance	X06590	3-hydroxy-	Radius_SOS	-0,04382	0,054112	NA
3	abundance	X06590	3-hydroxy-	Tibia_SOS	0,003062	0,057719	NA
3	abundance	X06590	3-hydroxy-	Handgrip	-0,10993	0,063298	NA
3	abundance	X06600	MFCD0014	Tibia_length	0,034702	0,053978	NA
3	abundance	X06600	MFCD0014	Radius_length	-0,00478	0,073413	NA
3	abundance	X06600	MFCD0014	Radius_SOS	0,052592	0,051567	NA
3	abundance	X06600	MFCD0014	Tibia_SOS	-0,01328	0,055262	NA
3	abundance	X06600	MFCD0014	Handgrip	0,076002	0,063528	NA
3	abundance	X06606	Leu-Val	Tibia_length	0,083745	0,054077	NA
3	abundance	X06606	Leu-Val	Radius_length	0,037899	0,073488	NA
3	abundance	X06606	Leu-Val	Radius_SOS	0,093234	0,051888	NA
3	abundance	X06606	Leu-Val	Tibia_SOS	0,045525	0,055634	NA
3	abundance	X06606	Leu-Val	Handgrip	-0,10517	0,06315	NA
3	abundance	X06612	Spermic ac	Tibia_length	-0,11243	0,053635	NA
3	abundance	X06612	Spermic ac	Radius_length	0,040578	0,072905	NA
3	abundance	X06612	Spermic ac	Radius_SOS	-0,00546	0,051334	NA
3	abundance	X06612	Spermic ac	Tibia_SOS	-0,00334	0,055054	NA
3	abundance	X06612	Spermic ac	Handgrip	0,056369	0,060823	NA
3	abundance	X06622	Lys-Pro	Tibia_length	-0,01674	0,053387	NA
3	abundance	X06622	Lys-Pro	Radius_length	0,013556	0,072486	NA
3	abundance	X06622	Lys-Pro	Radius_SOS	-0,00924	0,051031	NA
3	abundance	X06622	Lys-Pro	Tibia_SOS	0,07917	0,054245	NA
3	abundance	X06622	Lys-Pro	Handgrip	0,089221	0,060697	NA
3	abundance	X06623	quinol sulf	Tibia_length	-0,09121	0,054187	NA
3	abundance	X06623	quinol sulf	Radius_length	-0,09329	0,073039	NA
3	abundance	X06623	quinol sulf	Radius_SOS	0,018626	0,051751	NA
3	abundance	X06623	quinol sulf	Tibia_SOS	-0,05295	0,05455	NA
3	abundance	X06623	quinol sulf	Handgrip	-0,03626	0,061874	NA
3	abundance	X06626	N-Butyryl-l	Tibia_length	0,083905	0,052171	NA
3	abundance	X06626	N-Butyryl-l	Radius_length	0,108349	0,070868	NA
3	abundance	X06626	N-Butyryl-l	Radius_SOS	0,026337	0,050151	NA
3	abundance	X06626	N-Butyryl-l	Tibia_SOS	0,117375	0,053354	NA
3	abundance	X06626	N-Butyryl-l	Handgrip	0,041711	0,060858	NA
3	abundance	X06630	4-(METHYL	Tibia_length	-0,02789	0,053959	NA
3	abundance	X06630	4-(METHYL	Radius_length	0,041762	0,072929	NA
3	abundance	X06630	4-(METHYL	Radius_SOS	0,043886	0,051434	NA
3	abundance	X06630	4-(METHYL	Tibia_SOS	0,091947	0,056172	NA
3	abundance	X06630	4-(METHYL	Handgrip	0,053172	0,063144	NA
2b	abundance	X06631	2-Isopropy	Tibia_length	-0,07738	0,054009	NA
2b	abundance	X06631	2-Isopropy	Radius_length	-0,09126	0,072521	NA
2b	abundance	X06631	2-Isopropy	Radius_SOS	-0,03017	0,051475	NA
2b	abundance	X06631	2-Isopropy	Tibia_SOS	0,002804	0,055211	NA
2b	abundance	X06631	2-Isopropy	Handgrip	-0,0028	0,0623	NA
3	abundance	X06634	Glycitein	Tibia_length	-0,00774	0,058922	NA
3	abundance	X06634	Glycitein	Radius_length	0,047477	0,075938	NA
3	abundance	X06634	Glycitein	Radius_SOS	-0,0787	0,056281	NA

3	abundance	X06634	Glycitein	Tibia_SOS	0,038897	0,05946	NA
3	abundance	X06634	Glycitein	Handgrip	0,103494	0,05987	NA
2a	abundance	X06642	Homocitru	Tibia_length	0,028947	0,049508	NA
2a	abundance	X06642	Homocitru	Radius_length	0,098153	0,066607	NA
2a	abundance	X06642	Homocitru	Radius_SOS	-0,03886	0,049004	NA
2a	abundance	X06642	Homocitru	Tibia_SOS	0,045314	0,052433	NA
2a	abundance	X06642	Homocitru	Handgrip	-0,00863	0,060382	NA
3	abundance	X06646	(S)-3-sulfo	Tibia_length	0,037842	0,058133	NA
3	abundance	X06646	(S)-3-sulfo	Radius_length	-0,11991	0,074671	NA
3	abundance	X06646	(S)-3-sulfo	Radius_SOS	-0,05032	0,055447	NA
3	abundance	X06646	(S)-3-sulfo	Tibia_SOS	0,011386	0,052483	NA
3	abundance	X06646	(S)-3-sulfo	Handgrip	-0,02312	0,060177	NA
3	abundance	X06655	Hydroxyca	Tibia_length	0,024806	0,052269	NA
3	abundance	X06655	Hydroxyca	Radius_length	0,01916	0,071731	NA
3	abundance	X06655	Hydroxyca	Radius_SOS	0,060133	0,050345	NA
3	abundance	X06655	Hydroxyca	Tibia_SOS	-0,01027	0,054244	NA
3	abundance	X06655	Hydroxyca	Handgrip	-0,08892	0,061912	NA
3	abundance	X06656	Valylvaline	Tibia_length	-0,0317	0,055095	NA
3	abundance	X06656	Valylvaline	Radius_length	-0,02478	0,073437	NA
3	abundance	X06656	Valylvaline	Radius_SOS	0,036989	0,052365	NA
3	abundance	X06656	Valylvaline	Tibia_SOS	-0,00816	0,056005	NA
3	abundance	X06656	Valylvaline	Handgrip	-0,05226	0,060111	NA
3	abundance	X06663	Leucyltyros	Tibia_length	-0,0709	0,051855	NA
3	abundance	X06663	Leucyltyros	Radius_length	0,010166	0,07104	NA
3	abundance	X06663	Leucyltyros	Radius_SOS	0,099586	0,049551	NA
3	abundance	X06663	Leucyltyros	Tibia_SOS	-0,05339	0,053558	NA
3	abundance	X06663	Leucyltyros	Handgrip	-0,01579	0,060849	NA
3	abundance	X06675	L-gamma-(	Tibia_length	0,036356	0,055886	NA
3	abundance	X06675	L-gamma-(	Radius_length	0,04521	0,074187	NA
3	abundance	X06675	L-gamma-(	Radius_SOS	0,101688	0,052696	NA
3	abundance	X06675	L-gamma-(	Tibia_SOS	3,96E-04	0,056606	NA
3	abundance	X06675	L-gamma-(	Handgrip	0,087877	0,062511	NA
2b	abundance	X06680	7-Hydroxy-	Tibia_length	-0,08058	0,053952	NA
2b	abundance	X06680	7-Hydroxy-	Radius_length	-0,14723	0,072008	NA
2b	abundance	X06680	7-Hydroxy-	Radius_SOS	0,034995	0,051514	NA
2b	abundance	X06680	7-Hydroxy-	Tibia_SOS	-0,06741	0,054174	NA
2b	abundance	X06680	7-Hydroxy-	Handgrip	-0,04877	0,061759	NA
3	abundance	X06681	Scymnol	Tibia_length	-0,06738	0,049478	NA
3	abundance	X06681	Scymnol	Radius_length	-0,10062	0,066608	NA
3	abundance	X06681	Scymnol	Radius_SOS	0,009776	0,049027	NA
3	abundance	X06681	Scymnol	Tibia_SOS	0,073858	0,052353	NA
3	abundance	X06681	Scymnol	Handgrip	-0,11106	0,060032	NA
3	abundance	X06684	Bromazine	Tibia_length	0,014308	0,057047	NA
3	abundance	X06684	Bromazine	Radius_length	0,100909	0,073986	NA
3	abundance	X06684	Bromazine	Radius_SOS	-0,01711	0,054296	NA
3	abundance	X06684	Bromazine	Tibia_SOS	0,109592	0,057288	NA
3	abundance	X06684	Bromazine	Handgrip	-0,01926	0,060182	NA
2b	abundance	X06690	4-Methylca	Tibia_length	-0,13185	0,056951	NA

2b	abundance	X06690	4-Methylca	Radius_length	-0,08969	0,075065	NA
2b	abundance	X06690	4-Methylca	Radius_SOS	-0,13296	0,054364	NA
2b	abundance	X06690	4-Methylca	Tibia_SOS	-0,06872	0,060499	NA
2b	abundance	X06690	4-Methylca	Handgrip	-0,01645	0,063435	NA
3	abundance	X06698	entecavir	Tibia_length	0,101358	0,053603	NA
3	abundance	X06698	entecavir	Radius_length	0,125455	0,073105	NA
3	abundance	X06698	entecavir	Radius_SOS	0,047574	0,051412	NA
3	abundance	X06698	entecavir	Tibia_SOS	0,146076	0,054626	NA
3	abundance	X06698	entecavir	Handgrip	0,103758	0,061238	NA
3	abundance	X06703	N6-METHY	Tibia_length	-0,01833	0,055883	NA
3	abundance	X06703	N6-METHY	Radius_length	-0,0327	0,073711	NA
3	abundance	X06703	N6-METHY	Radius_SOS	-0,03553	0,052985	NA
3	abundance	X06703	N6-METHY	Tibia_SOS	-0,14313	0,057121	NA
3	abundance	X06703	N6-METHY	Handgrip	-0,03742	0,060151	NA
3	abundance	X06704	feruloylagr	Tibia_length	-0,03691	0,051345	NA
3	abundance	X06704	feruloylagr	Radius_length	0,021049	0,070145	NA
3	abundance	X06704	feruloylagr	Radius_SOS	-0,02052	0,049663	NA
3	abundance	X06704	feruloylagr	Tibia_SOS	0,050821	0,052941	NA
3	abundance	X06704	feruloylagr	Handgrip	0,017247	0,061357	NA
3	abundance	X06709	Tetraacety	Tibia_length	-0,10496	0,052876	NA
3	abundance	X06709	Tetraacety	Radius_length	-0,02949	0,072277	NA
3	abundance	X06709	Tetraacety	Radius_SOS	0,010049	0,050712	NA
3	abundance	X06709	Tetraacety	Tibia_SOS	-0,09949	0,053939	NA
3	abundance	X06709	Tetraacety	Handgrip	-0,00132	0,061115	NA
3	abundance	X06722	LW800000	Tibia_length	-0,00675	0,053179	NA
3	abundance	X06722	LW800000	Radius_length	0,015904	0,071926	NA
3	abundance	X06722	LW800000	Radius_SOS	0,003364	0,050807	NA
3	abundance	X06722	LW800000	Tibia_SOS	0,060353	0,053865	NA
3	abundance	X06722	LW800000	Handgrip	0,040105	0,061282	NA
3	abundance	X06723	3-Hydroxy-	Tibia_length	-0,09098	0,054999	NA
3	abundance	X06723	3-Hydroxy-	Radius_length	-0,11703	0,072912	NA
3	abundance	X06723	3-Hydroxy-	Radius_SOS	-0,08827	0,052287	NA
3	abundance	X06723	3-Hydroxy-	Tibia_SOS	-0,05767	0,055261	NA
3	abundance	X06723	3-Hydroxy-	Handgrip	-0,14083	0,061739	NA
3	abundance	X06727	MFCD0087	Tibia_length	0,021897	0,054925	NA
3	abundance	X06727	MFCD0087	Radius_length	0,075461	0,073314	NA
3	abundance	X06727	MFCD0087	Radius_SOS	0,089648	0,052571	NA
3	abundance	X06727	MFCD0087	Tibia_SOS	0,027418	0,053738	NA
3	abundance	X06727	MFCD0087	Handgrip	-0,02834	0,061094	NA
2b	abundance	X06735	7-Methylxa	Tibia_length	-0,04275	0,054748	NA
2b	abundance	X06735	7-Methylxa	Radius_length	-0,0288	0,073545	NA
2b	abundance	X06735	7-Methylxa	Radius_SOS	0,068979	0,051909	NA
2b	abundance	X06735	7-Methylxa	Tibia_SOS	-0,01125	0,05434	NA
2b	abundance	X06735	7-Methylxa	Handgrip	0,021141	0,061705	NA
3	abundance	X06744	Diethylpyro	Tibia_length	-0,08562	0,053141	NA
3	abundance	X06744	Diethylpyro	Radius_length	-0,17959	0,072013	NA
3	abundance	X06744	Diethylpyro	Radius_SOS	-0,08754	0,050655	NA
3	abundance	X06744	Diethylpyro	Tibia_SOS	-0,09135	0,055114	NA

3	abundance	X06744	Diethylpyr	Handgrip	0,01781	0,063007	NA
3	abundance	X06750	1-PYRENYl	Tibia_length	-0,05846	0,054532	NA
3	abundance	X06750	1-PYRENYl	Radius_length	-0,0132	0,073379	NA
3	abundance	X06750	1-PYRENYl	Radius_SOS	0,091798	0,051646	NA
3	abundance	X06750	1-PYRENYl	Tibia_SOS	0,053935	0,054281	NA
3	abundance	X06750	1-PYRENYl	Handgrip	0,055804	0,061677	NA
3	abundance	X06764	N-(4-Hydro	Tibia_length	-0,16915	0,056521	NA
3	abundance	X06764	N-(4-Hydro	Radius_length	-0,14114	0,074985	NA
3	abundance	X06764	N-(4-Hydro	Radius_SOS	-0,05961	0,054297	NA
3	abundance	X06764	N-(4-Hydro	Tibia_SOS	-0,12962	0,056727	NA
3	abundance	X06764	N-(4-Hydro	Handgrip	-0,07431	0,061727	NA
1	abundance	X06767	2-Hydroxyb	Tibia_length	-0,04938	0,053012	NA
1	abundance	X06767	2-Hydroxyb	Radius_length	-0,05323	0,072316	NA
1	abundance	X06767	2-Hydroxyb	Radius_SOS	0,060691	0,050511	NA
1	abundance	X06767	2-Hydroxyb	Tibia_SOS	-0,02594	0,054107	NA
1	abundance	X06767	2-Hydroxyb	Handgrip	0,149222	0,060532	NA
3	abundance	X06768	Sinapyl alc	Tibia_length	0,043564	0,054993	NA
3	abundance	X06768	Sinapyl alc	Radius_length	0,076407	0,073781	NA
3	abundance	X06768	Sinapyl alc	Radius_SOS	0,07818	0,052158	NA
3	abundance	X06768	Sinapyl alc	Tibia_SOS	-0,11395	0,056292	NA
3	abundance	X06768	Sinapyl alc	Handgrip	0,149412	0,062158	NA
3	abundance	X06771	MFCD0015	Tibia_length	0,17044	0,053559	NA
3	abundance	X06771	MFCD0015	Radius_length	0,187557	0,071867	NA
3	abundance	X06771	MFCD0015	Radius_SOS	-0,00312	0,051758	NA
3	abundance	X06771	MFCD0015	Tibia_SOS	0,029434	0,055303	NA
3	abundance	X06771	MFCD0015	Handgrip	0,0503	0,062068	NA
3	abundance	X06783	N~6~-Octa	Tibia_length	-0,02314	0,053407	NA
3	abundance	X06783	N~6~-Octa	Radius_length	-8,32E-04	0,072051	NA
3	abundance	X06783	N~6~-Octa	Radius_SOS	0,001686	0,050984	NA
3	abundance	X06783	N~6~-Octa	Tibia_SOS	0,018038	0,054885	NA
3	abundance	X06783	N~6~-Octa	Handgrip	0,046904	0,061478	NA
3	abundance	X06797	pro-gln	Tibia_length	-0,0966	0,05381	NA
3	abundance	X06797	pro-gln	Radius_length	-0,04364	0,072664	NA
3	abundance	X06797	pro-gln	Radius_SOS	-0,04105	0,051192	NA
3	abundance	X06797	pro-gln	Tibia_SOS	-0,11199	0,054305	NA
3	abundance	X06797	pro-gln	Handgrip	-0,08485	0,061934	NA
3	abundance	X06805	(9cis)-O~1	Tibia_length	-0,00244	0,053064	NA
3	abundance	X06805	(9cis)-O~1	Radius_length	0,026608	0,072132	NA
3	abundance	X06805	(9cis)-O~1	Radius_SOS	0,018717	0,050656	NA
3	abundance	X06805	(9cis)-O~1	Tibia_SOS	0,056511	0,053544	NA
3	abundance	X06805	(9cis)-O~1	Handgrip	0,038791	0,061391	NA
3	abundance	X06807	1,1'-[1,12-	Tibia_length	0,049484	0,050797	NA
3	abundance	X06807	1,1'-[1,12-	Radius_length	0,062061	0,0694	NA
3	abundance	X06807	1,1'-[1,12-	Radius_SOS	0,039404	0,049322	NA
3	abundance	X06807	1,1'-[1,12-	Tibia_SOS	-0,06482	0,05295	NA
3	abundance	X06807	1,1'-[1,12-	Handgrip	0,017726	0,061034	NA
3	abundance	X06812	2-Hydroxy-	Tibia_length	0,065471	0,051536	NA
3	abundance	X06812	2-Hydroxy-	Radius_length	0,121866	0,070334	NA

3	abundance	X06812	2-Hydroxy-	Radius_SOS	0,003955	0,049757	NA
3	abundance	X06812	2-Hydroxy-	Tibia_SOS	0,092413	0,05281	NA
3	abundance	X06812	2-Hydroxy-	Handgrip	0,022469	0,06093	NA
3	abundance	X06814	N-Benzoyla	Tibia_length	-0,13136	0,051254	NA
3	abundance	X06814	N-Benzoyla	Radius_length	-0,15255	0,069824	NA
3	abundance	X06814	N-Benzoyla	Radius_SOS	-0,02911	0,049639	NA
3	abundance	X06814	N-Benzoyla	Tibia_SOS	-0,13185	0,052896	NA
3	abundance	X06814	N-Benzoyla	Handgrip	-0,09298	0,060525	NA
3	abundance	X06818	Homoanse	Tibia_length	0,032353	0,052677	NA
3	abundance	X06818	Homoanse	Radius_length	0,08255	0,071432	NA
3	abundance	X06818	Homoanse	Radius_SOS	0,046599	0,050538	NA
3	abundance	X06818	Homoanse	Tibia_SOS	0,009381	0,053944	NA
3	abundance	X06818	Homoanse	Handgrip	-0,11111	0,060978	NA
3	abundance	X06819	Primaquin	Tibia_length	0,069175	0,050275	NA
3	abundance	X06819	Primaquin	Radius_length	-0,02637	0,068934	NA
3	abundance	X06819	Primaquin	Radius_SOS	-0,04668	0,049187	NA
3	abundance	X06819	Primaquin	Tibia_SOS	-0,0263	0,052827	NA
3	abundance	X06819	Primaquin	Handgrip	0,10708	0,060206	NA
2b	abundance	X06820	1-(3,4-dim	Tibia_length	-0,10595	0,050716	NA
2b	abundance	X06820	1-(3,4-dim	Radius_length	-0,1193	0,069003	NA
2b	abundance	X06820	1-(3,4-dim	Radius_SOS	0,002599	0,049341	NA
2b	abundance	X06820	1-(3,4-dim	Tibia_SOS	-0,0508	0,052711	NA
2b	abundance	X06820	1-(3,4-dim	Handgrip	-0,05757	0,060129	NA
3	abundance	X06836	asn-lys	Tibia_length	-0,11015	0,057788	NA
3	abundance	X06836	asn-lys	Radius_length	-0,05364	0,07541	NA
3	abundance	X06836	asn-lys	Radius_SOS	-0,13845	0,055073	NA
3	abundance	X06836	asn-lys	Tibia_SOS	0,070933	0,057623	NA
3	abundance	X06836	asn-lys	Handgrip	0,059631	0,063449	NA
3	abundance	X06850	Leu-Val	Tibia_length	0,00905	0,05286	NA
3	abundance	X06850	Leu-Val	Radius_length	-0,02058	0,072052	NA
3	abundance	X06850	Leu-Val	Radius_SOS	-0,08239	0,050239	NA
3	abundance	X06850	Leu-Val	Tibia_SOS	-0,0039	0,054078	NA
3	abundance	X06850	Leu-Val	Handgrip	0,005546	0,061803	NA
2b	abundance	X06856	Tiglic acid	Tibia_length	-0,02767	0,054759	NA
2b	abundance	X06856	Tiglic acid	Radius_length	-0,00171	0,07342	NA
2b	abundance	X06856	Tiglic acid	Radius_SOS	-0,05072	0,052061	NA
2b	abundance	X06856	Tiglic acid	Tibia_SOS	0,024569	0,056093	NA
2b	abundance	X06856	Tiglic acid	Handgrip	0,029463	0,062545	NA
3	abundance	X06858	tert-Butyl 3	Tibia_length	-0,00153	0,052093	NA
3	abundance	X06858	tert-Butyl 3	Radius_length	0,094369	0,071222	NA
3	abundance	X06858	tert-Butyl 3	Radius_SOS	-0,00341	0,050089	NA
3	abundance	X06858	tert-Butyl 3	Tibia_SOS	0,072174	0,053817	NA
3	abundance	X06858	tert-Butyl 3	Handgrip	0,07778	0,060849	NA
3	abundance	X06859	Methylol D	Tibia_length	0,013763	0,052407	NA
3	abundance	X06859	Methylol D	Radius_length	0,147736	0,070831	NA
3	abundance	X06859	Methylol D	Radius_SOS	0,033363	0,050311	NA
3	abundance	X06859	Methylol D	Tibia_SOS	-0,02246	0,054224	NA
3	abundance	X06859	Methylol D	Handgrip	-0,09627	0,061282	NA

3	abundance	X06861	4-(4-Deoxy	Tibia_length	0,089862	0,051573	NA
3	abundance	X06861	4-(4-Deoxy	Radius_length	0,161718	0,069556	NA
3	abundance	X06861	4-(4-Deoxy	Radius_SOS	-0,0367	0,049691	NA
3	abundance	X06861	4-(4-Deoxy	Tibia_SOS	-0,02612	0,053247	NA
3	abundance	X06861	4-(4-Deoxy	Handgrip	0,108632	0,061093	NA
3	abundance	X06866	tert-Butyl 3	Tibia_length	-0,04579	0,052775	NA
3	abundance	X06866	tert-Butyl 3	Radius_length	0,026096	0,07201	NA
3	abundance	X06866	tert-Butyl 3	Radius_SOS	0,020269	0,050582	NA
3	abundance	X06866	tert-Butyl 3	Tibia_SOS	-0,02372	0,054468	NA
3	abundance	X06866	tert-Butyl 3	Handgrip	0,026373	0,062104	NA
3	abundance	X06870	Menadiol	Tibia_length	-0,02258	0,056304	NA
3	abundance	X06870	Menadiol	Radius_length	0,037226	0,074458	NA
3	abundance	X06870	Menadiol	Radius_SOS	-0,03109	0,05371	NA
3	abundance	X06870	Menadiol	Tibia_SOS	-0,09844	0,057728	NA
3	abundance	X06870	Menadiol	Handgrip	-0,02768	0,062667	NA
3	abundance	X06874	N-Acetyl-5	Tibia_length	0,038152	0,055535	NA
3	abundance	X06874	N-Acetyl-5	Radius_length	0,061546	0,073755	NA
3	abundance	X06874	N-Acetyl-5	Radius_SOS	-0,08377	0,052462	NA
3	abundance	X06874	N-Acetyl-5	Tibia_SOS	-0,07474	0,057796	NA
3	abundance	X06874	N-Acetyl-5	Handgrip	0,011704	0,063472	NA
3	abundance	X06875	Nicotine gl	Tibia_length	0,043837	0,053253	NA
3	abundance	X06875	Nicotine gl	Radius_length	0,043331	0,072177	NA
3	abundance	X06875	Nicotine gl	Radius_SOS	0,030089	0,050966	NA
3	abundance	X06875	Nicotine gl	Tibia_SOS	0,076538	0,054199	NA
3	abundance	X06875	Nicotine gl	Handgrip	-0,05668	0,061503	NA
3	abundance	X06880	pentobarbi	Tibia_length	0,056551	0,051559	NA
3	abundance	X06880	pentobarbi	Radius_length	0,070223	0,070739	NA
3	abundance	X06880	pentobarbi	Radius_SOS	-0,0039	0,049824	NA
3	abundance	X06880	pentobarbi	Tibia_SOS	-0,01054	0,053448	NA
3	abundance	X06880	pentobarbi	Handgrip	0,048896	0,061211	NA
3	abundance	X06883	Agomelatir	Tibia_length	-0,09117	0,053593	NA
3	abundance	X06883	Agomelatir	Radius_length	-0,10354	0,072102	NA
3	abundance	X06883	Agomelatir	Radius_SOS	-0,01737	0,051079	NA
3	abundance	X06883	Agomelatir	Tibia_SOS	-0,09055	0,054464	NA
3	abundance	X06883	Agomelatir	Handgrip	0,005076	0,061538	NA
2a	abundance	X06889	3,4-Dimetl	Tibia_length	0,076816	0,052617	NA
2a	abundance	X06889	3,4-Dimetl	Radius_length	0,122478	0,071219	NA
2a	abundance	X06889	3,4-Dimetl	Radius_SOS	0,033918	0,050469	NA
2a	abundance	X06889	3,4-Dimetl	Tibia_SOS	-0,00998	0,053909	NA
2a	abundance	X06889	3,4-Dimetl	Handgrip	0,120551	0,061114	NA
3	abundance	X06896	Pirbuterol	Tibia_length	-0,11454	0,055738	NA
3	abundance	X06896	Pirbuterol	Radius_length	-0,12466	0,073643	NA
3	abundance	X06896	Pirbuterol	Radius_SOS	-0,00712	0,053037	NA
3	abundance	X06896	Pirbuterol	Tibia_SOS	-0,00527	0,056137	NA
3	abundance	X06896	Pirbuterol	Handgrip	0,001672	0,062864	NA
3	abundance	X06902	Homocyst	Tibia_length	0,081324	0,055105	NA
3	abundance	X06902	Homocyst	Radius_length	0,104777	0,074082	NA
3	abundance	X06902	Homocyst	Radius_SOS	0,089676	0,052168	NA

3	abundance	X06902	Homocyst	Tibia_SOS	0,102947	0,05573	NA
3	abundance	X06902	Homocyst	Handgrip	0,045326	0,063152	NA
2b	abundance	X06903	Esculin	Tibia_length	-0,05818	0,055993	NA
2b	abundance	X06903	Esculin	Radius_length	-0,08847	0,073603	NA
2b	abundance	X06903	Esculin	Radius_SOS	-0,09378	0,053585	NA
2b	abundance	X06903	Esculin	Tibia_SOS	-0,04748	0,056707	NA
2b	abundance	X06903	Esculin	Handgrip	-0,07039	0,062551	NA
3	abundance	X06905	Ectoine	Tibia_length	0,001601	0,055002	NA
3	abundance	X06905	Ectoine	Radius_length	-0,02741	0,073509	NA
3	abundance	X06905	Ectoine	Radius_SOS	-0,03201	0,052243	NA
3	abundance	X06905	Ectoine	Tibia_SOS	-0,06419	0,055673	NA
3	abundance	X06905	Ectoine	Handgrip	-0,05445	0,062132	NA
3	abundance	X06906	Arg-pro	Tibia_length	0,025879	0,051776	NA
3	abundance	X06906	Arg-pro	Radius_length	0,072553	0,070589	NA
3	abundance	X06906	Arg-pro	Radius_SOS	0,012145	0,049839	NA
3	abundance	X06906	Arg-pro	Tibia_SOS	0,06442	0,053373	NA
3	abundance	X06906	Arg-pro	Handgrip	0,042568	0,060565	NA
3	abundance	X06917	Alanyltrypt	Tibia_length	-0,10399	0,055014	NA
3	abundance	X06917	Alanyltrypt	Radius_length	-0,04648	0,073905	NA
3	abundance	X06917	Alanyltrypt	Radius_SOS	0,024889	0,05233	NA
3	abundance	X06917	Alanyltrypt	Tibia_SOS	0,009431	0,055804	NA
3	abundance	X06917	Alanyltrypt	Handgrip	0,061714	0,061015	NA
3	abundance	X06933	3-Methoxy-	Tibia_length	-0,09044	0,052294	NA
3	abundance	X06933	3-Methoxy-	Radius_length	-0,08699	0,071566	NA
3	abundance	X06933	3-Methoxy-	Radius_SOS	0,018143	0,050061	NA
3	abundance	X06933	3-Methoxy-	Tibia_SOS	-0,09548	0,053282	NA
3	abundance	X06933	3-Methoxy-	Handgrip	-0,13456	0,060573	NA
3	abundance	X06943	NSC 9277	Tibia_length	-0,11298	0,054299	NA
3	abundance	X06943	NSC 9277	Radius_length	0,024543	0,073394	NA
3	abundance	X06943	NSC 9277	Radius_SOS	0,068491	0,051798	NA
3	abundance	X06943	NSC 9277	Tibia_SOS	0,080848	0,055383	NA
3	abundance	X06943	NSC 9277	Handgrip	0,0717	0,061844	NA
3	abundance	X06946	SECONAL	Tibia_length	-0,11713	0,054175	NA
3	abundance	X06946	SECONAL	Radius_length	-0,12585	0,072506	NA
3	abundance	X06946	SECONAL	Radius_SOS	-0,04603	0,051903	NA
3	abundance	X06946	SECONAL	Tibia_SOS	-0,09318	0,055212	NA
3	abundance	X06946	SECONAL	Handgrip	-0,15414	0,061152	NA
3	abundance	X06948	(1R,3R,5R)	Tibia_length	0,023472	0,054973	NA
3	abundance	X06948	(1R,3R,5R)	Radius_length	0,119594	0,072886	NA
3	abundance	X06948	(1R,3R,5R)	Radius_SOS	0,024257	0,052007	NA
3	abundance	X06948	(1R,3R,5R)	Tibia_SOS	0,060276	0,05574	NA
3	abundance	X06948	(1R,3R,5R)	Handgrip	-0,07682	0,063576	NA
2b	abundance	X06958	2-[(carbox	Tibia_length	-0,02865	0,051944	NA
2b	abundance	X06958	2-[(carbox	Radius_length	0,114578	0,070477	NA
2b	abundance	X06958	2-[(carbox	Radius_SOS	0,083662	0,049763	NA
2b	abundance	X06958	2-[(carbox	Tibia_SOS	0,114901	0,053475	NA
2b	abundance	X06958	2-[(carbox	Handgrip	-0,04664	0,061802	NA
3	abundance	X06961	Zalcitabine	Tibia_length	-0,15419	0,056592	NA

3	abundance	X06961	Zalcitabine	Radius_length	-0,17899	0,074107	NA
3	abundance	X06961	Zalcitabine	Radius_SOS	-0,03748	0,053851	NA
3	abundance	X06961	Zalcitabine	Tibia_SOS	-0,24567	0,056158	NA
3	abundance	X06961	Zalcitabine	Handgrip	-0,14281	0,06285	NA
3	abundance	X06977	4-Hydroxyp	Tibia_length	-0,0686	0,055717	NA
3	abundance	X06977	4-Hydroxyp	Radius_length	-0,03907	0,074514	NA
3	abundance	X06977	4-Hydroxyp	Radius_SOS	-0,02258	0,052958	NA
3	abundance	X06977	4-Hydroxyp	Tibia_SOS	0,104063	0,055155	NA
3	abundance	X06977	4-Hydroxyp	Handgrip	0,026248	0,062609	NA
3	abundance	X06985	N-Ethylpro	Tibia_length	-0,03885	0,053908	NA
3	abundance	X06985	N-Ethylpro	Radius_length	-0,01268	0,07311	NA
3	abundance	X06985	N-Ethylpro	Radius_SOS	-0,03012	0,051307	NA
3	abundance	X06985	N-Ethylpro	Tibia_SOS	0,065901	0,055055	NA
3	abundance	X06985	N-Ethylpro	Handgrip	0,03299	0,061223	NA
3	abundance	X07002	Paraldehyc	Tibia_length	0,021049	0,05586	NA
3	abundance	X07002	Paraldehyc	Radius_length	0,060662	0,073961	NA
3	abundance	X07002	Paraldehyc	Radius_SOS	-0,00641	0,053165	NA
3	abundance	X07002	Paraldehyc	Tibia_SOS	0,05237	0,056911	NA
3	abundance	X07002	Paraldehyc	Handgrip	0,006827	0,063346	NA
3	abundance	X07013	pimethixer	Tibia_length	0,052023	0,051502	NA
3	abundance	X07013	pimethixer	Radius_length	0,022907	0,07041	NA
3	abundance	X07013	pimethixer	Radius_SOS	-4,30E-04	0,049738	NA
3	abundance	X07013	pimethixer	Tibia_SOS	-0,00665	0,053202	NA
3	abundance	X07013	pimethixer	Handgrip	-0,00722	0,061181	NA
3	abundance	X07014	Octyl benz	Tibia_length	0,050226	0,053022	NA
3	abundance	X07014	Octyl benz	Radius_length	0,08625	0,071601	NA
3	abundance	X07014	Octyl benz	Radius_SOS	-0,03123	0,050708	NA
3	abundance	X07014	Octyl benz	Tibia_SOS	0,018126	0,053493	NA
3	abundance	X07014	Octyl benz	Handgrip	-0,01663	0,061499	NA
2b	abundance	X07026	7Î±-Hydrox	Tibia_length	0,029098	0,052006	NA
2b	abundance	X07026	7Î±-Hydrox	Radius_length	0,107971	0,070796	NA
2b	abundance	X07026	7Î±-Hydrox	Radius_SOS	0,097288	0,049809	NA
2b	abundance	X07026	7Î±-Hydrox	Tibia_SOS	0,13538	0,05389	NA
2b	abundance	X07026	7Î±-Hydrox	Handgrip	0,063795	0,061052	NA
3	abundance	X07027	Allyl merca	Tibia_length	-0,11209	0,055583	NA
3	abundance	X07027	Allyl merca	Radius_length	-0,06685	0,073822	NA
3	abundance	X07027	Allyl merca	Radius_SOS	-0,02035	0,052721	NA
3	abundance	X07027	Allyl merca	Tibia_SOS	0,05716	0,055942	NA
3	abundance	X07027	Allyl merca	Handgrip	0,051205	0,060114	NA
3	abundance	X07028	N-Pentano	Tibia_length	0,193979	0,053041	NA
3	abundance	X07028	N-Pentano	Radius_length	0,145861	0,07263	NA
3	abundance	X07028	N-Pentano	Radius_SOS	0,095882	0,051335	NA
3	abundance	X07028	N-Pentano	Tibia_SOS	0,156631	0,054944	NA
3	abundance	X07028	N-Pentano	Handgrip	0,070124	0,061043	NA
3	abundance	X07038	Piperidine	Tibia_length	-0,01698	0,05044	NA
3	abundance	X07038	Piperidine	Radius_length	-0,02204	0,068842	NA
3	abundance	X07038	Piperidine	Radius_SOS	-0,03273	0,049187	NA
3	abundance	X07038	Piperidine	Tibia_SOS	-0,02928	0,052777	NA

	3	abundance	X07038	Piperidine	Handgrip	0,044143	0,060733	NA
	3	abundance	X07040	6-Hydroxyp	Tibia_length	0,083608	0,050118	NA
	3	abundance	X07040	6-Hydroxyp	Radius_length	0,141239	0,068021	NA
	3	abundance	X07040	6-Hydroxyp	Radius_SOS	0,036518	0,049112	NA
	3	abundance	X07040	6-Hydroxyp	Tibia_SOS	-0,00307	0,052568	NA
	3	abundance	X07040	6-Hydroxyp	Handgrip	0,045804	0,060312	NA
	3	abundance	X07050	2-Methylth	Tibia_length	0,090124	0,051078	NA
	3	abundance	X07050	2-Methylth	Radius_length	0,167208	0,068944	NA
	3	abundance	X07050	2-Methylth	Radius_SOS	0,02503	0,049449	NA
	3	abundance	X07050	2-Methylth	Tibia_SOS	0,132958	0,052524	NA
	3	abundance	X07050	2-Methylth	Handgrip	0,105814	0,060213	NA
	3	abundance	X07051	O-succinyl	Tibia_length	0,012573	0,049856	NA
	3	abundance	X07051	O-succinyl	Radius_length	0,119294	0,06732	NA
	3	abundance	X07051	O-succinyl	Radius_SOS	1,45E-04	0,049048	NA
	3	abundance	X07051	O-succinyl	Tibia_SOS	0,010989	0,052879	NA
	3	abundance	X07051	O-succinyl	Handgrip	-0,053	0,060217	NA
2b		abundance	X07054	Valylprolin	Tibia_length	-0,02519	0,053275	NA
2b		abundance	X07054	Valylprolin	Radius_length	0,008665	0,072453	NA
2b		abundance	X07054	Valylprolin	Radius_SOS	-0,01655	0,050965	NA
2b		abundance	X07054	Valylprolin	Tibia_SOS	0,06465	0,054966	NA
2b		abundance	X07054	Valylprolin	Handgrip	0,101705	0,061576	NA
	3	abundance	X07057	Leu-Leu	Tibia_length	-0,00783	0,053728	NA
	3	abundance	X07057	Leu-Leu	Radius_length	0,0819	0,072631	NA
	3	abundance	X07057	Leu-Leu	Radius_SOS	-0,01967	0,05121	NA
	3	abundance	X07057	Leu-Leu	Tibia_SOS	0,043505	0,056063	NA
	3	abundance	X07057	Leu-Leu	Handgrip	-0,00326	0,062931	NA
	3	abundance	X07073	asn-val	Tibia_length	-0,01297	0,052478	NA
	3	abundance	X07073	asn-val	Radius_length	0,125858	0,071428	NA
	3	abundance	X07073	asn-val	Radius_SOS	0,031459	0,050435	NA
	3	abundance	X07073	asn-val	Tibia_SOS	0,041468	0,053417	NA
	3	abundance	X07073	asn-val	Handgrip	-0,08298	0,06115	NA
	1	abundance	X07077	Hyodeoxyc	Tibia_length	-0,0031	0,051544	NA
	1	abundance	X07077	Hyodeoxyc	Radius_length	-0,04374	0,070861	NA
	1	abundance	X07077	Hyodeoxyc	Radius_SOS	0,094373	0,04963	NA
	1	abundance	X07077	Hyodeoxyc	Tibia_SOS	0,021679	0,053424	NA
	1	abundance	X07077	Hyodeoxyc	Handgrip	0,048467	0,060696	NA
	3	abundance	X07079	N-Pentano	Tibia_length	0,187092	0,052438	NA
	3	abundance	X07079	N-Pentano	Radius_length	0,130749	0,072105	NA
	3	abundance	X07079	N-Pentano	Radius_SOS	0,082246	0,050879	NA
	3	abundance	X07079	N-Pentano	Tibia_SOS	0,157963	0,054173	NA
	3	abundance	X07079	N-Pentano	Handgrip	0,073031	0,060752	NA
	3	abundance	X07081	N-LACTOYI	Tibia_length	-0,03171	0,053697	NA
	3	abundance	X07081	N-LACTOYI	Radius_length	-0,07256	0,072201	NA
	3	abundance	X07081	N-LACTOYI	Radius_SOS	0,012966	0,051154	NA
	3	abundance	X07081	N-LACTOYI	Tibia_SOS	-0,0183	0,054973	NA
	3	abundance	X07081	N-LACTOYI	Handgrip	-0,07126	0,062295	NA
	3	abundance	X07084	heptabarbi	Tibia_length	0,029443	0,052254	NA
	3	abundance	X07084	heptabarbi	Radius_length	0,071104	0,07128	NA

3	abundance	X07084	heptabarbi	Radius_SOS	0,034692	0,050223	NA
3	abundance	X07084	heptabarbi	Tibia_SOS	0,019146	0,053361	NA
3	abundance	X07084	heptabarbi	Handgrip	-0,02888	0,061371	NA
3	abundance	X07089	Pregabalin	Tibia_length	-0,14134	0,052381	NA
3	abundance	X07089	Pregabalin	Radius_length	-0,16872	0,070773	NA
3	abundance	X07089	Pregabalin	Radius_SOS	-0,11396	0,050174	NA
3	abundance	X07089	Pregabalin	Tibia_SOS	-0,0663	0,054416	NA
3	abundance	X07089	Pregabalin	Handgrip	-0,10563	0,0605	NA
3	abundance	X07092	Dibutyl ma	Tibia_length	0,095306	0,050728	NA
3	abundance	X07092	Dibutyl ma	Radius_length	0,05183	0,06981	NA
3	abundance	X07092	Dibutyl ma	Radius_SOS	0,073963	0,049361	NA
3	abundance	X07092	Dibutyl ma	Tibia_SOS	0,008564	0,053103	NA
3	abundance	X07092	Dibutyl ma	Handgrip	0,002374	0,061292	NA
3	abundance	X07107	Lysylvaline	Tibia_length	0,062336	0,051776	NA
3	abundance	X07107	Lysylvaline	Radius_length	0,033788	0,070941	NA
3	abundance	X07107	Lysylvaline	Radius_SOS	-0,00102	0,050008	NA
3	abundance	X07107	Lysylvaline	Tibia_SOS	-0,03893	0,054016	NA
3	abundance	X07107	Lysylvaline	Handgrip	0,068033	0,062356	NA
3	abundance	X07112	MFCD0272	Tibia_length	-0,08099	0,053333	NA
3	abundance	X07112	MFCD0272	Radius_length	-0,08935	0,072374	NA
3	abundance	X07112	MFCD0272	Radius_SOS	0,014578	0,051134	NA
3	abundance	X07112	MFCD0272	Tibia_SOS	-0,06979	0,054402	NA
3	abundance	X07112	MFCD0272	Handgrip	-0,10836	0,062826	NA
3	abundance	X07113	Sulfoaceta	Tibia_length	0,021406	0,054894	NA
3	abundance	X07113	Sulfoaceta	Radius_length	-0,16059	0,072979	NA
3	abundance	X07113	Sulfoaceta	Radius_SOS	-0,11149	0,052573	NA
3	abundance	X07113	Sulfoaceta	Tibia_SOS	-0,01553	0,056807	NA
3	abundance	X07113	Sulfoaceta	Handgrip	-0,01974	0,062705	NA
3	abundance	X07125	Zalcitabine	Tibia_length	-0,05401	0,057689	NA
3	abundance	X07125	Zalcitabine	Radius_length	-0,0093	0,075344	NA
3	abundance	X07125	Zalcitabine	Radius_SOS	0,048086	0,054877	NA
3	abundance	X07125	Zalcitabine	Tibia_SOS	-0,06863	0,057305	NA
3	abundance	X07125	Zalcitabine	Handgrip	-0,06472	0,063179	NA
3	abundance	X07126	Leu-Val	Tibia_length	-0,03713	0,050849	NA
3	abundance	X07126	Leu-Val	Radius_length	-0,09651	0,069209	NA
3	abundance	X07126	Leu-Val	Radius_SOS	0,006319	0,049411	NA
3	abundance	X07126	Leu-Val	Tibia_SOS	-0,00444	0,052768	NA
3	abundance	X07126	Leu-Val	Handgrip	-0,07313	0,060875	NA
3	abundance	X07127	N~5~-[P-Ar	Tibia_length	0,033681	0,056274	NA
3	abundance	X07127	N~5~-[P-Ar	Radius_length	0,08383	0,074322	NA
3	abundance	X07127	N~5~-[P-Ar	Radius_SOS	-0,02498	0,053473	NA
3	abundance	X07127	N~5~-[P-Ar	Tibia_SOS	0,005774	0,056538	NA
3	abundance	X07127	N~5~-[P-Ar	Handgrip	0,121057	0,062138	NA
2b	abundance	X07134	4-Phenolsu	Tibia_length	-0,09771	0,051685	NA
2b	abundance	X07134	4-Phenolsu	Radius_length	-0,06719	0,070723	NA
2b	abundance	X07134	4-Phenolsu	Radius_SOS	-0,09754	0,049722	NA
2b	abundance	X07134	4-Phenolsu	Tibia_SOS	0,035396	0,053604	NA
2b	abundance	X07134	4-Phenolsu	Handgrip	-0,05085	0,061344	NA

3	abundance	X07136	hexobarbit	Tibia_length	-0,19506	0,053667	NA
3	abundance	X07136	hexobarbit	Radius_length	-0,10116	0,072686	NA
3	abundance	X07136	hexobarbit	Radius_SOS	-0,06975	0,051604	NA
3	abundance	X07136	hexobarbit	Tibia_SOS	-0,06928	0,055688	NA
3	abundance	X07136	hexobarbit	Handgrip	0,043566	0,061805	NA
3	abundance	X07139	N,N-Diethy	Tibia_length	-0,00163	0,052542	NA
3	abundance	X07139	N,N-Diethy	Radius_length	-0,05084	0,071296	NA
3	abundance	X07139	N,N-Diethy	Radius_SOS	0,009261	0,050304	NA
3	abundance	X07139	N,N-Diethy	Tibia_SOS	-0,02563	0,053711	NA
3	abundance	X07139	N,N-Diethy	Handgrip	0,08475	0,060803	NA
3	abundance	X07146	meprobam	Tibia_length	-0,07175	0,056424	NA
3	abundance	X07146	meprobam	Radius_length	0,041712	0,074657	NA
3	abundance	X07146	meprobam	Radius_SOS	-0,03676	0,053743	NA
3	abundance	X07146	meprobam	Tibia_SOS	-0,00664	0,058833	NA
3	abundance	X07146	meprobam	Handgrip	0,007537	0,063789	NA
3	abundance	X07155	Naphthale	Tibia_length	0,049107	0,051611	NA
3	abundance	X07155	Naphthale	Radius_length	0,10071	0,069797	NA
3	abundance	X07155	Naphthale	Radius_SOS	-0,03361	0,049561	NA
3	abundance	X07155	Naphthale	Tibia_SOS	-0,00703	0,053064	NA
3	abundance	X07155	Naphthale	Handgrip	-0,05313	0,060803	NA
3	abundance	X07164	N-Acetyl-5	Tibia_length	-0,02619	0,052612	NA
3	abundance	X07164	N-Acetyl-5	Radius_length	0,068938	0,071458	NA
3	abundance	X07164	N-Acetyl-5	Radius_SOS	0,030908	0,050222	NA
3	abundance	X07164	N-Acetyl-5	Tibia_SOS	0,026429	0,054934	NA
3	abundance	X07164	N-Acetyl-5	Handgrip	0,058975	0,061378	NA
3	abundance	X07165	pro-gln	Tibia_length	0,062673	0,051595	NA
3	abundance	X07165	pro-gln	Radius_length	-0,01495	0,070923	NA
3	abundance	X07165	pro-gln	Radius_SOS	0,031768	0,049883	NA
3	abundance	X07165	pro-gln	Tibia_SOS	0,01233	0,053225	NA
3	abundance	X07165	pro-gln	Handgrip	-0,09777	0,060721	NA
3	abundance	X07168	L-(+)-Eryth	Tibia_length	0,043361	0,05447	NA
3	abundance	X07168	L-(+)-Eryth	Radius_length	0,073138	0,073465	NA
3	abundance	X07168	L-(+)-Eryth	Radius_SOS	-0,02762	0,051971	NA
3	abundance	X07168	L-(+)-Eryth	Tibia_SOS	0,037731	0,055703	NA
3	abundance	X07168	L-(+)-Eryth	Handgrip	0,109726	0,062916	NA
3	abundance	X07193	N-(1-Methy	Tibia_length	-0,02535	0,053437	NA
3	abundance	X07193	N-(1-Methy	Radius_length	0,007394	0,072298	NA
3	abundance	X07193	N-(1-Methy	Radius_SOS	-0,02202	0,051028	NA
3	abundance	X07193	N-(1-Methy	Tibia_SOS	-0,00385	0,054115	NA
3	abundance	X07193	N-(1-Methy	Handgrip	-0,08663	0,061651	NA
2b	abundance	X07201	2,4-Quinol	Tibia_length	0,012319	0,053162	NA
2b	abundance	X07201	2,4-Quinol	Radius_length	-0,03458	0,072374	NA
2b	abundance	X07201	2,4-Quinol	Radius_SOS	0,006306	0,050926	NA
2b	abundance	X07201	2,4-Quinol	Tibia_SOS	-0,02751	0,05431	NA
2b	abundance	X07201	2,4-Quinol	Handgrip	0,07342	0,062223	NA
3	abundance	X07216	pentobarbi	Tibia_length	0,074128	0,051686	NA
3	abundance	X07216	pentobarbi	Radius_length	0,104118	0,070325	NA
3	abundance	X07216	pentobarbi	Radius_SOS	0,01832	0,04984	NA

	3	abundance	X07216	pentobarbi	Tibia_SOS	0,034764	0,053311	NA
	3	abundance	X07216	pentobarbi	Handgrip	-0,1044	0,060999	NA
2b		abundance	X07219	3,3-Dimetł	Tibia_length	0,024001	0,054467	NA
2b		abundance	X07219	3,3-Dimetł	Radius_length	-0,04157	0,073401	NA
2b		abundance	X07219	3,3-Dimetł	Radius_SOS	-0,00463	0,052007	NA
2b		abundance	X07219	3,3-Dimetł	Tibia_SOS	7,53E-04	0,055928	NA
2b		abundance	X07219	3,3-Dimetł	Handgrip	0,141138	0,061746	NA
	3	abundance	X07220	Glycylproly	Tibia_length	-0,06881	0,052534	NA
	3	abundance	X07220	Glycylproly	Radius_length	-0,06508	0,071465	NA
	3	abundance	X07220	Glycylproly	Radius_SOS	-0,05053	0,050348	NA
	3	abundance	X07220	Glycylproly	Tibia_SOS	-0,04178	0,053994	NA
	3	abundance	X07220	Glycylproly	Handgrip	-0,09286	0,060541	NA
	3	abundance	X07226	threonylph	Tibia_length	-0,06391	0,054466	NA
	3	abundance	X07226	threonylph	Radius_length	-0,10517	0,072854	NA
	3	abundance	X07226	threonylph	Radius_SOS	-0,04593	0,051901	NA
	3	abundance	X07226	threonylph	Tibia_SOS	-0,03235	0,055616	NA
	3	abundance	X07226	threonylph	Handgrip	-0,17228	0,062061	NA
	3	abundance	X07236	2-methylci	Tibia_length	-0,09624	0,053628	NA
	3	abundance	X07236	2-methylci	Radius_length	-0,05269	0,072974	NA
	3	abundance	X07236	2-methylci	Radius_SOS	0,036082	0,051342	NA
	3	abundance	X07236	2-methylci	Tibia_SOS	-0,00887	0,054608	NA
	3	abundance	X07236	2-methylci	Handgrip	-0,04404	0,0612	NA
	3	abundance	X07250	Leucylaspa	Tibia_length	-3,34E-04	0,054511	NA
	3	abundance	X07250	Leucylaspa	Radius_length	0,073979	0,073518	NA
	3	abundance	X07250	Leucylaspa	Radius_SOS	-0,00485	0,051865	NA
	3	abundance	X07250	Leucylaspa	Tibia_SOS	-0,01181	0,056172	NA
	3	abundance	X07250	Leucylaspa	Handgrip	-0,00689	0,062196	NA
	3	abundance	X07260	(7E,7'E)-5,	Tibia_length	-0,09821	0,056169	NA
	3	abundance	X07260	(7E,7'E)-5,	Radius_length	-0,0853	0,074181	NA
	3	abundance	X07260	(7E,7'E)-5,	Radius_SOS	-0,15478	0,053552	NA
	3	abundance	X07260	(7E,7'E)-5,	Tibia_SOS	-0,06095	0,057946	NA
	3	abundance	X07260	(7E,7'E)-5,	Handgrip	-0,11503	0,059793	NA
2b		abundance	X07263	3,3-Dimetł	Tibia_length	0,004582	0,055249	NA
2b		abundance	X07263	3,3-Dimetł	Radius_length	0,014736	0,073308	NA
2b		abundance	X07263	3,3-Dimetł	Radius_SOS	0,011365	0,052584	NA
2b		abundance	X07263	3,3-Dimetł	Tibia_SOS	0,033287	0,056536	NA
2b		abundance	X07263	3,3-Dimetł	Handgrip	0,136461	0,061024	NA
	3	abundance	X07269	(3aR,4R,5F	Tibia_length	0,033666	0,050381	NA
	3	abundance	X07269	(3aR,4R,5F	Radius_length	-0,00308	0,068819	NA
	3	abundance	X07269	(3aR,4R,5F	Radius_SOS	0,008457	0,049229	NA
	3	abundance	X07269	(3aR,4R,5F	Tibia_SOS	-0,011	0,052833	NA
	3	abundance	X07269	(3aR,4R,5F	Handgrip	-0,00759	0,061178	NA
	3	abundance	X07270	5-(2-Carbc	Tibia_length	-0,00159	0,055706	NA
	3	abundance	X07270	5-(2-Carbc	Radius_length	0,057529	0,073787	NA
	3	abundance	X07270	5-(2-Carbc	Radius_SOS	0,148391	0,052624	NA
	3	abundance	X07270	5-(2-Carbc	Tibia_SOS	0,126037	0,056299	NA
	3	abundance	X07270	5-(2-Carbc	Handgrip	0,120646	0,062758	NA
	3	abundance	X07278	5-Phospho	Tibia_length	-0,02866	0,051177	NA

3	abundance	X07278	5-Phospho	Radius_length	-0,09963	0,06979	NA
3	abundance	X07278	5-Phospho	Radius_SOS	0,070246	0,049395	NA
3	abundance	X07278	5-Phospho	Tibia_SOS	-0,05923	0,053042	NA
3	abundance	X07278	5-Phospho	Handgrip	-0,00151	0,061163	NA
3	abundance	X07283	8-Hydroxyl	Tibia_length	0,060744	0,054079	NA
3	abundance	X07283	8-Hydroxyl	Radius_length	0,055447	0,072865	NA
3	abundance	X07283	8-Hydroxyl	Radius_SOS	0,021132	0,051621	NA
3	abundance	X07283	8-Hydroxyl	Tibia_SOS	-0,03535	0,054907	NA
3	abundance	X07283	8-Hydroxyl	Handgrip	-0,0526	0,061994	NA
3	abundance	X07285	2-(Carboxy	Tibia_length	-0,01644	0,058426	NA
3	abundance	X07285	2-(Carboxy	Radius_length	-0,03809	0,075218	NA
3	abundance	X07285	2-(Carboxy	Radius_SOS	-0,05306	0,055669	NA
3	abundance	X07285	2-(Carboxy	Tibia_SOS	0,020046	0,059538	NA
3	abundance	X07285	2-(Carboxy	Handgrip	-0,07295	0,060033	NA
3	abundance	X07294	6-Acetami	Tibia_length	-0,13891	0,056614	NA
3	abundance	X07294	6-Acetami	Radius_length	-0,07191	0,074958	NA
3	abundance	X07294	6-Acetami	Radius_SOS	-0,0677	0,054176	NA
3	abundance	X07294	6-Acetami	Tibia_SOS	-0,01523	0,057384	NA
3	abundance	X07294	6-Acetami	Handgrip	-0,08632	0,062543	NA
3	abundance	X07303	Triacetin	Tibia_length	-0,10421	0,053858	NA
3	abundance	X07303	Triacetin	Radius_length	-0,1342	0,072507	NA
3	abundance	X07303	Triacetin	Radius_SOS	-0,02548	0,051401	NA
3	abundance	X07303	Triacetin	Tibia_SOS	-7,66E-05	0,054894	NA
3	abundance	X07303	Triacetin	Handgrip	-0,09232	0,061381	NA
2a	abundance	X07308	Nicotinic a	Tibia_length	-0,07122	0,053639	NA
2a	abundance	X07308	Nicotinic a	Radius_length	-0,02484	0,073068	NA
2a	abundance	X07308	Nicotinic a	Radius_SOS	-0,13017	0,051365	NA
2a	abundance	X07308	Nicotinic a	Tibia_SOS	-0,13351	0,055965	NA
2a	abundance	X07308	Nicotinic a	Handgrip	-0,03495	0,062574	NA
3	abundance	X07309	3,4,15-Trih	Tibia_length	-0,04257	0,054518	NA
3	abundance	X07309	3,4,15-Trih	Radius_length	-0,04779	0,07292	NA
3	abundance	X07309	3,4,15-Trih	Radius_SOS	0,02824	0,051999	NA
3	abundance	X07309	3,4,15-Trih	Tibia_SOS	-0,0067	0,055285	NA
3	abundance	X07309	3,4,15-Trih	Handgrip	-0,00228	0,062056	NA
3	abundance	X07315	Rivastigmi	Tibia_length	-0,08064	0,052693	NA
3	abundance	X07315	Rivastigmi	Radius_length	-0,11685	0,071424	NA
3	abundance	X07315	Rivastigmi	Radius_SOS	-0,03098	0,050575	NA
3	abundance	X07315	Rivastigmi	Tibia_SOS	-0,13228	0,054427	NA
3	abundance	X07315	Rivastigmi	Handgrip	-0,01874	0,06189	NA
3	abundance	X07320	asn-pro	Tibia_length	-0,16499	0,053347	NA
3	abundance	X07320	asn-pro	Radius_length	-0,14969	0,071856	NA
3	abundance	X07320	asn-pro	Radius_SOS	-0,04432	0,051119	NA
3	abundance	X07320	asn-pro	Tibia_SOS	-0,09762	0,05417	NA
3	abundance	X07320	asn-pro	Handgrip	-0,13636	0,060222	NA
3	abundance	X07327	Aspartyl-L-	Tibia_length	-0,09795	0,056207	NA
3	abundance	X07327	Aspartyl-L-	Radius_length	-0,07506	0,074316	NA
3	abundance	X07327	Aspartyl-L-	Radius_SOS	0,030902	0,053579	NA
3	abundance	X07327	Aspartyl-L-	Tibia_SOS	-0,03313	0,05824	NA

3	abundance	X07327	Aspartyl-L-	Handgrip	-0,0027	0,06283	NA
3	abundance	X07334	2,2-Bis(hy(	Tibia_length	-0,02963	0,055111	NA
3	abundance	X07334	2,2-Bis(hy(	Radius_length	-0,05214	0,074267	NA
3	abundance	X07334	2,2-Bis(hy(	Radius_SOS	-0,10606	0,052668	NA
3	abundance	X07334	2,2-Bis(hy(	Tibia_SOS	-0,00682	0,056498	NA
3	abundance	X07334	2,2-Bis(hy(	Handgrip	0,133614	0,061762	NA
3	abundance	X07336	Threonylse	Tibia_length	-0,04995	0,055804	NA
3	abundance	X07336	Threonylse	Radius_length	0,05304	0,074246	NA
3	abundance	X07336	Threonylse	Radius_SOS	0,012411	0,053052	NA
3	abundance	X07336	Threonylse	Tibia_SOS	0,002745	0,058112	NA
3	abundance	X07336	Threonylse	Handgrip	0,004495	0,063176	NA
3	abundance	X07344	(âˆ’)-nabil(	Tibia_length	-0,02115	0,053851	NA
3	abundance	X07344	(âˆ’)-nabil(	Radius_length	-0,04912	0,072744	NA
3	abundance	X07344	(âˆ’)-nabil(	Radius_SOS	0,083069	0,051079	NA
3	abundance	X07344	(âˆ’)-nabil(	Tibia_SOS	0,008266	0,054675	NA
3	abundance	X07344	(âˆ’)-nabil(	Handgrip	0,06473	0,060861	NA
3	abundance	X07345	Phenyl D-g	Tibia_length	-0,00572	0,055382	NA
3	abundance	X07345	Phenyl D-g	Radius_length	-0,13536	0,072941	NA
3	abundance	X07345	Phenyl D-g	Radius_SOS	-0,06256	0,052534	NA
3	abundance	X07345	Phenyl D-g	Tibia_SOS	-0,05937	0,055919	NA
3	abundance	X07345	Phenyl D-g	Handgrip	-0,0791	0,062799	NA
3	abundance	X07375	porphobilir	Tibia_length	-0,02991	0,050341	NA
3	abundance	X07375	porphobilir	Radius_length	-0,04556	0,068777	NA
3	abundance	X07375	porphobilir	Radius_SOS	-0,06814	0,049044	NA
3	abundance	X07375	porphobilir	Tibia_SOS	-0,01425	0,052658	NA
3	abundance	X07375	porphobilir	Handgrip	-0,08516	0,0601	NA
3	abundance	X07381	Bis-D-fruct	Tibia_length	-0,10447	0,05486	NA
3	abundance	X07381	Bis-D-fruct	Radius_length	-0,07034	0,07377	NA
3	abundance	X07381	Bis-D-fruct	Radius_SOS	-0,05361	0,052291	NA
3	abundance	X07381	Bis-D-fruct	Tibia_SOS	0,066094	0,055991	NA
3	abundance	X07381	Bis-D-fruct	Handgrip	-0,01883	0,062417	NA
3	abundance	X07394	Hydroxypr(	Tibia_length	0,109409	0,056658	NA
3	abundance	X07394	Hydroxypr(	Radius_length	0,216922	0,07316	NA
3	abundance	X07394	Hydroxypr(	Radius_SOS	0,062831	0,053934	NA
3	abundance	X07394	Hydroxypr(	Tibia_SOS	0,039003	0,059304	NA
3	abundance	X07394	Hydroxypr(	Handgrip	0,177365	0,062821	NA
3	abundance	X07399	Homoanse	Tibia_length	-0,03375	0,054761	NA
3	abundance	X07399	Homoanse	Radius_length	-0,0021	0,073531	NA
3	abundance	X07399	Homoanse	Radius_SOS	-0,09373	0,051837	NA
3	abundance	X07399	Homoanse	Tibia_SOS	0,059337	0,055391	NA
3	abundance	X07399	Homoanse	Handgrip	-0,01181	0,062513	NA
2b	abundance	X07400	Tranexami(	Tibia_length	0,006156	0,049686	NA
2b	abundance	X07400	Tranexami(	Radius_length	0,108297	0,067363	NA
2b	abundance	X07400	Tranexami(	Radius_SOS	-0,01192	0,049028	NA
2b	abundance	X07400	Tranexami(	Tibia_SOS	0,087307	0,052313	NA
2b	abundance	X07400	Tranexami(	Handgrip	0,079096	0,060196	NA
1	abundance	X07413	Chenodeo:	Tibia_length	-0,02875	0,050302	NA
1	abundance	X07413	Chenodeo:	Radius_length	0,051037	0,06869	NA

1	abundance	X07413	Chenodeo:	Radius_SOS	-0,03698	0,049185	NA
1	abundance	X07413	Chenodeo:	Tibia_SOS	-0,05206	0,052631	NA
1	abundance	X07413	Chenodeo:	Handgrip	0,012682	0,060809	NA
3	abundance	X07414	2,2-Bis(hy(	Tibia_length	-0,03858	0,053783	NA
3	abundance	X07414	2,2-Bis(hy(	Radius_length	0,031799	0,073337	NA
3	abundance	X07414	2,2-Bis(hy(	Radius_SOS	0,013659	0,051182	NA
3	abundance	X07414	2,2-Bis(hy(	Tibia_SOS	0,013899	0,055892	NA
3	abundance	X07414	2,2-Bis(hy(	Handgrip	0,089777	0,06125	NA
2b	abundance	X07417	Î´-Valerola	Tibia_length	0,027512	0,054467	NA
2b	abundance	X07417	Î´-Valerola	Radius_length	-0,00213	0,073212	NA
2b	abundance	X07417	Î´-Valerola	Radius_SOS	-0,03751	0,051933	NA
2b	abundance	X07417	Î´-Valerola	Tibia_SOS	-0,01274	0,054755	NA
2b	abundance	X07417	Î´-Valerola	Handgrip	0,030278	0,061712	NA
3	abundance	X07420	DNOP	Tibia_length	0,057327	0,052776	NA
3	abundance	X07420	DNOP	Radius_length	0,079998	0,072035	NA
3	abundance	X07420	DNOP	Radius_SOS	0,009899	0,050448	NA
3	abundance	X07420	DNOP	Tibia_SOS	0,009909	0,053878	NA
3	abundance	X07420	DNOP	Handgrip	0,033313	0,061197	NA
3	abundance	X07424	Rutinose (I	Tibia_length	0,06221	0,057132	NA
3	abundance	X07424	Rutinose (I	Radius_length	0,13216	0,074508	NA
3	abundance	X07424	Rutinose (I	Radius_SOS	0,020259	0,054419	NA
3	abundance	X07424	Rutinose (I	Tibia_SOS	0,0644	0,057913	NA
3	abundance	X07424	Rutinose (I	Handgrip	0,0548	0,062901	NA
2a	abundance	X07425	N-Acetylas	Tibia_length	0,164971	0,0526	NA
2a	abundance	X07425	N-Acetylas	Radius_length	0,067226	0,072696	NA
2a	abundance	X07425	N-Acetylas	Radius_SOS	0,033216	0,051014	NA
2a	abundance	X07425	N-Acetylas	Tibia_SOS	0,098075	0,055218	NA
2a	abundance	X07425	N-Acetylas	Handgrip	-0,03629	0,061877	NA
3	abundance	X07428	N6-METHY	Tibia_length	-0,02026	0,049214	NA
3	abundance	X07428	N6-METHY	Radius_length	-0,03843	0,06548	NA
3	abundance	X07428	N6-METHY	Radius_SOS	0,019345	0,049118	NA
3	abundance	X07428	N6-METHY	Tibia_SOS	-0,07451	0,05239	NA
3	abundance	X07428	N6-METHY	Handgrip	-0,01044	0,060191	NA
3	abundance	X07429	DNOP	Tibia_length	0,019673	0,049922	NA
3	abundance	X07429	DNOP	Radius_length	-0,01385	0,067886	NA
3	abundance	X07429	DNOP	Radius_SOS	0,044535	0,049021	NA
3	abundance	X07429	DNOP	Tibia_SOS	-0,08315	0,052475	NA
3	abundance	X07429	DNOP	Handgrip	0,017707	0,061235	NA
2b	abundance	X07434	N'-Hydroxy	Tibia_length	-0,05275	0,052203	NA
2b	abundance	X07434	N'-Hydroxy	Radius_length	-0,12844	0,070667	NA
2b	abundance	X07434	N'-Hydroxy	Radius_SOS	-0,06424	0,050241	NA
2b	abundance	X07434	N'-Hydroxy	Tibia_SOS	0,013957	0,053971	NA
2b	abundance	X07434	N'-Hydroxy	Handgrip	-0,04044	0,061284	NA
1	abundance	X07439	4-pyridoxic	Tibia_length	-0,02306	0,049463	NA
1	abundance	X07439	4-pyridoxic	Radius_length	0,10742	0,066694	NA
1	abundance	X07439	4-pyridoxic	Radius_SOS	0,04012	0,048991	NA
1	abundance	X07439	4-pyridoxic	Tibia_SOS	0,076675	0,052353	NA
1	abundance	X07439	4-pyridoxic	Handgrip	0,060765	0,060097	NA

3	abundance	X07442	coenzyme	Tibia_length	0,021261	0,049326	NA
3	abundance	X07442	coenzyme	Radius_length	-0,01147	0,066216	NA
3	abundance	X07442	coenzyme	Radius_SOS	0,009681	0,049059	NA
3	abundance	X07442	coenzyme	Tibia_SOS	-5,71E-04	0,052711	NA
3	abundance	X07442	coenzyme	Handgrip	0,084291	0,060015	NA
3	abundance	X07443	Butylparab	Tibia_length	-0,01092	0,052098	NA
3	abundance	X07443	Butylparab	Radius_length	0,016982	0,071145	NA
3	abundance	X07443	Butylparab	Radius_SOS	0,071953	0,050183	NA
3	abundance	X07443	Butylparab	Tibia_SOS	-0,04233	0,053398	NA
3	abundance	X07443	Butylparab	Handgrip	-0,10195	0,060874	NA
3	abundance	X07445	3-(Sulfoox)	Tibia_length	0,007346	0,053667	NA
3	abundance	X07445	3-(Sulfoox)	Radius_length	-0,16235	0,071541	NA
3	abundance	X07445	3-(Sulfoox)	Radius_SOS	-0,08105	0,050872	NA
3	abundance	X07445	3-(Sulfoox)	Tibia_SOS	-0,06188	0,055151	NA
3	abundance	X07445	3-(Sulfoox)	Handgrip	-0,11909	0,06143	NA
3	abundance	X07451	Valylvaline	Tibia_length	0,068765	0,052839	NA
3	abundance	X07451	Valylvaline	Radius_length	0,085293	0,071974	NA
3	abundance	X07451	Valylvaline	Radius_SOS	0,071869	0,050431	NA
3	abundance	X07451	Valylvaline	Tibia_SOS	0,113124	0,053911	NA
3	abundance	X07451	Valylvaline	Handgrip	0,040499	0,061041	NA
3	abundance	X07452	4-Amino-1	Tibia_length	-0,10505	0,0539	NA
3	abundance	X07452	4-Amino-1	Radius_length	-0,12597	0,072031	NA
3	abundance	X07452	4-Amino-1	Radius_SOS	-0,00753	0,051071	NA
3	abundance	X07452	4-Amino-1	Tibia_SOS	0,02315	0,0553	NA
3	abundance	X07452	4-Amino-1	Handgrip	-0,07792	0,061803	NA
2b	abundance	X07454	2,4-Quinol	Tibia_length	-0,13414	0,051138	NA
2b	abundance	X07454	2,4-Quinol	Radius_length	-0,00302	0,071023	NA
2b	abundance	X07454	2,4-Quinol	Radius_SOS	-0,02422	0,04997	NA
2b	abundance	X07454	2,4-Quinol	Tibia_SOS	0,100593	0,053046	NA
2b	abundance	X07454	2,4-Quinol	Handgrip	-0,05915	0,061272	NA
1	abundance	X07457	Glucosami	Tibia_length	-0,03176	0,056103	NA
1	abundance	X07457	Glucosami	Radius_length	0,01984	0,074996	NA
1	abundance	X07457	Glucosami	Radius_SOS	-0,03791	0,053334	NA
1	abundance	X07457	Glucosami	Tibia_SOS	-0,02765	0,057629	NA
1	abundance	X07457	Glucosami	Handgrip	0,093792	0,063536	NA
3	abundance	X07460	1-pyrroline	Tibia_length	0,06433	0,052463	NA
3	abundance	X07460	1-pyrroline	Radius_length	0,080005	0,071889	NA
3	abundance	X07460	1-pyrroline	Radius_SOS	-0,00522	0,050291	NA
3	abundance	X07460	1-pyrroline	Tibia_SOS	0,144975	0,052954	NA
3	abundance	X07460	1-pyrroline	Handgrip	0,072097	0,060421	NA
3	abundance	X07465	TO012790	Tibia_length	-0,05961	0,051863	NA
3	abundance	X07465	TO012790	Radius_length	-0,09961	0,070328	NA
3	abundance	X07465	TO012790	Radius_SOS	-0,07033	0,049845	NA
3	abundance	X07465	TO012790	Tibia_SOS	-0,05583	0,05361	NA
3	abundance	X07465	TO012790	Handgrip	-0,05997	0,061451	NA
3	abundance	X07469	1,1'-[1,12-	Tibia_length	0,02059	0,050088	NA
3	abundance	X07469	1,1'-[1,12-	Radius_length	0,06552	0,06823	NA
3	abundance	X07469	1,1'-[1,12-	Radius_SOS	-0,00101	0,049114	NA

	3	abundance	X07469	1,1'-[1,12-I	Tibia_SOS	-0,07406	0,052454	NA
	3	abundance	X07469	1,1'-[1,12-I	Handgrip	0,049803	0,060684	NA
	3	abundance	X07475	2'-Deoxy-5	Tibia_length	-4,68E-04	0,053589	NA
	3	abundance	X07475	2'-Deoxy-5	Radius_length	-0,01699	0,072445	NA
	3	abundance	X07475	2'-Deoxy-5	Radius_SOS	0,045056	0,051176	NA
	3	abundance	X07475	2'-Deoxy-5	Tibia_SOS	-0,00836	0,054775	NA
	3	abundance	X07475	2'-Deoxy-5	Handgrip	0,022658	0,062278	NA
	1	abundance	X07476	4-Hydroxyb	Tibia_length	0,183258	0,052349	NA
	1	abundance	X07476	4-Hydroxyb	Radius_length	0,139486	0,071645	NA
	1	abundance	X07476	4-Hydroxyb	Radius_SOS	0,018813	0,050741	NA
	1	abundance	X07476	4-Hydroxyb	Tibia_SOS	0,126187	0,053634	NA
	1	abundance	X07476	4-Hydroxyb	Handgrip	0,093218	0,060526	NA
2a		abundance	X07480	Naringenin	Tibia_length	-0,01142	0,055726	NA
2a		abundance	X07480	Naringenin	Radius_length	0,045111	0,074188	NA
2a		abundance	X07480	Naringenin	Radius_SOS	-0,05585	0,052786	NA
2a		abundance	X07480	Naringenin	Tibia_SOS	-0,03558	0,056421	NA
2a		abundance	X07480	Naringenin	Handgrip	0,046752	0,061681	NA
	3	abundance	X07484	MFCD0002	Tibia_length	0,013285	0,05087	NA
	3	abundance	X07484	MFCD0002	Radius_length	-0,07857	0,069269	NA
	3	abundance	X07484	MFCD0002	Radius_SOS	-0,02073	0,049375	NA
	3	abundance	X07484	MFCD0002	Tibia_SOS	0,049416	0,052748	NA
	3	abundance	X07484	MFCD0002	Handgrip	-0,02123	0,060325	NA
	3	abundance	X07487	Oxypeucec	Tibia_length	0,001246	0,05503	NA
	3	abundance	X07487	Oxypeucec	Radius_length	0,004789	0,073591	NA
	3	abundance	X07487	Oxypeucec	Radius_SOS	-0,04462	0,05236	NA
	3	abundance	X07487	Oxypeucec	Tibia_SOS	0,016047	0,055442	NA
	3	abundance	X07487	Oxypeucec	Handgrip	0,032968	0,061837	NA
2b		abundance	X07491	1-Vinylimic	Tibia_length	-0,09577	0,050267	NA
2b		abundance	X07491	1-Vinylimic	Radius_length	-0,13613	0,068523	NA
2b		abundance	X07491	1-Vinylimic	Radius_SOS	-0,04581	0,04914	NA
2b		abundance	X07491	1-Vinylimic	Tibia_SOS	-0,08339	0,052499	NA
2b		abundance	X07491	1-Vinylimic	Handgrip	0,007301	0,060623	NA
2b		abundance	X07504	4-Acetamid	Tibia_length	0,03474	0,0506	NA
2b		abundance	X07504	4-Acetamid	Radius_length	0,056238	0,069626	NA
2b		abundance	X07504	4-Acetamid	Radius_SOS	0,042019	0,049259	NA
2b		abundance	X07504	4-Acetamid	Tibia_SOS	0,110632	0,052734	NA
2b		abundance	X07504	4-Acetamid	Handgrip	0,031441	0,061011	NA
2b		abundance	X07508	Procaine	Tibia_length	-0,12238	0,051995	NA
2b		abundance	X07508	Procaine	Radius_length	-0,08601	0,070867	NA
2b		abundance	X07508	Procaine	Radius_SOS	0,035632	0,050102	NA
2b		abundance	X07508	Procaine	Tibia_SOS	-0,03999	0,053981	NA
2b		abundance	X07508	Procaine	Handgrip	0,006215	0,061378	NA
	3	abundance	X07511	Piperidine	Tibia_length	-0,05128	0,049841	NA
	3	abundance	X07511	Piperidine	Radius_length	-0,0322	0,06781	NA
	3	abundance	X07511	Piperidine	Radius_SOS	0,008508	0,049065	NA
	3	abundance	X07511	Piperidine	Tibia_SOS	0,044032	0,052543	NA
	3	abundance	X07511	Piperidine	Handgrip	-0,00571	0,060269	NA
	3	abundance	X07512	4-{[(3-Hydr	Tibia_length	-0,10488	0,055778	NA

	3	abundance	X07512	4-{[(3-Hydr	Radius_length	0,06503	0,074429	NA
	3	abundance	X07512	4-{[(3-Hydr	Radius_SOS	0,018602	0,053086	NA
	3	abundance	X07512	4-{[(3-Hydr	Tibia_SOS	0,101849	0,057316	NA
	3	abundance	X07512	4-{[(3-Hydr	Handgrip	0,047789	0,06314	NA
	3	abundance	X07513	(âˆ’)-nabil	Tibia_length	0,009467	0,051798	NA
	3	abundance	X07513	(âˆ’)-nabil	Radius_length	0,082794	0,070732	NA
	3	abundance	X07513	(âˆ’)-nabil	Radius_SOS	0,032348	0,049891	NA
	3	abundance	X07513	(âˆ’)-nabil	Tibia_SOS	-0,06681	0,053567	NA
	3	abundance	X07513	(âˆ’)-nabil	Handgrip	-0,0237	0,061387	NA
	3	abundance	X07514	L-(+)-Eryth	Tibia_length	-0,03831	0,055745	NA
	3	abundance	X07514	L-(+)-Eryth	Radius_length	0,019335	0,074647	NA
	3	abundance	X07514	L-(+)-Eryth	Radius_SOS	-0,03769	0,053031	NA
	3	abundance	X07514	L-(+)-Eryth	Tibia_SOS	0,004369	0,056982	NA
	3	abundance	X07514	L-(+)-Eryth	Handgrip	0,099953	0,06309	NA
	3	abundance	X07518	(+/-)-2-Hyc	Tibia_length	-0,09824	0,053713	NA
	3	abundance	X07518	(+/-)-2-Hyc	Radius_length	-0,0266	0,073408	NA
	3	abundance	X07518	(+/-)-2-Hyc	Radius_SOS	-0,01623	0,051242	NA
	3	abundance	X07518	(+/-)-2-Hyc	Tibia_SOS	0,032752	0,054626	NA
	3	abundance	X07518	(+/-)-2-Hyc	Handgrip	-0,02391	0,061791	NA
	3	abundance	X07519	Pyrrolidine	Tibia_length	-0,12554	0,051539	NA
	3	abundance	X07519	Pyrrolidine	Radius_length	-0,1061	0,070376	NA
	3	abundance	X07519	Pyrrolidine	Radius_SOS	0,010469	0,049788	NA
	3	abundance	X07519	Pyrrolidine	Tibia_SOS	0,03124	0,053453	NA
	3	abundance	X07519	Pyrrolidine	Handgrip	0,012938	0,061032	NA
	1	abundance	X07523	Ursodeoxy	Tibia_length	0,030724	0,049207	NA
	1	abundance	X07523	Ursodeoxy	Radius_length	0,060665	0,065524	NA
	1	abundance	X07523	Ursodeoxy	Radius_SOS	0,017946	0,049096	NA
	1	abundance	X07523	Ursodeoxy	Tibia_SOS	-0,00882	0,052554	NA
	1	abundance	X07523	Ursodeoxy	Handgrip	0,025276	0,060305	NA
2b		abundance	X07524	Prolinamid	Tibia_length	-0,06045	0,051613	NA
2b		abundance	X07524	Prolinamid	Radius_length	-0,01289	0,070786	NA
2b		abundance	X07524	Prolinamid	Radius_SOS	0,018226	0,04972	NA
2b		abundance	X07524	Prolinamid	Tibia_SOS	0,017635	0,052984	NA
2b		abundance	X07524	Prolinamid	Handgrip	0,032048	0,060625	NA
2b		abundance	X07527	1,7-Dimetl	Tibia_length	-0,07081	0,051452	NA
2b		abundance	X07527	1,7-Dimetl	Radius_length	-0,11553	0,070108	NA
2b		abundance	X07527	1,7-Dimetl	Radius_SOS	0,007157	0,049766	NA
2b		abundance	X07527	1,7-Dimetl	Tibia_SOS	-0,06318	0,052851	NA
2b		abundance	X07527	1,7-Dimetl	Handgrip	-0,02104	0,061139	NA
	1	abundance	X07530	Alanine	Tibia_length	0,143892	0,053473	NA
	1	abundance	X07530	Alanine	Radius_length	0,184118	0,07201	NA
	1	abundance	X07530	Alanine	Radius_SOS	0,032143	0,05138	NA
	1	abundance	X07530	Alanine	Tibia_SOS	0,145468	0,054344	NA
	1	abundance	X07530	Alanine	Handgrip	0,166331	0,059862	NA
	3	abundance	X07537	alpha-Cha	Tibia_length	0,026371	0,05629	NA
	3	abundance	X07537	alpha-Cha	Radius_length	0,210586	0,072524	NA
	3	abundance	X07537	alpha-Cha	Radius_SOS	-6,38E-04	0,05328	NA
	3	abundance	X07537	alpha-Cha	Tibia_SOS	0,017049	0,05593	NA

	3	abundance	X07537	alpha-Cha	Handgrip	0,100769	0,062753	NA
	3	abundance	X07538	Homoanse	Tibia_length	0,014205	0,049723	NA
	3	abundance	X07538	Homoanse	Radius_length	0,160203	0,066597	NA
	3	abundance	X07538	Homoanse	Radius_SOS	0,048674	0,048985	NA
	3	abundance	X07538	Homoanse	Tibia_SOS	0,004652	0,052488	NA
	3	abundance	X07538	Homoanse	Handgrip	0,048039	0,060754	NA
	1	abundance	X07541	Saccharin	Tibia_length	-0,01314	0,054871	NA
	1	abundance	X07541	Saccharin	Radius_length	0,119113	0,072913	NA
	1	abundance	X07541	Saccharin	Radius_SOS	0,010357	0,052286	NA
	1	abundance	X07541	Saccharin	Tibia_SOS	0,061427	0,055635	NA
	1	abundance	X07541	Saccharin	Handgrip	0,057012	0,061181	NA
	3	abundance	X07544	Ethyl sulfat	Tibia_length	-0,0172	0,054959	NA
	3	abundance	X07544	Ethyl sulfat	Radius_length	-0,0587	0,073363	NA
	3	abundance	X07544	Ethyl sulfat	Radius_SOS	-0,02788	0,052368	NA
	3	abundance	X07544	Ethyl sulfat	Tibia_SOS	0,049725	0,055571	NA
	3	abundance	X07544	Ethyl sulfat	Handgrip	0,063832	0,062363	NA
2b		abundance	X07558	Leucylproli	Tibia_length	-0,03427	0,051824	NA
2b		abundance	X07558	Leucylproli	Radius_length	0,017519	0,071082	NA
2b		abundance	X07558	Leucylproli	Radius_SOS	-0,01007	0,049968	NA
2b		abundance	X07558	Leucylproli	Tibia_SOS	0,063969	0,053955	NA
2b		abundance	X07558	Leucylproli	Handgrip	0,05206	0,06038	NA
	1	abundance	X07563	Xanthine	Tibia_length	0,071987	0,051585	NA
	1	abundance	X07563	Xanthine	Radius_length	0,106026	0,071412	NA
	1	abundance	X07563	Xanthine	Radius_SOS	0,023315	0,049821	NA
	1	abundance	X07563	Xanthine	Tibia_SOS	0,138707	0,052851	NA
	1	abundance	X07563	Xanthine	Handgrip	0,131496	0,059798	NA
2b		abundance	X07565	5-Hydroxyi	Tibia_length	-0,03121	0,049103	NA
2b		abundance	X07565	5-Hydroxyi	Radius_length	-0,00647	0,065351	NA
2b		abundance	X07565	5-Hydroxyi	Radius_SOS	-0,01395	0,049184	NA
2b		abundance	X07565	5-Hydroxyi	Tibia_SOS	0,059275	0,052438	NA
2b		abundance	X07565	5-Hydroxyi	Handgrip	-0,00956	0,06037	NA
	3	abundance	X07566	5-Allyl-5-se	Tibia_length	-0,12387	0,055157	NA
	3	abundance	X07566	5-Allyl-5-se	Radius_length	-0,19045	0,072298	NA
	3	abundance	X07566	5-Allyl-5-se	Radius_SOS	0,030126	0,052787	NA
	3	abundance	X07566	5-Allyl-5-se	Tibia_SOS	-0,00865	0,055103	NA
	3	abundance	X07566	5-Allyl-5-se	Handgrip	-0,06986	0,062441	NA
	3	abundance	X07572	8-(3-Furyl)-	Tibia_length	-0,02294	0,056769	NA
	3	abundance	X07572	8-(3-Furyl)-	Radius_length	0,062042	0,074688	NA
	3	abundance	X07572	8-(3-Furyl)-	Radius_SOS	-0,00328	0,053926	NA
	3	abundance	X07572	8-(3-Furyl)-	Tibia_SOS	0,050518	0,057465	NA
	3	abundance	X07572	8-(3-Furyl)-	Handgrip	0,053135	0,063524	NA
	3	abundance	X07574	7-ketodeox	Tibia_length	0,035202	0,050924	NA
	3	abundance	X07574	7-ketodeox	Radius_length	0,030711	0,069549	NA
	3	abundance	X07574	7-ketodeox	Radius_SOS	0,044063	0,049357	NA
	3	abundance	X07574	7-ketodeox	Tibia_SOS	-0,04687	0,05333	NA
	3	abundance	X07574	7-ketodeox	Handgrip	-0,04052	0,061247	NA
	3	abundance	X07577	2-[(Sulfoox	Tibia_length	-0,06084	0,054836	NA
	3	abundance	X07577	2-[(Sulfoox	Radius_length	-0,19313	0,071953	NA

3	abundance	X07577	2-[(Sulfoox	Radius_SOS	-0,05653	0,051854	NA
3	abundance	X07577	2-[(Sulfoox	Tibia_SOS	-0,07707	0,056116	NA
3	abundance	X07577	2-[(Sulfoox	Handgrip	-0,13484	0,062371	NA
3	abundance	X07578	5-Allyl-5-se	Tibia_length	-0,15716	0,053919	NA
3	abundance	X07578	5-Allyl-5-se	Radius_length	-0,0692	0,072856	NA
3	abundance	X07578	5-Allyl-5-se	Radius_SOS	0,023987	0,051651	NA
3	abundance	X07578	5-Allyl-5-se	Tibia_SOS	-0,07424	0,054634	NA
3	abundance	X07578	5-Allyl-5-se	Handgrip	0,010047	0,061869	NA
3	abundance	X07580	N-Pentano	Tibia_length	0,099508	0,053614	NA
3	abundance	X07580	N-Pentano	Radius_length	0,100006	0,072693	NA
3	abundance	X07580	N-Pentano	Radius_SOS	0,06809	0,051473	NA
3	abundance	X07580	N-Pentano	Tibia_SOS	0,037046	0,055154	NA
3	abundance	X07580	N-Pentano	Handgrip	-0,04805	0,06239	NA
3	abundance	X07581	N-Acetylva	Tibia_length	-0,0075	0,054011	NA
3	abundance	X07581	N-Acetylva	Radius_length	0,014107	0,072885	NA
3	abundance	X07581	N-Acetylva	Radius_SOS	-0,02851	0,051404	NA
3	abundance	X07581	N-Acetylva	Tibia_SOS	-8,29E-04	0,055257	NA
3	abundance	X07581	N-Acetylva	Handgrip	0,044225	0,062285	NA
2b	abundance	X07582	4-methylpy	Tibia_length	-0,1037	0,055169	NA
2b	abundance	X07582	4-methylpy	Radius_length	-0,05071	0,073782	NA
2b	abundance	X07582	4-methylpy	Radius_SOS	0,108528	0,052087	NA
2b	abundance	X07582	4-methylpy	Tibia_SOS	0,036407	0,054558	NA
2b	abundance	X07582	4-methylpy	Handgrip	0,066724	0,061572	NA
2b	abundance	X07596	5-amino-2-	Tibia_length	0,017124	0,051352	NA
2b	abundance	X07596	5-amino-2-	Radius_length	0,076829	0,070271	NA
2b	abundance	X07596	5-amino-2-	Radius_SOS	-0,03027	0,049605	NA
2b	abundance	X07596	5-amino-2-	Tibia_SOS	0,045954	0,053464	NA
2b	abundance	X07596	5-amino-2-	Handgrip	0,014391	0,060605	NA
3	abundance	X07597	p-Cresylsu	Tibia_length	-0,00953	0,054926	NA
3	abundance	X07597	p-Cresylsu	Radius_length	-0,04657	0,073468	NA
3	abundance	X07597	p-Cresylsu	Radius_SOS	0,001611	0,052292	NA
3	abundance	X07597	p-Cresylsu	Tibia_SOS	0,018762	0,056116	NA
3	abundance	X07597	p-Cresylsu	Handgrip	-0,20319	0,06105	NA
3	abundance	X07601	Asp-lys	Tibia_length	-0,03641	0,051775	NA
3	abundance	X07601	Asp-lys	Radius_length	0,00275	0,071087	NA
3	abundance	X07601	Asp-lys	Radius_SOS	0,047715	0,04968	NA
3	abundance	X07601	Asp-lys	Tibia_SOS	0,1437	0,052482	NA
3	abundance	X07601	Asp-lys	Handgrip	-0,05065	0,061229	NA
3	abundance	X07613	1-(4-Aminc	Tibia_length	-0,08107	0,052174	NA
3	abundance	X07613	1-(4-Aminc	Radius_length	-0,03026	0,071169	NA
3	abundance	X07613	1-(4-Aminc	Radius_SOS	0,003101	0,050062	NA
3	abundance	X07613	1-(4-Aminc	Tibia_SOS	-0,00915	0,053345	NA
3	abundance	X07613	1-(4-Aminc	Handgrip	0,041526	0,061067	NA
3	abundance	X07618	N-(3-aceta	Tibia_length	0,042082	0,049628	NA
3	abundance	X07618	N-(3-aceta	Radius_length	0,123032	0,067112	NA
3	abundance	X07618	N-(3-aceta	Radius_SOS	0,012801	0,049026	NA
3	abundance	X07618	N-(3-aceta	Tibia_SOS	0,07934	0,052429	NA
3	abundance	X07618	N-(3-aceta	Handgrip	0,100117	0,060267	NA

	1	abundance	X07630	Methionine	Tibia_length	0,049281	0,056188	NA
	1	abundance	X07630	Methionine	Radius_length	0,090601	0,073718	NA
	1	abundance	X07630	Methionine	Radius_SOS	-0,02008	0,053401	NA
	1	abundance	X07630	Methionine	Tibia_SOS	0,101076	0,055453	NA
	1	abundance	X07630	Methionine	Handgrip	0,076788	0,061668	NA
2b		abundance	X07634	3,8,9-trihy	Tibia_length	-0,02141	0,051023	NA
2b		abundance	X07634	3,8,9-trihy	Radius_length	0,036262	0,069883	NA
2b		abundance	X07634	3,8,9-trihy	Radius_SOS	0,055101	0,049494	NA
2b		abundance	X07634	3,8,9-trihy	Tibia_SOS	-0,00641	0,052866	NA
2b		abundance	X07634	3,8,9-trihy	Handgrip	-0,03531	0,060787	NA
	3	abundance	X07638	7-ketodeo	Tibia_length	0,073864	0,050429	NA
	3	abundance	X07638	7-ketodeo	Radius_length	0,099891	0,068602	NA
	3	abundance	X07638	7-ketodeo	Radius_SOS	0,017825	0,049213	NA
	3	abundance	X07638	7-ketodeo	Tibia_SOS	-0,04804	0,052771	NA
	3	abundance	X07638	7-ketodeo	Handgrip	0,00999	0,060632	NA
	3	abundance	X07645	O-propeno	Tibia_length	-0,02676	0,058818	NA
	3	abundance	X07645	O-propeno	Radius_length	0,109392	0,075328	NA
	3	abundance	X07645	O-propeno	Radius_SOS	0,077666	0,056191	NA
	3	abundance	X07645	O-propeno	Tibia_SOS	0,006664	0,057979	NA
	3	abundance	X07645	O-propeno	Handgrip	-0,09383	0,062997	NA
	3	abundance	X07646	(2Z)-2-({[(6	Tibia_length	-0,05021	0,05902	NA
	3	abundance	X07646	(2Z)-2-({[(6	Radius_length	0,033899	0,075926	NA
	3	abundance	X07646	(2Z)-2-({[(6	Radius_SOS	0,028459	0,056215	NA
	3	abundance	X07646	(2Z)-2-({[(6	Tibia_SOS	0,142858	0,058733	NA
	3	abundance	X07646	(2Z)-2-({[(6	Handgrip	0,050734	0,060115	NA
	1	abundance	X07647	Genistein	Tibia_length	-0,02173	0,052035	NA
	1	abundance	X07647	Genistein	Radius_length	-0,01071	0,071285	NA
	1	abundance	X07647	Genistein	Radius_SOS	0,004726	0,050063	NA
	1	abundance	X07647	Genistein	Tibia_SOS	0,071723	0,054428	NA
	1	abundance	X07647	Genistein	Handgrip	0,003108	0,062105	NA
	3	abundance	X07648	NL851300	Tibia_length	0,098866	0,049884	NA
	3	abundance	X07648	NL851300	Radius_length	0,1343	0,067735	NA
	3	abundance	X07648	NL851300	Radius_SOS	0,055516	0,049044	NA
	3	abundance	X07648	NL851300	Tibia_SOS	0,088653	0,052377	NA
	3	abundance	X07648	NL851300	Handgrip	0,0383	0,060297	NA
2b		abundance	X07657	Docosahe	Tibia_length	0,023293	0,051566	NA
2b		abundance	X07657	Docosahe	Radius_length	-0,03085	0,070911	NA
2b		abundance	X07657	Docosahe	Radius_SOS	0,103904	0,049609	NA
2b		abundance	X07657	Docosahe	Tibia_SOS	-0,00716	0,05362	NA
2b		abundance	X07657	Docosahe	Handgrip	0,053012	0,060937	NA
	1	abundance	X07658	Î´-Glucono	Tibia_length	-0,03725	0,056502	NA
	1	abundance	X07658	Î´-Glucono	Radius_length	-0,00848	0,075058	NA
	1	abundance	X07658	Î´-Glucono	Radius_SOS	0,021002	0,05368	NA
	1	abundance	X07658	Î´-Glucono	Tibia_SOS	0,019914	0,058023	NA
	1	abundance	X07658	Î´-Glucono	Handgrip	0,053244	0,062783	NA
	1	abundance	X07674	Lysine	Tibia_length	0,144023	0,049801	NA
	1	abundance	X07674	Lysine	Radius_length	0,208921	0,066996	NA
	1	abundance	X07674	Lysine	Radius_SOS	0,035407	0,04906	NA

	1	abundance	X07674	Lysine	Tibia_SOS	0,141824	0,052233	NA
	1	abundance	X07674	Lysine	Handgrip	0,089096	0,060446	NA
2b		abundance	X07678	(2E)-3-(3,4	Tibia_length	-0,16359	0,054599	NA
2b		abundance	X07678	(2E)-3-(3,4	Radius_length	-0,14041	0,072891	NA
2b		abundance	X07678	(2E)-3-(3,4	Radius_SOS	-0,04674	0,05264	NA
2b		abundance	X07678	(2E)-3-(3,4	Tibia_SOS	-0,12755	0,05485	NA
2b		abundance	X07678	(2E)-3-(3,4	Handgrip	-0,07404	0,062075	NA
	1	abundance	X07680	Indole-3-ac	Tibia_length	0,029684	0,053994	NA
	1	abundance	X07680	Indole-3-ac	Radius_length	-0,09566	0,072534	NA
	1	abundance	X07680	Indole-3-ac	Radius_SOS	-0,15769	0,050784	NA
	1	abundance	X07680	Indole-3-ac	Tibia_SOS	0,064672	0,0544	NA
	1	abundance	X07680	Indole-3-ac	Handgrip	-0,0112	0,061873	NA
	3	abundance	X07694	DNOP	Tibia_length	0,027987	0,052985	NA
	3	abundance	X07694	DNOP	Radius_length	-0,06337	0,071656	NA
	3	abundance	X07694	DNOP	Radius_SOS	0,097625	0,050277	NA
	3	abundance	X07694	DNOP	Tibia_SOS	-0,0333	0,055035	NA
	3	abundance	X07694	DNOP	Handgrip	0,042618	0,061444	NA
	3	abundance	X07699	N-(1-Methy	Tibia_length	-0,0735	0,054815	NA
	3	abundance	X07699	N-(1-Methy	Radius_length	-0,05673	0,07339	NA
	3	abundance	X07699	N-(1-Methy	Radius_SOS	-0,00409	0,052279	NA
	3	abundance	X07699	N-(1-Methy	Tibia_SOS	0,020856	0,054751	NA
	3	abundance	X07699	N-(1-Methy	Handgrip	-0,07158	0,061763	NA
	3	abundance	X07701	3-Benzyl-6	Tibia_length	-0,10104	0,054205	NA
	3	abundance	X07701	3-Benzyl-6	Radius_length	-0,07226	0,072799	NA
	3	abundance	X07701	3-Benzyl-6	Radius_SOS	0,006438	0,051632	NA
	3	abundance	X07701	3-Benzyl-6	Tibia_SOS	0,058094	0,055211	NA
	3	abundance	X07701	3-Benzyl-6	Handgrip	0,09431	0,061885	NA
	3	abundance	X07710	3-O-beta-D	Tibia_length	0,0578	0,053809	NA
	3	abundance	X07710	3-O-beta-D	Radius_length	0,065841	0,073246	NA
	3	abundance	X07710	3-O-beta-D	Radius_SOS	0,002371	0,051415	NA
	3	abundance	X07710	3-O-beta-D	Tibia_SOS	0,061044	0,054752	NA
	3	abundance	X07710	3-O-beta-D	Handgrip	0,026279	0,061472	NA
	3	abundance	X07712	paracetam	Tibia_length	-0,07069	0,054783	NA
	3	abundance	X07712	paracetam	Radius_length	-0,08983	0,073176	NA
	3	abundance	X07712	paracetam	Radius_SOS	-0,00446	0,052202	NA
	3	abundance	X07712	paracetam	Tibia_SOS	-0,07439	0,055036	NA
	3	abundance	X07712	paracetam	Handgrip	-0,01548	0,061746	NA
2b		abundance	X07731	3-[4-methy	Tibia_length	-0,08961	0,053252	NA
2b		abundance	X07731	3-[4-methy	Radius_length	-0,1516	0,071494	NA
2b		abundance	X07731	3-[4-methy	Radius_SOS	0,053957	0,051045	NA
2b		abundance	X07731	3-[4-methy	Tibia_SOS	-0,04324	0,054048	NA
2b		abundance	X07731	3-[4-methy	Handgrip	-0,03255	0,061598	NA
2b		abundance	X07732	N-Acetylva	Tibia_length	0,034365	0,05398	NA
2b		abundance	X07732	N-Acetylva	Radius_length	0,086872	0,072508	NA
2b		abundance	X07732	N-Acetylva	Radius_SOS	0,023053	0,051357	NA
2b		abundance	X07732	N-Acetylva	Tibia_SOS	0,126213	0,054626	NA
2b		abundance	X07732	N-Acetylva	Handgrip	0,00865	0,061599	NA
2b		abundance	X07743	D-Alanine	Tibia_length	-0,01959	0,049591	NA

2b	abundance	X07743	D-Alanine ı	Radius_length	-0,04137	0,067684	NA
2b	abundance	X07743	D-Alanine ı	Radius_SOS	0,003469	0,049029	NA
2b	abundance	X07743	D-Alanine ı	Tibia_SOS	-0,02225	0,05248	NA
2b	abundance	X07743	D-Alanine ı	Handgrip	0,126175	0,06002	NA
3	abundance	X07745	APM	Tibia_length	-0,09503	0,05007	NA
3	abundance	X07745	APM	Radius_length	-0,12331	0,068075	NA
3	abundance	X07745	APM	Radius_SOS	-0,02999	0,049133	NA
3	abundance	X07745	APM	Tibia_SOS	-0,05713	0,052723	NA
3	abundance	X07745	APM	Handgrip	-0,04308	0,06045	NA
2b	abundance	X07746	3-(2-Oxo-2	Tibia_length	-0,03166	0,05117	NA
2b	abundance	X07746	3-(2-Oxo-2	Radius_length	-0,09216	0,069801	NA
2b	abundance	X07746	3-(2-Oxo-2	Radius_SOS	0,078653	0,049347	NA
2b	abundance	X07746	3-(2-Oxo-2	Tibia_SOS	-0,05417	0,053052	NA
2b	abundance	X07746	3-(2-Oxo-2	Handgrip	0,016932	0,060915	NA
3	abundance	X07747	Diethylpyr(	Tibia_length	-0,02123	0,055614	NA
3	abundance	X07747	Diethylpyr(	Radius_length	0,041428	0,074433	NA
3	abundance	X07747	Diethylpyr(	Radius_SOS	-0,02222	0,05289	NA
3	abundance	X07747	Diethylpyr(	Tibia_SOS	-0,0168	0,056882	NA
3	abundance	X07747	Diethylpyr(	Handgrip	0,104346	0,063468	NA
3	abundance	X07749	(DL)-3-O-M	Tibia_length	-0,11832	0,056045	NA
3	abundance	X07749	(DL)-3-O-M	Radius_length	-0,20034	0,073456	NA
3	abundance	X07749	(DL)-3-O-M	Radius_SOS	-0,06357	0,053412	NA
3	abundance	X07749	(DL)-3-O-M	Tibia_SOS	-0,14097	0,056261	NA
3	abundance	X07749	(DL)-3-O-M	Handgrip	-0,13088	0,061163	NA
3	abundance	X07753	Lovastatin	Tibia_length	0,052226	0,052936	NA
3	abundance	X07753	Lovastatin	Radius_length	0,071328	0,071513	NA
3	abundance	X07753	Lovastatin	Radius_SOS	0,019761	0,050601	NA
3	abundance	X07753	Lovastatin	Tibia_SOS	0,018003	0,055041	NA
3	abundance	X07753	Lovastatin	Handgrip	-0,08845	0,061769	NA
3	abundance	X07754	hexobarbit	Tibia_length	0,025421	0,056431	NA
3	abundance	X07754	hexobarbit	Radius_length	0,091417	0,074169	NA
3	abundance	X07754	hexobarbit	Radius_SOS	0,055307	0,053521	NA
3	abundance	X07754	hexobarbit	Tibia_SOS	-0,05278	0,057532	NA
3	abundance	X07754	hexobarbit	Handgrip	0,061091	0,063013	NA
3	abundance	X07764	(S)-2-meth	Tibia_length	0,101078	0,054404	NA
3	abundance	X07764	(S)-2-meth	Radius_length	0,138237	0,073259	NA
3	abundance	X07764	(S)-2-meth	Radius_SOS	0,072107	0,051534	NA
3	abundance	X07764	(S)-2-meth	Tibia_SOS	0,06622	0,055853	NA
3	abundance	X07764	(S)-2-meth	Handgrip	0,183512	0,060648	NA
1	abundance	X07770	Piperine	Tibia_length	-0,03421	0,056851	NA
1	abundance	X07770	Piperine	Radius_length	0,123031	0,073999	NA
1	abundance	X07770	Piperine	Radius_SOS	0,036006	0,053879	NA
1	abundance	X07770	Piperine	Tibia_SOS	0,00638	0,057426	NA
1	abundance	X07770	Piperine	Handgrip	0,060569	0,063447	NA
2b	abundance	X07771	Caprolacta	Tibia_length	-0,08884	0,051143	NA
2b	abundance	X07771	Caprolacta	Radius_length	-0,10722	0,069904	NA
2b	abundance	X07771	Caprolacta	Radius_SOS	-0,1099	0,049483	NA
2b	abundance	X07771	Caprolacta	Tibia_SOS	-0,03366	0,052887	NA

2b		abundance	X07771	Caprolacta	Handgrip	0,017919	0,06094	NA
	3	abundance	X07774	7alpha-Hy	Tibia_length	0,039275	0,054984	NA
	3	abundance	X07774	7alpha-Hy	Radius_length	0,026382	0,073505	NA
	3	abundance	X07774	7alpha-Hy	Radius_SOS	0,001584	0,052212	NA
	3	abundance	X07774	7alpha-Hy	Tibia_SOS	-0,01003	0,056185	NA
	3	abundance	X07774	7alpha-Hy	Handgrip	-0,00559	0,061933	NA
	1	abundance	X07777	Arginine	Tibia_length	-0,0082	0,052496	NA
	1	abundance	X07777	Arginine	Radius_length	0,10463	0,071004	NA
	1	abundance	X07777	Arginine	Radius_SOS	-0,00415	0,050346	NA
	1	abundance	X07777	Arginine	Tibia_SOS	0,050424	0,0536	NA
	1	abundance	X07777	Arginine	Handgrip	0,026025	0,061871	NA
	1	abundance	X07782	Cytosine	Tibia_length	-0,01612	0,053325	NA
	1	abundance	X07782	Cytosine	Radius_length	0,083246	0,072283	NA
	1	abundance	X07782	Cytosine	Radius_SOS	0,029968	0,050883	NA
	1	abundance	X07782	Cytosine	Tibia_SOS	0,061425	0,05452	NA
	1	abundance	X07782	Cytosine	Handgrip	0,089316	0,061724	NA
	3	abundance	X07783	1,3-dimeth	Tibia_length	-0,06265	0,054093	NA
	3	abundance	X07783	1,3-dimeth	Radius_length	0,040697	0,072841	NA
	3	abundance	X07783	1,3-dimeth	Radius_SOS	0,047506	0,051469	NA
	3	abundance	X07783	1,3-dimeth	Tibia_SOS	0,010687	0,054898	NA
	3	abundance	X07783	1,3-dimeth	Handgrip	-0,01109	0,06181	NA
2b		abundance	X07792	N-Acetylhi:	Tibia_length	-0,07059	0,051037	NA
2b		abundance	X07792	N-Acetylhi:	Radius_length	-0,18816	0,068797	NA
2b		abundance	X07792	N-Acetylhi:	Radius_SOS	-0,09751	0,049295	NA
2b		abundance	X07792	N-Acetylhi:	Tibia_SOS	-0,09188	0,053142	NA
2b		abundance	X07792	N-Acetylhi:	Handgrip	0,039776	0,060822	NA
2b		abundance	X07794	Prolylleuci	Tibia_length	-0,06367	0,052555	NA
2b		abundance	X07794	Prolylleuci	Radius_length	-0,11277	0,071348	NA
2b		abundance	X07794	Prolylleuci	Radius_SOS	-0,08908	0,050242	NA
2b		abundance	X07794	Prolylleuci	Tibia_SOS	0,028622	0,054341	NA
2b		abundance	X07794	Prolylleuci	Handgrip	0,01926	0,062004	NA
	3	abundance	X07798	N-lauroylgl	Tibia_length	-0,06543	0,053664	NA
	3	abundance	X07798	N-lauroylgl	Radius_length	-0,0779	0,072217	NA
	3	abundance	X07798	N-lauroylgl	Radius_SOS	-0,04995	0,051119	NA
	3	abundance	X07798	N-lauroylgl	Tibia_SOS	-0,08959	0,055371	NA
	3	abundance	X07798	N-lauroylgl	Handgrip	-0,13977	0,060758	NA
2b		abundance	X07799	Capryloylg	Tibia_length	0,096043	0,051877	NA
2b		abundance	X07799	Capryloylg	Radius_length	0,098338	0,071017	NA
2b		abundance	X07799	Capryloylg	Radius_SOS	0,052412	0,049938	NA
2b		abundance	X07799	Capryloylg	Tibia_SOS	0,048435	0,053343	NA
2b		abundance	X07799	Capryloylg	Handgrip	-0,00411	0,061303	NA
	3	abundance	X07811	Metirosine	Tibia_length	-0,07846	0,051586	NA
	3	abundance	X07811	Metirosine	Radius_length	-0,02434	0,07071	NA
	3	abundance	X07811	Metirosine	Radius_SOS	-0,00483	0,049788	NA
	3	abundance	X07811	Metirosine	Tibia_SOS	0,006147	0,053729	NA
	3	abundance	X07811	Metirosine	Handgrip	-0,17638	0,06022	NA
	3	abundance	X07812	Triethyl citı	Tibia_length	-0,11385	0,057643	NA
	3	abundance	X07812	Triethyl citı	Radius_length	0,001445	0,075244	NA

	3	abundance	X07812	Triethyl citı	Radius_SOS	-0,11366	0,055313	NA
	3	abundance	X07812	Triethyl citı	Tibia_SOS	0,007905	0,057768	NA
	3	abundance	X07812	Triethyl citı	Handgrip	0,067781	0,063421	NA
	3	abundance	X07815	3-[2-[(Z)-[3	Tibia_length	-0,01148	0,051443	NA
	3	abundance	X07815	3-[2-[(Z)-[3	Radius_length	0,068352	0,070591	NA
	3	abundance	X07815	3-[2-[(Z)-[3	Radius_SOS	0,03638	0,049632	NA
	3	abundance	X07815	3-[2-[(Z)-[3	Tibia_SOS	-0,00331	0,053089	NA
	3	abundance	X07815	3-[2-[(Z)-[3	Handgrip	-0,00873	0,061033	NA
	1	abundance	X07820	Creatinine	Tibia_length	-0,12657	0,053633	NA
	1	abundance	X07820	Creatinine	Radius_length	-0,12764	0,072771	NA
	1	abundance	X07820	Creatinine	Radius_SOS	-0,12413	0,050986	NA
	1	abundance	X07820	Creatinine	Tibia_SOS	-0,0939	0,05468	NA
	1	abundance	X07820	Creatinine	Handgrip	0,040661	0,061327	NA
	1	abundance	X07825	Maltotriose	Tibia_length	-0,06289	0,055038	NA
	1	abundance	X07825	Maltotriose	Radius_length	0,063697	0,073798	NA
	1	abundance	X07825	Maltotriose	Radius_SOS	-0,00425	0,052399	NA
	1	abundance	X07825	Maltotriose	Tibia_SOS	-0,016	0,05696	NA
	1	abundance	X07825	Maltotriose	Handgrip	0,032563	0,060161	NA
2b		abundance	X07830	1,5-Isoquir	Tibia_length	-0,05407	0,057577	NA
2b		abundance	X07830	1,5-Isoquir	Radius_length	-0,05937	0,074697	NA
2b		abundance	X07830	1,5-Isoquir	Radius_SOS	-0,05678	0,054778	NA
2b		abundance	X07830	1,5-Isoquir	Tibia_SOS	0,008627	0,058477	NA
2b		abundance	X07830	1,5-Isoquir	Handgrip	-0,06868	0,060051	NA
	3	abundance	X07834	Choline su	Tibia_length	0,005045	0,05274	NA
	3	abundance	X07834	Choline su	Radius_length	-0,07703	0,07163	NA
	3	abundance	X07834	Choline su	Radius_SOS	-0,00982	0,050678	NA
	3	abundance	X07834	Choline su	Tibia_SOS	-0,05752	0,054798	NA
	3	abundance	X07834	Choline su	Handgrip	0,027126	0,061858	NA
	3	abundance	X07835	7-ketodeo›	Tibia_length	0,010405	0,050301	NA
	3	abundance	X07835	7-ketodeo›	Radius_length	0,062094	0,068687	NA
	3	abundance	X07835	7-ketodeo›	Radius_SOS	-0,01452	0,049174	NA
	3	abundance	X07835	7-ketodeo›	Tibia_SOS	-0,06937	0,052563	NA
	3	abundance	X07835	7-ketodeo›	Handgrip	-5,32E-04	0,060765	NA
	3	abundance	X07841	2-(5-Benzy	Tibia_length	-0,06786	0,053771	NA
	3	abundance	X07841	2-(5-Benzy	Radius_length	-0,08999	0,07311	NA
	3	abundance	X07841	2-(5-Benzy	Radius_SOS	-0,00107	0,051259	NA
	3	abundance	X07841	2-(5-Benzy	Tibia_SOS	7,25E-04	0,05429	NA
	3	abundance	X07841	2-(5-Benzy	Handgrip	-0,11288	0,061396	NA
	3	abundance	X07843	Piperidine	Tibia_length	-0,05491	0,049581	NA
	3	abundance	X07843	Piperidine	Radius_length	-0,04331	0,067069	NA
	3	abundance	X07843	Piperidine	Radius_SOS	-0,03165	0,049008	NA
	3	abundance	X07843	Piperidine	Tibia_SOS	-0,10448	0,052201	NA
	3	abundance	X07843	Piperidine	Handgrip	0,035734	0,060195	NA
	3	abundance	X07846	Carbofurar	Tibia_length	0,054662	0,053533	NA
	3	abundance	X07846	Carbofurar	Radius_length	0,006792	0,072815	NA
	3	abundance	X07846	Carbofurar	Radius_SOS	-0,00653	0,051175	NA
	3	abundance	X07846	Carbofurar	Tibia_SOS	0,098382	0,0551	NA
	3	abundance	X07846	Carbofurar	Handgrip	-0,02182	0,06346	NA

3	abundance	X07848	(âˆ’)-nabil	Tibia_length	0,010463	0,049541	NA
3	abundance	X07848	(âˆ’)-nabil	Radius_length	-0,02487	0,066858	NA
3	abundance	X07848	(âˆ’)-nabil	Radius_SOS	0,030418	0,049007	NA
3	abundance	X07848	(âˆ’)-nabil	Tibia_SOS	-0,09229	0,052351	NA
3	abundance	X07848	(âˆ’)-nabil	Handgrip	0,007796	0,061017	NA
3	abundance	X07849	Crotamitor	Tibia_length	0,014823	0,050701	NA
3	abundance	X07849	Crotamitor	Radius_length	0,003259	0,069471	NA
3	abundance	X07849	Crotamitor	Radius_SOS	-0,02841	0,049323	NA
3	abundance	X07849	Crotamitor	Tibia_SOS	0,025134	0,052972	NA
3	abundance	X07849	Crotamitor	Handgrip	-0,00751	0,060987	NA
3	abundance	X07852	N6,N6,N6-	Tibia_length	-0,06029	0,05155	NA
3	abundance	X07852	N6,N6,N6-	Radius_length	-0,12041	0,06979	NA
3	abundance	X07852	N6,N6,N6-	Radius_SOS	-0,06969	0,049645	NA
3	abundance	X07852	N6,N6,N6-	Tibia_SOS	-0,05821	0,053371	NA
3	abundance	X07852	N6,N6,N6-	Handgrip	-0,05672	0,061224	NA
3	abundance	X07854	TO012790	Tibia_length	-0,01418	0,05041	NA
3	abundance	X07854	TO012790	Radius_length	-0,01292	0,069178	NA
3	abundance	X07854	TO012790	Radius_SOS	-0,07672	0,049039	NA
3	abundance	X07854	TO012790	Tibia_SOS	-0,03018	0,052751	NA
3	abundance	X07854	TO012790	Handgrip	0,0189	0,06069	NA

3	abundance	X07856	[illegible],6R,7R,9R,11R,12R,13S,14R)-6-{[(2S,3R,4S,6R)-4-(Dimethylamino)-3-hydroxy-6-methyltetrahydro-2H-pyran-2-yl]oxy}-12,13-dihydroxy-14-[(1S)-1-hydroxyethyl]-4-{[(2R,4R,5S,6S)-5-hydroxy-4-methoxy-4,6-dimethyltetrahydro-2H-pyran-2-yl]oxy}-7	Tibia_length	0,015311	0,058575	NA

3	abundance	X07856	[illegible],6R,7R,9R,11R,12R,13S,14R)-6-{[(2S,3R,4S,6R)-4-(Dimethylamino)-3-hydroxy-6-methyltetrahydro-2H-pyran-2-yl]oxy}-12,13-dihydroxy-14-[(1S)-1-hydroxyethyl]-4-{[(2R,4R,5S,6S)-5-hydroxy-4-methoxy-4,6-dimethyltetrahydro-2H-pyran-2-yl]oxy}-7	Radius_length	0,182576	0,073966	NA

3	abundance	X07856	,6R,7R,9R,11R,12R,13S,14R)-6-{[(2S,3R,4S,6R)-4-(Dimethylamino)-3-hydroxy-6-methyltetrahydro-2H-pyran-2-yl]oxy}-12,13-dihydroxy-14-[(1S)-1-hydroxyethyl]-4-{[(2R,4R,5S,6S)-5-hydroxy-4-methoxy-4,6-dimethyltetrahydro-2H-pyran-2-yl]oxy}-7	Radius_SOS	0,013029	0,055562	NA

3	abundance	X07856	,6R,7R,9R ,11R,12R, 13S,14R)- 6- {[(2S,3R,4 S,6R)-4- (Dimethyl amino)-3- hydroxy-6- methyltetr ahydro- 2H-pyran- 2-yl]oxy}- 12,13- dihydroxy- 14-[(1S)-1- hydroxyet hyl]-4- {[(2R,4R,5 S,6S)-5- hydroxy-4- methoxy- 4 ,6- dimethylt etrahydro- 2H-pyran- 2-yl]oxy}-7	Tibia_SOS	0,089392	0,058444	NA

	3	abundance	X07856	[illegible],6R,7R,9R,11R,12R,13S,14R)-6-{[(2S,3R,4S,6R)-4-(Dimethylamino)-3-hydroxy-6-methyltetrahydro-2H-pyran-2-yl]oxy}-12,13-dihydroxy-14-[(1S)-1-hydroxyethyl]-4-{[(2R,4R,5S,6S)-5-hydroxy-4-methoxy-4,6-dimethyltetrahydro-2H-pyran-2-yl]oxy}-7	Handgrip	0,090305	0,059947	NA
2b		abundance	X07866	3,3,5,5-Tet	Tibia_length	-0,0115	0,051055	NA
2b		abundance	X07866	3,3,5,5-Tet	Radius_length	-0,05724	0,069643	NA
2b		abundance	X07866	3,3,5,5-Tet	Radius_SOS	-0,0417	0,049405	NA
2b		abundance	X07866	3,3,5,5-Tet	Tibia_SOS	-0,02183	0,05342	NA
2b		abundance	X07866	3,3,5,5-Tet	Handgrip	0,018504	0,061341	NA
	3	abundance	X07867	N-Acetylva	Tibia_length	-0,04367	0,057069	NA
	3	abundance	X07867	N-Acetylva	Radius_length	-0,12125	0,074199	NA
	3	abundance	X07867	N-Acetylva	Radius_SOS	-0,02087	0,05396	NA
	3	abundance	X07867	N-Acetylva	Tibia_SOS	-0,00616	0,057578	NA
	3	abundance	X07867	N-Acetylva	Handgrip	-0,04629	0,062294	NA
2b		abundance	X07868	Biotin	Tibia_length	0,053104	0,054089	NA
2b		abundance	X07868	Biotin	Radius_length	0,019804	0,072976	NA
2b		abundance	X07868	Biotin	Radius_SOS	0,022732	0,051705	NA
2b		abundance	X07868	Biotin	Tibia_SOS	-0,03283	0,054604	NA
2b		abundance	X07868	Biotin	Handgrip	-0,15687	0,061445	NA
2a		abundance	X07869	Hexamethy	Tibia_length	0,054785	0,052011	NA
2a		abundance	X07869	Hexamethy	Radius_length	0,032519	0,071196	NA
2a		abundance	X07869	Hexamethy	Radius_SOS	0,012406	0,050129	NA
2a		abundance	X07869	Hexamethy	Tibia_SOS	-0,00824	0,053913	NA

2a		abundance	X07869	Hexamethy	Handgrip	0,063074	0,061539	NA
	3	abundance	X07873	(3Z,6Z,9Z,:	Tibia_length	0,081502	0,054887	NA
	3	abundance	X07873	(3Z,6Z,9Z,:	Radius_length	0,178263	0,07243	NA
	3	abundance	X07873	(3Z,6Z,9Z,:	Radius_SOS	-0,05146	0,052033	NA
	3	abundance	X07873	(3Z,6Z,9Z,:	Tibia_SOS	0,049316	0,055146	NA
	3	abundance	X07873	(3Z,6Z,9Z,:	Handgrip	0,00541	0,061955	NA
2b		abundance	X07874	Î´-Valerola	Tibia_length	-0,06967	0,053512	NA
2b		abundance	X07874	Î´-Valerola	Radius_length	-0,0747	0,072546	NA
2b		abundance	X07874	Î´-Valerola	Radius_SOS	-0,06919	0,051173	NA
2b		abundance	X07874	Î´-Valerola	Tibia_SOS	0,024973	0,054579	NA
2b		abundance	X07874	Î´-Valerola	Handgrip	0,005603	0,061503	NA
	3	abundance	X07886	L-Urobilin	Tibia_length	-0,00851	0,049539	NA
	3	abundance	X07886	L-Urobilin	Radius_length	0,060657	0,067216	NA
	3	abundance	X07886	L-Urobilin	Radius_SOS	0,012218	0,049025	NA
	3	abundance	X07886	L-Urobilin	Tibia_SOS	0,012529	0,052499	NA
	3	abundance	X07886	L-Urobilin	Handgrip	0,064326	0,0603	NA
	3	abundance	X07889	UROBILIN,	Tibia_length	0,001385	0,050836	NA
	3	abundance	X07889	UROBILIN,	Radius_length	0,063977	0,069607	NA
	3	abundance	X07889	UROBILIN,	Radius_SOS	0,041284	0,049389	NA
	3	abundance	X07889	UROBILIN,	Tibia_SOS	0,06172	0,052693	NA
	3	abundance	X07889	UROBILIN,	Handgrip	-0,07384	0,060702	NA
2b		abundance	X07890	N-(5-aceta	Tibia_length	0,060732	0,050581	NA
2b		abundance	X07890	N-(5-aceta	Radius_length	0,072368	0,069251	NA
2b		abundance	X07890	N-(5-aceta	Radius_SOS	0,016611	0,049335	NA
2b		abundance	X07890	N-(5-aceta	Tibia_SOS	-0,05008	0,052766	NA
2b		abundance	X07890	N-(5-aceta	Handgrip	0,054902	0,060998	NA
2b		abundance	X07891	Cyclamic a	Tibia_length	-0,04836	0,056007	NA
2b		abundance	X07891	Cyclamic a	Radius_length	-0,06668	0,074116	NA
2b		abundance	X07891	Cyclamic a	Radius_SOS	-0,00232	0,0534	NA
2b		abundance	X07891	Cyclamic a	Tibia_SOS	-0,03135	0,055491	NA
2b		abundance	X07891	Cyclamic a	Handgrip	-0,06045	0,061825	NA
	3	abundance	X07892	urobilinoge	Tibia_length	0,001871	0,051819	NA
	3	abundance	X07892	urobilinoge	Radius_length	0,052779	0,071636	NA
	3	abundance	X07892	urobilinoge	Radius_SOS	0,051592	0,049982	NA
	3	abundance	X07892	urobilinoge	Tibia_SOS	0,131447	0,053844	NA
	3	abundance	X07892	urobilinoge	Handgrip	-0,0179	0,061364	NA
2b		abundance	X07899	N-Acetylpu	Tibia_length	-0,09754	0,050307	NA
2b		abundance	X07899	N-Acetylpu	Radius_length	-0,11089	0,068686	NA
2b		abundance	X07899	N-Acetylpu	Radius_SOS	-0,08572	0,049127	NA
2b		abundance	X07899	N-Acetylpu	Tibia_SOS	-0,01433	0,052638	NA
2b		abundance	X07899	N-Acetylpu	Handgrip	0,021191	0,060944	NA
	3	abundance	X07900	presqualer	Tibia_length	0,032448	0,057039	NA
	3	abundance	X07900	presqualer	Radius_length	0,159619	0,073869	NA
	3	abundance	X07900	presqualer	Radius_SOS	0,031137	0,054094	NA
	3	abundance	X07900	presqualer	Tibia_SOS	0,090225	0,05646	NA
	3	abundance	X07900	presqualer	Handgrip	2,14E-04	0,063294	NA
2b		abundance	X07903	Acetophen	Tibia_length	-0,12125	0,049417	NA
2b		abundance	X07903	Acetophen	Radius_length	-0,15505	0,066799	NA

2b	abundance	X07903	Acetophen	Radius_SOS	-0,15903	0,048426	NA
2b	abundance	X07903	Acetophen	Tibia_SOS	-0,07237	0,052371	NA
2b	abundance	X07903	Acetophen	Handgrip	-0,03693	0,060655	NA
2b	abundance	X07905	Prilocaine	Tibia_length	0,049833	0,050538	NA
2b	abundance	X07905	Prilocaine	Radius_length	0,003622	0,069423	NA
2b	abundance	X07905	Prilocaine	Radius_SOS	-0,01322	0,049291	NA
2b	abundance	X07905	Prilocaine	Tibia_SOS	0,008649	0,053007	NA
2b	abundance	X07905	Prilocaine	Handgrip	-0,02725	0,060869	NA
1	abundance	X07907	4-Acetami	Tibia_length	-0,02026	0,04896	NA
1	abundance	X07907	4-Acetami	Radius_length	0,100265	0,048723	NA
1	abundance	X07907	4-Acetami	Radius_SOS	-0,04723	0,048974	NA
1	abundance	X07907	4-Acetami	Tibia_SOS	0,064564	0,059731	NA
1	abundance	X07907	4-Acetami	Handgrip	0,105677	0,059856	NA
2b	abundance	X07909	(Â±)-Albute	Tibia_length	-0,01962	0,057889	NA
2b	abundance	X07909	(Â±)-Albute	Radius_length	-1,32E-04	0,075551	NA
2b	abundance	X07909	(Â±)-Albute	Radius_SOS	-0,07314	0,054402	NA
2b	abundance	X07909	(Â±)-Albute	Tibia_SOS	-0,04093	0,060059	NA
2b	abundance	X07909	(Â±)-Albute	Handgrip	0,112937	0,062621	NA
3	abundance	X07914	(-)-Erythro	Tibia_length	-0,03898	0,055847	NA
3	abundance	X07914	(-)-Erythro	Radius_length	0,149645	0,073403	NA
3	abundance	X07914	(-)-Erythro	Radius_SOS	0,03203	0,053029	NA
3	abundance	X07914	(-)-Erythro	Tibia_SOS	0,119384	0,055666	NA
3	abundance	X07914	(-)-Erythro	Handgrip	0,126326	0,061687	NA
3	abundance	X07916	N6,N6,N6-	Tibia_length	-0,02816	0,050432	NA
3	abundance	X07916	N6,N6,N6-	Radius_length	-0,0357	0,069068	NA
3	abundance	X07916	N6,N6,N6-	Radius_SOS	-0,0693	0,049081	NA
3	abundance	X07916	N6,N6,N6-	Tibia_SOS	-0,04373	0,052773	NA
3	abundance	X07916	N6,N6,N6-	Handgrip	-0,00803	0,060777	NA
1	abundance	X07921	Pipecolinic	Tibia_length	0,054872	0,051332	NA
1	abundance	X07921	Pipecolinic	Radius_length	0,203248	0,06902	NA
1	abundance	X07921	Pipecolinic	Radius_SOS	0,13199	0,049246	NA
1	abundance	X07921	Pipecolinic	Tibia_SOS	0,077302	0,053117	NA
1	abundance	X07921	Pipecolinic	Handgrip	0,027809	0,061031	NA
3	abundance	X07925	DNOP	Tibia_length	0,020695	0,052845	NA
3	abundance	X07925	DNOP	Radius_length	-0,00199	0,072123	NA
3	abundance	X07925	DNOP	Radius_SOS	0,002203	0,050532	NA
3	abundance	X07925	DNOP	Tibia_SOS	0,002927	0,054357	NA
3	abundance	X07925	DNOP	Handgrip	0,014263	0,061303	NA
2b	abundance	X07930	N-Methylc	Tibia_length	-0,00359	0,050141	NA
2b	abundance	X07930	N-Methylc	Radius_length	-0,03671	0,068252	NA
2b	abundance	X07930	N-Methylc	Radius_SOS	-0,04212	0,049071	NA
2b	abundance	X07930	N-Methylc	Tibia_SOS	-0,0636	0,052707	NA
2b	abundance	X07930	N-Methylc	Handgrip	0,011812	0,060848	NA
3	abundance	X07935	Limonin	Tibia_length	0,017767	0,05816	NA
3	abundance	X07935	Limonin	Radius_length	0,08559	0,074802	NA
3	abundance	X07935	Limonin	Radius_SOS	-0,01328	0,055372	NA
3	abundance	X07935	Limonin	Tibia_SOS	-0,02916	0,058934	NA
3	abundance	X07935	Limonin	Handgrip	0,105448	0,059857	NA

	1	abundance	X07937	Atenolol	Tibia_length	-0,06213	0,051585	NA
	1	abundance	X07937	Atenolol	Radius_length	-0,09534	0,070475	NA
	1	abundance	X07937	Atenolol	Radius_SOS	4,62E-04	0,04984	NA
	1	abundance	X07937	Atenolol	Tibia_SOS	-0,02303	0,053009	NA
	1	abundance	X07937	Atenolol	Handgrip	0,038839	0,06105	NA
	3	abundance	X07944	Styrene	Tibia_length	-0,0162	0,050897	NA
	3	abundance	X07944	Styrene	Radius_length	-0,15548	0,068925	NA
	3	abundance	X07944	Styrene	Radius_SOS	-0,19338	0,048441	NA
	3	abundance	X07944	Styrene	Tibia_SOS	-0,04855	0,052973	NA
	3	abundance	X07944	Styrene	Handgrip	-0,15638	0,06047	NA
	3	abundance	X07955	Valylvaline	Tibia_length	-0,10289	0,054945	NA
	3	abundance	X07955	Valylvaline	Radius_length	-0,03377	0,073842	NA
	3	abundance	X07955	Valylvaline	Radius_SOS	-0,05754	0,052641	NA
	3	abundance	X07955	Valylvaline	Tibia_SOS	0,00482	0,056956	NA
	3	abundance	X07955	Valylvaline	Handgrip	-0,07991	0,062236	NA
	3	abundance	X07961	2-(Hydroxy	Tibia_length	0,076885	0,049801	NA
	3	abundance	X07961	2-(Hydroxy	Radius_length	0,158449	0,067268	NA
	3	abundance	X07961	2-(Hydroxy	Radius_SOS	0,014108	0,04905	NA
	3	abundance	X07961	2-(Hydroxy	Tibia_SOS	0,100084	0,052298	NA
	3	abundance	X07961	2-(Hydroxy	Handgrip	0,009772	0,06045	NA
	1	abundance	X07963	Theobromi	Tibia_length	-0,07634	0,055423	NA
	1	abundance	X07963	Theobromi	Radius_length	-0,01924	0,073997	NA
	1	abundance	X07963	Theobromi	Radius_SOS	0,033707	0,05279	NA
	1	abundance	X07963	Theobromi	Tibia_SOS	-0,07457	0,055741	NA
	1	abundance	X07963	Theobromi	Handgrip	-0,02957	0,063627	NA
	3	abundance	X07969	6-hydroxyp	Tibia_length	0,015869	0,049659	NA
	3	abundance	X07969	6-hydroxyp	Radius_length	0,04797	0,067164	NA
	3	abundance	X07969	6-hydroxyp	Radius_SOS	-0,03278	0,049005	NA
	3	abundance	X07969	6-hydroxyp	Tibia_SOS	-0,01121	0,05253	NA
	3	abundance	X07969	6-hydroxyp	Handgrip	0,007499	0,060913	NA
	3	abundance	X07974	Isoprene	Tibia_length	0,083101	0,050614	NA
	3	abundance	X07974	Isoprene	Radius_length	0,138028	0,06878	NA
	3	abundance	X07974	Isoprene	Radius_SOS	0,020789	0,049277	NA
	3	abundance	X07974	Isoprene	Tibia_SOS	0,146559	0,052199	NA
	3	abundance	X07974	Isoprene	Handgrip	0,066748	0,060235	NA
2b		abundance	X07977	Tyramine	Tibia_length	-0,13145	0,049771	NA
2b		abundance	X07977	Tyramine	Radius_length	-0,1574	0,067714	NA
2b		abundance	X07977	Tyramine	Radius_SOS	-0,16095	0,048516	NA
2b		abundance	X07977	Tyramine	Tibia_SOS	-0,0739	0,052473	NA
2b		abundance	X07977	Tyramine	Handgrip	-0,04435	0,060901	NA
	3	abundance	X07978	1-[(4E)-4-(	Tibia_length	0,02052	0,054118	NA
	3	abundance	X07978	1-[(4E)-4-(	Radius_length	0,020312	0,073111	NA
	3	abundance	X07978	1-[(4E)-4-(	Radius_SOS	0,045207	0,051503	NA
	3	abundance	X07978	1-[(4E)-4-(	Tibia_SOS	0,063832	0,055475	NA
	3	abundance	X07978	1-[(4E)-4-(	Handgrip	0,002478	0,062851	NA
2b		abundance	X07981	Methylimic	Tibia_length	0,03825	0,053222	NA
2b		abundance	X07981	Methylimic	Radius_length	0,067335	0,072175	NA
2b		abundance	X07981	Methylimic	Radius_SOS	0,115759	0,050676	NA

2b	abundance	X07981	Methylimic	Tibia_SOS	0,045006	0,054243	NA
2b	abundance	X07981	Methylimic	Handgrip	0,071448	0,061845	NA
3	abundance	X07983	butalbital	Tibia_length	-0,12521	0,052393	NA
3	abundance	X07983	butalbital	Radius_length	-0,10612	0,071829	NA
3	abundance	X07983	butalbital	Radius_SOS	-0,00494	0,050404	NA
3	abundance	X07983	butalbital	Tibia_SOS	-0,05652	0,054624	NA
3	abundance	X07983	butalbital	Handgrip	0,040035	0,062194	NA
3	abundance	X07989	Piperidine	Tibia_length	0,094505	0,050348	NA
3	abundance	X07989	Piperidine	Radius_length	0,141343	0,068485	NA
3	abundance	X07989	Piperidine	Radius_SOS	0,004185	0,049224	NA
3	abundance	X07989	Piperidine	Tibia_SOS	0,124752	0,052323	NA
3	abundance	X07989	Piperidine	Handgrip	0,088217	0,060079	NA
3	abundance	X07993	UROBILIN,	Tibia_length	0,039627	0,052289	NA
3	abundance	X07993	UROBILIN,	Radius_length	0,108659	0,071662	NA
3	abundance	X07993	UROBILIN,	Radius_SOS	0,099417	0,050047	NA
3	abundance	X07993	UROBILIN,	Tibia_SOS	0,093939	0,054427	NA
3	abundance	X07993	UROBILIN,	Handgrip	-0,02083	0,062674	NA
3	abundance	X08002	(2S)-6-Ami	Tibia_length	0,033618	0,053605	NA
3	abundance	X08002	(2S)-6-Ami	Radius_length	0,108596	0,072786	NA
3	abundance	X08002	(2S)-6-Ami	Radius_SOS	0,095517	0,051441	NA
3	abundance	X08002	(2S)-6-Ami	Tibia_SOS	0,009348	0,055902	NA
3	abundance	X08002	(2S)-6-Ami	Handgrip	-0,02625	0,062777	NA
2b	abundance	X08004	Î´-Valerola	Tibia_length	-0,02357	0,053294	NA
2b	abundance	X08004	Î´-Valerola	Radius_length	-0,05455	0,072289	NA
2b	abundance	X08004	Î´-Valerola	Radius_SOS	-0,05305	0,050978	NA
2b	abundance	X08004	Î´-Valerola	Tibia_SOS	-0,00882	0,054282	NA
2b	abundance	X08004	Î´-Valerola	Handgrip	-0,00677	0,060906	NA
3	abundance	X08007	MFCD0002	Tibia_length	0,010915	0,051021	NA
3	abundance	X08007	MFCD0002	Radius_length	-0,03588	0,069847	NA
3	abundance	X08007	MFCD0002	Radius_SOS	-0,07342	0,049316	NA
3	abundance	X08007	MFCD0002	Tibia_SOS	-0,05581	0,052972	NA
3	abundance	X08007	MFCD0002	Handgrip	0,04028	0,061083	NA
1	abundance	X08008	N-Acetylne	Tibia_length	0,005488	0,053453	NA
1	abundance	X08008	N-Acetylne	Radius_length	0,064249	0,072828	NA
1	abundance	X08008	N-Acetylne	Radius_SOS	-0,03463	0,051076	NA
1	abundance	X08008	N-Acetylne	Tibia_SOS	0,010702	0,054264	NA
1	abundance	X08008	N-Acetylne	Handgrip	0,090081	0,062474	NA
3	abundance	X08012	Methylol D	Tibia_length	0,151391	0,049991	NA
3	abundance	X08012	Methylol D	Radius_length	0,28193	0,066276	NA
3	abundance	X08012	Methylol D	Radius_SOS	0,056176	0,049103	NA
3	abundance	X08012	Methylol D	Tibia_SOS	0,156098	0,052071	NA
3	abundance	X08012	Methylol D	Handgrip	0,109775	0,059871	NA
1	abundance	X08013	Deoxysuga	Tibia_length	-0,07156	0,054368	NA
1	abundance	X08013	Deoxysuga	Radius_length	-0,04444	0,073608	NA
1	abundance	X08013	Deoxysuga	Radius_SOS	-0,03742	0,052063	NA
1	abundance	X08013	Deoxysuga	Tibia_SOS	-0,03504	0,055391	NA
1	abundance	X08013	Deoxysuga	Handgrip	0,049928	0,062065	NA
3	abundance	X08014	DNOP	Tibia_length	0,027763	0,052965	NA

3	abundance	X08014	DNOP	Radius_length	0,015316	0,072287	NA
3	abundance	X08014	DNOP	Radius_SOS	0,018523	0,050675	NA
3	abundance	X08014	DNOP	Tibia_SOS	0,017422	0,054422	NA
3	abundance	X08014	DNOP	Handgrip	-0,01667	0,061435	NA
2b	abundance	X08017	Tropinone	Tibia_length	0,053118	0,052838	NA
2b	abundance	X08017	Tropinone	Radius_length	0,055309	0,071998	NA
2b	abundance	X08017	Tropinone	Radius_SOS	0,011145	0,050613	NA
2b	abundance	X08017	Tropinone	Tibia_SOS	0,041884	0,054098	NA
2b	abundance	X08017	Tropinone	Handgrip	-0,02755	0,061482	NA
1	abundance	X08019	Thymidine	Tibia_length	0,012586	0,050637	NA
1	abundance	X08019	Thymidine	Radius_length	0,073271	0,069077	NA
1	abundance	X08019	Thymidine	Radius_SOS	-0,08013	0,049126	NA
1	abundance	X08019	Thymidine	Tibia_SOS	0,059926	0,052619	NA
1	abundance	X08019	Thymidine	Handgrip	0,043257	0,060832	NA
3	abundance	X08024	8-Methyl-8	Tibia_length	-0,08384	0,055201	NA
3	abundance	X08024	8-Methyl-8	Radius_length	0,003353	0,074301	NA
3	abundance	X08024	8-Methyl-8	Radius_SOS	-0,06499	0,052809	NA
3	abundance	X08024	8-Methyl-8	Tibia_SOS	-0,00237	0,056374	NA
3	abundance	X08024	8-Methyl-8	Handgrip	-0,00487	0,062615	NA
3	abundance	X08028	Solanidine	Tibia_length	-0,00924	0,054843	NA
3	abundance	X08028	Solanidine	Radius_length	0,155234	0,072574	NA
3	abundance	X08028	Solanidine	Radius_SOS	0,017579	0,052139	NA
3	abundance	X08028	Solanidine	Tibia_SOS	-0,01124	0,055502	NA
3	abundance	X08028	Solanidine	Handgrip	0,119661	0,06245	NA
3	abundance	X08035	Tetrahydro	Tibia_length	0,092631	0,053142	NA
3	abundance	X08035	Tetrahydro	Radius_length	0,147901	0,07205	NA
3	abundance	X08035	Tetrahydro	Radius_SOS	0,069843	0,050566	NA
3	abundance	X08035	Tetrahydro	Tibia_SOS	0,066032	0,054551	NA
3	abundance	X08035	Tetrahydro	Handgrip	0,179606	0,059914	NA
2b	abundance	X08037	Crotonic a	Tibia_length	-0,16031	0,050598	NA
2b	abundance	X08037	Crotonic a	Radius_length	-0,10625	0,069895	NA
2b	abundance	X08037	Crotonic a	Radius_SOS	-0,04215	0,049331	NA
2b	abundance	X08037	Crotonic a	Tibia_SOS	-0,03246	0,052803	NA
2b	abundance	X08037	Crotonic a	Handgrip	-0,02522	0,060399	NA
2b	abundance	X08038	N-{6-[(7-Cl	Tibia_length	-5,15E-04	0,054161	NA
2b	abundance	X08038	N-{6-[(7-Cl	Radius_length	-0,00622	0,072749	NA
2b	abundance	X08038	N-{6-[(7-Cl	Radius_SOS	0,051797	0,05162	NA
2b	abundance	X08038	N-{6-[(7-Cl	Tibia_SOS	0,014529	0,055364	NA
2b	abundance	X08038	N-{6-[(7-Cl	Handgrip	0,039477	0,062795	NA
2b	abundance	X08040	Prolylleuci	Tibia_length	-0,06209	0,053351	NA
2b	abundance	X08040	Prolylleuci	Radius_length	-0,09382	0,072143	NA
2b	abundance	X08040	Prolylleuci	Radius_SOS	-0,06668	0,050837	NA
2b	abundance	X08040	Prolylleuci	Tibia_SOS	0,031689	0,055002	NA
2b	abundance	X08040	Prolylleuci	Handgrip	0,046009	0,061882	NA
3	abundance	X08044	3-[2-[(Z)-[3	Tibia_length	0,007606	0,050235	NA
3	abundance	X08044	3-[2-[(Z)-[3	Radius_length	0,06867	0,06866	NA
3	abundance	X08044	3-[2-[(Z)-[3	Radius_SOS	0,060385	0,049099	NA
3	abundance	X08044	3-[2-[(Z)-[3	Tibia_SOS	0,029321	0,052525	NA

	3	abundance	X08044	3-[2-[(Z)-[3	Handgrip	-0,04912	0,060381	NA
	3	abundance	X08046	3-Hydroxy-	Tibia_length	-3,39E-04	0,056525	NA
	3	abundance	X08046	3-Hydroxy-	Radius_length	-0,05195	0,074216	NA
	3	abundance	X08046	3-Hydroxy-	Radius_SOS	-0,10584	0,053416	NA
	3	abundance	X08046	3-Hydroxy-	Tibia_SOS	-0,02336	0,058018	NA
	3	abundance	X08046	3-Hydroxy-	Handgrip	-0,10396	0,062355	NA
2b		abundance	X08054	Docosahe›	Tibia_length	0,043228	0,052621	NA
2b		abundance	X08054	Docosahe›	Radius_length	0,035214	0,071942	NA
2b		abundance	X08054	Docosahe›	Radius_SOS	0,115473	0,050268	NA
2b		abundance	X08054	Docosahe›	Tibia_SOS	-0,01595	0,053862	NA
2b		abundance	X08054	Docosahe›	Handgrip	0,034277	0,060759	NA
2b		abundance	X08056	3-(2-Oxo-2	Tibia_length	-0,05403	0,056664	NA
2b		abundance	X08056	3-(2-Oxo-2	Radius_length	-0,04941	0,074242	NA
2b		abundance	X08056	3-(2-Oxo-2	Radius_SOS	-0,0452	0,053871	NA
2b		abundance	X08056	3-(2-Oxo-2	Tibia_SOS	0,024144	0,057619	NA
2b		abundance	X08056	3-(2-Oxo-2	Handgrip	-0,05051	0,060116	NA
	3	abundance	X08057	N-{3-Carbo	Tibia_length	-0,08005	0,053583	NA
	3	abundance	X08057	N-{3-Carbo	Radius_length	-0,07852	0,072584	NA
	3	abundance	X08057	N-{3-Carbo	Radius_SOS	-0,09244	0,051233	NA
	3	abundance	X08057	N-{3-Carbo	Tibia_SOS	-0,03424	0,054999	NA
	3	abundance	X08057	N-{3-Carbo	Handgrip	-0,03315	0,06259	NA
	3	abundance	X08058	2-Hydroxy-	Tibia_length	-0,01284	0,056783	NA
	3	abundance	X08058	2-Hydroxy-	Radius_length	0,055006	0,074562	NA
	3	abundance	X08058	2-Hydroxy-	Radius_SOS	-0,02271	0,05398	NA
	3	abundance	X08058	2-Hydroxy-	Tibia_SOS	-0,02647	0,05704	NA
	3	abundance	X08058	2-Hydroxy-	Handgrip	0,041845	0,062604	NA
	3	abundance	X08059	Cadaverine	Tibia_length	-0,05192	0,049605	NA
	3	abundance	X08059	Cadaverine	Radius_length	-0,04483	0,067097	NA
	3	abundance	X08059	Cadaverine	Radius_SOS	-0,03426	0,049005	NA
	3	abundance	X08059	Cadaverine	Tibia_SOS	-0,10757	0,052182	NA
	3	abundance	X08059	Cadaverine	Handgrip	0,032556	0,060213	NA
	1	abundance	X08062	Indole-3-ca	Tibia_length	0,178147	0,050241	NA
	1	abundance	X08062	Indole-3-ca	Radius_length	0,124814	0,069358	NA
	1	abundance	X08062	Indole-3-ca	Radius_SOS	-0,04193	0,049334	NA
	1	abundance	X08062	Indole-3-ca	Tibia_SOS	0,127775	0,052431	NA
	1	abundance	X08062	Indole-3-ca	Handgrip	0,112211	0,059936	NA
	3	abundance	X08072	4-Hydroxyp	Tibia_length	0,062423	0,051159	NA
	3	abundance	X08072	4-Hydroxyp	Radius_length	0,074762	0,070361	NA
	3	abundance	X08072	4-Hydroxyp	Radius_SOS	-0,01775	0,049597	NA
	3	abundance	X08072	4-Hydroxyp	Tibia_SOS	-0,02114	0,053423	NA
	3	abundance	X08072	4-Hydroxyp	Handgrip	0,050254	0,06153	NA
2b		abundance	X08076	3',5,7-Trihy	Tibia_length	-0,07474	0,057627	NA
2b		abundance	X08076	3',5,7-Trihy	Radius_length	-0,01745	0,075493	NA
2b		abundance	X08076	3',5,7-Trihy	Radius_SOS	-0,05843	0,055055	NA
2b		abundance	X08076	3',5,7-Trihy	Tibia_SOS	0,013226	0,058912	NA
2b		abundance	X08076	3',5,7-Trihy	Handgrip	0,068207	0,061597	NA
	3	abundance	X08077	6-(alpha-D	Tibia_length	-0,15738	0,054861	NA
	3	abundance	X08077	6-(alpha-D	Radius_length	-0,11328	0,073345	NA

3	abundance	X08077	6-(alpha-D	Radius_SOS	-0,04479	0,052886	NA
3	abundance	X08077	6-(alpha-D	Tibia_SOS	-0,00442	0,055736	NA
3	abundance	X08077	6-(alpha-D	Handgrip	-0,00131	0,062416	NA
3	abundance	X08078	Ethyl mala	Tibia_length	-0,06439	0,056866	NA
3	abundance	X08078	Ethyl mala	Radius_length	0,005186	0,074482	NA
3	abundance	X08078	Ethyl mala	Radius_SOS	-0,06559	0,054379	NA
3	abundance	X08078	Ethyl mala	Tibia_SOS	0,072512	0,057832	NA
3	abundance	X08078	Ethyl mala	Handgrip	-0,02742	0,062983	NA
3	abundance	X08092	Safrole	Tibia_length	-0,05582	0,0571	NA
3	abundance	X08092	Safrole	Radius_length	0,005262	0,075596	NA
3	abundance	X08092	Safrole	Radius_SOS	-0,02605	0,054457	NA
3	abundance	X08092	Safrole	Tibia_SOS	0,018042	0,05731	NA
3	abundance	X08092	Safrole	Handgrip	0,057867	0,062464	NA
3	abundance	X08094	1,1'-[1,12-	Tibia_length	0,040189	0,049385	NA
3	abundance	X08094	1,1'-[1,12-	Radius_length	0,01234	0,066443	NA
3	abundance	X08094	1,1'-[1,12-	Radius_SOS	0,021171	0,049031	NA
3	abundance	X08094	1,1'-[1,12-	Tibia_SOS	-0,06605	0,05238	NA
3	abundance	X08094	1,1'-[1,12-	Handgrip	0,068819	0,060398	NA
3	abundance	X08095	DNOP	Tibia_length	-0,0024	0,050493	NA
3	abundance	X08095	DNOP	Radius_length	0,109271	0,068679	NA
3	abundance	X08095	DNOP	Radius_SOS	0,001067	0,049247	NA
3	abundance	X08095	DNOP	Tibia_SOS	-0,01033	0,052756	NA
3	abundance	X08095	DNOP	Handgrip	0,055974	0,060533	NA
3	abundance	X08096	(2E)-3-Met	Tibia_length	-0,08032	0,057747	NA
3	abundance	X08096	(2E)-3-Met	Radius_length	-0,08933	0,075273	NA
3	abundance	X08096	(2E)-3-Met	Radius_SOS	-0,12773	0,05486	NA
3	abundance	X08096	(2E)-3-Met	Tibia_SOS	-0,08869	0,060057	NA
3	abundance	X08096	(2E)-3-Met	Handgrip	-0,06278	0,060074	NA
1	abundance	X08098	Citrulline	Tibia_length	0,173684	0,049491	NA
1	abundance	X08098	Citrulline	Radius_length	0,232945	0,06668	NA
1	abundance	X08098	Citrulline	Radius_SOS	0,025595	0,049091	NA
1	abundance	X08098	Citrulline	Tibia_SOS	0,148024	0,052125	NA
1	abundance	X08098	Citrulline	Handgrip	0,05298	0,06024	NA
3	abundance	X08099	g-Butyrobe	Tibia_length	0,010104	0,051802	NA
3	abundance	X08099	g-Butyrobe	Radius_length	0,066637	0,070943	NA
3	abundance	X08099	g-Butyrobe	Radius_SOS	0,011406	0,049927	NA
3	abundance	X08099	g-Butyrobe	Tibia_SOS	-0,05505	0,053168	NA
3	abundance	X08099	g-Butyrobe	Handgrip	-0,10231	0,060674	NA
3	abundance	X08100	Piperidine	Tibia_length	-0,00278	0,051312	NA
3	abundance	X08100	Piperidine	Radius_length	-0,05694	0,070048	NA
3	abundance	X08100	Piperidine	Radius_SOS	-0,04154	0,049532	NA
3	abundance	X08100	Piperidine	Tibia_SOS	-0,01394	0,053544	NA
3	abundance	X08100	Piperidine	Handgrip	0,03772	0,061549	NA
3	abundance	X08103	Isopelletie	Tibia_length	-0,0343	0,051604	NA
3	abundance	X08103	Isopelletie	Radius_length	-0,13674	0,069827	NA
3	abundance	X08103	Isopelletie	Radius_SOS	-0,11548	0,049523	NA
3	abundance	X08103	Isopelletie	Tibia_SOS	-0,08121	0,05348	NA
3	abundance	X08103	Isopelletie	Handgrip	-0,0078	0,062063	NA

3	abundance	X08105	2-Acetami	Tibia_length	0,013215	0,053831	NA
3	abundance	X08105	2-Acetami	Radius_length	0,11495	0,072169	NA
3	abundance	X08105	2-Acetami	Radius_SOS	0,054813	0,051446	NA
3	abundance	X08105	2-Acetami	Tibia_SOS	-0,02644	0,055204	NA
3	abundance	X08105	2-Acetami	Handgrip	-0,06031	0,062824	NA
1	abundance	X08106	4-Vinylphe	Tibia_length	4,47E-04	0,051333	NA
1	abundance	X08106	4-Vinylphe	Radius_length	-0,15825	0,069636	NA
1	abundance	X08106	4-Vinylphe	Radius_SOS	0,017349	0,049633	NA
1	abundance	X08106	4-Vinylphe	Tibia_SOS	-0,03519	0,05414	NA
1	abundance	X08106	4-Vinylphe	Handgrip	-0,01429	0,061315	NA
2b	abundance	X08111	2-Hydroxy	Tibia_length	0,142868	0,049517	NA
2b	abundance	X08111	2-Hydroxy	Radius_length	0,136073	0,067227	NA
2b	abundance	X08111	2-Hydroxy	Radius_SOS	0,036791	0,049032	NA
2b	abundance	X08111	2-Hydroxy	Tibia_SOS	0,096249	0,052251	NA
2b	abundance	X08111	2-Hydroxy	Handgrip	0,059261	0,06013	NA
3	abundance	X08116	Methyl (2Z,	Tibia_length	0,047458	0,05711	NA
3	abundance	X08116	Methyl (2Z,	Radius_length	0,290935	0,072176	NA
3	abundance	X08116	Methyl (2Z,	Radius_SOS	0,063529	0,054142	NA
3	abundance	X08116	Methyl (2Z,	Tibia_SOS	0,13439	0,056402	NA
3	abundance	X08116	Methyl (2Z,	Handgrip	0,125533	0,063158	NA
2b	abundance	X08123	Tiglic acid	Tibia_length	-0,10287	0,054242	NA
2b	abundance	X08123	Tiglic acid	Radius_length	-0,07271	0,073593	NA
2b	abundance	X08123	Tiglic acid	Radius_SOS	-0,08381	0,051962	NA
2b	abundance	X08123	Tiglic acid	Tibia_SOS	0,027944	0,055935	NA
2b	abundance	X08123	Tiglic acid	Handgrip	0,016682	0,062013	NA
3	abundance	X08146	O-Ethyl (4-	Tibia_length	0,07138	0,054262	NA
3	abundance	X08146	O-Ethyl (4-	Radius_length	0,028869	0,073125	NA
3	abundance	X08146	O-Ethyl (4-	Radius_SOS	-0,03969	0,051681	NA
3	abundance	X08146	O-Ethyl (4-	Tibia_SOS	0,051507	0,05489	NA
3	abundance	X08146	O-Ethyl (4-	Handgrip	-0,05716	0,062585	NA
3	abundance	X08147	porphobili	Tibia_length	-0,14218	0,056419	NA
3	abundance	X08147	porphobili	Radius_length	-0,14644	0,07411	NA
3	abundance	X08147	porphobili	Radius_SOS	-0,05853	0,053456	NA
3	abundance	X08147	porphobili	Tibia_SOS	-0,10019	0,055826	NA
3	abundance	X08147	porphobili	Handgrip	-0,09824	0,06042	NA
2b	abundance	X08150	DL-4-Hydr	Tibia_length	0,133515	0,052014	NA
2b	abundance	X08150	DL-4-Hydr	Radius_length	0,22174	0,070295	NA
2b	abundance	X08150	DL-4-Hydr	Radius_SOS	0,17869	0,048993	NA
2b	abundance	X08150	DL-4-Hydr	Tibia_SOS	0,100249	0,053351	NA
2b	abundance	X08150	DL-4-Hydr	Handgrip	0,190159	0,059685	NA
3	abundance	X08154	4-(METHYL	Tibia_length	-0,03205	0,052878	NA
3	abundance	X08154	4-(METHYL	Radius_length	0,012608	0,071727	NA
3	abundance	X08154	4-(METHYL	Radius_SOS	0,020472	0,050676	NA
3	abundance	X08154	4-(METHYL	Tibia_SOS	-0,02793	0,054184	NA
3	abundance	X08154	4-(METHYL	Handgrip	-0,06836	0,06175	NA
3	abundance	X08157	Leu-Val	Tibia_length	0,079543	0,053801	NA
3	abundance	X08157	Leu-Val	Radius_length	0,099715	0,072419	NA
3	abundance	X08157	Leu-Val	Radius_SOS	0,082724	0,051617	NA

3	abundance	X08157	Leu-Val	Tibia_SOS	0,026612	0,054906	NA
3	abundance	X08157	Leu-Val	Handgrip	-0,03994	0,061765	NA
3	abundance	X08162	Valylvaline	Tibia_length	0,028177	0,052496	NA
3	abundance	X08162	Valylvaline	Radius_length	0,101111	0,071287	NA
3	abundance	X08162	Valylvaline	Radius_SOS	-0,02423	0,05034	NA
3	abundance	X08162	Valylvaline	Tibia_SOS	0,008968	0,054458	NA
3	abundance	X08162	Valylvaline	Handgrip	-0,00206	0,061131	NA
3	abundance	X08163	Phenyl D-g	Tibia_length	-0,13879	0,052032	NA
3	abundance	X08163	Phenyl D-g	Radius_length	-0,11946	0,070958	NA
3	abundance	X08163	Phenyl D-g	Radius_SOS	-0,06064	0,050363	NA
3	abundance	X08163	Phenyl D-g	Tibia_SOS	-0,0378	0,053649	NA
3	abundance	X08163	Phenyl D-g	Handgrip	-0,06591	0,061032	NA
3	abundance	X08167	Lanthionin	Tibia_length	-0,04868	0,056008	NA
3	abundance	X08167	Lanthionin	Radius_length	-0,06812	0,074178	NA
3	abundance	X08167	Lanthionin	Radius_SOS	-0,09205	0,053105	NA
3	abundance	X08167	Lanthionin	Tibia_SOS	-0,05797	0,057582	NA
3	abundance	X08167	Lanthionin	Handgrip	-0,08434	0,062198	NA
2b	abundance	X08173	9-Methylur	Tibia_length	-0,07874	0,054016	NA
2b	abundance	X08173	9-Methylur	Radius_length	-0,11465	0,072582	NA
2b	abundance	X08173	9-Methylur	Radius_SOS	4,19E-04	0,051663	NA
2b	abundance	X08173	9-Methylur	Tibia_SOS	-0,05068	0,054818	NA
2b	abundance	X08173	9-Methylur	Handgrip	-0,13649	0,062226	NA
2b	abundance	X08188	2-Hydroxy-	Tibia_length	0,121372	0,054696	NA
2b	abundance	X08188	2-Hydroxy-	Radius_length	0,17188	0,073077	NA
2b	abundance	X08188	2-Hydroxy-	Radius_SOS	0,088412	0,051731	NA
2b	abundance	X08188	2-Hydroxy-	Tibia_SOS	0,1031	0,056616	NA
2b	abundance	X08188	2-Hydroxy-	Handgrip	0,147217	0,061033	NA
3	abundance	X08193	Seryltyrosi	Tibia_length	-0,14022	0,051431	NA
3	abundance	X08193	Seryltyrosi	Radius_length	-0,16715	0,069521	NA
3	abundance	X08193	Seryltyrosi	Radius_SOS	-0,10794	0,049398	NA
3	abundance	X08193	Seryltyrosi	Tibia_SOS	-0,09366	0,05333	NA
3	abundance	X08193	Seryltyrosi	Handgrip	-0,06576	0,060683	NA
2b	abundance	X08204	2-Amino-4	Tibia_length	0,048487	0,049489	NA
2b	abundance	X08204	2-Amino-4	Radius_length	0,242443	0,064975	NA
2b	abundance	X08204	2-Amino-4	Radius_SOS	0,024904	0,049017	NA
2b	abundance	X08204	2-Amino-4	Tibia_SOS	0,110306	0,052166	NA
2b	abundance	X08204	2-Amino-4	Handgrip	0,103103	0,059873	NA
3	abundance	X08212	LU345300	Tibia_length	0,018436	0,055477	NA
3	abundance	X08212	LU345300	Radius_length	0,068327	0,074609	NA
3	abundance	X08212	LU345300	Radius_SOS	-0,00218	0,052782	NA
3	abundance	X08212	LU345300	Tibia_SOS	0,026175	0,057022	NA
3	abundance	X08212	LU345300	Handgrip	0,124035	0,063414	NA
2b	abundance	X08221	Brilliant blu	Tibia_length	-0,10183	0,054113	NA
2b	abundance	X08221	Brilliant blu	Radius_length	-0,0729	0,073024	NA
2b	abundance	X08221	Brilliant blu	Radius_SOS	-0,02722	0,051973	NA
2b	abundance	X08221	Brilliant blu	Tibia_SOS	3,64E-04	0,054782	NA
2b	abundance	X08221	Brilliant blu	Handgrip	-0,12142	0,061922	NA
3	abundance	X08230	Coumaron	Tibia_length	0,122435	0,049228	NA

	3	abundance	X08230	Coumaron	Radius_length	0,174115	0,065867	NA
	3	abundance	X08230	Coumaron	Radius_SOS	0,072636	0,048901	NA
	3	abundance	X08230	Coumaron	Tibia_SOS	0,13205	0,052065	NA
	3	abundance	X08230	Coumaron	Handgrip	0,068961	0,060058	NA
2b		abundance	X08235	1-Methylhi	Tibia_length	-0,07755	0,054589	NA
2b		abundance	X08235	1-Methylhi	Radius_length	-0,04677	0,073723	NA
2b		abundance	X08235	1-Methylhi	Radius_SOS	-0,02919	0,052192	NA
2b		abundance	X08235	1-Methylhi	Tibia_SOS	-0,0752	0,05546	NA
2b		abundance	X08235	1-Methylhi	Handgrip	0,04851	0,063654	NA
	3	abundance	X08244	Setoclavin	Tibia_length	-0,00506	0,050202	NA
	3	abundance	X08244	Setoclavin	Radius_length	0,087587	0,068815	NA
	3	abundance	X08244	Setoclavin	Radius_SOS	0,026542	0,049133	NA
	3	abundance	X08244	Setoclavin	Tibia_SOS	0,048187	0,052507	NA
	3	abundance	X08244	Setoclavin	Handgrip	0,045502	0,06077	NA
	3	abundance	X08270	Butabarbit	Tibia_length	-0,11266	0,052964	NA
	3	abundance	X08270	Butabarbit	Radius_length	-0,15804	0,071438	NA
	3	abundance	X08270	Butabarbit	Radius_SOS	-0,02852	0,050633	NA
	3	abundance	X08270	Butabarbit	Tibia_SOS	-0,04872	0,054672	NA
	3	abundance	X08270	Butabarbit	Handgrip	-0,01779	0,061745	NA
2b		abundance	X08274	Mevalonic	Tibia_length	-0,06393	0,05403	NA
2b		abundance	X08274	Mevalonic	Radius_length	-6,00E-04	0,073239	NA
2b		abundance	X08274	Mevalonic	Radius_SOS	0,015856	0,051418	NA
2b		abundance	X08274	Mevalonic	Tibia_SOS	0,02605	0,056307	NA
2b		abundance	X08274	Mevalonic	Handgrip	0,064517	0,061424	NA
	3	abundance	X08277	adrenaline	Tibia_length	0,049735	0,056359	NA
	3	abundance	X08277	adrenaline	Radius_length	0,096629	0,073917	NA
	3	abundance	X08277	adrenaline	Radius_SOS	-0,03784	0,053406	NA
	3	abundance	X08277	adrenaline	Tibia_SOS	-0,04926	0,057378	NA
	3	abundance	X08277	adrenaline	Handgrip	-0,01728	0,062098	NA
	3	abundance	X08279	DNOP	Tibia_length	0,042791	0,052578	NA
	3	abundance	X08279	DNOP	Radius_length	0,172601	0,070401	NA
	3	abundance	X08279	DNOP	Radius_SOS	0,055804	0,050246	NA
	3	abundance	X08279	DNOP	Tibia_SOS	-0,01718	0,05376	NA
	3	abundance	X08279	DNOP	Handgrip	0,055043	0,062092	NA
	3	abundance	X08288	Capryloylg	Tibia_length	-0,12484	0,05771	NA
	3	abundance	X08288	Capryloylg	Radius_length	-0,03818	0,075222	NA
	3	abundance	X08288	Capryloylg	Radius_SOS	-0,08278	0,054739	NA
	3	abundance	X08288	Capryloylg	Tibia_SOS	0,044086	0,06052	NA
	3	abundance	X08288	Capryloylg	Handgrip	-0,04358	0,060136	NA
2b		abundance	X08292	Lidocaine	Tibia_length	-0,05811	0,051232	NA
2b		abundance	X08292	Lidocaine	Radius_length	0,033499	0,070092	NA
2b		abundance	X08292	Lidocaine	Radius_SOS	-0,03218	0,04957	NA
2b		abundance	X08292	Lidocaine	Tibia_SOS	0,040178	0,053515	NA
2b		abundance	X08292	Lidocaine	Handgrip	0,013338	0,060837	NA
2b		abundance	X08298	N6,N6,N6-	Tibia_length	-0,05541	0,050295	NA
2b		abundance	X08298	N6,N6,N6-	Radius_length	-0,03348	0,068608	NA
2b		abundance	X08298	N6,N6,N6-	Radius_SOS	0,010431	0,049151	NA
2b		abundance	X08298	N6,N6,N6-	Tibia_SOS	-0,07537	0,052353	NA

2b		abundance	X08298	N6,N6,N6-	Handgrip	-0,02558	0,060288	NA
	3	abundance	X08300	Indole-3-ac	Tibia_length	0,044443	0,050343	NA
	3	abundance	X08300	Indole-3-ac	Radius_length	-0,03738	0,068725	NA
	3	abundance	X08300	Indole-3-ac	Radius_SOS	-0,05459	0,049099	NA
	3	abundance	X08300	Indole-3-ac	Tibia_SOS	-0,00714	0,052838	NA
	3	abundance	X08300	Indole-3-ac	Handgrip	-0,09283	0,060127	NA
2a		abundance	X08305	Prolylhydrc	Tibia_length	-0,07696	0,055202	NA
2a		abundance	X08305	Prolylhydrc	Radius_length	-0,15321	0,073273	NA
2a		abundance	X08305	Prolylhydrc	Radius_SOS	-0,09079	0,052356	NA
2a		abundance	X08305	Prolylhydrc	Tibia_SOS	-0,09629	0,055258	NA
2a		abundance	X08305	Prolylhydrc	Handgrip	-0,07414	0,061585	NA
2b		abundance	X08306	1-Methylxa	Tibia_length	-0,03353	0,054302	NA
2b		abundance	X08306	1-Methylxa	Radius_length	-0,14563	0,072435	NA
2b		abundance	X08306	1-Methylxa	Radius_SOS	0,008471	0,051427	NA
2b		abundance	X08306	1-Methylxa	Tibia_SOS	-0,01217	0,05471	NA
2b		abundance	X08306	1-Methylxa	Handgrip	-0,00907	0,061973	NA
	3	abundance	X08311	Methyl 3-fc	Tibia_length	-0,16881	0,054349	NA
	3	abundance	X08311	Methyl 3-fc	Radius_length	-0,08297	0,073049	NA
	3	abundance	X08311	Methyl 3-fc	Radius_SOS	0,04738	0,05185	NA
	3	abundance	X08311	Methyl 3-fc	Tibia_SOS	0,010144	0,054502	NA
	3	abundance	X08311	Methyl 3-fc	Handgrip	-0,01905	0,061306	NA
2b		abundance	X08317	8-Hydroxyc	Tibia_length	-2,42E-04	0,05573	NA
2b		abundance	X08317	8-Hydroxyc	Radius_length	-0,01227	0,073825	NA
2b		abundance	X08317	8-Hydroxyc	Radius_SOS	0,007313	0,052913	NA
2b		abundance	X08317	8-Hydroxyc	Tibia_SOS	0,050635	0,055926	NA
2b		abundance	X08317	8-Hydroxyc	Handgrip	-0,02061	0,06295	NA
	3	abundance	X08322	Glycylproly	Tibia_length	-0,10256	0,052131	NA
	3	abundance	X08322	Glycylproly	Radius_length	-0,11943	0,070735	NA
	3	abundance	X08322	Glycylproly	Radius_SOS	0,030865	0,050057	NA
	3	abundance	X08322	Glycylproly	Tibia_SOS	-0,04206	0,053357	NA
	3	abundance	X08322	Glycylproly	Handgrip	0,060435	0,060792	NA
2b		abundance	X08323	(15Z)-9,12	Tibia_length	0,102356	0,049525	NA
2b		abundance	X08323	(15Z)-9,12	Radius_length	0,148534	0,067078	NA
2b		abundance	X08323	(15Z)-9,12	Radius_SOS	0,016637	0,049035	NA
2b		abundance	X08323	(15Z)-9,12	Tibia_SOS	-0,02269	0,05256	NA
2b		abundance	X08323	(15Z)-9,12	Handgrip	0,066436	0,060119	NA
	3	abundance	X08326	2-(1,3-Ben	Tibia_length	-0,06568	0,051452	NA
	3	abundance	X08326	2-(1,3-Ben	Radius_length	-3,35E-04	0,070532	NA
	3	abundance	X08326	2-(1,3-Ben	Radius_SOS	0,015755	0,049815	NA
	3	abundance	X08326	2-(1,3-Ben	Tibia_SOS	-0,05687	0,052938	NA
	3	abundance	X08326	2-(1,3-Ben	Handgrip	-0,0192	0,061573	NA
	3	abundance	X08338	Methyl [9-(	Tibia_length	0,012541	0,059461	NA
	3	abundance	X08338	Methyl [9-(	Radius_length	0,084289	0,075456	NA
	3	abundance	X08338	Methyl [9-(	Radius_SOS	-0,02255	0,056628	NA
	3	abundance	X08338	Methyl [9-(	Tibia_SOS	0,129346	0,058851	NA
	3	abundance	X08338	Methyl [9-(	Handgrip	0,019951	0,062927	NA
	3	abundance	X08341	Midodrine	Tibia_length	-0,06762	0,055373	NA
	3	abundance	X08341	Midodrine	Radius_length	-0,08928	0,074007	NA

	3	abundance	X08341	Midodrine	Radius_SOS	0,053699	0,052698	NA
	3	abundance	X08341	Midodrine	Tibia_SOS	-0,09865	0,055512	NA
	3	abundance	X08341	Midodrine	Handgrip	-0,10156	0,062973	NA
2b		abundance	X08352	7-[4-(tert-b	Tibia_length	0,05658	0,056834	NA
2b		abundance	X08352	7-[4-(tert-b	Radius_length	0,034342	0,074735	NA
2b		abundance	X08352	7-[4-(tert-b	Radius_SOS	-0,01964	0,054273	NA
2b		abundance	X08352	7-[4-(tert-b	Tibia_SOS	-0,01872	0,058151	NA
2b		abundance	X08352	7-[4-(tert-b	Handgrip	-0,0152	0,063566	NA
	3	abundance	X08365	Lysylvaline	Tibia_length	-0,03871	0,051214	NA
	3	abundance	X08365	Lysylvaline	Radius_length	-0,07481	0,070006	NA
	3	abundance	X08365	Lysylvaline	Radius_SOS	-0,00329	0,049586	NA
	3	abundance	X08365	Lysylvaline	Tibia_SOS	-0,07525	0,053233	NA
	3	abundance	X08365	Lysylvaline	Handgrip	0,011568	0,06076	NA
2b		abundance	X08367	11-dehydro	Tibia_length	-0,03525	0,057438	NA
2b		abundance	X08367	11-dehydro	Radius_length	0,053907	0,074711	NA
2b		abundance	X08367	11-dehydro	Radius_SOS	0,072632	0,054237	NA
2b		abundance	X08367	11-dehydro	Tibia_SOS	-0,05084	0,057899	NA
2b		abundance	X08367	11-dehydro	Handgrip	-0,03451	0,063629	NA
	3	abundance	X08373	2-Hydroxy-	Tibia_length	-0,00621	0,056722	NA
	3	abundance	X08373	2-Hydroxy-	Radius_length	0,068745	0,074253	NA
	3	abundance	X08373	2-Hydroxy-	Radius_SOS	0,033771	0,053842	NA
	3	abundance	X08373	2-Hydroxy-	Tibia_SOS	0,098109	0,056104	NA
	3	abundance	X08373	2-Hydroxy-	Handgrip	0,038367	0,063442	NA
	3	abundance	X08386	3b-Hydroxy	Tibia_length	-0,02409	0,051484	NA
	3	abundance	X08386	3b-Hydroxy	Radius_length	-0,03328	0,070861	NA
	3	abundance	X08386	3b-Hydroxy	Radius_SOS	0,114054	0,049557	NA
	3	abundance	X08386	3b-Hydroxy	Tibia_SOS	0,022454	0,053425	NA
	3	abundance	X08386	3b-Hydroxy	Handgrip	0,053688	0,060896	NA
	3	abundance	X08387	4-[(2E,4Z)-	Tibia_length	0,005654	0,05721	NA
	3	abundance	X08387	4-[(2E,4Z)-	Radius_length	-0,00407	0,074683	NA
	3	abundance	X08387	4-[(2E,4Z)-	Radius_SOS	0,02952	0,054418	NA
	3	abundance	X08387	4-[(2E,4Z)-	Tibia_SOS	0,10648	0,057283	NA
	3	abundance	X08387	4-[(2E,4Z)-	Handgrip	-0,01543	0,060186	NA
2b		abundance	X08412	2-Hydroxyp	Tibia_length	0,053205	0,051049	NA
2b		abundance	X08412	2-Hydroxyp	Radius_length	0,054675	0,07036	NA
2b		abundance	X08412	2-Hydroxyp	Radius_SOS	0,061382	0,04944	NA
2b		abundance	X08412	2-Hydroxyp	Tibia_SOS	0,11376	0,052907	NA
2b		abundance	X08412	2-Hydroxyp	Handgrip	0,023222	0,061626	NA
	3	abundance	X08417	asn-pro	Tibia_length	-0,05651	0,054612	NA
	3	abundance	X08417	asn-pro	Radius_length	-0,01856	0,073274	NA
	3	abundance	X08417	asn-pro	Radius_SOS	0,017093	0,052088	NA
	3	abundance	X08417	asn-pro	Tibia_SOS	0,010722	0,055199	NA
	3	abundance	X08417	asn-pro	Handgrip	-0,08079	0,063	NA
	1	abundance	X08420	Dodecane	Tibia_length	0,086289	0,05856	NA
	1	abundance	X08420	Dodecane	Radius_length	0,08195	0,075326	NA
	1	abundance	X08420	Dodecane	Radius_SOS	0,105434	0,055697	NA
	1	abundance	X08420	Dodecane	Tibia_SOS	0,038174	0,058303	NA
	1	abundance	X08420	Dodecane	Handgrip	0,02143	0,062871	NA

3	abundance	X08421	Cys-tyr	Tibia_length	-0,06938	0,058293	NA
3	abundance	X08421	Cys-tyr	Radius_length	0,034139	0,075425	NA
3	abundance	X08421	Cys-tyr	Radius_SOS	-0,16071	0,055613	NA
3	abundance	X08421	Cys-tyr	Tibia_SOS	-0,05501	0,058722	NA
3	abundance	X08421	Cys-tyr	Handgrip	0,073757	0,063418	NA
3	abundance	X08422	N-(3-aceta	Tibia_length	-0,1235	0,051033	NA
3	abundance	X08422	N-(3-aceta	Radius_length	-0,08204	0,070524	NA
3	abundance	X08422	N-(3-aceta	Radius_SOS	-0,05349	0,049798	NA
3	abundance	X08422	N-(3-aceta	Tibia_SOS	0,073877	0,052635	NA
3	abundance	X08422	N-(3-aceta	Handgrip	0,055945	0,061442	NA
3	abundance	X08425	MFCD1297	Tibia_length	0,042021	0,052629	NA
3	abundance	X08425	MFCD1297	Radius_length	0,008149	0,071705	NA
3	abundance	X08425	MFCD1297	Radius_SOS	0,027764	0,050475	NA
3	abundance	X08425	MFCD1297	Tibia_SOS	-0,00482	0,054026	NA
3	abundance	X08425	MFCD1297	Handgrip	-0,07118	0,061908	NA
3	abundance	X08436	N,N-Dimet	Tibia_length	0,080413	0,054827	NA
3	abundance	X08436	N,N-Dimet	Radius_length	0,095793	0,073082	NA
3	abundance	X08436	N,N-Dimet	Radius_SOS	0,032614	0,05187	NA
3	abundance	X08436	N,N-Dimet	Tibia_SOS	0,026321	0,055864	NA
3	abundance	X08436	N,N-Dimet	Handgrip	0,116785	0,062483	NA
3	abundance	X08439	Leucyltyros	Tibia_length	-0,0441	0,050684	NA
3	abundance	X08439	Leucyltyros	Radius_length	-0,14423	0,068769	NA
3	abundance	X08439	Leucyltyros	Radius_SOS	9,73E-04	0,049321	NA
3	abundance	X08439	Leucyltyros	Tibia_SOS	-0,07756	0,052612	NA
3	abundance	X08439	Leucyltyros	Handgrip	-0,09822	0,060888	NA
3	abundance	X08451	L-gamma-(	Tibia_length	-0,08127	0,053098	NA
3	abundance	X08451	L-gamma-(	Radius_length	-0,00382	0,072147	NA
3	abundance	X08451	L-gamma-(	Radius_SOS	-0,01026	0,050896	NA
3	abundance	X08451	L-gamma-(	Tibia_SOS	-0,02356	0,054521	NA
3	abundance	X08451	L-gamma-(	Handgrip	-0,02657	0,061225	NA
1	abundance	X08460	Î±-Muricho	Tibia_length	0,002963	0,052972	NA
1	abundance	X08460	Î±-Muricho	Radius_length	0,023307	0,072136	NA
1	abundance	X08460	Î±-Muricho	Radius_SOS	0,088061	0,05052	NA
1	abundance	X08460	Î±-Muricho	Tibia_SOS	-0,03573	0,054545	NA
1	abundance	X08460	Î±-Muricho	Handgrip	0,011276	0,061313	NA
3	abundance	X08461	Dihydrouri	Tibia_length	-0,13522	0,056541	NA
3	abundance	X08461	Dihydrouri	Radius_length	-0,04837	0,075387	NA
3	abundance	X08461	Dihydrouri	Radius_SOS	-0,09602	0,054209	NA
3	abundance	X08461	Dihydrouri	Tibia_SOS	-0,0618	0,057454	NA
3	abundance	X08461	Dihydrouri	Handgrip	-0,02031	0,063398	NA
3	abundance	X08475	1-(4-Aminc	Tibia_length	-0,11308	0,053297	NA
3	abundance	X08475	1-(4-Aminc	Radius_length	-0,15509	0,071536	NA
3	abundance	X08475	1-(4-Aminc	Radius_SOS	-0,0849	0,050754	NA
3	abundance	X08475	1-(4-Aminc	Tibia_SOS	-0,08929	0,053909	NA
3	abundance	X08475	1-(4-Aminc	Handgrip	-0,02835	0,060428	NA
3	abundance	X08476	Coprine	Tibia_length	0,070297	0,053869	NA
3	abundance	X08476	Coprine	Radius_length	0,07389	0,072496	NA
3	abundance	X08476	Coprine	Radius_SOS	0,071998	0,05141	NA

3	abundance	X08476	Coprine	Tibia_SOS	0,042757	0,054555	NA
3	abundance	X08476	Coprine	Handgrip	0,1153	0,061216	NA
3	abundance	X08478	1-(beta-D-I	Tibia_length	-0,1019	0,05641	NA
3	abundance	X08478	1-(beta-D-I	Radius_length	-0,10432	0,073979	NA
3	abundance	X08478	1-(beta-D-I	Radius_SOS	-0,07437	0,053683	NA
3	abundance	X08478	1-(beta-D-I	Tibia_SOS	-0,09458	0,055549	NA
3	abundance	X08478	1-(beta-D-I	Handgrip	-0,10999	0,063257	NA
3	abundance	X08485	Isoprenalir	Tibia_length	0,105656	0,052613	NA
3	abundance	X08485	Isoprenalir	Radius_length	0,082483	0,071457	NA
3	abundance	X08485	Isoprenalir	Radius_SOS	0,005511	0,050504	NA
3	abundance	X08485	Isoprenalir	Tibia_SOS	0,043541	0,054141	NA
3	abundance	X08485	Isoprenalir	Handgrip	0,021274	0,06178	NA
3	abundance	X08486	(2E,6E)-9-[	Tibia_length	0,100846	0,05286	NA
3	abundance	X08486	(2E,6E)-9-[	Radius_length	0,066597	0,072106	NA
3	abundance	X08486	(2E,6E)-9-[	Radius_SOS	0,007055	0,050552	NA
3	abundance	X08486	(2E,6E)-9-[	Tibia_SOS	0,06931	0,0543	NA
3	abundance	X08486	(2E,6E)-9-[	Handgrip	-0,06029	0,060797	NA
2a	abundance	X08504	Indole-3-ca	Tibia_length	-0,09774	0,051542	NA
2a	abundance	X08504	Indole-3-ca	Radius_length	0,033397	0,071118	NA
2a	abundance	X08504	Indole-3-ca	Radius_SOS	-0,02305	0,050003	NA
2a	abundance	X08504	Indole-3-ca	Tibia_SOS	0,106862	0,052899	NA
2a	abundance	X08504	Indole-3-ca	Handgrip	-0,02879	0,061603	NA
3	abundance	X08509	Piceid	Tibia_length	-0,01764	0,050602	NA
3	abundance	X08509	Piceid	Radius_length	-0,07442	0,068996	NA
3	abundance	X08509	Piceid	Radius_SOS	0,022225	0,049279	NA
3	abundance	X08509	Piceid	Tibia_SOS	0,016067	0,053037	NA
3	abundance	X08509	Piceid	Handgrip	0,002182	0,061003	NA
2b	abundance	X08512	Bile acid I (	Tibia_length	0,026762	0,052313	NA
2b	abundance	X08512	Bile acid I (	Radius_length	1,74E-04	0,071691	NA
2b	abundance	X08512	Bile acid I (	Radius_SOS	0,114951	0,050089	NA
2b	abundance	X08512	Bile acid I (	Tibia_SOS	-0,00187	0,053625	NA
2b	abundance	X08512	Bile acid I (	Handgrip	0,039189	0,061097	NA
3	abundance	X08514	N-Ethylpro	Tibia_length	-0,05126	0,049541	NA
3	abundance	X08514	N-Ethylpro	Radius_length	0,015882	0,066989	NA
3	abundance	X08514	N-Ethylpro	Radius_SOS	0,023494	0,049016	NA
3	abundance	X08514	N-Ethylpro	Tibia_SOS	-0,08299	0,052311	NA
3	abundance	X08514	N-Ethylpro	Handgrip	0,067024	0,060058	NA
3	abundance	X08525	MFCD1869	Tibia_length	-0,08698	0,051972	NA
3	abundance	X08525	MFCD1869	Radius_length	0,007672	0,071123	NA
3	abundance	X08525	MFCD1869	Radius_SOS	0,035193	0,050126	NA
3	abundance	X08525	MFCD1869	Tibia_SOS	0,016873	0,053242	NA
3	abundance	X08525	MFCD1869	Handgrip	0,038093	0,061281	NA
3	abundance	X08529	7,8-Diamir	Tibia_length	-0,04388	0,049768	NA
3	abundance	X08529	7,8-Diamir	Radius_length	-0,03323	0,067502	NA
3	abundance	X08529	7,8-Diamir	Radius_SOS	-0,01618	0,049037	NA
3	abundance	X08529	7,8-Diamir	Tibia_SOS	-0,07977	0,05232	NA
3	abundance	X08529	7,8-Diamir	Handgrip	-0,02716	0,060293	NA
3	abundance	X08533	tyramine si	Tibia_length	-0,11943	0,055846	NA

	3	abundance	X08533	tyramine sı	Radius_length	-0,15901	0,073522	NA
	3	abundance	X08533	tyramine sı	Radius_SOS	-0,01407	0,053488	NA
	3	abundance	X08533	tyramine sı	Tibia_SOS	-0,03323	0,056566	NA
	3	abundance	X08533	tyramine sı	Handgrip	-0,094	0,061636	NA
2b		abundance	X08535	Nitrosohep	Tibia_length	-0,00909	0,049124	NA
2b		abundance	X08535	Nitrosohep	Radius_length	0,037569	0,065028	NA
2b		abundance	X08535	Nitrosohep	Radius_SOS	0,03593	0,04916	NA
2b		abundance	X08535	Nitrosohep	Tibia_SOS	-0,04315	0,052645	NA
2b		abundance	X08535	Nitrosohep	Handgrip	0,019273	0,060268	NA
2b		abundance	X08546	1,3,7-Trim	Tibia_length	-0,09986	0,051618	NA
2b		abundance	X08546	1,3,7-Trim	Radius_length	-0,06316	0,070617	NA
2b		abundance	X08546	1,3,7-Trim	Radius_SOS	0,013394	0,049947	NA
2b		abundance	X08546	1,3,7-Trim	Tibia_SOS	-0,04789	0,053142	NA
2b		abundance	X08546	1,3,7-Trim	Handgrip	-0,05072	0,061014	NA
	1	abundance	X08548	N2-Acetyll	Tibia_length	0,097855	0,050775	NA
	1	abundance	X08548	N2-Acetyll	Radius_length	0,142857	0,069007	NA
	1	abundance	X08548	N2-Acetyll	Radius_SOS	0,021342	0,049362	NA
	1	abundance	X08548	N2-Acetyll	Tibia_SOS	0,098081	0,052408	NA
	1	abundance	X08548	N2-Acetyll	Handgrip	0,053602	0,060123	NA
	3	abundance	X08549	3-Methylsu	Tibia_length	0,08809	0,051064	NA
	3	abundance	X08549	3-Methylsu	Radius_length	0,166508	0,068927	NA
	3	abundance	X08549	3-Methylsu	Radius_SOS	0,025681	0,049438	NA
	3	abundance	X08549	3-Methylsu	Tibia_SOS	0,131949	0,052548	NA
	3	abundance	X08549	3-Methylsu	Handgrip	0,108729	0,060195	NA
	3	abundance	X08561	N-(Carboxy	Tibia_length	-0,06835	0,052997	NA
	3	abundance	X08561	N-(Carboxy	Radius_length	0,122568	0,071905	NA
	3	abundance	X08561	N-(Carboxy	Radius_SOS	0,081871	0,050662	NA
	3	abundance	X08561	N-(Carboxy	Tibia_SOS	0,078007	0,054776	NA
	3	abundance	X08561	N-(Carboxy	Handgrip	0,060315	0,062232	NA
	3	abundance	X08578	tert-Butyl 3	Tibia_length	0,020583	0,054508	NA
	3	abundance	X08578	tert-Butyl 3	Radius_length	0,066164	0,072951	NA
	3	abundance	X08578	tert-Butyl 3	Radius_SOS	0,006159	0,05177	NA
	3	abundance	X08578	tert-Butyl 3	Tibia_SOS	-0,01966	0,055236	NA
	3	abundance	X08578	tert-Butyl 3	Handgrip	0,057245	0,061797	NA
	3	abundance	X08584	meticillin	Tibia_length	-0,04979	0,056601	NA
	3	abundance	X08584	meticillin	Radius_length	-0,03805	0,074587	NA
	3	abundance	X08584	meticillin	Radius_SOS	-0,03673	0,054154	NA
	3	abundance	X08584	meticillin	Tibia_SOS	-0,02036	0,055754	NA
	3	abundance	X08584	meticillin	Handgrip	-0,03735	0,062185	NA
	3	abundance	X08588	2-Acetamid	Tibia_length	0,06508	0,053645	NA
	3	abundance	X08588	2-Acetamid	Radius_length	0,111555	0,072632	NA
	3	abundance	X08588	2-Acetamid	Radius_SOS	-0,01325	0,051293	NA
	3	abundance	X08588	2-Acetamid	Tibia_SOS	0,055023	0,054585	NA
	3	abundance	X08588	2-Acetamid	Handgrip	0,103506	0,062641	NA
	1	abundance	X08593	Uric acid	Tibia_length	0,095544	0,052993	NA
	1	abundance	X08593	Uric acid	Radius_length	0,065408	0,072175	NA
	1	abundance	X08593	Uric acid	Radius_SOS	0,029681	0,050865	NA
	1	abundance	X08593	Uric acid	Tibia_SOS	0,089129	0,054408	NA

	1	abundance	X08593	Uric acid	Handgrip	-0,03096	0,061278	NA
	3	abundance	X08594	7-ketodeo›	Tibia_length	0,007638	0,054571	NA
	3	abundance	X08594	7-ketodeo›	Radius_length	-0,044	0,073011	NA
	3	abundance	X08594	7-ketodeo›	Radius_SOS	0,044125	0,051893	NA
	3	abundance	X08594	7-ketodeo›	Tibia_SOS	-0,06891	0,05513	NA
	3	abundance	X08594	7-ketodeo›	Handgrip	-0,04881	0,062423	NA
	3	abundance	X08600	N-Propiony	Tibia_length	0,104381	0,051681	NA
	3	abundance	X08600	N-Propiony	Radius_length	0,032975	0,070747	NA
	3	abundance	X08600	N-Propiony	Radius_SOS	-0,01462	0,049947	NA
	3	abundance	X08600	N-Propiony	Tibia_SOS	0,070451	0,053319	NA
	3	abundance	X08600	N-Propiony	Handgrip	-0,05108	0,061508	NA
	3	abundance	X08606	Butabarbit	Tibia_length	-0,03719	0,051246	NA
	3	abundance	X08606	Butabarbit	Radius_length	-0,0693	0,070183	NA
	3	abundance	X08606	Butabarbit	Radius_SOS	-0,05221	0,049585	NA
	3	abundance	X08606	Butabarbit	Tibia_SOS	-0,02708	0,053671	NA
	3	abundance	X08606	Butabarbit	Handgrip	-0,00988	0,060664	NA
	3	abundance	X08610	Leucyltyros	Tibia_length	-0,12827	0,05042	NA
	3	abundance	X08610	Leucyltyros	Radius_length	-0,0675	0,069047	NA
	3	abundance	X08610	Leucyltyros	Radius_SOS	0,022208	0,049295	NA
	3	abundance	X08610	Leucyltyros	Tibia_SOS	-0,09084	0,052632	NA
	3	abundance	X08610	Leucyltyros	Handgrip	-0,01089	0,060395	NA
	3	abundance	X08616	MFCD0002	Tibia_length	0,004225	0,05044	NA
	3	abundance	X08616	MFCD0002	Radius_length	-0,03195	0,068972	NA
	3	abundance	X08616	MFCD0002	Radius_SOS	0,005928	0,049217	NA
	3	abundance	X08616	MFCD0002	Tibia_SOS	0,076103	0,052572	NA
	3	abundance	X08616	MFCD0002	Handgrip	0,150695	0,060157	NA
	3	abundance	X08625	Hept-2-ulo	Tibia_length	-0,02249	0,054553	NA
	3	abundance	X08625	Hept-2-ulo	Radius_length	0,099938	0,073273	NA
	3	abundance	X08625	Hept-2-ulo	Radius_SOS	0,038171	0,051843	NA
	3	abundance	X08625	Hept-2-ulo	Tibia_SOS	0,004525	0,055811	NA
	3	abundance	X08625	Hept-2-ulo	Handgrip	0,007046	0,061731	NA
2b		abundance	X08634	1,5-Isoquir	Tibia_length	-0,03348	0,051237	NA
2b		abundance	X08634	1,5-Isoquir	Radius_length	-0,08654	0,069907	NA
2b		abundance	X08634	1,5-Isoquir	Radius_SOS	0,078391	0,049386	NA
2b		abundance	X08634	1,5-Isoquir	Tibia_SOS	-0,05234	0,05315	NA
2b		abundance	X08634	1,5-Isoquir	Handgrip	0,010771	0,060926	NA
	3	abundance	X08639	hypaphorir	Tibia_length	-0,13346	0,048768	NA
	3	abundance	X08639	hypaphorir	Radius_length	-0,151	0,064992	NA
	3	abundance	X08639	hypaphorir	Radius_SOS	-0,03314	0,049091	NA
	3	abundance	X08639	hypaphorir	Tibia_SOS	0,059356	0,052567	NA
	3	abundance	X08639	hypaphorir	Handgrip	0,049493	0,060138	NA
	3	abundance	X08641	meprobam	Tibia_length	-0,07479	0,055189	NA
	3	abundance	X08641	meprobam	Radius_length	-0,00709	0,07407	NA
	3	abundance	X08641	meprobam	Radius_SOS	-0,00521	0,052761	NA
	3	abundance	X08641	meprobam	Tibia_SOS	-0,00203	0,057322	NA
	3	abundance	X08641	meprobam	Handgrip	-0,07826	0,063269	NA
2b		abundance	X08644	2,5-Dimetl	Tibia_length	0,006138	0,051283	NA
2b		abundance	X08644	2,5-Dimetl	Radius_length	-0,17277	0,069305	NA

2b	abundance	X08644	2,5-Dimetl	Radius_SOS	-0,0122	0,049611	NA
2b	abundance	X08644	2,5-Dimetl	Tibia_SOS	-0,07197	0,053611	NA
2b	abundance	X08644	2,5-Dimetl	Handgrip	0,003841	0,060887	NA
3	abundance	X08646	N-Stearoyl	Tibia_length	-0,00476	0,04897	NA
3	abundance	X08646	N-Stearoyl	Radius_length	0,067476	0,048859	NA
3	abundance	X08646	N-Stearoyl	Radius_SOS	-0,01532	0,049023	NA
3	abundance	X08646	N-Stearoyl	Tibia_SOS	0,003852	0,052486	NA
3	abundance	X08646	N-Stearoyl	Handgrip	-0,01639	0,060185	NA
3	abundance	X08657	1-(4-Aminc	Tibia_length	-0,06285	0,049178	NA
3	abundance	X08657	1-(4-Aminc	Radius_length	0,027631	0,065953	NA
3	abundance	X08657	1-(4-Aminc	Radius_SOS	-0,04473	0,049022	NA
3	abundance	X08657	1-(4-Aminc	Tibia_SOS	-0,09281	0,052269	NA
3	abundance	X08657	1-(4-Aminc	Handgrip	0,055599	0,060141	NA
2b	abundance	X08659	3,3-Dimetl	Tibia_length	-0,04302	0,049152	NA
2b	abundance	X08659	3,3-Dimetl	Radius_length	0,008955	0,065459	NA
2b	abundance	X08659	3,3-Dimetl	Radius_SOS	0,044301	0,049065	NA
2b	abundance	X08659	3,3-Dimetl	Tibia_SOS	0,04621	0,052557	NA
2b	abundance	X08659	3,3-Dimetl	Handgrip	-0,05597	0,060117	NA
2b	abundance	X08663	Pilocarpine	Tibia_length	0,004847	0,050267	NA
2b	abundance	X08663	Pilocarpine	Radius_length	-0,08905	0,068528	NA
2b	abundance	X08663	Pilocarpine	Radius_SOS	-0,0705	0,049018	NA
2b	abundance	X08663	Pilocarpine	Tibia_SOS	-0,08878	0,052677	NA
2b	abundance	X08663	Pilocarpine	Handgrip	-0,00115	0,060998	NA
3	abundance	X08670	Hydroxypro	Tibia_length	-0,00213	0,054294	NA
3	abundance	X08670	Hydroxypro	Radius_length	0,046983	0,073303	NA
3	abundance	X08670	Hydroxypro	Radius_SOS	-0,03422	0,051867	NA
3	abundance	X08670	Hydroxypro	Tibia_SOS	-3,17E-04	0,054394	NA
3	abundance	X08670	Hydroxypro	Handgrip	-0,01488	0,061604	NA
3	abundance	X08673	NPC	Tibia_length	0,013646	0,054438	NA
3	abundance	X08673	NPC	Radius_length	0,084763	0,073221	NA
3	abundance	X08673	NPC	Radius_SOS	-0,02542	0,051709	NA
3	abundance	X08673	NPC	Tibia_SOS	0,072424	0,055114	NA
3	abundance	X08673	NPC	Handgrip	0,06369	0,061766	NA
3	abundance	X08677	1-methylhy	Tibia_length	-0,00827	0,053627	NA
3	abundance	X08677	1-methylhy	Radius_length	0,077453	0,072641	NA
3	abundance	X08677	1-methylhy	Radius_SOS	0,09538	0,05062	NA
3	abundance	X08677	1-methylhy	Tibia_SOS	0,098411	0,053962	NA
3	abundance	X08677	1-methylhy	Handgrip	0,045441	0,061345	NA
3	abundance	X08690	Ethanoic a	Tibia_length	-0,02787	0,055952	NA
3	abundance	X08690	Ethanoic a	Radius_length	0,034562	0,074718	NA
3	abundance	X08690	Ethanoic a	Radius_SOS	-0,04866	0,053188	NA
3	abundance	X08690	Ethanoic a	Tibia_SOS	0,018138	0,056652	NA
3	abundance	X08690	Ethanoic a	Handgrip	0,097074	0,063294	NA
3	abundance	X08693	7-Sulfocho	Tibia_length	-0,06003	0,050303	NA
3	abundance	X08693	7-Sulfocho	Radius_length	-0,12802	0,068307	NA
3	abundance	X08693	7-Sulfocho	Radius_SOS	0,021287	0,049146	NA
3	abundance	X08693	7-Sulfocho	Tibia_SOS	-0,00883	0,052636	NA
3	abundance	X08693	7-Sulfocho	Handgrip	0,003903	0,060681	NA

3	abundance	X08695	3b-Hydroxy	Tibia_length	0,023133	0,050348	NA
3	abundance	X08695	3b-Hydroxy	Radius_length	0,080285	0,068619	NA
3	abundance	X08695	3b-Hydroxy	Radius_SOS	-0,02308	0,049201	NA
3	abundance	X08695	3b-Hydroxy	Tibia_SOS	-0,03838	0,052675	NA
3	abundance	X08695	3b-Hydroxy	Handgrip	0,063025	0,060887	NA
3	abundance	X08699	Aurorix	Tibia_length	-0,00486	0,05643	NA
3	abundance	X08699	Aurorix	Radius_length	-0,00592	0,07436	NA
3	abundance	X08699	Aurorix	Radius_SOS	-0,06823	0,05352	NA
3	abundance	X08699	Aurorix	Tibia_SOS	-0,02244	0,05638	NA
3	abundance	X08699	Aurorix	Handgrip	0,00802	0,062236	NA
3	abundance	X08707	2-(1-Ethoxy	Tibia_length	-0,04683	0,052097	NA
3	abundance	X08707	2-(1-Ethoxy	Radius_length	-0,11993	0,070825	NA
3	abundance	X08707	2-(1-Ethoxy	Radius_SOS	-0,07561	0,05	NA
3	abundance	X08707	2-(1-Ethoxy	Tibia_SOS	0,008055	0,053992	NA
3	abundance	X08707	2-(1-Ethoxy	Handgrip	0,020816	0,061641	NA
3	abundance	X08711	2-Acetami	Tibia_length	0,165222	0,055213	NA
3	abundance	X08711	2-Acetami	Radius_length	0,196857	0,072706	NA
3	abundance	X08711	2-Acetami	Radius_SOS	0,047479	0,053208	NA
3	abundance	X08711	2-Acetami	Tibia_SOS	0,125135	0,055586	NA
3	abundance	X08711	2-Acetami	Handgrip	-0,03051	0,062931	NA
3	abundance	X08712	4-Iodoanis	Tibia_length	0,076453	0,050953	NA
3	abundance	X08712	4-Iodoanis	Radius_length	0,210508	0,068449	NA
3	abundance	X08712	4-Iodoanis	Radius_SOS	0,056156	0,049355	NA
3	abundance	X08712	4-Iodoanis	Tibia_SOS	0,084398	0,052604	NA
3	abundance	X08712	4-Iodoanis	Handgrip	0,155118	0,059918	NA
2b	abundance	X08713	Phenethyla	Tibia_length	-0,03502	0,050664	NA
2b	abundance	X08713	Phenethyla	Radius_length	-0,15883	0,068576	NA
2b	abundance	X08713	Phenethyla	Radius_SOS	-0,18975	0,048374	NA
2b	abundance	X08713	Phenethyla	Tibia_SOS	-0,03903	0,052816	NA
2b	abundance	X08713	Phenethyla	Handgrip	-0,16303	0,060045	NA
3	abundance	X08714	Arabic acic	Tibia_length	-0,08174	0,05581	NA
3	abundance	X08714	Arabic acic	Radius_length	-0,03061	0,075095	NA
3	abundance	X08714	Arabic acic	Radius_SOS	-0,04959	0,053177	NA
3	abundance	X08714	Arabic acic	Tibia_SOS	-0,0147	0,056301	NA
3	abundance	X08714	Arabic acic	Handgrip	0,038692	0,063221	NA
2b	abundance	X08719	6-Methylqu	Tibia_length	0,054349	0,049302	NA
2b	abundance	X08719	6-Methylqu	Radius_length	-0,00369	0,066372	NA
2b	abundance	X08719	6-Methylqu	Radius_SOS	-0,0575	0,048977	NA
2b	abundance	X08719	6-Methylqu	Tibia_SOS	-0,00961	0,052489	NA
2b	abundance	X08719	6-Methylqu	Handgrip	-0,08956	0,059966	NA
3	abundance	X08723	S-Allylcyst	Tibia_length	-0,02273	0,053547	NA
3	abundance	X08723	S-Allylcyst	Radius_length	-0,04185	0,072786	NA
3	abundance	X08723	S-Allylcyst	Radius_SOS	-0,07394	0,051179	NA
3	abundance	X08723	S-Allylcyst	Tibia_SOS	-0,01336	0,054575	NA
3	abundance	X08723	S-Allylcyst	Handgrip	0,071257	0,061615	NA
3	abundance	X08726	YV8195000	Tibia_length	0,022069	0,051977	NA
3	abundance	X08726	YV8195000	Radius_length	0,195819	0,069748	NA
3	abundance	X08726	YV8195000	Radius_SOS	0,063843	0,049899	NA

3	abundance	X08726	YV819500(	Tibia_SOS	0,100613	0,053414	NA
3	abundance	X08726	YV819500(	Handgrip	0,036541	0,061638	NA
3	abundance	X08733	N-Methyl-1	Tibia_length	0,041542	0,050781	NA
3	abundance	X08733	N-Methyl-1	Radius_length	0,114327	0,068625	NA
3	abundance	X08733	N-Methyl-1	Radius_SOS	-0,01544	0,049236	NA
3	abundance	X08733	N-Methyl-1	Tibia_SOS	-0,0062	0,052764	NA
3	abundance	X08733	N-Methyl-1	Handgrip	-0,04266	0,060667	NA
3	abundance	X08739	1,1'-[1,12-I	Tibia_length	0,025925	0,049905	NA
3	abundance	X08739	1,1'-[1,12-I	Radius_length	0,007226	0,067823	NA
3	abundance	X08739	1,1'-[1,12-I	Radius_SOS	0,00216	0,049075	NA
3	abundance	X08739	1,1'-[1,12-I	Tibia_SOS	-0,04288	0,052654	NA
3	abundance	X08739	1,1'-[1,12-I	Handgrip	0,072572	0,060401	NA
3	abundance	X08758	Methohexi	Tibia_length	-0,04856	0,050953	NA
3	abundance	X08758	Methohexi	Radius_length	0,023059	0,070082	NA
3	abundance	X08758	Methohexi	Radius_SOS	-0,02336	0,049505	NA
3	abundance	X08758	Methohexi	Tibia_SOS	0,040455	0,05295	NA
3	abundance	X08758	Methohexi	Handgrip	0,079454	0,060273	NA
2b	abundance	X08777	3-Aminosa	Tibia_length	-0,02776	0,049111	NA
2b	abundance	X08777	3-Aminosa	Radius_length	0,061043	0,065245	NA
2b	abundance	X08777	3-Aminosa	Radius_SOS	-0,04588	0,049109	NA
2b	abundance	X08777	3-Aminosa	Tibia_SOS	0,123172	0,052114	NA
2b	abundance	X08777	3-Aminosa	Handgrip	-0,08612	0,060109	NA
2b	abundance	X08779	3-(1-hydro:	Tibia_length	-0,1039	0,054218	NA
2b	abundance	X08779	3-(1-hydro:	Radius_length	-0,22238	0,07215	NA
2b	abundance	X08779	3-(1-hydro:	Radius_SOS	-0,07405	0,051568	NA
2b	abundance	X08779	3-(1-hydro:	Tibia_SOS	-0,10464	0,054312	NA
2b	abundance	X08779	3-(1-hydro:	Handgrip	-0,03226	0,062169	NA
3	abundance	X08781	Procaine	Tibia_length	-0,0265	0,051618	NA
3	abundance	X08781	Procaine	Radius_length	-0,02269	0,070484	NA
3	abundance	X08781	Procaine	Radius_SOS	-0,01492	0,049717	NA
3	abundance	X08781	Procaine	Tibia_SOS	0,003045	0,05376	NA
3	abundance	X08781	Procaine	Handgrip	-0,04272	0,061558	NA
2b	abundance	X08789	Acrylic aci	Tibia_length	-0,01858	0,056008	NA
2b	abundance	X08789	Acrylic aci	Radius_length	0,031532	0,074842	NA
2b	abundance	X08789	Acrylic aci	Radius_SOS	-0,03472	0,053225	NA
2b	abundance	X08789	Acrylic aci	Tibia_SOS	0,010509	0,05684	NA
2b	abundance	X08789	Acrylic aci	Handgrip	0,112628	0,063152	NA
3	abundance	X08792	3,4-Methyl	Tibia_length	0,05785	0,059142	NA
3	abundance	X08792	3,4-Methyl	Radius_length	0,162772	0,075082	NA
3	abundance	X08792	3,4-Methyl	Radius_SOS	0,060053	0,056187	NA
3	abundance	X08792	3,4-Methyl	Tibia_SOS	-0,03731	0,058941	NA
3	abundance	X08792	3,4-Methyl	Handgrip	0,043709	0,063724	NA
3	abundance	X08795	N-Acetylva	Tibia_length	0,045009	0,054602	NA
3	abundance	X08795	N-Acetylva	Radius_length	0,028171	0,073175	NA
3	abundance	X08795	N-Acetylva	Radius_SOS	0,04974	0,05206	NA
3	abundance	X08795	N-Acetylva	Tibia_SOS	-0,01346	0,056093	NA
3	abundance	X08795	N-Acetylva	Handgrip	0,001905	0,062067	NA
2b	abundance	X08801	3,7-Dimetl	Tibia_length	-0,06149	0,051311	NA

2b	abundance	X08801	3,7-Dimetł	Radius_length	-0,0936	0,070158	NA
2b	abundance	X08801	3,7-Dimetł	Radius_SOS	0,042512	0,049612	NA
2b	abundance	X08801	3,7-Dimetł	Tibia_SOS	-0,15674	0,052662	NA
2b	abundance	X08801	3,7-Dimetł	Handgrip	-0,07427	0,060885	NA
3	abundance	X08810	N~6~,N~6	Tibia_length	-0,02811	0,049102	NA
3	abundance	X08810	N~6~,N~6	Radius_length	-0,05543	0,064922	NA
3	abundance	X08810	N~6~,N~6	Radius_SOS	-0,01286	0,04919	NA
3	abundance	X08810	N~6~,N~6	Tibia_SOS	-0,07475	0,052529	NA
3	abundance	X08810	N~6~,N~6	Handgrip	-0,02882	0,060185	NA
3	abundance	X08822	CYS-ASP	Tibia_length	-0,08206	0,05706	NA
3	abundance	X08822	CYS-ASP	Radius_length	-0,06831	0,074648	NA
3	abundance	X08822	CYS-ASP	Radius_SOS	-0,11136	0,054203	NA
3	abundance	X08822	CYS-ASP	Tibia_SOS	-0,00865	0,058184	NA
3	abundance	X08822	CYS-ASP	Handgrip	-0,15072	0,062704	NA
3	abundance	X08825	Chenodeo:	Tibia_length	0,091741	0,052253	NA
3	abundance	X08825	Chenodeo:	Radius_length	0,094265	0,071086	NA
3	abundance	X08825	Chenodeo:	Radius_SOS	0,047064	0,050233	NA
3	abundance	X08825	Chenodeo:	Tibia_SOS	-0,05111	0,053679	NA
3	abundance	X08825	Chenodeo:	Handgrip	0,097845	0,061432	NA
3	abundance	X08842	Spermic ac	Tibia_length	-0,09026	0,054478	NA
3	abundance	X08842	Spermic ac	Radius_length	0,042207	0,073833	NA
3	abundance	X08842	Spermic ac	Radius_SOS	-0,02152	0,05218	NA
3	abundance	X08842	Spermic ac	Tibia_SOS	0,006641	0,05703	NA
3	abundance	X08842	Spermic ac	Handgrip	-0,10503	0,062577	NA
3	abundance	X08843	(2R,3S)-3-I	Tibia_length	0,006324	0,050976	NA
3	abundance	X08843	(2R,3S)-3-I	Radius_length	0,117945	0,069379	NA
3	abundance	X08843	(2R,3S)-3-I	Radius_SOS	0,032254	0,049463	NA
3	abundance	X08843	(2R,3S)-3-I	Tibia_SOS	0,157639	0,052555	NA
3	abundance	X08843	(2R,3S)-3-I	Handgrip	0,035096	0,060737	NA
3	abundance	X08844	4-Hydroxy-	Tibia_length	-0,0188	0,05632	NA
3	abundance	X08844	4-Hydroxy-	Radius_length	0,025766	0,074869	NA
3	abundance	X08844	4-Hydroxy-	Radius_SOS	-0,04569	0,053508	NA
3	abundance	X08844	4-Hydroxy-	Tibia_SOS	0,019409	0,05684	NA
3	abundance	X08844	4-Hydroxy-	Handgrip	0,136446	0,063032	NA
3	abundance	X08845	14-Hydroxy	Tibia_length	-0,02646	0,054533	NA
3	abundance	X08845	14-Hydroxy	Radius_length	-0,09553	0,072988	NA
3	abundance	X08845	14-Hydroxy	Radius_SOS	-0,02076	0,051771	NA
3	abundance	X08845	14-Hydroxy	Tibia_SOS	0,00602	0,054882	NA
3	abundance	X08845	14-Hydroxy	Handgrip	-0,06082	0,061407	NA
2b	abundance	X08847	1-Vinylimic	Tibia_length	-0,09546	0,051969	NA
2b	abundance	X08847	1-Vinylimic	Radius_length	-0,18639	0,069914	NA
2b	abundance	X08847	1-Vinylimic	Radius_SOS	-0,12138	0,049764	NA
2b	abundance	X08847	1-Vinylimic	Tibia_SOS	-0,10612	0,053416	NA
2b	abundance	X08847	1-Vinylimic	Handgrip	0,042198	0,060851	NA
3	abundance	X08869	Lanthionin	Tibia_length	-0,05365	0,055366	NA
3	abundance	X08869	Lanthionin	Radius_length	-0,06518	0,073708	NA
3	abundance	X08869	Lanthionin	Radius_SOS	-0,08191	0,052851	NA
3	abundance	X08869	Lanthionin	Tibia_SOS	-0,06136	0,056532	NA

3	abundance	X08869	Lanthionin	Handgrip	-0,05779	0,062361	NA
3	abundance	X08870	Tetraacety	Tibia_length	-0,0571	0,058432	NA
3	abundance	X08870	Tetraacety	Radius_length	0,008388	0,075711	NA
3	abundance	X08870	Tetraacety	Radius_SOS	-0,03028	0,055857	NA
3	abundance	X08870	Tetraacety	Tibia_SOS	-0,05001	0,058601	NA
3	abundance	X08870	Tetraacety	Handgrip	0,042452	0,063326	NA
3	abundance	X08883	3-Phenylpr	Tibia_length	0,006676	0,054607	NA
3	abundance	X08883	3-Phenylpr	Radius_length	-0,01075	0,072972	NA
3	abundance	X08883	3-Phenylpr	Radius_SOS	-0,03068	0,051936	NA
3	abundance	X08883	3-Phenylpr	Tibia_SOS	-0,04544	0,055021	NA
3	abundance	X08883	3-Phenylpr	Handgrip	-0,00515	0,061234	NA
3	abundance	X08893	5beta-Cho	Tibia_length	-0,17351	0,052632	NA
3	abundance	X08893	5beta-Cho	Radius_length	-0,15215	0,072163	NA
3	abundance	X08893	5beta-Cho	Radius_SOS	-0,02894	0,051072	NA
3	abundance	X08893	5beta-Cho	Tibia_SOS	-0,07613	0,054544	NA
3	abundance	X08893	5beta-Cho	Handgrip	-0,18992	0,060926	NA
3	abundance	X08895	pentoxyl	Tibia_length	-0,15378	0,051761	NA
3	abundance	X08895	pentoxyl	Radius_length	-0,01062	0,071552	NA
3	abundance	X08895	pentoxyl	Radius_SOS	-0,03398	0,050303	NA
3	abundance	X08895	pentoxyl	Tibia_SOS	-0,0603	0,053965	NA
3	abundance	X08895	pentoxyl	Handgrip	0,064942	0,062131	NA
3	abundance	X08908	Vorinostat	Tibia_length	-0,00106	0,053053	NA
3	abundance	X08908	Vorinostat	Radius_length	-0,00703	0,072306	NA
3	abundance	X08908	Vorinostat	Radius_SOS	0,032611	0,050837	NA
3	abundance	X08908	Vorinostat	Tibia_SOS	-0,0053	0,054466	NA
3	abundance	X08908	Vorinostat	Handgrip	-0,00177	0,062179	NA
2b	abundance	X08909	GLK (Pepti	Tibia_length	-0,05759	0,054948	NA
2b	abundance	X08909	GLK (Pepti	Radius_length	0,005018	0,074014	NA
2b	abundance	X08909	GLK (Pepti	Radius_SOS	0,032855	0,052162	NA
2b	abundance	X08909	GLK (Pepti	Tibia_SOS	-0,0023	0,054965	NA
2b	abundance	X08909	GLK (Pepti	Handgrip	0,09963	0,060417	NA
3	abundance	X08923	Semilicois	Tibia_length	-0,00225	0,055437	NA
3	abundance	X08923	Semilicois	Radius_length	-0,12814	0,073259	NA
3	abundance	X08923	Semilicois	Radius_SOS	-0,11687	0,052794	NA
3	abundance	X08923	Semilicois	Tibia_SOS	-0,08433	0,056647	NA
3	abundance	X08923	Semilicois	Handgrip	-0,10636	0,060971	NA
3	abundance	X08938	Nonivamid	Tibia_length	0,093284	0,050684	NA
3	abundance	X08938	Nonivamid	Radius_length	0,14699	0,069082	NA
3	abundance	X08938	Nonivamid	Radius_SOS	0,081355	0,049201	NA
3	abundance	X08938	Nonivamid	Tibia_SOS	0,142421	0,052613	NA
3	abundance	X08938	Nonivamid	Handgrip	0,092842	0,060287	NA
3	abundance	X08944	Dipivefrin	Tibia_length	0,153598	0,053922	NA
3	abundance	X08944	Dipivefrin	Radius_length	0,210937	0,072185	NA
3	abundance	X08944	Dipivefrin	Radius_SOS	0,096094	0,051822	NA
3	abundance	X08944	Dipivefrin	Tibia_SOS	0,164985	0,055135	NA
3	abundance	X08944	Dipivefrin	Handgrip	0,102028	0,061934	NA
3	abundance	X08965	1,9-Nonan	Tibia_length	-0,09059	0,055059	NA
3	abundance	X08965	1,9-Nonan	Radius_length	-0,01841	0,074199	NA

3	abundance	X08965	1,9-Nonan	Radius_SOS	0,054925	0,052208	NA
3	abundance	X08965	1,9-Nonan	Tibia_SOS	0,111736	0,054684	NA
3	abundance	X08965	1,9-Nonan	Handgrip	0,151883	0,061106	NA
3	abundance	X08973	Aminohipp	Tibia_length	0,080595	0,054533	NA
3	abundance	X08973	Aminohipp	Radius_length	-0,00388	0,07313	NA
3	abundance	X08973	Aminohipp	Radius_SOS	-0,02284	0,052111	NA
3	abundance	X08973	Aminohipp	Tibia_SOS	-0,01088	0,057049	NA
3	abundance	X08973	Aminohipp	Handgrip	-0,02156	0,060179	NA
3	abundance	X08983	IN00150	Tibia_length	-0,05538	0,051893	NA
3	abundance	X08983	IN00150	Radius_length	-0,06949	0,071257	NA
3	abundance	X08983	IN00150	Radius_SOS	0,106715	0,049829	NA
3	abundance	X08983	IN00150	Tibia_SOS	0,045312	0,053537	NA
3	abundance	X08983	IN00150	Handgrip	-0,01214	0,061863	NA
3	abundance	X08987	feruloylser	Tibia_length	-0,10547	0,055395	NA
3	abundance	X08987	feruloylser	Radius_length	0,008676	0,073896	NA
3	abundance	X08987	feruloylser	Radius_SOS	-0,05471	0,052935	NA
3	abundance	X08987	feruloylser	Tibia_SOS	0,011178	0,056354	NA
3	abundance	X08987	feruloylser	Handgrip	-0,01821	0,062205	NA
3	abundance	X08990	Procaine	Tibia_length	-0,14917	0,052915	NA
3	abundance	X08990	Procaine	Radius_length	-0,12071	0,071672	NA
3	abundance	X08990	Procaine	Radius_SOS	-0,02578	0,050893	NA
3	abundance	X08990	Procaine	Tibia_SOS	-0,07543	0,054209	NA
3	abundance	X08990	Procaine	Handgrip	-0,18563	0,060615	NA
3	abundance	X08992	N(2)-succi	Tibia_length	-0,17791	0,051354	NA
3	abundance	X08992	N(2)-succi	Radius_length	-0,05434	0,071053	NA
3	abundance	X08992	N(2)-succi	Radius_SOS	-0,02348	0,049948	NA
3	abundance	X08992	N(2)-succi	Tibia_SOS	0,016387	0,053962	NA
3	abundance	X08992	N(2)-succi	Handgrip	0,012516	0,061205	NA
3	abundance	X09001	Varanic ac	Tibia_length	-0,00164	0,052121	NA
3	abundance	X09001	Varanic ac	Radius_length	-0,01188	0,071126	NA
3	abundance	X09001	Varanic ac	Radius_SOS	-0,06526	0,04993	NA
3	abundance	X09001	Varanic ac	Tibia_SOS	-0,06027	0,053673	NA
3	abundance	X09001	Varanic ac	Handgrip	-0,11252	0,060589	NA
3	abundance	X09004	Arenaine	Tibia_length	0,010785	0,05028	NA
3	abundance	X09004	Arenaine	Radius_length	0,03267	0,068489	NA
3	abundance	X09004	Arenaine	Radius_SOS	-0,06931	0,049007	NA
3	abundance	X09004	Arenaine	Tibia_SOS	0,064287	0,052509	NA
3	abundance	X09004	Arenaine	Handgrip	0,0775	0,060215	NA
3	abundance	X09008	5-guanidin	Tibia_length	0,125014	0,050463	NA
3	abundance	X09008	5-guanidin	Radius_length	0,131771	0,068999	NA
3	abundance	X09008	5-guanidin	Radius_SOS	0,074697	0,049209	NA
3	abundance	X09008	5-guanidin	Tibia_SOS	0,104171	0,052651	NA
3	abundance	X09008	5-guanidin	Handgrip	0,157024	0,060219	NA
1	abundance	X09010	4-Hydroxyp	Tibia_length	0,019335	0,053752	NA
1	abundance	X09010	4-Hydroxyp	Radius_length	0,1638	0,072475	NA
1	abundance	X09010	4-Hydroxyp	Radius_SOS	0,018221	0,051203	NA
1	abundance	X09010	4-Hydroxyp	Tibia_SOS	0,085339	0,053884	NA
1	abundance	X09010	4-Hydroxyp	Handgrip	0,232762	0,059999	NA

3	abundance	X09055	N-[(1R,2S,(	Tibia_length	0,032809	0,052749	NA
3	abundance	X09055	N-[(1R,2S,(	Radius_length	0,124025	0,071114	NA
3	abundance	X09055	N-[(1R,2S,(	Radius_SOS	-0,02043	0,050463	NA
3	abundance	X09055	N-[(1R,2S,(	Tibia_SOS	0,109438	0,05362	NA
3	abundance	X09055	N-[(1R,2S,(	Handgrip	0,1121	0,061078	NA
3	abundance	X09064	Tetrahydro	Tibia_length	-0,1504	0,053156	NA
3	abundance	X09064	Tetrahydro	Radius_length	-0,14616	0,071629	NA
3	abundance	X09064	Tetrahydro	Radius_SOS	-0,07948	0,050868	NA
3	abundance	X09064	Tetrahydro	Tibia_SOS	-0,07914	0,054435	NA
3	abundance	X09064	Tetrahydro	Handgrip	-0,02844	0,061494	NA
3	abundance	X09068	N,N-Diethy	Tibia_length	0,04333	0,053282	NA
3	abundance	X09068	N,N-Diethy	Radius_length	0,013416	0,072437	NA
3	abundance	X09068	N,N-Diethy	Radius_SOS	-0,01365	0,050951	NA
3	abundance	X09068	N,N-Diethy	Tibia_SOS	-0,01189	0,055695	NA
3	abundance	X09068	N,N-Diethy	Handgrip	0,062932	0,063076	NA
3	abundance	X09082	1_2-Dihydr	Tibia_length	-0,09956	0,055877	NA
3	abundance	X09082	1_2-Dihydr	Radius_length	-0,05037	0,074708	NA
3	abundance	X09082	1_2-Dihydr	Radius_SOS	-0,10081	0,05336	NA
3	abundance	X09082	1_2-Dihydr	Tibia_SOS	0,046593	0,055854	NA
3	abundance	X09082	1_2-Dihydr	Handgrip	0,139681	0,063241	NA
3	abundance	X09091	N-(3-aceta	Tibia_length	-0,01706	0,055433	NA
3	abundance	X09091	N-(3-aceta	Radius_length	0,055711	0,073703	NA
3	abundance	X09091	N-(3-aceta	Radius_SOS	-0,02055	0,052616	NA
3	abundance	X09091	N-(3-aceta	Tibia_SOS	0,033633	0,056606	NA
3	abundance	X09091	N-(3-aceta	Handgrip	-0,04342	0,062297	NA
3	abundance	X09092	LysoSM(d1	Tibia_length	0,032399	0,052334	NA
3	abundance	X09092	LysoSM(d1	Radius_length	0,047097	0,071723	NA
3	abundance	X09092	LysoSM(d1	Radius_SOS	0,032005	0,050264	NA
3	abundance	X09092	LysoSM(d1	Tibia_SOS	0,059106	0,053614	NA
3	abundance	X09092	LysoSM(d1	Handgrip	-0,10647	0,060478	NA
3	abundance	X09099	1-(2-Hydro	Tibia_length	-0,00389	0,049462	NA
3	abundance	X09099	1-(2-Hydro	Radius_length	0,009815	0,066785	NA
3	abundance	X09099	1-(2-Hydro	Radius_SOS	0,067797	0,048916	NA
3	abundance	X09099	1-(2-Hydro	Tibia_SOS	0,001563	0,0525	NA
3	abundance	X09099	1-(2-Hydro	Handgrip	-0,05713	0,060194	NA
3	abundance	X09100	Ethyl aceta	Tibia_length	0,05081	0,050971	NA
3	abundance	X09100	Ethyl aceta	Radius_length	0,017473	0,06968	NA
3	abundance	X09100	Ethyl aceta	Radius_SOS	0,055077	0,04934	NA
3	abundance	X09100	Ethyl aceta	Tibia_SOS	0,065691	0,052677	NA
3	abundance	X09100	Ethyl aceta	Handgrip	0,038334	0,060482	NA
3	abundance	X09110	Valylvaline	Tibia_length	-0,07295	0,051097	NA
3	abundance	X09110	Valylvaline	Radius_length	-0,10202	0,069589	NA
3	abundance	X09110	Valylvaline	Radius_SOS	-0,0489	0,049582	NA
3	abundance	X09110	Valylvaline	Tibia_SOS	-0,01317	0,052884	NA
3	abundance	X09110	Valylvaline	Handgrip	-0,03796	0,061104	NA
3	abundance	X09122	Valyl-4-hyc	Tibia_length	-0,05255	0,053157	NA
3	abundance	X09122	Valyl-4-hyc	Radius_length	-0,06173	0,071933	NA
3	abundance	X09122	Valyl-4-hyc	Radius_SOS	0,057459	0,05086	NA

3	abundance	X09122	Valyl-4-hyc	Tibia_SOS	0,037745	0,055062	NA
3	abundance	X09122	Valyl-4-hyc	Handgrip	-0,03158	0,062588	NA
3	abundance	X09129	Coprine	Tibia_length	-0,01123	0,05763	NA
3	abundance	X09129	Coprine	Radius_length	-0,00522	0,075388	NA
3	abundance	X09129	Coprine	Radius_SOS	-0,0232	0,054753	NA
3	abundance	X09129	Coprine	Tibia_SOS	0,056568	0,05748	NA
3	abundance	X09129	Coprine	Handgrip	-0,02927	0,062575	NA
1	abundance	X09152	Ferulic aci	Tibia_length	0,016195	0,053974	NA
1	abundance	X09152	Ferulic aci	Radius_length	0,099672	0,072966	NA
1	abundance	X09152	Ferulic aci	Radius_SOS	-0,02513	0,051337	NA
1	abundance	X09152	Ferulic aci	Tibia_SOS	-0,03024	0,05457	NA
1	abundance	X09152	Ferulic aci	Handgrip	0,068673	0,062816	NA
3	abundance	X09163	Prenisteine	Tibia_length	-0,00523	0,05106	NA
3	abundance	X09163	Prenisteine	Radius_length	-0,0235	0,069784	NA
3	abundance	X09163	Prenisteine	Radius_SOS	0,049264	0,049365	NA
3	abundance	X09163	Prenisteine	Tibia_SOS	0,058154	0,05263	NA
3	abundance	X09163	Prenisteine	Handgrip	-0,09294	0,060802	NA
3	abundance	X09165	N-[1-Carbc	Tibia_length	0,023196	0,051924	NA
3	abundance	X09165	N-[1-Carbc	Radius_length	0,076971	0,070676	NA
3	abundance	X09165	N-[1-Carbc	Radius_SOS	-0,05251	0,049928	NA
3	abundance	X09165	N-[1-Carbc	Tibia_SOS	0,090101	0,05332	NA
3	abundance	X09165	N-[1-Carbc	Handgrip	0,043483	0,061432	NA
1	abundance	X09187	Salicylic ac	Tibia_length	-0,03499	0,054637	NA
1	abundance	X09187	Salicylic ac	Radius_length	-0,06202	0,074124	NA
1	abundance	X09187	Salicylic ac	Radius_SOS	0,028093	0,051999	NA
1	abundance	X09187	Salicylic ac	Tibia_SOS	-0,03304	0,055777	NA
1	abundance	X09187	Salicylic ac	Handgrip	0,119471	0,063071	NA
3	abundance	X09189	4-[(E)-2-(3,	Tibia_length	-0,02417	0,051564	NA
3	abundance	X09189	4-[(E)-2-(3,	Radius_length	0,035037	0,070932	NA
3	abundance	X09189	4-[(E)-2-(3,	Radius_SOS	-0,00237	0,04982	NA
3	abundance	X09189	4-[(E)-2-(3,	Tibia_SOS	0,043818	0,053261	NA
3	abundance	X09189	4-[(E)-2-(3,	Handgrip	-0,04949	0,061682	NA
3	abundance	X09207	AAMU	Tibia_length	-0,12997	0,051601	NA
3	abundance	X09207	AAMU	Radius_length	-0,1368	0,070464	NA
3	abundance	X09207	AAMU	Radius_SOS	0,016058	0,04994	NA
3	abundance	X09207	AAMU	Tibia_SOS	-0,13905	0,053386	NA
3	abundance	X09207	AAMU	Handgrip	-0,19252	0,060076	NA
3	abundance	X09208	IN00260	Tibia_length	0,04151	0,054946	NA
3	abundance	X09208	IN00260	Radius_length	0,04349	0,073386	NA
3	abundance	X09208	IN00260	Radius_SOS	-0,13431	0,051849	NA
3	abundance	X09208	IN00260	Tibia_SOS	-0,03154	0,055544	NA
3	abundance	X09208	IN00260	Handgrip	-0,05846	0,061867	NA
3	abundance	X09222	L-gamma-(	Tibia_length	0,001097	0,049889	NA
3	abundance	X09222	L-gamma-(	Radius_length	0,051996	0,068052	NA
3	abundance	X09222	L-gamma-(	Radius_SOS	-0,03126	0,049038	NA
3	abundance	X09222	L-gamma-(	Tibia_SOS	0,025226	0,052511	NA
3	abundance	X09222	L-gamma-(	Handgrip	-0,06808	0,060344	NA
3	abundance	X09231	2-Keto-glut	Tibia_length	0,120889	0,049899	NA

3	abundance	X09231	2-Keto-glut	Radius_length	0,155821	0,067633	NA
3	abundance	X09231	2-Keto-glut	Radius_SOS	-0,06299	0,048965	NA
3	abundance	X09231	2-Keto-glut	Tibia_SOS	0,06173	0,052583	NA
3	abundance	X09231	2-Keto-glut	Handgrip	-5,90E-04	0,060499	NA
3	abundance	X09244	gamma-Gl	Tibia_length	-1,16E-04	0,056372	NA
3	abundance	X09244	gamma-Gl	Radius_length	0,091002	0,074635	NA
3	abundance	X09244	gamma-Gl	Radius_SOS	0,006474	0,053527	NA
3	abundance	X09244	gamma-Gl	Tibia_SOS	0,024224	0,056804	NA
3	abundance	X09244	gamma-Gl	Handgrip	0,079191	0,062961	NA
3	abundance	X09245	L-gamma-(	Tibia_length	-0,11806	0,0546	NA
3	abundance	X09245	L-gamma-(	Radius_length	-0,01379	0,073548	NA
3	abundance	X09245	L-gamma-(	Radius_SOS	-0,0295	0,052061	NA
3	abundance	X09245	L-gamma-(	Tibia_SOS	0,021986	0,055222	NA
3	abundance	X09245	L-gamma-(	Handgrip	0,004639	0,060997	NA
3	abundance	X09256	Aspartyl-L-	Tibia_length	0,114101	0,049696	NA
3	abundance	X09256	Aspartyl-L-	Radius_length	0,113718	0,067407	NA
3	abundance	X09256	Aspartyl-L-	Radius_SOS	-0,05563	0,048975	NA
3	abundance	X09256	Aspartyl-L-	Tibia_SOS	0,10126	0,052412	NA
3	abundance	X09256	Aspartyl-L-	Handgrip	0,089089	0,060893	NA
3	abundance	X09260	(4S)-4-[(2E	Tibia_length	0,22391	0,053544	NA
3	abundance	X09260	(4S)-4-[(2E	Radius_length	0,291636	0,071435	NA
3	abundance	X09260	(4S)-4-[(2E	Radius_SOS	0,14566	0,052031	NA
3	abundance	X09260	(4S)-4-[(2E	Tibia_SOS	0,144969	0,054893	NA
3	abundance	X09260	(4S)-4-[(2E	Handgrip	0,189808	0,060209	NA
3	abundance	X09284	4-(Nitrosoa	Tibia_length	-0,03791	0,055657	NA
3	abundance	X09284	4-(Nitrosoa	Radius_length	-0,03807	0,073912	NA
3	abundance	X09284	4-(Nitrosoa	Radius_SOS	0,079449	0,052851	NA
3	abundance	X09284	4-(Nitrosoa	Tibia_SOS	0,020502	0,05572	NA
3	abundance	X09284	4-(Nitrosoa	Handgrip	0,035479	0,062683	NA
3	abundance	X09285	MFCD0272	Tibia_length	-0,06365	0,052772	NA
3	abundance	X09285	MFCD0272	Radius_length	-0,17256	0,070936	NA
3	abundance	X09285	MFCD0272	Radius_SOS	0,003296	0,050577	NA
3	abundance	X09285	MFCD0272	Tibia_SOS	-0,09389	0,053381	NA
3	abundance	X09285	MFCD0272	Handgrip	-0,13094	0,061001	NA
3	abundance	X09302	Sular	Tibia_length	0,023048	0,053652	NA
3	abundance	X09302	Sular	Radius_length	0,071358	0,072228	NA
3	abundance	X09302	Sular	Radius_SOS	0,057669	0,051134	NA
3	abundance	X09302	Sular	Tibia_SOS	0,125785	0,055429	NA
3	abundance	X09302	Sular	Handgrip	0,045917	0,061578	NA
3	abundance	X09318	butalbital	Tibia_length	-0,03481	0,051974	NA
3	abundance	X09318	butalbital	Radius_length	-0,02059	0,071195	NA
3	abundance	X09318	butalbital	Radius_SOS	0,081311	0,049883	NA
3	abundance	X09318	butalbital	Tibia_SOS	-0,0651	0,053284	NA
3	abundance	X09318	butalbital	Handgrip	-0,16799	0,060424	NA
3	abundance	X09336	1,1'-[1,12-I	Tibia_length	0,054987	0,05447	NA
3	abundance	X09336	1,1'-[1,12-I	Radius_length	0,056651	0,073172	NA
3	abundance	X09336	1,1'-[1,12-I	Radius_SOS	0,056608	0,051787	NA
3	abundance	X09336	1,1'-[1,12-I	Tibia_SOS	0,012368	0,056029	NA

3	abundance	X09336	1,1'-[1,12-I	Handgrip	0,027993	0,062084	NA
3	abundance	X09357	5-(5-Methy	Tibia_length	-0,06289	0,05421	NA
3	abundance	X09357	5-(5-Methy	Radius_length	-0,09318	0,072819	NA
3	abundance	X09357	5-(5-Methy	Radius_SOS	-0,06997	0,051322	NA
3	abundance	X09357	5-(5-Methy	Tibia_SOS	-0,05033	0,055521	NA
3	abundance	X09357	5-(5-Methy	Handgrip	0,020731	0,062383	NA
3	abundance	X09374	3-Deoxy-D	Tibia_length	-0,15367	0,054473	NA
3	abundance	X09374	3-Deoxy-D	Radius_length	-0,13516	0,073218	NA
3	abundance	X09374	3-Deoxy-D	Radius_SOS	-0,08846	0,052378	NA
3	abundance	X09374	3-Deoxy-D	Tibia_SOS	0,010256	0,056555	NA
3	abundance	X09374	3-Deoxy-D	Handgrip	-0,08406	0,061961	NA
3	abundance	X09385	2-(1-Napht	Tibia_length	-0,02321	0,048957	NA
3	abundance	X09385	2-(1-Napht	Radius_length	0,079437	0,048815	NA
3	abundance	X09385	2-(1-Napht	Radius_SOS	0,015975	0,049023	NA
3	abundance	X09385	2-(1-Napht	Tibia_SOS	0,05937	0,052394	NA
3	abundance	X09385	2-(1-Napht	Handgrip	0,049917	0,060118	NA
3	abundance	X09390	asn-pro	Tibia_length	-0,03318	0,052923	NA
3	abundance	X09390	asn-pro	Radius_length	0,066753	0,071994	NA
3	abundance	X09390	asn-pro	Radius_SOS	0,013789	0,050672	NA
3	abundance	X09390	asn-pro	Tibia_SOS	0,061727	0,054244	NA
3	abundance	X09390	asn-pro	Handgrip	0,100582	0,060898	NA
3	abundance	X09391	ophthalmi	Tibia_length	-0,12184	0,052075	NA
3	abundance	X09391	ophthalmi	Radius_length	-0,00216	0,071524	NA
3	abundance	X09391	ophthalmi	Radius_SOS	0,040285	0,050067	NA
3	abundance	X09391	ophthalmi	Tibia_SOS	0,069454	0,0538	NA
3	abundance	X09391	ophthalmi	Handgrip	0,117022	0,061133	NA
3	abundance	X09393	2-Isopropy	Tibia_length	0,091471	0,055562	NA
3	abundance	X09393	2-Isopropy	Radius_length	0,046083	0,073934	NA
3	abundance	X09393	2-Isopropy	Radius_SOS	-0,01992	0,053143	NA
3	abundance	X09393	2-Isopropy	Tibia_SOS	0,033524	0,056881	NA
3	abundance	X09393	2-Isopropy	Handgrip	0,117566	0,062678	NA
3	abundance	X09399	L-gamma-(	Tibia_length	-0,11651	0,053089	NA
3	abundance	X09399	L-gamma-(	Radius_length	-0,06751	0,072048	NA
3	abundance	X09399	L-gamma-(	Radius_SOS	0,00477	0,050854	NA
3	abundance	X09399	L-gamma-(	Tibia_SOS	0,019591	0,054596	NA
3	abundance	X09399	L-gamma-(	Handgrip	0,050466	0,06141	NA
3	abundance	X09454	2-(2-Hydro	Tibia_length	-0,09633	0,053026	NA
3	abundance	X09454	2-(2-Hydro	Radius_length	-0,16088	0,071253	NA
3	abundance	X09454	2-(2-Hydro	Radius_SOS	0,012904	0,050746	NA
3	abundance	X09454	2-(2-Hydro	Tibia_SOS	-0,03631	0,054184	NA
3	abundance	X09454	2-(2-Hydro	Handgrip	-0,08002	0,061512	NA
3	abundance	X09476	N(1),N(8)-I	Tibia_length	0,013932	0,051418	NA
3	abundance	X09476	N(1),N(8)-I	Radius_length	0,089192	0,070631	NA
3	abundance	X09476	N(1),N(8)-I	Radius_SOS	0,060564	0,049577	NA
3	abundance	X09476	N(1),N(8)-I	Tibia_SOS	0,085512	0,053091	NA
3	abundance	X09476	N(1),N(8)-I	Handgrip	0,131477	0,061439	NA
3	abundance	X09480	Spermic ac	Tibia_length	0,037957	0,051973	NA
3	abundance	X09480	Spermic ac	Radius_length	0,032369	0,071013	NA

3	abundance	X09480	Spermic ac	Radius_SOS	0,01524	0,050086	NA
3	abundance	X09480	Spermic ac	Tibia_SOS	-0,05839	0,053498	NA
3	abundance	X09480	Spermic ac	Handgrip	0,047416	0,061792	NA
3	abundance	X09490	2-Hydroxy-	Tibia_length	-0,08906	0,056099	NA
3	abundance	X09490	2-Hydroxy-	Radius_length	-0,11697	0,07398	NA
3	abundance	X09490	2-Hydroxy-	Radius_SOS	-0,09002	0,054076	NA
3	abundance	X09490	2-Hydroxy-	Tibia_SOS	-0,11517	0,056856	NA
3	abundance	X09490	2-Hydroxy-	Handgrip	-0,06445	0,062227	NA
3	abundance	X09503	4-(METHYL	Tibia_length	-0,10324	0,052917	NA
3	abundance	X09503	4-(METHYL	Radius_length	0,017356	0,072326	NA
3	abundance	X09503	4-(METHYL	Radius_SOS	-0,00228	0,050872	NA
3	abundance	X09503	4-(METHYL	Tibia_SOS	0,036867	0,054131	NA
3	abundance	X09503	4-(METHYL	Handgrip	-0,05164	0,062635	NA
3	abundance	X09505	(-)-Physost	Tibia_length	-0,02631	0,054803	NA
3	abundance	X09505	(-)-Physost	Radius_length	-0,04862	0,073543	NA
3	abundance	X09505	(-)-Physost	Radius_SOS	-0,02538	0,052229	NA
3	abundance	X09505	(-)-Physost	Tibia_SOS	0,02696	0,054465	NA
3	abundance	X09505	(-)-Physost	Handgrip	-0,10846	0,061634	NA
3	abundance	X09523	N-(3,5-Din	Tibia_length	-0,16996	0,056196	NA
3	abundance	X09523	N-(3,5-Din	Radius_length	-0,11048	0,074461	NA
3	abundance	X09523	N-(3,5-Din	Radius_SOS	-0,00894	0,054149	NA
3	abundance	X09523	N-(3,5-Din	Tibia_SOS	-0,05174	0,057451	NA
3	abundance	X09523	N-(3,5-Din	Handgrip	0,051117	0,062687	NA
3	abundance	X09525	Homoanse	Tibia_length	-0,04461	0,055883	NA
3	abundance	X09525	Homoanse	Radius_length	0,047628	0,074708	NA
3	abundance	X09525	Homoanse	Radius_SOS	-0,05342	0,05317	NA
3	abundance	X09525	Homoanse	Tibia_SOS	-0,01402	0,056184	NA
3	abundance	X09525	Homoanse	Handgrip	0,072824	0,062361	NA
3	abundance	X09533	S(6)-acetyl	Tibia_length	0,012014	0,052325	NA
3	abundance	X09533	S(6)-acetyl	Radius_length	0,042381	0,071455	NA
3	abundance	X09533	S(6)-acetyl	Radius_SOS	0,054809	0,050173	NA
3	abundance	X09533	S(6)-acetyl	Tibia_SOS	0,076803	0,054056	NA
3	abundance	X09533	S(6)-acetyl	Handgrip	-0,14206	0,061505	NA
3	abundance	X09545	4-Methylen	Tibia_length	0,057747	0,052541	NA
3	abundance	X09545	4-Methylen	Radius_length	0,00568	0,07158	NA
3	abundance	X09545	4-Methylen	Radius_SOS	0,074496	0,050405	NA
3	abundance	X09545	4-Methylen	Tibia_SOS	-0,02317	0,053882	NA
3	abundance	X09545	4-Methylen	Handgrip	-0,14228	0,06021	NA
3	abundance	X09546	asn-phe	Tibia_length	-0,03136	0,053712	NA
3	abundance	X09546	asn-phe	Radius_length	-0,04323	0,072319	NA
3	abundance	X09546	asn-phe	Radius_SOS	-0,07883	0,050985	NA
3	abundance	X09546	asn-phe	Tibia_SOS	-0,12011	0,054511	NA
3	abundance	X09546	asn-phe	Handgrip	-0,05289	0,062018	NA
3	abundance	X09548	2-O-beta-D	Tibia_length	-0,07417	0,056306	NA
3	abundance	X09548	2-O-beta-D	Radius_length	0,017916	0,07518	NA
3	abundance	X09548	2-O-beta-D	Radius_SOS	-0,0222	0,053606	NA
3	abundance	X09548	2-O-beta-D	Tibia_SOS	-0,00854	0,05731	NA
3	abundance	X09548	2-O-beta-D	Handgrip	0,00403	0,062877	NA

3	abundance	X09554	N-Propiony	Tibia_length	0,052106	0,053563	NA
3	abundance	X09554	N-Propiony	Radius_length	0,01105	0,072239	NA
3	abundance	X09554	N-Propiony	Radius_SOS	0,102164	0,051013	NA
3	abundance	X09554	N-Propiony	Tibia_SOS	-0,06708	0,053692	NA
3	abundance	X09554	N-Propiony	Handgrip	-0,07217	0,061185	NA
3	abundance	X09555	N4-(beta-N	Tibia_length	0,058077	0,051727	NA
3	abundance	X09555	N4-(beta-N	Radius_length	0,011539	0,070824	NA
3	abundance	X09555	N4-(beta-N	Radius_SOS	-0,10539	0,049565	NA
3	abundance	X09555	N4-(beta-N	Tibia_SOS	0,003575	0,053467	NA
3	abundance	X09555	N4-(beta-N	Handgrip	0,043789	0,061197	NA
3	abundance	X09565	3-Methoxy-	Tibia_length	-0,07543	0,051416	NA
3	abundance	X09565	3-Methoxy-	Radius_length	-0,11119	0,069911	NA
3	abundance	X09565	3-Methoxy-	Radius_SOS	-0,02268	0,049656	NA
3	abundance	X09565	3-Methoxy-	Tibia_SOS	-0,09218	0,052867	NA
3	abundance	X09565	3-Methoxy-	Handgrip	-0,08141	0,060431	NA
3	abundance	X09577	Spermic ac	Tibia_length	-0,09471	0,052897	NA
3	abundance	X09577	Spermic ac	Radius_length	-0,05197	0,071829	NA
3	abundance	X09577	Spermic ac	Radius_SOS	-0,0189	0,050786	NA
3	abundance	X09577	Spermic ac	Tibia_SOS	-0,06152	0,053867	NA
3	abundance	X09577	Spermic ac	Handgrip	0,010362	0,061443	NA
3	abundance	X09581	L-gamma-(	Tibia_length	-0,03065	0,04969	NA
3	abundance	X09581	L-gamma-(	Radius_length	0,048777	0,067801	NA
3	abundance	X09581	L-gamma-(	Radius_SOS	-8,03E-04	0,049038	NA
3	abundance	X09581	L-gamma-(	Tibia_SOS	0,03377	0,052513	NA
3	abundance	X09581	L-gamma-(	Handgrip	-0,05586	0,060609	NA
3	abundance	X09607	N-(1H-Pyrr	Tibia_length	-0,05013	0,053151	NA
3	abundance	X09607	N-(1H-Pyrr	Radius_length	-0,02657	0,072323	NA
3	abundance	X09607	N-(1H-Pyrr	Radius_SOS	-0,01416	0,050879	NA
3	abundance	X09607	N-(1H-Pyrr	Tibia_SOS	-0,03182	0,054808	NA
3	abundance	X09607	N-(1H-Pyrr	Handgrip	-0,122	0,061417	NA
3	abundance	X09608	N-(3,5-Dim	Tibia_length	-0,10328	0,056088	NA
3	abundance	X09608	N-(3,5-Dim	Radius_length	-0,15028	0,074177	NA
3	abundance	X09608	N-(3,5-Dim	Radius_SOS	-0,06	0,053487	NA
3	abundance	X09608	N-(3,5-Dim	Tibia_SOS	-0,15154	0,055174	NA
3	abundance	X09608	N-(3,5-Dim	Handgrip	-0,04706	0,061301	NA
3	abundance	X09609	N-{3-[(4-Ac	Tibia_length	-0,07881	0,053549	NA
3	abundance	X09609	N-{3-[(4-Ac	Radius_length	-0,119	0,071804	NA
3	abundance	X09609	N-{3-[(4-Ac	Radius_SOS	-0,04475	0,05133	NA
3	abundance	X09609	N-{3-[(4-Ac	Tibia_SOS	0,017759	0,05438	NA
3	abundance	X09609	N-{3-[(4-Ac	Handgrip	-0,00761	0,061118	NA
3	abundance	X09612	3-hydroxy-	Tibia_length	-0,0333	0,051325	NA
3	abundance	X09612	3-hydroxy-	Radius_length	-0,10562	0,06998	NA
3	abundance	X09612	3-hydroxy-	Radius_SOS	0,077214	0,049421	NA
3	abundance	X09612	3-hydroxy-	Tibia_SOS	-0,05733	0,053207	NA
3	abundance	X09612	3-hydroxy-	Handgrip	0,019681	0,061218	NA
3	abundance	X09615	tyramine s	Tibia_length	-0,11941	0,051315	NA
3	abundance	X09615	tyramine s	Radius_length	-0,14484	0,070097	NA
3	abundance	X09615	tyramine s	Radius_SOS	-0,12958	0,049565	NA

	3	abundance	X09615	tyramine sı	Tibia_SOS	-0,06182	0,053178	NA
	3	abundance	X09615	tyramine sı	Handgrip	-0,03043	0,061275	NA
	3	abundance	X09622	N-Hydroxy-	Tibia_length	-0,00748	0,049049	NA
	3	abundance	X09622	N-Hydroxy-	Radius_length	0,023812	0,061829	NA
	3	abundance	X09622	N-Hydroxy-	Radius_SOS	-0,04725	0,050009	NA
	3	abundance	X09622	N-Hydroxy-	Tibia_SOS	0,126369	0,053019	NA
	3	abundance	X09622	N-Hydroxy-	Handgrip	0,012226	0,060405	NA
2b		abundance	X09623	Endothal	Tibia_length	-0,17515	0,053373	NA
2b		abundance	X09623	Endothal	Radius_length	-0,23975	0,070523	NA
2b		abundance	X09623	Endothal	Radius_SOS	-0,02113	0,051072	NA
2b		abundance	X09623	Endothal	Tibia_SOS	-0,16373	0,054881	NA
2b		abundance	X09623	Endothal	Handgrip	-0,15493	0,060564	NA
	3	abundance	X09635	2,4,6-Triisc	Tibia_length	0,063804	0,054969	NA
	3	abundance	X09635	2,4,6-Triisc	Radius_length	0,11365	0,07304	NA
	3	abundance	X09635	2,4,6-Triisc	Radius_SOS	0,075456	0,052315	NA
	3	abundance	X09635	2,4,6-Triisc	Tibia_SOS	0,033625	0,056943	NA
	3	abundance	X09635	2,4,6-Triisc	Handgrip	0,02946	0,06274	NA
	3	abundance	X09643	N-(3,5-Dim	Tibia_length	-0,10778	0,05579	NA
	3	abundance	X09643	N-(3,5-Dim	Radius_length	-0,13317	0,073612	NA
	3	abundance	X09643	N-(3,5-Dim	Radius_SOS	0,042264	0,053099	NA
	3	abundance	X09643	N-(3,5-Dim	Tibia_SOS	-0,10677	0,055991	NA
	3	abundance	X09643	N-(3,5-Dim	Handgrip	-0,15283	0,061809	NA
	3	abundance	X09645	TOLMETIN	Tibia_length	-0,14668	0,053524	NA
	3	abundance	X09645	TOLMETIN	Radius_length	-0,11528	0,072179	NA
	3	abundance	X09645	TOLMETIN	Radius_SOS	-0,04365	0,051132	NA
	3	abundance	X09645	TOLMETIN	Tibia_SOS	-0,06112	0,054299	NA
	3	abundance	X09645	TOLMETIN	Handgrip	-0,00758	0,061228	NA
	3	abundance	X09654	linatine	Tibia_length	0,056995	0,051815	NA
	3	abundance	X09654	linatine	Radius_length	0,110159	0,070439	NA
	3	abundance	X09654	linatine	Radius_SOS	0,042926	0,049962	NA
	3	abundance	X09654	linatine	Tibia_SOS	0,029559	0,053015	NA
	3	abundance	X09654	linatine	Handgrip	-0,03676	0,061317	NA
	3	abundance	X09657	2,3,4,5,6-F	Tibia_length	0,082339	0,051033	NA
	3	abundance	X09657	2,3,4,5,6-F	Radius_length	0,150403	0,069233	NA
	3	abundance	X09657	2,3,4,5,6-F	Radius_SOS	0,068291	0,04943	NA
	3	abundance	X09657	2,3,4,5,6-F	Tibia_SOS	0,031701	0,053078	NA
	3	abundance	X09657	2,3,4,5,6-F	Handgrip	0,056335	0,060764	NA
	3	abundance	X09675	UK387000	Tibia_length	0,07886	0,052647	NA
	3	abundance	X09675	UK387000	Radius_length	0,08252	0,072073	NA
	3	abundance	X09675	UK387000	Radius_SOS	0,04474	0,050516	NA
	3	abundance	X09675	UK387000	Tibia_SOS	0,076291	0,055319	NA
	3	abundance	X09675	UK387000	Handgrip	0,093847	0,060851	NA
	3	abundance	X09684	(+)-Etomid	Tibia_length	-0,0415	0,049737	NA
	3	abundance	X09684	(+)-Etomid	Radius_length	0,059324	0,067937	NA
	3	abundance	X09684	(+)-Etomid	Radius_SOS	0,004625	0,049047	NA
	3	abundance	X09684	(+)-Etomid	Tibia_SOS	0,042586	0,052499	NA
	3	abundance	X09684	(+)-Etomid	Handgrip	-0,07374	0,060492	NA
	3	abundance	X09703	7,8-Didehy	Tibia_length	-0,14044	0,052953	NA

	3	abundance	X09703	7,8-Didehy	Radius_length	-0,12126	0,071684	NA
	3	abundance	X09703	7,8-Didehy	Radius_SOS	0,042648	0,050718	NA
	3	abundance	X09703	7,8-Didehy	Tibia_SOS	-0,10022	0,053747	NA
	3	abundance	X09703	7,8-Didehy	Handgrip	-0,13211	0,061305	NA
	3	abundance	X09710	LW800000	Tibia_length	-0,07642	0,054709	NA
	3	abundance	X09710	LW800000	Radius_length	-0,04915	0,07351	NA
	3	abundance	X09710	LW800000	Radius_SOS	0,033147	0,05212	NA
	3	abundance	X09710	LW800000	Tibia_SOS	0,017519	0,055337	NA
	3	abundance	X09710	LW800000	Handgrip	0,07235	0,062483	NA
	3	abundance	X09713	N(2)-succi	Tibia_length	0,025192	0,050978	NA
	3	abundance	X09713	N(2)-succi	Radius_length	0,018359	0,069968	NA
	3	abundance	X09713	N(2)-succi	Radius_SOS	0,001834	0,049452	NA
	3	abundance	X09713	N(2)-succi	Tibia_SOS	0,048009	0,052909	NA
	3	abundance	X09713	N(2)-succi	Handgrip	-0,06953	0,061184	NA
	3	abundance	X09721	3-(2,3-Dihy	Tibia_length	-0,09914	0,056523	NA
	3	abundance	X09721	3-(2,3-Dihy	Radius_length	-0,16646	0,073559	NA
	3	abundance	X09721	3-(2,3-Dihy	Radius_SOS	-0,0019	0,053479	NA
	3	abundance	X09721	3-(2,3-Dihy	Tibia_SOS	0,009787	0,057603	NA
	3	abundance	X09721	3-(2,3-Dihy	Handgrip	0,023364	0,063407	NA
	3	abundance	X09724	Formylkynu	Tibia_length	0,14262	0,052547	NA
	3	abundance	X09724	Formylkynu	Radius_length	0,104316	0,071817	NA
	3	abundance	X09724	Formylkynu	Radius_SOS	-0,0616	0,05083	NA
	3	abundance	X09724	Formylkynu	Tibia_SOS	0,036885	0,053869	NA
	3	abundance	X09724	Formylkynu	Handgrip	0,010562	0,061093	NA
2b		abundance	X09726	indoline-2-	Tibia_length	0,053272	0,052429	NA
2b		abundance	X09726	indoline-2-	Radius_length	0,095185	0,07152	NA
2b		abundance	X09726	indoline-2-	Radius_SOS	-0,05069	0,05028	NA
2b		abundance	X09726	indoline-2-	Tibia_SOS	0,08365	0,053365	NA
2b		abundance	X09726	indoline-2-	Handgrip	0,045384	0,060598	NA
	3	abundance	X09728	{[(15-Hydro	Tibia_length	-0,03691	0,050741	NA
	3	abundance	X09728	{[(15-Hydro	Radius_length	-0,09145	0,069485	NA
	3	abundance	X09728	{[(15-Hydro	Radius_SOS	-0,00874	0,049328	NA
	3	abundance	X09728	{[(15-Hydro	Tibia_SOS	0,079847	0,053296	NA
	3	abundance	X09728	{[(15-Hydro	Handgrip	0,032694	0,060999	NA
	3	abundance	X09738	(7E,7'E)-5,	Tibia_length	-0,09714	0,051877	NA
	3	abundance	X09738	(7E,7'E)-5,	Radius_length	-0,20403	0,069825	NA
	3	abundance	X09738	(7E,7'E)-5,	Radius_SOS	-0,0622	0,050189	NA
	3	abundance	X09738	(7E,7'E)-5,	Tibia_SOS	-0,12973	0,053255	NA
	3	abundance	X09738	(7E,7'E)-5,	Handgrip	-0,06307	0,061205	NA
	3	abundance	X09740	MFCD0166	Tibia_length	-0,05057	0,056308	NA
	3	abundance	X09740	MFCD0166	Radius_length	-0,00958	0,075055	NA
	3	abundance	X09740	MFCD0166	Radius_SOS	0,020661	0,053519	NA
	3	abundance	X09740	MFCD0166	Tibia_SOS	0,045421	0,057661	NA
	3	abundance	X09740	MFCD0166	Handgrip	0,055839	0,062541	NA
	3	abundance	X09741	Estrone glu	Tibia_length	0,106268	0,051299	NA
	3	abundance	X09741	Estrone glu	Radius_length	0,082117	0,070347	NA
	3	abundance	X09741	Estrone glu	Radius_SOS	-0,01356	0,049701	NA
	3	abundance	X09741	Estrone glu	Tibia_SOS	0,08029	0,053529	NA

3	abundance	X09741	Estrone glu	Handgrip	0,014015	0,061108	NA
2b	abundance	X09746	ELK (Peptic	Tibia_length	-0,03112	0,053008	NA
2b	abundance	X09746	ELK (Peptic	Radius_length	0,081738	0,072026	NA
2b	abundance	X09746	ELK (Peptic	Radius_SOS	0,041496	0,050715	NA
2b	abundance	X09746	ELK (Peptic	Tibia_SOS	-0,00372	0,054589	NA
2b	abundance	X09746	ELK (Peptic	Handgrip	0,065917	0,060553	NA
3	abundance	X09765	Tetrahydro	Tibia_length	0,017826	0,052768	NA
3	abundance	X09765	Tetrahydro	Radius_length	0,036745	0,071961	NA
3	abundance	X09765	Tetrahydro	Radius_SOS	-0,01215	0,050548	NA
3	abundance	X09765	Tetrahydro	Tibia_SOS	-0,05471	0,053299	NA
3	abundance	X09765	Tetrahydro	Handgrip	-0,15451	0,06102	NA
3	abundance	X09771	2-(3-CARB	Tibia_length	-0,12049	0,052887	NA
3	abundance	X09771	2-(3-CARB	Radius_length	-0,11901	0,071553	NA
3	abundance	X09771	2-(3-CARB	Radius_SOS	-0,04373	0,050975	NA
3	abundance	X09771	2-(3-CARB	Tibia_SOS	-0,05583	0,053949	NA
3	abundance	X09771	2-(3-CARB	Handgrip	-0,04683	0,061112	NA
3	abundance	X09787	Queuosine	Tibia_length	-0,12219	0,053289	NA
3	abundance	X09787	Queuosine	Radius_length	-0,1596	0,071832	NA
3	abundance	X09787	Queuosine	Radius_SOS	-0,20754	0,050106	NA
3	abundance	X09787	Queuosine	Tibia_SOS	0,02477	0,055194	NA
3	abundance	X09787	Queuosine	Handgrip	0,023877	0,062421	NA
3	abundance	X09805	Methyl alpl	Tibia_length	0,038465	0,055047	NA
3	abundance	X09805	Methyl alpl	Radius_length	-0,00978	0,073723	NA
3	abundance	X09805	Methyl alpl	Radius_SOS	-0,09859	0,052127	NA
3	abundance	X09805	Methyl alpl	Tibia_SOS	0,123584	0,056581	NA
3	abundance	X09805	Methyl alpl	Handgrip	0,018596	0,062297	NA
3	abundance	X09810	Zalcitabine	Tibia_length	-0,11719	0,056275	NA
3	abundance	X09810	Zalcitabine	Radius_length	0,034666	0,074336	NA
3	abundance	X09810	Zalcitabine	Radius_SOS	0,006894	0,054004	NA
3	abundance	X09810	Zalcitabine	Tibia_SOS	-0,07127	0,058724	NA
3	abundance	X09810	Zalcitabine	Handgrip	-0,02774	0,06017	NA
3	abundance	X09825	(2S)-6-Ami	Tibia_length	-0,03425	0,053792	NA
3	abundance	X09825	(2S)-6-Ami	Radius_length	-0,05791	0,072594	NA
3	abundance	X09825	(2S)-6-Ami	Radius_SOS	-0,00944	0,051327	NA
3	abundance	X09825	(2S)-6-Ami	Tibia_SOS	-0,00856	0,055386	NA
3	abundance	X09825	(2S)-6-Ami	Handgrip	0,130958	0,061754	NA
3	abundance	X09849	3-Methylac	Tibia_length	-0,01873	0,051241	NA
3	abundance	X09849	3-Methylac	Radius_length	0,025421	0,070322	NA
3	abundance	X09849	3-Methylac	Radius_SOS	-0,08903	0,049466	NA
3	abundance	X09849	3-Methylac	Tibia_SOS	0,052152	0,053395	NA
3	abundance	X09849	3-Methylac	Handgrip	0,008331	0,061591	NA
2b	abundance	X09854	N-Benzyl-3	Tibia_length	0,040976	0,05582	NA
2b	abundance	X09854	N-Benzyl-3	Radius_length	0,037878	0,074317	NA
2b	abundance	X09854	N-Benzyl-3	Radius_SOS	-0,01559	0,052746	NA
2b	abundance	X09854	N-Benzyl-3	Tibia_SOS	0,010895	0,055953	NA
2b	abundance	X09854	N-Benzyl-3	Handgrip	-0,02859	0,062235	NA
3	abundance	X09875	4-[(E)-2-(3,	Tibia_length	-0,03949	0,053	NA
3	abundance	X09875	4-[(E)-2-(3,	Radius_length	0,053591	0,071787	NA

3	abundance	X09875	4-[(E)-2-(3,	Radius_SOS	0,047118	0,050807	NA
3	abundance	X09875	4-[(E)-2-(3,	Tibia_SOS	-0,01369	0,054012	NA
3	abundance	X09875	4-[(E)-2-(3,	Handgrip	0,007423	0,0626	NA
3	abundance	X09882	Harmane	Tibia_length	0,079326	0,049754	NA
3	abundance	X09882	Harmane	Radius_length	0,096156	0,067561	NA
3	abundance	X09882	Harmane	Radius_SOS	0,018642	0,049055	NA
3	abundance	X09882	Harmane	Tibia_SOS	0,070531	0,052385	NA
3	abundance	X09882	Harmane	Handgrip	0,005329	0,060517	NA
3	abundance	X09895	1-(beta-D-I	Tibia_length	0,061678	0,055282	NA
3	abundance	X09895	1-(beta-D-I	Radius_length	0,054948	0,07432	NA
3	abundance	X09895	1-(beta-D-I	Radius_SOS	-0,07189	0,053154	NA
3	abundance	X09895	1-(beta-D-I	Tibia_SOS	0,081836	0,056845	NA
3	abundance	X09895	1-(beta-D-I	Handgrip	0,082202	0,061418	NA
3	abundance	X09898	N(6),N(6)-I	Tibia_length	-0,02736	0,053694	NA
3	abundance	X09898	N(6),N(6)-I	Radius_length	0,057382	0,072405	NA
3	abundance	X09898	N(6),N(6)-I	Radius_SOS	0,023504	0,051159	NA
3	abundance	X09898	N(6),N(6)-I	Tibia_SOS	0,080266	0,054252	NA
3	abundance	X09898	N(6),N(6)-I	Handgrip	-0,01973	0,061638	NA
3	abundance	X09912	MC05553C	Tibia_length	-0,10637	0,053384	NA
3	abundance	X09912	MC05553C	Radius_length	-0,11019	0,072053	NA
3	abundance	X09912	MC05553C	Radius_SOS	-0,03573	0,051074	NA
3	abundance	X09912	MC05553C	Tibia_SOS	-0,07495	0,054832	NA
3	abundance	X09912	MC05553C	Handgrip	-0,02442	0,061485	NA
3	abundance	X09919	4-ethylphe	Tibia_length	-0,14342	0,054385	NA
3	abundance	X09919	4-ethylphe	Radius_length	-0,13605	0,072706	NA
3	abundance	X09919	4-ethylphe	Radius_SOS	-0,01519	0,05219	NA
3	abundance	X09919	4-ethylphe	Tibia_SOS	0,050688	0,055085	NA
3	abundance	X09919	4-ethylphe	Handgrip	-0,05092	0,061786	NA
3	abundance	X09921	5-Hydroxy-	Tibia_length	0,057506	0,05029	NA
3	abundance	X09921	5-Hydroxy-	Radius_length	0,101428	0,068519	NA
3	abundance	X09921	5-Hydroxy-	Radius_SOS	0,025798	0,049171	NA
3	abundance	X09921	5-Hydroxy-	Tibia_SOS	-0,0527	0,052538	NA
3	abundance	X09921	5-Hydroxy-	Handgrip	0,013386	0,060265	NA
3	abundance	X09932	g-Aminobu	Tibia_length	0,050897	0,051846	NA
3	abundance	X09932	g-Aminobu	Radius_length	0,012208	0,070822	NA
3	abundance	X09932	g-Aminobu	Radius_SOS	-3,23E-04	0,049978	NA
3	abundance	X09932	g-Aminobu	Tibia_SOS	-0,03792	0,053798	NA
3	abundance	X09932	g-Aminobu	Handgrip	0,037076	0,061389	NA
3	abundance	X09940	pentobarbi	Tibia_length	0,011887	0,057789	NA
3	abundance	X09940	pentobarbi	Radius_length	0,134566	0,075011	NA
3	abundance	X09940	pentobarbi	Radius_SOS	0,095833	0,05476	NA
3	abundance	X09940	pentobarbi	Tibia_SOS	-0,0448	0,055834	NA
3	abundance	X09940	pentobarbi	Handgrip	-0,10466	0,062345	NA
3	abundance	X09942	Spermic ac	Tibia_length	-0,04508	0,054967	NA
3	abundance	X09942	Spermic ac	Radius_length	-0,0434	0,07333	NA
3	abundance	X09942	Spermic ac	Radius_SOS	-0,05746	0,052105	NA
3	abundance	X09942	Spermic ac	Tibia_SOS	-0,06799	0,055301	NA
3	abundance	X09942	Spermic ac	Handgrip	-0,07975	0,062242	NA

	3	abundance	X09950	3-Hydroxyl	Tibia_length	0,003243	0,050373	NA
	3	abundance	X09950	3-Hydroxyl	Radius_length	0,047149	0,068605	NA
	3	abundance	X09950	3-Hydroxyl	Radius_SOS	0,069859	0,04906	NA
	3	abundance	X09950	3-Hydroxyl	Tibia_SOS	-0,02221	0,052785	NA
	3	abundance	X09950	3-Hydroxyl	Handgrip	-0,04647	0,060442	NA
	3	abundance	X09951	5beta-Cypr	Tibia_length	-0,05758	0,050821	NA
	3	abundance	X09951	5beta-Cypr	Radius_length	-0,05984	0,06951	NA
	3	abundance	X09951	5beta-Cypr	Radius_SOS	-0,01319	0,04947	NA
	3	abundance	X09951	5beta-Cypr	Tibia_SOS	-0,02615	0,053442	NA
	3	abundance	X09951	5beta-Cypr	Handgrip	-0,16171	0,060447	NA
	3	abundance	X09963	Iminoglycin	Tibia_length	0,09566	0,049707	NA
	3	abundance	X09963	Iminoglycin	Radius_length	0,199129	0,06643	NA
	3	abundance	X09963	Iminoglycin	Radius_SOS	0,017495	0,049044	NA
	3	abundance	X09963	Iminoglycin	Tibia_SOS	0,117733	0,052221	NA
	3	abundance	X09963	Iminoglycin	Handgrip	0,074615	0,060269	NA
2b		abundance	X09965	Biocytin	Tibia_length	0,087623	0,051692	NA
2b		abundance	X09965	Biocytin	Radius_length	-0,06527	0,070809	NA
2b		abundance	X09965	Biocytin	Radius_SOS	-0,04714	0,049921	NA
2b		abundance	X09965	Biocytin	Tibia_SOS	0,01204	0,054054	NA
2b		abundance	X09965	Biocytin	Handgrip	-0,12002	0,062086	NA
	3	abundance	X09969	3-Methylac	Tibia_length	0,049117	0,05345	NA
	3	abundance	X09969	3-Methylac	Radius_length	0,127273	0,072513	NA
	3	abundance	X09969	3-Methylac	Radius_SOS	0,016399	0,050973	NA
	3	abundance	X09969	3-Methylac	Tibia_SOS	0,144866	0,05383	NA
	3	abundance	X09969	3-Methylac	Handgrip	0,101968	0,061722	NA
2b		abundance	X09977	TLK (Peptic	Tibia_length	0,018372	0,055264	NA
2b		abundance	X09977	TLK (Peptic	Radius_length	0,104415	0,073456	NA
2b		abundance	X09977	TLK (Peptic	Radius_SOS	-0,00283	0,052516	NA
2b		abundance	X09977	TLK (Peptic	Tibia_SOS	0,074474	0,056214	NA
2b		abundance	X09977	TLK (Peptic	Handgrip	0,130429	0,061735	NA
	3	abundance	X09980	N-(4-Hepta	Tibia_length	0,093606	0,050513	NA
	3	abundance	X09980	N-(4-Hepta	Radius_length	0,138913	0,069006	NA
	3	abundance	X09980	N-(4-Hepta	Radius_SOS	0,05949	0,049226	NA
	3	abundance	X09980	N-(4-Hepta	Tibia_SOS	0,121126	0,052666	NA
	3	abundance	X09980	N-(4-Hepta	Handgrip	0,062811	0,060668	NA
	3	abundance	X09981	N-Acetylva	Tibia_length	0,106002	0,049654	NA
	3	abundance	X09981	N-Acetylva	Radius_length	0,130934	0,067437	NA
	3	abundance	X09981	N-Acetylva	Radius_SOS	0,029732	0,049028	NA
	3	abundance	X09981	N-Acetylva	Tibia_SOS	0,125416	0,052192	NA
	3	abundance	X09981	N-Acetylva	Handgrip	0,020334	0,060518	NA
	3	abundance	X09998	N-[(10Z)-7-	Tibia_length	0,029392	0,049888	NA
	3	abundance	X09998	N-[(10Z)-7-	Radius_length	-0,0105	0,068117	NA
	3	abundance	X09998	N-[(10Z)-7-	Radius_SOS	0,027418	0,049056	NA
	3	abundance	X09998	N-[(10Z)-7-	Tibia_SOS	-0,06236	0,052531	NA
	3	abundance	X09998	N-[(10Z)-7-	Handgrip	-0,06794	0,06057	NA
	3	abundance	X10011	Midodrine	Tibia_length	-0,191	0,056455	NA
	3	abundance	X10011	Midodrine	Radius_length	-0,14845	0,075068	NA
	3	abundance	X10011	Midodrine	Radius_SOS	-0,06253	0,054364	NA

	3	abundance	X10011	Midodrine	Tibia_SOS	-0,11074	0,058012	NA
	3	abundance	X10011	Midodrine	Handgrip	-0,14464	0,062403	NA
	3	abundance	X10025	2-[4-(3-Hy(	Tibia_length	-0,01103	0,051462	NA
	3	abundance	X10025	2-[4-(3-Hy(	Radius_length	0,012686	0,070443	NA
	3	abundance	X10025	2-[4-(3-Hy(	Radius_SOS	0,035632	0,049681	NA
	3	abundance	X10025	2-[4-(3-Hy(	Tibia_SOS	-0,03627	0,053838	NA
	3	abundance	X10025	2-[4-(3-Hy(	Handgrip	0,120878	0,061198	NA
	3	abundance	X10032	4,9a-Dime	Tibia_length	-0,02941	0,058571	NA
	3	abundance	X10032	4,9a-Dime	Radius_length	-0,04128	0,075603	NA
	3	abundance	X10032	4,9a-Dime	Radius_SOS	-0,1239	0,055862	NA
	3	abundance	X10032	4,9a-Dime	Tibia_SOS	-0,02472	0,05247	NA
	3	abundance	X10032	4,9a-Dime	Handgrip	0,032883	0,06016	NA
	3	abundance	X10033	epsilon-(ga	Tibia_length	-0,04098	0,053716	NA
	3	abundance	X10033	epsilon-(ga	Radius_length	-0,09118	0,072228	NA
	3	abundance	X10033	epsilon-(ga	Radius_SOS	-0,02517	0,051144	NA
	3	abundance	X10033	epsilon-(ga	Tibia_SOS	-0,03811	0,054473	NA
	3	abundance	X10033	epsilon-(ga	Handgrip	-0,05871	0,061681	NA
	3	abundance	X10036	Nimodipin	Tibia_length	-0,05748	0,055397	NA
	3	abundance	X10036	Nimodipin	Radius_length	-0,01665	0,073729	NA
	3	abundance	X10036	Nimodipin	Radius_SOS	-0,00886	0,052704	NA
	3	abundance	X10036	Nimodipin	Tibia_SOS	0,099531	0,056188	NA
	3	abundance	X10036	Nimodipin	Handgrip	-0,05835	0,061673	NA
	3	abundance	X10037	his-gln	Tibia_length	-0,09452	0,051956	NA
	3	abundance	X10037	his-gln	Radius_length	-0,10742	0,070637	NA
	3	abundance	X10037	his-gln	Radius_SOS	0,030657	0,049951	NA
	3	abundance	X10037	his-gln	Tibia_SOS	-0,04252	0,053275	NA
	3	abundance	X10037	his-gln	Handgrip	0,087995	0,060459	NA
2b		abundance	X10046	Dehydroac	Tibia_length	-0,07816	0,05681	NA
2b		abundance	X10046	Dehydroac	Radius_length	-0,01319	0,075527	NA
2b		abundance	X10046	Dehydroac	Radius_SOS	-0,05365	0,054121	NA
2b		abundance	X10046	Dehydroac	Tibia_SOS	-0,05608	0,057977	NA
2b		abundance	X10046	Dehydroac	Handgrip	0,011659	0,060189	NA
	3	abundance	X10076	trimethadi	Tibia_length	-8,80E-04	0,049592	NA
	3	abundance	X10076	trimethadi	Radius_length	-0,03036	0,067512	NA
	3	abundance	X10076	trimethadi	Radius_SOS	0,028374	0,049012	NA
	3	abundance	X10076	trimethadi	Tibia_SOS	0,065704	0,052424	NA
	3	abundance	X10076	trimethadi	Handgrip	0,078664	0,060125	NA
	3	abundance	X10091	(2E,6E)-9-[	Tibia_length	-0,00858	0,052253	NA
	3	abundance	X10091	(2E,6E)-9-[	Radius_length	0,065677	0,071111	NA
	3	abundance	X10091	(2E,6E)-9-[	Radius_SOS	-0,0301	0,050207	NA
	3	abundance	X10091	(2E,6E)-9-[	Tibia_SOS	0,021258	0,054192	NA
	3	abundance	X10091	(2E,6E)-9-[	Handgrip	-0,0271	0,061348	NA
	3	abundance	X10097	Coixol	Tibia_length	-0,00938	0,054503	NA
	3	abundance	X10097	Coixol	Radius_length	-0,11519	0,072472	NA
	3	abundance	X10097	Coixol	Radius_SOS	-0,04026	0,051783	NA
	3	abundance	X10097	Coixol	Tibia_SOS	0,026316	0,054938	NA
	3	abundance	X10097	Coixol	Handgrip	-0,07837	0,06131	NA
	3	abundance	X10098	Methyl 2,3-	Tibia_length	-0,12073	0,055423	NA

	3	abundance	X10098	Methyl 2,3-	Radius_length	-0,05617	0,07373	NA
	3	abundance	X10098	Methyl 2,3-	Radius_SOS	-0,0424	0,052918	NA
	3	abundance	X10098	Methyl 2,3-	Tibia_SOS	-0,0189	0,057397	NA
	3	abundance	X10098	Methyl 2,3-	Handgrip	-0,05782	0,062591	NA
	3	abundance	X10099	ala-met	Tibia_length	0,15334	0,05631	NA
	3	abundance	X10099	ala-met	Radius_length	0,197652	0,07357	NA
	3	abundance	X10099	ala-met	Radius_SOS	0,071959	0,0538	NA
	3	abundance	X10099	ala-met	Tibia_SOS	0,126119	0,058209	NA
	3	abundance	X10099	ala-met	Handgrip	0,038361	0,062978	NA
	3	abundance	X10121	Leu-pro	Tibia_length	-0,17913	0,053933	NA
	3	abundance	X10121	Leu-pro	Radius_length	-0,28939	0,069625	NA
	3	abundance	X10121	Leu-pro	Radius_SOS	-0,02526	0,051081	NA
	3	abundance	X10121	Leu-pro	Tibia_SOS	-0,13494	0,053404	NA
	3	abundance	X10121	Leu-pro	Handgrip	-0,23066	0,060063	NA
	3	abundance	X10129	SECONAL	Tibia_length	-0,05783	0,054075	NA
	3	abundance	X10129	SECONAL	Radius_length	-0,05934	0,072812	NA
	3	abundance	X10129	SECONAL	Radius_SOS	0,043489	0,051451	NA
	3	abundance	X10129	SECONAL	Tibia_SOS	-0,05072	0,054467	NA
	3	abundance	X10129	SECONAL	Handgrip	-0,11527	0,061593	NA
	3	abundance	X10130	N-(4-Hydro	Tibia_length	-0,15984	0,054111	NA
	3	abundance	X10130	N-(4-Hydro	Radius_length	-0,1305	0,0729	NA
	3	abundance	X10130	N-(4-Hydro	Radius_SOS	-0,04283	0,0519	NA
	3	abundance	X10130	N-(4-Hydro	Tibia_SOS	-0,09418	0,054984	NA
	3	abundance	X10130	N-(4-Hydro	Handgrip	-0,085	0,061895	NA
	3	abundance	X10137	1-Methyl-1	Tibia_length	-0,0112	0,055082	NA
	3	abundance	X10137	1-Methyl-1	Radius_length	0,092025	0,073061	NA
	3	abundance	X10137	1-Methyl-1	Radius_SOS	0,02183	0,052452	NA
	3	abundance	X10137	1-Methyl-1	Tibia_SOS	0,134014	0,056539	NA
	3	abundance	X10137	1-Methyl-1	Handgrip	-0,00551	0,063011	NA
	3	abundance	X10144	imazameth	Tibia_length	0,128195	0,053041	NA
	3	abundance	X10144	imazameth	Radius_length	0,135141	0,072082	NA
	3	abundance	X10144	imazameth	Radius_SOS	0,113969	0,050917	NA
	3	abundance	X10144	imazameth	Tibia_SOS	0,087394	0,054805	NA
	3	abundance	X10144	imazameth	Handgrip	0,105309	0,060635	NA
	3	abundance	X10148	cys-met	Tibia_length	-0,05984	0,054967	NA
	3	abundance	X10148	cys-met	Radius_length	-0,03052	0,073764	NA
	3	abundance	X10148	cys-met	Radius_SOS	-0,01989	0,052465	NA
	3	abundance	X10148	cys-met	Tibia_SOS	0,052811	0,05584	NA
	3	abundance	X10148	cys-met	Handgrip	0,102324	0,062038	NA
	3	abundance	X10151	2-(3-CARB	Tibia_length	-0,16704	0,053486	NA
	3	abundance	X10151	2-(3-CARB	Radius_length	-0,16255	0,071944	NA
	3	abundance	X10151	2-(3-CARB	Radius_SOS	-0,03753	0,051402	NA
	3	abundance	X10151	2-(3-CARB	Tibia_SOS	-0,05614	0,054432	NA
	3	abundance	X10151	2-(3-CARB	Handgrip	-0,00857	0,062027	NA
2a		abundance	X10162	Quinaldic a	Tibia_length	0,105264	0,05229	NA
2a		abundance	X10162	Quinaldic a	Radius_length	0,108218	0,070861	NA
2a		abundance	X10162	Quinaldic a	Radius_SOS	0,101926	0,049934	NA
2a		abundance	X10162	Quinaldic a	Tibia_SOS	-0,01328	0,053025	NA

2a		abundance	X10162	Quinaldic a	Handgrip	0,041163	0,060196	NA
	3	abundance	X10177	Gly-DL-Phe	Tibia_length	-0,16268	0,05653	NA
	3	abundance	X10177	Gly-DL-Phe	Radius_length	-0,28107	0,071815	NA
	3	abundance	X10177	Gly-DL-Phe	Radius_SOS	-0,0898	0,053812	NA
	3	abundance	X10177	Gly-DL-Phe	Tibia_SOS	-0,02548	0,057844	NA
	3	abundance	X10177	Gly-DL-Phe	Handgrip	-0,10767	0,062559	NA
	3	abundance	X10180	4-Formyl-2	Tibia_length	-0,07941	0,053448	NA
	3	abundance	X10180	4-Formyl-2	Radius_length	-0,11344	0,072323	NA
	3	abundance	X10180	4-Formyl-2	Radius_SOS	-0,09591	0,051181	NA
	3	abundance	X10180	4-Formyl-2	Tibia_SOS	-0,04384	0,05449	NA
	3	abundance	X10180	4-Formyl-2	Handgrip	-0,04385	0,062431	NA
	3	abundance	X10203	L-gamma-(	Tibia_length	-0,06057	0,049904	NA
	3	abundance	X10203	L-gamma-(	Radius_length	-0,0801	0,06765	NA
	3	abundance	X10203	L-gamma-(	Radius_SOS	0,020111	0,04902	NA
	3	abundance	X10203	L-gamma-(	Tibia_SOS	-0,05334	0,052422	NA
	3	abundance	X10203	L-gamma-(	Handgrip	0,006506	0,060503	NA
2b		abundance	X10224	Imidazolela	Tibia_length	0,097378	0,052147	NA
2b		abundance	X10224	Imidazolela	Radius_length	0,136559	0,070874	NA
2b		abundance	X10224	Imidazolela	Radius_SOS	0,054781	0,050089	NA
2b		abundance	X10224	Imidazolela	Tibia_SOS	0,096909	0,054258	NA
2b		abundance	X10224	Imidazolela	Handgrip	0,024936	0,060552	NA
	3	abundance	X10246	Ethynodiol	Tibia_length	-0,00572	0,0556	NA
	3	abundance	X10246	Ethynodiol	Radius_length	-0,02426	0,073912	NA
	3	abundance	X10246	Ethynodiol	Radius_SOS	-0,0445	0,052781	NA
	3	abundance	X10246	Ethynodiol	Tibia_SOS	0,040606	0,056783	NA
	3	abundance	X10246	Ethynodiol	Handgrip	-0,05323	0,061891	NA
	3	abundance	X10248	(+)-Etomid	Tibia_length	0,053662	0,054665	NA
	3	abundance	X10248	(+)-Etomid	Radius_length	0,025848	0,07336	NA
	3	abundance	X10248	(+)-Etomid	Radius_SOS	-0,11431	0,051785	NA
	3	abundance	X10248	(+)-Etomid	Tibia_SOS	0,170121	0,056204	NA
	3	abundance	X10248	(+)-Etomid	Handgrip	0,048821	0,062272	NA
	3	abundance	X10265	3-methyl-4	Tibia_length	0,093829	0,056245	NA
	3	abundance	X10265	3-methyl-4	Radius_length	0,231717	0,07288	NA
	3	abundance	X10265	3-methyl-4	Radius_SOS	0,067941	0,05321	NA
	3	abundance	X10265	3-methyl-4	Tibia_SOS	0,08131	0,056662	NA
	3	abundance	X10265	3-methyl-4	Handgrip	0,141105	0,062218	NA
	3	abundance	X10276	Maleamate	Tibia_length	0,099216	0,049533	NA
	3	abundance	X10276	Maleamate	Radius_length	0,209397	0,065932	NA
	3	abundance	X10276	Maleamate	Radius_SOS	0,015183	0,049028	NA
	3	abundance	X10276	Maleamate	Tibia_SOS	0,110757	0,052229	NA
	3	abundance	X10276	Maleamate	Handgrip	0,068693	0,060189	NA
	3	abundance	X10279	Lys-Pro	Tibia_length	0,068173	0,05165	NA
	3	abundance	X10279	Lys-Pro	Radius_length	0,061977	0,070759	NA
	3	abundance	X10279	Lys-Pro	Radius_SOS	0,050258	0,049954	NA
	3	abundance	X10279	Lys-Pro	Tibia_SOS	-0,11218	0,053388	NA
	3	abundance	X10279	Lys-Pro	Handgrip	0,010282	0,062535	NA
	3	abundance	X10304	2,3,8,9-Tet	Tibia_length	0,017223	0,05322	NA
	3	abundance	X10304	2,3,8,9-Tet	Radius_length	0,053191	0,072358	NA

3	abundance	X10304	2,3,8,9-Tet	Radius_SOS	-0,02524	0,050748	NA
3	abundance	X10304	2,3,8,9-Tet	Tibia_SOS	0,082596	0,053318	NA
3	abundance	X10304	2,3,8,9-Tet	Handgrip	-0,03372	0,060877	NA
2b	abundance	X10314	4-morphol	Tibia_length	-0,08555	0,052946	NA
2b	abundance	X10314	4-morphol	Radius_length	-0,14646	0,071511	NA
2b	abundance	X10314	4-morphol	Radius_SOS	0,029972	0,050669	NA
2b	abundance	X10314	4-morphol	Tibia_SOS	0,027701	0,053745	NA
2b	abundance	X10314	4-morphol	Handgrip	0,00429	0,060885	NA
3	abundance	X10319	1-Methyl-1	Tibia_length	-0,04898	0,052849	NA
3	abundance	X10319	1-Methyl-1	Radius_length	0,029399	0,071895	NA
3	abundance	X10319	1-Methyl-1	Radius_SOS	-0,02592	0,050662	NA
3	abundance	X10319	1-Methyl-1	Tibia_SOS	-0,07461	0,053792	NA
3	abundance	X10319	1-Methyl-1	Handgrip	-0,11527	0,061891	NA
3	abundance	X10325	Lovastatin	Tibia_length	0,044831	0,051413	NA
3	abundance	X10325	Lovastatin	Radius_length	-0,0094	0,070892	NA
3	abundance	X10325	Lovastatin	Radius_SOS	-0,01154	0,049752	NA
3	abundance	X10325	Lovastatin	Tibia_SOS	0,016759	0,053289	NA
3	abundance	X10325	Lovastatin	Handgrip	-0,07421	0,06208	NA
3	abundance	X10330	Sulfurous a	Tibia_length	-0,07706	0,052603	NA
3	abundance	X10330	Sulfurous a	Radius_length	-0,18794	0,070371	NA
3	abundance	X10330	Sulfurous a	Radius_SOS	-0,0797	0,050418	NA
3	abundance	X10330	Sulfurous a	Tibia_SOS	-0,04767	0,054185	NA
3	abundance	X10330	Sulfurous a	Handgrip	-0,03842	0,061614	NA
2b	abundance	X10339	4H-1-Benz	Tibia_length	0,009642	0,055517	NA
2b	abundance	X10339	4H-1-Benz	Radius_length	-0,05303	0,074428	NA
2b	abundance	X10339	4H-1-Benz	Radius_SOS	0,043082	0,052623	NA
2b	abundance	X10339	4H-1-Benz	Tibia_SOS	0,030176	0,056535	NA
2b	abundance	X10339	4H-1-Benz	Handgrip	0,044027	0,062501	NA
3	abundance	X10345	S-Allylcyst	Tibia_length	-0,02148	0,051249	NA
3	abundance	X10345	S-Allylcyst	Radius_length	0,047166	0,069974	NA
3	abundance	X10345	S-Allylcyst	Radius_SOS	-0,10033	0,049542	NA
3	abundance	X10345	S-Allylcyst	Tibia_SOS	-0,01031	0,052969	NA
3	abundance	X10345	S-Allylcyst	Handgrip	0,015073	0,060479	NA
3	abundance	X10353	L-gamma-(	Tibia_length	-0,13451	0,051286	NA
3	abundance	X10353	L-gamma-(	Radius_length	0,012845	0,07057	NA
3	abundance	X10353	L-gamma-(	Radius_SOS	0,01902	0,049714	NA
3	abundance	X10353	L-gamma-(	Tibia_SOS	-0,02432	0,053021	NA
3	abundance	X10353	L-gamma-(	Handgrip	-0,02644	0,060172	NA
3	abundance	X10367	2-(1-Napht	Tibia_length	-0,07371	0,056755	NA
3	abundance	X10367	2-(1-Napht	Radius_length	0,010875	0,074491	NA
3	abundance	X10367	2-(1-Napht	Radius_SOS	0,015312	0,053823	NA
3	abundance	X10367	2-(1-Napht	Tibia_SOS	0,038281	0,057067	NA
3	abundance	X10367	2-(1-Napht	Handgrip	0,046293	0,063195	NA
3	abundance	X10370	L-Arogenat	Tibia_length	-0,01487	0,056491	NA
3	abundance	X10370	L-Arogenat	Radius_length	0,023988	0,075024	NA
3	abundance	X10370	L-Arogenat	Radius_SOS	-0,03363	0,053643	NA
3	abundance	X10370	L-Arogenat	Tibia_SOS	-0,02154	0,055882	NA
3	abundance	X10370	L-Arogenat	Handgrip	-0,08589	0,062869	NA

2b	abundance	X10375	4-Indoleca	Tibia_length	0,053272	0,052429	NA
2b	abundance	X10375	4-Indoleca	Radius_length	0,095185	0,07152	NA
2b	abundance	X10375	4-Indoleca	Radius_SOS	-0,05069	0,05028	NA
2b	abundance	X10375	4-Indoleca	Tibia_SOS	0,08365	0,053365	NA
2b	abundance	X10375	4-Indoleca	Handgrip	0,045384	0,060598	NA
3	abundance	X10379	Phenyl D-g	Tibia_length	-0,06079	0,053844	NA
3	abundance	X10379	Phenyl D-g	Radius_length	-0,16079	0,071758	NA
3	abundance	X10379	Phenyl D-g	Radius_SOS	-0,10268	0,051169	NA
3	abundance	X10379	Phenyl D-g	Tibia_SOS	-0,05134	0,055774	NA
3	abundance	X10379	Phenyl D-g	Handgrip	-0,09801	0,062303	NA
3	abundance	X10382	Zalcitabine	Tibia_length	-0,08393	0,05116	NA
3	abundance	X10382	Zalcitabine	Radius_length	-0,13333	0,069331	NA
3	abundance	X10382	Zalcitabine	Radius_SOS	0,014543	0,049421	NA
3	abundance	X10382	Zalcitabine	Tibia_SOS	-0,06058	0,052823	NA
3	abundance	X10382	Zalcitabine	Handgrip	0,058339	0,060515	NA
3	abundance	X10384	L-N2-(2-Ca	Tibia_length	-0,0014	0,059206	NA
3	abundance	X10384	L-N2-(2-Ca	Radius_length	0,105062	0,075741	NA
3	abundance	X10384	L-N2-(2-Ca	Radius_SOS	-0,02547	0,056479	NA
3	abundance	X10384	L-N2-(2-Ca	Tibia_SOS	0,179291	0,057935	NA
3	abundance	X10384	L-N2-(2-Ca	Handgrip	0,078421	0,060008	NA
3	abundance	X10387	Coenzyme	Tibia_length	0,089777	0,05019	NA
3	abundance	X10387	Coenzyme	Radius_length	0,197456	0,068161	NA
3	abundance	X10387	Coenzyme	Radius_SOS	0,050677	0,04914	NA
3	abundance	X10387	Coenzyme	Tibia_SOS	-0,01225	0,052705	NA
3	abundance	X10387	Coenzyme	Handgrip	-0,00607	0,060372	NA
3	abundance	X10389	Leucylaspa	Tibia_length	-0,06426	0,054758	NA
3	abundance	X10389	Leucylaspa	Radius_length	0,033346	0,073368	NA
3	abundance	X10389	Leucylaspa	Radius_SOS	-0,00538	0,052377	NA
3	abundance	X10389	Leucylaspa	Tibia_SOS	-9,50E-04	0,05652	NA
3	abundance	X10389	Leucylaspa	Handgrip	-0,10897	0,062004	NA
3	abundance	X10393	Ro 20-1724	Tibia_length	-0,05413	0,051886	NA
3	abundance	X10393	Ro 20-1724	Radius_length	-0,04712	0,070816	NA
3	abundance	X10393	Ro 20-1724	Radius_SOS	-0,01191	0,050061	NA
3	abundance	X10393	Ro 20-1724	Tibia_SOS	0,005242	0,053424	NA
3	abundance	X10393	Ro 20-1724	Handgrip	-0,00721	0,061103	NA
1	abundance	X10401	3-Methylxa	Tibia_length	-0,1079	0,0516	NA
1	abundance	X10401	3-Methylxa	Radius_length	-0,09416	0,070689	NA
1	abundance	X10401	3-Methylxa	Radius_SOS	0,017363	0,049896	NA
1	abundance	X10401	3-Methylxa	Tibia_SOS	-0,07214	0,053348	NA
1	abundance	X10401	3-Methylxa	Handgrip	-0,0024	0,061597	NA
3	abundance	X10415	beta-D-Glc	Tibia_length	0,004523	0,054215	NA
3	abundance	X10415	beta-D-Glc	Radius_length	0,050039	0,072972	NA
3	abundance	X10415	beta-D-Glc	Radius_SOS	0,034281	0,051533	NA
3	abundance	X10415	beta-D-Glc	Tibia_SOS	0,112194	0,055152	NA
3	abundance	X10415	beta-D-Glc	Handgrip	0,059216	0,061296	NA
2b	abundance	X10417	L-Sacchar	Tibia_length	-0,09847	0,056041	NA
2b	abundance	X10417	L-Sacchar	Radius_length	0,045416	0,075369	NA
2b	abundance	X10417	L-Sacchar	Radius_SOS	-0,01841	0,053618	NA

2b	abundance	X10417	L-Sacchar	Tibia_SOS	-0,06303	0,056695	NA
2b	abundance	X10417	L-Sacchar	Handgrip	-0,17289	0,060191	NA
2b	abundance	X10429	1,5-Isoqui	Tibia_length	-0,01101	0,059516	NA
2b	abundance	X10429	1,5-Isoqui	Radius_length	-0,00706	0,075892	NA
2b	abundance	X10429	1,5-Isoqui	Radius_SOS	-0,03733	0,056858	NA
2b	abundance	X10429	1,5-Isoqui	Tibia_SOS	0,036982	0,05245	NA
2b	abundance	X10429	1,5-Isoqui	Handgrip	-0,02678	0,060171	NA
3	abundance	X10442	(6alpha,11	Tibia_length	-0,03154	0,051056	NA
3	abundance	X10442	(6alpha,11	Radius_length	0,030258	0,070131	NA
3	abundance	X10442	(6alpha,11	Radius_SOS	-0,04916	0,049467	NA
3	abundance	X10442	(6alpha,11	Tibia_SOS	0,041885	0,05358	NA
3	abundance	X10442	(6alpha,11	Handgrip	0,07838	0,060962	NA
3	abundance	X10445	2-(Carboxy	Tibia_length	0,030718	0,053434	NA
3	abundance	X10445	2-(Carboxy	Radius_length	-0,04759	0,072521	NA
3	abundance	X10445	2-(Carboxy	Radius_SOS	0,025369	0,051051	NA
3	abundance	X10445	2-(Carboxy	Tibia_SOS	-0,0429	0,054817	NA
3	abundance	X10445	2-(Carboxy	Handgrip	0,073507	0,062759	NA
3	abundance	X10451	Ro 20-172	Tibia_length	0,025152	0,053592	NA
3	abundance	X10451	Ro 20-172	Radius_length	0,101712	0,072471	NA
3	abundance	X10451	Ro 20-172	Radius_SOS	0,069148	0,051008	NA
3	abundance	X10451	Ro 20-172	Tibia_SOS	0,050442	0,055241	NA
3	abundance	X10451	Ro 20-172	Handgrip	1,16E-04	0,062184	NA
3	abundance	X10454	8-Amino-7	Tibia_length	-0,15379	0,053033	NA
3	abundance	X10454	8-Amino-7	Radius_length	-0,04634	0,072644	NA
3	abundance	X10454	8-Amino-7	Radius_SOS	0,007134	0,050833	NA
3	abundance	X10454	8-Amino-7	Tibia_SOS	-0,07555	0,05473	NA
3	abundance	X10454	8-Amino-7	Handgrip	-0,14514	0,060624	NA
3	abundance	X10457	glu-ser	Tibia_length	-0,07871	0,05516	NA
3	abundance	X10457	glu-ser	Radius_length	-0,04788	0,073937	NA
3	abundance	X10457	glu-ser	Radius_SOS	-0,01213	0,052319	NA
3	abundance	X10457	glu-ser	Tibia_SOS	0,045789	0,056751	NA
3	abundance	X10457	glu-ser	Handgrip	-0,03381	0,062247	NA
3	abundance	X10476	GAMMA-H	Tibia_length	-0,05669	0,057587	NA
3	abundance	X10476	GAMMA-H	Radius_length	0,029901	0,075606	NA
3	abundance	X10476	GAMMA-H	Radius_SOS	-0,03352	0,05517	NA
3	abundance	X10476	GAMMA-H	Tibia_SOS	0,095762	0,057459	NA
3	abundance	X10476	GAMMA-H	Handgrip	0,083393	0,059983	NA
2b	abundance	X10486	Coumarin	Tibia_length	0,128856	0,049179	NA
2b	abundance	X10486	Coumarin	Radius_length	0,165877	0,065935	NA
2b	abundance	X10486	Coumarin	Radius_SOS	0,08044	0,048872	NA
2b	abundance	X10486	Coumarin	Tibia_SOS	0,134742	0,052036	NA
2b	abundance	X10486	Coumarin	Handgrip	0,078694	0,060013	NA
1	abundance	X10488	4-Guanidir	Tibia_length	-0,09908	0,049896	NA
1	abundance	X10488	4-Guanidir	Radius_length	-0,09338	0,068167	NA
1	abundance	X10488	4-Guanidir	Radius_SOS	-0,01845	0,0491	NA
1	abundance	X10488	4-Guanidir	Tibia_SOS	-0,01441	0,052548	NA
1	abundance	X10488	4-Guanidir	Handgrip	0,006416	0,060527	NA
3	abundance	X10494	26Q0EO75	Tibia_length	-0,05805	0,054548	NA

	3	abundance	X10494	26Q0EO75	Radius_length	-0,01284	0,073389	NA
	3	abundance	X10494	26Q0EO75	Radius_SOS	0,091345	0,051659	NA
	3	abundance	X10494	26Q0EO75	Tibia_SOS	0,053464	0,054289	NA
	3	abundance	X10494	26Q0EO75	Handgrip	0,056562	0,061672	NA
	3	abundance	X10497	4-Guanidir	Tibia_length	-0,10038	0,048723	NA
	3	abundance	X10497	4-Guanidir	Radius_length	-0,01293	0,048966	NA
	3	abundance	X10497	4-Guanidir	Radius_SOS	-0,00935	0,049027	NA
	3	abundance	X10497	4-Guanidir	Tibia_SOS	-0,04522	0,052433	NA
	3	abundance	X10497	4-Guanidir	Handgrip	0,053975	0,060105	NA
	3	abundance	X10503	APM	Tibia_length	-0,17016	0,05907	NA
	3	abundance	X10503	APM	Radius_length	-0,13001	0,075729	NA
	3	abundance	X10503	APM	Radius_SOS	-0,08946	0,056259	NA
	3	abundance	X10503	APM	Tibia_SOS	-0,15184	0,058495	NA
	3	abundance	X10503	APM	Handgrip	-0,01218	0,060188	NA
	3	abundance	X10511	dopaquino	Tibia_length	-0,02922	0,050748	NA
	3	abundance	X10511	dopaquino	Radius_length	-0,06309	0,06934	NA
	3	abundance	X10511	dopaquino	Radius_SOS	0,032571	0,049307	NA
	3	abundance	X10511	dopaquino	Tibia_SOS	-0,10259	0,05231	NA
	3	abundance	X10511	dopaquino	Handgrip	-0,10363	0,060331	NA
	3	abundance	X10513	ophthalmic	Tibia_length	6,24E-04	0,054121	NA
	3	abundance	X10513	ophthalmic	Radius_length	0,022431	0,073246	NA
	3	abundance	X10513	ophthalmic	Radius_SOS	0,01673	0,051605	NA
	3	abundance	X10513	ophthalmic	Tibia_SOS	0,030784	0,056034	NA
	3	abundance	X10513	ophthalmic	Handgrip	0,125514	0,062753	NA
	3	abundance	X10518	MFCD0002	Tibia_length	-0,11893	0,057908	NA
	3	abundance	X10518	MFCD0002	Radius_length	0,02933	0,075097	NA
	3	abundance	X10518	MFCD0002	Radius_SOS	-0,02112	0,05524	NA
	3	abundance	X10518	MFCD0002	Tibia_SOS	-0,00342	0,057342	NA
	3	abundance	X10518	MFCD0002	Handgrip	0,025594	0,060173	NA
	3	abundance	X10534	L-N2-(2-Ca	Tibia_length	9,25E-04	0,050151	NA
	3	abundance	X10534	L-N2-(2-Ca	Radius_length	-0,04488	0,06853	NA
	3	abundance	X10534	L-N2-(2-Ca	Radius_SOS	-0,08561	0,048933	NA
	3	abundance	X10534	L-N2-(2-Ca	Tibia_SOS	-0,05926	0,052682	NA
	3	abundance	X10534	L-N2-(2-Ca	Handgrip	-0,06491	0,060205	NA
	3	abundance	X10536	CMPF	Tibia_length	-0,06267	0,050801	NA
	3	abundance	X10536	CMPF	Radius_length	-0,0237	0,069951	NA
	3	abundance	X10536	CMPF	Radius_SOS	0,058201	0,04928	NA
	3	abundance	X10536	CMPF	Tibia_SOS	-0,09314	0,052828	NA
	3	abundance	X10536	CMPF	Handgrip	-0,02771	0,060973	NA
2b		abundance	X10549	Glaucine	Tibia_length	-0,06753	0,056005	NA
2b		abundance	X10549	Glaucine	Radius_length	-0,0061	0,074098	NA
2b		abundance	X10549	Glaucine	Radius_SOS	0,009982	0,053083	NA
2b		abundance	X10549	Glaucine	Tibia_SOS	-0,03206	0,05628	NA
2b		abundance	X10549	Glaucine	Handgrip	-0,0245	0,063102	NA
	3	abundance	X10550	pentobarbi	Tibia_length	-0,09755	0,053255	NA
	3	abundance	X10550	pentobarbi	Radius_length	-0,15985	0,071562	NA
	3	abundance	X10550	pentobarbi	Radius_SOS	0,010467	0,050859	NA
	3	abundance	X10550	pentobarbi	Tibia_SOS	-0,14903	0,053994	NA

	3	abundance	X10550	pentobarbi	Handgrip	-0,07611	0,06169	NA
	1	abundance	X10554	Glycine	Tibia_length	0,088119	0,05012	NA
	1	abundance	X10554	Glycine	Radius_length	0,092077	0,068395	NA
	1	abundance	X10554	Glycine	Radius_SOS	-0,01614	0,049138	NA
	1	abundance	X10554	Glycine	Tibia_SOS	0,079014	0,05251	NA
	1	abundance	X10554	Glycine	Handgrip	0,08703	0,060138	NA
	3	abundance	X10564	2-(1-Ethox	Tibia_length	4,29E-04	0,051713	NA
	3	abundance	X10564	2-(1-Ethox	Radius_length	0,124221	0,070313	NA
	3	abundance	X10564	2-(1-Ethox	Radius_SOS	0,050264	0,049814	NA
	3	abundance	X10564	2-(1-Ethox	Tibia_SOS	0,058835	0,053609	NA
	3	abundance	X10564	2-(1-Ethox	Handgrip	0,062087	0,061427	NA
	3	abundance	X10580	Leu-Leu	Tibia_length	0,041398	0,054631	NA
	3	abundance	X10580	Leu-Leu	Radius_length	0,058291	0,07306	NA
	3	abundance	X10580	Leu-Leu	Radius_SOS	0,058362	0,052309	NA
	3	abundance	X10580	Leu-Leu	Tibia_SOS	0,033963	0,05501	NA
	3	abundance	X10580	Leu-Leu	Handgrip	-0,04665	0,062227	NA
	3	abundance	X10588	(8)-Ginger	Tibia_length	0,044372	0,051331	NA
	3	abundance	X10588	(8)-Ginger	Radius_length	0,093985	0,070089	NA
	3	abundance	X10588	(8)-Ginger	Radius_SOS	0,100839	0,049488	NA
	3	abundance	X10588	(8)-Ginger	Tibia_SOS	0,123579	0,053329	NA
	3	abundance	X10588	(8)-Ginger	Handgrip	0,057646	0,060875	NA
	3	abundance	X10593	Vorinostat	Tibia_length	-0,17411	0,053162	NA
	3	abundance	X10593	Vorinostat	Radius_length	-0,1249	0,072388	NA
	3	abundance	X10593	Vorinostat	Radius_SOS	0,027436	0,051104	NA
	3	abundance	X10593	Vorinostat	Tibia_SOS	0,001475	0,055781	NA
	3	abundance	X10593	Vorinostat	Handgrip	-0,00711	0,06203	NA
	3	abundance	X10600	Glycocyam	Tibia_length	-0,00129	0,05456	NA
	3	abundance	X10600	Glycocyam	Radius_length	0,083033	0,073224	NA
	3	abundance	X10600	Glycocyam	Radius_SOS	0,022204	0,051949	NA
	3	abundance	X10600	Glycocyam	Tibia_SOS	-0,06994	0,055378	NA
	3	abundance	X10600	Glycocyam	Handgrip	0,191995	0,061366	NA
	3	abundance	X10611	2-Acrylami	Tibia_length	0,155823	0,052199	NA
	3	abundance	X10611	2-Acrylami	Radius_length	0,105456	0,071249	NA
	3	abundance	X10611	2-Acrylami	Radius_SOS	0,011709	0,050408	NA
	3	abundance	X10611	2-Acrylami	Tibia_SOS	0,138738	0,053222	NA
	3	abundance	X10611	2-Acrylami	Handgrip	0,102412	0,06042	NA
2a		abundance	X10614	Monometh	Tibia_length	-0,04283	0,053115	NA
2a		abundance	X10614	Monometh	Radius_length	-0,04734	0,071954	NA
2a		abundance	X10614	Monometh	Radius_SOS	-0,04376	0,050659	NA
2a		abundance	X10614	Monometh	Tibia_SOS	-0,1403	0,053638	NA
2a		abundance	X10614	Monometh	Handgrip	-0,09461	0,061393	NA
2b		abundance	X10617	2-methyl-1	Tibia_length	0,117428	0,050984	NA
2b		abundance	X10617	2-methyl-1	Radius_length	0,206438	0,068777	NA
2b		abundance	X10617	2-methyl-1	Radius_SOS	0,03117	0,049521	NA
2b		abundance	X10617	2-methyl-1	Tibia_SOS	0,124557	0,05272	NA
2b		abundance	X10617	2-methyl-1	Handgrip	0,067617	0,060956	NA
	3	abundance	X10623	N-Nonano	Tibia_length	0,114239	0,058213	NA
	3	abundance	X10623	N-Nonano	Radius_length	0,122755	0,075569	NA

	3	abundance	X10623	N-Nonanoy	Radius_SOS	-0,03999	0,055845	NA
	3	abundance	X10623	N-Nonanoy	Tibia_SOS	0,021846	0,058851	NA
	3	abundance	X10623	N-Nonanoy	Handgrip	0,008008	0,063794	NA
2b		abundance	X10636	8-hydroxy-	Tibia_length	0,041204	0,054428	NA
2b		abundance	X10636	8-hydroxy-	Radius_length	0,084032	0,072663	NA
2b		abundance	X10636	8-hydroxy-	Radius_SOS	0,026512	0,051903	NA
2b		abundance	X10636	8-hydroxy-	Tibia_SOS	0,022472	0,055924	NA
2b		abundance	X10636	8-hydroxy-	Handgrip	0,030428	0,06238	NA
	3	abundance	X10647	4-Hydroxyp	Tibia_length	-0,01703	0,055579	NA
	3	abundance	X10647	4-Hydroxyp	Radius_length	-0,08099	0,073287	NA
	3	abundance	X10647	4-Hydroxyp	Radius_SOS	0,029216	0,05262	NA
	3	abundance	X10647	4-Hydroxyp	Tibia_SOS	0,063316	0,056941	NA
	3	abundance	X10647	4-Hydroxyp	Handgrip	0,04325	0,062979	NA
	3	abundance	X10651	Methyl 1-h	Tibia_length	-0,11102	0,053096	NA
	3	abundance	X10651	Methyl 1-h	Radius_length	-0,17179	0,071081	NA
	3	abundance	X10651	Methyl 1-h	Radius_SOS	-0,02781	0,050738	NA
	3	abundance	X10651	Methyl 1-h	Tibia_SOS	-0,13543	0,053835	NA
	3	abundance	X10651	Methyl 1-h	Handgrip	-0,10799	0,061137	NA
	3	abundance	X10660	2-Methylth	Tibia_length	0,179829	0,050915	NA
	3	abundance	X10660	2-Methylth	Radius_length	0,132699	0,069745	NA
	3	abundance	X10660	2-Methylth	Radius_SOS	-0,02165	0,049609	NA
	3	abundance	X10660	2-Methylth	Tibia_SOS	0,140965	0,052699	NA
	3	abundance	X10660	2-Methylth	Handgrip	0,047523	0,060445	NA
2b		abundance	X10664	N1-(5-met	Tibia_length	-0,11855	0,056149	NA
2b		abundance	X10664	N1-(5-met	Radius_length	-0,19907	0,072891	NA
2b		abundance	X10664	N1-(5-met	Radius_SOS	0,009345	0,05306	NA
2b		abundance	X10664	N1-(5-met	Tibia_SOS	-0,00457	0,055554	NA
2b		abundance	X10664	N1-(5-met	Handgrip	-0,09386	0,062056	NA
	3	abundance	X10665	Aprobarbit	Tibia_length	-0,04554	0,053021	NA
	3	abundance	X10665	Aprobarbit	Radius_length	0,088063	0,07175	NA
	3	abundance	X10665	Aprobarbit	Radius_SOS	-0,02951	0,05076	NA
	3	abundance	X10665	Aprobarbit	Tibia_SOS	0,091309	0,054783	NA
	3	abundance	X10665	Aprobarbit	Handgrip	-0,0453	0,061967	NA
	3	abundance	X10667	N-Phenyla	Tibia_length	-0,06766	0,057209	NA
	3	abundance	X10667	N-Phenyla	Radius_length	0,109225	0,074367	NA
	3	abundance	X10667	N-Phenyla	Radius_SOS	0,038978	0,054582	NA
	3	abundance	X10667	N-Phenyla	Tibia_SOS	0,031039	0,058651	NA
	3	abundance	X10667	N-Phenyla	Handgrip	-0,07506	0,060023	NA
	3	abundance	X10676	6-(1-Hydro	Tibia_length	-0,12521	0,054866	NA
	3	abundance	X10676	6-(1-Hydro	Radius_length	-0,08425	0,074795	NA
	3	abundance	X10676	6-(1-Hydro	Radius_SOS	-0,02035	0,052625	NA
	3	abundance	X10676	6-(1-Hydro	Tibia_SOS	-0,06248	0,056025	NA
	3	abundance	X10676	6-(1-Hydro	Handgrip	-0,22646	0,060833	NA
	3	abundance	X10680	8-hydroxy-	Tibia_length	-0,00494	0,05068	NA
	3	abundance	X10680	8-hydroxy-	Radius_length	0,031112	0,069375	NA
	3	abundance	X10680	8-hydroxy-	Radius_SOS	-0,00783	0,049324	NA
	3	abundance	X10680	8-hydroxy-	Tibia_SOS	0,010911	0,052878	NA
	3	abundance	X10680	8-hydroxy-	Handgrip	-0,00199	0,061031	NA

2a	abundance	X10685	Glycylvalin	Tibia_length	0,059206	0,056986	NA
2a	abundance	X10685	Glycylvalin	Radius_length	-0,06876	0,074864	NA
2a	abundance	X10685	Glycylvalin	Radius_SOS	0,021048	0,054213	NA
2a	abundance	X10685	Glycylvalin	Tibia_SOS	0,043454	0,057814	NA
2a	abundance	X10685	Glycylvalin	Handgrip	0,084574	0,06238	NA
3	abundance	X10687	Gln-Gln	Tibia_length	-0,02574	0,053195	NA
3	abundance	X10687	Gln-Gln	Radius_length	-0,01691	0,072106	NA
3	abundance	X10687	Gln-Gln	Radius_SOS	-0,02965	0,050873	NA
3	abundance	X10687	Gln-Gln	Tibia_SOS	0,017694	0,054146	NA
3	abundance	X10687	Gln-Gln	Handgrip	0,135337	0,060791	NA
3	abundance	X10691	(âˆ’)-nabil	Tibia_length	0,03596	0,05305	NA
3	abundance	X10691	(âˆ’)-nabil	Radius_length	0,048387	0,072151	NA
3	abundance	X10691	(âˆ’)-nabil	Radius_SOS	0,048786	0,050729	NA
3	abundance	X10691	(âˆ’)-nabil	Tibia_SOS	0,05104	0,054473	NA
3	abundance	X10691	(âˆ’)-nabil	Handgrip	0,018515	0,06153	NA
3	abundance	X10706	Furaneol	Tibia_length	0,062299	0,051773	NA
3	abundance	X10706	Furaneol	Radius_length	0,157073	0,069902	NA
3	abundance	X10706	Furaneol	Radius_SOS	0,007466	0,04982	NA
3	abundance	X10706	Furaneol	Tibia_SOS	0,198195	0,052305	NA
3	abundance	X10706	Furaneol	Handgrip	0,075002	0,060748	NA
3	abundance	X10713	norhaman	Tibia_length	-0,06412	0,056636	NA
3	abundance	X10713	norhaman	Radius_length	-0,05613	0,075	NA
3	abundance	X10713	norhaman	Radius_SOS	-0,12552	0,053759	NA
3	abundance	X10713	norhaman	Tibia_SOS	0,008521	0,05686	NA
3	abundance	X10713	norhaman	Handgrip	-0,04504	0,063131	NA
2b	abundance	X10722	Acetanilide	Tibia_length	0,128947	0,049257	NA
2b	abundance	X10722	Acetanilide	Radius_length	0,175492	0,066015	NA
2b	abundance	X10722	Acetanilide	Radius_SOS	0,076415	0,048886	NA
2b	abundance	X10722	Acetanilide	Tibia_SOS	0,143786	0,051963	NA
2b	abundance	X10722	Acetanilide	Handgrip	0,069061	0,060055	NA
3	abundance	X10725	Ethyl mala	Tibia_length	-0,05379	0,051701	NA
3	abundance	X10725	Ethyl mala	Radius_length	-0,0165	0,071009	NA
3	abundance	X10725	Ethyl mala	Radius_SOS	0,019486	0,049837	NA
3	abundance	X10725	Ethyl mala	Tibia_SOS	0,046422	0,05334	NA
3	abundance	X10725	Ethyl mala	Handgrip	0,119242	0,060927	NA
3	abundance	X10736	Toxopyrimi	Tibia_length	-0,05254	0,05209	NA
3	abundance	X10736	Toxopyrimi	Radius_length	-0,11492	0,070947	NA
3	abundance	X10736	Toxopyrimi	Radius_SOS	0,049583	0,04989	NA
3	abundance	X10736	Toxopyrimi	Tibia_SOS	0,040079	0,053586	NA
3	abundance	X10736	Toxopyrimi	Handgrip	0,075057	0,060849	NA
3	abundance	X10741	5-Nitro-2-p	Tibia_length	0,002812	0,056544	NA
3	abundance	X10741	5-Nitro-2-p	Radius_length	-0,07672	0,074805	NA
3	abundance	X10741	5-Nitro-2-p	Radius_SOS	-0,0287	0,053534	NA
3	abundance	X10741	5-Nitro-2-p	Tibia_SOS	-0,00959	0,059506	NA
3	abundance	X10741	5-Nitro-2-p	Handgrip	-0,10399	0,061231	NA
3	abundance	X10757	Glycyrin	Tibia_length	-0,00565	0,051732	NA
3	abundance	X10757	Glycyrin	Radius_length	-0,12583	0,070149	NA
3	abundance	X10757	Glycyrin	Radius_SOS	-0,00941	0,049835	NA

	3	abundance	X10757	Glycyrin	Tibia_SOS	-0,05387	0,054123	NA
	3	abundance	X10757	Glycyrin	Handgrip	-0,02368	0,061502	NA
	1	abundance	X10769	Cystine	Tibia_length	0,062309	0,049407	NA
	1	abundance	X10769	Cystine	Radius_length	0,051246	0,06679	NA
	1	abundance	X10769	Cystine	Radius_SOS	0,025553	0,049016	NA
	1	abundance	X10769	Cystine	Tibia_SOS	0,053068	0,052413	NA
	1	abundance	X10769	Cystine	Handgrip	0,082813	0,059998	NA
	3	abundance	X10770	2-(4-Isopro	Tibia_length	0,006987	0,056164	NA
	3	abundance	X10770	2-(4-Isopro	Radius_length	0,027084	0,074345	NA
	3	abundance	X10770	2-(4-Isopro	Radius_SOS	0,103392	0,053336	NA
	3	abundance	X10770	2-(4-Isopro	Tibia_SOS	0,009668	0,057353	NA
	3	abundance	X10770	2-(4-Isopro	Handgrip	-0,01218	0,060188	NA
	3	abundance	X10772	Bicine	Tibia_length	0,032505	0,056634	NA
	3	abundance	X10772	Bicine	Radius_length	0,029193	0,074769	NA
	3	abundance	X10772	Bicine	Radius_SOS	0,020097	0,053814	NA
	3	abundance	X10772	Bicine	Tibia_SOS	0,024178	0,057133	NA
	3	abundance	X10772	Bicine	Handgrip	0,098589	0,062753	NA
	3	abundance	X10776	Histidylgly	Tibia_length	-0,03711	0,05514	NA
	3	abundance	X10776	Histidylgly	Radius_length	0,085366	0,0739	NA
	3	abundance	X10776	Histidylgly	Radius_SOS	0,001646	0,052389	NA
	3	abundance	X10776	Histidylgly	Tibia_SOS	0,111027	0,055519	NA
	3	abundance	X10776	Histidylgly	Handgrip	0,116692	0,062416	NA
	3	abundance	X10793	Zalcitabine	Tibia_length	-0,09575	0,055985	NA
	3	abundance	X10793	Zalcitabine	Radius_length	0,025594	0,074459	NA
	3	abundance	X10793	Zalcitabine	Radius_SOS	0,037743	0,053532	NA
	3	abundance	X10793	Zalcitabine	Tibia_SOS	0,081892	0,056274	NA
	3	abundance	X10793	Zalcitabine	Handgrip	0,081323	0,061287	NA
	3	abundance	X10818	butalbital	Tibia_length	0,106893	0,053869	NA
	3	abundance	X10818	butalbital	Radius_length	0,076143	0,073081	NA
	3	abundance	X10818	butalbital	Radius_SOS	0,108841	0,051581	NA
	3	abundance	X10818	butalbital	Tibia_SOS	0,156525	0,054767	NA
	3	abundance	X10818	butalbital	Handgrip	-0,02949	0,062254	NA
2a		abundance	X10819	Hyocholic	Tibia_length	0,038143	0,049433	NA
2a		abundance	X10819	Hyocholic	Radius_length	0,060945	0,066462	NA
2a		abundance	X10819	Hyocholic	Radius_SOS	-0,00398	0,049033	NA
2a		abundance	X10819	Hyocholic	Tibia_SOS	-0,04949	0,052434	NA
2a		abundance	X10819	Hyocholic	Handgrip	-0,03289	0,060519	NA
2b		abundance	X10864	5-amino-2	Tibia_length	-0,07987	0,055059	NA
2b		abundance	X10864	5-amino-2	Radius_length	-0,07844	0,073284	NA
2b		abundance	X10864	5-amino-2	Radius_SOS	0,038973	0,052458	NA
2b		abundance	X10864	5-amino-2	Tibia_SOS	-0,01342	0,054744	NA
2b		abundance	X10864	5-amino-2	Handgrip	-0,15264	0,061085	NA
	3	abundance	X10865	Melatonin	Tibia_length	-0,04615	0,054208	NA
	3	abundance	X10865	Melatonin	Radius_length	0,005964	0,072881	NA
	3	abundance	X10865	Melatonin	Radius_SOS	-0,0184	0,051581	NA
	3	abundance	X10865	Melatonin	Tibia_SOS	0,022843	0,055187	NA
	3	abundance	X10865	Melatonin	Handgrip	0,051998	0,061947	NA
	3	abundance	X10881	L-gamma-(	Tibia_length	-0,02533	0,053342	NA

	3	abundance	X10881	L-gamma-(	Radius_length	-0,02522	0,072749	NA
	3	abundance	X10881	L-gamma-(	Radius_SOS	0,016428	0,050973	NA
	3	abundance	X10881	L-gamma-(	Tibia_SOS	-0,01295	0,054953	NA
	3	abundance	X10881	L-gamma-(	Handgrip	-0,0218	0,060712	NA
	3	abundance	X10886	L-gamma-(	Tibia_length	-0,05113	0,05099	NA
	3	abundance	X10886	L-gamma-(	Radius_length	-0,13952	0,068901	NA
	3	abundance	X10886	L-gamma-(	Radius_SOS	0,004486	0,049336	NA
	3	abundance	X10886	L-gamma-(	Tibia_SOS	-0,03701	0,052809	NA
	3	abundance	X10886	L-gamma-(	Handgrip	-0,08308	0,061035	NA
	3	abundance	X10889	N-acetyl-b	Tibia_length	-0,09487	0,056135	NA
	3	abundance	X10889	N-acetyl-b	Radius_length	0,014562	0,074753	NA
	3	abundance	X10889	N-acetyl-b	Radius_SOS	-0,0155	0,053699	NA
	3	abundance	X10889	N-acetyl-b	Tibia_SOS	0,006646	0,058374	NA
	3	abundance	X10889	N-acetyl-b	Handgrip	-0,0266	0,063209	NA
2b		abundance	X10896	6-Hydroxyr	Tibia_length	0,014688	0,054222	NA
2b		abundance	X10896	6-Hydroxyr	Radius_length	0,030253	0,073073	NA
2b		abundance	X10896	6-Hydroxyr	Radius_SOS	0,042061	0,051616	NA
2b		abundance	X10896	6-Hydroxyr	Tibia_SOS	0,009368	0,054838	NA
2b		abundance	X10896	6-Hydroxyr	Handgrip	-0,04056	0,06143	NA
	3	abundance	X10901	bis-noryan	Tibia_length	0,105883	0,049847	NA
	3	abundance	X10901	bis-noryan	Radius_length	0,242208	0,06637	NA
	3	abundance	X10901	bis-noryan	Radius_SOS	0,062125	0,04896	NA
	3	abundance	X10901	bis-noryan	Tibia_SOS	0,135274	0,052029	NA
	3	abundance	X10901	bis-noryan	Handgrip	0,155631	0,05966	NA
2b		abundance	X10902	Isoquinolir	Tibia_length	0,094877	0,056281	NA
2b		abundance	X10902	Isoquinolir	Radius_length	0,072258	0,074296	NA
2b		abundance	X10902	Isoquinolir	Radius_SOS	-0,0854	0,053906	NA
2b		abundance	X10902	Isoquinolir	Tibia_SOS	0,118345	0,056815	NA
2b		abundance	X10902	Isoquinolir	Handgrip	0,145014	0,062382	NA
	3	abundance	X10918	meprobam	Tibia_length	0,038575	0,050403	NA
	3	abundance	X10918	meprobam	Radius_length	0,091911	0,068864	NA
	3	abundance	X10918	meprobam	Radius_SOS	-5,96E-04	0,049206	NA
	3	abundance	X10918	meprobam	Tibia_SOS	-0,06668	0,052572	NA
	3	abundance	X10918	meprobam	Handgrip	-0,19735	0,059587	NA
	3	abundance	X10929	Temozolon	Tibia_length	-0,04592	0,05636	NA
	3	abundance	X10929	Temozolon	Radius_length	0,047465	0,074883	NA
	3	abundance	X10929	Temozolon	Radius_SOS	0,014445	0,053551	NA
	3	abundance	X10929	Temozolon	Tibia_SOS	0,093054	0,057321	NA
	3	abundance	X10929	Temozolon	Handgrip	0,094927	0,062323	NA
	3	abundance	X10930	MFCD0995	Tibia_length	0,03017	0,054567	NA
	3	abundance	X10930	MFCD0995	Radius_length	0,049861	0,073123	NA
	3	abundance	X10930	MFCD0995	Radius_SOS	0,068189	0,052157	NA
	3	abundance	X10930	MFCD0995	Tibia_SOS	-0,04696	0,054807	NA
	3	abundance	X10930	MFCD0995	Handgrip	-0,1036	0,062953	NA
	3	abundance	X10936	clavulanic	Tibia_length	0,003886	0,05182	NA
	3	abundance	X10936	clavulanic	Radius_length	-0,04436	0,070941	NA
	3	abundance	X10936	clavulanic	Radius_SOS	0,040443	0,049844	NA
	3	abundance	X10936	clavulanic	Tibia_SOS	-0,0234	0,053408	NA

	3	abundance	X10936	clavulanic	Handgrip	-0,07214	0,060639	NA
	3	abundance	X10947	1,1'-[1,12-I	Tibia_length	0,07165	0,050332	NA
	3	abundance	X10947	1,1'-[1,12-I	Radius_length	0,093059	0,068396	NA
	3	abundance	X10947	1,1'-[1,12-I	Radius_SOS	0,005253	0,049176	NA
	3	abundance	X10947	1,1'-[1,12-I	Tibia_SOS	-0,01685	0,052742	NA
	3	abundance	X10947	1,1'-[1,12-I	Handgrip	0,008886	0,060584	NA
	3	abundance	X10951	8-Methyl-8	Tibia_length	-0,03872	0,049372	NA
	3	abundance	X10951	8-Methyl-8	Radius_length	0,030798	0,066519	NA
	3	abundance	X10951	8-Methyl-8	Radius_SOS	-0,05605	0,048968	NA
	3	abundance	X10951	8-Methyl-8	Tibia_SOS	-0,03112	0,052593	NA
	3	abundance	X10951	8-Methyl-8	Handgrip	-0,02624	0,06018	NA
	3	abundance	X10953	Vorinostat	Tibia_length	-0,03339	0,051647	NA
	3	abundance	X10953	Vorinostat	Radius_length	-0,04915	0,070354	NA
	3	abundance	X10953	Vorinostat	Radius_SOS	-5,24E-04	0,04976	NA
	3	abundance	X10953	Vorinostat	Tibia_SOS	0,026273	0,053178	NA
	3	abundance	X10953	Vorinostat	Handgrip	0,030962	0,061654	NA
	3	abundance	X10974	Threonylgl	Tibia_length	-0,04208	0,056882	NA
	3	abundance	X10974	Threonylgl	Radius_length	0,147572	0,074226	NA
	3	abundance	X10974	Threonylgl	Radius_SOS	-0,02341	0,054196	NA
	3	abundance	X10974	Threonylgl	Tibia_SOS	0,006775	0,057345	NA
	3	abundance	X10974	Threonylgl	Handgrip	-0,03421	0,062758	NA
	3	abundance	X10977	pentobarbi	Tibia_length	-0,08498	0,055257	NA
	3	abundance	X10977	pentobarbi	Radius_length	-0,17664	0,072591	NA
	3	abundance	X10977	pentobarbi	Radius_SOS	-7,15E-04	0,05233	NA
	3	abundance	X10977	pentobarbi	Tibia_SOS	-0,0589	0,056745	NA
	3	abundance	X10977	pentobarbi	Handgrip	-0,04888	0,063438	NA
	3	abundance	X10979	acetyltauri	Tibia_length	-0,02733	0,053843	NA
	3	abundance	X10979	acetyltauri	Radius_length	-0,1109	0,072916	NA
	3	abundance	X10979	acetyltauri	Radius_SOS	-0,0386	0,051644	NA
	3	abundance	X10979	acetyltauri	Tibia_SOS	-0,01852	0,055653	NA
	3	abundance	X10979	acetyltauri	Handgrip	-0,00162	0,062145	NA
	3	abundance	X10982	8-Methyl-8	Tibia_length	-0,04096	0,049941	NA
	3	abundance	X10982	8-Methyl-8	Radius_length	0,004285	0,068592	NA
	3	abundance	X10982	8-Methyl-8	Radius_SOS	-0,05465	0,048989	NA
	3	abundance	X10982	8-Methyl-8	Tibia_SOS	-0,01959	0,052593	NA
	3	abundance	X10982	8-Methyl-8	Handgrip	-0,14553	0,060037	NA
	3	abundance	X10987	2-BUTYL PI	Tibia_length	0,010237	0,051698	NA
	3	abundance	X10987	2-BUTYL PI	Radius_length	0,023515	0,070827	NA
	3	abundance	X10987	2-BUTYL PI	Radius_SOS	-0,00316	0,049827	NA
	3	abundance	X10987	2-BUTYL PI	Tibia_SOS	0,109739	0,053079	NA
	3	abundance	X10987	2-BUTYL PI	Handgrip	0,014175	0,061001	NA
2b		abundance	X11001	2-[(carboxy	Tibia_length	-0,01399	0,053498	NA
2b		abundance	X11001	2-[(carboxy	Radius_length	0,084887	0,072483	NA
2b		abundance	X11001	2-[(carboxy	Radius_SOS	0,065543	0,050977	NA
2b		abundance	X11001	2-[(carboxy	Tibia_SOS	0,072588	0,054805	NA
2b		abundance	X11001	2-[(carboxy	Handgrip	-0,05231	0,062091	NA
	3	abundance	X11012	Furan	Tibia_length	0,005922	0,054961	NA
	3	abundance	X11012	Furan	Radius_length	-0,01515	0,073789	NA

	3	abundance	X11012	Furan	Radius_SOS	-0,07174	0,052307	NA
	3	abundance	X11012	Furan	Tibia_SOS	0,107196	0,056423	NA
	3	abundance	X11012	Furan	Handgrip	0,060533	0,062823	NA
	3	abundance	X11028	butalbital	Tibia_length	-0,11738	0,054105	NA
	3	abundance	X11028	butalbital	Radius_length	-0,09058	0,072769	NA
	3	abundance	X11028	butalbital	Radius_SOS	0,041577	0,051958	NA
	3	abundance	X11028	butalbital	Tibia_SOS	-0,02498	0,055062	NA
	3	abundance	X11028	butalbital	Handgrip	-0,07645	0,061585	NA
	3	abundance	X11032	2,4-Bis(3-r	Tibia_length	0,05747	0,050319	NA
	3	abundance	X11032	2,4-Bis(3-r	Radius_length	0,201492	0,067469	NA
	3	abundance	X11032	2,4-Bis(3-r	Radius_SOS	0,116883	0,048836	NA
	3	abundance	X11032	2,4-Bis(3-r	Tibia_SOS	0,048286	0,052499	NA
	3	abundance	X11032	2,4-Bis(3-r	Handgrip	0,039523	0,060431	NA
	3	abundance	X11033	N-Acetyl-5	Tibia_length	-6,69E-05	0,052785	NA
	3	abundance	X11033	N-Acetyl-5	Radius_length	0,079828	0,072253	NA
	3	abundance	X11033	N-Acetyl-5	Radius_SOS	0,036202	0,050683	NA
	3	abundance	X11033	N-Acetyl-5	Tibia_SOS	-0,05233	0,053333	NA
	3	abundance	X11033	N-Acetyl-5	Handgrip	-0,11752	0,060882	NA
	3	abundance	X11034	1-(3-Aminc	Tibia_length	-0,12681	0,053672	NA
	3	abundance	X11034	1-(3-Aminc	Radius_length	-0,05969	0,072663	NA
	3	abundance	X11034	1-(3-Aminc	Radius_SOS	-0,02466	0,051459	NA
	3	abundance	X11034	1-(3-Aminc	Tibia_SOS	-0,06057	0,055662	NA
	3	abundance	X11034	1-(3-Aminc	Handgrip	-0,04432	0,062609	NA
2b		abundance	X11036	3-Hydroxyp	Tibia_length	0,154582	0,049942	NA
2b		abundance	X11036	3-Hydroxyp	Radius_length	0,283469	0,066193	NA
2b		abundance	X11036	3-Hydroxyp	Radius_SOS	0,044317	0,049114	NA
2b		abundance	X11036	3-Hydroxyp	Tibia_SOS	0,162141	0,051953	NA
2b		abundance	X11036	3-Hydroxyp	Handgrip	0,093731	0,060032	NA
	3	abundance	X11038	(2E)-3-(3,4	Tibia_length	0,021091	0,055342	NA
	3	abundance	X11038	(2E)-3-(3,4	Radius_length	0,010551	0,073882	NA
	3	abundance	X11038	(2E)-3-(3,4	Radius_SOS	0,130081	0,052342	NA
	3	abundance	X11038	(2E)-3-(3,4	Tibia_SOS	-0,0317	0,055508	NA
	3	abundance	X11038	(2E)-3-(3,4	Handgrip	0,052698	0,062053	NA
	3	abundance	X11042	Leu-arg	Tibia_length	-0,15314	0,055276	NA
	3	abundance	X11042	Leu-arg	Radius_length	-0,04031	0,073802	NA
	3	abundance	X11042	Leu-arg	Radius_SOS	-0,05549	0,05233	NA
	3	abundance	X11042	Leu-arg	Tibia_SOS	-0,06943	0,056619	NA
	3	abundance	X11042	Leu-arg	Handgrip	0,032876	0,062707	NA
	3	abundance	X11059	Dihydrocoı	Tibia_length	0,021791	0,050081	NA
	3	abundance	X11059	Dihydrocoı	Radius_length	0,058686	0,068159	NA
	3	abundance	X11059	Dihydrocoı	Radius_SOS	-0,01914	0,049071	NA
	3	abundance	X11059	Dihydrocoı	Tibia_SOS	0,041161	0,052453	NA
	3	abundance	X11059	Dihydrocoı	Handgrip	0,088915	0,059976	NA
	3	abundance	X11067	Resveratro	Tibia_length	0,123911	0,051632	NA
	3	abundance	X11067	Resveratro	Radius_length	0,17658	0,070064	NA
	3	abundance	X11067	Resveratro	Radius_SOS	0,031288	0,049892	NA
	3	abundance	X11067	Resveratro	Tibia_SOS	0,14165	0,052632	NA
	3	abundance	X11067	Resveratro	Handgrip	0,004884	0,060766	NA

	3	abundance	X11068	N,N'-Bis[4-	Tibia_length	-0,01527	0,052744	NA
	3	abundance	X11068	N,N'-Bis[4-	Radius_length	-0,02427	0,071838	NA
	3	abundance	X11068	N,N'-Bis[4-	Radius_SOS	0,030367	0,050734	NA
	3	abundance	X11068	N,N'-Bis[4-	Tibia_SOS	0,078151	0,053998	NA
	3	abundance	X11068	N,N'-Bis[4-	Handgrip	-0,01289	0,061604	NA
	3	abundance	X11110	Tauropine	Tibia_length	-0,1275	0,056303	NA
	3	abundance	X11110	Tauropine	Radius_length	-0,11531	0,074153	NA
	3	abundance	X11110	Tauropine	Radius_SOS	0,04279	0,05323	NA
	3	abundance	X11110	Tauropine	Tibia_SOS	-0,03995	0,0571	NA
	3	abundance	X11110	Tauropine	Handgrip	0,040631	0,061847	NA
	3	abundance	X11115	Hydroxycai	Tibia_length	-0,15206	0,054479	NA
	3	abundance	X11115	Hydroxycai	Radius_length	-0,05765	0,073295	NA
	3	abundance	X11115	Hydroxycai	Radius_SOS	-0,00158	0,052031	NA
	3	abundance	X11115	Hydroxycai	Tibia_SOS	-0,03997	0,055322	NA
	3	abundance	X11115	Hydroxycai	Handgrip	0,049654	0,06181	NA
	3	abundance	X11117	Coprine	Tibia_length	7,70E-04	0,050897	NA
	3	abundance	X11117	Coprine	Radius_length	0,110585	0,069489	NA
	3	abundance	X11117	Coprine	Radius_SOS	-0,00908	0,049409	NA
	3	abundance	X11117	Coprine	Tibia_SOS	0,03715	0,053233	NA
	3	abundance	X11117	Coprine	Handgrip	0,033942	0,060448	NA
	3	abundance	X11125	5,8,12-Trih	Tibia_length	-0,08103	0,050469	NA
	3	abundance	X11125	5,8,12-Trih	Radius_length	-0,09259	0,068834	NA
	3	abundance	X11125	5,8,12-Trih	Radius_SOS	0,044237	0,049182	NA
	3	abundance	X11125	5,8,12-Trih	Tibia_SOS	-0,01126	0,052663	NA
	3	abundance	X11125	5,8,12-Trih	Handgrip	0,001447	0,060754	NA
	3	abundance	X11140	Leu-Leu	Tibia_length	-0,02843	0,053445	NA
	3	abundance	X11140	Leu-Leu	Radius_length	-0,02712	0,072422	NA
	3	abundance	X11140	Leu-Leu	Radius_SOS	-0,05183	0,051209	NA
	3	abundance	X11140	Leu-Leu	Tibia_SOS	-0,07024	0,053968	NA
	3	abundance	X11140	Leu-Leu	Handgrip	-0,05668	0,06143	NA
	3	abundance	X11163	Lys-Pro	Tibia_length	0,055175	0,049348	NA
	3	abundance	X11163	Lys-Pro	Radius_length	0,126984	0,06626	NA
	3	abundance	X11163	Lys-Pro	Radius_SOS	0,015498	0,049033	NA
	3	abundance	X11163	Lys-Pro	Tibia_SOS	-0,06483	0,052485	NA
	3	abundance	X11163	Lys-Pro	Handgrip	-0,03495	0,060175	NA
	3	abundance	X11170	QJ972000(	Tibia_length	0,164479	0,051997	NA
	3	abundance	X11170	QJ972000(	Radius_length	0,115252	0,071309	NA
	3	abundance	X11170	QJ972000(	Radius_SOS	-0,05064	0,050367	NA
	3	abundance	X11170	QJ972000(	Tibia_SOS	0,112584	0,053292	NA
	3	abundance	X11170	QJ972000(	Handgrip	0,120497	0,06039	NA
2b		abundance	X11186	Fluocinolo	Tibia_length	-0,00747	0,055504	NA
2b		abundance	X11186	Fluocinolo	Radius_length	-0,0312	0,073521	NA
2b		abundance	X11186	Fluocinolo	Radius_SOS	0,005775	0,052835	NA
2b		abundance	X11186	Fluocinolo	Tibia_SOS	-0,02304	0,056185	NA
2b		abundance	X11186	Fluocinolo	Handgrip	0,019418	0,063137	NA
	3	abundance	X11215	Leu-Leu	Tibia_length	-0,02857	0,052224	NA
	3	abundance	X11215	Leu-Leu	Radius_length	-0,13999	0,070634	NA
	3	abundance	X11215	Leu-Leu	Radius_SOS	-0,03474	0,050065	NA

	3	abundance	X11215	Leu-Leu	Tibia_SOS	-0,00671	0,054141	NA
	3	abundance	X11215	Leu-Leu	Handgrip	-0,07461	0,061256	NA
	3	abundance	X11223	Ethyl mala	Tibia_length	-0,0855	0,058623	NA
	3	abundance	X11223	Ethyl mala	Radius_length	-0,00473	0,075831	NA
	3	abundance	X11223	Ethyl mala	Radius_SOS	0,045072	0,056106	NA
	3	abundance	X11223	Ethyl mala	Tibia_SOS	-0,13618	0,057606	NA
	3	abundance	X11223	Ethyl mala	Handgrip	0,00638	0,063408	NA
	3	abundance	X11245	MFCD0003	Tibia_length	-0,13827	0,055616	NA
	3	abundance	X11245	MFCD0003	Radius_length	-0,0312	0,07455	NA
	3	abundance	X11245	MFCD0003	Radius_SOS	-0,02675	0,053522	NA
	3	abundance	X11245	MFCD0003	Tibia_SOS	-0,06403	0,057654	NA
	3	abundance	X11245	MFCD0003	Handgrip	0,025606	0,062971	NA
	3	abundance	X11251	N-Nonano	Tibia_length	-0,04286	0,052558	NA
	3	abundance	X11251	N-Nonano	Radius_length	-0,01734	0,071696	NA
	3	abundance	X11251	N-Nonano	Radius_SOS	-0,02683	0,050399	NA
	3	abundance	X11251	N-Nonano	Tibia_SOS	0,096789	0,054557	NA
	3	abundance	X11251	N-Nonano	Handgrip	0,039805	0,061807	NA
	3	abundance	X11254	(4R,5S,9S,	Tibia_length	-0,03232	0,049804	NA
	3	abundance	X11254	(4R,5S,9S,	Radius_length	-0,03125	0,067627	NA
	3	abundance	X11254	(4R,5S,9S,	Radius_SOS	0,079106	0,048885	NA
	3	abundance	X11254	(4R,5S,9S,	Tibia_SOS	0,034428	0,052462	NA
	3	abundance	X11254	(4R,5S,9S,	Handgrip	-0,02885	0,06018	NA
2b		abundance	X11257	1-Methylgu	Tibia_length	-0,05844	0,052625	NA
2b		abundance	X11257	1-Methylgu	Radius_length	0,007934	0,071654	NA
2b		abundance	X11257	1-Methylgu	Radius_SOS	-0,1174	0,050527	NA
2b		abundance	X11257	1-Methylgu	Tibia_SOS	0,052529	0,053639	NA
2b		abundance	X11257	1-Methylgu	Handgrip	0,03968	0,061718	NA
	3	abundance	X11258	Valyl-4-hyc	Tibia_length	0,049789	0,053947	NA
	3	abundance	X11258	Valyl-4-hyc	Radius_length	0,028295	0,07267	NA
	3	abundance	X11258	Valyl-4-hyc	Radius_SOS	0,04049	0,05153	NA
	3	abundance	X11258	Valyl-4-hyc	Tibia_SOS	0,022851	0,054707	NA
	3	abundance	X11258	Valyl-4-hyc	Handgrip	-0,03503	0,061812	NA
	3	abundance	X11265	Sulfurol	Tibia_length	-0,06036	0,055286	NA
	3	abundance	X11265	Sulfurol	Radius_length	-0,0806	0,073367	NA
	3	abundance	X11265	Sulfurol	Radius_SOS	-0,03619	0,05258	NA
	3	abundance	X11265	Sulfurol	Tibia_SOS	0,076923	0,055348	NA
	3	abundance	X11265	Sulfurol	Handgrip	-0,06465	0,062023	NA
	3	abundance	X11273	trp-pro	Tibia_length	-0,01046	0,051215	NA
	3	abundance	X11273	trp-pro	Radius_length	0,05933	0,070151	NA
	3	abundance	X11273	trp-pro	Radius_SOS	-0,05671	0,049468	NA
	3	abundance	X11273	trp-pro	Tibia_SOS	0,024148	0,053354	NA
	3	abundance	X11273	trp-pro	Handgrip	0,047567	0,06069	NA
	3	abundance	X11277	lys-leu	Tibia_length	0,082731	0,053552	NA
	3	abundance	X11277	lys-leu	Radius_length	0,042809	0,072576	NA
	3	abundance	X11277	lys-leu	Radius_SOS	0,01048	0,051372	NA
	3	abundance	X11277	lys-leu	Tibia_SOS	0,035179	0,055896	NA
	3	abundance	X11277	lys-leu	Handgrip	0,122517	0,062819	NA
	3	abundance	X11281	Penbutolol	Tibia_length	0,050149	0,057084	NA

3	abundance	X11281	Penbutolol	Radius_length	0,135145	0,074786	NA
3	abundance	X11281	Penbutolol	Radius_SOS	0,003582	0,054273	NA
3	abundance	X11281	Penbutolol	Tibia_SOS	-0,04431	0,057266	NA
3	abundance	X11281	Penbutolol	Handgrip	0,082006	0,062885	NA
3	abundance	X11301	(6S)-2-Ami	Tibia_length	-0,04977	0,053886	NA
3	abundance	X11301	(6S)-2-Ami	Radius_length	-0,06583	0,072815	NA
3	abundance	X11301	(6S)-2-Ami	Radius_SOS	-0,07793	0,0511	NA
3	abundance	X11301	(6S)-2-Ami	Tibia_SOS	-0,04428	0,053923	NA
3	abundance	X11301	(6S)-2-Ami	Handgrip	0,019104	0,061905	NA
3	abundance	X11310	Leu-Val	Tibia_length	0,15967	0,05192	NA
3	abundance	X11310	Leu-Val	Radius_length	0,103905	0,071555	NA
3	abundance	X11310	Leu-Val	Radius_SOS	0,079713	0,050278	NA
3	abundance	X11310	Leu-Val	Tibia_SOS	0,090358	0,053965	NA
3	abundance	X11310	Leu-Val	Handgrip	0,075329	0,061548	NA
3	abundance	X11319	Methohexit	Tibia_length	0,037731	0,054617	NA
3	abundance	X11319	Methohexit	Radius_length	0,041965	0,074187	NA
3	abundance	X11319	Methohexit	Radius_SOS	-0,02423	0,051814	NA
3	abundance	X11319	Methohexit	Tibia_SOS	-0,02155	0,054849	NA
3	abundance	X11319	Methohexit	Handgrip	0,01476	0,061295	NA
3	abundance	X11323	dihydroxyb	Tibia_length	-0,06516	0,049668	NA
3	abundance	X11323	dihydroxyb	Radius_length	0,073088	0,068072	NA
3	abundance	X11323	dihydroxyb	Radius_SOS	-1,77E-04	0,049064	NA
3	abundance	X11323	dihydroxyb	Tibia_SOS	0,01664	0,052522	NA
3	abundance	X11323	dihydroxyb	Handgrip	-0,00423	0,0606	NA
3	abundance	X11331	N~6~-Octa	Tibia_length	-0,01528	0,049689	NA
3	abundance	X11331	N~6~-Octa	Radius_length	-0,06255	0,067199	NA
3	abundance	X11331	N~6~-Octa	Radius_SOS	0,003637	0,049033	NA
3	abundance	X11331	N~6~-Octa	Tibia_SOS	-0,0907	0,052282	NA
3	abundance	X11331	N~6~-Octa	Handgrip	-0,07715	0,060069	NA
3	abundance	X11339	L-gamma-(	Tibia_length	-0,03176	0,051125	NA
3	abundance	X11339	L-gamma-(	Radius_length	-0,0691	0,070062	NA
3	abundance	X11339	L-gamma-(	Radius_SOS	-0,01232	0,049565	NA
3	abundance	X11339	L-gamma-(	Tibia_SOS	0,00387	0,05336	NA
3	abundance	X11339	L-gamma-(	Handgrip	-0,04464	0,061119	NA
3	abundance	X11347	Voglibose	Tibia_length	-0,0196	0,052181	NA
3	abundance	X11347	Voglibose	Radius_length	-0,08421	0,071951	NA
3	abundance	X11347	Voglibose	Radius_SOS	-0,04241	0,049997	NA
3	abundance	X11347	Voglibose	Tibia_SOS	-0,07152	0,053378	NA
3	abundance	X11347	Voglibose	Handgrip	-0,17173	0,060593	NA
3	abundance	X11349	Butabarbit	Tibia_length	-0,08249	0,053622	NA
3	abundance	X11349	Butabarbit	Radius_length	-0,11782	0,071922	NA
3	abundance	X11349	Butabarbit	Radius_SOS	-0,01501	0,051275	NA
3	abundance	X11349	Butabarbit	Tibia_SOS	-0,07096	0,053109	NA
3	abundance	X11349	Butabarbit	Handgrip	-0,17799	0,060289	NA
3	abundance	X11354	3-Oxo-4,6-	Tibia_length	0,045547	0,050951	NA
3	abundance	X11354	3-Oxo-4,6-	Radius_length	0,070868	0,069616	NA
3	abundance	X11354	3-Oxo-4,6-	Radius_SOS	0,09886	0,049199	NA
3	abundance	X11354	3-Oxo-4,6-	Tibia_SOS	-0,02946	0,0531	NA

3	abundance	X11354	3-Oxo-4,6-	Handgrip	-0,04925	0,061154	NA
3	abundance	X11361	1-Methylhi	Tibia_length	-0,138	0,048774	NA
3	abundance	X11361	1-Methylhi	Radius_length	-0,19979	0,064294	NA
3	abundance	X11361	1-Methylhi	Radius_SOS	-0,04799	0,049113	NA
3	abundance	X11361	1-Methylhi	Tibia_SOS	-0,0758	0,052655	NA
3	abundance	X11361	1-Methylhi	Handgrip	-0,06356	0,060083	NA
3	abundance	X11366	3-Sulfino-L	Tibia_length	-0,05369	0,054639	NA
3	abundance	X11366	3-Sulfino-L	Radius_length	-0,11181	0,073327	NA
3	abundance	X11366	3-Sulfino-L	Radius_SOS	-0,06479	0,052338	NA
3	abundance	X11366	3-Sulfino-L	Tibia_SOS	-0,0365	0,056494	NA
3	abundance	X11366	3-Sulfino-L	Handgrip	0,016662	0,062185	NA
2b	abundance	X11373	3-Succinoy	Tibia_length	-0,08815	0,052017	NA
2b	abundance	X11373	3-Succinoy	Radius_length	-0,14358	0,070384	NA
2b	abundance	X11373	3-Succinoy	Radius_SOS	-0,04443	0,049868	NA
2b	abundance	X11373	3-Succinoy	Tibia_SOS	-0,03881	0,053791	NA
2b	abundance	X11373	3-Succinoy	Handgrip	-0,05022	0,060964	NA
3	abundance	X11375	L-gamma-(	Tibia_length	0,063048	0,054491	NA
3	abundance	X11375	L-gamma-(	Radius_length	0,041386	0,07344	NA
3	abundance	X11375	L-gamma-(	Radius_SOS	-0,08991	0,051708	NA
3	abundance	X11375	L-gamma-(	Tibia_SOS	0,178839	0,055505	NA
3	abundance	X11375	L-gamma-(	Handgrip	0,053698	0,062104	NA
3	abundance	X11383	N-{3-[(4-Ac	Tibia_length	0,00298	0,055386	NA
3	abundance	X11383	N-{3-[(4-Ac	Radius_length	-0,04597	0,073342	NA
3	abundance	X11383	N-{3-[(4-Ac	Radius_SOS	-0,05043	0,052701	NA
3	abundance	X11383	N-{3-[(4-Ac	Tibia_SOS	-0,04596	0,057415	NA
3	abundance	X11383	N-{3-[(4-Ac	Handgrip	-0,06556	0,060063	NA
3	abundance	X11408	5-Amino-6	Tibia_length	-0,12883	0,053115	NA
3	abundance	X11408	5-Amino-6	Radius_length	-0,13989	0,071446	NA
3	abundance	X11408	5-Amino-6	Radius_SOS	-0,11937	0,050485	NA
3	abundance	X11408	5-Amino-6	Tibia_SOS	-0,07694	0,053388	NA
3	abundance	X11408	5-Amino-6	Handgrip	-0,04392	0,06085	NA
3	abundance	X11421	8-Amino-7	Tibia_length	-0,03937	0,053097	NA
3	abundance	X11421	8-Amino-7	Radius_length	0,028334	0,072032	NA
3	abundance	X11421	8-Amino-7	Radius_SOS	0,053583	0,050742	NA
3	abundance	X11421	8-Amino-7	Tibia_SOS	-0,03173	0,054387	NA
3	abundance	X11421	8-Amino-7	Handgrip	-0,105	0,060766	NA
3	abundance	X11438	pro-met	Tibia_length	-0,02797	0,054013	NA
3	abundance	X11438	pro-met	Radius_length	0,025082	0,072991	NA
3	abundance	X11438	pro-met	Radius_SOS	-0,02456	0,051506	NA
3	abundance	X11438	pro-met	Tibia_SOS	-0,05996	0,055043	NA
3	abundance	X11438	pro-met	Handgrip	0,02725	0,061204	NA
3	abundance	X11441	beta-D-Eth	Tibia_length	-0,10641	0,053195	NA
3	abundance	X11441	beta-D-Eth	Radius_length	-0,1006	0,07253	NA
3	abundance	X11441	beta-D-Eth	Radius_SOS	-0,05544	0,050873	NA
3	abundance	X11441	beta-D-Eth	Tibia_SOS	0,017786	0,054969	NA
3	abundance	X11441	beta-D-Eth	Handgrip	-0,15857	0,061543	NA
3	abundance	X11477	gamma-Gl	Tibia_length	-0,07708	0,055681	NA
3	abundance	X11477	gamma-Gl	Radius_length	-0,09242	0,073939	NA

3	abundance	X11477	gamma-Gl	Radius_SOS	-0,0478	0,053228	NA
3	abundance	X11477	gamma-Gl	Tibia_SOS	-0,03769	0,056207	NA
3	abundance	X11477	gamma-Gl	Handgrip	-0,05539	0,060101	NA
3	abundance	X11481	his-gln	Tibia_length	-0,07784	0,052138	NA
3	abundance	X11481	his-gln	Radius_length	-0,06042	0,070812	NA
3	abundance	X11481	his-gln	Radius_SOS	-0,04276	0,049924	NA
3	abundance	X11481	his-gln	Tibia_SOS	-0,05205	0,054098	NA
3	abundance	X11481	his-gln	Handgrip	0,056883	0,062025	NA
3	abundance	X11494	3-Hydroxys	Tibia_length	-0,09187	0,053335	NA
3	abundance	X11494	3-Hydroxys	Radius_length	-0,11454	0,07192	NA
3	abundance	X11494	3-Hydroxys	Radius_SOS	0,0612	0,050779	NA
3	abundance	X11494	3-Hydroxys	Tibia_SOS	-0,14401	0,053197	NA
3	abundance	X11494	3-Hydroxys	Handgrip	-0,12054	0,061196	NA
3	abundance	X11498	Valylvaline	Tibia_length	-0,06828	0,054397	NA
3	abundance	X11498	Valylvaline	Radius_length	-0,13512	0,072368	NA
3	abundance	X11498	Valylvaline	Radius_SOS	0,014875	0,051842	NA
3	abundance	X11498	Valylvaline	Tibia_SOS	0,051739	0,057604	NA
3	abundance	X11498	Valylvaline	Handgrip	-0,00372	0,062693	NA
3	abundance	X11505	FB950000(	Tibia_length	-0,006	0,055718	NA
3	abundance	X11505	FB950000(	Radius_length	0,002042	0,073806	NA
3	abundance	X11505	FB950000(	Radius_SOS	0,037275	0,05309	NA
3	abundance	X11505	FB950000(	Tibia_SOS	-0,01138	0,055064	NA
3	abundance	X11505	FB950000(	Handgrip	-0,02719	0,061936	NA
2b	abundance	X11515	13(S)-HOT	Tibia_length	0,078608	0,054567	NA
2b	abundance	X11515	13(S)-HOT	Radius_length	0,110711	0,073306	NA
2b	abundance	X11515	13(S)-HOT	Radius_SOS	0,024304	0,051932	NA
2b	abundance	X11515	13(S)-HOT	Tibia_SOS	0,024247	0,056115	NA
2b	abundance	X11515	13(S)-HOT	Handgrip	-0,04113	0,062716	NA
3	abundance	X11522	thr-trp	Tibia_length	-0,09404	0,054379	NA
3	abundance	X11522	thr-trp	Radius_length	-0,13636	0,072342	NA
3	abundance	X11522	thr-trp	Radius_SOS	-0,10162	0,05128	NA
3	abundance	X11522	thr-trp	Tibia_SOS	-0,03325	0,056232	NA
3	abundance	X11522	thr-trp	Handgrip	0,041312	0,062715	NA
3	abundance	X11524	6-hydroxyp	Tibia_length	-0,09517	0,057523	NA
3	abundance	X11524	6-hydroxyp	Radius_length	-0,06374	0,074603	NA
3	abundance	X11524	6-hydroxyp	Radius_SOS	-0,02021	0,055202	NA
3	abundance	X11524	6-hydroxyp	Tibia_SOS	-0,06879	0,057552	NA
3	abundance	X11524	6-hydroxyp	Handgrip	-0,08084	0,059996	NA
2b	abundance	X11529	2-Aminooc	Tibia_length	-0,04954	0,054866	NA
2b	abundance	X11529	2-Aminooc	Radius_length	-0,01834	0,073182	NA
2b	abundance	X11529	2-Aminooc	Radius_SOS	-0,03451	0,052209	NA
2b	abundance	X11529	2-Aminooc	Tibia_SOS	0,038232	0,055951	NA
2b	abundance	X11529	2-Aminooc	Handgrip	-0,07999	0,061521	NA
3	abundance	X11549	1,1'-[1,12-I	Tibia_length	-0,00803	0,051711	NA
3	abundance	X11549	1,1'-[1,12-I	Radius_length	-0,03138	0,070721	NA
3	abundance	X11549	1,1'-[1,12-I	Radius_SOS	0,113276	0,049445	NA
3	abundance	X11549	1,1'-[1,12-I	Tibia_SOS	-7,27E-04	0,053353	NA
3	abundance	X11549	1,1'-[1,12-I	Handgrip	0,118909	0,060228	NA

3	abundance	X11556	N-[(2E)-3-(	Tibia_length	0,102347	0,050752	NA
3	abundance	X11556	N-[(2E)-3-(	Radius_length	0,115514	0,069545	NA
3	abundance	X11556	N-[(2E)-3-(	Radius_SOS	0,010461	0,049411	NA
3	abundance	X11556	N-[(2E)-3-(	Tibia_SOS	0,123794	0,052364	NA
3	abundance	X11556	N-[(2E)-3-(	Handgrip	0,059561	0,060136	NA
3	abundance	X11562	ALA-PRO	Tibia_length	0,018269	0,056659	NA
3	abundance	X11562	ALA-PRO	Radius_length	0,002278	0,074568	NA
3	abundance	X11562	ALA-PRO	Radius_SOS	0,083006	0,053723	NA
3	abundance	X11562	ALA-PRO	Tibia_SOS	0,01613	0,05714	NA
3	abundance	X11562	ALA-PRO	Handgrip	-0,01191	0,060189	NA
3	abundance	X11563	threonylph	Tibia_length	-0,08928	0,051104	NA
3	abundance	X11563	threonylph	Radius_length	-0,14826	0,069605	NA
3	abundance	X11563	threonylph	Radius_SOS	-0,00359	0,049621	NA
3	abundance	X11563	threonylph	Tibia_SOS	-0,06253	0,052621	NA
3	abundance	X11563	threonylph	Handgrip	-0,1745	0,060415	NA
3	abundance	X11576	Phenyl D-g	Tibia_length	-0,0362	0,055726	NA
3	abundance	X11576	Phenyl D-g	Radius_length	-0,20399	0,072257	NA
3	abundance	X11576	Phenyl D-g	Radius_SOS	-0,10155	0,052928	NA
3	abundance	X11576	Phenyl D-g	Tibia_SOS	-0,0862	0,056726	NA
3	abundance	X11576	Phenyl D-g	Handgrip	-0,07188	0,063099	NA
3	abundance	X11605	3-Methyl-2	Tibia_length	0,12315	0,052268	NA
3	abundance	X11605	3-Methyl-2	Radius_length	0,148259	0,071401	NA
3	abundance	X11605	3-Methyl-2	Radius_SOS	0,066732	0,05014	NA
3	abundance	X11605	3-Methyl-2	Tibia_SOS	0,093133	0,05395	NA
3	abundance	X11605	3-Methyl-2	Handgrip	0,181612	0,05966	NA
3	abundance	X11614	2-Methylth	Tibia_length	0,170678	0,050023	NA
3	abundance	X11614	2-Methylth	Radius_length	0,176449	0,067733	NA
3	abundance	X11614	2-Methylth	Radius_SOS	0,058042	0,049085	NA
3	abundance	X11614	2-Methylth	Tibia_SOS	0,145524	0,052229	NA
3	abundance	X11614	2-Methylth	Handgrip	0,120413	0,059757	NA
3	abundance	X11627	N-(3-aceta	Tibia_length	0,017375	0,050788	NA
3	abundance	X11627	N-(3-aceta	Radius_length	0,096769	0,069074	NA
3	abundance	X11627	N-(3-aceta	Radius_SOS	0,068749	0,049236	NA
3	abundance	X11627	N-(3-aceta	Tibia_SOS	-0,03225	0,052902	NA
3	abundance	X11627	N-(3-aceta	Handgrip	0,035361	0,061098	NA
3	abundance	X11639	Midodrine	Tibia_length	-0,14048	0,057077	NA
3	abundance	X11639	Midodrine	Radius_length	-0,08227	0,075202	NA
3	abundance	X11639	Midodrine	Radius_SOS	-0,03211	0,054011	NA
3	abundance	X11639	Midodrine	Tibia_SOS	-0,17998	0,056494	NA
3	abundance	X11639	Midodrine	Handgrip	-0,13347	0,06216	NA
3	abundance	X11645	glu-thr	Tibia_length	-0,02041	0,055833	NA
3	abundance	X11645	glu-thr	Radius_length	0,021987	0,074674	NA
3	abundance	X11645	glu-thr	Radius_SOS	-0,00421	0,053039	NA
3	abundance	X11645	glu-thr	Tibia_SOS	0,0327	0,056886	NA
3	abundance	X11645	glu-thr	Handgrip	-0,00615	0,062272	NA
3	abundance	X11666	N-(4-Hydro	Tibia_length	-0,15223	0,050254	NA
3	abundance	X11666	N-(4-Hydro	Radius_length	-0,12625	0,069036	NA
3	abundance	X11666	N-(4-Hydro	Radius_SOS	-0,03871	0,049291	NA

3	abundance	X11666	N-(4-Hydrc	Tibia_SOS	-0,09268	0,052491	NA
3	abundance	X11666	N-(4-Hydrc	Handgrip	-0,11245	0,05999	NA
3	abundance	X11667	Tetraacety	Tibia_length	-0,0303	0,05296	NA
3	abundance	X11667	Tetraacety	Radius_length	-0,03054	0,072531	NA
3	abundance	X11667	Tetraacety	Radius_SOS	-0,01102	0,050721	NA
3	abundance	X11667	Tetraacety	Tibia_SOS	0,02071	0,054975	NA
3	abundance	X11667	Tetraacety	Handgrip	-0,05568	0,061777	NA
3	abundance	X11692	2_7-Anhyd	Tibia_length	-0,00575	0,053459	NA
3	abundance	X11692	2_7-Anhyd	Radius_length	0,056315	0,072835	NA
3	abundance	X11692	2_7-Anhyd	Radius_SOS	-0,01301	0,051093	NA
3	abundance	X11692	2_7-Anhyd	Tibia_SOS	0,022063	0,054457	NA
3	abundance	X11692	2_7-Anhyd	Handgrip	0,084921	0,062466	NA
3	abundance	X11698	1-(beta-D-I	Tibia_length	-0,00738	0,053302	NA
3	abundance	X11698	1-(beta-D-I	Radius_length	0,187096	0,070924	NA
3	abundance	X11698	1-(beta-D-I	Radius_SOS	0,090466	0,050779	NA
3	abundance	X11698	1-(beta-D-I	Tibia_SOS	0,163723	0,053974	NA
3	abundance	X11698	1-(beta-D-I	Handgrip	0,092677	0,062939	NA
3	abundance	X11699	{2-[2-(Isob	Tibia_length	-0,006	0,050013	NA
3	abundance	X11699	{2-[2-(Isob	Radius_length	0,218077	0,066862	NA
3	abundance	X11699	{2-[2-(Isob	Radius_SOS	0,039483	0,049055	NA
3	abundance	X11699	{2-[2-(Isob	Tibia_SOS	0,066048	0,052429	NA
3	abundance	X11699	{2-[2-(Isob	Handgrip	-0,01913	0,060486	NA
3	abundance	X11716	ala-ser	Tibia_length	0,03907	0,055341	NA
3	abundance	X11716	ala-ser	Radius_length	0,029627	0,074162	NA
3	abundance	X11716	ala-ser	Radius_SOS	-0,01958	0,052696	NA
3	abundance	X11716	ala-ser	Tibia_SOS	0,037359	0,055811	NA
3	abundance	X11716	ala-ser	Handgrip	0,044096	0,062573	NA
3	abundance	X11717	nicotianan	Tibia_length	-0,00639	0,055566	NA
3	abundance	X11717	nicotianan	Radius_length	0,147162	0,073129	NA
3	abundance	X11717	nicotianan	Radius_SOS	0,050297	0,052768	NA
3	abundance	X11717	nicotianan	Tibia_SOS	0,015448	0,056333	NA
3	abundance	X11717	nicotianan	Handgrip	0,021625	0,062949	NA
3	abundance	X11733	1,3-Dihydr	Tibia_length	7,41E-04	0,052794	NA
3	abundance	X11733	1,3-Dihydr	Radius_length	0,00134	0,071987	NA
3	abundance	X11733	1,3-Dihydr	Radius_SOS	-0,02389	0,050455	NA
3	abundance	X11733	1,3-Dihydr	Tibia_SOS	-0,01644	0,054268	NA
3	abundance	X11733	1,3-Dihydr	Handgrip	6,04E-04	0,061814	NA
3	abundance	X11745	2-Despipe	Tibia_length	-0,08914	0,054487	NA
3	abundance	X11745	2-Despipe	Radius_length	-0,06255	0,072948	NA
3	abundance	X11745	2-Despipe	Radius_SOS	-0,06091	0,051936	NA
3	abundance	X11745	2-Despipe	Tibia_SOS	-0,07181	0,055348	NA
3	abundance	X11745	2-Despipe	Handgrip	0,022906	0,062301	NA
3	abundance	X11755	Furfuranol	Tibia_length	-0,00726	0,055152	NA
3	abundance	X11755	Furfuranol	Radius_length	-0,00384	0,074024	NA
3	abundance	X11755	Furfuranol	Radius_SOS	-0,04027	0,052448	NA
3	abundance	X11755	Furfuranol	Tibia_SOS	0,123617	0,056687	NA
3	abundance	X11755	Furfuranol	Handgrip	0,044696	0,062896	NA
1	abundance	p_cresol	p-Cresol	Tibia_length	0,078042	0,054031	NA

1	abundance	p_cresol	p-Cresol	Radius_length	0,019396	0,073198	NA
1	abundance	p_cresol	p-Cresol	Radius_SOS	0,063676	0,051886	NA
1	abundance	p_cresol	p-Cresol	Tibia_SOS	0,012656	0,055272	NA
1	abundance	p_cresol	p-Cresol	Handgrip	-0,15135	0,061875	NA

Test statist	Sample siz	Degrees of	P-value	FDR	FDR featur
0,940161	417	340,954	0,347801	1	0,637437
-0,53498	417	189,7765	0,593288	1	0,808395
0,478916	416	372,5932	0,632279	1	0,964878
-0,70316	364	336,4325	0,482441	1	0,785988
-2,06712	278	254,7753	0,039733	0,238397	0,360707
0,598382	409	336,793	0,549987	1	0,786367
-0,46823	409	197,1044	0,640138	1	0,838647
0,795436	408	368,0154	0,426873	1	0,95743
-0,5744	358	327,3629	0,566089	1	0,837838
-2,68769	274	250,6664	0,007677	0,053737	0,199202
-0,21408	421	325,5488	0,830615	1	0,936611
0,006491	421	183,3901	0,994828	1	0,997935
0,583166	420	361,0078	0,560146	1	0,960096
0,363202	367	316,4288	0,716697	1	0,913008
0,483672	280	257,2008	0,62903	1	0,901091
-1,25644	420	354,6647	0,209785	1	0,498652
-0,61736	420	193,7982	0,537725	1	0,771054
0,50878	419	386,6837	0,611197	1	0,964878
0,23152	367	334,1406	0,817052	1	0,95095
-0,15297	280	262,5589	0,878539	1	0,973498
-0,26556	242	221,8572	0,790822	1	0,919136
-0,08735	242	145,0154	0,930513	1	0,97433
1,283233	241	221,3849	0,200752	1	0,928589
-1,10253	214	207,0998	0,271509	1	0,609149
0,720072	156	150,7491	0,472596	1	0,824256
-1,22492	421	351,1787	0,221427	1	0,51655
-0,35925	421	187,4574	0,719812	1	0,875826
-1,07603	420	382,4549	0,282592	1	0,943897
0,006183	367	335,4267	0,995071	1	0,997335
1,252247	280	257,7	0,211615	1	0,644251
-0,23342	421	372,5824	0,815563	1	0,928288
-0,4537	421	195,8242	0,650544	1	0,839358
-0,77418	420	400,5356	0,439282	1	0,960096
2,206809	367	353,1883	0,02797	0,811136	0,257802
1,86746	280	264,5884	0,062943	1	0,422605
-1,96618	421	385,4299	0,049995	1	0,246877
-1,65893	421	206,0306	0,09865	1	0,379251
0,795725	420	410,4416	0,426652	1	0,95743
-2,80953	367	360,0738	0,005232	0,172665	0,178183
-3,05846	280	270,8291	0,002448	0,083218	0,135787
2,366297	421	402,303	0,01844	0,516322	0,158072
2,808307	421	215,5692	0,005438	0,174001	0,1403
1,407476	420	415,7315	0,160034	1	0,914005
1,707782	367	362,4631	0,088533	1	0,394732
1,432094	280	275,2263	0,153252	1	0,565026
3,424119	421	359,6481	6,88E-04	0,02339	0,051145
1,05236	421	192,1179	0,293956	1	0,590656

0,155587	420	392,2919	0,876438	1	0,982463
2,54507	367	331,5024	0,011378	0,364105	0,189546
1,007772	280	268,99	0,314469	1	0,734737
0,483361	421	371,3837	0,629124	1	0,833119
0,240464	421	196,9791	0,81022	1	0,926063
0,934101	420	400,4943	0,350815	1	0,947373
0,189168	367	348,1538	0,850071	1	0,956935
0,023022	280	269,5658	0,981649	1	0,99236
-1,70657	421	352,8029	0,088782	1	0,319007
0,253945	421	191,287	0,799811	1	0,921861
0,23943	420	388,2938	0,810898	1	0,969427
-0,39089	367	338,221	0,696124	1	0,905141
0,589659	280	271,5839	0,55591	1	0,856601
2,04899	421	349,6849	0,041209	0,989015	0,22748
2,343653	421	187,7855	0,020142	0,552641	0,198392
1,806514	420	389,22	0,07161	1	0,848246
1,434686	367	332,1031	0,152318	1	0,500093
3,033948	280	270,3887	0,002649	0,087406	0,135787
-1,06506	421	315,9777	0,287664	1	0,584852
0,171008	421	178,7707	0,864411	1	0,947215
-0,33363	420	349,3103	0,738862	1	0,969427
0,063846	367	296,4374	0,949136	1	0,979539
-0,00323	280	253,2259	0,997425	1	0,998213
-2,16886	421	362,1765	0,030743	0,79933	0,200565
-2,7401	421	195,8886	0,00671	0,201289	0,141161
-1,50798	420	395,1664	0,132359	1	0,888479
-1,74521	367	343,5403	0,081843	1	0,381841
-2,20158	280	264,5103	0,028559	0,913879	0,326641
0,263973	421	410,526	0,791933	1	0,919136
0,862549	421	224,4208	0,389306	1	0,664422
0,813359	420	415,754	0,416478	1	0,95743
1,250407	367	362,9911	0,211956	1	0,563532
-0,12742	280	274,4961	0,898704	1	0,975243
2,516964	421	407,9843	0,01222	0,366585	0,131857
2,953631	421	225,9277	0,003473	0,118069	0,118336
0,448991	420	415,7268	0,653672	1	0,966867
2,948846	367	362,7826	0,003396	0,115475	0,176492
1,621911	280	275,9916	0,105964	1	0,492865
-0,41496	421	415,4472	0,678388	1	0,861268
1,688314	421	237,3741	0,092664	1	0,373113
-0,26217	420	412,3401	0,793324	1	0,969427
0,902331	367	359,1509	0,367486	1	0,678946
0,329149	280	275,9935	0,742293	1	0,94096
-0,99344	421	322,885	0,321241	1	0,615134
-1,64204	421	183,136	0,102298	1	0,383322
-2,63786	420	352,5412	0,008713	0,296237	0,582436
-1,29375	367	299,1008	0,196749	1	0,549567
-1,56517	280	255,9602	0,118779	1	0,521866

-0,60232	421	317,3493	0,54739	1	0,784719
1,294711	421	179,9817	0,197078	1	0,486881
-0,52033	420	351,0539	0,60316	1	0,964878
1,603312	367	314,9177	0,109868	1	0,435936
1,527912	280	252,3714	0,127787	1	0,536498
1,655188	421	406,2959	0,098658	1	0,339344
1,443921	421	221,1176	0,150177	1	0,430627
0,150628	420	415,9924	0,880343	1	0,982463
1,262222	367	362,9999	0,207679	1	0,560883
-0,24283	280	275,2554	0,808318	1	0,95747
-1,70436	421	385,8626	0,089119	1	0,319007
-1,49657	421	206,2703	0,136032	1	0,419225
-1,7427	420	408,6207	0,082138	1	0,848246
-0,79118	367	359,5352	0,42936	1	0,737794
0,068765	280	270,1818	0,945228	1	0,982385
1,060343	421	336,9783	0,289748	1	0,586895
2,614072	421	187,7901	0,009673	0,28051	0,149771
-0,27911	420	376,9109	0,780312	1	0,969427
1,052621	367	332,1272	0,29328	1	0,627013
0,403816	280	253,6556	0,686688	1	0,915426
-1,62472	421	397,1563	0,105016	1	0,347821
-1,97115	421	214,2528	0,049994	1	0,289774
-0,94915	420	414,9125	0,343098	1	0,947373
-1,9049	367	362,1968	0,057585	1	0,338985
-1,48843	280	275,2255	0,137783	1	0,54931
-0,96941	421	390,2172	0,33294	1	0,62159
-1,38648	421	208,0464	0,167085	1	0,453987
1,512837	420	412,2967	0,131087	1	0,888479
-0,85037	367	357,8837	0,395689	1	0,707784
-0,18545	280	269,6694	0,853014	1	0,966655
-2,68048	421	416,8588	0,007643	0,244585	0,108664
-2,82547	421	247,0705	0,005107	0,168528	0,137431
-1,74236	420	407,8797	0,082199	1	0,848246
-1,89804	367	354,409	0,058503	1	0,339097
0,020324	280	274,5196	0,9838	1	0,99236
-0,34777	421	302,5929	0,728251	1	0,890807
-0,46954	421	178,9269	0,639256	1	0,838432
-0,45581	420	334,6956	0,648824	1	0,964878
0,363203	367	293,6671	0,716715	1	0,913008
-0,78317	280	276	0,434201	1	0,803161
-2,44696	421	372,3889	0,014868	0,431181	0,147897
-2,07109	421	199,5111	0,039637	1	0,26998
-0,5178	420	402,972	0,604879	1	0,964878
-2,505	367	353,5256	0,012694	0,393522	0,189546
-2,13344	280	268,3371	0,033796	1	0,329568
-1,44548	421	406,113	0,149096	1	0,422053
-1,66412	421	223,449	0,09749	1	0,379251
0,033953	420	415,9847	0,972931	1	0,997342

-0,91906	367	362,8223	0,358677	1	0,674813
-0,71913	280	275,3646	0,472672	1	0,824256
-1,04844	421	355,155	0,295151	1	0,593552
0,288637	421	191,804	0,773171	1	0,909577
0,010961	420	386,7903	0,99126	1	0,997342
-0,06582	367	353,1487	0,947561	1	0,979539
-1,27541	280	266,0574	0,203276	1	0,632429
-2,2182	421	351,1607	0,027181	0,733878	0,192509
-2,3514	421	188,2281	0,019737	0,552641	0,195731
0,142906	420	387,8317	0,886439	1	0,982463
-2,34106	367	344,5821	0,019799	0,593956	0,221237
-1,85548	280	259,8525	0,06466	1	0,423666
-0,58791	421	414,462	0,556912	1	0,791015
0,013211	421	236,8894	0,98947	1	0,997412
0,498721	420	413,2641	0,618241	1	0,964878
-1,30821	367	360,0974	0,191637	1	0,542851
-0,10244	280	275,5809	0,918479	1	0,977051
0,016815	421	354,8043	0,986594	1	0,998043
-0,02817	421	192,4406	0,977556	1	0,991789
1,134841	420	386,8471	0,257145	1	0,943897
-0,11695	367	336,7968	0,906971	1	0,970486
0,565797	280	260,2059	0,572019	1	0,869862
0,268707	421	416,653	0,788288	1	0,918755
0,879811	421	257,6631	0,379782	1	0,657096
0,310836	420	403,8049	0,756086	1	0,969427
-0,6704	367	356,1869	0,503039	1	0,793601
0,210913	280	275,4307	0,833111	1	0,964667
0,528765	421	386,8889	0,597272	1	0,815122
0,090983	421	205,8635	0,927594	1	0,972749
-0,48389	420	410,0513	0,628725	1	0,964878
-0,414	367	353,8331	0,679123	1	0,899694
0,676458	280	266,6874	0,499336	1	0,831491
-2,6456	421	365,7279	0,008507	0,263703	0,112451
-2,75665	421	198,2909	0,006385	0,197924	0,141161
-1,09554	420	401,7213	0,273936	1	0,943897
-2,16331	367	348,7886	0,031196	0,873493	0,266905
-1,46462	280	271,6023	0,144181	1	0,555466
2,10593	421	360,3432	0,035902	0,897543	0,216416
1,322744	421	192,6863	0,187488	1	0,475664
1,421111	420	390,0283	0,156083	1	0,914005
0,839448	367	341,739	0,401805	1	0,715745
-0,95587	280	260,0617	0,340025	1	0,756894
-3,19769	421	377,6591	0,001502	0,049572	0,069718
-1,53722	421	200,3647	0,125818	1	0,410224
-0,48511	420	406,6734	0,627857	1	0,964878
-1,36002	367	349,9599	0,174699	1	0,523018
0,131238	280	270,0609	0,895684	1	0,975243
-0,54607	421	391,3521	0,585329	1	0,807036

0,799957	421	207,0572	0,424652	1	0,693378
-0,27468	420	411,7471	0,783699	1	0,969427
1,106627	367	355,1849	0,269204	1	0,609149
0,737109	280	271,634	0,461692	1	0,821452
-0,31533	405	349,9665	0,752702	1	0,898125
-0,12882	405	198,5898	0,897627	1	0,960059
0,522575	404	381,8159	0,601573	1	0,964878
-1,31522	355	335,5575	0,189334	1	0,53937
-1,09358	270	251,2068	0,275188	1	0,701156
0,105238	421	378,0441	0,916243	1	0,966828
-0,81101	421	202,0244	0,418313	1	0,688959
-0,49563	420	405,1165	0,620424	1	0,964878
0,506918	367	352,4692	0,612529	1	0,871158
-0,36104	280	266,1066	0,718352	1	0,931633
-2,09567	421	332,5915	0,036867	1	0,217944
-1,00441	421	184,2914	0,3165	1	0,614646
-1,12922	420	370,6449	0,259535	1	0,943897
-0,75407	367	336,5668	0,451336	1	0,756578
-2,10247	280	258,1964	0,036481	1	0,34044
0,219369	421	343,0603	0,826493	1	0,936485
0,61945	421	187,1775	0,536373	1	0,770962
0,061319	420	377,6697	0,951138	1	0,992811
1,011641	367	320,9083	0,312472	1	0,646834
1,256006	280	260,1753	0,210241	1	0,641361
2,18004	421	399,4892	0,029838	1	0,200565
3,222267	421	216,3532	0,001468	0,145341	0,093671
0,297922	420	415,3334	0,765912	1	0,969427
2,249468	367	360,3696	0,025086	1	0,247087
1,265257	280	273,9733	0,206854	1	0,63879
0,50522	421	365,9535	0,613708	1	0,825172
-0,17879	421	196,814	0,858289	1	0,946189
-1,22958	420	395,6222	0,219586	1	0,943055
-1,27026	367	346,1827	0,204847	1	0,558434
0,085236	280	263,371	0,932138	1	0,979504
-1,26388	421	339,1022	0,207142	1	0,496728
-1,12648	421	185,7002	0,261416	1	0,557979
-1,39515	420	373,0307	0,1638	1	0,914005
-0,09345	367	320,5106	0,925603	1	0,976733
0,431779	280	260,773	0,666259	1	0,908644
0,520947	421	335,7481	0,602747	1	0,820542
0,624449	421	184,572	0,533104	1	0,769894
-0,19816	420	370,2978	0,843026	1	0,973894
0,997844	367	324,3788	0,319099	1	0,653184
0,508948	280	256,6814	0,611226	1	0,888252
-0,77874	421	388,6902	0,436607	1	0,707193
-1,43008	421	206,8234	0,154202	1	0,435989
-1,53467	420	412,0431	0,125631	1	0,880587
-1,41461	367	358,3053	0,158051	1	0,504558

-1,62283	280	273,192	0,105779	1	0,492865
1,293491	421	359,2499	0,196673	1	0,482712
1,135606	421	195,4154	0,257512	1	0,556025
0,037741	420	398,3943	0,969913	1	0,997342
1,210457	367	345,4793	0,226931	1	0,576775
1,134934	280	270,7141	0,257407	1	0,680548
1,823692	421	324,469	0,069119	1	0,282663
2,778855	421	182,6351	0,006026	0,584682	0,141161
1,186346	420	358,8557	0,23627	1	0,943055
0,904294	367	316,5429	0,366527	1	0,678571
-0,20468	280	247,2618	0,837987	1	0,964667
2,023809	421	362,364	0,043723	1	0,235324
1,431568	421	192,2246	0,153891	1	0,435929
2,096976	420	398,7585	0,036625	1	0,828713
1,818242	367	343,4417	0,069898	1	0,364488
-0,26949	280	265,4449	0,787761	1	0,953235
2,174047	421	368,069	0,030338	1	0,200565
0,986	421	194,3918	0,325359	1	0,619085
-0,11214	420	399,73	0,910765	1	0,987181
2,18841	367	345,4108	0,029309	1	0,260082
1,488737	280	273,0983	0,137711	1	0,54931
0,112108	421	369,4192	0,910799	1	0,966828
1,224323	421	197,099	0,222292	1	0,518566
-0,66682	420	399,9273	0,505269	1	0,960096
1,635519	367	349,746	0,10284	1	0,426647
-0,41529	280	263,5783	0,678267	1	0,909385
0,34572	421	351,7017	0,72976	1	0,891206
0,678194	421	189,2662	0,498477	1	0,749329
-1,40236	420	383,0811	0,161617	1	0,914005
2,126863	367	339,7339	0,034152	1	0,279197
1,626715	280	267,4116	0,104975	1	0,492014
-1,63291	421	371,9155	0,103334	1	0,345287
0,505877	421	197,1577	0,613508	1	0,824026
-0,32299	420	400,0619	0,74687	1	0,969427
-0,02707	367	352,4483	0,978421	1	0,99209
0,531264	280	269,4822	0,595674	1	0,880079
2,040286	421	305,7404	0,042181	1	0,229482
2,297106	421	171,053	0,022824	1	0,212324
3,044897	420	345,1777	0,002506	0,333362	0,314772
1,481493	367	297,7674	0,139533	1	0,487879
2,26903	280	255,2685	0,024102	1	0,313396
1,29524	421	371,2048	0,196042	1	0,481951
1,915094	421	195,3424	0,056941	1	0,312039
-0,6769	420	401,7833	0,498858	1	0,960096
-0,26311	367	351,8518	0,792617	1	0,944997
0,074212	280	268,9521	0,940897	1	0,982175
-2,86587	421	328,1325	0,004427	0,562235	0,087964
-0,9942	421	179,3376	0,321464	1	0,617772

-2,22225	420	357,0618	0,026893	1	0,767829
-1,41067	367	318,5754	0,159319	1	0,504558
-2,70978	280	256,9615	0,007186	0,919788	0,195931
-0,19793	421	322,8182	0,843224	1	0,939735
0,958829	421	180,0878	0,33893	1	0,632922
-0,41404	420	357,6658	0,679092	1	0,96781
0,361626	367	313,6702	0,717875	1	0,913715
1,520321	280	254,9178	0,12967	1	0,536847
0,77257	421	347,9701	0,440301	1	0,710422
0,567056	421	187,719	0,571354	1	0,792729
0,3025	420	381,7471	0,762436	1	0,969427
0,831038	367	337,99	0,406539	1	0,719923
1,699857	280	263,8898	0,090336	1	0,468147
-0,46402	421	317,5423	0,642949	1	0,836
0,208644	421	178,5696	0,834964	1	0,937624
-0,82744	420	350,8033	0,40855	1	0,95743
-0,03876	367	304,1579	0,96911	1	0,988125
1,437854	280	247,0248	0,151741	1	0,565026
-2,53589	421	348,8497	0,011652	1	0,130072
-0,49933	421	190,2061	0,618121	1	0,825805
-1,64392	420	378,2835	0,101023	1	0,848246
-0,23136	367	346,0079	0,817173	1	0,95095
1,39943	280	263,5318	0,16286	1	0,584818
-2,34227	421	329,2059	0,019762	1	0,162476
-0,32307	421	182,3012	0,747011	1	0,889215
-0,5942	420	366,0168	0,552742	1	0,960096
0,243801	367	321,2	0,80754	1	0,950149
0,611048	280	252,2009	0,541718	1	0,849499
0,02837	421	361,9689	0,977383	1	0,995982
0,677763	421	195,3553	0,498724	1	0,749329
0,240685	420	393,4231	0,809925	1	0,969427
1,902688	367	336,9528	0,057934	1	0,339097
-0,20986	280	262,8698	0,83394	1	0,964667
1,555678	421	355,0834	0,120675	1	0,382054
0,402085	421	194,3528	0,688063	1	0,863373
-0,93801	420	391,3075	0,348818	1	0,947373
-0,77921	367	343,5162	0,436394	1	0,742744
0,259119	280	264,65	0,795745	1	0,954269
-0,14568	421	318,7034	0,884267	1	0,961484
-1,04272	421	181,2629	0,298466	1	0,597214
0,975057	420	354,0481	0,330198	1	0,947373
-1,43598	367	317,5324	0,151994	1	0,500093
-0,05208	280	252,6431	0,958508	1	0,986271
1,890656	421	366,2263	0,059459	1	0,268966
2,726289	421	193,9287	0,006992	0,887979	0,141161
1,112543	420	398,1068	0,266576	1	0,943897
2,681418	367	349,7048	0,007679	0,975186	0,180403
1,083103	280	274,9071	0,279712	1	0,703716

0,238024	421	353,9064	0,812	1	0,924931
1,125186	421	189,8061	0,261931	1	0,557979
-0,48754	420	387,8689	0,626148	1	0,964878
-0,35129	367	336,6047	0,725591	1	0,919068
0,113835	280	262,685	0,909456	1	0,976008
-0,68396	421	320,6954	0,494496	1	0,74916
0,611718	421	180,2178	0,541495	1	0,773814
0,316911	420	354,6829	0,751498	1	0,969427
0,216698	367	312,0687	0,828585	1	0,956109
1,51589	280	252,323	0,130799	1	0,538563
0,481095	421	367,6657	0,630735	1	0,834519
1,021089	421	190,8293	0,308505	1	0,60631
0,169237	420	398,4033	0,865696	1	0,979433
2,538125	367	354,4637	0,011572	1	0,189546
2,505076	280	269,603	0,012833	1	0,2448
0,154051	421	364,5277	0,877655	1	0,95861
1,117277	421	194,7009	0,265253	1	0,562747
-1,42696	420	395,3386	0,15438	1	0,914005
-0,06239	367	346,0356	0,950289	1	0,979539
-0,01861	280	266,1745	0,985167	1	0,992411
0,246459	421	304,0021	0,805493	1	0,922616
-2,06574	421	177,1111	0,040309	1	0,26998
-1,59098	420	332,4871	0,112564	1	0,848246
0,443184	367	295,5441	0,657957	1	0,892476
-0,0618	280	247,7695	0,950774	1	0,982409
-0,43251	421	327,3567	0,665656	1	0,853014
-0,84954	421	184,0767	0,396686	1	0,667939
-0,5136	420	365,3298	0,607843	1	0,964878
-0,95182	367	323,1393	0,3419	1	0,664831
0,333058	280	249,885	0,73937	1	0,940183
2,677691	421	315,6563	0,007801	0,741091	0,108852
0,278583	421	178,4785	0,780888	1	0,91393
-0,84939	420	349,8294	0,396245	1	0,95743
2,05148	367	300,0478	0,041088	1	0,289956
1,117873	280	255,6261	0,264671	1	0,688875
-1,36727	421	366,794	0,172379	1	0,444059
-0,43612	421	195,7272	0,663234	1	0,845595
-1,96851	420	396,743	0,049705	1	0,846627
-0,19845	367	344,7757	0,842811	1	0,956935
1,373416	280	263,1554	0,170792	1	0,59737
0,681756	421	361,3531	0,49583	1	0,750117
0,755298	421	192,2886	0,450994	1	0,713861
0,306096	420	392,2657	0,759694	1	0,969427
0,648816	367	333,2407	0,516904	1	0,80059
0,75648	280	257,0096	0,450054	1	0,813228
0,156795	421	368,2376	0,875492	1	0,95861
1,938991	421	193,8709	0,053954	1	0,303391
0,349756	420	399,6188	0,726706	1	0,969427

1,5967	367	354,5759	0,111224	1	0,437634
2,704292	280	269,432	0,007281	0,698956	0,195931
-0,75069	421	307,8076	0,453413	1	0,717744
-0,26704	421	176,9498	0,78975	1	0,918329
-1,36137	420	337,6271	0,174304	1	0,9199
-1,27814	367	306,7889	0,202167	1	0,556171
0,242903	280	253,1775	0,808278	1	0,95747
-0,91076	421	313,5594	0,363119	1	0,653008
-0,6968	421	178,0402	0,486836	1	0,741822
-2,68019	420	334,7233	0,007722	0,764482	0,554149
-0,47676	367	309,7686	0,633869	1	0,880707
1,68325	280	244,6705	0,093602	1	0,47335
-0,71907	421	378,7233	0,472544	1	0,731548
-2,74148	421	202,5446	0,006664	0,639698	0,141161
-0,41615	420	406,3692	0,677523	1	0,96781
-2,88708	367	353,5432	0,004127	0,404487	0,178183
-2,56863	280	271,0657	0,010746	1	0,231345
1,383947	421	324,8093	0,167325	1	0,441901
2,553414	421	182,462	0,011485	1	0,163285
0,767577	420	363,6186	0,443237	1	0,960096
2,544432	367	309,008	0,011432	1	0,189546
1,019199	280	257,7083	0,309064	1	0,730032
-0,87694	421	350,3942	0,38112	1	0,671614
-0,75966	421	187,8477	0,448409	1	0,711318
-0,7328	420	382,3271	0,46413	1	0,960096
1,104689	367	331,1227	0,270096	1	0,609149
2,03762	280	257,62	0,04261	1	0,377723
-0,55492	421	321,4842	0,579335	1	0,803921
0,04868	421	181,6032	0,961228	1	0,985228
-0,43067	420	351,8058	0,666975	1	0,96781
-0,238	367	312,5972	0,812039	1	0,950149
2,531838	280	261,5847	0,011933	1	0,239766
1,796802	421	340,9179	0,073252	1	0,292681
2,16823	421	188,3647	0,031395	1	0,241423
-0,11555	420	376,7942	0,908068	1	0,986632
1,754209	367	317,0658	0,080361	1	0,381841
0,93858	280	259,6001	0,348819	1	0,761841
-1,74966	421	326,7572	0,081115	1	0,30376
-0,5031	421	184,7705	0,615492	1	0,825219
0,469305	420	364,3207	0,639132	1	0,964878
-1,15713	367	313,8578	0,2481	1	0,602072
-0,95744	280	247,8113	0,33928	1	0,756354
1,413861	421	323,6046	0,158364	1	0,431264
0,402797	421	180,7602	0,687574	1	0,863373
-1,12978	420	352,3242	0,259336	1	0,943897
1,222055	367	316,5402	0,222596	1	0,574455
3,479524	280	259,6851	5,89E-04	0,077738	0,093926
0,102612	421	335,8349	0,918332	1	0,966828

1,160249	421	181,8157	0,247469	1	0,548314
1,03394	420	372,4143	0,301835	1	0,943897
0,104571	367	330,7437	0,91678	1	0,973634
2,274834	280	262,4567	0,023723	1	0,313396
0,182518	421	300,1993	0,8553	1	0,947051
1,074721	421	171,8375	0,284006	1	0,582654
1,445104	420	335,5285	0,149362	1	0,914005
0,169278	367	297,304	0,865693	1	0,959636
2,768545	280	260,0171	0,006036	0,585479	0,186692
-0,20358	421	363,6472	0,838796	1	0,93721
0,327029	421	191,5071	0,744003	1	0,888441
0,125969	420	395,6891	0,89982	1	0,98503
0,677286	367	345,219	0,498678	1	0,793601
1,753243	280	263,2	0,080724	1	0,455624
0,211007	421	289,6284	0,83303	1	0,936611
-0,88783	421	175,0932	0,375851	1	0,654686
-1,01517	420	315,1101	0,310803	1	0,943897
1,089042	367	274,553	0,27709	1	0,611884
0,161264	280	254,6609	0,872014	1	0,969656
0,177738	421	347,3956	0,859032	1	0,949789
1,914007	421	185,84	0,057156	1	0,312079
-0,0065	420	382,7002	0,994821	1	0,997342
1,290757	367	344,0779	0,197654	1	0,549567
1,84142	280	260,5525	0,066697	1	0,425749
-0,3814	421	330,1591	0,703152	1	0,874567
0,690847	421	182,4941	0,49054	1	0,744768
-0,77927	420	364,2023	0,436327	1	0,960096
0,05129	367	322,5202	0,959126	1	0,981063
1,837023	280	251,6783	0,067386	1	0,425749
-2,04279	421	331,8556	0,041863	1	0,228575
-0,38767	421	184,6645	0,698704	1	0,865528
-1,0508	420	366,3947	0,294042	1	0,943897
-2,23248	367	311,1579	0,026295	1	0,252225
-0,49152	280	254,6783	0,623484	1	0,89827
2,062161	421	355,776	0,039918	1	0,224527
1,814842	421	193,5021	0,071097	1	0,339864
0,554638	420	392,7085	0,579458	1	0,964878
1,807911	367	338,3942	0,071508	1	0,367791
-0,48805	280	263,6945	0,625918	1	0,899494
0,326905	421	333,0427	0,743945	1	0,895468
1,463385	421	183,958	0,145069	1	0,426157
0,635791	420	370,0009	0,525306	1	0,960096
0,677798	367	319,014	0,498391	1	0,793601
1,933197	280	261,2428	0,054292	1	0,40654
0,303503	421	342,0107	0,761691	1	0,902412
-0,18778	421	185,8799	0,851252	1	0,944105
-0,45902	420	376,2929	0,646488	1	0,964878
2,78676	367	308,8542	0,005653	0,740582	0,178183

0,338204	280	246,5634	0,735498	1	0,938189
-1,48201	421	353,856	0,139228	1	0,4106
0,352512	421	193,145	0,724839	1	0,878959
0,741833	420	386,5738	0,458639	1	0,960096
-0,36649	367	350,4557	0,714219	1	0,912652
0,296825	280	269,1122	0,766829	1	0,945436
-2,92438	421	340,5357	0,003683	0,3609	0,087964
-1,81759	421	190,8139	0,070695	1	0,339864
-1,79882	420	379,9496	0,07284	1	0,848246
-1,67132	367	336,7584	0,095587	1	0,412696
-0,54628	280	263,9551	0,585336	1	0,87561
-2,61141	421	286,9843	0,009491	0,88267	0,114424
-2,23936	421	173,5228	0,026403	1	0,222284
-0,88798	420	320,9434	0,375216	1	0,95743
-2,64297	367	363	0,008574	0,823129	0,183886
-3,01429	280	258,1043	0,002832	0,280405	0,135787
-0,64993	421	357,9475	0,516153	1	0,767364
0,486269	421	194,1613	0,627325	1	0,832379
-0,75682	420	389,5051	0,449614	1	0,960096
-2,20705	367	331,5724	0,027995	1	0,257802
2,000972	280	252,8934	0,046464	1	0,387749
-0,82586	421	383,0917	0,409398	1	0,695339
-1,0649	421	205,5979	0,28817	1	0,585544
-0,04269	420	409,8801	0,965972	1	0,996924
-1,89791	367	361,483	0,058504	1	0,339097
-1,69315	280	271,9759	0,091572	1	0,468147
-0,24683	421	289,3452	0,805215	1	0,922616
0,239893	421	169,4393	0,810703	1	0,926063
1,524263	420	321,0578	0,128427	1	0,887798
0,146229	367	298,142	0,883839	1	0,962871
0,020349	280	276	0,98378	1	0,99236
0,315144	421	336,3509	0,752847	1	0,898125
0,828026	421	182,9941	0,408734	1	0,681984
-0,6286	420	369,4799	0,53	1	0,960096
0,411411	367	325,608	0,681042	1	0,900574
1,456168	280	252,9048	0,146587	1	0,555466
-0,53449	421	299,1761	0,5934	1	0,812958
1,290825	421	176,8942	0,198449	1	0,488664
0,42461	420	329,8427	0,671398	1	0,96781
1,502617	367	301,058	0,133986	1	0,477542
-0,17874	280	254,7183	0,858281	1	0,966655
0,757373	421	341,9906	0,449348	1	0,714643
-0,09703	421	186,386	0,922808	1	0,971139
-1,84198	420	378,32	0,066261	1	0,848246
1,395137	367	338,1779	0,16389	1	0,510294
0,072315	280	261,8677	0,942407	1	0,982175
-0,00593	421	408,5344	0,995275	1	0,998054
-1,17188	421	222,7089	0,242497	1	0,542422

-0,1558	420	415,8361	0,876263	1	0,982463
1,048421	367	361,4775	0,295145	1	0,62734
0,554411	280	274,663	0,579749	1	0,874212
0,733434	421	388,989	0,463736	1	0,727964
1,636918	421	207,8753	0,103161	1	0,383322
1,470739	420	412,2279	0,142125	1	0,90372
-0,66841	367	352,6374	0,504312	1	0,793601
0,507514	280	264,0923	0,612218	1	0,888837
1,740403	421	383,4825	0,08259	1	0,305058
2,503938	421	205,6	0,013059	1	0,171643
0,54747	420	409,7699	0,584354	1	0,964878
3,09924	367	356,6416	0,002094	0,207339	0,176492
1,692793	280	271,6763	0,091641	1	0,468147
-1,77644	421	345,9665	0,07654	1	0,300378
-0,80249	421	188,8458	0,423279	1	0,693349
0,10971	420	381,398	0,912697	1	0,987181
0,234481	367	335,0449	0,814754	1	0,95095
-0,11096	280	257,7267	0,911736	1	0,976008
-1,91066	421	327,417	0,056922	1	0,266382
-0,70276	421	181,7787	0,483103	1	0,739236
-1,26295	420	365,4105	0,207414	1	0,930277
-1,67323	367	310,9175	0,095287	1	0,412696
-1,14134	280	269,7576	0,254739	1	0,679673
1,507622	421	402,5399	0,132436	1	0,404829
1,900044	421	218,6299	0,058743	1	0,314262
0,266809	420	415,6339	0,789749	1	0,969427
0,615437	367	362,9876	0,538652	1	0,819758
2,148593	280	275,9522	0,032537	1	0,329568
-2,84416	421	346,772	0,004717	0,594351	0,087964
-3,19874	421	189,1967	0,001618	0,212013	0,093671
-0,26119	420	387,2982	0,794081	1	0,969427
-1,05438	367	335,7133	0,292468	1	0,627013
-1,12437	280	261,8719	0,261888	1	0,687569
-2,3674	421	347,3745	0,018461	1	0,158072
-1,23575	421	190,6404	0,21807	1	0,514565
0,275231	420	384,2739	0,783287	1	0,969427
-1,85939	367	340,3772	0,063834	1	0,350852
-1,2144	280	265,6953	0,225672	1	0,654014
-0,23824	421	313,2693	0,811851	1	0,924931
0,631625	421	181,043	0,528429	1	0,768085
0,544819	420	347,1096	0,586228	1	0,964878
0,926256	367	307,426	0,35504	1	0,674813
-0,853	280	252,5056	0,394467	1	0,782194
1,415104	421	379,9243	0,157857	1	0,430962
0,019373	421	201,1462	0,984563	1	0,995074
0,379744	420	406,2992	0,704334	1	0,968866
-0,84995	367	352,7214	0,395927	1	0,707784
-0,28649	280	266,9731	0,774722	1	0,947651

1,761045	421	344,3668	0,079118	1	0,302838
1,90802	421	186,4937	0,057925	1	0,312877
1,282994	420	383,0896	0,20027	1	0,928589
1,053424	367	331,4512	0,292914	1	0,627013
2,998861	280	267,8608	0,002965	0,290544	0,135787
0,479246	421	316,4877	0,632095	1	0,83455
1,057293	421	181,7838	0,291781	1	0,588663
1,62149	420	351,7227	0,105809	1	0,848246
0,469665	367	308,4848	0,638926	1	0,880707
1,187079	280	246,0053	0,236342	1	0,659852
-1,36471	421	330,3627	0,173273	1	0,445601
-2,23987	421	183,6588	0,026299	1	0,222284
-1,38305	420	370,6997	0,167481	1	0,914005
-1,02686	367	335,3703	0,305225	1	0,637666
-0,4522	280	269,2347	0,651488	1	0,908644
0,537377	421	377,0488	0,591324	1	0,81147
-0,7933	421	201,4207	0,428539	1	0,694836
0,164169	420	403,7117	0,869681	1	0,980994
0,179495	367	355,9029	0,857651	1	0,958813
-0,31878	280	261,2172	0,750149	1	0,942071
-1,06914	421	373,9378	0,285695	1	0,583534
-0,45669	421	200,4989	0,648389	1	0,839358
-1,24739	420	402,3766	0,212978	1	0,943055
-0,98111	367	349,2488	0,327217	1	0,655788
0,89123	280	272,2392	0,373593	1	0,763914
0,132662	421	382,9723	0,894531	1	0,962
1,372465	421	203,4823	0,17143	1	0,456365
-0,17314	420	407,9182	0,862631	1	0,977432
-0,69888	367	355,4422	0,485083	1	0,786044
-1,01419	280	266,6888	0,311413	1	0,733729
-0,09935	421	397,9423	0,920909	1	0,966828
-0,5517	421	214,5748	0,581727	1	0,798417
-0,85584	420	414,6128	0,392583	1	0,95743
-1,20728	367	358,5152	0,22812	1	0,577307
0,162446	280	270,0312	0,871076	1	0,969656
-0,66885	421	297,9486	0,504109	1	0,75707
-0,57207	421	178,1536	0,567994	1	0,78927
-0,26157	420	328,848	0,793813	1	0,969427
1,256805	367	293,3996	0,209824	1	0,563436
0,273906	280	276	0,784362	1	0,953235
0,77003	421	416,6381	0,441718	1	0,711185
2,42621	421	244,2952	0,015982	1	0,179127
1,068849	420	409,4399	0,285767	1	0,943897
-0,00879	367	359,7972	0,992988	1	0,997335
2,210226	280	275,5855	0,027911	1	0,326641
-2,6185	421	307,4641	0,009269	0,871303	0,113566
-0,87955	421	180,7994	0,380269	1	0,657185
-1,75005	420	348,757	0,080989	1	0,848246

-1,81872	367	316,8158	0,069898	1	0,364488
-1,71567	280	256,1882	0,087431	1	0,460069
0,641437	421	376,9035	0,521629	1	0,769172
2,061612	421	198,9196	0,040545	1	0,270359
0,314452	420	404,0615	0,75334	1	0,969427
0,365956	367	351,0396	0,714618	1	0,912652
-0,23619	280	262,6273	0,813467	1	0,95747
0,755926	421	346,4492	0,450207	1	0,714643
0,369028	421	189,5066	0,712519	1	0,872396
1,363773	420	381,7732	0,173443	1	0,9199
1,934065	367	321,1918	0,053983	1	0,334402
0,026921	280	258,5065	0,978543	1	0,99236
1,089249	421	361,2544	0,27677	1	0,57291
-0,7926	421	195,0737	0,428972	1	0,694836
-1,36536	420	394,2329	0,172918	1	0,9199
-0,66717	367	339,1857	0,505116	1	0,793601
-0,38982	280	253,1574	0,696997	1	0,92034
-2,67978	421	349,7835	0,007715	0,740677	0,108664
-1,23679	421	190,1101	0,217692	1	0,514565
-0,73399	420	386,0862	0,463401	1	0,960096
-1,89235	367	340,6708	0,059292	1	0,340409
-0,91016	280	263,2884	0,36357	1	0,762031
2,259586	421	364,4617	0,024437	1	0,182309
1,47822	421	195,8642	0,140955	1	0,423499
1,038336	420	397,0482	0,299746	1	0,943897
1,241084	367	340,9545	0,215428	1	0,56658
-0,11218	280	261,8314	0,910765	1	0,976008
-1,14691	421	332,2245	0,252245	1	0,546261
-1,48335	421	185,333	0,13968	1	0,423351
-0,15214	420	365,6514	0,879162	1	0,982463
-0,36997	367	331,8533	0,711644	1	0,912652
-0,90117	280	262,6047	0,368322	1	0,763914
-1,02783	421	376,161	0,304692	1	0,601009
-1,75388	421	200,1319	0,080981	1	0,360416
-0,87652	420	404,2818	0,381268	1	0,95743
-1,36521	367	348,4898	0,173069	1	0,522676
-0,34471	280	272,9637	0,730577	1	0,936637
-0,96124	421	334,1762	0,337124	1	0,627993
-2,40358	421	184,0963	0,017228	1	0,181988
-1,40128	420	369,87	0,161968	1	0,914005
-2,04286	367	326,5031	0,041869	1	0,291798
-2,25647	280	258,1614	0,024878	1	0,315049
-2,71607	421	409,5691	0,006886	0,667938	0,10482
-2,42689	421	224,4612	0,016018	1	0,179127
-1,4337	420	415,9468	0,15241	1	0,914005
-2,16101	367	362,6483	0,031348	1	0,266905
-0,34292	280	274,2284	0,731922	1	0,936637
-1,06487	421	349,1616	0,287669	1	0,584852

-0,23547	421	190,7353	0,814094	1	0,926063
0,484273	420	383,4703	0,628468	1	0,964878
0,240255	367	336,9295	0,810279	1	0,950149
-0,21962	280	258,6026	0,826339	1	0,96121
-1,27213	421	349,6439	0,204173	1	0,494095
-2,39569	421	189,4028	0,017563	1	0,182536
-0,14913	420	384,2939	0,881528	1	0,982463
-2,87197	367	339,5721	0,004335	0,420541	0,178183
-1,88633	280	258,1757	0,060373	1	0,422605
-1,5576	421	346,5078	0,12024	1	0,381479
1,683495	421	186,7694	0,09395	1	0,373113
-0,44466	420	379,8227	0,656816	1	0,96781
-0,42933	367	335,8906	0,667957	1	0,894765
0,239561	280	260,1924	0,81086	1	0,95747
0,316286	421	346,1685	0,751976	1	0,898125
1,242218	421	190,3798	0,215684	1	0,514565
1,628376	420	381,2408	0,104271	1	0,848246
1,215799	367	341,5284	0,224901	1	0,576775
0,51618	280	268,0465	0,606154	1	0,88515
-0,15188	421	341,6175	0,87937	1	0,95861
-0,28367	421	184,5822	0,776981	1	0,911265
0,213856	420	374,9309	0,830775	1	0,973894
0,797509	367	326,6733	0,425735	1	0,733464
0,264908	280	248,9713	0,7913	1	0,953435
0,329055	421	358,7989	0,742306	1	0,894925
2,780287	421	193,9782	0,005966	0,584682	0,141161
0,223391	420	391,755	0,823348	1	0,972623
1,59096	367	337,3743	0,112555	1	0,438296
2,005076	280	268,4246	0,045959	1	0,387749
-1,55896	421	317,3176	0,120003	1	0,381479
-0,87481	421	180,7585	0,382839	1	0,659083
-1,84507	420	345,405	0,065883	1	0,848246
-0,44048	367	313,7942	0,659892	1	0,893492
0,024636	280	252,7957	0,980365	1	0,99236
0,406695	421	343,2761	0,684485	1	0,863919
-0,04497	421	185,9522	0,964177	1	0,985228
-1,03722	420	375,315	0,300301	1	0,943897
-0,39001	367	313,5799	0,696796	1	0,905235
1,446168	280	252,0211	0,149373	1	0,561358
2,060189	421	370,6119	0,040078	1	0,224527
2,518889	421	199,9442	0,012556	1	0,169272
0,811017	420	403,5289	0,417834	1	0,95743
0,27603	367	354,1688	0,782686	1	0,94337
0,346726	280	270,6415	0,729066	1	0,936637
-0,27672	421	346,3147	0,782158	1	0,915149
-0,38554	421	189,8947	0,700267	1	0,865528
0,94199	420	379,233	0,346798	1	0,947373
-0,0265	367	332,8107	0,978876	1	0,99209

0,653619	280	257,1646	0,513941	1	0,831912
0,761286	421	373,7025	0,446966	1	0,714643
-0,73584	421	198,4296	0,4627	1	0,721079
0,401186	420	402,024	0,688496	1	0,968866
1,186262	367	340,4124	0,236346	1	0,585627
0,651635	280	267,7769	0,515195	1	0,83211
1,596003	421	364,7889	0,111354	1	0,358728
1,56664	421	197,3491	0,118801	1	0,396436
-0,15115	420	399,1967	0,879934	1	0,982463
2,565891	367	350,6723	0,010706	1	0,189546
0,516182	280	271,2229	0,606148	1	0,88515
0,683739	421	320,4866	0,494634	1	0,74916
0,447004	421	182,3297	0,655403	1	0,841107
0,662791	420	355,68	0,507894	1	0,960096
-0,67382	367	318,5946	0,500917	1	0,793601
2,096842	280	260,2183	0,036973	1	0,34183
-1,16646	421	298,8357	0,244357	1	0,541538
-0,87276	421	175,029	0,383991	1	0,659083
0,594196	420	337,2803	0,552779	1	0,960096
-0,34634	367	299,3205	0,729329	1	0,922837
0,913383	280	251,9268	0,361914	1	0,762031
2,282877	421	375,1051	0,022997	1	0,177419
3,231	421	200,8891	0,001441	0,190274	0,093671
0,988044	420	406,8304	0,323718	1	0,945573
3,369421	367	355,2434	8,36E-04	0,112016	0,139974
1,226673	280	272,3915	0,221005	1	0,653047
-1,26509	421	386,5772	0,2066	1	0,496566
-1,4215	421	202,5397	0,156708	1	0,438625
-0,70213	420	409,6853	0,482995	1	0,960096
-0,92302	367	357,7803	0,356621	1	0,674813
-2,45016	280	274,1624	0,014905	1	0,263522
-0,95319	421	358,9711	0,341136	1	0,63164
-1,96442	421	191,5368	0,050927	1	0,294052
0,681747	420	393,3773	0,4958	1	0,960096
-0,20799	367	332,335	0,835362	1	0,956935
-0,78959	280	263,7572	0,430475	1	0,801886
-0,57102	421	320,164	0,568383	1	0,796637
-0,87142	421	181,8357	0,384674	1	0,659504
-2,16828	420	353,221	0,030805	1	0,80141
0,988245	367	316,515	0,323788	1	0,655597
0,461243	280	256,6757	0,645015	1	0,907804
2,834002	421	359,8424	0,004856	0,607022	0,087964
2,78222	421	195,9348	0,005927	0,758613	0,141161
0,596319	420	396,9399	0,551302	1	0,960096
1,346105	367	355,9509	0,179125	1	0,526202
0,144776	280	272,9712	0,884995	1	0,973784
0,122606	421	349,8235	0,90249	1	0,965517
-1,50292	421	188,1665	0,134535	1	0,417169

-0,00853	420	383,5007	0,993197	1	0,997342
2,080609	367	325,8065	0,038251	1	0,28956
0,934912	280	250,5371	0,350734	1	0,761846
-3,02766	421	345,7989	0,00265	0,34445	0,073944
-0,88713	421	190,1779	0,376128	1	0,654686
0,131268	420	384,3475	0,895632	1	0,98503
-0,96414	367	339,5607	0,335664	1	0,662363
0,675014	280	255,4503	0,500277	1	0,831912
0,412868	421	335,2299	0,679967	1	0,861268
0,974545	421	186,0173	0,331051	1	0,626331
0,77193	420	370,8254	0,440647	1	0,960096
1,109686	367	325,7066	0,267953	1	0,609149
1,944799	280	251,1354	0,052915	1	0,402746
0,499732	421	367,058	0,617564	1	0,826526
0,252733	421	195,6396	0,80074	1	0,921861
1,95664	420	395,4648	0,051093	1	0,846627
-0,0931	367	347,9994	0,925875	1	0,976733
0,461561	280	270,5513	0,644767	1	0,907804
2,076724	421	350,3698	0,038555	1	0,221767
1,625023	421	190,12	0,105814	1	0,385174
0,831027	420	385,6183	0,406472	1	0,95743
1,398657	367	331,3182	0,162851	1	0,510222
1,386404	280	272,5164	0,166757	1	0,5913
-1,27946	421	340,6521	0,201607	1	0,490826
-1,47116	421	187,4123	0,142925	1	0,423993
-1,77772	420	366,5003	0,076278	1	0,848246
-0,57359	367	327,9016	0,566636	1	0,837838
-0,07228	280	258,542	0,942438	1	0,982175
0,493649	421	374,8659	0,621843	1	0,828413
0,940548	421	200,82	0,348066	1	0,642814
-0,69383	420	402,7754	0,488188	1	0,960096
1,35665	367	351,703	0,175763	1	0,523018
1,852253	280	262,1248	0,065114	1	0,424733
-2,1958	421	414,7276	0,028659	1	0,197211
-2,48118	421	237,6112	0,013788	1	0,17172
-0,47479	420	411,597	0,635188	1	0,964878
-2,55936	367	360,4131	0,010894	1	0,189546
0,435822	280	274,655	0,663308	1	0,908644
1,758954	421	363,7784	0,079426	1	0,302838
1,373401	421	195,7659	0,171199	1	0,456365
1,752774	420	396,643	0,080413	1	0,848246
1,577505	367	350,2264	0,115582	1	0,443225
-0,60524	280	268,991	0,545533	1	0,851507
-0,2169	421	315,9284	0,828426	1	0,936611
-0,21978	421	177,4269	0,826299	1	0,932758
-0,90875	420	351,1132	0,364106	1	0,956033
0,589492	367	314,432	0,555955	1	0,830257
2,060237	280	246,6695	0,040425	1	0,364793

1,435106	421	377,9844	0,152084	1	0,42445
1,656072	421	201,9085	0,09926	1	0,379659
-0,45653	420	405,972	0,648251	1	0,964878
1,472753	367	354,264	0,141706	1	0,489794
1,76418	280	271,4847	0,078826	1	0,453386
-1,15474	421	353,7791	0,248977	1	0,545362
0,006725	421	192,1043	0,994641	1	0,997935
-1,06176	420	386,2803	0,28901	1	0,943897
0,497143	367	340,1701	0,619409	1	0,875495
0,7167	280	261,8411	0,474198	1	0,824256
0,262689	421	306,463	0,792967	1	0,919136
0,189944	421	175,6259	0,849572	1	0,944105
-0,11119	420	340,1732	0,91153	1	0,987181
1,007129	367	292,3943	0,314706	1	0,647568
2,200574	280	276	0,028595	1	0,326641
0,602088	421	347,5032	0,547508	1	0,784719
-0,64957	421	188,5686	0,516761	1	0,76125
1,222422	420	383,6817	0,222299	1	0,943055
0,134372	367	318,6362	0,893193	1	0,969073
0,851167	280	258,7232	0,395464	1	0,782194
0,012854	421	340,3181	0,989752	1	0,998054
0,685944	421	185,8364	0,493603	1	0,746291
0,011601	420	374,9416	0,99075	1	0,997342
0,729	367	328,1466	0,466522	1	0,771623
-0,66912	280	263,9313	0,504007	1	0,831912
-2,64757	421	330,376	0,008496	1	0,112451
-0,55862	421	183,7565	0,577103	1	0,794719
-1,08055	420	367,2509	0,280608	1	0,943897
-1,92471	367	325,2589	0,055138	1	0,335053
1,789363	280	267,5238	0,074688	1	0,447755
-0,06239	421	334,0884	0,950289	1	0,984251
0,479458	421	186,1747	0,632175	1	0,835691
1,324237	420	369,8185	0,186242	1	0,923408
-1,15952	367	330,9593	0,247081	1	0,600675
-0,91421	280	255,4472	0,361469	1	0,762031
-0,02977	421	398,3421	0,976264	1	0,995982
-0,60979	421	214,2749	0,542645	1	0,774002
-1,36486	420	414,3595	0,173039	1	0,9199
1,111662	367	357,7998	0,26703	1	0,609149
-0,8861	280	269,6596	0,376352	1	0,767473
1,92845	421	339,255	0,054633	1	0,259162
1,900368	421	184,618	0,058943	1	0,314262
1,337016	420	378,6396	0,18202	1	0,923408
1,287005	367	321,0334	0,19902	1	0,551573
3,1135	280	264,0337	0,002052	0,266792	0,135787
1,227599	421	343,4434	0,220438	1	0,515839
1,028797	421	187,3841	0,304901	1	0,602952
1,324899	420	377,3163	0,186006	1	0,923408

1,443458	367	340,2888	0,149811	1	0,499998
-0,05655	280	262,8967	0,95495	1	0,984343
0,757787	421	408,521	0,449016	1	0,714643
-0,067	421	224,4329	0,94664	1	0,980846
-1,0104	420	415,8334	0,312893	1	0,943897
-0,47138	367	362,6481	0,637655	1	0,880707
-1,52928	280	275,7835	0,127342	1	0,536498
-1,76382	421	326,4084	0,078697	1	0,302838
-0,12963	421	180,4628	0,897007	1	0,960059
-1,33856	420	359,0741	0,181559	1	0,923408
1,493816	367	320,578	0,136207	1	0,479589
1,470449	280	251,305	0,142691	1	0,554427
2,821922	421	387,5174	0,00502	0,622425	0,087964
2,357531	421	210,1825	0,019315	1	0,195731
0,663607	420	412,7806	0,507312	1	0,960096
2,055167	367	358,6988	0,040587	1	0,289956
1,143702	280	275,3496	0,25374	1	0,679673
-2,91662	421	363,5551	0,003758	0,484802	0,087964
-1,68717	421	194,0786	0,093178	1	0,373113
-0,7458	420	399,3941	0,456229	1	0,960096
-1,17163	367	349,6307	0,242143	1	0,59405
0,092755	280	274,0546	0,926166	1	0,979504
3,532367	421	409,1716	4,59E-04	0,061017	0,04642
2,214153	421	227,149	0,027812	1	0,226812
1,210112	420	415,7238	0,226924	1	0,943055
1,974572	367	362,3363	0,049076	1	0,314711
0,595531	280	275,9185	0,551976	1	0,854033
-1,88597	421	329,0351	0,06018	1	0,270611
-0,68273	421	184,2899	0,495635	1	0,747712
-1,77191	420	360,9004	0,077253	1	0,848246
-0,67889	367	328,4753	0,497685	1	0,793601
-0,16181	280	254,024	0,871582	1	0,969656
1,866546	421	356,3081	0,062786	1	0,273465
2,439122	421	191,2629	0,015636	1	0,179127
1,289924	420	395,2622	0,197831	1	0,928589
1,759116	367	337,6802	0,079464	1	0,381841
3,968968	280	268,4838	9,27E-05	0,012424	0,046455
0,849953	421	383,4022	0,395882	1	0,686065
0,086678	421	205,4958	0,931012	1	0,97433
1,059055	420	409,1698	0,2902	1	0,943897
1,131329	367	358,1613	0,258674	1	0,609149
0,593773	280	272,3697	0,553157	1	0,854225
2,380993	421	391,6578	0,017743	1	0,15546
2,15866	421	210,3719	0,032009	1	0,241423
0,233176	420	413,337	0,81574	1	0,969427
2,777008	367	360,1829	0,005773	0,750497	0,178183
1,303998	280	275,28	0,193324	1	0,62543
0,016574	421	392,1099	0,986785	1	0,998043

-0,93092	421	208,1287	0,352971	1	0,643758
-1,63568	420	412,1823	0,102669	1	0,848246
-0,97986	367	356,7202	0,32782	1	0,655788
0,186827	280	271,3233	0,851936	1	0,966655
-8,80E-04	421	399,495	0,999298	1	0,999298
0,887466	421	214,7017	0,375821	1	0,654686
-0,13849	420	414,7987	0,889921	1	0,983483
-1,31677	367	361,5123	0,188749	1	0,538722
0,437764	280	270,7268	0,661906	1	0,908644
-1,21373	421	390,2789	0,225583	1	0,518147
-1,15202	421	208,6058	0,250633	1	0,548794
0,559135	420	412,0877	0,576373	1	0,964878
-0,99195	367	362,7919	0,321885	1	0,653748
0,087227	280	274,5375	0,930554	1	0,979504
-0,64252	421	352,9978	0,520952	1	0,769172
-1,18318	421	192,5727	0,238196	1	0,538172
-0,52507	420	386,9614	0,599836	1	0,964878
-1,59245	367	346,1897	0,112197	1	0,438296
-0,37789	280	262,3846	0,705817	1	0,922762
-0,93373	421	320,8366	0,351144	1	0,640439
-1,49876	421	182,5074	0,135664	1	0,418945
-1,46835	420	347,6517	0,142913	1	0,904916
-0,14172	367	316,0889	0,887389	1	0,964898
-0,38643	280	258,5706	0,699494	1	0,92034
-0,57034	421	363,4435	0,5688	1	0,796637
-0,37979	421	194,4411	0,704514	1	0,867404
-1,31467	420	394,3205	0,189383	1	0,925729
0,550713	367	355,1226	0,582177	1	0,845222
3,462242	280	268,1811	6,23E-04	0,081647	0,093926
-0,55707	421	351,9632	0,577836	1	0,802579
-1,59608	421	188,8067	0,112144	1	0,391524
-1,10563	420	384,2693	0,269577	1	0,943897
0,569782	367	335,0732	0,569208	1	0,837838
1,523638	280	252,4748	0,128851	1	0,536498
-2,00596	421	376,732	0,045575	1	0,237403
-0,26832	421	200,9375	0,78873	1	0,918329
0,009638	420	403,9462	0,992315	1	0,997342
0,670983	367	351,6451	0,502672	1	0,793601
-0,03549	280	266,1456	0,971715	1	0,992124
0,313466	421	372,1573	0,754103	1	0,898125
0,736199	421	199,4363	0,462475	1	0,721079
-1,39762	420	401,8831	0,162997	1	0,914005
0,767889	367	343,9403	0,44308	1	0,747728
1,636696	280	263,3375	0,102888	1	0,486059
1,99343	421	385,2465	0,046919	1	0,239762
2,623871	421	207,6318	0,009339	1	0,14815
0,481569	420	411,0817	0,630368	1	0,964878
2,590987	367	357,7162	0,009962	1	0,183886

1,864553	280	274,0769	0,063313	1	0,422605
-2,2262	421	380,0743	0,026586	1	0,1917
-1,37763	421	200,5801	0,169852	1	0,456365
-0,66331	420	406,1307	0,507508	1	0,960096
-1,35838	367	352,7254	0,175213	1	0,523018
0,058076	280	271,5206	0,953731	1	0,983759
-1,99516	421	371,1883	0,046756	1	0,239762
-1,62866	421	197,3732	0,104981	1	0,38493
-0,93935	420	398,2066	0,348122	1	0,947373
-0,0637	367	347,05	0,949246	1	0,979539
-0,44743	280	256,4882	0,65494	1	0,908644
0,375108	421	366,2215	0,707798	1	0,875021
1,008285	421	194,0451	0,314573	1	0,61407
1,021226	420	397,9055	0,307768	1	0,943897
0,825235	367	345,9097	0,409807	1	0,722315
1,602627	280	270,712	0,110183	1	0,50165
0,911571	421	356,7666	0,36261	1	0,652872
1,541786	421	190,5442	0,124785	1	0,409697
-0,64945	420	389,4323	0,516433	1	0,960096
1,81869	367	343,3177	0,06983	1	0,364488
0,484826	280	271,5365	0,628191	1	0,900874
-0,35601	421	306,5804	0,72208	1	0,886136
-0,69541	421	176,3063	0,487714	1	0,742408
-0,51934	420	339,0085	0,603863	1	0,964878
0,270179	367	307,0635	0,787204	1	0,943768
-0,20474	280	249,1058	0,837946	1	0,964667
2,148605	421	367,8403	0,032318	1	0,20468
2,644373	421	198,0905	0,00884	1	0,14815
1,940205	420	399,5724	0,053058	1	0,846627
2,095809	367	347,9021	0,036821	1	0,28464
0,974942	280	269,0204	0,330465	1	0,752282
0,141743	421	348,7469	0,887365	1	0,962
-0,99975	421	188,7404	0,318711	1	0,617081
-1,18078	420	382,9329	0,238423	1	0,943055
1,718107	367	338,1913	0,086692	1	0,391959
-0,08373	280	255,7537	0,933333	1	0,979504
0,132986	421	324,5744	0,894287	1	0,962
-0,68218	421	183,0403	0,49599	1	0,747712
0,734122	420	358,9402	0,463353	1	0,960096
1,17111	367	312,5532	0,242447	1	0,59405
-0,54648	280	247,4966	0,585231	1	0,87561
1,806316	421	309,1336	0,071841	1	0,288707
1,196105	421	179,5539	0,233233	1	0,533704
-1,33288	420	339,2819	0,183467	1	0,923408
2,436245	367	307,3588	0,015408	1	0,204372
2,392986	280	251,4394	0,017446	1	0,26828
1,016579	421	407,6866	0,309957	1	0,605668
3,690145	421	223,4056	2,82E-04	0,037736	0,035752

0,455165	420	415,9149	0,649228	1	0,964878
2,069324	367	363	0,039223	1	0,28956
1,735315	280	275,9977	0,083801	1	0,456738
-0,79218	421	331,1414	0,428824	1	0,700922
0,592712	421	185,8598	0,554094	1	0,778237
-1,70282	420	362,6613	0,089458	1	0,848246
0,195511	367	318,2352	0,845117	1	0,956935
-0,80533	280	254,4483	0,421379	1	0,796701
-0,63983	421	377,0304	0,522669	1	0,769202
0,383523	421	202,5454	0,701734	1	0,866104
0,590918	420	405,7996	0,554904	1	0,960096
-1,87002	367	355,2656	0,062303	1	0,347744
0,23337	280	271,8122	0,81565	1	0,95747
2,296635	421	391,4916	0,022167	1	0,176042
1,64528	421	210,0306	0,101408	1	0,383011
-0,14694	420	412,6549	0,883253	1	0,982463
2,053866	367	360,4578	0,040709	1	0,289956
0,850998	280	275,7039	0,395509	1	0,782194
2,005333	421	389,4155	0,045619	1	0,237403
2,128965	421	208,3466	0,034431	1	0,252759
0,051653	420	412,0718	0,95883	1	0,996522
2,407072	367	358,8386	0,016585	1	0,210035
1,457139	280	274,9854	0,146219	1	0,555466
-0,69784	421	343,9624	0,485751	1	0,745445
-1,37096	421	185,208	0,172047	1	0,456365
-0,94649	420	372,6044	0,344511	1	0,947373
-0,27226	367	324,5151	0,785593	1	0,94337
0,579383	280	254,6474	0,562843	1	0,861728
-0,27938	421	317,7411	0,780134	1	0,9142
0,602313	421	179,7743	0,547725	1	0,776424
-0,41993	420	347,759	0,674798	1	0,96781
1,408394	367	314,5385	0,160002	1	0,504558
1,295506	280	250,3155	0,196339	1	0,626871
-0,6964	421	360,2955	0,486627	1	0,74603
-1,13238	421	194,1037	0,258871	1	0,556516
-2,10898	420	391,4758	0,035581	1	0,828713
-3,11274	367	323,801	0,002019	0,268504	0,176492
-0,87701	280	268,5395	0,381263	1	0,770948
0,54639	421	384,8449	0,585115	1	0,807036
1,194291	421	205,8623	0,233738	1	0,533704
-1,48475	420	409,5319	0,138378	1	0,891179
1,169882	367	359,3835	0,242824	1	0,59405
0,685823	280	270,8531	0,493411	1	0,827962
0,386603	421	361,7141	0,699277	1	0,873083
0,952991	421	193,0779	0,341786	1	0,635891
0,016149	420	394,4707	0,987124	1	0,997342
0,620927	367	346,6334	0,535055	1	0,816122
0,617856	280	266,8046	0,537197	1	0,845931

0,21005	421	360,3783	0,833747	1	0,936611
0,690447	421	192,0819	0,490747	1	0,744768
-0,48561	420	391,5192	0,627513	1	0,964878
0,599507	367	341,8858	0,549232	1	0,828521
1,17947	280	255,3491	0,239309	1	0,662939
4,209855	421	335,9773	3,28E-05	0,035439	0,019267
1,803898	421	185,0828	0,072873	1	0,342119
1,66888	420	370,0816	0,095987	1	0,848246
1,614232	367	320,9227	0,10746	1	0,43416
1,365828	280	257,8501	0,173183	1	0,598625
0,603881	421	343,2753	0,546321	1	0,784719
-0,06305	421	184,7521	0,949793	1	0,980953
-1,82658	420	378,4254	0,068551	1	0,848246
1,162811	367	325,2963	0,245758	1	0,599343
0,278598	280	259,7905	0,780775	1	0,951006
-2,17097	421	318,3981	0,030672	1	0,200565
-1,54986	421	179,3698	0,122939	1	0,405402
-1,18507	420	354,9007	0,236783	1	0,943055
-1,51116	367	317,56	0,131742	1	0,475026
-2,22797	280	259,9821	0,026739	1	0,324814
1,680079	421	368,6619	0,093789	1	0,328698
0,714594	421	194,4679	0,475717	1	0,734136
0,683316	420	397,9333	0,494805	1	0,960096
-0,36661	367	351,5161	0,714128	1	0,912652
-3,05591	280	266,4202	0,002472	1	0,135787
-1,48988	421	331,2379	0,137208	1	0,409449
-1,71079	421	185,1226	0,088794	1	0,370674
-1,30045	420	365,4765	0,194267	1	0,928589
-1,35935	367	304,9009	0,175039	1	0,523018
-1,45712	280	254,9576	0,146315	1	0,555466
-0,34045	421	389,5407	0,733705	1	0,893273
1,729944	421	207,594	0,085127	1	0,365475
0,929591	420	411,3676	0,353128	1	0,947373
1,661508	367	356,0661	0,097492	1	0,412696
1,312479	280	268,8923	0,190478	1	0,625249
1,152477	421	405,5012	0,249804	1	0,545586
2,241231	421	222,1854	0,026	1	0,222284
-0,77546	420	415,9538	0,438506	1	0,960096
1,123594	367	362,8397	0,261928	1	0,609149
0,562918	280	274,1923	0,573951	1	0,870165
0,028254	421	341,6188	0,977476	1	0,995982
-0,20081	421	186,4356	0,841065	1	0,940271
-0,50773	420	374,5741	0,611941	1	0,964878
0,068016	367	339,1871	0,945813	1	0,979539
-1,3619	280	257,4537	0,174419	1	0,598625
0,787873	421	343,8283	0,431314	1	0,704214
0,722057	421	188,0622	0,471156	1	0,731044
-0,02652	420	376,5545	0,978857	1	0,997342

-0,95333	367	329,9666	0,341122	1	0,664831
1,24191	280	252,7421	0,215421	1	0,648472
0,092829	421	378,9364	0,926088	1	0,968505
0,224185	421	202,6447	0,82284	1	0,930247
0,963537	420	406,1514	0,335851	1	0,947373
0,663308	367	354,4386	0,507565	1	0,795942
-0,89322	280	267,825	0,372544	1	0,763914
-1,98795	421	314,0213	0,047685	1	0,241202
0,37477	421	178,7183	0,708276	1	0,870812
-1,3022	420	344,4671	0,193717	1	0,928589
1,10226	367	302,2262	0,271226	1	0,609149
0,698695	280	251,3969	0,485389	1	0,826532
-1,01839	421	377,6997	0,309143	1	0,605668
0,007282	421	200,3375	0,994197	1	0,997935
0,829221	420	401,9973	0,407471	1	0,95743
0,324896	367	355,5222	0,745451	1	0,923669
-2,02784	280	270,5668	0,043557	1	0,383858
-1,20409	421	320,2351	0,229444	1	0,520357
-0,68052	421	182,2466	0,49704	1	0,748291
-0,25048	420	357,6387	0,802361	1	0,969427
-0,06079	367	309,9695	0,951565	1	0,979686
1,359281	280	257,4192	0,175247	1	0,598625
0,78656	421	316,4432	0,432128	1	0,70478
1,894022	421	181,7821	0,05981	1	0,314727
-1,26405	420	348,4078	0,207056	1	0,930277
0,262319	367	316,2982	0,793246	1	0,944997
-0,14311	280	249,2967	0,886317	1	0,973784
1,586009	421	332,3537	0,113688	1	0,363754
2,027039	421	186,0862	0,044085	1	0,277922
0,619715	420	367,6693	0,53583	1	0,960096
0,129713	367	315,8309	0,896876	1	0,970443
0,331362	280	248,7716	0,74065	1	0,94032
1,756342	421	350,9696	0,079902	1	0,302838
0,79216	421	189,4407	0,429258	1	0,694836
-1,09403	420	383,0714	0,274627	1	0,943897
-0,46822	367	335,3083	0,63993	1	0,880707
-0,51254	280	254,6599	0,608716	1	0,886655
-0,94868	421	379,2157	0,343388	1	0,63185
-1,0893	421	202,3555	0,277316	1	0,578094
1,242551	420	406,6358	0,214749	1	0,943055
-0,00478	367	355,7578	0,996192	1	0,997516
-0,94077	280	267,9901	0,347671	1	0,761841
-1,76172	421	380,0784	0,07892	1	0,302838
-2,43475	421	201,5687	0,015772	1	0,179127
-0,64598	420	405,4417	0,518656	1	0,960096
-0,70122	367	355,8835	0,483625	1	0,786044
-0,55903	280	268,4008	0,57661	1	0,871183
-1,46608	421	321,944	0,143603	1	0,412154

-0,51185	421	182,4439	0,609376	1	0,82272
1,033199	420	356,3226	0,302212	1	0,943897
-1,11625	367	319,5807	0,265154	1	0,609149
-1,91559	280	251,5664	0,056551	1	0,406703
0,959016	421	339,3175	0,338233	1	0,629281
2,135883	421	186,7939	0,033991	1	0,252338
0,435029	420	380,2635	0,663788	1	0,96781
1,379469	367	332,8091	0,168677	1	0,514971
1,664512	280	251,6949	0,097254	1	0,480557
-0,95294	421	378,8882	0,341227	1	0,63164
-0,04686	421	201,9939	0,962671	1	0,985228
0,702573	420	404,7767	0,482726	1	0,960096
-0,56394	367	342,9856	0,573164	1	0,840232
-1,83645	280	265,457	0,067409	1	0,425749
-1,70624	421	321,0485	0,08893	1	0,319007
-1,64082	421	185,1371	0,102532	1	0,383322
-0,28002	420	359,8406	0,779623	1	0,969427
-1,62405	367	312,1372	0,105374	1	0,429907
-1,16341	280	248,1266	0,24578	1	0,676332
0,578688	421	323,3547	0,563203	1	0,796197
2,08945	421	182,3577	0,038055	1	0,267984
-0,33465	420	358,499	0,738086	1	0,969427
1,486307	367	308,6549	0,138219	1	0,484408
1,788445	280	258,8964	0,074873	1	0,447755
-0,84968	421	360,004	0,396069	1	0,686065
-1,24564	421	195,5003	0,214389	1	0,514565
0,346272	420	394,5267	0,729323	1	0,969427
-1,06599	367	351,4225	0,287161	1	0,623561
-0,68543	280	258,2894	0,493688	1	0,827962
-1,6727	421	318,4072	0,095368	1	0,331916
-1,89473	421	182,0867	0,059713	1	0,314727
-1,90265	420	348,5499	0,057909	1	0,848246
-0,72932	367	320,5617	0,466338	1	0,771623
-1,41243	280	259,5882	0,159022	1	0,578854
1,022258	421	340,9489	0,307384	1	0,604218
0,983511	421	188,8085	0,326614	1	0,620691
-0,23161	420	377,0743	0,816969	1	0,969427
-0,39994	367	323,5064	0,689465	1	0,903032
0,428494	280	266,5982	0,668638	1	0,908644
-2,49486	421	321,1813	0,013102	1	0,138079
-2,43274	421	183,4668	0,015945	1	0,179127
-0,80887	420	357,545	0,419127	1	0,95743
-1,88802	367	326,0117	0,059911	1	0,340409
-1,96901	280	266,1366	0,04999	1	0,39442
-1,63236	421	318,8708	0,103591	1	0,345379
-1,58781	421	183,0965	0,114054	1	0,391524
-0,03853	420	354,7722	0,96929	1	0,997342
-0,94971	367	313,29	0,342992	1	0,665683

-2,31822	280	253,7659	0,021233	1	0,30229
-1,71172	421	338,3697	0,087864	1	0,317534
-0,15672	421	186,59	0,875632	1	0,953452
-0,51056	420	373,2939	0,60996	1	0,964878
-0,32812	367	328,8679	0,743028	1	0,923669
1,059226	280	253,2045	0,290506	1	0,720054
0,313388	421	324,7982	0,754187	1	0,898125
0,230964	421	181,7099	0,817603	1	0,926377
-1,03421	420	355,1533	0,301743	1	0,943897
-1,00645	367	313,5493	0,314975	1	0,647568
-0,96053	280	259,5011	0,337681	1	0,755731
2,375303	421	359,5553	0,018058	1	0,156401
1,647058	421	194,3969	0,101162	1	0,383011
0,87544	420	392,5764	0,38187	1	0,95743
-1,15933	367	343,5731	0,247126	1	0,600675
2,101036	280	261,0489	0,036597	1	0,34044
0,626488	421	402,4946	0,53135	1	0,776668
-0,32582	421	218,9282	0,744868	1	0,888542
-0,70323	420	415,6989	0,482304	1	0,960096
-0,43534	367	362,8329	0,663574	1	0,894765
-0,41553	280	272,0988	0,678079	1	0,909385
-0,99222	421	332,2909	0,32181	1	0,615441
-1,46406	421	186,096	0,144864	1	0,426157
0,70045	420	366,843	0,48409	1	0,960096
-0,96787	367	331,0009	0,333816	1	0,661796
-0,41619	280	261,0038	0,677616	1	0,909385
-1,31962	421	373,1427	0,187772	1	0,472953
-2,39594	421	198,8033	0,017505	1	0,182536
-1,07329	420	402,1359	0,283786	1	0,943897
-2,74337	367	348,2813	0,006396	1	0,178183
-1,86028	280	268,733	0,063938	1	0,422605
-0,84208	421	366,9098	0,400291	1	0,689415
-1,34242	421	196,2058	0,181013	1	0,470641
0,405935	420	399,3875	0,685007	1	0,968613
-1,65386	367	339,9432	0,099079	1	0,415911
-1,18588	280	258,8139	0,236756	1	0,659852
-0,40215	421	323,9427	0,687842	1	0,865979
0,407585	421	183,9853	0,684053	1	0,859821
-0,01355	420	356,8221	0,989193	1	0,997342
-1,71074	367	305,6033	0,088144	1	0,394165
1,481193	280	253,7702	0,139796	1	0,552945
0,211478	421	355,852	0,832635	1	0,936611
1,218693	421	192,2684	0,224454	1	0,521994
0,462802	420	388,7593	0,643765	1	0,964878
2,303223	367	338,9246	0,021871	1	0,230489
-0,38557	280	264,8673	0,700126	1	0,92034
-0,46512	421	409,4036	0,642096	1	0,835611
-0,2949	421	226,7995	0,768339	1	0,905801

0,284916	420	415,682	0,77585	1	0,969427
-0,89292	367	362,4629	0,37249	1	0,682069
-0,62873	280	275,9965	0,530044	1	0,840949
-0,19652	421	357,3876	0,844317	1	0,939735
-0,75207	421	194,245	0,452919	1	0,71471
0,721604	420	390,1659	0,47097	1	0,960096
-2,66461	367	348,7368	0,008066	1	0,181422
-2,18308	280	269,8707	0,029892	1	0,328807
-0,36535	421	313,5694	0,715093	1	0,881149
0,445062	421	181,0622	0,656807	1	0,841515
-0,42673	420	348,7287	0,669836	1	0,96781
-0,46484	367	305,8107	0,642378	1	0,882539
2,010098	280	249,0282	0,045499	1	0,387749
-2,44553	421	330,5268	0,014985	1	0,147897
-1,41803	421	184,9643	0,157863	1	0,43893
-0,41727	420	368,4116	0,676724	1	0,96781
0,313076	367	322,1756	0,754426	1	0,92504
-0,91251	280	246,984	0,362389	1	0,762031
0,720655	421	310,012	0,471665	1	0,731548
1,08517	421	178,9461	0,279306	1	0,578974
-0,32786	420	343,0449	0,743215	1	0,969427
-2,2033	367	308,9442	0,028312	1	0,257802
1,500053	280	248,7848	0,134869	1	0,544898
-0,68782	421	331,1258	0,492051	1	0,748579
-1,69961	421	185,3088	0,090882	1	0,373113
0,834571	420	364,0105	0,404507	1	0,95743
-1,12769	367	334,1037	0,260258	1	0,609149
0,13066	280	261,9096	0,896144	1	0,975243
0,376313	421	367,8797	0,706901	1	0,875021
1,300459	421	197,9817	0,194956	1	0,483337
-0,753	420	398,5073	0,451893	1	0,960096
1,51686	367	348,7579	0,130208	1	0,47397
-0,17121	280	267,8193	0,864186	1	0,968274
-0,48687	421	324,1637	0,626678	1	0,832808
0,973078	421	181,0788	0,331813	1	0,626331
-0,75662	420	357,9055	0,449774	1	0,960096
0,756317	367	312,1434	0,45003	1	0,755345
1,187894	280	276	0,235896	1	0,659852
0,618086	421	367,2654	0,536902	1	0,778195
0,620274	421	196,8047	0,535795	1	0,770962
0,642188	420	397,6632	0,521121	1	0,960096
2,827545	367	347,931	0,004962	1	0,178183
1,088708	280	262,4767	0,277281	1	0,701156
-1,84252	421	333,9214	0,066286	1	0,278382
-2,25643	421	185,4448	0,025211	1	0,218351
-1,45312	420	375,1536	0,147025	1	0,914005
-1,14426	367	312,1015	0,253393	1	0,609031
-1,48587	280	256,9764	0,138539	1	0,550865

0,529596	421	301,9574	0,596781	1	0,815122
0,837295	421	178,1524	0,403549	1	0,677774
-1,86416	420	332,673	0,06318	1	0,848246
0,978006	367	307,3658	0,32884	1	0,656374
-0,329	280	276	0,742403	1	0,94096
-2,41731	421	320,7851	0,016193	1	0,147897
-2,42771	421	184,0791	0,016157	1	0,179127
0,997229	420	363,4896	0,319317	1	0,945573
-1,88722	367	329,5118	0,060009	1	0,340409
-1,12508	280	258,9151	0,261598	1	0,687569
-3,12277	421	326,4639	0,001952	1	0,069718
-1,89977	421	185,36	0,059015	1	0,314262
-0,73018	420	361,2446	0,465755	1	0,960096
0,322932	367	324,1215	0,746955	1	0,923669
-2,13259	280	259,4661	0,033897	1	0,329568
-0,09187	421	382,7967	0,92685	1	0,968629
1,530861	421	203,55	0,127356	1	0,410224
0,690607	420	407,5104	0,490206	1	0,960096
-1,22858	367	359,4888	0,220034	1	0,572696
-0,23638	280	270,0793	0,813315	1	0,95747
-2,62452	421	344,7036	0,009063	1	0,113397
-2,2628	421	186,3847	0,024802	1	0,218351
-0,77577	420	376,662	0,43837	1	0,960096
-0,77932	367	322,9821	0,436365	1	0,742744
-3,03086	280	256,4497	0,002688	1	0,135787
-0,1005	421	365,0038	0,920001	1	0,966828
0,819328	421	195,7369	0,413596	1	0,684933
1,225456	420	393,2492	0,221137	1	0,943055
0,646297	367	347,6258	0,518513	1	0,801766
-0,00966	280	260,1511	0,992303	1	0,996269
1,503396	421	386,3545	0,133554	1	0,406597
1,304988	421	204,6189	0,193363	1	0,481649
1,491172	420	409,6761	0,136686	1	0,891179
1,122793	367	356,4507	0,262281	1	0,609149
-0,52207	280	273,7215	0,602044	1	0,882569
-1,55896	421	417	0,119765	1	0,381479
-1,23244	421	417	0,218479	1	0,514565
-0,63112	420	416	0,528308	1	0,960096
-0,68944	367	283,1911	0,491111	1	0,792348
-1,05505	280	276	0,292327	1	0,722191
-0,90693	421	323,0142	0,365121	1	0,655826
-0,38694	421	183,4845	0,69925	1	0,865528
2,054155	420	360,807	0,040681	1	0,846132
1,815338	367	318,0271	0,070414	1	0,36493
1,190582	280	251,377	0,234941	1	0,659852
-0,8027	421	336,5846	0,422715	1	0,697735
-1,37104	421	187,328	0,172002	1	0,456365
-2,27848	420	366,2969	0,023273	1	0,75904

-0,52477	367	325,8241	0,600097	1	0,862104
-0,66422	280	261,7969	0,507135	1	0,831912
-0,38201	421	346,1037	0,702685	1	0,874567
-1,73564	421	187,8224	0,084267	1	0,365475
-0,17624	420	381,5928	0,860201	1	0,976988
0,920822	367	337,7545	0,357801	1	0,674813
0,130065	280	262,8495	0,896614	1	0,975243
1,670649	421	345,0164	0,095698	1	0,332296
0,465191	421	191,2167	0,642324	1	0,838647
-0,14622	420	382,804	0,883827	1	0,982463
1,747694	367	333,1962	0,081438	1	0,381841
0,904271	280	259,3109	0,366691	1	0,763914
-1,41281	421	375,1203	0,15854	1	0,431264
-1,37881	421	200,6188	0,169489	1	0,456365
-0,26761	420	404,9385	0,789134	1	0,969427
-3,01075	367	355,4673	0,002792	1	0,176492
-2,98635	280	269,8125	0,003083	1	0,136636
3,13647	421	349,7123	0,001855	1	0,069718
1,316958	421	189,7112	0,189441	1	0,476746
0,9521	420	385,652	0,341643	1	0,947373
-0,20925	367	333,7097	0,834379	1	0,956935
0,377181	280	258,3543	0,706349	1	0,922762
-0,39245	421	318,7575	0,694985	1	0,870609
-0,3735	421	178,072	0,709222	1	0,870869
-0,9418	420	353,7145	0,346938	1	0,947373
1,34051	367	295,6478	0,181109	1	0,527769
-3,14388	280	246,0309	0,001872	1	0,135787
2,830827	421	358,8424	0,004904	1	0,087964
2,9197	421	190,7183	0,003926	1	0,118336
1,657965	420	388,6881	0,098131	1	0,848246
1,62017	367	341,1268	0,10612	1	0,431058
1,588718	280	269,6216	0,113296	1	0,508687
-1,87296	421	353,671	0,061898	1	0,271708
-2,9195	421	191,5542	0,003926	1	0,118336
-1,14094	420	389,5524	0,254594	1	0,943897
-3,94941	367	341,3783	9,52E-05	0,102617	0,053579
-3,86937	280	260,9652	1,38E-04	0,148664	0,046455
0,819369	421	350,3365	0,413133	1	0,695339
1,078272	421	188,9859	0,282287	1	0,581951
-0,02208	420	384,1508	0,982397	1	0,997342
1,086491	367	330,1871	0,278054	1	0,612614
0,509712	280	260,6841	0,610684	1	0,888252
-1,93204	421	341,7002	0,054181	1	0,259162
-2,84503	421	189,7	0,004928	1	0,137431
-1,07326	420	382,1213	0,283833	1	0,943897
-2,5615	367	343,6309	0,010848	1	0,189546
-0,27811	280	265,5697	0,781142	1	0,951006
-0,19644	421	340,2744	0,844381	1	0,939735

-1,07712	421	185,7399	0,282824	1	0,582262
-1,79315	420	375,0219	0,073755	1	0,848246
-1,12487	367	315,1952	0,261499	1	0,609149
-1,82641	280	252,9107	0,068968	1	0,429481
-2,42158	421	279,4383	0,01609	1	0,147897
-0,58919	421	171,5692	0,556507	1	0,778696
0,832472	420	313,8818	0,405775	1	0,95743
0,836324	367	277,3579	0,403692	1	0,716566
1,737018	280	245,9324	0,083636	1	0,456738
1,151216	421	294,4045	0,250577	1	0,546261
3,416893	421	175,2191	7,87E-04	0,840194	0,065272
-1,47194	420	326,1456	0,142	1	0,90372
0,656943	367	296,401	0,511727	1	0,797829
1,999487	280	276	0,046536	1	0,387749
-0,36015	421	344,8103	0,718952	1	0,884157
0,587443	421	189,5389	0,557605	1	0,779138
0,286841	420	379,1988	0,774391	1	0,969427
1,030418	367	329,4693	0,30357	1	0,636272
0,677818	280	252,6134	0,498508	1	0,83103
-3,61478	421	318,5376	3,49E-04	0,373728	0,04642
-1,66475	421	184,0682	0,097664	1	0,379251
0,055439	420	361,283	0,955819	1	0,995923
-0,96816	367	316,9403	0,333703	1	0,661796
-0,80637	280	259,0303	0,420767	1	0,796701
0,733449	421	322,5398	0,463817	1	0,727964
-0,45756	421	182,4174	0,647814	1	0,839358
-0,84899	420	355,0611	0,396459	1	0,95743
1,493995	367	323,1178	0,136153	1	0,479589
0,692082	280	259,5249	0,489505	1	0,826999
-1,83188	421	370,0954	0,067773	1	0,280051
-1,6877	421	197,7994	0,093046	1	0,373113
1,270197	420	399,5515	0,204754	1	0,928589
-1,49848	367	347,4335	0,134917	1	0,477542
-2,73515	280	269,3672	0,006649	1	0,192695
-1,43182	421	365,4	0,153049	1	0,42476
0,309091	421	197,3956	0,757578	1	0,898246
-0,22315	420	398,1452	0,823534	1	0,972623
-1,30206	367	344,7814	0,193766	1	0,547402
-0,82177	280	266,9322	0,411943	1	0,788151
1,476219	421	386,1244	0,1407	1	0,410878
1,276603	421	207,1804	0,203172	1	0,497853
0,155193	420	410,1018	0,876745	1	0,982463
0,335749	367	360,4973	0,737255	1	0,923669
0,02671	280	272,7211	0,978711	1	0,99236
-0,7129	421	297,7232	0,476468	1	0,734941
-1,56543	421	174,6099	0,119294	1	0,396436
0,531895	420	330,8111	0,595156	1	0,964878
-2,83744	367	309,7026	0,004848	1	0,178183

-1,81974	280	251,2013	0,069989	1	0,432271
-2,83866	421	309,206	0,00483	1	0,087964
-1,79625	421	176,9731	0,074161	1	0,344631
0,318201	420	342,0608	0,750526	1	0,969427
-2,46768	367	305,8839	0,014146	1	0,201413
-0,80041	280	248,779	0,424235	1	0,797636
-0,68845	421	316,3922	0,491676	1	0,748579
-0,98851	421	181,5591	0,32422	1	0,619085
-1,27431	420	350,5728	0,203397	1	0,928589
-1,64704	367	304,1144	0,100583	1	0,42105
-0,11746	280	257,3637	0,906588	1	0,975308
-2,20387	421	332,7127	0,028218	1	0,19529
-3,63941	421	186,1064	3,54E-04	0,378683	0,038096
-0,80269	420	371,8112	0,422664	1	0,95743
-1,95445	367	332,2506	0,051486	1	0,32195
-2,16883	280	255,7904	0,031017	1	0,329568
-0,95025	421	310,5259	0,342723	1	0,63164
-0,39133	421	177,1927	0,696023	1	0,865526
0,41916	420	343,5978	0,675361	1	0,96781
0,376087	367	309,1963	0,70711	1	0,91071
-0,91923	280	245,7744	0,358878	1	0,761846
-1,14336	421	369,4957	0,253628	1	0,546261
-1,59923	421	197,6717	0,111367	1	0,391524
0,025644	420	398,8749	0,979554	1	0,997342
-2,45559	367	355,1585	0,014544	1	0,204372
-1,8718	280	271,39	0,06231	1	0,422605
-1,4345	421	331,0548	0,152374	1	0,42445
-0,39375	421	182,9819	0,694223	1	0,865526
-0,48556	420	363,6066	0,627573	1	0,964878
-0,43032	367	307,4014	0,667264	1	0,894765
-0,25396	280	259,8997	0,799728	1	0,954985
1,849456	421	377,7584	0,065173	1	0,276236
1,332861	421	199,6916	0,184096	1	0,473593
1,711076	420	405,9387	0,087831	1	0,848246
0,070812	367	352,8454	0,943587	1	0,979539
-0,59902	280	267,2306	0,549665	1	0,852208
-0,64691	421	348,8799	0,518115	1	0,768119
-1,3907	421	189,0606	0,16595	1	0,453055
-0,98546	420	382,3141	0,325021	1	0,945573
-0,0906	367	331,7287	0,927865	1	0,976733
0,351922	280	264,0023	0,725177	1	0,936493
3,157493	421	303,0739	0,001752	1	0,069718
3,986601	421	173,5941	9,86E-05	0,105979	0,021224
1,82159	420	339,3723	0,069397	1	0,848246
1,504513	367	286,6263	0,133551	1	0,477542
2,416704	280	276	0,016311	1	0,266517
1,24994	421	315,4375	0,212248	1	0,502921
2,605084	421	174,3588	0,009979	1	0,150377

1,923362	420	353,0493	0,055239	1	0,848246
0,878495	367	301,9481	0,380374	1	0,691076
2,585688	280	248,9666	0,010288	1	0,229419
-0,34679	421	297,478	0,728993	1	0,890992
0,564848	421	176,0082	0,572897	1	0,792796
0,029504	420	328,8258	0,97648	1	0,997342
0,688644	367	287,696	0,491603	1	0,792348
0,132824	280	246,8869	0,894441	1	0,975243
0,777576	421	338,8497	0,437362	1	0,707193
1,481011	421	186,8909	0,140288	1	0,423351
-0,65022	420	372,1548	0,515948	1	0,960096
0,358039	367	324,9923	0,720547	1	0,914797
0,893851	280	252,5624	0,372253	1	0,763914
-0,12149	421	356,7697	0,90337	1	0,965517
-0,4909	421	193,687	0,624053	1	0,830785
0,698649	420	390,8116	0,485187	1	0,960096
0,121632	367	343,3179	0,903262	1	0,970486
-0,38952	280	256,4036	0,697216	1	0,92034
-2,05563	421	337,072	0,040588	1	0,226301
-1,37139	421	188,047	0,171886	1	0,456365
0,312302	420	375,8693	0,754984	1	0,969427
-1,15276	367	321,4095	0,249865	1	0,604409
-1,41945	280	257,3723	0,156979	1	0,575588
-2,04311	421	393,6028	0,041707	1	0,228553
-2,63391	421	210,433	0,009068	1	0,14815
0,231676	420	413,9775	0,816904	1	0,969427
-3,82453	367	362,867	1,54E-04	0,165916	0,053579
-3,6329	280	275,8427	3,34E-04	0,358847	0,06294
-1,49294	421	367,6288	0,13631	1	0,407576
-1,76534	421	197,9025	0,079048	1	0,355599
0,184743	420	401,8197	0,853524	1	0,973894
-2,50116	367	354,4778	0,012829	1	0,189546
-1,64635	280	273,2807	0,10084	1	0,480557
0,043306	421	333,3528	0,965483	1	0,992572
-1,16405	421	183,1662	0,24592	1	0,546224
-0,6852	420	369,1802	0,493645	1	0,960096
1,917739	367	323,7002	0,056024	1	0,337523
-0,18097	280	268,3374	0,856531	1	0,966655
-2,83032	421	347,0363	0,004921	1	0,087964
-3,03172	421	191,0084	0,002769	1	0,114447
-1,48614	420	385,9621	0,138059	1	0,891179
-2,60101	367	335,9949	0,009706	1	0,183886
-1,72921	280	267,6715	0,084924	1	0,45831
0,077166	421	353,322	0,938535	1	0,97543
2,660864	421	192,5582	0,008452	1	0,148102
-0,89782	420	388,8481	0,369838	1	0,95743
-0,98445	367	331,3493	0,325611	1	0,655597
-0,31455	280	263,3819	0,753353	1	0,942071

2,52588	421	370,4558	0,011957	1	0,131857
2,190993	421	198,2817	0,029618	1	0,236159
-0,23365	420	403,0765	0,815371	1	0,969427
1,421168	367	346,4986	0,156167	1	0,502197
0,537957	280	263,9155	0,59106	1	0,8767
-1,36772	421	397,0033	0,172174	1	0,444059
-1,1277	421	212,6171	0,260719	1	0,557979
-1,19055	420	414,0841	0,234511	1	0,943055
-0,57051	367	361,5091	0,568684	1	0,837838
-0,12688	280	272,8679	0,899131	1	0,975243
-2,17353	421	344,6354	0,030421	1	0,200565
-2,0951	421	191,1915	0,037479	1	0,266419
0,097539	420	385,2307	0,922349	1	0,989204
-1,92893	367	343,0807	0,054564	1	0,335053
-1,14904	280	268,2209	0,251565	1	0,679007
-1,39989	421	308,3024	0,162553	1	0,436662
0,561022	421	178,7227	0,575485	1	0,794719
1,036318	420	343,0789	0,300784	1	0,943897
1,603372	367	294,5728	0,109924	1	0,435936
0,868683	280	262,6821	0,385813	1	0,773166
0,493081	421	342,0149	0,622272	1	0,828413
1,998569	421	188,7997	0,04709	1	0,284997
-0,20377	420	380,7065	0,838645	1	0,973894
0,473138	367	334,3866	0,636424	1	0,880707
0,453982	280	251,9935	0,650233	1	0,908644
-1,02176	421	299,1653	0,307719	1	0,604218
0,133803	421	178,0153	0,893709	1	0,958882
-0,61909	420	331,7532	0,536279	1	0,960096
0,186374	367	302,3193	0,852277	1	0,956935
-0,79813	280	252,6953	0,425547	1	0,797636
-2,35125	421	339,1889	0,019282	1	0,159771
-2,16907	421	184,0264	0,03136	1	0,241423
-1,69132	420	372,1633	0,091614	1	0,848246
-0,48031	367	319,693	0,631338	1	0,880707
-2,0441	280	251,7894	0,041984	1	0,374382
0,178164	421	363,5919	0,858693	1	0,949789
-0,75111	421	196,3262	0,453486	1	0,714856
0,636561	420	394,944	0,524779	1	0,960096
-2,1621	367	344,1308	0,031299	1	0,266905
-1,80109	280	260,7785	0,072844	1	0,44087
2,390153	421	365,5677	0,017347	1	0,153976
2,021274	421	193,7573	0,044627	1	0,279059
1,03627	420	398,5347	0,300705	1	0,943897
1,92514	367	344,733	0,055034	1	0,335053
2,921402	280	272,2313	0,003777	1	0,147267
-1,93258	421	324,5681	0,054158	1	0,259162
0,462774	421	184,5241	0,644072	1	0,838647
0,186027	420	358,7072	0,852529	1	0,973894

0,413668	367	316,6939	0,679397	1	0,899694
-0,52646	280	270,5825	0,598997	1	0,881231
-0,81417	421	351,6625	0,416099	1	0,695339
-1,47193	421	189,9476	0,142695	1	0,423993
-1,54075	420	385,7334	0,124198	1	0,87461
-1,92116	367	340,5723	0,055546	1	0,336177
-0,64124	280	261,1722	0,521931	1	0,833721
-1,83526	421	341,1048	0,067337	1	0,279551
-1,64896	421	187,8157	0,100828	1	0,383011
0,324786	420	375,697	0,745524	1	0,969427
0,165765	367	336,2388	0,868441	1	0,959978
-2,69026	280	249,435	0,007622	1	0,199202
-1,37044	421	340,7435	0,171452	1	0,443949
0,170025	421	187,9446	0,865173	1	0,947215
-0,01491	420	375,7791	0,988115	1	0,997342
-0,6251	367	324,4585	0,532343	1	0,814457
0,194304	280	265,5515	0,846086	1	0,965181
-0,60462	421	330,2331	0,545847	1	0,784719
0,619042	421	183,8363	0,536655	1	0,770962
1,230447	420	363,0291	0,219326	1	0,943055
0,912338	367	319,112	0,36228	1	0,677287
0,758812	280	263,159	0,448644	1	0,813228
1,242346	421	385,2484	0,214865	1	0,506731
1,255644	421	205,2037	0,210673	1	0,509968
0,338467	420	410,0009	0,735185	1	0,969427
2,502283	367	352,3091	0,012792	1	0,189546
0,233111	280	271,4693	0,815851	1	0,95747
2,704879	421	379,9553	0,00714	1	0,105763
2,542918	421	204,2296	0,011734	1	0,165261
-1,12701	420	410,6461	0,260398	1	0,943897
1,407882	367	355,8247	0,160039	1	0,504558
0,663477	280	273,2936	0,507584	1	0,831912
-2,20796	421	365,8455	0,027867	1	0,19529
-1,37254	421	195,246	0,17147	1	0,456365
-0,2955	420	397,009	0,767764	1	0,969427
-0,9554	367	346,2988	0,340042	1	0,664831
-0,42765	280	259,0374	0,669263	1	0,908644
0,487919	421	326,3473	0,625935	1	0,832554
-0,19335	421	184,2414	0,846898	1	0,943293
-0,21759	420	360,9658	0,82787	1	0,973894
-1,39401	367	317,5757	0,164289	1	0,510483
-1,65737	280	252,3712	0,098688	1	0,480557
-1,54976	421	329,3391	0,122159	1	0,383489
-1,5681	421	186,8358	0,118551	1	0,396436
-1,76034	420	371,0984	0,079173	1	0,848246
-0,66863	367	321,5754	0,504214	1	0,793601
-0,0453	280	266,1343	0,963899	1	0,989749
-1,60308	421	352,1827	0,109814	1	0,35666

-1,71434	421	192,9852	0,088071	1	0,368676
-0,9678	420	387,0991	0,333748	1	0,947373
-2,06454	367	333,4073	0,03974	1	0,28956
-0,22001	280	258,3268	0,826039	1	0,96121
-0,80606	421	370,856	0,420724	1	0,697735
-0,66426	421	198,8657	0,507292	1	0,754678
0,475521	420	402,1602	0,634674	1	0,964878
0,127059	367	350,1052	0,898967	1	0,970486
-0,29626	280	263,7521	0,767265	1	0,945436
1,055552	421	406,0371	0,291801	1	0,589468
1,235726	421	221,4694	0,21787	1	0,514565
-0,87508	420	415,9932	0,382035	1	0,95743
0,446296	367	362,9171	0,655649	1	0,890147
1,924581	280	273,3819	0,055319	1	0,40654
-2,28795	421	324,5218	0,022783	1	0,177419
-1,56553	421	184,7853	0,11917	1	0,396436
-0,48558	420	367,8543	0,627551	1	0,964878
-2,1258	367	324,6972	0,034274	1	0,279197
-1,31522	280	257,1582	0,189608	1	0,625017
-0,2174	421	382,5044	0,828014	1	0,936611
0,169745	421	203,9768	0,865379	1	0,947215
-0,43625	420	407,4436	0,662886	1	0,96781
-0,626	367	356,7739	0,531712	1	0,814457
0,872762	280	270,0102	0,383568	1	0,772778
-1,48232	421	370,5601	0,139106	1	0,4106
-1,12454	421	198,4352	0,262143	1	0,557979
-0,68156	420	401,5117	0,49591	1	0,960096
-1,72174	367	343,7475	0,086016	1	0,390442
0,775282	280	261,7586	0,438873	1	0,808534
-1,61251	421	332,2817	0,1078	1	0,353931
-1,13792	421	183,7655	0,256634	1	0,556025
-0,93996	420	368,5727	0,347851	1	0,947373
-0,60754	367	323,6895	0,543915	1	0,824628
-0,98027	280	252,4947	0,327892	1	0,752105
-2,57868	421	328,9734	0,010352	1	0,120936
-1,73178	421	184,8265	0,084982	1	0,365475
-1,5083	420	368,317	0,132335	1	0,888479
-1,96929	367	316,1629	0,049793	1	0,315285
-1,13917	280	263,2284	0,25567	1	0,679673
0,312998	421	304,4939	0,754497	1	0,898125
1,176804	421	179,1003	0,240835	1	0,5417
0,340504	420	337,8843	0,733688	1	0,969427
-0,44708	367	300,7289	0,655142	1	0,890147
0,103658	280	276	0,917516	1	0,977051
1,077046	421	363,571	0,282174	1	0,581718
0,306291	421	196,0871	0,759708	1	0,899424
0,331435	420	394,7194	0,740492	1	0,969427
-0,53668	367	338,2129	0,591844	1	0,855959

0,642557	280	267,2102	0,521062	1	0,833721
-2,2076	421	356,9542	0,027908	1	0,19529
-2,4016	421	193,6414	0,017269	1	0,181988
-1,44462	420	389,8563	0,149367	1	0,914005
-2,3323	367	347,2841	0,020256	1	0,224016
-1,21733	280	270,1011	0,224543	1	0,654014
-3,38948	421	328,8874	7,85E-04	0,834891	0,053127
-0,81523	421	184,958	0,415988	1	0,686888
-0,81295	420	361,7333	0,41678	1	0,95743
-0,3511	367	322,1168	0,725741	1	0,919068
-1,0516	280	262,6315	0,293948	1	0,723324
-0,16581	421	298,7658	0,868423	1	0,954568
-0,15829	421	178,0556	0,874404	1	0,952803
-0,35033	420	327,2023	0,726313	1	0,969427
-0,10343	367	296,2925	0,91769	1	0,973915
-0,65037	280	256,6589	0,516038	1	0,83211
0,271233	421	379,7695	0,786359	1	0,917552
1,18037	421	203,1832	0,239233	1	0,539707
1,135573	420	406,9419	0,256804	1	0,943897
1,271348	367	344,7605	0,204462	1	0,558434
-0,89843	280	269,1909	0,369758	1	0,763914
-2,64879	421	318,3992	0,008481	1	0,112451
-0,92862	421	179,7624	0,354329	1	0,643758
-1,38669	420	345,7572	0,16643	1	0,914005
-1,53776	367	331,8663	0,125059	1	0,462104
-0,23507	280	268,7875	0,814337	1	0,95747
-1,50487	421	328,6952	0,133319	1	0,406597
0,051071	421	184,0634	0,959324	1	0,98414
-0,18505	420	366,5594	0,853293	1	0,973894
-1,26713	367	319,1893	0,206031	1	0,558434
-0,07126	280	252,3108	0,943246	1	0,982175
-2,8188	421	304,0327	0,005137	1	0,087964
-0,88715	421	175,1928	0,376216	1	0,654686
-1,28362	420	332,524	0,200168	1	0,928589
-0,96122	367	289,5257	0,337244	1	0,662616
-0,69411	280	276	0,488196	1	0,826999
-1,67666	421	357,278	0,094483	1	0,330363
-1,17763	421	192,5397	0,240397	1	0,541523
-0,95832	420	390,6535	0,338496	1	0,947373
-1,51914	367	344,8206	0,129644	1	0,473621
-1,50696	280	267,9769	0,132998	1	0,543276
2,063454	421	347,5333	0,039812	1	0,224527
3,697873	421	189,3841	2,85E-04	0,305187	0,035752
0,131632	420	382,6881	0,895345	1	0,98503
0,088827	367	326,7499	0,929274	1	0,976733
0,230043	280	257,0787	0,818241	1	0,95778
-0,24622	421	352,5275	0,805656	1	0,922616
-0,02614	421	191,5255	0,979174	1	0,992344

0,339584	420	386,0545	0,734354	1	0,969427
1,569531	367	343,8313	0,117444	1	0,445308
-0,26173	280	261,8226	0,793736	1	0,954269
-2,59143	421	307,3621	0,010014	1	0,117898
-0,82081	421	174,7247	0,412874	1	0,684933
0,795007	420	337,7918	0,427167	1	0,95743
0,507974	367	283,2266	0,611867	1	0,871158
-2,00369	280	251,0358	0,046178	1	0,387749
0,367442	421	395,1869	0,713486	1	0,881149
1,025759	421	210,8341	0,30618	1	0,603743
0,780164	420	414,0113	0,43574	1	0,960096
2,265093	367	357,0774	0,024106	1	0,245454
0,732566	280	274,9309	0,464447	1	0,824256
-1,00398	421	337,9406	0,316107	1	0,609953
-1,19064	421	181,584	0,235348	1	0,535889
0,554702	420	376,1105	0,579428	1	0,964878
-0,49402	367	329,9276	0,621619	1	0,875495
1,539913	280	264,2012	0,124778	1	0,535729
-1,52943	421	307,5539	0,127186	1	0,392764
-2,56713	421	180,4428	0,011065	1	0,161895
-1,17274	420	345,7413	0,241707	1	0,943659
-2,12972	367	302,9926	0,034	1	0,279197
-1,45795	280	258,6428	0,146066	1	0,555466
-2,3502	421	364,7326	0,019295	1	0,159771
-2,47808	421	195,9251	0,014055	1	0,173612
-2,13995	420	397,864	0,032966	1	0,828006
-1,42264	367	347,9718	0,155735	1	0,502197
-0,68588	280	271,1955	0,493374	1	0,827962
0,59227	421	348,5409	0,554054	1	0,790572
1,511267	421	189,5888	0,132385	1	0,414338
0,69136	420	381,6753	0,48976	1	0,960096
0,229297	367	340,5827	0,818776	1	0,951644
-0,7676	280	252,286	0,44344	1	0,810247
3,044085	421	322,4072	0,002526	1	0,073944
2,231628	421	181,9878	0,02686	1	0,223635
1,546927	420	354,1193	0,122774	1	0,87201
1,749128	367	291,6567	0,081321	1	0,381841
1,038691	280	254,3762	0,299935	1	0,723324
1,75072	421	409,9064	0,080742	1	0,30376
1,804162	421	226,6301	0,072534	1	0,341588
0,112327	420	415,6615	0,910619	1	0,987181
1,478144	367	362,6358	0,140237	1	0,488078
0,178244	280	275,589	0,858663	1	0,966655
-1,15907	421	355,5068	0,247205	1	0,544195
-0,93228	421	193,6202	0,352354	1	0,643758
1,42364	420	388,6646	0,155353	1	0,914005
-0,65973	367	334,1106	0,509885	1	0,797092
-0,82221	280	261,0238	0,411709	1	0,788151

-1,20392	421	300,8466	0,229567	1	0,520357
-0,48142	421	175,8267	0,630815	1	0,835359
-1,37261	420	329,5722	0,170805	1	0,9199
0,595491	367	290,0498	0,55198	1	0,830257
0,143129	280	248,7619	0,886304	1	0,973784
2,223839	421	328,3967	0,026839	1	0,192509
1,989322	421	184,0322	0,048147	1	0,286754
0,836765	420	362,6318	0,403276	1	0,95743
1,285694	367	321,9245	0,199474	1	0,551573
0,301785	280	252,2805	0,763065	1	0,944296
0,476684	421	346,9768	0,633887	1	0,83455
1,461055	421	190,6178	0,145647	1	0,426194
0,7969	420	381,3761	0,426005	1	0,95743
0,875269	367	341,649	0,382042	1	0,692825
1,042046	280	257,9818	0,298366	1	0,723324
-0,25368	421	336,6902	0,799901	1	0,922616
-0,89773	421	184,283	0,370504	1	0,649999
-0,27344	420	370,802	0,784667	1	0,969427
1,268315	367	337,5372	0,20556	1	0,558434
1,227428	280	266,9377	0,220743	1	0,653047
1,406248	421	319,1545	0,160623	1	0,434577
1,897255	421	180,9546	0,059387	1	0,314727
0,360255	420	351,9323	0,718873	1	0,969427
1,444284	367	315,0529	0,149653	1	0,499998
1,700154	280	262,9977	0,090284	1	0,468147
-1,28382	421	314,7783	0,200151	1	0,488069
-0,16881	421	179,5217	0,866133	1	0,947215
-0,48086	420	347,5893	0,630921	1	0,964878
0,078763	367	291,1824	0,937275	1	0,978845
0,222499	280	249,6737	0,824108	1	0,96121
-2,87614	421	336,6441	0,004282	1	0,087964
-1,50666	421	183,0495	0,133621	1	0,415189
-1,28865	420	372,0179	0,198322	1	0,928589
-0,19483	367	314,8202	0,845653	1	0,956935
-2,13686	280	257,6591	0,033551	1	0,329568
-2,6294	421	312,3496	0,008977	1	0,113397
-0,27345	421	178,2001	0,784826	1	0,916847
-1,35948	420	341,15	0,174894	1	0,9199
-0,39443	367	310,2691	0,693537	1	0,904597
1,595205	280	252,7417	0,111916	1	0,506479
1,468264	421	330,9759	0,142982	1	0,412154
1,982386	421	185,6181	0,048911	1	0,286808
-1,14665	420	365,9345	0,252275	1	0,943897
0,267765	367	330,2005	0,789048	1	0,944997
0,099483	280	261,8677	0,920831	1	0,977136
0,173471	421	362,7947	0,862378	1	0,951261
-0,62355	421	195,6754	0,53365	1	0,769894
-0,81455	420	393,5188	0,415822	1	0,95743

-1,12544	367	353,6949	0,261164	1	0,609149
0,153554	280	273,522	0,878074	1	0,973498
0,43063	421	317,0663	0,66703	1	0,853323
0,516554	421	180,3992	0,6061	1	0,821338
1,095079	420	353,5535	0,274228	1	0,943897
-0,33128	367	312,9173	0,740655	1	0,923669
2,261858	280	257,8279	0,024539	1	0,313396
-0,15382	421	353,0516	0,877837	1	0,95861
-0,71425	421	189,9915	0,475946	1	0,734136
-0,81848	420	382,2276	0,413594	1	0,95743
-0,95333	367	334,5098	0,341109	1	0,664831
0,237278	280	265,9415	0,812624	1	0,95747
-0,57691	421	355,2722	0,564364	1	0,796637
0,21654	421	192,2738	0,828796	1	0,934178
-0,83605	420	388,8824	0,403643	1	0,95743
-1,13407	367	345,417	0,257551	1	0,609149
0,410908	280	267,436	0,681469	1	0,911245
-0,60389	421	350,2468	0,546306	1	0,784719
-0,97242	421	191,8077	0,332067	1	0,626331
1,187071	420	384,8418	0,235932	1	0,943055
0,185955	367	342,9693	0,85259	1	0,956935
-1,22096	280	264,3868	0,223188	1	0,654014
1,221865	421	376,474	0,222523	1	0,51676
0,792637	421	203,1517	0,428914	1	0,694836
0,246164	420	405,3608	0,80568	1	0,969427
0,81027	367	346,7845	0,418341	1	0,729676
-1,58812	280	273,0388	0,113417	1	0,508687
-1,49833	421	345,1067	0,134961	1	0,407576
-2,8388	421	188,1138	0,005026	1	0,137431
-0,29867	420	381,0731	0,765357	1	0,969427
-2,84956	367	338,1146	0,004646	1	0,178183
-1,49992	280	260,4532	0,134847	1	0,544898
2,265197	421	399,903	0,024035	1	0,181103
3,755445	421	215,3338	2,23E-04	0,238948	0,035752
0,95849	420	415,8525	0,338372	1	0,947373
2,296039	367	362,9771	0,022243	1	0,231173
2,322369	280	274,7461	0,020944	1	0,30229
0,972896	421	350,8692	0,331275	1	0,619505
-0,05547	421	189,0128	0,955822	1	0,98414
-1,00857	420	385,0306	0,313815	1	0,943897
1,814623	367	338,2463	0,070468	1	0,36493
-0,32335	280	264,7844	0,746684	1	0,942071
-0,83001	421	344,7569	0,40711	1	0,695339
-0,35623	421	189,1993	0,722066	1	0,877543
1,027802	420	379,3862	0,304698	1	0,943897
0,33031	367	334,0406	0,741373	1	0,923669
-0,7264	280	267,318	0,468232	1	0,824256
0,915644	421	316,3673	0,360551	1	0,650719

-0,60006	421	180,634	0,549216	1	0,776424
0,388867	420	350,8987	0,69761	1	0,968866
0,941422	367	303,3749	0,347238	1	0,668671
1,394722	280	255,4563	0,164311	1	0,586115
-0,79551	421	292,2703	0,426963	1	0,700145
-1,14079	421	174,1687	0,255526	1	0,555667
0,957303	420	322,6233	0,339131	1	0,947373
-0,84249	367	290,4507	0,400209	1	0,713745
0,200164	280	276	0,8415	1	0,965181
-1,14525	421	323,7758	0,252949	1	0,546261
-0,12886	421	184,0211	0,897609	1	0,960059
0,003438	420	357,149	0,997259	1	0,997643
0,540192	367	327,8438	0,589431	1	0,853288
-0,26552	280	263,5144	0,790817	1	0,953435
-1,76731	421	342,3672	0,078068	1	0,302838
-2,04183	421	188,0219	0,042566	1	0,273775
-0,81619	420	377,2068	0,414909	1	0,95743
-2,04951	367	341,9874	0,041175	1	0,289956
-1,23135	280	262,8233	0,219293	1	0,650539
-2,41653	421	361,2736	0,016164	1	0,147897
-2,55457	421	193,6597	0,011401	1	0,163285
-1,00814	420	396,1296	0,314	1	0,943897
-1,38057	367	339,0433	0,168321	1	0,514971
-0,30076	280	263,4825	0,763833	1	0,944296
0,437255	421	351,9221	0,662195	1	0,851474
0,701773	421	192,6609	0,483667	1	0,739236
-0,7208	420	385,2341	0,471468	1	0,960096
0,38001	367	346,6155	0,704171	1	0,908549
-0,62321	280	261,8718	0,533686	1	0,84326
-1,31628	421	361,7638	0,188913	1	0,472953
-1,35563	421	193,9234	0,176792	1	0,46335
-0,14266	420	396,1548	0,886629	1	0,982463
-2,56854	367	339,6392	0,01064	1	0,189546
-2,13966	280	267,865	0,033286	1	0,329568
0,314686	421	403,9728	0,753162	1	0,898125
2,322353	421	219,2961	0,021131	1	0,2022
0,642201	420	415,7287	0,521096	1	0,960096
0,10519	367	362,9487	0,916283	1	0,973634
0,722389	280	271,485	0,470677	1	0,824256
-3,24328	421	337,0501	0,0013	1	0,066922
-1,21056	421	186,8036	0,227593	1	0,526855
-0,49857	420	376,6277	0,618372	1	0,964878
-0,01039	367	325,0225	0,991714	1	0,997007
-0,65978	280	260,5449	0,509975	1	0,831912
-1,10734	421	375,0698	0,268857	1	0,563516
-0,19251	421	201,4401	0,84754	1	0,94331
1,613309	420	404,3494	0,107457	1	0,848246
-1,9778	367	355,3456	0,048723	1	0,313787

-1,73466	280	267,7038	0,083953	1	0,456738
-0,53738	421	352,3344	0,591347	1	0,81147
0,352377	421	188,7412	0,724949	1	0,878959
-0,25823	420	386,0151	0,796369	1	0,969427
-0,09249	367	333,1906	0,926362	1	0,976733
0,199451	280	260,08	0,842065	1	0,965181
0,58271	421	368,2211	0,560445	1	0,793043
0,446546	421	197,8807	0,655692	1	0,841107
-0,30628	420	398,9172	0,759552	1	0,969427
-0,55165	367	348,5255	0,581543	1	0,845115
-0,18874	280	268,081	0,850438	1	0,966155
-2,59845	421	306,9295	0,009816	1	0,116484
-1,41948	421	180,2446	0,157485	1	0,43893
0,235028	420	342,9493	0,814327	1	0,969427
1,333782	367	304,4131	0,183272	1	0,529103
-1,59941	280	251,6728	0,110984	1	0,503775
-2,15974	421	413,8844	0,031366	1	0,201145
-1,67395	421	232,1096	0,095487	1	0,377685
-1,5993	420	414,606	0,110516	1	0,848246
0,246336	367	361,3695	0,805562	1	0,950149
0,350038	280	275,3377	0,726578	1	0,936637
-0,19297	421	319,582	0,847101	1	0,940738
0,902091	421	182,8545	0,368195	1	0,648213
-1,21988	420	354,59	0,223319	1	0,943055
-1,09756	367	310,5712	0,273248	1	0,609149
-0,85166	280	247,4151	0,395224	1	0,782194
-0,24543	421	294,3827	0,806294	1	0,922616
-1,37047	421	173,4082	0,17231	1	0,456365
0,84768	420	324,4674	0,397241	1	0,95743
0,238615	367	277,5116	0,811581	1	0,950149
0,452458	280	252,25	0,651328	1	0,908644
0,332037	421	343,8283	0,740064	1	0,893273
1,812636	421	189,538	0,07147	1	0,339864
1,022683	420	379,4566	0,307109	1	0,943897
1,431208	367	332,1369	0,153311	1	0,501171
-0,08406	280	265,9715	0,933069	1	0,979504
-2,21696	421	385,2208	0,027209	1	0,192509
-0,94716	421	204,1221	0,344677	1	0,638117
-0,2865	420	409,1951	0,774637	1	0,969427
1,277548	367	354,8555	0,202244	1	0,556171
-0,66375	280	265,3222	0,507428	1	0,831912
1,702968	421	331,3874	0,089512	1	0,319654
0,800614	421	181,6787	0,424401	1	0,693378
0,118295	420	369,4845	0,905898	1	0,98503
1,537956	367	301,4333	0,125108	1	0,462104
0,439853	280	261,4471	0,660407	1	0,908644
0,025268	421	328,1935	0,979857	1	0,997025
-0,50038	421	185,9982	0,617402	1	0,825805

0,861823	420	364,1205	0,389352	1	0,95743
0,717318	367	317,0785	0,473706	1	0,776795
-0,22878	280	250,3821	0,819229	1	0,95778
2,41113	421	370,6734	0,016389	1	0,148783
2,530106	421	197,1841	0,012185	1	0,169272
0,784971	420	402,961	0,432932	1	0,960096
0,946293	367	346,9818	0,344658	1	0,667378
1,691056	280	266,5653	0,091995	1	0,468366
-0,14785	421	349,7315	0,882544	1	0,960978
-0,14	421	187,6219	0,888812	1	0,958798
-0,92213	420	385,3348	0,357037	1	0,947373
-1,21208	367	332,3278	0,226344	1	0,576775
-1,96171	280	260,0599	0,050863	1	0,398004
-3,07373	421	361,2447	0,002275	1	0,069718
-0,92879	421	193,8867	0,354154	1	0,643758
0,44398	420	391,6803	0,657302	1	0,96781
0,517999	367	353,4452	0,604783	1	0,867182
-0,7097	280	270,7356	0,478502	1	0,824256
1,64678	421	311,6226	0,100611	1	0,342121
0,104473	421	180,8732	0,91691	1	0,969673
-0,20482	420	345,0799	0,837836	1	0,973894
0,077081	367	295,6017	0,938611	1	0,979214
-1,92865	280	276	0,054799	1	0,40654
0,843585	421	364,9668	0,399454	1	0,688761
1,474017	421	196,0952	0,142081	1	0,423499
0,211517	420	396,7369	0,832592	1	0,973894
2,817298	367	343,6727	0,005123	1	0,178183
0,093361	280	268,421	0,925687	1	0,979504
0,837817	421	312,6603	0,402774	1	0,692899
1,728248	421	178,419	0,085674	1	0,365475
0,66433	420	347,4099	0,50692	1	0,960096
0,382791	367	299,625	0,702146	1	0,908269
-0,83984	280	260,6694	0,401767	1	0,784757
-0,86094	421	338,9724	0,389882	1	0,679251
-0,45411	421	188,1759	0,65027	1	0,839358
-0,7458	420	374,7178	0,456259	1	0,960096
0,973373	367	328,2792	0,331085	1	0,659108
-0,65855	280	261,3574	0,510765	1	0,831912
-0,27939	421	376,207	0,780101	1	0,9142
-2,62076	421	200,1097	0,009447	1	0,148297
-0,09631	420	403,9972	0,923322	1	0,989204
-1,42653	367	337,2908	0,154639	1	0,502197
-1,08091	280	268,4171	0,280706	1	0,703928
-2,61201	421	332,1377	0,00941	1	0,114362
-1,19714	421	185,0569	0,232784	1	0,533704
-0,69229	420	368,883	0,489193	1	0,960096
-1,44249	367	326,0981	0,150125	1	0,499998
-2,08657	280	259,5624	0,037903	1	0,348289

-1,38478	421	354,4678	0,16699	1	0,441901
-2,63974	421	191,6176	0,00898	1	0,14815
-0,1599	420	388,8855	0,873041	1	0,982463
-2,96436	367	343,9035	0,003245	1	0,176492
-1,48865	280	263,788	0,137775	1	0,54931
-1,41941	421	359,4645	0,156647	1	0,429212
0,648244	421	192,042	0,517602	1	0,761675
1,306154	420	389,2108	0,192272	1	0,925729
2,2244	367	339,2876	0,026779	1	0,252225
1,29389	280	259,0694	0,196856	1	0,627192
-0,47677	421	364,0687	0,633812	1	0,83455
0,25478	421	195,6456	0,799161	1	0,921861
0,250566	420	396,3991	0,80228	1	0,969427
0,55346	367	342,6254	0,580309	1	0,844137
0,43761	280	262,7544	0,662029	1	0,908644
-0,27683	421	340,3201	0,782077	1	0,915149
-0,95071	421	187,6892	0,342976	1	0,636552
-1,34457	420	367,9278	0,179594	1	0,923408
-0,12126	367	325,3888	0,903559	1	0,970486
0,793348	280	253,8487	0,428316	1	0,800834
-2,28044	421	328,9448	0,023221	1	0,177419
-2,06556	421	184,0897	0,040271	1	0,26998
-0,32646	420	368,937	0,744258	1	0,969427
-0,51098	367	333,3934	0,6097	1	0,870021
-2,66253	280	265,741	0,008229	1	0,203287
1,906079	421	370,3544	0,057414	1	0,266382
0,673313	421	198,9124	0,50153	1	0,750551
-0,12723	420	403,5943	0,898821	1	0,98503
0,510241	367	352,1793	0,610202	1	0,870021
-1,26711	280	267,1992	0,20622	1	0,638139
-3,68508	421	341,2196	2,66E-04	0,285222	0,04642
-3,21426	421	185,8272	0,001542	1	0,093671
-0,12578	420	381,2044	0,899972	1	0,98503
-0,71163	367	338,1057	0,477186	1	0,780803
-3,20901	280	261,4328	0,001498	1	0,135787
-1,62974	421	282,838	0,10427	1	0,346875
0,247491	421	168,9577	0,804828	1	0,923205
-0,66248	420	308,8002	0,508158	1	0,960096
-2,23628	367	281,2352	0,026118	1	0,252225
-1,02423	280	276	0,306625	1	0,72861
-0,54676	421	333,4453	0,584908	1	0,807036
0,396114	421	186,0925	0,692475	1	0,865526
-1,02047	420	368,6294	0,308177	1	0,943897
0,017076	367	332,2993	0,986386	1	0,996248
-0,67327	280	261,829	0,501372	1	0,831912
1,825221	421	328,1839	0,068876	1	0,282663
-0,70835	421	182,4859	0,479634	1	0,736057
-0,33783	420	362,5049	0,735686	1	0,969427

2,016276	367	319,9469	0,044606	1	0,302719
-0,39928	280	264,8949	0,690009	1	0,91859
0,315713	421	339,8613	0,752414	1	0,898125
2,004891	421	186,652	0,04642	1	0,282075
0,243454	420	375,0562	0,807787	1	0,969427
-0,14978	367	337,2362	0,881025	1	0,962453
1,095201	280	261,2703	0,274437	1	0,701156
0,79369	421	402,7467	0,427844	1	0,700826
1,482785	421	218,1445	0,139575	1	0,423351
0,291248	420	415,608	0,771007	1	0,969427
1,199619	367	362,3068	0,231071	1	0,582149
1,247227	280	273,5889	0,213381	1	0,646827
-0,4845	421	352,7497	0,628329	1	0,833119
-1,0571	421	192,4276	0,291792	1	0,588663
-1,28592	420	385,9596	0,199241	1	0,928589
-1,35587	367	339,2905	0,176042	1	0,523018
-0,25781	280	265,6046	0,796755	1	0,954269
-1,4947	421	361,6268	0,135865	1	0,407576
-1,25559	421	195,9639	0,210761	1	0,509968
0,287344	420	393,9912	0,774	1	0,969427
-1,66297	367	351,5307	0,09721	1	0,412696
-1,74412	280	266,0778	0,082293	1	0,456738
-1,40095	421	351,3243	0,162113	1	0,436257
-2,74102	421	187,7683	0,006717	1	0,141161
-1,19102	420	383,4993	0,234383	1	0,943055
-1,2925	367	326,0875	0,197098	1	0,549567
-0,27716	280	255,0389	0,781882	1	0,951006
-2,43618	421	353,4075	0,015337	1	0,147897
-1,08774	421	193,6705	0,278063	1	0,578094
-0,04037	420	390,4993	0,967817	1	0,996924
-1,62609	367	343,5077	0,104848	1	0,429852
-0,72053	280	262,0792	0,471839	1	0,824256
-2,37765	421	354,1991	0,017953	1	0,156391
-1,68261	421	191,2206	0,094083	1	0,373113
-1,13848	420	382,3083	0,255634	1	0,943897
1,353854	367	351,5076	0,176653	1	0,523018
0,717463	280	267,3417	0,473715	1	0,824256
-2,65385	421	332,1598	0,008341	1	0,112451
-2,6795	421	185,0808	0,008038	1	0,148102
-0,0632	420	374,0378	0,949637	1	0,992811
-2,09568	367	327,838	0,036877	1	0,28464
-1,10782	280	263,0191	0,268951	1	0,693794
2,561384	421	334,4227	0,010864	1	0,124028
1,733155	421	184,9741	0,084735	1	0,365475
4,118544	420	361,5284	4,73E-05	0,050981	0,035635
1,634724	367	329,5951	0,103062	1	0,426647
0,67844	280	258,2769	0,4981	1	0,83103
-2,82673	421	366,5124	0,004961	1	0,087964

-1,53553	421	198,9888	0,126242	1	0,410224
-0,15222	420	399,9683	0,879087	1	0,982463
-0,013	367	355,4261	0,989638	1	0,996248
-1,84257	280	272,9969	0,066476	1	0,425749
0,474696	421	341,65	0,635307	1	0,83455
-0,70582	421	188,6666	0,481169	1	0,737662
0,640589	420	376,5051	0,52218	1	0,960096
-0,3123	367	327,2007	0,75501	1	0,92504
-2,95961	280	265,3607	0,003359	1	0,143818
1,242899	421	369,8708	0,214692	1	0,506731
1,861885	421	197,6156	0,064104	1	0,324177
0,437411	420	400,4108	0,662049	1	0,96781
1,201782	367	350,4851	0,23026	1	0,581242
1,11817	280	265,8394	0,264504	1	0,688875
-2,1929	421	320,5476	0,029033	1	0,197976
-1,18456	421	183,8233	0,237719	1	0,537903
1,507196	420	353,8233	0,132653	1	0,888479
0,033845	367	314,9432	0,973022	1	0,989436
-0,29244	280	259,8642	0,770185	1	0,946609
-0,28893	421	357,7741	0,772799	1	0,90985
-1,23808	421	191,055	0,217206	1	0,514565
-0,33094	420	390,6472	0,740868	1	0,969427
2,657932	367	348,3803	0,008225	1	0,182271
-2,41455	280	262,2774	0,01644	1	0,266517
-1,43665	421	334,4396	0,151752	1	0,42445
0,09964	421	188,3188	0,920736	1	0,970834
0,857526	420	375,8376	0,391701	1	0,95743
1,51475	367	329,0314	0,130796	1	0,47485
1,271219	280	260,9225	0,204783	1	0,634995
0,140836	421	320,3564	0,888088	1	0,962
1,095717	421	181,7904	0,274652	1	0,576463
1,055878	420	356,1726	0,29174	1	0,943897
1,966449	367	290,0909	0,0502	1	0,316533
0,933786	280	251,1464	0,351311	1	0,761846
1,333402	421	352,9918	0,18326	1	0,465721
0,479519	421	192,664	0,632113	1	0,835691
-1,26511	420	388,8842	0,206591	1	0,930277
0,47419	367	338,9025	0,63567	1	0,880707
-1,4725	280	264,1437	0,142078	1	0,554427
-0,75326	421	288,4321	0,451909	1	0,716117
-0,57988	421	175,4626	0,562742	1	0,785233
-0,63622	420	317,5329	0,525091	1	0,960096
-1,55564	367	289,7173	0,120885	1	0,452158
-0,6407	280	276	0,522251	1	0,833721
-0,24009	421	361,3212	0,810396	1	0,924931
-0,63138	421	193,5405	0,528536	1	0,768085
-1,19093	420	393,3309	0,234399	1	0,943055
1,610086	367	339,9283	0,108307	1	0,435251

-2,10269	280	258,5387	0,03646	1	0,34044
-3,21771	421	311,4031	0,001428	1	0,069432
-2,58662	421	180,4913	0,01048	1	0,156367
-2,02091	420	355,9149	0,044037	1	0,846132
-0,85468	367	310,1065	0,393386	1	0,705753
-0,21409	280	257,4297	0,830647	1	0,964667
-2,97173	421	391,8044	0,003144	1	0,078203
-2,64159	421	210,5343	0,008871	1	0,14815
-0,24242	420	413,8821	0,808575	1	0,969427
-1,91434	367	360,1598	0,056368	1	0,337523
-2,151	280	274,1069	0,03235	1	0,329568
-2,5166	421	340,2185	0,012309	1	0,131857
-2,51222	421	186,5418	0,012845	1	0,171309
0,721021	420	375,2842	0,471345	1	0,960096
-2,19921	367	324,0974	0,028569	1	0,257802
-0,94693	280	256,6152	0,344566	1	0,758934
-0,80334	421	353,8382	0,422316	1	0,697735
-1,62751	421	191,5444	0,105273	1	0,385063
-0,92837	420	381,928	0,353804	1	0,947373
-1,55662	367	342,3388	0,120486	1	0,452158
-0,2114	280	263,8818	0,832736	1	0,964667
-1,19542	421	345,1052	0,232743	1	0,525066
-1,40359	421	187,757	0,162094	1	0,445827
1,124254	420	382,1834	0,261611	1	0,943897
-2,06963	367	338,0905	0,039246	1	0,28956
-0,42477	280	264,9047	0,67135	1	0,908644
-0,65793	421	324,6232	0,511047	1	0,764035
0,91833	421	178,6801	0,359684	1	0,643758
-0,31142	420	359,8456	0,755661	1	0,969427
1,543917	367	317,7866	0,123604	1	0,458795
1,517571	280	261,1476	0,130332	1	0,538112
-1,22002	421	344,3522	0,223292	1	0,51676
0,59034	421	186,7254	0,555676	1	0,778696
0,123919	420	377,9368	0,901445	1	0,98503
0,266359	367	316,6727	0,790136	1	0,944997
-1,83047	280	248,5072	0,068377	1	0,427568
1,17268	421	370,7724	0,241677	1	0,538768
1,170726	421	197,3204	0,243121	1	0,542422
-0,30386	420	403,0993	0,761389	1	0,969427
2,590317	367	336,5089	0,010006	1	0,183886
2,280324	280	265,8545	0,02338	1	0,313396
1,375093	421	372,0781	0,16993	1	0,442288
1,58384	421	197,0385	0,114834	1	0,392379
0,423229	420	403,073	0,672354	1	0,96781
1,374361	367	346,2447	0,170219	1	0,518221
0,251469	280	265,9479	0,801646	1	0,956473
1,484697	421	323,9288	0,138597	1	0,410344
1,769321	421	183,3842	0,078502	1	0,355264

-1,11198	420	359,3012	0,266891	1	0,943897
0,900899	367	319,3974	0,368321	1	0,678946
-0,19721	280	257,7169	0,84382	1	0,965181
-1,3418	421	310,1417	0,180641	1	0,46049
-3,53117	421	179,702	5,26E-04	0,561433	0,049513
-1,93894	420	343,1743	0,053328	1	0,846627
-2,76726	367	302,8416	0,006001	1	0,178183
-2,40308	280	253,1115	0,016978	1	0,266517
-2,28112	421	351,2858	0,02314	1	0,177419
-0,69403	421	188,188	0,488522	1	0,742889
-1,0504	420	384,5554	0,294194	1	0,943897
-1,68615	367	333,8723	0,0927	1	0,40529
0,104941	280	264,0295	0,916503	1	0,977051
-1,82214	421	383,9974	0,069212	1	0,282663
-0,9329	421	204,0917	0,351975	1	0,643758
-0,24472	420	408,5996	0,806796	1	0,969427
-0,79723	367	344,2293	0,425867	1	0,733464
0,206255	280	257,2509	0,836755	1	0,964667
-1,05929	421	339,5734	0,290219	1	0,587061
-1,56765	421	188,5195	0,118639	1	0,396436
-2,00171	420	372,8781	0,04604	1	0,846132
-1,44808	367	323,1604	0,148565	1	0,499998
-0,65589	280	262,107	0,512471	1	0,831912
-1,7132	421	414,3569	0,087423	1	0,316698
-1,83519	421	236,1399	0,067735	1	0,334428
-2,57122	420	413,5714	0,010483	1	0,607592
-1,28596	367	361,9373	0,19928	1	0,551573
0,567064	280	274,4987	0,571134	1	0,869862
-1,30692	421	303,8349	0,192229	1	0,478035
-0,38533	421	177,5437	0,700458	1	0,865528
0,134077	420	335,4814	0,893422	1	0,98503
-0,4692	367	292,2101	0,639275	1	0,880707
-0,16336	280	250,9552	0,870363	1	0,969656
0,290923	421	382,1449	0,771268	1	0,909469
1,83947	421	203,0166	0,067306	1	0,333654
-0,18967	420	407,573	0,849664	1	0,973894
2,234847	367	351,892	0,026055	1	0,252225
-1,6133	280	266,7363	0,107862	1	0,497151
-0,78511	421	306,5442	0,432993	1	0,705289
-1,69129	421	177,9168	0,092532	1	0,373113
-3,2544	420	334,4514	0,001252	1	0,235929
-2,00594	367	284,9618	0,045808	1	0,306809
-1,21538	280	251,4584	0,225362	1	0,654014
1,037783	421	326,9855	0,300138	1	0,596172
2,226885	421	181,7493	0,027183	1	0,22508
1,643208	420	360,6037	0,101212	1	0,848246
2,414894	367	315,0385	0,016309	1	0,208286
1,610713	280	253,7666	0,108485	1	0,497151

-0,8214	421	384,2025	0,41193	1	0,695339
-1,03289	421	205,6945	0,302867	1	0,601345
-0,00598	420	409,1586	0,995233	1	0,997342
-1,56751	367	358,1625	0,117879	1	0,445308
-1,08918	280	271,1692	0,277042	1	0,701156
-0,80342	421	402,6236	0,422208	1	0,697735
0,850177	421	215,5797	0,39617	1	0,667817
-1,05928	420	415,849	0,290085	1	0,943897
1,198274	367	361,6584	0,231595	1	0,582149
-0,95993	280	274,6377	0,337935	1	0,755731
-2,44073	421	307,2961	0,015222	1	0,147897
-1,58155	421	177,2676	0,115535	1	0,392379
-0,55438	420	340,9017	0,579685	1	0,964878
-2,51779	367	303,5945	0,012324	1	0,189546
-2,28634	280	264,1806	0,023026	1	0,313396
-1,15281	421	342,5148	0,249793	1	0,545586
-1,58866	421	188,6339	0,113813	1	0,391524
-1,6743	420	375,239	0,094905	1	0,848246
0,606107	367	342,4683	0,544845	1	0,825208
0,243352	280	268,1218	0,807919	1	0,95747
-1,03152	421	361,7155	0,302984	1	0,599209
-1,82928	421	193,5247	0,068896	1	0,335504
-0,49549	420	394,0363	0,62053	1	0,964878
-1,23541	367	354,2353	0,217498	1	0,569492
-0,91085	280	262,6828	0,36321	1	0,762031
-0,29535	421	378,7062	0,767887	1	0,906901
-1,07291	421	200,6995	0,2846	1	0,582733
0,56652	420	405,5602	0,571354	1	0,964199
-0,21724	367	354,2365	0,828147	1	0,956109
-0,59978	280	265,2129	0,549162	1	0,852208
-0,33713	421	404,8228	0,736192	1	0,893273
-0,60786	421	220,0781	0,543908	1	0,774002
-2,20468	420	415,9345	0,028023	1	0,767829
-2,05778	367	362,9797	0,040325	1	0,289956
-1,00923	280	274,1726	0,313755	1	0,734206
0,826612	421	344,1152	0,40903	1	0,695339
0,804139	421	187,7657	0,422334	1	0,692554
-0,29564	420	377,2966	0,767669	1	0,969427
0,409097	367	329,6813	0,682734	1	0,900574
0,746736	280	254,9534	0,455911	1	0,815983
0,765554	421	379,5849	0,444418	1	0,713799
0,30358	421	197,8764	0,761767	1	0,901085
0,009039	420	405,1122	0,992792	1	0,997342
2,318869	367	355,1562	0,020969	1	0,225192
2,178623	280	267,2342	0,030234	1	0,329568
0,729525	421	333,5225	0,466193	1	0,728034
2,121008	421	185,8498	0,035247	1	0,255449
1,697462	420	367,7605	0,090455	1	0,848246

1,293785	367	312,8648	0,196694	1	0,549567
-0,1031	280	263,0148	0,917965	1	0,977051
-0,87112	421	295,5786	0,384394	1	0,674247
-0,96299	421	175,7601	0,336874	1	0,63143
-0,14903	420	325,1485	0,881622	1	0,982463
-0,77619	367	300,1448	0,438248	1	0,742744
-1,30428	280	246,2271	0,193354	1	0,62543
0,807442	421	348,1858	0,419963	1	0,697735
0,821702	421	191,2795	0,412269	1	0,684933
0,616027	420	383,3723	0,538242	1	0,960096
0,689377	367	342,429	0,491053	1	0,792348
-0,08207	280	256,342	0,934657	1	0,979504
2,203356	421	353,7368	0,028214	1	0,19529
-1,61156	421	191,783	0,108702	1	0,388185
-0,67875	420	387,9316	0,497699	1	0,960096
0,330706	367	342,2205	0,741069	1	0,923669
-2,61302	280	266,1754	0,009485	1	0,226737
-0,404	421	360,0912	0,686454	1	0,864986
-0,7711	421	193,9526	0,441587	1	0,706446
0,22918	420	390,9227	0,818849	1	0,97003
0,287446	367	338,406	0,773947	1	0,93908
-0,82055	280	264,6524	0,412642	1	0,788151
0,133827	421	352,8096	0,893616	1	0,962
0,542169	421	192,417	0,58833	1	0,80382
-0,19265	420	385,91	0,847335	1	0,973894
0,072297	367	343,6499	0,942408	1	0,979539
-1,43328	280	257,5229	0,152991	1	0,565026
-0,33742	421	387,8776	0,73598	1	0,893273
0,544098	421	206,8705	0,58696	1	0,802811
1,102994	420	410,9008	0,270675	1	0,943897
0,09169	367	358,913	0,926995	1	0,976733
0,380465	280	274,3266	0,703894	1	0,921995
-2,76215	421	365,3872	0,006032	1	0,097752
0,090211	421	196,9369	0,928211	1	0,972749
-0,18387	420	394,718	0,85421	1	0,973894
0,819078	367	339,6692	0,413316	1	0,724248
0,512199	280	258,0596	0,60895	1	0,886655
-1,61869	421	378,4524	0,106347	1	0,351457
-1,65106	421	201,8408	0,100282	1	0,382595
0,267656	420	405,0932	0,789101	1	0,969427
-1,05598	367	357,1198	0,29169	1	0,627013
-0,27143	280	267,7044	0,786268	1	0,953235
0,331654	421	360,7466	0,740343	1	0,893273
1,582441	421	194,4827	0,115174	1	0,392379
0,474097	420	393,0618	0,635694	1	0,964878
3,157861	367	352,0154	0,001727	1	0,176492
2,193544	280	263,9148	0,029141	1	0,326641
-1,17034	421	328,8754	0,242712	1	0,53918

-1,5263	421	184,2919	0,12865	1	0,410224
-2,83062	420	364,7921	0,004903	1	0,449965
-1,10889	367	321,0355	0,268308	1	0,609149
-2,91947	280	262,0047	0,003811	1	0,147267
-0,80973	421	358,2337	0,418633	1	0,697735
-0,48518	421	195,0391	0,628091	1	0,832483
0,67109	420	391,9953	0,502558	1	0,960096
0,190256	367	326,9406	0,849226	1	0,956935
0,437683	280	262,9328	0,661976	1	0,908644
-2,11532	421	356,1883	0,035097	1	0,215003
-1,99095	421	192,6606	0,047899	1	0,286754
0,966472	420	392,2993	0,334404	1	0,947373
-1,98782	367	348,067	0,047615	1	0,311448
-3,31455	280	263,3553	0,001047	1	0,121989
-1,62552	421	331,8978	0,105001	1	0,347821
-0,637	421	183,7845	0,52492	1	0,766525
0,866757	420	370,5091	0,386636	1	0,95743
0,018831	367	329,5335	0,984987	1	0,996248
0,466337	280	264,547	0,641358	1	0,907537
0,13197	421	353,2736	0,895083	1	0,962
-0,62155	421	190,7693	0,534982	1	0,770763
-0,94946	420	386,3412	0,34298	1	0,947373
2,432857	367	348,6969	0,015482	1	0,204372
0,074049	280	257,4162	0,941029	1	0,982175
1,857883	421	393,0786	0,063933	1	0,275801
2,094054	421	210,1981	0,037454	1	0,266419
0,78516	420	413,5489	0,43281	1	0,960096
-0,27916	367	359,2735	0,780283	1	0,94337
0,52577	280	273,5221	0,599474	1	0,881231
-2,25354	421	368,3868	0,024813	1	0,183798
-0,75334	421	196,1169	0,452147	1	0,714241
0,350172	420	398,2541	0,726395	1	0,969427
-0,20051	367	352,9896	0,841199	1	0,956935
-1,05307	280	270,209	0,293248	1	0,723282
-0,88677	421	287,1334	0,375947	1	0,669684
0,165881	421	176,2616	0,868441	1	0,947676
-0,14444	420	319,9572	0,885247	1	0,982463
-0,15673	367	288,8031	0,875564	1	0,962417
2,719482	280	247,4749	0,007002	1	0,195931
-0,93311	421	325,4759	0,351455	1	0,640439
-1,25056	421	184,1218	0,212682	1	0,512644
-2,36161	420	353,1087	0,018738	1	0,75904
-0,27241	367	307,9662	0,785487	1	0,94337
-0,77767	280	254,032	0,43749	1	0,806974
-0,34802	421	352,9749	0,728036	1	0,890807
-1,32938	421	191,8993	0,1853	1	0,473945
-1,05726	420	384,5791	0,291058	1	0,943897
0,427824	367	342,6991	0,669048	1	0,895431

1,816641	280	262,7143	0,070412	1	0,433103
0,141296	421	361,3676	0,887715	1	0,962
0,597348	421	193,3846	0,550974	1	0,777451
-0,10113	420	393,4093	0,919495	1	0,989204
0,375322	367	334,8981	0,707658	1	0,91071
0,793843	280	266,5049	0,427993	1	0,800834
-0,7213	421	375,5647	0,471171	1	0,731548
-0,1122	421	199,4544	0,910775	1	0,96646
-0,44042	420	403,4081	0,659868	1	0,96781
-0,40195	367	343,0286	0,687975	1	0,903032
-1,87233	280	265,1789	0,062261	1	0,422605
-1,132	421	390,8249	0,258331	1	0,550898
-1,90784	421	209,8052	0,057777	1	0,312877
-0,76264	420	412,3269	0,446114	1	0,960096
-2,63408	367	359,6659	0,008801	1	0,183886
-2,22176	280	273,5982	0,027118	1	0,324814
-0,19993	421	335,3791	0,841657	1	0,939539
1,835992	421	186,84	0,067948	1	0,334428
0,312559	420	370,6029	0,754792	1	0,969427
2,341363	367	324,207	0,019819	1	0,221237
2,338954	280	255,4978	0,020109	1	0,297103
-2,69925	421	344,4485	0,007292	1	0,105763
-1,8651	421	190,6514	0,063704	1	0,323237
0,777354	420	383,0462	0,43743	1	0,960096
-2,36772	367	347,232	0,018446	1	0,217108
-2,01922	280	264,8427	0,044471	1	0,387749
0,731426	421	283,8612	0,465123	1	0,727964
1,135226	421	172,7288	0,257853	1	0,556025
0,904263	420	308,3707	0,366562	1	0,95743
0,671699	367	279,0193	0,502331	1	0,793601
1,302009	280	276	0,193999	1	0,62543
-0,50257	421	350,8591	0,615581	1	0,826526
1,668154	421	189,6227	0,096936	1	0,379251
1,098814	420	385,5456	0,272535	1	0,943897
0,765494	367	334,071	0,444518	1	0,749316
1,246095	280	266,3134	0,213825	1	0,646827
-0,81864	421	316,9526	0,413607	1	0,695339
1,035505	421	181,2862	0,301812	1	0,600041
2,003062	420	348,4646	0,045945	1	0,846132
-0,19055	367	299,8225	0,849003	1	0,956935
0,061364	280	251,5822	0,951118	1	0,982409
-0,04278	421	371,5628	0,965898	1	0,992572
0,408421	421	198,1204	0,683406	1	0,859821
0,31469	420	400,3488	0,753161	1	0,969427
-0,39265	367	350,7233	0,694815	1	0,904597
0,659131	280	271,3076	0,51037	1	0,831912
-2,25331	421	337,8528	0,02488	1	0,183798
-3,26601	421	188,6784	0,001296	1	0,092971

-0,40683	420	378,1723	0,68436	1	0,968613
-3,38045	367	324,2815	8,12E-04	0,871337	0,139974
-2,20696	280	266,978	0,028168	1	0,326641
-0,47098	421	346,4216	0,637952	1	0,834842
-1,70687	421	187,6887	0,0895	1	0,37156
-0,69982	420	380,8897	0,484466	1	0,960096
-1,26788	367	325,1377	0,205746	1	0,558434
-2,44371	280	255,8553	0,015213	1	0,263522
-1,41966	421	357,6599	0,156577	1	0,429212
-1,04061	421	192,9375	0,299358	1	0,597527
-0,03483	420	391,7902	0,972232	1	0,997342
-0,08422	367	335,774	0,932929	1	0,977109
-1,77101	280	256,7253	0,077746	1	0,453386
-0,84507	421	354,6055	0,398644	1	0,688151
-0,29876	421	191,6305	0,765444	1	0,90393
-0,15656	420	387,1451	0,875674	1	0,982463
-0,45004	367	333,1684	0,652975	1	0,888919
-1,71451	280	261,7792	0,087618	1	0,460069
-1,38948	421	367,0063	0,165529	1	0,440727
-1,88933	421	196,7056	0,060319	1	0,314727
-0,04496	420	398,7057	0,964162	1	0,996924
-1,88657	367	345,1932	0,060057	1	0,340409
-1,34108	280	264,9777	0,181042	1	0,607861
0,261922	421	395,7989	0,793518	1	0,919136
0,76316	421	213,1282	0,446212	1	0,710076
1,495627	420	414,0236	0,135513	1	0,891179
-0,38572	367	359,3718	0,69993	1	0,906491
-0,93881	280	272,2895	0,348663	1	0,761841
-0,35763	421	314,6208	0,720858	1	0,885357
-0,53023	421	182,3682	0,596596	1	0,811435
-0,52817	420	349,0733	0,597715	1	0,964878
0,857308	367	311,627	0,391933	1	0,705088
1,000655	280	276	0,31787	1	0,741533
-0,91895	421	368,8713	0,358721	1	0,648267
-0,14687	421	197,9958	0,883383	1	0,957051
0,941619	420	398,6145	0,346958	1	0,947373
0,909762	367	343,072	0,363587	1	0,677287
-0,25474	280	265,1992	0,799121	1	0,954985
0,409473	421	323,9817	0,682463	1	0,862088
0,076739	421	181,9285	0,938915	1	0,977847
1,762433	420	359,2124	0,078847	1	0,848246
0,462136	367	302,9838	0,644316	1	0,882712
0,243487	280	251,6219	0,807827	1	0,95747
1,130128	421	335,0888	0,25923	1	0,55178
1,572576	421	187,07	0,117507	1	0,396436
0,190135	420	370,3822	0,849308	1	0,973894
0,935356	367	319,5188	0,350311	1	0,669099
1,040195	280	255,4586	0,299233	1	0,723324

0,56233	421	366,6219	0,574235	1	0,800529
0,143955	421	197,6738	0,885682	1	0,957782
0,766971	420	397,0717	0,443555	1	0,960096
-1,96964	367	346,9822	0,049675	1	0,315285
-0,54379	280	258,9062	0,587052	1	0,87561
-1,04371	421	340,3536	0,297362	1	0,59543
-0,11539	421	186,4953	0,908258	1	0,96646
0,990169	420	372,862	0,322733	1	0,945573
1,088558	367	325,1329	0,277155	1	0,611884
-0,97657	280	260,3237	0,329689	1	0,752282
-4,04619	421	328,9785	6,49E-05	0,069863	0,024462
-2,52768	421	186,1954	0,012313	1	0,169272
-1,71385	420	375,152	0,087382	1	0,848246
-2,82787	367	337,4379	0,004966	1	0,178183
-2,5821	280	267,7389	0,010352	1	0,229419
-0,67424	421	335,5566	0,50062	1	0,754435
-1,78988	421	185,4961	0,075104	1	0,345067
-0,69	420	369,4851	0,490629	1	0,960096
-2,18869	367	321,0782	0,029339	1	0,260082
-2,23974	280	251,7987	0,025981	1	0,324814
0,414468	421	330,0091	0,678801	1	0,861268
0,236575	421	183,9565	0,81325	1	0,926063
-0,10138	420	364,9369	0,919302	1	0,989204
0,558465	367	316,7155	0,576921	1	0,843313
-1,26083	280	266,186	0,208475	1	0,64091
-1,97497	421	350,5067	0,049056	1	0,244581
-2,50129	421	191,5965	0,013212	1	0,171643
-1,22303	420	387,5586	0,222063	1	0,943055
-1,7282	367	342,9994	0,084853	1	0,388246
-1,64905	280	269,9372	0,100301	1	0,480557
-0,67948	421	309,432	0,49734	1	0,750993
-0,13363	421	178,9866	0,893842	1	0,958882
-0,36607	420	341,1694	0,714536	1	0,968866
-0,80493	367	323,2478	0,421451	1	0,731532
-0,38009	280	256,2867	0,704191	1	0,921995
-1,64476	421	312,5773	0,101024	1	0,342121
-1,88999	421	181,2535	0,060356	1	0,314727
-0,62005	420	347,4214	0,535631	1	0,960096
-2,01486	367	296,1231	0,044822	1	0,302719
-2,51577	280	253,2756	0,012497	1	0,244579
0,602403	421	357,4946	0,547288	1	0,784719
-1,2555	421	191,8172	0,210823	1	0,509968
0,200964	420	389,9363	0,840832	1	0,973894
0,937836	367	341,4636	0,348992	1	0,668671
1,415705	280	265,2392	0,158035	1	0,576656
-0,51392	421	350,8104	0,60763	1	0,824024
-0,50199	421	190,7923	0,616252	1	0,825504
-0,81076	420	383,8373	0,418005	1	0,95743

-0,12415	367	338,7143	0,901267	1	0,970486
-0,22594	280	264,4403	0,821422	1	0,9596
1,774516	421	320,6921	0,076927	1	0,301112
0,331245	421	181,8622	0,740841	1	0,886773
-0,9582	420	355,521	0,338611	1	0,947373
1,592177	367	305,9295	0,112378	1	0,438296
0,750009	280	255,7471	0,453939	1	0,814707
2,228716	421	374,7479	0,026425	1	0,191457
1,3156	421	200,2181	0,189813	1	0,476746
0,201009	420	403,7613	0,840793	1	0,973894
2,325808	367	354,8049	0,020592	1	0,224016
1,042974	280	271,524	0,297888	1	0,723324
2,270672	421	346,1407	0,023781	1	0,180089
1,66235	421	185,3619	0,098133	1	0,379251
0,923318	420	379,0737	0,356429	1	0,947373
2,486971	367	331,3743	0,013375	1	0,193812
1,407606	280	263,9471	0,160424	1	0,581516
-1,26923	421	333,3075	0,205244	1	0,494095
-1,7355	421	184,2122	0,084325	1	0,365475
-2,71969	420	362,0129	0,006849	1	0,543263
-2,2295	367	312,4065	0,026492	1	0,252225
-0,54711	280	254,1161	0,58478	1	0,87561
-0,08927	421	408,2323	0,928908	1	0,970107
-1,12143	421	221,7567	0,263319	1	0,559693
0,646482	420	415,8196	0,518324	1	0,960096
1,012041	367	362,6459	0,312193	1	0,646834
1,361298	280	274,4279	0,174536	1	0,598625
-0,79841	421	366,6066	0,425151	1	0,6997
-1,85637	421	193,8972	0,064918	1	0,325826
-0,49937	420	401,4341	0,617794	1	0,964878
-0,96388	367	347,479	0,335776	1	0,662363
0,054842	280	263,2025	0,956306	1	0,985067
1,328401	421	337,9206	0,184942	1	0,46885
1,643514	421	187,5851	0,101952	1	0,383322
-0,06109	420	372,0542	0,951317	1	0,992811
0,996303	367	323,2337	0,319848	1	0,653184
-0,14281	280	255,5575	0,886551	1	0,973784
1,81519	421	335,9588	0,070386	1	0,28514
-1,01736	421	185,8871	0,310302	1	0,607306
-0,59288	420	368,827	0,553628	1	0,960096
0,986939	367	330,8602	0,324394	1	0,655597
0,621728	280	261,28	0,534663	1	0,84326
-1,32312	421	354,9258	0,186647	1	0,471942
-1,71948	421	193,0714	0,08713	1	0,3678
1,577949	420	389,854	0,115388	1	0,848246
-0,99588	367	340,7807	0,320014	1	0,653184
-0,12937	280	265,8672	0,897165	1	0,975243
-0,81829	421	368,6026	0,413722	1	0,695339

0,472468	421	197,3467	0,637115	1	0,837812
-0,07773	420	398,5184	0,938078	1	0,992059
1,811214	367	340,8888	0,070988	1	0,366365
0,90007	280	261,9436	0,36891	1	0,763914
0,949901	421	331,5542	0,342854	1	0,63164
-0,31516	421	185,1068	0,752993	1	0,893512
0,828022	420	364,5783	0,4082	1	0,95743
-0,74097	367	319,457	0,459258	1	0,763917
-0,35733	280	276	0,721119	1	0,932812
0,058531	421	339,5915	0,95336	1	0,986076
0,09117	421	185,8756	0,927455	1	0,972749
-0,36842	420	375,1218	0,712767	1	0,968866
1,292625	367	306,3794	0,197115	1	0,549567
-0,98334	280	257,2743	0,326365	1	0,749743
1,121052	421	341,3032	0,263054	1	0,556772
-0,16887	421	188,1056	0,86608	1	0,947215
-1,69635	420	377,4387	0,090645	1	0,848246
-1,62576	367	325,7861	0,104967	1	0,429852
0,155597	280	260,398	0,876471	1	0,972638
-0,3761	421	381,2359	0,707054	1	0,875021
0,138103	421	201,3449	0,890297	1	0,958882
-0,46836	420	406,6291	0,639778	1	0,964878
1,091093	367	357,6018	0,275966	1	0,611884
-1,09782	280	268,7893	0,273267	1	0,701156
0,067608	421	368,7192	0,946134	1	0,981297
1,922494	421	197,3852	0,055983	1	0,309036
1,274761	420	397,3238	0,203139	1	0,928589
2,051097	367	340,1804	0,041023	1	0,289956
0,468518	280	266,0078	0,639798	1	0,907449
-2,0897	421	311,0395	0,037457	1	0,217944
-0,62915	421	179,9116	0,530046	1	0,768955
0,514305	420	347,2677	0,607366	1	0,964878
-1,46784	367	320,5722	0,143129	1	0,493583
-0,60402	280	249,9751	0,54638	1	0,851507
-2,54382	421	332,2248	0,011417	1	0,128401
-2,05372	421	185,0155	0,041409	1	0,272972
-0,32632	420	371,2088	0,744367	1	0,969427
-1,74557	367	325,5745	0,081828	1	0,381841
-2,76723	280	265,4731	0,006051	1	0,186692
-2,48777	421	354,9916	0,013313	1	0,138362
-2,25622	421	189,6077	0,025199	1	0,218351
-1,61397	420	382,154	0,107358	1	0,848246
-3,82905	367	331,8493	1,54E-04	0,165612	0,053579
-2,18667	280	269,9561	0,029625	1	0,32827
-0,07855	421	344,426	0,937433	1	0,974957
-0,53516	421	186,4229	0,59318	1	0,808395
-1,12301	420	374,5326	0,262154	1	0,943897
2,951486	367	349,0045	0,003376	1	0,176492

2,463375	280	259,5321	0,014413	1	0,258576
1,288869	421	341,2887	0,198317	1	0,484381
0,338544	421	187,3842	0,735332	1	0,884613
-1,46049	420	377,2333	0,144988	1	0,914005
1,629345	367	321,3932	0,10422	1	0,429123
-0,26651	280	255,8155	0,790063	1	0,953435
0,79686	421	365,4331	0,42605	1	0,700145
0,246833	421	196,4325	0,805296	1	0,923205
0,060849	420	395,3108	0,95151	1	0,992811
-0,14167	367	342,7618	0,887424	1	0,964898
-1,02082	280	261,4639	0,308286	1	0,729704
0,691906	421	396,9272	0,489401	1	0,748388
2,14789	421	213,0248	0,032851	1	0,246304
1,220487	420	413,5438	0,222976	1	0,943055
0,410183	367	362,1412	0,681914	1	0,900574
1,861249	280	270,4601	0,063794	1	0,422605
-0,64576	421	330,4593	0,518882	1	0,768129
-0,00884	421	185,1619	0,992956	1	0,997935
-0,38656	420	363,4756	0,699305	1	0,968866
-0,05747	367	320,5735	0,95421	1	0,980228
0,028636	280	257,7767	0,977177	1	0,99236
-0,59036	421	353,0911	0,555324	1	0,790572
0,655302	421	190,7982	0,513063	1	0,756803
0,174959	420	386,5669	0,861204	1	0,977284
1,097705	367	335,0321	0,273121	1	0,609149
1,574665	280	265,1352	0,116526	1	0,518958
-0,25475	421	348,4399	0,799064	1	0,922616
-1,53221	421	190,8108	0,127127	1	0,410224
-0,7304	420	383,0725	0,46559	1	0,960096
0,56063	367	340,0523	0,575419	1	0,842717
-1,53482	280	269,8638	0,125999	1	0,536498
1,854748	421	386,1749	0,064394	1	0,275801
2,014013	421	204,1704	0,045321	1	0,281076
1,082317	420	409,0654	0,279749	1	0,943897
0,3872	367	360,1351	0,698837	1	0,906323
-0,9201	280	273,6399	0,358332	1	0,761846
-3,60145	421	338,47	3,64E-04	0,38893	0,04642
-0,80498	421	187,0223	0,421851	1	0,692515
0,295499	420	373,8959	0,767777	1	0,969427
-1,43354	367	315,6348	0,152692	1	0,500233
-1,11893	280	259,1323	0,264207	1	0,688875
-1,839	421	341,9035	0,066782	1	0,278784
-1,32722	421	187,2556	0,18605	1	0,474401
-1,74921	420	373,5219	0,081075	1	0,848246
2,808777	367	348,2005	0,005254	1	0,178183
1,443464	280	260,1617	0,150093	1	0,562661
-1,00797	421	350,6983	0,314162	1	0,608538
-0,23198	421	190,4658	0,816803	1	0,926377

-0,68365	420	383,0028	0,49461	1	0,960096
2,076894	367	349,263	0,038542	1	0,28956
0,900325	280	261,5252	0,368776	1	0,763914
-2,02497	421	347,2718	0,043635	1	0,235324
-2,75287	421	191,0249	0,006477	1	0,141161
-0,5019	420	384,0948	0,616028	1	0,964878
0,544559	367	345,2714	0,586408	1	0,849728
-0,18188	280	266,8528	0,855814	1	0,966655
-0,24068	421	348,2149	0,809946	1	0,924931
1,064845	421	189,1134	0,288304	1	0,585544
-0,01087	420	381,7734	0,991334	1	0,997342
-1,25402	367	329,1897	0,210723	1	0,563436
0,903259	280	260,9181	0,367222	1	0,763914
-1,1459	421	311,0884	0,252718	1	0,546261
-0,97123	421	178,6671	0,332749	1	0,626816
-0,46823	420	347,3977	0,639914	1	0,964878
-1,21364	367	313,4474	0,225798	1	0,576775
-2,139	280	247,9397	0,033413	1	0,329568
-0,80571	421	416,9813	0,420871	1	0,697735
-1,59079	421	251,0584	0,112916	1	0,391524
0,130622	420	405,752	0,896139	1	0,98503
0,185995	367	359,1042	0,852554	1	0,956935
0,683029	280	275,8965	0,495161	1	0,8282
-2,27906	421	359,3757	0,023249	1	0,177419
-3,02072	421	194,3238	0,002861	1	0,114447
-0,09093	420	395,464	0,927592	1	0,990001
-2,22442	367	339,1708	0,026778	1	0,252225
-1,32245	280	265,8999	0,187156	1	0,621241
-2,04399	421	351,2835	0,0417	1	0,228553
-1,94168	421	191,4843	0,053644	1	0,302776
-0,41327	420	383,6531	0,679641	1	0,96781
-0,5934	367	346,8728	0,5533	1	0,830257
-1,20314	280	268,6839	0,229982	1	0,657169
0,445833	421	334,1353	0,656007	1	0,846936
1,635339	421	185,5829	0,103673	1	0,383322
-0,89268	420	369,4789	0,372611	1	0,95743
1,338663	367	319,1379	0,181633	1	0,527769
0,459503	280	260,2188	0,646257	1	0,907804
2,841663	421	330,7366	0,004766	1	0,087964
1,404726	421	182,9058	0,161799	1	0,445827
1,38226	420	364,1698	0,167739	1	0,914005
1,559802	367	309,5582	0,119828	1	0,451453
-0,12947	280	260,8681	0,897089	1	0,975243
-3,23705	421	340,4617	0,001326	1	0,066922
-2,25766	421	188,3073	0,025115	1	0,218351
-0,70875	420	378,5973	0,478913	1	0,960096
-1,05774	367	336,9474	0,290931	1	0,627013
-0,1479	280	260,5063	0,882538	1	0,973784

-0,53218	421	329,1862	0,594961	1	0,813756
-1,44767	421	184,6275	0,149406	1	0,430506
0,224736	420	364,1976	0,822311	1	0,972623
-1,84041	367	324,3387	0,066621	1	0,359849
-4,11389	280	251,8654	5,27E-05	0,056893	0,046455
0,505663	421	332,9748	0,613428	1	0,825172
-0,61154	421	180,3442	0,541611	1	0,773814
0,186718	420	367,1156	0,851985	1	0,973894
0,756614	367	317,8236	0,449842	1	0,755345
0,190779	280	251,9783	0,848852	1	0,966155
1,193628	421	383,3443	0,233362	1	0,525147
2,502968	421	203,9761	0,0131	1	0,171643
2,106401	420	406,3069	0,035782	1	0,828713
3,314459	367	348,0373	0,001015	1	0,152933
1,920537	280	270,3348	0,055842	1	0,40654
-0,15206	421	356,1079	0,879223	1	0,95861
-0,09922	421	192,7918	0,92107	1	0,970834
-1,23417	420	386,9998	0,21789	1	0,943055
-0,95535	367	334,1067	0,340092	1	0,664831
0,836071	280	262,2781	0,403876	1	0,786359
-1,0754	421	347,6763	0,282941	1	0,582502
0,747878	421	190,0701	0,455458	1	0,716467
0,26888	420	385,3763	0,788166	1	0,969427
1,510875	367	336,4488	0,131759	1	0,475026
0,425472	280	260,0613	0,670844	1	0,908644
-0,98551	421	344,0562	0,325064	1	0,61749
0,256858	421	188,7735	0,797568	1	0,921861
0,628538	420	378,1029	0,530031	1	0,960096
0,75618	367	318,9055	0,450099	1	0,755345
-0,41271	280	259,0036	0,680158	1	0,91111
-0,80376	421	293,4985	0,422185	1	0,697735
-1,29284	421	177,5398	0,197746	1	0,48773
-1,03857	420	321,8928	0,299786	1	0,943897
-0,11671	367	294,1079	0,907169	1	0,970486
-2,07329	280	276	0,039073	1	0,356865
-2,24739	421	335,7226	0,025264	1	0,184916
-1,47544	421	185,8554	0,141785	1	0,423499
-1,35067	420	368,415	0,177632	1	0,9199
-0,66652	367	331,6858	0,505545	1	0,793601
-0,3756	280	263,4639	0,70752	1	0,923145
-1,38592	421	342,4892	0,166674	1	0,441901
-1,08946	421	189,4463	0,277336	1	0,578094
-1,29383	420	383,0216	0,196502	1	0,928589
-1,12106	367	340,8719	0,26305	1	0,609149
-0,96621	280	264,5862	0,334821	1	0,755731
2,016783	421	340,4293	0,044503	1	0,236315
2,627433	421	185,189	0,009324	1	0,14815
1,655644	420	380,9008	0,098617	1	0,848246

2,352756	367	311,036	0,019257	1	0,218203
2,739114	280	264,7494	0,006579	1	0,192695
-0,46101	421	385,2281	0,645049	1	0,838007
0,486037	421	204,9414	0,62746	1	0,832379
0,714841	420	409,9869	0,475114	1	0,960096
-0,33216	367	359,1034	0,73996	1	0,923669
1,191517	280	274,4615	0,23448	1	0,659852
-1,22313	421	378,6531	0,222043	1	0,51676
-0,39474	421	201,3339	0,693456	1	0,865526
0,404245	420	406,8684	0,686245	1	0,968613
-0,19351	367	358,9423	0,846667	1	0,956935
-0,01372	280	266,9092	0,989062	1	0,995004
0,896184	421	385,0552	0,370714	1	0,662712
0,218335	421	206,4919	0,827384	1	0,933284
-0,98746	420	412,1729	0,323998	1	0,945573
-0,24371	367	357,3679	0,807598	1	0,950149
0,517874	280	269,3695	0,604971	1	0,885137
-2,59565	421	372,2138	0,009815	1	0,116484
-2,9746	421	198,7648	0,003297	1	0,118304
-0,85424	420	402,1128	0,393479	1	0,95743
-1,57744	367	355,2218	0,115585	1	0,443225
-1,91159	280	270,3312	0,056986	1	0,407001
-1,71732	421	316,6202	0,086899	1	0,315558
-2,75476	421	180,3404	0,006476	1	0,141161
0,337297	420	353,7674	0,736093	1	0,969427
-0,01528	367	304,6741	0,987822	1	0,996248
0,866087	280	256,8101	0,387251	1	0,775016
-0,98522	421	417	0,325086	1	0,61749
-0,92489	421	417	0,355556	1	0,643758
-1,17847	420	416	0,239283	1	0,943659
-0,11704	367	363	0,906892	1	0,970486
-0,43016	280	276	0,667413	1	0,908644
0,136034	421	313,9816	0,891882	1	0,962
0,335571	421	179,5793	0,737586	1	0,884986
1,079717	420	342,7709	0,281027	1	0,943897
-0,28496	367	302,3181	0,775868	1	0,940654
-1,04091	280	251,2998	0,298918	1	0,723324
-2,43121	421	357,5105	0,015539	1	0,147897
-2,6774	421	193,2622	0,008058	1	0,148102
-0,26839	420	397,533	0,788542	1	0,969427
-0,71777	367	345,0119	0,473387	1	0,776795
0,17632	280	267,3742	0,860176	1	0,966655
-1,14309	421	332,1235	0,253825	1	0,546261
0,895732	421	187,1468	0,371546	1	0,650743
-0,21543	420	367,3245	0,82955	1	0,973894
0,247002	367	308,7536	0,805071	1	0,950149
0,753467	280	250,9084	0,451876	1	0,814566
-0,50846	421	383,5481	0,611426	1	0,825172

0,037869	421	203,9601	0,969829	1	0,988858
-0,30446	420	408,295	0,760935	1	0,969427
0,222499	367	355,7606	0,824053	1	0,953906
0,572241	280	270,2938	0,567634	1	0,866692
2,177367	421	352,6047	0,030116	1	0,200565
1,775967	421	189,9463	0,077339	1	0,353183
0,231808	420	390,3719	0,816809	1	0,969427
2,353985	367	330,026	0,019159	1	0,218203
1,654996	280	265,9815	0,099105	1	0,480557
0,635685	421	330,4744	0,525422	1	0,770244
1,312348	421	183,2841	0,191044	1	0,47904
0,422633	420	366,0963	0,672811	1	0,96781
1,599618	367	318,3571	0,110675	1	0,437634
2,364481	280	276	0,018748	1	0,281002
-1,47718	421	350,8023	0,140525	1	0,410878
-1,42349	421	192,9245	0,15621	1	0,438625
-0,5451	420	386,211	0,586002	1	0,964878
-1,21063	367	340,454	0,226877	1	0,576775
0,728746	280	262,3963	0,466807	1	0,824256
2,787513	421	354,3984	0,005598	1	0,09373
3,205793	421	193,8638	0,001575	1	0,093671
1,593169	420	391,7459	0,111929	1	0,848246
2,671934	367	343,5724	0,007901	1	0,180403
2,680192	280	266,6882	0,007816	1	0,199202
1,109044	421	309,7371	0,268272	1	0,563516
-0,93296	421	178,4268	0,352103	1	0,643758
-0,57348	420	340,8861	0,5667	1	0,962539
1,269887	367	291,5845	0,205137	1	0,558434
0,102288	280	245,4368	0,918612	1	0,977051
-0,6187	421	357,7327	0,536509	1	0,778195
-0,00592	421	193,3238	0,995287	1	0,997935
-0,9445	420	389,3517	0,345498	1	0,947373
0,625226	367	344,6604	0,532236	1	0,814457
1,398983	280	264,7469	0,162988	1	0,584818
1,776817	421	372,8364	0,076414	1	0,300378
1,483473	421	198,0207	0,139539	1	0,423351
-0,50614	420	402,4499	0,613035	1	0,964878
1,711079	367	354,5261	0,087941	1	0,394165
1,984144	280	263,4779	0,048277	1	0,393265
0,383353	421	390,1686	0,701667	1	0,874567
0,205594	421	209,0447	0,837308	1	0,938419
0,254759	420	412,3054	0,799037	1	0,969427
0,469004	367	353,8999	0,639356	1	0,880707
-0,20502	280	265,878	0,83771	1	0,964667
-2,24642	421	301,9517	0,0254	1	0,184916
0,059622	421	175,3099	0,952525	1	0,982515
-1,26974	420	335,2816	0,205059	1	0,928589
-0,30835	367	284,5844	0,758041	1	0,928

-0,4925	280	254,1714	0,622793	1	0,898132
-1,48828	421	344,1144	0,137592	1	0,409785
-2,48714	421	188,565	0,013746	1	0,17172
0,043002	420	378,7131	0,965723	1	0,996924
-1,11697	367	331,7385	0,264815	1	0,609149
-1,80626	280	263,8475	0,072017	1	0,439393
-2,11748	421	340,5811	0,034943	1	0,214936
-2,46154	421	187,2494	0,014739	1	0,178042
-2,16751	420	363,9452	0,030844	1	0,80141
0,084178	367	324,1735	0,932967	1	0,977109
-0,94921	280	259,8742	0,343395	1	0,758791
2,20327	421	337,8039	0,02825	1	0,19529
1,437635	421	185,3758	0,152224	1	0,432832
2,247102	420	371,1185	0,025221	1	0,767829
1,992506	367	319,6085	0,047166	1	0,311448
1,024937	280	259,5543	0,306347	1	0,72861
-1,40338	421	299,1771	0,161541	1	0,435497
-1,98984	421	175,2359	0,048164	1	0,286754
1,006115	420	332,5421	0,315092	1	0,943897
0,105592	367	285,2913	0,91598	1	0,973634
-0,45716	280	255,8414	0,647945	1	0,908644
1,735607	421	365,4671	0,083476	1	0,306826
1,484653	421	197,427	0,139231	1	0,423351
0,747733	420	399,4167	0,455061	1	0,960096
1,941057	367	341,3394	0,053074	1	0,330508
-0,25708	280	261,4063	0,79732	1	0,954269
-2,09225	421	335,4755	0,037167	1	0,217944
-2,78946	421	187,2142	0,005826	1	0,141161
-0,19057	420	379,1877	0,848965	1	0,973894
-1,9708	367	331,9634	0,049578	1	0,315285
-1,38006	280	265,9072	0,168728	1	0,59271
-2,69871	421	349,5087	0,007299	0,897761	0,105763
-1,19028	421	191,7593	0,235407	1	0,535889
-0,97386	420	385,5642	0,330739	1	0,947373
-2,32517	367	342,5811	0,020646	1	0,224016
-0,50498	280	265,9049	0,613992	1	0,889365
-1,44473	421	340,9231	0,149452	1	0,422053
0,211172	421	184,6058	0,832986	1	0,936588
-1,97246	420	370,5686	0,0493	1	0,846627
0,291716	367	334,0298	0,770685	1	0,938144
1,185555	280	259,79	0,236881	1	0,659852
3,104908	421	368,1334	0,002051	1	0,069718
2,854272	421	199,336	0,004769	1	0,135614
1,680731	420	404,0578	0,093588	1	0,848246
2,423107	367	351,6556	0,015893	1	0,204703
2,191546	280	271,7379	0,029261	1	0,326641
1,463794	421	356,8069	0,144131	1	0,412154
1,779492	421	191,9507	0,076741	1	0,351518

1,00055	420	389,7155	0,317665	1	0,945573
2,132069	367	339,4262	0,033719	1	0,279197
2,763225	280	267,3248	0,006121	1	0,186692
-2,09142	421	298,0564	0,037337	1	0,217944
-0,64664	421	177,8698	0,518696	1	0,761752
-0,63165	420	327,6936	0,528053	1	0,960096
0,263114	367	295,9256	0,792646	1	0,944997
0,20532	280	262,105	0,837482	1	0,964667
-2,11129	421	332,9125	0,035492	1	0,215166
-1,24976	421	187,098	0,21295	1	0,512644
-0,79741	420	369,9738	0,425723	1	0,95743
-2,7501	367	320,0586	0,006297	1	0,178183
-1,29814	280	263,5185	0,195375	1	0,626244
-0,00576	421	294,2101	0,995405	1	0,998054
0,013265	421	173,8489	0,989431	1	0,997412
-0,33954	420	324,7589	0,734421	1	0,969427
0,277554	367	296,2535	0,781549	1	0,94337
-1,23225	280	276	0,218906	1	0,650539
-1,41796	421	366,7558	0,157052	1	0,429541
-0,96553	421	197,6061	0,335459	1	0,630335
0,005824	420	400,3212	0,995356	1	0,997342
-1,54554	367	345,2693	0,123131	1	0,458167
-0,14358	280	266,4847	0,885938	1	0,973784
-1,06946	421	323,515	0,285658	1	0,583534
0,073696	421	183,4106	0,941333	1	0,979688
0,654063	420	357,4206	0,513492	1	0,960096
0,237951	367	320,8301	0,812071	1	0,950149
0,50332	280	258,785	0,615168	1	0,889691
0,247477	421	391,5689	0,804668	1	0,922616
1,236957	421	208,793	0,217493	1	0,514565
0,595766	420	412,6839	0,551658	1	0,960096
-1,05433	367	362,0564	0,292435	1	0,627013
0,150694	280	275,0184	0,880328	1	0,973784
0,131436	421	349,9535	0,895506	1	0,962
-1,52902	421	188,2734	0,127939	1	0,410224
0,040653	420	383,6573	0,967593	1	0,996924
1,794474	367	324,6863	0,073668	1	0,373795
0,963467	280	251,0118	0,33624	1	0,755731
2,14803	421	391,0987	0,032325	1	0,20468
1,798873	421	209,711	0,073477	1	0,342815
0,145484	420	413,2983	0,8844	1	0,982463
1,664766	367	359,1265	0,096832	1	0,412696
1,397242	280	275,3984	0,163465	1	0,585137
-1,80839	421	319,8613	0,071485	1	0,288707
-0,43814	421	183,0364	0,661801	1	0,845199
0,054872	420	355,044	0,956271	1	0,995923
0,980704	367	322,0019	0,327475	1	0,655788
-0,11731	280	263,2927	0,906706	1	0,975308

0,118566	421	339,6284	0,905689	1	0,96594
-0,02305	421	187,1001	0,981635	1	0,993502
0,550352	420	373,4536	0,582407	1	0,964878
-0,32544	367	327,1511	0,745056	1	0,923669
0,388061	280	255,5744	0,698294	1	0,92034
-0,36246	421	313,7936	0,71725	1	0,883085
-0,86546	421	180,7511	0,387933	1	0,663581
0,630217	420	347,4243	0,528967	1	0,960096
-1,57236	367	318,2725	0,116861	1	0,445308
0,524878	280	253,8829	0,600126	1	0,881231
-2,77537	421	300,0448	0,00586	1	0,097046
-0,2775	421	177,436	0,781723	1	0,91393
-0,20012	420	331,4281	0,841508	1	0,973894
-1,8063	367	296,0061	0,071887	1	0,36848
-1,3583	280	247,1764	0,175607	1	0,598625
-1,60514	421	387,6563	0,109277	1	0,355682
-0,08327	421	205,7696	0,933721	1	0,975655
-0,4303	420	408,5057	0,667206	1	0,96781
-0,77533	367	358,7269	0,438658	1	0,742744
0,25647	280	268,3965	0,797784	1	0,954269
2,003597	421	377,9942	0,045827	1	0,237403
1,02077	421	201,6368	0,308587	1	0,60631
-0,51822	420	406,0814	0,604589	1	0,964878
1,505348	367	348,8435	0,13314	1	0,477542
-0,00786	280	267,6442	0,993737	1	0,996381
-0,49608	421	314,1759	0,620184	1	0,827828
-0,03526	421	178,3823	0,971909	1	0,990309
0,490624	420	350,122	0,624	1	0,964878
-0,8059	367	306,8497	0,420923	1	0,731532
2,114321	280	276	0,035385	1	0,339407
-3,15818	421	356,485	0,001723	1	0,069718
-2,19852	421	193,288	0,029099	1	0,233259
-0,54448	420	394,9482	0,586415	1	0,964878
-2,06683	367	339,2602	0,039508	1	0,28956
-0,76839	280	268,4748	0,442932	1	0,810247
-2,01457	421	300,3767	0,044841	1	0,236315
-2,37161	421	174,3022	0,018802	1	0,19275
-0,90807	420	330,0592	0,364504	1	0,956033
-2,26004	367	293,0875	0,024553	1	0,247087
-0,19521	280	258,655	0,845378	1	0,965181
-1,01272	421	344,9963	0,311904	1	0,608071
-1,32868	421	189,4394	0,185552	1	0,473945
-0,26328	420	380,6908	0,792474	1	0,969427
0,688925	367	336,1731	0,491346	1	0,792348
-1,29715	280	260,164	0,195727	1	0,626244
-3,07784	421	348,1212	0,00225	1	0,069718
-1,92977	421	190,6577	0,05512	1	0,308795
-1,03892	420	386,9536	0,299491	1	0,943897

-1,98032	367	343,5354	0,048465	1	0,313787
-1,09093	280	265,3202	0,276292	1	0,701156
0,925662	421	313,4494	0,355334	1	0,645166
2,278151	421	175,7284	0,023922	1	0,218351
0,118248	420	347,6876	0,90594	1	0,98503
0,621839	367	301,4892	0,534518	1	0,816122
1,338742	280	246,1816	0,18189	1	0,607861
-1,55378	421	326,3537	0,121206	1	0,382129
-2,49035	421	185,0481	0,013643	1	0,17172
-1,14572	420	365,6615	0,252661	1	0,943897
-2,07042	367	319,2825	0,039216	1	0,28956
-0,71127	280	260,2967	0,477554	1	0,824256
1,256523	421	403,1434	0,209654	1	0,498652
2,756097	421	217,4838	0,006346	1	0,141161
-0,19511	420	415,8568	0,845401	1	0,973894
0,723908	367	362,6772	0,469589	1	0,775105
0,712338	280	273,2196	0,476863	1	0,824256
1,764967	421	332,2078	0,078488	1	0,302838
-0,8546	421	183,8051	0,393886	1	0,665792
-0,63352	420	366,9345	0,526791	1	0,960096
1,861031	367	323,7272	0,063646	1	0,350852
-0,32576	280	264,1936	0,744865	1	0,942071
-0,25361	421	310,2111	0,799967	1	0,922616
1,440551	421	180,083	0,151448	1	0,432258
-0,50205	420	342,3161	0,615953	1	0,964878
0,097349	367	300,1546	0,922514	1	0,975687
0,341579	280	263,8036	0,73294	1	0,936751
-2,15429	421	326,5393	0,031949	1	0,204011
-1,23698	421	181,4133	0,217693	1	0,514565
-0,41276	420	364,1677	0,680024	1	0,96781
-1,385	367	324,0824	0,167005	1	0,514677
-2,76056	280	252,1016	0,006194	1	0,186692
-1,37945	421	300,7472	0,168781	1	0,442288
0,280614	421	177,3233	0,779334	1	0,913263
1,88485	420	330,0334	0,060329	1	0,848246
2,078287	367	290,1529	0,038562	1	0,28956
-0,82649	280	254,1675	0,409299	1	0,788151
-0,15607	421	375,1347	0,876059	1	0,95861
2,354056	421	200,4432	0,019537	1	0,195731
1,125393	420	401,6818	0,261095	1	0,943897
1,026177	367	356,4303	0,305504	1	0,637666
0,317193	280	273,2205	0,751339	1	0,942071
-0,68483	421	350,4071	0,493906	1	0,74916
-0,53077	421	189,267	0,596202	1	0,811435
0,717397	420	382,8844	0,473567	1	0,960096
-1,07211	367	340,4578	0,284431	1	0,619419
-0,39236	280	261,6402	0,695108	1	0,92034
-0,74191	421	334,3437	0,458665	1	0,724077

0,781935	421	186,6743	0,435243	1	0,702261
0,100871	420	371,9425	0,919707	1	0,989204
-0,47036	367	327,6904	0,638408	1	0,880707
-0,80654	280	254,9929	0,420682	1	0,796701
2,171441	421	354,3953	0,030561	1	0,200565
2,165402	421	192,6064	0,031586	1	0,241423
1,250758	420	390,9582	0,211771	1	0,943055
2,465447	367	347,3508	0,014167	1	0,201413
2,413806	280	267,0065	0,01646	1	0,266517
-2,30311	421	347,3127	0,021863	1	0,175251
-1,89004	421	190,2733	0,060273	1	0,314727
-1,64861	420	381,9723	0,10005	1	0,848246
1,23501	367	344,1935	0,217669	1	0,569492
0,478897	280	263,6875	0,632409	1	0,903355
0,815993	421	367,017	0,415033	1	0,695339
1,341396	421	194,4026	0,181357	1	0,470641
0,588001	420	398,6902	0,556864	1	0,960096
0,856065	367	350,1923	0,392547	1	0,705088
0,310527	280	261,9976	0,756407	1	0,942071
-1,63675	421	320,6977	0,102663	1	0,344557
0,349604	421	180,6034	0,727044	1	0,880751
0,336816	420	359,6004	0,736452	1	0,969427
0,223928	367	311,1297	0,82296	1	0,953906
0,896378	280	252,4439	0,370905	1	0,763914
1,428974	421	384,8082	0,153823	1	0,426123
0,87301	421	205,5186	0,383677	1	0,659083
1,076934	420	408,139	0,282146	1	0,943897
1,097581	367	355,556	0,27313	1	0,609149
-0,39501	280	268,4455	0,693152	1	0,920335
-0,46751	421	390,3317	0,640397	1	0,834842
-1,61846	421	208,6723	0,107074	1	0,386957
0,770462	420	412,2367	0,441467	1	0,960096
-0,38126	367	357,7271	0,703236	1	0,908463
-0,7601	280	271,9526	0,447851	1	0,813228
-1,40288	421	367,4394	0,161496	1	0,435497
-1,06059	421	197,7566	0,29017	1	0,586961
0,791131	420	397,9857	0,429339	1	0,958538
0,050717	367	353,1519	0,95958	1	0,981063
-2,54801	280	274,2662	0,01138	1	0,234919
-0,58923	421	374,4911	0,556066	1	0,790572
-1,51	421	199,5536	0,132624	1	0,414338
0,725128	420	403,1821	0,468794	1	0,960096
-1,04882	367	350,6692	0,294985	1	0,62734
-0,43	280	267,9841	0,667541	1	0,908644
-1,0886	421	323,0973	0,277141	1	0,57291
-0,44646	421	185,5571	0,655783	1	0,841107
-1,85718	420	361,7638	0,064097	1	0,848246
0,329817	367	325,9364	0,74175	1	0,923669

0,365635	280	257,7401	0,714938	1	0,929604
-1,00668	421	410,2239	0,31468	1	0,608758
-0,50406	421	225,5208	0,614712	1	0,824908
-0,58477	420	415,8466	0,559018	1	0,960096
-1,09841	367	362,9384	0,272752	1	0,609149
-0,57899	280	274,2406	0,563073	1	0,861728
0,882819	421	393,6035	0,377873	1	0,670163
-0,55717	421	211,2744	0,578	1	0,794719
-1,1601	420	413,529	0,246675	1	0,943897
-0,17299	367	358,2578	0,862759	1	0,959636
-1,5681	280	274,1506	0,118011	1	0,520006
-0,77819	421	353,7413	0,43698	1	0,707193
-2,18332	421	193,3777	0,030216	1	0,238409
-0,61255	420	387,4998	0,540531	1	0,960096
-1,82519	367	344,4365	0,068838	1	0,364488
-0,65217	280	267,8368	0,514848	1	0,83211
-1,24755	421	362,1201	0,213002	1	0,503916
-2,05245	421	195,0196	0,041461	1	0,272972
-0,14592	420	396,6855	0,884058	1	0,982463
-2,42696	367	349,1786	0,015731	1	0,204372
-3,69498	280	267,5443	2,67E-04	0,286655	0,057407
-2,18743	421	374,2763	0,029329	1	0,199094
-2,04294	421	201,4692	0,042361	1	0,273775
-0,6421	420	405,2657	0,521174	1	0,960096
-1,86388	367	356,2399	0,063161	1	0,34994
-1,72586	280	270,8237	0,085513	1	0,458532
-1,59554	421	331,7831	0,111543	1	0,358728
0,111514	421	184,3379	0,91133	1	0,96646
0,35455	420	364,4432	0,723132	1	0,969427
-0,02553	367	329,8586	0,979652	1	0,992161
-0,87678	280	258,5254	0,381419	1	0,770948
0,914151	421	360,6026	0,361248	1	0,651198
0,25275	421	195,3443	0,800727	1	0,921861
-0,45927	420	392,7153	0,646294	1	0,964878
-0,91113	367	337,6143	0,362876	1	0,677287
0,477505	280	261,0874	0,633402	1	0,903918
-0,9731	421	332,3109	0,331214	1	0,619505
-1,61338	421	184,5704	0,10837	1	0,388185
-3,09207	420	351,2598	0,002147	1	0,294094
-0,52551	367	320,2622	0,599594	1	0,862104
-0,91899	280	255,5915	0,358966	1	0,761846
0,542308	421	375,2195	0,587929	1	0,80988
0,454043	421	198,4796	0,650294	1	0,839358
0,794155	420	404,216	0,427571	1	0,95743
1,009937	367	357,4538	0,313209	1	0,64747
-1,02041	280	271,0822	0,308442	1	0,729704
-0,2859	421	305,5089	0,77515	1	0,911337
1,051665	421	178,5826	0,294374	1	0,590708

1,834708	420	331,865	0,067444	1	0,848246
-0,37238	367	282,4456	0,709888	1	0,912021
0,437857	280	249,0717	0,661869	1	0,908644
-4,17356	421	331,6505	3,84E-05	0,041309	0,019267
-1,037	421	185,7217	0,301085	1	0,600041
-0,65721	420	368,2547	0,511459	1	0,960096
-1,35438	367	318,995	0,176574	1	0,523018
0,783421	280	254,5124	0,434108	1	0,803161
-0,72847	421	392,2095	0,466762	1	0,728168
-1,6986	421	209,3658	0,09088	1	0,373113
0,659897	420	413,2626	0,509687	1	0,960096
-0,06783	367	360,6228	0,945957	1	0,979539
0,323504	280	271,9814	0,746562	1	0,942071
0,132172	421	353,4875	0,894924	1	0,962
0,613068	421	191,8765	0,540557	1	0,773814
-1,67815	420	382,7339	0,094133	1	0,848246
0,476848	367	332,9033	0,633784	1	0,880707
-0,42073	280	265,216	0,67429	1	0,909385
-0,88545	421	354,8238	0,376513	1	0,669899
-1,20585	421	192,8986	0,229353	1	0,529303
-0,48051	420	388,9081	0,631134	1	0,964878
-1,4594	367	335,0252	0,145391	1	0,496835
-0,13143	280	259,5556	0,895539	1	0,975243
-0,81638	421	341,213	0,414855	1	0,695339
-2,29602	421	189,3688	0,02277	1	0,212324
-0,5716	420	378,9455	0,567929	1	0,962723
-1,42455	367	323,2224	0,155252	1	0,502197
-0,70919	280	264,0791	0,478835	1	0,824256
-1,87988	421	338,3047	0,060984	1	0,270611
-2,94593	421	182,2596	0,00364	1	0,118336
-1,62643	420	377,3342	0,104693	1	0,848246
-1,99022	367	337,8808	0,047372	1	0,311448
-0,95371	280	258,3731	0,341121	1	0,757098
-3,09451	421	310,9309	0,002151	1	0,069718
-2,76938	421	178,0919	0,006211	1	0,141161
-0,21682	420	350,1947	0,828475	1	0,973894
0,173562	367	306,8567	0,862324	1	0,959636
-0,99525	280	246,8638	0,320588	1	0,744349
0,445207	421	389,0207	0,656418	1	0,846936
-0,83264	421	206,2301	0,406013	1	0,679847
1,909847	420	411,512	0,056848	1	0,848246
-0,06524	367	357,6274	0,948022	1	0,979539
0,396668	280	266,6567	0,69193	1	0,919522
0,804803	421	332,9161	0,421508	1	0,697735
2,574914	421	185,2094	0,010807	1	0,159671
-0,40864	420	370,7524	0,683041	1	0,968338
0,678043	367	335,3696	0,498212	1	0,793601
-0,08211	280	256,1976	0,934625	1	0,979504

1,848761	421	386,7111	0,065255	1	0,276236
1,299213	421	205,9914	0,195323	1	0,483337
0,464228	420	409,6476	0,642731	1	0,964878
1,664842	367	358,3256	0,096818	1	0,412696
0,549278	280	273,772	0,583262	1	0,87561
-0,27114	421	353,7442	0,786444	1	0,917552
0,773556	421	190,8193	0,440151	1	0,705407
-1,30659	420	384,2582	0,192134	1	0,925729
1,308901	367	338,2023	0,191456	1	0,542851
0,264663	280	269,2261	0,791471	1	0,953435
-1,23642	421	331,8998	0,217178	1	0,510588
0,113702	421	184,2327	0,909598	1	0,96646
0,581925	420	368,9704	0,560973	1	0,960096
0,150454	367	311,426	0,880504	1	0,962453
1,155998	280	271,5414	0,248698	1	0,677438
0,108547	421	335,4657	0,913627	1	0,966828
-0,78242	421	184,4023	0,434973	1	0,702261
0,470089	420	369,8161	0,638569	1	0,964878
0,607657	367	314,962	0,543853	1	0,824628
-0,60063	280	276	0,548581	1	0,852208
-1,29584	421	314,4034	0,19598	1	0,481951
-1,73974	421	181,6289	0,083598	1	0,365475
-0,64954	420	347,8617	0,51642	1	0,960096
-0,89879	367	313,7171	0,369452	1	0,679634
-0,29705	280	260,6987	0,766666	1	0,945436
0,539731	421	329,0559	0,589748	1	0,81147
0,806805	421	183,6249	0,420823	1	0,691581
3,137613	420	366,2428	0,001841	1	0,294094
-0,50385	367	328,0369	0,614707	1	0,872281
1,041883	280	258,4483	0,298439	1	0,723324
2,127428	421	356,8182	0,03407	1	0,212429
0,59455	421	194,4883	0,552835	1	0,778237
-0,46371	420	389,6139	0,643118	1	0,964878
3,536237	367	339,3541	4,62E-04	0,49647	0,116105
0,174992	280	267,4771	0,861219	1	0,967106
-0,30853	421	350,8809	0,757865	1	0,900919
2,673201	421	191,4203	0,008162	1	0,148102
1,764046	420	383,6445	0,07852	1	0,848246
2,983479	367	334,2325	0,00306	1	0,176492
1,52216	280	250,6968	0,129229	1	0,536498
-1,99381	421	356,429	0,046934	1	0,239762
-1,42142	421	192,6346	0,156812	1	0,438625
-1,14827	420	391,1713	0,25156	1	0,943897
-2,10369	367	333,5381	0,036154	1	0,28464
-2,16745	280	258,4584	0,031114	1	0,329568
1,764429	421	380,1779	0,078463	1	0,302838
2,425267	421	204,4994	0,016165	1	0,179127
0,506182	420	408,7094	0,613002	1	0,964878

2,531366	367	356,0689	0,011792	1	0,189546
1,757324	280	272,7278	0,079985	1	0,453386
1,476119	421	355,2789	0,140798	1	0,410878
2,715323	421	193,1493	0,007221	1	0,14319
1,108885	420	392,5552	0,268159	1	0,943897
2,755089	367	340,3055	0,006183	1	0,178183
1,460624	280	260,3698	0,145324	1	0,555466
-0,99378	421	368,0918	0,320981	1	0,615134
-1,46102	421	198,0804	0,145593	1	0,426194
-1,57992	420	402,3492	0,114912	1	0,848246
1,757413	367	353,813	0,079712	1	0,381841
-1,97397	280	271,8612	0,049398	1	0,39442
-1,34403	421	332,133	0,179857	1	0,459396
-0,28362	421	182,2297	0,777024	1	0,911265
-0,3748	420	366,6533	0,708024	1	0,968866
-0,36725	367	312,7257	0,713684	1	0,912652
0,243239	280	264,427	0,808009	1	0,95747
-0,82907	421	322,889	0,407677	1	0,695339
-1,45459	421	181,8776	0,147506	1	0,428784
-1,09683	420	359,3092	0,273452	1	0,943897
-2,18514	367	298,8422	0,029655	1	0,260259
-3,06462	280	258,3638	0,00241	1	0,135787
-2,09494	421	377,0283	0,036843	1	0,217944
-1,31818	421	202,719	0,188931	1	0,476746
-0,58309	420	407,0911	0,560157	1	0,960096
-2,01182	367	354,0302	0,044996	1	0,302719
-1,99935	280	270,5338	0,046571	1	0,387749
-1,1572	421	322,7897	0,248045	1	0,544195
0,069795	421	181,4111	0,944434	1	0,980711
-1,01466	420	353,4149	0,31096	1	0,943897
-0,27275	367	315,6962	0,785227	1	0,94337
1,786653	280	252,2671	0,075194	1	0,447896
-3,45908	421	352,8876	6,08E-04	0,647889	0,048251
-1,5248	421	193,1103	0,128945	1	0,410224
-1,4537	420	389,9518	0,146835	1	0,914005
-0,74964	367	341,1346	0,453987	1	0,758491
1,038513	280	263,0336	0,299985	1	0,723324
1,375102	421	404,8294	0,16986	1	0,442288
1,448248	421	218,794	0,148979	1	0,430099
0,256685	420	415,9813	0,797549	1	0,969427
1,335775	367	362,661	0,18246	1	0,527769
1,33932	280	274,2278	0,181575	1	0,607861
1,439306	421	407,6197	0,150831	1	0,424072
1,105237	421	221,4736	0,270256	1	0,569587
-1,19815	420	415,995	0,231542	1	0,943055
-0,66073	367	360,7976	0,509206	1	0,797092
-1,84051	280	274,2962	0,066774	1	0,425749
-1,34606	421	324,0802	0,179222	1	0,458554

0,133899	421	183,9055	0,893629	1	0,958882
0,084959	420	360,385	0,932342	1	0,991559
0,204756	367	319,0842	0,837893	1	0,956935
-1,27557	280	251,6338	0,203283	1	0,632429
-1,74737	421	357,3226	0,081433	1	0,30376
-0,10286	421	193,7725	0,918177	1	0,970154
-0,69747	420	390,2242	0,485925	1	0,960096
0,531157	367	341,6848	0,595655	1	0,858998
0,062162	280	266,9863	0,95048	1	0,982409
-0,09774	421	316,6862	0,9222	1	0,966828
0,926396	421	181,2171	0,355472	1	0,643758
0,112562	420	350,15	0,910442	1	0,987181
0,101182	367	309,3521	0,919472	1	0,974433
-0,14451	280	254,6888	0,885215	1	0,973784
0,739718	421	365,6651	0,459946	1	0,724425
-0,12613	421	196,8217	0,899758	1	0,960975
0,178927	420	396,2287	0,858087	1	0,976689
1,044669	367	346,545	0,296904	1	0,629303
-2,40893	280	271,9799	0,016665	1	0,266517
-0,43461	421	361,2108	0,664107	1	0,852478
0,592268	421	193,6737	0,554362	1	0,778237
0,119505	420	392,1639	0,904936	1	0,98503
0,263184	367	346,4404	0,792565	1	0,944997
-0,5611	280	258,0386	0,575219	1	0,870337
0,243827	421	365,4042	0,807502	1	0,923297
-0,00834	421	195,0437	0,993356	1	0,997935
0,253454	420	396,1555	0,800049	1	0,969427
1,825925	367	345,0026	0,068726	1	0,364488
0,640792	280	261,5653	0,522219	1	0,833721
-1,05051	421	322,754	0,294268	1	0,592864
0,285975	421	180,5052	0,775225	1	0,910824
1,190094	420	360,6732	0,234792	1	0,943055
0,993236	367	301,289	0,321392	1	0,653627
1,079893	280	258,2582	0,281198	1	0,703928
-1,37352	421	339,1428	0,170499	1	0,443003
0,210614	421	184,9108	0,833421	1	0,936588
-1,08426	420	371,1376	0,278954	1	0,943897
0,388174	367	312,3804	0,698152	1	0,906214
-0,40334	280	261,267	0,687025	1	0,915426
-0,77316	421	353,5508	0,439945	1	0,710422
0,436904	421	191,1314	0,662674	1	0,845595
0,047689	420	387,1524	0,961989	1	0,996924
-0,48678	367	335,3839	0,626733	1	0,88023
0,017092	280	267,7621	0,986376	1	0,992965
0,29681	421	405,4609	0,766764	1	0,906285
1,235433	421	221,5585	0,217978	1	0,514565
0,605813	420	415,9996	0,544969	1	0,960096
0,494374	367	362,7965	0,621341	1	0,875495

0,685012	280	271,6957	0,49392	1	0,827962
-1,2597	421	338,0487	0,208645	1	0,497513
-0,39955	421	188,3719	0,689943	1	0,864363
0,21202	420	372,3247	0,832208	1	0,973894
0,122801	367	319,9117	0,902342	1	0,970486
-0,34976	280	253,266	0,726812	1	0,936637
-0,04238	421	308,6554	0,966226	1	0,992572
0,859394	421	177,0876	0,391285	1	0,665538
1,011473	420	341,5403	0,312506	1	0,943897
-2,20492	367	312,0316	0,028189	1	0,257802
0,999444	280	260,7078	0,318506	1	0,741869
-1,59672	421	345,2905	0,111243	1	0,358728
-1,36119	421	189,0631	0,175074	1	0,459682
0,291399	420	381,0515	0,770904	1	0,969427
-2,15487	367	331,301	0,031892	1	0,27001
-0,3906	280	264,172	0,696405	1	0,92034
-1,59873	421	341,5218	0,110806	1	0,358728
-0,2302	421	187,2567	0,818187	1	0,926377
0,190299	420	377,0735	0,849178	1	0,973894
-0,51248	367	329,4915	0,608661	1	0,870021
0,894863	280	266,8139	0,371667	1	0,763914
-2,49734	421	325,9916	0,013006	1	0,138025
-1,49981	421	183,2114	0,135385	1	0,418943
-0,9332	420	365,6511	0,351334	1	0,947373
-1,25261	367	327,0069	0,211242	1	0,563436
-0,8426	280	273,4527	0,400187	1	0,784757
-1,96565	421	350,386	0,050129	1	0,246877
-0,19783	421	189,8298	0,843392	1	0,941476
-1,35385	420	378,5044	0,176593	1	0,9199
-0,92331	367	319,1825	0,356544	1	0,674813
-0,06342	280	252,0714	0,949478	1	0,982409
0,101841	421	373,4761	0,918938	1	0,966828
0,206073	421	199,0011	0,836945	1	0,938419
-0,07641	420	402,2618	0,939132	1	0,992477
1,132207	367	343,7978	0,258336	1	0,609149
1,012232	280	269,566	0,312335	1	0,733729
-2,51636	421	326,0781	0,012337	1	0,131857
-4,61072	421	183,9132	7,49E-06	0,001154	0,011291
-2,83382	420	362,8293	0,004857	0,728496	0,449965
-3,40148	367	322,5557	7,55E-04	0,116201	0,139974
-3,25997	280	253,0388	0,001267	0,19514	0,135787
-1,23869	421	335,0949	0,216327	1	0,509382
-1,6051	421	187,2957	0,110158	1	0,389736
-1,21407	420	370,1305	0,225495	1	0,943055
-0,46393	367	316,4809	0,643018	1	0,882539
-1,13717	280	256,5577	0,256529	1	0,680087
-0,95121	421	324,0651	0,342206	1	0,63164
-0,94154	421	181,2303	0,34768	1	0,642814

-1,05211	420	354,5117	0,293464	1	0,943897
-0,24243	367	309,6337	0,808608	1	0,950149
0,563923	280	247,9358	0,573317	1	0,870079
0,347958	421	330,0697	0,728094	1	0,890807
0,365005	421	183,4029	0,715528	1	0,874476
0,832742	420	364,3883	0,405536	1	0,95743
0,941166	367	321,3783	0,347327	1	0,668671
0,984635	280	258,6741	0,325723	1	0,749412
0,206866	421	345,9339	0,836236	1	0,93721
-0,18588	421	188,5988	0,852737	1	0,944105
0,154957	420	379,1685	0,876938	1	0,982463
-0,32548	367	320,3805	0,745031	1	0,923669
0,536752	280	248,4581	0,591919	1	0,876993
-0,44191	421	334,2153	0,658838	1	0,848606
-1,4596	421	184,3089	0,146102	1	0,426698
-0,6551	420	371,1214	0,512812	1	0,960096
-1,74511	367	321,6483	0,081921	1	0,381841
-2,22598	280	255,9396	0,026886	1	0,324814
-0,84549	421	314,9812	0,398477	1	0,688151
-0,85476	421	178,5192	0,393832	1	0,665792
-1,21506	420	349,5313	0,225163	1	0,943055
-2,09772	367	329,9933	0,03669	1	0,28464
-2,36838	280	262,145	0,018592	1	0,281002
0,108661	421	401,1812	0,913525	1	0,966828
0,869479	421	215,5778	0,385552	1	0,660258
-0,86277	420	415,1901	0,388763	1	0,95743
0,053777	367	361,5639	0,957142	1	0,981063
0,188333	280	274,7782	0,850755	1	0,966155
0,64197	421	342,6873	0,521322	1	0,769172
-0,06493	421	185,5	0,948303	1	0,980846
1,019276	420	374,9285	0,308729	1	0,943897
-0,24525	367	327,3245	0,806416	1	0,950149
1,197285	280	246,3251	0,232346	1	0,659852
0,388349	421	399,661	0,697965	1	0,872167
0,922262	421	215,1303	0,357425	1	0,643758
-0,60527	420	415,415	0,54533	1	0,960096
0,961399	367	362,9631	0,336992	1	0,662616
1,040485	280	275,7698	0,299026	1	0,723324
0,103522	421	300,2391	0,917618	1	0,966828
0,270287	421	175,0153	0,787258	1	0,917996
-0,50336	420	331,7107	0,615047	1	0,964878
-0,39951	367	278,5258	0,689825	1	0,903032
0,447709	280	276	0,654714	1	0,908644
-1,84487	421	346,7801	0,06591	1	0,278224
-1,58845	421	190,3869	0,113843	1	0,391524
-0,54706	420	383,0963	0,584658	1	0,964878
-1,40143	367	345,617	0,161983	1	0,508558
-0,39139	280	267,247	0,695821	1	0,92034

0,829432	421	340,1244	0,407442	1	0,695339
-1,15401	421	187,737	0,249961	1	0,548314
-2,6253	420	374,8251	0,009012	1	0,582436
1,047481	367	332,7619	0,295638	1	0,627502
-0,22894	280	256,0328	0,819099	1	0,95778
1,132594	421	346,4609	0,258168	1	0,550898
1,00629	421	189,2106	0,315561	1	0,614499
0,461719	420	378,8452	0,644548	1	0,964878
-0,95203	367	331,7855	0,341773	1	0,664831
0,314982	280	256,6433	0,753032	1	0,942071
0,781893	421	371,248	0,434776	1	0,706341
-0,1352	421	199,5834	0,892588	1	0,958882
0,41175	420	400,9927	0,680743	1	0,96781
-1,43818	367	343,5719	0,151293	1	0,499998
-0,40809	280	262,8844	0,683538	1	0,913202
-1,38026	421	327,5002	0,168448	1	0,442288
-0,71109	421	185,3717	0,477925	1	0,734183
-0,48982	420	364,2548	0,624559	1	0,964878
-0,92881	367	308,457	0,353714	1	0,673889
-0,70161	280	258,5602	0,483554	1	0,824339
-0,50039	421	364,4945	0,617106	1	0,826526
-0,26975	421	193,9945	0,787637	1	0,917996
-0,59902	420	395,8895	0,549503	1	0,960096
1,363281	367	347,5077	0,173677	1	0,523018
-0,42467	280	257,1062	0,671431	1	0,908644
-0,9387	421	347,4999	0,348539	1	0,637437
-0,19813	421	188,7908	0,843154	1	0,941476
0,130588	420	382,5389	0,89617	1	0,98503
-1,50045	367	348,1882	0,134403	1	0,477542
-1,28591	280	255,299	0,199639	1	0,631529
-2,0742	421	311,356	0,038882	1	0,222577
-1,72853	421	182,9966	0,085581	1	0,365475
-0,92737	420	356,7293	0,354362	1	0,947373
-0,33812	367	317,8817	0,735498	1	0,923669
0,030715	280	255,9556	0,975521	1	0,99236
3,901761	421	400,7965	1,12E-04	0,120378	0,033751
2,99969	421	214,476	0,003022	1	0,114447
1,328991	420	415,4531	0,18458	1	0,923408
2,250173	367	361,0465	0,025039	1	0,247087
1,796358	280	274,9372	0,073535	1	0,443271
1,060486	421	385,3988	0,289587	1	0,586895
0,81751	421	205,2503	0,414585	1	0,685818
0,384404	420	408,7972	0,700879	1	0,968866
1,767738	367	359,833	0,077952	1	0,381409
-0,54766	280	274,4276	0,584368	1	0,87561
-1,63572	421	317,9097	0,102887	1	0,344557
-0,9115	421	180,5143	0,363249	1	0,646044
-0,14839	420	354,8761	0,882116	1	0,982463

0,129521	367	301,6522	0,897032	1	0,970443
-0,36216	280	257,064	0,717527	1	0,931363
1,410462	421	380,0976	0,159221	1	0,432185
1,565061	421	203,3604	0,119123	1	0,396436
-0,22931	420	407,2742	0,818742	1	0,97003
2,436802	367	357,9244	0,015304	1	0,204372
1,318762	280	267,3297	0,188377	1	0,623041
-3,73855	421	417	2,11E-04	0,226527	0,04642
-3,51997	421	417	4,79E-04	0,51221	0,048139
-0,37198	420	416	0,710097	1	0,968866
-1,82824	367	363	0,068334	1	0,364488
-1,42348	280	276	0,155728	1	0,572395
-2,43442	421	341,3735	0,015428	1	0,147897
-1,43895	421	188,218	0,151824	1	0,432513
-2,28084	420	378,1639	0,023113	1	0,75904
-2,71173	367	330,6376	0,007043	0,901534	0,180403
0,597378	280	261,1071	0,550772	1	0,853046
0,993199	421	399,3848	0,321214	1	0,615134
-0,2552	421	215,767	0,798815	1	0,921861
-0,70265	420	415,2057	0,482668	1	0,960096
-1,09968	367	360,8198	0,272203	1	0,609149
-0,69219	280	270,5742	0,48941	1	0,826999
-3,06703	421	389,0227	0,002313	0,349283	0,069718
-1,82436	421	207,9554	0,069533	1	0,336932
-0,3212	420	412,2113	0,748221	1	0,969427
-1,60329	367	361,405	0,109744	1	0,435936
-1,87327	280	275,0017	0,062092	1	0,422605
0,469462	421	355,1818	0,639028	1	0,834842
0,49115	421	194,0539	0,623875	1	0,830785
0,595835	420	388,2638	0,551633	1	0,960096
1,21411	367	329,9456	0,225574	1	0,576775
-0,78293	280	269,321	0,434357	1	0,803161
-1,40986	421	356,8934	0,159453	1	0,432185
-1,05242	421	192,0844	0,293931	1	0,590656
-0,83925	420	391,3615	0,40184	1	0,95743
-1,3716	367	352,3526	0,171061	1	0,51869
-1,30659	280	266,6104	0,192478	1	0,62543
1,31624	421	357,9134	0,188936	1	0,472953
0,13414	421	195,1166	0,89343	1	0,958882
-0,73493	420	392,1041	0,462824	1	0,960096
1,82822	367	324,3768	0,068435	1	0,364488
1,201864	280	259,2291	0,230513	1	0,657169
1,55234	421	311,4504	0,121597	1	0,382559
0,66257	421	180,1974	0,508452	1	0,754914
-0,62679	420	346,1786	0,531213	1	0,960096
1,132892	367	308,4693	0,258139	1	0,609149
-1,27482	280	254,4733	0,203536	1	0,632429
2,386537	421	400,2074	0,017472	1	0,153976

1,445398	421	215,9583	0,149796	1	0,430627
0,456734	420	415,2025	0,648101	1	0,964878
1,789409	367	361,9827	0,074385	1	0,374909
0,655244	280	274,8551	0,512858	1	0,831912
-1,37212	421	339,3431	0,170932	1	0,443364
0,921374	421	186,4666	0,358046	1	0,643758
-0,38192	420	373,7545	0,702736	1	0,968866
-0,19896	367	316,362	0,84242	1	0,956935
0,307367	280	256,6904	0,758813	1	0,94273
-1,5225	421	382,7633	0,128709	1	0,396656
-0,91079	421	202,9694	0,363484	1	0,646044
-0,08561	420	408,1738	0,931816	1	0,991559
1,57007	367	357,4785	0,117283	1	0,445308
0,038908	280	263,2134	0,968993	1	0,991502
1,142523	421	374,1848	0,253967	1	0,546261
0,827211	421	200,6952	0,4091	1	0,681984
1,126745	420	404,3656	0,260519	1	0,943897
0,397955	367	349,7055	0,690906	1	0,903032
-0,23379	280	267,9997	0,815328	1	0,95747
1,646284	421	317,8058	0,100694	1	0,342121
1,158926	421	178,7691	0,248032	1	0,548314
-0,93997	420	349,4337	0,347885	1	0,947373
0,02995	367	305,3103	0,976127	1	0,9914
1,329027	280	247,7589	0,185062	1	0,617008
-0,51097	421	338,9512	0,609703	1	0,824796
0,827344	421	186,6249	0,409099	1	0,681984
1,205747	420	371,8745	0,228682	1	0,943055
-0,43148	367	328,6805	0,666404	1	0,894765
-1,47519	280	259,6337	0,141374	1	0,554427
-2,44152	421	356,1655	0,015112	1	0,147897
0,141632	421	193,3078	0,887518	1	0,958088
0,267525	420	389,4319	0,789206	1	0,969427
0,036631	367	340,92	0,970801	1	0,98918
-1,63462	280	264,0905	0,10332	1	0,486574
0,757773	421	338,1336	0,449115	1	0,714643
1,055934	421	186,5458	0,292364	1	0,589027
0,880021	420	371,6187	0,379417	1	0,95743
-1,39572	367	305,2818	0,163812	1	0,510294
0,219263	280	262,5117	0,826616	1	0,96121
0,73206	421	395,133	0,464566	1	0,727964
1,533859	421	212,1401	0,126554	1	0,410224
1,186919	420	414,2784	0,23594	1	0,943055
-0,81851	367	358,9971	0,41361	1	0,724248
-0,00307	280	269,7969	0,997551	1	0,998213
-1,85657	421	280,4075	0,064421	1	0,275801
-1,46944	421	173,1969	0,143528	1	0,42451
-0,8907	420	309,8846	0,373779	1	0,95743
-0,0874	367	363	0,930402	1	0,977084

0,176781	280	276	0,85981	1	0,966655
1,768792	421	343,0908	0,077817	1	0,302838
1,333883	421	187,0438	0,183864	1	0,473593
1,856176	420	375,8585	0,064211	1	0,848246
1,605338	367	325,0931	0,10939	1	0,435936
0,879154	280	261,4382	0,380125	1	0,770948
2,03357	421	324,4714	0,042808	1	0,232058
1,708226	421	183,3573	0,089287	1	0,37156
1,529995	420	362,7914	0,126889	1	0,885289
1,633506	367	323,5534	0,103335	1	0,426647
0,160648	280	255,9832	0,872497	1	0,969656
-0,03	421	311,1052	0,976083	1	0,995982
3,134364	421	179,1211	0,002013	1	0,099921
0,391431	420	346,4114	0,695719	1	0,968866
0,451696	367	322,2967	0,651792	1	0,88811
0,062657	280	259,6513	0,950088	1	0,982409
-0,82894	421	317,7811	0,407763	1	0,695339
-1,67399	421	182,4239	0,095847	1	0,378119
-1,31168	420	352,0339	0,190484	1	0,925729
-1,06381	367	313,6698	0,288234	1	0,624991
-1,50483	280	251,0812	0,133625	1	0,543276
-1,26936	421	350,4908	0,205156	1	0,494095
-1,85984	421	191,7577	0,06444	1	0,324784
-1,59492	420	385,8178	0,11155	1	0,848246
-1,14985	367	338,8576	0,251016	1	0,605249
-1,3819	280	263,6373	0,168173	1	0,59271
-0,24899	421	353,5061	0,803511	1	0,922616
-1,06955	421	191,5343	0,286169	1	0,584263
0,430243	420	387,728	0,667258	1	0,96781
0,74817	367	327,067	0,454895	1	0,758509
-0,25801	280	256,0992	0,796607	1	0,954269
-0,34374	421	317,2345	0,731271	1	0,892329
2,54E-04	421	180,765	0,999797	1	0,999797
0,666388	420	347,1445	0,505606	1	0,960096
-0,71776	367	304,3181	0,473458	1	0,776795
-1,25768	280	253,1803	0,209667	1	0,64091
-1,21324	421	374,601	0,225801	1	0,518147
-1,0849	421	199,5437	0,279274	1	0,578974
-0,19418	420	405,9683	0,846134	1	0,973894
-0,28893	367	352,4269	0,772806	1	0,938452
0,425654	280	265,5601	0,670705	1	0,908644
1,023192	421	340,636	0,306943	1	0,604218
0,993666	421	188,9934	0,321656	1	0,617772
1,232569	420	375,9061	0,218507	1	0,943055
0,001998	367	329,1846	0,998407	1	0,998887
0,193537	280	250,9208	0,846695	1	0,965181
-0,05525	421	320,6585	0,955976	1	0,987258
-0,29809	421	182,0485	0,765972	1	0,90393

1,306669	420	355,4055	0,19217	1	0,925729
0,730374	367	306,3831	0,46572	1	0,771623
0,613711	280	247,1576	0,539971	1	0,84937
-1,56241	421	327,0597	0,119158	1	0,380448
-1,66084	421	182,9281	0,098459	1	0,379251
-0,70573	420	363,1676	0,48081	1	0,960096
-1,55484	367	339,6815	0,120916	1	0,452158
-1,21065	280	262,1937	0,227118	1	0,656943
-0,87553	421	352,9236	0,381881	1	0,671614
0,381297	421	189,2242	0,70341	1	0,866753
0,074274	420	386,5936	0,940831	1	0,992811
0,28974	367	336,8625	0,772194	1	0,938452
1,557989	280	266,2874	0,120424	1	0,526024
-0,09615	421	324,005	0,923459	1	0,966828
0,95518	421	184,874	0,340734	1	0,635503
0,572745	420	358,3917	0,567176	1	0,962539
2,094393	367	316,4171	0,03702	1	0,28464
0,89314	280	261,6259	0,372603	1	0,763914
-0,82293	421	373,8249	0,411071	1	0,695339
-0,88489	421	199,821	0,377282	1	0,655017
-0,61746	420	403,3481	0,537282	1	0,960096
-0,33316	367	354,2265	0,739211	1	0,923669
-1,34137	280	273,7954	0,180911	1	0,607861
-0,01213	421	363,0178	0,990332	1	0,998054
0,426692	421	196,0177	0,670072	1	0,851432
-0,35669	420	394,827	0,721515	1	0,969427
-0,52163	367	340,2119	0,602271	1	0,864402
0,650026	280	249,7554	0,516273	1	0,83211
-1,493	421	362,518	0,136306	1	0,407576
0,017369	421	195,6885	0,98616	1	0,995407
-0,06048	420	394,699	0,951801	1	0,992811
-0,17366	367	349,0866	0,862231	1	0,959636
0,977115	280	263,5786	0,329408	1	0,752282
0,457642	421	309,7943	0,64753	1	0,839784
0,777003	421	178,5968	0,438185	1	0,704743
-0,06646	420	343,395	0,947053	1	0,992811
-0,0688	367	311,0989	0,945196	1	0,979539
0,287794	280	253,2867	0,77374	1	0,947219
-3,23632	421	333,5868	0,001332	1	0,066922
-1,82884	421	187,1584	0,069015	1	0,335504
-1,82049	420	371,2255	0,069489	1	0,848246
-2,62787	367	325,9329	0,008999	1	0,183886
-2,01893	280	256,434	0,044535	1	0,387749
-2,84947	421	412,4442	0,004599	0,680607	0,087964
-3,09432	421	232,4301	0,002214	0,329944	0,101124
-0,94628	420	413,5522	0,344558	1	0,947373
-1,44648	367	358,4311	0,148917	1	0,499998
-1,06444	280	275,8935	0,288058	1	0,71621

-1,26197	421	307,7015	0,207915	1	0,496728
-1,24302	421	180,3033	0,215474	1	0,514565
-0,80977	420	340,8617	0,418634	1	0,95743
0,053043	367	300,1645	0,957733	1	0,981063
-1,73668	280	246,5673	0,083693	1	0,456738
0,642878	421	342,7956	0,520733	1	0,769172
-0,0651	421	185,5431	0,948162	1	0,980846
1,019885	420	375,0228	0,308441	1	0,943897
-0,24022	367	327,39	0,81031	1	0,950149
1,19635	280	246,3481	0,23271	1	0,659852
1,548635	421	339,5633	0,122401	1	0,383489
0,515723	421	184,9021	0,606664	1	0,821338
1,796823	420	368,1854	0,073183	1	0,848246
0,818305	367	322,4226	0,413787	1	0,724248
-1,66544	280	247,9866	0,097088	1	0,480557
-2,09611	421	343,2201	0,036805	1	0,217944
0,556581	421	187,8317	0,578476	1	0,794719
-0,10644	420	379,4677	0,915292	1	0,988069
-0,06058	367	329,9245	0,951732	1	0,979686
0,926773	280	269,4529	0,354874	1	0,761846
-0,3135	421	350,7521	0,754087	1	0,898125
0,187014	421	190,2869	0,851849	1	0,944105
-0,18102	420	383,9701	0,856448	1	0,975561
1,459506	367	337,7185	0,145355	1	0,496835
1,46994	280	269,276	0,142746	1	0,554427
-1,68325	421	337,7353	0,093251	1	0,327788
-1,27726	421	185,8204	0,203103	1	0,497853
0,359925	420	373,2647	0,719106	1	0,969427
-0,97064	367	335,1133	0,332428	1	0,660908
-0,58598	280	260,8652	0,558396	1	0,858676
1,608267	421	364,8168	0,108642	1	0,355148
1,528897	421	196,7771	0,127896	1	0,410224
0,525146	420	397,3139	0,599775	1	0,964878
2,199953	367	346,456	0,02847	1	0,257802
0,685383	280	269,5274	0,493691	1	0,827962
-0,5169	421	343,1947	0,605556	1	0,822137
0,572641	421	187,6892	0,567573	1	0,78927
0,853253	420	377,2842	0,39406	1	0,95743
1,636877	367	314,2438	0,102657	1	0,426647
0,842065	280	250,094	0,400556	1	0,784757
-1,43279	421	340,7678	0,152834	1	0,42476
-1,2584	421	188,5543	0,209801	1	0,509953
-0,58606	420	377,0662	0,558186	1	0,960096
0,050782	367	328,0538	0,95953	1	0,981063
-0,04501	280	257,6472	0,964135	1	0,989749
-0,13132	421	288,0161	0,895612	1	0,962
0,625201	421	173,0203	0,532663	1	0,769894
-1,39833	420	313,7467	0,163	1	0,914005

0,654173	367	282,4149	0,513533	1	0,797829
1,728653	280	276	0,084989	1	0,45831
0,584696	421	407,6488	0,559076	1	0,792594
1,473617	421	223,2317	0,141993	1	0,423499
-0,79298	420	415,8011	0,428239	1	0,957502
0,864223	367	362,9965	0,388036	1	0,702006
-0,14288	280	274,2517	0,886492	1	0,973784
0,650955	421	295,4838	0,515582	1	0,767364
-1,60585	421	176,7681	0,110093	1	0,389736
-0,90757	420	324,441	0,364777	1	0,956033
0,216944	367	363	0,828374	1	0,956109
-0,38424	280	276	0,701095	1	0,92034
0,474591	421	365,7979	0,635362	1	0,83455
0,267114	421	194,282	0,789665	1	0,918329
1,194426	420	393,1131	0,233031	1	0,943055
-0,18938	367	339,8265	0,84991	1	0,956935
-1,43621	280	258,8195	0,15215	1	0,565026
-0,57532	421	329,1036	0,565469	1	0,796637
-0,33748	421	185,3144	0,736138	1	0,884613
0,706358	420	364,1794	0,480417	1	0,960096
-0,14566	367	318,7942	0,884282	1	0,962871
-0,86947	280	276	0,385345	1	0,773166
-1,36725	421	370,0179	0,172377	1	0,444059
0,143105	421	198,1271	0,886353	1	0,957782
2,009772	420	403,2478	0,045121	1	0,846132
-0,99687	367	347,621	0,31952	1	0,653184
-0,25949	280	270,0145	0,795457	1	0,954269
0,650544	421	319,7623	0,515808	1	0,767364
0,609407	421	181,3252	0,543018	1	0,774002
1,92972	420	356,3966	0,054435	1	0,848246
0,006995	367	312,0876	0,994423	1	0,997335
1,405782	280	253,9344	0,161011	1	0,581516
-1,49361	421	341,318	0,136202	1	0,407576
-2,04462	421	188,6768	0,042281	1	0,273775
0,679328	420	376,3736	0,497348	1	0,960096
-1,24423	367	339,1844	0,214274	1	0,564902
-0,78975	280	261,5524	0,430387	1	0,801886
-1,36174	421	406,6334	0,174033	1	0,446794
-1,51066	421	223,1146	0,13229	1	0,414338
0,199394	420	415,9963	0,842052	1	0,973894
1,410763	367	362,8602	0,159171	1	0,504558
-1,85002	280	274,0597	0,065387	1	0,424733
0,250808	421	307,2213	0,802131	1	0,922616
1,363901	421	180,8256	0,174294	1	0,459198
-0,3151	420	339,1034	0,75288	1	0,969427
1,913014	367	301,0442	0,056695	1	0,337704
-0,32002	280	276	0,749198	1	0,942071
-2,31516	421	302,9563	0,021272	1	0,172351

-1,19489	421	176,0421	0,233739	1	0,533704
-2,44576	420	332,3802	0,014973	1	0,723749
-1,13587	367	271,9282	0,257011	1	0,609149
-0,2593	280	248,4418	0,795622	1	0,954269
1,890901	421	344,4608	0,059477	1	0,268966
1,716092	421	184,1687	0,087827	1	0,368676
0,925355	420	377,4746	0,355372	1	0,947373
2,674114	367	327,969	0,007868	1	0,180403
1,694345	280	263,7913	0,09138	1	0,468147
-0,328	421	320,1103	0,743123	1	0,895193
-0,44367	421	183,8531	0,6578	1	0,841515
-0,67058	420	355,7484	0,502926	1	0,960096
-2,50566	367	300,2041	0,012752	1	0,189546
-0,62212	280	276	0,534378	1	0,84326
-0,7189	421	378,8003	0,472647	1	0,731548
0,300073	421	203,1476	0,764428	1	0,903524
-0,41314	420	405,274	0,679724	1	0,96781
0,959957	367	355,8699	0,337729	1	0,662705
0,281089	280	265,5483	0,778861	1	0,951006
-1,98494	421	353,7277	0,047923	1	0,241202
-0,40808	421	191,2606	0,683674	1	0,859821
0,198156	420	388,8078	0,843026	1	0,973894
-1,84448	367	340,3121	0,065982	1	0,358968
-0,02165	280	267,7366	0,982744	1	0,99236
-0,12693	421	353,5926	0,899066	1	0,965023
0,22111	421	193,2482	0,825241	1	0,932262
0,066216	420	387,3938	0,94724	1	0,992811
1,120453	367	343,4063	0,263304	1	0,609149
0,654433	280	265,8496	0,513399	1	0,831912
-1,65427	421	327,8577	0,099029	1	0,339344
-1,60511	421	185,5298	0,110171	1	0,389736
-1,68817	420	362,9176	0,092237	1	0,848246
-1,04353	367	326,376	0,297477	1	0,629631
-2,28104	280	257,1461	0,023364	1	0,313396
0,39868	421	331,3244	0,690386	1	0,867733
1,029279	421	184,9883	0,304692	1	0,602952
1,705259	420	358,9219	0,089011	1	0,848246
0,510213	367	346,0332	0,610227	1	0,870021
-0,4639	280	267,7003	0,643094	1	0,907804
-0,78088	421	333,0233	0,435428	1	0,706341
-0,39153	421	184,7259	0,695854	1	0,865526
1,328841	420	369,355	0,184721	1	0,923408
-0,20698	367	338,616	0,836151	1	0,956935
0,342624	280	262,5246	0,732155	1	0,936637
-1,61117	421	351,5163	0,10804	1	0,353949
-2,49391	421	186,6105	0,013504	1	0,17172
-1,72815	420	386,7296	0,08476	1	0,848246
-1,65756	367	326,469	0,098367	1	0,414924

0,282665	280	251,8163	0,777666	1	0,950481
-1,0721	421	335,1256	0,284448	1	0,583534
-0,17983	421	185,6871	0,857481	1	0,945992
1,777447	420	371,7523	0,076312	1	0,848246
0,993627	367	338,4095	0,321114	1	0,653627
0,904791	280	262,0627	0,366407	1	0,763914
-2,99271	421	304,0653	0,002992	1	0,077359
-1,88228	421	174,3063	0,061465	1	0,317217
-1,09787	420	337,9902	0,273042	1	0,943897
-2,2849	367	305,5373	0,023004	1	0,237443
-1,2039	280	261,0026	0,229721	1	0,657169
-0,93156	421	354,9687	0,352195	1	0,641012
-0,73605	421	190,677	0,462606	1	0,721079
1,201544	420	390,5083	0,230268	1	0,943055
-0,47939	367	341,3564	0,631967	1	0,880707
2,465167	280	266,8391	0,014325	1	0,258576
0,792167	421	330,0358	0,428832	1	0,700922
1,035602	421	182,6297	0,301757	1	0,600041
1,498908	420	365,342	0,134761	1	0,890998
-2,02428	367	311,4797	0,043795	1	0,299994
2,403749	280	253,0488	0,016948	1	0,266517
3,182281	421	338,4767	0,001597	1	0,069718
2,609771	421	186,8045	0,009795	1	0,149771
-0,06028	420	373,2857	0,951966	1	0,992811
0,532222	367	326,6779	0,594934	1	0,85878
0,810406	280	258,9201	0,418451	1	0,795216
-0,43325	421	350,4119	0,665098	1	0,853014
-0,01155	421	192,6258	0,990795	1	0,997935
0,033063	420	384,7007	0,973642	1	0,997342
0,328657	367	331,8539	0,742622	1	0,923669
0,76294	280	264,0028	0,44618	1	0,813051
-1,79525	421	342,1423	0,073497	1	0,292681
-0,60055	421	189,0311	0,548859	1	0,776424
-0,80185	420	380,9479	0,42314	1	0,95743
-2,06229	367	334,8353	0,039952	1	0,28956
-1,36996	280	258,8216	0,171886	1	0,598625
-0,04601	421	355,1398	0,963331	1	0,991625
0,368883	421	192,06	0,712621	1	0,872396
0,369499	420	389,5752	0,711957	1	0,968866
1,055424	367	347,6904	0,291964	1	0,627013
0,631872	280	264,935	0,528015	1	0,839366
0,974149	421	386,599	0,330592	1	0,619505
0,894258	421	206,8264	0,372223	1	0,650743
0,798914	420	410,4331	0,424803	1	0,95743
-1,22425	367	355,1665	0,221669	1	0,573978
0,290436	280	268,3605	0,771707	1	0,947038
1,270408	421	374,904	0,204727	1	0,494095
1,732662	421	199,1435	0,084704	1	0,365475

0,079496	420	403,9098	0,936678	1	0,992059
1,749937	367	355,5087	0,080992	1	0,381841
0,368772	280	269,2275	0,712588	1	0,928432
-2,56285	421	374,0967	0,010772	1	0,12392
-2,18485	421	200,3399	0,03006	1	0,238409
-0,58644	420	405,4949	0,557905	1	0,960096
-2,49256	367	351,1838	0,013143	1	0,192294
-1,53629	280	270,6232	0,125635	1	0,536498
0,614188	421	360,004	0,539479	1	0,779259
1,15564	421	194,6438	0,249245	1	0,548314
0,922049	420	390,6767	0,357072	1	0,947373
0,1739	367	343,6219	0,862047	1	0,959636
-1,82211	280	265,6231	0,069563	1	0,431405
1,375922	421	393,7408	0,169628	1	0,442288
-0,38258	421	210,293	0,70242	1	0,866241
-0,94912	420	412,4237	0,343115	1	0,947373
-0,49777	367	358,0859	0,61895	1	0,875495
1,77856	280	272,7185	0,076426	1	0,450485
-2,08912	421	384,418	0,037354	1	0,217944
-1,72889	421	207,0354	0,085319	1	0,365475
0,052675	420	410,7598	0,958017	1	0,996364
-0,9638	367	358,9845	0,335796	1	0,662363
-0,95743	280	275,6717	0,339189	1	0,756354
-1,90609	421	295,8189	0,057608	1	0,266382
-0,7113	421	175,3431	0,477842	1	0,734183
-2,51393	420	323,3786	0,012425	1	0,668711
1,230984	367	299,6558	0,219294	1	0,572696
0,939831	280	247,5153	0,348221	1	0,761841
0,171215	421	357,8587	0,864151	1	0,951261
-0,28567	421	192,5425	0,775439	1	0,910824
-1,64002	420	393,5181	0,101801	1	0,848246
-0,0721	367	341,9435	0,942565	1	0,979539
0,089733	280	261,7959	0,928568	1	0,979504
-0,50526	421	333,2452	0,613709	1	0,825172
-0,02332	421	185,5094	0,981424	1	0,993502
-0,97425	420	368,0114	0,330571	1	0,947373
0,438009	367	317,632	0,661677	1	0,894303
0,471065	280	255,4135	0,637996	1	0,907449
-0,02934	421	368,506	0,976611	1	0,995982
1,325009	421	195,3846	0,186715	1	0,474503
-0,0681	420	398,5799	0,945737	1	0,992811
1,341111	367	343,4758	0,180771	1	0,527769
1,278247	280	268,4472	0,202266	1	0,632429
0,262623	421	364,0359	0,79299	1	0,919136
2,085738	421	194,9685	0,038304	1	0,268483
0,663133	420	394,6352	0,507633	1	0,960096
-0,41428	367	339,9382	0,678931	1	0,899694
-1,57093	280	263,811	0,117398	1	0,518958

1,742399	421	372,9288	0,082263	1	0,304997
2,325012	421	201,2914	0,021068	1	0,2022
-0,73861	420	404,4445	0,460571	1	0,960096
-0,49057	367	352,4651	0,624039	1	0,878083
1,778157	280	264,7689	0,076526	1	0,450485
-0,86763	421	358,2867	0,386177	1	0,675138
0,362399	421	192,7181	0,717451	1	0,874811
0,40071	420	390,6935	0,688853	1	0,968866
-0,4354	367	336,8814	0,663551	1	0,894765
0,424651	280	259,0931	0,671444	1	0,908644
-0,40111	421	315,2803	0,688609	1	0,866222
0,499962	421	180,1274	0,617712	1	0,825805
-0,57878	420	346,3186	0,563111	1	0,961052
-1,70532	367	297,165	0,08918	1	0,396441
-0,44163	280	254,444	0,659132	1	0,908644
0,686989	421	323,7626	0,492582	1	0,748579
0,83446	421	183,1326	0,405109	1	0,679087
-1,59683	420	360,7834	0,111179	1	0,848246
-1,29317	367	297,6946	0,196953	1	0,549567
0,1844	280	248,1834	0,85385	1	0,966655
0,823173	421	351,944	0,410967	1	0,695339
0,600347	421	191,598	0,548985	1	0,776424
0,59038	420	384,6331	0,555283	1	0,960096
1,412162	367	338,4243	0,158821	1	0,504558
-0,92161	280	263,5172	0,357577	1	0,761846
1,096809	421	374,967	0,273429	1	0,56914
0,99271	421	198,8565	0,322057	1	0,617772
-0,07819	420	402,8216	0,937714	1	0,992059
-0,19727	367	350,0211	0,843731	1	0,956935
0,79881	280	266,2552	0,425112	1	0,797636
-1,7011	421	345,2754	0,089825	1	0,320014
-1,43601	421	190,2963	0,152641	1	0,433201
-0,34016	420	383,1698	0,733925	1	0,969427
-1,66262	367	334,3484	0,097325	1	0,412696
0,082493	280	264,0634	0,934318	1	0,979504
1,459889	421	359,0631	0,145195	1	0,41441
1,719747	421	194,1986	0,087072	1	0,3678
0,672058	420	392,1466	0,501943	1	0,960096
-0,18516	367	344,0602	0,853211	1	0,956935
1,972555	280	263,8532	0,04959	1	0,39442
-2,05501	421	317,6547	0,040695	1	0,226301
-1,69273	421	181,5234	0,092223	1	0,373113
-0,13433	420	355,4875	0,893219	1	0,98503
-0,0938	367	317,3168	0,925328	1	0,976733
0,026597	280	253,043	0,978802	1	0,99236
1,475805	421	327,1426	0,140958	1	0,410878
1,414349	421	180,2123	0,158985	1	0,440423
1,718971	420	364,4826	0,086469	1	0,848246

1,847244	367	318,5603	0,065639	1	0,358398
0,717722	280	250,2226	0,473599	1	0,824256
-1,03911	421	317,8728	0,299542	1	0,596172
-1,20204	421	183,1454	0,230899	1	0,531243
-1,75019	420	345,2078	0,080974	1	0,848246
-0,8373	367	310,2765	0,403066	1	0,716298
-1,12526	280	254,3141	0,261539	1	0,687569
0,029112	421	330,559	0,976793	1	0,995982
-0,37288	421	184,9241	0,709666	1	0,870869
-0,61277	420	366,0132	0,540411	1	0,960096
-1,15306	367	321,3062	0,249741	1	0,604409
-0,87638	280	258,2751	0,381637	1	0,770948
0,499815	421	372,7772	0,6175	1	0,826526
1,027822	421	199,6325	0,305277	1	0,602952
0,24368	420	402,5273	0,807603	1	0,969427
1,206984	367	349,5841	0,228254	1	0,577307
0,702857	280	272,1276	0,482746	1	0,824339
-1,89023	421	326,8418	0,059612	1	0,268966
-0,62897	421	182,6896	0,530155	1	0,768955
0,475617	420	364,9529	0,634632	1	0,964878
0,169009	367	321,0901	0,865896	1	0,959636
1,011451	280	267,5863	0,312714	1	0,733729
-1,7294	421	362,6804	0,084589	1	0,309688
-1,21549	421	193,7695	0,22566	1	0,523989
0,362409	420	398,8937	0,717238	1	0,969427
-1,79205	367	349,0268	0,07399	1	0,374173
-2,22145	280	267,6123	0,027158	1	0,324814
-2,08064	421	334,834	0,038227	1	0,220722
0,334403	421	185,5297	0,738453	1	0,885321
1,322287	420	370,9711	0,186887	1	0,923408
1,459815	367	323,8962	0,14531	1	0,496835
1,159368	280	260,1167	0,247369	1	0,677438
-2,16214	421	336,0445	0,031312	1	0,201145
-1,73574	421	187,2069	0,084254	1	0,365475
-0,88681	420	370,416	0,375755	1	0,95743
-1,68767	367	325,2019	0,092434	1	0,40529
-2,52055	280	261,0618	0,012314	1	0,244171
0,426978	421	330,7159	0,669673	1	0,855252
1,640834	421	185,5487	0,102526	1	0,383322
0,46642	420	369,5026	0,64119	1	0,964878
1,081379	367	320,6949	0,280341	1	0,614784
-1,2083	280	245,9501	0,228094	1	0,657169
-0,55154	421	370,3118	0,581595	1	0,805573
1,625748	421	198,6856	0,105588	1	0,385174
1,681223	420	400,9986	0,093498	1	0,848246
2,148697	367	345,0875	0,032354	1	0,270873
-0,75466	280	261,2424	0,451134	1	0,814202
-2,72456	421	304,8228	0,00681	1	0,10482

-2,41532	421	176,2544	0,016743	1	0,181525
-0,69606	420	344,3483	0,486863	1	0,960096
-4,37463	367	297,9442	1,68E-05	0,018171	0,025378
-2,2722	280	247,995	0,023931	1	0,313396
-1,23122	421	320,6098	0,219142	1	0,513603
-0,52434	421	179,8288	0,600689	1	0,816265
-0,42643	420	356,3854	0,670054	1	0,96781
1,886745	367	325,165	0,060085	1	0,340409
0,419228	280	254,9317	0,675403	1	0,909385
-0,72074	421	343,5882	0,471557	1	0,731548
-0,17346	421	187,0588	0,862479	1	0,947215
-0,58698	420	379,5393	0,557566	1	0,960096
1,197014	367	328,4904	0,232164	1	0,582149
0,538861	280	266,5035	0,590433	1	0,876633
0,376817	421	320,3405	0,706559	1	0,875021
0,820191	421	182,1355	0,413179	1	0,684933
-0,12066	420	353,7744	0,90403	1	0,98503
0,920207	367	307,902	0,358185	1	0,674813
0,107771	280	249,196	0,914264	1	0,977051
1,010111	421	375,992	0,313092	1	0,608538
0,325333	421	201,6062	0,745267	1	0,888542
-0,00865	420	404,223	0,993105	1	0,997342
-0,12491	367	353,2795	0,900669	1	0,970486
-0,11796	280	267,1453	0,906189	1	0,975308
0,947264	421	354,8071	0,344149	1	0,632479
1,204606	421	193,6086	0,229826	1	0,529584
-0,61589	420	388,5275	0,538326	1	0,960096
0,338845	367	349,3502	0,73493	1	0,923669
-0,27049	280	264,3323	0,786996	1	0,953235
0,559511	421	369,4289	0,576152	1	0,801719
1,525111	421	197,194	0,128834	1	0,410224
1,953239	420	399,262	0,051489	1	0,846627
2,512173	367	338,0323	0,012464	1	0,189546
1,044928	280	267,198	0,297001	1	0,723324
-2,01662	421	319,6165	0,044571	1	0,236315
-0,90559	421	182,6781	0,366346	1	0,647779
-0,38598	420	359,6301	0,699741	1	0,968866
1,021763	367	318,492	0,307668	1	0,640947
0,851805	280	276	0,395061	1	0,782194
3,657168	421	342,0778	2,95E-04	0,3164	0,04642
2,008284	421	185,5373	0,046063	1	0,281076
1,867768	420	375,9797	0,062572	1	0,848246
2,850752	367	323,1288	0,004642	1	0,178183
1,148764	280	267,0478	0,251681	1	0,679007
-0,33656	421	392,9457	0,736629	1	0,893273
-0,32021	421	210,9018	0,749126	1	0,890124
-0,66536	420	412,8956	0,506194	1	0,960096
-0,55486	367	358,7001	0,579336	1	0,844137

0,726832	280	270,5849	0,467957	1	0,824256
1,668227	421	395,3336	0,096063	1	0,332744
2,076396	421	211,8173	0,039064	1	0,26998
0,743562	420	414,0492	0,457563	1	0,960096
-0,05836	367	361,8688	0,953491	1	0,980157
0,759448	280	274,33	0,448237	1	0,813228
1,764429	421	380,1779	0,078463	1	0,302838
2,425267	421	204,4994	0,016165	1	0,179127
0,506182	420	408,7094	0,613002	1	0,964878
2,531366	367	356,0689	0,011792	1	0,189546
1,757324	280	272,7278	0,079985	1	0,453386
0,252194	421	402,2566	0,80102	1	0,922616
1,772041	421	217,5131	0,077788	1	0,353243
0,002956	420	415,6822	0,997643	1	0,997643
0,207812	367	357,5851	0,835494	1	0,956935
-0,88013	280	275,0072	0,379557	1	0,770948
-0,47275	421	352,1126	0,636688	1	0,834842
0,119596	421	190,4803	0,904929	1	0,96513
-0,32474	420	384,8961	0,745551	1	0,969427
1,176174	367	329,6029	0,240374	1	0,59287
1,651686	280	261,0102	0,099801	1	0,480557
-0,14565	421	346,3988	0,884285	1	0,961484
1,127619	421	188,2942	0,260916	1	0,557979
-0,38415	420	381,1751	0,701081	1	0,968866
0,776005	367	317,5612	0,438324	1	0,742744
-0,05173	280	252,5041	0,958784	1	0,986271
-0,2471	421	363,0577	0,80497	1	0,922616
1,762039	421	192,901	0,079647	1	0,357225
0,623758	420	392,7457	0,533149	1	0,960096
0,776301	367	349,8565	0,438096	1	0,742744
-1,35694	280	265,5846	0,175951	1	0,598625
-0,06023	421	376,3953	0,952002	1	0,985348
-0,6173	421	198,7701	0,537743	1	0,771054
1,901504	420	402,363	0,05795	1	0,848246
0,405795	367	350,204	0,685141	1	0,902395
0,798518	280	270,8043	0,42527	1	0,797636
3,567894	421	350,945	4,10E-04	0,437735	0,04642
1,81333	421	189,054	0,071367	1	0,339864
1,616497	420	383,6865	0,106809	1	0,848246
2,915876	367	332,2422	0,003788	1	0,178183
1,202108	280	269,4946	0,230377	1	0,657169
-0,59051	421	346,4633	0,555235	1	0,790572
-1,00497	421	190,819	0,316183	1	0,614646
0,253477	420	382,09	0,800036	1	0,969427
-0,33281	367	330,7953	0,739486	1	0,923669
-1,14397	280	256,3758	0,253704	1	0,679673
0,563463	421	365,919	0,573465	1	0,800196
0,997539	421	195,8241	0,319734	1	0,617081

0,690747	420	395,9741	0,490129	1	0,960096
0,358802	367	351,0641	0,719959	1	0,914797
-0,47064	280	265,2804	0,638284	1	0,907449
-2,69837	421	357,1825	0,007299	1	0,105763
-2,38393	421	193,9674	0,018093	1	0,186759
-2,27126	420	392,0644	0,023673	1	0,75904
-1,21832	367	336,2233	0,223956	1	0,576775
-1,74596	280	270,1559	0,081955	1	0,456738
1,878767	421	385,0737	0,061031	1	0,270611
0,742443	421	204,6428	0,45867	1	0,719268
1,498399	420	408,1737	0,134803	1	0,890998
0,161269	367	354,599	0,871974	1	0,959978
0,038733	280	266,1884	0,969132	1	0,991502
1,203944	421	371,5746	0,229377	1	0,520357
0,476276	421	198,4753	0,634402	1	0,835703
-0,02044	420	399,8789	0,983706	1	0,997342
-0,72079	367	342,2166	0,471531	1	0,77576
1,091054	280	255,9953	0,276275	1	0,701156
-1,51849	421	349,2605	0,129795	1	0,397564
-1,23455	421	189,3868	0,218528	1	0,514565
0,285101	420	382,3767	0,775721	1	0,969427
-1,28286	367	336,2429	0,200424	1	0,553185
-1,72477	280	250,3734	0,085804	1	0,458532
0,389944	421	331,7016	0,696828	1	0,871469
-2,20046	421	182,9167	0,029026	1	0,233259
-2,12074	420	357,3108	0,034632	1	0,828713
-0,27338	367	309,8053	0,784741	1	0,94337
-0,31477	280	254,2294	0,753193	1	0,942071
-0,93616	421	299,6033	0,349947	1	0,639237
-0,1235	421	176,145	0,901854	1	0,962531
0,876245	420	331,2963	0,381532	1	0,95743
-1,19759	367	303,0825	0,232013	1	0,582149
-1,02432	280	249,4785	0,306675	1	0,72861
-0,73028	421	386,2254	0,465665	1	0,727964
-1,39447	421	206,8313	0,164671	1	0,451197
0,127878	420	409,5715	0,898308	1	0,98503
-0,08411	367	359,1235	0,933019	1	0,977109
-1,20132	280	268,4023	0,230685	1	0,657169
0,59851	421	315,4176	0,549929	1	0,786367
1,127934	421	179,7646	0,260851	1	0,557979
-0,46715	420	349,5044	0,64068	1	0,964878
0,102121	367	312,8228	0,918726	1	0,974328
1,948176	280	255,1928	0,05249	1	0,401537
-1,8905	421	370,7732	0,059471	1	0,268966
-0,9501	421	199,0273	0,343214	1	0,636552
-1,96179	420	400,6439	0,050479	1	0,846627
0,660322	367	347,5813	0,509485	1	0,797092
-0,82889	280	265,0519	0,407912	1	0,788151

-3,63462	421	333,9909	3,22E-04	0,345098	0,04642
-1,39179	421	187,3385	0,165635	1	0,453017
-1,35161	420	373,688	0,177317	1	0,9199
-1,24398	367	320,9077	0,214415	1	0,564902
0,704891	280	261,2946	0,481506	1	0,824339
-0,03111	421	362,2349	0,975201	1	0,995982
-0,71311	421	196,2191	0,476624	1	0,734183
0,184106	420	395,1493	0,854025	1	0,973894
-0,4772	367	346,4075	0,633518	1	0,880707
1,393845	280	268,5456	0,164517	1	0,586115
-1,27155	421	312,4841	0,204478	1	0,494095
0,558709	421	179,1029	0,577058	1	0,794719
-0,68402	420	345,7489	0,494423	1	0,960096
-0,11284	367	288,8904	0,910234	1	0,970788
0,118162	280	245,7461	0,906035	1	0,975308
0,951485	421	374,5093	0,341972	1	0,63164
1,442892	421	203,187	0,150591	1	0,430627
-0,67806	420	406,6629	0,498118	1	0,960096
-0,13246	367	355,1276	0,894696	1	0,969307
-0,8738	280	269,7214	0,383002	1	0,772669
-0,49778	421	361,0213	0,618944	1	0,826904
0,964737	421	194,9093	0,335872	1	0,630335
0,615431	420	396,0853	0,538624	1	0,960096
0,481111	367	331,1413	0,630755	1	0,880707
0,960851	280	264,5244	0,337505	1	0,755731
1,214711	421	374,1808	0,225242	1	0,518147
-0,21085	421	198,7573	0,83322	1	0,936588
0,636842	420	401,4665	0,52459	1	0,960096
0,231664	367	352,9372	0,816933	1	0,95095
-1,61017	280	268,6269	0,108535	1	0,497151
0,796063	421	336,4126	0,426557	1	0,700145
0,995547	421	184,295	0,320776	1	0,617772
-0,53149	420	369,9564	0,595395	1	0,964878
0,677359	367	321,8288	0,498665	1	0,793601
1,743996	280	249,5827	0,082391	1	0,456738
-0,47443	421	349,9743	0,635493	1	0,83455
0,102266	421	191,304	0,918653	1	0,970154
-0,43151	420	383,8574	0,666337	1	0,96781
-0,07119	367	341,4783	0,943289	1	0,979539
-1,40518	280	261,1267	0,161156	1	0,581516
0,231725	421	353,7755	0,816886	1	0,929092
-0,47782	421	190,685	0,633327	1	0,835702
0,123828	420	385,5682	0,901516	1	0,98503
-0,50661	367	338,7776	0,612759	1	0,871158
1,179952	280	256,8916	0,239111	1	0,662939
1,434203	421	372,2729	0,152354	1	0,42445
1,480541	421	200,0103	0,140302	1	0,423351
0,367566	420	402,4326	0,71339	1	0,968866

0,652104	367	351,436	0,51476	1	0,798912
-1,71142	280	265,824	0,08817	1	0,461362
0,440656	421	336,8893	0,659744	1	0,849048
-0,5663	421	185,2886	0,571875	1	0,792729
-0,089	420	369,7122	0,929131	1	0,990942
0,013462	367	319,7039	0,989267	1	0,996248
2,285796	280	257,069	0,02308	1	0,313396
-1,30979	421	360,6209	0,191099	1	0,47601
-0,91059	421	194,9707	0,363636	1	0,646044
-1,00356	420	393,4867	0,31621	1	0,943897
-0,77376	367	342,4088	0,439607	1	0,742744
-1,53382	280	270,4866	0,126242	1	0,536498
-1,17336	421	335,7163	0,241484	1	0,538768
-1,44355	421	186,3218	0,150544	1	0,430627
-0,88498	420	370,4524	0,376739	1	0,95743
-0,58171	367	322,9591	0,561165	1	0,834
-2,77604	280	251,9303	0,005915	1	0,186692
-1,79452	421	344,4921	0,073607	1	0,292681
-0,72197	421	187,2652	0,471213	1	0,731044
0,702781	420	378,8656	0,482624	1	0,960096
-0,16249	367	335,3115	0,871017	1	0,959978
-0,71964	280	266,472	0,472375	1	0,824256
-0,00613	421	336,533	0,99511	1	0,998054
1,006267	421	184,0047	0,315609	1	0,614499
-0,09353	420	371,7411	0,925533	1	0,989204
-0,21024	367	316,8825	0,833619	1	0,956935
-0,11076	280	258,4969	0,911891	1	0,976008
-1,74851	421	313,9047	0,081353	1	0,30376
-1,14994	421	180,4036	0,251691	1	0,549707
-2,89026	420	340,3417	0,004096	1	0,443819
-1,05181	367	296,7096	0,293743	1	0,627013
-1,92376	280	276	0,055413	1	0,40654
0,08293	421	327,6037	0,933958	1	0,972684
0,201015	421	186,0393	0,840907	1	0,940271
0,21613	420	361,6084	0,829009	1	0,973894
0,588767	367	312,5094	0,556443	1	0,830257
2,236184	280	263,5309	0,026177	1	0,324814
0,66823	421	393,5269	0,504378	1	0,75707
-0,0448	421	211,1424	0,964308	1	0,985228
0,171788	420	412,5987	0,863688	1	0,977895
-0,20814	367	358,2115	0,835237	1	0,956935
-0,12406	280	267,1709	0,901358	1	0,975243
-0,02853	421	322,2548	0,97726	1	0,995982
0,779664	421	183,064	0,436595	1	0,703689
2,819839	420	353,1527	0,005076	1	0,449965
2,238706	367	310,4892	0,025883	1	0,252225
1,922397	280	250,205	0,055691	1	0,40654
-0,55997	421	381,5023	0,575828	1	0,801719

-1,42764	421	203,2732	0,15493	1	0,437229
1,42213	420	407,8369	0,155753	1	0,914005
-1,11663	367	354,1847	0,264911	1	0,609149
-0,0247	280	267,3132	0,980314	1	0,99236
1,123239	421	340,6675	0,262127	1	0,555878
0,760962	421	187,7712	0,447634	1	0,710837
0,409374	420	375,0993	0,682499	1	0,968338
-0,64384	367	331,287	0,520122	1	0,802988
-0,84851	280	259,474	0,396935	1	0,782458
-0,28135	421	292,8697	0,77864	1	0,913934
-0,50637	421	176,4939	0,61323	1	0,824026
-0,95311	420	321,7744	0,341248	1	0,947373
0,336688	367	281,9933	0,736602	1	0,923669
-1,21512	280	276	0,225358	1	0,654014
-2,45368	421	305,9826	0,014698	1	0,147663
-0,95929	421	177,0555	0,338718	1	0,632922
-1,24964	420	339,1497	0,212292	1	0,943055
-0,26533	367	303,6077	0,790937	1	0,944997
-1,38024	280	253,744	0,168727	1	0,59271
-1,93487	421	341,0001	0,053833	1	0,259162
-1,85084	421	186,7895	0,065771	1	0,328203
-0,4957	420	378,2412	0,620394	1	0,964878
-0,0014	367	331,8519	0,998887	1	0,998887
-1,50413	280	263,159	0,133746	1	0,543276
-1,32782	421	345,8028	0,185113	1	0,46885
-0,34003	421	187,19	0,734218	1	0,884613
-2,53426	420	372,6023	0,011677	1	0,651769
-2,38566	367	313,5884	0,017642	1	0,216225
-0,55859	280	255,0801	0,576935	1	0,871183
-0,78088	421	335,8358	0,435425	1	0,706341
-0,65542	421	187,6372	0,512997	1	0,756803
0,543079	420	369,5434	0,587402	1	0,964878
-0,1212	367	327,1632	0,90361	1	0,970486
-0,0368	280	259,6716	0,970674	1	0,992124
-1,53033	421	357,8201	0,126817	1	0,392431
-1,63597	421	193,348	0,103472	1	0,383322
-0,61264	420	390,5886	0,54047	1	0,960096
-2,43047	367	331,6669	0,015609	1	0,204372
-0,30283	280	260,9803	0,762263	1	0,944296
-3,09276	421	341,8152	0,002146	1	0,069718
-2,08322	421	189,3348	0,038574	1	0,269128
-0,867	420	381,9326	0,386485	1	0,95743
-1,80206	367	337,5367	0,072428	1	0,369998
-2,26421	280	270,6078	0,024353	1	0,313396
-1,74267	421	313,4994	0,082371	1	0,304997
-1,01	421	180,0432	0,31385	1	0,613453
0,576759	420	348,009	0,564475	1	0,961448
-0,56881	367	294,4921	0,569916	1	0,837915

-0,04293	280	253,3187	0,965788	1	0,990771
-0,5376	421	328,9598	0,591215	1	0,81147
-0,7021	421	180,8114	0,48352	1	0,739236
-2,01367	420	356,4401	0,044795	1	0,846132
-0,12067	367	313,2687	0,904029	1	0,970486
2,16336	280	257,4719	0,031434	1	0,329568
-0,89503	421	320,3257	0,371446	1	0,663233
0,714381	421	180,8945	0,475912	1	0,734136
0,233944	420	355,2492	0,815163	1	0,969427
0,04724	367	296,1207	0,962354	1	0,983232
0,071149	280	250,5472	0,943336	1	0,982175
-0,39281	421	344,6844	0,694702	1	0,870609
-0,6752	421	188,5175	0,500378	1	0,750551
1,6263	420	380,6367	0,104714	1	0,848246
0,151189	367	334,4954	0,879918	1	0,962453
1,063565	280	268,8416	0,28848	1	0,71621
-0,10334	421	326,0262	0,917755	1	0,966828
-1,85579	421	184,5104	0,065079	1	0,325826
-1,19088	420	360,9288	0,234485	1	0,943055
-1,06167	367	318,6758	0,289191	1	0,626164
-1,25959	280	251,9792	0,208982	1	0,64091
-0,59407	421	394,2501	0,552807	1	0,78965
-0,66243	421	210,9649	0,508421	1	0,754914
-1,38926	420	413,813	0,1655	1	0,914005
-0,27063	367	360,5593	0,786827	1	0,943768
-1,41695	280	274,8464	0,157631	1	0,576579
-1,90423	421	328,638	0,057753	1	0,266382
-0,95353	421	182,8482	0,341579	1	0,635891
-1,02514	420	364,6701	0,305979	1	0,943897
1,180437	367	317,5824	0,23871	1	0,589731
-0,30166	280	256,5904	0,763153	1	0,944296
1,931031	421	307,7807	0,054397	1	0,259162
2,965019	421	178,0393	0,003442	1	0,118336
1,164958	420	342,417	0,244847	1	0,943897
0,657667	367	283,8996	0,511285	1	0,797829
2,823341	280	245,4197	0,005143	1	0,180228
-0,61636	421	333,0931	0,538075	1	0,778195
-0,02853	421	184,9489	0,977269	1	0,991789
-1,80813	420	368,8824	0,071401	1	0,848246
1,071239	367	324,7762	0,284857	1	0,619451
-0,18899	280	255,8609	0,850248	1	0,966155
0,123888	421	405,0566	0,901465	1	0,965517
1,607667	421	217,789	0,109357	1	0,3896
-0,24312	420	415,9567	0,808029	1	0,969427
1,668917	367	362,619	0,095997	1	0,412696
1,313976	280	274,2432	0,189952	1	0,625017
-0,57155	421	394,8918	0,567954	1	0,796637
0,743	421	211,3887	0,458307	1	0,719268

-0,75178	420	412,8043	0,452611	1	0,960096
-0,98917	367	360,0261	0,323243	1	0,655597
0,208555	280	270,3955	0,834953	1	0,964667
-0,71741	421	345,1912	0,473607	1	0,732026
0,433604	421	185,7417	0,665079	1	0,846515
0,266868	420	381,6678	0,789715	1	0,969427
0,248683	367	320,0521	0,803766	1	0,950149
1,465763	280	264,411	0,143901	1	0,555466
0,505107	421	336,8254	0,613814	1	0,825172
-0,02909	421	186,5674	0,976821	1	0,991789
-0,72233	420	370,2538	0,470548	1	0,960096
-0,23273	367	333,4949	0,816112	1	0,95095
0,490627	280	262,3369	0,624101	1	0,898299
1,086233	421	357,8508	0,278107	1	0,57412
1,110541	421	191,4802	0,268159	1	0,566781
0,196228	420	392,895	0,844533	1	0,973894
0,18392	367	344,4588	0,854184	1	0,957067
0,544366	280	266,7237	0,586645	1	0,87561
1,088896	421	305,1849	0,277059	1	0,57291
1,773766	421	176,987	0,077821	1	0,353243
0,372279	420	337,5377	0,709919	1	0,968866
1,112019	367	296,9256	0,267029	1	0,609149
0,871212	280	251,9845	0,384468	1	0,773166
3,136308	421	351,5939	0,001855	0,183661	0,069718
0,924748	421	188,3698	0,35628	1	0,643758
0,651116	420	383,8379	0,515361	1	0,960096
1,776127	367	324,8138	0,076648	1	0,378716
-0,58656	280	260,8385	0,558008	1	0,858676
-0,41175	421	412,71	0,680733	1	0,861268
-0,58687	421	232,8864	0,557857	1	0,779138
0,393849	420	414,3397	0,693895	1	0,968866
-1,4223	367	362,3139	0,155801	1	0,502197
-0,17338	280	275,9886	0,862479	1	0,967527
0,394076	421	401,0993	0,693734	1	0,870489
-0,20409	421	216,9477	0,838473	1	0,938766
0,90849	420	415,3158	0,364146	1	0,956033
-1,58461	367	360,6405	0,113931	1	0,440054
0,289161	280	266,5999	0,772683	1	0,947219
-1,01051	421	365,936	0,312918	1	0,608538
-1,81748	421	196,9419	0,070663	1	0,339864
-1,27866	420	394,5399	0,201768	1	0,928589
0,258601	367	343,2328	0,796098	1	0,946151
-0,65991	280	265,8256	0,509882	1	0,831912
-0,46629	421	408,5203	0,641258	1	0,835242
1,610641	421	222,2229	0,108678	1	0,388185
0,818929	420	415,9696	0,413296	1	0,95743
1,464593	367	362,7114	0,143898	1	0,495102
1,011114	280	275,8561	0,312848	1	0,733729

0,431028	421	410,8255	0,666674	1	0,853323
-0,17318	421	228,0461	0,862663	1	0,947215
0,197335	420	415,4462	0,843662	1	0,973894
-0,01084	367	359,9192	0,991357	1	0,997007
1,404497	280	275,6635	0,161297	1	0,581516
-0,20964	421	368,3881	0,834062	1	0,936611
0,238691	421	197,508	0,811593	1	0,926063
1,433812	420	395,0291	0,152417	1	0,914005
-0,79277	367	350,0884	0,428451	1	0,737072
-1,67474	280	267,0513	0,095156	1	0,476412
0,136873	421	347,187	0,89121	1	0,962
-2,26932	421	190,2324	0,024371	1	0,218351
-1,59327	420	383,867	0,111923	1	0,848246
-1,12205	367	327,5109	0,262663	1	0,609149
-1,93866	280	261,2396	0,05362	1	0,406056
1,301413	421	356,4785	0,193958	1	0,479958
1,185057	421	191,6386	0,237462	1	0,537903
1,425091	420	391,1562	0,154928	1	0,914005
2,098353	367	339,6705	0,036612	1	0,28464
0,663467	280	267,9418	0,507602	1	0,831912
-1,94894	421	340,4102	0,052124	1	0,254208
-1,74886	421	189,6744	0,081933	1	0,362173
-0,14746	420	383,3842	0,88285	1	0,982463
0,418624	367	326,8245	0,675766	1	0,898834
-1,26085	280	260,2209	0,208493	1	0,64091
-2,62305	421	375,5129	0,00907	1	0,113397
-0,04254	421	198,2413	0,966107	1	0,986398
-0,48474	420	400,2456	0,628127	1	0,964878
1,896335	367	351,7866	0,058735	1	0,339132
-0,96537	280	265,4288	0,335238	1	0,755731
-0,56604	421	317,3923	0,571764	1	0,79928
0,264547	421	177,7291	0,791665	1	0,918572
-0,71088	420	351,0529	0,477632	1	0,960096
-0,47982	367	300,8707	0,631703	1	0,880707
1,476215	280	245,5421	0,141168	1	0,554427
1,226187	421	361,8154	0,220926	1	0,516179
1,112883	421	192,2564	0,267148	1	0,565438
-0,10376	420	395,3774	0,917409	1	0,989204
2,737749	367	349,1204	0,006503	1	0,178183
1,193239	280	272,4927	0,233814	1	0,659852
-1,14946	421	370,4547	0,251108	1	0,546261
-1,41636	421	200,1758	0,158226	1	0,439127
-1,41104	420	400,5014	0,159008	1	0,914005
-1,04147	367	346,8529	0,298382	1	0,630662
-0,97597	280	263,8663	0,329973	1	0,752282
0,41107	421	398,4215	0,681242	1	0,861268
0,960274	421	213,8849	0,338001	1	0,632756
-0,02063	420	414,5592	0,983555	1	0,997342

-1,4119	367	361,4611	0,158839	1	0,504558
0,820705	280	270,8814	0,412536	1	0,788151
-0,00873	421	348,2107	0,99304	1	0,998054
-0,23454	421	190,4812	0,814818	1	0,926063
0,880398	420	381,0458	0,379199	1	0,95743
-0,15259	367	333,274	0,878814	1	0,962453
0,363824	280	257,6988	0,716288	1	0,930557
3,500663	421	352,6467	5,24E-04	0,068598	0,04642
1,946898	421	191,0247	0,053013	1	0,302617
0,370758	420	388,2622	0,71102	1	0,968866
2,352714	367	342,0903	0,019203	1	0,218203
1,540145	280	270,6019	0,124694	1	0,535729
-0,20487	421	321,9835	0,837806	1	0,93721
0,608072	421	181,3233	0,5439	1	0,774002
-1,05797	420	357,7683	0,290784	1	0,943897
-0,63061	367	313,7425	0,528755	1	0,812268
0,757959	280	262,2655	0,449156	1	0,813228
0,261165	421	386,3657	0,794104	1	0,919136
-1,13433	421	207,1278	0,257968	1	0,556025
-0,41976	420	410,0164	0,67488	1	0,96781
0,936837	367	358,5345	0,349473	1	0,668671
-0,35187	280	274,6652	0,725207	1	0,936493
0,022648	421	330,2203	0,981945	1	0,997025
0,065079	421	184,6457	0,948182	1	0,980846
-0,85223	420	364,0253	0,394649	1	0,95743
0,289442	367	325,2496	0,772427	1	0,938452
0,533141	280	261,2364	0,594389	1	0,879042
-1,90517	421	392,1294	0,057489	1	0,266382
-1,98655	421	209,0257	0,048278	1	0,286754
-0,9323	420	413,2587	0,351725	1	0,947373
-1,58835	367	360,2988	0,113085	1	0,439223
0,120433	280	272,087	0,904229	1	0,975308
0,686571	421	390,0994	0,492761	1	0,748579
0,807713	421	205,6247	0,420189	1	0,691294
0,853028	420	411,4056	0,39414	1	0,95743
2,097903	367	355,1923	0,03662	1	0,28464
0,515337	280	268,3817	0,606742	1	0,88515
-2,35376	421	364,3576	0,019115	1	0,159771
-1,21364	421	197,6429	0,226332	1	0,524743
0,711196	420	397,8642	0,477379	1	0,960096
-0,7409	367	342,6329	0,459263	1	0,763917
0,101256	280	265,4341	0,919423	1	0,977051
-1,02887	421	401,4932	0,30416	1	0,600745
-0,4748	421	217,251	0,635404	1	0,836291
0,173409	420	415,3675	0,862415	1	0,977432
0,838019	367	361,5132	0,402574	1	0,716268
-0,09469	280	275,2942	0,924634	1	0,979504
-1,8804	421	317,8854	0,060968	1	0,270611

0,873707	421	179,7506	0,383443	1	0,659083
0,350411	420	354,728	0,726238	1	0,969427
1,776954	367	301,2407	0,076585	1	0,378716
0,756872	280	250,2639	0,449839	1	0,813228
0,182764	421	372,6793	0,855083	1	0,947051
1,170541	421	198,5105	0,243186	1	0,542422
0,648385	420	401,3336	0,517107	1	0,960096
-1,24729	367	346,9433	0,213131	1	0,563645
-0,38602	280	265,2199	0,69979	1	0,92034
-0,68723	421	321,3329	0,492436	1	0,748579
0,259025	421	179,3958	0,795913	1	0,92123
-0,7107	420	355,0785	0,477736	1	0,960096
0,07668	367	307,9784	0,938928	1	0,979214
1,584287	280	248,7221	0,114399	1	0,51157
-1,82899	421	343,2689	0,068269	1	0,281097
-0,36236	421	185,4395	0,717496	1	0,874811
-0,31674	420	380,7448	0,751611	1	0,969427
0,599573	367	334,7574	0,549196	1	0,828521
-0,38696	280	261,7585	0,699101	1	0,92034
-2,43574	421	370,5323	0,015332	1	0,147897
-1,50756	421	199,6353	0,133247	1	0,414884
0,210266	420	403,3709	0,833567	1	0,973894
0,58445	367	349,6542	0,559294	1	0,832862
0,211992	280	268,4182	0,832274	1	0,964667
0,624397	421	412,6149	0,532712	1	0,777904
0,925843	421	232,0588	0,35549	1	0,643758
0,365523	420	414,7357	0,714907	1	0,968866
-0,16779	367	362,0322	0,866845	1	0,959636
0,419143	280	274,799	0,675439	1	0,909385
-1,17129	421	374,0104	0,242227	1	0,53918
-0,18206	421	199,539	0,855718	1	0,945471
0,366584	420	404,3884	0,714121	1	0,968866
0,332836	367	356,1043	0,739454	1	0,923669
0,528617	280	271,7961	0,597503	1	0,880334
-1,37618	421	375,8516	0,169584	1	0,442288
-1,64794	421	200,7389	0,100929	1	0,383011
0,143821	420	403,7468	0,885714	1	0,982463
-1,19542	367	356,5785	0,232719	1	0,582158
-0,34421	280	267,4034	0,730958	1	0,936637
2,690951	421	342,4931	0,007474	0,911863	0,107274
2,556827	421	186,3078	0,01136	1	0,163285
0,625596	420	378,4146	0,531957	1	0,960096
2,676773	367	331,4374	0,007803	0,983202	0,180403
2,77857	280	271,3374	0,005841	0,753505	0,186692
0,46849	421	315,38	0,639758	1	0,834842
2,903664	421	181,6914	0,004146	1	0,120964
-0,01198	420	352,2675	0,990447	1	0,997342
0,304833	367	319,5874	0,760691	1	0,929966

1,605806	280	251,3611	0,109572	1	0,500379
0,285677	421	404,3918	0,775271	1	0,911337
2,405566	421	219,6866	0,016976	1	0,181988
0,993658	420	415,7624	0,320968	1	0,945573
0,088637	367	362,9668	0,929419	1	0,976733
0,790709	280	270,2979	0,429807	1	0,801886
-0,23955	421	332,0782	0,810827	1	0,924931
1,633628	421	185,4318	0,104034	1	0,383322
0,198086	420	365,7437	0,843088	1	0,973894
1,1041	367	321,853	0,270375	1	0,609149
0,931865	280	266,2887	0,35225	1	0,761846
-0,313	421	330,9683	0,754479	1	0,898125
-0,8001	421	185,1617	0,424677	1	0,693378
-0,53235	420	364,3543	0,59481	1	0,964878
0,894798	367	323,0218	0,371561	1	0,68168
1,023553	280	256,0784	0,307012	1	0,72861
-0,66135	421	371,9074	0,508797	1	0,762184
0,246458	421	197,8571	0,805583	1	0,923205
-0,20147	420	400,4687	0,840436	1	0,973894
1,185613	367	342,1052	0,236598	1	0,585627
0,862219	280	273,5532	0,389322	1	0,777098
1,395483	421	373,8429	0,163699	1	0,438177
1,484709	421	193,8851	0,139245	1	0,423351
0,46798	420	402,6647	0,640052	1	0,964878
2,62447	367	351,1159	0,009057	1	0,183886
2,198986	280	274,8183	0,028712	1	0,326641
-0,63561	421	414,3484	0,525382	1	0,770244
-0,09899	421	234,1414	0,921229	1	0,970834
-0,28366	420	413,3083	0,776809	1	0,969427
1,130387	367	362,3904	0,259061	1	0,609149
-0,15828	280	274,3616	0,874351	1	0,971
-2,24576	421	323,6558	0,025394	1	0,184916
-2,63423	421	184,3765	0,00915	1	0,14815
0,570698	420	358,5467	0,568562	1	0,962723
-0,15702	367	329,3156	0,875325	1	0,962417
-1,11876	280	255,2349	0,264296	1	0,688875
-0,40402	421	310,1323	0,686479	1	0,864986
0,830679	421	178,5766	0,407264	1	0,681185
-0,06089	420	343,8757	0,951482	1	0,992811
0,879105	367	302,0558	0,380043	1	0,691076
0,836456	280	247,1131	0,403706	1	0,786359
0,691255	421	385,1319	0,489822	1	0,748388
0,441574	421	206,5433	0,65926	1	0,842667
0,89274	420	409,6856	0,37252	1	0,95743
-0,87895	367	350,83	0,380031	1	0,691076
-0,66165	280	266,1438	0,508767	1	0,831912
-1,10947	421	331,3281	0,268034	1	0,563516
-2,68415	421	185,9491	0,007928	1	0,148102

-1,09013	420	370,7165	0,276363	1	0,943897
-1,37333	367	315,68	0,170625	1	0,51841
-2,16186	280	252,3862	0,031569	1	0,329568
-2,9147	421	335,4656	0,0038	1	0,087964
-0,94986	421	187,4911	0,343408	1	0,636552
0,4644	420	374,6156	0,642631	1	0,964878
-1,35883	367	333,1804	0,175119	1	0,523018
0,162399	280	261,2224	0,871117	1	0,969656
1,856013	421	344,4514	0,064306	1	0,275801
1,375729	421	187,3477	0,170548	1	0,456365
1,322835	420	375,6871	0,186695	1	0,923408
0,67168	367	328,2808	0,50226	1	0,793601
-0,77021	280	256,3085	0,441883	1	0,810247
-0,13894	421	342,7796	0,889582	1	0,962
0,193557	421	188,2083	0,846731	1	0,943293
-0,55459	420	378,1352	0,579503	1	0,964878
-0,015	367	327,51	0,988038	1	0,996248
0,710041	280	257,2632	0,478322	1	0,824256
-1,87963	421	325,0274	0,061054	1	0,270611
-0,68735	421	183,2253	0,492729	1	0,746275
2,083572	420	364,242	0,037896	1	0,83984
0,667308	367	335,5129	0,505034	1	0,793601
1,083664	280	262,598	0,279508	1	0,703716
0,333467	421	379,1098	0,738966	1	0,893273
1,093324	421	201,3152	0,275558	1	0,576759
-0,61027	420	406,0298	0,542024	1	0,960096
0,859528	367	349,1095	0,390639	1	0,705022
0,237455	280	272,2022	0,812483	1	0,95747
-0,17359	421	331,4373	0,862296	1	0,951261
-0,63385	421	184,8652	0,526963	1	0,767321
0,030801	420	365,6975	0,975445	1	0,997342
0,33434	367	317,4446	0,738344	1	0,923669
-3,32828	280	257,2297	0,001002	1	0,121989
-0,70322	421	372,5466	0,48236	1	0,74251
0,038687	421	197,8867	0,969179	1	0,988858
0,960441	420	404,2441	0,337407	1	0,947373
2,738083	367	355,5653	0,006491	1	0,178183
-0,82721	280	266,0567	0,408858	1	0,788151
-1,55378	421	364,9421	0,121103	1	0,382129
-0,42516	421	197,2531	0,67118	1	0,852122
0,061942	420	399,0087	0,95064	1	0,992811
-0,1715	367	351,3819	0,863929	1	0,959636
0,680014	280	267,695	0,497083	1	0,830492
0,847958	421	405,3011	0,396962	1	0,686821
1,833227	421	218,6602	0,068128	1	0,334428
0,261099	420	415,9756	0,794146	1	0,969427
1,513289	367	361,5083	0,13108	1	0,47485
1,661235	280	272,5664	0,097816	1	0,480557

0,877074	421	315,9817	0,381113	1	0,671614
1,229017	421	182,5045	0,220648	1	0,516786
-0,37606	420	350,5334	0,707098	1	0,968866
1,822729	367	321,877	0,069272	1	0,364488
1,245187	280	261,4069	0,214178	1	0,646827
-0,41962	421	383,9488	0,674997	1	0,859543
0,518896	421	204,4937	0,604394	1	0,820561
1,113288	420	406,9765	0,266242	1	0,943897
-0,12126	367	357,7856	0,903552	1	0,970486
-0,58085	280	270,2957	0,561828	1	0,861728
1,464725	421	391,0827	0,143799	1	0,412154
1,45609	421	210,3614	0,146859	1	0,428077
0,362201	420	412,7593	0,717387	1	0,969427
-0,91044	367	358,2706	0,363203	1	0,677287
0,164772	280	271,989	0,869246	1	0,969656
-0,45496	421	288,8439	0,649477	1	0,841583
1,452222	421	174,1257	0,148239	1	0,428784
1,38218	420	314,8003	0,167896	1	0,914005
0,114938	367	297,4677	0,908572	1	0,970624
-1,4895	280	249,7545	0,137618	1	0,54931
-0,85075	421	286,3578	0,395618	1	0,686065
0,446482	421	173,2704	0,655806	1	0,841107
0,506261	420	316,1905	0,613026	1	0,964878
2,432326	367	283,9737	0,01562	1	0,204372
0,84395	280	276	0,399428	1	0,784757
-0,41756	421	369,1451	0,676512	1	0,860341
-0,15024	421	196,7671	0,880732	1	0,956931
0,094398	420	398,9898	0,924841	1	0,989204
1,317779	367	335,8324	0,188476	1	0,538722
0,050049	280	259,2614	0,960122	1	0,986974
1,981915	421	397,9303	0,048176	1	0,241202
1,982732	421	214,0287	0,048676	1	0,286808
1,131957	420	414,4592	0,258307	1	0,943897
1,692593	367	361,651	0,091394	1	0,403903
0,635189	280	274,6457	0,525834	1	0,837665
0,451715	421	375,8685	0,651735	1	0,843783
-0,43503	421	198,6833	0,664015	1	0,845876
2,094468	420	401,9486	0,036844	1	0,828713
-0,13352	367	347,796	0,893857	1	0,969095
0,869949	280	268,5469	0,385105	1	0,773166
-0,65935	421	312,8036	0,510156	1	0,763461
-0,11298	421	177,4882	0,910176	1	0,96646
0,391235	420	346,8796	0,695864	1	0,968866
0,343206	367	296,9155	0,731687	1	0,923669
0,848071	280	252,9802	0,3972	1	0,782458
2,891958	421	394,8344	0,00404	0,517157	0,087964
3,118397	421	213,0665	0,00207	0,26904	0,099921
0,721708	420	414,9563	0,47088	1	0,960096

2,715232	367	359,1638	0,006943	0,895625	0,180403
1,473967	280	271,5174	0,141649	1	0,554427
-2,99615	421	326,4726	0,002943	0,441515	0,077359
-1,9263	421	184,5061	0,055604	1	0,309036
-0,8879	420	360,0954	0,375185	1	0,95743
-2,32539	367	326,978	0,020662	1	0,224016
-1,1928	280	258,0987	0,234042	1	0,659852
0,549767	421	342,7123	0,582837	1	0,806553
-1,31886	421	188,3332	0,188816	1	0,476746
-3,10504	420	378,1066	0,002046	0,274155	0,294094
1,188808	367	336,4936	0,235353	1	0,585627
-0,18103	280	261,1816	0,856487	1	0,966655
0,528209	421	355,9146	0,597684	1	0,815122
-0,88437	421	193,9782	0,377593	1	0,655017
1,941739	420	391,8351	0,052885	1	0,846627
-0,60498	367	329,7922	0,545605	1	0,825529
0,693602	280	264,3906	0,488541	1	0,826999
-1,34083	421	331,019	0,180896	1	0,46049
-0,77307	421	185,0679	0,44047	1	0,705407
-0,07814	420	365,8752	0,937757	1	0,992059
0,380925	367	333,4454	0,703501	1	0,908463
-1,15887	280	260,8006	0,24757	1	0,677438
-1,86395	421	336,8716	0,063199	1	0,274468
-0,99255	421	187,7066	0,322209	1	0,617772
0,12469	420	375,0966	0,900836	1	0,98503
1,052233	367	326,9544	0,29347	1	0,627013
1,523959	280	258,7944	0,12874	1	0,536498
1,074185	421	344,2251	0,283492	1	0,582841
0,898905	421	185,5869	0,369868	1	0,64964
0,046109	420	378,2789	0,963247	1	0,996924
1,114921	367	332,3354	0,26569	1	0,609149
0,427499	280	264,447	0,669364	1	0,908644
-1,2904	421	331,5352	0,197811	1	0,484381
-1,22763	421	185,2451	0,221142	1	0,516786
-0,0855	420	366,9638	0,931907	1	0,991559
-1,35175	367	328,3201	0,177385	1	0,523131
-0,2507	280	262,23	0,802244	1	0,956473
-1,68277	421	349,8119	0,093312	1	0,327788
-2,12045	421	191,1459	0,035258	1	0,255449
1,057054	420	382,6747	0,291154	1	0,943897
-0,79996	367	341,6848	0,424292	1	0,733267
-0,5285	280	263,2729	0,597599	1	0,880334
0,636628	421	342,7812	0,524792	1	0,770244
1,198095	421	188,7721	0,232382	1	0,533704
0,448881	420	378,943	0,653774	1	0,966867
2,310479	367	329,7775	0,021478	1	0,227943
0,140418	280	263,5209	0,888437	1	0,975145
-0,39508	421	406,4711	0,692994	1	0,870285

-0,61117	421	217,9119	0,541721	1	0,773814
0,070762	420	415,9991	0,943621	1	0,992811
-0,42406	367	362,9048	0,671772	1	0,897482
2,102209	280	273,1731	0,03645	1	0,34044
-1,89803	421	395,2871	0,05842	1	0,268334
-1,8114	421	212,5055	0,071491	1	0,339864
-0,61045	420	413,8736	0,541897	1	0,960096
-1,08365	367	358,5795	0,279246	1	0,613446
-0,71274	280	273,1506	0,476618	1	0,824256
-0,61872	421	381,5365	0,536469	1	0,778195
-1,32036	421	203,5046	0,188198	1	0,476663
1,593883	420	408,1208	0,111736	1	0,848246
-1,02105	367	354,2529	0,307927	1	0,640947
0,27796	280	269,4141	0,781257	1	0,951006
-0,38165	421	323,1705	0,702971	1	0,874567
0,55658	421	180,1846	0,578505	1	0,794719
-0,42012	420	357,3044	0,674653	1	0,96781
-0,29526	367	308,9778	0,767992	1	0,935621
1,644059	280	245,5458	0,101444	1	0,480741
-2,11113	421	313,9074	0,035552	1	0,215166
-2,7273	421	177,8901	0,007025	1	0,141161
-1,19014	420	349,1058	0,234801	1	0,943055
-2,5056	367	309,6481	0,012737	1	0,189546
-2,13983	280	262,7331	0,03329	1	0,329568
0,986581	421	355,8835	0,324518	1	0,61749
0,997417	421	194,5439	0,319801	1	0,617081
0,390526	420	390,4039	0,696361	1	0,968866
0,327085	367	329,9738	0,743811	1	0,923669
-1,43199	280	260,0426	0,153348	1	0,565026
0,450482	421	313,8198	0,652674	1	0,844274
1,232546	421	180,265	0,21935	1	0,515696
1,033357	420	348,0289	0,302154	1	0,943897
-0,91749	367	301,2833	0,359622	1	0,674813
0,969503	280	250,9081	0,333228	1	0,755731
1,85793	421	334,4123	0,064058	1	0,275801
1,886957	421	182,7652	0,060751	1	0,315698
1,399198	420	374,5787	0,162581	1	0,914005
1,185606	367	319,1515	0,23666	1	0,585627
3,025839	280	262,7156	0,002725	1	0,135787
-0,60173	421	309,0412	0,547793	1	0,784719
1,662595	421	179,8539	0,098135	1	0,379251
0,668271	420	344,0325	0,504409	1	0,960096
0,111102	367	303,2259	0,911609	1	0,971566
0,954635	280	247,5013	0,340694	1	0,757098
-1,73717	421	379,3001	0,08317	1	0,306447
-1,53384	421	202,2884	0,126632	1	0,410224
-2,22101	420	403,4673	0,026905	1	0,767829
-0,63641	367	357,119	0,524916	1	0,808843

0,294036	280	269,1857	0,768957	1	0,946609
0,714285	421	330,2562	0,475556	1	0,734285
0,358909	421	184,9523	0,720072	1	0,875826
0,030339	420	366,8282	0,975813	1	0,997342
-0,17853	367	316,7513	0,858423	1	0,958964
-0,09025	280	260,7022	0,92816	1	0,979504
-0,15627	421	362,8407	0,875905	1	0,95861
1,473583	421	196,1804	0,142197	1	0,423499
-0,08235	420	394,522	0,934406	1	0,992059
0,940739	367	347,1827	0,347493	1	0,668671
0,420639	280	261,0522	0,674365	1	0,909385
-0,30224	421	351,5805	0,762645	1	0,902833
1,151658	421	190,0652	0,250908	1	0,548794
0,588959	420	385,8864	0,556233	1	0,960096
1,126654	367	335,1554	0,260695	1	0,609149
1,447031	280	260,387	0,149091	1	0,561358
-1,15821	421	340,4153	0,247591	1	0,544195
0,558711	421	188,1593	0,577023	1	0,794719
0,923003	420	376,6377	0,356596	1	0,947373
0,194663	367	331,7747	0,845776	1	0,956935
-0,17946	280	261,7192	0,857719	1	0,966655
-1,38312	421	382,0044	0,167435	1	0,441901
-2,73504	421	203,7986	0,006787	0,990894	0,141161
-1,97806	420	407,6153	0,048595	1	0,846627
-1,729	367	351,1092	0,084689	1	0,388246
0,653971	280	269,8911	0,513687	1	0,831912
-1,21147	421	360,589	0,226509	1	0,518767
-1,58061	421	193,9419	0,115597	1	0,392379
-1,77305	420	393,0112	0,076996	1	0,848246
0,526704	367	338,3657	0,598744	1	0,861803
0,310621	280	260,012	0,756338	1	0,942071
-1,21926	421	345,7544	0,223575	1	0,51676
-1,0787	421	190,5779	0,282085	1	0,581951
-0,97708	420	381,724	0,329151	1	0,947373
-1,61793	367	323,5469	0,106653	1	0,432057
-2,30043	280	265,6023	0,022199	1	0,309763
1,851372	421	368,1565	0,064917	1	0,276236
1,384711	421	196,3607	0,167712	1	0,45454
1,049549	420	399,8968	0,294559	1	0,943897
0,908003	367	350,6145	0,3645	1	0,677906
-0,06699	280	266,0934	0,946637	1	0,982409
-1,521	421	373,4741	0,129105	1	0,397064
-0,34427	421	199,8846	0,731004	1	0,882616
-0,09699	420	403,3985	0,922781	1	0,989204
0,114409	367	346,3845	0,90898	1	0,970624
-2,92896	280	267,1698	0,003694	1	0,147267
-1,97516	421	297,0574	0,049176	1	0,244581
0,019207	421	176,6269	0,984698	1	0,995074

-2,05477	420	322,6269	0,040706	1	0,846132
0,136848	367	299,644	0,891243	1	0,968351
1,068752	280	247,4777	0,286223	1	0,714135
-0,22307	421	377,8307	0,823603	1	0,933913
0,968289	421	199,7419	0,334071	1	0,62852
0,733	420	405,4129	0,463982	1	0,960096
-0,06242	367	354,8035	0,950262	1	0,979539
-0,143	280	268,4367	0,886398	1	0,973784
-2,35998	421	342,0787	0,018837	1	0,15948
-1,75402	421	185,7604	0,081076	1	0,360416
-2,43463	420	378,7515	0,015368	1	0,723749
-1,71723	367	331,5114	0,086871	1	0,391959
0,663031	280	265,4504	0,507886	1	0,831912
-1,14259	421	328,8185	0,25404	1	0,546261
0,863129	421	182,8703	0,389197	1	0,664422
-0,08119	420	364,2012	0,935337	1	0,992059
-0,28086	367	308,1366	0,779007	1	0,942943
0,541267	280	276	0,58876	1	0,87561
-0,93905	421	300,7722	0,348458	1	0,637437
-0,79487	421	178,5929	0,427747	1	0,694836
-1,0365	420	332,1866	0,300723	1	0,943897
0,147527	367	292,4143	0,882818	1	0,962667
-1,14367	280	276	0,253751	1	0,679673
0,09566	421	359,5069	0,923844	1	0,966828
-1,0754	421	193,7452	0,283531	1	0,582654
-0,19378	420	389,3374	0,846451	1	0,973894
-1,04971	367	331,9234	0,294614	1	0,62734
0,438521	280	261,1523	0,661372	1	0,908644
0,206847	421	395,1825	0,836236	1	0,93721
0,904012	421	211,143	0,367019	1	0,647779
-0,29531	420	413,4716	0,767903	1	0,969427
-1,31966	367	360,1996	0,187787	1	0,538014
-0,00875	280	270,8259	0,993025	1	0,996331
-1,262	421	344,2699	0,207802	1	0,496728
-1,23087	421	185,5748	0,219931	1	0,516256
-0,02084	420	380,5925	0,983383	1	0,997342
0,013363	367	339,2774	0,989346	1	0,996248
-1,83855	280	261,9063	0,067113	1	0,425749
-1,10745	421	405,5686	0,268756	1	0,563516
-0,64573	421	221,8911	0,519124	1	0,761752
-0,6458	420	415,9446	0,518763	1	0,960096
-2,00143	367	362,968	0,04609	1	0,307337
0,593642	280	275,6302	0,553238	1	0,854225
1,021087	421	347,9017	0,307923	1	0,604218
0,09328	421	188,5995	0,92578	1	0,972483
-0,12752	420	381,8207	0,898599	1	0,98503
1,785532	367	326,1943	0,075104	1	0,376019
-0,34378	280	248,1955	0,731306	1	0,936637

0,211196	421	407,4088	0,83284	1	0,936611
-0,37204	421	223,5729	0,710218	1	0,870869
0,620685	420	415,9933	0,535147	1	0,960096
-1,763	367	361,7733	0,078745	1	0,381638
0,127765	280	268,5809	0,898431	1	0,975243
0,292369	421	388,9313	0,77016	1	0,908874
0,046912	421	207,1991	0,962628	1	0,985228
-0,57599	420	410,7217	0,56494	1	0,961448
0,474481	367	356,1453	0,635448	1	0,880707
-0,12315	280	268,8432	0,90208	1	0,975243
-1,16952	421	374,9388	0,242935	1	0,53918
-1,72528	421	202,3339	0,086005	1	0,365475
-1,40368	420	403,7631	0,161184	1	0,914005
-1,0907	367	349,8801	0,276155	1	0,611884
-0,92651	280	265,9239	0,355021	1	0,761846
-0,28122	421	393,4367	0,778693	1	0,913934
-0,18671	421	208,9251	0,852072	1	0,944105
-1,56444	420	413,3765	0,11848	1	0,858144
-0,57207	367	359,0357	0,567635	1	0,837838
0,31141	280	271,3999	0,755728	1	0,942071

0,261387		421	291,3901	0,793979	1	0,919136

2,468389		421	176,6911	0,014524	1	0,177943

0,234489	420	323,8752	0,814753	1	0,969427

1,529538	367	290,4254	0,12722	1	0,468753

1,506406	280	276	0,133107	1	0,543276
-0,22518	421	383,5916	0,821962	1	0,932754
-0,82185	421	205,5047	0,412118	1	0,684933
-0,84402	420	408,9753	0,399154	1	0,95743
-0,40865	367	350,2533	0,68305	1	0,900574
0,301654	280	265,6756	0,763152	1	0,944296
-0,76516	421	306,4555	0,444763	1	0,713799
-1,63416	421	178,965	0,103984	1	0,383322
-0,38674	420	343,3008	0,69919	1	0,968866
-0,10693	367	301,6272	0,914918	1	0,973634
-0,74309	280	257,1426	0,458109	1	0,817005
0,981789	421	340,8426	0,3269	1	0,61749
0,271377	421	187,7001	0,786399	1	0,917973
0,439635	420	373,8547	0,660455	1	0,96781
-0,60126	367	335,0321	0,548076	1	0,828435
-2,55307	280	258,3456	0,011253	1	0,234919
1,053335	421	368,5601	0,292878	1	0,590852
0,456747	421	197,0735	0,648356	1	0,839358
0,247483	420	397,8762	0,804662	1	0,969427
-0,15276	367	344,0178	0,878675	1	0,962453

1,024944	280	263,0076	0,306332	1	0,72861
1,484916	421	329,7369	0,138522	1	0,410344
2,461161	421	184,5584	0,014768	1	0,178042
-0,98898	420	368,3697	0,323323	1	0,945573
0,894284	367	328,0314	0,371826	1	0,68168
0,087328	280	260,5172	0,930478	1	0,979504
-1,30203	421	347,5191	0,193768	1	0,479958
-1,0297	421	188,9502	0,304467	1	0,602952
-1,35203	420	380,0379	0,177169	1	0,9199
0,457548	367	335,4884	0,647573	1	0,884762
0,091101	280	264,363	0,927481	1	0,979504
-0,17183	421	407,4554	0,863654	1	0,951261
0,902428	421	220,524	0,367814	1	0,648213
0,249209	420	415,9997	0,803322	1	0,969427
0,238646	367	362,7635	0,811515	1	0,950149
1,066772	280	273,8836	0,287014	1	0,714926
0,02724	421	386,948	0,978283	1	0,99613
0,919114	421	205,546	0,359113	1	0,643758
0,835882	420	409,2553	0,403709	1	0,95743
1,171314	367	358,7931	0,24225	1	0,59405
-1,21638	280	269,9085	0,224901	1	0,654014
1,200699	421	389,4238	0,230598	1	0,521788
1,045011	421	207,4269	0,297234	1	0,595654
0,336691	420	410,7426	0,736522	1	0,969427
-0,94907	367	358,2581	0,343222	1	0,665683
0,900072	280	267,9561	0,36889	1	0,763914
-0,86343	421	318,0471	0,388551	1	0,677715
-0,89969	421	181,236	0,369479	1	0,64964
-0,04341	420	350,6882	0,965403	1	0,996924
-0,56488	367	324,4311	0,572547	1	0,840144
-0,97771	280	260,6618	0,329122	1	0,752282
0,036102	421	372,4119	0,97122	1	0,995666
0,736772	421	194,3263	0,46215	1	0,721079
1,032202	420	399,2189	0,302602	1	0,943897
2,441265	367	338,9704	0,015147	1	0,204372
-0,29172	280	265,4827	0,770729	1	0,946609
-1,93881	421	391,3723	0,053242	1	0,258827
-1,61449	421	209,3561	0,107928	1	0,388181
-1,74491	420	411,3007	0,081748	1	0,848246
-0,27221	367	360,8366	0,78562	1	0,94337
0,347723	280	269,1211	0,72832	1	0,936637
0,568884	421	307,0467	0,569851	1	0,797368
2,160832	421	178,5938	0,03204	1	0,241423
0,575611	420	341,4071	0,565257	1	0,961448
1,598041	367	311,15	0,111049	1	0,437634
0,003387	280	249,6167	0,9973	1	0,998213
-2,45357	421	403,4711	0,014567	1	0,14733
-2,32115	421	218,723	0,021199	1	0,2022

-3,28401	420	415,6324	0,00111	0,167613	0,235929
-1,3819	367	362,6856	0,167854	1	0,514971
-0,60891	280	271,4431	0,543092	1	0,849885
0,986053	421	390,5526	0,324718	1	0,61749
0,05217	421	207,4835	0,958444	1	0,98414
-0,26814	420	411,5228	0,788722	1	0,969427
0,163165	367	355,8754	0,870481	1	0,959978
-0,44763	280	269,7018	0,654777	1	0,908644
-0,41385	421	417	0,679195	1	0,861268
2,057842	421	417	0,040226	1	0,26998
-0,96448	420	416	0,335364	1	0,947373
1,080908	367	279,1165	0,280671	1	0,614784
1,765519	280	276	0,078582	1	0,453386
-0,33884	421	298,2922	0,734969	1	0,893273
-0,00175	421	175,1964	0,998603	1	0,999267
-1,34441	420	336,0726	0,179724	1	0,923408
-0,68157	367	276,7699	0,496082	1	0,793601
1,803485	280	251,756	0,072508	1	0,4406
-0,69796	421	320,1413	0,485712	1	0,745445
2,038677	421	181,4417	0,042933	1	0,274154
0,604012	420	355,2417	0,546221	1	0,960096
2,144633	367	318,1115	0,032739	1	0,272587
2,047839	280	258,596	0,041586	1	0,373036
-0,55829	421	392,8603	0,576962	1	0,802105
-0,51689	421	209,3615	0,605779	1	0,821338
-1,41197	420	413,1194	0,15871	1	0,914005
-0,82874	367	358,3838	0,407804	1	0,72047
-0,13212	280	270,6998	0,89499	1	0,975243
1,068969	421	378,3651	0,285765	1	0,583534
2,944786	421	201,2488	0,003612	0,465976	0,118336
2,680185	420	405,1516	0,007658	1	0,554149
1,455316	367	352,3125	0,146472	1	0,498729
0,45566	280	268,2637	0,649003	1	0,908644
0,39162	421	357,9396	0,695572	1	0,870621
-0,02765	421	192,2457	0,977969	1	0,991789
0,043597	420	391,6265	0,965248	1	0,996924
0,053852	367	338,4467	0,957085	1	0,981063
0,232663	280	266,0413	0,816202	1	0,95747
-0,07155	421	397,7491	0,942999	1	0,978719
-0,5378	421	214,3812	0,591275	1	0,807111
-0,85839	420	414,5491	0,391171	1	0,95743
-1,20677	367	358,514	0,228318	1	0,577307
0,194122	280	270,0533	0,846226	1	0,965181
0,30548	421	295,5399	0,760215	1	0,901373
1,144227	421	177,4109	0,254071	1	0,553302
-0,23978	420	326,0899	0,81065	1	0,969427
-0,4948	367	287,6717	0,621117	1	0,875495
1,761658	280	276	0,079234	1	0,453386

-1,20451	421	374,344	0,229152	1	0,520357
-1,35284	421	199,511	0,177637	1	0,464755
0,009274	420	402,5761	0,992605	1	0,997342
-0,43449	367	355,6837	0,664199	1	0,894765
0,636188	280	267,9003	0,525197	1	0,837536
-0,31827	421	385,9218	0,750454	1	0,898125
-2,25585	421	205,4097	0,025134	1	0,218351
-3,99208	420	410,2187	7,76E-05	0,083572	0,038681
-0,91651	367	355,5264	0,36002	1	0,674813
-2,58608	280	266,7874	0,010238	1	0,229419
-1,87254	421	327,7354	0,062022	1	0,271708
-0,4573	421	183,1878	0,648001	1	0,839358
-1,09308	420	359,6744	0,275091	1	0,943897
0,084636	367	308,2607	0,932606	1	0,977109
-1,28397	280	256,5265	0,200312	1	0,631529
1,543822	421	400,8131	0,123421	1	0,385082
2,355496	421	215,4486	0,019396	1	0,195731
0,287633	420	415,5648	0,773771	1	0,969427
1,91375	367	361,9636	0,056441	1	0,337523
0,161652	280	273,6316	0,871699	1	0,969656
-1,3774	421	323,651	0,16934	1	0,442288
-0,26004	421	182,5619	0,79513	1	0,92103
0,638508	420	358,434	0,523551	1	0,960096
-1,33784	367	320,0573	0,181899	1	0,527769
-0,4647	280	246,7956	0,642558	1	0,907804
0,319559	421	405,4088	0,749468	1	0,898125
0,714218	421	221,1722	0,475846	1	0,734136
-0,66889	420	415,9536	0,503938	1	0,960096
-0,21341	367	362,348	0,831126	1	0,956843
0,123105	280	269,5017	0,902116	1	0,975243
1,641852	421	387,6569	0,101432	1	0,34273
2,006807	421	207,3597	0,046069	1	0,281076
0,42187	420	411,6427	0,67334	1	0,96781
2,807719	367	359,1298	0,005262	1	0,178183
1,108121	280	274,3846	0,26878	1	0,693794
-2,64104	421	396,7151	0,008592	1	0,112587
-2,32453	421	212,6916	0,02104	1	0,2022
-3,31741	420	413,8376	9,89E-04	0,150303	0,235929
-1,40836	367	361,2028	0,159886	1	0,504558
-0,72824	280	269,0903	0,467102	1	0,824256
0,379169	421	341,2996	0,704798	1	0,875021
0,277827	421	187,0082	0,781452	1	0,91393
0,877742	420	376,2167	0,380644	1	0,95743
1,150634	367	323,617	0,250732	1	0,605249
0,039433	280	253,1467	0,968576	1	0,991502
0,718684	421	352,5149	0,472812	1	0,731548
0,932942	421	191,0964	0,352027	1	0,643758
2,284305	420	384,185	0,022898	1	0,75904

0,829703	367	339,1757	0,40729	1	0,720407
1,155274	280	260,1165	0,249038	1	0,677438
-2,38988	421	358,5878	0,017369	1	0,153976
-1,47733	421	191,6376	0,14123	1	0,423499
-0,09809	420	393,6064	0,921908	1	0,989204
-1,03469	367	334,076	0,301561	1	0,633826
0,643702	280	258,1095	0,52034	1	0,833721
1,877061	421	390,9727	0,061254	1	0,270704
2,063846	421	208,9483	0,040268	1	0,26998
0,085025	420	412,6984	0,932283	1	0,991559
2,384285	367	359,5889	0,01763	1	0,216225
1,468356	280	274,894	0,143151	1	0,554571
0,757829	421	365,1647	0,449042	1	0,714643
1,516282	421	192,4284	0,131089	1	0,413287
1,986494	420	395,3095	0,047669	1	0,846627
1,725976	367	334,6011	0,085275	1	0,388246
-0,33234	280	254,4738	0,739903	1	0,940183
0,627153	421	347,6161	0,53097	1	0,776668
1,491998	421	186,534	0,137389	1	0,422541
1,856821	420	374,4558	0,064122	1	0,848246
0,167221	367	319,9663	0,867302	1	0,959636
-0,41814	280	253,5674	0,676202	1	0,909385
-0,44234	421	351,8812	0,658513	1	0,848606
-0,75458	421	190,7904	0,451433	1	0,713861
-1,0407	420	383,719	0,29867	1	0,943897
-0,1625	367	339,3567	0,871011	1	0,959978
-0,11114	280	269,5606	0,911588	1	0,976008
0,213922	421	384,1044	0,830721	1	0,936611
-0,51374	421	204,7112	0,607988	1	0,821738
-1,48869	420	408,9587	0,137341	1	0,891179
-1,05354	367	355,2693	0,29281	1	0,627013
0,659423	280	267,5771	0,510191	1	0,831912
0,102664	421	349,9846	0,918288	1	0,966828
0,882204	421	187,7631	0,378794	1	0,656141
-0,67794	420	382,8639	0,498221	1	0,960096
0,197227	367	339,562	0,843768	1	0,956935
1,441899	280	254,1342	0,150562	1	0,56302
3,028396	421	390,9804	0,002622	1	0,073944
4,253844	421	209,562	3,17E-05	0,034167	0,015907
1,144032	420	413,436	0,253272	1	0,943897
2,997789	367	359,8282	0,002908	1	0,176492
1,833521	280	275,6126	0,067803	1	0,425749
-1,31621	421	336,5821	0,188999	1	0,472953
-0,60372	421	184,1998	0,546772	1	0,776424
-0,71867	420	368,414	0,472803	1	0,960096
-0,63259	367	325,5314	0,527447	1	0,811085
0,804448	280	258,9554	0,421876	1	0,796701
0,524183	421	356,1993	0,600477	1	0,818191

0,211878	421	191,3281	0,832428	1	0,936588
0,365528	420	389,2739	0,714916	1	0,968866
0,320123	367	337,5396	0,749073	1	0,923669
-0,27134	280	264,876	0,786338	1	0,953235
1,005292	421	357,1757	0,315437	1	0,60944
0,768197	421	192,3221	0,443312	1	0,708362
0,220208	420	390,317	0,825825	1	0,973894
0,774219	367	341,0887	0,439338	1	0,742744
-0,44808	280	264,3438	0,654464	1	0,908644
0,248551	421	389,9447	0,803839	1	0,922616
1,060718	421	208,4461	0,290045	1	0,586961
-1,6311	420	411,7016	0,103635	1	0,848246
1,138858	367	359,8762	0,25552	1	0,609149
0,711099	280	269,7281	0,477637	1	0,824256
-1,51888	421	325,8689	0,129762	1	0,397564
0,045122	421	181,1358	0,964059	1	0,985228
-1,23071	420	357,0648	0,219242	1	0,943055
-0,04201	367	314,6606	0,96652	1	0,986276
-0,0777	280	255,0582	0,938129	1	0,981778
-0,16857	421	332,446	0,866237	1	0,95286
2,138968	421	185,286	0,033747	1	0,251769
0,337166	420	367,7441	0,736184	1	0,969427
-0,20246	367	324,5857	0,839688	1	0,956935
1,916111	280	252,7381	0,056479	1	0,406703
1,743078	421	351,0605	0,082196	1	0,304997
2,05274	421	188,4177	0,04148	1	0,272972
1,381225	420	389,1861	0,168002	1	0,914005
1,210453	367	334,5755	0,226959	1	0,576775
2,997761	280	269,5935	0,002973	1	0,135787
-3,1684	421	380,566	0,001657	0,253473	0,069718
-1,52009	421	202,3843	0,130049	1	0,410868
-0,85445	420	410,1934	0,393356	1	0,95743
-0,61481	367	358,2799	0,539071	1	0,819758
-0,41752	280	273,9461	0,676627	1	0,909385
-0,0095	421	340,9034	0,992422	1	0,998054
-0,08555	421	188,9444	0,931916	1	0,974599
1,003428	420	374,2851	0,316302	1	0,943897
0,26242	367	326,1826	0,793163	1	0,944997
0,628677	280	253,2086	0,530127	1	0,840949
-1,16376	421	349,9736	0,245315	1	0,542863
-1,30041	421	190,4479	0,195032	1	0,483337
-1,31167	420	385,2089	0,190413	1	0,925729
0,576152	367	330,2263	0,564905	1	0,837838
0,743493	280	260,5862	0,457853	1	0,817005
0,151405	421	396,2405	0,879733	1	0,95861
1,000152	421	211,1251	0,318382	1	0,617081
1,22988	420	413,3089	0,219442	1	0,943055
0,558224	367	362,1551	0,577036	1	0,843313

-0,81354	280	273,6199	0,416613	1	0,792723
-0,00599	421	312,9761	0,995224	1	0,998054
-0,69995	421	181,0641	0,484859	1	0,740307
-1,98144	420	346,5524	0,048332	1	0,846627
-0,40261	367	296,9141	0,687525	1	0,903032
-1,66723	280	254,4096	0,096698	1	0,480557
0,821506	421	360,4764	0,411901	1	0,695339
0,489487	421	192,975	0,625053	1	0,831381
2,297159	420	390,4707	0,022138	1	0,75904
-0,2961	367	344,6023	0,767329	1	0,93557
0,564152	280	270,5633	0,573118	1	0,870079
-0,95348	421	310,5382	0,341087	1	0,63164
-0,66551	421	180,9834	0,506572	1	0,754351
-0,83913	420	343,8698	0,401981	1	0,95743
0,419038	367	301,0386	0,675487	1	0,898834
-0,84014	280	276	0,401556	1	0,784757
-1,4939	421	346,0635	0,136112	1	0,407576
-1,08175	421	188,6381	0,280743	1	0,581153
-1,80426	420	377,7193	0,071986	1	0,848246
-0,62257	367	330,2062	0,533998	1	0,816122
-0,52967	280	254,9807	0,596798	1	0,880334
-0,22618	421	310,0929	0,821208	1	0,932601
0,737725	421	179,3271	0,461646	1	0,721079
-0,42066	420	343,0103	0,674269	1	0,96781
-0,46402	367	307,1363	0,642962	1	0,882539
0,668412	280	254,7067	0,504476	1	0,831912
-1,04661	421	405,2993	0,295902	1	0,593774
-0,6681	421	221,6749	0,504762	1	0,753145
-0,69918	420	415,9228	0,484831	1	0,960096
-2,06151	367	362,9919	0,039966	1	0,28956
0,540683	280	275,5251	0,589163	1	0,87561
3,54586	421	383,6023	4,40E-04	0,058922	0,04642
1,799562	421	204,639	0,073403	1	0,342815
-0,84983	420	410,151	0,395917	1	0,95743
2,437019	367	357,8285	0,015296	1	0,204372
1,872165	280	274,8617	0,062246	1	0,422605
1,220187	421	380,6	0,22315	1	0,51676
1,062562	421	200,8663	0,289256	1	0,586688
-0,35796	420	406,398	0,720559	1	0,969427
-0,39577	367	350,2247	0,692516	1	0,904352
0,816737	280	263,4705	0,414817	1	0,791302
-1,29701	421	299,4393	0,195627	1	0,481951
-0,23121	421	175,4113	0,81742	1	0,926377
-1,06121	420	328,7896	0,289373	1	0,943897
0,224505	367	288,0811	0,822524	1	0,953906
1,107317	280	262,3382	0,269171	1	0,693794
-2,86861	421	324,0245	0,004393	1	0,087964
-1,54453	421	183,5082	0,124182	1	0,408607

-0,847	420	356,8136	0,397561	1	0,95743
-0,07928	367	321,8976	0,936856	1	0,978845
-0,02102	280	256,6897	0,983248	1	0,99236
-1,13229	421	307,9593	0,258395	1	0,550898
0,069627	421	180,2539	0,944568	1	0,980711
-1,20623	420	336,716	0,228576	1	0,943055
1,253844	367	297,4254	0,210883	1	0,563436
-0,43534	280	251,898	0,663687	1	0,908644
-0,97762	421	305,7499	0,329037	1	0,618807
0,069605	421	174,9801	0,944588	1	0,980711
-0,47844	420	336,9751	0,632644	1	0,964878
0,314814	367	304,3636	0,753119	1	0,924494
0,926408	280	255,4391	0,355108	1	0,761846
0,813793	421	409,3638	0,416236	1	0,695339
0,185715	421	226,4821	0,852834	1	0,944105
0,431789	420	415,7797	0,666118	1	0,96781
-1,26105	367	362,8911	0,2081	1	0,561015
1,139435	280	272,8319	0,255521	1	0,679673
-0,04746	421	392,2278	0,962167	1	0,991104
1,591034	421	209,4748	0,11311	1	0,391524
0,021671	420	412,3311	0,982721	1	0,997342
-0,19579	367	359,2615	0,844887	1	0,956935
0,924688	280	272,052	0,355948	1	0,761846
-1,39096	421	297,9361	0,165276	1	0,440727
-1,18676	421	175,0805	0,236931	1	0,537903
-2,32827	420	326,8464	0,020507	1	0,75904
-1,47677	367	275,0715	0,140882	1	0,48919
-1,0451	280	276	0,296892	1	0,723324
3,509408	421	395,9528	5,01E-04	0,066094	0,04642
3,49346	421	212,7027	5,80E-04	0,077134	0,051411
0,521372	420	414,6819	0,602386	1	0,964878
2,839781	367	359,9863	0,004771	0,629756	0,178183
0,879478	280	274,7973	0,379911	1	0,770948
0,195054	421	372,6211	0,845457	1	0,939735
0,939294	421	197,8084	0,348725	1	0,643243
0,228456	420	401,1166	0,819408	1	0,97003
-1,03536	367	352,6744	0,301212	1	0,633826
-1,68627	280	268,7947	0,092904	1	0,471402
-0,05409	421	379,8092	0,956889	1	0,987258
-0,81287	421	203,1417	0,417247	1	0,687955
-0,83857	420	406,889	0,402201	1	0,95743
-0,26035	367	348,7312	0,79475	1	0,945294
0,612844	280	263,598	0,540508	1	0,84937
-0,66459	421	375,0804	0,506721	1	0,75983
-1,95827	421	201,2586	0,051581	1	0,296106
-2,33192	420	402,3088	0,020198	1	0,75904
-1,5185	367	347,3267	0,129798	1	0,473621
-0,12572	280	259,6033	0,900054	1	0,975243

0,245494	421	345,0306	0,80622	1	0,922616
1,592796	421	189,4635	0,112873	1	0,391524
1,065449	420	376,6948	0,287355	1	0,943897
-0,47899	367	327,9068	0,632262	1	0,880707
-0,95995	280	252,4485	0,337998	1	0,755731
0,008714	421	379,4923	0,993052	1	0,998054
-2,27251	421	201,0573	0,024114	1	0,218351
0,349547	420	405,8121	0,72686	1	0,969427
-0,65	367	340,7467	0,516131	1	0,800216
-0,23304	280	265,9341	0,81591	1	0,95747
2,885226	421	399,5137	0,004123	0,614373	0,087964
2,024083	421	217,1692	0,044186	1	0,277922
0,750363	420	415,3932	0,453461	1	0,960096
1,84204	367	362,8789	0,066285	1	0,359322
0,985549	280	275,6088	0,325219	1	0,749395
0,830989	421	305,9147	0,406628	1	0,695339
4,030909	421	175,7127	8,27E-05	0,088941	0,020761
1,173375	420	339,7661	0,241467	1	0,943659
2,382694	367	308,6664	0,017792	1	0,216225
1,987623	280	246,7465	0,047959	1	0,393265
-1,89644	421	336,2814	0,058759	1	0,268334
-0,98798	421	183,6667	0,324464	1	0,619085
-1,61292	420	367,7599	0,10762	1	0,848246
0,499582	367	319,3752	0,617714	1	0,875495
0,269001	280	259,961	0,788142	1	0,953235
1,31547	421	337,9061	0,189244	1	0,472953
0,394785	421	186,8545	0,693452	1	0,865526
-0,76793	420	373,8101	0,443015	1	0,960096
0,938369	367	331,0257	0,348739	1	0,668671
-0,91331	280	254,471	0,361942	1	0,762031
-2,52002	421	307,8054	0,012241	1	0,131857
-1,97596	421	178,1669	0,049703	1	0,289197
-1,09499	420	348,753	0,274279	1	0,943897
-1,79476	367	317,6477	0,073642	1	0,373795
-1,62592	280	271,2856	0,105128	1	0,492014
2,566926	421	363,041	0,01066	1	0,12358
3,154432	421	192,4239	0,001866	0,279939	0,096981
3,647284	420	403,3152	3,00E-04	0,045865	0,09035
1,879057	367	347,8042	0,061073	1	0,34342
3,186029	280	270,5652	0,001611	0,246546	0,135787
-0,60608	421	357,2729	0,544849	1	0,784719
0,175784	421	194,3411	0,860647	1	0,947215
0,40398	420	389,2325	0,686449	1	0,968613
-0,51547	367	340,349	0,606559	1	0,868127
-1,10698	280	261,0312	0,269323	1	0,693794
1,47846	421	343,2893	0,140202	1	0,410878
1,376929	421	188,7815	0,170165	1	0,456365
1,602655	420	372,7658	0,109858	1	0,848246

0,484678	367	331,4785	0,628225	1	0,880707
-0,64671	280	261,7128	0,518387	1	0,833721
0,536741	421	362,5745	0,591776	1	0,81147
1,418365	421	194,7665	0,157682	1	0,43893
-0,48133	420	394,3769	0,630548	1	0,964878
0,164686	367	337,1633	0,86929	1	0,959978
-0,03374	280	267,5907	0,973111	1	0,99236
-2,66741	421	362,252	0,007987	1	0,110425
-1,68347	421	195,7724	0,093878	1	0,373113
-1,20414	420	392,8096	0,22926	1	0,943055
-0,70454	367	346,9449	0,481568	1	0,785415
-1,07998	280	267,2926	0,281126	1	0,703928
-0,86919	421	318,027	0,385396	1	0,674555
-0,9184	421	180,8952	0,359635	1	0,643758
-1,73335	420	351,5893	0,08391	1	0,848246
-1,00679	367	300,5845	0,314847	1	0,647568
-1,35591	280	256,6522	0,176319	1	0,598625
-1,45768	421	340,6136	0,145851	1	0,414711
-1,57954	421	187,3225	0,115899	1	0,392493
0,00812	420	374,6698	0,993526	1	0,997342
-0,92446	367	331,9262	0,355919	1	0,674813
-2,19339	280	253,4477	0,029188	1	0,326641
2,219006	421	329,3339	0,027168	1	0,192509
2,352028	421	181,7229	0,019742	1	0,195731
1,709059	420	370,755	0,088277	1	0,848246
1,821036	367	308,6597	0,069569	1	0,364488
2,4121	280	262,6387	0,016547	1	0,266517
-2,72628	421	370,6193	0,006709	1	0,10482
-2,40439	421	201,1242	0,017106	1	0,181988
-2,18507	420	405,0367	0,029456	1	0,792693
-1,75628	367	348,52	0,079919	1	0,381841
-1,08369	280	270,3853	0,279466	1	0,703716
0,979748	421	407,3453	0,327792	1	0,618251
3,731326	421	222,9463	2,42E-04	0,036743	0,035752
0,508068	420	415,9424	0,611675	1	0,964878
2,114511	367	362,9999	0,035153	1	0,283291
1,722044	280	275,9961	0,086182	1	0,458713
0,332315	421	324,8042	0,739866	1	0,893273
0,915802	421	178,807	0,361004	1	0,64452
-0,04128	420	358,9438	0,967099	1	0,996924
0,459044	367	307,3436	0,646527	1	0,884339
1,955964	280	244,8488	0,051607	1	0,398826
-1,88187	421	337,9586	0,060713	1	0,270611
-0,99833	421	186,5324	0,319411	1	0,617081
-0,52376	420	369,9299	0,600757	1	0,964878
0,006645	367	333,2143	0,994702	1	0,997335
-1,96089	280	256,959	0,050972	1	0,398004
2,487107	421	406,4633	0,013279	1	0,138362

2,64343	421	223,5071	0,008788	1	0,14815
1,485364	420	415,9745	0,138205	1	0,891179
2,536243	367	362,4622	0,011624	1	0,189546
1,148231	280	275,9216	0,251868	1	0,679007
-1,42061	421	333,5531	0,156364	1	0,429212
-0,63443	421	183,5899	0,526587	1	0,767321
-0,5593	420	366,7885	0,5763	1	0,964878
-1,35591	367	323,277	0,176073	1	0,523018
0,762081	280	246,2202	0,446741	1	0,81309
-0,10083	421	396,7702	0,919739	1	0,966828
1,2728	421	209,5537	0,204499	1	0,500293
0,540207	420	413,9528	0,589344	1	0,964878
0,917737	367	361,8776	0,359368	1	0,674813
0,748753	280	270,2197	0,454657	1	0,814707
-2,12703	421	351,9591	0,034113	1	0,212429
-2,21228	421	191,0521	0,028132	1	0,227932
-0,5633	420	389,74	0,573555	1	0,964878
-0,89118	367	333,7622	0,373472	1	0,683037
-0,28805	280	262,2157	0,773539	1	0,947219
-1,1833	421	341,1559	0,237513	1	0,532577
-0,00819	421	186,4307	0,993474	1	0,997935
0,308363	420	378,1432	0,757976	1	0,969427
0,462637	367	315,1921	0,643944	1	0,882712
1,050352	280	263,9401	0,294517	1	0,723324
0,882459	421	314,049	0,378204	1	0,670163
1,307266	421	181,318	0,192778	1	0,480987
-0,70855	420	350,1026	0,479076	1	0,960096
-0,85849	367	303,0081	0,391298	1	0,705088
-0,27828	280	259,2493	0,781021	1	0,951006
0,813855	421	361,0768	0,416264	1	0,695339
2,4517	421	195,7548	0,015095	1	0,179127
1,110631	420	394,865	0,267403	1	0,943897
-0,3195	367	345,8988	0,749537	1	0,923669
0,88647	280	258,5875	0,376188	1	0,767473
-2,16321	421	295,5778	0,031326	1	0,201145
-0,50759	421	176,4738	0,612376	1	0,823974
-1,51229	420	331,4502	0,131414	1	0,888479
0,728444	367	272,4924	0,466967	1	0,771623
-0,72465	280	276	0,469281	1	0,824256
-1,1342	421	379,7111	0,257427	1	0,550898
0,477931	421	203,3197	0,633213	1	0,835702
-0,64927	420	406,5461	0,516528	1	0,960096
0,750795	367	348,622	0,453282	1	0,758154
0,219241	280	270,1373	0,826628	1	0,96121
-1,10176	421	394,1102	0,271238	1	0,56693
-0,48805	421	212,2084	0,626015	1	0,831927
0,212218	420	413,8968	0,832041	1	0,973894
-1,43969	367	362,7802	0,150818	1	0,499998

-0,42429	280	274,9512	0,671685	1	0,908644
0,882815	421	393,7944	0,377875	1	0,670163
-0,54394	421	211,4274	0,587059	1	0,802811
-1,11193	420	413,5817	0,266813	1	0,943897
-0,13521	367	358,173	0,892522	1	0,969042
-1,54396	280	274,2203	0,12375	1	0,535729
-1,39423	421	326,2192	0,164196	1	0,438729
-2,091	421	181,8849	0,037918	1	0,267984
-1,73405	420	361,7979	0,08376	1	0,848246
-1,7426	367	324,4584	0,082351	1	0,381856
-1,20378	280	262,2113	0,229759	1	0,657169
-0,61746	421	338,747	0,537346	1	0,778195
-2,01046	421	186,5478	0,045823	1	0,281076
0,164716	420	378,088	0,869256	1	0,980994
-0,22239	367	334,0429	0,824144	1	0,953906
-0,14637	280	260,352	0,883744	1	0,973784
-3,10603	421	328,9033	0,002061	1	0,069718
-1,13582	421	186,1122	0,257494	1	0,556025
0,913789	420	371,1248	0,361421	1	0,955547
0,186114	367	336,6115	0,852467	1	0,956935
-0,31075	280	265,9721	0,756232	1	0,942071
-0,00434	421	321,9759	0,996538	1	0,998526
-0,1662	421	183,4552	0,868185	1	0,947676
0,1382	420	357,1451	0,89016	1	0,983483
0,90539	367	318,903	0,365942	1	0,678321
-0,32746	280	252,2478	0,743591	1	0,941674
-1,96735	421	364,0957	0,049902	1	0,246877
-1,68839	421	197,0104	0,092919	1	0,373113
0,616596	420	398,7091	0,537853	1	0,960096
-0,78822	367	350,6341	0,431102	1	0,73975
0,994126	280	269,6008	0,321053	1	0,744349
2,066749	421	403,4324	0,039395	1	0,224033
2,214349	421	217,3464	0,027844	1	0,226812
0,339286	420	415,7851	0,734565	1	0,969427
-0,43177	367	361,7941	0,666163	1	0,894765
1,105078	280	275,4601	0,27009	1	0,694583
-1,27656	421	376,1125	0,202545	1	0,491523
-0,00475	421	201,0136	0,996212	1	0,998199
0,316271	420	402,8773	0,751961	1	0,969427
-1,07425	367	355,6843	0,283438	1	0,61815
-0,31191	280	263,6728	0,755357	1	0,942071
0,210908	421	282,7875	0,833111	1	0,936611
1,117066	421	174,3888	0,265503	1	0,562747
-0,3982	420	311,6847	0,690758	1	0,968866
2,197866	367	283,9018	0,028766	1	0,258038
0,317049	280	252,441	0,751468	1	0,942071
-1,22112	421	324,6545	0,222925	1	0,51676
-1,20636	421	181,1243	0,229252	1	0,529303

1,018992	420	359,0568	0,308893	1	0,943897
-1,77714	367	321,3469	0,076491	1	0,378716
-1,61282	280	249,5713	0,108048	1	0,497151
0,995521	421	308,5914	0,320263	1	0,615134
0,459521	421	178,8293	0,646418	1	0,839358
-0,36196	420	339,3613	0,717608	1	0,969427
-0,32195	367	295,6209	0,74772	1	0,923669
-0,23912	280	247,4281	0,811212	1	0,95747
-0,75593	421	380,6889	0,450161	1	0,714643
-1,06862	421	202,9021	0,28651	1	0,584263
-0,06644	420	406,7078	0,947059	1	0,992811
-1,41354	367	350,8966	0,158384	1	0,504558
0,190381	280	270,8356	0,849153	1	0,966155
-0,61374	421	302,7342	0,539845	1	0,779259
0,721547	421	178,6353	0,471516	1	0,731044
1,339159	420	338,1481	0,181418	1	0,923408
-0,87806	367	297,5303	0,380619	1	0,691076
-0,54244	280	246,7019	0,588007	1	0,87561
-0,10942	421	310,8023	0,912939	1	0,966828
0,925821	421	180,5162	0,355775	1	0,643758
0,627229	420	344,5587	0,530924	1	0,960096
1,748707	367	314,6422	0,081317	1	0,381841
0,604762	280	248,0912	0,54589	1	0,851507
-0,46795	421	377,0561	0,640092	1	0,834842
-0,4697	421	198,9307	0,639082	1	0,838432
2,30146	420	401,8864	0,021876	1	0,75904
0,420289	367	350,1798	0,674532	1	0,898779
0,881645	280	268,8894	0,378756	1	0,770948
0,098837	421	305,5179	0,921333	1	0,966828
-0,0545	421	179,2872	0,956601	1	0,98414
0,542465	420	337,3918	0,587857	1	0,964878
1,858845	367	301,3004	0,064024	1	0,350852
-0,25636	280	276	0,797863	1	0,954269
1,042232	421	382,6393	0,297962	1	0,595529
0,777071	421	201,3922	0,438029	1	0,704743
1,241535	420	407,5687	0,215122	1	0,943055
2,150203	367	352,634	0,032219	1	0,270873
0,376815	280	263,1696	0,706614	1	0,922762
-1,03477	421	334,2196	0,301525	1	0,597107
-0,25332	421	186,1856	0,800297	1	0,921861
0,32816	420	368,4632	0,742977	1	0,969427
0,194247	367	328,1625	0,846103	1	0,956935
-1,2824	280	250,3066	0,200888	1	0,631761
1,473508	421	289,4359	0,141701	1	0,412154
1,087946	421	175,0608	0,278114	1	0,578094
1,892998	420	318,7735	0,059264	1	0,848246
0,654758	367	293,7578	0,513136	1	0,797829
0,340865	280	252,8739	0,733488	1	0,936751

-1,19027	421	292,8686	0,234905	1	0,527573
0,452621	421	175,5733	0,65138	1	0,839718
-2,88974	420	314,9845	0,004123	1	0,443819
-0,9368	367	289,1259	0,349644	1	0,668671
1,163029	280	247,2901	0,245939	1	0,676332
-2,42003	421	378,1189	0,01599	1	0,147897
-1,16327	421	199,7098	0,246109	1	0,546224
-1,0741	420	402,102	0,28342	1	0,943897
1,403579	367	358,9895	0,161309	1	0,5075
0,910538	280	264,0638	0,363369	1	0,762031
0,798442	421	360,396	0,42514	1	0,6997
0,113653	421	194,4798	0,90963	1	0,96646
0,550056	420	392,2053	0,582594	1	0,964878
-0,08915	367	342,5975	0,929011	1	0,976733
-1,14979	280	259,5988	0,251289	1	0,679007
1,466671	421	330,5182	0,143416	1	0,412154
1,310765	421	185,5137	0,191558	1	0,47953
0,628765	420	371,2824	0,529889	1	0,960096
0,471171	367	320,2125	0,63784	1	0,880707
1,869089	280	252,6494	0,062767	1	0,422605
-0,87012	421	388,5162	0,384773	1	0,674247
-2,0973	421	207,0535	0,037181	1	0,266419
0,019732	420	411,0897	0,984267	1	0,997342
-1,47411	367	359,102	0,141329	1	0,489614
-1,6132	280	267,1303	0,107883	1	0,497151
-1,53059	421	352,3386	0,126768	1	0,392431
-0,05301	421	192,1116	0,957783	1	0,98414
-0,20153	420	386,0007	0,840389	1	0,973894
-0,43213	367	336,2254	0,665921	1	0,894765
-0,43404	280	266,5869	0,664612	1	0,908644
0,05594	421	356,3778	0,955421	1	0,987258
0,323102	421	192,0701	0,74697	1	0,889215
1,743096	420	388,7691	0,082107	1	0,848246
-0,65512	367	335,6835	0,51284	1	0,797829
0,183905	280	265,9766	0,854228	1	0,966655
-2,3915	421	307,0811	0,017381	1	0,153976
-0,64165	421	175,5478	0,521938	1	0,765001
-1,77124	420	337,1581	0,077424	1	0,848246
-1,07561	367	301,7824	0,282963	1	0,618008
-0,32028	280	248,6943	0,749023	1	0,942071
-2,12171	421	347,5352	0,034569	1	0,213506
-2,16797	421	190,7107	0,0314	1	0,241423
-1,67267	420	385,403	0,095203	1	0,848246
-1,65636	367	341,352	0,098569	1	0,414924
-0,46912	280	273,6323	0,63936	1	0,907449
1,304958	421	342,9018	0,192782	1	0,478621
1,019239	421	189,234	0,309391	1	0,607099
1,400466	420	376,3928	0,162198	1	0,914005

0,783736	367	335,3732	0,433748	1	0,74195
1,88348	280	263,3022	0,060737	1	0,422605
-1,80648	421	310,9924	0,07181	1	0,288707
-1,41019	421	180,7308	0,160203	1	0,442985
-1,3853	420	345,0766	0,166855	1	0,914005
-1,70263	367	321,1735	0,089605	1	0,397163
-1,7387	280	246,8828	0,083335	1	0,456738
2,008169	421	357,2193	0,045378	1	0,237403
1,154308	421	194,5124	0,24979	1	0,548314
0,109125	420	392,0386	0,913159	1	0,987181
0,804218	367	340,5037	0,421832	1	0,731532
0,344357	280	261,8799	0,730854	1	0,936637
1,907792	421	354,2446	0,057226	1	0,266382
0,9236	421	191,4797	0,356857	1	0,643758
0,139552	420	391,292	0,889086	1	0,983483
1,276448	367	337,5333	0,202675	1	0,556341
-0,99171	280	269,5601	0,322227	1	0,745172
-1,89629	421	372,8233	0,058695	1	0,268334
0,469599	421	197,4951	0,639159	1	0,838432
-0,46107	420	399,7383	0,645	1	0,964878
2,020108	367	353,2732	0,044127	1	0,300899
-0,46727	280	263,2893	0,640694	1	0,907449
-0,34867	421	390,42	0,727526	1	0,890807
-1,07858	421	208,9035	0,28202	1	0,581951
0,450995	420	411,5854	0,652231	1	0,966867
0,302936	367	355,4145	0,762116	1	0,929966
0,035767	280	268,7134	0,971495	1	0,992124
0,51158	421	365,1446	0,609255	1	0,824796
0,002427	421	194,567	0,998066	1	0,999267
2,29493	420	393,3081	0,022263	1	0,75904
-0,03492	367	347,7534	0,972162	1	0,989229
0,641425	280	267,4801	0,521796	1	0,833721
-1,03467	421	406,3705	0,301436	1	0,597107
0,237089	421	222,7826	0,812806	1	0,926063
0,479318	420	415,9938	0,631964	1	0,964878
-1,58645	367	362,9189	0,113508	1	0,439732
1,115989	280	275,9997	0,265397	1	0,689576
-1,67358	421	367,4248	0,095065	1	0,331627
0,107868	421	197,6753	0,91421	1	0,968176
0,702088	420	397,5062	0,483035	1	0,960096
0,316907	367	352,6718	0,751502	1	0,924056
0,621619	280	265,9026	0,534725	1	0,84326
-0,88175	421	402,9676	0,37844	1	0,670163
-0,49236	421	219,2249	0,62296	1	0,830785
-0,32996	420	415,7575	0,741598	1	0,969427
-1,52463	367	362,9943	0,128222	1	0,471294
-0,45046	280	274,8785	0,652732	1	0,908644
-2,13861	421	316,0605	0,033233	1	0,209551

-2,16275	421	180,321	0,031877	1	0,241423
-0,26312	420	349,4618	0,792612	1	0,969427
-0,58749	367	312,1822	0,557298	1	0,83071
-1,52504	280	260,9048	0,12846	1	0,536498
-0,18498	421	414,3569	0,853337	1	0,946355
0,57774	421	236,1468	0,56399	1	0,786247
0,730886	420	413,2583	0,465263	1	0,960096
-0,81969	367	360,1392	0,412937	1	0,724248
0,319792	280	275,2126	0,749369	1	0,942071
-1,9346	421	371,5745	0,053798	1	0,259162
-0,89442	421	199,7327	0,372173	1	0,650743
0,268176	420	400,7849	0,788702	1	0,969427
-0,90114	367	353,2878	0,368126	1	0,678946
-0,83131	280	267,9322	0,406538	1	0,788151
1,927238	421	384,1736	0,054687	1	0,259162
2,07018	421	205,712	0,039684	1	0,26998
0,432348	420	410,2208	0,665716	1	0,96781
1,871484	367	360,582	0,062087	1	0,347744
0,891537	280	275,8465	0,373418	1	0,763914
1,725094	421	380,5282	0,085322	1	0,311334
2,415715	421	204,6496	0,016583	1	0,181207
0,519447	420	408,8722	0,60373	1	0,964878
2,511035	367	355,8507	0,01248	1	0,189546
1,806268	280	272,7164	0,071979	1	0,439393
-1,28968	421	354,378	0,198004	1	0,484381
1,704589	421	190,5058	0,089901	1	0,372201
1,616025	420	387,001	0,106904	1	0,848246
1,424106	367	331,2547	0,155357	1	0,502197
0,969194	280	257,2669	0,333359	1	0,755731
0,377612	421	336,4324	0,705957	1	0,875021
0,906961	421	187,0831	0,365594	1	0,647779
0,118977	420	373,1014	0,905358	1	0,98503
-0,35597	367	327,6356	0,722095	1	0,91599
0,926337	280	260,9998	0,355126	1	0,761846
-0,87966	421	311,3713	0,37972	1	0,671614
-0,51011	421	179,4903	0,610601	1	0,82272
-0,67833	420	340,532	0,498021	1	0,960096
-0,36511	367	321,5677	0,715266	1	0,912706
-0,60065	280	258,2362	0,5486	1	0,852208
1,213165	421	346,0224	0,225894	1	0,518147
1,535897	421	187,2004	0,126252	1	0,410224
-0,25825	420	380,0149	0,796352	1	0,969427
1,008016	367	334,6069	0,314175	1	0,647568
1,652362	280	252,1158	0,099706	1	0,480557
1,802947	421	352,8395	0,07225	1	0,289575
0,906243	421	191,148	0,365948	1	0,647779
0,583518	420	386,1709	0,559885	1	0,960096
1,638155	367	335,1298	0,102328	1	0,426647

-0,50522	280	266,0531	0,61382	1	0,889365
0,139971	421	335,7723	0,888767	1	0,962
-0,60269	421	187,2342	0,547447	1	0,776424
0,850309	420	370,6216	0,395702	1	0,95743
-1,25001	367	327,459	0,212188	1	0,563532
-0,782	280	256,0227	0,434939	1	0,803251
2,019707	421	370,32	0,044134	1	0,236315
0,466091	421	199,5763	0,641658	1	0,838647
-0,29275	420	400,7636	0,769863	1	0,969427
1,321325	367	350,0102	0,187256	1	0,537514
-0,83047	280	263,6337	0,407025	1	0,788151
-0,72566	421	380,2603	0,468493	1	0,729358
-0,9874	421	202,0416	0,324627	1	0,619085
-1,05301	420	405,6128	0,292963	1	0,943897
-0,50454	367	346,9015	0,614205	1	0,872281
-0,1628	280	271,7066	0,870798	1	0,969656
-2,54395	421	386,8959	0,011349	1	0,128401
-0,97765	421	208,7987	0,329379	1	0,625156
0,450504	420	411,3172	0,652585	1	0,966867
-1,72602	367	358,0146	0,085207	1	0,388246
-0,18034	280	274,1267	0,857017	1	0,966655
0,083753	421	393,0394	0,933295	1	0,972684
-0,4632	421	209,9942	0,643698	1	0,838647
0,120449	420	412,8067	0,904186	1	0,98503
1,447597	367	359,7225	0,148601	1	0,499998
2,505044	280	270,0583	0,012833	1	0,2448
-0,41226	421	335,8438	0,680411	1	0,861268
1,363905	421	184,395	0,17426	1	0,459198
0,736273	420	371,5189	0,462029	1	0,960096
0,081074	367	321,0303	0,935434	1	0,978278
0,114145	280	262,4054	0,90921	1	0,976008
-0,65337	421	380,4995	0,513914	1	0,767364
-1,23795	421	203,0941	0,217162	1	0,514565
1,587312	420	407,4932	0,113218	1	0,848246
-0,98477	367	353,0264	0,32541	1	0,655597
0,176786	280	269,3633	0,85981	1	0,966655
-2,7366	421	412,9718	0,006476	1	0,102724
-2,32341	421	231,343	0,021025	1	0,2022
-0,67507	420	414,4896	0,500009	1	0,960096
1,129159	367	360,6162	0,259581	1	0,609149
0,822985	280	275,8235	0,411228	1	0,788151
-1,35522	421	326,4788	0,176284	1	0,451804
-0,09578	421	182,2599	0,923804	1	0,971509
-0,09884	420	359,2158	0,921323	1	0,989204
-0,03536	367	304,3403	0,971816	1	0,989229
-1,23701	280	248,2874	0,217251	1	0,650539
0,119683	421	380,2272	0,904798	1	0,965673
-2,4929	421	201,9798	0,013474	1	0,17172

-0,24583	420	406,2342	0,805938	1	0,969427
-1,34252	367	346,1269	0,180307	1	0,527769
0,063076	280	269,743	0,949752	1	0,982409
-0,09717	421	417	0,922635	1	0,966828
1,381054	421	417	0,168002	1	0,45454
-0,31254	420	416	0,754785	1	0,969427
0,073382	367	363	0,941542	1	0,979539
-0,27237	280	276	0,785541	1	0,953235
-1,27801	421	411,8544	0,201965	1	0,490904
0,418944	421	229,7203	0,675649	1	0,854676
-0,91239	420	415,2821	0,36209	1	0,955639
-1,77564	367	362,8715	0,07663	1	0,378716
0,924486	280	275,6225	0,356042	1	0,761846
-0,87527	421	413,1565	0,381933	1	0,671614
0,136809	421	233,3615	0,8913	1	0,958882
0,902906	420	414,5719	0,3671	1	0,95743
0,879235	367	361,2532	0,379858	1	0,691076
-0,93096	280	275,829	0,35269	1	0,761846
0,096429	421	395,7551	0,923229	1	0,966828
-1,29952	421	211,2546	0,195182	1	0,483337
-1,43818	420	414,1136	0,151139	1	0,914005
-1,6854	367	357,543	0,092784	1	0,40529
-0,0189	280	268,7598	0,984931	1	0,992411
-0,03919	421	339,2272	0,968759	1	0,993819
0,640939	421	185,6914	0,522353	1	0,765001
-0,65969	420	371,2818	0,509863	1	0,960096
-0,00583	367	337,9864	0,99535	1	0,997335
-0,24155	280	263,443	0,80932	1	0,95747
0,250671	421	337,3721	0,802221	1	0,922616
1,157628	421	185,1788	0,248507	1	0,548314
-0,49152	420	373,7519	0,623345	1	0,964878
1,314068	367	327,485	0,189743	1	0,539515
1,031157	280	261,0599	0,303421	1	0,726446
-0,15425	421	347,7046	0,877502	1	0,95861
1,066234	421	188,3727	0,287683	1	0,585544
1,884235	420	386,7104	0,060283	1	0,848246
1,823715	367	340,0921	0,069073	1	0,364488
0,740736	280	265,1779	0,459509	1	0,818534
-0,49805	421	319,1808	0,61879	1	0,826904
0,462571	421	178,9106	0,644234	1	0,838647
-0,91485	420	352,6521	0,360893	1	0,955547
0,320159	367	311,481	0,749062	1	0,923669
1,533685	280	247,2629	0,126386	1	0,536498
-1,19328	421	393,7655	0,233476	1	0,525147
-1,87427	421	210,8132	0,062277	1	0,319638
0,433149	420	413,8421	0,665132	1	0,96781
-0,16767	367	360,9151	0,866937	1	0,959636
0,064315	280	271,5771	0,948767	1	0,982409

0,459466	421	394,2861	0,646153	1	0,838719
1,170012	421	211,0092	0,243316	1	0,542422
-0,46911	420	412,8733	0,639236	1	0,964878
-0,72855	367	359,8699	0,466753	1	0,771623
1,035113	280	268,6732	0,301547	1	0,723617
-0,08612	421	314,0301	0,931426	1	0,972063
-0,07967	421	180,844	0,936592	1	0,97636
-1,27484	420	347,4931	0,203216	1	0,928589
-0,39804	367	314,434	0,69087	1	0,903032
0,128866	280	258,164	0,897564	1	0,975243
-0,89894	421	367,6415	0,369272	1	0,661703
-1,6934	421	196,4882	0,091964	1	0,373113
-1,51213	420	397,7111	0,131296	1	0,888479
0,14919	367	343,0198	0,881491	1	0,962453
0,3377	280	263,0734	0,735859	1	0,938189
2,992439	421	319,0757	0,002983	1	0,077359
2,707564	421	181,8404	0,007424	1	0,145306
0,892338	420	352,4297	0,37282	1	0,95743
2,251196	367	318,5761	0,025054	1	0,247087
-0,48474	280	252,2678	0,62828	1	0,900874
1,500458	421	382,92	0,13432	1	0,407576
3,075385	421	203,9758	0,00239	1	0,10292
1,137801	420	409,2253	0,255869	1	0,943897
1,604412	367	358,8058	0,109503	1	0,435936
2,588832	280	271,8358	0,010149	1	0,229419
-0,69126	421	389,1036	0,489816	1	0,748388
-2,31607	421	207,2808	0,021532	1	0,204081
-3,92244	420	411,9522	1,03E-04	0,015811	0,038681
-0,73889	367	357,9356	0,460457	1	0,765059
-2,71519	280	269,991	0,007051	1	0,195931
-1,46461	421	318,9094	0,144013	1	0,412154
-0,40756	421	177,1622	0,684092	1	0,859821
-0,93252	420	352,7583	0,351706	1	0,947373
-0,26116	367	315,4071	0,794142	1	0,945294
0,612012	280	249,8235	0,541087	1	0,849393
1,102367	421	410,1829	0,270949	1	0,56693
-0,05554	421	226,9993	0,95576	1	0,98414
-1,17393	420	415,514	0,241096	1	0,943659
-0,183	367	362,9295	0,854899	1	0,957157
-1,49358	280	275,8614	0,136428	1	0,54931
-0,42443	421	348,5768	0,671517	1	0,856155
-0,5749	421	188,4259	0,566043	1	0,788379
-1,44467	420	379,6967	0,149377	1	0,914005
-0,24473	367	335,6903	0,806817	1	0,950149
1,156474	280	262,0665	0,24854	1	0,677438
0,424598	421	369,9654	0,671376	1	0,856155
2,807515	421	197,6762	0,005493	1	0,1403
1,279435	420	399,9807	0,201486	1	0,928589

1,883631	367	346,9476	0,060451	1	0,341198
0,592836	280	262,8606	0,553801	1	0,854225
0,818056	421	387,1151	0,413828	1	0,695339
1,665971	421	209,5689	0,097213	1	0,379251
-0,31353	420	412,4163	0,754034	1	0,969427
-0,11755	367	359,1707	0,906492	1	0,970486
-0,70326	280	271,2127	0,4825	1	0,824339
0,519487	421	401,259	0,603708	1	0,821108
0,106535	421	217,3806	0,915256	1	0,968604
0,044014	420	415,2273	0,964914	1	0,996924
-0,81441	367	360,0284	0,41595	1	0,727188
1,201512	280	272,6588	0,230595	1	0,657169
-0,95303	421	384,2729	0,341176	1	0,63164
0,329027	421	203,4946	0,742474	1	0,888022
-0,47193	420	407,8185	0,63723	1	0,964878
0,764031	367	356,0909	0,445355	1	0,749888
1,318245	280	273,5321	0,188525	1	0,623041
-0,56515	421	414,2937	0,572278	1	0,79928
0,935605	421	234,0395	0,350441	1	0,643758
-0,93417	420	413,7813	0,35076	1	0,947373
2,363507	367	362,6169	0,01863	1	0,217108
-1,43271	280	274,7186	0,153079	1	0,565026
-1,91637	421	336,5146	0,056165	1	0,2645
-3,08218	421	182,6014	0,002373	0,351256	0,10292
-1,43604	420	373,9794	0,151825	1	0,914005
-1,92657	367	335,2943	0,054879	1	0,335053
-0,51892	280	258,4616	0,60426	1	0,884956
-0,51331	421	375,056	0,608039	1	0,824024
-0,32188	421	201,1877	0,747876	1	0,889542
-0,30012	420	404,4742	0,764242	1	0,969427
0,056636	367	346,0006	0,954867	1	0,980235
-0,694	280	263,4167	0,488296	1	0,826999
-0,33179	421	318,6714	0,740269	1	0,893273
0,421313	421	178,3538	0,674034	1	0,853746
-0,65225	420	352,5703	0,514664	1	0,960096
0,184897	367	309,4924	0,853431	1	0,956935
1,78346	280	247,564	0,075736	1	0,449349
0,978156	421	284,9411	0,328827	1	0,618807
2,167925	421	172,691	0,031533	1	0,241423
1,068806	420	315,6195	0,285974	1	0,943897
-0,63297	367	287,4459	0,527258	1	0,811085
0,685908	280	245,7876	0,493417	1	0,827962
0,824307	421	334,7309	0,410352	1	0,695339
0,384977	421	186,6071	0,700693	1	0,865528
0,955426	420	368,0531	0,339989	1	0,947373
-0,24003	367	317,7672	0,810465	1	0,950149
0,0307	280	259,5813	0,975532	1	0,99236
-1,1984	421	378,384	0,231511	1	0,523069

-1,3341	421	201,3864	0,183679	1	0,473593
0,856885	420	405,5458	0,392014	1	0,95743
-2,97624	367	351,7199	0,00312	0,471125	0,176492
-1,2199	280	268,2771	0,223573	1	0,654014
-0,57248	421	414,4287	0,56731	1	0,796637
-0,85379	421	236,5252	0,394085	1	0,665792
-0,26135	420	413,2087	0,793949	1	0,969427
-1,42304	367	360,391	0,15559	1	0,502197
-0,47892	280	275,8408	0,632374	1	0,903355
-1,43822	421	305,0721	0,151397	1	0,42445
-0,91506	421	178,6192	0,361393	1	0,64452
-2,05455	420	336,1562	0,040695	1	0,846132
-0,14859	367	295,3664	0,881982	1	0,962453
-2,40365	280	248,5584	0,016965	1	0,266517
1,755717	421	363,1739	0,07998	1	0,302838
1,326064	421	196,1346	0,186361	1	0,474401
0,936926	420	395,4276	0,349369	1	0,947373
-0,95211	367	346,1415	0,341707	1	0,664831
1,592727	280	262,4411	0,112425	1	0,50726
-1,65675	421	334,1975	0,098508	1	0,339344
0,57166	421	183,1155	0,568254	1	0,78927
-0,41234	420	367,1048	0,680331	1	0,96781
0,116446	367	307,4477	0,907375	1	0,970486
-1,67836	280	252,5562	0,094514	1	0,474774
0,124058	421	384,8157	0,901334	1	0,965517
1,700011	421	204,8602	0,090646	1	0,373113
0,652078	420	408,308	0,514718	1	0,960096
2,999476	367	353,0503	0,002896	1	0,176492
0,577843	280	270,7444	0,56385	1	0,861787
-0,33375	421	315,1521	0,738793	1	0,893273
0,34415	421	178,2797	0,731139	1	0,882616
-0,85389	420	348,5427	0,393751	1	0,95743
0,341465	367	309,4003	0,732985	1	0,923669
2,164711	280	247,0127	0,031368	1	0,329568
-0,48517	421	336,0256	0,627875	1	0,833119
-1,30892	421	186,0032	0,192176	1	0,48028
-0,40102	420	372,935	0,688638	1	0,968866
0,109696	367	331,99	0,912717	1	0,972059
-0,99039	280	264,2102	0,322891	1	0,745172
-1,83682	421	366,8837	0,067046	1	0,279111
-2,66601	421	197,4745	0,008312	1	0,148102
-2,43915	420	397,8489	0,015158	1	0,723749
-1,98672	367	346,5264	0,04774	1	0,311448
0,693474	280	269,5858	0,488609	1	0,826999
-0,96898	421	325,286	0,333275	1	0,62159
-0,88427	421	183,2823	0,377711	1	0,655017
-1,54984	420	355,6058	0,122069	1	0,87201
-1,08545	367	311,7266	0,278563	1	0,612838

-0,92677	280	256,2846	0,354919	1	0,761846
-0,97712	421	291,9268	0,329319	1	0,618807
0,110787	421	174,4399	0,911913	1	0,96646
-0,5421	420	320,2197	0,588126	1	0,964878
-0,85342	367	290,4671	0,39413	1	0,706248
0,670369	280	248,9153	0,503244	1	0,831912
0,12225	421	335,3351	0,902774	1	0,965517
-0,14735	421	187,7766	0,883016	1	0,957051
-0,59068	420	370,3786	0,555093	1	0,960096
-0,82578	367	329,6482	0,409524	1	0,722315
-0,08404	280	266,6837	0,933092	1	0,979504
-3,29672	421	350,1292	0,001078	1	0,064989
-2,10838	421	187,5832	0,036326	1	0,261928
-0,56673	420	383,0667	0,571232	1	0,964199
-1,39579	367	334,1796	0,163703	1	0,510294
-3,11726	280	259,6821	0,002031	1	0,135787
-2,97097	421	364,4191	0,003165	1	0,078203
-0,14838	421	195,3024	0,882195	1	0,957051
-0,67548	420	394,745	0,499764	1	0,960096
-1,11736	367	342,1266	0,264625	1	0,609149
1,045235	280	257,9539	0,296893	1	0,723324
-0,01992	421	355,2843	0,984121	1	0,998028
-0,09721	421	191,2627	0,922658	1	0,971139
0,641486	420	386,5271	0,521587	1	0,960096
-0,09724	367	337,0796	0,922597	1	0,975687
-0,02855	280	258,648	0,977249	1	0,99236
-1,04802	421	330,1023	0,295397	1	0,593552
0,067802	421	182,5403	0,946018	1	0,980846
0,629857	420	367,1333	0,52918	1	0,960096
-0,04185	367	331,0031	0,966642	1	0,986276
1,649038	280	271,2348	0,100298	1	0,480557
-0,0405	421	325,3836	0,967717	1	0,993427
-1,74908	421	183,2684	0,081952	1	0,362173
-2,21372	420	353,8769	0,027485	1	0,767829
-1,4886	367	309,4171	0,13761	1	0,4834
-1,74442	280	265,9595	0,082242	1	0,456738
1,84051	421	385,8965	0,066461	1	0,278382
2,127765	421	205,0155	0,034551	1	0,252759
1,653506	420	410,3586	0,098993	1	0,848246
2,706974	367	353,9312	0,007119	1	0,180403
1,539998	280	272,7661	0,12472	1	0,535729
2,848518	421	335,8126	0,004663	1	0,087964
2,922194	421	183,3767	0,003913	1	0,118336
1,85433	420	368,934	0,06449	1	0,848246
2,992389	367	320,0069	0,002983	1	0,176492
1,647364	280	257,9854	0,100701	1	0,480557
-1,64528	421	327,1648	0,100873	1	0,342121
-0,24808	421	181,576	0,804353	1	0,923205

1,052041	420	365,7809	0,293475	1	0,943897
2,043324	367	330,2396	0,041814	1	0,291798
2,485578	280	261,6365	0,013559	1	0,252268
1,477914	421	334,0784	0,140373	1	0,410878
-0,05312	421	186,9832	0,95769	1	0,98414
-0,43828	420	368,0537	0,66144	1	0,96781
-0,1907	367	307,219	0,848886	1	0,956935
-0,35823	280	276	0,720448	1	0,932745
-1,06721	421	370,2052	0,286571	1	0,584387
-0,97525	421	195,9932	0,330637	1	0,626331
2,141617	420	398,1637	0,032831	1	0,828006
0,846375	367	348,1776	0,397925	1	0,710512
-0,19624	280	261,2605	0,844577	1	0,965181
-1,90399	421	322,2538	0,057802	1	0,266382
0,117403	421	183,1154	0,906669	1	0,966302
-1,03345	420	355,8029	0,302093	1	0,943897
0,19836	367	314,8494	0,842891	1	0,956935
-0,2928	280	258,3483	0,76991	1	0,946609
-2,81899	421	349,2007	0,005092	1	0,087964
-1,68421	421	191,8326	0,093768	1	0,373113
-0,50659	420	385,8319	0,612735	1	0,964878
-1,39147	367	338,3561	0,164997	1	0,511628
-3,06244	280	262,7949	0,002423	1	0,135787
-3,4644	421	367,1807	5,94E-04	0,633427	0,048251
-0,76484	421	197,4901	0,445282	1	0,709954
-0,47016	420	400,615	0,638495	1	0,964878
0,303677	367	343,3279	0,761558	1	0,929966
0,204493	280	266,9101	0,838124	1	0,964667
-0,03149	421	368,104	0,974893	1	0,995982
-0,16707	421	197,6424	0,867486	1	0,947676
-1,30695	420	399,4146	0,191983	1	0,925729
-1,12293	367	345,8713	0,262247	1	0,609149
-1,85711	280	268,9587	0,064389	1	0,423666
0,214507	421	395,5173	0,830262	1	0,936611
0,477007	421	212,9607	0,633847	1	0,835702
-1,41439	420	414,376	0,158	1	0,914005
1,224302	367	361,1857	0,221636	1	0,573978
1,287053	280	274,1416	0,199161	1	0,631529
2,47734	421	386,559	0,013663	1	0,140071
1,909756	421	206,3998	0,057551	1	0,312877
1,517966	420	410,6613	0,129792	1	0,888479
1,978496	367	356,8142	0,048641	1	0,313787
2,607528	280	268,9589	0,009629	1	0,226737
0,359696	421	345,9731	0,719294	1	0,884157
2,260095	421	185,2741	0,024979	1	0,218351
0,355868	420	381,302	0,722136	1	0,969427
1,583752	367	341,9041	0,114175	1	0,440054
3,879423	280	262,7357	1,32E-04	0,017612	0,046455

0,621981	421	359,0061	0,534349	1	0,778195
1,744021	421	194,6942	0,082734	1	0,36456
-0,40486	420	392,5231	0,6858	1	0,968613
2,040971	367	343,6427	0,042017	1	0,291798
1,83535	280	264,6864	0,067576	1	0,425749
-2,82935	421	345,9053	0,004936	1	0,087964
-2,0405	421	190,7387	0,042678	1	0,273775
-1,56242	420	384,022	0,119013	1	0,858144
-1,45379	367	335,3668	0,14694	1	0,498736
-0,46254	280	264,2323	0,644074	1	0,907804
0,813207	421	351,5726	0,41665	1	0,695339
0,185204	421	190,5475	0,853266	1	0,944105
-0,26793	420	385,1346	0,788898	1	0,969427
-0,21353	367	322,3311	0,831052	1	0,956843
0,997724	280	250,3507	0,319377	1	0,742748
-1,78179	421	317,1114	0,075741	1	0,2988
-0,6742	421	178,7173	0,501056	1	0,750551
-1,8892	420	347,6485	0,059697	1	0,848246
0,834203	367	319,8536	0,404789	1	0,717667
2,208721	280	245,1604	0,02812	1	0,326641
-0,30768	421	325,3374	0,758526	1	0,900919
0,755883	421	183,5188	0,450688	1	0,713861
-0,39058	420	361,0623	0,696339	1	0,968866
0,594164	367	311,7336	0,552833	1	0,830257
-0,69703	280	257,183	0,486413	1	0,826999
0,619087	421	364,738	0,536245	1	0,778195
0,656643	421	193,9603	0,512189	1	0,756803
0,636734	420	395,4068	0,524667	1	0,960096
1,102426	367	346,6711	0,271041	1	0,609149
-1,76051	280	270,3014	0,079452	1	0,453386
-0,07857	421	408,7492	0,937414	1	0,974957
0,146962	421	224,1845	0,883294	1	0,957051
1,385988	420	415,9996	0,166493	1	0,914005
0,029768	367	362,8119	0,976269	1	0,9914
-0,94916	280	275,0885	0,343372	1	0,758791
0,996848	421	383,9121	0,319466	1	0,61486
0,250764	421	205,8988	0,802246	1	0,922323
1,116285	420	409,5273	0,264955	1	0,943897
1,247056	367	358,8285	0,21319	1	0,563645
0,633805	280	272,9639	0,526739	1	0,838222
-1,42772	421	380,9707	0,154192	1	0,426363
-1,46606	421	204,3498	0,144169	1	0,425172
-0,98617	420	405,7957	0,324636	1	0,945573
-0,24904	367	357,4966	0,803477	1	0,950149
-0,62128	280	267,4477	0,534942	1	0,84326
-0,98852	421	352,9246	0,323576	1	0,61749
-0,85811	421	192,5222	0,391901	1	0,665733
1,129759	420	385,3133	0,259281	1	0,943897

0,685497	367	329,3661	0,493512	1	0,793601
-0,50449	280	255,022	0,614353	1	0,889365
-0,19494	421	301,058	0,845574	1	0,939735
-0,06919	421	175,9478	0,944918	1	0,980711
-0,42378	420	333,3917	0,671998	1	0,96781
0,984128	367	301,6995	0,325841	1	0,655597
-0,46776	280	255,1679	0,640358	1	0,907449
0,300054	421	343,1802	0,764317	1	0,904102
1,366007	421	185,9598	0,173586	1	0,458938
-0,48955	420	379,1952	0,624735	1	0,964878
-0,55411	367	335,4992	0,579872	1	0,844137
1,093233	280	252,2335	0,275335	1	0,701156
-0,10235	421	383,5601	0,91853	1	0,966828
-0,3367	421	205,2325	0,736688	1	0,884613
0,997952	420	409,3639	0,318892	1	0,945573
1,104959	367	359,7997	0,269916	1	0,609149
-1,52849	280	268,1604	0,12757	1	0,536498
0,446718	421	370,6997	0,655339	1	0,846936
1,089064	421	199,0082	0,277443	1	0,578094
-1,05175	420	400,0411	0,293551	1	0,943897
1,689807	367	348,88	0,091958	1	0,405208
0,707826	280	264,4742	0,479676	1	0,824256
-0,64045	421	334,581	0,522316	1	0,769202
-0,83668	421	181,3066	0,403876	1	0,677774
0,540266	420	369,5408	0,589339	1	0,964878
-0,59244	367	321,0848	0,553975	1	0,830257
1,894228	280	247,7957	0,059359	1	0,421955
-0,46871	421	375,8798	0,639549	1	0,834842
0,493956	421	198,5118	0,621883	1	0,830096
-0,04758	420	402,8887	0,962076	1	0,996924
0,82271	367	351,8395	0,41123	1	0,723976
-0,80227	280	262,1887	0,423122	1	0,797055
-2,51882	421	369,2126	0,012197	1	0,131857
-1,94146	421	197,6358	0,053624	1	0,302776
0,321551	420	400,8507	0,747961	1	0,969427
-2,60465	367	344,0899	0,009596	1	0,183886
-3,20453	280	266,8068	0,001517	1	0,135787
0,755469	421	330,6574	0,450505	1	0,714643
0,592623	421	185,3343	0,554156	1	0,778237
-2,59041	420	365,2649	0,00997	1	0,600995
-0,5679	367	323,8067	0,570493	1	0,837947
-0,9449	280	260,3684	0,345587	1	0,759183
0,021986	421	401,7804	0,98247	1	0,997025
0,764065	421	215,3475	0,445665	1	0,709954
-0,63754	420	415,4345	0,524125	1	0,960096
0,480395	367	362,4251	0,631236	1	0,880707
-1,12816	280	273,3512	0,260243	1	0,686841
2,42267	421	395,7527	0,015855	1	0,147897

2,303897	421	213,3054	0,022191	1	0,209014
-1,28642	420	415,441	0,199012	1	0,928589
1,173957	367	360,2875	0,241188	1	0,593905
-0,00975	280	273,2123	0,992226	1	0,996269
-0,00206	421	314,6823	0,998355	1	0,999298
1,2193	421	178,036	0,224343	1	0,521994
0,120944	420	349,0044	0,903805	1	0,98503
0,426453	367	309,7382	0,670074	1	0,896008
1,257791	280	250,6857	0,209638	1	0,64091
-2,16233	421	330,7598	0,031309	1	0,201145
-0,18749	421	184,8335	0,851481	1	0,944105
-0,56671	420	368,6302	0,571259	1	0,964199
0,398141	367	327,7669	0,690785	1	0,903032
0,076045	280	268,7624	0,93944	1	0,982175
2,29596	421	399,6308	0,022195	1	0,176042
1,687042	421	217,2391	0,093031	1	0,373113
-1,13581	420	415,6295	0,25669	1	0,943897
1,931999	367	360,2983	0,054143	1	0,334402
1,463045	280	267,5481	0,144629	1	0,555466
4,181797	421	331,3137	3,71E-05	0,039958	0,019267
4,082518	421	179,2959	6,70E-05	0,072182	0,0202
2,799487	420	361,5461	0,005393	1	0,451515
2,640948	367	324,8985	0,008667	1	0,183886
3,152494	280	265,9154	0,001804	1	0,135787
-0,68116	421	322,3574	0,496262	1	0,750117
-0,51504	421	182,7857	0,607147	1	0,821338
1,503257	420	355,7423	0,13366	1	0,890998
0,367948	367	321,9551	0,713154	1	0,912652
0,566006	280	254,1897	0,571889	1	0,869862
-1,20608	421	357,6289	0,228586	1	0,520357
-2,43263	421	192,8162	0,015902	1	0,179127
0,06516	420	390,9262	0,94808	1	0,992811
-1,75894	367	347,8348	0,079467	1	0,381841
-2,14653	280	264,1258	0,032741	1	0,329568
0,429584	421	347,2162	0,667765	1	0,853538
0,987964	421	190,711	0,324422	1	0,619085
1,127803	420	381,1829	0,260113	1	0,943897
2,269306	367	320,3353	0,023914	1	0,245162
0,745671	280	263,1695	0,456532	1	0,816125
-0,66977	421	369,7436	0,50342	1	0,75707
-0,28914	421	197,206	0,772774	1	0,909577
1,630028	420	399,2188	0,103884	1	0,848246
-1,22177	367	350,7193	0,222616	1	0,574455
-2,78016	280	266,1668	0,005821	1	0,186692
1,009491	421	336,0295	0,313466	1	0,608538
0,774211	421	186,1702	0,439788	1	0,705407
1,093104	420	371,6823	0,275056	1	0,943897
0,220749	367	318,4978	0,825429	1	0,95466

0,450894	280	259,238	0,652443	1	0,908644
-1,16003	421	338,9363	0,246852	1	0,544195
-1,27954	421	186,9494	0,202292	1	0,497314
-1,36332	420	377,7971	0,173593	1	0,9199
-0,9065	367	323,5761	0,365346	1	0,678049
0,332321	280	256,8531	0,739918	1	0,940183
-2,82097	421	329,0503	0,005079	1	0,087964
-1,84595	421	183,1302	0,066513	1	0,330811
-1,68889	420	361,6466	0,092103	1	0,848246
0,18134	367	312,6213	0,856218	1	0,957922
-1,35663	280	258,63	0,176083	1	0,598625
-0,47401	421	417	0,635742	1	0,83455
1,627285	421	417	0,104432	1	0,383851
0,325876	420	416	0,744682	1	0,969427
1,133141	367	363	0,257903	1	0,609149
0,830326	280	276	0,407072	1	0,788151
-0,62694	421	356,643	0,531102	1	0,776668
0,927192	421	192,0711	0,354991	1	0,643758
0,272119	420	389,3929	0,785674	1	0,969427
1,137938	367	338,5565	0,255951	1	0,609149
1,651643	280	266,9158	0,099783	1	0,480557
-2,33975	421	363,2789	0,019838	1	0,162476
-0,03025	421	195,4758	0,975896	1	0,991789
0,804634	420	398,2893	0,421511	1	0,95743
1,290983	367	343,8297	0,197577	1	0,549567
1,914216	280	263,9112	0,056674	1	0,406703
1,646289	421	321,2157	0,100682	1	0,342121
0,623296	421	182,5525	0,533868	1	0,769894
-0,37475	420	353,9479	0,708067	1	0,968866
0,589373	367	308,7314	0,556042	1	0,830257
1,875705	280	251,0287	0,061856	1	0,422605
-2,19463	421	349,9881	0,028846	1	0,197598
-0,93698	421	191,7648	0,349949	1	0,643758
0,093804	420	386,6665	0,925313	1	0,989204
0,358844	367	335,3655	0,719938	1	0,914797
0,821791	280	264,4961	0,411936	1	0,788151
-1,81672	421	352,3461	0,07011	1	0,284787
-2,25783	421	191,8717	0,025083	1	0,218351
0,254294	420	388,2592	0,799403	1	0,969427
-0,67014	367	340,1654	0,503223	1	0,793601
-1,30083	280	262,5982	0,194458	1	0,62543
0,27096	421	378,1717	0,786569	1	0,917552
1,262787	421	198,8577	0,208145	1	0,508069
1,221622	420	405,3664	0,222561	1	0,943055
1,610653	367	352,1818	0,108151	1	0,435251
2,139969	280	260,341	0,033287	1	0,329568
0,730309	421	369,6667	0,465664	1	0,727964
0,455823	421	198,0936	0,649017	1	0,839358

0,304282	420	398,5312	0,761072	1	0,969427
-1,0914	367	348,2116	0,275852	1	0,611884
0,76735	280	261,3146	0,443566	1	0,810247
-1,58763	421	315,2332	0,113373	1	0,363517
-1,58112	421	180,213	0,115605	1	0,392379
-1,66474	420	339,1952	0,096888	1	0,848246
-2,02569	367	305,2445	0,043666	1	0,299994
-1,03573	280	257,1793	0,3013	1	0,723617
-1,95092	421	353,3063	0,051857	1	0,253727
0,239969	421	191,1091	0,810611	1	0,926063
-0,04486	420	386,3956	0,96424	1	0,996924
0,681067	367	340,8079	0,496291	1	0,793601
-0,82446	280	254,2183	0,410449	1	0,788151
-0,48015	421	332,7248	0,631436	1	0,83455
-0,66118	421	184,4558	0,509324	1	0,755464
-0,486	420	366,3563	0,627254	1	0,964878
0,494987	367	336,8544	0,620932	1	0,875495
-1,75981	280	260,1482	0,079616	1	0,453386
-3,02448	421	307,5152	0,002701	1	0,073997
-1,48378	421	178,1607	0,139634	1	0,423351
-0,16519	420	341,0265	0,868894	1	0,980994
-0,90057	367	302,1581	0,368532	1	0,678946
0,815442	280	253,8128	0,415584	1	0,791764
-0,79828	421	319,5741	0,425298	1	0,6997
0,637518	421	178,7637	0,524603	1	0,766525
-1,00467	420	352,7199	0,315743	1	0,943897
-0,2495	367	316,7267	0,803139	1	0,950149
1,16777	280	255,7753	0,243987	1	0,673421
0,229613	421	365,1928	0,818521	1	0,93025
0,593121	421	195,503	0,553786	1	0,778237
1,092409	420	396,058	0,275317	1	0,943897
1,420799	367	340,2012	0,156291	1	0,502197
-2,30972	280	259,0165	0,02169	1	0,305489
1,099099	421	361,0444	0,272457	1	0,567902
0,079346	421	195,1642	0,936839	1	0,97636
1,477949	420	391,41	0,140226	1	0,899233
-0,43003	367	344,2559	0,667445	1	0,894765
-2,36306	280	270,2607	0,018833	1	0,281002
-0,5839	421	346,2844	0,559669	1	0,79269
-0,59781	421	190,8458	0,550673	1	0,777451
-1,54606	420	382,3073	0,122918	1	0,87201
-2,20349	367	331,6803	0,028247	1	0,257802
-0,85277	280	259,2637	0,394573	1	0,782194
-1,31727	421	313,6879	0,188709	1	0,472953
0,238304	421	176,8705	0,811921	1	0,926063
-0,4142	420	347,8237	0,678985	1	0,96781
-0,1491	367	304,4482	0,881573	1	0,962453
0,0641	280	252,9358	0,948941	1	0,982409

0,972789	421	347,6045	0,331335	1	0,619505
0,152964	421	191,6013	0,878587	1	0,955289
2,002702	420	380,2604	0,045919	1	0,846132
-1,24931	367	345,3198	0,212399	1	0,563532
-1,17961	280	265,7326	0,239209	1	0,662939
1,122761	421	372,4808	0,262262	1	0,555878
0,162918	421	199,333	0,870748	1	0,949506
-2,1264	420	402,5338	0,034078	1	0,828713
0,066857	367	349,7993	0,946734	1	0,979539
0,715536	280	266,5051	0,474904	1	0,824256
-1,46712	421	376,1211	0,143179	1	0,412154
-1,59048	421	202,0726	0,11329	1	0,391524
-0,45664	420	405,346	0,648174	1	0,964878
-1,7436	367	354,7459	0,082094	1	0,381841
-1,34708	280	272,0142	0,179075	1	0,605081
-1,7904	421	354,1745	0,074244	1	0,294383
-0,7235	421	193,2991	0,470249	1	0,731044
-0,37224	420	387,5692	0,709916	1	0,968866
-1,14199	367	343,3225	0,254255	1	0,609149
0,168636	280	264,8536	0,866212	1	0,969656
-0,61678	421	404,6322	0,537724	1	0,778195
0,719411	421	217,0148	0,472662	1	0,732067
-0,01637	420	415,8555	0,98695	1	0,997342
0,643073	367	362,2158	0,520584	1	0,802988
-0,92173	280	271,3743	0,35749	1	0,761846
-0,94312	421	353,0844	0,346265	1	0,635592
-0,36732	421	191,0447	0,713787	1	0,873114
-0,2784	420	386,2254	0,780852	1	0,969427
-0,58062	367	332,5641	0,561889	1	0,834252
-1,98643	280	261,159	0,048031	1	0,393265
-1,84142	421	314,4863	0,066501	1	0,278382
-2,026	421	177,6396	0,044261	1	0,277922
-1,12171	420	348,2901	0,262758	1	0,943897
-2,74663	367	320,9487	0,006361	1	0,178183
-0,76765	280	265,5215	0,443375	1	0,810247
-1,47178	421	346,5676	0,141989	1	0,412154
-1,65731	421	191,2069	0,099097	1	0,379659
-0,87178	420	378,7759	0,38388	1	0,95743
0,326565	367	338,0557	0,744199	1	0,923669
-0,12453	280	267,6921	0,90099	1	0,975243
-0,64883	421	379,2001	0,516838	1	0,767364
-1,50925	421	201,9176	0,132797	1	0,414338
1,562348	420	406,9784	0,118983	1	0,858144
-1,07754	367	352,0664	0,281978	1	0,61675
0,321498	280	266,7351	0,748085	1	0,942071
-2,32704	421	374,352	0,020496	1	0,16696
-2,06635	421	199,2497	0,040088	1	0,26998
-2,61441	420	400,2246	0,009276	1	0,582436

-1,16259	367	352,2621	0,245782	1	0,599343
-0,49668	280	266,0922	0,619826	1	0,894711
-0,15257	421	415,6387	0,878813	1	0,95861
0,38512	421	261,4344	0,700462	1	0,865528
-0,94479	420	398,9652	0,345336	1	0,947373
2,383476	367	350,0643	0,017683	1	0,216225
0,202392	280	274,021	0,83976	1	0,964667
-3,28165	421	340,276	0,001139	0,1754	0,064989
-3,39954	421	189,5102	8,23E-04	0,124263	0,065272
-0,41378	420	383,2107	0,679269	1	0,96781
-2,98335	367	323,1125	0,003068	0,466364	0,176492
-2,55813	280	266,0883	0,011078	1	0,234919
1,160722	421	329,6004	0,246595	1	0,544195
1,55599	421	185,0252	0,121419	1	0,402151
1,442342	420	363,3061	0,150067	1	0,914005
0,590506	367	308,0536	0,555284	1	0,830257
0,469567	280	253,8273	0,639068	1	0,907449
-1,93196	421	317,5547	0,054254	1	0,259162
-1,80913	421	181,2702	0,072087	1	0,341588
0,795946	420	354,0451	0,426597	1	0,95743
-1,90695	367	315,3404	0,057435	1	0,338985
-2,47258	280	255,6438	0,014065	1	0,258495
-2,74038	421	341,5526	0,006459	1	0,102724
-1,59712	421	189,3938	0,111905	1	0,391524
-0,85369	420	381,7627	0,39381	1	0,95743
-1,12562	367	337,8966	0,261124	1	0,609149
-0,12375	280	266,7344	0,901604	1	0,975243
1,099963	421	371,2548	0,272061	1	0,567861
1,563893	421	199,0991	0,119431	1	0,396436
0,859176	420	399,8722	0,390758	1	0,95743
0,557547	367	355,4802	0,577504	1	0,843313
-0,59949	280	265,618	0,549359	1	0,852208
1,613448	421	381,372	0,107474	1	0,353632
2,17241	421	203,9067	0,030978	1	0,241423
1,381552	420	407,364	0,167867	1	0,914005
0,597262	367	354,6015	0,550713	1	0,829925
0,927101	280	269,9736	0,354702	1	0,761846
1,497911	421	358,5509	0,135036	1	0,407576
1,144961	421	191,2021	0,253656	1	0,553199
0,885668	420	391,0911	0,376341	1	0,95743
1,37911	367	324,8728	0,168809	1	0,514971
1,542249	280	267,6845	0,124194	1	0,535729
-0,83445	421	403,5479	0,404518	1	0,695107
0,873218	421	215,8994	0,383514	1	0,659083
0,094293	420	415,6888	0,924922	1	0,989204
0,811175	367	362,1643	0,417798	1	0,729573
-1,21903	280	271,7878	0,223889	1	0,654014
-2,65214	421	349,6009	0,008363	1	0,112451

-1,69163	421	191,7417	0,092341	1	0,373113
0,840876	420	388,0467	0,400935	1	0,95743
-1,86467	367	342,7001	0,063083	1	0,34994
-2,15499	280	261,4301	0,032076	1	0,329568
-1,39693	421	332,1548	0,163366	1	0,438064
-0,66862	421	184,61	0,504575	1	0,753145
0,63597	420	367,7207	0,525191	1	0,960096
0,316595	367	326,4676	0,751754	1	0,924056
1,157922	280	254,8011	0,24798	1	0,677438
0,494183	421	384,5589	0,621459	1	0,828413
0,262392	421	204,2007	0,793284	1	0,919599
0,037084	420	408,9146	0,970436	1	0,997342
0,907381	367	356,4008	0,364819	1	0,677906
-1,13647	280	265,8415	0,256781	1	0,680087
-1,75403	421	309,9246	0,080414	1	0,303719
-2,26289	421	179,6919	0,024839	1	0,218351
-0,03545	420	349,6466	0,971743	1	0,997342
0,169905	367	301,3502	0,865199	1	0,959636
0,368474	280	248,5892	0,712833	1	0,928432
2,714147	421	354,7978	0,006969	1	0,105025
1,452537	421	191,7772	0,147986	1	0,428784
-1,21191	420	385,582	0,226288	1	0,943055
0,684721	367	344,1366	0,493981	1	0,793601
0,172879	280	267,8989	0,862877	1	0,967527
1,016078	421	362,7644	0,310269	1	0,605668
1,3309	421	193,7298	0,184786	1	0,473593
-1,00811	420	394,5444	0,314018	1	0,943897
1,567514	367	348,6905	0,117902	1	0,445308
0,748935	280	271,7611	0,454544	1	0,814707
-0,72744	421	387,8656	0,467395	1	0,728402
-1,31611	421	205,3847	0,189603	1	0,476746
-0,17728	420	410,9483	0,859377	1	0,976988
1,498166	367	349,807	0,134992	1	0,477542
0,535972	280	268,4681	0,592421	1	0,876993
-1,87243	421	368,0659	0,06194	1	0,271708
-2,92195	421	196,5668	0,003886	1	0,118336
-1,23929	420	395,4533	0,215971	1	0,943055
-2,43606	367	346,6591	0,015351	1	0,204372
-1,03055	280	265,8875	0,30369	1	0,726446
-0,8981	421	314,5915	0,369819	1	0,661896
-0,12768	421	177,5032	0,898545	1	0,960359
0,386054	420	348,9735	0,699692	1	0,968866
0,787723	367	300,1543	0,43148	1	0,73975
0,892836	280	254,865	0,372787	1	0,763914
2,071528	421	375,7018	0,038992	1	0,222577
1,16732	421	200,7128	0,244466	1	0,54418
-0,27274	420	404,7535	0,785192	1	0,969427
1,499933	367	346,7505	0,134542	1	0,477542

0,229347	280	267,747	0,818774	1	0,95778
-0,58702	421	355,5486	0,557564	1	0,791195
1,134843	421	191,4729	0,25786	1	0,556025
0,818212	420	388,1265	0,413738	1	0,95743
-0,06821	367	335,5662	0,945663	1	0,979539
1,088593	280	271,5438	0,277299	1	0,701156
0,337818	421	359,0232	0,735698	1	0,893273
0,510615	421	192,8472	0,610205	1	0,82272
-0,2404	420	391,3205	0,810147	1	0,969427
-1,02657	367	350,962	0,305332	1	0,637666
-2,53213	280	262,1566	0,011922	1	0,239766
-2,27824	421	352,3344	0,02331	1	0,177419
-1,66332	421	192,555	0,097875	1	0,379251
-0,85795	420	384,1122	0,391455	1	0,95743
-1,03494	367	342,512	0,301426	1	0,633826
-0,76631	280	267,1718	0,444167	1	0,810362
-2,29288	421	346,8862	0,022453	1	0,177154
-2,22189	421	188,8651	0,027478	1	0,22628
-4,14205	420	381,1527	4,24E-05	0,045775	0,035635
0,448782	367	328,0538	0,653885	1	0,889355
0,38252	280	256,5007	0,702392	1	0,92124
0,698778	421	329,5283	0,485184	1	0,745445
-0,1327	421	183,9718	0,894579	1	0,958882
-1,8914	420	364,445	0,059364	1	0,848246
2,184174	367	307,5871	0,029704	1	0,260259
0,298507	280	257,5821	0,765557	1	0,945436
-2,08248	421	311,4314	0,038114	1	0,220722
0,46634	421	180,7495	0,641534	1	0,838647
0,127657	420	342,8673	0,898495	1	0,98503
-1,21367	367	288,5071	0,225867	1	0,576775
-0,46099	280	276	0,64517	1	0,907804
-0,63665	421	345,1906	0,524772	1	0,770244
-0,79767	421	189,1186	0,42606	1	0,694133
-0,184	420	379,5538	0,854114	1	0,973894
-0,15455	367	325,9635	0,87727	1	0,962453
2,120631	280	257,7218	0,034908	1	0,337221
-0,36554	421	380,7206	0,714911	1	0,881149
0,361491	421	202,0871	0,71811	1	0,874852
-1,79991	420	405,4388	0,072618	1	0,848246
0,976722	367	349,8028	0,329382	1	0,656585
0,135269	280	263,5934	0,892502	1	0,975243
0,734081	421	320,3989	0,463436	1	0,727964
0,509678	421	180,8014	0,610898	1	0,82272
-0,29565	420	359,3506	0,767664	1	0,969427
0,194709	367	319,3758	0,845745	1	0,956935
-0,45935	280	257,975	0,646366	1	0,907804
-0,74517	421	355,4466	0,456661	1	0,722128
0,746518	421	193,4883	0,456261	1	0,716981

0,927387	420	386,5321	0,354304	1	0,947373
-0,25338	367	342,7167	0,800126	1	0,950149
0,118571	280	255,1671	0,905709	1	0,975308
1,594365	421	401,4231	0,111641	1	0,358728
1,423244	421	217,054	0,156101	1	0,438625
0,380026	420	415,4158	0,70412	1	0,968866
1,346384	367	362,5874	0,17902	1	0,526202
0,088059	280	273,046	0,929894	1	0,979504
1,11571	421	325,9717	0,265368	1	0,560098
0,739333	421	180,4969	0,460665	1	0,721079
-1,35242	420	352,1151	0,177108	1	0,9199
1,439621	367	307,3909	0,150992	1	0,499998
1,338419	280	263,3115	0,181915	1	0,607861
-0,50953	421	346,5986	0,610708	1	0,825172
0,79251	421	190,1199	0,429051	1	0,694836
0,459433	420	381,8699	0,646185	1	0,964878
1,479507	367	337,57	0,139937	1	0,488078
-0,32012	280	263,1112	0,749134	1	0,942071
-1,99246	421	346,9233	0,047104	1	0,239816
-1,52922	421	190,2785	0,127869	1	0,410224
-0,69949	420	382,8631	0,484673	1	0,960096
-1,36691	367	330,7436	0,172582	1	0,522252
-0,39715	280	264,3599	0,691576	1	0,919522
-2,63715	421	331,1404	0,008755	1	0,113397
-1,87126	421	185,6709	0,062882	1	0,321231
-0,29101	420	367,0561	0,771205	1	0,969427
0,920181	367	328,717	0,358153	1	0,674813
-0,82416	280	261,2734	0,410602	1	0,788151
1,143492	421	394,0953	0,253528	1	0,546261
1,480288	421	210,8077	0,140289	1	0,423351
0,524664	420	413,3342	0,600098	1	0,964878
-1,00308	367	361,2801	0,316491	1	0,649799
0,222112	280	275,2924	0,824392	1	0,96121
0,981687	421	371,0562	0,326894	1	0,61749
0,172379	421	199,343	0,863315	1	0,947215
-0,00646	420	400,3528	0,994846	1	0,997342
-0,70487	367	345,0138	0,481366	1	0,785415
0,603958	280	264,9879	0,546388	1	0,851507
0,205692	421	299,4013	0,837171	1	0,93721
1,793954	421	174,5084	0,074552	1	0,344631
1,750046	420	330,4159	0,081039	1	0,848246
-0,80242	367	320,1358	0,422903	1	0,732546
-1,67875	280	254,4562	0,094428	1	0,474774
-0,82014	421	330,3024	0,412726	1	0,695339
-0,59191	421	185,6175	0,554629	1	0,778237
-1,10268	420	367,1121	0,270889	1	0,943897
-1,22952	367	325,4803	0,219765	1	0,572696
-1,28136	280	256,4836	0,201224	1	0,631761

0,064371	421	394,0978	0,948707	1	0,983289
0,68725	421	211,9926	0,492676	1	0,746275
1,423952	420	413,4498	0,155215	1	0,914005
-0,4208	367	358,7325	0,674151	1	0,898779
-0,7689	280	273,1425	0,442617	1	0,810247
-1,13292	421	385,8972	0,257951	1	0,550898
-0,86084	421	206,2284	0,390323	1	0,664652
-0,26663	420	408,5456	0,789892	1	0,969427
-0,48931	367	349,8927	0,624928	1	0,878514
-2,67518	280	266,5249	0,007931	1	0,199202
1,92449	421	401,0312	0,055	1	0,259829
2,997557	421	217,6185	0,003038	1	0,114447
0,356727	420	415,6213	0,721477	1	0,969427
2,254509	367	361,6155	0,024762	1	0,247087
1,238043	280	273,772	0,216761	1	0,650539
1,695075	421	371,3632	0,090899	1	0,32265
-0,92185	421	198,5972	0,357725	1	0,643758
-0,94438	420	400,3706	0,345543	1	0,947373
0,222736	367	342,2036	0,823874	1	0,953906
-1,93314	280	255,6907	0,054322	1	0,40654
0,918938	421	349,1898	0,358762	1	0,648267
1,755169	421	187,0995	0,080867	1	0,360416
0,321723	420	384,7728	0,747837	1	0,969427
2,691169	367	337,8622	0,007474	1	0,180403
1,652043	280	259,7634	0,099734	1	0,480557
0,332436	421	327,3152	0,739773	1	0,893273
1,421466	421	183,3112	0,15688	1	0,438625
-0,05386	420	362,5821	0,957077	1	0,996074
1,324826	367	314,6971	0,18619	1	0,535663
2,112732	280	257,923	0,035585	1	0,339407
1,85311	421	388,4864	0,064625	1	0,27589
2,013058	421	205,9498	0,04541	1	0,281076
1,208527	420	411,2248	0,227539	1	0,943055
2,299863	367	355,2335	0,022035	1	0,230605
1,035334	280	270,6263	0,301437	1	0,723617
2,134795	421	401,0287	0,033383	1	0,20962
1,941579	421	216,1203	0,053488	1	0,302776
0,606428	420	415,6551	0,544562	1	0,960096
2,402973	367	361,3296	0,016766	1	0,210552
0,336005	280	272,9266	0,737125	1	0,939009
0,589174	421	401,4582	0,556076	1	0,790572
-0,15414	421	215,4966	0,877643	1	0,954952
0,558898	420	415,2225	0,576533	1	0,964878
-1,18715	367	360,9753	0,235949	1	0,585627
-1,12168	280	271,3139	0,262992	1	0,688875
-3,38322	421	302,3081	8,11E-04	0,861102	0,053127
-1,9775	421	173,5462	0,049569	1	0,289197
-1,1503	420	337,0392	0,250834	1	0,943897

-1,90897	367	293,4957	0,057241	1	0,338985
-2,31782	280	251,4259	0,021263	1	0,30229
-0,21439	421	377,5464	0,830362	1	0,936611
0,180092	421	201,4922	0,857261	1	0,945992
0,717212	420	404,6395	0,473657	1	0,960096
-0,67373	367	344,5443	0,500935	1	0,793601
1,975203	280	263,1081	0,04929	1	0,39442
-0,50207	421	291,2411	0,615996	1	0,826526
-0,546	421	174,6541	0,585763	1	0,802496
-2,21804	420	315,5389	0,027264	1	0,767829
-0,47107	367	363	0,637872	1	0,880707
0,546597	280	276	0,585097	1	0,87561
-0,76291	421	345,9956	0,446037	1	0,714643
-1,2624	421	190,0914	0,208352	1	0,508069
-0,49219	420	382,0686	0,622866	1	0,964878
-0,6996	367	336,5119	0,484661	1	0,786044
-0,95178	280	261,9341	0,342084	1	0,75812
-1,03753	421	324,7844	0,300262	1	0,596172
-0,22584	421	183,9069	0,821573	1	0,929512
-0,16804	420	359,9738	0,866649	1	0,979775
1,771398	367	313,6095	0,077466	1	0,380263
-0,9461	280	262,0198	0,34497	1	0,758934
-1,8193	421	367,1458	0,069681	1	0,283808
-1,52072	421	198,1063	0,129924	1	0,410868
0,613731	420	400,4036	0,539742	1	0,960096
-0,79811	367	351,6991	0,425347	1	0,733464
1,455454	280	271,4612	0,146699	1	0,555466
-1,37575	421	307,9567	0,169898	1	0,442288
-0,1746	421	175,2748	0,861599	1	0,947215
-0,99132	420	340,4193	0,322233	1	0,945573
-0,96728	367	296,5628	0,334192	1	0,661796
0,193714	280	276	0,846542	1	0,965181
-0,01774	421	406,6098	0,985857	1	0,998043
-0,44963	421	219,199	0,653419	1	0,841107
0,578928	420	415,9553	0,562951	1	0,961052
1,253318	367	362,2971	0,210898	1	0,563436
1,30834	280	274,9114	0,191851	1	0,62543
-0,16423	421	366,223	0,869643	1	0,955213
0,923578	421	196,9014	0,356836	1	0,643758
-0,59951	420	396,3547	0,549176	1	0,960096
0,392269	367	340,3608	0,695105	1	0,904597
-0,4417	280	265,5092	0,659066	1	0,908644
-0,17217	421	336,603	0,863407	1	0,951261
-1,58942	421	187,868	0,113646	1	0,391524
-0,77753	420	372,3283	0,43734	1	0,960096
0,479009	367	331,1014	0,632249	1	0,880707
-1,27821	280	264,4032	0,202295	1	0,632429
-2,17828	421	320,8056	0,030113	1	0,200565

-0,76177	421	183,3768	0,447175	1	0,710837
-0,8012	420	356,4557	0,423548	1	0,95743
-0,32924	367	303,4355	0,742205	1	0,923669
-0,92381	280	254,4028	0,356458	1	0,761846
2,723148	421	307,9625	0,006835	1	0,10482
2,686584	421	177,5377	0,007904	1	0,148102
1,337537	420	343,7054	0,181932	1	0,923408
2,16665	367	290,4388	0,031075	1	0,266905
0,609109	280	251,7549	0,543001	1	0,849885
-3,32139	421	332,7553	9,95E-04	1	0,062491
-4,1564	421	189,0096	4,90E-05	0,052783	0,018447
-0,49448	420	383,0098	0,621254	1	0,964878
-2,52674	367	344,2422	0,01196	1	0,189546
-3,84027	280	262,4497	1,54E-04	0,165998	0,046455
-1,06946	421	340,8429	0,285621	1	0,583534
-0,81493	421	187,9591	0,416144	1	0,686888
0,845242	420	377,0386	0,398512	1	0,95743
-0,93113	367	336,2102	0,352454	1	0,67234
-1,87146	280	260,094	0,062405	1	0,422605
-2,95403	421	332,8081	0,00336	1	0,081668
-1,79019	421	184,9633	0,07506	1	0,345067
-0,82532	420	370,5713	0,409721	1	0,95743
-1,71287	367	327,8354	0,087681	1	0,394165
-1,3733	280	259,1397	0,170847	1	0,59737
-0,2034	421	329,5572	0,838949	1	0,93721
1,259562	421	185,753	0,209408	1	0,509818
0,416194	420	363,3091	0,677514	1	0,96781
2,370316	367	307,2135	0,01839	1	0,217108
-0,08746	280	251,854	0,930373	1	0,979504
2,416907	421	349,6098	0,016164	1	0,147897
1,874837	421	188,9497	0,062358	1	0,319638
2,238326	420	380,7107	0,025777	1	0,767829
1,594632	367	330,3913	0,111751	1	0,438296
1,736774	280	268,9747	0,083572	1	0,456738
-1,0886	421	329,7888	0,277125	1	0,57291
-0,41379	421	183,6144	0,679509	1	0,857638
-0,37903	420	363,1531	0,704887	1	0,968866
0,945766	367	319,8187	0,344982	1	0,667378
1,64937	280	257,1045	0,100293	1	0,480557
-3,12306	421	339,8107	0,001944	1	0,069718
-2,25939	421	188,0962	0,025006	1	0,218351
-0,73018	420	377,9418	0,465732	1	0,960096
-1,03139	367	336,4474	0,3031	1	0,636172
-0,13812	280	259,9025	0,890256	1	0,975243
2,013068	421	361,6752	0,044848	1	0,236315
1,527187	421	196,8188	0,12832	1	0,410224
2,041224	420	396,8918	0,041889	1	0,846132
-0,25039	367	355,6048	0,802434	1	0,950149

0,683814	280	275,5021	0,494668	1	0,8282
-2,87777	421	304,6496	0,004288	1	0,087964
-3,91382	421	178,5792	1,29E-04	0,138609	0,024311
-1,6688	420	342,5535	0,09607	1	0,848246
-0,44056	367	298,678	0,659849	1	0,893492
-1,72116	280	252,5533	0,086446	1	0,458713
-1,48566	421	347,8447	0,138274	1	0,410344
-1,56854	421	188,7222	0,11843	1	0,396436
-1,87394	420	378,2358	0,061709	1	0,848246
-0,80451	367	336,1424	0,42167	1	0,731532
-0,70231	280	256,0761	0,483124	1	0,824339
-1,21371	421	400,0734	0,225574	1	0,518147
-1,18403	421	217,1047	0,237696	1	0,537903
0,410251	420	415,9799	0,681833	1	0,968338
-1,01757	367	362,8528	0,309559	1	0,643456
0,107536	280	273,1645	0,914443	1	0,977051
1,867384	421	364,2545	0,062651	1	0,273465
1,926779	421	195,3661	0,055458	1	0,309036
1,093673	420	397,3763	0,274761	1	0,943897
1,78608	367	336,4938	0,074987	1	0,376019
0,411819	280	272,5694	0,680796	1	0,911154
-0,10295	421	323,4754	0,918068	1	0,966828
-0,32819	421	182,9434	0,743143	1	0,888118
-0,84312	420	358,2434	0,399727	1	0,95743
0,715112	367	309,6287	0,475079	1	0,7782
-0,86004	280	260,323	0,390556	1	0,778529
0,981659	421	333,6805	0,326979	1	0,61749
0,352343	421	185,6913	0,724981	1	0,878959
-2,2075	420	368,0317	0,027895	1	0,767829
3,026825	367	307,4008	0,00268	1	0,176492
0,784004	280	257,2645	0,433759	1	0,803161
1,668234	421	313,3276	0,096268	1	0,332744
3,179432	421	178,1614	0,00174	1	0,093671
1,276857	420	351,566	0,202495	1	0,928589
1,434997	367	309,411	0,152298	1	0,500093
2,267908	280	253,1815	0,024178	1	0,313396
2,003032	421	403,571	0,045842	1	0,237403
3,175973	421	219,9582	0,001708	1	0,093671
0,309673	420	415,9273	0,756965	1	0,969427
2,120593	367	362,0875	0,034635	1	0,280622
1,141304	280	274,7378	0,254737	1	0,679673
1,31989	421	373,107	0,187681	1	0,472953
0,875898	421	198,9616	0,382142	1	0,659083
1,006091	420	399,7217	0,31498	1	0,943897
-2,10128	367	346,4253	0,036338	1	0,28464
0,164413	280	255,6848	0,869536	1	0,969656
0,32362	421	352,9531	0,746417	1	0,897726
0,735113	421	190,4565	0,463175	1	0,721079

-0,49742	420	388,0439	0,619174	1	0,964878
1,549112	367	349,359	0,12226	1	0,456054
-0,55391	280	269,5249	0,580101	1	0,874212
-1,61574	421	354,1206	0,107041	1	0,352976
-2,0481	421	191,3557	0,041916	1	0,273775
0,591525	420	389,1596	0,554512	1	0,960096
0,515412	367	345,9292	0,606594	1	0,868127
0,070468	280	269,757	0,943873	1	0,982175
-0,92671	421	357,1822	0,354704	1	0,644799
0,408918	421	193,2975	0,683052	1	0,859821
-0,51164	420	389,3511	0,609195	1	0,964878
-1,3871	367	343,6699	0,16631	1	0,513583
-1,8624	280	257,5908	0,063685	1	0,422605
0,871978	421	377,5489	0,383775	1	0,674066
-0,13263	421	198,9619	0,894617	1	0,958882
-0,23195	420	403,9464	0,816691	1	0,969427
0,314488	367	352,0458	0,753337	1	0,924494
-1,19544	280	258,0438	0,233011	1	0,659852
-1,46484	421	359,2408	0,143838	1	0,412154
-2,67068	421	194,8003	0,00821	1	0,148102
-1,58076	420	390,9006	0,114741	1	0,848246
-0,8798	367	339,8245	0,379589	1	0,691076
-0,62361	280	263,0257	0,533426	1	0,84326
0,173685	421	324,422	0,862221	1	0,951261
-0,71251	421	180,0148	0,477071	1	0,734183
0,818694	420	360,4515	0,413502	1	0,95743
0,533762	367	312,5857	0,593885	1	0,858088
0,704413	280	255,4924	0,481817	1	0,824339
-0,41919	421	380,5626	0,675315	1	0,859543
0,674045	421	203,7788	0,501047	1	0,750551
-2,02518	420	403,3258	0,043507	1	0,846132
-0,19466	367	356,3754	0,845771	1	0,956935
0,249228	280	273,3318	0,803371	1	0,95706
-2,62279	421	373,3134	0,009079	1	0,113397
0,182014	421	200,7633	0,855755	1	0,945471
0,382598	420	404,4702	0,702219	1	0,968866
-0,45877	367	355,5084	0,646676	1	0,884339
-0,43934	280	275,9994	0,660762	1	0,908644
-1,2988	421	308,7635	0,194983	1	0,481703
0,145994	421	180,196	0,884089	1	0,957128
0,284489	420	345,1151	0,776206	1	0,969427
0,670807	367	306,6172	0,502848	1	0,793601
0,73254	280	249,8626	0,464525	1	0,824256
-0,26326	421	313,2854	0,79252	1	0,919136
0,319732	421	177,5624	0,749547	1	0,890124
-0,62688	420	347,1274	0,531153	1	0,960096
-0,38544	367	320,0758	0,700169	1	0,906491
-1,36617	280	251,1344	0,173108	1	0,598625

1,016078	421	362,7644	0,310269	1	0,605668
1,3309	421	193,7298	0,184786	1	0,473593
-1,00811	420	394,5444	0,314018	1	0,943897
1,567514	367	348,6905	0,117902	1	0,445308
0,748935	280	271,7611	0,454544	1	0,814707
-1,12899	421	343,6453	0,25969	1	0,551979
-2,24069	421	189,1831	0,026209	1	0,222284
-2,00673	420	377,9106	0,045491	1	0,846132
-0,92054	367	320,6143	0,357981	1	0,674813
-1,57311	280	255,1481	0,116933	1	0,518958
-1,64051	421	379,3701	0,101728	1	0,342962
-1,92314	421	204,3405	0,055852	1	0,309036
0,294264	420	409,3468	0,768706	1	0,969427
-1,1468	367	357,0693	0,252231	1	0,607208
0,964037	280	272,1374	0,335883	1	0,755731
-0,02365	421	285,2787	0,981148	1	0,997025
1,387123	421	172,3926	0,167195	1	0,453987
-0,45099	420	313,291	0,652306	1	0,966867
3,094708	367	288,36	0,002164	1	0,176492
1,306846	280	276	0,192353	1	0,62543
1,788736	421	393,7764	0,074426	1	0,294383
2,896915	421	206,8525	0,004174	1	0,120964
1,031289	420	413,0626	0,303009	1	0,943897
-0,23245	367	359,9392	0,816323	1	0,95095
-0,10053	280	274,351	0,919997	1	0,977051
-1,17346	421	332,1243	0,241454	1	0,538768
0,454509	421	185,5693	0,649994	1	0,839358
-0,10267	420	364,5006	0,918283	1	0,989204
-0,01682	367	313,0363	0,986594	1	0,996248
-1,75748	280	257,0206	0,080027	1	0,453386
-1,04324	421	370,3571	0,297518	1	0,59543
-0,6654	421	198,9601	0,506566	1	0,754351
-0,23789	420	398,9712	0,812086	1	0,969427
0,09812	367	350,3663	0,921893	1	0,975687
-0,11806	280	267,8253	0,906108	1	0,975308
-2,09119	421	371,2105	0,037191	1	0,217944
-1,33208	421	198,3502	0,184364	1	0,473593
0,347981	420	401,5561	0,728037	1	0,969427
-1,35228	367	349,5394	0,17716	1	0,523131
-0,03899	280	263,5582	0,968931	1	0,991502
0,083435	421	340,2112	0,933554	1	0,972684
0,685731	421	187,326	0,493731	1	0,746291
0,665221	420	376,1094	0,506317	1	0,960096
2,034271	367	324,6208	0,042737	1	0,295433
0,966063	280	265,2183	0,334893	1	0,755731
-1,75712	421	315,3212	0,079868	1	0,302838
0,602585	421	175,6794	0,547562	1	0,776424
-0,34333	420	347,7171	0,731557	1	0,969427

-1,11172	367	309,8666	0,267121	1	0,609149
-2,87228	280	267,7646	0,004401	0,669004	0,161776
-0,18493	421	282,2774	0,853415	1	0,946355
-0,09299	421	173,6144	0,926021	1	0,972483
-0,65657	420	308,8926	0,511949	1	0,960096
0,705087	367	363	0,481208	1	0,785415
-0,44499	280	276	0,656676	1	0,908644
-0,61777	421	383,2461	0,537096	1	0,778195
0,431454	421	203,1355	0,666596	1	0,84773
-0,99382	420	407,6765	0,320898	1	0,945573
0,781729	367	347,7221	0,434906	1	0,742246
1,285723	280	267,4258	0,199652	1	0,631529
0,574883	421	349,9134	0,56574	1	0,796637
-0,6562	421	189,7115	0,512488	1	0,756803
0,49694	420	383,4588	0,619516	1	0,964878
-0,78262	367	332,1749	0,434409	1	0,742238
1,171262	280	252,5193	0,242597	1	0,670815
0,469317	421	347,9504	0,639137	1	0,834842
1,403482	421	188,4297	0,162119	1	0,445827
1,355623	420	382,5092	0,176019	1	0,9199
0,913122	367	326,8698	0,361851	1	0,677287
0,001865	280	258,6067	0,998514	1	0,998514
-2,89986	421	347,1431	0,003971	1	0,087964
-0,63795	421	189,0895	0,524278	1	0,766525
0,140335	420	386,971	0,888468	1	0,983483
-1,38048	367	331,9461	0,168369	1	0,514971
-2,39417	280	266,3586	0,01735	1	0,26828
-1,42702	421	326,6326	0,154531	1	0,426516
-0,6476	421	182,5051	0,518061	1	0,761675
-0,23179	420	365,2731	0,816832	1	0,969427
0,806849	367	309,8468	0,420372	1	0,731532
-0,54321	280	257,7876	0,587454	1	0,87561
-0,98441	421	300,5785	0,325708	1	0,61749
0,39549	421	174,7822	0,692963	1	0,865526
-0,60767	420	328,1797	0,543826	1	0,960096
1,666609	367	300,1084	0,096635	1	0,412696
1,390275	280	276	0,165566	1	0,588461
2,620137	421	406,6027	0,009119	1	0,113397
2,515746	421	223,6889	0,01258	1	0,169272
1,64592	420	415,9667	0,100536	1	0,848246
2,589374	367	362,6008	0,010002	1	0,183886
1,311282	280	275,9407	0,190852	1	0,625249
-1,9857	421	397,731	0,047753	1	0,241202
-1,36984	421	213,3285	0,172178	1	0,456365
-0,37575	420	414,6632	0,707293	1	0,968866
-0,27417	367	362,0687	0,784114	1	0,94337
0,105998	280	272,9487	0,915662	1	0,977051
-1,06429	421	334,9518	0,287963	1	0,584852

-0,17493	421	185,6366	0,861328	1	0,947215
1,768217	420	371,5872	0,077845	1	0,848246
0,984812	367	338,3289	0,32542	1	0,655597
0,917145	280	262,0799	0,35991	1	0,761846
-2,06019	421	417	0,040001	1	0,224527
-0,26416	421	417	0,791788	1	0,918572
-0,19073	420	416	0,848832	1	0,973894
-0,86237	367	363	0,389055	1	0,703005
0,89801	280	276	0,369963	1	0,763914
-2,88065	421	278,2977	0,004277	1	0,087964
-1,7168	421	171,422	0,087821	1	0,368676
-1,59009	420	313,4189	0,112822	1	0,848246
-2,59579	367	285,5201	0,009925	1	0,183886
-0,20229	280	276	0,839842	1	0,964667
-0,57573	421	387,959	0,565134	1	0,796637
-0,90984	421	207,1596	0,363962	1	0,646044
0,660586	420	410,8894	0,509248	1	0,960096
-1,96121	367	361,6017	0,050622	1	0,317862
-1,71765	280	271,7894	0,087	1	0,460033
0,01153	421	341,4078	0,990807	1	0,998054
0,306238	421	186,2987	0,759765	1	0,899424
0,324188	420	375,4016	0,745976	1	0,969427
0,549386	367	318,1933	0,583126	1	0,845786
2,000122	280	249,9399	0,046569	1	0,387749
-2,05385	421	293,9954	0,040875	1	0,226465
0,390555	421	177,1656	0,696596	1	0,865526
-0,38242	420	327,5654	0,702399	1	0,968866
-0,05966	367	304,1177	0,952465	1	0,979771
0,425343	280	276	0,670918	1	0,908644
0,018438	421	397,5987	0,985298	1	0,998043
-0,6549	421	212,5027	0,51324	1	0,756803
-1,74948	420	414,5734	0,080948	1	0,848246
-1,12484	367	359,0384	0,261406	1	0,609149
-1,07817	280	274,7254	0,281905	1	0,70453
-1,23359	421	385,9616	0,218107	1	0,511974
-0,33887	421	204,2532	0,735054	1	0,884613
1,18102	420	410,3779	0,238279	1	0,943055
-1,76301	367	355,2118	0,078759	1	0,381638
-0,45445	280	268,7791	0,649875	1	0,908644
-1,20582	421	317,3667	0,228784	1	0,520357
-0,08238	421	182,1247	0,934435	1	0,975655
0,188053	420	354,853	0,850942	1	0,973894
-0,56967	367	315,3903	0,569307	1	0,837838
-0,38834	280	250,9889	0,698094	1	0,92034
-1,83182	421	349,2376	0,067829	1	0,280051
-2,23371	421	190,282	0,026667	1	0,223261
0,2058	420	386,5606	0,837056	1	0,973894
-2,76018	367	335,3899	0,006094	1	0,178183

-1,23371	280	261,245	0,218418	1	0,650539
1,758154	421	394,9928	0,079496	1	0,302838
1,346257	421	211,9613	0,179658	1	0,469227
-0,32845	420	414,0429	0,742738	1	0,969427
1,504746	367	360,4147	0,133265	1	0,477542
1,447158	280	274,4071	0,148994	1	0,561358
0,008293	421	373,9399	0,993387	1	0,998054
1,766687	421	199,1463	0,078813	1	0,355599
1,009037	420	401,9757	0,313564	1	0,943897
1,09748	367	346,7496	0,273193	1	0,609149
1,010744	280	263,9966	0,313064	1	0,733729
0,757776	421	334,4863	0,449118	1	0,714643
0,797855	421	186,7079	0,425968	1	0,694133
1,115715	420	364,2158	0,26528	1	0,943897
0,617399	367	330,0824	0,537397	1	0,818865
-0,74961	280	257,6896	0,454171	1	0,814707
0,864428	421	378,7754	0,3879	1	0,677364
1,340938	421	201,764	0,181448	1	0,470641
2,037659	420	404,1736	0,042235	1	0,846132
2,317307	367	346,2531	0,02107	1	0,225192
0,946964	280	268,9526	0,344507	1	0,758934
-3,275	421	343,1078	0,001164	1	0,064989
-1,72543	421	187,8591	0,086094	1	0,365475
0,536854	420	382,6113	0,591681	1	0,964878
0,026439	367	321,3857	0,978923	1	0,99209
-0,1147	280	259,8847	0,90877	1	0,976008
-0,02373	421	335,9261	0,981084	1	0,997025
1,133969	421	185,2216	0,258273	1	0,556025
0,427416	420	370,3629	0,669325	1	0,96781
-1,2629	367	324,4849	0,207532	1	0,560883
3,128716	280	255,764	0,001959	1	0,135787
2,985205	421	358,103	0,003029	1	0,077359
1,480102	421	194,7995	0,140461	1	0,423351
0,232294	420	393,4951	0,816431	1	0,969427
2,606769	367	346,2388	0,009535	1	0,183886
1,695005	280	271,055	0,091223	1	0,468147
-0,80645	421	353,8044	0,420525	1	0,697735
-0,65792	421	192,7128	0,511376	1	0,756803
-0,86381	420	388,9131	0,388227	1	0,95743
-2,61577	367	340,7399	0,009299	0,883377	0,183886
-1,5411	280	262,9409	0,124494	1	0,535729
2,303236	421	379,4055	0,021806	1	0,175251
3,001541	421	202,3938	0,003024	0,444555	0,114447
0,629418	420	407,3718	0,529428	1	0,960096
2,362603	367	354,2021	0,018687	1	0,217108
1,109279	280	267,9047	0,268304	1	0,693794
1,962421	421	291,2412	0,050665	1	0,248706
1,624413	421	172,473	0,106114	1	0,385334

-0,71605	420	320,134	0,474482	1	0,960096
0,371198	367	288,5893	0,710762	1	0,912367
0,125525	280	245,7077	0,90021	1	0,975243
0,757032	421	336,9901	0,44956	1	0,714643
1,156455	421	188,0579	0,248962	1	0,548314
0,510793	420	370,9503	0,6098	1	0,964878
0,401837	367	319,5815	0,688073	1	0,903032
0,487777	280	256,7452	0,626124	1	0,899494
-0,30638	421	323,6296	0,759515	1	0,901251
-1,10511	421	184,9624	0,270548	1	0,569587
0,555228	420	360,8503	0,579083	1	0,964878
1,111962	367	307,1866	0,267024	1	0,609149
0,686729	280	251,6479	0,492886	1	0,827962
-2,09089	421	350,339	0,037258	1	0,217944
-2,41678	421	192,0827	0,016594	1	0,181207
-0,54809	420	388,1487	0,583944	1	0,964878
-2,5156	367	338,7137	0,012345	1	0,189546
-1,76644	280	264,4217	0,078476	1	0,453386
3,531914	421	373,2717	4,64E-04	0,495334	0,04642
1,902644	421	201,9585	0,05851	1	0,314262
-0,43644	420	406,1375	0,66275	1	0,96781
2,674924	367	352,9248	0,007823	1	0,180403
0,786215	280	273,0843	0,432423	1	0,803161
-2,11135	421	312,7291	0,035535	1	0,215166
-2,73103	421	180,7543	0,006939	1	0,141161
0,176127	420	355,1658	0,860294	1	0,976988
-0,08223	367	324,0058	0,934517	1	0,977998
-1,51246	280	257,3867	0,131643	1	0,540563
-0,85888	421	354,9827	0,390987	1	0,68039
1,227356	421	192,741	0,221186	1	0,516786
-0,58145	420	387,7802	0,561277	1	0,960096
1,666734	367	330,4253	0,096515	1	0,412696
-0,73101	280	259,8915	0,465434	1	0,824256
-1,18273	421	304,1407	0,237839	1	0,532577
1,468736	421	178,6605	0,143663	1	0,42451
0,714124	420	335,1542	0,475647	1	0,960096
0,529207	367	290,4182	0,597066	1	0,860209
-1,25045	280	276	0,212193	1	0,644706
-2,28216	421	326,9886	0,023122	1	0,177419
-1,12642	421	177,4842	0,261507	1	0,557979
-0,38665	420	360,9378	0,699244	1	0,968866
-1,11516	367	317,3483	0,265625	1	0,609149
-3,72258	280	256,3634	2,42E-04	0,260853	0,057407
-0,09753	421	389,3311	0,922355	1	0,966828
0,448468	421	207,5758	0,654284	1	0,841107
-0,15877	420	411,0151	0,873927	1	0,982463
0,206334	367	357,5965	0,836648	1	0,956935
-0,0326	280	268,4728	0,974021	1	0,99236

1,038949	421	306,8549	0,299646	1	0,596172
-0,91851	421	177,5783	0,359598	1	0,643758
0,388251	420	340,0944	0,698073	1	0,968866
0,75161	367	298,6131	0,452877	1	0,758154
1,355774	280	255,1447	0,17637	1	0,598625
-0,48389	421	353,1639	0,628767	1	0,833119
-0,23451	421	192,2805	0,814837	1	0,926063
-0,58282	420	386,0532	0,560358	1	0,960096
0,32678	367	340,9857	0,744034	1	0,923669
2,226245	280	265,636	0,026837	1	0,324814
0,677848	421	354,8659	0,49831	1	0,751705
0,670639	421	191,6469	0,503258	1	0,75239
0,961684	420	387,6559	0,336808	1	0,947373
0,936971	367	336,1274	0,349446	1	0,668671
0,300908	280	264,046	0,763722	1	0,944296
1,203316	421	371,6277	0,22962	1	0,520357
2,247047	421	199,6042	0,025732	1	0,221586
0,149857	420	402,8782	0,880952	1	0,982463
3,78924	367	351,1683	1,78E-04	0,191099	0,053579
1,234639	280	269,4521	0,21804	1	0,650539
-1,13214	421	310,4769	0,258451	1	0,550898
-0,74836	421	177,22	0,455233	1	0,716467
-2,33488	420	340,5717	0,02013	1	0,75904
0,149859	367	309,2791	0,880973	1	0,962453
-0,7134	280	250,3957	0,476263	1	0,824256
2,617844	421	405,3053	0,00918	1	0,113397
2,65837	421	222,397	0,008422	1	0,148102
1,563141	420	415,9996	0,11878	1	0,858144
2,767067	367	362,6869	0,005946	0,891928	0,178183
1,14996	280	275,9482	0,251156	1	0,679007
-1,04051	421	373,0368	0,298776	1	0,596172
-0,23236	421	198,2694	0,816501	1	0,926377
0,390982	420	402,46	0,696018	1	0,968866
0,870296	367	350,714	0,384734	1	0,696868
1,957127	280	265,5605	0,05138	1	0,398826
-1,00855	421	367,5238	0,313856	1	0,608538
-1,61982	421	196,0447	0,106878	1	0,386957
0,993852	420	400,7841	0,320895	1	0,945573
0,747935	367	347,6894	0,455005	1	0,758509
1,233509	280	268,5619	0,218465	1	0,650539
0,049739	421	312,7677	0,960362	1	0,989922
-1,02559	421	177,6542	0,306479	1	0,603743
-0,53607	420	348,645	0,59225	1	0,964878
-0,16118	367	282,3824	0,87207	1	0,959978
-1,6983	280	263,8357	0,09063	1	0,468147
-0,10912	421	373,6465	0,913166	1	0,966828
-1,79379	421	199,9963	0,074357	1	0,344631
-0,18884	420	402,6104	0,850316	1	0,973894

-0,99528	367	340,3847	0,320307	1	0,653184
-0,38496	280	264,2318	0,700575	1	0,92034
1,26112	421	408,0635	0,207986	1	0,496728
0,767279	421	223,5844	0,443725	1	0,708362
0,521315	420	415,9492	0,602425	1	0,964878
1,012493	367	362,9897	0,311977	1	0,646834
1,380274	280	275,8943	0,168619	1	0,59271
0,124398	421	316,9992	0,901079	1	0,965517
0,364298	421	180,7908	0,716061	1	0,874476
1,938504	420	347,7671	0,053371	1	0,846627
0,168565	367	303,9793	0,866251	1	0,959636
-0,20242	280	276	0,839735	1	0,964667
0,573957	421	311,4499	0,566411	1	0,796637
0,390447	421	178,7275	0,696671	1	0,865526
0,37345	420	345,1683	0,709043	1	0,968866
0,423193	367	306,1815	0,672451	1	0,897595
1,571049	280	251,4695	0,117429	1	0,518958
-0,673	421	328,4477	0,501422	1	0,754888
1,155156	421	181,7753	0,249543	1	0,548314
0,031416	420	364,3497	0,974955	1	0,997342
1,999807	367	320,429	0,046365	1	0,307806
1,869604	280	253,1978	0,062693	1	0,422605
-1,71038	421	316,1281	0,088177	1	0,3179
0,343736	421	180,2503	0,731445	1	0,882616
0,705051	420	348,4586	0,481249	1	0,960096
1,455224	367	313,658	0,146607	1	0,498729
1,326916	280	264,472	0,185681	1	0,617707
1,984295	421	340,6618	0,048025	1	0,241202
1,041909	421	186,1531	0,298805	1	0,597214
2,110098	420	371,4043	0,035518	1	0,828713
2,858008	367	325,2263	0,004538	1	0,178183
-0,47363	280	257,8011	0,636161	1	0,906996
0,771626	421	408,64	0,440782	1	0,710437
0,917003	421	225,5502	0,360121	1	0,643774
-0,08109	420	415,9306	0,935411	1	0,992059
-0,94381	367	362,8344	0,345897	1	0,66829
-0,54352	280	272,7383	0,587215	1	0,87561
-1,45058	421	327,7656	0,147853	1	0,419613
-1,07037	421	185,0551	0,285846	1	0,584263
0,742937	420	362,8403	0,458001	1	0,960096
-0,2452	367	333,6156	0,80645	1	0,950149
-2,49877	280	261,7576	0,013076	1	0,246323
-0,85129	421	339,5798	0,39521	1	0,686065
0,081831	421	188,2587	0,934868	1	0,975655
-0,35668	420	375,7325	0,721535	1	0,969427
0,413918	367	328,1653	0,679205	1	0,899694
0,839407	280	259,8894	0,402012	1	0,784757
-0,47492	421	351,2221	0,635138	1	0,83455

-0,34673	421	188,8305	0,729181	1	0,882616
0,322295	420	384,7745	0,747404	1	0,969427
-0,23562	367	331,0932	0,813877	1	0,95095
-0,35913	280	271,1695	0,719774	1	0,932673
-1,00271	421	383,6152	0,316633	1	0,610186
-2,02501	421	206,545	0,044153	1	0,277922
0,090936	420	410,8395	0,927588	1	0,990001
-0,70085	367	358,0886	0,48385	1	0,786044
-1,36127	280	266,5857	0,174579	1	0,598625
-1,68997	421	314,4926	0,092024	1	0,325541
0,194797	421	178,9155	0,845773	1	0,943293
-0,28862	420	346,7018	0,773042	1	0,969427
0,113847	367	293,4507	0,909437	1	0,970624
-0,42085	280	250,1124	0,674229	1	0,909385
0,27088	421	340,0564	0,786647	1	0,917552
0,414018	421	187,1087	0,679335	1	0,857638
0,814868	420	374,6764	0,415666	1	0,95743
0,17083	367	332,5113	0,864462	1	0,959636
-0,66029	280	264,5588	0,509642	1	0,831912
2,124161	421	397,9495	0,034273	1	0,212548
3,649374	421	213,6998	3,30E-04	0,353916	0,038096
1,268893	420	415,5606	0,205189	1	0,928589
2,600003	367	362,6563	0,009703	1	0,183886
2,608654	280	274,1512	0,009589	1	0,226737
1,685779	421	312,862	0,092835	1	0,327642
0,97256	421	180,2151	0,332076	1	0,626331
-1,58427	420	341,6218	0,114057	1	0,848246
2,082992	367	305,4559	0,038084	1	0,28956
2,324614	280	251,5674	0,020889	1	0,30229
0,765341	421	393,0499	0,444528	1	0,713799
1,334679	421	209,0897	0,183433	1	0,473593
-0,01212	420	413,0092	0,990339	1	0,997342
-1,26833	367	360,2167	0,205501	1	0,558434
-3,31201	280	270,669	0,001052	1	0,121989
-0,81484	421	314,1524	0,415784	1	0,695339
0,63385	421	177,9319	0,526992	1	0,767321
0,269746	420	348,6396	0,787515	1	0,969427
1,623386	367	301,7135	0,105551	1	0,429907
1,523149	280	255,1368	0,12896	1	0,536498
0,552896	421	335,5372	0,580703	1	0,805078
0,681882	421	186,5567	0,496159	1	0,747712
1,307386	420	365,8961	0,191903	1	0,925729
-0,85676	367	332,1748	0,392193	1	0,705088
-1,64569	280	249,6176	0,101086	1	0,480557
0,074991	421	372,3887	0,940262	1	0,97655
-0,62532	421	198,3139	0,532482	1	0,769894
0,811393	420	401,8454	0,41762	1	0,95743
-0,43821	367	350,3945	0,661503	1	0,894303

-1,18964	280	270,5414	0,235232	1	0,659852
1,423561	421	392,7209	0,155367	1	0,428042
1,360592	421	211,9163	0,175088	1	0,459682
0,106825	420	413,5082	0,914979	1	0,988069
-0,31941	367	359,3819	0,7496	1	0,923669
0,146671	280	272,4257	0,8835	1	0,973784
-0,7842	421	409,6316	0,433376	1	0,705289
0,463003	421	225,787	0,643808	1	0,838647
-1,14463	420	415,7317	0,253021	1	0,943897
-0,59163	367	361,1787	0,554468	1	0,830257
-0,43595	280	275,9257	0,663214	1	0,908644
-0,64648	421	374,482	0,518366	1	0,768119
-0,69859	421	201,5468	0,485616	1	0,740711
-0,01052	420	403,8725	0,991608	1	0,997342
0,494058	367	353,3777	0,621572	1	0,875495
0,502187	280	262,8239	0,615956	1	0,889977
-0,73971	421	308,5217	0,460036	1	0,724425
1,988153	421	177,5532	0,048331	1	0,286754
-0,43192	420	340,2697	0,666072	1	0,96781
0,11815	367	304,0849	0,906027	1	0,970486
-0,54513	280	253,6061	0,58614	1	0,87561
-1,53786	421	325,144	0,125054	1	0,38857
-2,43332	421	183,8519	0,015918	1	0,179127
-0,01365	420	365,1663	0,989113	1	0,997342
-1,03797	367	309,4796	0,300094	1	0,633391
-0,77048	280	247,892	0,441751	1	0,810247
-0,50761	421	344,6782	0,61205	1	0,825172
-1,52088	421	185,7719	0,129989	1	0,410868
-0,74749	420	374,3751	0,455236	1	0,960096
-0,33276	367	322,751	0,739535	1	0,923669
-0,02607	280	258,9337	0,979225	1	0,99236
-0,82023	421	400,2724	0,412571	1	0,695339
0,062477	421	212,5393	0,950242	1	0,980953
-1,11556	420	415,4417	0,265255	1	0,943897
-0,37257	367	361,3899	0,709683	1	0,912021
-2,42401	280	271,5589	0,016004	1	0,266517
0,198019	421	374,1179	0,843138	1	0,939735
0,332002	421	199,2335	0,740237	1	0,886754
-0,06338	420	402,7728	0,949494	1	0,992811
2,067488	367	350,6706	0,039421	1	0,28956
0,232376	280	268,6849	0,816423	1	0,95747
-0,26146	421	349,3311	0,793889	1	0,919136
1,17113	421	188,9668	0,243021	1	0,542422
1,285755	420	383,1674	0,199305	1	0,928589
1,324483	367	331,1853	0,186256	1	0,535663
-0,84247	280	258,6771	0,400302	1	0,784757
0,107756	421	331,0368	0,914255	1	0,966828
-0,20533	421	183,6212	0,837541	1	0,938419

-1,37147	420	363,6153	0,171073	1	0,9199
1,899866	367	310,5056	0,058377	1	0,339097
0,963557	280	252,4494	0,33619	1	0,755731
-2,1695	421	336,9031	0,030744	1	0,200565
-1,24482	421	187,2978	0,214754	1	0,514565
0,800207	420	369,7844	0,424105	1	0,95743
-0,45367	367	329,6274	0,650365	1	0,887772
-1,24136	280	262,1244	0,215584	1	0,648472
1,142115	421	393,6404	0,2541	1	0,546261
2,986443	421	210,763	0,003157	1	0,116024
2,393397	420	413,5717	0,017138	1	0,75904
0,919739	367	361,977	0,358322	1	0,674813
0,654008	280	273,399	0,513657	1	0,831912
-0,00127	421	358,9014	0,998989	1	0,999298
1,104849	421	190,3343	0,27062	1	0,569587
0,71429	420	388,7824	0,475477	1	0,960096
-0,9811	367	350,6026	0,32722	1	0,655788
-1,93023	280	266,0587	0,054641	1	0,40654
-2,36273	421	341,5615	0,018701	1	0,159223
-0,82141	421	188,722	0,412449	1	0,684933
-0,47922	420	377,4115	0,632062	1	0,964878
-1,08821	367	321,5757	0,277317	1	0,611884
-0,70787	280	254,6096	0,479673	1	0,824256
3,095249	421	391,3564	0,002108	0,320402	0,069718
4,282465	421	209,8918	2,81E-05	0,004303	0,015907
0,902333	420	413,7445	0,367405	1	0,95743
3,120889	367	360,7462	0,001948	0,298109	0,176492
1,561356	280	275,0465	0,11959	1	0,5239
0,381113	421	326,362	0,703368	1	0,874567
0,142806	421	183,1809	0,8866	1	0,957782
2,485227	420	358,8325	0,013402	1	0,69643
-0,57102	367	324,2271	0,568384	1	0,837838
0,849233	280	258,9782	0,396536	1	0,782458
-2,77049	421	319,611	0,005925	1	0,09705
-0,54624	421	183,2975	0,585568	1	0,802496
-1,0603	420	364,0426	0,28971	1	0,943897
-1,22625	367	310,4442	0,221033	1	0,573978
0,524271	280	254,0357	0,600547	1	0,881231
0,435114	421	398,5157	0,663716	1	0,852478
0,861007	421	214,511	0,390196	1	0,664652
-0,38998	420	415,1436	0,696753	1	0,968866
0,784719	367	362,8528	0,43313	1	0,741735
1,482501	280	275,7997	0,139349	1	0,552627
2,399886	421	369,3532	0,016895	1	0,152456
2,520281	421	197,3595	0,012518	1	0,169272
0,627125	420	401,344	0,530934	1	0,960096
2,691347	367	353,7537	0,007454	1	0,180403
0,080379	280	270,8129	0,935995	1	0,980226

-0,28951	421	359,377	0,772356	1	0,90985
-0,33789	421	193,6585	0,735811	1	0,884613
0,598547	420	388,145	0,549824	1	0,960096
1,447284	367	340,8629	0,148737	1	0,499998
-0,2093	280	263,4609	0,834379	1	0,964667
-2,26445	421	310,3259	0,024236	1	0,181711
-1,555	421	179,4424	0,121707	1	0,402221
0,803883	420	352,2882	0,422007	1	0,95743
-0,6997	367	306,2249	0,484644	1	0,786044
0,656965	280	261,0067	0,511782	1	0,831912
-2,79114	421	329,1384	0,005559	1	0,09373
-0,7866	421	185,5281	0,432517	1	0,69936
-0,03043	420	369,3743	0,975743	1	0,997342
-0,72257	367	326,2228	0,470459	1	0,775692
0,803332	280	261,0983	0,422514	1	0,796907
0,015129	421	386,0305	0,987937	1	0,998054
1,591394	421	204,5589	0,113065	1	0,391524
-0,1837	420	409,5978	0,854338	1	0,973894
0,697874	367	352,4001	0,485716	1	0,786224
0,561497	280	273,3581	0,574919	1	0,870337
-1,60553	421	390,0292	0,109186	1	0,355682
-1,34509	421	209,2436	0,180052	1	0,469443
0,89944	420	412,6017	0,368943	1	0,95743
-0,21388	367	360,5264	0,830761	1	0,956843
0,023815	280	270,9258	0,981017	1	0,99236
-0,532	421	349,812	0,595062	1	0,813756
-0,37451	421	190,5176	0,708438	1	0,870812
-1,01203	420	380,3103	0,312165	1	0,943897
-1,30148	367	341,646	0,19397	1	0,547402
-0,92266	280	264,1439	0,357026	1	0,761846
1,11807	421	409,3852	0,264193	1	0,558399
1,916457	421	224,0978	0,056579	1	0,311185
0,316079	420	415,8333	0,752101	1	0,969427
-1,23521	367	361,4998	0,217555	1	0,569492
-0,58089	280	275,8305	0,561787	1	0,861728
3,163233	421	359,8592	0,001693	1	0,069718
1,616235	421	194,0476	0,107668	1	0,388172
-1,00534	420	393,1771	0,315352	1	0,943897
2,112594	367	347,6468	0,035348	1	0,283349
1,995314	280	270,219	0,047013	1	0,389275
-0,13456	421	324,58	0,893044	1	0,962
-0,42439	421	184,8218	0,671772	1	0,852155
0,109303	420	358,2178	0,913023	1	0,987181
-0,41011	367	316,612	0,682002	1	0,900574
0,30755	280	250,7635	0,75868	1	0,94273
-0,54703	421	366,3515	0,584691	1	0,807036
-1,98186	421	196,5041	0,048889	1	0,286808
-0,69387	420	398,4784	0,488168	1	0,960096

-0,1239	367	341,1398	0,901465	1	0,970486
-1,21794	280	265,0216	0,224331	1	0,654014
-1,45849	421	288,8507	0,145792	1	0,414711
-0,06235	421	173,8982	0,950359	1	0,980953
0,803342	420	317,0324	0,422379	1	0,95743
-2,36393	367	295,761	0,018729	1	0,217108
0,100611	280	248,7142	0,91994	1	0,977051
-2,48624	421	317,1169	0,013424	1	0,138566
-0,41857	421	179,7545	0,676028	1	0,854676
-0,49984	420	348,8382	0,617504	1	0,964878
-1,11062	367	299,6148	0,267621	1	0,609149
0,40663	280	252,0154	0,684625	1	0,913844
-0,81548	421	361,3447	0,415336	1	0,695339
-0,24181	421	194,4808	0,809186	1	0,926063
-0,53232	420	393,4155	0,594803	1	0,964878
1,774089	367	332,8189	0,076963	1	0,379028
0,64402	280	261,3544	0,520127	1	0,833721
-0,64886	421	402,7289	0,516799	1	0,767364
-0,46216	421	218,4403	0,644428	1	0,838647
1,61821	420	415,8375	0,106375	1	0,848246
0,65624	367	362,9035	0,512086	1	0,797829
-0,47944	280	275,8884	0,632009	1	0,903355
-1,11059	421	359,8615	0,267488	1	0,563516
0,110726	421	194,7577	0,911948	1	0,96646
-2,32362	420	386,3033	0,020664	1	0,75904
0,979306	367	346,6134	0,328112	1	0,655788
0,642914	280	262,1129	0,520842	1	0,833721
0,922934	421	342,7607	0,356691	1	0,646073
0,389368	421	189,2085	0,697442	1	0,865528
0,785752	420	375,9867	0,432508	1	0,960096
0,417705	367	333,9545	0,676431	1	0,898926
-0,56678	280	261,4124	0,571352	1	0,869862
-1,09184	421	325,9774	0,275709	1	0,57291
-1,09862	421	184,5723	0,273366	1	0,574564
-0,68831	420	361,2318	0,491698	1	0,960096
1,389816	367	324,5064	0,165537	1	0,512248
-1,04233	280	258,8689	0,298232	1	0,723324
-0,20428	421	381,2053	0,838241	1	0,93721
0,845743	421	202,4886	0,398694	1	0,670572
-1,14639	420	407,333	0,252305	1	0,943897
0,452599	367	351,0887	0,651117	1	0,887994
0,783773	280	270,8801	0,433858	1	0,803161
1,544888	421	346,3149	0,123287	1	0,385082
0,589855	421	189,5034	0,555991	1	0,778696
0,204009	420	378,8723	0,838456	1	0,973894
0,629373	367	319,6714	0,529554	1	0,812666
1,950312	280	249,6034	0,052258	1	0,401537
0,878517	421	306,1091	0,380352	1	0,671614

1,807084	421	175,5292	0,072462	1	0,341588
0,065993	420	339,4882	0,947422	1	0,992811
-0,77383	367	304,3371	0,439633	1	0,742744
1,304061	280	251,1761	0,193407	1	0,62543
-0,92358	421	343,5314	0,356353	1	0,646073
-0,90412	421	187,7874	0,367089	1	0,647779
-1,52497	420	380,6371	0,128097	1	0,887798
-0,82114	367	343,2455	0,412136	1	0,724248
0,308608	280	260,8469	0,757866	1	0,94273
3,075338	421	361,5109	0,002263	1	0,069718
1,45211	421	193,2008	0,148093	1	0,428784
1,585437	420	393,0718	0,113671	1	0,848246
1,674371	367	340,571	0,094976	1	0,412696
1,223912	280	262,4858	0,222083	1	0,654014
0,690828	421	334,752	0,490152	1	0,748388
0,565663	421	181,3768	0,572322	1	0,792729
-0,46757	420	372,2681	0,640365	1	0,964878
-0,39295	367	332,2464	0,694612	1	0,904597
0,240806	280	266,1087	0,809891	1	0,95747
-1,31201	421	403,6511	0,190263	1	0,474713
1,073674	421	214,6501	0,284174	1	0,582654
-0,0036	420	415,4142	0,99713	1	0,997643
0,316823	367	362,4135	0,751561	1	0,924056
-0,06984	280	272,2956	0,944374	1	0,982175
-0,30748	421	404,9282	0,758637	1	0,900919
-0,9308	421	220,5825	0,352974	1	0,643758
0,074184	420	415,9231	0,9409	1	0,992811
-1,7348	367	362,8302	0,083625	1	0,386574
-1,28441	280	275,4935	0,200076	1	0,631529
-0,6213	421	382,2091	0,534774	1	0,778195
-0,98632	421	202,7481	0,325153	1	0,619085
-0,24848	420	406,9909	0,803887	1	0,969427
0,072518	367	351,2125	0,942231	1	0,979539
-0,73039	280	267,1675	0,465791	1	0,824256
-0,3757	421	367,1192	0,707355	1	0,875021
-1,17041	421	191,7944	0,243288	1	0,542422
-0,84835	420	399,3326	0,396753	1	0,95743
-1,33985	367	349,1757	0,181166	1	0,527769
-2,8342	280	264,3323	0,004949	1	0,177558
-1,53845	421	345,4213	0,124854	1	0,38857
-1,63819	421	190,6362	0,103031	1	0,383322
-0,29265	420	380,2743	0,769953	1	0,969427
-1,33616	367	352,7469	0,182358	1	0,527769
-2,95228	280	266,4006	0,003436	1	0,143818
0,893946	421	384,4086	0,37191	1	0,663276
1,017986	421	205,305	0,309883	1	0,607274
2,009395	420	409,0968	0,045152	1	0,846132
-0,55479	367	354,3473	0,579386	1	0,844137

-0,8053	280	266,746	0,421366	1	0,796701
-2,82945	421	412,3571	0,004891	1	0,087964
-3,10753	421	232,2592	0,002122	1	0,099921
-0,97716	420	413,622	0,329061	1	0,947373
-1,43961	367	358,6006	0,150851	1	0,499998
-1,05782	280	275,8943	0,291062	1	0,720248
-0,98261	421	333,9896	0,326512	1	0,61749
-1,5248	421	183,6554	0,129029	1	0,410224
-1,23799	420	363,532	0,216517	1	0,943055
-0,64604	367	312,9026	0,518727	1	0,801766
0,267938	280	258,527	0,788961	1	0,953435
-1,69464	421	366,7113	0,090993	1	0,32265
-2,03988	421	197,6985	0,042692	1	0,273775
-0,89086	420	401,3289	0,37354	1	0,95743
-0,7215	367	345,0804	0,471091	1	0,77576
-0,82375	280	268,3824	0,410811	1	0,788151
1,15703	421	335,4402	0,248083	1	0,544195
0,563527	421	185,0919	0,573758	1	0,79326
-1,73879	420	370,9826	0,082902	1	0,848246
3,222047	367	314,211	0,001406	1	0,176492
0,864642	280	258,5248	0,388037	1	0,775559
0,053806	421	325,9849	0,957123	1	0,987258
-0,62679	421	185,5134	0,531568	1	0,769894
-0,95691	420	359,1352	0,339257	1	0,947373
-0,80045	367	302,716	0,424081	1	0,733267
-1,09155	280	276	0,275984	1	0,701156
-2,4255	421	348,5788	0,015795	1	0,147897
-1,95802	421	192,0726	0,051676	1	0,296106
-2,36439	420	386,7677	0,018553	1	0,75904
-1,44106	367	348,7639	0,150465	1	0,499998
-0,7218	280	269,5498	0,471045	1	0,824256
-0,74155	421	354,1529	0,458854	1	0,724077
0,393357	421	192,5742	0,694491	1	0,865526
1,055988	420	387,2666	0,291632	1	0,943897
-0,5835	367	337,7339	0,559948	1	0,833012
-1,72793	280	267,8287	0,085154	1	0,45831
-0,51776	421	342,5019	0,604961	1	0,822071
0,343628	421	187,5824	0,731511	1	0,882616
-0,4769	420	376,7183	0,63371	1	0,964878
-1,0894	367	328,8757	0,276774	1	0,611884
0,445242	280	266,7619	0,656506	1	0,908644
-2,00042	421	349,3967	0,046228	1	0,238583
-1,38701	421	188,1661	0,167079	1	0,453987
-1,0897	420	385,199	0,276528	1	0,943897
0,323572	367	330,8476	0,746466	1	0,923669
-2,57661	280	257,3843	0,010535	1	0,230098
-1,38434	421	320,6223	0,167217	1	0,441901
-1,2499	421	181,3554	0,212946	1	0,512644

-0,89811	420	352,1508	0,369742	1	0,95743
-0,67051	367	316,0825	0,50302	1	0,793601
-0,92158	280	276	0,357554	1	0,761846
-1,49305	421	365,64	0,136288	1	0,407576
-0,85318	421	198,6994	0,394588	1	0,665895
-0,85642	420	400,4861	0,392279	1	0,95743
-0,96219	367	340,7691	0,336637	1	0,662616
0,9171	280	259,0943	0,359943	1	0,761846
-1,72241	421	348,5686	0,085882	1	0,31262
-1,59259	421	190,796	0,112907	1	0,391524
1,205212	420	386,3655	0,228859	1	0,943055
-2,70722	367	346,043	0,007122	1	0,180403
-1,96978	280	263,1488	0,049912	1	0,39442
-1,25519	421	336,3748	0,21028	1	0,499043
-1,86708	421	187,4607	0,063452	1	0,323047
0,286922	420	371,9979	0,774332	1	0,969427
0,898182	367	300,5555	0,369808	1	0,679634
-0,05934	280	254,4199	0,952726	1	0,983396
-0,10769	421	322,1036	0,914312	1	0,966828
0,027662	421	183,5769	0,977962	1	0,991789
0,702118	420	354,3023	0,483066	1	0,960096
-0,20666	367	329,7669	0,836405	1	0,956935
-0,43899	280	260,495	0,661036	1	0,908644
1,440573	421	333,7691	0,150643	1	0,424072
1,510256	421	183,809	0,132695	1	0,414338
0,468002	420	370,5698	0,640058	1	0,964878
0,432093	367	317,3803	0,665968	1	0,894765
-0,65576	280	253,8079	0,51257	1	0,831912
-1,72936	421	335,1813	0,084666	1	0,309688
-1,8849	421	187,5289	0,06099	1	0,31585
-1,98164	420	376,3541	0,048247	1	0,846627
-0,59126	367	315,9076	0,554769	1	0,830257
0,658733	280	253,816	0,510664	1	0,831912
-1,65448	421	299,4747	0,099079	1	0,339344
-0,85433	421	178,9454	0,394065	1	0,665792
-0,36615	420	328,0285	0,714489	1	0,968866
-1,19523	367	300,4799	0,23294	1	0,582158
-1,34747	280	276	0,178935	1	0,605081
-0,90297	421	331,3802	0,367198	1	0,658771
-0,25064	421	186,6549	0,802366	1	0,922323
-0,66106	420	366,4243	0,508989	1	0,960096
0,683311	367	318,9748	0,494906	1	0,793601
-1,30029	280	262,5247	0,194643	1	0,62543
-0,15526	421	373,9497	0,876698	1	0,95861
-0,44367	421	199,7456	0,657763	1	0,841515
2,290948	420	403,7838	0,022481	1	0,75904
-0,01363	367	351,2969	0,989135	1	0,996248
1,974315	280	271,7826	0,049358	1	0,39442

2,016618	421	384,1685	0,044431	1	0,236315
1,660994	421	204,0019	0,098251	1	0,379251
0,211717	420	409,5479	0,832433	1	0,973894
2,364092	367	359,104	0,018606	1	0,217108
0,990429	280	275,539	0,322833	1	0,745172
0,322431	421	311,3955	0,747343	1	0,898122
0,030555	421	179,8437	0,975658	1	0,991789
1,545063	420	344,0874	0,12325	1	0,87201
0,282296	367	306,2039	0,777907	1	0,942368
-0,19788	280	276	0,843283	1	0,965181
-1,74711	421	379,8534	0,081426	1	0,30376
-2,13005	421	201,8678	0,034378	1	0,252759
-0,07232	420	406,1289	0,942381	1	0,992811
-1,18833	367	359,7339	0,235487	1	0,585627
-2,8884	280	265,6284	0,004191	1	0,157901
-0,64952	421	321,5999	0,516467	1	0,767364
-2,82307	421	183,563	0,005282	1	0,139638
-1,91871	420	353,2891	0,055827	1	0,848246
-1,51964	367	308,4635	0,129627	1	0,473621
-1,13917	280	249,864	0,255723	1	0,679673
2,356133	421	360,4913	0,019001	1	0,159771
2,076425	421	191,8405	0,039187	1	0,26998
1,330916	420	395,9954	0,183982	1	0,923408
1,726266	367	340,5851	0,085206	1	0,388246
3,044095	280	271,6828	0,002563	1	0,135787
3,412009	421	387,9929	7,13E-04	0,758315	0,051145
2,60506	421	211,185	0,009839	1	0,149771
1,182479	420	413,6583	0,237695	1	0,943055
2,786282	367	358,8268	0,005615	1	0,178183
2,015045	280	275,9795	0,044869	1	0,387749
0,3421	421	387,5638	0,732461	1	0,893058
1,400941	421	207,6276	0,162724	1	0,446677
1,396311	420	410,5616	0,163375	1	0,914005
-0,60964	367	356,9467	0,542487	1	0,824121
0,578757	280	267,5444	0,56324	1	0,861728
-2,46131	421	300,8974	0,014404	1	0,146671
-1,09399	421	175,6288	0,275456	1	0,576759
-0,59448	420	342,4395	0,552583	1	0,960096
-3,18578	367	303,1749	0,001594	1	0,176492
-2,14713	280	254,1944	0,032729	1	0,329568
-0,36548	421	320,6501	0,714996	1	0,881149
0,294444	421	179,2458	0,768759	1	0,905801
-0,07947	420	355,4728	0,936706	1	0,992059
0,574832	367	308,6863	0,565824	1	0,837838
-0,0988	280	257,8714	0,921374	1	0,977136
-3,0293	421	386,7898	0,002616	1	0,073944
-1,82879	421	206,4779	0,068874	1	0,335504
-0,78531	420	410,9803	0,432726	1	0,960096

-1,76559	367	359,8192	0,078313	1	0,381638
-1,87443	280	274,3596	0,061933	1	0,422605
-0,57211	421	356,2084	0,56761	1	0,796637
-0,4211	421	189,9094	0,674159	1	0,853746
-0,21736	420	388,6587	0,828045	1	0,973894
0,376707	367	330,735	0,706633	1	0,91071
-0,90131	280	261,2182	0,368255	1	0,763914
-0,10753	421	349,9041	0,914431	1	0,966828
0,773194	421	187,9072	0,440379	1	0,705407
-0,25458	420	382,9977	0,799186	1	0,969427
0,405144	367	337,0403	0,685629	1	0,902395
1,359468	280	254,4292	0,175202	1	0,598625
-0,13847	421	351,9567	0,889952	1	0,962
2,637997	421	191,8419	0,009024	1	0,14815
1,781552	420	384,6462	0,075611	1	0,848246
3,033374	367	334,0667	0,002608	1	0,176492
1,472479	280	250,2707	0,142148	1	0,554427
-0,1199	421	399,7748	0,904624	1	0,965673
3,26158	421	213,0474	0,00129	1	0,092971
0,804868	420	414,9107	0,421357	1	0,95743
1,259769	367	362,2098	0,208564	1	0,56126
-0,31628	280	273,2312	0,752031	1	0,942071
0,705982	421	326,0142	0,480703	1	0,740716
0,399493	421	181,6587	0,69	1	0,864363
-0,37164	420	359,9845	0,710377	1	0,968866
0,669377	367	320,5871	0,503737	1	0,793601
0,704706	280	254,9061	0,481637	1	0,824339
-0,11508	421	323,8627	0,90845	1	0,966828
2,012363	421	182,9412	0,045648	1	0,281076
0,953172	420	358,2234	0,341146	1	0,947373
0,274221	367	315,0477	0,784094	1	0,94337
0,343537	280	252,2405	0,731481	1	0,936637
0,014042	421	358,7818	0,988804	1	0,998054
0,018613	421	192,9728	0,985169	1	0,995074
-0,47339	420	392,5891	0,636198	1	0,964878
-0,30297	367	339,4662	0,762096	1	0,929966
0,009766	280	261,7127	0,992215	1	0,996269
-1,63598	421	334,1571	0,102785	1	0,344557
-0,85747	421	187,1859	0,392284	1	0,665733
-1,17277	420	369,3536	0,241643	1	0,943659
-1,29743	367	324,748	0,195405	1	0,549567
0,367672	280	257,5062	0,71342	1	0,928432
-0,13171	421	328,7364	0,895291	1	0,962
-0,05187	421	182,4923	0,958691	1	0,98414
-0,76787	420	362,9392	0,443064	1	0,960096
2,180701	367	306,4423	0,029966	1	0,261032
0,710641	280	252,2844	0,477963	1	0,824256
1,444376	421	340,4516	0,149553	1	0,422053

0,264981	421	186,5692	0,791317	1	0,918572
1,227244	420	369,9486	0,220511	1	0,943055
0,228973	367	327,2746	0,819033	1	0,951644
-2,44601	280	255,211	0,015121	1	0,263522

Model formula fit

y ~ current_feature + Sex + (1 | Subject) + storage_time
y ~ current_feature + Sex + (1 | Subject) + storage_time
y ~ current_feature + Sex + (1 | Subject) + storage_time
y ~ current_feature + Sex + (1 | Subject) + storage_time
y ~ current_feature + Sex + (1 | Subject) + storage_time
y ~ current_feature + Sex + (1 | Subject) + storage_time
y ~ current_feature + Sex + (1 | Subject) + storage_time
y ~ current_feature + Sex + (1 | Subject) + storage_time
y ~ current_feature + Sex + (1 | Subject) + storage_time
y ~ current_feature + Sex + (1 | Subject) + storage_time
y ~ current_feature + Sex + (1 | Subject) + storage_time
y ~ current_feature + Sex + (1 | Subject) + storage_time
y ~ current_feature + Sex + (1 | Subject) + storage_time
y ~ current_feature + Sex + (1 | Subject) + storage_time
y ~ current_feature + Sex + (1 | Subject) + storage_time
y ~ current_feature + Sex + (1 | Subject) + storage_time
y ~ current_feature + Sex + (1 | Subject) + storage_time
y ~ current_feature + Sex + (1 | Subject) + storage_time
y ~ current_feature + Sex + (1 | Subject) + storage_time
y ~ current_feature + Sex + (1 | Subject) + storage_time
y ~ current_feature + Sex + (1 | Subject) + storage_time
y ~ current_feature + Sex + (1 | Subject) + storage_time
y ~ current_feature + Sex + (1 | Subject) + storage_time
y ~ current_feature + Sex + (1 | Subject) + storage_time
y ~ current_feature + Sex + (1 | Subject) + storage_time
y ~ current_feature + Sex + (1 | Subject) + storage_time
y ~ current_feature + Sex + (1 | Subject) + storage_time
y ~ current_feature + Sex + (1 | Subject) + storage_time
y ~ current_feature + Sex + (1 | Subject) + storage_time
y ~ current_feature + Sex + (1 | Subject) + storage_time
y ~ current_feature + Sex + (1 | Subject) + storage_time
y ~ current_feature + Sex + (1 | Subject) + storage_time
y ~ current_feature + Sex + (1 | Subject) + storage_time
y ~ current_feature + Sex + (1 | Subject) + storage_time
y ~ current_feature + Sex + (1 | Subject) + storage_time
y ~ current_feature + Sex + (1 | Subject) + storage_time
y ~ current_feature + Sex + (1 | Subject) + storage_time
y ~ current_feature + Sex + (1 | Subject) + storage_time
y ~ current_feature + Sex + (1 | Subject) + storage_time
y ~ current_feature + Sex + (1 | Subject) + storage_time
y ~ current_feature + Sex + (1 | Subject) + storage_time
y ~ current_feature + Sex + (1 | Subject) + storage_time
y ~ current_feature + Sex + (1 | Subject) + storage_time
y ~ current_feature + Sex + (1 | Subject) + storage_time
y ~ current_feature + Sex + (1 | Subject) + storage_time
y ~ current_feature + Sex + (1 | Subject) + storage_time
y ~ current_feature + Sex + (1 | Subject) + storage_time

```
y ~ current_feature + Sex + (1 | Subject) + storage_time
y ~ current_feature + Sex + (1 | Subject) + storage_time
y ~ current_feature + Sex + (1 | Subject) + storage_time
y ~ current_feature + Sex + (1 | Subject) + storage_time
y ~ current_feature + Sex + (1 | Subject) + storage_time
y ~ current_feature + Sex + (1 | Subject) + storage_time
y ~ current_feature + Sex + (1 | Subject) + storage_time
y ~ current_feature + Sex + (1 | Subject) + storage_time
y ~ current_feature + Sex + (1 | Subject) + storage_time
y ~ current_feature + Sex + (1 | Subject) + storage_time
y ~ current_feature + Sex + (1 | Subject) + storage_time
y ~ current_feature + Sex + (1 | Subject) + storage_time
y ~ current_feature + Sex + (1 | Subject) + storage_time
y ~ current_feature + Sex + (1 | Subject) + storage_time
y ~ current_feature + Sex + (1 | Subject) + storage_time
y ~ current_feature + Sex + (1 | Subject) + storage_time
y ~ current_feature + Sex + (1 | Subject) + storage_time
y ~ current_feature + Sex + (1 | Subject) + storage_time
y ~ current_feature + Sex + (1 | Subject) + storage_time
y ~ current_feature + Sex + (1 | Subject) + storage_time
y ~ current_feature + Sex + (1 | Subject) + storage_time
y ~ current_feature + Sex + (1 | Subject) + storage_time
y ~ current_feature + Sex + (1 | Subject) + storage_time
y ~ current_feature + Sex + (1 | Subject) + storage_time
y ~ current_feature + Sex + (1 | Subject) + storage_time
y ~ current_feature + Sex + (1 | Subject) + storage_time
y ~ current_feature + Sex + (1 | Subject) + storage_time
y ~ current_feature + Sex + (1 | Subject) + storage_time
y ~ current_feature + Sex + (1 | Subject) + storage_time
y ~ current_feature + Sex + (1 | Subject) + storage_time
y ~ current_feature + Sex + (1 | Subject) + storage_time
y ~ current_feature + Sex + (1 | Subject) + storage_time
y ~ current_feature + Sex + (1 | Subject) + storage_time
y ~ current_feature + Sex + (1 | Subject) + storage_time
y ~ current_feature + Sex + (1 | Subject) + storage_time
y ~ current_feature + Sex + (1 | Subject) + storage_time
y ~ current_feature + Sex + (1 | Subject) + storage_time
y ~ current_feature + Sex + (1 | Subject) + storage_time
y ~ current_feature + Sex + (1 | Subject) + storage_time
y ~ current_feature + Sex + (1 | Subject) + storage_time
y ~ current_feature + Sex + (1 | Subject) + storage_time
y ~ current_feature + Sex + (1 | Subject) + storage_time
y ~ current_feature + Sex + (1 | Subject) + storage_time
y ~ current_feature + Sex + (1 | Subject) + storage_time
y ~ current_feature + Sex + (1 | Subject) + storage_time
y ~ current_feature + Sex + (1 | Subject) + storage_time
y ~ current_feature + Sex + (1 | Subject) + storage_time
y ~ current_feature + Sex + (1 | Subject) + storage_time
```

y ~ current_feature + Sex + (1 | Subject) + storage_time
y ~ current_feature + Sex + (1 | Subject) + storage_time
y ~ current_feature + Sex + (1 | Subject) + storage_time
y ~ current_feature + Sex + (1 | Subject) + storage_time
y ~ current_feature + Sex + (1 | Subject) + storage_time
y ~ current_feature + Sex + (1 | Subject) + storage_time
y ~ current_feature + Sex + (1 | Subject) + storage_time
y ~ current_feature + Sex + (1 | Subject) + storage_time
y ~ current_feature + Sex + (1 | Subject) + storage_time
y ~ current_feature + Sex + (1 | Subject) + storage_time
y ~ current_feature + Sex + (1 | Subject) + storage_time
y ~ current_feature + Sex + (1 | Subject) + storage_time
y ~ current_feature + Sex + (1 | Subject) + storage_time
y ~ current_feature + Sex + (1 | Subject) + storage_time
y ~ current_feature + Sex + (1 | Subject) + storage_time
y ~ current_feature + Sex + (1 | Subject) + storage_time
y ~ current_feature + Sex + (1 | Subject) + storage_time
y ~ current_feature + Sex + (1 | Subject) + storage_time
y ~ current_feature + Sex + (1 | Subject) + storage_time
y ~ current_feature + Sex + (1 | Subject) + storage_time
y ~ current_feature + Sex + (1 | Subject) + storage_time
y ~ current_feature + Sex + (1 | Subject) + storage_time
y ~ current_feature + Sex + (1 | Subject) + storage_time
y ~ current_feature + Sex + (1 | Subject) + storage_time
y ~ current_feature + Sex + (1 | Subject) + storage_time
y ~ current_feature + Sex + (1 | Subject) + storage_time
y ~ current_feature + Sex + (1 | Subject) + storage_time
y ~ current_feature + Sex + (1 | Subject) + storage_time
y ~ current_feature + Sex + (1 | Subject) + storage_time
y ~ current_feature + Sex + (1 | Subject) + storage_time
y ~ current_feature + Sex + (1 | Subject) + storage_time
y ~ current_feature + Sex + (1 | Subject) + storage_time
y ~ current_feature + Sex + (1 | Subject) + storage_time
y ~ current_feature + Sex + (1 | Subject) + storage_time
y ~ current_feature + Sex + (1 | Subject) + storage_time
y ~ current_feature + Sex + (1 | Subject) + storage_time
y ~ current_feature + Sex + (1 | Subject) + storage_time
y ~ current_feature + Sex + (1 | Subject) + storage_time
y ~ current_feature + Sex + (1 | Subject) + storage_time
y ~ current_feature + Sex + (1 | Subject) + storage_time
y ~ current_feature + Sex + (1 | Subject) + storage_time
y ~ current_feature + Sex + (1 | Subject) + storage_time
y ~ current_feature + Sex + (1 | Subject) + storage_time
y ~ current_feature + Sex + (1 | Subject) + storage_time
y ~ current_feature + Sex + (1 | Subject) + storage_time
y ~ current_feature + Sex + (1 | Subject) + storage_time
y ~ current_feature + Sex + (1 | Subject) + storage_time
y ~ current_feature + Sex + (1 | Subject) + storage_time

y ~ current_feature + Sex + (1 | Subject) + storage_time
y ~ current_feature + Sex + (1 | Subject) + storage_time
y ~ current_feature + Sex + (1 | Subject) + storage_time
y ~ current_feature + Sex + (1 | Subject) + storage_time
y ~ current_feature + Sex + (1 | Subject) + storage_time
y ~ current_feature + Sex + (1 | Subject) + storage_time
y ~ current_feature + Sex + (1 | Subject) + storage_time
y ~ current_feature + Sex + (1 | Subject) + storage_time
y ~ current_feature + Sex + (1 | Subject) + storage_time
y ~ current_feature + Sex + (1 | Subject) + storage_time
y ~ current_feature + Sex + (1 | Subject) + storage_time
y ~ current_feature + Sex + (1 | Subject) + storage_time
y ~ current_feature + Sex + (1 | Subject) + storage_time
y ~ current_feature + Sex + (1 | Subject) + storage_time
y ~ current_feature + Sex + (1 | Subject) + storage_time
y ~ current_feature + Sex + (1 | Subject) + storage_time
y ~ current_feature + Sex + (1 | Subject) + storage_time
y ~ current_feature + Sex + (1 | Subject) + storage_time
y ~ current_feature + Sex + (1 | Subject) + storage_time
y ~ current_feature + Sex + (1 | Subject) + storage_time
y ~ current_feature + Sex + (1 | Subject) + storage_time
y ~ current_feature + Sex + (1 | Subject) + storage_time
y ~ current_feature + Sex + (1 | Subject) + storage_time
y ~ current_feature + Sex + (1 | Subject) + storage_time
y ~ current_feature + Sex + (1 | Subject) + storage_time
y ~ current_feature + Sex + (1 | Subject) + storage_time
y ~ current_feature + Sex + (1 | Subject) + storage_time
y ~ current_feature + Sex + (1 | Subject) + storage_time
y ~ current_feature + Sex + (1 | Subject) + storage_time
y ~ current_feature + Sex + (1 | Subject) + storage_time
y ~ current_feature + Sex + (1 | Subject) + storage_time
y ~ current_feature + Sex + (1 | Subject) + storage_time
y ~ current_feature + Sex + (1 | Subject) + storage_time
y ~ current_feature + Sex + (1 | Subject) + storage_time
y ~ current_feature + Sex + (1 | Subject) + storage_time
y ~ current_feature + Sex + (1 | Subject) + storage_time
y ~ current_feature + Sex + (1 | Subject) + storage_time
y ~ current_feature + Sex + (1 | Subject) + storage_time
y ~ current_feature + Sex + (1 | Subject) + storage_time
y ~ current_feature + Sex + (1 | Subject) + storage_time
y ~ current_feature + Sex + (1 | Subject) + storage_time
y ~ current_feature + Sex + (1 | Subject) + storage_time
y ~ current_feature + Sex + (1 | Subject) + storage_time
y ~ current_feature + Sex + (1 | Subject) + storage_time
y ~ current_feature + Sex + (1 | Subject) + storage_time
y ~ current_feature + Sex + (1 | Subject) + storage_time
y ~ current_feature + Sex + (1 | Subject) + storage_time
y ~ current_feature + Sex + (1 | Subject) + storage_time

```
y ~ current_feature + Sex + (1 | Subject) + storage_time
y ~ current_feature + Sex + (1 | Subject) + storage_time
y ~ current_feature + Sex + (1 | Subject) + storage_time
y ~ current_feature + Sex + (1 | Subject) + storage_time
y ~ current_feature + Sex + (1 | Subject) + storage_time
y ~ current_feature + Sex + (1 | Subject) + storage_time
y ~ current_feature + Sex + (1 | Subject) + storage_time
y ~ current_feature + Sex + (1 | Subject) + storage_time
y ~ current_feature + Sex + (1 | Subject) + storage_time
y ~ current_feature + Sex + (1 | Subject) + storage_time
y ~ current_feature + Sex + (1 | Subject) + storage_time
y ~ current_feature + Sex + (1 | Subject) + storage_time
y ~ current_feature + Sex + (1 | Subject) + storage_time
y ~ current_feature + Sex + (1 | Subject) + storage_time
y ~ current_feature + Sex + (1 | Subject) + storage_time
y ~ current_feature + Sex + (1 | Subject) + storage_time
y ~ current_feature + Sex + (1 | Subject) + storage_time
y ~ current_feature + Sex + (1 | Subject) + storage_time
y ~ current_feature + Sex + (1 | Subject) + storage_time
y ~ current_feature + Sex + (1 | Subject) + storage_time
y ~ current_feature + Sex + (1 | Subject) + storage_time
y ~ current_feature + Sex + (1 | Subject) + storage_time
y ~ current_feature + Sex + (1 | Subject) + storage_time
y ~ current_feature + Sex + (1 | Subject) + storage_time
y ~ current_feature + Sex + (1 | Subject) + storage_time
y ~ current_feature + Sex + (1 | Subject) + storage_time
y ~ current_feature + Sex + (1 | Subject) + storage_time
y ~ current_feature + Sex + (1 | Subject) + storage_time
y ~ current_feature + Sex + (1 | Subject) + storage_time
y ~ current_feature + Sex + (1 | Subject) + storage_time
y ~ current_feature + Sex + (1 | Subject) + storage_time
y ~ current_feature + Sex + (1 | Subject) + storage_time
y ~ current_feature + Sex + (1 | Subject) + storage_time
y ~ current_feature + Sex + (1 | Subject) + storage_time
y ~ current_feature + Sex + (1 | Subject) + storage_time
y ~ current_feature + Sex + (1 | Subject) + storage_time
y ~ current_feature + Sex + (1 | Subject) + storage_time
y ~ current_feature + Sex + (1 | Subject) + storage_time
y ~ current_feature + Sex + (1 | Subject) + storage_time
y ~ current_feature + Sex + (1 | Subject) + storage_time
y ~ current_feature + Sex + (1 | Subject) + storage_time
y ~ current_feature + Sex + (1 | Subject) + storage_time
y ~ current_feature + Sex + (1 | Subject) + storage_time
y ~ current_feature + Sex + (1 | Subject) + storage_time
y ~ current_feature + Sex + (1 | Subject) + storage_time
y ~ current_feature + Sex + (1 | Subject) + storage_time
y ~ current_feature + Sex + (1 | Subject) + storage_time
y ~ current_feature + Sex + (1 | Subject) + storage_time
```

y ~ current_feature + Sex + (1 | Subject) + storage_time
y ~ current_feature + Sex + (1 | Subject) + storage_time
y ~ current_feature + Sex + (1 | Subject) + storage_time
y ~ current_feature + Sex + (1 | Subject) + storage_time
y ~ current_feature + Sex + (1 | Subject) + storage_time
y ~ current_feature + Sex + (1 | Subject) + storage_time
y ~ current_feature + Sex + (1 | Subject) + storage_time
y ~ current_feature + Sex + (1 | Subject) + storage_time
y ~ current_feature + Sex + (1 | Subject) + storage_time
y ~ current_feature + Sex + (1 | Subject) + storage_time
y ~ current_feature + Sex + (1 | Subject) + storage_time
y ~ current_feature + Sex + (1 | Subject) + storage_time
y ~ current_feature + Sex + (1 | Subject) + storage_time
y ~ current_feature + Sex + (1 | Subject) + storage_time
y ~ current_feature + Sex + (1 | Subject) + storage_time
y ~ current_feature + Sex + (1 | Subject) + storage_time
y ~ current_feature + Sex + (1 | Subject) + storage_time
y ~ current_feature + Sex + (1 | Subject) + storage_time
y ~ current_feature + Sex + (1 | Subject) + storage_time
y ~ current_feature + Sex + (1 | Subject) + storage_time
y ~ current_feature + Sex + (1 | Subject) + storage_time
y ~ current_feature + Sex + (1 | Subject) + storage_time
y ~ current_feature + Sex + (1 | Subject) + storage_time
y ~ current_feature + Sex + (1 | Subject) + storage_time
y ~ current_feature + Sex + (1 | Subject) + storage_time
y ~ current_feature + Sex + (1 | Subject) + storage_time
y ~ current_feature + Sex + (1 | Subject) + storage_time
y ~ current_feature + Sex + (1 | Subject) + storage_time
y ~ current_feature + Sex + (1 | Subject) + storage_time
y ~ current_feature + Sex + (1 | Subject) + storage_time
y ~ current_feature + Sex + (1 | Subject) + storage_time
y ~ current_feature + Sex + (1 | Subject) + storage_time
y ~ current_feature + Sex + (1 | Subject) + storage_time
y ~ current_feature + Sex + (1 | Subject) + storage_time
y ~ current_feature + Sex + (1 | Subject) + storage_time
y ~ current_feature + Sex + (1 | Subject) + storage_time
y ~ current_feature + Sex + (1 | Subject) + storage_time
y ~ current_feature + Sex + (1 | Subject) + storage_time
y ~ current_feature + Sex + (1 | Subject) + storage_time
y ~ current_feature + Sex + (1 | Subject) + storage_time
y ~ current_feature + Sex + (1 | Subject) + storage_time
y ~ current_feature + Sex + (1 | Subject) + storage_time
y ~ current_feature + Sex + (1 | Subject) + storage_time
y ~ current_feature + Sex + (1 | Subject) + storage_time
y ~ current_feature + Sex + (1 | Subject) + storage_time
y ~ current_feature + Sex + (1 | Subject) + storage_time
y ~ current_feature + Sex + (1 | Subject) + storage_time
y ~ current_feature + Sex + (1 | Subject) + storage_time

y ~ current_feature + Sex + (1 | Subject) + storage_time
y ~ current_feature + Sex + (1 | Subject) + storage_time
y ~ current_feature + Sex + (1 | Subject) + storage_time
y ~ current_feature + Sex + (1 | Subject) + storage_time
y ~ current_feature + Sex + (1 | Subject) + storage_time
y ~ current_feature + Sex + (1 | Subject) + storage_time
y ~ current_feature + Sex + (1 | Subject) + storage_time
y ~ current_feature + Sex + (1 | Subject) + storage_time
y ~ current_feature + Sex + (1 | Subject) + storage_time
y ~ current_feature + Sex + (1 | Subject) + storage_time
y ~ current_feature + Sex + (1 | Subject) + storage_time
y ~ current_feature + Sex + (1 | Subject) + storage_time
y ~ current_feature + Sex + (1 | Subject) + storage_time
y ~ current_feature + Sex + (1 | Subject) + storage_time
y ~ current_feature + Sex + (1 | Subject) + storage_time
y ~ current_feature + Sex + (1 | Subject) + storage_time
y ~ current_feature + Sex + (1 | Subject) + storage_time
y ~ current_feature + Sex + (1 | Subject) + storage_time
y ~ current_feature + Sex + (1 | Subject) + storage_time
y ~ current_feature + Sex + (1 | Subject) + storage_time
y ~ current_feature + Sex + (1 | Subject) + storage_time
y ~ current_feature + Sex + (1 | Subject) + storage_time
y ~ current_feature + Sex + (1 | Subject) + storage_time
y ~ current_feature + Sex + (1 | Subject) + storage_time
y ~ current_feature + Sex + (1 | Subject) + storage_time
y ~ current_feature + Sex + (1 | Subject) + storage_time
y ~ current_feature + Sex + (1 | Subject) + storage_time
y ~ current_feature + Sex + (1 | Subject) + storage_time
y ~ current_feature + Sex + (1 | Subject) + storage_time
y ~ current_feature + Sex + (1 | Subject) + storage_time
y ~ current_feature + Sex + (1 | Subject) + storage_time
y ~ current_feature + Sex + (1 | Subject) + storage_time
y ~ current_feature + Sex + (1 | Subject) + storage_time
y ~ current_feature + Sex + (1 | Subject) + storage_time
y ~ current_feature + Sex + (1 | Subject) + storage_time
y ~ current_feature + Sex + (1 | Subject) + storage_time
y ~ current_feature + Sex + (1 | Subject) + storage_time
y ~ current_feature + Sex + (1 | Subject) + storage_time
y ~ current_feature + Sex + (1 | Subject) + storage_time
y ~ current_feature + Sex + (1 | Subject) + storage_time
y ~ current_feature + Sex + (1 | Subject) + storage_time
y ~ current_feature + Sex + (1 | Subject) + storage_time
y ~ current_feature + Sex + (1 | Subject) + storage_time
y ~ current_feature + Sex + (1 | Subject) + storage_time
y ~ current_feature + Sex + (1 | Subject) + storage_time
y ~ current_feature + Sex + (1 | Subject) + storage_time
y ~ current_feature + Sex + (1 | Subject) + storage_time
y ~ current_feature + Sex + (1 | Subject) + storage_time

y ~ current_feature + Sex + (1 | Subject) + storage_time
y ~ current_feature + Sex + (1 | Subject) + storage_time
y ~ current_feature + Sex + (1 | Subject) + storage_time
y ~ current_feature + Sex + (1 | Subject) + storage_time
y ~ current_feature + Sex + (1 | Subject) + storage_time
y ~ current_feature + Sex + (1 | Subject) + storage_time
y ~ current_feature + Sex + (1 | Subject) + storage_time
y ~ current_feature + Sex + (1 | Subject) + storage_time
y ~ current_feature + Sex + (1 | Subject) + storage_time
y ~ current_feature + Sex + (1 | Subject) + storage_time
y ~ current_feature + Sex + (1 | Subject) + storage_time
y ~ current_feature + Sex + (1 | Subject) + storage_time
y ~ current_feature + Sex + (1 | Subject) + storage_time
y ~ current_feature + Sex + (1 | Subject) + storage_time
y ~ current_feature + Sex + (1 | Subject) + storage_time
y ~ current_feature + Sex + (1 | Subject) + storage_time
y ~ current_feature + Sex + (1 | Subject) + storage_time
y ~ current_feature + Sex + (1 | Subject) + storage_time
y ~ current_feature + Sex + (1 | Subject) + storage_time
y ~ current_feature + Sex + (1 | Subject) + storage_time
y ~ current_feature + Sex + (1 | Subject) + storage_time
y ~ current_feature + Sex + (1 | Subject) + storage_time
y ~ current_feature + Sex + (1 | Subject) + storage_time
y ~ current_feature + Sex + (1 | Subject) + storage_time
y ~ current_feature + Sex + (1 | Subject) + storage_time
y ~ current_feature + Sex + (1 | Subject) + storage_time
y ~ current_feature + Sex + (1 | Subject) + storage_time
y ~ current_feature + Sex + (1 | Subject) + storage_time
y ~ current_feature + Sex + (1 | Subject) + storage_time
y ~ current_feature + Sex + (1 | Subject) + storage_time
y ~ current_feature + Sex + (1 | Subject) + storage_time
y ~ current_feature + Sex + (1 | Subject) + storage_time
y ~ current_feature + Sex + (1 | Subject) + storage_time
y ~ current_feature + Sex + (1 | Subject) + storage_time
y ~ current_feature + Sex + (1 | Subject) + storage_time
y ~ current_feature + Sex + (1 | Subject) + storage_time
y ~ current_feature + Sex + (1 | Subject) + storage_time
y ~ current_feature + Sex + (1 | Subject) + storage_time
y ~ current_feature + Sex + (1 | Subject) + storage_time
y ~ current_feature + Sex + (1 | Subject) + storage_time
y ~ current_feature + Sex + (1 | Subject) + storage_time
y ~ current_feature + Sex + (1 | Subject) + storage_time
y ~ current_feature + Sex + (1 | Subject) + storage_time
y ~ current_feature + Sex + (1 | Subject) + storage_time
y ~ current_feature + Sex + (1 | Subject) + storage_time
y ~ current_feature + Sex + (1 | Subject) + storage_time
y ~ current_feature + Sex + (1 | Subject) + storage_time
y ~ current_feature + Sex + (1 | Subject) + storage_time

y ~ current_feature + Sex + (1 | Subject) + storage_time
y ~ current_feature + Sex + (1 | Subject) + storage_time
y ~ current_feature + Sex + (1 | Subject) + storage_time
y ~ current_feature + Sex + (1 | Subject) + storage_time
y ~ current_feature + Sex + (1 | Subject) + storage_time
y ~ current_feature + Sex + (1 | Subject) + storage_time
y ~ current_feature + Sex + (1 | Subject) + storage_time
y ~ current_feature + Sex + (1 | Subject) + storage_time
y ~ current_feature + Sex + (1 | Subject) + storage_time
y ~ current_feature + Sex + (1 | Subject) + storage_time
y ~ current_feature + Sex + (1 | Subject) + storage_time
y ~ current_feature + Sex + (1 | Subject) + storage_time
y ~ current_feature + Sex + (1 | Subject) + storage_time
y ~ current_feature + Sex + (1 | Subject) + storage_time
y ~ current_feature + Sex + (1 | Subject) + storage_time
y ~ current_feature + Sex + (1 | Subject) + storage_time
y ~ current_feature + Sex + (1 | Subject) + storage_time
y ~ current_feature + Sex + (1 | Subject) + storage_time
y ~ current_feature + Sex + (1 | Subject) + storage_time
y ~ current_feature + Sex + (1 | Subject) + storage_time
y ~ current_feature + Sex + (1 | Subject) + storage_time
y ~ current_feature + Sex + (1 | Subject) + storage_time
y ~ current_feature + Sex + (1 | Subject) + storage_time
y ~ current_feature + Sex + (1 | Subject) + storage_time
y ~ current_feature + Sex + (1 | Subject) + storage_time
y ~ current_feature + Sex + (1 | Subject) + storage_time
y ~ current_feature + Sex + (1 | Subject) + storage_time
y ~ current_feature + Sex + (1 | Subject) + storage_time
y ~ current_feature + Sex + (1 | Subject) + storage_time
y ~ current_feature + Sex + (1 | Subject) + storage_time
y ~ current_feature + Sex + (1 | Subject) + storage_time
y ~ current_feature + Sex + (1 | Subject) + storage_time
y ~ current_feature + Sex + (1 | Subject) + storage_time
y ~ current_feature + Sex + (1 | Subject) + storage_time
y ~ current_feature + Sex + (1 | Subject) + storage_time
y ~ current_feature + Sex + (1 | Subject) + storage_time
y ~ current_feature + Sex + (1 | Subject) + storage_time
y ~ current_feature + Sex + (1 | Subject) + storage_time
y ~ current_feature + Sex + (1 | Subject) + storage_time
y ~ current_feature + Sex + (1 | Subject) + storage_time
y ~ current_feature + Sex + (1 | Subject) + storage_time
y ~ current_feature + Sex + (1 | Subject) + storage_time
y ~ current_feature + Sex + (1 | Subject) + storage_time
y ~ current_feature + Sex + (1 | Subject) + storage_time
y ~ current_feature + Sex + (1 | Subject) + storage_time
y ~ current_feature + Sex + (1 | Subject) + storage_time
y ~ current_feature + Sex + (1 | Subject) + storage_time
y ~ current_feature + Sex + (1 | Subject) + storage_time

y ~ current_feature + Sex + (1 | Subject) + storage_time
y ~ current_feature + Sex + (1 | Subject) + storage_time
y ~ current_feature + Sex + (1 | Subject) + storage_time
y ~ current_feature + Sex + (1 | Subject) + storage_time
y ~ current_feature + Sex + (1 | Subject) + storage_time
y ~ current_feature + Sex + (1 | Subject) + storage_time
y ~ current_feature + Sex + (1 | Subject) + storage_time
y ~ current_feature + Sex + (1 | Subject) + storage_time
y ~ current_feature + Sex + (1 | Subject) + storage_time
y ~ current_feature + Sex + (1 | Subject) + storage_time
y ~ current_feature + Sex + (1 | Subject) + storage_time
y ~ current_feature + Sex + (1 | Subject) + storage_time
y ~ current_feature + Sex + (1 | Subject) + storage_time
y ~ current_feature + Sex + (1 | Subject) + storage_time
y ~ current_feature + Sex + (1 | Subject) + storage_time
y ~ current_feature + Sex + (1 | Subject) + storage_time
y ~ current_feature + Sex + (1 | Subject) + storage_time
y ~ current_feature + Sex + (1 | Subject) + storage_time
y ~ current_feature + Sex + (1 | Subject) + storage_time
y ~ current_feature + Sex + (1 | Subject) + storage_time
y ~ current_feature + Sex + (1 | Subject) + storage_time
y ~ current_feature + Sex + (1 | Subject) + storage_time
y ~ current_feature + Sex + (1 | Subject) + storage_time
y ~ current_feature + Sex + (1 | Subject) + storage_time
y ~ current_feature + Sex + (1 | Subject) + storage_time
y ~ current_feature + Sex + (1 | Subject) + storage_time
y ~ current_feature + Sex + (1 | Subject) + storage_time
y ~ current_feature + Sex + (1 | Subject) + storage_time
y ~ current_feature + Sex + (1 | Subject) + storage_time
y ~ current_feature + Sex + (1 | Subject) + storage_time
y ~ current_feature + Sex + (1 | Subject) + storage_time
y ~ current_feature + Sex + (1 | Subject) + storage_time
y ~ current_feature + Sex + (1 | Subject) + storage_time
y ~ current_feature + Sex + (1 | Subject) + storage_time
y ~ current_feature + Sex + (1 | Subject) + storage_time
y ~ current_feature + Sex + (1 | Subject) + storage_time
y ~ current_feature + Sex + (1 | Subject) + storage_time
y ~ current_feature + Sex + (1 | Subject) + storage_time
y ~ current_feature + Sex + (1 | Subject) + storage_time
y ~ current_feature + Sex + (1 | Subject) + storage_time
y ~ current_feature + Sex + (1 | Subject) + storage_time
y ~ current_feature + Sex + (1 | Subject) + storage_time
y ~ current_feature + Sex + (1 | Subject) + storage_time
y ~ current_feature + Sex + (1 | Subject) + storage_time
y ~ current_feature + Sex + (1 | Subject) + storage_time
y ~ current_feature + Sex + (1 | Subject) + storage_time
y ~ current_feature + Sex + (1 | Subject) + storage_time
y ~ current_feature + Sex + (1 | Subject) + storage_time

y ~ current_feature + Sex + (1 | Subject) + storage_time
y ~ current_feature + Sex + (1 | Subject) + storage_time
y ~ current_feature + Sex + (1 | Subject) + storage_time
y ~ current_feature + Sex + (1 | Subject) + storage_time
y ~ current_feature + Sex + (1 | Subject) + storage_time
y ~ current_feature + Sex + (1 | Subject) + storage_time
y ~ current_feature + Sex + (1 | Subject) + storage_time
y ~ current_feature + Sex + (1 | Subject) + storage_time
y ~ current_feature + Sex + (1 | Subject) + storage_time
y ~ current_feature + Sex + (1 | Subject) + storage_time
y ~ current_feature + Sex + (1 | Subject) + storage_time
y ~ current_feature + Sex + (1 | Subject) + storage_time
y ~ current_feature + Sex + (1 | Subject) + storage_time
y ~ current_feature + Sex + (1 | Subject) + storage_time
y ~ current_feature + Sex + (1 | Subject) + storage_time
y ~ current_feature + Sex + (1 | Subject) + storage_time
y ~ current_feature + Sex + (1 | Subject) + storage_time
y ~ current_feature + Sex + (1 | Subject) + storage_time
y ~ current_feature + Sex + (1 | Subject) + storage_time
y ~ current_feature + Sex + (1 | Subject) + storage_time
y ~ current_feature + Sex + (1 | Subject) + storage_time
y ~ current_feature + Sex + (1 | Subject) + storage_time
y ~ current_feature + Sex + (1 | Subject) + storage_time
y ~ current_feature + Sex + (1 | Subject) + storage_time
y ~ current_feature + Sex + (1 | Subject) + storage_time
y ~ current_feature + Sex + (1 | Subject) + storage_time
y ~ current_feature + Sex + (1 | Subject) + storage_time
y ~ current_feature + Sex + (1 | Subject) + storage_time
y ~ current_feature + Sex + (1 | Subject) + storage_time
y ~ current_feature + Sex + (1 | Subject) + storage_time
y ~ current_feature + Sex + (1 | Subject) + storage_time
y ~ current_feature + Sex + (1 | Subject) + storage_time
y ~ current_feature + Sex + (1 | Subject) + storage_time
y ~ current_feature + Sex + (1 | Subject) + storage_time
y ~ current_feature + Sex + (1 | Subject) + storage_time
y ~ current_feature + Sex + (1 | Subject) + storage_time
y ~ current_feature + Sex + (1 | Subject) + storage_time
y ~ current_feature + Sex + (1 | Subject) + storage_time
y ~ current_feature + Sex + (1 | Subject) + storage_time
y ~ current_feature + Sex + (1 | Subject) + storage_time
y ~ current_feature + Sex + (1 | Subject) + storage_time
y ~ current_feature + Sex + (1 | Subject) + storage_time
y ~ current_feature + Sex + (1 | Subject) + storage_time
y ~ current_feature + Sex + (1 | Subject) + storage_time
y ~ current_feature + Sex + (1 | Subject) + storage_time
y ~ current_feature + Sex + (1 | Subject) + storage_time
y ~ current_feature + Sex + (1 | Subject) + storage_time
y ~ current_feature + Sex + (1 | Subject) + storage_time

y ~ current_feature + Sex + (1 | Subject) + storage_time
y ~ current_feature + Sex + (1 | Subject) + storage_time
y ~ current_feature + Sex + (1 | Subject) + storage_time
y ~ current_feature + Sex + (1 | Subject) + storage_time
y ~ current_feature + Sex + (1 | Subject) + storage_time
y ~ current_feature + Sex + (1 | Subject) + storage_time
y ~ current_feature + Sex + (1 | Subject) + storage_time
y ~ current_feature + Sex + (1 | Subject) + storage_time
y ~ current_feature + Sex + (1 | Subject) + storage_time
y ~ current_feature + Sex + (1 | Subject) + storage_time
y ~ current_feature + Sex + (1 | Subject) + storage_time
y ~ current_feature + Sex + (1 | Subject) + storage_time
y ~ current_feature + Sex + (1 | Subject) + storage_time
y ~ current_feature + Sex + (1 | Subject) + storage_time
y ~ current_feature + Sex + (1 | Subject) + storage_time
y ~ current_feature + Sex + (1 | Subject) + storage_time
y ~ current_feature + Sex + (1 | Subject) + storage_time
y ~ current_feature + Sex + (1 | Subject) + storage_time
y ~ current_feature + Sex + (1 | Subject) + storage_time
y ~ current_feature + Sex + (1 | Subject) + storage_time
y ~ current_feature + Sex + (1 | Subject) + storage_time
y ~ current_feature + Sex + (1 | Subject) + storage_time
y ~ current_feature + Sex + (1 | Subject) + storage_time
y ~ current_feature + Sex + (1 | Subject) + storage_time
y ~ current_feature + Sex + (1 | Subject) + storage_time
y ~ current_feature + Sex + (1 | Subject) + storage_time
y ~ current_feature + Sex + (1 | Subject) + storage_time
y ~ current_feature + Sex + (1 | Subject) + storage_time
y ~ current_feature + Sex + (1 | Subject) + storage_time
y ~ current_feature + Sex + (1 | Subject) + storage_time
y ~ current_feature + Sex + (1 | Subject) + storage_time
y ~ current_feature + Sex + (1 | Subject) + storage_time
y ~ current_feature + Sex + (1 | Subject) + storage_time
y ~ current_feature + Sex + (1 | Subject) + storage_time
y ~ current_feature + Sex + (1 | Subject) + storage_time
y ~ current_feature + Sex + (1 | Subject) + storage_time
y ~ current_feature + Sex + (1 | Subject) + storage_time
y ~ current_feature + Sex + (1 | Subject) + storage_time
y ~ current_feature + Sex + (1 | Subject) + storage_time
y ~ current_feature + Sex + (1 | Subject) + storage_time
y ~ current_feature + Sex + (1 | Subject) + storage_time
y ~ current_feature + Sex + (1 | Subject) + storage_time
y ~ current_feature + Sex + (1 | Subject) + storage_time
y ~ current_feature + Sex + (1 | Subject) + storage_time
y ~ current_feature + Sex + (1 | Subject) + storage_time
y ~ current_feature + Sex + (1 | Subject) + storage_time
y ~ current_feature + Sex + (1 | Subject) + storage_time
y ~ current_feature + Sex + (1 | Subject) + storage_time

y ~ current_feature + Sex + (1 | Subject) + storage_time
y ~ current_feature + Sex + (1 | Subject) + storage_time
y ~ current_feature + Sex + (1 | Subject) + storage_time
y ~ current_feature + Sex + (1 | Subject) + storage_time
y ~ current_feature + Sex + (1 | Subject) + storage_time
y ~ current_feature + Sex + (1 | Subject) + storage_time
y ~ current_feature + Sex + (1 | Subject) + storage_time
y ~ current_feature + Sex + (1 | Subject) + storage_time
y ~ current_feature + Sex + (1 | Subject) + storage_time
y ~ current_feature + Sex + (1 | Subject) + storage_time
y ~ current_feature + Sex + (1 | Subject) + storage_time
y ~ current_feature + Sex + (1 | Subject) + storage_time
y ~ current_feature + Sex + (1 | Subject) + storage_time
y ~ current_feature + Sex + (1 | Subject) + storage_time
y ~ current_feature + Sex + (1 | Subject) + storage_time
y ~ current_feature + Sex + (1 | Subject) + storage_time
y ~ current_feature + Sex + (1 | Subject) + storage_time
y ~ current_feature + Sex + (1 | Subject) + storage_time
y ~ current_feature + Sex + (1 | Subject) + storage_time
y ~ current_feature + Sex + (1 | Subject) + storage_time
y ~ current_feature + Sex + (1 | Subject) + storage_time
y ~ current_feature + Sex + (1 | Subject) + storage_time
y ~ current_feature + Sex + (1 | Subject) + storage_time
y ~ current_feature + Sex + (1 | Subject) + storage_time
y ~ current_feature + Sex + (1 | Subject) + storage_time
y ~ current_feature + Sex + (1 | Subject) + storage_time
y ~ current_feature + Sex + (1 | Subject) + storage_time
y ~ current_feature + Sex + (1 | Subject) + storage_time
y ~ current_feature + Sex + (1 | Subject) + storage_time
y ~ current_feature + Sex + (1 | Subject) + storage_time
y ~ current_feature + Sex + (1 | Subject) + storage_time
y ~ current_feature + Sex + (1 | Subject) + storage_time
y ~ current_feature + Sex + (1 | Subject) + storage_time
y ~ current_feature + Sex + (1 | Subject) + storage_time
y ~ current_feature + Sex + (1 | Subject) + storage_time
y ~ current_feature + Sex + (1 | Subject) + storage_time
y ~ current_feature + Sex + (1 | Subject) + storage_time
y ~ current_feature + Sex + (1 | Subject) + storage_time
y ~ current_feature + Sex + (1 | Subject) + storage_time
y ~ current_feature + Sex + (1 | Subject) + storage_time
y ~ current_feature + Sex + (1 | Subject) + storage_time
y ~ current_feature + Sex + (1 | Subject) + storage_time
y ~ current_feature + Sex + (1 | Subject) + storage_time
y ~ current_feature + Sex + (1 | Subject) + storage_time
y ~ current_feature + Sex + (1 | Subject) + storage_time
y ~ current_feature + Sex + (1 | Subject) + storage_time
y ~ current_feature + Sex + (1 | Subject) + storage_time
y ~ current_feature + Sex + (1 | Subject) + storage_time

y ~ current_feature + Sex + (1 | Subject) + storage_time
y ~ current_feature + Sex + (1 | Subject) + storage_time
y ~ current_feature + Sex + (1 | Subject) + storage_time
y ~ current_feature + Sex + (1 | Subject) + storage_time
y ~ current_feature + Sex + (1 | Subject) + storage_time
y ~ current_feature + Sex + (1 | Subject) + storage_time
y ~ current_feature + Sex + (1 | Subject) + storage_time
y ~ current_feature + Sex + (1 | Subject) + storage_time
y ~ current_feature + Sex + (1 | Subject) + storage_time
y ~ current_feature + Sex + (1 | Subject) + storage_time
y ~ current_feature + Sex + (1 | Subject) + storage_time
y ~ current_feature + Sex + (1 | Subject) + storage_time
y ~ current_feature + Sex + (1 | Subject) + storage_time
y ~ current_feature + Sex + (1 | Subject) + storage_time
y ~ current_feature + Sex + (1 | Subject) + storage_time
y ~ current_feature + Sex + (1 | Subject) + storage_time
y ~ current_feature + Sex + (1 | Subject) + storage_time
y ~ current_feature + Sex + (1 | Subject) + storage_time
y ~ current_feature + Sex + (1 | Subject) + storage_time
y ~ current_feature + Sex + (1 | Subject) + storage_time
y ~ current_feature + Sex + (1 | Subject) + storage_time
y ~ current_feature + Sex + (1 | Subject) + storage_time
y ~ current_feature + Sex + (1 | Subject) + storage_time
y ~ current_feature + Sex + (1 | Subject) + storage_time
y ~ current_feature + Sex + (1 | Subject) + storage_time
y ~ current_feature + Sex + (1 | Subject) + storage_time
y ~ current_feature + Sex + (1 | Subject) + storage_time
y ~ current_feature + Sex + (1 | Subject) + storage_time
y ~ current_feature + Sex + (1 | Subject) + storage_time
y ~ current_feature + Sex + (1 | Subject) + storage_time
y ~ current_feature + Sex + (1 | Subject) + storage_time
y ~ current_feature + Sex + (1 | Subject) + storage_time
y ~ current_feature + Sex + (1 | Subject) + storage_time
y ~ current_feature + Sex + (1 | Subject) + storage_time
y ~ current_feature + Sex + (1 | Subject) + storage_time
y ~ current_feature + Sex + (1 | Subject) + storage_time
y ~ current_feature + Sex + (1 | Subject) + storage_time
y ~ current_feature + Sex + (1 | Subject) + storage_time
y ~ current_feature + Sex + (1 | Subject) + storage_time
y ~ current_feature + Sex + (1 | Subject) + storage_time
y ~ current_feature + Sex + (1 | Subject) + storage_time
y ~ current_feature + Sex + (1 | Subject) + storage_time
y ~ current_feature + Sex + (1 | Subject) + storage_time
y ~ current_feature + Sex + (1 | Subject) + storage_time
y ~ current_feature + Sex + (1 | Subject) + storage_time
y ~ current_feature + Sex + (1 | Subject) + storage_time
y ~ current_feature + Sex + (1 | Subject) + storage_time
y ~ current_feature + Sex + (1 | Subject) + storage_time

y ~ current_feature + Sex + (1 | Subject) + storage_time
y ~ current_feature + Sex + (1 | Subject) + storage_time
y ~ current_feature + Sex + (1 | Subject) + storage_time
y ~ current_feature + Sex + (1 | Subject) + storage_time
y ~ current_feature + Sex + (1 | Subject) + storage_time
y ~ current_feature + Sex + (1 | Subject) + storage_time
y ~ current_feature + Sex + (1 | Subject) + storage_time
y ~ current_feature + Sex + (1 | Subject) + storage_time
y ~ current_feature + Sex + (1 | Subject) + storage_time
y ~ current_feature + Sex + (1 | Subject) + storage_time
y ~ current_feature + Sex + (1 | Subject) + storage_time
y ~ current_feature + Sex + (1 | Subject) + storage_time
y ~ current_feature + Sex + (1 | Subject) + storage_time
y ~ current_feature + Sex + (1 | Subject) + storage_time
y ~ current_feature + Sex + (1 | Subject) + storage_time
y ~ current_feature + Sex + (1 | Subject) + storage_time
y ~ current_feature + Sex + (1 | Subject) + storage_time
y ~ current_feature + Sex + (1 | Subject) + storage_time
y ~ current_feature + Sex + (1 | Subject) + storage_time
y ~ current_feature + Sex + (1 | Subject) + storage_time
y ~ current_feature + Sex + (1 | Subject) + storage_time
y ~ current_feature + Sex + (1 | Subject) + storage_time
y ~ current_feature + Sex + (1 | Subject) + storage_time
y ~ current_feature + Sex + (1 | Subject) + storage_time
y ~ current_feature + Sex + (1 | Subject) + storage_time
y ~ current_feature + Sex + (1 | Subject) + storage_time
y ~ current_feature + Sex + (1 | Subject) + storage_time
y ~ current_feature + Sex + (1 | Subject) + storage_time
y ~ current_feature + Sex + (1 | Subject) + storage_time
y ~ current_feature + Sex + (1 | Subject) + storage_time
y ~ current_feature + Sex + (1 | Subject) + storage_time
y ~ current_feature + Sex + (1 | Subject) + storage_time
y ~ current_feature + Sex + (1 | Subject) + storage_time
y ~ current_feature + Sex + (1 | Subject) + storage_time
y ~ current_feature + Sex + (1 | Subject) + storage_time
y ~ current_feature + Sex + (1 | Subject) + storage_time
y ~ current_feature + Sex + (1 | Subject) + storage_time
y ~ current_feature + Sex + (1 | Subject) + storage_time
y ~ current_feature + Sex + (1 | Subject) + storage_time
y ~ current_feature + Sex + (1 | Subject) + storage_time
y ~ current_feature + Sex + (1 | Subject) + storage_time
y ~ current_feature + Sex + (1 | Subject) + storage_time
y ~ current_feature + Sex + (1 | Subject) + storage_time
y ~ current_feature + Sex + (1 | Subject) + storage_time
y ~ current_feature + Sex + (1 | Subject) + storage_time
y ~ current_feature + Sex + (1 | Subject) + storage_time
y ~ current_feature + Sex + (1 | Subject) + storage_time
y ~ current_feature + Sex + (1 | Subject) + storage_time

y ~ current_feature + Sex + (1 | Subject) + storage_time
y ~ current_feature + Sex + (1 | Subject) + storage_time
y ~ current_feature + Sex + (1 | Subject) + storage_time
y ~ current_feature + Sex + (1 | Subject) + storage_time
y ~ current_feature + Sex + (1 | Subject) + storage_time
y ~ current_feature + Sex + (1 | Subject) + storage_time
y ~ current_feature + Sex + (1 | Subject) + storage_time
y ~ current_feature + Sex + (1 | Subject) + storage_time
y ~ current_feature + Sex + (1 | Subject) + storage_time
y ~ current_feature + Sex + (1 | Subject) + storage_time
y ~ current_feature + Sex + (1 | Subject) + storage_time
y ~ current_feature + Sex + (1 | Subject) + storage_time
y ~ current_feature + Sex + (1 | Subject) + storage_time
y ~ current_feature + Sex + (1 | Subject) + storage_time
y ~ current_feature + Sex + (1 | Subject) + storage_time
y ~ current_feature + Sex + (1 | Subject) + storage_time
y ~ current_feature + Sex + (1 | Subject) + storage_time
y ~ current_feature + Sex + (1 | Subject) + storage_time
y ~ current_feature + Sex + (1 | Subject) + storage_time
y ~ current_feature + Sex + (1 | Subject) + storage_time
y ~ current_feature + Sex + (1 | Subject) + storage_time
y ~ current_feature + Sex + (1 | Subject) + storage_time
y ~ current_feature + Sex + (1 | Subject) + storage_time
y ~ current_feature + Sex + (1 | Subject) + storage_time
y ~ current_feature + Sex + (1 | Subject) + storage_time
y ~ current_feature + Sex + (1 | Subject) + storage_time
y ~ current_feature + Sex + (1 | Subject) + storage_time
y ~ current_feature + Sex + (1 | Subject) + storage_time
y ~ current_feature + Sex + (1 | Subject) + storage_time
y ~ current_feature + Sex + (1 | Subject) + storage_time
y ~ current_feature + Sex + (1 | Subject) + storage_time
y ~ current_feature + Sex + (1 | Subject) + storage_time
y ~ current_feature + Sex + (1 | Subject) + storage_time
y ~ current_feature + Sex + (1 | Subject) + storage_time
y ~ current_feature + Sex + (1 | Subject) + storage_time
y ~ current_feature + Sex + (1 | Subject) + storage_time
y ~ current_feature + Sex + (1 | Subject) + storage_time
y ~ current_feature + Sex + (1 | Subject) + storage_time
y ~ current_feature + Sex + (1 | Subject) + storage_time
y ~ current_feature + Sex + (1 | Subject) + storage_time
y ~ current_feature + Sex + (1 | Subject) + storage_time
y ~ current_feature + Sex + (1 | Subject) + storage_time
y ~ current_feature + Sex + (1 | Subject) + storage_time
y ~ current_feature + Sex + (1 | Subject) + storage_time
y ~ current_feature + Sex + (1 | Subject) + storage_time
y ~ current_feature + Sex + (1 | Subject) + storage_time
y ~ current_feature + Sex + (1 | Subject) + storage_time
y ~ current_feature + Sex + (1 | Subject) + storage_time

y ~ current_feature + Sex + (1 | Subject) + storage_time
y ~ current_feature + Sex + (1 | Subject) + storage_time
y ~ current_feature + Sex + (1 | Subject) + storage_time
y ~ current_feature + Sex + (1 | Subject) + storage_time
y ~ current_feature + Sex + (1 | Subject) + storage_time
y ~ current_feature + Sex + (1 | Subject) + storage_time
y ~ current_feature + Sex + (1 | Subject) + storage_time
y ~ current_feature + Sex + (1 | Subject) + storage_time
y ~ current_feature + Sex + (1 | Subject) + storage_time
y ~ current_feature + Sex + (1 | Subject) + storage_time
y ~ current_feature + Sex + (1 | Subject) + storage_time
y ~ current_feature + Sex + (1 | Subject) + storage_time
y ~ current_feature + Sex + (1 | Subject) + storage_time
y ~ current_feature + Sex + (1 | Subject) + storage_time
y ~ current_feature + Sex + (1 | Subject) + storage_time
y ~ current_feature + Sex + (1 | Subject) + storage_time
y ~ current_feature + Sex + (1 | Subject) + storage_time
y ~ current_feature + Sex + (1 | Subject) + storage_time
y ~ current_feature + Sex + (1 | Subject) + storage_time
y ~ current_feature + Sex + (1 | Subject) + storage_time
y ~ current_feature + Sex + (1 | Subject) + storage_time
y ~ current_feature + Sex + (1 | Subject) + storage_time
y ~ current_feature + Sex + (1 | Subject) + storage_time
y ~ current_feature + Sex + (1 | Subject) + storage_time
y ~ current_feature + Sex + (1 | Subject) + storage_time
y ~ current_feature + Sex + (1 | Subject) + storage_time
y ~ current_feature + Sex + (1 | Subject) + storage_time
y ~ current_feature + Sex + (1 | Subject) + storage_time
y ~ current_feature + Sex + (1 | Subject) + storage_time
y ~ current_feature + Sex + (1 | Subject) + storage_time
y ~ current_feature + Sex + (1 | Subject) + storage_time
y ~ current_feature + Sex + (1 | Subject) + storage_time
y ~ current_feature + Sex + (1 | Subject) + storage_time
y ~ current_feature + Sex + (1 | Subject) + storage_time
y ~ current_feature + Sex + (1 | Subject) + storage_time
y ~ current_feature + Sex + (1 | Subject) + storage_time
y ~ current_feature + Sex + (1 | Subject) + storage_time
y ~ current_feature + Sex + (1 | Subject) + storage_time
y ~ current_feature + Sex + (1 | Subject) + storage_time
y ~ current_feature + Sex + (1 | Subject) + storage_time
y ~ current_feature + Sex + (1 | Subject) + storage_time
y ~ current_feature + Sex + (1 | Subject) + storage_time
y ~ current_feature + Sex + (1 | Subject) + storage_time
y ~ current_feature + Sex + (1 | Subject) + storage_time
y ~ current_feature + Sex + (1 | Subject) + storage_time
y ~ current_feature + Sex + (1 | Subject) + storage_time
y ~ current_feature + Sex + (1 | Subject) + storage_time
y ~ current_feature + Sex + (1 | Subject) + storage_time

y ~ current_feature + Sex + (1 | Subject) + storage_time
y ~ current_feature + Sex + (1 | Subject) + storage_time
y ~ current_feature + Sex + (1 | Subject) + storage_time
y ~ current_feature + Sex + (1 | Subject) + storage_time
y ~ current_feature + Sex + (1 | Subject) + storage_time
y ~ current_feature + Sex + (1 | Subject) + storage_time
y ~ current_feature + Sex + (1 | Subject) + storage_time
y ~ current_feature + Sex + (1 | Subject) + storage_time
y ~ current_feature + Sex + (1 | Subject) + storage_time
y ~ current_feature + Sex + (1 | Subject) + storage_time
y ~ current_feature + Sex + (1 | Subject) + storage_time
y ~ current_feature + Sex + (1 | Subject) + storage_time
y ~ current_feature + Sex + (1 | Subject) + storage_time
y ~ current_feature + Sex + (1 | Subject) + storage_time
y ~ current_feature + Sex + (1 | Subject) + storage_time
y ~ current_feature + Sex + (1 | Subject) + storage_time
y ~ current_feature + Sex + (1 | Subject) + storage_time
y ~ current_feature + Sex + (1 | Subject) + storage_time
y ~ current_feature + Sex + (1 | Subject) + storage_time
y ~ current_feature + Sex + (1 | Subject) + storage_time
y ~ current_feature + Sex + (1 | Subject) + storage_time
y ~ current_feature + Sex + (1 | Subject) + storage_time
y ~ current_feature + Sex + (1 | Subject) + storage_time
y ~ current_feature + Sex + (1 | Subject) + storage_time
y ~ current_feature + Sex + (1 | Subject) + storage_time
y ~ current_feature + Sex + (1 | Subject) + storage_time
y ~ current_feature + Sex + (1 | Subject) + storage_time
y ~ current_feature + Sex + (1 | Subject) + storage_time
y ~ current_feature + Sex + (1 | Subject) + storage_time
y ~ current_feature + Sex + (1 | Subject) + storage_time
y ~ current_feature + Sex + (1 | Subject) + storage_time
y ~ current_feature + Sex + (1 | Subject) + storage_time
y ~ current_feature + Sex + (1 | Subject) + storage_time
y ~ current_feature + Sex + (1 | Subject) + storage_time
y ~ current_feature + Sex + (1 | Subject) + storage_time
y ~ current_feature + Sex + (1 | Subject) + storage_time
y ~ current_feature + Sex + (1 | Subject) + storage_time
y ~ current_feature + Sex + (1 | Subject) + storage_time
y ~ current_feature + Sex + (1 | Subject) + storage_time
y ~ current_feature + Sex + (1 | Subject) + storage_time
y ~ current_feature + Sex + (1 | Subject) + storage_time
y ~ current_feature + Sex + (1 | Subject) + storage_time
y ~ current_feature + Sex + (1 | Subject) + storage_time
y ~ current_feature + Sex + (1 | Subject) + storage_time
y ~ current_feature + Sex + (1 | Subject) + storage_time
y ~ current_feature + Sex + (1 | Subject) + storage_time
y ~ current_feature + Sex + (1 | Subject) + storage_time
y ~ current_feature + Sex + (1 | Subject) + storage_time

y ~ current_feature + Sex + (1 | Subject) + storage_time
y ~ current_feature + Sex + (1 | Subject) + storage_time
y ~ current_feature + Sex + (1 | Subject) + storage_time
y ~ current_feature + Sex + (1 | Subject) + storage_time
y ~ current_feature + Sex + (1 | Subject) + storage_time
y ~ current_feature + Sex + (1 | Subject) + storage_time
y ~ current_feature + Sex + (1 | Subject) + storage_time
y ~ current_feature + Sex + (1 | Subject) + storage_time
y ~ current_feature + Sex + (1 | Subject) + storage_time
y ~ current_feature + Sex + (1 | Subject) + storage_time
y ~ current_feature + Sex + (1 | Subject) + storage_time
y ~ current_feature + Sex + (1 | Subject) + storage_time
y ~ current_feature + Sex + (1 | Subject) + storage_time
y ~ current_feature + Sex + (1 | Subject) + storage_time
y ~ current_feature + Sex + (1 | Subject) + storage_time
y ~ current_feature + Sex + (1 | Subject) + storage_time
y ~ current_feature + Sex + (1 | Subject) + storage_time
y ~ current_feature + Sex + (1 | Subject) + storage_time
y ~ current_feature + Sex + (1 | Subject) + storage_time
y ~ current_feature + Sex + (1 | Subject) + storage_time
y ~ current_feature + Sex + (1 | Subject) + storage_time
y ~ current_feature + Sex + (1 | Subject) + storage_time
y ~ current_feature + Sex + (1 | Subject) + storage_time
y ~ current_feature + Sex + (1 | Subject) + storage_time
y ~ current_feature + Sex + (1 | Subject) + storage_time
y ~ current_feature + Sex + (1 | Subject) + storage_time
y ~ current_feature + Sex + (1 | Subject) + storage_time
y ~ current_feature + Sex + (1 | Subject) + storage_time
y ~ current_feature + Sex + (1 | Subject) + storage_time
y ~ current_feature + Sex + (1 | Subject) + storage_time
y ~ current_feature + Sex + (1 | Subject) + storage_time
y ~ current_feature + Sex + (1 | Subject) + storage_time
y ~ current_feature + Sex + (1 | Subject) + storage_time
y ~ current_feature + Sex + (1 | Subject) + storage_time
y ~ current_feature + Sex + (1 | Subject) + storage_time
y ~ current_feature + Sex + (1 | Subject) + storage_time
y ~ current_feature + Sex + (1 | Subject) + storage_time
y ~ current_feature + Sex + (1 | Subject) + storage_time
y ~ current_feature + Sex + (1 | Subject) + storage_time
y ~ current_feature + Sex + (1 | Subject) + storage_time
y ~ current_feature + Sex + (1 | Subject) + storage_time
y ~ current_feature + Sex + (1 | Subject) + storage_time
y ~ current_feature + Sex + (1 | Subject) + storage_time
y ~ current_feature + Sex + (1 | Subject) + storage_time
y ~ current_feature + Sex + (1 | Subject) + storage_time
y ~ current_feature + Sex + (1 | Subject) + storage_time
y ~ current_feature + Sex + (1 | Subject) + storage_time
y ~ current_feature + Sex + (1 | Subject) + storage_time

y ~ current_feature + Sex + (1 | Subject) + storage_time
y ~ current_feature + Sex + (1 | Subject) + storage_time
y ~ current_feature + Sex + (1 | Subject) + storage_time
y ~ current_feature + Sex + (1 | Subject) + storage_time
y ~ current_feature + Sex + (1 | Subject) + storage_time
y ~ current_feature + Sex + (1 | Subject) + storage_time
y ~ current_feature + Sex + (1 | Subject) + storage_time
y ~ current_feature + Sex + (1 | Subject) + storage_time
y ~ current_feature + Sex + (1 | Subject) + storage_time
y ~ current_feature + Sex + (1 | Subject) + storage_time
y ~ current_feature + Sex + (1 | Subject) + storage_time
y ~ current_feature + Sex + (1 | Subject) + storage_time
y ~ current_feature + Sex + (1 | Subject) + storage_time
y ~ current_feature + Sex + (1 | Subject) + storage_time
y ~ current_feature + Sex + (1 | Subject) + storage_time
y ~ current_feature + Sex + (1 | Subject) + storage_time
y ~ current_feature + Sex + (1 | Subject) + storage_time
y ~ current_feature + Sex + (1 | Subject) + storage_time
y ~ current_feature + Sex + (1 | Subject) + storage_time
y ~ current_feature + Sex + (1 | Subject) + storage_time
y ~ current_feature + Sex + (1 | Subject) + storage_time
y ~ current_feature + Sex + (1 | Subject) + storage_time
y ~ current_feature + Sex + (1 | Subject) + storage_time
y ~ current_feature + Sex + (1 | Subject) + storage_time
y ~ current_feature + Sex + (1 | Subject) + storage_time
y ~ current_feature + Sex + (1 | Subject) + storage_time
y ~ current_feature + Sex + (1 | Subject) + storage_time
y ~ current_feature + Sex + (1 | Subject) + storage_time
y ~ current_feature + Sex + (1 | Subject) + storage_time
y ~ current_feature + Sex + (1 | Subject) + storage_time
y ~ current_feature + Sex + (1 | Subject) + storage_time
y ~ current_feature + Sex + (1 | Subject) + storage_time
y ~ current_feature + Sex + (1 | Subject) + storage_time
y ~ current_feature + Sex + (1 | Subject) + storage_time
y ~ current_feature + Sex + (1 | Subject) + storage_time
y ~ current_feature + Sex + (1 | Subject) + storage_time
y ~ current_feature + Sex + (1 | Subject) + storage_time
y ~ current_feature + Sex + (1 | Subject) + storage_time
y ~ current_feature + Sex + (1 | Subject) + storage_time
y ~ current_feature + Sex + (1 | Subject) + storage_time
y ~ current_feature + Sex + (1 | Subject) + storage_time
y ~ current_feature + Sex + (1 | Subject) + storage_time
y ~ current_feature + Sex + (1 | Subject) + storage_time
y ~ current_feature + Sex + (1 | Subject) + storage_time
y ~ current_feature + Sex + (1 | Subject) + storage_time
y ~ current_feature + Sex + (1 | Subject) + storage_time
y ~ current_feature + Sex + (1 | Subject) + storage_time
y ~ current_feature + Sex + (1 | Subject) + storage_time

y ~ current_feature + Sex + (1 | Subject) + storage_time
y ~ current_feature + Sex + (1 | Subject) + storage_time
y ~ current_feature + Sex + (1 | Subject) + storage_time
y ~ current_feature + Sex + (1 | Subject) + storage_time
y ~ current_feature + Sex + (1 | Subject) + storage_time
y ~ current_feature + Sex + (1 | Subject) + storage_time
y ~ current_feature + Sex + (1 | Subject) + storage_time
y ~ current_feature + Sex + (1 | Subject) + storage_time
y ~ current_feature + Sex + (1 | Subject) + storage_time
y ~ current_feature + Sex + (1 | Subject) + storage_time
y ~ current_feature + Sex + (1 | Subject) + storage_time
y ~ current_feature + Sex + (1 | Subject) + storage_time
y ~ current_feature + Sex + (1 | Subject) + storage_time
y ~ current_feature + Sex + (1 | Subject) + storage_time
y ~ current_feature + Sex + (1 | Subject) + storage_time
y ~ current_feature + Sex + (1 | Subject) + storage_time
y ~ current_feature + Sex + (1 | Subject) + storage_time
y ~ current_feature + Sex + (1 | Subject) + storage_time
y ~ current_feature + Sex + (1 | Subject) + storage_time
y ~ current_feature + Sex + (1 | Subject) + storage_time
y ~ current_feature + Sex + (1 | Subject) + storage_time
y ~ current_feature + Sex + (1 | Subject) + storage_time
y ~ current_feature + Sex + (1 | Subject) + storage_time
y ~ current_feature + Sex + (1 | Subject) + storage_time
y ~ current_feature + Sex + (1 | Subject) + storage_time
y ~ current_feature + Sex + (1 | Subject) + storage_time
y ~ current_feature + Sex + (1 | Subject) + storage_time
y ~ current_feature + Sex + (1 | Subject) + storage_time
y ~ current_feature + Sex + (1 | Subject) + storage_time
y ~ current_feature + Sex + (1 | Subject) + storage_time
y ~ current_feature + Sex + (1 | Subject) + storage_time
y ~ current_feature + Sex + (1 | Subject) + storage_time
y ~ current_feature + Sex + (1 | Subject) + storage_time
y ~ current_feature + Sex + (1 | Subject) + storage_time
y ~ current_feature + Sex + (1 | Subject) + storage_time
y ~ current_feature + Sex + (1 | Subject) + storage_time
y ~ current_feature + Sex + (1 | Subject) + storage_time
y ~ current_feature + Sex + (1 | Subject) + storage_time
y ~ current_feature + Sex + (1 | Subject) + storage_time
y ~ current_feature + Sex + (1 | Subject) + storage_time
y ~ current_feature + Sex + (1 | Subject) + storage_time
y ~ current_feature + Sex + (1 | Subject) + storage_time
y ~ current_feature + Sex + (1 | Subject) + storage_time
y ~ current_feature + Sex + (1 | Subject) + storage_time
y ~ current_feature + Sex + (1 | Subject) + storage_time
y ~ current_feature + Sex + (1 | Subject) + storage_time
y ~ current_feature + Sex + (1 | Subject) + storage_time
y ~ current_feature + Sex + (1 | Subject) + storage_time

y ~ current_feature + Sex + (1 | Subject) + storage_time
y ~ current_feature + Sex + (1 | Subject) + storage_time
y ~ current_feature + Sex + (1 | Subject) + storage_time
y ~ current_feature + Sex + (1 | Subject) + storage_time
y ~ current_feature + Sex + (1 | Subject) + storage_time
y ~ current_feature + Sex + (1 | Subject) + storage_time
y ~ current_feature + Sex + (1 | Subject) + storage_time
y ~ current_feature + Sex + (1 | Subject) + storage_time
y ~ current_feature + Sex + (1 | Subject) + storage_time
y ~ current_feature + Sex + (1 | Subject) + storage_time
y ~ current_feature + Sex + (1 | Subject) + storage_time
y ~ current_feature + Sex + (1 | Subject) + storage_time
y ~ current_feature + Sex + (1 | Subject) + storage_time
y ~ current_feature + Sex + (1 | Subject) + storage_time
y ~ current_feature + Sex + (1 | Subject) + storage_time
y ~ current_feature + Sex + (1 | Subject) + storage_time
y ~ current_feature + Sex + (1 | Subject) + storage_time
y ~ current_feature + Sex + (1 | Subject) + storage_time
y ~ current_feature + Sex + (1 | Subject) + storage_time
y ~ current_feature + Sex + (1 | Subject) + storage_time
y ~ current_feature + Sex + (1 | Subject) + storage_time
y ~ current_feature + Sex + (1 | Subject) + storage_time
y ~ current_feature + Sex + (1 | Subject) + storage_time
y ~ current_feature + Sex + (1 | Subject) + storage_time
y ~ current_feature + Sex + (1 | Subject) + storage_time
y ~ current_feature + Sex + (1 | Subject) + storage_time
y ~ current_feature + Sex + (1 | Subject) + storage_time
y ~ current_feature + Sex + (1 | Subject) + storage_time
y ~ current_feature + Sex + (1 | Subject) + storage_time
y ~ current_feature + Sex + (1 | Subject) + storage_time
y ~ current_feature + Sex + (1 | Subject) + storage_time
y ~ current_feature + Sex + (1 | Subject) + storage_time
y ~ current_feature + Sex + (1 | Subject) + storage_time
y ~ current_feature + Sex + (1 | Subject) + storage_time
y ~ current_feature + Sex + (1 | Subject) + storage_time
y ~ current_feature + Sex + (1 | Subject) + storage_time
y ~ current_feature + Sex + (1 | Subject) + storage_time
y ~ current_feature + Sex + (1 | Subject) + storage_time
y ~ current_feature + Sex + (1 | Subject) + storage_time
y ~ current_feature + Sex + (1 | Subject) + storage_time
y ~ current_feature + Sex + (1 | Subject) + storage_time
y ~ current_feature + Sex + (1 | Subject) + storage_time
y ~ current_feature + Sex + (1 | Subject) + storage_time
y ~ current_feature + Sex + (1 | Subject) + storage_time
y ~ current_feature + Sex + (1 | Subject) + storage_time
y ~ current_feature + Sex + (1 | Subject) + storage_time
y ~ current_feature + Sex + (1 | Subject) + storage_time
y ~ current_feature + Sex + (1 | Subject) + storage_time

y ~ current_feature + Sex + (1 | Subject) + storage_time
y ~ current_feature + Sex + (1 | Subject) + storage_time
y ~ current_feature + Sex + (1 | Subject) + storage_time
y ~ current_feature + Sex + (1 | Subject) + storage_time
y ~ current_feature + Sex + (1 | Subject) + storage_time
y ~ current_feature + Sex + (1 | Subject) + storage_time
y ~ current_feature + Sex + (1 | Subject) + storage_time
y ~ current_feature + Sex + (1 | Subject) + storage_time
y ~ current_feature + Sex + (1 | Subject) + storage_time
y ~ current_feature + Sex + (1 | Subject) + storage_time
y ~ current_feature + Sex + (1 | Subject) + storage_time
y ~ current_feature + Sex + (1 | Subject) + storage_time
y ~ current_feature + Sex + (1 | Subject) + storage_time
y ~ current_feature + Sex + (1 | Subject) + storage_time
y ~ current_feature + Sex + (1 | Subject) + storage_time
y ~ current_feature + Sex + (1 | Subject) + storage_time
y ~ current_feature + Sex + (1 | Subject) + storage_time
y ~ current_feature + Sex + (1 | Subject) + storage_time
y ~ current_feature + Sex + (1 | Subject) + storage_time
y ~ current_feature + Sex + (1 | Subject) + storage_time
y ~ current_feature + Sex + (1 | Subject) + storage_time
y ~ current_feature + Sex + (1 | Subject) + storage_time
y ~ current_feature + Sex + (1 | Subject) + storage_time
y ~ current_feature + Sex + (1 | Subject) + storage_time
y ~ current_feature + Sex + (1 | Subject) + storage_time
y ~ current_feature + Sex + (1 | Subject) + storage_time
y ~ current_feature + Sex + (1 | Subject) + storage_time
y ~ current_feature + Sex + (1 | Subject) + storage_time
y ~ current_feature + Sex + (1 | Subject) + storage_time
y ~ current_feature + Sex + (1 | Subject) + storage_time
y ~ current_feature + Sex + (1 | Subject) + storage_time
y ~ current_feature + Sex + (1 | Subject) + storage_time
y ~ current_feature + Sex + (1 | Subject) + storage_time
y ~ current_feature + Sex + (1 | Subject) + storage_time
y ~ current_feature + Sex + (1 | Subject) + storage_time
y ~ current_feature + Sex + (1 | Subject) + storage_time
y ~ current_feature + Sex + (1 | Subject) + storage_time
y ~ current_feature + Sex + (1 | Subject) + storage_time
y ~ current_feature + Sex + (1 | Subject) + storage_time
y ~ current_feature + Sex + (1 | Subject) + storage_time
y ~ current_feature + Sex + (1 | Subject) + storage_time
y ~ current_feature + Sex + (1 | Subject) + storage_time
y ~ current_feature + Sex + (1 | Subject) + storage_time
y ~ current_feature + Sex + (1 | Subject) + storage_time
y ~ current_feature + Sex + (1 | Subject) + storage_time
y ~ current_feature + Sex + (1 | Subject) + storage_time
y ~ current_feature + Sex + (1 | Subject) + storage_time
y ~ current_feature + Sex + (1 | Subject) + storage_time

y ~ current_feature + Sex + (1 | Subject) + storage_time
y ~ current_feature + Sex + (1 | Subject) + storage_time
y ~ current_feature + Sex + (1 | Subject) + storage_time
y ~ current_feature + Sex + (1 | Subject) + storage_time
y ~ current_feature + Sex + (1 | Subject) + storage_time
y ~ current_feature + Sex + (1 | Subject) + storage_time
y ~ current_feature + Sex + (1 | Subject) + storage_time
y ~ current_feature + Sex + (1 | Subject) + storage_time
y ~ current_feature + Sex + (1 | Subject) + storage_time
y ~ current_feature + Sex + (1 | Subject) + storage_time
y ~ current_feature + Sex + (1 | Subject) + storage_time
y ~ current_feature + Sex + (1 | Subject) + storage_time
y ~ current_feature + Sex + (1 | Subject) + storage_time
y ~ current_feature + Sex + (1 | Subject) + storage_time
y ~ current_feature + Sex + (1 | Subject) + storage_time
y ~ current_feature + Sex + (1 | Subject) + storage_time
y ~ current_feature + Sex + (1 | Subject) + storage_time
y ~ current_feature + Sex + (1 | Subject) + storage_time
y ~ current_feature + Sex + (1 | Subject) + storage_time
y ~ current_feature + Sex + (1 | Subject) + storage_time
y ~ current_feature + Sex + (1 | Subject) + storage_time
y ~ current_feature + Sex + (1 | Subject) + storage_time
y ~ current_feature + Sex + (1 | Subject) + storage_time
y ~ current_feature + Sex + (1 | Subject) + storage_time
y ~ current_feature + Sex + (1 | Subject) + storage_time
y ~ current_feature + Sex + (1 | Subject) + storage_time
y ~ current_feature + Sex + (1 | Subject) + storage_time
y ~ current_feature + Sex + (1 | Subject) + storage_time
y ~ current_feature + Sex + (1 | Subject) + storage_time
y ~ current_feature + Sex + (1 | Subject) + storage_time
y ~ current_feature + Sex + (1 | Subject) + storage_time
y ~ current_feature + Sex + (1 | Subject) + storage_time
y ~ current_feature + Sex + (1 | Subject) + storage_time
y ~ current_feature + Sex + (1 | Subject) + storage_time
y ~ current_feature + Sex + (1 | Subject) + storage_time
y ~ current_feature + Sex + (1 | Subject) + storage_time
y ~ current_feature + Sex + (1 | Subject) + storage_time
y ~ current_feature + Sex + (1 | Subject) + storage_time
y ~ current_feature + Sex + (1 | Subject) + storage_time
y ~ current_feature + Sex + (1 | Subject) + storage_time
y ~ current_feature + Sex + (1 | Subject) + storage_time
y ~ current_feature + Sex + (1 | Subject) + storage_time
y ~ current_feature + Sex + (1 | Subject) + storage_time
y ~ current_feature + Sex + (1 | Subject) + storage_time
y ~ current_feature + Sex + (1 | Subject) + storage_time
y ~ current_feature + Sex + (1 | Subject) + storage_time
y ~ current_feature + Sex + (1 | Subject) + storage_time
y ~ current_feature + Sex + (1 | Subject) + storage_time

y ~ current_feature + Sex + (1 | Subject) + storage_time
y ~ current_feature + Sex + (1 | Subject) + storage_time
y ~ current_feature + Sex + (1 | Subject) + storage_time
y ~ current_feature + Sex + (1 | Subject) + storage_time
y ~ current_feature + Sex + (1 | Subject) + storage_time
y ~ current_feature + Sex + (1 | Subject) + storage_time
y ~ current_feature + Sex + (1 | Subject) + storage_time
y ~ current_feature + Sex + (1 | Subject) + storage_time
y ~ current_feature + Sex + (1 | Subject) + storage_time
y ~ current_feature + Sex + (1 | Subject) + storage_time
y ~ current_feature + Sex + (1 | Subject) + storage_time
y ~ current_feature + Sex + (1 | Subject) + storage_time
y ~ current_feature + Sex + (1 | Subject) + storage_time
y ~ current_feature + Sex + (1 | Subject) + storage_time
y ~ current_feature + Sex + (1 | Subject) + storage_time
y ~ current_feature + Sex + (1 | Subject) + storage_time
y ~ current_feature + Sex + (1 | Subject) + storage_time
y ~ current_feature + Sex + (1 | Subject) + storage_time
y ~ current_feature + Sex + (1 | Subject) + storage_time
y ~ current_feature + Sex + (1 | Subject) + storage_time
y ~ current_feature + Sex + (1 | Subject) + storage_time
y ~ current_feature + Sex + (1 | Subject) + storage_time
y ~ current_feature + Sex + (1 | Subject) + storage_time
y ~ current_feature + Sex + (1 | Subject) + storage_time
y ~ current_feature + Sex + (1 | Subject) + storage_time
y ~ current_feature + Sex + (1 | Subject) + storage_time
y ~ current_feature + Sex + (1 | Subject) + storage_time
y ~ current_feature + Sex + (1 | Subject) + storage_time
y ~ current_feature + Sex + (1 | Subject) + storage_time
y ~ current_feature + Sex + (1 | Subject) + storage_time
y ~ current_feature + Sex + (1 | Subject) + storage_time
y ~ current_feature + Sex + (1 | Subject) + storage_time
y ~ current_feature + Sex + (1 | Subject) + storage_time
y ~ current_feature + Sex + (1 | Subject) + storage_time
y ~ current_feature + Sex + (1 | Subject) + storage_time
y ~ current_feature + Sex + (1 | Subject) + storage_time
y ~ current_feature + Sex + (1 | Subject) + storage_time
y ~ current_feature + Sex + (1 | Subject) + storage_time
y ~ current_feature + Sex + (1 | Subject) + storage_time
y ~ current_feature + Sex + (1 | Subject) + storage_time
y ~ current_feature + Sex + (1 | Subject) + storage_time
y ~ current_feature + Sex + (1 | Subject) + storage_time
y ~ current_feature + Sex + (1 | Subject) + storage_time
y ~ current_feature + Sex + (1 | Subject) + storage_time
y ~ current_feature + Sex + (1 | Subject) + storage_time
y ~ current_feature + Sex + (1 | Subject) + storage_time
y ~ current_feature + Sex + (1 | Subject) + storage_time
y ~ current_feature + Sex + (1 | Subject) + storage_time

y ~ current_feature + Sex + (1 | Subject) + storage_time
y ~ current_feature + Sex + (1 | Subject) + storage_time
y ~ current_feature + Sex + (1 | Subject) + storage_time
y ~ current_feature + Sex + (1 | Subject) + storage_time
y ~ current_feature + Sex + (1 | Subject) + storage_time
y ~ current_feature + Sex + (1 | Subject) + storage_time
y ~ current_feature + Sex + (1 | Subject) + storage_time
y ~ current_feature + Sex + (1 | Subject) + storage_time
y ~ current_feature + Sex + (1 | Subject) + storage_time
y ~ current_feature + Sex + (1 | Subject) + storage_time
y ~ current_feature + Sex + (1 | Subject) + storage_time
y ~ current_feature + Sex + (1 | Subject) + storage_time
y ~ current_feature + Sex + (1 | Subject) + storage_time
y ~ current_feature + Sex + (1 | Subject) + storage_time
y ~ current_feature + Sex + (1 | Subject) + storage_time
y ~ current_feature + Sex + (1 | Subject) + storage_time
y ~ current_feature + Sex + (1 | Subject) + storage_time
y ~ current_feature + Sex + (1 | Subject) + storage_time
y ~ current_feature + Sex + (1 | Subject) + storage_time
y ~ current_feature + Sex + (1 | Subject) + storage_time
y ~ current_feature + Sex + (1 | Subject) + storage_time
y ~ current_feature + Sex + (1 | Subject) + storage_time
y ~ current_feature + Sex + (1 | Subject) + storage_time
y ~ current_feature + Sex + (1 | Subject) + storage_time
y ~ current_feature + Sex + (1 | Subject) + storage_time
y ~ current_feature + Sex + (1 | Subject) + storage_time
y ~ current_feature + Sex + (1 | Subject) + storage_time
y ~ current_feature + Sex + (1 | Subject) + storage_time
y ~ current_feature + Sex + (1 | Subject) + storage_time
y ~ current_feature + Sex + (1 | Subject) + storage_time
y ~ current_feature + Sex + (1 | Subject) + storage_time
y ~ current_feature + Sex + (1 | Subject) + storage_time
y ~ current_feature + Sex + (1 | Subject) + storage_time
y ~ current_feature + Sex + (1 | Subject) + storage_time
y ~ current_feature + Sex + (1 | Subject) + storage_time
y ~ current_feature + Sex + (1 | Subject) + storage_time
y ~ current_feature + Sex + (1 | Subject) + storage_time
y ~ current_feature + Sex + (1 | Subject) + storage_time
y ~ current_feature + Sex + (1 | Subject) + storage_time
y ~ current_feature + Sex + (1 | Subject) + storage_time
y ~ current_feature + Sex + (1 | Subject) + storage_time
y ~ current_feature + Sex + (1 | Subject) + storage_time
y ~ current_feature + Sex + (1 | Subject) + storage_time
y ~ current_feature + Sex + (1 | Subject) + storage_time
y ~ current_feature + Sex + (1 | Subject) + storage_time
y ~ current_feature + Sex + (1 | Subject) + storage_time
y ~ current_feature + Sex + (1 | Subject) + storage_time
y ~ current_feature + Sex + (1 | Subject) + storage_time

y ~ current_feature + Sex + (1 | Subject) + storage_time
y ~ current_feature + Sex + (1 | Subject) + storage_time
y ~ current_feature + Sex + (1 | Subject) + storage_time
y ~ current_feature + Sex + (1 | Subject) + storage_time
y ~ current_feature + Sex + (1 | Subject) + storage_time
y ~ current_feature + Sex + (1 | Subject) + storage_time
y ~ current_feature + Sex + (1 | Subject) + storage_time
y ~ current_feature + Sex + (1 | Subject) + storage_time
y ~ current_feature + Sex + (1 | Subject) + storage_time
y ~ current_feature + Sex + (1 | Subject) + storage_time
y ~ current_feature + Sex + (1 | Subject) + storage_time
y ~ current_feature + Sex + (1 | Subject) + storage_time
y ~ current_feature + Sex + (1 | Subject) + storage_time
y ~ current_feature + Sex + (1 | Subject) + storage_time
y ~ current_feature + Sex + (1 | Subject) + storage_time
y ~ current_feature + Sex + (1 | Subject) + storage_time
y ~ current_feature + Sex + (1 | Subject) + storage_time
y ~ current_feature + Sex + (1 | Subject) + storage_time
y ~ current_feature + Sex + (1 | Subject) + storage_time
y ~ current_feature + Sex + (1 | Subject) + storage_time
y ~ current_feature + Sex + (1 | Subject) + storage_time
y ~ current_feature + Sex + (1 | Subject) + storage_time
y ~ current_feature + Sex + (1 | Subject) + storage_time
y ~ current_feature + Sex + (1 | Subject) + storage_time
y ~ current_feature + Sex + (1 | Subject) + storage_time
y ~ current_feature + Sex + (1 | Subject) + storage_time
y ~ current_feature + Sex + (1 | Subject) + storage_time
y ~ current_feature + Sex + (1 | Subject) + storage_time
y ~ current_feature + Sex + (1 | Subject) + storage_time
y ~ current_feature + Sex + (1 | Subject) + storage_time
y ~ current_feature + Sex + (1 | Subject) + storage_time
y ~ current_feature + Sex + (1 | Subject) + storage_time
y ~ current_feature + Sex + (1 | Subject) + storage_time
y ~ current_feature + Sex + (1 | Subject) + storage_time
y ~ current_feature + Sex + (1 | Subject) + storage_time
y ~ current_feature + Sex + (1 | Subject) + storage_time
y ~ current_feature + Sex + (1 | Subject) + storage_time
y ~ current_feature + Sex + (1 | Subject) + storage_time
y ~ current_feature + Sex + (1 | Subject) + storage_time
y ~ current_feature + Sex + (1 | Subject) + storage_time
y ~ current_feature + Sex + (1 | Subject) + storage_time
y ~ current_feature + Sex + (1 | Subject) + storage_time
y ~ current_feature + Sex + (1 | Subject) + storage_time
y ~ current_feature + Sex + (1 | Subject) + storage_time
y ~ current_feature + Sex + (1 | Subject) + storage_time
y ~ current_feature + Sex + (1 | Subject) + storage_time
y ~ current_feature + Sex + (1 | Subject) + storage_time
y ~ current_feature + Sex + (1 | Subject) + storage_time

y ~ current_feature + Sex + (1 | Subject) + storage_time
y ~ current_feature + Sex + (1 | Subject) + storage_time
y ~ current_feature + Sex + (1 | Subject) + storage_time
y ~ current_feature + Sex + (1 | Subject) + storage_time
y ~ current_feature + Sex + (1 | Subject) + storage_time
y ~ current_feature + Sex + (1 | Subject) + storage_time
y ~ current_feature + Sex + (1 | Subject) + storage_time
y ~ current_feature + Sex + (1 | Subject) + storage_time
y ~ current_feature + Sex + (1 | Subject) + storage_time
y ~ current_feature + Sex + (1 | Subject) + storage_time
y ~ current_feature + Sex + (1 | Subject) + storage_time
y ~ current_feature + Sex + (1 | Subject) + storage_time
y ~ current_feature + Sex + (1 | Subject) + storage_time
y ~ current_feature + Sex + (1 | Subject) + storage_time
y ~ current_feature + Sex + (1 | Subject) + storage_time
y ~ current_feature + Sex + (1 | Subject) + storage_time
y ~ current_feature + Sex + (1 | Subject) + storage_time
y ~ current_feature + Sex + (1 | Subject) + storage_time
y ~ current_feature + Sex + (1 | Subject) + storage_time
y ~ current_feature + Sex + (1 | Subject) + storage_time
y ~ current_feature + Sex + (1 | Subject) + storage_time
y ~ current_feature + Sex + (1 | Subject) + storage_time
y ~ current_feature + Sex + (1 | Subject) + storage_time
y ~ current_feature + Sex + (1 | Subject) + storage_time
y ~ current_feature + Sex + (1 | Subject) + storage_time
y ~ current_feature + Sex + (1 | Subject) + storage_time
y ~ current_feature + Sex + (1 | Subject) + storage_time
y ~ current_feature + Sex + (1 | Subject) + storage_time
y ~ current_feature + Sex + (1 | Subject) + storage_time
y ~ current_feature + Sex + (1 | Subject) + storage_time
y ~ current_feature + Sex + (1 | Subject) + storage_time
y ~ current_feature + Sex + (1 | Subject) + storage_time
y ~ current_feature + Sex + (1 | Subject) + storage_time
y ~ current_feature + Sex + (1 | Subject) + storage_time
y ~ current_feature + Sex + (1 | Subject) + storage_time
y ~ current_feature + Sex + (1 | Subject) + storage_time
y ~ current_feature + Sex + (1 | Subject) + storage_time
y ~ current_feature + Sex + (1 | Subject) + storage_time
y ~ current_feature + Sex + (1 | Subject) + storage_time
y ~ current_feature + Sex + (1 | Subject) + storage_time
y ~ current_feature + Sex + (1 | Subject) + storage_time
y ~ current_feature + Sex + (1 | Subject) + storage_time
y ~ current_feature + Sex + (1 | Subject) + storage_time
y ~ current_feature + Sex + (1 | Subject) + storage_time
y ~ current_feature + Sex + (1 | Subject) + storage_time
y ~ current_feature + Sex + (1 | Subject) + storage_time
y ~ current_feature + Sex + (1 | Subject) + storage_time
y ~ current_feature + Sex + (1 | Subject) + storage_time

y ~ current_feature + Sex + (1 | Subject) + storage_time
y ~ current_feature + Sex + (1 | Subject) + storage_time
y ~ current_feature + Sex + (1 | Subject) + storage_time
y ~ current_feature + Sex + (1 | Subject) + storage_time
y ~ current_feature + Sex + (1 | Subject) + storage_time
y ~ current_feature + Sex + (1 | Subject) + storage_time
y ~ current_feature + Sex + (1 | Subject) + storage_time
y ~ current_feature + Sex + (1 | Subject) + storage_time
y ~ current_feature + Sex + (1 | Subject) + storage_time
y ~ current_feature + Sex + (1 | Subject) + storage_time
y ~ current_feature + Sex + (1 | Subject) + storage_time
y ~ current_feature + Sex + (1 | Subject) + storage_time
y ~ current_feature + Sex + (1 | Subject) + storage_time
y ~ current_feature + Sex + (1 | Subject) + storage_time
y ~ current_feature + Sex + (1 | Subject) + storage_time
y ~ current_feature + Sex + (1 | Subject) + storage_time
y ~ current_feature + Sex + (1 | Subject) + storage_time
y ~ current_feature + Sex + (1 | Subject) + storage_time
y ~ current_feature + Sex + (1 | Subject) + storage_time
y ~ current_feature + Sex + (1 | Subject) + storage_time
y ~ current_feature + Sex + (1 | Subject) + storage_time
y ~ current_feature + Sex + (1 | Subject) + storage_time
y ~ current_feature + Sex + (1 | Subject) + storage_time
y ~ current_feature + Sex + (1 | Subject) + storage_time
y ~ current_feature + Sex + (1 | Subject) + storage_time
y ~ current_feature + Sex + (1 | Subject) + storage_time
y ~ current_feature + Sex + (1 | Subject) + storage_time
y ~ current_feature + Sex + (1 | Subject) + storage_time
y ~ current_feature + Sex + (1 | Subject) + storage_time
y ~ current_feature + Sex + (1 | Subject) + storage_time
y ~ current_feature + Sex + (1 | Subject) + storage_time
y ~ current_feature + Sex + (1 | Subject) + storage_time
y ~ current_feature + Sex + (1 | Subject) + storage_time
y ~ current_feature + Sex + (1 | Subject) + storage_time
y ~ current_feature + Sex + (1 | Subject) + storage_time
y ~ current_feature + Sex + (1 | Subject) + storage_time
y ~ current_feature + Sex + (1 | Subject) + storage_time
y ~ current_feature + Sex + (1 | Subject) + storage_time
y ~ current_feature + Sex + (1 | Subject) + storage_time
y ~ current_feature + Sex + (1 | Subject) + storage_time
y ~ current_feature + Sex + (1 | Subject) + storage_time
y ~ current_feature + Sex + (1 | Subject) + storage_time
y ~ current_feature + Sex + (1 | Subject) + storage_time
y ~ current_feature + Sex + (1 | Subject) + storage_time
y ~ current_feature + Sex + (1 | Subject) + storage_time
y ~ current_feature + Sex + (1 | Subject) + storage_time
y ~ current_feature + Sex + (1 | Subject) + storage_time
y ~ current_feature + Sex + (1 | Subject) + storage_time

y ~ current_feature + Sex + (1 | Subject) + storage_time
y ~ current_feature + Sex + (1 | Subject) + storage_time
y ~ current_feature + Sex + (1 | Subject) + storage_time
y ~ current_feature + Sex + (1 | Subject) + storage_time
y ~ current_feature + Sex + (1 | Subject) + storage_time
y ~ current_feature + Sex + (1 | Subject) + storage_time
y ~ current_feature + Sex + (1 | Subject) + storage_time
y ~ current_feature + Sex + (1 | Subject) + storage_time
y ~ current_feature + Sex + (1 | Subject) + storage_time
y ~ current_feature + Sex + (1 | Subject) + storage_time
y ~ current_feature + Sex + (1 | Subject) + storage_time
y ~ current_feature + Sex + (1 | Subject) + storage_time
y ~ current_feature + Sex + (1 | Subject) + storage_time
y ~ current_feature + Sex + (1 | Subject) + storage_time
y ~ current_feature + Sex + (1 | Subject) + storage_time
y ~ current_feature + Sex + (1 | Subject) + storage_time
y ~ current_feature + Sex + (1 | Subject) + storage_time
y ~ current_feature + Sex + (1 | Subject) + storage_time
y ~ current_feature + Sex + (1 | Subject) + storage_time
y ~ current_feature + Sex + (1 | Subject) + storage_time
y ~ current_feature + Sex + (1 | Subject) + storage_time
y ~ current_feature + Sex + (1 | Subject) + storage_time
y ~ current_feature + Sex + (1 | Subject) + storage_time
y ~ current_feature + Sex + (1 | Subject) + storage_time
y ~ current_feature + Sex + (1 | Subject) + storage_time
y ~ current_feature + Sex + (1 | Subject) + storage_time
y ~ current_feature + Sex + (1 | Subject) + storage_time
y ~ current_feature + Sex + (1 | Subject) + storage_time
y ~ current_feature + Sex + (1 | Subject) + storage_time
y ~ current_feature + Sex + (1 | Subject) + storage_time
y ~ current_feature + Sex + (1 | Subject) + storage_time
y ~ current_feature + Sex + (1 | Subject) + storage_time
y ~ current_feature + Sex + (1 | Subject) + storage_time
y ~ current_feature + Sex + (1 | Subject) + storage_time
y ~ current_feature + Sex + (1 | Subject) + storage_time
y ~ current_feature + Sex + (1 | Subject) + storage_time
y ~ current_feature + Sex + (1 | Subject) + storage_time
y ~ current_feature + Sex + (1 | Subject) + storage_time
y ~ current_feature + Sex + (1 | Subject) + storage_time
y ~ current_feature + Sex + (1 | Subject) + storage_time
y ~ current_feature + Sex + (1 | Subject) + storage_time
y ~ current_feature + Sex + (1 | Subject) + storage_time
y ~ current_feature + Sex + (1 | Subject) + storage_time
y ~ current_feature + Sex + (1 | Subject) + storage_time
y ~ current_feature + Sex + (1 | Subject) + storage_time
y ~ current_feature + Sex + (1 | Subject) + storage_time
y ~ current_feature + Sex + (1 | Subject) + storage_time
y ~ current_feature + Sex + (1 | Subject) + storage_time

y ~ current_feature + Sex + (1 | Subject) + storage_time
y ~ current_feature + Sex + (1 | Subject) + storage_time
y ~ current_feature + Sex + (1 | Subject) + storage_time
y ~ current_feature + Sex + (1 | Subject) + storage_time
y ~ current_feature + Sex + (1 | Subject) + storage_time
y ~ current_feature + Sex + (1 | Subject) + storage_time
y ~ current_feature + Sex + (1 | Subject) + storage_time
y ~ current_feature + Sex + (1 | Subject) + storage_time
y ~ current_feature + Sex + (1 | Subject) + storage_time
y ~ current_feature + Sex + (1 | Subject) + storage_time
y ~ current_feature + Sex + (1 | Subject) + storage_time
y ~ current_feature + Sex + (1 | Subject) + storage_time
y ~ current_feature + Sex + (1 | Subject) + storage_time
y ~ current_feature + Sex + (1 | Subject) + storage_time
y ~ current_feature + Sex + (1 | Subject) + storage_time
y ~ current_feature + Sex + (1 | Subject) + storage_time
y ~ current_feature + Sex + (1 | Subject) + storage_time
y ~ current_feature + Sex + (1 | Subject) + storage_time
y ~ current_feature + Sex + (1 | Subject) + storage_time
y ~ current_feature + Sex + (1 | Subject) + storage_time
y ~ current_feature + Sex + (1 | Subject) + storage_time
y ~ current_feature + Sex + (1 | Subject) + storage_time
y ~ current_feature + Sex + (1 | Subject) + storage_time
y ~ current_feature + Sex + (1 | Subject) + storage_time
y ~ current_feature + Sex + (1 | Subject) + storage_time
y ~ current_feature + Sex + (1 | Subject) + storage_time
y ~ current_feature + Sex + (1 | Subject) + storage_time
y ~ current_feature + Sex + (1 | Subject) + storage_time
y ~ current_feature + Sex + (1 | Subject) + storage_time
y ~ current_feature + Sex + (1 | Subject) + storage_time
y ~ current_feature + Sex + (1 | Subject) + storage_time
y ~ current_feature + Sex + (1 | Subject) + storage_time
y ~ current_feature + Sex + (1 | Subject) + storage_time
y ~ current_feature + Sex + (1 | Subject) + storage_time
y ~ current_feature + Sex + (1 | Subject) + storage_time
y ~ current_feature + Sex + (1 | Subject) + storage_time
y ~ current_feature + Sex + (1 | Subject) + storage_time
y ~ current_feature + Sex + (1 | Subject) + storage_time
y ~ current_feature + Sex + (1 | Subject) + storage_time
y ~ current_feature + Sex + (1 | Subject) + storage_time
y ~ current_feature + Sex + (1 | Subject) + storage_time
y ~ current_feature + Sex + (1 | Subject) + storage_time
y ~ current_feature + Sex + (1 | Subject) + storage_time
y ~ current_feature + Sex + (1 | Subject) + storage_time
y ~ current_feature + Sex + (1 | Subject) + storage_time
y ~ current_feature + Sex + (1 | Subject) + storage_time
y ~ current_feature + Sex + (1 | Subject) + storage_time
y ~ current_feature + Sex + (1 | Subject) + storage_time

y ~ current_feature + Sex + (1 | Subject) + storage_time
y ~ current_feature + Sex + (1 | Subject) + storage_time
y ~ current_feature + Sex + (1 | Subject) + storage_time
y ~ current_feature + Sex + (1 | Subject) + storage_time
y ~ current_feature + Sex + (1 | Subject) + storage_time
y ~ current_feature + Sex + (1 | Subject) + storage_time
y ~ current_feature + Sex + (1 | Subject) + storage_time
y ~ current_feature + Sex + (1 | Subject) + storage_time
y ~ current_feature + Sex + (1 | Subject) + storage_time
y ~ current_feature + Sex + (1 | Subject) + storage_time
y ~ current_feature + Sex + (1 | Subject) + storage_time
y ~ current_feature + Sex + (1 | Subject) + storage_time
y ~ current_feature + Sex + (1 | Subject) + storage_time
y ~ current_feature + Sex + (1 | Subject) + storage_time
y ~ current_feature + Sex + (1 | Subject) + storage_time
y ~ current_feature + Sex + (1 | Subject) + storage_time
y ~ current_feature + Sex + (1 | Subject) + storage_time
y ~ current_feature + Sex + (1 | Subject) + storage_time
y ~ current_feature + Sex + (1 | Subject) + storage_time
y ~ current_feature + Sex + (1 | Subject) + storage_time
y ~ current_feature + Sex + (1 | Subject) + storage_time
y ~ current_feature + Sex + (1 | Subject) + storage_time
y ~ current_feature + Sex + (1 | Subject) + storage_time
y ~ current_feature + Sex + (1 | Subject) + storage_time
y ~ current_feature + Sex + (1 | Subject) + storage_time
y ~ current_feature + Sex + (1 | Subject) + storage_time
y ~ current_feature + Sex + (1 | Subject) + storage_time
y ~ current_feature + Sex + (1 | Subject) + storage_time
y ~ current_feature + Sex + (1 | Subject) + storage_time
y ~ current_feature + Sex + (1 | Subject) + storage_time
y ~ current_feature + Sex + (1 | Subject) + storage_time
y ~ current_feature + Sex + (1 | Subject) + storage_time
y ~ current_feature + Sex + (1 | Subject) + storage_time
y ~ current_feature + Sex + (1 | Subject) + storage_time
y ~ current_feature + Sex + (1 | Subject) + storage_time
y ~ current_feature + Sex + (1 | Subject) + storage_time
y ~ current_feature + Sex + (1 | Subject) + storage_time
y ~ current_feature + Sex + (1 | Subject) + storage_time
y ~ current_feature + Sex + (1 | Subject) + storage_time
y ~ current_feature + Sex + (1 | Subject) + storage_time
y ~ current_feature + Sex + (1 | Subject) + storage_time
y ~ current_feature + Sex + (1 | Subject) + storage_time
y ~ current_feature + Sex + (1 | Subject) + storage_time
y ~ current_feature + Sex + (1 | Subject) + storage_time
y ~ current_feature + Sex + (1 | Subject) + storage_time
y ~ current_feature + Sex + (1 | Subject) + storage_time
y ~ current_feature + Sex + (1 | Subject) + storage_time
y ~ current_feature + Sex + (1 | Subject) + storage_time

y ~ current_feature + Sex + (1 | Subject) + storage_time
y ~ current_feature + Sex + (1 | Subject) + storage_time
y ~ current_feature + Sex + (1 | Subject) + storage_time
y ~ current_feature + Sex + (1 | Subject) + storage_time
y ~ current_feature + Sex + (1 | Subject) + storage_time
y ~ current_feature + Sex + (1 | Subject) + storage_time
y ~ current_feature + Sex + (1 | Subject) + storage_time
y ~ current_feature + Sex + (1 | Subject) + storage_time
y ~ current_feature + Sex + (1 | Subject) + storage_time
y ~ current_feature + Sex + (1 | Subject) + storage_time
y ~ current_feature + Sex + (1 | Subject) + storage_time
y ~ current_feature + Sex + (1 | Subject) + storage_time
y ~ current_feature + Sex + (1 | Subject) + storage_time
y ~ current_feature + Sex + (1 | Subject) + storage_time
y ~ current_feature + Sex + (1 | Subject) + storage_time
y ~ current_feature + Sex + (1 | Subject) + storage_time
y ~ current_feature + Sex + (1 | Subject) + storage_time
y ~ current_feature + Sex + (1 | Subject) + storage_time
y ~ current_feature + Sex + (1 | Subject) + storage_time
y ~ current_feature + Sex + (1 | Subject) + storage_time
y ~ current_feature + Sex + (1 | Subject) + storage_time
y ~ current_feature + Sex + (1 | Subject) + storage_time
y ~ current_feature + Sex + (1 | Subject) + storage_time
y ~ current_feature + Sex + (1 | Subject) + storage_time
y ~ current_feature + Sex + (1 | Subject) + storage_time
y ~ current_feature + Sex + (1 | Subject) + storage_time
y ~ current_feature + Sex + (1 | Subject) + storage_time
y ~ current_feature + Sex + (1 | Subject) + storage_time
y ~ current_feature + Sex + (1 | Subject) + storage_time
y ~ current_feature + Sex + (1 | Subject) + storage_time
y ~ current_feature + Sex + (1 | Subject) + storage_time
y ~ current_feature + Sex + (1 | Subject) + storage_time
y ~ current_feature + Sex + (1 | Subject) + storage_time
y ~ current_feature + Sex + (1 | Subject) + storage_time
y ~ current_feature + Sex + (1 | Subject) + storage_time
y ~ current_feature + Sex + (1 | Subject) + storage_time
y ~ current_feature + Sex + (1 | Subject) + storage_time
y ~ current_feature + Sex + (1 | Subject) + storage_time
y ~ current_feature + Sex + (1 | Subject) + storage_time
y ~ current_feature + Sex + (1 | Subject) + storage_time
y ~ current_feature + Sex + (1 | Subject) + storage_time
y ~ current_feature + Sex + (1 | Subject) + storage_time
y ~ current_feature + Sex + (1 | Subject) + storage_time
y ~ current_feature + Sex + (1 | Subject) + storage_time
y ~ current_feature + Sex + (1 | Subject) + storage_time
y ~ current_feature + Sex + (1 | Subject) + storage_time
y ~ current_feature + Sex + (1 | Subject) + storage_time
y ~ current_feature + Sex + (1 | Subject) + storage_time

y ~ current_feature + Sex + (1 | Subject) + storage_time
y ~ current_feature + Sex + (1 | Subject) + storage_time
y ~ current_feature + Sex + (1 | Subject) + storage_time
y ~ current_feature + Sex + (1 | Subject) + storage_time
y ~ current_feature + Sex + (1 | Subject) + storage_time
y ~ current_feature + Sex + (1 | Subject) + storage_time
y ~ current_feature + Sex + (1 | Subject) + storage_time
y ~ current_feature + Sex + (1 | Subject) + storage_time
y ~ current_feature + Sex + (1 | Subject) + storage_time
y ~ current_feature + Sex + (1 | Subject) + storage_time
y ~ current_feature + Sex + (1 | Subject) + storage_time
y ~ current_feature + Sex + (1 | Subject) + storage_time
y ~ current_feature + Sex + (1 | Subject) + storage_time
y ~ current_feature + Sex + (1 | Subject) + storage_time
y ~ current_feature + Sex + (1 | Subject) + storage_time
y ~ current_feature + Sex + (1 | Subject) + storage_time
y ~ current_feature + Sex + (1 | Subject) + storage_time
y ~ current_feature + Sex + (1 | Subject) + storage_time
y ~ current_feature + Sex + (1 | Subject) + storage_time
y ~ current_feature + Sex + (1 | Subject) + storage_time
y ~ current_feature + Sex + (1 | Subject) + storage_time
y ~ current_feature + Sex + (1 | Subject) + storage_time
y ~ current_feature + Sex + (1 | Subject) + storage_time
y ~ current_feature + Sex + (1 | Subject) + storage_time
y ~ current_feature + Sex + (1 | Subject) + storage_time
y ~ current_feature + Sex + (1 | Subject) + storage_time
y ~ current_feature + Sex + (1 | Subject) + storage_time
y ~ current_feature + Sex + (1 | Subject) + storage_time
y ~ current_feature + Sex + (1 | Subject) + storage_time
y ~ current_feature + Sex + (1 | Subject) + storage_time
y ~ current_feature + Sex + (1 | Subject) + storage_time
y ~ current_feature + Sex + (1 | Subject) + storage_time
y ~ current_feature + Sex + (1 | Subject) + storage_time
y ~ current_feature + Sex + (1 | Subject) + storage_time
y ~ current_feature + Sex + (1 | Subject) + storage_time
y ~ current_feature + Sex + (1 | Subject) + storage_time
y ~ current_feature + Sex + (1 | Subject) + storage_time
y ~ current_feature + Sex + (1 | Subject) + storage_time
y ~ current_feature + Sex + (1 | Subject) + storage_time
y ~ current_feature + Sex + (1 | Subject) + storage_time
y ~ current_feature + Sex + (1 | Subject) + storage_time
y ~ current_feature + Sex + (1 | Subject) + storage_time
y ~ current_feature + Sex + (1 | Subject) + storage_time
y ~ current_feature + Sex + (1 | Subject) + storage_time
y ~ current_feature + Sex + (1 | Subject) + storage_time
y ~ current_feature + Sex + (1 | Subject) + storage_time
y ~ current_feature + Sex + (1 | Subject) + storage_time
y ~ current_feature + Sex + (1 | Subject) + storage_time

y ~ current_feature + Sex + (1 | Subject) + storage_time
y ~ current_feature + Sex + (1 | Subject) + storage_time
y ~ current_feature + Sex + (1 | Subject) + storage_time
y ~ current_feature + Sex + (1 | Subject) + storage_time
y ~ current_feature + Sex + (1 | Subject) + storage_time
y ~ current_feature + Sex + (1 | Subject) + storage_time
y ~ current_feature + Sex + (1 | Subject) + storage_time
y ~ current_feature + Sex + (1 | Subject) + storage_time
y ~ current_feature + Sex + (1 | Subject) + storage_time
y ~ current_feature + Sex + (1 | Subject) + storage_time
y ~ current_feature + Sex + (1 | Subject) + storage_time
y ~ current_feature + Sex + (1 | Subject) + storage_time
y ~ current_feature + Sex + (1 | Subject) + storage_time
y ~ current_feature + Sex + (1 | Subject) + storage_time
y ~ current_feature + Sex + (1 | Subject) + storage_time
y ~ current_feature + Sex + (1 | Subject) + storage_time
y ~ current_feature + Sex + (1 | Subject) + storage_time
y ~ current_feature + Sex + (1 | Subject) + storage_time
y ~ current_feature + Sex + (1 | Subject) + storage_time
y ~ current_feature + Sex + (1 | Subject) + storage_time
y ~ current_feature + Sex + (1 | Subject) + storage_time
y ~ current_feature + Sex + (1 | Subject) + storage_time
y ~ current_feature + Sex + (1 | Subject) + storage_time
y ~ current_feature + Sex + (1 | Subject) + storage_time
y ~ current_feature + Sex + (1 | Subject) + storage_time
y ~ current_feature + Sex + (1 | Subject) + storage_time
y ~ current_feature + Sex + (1 | Subject) + storage_time
y ~ current_feature + Sex + (1 | Subject) + storage_time
y ~ current_feature + Sex + (1 | Subject) + storage_time
y ~ current_feature + Sex + (1 | Subject) + storage_time
y ~ current_feature + Sex + (1 | Subject) + storage_time
y ~ current_feature + Sex + (1 | Subject) + storage_time
y ~ current_feature + Sex + (1 | Subject) + storage_time
y ~ current_feature + Sex + (1 | Subject) + storage_time
y ~ current_feature + Sex + (1 | Subject) + storage_time
y ~ current_feature + Sex + (1 | Subject) + storage_time
y ~ current_feature + Sex + (1 | Subject) + storage_time
y ~ current_feature + Sex + (1 | Subject) + storage_time
y ~ current_feature + Sex + (1 | Subject) + storage_time
y ~ current_feature + Sex + (1 | Subject) + storage_time
y ~ current_feature + Sex + (1 | Subject) + storage_time
y ~ current_feature + Sex + (1 | Subject) + storage_time
y ~ current_feature + Sex + (1 | Subject) + storage_time
y ~ current_feature + Sex + (1 | Subject) + storage_time
y ~ current_feature + Sex + (1 | Subject) + storage_time
y ~ current_feature + Sex + (1 | Subject) + storage_time
y ~ current_feature + Sex + (1 | Subject) + storage_time
y ~ current_feature + Sex + (1 | Subject) + storage_time

y ~ current_feature + Sex + (1 | Subject) + storage_time
y ~ current_feature + Sex + (1 | Subject) + storage_time
y ~ current_feature + Sex + (1 | Subject) + storage_time
y ~ current_feature + Sex + (1 | Subject) + storage_time
y ~ current_feature + Sex + (1 | Subject) + storage_time
y ~ current_feature + Sex + (1 | Subject) + storage_time
y ~ current_feature + Sex + (1 | Subject) + storage_time
y ~ current_feature + Sex + (1 | Subject) + storage_time
y ~ current_feature + Sex + (1 | Subject) + storage_time
y ~ current_feature + Sex + (1 | Subject) + storage_time
y ~ current_feature + Sex + (1 | Subject) + storage_time
y ~ current_feature + Sex + (1 | Subject) + storage_time
y ~ current_feature + Sex + (1 | Subject) + storage_time
y ~ current_feature + Sex + (1 | Subject) + storage_time
y ~ current_feature + Sex + (1 | Subject) + storage_time
y ~ current_feature + Sex + (1 | Subject) + storage_time
y ~ current_feature + Sex + (1 | Subject) + storage_time
y ~ current_feature + Sex + (1 | Subject) + storage_time
y ~ current_feature + Sex + (1 | Subject) + storage_time
y ~ current_feature + Sex + (1 | Subject) + storage_time
y ~ current_feature + Sex + (1 | Subject) + storage_time
y ~ current_feature + Sex + (1 | Subject) + storage_time
y ~ current_feature + Sex + (1 | Subject) + storage_time
y ~ current_feature + Sex + (1 | Subject) + storage_time
y ~ current_feature + Sex + (1 | Subject) + storage_time
y ~ current_feature + Sex + (1 | Subject) + storage_time
y ~ current_feature + Sex + (1 | Subject) + storage_time
y ~ current_feature + Sex + (1 | Subject) + storage_time
y ~ current_feature + Sex + (1 | Subject) + storage_time
y ~ current_feature + Sex + (1 | Subject) + storage_time
y ~ current_feature + Sex + (1 | Subject) + storage_time
y ~ current_feature + Sex + (1 | Subject) + storage_time
y ~ current_feature + Sex + (1 | Subject) + storage_time
y ~ current_feature + Sex + (1 | Subject) + storage_time
y ~ current_feature + Sex + (1 | Subject) + storage_time
y ~ current_feature + Sex + (1 | Subject) + storage_time
y ~ current_feature + Sex + (1 | Subject) + storage_time
y ~ current_feature + Sex + (1 | Subject) + storage_time
y ~ current_feature + Sex + (1 | Subject) + storage_time
y ~ current_feature + Sex + (1 | Subject) + storage_time
y ~ current_feature + Sex + (1 | Subject) + storage_time
y ~ current_feature + Sex + (1 | Subject) + storage_time
y ~ current_feature + Sex + (1 | Subject) + storage_time
y ~ current_feature + Sex + (1 | Subject) + storage_time
y ~ current_feature + Sex + (1 | Subject) + storage_time
y ~ current_feature + Sex + (1 | Subject) + storage_time
y ~ current_feature + Sex + (1 | Subject) + storage_time
y ~ current_feature + Sex + (1 | Subject) + storage_time

y ~ current_feature + Sex + (1 | Subject) + storage_time
y ~ current_feature + Sex + (1 | Subject) + storage_time
y ~ current_feature + Sex + (1 | Subject) + storage_time
y ~ current_feature + Sex + (1 | Subject) + storage_time
y ~ current_feature + Sex + (1 | Subject) + storage_time
y ~ current_feature + Sex + (1 | Subject) + storage_time
y ~ current_feature + Sex + (1 | Subject) + storage_time
y ~ current_feature + Sex + (1 | Subject) + storage_time
y ~ current_feature + Sex + (1 | Subject) + storage_time
y ~ current_feature + Sex + (1 | Subject) + storage_time
y ~ current_feature + Sex + (1 | Subject) + storage_time
y ~ current_feature + Sex + (1 | Subject) + storage_time
y ~ current_feature + Sex + (1 | Subject) + storage_time
y ~ current_feature + Sex + (1 | Subject) + storage_time
y ~ current_feature + Sex + (1 | Subject) + storage_time
y ~ current_feature + Sex + (1 | Subject) + storage_time
y ~ current_feature + Sex + (1 | Subject) + storage_time
y ~ current_feature + Sex + (1 | Subject) + storage_time
y ~ current_feature + Sex + (1 | Subject) + storage_time
y ~ current_feature + Sex + (1 | Subject) + storage_time
y ~ current_feature + Sex + (1 | Subject) + storage_time
y ~ current_feature + Sex + (1 | Subject) + storage_time
y ~ current_feature + Sex + (1 | Subject) + storage_time
y ~ current_feature + Sex + (1 | Subject) + storage_time
y ~ current_feature + Sex + (1 | Subject) + storage_time
y ~ current_feature + Sex + (1 | Subject) + storage_time
y ~ current_feature + Sex + (1 | Subject) + storage_time
y ~ current_feature + Sex + (1 | Subject) + storage_time
y ~ current_feature + Sex + (1 | Subject) + storage_time
y ~ current_feature + Sex + (1 | Subject) + storage_time
y ~ current_feature + Sex + (1 | Subject) + storage_time
y ~ current_feature + Sex + (1 | Subject) + storage_time
y ~ current_feature + Sex + (1 | Subject) + storage_time
y ~ current_feature + Sex + (1 | Subject) + storage_time
y ~ current_feature + Sex + (1 | Subject) + storage_time
y ~ current_feature + Sex + (1 | Subject) + storage_time
y ~ current_feature + Sex + (1 | Subject) + storage_time
y ~ current_feature + Sex + (1 | Subject) + storage_time
y ~ current_feature + Sex + (1 | Subject) + storage_time
y ~ current_feature + Sex + (1 | Subject) + storage_time
y ~ current_feature + Sex + (1 | Subject) + storage_time
y ~ current_feature + Sex + (1 | Subject) + storage_time
y ~ current_feature + Sex + (1 | Subject) + storage_time
y ~ current_feature + Sex + (1 | Subject) + storage_time
y ~ current_feature + Sex + (1 | Subject) + storage_time
y ~ current_feature + Sex + (1 | Subject) + storage_time
y ~ current_feature + Sex + (1 | Subject) + storage_time
y ~ current_feature + Sex + (1 | Subject) + storage_time

y ~ current_feature + Sex + (1 | Subject) + storage_time
y ~ current_feature + Sex + (1 | Subject) + storage_time
y ~ current_feature + Sex + (1 | Subject) + storage_time
y ~ current_feature + Sex + (1 | Subject) + storage_time
y ~ current_feature + Sex + (1 | Subject) + storage_time
y ~ current_feature + Sex + (1 | Subject) + storage_time
y ~ current_feature + Sex + (1 | Subject) + storage_time
y ~ current_feature + Sex + (1 | Subject) + storage_time
y ~ current_feature + Sex + (1 | Subject) + storage_time
y ~ current_feature + Sex + (1 | Subject) + storage_time
y ~ current_feature + Sex + (1 | Subject) + storage_time
y ~ current_feature + Sex + (1 | Subject) + storage_time
y ~ current_feature + Sex + (1 | Subject) + storage_time
y ~ current_feature + Sex + (1 | Subject) + storage_time
y ~ current_feature + Sex + (1 | Subject) + storage_time
y ~ current_feature + Sex + (1 | Subject) + storage_time
y ~ current_feature + Sex + (1 | Subject) + storage_time
y ~ current_feature + Sex + (1 | Subject) + storage_time
y ~ current_feature + Sex + (1 | Subject) + storage_time
y ~ current_feature + Sex + (1 | Subject) + storage_time
y ~ current_feature + Sex + (1 | Subject) + storage_time
y ~ current_feature + Sex + (1 | Subject) + storage_time
y ~ current_feature + Sex + (1 | Subject) + storage_time
y ~ current_feature + Sex + (1 | Subject) + storage_time
y ~ current_feature + Sex + (1 | Subject) + storage_time
y ~ current_feature + Sex + (1 | Subject) + storage_time
y ~ current_feature + Sex + (1 | Subject) + storage_time
y ~ current_feature + Sex + (1 | Subject) + storage_time
y ~ current_feature + Sex + (1 | Subject) + storage_time
y ~ current_feature + Sex + (1 | Subject) + storage_time
y ~ current_feature + Sex + (1 | Subject) + storage_time
y ~ current_feature + Sex + (1 | Subject) + storage_time
y ~ current_feature + Sex + (1 | Subject) + storage_time
y ~ current_feature + Sex + (1 | Subject) + storage_time
y ~ current_feature + Sex + (1 | Subject) + storage_time
y ~ current_feature + Sex + (1 | Subject) + storage_time
y ~ current_feature + Sex + (1 | Subject) + storage_time
y ~ current_feature + Sex + (1 | Subject) + storage_time
y ~ current_feature + Sex + (1 | Subject) + storage_time
y ~ current_feature + Sex + (1 | Subject) + storage_time
y ~ current_feature + Sex + (1 | Subject) + storage_time
y ~ current_feature + Sex + (1 | Subject) + storage_time
y ~ current_feature + Sex + (1 | Subject) + storage_time
y ~ current_feature + Sex + (1 | Subject) + storage_time
y ~ current_feature + Sex + (1 | Subject) + storage_time
y ~ current_feature + Sex + (1 | Subject) + storage_time
y ~ current_feature + Sex + (1 | Subject) + storage_time
y ~ current_feature + Sex + (1 | Subject) + storage_time

y ~ current_feature + Sex + (1 | Subject) + storage_time
y ~ current_feature + Sex + (1 | Subject) + storage_time
y ~ current_feature + Sex + (1 | Subject) + storage_time
y ~ current_feature + Sex + (1 | Subject) + storage_time
y ~ current_feature + Sex + (1 | Subject) + storage_time
y ~ current_feature + Sex + (1 | Subject) + storage_time
y ~ current_feature + Sex + (1 | Subject) + storage_time
y ~ current_feature + Sex + (1 | Subject) + storage_time
y ~ current_feature + Sex + (1 | Subject) + storage_time
y ~ current_feature + Sex + (1 | Subject) + storage_time
y ~ current_feature + Sex + (1 | Subject) + storage_time
y ~ current_feature + Sex + (1 | Subject) + storage_time
y ~ current_feature + Sex + (1 | Subject) + storage_time
y ~ current_feature + Sex + (1 | Subject) + storage_time
y ~ current_feature + Sex + (1 | Subject) + storage_time
y ~ current_feature + Sex + (1 | Subject) + storage_time
y ~ current_feature + Sex + (1 | Subject) + storage_time
y ~ current_feature + Sex + (1 | Subject) + storage_time
y ~ current_feature + Sex + (1 | Subject) + storage_time
y ~ current_feature + Sex + (1 | Subject) + storage_time
y ~ current_feature + Sex + (1 | Subject) + storage_time
y ~ current_feature + Sex + (1 | Subject) + storage_time
y ~ current_feature + Sex + (1 | Subject) + storage_time
y ~ current_feature + Sex + (1 | Subject) + storage_time
y ~ current_feature + Sex + (1 | Subject) + storage_time
y ~ current_feature + Sex + (1 | Subject) + storage_time
y ~ current_feature + Sex + (1 | Subject) + storage_time
y ~ current_feature + Sex + (1 | Subject) + storage_time
y ~ current_feature + Sex + (1 | Subject) + storage_time
y ~ current_feature + Sex + (1 | Subject) + storage_time
y ~ current_feature + Sex + (1 | Subject) + storage_time
y ~ current_feature + Sex + (1 | Subject) + storage_time
y ~ current_feature + Sex + (1 | Subject) + storage_time
y ~ current_feature + Sex + (1 | Subject) + storage_time
y ~ current_feature + Sex + (1 | Subject) + storage_time
y ~ current_feature + Sex + (1 | Subject) + storage_time
y ~ current_feature + Sex + (1 | Subject) + storage_time
y ~ current_feature + Sex + (1 | Subject) + storage_time
y ~ current_feature + Sex + (1 | Subject) + storage_time
y ~ current_feature + Sex + (1 | Subject) + storage_time
y ~ current_feature + Sex + (1 | Subject) + storage_time
y ~ current_feature + Sex + (1 | Subject) + storage_time
y ~ current_feature + Sex + (1 | Subject) + storage_time
y ~ current_feature + Sex + (1 | Subject) + storage_time
y ~ current_feature + Sex + (1 | Subject) + storage_time
y ~ current_feature + Sex + (1 | Subject) + storage_time
y ~ current_feature + Sex + (1 | Subject) + storage_time
y ~ current_feature + Sex + (1 | Subject) + storage_time

y ~ current_feature + Sex + (1 | Subject) + storage_time
y ~ current_feature + Sex + (1 | Subject) + storage_time
y ~ current_feature + Sex + (1 | Subject) + storage_time
y ~ current_feature + Sex + (1 | Subject) + storage_time
y ~ current_feature + Sex + (1 | Subject) + storage_time
y ~ current_feature + Sex + (1 | Subject) + storage_time
y ~ current_feature + Sex + (1 | Subject) + storage_time
y ~ current_feature + Sex + (1 | Subject) + storage_time
y ~ current_feature + Sex + (1 | Subject) + storage_time
y ~ current_feature + Sex + (1 | Subject) + storage_time
y ~ current_feature + Sex + (1 | Subject) + storage_time
y ~ current_feature + Sex + (1 | Subject) + storage_time
y ~ current_feature + Sex + (1 | Subject) + storage_time
y ~ current_feature + Sex + (1 | Subject) + storage_time
y ~ current_feature + Sex + (1 | Subject) + storage_time
y ~ current_feature + Sex + (1 | Subject) + storage_time
y ~ current_feature + Sex + (1 | Subject) + storage_time
y ~ current_feature + Sex + (1 | Subject) + storage_time
y ~ current_feature + Sex + (1 | Subject) + storage_time
y ~ current_feature + Sex + (1 | Subject) + storage_time
y ~ current_feature + Sex + (1 | Subject) + storage_time
y ~ current_feature + Sex + (1 | Subject) + storage_time
y ~ current_feature + Sex + (1 | Subject) + storage_time
y ~ current_feature + Sex + (1 | Subject) + storage_time
y ~ current_feature + Sex + (1 | Subject) + storage_time
y ~ current_feature + Sex + (1 | Subject) + storage_time
y ~ current_feature + Sex + (1 | Subject) + storage_time
y ~ current_feature + Sex + (1 | Subject) + storage_time
y ~ current_feature + Sex + (1 | Subject) + storage_time
y ~ current_feature + Sex + (1 | Subject) + storage_time
y ~ current_feature + Sex + (1 | Subject) + storage_time
y ~ current_feature + Sex + (1 | Subject) + storage_time
y ~ current_feature + Sex + (1 | Subject) + storage_time
y ~ current_feature + Sex + (1 | Subject) + storage_time
y ~ current_feature + Sex + (1 | Subject) + storage_time
y ~ current_feature + Sex + (1 | Subject) + storage_time
y ~ current_feature + Sex + (1 | Subject) + storage_time
y ~ current_feature + Sex + (1 | Subject) + storage_time
y ~ current_feature + Sex + (1 | Subject) + storage_time
y ~ current_feature + Sex + (1 | Subject) + storage_time
y ~ current_feature + Sex + (1 | Subject) + storage_time
y ~ current_feature + Sex + (1 | Subject) + storage_time
y ~ current_feature + Sex + (1 | Subject) + storage_time
y ~ current_feature + Sex + (1 | Subject) + storage_time
y ~ current_feature + Sex + (1 | Subject) + storage_time
y ~ current_feature + Sex + (1 | Subject) + storage_time
y ~ current_feature + Sex + (1 | Subject) + storage_time
y ~ current_feature + Sex + (1 | Subject) + storage_time

y ~ current_feature + Sex + (1 | Subject) + storage_time
y ~ current_feature + Sex + (1 | Subject) + storage_time
y ~ current_feature + Sex + (1 | Subject) + storage_time
y ~ current_feature + Sex + (1 | Subject) + storage_time
y ~ current_feature + Sex + (1 | Subject) + storage_time
y ~ current_feature + Sex + (1 | Subject) + storage_time
y ~ current_feature + Sex + (1 | Subject) + storage_time
y ~ current_feature + Sex + (1 | Subject) + storage_time
y ~ current_feature + Sex + (1 | Subject) + storage_time
y ~ current_feature + Sex + (1 | Subject) + storage_time
y ~ current_feature + Sex + (1 | Subject) + storage_time
y ~ current_feature + Sex + (1 | Subject) + storage_time
y ~ current_feature + Sex + (1 | Subject) + storage_time
y ~ current_feature + Sex + (1 | Subject) + storage_time
y ~ current_feature + Sex + (1 | Subject) + storage_time
y ~ current_feature + Sex + (1 | Subject) + storage_time
y ~ current_feature + Sex + (1 | Subject) + storage_time
y ~ current_feature + Sex + (1 | Subject) + storage_time
y ~ current_feature + Sex + (1 | Subject) + storage_time
y ~ current_feature + Sex + (1 | Subject) + storage_time
y ~ current_feature + Sex + (1 | Subject) + storage_time
y ~ current_feature + Sex + (1 | Subject) + storage_time
y ~ current_feature + Sex + (1 | Subject) + storage_time
y ~ current_feature + Sex + (1 | Subject) + storage_time
y ~ current_feature + Sex + (1 | Subject) + storage_time
y ~ current_feature + Sex + (1 | Subject) + storage_time
y ~ current_feature + Sex + (1 | Subject) + storage_time
y ~ current_feature + Sex + (1 | Subject) + storage_time
y ~ current_feature + Sex + (1 | Subject) + storage_time
y ~ current_feature + Sex + (1 | Subject) + storage_time
y ~ current_feature + Sex + (1 | Subject) + storage_time
y ~ current_feature + Sex + (1 | Subject) + storage_time
y ~ current_feature + Sex + (1 | Subject) + storage_time
y ~ current_feature + Sex + (1 | Subject) + storage_time
y ~ current_feature + Sex + (1 | Subject) + storage_time
y ~ current_feature + Sex + (1 | Subject) + storage_time
y ~ current_feature + Sex + (1 | Subject) + storage_time
y ~ current_feature + Sex + (1 | Subject) + storage_time
y ~ current_feature + Sex + (1 | Subject) + storage_time
y ~ current_feature + Sex + (1 | Subject) + storage_time
y ~ current_feature + Sex + (1 | Subject) + storage_time
y ~ current_feature + Sex + (1 | Subject) + storage_time
y ~ current_feature + Sex + (1 | Subject) + storage_time
y ~ current_feature + Sex + (1 | Subject) + storage_time
y ~ current_feature + Sex + (1 | Subject) + storage_time
y ~ current_feature + Sex + (1 | Subject) + storage_time
y ~ current_feature + Sex + (1 | Subject) + storage_time
y ~ current_feature + Sex + (1 | Subject) + storage_time

y ~ current_feature + Sex + (1 | Subject) + storage_time
y ~ current_feature + Sex + (1 | Subject) + storage_time
y ~ current_feature + Sex + (1 | Subject) + storage_time
y ~ current_feature + Sex + (1 | Subject) + storage_time
y ~ current_feature + Sex + (1 | Subject) + storage_time
y ~ current_feature + Sex + (1 | Subject) + storage_time
y ~ current_feature + Sex + (1 | Subject) + storage_time
y ~ current_feature + Sex + (1 | Subject) + storage_time
y ~ current_feature + Sex + (1 | Subject) + storage_time
y ~ current_feature + Sex + (1 | Subject) + storage_time
y ~ current_feature + Sex + (1 | Subject) + storage_time
y ~ current_feature + Sex + (1 | Subject) + storage_time
y ~ current_feature + Sex + (1 | Subject) + storage_time
y ~ current_feature + Sex + (1 | Subject) + storage_time
y ~ current_feature + Sex + (1 | Subject) + storage_time
y ~ current_feature + Sex + (1 | Subject) + storage_time
y ~ current_feature + Sex + (1 | Subject) + storage_time
y ~ current_feature + Sex + (1 | Subject) + storage_time
y ~ current_feature + Sex + (1 | Subject) + storage_time
y ~ current_feature + Sex + (1 | Subject) + storage_time
y ~ current_feature + Sex + (1 | Subject) + storage_time
y ~ current_feature + Sex + (1 | Subject) + storage_time
y ~ current_feature + Sex + (1 | Subject) + storage_time
y ~ current_feature + Sex + (1 | Subject) + storage_time
y ~ current_feature + Sex + (1 | Subject) + storage_time
y ~ current_feature + Sex + (1 | Subject) + storage_time
y ~ current_feature + Sex + (1 | Subject) + storage_time
y ~ current_feature + Sex + (1 | Subject) + storage_time
y ~ current_feature + Sex + (1 | Subject) + storage_time
y ~ current_feature + Sex + (1 | Subject) + storage_time
y ~ current_feature + Sex + (1 | Subject) + storage_time
y ~ current_feature + Sex + (1 | Subject) + storage_time
y ~ current_feature + Sex + (1 | Subject) + storage_time
y ~ current_feature + Sex + (1 | Subject) + storage_time
y ~ current_feature + Sex + (1 | Subject) + storage_time
y ~ current_feature + Sex + (1 | Subject) + storage_time
y ~ current_feature + Sex + (1 | Subject) + storage_time
y ~ current_feature + Sex + (1 | Subject) + storage_time
y ~ current_feature + Sex + (1 | Subject) + storage_time
y ~ current_feature + Sex + (1 | Subject) + storage_time
y ~ current_feature + Sex + (1 | Subject) + storage_time
y ~ current_feature + Sex + (1 | Subject) + storage_time
y ~ current_feature + Sex + (1 | Subject) + storage_time
y ~ current_feature + Sex + (1 | Subject) + storage_time
y ~ current_feature + Sex + (1 | Subject) + storage_time
y ~ current_feature + Sex + (1 | Subject) + storage_time
y ~ current_feature + Sex + (1 | Subject) + storage_time
y ~ current_feature + Sex + (1 | Subject) + storage_time

y ~ current_feature + Sex + (1 | Subject) + storage_time
y ~ current_feature + Sex + (1 | Subject) + storage_time
y ~ current_feature + Sex + (1 | Subject) + storage_time
y ~ current_feature + Sex + (1 | Subject) + storage_time
y ~ current_feature + Sex + (1 | Subject) + storage_time
y ~ current_feature + Sex + (1 | Subject) + storage_time
y ~ current_feature + Sex + (1 | Subject) + storage_time
y ~ current_feature + Sex + (1 | Subject) + storage_time
y ~ current_feature + Sex + (1 | Subject) + storage_time
y ~ current_feature + Sex + (1 | Subject) + storage_time
y ~ current_feature + Sex + (1 | Subject) + storage_time
y ~ current_feature + Sex + (1 | Subject) + storage_time
y ~ current_feature + Sex + (1 | Subject) + storage_time
y ~ current_feature + Sex + (1 | Subject) + storage_time
y ~ current_feature + Sex + (1 | Subject) + storage_time
y ~ current_feature + Sex + (1 | Subject) + storage_time
y ~ current_feature + Sex + (1 | Subject) + storage_time
y ~ current_feature + Sex + (1 | Subject) + storage_time
y ~ current_feature + Sex + (1 | Subject) + storage_time
y ~ current_feature + Sex + (1 | Subject) + storage_time
y ~ current_feature + Sex + (1 | Subject) + storage_time
y ~ current_feature + Sex + (1 | Subject) + storage_time
y ~ current_feature + Sex + (1 | Subject) + storage_time
y ~ current_feature + Sex + (1 | Subject) + storage_time
y ~ current_feature + Sex + (1 | Subject) + storage_time
y ~ current_feature + Sex + (1 | Subject) + storage_time
y ~ current_feature + Sex + (1 | Subject) + storage_time
y ~ current_feature + Sex + (1 | Subject) + storage_time
y ~ current_feature + Sex + (1 | Subject) + storage_time
y ~ current_feature + Sex + (1 | Subject) + storage_time
y ~ current_feature + Sex + (1 | Subject) + storage_time
y ~ current_feature + Sex + (1 | Subject) + storage_time
y ~ current_feature + Sex + (1 | Subject) + storage_time
y ~ current_feature + Sex + (1 | Subject) + storage_time
y ~ current_feature + Sex + (1 | Subject) + storage_time
y ~ current_feature + Sex + (1 | Subject) + storage_time
y ~ current_feature + Sex + (1 | Subject) + storage_time
y ~ current_feature + Sex + (1 | Subject) + storage_time
y ~ current_feature + Sex + (1 | Subject) + storage_time
y ~ current_feature + Sex + (1 | Subject) + storage_time
y ~ current_feature + Sex + (1 | Subject) + storage_time
y ~ current_feature + Sex + (1 | Subject) + storage_time
y ~ current_feature + Sex + (1 | Subject) + storage_time
y ~ current_feature + Sex + (1 | Subject) + storage_time
y ~ current_feature + Sex + (1 | Subject) + storage_time
y ~ current_feature + Sex + (1 | Subject) + storage_time
y ~ current_feature + Sex + (1 | Subject) + storage_time
y ~ current_feature + Sex + (1 | Subject) + storage_time

y ~ current_feature + Sex + (1 | Subject) + storage_time
y ~ current_feature + Sex + (1 | Subject) + storage_time
y ~ current_feature + Sex + (1 | Subject) + storage_time
y ~ current_feature + Sex + (1 | Subject) + storage_time
y ~ current_feature + Sex + (1 | Subject) + storage_time
y ~ current_feature + Sex + (1 | Subject) + storage_time
y ~ current_feature + Sex + (1 | Subject) + storage_time
y ~ current_feature + Sex + (1 | Subject) + storage_time
y ~ current_feature + Sex + (1 | Subject) + storage_time
y ~ current_feature + Sex + (1 | Subject) + storage_time
y ~ current_feature + Sex + (1 | Subject) + storage_time
y ~ current_feature + Sex + (1 | Subject) + storage_time
y ~ current_feature + Sex + (1 | Subject) + storage_time
y ~ current_feature + Sex + (1 | Subject) + storage_time
y ~ current_feature + Sex + (1 | Subject) + storage_time
y ~ current_feature + Sex + (1 | Subject) + storage_time
y ~ current_feature + Sex + (1 | Subject) + storage_time
y ~ current_feature + Sex + (1 | Subject) + storage_time
y ~ current_feature + Sex + (1 | Subject) + storage_time
y ~ current_feature + Sex + (1 | Subject) + storage_time
y ~ current_feature + Sex + (1 | Subject) + storage_time
y ~ current_feature + Sex + (1 | Subject) + storage_time
y ~ current_feature + Sex + (1 | Subject) + storage_time
y ~ current_feature + Sex + (1 | Subject) + storage_time
y ~ current_feature + Sex + (1 | Subject) + storage_time
y ~ current_feature + Sex + (1 | Subject) + storage_time
y ~ current_feature + Sex + (1 | Subject) + storage_time
y ~ current_feature + Sex + (1 | Subject) + storage_time
y ~ current_feature + Sex + (1 | Subject) + storage_time
y ~ current_feature + Sex + (1 | Subject) + storage_time
y ~ current_feature + Sex + (1 | Subject) + storage_time
y ~ current_feature + Sex + (1 | Subject) + storage_time
y ~ current_feature + Sex + (1 | Subject) + storage_time
y ~ current_feature + Sex + (1 | Subject) + storage_time
y ~ current_feature + Sex + (1 | Subject) + storage_time
y ~ current_feature + Sex + (1 | Subject) + storage_time
y ~ current_feature + Sex + (1 | Subject) + storage_time
y ~ current_feature + Sex + (1 | Subject) + storage_time
y ~ current_feature + Sex + (1 | Subject) + storage_time
y ~ current_feature + Sex + (1 | Subject) + storage_time
y ~ current_feature + Sex + (1 | Subject) + storage_time
y ~ current_feature + Sex + (1 | Subject) + storage_time
y ~ current_feature + Sex + (1 | Subject) + storage_time
y ~ current_feature + Sex + (1 | Subject) + storage_time
y ~ current_feature + Sex + (1 | Subject) + storage_time
y ~ current_feature + Sex + (1 | Subject) + storage_time
y ~ current_feature + Sex + (1 | Subject) + storage_time
y ~ current_feature + Sex + (1 | Subject) + storage_time

y ~ current_feature + Sex + (1 | Subject) + storage_time
y ~ current_feature + Sex + (1 | Subject) + storage_time
y ~ current_feature + Sex + (1 | Subject) + storage_time
y ~ current_feature + Sex + (1 | Subject) + storage_time
y ~ current_feature + Sex + (1 | Subject) + storage_time
y ~ current_feature + Sex + (1 | Subject) + storage_time
y ~ current_feature + Sex + (1 | Subject) + storage_time
y ~ current_feature + Sex + (1 | Subject) + storage_time
y ~ current_feature + Sex + (1 | Subject) + storage_time
y ~ current_feature + Sex + (1 | Subject) + storage_time
y ~ current_feature + Sex + (1 | Subject) + storage_time
y ~ current_feature + Sex + (1 | Subject) + storage_time
y ~ current_feature + Sex + (1 | Subject) + storage_time
y ~ current_feature + Sex + (1 | Subject) + storage_time
y ~ current_feature + Sex + (1 | Subject) + storage_time
y ~ current_feature + Sex + (1 | Subject) + storage_time
y ~ current_feature + Sex + (1 | Subject) + storage_time
y ~ current_feature + Sex + (1 | Subject) + storage_time
y ~ current_feature + Sex + (1 | Subject) + storage_time
y ~ current_feature + Sex + (1 | Subject) + storage_time
y ~ current_feature + Sex + (1 | Subject) + storage_time
y ~ current_feature + Sex + (1 | Subject) + storage_time
y ~ current_feature + Sex + (1 | Subject) + storage_time
y ~ current_feature + Sex + (1 | Subject) + storage_time
y ~ current_feature + Sex + (1 | Subject) + storage_time
y ~ current_feature + Sex + (1 | Subject) + storage_time
y ~ current_feature + Sex + (1 | Subject) + storage_time
y ~ current_feature + Sex + (1 | Subject) + storage_time
y ~ current_feature + Sex + (1 | Subject) + storage_time
y ~ current_feature + Sex + (1 | Subject) + storage_time
y ~ current_feature + Sex + (1 | Subject) + storage_time
y ~ current_feature + Sex + (1 | Subject) + storage_time
y ~ current_feature + Sex + (1 | Subject) + storage_time
y ~ current_feature + Sex + (1 | Subject) + storage_time
y ~ current_feature + Sex + (1 | Subject) + storage_time
y ~ current_feature + Sex + (1 | Subject) + storage_time
y ~ current_feature + Sex + (1 | Subject) + storage_time
y ~ current_feature + Sex + (1 | Subject) + storage_time
y ~ current_feature + Sex + (1 | Subject) + storage_time
y ~ current_feature + Sex + (1 | Subject) + storage_time
y ~ current_feature + Sex + (1 | Subject) + storage_time
y ~ current_feature + Sex + (1 | Subject) + storage_time
y ~ current_feature + Sex + (1 | Subject) + storage_time
y ~ current_feature + Sex + (1 | Subject) + storage_time
y ~ current_feature + Sex + (1 | Subject) + storage_time
y ~ current_feature + Sex + (1 | Subject) + storage_time
y ~ current_feature + Sex + (1 | Subject) + storage_time
y ~ current_feature + Sex + (1 | Subject) + storage_time

y ~ current_feature + Sex + (1 | Subject) + storage_time
y ~ current_feature + Sex + (1 | Subject) + storage_time
y ~ current_feature + Sex + (1 | Subject) + storage_time
y ~ current_feature + Sex + (1 | Subject) + storage_time
y ~ current_feature + Sex + (1 | Subject) + storage_time
y ~ current_feature + Sex + (1 | Subject) + storage_time
y ~ current_feature + Sex + (1 | Subject) + storage_time
y ~ current_feature + Sex + (1 | Subject) + storage_time
y ~ current_feature + Sex + (1 | Subject) + storage_time
y ~ current_feature + Sex + (1 | Subject) + storage_time
y ~ current_feature + Sex + (1 | Subject) + storage_time
y ~ current_feature + Sex + (1 | Subject) + storage_time
y ~ current_feature + Sex + (1 | Subject) + storage_time
y ~ current_feature + Sex + (1 | Subject) + storage_time
y ~ current_feature + Sex + (1 | Subject) + storage_time
y ~ current_feature + Sex + (1 | Subject) + storage_time
y ~ current_feature + Sex + (1 | Subject) + storage_time
y ~ current_feature + Sex + (1 | Subject) + storage_time
y ~ current_feature + Sex + (1 | Subject) + storage_time
y ~ current_feature + Sex + (1 | Subject) + storage_time
y ~ current_feature + Sex + (1 | Subject) + storage_time
y ~ current_feature + Sex + (1 | Subject) + storage_time
y ~ current_feature + Sex + (1 | Subject) + storage_time
y ~ current_feature + Sex + (1 | Subject) + storage_time
y ~ current_feature + Sex + (1 | Subject) + storage_time
y ~ current_feature + Sex + (1 | Subject) + storage_time
y ~ current_feature + Sex + (1 | Subject) + storage_time
y ~ current_feature + Sex + (1 | Subject) + storage_time
y ~ current_feature + Sex + (1 | Subject) + storage_time
y ~ current_feature + Sex + (1 | Subject) + storage_time
y ~ current_feature + Sex + (1 | Subject) + storage_time
y ~ current_feature + Sex + (1 | Subject) + storage_time
y ~ current_feature + Sex + (1 | Subject) + storage_time
y ~ current_feature + Sex + (1 | Subject) + storage_time
y ~ current_feature + Sex + (1 | Subject) + storage_time
y ~ current_feature + Sex + (1 | Subject) + storage_time
y ~ current_feature + Sex + (1 | Subject) + storage_time
y ~ current_feature + Sex + (1 | Subject) + storage_time
y ~ current_feature + Sex + (1 | Subject) + storage_time
y ~ current_feature + Sex + (1 | Subject) + storage_time
y ~ current_feature + Sex + (1 | Subject) + storage_time
y ~ current_feature + Sex + (1 | Subject) + storage_time
y ~ current_feature + Sex + (1 | Subject) + storage_time
y ~ current_feature + Sex + (1 | Subject) + storage_time
y ~ current_feature + Sex + (1 | Subject) + storage_time
y ~ current_feature + Sex + (1 | Subject) + storage_time
y ~ current_feature + Sex + (1 | Subject) + storage_time
y ~ current_feature + Sex + (1 | Subject) + storage_time

y ~ current_feature + Sex + (1 | Subject) + storage_time
y ~ current_feature + Sex + (1 | Subject) + storage_time
y ~ current_feature + Sex + (1 | Subject) + storage_time
y ~ current_feature + Sex + (1 | Subject) + storage_time
y ~ current_feature + Sex + (1 | Subject) + storage_time
y ~ current_feature + Sex + (1 | Subject) + storage_time
y ~ current_feature + Sex + (1 | Subject) + storage_time
y ~ current_feature + Sex + (1 | Subject) + storage_time
y ~ current_feature + Sex + (1 | Subject) + storage_time
y ~ current_feature + Sex + (1 | Subject) + storage_time
y ~ current_feature + Sex + (1 | Subject) + storage_time
y ~ current_feature + Sex + (1 | Subject) + storage_time
y ~ current_feature + Sex + (1 | Subject) + storage_time
y ~ current_feature + Sex + (1 | Subject) + storage_time
y ~ current_feature + Sex + (1 | Subject) + storage_time
y ~ current_feature + Sex + (1 | Subject) + storage_time
y ~ current_feature + Sex + (1 | Subject) + storage_time
y ~ current_feature + Sex + (1 | Subject) + storage_time
y ~ current_feature + Sex + (1 | Subject) + storage_time
y ~ current_feature + Sex + (1 | Subject) + storage_time
y ~ current_feature + Sex + (1 | Subject) + storage_time
y ~ current_feature + Sex + (1 | Subject) + storage_time
y ~ current_feature + Sex + (1 | Subject) + storage_time
y ~ current_feature + Sex + (1 | Subject) + storage_time
y ~ current_feature + Sex + (1 | Subject) + storage_time
y ~ current_feature + Sex + (1 | Subject) + storage_time
y ~ current_feature + Sex + (1 | Subject) + storage_time
y ~ current_feature + Sex + (1 | Subject) + storage_time
y ~ current_feature + Sex + (1 | Subject) + storage_time
y ~ current_feature + Sex + (1 | Subject) + storage_time
y ~ current_feature + Sex + (1 | Subject) + storage_time
y ~ current_feature + Sex + (1 | Subject) + storage_time
y ~ current_feature + Sex + (1 | Subject) + storage_time
y ~ current_feature + Sex + (1 | Subject) + storage_time
y ~ current_feature + Sex + (1 | Subject) + storage_time
y ~ current_feature + Sex + (1 | Subject) + storage_time
y ~ current_feature + Sex + (1 | Subject) + storage_time
y ~ current_feature + Sex + (1 | Subject) + storage_time
y ~ current_feature + Sex + (1 | Subject) + storage_time
y ~ current_feature + Sex + (1 | Subject) + storage_time
y ~ current_feature + Sex + (1 | Subject) + storage_time
y ~ current_feature + Sex + (1 | Subject) + storage_time
y ~ current_feature + Sex + (1 | Subject) + storage_time
y ~ current_feature + Sex + (1 | Subject) + storage_time
y ~ current_feature + Sex + (1 | Subject) + storage_time
y ~ current_feature + Sex + (1 | Subject) + storage_time
y ~ current_feature + Sex + (1 | Subject) + storage_time
y ~ current_feature + Sex + (1 | Subject) + storage_time

y ~ current_feature + Sex + (1 | Subject) + storage_time
y ~ current_feature + Sex + (1 | Subject) + storage_time
y ~ current_feature + Sex + (1 | Subject) + storage_time
y ~ current_feature + Sex + (1 | Subject) + storage_time
y ~ current_feature + Sex + (1 | Subject) + storage_time
y ~ current_feature + Sex + (1 | Subject) + storage_time
y ~ current_feature + Sex + (1 | Subject) + storage_time
y ~ current_feature + Sex + (1 | Subject) + storage_time
y ~ current_feature + Sex + (1 | Subject) + storage_time
y ~ current_feature + Sex + (1 | Subject) + storage_time
y ~ current_feature + Sex + (1 | Subject) + storage_time
y ~ current_feature + Sex + (1 | Subject) + storage_time
y ~ current_feature + Sex + (1 | Subject) + storage_time
y ~ current_feature + Sex + (1 | Subject) + storage_time
y ~ current_feature + Sex + (1 | Subject) + storage_time
y ~ current_feature + Sex + (1 | Subject) + storage_time
y ~ current_feature + Sex + (1 | Subject) + storage_time
y ~ current_feature + Sex + (1 | Subject) + storage_time
y ~ current_feature + Sex + (1 | Subject) + storage_time
y ~ current_feature + Sex + (1 | Subject) + storage_time
y ~ current_feature + Sex + (1 | Subject) + storage_time
y ~ current_feature + Sex + (1 | Subject) + storage_time
y ~ current_feature + Sex + (1 | Subject) + storage_time
y ~ current_feature + Sex + (1 | Subject) + storage_time
y ~ current_feature + Sex + (1 | Subject) + storage_time
y ~ current_feature + Sex + (1 | Subject) + storage_time
y ~ current_feature + Sex + (1 | Subject) + storage_time
y ~ current_feature + Sex + (1 | Subject) + storage_time
y ~ current_feature + Sex + (1 | Subject) + storage_time
y ~ current_feature + Sex + (1 | Subject) + storage_time
y ~ current_feature + Sex + (1 | Subject) + storage_time
y ~ current_feature + Sex + (1 | Subject) + storage_time
y ~ current_feature + Sex + (1 | Subject) + storage_time
y ~ current_feature + Sex + (1 | Subject) + storage_time
y ~ current_feature + Sex + (1 | Subject) + storage_time
y ~ current_feature + Sex + (1 | Subject) + storage_time
y ~ current_feature + Sex + (1 | Subject) + storage_time
y ~ current_feature + Sex + (1 | Subject) + storage_time
y ~ current_feature + Sex + (1 | Subject) + storage_time
y ~ current_feature + Sex + (1 | Subject) + storage_time
y ~ current_feature + Sex + (1 | Subject) + storage_time
y ~ current_feature + Sex + (1 | Subject) + storage_time
y ~ current_feature + Sex + (1 | Subject) + storage_time
y ~ current_feature + Sex + (1 | Subject) + storage_time
y ~ current_feature + Sex + (1 | Subject) + storage_time
y ~ current_feature + Sex + (1 | Subject) + storage_time
y ~ current_feature + Sex + (1 | Subject) + storage_time
y ~ current_feature + Sex + (1 | Subject) + storage_time

y ~ current_feature + Sex + (1 | Subject) + storage_time
y ~ current_feature + Sex + (1 | Subject) + storage_time
y ~ current_feature + Sex + (1 | Subject) + storage_time
y ~ current_feature + Sex + (1 | Subject) + storage_time
y ~ current_feature + Sex + (1 | Subject) + storage_time
y ~ current_feature + Sex + (1 | Subject) + storage_time
y ~ current_feature + Sex + (1 | Subject) + storage_time
y ~ current_feature + Sex + (1 | Subject) + storage_time
y ~ current_feature + Sex + (1 | Subject) + storage_time
y ~ current_feature + Sex + (1 | Subject) + storage_time
y ~ current_feature + Sex + (1 | Subject) + storage_time
y ~ current_feature + Sex + (1 | Subject) + storage_time
y ~ current_feature + Sex + (1 | Subject) + storage_time
y ~ current_feature + Sex + (1 | Subject) + storage_time
y ~ current_feature + Sex + (1 | Subject) + storage_time
y ~ current_feature + Sex + (1 | Subject) + storage_time
y ~ current_feature + Sex + (1 | Subject) + storage_time
y ~ current_feature + Sex + (1 | Subject) + storage_time
y ~ current_feature + Sex + (1 | Subject) + storage_time
y ~ current_feature + Sex + (1 | Subject) + storage_time
y ~ current_feature + Sex + (1 | Subject) + storage_time
y ~ current_feature + Sex + (1 | Subject) + storage_time
y ~ current_feature + Sex + (1 | Subject) + storage_time
y ~ current_feature + Sex + (1 | Subject) + storage_time
y ~ current_feature + Sex + (1 | Subject) + storage_time
y ~ current_feature + Sex + (1 | Subject) + storage_time
y ~ current_feature + Sex + (1 | Subject) + storage_time
y ~ current_feature + Sex + (1 | Subject) + storage_time
y ~ current_feature + Sex + (1 | Subject) + storage_time
y ~ current_feature + Sex + (1 | Subject) + storage_time
y ~ current_feature + Sex + (1 | Subject) + storage_time
y ~ current_feature + Sex + (1 | Subject) + storage_time
y ~ current_feature + Sex + (1 | Subject) + storage_time
y ~ current_feature + Sex + (1 | Subject) + storage_time
y ~ current_feature + Sex + (1 | Subject) + storage_time
y ~ current_feature + Sex + (1 | Subject) + storage_time
y ~ current_feature + Sex + (1 | Subject) + storage_time
y ~ current_feature + Sex + (1 | Subject) + storage_time
y ~ current_feature + Sex + (1 | Subject) + storage_time
y ~ current_feature + Sex + (1 | Subject) + storage_time
y ~ current_feature + Sex + (1 | Subject) + storage_time
y ~ current_feature + Sex + (1 | Subject) + storage_time
y ~ current_feature + Sex + (1 | Subject) + storage_time
y ~ current_feature + Sex + (1 | Subject) + storage_time
y ~ current_feature + Sex + (1 | Subject) + storage_time
y ~ current_feature + Sex + (1 | Subject) + storage_time
y ~ current_feature + Sex + (1 | Subject) + storage_time
y ~ current_feature + Sex + (1 | Subject) + storage_time

y ~ current_feature + Sex + (1 | Subject) + storage_time
y ~ current_feature + Sex + (1 | Subject) + storage_time
y ~ current_feature + Sex + (1 | Subject) + storage_time
y ~ current_feature + Sex + (1 | Subject) + storage_time
y ~ current_feature + Sex + (1 | Subject) + storage_time
y ~ current_feature + Sex + (1 | Subject) + storage_time
y ~ current_feature + Sex + (1 | Subject) + storage_time
y ~ current_feature + Sex + (1 | Subject) + storage_time
y ~ current_feature + Sex + (1 | Subject) + storage_time
y ~ current_feature + Sex + (1 | Subject) + storage_time
y ~ current_feature + Sex + (1 | Subject) + storage_time
y ~ current_feature + Sex + (1 | Subject) + storage_time
y ~ current_feature + Sex + (1 | Subject) + storage_time
y ~ current_feature + Sex + (1 | Subject) + storage_time
y ~ current_feature + Sex + (1 | Subject) + storage_time
y ~ current_feature + Sex + (1 | Subject) + storage_time
y ~ current_feature + Sex + (1 | Subject) + storage_time
y ~ current_feature + Sex + (1 | Subject) + storage_time
y ~ current_feature + Sex + (1 | Subject) + storage_time
y ~ current_feature + Sex + (1 | Subject) + storage_time
y ~ current_feature + Sex + (1 | Subject) + storage_time
y ~ current_feature + Sex + (1 | Subject) + storage_time
y ~ current_feature + Sex + (1 | Subject) + storage_time
y ~ current_feature + Sex + (1 | Subject) + storage_time
y ~ current_feature + Sex + (1 | Subject) + storage_time
y ~ current_feature + Sex + (1 | Subject) + storage_time
y ~ current_feature + Sex + (1 | Subject) + storage_time
y ~ current_feature + Sex + (1 | Subject) + storage_time
y ~ current_feature + Sex + (1 | Subject) + storage_time
y ~ current_feature + Sex + (1 | Subject) + storage_time
y ~ current_feature + Sex + (1 | Subject) + storage_time
y ~ current_feature + Sex + (1 | Subject) + storage_time
y ~ current_feature + Sex + (1 | Subject) + storage_time
y ~ current_feature + Sex + (1 | Subject) + storage_time
y ~ current_feature + Sex + (1 | Subject) + storage_time
y ~ current_feature + Sex + (1 | Subject) + storage_time
y ~ current_feature + Sex + (1 | Subject) + storage_time
y ~ current_feature + Sex + (1 | Subject) + storage_time
y ~ current_feature + Sex + (1 | Subject) + storage_time
y ~ current_feature + Sex + (1 | Subject) + storage_time
y ~ current_feature + Sex + (1 | Subject) + storage_time
y ~ current_feature + Sex + (1 | Subject) + storage_time
y ~ current_feature + Sex + (1 | Subject) + storage_time
y ~ current_feature + Sex + (1 | Subject) + storage_time
y ~ current_feature + Sex + (1 | Subject) + storage_time
y ~ current_feature + Sex + (1 | Subject) + storage_time
y ~ current_feature + Sex + (1 | Subject) + storage_time
y ~ current_feature + Sex + (1 | Subject) + storage_time

y ~ current_feature + Sex + (1 | Subject) + storage_time
y ~ current_feature + Sex + (1 | Subject) + storage_time
y ~ current_feature + Sex + (1 | Subject) + storage_time
y ~ current_feature + Sex + (1 | Subject) + storage_time
y ~ current_feature + Sex + (1 | Subject) + storage_time
y ~ current_feature + Sex + (1 | Subject) + storage_time
y ~ current_feature + Sex + (1 | Subject) + storage_time
y ~ current_feature + Sex + (1 | Subject) + storage_time
y ~ current_feature + Sex + (1 | Subject) + storage_time
y ~ current_feature + Sex + (1 | Subject) + storage_time
y ~ current_feature + Sex + (1 | Subject) + storage_time
y ~ current_feature + Sex + (1 | Subject) + storage_time
y ~ current_feature + Sex + (1 | Subject) + storage_time
y ~ current_feature + Sex + (1 | Subject) + storage_time
y ~ current_feature + Sex + (1 | Subject) + storage_time
y ~ current_feature + Sex + (1 | Subject) + storage_time
y ~ current_feature + Sex + (1 | Subject) + storage_time
y ~ current_feature + Sex + (1 | Subject) + storage_time
y ~ current_feature + Sex + (1 | Subject) + storage_time
y ~ current_feature + Sex + (1 | Subject) + storage_time
y ~ current_feature + Sex + (1 | Subject) + storage_time
y ~ current_feature + Sex + (1 | Subject) + storage_time
y ~ current_feature + Sex + (1 | Subject) + storage_time
y ~ current_feature + Sex + (1 | Subject) + storage_time
y ~ current_feature + Sex + (1 | Subject) + storage_time
y ~ current_feature + Sex + (1 | Subject) + storage_time
y ~ current_feature + Sex + (1 | Subject) + storage_time
y ~ current_feature + Sex + (1 | Subject) + storage_time
y ~ current_feature + Sex + (1 | Subject) + storage_time
y ~ current_feature + Sex + (1 | Subject) + storage_time
y ~ current_feature + Sex + (1 | Subject) + storage_time
y ~ current_feature + Sex + (1 | Subject) + storage_time
y ~ current_feature + Sex + (1 | Subject) + storage_time
y ~ current_feature + Sex + (1 | Subject) + storage_time
y ~ current_feature + Sex + (1 | Subject) + storage_time
y ~ current_feature + Sex + (1 | Subject) + storage_time
y ~ current_feature + Sex + (1 | Subject) + storage_time
y ~ current_feature + Sex + (1 | Subject) + storage_time
y ~ current_feature + Sex + (1 | Subject) + storage_time
y ~ current_feature + Sex + (1 | Subject) + storage_time
y ~ current_feature + Sex + (1 | Subject) + storage_time
y ~ current_feature + Sex + (1 | Subject) + storage_time
y ~ current_feature + Sex + (1 | Subject) + storage_time
y ~ current_feature + Sex + (1 | Subject) + storage_time
y ~ current_feature + Sex + (1 | Subject) + storage_time
y ~ current_feature + Sex + (1 | Subject) + storage_time
y ~ current_feature + Sex + (1 | Subject) + storage_time
y ~ current_feature + Sex + (1 | Subject) + storage_time

y ~ current_feature + Sex + (1 | Subject) + storage_time
y ~ current_feature + Sex + (1 | Subject) + storage_time
y ~ current_feature + Sex + (1 | Subject) + storage_time
y ~ current_feature + Sex + (1 | Subject) + storage_time
y ~ current_feature + Sex + (1 | Subject) + storage_time
y ~ current_feature + Sex + (1 | Subject) + storage_time
y ~ current_feature + Sex + (1 | Subject) + storage_time
y ~ current_feature + Sex + (1 | Subject) + storage_time
y ~ current_feature + Sex + (1 | Subject) + storage_time
y ~ current_feature + Sex + (1 | Subject) + storage_time
y ~ current_feature + Sex + (1 | Subject) + storage_time
y ~ current_feature + Sex + (1 | Subject) + storage_time
y ~ current_feature + Sex + (1 | Subject) + storage_time
y ~ current_feature + Sex + (1 | Subject) + storage_time
y ~ current_feature + Sex + (1 | Subject) + storage_time
y ~ current_feature + Sex + (1 | Subject) + storage_time
y ~ current_feature + Sex + (1 | Subject) + storage_time
y ~ current_feature + Sex + (1 | Subject) + storage_time
y ~ current_feature + Sex + (1 | Subject) + storage_time
y ~ current_feature + Sex + (1 | Subject) + storage_time
y ~ current_feature + Sex + (1 | Subject) + storage_time
y ~ current_feature + Sex + (1 | Subject) + storage_time
y ~ current_feature + Sex + (1 | Subject) + storage_time
y ~ current_feature + Sex + (1 | Subject) + storage_time
y ~ current_feature + Sex + (1 | Subject) + storage_time
y ~ current_feature + Sex + (1 | Subject) + storage_time
y ~ current_feature + Sex + (1 | Subject) + storage_time
y ~ current_feature + Sex + (1 | Subject) + storage_time
y ~ current_feature + Sex + (1 | Subject) + storage_time
y ~ current_feature + Sex + (1 | Subject) + storage_time
y ~ current_feature + Sex + (1 | Subject) + storage_time
y ~ current_feature + Sex + (1 | Subject) + storage_time
y ~ current_feature + Sex + (1 | Subject) + storage_time
y ~ current_feature + Sex + (1 | Subject) + storage_time
y ~ current_feature + Sex + (1 | Subject) + storage_time
y ~ current_feature + Sex + (1 | Subject) + storage_time
y ~ current_feature + Sex + (1 | Subject) + storage_time
y ~ current_feature + Sex + (1 | Subject) + storage_time
y ~ current_feature + Sex + (1 | Subject) + storage_time
y ~ current_feature + Sex + (1 | Subject) + storage_time
y ~ current_feature + Sex + (1 | Subject) + storage_time
y ~ current_feature + Sex + (1 | Subject) + storage_time
y ~ current_feature + Sex + (1 | Subject) + storage_time
y ~ current_feature + Sex + (1 | Subject) + storage_time
y ~ current_feature + Sex + (1 | Subject) + storage_time
y ~ current_feature + Sex + (1 | Subject) + storage_time
y ~ current_feature + Sex + (1 | Subject) + storage_time
y ~ current_feature + Sex + (1 | Subject) + storage_time

y ~ current_feature + Sex + (1 | Subject) + storage_time
y ~ current_feature + Sex + (1 | Subject) + storage_time
y ~ current_feature + Sex + (1 | Subject) + storage_time
y ~ current_feature + Sex + (1 | Subject) + storage_time
y ~ current_feature + Sex + (1 | Subject) + storage_time
y ~ current_feature + Sex + (1 | Subject) + storage_time
y ~ current_feature + Sex + (1 | Subject) + storage_time
y ~ current_feature + Sex + (1 | Subject) + storage_time
y ~ current_feature + Sex + (1 | Subject) + storage_time
y ~ current_feature + Sex + (1 | Subject) + storage_time
y ~ current_feature + Sex + (1 | Subject) + storage_time
y ~ current_feature + Sex + (1 | Subject) + storage_time
y ~ current_feature + Sex + (1 | Subject) + storage_time
y ~ current_feature + Sex + (1 | Subject) + storage_time
y ~ current_feature + Sex + (1 | Subject) + storage_time
y ~ current_feature + Sex + (1 | Subject) + storage_time
y ~ current_feature + Sex + (1 | Subject) + storage_time
y ~ current_feature + Sex + (1 | Subject) + storage_time
y ~ current_feature + Sex + (1 | Subject) + storage_time
y ~ current_feature + Sex + (1 | Subject) + storage_time
y ~ current_feature + Sex + (1 | Subject) + storage_time
y ~ current_feature + Sex + (1 | Subject) + storage_time
y ~ current_feature + Sex + (1 | Subject) + storage_time
y ~ current_feature + Sex + (1 | Subject) + storage_time
y ~ current_feature + Sex + (1 | Subject) + storage_time
y ~ current_feature + Sex + (1 | Subject) + storage_time
y ~ current_feature + Sex + (1 | Subject) + storage_time
y ~ current_feature + Sex + (1 | Subject) + storage_time
y ~ current_feature + Sex + (1 | Subject) + storage_time
y ~ current_feature + Sex + (1 | Subject) + storage_time
y ~ current_feature + Sex + (1 | Subject) + storage_time
y ~ current_feature + Sex + (1 | Subject) + storage_time
y ~ current_feature + Sex + (1 | Subject) + storage_time
y ~ current_feature + Sex + (1 | Subject) + storage_time
y ~ current_feature + Sex + (1 | Subject) + storage_time
y ~ current_feature + Sex + (1 | Subject) + storage_time
y ~ current_feature + Sex + (1 | Subject) + storage_time
y ~ current_feature + Sex + (1 | Subject) + storage_time
y ~ current_feature + Sex + (1 | Subject) + storage_time
y ~ current_feature + Sex + (1 | Subject) + storage_time
y ~ current_feature + Sex + (1 | Subject) + storage_time
y ~ current_feature + Sex + (1 | Subject) + storage_time
y ~ current_feature + Sex + (1 | Subject) + storage_time
y ~ current_feature + Sex + (1 | Subject) + storage_time
y ~ current_feature + Sex + (1 | Subject) + storage_time
y ~ current_feature + Sex + (1 | Subject) + storage_time
y ~ current_feature + Sex + (1 | Subject) + storage_time
y ~ current_feature + Sex + (1 | Subject) + storage_time

y ~ current_feature + Sex + (1 | Subject) + storage_time
y ~ current_feature + Sex + (1 | Subject) + storage_time
y ~ current_feature + Sex + (1 | Subject) + storage_time
y ~ current_feature + Sex + (1 | Subject) + storage_time
y ~ current_feature + Sex + (1 | Subject) + storage_time
y ~ current_feature + Sex + (1 | Subject) + storage_time
y ~ current_feature + Sex + (1 | Subject) + storage_time
y ~ current_feature + Sex + (1 | Subject) + storage_time
y ~ current_feature + Sex + (1 | Subject) + storage_time
y ~ current_feature + Sex + (1 | Subject) + storage_time
y ~ current_feature + Sex + (1 | Subject) + storage_time
y ~ current_feature + Sex + (1 | Subject) + storage_time
y ~ current_feature + Sex + (1 | Subject) + storage_time
y ~ current_feature + Sex + (1 | Subject) + storage_time
y ~ current_feature + Sex + (1 | Subject) + storage_time
y ~ current_feature + Sex + (1 | Subject) + storage_time
y ~ current_feature + Sex + (1 | Subject) + storage_time
y ~ current_feature + Sex + (1 | Subject) + storage_time
y ~ current_feature + Sex + (1 | Subject) + storage_time
y ~ current_feature + Sex + (1 | Subject) + storage_time
y ~ current_feature + Sex + (1 | Subject) + storage_time
y ~ current_feature + Sex + (1 | Subject) + storage_time
y ~ current_feature + Sex + (1 | Subject) + storage_time
y ~ current_feature + Sex + (1 | Subject) + storage_time
y ~ current_feature + Sex + (1 | Subject) + storage_time
y ~ current_feature + Sex + (1 | Subject) + storage_time
y ~ current_feature + Sex + (1 | Subject) + storage_time
y ~ current_feature + Sex + (1 | Subject) + storage_time
y ~ current_feature + Sex + (1 | Subject) + storage_time
y ~ current_feature + Sex + (1 | Subject) + storage_time
y ~ current_feature + Sex + (1 | Subject) + storage_time
y ~ current_feature + Sex + (1 | Subject) + storage_time
y ~ current_feature + Sex + (1 | Subject) + storage_time
y ~ current_feature + Sex + (1 | Subject) + storage_time
y ~ current_feature + Sex + (1 | Subject) + storage_time
y ~ current_feature + Sex + (1 | Subject) + storage_time
y ~ current_feature + Sex + (1 | Subject) + storage_time
y ~ current_feature + Sex + (1 | Subject) + storage_time
y ~ current_feature + Sex + (1 | Subject) + storage_time
y ~ current_feature + Sex + (1 | Subject) + storage_time
y ~ current_feature + Sex + (1 | Subject) + storage_time
y ~ current_feature + Sex + (1 | Subject) + storage_time
y ~ current_feature + Sex + (1 | Subject) + storage_time
y ~ current_feature + Sex + (1 | Subject) + storage_time
y ~ current_feature + Sex + (1 | Subject) + storage_time
y ~ current_feature + Sex + (1 | Subject) + storage_time
y ~ current_feature + Sex + (1 | Subject) + storage_time
y ~ current_feature + Sex + (1 | Subject) + storage_time

y ~ current_feature + Sex + (1 | Subject) + storage_time
y ~ current_feature + Sex + (1 | Subject) + storage_time
y ~ current_feature + Sex + (1 | Subject) + storage_time
y ~ current_feature + Sex + (1 | Subject) + storage_time
y ~ current_feature + Sex + (1 | Subject) + storage_time
y ~ current_feature + Sex + (1 | Subject) + storage_time
y ~ current_feature + Sex + (1 | Subject) + storage_time
y ~ current_feature + Sex + (1 | Subject) + storage_time
y ~ current_feature + Sex + (1 | Subject) + storage_time
y ~ current_feature + Sex + (1 | Subject) + storage_time
y ~ current_feature + Sex + (1 | Subject) + storage_time
y ~ current_feature + Sex + (1 | Subject) + storage_time
y ~ current_feature + Sex + (1 | Subject) + storage_time
y ~ current_feature + Sex + (1 | Subject) + storage_time
y ~ current_feature + Sex + (1 | Subject) + storage_time
y ~ current_feature + Sex + (1 | Subject) + storage_time
y ~ current_feature + Sex + (1 | Subject) + storage_time
y ~ current_feature + Sex + (1 | Subject) + storage_time
y ~ current_feature + Sex + (1 | Subject) + storage_time
y ~ current_feature + Sex + (1 | Subject) + storage_time
y ~ current_feature + Sex + (1 | Subject) + storage_time
y ~ current_feature + Sex + (1 | Subject) + storage_time
y ~ current_feature + Sex + (1 | Subject) + storage_time
y ~ current_feature + Sex + (1 | Subject) + storage_time
y ~ current_feature + Sex + (1 | Subject) + storage_time
y ~ current_feature + Sex + (1 | Subject) + storage_time
y ~ current_feature + Sex + (1 | Subject) + storage_time
y ~ current_feature + Sex + (1 | Subject) + storage_time
y ~ current_feature + Sex + (1 | Subject) + storage_time
y ~ current_feature + Sex + (1 | Subject) + storage_time
y ~ current_feature + Sex + (1 | Subject) + storage_time
y ~ current_feature + Sex + (1 | Subject) + storage_time
y ~ current_feature + Sex + (1 | Subject) + storage_time
y ~ current_feature + Sex + (1 | Subject) + storage_time
y ~ current_feature + Sex + (1 | Subject) + storage_time
y ~ current_feature + Sex + (1 | Subject) + storage_time
y ~ current_feature + Sex + (1 | Subject) + storage_time
y ~ current_feature + Sex + (1 | Subject) + storage_time
y ~ current_feature + Sex + (1 | Subject) + storage_time
y ~ current_feature + Sex + (1 | Subject) + storage_time
y ~ current_feature + Sex + (1 | Subject) + storage_time
y ~ current_feature + Sex + (1 | Subject) + storage_time
y ~ current_feature + Sex + (1 | Subject) + storage_time
y ~ current_feature + Sex + (1 | Subject) + storage_time
y ~ current_feature + Sex + (1 | Subject) + storage_time
y ~ current_feature + Sex + (1 | Subject) + storage_time
y ~ current_feature + Sex + (1 | Subject) + storage_time
y ~ current_feature + Sex + (1 | Subject) + storage_time

y ~ current_feature + Sex + (1 | Subject) + storage_time
y ~ current_feature + Sex + (1 | Subject) + storage_time
y ~ current_feature + Sex + (1 | Subject) + storage_time
y ~ current_feature + Sex + (1 | Subject) + storage_time
y ~ current_feature + Sex + (1 | Subject) + storage_time
y ~ current_feature + Sex + (1 | Subject) + storage_time
y ~ current_feature + Sex + (1 | Subject) + storage_time
y ~ current_feature + Sex + (1 | Subject) + storage_time
y ~ current_feature + Sex + (1 | Subject) + storage_time
y ~ current_feature + Sex + (1 | Subject) + storage_time
y ~ current_feature + Sex + (1 | Subject) + storage_time
y ~ current_feature + Sex + (1 | Subject) + storage_time
y ~ current_feature + Sex + (1 | Subject) + storage_time
y ~ current_feature + Sex + (1 | Subject) + storage_time
y ~ current_feature + Sex + (1 | Subject) + storage_time
y ~ current_feature + Sex + (1 | Subject) + storage_time
y ~ current_feature + Sex + (1 | Subject) + storage_time
y ~ current_feature + Sex + (1 | Subject) + storage_time
y ~ current_feature + Sex + (1 | Subject) + storage_time
y ~ current_feature + Sex + (1 | Subject) + storage_time
y ~ current_feature + Sex + (1 | Subject) + storage_time
y ~ current_feature + Sex + (1 | Subject) + storage_time
y ~ current_feature + Sex + (1 | Subject) + storage_time
y ~ current_feature + Sex + (1 | Subject) + storage_time
y ~ current_feature + Sex + (1 | Subject) + storage_time
y ~ current_feature + Sex + (1 | Subject) + storage_time
y ~ current_feature + Sex + (1 | Subject) + storage_time
y ~ current_feature + Sex + (1 | Subject) + storage_time
y ~ current_feature + Sex + (1 | Subject) + storage_time
y ~ current_feature + Sex + (1 | Subject) + storage_time
y ~ current_feature + Sex + (1 | Subject) + storage_time
y ~ current_feature + Sex + (1 | Subject) + storage_time
y ~ current_feature + Sex + (1 | Subject) + storage_time
y ~ current_feature + Sex + (1 | Subject) + storage_time
y ~ current_feature + Sex + (1 | Subject) + storage_time
y ~ current_feature + Sex + (1 | Subject) + storage_time
y ~ current_feature + Sex + (1 | Subject) + storage_time
y ~ current_feature + Sex + (1 | Subject) + storage_time
y ~ current_feature + Sex + (1 | Subject) + storage_time
y ~ current_feature + Sex + (1 | Subject) + storage_time
y ~ current_feature + Sex + (1 | Subject) + storage_time
y ~ current_feature + Sex + (1 | Subject) + storage_time
y ~ current_feature + Sex + (1 | Subject) + storage_time
y ~ current_feature + Sex + (1 | Subject) + storage_time
y ~ current_feature + Sex + (1 | Subject) + storage_time
y ~ current_feature + Sex + (1 | Subject) + storage_time
y ~ current_feature + Sex + (1 | Subject) + storage_time
y ~ current_feature + Sex + (1 | Subject) + storage_time

y ~ current_feature + Sex + (1 | Subject) + storage_time
y ~ current_feature + Sex + (1 | Subject) + storage_time
y ~ current_feature + Sex + (1 | Subject) + storage_time
y ~ current_feature + Sex + (1 | Subject) + storage_time
y ~ current_feature + Sex + (1 | Subject) + storage_time
y ~ current_feature + Sex + (1 | Subject) + storage_time
y ~ current_feature + Sex + (1 | Subject) + storage_time
y ~ current_feature + Sex + (1 | Subject) + storage_time
y ~ current_feature + Sex + (1 | Subject) + storage_time
y ~ current_feature + Sex + (1 | Subject) + storage_time
y ~ current_feature + Sex + (1 | Subject) + storage_time
y ~ current_feature + Sex + (1 | Subject) + storage_time
y ~ current_feature + Sex + (1 | Subject) + storage_time
y ~ current_feature + Sex + (1 | Subject) + storage_time
y ~ current_feature + Sex + (1 | Subject) + storage_time
y ~ current_feature + Sex + (1 | Subject) + storage_time
y ~ current_feature + Sex + (1 | Subject) + storage_time
y ~ current_feature + Sex + (1 | Subject) + storage_time
y ~ current_feature + Sex + (1 | Subject) + storage_time
y ~ current_feature + Sex + (1 | Subject) + storage_time
y ~ current_feature + Sex + (1 | Subject) + storage_time
y ~ current_feature + Sex + (1 | Subject) + storage_time
y ~ current_feature + Sex + (1 | Subject) + storage_time
y ~ current_feature + Sex + (1 | Subject) + storage_time
y ~ current_feature + Sex + (1 | Subject) + storage_time
y ~ current_feature + Sex + (1 | Subject) + storage_time
y ~ current_feature + Sex + (1 | Subject) + storage_time
y ~ current_feature + Sex + (1 | Subject) + storage_time
y ~ current_feature + Sex + (1 | Subject) + storage_time
y ~ current_feature + Sex + (1 | Subject) + storage_time
y ~ current_feature + Sex + (1 | Subject) + storage_time
y ~ current_feature + Sex + (1 | Subject) + storage_time
y ~ current_feature + Sex + (1 | Subject) + storage_time
y ~ current_feature + Sex + (1 | Subject) + storage_time
y ~ current_feature + Sex + (1 | Subject) + storage_time
y ~ current_feature + Sex + (1 | Subject) + storage_time
y ~ current_feature + Sex + (1 | Subject) + storage_time
y ~ current_feature + Sex + (1 | Subject) + storage_time
y ~ current_feature + Sex + (1 | Subject) + storage_time
y ~ current_feature + Sex + (1 | Subject) + storage_time
y ~ current_feature + Sex + (1 | Subject) + storage_time
y ~ current_feature + Sex + (1 | Subject) + storage_time
y ~ current_feature + Sex + (1 | Subject) + storage_time
y ~ current_feature + Sex + (1 | Subject) + storage_time
y ~ current_feature + Sex + (1 | Subject) + storage_time
y ~ current_feature + Sex + (1 | Subject) + storage_time
y ~ current_feature + Sex + (1 | Subject) + storage_time
y ~ current_feature + Sex + (1 | Subject) + storage_time

y ~ current_feature + Sex + (1 | Subject) + storage_time
y ~ current_feature + Sex + (1 | Subject) + storage_time
y ~ current_feature + Sex + (1 | Subject) + storage_time
y ~ current_feature + Sex + (1 | Subject) + storage_time
y ~ current_feature + Sex + (1 | Subject) + storage_time
y ~ current_feature + Sex + (1 | Subject) + storage_time
y ~ current_feature + Sex + (1 | Subject) + storage_time
y ~ current_feature + Sex + (1 | Subject) + storage_time
y ~ current_feature + Sex + (1 | Subject) + storage_time
y ~ current_feature + Sex + (1 | Subject) + storage_time
y ~ current_feature + Sex + (1 | Subject) + storage_time
y ~ current_feature + Sex + (1 | Subject) + storage_time
y ~ current_feature + Sex + (1 | Subject) + storage_time
y ~ current_feature + Sex + (1 | Subject) + storage_time
y ~ current_feature + Sex + (1 | Subject) + storage_time
y ~ current_feature + Sex + (1 | Subject) + storage_time
y ~ current_feature + Sex + (1 | Subject) + storage_time
y ~ current_feature + Sex + (1 | Subject) + storage_time
y ~ current_feature + Sex + (1 | Subject) + storage_time
y ~ current_feature + Sex + (1 | Subject) + storage_time
y ~ current_feature + Sex + (1 | Subject) + storage_time
y ~ current_feature + Sex + (1 | Subject) + storage_time
y ~ current_feature + Sex + (1 | Subject) + storage_time
y ~ current_feature + Sex + (1 | Subject) + storage_time
y ~ current_feature + Sex + (1 | Subject) + storage_time
y ~ current_feature + Sex + (1 | Subject) + storage_time
y ~ current_feature + Sex + (1 | Subject) + storage_time
y ~ current_feature + Sex + (1 | Subject) + storage_time
y ~ current_feature + Sex + (1 | Subject) + storage_time
y ~ current_feature + Sex + (1 | Subject) + storage_time
y ~ current_feature + Sex + (1 | Subject) + storage_time
y ~ current_feature + Sex + (1 | Subject) + storage_time
y ~ current_feature + Sex + (1 | Subject) + storage_time
y ~ current_feature + Sex + (1 | Subject) + storage_time
y ~ current_feature + Sex + (1 | Subject) + storage_time
y ~ current_feature + Sex + (1 | Subject) + storage_time
y ~ current_feature + Sex + (1 | Subject) + storage_time
y ~ current_feature + Sex + (1 | Subject) + storage_time
y ~ current_feature + Sex + (1 | Subject) + storage_time
y ~ current_feature + Sex + (1 | Subject) + storage_time
y ~ current_feature + Sex + (1 | Subject) + storage_time
y ~ current_feature + Sex + (1 | Subject) + storage_time
y ~ current_feature + Sex + (1 | Subject) + storage_time
y ~ current_feature + Sex + (1 | Subject) + storage_time
y ~ current_feature + Sex + (1 | Subject) + storage_time
y ~ current_feature + Sex + (1 | Subject) + storage_time
y ~ current_feature + Sex + (1 | Subject) + storage_time
y ~ current_feature + Sex + (1 | Subject) + storage_time

```
y ~ current_feature + Sex + (1 | Subject) + storage_time
y ~ current_feature + Sex + (1 | Subject) + storage_time
y ~ current_feature + Sex + (1 | Subject) + storage_time
y ~ current_feature + Sex + (1 | Subject) + storage_time
y ~ current_feature + Sex + (1 | Subject) + storage_time
y ~ current_feature + Sex + (1 | Subject) + storage_time
y ~ current_feature + Sex + (1 | Subject) + storage_time
y ~ current_feature + Sex + (1 | Subject) + storage_time
y ~ current_feature + Sex + (1 | Subject) + storage_time
y ~ current_feature + Sex + (1 | Subject) + storage_time
y ~ current_feature + Sex + (1 | Subject) + storage_time
y ~ current_feature + Sex + (1 | Subject) + storage_time
y ~ current_feature + Sex + (1 | Subject) + storage_time
y ~ current_feature + Sex + (1 | Subject) + storage_time
y ~ current_feature + Sex + (1 | Subject) + storage_time
y ~ current_feature + Sex + (1 | Subject) + storage_time
y ~ current_feature + Sex + (1 | Subject) + storage_time
y ~ current_feature + Sex + (1 | Subject) + storage_time
y ~ current_feature + Sex + (1 | Subject) + storage_time
y ~ current_feature + Sex + (1 | Subject) + storage_time
y ~ current_feature + Sex + (1 | Subject) + storage_time
y ~ current_feature + Sex + (1 | Subject) + storage_time
y ~ current_feature + Sex + (1 | Subject) + storage_time
y ~ current_feature + Sex + (1 | Subject) + storage_time
y ~ current_feature + Sex + (1 | Subject) + storage_time
y ~ current_feature + Sex + (1 | Subject) + storage_time
y ~ current_feature + Sex + (1 | Subject) + storage_time
y ~ current_feature + Sex + (1 | Subject) + storage_time
y ~ current_feature + Sex + (1 | Subject) + storage_time
y ~ current_feature + Sex + (1 | Subject) + storage_time
y ~ current_feature + Sex + (1 | Subject) + storage_time
y ~ current_feature + Sex + (1 | Subject) + storage_time
y ~ current_feature + Sex + (1 | Subject) + storage_time
y ~ current_feature + Sex + (1 | Subject) + storage_time
y ~ current_feature + Sex + (1 | Subject) + storage_time
y ~ current_feature + Sex + (1 | Subject) + storage_time
y ~ current_feature + Sex + (1 | Subject) + storage_time
y ~ current_feature + Sex + (1 | Subject) + storage_time
y ~ current_feature + Sex + (1 | Subject) + storage_time
y ~ current_feature + Sex + (1 | Subject) + storage_time
y ~ current_feature + Sex + (1 | Subject) + storage_time
y ~ current_feature + Sex + (1 | Subject) + storage_time
y ~ current_feature + Sex + (1 | Subject) + storage_time
y ~ current_feature + Sex + (1 | Subject) + storage_time
y ~ current_feature + Sex + (1 | Subject) + storage_time
y ~ current_feature + Sex + (1 | Subject) + storage_time
y ~ current_feature + Sex + (1 | Subject) + storage_time
y ~ current_feature + Sex + (1 | Subject) + storage_time
```

y ~ current_feature + Sex + (1 | Subject) + storage_time
y ~ current_feature + Sex + (1 | Subject) + storage_time
y ~ current_feature + Sex + (1 | Subject) + storage_time
y ~ current_feature + Sex + (1 | Subject) + storage_time
y ~ current_feature + Sex + (1 | Subject) + storage_time
y ~ current_feature + Sex + (1 | Subject) + storage_time
y ~ current_feature + Sex + (1 | Subject) + storage_time
y ~ current_feature + Sex + (1 | Subject) + storage_time
y ~ current_feature + Sex + (1 | Subject) + storage_time
y ~ current_feature + Sex + (1 | Subject) + storage_time
y ~ current_feature + Sex + (1 | Subject) + storage_time
y ~ current_feature + Sex + (1 | Subject) + storage_time
y ~ current_feature + Sex + (1 | Subject) + storage_time
y ~ current_feature + Sex + (1 | Subject) + storage_time
y ~ current_feature + Sex + (1 | Subject) + storage_time
y ~ current_feature + Sex + (1 | Subject) + storage_time
y ~ current_feature + Sex + (1 | Subject) + storage_time
y ~ current_feature + Sex + (1 | Subject) + storage_time
y ~ current_feature + Sex + (1 | Subject) + storage_time
y ~ current_feature + Sex + (1 | Subject) + storage_time
y ~ current_feature + Sex + (1 | Subject) + storage_time
y ~ current_feature + Sex + (1 | Subject) + storage_time
y ~ current_feature + Sex + (1 | Subject) + storage_time
y ~ current_feature + Sex + (1 | Subject) + storage_time
y ~ current_feature + Sex + (1 | Subject) + storage_time
y ~ current_feature + Sex + (1 | Subject) + storage_time
y ~ current_feature + Sex + (1 | Subject) + storage_time
y ~ current_feature + Sex + (1 | Subject) + storage_time
y ~ current_feature + Sex + (1 | Subject) + storage_time
y ~ current_feature + Sex + (1 | Subject) + storage_time
y ~ current_feature + Sex + (1 | Subject) + storage_time
y ~ current_feature + Sex + (1 | Subject) + storage_time
y ~ current_feature + Sex + (1 | Subject) + storage_time
y ~ current_feature + Sex + (1 | Subject) + storage_time
y ~ current_feature + Sex + (1 | Subject) + storage_time
y ~ current_feature + Sex + (1 | Subject) + storage_time
y ~ current_feature + Sex + (1 | Subject) + storage_time
y ~ current_feature + Sex + (1 | Subject) + storage_time
y ~ current_feature + Sex + (1 | Subject) + storage_time
y ~ current_feature + Sex + (1 | Subject) + storage_time
y ~ current_feature + Sex + (1 | Subject) + storage_time
y ~ current_feature + Sex + (1 | Subject) + storage_time
y ~ current_feature + Sex + (1 | Subject) + storage_time
y ~ current_feature + Sex + (1 | Subject) + storage_time
y ~ current_feature + Sex + (1 | Subject) + storage_time
y ~ current_feature + Sex + (1 | Subject) + storage_time
y ~ current_feature + Sex + (1 | Subject) + storage_time
y ~ current_feature + Sex + (1 | Subject) + storage_time

y ~ current_feature + Sex + (1 | Subject) + storage_time
y ~ current_feature + Sex + (1 | Subject) + storage_time
y ~ current_feature + Sex + (1 | Subject) + storage_time
y ~ current_feature + Sex + (1 | Subject) + storage_time
y ~ current_feature + Sex + (1 | Subject) + storage_time
y ~ current_feature + Sex + (1 | Subject) + storage_time
y ~ current_feature + Sex + (1 | Subject) + storage_time
y ~ current_feature + Sex + (1 | Subject) + storage_time
y ~ current_feature + Sex + (1 | Subject) + storage_time
y ~ current_feature + Sex + (1 | Subject) + storage_time
y ~ current_feature + Sex + (1 | Subject) + storage_time
y ~ current_feature + Sex + (1 | Subject) + storage_time
y ~ current_feature + Sex + (1 | Subject) + storage_time
y ~ current_feature + Sex + (1 | Subject) + storage_time
y ~ current_feature + Sex + (1 | Subject) + storage_time
y ~ current_feature + Sex + (1 | Subject) + storage_time
y ~ current_feature + Sex + (1 | Subject) + storage_time
y ~ current_feature + Sex + (1 | Subject) + storage_time
y ~ current_feature + Sex + (1 | Subject) + storage_time
y ~ current_feature + Sex + (1 | Subject) + storage_time
y ~ current_feature + Sex + (1 | Subject) + storage_time
y ~ current_feature + Sex + (1 | Subject) + storage_time
y ~ current_feature + Sex + (1 | Subject) + storage_time
y ~ current_feature + Sex + (1 | Subject) + storage_time
y ~ current_feature + Sex + (1 | Subject) + storage_time
y ~ current_feature + Sex + (1 | Subject) + storage_time
y ~ current_feature + Sex + (1 | Subject) + storage_time
y ~ current_feature + Sex + (1 | Subject) + storage_time
y ~ current_feature + Sex + (1 | Subject) + storage_time
y ~ current_feature + Sex + (1 | Subject) + storage_time
y ~ current_feature + Sex + (1 | Subject) + storage_time
y ~ current_feature + Sex + (1 | Subject) + storage_time
y ~ current_feature + Sex + (1 | Subject) + storage_time
y ~ current_feature + Sex + (1 | Subject) + storage_time
y ~ current_feature + Sex + (1 | Subject) + storage_time
y ~ current_feature + Sex + (1 | Subject) + storage_time
y ~ current_feature + Sex + (1 | Subject) + storage_time
y ~ current_feature + Sex + (1 | Subject) + storage_time
y ~ current_feature + Sex + (1 | Subject) + storage_time
y ~ current_feature + Sex + (1 | Subject) + storage_time
y ~ current_feature + Sex + (1 | Subject) + storage_time
y ~ current_feature + Sex + (1 | Subject) + storage_time
y ~ current_feature + Sex + (1 | Subject) + storage_time
y ~ current_feature + Sex + (1 | Subject) + storage_time
y ~ current_feature + Sex + (1 | Subject) + storage_time
y ~ current_feature + Sex + (1 | Subject) + storage_time
y ~ current_feature + Sex + (1 | Subject) + storage_time
y ~ current_feature + Sex + (1 | Subject) + storage_time

y ~ current_feature + Sex + (1 | Subject) + storage_time
y ~ current_feature + Sex + (1 | Subject) + storage_time
y ~ current_feature + Sex + (1 | Subject) + storage_time
y ~ current_feature + Sex + (1 | Subject) + storage_time
y ~ current_feature + Sex + (1 | Subject) + storage_time
y ~ current_feature + Sex + (1 | Subject) + storage_time
y ~ current_feature + Sex + (1 | Subject) + storage_time
y ~ current_feature + Sex + (1 | Subject) + storage_time
y ~ current_feature + Sex + (1 | Subject) + storage_time
y ~ current_feature + Sex + (1 | Subject) + storage_time
y ~ current_feature + Sex + (1 | Subject) + storage_time
y ~ current_feature + Sex + (1 | Subject) + storage_time
y ~ current_feature + Sex + (1 | Subject) + storage_time
y ~ current_feature + Sex + (1 | Subject) + storage_time
y ~ current_feature + Sex + (1 | Subject) + storage_time
y ~ current_feature + Sex + (1 | Subject) + storage_time
y ~ current_feature + Sex + (1 | Subject) + storage_time
y ~ current_feature + Sex + (1 | Subject) + storage_time
y ~ current_feature + Sex + (1 | Subject) + storage_time
y ~ current_feature + Sex + (1 | Subject) + storage_time
y ~ current_feature + Sex + (1 | Subject) + storage_time
y ~ current_feature + Sex + (1 | Subject) + storage_time
y ~ current_feature + Sex + (1 | Subject) + storage_time
y ~ current_feature + Sex + (1 | Subject) + storage_time
y ~ current_feature + Sex + (1 | Subject) + storage_time
y ~ current_feature + Sex + (1 | Subject) + storage_time
y ~ current_feature + Sex + (1 | Subject) + storage_time
y ~ current_feature + Sex + (1 | Subject) + storage_time
y ~ current_feature + Sex + (1 | Subject) + storage_time
y ~ current_feature + Sex + (1 | Subject) + storage_time
y ~ current_feature + Sex + (1 | Subject) + storage_time
y ~ current_feature + Sex + (1 | Subject) + storage_time
y ~ current_feature + Sex + (1 | Subject) + storage_time
y ~ current_feature + Sex + (1 | Subject) + storage_time
y ~ current_feature + Sex + (1 | Subject) + storage_time
y ~ current_feature + Sex + (1 | Subject) + storage_time
y ~ current_feature + Sex + (1 | Subject) + storage_time
y ~ current_feature + Sex + (1 | Subject) + storage_time
y ~ current_feature + Sex + (1 | Subject) + storage_time
y ~ current_feature + Sex + (1 | Subject) + storage_time
y ~ current_feature + Sex + (1 | Subject) + storage_time
y ~ current_feature + Sex + (1 | Subject) + storage_time
y ~ current_feature + Sex + (1 | Subject) + storage_time
y ~ current_feature + Sex + (1 | Subject) + storage_time
y ~ current_feature + Sex + (1 | Subject) + storage_time
y ~ current_feature + Sex + (1 | Subject) + storage_time
y ~ current_feature + Sex + (1 | Subject) + storage_time
y ~ current_feature + Sex + (1 | Subject) + storage_time

y ~ current_feature + Sex + (1 | Subject) + storage_time
y ~ current_feature + Sex + (1 | Subject) + storage_time
y ~ current_feature + Sex + (1 | Subject) + storage_time
y ~ current_feature + Sex + (1 | Subject) + storage_time
y ~ current_feature + Sex + (1 | Subject) + storage_time
y ~ current_feature + Sex + (1 | Subject) + storage_time
y ~ current_feature + Sex + (1 | Subject) + storage_time
y ~ current_feature + Sex + (1 | Subject) + storage_time
y ~ current_feature + Sex + (1 | Subject) + storage_time
y ~ current_feature + Sex + (1 | Subject) + storage_time
y ~ current_feature + Sex + (1 | Subject) + storage_time
y ~ current_feature + Sex + (1 | Subject) + storage_time
y ~ current_feature + Sex + (1 | Subject) + storage_time
y ~ current_feature + Sex + (1 | Subject) + storage_time
y ~ current_feature + Sex + (1 | Subject) + storage_time
y ~ current_feature + Sex + (1 | Subject) + storage_time
y ~ current_feature + Sex + (1 | Subject) + storage_time
y ~ current_feature + Sex + (1 | Subject) + storage_time
y ~ current_feature + Sex + (1 | Subject) + storage_time
y ~ current_feature + Sex + (1 | Subject) + storage_time
y ~ current_feature + Sex + (1 | Subject) + storage_time
y ~ current_feature + Sex + (1 | Subject) + storage_time
y ~ current_feature + Sex + (1 | Subject) + storage_time
y ~ current_feature + Sex + (1 | Subject) + storage_time
y ~ current_feature + Sex + (1 | Subject) + storage_time
y ~ current_feature + Sex + (1 | Subject) + storage_time
y ~ current_feature + Sex + (1 | Subject) + storage_time
y ~ current_feature + Sex + (1 | Subject) + storage_time
y ~ current_feature + Sex + (1 | Subject) + storage_time
y ~ current_feature + Sex + (1 | Subject) + storage_time
y ~ current_feature + Sex + (1 | Subject) + storage_time
y ~ current_feature + Sex + (1 | Subject) + storage_time
y ~ current_feature + Sex + (1 | Subject) + storage_time
y ~ current_feature + Sex + (1 | Subject) + storage_time
y ~ current_feature + Sex + (1 | Subject) + storage_time
y ~ current_feature + Sex + (1 | Subject) + storage_time
y ~ current_feature + Sex + (1 | Subject) + storage_time
y ~ current_feature + Sex + (1 | Subject) + storage_time
y ~ current_feature + Sex + (1 | Subject) + storage_time
y ~ current_feature + Sex + (1 | Subject) + storage_time
y ~ current_feature + Sex + (1 | Subject) + storage_time
y ~ current_feature + Sex + (1 | Subject) + storage_time
y ~ current_feature + Sex + (1 | Subject) + storage_time
y ~ current_feature + Sex + (1 | Subject) + storage_time
y ~ current_feature + Sex + (1 | Subject) + storage_time
y ~ current_feature + Sex + (1 | Subject) + storage_time
y ~ current_feature + Sex + (1 | Subject) + storage_time
y ~ current_feature + Sex + (1 | Subject) + storage_time

y ~ current_feature + Sex + (1 | Subject) + storage_time
y ~ current_feature + Sex + (1 | Subject) + storage_time
y ~ current_feature + Sex + (1 | Subject) + storage_time
y ~ current_feature + Sex + (1 | Subject) + storage_time
y ~ current_feature + Sex + (1 | Subject) + storage_time
y ~ current_feature + Sex + (1 | Subject) + storage_time
y ~ current_feature + Sex + (1 | Subject) + storage_time
y ~ current_feature + Sex + (1 | Subject) + storage_time
y ~ current_feature + Sex + (1 | Subject) + storage_time
y ~ current_feature + Sex + (1 | Subject) + storage_time
y ~ current_feature + Sex + (1 | Subject) + storage_time
y ~ current_feature + Sex + (1 | Subject) + storage_time
y ~ current_feature + Sex + (1 | Subject) + storage_time
y ~ current_feature + Sex + (1 | Subject) + storage_time
y ~ current_feature + Sex + (1 | Subject) + storage_time
y ~ current_feature + Sex + (1 | Subject) + storage_time
y ~ current_feature + Sex + (1 | Subject) + storage_time
y ~ current_feature + Sex + (1 | Subject) + storage_time
y ~ current_feature + Sex + (1 | Subject) + storage_time
y ~ current_feature + Sex + (1 | Subject) + storage_time
y ~ current_feature + Sex + (1 | Subject) + storage_time
y ~ current_feature + Sex + (1 | Subject) + storage_time
y ~ current_feature + Sex + (1 | Subject) + storage_time
y ~ current_feature + Sex + (1 | Subject) + storage_time
y ~ current_feature + Sex + (1 | Subject) + storage_time
y ~ current_feature + Sex + (1 | Subject) + storage_time
y ~ current_feature + Sex + (1 | Subject) + storage_time
y ~ current_feature + Sex + (1 | Subject) + storage_time
y ~ current_feature + Sex + (1 | Subject) + storage_time
y ~ current_feature + Sex + (1 | Subject) + storage_time
y ~ current_feature + Sex + (1 | Subject) + storage_time
y ~ current_feature + Sex + (1 | Subject) + storage_time
y ~ current_feature + Sex + (1 | Subject) + storage_time
y ~ current_feature + Sex + (1 | Subject) + storage_time
y ~ current_feature + Sex + (1 | Subject) + storage_time
y ~ current_feature + Sex + (1 | Subject) + storage_time
y ~ current_feature + Sex + (1 | Subject) + storage_time
y ~ current_feature + Sex + (1 | Subject) + storage_time
y ~ current_feature + Sex + (1 | Subject) + storage_time
y ~ current_feature + Sex + (1 | Subject) + storage_time
y ~ current_feature + Sex + (1 | Subject) + storage_time
y ~ current_feature + Sex + (1 | Subject) + storage_time
y ~ current_feature + Sex + (1 | Subject) + storage_time
y ~ current_feature + Sex + (1 | Subject) + storage_time
y ~ current_feature + Sex + (1 | Subject) + storage_time
y ~ current_feature + Sex + (1 | Subject) + storage_time
y ~ current_feature + Sex + (1 | Subject) + storage_time
y ~ current_feature + Sex + (1 | Subject) + storage_time

y ~ current_feature + Sex + (1 | Subject) + storage_time
y ~ current_feature + Sex + (1 | Subject) + storage_time
y ~ current_feature + Sex + (1 | Subject) + storage_time
y ~ current_feature + Sex + (1 | Subject) + storage_time
y ~ current_feature + Sex + (1 | Subject) + storage_time
y ~ current_feature + Sex + (1 | Subject) + storage_time
y ~ current_feature + Sex + (1 | Subject) + storage_time
y ~ current_feature + Sex + (1 | Subject) + storage_time
y ~ current_feature + Sex + (1 | Subject) + storage_time
y ~ current_feature + Sex + (1 | Subject) + storage_time
y ~ current_feature + Sex + (1 | Subject) + storage_time
y ~ current_feature + Sex + (1 | Subject) + storage_time
y ~ current_feature + Sex + (1 | Subject) + storage_time
y ~ current_feature + Sex + (1 | Subject) + storage_time
y ~ current_feature + Sex + (1 | Subject) + storage_time
y ~ current_feature + Sex + (1 | Subject) + storage_time
y ~ current_feature + Sex + (1 | Subject) + storage_time
y ~ current_feature + Sex + (1 | Subject) + storage_time
y ~ current_feature + Sex + (1 | Subject) + storage_time
y ~ current_feature + Sex + (1 | Subject) + storage_time
y ~ current_feature + Sex + (1 | Subject) + storage_time
y ~ current_feature + Sex + (1 | Subject) + storage_time
y ~ current_feature + Sex + (1 | Subject) + storage_time
y ~ current_feature + Sex + (1 | Subject) + storage_time
y ~ current_feature + Sex + (1 | Subject) + storage_time
y ~ current_feature + Sex + (1 | Subject) + storage_time
y ~ current_feature + Sex + (1 | Subject) + storage_time
y ~ current_feature + Sex + (1 | Subject) + storage_time
y ~ current_feature + Sex + (1 | Subject) + storage_time
y ~ current_feature + Sex + (1 | Subject) + storage_time
y ~ current_feature + Sex + (1 | Subject) + storage_time
y ~ current_feature + Sex + (1 | Subject) + storage_time
y ~ current_feature + Sex + (1 | Subject) + storage_time
y ~ current_feature + Sex + (1 | Subject) + storage_time
y ~ current_feature + Sex + (1 | Subject) + storage_time
y ~ current_feature + Sex + (1 | Subject) + storage_time
y ~ current_feature + Sex + (1 | Subject) + storage_time
y ~ current_feature + Sex + (1 | Subject) + storage_time
y ~ current_feature + Sex + (1 | Subject) + storage_time
y ~ current_feature + Sex + (1 | Subject) + storage_time
y ~ current_feature + Sex + (1 | Subject) + storage_time
y ~ current_feature + Sex + (1 | Subject) + storage_time
y ~ current_feature + Sex + (1 | Subject) + storage_time
y ~ current_feature + Sex + (1 | Subject) + storage_time
y ~ current_feature + Sex + (1 | Subject) + storage_time
y ~ current_feature + Sex + (1 | Subject) + storage_time
y ~ current_feature + Sex + (1 | Subject) + storage_time
y ~ current_feature + Sex + (1 | Subject) + storage_time

y ~ current_feature + Sex + (1 | Subject) + storage_time
y ~ current_feature + Sex + (1 | Subject) + storage_time
y ~ current_feature + Sex + (1 | Subject) + storage_time
y ~ current_feature + Sex + (1 | Subject) + storage_time
y ~ current_feature + Sex + (1 | Subject) + storage_time
y ~ current_feature + Sex + (1 | Subject) + storage_time
y ~ current_feature + Sex + (1 | Subject) + storage_time
y ~ current_feature + Sex + (1 | Subject) + storage_time
y ~ current_feature + Sex + (1 | Subject) + storage_time
y ~ current_feature + Sex + (1 | Subject) + storage_time
y ~ current_feature + Sex + (1 | Subject) + storage_time
y ~ current_feature + Sex + (1 | Subject) + storage_time
y ~ current_feature + Sex + (1 | Subject) + storage_time
y ~ current_feature + Sex + (1 | Subject) + storage_time
y ~ current_feature + Sex + (1 | Subject) + storage_time
y ~ current_feature + Sex + (1 | Subject) + storage_time
y ~ current_feature + Sex + (1 | Subject) + storage_time
y ~ current_feature + Sex + (1 | Subject) + storage_time
y ~ current_feature + Sex + (1 | Subject) + storage_time
y ~ current_feature + Sex + (1 | Subject) + storage_time
y ~ current_feature + Sex + (1 | Subject) + storage_time
y ~ current_feature + Sex + (1 | Subject) + storage_time
y ~ current_feature + Sex + (1 | Subject) + storage_time
y ~ current_feature + Sex + (1 | Subject) + storage_time
y ~ current_feature + Sex + (1 | Subject) + storage_time
y ~ current_feature + Sex + (1 | Subject) + storage_time
y ~ current_feature + Sex + (1 | Subject) + storage_time
y ~ current_feature + Sex + (1 | Subject) + storage_time
y ~ current_feature + Sex + (1 | Subject) + storage_time
y ~ current_feature + Sex + (1 | Subject) + storage_time
y ~ current_feature + Sex + (1 | Subject) + storage_time
y ~ current_feature + Sex + (1 | Subject) + storage_time
y ~ current_feature + Sex + (1 | Subject) + storage_time
y ~ current_feature + Sex + (1 | Subject) + storage_time
y ~ current_feature + Sex + (1 | Subject) + storage_time
y ~ current_feature + Sex + (1 | Subject) + storage_time
y ~ current_feature + Sex + (1 | Subject) + storage_time
y ~ current_feature + Sex + (1 | Subject) + storage_time
y ~ current_feature + Sex + (1 | Subject) + storage_time
y ~ current_feature + Sex + (1 | Subject) + storage_time
y ~ current_feature + Sex + (1 | Subject) + storage_time
y ~ current_feature + Sex + (1 | Subject) + storage_time
y ~ current_feature + Sex + (1 | Subject) + storage_time
y ~ current_feature + Sex + (1 | Subject) + storage_time
y ~ current_feature + Sex + (1 | Subject) + storage_time
y ~ current_feature + Sex + (1 | Subject) + storage_time
y ~ current_feature + Sex + (1 | Subject) + storage_time
y ~ current_feature + Sex + (1 | Subject) + storage_time

y ~ current_feature + Sex + (1 | Subject) + storage_time
y ~ current_feature + Sex + (1 | Subject) + storage_time
y ~ current_feature + Sex + (1 | Subject) + storage_time
y ~ current_feature + Sex + (1 | Subject) + storage_time
y ~ current_feature + Sex + (1 | Subject) + storage_time
y ~ current_feature + Sex + (1 | Subject) + storage_time
y ~ current_feature + Sex + (1 | Subject) + storage_time
y ~ current_feature + Sex + (1 | Subject) + storage_time
y ~ current_feature + Sex + (1 | Subject) + storage_time
y ~ current_feature + Sex + (1 | Subject) + storage_time
y ~ current_feature + Sex + (1 | Subject) + storage_time
y ~ current_feature + Sex + (1 | Subject) + storage_time
y ~ current_feature + Sex + (1 | Subject) + storage_time
y ~ current_feature + Sex + (1 | Subject) + storage_time
y ~ current_feature + Sex + (1 | Subject) + storage_time
y ~ current_feature + Sex + (1 | Subject) + storage_time
y ~ current_feature + Sex + (1 | Subject) + storage_time
y ~ current_feature + Sex + (1 | Subject) + storage_time
y ~ current_feature + Sex + (1 | Subject) + storage_time
y ~ current_feature + Sex + (1 | Subject) + storage_time
y ~ current_feature + Sex + (1 | Subject) + storage_time
y ~ current_feature + Sex + (1 | Subject) + storage_time
y ~ current_feature + Sex + (1 | Subject) + storage_time
y ~ current_feature + Sex + (1 | Subject) + storage_time
y ~ current_feature + Sex + (1 | Subject) + storage_time
y ~ current_feature + Sex + (1 | Subject) + storage_time
y ~ current_feature + Sex + (1 | Subject) + storage_time
y ~ current_feature + Sex + (1 | Subject) + storage_time
y ~ current_feature + Sex + (1 | Subject) + storage_time
y ~ current_feature + Sex + (1 | Subject) + storage_time
y ~ current_feature + Sex + (1 | Subject) + storage_time
y ~ current_feature + Sex + (1 | Subject) + storage_time
y ~ current_feature + Sex + (1 | Subject) + storage_time
y ~ current_feature + Sex + (1 | Subject) + storage_time
y ~ current_feature + Sex + (1 | Subject) + storage_time
y ~ current_feature + Sex + (1 | Subject) + storage_time
y ~ current_feature + Sex + (1 | Subject) + storage_time
y ~ current_feature + Sex + (1 | Subject) + storage_time
y ~ current_feature + Sex + (1 | Subject) + storage_time
y ~ current_feature + Sex + (1 | Subject) + storage_time
y ~ current_feature + Sex + (1 | Subject) + storage_time
y ~ current_feature + Sex + (1 | Subject) + storage_time
y ~ current_feature + Sex + (1 | Subject) + storage_time
y ~ current_feature + Sex + (1 | Subject) + storage_time
y ~ current_feature + Sex + (1 | Subject) + storage_time
y ~ current_feature + Sex + (1 | Subject) + storage_time
y ~ current_feature + Sex + (1 | Subject) + storage_time
y ~ current_feature + Sex + (1 | Subject) + storage_time

y ~ current_feature + Sex + (1 | Subject) + storage_time
y ~ current_feature + Sex + (1 | Subject) + storage_time
y ~ current_feature + Sex + (1 | Subject) + storage_time
y ~ current_feature + Sex + (1 | Subject) + storage_time
y ~ current_feature + Sex + (1 | Subject) + storage_time
y ~ current_feature + Sex + (1 | Subject) + storage_time
y ~ current_feature + Sex + (1 | Subject) + storage_time
y ~ current_feature + Sex + (1 | Subject) + storage_time
y ~ current_feature + Sex + (1 | Subject) + storage_time
y ~ current_feature + Sex + (1 | Subject) + storage_time
y ~ current_feature + Sex + (1 | Subject) + storage_time
y ~ current_feature + Sex + (1 | Subject) + storage_time
y ~ current_feature + Sex + (1 | Subject) + storage_time
y ~ current_feature + Sex + (1 | Subject) + storage_time
y ~ current_feature + Sex + (1 | Subject) + storage_time
y ~ current_feature + Sex + (1 | Subject) + storage_time
y ~ current_feature + Sex + (1 | Subject) + storage_time
y ~ current_feature + Sex + (1 | Subject) + storage_time
y ~ current_feature + Sex + (1 | Subject) + storage_time
y ~ current_feature + Sex + (1 | Subject) + storage_time
y ~ current_feature + Sex + (1 | Subject) + storage_time
y ~ current_feature + Sex + (1 | Subject) + storage_time
y ~ current_feature + Sex + (1 | Subject) + storage_time
y ~ current_feature + Sex + (1 | Subject) + storage_time
y ~ current_feature + Sex + (1 | Subject) + storage_time
y ~ current_feature + Sex + (1 | Subject) + storage_time
y ~ current_feature + Sex + (1 | Subject) + storage_time
y ~ current_feature + Sex + (1 | Subject) + storage_time
y ~ current_feature + Sex + (1 | Subject) + storage_time
y ~ current_feature + Sex + (1 | Subject) + storage_time
y ~ current_feature + Sex + (1 | Subject) + storage_time
y ~ current_feature + Sex + (1 | Subject) + storage_time
y ~ current_feature + Sex + (1 | Subject) + storage_time
y ~ current_feature + Sex + (1 | Subject) + storage_time
y ~ current_feature + Sex + (1 | Subject) + storage_time
y ~ current_feature + Sex + (1 | Subject) + storage_time
y ~ current_feature + Sex + (1 | Subject) + storage_time
y ~ current_feature + Sex + (1 | Subject) + storage_time
y ~ current_feature + Sex + (1 | Subject) + storage_time
y ~ current_feature + Sex + (1 | Subject) + storage_time
y ~ current_feature + Sex + (1 | Subject) + storage_time
y ~ current_feature + Sex + (1 | Subject) + storage_time
y ~ current_feature + Sex + (1 | Subject) + storage_time
y ~ current_feature + Sex + (1 | Subject) + storage_time
y ~ current_feature + Sex + (1 | Subject) + storage_time
y ~ current_feature + Sex + (1 | Subject) + storage_time
y ~ current_feature + Sex + (1 | Subject) + storage_time
y ~ current_feature + Sex + (1 | Subject) + storage_time

y ~ current_feature + Sex + (1 | Subject) + storage_time
y ~ current_feature + Sex + (1 | Subject) + storage_time
y ~ current_feature + Sex + (1 | Subject) + storage_time
y ~ current_feature + Sex + (1 | Subject) + storage_time
y ~ current_feature + Sex + (1 | Subject) + storage_time
y ~ current_feature + Sex + (1 | Subject) + storage_time
y ~ current_feature + Sex + (1 | Subject) + storage_time
y ~ current_feature + Sex + (1 | Subject) + storage_time
y ~ current_feature + Sex + (1 | Subject) + storage_time
y ~ current_feature + Sex + (1 | Subject) + storage_time
y ~ current_feature + Sex + (1 | Subject) + storage_time
y ~ current_feature + Sex + (1 | Subject) + storage_time
y ~ current_feature + Sex + (1 | Subject) + storage_time
y ~ current_feature + Sex + (1 | Subject) + storage_time
y ~ current_feature + Sex + (1 | Subject) + storage_time
y ~ current_feature + Sex + (1 | Subject) + storage_time
y ~ current_feature + Sex + (1 | Subject) + storage_time
y ~ current_feature + Sex + (1 | Subject) + storage_time
y ~ current_feature + Sex + (1 | Subject) + storage_time
y ~ current_feature + Sex + (1 | Subject) + storage_time
y ~ current_feature + Sex + (1 | Subject) + storage_time
y ~ current_feature + Sex + (1 | Subject) + storage_time
y ~ current_feature + Sex + (1 | Subject) + storage_time
y ~ current_feature + Sex + (1 | Subject) + storage_time
y ~ current_feature + Sex + (1 | Subject) + storage_time
y ~ current_feature + Sex + (1 | Subject) + storage_time
y ~ current_feature + Sex + (1 | Subject) + storage_time
y ~ current_feature + Sex + (1 | Subject) + storage_time
y ~ current_feature + Sex + (1 | Subject) + storage_time
y ~ current_feature + Sex + (1 | Subject) + storage_time
y ~ current_feature + Sex + (1 | Subject) + storage_time
y ~ current_feature + Sex + (1 | Subject) + storage_time
y ~ current_feature + Sex + (1 | Subject) + storage_time
y ~ current_feature + Sex + (1 | Subject) + storage_time
y ~ current_feature + Sex + (1 | Subject) + storage_time
y ~ current_feature + Sex + (1 | Subject) + storage_time
y ~ current_feature + Sex + (1 | Subject) + storage_time
y ~ current_feature + Sex + (1 | Subject) + storage_time
y ~ current_feature + Sex + (1 | Subject) + storage_time
y ~ current_feature + Sex + (1 | Subject) + storage_time
y ~ current_feature + Sex + (1 | Subject) + storage_time
y ~ current_feature + Sex + (1 | Subject) + storage_time
y ~ current_feature + Sex + (1 | Subject) + storage_time
y ~ current_feature + Sex + (1 | Subject) + storage_time
y ~ current_feature + Sex + (1 | Subject) + storage_time
y ~ current_feature + Sex + (1 | Subject) + storage_time
y ~ current_feature + Sex + (1 | Subject) + storage_time
y ~ current_feature + Sex + (1 | Subject) + storage_time

y ~ current_feature + Sex + (1 | Subject) + storage_time
y ~ current_feature + Sex + (1 | Subject) + storage_time
y ~ current_feature + Sex + (1 | Subject) + storage_time
y ~ current_feature + Sex + (1 | Subject) + storage_time
y ~ current_feature + Sex + (1 | Subject) + storage_time
y ~ current_feature + Sex + (1 | Subject) + storage_time
y ~ current_feature + Sex + (1 | Subject) + storage_time
y ~ current_feature + Sex + (1 | Subject) + storage_time
y ~ current_feature + Sex + (1 | Subject) + storage_time
y ~ current_feature + Sex + (1 | Subject) + storage_time
y ~ current_feature + Sex + (1 | Subject) + storage_time
y ~ current_feature + Sex + (1 | Subject) + storage_time
y ~ current_feature + Sex + (1 | Subject) + storage_time
y ~ current_feature + Sex + (1 | Subject) + storage_time
y ~ current_feature + Sex + (1 | Subject) + storage_time
y ~ current_feature + Sex + (1 | Subject) + storage_time
y ~ current_feature + Sex + (1 | Subject) + storage_time
y ~ current_feature + Sex + (1 | Subject) + storage_time
y ~ current_feature + Sex + (1 | Subject) + storage_time
y ~ current_feature + Sex + (1 | Subject) + storage_time
y ~ current_feature + Sex + (1 | Subject) + storage_time
y ~ current_feature + Sex + (1 | Subject) + storage_time
y ~ current_feature + Sex + (1 | Subject) + storage_time
y ~ current_feature + Sex + (1 | Subject) + storage_time
y ~ current_feature + Sex + (1 | Subject) + storage_time
y ~ current_feature + Sex + (1 | Subject) + storage_time
y ~ current_feature + Sex + (1 | Subject) + storage_time
y ~ current_feature + Sex + (1 | Subject) + storage_time
y ~ current_feature + Sex + (1 | Subject) + storage_time
y ~ current_feature + Sex + (1 | Subject) + storage_time
y ~ current_feature + Sex + (1 | Subject) + storage_time
y ~ current_feature + Sex + (1 | Subject) + storage_time
y ~ current_feature + Sex + (1 | Subject) + storage_time
y ~ current_feature + Sex + (1 | Subject) + storage_time
y ~ current_feature + Sex + (1 | Subject) + storage_time
y ~ current_feature + Sex + (1 | Subject) + storage_time
y ~ current_feature + Sex + (1 | Subject) + storage_time
y ~ current_feature + Sex + (1 | Subject) + storage_time
y ~ current_feature + Sex + (1 | Subject) + storage_time
y ~ current_feature + Sex + (1 | Subject) + storage_time
y ~ current_feature + Sex + (1 | Subject) + storage_time
y ~ current_feature + Sex + (1 | Subject) + storage_time
y ~ current_feature + Sex + (1 | Subject) + storage_time
y ~ current_feature + Sex + (1 | Subject) + storage_time
y ~ current_feature + Sex + (1 | Subject) + storage_time
y ~ current_feature + Sex + (1 | Subject) + storage_time
y ~ current_feature + Sex + (1 | Subject) + storage_time
y ~ current_feature + Sex + (1 | Subject) + storage_time

y ~ current_feature + Sex + (1 | Subject) + storage_time
y ~ current_feature + Sex + (1 | Subject) + storage_time
y ~ current_feature + Sex + (1 | Subject) + storage_time
y ~ current_feature + Sex + (1 | Subject) + storage_time
y ~ current_feature + Sex + (1 | Subject) + storage_time
y ~ current_feature + Sex + (1 | Subject) + storage_time
y ~ current_feature + Sex + (1 | Subject) + storage_time
y ~ current_feature + Sex + (1 | Subject) + storage_time
y ~ current_feature + Sex + (1 | Subject) + storage_time
y ~ current_feature + Sex + (1 | Subject) + storage_time
y ~ current_feature + Sex + (1 | Subject) + storage_time
y ~ current_feature + Sex + (1 | Subject) + storage_time
y ~ current_feature + Sex + (1 | Subject) + storage_time
y ~ current_feature + Sex + (1 | Subject) + storage_time
y ~ current_feature + Sex + (1 | Subject) + storage_time
y ~ current_feature + Sex + (1 | Subject) + storage_time
y ~ current_feature + Sex + (1 | Subject) + storage_time
y ~ current_feature + Sex + (1 | Subject) + storage_time
y ~ current_feature + Sex + (1 | Subject) + storage_time
y ~ current_feature + Sex + (1 | Subject) + storage_time
y ~ current_feature + Sex + (1 | Subject) + storage_time
y ~ current_feature + Sex + (1 | Subject) + storage_time
y ~ current_feature + Sex + (1 | Subject) + storage_time
y ~ current_feature + Sex + (1 | Subject) + storage_time
y ~ current_feature + Sex + (1 | Subject) + storage_time
y ~ current_feature + Sex + (1 | Subject) + storage_time
y ~ current_feature + Sex + (1 | Subject) + storage_time
y ~ current_feature + Sex + (1 | Subject) + storage_time
y ~ current_feature + Sex + (1 | Subject) + storage_time
y ~ current_feature + Sex + (1 | Subject) + storage_time
y ~ current_feature + Sex + (1 | Subject) + storage_time
y ~ current_feature + Sex + (1 | Subject) + storage_time
y ~ current_feature + Sex + (1 | Subject) + storage_time
y ~ current_feature + Sex + (1 | Subject) + storage_time
y ~ current_feature + Sex + (1 | Subject) + storage_time
y ~ current_feature + Sex + (1 | Subject) + storage_time
y ~ current_feature + Sex + (1 | Subject) + storage_time
y ~ current_feature + Sex + (1 | Subject) + storage_time
y ~ current_feature + Sex + (1 | Subject) + storage_time
y ~ current_feature + Sex + (1 | Subject) + storage_time
y ~ current_feature + Sex + (1 | Subject) + storage_time
y ~ current_feature + Sex + (1 | Subject) + storage_time
y ~ current_feature + Sex + (1 | Subject) + storage_time
y ~ current_feature + Sex + (1 | Subject) + storage_time
y ~ current_feature + Sex + (1 | Subject) + storage_time
y ~ current_feature + Sex + (1 | Subject) + storage_time
y ~ current_feature + Sex + (1 | Subject) + storage_time
y ~ current_feature + Sex + (1 | Subject) + storage_time

y ~ current_feature + Sex + (1 | Subject) + storage_time
y ~ current_feature + Sex + (1 | Subject) + storage_time
y ~ current_feature + Sex + (1 | Subject) + storage_time
y ~ current_feature + Sex + (1 | Subject) + storage_time
y ~ current_feature + Sex + (1 | Subject) + storage_time
y ~ current_feature + Sex + (1 | Subject) + storage_time
y ~ current_feature + Sex + (1 | Subject) + storage_time
y ~ current_feature + Sex + (1 | Subject) + storage_time
y ~ current_feature + Sex + (1 | Subject) + storage_time
y ~ current_feature + Sex + (1 | Subject) + storage_time
y ~ current_feature + Sex + (1 | Subject) + storage_time
y ~ current_feature + Sex + (1 | Subject) + storage_time
y ~ current_feature + Sex + (1 | Subject) + storage_time
y ~ current_feature + Sex + (1 | Subject) + storage_time
y ~ current_feature + Sex + (1 | Subject) + storage_time
y ~ current_feature + Sex + (1 | Subject) + storage_time
y ~ current_feature + Sex + (1 | Subject) + storage_time
y ~ current_feature + Sex + (1 | Subject) + storage_time
y ~ current_feature + Sex + (1 | Subject) + storage_time
y ~ current_feature + Sex + (1 | Subject) + storage_time
y ~ current_feature + Sex + (1 | Subject) + storage_time
y ~ current_feature + Sex + (1 | Subject) + storage_time
y ~ current_feature + Sex + (1 | Subject) + storage_time
y ~ current_feature + Sex + (1 | Subject) + storage_time
y ~ current_feature + Sex + (1 | Subject) + storage_time
y ~ current_feature + Sex + (1 | Subject) + storage_time
y ~ current_feature + Sex + (1 | Subject) + storage_time
y ~ current_feature + Sex + (1 | Subject) + storage_time
y ~ current_feature + Sex + (1 | Subject) + storage_time
y ~ current_feature + Sex + (1 | Subject) + storage_time
y ~ current_feature + Sex + (1 | Subject) + storage_time
y ~ current_feature + Sex + (1 | Subject) + storage_time
y ~ current_feature + Sex + (1 | Subject) + storage_time
y ~ current_feature + Sex + (1 | Subject) + storage_time
y ~ current_feature + Sex + (1 | Subject) + storage_time
y ~ current_feature + Sex + (1 | Subject) + storage_time
y ~ current_feature + Sex + (1 | Subject) + storage_time
y ~ current_feature + Sex + (1 | Subject) + storage_time
y ~ current_feature + Sex + (1 | Subject) + storage_time
y ~ current_feature + Sex + (1 | Subject) + storage_time
y ~ current_feature + Sex + (1 | Subject) + storage_time
y ~ current_feature + Sex + (1 | Subject) + storage_time
y ~ current_feature + Sex + (1 | Subject) + storage_time
y ~ current_feature + Sex + (1 | Subject) + storage_time
y ~ current_feature + Sex + (1 | Subject) + storage_time
y ~ current_feature + Sex + (1 | Subject) + storage_time
y ~ current_feature + Sex + (1 | Subject) + storage_time
y ~ current_feature + Sex + (1 | Subject) + storage_time

y ~ current_feature + Sex + (1 | Subject) + storage_time
y ~ current_feature + Sex + (1 | Subject) + storage_time
y ~ current_feature + Sex + (1 | Subject) + storage_time
y ~ current_feature + Sex + (1 | Subject) + storage_time
y ~ current_feature + Sex + (1 | Subject) + storage_time
y ~ current_feature + Sex + (1 | Subject) + storage_time
y ~ current_feature + Sex + (1 | Subject) + storage_time
y ~ current_feature + Sex + (1 | Subject) + storage_time
y ~ current_feature + Sex + (1 | Subject) + storage_time
y ~ current_feature + Sex + (1 | Subject) + storage_time
y ~ current_feature + Sex + (1 | Subject) + storage_time
y ~ current_feature + Sex + (1 | Subject) + storage_time
y ~ current_feature + Sex + (1 | Subject) + storage_time
y ~ current_feature + Sex + (1 | Subject) + storage_time
y ~ current_feature + Sex + (1 | Subject) + storage_time
y ~ current_feature + Sex + (1 | Subject) + storage_time
y ~ current_feature + Sex + (1 | Subject) + storage_time
y ~ current_feature + Sex + (1 | Subject) + storage_time
y ~ current_feature + Sex + (1 | Subject) + storage_time
y ~ current_feature + Sex + (1 | Subject) + storage_time
y ~ current_feature + Sex + (1 | Subject) + storage_time
y ~ current_feature + Sex + (1 | Subject) + storage_time
y ~ current_feature + Sex + (1 | Subject) + storage_time
y ~ current_feature + Sex + (1 | Subject) + storage_time
y ~ current_feature + Sex + (1 | Subject) + storage_time
y ~ current_feature + Sex + (1 | Subject) + storage_time
y ~ current_feature + Sex + (1 | Subject) + storage_time
y ~ current_feature + Sex + (1 | Subject) + storage_time
y ~ current_feature + Sex + (1 | Subject) + storage_time
y ~ current_feature + Sex + (1 | Subject) + storage_time
y ~ current_feature + Sex + (1 | Subject) + storage_time
y ~ current_feature + Sex + (1 | Subject) + storage_time
y ~ current_feature + Sex + (1 | Subject) + storage_time
y ~ current_feature + Sex + (1 | Subject) + storage_time
y ~ current_feature + Sex + (1 | Subject) + storage_time
y ~ current_feature + Sex + (1 | Subject) + storage_time
y ~ current_feature + Sex + (1 | Subject) + storage_time
y ~ current_feature + Sex + (1 | Subject) + storage_time
y ~ current_feature + Sex + (1 | Subject) + storage_time
y ~ current_feature + Sex + (1 | Subject) + storage_time
y ~ current_feature + Sex + (1 | Subject) + storage_time
y ~ current_feature + Sex + (1 | Subject) + storage_time
y ~ current_feature + Sex + (1 | Subject) + storage_time
y ~ current_feature + Sex + (1 | Subject) + storage_time
y ~ current_feature + Sex + (1 | Subject) + storage_time
y ~ current_feature + Sex + (1 | Subject) + storage_time
y ~ current_feature + Sex + (1 | Subject) + storage_time
y ~ current_feature + Sex + (1 | Subject) + storage_time

y ~ current_feature + Sex + (1 | Subject) + storage_time
y ~ current_feature + Sex + (1 | Subject) + storage_time
y ~ current_feature + Sex + (1 | Subject) + storage_time
y ~ current_feature + Sex + (1 | Subject) + storage_time
y ~ current_feature + Sex + (1 | Subject) + storage_time
y ~ current_feature + Sex + (1 | Subject) + storage_time
y ~ current_feature + Sex + (1 | Subject) + storage_time
y ~ current_feature + Sex + (1 | Subject) + storage_time
y ~ current_feature + Sex + (1 | Subject) + storage_time
y ~ current_feature + Sex + (1 | Subject) + storage_time
y ~ current_feature + Sex + (1 | Subject) + storage_time
y ~ current_feature + Sex + (1 | Subject) + storage_time
y ~ current_feature + Sex + (1 | Subject) + storage_time
y ~ current_feature + Sex + (1 | Subject) + storage_time
y ~ current_feature + Sex + (1 | Subject) + storage_time
y ~ current_feature + Sex + (1 | Subject) + storage_time
y ~ current_feature + Sex + (1 | Subject) + storage_time
y ~ current_feature + Sex + (1 | Subject) + storage_time
y ~ current_feature + Sex + (1 | Subject) + storage_time
y ~ current_feature + Sex + (1 | Subject) + storage_time
y ~ current_feature + Sex + (1 | Subject) + storage_time
y ~ current_feature + Sex + (1 | Subject) + storage_time
y ~ current_feature + Sex + (1 | Subject) + storage_time
y ~ current_feature + Sex + (1 | Subject) + storage_time
y ~ current_feature + Sex + (1 | Subject) + storage_time
y ~ current_feature + Sex + (1 | Subject) + storage_time
y ~ current_feature + Sex + (1 | Subject) + storage_time
y ~ current_feature + Sex + (1 | Subject) + storage_time
y ~ current_feature + Sex + (1 | Subject) + storage_time
y ~ current_feature + Sex + (1 | Subject) + storage_time
y ~ current_feature + Sex + (1 | Subject) + storage_time
y ~ current_feature + Sex + (1 | Subject) + storage_time
y ~ current_feature + Sex + (1 | Subject) + storage_time
y ~ current_feature + Sex + (1 | Subject) + storage_time
y ~ current_feature + Sex + (1 | Subject) + storage_time
y ~ current_feature + Sex + (1 | Subject) + storage_time
y ~ current_feature + Sex + (1 | Subject) + storage_time
y ~ current_feature + Sex + (1 | Subject) + storage_time
y ~ current_feature + Sex + (1 | Subject) + storage_time
y ~ current_feature + Sex + (1 | Subject) + storage_time
y ~ current_feature + Sex + (1 | Subject) + storage_time
y ~ current_feature + Sex + (1 | Subject) + storage_time
y ~ current_feature + Sex + (1 | Subject) + storage_time
y ~ current_feature + Sex + (1 | Subject) + storage_time
y ~ current_feature + Sex + (1 | Subject) + storage_time
y ~ current_feature + Sex + (1 | Subject) + storage_time
y ~ current_feature + Sex + (1 | Subject) + storage_time
y ~ current_feature + Sex + (1 | Subject) + storage_time

y ~ current_feature + Sex + (1 | Subject) + storage_time
y ~ current_feature + Sex + (1 | Subject) + storage_time
y ~ current_feature + Sex + (1 | Subject) + storage_time
y ~ current_feature + Sex + (1 | Subject) + storage_time
y ~ current_feature + Sex + (1 | Subject) + storage_time
y ~ current_feature + Sex + (1 | Subject) + storage_time
y ~ current_feature + Sex + (1 | Subject) + storage_time
y ~ current_feature + Sex + (1 | Subject) + storage_time
y ~ current_feature + Sex + (1 | Subject) + storage_time
y ~ current_feature + Sex + (1 | Subject) + storage_time
y ~ current_feature + Sex + (1 | Subject) + storage_time
y ~ current_feature + Sex + (1 | Subject) + storage_time
y ~ current_feature + Sex + (1 | Subject) + storage_time
y ~ current_feature + Sex + (1 | Subject) + storage_time
y ~ current_feature + Sex + (1 | Subject) + storage_time
y ~ current_feature + Sex + (1 | Subject) + storage_time
y ~ current_feature + Sex + (1 | Subject) + storage_time
y ~ current_feature + Sex + (1 | Subject) + storage_time
y ~ current_feature + Sex + (1 | Subject) + storage_time
y ~ current_feature + Sex + (1 | Subject) + storage_time
y ~ current_feature + Sex + (1 | Subject) + storage_time
y ~ current_feature + Sex + (1 | Subject) + storage_time
y ~ current_feature + Sex + (1 | Subject) + storage_time
y ~ current_feature + Sex + (1 | Subject) + storage_time
y ~ current_feature + Sex + (1 | Subject) + storage_time
y ~ current_feature + Sex + (1 | Subject) + storage_time
y ~ current_feature + Sex + (1 | Subject) + storage_time
y ~ current_feature + Sex + (1 | Subject) + storage_time
y ~ current_feature + Sex + (1 | Subject) + storage_time
y ~ current_feature + Sex + (1 | Subject) + storage_time
y ~ current_feature + Sex + (1 | Subject) + storage_time
y ~ current_feature + Sex + (1 | Subject) + storage_time
y ~ current_feature + Sex + (1 | Subject) + storage_time
y ~ current_feature + Sex + (1 | Subject) + storage_time
y ~ current_feature + Sex + (1 | Subject) + storage_time
y ~ current_feature + Sex + (1 | Subject) + storage_time
y ~ current_feature + Sex + (1 | Subject) + storage_time
y ~ current_feature + Sex + (1 | Subject) + storage_time
y ~ current_feature + Sex + (1 | Subject) + storage_time
y ~ current_feature + Sex + (1 | Subject) + storage_time
y ~ current_feature + Sex + (1 | Subject) + storage_time
y ~ current_feature + Sex + (1 | Subject) + storage_time
y ~ current_feature + Sex + (1 | Subject) + storage_time
y ~ current_feature + Sex + (1 | Subject) + storage_time
y ~ current_feature + Sex + (1 | Subject) + storage_time
y ~ current_feature + Sex + (1 | Subject) + storage_time
y ~ current_feature + Sex + (1 | Subject) + storage_time
y ~ current_feature + Sex + (1 | Subject) + storage_time

y ~ current_feature + Sex + (1 | Subject) + storage_time
y ~ current_feature + Sex + (1 | Subject) + storage_time
y ~ current_feature + Sex + (1 | Subject) + storage_time
y ~ current_feature + Sex + (1 | Subject) + storage_time
y ~ current_feature + Sex + (1 | Subject) + storage_time
y ~ current_feature + Sex + (1 | Subject) + storage_time
y ~ current_feature + Sex + (1 | Subject) + storage_time
y ~ current_feature + Sex + (1 | Subject) + storage_time
y ~ current_feature + Sex + (1 | Subject) + storage_time
y ~ current_feature + Sex + (1 | Subject) + storage_time
y ~ current_feature + Sex + (1 | Subject) + storage_time
y ~ current_feature + Sex + (1 | Subject) + storage_time
y ~ current_feature + Sex + (1 | Subject) + storage_time
y ~ current_feature + Sex + (1 | Subject) + storage_time
y ~ current_feature + Sex + (1 | Subject) + storage_time
y ~ current_feature + Sex + (1 | Subject) + storage_time
y ~ current_feature + Sex + (1 | Subject) + storage_time
y ~ current_feature + Sex + (1 | Subject) + storage_time
y ~ current_feature + Sex + (1 | Subject) + storage_time
y ~ current_feature + Sex + (1 | Subject) + storage_time
y ~ current_feature + Sex + (1 | Subject) + storage_time
y ~ current_feature + Sex + (1 | Subject) + storage_time
y ~ current_feature + Sex + (1 | Subject) + storage_time
y ~ current_feature + Sex + (1 | Subject) + storage_time
y ~ current_feature + Sex + (1 | Subject) + storage_time
y ~ current_feature + Sex + (1 | Subject) + storage_time
y ~ current_feature + Sex + (1 | Subject) + storage_time
y ~ current_feature + Sex + (1 | Subject) + storage_time
y ~ current_feature + Sex + (1 | Subject) + storage_time
y ~ current_feature + Sex + (1 | Subject) + storage_time
y ~ current_feature + Sex + (1 | Subject) + storage_time
y ~ current_feature + Sex + (1 | Subject) + storage_time
y ~ current_feature + Sex + (1 | Subject) + storage_time
y ~ current_feature + Sex + (1 | Subject) + storage_time
y ~ current_feature + Sex + (1 | Subject) + storage_time
y ~ current_feature + Sex + (1 | Subject) + storage_time
y ~ current_feature + Sex + (1 | Subject) + storage_time
y ~ current_feature + Sex + (1 | Subject) + storage_time
y ~ current_feature + Sex + (1 | Subject) + storage_time
y ~ current_feature + Sex + (1 | Subject) + storage_time
y ~ current_feature + Sex + (1 | Subject) + storage_time
y ~ current_feature + Sex + (1 | Subject) + storage_time
y ~ current_feature + Sex + (1 | Subject) + storage_time
y ~ current_feature + Sex + (1 | Subject) + storage_time
y ~ current_feature + Sex + (1 | Subject) + storage_time
y ~ current_feature + Sex + (1 | Subject) + storage_time
y ~ current_feature + Sex + (1 | Subject) + storage_time
y ~ current_feature + Sex + (1 | Subject) + storage_time

y ~ current_feature + Sex + (1 | Subject) + storage_time
y ~ current_feature + Sex + (1 | Subject) + storage_time
y ~ current_feature + Sex + (1 | Subject) + storage_time
y ~ current_feature + Sex + (1 | Subject) + storage_time
y ~ current_feature + Sex + (1 | Subject) + storage_time
y ~ current_feature + Sex + (1 | Subject) + storage_time
y ~ current_feature + Sex + (1 | Subject) + storage_time
y ~ current_feature + Sex + (1 | Subject) + storage_time
y ~ current_feature + Sex + (1 | Subject) + storage_time
y ~ current_feature + Sex + (1 | Subject) + storage_time
y ~ current_feature + Sex + (1 | Subject) + storage_time
y ~ current_feature + Sex + (1 | Subject) + storage_time
y ~ current_feature + Sex + (1 | Subject) + storage_time
y ~ current_feature + Sex + (1 | Subject) + storage_time
y ~ current_feature + Sex + (1 | Subject) + storage_time
y ~ current_feature + Sex + (1 | Subject) + storage_time
y ~ current_feature + Sex + (1 | Subject) + storage_time
y ~ current_feature + Sex + (1 | Subject) + storage_time
y ~ current_feature + Sex + (1 | Subject) + storage_time
y ~ current_feature + Sex + (1 | Subject) + storage_time
y ~ current_feature + Sex + (1 | Subject) + storage_time
y ~ current_feature + Sex + (1 | Subject) + storage_time
y ~ current_feature + Sex + (1 | Subject) + storage_time
y ~ current_feature + Sex + (1 | Subject) + storage_time
y ~ current_feature + Sex + (1 | Subject) + storage_time
y ~ current_feature + Sex + (1 | Subject) + storage_time
y ~ current_feature + Sex + (1 | Subject) + storage_time
y ~ current_feature + Sex + (1 | Subject) + storage_time
y ~ current_feature + Sex + (1 | Subject) + storage_time
y ~ current_feature + Sex + (1 | Subject) + storage_time
y ~ current_feature + Sex + (1 | Subject) + storage_time
y ~ current_feature + Sex + (1 | Subject) + storage_time
y ~ current_feature + Sex + (1 | Subject) + storage_time
y ~ current_feature + Sex + (1 | Subject) + storage_time
y ~ current_feature + Sex + (1 | Subject) + storage_time
y ~ current_feature + Sex + (1 | Subject) + storage_time
y ~ current_feature + Sex + (1 | Subject) + storage_time
y ~ current_feature + Sex + (1 | Subject) + storage_time
y ~ current_feature + Sex + (1 | Subject) + storage_time
y ~ current_feature + Sex + (1 | Subject) + storage_time
y ~ current_feature + Sex + (1 | Subject) + storage_time
y ~ current_feature + Sex + (1 | Subject) + storage_time
y ~ current_feature + Sex + (1 | Subject) + storage_time
y ~ current_feature + Sex + (1 | Subject) + storage_time
y ~ current_feature + Sex + (1 | Subject) + storage_time
y ~ current_feature + Sex + (1 | Subject) + storage_time
y ~ current_feature + Sex + (1 | Subject) + storage_time
y ~ current_feature + Sex + (1 | Subject) + storage_time

y ~ current_feature + Sex + (1 | Subject) + storage_time
y ~ current_feature + Sex + (1 | Subject) + storage_time
y ~ current_feature + Sex + (1 | Subject) + storage_time
y ~ current_feature + Sex + (1 | Subject) + storage_time
y ~ current_feature + Sex + (1 | Subject) + storage_time
y ~ current_feature + Sex + (1 | Subject) + storage_time
y ~ current_feature + Sex + (1 | Subject) + storage_time
y ~ current_feature + Sex + (1 | Subject) + storage_time
y ~ current_feature + Sex + (1 | Subject) + storage_time
y ~ current_feature + Sex + (1 | Subject) + storage_time
y ~ current_feature + Sex + (1 | Subject) + storage_time
y ~ current_feature + Sex + (1 | Subject) + storage_time
y ~ current_feature + Sex + (1 | Subject) + storage_time
y ~ current_feature + Sex + (1 | Subject) + storage_time
y ~ current_feature + Sex + (1 | Subject) + storage_time
y ~ current_feature + Sex + (1 | Subject) + storage_time
y ~ current_feature + Sex + (1 | Subject) + storage_time
y ~ current_feature + Sex + (1 | Subject) + storage_time
y ~ current_feature + Sex + (1 | Subject) + storage_time
y ~ current_feature + Sex + (1 | Subject) + storage_time
y ~ current_feature + Sex + (1 | Subject) + storage_time
y ~ current_feature + Sex + (1 | Subject) + storage_time
y ~ current_feature + Sex + (1 | Subject) + storage_time
y ~ current_feature + Sex + (1 | Subject) + storage_time
y ~ current_feature + Sex + (1 | Subject) + storage_time
y ~ current_feature + Sex + (1 | Subject) + storage_time
y ~ current_feature + Sex + (1 | Subject) + storage_time
y ~ current_feature + Sex + (1 | Subject) + storage_time
y ~ current_feature + Sex + (1 | Subject) + storage_time
y ~ current_feature + Sex + (1 | Subject) + storage_time
y ~ current_feature + Sex + (1 | Subject) + storage_time
y ~ current_feature + Sex + (1 | Subject) + storage_time
y ~ current_feature + Sex + (1 | Subject) + storage_time
y ~ current_feature + Sex + (1 | Subject) + storage_time
y ~ current_feature + Sex + (1 | Subject) + storage_time
y ~ current_feature + Sex + (1 | Subject) + storage_time
y ~ current_feature + Sex + (1 | Subject) + storage_time
y ~ current_feature + Sex + (1 | Subject) + storage_time
y ~ current_feature + Sex + (1 | Subject) + storage_time
y ~ current_feature + Sex + (1 | Subject) + storage_time
y ~ current_feature + Sex + (1 | Subject) + storage_time
y ~ current_feature + Sex + (1 | Subject) + storage_time
y ~ current_feature + Sex + (1 | Subject) + storage_time
y ~ current_feature + Sex + (1 | Subject) + storage_time
y ~ current_feature + Sex + (1 | Subject) + storage_time
y ~ current_feature + Sex + (1 | Subject) + storage_time
y ~ current_feature + Sex + (1 | Subject) + storage_time
y ~ current_feature + Sex + (1 | Subject) + storage_time

y ~ current_feature + Sex + (1 | Subject) + storage_time
y ~ current_feature + Sex + (1 | Subject) + storage_time
y ~ current_feature + Sex + (1 | Subject) + storage_time
y ~ current_feature + Sex + (1 | Subject) + storage_time
y ~ current_feature + Sex + (1 | Subject) + storage_time
y ~ current_feature + Sex + (1 | Subject) + storage_time
y ~ current_feature + Sex + (1 | Subject) + storage_time
y ~ current_feature + Sex + (1 | Subject) + storage_time
y ~ current_feature + Sex + (1 | Subject) + storage_time
y ~ current_feature + Sex + (1 | Subject) + storage_time
y ~ current_feature + Sex + (1 | Subject) + storage_time
y ~ current_feature + Sex + (1 | Subject) + storage_time
y ~ current_feature + Sex + (1 | Subject) + storage_time
y ~ current_feature + Sex + (1 | Subject) + storage_time
y ~ current_feature + Sex + (1 | Subject) + storage_time
y ~ current_feature + Sex + (1 | Subject) + storage_time
y ~ current_feature + Sex + (1 | Subject) + storage_time
y ~ current_feature + Sex + (1 | Subject) + storage_time
y ~ current_feature + Sex + (1 | Subject) + storage_time
y ~ current_feature + Sex + (1 | Subject) + storage_time
y ~ current_feature + Sex + (1 | Subject) + storage_time
y ~ current_feature + Sex + (1 | Subject) + storage_time
y ~ current_feature + Sex + (1 | Subject) + storage_time
y ~ current_feature + Sex + (1 | Subject) + storage_time
y ~ current_feature + Sex + (1 | Subject) + storage_time
y ~ current_feature + Sex + (1 | Subject) + storage_time
y ~ current_feature + Sex + (1 | Subject) + storage_time
y ~ current_feature + Sex + (1 | Subject) + storage_time
y ~ current_feature + Sex + (1 | Subject) + storage_time
y ~ current_feature + Sex + (1 | Subject) + storage_time
y ~ current_feature + Sex + (1 | Subject) + storage_time
y ~ current_feature + Sex + (1 | Subject) + storage_time
y ~ current_feature + Sex + (1 | Subject) + storage_time
y ~ current_feature + Sex + (1 | Subject) + storage_time
y ~ current_feature + Sex + (1 | Subject) + storage_time
y ~ current_feature + Sex + (1 | Subject) + storage_time
y ~ current_feature + Sex + (1 | Subject) + storage_time
y ~ current_feature + Sex + (1 | Subject) + storage_time
y ~ current_feature + Sex + (1 | Subject) + storage_time
y ~ current_feature + Sex + (1 | Subject) + storage_time
y ~ current_feature + Sex + (1 | Subject) + storage_time
y ~ current_feature + Sex + (1 | Subject) + storage_time
y ~ current_feature + Sex + (1 | Subject) + storage_time
y ~ current_feature + Sex + (1 | Subject) + storage_time
y ~ current_feature + Sex + (1 | Subject) + storage_time
y ~ current_feature + Sex + (1 | Subject) + storage_time
y ~ current_feature + Sex + (1 | Subject) + storage_time
y ~ current_feature + Sex + (1 | Subject) + storage_time

y ~ current_feature + Sex + (1 | Subject) + storage_time
y ~ current_feature + Sex + (1 | Subject) + storage_time
y ~ current_feature + Sex + (1 | Subject) + storage_time
y ~ current_feature + Sex + (1 | Subject) + storage_time
y ~ current_feature + Sex + (1 | Subject) + storage_time
y ~ current_feature + Sex + (1 | Subject) + storage_time
y ~ current_feature + Sex + (1 | Subject) + storage_time
y ~ current_feature + Sex + (1 | Subject) + storage_time
y ~ current_feature + Sex + (1 | Subject) + storage_time
y ~ current_feature + Sex + (1 | Subject) + storage_time
y ~ current_feature + Sex + (1 | Subject) + storage_time
y ~ current_feature + Sex + (1 | Subject) + storage_time
y ~ current_feature + Sex + (1 | Subject) + storage_time
y ~ current_feature + Sex + (1 | Subject) + storage_time
y ~ current_feature + Sex + (1 | Subject) + storage_time
y ~ current_feature + Sex + (1 | Subject) + storage_time
y ~ current_feature + Sex + (1 | Subject) + storage_time
y ~ current_feature + Sex + (1 | Subject) + storage_time
y ~ current_feature + Sex + (1 | Subject) + storage_time
y ~ current_feature + Sex + (1 | Subject) + storage_time
y ~ current_feature + Sex + (1 | Subject) + storage_time
y ~ current_feature + Sex + (1 | Subject) + storage_time
y ~ current_feature + Sex + (1 | Subject) + storage_time
y ~ current_feature + Sex + (1 | Subject) + storage_time
y ~ current_feature + Sex + (1 | Subject) + storage_time
y ~ current_feature + Sex + (1 | Subject) + storage_time
y ~ current_feature + Sex + (1 | Subject) + storage_time
y ~ current_feature + Sex + (1 | Subject) + storage_time
y ~ current_feature + Sex + (1 | Subject) + storage_time
y ~ current_feature + Sex + (1 | Subject) + storage_time
y ~ current_feature + Sex + (1 | Subject) + storage_time
y ~ current_feature + Sex + (1 | Subject) + storage_time
y ~ current_feature + Sex + (1 | Subject) + storage_time
y ~ current_feature + Sex + (1 | Subject) + storage_time
y ~ current_feature + Sex + (1 | Subject) + storage_time
y ~ current_feature + Sex + (1 | Subject) + storage_time
y ~ current_feature + Sex + (1 | Subject) + storage_time
y ~ current_feature + Sex + (1 | Subject) + storage_time
y ~ current_feature + Sex + (1 | Subject) + storage_time
y ~ current_feature + Sex + (1 | Subject) + storage_time
y ~ current_feature + Sex + (1 | Subject) + storage_time
y ~ current_feature + Sex + (1 | Subject) + storage_time
y ~ current_feature + Sex + (1 | Subject) + storage_time
y ~ current_feature + Sex + (1 | Subject) + storage_time
y ~ current_feature + Sex + (1 | Subject) + storage_time
y ~ current_feature + Sex + (1 | Subject) + storage_time
y ~ current_feature + Sex + (1 | Subject) + storage_time
y ~ current_feature + Sex + (1 | Subject) + storage_time

y ~ current_feature + Sex + (1 | Subject) + storage_time
y ~ current_feature + Sex + (1 | Subject) + storage_time
y ~ current_feature + Sex + (1 | Subject) + storage_time
y ~ current_feature + Sex + (1 | Subject) + storage_time
y ~ current_feature + Sex + (1 | Subject) + storage_time
y ~ current_feature + Sex + (1 | Subject) + storage_time
y ~ current_feature + Sex + (1 | Subject) + storage_time
y ~ current_feature + Sex + (1 | Subject) + storage_time
y ~ current_feature + Sex + (1 | Subject) + storage_time
y ~ current_feature + Sex + (1 | Subject) + storage_time
y ~ current_feature + Sex + (1 | Subject) + storage_time
y ~ current_feature + Sex + (1 | Subject) + storage_time
y ~ current_feature + Sex + (1 | Subject) + storage_time
y ~ current_feature + Sex + (1 | Subject) + storage_time
y ~ current_feature + Sex + (1 | Subject) + storage_time
y ~ current_feature + Sex + (1 | Subject) + storage_time
y ~ current_feature + Sex + (1 | Subject) + storage_time
y ~ current_feature + Sex + (1 | Subject) + storage_time
y ~ current_feature + Sex + (1 | Subject) + storage_time
y ~ current_feature + Sex + (1 | Subject) + storage_time
y ~ current_feature + Sex + (1 | Subject) + storage_time
y ~ current_feature + Sex + (1 | Subject) + storage_time
y ~ current_feature + Sex + (1 | Subject) + storage_time
y ~ current_feature + Sex + (1 | Subject) + storage_time
y ~ current_feature + Sex + (1 | Subject) + storage_time
y ~ current_feature + Sex + (1 | Subject) + storage_time
y ~ current_feature + Sex + (1 | Subject) + storage_time
y ~ current_feature + Sex + (1 | Subject) + storage_time
y ~ current_feature + Sex + (1 | Subject) + storage_time
y ~ current_feature + Sex + (1 | Subject) + storage_time
y ~ current_feature + Sex + (1 | Subject) + storage_time
y ~ current_feature + Sex + (1 | Subject) + storage_time
y ~ current_feature + Sex + (1 | Subject) + storage_time
y ~ current_feature + Sex + (1 | Subject) + storage_time
y ~ current_feature + Sex + (1 | Subject) + storage_time
y ~ current_feature + Sex + (1 | Subject) + storage_time
y ~ current_feature + Sex + (1 | Subject) + storage_time
y ~ current_feature + Sex + (1 | Subject) + storage_time
y ~ current_feature + Sex + (1 | Subject) + storage_time
y ~ current_feature + Sex + (1 | Subject) + storage_time
y ~ current_feature + Sex + (1 | Subject) + storage_time
y ~ current_feature + Sex + (1 | Subject) + storage_time
y ~ current_feature + Sex + (1 | Subject) + storage_time
y ~ current_feature + Sex + (1 | Subject) + storage_time
y ~ current_feature + Sex + (1 | Subject) + storage_time
y ~ current_feature + Sex + (1 | Subject) + storage_time
y ~ current_feature + Sex + (1 | Subject) + storage_time
y ~ current_feature + Sex + (1 | Subject) + storage_time

y ~ current_feature + Sex + (1 | Subject) + storage_time
y ~ current_feature + Sex + (1 | Subject) + storage_time
y ~ current_feature + Sex + (1 | Subject) + storage_time
y ~ current_feature + Sex + (1 | Subject) + storage_time
y ~ current_feature + Sex + (1 | Subject) + storage_time
y ~ current_feature + Sex + (1 | Subject) + storage_time
y ~ current_feature + Sex + (1 | Subject) + storage_time
y ~ current_feature + Sex + (1 | Subject) + storage_time
y ~ current_feature + Sex + (1 | Subject) + storage_time
y ~ current_feature + Sex + (1 | Subject) + storage_time
y ~ current_feature + Sex + (1 | Subject) + storage_time
y ~ current_feature + Sex + (1 | Subject) + storage_time
y ~ current_feature + Sex + (1 | Subject) + storage_time
y ~ current_feature + Sex + (1 | Subject) + storage_time
y ~ current_feature + Sex + (1 | Subject) + storage_time
y ~ current_feature + Sex + (1 | Subject) + storage_time
y ~ current_feature + Sex + (1 | Subject) + storage_time
y ~ current_feature + Sex + (1 | Subject) + storage_time
y ~ current_feature + Sex + (1 | Subject) + storage_time
y ~ current_feature + Sex + (1 | Subject) + storage_time
y ~ current_feature + Sex + (1 | Subject) + storage_time
y ~ current_feature + Sex + (1 | Subject) + storage_time
y ~ current_feature + Sex + (1 | Subject) + storage_time
y ~ current_feature + Sex + (1 | Subject) + storage_time
y ~ current_feature + Sex + (1 | Subject) + storage_time
y ~ current_feature + Sex + (1 | Subject) + storage_time
y ~ current_feature + Sex + (1 | Subject) + storage_time
y ~ current_feature + Sex + (1 | Subject) + storage_time
y ~ current_feature + Sex + (1 | Subject) + storage_time
y ~ current_feature + Sex + (1 | Subject) + storage_time
y ~ current_feature + Sex + (1 | Subject) + storage_time
y ~ current_feature + Sex + (1 | Subject) + storage_time
y ~ current_feature + Sex + (1 | Subject) + storage_time
y ~ current_feature + Sex + (1 | Subject) + storage_time
y ~ current_feature + Sex + (1 | Subject) + storage_time
y ~ current_feature + Sex + (1 | Subject) + storage_time
y ~ current_feature + Sex + (1 | Subject) + storage_time
y ~ current_feature + Sex + (1 | Subject) + storage_time
y ~ current_feature + Sex + (1 | Subject) + storage_time
y ~ current_feature + Sex + (1 | Subject) + storage_time
y ~ current_feature + Sex + (1 | Subject) + storage_time
y ~ current_feature + Sex + (1 | Subject) + storage_time
y ~ current_feature + Sex + (1 | Subject) + storage_time
y ~ current_feature + Sex + (1 | Subject) + storage_time
y ~ current_feature + Sex + (1 | Subject) + storage_time
y ~ current_feature + Sex + (1 | Subject) + storage_time
y ~ current_feature + Sex + (1 | Subject) + storage_time
y ~ current_feature + Sex + (1 | Subject) + storage_time

y ~ current_feature + Sex + (1 | Subject) + storage_time
y ~ current_feature + Sex + (1 | Subject) + storage_time
y ~ current_feature + Sex + (1 | Subject) + storage_time
y ~ current_feature + Sex + (1 | Subject) + storage_time
y ~ current_feature + Sex + (1 | Subject) + storage_time
y ~ current_feature + Sex + (1 | Subject) + storage_time
y ~ current_feature + Sex + (1 | Subject) + storage_time
y ~ current_feature + Sex + (1 | Subject) + storage_time
y ~ current_feature + Sex + (1 | Subject) + storage_time
y ~ current_feature + Sex + (1 | Subject) + storage_time
y ~ current_feature + Sex + (1 | Subject) + storage_time
y ~ current_feature + Sex + (1 | Subject) + storage_time
y ~ current_feature + Sex + (1 | Subject) + storage_time
y ~ current_feature + Sex + (1 | Subject) + storage_time
y ~ current_feature + Sex + (1 | Subject) + storage_time
y ~ current_feature + Sex + (1 | Subject) + storage_time
y ~ current_feature + Sex + (1 | Subject) + storage_time
y ~ current_feature + Sex + (1 | Subject) + storage_time
y ~ current_feature + Sex + (1 | Subject) + storage_time
y ~ current_feature + Sex + (1 | Subject) + storage_time
y ~ current_feature + Sex + (1 | Subject) + storage_time
y ~ current_feature + Sex + (1 | Subject) + storage_time
y ~ current_feature + Sex + (1 | Subject) + storage_time
y ~ current_feature + Sex + (1 | Subject) + storage_time
y ~ current_feature + Sex + (1 | Subject) + storage_time
y ~ current_feature + Sex + (1 | Subject) + storage_time
y ~ current_feature + Sex + (1 | Subject) + storage_time
y ~ current_feature + Sex + (1 | Subject) + storage_time
y ~ current_feature + Sex + (1 | Subject) + storage_time
y ~ current_feature + Sex + (1 | Subject) + storage_time
y ~ current_feature + Sex + (1 | Subject) + storage_time
y ~ current_feature + Sex + (1 | Subject) + storage_time
y ~ current_feature + Sex + (1 | Subject) + storage_time
y ~ current_feature + Sex + (1 | Subject) + storage_time
y ~ current_feature + Sex + (1 | Subject) + storage_time
y ~ current_feature + Sex + (1 | Subject) + storage_time
y ~ current_feature + Sex + (1 | Subject) + storage_time
y ~ current_feature + Sex + (1 | Subject) + storage_time
y ~ current_feature + Sex + (1 | Subject) + storage_time
y ~ current_feature + Sex + (1 | Subject) + storage_time
y ~ current_feature + Sex + (1 | Subject) + storage_time
y ~ current_feature + Sex + (1 | Subject) + storage_time
y ~ current_feature + Sex + (1 | Subject) + storage_time
y ~ current_feature + Sex + (1 | Subject) + storage_time
y ~ current_feature + Sex + (1 | Subject) + storage_time
y ~ current_feature + Sex + (1 | Subject) + storage_time
y ~ current_feature + Sex + (1 | Subject) + storage_time
y ~ current_feature + Sex + (1 | Subject) + storage_time

y ~ current_feature + Sex + (1 | Subject) + storage_time
y ~ current_feature + Sex + (1 | Subject) + storage_time
y ~ current_feature + Sex + (1 | Subject) + storage_time
y ~ current_feature + Sex + (1 | Subject) + storage_time
y ~ current_feature + Sex + (1 | Subject) + storage_time
y ~ current_feature + Sex + (1 | Subject) + storage_time
y ~ current_feature + Sex + (1 | Subject) + storage_time
y ~ current_feature + Sex + (1 | Subject) + storage_time
y ~ current_feature + Sex + (1 | Subject) + storage_time
y ~ current_feature + Sex + (1 | Subject) + storage_time
y ~ current_feature + Sex + (1 | Subject) + storage_time
y ~ current_feature + Sex + (1 | Subject) + storage_time
y ~ current_feature + Sex + (1 | Subject) + storage_time
y ~ current_feature + Sex + (1 | Subject) + storage_time
y ~ current_feature + Sex + (1 | Subject) + storage_time
y ~ current_feature + Sex + (1 | Subject) + storage_time
y ~ current_feature + Sex + (1 | Subject) + storage_time
y ~ current_feature + Sex + (1 | Subject) + storage_time
y ~ current_feature + Sex + (1 | Subject) + storage_time
y ~ current_feature + Sex + (1 | Subject) + storage_time
y ~ current_feature + Sex + (1 | Subject) + storage_time
y ~ current_feature + Sex + (1 | Subject) + storage_time
y ~ current_feature + Sex + (1 | Subject) + storage_time
y ~ current_feature + Sex + (1 | Subject) + storage_time
y ~ current_feature + Sex + (1 | Subject) + storage_time
y ~ current_feature + Sex + (1 | Subject) + storage_time
y ~ current_feature + Sex + (1 | Subject) + storage_time
y ~ current_feature + Sex + (1 | Subject) + storage_time
y ~ current_feature + Sex + (1 | Subject) + storage_time
y ~ current_feature + Sex + (1 | Subject) + storage_time
y ~ current_feature + Sex + (1 | Subject) + storage_time
y ~ current_feature + Sex + (1 | Subject) + storage_time
y ~ current_feature + Sex + (1 | Subject) + storage_time
y ~ current_feature + Sex + (1 | Subject) + storage_time
y ~ current_feature + Sex + (1 | Subject) + storage_time
y ~ current_feature + Sex + (1 | Subject) + storage_time
y ~ current_feature + Sex + (1 | Subject) + storage_time
y ~ current_feature + Sex + (1 | Subject) + storage_time
y ~ current_feature + Sex + (1 | Subject) + storage_time
y ~ current_feature + Sex + (1 | Subject) + storage_time
y ~ current_feature + Sex + (1 | Subject) + storage_time
y ~ current_feature + Sex + (1 | Subject) + storage_time
y ~ current_feature + Sex + (1 | Subject) + storage_time
y ~ current_feature + Sex + (1 | Subject) + storage_time
y ~ current_feature + Sex + (1 | Subject) + storage_time
y ~ current_feature + Sex + (1 | Subject) + storage_time
y ~ current_feature + Sex + (1 | Subject) + storage_time
y ~ current_feature + Sex + (1 | Subject) + storage_time

y ~ current_feature + Sex + (1 | Subject) + storage_time
y ~ current_feature + Sex + (1 | Subject) + storage_time
y ~ current_feature + Sex + (1 | Subject) + storage_time
y ~ current_feature + Sex + (1 | Subject) + storage_time
y ~ current_feature + Sex + (1 | Subject) + storage_time
y ~ current_feature + Sex + (1 | Subject) + storage_time
y ~ current_feature + Sex + (1 | Subject) + storage_time
y ~ current_feature + Sex + (1 | Subject) + storage_time
y ~ current_feature + Sex + (1 | Subject) + storage_time
y ~ current_feature + Sex + (1 | Subject) + storage_time
y ~ current_feature + Sex + (1 | Subject) + storage_time
y ~ current_feature + Sex + (1 | Subject) + storage_time
y ~ current_feature + Sex + (1 | Subject) + storage_time
y ~ current_feature + Sex + (1 | Subject) + storage_time
y ~ current_feature + Sex + (1 | Subject) + storage_time
y ~ current_feature + Sex + (1 | Subject) + storage_time
y ~ current_feature + Sex + (1 | Subject) + storage_time
y ~ current_feature + Sex + (1 | Subject) + storage_time
y ~ current_feature + Sex + (1 | Subject) + storage_time
y ~ current_feature + Sex + (1 | Subject) + storage_time
y ~ current_feature + Sex + (1 | Subject) + storage_time
y ~ current_feature + Sex + (1 | Subject) + storage_time
y ~ current_feature + Sex + (1 | Subject) + storage_time
y ~ current_feature + Sex + (1 | Subject) + storage_time
y ~ current_feature + Sex + (1 | Subject) + storage_time
y ~ current_feature + Sex + (1 | Subject) + storage_time
y ~ current_feature + Sex + (1 | Subject) + storage_time
y ~ current_feature + Sex + (1 | Subject) + storage_time
y ~ current_feature + Sex + (1 | Subject) + storage_time
y ~ current_feature + Sex + (1 | Subject) + storage_time
y ~ current_feature + Sex + (1 | Subject) + storage_time
y ~ current_feature + Sex + (1 | Subject) + storage_time
y ~ current_feature + Sex + (1 | Subject) + storage_time
y ~ current_feature + Sex + (1 | Subject) + storage_time
y ~ current_feature + Sex + (1 | Subject) + storage_time
y ~ current_feature + Sex + (1 | Subject) + storage_time
y ~ current_feature + Sex + (1 | Subject) + storage_time
y ~ current_feature + Sex + (1 | Subject) + storage_time
y ~ current_feature + Sex + (1 | Subject) + storage_time
y ~ current_feature + Sex + (1 | Subject) + storage_time
y ~ current_feature + Sex + (1 | Subject) + storage_time
y ~ current_feature + Sex + (1 | Subject) + storage_time
y ~ current_feature + Sex + (1 | Subject) + storage_time
y ~ current_feature + Sex + (1 | Subject) + storage_time
y ~ current_feature + Sex + (1 | Subject) + storage_time
y ~ current_feature + Sex + (1 | Subject) + storage_time
y ~ current_feature + Sex + (1 | Subject) + storage_time
y ~ current_feature + Sex + (1 | Subject) + storage_time

y ~ current_feature + Sex + (1 | Subject) + storage_time
y ~ current_feature + Sex + (1 | Subject) + storage_time
y ~ current_feature + Sex + (1 | Subject) + storage_time
y ~ current_feature + Sex + (1 | Subject) + storage_time
y ~ current_feature + Sex + (1 | Subject) + storage_time
y ~ current_feature + Sex + (1 | Subject) + storage_time
y ~ current_feature + Sex + (1 | Subject) + storage_time
y ~ current_feature + Sex + (1 | Subject) + storage_time
y ~ current_feature + Sex + (1 | Subject) + storage_time
y ~ current_feature + Sex + (1 | Subject) + storage_time
y ~ current_feature + Sex + (1 | Subject) + storage_time
y ~ current_feature + Sex + (1 | Subject) + storage_time
y ~ current_feature + Sex + (1 | Subject) + storage_time
y ~ current_feature + Sex + (1 | Subject) + storage_time
y ~ current_feature + Sex + (1 | Subject) + storage_time
y ~ current_feature + Sex + (1 | Subject) + storage_time
y ~ current_feature + Sex + (1 | Subject) + storage_time
y ~ current_feature + Sex + (1 | Subject) + storage_time
y ~ current_feature + Sex + (1 | Subject) + storage_time
y ~ current_feature + Sex + (1 | Subject) + storage_time
y ~ current_feature + Sex + (1 | Subject) + storage_time
y ~ current_feature + Sex + (1 | Subject) + storage_time
y ~ current_feature + Sex + (1 | Subject) + storage_time
y ~ current_feature + Sex + (1 | Subject) + storage_time
y ~ current_feature + Sex + (1 | Subject) + storage_time
y ~ current_feature + Sex + (1 | Subject) + storage_time
y ~ current_feature + Sex + (1 | Subject) + storage_time
y ~ current_feature + Sex + (1 | Subject) + storage_time
y ~ current_feature + Sex + (1 | Subject) + storage_time
y ~ current_feature + Sex + (1 | Subject) + storage_time
y ~ current_feature + Sex + (1 | Subject) + storage_time
y ~ current_feature + Sex + (1 | Subject) + storage_time
y ~ current_feature + Sex + (1 | Subject) + storage_time
y ~ current_feature + Sex + (1 | Subject) + storage_time
y ~ current_feature + Sex + (1 | Subject) + storage_time
y ~ current_feature + Sex + (1 | Subject) + storage_time
y ~ current_feature + Sex + (1 | Subject) + storage_time
y ~ current_feature + Sex + (1 | Subject) + storage_time
y ~ current_feature + Sex + (1 | Subject) + storage_time
y ~ current_feature + Sex + (1 | Subject) + storage_time
y ~ current_feature + Sex + (1 | Subject) + storage_time
y ~ current_feature + Sex + (1 | Subject) + storage_time
y ~ current_feature + Sex + (1 | Subject) + storage_time
y ~ current_feature + Sex + (1 | Subject) + storage_time
y ~ current_feature + Sex + (1 | Subject) + storage_time
y ~ current_feature + Sex + (1 | Subject) + storage_time
y ~ current_feature + Sex + (1 | Subject) + storage_time
y ~ current_feature + Sex + (1 | Subject) + storage_time

y ~ current_feature + Sex + (1 | Subject) + storage_time
y ~ current_feature + Sex + (1 | Subject) + storage_time
y ~ current_feature + Sex + (1 | Subject) + storage_time
y ~ current_feature + Sex + (1 | Subject) + storage_time
y ~ current_feature + Sex + (1 | Subject) + storage_time
y ~ current_feature + Sex + (1 | Subject) + storage_time
y ~ current_feature + Sex + (1 | Subject) + storage_time
y ~ current_feature + Sex + (1 | Subject) + storage_time
y ~ current_feature + Sex + (1 | Subject) + storage_time
y ~ current_feature + Sex + (1 | Subject) + storage_time
y ~ current_feature + Sex + (1 | Subject) + storage_time
y ~ current_feature + Sex + (1 | Subject) + storage_time
y ~ current_feature + Sex + (1 | Subject) + storage_time
y ~ current_feature + Sex + (1 | Subject) + storage_time
y ~ current_feature + Sex + (1 | Subject) + storage_time
y ~ current_feature + Sex + (1 | Subject) + storage_time
y ~ current_feature + Sex + (1 | Subject) + storage_time
y ~ current_feature + Sex + (1 | Subject) + storage_time
y ~ current_feature + Sex + (1 | Subject) + storage_time
y ~ current_feature + Sex + (1 | Subject) + storage_time
y ~ current_feature + Sex + (1 | Subject) + storage_time
y ~ current_feature + Sex + (1 | Subject) + storage_time
y ~ current_feature + Sex + (1 | Subject) + storage_time
y ~ current_feature + Sex + (1 | Subject) + storage_time
y ~ current_feature + Sex + (1 | Subject) + storage_time
y ~ current_feature + Sex + (1 | Subject) + storage_time
y ~ current_feature + Sex + (1 | Subject) + storage_time
y ~ current_feature + Sex + (1 | Subject) + storage_time
y ~ current_feature + Sex + (1 | Subject) + storage_time
y ~ current_feature + Sex + (1 | Subject) + storage_time
y ~ current_feature + Sex + (1 | Subject) + storage_time
y ~ current_feature + Sex + (1 | Subject) + storage_time
y ~ current_feature + Sex + (1 | Subject) + storage_time
y ~ current_feature + Sex + (1 | Subject) + storage_time
y ~ current_feature + Sex + (1 | Subject) + storage_time
y ~ current_feature + Sex + (1 | Subject) + storage_time
y ~ current_feature + Sex + (1 | Subject) + storage_time
y ~ current_feature + Sex + (1 | Subject) + storage_time
y ~ current_feature + Sex + (1 | Subject) + storage_time
y ~ current_feature + Sex + (1 | Subject) + storage_time
y ~ current_feature + Sex + (1 | Subject) + storage_time
y ~ current_feature + Sex + (1 | Subject) + storage_time
y ~ current_feature + Sex + (1 | Subject) + storage_time
y ~ current_feature + Sex + (1 | Subject) + storage_time
y ~ current_feature + Sex + (1 | Subject) + storage_time
y ~ current_feature + Sex + (1 | Subject) + storage_time
y ~ current_feature + Sex + (1 | Subject) + storage_time
y ~ current_feature + Sex + (1 | Subject) + storage_time

y ~ current_feature + Sex + (1 | Subject) + storage_time
y ~ current_feature + Sex + (1 | Subject) + storage_time
y ~ current_feature + Sex + (1 | Subject) + storage_time
y ~ current_feature + Sex + (1 | Subject) + storage_time
y ~ current_feature + Sex + (1 | Subject) + storage_time
y ~ current_feature + Sex + (1 | Subject) + storage_time
y ~ current_feature + Sex + (1 | Subject) + storage_time
y ~ current_feature + Sex + (1 | Subject) + storage_time
y ~ current_feature + Sex + (1 | Subject) + storage_time
y ~ current_feature + Sex + (1 | Subject) + storage_time
y ~ current_feature + Sex + (1 | Subject) + storage_time
y ~ current_feature + Sex + (1 | Subject) + storage_time
y ~ current_feature + Sex + (1 | Subject) + storage_time
y ~ current_feature + Sex + (1 | Subject) + storage_time
y ~ current_feature + Sex + (1 | Subject) + storage_time
y ~ current_feature + Sex + (1 | Subject) + storage_time
y ~ current_feature + Sex + (1 | Subject) + storage_time
y ~ current_feature + Sex + (1 | Subject) + storage_time
y ~ current_feature + Sex + (1 | Subject) + storage_time
y ~ current_feature + Sex + (1 | Subject) + storage_time
y ~ current_feature + Sex + (1 | Subject) + storage_time
y ~ current_feature + Sex + (1 | Subject) + storage_time
y ~ current_feature + Sex + (1 | Subject) + storage_time
y ~ current_feature + Sex + (1 | Subject) + storage_time
y ~ current_feature + Sex + (1 | Subject) + storage_time
y ~ current_feature + Sex + (1 | Subject) + storage_time
y ~ current_feature + Sex + (1 | Subject) + storage_time
y ~ current_feature + Sex + (1 | Subject) + storage_time
y ~ current_feature + Sex + (1 | Subject) + storage_time
y ~ current_feature + Sex + (1 | Subject) + storage_time
y ~ current_feature + Sex + (1 | Subject) + storage_time
y ~ current_feature + Sex + (1 | Subject) + storage_time
y ~ current_feature + Sex + (1 | Subject) + storage_time
y ~ current_feature + Sex + (1 | Subject) + storage_time
y ~ current_feature + Sex + (1 | Subject) + storage_time
y ~ current_feature + Sex + (1 | Subject) + storage_time
y ~ current_feature + Sex + (1 | Subject) + storage_time
y ~ current_feature + Sex + (1 | Subject) + storage_time
y ~ current_feature + Sex + (1 | Subject) + storage_time
y ~ current_feature + Sex + (1 | Subject) + storage_time
y ~ current_feature + Sex + (1 | Subject) + storage_time
y ~ current_feature + Sex + (1 | Subject) + storage_time
y ~ current_feature + Sex + (1 | Subject) + storage_time
y ~ current_feature + Sex + (1 | Subject) + storage_time
y ~ current_feature + Sex + (1 | Subject) + storage_time
y ~ current_feature + Sex + (1 | Subject) + storage_time
y ~ current_feature + Sex + (1 | Subject) + storage_time
y ~ current_feature + Sex + (1 | Subject) + storage_time

y ~ current_feature + Sex + (1 | Subject) + storage_time
y ~ current_feature + Sex + (1 | Subject) + storage_time
y ~ current_feature + Sex + (1 | Subject) + storage_time
y ~ current_feature + Sex + (1 | Subject) + storage_time
y ~ current_feature + Sex + (1 | Subject) + storage_time
y ~ current_feature + Sex + (1 | Subject) + storage_time
y ~ current_feature + Sex + (1 | Subject) + storage_time
y ~ current_feature + Sex + (1 | Subject) + storage_time
y ~ current_feature + Sex + (1 | Subject) + storage_time
y ~ current_feature + Sex + (1 | Subject) + storage_time
y ~ current_feature + Sex + (1 | Subject) + storage_time
y ~ current_feature + Sex + (1 | Subject) + storage_time
y ~ current_feature + Sex + (1 | Subject) + storage_time
y ~ current_feature + Sex + (1 | Subject) + storage_time
y ~ current_feature + Sex + (1 | Subject) + storage_time
y ~ current_feature + Sex + (1 | Subject) + storage_time
y ~ current_feature + Sex + (1 | Subject) + storage_time
y ~ current_feature + Sex + (1 | Subject) + storage_time
y ~ current_feature + Sex + (1 | Subject) + storage_time
y ~ current_feature + Sex + (1 | Subject) + storage_time
y ~ current_feature + Sex + (1 | Subject) + storage_time
y ~ current_feature + Sex + (1 | Subject) + storage_time
y ~ current_feature + Sex + (1 | Subject) + storage_time
y ~ current_feature + Sex + (1 | Subject) + storage_time
y ~ current_feature + Sex + (1 | Subject) + storage_time
y ~ current_feature + Sex + (1 | Subject) + storage_time
y ~ current_feature + Sex + (1 | Subject) + storage_time
y ~ current_feature + Sex + (1 | Subject) + storage_time
y ~ current_feature + Sex + (1 | Subject) + storage_time
y ~ current_feature + Sex + (1 | Subject) + storage_time
y ~ current_feature + Sex + (1 | Subject) + storage_time
y ~ current_feature + Sex + (1 | Subject) + storage_time
y ~ current_feature + Sex + (1 | Subject) + storage_time
y ~ current_feature + Sex + (1 | Subject) + storage_time
y ~ current_feature + Sex + (1 | Subject) + storage_time
y ~ current_feature + Sex + (1 | Subject) + storage_time
y ~ current_feature + Sex + (1 | Subject) + storage_time
y ~ current_feature + Sex + (1 | Subject) + storage_time
y ~ current_feature + Sex + (1 | Subject) + storage_time
y ~ current_feature + Sex + (1 | Subject) + storage_time
y ~ current_feature + Sex + (1 | Subject) + storage_time
y ~ current_feature + Sex + (1 | Subject) + storage_time
y ~ current_feature + Sex + (1 | Subject) + storage_time
y ~ current_feature + Sex + (1 | Subject) + storage_time
y ~ current_feature + Sex + (1 | Subject) + storage_time
y ~ current_feature + Sex + (1 | Subject) + storage_time
y ~ current_feature + Sex + (1 | Subject) + storage_time
y ~ current_feature + Sex + (1 | Subject) + storage_time

y ~ current_feature + Sex + (1 | Subject) + storage_time
y ~ current_feature + Sex + (1 | Subject) + storage_time
y ~ current_feature + Sex + (1 | Subject) + storage_time
y ~ current_feature + Sex + (1 | Subject) + storage_time
y ~ current_feature + Sex + (1 | Subject) + storage_time
y ~ current_feature + Sex + (1 | Subject) + storage_time
y ~ current_feature + Sex + (1 | Subject) + storage_time
y ~ current_feature + Sex + (1 | Subject) + storage_time
y ~ current_feature + Sex + (1 | Subject) + storage_time
y ~ current_feature + Sex + (1 | Subject) + storage_time
y ~ current_feature + Sex + (1 | Subject) + storage_time
y ~ current_feature + Sex + (1 | Subject) + storage_time
y ~ current_feature + Sex + (1 | Subject) + storage_time
y ~ current_feature + Sex + (1 | Subject) + storage_time
y ~ current_feature + Sex + (1 | Subject) + storage_time
y ~ current_feature + Sex + (1 | Subject) + storage_time
y ~ current_feature + Sex + (1 | Subject) + storage_time
y ~ current_feature + Sex + (1 | Subject) + storage_time
y ~ current_feature + Sex + (1 | Subject) + storage_time
y ~ current_feature + Sex + (1 | Subject) + storage_time
y ~ current_feature + Sex + (1 | Subject) + storage_time
y ~ current_feature + Sex + (1 | Subject) + storage_time
y ~ current_feature + Sex + (1 | Subject) + storage_time
y ~ current_feature + Sex + (1 | Subject) + storage_time
y ~ current_feature + Sex + (1 | Subject) + storage_time
y ~ current_feature + Sex + (1 | Subject) + storage_time
y ~ current_feature + Sex + (1 | Subject) + storage_time
y ~ current_feature + Sex + (1 | Subject) + storage_time
y ~ current_feature + Sex + (1 | Subject) + storage_time
y ~ current_feature + Sex + (1 | Subject) + storage_time
y ~ current_feature + Sex + (1 | Subject) + storage_time
y ~ current_feature + Sex + (1 | Subject) + storage_time
y ~ current_feature + Sex + (1 | Subject) + storage_time
y ~ current_feature + Sex + (1 | Subject) + storage_time
y ~ current_feature + Sex + (1 | Subject) + storage_time
y ~ current_feature + Sex + (1 | Subject) + storage_time
y ~ current_feature + Sex + (1 | Subject) + storage_time
y ~ current_feature + Sex + (1 | Subject) + storage_time
y ~ current_feature + Sex + (1 | Subject) + storage_time
y ~ current_feature + Sex + (1 | Subject) + storage_time
y ~ current_feature + Sex + (1 | Subject) + storage_time
y ~ current_feature + Sex + (1 | Subject) + storage_time
y ~ current_feature + Sex + (1 | Subject) + storage_time
y ~ current_feature + Sex + (1 | Subject) + storage_time
y ~ current_feature + Sex + (1 | Subject) + storage_time
y ~ current_feature + Sex + (1 | Subject) + storage_time
y ~ current_feature + Sex + (1 | Subject) + storage_time
y ~ current_feature + Sex + (1 | Subject) + storage_time

y ~ current_feature + Sex + (1 | Subject) + storage_time
y ~ current_feature + Sex + (1 | Subject) + storage_time
y ~ current_feature + Sex + (1 | Subject) + storage_time
y ~ current_feature + Sex + (1 | Subject) + storage_time
y ~ current_feature + Sex + (1 | Subject) + storage_time
y ~ current_feature + Sex + (1 | Subject) + storage_time
y ~ current_feature + Sex + (1 | Subject) + storage_time
y ~ current_feature + Sex + (1 | Subject) + storage_time
y ~ current_feature + Sex + (1 | Subject) + storage_time
y ~ current_feature + Sex + (1 | Subject) + storage_time
y ~ current_feature + Sex + (1 | Subject) + storage_time
y ~ current_feature + Sex + (1 | Subject) + storage_time
y ~ current_feature + Sex + (1 | Subject) + storage_time
y ~ current_feature + Sex + (1 | Subject) + storage_time
y ~ current_feature + Sex + (1 | Subject) + storage_time
y ~ current_feature + Sex + (1 | Subject) + storage_time
y ~ current_feature + Sex + (1 | Subject) + storage_time
y ~ current_feature + Sex + (1 | Subject) + storage_time
y ~ current_feature + Sex + (1 | Subject) + storage_time
y ~ current_feature + Sex + (1 | Subject) + storage_time
y ~ current_feature + Sex + (1 | Subject) + storage_time
y ~ current_feature + Sex + (1 | Subject) + storage_time
y ~ current_feature + Sex + (1 | Subject) + storage_time
y ~ current_feature + Sex + (1 | Subject) + storage_time
y ~ current_feature + Sex + (1 | Subject) + storage_time
y ~ current_feature + Sex + (1 | Subject) + storage_time
y ~ current_feature + Sex + (1 | Subject) + storage_time
y ~ current_feature + Sex + (1 | Subject) + storage_time
y ~ current_feature + Sex + (1 | Subject) + storage_time
y ~ current_feature + Sex + (1 | Subject) + storage_time
y ~ current_feature + Sex + (1 | Subject) + storage_time
y ~ current_feature + Sex + (1 | Subject) + storage_time
y ~ current_feature + Sex + (1 | Subject) + storage_time
y ~ current_feature + Sex + (1 | Subject) + storage_time
y ~ current_feature + Sex + (1 | Subject) + storage_time
y ~ current_feature + Sex + (1 | Subject) + storage_time
y ~ current_feature + Sex + (1 | Subject) + storage_time
y ~ current_feature + Sex + (1 | Subject) + storage_time
y ~ current_feature + Sex + (1 | Subject) + storage_time
y ~ current_feature + Sex + (1 | Subject) + storage_time
y ~ current_feature + Sex + (1 | Subject) + storage_time
y ~ current_feature + Sex + (1 | Subject) + storage_time
y ~ current_feature + Sex + (1 | Subject) + storage_time
y ~ current_feature + Sex + (1 | Subject) + storage_time
y ~ current_feature + Sex + (1 | Subject) + storage_time
y ~ current_feature + Sex + (1 | Subject) + storage_time
y ~ current_feature + Sex + (1 | Subject) + storage_time
y ~ current_feature + Sex + (1 | Subject) + storage_time

y ~ current_feature + Sex + (1 | Subject) + storage_time
y ~ current_feature + Sex + (1 | Subject) + storage_time
y ~ current_feature + Sex + (1 | Subject) + storage_time
y ~ current_feature + Sex + (1 | Subject) + storage_time
y ~ current_feature + Sex + (1 | Subject) + storage_time
y ~ current_feature + Sex + (1 | Subject) + storage_time
y ~ current_feature + Sex + (1 | Subject) + storage_time
y ~ current_feature + Sex + (1 | Subject) + storage_time
y ~ current_feature + Sex + (1 | Subject) + storage_time
y ~ current_feature + Sex + (1 | Subject) + storage_time
y ~ current_feature + Sex + (1 | Subject) + storage_time
y ~ current_feature + Sex + (1 | Subject) + storage_time
y ~ current_feature + Sex + (1 | Subject) + storage_time
y ~ current_feature + Sex + (1 | Subject) + storage_time
y ~ current_feature + Sex + (1 | Subject) + storage_time
y ~ current_feature + Sex + (1 | Subject) + storage_time
y ~ current_feature + Sex + (1 | Subject) + storage_time
y ~ current_feature + Sex + (1 | Subject) + storage_time
y ~ current_feature + Sex + (1 | Subject) + storage_time
y ~ current_feature + Sex + (1 | Subject) + storage_time
y ~ current_feature + Sex + (1 | Subject) + storage_time
y ~ current_feature + Sex + (1 | Subject) + storage_time
y ~ current_feature + Sex + (1 | Subject) + storage_time
y ~ current_feature + Sex + (1 | Subject) + storage_time
y ~ current_feature + Sex + (1 | Subject) + storage_time
y ~ current_feature + Sex + (1 | Subject) + storage_time
y ~ current_feature + Sex + (1 | Subject) + storage_time
y ~ current_feature + Sex + (1 | Subject) + storage_time
y ~ current_feature + Sex + (1 | Subject) + storage_time
y ~ current_feature + Sex + (1 | Subject) + storage_time
y ~ current_feature + Sex + (1 | Subject) + storage_time
y ~ current_feature + Sex + (1 | Subject) + storage_time
y ~ current_feature + Sex + (1 | Subject) + storage_time
y ~ current_feature + Sex + (1 | Subject) + storage_time
y ~ current_feature + Sex + (1 | Subject) + storage_time
y ~ current_feature + Sex + (1 | Subject) + storage_time
y ~ current_feature + Sex + (1 | Subject) + storage_time
y ~ current_feature + Sex + (1 | Subject) + storage_time
y ~ current_feature + Sex + (1 | Subject) + storage_time
y ~ current_feature + Sex + (1 | Subject) + storage_time
y ~ current_feature + Sex + (1 | Subject) + storage_time
y ~ current_feature + Sex + (1 | Subject) + storage_time
y ~ current_feature + Sex + (1 | Subject) + storage_time
y ~ current_feature + Sex + (1 | Subject) + storage_time
y ~ current_feature + Sex + (1 | Subject) + storage_time
y ~ current_feature + Sex + (1 | Subject) + storage_time
y ~ current_feature + Sex + (1 | Subject) + storage_time
y ~ current_feature + Sex + (1 | Subject) + storage_time

y ~ current_feature + Sex + (1 | Subject) + storage_time
y ~ current_feature + Sex + (1 | Subject) + storage_time
y ~ current_feature + Sex + (1 | Subject) + storage_time
y ~ current_feature + Sex + (1 | Subject) + storage_time
y ~ current_feature + Sex + (1 | Subject) + storage_time
y ~ current_feature + Sex + (1 | Subject) + storage_time
y ~ current_feature + Sex + (1 | Subject) + storage_time
y ~ current_feature + Sex + (1 | Subject) + storage_time
y ~ current_feature + Sex + (1 | Subject) + storage_time
y ~ current_feature + Sex + (1 | Subject) + storage_time
y ~ current_feature + Sex + (1 | Subject) + storage_time
y ~ current_feature + Sex + (1 | Subject) + storage_time
y ~ current_feature + Sex + (1 | Subject) + storage_time
y ~ current_feature + Sex + (1 | Subject) + storage_time
y ~ current_feature + Sex + (1 | Subject) + storage_time
y ~ current_feature + Sex + (1 | Subject) + storage_time
y ~ current_feature + Sex + (1 | Subject) + storage_time
y ~ current_feature + Sex + (1 | Subject) + storage_time
y ~ current_feature + Sex + (1 | Subject) + storage_time
y ~ current_feature + Sex + (1 | Subject) + storage_time
y ~ current_feature + Sex + (1 | Subject) + storage_time
y ~ current_feature + Sex + (1 | Subject) + storage_time
y ~ current_feature + Sex + (1 | Subject) + storage_time
y ~ current_feature + Sex + (1 | Subject) + storage_time
y ~ current_feature + Sex + (1 | Subject) + storage_time
y ~ current_feature + Sex + (1 | Subject) + storage_time
y ~ current_feature + Sex + (1 | Subject) + storage_time
y ~ current_feature + Sex + (1 | Subject) + storage_time
y ~ current_feature + Sex + (1 | Subject) + storage_time
y ~ current_feature + Sex + (1 | Subject) + storage_time
y ~ current_feature + Sex + (1 | Subject) + storage_time
y ~ current_feature + Sex + (1 | Subject) + storage_time
y ~ current_feature + Sex + (1 | Subject) + storage_time
y ~ current_feature + Sex + (1 | Subject) + storage_time
y ~ current_feature + Sex + (1 | Subject) + storage_time
y ~ current_feature + Sex + (1 | Subject) + storage_time
y ~ current_feature + Sex + (1 | Subject) + storage_time
y ~ current_feature + Sex + (1 | Subject) + storage_time
y ~ current_feature + Sex + (1 | Subject) + storage_time
y ~ current_feature + Sex + (1 | Subject) + storage_time
y ~ current_feature + Sex + (1 | Subject) + storage_time
y ~ current_feature + Sex + (1 | Subject) + storage_time
y ~ current_feature + Sex + (1 | Subject) + storage_time
y ~ current_feature + Sex + (1 | Subject) + storage_time
y ~ current_feature + Sex + (1 | Subject) + storage_time
y ~ current_feature + Sex + (1 | Subject) + storage_time
y ~ current_feature + Sex + (1 | Subject) + storage_time
y ~ current_feature + Sex + (1 | Subject) + storage_time

y ~ current_feature + Sex + (1 | Subject) + storage_time
y ~ current_feature + Sex + (1 | Subject) + storage_time
y ~ current_feature + Sex + (1 | Subject) + storage_time
y ~ current_feature + Sex + (1 | Subject) + storage_time
y ~ current_feature + Sex + (1 | Subject) + storage_time
y ~ current_feature + Sex + (1 | Subject) + storage_time
y ~ current_feature + Sex + (1 | Subject) + storage_time
y ~ current_feature + Sex + (1 | Subject) + storage_time
y ~ current_feature + Sex + (1 | Subject) + storage_time
y ~ current_feature + Sex + (1 | Subject) + storage_time
y ~ current_feature + Sex + (1 | Subject) + storage_time
y ~ current_feature + Sex + (1 | Subject) + storage_time
y ~ current_feature + Sex + (1 | Subject) + storage_time
y ~ current_feature + Sex + (1 | Subject) + storage_time
y ~ current_feature + Sex + (1 | Subject) + storage_time
y ~ current_feature + Sex + (1 | Subject) + storage_time
y ~ current_feature + Sex + (1 | Subject) + storage_time
y ~ current_feature + Sex + (1 | Subject) + storage_time
y ~ current_feature + Sex + (1 | Subject) + storage_time
y ~ current_feature + Sex + (1 | Subject) + storage_time
y ~ current_feature + Sex + (1 | Subject) + storage_time
y ~ current_feature + Sex + (1 | Subject) + storage_time
y ~ current_feature + Sex + (1 | Subject) + storage_time
y ~ current_feature + Sex + (1 | Subject) + storage_time
y ~ current_feature + Sex + (1 | Subject) + storage_time
y ~ current_feature + Sex + (1 | Subject) + storage_time
y ~ current_feature + Sex + (1 | Subject) + storage_time
y ~ current_feature + Sex + (1 | Subject) + storage_time
y ~ current_feature + Sex + (1 | Subject) + storage_time
y ~ current_feature + Sex + (1 | Subject) + storage_time
y ~ current_feature + Sex + (1 | Subject) + storage_time
y ~ current_feature + Sex + (1 | Subject) + storage_time
y ~ current_feature + Sex + (1 | Subject) + storage_time
y ~ current_feature + Sex + (1 | Subject) + storage_time
y ~ current_feature + Sex + (1 | Subject) + storage_time
y ~ current_feature + Sex + (1 | Subject) + storage_time
y ~ current_feature + Sex + (1 | Subject) + storage_time
y ~ current_feature + Sex + (1 | Subject) + storage_time
y ~ current_feature + Sex + (1 | Subject) + storage_time
y ~ current_feature + Sex + (1 | Subject) + storage_time
y ~ current_feature + Sex + (1 | Subject) + storage_time
y ~ current_feature + Sex + (1 | Subject) + storage_time
y ~ current_feature + Sex + (1 | Subject) + storage_time
y ~ current_feature + Sex + (1 | Subject) + storage_time
y ~ current_feature + Sex + (1 | Subject) + storage_time
y ~ current_feature + Sex + (1 | Subject) + storage_time
y ~ current_feature + Sex + (1 | Subject) + storage_time
y ~ current_feature + Sex + (1 | Subject) + storage_time

y ~ current_feature + Sex + (1 | Subject) + storage_time
y ~ current_feature + Sex + (1 | Subject) + storage_time
y ~ current_feature + Sex + (1 | Subject) + storage_time
y ~ current_feature + Sex + (1 | Subject) + storage_time
y ~ current_feature + Sex + (1 | Subject) + storage_time
y ~ current_feature + Sex + (1 | Subject) + storage_time
y ~ current_feature + Sex + (1 | Subject) + storage_time
y ~ current_feature + Sex + (1 | Subject) + storage_time
y ~ current_feature + Sex + (1 | Subject) + storage_time
y ~ current_feature + Sex + (1 | Subject) + storage_time
y ~ current_feature + Sex + (1 | Subject) + storage_time
y ~ current_feature + Sex + (1 | Subject) + storage_time
y ~ current_feature + Sex + (1 | Subject) + storage_time
y ~ current_feature + Sex + (1 | Subject) + storage_time
y ~ current_feature + Sex + (1 | Subject) + storage_time
y ~ current_feature + Sex + (1 | Subject) + storage_time
y ~ current_feature + Sex + (1 | Subject) + storage_time
y ~ current_feature + Sex + (1 | Subject) + storage_time
y ~ current_feature + Sex + (1 | Subject) + storage_time
y ~ current_feature + Sex + (1 | Subject) + storage_time
y ~ current_feature + Sex + (1 | Subject) + storage_time
y ~ current_feature + Sex + (1 | Subject) + storage_time
y ~ current_feature + Sex + (1 | Subject) + storage_time
y ~ current_feature + Sex + (1 | Subject) + storage_time
y ~ current_feature + Sex + (1 | Subject) + storage_time
y ~ current_feature + Sex + (1 | Subject) + storage_time
y ~ current_feature + Sex + (1 | Subject) + storage_time
y ~ current_feature + Sex + (1 | Subject) + storage_time
y ~ current_feature + Sex + (1 | Subject) + storage_time
y ~ current_feature + Sex + (1 | Subject) + storage_time
y ~ current_feature + Sex + (1 | Subject) + storage_time
y ~ current_feature + Sex + (1 | Subject) + storage_time
y ~ current_feature + Sex + (1 | Subject) + storage_time
y ~ current_feature + Sex + (1 | Subject) + storage_time
y ~ current_feature + Sex + (1 | Subject) + storage_time
y ~ current_feature + Sex + (1 | Subject) + storage_time
y ~ current_feature + Sex + (1 | Subject) + storage_time
y ~ current_feature + Sex + (1 | Subject) + storage_time
y ~ current_feature + Sex + (1 | Subject) + storage_time
y ~ current_feature + Sex + (1 | Subject) + storage_time
y ~ current_feature + Sex + (1 | Subject) + storage_time
y ~ current_feature + Sex + (1 | Subject) + storage_time
y ~ current_feature + Sex + (1 | Subject) + storage_time
y ~ current_feature + Sex + (1 | Subject) + storage_time
y ~ current_feature + Sex + (1 | Subject) + storage_time
y ~ current_feature + Sex + (1 | Subject) + storage_time
y ~ current_feature + Sex + (1 | Subject) + storage_time
y ~ current_feature + Sex + (1 | Subject) + storage_time

y ~ current_feature + Sex + (1 | Subject) + storage_time
y ~ current_feature + Sex + (1 | Subject) + storage_time
y ~ current_feature + Sex + (1 | Subject) + storage_time
y ~ current_feature + Sex + (1 | Subject) + storage_time
y ~ current_feature + Sex + (1 | Subject) + storage_time
y ~ current_feature + Sex + (1 | Subject) + storage_time
y ~ current_feature + Sex + (1 | Subject) + storage_time
y ~ current_feature + Sex + (1 | Subject) + storage_time
y ~ current_feature + Sex + (1 | Subject) + storage_time
y ~ current_feature + Sex + (1 | Subject) + storage_time
y ~ current_feature + Sex + (1 | Subject) + storage_time
y ~ current_feature + Sex + (1 | Subject) + storage_time
y ~ current_feature + Sex + (1 | Subject) + storage_time
y ~ current_feature + Sex + (1 | Subject) + storage_time
y ~ current_feature + Sex + (1 | Subject) + storage_time
y ~ current_feature + Sex + (1 | Subject) + storage_time
y ~ current_feature + Sex + (1 | Subject) + storage_time
y ~ current_feature + Sex + (1 | Subject) + storage_time
y ~ current_feature + Sex + (1 | Subject) + storage_time
y ~ current_feature + Sex + (1 | Subject) + storage_time
y ~ current_feature + Sex + (1 | Subject) + storage_time
y ~ current_feature + Sex + (1 | Subject) + storage_time
y ~ current_feature + Sex + (1 | Subject) + storage_time
y ~ current_feature + Sex + (1 | Subject) + storage_time
y ~ current_feature + Sex + (1 | Subject) + storage_time
y ~ current_feature + Sex + (1 | Subject) + storage_time
y ~ current_feature + Sex + (1 | Subject) + storage_time
y ~ current_feature + Sex + (1 | Subject) + storage_time
y ~ current_feature + Sex + (1 | Subject) + storage_time
y ~ current_feature + Sex + (1 | Subject) + storage_time
y ~ current_feature + Sex + (1 | Subject) + storage_time
y ~ current_feature + Sex + (1 | Subject) + storage_time
y ~ current_feature + Sex + (1 | Subject) + storage_time
y ~ current_feature + Sex + (1 | Subject) + storage_time
y ~ current_feature + Sex + (1 | Subject) + storage_time
y ~ current_feature + Sex + (1 | Subject) + storage_time
y ~ current_feature + Sex + (1 | Subject) + storage_time
y ~ current_feature + Sex + (1 | Subject) + storage_time
y ~ current_feature + Sex + (1 | Subject) + storage_time
y ~ current_feature + Sex + (1 | Subject) + storage_time
y ~ current_feature + Sex + (1 | Subject) + storage_time
y ~ current_feature + Sex + (1 | Subject) + storage_time
y ~ current_feature + Sex + (1 | Subject) + storage_time
y ~ current_feature + Sex + (1 | Subject) + storage_time
y ~ current_feature + Sex + (1 | Subject) + storage_time
y ~ current_feature + Sex + (1 | Subject) + storage_time
y ~ current_feature + Sex + (1 | Subject) + storage_time
y ~ current_feature + Sex + (1 | Subject) + storage_time

y ~ current_feature + Sex + (1 | Subject) + storage_time
y ~ current_feature + Sex + (1 | Subject) + storage_time
y ~ current_feature + Sex + (1 | Subject) + storage_time
y ~ current_feature + Sex + (1 | Subject) + storage_time
y ~ current_feature + Sex + (1 | Subject) + storage_time
y ~ current_feature + Sex + (1 | Subject) + storage_time
y ~ current_feature + Sex + (1 | Subject) + storage_time
y ~ current_feature + Sex + (1 | Subject) + storage_time
y ~ current_feature + Sex + (1 | Subject) + storage_time
y ~ current_feature + Sex + (1 | Subject) + storage_time
y ~ current_feature + Sex + (1 | Subject) + storage_time
y ~ current_feature + Sex + (1 | Subject) + storage_time
y ~ current_feature + Sex + (1 | Subject) + storage_time
y ~ current_feature + Sex + (1 | Subject) + storage_time
y ~ current_feature + Sex + (1 | Subject) + storage_time
y ~ current_feature + Sex + (1 | Subject) + storage_time
y ~ current_feature + Sex + (1 | Subject) + storage_time
y ~ current_feature + Sex + (1 | Subject) + storage_time
y ~ current_feature + Sex + (1 | Subject) + storage_time
y ~ current_feature + Sex + (1 | Subject) + storage_time
y ~ current_feature + Sex + (1 | Subject) + storage_time
y ~ current_feature + Sex + (1 | Subject) + storage_time
y ~ current_feature + Sex + (1 | Subject) + storage_time
y ~ current_feature + Sex + (1 | Subject) + storage_time
y ~ current_feature + Sex + (1 | Subject) + storage_time
y ~ current_feature + Sex + (1 | Subject) + storage_time
y ~ current_feature + Sex + (1 | Subject) + storage_time
y ~ current_feature + Sex + (1 | Subject) + storage_time
y ~ current_feature + Sex + (1 | Subject) + storage_time
y ~ current_feature + Sex + (1 | Subject) + storage_time
y ~ current_feature + Sex + (1 | Subject) + storage_time
y ~ current_feature + Sex + (1 | Subject) + storage_time
y ~ current_feature + Sex + (1 | Subject) + storage_time
y ~ current_feature + Sex + (1 | Subject) + storage_time
y ~ current_feature + Sex + (1 | Subject) + storage_time
y ~ current_feature + Sex + (1 | Subject) + storage_time
y ~ current_feature + Sex + (1 | Subject) + storage_time
y ~ current_feature + Sex + (1 | Subject) + storage_time
y ~ current_feature + Sex + (1 | Subject) + storage_time
y ~ current_feature + Sex + (1 | Subject) + storage_time
y ~ current_feature + Sex + (1 | Subject) + storage_time
y ~ current_feature + Sex + (1 | Subject) + storage_time
y ~ current_feature + Sex + (1 | Subject) + storage_time
y ~ current_feature + Sex + (1 | Subject) + storage_time
y ~ current_feature + Sex + (1 | Subject) + storage_time
y ~ current_feature + Sex + (1 | Subject) + storage_time
y ~ current_feature + Sex + (1 | Subject) + storage_time
y ~ current_feature + Sex + (1 | Subject) + storage_time

y ~ current_feature + Sex + (1 | Subject) + storage_time
y ~ current_feature + Sex + (1 | Subject) + storage_time
y ~ current_feature + Sex + (1 | Subject) + storage_time
y ~ current_feature + Sex + (1 | Subject) + storage_time
y ~ current_feature + Sex + (1 | Subject) + storage_time
y ~ current_feature + Sex + (1 | Subject) + storage_time
y ~ current_feature + Sex + (1 | Subject) + storage_time
y ~ current_feature + Sex + (1 | Subject) + storage_time
y ~ current_feature + Sex + (1 | Subject) + storage_time
y ~ current_feature + Sex + (1 | Subject) + storage_time
y ~ current_feature + Sex + (1 | Subject) + storage_time
y ~ current_feature + Sex + (1 | Subject) + storage_time
y ~ current_feature + Sex + (1 | Subject) + storage_time
y ~ current_feature + Sex + (1 | Subject) + storage_time
y ~ current_feature + Sex + (1 | Subject) + storage_time
y ~ current_feature + Sex + (1 | Subject) + storage_time
y ~ current_feature + Sex + (1 | Subject) + storage_time
y ~ current_feature + Sex + (1 | Subject) + storage_time
y ~ current_feature + Sex + (1 | Subject) + storage_time
y ~ current_feature + Sex + (1 | Subject) + storage_time
y ~ current_feature + Sex + (1 | Subject) + storage_time
y ~ current_feature + Sex + (1 | Subject) + storage_time
y ~ current_feature + Sex + (1 | Subject) + storage_time
y ~ current_feature + Sex + (1 | Subject) + storage_time
y ~ current_feature + Sex + (1 | Subject) + storage_time
y ~ current_feature + Sex + (1 | Subject) + storage_time
y ~ current_feature + Sex + (1 | Subject) + storage_time
y ~ current_feature + Sex + (1 | Subject) + storage_time
y ~ current_feature + Sex + (1 | Subject) + storage_time
y ~ current_feature + Sex + (1 | Subject) + storage_time
y ~ current_feature + Sex + (1 | Subject) + storage_time
y ~ current_feature + Sex + (1 | Subject) + storage_time
y ~ current_feature + Sex + (1 | Subject) + storage_time
y ~ current_feature + Sex + (1 | Subject) + storage_time
y ~ current_feature + Sex + (1 | Subject) + storage_time
y ~ current_feature + Sex + (1 | Subject) + storage_time
y ~ current_feature + Sex + (1 | Subject) + storage_time
y ~ current_feature + Sex + (1 | Subject) + storage_time
y ~ current_feature + Sex + (1 | Subject) + storage_time
y ~ current_feature + Sex + (1 | Subject) + storage_time
y ~ current_feature + Sex + (1 | Subject) + storage_time
y ~ current_feature + Sex + (1 | Subject) + storage_time
y ~ current_feature + Sex + (1 | Subject) + storage_time
y ~ current_feature + Sex + (1 | Subject) + storage_time
y ~ current_feature + Sex + (1 | Subject) + storage_time
y ~ current_feature + Sex + (1 | Subject) + storage_time
y ~ current_feature + Sex + (1 | Subject) + storage_time
y ~ current_feature + Sex + (1 | Subject) + storage_time

y ~ current_feature + Sex + (1 | Subject) + storage_time
y ~ current_feature + Sex + (1 | Subject) + storage_time
y ~ current_feature + Sex + (1 | Subject) + storage_time
y ~ current_feature + Sex + (1 | Subject) + storage_time
y ~ current_feature + Sex + (1 | Subject) + storage_time
y ~ current_feature + Sex + (1 | Subject) + storage_time
y ~ current_feature + Sex + (1 | Subject) + storage_time
y ~ current_feature + Sex + (1 | Subject) + storage_time
y ~ current_feature + Sex + (1 | Subject) + storage_time
y ~ current_feature + Sex + (1 | Subject) + storage_time
y ~ current_feature + Sex + (1 | Subject) + storage_time
y ~ current_feature + Sex + (1 | Subject) + storage_time
y ~ current_feature + Sex + (1 | Subject) + storage_time
y ~ current_feature + Sex + (1 | Subject) + storage_time
y ~ current_feature + Sex + (1 | Subject) + storage_time
y ~ current_feature + Sex + (1 | Subject) + storage_time
y ~ current_feature + Sex + (1 | Subject) + storage_time
y ~ current_feature + Sex + (1 | Subject) + storage_time
y ~ current_feature + Sex + (1 | Subject) + storage_time
y ~ current_feature + Sex + (1 | Subject) + storage_time
y ~ current_feature + Sex + (1 | Subject) + storage_time
y ~ current_feature + Sex + (1 | Subject) + storage_time
y ~ current_feature + Sex + (1 | Subject) + storage_time
y ~ current_feature + Sex + (1 | Subject) + storage_time
y ~ current_feature + Sex + (1 | Subject) + storage_time
y ~ current_feature + Sex + (1 | Subject) + storage_time
y ~ current_feature + Sex + (1 | Subject) + storage_time
y ~ current_feature + Sex + (1 | Subject) + storage_time
y ~ current_feature + Sex + (1 | Subject) + storage_time
y ~ current_feature + Sex + (1 | Subject) + storage_time
y ~ current_feature + Sex + (1 | Subject) + storage_time
y ~ current_feature + Sex + (1 | Subject) + storage_time
y ~ current_feature + Sex + (1 | Subject) + storage_time
y ~ current_feature + Sex + (1 | Subject) + storage_time
y ~ current_feature + Sex + (1 | Subject) + storage_time
y ~ current_feature + Sex + (1 | Subject) + storage_time
y ~ current_feature + Sex + (1 | Subject) + storage_time
y ~ current_feature + Sex + (1 | Subject) + storage_time
y ~ current_feature + Sex + (1 | Subject) + storage_time
y ~ current_feature + Sex + (1 | Subject) + storage_time
y ~ current_feature + Sex + (1 | Subject) + storage_time
y ~ current_feature + Sex + (1 | Subject) + storage_time
y ~ current_feature + Sex + (1 | Subject) + storage_time
y ~ current_feature + Sex + (1 | Subject) + storage_time
y ~ current_feature + Sex + (1 | Subject) + storage_time
y ~ current_feature + Sex + (1 | Subject) + storage_time
y ~ current_feature + Sex + (1 | Subject) + storage_time
y ~ current_feature + Sex + (1 | Subject) + storage_time

y ~ current_feature + Sex + (1 | Subject) + storage_time
y ~ current_feature + Sex + (1 | Subject) + storage_time
y ~ current_feature + Sex + (1 | Subject) + storage_time
y ~ current_feature + Sex + (1 | Subject) + storage_time
y ~ current_feature + Sex + (1 | Subject) + storage_time
y ~ current_feature + Sex + (1 | Subject) + storage_time
y ~ current_feature + Sex + (1 | Subject) + storage_time
y ~ current_feature + Sex + (1 | Subject) + storage_time
y ~ current_feature + Sex + (1 | Subject) + storage_time
y ~ current_feature + Sex + (1 | Subject) + storage_time
y ~ current_feature + Sex + (1 | Subject) + storage_time
y ~ current_feature + Sex + (1 | Subject) + storage_time
y ~ current_feature + Sex + (1 | Subject) + storage_time
y ~ current_feature + Sex + (1 | Subject) + storage_time
y ~ current_feature + Sex + (1 | Subject) + storage_time
y ~ current_feature + Sex + (1 | Subject) + storage_time
y ~ current_feature + Sex + (1 | Subject) + storage_time
y ~ current_feature + Sex + (1 | Subject) + storage_time
y ~ current_feature + Sex + (1 | Subject) + storage_time
y ~ current_feature + Sex + (1 | Subject) + storage_time
y ~ current_feature + Sex + (1 | Subject) + storage_time
y ~ current_feature + Sex + (1 | Subject) + storage_time
y ~ current_feature + Sex + (1 | Subject) + storage_time
y ~ current_feature + Sex + (1 | Subject) + storage_time
y ~ current_feature + Sex + (1 | Subject) + storage_time
y ~ current_feature + Sex + (1 | Subject) + storage_time
y ~ current_feature + Sex + (1 | Subject) + storage_time
y ~ current_feature + Sex + (1 | Subject) + storage_time
y ~ current_feature + Sex + (1 | Subject) + storage_time
y ~ current_feature + Sex + (1 | Subject) + storage_time
y ~ current_feature + Sex + (1 | Subject) + storage_time
y ~ current_feature + Sex + (1 | Subject) + storage_time
y ~ current_feature + Sex + (1 | Subject) + storage_time
y ~ current_feature + Sex + (1 | Subject) + storage_time
y ~ current_feature + Sex + (1 | Subject) + storage_time
y ~ current_feature + Sex + (1 | Subject) + storage_time
y ~ current_feature + Sex + (1 | Subject) + storage_time
y ~ current_feature + Sex + (1 | Subject) + storage_time
y ~ current_feature + Sex + (1 | Subject) + storage_time
y ~ current_feature + Sex + (1 | Subject) + storage_time
y ~ current_feature + Sex + (1 | Subject) + storage_time
y ~ current_feature + Sex + (1 | Subject) + storage_time
y ~ current_feature + Sex + (1 | Subject) + storage_time
y ~ current_feature + Sex + (1 | Subject) + storage_time
y ~ current_feature + Sex + (1 | Subject) + storage_time
y ~ current_feature + Sex + (1 | Subject) + storage_time
y ~ current_feature + Sex + (1 | Subject) + storage_time
y ~ current_feature + Sex + (1 | Subject) + storage_time

y ~ current_feature + Sex + (1 | Subject) + storage_time
y ~ current_feature + Sex + (1 | Subject) + storage_time
y ~ current_feature + Sex + (1 | Subject) + storage_time
y ~ current_feature + Sex + (1 | Subject) + storage_time
y ~ current_feature + Sex + (1 | Subject) + storage_time
y ~ current_feature + Sex + (1 | Subject) + storage_time
y ~ current_feature + Sex + (1 | Subject) + storage_time
y ~ current_feature + Sex + (1 | Subject) + storage_time
y ~ current_feature + Sex + (1 | Subject) + storage_time
y ~ current_feature + Sex + (1 | Subject) + storage_time
y ~ current_feature + Sex + (1 | Subject) + storage_time
y ~ current_feature + Sex + (1 | Subject) + storage_time
y ~ current_feature + Sex + (1 | Subject) + storage_time
y ~ current_feature + Sex + (1 | Subject) + storage_time
y ~ current_feature + Sex + (1 | Subject) + storage_time
y ~ current_feature + Sex + (1 | Subject) + storage_time
y ~ current_feature + Sex + (1 | Subject) + storage_time
y ~ current_feature + Sex + (1 | Subject) + storage_time
y ~ current_feature + Sex + (1 | Subject) + storage_time
y ~ current_feature + Sex + (1 | Subject) + storage_time
y ~ current_feature + Sex + (1 | Subject) + storage_time
y ~ current_feature + Sex + (1 | Subject) + storage_time
y ~ current_feature + Sex + (1 | Subject) + storage_time
y ~ current_feature + Sex + (1 | Subject) + storage_time
y ~ current_feature + Sex + (1 | Subject) + storage_time
y ~ current_feature + Sex + (1 | Subject) + storage_time
y ~ current_feature + Sex + (1 | Subject) + storage_time
y ~ current_feature + Sex + (1 | Subject) + storage_time
y ~ current_feature + Sex + (1 | Subject) + storage_time
y ~ current_feature + Sex + (1 | Subject) + storage_time
y ~ current_feature + Sex + (1 | Subject) + storage_time
y ~ current_feature + Sex + (1 | Subject) + storage_time
y ~ current_feature + Sex + (1 | Subject) + storage_time
y ~ current_feature + Sex + (1 | Subject) + storage_time
y ~ current_feature + Sex + (1 | Subject) + storage_time
y ~ current_feature + Sex + (1 | Subject) + storage_time
y ~ current_feature + Sex + (1 | Subject) + storage_time
y ~ current_feature + Sex + (1 | Subject) + storage_time
y ~ current_feature + Sex + (1 | Subject) + storage_time
y ~ current_feature + Sex + (1 | Subject) + storage_time
y ~ current_feature + Sex + (1 | Subject) + storage_time
y ~ current_feature + Sex + (1 | Subject) + storage_time
y ~ current_feature + Sex + (1 | Subject) + storage_time
y ~ current_feature + Sex + (1 | Subject) + storage_time
y ~ current_feature + Sex + (1 | Subject) + storage_time
y ~ current_feature + Sex + (1 | Subject) + storage_time
y ~ current_feature + Sex + (1 | Subject) + storage_time
y ~ current_feature + Sex + (1 | Subject) + storage_time

y ~ current_feature + Sex + (1 | Subject) + storage_time
y ~ current_feature + Sex + (1 | Subject) + storage_time
y ~ current_feature + Sex + (1 | Subject) + storage_time
y ~ current_feature + Sex + (1 | Subject) + storage_time
y ~ current_feature + Sex + (1 | Subject) + storage_time
y ~ current_feature + Sex + (1 | Subject) + storage_time
y ~ current_feature + Sex + (1 | Subject) + storage_time
y ~ current_feature + Sex + (1 | Subject) + storage_time
y ~ current_feature + Sex + (1 | Subject) + storage_time
y ~ current_feature + Sex + (1 | Subject) + storage_time
y ~ current_feature + Sex + (1 | Subject) + storage_time
y ~ current_feature + Sex + (1 | Subject) + storage_time
y ~ current_feature + Sex + (1 | Subject) + storage_time
y ~ current_feature + Sex + (1 | Subject) + storage_time
y ~ current_feature + Sex + (1 | Subject) + storage_time
y ~ current_feature + Sex + (1 | Subject) + storage_time
y ~ current_feature + Sex + (1 | Subject) + storage_time
y ~ current_feature + Sex + (1 | Subject) + storage_time
y ~ current_feature + Sex + (1 | Subject) + storage_time
y ~ current_feature + Sex + (1 | Subject) + storage_time
y ~ current_feature + Sex + (1 | Subject) + storage_time
y ~ current_feature + Sex + (1 | Subject) + storage_time
y ~ current_feature + Sex + (1 | Subject) + storage_time
y ~ current_feature + Sex + (1 | Subject) + storage_time
y ~ current_feature + Sex + (1 | Subject) + storage_time
y ~ current_feature + Sex + (1 | Subject) + storage_time
y ~ current_feature + Sex + (1 | Subject) + storage_time
y ~ current_feature + Sex + (1 | Subject) + storage_time
y ~ current_feature + Sex + (1 | Subject) + storage_time
y ~ current_feature + Sex + (1 | Subject) + storage_time
y ~ current_feature + Sex + (1 | Subject) + storage_time
y ~ current_feature + Sex + (1 | Subject) + storage_time
y ~ current_feature + Sex + (1 | Subject) + storage_time
y ~ current_feature + Sex + (1 | Subject) + storage_time
y ~ current_feature + Sex + (1 | Subject) + storage_time
y ~ current_feature + Sex + (1 | Subject) + storage_time
y ~ current_feature + Sex + (1 | Subject) + storage_time
y ~ current_feature + Sex + (1 | Subject) + storage_time
y ~ current_feature + Sex + (1 | Subject) + storage_time
y ~ current_feature + Sex + (1 | Subject) + storage_time
y ~ current_feature + Sex + (1 | Subject) + storage_time
y ~ current_feature + Sex + (1 | Subject) + storage_time
y ~ current_feature + Sex + (1 | Subject) + storage_time
y ~ current_feature + Sex + (1 | Subject) + storage_time
y ~ current_feature + Sex + (1 | Subject) + storage_time
y ~ current_feature + Sex + (1 | Subject) + storage_time
y ~ current_feature + Sex + (1 | Subject) + storage_time
y ~ current_feature + Sex + (1 | Subject) + storage_time

y ~ current_feature + Sex + (1 | Subject) + storage_time
y ~ current_feature + Sex + (1 | Subject) + storage_time
y ~ current_feature + Sex + (1 | Subject) + storage_time
y ~ current_feature + Sex + (1 | Subject) + storage_time
y ~ current_feature + Sex + (1 | Subject) + storage_time
y ~ current_feature + Sex + (1 | Subject) + storage_time
y ~ current_feature + Sex + (1 | Subject) + storage_time
y ~ current_feature + Sex + (1 | Subject) + storage_time
y ~ current_feature + Sex + (1 | Subject) + storage_time
y ~ current_feature + Sex + (1 | Subject) + storage_time
y ~ current_feature + Sex + (1 | Subject) + storage_time
y ~ current_feature + Sex + (1 | Subject) + storage_time
y ~ current_feature + Sex + (1 | Subject) + storage_time
y ~ current_feature + Sex + (1 | Subject) + storage_time
y ~ current_feature + Sex + (1 | Subject) + storage_time
y ~ current_feature + Sex + (1 | Subject) + storage_time
y ~ current_feature + Sex + (1 | Subject) + storage_time
y ~ current_feature + Sex + (1 | Subject) + storage_time
y ~ current_feature + Sex + (1 | Subject) + storage_time
y ~ current_feature + Sex + (1 | Subject) + storage_time

y ~ current_feature + Sex + (1 | Subject) + storage_time

y ~ current_feature + Sex + (1 | Subject) + storage_time

y ~ current_feature + Sex + (1 | Subject) + storage_time

y ~ current_feature + Sex + (1 | Subject) + storage_time

```
y ~ current_feature + Sex + (1 | Subject) + storage_time
y ~ current_feature + Sex + (1 | Subject) + storage_time
y ~ current_feature + Sex + (1 | Subject) + storage_time
y ~ current_feature + Sex + (1 | Subject) + storage_time
y ~ current_feature + Sex + (1 | Subject) + storage_time
y ~ current_feature + Sex + (1 | Subject) + storage_time
y ~ current_feature + Sex + (1 | Subject) + storage_time
y ~ current_feature + Sex + (1 | Subject) + storage_time
y ~ current_feature + Sex + (1 | Subject) + storage_time
y ~ current_feature + Sex + (1 | Subject) + storage_time
y ~ current_feature + Sex + (1 | Subject) + storage_time
y ~ current_feature + Sex + (1 | Subject) + storage_time
y ~ current_feature + Sex + (1 | Subject) + storage_time
y ~ current_feature + Sex + (1 | Subject) + storage_time
y ~ current_feature + Sex + (1 | Subject) + storage_time
y ~ current_feature + Sex + (1 | Subject) + storage_time
y ~ current_feature + Sex + (1 | Subject) + storage_time
y ~ current_feature + Sex + (1 | Subject) + storage_time
y ~ current_feature + Sex + (1 | Subject) + storage_time
y ~ current_feature + Sex + (1 | Subject) + storage_time
```

y ~ current_feature + Sex + (1 | Subject) + storage_time
y ~ current_feature + Sex + (1 | Subject) + storage_time
y ~ current_feature + Sex + (1 | Subject) + storage_time
y ~ current_feature + Sex + (1 | Subject) + storage_time
y ~ current_feature + Sex + (1 | Subject) + storage_time
y ~ current_feature + Sex + (1 | Subject) + storage_time
y ~ current_feature + Sex + (1 | Subject) + storage_time
y ~ current_feature + Sex + (1 | Subject) + storage_time
y ~ current_feature + Sex + (1 | Subject) + storage_time
y ~ current_feature + Sex + (1 | Subject) + storage_time
y ~ current_feature + Sex + (1 | Subject) + storage_time
y ~ current_feature + Sex + (1 | Subject) + storage_time
y ~ current_feature + Sex + (1 | Subject) + storage_time
y ~ current_feature + Sex + (1 | Subject) + storage_time
y ~ current_feature + Sex + (1 | Subject) + storage_time
y ~ current_feature + Sex + (1 | Subject) + storage_time
y ~ current_feature + Sex + (1 | Subject) + storage_time
y ~ current_feature + Sex + (1 | Subject) + storage_time
y ~ current_feature + Sex + (1 | Subject) + storage_time
y ~ current_feature + Sex + (1 | Subject) + storage_time
y ~ current_feature + Sex + (1 | Subject) + storage_time
y ~ current_feature + Sex + (1 | Subject) + storage_time
y ~ current_feature + Sex + (1 | Subject) + storage_time
y ~ current_feature + Sex + (1 | Subject) + storage_time
y ~ current_feature + Sex + (1 | Subject) + storage_time
y ~ current_feature + Sex + (1 | Subject) + storage_time
y ~ current_feature + Sex + (1 | Subject) + storage_time
y ~ current_feature + Sex + (1 | Subject) + storage_time
y ~ current_feature + Sex + (1 | Subject) + storage_time
y ~ current_feature + Sex + (1 | Subject) + storage_time
y ~ current_feature + Sex + (1 | Subject) + storage_time
y ~ current_feature + Sex + (1 | Subject) + storage_time
y ~ current_feature + Sex + (1 | Subject) + storage_time
y ~ current_feature + Sex + (1 | Subject) + storage_time
y ~ current_feature + Sex + (1 | Subject) + storage_time
y ~ current_feature + Sex + (1 | Subject) + storage_time
y ~ current_feature + Sex + (1 | Subject) + storage_time
y ~ current_feature + Sex + (1 | Subject) + storage_time
y ~ current_feature + Sex + (1 | Subject) + storage_time
y ~ current_feature + Sex + (1 | Subject) + storage_time
y ~ current_feature + Sex + (1 | Subject) + storage_time
y ~ current_feature + Sex + (1 | Subject) + storage_time
y ~ current_feature + Sex + (1 | Subject) + storage_time
y ~ current_feature + Sex + (1 | Subject) + storage_time
y ~ current_feature + Sex + (1 | Subject) + storage_time
y ~ current_feature + Sex + (1 | Subject) + storage_time
y ~ current_feature + Sex + (1 | Subject) + storage_time
y ~ current_feature + Sex + (1 | Subject) + storage_time

y ~ current_feature + Sex + (1 | Subject) + storage_time
y ~ current_feature + Sex + (1 | Subject) + storage_time
y ~ current_feature + Sex + (1 | Subject) + storage_time
y ~ current_feature + Sex + (1 | Subject) + storage_time
y ~ current_feature + Sex + (1 | Subject) + storage_time
y ~ current_feature + Sex + (1 | Subject) + storage_time
y ~ current_feature + Sex + (1 | Subject) + storage_time
y ~ current_feature + Sex + (1 | Subject) + storage_time
y ~ current_feature + Sex + (1 | Subject) + storage_time
y ~ current_feature + Sex + (1 | Subject) + storage_time
y ~ current_feature + Sex + (1 | Subject) + storage_time
y ~ current_feature + Sex + (1 | Subject) + storage_time
y ~ current_feature + Sex + (1 | Subject) + storage_time
y ~ current_feature + Sex + (1 | Subject) + storage_time
y ~ current_feature + Sex + (1 | Subject) + storage_time
y ~ current_feature + Sex + (1 | Subject) + storage_time
y ~ current_feature + Sex + (1 | Subject) + storage_time
y ~ current_feature + Sex + (1 | Subject) + storage_time
y ~ current_feature + Sex + (1 | Subject) + storage_time
y ~ current_feature + Sex + (1 | Subject) + storage_time
y ~ current_feature + Sex + (1 | Subject) + storage_time
y ~ current_feature + Sex + (1 | Subject) + storage_time
y ~ current_feature + Sex + (1 | Subject) + storage_time
y ~ current_feature + Sex + (1 | Subject) + storage_time
y ~ current_feature + Sex + (1 | Subject) + storage_time
y ~ current_feature + Sex + (1 | Subject) + storage_time
y ~ current_feature + Sex + (1 | Subject) + storage_time
y ~ current_feature + Sex + (1 | Subject) + storage_time
y ~ current_feature + Sex + (1 | Subject) + storage_time
y ~ current_feature + Sex + (1 | Subject) + storage_time
y ~ current_feature + Sex + (1 | Subject) + storage_time
y ~ current_feature + Sex + (1 | Subject) + storage_time
y ~ current_feature + Sex + (1 | Subject) + storage_time
y ~ current_feature + Sex + (1 | Subject) + storage_time
y ~ current_feature + Sex + (1 | Subject) + storage_time
y ~ current_feature + Sex + (1 | Subject) + storage_time
y ~ current_feature + Sex + (1 | Subject) + storage_time
y ~ current_feature + Sex + (1 | Subject) + storage_time
y ~ current_feature + Sex + (1 | Subject) + storage_time
y ~ current_feature + Sex + (1 | Subject) + storage_time
y ~ current_feature + Sex + (1 | Subject) + storage_time
y ~ current_feature + Sex + (1 | Subject) + storage_time
y ~ current_feature + Sex + (1 | Subject) + storage_time
y ~ current_feature + Sex + (1 | Subject) + storage_time
y ~ current_feature + Sex + (1 | Subject) + storage_time
y ~ current_feature + Sex + (1 | Subject) + storage_time
y ~ current_feature + Sex + (1 | Subject) + storage_time
y ~ current_feature + Sex + (1 | Subject) + storage_time

y ~ current_feature + Sex + (1 | Subject) + storage_time
y ~ current_feature + Sex + (1 | Subject) + storage_time
y ~ current_feature + Sex + (1 | Subject) + storage_time
y ~ current_feature + Sex + (1 | Subject) + storage_time
y ~ current_feature + Sex + (1 | Subject) + storage_time
y ~ current_feature + Sex + (1 | Subject) + storage_time
y ~ current_feature + Sex + (1 | Subject) + storage_time
y ~ current_feature + Sex + (1 | Subject) + storage_time
y ~ current_feature + Sex + (1 | Subject) + storage_time
y ~ current_feature + Sex + (1 | Subject) + storage_time
y ~ current_feature + Sex + (1 | Subject) + storage_time
y ~ current_feature + Sex + (1 | Subject) + storage_time
y ~ current_feature + Sex + (1 | Subject) + storage_time
y ~ current_feature + Sex + (1 | Subject) + storage_time
y ~ current_feature + Sex + (1 | Subject) + storage_time
y ~ current_feature + Sex + (1 | Subject) + storage_time
y ~ current_feature + Sex + (1 | Subject) + storage_time
y ~ current_feature + Sex + (1 | Subject) + storage_time
y ~ current_feature + Sex + (1 | Subject) + storage_time
y ~ current_feature + Sex + (1 | Subject) + storage_time
y ~ current_feature + Sex + (1 | Subject) + storage_time
y ~ current_feature + Sex + (1 | Subject) + storage_time
y ~ current_feature + Sex + (1 | Subject) + storage_time
y ~ current_feature + Sex + (1 | Subject) + storage_time
y ~ current_feature + Sex + (1 | Subject) + storage_time
y ~ current_feature + Sex + (1 | Subject) + storage_time
y ~ current_feature + Sex + (1 | Subject) + storage_time
y ~ current_feature + Sex + (1 | Subject) + storage_time
y ~ current_feature + Sex + (1 | Subject) + storage_time
y ~ current_feature + Sex + (1 | Subject) + storage_time
y ~ current_feature + Sex + (1 | Subject) + storage_time
y ~ current_feature + Sex + (1 | Subject) + storage_time
y ~ current_feature + Sex + (1 | Subject) + storage_time
y ~ current_feature + Sex + (1 | Subject) + storage_time
y ~ current_feature + Sex + (1 | Subject) + storage_time
y ~ current_feature + Sex + (1 | Subject) + storage_time
y ~ current_feature + Sex + (1 | Subject) + storage_time
y ~ current_feature + Sex + (1 | Subject) + storage_time
y ~ current_feature + Sex + (1 | Subject) + storage_time
y ~ current_feature + Sex + (1 | Subject) + storage_time
y ~ current_feature + Sex + (1 | Subject) + storage_time
y ~ current_feature + Sex + (1 | Subject) + storage_time
y ~ current_feature + Sex + (1 | Subject) + storage_time
y ~ current_feature + Sex + (1 | Subject) + storage_time
y ~ current_feature + Sex + (1 | Subject) + storage_time
y ~ current_feature + Sex + (1 | Subject) + storage_time
y ~ current_feature + Sex + (1 | Subject) + storage_time
y ~ current_feature + Sex + (1 | Subject) + storage_time

y ~ current_feature + Sex + (1 | Subject) + storage_time
y ~ current_feature + Sex + (1 | Subject) + storage_time
y ~ current_feature + Sex + (1 | Subject) + storage_time
y ~ current_feature + Sex + (1 | Subject) + storage_time
y ~ current_feature + Sex + (1 | Subject) + storage_time
y ~ current_feature + Sex + (1 | Subject) + storage_time
y ~ current_feature + Sex + (1 | Subject) + storage_time
y ~ current_feature + Sex + (1 | Subject) + storage_time
y ~ current_feature + Sex + (1 | Subject) + storage_time
y ~ current_feature + Sex + (1 | Subject) + storage_time
y ~ current_feature + Sex + (1 | Subject) + storage_time
y ~ current_feature + Sex + (1 | Subject) + storage_time
y ~ current_feature + Sex + (1 | Subject) + storage_time
y ~ current_feature + Sex + (1 | Subject) + storage_time
y ~ current_feature + Sex + (1 | Subject) + storage_time
y ~ current_feature + Sex + (1 | Subject) + storage_time
y ~ current_feature + Sex + (1 | Subject) + storage_time
y ~ current_feature + Sex + (1 | Subject) + storage_time
y ~ current_feature + Sex + (1 | Subject) + storage_time
y ~ current_feature + Sex + (1 | Subject) + storage_time
y ~ current_feature + Sex + (1 | Subject) + storage_time
y ~ current_feature + Sex + (1 | Subject) + storage_time
y ~ current_feature + Sex + (1 | Subject) + storage_time
y ~ current_feature + Sex + (1 | Subject) + storage_time
y ~ current_feature + Sex + (1 | Subject) + storage_time
y ~ current_feature + Sex + (1 | Subject) + storage_time
y ~ current_feature + Sex + (1 | Subject) + storage_time
y ~ current_feature + Sex + (1 | Subject) + storage_time
y ~ current_feature + Sex + (1 | Subject) + storage_time
y ~ current_feature + Sex + (1 | Subject) + storage_time
y ~ current_feature + Sex + (1 | Subject) + storage_time
y ~ current_feature + Sex + (1 | Subject) + storage_time
y ~ current_feature + Sex + (1 | Subject) + storage_time
y ~ current_feature + Sex + (1 | Subject) + storage_time
y ~ current_feature + Sex + (1 | Subject) + storage_time
y ~ current_feature + Sex + (1 | Subject) + storage_time
y ~ current_feature + Sex + (1 | Subject) + storage_time
y ~ current_feature + Sex + (1 | Subject) + storage_time
y ~ current_feature + Sex + (1 | Subject) + storage_time
y ~ current_feature + Sex + (1 | Subject) + storage_time
y ~ current_feature + Sex + (1 | Subject) + storage_time
y ~ current_feature + Sex + (1 | Subject) + storage_time
y ~ current_feature + Sex + (1 | Subject) + storage_time
y ~ current_feature + Sex + (1 | Subject) + storage_time
y ~ current_feature + Sex + (1 | Subject) + storage_time
y ~ current_feature + Sex + (1 | Subject) + storage_time
y ~ current_feature + Sex + (1 | Subject) + storage_time
y ~ current_feature + Sex + (1 | Subject) + storage_time

y ~ current_feature + Sex + (1 | Subject) + storage_time
y ~ current_feature + Sex + (1 | Subject) + storage_time
y ~ current_feature + Sex + (1 | Subject) + storage_time
y ~ current_feature + Sex + (1 | Subject) + storage_time
y ~ current_feature + Sex + (1 | Subject) + storage_time
y ~ current_feature + Sex + (1 | Subject) + storage_time
y ~ current_feature + Sex + (1 | Subject) + storage_time
y ~ current_feature + Sex + (1 | Subject) + storage_time
y ~ current_feature + Sex + (1 | Subject) + storage_time
y ~ current_feature + Sex + (1 | Subject) + storage_time
y ~ current_feature + Sex + (1 | Subject) + storage_time
y ~ current_feature + Sex + (1 | Subject) + storage_time
y ~ current_feature + Sex + (1 | Subject) + storage_time
y ~ current_feature + Sex + (1 | Subject) + storage_time
y ~ current_feature + Sex + (1 | Subject) + storage_time
y ~ current_feature + Sex + (1 | Subject) + storage_time
y ~ current_feature + Sex + (1 | Subject) + storage_time
y ~ current_feature + Sex + (1 | Subject) + storage_time
y ~ current_feature + Sex + (1 | Subject) + storage_time
y ~ current_feature + Sex + (1 | Subject) + storage_time
y ~ current_feature + Sex + (1 | Subject) + storage_time
y ~ current_feature + Sex + (1 | Subject) + storage_time
y ~ current_feature + Sex + (1 | Subject) + storage_time
y ~ current_feature + Sex + (1 | Subject) + storage_time
y ~ current_feature + Sex + (1 | Subject) + storage_time
y ~ current_feature + Sex + (1 | Subject) + storage_time
y ~ current_feature + Sex + (1 | Subject) + storage_time
y ~ current_feature + Sex + (1 | Subject) + storage_time
y ~ current_feature + Sex + (1 | Subject) + storage_time
y ~ current_feature + Sex + (1 | Subject) + storage_time
y ~ current_feature + Sex + (1 | Subject) + storage_time
y ~ current_feature + Sex + (1 | Subject) + storage_time
y ~ current_feature + Sex + (1 | Subject) + storage_time
y ~ current_feature + Sex + (1 | Subject) + storage_time
y ~ current_feature + Sex + (1 | Subject) + storage_time
y ~ current_feature + Sex + (1 | Subject) + storage_time
y ~ current_feature + Sex + (1 | Subject) + storage_time
y ~ current_feature + Sex + (1 | Subject) + storage_time
y ~ current_feature + Sex + (1 | Subject) + storage_time
y ~ current_feature + Sex + (1 | Subject) + storage_time
y ~ current_feature + Sex + (1 | Subject) + storage_time
y ~ current_feature + Sex + (1 | Subject) + storage_time
y ~ current_feature + Sex + (1 | Subject) + storage_time
y ~ current_feature + Sex + (1 | Subject) + storage_time
y ~ current_feature + Sex + (1 | Subject) + storage_time
y ~ current_feature + Sex + (1 | Subject) + storage_time
y ~ current_feature + Sex + (1 | Subject) + storage_time
y ~ current_feature + Sex + (1 | Subject) + storage_time

y ~ current_feature + Sex + (1 | Subject) + storage_time
y ~ current_feature + Sex + (1 | Subject) + storage_time
y ~ current_feature + Sex + (1 | Subject) + storage_time
y ~ current_feature + Sex + (1 | Subject) + storage_time
y ~ current_feature + Sex + (1 | Subject) + storage_time
y ~ current_feature + Sex + (1 | Subject) + storage_time
y ~ current_feature + Sex + (1 | Subject) + storage_time
y ~ current_feature + Sex + (1 | Subject) + storage_time
y ~ current_feature + Sex + (1 | Subject) + storage_time
y ~ current_feature + Sex + (1 | Subject) + storage_time
y ~ current_feature + Sex + (1 | Subject) + storage_time
y ~ current_feature + Sex + (1 | Subject) + storage_time
y ~ current_feature + Sex + (1 | Subject) + storage_time
y ~ current_feature + Sex + (1 | Subject) + storage_time
y ~ current_feature + Sex + (1 | Subject) + storage_time
y ~ current_feature + Sex + (1 | Subject) + storage_time
y ~ current_feature + Sex + (1 | Subject) + storage_time
y ~ current_feature + Sex + (1 | Subject) + storage_time
y ~ current_feature + Sex + (1 | Subject) + storage_time
y ~ current_feature + Sex + (1 | Subject) + storage_time
y ~ current_feature + Sex + (1 | Subject) + storage_time
y ~ current_feature + Sex + (1 | Subject) + storage_time
y ~ current_feature + Sex + (1 | Subject) + storage_time
y ~ current_feature + Sex + (1 | Subject) + storage_time
y ~ current_feature + Sex + (1 | Subject) + storage_time
y ~ current_feature + Sex + (1 | Subject) + storage_time
y ~ current_feature + Sex + (1 | Subject) + storage_time
y ~ current_feature + Sex + (1 | Subject) + storage_time
y ~ current_feature + Sex + (1 | Subject) + storage_time
y ~ current_feature + Sex + (1 | Subject) + storage_time
y ~ current_feature + Sex + (1 | Subject) + storage_time
y ~ current_feature + Sex + (1 | Subject) + storage_time
y ~ current_feature + Sex + (1 | Subject) + storage_time
y ~ current_feature + Sex + (1 | Subject) + storage_time
y ~ current_feature + Sex + (1 | Subject) + storage_time
y ~ current_feature + Sex + (1 | Subject) + storage_time
y ~ current_feature + Sex + (1 | Subject) + storage_time
y ~ current_feature + Sex + (1 | Subject) + storage_time
y ~ current_feature + Sex + (1 | Subject) + storage_time
y ~ current_feature + Sex + (1 | Subject) + storage_time
y ~ current_feature + Sex + (1 | Subject) + storage_time
y ~ current_feature + Sex + (1 | Subject) + storage_time
y ~ current_feature + Sex + (1 | Subject) + storage_time
y ~ current_feature + Sex + (1 | Subject) + storage_time
y ~ current_feature + Sex + (1 | Subject) + storage_time
y ~ current_feature + Sex + (1 | Subject) + storage_time
y ~ current_feature + Sex + (1 | Subject) + storage_time
y ~ current_feature + Sex + (1 | Subject) + storage_time

y ~ current_feature + Sex + (1 | Subject) + storage_time
y ~ current_feature + Sex + (1 | Subject) + storage_time
y ~ current_feature + Sex + (1 | Subject) + storage_time
y ~ current_feature + Sex + (1 | Subject) + storage_time
y ~ current_feature + Sex + (1 | Subject) + storage_time
y ~ current_feature + Sex + (1 | Subject) + storage_time
y ~ current_feature + Sex + (1 | Subject) + storage_time
y ~ current_feature + Sex + (1 | Subject) + storage_time
y ~ current_feature + Sex + (1 | Subject) + storage_time
y ~ current_feature + Sex + (1 | Subject) + storage_time
y ~ current_feature + Sex + (1 | Subject) + storage_time
y ~ current_feature + Sex + (1 | Subject) + storage_time
y ~ current_feature + Sex + (1 | Subject) + storage_time
y ~ current_feature + Sex + (1 | Subject) + storage_time
y ~ current_feature + Sex + (1 | Subject) + storage_time
y ~ current_feature + Sex + (1 | Subject) + storage_time
y ~ current_feature + Sex + (1 | Subject) + storage_time
y ~ current_feature + Sex + (1 | Subject) + storage_time
y ~ current_feature + Sex + (1 | Subject) + storage_time
y ~ current_feature + Sex + (1 | Subject) + storage_time
y ~ current_feature + Sex + (1 | Subject) + storage_time
y ~ current_feature + Sex + (1 | Subject) + storage_time
y ~ current_feature + Sex + (1 | Subject) + storage_time
y ~ current_feature + Sex + (1 | Subject) + storage_time
y ~ current_feature + Sex + (1 | Subject) + storage_time
y ~ current_feature + Sex + (1 | Subject) + storage_time
y ~ current_feature + Sex + (1 | Subject) + storage_time
y ~ current_feature + Sex + (1 | Subject) + storage_time
y ~ current_feature + Sex + (1 | Subject) + storage_time
y ~ current_feature + Sex + (1 | Subject) + storage_time
y ~ current_feature + Sex + (1 | Subject) + storage_time
y ~ current_feature + Sex + (1 | Subject) + storage_time
y ~ current_feature + Sex + (1 | Subject) + storage_time
y ~ current_feature + Sex + (1 | Subject) + storage_time
y ~ current_feature + Sex + (1 | Subject) + storage_time
y ~ current_feature + Sex + (1 | Subject) + storage_time
y ~ current_feature + Sex + (1 | Subject) + storage_time
y ~ current_feature + Sex + (1 | Subject) + storage_time
y ~ current_feature + Sex + (1 | Subject) + storage_time
y ~ current_feature + Sex + (1 | Subject) + storage_time
y ~ current_feature + Sex + (1 | Subject) + storage_time
y ~ current_feature + Sex + (1 | Subject) + storage_time
y ~ current_feature + Sex + (1 | Subject) + storage_time
y ~ current_feature + Sex + (1 | Subject) + storage_time
y ~ current_feature + Sex + (1 | Subject) + storage_time
y ~ current_feature + Sex + (1 | Subject) + storage_time
y ~ current_feature + Sex + (1 | Subject) + storage_time
y ~ current_feature + Sex + (1 | Subject) + storage_time

y ~ current_feature + Sex + (1 | Subject) + storage_time
y ~ current_feature + Sex + (1 | Subject) + storage_time
y ~ current_feature + Sex + (1 | Subject) + storage_time
y ~ current_feature + Sex + (1 | Subject) + storage_time
y ~ current_feature + Sex + (1 | Subject) + storage_time
y ~ current_feature + Sex + (1 | Subject) + storage_time
y ~ current_feature + Sex + (1 | Subject) + storage_time
y ~ current_feature + Sex + (1 | Subject) + storage_time
y ~ current_feature + Sex + (1 | Subject) + storage_time
y ~ current_feature + Sex + (1 | Subject) + storage_time
y ~ current_feature + Sex + (1 | Subject) + storage_time
y ~ current_feature + Sex + (1 | Subject) + storage_time
y ~ current_feature + Sex + (1 | Subject) + storage_time
y ~ current_feature + Sex + (1 | Subject) + storage_time
y ~ current_feature + Sex + (1 | Subject) + storage_time
y ~ current_feature + Sex + (1 | Subject) + storage_time
y ~ current_feature + Sex + (1 | Subject) + storage_time
y ~ current_feature + Sex + (1 | Subject) + storage_time
y ~ current_feature + Sex + (1 | Subject) + storage_time
y ~ current_feature + Sex + (1 | Subject) + storage_time
y ~ current_feature + Sex + (1 | Subject) + storage_time
y ~ current_feature + Sex + (1 | Subject) + storage_time
y ~ current_feature + Sex + (1 | Subject) + storage_time
y ~ current_feature + Sex + (1 | Subject) + storage_time
y ~ current_feature + Sex + (1 | Subject) + storage_time
y ~ current_feature + Sex + (1 | Subject) + storage_time
y ~ current_feature + Sex + (1 | Subject) + storage_time
y ~ current_feature + Sex + (1 | Subject) + storage_time
y ~ current_feature + Sex + (1 | Subject) + storage_time
y ~ current_feature + Sex + (1 | Subject) + storage_time
y ~ current_feature + Sex + (1 | Subject) + storage_time
y ~ current_feature + Sex + (1 | Subject) + storage_time
y ~ current_feature + Sex + (1 | Subject) + storage_time
y ~ current_feature + Sex + (1 | Subject) + storage_time
y ~ current_feature + Sex + (1 | Subject) + storage_time
y ~ current_feature + Sex + (1 | Subject) + storage_time
y ~ current_feature + Sex + (1 | Subject) + storage_time
y ~ current_feature + Sex + (1 | Subject) + storage_time
y ~ current_feature + Sex + (1 | Subject) + storage_time
y ~ current_feature + Sex + (1 | Subject) + storage_time
y ~ current_feature + Sex + (1 | Subject) + storage_time
y ~ current_feature + Sex + (1 | Subject) + storage_time
y ~ current_feature + Sex + (1 | Subject) + storage_time
y ~ current_feature + Sex + (1 | Subject) + storage_time
y ~ current_feature + Sex + (1 | Subject) + storage_time
y ~ current_feature + Sex + (1 | Subject) + storage_time
y ~ current_feature + Sex + (1 | Subject) + storage_time
y ~ current_feature + Sex + (1 | Subject) + storage_time

y ~ current_feature + Sex + (1 | Subject) + storage_time
y ~ current_feature + Sex + (1 | Subject) + storage_time
y ~ current_feature + Sex + (1 | Subject) + storage_time
y ~ current_feature + Sex + (1 | Subject) + storage_time
y ~ current_feature + Sex + (1 | Subject) + storage_time
y ~ current_feature + Sex + (1 | Subject) + storage_time
y ~ current_feature + Sex + (1 | Subject) + storage_time
y ~ current_feature + Sex + (1 | Subject) + storage_time
y ~ current_feature + Sex + (1 | Subject) + storage_time
y ~ current_feature + Sex + (1 | Subject) + storage_time
y ~ current_feature + Sex + (1 | Subject) + storage_time
y ~ current_feature + Sex + (1 | Subject) + storage_time
y ~ current_feature + Sex + (1 | Subject) + storage_time
y ~ current_feature + Sex + (1 | Subject) + storage_time
y ~ current_feature + Sex + (1 | Subject) + storage_time
y ~ current_feature + Sex + (1 | Subject) + storage_time
y ~ current_feature + Sex + (1 | Subject) + storage_time
y ~ current_feature + Sex + (1 | Subject) + storage_time
y ~ current_feature + Sex + (1 | Subject) + storage_time
y ~ current_feature + Sex + (1 | Subject) + storage_time
y ~ current_feature + Sex + (1 | Subject) + storage_time
y ~ current_feature + Sex + (1 | Subject) + storage_time
y ~ current_feature + Sex + (1 | Subject) + storage_time
y ~ current_feature + Sex + (1 | Subject) + storage_time
y ~ current_feature + Sex + (1 | Subject) + storage_time
y ~ current_feature + Sex + (1 | Subject) + storage_time
y ~ current_feature + Sex + (1 | Subject) + storage_time
y ~ current_feature + Sex + (1 | Subject) + storage_time
y ~ current_feature + Sex + (1 | Subject) + storage_time
y ~ current_feature + Sex + (1 | Subject) + storage_time
y ~ current_feature + Sex + (1 | Subject) + storage_time
y ~ current_feature + Sex + (1 | Subject) + storage_time
y ~ current_feature + Sex + (1 | Subject) + storage_time
y ~ current_feature + Sex + (1 | Subject) + storage_time
y ~ current_feature + Sex + (1 | Subject) + storage_time
y ~ current_feature + Sex + (1 | Subject) + storage_time
y ~ current_feature + Sex + (1 | Subject) + storage_time
y ~ current_feature + Sex + (1 | Subject) + storage_time
y ~ current_feature + Sex + (1 | Subject) + storage_time
y ~ current_feature + Sex + (1 | Subject) + storage_time
y ~ current_feature + Sex + (1 | Subject) + storage_time
y ~ current_feature + Sex + (1 | Subject) + storage_time
y ~ current_feature + Sex + (1 | Subject) + storage_time
y ~ current_feature + Sex + (1 | Subject) + storage_time
y ~ current_feature + Sex + (1 | Subject) + storage_time
y ~ current_feature + Sex + (1 | Subject) + storage_time
y ~ current_feature + Sex + (1 | Subject) + storage_time
y ~ current_feature + Sex + (1 | Subject) + storage_time

y ~ current_feature + Sex + (1 | Subject) + storage_time
y ~ current_feature + Sex + (1 | Subject) + storage_time
y ~ current_feature + Sex + (1 | Subject) + storage_time
y ~ current_feature + Sex + (1 | Subject) + storage_time
y ~ current_feature + Sex + (1 | Subject) + storage_time
y ~ current_feature + Sex + (1 | Subject) + storage_time
y ~ current_feature + Sex + (1 | Subject) + storage_time
y ~ current_feature + Sex + (1 | Subject) + storage_time
y ~ current_feature + Sex + (1 | Subject) + storage_time
y ~ current_feature + Sex + (1 | Subject) + storage_time
y ~ current_feature + Sex + (1 | Subject) + storage_time
y ~ current_feature + Sex + (1 | Subject) + storage_time
y ~ current_feature + Sex + (1 | Subject) + storage_time
y ~ current_feature + Sex + (1 | Subject) + storage_time
y ~ current_feature + Sex + (1 | Subject) + storage_time
y ~ current_feature + Sex + (1 | Subject) + storage_time
y ~ current_feature + Sex + (1 | Subject) + storage_time
y ~ current_feature + Sex + (1 | Subject) + storage_time
y ~ current_feature + Sex + (1 | Subject) + storage_time
y ~ current_feature + Sex + (1 | Subject) + storage_time
y ~ current_feature + Sex + (1 | Subject) + storage_time
y ~ current_feature + Sex + (1 | Subject) + storage_time
y ~ current_feature + Sex + (1 | Subject) + storage_time
y ~ current_feature + Sex + (1 | Subject) + storage_time
y ~ current_feature + Sex + (1 | Subject) + storage_time
y ~ current_feature + Sex + (1 | Subject) + storage_time
y ~ current_feature + Sex + (1 | Subject) + storage_time
y ~ current_feature + Sex + (1 | Subject) + storage_time
y ~ current_feature + Sex + (1 | Subject) + storage_time
y ~ current_feature + Sex + (1 | Subject) + storage_time
y ~ current_feature + Sex + (1 | Subject) + storage_time
y ~ current_feature + Sex + (1 | Subject) + storage_time
y ~ current_feature + Sex + (1 | Subject) + storage_time
y ~ current_feature + Sex + (1 | Subject) + storage_time
y ~ current_feature + Sex + (1 | Subject) + storage_time
y ~ current_feature + Sex + (1 | Subject) + storage_time
y ~ current_feature + Sex + (1 | Subject) + storage_time
y ~ current_feature + Sex + (1 | Subject) + storage_time
y ~ current_feature + Sex + (1 | Subject) + storage_time
y ~ current_feature + Sex + (1 | Subject) + storage_time
y ~ current_feature + Sex + (1 | Subject) + storage_time
y ~ current_feature + Sex + (1 | Subject) + storage_time
y ~ current_feature + Sex + (1 | Subject) + storage_time
y ~ current_feature + Sex + (1 | Subject) + storage_time
y ~ current_feature + Sex + (1 | Subject) + storage_time
y ~ current_feature + Sex + (1 | Subject) + storage_time
y ~ current_feature + Sex + (1 | Subject) + storage_time
y ~ current_feature + Sex + (1 | Subject) + storage_time

y ~ current_feature + Sex + (1 | Subject) + storage_time
y ~ current_feature + Sex + (1 | Subject) + storage_time
y ~ current_feature + Sex + (1 | Subject) + storage_time
y ~ current_feature + Sex + (1 | Subject) + storage_time
y ~ current_feature + Sex + (1 | Subject) + storage_time
y ~ current_feature + Sex + (1 | Subject) + storage_time
y ~ current_feature + Sex + (1 | Subject) + storage_time
y ~ current_feature + Sex + (1 | Subject) + storage_time
y ~ current_feature + Sex + (1 | Subject) + storage_time
y ~ current_feature + Sex + (1 | Subject) + storage_time
y ~ current_feature + Sex + (1 | Subject) + storage_time
y ~ current_feature + Sex + (1 | Subject) + storage_time
y ~ current_feature + Sex + (1 | Subject) + storage_time
y ~ current_feature + Sex + (1 | Subject) + storage_time
y ~ current_feature + Sex + (1 | Subject) + storage_time
y ~ current_feature + Sex + (1 | Subject) + storage_time
y ~ current_feature + Sex + (1 | Subject) + storage_time
y ~ current_feature + Sex + (1 | Subject) + storage_time
y ~ current_feature + Sex + (1 | Subject) + storage_time
y ~ current_feature + Sex + (1 | Subject) + storage_time
y ~ current_feature + Sex + (1 | Subject) + storage_time
y ~ current_feature + Sex + (1 | Subject) + storage_time
y ~ current_feature + Sex + (1 | Subject) + storage_time
y ~ current_feature + Sex + (1 | Subject) + storage_time
y ~ current_feature + Sex + (1 | Subject) + storage_time
y ~ current_feature + Sex + (1 | Subject) + storage_time
y ~ current_feature + Sex + (1 | Subject) + storage_time
y ~ current_feature + Sex + (1 | Subject) + storage_time
y ~ current_feature + Sex + (1 | Subject) + storage_time
y ~ current_feature + Sex + (1 | Subject) + storage_time
y ~ current_feature + Sex + (1 | Subject) + storage_time
y ~ current_feature + Sex + (1 | Subject) + storage_time
y ~ current_feature + Sex + (1 | Subject) + storage_time
y ~ current_feature + Sex + (1 | Subject) + storage_time
y ~ current_feature + Sex + (1 | Subject) + storage_time
y ~ current_feature + Sex + (1 | Subject) + storage_time
y ~ current_feature + Sex + (1 | Subject) + storage_time
y ~ current_feature + Sex + (1 | Subject) + storage_time
y ~ current_feature + Sex + (1 | Subject) + storage_time
y ~ current_feature + Sex + (1 | Subject) + storage_time
y ~ current_feature + Sex + (1 | Subject) + storage_time
y ~ current_feature + Sex + (1 | Subject) + storage_time
y ~ current_feature + Sex + (1 | Subject) + storage_time
y ~ current_feature + Sex + (1 | Subject) + storage_time
y ~ current_feature + Sex + (1 | Subject) + storage_time
y ~ current_feature + Sex + (1 | Subject) + storage_time
y ~ current_feature + Sex + (1 | Subject) + storage_time
y ~ current_feature + Sex + (1 | Subject) + storage_time

y ~ current_feature + Sex + (1 | Subject) + storage_time
y ~ current_feature + Sex + (1 | Subject) + storage_time
y ~ current_feature + Sex + (1 | Subject) + storage_time
y ~ current_feature + Sex + (1 | Subject) + storage_time
y ~ current_feature + Sex + (1 | Subject) + storage_time
y ~ current_feature + Sex + (1 | Subject) + storage_time
y ~ current_feature + Sex + (1 | Subject) + storage_time
y ~ current_feature + Sex + (1 | Subject) + storage_time
y ~ current_feature + Sex + (1 | Subject) + storage_time
y ~ current_feature + Sex + (1 | Subject) + storage_time
y ~ current_feature + Sex + (1 | Subject) + storage_time
y ~ current_feature + Sex + (1 | Subject) + storage_time
y ~ current_feature + Sex + (1 | Subject) + storage_time
y ~ current_feature + Sex + (1 | Subject) + storage_time
y ~ current_feature + Sex + (1 | Subject) + storage_time
y ~ current_feature + Sex + (1 | Subject) + storage_time
y ~ current_feature + Sex + (1 | Subject) + storage_time
y ~ current_feature + Sex + (1 | Subject) + storage_time
y ~ current_feature + Sex + (1 | Subject) + storage_time
y ~ current_feature + Sex + (1 | Subject) + storage_time
y ~ current_feature + Sex + (1 | Subject) + storage_time
y ~ current_feature + Sex + (1 | Subject) + storage_time
y ~ current_feature + Sex + (1 | Subject) + storage_time
y ~ current_feature + Sex + (1 | Subject) + storage_time
y ~ current_feature + Sex + (1 | Subject) + storage_time
y ~ current_feature + Sex + (1 | Subject) + storage_time
y ~ current_feature + Sex + (1 | Subject) + storage_time
y ~ current_feature + Sex + (1 | Subject) + storage_time
y ~ current_feature + Sex + (1 | Subject) + storage_time
y ~ current_feature + Sex + (1 | Subject) + storage_time
y ~ current_feature + Sex + (1 | Subject) + storage_time
y ~ current_feature + Sex + (1 | Subject) + storage_time
y ~ current_feature + Sex + (1 | Subject) + storage_time
y ~ current_feature + Sex + (1 | Subject) + storage_time
y ~ current_feature + Sex + (1 | Subject) + storage_time
y ~ current_feature + Sex + (1 | Subject) + storage_time
y ~ current_feature + Sex + (1 | Subject) + storage_time
y ~ current_feature + Sex + (1 | Subject) + storage_time
y ~ current_feature + Sex + (1 | Subject) + storage_time
y ~ current_feature + Sex + (1 | Subject) + storage_time
y ~ current_feature + Sex + (1 | Subject) + storage_time
y ~ current_feature + Sex + (1 | Subject) + storage_time
y ~ current_feature + Sex + (1 | Subject) + storage_time
y ~ current_feature + Sex + (1 | Subject) + storage_time
y ~ current_feature + Sex + (1 | Subject) + storage_time
y ~ current_feature + Sex + (1 | Subject) + storage_time
y ~ current_feature + Sex + (1 | Subject) + storage_time
y ~ current_feature + Sex + (1 | Subject) + storage_time

y ~ current_feature + Sex + (1 | Subject) + storage_time
y ~ current_feature + Sex + (1 | Subject) + storage_time
y ~ current_feature + Sex + (1 | Subject) + storage_time
y ~ current_feature + Sex + (1 | Subject) + storage_time
y ~ current_feature + Sex + (1 | Subject) + storage_time
y ~ current_feature + Sex + (1 | Subject) + storage_time
y ~ current_feature + Sex + (1 | Subject) + storage_time
y ~ current_feature + Sex + (1 | Subject) + storage_time
y ~ current_feature + Sex + (1 | Subject) + storage_time
y ~ current_feature + Sex + (1 | Subject) + storage_time
y ~ current_feature + Sex + (1 | Subject) + storage_time
y ~ current_feature + Sex + (1 | Subject) + storage_time
y ~ current_feature + Sex + (1 | Subject) + storage_time
y ~ current_feature + Sex + (1 | Subject) + storage_time
y ~ current_feature + Sex + (1 | Subject) + storage_time
y ~ current_feature + Sex + (1 | Subject) + storage_time
y ~ current_feature + Sex + (1 | Subject) + storage_time
y ~ current_feature + Sex + (1 | Subject) + storage_time
y ~ current_feature + Sex + (1 | Subject) + storage_time
y ~ current_feature + Sex + (1 | Subject) + storage_time
y ~ current_feature + Sex + (1 | Subject) + storage_time
y ~ current_feature + Sex + (1 | Subject) + storage_time
y ~ current_feature + Sex + (1 | Subject) + storage_time
y ~ current_feature + Sex + (1 | Subject) + storage_time
y ~ current_feature + Sex + (1 | Subject) + storage_time
y ~ current_feature + Sex + (1 | Subject) + storage_time
y ~ current_feature + Sex + (1 | Subject) + storage_time
y ~ current_feature + Sex + (1 | Subject) + storage_time
y ~ current_feature + Sex + (1 | Subject) + storage_time
y ~ current_feature + Sex + (1 | Subject) + storage_time
y ~ current_feature + Sex + (1 | Subject) + storage_time
y ~ current_feature + Sex + (1 | Subject) + storage_time
y ~ current_feature + Sex + (1 | Subject) + storage_time
y ~ current_feature + Sex + (1 | Subject) + storage_time
y ~ current_feature + Sex + (1 | Subject) + storage_time
y ~ current_feature + Sex + (1 | Subject) + storage_time
y ~ current_feature + Sex + (1 | Subject) + storage_time
y ~ current_feature + Sex + (1 | Subject) + storage_time
y ~ current_feature + Sex + (1 | Subject) + storage_time
y ~ current_feature + Sex + (1 | Subject) + storage_time
y ~ current_feature + Sex + (1 | Subject) + storage_time
y ~ current_feature + Sex + (1 | Subject) + storage_time
y ~ current_feature + Sex + (1 | Subject) + storage_time
y ~ current_feature + Sex + (1 | Subject) + storage_time
y ~ current_feature + Sex + (1 | Subject) + storage_time
y ~ current_feature + Sex + (1 | Subject) + storage_time
y ~ current_feature + Sex + (1 | Subject) + storage_time
y ~ current_feature + Sex + (1 | Subject) + storage_time

y ~ current_feature + Sex + (1 | Subject) + storage_time
y ~ current_feature + Sex + (1 | Subject) + storage_time
y ~ current_feature + Sex + (1 | Subject) + storage_time
y ~ current_feature + Sex + (1 | Subject) + storage_time
y ~ current_feature + Sex + (1 | Subject) + storage_time
y ~ current_feature + Sex + (1 | Subject) + storage_time
y ~ current_feature + Sex + (1 | Subject) + storage_time
y ~ current_feature + Sex + (1 | Subject) + storage_time
y ~ current_feature + Sex + (1 | Subject) + storage_time
y ~ current_feature + Sex + (1 | Subject) + storage_time
y ~ current_feature + Sex + (1 | Subject) + storage_time
y ~ current_feature + Sex + (1 | Subject) + storage_time
y ~ current_feature + Sex + (1 | Subject) + storage_time
y ~ current_feature + Sex + (1 | Subject) + storage_time
y ~ current_feature + Sex + (1 | Subject) + storage_time
y ~ current_feature + Sex + (1 | Subject) + storage_time
y ~ current_feature + Sex + (1 | Subject) + storage_time
y ~ current_feature + Sex + (1 | Subject) + storage_time
y ~ current_feature + Sex + (1 | Subject) + storage_time
y ~ current_feature + Sex + (1 | Subject) + storage_time
y ~ current_feature + Sex + (1 | Subject) + storage_time
y ~ current_feature + Sex + (1 | Subject) + storage_time
y ~ current_feature + Sex + (1 | Subject) + storage_time
y ~ current_feature + Sex + (1 | Subject) + storage_time
y ~ current_feature + Sex + (1 | Subject) + storage_time
y ~ current_feature + Sex + (1 | Subject) + storage_time
y ~ current_feature + Sex + (1 | Subject) + storage_time
y ~ current_feature + Sex + (1 | Subject) + storage_time
y ~ current_feature + Sex + (1 | Subject) + storage_time
y ~ current_feature + Sex + (1 | Subject) + storage_time
y ~ current_feature + Sex + (1 | Subject) + storage_time
y ~ current_feature + Sex + (1 | Subject) + storage_time
y ~ current_feature + Sex + (1 | Subject) + storage_time
y ~ current_feature + Sex + (1 | Subject) + storage_time
y ~ current_feature + Sex + (1 | Subject) + storage_time
y ~ current_feature + Sex + (1 | Subject) + storage_time
y ~ current_feature + Sex + (1 | Subject) + storage_time
y ~ current_feature + Sex + (1 | Subject) + storage_time
y ~ current_feature + Sex + (1 | Subject) + storage_time
y ~ current_feature + Sex + (1 | Subject) + storage_time
y ~ current_feature + Sex + (1 | Subject) + storage_time
y ~ current_feature + Sex + (1 | Subject) + storage_time
y ~ current_feature + Sex + (1 | Subject) + storage_time
y ~ current_feature + Sex + (1 | Subject) + storage_time
y ~ current_feature + Sex + (1 | Subject) + storage_time
y ~ current_feature + Sex + (1 | Subject) + storage_time
y ~ current_feature + Sex + (1 | Subject) + storage_time
y ~ current_feature + Sex + (1 | Subject) + storage_time

y ~ current_feature + Sex + (1 | Subject) + storage_time
y ~ current_feature + Sex + (1 | Subject) + storage_time
y ~ current_feature + Sex + (1 | Subject) + storage_time
y ~ current_feature + Sex + (1 | Subject) + storage_time
y ~ current_feature + Sex + (1 | Subject) + storage_time
y ~ current_feature + Sex + (1 | Subject) + storage_time
y ~ current_feature + Sex + (1 | Subject) + storage_time
y ~ current_feature + Sex + (1 | Subject) + storage_time
y ~ current_feature + Sex + (1 | Subject) + storage_time
y ~ current_feature + Sex + (1 | Subject) + storage_time
y ~ current_feature + Sex + (1 | Subject) + storage_time
y ~ current_feature + Sex + (1 | Subject) + storage_time
y ~ current_feature + Sex + (1 | Subject) + storage_time
y ~ current_feature + Sex + (1 | Subject) + storage_time
y ~ current_feature + Sex + (1 | Subject) + storage_time
y ~ current_feature + Sex + (1 | Subject) + storage_time
y ~ current_feature + Sex + (1 | Subject) + storage_time
y ~ current_feature + Sex + (1 | Subject) + storage_time
y ~ current_feature + Sex + (1 | Subject) + storage_time
y ~ current_feature + Sex + (1 | Subject) + storage_time
y ~ current_feature + Sex + (1 | Subject) + storage_time
y ~ current_feature + Sex + (1 | Subject) + storage_time
y ~ current_feature + Sex + (1 | Subject) + storage_time
y ~ current_feature + Sex + (1 | Subject) + storage_time
y ~ current_feature + Sex + (1 | Subject) + storage_time
y ~ current_feature + Sex + (1 | Subject) + storage_time
y ~ current_feature + Sex + (1 | Subject) + storage_time
y ~ current_feature + Sex + (1 | Subject) + storage_time
y ~ current_feature + Sex + (1 | Subject) + storage_time
y ~ current_feature + Sex + (1 | Subject) + storage_time
y ~ current_feature + Sex + (1 | Subject) + storage_time
y ~ current_feature + Sex + (1 | Subject) + storage_time
y ~ current_feature + Sex + (1 | Subject) + storage_time
y ~ current_feature + Sex + (1 | Subject) + storage_time
y ~ current_feature + Sex + (1 | Subject) + storage_time
y ~ current_feature + Sex + (1 | Subject) + storage_time
y ~ current_feature + Sex + (1 | Subject) + storage_time
y ~ current_feature + Sex + (1 | Subject) + storage_time
y ~ current_feature + Sex + (1 | Subject) + storage_time
y ~ current_feature + Sex + (1 | Subject) + storage_time
y ~ current_feature + Sex + (1 | Subject) + storage_time
y ~ current_feature + Sex + (1 | Subject) + storage_time
y ~ current_feature + Sex + (1 | Subject) + storage_time
y ~ current_feature + Sex + (1 | Subject) + storage_time
y ~ current_feature + Sex + (1 | Subject) + storage_time
y ~ current_feature + Sex + (1 | Subject) + storage_time
y ~ current_feature + Sex + (1 | Subject) + storage_time
y ~ current_feature + Sex + (1 | Subject) + storage_time

y ~ current_feature + Sex + (1 | Subject) + storage_time
y ~ current_feature + Sex + (1 | Subject) + storage_time
y ~ current_feature + Sex + (1 | Subject) + storage_time
y ~ current_feature + Sex + (1 | Subject) + storage_time
y ~ current_feature + Sex + (1 | Subject) + storage_time
y ~ current_feature + Sex + (1 | Subject) + storage_time
y ~ current_feature + Sex + (1 | Subject) + storage_time
y ~ current_feature + Sex + (1 | Subject) + storage_time
y ~ current_feature + Sex + (1 | Subject) + storage_time
y ~ current_feature + Sex + (1 | Subject) + storage_time
y ~ current_feature + Sex + (1 | Subject) + storage_time
y ~ current_feature + Sex + (1 | Subject) + storage_time
y ~ current_feature + Sex + (1 | Subject) + storage_time
y ~ current_feature + Sex + (1 | Subject) + storage_time
y ~ current_feature + Sex + (1 | Subject) + storage_time
y ~ current_feature + Sex + (1 | Subject) + storage_time
y ~ current_feature + Sex + (1 | Subject) + storage_time
y ~ current_feature + Sex + (1 | Subject) + storage_time
y ~ current_feature + Sex + (1 | Subject) + storage_time
y ~ current_feature + Sex + (1 | Subject) + storage_time
y ~ current_feature + Sex + (1 | Subject) + storage_time
y ~ current_feature + Sex + (1 | Subject) + storage_time
y ~ current_feature + Sex + (1 | Subject) + storage_time
y ~ current_feature + Sex + (1 | Subject) + storage_time
y ~ current_feature + Sex + (1 | Subject) + storage_time
y ~ current_feature + Sex + (1 | Subject) + storage_time
y ~ current_feature + Sex + (1 | Subject) + storage_time
y ~ current_feature + Sex + (1 | Subject) + storage_time
y ~ current_feature + Sex + (1 | Subject) + storage_time
y ~ current_feature + Sex + (1 | Subject) + storage_time
y ~ current_feature + Sex + (1 | Subject) + storage_time
y ~ current_feature + Sex + (1 | Subject) + storage_time
y ~ current_feature + Sex + (1 | Subject) + storage_time
y ~ current_feature + Sex + (1 | Subject) + storage_time
y ~ current_feature + Sex + (1 | Subject) + storage_time
y ~ current_feature + Sex + (1 | Subject) + storage_time
y ~ current_feature + Sex + (1 | Subject) + storage_time
y ~ current_feature + Sex + (1 | Subject) + storage_time
y ~ current_feature + Sex + (1 | Subject) + storage_time
y ~ current_feature + Sex + (1 | Subject) + storage_time
y ~ current_feature + Sex + (1 | Subject) + storage_time
y ~ current_feature + Sex + (1 | Subject) + storage_time
y ~ current_feature + Sex + (1 | Subject) + storage_time
y ~ current_feature + Sex + (1 | Subject) + storage_time
y ~ current_feature + Sex + (1 | Subject) + storage_time
y ~ current_feature + Sex + (1 | Subject) + storage_time
y ~ current_feature + Sex + (1 | Subject) + storage_time
y ~ current_feature + Sex + (1 | Subject) + storage_time

y ~ current_feature + Sex + (1 | Subject) + storage_time
y ~ current_feature + Sex + (1 | Subject) + storage_time
y ~ current_feature + Sex + (1 | Subject) + storage_time
y ~ current_feature + Sex + (1 | Subject) + storage_time
y ~ current_feature + Sex + (1 | Subject) + storage_time
y ~ current_feature + Sex + (1 | Subject) + storage_time
y ~ current_feature + Sex + (1 | Subject) + storage_time
y ~ current_feature + Sex + (1 | Subject) + storage_time
y ~ current_feature + Sex + (1 | Subject) + storage_time
y ~ current_feature + Sex + (1 | Subject) + storage_time
y ~ current_feature + Sex + (1 | Subject) + storage_time
y ~ current_feature + Sex + (1 | Subject) + storage_time
y ~ current_feature + Sex + (1 | Subject) + storage_time
y ~ current_feature + Sex + (1 | Subject) + storage_time
y ~ current_feature + Sex + (1 | Subject) + storage_time
y ~ current_feature + Sex + (1 | Subject) + storage_time
y ~ current_feature + Sex + (1 | Subject) + storage_time
y ~ current_feature + Sex + (1 | Subject) + storage_time
y ~ current_feature + Sex + (1 | Subject) + storage_time
y ~ current_feature + Sex + (1 | Subject) + storage_time
y ~ current_feature + Sex + (1 | Subject) + storage_time
y ~ current_feature + Sex + (1 | Subject) + storage_time
y ~ current_feature + Sex + (1 | Subject) + storage_time
y ~ current_feature + Sex + (1 | Subject) + storage_time
y ~ current_feature + Sex + (1 | Subject) + storage_time
y ~ current_feature + Sex + (1 | Subject) + storage_time
y ~ current_feature + Sex + (1 | Subject) + storage_time
y ~ current_feature + Sex + (1 | Subject) + storage_time
y ~ current_feature + Sex + (1 | Subject) + storage_time
y ~ current_feature + Sex + (1 | Subject) + storage_time
y ~ current_feature + Sex + (1 | Subject) + storage_time
y ~ current_feature + Sex + (1 | Subject) + storage_time
y ~ current_feature + Sex + (1 | Subject) + storage_time
y ~ current_feature + Sex + (1 | Subject) + storage_time
y ~ current_feature + Sex + (1 | Subject) + storage_time
y ~ current_feature + Sex + (1 | Subject) + storage_time
y ~ current_feature + Sex + (1 | Subject) + storage_time
y ~ current_feature + Sex + (1 | Subject) + storage_time
y ~ current_feature + Sex + (1 | Subject) + storage_time
y ~ current_feature + Sex + (1 | Subject) + storage_time
y ~ current_feature + Sex + (1 | Subject) + storage_time
y ~ current_feature + Sex + (1 | Subject) + storage_time
y ~ current_feature + Sex + (1 | Subject) + storage_time
y ~ current_feature + Sex + (1 | Subject) + storage_time
y ~ current_feature + Sex + (1 | Subject) + storage_time
y ~ current_feature + Sex + (1 | Subject) + storage_time
y ~ current_feature + Sex + (1 | Subject) + storage_time
y ~ current_feature + Sex + (1 | Subject) + storage_time

y ~ current_feature + Sex + (1 | Subject) + storage_time
y ~ current_feature + Sex + (1 | Subject) + storage_time
y ~ current_feature + Sex + (1 | Subject) + storage_time
y ~ current_feature + Sex + (1 | Subject) + storage_time
y ~ current_feature + Sex + (1 | Subject) + storage_time
y ~ current_feature + Sex + (1 | Subject) + storage_time
y ~ current_feature + Sex + (1 | Subject) + storage_time
y ~ current_feature + Sex + (1 | Subject) + storage_time
y ~ current_feature + Sex + (1 | Subject) + storage_time
y ~ current_feature + Sex + (1 | Subject) + storage_time
y ~ current_feature + Sex + (1 | Subject) + storage_time
y ~ current_feature + Sex + (1 | Subject) + storage_time
y ~ current_feature + Sex + (1 | Subject) + storage_time
y ~ current_feature + Sex + (1 | Subject) + storage_time
y ~ current_feature + Sex + (1 | Subject) + storage_time
y ~ current_feature + Sex + (1 | Subject) + storage_time
y ~ current_feature + Sex + (1 | Subject) + storage_time
y ~ current_feature + Sex + (1 | Subject) + storage_time
y ~ current_feature + Sex + (1 | Subject) + storage_time
y ~ current_feature + Sex + (1 | Subject) + storage_time
y ~ current_feature + Sex + (1 | Subject) + storage_time
y ~ current_feature + Sex + (1 | Subject) + storage_time
y ~ current_feature + Sex + (1 | Subject) + storage_time
y ~ current_feature + Sex + (1 | Subject) + storage_time
y ~ current_feature + Sex + (1 | Subject) + storage_time
y ~ current_feature + Sex + (1 | Subject) + storage_time
y ~ current_feature + Sex + (1 | Subject) + storage_time
y ~ current_feature + Sex + (1 | Subject) + storage_time
y ~ current_feature + Sex + (1 | Subject) + storage_time
y ~ current_feature + Sex + (1 | Subject) + storage_time
y ~ current_feature + Sex + (1 | Subject) + storage_time
y ~ current_feature + Sex + (1 | Subject) + storage_time
y ~ current_feature + Sex + (1 | Subject) + storage_time
y ~ current_feature + Sex + (1 | Subject) + storage_time
y ~ current_feature + Sex + (1 | Subject) + storage_time
y ~ current_feature + Sex + (1 | Subject) + storage_time
y ~ current_feature + Sex + (1 | Subject) + storage_time
y ~ current_feature + Sex + (1 | Subject) + storage_time
y ~ current_feature + Sex + (1 | Subject) + storage_time
y ~ current_feature + Sex + (1 | Subject) + storage_time
y ~ current_feature + Sex + (1 | Subject) + storage_time
y ~ current_feature + Sex + (1 | Subject) + storage_time
y ~ current_feature + Sex + (1 | Subject) + storage_time
y ~ current_feature + Sex + (1 | Subject) + storage_time
y ~ current_feature + Sex + (1 | Subject) + storage_time
y ~ current_feature + Sex + (1 | Subject) + storage_time
y ~ current_feature + Sex + (1 | Subject) + storage_time
y ~ current_feature + Sex + (1 | Subject) + storage_time

y ~ current_feature + Sex + (1 | Subject) + storage_time
y ~ current_feature + Sex + (1 | Subject) + storage_time
y ~ current_feature + Sex + (1 | Subject) + storage_time
y ~ current_feature + Sex + (1 | Subject) + storage_time
y ~ current_feature + Sex + (1 | Subject) + storage_time
y ~ current_feature + Sex + (1 | Subject) + storage_time
y ~ current_feature + Sex + (1 | Subject) + storage_time
y ~ current_feature + Sex + (1 | Subject) + storage_time
y ~ current_feature + Sex + (1 | Subject) + storage_time
y ~ current_feature + Sex + (1 | Subject) + storage_time
y ~ current_feature + Sex + (1 | Subject) + storage_time
y ~ current_feature + Sex + (1 | Subject) + storage_time
y ~ current_feature + Sex + (1 | Subject) + storage_time
y ~ current_feature + Sex + (1 | Subject) + storage_time
y ~ current_feature + Sex + (1 | Subject) + storage_time
y ~ current_feature + Sex + (1 | Subject) + storage_time
y ~ current_feature + Sex + (1 | Subject) + storage_time
y ~ current_feature + Sex + (1 | Subject) + storage_time
y ~ current_feature + Sex + (1 | Subject) + storage_time
y ~ current_feature + Sex + (1 | Subject) + storage_time
y ~ current_feature + Sex + (1 | Subject) + storage_time
y ~ current_feature + Sex + (1 | Subject) + storage_time
y ~ current_feature + Sex + (1 | Subject) + storage_time
y ~ current_feature + Sex + (1 | Subject) + storage_time
y ~ current_feature + Sex + (1 | Subject) + storage_time
y ~ current_feature + Sex + (1 | Subject) + storage_time
y ~ current_feature + Sex + (1 | Subject) + storage_time
y ~ current_feature + Sex + (1 | Subject) + storage_time
y ~ current_feature + Sex + (1 | Subject) + storage_time
y ~ current_feature + Sex + (1 | Subject) + storage_time
y ~ current_feature + Sex + (1 | Subject) + storage_time
y ~ current_feature + Sex + (1 | Subject) + storage_time
y ~ current_feature + Sex + (1 | Subject) + storage_time
y ~ current_feature + Sex + (1 | Subject) + storage_time
y ~ current_feature + Sex + (1 | Subject) + storage_time
y ~ current_feature + Sex + (1 | Subject) + storage_time
y ~ current_feature + Sex + (1 | Subject) + storage_time
y ~ current_feature + Sex + (1 | Subject) + storage_time
y ~ current_feature + Sex + (1 | Subject) + storage_time
y ~ current_feature + Sex + (1 | Subject) + storage_time
y ~ current_feature + Sex + (1 | Subject) + storage_time
y ~ current_feature + Sex + (1 | Subject) + storage_time
y ~ current_feature + Sex + (1 | Subject) + storage_time
y ~ current_feature + Sex + (1 | Subject) + storage_time
y ~ current_feature + Sex + (1 | Subject) + storage_time
y ~ current_feature + Sex + (1 | Subject) + storage_time
y ~ current_feature + Sex + (1 | Subject) + storage_time
y ~ current_feature + Sex + (1 | Subject) + storage_time

```
y ~ current_feature + Sex + (1 | Subject) + storage_time
y ~ current_feature + Sex + (1 | Subject) + storage_time
y ~ current_feature + Sex + (1 | Subject) + storage_time
y ~ current_feature + Sex + (1 | Subject) + storage_time
y ~ current_feature + Sex + (1 | Subject) + storage_time
y ~ current_feature + Sex + (1 | Subject) + storage_time
y ~ current_feature + Sex + (1 | Subject) + storage_time
y ~ current_feature + Sex + (1 | Subject) + storage_time
y ~ current_feature + Sex + (1 | Subject) + storage_time
y ~ current_feature + Sex + (1 | Subject) + storage_time
y ~ current_feature + Sex + (1 | Subject) + storage_time
y ~ current_feature + Sex + (1 | Subject) + storage_time
y ~ current_feature + Sex + (1 | Subject) + storage_time
y ~ current_feature + Sex + (1 | Subject) + storage_time
y ~ current_feature + Sex + (1 | Subject) + storage_time
y ~ current_feature + Sex + (1 | Subject) + storage_time
y ~ current_feature + Sex + (1 | Subject) + storage_time
y ~ current_feature + Sex + (1 | Subject) + storage_time
y ~ current_feature + Sex + (1 | Subject) + storage_time
y ~ current_feature + Sex + (1 | Subject) + storage_time
y ~ current_feature + Sex + (1 | Subject) + storage_time
y ~ current_feature + Sex + (1 | Subject) + storage_time
y ~ current_feature + Sex + (1 | Subject) + storage_time
y ~ current_feature + Sex + (1 | Subject) + storage_time
y ~ current_feature + Sex + (1 | Subject) + storage_time
y ~ current_feature + Sex + (1 | Subject) + storage_time
y ~ current_feature + Sex + (1 | Subject) + storage_time
y ~ current_feature + Sex + (1 | Subject) + storage_time
y ~ current_feature + Sex + (1 | Subject) + storage_time
y ~ current_feature + Sex + (1 | Subject) + storage_time
y ~ current_feature + Sex + (1 | Subject) + storage_time
y ~ current_feature + Sex + (1 | Subject) + storage_time
y ~ current_feature + Sex + (1 | Subject) + storage_time
y ~ current_feature + Sex + (1 | Subject) + storage_time
y ~ current_feature + Sex + (1 | Subject) + storage_time
y ~ current_feature + Sex + (1 | Subject) + storage_time
y ~ current_feature + Sex + (1 | Subject) + storage_time
y ~ current_feature + Sex + (1 | Subject) + storage_time
y ~ current_feature + Sex + (1 | Subject) + storage_time
y ~ current_feature + Sex + (1 | Subject) + storage_time
y ~ current_feature + Sex + (1 | Subject) + storage_time
y ~ current_feature + Sex + (1 | Subject) + storage_time
y ~ current_feature + Sex + (1 | Subject) + storage_time
y ~ current_feature + Sex + (1 | Subject) + storage_time
y ~ current_feature + Sex + (1 | Subject) + storage_time
y ~ current_feature + Sex + (1 | Subject) + storage_time
y ~ current_feature + Sex + (1 | Subject) + storage_time
y ~ current_feature + Sex + (1 | Subject) + storage_time
```

y ~ current_feature + Sex + (1 | Subject) + storage_time
y ~ current_feature + Sex + (1 | Subject) + storage_time
y ~ current_feature + Sex + (1 | Subject) + storage_time
y ~ current_feature + Sex + (1 | Subject) + storage_time
y ~ current_feature + Sex + (1 | Subject) + storage_time
y ~ current_feature + Sex + (1 | Subject) + storage_time
y ~ current_feature + Sex + (1 | Subject) + storage_time
y ~ current_feature + Sex + (1 | Subject) + storage_time
y ~ current_feature + Sex + (1 | Subject) + storage_time
y ~ current_feature + Sex + (1 | Subject) + storage_time
y ~ current_feature + Sex + (1 | Subject) + storage_time
y ~ current_feature + Sex + (1 | Subject) + storage_time
y ~ current_feature + Sex + (1 | Subject) + storage_time
y ~ current_feature + Sex + (1 | Subject) + storage_time
y ~ current_feature + Sex + (1 | Subject) + storage_time
y ~ current_feature + Sex + (1 | Subject) + storage_time
y ~ current_feature + Sex + (1 | Subject) + storage_time
y ~ current_feature + Sex + (1 | Subject) + storage_time
y ~ current_feature + Sex + (1 | Subject) + storage_time
y ~ current_feature + Sex + (1 | Subject) + storage_time
y ~ current_feature + Sex + (1 | Subject) + storage_time
y ~ current_feature + Sex + (1 | Subject) + storage_time
y ~ current_feature + Sex + (1 | Subject) + storage_time
y ~ current_feature + Sex + (1 | Subject) + storage_time
y ~ current_feature + Sex + (1 | Subject) + storage_time
y ~ current_feature + Sex + (1 | Subject) + storage_time
y ~ current_feature + Sex + (1 | Subject) + storage_time
y ~ current_feature + Sex + (1 | Subject) + storage_time
y ~ current_feature + Sex + (1 | Subject) + storage_time
y ~ current_feature + Sex + (1 | Subject) + storage_time
y ~ current_feature + Sex + (1 | Subject) + storage_time
y ~ current_feature + Sex + (1 | Subject) + storage_time
y ~ current_feature + Sex + (1 | Subject) + storage_time
y ~ current_feature + Sex + (1 | Subject) + storage_time
y ~ current_feature + Sex + (1 | Subject) + storage_time
y ~ current_feature + Sex + (1 | Subject) + storage_time
y ~ current_feature + Sex + (1 | Subject) + storage_time
y ~ current_feature + Sex + (1 | Subject) + storage_time
y ~ current_feature + Sex + (1 | Subject) + storage_time
y ~ current_feature + Sex + (1 | Subject) + storage_time
y ~ current_feature + Sex + (1 | Subject) + storage_time
y ~ current_feature + Sex + (1 | Subject) + storage_time
y ~ current_feature + Sex + (1 | Subject) + storage_time
y ~ current_feature + Sex + (1 | Subject) + storage_time
y ~ current_feature + Sex + (1 | Subject) + storage_time
y ~ current_feature + Sex + (1 | Subject) + storage_time
y ~ current_feature + Sex + (1 | Subject) + storage_time
y ~ current_feature + Sex + (1 | Subject) + storage_time

y ~ current_feature + Sex + (1 | Subject) + storage_time
y ~ current_feature + Sex + (1 | Subject) + storage_time
y ~ current_feature + Sex + (1 | Subject) + storage_time
y ~ current_feature + Sex + (1 | Subject) + storage_time
y ~ current_feature + Sex + (1 | Subject) + storage_time
y ~ current_feature + Sex + (1 | Subject) + storage_time
y ~ current_feature + Sex + (1 | Subject) + storage_time
y ~ current_feature + Sex + (1 | Subject) + storage_time
y ~ current_feature + Sex + (1 | Subject) + storage_time
y ~ current_feature + Sex + (1 | Subject) + storage_time
y ~ current_feature + Sex + (1 | Subject) + storage_time
y ~ current_feature + Sex + (1 | Subject) + storage_time
y ~ current_feature + Sex + (1 | Subject) + storage_time
y ~ current_feature + Sex + (1 | Subject) + storage_time
y ~ current_feature + Sex + (1 | Subject) + storage_time
y ~ current_feature + Sex + (1 | Subject) + storage_time
y ~ current_feature + Sex + (1 | Subject) + storage_time
y ~ current_feature + Sex + (1 | Subject) + storage_time
y ~ current_feature + Sex + (1 | Subject) + storage_time
y ~ current_feature + Sex + (1 | Subject) + storage_time
y ~ current_feature + Sex + (1 | Subject) + storage_time
y ~ current_feature + Sex + (1 | Subject) + storage_time
y ~ current_feature + Sex + (1 | Subject) + storage_time
y ~ current_feature + Sex + (1 | Subject) + storage_time
y ~ current_feature + Sex + (1 | Subject) + storage_time
y ~ current_feature + Sex + (1 | Subject) + storage_time
y ~ current_feature + Sex + (1 | Subject) + storage_time
y ~ current_feature + Sex + (1 | Subject) + storage_time
y ~ current_feature + Sex + (1 | Subject) + storage_time
y ~ current_feature + Sex + (1 | Subject) + storage_time
y ~ current_feature + Sex + (1 | Subject) + storage_time
y ~ current_feature + Sex + (1 | Subject) + storage_time
y ~ current_feature + Sex + (1 | Subject) + storage_time
y ~ current_feature + Sex + (1 | Subject) + storage_time
y ~ current_feature + Sex + (1 | Subject) + storage_time
y ~ current_feature + Sex + (1 | Subject) + storage_time
y ~ current_feature + Sex + (1 | Subject) + storage_time
y ~ current_feature + Sex + (1 | Subject) + storage_time
y ~ current_feature + Sex + (1 | Subject) + storage_time
y ~ current_feature + Sex + (1 | Subject) + storage_time
y ~ current_feature + Sex + (1 | Subject) + storage_time
y ~ current_feature + Sex + (1 | Subject) + storage_time
y ~ current_feature + Sex + (1 | Subject) + storage_time
y ~ current_feature + Sex + (1 | Subject) + storage_time
y ~ current_feature + Sex + (1 | Subject) + storage_time
y ~ current_feature + Sex + (1 | Subject) + storage_time
y ~ current_feature + Sex + (1 | Subject) + storage_time
y ~ current_feature + Sex + (1 | Subject) + storage_time

y ~ current_feature + Sex + (1 | Subject) + storage_time
y ~ current_feature + Sex + (1 | Subject) + storage_time
y ~ current_feature + Sex + (1 | Subject) + storage_time
y ~ current_feature + Sex + (1 | Subject) + storage_time
y ~ current_feature + Sex + (1 | Subject) + storage_time
y ~ current_feature + Sex + (1 | Subject) + storage_time
y ~ current_feature + Sex + (1 | Subject) + storage_time
y ~ current_feature + Sex + (1 | Subject) + storage_time
y ~ current_feature + Sex + (1 | Subject) + storage_time
y ~ current_feature + Sex + (1 | Subject) + storage_time
y ~ current_feature + Sex + (1 | Subject) + storage_time
y ~ current_feature + Sex + (1 | Subject) + storage_time
y ~ current_feature + Sex + (1 | Subject) + storage_time
y ~ current_feature + Sex + (1 | Subject) + storage_time
y ~ current_feature + Sex + (1 | Subject) + storage_time
y ~ current_feature + Sex + (1 | Subject) + storage_time
y ~ current_feature + Sex + (1 | Subject) + storage_time
y ~ current_feature + Sex + (1 | Subject) + storage_time
y ~ current_feature + Sex + (1 | Subject) + storage_time
y ~ current_feature + Sex + (1 | Subject) + storage_time
y ~ current_feature + Sex + (1 | Subject) + storage_time
y ~ current_feature + Sex + (1 | Subject) + storage_time
y ~ current_feature + Sex + (1 | Subject) + storage_time
y ~ current_feature + Sex + (1 | Subject) + storage_time
y ~ current_feature + Sex + (1 | Subject) + storage_time
y ~ current_feature + Sex + (1 | Subject) + storage_time
y ~ current_feature + Sex + (1 | Subject) + storage_time
y ~ current_feature + Sex + (1 | Subject) + storage_time
y ~ current_feature + Sex + (1 | Subject) + storage_time
y ~ current_feature + Sex + (1 | Subject) + storage_time
y ~ current_feature + Sex + (1 | Subject) + storage_time
y ~ current_feature + Sex + (1 | Subject) + storage_time
y ~ current_feature + Sex + (1 | Subject) + storage_time
y ~ current_feature + Sex + (1 | Subject) + storage_time
y ~ current_feature + Sex + (1 | Subject) + storage_time
y ~ current_feature + Sex + (1 | Subject) + storage_time
y ~ current_feature + Sex + (1 | Subject) + storage_time
y ~ current_feature + Sex + (1 | Subject) + storage_time
y ~ current_feature + Sex + (1 | Subject) + storage_time
y ~ current_feature + Sex + (1 | Subject) + storage_time
y ~ current_feature + Sex + (1 | Subject) + storage_time
y ~ current_feature + Sex + (1 | Subject) + storage_time
y ~ current_feature + Sex + (1 | Subject) + storage_time
y ~ current_feature + Sex + (1 | Subject) + storage_time
y ~ current_feature + Sex + (1 | Subject) + storage_time
y ~ current_feature + Sex + (1 | Subject) + storage_time
y ~ current_feature + Sex + (1 | Subject) + storage_time
y ~ current_feature + Sex + (1 | Subject) + storage_time

y ~ current_feature + Sex + (1 | Subject) + storage_time
y ~ current_feature + Sex + (1 | Subject) + storage_time
y ~ current_feature + Sex + (1 | Subject) + storage_time
y ~ current_feature + Sex + (1 | Subject) + storage_time
y ~ current_feature + Sex + (1 | Subject) + storage_time
y ~ current_feature + Sex + (1 | Subject) + storage_time
y ~ current_feature + Sex + (1 | Subject) + storage_time
y ~ current_feature + Sex + (1 | Subject) + storage_time
y ~ current_feature + Sex + (1 | Subject) + storage_time
y ~ current_feature + Sex + (1 | Subject) + storage_time
y ~ current_feature + Sex + (1 | Subject) + storage_time
y ~ current_feature + Sex + (1 | Subject) + storage_time
y ~ current_feature + Sex + (1 | Subject) + storage_time
y ~ current_feature + Sex + (1 | Subject) + storage_time
y ~ current_feature + Sex + (1 | Subject) + storage_time
y ~ current_feature + Sex + (1 | Subject) + storage_time
y ~ current_feature + Sex + (1 | Subject) + storage_time
y ~ current_feature + Sex + (1 | Subject) + storage_time
y ~ current_feature + Sex + (1 | Subject) + storage_time
y ~ current_feature + Sex + (1 | Subject) + storage_time
y ~ current_feature + Sex + (1 | Subject) + storage_time
y ~ current_feature + Sex + (1 | Subject) + storage_time
y ~ current_feature + Sex + (1 | Subject) + storage_time
y ~ current_feature + Sex + (1 | Subject) + storage_time
y ~ current_feature + Sex + (1 | Subject) + storage_time
y ~ current_feature + Sex + (1 | Subject) + storage_time
y ~ current_feature + Sex + (1 | Subject) + storage_time
y ~ current_feature + Sex + (1 | Subject) + storage_time
y ~ current_feature + Sex + (1 | Subject) + storage_time
y ~ current_feature + Sex + (1 | Subject) + storage_time
y ~ current_feature + Sex + (1 | Subject) + storage_time
y ~ current_feature + Sex + (1 | Subject) + storage_time
y ~ current_feature + Sex + (1 | Subject) + storage_time
y ~ current_feature + Sex + (1 | Subject) + storage_time
y ~ current_feature + Sex + (1 | Subject) + storage_time
y ~ current_feature + Sex + (1 | Subject) + storage_time
y ~ current_feature + Sex + (1 | Subject) + storage_time
y ~ current_feature + Sex + (1 | Subject) + storage_time
y ~ current_feature + Sex + (1 | Subject) + storage_time
y ~ current_feature + Sex + (1 | Subject) + storage_time
y ~ current_feature + Sex + (1 | Subject) + storage_time
y ~ current_feature + Sex + (1 | Subject) + storage_time
y ~ current_feature + Sex + (1 | Subject) + storage_time
y ~ current_feature + Sex + (1 | Subject) + storage_time
y ~ current_feature + Sex + (1 | Subject) + storage_time
y ~ current_feature + Sex + (1 | Subject) + storage_time
y ~ current_feature + Sex + (1 | Subject) + storage_time
y ~ current_feature + Sex + (1 | Subject) + storage_time

y ~ current_feature + Sex + (1 | Subject) + storage_time
y ~ current_feature + Sex + (1 | Subject) + storage_time
y ~ current_feature + Sex + (1 | Subject) + storage_time
y ~ current_feature + Sex + (1 | Subject) + storage_time
y ~ current_feature + Sex + (1 | Subject) + storage_time
y ~ current_feature + Sex + (1 | Subject) + storage_time
y ~ current_feature + Sex + (1 | Subject) + storage_time
y ~ current_feature + Sex + (1 | Subject) + storage_time
y ~ current_feature + Sex + (1 | Subject) + storage_time
y ~ current_feature + Sex + (1 | Subject) + storage_time
y ~ current_feature + Sex + (1 | Subject) + storage_time
y ~ current_feature + Sex + (1 | Subject) + storage_time
y ~ current_feature + Sex + (1 | Subject) + storage_time
y ~ current_feature + Sex + (1 | Subject) + storage_time
y ~ current_feature + Sex + (1 | Subject) + storage_time
y ~ current_feature + Sex + (1 | Subject) + storage_time
y ~ current_feature + Sex + (1 | Subject) + storage_time
y ~ current_feature + Sex + (1 | Subject) + storage_time
y ~ current_feature + Sex + (1 | Subject) + storage_time
y ~ current_feature + Sex + (1 | Subject) + storage_time
y ~ current_feature + Sex + (1 | Subject) + storage_time
y ~ current_feature + Sex + (1 | Subject) + storage_time
y ~ current_feature + Sex + (1 | Subject) + storage_time
y ~ current_feature + Sex + (1 | Subject) + storage_time
y ~ current_feature + Sex + (1 | Subject) + storage_time
y ~ current_feature + Sex + (1 | Subject) + storage_time
y ~ current_feature + Sex + (1 | Subject) + storage_time
y ~ current_feature + Sex + (1 | Subject) + storage_time
y ~ current_feature + Sex + (1 | Subject) + storage_time
y ~ current_feature + Sex + (1 | Subject) + storage_time
y ~ current_feature + Sex + (1 | Subject) + storage_time
y ~ current_feature + Sex + (1 | Subject) + storage_time
y ~ current_feature + Sex + (1 | Subject) + storage_time
y ~ current_feature + Sex + (1 | Subject) + storage_time
y ~ current_feature + Sex + (1 | Subject) + storage_time
y ~ current_feature + Sex + (1 | Subject) + storage_time
y ~ current_feature + Sex + (1 | Subject) + storage_time
y ~ current_feature + Sex + (1 | Subject) + storage_time
y ~ current_feature + Sex + (1 | Subject) + storage_time
y ~ current_feature + Sex + (1 | Subject) + storage_time
y ~ current_feature + Sex + (1 | Subject) + storage_time
y ~ current_feature + Sex + (1 | Subject) + storage_time
y ~ current_feature + Sex + (1 | Subject) + storage_time
y ~ current_feature + Sex + (1 | Subject) + storage_time
y ~ current_feature + Sex + (1 | Subject) + storage_time
y ~ current_feature + Sex + (1 | Subject) + storage_time
y ~ current_feature + Sex + (1 | Subject) + storage_time
y ~ current_feature + Sex + (1 | Subject) + storage_time

y ~ current_feature + Sex + (1 | Subject) + storage_time
y ~ current_feature + Sex + (1 | Subject) + storage_time
y ~ current_feature + Sex + (1 | Subject) + storage_time
y ~ current_feature + Sex + (1 | Subject) + storage_time
y ~ current_feature + Sex + (1 | Subject) + storage_time
y ~ current_feature + Sex + (1 | Subject) + storage_time
y ~ current_feature + Sex + (1 | Subject) + storage_time
y ~ current_feature + Sex + (1 | Subject) + storage_time
y ~ current_feature + Sex + (1 | Subject) + storage_time
y ~ current_feature + Sex + (1 | Subject) + storage_time
y ~ current_feature + Sex + (1 | Subject) + storage_time
y ~ current_feature + Sex + (1 | Subject) + storage_time
y ~ current_feature + Sex + (1 | Subject) + storage_time
y ~ current_feature + Sex + (1 | Subject) + storage_time
y ~ current_feature + Sex + (1 | Subject) + storage_time
y ~ current_feature + Sex + (1 | Subject) + storage_time
y ~ current_feature + Sex + (1 | Subject) + storage_time
y ~ current_feature + Sex + (1 | Subject) + storage_time
y ~ current_feature + Sex + (1 | Subject) + storage_time
y ~ current_feature + Sex + (1 | Subject) + storage_time
y ~ current_feature + Sex + (1 | Subject) + storage_time
y ~ current_feature + Sex + (1 | Subject) + storage_time
y ~ current_feature + Sex + (1 | Subject) + storage_time
y ~ current_feature + Sex + (1 | Subject) + storage_time
y ~ current_feature + Sex + (1 | Subject) + storage_time
y ~ current_feature + Sex + (1 | Subject) + storage_time
y ~ current_feature + Sex + (1 | Subject) + storage_time
y ~ current_feature + Sex + (1 | Subject) + storage_time
y ~ current_feature + Sex + (1 | Subject) + storage_time
y ~ current_feature + Sex + (1 | Subject) + storage_time
y ~ current_feature + Sex + (1 | Subject) + storage_time
y ~ current_feature + Sex + (1 | Subject) + storage_time
y ~ current_feature + Sex + (1 | Subject) + storage_time
y ~ current_feature + Sex + (1 | Subject) + storage_time
y ~ current_feature + Sex + (1 | Subject) + storage_time
y ~ current_feature + Sex + (1 | Subject) + storage_time
y ~ current_feature + Sex + (1 | Subject) + storage_time
y ~ current_feature + Sex + (1 | Subject) + storage_time
y ~ current_feature + Sex + (1 | Subject) + storage_time
y ~ current_feature + Sex + (1 | Subject) + storage_time
y ~ current_feature + Sex + (1 | Subject) + storage_time
y ~ current_feature + Sex + (1 | Subject) + storage_time
y ~ current_feature + Sex + (1 | Subject) + storage_time
y ~ current_feature + Sex + (1 | Subject) + storage_time
y ~ current_feature + Sex + (1 | Subject) + storage_time
y ~ current_feature + Sex + (1 | Subject) + storage_time
y ~ current_feature + Sex + (1 | Subject) + storage_time
y ~ current_feature + Sex + (1 | Subject) + storage_time

y ~ current_feature + Sex + (1 | Subject) + storage_time
y ~ current_feature + Sex + (1 | Subject) + storage_time
y ~ current_feature + Sex + (1 | Subject) + storage_time
y ~ current_feature + Sex + (1 | Subject) + storage_time
y ~ current_feature + Sex + (1 | Subject) + storage_time
y ~ current_feature + Sex + (1 | Subject) + storage_time
y ~ current_feature + Sex + (1 | Subject) + storage_time
y ~ current_feature + Sex + (1 | Subject) + storage_time
y ~ current_feature + Sex + (1 | Subject) + storage_time
y ~ current_feature + Sex + (1 | Subject) + storage_time
y ~ current_feature + Sex + (1 | Subject) + storage_time
y ~ current_feature + Sex + (1 | Subject) + storage_time
y ~ current_feature + Sex + (1 | Subject) + storage_time
y ~ current_feature + Sex + (1 | Subject) + storage_time
y ~ current_feature + Sex + (1 | Subject) + storage_time
y ~ current_feature + Sex + (1 | Subject) + storage_time
y ~ current_feature + Sex + (1 | Subject) + storage_time
y ~ current_feature + Sex + (1 | Subject) + storage_time
y ~ current_feature + Sex + (1 | Subject) + storage_time
y ~ current_feature + Sex + (1 | Subject) + storage_time
y ~ current_feature + Sex + (1 | Subject) + storage_time
y ~ current_feature + Sex + (1 | Subject) + storage_time
y ~ current_feature + Sex + (1 | Subject) + storage_time
y ~ current_feature + Sex + (1 | Subject) + storage_time
y ~ current_feature + Sex + (1 | Subject) + storage_time
y ~ current_feature + Sex + (1 | Subject) + storage_time
y ~ current_feature + Sex + (1 | Subject) + storage_time
y ~ current_feature + Sex + (1 | Subject) + storage_time
y ~ current_feature + Sex + (1 | Subject) + storage_time
y ~ current_feature + Sex + (1 | Subject) + storage_time
y ~ current_feature + Sex + (1 | Subject) + storage_time
y ~ current_feature + Sex + (1 | Subject) + storage_time
y ~ current_feature + Sex + (1 | Subject) + storage_time
y ~ current_feature + Sex + (1 | Subject) + storage_time
y ~ current_feature + Sex + (1 | Subject) + storage_time
y ~ current_feature + Sex + (1 | Subject) + storage_time
y ~ current_feature + Sex + (1 | Subject) + storage_time
y ~ current_feature + Sex + (1 | Subject) + storage_time
y ~ current_feature + Sex + (1 | Subject) + storage_time
y ~ current_feature + Sex + (1 | Subject) + storage_time
y ~ current_feature + Sex + (1 | Subject) + storage_time
y ~ current_feature + Sex + (1 | Subject) + storage_time
y ~ current_feature + Sex + (1 | Subject) + storage_time
y ~ current_feature + Sex + (1 | Subject) + storage_time
y ~ current_feature + Sex + (1 | Subject) + storage_time
y ~ current_feature + Sex + (1 | Subject) + storage_time
y ~ current_feature + Sex + (1 | Subject) + storage_time
y ~ current_feature + Sex + (1 | Subject) + storage_time

y ~ current_feature + Sex + (1 | Subject) + storage_time
y ~ current_feature + Sex + (1 | Subject) + storage_time
y ~ current_feature + Sex + (1 | Subject) + storage_time
y ~ current_feature + Sex + (1 | Subject) + storage_time
y ~ current_feature + Sex + (1 | Subject) + storage_time
y ~ current_feature + Sex + (1 | Subject) + storage_time
y ~ current_feature + Sex + (1 | Subject) + storage_time
y ~ current_feature + Sex + (1 | Subject) + storage_time
y ~ current_feature + Sex + (1 | Subject) + storage_time
y ~ current_feature + Sex + (1 | Subject) + storage_time
y ~ current_feature + Sex + (1 | Subject) + storage_time
y ~ current_feature + Sex + (1 | Subject) + storage_time
y ~ current_feature + Sex + (1 | Subject) + storage_time
y ~ current_feature + Sex + (1 | Subject) + storage_time
y ~ current_feature + Sex + (1 | Subject) + storage_time
y ~ current_feature + Sex + (1 | Subject) + storage_time
y ~ current_feature + Sex + (1 | Subject) + storage_time
y ~ current_feature + Sex + (1 | Subject) + storage_time
y ~ current_feature + Sex + (1 | Subject) + storage_time
y ~ current_feature + Sex + (1 | Subject) + storage_time
y ~ current_feature + Sex + (1 | Subject) + storage_time
y ~ current_feature + Sex + (1 | Subject) + storage_time
y ~ current_feature + Sex + (1 | Subject) + storage_time
y ~ current_feature + Sex + (1 | Subject) + storage_time
y ~ current_feature + Sex + (1 | Subject) + storage_time
y ~ current_feature + Sex + (1 | Subject) + storage_time
y ~ current_feature + Sex + (1 | Subject) + storage_time
y ~ current_feature + Sex + (1 | Subject) + storage_time
y ~ current_feature + Sex + (1 | Subject) + storage_time
y ~ current_feature + Sex + (1 | Subject) + storage_time
y ~ current_feature + Sex + (1 | Subject) + storage_time
y ~ current_feature + Sex + (1 | Subject) + storage_time
y ~ current_feature + Sex + (1 | Subject) + storage_time
y ~ current_feature + Sex + (1 | Subject) + storage_time
y ~ current_feature + Sex + (1 | Subject) + storage_time
y ~ current_feature + Sex + (1 | Subject) + storage_time
y ~ current_feature + Sex + (1 | Subject) + storage_time
y ~ current_feature + Sex + (1 | Subject) + storage_time
y ~ current_feature + Sex + (1 | Subject) + storage_time
y ~ current_feature + Sex + (1 | Subject) + storage_time
y ~ current_feature + Sex + (1 | Subject) + storage_time
y ~ current_feature + Sex + (1 | Subject) + storage_time
y ~ current_feature + Sex + (1 | Subject) + storage_time
y ~ current_feature + Sex + (1 | Subject) + storage_time
y ~ current_feature + Sex + (1 | Subject) + storage_time
y ~ current_feature + Sex + (1 | Subject) + storage_time
y ~ current_feature + Sex + (1 | Subject) + storage_time
y ~ current_feature + Sex + (1 | Subject) + storage_time

y ~ current_feature + Sex + (1 | Subject) + storage_time
y ~ current_feature + Sex + (1 | Subject) + storage_time
y ~ current_feature + Sex + (1 | Subject) + storage_time
y ~ current_feature + Sex + (1 | Subject) + storage_time
y ~ current_feature + Sex + (1 | Subject) + storage_time
y ~ current_feature + Sex + (1 | Subject) + storage_time
y ~ current_feature + Sex + (1 | Subject) + storage_time
y ~ current_feature + Sex + (1 | Subject) + storage_time
y ~ current_feature + Sex + (1 | Subject) + storage_time
y ~ current_feature + Sex + (1 | Subject) + storage_time
y ~ current_feature + Sex + (1 | Subject) + storage_time
y ~ current_feature + Sex + (1 | Subject) + storage_time
y ~ current_feature + Sex + (1 | Subject) + storage_time
y ~ current_feature + Sex + (1 | Subject) + storage_time
y ~ current_feature + Sex + (1 | Subject) + storage_time
y ~ current_feature + Sex + (1 | Subject) + storage_time
y ~ current_feature + Sex + (1 | Subject) + storage_time
y ~ current_feature + Sex + (1 | Subject) + storage_time
y ~ current_feature + Sex + (1 | Subject) + storage_time
y ~ current_feature + Sex + (1 | Subject) + storage_time
y ~ current_feature + Sex + (1 | Subject) + storage_time
y ~ current_feature + Sex + (1 | Subject) + storage_time
y ~ current_feature + Sex + (1 | Subject) + storage_time
y ~ current_feature + Sex + (1 | Subject) + storage_time
y ~ current_feature + Sex + (1 | Subject) + storage_time
y ~ current_feature + Sex + (1 | Subject) + storage_time
y ~ current_feature + Sex + (1 | Subject) + storage_time
y ~ current_feature + Sex + (1 | Subject) + storage_time
y ~ current_feature + Sex + (1 | Subject) + storage_time
y ~ current_feature + Sex + (1 | Subject) + storage_time
y ~ current_feature + Sex + (1 | Subject) + storage_time
y ~ current_feature + Sex + (1 | Subject) + storage_time
y ~ current_feature + Sex + (1 | Subject) + storage_time
y ~ current_feature + Sex + (1 | Subject) + storage_time
y ~ current_feature + Sex + (1 | Subject) + storage_time
y ~ current_feature + Sex + (1 | Subject) + storage_time
y ~ current_feature + Sex + (1 | Subject) + storage_time
y ~ current_feature + Sex + (1 | Subject) + storage_time
y ~ current_feature + Sex + (1 | Subject) + storage_time
y ~ current_feature + Sex + (1 | Subject) + storage_time
y ~ current_feature + Sex + (1 | Subject) + storage_time
y ~ current_feature + Sex + (1 | Subject) + storage_time
y ~ current_feature + Sex + (1 | Subject) + storage_time
y ~ current_feature + Sex + (1 | Subject) + storage_time
y ~ current_feature + Sex + (1 | Subject) + storage_time
y ~ current_feature + Sex + (1 | Subject) + storage_time
y ~ current_feature + Sex + (1 | Subject) + storage_time
y ~ current_feature + Sex + (1 | Subject) + storage_time

y ~ current_feature + Sex + (1 | Subject) + storage_time
y ~ current_feature + Sex + (1 | Subject) + storage_time
y ~ current_feature + Sex + (1 | Subject) + storage_time
y ~ current_feature + Sex + (1 | Subject) + storage_time
y ~ current_feature + Sex + (1 | Subject) + storage_time
y ~ current_feature + Sex + (1 | Subject) + storage_time
y ~ current_feature + Sex + (1 | Subject) + storage_time
y ~ current_feature + Sex + (1 | Subject) + storage_time
y ~ current_feature + Sex + (1 | Subject) + storage_time
y ~ current_feature + Sex + (1 | Subject) + storage_time
y ~ current_feature + Sex + (1 | Subject) + storage_time
y ~ current_feature + Sex + (1 | Subject) + storage_time
y ~ current_feature + Sex + (1 | Subject) + storage_time
y ~ current_feature + Sex + (1 | Subject) + storage_time
y ~ current_feature + Sex + (1 | Subject) + storage_time
y ~ current_feature + Sex + (1 | Subject) + storage_time
y ~ current_feature + Sex + (1 | Subject) + storage_time
y ~ current_feature + Sex + (1 | Subject) + storage_time
y ~ current_feature + Sex + (1 | Subject) + storage_time
y ~ current_feature + Sex + (1 | Subject) + storage_time
y ~ current_feature + Sex + (1 | Subject) + storage_time
y ~ current_feature + Sex + (1 | Subject) + storage_time
y ~ current_feature + Sex + (1 | Subject) + storage_time
y ~ current_feature + Sex + (1 | Subject) + storage_time
y ~ current_feature + Sex + (1 | Subject) + storage_time
y ~ current_feature + Sex + (1 | Subject) + storage_time
y ~ current_feature + Sex + (1 | Subject) + storage_time
y ~ current_feature + Sex + (1 | Subject) + storage_time
y ~ current_feature + Sex + (1 | Subject) + storage_time
y ~ current_feature + Sex + (1 | Subject) + storage_time
y ~ current_feature + Sex + (1 | Subject) + storage_time
y ~ current_feature + Sex + (1 | Subject) + storage_time
y ~ current_feature + Sex + (1 | Subject) + storage_time
y ~ current_feature + Sex + (1 | Subject) + storage_time
y ~ current_feature + Sex + (1 | Subject) + storage_time
y ~ current_feature + Sex + (1 | Subject) + storage_time
y ~ current_feature + Sex + (1 | Subject) + storage_time
y ~ current_feature + Sex + (1 | Subject) + storage_time
y ~ current_feature + Sex + (1 | Subject) + storage_time
y ~ current_feature + Sex + (1 | Subject) + storage_time
y ~ current_feature + Sex + (1 | Subject) + storage_time
y ~ current_feature + Sex + (1 | Subject) + storage_time
y ~ current_feature + Sex + (1 | Subject) + storage_time
y ~ current_feature + Sex + (1 | Subject) + storage_time
y ~ current_feature + Sex + (1 | Subject) + storage_time
y ~ current_feature + Sex + (1 | Subject) + storage_time
y ~ current_feature + Sex + (1 | Subject) + storage_time
y ~ current_feature + Sex + (1 | Subject) + storage_time

y ~ current_feature + Sex + (1 | Subject) + storage_time
y ~ current_feature + Sex + (1 | Subject) + storage_time
y ~ current_feature + Sex + (1 | Subject) + storage_time
y ~ current_feature + Sex + (1 | Subject) + storage_time
y ~ current_feature + Sex + (1 | Subject) + storage_time
y ~ current_feature + Sex + (1 | Subject) + storage_time
y ~ current_feature + Sex + (1 | Subject) + storage_time
y ~ current_feature + Sex + (1 | Subject) + storage_time
y ~ current_feature + Sex + (1 | Subject) + storage_time
y ~ current_feature + Sex + (1 | Subject) + storage_time
y ~ current_feature + Sex + (1 | Subject) + storage_time
y ~ current_feature + Sex + (1 | Subject) + storage_time
y ~ current_feature + Sex + (1 | Subject) + storage_time
y ~ current_feature + Sex + (1 | Subject) + storage_time
y ~ current_feature + Sex + (1 | Subject) + storage_time
y ~ current_feature + Sex + (1 | Subject) + storage_time
y ~ current_feature + Sex + (1 | Subject) + storage_time
y ~ current_feature + Sex + (1 | Subject) + storage_time
y ~ current_feature + Sex + (1 | Subject) + storage_time
y ~ current_feature + Sex + (1 | Subject) + storage_time
y ~ current_feature + Sex + (1 | Subject) + storage_time
y ~ current_feature + Sex + (1 | Subject) + storage_time
y ~ current_feature + Sex + (1 | Subject) + storage_time
y ~ current_feature + Sex + (1 | Subject) + storage_time
y ~ current_feature + Sex + (1 | Subject) + storage_time
y ~ current_feature + Sex + (1 | Subject) + storage_time
y ~ current_feature + Sex + (1 | Subject) + storage_time
y ~ current_feature + Sex + (1 | Subject) + storage_time
y ~ current_feature + Sex + (1 | Subject) + storage_time
y ~ current_feature + Sex + (1 | Subject) + storage_time
y ~ current_feature + Sex + (1 | Subject) + storage_time
y ~ current_feature + Sex + (1 | Subject) + storage_time
y ~ current_feature + Sex + (1 | Subject) + storage_time
y ~ current_feature + Sex + (1 | Subject) + storage_time
y ~ current_feature + Sex + (1 | Subject) + storage_time
y ~ current_feature + Sex + (1 | Subject) + storage_time
y ~ current_feature + Sex + (1 | Subject) + storage_time
y ~ current_feature + Sex + (1 | Subject) + storage_time
y ~ current_feature + Sex + (1 | Subject) + storage_time
y ~ current_feature + Sex + (1 | Subject) + storage_time
y ~ current_feature + Sex + (1 | Subject) + storage_time
y ~ current_feature + Sex + (1 | Subject) + storage_time
y ~ current_feature + Sex + (1 | Subject) + storage_time
y ~ current_feature + Sex + (1 | Subject) + storage_time
y ~ current_feature + Sex + (1 | Subject) + storage_time
y ~ current_feature + Sex + (1 | Subject) + storage_time
y ~ current_feature + Sex + (1 | Subject) + storage_time
y ~ current_feature + Sex + (1 | Subject) + storage_time

y ~ current_feature + Sex + (1 | Subject) + storage_time
y ~ current_feature + Sex + (1 | Subject) + storage_time
y ~ current_feature + Sex + (1 | Subject) + storage_time
y ~ current_feature + Sex + (1 | Subject) + storage_time
y ~ current_feature + Sex + (1 | Subject) + storage_time
y ~ current_feature + Sex + (1 | Subject) + storage_time
y ~ current_feature + Sex + (1 | Subject) + storage_time
y ~ current_feature + Sex + (1 | Subject) + storage_time
y ~ current_feature + Sex + (1 | Subject) + storage_time
y ~ current_feature + Sex + (1 | Subject) + storage_time
y ~ current_feature + Sex + (1 | Subject) + storage_time
y ~ current_feature + Sex + (1 | Subject) + storage_time
y ~ current_feature + Sex + (1 | Subject) + storage_time
y ~ current_feature + Sex + (1 | Subject) + storage_time
y ~ current_feature + Sex + (1 | Subject) + storage_time
y ~ current_feature + Sex + (1 | Subject) + storage_time
y ~ current_feature + Sex + (1 | Subject) + storage_time
y ~ current_feature + Sex + (1 | Subject) + storage_time
y ~ current_feature + Sex + (1 | Subject) + storage_time
y ~ current_feature + Sex + (1 | Subject) + storage_time
y ~ current_feature + Sex + (1 | Subject) + storage_time
y ~ current_feature + Sex + (1 | Subject) + storage_time
y ~ current_feature + Sex + (1 | Subject) + storage_time
y ~ current_feature + Sex + (1 | Subject) + storage_time
y ~ current_feature + Sex + (1 | Subject) + storage_time
y ~ current_feature + Sex + (1 | Subject) + storage_time
y ~ current_feature + Sex + (1 | Subject) + storage_time
y ~ current_feature + Sex + (1 | Subject) + storage_time
y ~ current_feature + Sex + (1 | Subject) + storage_time
y ~ current_feature + Sex + (1 | Subject) + storage_time
y ~ current_feature + Sex + (1 | Subject) + storage_time
y ~ current_feature + Sex + (1 | Subject) + storage_time
y ~ current_feature + Sex + (1 | Subject) + storage_time
y ~ current_feature + Sex + (1 | Subject) + storage_time
y ~ current_feature + Sex + (1 | Subject) + storage_time
y ~ current_feature + Sex + (1 | Subject) + storage_time
y ~ current_feature + Sex + (1 | Subject) + storage_time
y ~ current_feature + Sex + (1 | Subject) + storage_time
y ~ current_feature + Sex + (1 | Subject) + storage_time
y ~ current_feature + Sex + (1 | Subject) + storage_time
y ~ current_feature + Sex + (1 | Subject) + storage_time
y ~ current_feature + Sex + (1 | Subject) + storage_time
y ~ current_feature + Sex + (1 | Subject) + storage_time
y ~ current_feature + Sex + (1 | Subject) + storage_time
y ~ current_feature + Sex + (1 | Subject) + storage_time
y ~ current_feature + Sex + (1 | Subject) + storage_time
y ~ current_feature + Sex + (1 | Subject) + storage_time
y ~ current_feature + Sex + (1 | Subject) + storage_time

y ~ current_feature + Sex + (1 | Subject) + storage_time
y ~ current_feature + Sex + (1 | Subject) + storage_time
y ~ current_feature + Sex + (1 | Subject) + storage_time
y ~ current_feature + Sex + (1 | Subject) + storage_time
y ~ current_feature + Sex + (1 | Subject) + storage_time
y ~ current_feature + Sex + (1 | Subject) + storage_time
y ~ current_feature + Sex + (1 | Subject) + storage_time
y ~ current_feature + Sex + (1 | Subject) + storage_time
y ~ current_feature + Sex + (1 | Subject) + storage_time
y ~ current_feature + Sex + (1 | Subject) + storage_time
y ~ current_feature + Sex + (1 | Subject) + storage_time
y ~ current_feature + Sex + (1 | Subject) + storage_time
y ~ current_feature + Sex + (1 | Subject) + storage_time
y ~ current_feature + Sex + (1 | Subject) + storage_time
y ~ current_feature + Sex + (1 | Subject) + storage_time
y ~ current_feature + Sex + (1 | Subject) + storage_time
y ~ current_feature + Sex + (1 | Subject) + storage_time
y ~ current_feature + Sex + (1 | Subject) + storage_time
y ~ current_feature + Sex + (1 | Subject) + storage_time
y ~ current_feature + Sex + (1 | Subject) + storage_time
y ~ current_feature + Sex + (1 | Subject) + storage_time
y ~ current_feature + Sex + (1 | Subject) + storage_time
y ~ current_feature + Sex + (1 | Subject) + storage_time
y ~ current_feature + Sex + (1 | Subject) + storage_time
y ~ current_feature + Sex + (1 | Subject) + storage_time
y ~ current_feature + Sex + (1 | Subject) + storage_time
y ~ current_feature + Sex + (1 | Subject) + storage_time
y ~ current_feature + Sex + (1 | Subject) + storage_time
y ~ current_feature + Sex + (1 | Subject) + storage_time
y ~ current_feature + Sex + (1 | Subject) + storage_time
y ~ current_feature + Sex + (1 | Subject) + storage_time
y ~ current_feature + Sex + (1 | Subject) + storage_time
y ~ current_feature + Sex + (1 | Subject) + storage_time
y ~ current_feature + Sex + (1 | Subject) + storage_time
y ~ current_feature + Sex + (1 | Subject) + storage_time
y ~ current_feature + Sex + (1 | Subject) + storage_time
y ~ current_feature + Sex + (1 | Subject) + storage_time
y ~ current_feature + Sex + (1 | Subject) + storage_time
y ~ current_feature + Sex + (1 | Subject) + storage_time
y ~ current_feature + Sex + (1 | Subject) + storage_time
y ~ current_feature + Sex + (1 | Subject) + storage_time
y ~ current_feature + Sex + (1 | Subject) + storage_time
y ~ current_feature + Sex + (1 | Subject) + storage_time
y ~ current_feature + Sex + (1 | Subject) + storage_time
y ~ current_feature + Sex + (1 | Subject) + storage_time
y ~ current_feature + Sex + (1 | Subject) + storage_time
y ~ current_feature + Sex + (1 | Subject) + storage_time
y ~ current_feature + Sex + (1 | Subject) + storage_time

y ~ current_feature + Sex + (1 | Subject) + storage_time
y ~ current_feature + Sex + (1 | Subject) + storage_time
y ~ current_feature + Sex + (1 | Subject) + storage_time
y ~ current_feature + Sex + (1 | Subject) + storage_time
y ~ current_feature + Sex + (1 | Subject) + storage_time
y ~ current_feature + Sex + (1 | Subject) + storage_time
y ~ current_feature + Sex + (1 | Subject) + storage_time
y ~ current_feature + Sex + (1 | Subject) + storage_time
y ~ current_feature + Sex + (1 | Subject) + storage_time
y ~ current_feature + Sex + (1 | Subject) + storage_time
y ~ current_feature + Sex + (1 | Subject) + storage_time
y ~ current_feature + Sex + (1 | Subject) + storage_time
y ~ current_feature + Sex + (1 | Subject) + storage_time
y ~ current_feature + Sex + (1 | Subject) + storage_time
y ~ current_feature + Sex + (1 | Subject) + storage_time
y ~ current_feature + Sex + (1 | Subject) + storage_time
y ~ current_feature + Sex + (1 | Subject) + storage_time
y ~ current_feature + Sex + (1 | Subject) + storage_time
y ~ current_feature + Sex + (1 | Subject) + storage_time
y ~ current_feature + Sex + (1 | Subject) + storage_time
y ~ current_feature + Sex + (1 | Subject) + storage_time
y ~ current_feature + Sex + (1 | Subject) + storage_time
y ~ current_feature + Sex + (1 | Subject) + storage_time
y ~ current_feature + Sex + (1 | Subject) + storage_time
y ~ current_feature + Sex + (1 | Subject) + storage_time
y ~ current_feature + Sex + (1 | Subject) + storage_time
y ~ current_feature + Sex + (1 | Subject) + storage_time
y ~ current_feature + Sex + (1 | Subject) + storage_time
y ~ current_feature + Sex + (1 | Subject) + storage_time
y ~ current_feature + Sex + (1 | Subject) + storage_time
y ~ current_feature + Sex + (1 | Subject) + storage_time
y ~ current_feature + Sex + (1 | Subject) + storage_time
y ~ current_feature + Sex + (1 | Subject) + storage_time
y ~ current_feature + Sex + (1 | Subject) + storage_time
y ~ current_feature + Sex + (1 | Subject) + storage_time
y ~ current_feature + Sex + (1 | Subject) + storage_time
y ~ current_feature + Sex + (1 | Subject) + storage_time
y ~ current_feature + Sex + (1 | Subject) + storage_time
y ~ current_feature + Sex + (1 | Subject) + storage_time
y ~ current_feature + Sex + (1 | Subject) + storage_time
y ~ current_feature + Sex + (1 | Subject) + storage_time
y ~ current_feature + Sex + (1 | Subject) + storage_time
y ~ current_feature + Sex + (1 | Subject) + storage_time
y ~ current_feature + Sex + (1 | Subject) + storage_time
y ~ current_feature + Sex + (1 | Subject) + storage_time
y ~ current_feature + Sex + (1 | Subject) + storage_time
y ~ current_feature + Sex + (1 | Subject) + storage_time
y ~ current_feature + Sex + (1 | Subject) + storage_time

y ~ current_feature + Sex + (1 | Subject) + storage_time
y ~ current_feature + Sex + (1 | Subject) + storage_time
y ~ current_feature + Sex + (1 | Subject) + storage_time
y ~ current_feature + Sex + (1 | Subject) + storage_time
y ~ current_feature + Sex + (1 | Subject) + storage_time
y ~ current_feature + Sex + (1 | Subject) + storage_time
y ~ current_feature + Sex + (1 | Subject) + storage_time
y ~ current_feature + Sex + (1 | Subject) + storage_time
y ~ current_feature + Sex + (1 | Subject) + storage_time
y ~ current_feature + Sex + (1 | Subject) + storage_time
y ~ current_feature + Sex + (1 | Subject) + storage_time
y ~ current_feature + Sex + (1 | Subject) + storage_time
y ~ current_feature + Sex + (1 | Subject) + storage_time
y ~ current_feature + Sex + (1 | Subject) + storage_time
y ~ current_feature + Sex + (1 | Subject) + storage_time
y ~ current_feature + Sex + (1 | Subject) + storage_time
y ~ current_feature + Sex + (1 | Subject) + storage_time
y ~ current_feature + Sex + (1 | Subject) + storage_time
y ~ current_feature + Sex + (1 | Subject) + storage_time
y ~ current_feature + Sex + (1 | Subject) + storage_time
y ~ current_feature + Sex + (1 | Subject) + storage_time
y ~ current_feature + Sex + (1 | Subject) + storage_time
y ~ current_feature + Sex + (1 | Subject) + storage_time
y ~ current_feature + Sex + (1 | Subject) + storage_time
y ~ current_feature + Sex + (1 | Subject) + storage_time
y ~ current_feature + Sex + (1 | Subject) + storage_time
y ~ current_feature + Sex + (1 | Subject) + storage_time
y ~ current_feature + Sex + (1 | Subject) + storage_time
y ~ current_feature + Sex + (1 | Subject) + storage_time
y ~ current_feature + Sex + (1 | Subject) + storage_time
y ~ current_feature + Sex + (1 | Subject) + storage_time
y ~ current_feature + Sex + (1 | Subject) + storage_time
y ~ current_feature + Sex + (1 | Subject) + storage_time
y ~ current_feature + Sex + (1 | Subject) + storage_time
y ~ current_feature + Sex + (1 | Subject) + storage_time
y ~ current_feature + Sex + (1 | Subject) + storage_time
y ~ current_feature + Sex + (1 | Subject) + storage_time
y ~ current_feature + Sex + (1 | Subject) + storage_time
y ~ current_feature + Sex + (1 | Subject) + storage_time
y ~ current_feature + Sex + (1 | Subject) + storage_time
y ~ current_feature + Sex + (1 | Subject) + storage_time
y ~ current_feature + Sex + (1 | Subject) + storage_time
y ~ current_feature + Sex + (1 | Subject) + storage_time
y ~ current_feature + Sex + (1 | Subject) + storage_time
y ~ current_feature + Sex + (1 | Subject) + storage_time
y ~ current_feature + Sex + (1 | Subject) + storage_time
y ~ current_feature + Sex + (1 | Subject) + storage_time
y ~ current_feature + Sex + (1 | Subject) + storage_time

y ~ current_feature + Sex + (1 | Subject) + storage_time
y ~ current_feature + Sex + (1 | Subject) + storage_time
y ~ current_feature + Sex + (1 | Subject) + storage_time
y ~ current_feature + Sex + (1 | Subject) + storage_time
y ~ current_feature + Sex + (1 | Subject) + storage_time
y ~ current_feature + Sex + (1 | Subject) + storage_time
y ~ current_feature + Sex + (1 | Subject) + storage_time
y ~ current_feature + Sex + (1 | Subject) + storage_time
y ~ current_feature + Sex + (1 | Subject) + storage_time
y ~ current_feature + Sex + (1 | Subject) + storage_time
y ~ current_feature + Sex + (1 | Subject) + storage_time
y ~ current_feature + Sex + (1 | Subject) + storage_time
y ~ current_feature + Sex + (1 | Subject) + storage_time
y ~ current_feature + Sex + (1 | Subject) + storage_time
y ~ current_feature + Sex + (1 | Subject) + storage_time
y ~ current_feature + Sex + (1 | Subject) + storage_time
y ~ current_feature + Sex + (1 | Subject) + storage_time
y ~ current_feature + Sex + (1 | Subject) + storage_time
y ~ current_feature + Sex + (1 | Subject) + storage_time
y ~ current_feature + Sex + (1 | Subject) + storage_time
y ~ current_feature + Sex + (1 | Subject) + storage_time
y ~ current_feature + Sex + (1 | Subject) + storage_time
y ~ current_feature + Sex + (1 | Subject) + storage_time
y ~ current_feature + Sex + (1 | Subject) + storage_time
y ~ current_feature + Sex + (1 | Subject) + storage_time
y ~ current_feature + Sex + (1 | Subject) + storage_time
y ~ current_feature + Sex + (1 | Subject) + storage_time
y ~ current_feature + Sex + (1 | Subject) + storage_time
y ~ current_feature + Sex + (1 | Subject) + storage_time
y ~ current_feature + Sex + (1 | Subject) + storage_time
y ~ current_feature + Sex + (1 | Subject) + storage_time
y ~ current_feature + Sex + (1 | Subject) + storage_time
y ~ current_feature + Sex + (1 | Subject) + storage_time
y ~ current_feature + Sex + (1 | Subject) + storage_time
y ~ current_feature + Sex + (1 | Subject) + storage_time
y ~ current_feature + Sex + (1 | Subject) + storage_time
y ~ current_feature + Sex + (1 | Subject) + storage_time
y ~ current_feature + Sex + (1 | Subject) + storage_time
y ~ current_feature + Sex + (1 | Subject) + storage_time
y ~ current_feature + Sex + (1 | Subject) + storage_time
y ~ current_feature + Sex + (1 | Subject) + storage_time
y ~ current_feature + Sex + (1 | Subject) + storage_time
y ~ current_feature + Sex + (1 | Subject) + storage_time
y ~ current_feature + Sex + (1 | Subject) + storage_time
y ~ current_feature + Sex + (1 | Subject) + storage_time
y ~ current_feature + Sex + (1 | Subject) + storage_time
y ~ current_feature + Sex + (1 | Subject) + storage_time
y ~ current_feature + Sex + (1 | Subject) + storage_time

y ~ current_feature + Sex + (1 | Subject) + storage_time
y ~ current_feature + Sex + (1 | Subject) + storage_time
y ~ current_feature + Sex + (1 | Subject) + storage_time
y ~ current_feature + Sex + (1 | Subject) + storage_time
y ~ current_feature + Sex + (1 | Subject) + storage_time
y ~ current_feature + Sex + (1 | Subject) + storage_time
y ~ current_feature + Sex + (1 | Subject) + storage_time
y ~ current_feature + Sex + (1 | Subject) + storage_time
y ~ current_feature + Sex + (1 | Subject) + storage_time
y ~ current_feature + Sex + (1 | Subject) + storage_time
y ~ current_feature + Sex + (1 | Subject) + storage_time
y ~ current_feature + Sex + (1 | Subject) + storage_time
y ~ current_feature + Sex + (1 | Subject) + storage_time
y ~ current_feature + Sex + (1 | Subject) + storage_time
y ~ current_feature + Sex + (1 | Subject) + storage_time
y ~ current_feature + Sex + (1 | Subject) + storage_time
y ~ current_feature + Sex + (1 | Subject) + storage_time
y ~ current_feature + Sex + (1 | Subject) + storage_time
y ~ current_feature + Sex + (1 | Subject) + storage_time
y ~ current_feature + Sex + (1 | Subject) + storage_time
y ~ current_feature + Sex + (1 | Subject) + storage_time
y ~ current_feature + Sex + (1 | Subject) + storage_time
y ~ current_feature + Sex + (1 | Subject) + storage_time
y ~ current_feature + Sex + (1 | Subject) + storage_time
y ~ current_feature + Sex + (1 | Subject) + storage_time
y ~ current_feature + Sex + (1 | Subject) + storage_time
y ~ current_feature + Sex + (1 | Subject) + storage_time
y ~ current_feature + Sex + (1 | Subject) + storage_time
y ~ current_feature + Sex + (1 | Subject) + storage_time
y ~ current_feature + Sex + (1 | Subject) + storage_time
y ~ current_feature + Sex + (1 | Subject) + storage_time
y ~ current_feature + Sex + (1 | Subject) + storage_time
y ~ current_feature + Sex + (1 | Subject) + storage_time
y ~ current_feature + Sex + (1 | Subject) + storage_time
y ~ current_feature + Sex + (1 | Subject) + storage_time
y ~ current_feature + Sex + (1 | Subject) + storage_time
y ~ current_feature + Sex + (1 | Subject) + storage_time
y ~ current_feature + Sex + (1 | Subject) + storage_time
y ~ current_feature + Sex + (1 | Subject) + storage_time
y ~ current_feature + Sex + (1 | Subject) + storage_time
y ~ current_feature + Sex + (1 | Subject) + storage_time
y ~ current_feature + Sex + (1 | Subject) + storage_time
y ~ current_feature + Sex + (1 | Subject) + storage_time
y ~ current_feature + Sex + (1 | Subject) + storage_time
y ~ current_feature + Sex + (1 | Subject) + storage_time
y ~ current_feature + Sex + (1 | Subject) + storage_time
y ~ current_feature + Sex + (1 | Subject) + storage_time
y ~ current_feature + Sex + (1 | Subject) + storage_time

y ~ current_feature + Sex + (1 | Subject) + storage_time
y ~ current_feature + Sex + (1 | Subject) + storage_time
y ~ current_feature + Sex + (1 | Subject) + storage_time
y ~ current_feature + Sex + (1 | Subject) + storage_time
y ~ current_feature + Sex + (1 | Subject) + storage_time
y ~ current_feature + Sex + (1 | Subject) + storage_time
y ~ current_feature + Sex + (1 | Subject) + storage_time
y ~ current_feature + Sex + (1 | Subject) + storage_time
y ~ current_feature + Sex + (1 | Subject) + storage_time
y ~ current_feature + Sex + (1 | Subject) + storage_time
y ~ current_feature + Sex + (1 | Subject) + storage_time
y ~ current_feature + Sex + (1 | Subject) + storage_time
y ~ current_feature + Sex + (1 | Subject) + storage_time
y ~ current_feature + Sex + (1 | Subject) + storage_time
y ~ current_feature + Sex + (1 | Subject) + storage_time
y ~ current_feature + Sex + (1 | Subject) + storage_time
y ~ current_feature + Sex + (1 | Subject) + storage_time
y ~ current_feature + Sex + (1 | Subject) + storage_time
y ~ current_feature + Sex + (1 | Subject) + storage_time
y ~ current_feature + Sex + (1 | Subject) + storage_time
y ~ current_feature + Sex + (1 | Subject) + storage_time
y ~ current_feature + Sex + (1 | Subject) + storage_time
y ~ current_feature + Sex + (1 | Subject) + storage_time
y ~ current_feature + Sex + (1 | Subject) + storage_time
y ~ current_feature + Sex + (1 | Subject) + storage_time
y ~ current_feature + Sex + (1 | Subject) + storage_time
y ~ current_feature + Sex + (1 | Subject) + storage_time
y ~ current_feature + Sex + (1 | Subject) + storage_time
y ~ current_feature + Sex + (1 | Subject) + storage_time
y ~ current_feature + Sex + (1 | Subject) + storage_time
y ~ current_feature + Sex + (1 | Subject) + storage_time
y ~ current_feature + Sex + (1 | Subject) + storage_time
y ~ current_feature + Sex + (1 | Subject) + storage_time
y ~ current_feature + Sex + (1 | Subject) + storage_time
y ~ current_feature + Sex + (1 | Subject) + storage_time
y ~ current_feature + Sex + (1 | Subject) + storage_time
y ~ current_feature + Sex + (1 | Subject) + storage_time
y ~ current_feature + Sex + (1 | Subject) + storage_time
y ~ current_feature + Sex + (1 | Subject) + storage_time
y ~ current_feature + Sex + (1 | Subject) + storage_time
y ~ current_feature + Sex + (1 | Subject) + storage_time
y ~ current_feature + Sex + (1 | Subject) + storage_time
y ~ current_feature + Sex + (1 | Subject) + storage_time
y ~ current_feature + Sex + (1 | Subject) + storage_time
y ~ current_feature + Sex + (1 | Subject) + storage_time
y ~ current_feature + Sex + (1 | Subject) + storage_time
y ~ current_feature + Sex + (1 | Subject) + storage_time
y ~ current_feature + Sex + (1 | Subject) + storage_time

```
y ~ current_feature + Sex + (1 | Subject) + storage_time
y ~ current_feature + Sex + (1 | Subject) + storage_time
y ~ current_feature + Sex + (1 | Subject) + storage_time
y ~ current_feature + Sex + (1 | Subject) + storage_time
y ~ current_feature + Sex + (1 | Subject) + storage_time
y ~ current_feature + Sex + (1 | Subject) + storage_time
y ~ current_feature + Sex + (1 | Subject) + storage_time
y ~ current_feature + Sex + (1 | Subject) + storage_time
y ~ current_feature + Sex + (1 | Subject) + storage_time
y ~ current_feature + Sex + (1 | Subject) + storage_time
y ~ current_feature + Sex + (1 | Subject) + storage_time
y ~ current_feature + Sex + (1 | Subject) + storage_time
y ~ current_feature + Sex + (1 | Subject) + storage_time
y ~ current_feature + Sex + (1 | Subject) + storage_time
y ~ current_feature + Sex + (1 | Subject) + storage_time
y ~ current_feature + Sex + (1 | Subject) + storage_time
y ~ current_feature + Sex + (1 | Subject) + storage_time
y ~ current_feature + Sex + (1 | Subject) + storage_time
y ~ current_feature + Sex + (1 | Subject) + storage_time
y ~ current_feature + Sex + (1 | Subject) + storage_time
y ~ current_feature + Sex + (1 | Subject) + storage_time
y ~ current_feature + Sex + (1 | Subject) + storage_time
y ~ current_feature + Sex + (1 | Subject) + storage_time
y ~ current_feature + Sex + (1 | Subject) + storage_time
y ~ current_feature + Sex + (1 | Subject) + storage_time
y ~ current_feature + Sex + (1 | Subject) + storage_time
y ~ current_feature + Sex + (1 | Subject) + storage_time
y ~ current_feature + Sex + (1 | Subject) + storage_time
y ~ current_feature + Sex + (1 | Subject) + storage_time
y ~ current_feature + Sex + (1 | Subject) + storage_time
y ~ current_feature + Sex + (1 | Subject) + storage_time
y ~ current_feature + Sex + (1 | Subject) + storage_time
y ~ current_feature + Sex + (1 | Subject) + storage_time
y ~ current_feature + Sex + (1 | Subject) + storage_time
y ~ current_feature + Sex + (1 | Subject) + storage_time
y ~ current_feature + Sex + (1 | Subject) + storage_time
y ~ current_feature + Sex + (1 | Subject) + storage_time
y ~ current_feature + Sex + (1 | Subject) + storage_time
y ~ current_feature + Sex + (1 | Subject) + storage_time
y ~ current_feature + Sex + (1 | Subject) + storage_time
y ~ current_feature + Sex + (1 | Subject) + storage_time
y ~ current_feature + Sex + (1 | Subject) + storage_time
y ~ current_feature + Sex + (1 | Subject) + storage_time
y ~ current_feature + Sex + (1 | Subject) + storage_time
y ~ current_feature + Sex + (1 | Subject) + storage_time
y ~ current_feature + Sex + (1 | Subject) + storage_time
y ~ current_feature + Sex + (1 | Subject) + storage_time
y ~ current_feature + Sex + (1 | Subject) + storage_time
```

y ~ current_feature + Sex + (1 | Subject) + storage_time
y ~ current_feature + Sex + (1 | Subject) + storage_time
y ~ current_feature + Sex + (1 | Subject) + storage_time
y ~ current_feature + Sex + (1 | Subject) + storage_time
y ~ current_feature + Sex + (1 | Subject) + storage_time
y ~ current_feature + Sex + (1 | Subject) + storage_time
y ~ current_feature + Sex + (1 | Subject) + storage_time
y ~ current_feature + Sex + (1 | Subject) + storage_time
y ~ current_feature + Sex + (1 | Subject) + storage_time
y ~ current_feature + Sex + (1 | Subject) + storage_time
y ~ current_feature + Sex + (1 | Subject) + storage_time
y ~ current_feature + Sex + (1 | Subject) + storage_time
y ~ current_feature + Sex + (1 | Subject) + storage_time
y ~ current_feature + Sex + (1 | Subject) + storage_time
y ~ current_feature + Sex + (1 | Subject) + storage_time
y ~ current_feature + Sex + (1 | Subject) + storage_time
y ~ current_feature + Sex + (1 | Subject) + storage_time
y ~ current_feature + Sex + (1 | Subject) + storage_time
y ~ current_feature + Sex + (1 | Subject) + storage_time
y ~ current_feature + Sex + (1 | Subject) + storage_time
y ~ current_feature + Sex + (1 | Subject) + storage_time
y ~ current_feature + Sex + (1 | Subject) + storage_time
y ~ current_feature + Sex + (1 | Subject) + storage_time
y ~ current_feature + Sex + (1 | Subject) + storage_time
y ~ current_feature + Sex + (1 | Subject) + storage_time
y ~ current_feature + Sex + (1 | Subject) + storage_time
y ~ current_feature + Sex + (1 | Subject) + storage_time
y ~ current_feature + Sex + (1 | Subject) + storage_time
y ~ current_feature + Sex + (1 | Subject) + storage_time
y ~ current_feature + Sex + (1 | Subject) + storage_time
y ~ current_feature + Sex + (1 | Subject) + storage_time
y ~ current_feature + Sex + (1 | Subject) + storage_time
y ~ current_feature + Sex + (1 | Subject) + storage_time
y ~ current_feature + Sex + (1 | Subject) + storage_time
y ~ current_feature + Sex + (1 | Subject) + storage_time
y ~ current_feature + Sex + (1 | Subject) + storage_time
y ~ current_feature + Sex + (1 | Subject) + storage_time
y ~ current_feature + Sex + (1 | Subject) + storage_time
y ~ current_feature + Sex + (1 | Subject) + storage_time
y ~ current_feature + Sex + (1 | Subject) + storage_time
y ~ current_feature + Sex + (1 | Subject) + storage_time
y ~ current_feature + Sex + (1 | Subject) + storage_time
y ~ current_feature + Sex + (1 | Subject) + storage_time
y ~ current_feature + Sex + (1 | Subject) + storage_time
y ~ current_feature + Sex + (1 | Subject) + storage_time
y ~ current_feature + Sex + (1 | Subject) + storage_time
y ~ current_feature + Sex + (1 | Subject) + storage_time
y ~ current_feature + Sex + (1 | Subject) + storage_time

y ~ current_feature + Sex + (1 | Subject) + storage_time
y ~ current_feature + Sex + (1 | Subject) + storage_time
y ~ current_feature + Sex + (1 | Subject) + storage_time
y ~ current_feature + Sex + (1 | Subject) + storage_time
y ~ current_feature + Sex + (1 | Subject) + storage_time
y ~ current_feature + Sex + (1 | Subject) + storage_time
y ~ current_feature + Sex + (1 | Subject) + storage_time
y ~ current_feature + Sex + (1 | Subject) + storage_time
y ~ current_feature + Sex + (1 | Subject) + storage_time
y ~ current_feature + Sex + (1 | Subject) + storage_time
y ~ current_feature + Sex + (1 | Subject) + storage_time
y ~ current_feature + Sex + (1 | Subject) + storage_time
y ~ current_feature + Sex + (1 | Subject) + storage_time
y ~ current_feature + Sex + (1 | Subject) + storage_time
y ~ current_feature + Sex + (1 | Subject) + storage_time
y ~ current_feature + Sex + (1 | Subject) + storage_time
y ~ current_feature + Sex + (1 | Subject) + storage_time
y ~ current_feature + Sex + (1 | Subject) + storage_time
y ~ current_feature + Sex + (1 | Subject) + storage_time
y ~ current_feature + Sex + (1 | Subject) + storage_time
y ~ current_feature + Sex + (1 | Subject) + storage_time
y ~ current_feature + Sex + (1 | Subject) + storage_time
y ~ current_feature + Sex + (1 | Subject) + storage_time
y ~ current_feature + Sex + (1 | Subject) + storage_time
y ~ current_feature + Sex + (1 | Subject) + storage_time
y ~ current_feature + Sex + (1 | Subject) + storage_time
y ~ current_feature + Sex + (1 | Subject) + storage_time
y ~ current_feature + Sex + (1 | Subject) + storage_time
y ~ current_feature + Sex + (1 | Subject) + storage_time
y ~ current_feature + Sex + (1 | Subject) + storage_time
y ~ current_feature + Sex + (1 | Subject) + storage_time
y ~ current_feature + Sex + (1 | Subject) + storage_time
y ~ current_feature + Sex + (1 | Subject) + storage_time
y ~ current_feature + Sex + (1 | Subject) + storage_time
y ~ current_feature + Sex + (1 | Subject) + storage_time
y ~ current_feature + Sex + (1 | Subject) + storage_time
y ~ current_feature + Sex + (1 | Subject) + storage_time
y ~ current_feature + Sex + (1 | Subject) + storage_time
y ~ current_feature + Sex + (1 | Subject) + storage_time
y ~ current_feature + Sex + (1 | Subject) + storage_time
y ~ current_feature + Sex + (1 | Subject) + storage_time
y ~ current_feature + Sex + (1 | Subject) + storage_time
y ~ current_feature + Sex + (1 | Subject) + storage_time
y ~ current_feature + Sex + (1 | Subject) + storage_time
y ~ current_feature + Sex + (1 | Subject) + storage_time
y ~ current_feature + Sex + (1 | Subject) + storage_time
y ~ current_feature + Sex + (1 | Subject) + storage_time
y ~ current_feature + Sex + (1 | Subject) + storage_time

y ~ current_feature + Sex + (1 | Subject) + storage_time
y ~ current_feature + Sex + (1 | Subject) + storage_time
y ~ current_feature + Sex + (1 | Subject) + storage_time
y ~ current_feature + Sex + (1 | Subject) + storage_time
y ~ current_feature + Sex + (1 | Subject) + storage_time
y ~ current_feature + Sex + (1 | Subject) + storage_time
y ~ current_feature + Sex + (1 | Subject) + storage_time
y ~ current_feature + Sex + (1 | Subject) + storage_time
y ~ current_feature + Sex + (1 | Subject) + storage_time
y ~ current_feature + Sex + (1 | Subject) + storage_time
y ~ current_feature + Sex + (1 | Subject) + storage_time
y ~ current_feature + Sex + (1 | Subject) + storage_time
y ~ current_feature + Sex + (1 | Subject) + storage_time
y ~ current_feature + Sex + (1 | Subject) + storage_time
y ~ current_feature + Sex + (1 | Subject) + storage_time
y ~ current_feature + Sex + (1 | Subject) + storage_time
y ~ current_feature + Sex + (1 | Subject) + storage_time
y ~ current_feature + Sex + (1 | Subject) + storage_time
y ~ current_feature + Sex + (1 | Subject) + storage_time
y ~ current_feature + Sex + (1 | Subject) + storage_time
y ~ current_feature + Sex + (1 | Subject) + storage_time
y ~ current_feature + Sex + (1 | Subject) + storage_time
y ~ current_feature + Sex + (1 | Subject) + storage_time
y ~ current_feature + Sex + (1 | Subject) + storage_time
y ~ current_feature + Sex + (1 | Subject) + storage_time
y ~ current_feature + Sex + (1 | Subject) + storage_time
y ~ current_feature + Sex + (1 | Subject) + storage_time
y ~ current_feature + Sex + (1 | Subject) + storage_time
y ~ current_feature + Sex + (1 | Subject) + storage_time
y ~ current_feature + Sex + (1 | Subject) + storage_time
y ~ current_feature + Sex + (1 | Subject) + storage_time
y ~ current_feature + Sex + (1 | Subject) + storage_time
y ~ current_feature + Sex + (1 | Subject) + storage_time
y ~ current_feature + Sex + (1 | Subject) + storage_time
y ~ current_feature + Sex + (1 | Subject) + storage_time
y ~ current_feature + Sex + (1 | Subject) + storage_time
y ~ current_feature + Sex + (1 | Subject) + storage_time
y ~ current_feature + Sex + (1 | Subject) + storage_time
y ~ current_feature + Sex + (1 | Subject) + storage_time
y ~ current_feature + Sex + (1 | Subject) + storage_time
y ~ current_feature + Sex + (1 | Subject) + storage_time
y ~ current_feature + Sex + (1 | Subject) + storage_time
y ~ current_feature + Sex + (1 | Subject) + storage_time
y ~ current_feature + Sex + (1 | Subject) + storage_time
y ~ current_feature + Sex + (1 | Subject) + storage_time
y ~ current_feature + Sex + (1 | Subject) + storage_time
y ~ current_feature + Sex + (1 | Subject) + storage_time
y ~ current_feature + Sex + (1 | Subject) + storage_time

```
y ~ current_feature + Sex + (1 | Subject) + storage_time
y ~ current_feature + Sex + (1 | Subject) + storage_time
y ~ current_feature + Sex + (1 | Subject) + storage_time
y ~ current_feature + Sex + (1 | Subject) + storage_time
y ~ current_feature + Sex + (1 | Subject) + storage_time
y ~ current_feature + Sex + (1 | Subject) + storage_time
y ~ current_feature + Sex + (1 | Subject) + storage_time
y ~ current_feature + Sex + (1 | Subject) + storage_time
y ~ current_feature + Sex + (1 | Subject) + storage_time
y ~ current_feature + Sex + (1 | Subject) + storage_time
y ~ current_feature + Sex + (1 | Subject) + storage_time
y ~ current_feature + Sex + (1 | Subject) + storage_time
y ~ current_feature + Sex + (1 | Subject) + storage_time
y ~ current_feature + Sex + (1 | Subject) + storage_time
y ~ current_feature + Sex + (1 | Subject) + storage_time
y ~ current_feature + Sex + (1 | Subject) + storage_time
y ~ current_feature + Sex + (1 | Subject) + storage_time
y ~ current_feature + Sex + (1 | Subject) + storage_time
y ~ current_feature + Sex + (1 | Subject) + storage_time
y ~ current_feature + Sex + (1 | Subject) + storage_time
y ~ current_feature + Sex + (1 | Subject) + storage_time
y ~ current_feature + Sex + (1 | Subject) + storage_time
y ~ current_feature + Sex + (1 | Subject) + storage_time
y ~ current_feature + Sex + (1 | Subject) + storage_time
y ~ current_feature + Sex + (1 | Subject) + storage_time
y ~ current_feature + Sex + (1 | Subject) + storage_time
y ~ current_feature + Sex + (1 | Subject) + storage_time
y ~ current_feature + Sex + (1 | Subject) + storage_time
y ~ current_feature + Sex + (1 | Subject) + storage_time
y ~ current_feature + Sex + (1 | Subject) + storage_time
y ~ current_feature + Sex + (1 | Subject) + storage_time
y ~ current_feature + Sex + (1 | Subject) + storage_time
y ~ current_feature + Sex + (1 | Subject) + storage_time
y ~ current_feature + Sex + (1 | Subject) + storage_time
y ~ current_feature + Sex + (1 | Subject) + storage_time
y ~ current_feature + Sex + (1 | Subject) + storage_time
y ~ current_feature + Sex + (1 | Subject) + storage_time
y ~ current_feature + Sex + (1 | Subject) + storage_time
y ~ current_feature + Sex + (1 | Subject) + storage_time
y ~ current_feature + Sex + (1 | Subject) + storage_time
y ~ current_feature + Sex + (1 | Subject) + storage_time
y ~ current_feature + Sex + (1 | Subject) + storage_time
y ~ current_feature + Sex + (1 | Subject) + storage_time
y ~ current_feature + Sex + (1 | Subject) + storage_time
y ~ current_feature + Sex + (1 | Subject) + storage_time
y ~ current_feature + Sex + (1 | Subject) + storage_time
y ~ current_feature + Sex + (1 | Subject) + storage_time
y ~ current_feature + Sex + (1 | Subject) + storage_time
```

y ~ current_feature + Sex + (1 | Subject) + storage_time
y ~ current_feature + Sex + (1 | Subject) + storage_time
y ~ current_feature + Sex + (1 | Subject) + storage_time
y ~ current_feature + Sex + (1 | Subject) + storage_time
y ~ current_feature + Sex + (1 | Subject) + storage_time
y ~ current_feature + Sex + (1 | Subject) + storage_time
y ~ current_feature + Sex + (1 | Subject) + storage_time
y ~ current_feature + Sex + (1 | Subject) + storage_time
y ~ current_feature + Sex + (1 | Subject) + storage_time
y ~ current_feature + Sex + (1 | Subject) + storage_time
y ~ current_feature + Sex + (1 | Subject) + storage_time
y ~ current_feature + Sex + (1 | Subject) + storage_time
y ~ current_feature + Sex + (1 | Subject) + storage_time
y ~ current_feature + Sex + (1 | Subject) + storage_time
y ~ current_feature + Sex + (1 | Subject) + storage_time
y ~ current_feature + Sex + (1 | Subject) + storage_time
y ~ current_feature + Sex + (1 | Subject) + storage_time
y ~ current_feature + Sex + (1 | Subject) + storage_time
y ~ current_feature + Sex + (1 | Subject) + storage_time
y ~ current_feature + Sex + (1 | Subject) + storage_time
y ~ current_feature + Sex + (1 | Subject) + storage_time
y ~ current_feature + Sex + (1 | Subject) + storage_time
y ~ current_feature + Sex + (1 | Subject) + storage_time
y ~ current_feature + Sex + (1 | Subject) + storage_time
y ~ current_feature + Sex + (1 | Subject) + storage_time
y ~ current_feature + Sex + (1 | Subject) + storage_time
y ~ current_feature + Sex + (1 | Subject) + storage_time
y ~ current_feature + Sex + (1 | Subject) + storage_time
y ~ current_feature + Sex + (1 | Subject) + storage_time
y ~ current_feature + Sex + (1 | Subject) + storage_time
y ~ current_feature + Sex + (1 | Subject) + storage_time
y ~ current_feature + Sex + (1 | Subject) + storage_time
y ~ current_feature + Sex + (1 | Subject) + storage_time
y ~ current_feature + Sex + (1 | Subject) + storage_time
y ~ current_feature + Sex + (1 | Subject) + storage_time
y ~ current_feature + Sex + (1 | Subject) + storage_time
y ~ current_feature + Sex + (1 | Subject) + storage_time
y ~ current_feature + Sex + (1 | Subject) + storage_time
y ~ current_feature + Sex + (1 | Subject) + storage_time
y ~ current_feature + Sex + (1 | Subject) + storage_time
y ~ current_feature + Sex + (1 | Subject) + storage_time
y ~ current_feature + Sex + (1 | Subject) + storage_time
y ~ current_feature + Sex + (1 | Subject) + storage_time
y ~ current_feature + Sex + (1 | Subject) + storage_time
y ~ current_feature + Sex + (1 | Subject) + storage_time
y ~ current_feature + Sex + (1 | Subject) + storage_time
y ~ current_feature + Sex + (1 | Subject) + storage_time
y ~ current_feature + Sex + (1 | Subject) + storage_time

y ~ current_feature + Sex + (1 | Subject) + storage_time
y ~ current_feature + Sex + (1 | Subject) + storage_time
y ~ current_feature + Sex + (1 | Subject) + storage_time
y ~ current_feature + Sex + (1 | Subject) + storage_time
y ~ current_feature + Sex + (1 | Subject) + storage_time
y ~ current_feature + Sex + (1 | Subject) + storage_time
y ~ current_feature + Sex + (1 | Subject) + storage_time
y ~ current_feature + Sex + (1 | Subject) + storage_time
y ~ current_feature + Sex + (1 | Subject) + storage_time
y ~ current_feature + Sex + (1 | Subject) + storage_time
y ~ current_feature + Sex + (1 | Subject) + storage_time
y ~ current_feature + Sex + (1 | Subject) + storage_time
y ~ current_feature + Sex + (1 | Subject) + storage_time
y ~ current_feature + Sex + (1 | Subject) + storage_time
y ~ current_feature + Sex + (1 | Subject) + storage_time
y ~ current_feature + Sex + (1 | Subject) + storage_time
y ~ current_feature + Sex + (1 | Subject) + storage_time
y ~ current_feature + Sex + (1 | Subject) + storage_time
y ~ current_feature + Sex + (1 | Subject) + storage_time
y ~ current_feature + Sex + (1 | Subject) + storage_time
y ~ current_feature + Sex + (1 | Subject) + storage_time
y ~ current_feature + Sex + (1 | Subject) + storage_time
y ~ current_feature + Sex + (1 | Subject) + storage_time
y ~ current_feature + Sex + (1 | Subject) + storage_time
y ~ current_feature + Sex + (1 | Subject) + storage_time
y ~ current_feature + Sex + (1 | Subject) + storage_time
y ~ current_feature + Sex + (1 | Subject) + storage_time
y ~ current_feature + Sex + (1 | Subject) + storage_time
y ~ current_feature + Sex + (1 | Subject) + storage_time
y ~ current_feature + Sex + (1 | Subject) + storage_time
y ~ current_feature + Sex + (1 | Subject) + storage_time
y ~ current_feature + Sex + (1 | Subject) + storage_time
y ~ current_feature + Sex + (1 | Subject) + storage_time
y ~ current_feature + Sex + (1 | Subject) + storage_time
y ~ current_feature + Sex + (1 | Subject) + storage_time
y ~ current_feature + Sex + (1 | Subject) + storage_time
y ~ current_feature + Sex + (1 | Subject) + storage_time
y ~ current_feature + Sex + (1 | Subject) + storage_time
y ~ current_feature + Sex + (1 | Subject) + storage_time
y ~ current_feature + Sex + (1 | Subject) + storage_time
y ~ current_feature + Sex + (1 | Subject) + storage_time
y ~ current_feature + Sex + (1 | Subject) + storage_time
y ~ current_feature + Sex + (1 | Subject) + storage_time
y ~ current_feature + Sex + (1 | Subject) + storage_time
y ~ current_feature + Sex + (1 | Subject) + storage_time
y ~ current_feature + Sex + (1 | Subject) + storage_time
y ~ current_feature + Sex + (1 | Subject) + storage_time
y ~ current_feature + Sex + (1 | Subject) + storage_time

y ~ current_feature + Sex + (1 | Subject) + storage_time
y ~ current_feature + Sex + (1 | Subject) + storage_time
y ~ current_feature + Sex + (1 | Subject) + storage_time
y ~ current_feature + Sex + (1 | Subject) + storage_time
y ~ current_feature + Sex + (1 | Subject) + storage_time
y ~ current_feature + Sex + (1 | Subject) + storage_time
y ~ current_feature + Sex + (1 | Subject) + storage_time
y ~ current_feature + Sex + (1 | Subject) + storage_time
y ~ current_feature + Sex + (1 | Subject) + storage_time
y ~ current_feature + Sex + (1 | Subject) + storage_time
y ~ current_feature + Sex + (1 | Subject) + storage_time
y ~ current_feature + Sex + (1 | Subject) + storage_time
y ~ current_feature + Sex + (1 | Subject) + storage_time
y ~ current_feature + Sex + (1 | Subject) + storage_time
y ~ current_feature + Sex + (1 | Subject) + storage_time
y ~ current_feature + Sex + (1 | Subject) + storage_time
y ~ current_feature + Sex + (1 | Subject) + storage_time
y ~ current_feature + Sex + (1 | Subject) + storage_time
y ~ current_feature + Sex + (1 | Subject) + storage_time
y ~ current_feature + Sex + (1 | Subject) + storage_time
y ~ current_feature + Sex + (1 | Subject) + storage_time
y ~ current_feature + Sex + (1 | Subject) + storage_time
y ~ current_feature + Sex + (1 | Subject) + storage_time
y ~ current_feature + Sex + (1 | Subject) + storage_time
y ~ current_feature + Sex + (1 | Subject) + storage_time
y ~ current_feature + Sex + (1 | Subject) + storage_time
y ~ current_feature + Sex + (1 | Subject) + storage_time
y ~ current_feature + Sex + (1 | Subject) + storage_time
y ~ current_feature + Sex + (1 | Subject) + storage_time
y ~ current_feature + Sex + (1 | Subject) + storage_time
y ~ current_feature + Sex + (1 | Subject) + storage_time
y ~ current_feature + Sex + (1 | Subject) + storage_time
y ~ current_feature + Sex + (1 | Subject) + storage_time
y ~ current_feature + Sex + (1 | Subject) + storage_time
y ~ current_feature + Sex + (1 | Subject) + storage_time
y ~ current_feature + Sex + (1 | Subject) + storage_time
y ~ current_feature + Sex + (1 | Subject) + storage_time
y ~ current_feature + Sex + (1 | Subject) + storage_time
y ~ current_feature + Sex + (1 | Subject) + storage_time
y ~ current_feature + Sex + (1 | Subject) + storage_time
y ~ current_feature + Sex + (1 | Subject) + storage_time
y ~ current_feature + Sex + (1 | Subject) + storage_time
y ~ current_feature + Sex + (1 | Subject) + storage_time
y ~ current_feature + Sex + (1 | Subject) + storage_time
y ~ current_feature + Sex + (1 | Subject) + storage_time
y ~ current_feature + Sex + (1 | Subject) + storage_time
y ~ current_feature + Sex + (1 | Subject) + storage_time
y ~ current_feature + Sex + (1 | Subject) + storage_time

y ~ current_feature + Sex + (1 | Subject) + storage_time
y ~ current_feature + Sex + (1 | Subject) + storage_time
y ~ current_feature + Sex + (1 | Subject) + storage_time
y ~ current_feature + Sex + (1 | Subject) + storage_time
y ~ current_feature + Sex + (1 | Subject) + storage_time
y ~ current_feature + Sex + (1 | Subject) + storage_time
y ~ current_feature + Sex + (1 | Subject) + storage_time
y ~ current_feature + Sex + (1 | Subject) + storage_time
y ~ current_feature + Sex + (1 | Subject) + storage_time
y ~ current_feature + Sex + (1 | Subject) + storage_time
y ~ current_feature + Sex + (1 | Subject) + storage_time
y ~ current_feature + Sex + (1 | Subject) + storage_time
y ~ current_feature + Sex + (1 | Subject) + storage_time
y ~ current_feature + Sex + (1 | Subject) + storage_time
y ~ current_feature + Sex + (1 | Subject) + storage_time
y ~ current_feature + Sex + (1 | Subject) + storage_time
y ~ current_feature + Sex + (1 | Subject) + storage_time
y ~ current_feature + Sex + (1 | Subject) + storage_time
y ~ current_feature + Sex + (1 | Subject) + storage_time
y ~ current_feature + Sex + (1 | Subject) + storage_time
y ~ current_feature + Sex + (1 | Subject) + storage_time
y ~ current_feature + Sex + (1 | Subject) + storage_time
y ~ current_feature + Sex + (1 | Subject) + storage_time
y ~ current_feature + Sex + (1 | Subject) + storage_time
y ~ current_feature + Sex + (1 | Subject) + storage_time
y ~ current_feature + Sex + (1 | Subject) + storage_time
y ~ current_feature + Sex + (1 | Subject) + storage_time
y ~ current_feature + Sex + (1 | Subject) + storage_time
y ~ current_feature + Sex + (1 | Subject) + storage_time
y ~ current_feature + Sex + (1 | Subject) + storage_time
y ~ current_feature + Sex + (1 | Subject) + storage_time
y ~ current_feature + Sex + (1 | Subject) + storage_time
y ~ current_feature + Sex + (1 | Subject) + storage_time
y ~ current_feature + Sex + (1 | Subject) + storage_time
y ~ current_feature + Sex + (1 | Subject) + storage_time
y ~ current_feature + Sex + (1 | Subject) + storage_time
y ~ current_feature + Sex + (1 | Subject) + storage_time
y ~ current_feature + Sex + (1 | Subject) + storage_time
y ~ current_feature + Sex + (1 | Subject) + storage_time
y ~ current_feature + Sex + (1 | Subject) + storage_time
y ~ current_feature + Sex + (1 | Subject) + storage_time
y ~ current_feature + Sex + (1 | Subject) + storage_time
y ~ current_feature + Sex + (1 | Subject) + storage_time
y ~ current_feature + Sex + (1 | Subject) + storage_time
y ~ current_feature + Sex + (1 | Subject) + storage_time
y ~ current_feature + Sex + (1 | Subject) + storage_time
y ~ current_feature + Sex + (1 | Subject) + storage_time
y ~ current_feature + Sex + (1 | Subject) + storage_time

y ~ current_feature + Sex + (1 | Subject) + storage_time
y ~ current_feature + Sex + (1 | Subject) + storage_time
y ~ current_feature + Sex + (1 | Subject) + storage_time
y ~ current_feature + Sex + (1 | Subject) + storage_time
y ~ current_feature + Sex + (1 | Subject) + storage_time
y ~ current_feature + Sex + (1 | Subject) + storage_time
y ~ current_feature + Sex + (1 | Subject) + storage_time
y ~ current_feature + Sex + (1 | Subject) + storage_time
y ~ current_feature + Sex + (1 | Subject) + storage_time
y ~ current_feature + Sex + (1 | Subject) + storage_time
y ~ current_feature + Sex + (1 | Subject) + storage_time
y ~ current_feature + Sex + (1 | Subject) + storage_time
y ~ current_feature + Sex + (1 | Subject) + storage_time
y ~ current_feature + Sex + (1 | Subject) + storage_time
y ~ current_feature + Sex + (1 | Subject) + storage_time
y ~ current_feature + Sex + (1 | Subject) + storage_time
y ~ current_feature + Sex + (1 | Subject) + storage_time
y ~ current_feature + Sex + (1 | Subject) + storage_time
y ~ current_feature + Sex + (1 | Subject) + storage_time
y ~ current_feature + Sex + (1 | Subject) + storage_time
y ~ current_feature + Sex + (1 | Subject) + storage_time
y ~ current_feature + Sex + (1 | Subject) + storage_time
y ~ current_feature + Sex + (1 | Subject) + storage_time
y ~ current_feature + Sex + (1 | Subject) + storage_time
y ~ current_feature + Sex + (1 | Subject) + storage_time
y ~ current_feature + Sex + (1 | Subject) + storage_time
y ~ current_feature + Sex + (1 | Subject) + storage_time
y ~ current_feature + Sex + (1 | Subject) + storage_time
y ~ current_feature + Sex + (1 | Subject) + storage_time
y ~ current_feature + Sex + (1 | Subject) + storage_time
y ~ current_feature + Sex + (1 | Subject) + storage_time
y ~ current_feature + Sex + (1 | Subject) + storage_time
y ~ current_feature + Sex + (1 | Subject) + storage_time
y ~ current_feature + Sex + (1 | Subject) + storage_time
y ~ current_feature + Sex + (1 | Subject) + storage_time
y ~ current_feature + Sex + (1 | Subject) + storage_time
y ~ current_feature + Sex + (1 | Subject) + storage_time
y ~ current_feature + Sex + (1 | Subject) + storage_time
y ~ current_feature + Sex + (1 | Subject) + storage_time
y ~ current_feature + Sex + (1 | Subject) + storage_time
y ~ current_feature + Sex + (1 | Subject) + storage_time
y ~ current_feature + Sex + (1 | Subject) + storage_time
y ~ current_feature + Sex + (1 | Subject) + storage_time
y ~ current_feature + Sex + (1 | Subject) + storage_time
y ~ current_feature + Sex + (1 | Subject) + storage_time
y ~ current_feature + Sex + (1 | Subject) + storage_time
y ~ current_feature + Sex + (1 | Subject) + storage_time
y ~ current_feature + Sex + (1 | Subject) + storage_time

y ~ current_feature + Sex + (1 | Subject) + storage_time
y ~ current_feature + Sex + (1 | Subject) + storage_time
y ~ current_feature + Sex + (1 | Subject) + storage_time
y ~ current_feature + Sex + (1 | Subject) + storage_time
y ~ current_feature + Sex + (1 | Subject) + storage_time
y ~ current_feature + Sex + (1 | Subject) + storage_time
y ~ current_feature + Sex + (1 | Subject) + storage_time
y ~ current_feature + Sex + (1 | Subject) + storage_time
y ~ current_feature + Sex + (1 | Subject) + storage_time
y ~ current_feature + Sex + (1 | Subject) + storage_time
y ~ current_feature + Sex + (1 | Subject) + storage_time
y ~ current_feature + Sex + (1 | Subject) + storage_time
y ~ current_feature + Sex + (1 | Subject) + storage_time
y ~ current_feature + Sex + (1 | Subject) + storage_time
y ~ current_feature + Sex + (1 | Subject) + storage_time
y ~ current_feature + Sex + (1 | Subject) + storage_time
y ~ current_feature + Sex + (1 | Subject) + storage_time
y ~ current_feature + Sex + (1 | Subject) + storage_time
y ~ current_feature + Sex + (1 | Subject) + storage_time
y ~ current_feature + Sex + (1 | Subject) + storage_time
y ~ current_feature + Sex + (1 | Subject) + storage_time
y ~ current_feature + Sex + (1 | Subject) + storage_time
y ~ current_feature + Sex + (1 | Subject) + storage_time
y ~ current_feature + Sex + (1 | Subject) + storage_time
y ~ current_feature + Sex + (1 | Subject) + storage_time
y ~ current_feature + Sex + (1 | Subject) + storage_time
y ~ current_feature + Sex + (1 | Subject) + storage_time
y ~ current_feature + Sex + (1 | Subject) + storage_time
y ~ current_feature + Sex + (1 | Subject) + storage_time
y ~ current_feature + Sex + (1 | Subject) + storage_time
y ~ current_feature + Sex + (1 | Subject) + storage_time
y ~ current_feature + Sex + (1 | Subject) + storage_time
y ~ current_feature + Sex + (1 | Subject) + storage_time
y ~ current_feature + Sex + (1 | Subject) + storage_time
y ~ current_feature + Sex + (1 | Subject) + storage_time
y ~ current_feature + Sex + (1 | Subject) + storage_time
y ~ current_feature + Sex + (1 | Subject) + storage_time
y ~ current_feature + Sex + (1 | Subject) + storage_time
y ~ current_feature + Sex + (1 | Subject) + storage_time
y ~ current_feature + Sex + (1 | Subject) + storage_time
y ~ current_feature + Sex + (1 | Subject) + storage_time
y ~ current_feature + Sex + (1 | Subject) + storage_time
y ~ current_feature + Sex + (1 | Subject) + storage_time
y ~ current_feature + Sex + (1 | Subject) + storage_time
y ~ current_feature + Sex + (1 | Subject) + storage_time
y ~ current_feature + Sex + (1 | Subject) + storage_time
y ~ current_feature + Sex + (1 | Subject) + storage_time
y ~ current_feature + Sex + (1 | Subject) + storage_time

y ~ current_feature + Sex + (1 | Subject) + storage_time
y ~ current_feature + Sex + (1 | Subject) + storage_time
y ~ current_feature + Sex + (1 | Subject) + storage_time
y ~ current_feature + Sex + (1 | Subject) + storage_time
y ~ current_feature + Sex + (1 | Subject) + storage_time
y ~ current_feature + Sex + (1 | Subject) + storage_time
y ~ current_feature + Sex + (1 | Subject) + storage_time
y ~ current_feature + Sex + (1 | Subject) + storage_time
y ~ current_feature + Sex + (1 | Subject) + storage_time
y ~ current_feature + Sex + (1 | Subject) + storage_time
y ~ current_feature + Sex + (1 | Subject) + storage_time
y ~ current_feature + Sex + (1 | Subject) + storage_time
y ~ current_feature + Sex + (1 | Subject) + storage_time
y ~ current_feature + Sex + (1 | Subject) + storage_time
y ~ current_feature + Sex + (1 | Subject) + storage_time
y ~ current_feature + Sex + (1 | Subject) + storage_time
y ~ current_feature + Sex + (1 | Subject) + storage_time
y ~ current_feature + Sex + (1 | Subject) + storage_time
y ~ current_feature + Sex + (1 | Subject) + storage_time
y ~ current_feature + Sex + (1 | Subject) + storage_time
y ~ current_feature + Sex + (1 | Subject) + storage_time
y ~ current_feature + Sex + (1 | Subject) + storage_time
y ~ current_feature + Sex + (1 | Subject) + storage_time
y ~ current_feature + Sex + (1 | Subject) + storage_time
y ~ current_feature + Sex + (1 | Subject) + storage_time
y ~ current_feature + Sex + (1 | Subject) + storage_time
y ~ current_feature + Sex + (1 | Subject) + storage_time
y ~ current_feature + Sex + (1 | Subject) + storage_time
y ~ current_feature + Sex + (1 | Subject) + storage_time
y ~ current_feature + Sex + (1 | Subject) + storage_time
y ~ current_feature + Sex + (1 | Subject) + storage_time
y ~ current_feature + Sex + (1 | Subject) + storage_time
y ~ current_feature + Sex + (1 | Subject) + storage_time
y ~ current_feature + Sex + (1 | Subject) + storage_time
y ~ current_feature + Sex + (1 | Subject) + storage_time
y ~ current_feature + Sex + (1 | Subject) + storage_time
y ~ current_feature + Sex + (1 | Subject) + storage_time
y ~ current_feature + Sex + (1 | Subject) + storage_time
y ~ current_feature + Sex + (1 | Subject) + storage_time
y ~ current_feature + Sex + (1 | Subject) + storage_time
y ~ current_feature + Sex + (1 | Subject) + storage_time
y ~ current_feature + Sex + (1 | Subject) + storage_time
y ~ current_feature + Sex + (1 | Subject) + storage_time
y ~ current_feature + Sex + (1 | Subject) + storage_time
y ~ current_feature + Sex + (1 | Subject) + storage_time
y ~ current_feature + Sex + (1 | Subject) + storage_time
y ~ current_feature + Sex + (1 | Subject) + storage_time
y ~ current_feature + Sex + (1 | Subject) + storage_time

y ~ current_feature + Sex + (1 | Subject) + storage_time
y ~ current_feature + Sex + (1 | Subject) + storage_time
y ~ current_feature + Sex + (1 | Subject) + storage_time
y ~ current_feature + Sex + (1 | Subject) + storage_time
y ~ current_feature + Sex + (1 | Subject) + storage_time
y ~ current_feature + Sex + (1 | Subject) + storage_time
y ~ current_feature + Sex + (1 | Subject) + storage_time
y ~ current_feature + Sex + (1 | Subject) + storage_time
y ~ current_feature + Sex + (1 | Subject) + storage_time
y ~ current_feature + Sex + (1 | Subject) + storage_time
y ~ current_feature + Sex + (1 | Subject) + storage_time
y ~ current_feature + Sex + (1 | Subject) + storage_time
y ~ current_feature + Sex + (1 | Subject) + storage_time
y ~ current_feature + Sex + (1 | Subject) + storage_time
y ~ current_feature + Sex + (1 | Subject) + storage_time
y ~ current_feature + Sex + (1 | Subject) + storage_time
y ~ current_feature + Sex + (1 | Subject) + storage_time
y ~ current_feature + Sex + (1 | Subject) + storage_time
y ~ current_feature + Sex + (1 | Subject) + storage_time
y ~ current_feature + Sex + (1 | Subject) + storage_time
y ~ current_feature + Sex + (1 | Subject) + storage_time
y ~ current_feature + Sex + (1 | Subject) + storage_time
y ~ current_feature + Sex + (1 | Subject) + storage_time
y ~ current_feature + Sex + (1 | Subject) + storage_time
y ~ current_feature + Sex + (1 | Subject) + storage_time
y ~ current_feature + Sex + (1 | Subject) + storage_time
y ~ current_feature + Sex + (1 | Subject) + storage_time
y ~ current_feature + Sex + (1 | Subject) + storage_time
y ~ current_feature + Sex + (1 | Subject) + storage_time
y ~ current_feature + Sex + (1 | Subject) + storage_time
y ~ current_feature + Sex + (1 | Subject) + storage_time
y ~ current_feature + Sex + (1 | Subject) + storage_time
y ~ current_feature + Sex + (1 | Subject) + storage_time
y ~ current_feature + Sex + (1 | Subject) + storage_time
y ~ current_feature + Sex + (1 | Subject) + storage_time
y ~ current_feature + Sex + (1 | Subject) + storage_time
y ~ current_feature + Sex + (1 | Subject) + storage_time
y ~ current_feature + Sex + (1 | Subject) + storage_time
y ~ current_feature + Sex + (1 | Subject) + storage_time
y ~ current_feature + Sex + (1 | Subject) + storage_time
y ~ current_feature + Sex + (1 | Subject) + storage_time
y ~ current_feature + Sex + (1 | Subject) + storage_time
y ~ current_feature + Sex + (1 | Subject) + storage_time
y ~ current_feature + Sex + (1 | Subject) + storage_time
y ~ current_feature + Sex + (1 | Subject) + storage_time
y ~ current_feature + Sex + (1 | Subject) + storage_time
y ~ current_feature + Sex + (1 | Subject) + storage_time
y ~ current_feature + Sex + (1 | Subject) + storage_time

y ~ current_feature + Sex + (1 | Subject) + storage_time
y ~ current_feature + Sex + (1 | Subject) + storage_time
y ~ current_feature + Sex + (1 | Subject) + storage_time
y ~ current_feature + Sex + (1 | Subject) + storage_time
y ~ current_feature + Sex + (1 | Subject) + storage_time
y ~ current_feature + Sex + (1 | Subject) + storage_time
y ~ current_feature + Sex + (1 | Subject) + storage_time
y ~ current_feature + Sex + (1 | Subject) + storage_time
y ~ current_feature + Sex + (1 | Subject) + storage_time
y ~ current_feature + Sex + (1 | Subject) + storage_time
y ~ current_feature + Sex + (1 | Subject) + storage_time
y ~ current_feature + Sex + (1 | Subject) + storage_time
y ~ current_feature + Sex + (1 | Subject) + storage_time
y ~ current_feature + Sex + (1 | Subject) + storage_time
y ~ current_feature + Sex + (1 | Subject) + storage_time
y ~ current_feature + Sex + (1 | Subject) + storage_time
y ~ current_feature + Sex + (1 | Subject) + storage_time
y ~ current_feature + Sex + (1 | Subject) + storage_time
y ~ current_feature + Sex + (1 | Subject) + storage_time
y ~ current_feature + Sex + (1 | Subject) + storage_time
y ~ current_feature + Sex + (1 | Subject) + storage_time
y ~ current_feature + Sex + (1 | Subject) + storage_time
y ~ current_feature + Sex + (1 | Subject) + storage_time
y ~ current_feature + Sex + (1 | Subject) + storage_time
y ~ current_feature + Sex + (1 | Subject) + storage_time
y ~ current_feature + Sex + (1 | Subject) + storage_time
y ~ current_feature + Sex + (1 | Subject) + storage_time
y ~ current_feature + Sex + (1 | Subject) + storage_time
y ~ current_feature + Sex + (1 | Subject) + storage_time
y ~ current_feature + Sex + (1 | Subject) + storage_time
y ~ current_feature + Sex + (1 | Subject) + storage_time
y ~ current_feature + Sex + (1 | Subject) + storage_time
y ~ current_feature + Sex + (1 | Subject) + storage_time
y ~ current_feature + Sex + (1 | Subject) + storage_time
y ~ current_feature + Sex + (1 | Subject) + storage_time
y ~ current_feature + Sex + (1 | Subject) + storage_time
y ~ current_feature + Sex + (1 | Subject) + storage_time
y ~ current_feature + Sex + (1 | Subject) + storage_time
y ~ current_feature + Sex + (1 | Subject) + storage_time
y ~ current_feature + Sex + (1 | Subject) + storage_time
y ~ current_feature + Sex + (1 | Subject) + storage_time
y ~ current_feature + Sex + (1 | Subject) + storage_time
y ~ current_feature + Sex + (1 | Subject) + storage_time
y ~ current_feature + Sex + (1 | Subject) + storage_time
y ~ current_feature + Sex + (1 | Subject) + storage_time
y ~ current_feature + Sex + (1 | Subject) + storage_time
y ~ current_feature + Sex + (1 | Subject) + storage_time
y ~ current_feature + Sex + (1 | Subject) + storage_time

y ~ current_feature + Sex + (1 | Subject) + storage_time
y ~ current_feature + Sex + (1 | Subject) + storage_time
y ~ current_feature + Sex + (1 | Subject) + storage_time
y ~ current_feature + Sex + (1 | Subject) + storage_time
y ~ current_feature + Sex + (1 | Subject) + storage_time
y ~ current_feature + Sex + (1 | Subject) + storage_time
y ~ current_feature + Sex + (1 | Subject) + storage_time
y ~ current_feature + Sex + (1 | Subject) + storage_time
y ~ current_feature + Sex + (1 | Subject) + storage_time
y ~ current_feature + Sex + (1 | Subject) + storage_time
y ~ current_feature + Sex + (1 | Subject) + storage_time
y ~ current_feature + Sex + (1 | Subject) + storage_time
y ~ current_feature + Sex + (1 | Subject) + storage_time
y ~ current_feature + Sex + (1 | Subject) + storage_time
y ~ current_feature + Sex + (1 | Subject) + storage_time
y ~ current_feature + Sex + (1 | Subject) + storage_time
y ~ current_feature + Sex + (1 | Subject) + storage_time
y ~ current_feature + Sex + (1 | Subject) + storage_time
y ~ current_feature + Sex + (1 | Subject) + storage_time
y ~ current_feature + Sex + (1 | Subject) + storage_time
y ~ current_feature + Sex + (1 | Subject) + storage_time
y ~ current_feature + Sex + (1 | Subject) + storage_time
y ~ current_feature + Sex + (1 | Subject) + storage_time
y ~ current_feature + Sex + (1 | Subject) + storage_time
y ~ current_feature + Sex + (1 | Subject) + storage_time
y ~ current_feature + Sex + (1 | Subject) + storage_time
y ~ current_feature + Sex + (1 | Subject) + storage_time
y ~ current_feature + Sex + (1 | Subject) + storage_time
y ~ current_feature + Sex + (1 | Subject) + storage_time
y ~ current_feature + Sex + (1 | Subject) + storage_time
y ~ current_feature + Sex + (1 | Subject) + storage_time
y ~ current_feature + Sex + (1 | Subject) + storage_time
y ~ current_feature + Sex + (1 | Subject) + storage_time
y ~ current_feature + Sex + (1 | Subject) + storage_time
y ~ current_feature + Sex + (1 | Subject) + storage_time
y ~ current_feature + Sex + (1 | Subject) + storage_time
y ~ current_feature + Sex + (1 | Subject) + storage_time
y ~ current_feature + Sex + (1 | Subject) + storage_time
y ~ current_feature + Sex + (1 | Subject) + storage_time
y ~ current_feature + Sex + (1 | Subject) + storage_time
y ~ current_feature + Sex + (1 | Subject) + storage_time
y ~ current_feature + Sex + (1 | Subject) + storage_time
y ~ current_feature + Sex + (1 | Subject) + storage_time
y ~ current_feature + Sex + (1 | Subject) + storage_time
y ~ current_feature + Sex + (1 | Subject) + storage_time
y ~ current_feature + Sex + (1 | Subject) + storage_time
y ~ current_feature + Sex + (1 | Subject) + storage_time
y ~ current_feature + Sex + (1 | Subject) + storage_time

y ~ current_feature + Sex + (1 | Subject) + storage_time
y ~ current_feature + Sex + (1 | Subject) + storage_time
y ~ current_feature + Sex + (1 | Subject) + storage_time
y ~ current_feature + Sex + (1 | Subject) + storage_time
y ~ current_feature + Sex + (1 | Subject) + storage_time
y ~ current_feature + Sex + (1 | Subject) + storage_time
y ~ current_feature + Sex + (1 | Subject) + storage_time
y ~ current_feature + Sex + (1 | Subject) + storage_time
y ~ current_feature + Sex + (1 | Subject) + storage_time
y ~ current_feature + Sex + (1 | Subject) + storage_time
y ~ current_feature + Sex + (1 | Subject) + storage_time
y ~ current_feature + Sex + (1 | Subject) + storage_time
y ~ current_feature + Sex + (1 | Subject) + storage_time
y ~ current_feature + Sex + (1 | Subject) + storage_time
y ~ current_feature + Sex + (1 | Subject) + storage_time
y ~ current_feature + Sex + (1 | Subject) + storage_time
y ~ current_feature + Sex + (1 | Subject) + storage_time
y ~ current_feature + Sex + (1 | Subject) + storage_time
y ~ current_feature + Sex + (1 | Subject) + storage_time
y ~ current_feature + Sex + (1 | Subject) + storage_time
y ~ current_feature + Sex + (1 | Subject) + storage_time
y ~ current_feature + Sex + (1 | Subject) + storage_time
y ~ current_feature + Sex + (1 | Subject) + storage_time
y ~ current_feature + Sex + (1 | Subject) + storage_time
y ~ current_feature + Sex + (1 | Subject) + storage_time
y ~ current_feature + Sex + (1 | Subject) + storage_time
y ~ current_feature + Sex + (1 | Subject) + storage_time
y ~ current_feature + Sex + (1 | Subject) + storage_time
y ~ current_feature + Sex + (1 | Subject) + storage_time
y ~ current_feature + Sex + (1 | Subject) + storage_time
y ~ current_feature + Sex + (1 | Subject) + storage_time
y ~ current_feature + Sex + (1 | Subject) + storage_time
y ~ current_feature + Sex + (1 | Subject) + storage_time
y ~ current_feature + Sex + (1 | Subject) + storage_time
y ~ current_feature + Sex + (1 | Subject) + storage_time
y ~ current_feature + Sex + (1 | Subject) + storage_time
y ~ current_feature + Sex + (1 | Subject) + storage_time
y ~ current_feature + Sex + (1 | Subject) + storage_time
y ~ current_feature + Sex + (1 | Subject) + storage_time
y ~ current_feature + Sex + (1 | Subject) + storage_time
y ~ current_feature + Sex + (1 | Subject) + storage_time
y ~ current_feature + Sex + (1 | Subject) + storage_time
y ~ current_feature + Sex + (1 | Subject) + storage_time
y ~ current_feature + Sex + (1 | Subject) + storage_time
y ~ current_feature + Sex + (1 | Subject) + storage_time
y ~ current_feature + Sex + (1 | Subject) + storage_time
y ~ current_feature + Sex + (1 | Subject) + storage_time
y ~ current_feature + Sex + (1 | Subject) + storage_time

y ~ current_feature + Sex + (1 | Subject) + storage_time
y ~ current_feature + Sex + (1 | Subject) + storage_time
y ~ current_feature + Sex + (1 | Subject) + storage_time
y ~ current_feature + Sex + (1 | Subject) + storage_time

Name unique
2-Methylpropanoic acid
2-Methylpropanoic acid
2-Methylpropanoic acid
2-Methylpropanoic acid
2-Methylpropanoic acid
3-Methylbutanoic acid
3-Methylbutanoic acid
3-Methylbutanoic acid
3-Methylbutanoic acid
3-Methylbutanoic acid
Acetic acid
Acetic acid
Acetic acid
Acetic acid
Acetic acid
Butanoic acid
Butanoic acid
Butanoic acid
Butanoic acid
Butanoic acid
Hexanoic acid
Hexanoic acid
Hexanoic acid
Hexanoic acid
Hexanoic acid
M1
M1
M1
M1
M1
M10
M10
M10
M10
M10
M11
M11
M11
M11
M11
M12
M12
M12
M12
M12
M13
M13

M13
M13
M13
M14
M14
M14
M14
M14
M15
M15
M15
M15
M15
M16
M16
M16
M16
M16
M17
M17
M17
M17
M17
M18
M18
M18
M18
M18
M19
M19
M19
M19
M19
M2
M2
M2
M2
M2
M20
M20
M20
M20
M20
M21
M21
M21
M21
M21

M22
M22
M22
M22
M22
M23
M23
M23
M23
M23
M24
M24
M24
M24
M24
M25
M25
M25
M25
M25
M26
M26
M26
M26
M26
M27
M27
M27
M27
M27
M28
M28
M28
M28
M28
M29
M29
M29
M29
M29
M3
M3
M3
M3
M3
M30
M30
M30

M30
M30
M31
M31
M31
M31
M31
M32
M32
M32
M32
M32
M33
M33
M33
M33
M33
M34
M34
M34
M34
M34
M4
M4
M4
M4
M4
M5
M5
M5
M5
M5
M6
M6
M6
M6
M6
M7
M7
M7
M7
M7
M8
M8
M8
M8
M8
M9

M9
M9
M9
M9
Pentanoic acid
Pentanoic acid
Pentanoic acid
Pentanoic acid
Pentanoic acid
Propanoic acid
Propanoic acid
Propanoic acid
Propanoic acid
Propanoic acid
Deoxycytidine
Deoxycytidine
Deoxycytidine
Deoxycytidine
Deoxycytidine
N-Acetylhistidine
N-Acetylhistidine
N-Acetylhistidine
N-Acetylhistidine
N-Acetylhistidine
Aspartic acid
Aspartic acid
Aspartic acid
Aspartic acid
Aspartic acid
Carnitine
Carnitine
Carnitine
Carnitine
Carnitine
Creatine
Creatine
Creatine
Creatine
Creatine
Glycylglycine
Glycylglycine
Glycylglycine
Glycylglycine
Glycylglycine
Kynurenine
Kynurenine
Kynurenine
Kynurenine

Kynurenine
N-Acetylarginine
N-Acetylarginine
N-Acetylarginine
N-Acetylarginine
N-Acetylarginine
N-Isovalerylglycine
N-Isovalerylglycine
N-Isovalerylglycine
N-Isovalerylglycine
N-Isovalerylglycine
Ornithine
Ornithine
Ornithine
Ornithine
Ornithine
Proline
Proline
Proline
Proline
Proline
Pyridoxal
Pyridoxal
Pyridoxal
Pyridoxal
Pyridoxal
Valine/ 5-Aminovaleric acid
Valine/ 5-Aminovaleric acid
Valine/ 5-Aminovaleric acid
Valine/ 5-Aminovaleric acid
Valine/ 5-Aminovaleric acid
5-Methylcytosine
5-Methylcytosine
5-Methylcytosine
5-Methylcytosine
5-Methylcytosine
Allopurinol
Allopurinol
Allopurinol
Allopurinol
Allopurinol
Dissaccarides I
Dissaccarides I
Dissaccarides I
Dissaccarides I
Dissaccarides I
Hexoses III
Hexoses III

Hexoses III
Hexoses III
Hexoses III
Hexoses II
Hexoses II
Hexoses II
Hexoses II
Hexoses II
Gulonic acid Î³-lactone
Gulonic acid Î³-lactone
Gulonic acid Î³-lactone
Gulonic acid Î³-lactone
Gulonic acid Î³-lactone
Hexoses I
Hexoses I
Hexoses I
Hexoses I
Hexoses I
Histidinol
Histidinol
Histidinol
Histidinol
Histidinol
Dissaccarides III
Dissaccarides III
Dissaccarides III
Dissaccarides III
Dissaccarides III
N-Acetylalanine
N-Acetylalanine
N-Acetylalanine
N-Acetylalanine
N-Acetylalanine
N-Acetylglutamine
N-Acetylglutamine
N-Acetylglutamine
N-Acetylglutamine
N-Acetylglutamine
Phenylacetylglutamine
Phenylacetylglutamine
Phenylacetylglutamine
Phenylacetylglutamine
Phenylacetylglutamine
Pyridoxamine
Pyridoxamine
Pyridoxamine
Pyridoxamine
Pyridoxamine

Dissaccarides II
Dissaccarides II
Dissaccarides II
Dissaccarides II
Dissaccarides II
Trigonelline
Trigonelline
Trigonelline
Trigonelline
Trigonelline
Uracil
Uracil
Uracil
Uracil
Uracil
Uridine
Uridine
Uridine
Uridine
Uridine
2-Oxo-3-phenylpropanoic acid
2-Oxo-3-phenylpropanoic acid
2-Oxo-3-phenylpropanoic acid
2-Oxo-3-phenylpropanoic acid
2-Oxo-3-phenylpropanoic acid
3-Hydroxybutyric acid
3-Hydroxybutyric acid
3-Hydroxybutyric acid
3-Hydroxybutyric acid
3-Hydroxybutyric acid
4-Methyl-2-oxovaleric acid
4-Methyl-2-oxovaleric acid
4-Methyl-2-oxovaleric acid
4-Methyl-2-oxovaleric acid
4-Methyl-2-oxovaleric acid
5-Hydroxyindole-3-acetic acid
5-Hydroxyindole-3-acetic acid
5-Hydroxyindole-3-acetic acid
5-Hydroxyindole-3-acetic acid
5-Hydroxyindole-3-acetic acid
8-Aminooctanoic acid
8-Aminooctanoic acid
8-Aminooctanoic acid
8-Aminooctanoic acid
8-Aminooctanoic acid
Pentose II
Pentose II
Pentose II

Pentose II
Pentose II
Citraconic acid
Citraconic acid
Citraconic acid
Citraconic acid
Citraconic acid
Galactonic acid
Galactonic acid
Galactonic acid
Galactonic acid
Galactonic acid
Galactosamine
Galactosamine
Galactosamine
Galactosamine
Galactosamine
Glycolic acid
Glycolic acid
Glycolic acid
Glycolic acid
Glycolic acid
Glyoxylic acid
Glyoxylic acid
Glyoxylic acid
Glyoxylic acid
Glyoxylic acid
Lactic acid
Lactic acid
Lactic acid
Lactic acid
Lactic acid
N-Acetylglycine
N-Acetylglycine
N-Acetylglycine
N-Acetylglycine
N-Acetylglycine
Pinitol
Pinitol
Pinitol
Pinitol
Pinitol
Pyruvic acid
Pyruvic acid
Pyruvic acid
Pyruvic acid
Pyruvic acid
Deoxysugar I (possibly Rhamnose/2-Deoxyglucose)

Deoxysugar I (possibly Rhamnose/2-Deoxyglucose)
Deoxysugar I (possibly Rhamnose/2-Deoxyglucose)
Deoxysugar I (possibly Rhamnose/2-Deoxyglucose)
Deoxysugar I (possibly Rhamnose/2-Deoxyglucose)
sugar alcohol (C5)
sugar alcohol (C5)
sugar alcohol (C5)
sugar alcohol (C5)
sugar alcohol (C5)
Pentose III
Pentose III
Pentose III
Pentose III
Pentose III
Shikimic acid
Shikimic acid
Shikimic acid
Shikimic acid
Shikimic acid
Gluconic acid
Gluconic acid
Gluconic acid
Gluconic acid
Gluconic acid
Pentose I
Pentose I
Pentose I
Pentose I
Pentose I
cis-Aconitic acid
cis-Aconitic acid
cis-Aconitic acid
cis-Aconitic acid
cis-Aconitic acid
Methyl acetoacetate
Methyl acetoacetate
Methyl acetoacetate
Methyl acetoacetate
Methyl acetoacetate
Purine
Purine
Purine
Purine
Purine
Thymine
Thymine
Thymine
Thymine

Thymine
3-hydroxy-4,5-dimethyl-3(5H)-furanone
3-hydroxy-4,5-dimethyl-3(5H)-furanone
3-hydroxy-4,5-dimethyl-3(5H)-furanone
3-hydroxy-4,5-dimethyl-3(5H)-furanone
3-hydroxy-4,5-dimethyl-3(5H)-furanone
Dopamine
Dopamine
Dopamine
Dopamine
Dopamine
3-Hydroxyhippuric acid
3-Hydroxyhippuric acid
3-Hydroxyhippuric acid
3-Hydroxyhippuric acid
3-Hydroxyhippuric acid
Dihydroferulic acid
Dihydroferulic acid
Dihydroferulic acid
Dihydroferulic acid
Dihydroferulic acid
DOPA
DOPA
DOPA
DOPA
DOPA
Propionylcarnitine
Propionylcarnitine
Propionylcarnitine
Propionylcarnitine
Propionylcarnitine
N-Acetylmannosamine
N-Acetylmannosamine
N-Acetylmannosamine
N-Acetylmannosamine
N-Acetylmannosamine
Butyrylcarnitine
Butyrylcarnitine
Butyrylcarnitine
Butyrylcarnitine
Butyrylcarnitine
5-Methyluridine
5-Methyluridine
5-Methyluridine
5-Methyluridine
5-Methyluridine
Acetylmuramic acid
Acetylmuramic acid

Acetylmuramic acid
Acetylmuramic acid
Acetylmuramic acid
Î²-Muricholic acid/ Ï‰-Muricholic acid
Î²-Muricholic acid/ Ï‰-Muricholic acid
Î²-Muricholic acid/ Ï‰-Muricholic acid
Î²-Muricholic acid/ Ï‰-Muricholic acid
Î²-Muricholic acid/ Ï‰-Muricholic acid
1-Aminocyclopropanecarboxylic acid
1-Aminocyclopropanecarboxylic acid
1-Aminocyclopropanecarboxylic acid
1-Aminocyclopropanecarboxylic acid
1-Aminocyclopropanecarboxylic acid
N,N-Dimethylglycine
N,N-Dimethylglycine
N,N-Dimethylglycine
N,N-Dimethylglycine
N,N-Dimethylglycine
2-Aminoisobutyric acid
2-Aminoisobutyric acid
2-Aminoisobutyric acid
2-Aminoisobutyric acid
2-Aminoisobutyric acid
Malonic acid
Malonic acid
Malonic acid
Malonic acid
Malonic acid
4-Aminophenol
4-Aminophenol
4-Aminophenol
4-Aminophenol
4-Aminophenol
Î³-Caprolactone
Î³-Caprolactone
Î³-Caprolactone
Î³-Caprolactone
Î³-Caprolactone
2-Methylmalonic acid
2-Methylmalonic acid
2-Methylmalonic acid
2-Methylmalonic acid
2-Methylmalonic acid
2-(hydroxymethyl)butanoic acid
2-(hydroxymethyl)butanoic acid
2-(hydroxymethyl)butanoic acid
2-(hydroxymethyl)butanoic acid
2-(hydroxymethyl)butanoic acid

2-Hydroxy-3-methylbutyric acid
2-Hydroxy-3-methylbutyric acid
2-Hydroxy-3-methylbutyric acid
2-Hydroxy-3-methylbutyric acid
2-Hydroxy-3-methylbutyric acid
Imidazoleacetic acid
Imidazoleacetic acid
Imidazoleacetic acid
Imidazoleacetic acid
Imidazoleacetic acid
N-Methylnicotinamide
N-Methylnicotinamide
N-Methylnicotinamide
N-Methylnicotinamide
N-Methylnicotinamide
1-Aminocyclohexanecarboxylic acid
1-Aminocyclohexanecarboxylic acid
1-Aminocyclohexanecarboxylic acid
1-Aminocyclohexanecarboxylic acid
1-Aminocyclohexanecarboxylic acid
Stachydrine
Stachydrine
Stachydrine
Stachydrine
Stachydrine
Adipic acid
Adipic acid
Adipic acid
Adipic acid
Adipic acid
N-(5-Aminopentyl)acetamide
N-(5-Aminopentyl)acetamide
N-(5-Aminopentyl)acetamide
N-(5-Aminopentyl)acetamide
N-(5-Aminopentyl)acetamide
N-Methyltyramine
N-Methyltyramine
N-Methyltyramine
N-Methyltyramine
N-Methyltyramine
N1-Methyl-2-pyridone-5-carboxamide
N1-Methyl-2-pyridone-5-carboxamide
N1-Methyl-2-pyridone-5-carboxamide
N1-Methyl-2-pyridone-5-carboxamide
N1-Methyl-2-pyridone-5-carboxamide
N-AcetylProline
N-AcetylProline
N-AcetylProline

N-AcetylProline
N-AcetylProline
2-Aminoadipic acid
2-Aminoadipic acid
2-Aminoadipic acid
2-Aminoadipic acid
2-Aminoadipic acid
3-(2-Hydroxyethyl)indole
3-(2-Hydroxyethyl)indole
3-(2-Hydroxyethyl)indole
3-(2-Hydroxyethyl)indole
3-(2-Hydroxyethyl)indole
p-Coumaric acid
p-Coumaric acid
p-Coumaric acid
p-Coumaric acid
p-Coumaric acid
Gallic acid
Gallic acid
Gallic acid
Gallic acid
Gallic acid
N-Acetylleucine/ Hexanoylglycine
N-Acetylleucine/ Hexanoylglycine
N-Acetylleucine/ Hexanoylglycine
N-Acetylleucine/ Hexanoylglycine
N-Acetylleucine/ Hexanoylglycine
Theophylline
Theophylline
Theophylline
Theophylline
Theophylline
Homovanilic acid
Homovanilic acid
Homovanilic acid
Homovanilic acid
Homovanilic acid
4-Hydroxy-3-methoxymandelic acid
4-Hydroxy-3-methoxymandelic acid
4-Hydroxy-3-methoxymandelic acid
4-Hydroxy-3-methoxymandelic acid
4-Hydroxy-3-methoxymandelic acid
Asymmetric dimethylarginine
Asymmetric dimethylarginine
Asymmetric dimethylarginine
Asymmetric dimethylarginine
Asymmetric dimethylarginine
Homocarnosine/ Anserine

Homocarnosine/ Anserine
Homocarnosine/ Anserine
Homocarnosine/ Anserine
Homocarnosine/ Anserine
Tiglylcarnitine
Tiglylcarnitine
Tiglylcarnitine
Tiglylcarnitine
Tiglylcarnitine
Isovalerylcarnitine
Isovalerylcarnitine
Isovalerylcarnitine
Isovalerylcarnitine
Isovalerylcarnitine
1-Carboxyethyltyrosine
1-Carboxyethyltyrosine
1-Carboxyethyltyrosine
1-Carboxyethyltyrosine
1-Carboxyethyltyrosine
Daidzein
Daidzein
Daidzein
Daidzein
Daidzein
5-Methylcytidine
5-Methylcytidine
5-Methylcytidine
5-Methylcytidine
5-Methylcytidine
Glucosamine-6-phosphate
Glucosamine-6-phosphate
Glucosamine-6-phosphate
Glucosamine-6-phosphate
Glucosamine-6-phosphate
Apigenin
Apigenin
Apigenin
Apigenin
Apigenin
12,13-DHOME
12,13-DHOME
12,13-DHOME
12,13-DHOME
12,13-DHOME
Sucralose
Sucralose
Sucralose
Sucralose

Sucralose
2-Ketobutyric acid
2-Ketobutyric acid
2-Ketobutyric acid
2-Ketobutyric acid
2-Ketobutyric acid
Glutamine
Glutamine
Glutamine
Glutamine
Glutamine
Succinic acid
Succinic acid
Succinic acid
Succinic acid
Succinic acid
sugar alcohol (C6)
sugar alcohol (C6)
sugar alcohol (C6)
sugar alcohol (C6)
sugar alcohol (C6)
N-Methylaspartic acid
N-Methylaspartic acid
N-Methylaspartic acid
N-Methylaspartic acid
N-Methylaspartic acid
N-Formylmethionine
N-Formylmethionine
N-Formylmethionine
N-Formylmethionine
N-Formylmethionine
Acesulfame
Acesulfame
Acesulfame
Acesulfame
Acesulfame
Inosine
Inosine
Inosine
Inosine
Inosine
N-Acetylglutamic acid
N-Acetylglutamic acid
N-Acetylglutamic acid
N-Acetylglutamic acid
N-Acetylglutamic acid
Urocanic acid
Urocanic acid

Urocanic acid
Urocanic acid
Urocanic acid
3,4-Dihydroxybenzoic acid
3,4-Dihydroxybenzoic acid
3,4-Dihydroxybenzoic acid
3,4-Dihydroxybenzoic acid
3,4-Dihydroxybenzoic acid
Indole-3-propionic acid
Indole-3-propionic acid
Indole-3-propionic acid
Indole-3-propionic acid
Indole-3-propionic acid
Deoxycholic acid
Deoxycholic acid
Deoxycholic acid
Deoxycholic acid
Deoxycholic acid
Pyroglutamic acid
Pyroglutamic acid
Pyroglutamic acid
Pyroglutamic acid
Pyroglutamic acid
Choline
Choline
Choline
Choline
Choline
Serine
Serine
Serine
Serine
Serine
Histamine
Histamine
Histamine
Histamine
Histamine
N-Acetyltyrosine
N-Acetyltyrosine
N-Acetyltyrosine
N-Acetyltyrosine
N-Acetyltyrosine
Glucuronic acid
Glucuronic acid
Glucuronic acid
Glucuronic acid
Glucuronic acid

Threonine
Threonine
Threonine
Threonine
Threonine
Î²-Hydroxyisovaleric acid
Î²-Hydroxyisovaleric acid
Î²-Hydroxyisovaleric acid
Î²-Hydroxyisovaleric acid
Î²-Hydroxyisovaleric acid
Î²-D-Glucopyranuronic acid
Î²-D-Glucopyranuronic acid
Î²-D-Glucopyranuronic acid
Î²-D-Glucopyranuronic acid
Î²-D-Glucopyranuronic acid
3-(4-hydroxyphenyl)propionic acid
3-(4-hydroxyphenyl)propionic acid
3-(4-hydroxyphenyl)propionic acid
3-(4-hydroxyphenyl)propionic acid
3-(4-hydroxyphenyl)propionic acid
N-Acetylornithine
N-Acetylornithine
N-Acetylornithine
N-Acetylornithine
N-Acetylornithine
3,4-Dihydroxyhydrocinnamic acid
3,4-Dihydroxyhydrocinnamic acid
3,4-Dihydroxyhydrocinnamic acid
3,4-Dihydroxyhydrocinnamic acid
3,4-Dihydroxyhydrocinnamic acid
Ethylmalonic acid
Ethylmalonic acid
Ethylmalonic acid
Ethylmalonic acid
Ethylmalonic acid
Thiamine
Thiamine
Thiamine
Thiamine
Thiamine
2-Hydroxycaproic acid
2-Hydroxycaproic acid
2-Hydroxycaproic acid
2-Hydroxycaproic acid
2-Hydroxycaproic acid
Traumatic acid
Traumatic acid
Traumatic acid

Traumatic acid
Traumatic acid
Tryptamine
Tryptamine
Tryptamine
Tryptamine
Tryptamine
Hypoxanthine
Hypoxanthine
Hypoxanthine
Hypoxanthine
Hypoxanthine
N-Acetylmethionine
N-Acetylmethionine
N-Acetylmethionine
N-Acetylmethionine
N-Acetylmethionine
Î±-aminobutyric acid
Î±-aminobutyric acid
Î±-aminobutyric acid
Î±-aminobutyric acid
Î±-aminobutyric acid
Tyrosine
Tyrosine
Tyrosine
Tyrosine
Tyrosine
3,5-Dihydroxybenzoic acid
3,5-Dihydroxybenzoic acid
3,5-Dihydroxybenzoic acid
3,5-Dihydroxybenzoic acid
3,5-Dihydroxybenzoic acid
Indole-3-lactic acid
Indole-3-lactic acid
Indole-3-lactic acid
Indole-3-lactic acid
Indole-3-lactic acid
Methylsuccinic acid
Methylsuccinic acid
Methylsuccinic acid
Methylsuccinic acid
Methylsuccinic acid
Isoleucine
Isoleucine
Isoleucine
Isoleucine
Isoleucine
Acetylagmatine

Acetylagmatine
Acetylagmatine
Acetylagmatine
Acetylagmatine
Cholic acid
Cholic acid
Cholic acid
Cholic acid
Cholic acid
1,7-Dimethylxanthine
1,7-Dimethylxanthine
1,7-Dimethylxanthine
1,7-Dimethylxanthine
1,7-Dimethylxanthine
Tricarballylic acid
Tricarballylic acid
Tricarballylic acid
Tricarballylic acid
Tricarballylic acid
Pantothenic acid
Pantothenic acid
Pantothenic acid
Pantothenic acid
Pantothenic acid
4-Hydroxybenzoic acid
4-Hydroxybenzoic acid
4-Hydroxybenzoic acid
4-Hydroxybenzoic acid
4-Hydroxybenzoic acid
Malic acid
Malic acid
Malic acid
Malic acid
Malic acid
3-Methylhistidine
3-Methylhistidine
3-Methylhistidine
3-Methylhistidine
3-Methylhistidine
Deoxyinosine
Deoxyinosine
Deoxyinosine
Deoxyinosine
Deoxyinosine
Methionine
Methionine
Methionine
Methionine

Methionine
2,6-Dihydroxybenzoic acid
2,6-Dihydroxybenzoic acid
2,6-Dihydroxybenzoic acid
2,6-Dihydroxybenzoic acid
2,6-Dihydroxybenzoic acid
7-Methylguanine
7-Methylguanine
7-Methylguanine
7-Methylguanine
7-Methylguanine
Threonic acid
Threonic acid
Threonic acid
Threonic acid
Threonic acid
Tryptophan
Tryptophan
Tryptophan
Tryptophan
Tryptophan
N-Acetyltryptophan
N-Acetyltryptophan
N-Acetyltryptophan
N-Acetyltryptophan
N-Acetyltryptophan
N6-Acetyllysine
N6-Acetyllysine
N6-Acetyllysine
N6-Acetyllysine
N6-Acetyllysine
Xanthosine
Xanthosine
Xanthosine
Xanthosine
Xanthosine
N-Acetylphenylalanine
N-Acetylphenylalanine
N-Acetylphenylalanine
N-Acetylphenylalanine
N-Acetylphenylalanine
Indole-3-methyl acetate
Indole-3-methyl acetate
Indole-3-methyl acetate
Indole-3-methyl acetate
Indole-3-methyl acetate
Histidine
Histidine

Histidine
Histidine
Histidine
Cytidine
Cytidine
Cytidine
Cytidine
Cytidine
Leucylalanine
Leucylalanine
Leucylalanine
Leucylalanine
Leucylalanine
Phenylalanine
Phenylalanine
Phenylalanine
Phenylalanine
Phenylalanine
Leucine
Leucine
Leucine
Leucine
Leucine
Taurine
Taurine
Taurine
Taurine
Taurine
Nicotinic acid
Nicotinic acid
Nicotinic acid
Nicotinic acid
Nicotinic acid
Quinic acid
Quinic acid
Quinic acid
Quinic acid
Quinic acid
Deoxyuridine
Deoxyuridine
Deoxyuridine
Deoxyuridine
Deoxyuridine
Glyceric acid
Glyceric acid
Glyceric acid
Glyceric acid
Glyceric acid

N-Acetylglucosamine
N-Acetylglucosamine
N-Acetylglucosamine
N-Acetylglucosamine
N-Acetylglucosamine
leu-gln_a
leu-gln_a
leu-gln_a
leu-gln_a
leu-gln_a
primidone_a
primidone_a
primidone_a
primidone_a
primidone_a
1-(2-Carboxyethyl)-2,3,4,9-tetrahydro-1H-beta-carboline-3-carboxylic acid_a
1-(2-Carboxyethyl)-2,3,4,9-tetrahydro-1H-beta-carboline-3-carboxylic acid_a
1-(2-Carboxyethyl)-2,3,4,9-tetrahydro-1H-beta-carboline-3-carboxylic acid_a
1-(2-Carboxyethyl)-2,3,4,9-tetrahydro-1H-beta-carboline-3-carboxylic acid_a
1-(2-Carboxyethyl)-2,3,4,9-tetrahydro-1H-beta-carboline-3-carboxylic acid_a
3-(Butylsulfinyl)-L-alanin
3-(Butylsulfinyl)-L-alanin
3-(Butylsulfinyl)-L-alanin
3-(Butylsulfinyl)-L-alanin
3-(Butylsulfinyl)-L-alanin
tert-Butyl 3-amino-1-methyl-2,3-dioxopropylcarbamate_b
tert-Butyl 3-amino-1-methyl-2,3-dioxopropylcarbamate_b
tert-Butyl 3-amino-1-methyl-2,3-dioxopropylcarbamate_b
tert-Butyl 3-amino-1-methyl-2,3-dioxopropylcarbamate_b
tert-Butyl 3-amino-1-methyl-2,3-dioxopropylcarbamate_b
4-O-{3-O-[(1S,2R,3S,4S,5R)-2,3,4-Trihydroxy-5-(hydroxymethyl)cyclohexyl]-beta-D-galactopyranosyl}-D-glucose
4-O-{3-O-[(1S,2R,3S,4S,5R)-2,3,4-Trihydroxy-5-(hydroxymethyl)cyclohexyl]-beta-D-galactopyranosyl}-D-glucose
4-O-{3-O-[(1S,2R,3S,4S,5R)-2,3,4-Trihydroxy-5-(hydroxymethyl)cyclohexyl]-beta-D-galactopyranosyl}-D-glucose
4-O-{3-O-[(1S,2R,3S,4S,5R)-2,3,4-Trihydroxy-5-(hydroxymethyl)cyclohexyl]-beta-D-galactopyranosyl}-D-glucose
4-O-{3-O-[(1S,2R,3S,4S,5R)-2,3,4-Trihydroxy-5-(hydroxymethyl)cyclohexyl]-beta-D-galactopyranosyl}-D-glucose
Astemizole_b
Astemizole_b
Astemizole_b
Astemizole_b
Astemizole_b
Nifedipine
Nifedipine
Nifedipine
Nifedipine
Nifedipine
g-Aminobutyryl-lysine_d
g-Aminobutyryl-lysine_d
g-Aminobutyryl-lysine_d

g-Aminobutyryl-lysine_d
g-Aminobutyryl-lysine_d
OI1700000
OI1700000
OI1700000
OI1700000
OI1700000
IN00258
IN00258
IN00258
IN00258
IN00258
TDP-2_a
TDP-2_a
TDP-2_a
TDP-2_a
TDP-2_a
Mexiletine
Mexiletine
Mexiletine
Mexiletine
Mexiletine
PEG n12
PEG n12
PEG n12
PEG n12
PEG n12
2-Oxo-3-(phosphonooxy)propyl decanoate
2-Oxo-3-(phosphonooxy)propyl decanoate
2-Oxo-3-(phosphonooxy)propyl decanoate
2-Oxo-3-(phosphonooxy)propyl decanoate
2-Oxo-3-(phosphonooxy)propyl decanoate
Formylkynurenine_b
Formylkynurenine_b
Formylkynurenine_b
Formylkynurenine_b
Formylkynurenine_b
Panthenol_b
Panthenol_b
Panthenol_b
Panthenol_b
Panthenol_b
3-Hydroxy-2-(hydroxymethyl)-2-[(sulfooxy)methyl]propanoic acid
3-Hydroxy-2-(hydroxymethyl)-2-[(sulfooxy)methyl]propanoic acid
3-Hydroxy-2-(hydroxymethyl)-2-[(sulfooxy)methyl]propanoic acid
3-Hydroxy-2-(hydroxymethyl)-2-[(sulfooxy)methyl]propanoic acid
3-Hydroxy-2-(hydroxymethyl)-2-[(sulfooxy)methyl]propanoic acid
4-(9H-beta-Carbolin-1-yl)-1,2,4-butanetriol_b

4-(9H-beta-Carbolin-1-yl)-1,2,4-butanetriol_b
4-(9H-beta-Carbolin-1-yl)-1,2,4-butanetriol_b
4-(9H-beta-Carbolin-1-yl)-1,2,4-butanetriol_b
4-(9H-beta-Carbolin-1-yl)-1,2,4-butanetriol_b
SECONAL_f
SECONAL_f
SECONAL_f
SECONAL_f
SECONAL_f
nitecapone
nitecapone
nitecapone
nitecapone
nitecapone
hexobarbital_a
hexobarbital_a
hexobarbital_a
hexobarbital_a
hexobarbital_a
N-[(2S)-2-Hydroxypropanoyl]methionine_c
N-[(2S)-2-Hydroxypropanoyl]methionine_c
N-[(2S)-2-Hydroxypropanoyl]methionine_c
N-[(2S)-2-Hydroxypropanoyl]methionine_c
N-[(2S)-2-Hydroxypropanoyl]methionine_c
pretazettine
pretazettine
pretazettine
pretazettine
pretazettine
3-Formyl-2a-methoxy-6,6,7b-trimethyl-2,2a,4a,5,6,7,7a,7b-octahydro-1H-cyclobuta[e]inden-2-yl 3-chloro-6-hyd
3-Formyl-2a-methoxy-6,6,7b-trimethyl-2,2a,4a,5,6,7,7a,7b-octahydro-1H-cyclobuta[e]inden-2-yl 3-chloro-6-hyd
3-Formyl-2a-methoxy-6,6,7b-trimethyl-2,2a,4a,5,6,7,7a,7b-octahydro-1H-cyclobuta[e]inden-2-yl 3-chloro-6-hyd
3-Formyl-2a-methoxy-6,6,7b-trimethyl-2,2a,4a,5,6,7,7a,7b-octahydro-1H-cyclobuta[e]inden-2-yl 3-chloro-6-hyd
3-Formyl-2a-methoxy-6,6,7b-trimethyl-2,2a,4a,5,6,7,7a,7b-octahydro-1H-cyclobuta[e]inden-2-yl 3-chloro-6-hyd
7alpha-Hydroxy-3-oxochol-4-en-24-oic acid_a
7alpha-Hydroxy-3-oxochol-4-en-24-oic acid_a
7alpha-Hydroxy-3-oxochol-4-en-24-oic acid_a
7alpha-Hydroxy-3-oxochol-4-en-24-oic acid_a
7alpha-Hydroxy-3-oxochol-4-en-24-oic acid_a
piscidic acid_a
piscidic acid_a
piscidic acid_a
piscidic acid_a
piscidic acid_a
8-(Methylsulfinyl)octyl isothiocyanate
8-(Methylsulfinyl)octyl isothiocyanate
8-(Methylsulfinyl)octyl isothiocyanate
8-(Methylsulfinyl)octyl isothiocyanate

8-(Methylsulfinyl)octyl isothiocyanate
Gly-Ser
Gly-Ser
Gly-Ser
Gly-Ser
Gly-Ser
Sparfloxacin
Sparfloxacin
Sparfloxacin
Sparfloxacin
Sparfloxacin
Nisinic acid_a
Nisinic acid_a
Nisinic acid_a
Nisinic acid_a
Nisinic acid_a
R-(+)-Etiracetam_b
R-(+)-Etiracetam_b
R-(+)-Etiracetam_b
R-(+)-Etiracetam_b
R-(+)-Etiracetam_b
3-hydroxyoctanoylcarnitine
3-hydroxyoctanoylcarnitine
3-hydroxyoctanoylcarnitine
3-hydroxyoctanoylcarnitine
3-hydroxyoctanoylcarnitine
3-[(2Z)-1-Oxo-2-buten-2-yl]pentanedioic acid_a
3-[(2Z)-1-Oxo-2-buten-2-yl]pentanedioic acid_a
3-[(2Z)-1-Oxo-2-buten-2-yl]pentanedioic acid_a
3-[(2Z)-1-Oxo-2-buten-2-yl]pentanedioic acid_a
3-[(2Z)-1-Oxo-2-buten-2-yl]pentanedioic acid_a
miglustat
miglustat
miglustat
miglustat
miglustat
Nicotinamide
Nicotinamide
Nicotinamide
Nicotinamide
Nicotinamide
4-(5,6-Dihydroxy-6-methyl-1-hepten-2-yl)-1-methyl-1,2-cyclohexanediol
4-(5,6-Dihydroxy-6-methyl-1-hepten-2-yl)-1-methyl-1,2-cyclohexanediol
4-(5,6-Dihydroxy-6-methyl-1-hepten-2-yl)-1-methyl-1,2-cyclohexanediol
4-(5,6-Dihydroxy-6-methyl-1-hepten-2-yl)-1-methyl-1,2-cyclohexanediol
4-(5,6-Dihydroxy-6-methyl-1-hepten-2-yl)-1-methyl-1,2-cyclohexanediol
N~6~,N~6~-Dimethyllysine_a
N~6~,N~6~-Dimethyllysine_a

N~6~,N~6~-Dimethyllysine_a
N~6~,N~6~-Dimethyllysine_a
N~6~,N~6~-Dimethyllysine_a
Deacetyldiltiazem
Deacetyldiltiazem
Deacetyldiltiazem
Deacetyldiltiazem
Deacetyldiltiazem
7-Chloro-5-(2-fluorophenyl)-1-(2-methoxyethyl)-1,3-dihydro-2H-1,4-benzodiazepin-2-one_b
7-Chloro-5-(2-fluorophenyl)-1-(2-methoxyethyl)-1,3-dihydro-2H-1,4-benzodiazepin-2-one_b
7-Chloro-5-(2-fluorophenyl)-1-(2-methoxyethyl)-1,3-dihydro-2H-1,4-benzodiazepin-2-one_b
7-Chloro-5-(2-fluorophenyl)-1-(2-methoxyethyl)-1,3-dihydro-2H-1,4-benzodiazepin-2-one_b
7-Chloro-5-(2-fluorophenyl)-1-(2-methoxyethyl)-1,3-dihydro-2H-1,4-benzodiazepin-2-one_b
Zinecard_a
Zinecard_a
Zinecard_a
Zinecard_a
Zinecard_a
n-Ribosylhistidine
n-Ribosylhistidine
n-Ribosylhistidine
n-Ribosylhistidine
n-Ribosylhistidine
valganciclovir
valganciclovir
valganciclovir
valganciclovir
valganciclovir
N-[(2S)-2-Hydroxypropanoyl]methionine_b
N-[(2S)-2-Hydroxypropanoyl]methionine_b
N-[(2S)-2-Hydroxypropanoyl]methionine_b
N-[(2S)-2-Hydroxypropanoyl]methionine_b
N-[(2S)-2-Hydroxypropanoyl]methionine_b
(3S,5R,6E)-7-[3-(4-Fluorophenyl)-5-hydroxy-1-isopropyl-1H-indol-2-yl]-3,5-dihydroxy-6-heptenoic acid
(3S,5R,6E)-7-[3-(4-Fluorophenyl)-5-hydroxy-1-isopropyl-1H-indol-2-yl]-3,5-dihydroxy-6-heptenoic acid
(3S,5R,6E)-7-[3-(4-Fluorophenyl)-5-hydroxy-1-isopropyl-1H-indol-2-yl]-3,5-dihydroxy-6-heptenoic acid
(3S,5R,6E)-7-[3-(4-Fluorophenyl)-5-hydroxy-1-isopropyl-1H-indol-2-yl]-3,5-dihydroxy-6-heptenoic acid
(3S,5R,6E)-7-[3-(4-Fluorophenyl)-5-hydroxy-1-isopropyl-1H-indol-2-yl]-3,5-dihydroxy-6-heptenoic acid
Aspartyl-L-proline_c
Aspartyl-L-proline_c
Aspartyl-L-proline_c
Aspartyl-L-proline_c
Aspartyl-L-proline_c
MFCD18695608_a
MFCD18695608_a
MFCD18695608_a
MFCD18695608_a
MFCD18695608_a

11beta,13-dihydro-8-deoxylactucin
11beta,13-dihydro-8-deoxylactucin
11beta,13-dihydro-8-deoxylactucin
11beta,13-dihydro-8-deoxylactucin
11beta,13-dihydro-8-deoxylactucin
(1R,2S)-1-(7,8-Dihydro-6-pteridinyl)-1,2-propanediol
(1R,2S)-1-(7,8-Dihydro-6-pteridinyl)-1,2-propanediol
(1R,2S)-1-(7,8-Dihydro-6-pteridinyl)-1,2-propanediol
(1R,2S)-1-(7,8-Dihydro-6-pteridinyl)-1,2-propanediol
(1R,2S)-1-(7,8-Dihydro-6-pteridinyl)-1,2-propanediol
3-[3-Methoxy-4-(sulfooxy)phenyl]propanoic acid
3-[3-Methoxy-4-(sulfooxy)phenyl]propanoic acid
3-[3-Methoxy-4-(sulfooxy)phenyl]propanoic acid
3-[3-Methoxy-4-(sulfooxy)phenyl]propanoic acid
3-[3-Methoxy-4-(sulfooxy)phenyl]propanoic acid
5-Allyl-5-sec-butyl-1,3-dimethyl-2,4,6(1H,3H,5H)-pyrimidinetrione_c
5-Allyl-5-sec-butyl-1,3-dimethyl-2,4,6(1H,3H,5H)-pyrimidinetrione_c
5-Allyl-5-sec-butyl-1,3-dimethyl-2,4,6(1H,3H,5H)-pyrimidinetrione_c
5-Allyl-5-sec-butyl-1,3-dimethyl-2,4,6(1H,3H,5H)-pyrimidinetrione_c
5-Allyl-5-sec-butyl-1,3-dimethyl-2,4,6(1H,3H,5H)-pyrimidinetrione_c
Midodrine_a
Midodrine_a
Midodrine_a
Midodrine_a
Midodrine_a
Fenoterol
Fenoterol
Fenoterol
Fenoterol
Fenoterol
3-Mercaptopropionic acid
3-Mercaptopropionic acid
3-Mercaptopropionic acid
3-Mercaptopropionic acid
3-Mercaptopropionic acid
4-O-beta-D-xylo-Hexopyranosyl-3-ulose-D-glucopyranose
4-O-beta-D-xylo-Hexopyranosyl-3-ulose-D-glucopyranose
4-O-beta-D-xylo-Hexopyranosyl-3-ulose-D-glucopyranose
4-O-beta-D-xylo-Hexopyranosyl-3-ulose-D-glucopyranose
4-O-beta-D-xylo-Hexopyranosyl-3-ulose-D-glucopyranose
LW8000000_a
LW8000000_a
LW8000000_a
LW8000000_a
LW8000000_a
N-(2,3,4-Trimethoxybenzoyl)glycine_a
N-(2,3,4-Trimethoxybenzoyl)glycine_a
N-(2,3,4-Trimethoxybenzoyl)glycine_a

N-(2,3,4-Trimethoxybenzoyl)glycine_a
N-(2,3,4-Trimethoxybenzoyl)glycine_a
1,2,3,4-Tetrahydro-ÃŽÂ²-carboline-3-carboxylic acid
1,2,3,4-Tetrahydro-ÃŽÂ²-carboline-3-carboxylic acid
1,2,3,4-Tetrahydro-ÃŽÂ²-carboline-3-carboxylic acid
1,2,3,4-Tetrahydro-ÃŽÂ²-carboline-3-carboxylic acid
1,2,3,4-Tetrahydro-ÃŽÂ²-carboline-3-carboxylic acid
(7R)-7-(5-carboxy-5-oxopentanamido)cephalosporanic acid
(7R)-7-(5-carboxy-5-oxopentanamido)cephalosporanic acid
(7R)-7-(5-carboxy-5-oxopentanamido)cephalosporanic acid
(7R)-7-(5-carboxy-5-oxopentanamido)cephalosporanic acid
(7R)-7-(5-carboxy-5-oxopentanamido)cephalosporanic acid
Linamarin
Linamarin
Linamarin
Linamarin
Linamarin
primidone_b
primidone_b
primidone_b
primidone_b
primidone_b
2-Phenylethyl ÃŽÂ²-D-glucopyranosiduronic acid
2-Phenylethyl ÃŽÂ²-D-glucopyranosiduronic acid
2-Phenylethyl ÃŽÂ²-D-glucopyranosiduronic acid
2-Phenylethyl ÃŽÂ²-D-glucopyranosiduronic acid
2-Phenylethyl ÃŽÂ²-D-glucopyranosiduronic acid
(4S)-4-[(2E)-2-Octenoyloxy]-4-(trimethylammonio)butanoate_b
(4S)-4-[(2E)-2-Octenoyloxy]-4-(trimethylammonio)butanoate_b
(4S)-4-[(2E)-2-Octenoyloxy]-4-(trimethylammonio)butanoate_b
(4S)-4-[(2E)-2-Octenoyloxy]-4-(trimethylammonio)butanoate_b
(4S)-4-[(2E)-2-Octenoyloxy]-4-(trimethylammonio)butanoate_b
1-(4-Amino-4-carboxybutanoyl)-2-piperidinecarboxylic acid_b
1-(4-Amino-4-carboxybutanoyl)-2-piperidinecarboxylic acid_b
1-(4-Amino-4-carboxybutanoyl)-2-piperidinecarboxylic acid_b
1-(4-Amino-4-carboxybutanoyl)-2-piperidinecarboxylic acid_b
1-(4-Amino-4-carboxybutanoyl)-2-piperidinecarboxylic acid_b
epsilon-(gamma-Glutamyl)-lysine_a
epsilon-(gamma-Glutamyl)-lysine_a
epsilon-(gamma-Glutamyl)-lysine_a
epsilon-(gamma-Glutamyl)-lysine_a
epsilon-(gamma-Glutamyl)-lysine_a
pentobarbital_c
pentobarbital_c
pentobarbital_c
pentobarbital_c
pentobarbital_c
DIBEHENIN

DIBEHENIN
DIBEHENIN
DIBEHENIN
DIBEHENIN
hexobarbital_b
hexobarbital_b
hexobarbital_b
hexobarbital_b
hexobarbital_b
3-(14-Ethyl-4,8,13,18-tetramethyl-20-oxo-9-vinyl-3-phorbinyl)propanoic acid
3-(14-Ethyl-4,8,13,18-tetramethyl-20-oxo-9-vinyl-3-phorbinyl)propanoic acid
3-(14-Ethyl-4,8,13,18-tetramethyl-20-oxo-9-vinyl-3-phorbinyl)propanoic acid
3-(14-Ethyl-4,8,13,18-tetramethyl-20-oxo-9-vinyl-3-phorbinyl)propanoic acid
3-(14-Ethyl-4,8,13,18-tetramethyl-20-oxo-9-vinyl-3-phorbinyl)propanoic acid
Ethyl maltol
Ethyl maltol
Ethyl maltol
Ethyl maltol
Ethyl maltol
coronatine
coronatine
coronatine
coronatine
coronatine
g-Aminobutyryl-lysine_a
g-Aminobutyryl-lysine_a
g-Aminobutyryl-lysine_a
g-Aminobutyryl-lysine_a
g-Aminobutyryl-lysine_a
Dihydrouracil_b
Dihydrouracil_b
Dihydrouracil_b
Dihydrouracil_b
Dihydrouracil_b
Methyl alpha-aspartylphenylalaninate_a
Methyl alpha-aspartylphenylalaninate_a
Methyl alpha-aspartylphenylalaninate_a
Methyl alpha-aspartylphenylalaninate_a
Methyl alpha-aspartylphenylalaninate_a
17,21-Dihydroxypregnenolone
17,21-Dihydroxypregnenolone
17,21-Dihydroxypregnenolone
17,21-Dihydroxypregnenolone
17,21-Dihydroxypregnenolone
(-)-Physostigmine_b
(-)-Physostigmine_b
(-)-Physostigmine_b
(-)-Physostigmine_b

(-)-Physostigmine_b
(betaS)-beta,3-Dihydroxy-D-tyrosine
(betaS)-beta,3-Dihydroxy-D-tyrosine
(betaS)-beta,3-Dihydroxy-D-tyrosine
(betaS)-beta,3-Dihydroxy-D-tyrosine
(betaS)-beta,3-Dihydroxy-D-tyrosine
N-(2,3,4-Trimethoxybenzoyl)glycine_c
N-(2,3,4-Trimethoxybenzoyl)glycine_c
N-(2,3,4-Trimethoxybenzoyl)glycine_c
N-(2,3,4-Trimethoxybenzoyl)glycine_c
N-(2,3,4-Trimethoxybenzoyl)glycine_c
Prephenic acid
Prephenic acid
Prephenic acid
Prephenic acid
Prephenic acid
DL-Carbocysteine
DL-Carbocysteine
DL-Carbocysteine
DL-Carbocysteine
DL-Carbocysteine
2-(3,4-Dimethoxyphenyl)-5,7-dihydroxy-6,8-dimethoxy-2,3-dihydro-4H-chromen-4-one
2-(3,4-Dimethoxyphenyl)-5,7-dihydroxy-6,8-dimethoxy-2,3-dihydro-4H-chromen-4-one
2-(3,4-Dimethoxyphenyl)-5,7-dihydroxy-6,8-dimethoxy-2,3-dihydro-4H-chromen-4-one
2-(3,4-Dimethoxyphenyl)-5,7-dihydroxy-6,8-dimethoxy-2,3-dihydro-4H-chromen-4-one
2-(3,4-Dimethoxyphenyl)-5,7-dihydroxy-6,8-dimethoxy-2,3-dihydro-4H-chromen-4-one
asn-val_b
asn-val_b
asn-val_b
asn-val_b
asn-val_b
Lys-Pro_c
Lys-Pro_c
Lys-Pro_c
Lys-Pro_c
Lys-Pro_c
DL-Mevalonic acid
DL-Mevalonic acid
DL-Mevalonic acid
DL-Mevalonic acid
DL-Mevalonic acid
3-[(3-Hydroxytridecanoyl)oxy]-4-(trimethylammonio)butanoate
3-[(3-Hydroxytridecanoyl)oxy]-4-(trimethylammonio)butanoate
3-[(3-Hydroxytridecanoyl)oxy]-4-(trimethylammonio)butanoate
3-[(3-Hydroxytridecanoyl)oxy]-4-(trimethylammonio)butanoate
3-[(3-Hydroxytridecanoyl)oxy]-4-(trimethylammonio)butanoate
bis(4-isothiocyanatobutyl) disulfide
bis(4-isothiocyanatobutyl) disulfide

bis(4-isothiocyanatobutyl) disulfide
bis(4-isothiocyanatobutyl) disulfide
bis(4-isothiocyanatobutyl) disulfide
3-Morpholino-4-tetrahydro-1H-pyrrol-1-ylcyclobut-3-ene-1,2-dione
3-Morpholino-4-tetrahydro-1H-pyrrol-1-ylcyclobut-3-ene-1,2-dione
3-Morpholino-4-tetrahydro-1H-pyrrol-1-ylcyclobut-3-ene-1,2-dione
3-Morpholino-4-tetrahydro-1H-pyrrol-1-ylcyclobut-3-ene-1,2-dione
3-Morpholino-4-tetrahydro-1H-pyrrol-1-ylcyclobut-3-ene-1,2-dione
(2R)-1-{[(2-Aminoethoxy)(hydroxy)phosphoryl]oxy}-3-hydroxy-2-propanyl formate
(2R)-1-{[(2-Aminoethoxy)(hydroxy)phosphoryl]oxy}-3-hydroxy-2-propanyl formate
(2R)-1-{[(2-Aminoethoxy)(hydroxy)phosphoryl]oxy}-3-hydroxy-2-propanyl formate
(2R)-1-{[(2-Aminoethoxy)(hydroxy)phosphoryl]oxy}-3-hydroxy-2-propanyl formate
(2R)-1-{[(2-Aminoethoxy)(hydroxy)phosphoryl]oxy}-3-hydroxy-2-propanyl formate
(âˆ’)-nabilone_a
(âˆ’)-nabilone_a
(âˆ’)-nabilone_a
(âˆ’)-nabilone_a
(âˆ’)-nabilone_a
FB9500000_a
FB9500000_a
FB9500000_a
FB9500000_a
FB9500000_a
(2,4-Dihydroxyphenyl)(oxo)acetic acid
(2,4-Dihydroxyphenyl)(oxo)acetic acid
(2,4-Dihydroxyphenyl)(oxo)acetic acid
(2,4-Dihydroxyphenyl)(oxo)acetic acid
(2,4-Dihydroxyphenyl)(oxo)acetic acid
L-gamma-Glutamyl-L-valine_i
L-gamma-Glutamyl-L-valine_i
L-gamma-Glutamyl-L-valine_i
L-gamma-Glutamyl-L-valine_i
L-gamma-Glutamyl-L-valine_i
5-Hydantoinpropionic acid
5-Hydantoinpropionic acid
5-Hydantoinpropionic acid
5-Hydantoinpropionic acid
5-Hydantoinpropionic acid
threonylphenylalanine_c
threonylphenylalanine_c
threonylphenylalanine_c
threonylphenylalanine_c
threonylphenylalanine_c
Cadralazine
Cadralazine
Cadralazine
Cadralazine
Cadralazine

metixene
metixene
metixene
metixene
metixene
IN00260_a
IN00260_a
IN00260_a
IN00260_a
IN00260_a
Seryltyrosine_a
Seryltyrosine_a
Seryltyrosine_a
Seryltyrosine_a
Seryltyrosine_a
Oleuropein aglycon
Oleuropein aglycon
Oleuropein aglycon
Oleuropein aglycon
Oleuropein aglycon
MFCD18695608_d
MFCD18695608_d
MFCD18695608_d
MFCD18695608_d
MFCD18695608_d
Spermic acid_a
Spermic acid_a
Spermic acid_a
Spermic acid_a
Spermic acid_a
Methyl 4-(4,6-dimethyl-9-oxo-4,9-dihydro-1H-imidazo[1,2-a]purin-7-yl)-3-hydroperoxy-2-[(methoxycarbonyl)amir
Methyl 4-(4,6-dimethyl-9-oxo-4,9-dihydro-1H-imidazo[1,2-a]purin-7-yl)-3-hydroperoxy-2-[(methoxycarbonyl)amir
Methyl 4-(4,6-dimethyl-9-oxo-4,9-dihydro-1H-imidazo[1,2-a]purin-7-yl)-3-hydroperoxy-2-[(methoxycarbonyl)amir
Methyl 4-(4,6-dimethyl-9-oxo-4,9-dihydro-1H-imidazo[1,2-a]purin-7-yl)-3-hydroperoxy-2-[(methoxycarbonyl)amir
Methyl 4-(4,6-dimethyl-9-oxo-4,9-dihydro-1H-imidazo[1,2-a]purin-7-yl)-3-hydroperoxy-2-[(methoxycarbonyl)amir
1-{3-Carboxy-3-[(3-carboxy-3-hydroxypropyl)amino]propyl}-2-azetidinecarboxylic acid
1-{3-Carboxy-3-[(3-carboxy-3-hydroxypropyl)amino]propyl}-2-azetidinecarboxylic acid
1-{3-Carboxy-3-[(3-carboxy-3-hydroxypropyl)amino]propyl}-2-azetidinecarboxylic acid
1-{3-Carboxy-3-[(3-carboxy-3-hydroxypropyl)amino]propyl}-2-azetidinecarboxylic acid
1-{3-Carboxy-3-[(3-carboxy-3-hydroxypropyl)amino]propyl}-2-azetidinecarboxylic acid
4-Thiapentanoic acid
4-Thiapentanoic acid
4-Thiapentanoic acid
4-Thiapentanoic acid
4-Thiapentanoic acid
Glycerophosphoglycerol
Glycerophosphoglycerol
Glycerophosphoglycerol

Glycerophosphoglycerol
Glycerophosphoglycerol
L-gamma-Glutamyl-L-leucine_g
L-gamma-Glutamyl-L-leucine_g
L-gamma-Glutamyl-L-leucine_g
L-gamma-Glutamyl-L-leucine_g
L-gamma-Glutamyl-L-leucine_g
Leucyltryptophan
Leucyltryptophan
Leucyltryptophan
Leucyltryptophan
Leucyltryptophan
butalbital_a
butalbital_a
butalbital_a
butalbital_a
butalbital_a
INK (Peptide Ile-Asn-Lys)
INK (Peptide Ile-Asn-Lys)
INK (Peptide Ile-Asn-Lys)
INK (Peptide Ile-Asn-Lys)
INK (Peptide Ile-Asn-Lys)
3-(Sulfooxy)-L-tyrosine_a
3-(Sulfooxy)-L-tyrosine_a
3-(Sulfooxy)-L-tyrosine_a
3-(Sulfooxy)-L-tyrosine_a
3-(Sulfooxy)-L-tyrosine_a
uridine 5â€^{2}-diphosphate
uridine 5â€^{2}-diphosphate
uridine 5â€^{2}-diphosphate
uridine 5â€^{2}-diphosphate
uridine 5â€^{2}-diphosphate
2-Furoylglycine
2-Furoylglycine
2-Furoylglycine
2-Furoylglycine
2-Furoylglycine
2-Ammonio-4-{4-[(2S)-2-ammonio-2-carboxylatoethyl]-1H-imidazol-2-yl}butanoate
2-Ammonio-4-{4-[(2S)-2-ammonio-2-carboxylatoethyl]-1H-imidazol-2-yl}butanoate
2-Ammonio-4-{4-[(2S)-2-ammonio-2-carboxylatoethyl]-1H-imidazol-2-yl}butanoate
2-Ammonio-4-{4-[(2S)-2-ammonio-2-carboxylatoethyl]-1H-imidazol-2-yl}butanoate
2-Ammonio-4-{4-[(2S)-2-ammonio-2-carboxylatoethyl]-1H-imidazol-2-yl}butanoate
carglumic acid
carglumic acid
carglumic acid
carglumic acid
carglumic acid
N-Acetylprocainamide_b

N-Acetylprocainamide_b
N-Acetylprocainamide_b
N-Acetylprocainamide_b
N-Acetylprocainamide_b
Prunasin
Prunasin
Prunasin
Prunasin
Prunasin
2-(2,4-Dihydroxyphenyl)-3-[(2Z)-3,7-dimethyl-2,6-octadien-1-yl]-5,7-dihydroxy-6-[(2E)-4-hydroxy-3-methyl-2-but
2-(2,4-Dihydroxyphenyl)-3-[(2Z)-3,7-dimethyl-2,6-octadien-1-yl]-5,7-dihydroxy-6-[(2E)-4-hydroxy-3-methyl-2-but
2-(2,4-Dihydroxyphenyl)-3-[(2Z)-3,7-dimethyl-2,6-octadien-1-yl]-5,7-dihydroxy-6-[(2E)-4-hydroxy-3-methyl-2-but
2-(2,4-Dihydroxyphenyl)-3-[(2Z)-3,7-dimethyl-2,6-octadien-1-yl]-5,7-dihydroxy-6-[(2E)-4-hydroxy-3-methyl-2-but
2-(2,4-Dihydroxyphenyl)-3-[(2Z)-3,7-dimethyl-2,6-octadien-1-yl]-5,7-dihydroxy-6-[(2E)-4-hydroxy-3-methyl-2-but
Ro 20-1724_b
Ro 20-1724_b
Ro 20-1724_b
Ro 20-1724_b
Ro 20-1724_b
Guanadrel
Guanadrel
Guanadrel
Guanadrel
Guanadrel
5-methylthioribose
5-methylthioribose
5-methylthioribose
5-methylthioribose
5-methylthioribose
N-(3,5-Dimethoxybenzoyl)glycine_d
N-(3,5-Dimethoxybenzoyl)glycine_d
N-(3,5-Dimethoxybenzoyl)glycine_d
N-(3,5-Dimethoxybenzoyl)glycine_d
N-(3,5-Dimethoxybenzoyl)glycine_d
Triethyl citrate_a
Triethyl citrate_a
Triethyl citrate_a
Triethyl citrate_a
Triethyl citrate_a
meprobamate_b
meprobamate_b
meprobamate_b
meprobamate_b
meprobamate_b
Ethyl malate_d
Ethyl malate_d
Ethyl malate_d
Ethyl malate_d

Ethyl malate_d
2-Hydroxy-3,4,5-trimethoxybenzoic acid
2-Hydroxy-3,4,5-trimethoxybenzoic acid
2-Hydroxy-3,4,5-trimethoxybenzoic acid
2-Hydroxy-3,4,5-trimethoxybenzoic acid
2-Hydroxy-3,4,5-trimethoxybenzoic acid
2-Acetamido-2-deoxy-3-O-(6-deoxy-alpha-L-galactopyranosyl)-D-glucose
2-Acetamido-2-deoxy-3-O-(6-deoxy-alpha-L-galactopyranosyl)-D-glucose
2-Acetamido-2-deoxy-3-O-(6-deoxy-alpha-L-galactopyranosyl)-D-glucose
2-Acetamido-2-deoxy-3-O-(6-deoxy-alpha-L-galactopyranosyl)-D-glucose
2-Acetamido-2-deoxy-3-O-(6-deoxy-alpha-L-galactopyranosyl)-D-glucose
Astemizole_a
Astemizole_a
Astemizole_a
Astemizole_a
Astemizole_a
Butabarbital_b
Butabarbital_b
Butabarbital_b
Butabarbital_b
Butabarbital_b
N-(Carboxymethyl)norleucine_c
N-(Carboxymethyl)norleucine_c
N-(Carboxymethyl)norleucine_c
N-(Carboxymethyl)norleucine_c
N-(Carboxymethyl)norleucine_c
Lys-phe_b
Lys-phe_b
Lys-phe_b
Lys-phe_b
Lys-phe_b
Erythorbic acid
Erythorbic acid
Erythorbic acid
Erythorbic acid
Erythorbic acid
2,3,4,9-Tetrahydro-1H-Î²-carboline-3-carboxylic acid
2,3,4,9-Tetrahydro-1H-Î²-carboline-3-carboxylic acid
2,3,4,9-Tetrahydro-1H-Î²-carboline-3-carboxylic acid
2,3,4,9-Tetrahydro-1H-Î²-carboline-3-carboxylic acid
2,3,4,9-Tetrahydro-1H-Î²-carboline-3-carboxylic acid
Methyl 2,3-dihydro-3-hydroxy-2-oxo-1H-indole-3-acetate_a
Methyl 2,3-dihydro-3-hydroxy-2-oxo-1H-indole-3-acetate_a
Methyl 2,3-dihydro-3-hydroxy-2-oxo-1H-indole-3-acetate_a
Methyl 2,3-dihydro-3-hydroxy-2-oxo-1H-indole-3-acetate_a
Methyl 2,3-dihydro-3-hydroxy-2-oxo-1H-indole-3-acetate_a
Tetramethylpyrazine
Tetramethylpyrazine

Tetramethylpyrazine
Tetramethylpyrazine
Tetramethylpyrazine
(2S)-3-Methyl-2-({[(3S,4S,5R)-2,3,4-trihydroxy-5-(hydroxymethyl)tetrahydro-2-furanyl]methyl}amino)butanoic aci
(2S)-3-Methyl-2-({[(3S,4S,5R)-2,3,4-trihydroxy-5-(hydroxymethyl)tetrahydro-2-furanyl]methyl}amino)butanoic aci
(2S)-3-Methyl-2-({[(3S,4S,5R)-2,3,4-trihydroxy-5-(hydroxymethyl)tetrahydro-2-furanyl]methyl}amino)butanoic aci
(2S)-3-Methyl-2-({[(3S,4S,5R)-2,3,4-trihydroxy-5-(hydroxymethyl)tetrahydro-2-furanyl]methyl}amino)butanoic aci
(2S)-3-Methyl-2-({[(3S,4S,5R)-2,3,4-trihydroxy-5-(hydroxymethyl)tetrahydro-2-furanyl]methyl}amino)butanoic aci
Roxane
Roxane
Roxane
Roxane
Roxane
lys-tyr_b
lys-tyr_b
lys-tyr_b
lys-tyr_b
lys-tyr_b
4-(METHYLNITROSAMINO)-1-(3-PYRIDYL-N-OXIDE)-1-BUTANOL_d
4-(METHYLNITROSAMINO)-1-(3-PYRIDYL-N-OXIDE)-1-BUTANOL_d
4-(METHYLNITROSAMINO)-1-(3-PYRIDYL-N-OXIDE)-1-BUTANOL_d
4-(METHYLNITROSAMINO)-1-(3-PYRIDYL-N-OXIDE)-1-BUTANOL_d
4-(METHYLNITROSAMINO)-1-(3-PYRIDYL-N-OXIDE)-1-BUTANOL_d
3-(2,3-Dihydroxy-3-methylbutyl)-4-methoxy-1-methyl-2(1H)-quinolinone_b
3-(2,3-Dihydroxy-3-methylbutyl)-4-methoxy-1-methyl-2(1H)-quinolinone_b
3-(2,3-Dihydroxy-3-methylbutyl)-4-methoxy-1-methyl-2(1H)-quinolinone_b
3-(2,3-Dihydroxy-3-methylbutyl)-4-methoxy-1-methyl-2(1H)-quinolinone_b
3-(2,3-Dihydroxy-3-methylbutyl)-4-methoxy-1-methyl-2(1H)-quinolinone_b
N-(Carboxymethyl)norleucine_b
N-(Carboxymethyl)norleucine_b
N-(Carboxymethyl)norleucine_b
N-(Carboxymethyl)norleucine_b
N-(Carboxymethyl)norleucine_b
asn-val_c
asn-val_c
asn-val_c
asn-val_c
asn-val_c
Tocainide
Tocainide
Tocainide
Tocainide
Tocainide
Histidylglycine_b
Histidylglycine_b
Histidylglycine_b
Histidylglycine_b
Histidylglycine_b

7-Chloro-5-(2-fluorophenyl)-1-(2-methoxyethyl)-1,3-dihydro-2H-1,4-benzodiazepin-2-one_a
7-Chloro-5-(2-fluorophenyl)-1-(2-methoxyethyl)-1,3-dihydro-2H-1,4-benzodiazepin-2-one_a
7-Chloro-5-(2-fluorophenyl)-1-(2-methoxyethyl)-1,3-dihydro-2H-1,4-benzodiazepin-2-one_a
7-Chloro-5-(2-fluorophenyl)-1-(2-methoxyethyl)-1,3-dihydro-2H-1,4-benzodiazepin-2-one_a
7-Chloro-5-(2-fluorophenyl)-1-(2-methoxyethyl)-1,3-dihydro-2H-1,4-benzodiazepin-2-one_a
3-Hydroxy-3-methyl-1-[(7-oxo-7H-furo[3,2-g]chromen-9-yl)oxy]-2-butanyl 3-methyl-2-butenoate
3-Hydroxy-3-methyl-1-[(7-oxo-7H-furo[3,2-g]chromen-9-yl)oxy]-2-butanyl 3-methyl-2-butenoate
3-Hydroxy-3-methyl-1-[(7-oxo-7H-furo[3,2-g]chromen-9-yl)oxy]-2-butanyl 3-methyl-2-butenoate
3-Hydroxy-3-methyl-1-[(7-oxo-7H-furo[3,2-g]chromen-9-yl)oxy]-2-butanyl 3-methyl-2-butenoate
3-Hydroxy-3-methyl-1-[(7-oxo-7H-furo[3,2-g]chromen-9-yl)oxy]-2-butanyl 3-methyl-2-butenoate
11-(4-Hydroxyphenyl)-3-methyl-6,7,8,9-tetrahydropyridazino[1,2-a]indazol-10-ium-1-olate
11-(4-Hydroxyphenyl)-3-methyl-6,7,8,9-tetrahydropyridazino[1,2-a]indazol-10-ium-1-olate
11-(4-Hydroxyphenyl)-3-methyl-6,7,8,9-tetrahydropyridazino[1,2-a]indazol-10-ium-1-olate
11-(4-Hydroxyphenyl)-3-methyl-6,7,8,9-tetrahydropyridazino[1,2-a]indazol-10-ium-1-olate
11-(4-Hydroxyphenyl)-3-methyl-6,7,8,9-tetrahydropyridazino[1,2-a]indazol-10-ium-1-olate
Arctiopicrin
Arctiopicrin
Arctiopicrin
Arctiopicrin
Arctiopicrin
Ile-cys
Ile-cys
Ile-cys
Ile-cys
Ile-cys
Val-Ser_b
Val-Ser_b
Val-Ser_b
Val-Ser_b
Val-Ser_b
beta-D-Ethyl glucuronide_a
beta-D-Ethyl glucuronide_a
beta-D-Ethyl glucuronide_a
beta-D-Ethyl glucuronide_a
beta-D-Ethyl glucuronide_a
N-acetyl-9-O-acetylneuraminic acid
N-acetyl-9-O-acetylneuraminic acid
N-acetyl-9-O-acetylneuraminic acid
N-acetyl-9-O-acetylneuraminic acid
N-acetyl-9-O-acetylneuraminic acid
(-)-Aspidospermine
(-)-Aspidospermine
(-)-Aspidospermine
(-)-Aspidospermine
(-)-Aspidospermine
6-Hydroxymelatonin
6-Hydroxymelatonin
6-Hydroxymelatonin

6-Hydroxymelatonin
6-Hydroxymelatonin
Succinic anhydride
Succinic anhydride
Succinic anhydride
Succinic anhydride
Succinic anhydride
Homovanillic acid sulfate
Homovanillic acid sulfate
Homovanillic acid sulfate
Homovanillic acid sulfate
Homovanillic acid sulfate
2-Acetamidohexanedioic acid_e
2-Acetamidohexanedioic acid_e
2-Acetamidohexanedioic acid_e
2-Acetamidohexanedioic acid_e
2-Acetamidohexanedioic acid_e
SECONAL_b
SECONAL_b
SECONAL_b
SECONAL_b
SECONAL_b
3'-Hydroxycotinine, Cis-
3'-Hydroxycotinine, Cis-
3'-Hydroxycotinine, Cis-
3'-Hydroxycotinine, Cis-
3'-Hydroxycotinine, Cis-
Asarone
Asarone
Asarone
Asarone
Asarone
YWA1
YWA1
YWA1
YWA1
YWA1
Piperonylonitrile
Piperonylonitrile
Piperonylonitrile
Piperonylonitrile
Piperonylonitrile
alliin
alliin
alliin
alliin
alliin
D-2-Amino-hexano-6-lactam

D-2-Amino-hexano-6-lactam
D-2-Amino-hexano-6-lactam
D-2-Amino-hexano-6-lactam
D-2-Amino-hexano-6-lactam
SECONAL_c
SECONAL_c
SECONAL_c
SECONAL_c
SECONAL_c
(E)-4-Methoxycinnamic acid
(E)-4-Methoxycinnamic acid
(E)-4-Methoxycinnamic acid
(E)-4-Methoxycinnamic acid
(E)-4-Methoxycinnamic acid
Homoanserine_c
Homoanserine_c
Homoanserine_c
Homoanserine_c
Homoanserine_c
Ala-Tyr
Ala-Tyr
Ala-Tyr
Ala-Tyr
Ala-Tyr
6-Myoporol
6-Myoporol
6-Myoporol
6-Myoporol
6-Myoporol
Hostmaniane
Hostmaniane
Hostmaniane
Hostmaniane
Hostmaniane
hexobarbital_c
hexobarbital_c
hexobarbital_c
hexobarbital_c
hexobarbital_c
ophthalmic acid_a
ophthalmic acid_a
ophthalmic acid_a
ophthalmic acid_a
ophthalmic acid_a
5-Hydroxy-6-[(2E)-4-hydroxy-3-methyl-2-buten-1-yl]-2-methyl-4-oxo-4H-chromen-7-yl hexopyranoside
5-Hydroxy-6-[(2E)-4-hydroxy-3-methyl-2-buten-1-yl]-2-methyl-4-oxo-4H-chromen-7-yl hexopyranoside
5-Hydroxy-6-[(2E)-4-hydroxy-3-methyl-2-buten-1-yl]-2-methyl-4-oxo-4H-chromen-7-yl hexopyranoside
5-Hydroxy-6-[(2E)-4-hydroxy-3-methyl-2-buten-1-yl]-2-methyl-4-oxo-4H-chromen-7-yl hexopyranoside

5-Hydroxy-6-[(2E)-4-hydroxy-3-methyl-2-buten-1-yl]-2-methyl-4-oxo-4H-chromen-7-yl hexopyranoside
tert-Butyl 3-amino-1-methyl-2,3-dioxopropylcarbamate_c
tert-Butyl 3-amino-1-methyl-2,3-dioxopropylcarbamate_c
tert-Butyl 3-amino-1-methyl-2,3-dioxopropylcarbamate_c
tert-Butyl 3-amino-1-methyl-2,3-dioxopropylcarbamate_c
tert-Butyl 3-amino-1-methyl-2,3-dioxopropylcarbamate_c
tert-Butyl 3-amino-1-methyl-2,3-dioxopropylcarbamate_f
tert-Butyl 3-amino-1-methyl-2,3-dioxopropylcarbamate_f
tert-Butyl 3-amino-1-methyl-2,3-dioxopropylcarbamate_f
tert-Butyl 3-amino-1-methyl-2,3-dioxopropylcarbamate_f
tert-Butyl 3-amino-1-methyl-2,3-dioxopropylcarbamate_f
9-Methyluric acid_a
9-Methyluric acid_a
9-Methyluric acid_a
9-Methyluric acid_a
9-Methyluric acid_a
Kynurenic acid
Kynurenic acid
Kynurenic acid
Kynurenic acid
Kynurenic acid
2-Aminooctanedioic acid_a
2-Aminooctanedioic acid_a
2-Aminooctanedioic acid_a
2-Aminooctanedioic acid_a
2-Aminooctanedioic acid_a
1H-Pyrazol-4-ylmethanol
1H-Pyrazol-4-ylmethanol
1H-Pyrazol-4-ylmethanol
1H-Pyrazol-4-ylmethanol
1H-Pyrazol-4-ylmethanol
N-Propionylmethionine_b
N-Propionylmethionine_b
N-Propionylmethionine_b
N-Propionylmethionine_b
N-Propionylmethionine_b
Methyl 1-hydroxy-7-(hydroxymethyl)-1,4a,5,7a-tetrahydrocyclopenta[c]pyran-4-carboxylate_a
Methyl 1-hydroxy-7-(hydroxymethyl)-1,4a,5,7a-tetrahydrocyclopenta[c]pyran-4-carboxylate_a
Methyl 1-hydroxy-7-(hydroxymethyl)-1,4a,5,7a-tetrahydrocyclopenta[c]pyran-4-carboxylate_a
Methyl 1-hydroxy-7-(hydroxymethyl)-1,4a,5,7a-tetrahydrocyclopenta[c]pyran-4-carboxylate_a
Methyl 1-hydroxy-7-(hydroxymethyl)-1,4a,5,7a-tetrahydrocyclopenta[c]pyran-4-carboxylate_a
N-Benzoylaspartic acid_a
N-Benzoylaspartic acid_a
N-Benzoylaspartic acid_a
N-Benzoylaspartic acid_a
N-Benzoylaspartic acid_a
Indole-3-carbidol
Indole-3-carbidol

Indole-3-carbidol
Indole-3-carbidol
Indole-3-carbidol
Propafenone
Propafenone
Propafenone
Propafenone
Propafenone
Glu-Glu
Glu-Glu
Glu-Glu
Glu-Glu
Glu-Glu
Raltitrexed
Raltitrexed
Raltitrexed
Raltitrexed
Raltitrexed
2-(3,5-dimethyl-1H-pyrazol-4-yl)-5-methoxybenzoic acid
2-(3,5-dimethyl-1H-pyrazol-4-yl)-5-methoxybenzoic acid
2-(3,5-dimethyl-1H-pyrazol-4-yl)-5-methoxybenzoic acid
2-(3,5-dimethyl-1H-pyrazol-4-yl)-5-methoxybenzoic acid
2-(3,5-dimethyl-1H-pyrazol-4-yl)-5-methoxybenzoic acid
3-(Sulfooxy)-L-tyrosine_b
3-(Sulfooxy)-L-tyrosine_b
3-(Sulfooxy)-L-tyrosine_b
3-(Sulfooxy)-L-tyrosine_b
3-(Sulfooxy)-L-tyrosine_b
Validamycin A
Validamycin A
Validamycin A
Validamycin A
Validamycin A
N,N-Diethyl-4-methyl-1-piperazinecarboxamide 4-oxide_a
N,N-Diethyl-4-methyl-1-piperazinecarboxamide 4-oxide_a
N,N-Diethyl-4-methyl-1-piperazinecarboxamide 4-oxide_a
N,N-Diethyl-4-methyl-1-piperazinecarboxamide 4-oxide_a
N,N-Diethyl-4-methyl-1-piperazinecarboxamide 4-oxide_a
n-Propyl Gallate
n-Propyl Gallate
n-Propyl Gallate
n-Propyl Gallate
n-Propyl Gallate
N-(3,5-Dimethoxybenzoyl)glycine_c
N-(3,5-Dimethoxybenzoyl)glycine_c
N-(3,5-Dimethoxybenzoyl)glycine_c
N-(3,5-Dimethoxybenzoyl)glycine_c
N-(3,5-Dimethoxybenzoyl)glycine_c

Butylphthalide
Butylphthalide
Butylphthalide
Butylphthalide
Butylphthalide
DLK (Peptide Asp-Leu-Lys)
DLK (Peptide Asp-Leu-Lys)
DLK (Peptide Asp-Leu-Lys)
DLK (Peptide Asp-Leu-Lys)
DLK (Peptide Asp-Leu-Lys)
7-Methyladenine
7-Methyladenine
7-Methyladenine
7-Methyladenine
7-Methyladenine
3-Ureidopropionic acid
3-Ureidopropionic acid
3-Ureidopropionic acid
3-Ureidopropionic acid
3-Ureidopropionic acid
6-(1-Hydroxyethyl)-3-(hydroxymethyl)-2,7-dioxabicyclo[4.1.0]hept-3-en-5-one_b
6-(1-Hydroxyethyl)-3-(hydroxymethyl)-2,7-dioxabicyclo[4.1.0]hept-3-en-5-one_b
6-(1-Hydroxyethyl)-3-(hydroxymethyl)-2,7-dioxabicyclo[4.1.0]hept-3-en-5-one_b
6-(1-Hydroxyethyl)-3-(hydroxymethyl)-2,7-dioxabicyclo[4.1.0]hept-3-en-5-one_b
6-(1-Hydroxyethyl)-3-(hydroxymethyl)-2,7-dioxabicyclo[4.1.0]hept-3-en-5-one_b
tenivastatin
tenivastatin
tenivastatin
tenivastatin
tenivastatin
Leu-pro_a
Leu-pro_a
Leu-pro_a
Leu-pro_a
Leu-pro_a
Dopamine 3-O-sulfate
Dopamine 3-O-sulfate
Dopamine 3-O-sulfate
Dopamine 3-O-sulfate
Dopamine 3-O-sulfate
Ethylvanillin propylene glycol acetal
Ethylvanillin propylene glycol acetal
Ethylvanillin propylene glycol acetal
Ethylvanillin propylene glycol acetal
Ethylvanillin propylene glycol acetal
Dihydrothymine
Dihydrothymine
Dihydrothymine

Dihydrothymine
Dihydrothymine
(7E,7'E)-5,5'-diferulic acid_c
(7E,7'E)-5,5'-diferulic acid_c
(7E,7'E)-5,5'-diferulic acid_c
(7E,7'E)-5,5'-diferulic acid_c
(7E,7'E)-5,5'-diferulic acid_c
4-Amino-1-(4-{[(3R)-3-amino-5-(N-methylcarbamimidamido)pentanoyl]amino}-2,3,4-trideoxy-beta-D-erythro-he[illegible]
4-Amino-1-(4-{[(3R)-3-amino-5-(N-methylcarbamimidamido)pentanoyl]amino}-2,3,4-trideoxy-beta-D-erythro-he[illegible]
4-Amino-1-(4-{[(3R)-3-amino-5-(N-methylcarbamimidamido)pentanoyl]amino}-2,3,4-trideoxy-beta-D-erythro-he[illegible]
4-Amino-1-(4-{[(3R)-3-amino-5-(N-methylcarbamimidamido)pentanoyl]amino}-2,3,4-trideoxy-beta-D-erythro-he[illegible]
4-Amino-1-(4-{[(3R)-3-amino-5-(N-methylcarbamimidamido)pentanoyl]amino}-2,3,4-trideoxy-beta-D-erythro-he[illegible]
6-APA_a
6-APA_a
6-APA_a
6-APA_a
6-APA_a
thyronine
thyronine
thyronine
thyronine
thyronine
L-gamma-Glutamyl-L-leucine_h
L-gamma-Glutamyl-L-leucine_h
L-gamma-Glutamyl-L-leucine_h
L-gamma-Glutamyl-L-leucine_h
L-gamma-Glutamyl-L-leucine_h
Leucyltyrosine_d
Leucyltyrosine_d
Leucyltyrosine_d
Leucyltyrosine_d
Leucyltyrosine_d
4-(3-Oxopentyl)phenyl hydrogen sulfate
4-(3-Oxopentyl)phenyl hydrogen sulfate
4-(3-Oxopentyl)phenyl hydrogen sulfate
4-(3-Oxopentyl)phenyl hydrogen sulfate
4-(3-Oxopentyl)phenyl hydrogen sulfate
Val-Trp_a
Val-Trp_a
Val-Trp_a
Val-Trp_a
Val-Trp_a
S-Propylcysteine
S-Propylcysteine
S-Propylcysteine
S-Propylcysteine
S-Propylcysteine
piscidic acid_b

piscidic acid_b
piscidic acid_b
piscidic acid_b
piscidic acid_b
R-(+)-Etiracetam_a
R-(+)-Etiracetam_a
R-(+)-Etiracetam_a
R-(+)-Etiracetam_a
R-(+)-Etiracetam_a
ala-ser_b
ala-ser_b
ala-ser_b
ala-ser_b
ala-ser_b
N-Phenylacetylglutamic acid_a
N-Phenylacetylglutamic acid_a
N-Phenylacetylglutamic acid_a
N-Phenylacetylglutamic acid_a
N-Phenylacetylglutamic acid_a
Calcitriol
Calcitriol
Calcitriol
Calcitriol
Calcitriol
N,N-Diethyl-4-methyl-1-piperazinecarboxamide 4-oxide_b
N,N-Diethyl-4-methyl-1-piperazinecarboxamide 4-oxide_b
N,N-Diethyl-4-methyl-1-piperazinecarboxamide 4-oxide_b
N,N-Diethyl-4-methyl-1-piperazinecarboxamide 4-oxide_b
N,N-Diethyl-4-methyl-1-piperazinecarboxamide 4-oxide_b
6-hydroxypseudooxynicotine_a
6-hydroxypseudooxynicotine_a
6-hydroxypseudooxynicotine_a
6-hydroxypseudooxynicotine_a
6-hydroxypseudooxynicotine_a
L-Pyrrolysine
L-Pyrrolysine
L-Pyrrolysine
L-Pyrrolysine
L-Pyrrolysine
6-APA_b
6-APA_b
6-APA_b
6-APA_b
6-APA_b
Tetraacetylethylenediamine_g
Tetraacetylethylenediamine_g
Tetraacetylethylenediamine_g
Tetraacetylethylenediamine_g

Tetraacetylethylenediamine_g
9-ribosylzeatin
9-ribosylzeatin
9-ribosylzeatin
9-ribosylzeatin
9-ribosylzeatin
3-Methoxy-4-hydroxyhippuric acid_e
3-Methoxy-4-hydroxyhippuric acid_e
3-Methoxy-4-hydroxyhippuric acid_e
3-Methoxy-4-hydroxyhippuric acid_e
3-Methoxy-4-hydroxyhippuric acid_e
FC2505000
FC2505000
FC2505000
FC2505000
FC2505000
6,8-Dimethoxy-1,2,3a,12c-tetrahydro-7H-furo[3',2':4,5]furo[2,3-c]xanthen-7-one
6,8-Dimethoxy-1,2,3a,12c-tetrahydro-7H-furo[3',2':4,5]furo[2,3-c]xanthen-7-one
6,8-Dimethoxy-1,2,3a,12c-tetrahydro-7H-furo[3',2':4,5]furo[2,3-c]xanthen-7-one
6,8-Dimethoxy-1,2,3a,12c-tetrahydro-7H-furo[3',2':4,5]furo[2,3-c]xanthen-7-one
6,8-Dimethoxy-1,2,3a,12c-tetrahydro-7H-furo[3',2':4,5]furo[2,3-c]xanthen-7-one
Eslicarbazepine
Eslicarbazepine
Eslicarbazepine
Eslicarbazepine
Eslicarbazepine
(+/-)-2-Hydroxyglutaric acid_b
(+/-)-2-Hydroxyglutaric acid_b
(+/-)-2-Hydroxyglutaric acid_b
(+/-)-2-Hydroxyglutaric acid_b
(+/-)-2-Hydroxyglutaric acid_b
Kyotorphin
Kyotorphin
Kyotorphin
Kyotorphin
Kyotorphin
4-(1-Hydroxy-2-{[6-(4-hydroxy-4-phenylbutoxy)hexyl]amino}ethyl)-2-(hydroxymethyl)phenol
4-(1-Hydroxy-2-{[6-(4-hydroxy-4-phenylbutoxy)hexyl]amino}ethyl)-2-(hydroxymethyl)phenol
4-(1-Hydroxy-2-{[6-(4-hydroxy-4-phenylbutoxy)hexyl]amino}ethyl)-2-(hydroxymethyl)phenol
4-(1-Hydroxy-2-{[6-(4-hydroxy-4-phenylbutoxy)hexyl]amino}ethyl)-2-(hydroxymethyl)phenol
4-(1-Hydroxy-2-{[6-(4-hydroxy-4-phenylbutoxy)hexyl]amino}ethyl)-2-(hydroxymethyl)phenol
7alpha-Hydroxy-3-oxochol-4-en-24-oic acid_d
7alpha-Hydroxy-3-oxochol-4-en-24-oic acid_d
7alpha-Hydroxy-3-oxochol-4-en-24-oic acid_d
7alpha-Hydroxy-3-oxochol-4-en-24-oic acid_d
7alpha-Hydroxy-3-oxochol-4-en-24-oic acid_d
Marimastat
Marimastat

Marimastat
Marimastat
Marimastat
2-(3-Hydroxy-3,4,5,6-tetrahydro-1H-cyclopenta[c]furan-4-yl)-3-methoxy-3-oxopropanoic acid
2-(3-Hydroxy-3,4,5,6-tetrahydro-1H-cyclopenta[c]furan-4-yl)-3-methoxy-3-oxopropanoic acid
2-(3-Hydroxy-3,4,5,6-tetrahydro-1H-cyclopenta[c]furan-4-yl)-3-methoxy-3-oxopropanoic acid
2-(3-Hydroxy-3,4,5,6-tetrahydro-1H-cyclopenta[c]furan-4-yl)-3-methoxy-3-oxopropanoic acid
2-(3-Hydroxy-3,4,5,6-tetrahydro-1H-cyclopenta[c]furan-4-yl)-3-methoxy-3-oxopropanoic acid
folinic acid
folinic acid
folinic acid
folinic acid
folinic acid
N~6~-[5-(1,2-Dithiolan-3-yl)pentanoyl]-D-lysine
N~6~-[5-(1,2-Dithiolan-3-yl)pentanoyl]-D-lysine
N~6~-[5-(1,2-Dithiolan-3-yl)pentanoyl]-D-lysine
N~6~-[5-(1,2-Dithiolan-3-yl)pentanoyl]-D-lysine
N~6~-[5-(1,2-Dithiolan-3-yl)pentanoyl]-D-lysine
N-(2,3,4-Trimethoxybenzoyl)glycine_b
N-(2,3,4-Trimethoxybenzoyl)glycine_b
N-(2,3,4-Trimethoxybenzoyl)glycine_b
N-(2,3,4-Trimethoxybenzoyl)glycine_b
N-(2,3,4-Trimethoxybenzoyl)glycine_b
Toluene_a
Toluene_a
Toluene_a
Toluene_a
Toluene_a
MFCD00055782
MFCD00055782
MFCD00055782
MFCD00055782
MFCD00055782
L-gamma-Glutamyl-L-leucine_a
L-gamma-Glutamyl-L-leucine_a
L-gamma-Glutamyl-L-leucine_a
L-gamma-Glutamyl-L-leucine_a
L-gamma-Glutamyl-L-leucine_a
(2E)-3-Methyl-4-(sulfooxy)-2-butenoic acid_b
(2E)-3-Methyl-4-(sulfooxy)-2-butenoic acid_b
(2E)-3-Methyl-4-(sulfooxy)-2-butenoic acid_b
(2E)-3-Methyl-4-(sulfooxy)-2-butenoic acid_b
(2E)-3-Methyl-4-(sulfooxy)-2-butenoic acid_b
N-Acetylprocainamide_a
N-Acetylprocainamide_a
N-Acetylprocainamide_a
N-Acetylprocainamide_a
N-Acetylprocainamide_a

3-Methoxy-4-hydroxyhippuric acid_a
3-Methoxy-4-hydroxyhippuric acid_a
3-Methoxy-4-hydroxyhippuric acid_a
3-Methoxy-4-hydroxyhippuric acid_a
3-Methoxy-4-hydroxyhippuric acid_a
Valylvaline_f
Valylvaline_f
Valylvaline_f
Valylvaline_f
Valylvaline_f
N-(2-Cyanoethyl)-L-glutamine
N-(2-Cyanoethyl)-L-glutamine
N-(2-Cyanoethyl)-L-glutamine
N-(2-Cyanoethyl)-L-glutamine
N-(2-Cyanoethyl)-L-glutamine
Vorinostat_b
Vorinostat_b
Vorinostat_b
Vorinostat_b
Vorinostat_b
Ro 20-1724_a
Ro 20-1724_a
Ro 20-1724_a
Ro 20-1724_a
Ro 20-1724_a
3-(Sulfooxy)benzenepropanoic acid_b
3-(Sulfooxy)benzenepropanoic acid_b
3-(Sulfooxy)benzenepropanoic acid_b
3-(Sulfooxy)benzenepropanoic acid_b
3-(Sulfooxy)benzenepropanoic acid_b
Diacetin_b
Diacetin_b
Diacetin_b
Diacetin_b
Diacetin_b
Pentoxifylline
Pentoxifylline
Pentoxifylline
Pentoxifylline
Pentoxifylline
Pseudouridine
Pseudouridine
Pseudouridine
Pseudouridine
Pseudouridine
Bicine_b
Bicine_b
Bicine_b

Bicine_b
Bicine_b
Pro-tyr
Pro-tyr
Pro-tyr
Pro-tyr
Pro-tyr
NPK (Peptide Asn-Pro-Lys)
NPK (Peptide Asn-Pro-Lys)
NPK (Peptide Asn-Pro-Lys)
NPK (Peptide Asn-Pro-Lys)
NPK (Peptide Asn-Pro-Lys)
Formiminoglutamic Acid
Formiminoglutamic Acid
Formiminoglutamic Acid
Formiminoglutamic Acid
Formiminoglutamic Acid
N-COUMAROYL-L-ASPARTIC ACID
N-COUMAROYL-L-ASPARTIC ACID
N-COUMAROYL-L-ASPARTIC ACID
N-COUMAROYL-L-ASPARTIC ACID
N-COUMAROYL-L-ASPARTIC ACID
2-Methoxy-2-methyldihydro-3(2H)-thiophenone_a
2-Methoxy-2-methyldihydro-3(2H)-thiophenone_a
2-Methoxy-2-methyldihydro-3(2H)-thiophenone_a
2-Methoxy-2-methyldihydro-3(2H)-thiophenone_a
2-Methoxy-2-methyldihydro-3(2H)-thiophenone_a
nicotianamine_b
nicotianamine_b
nicotianamine_b
nicotianamine_b
nicotianamine_b
6-hydroxymethylpterin
6-hydroxymethylpterin
6-hydroxymethylpterin
6-hydroxymethylpterin
6-hydroxymethylpterin
Guanidinoethyl methyl phosphate
Guanidinoethyl methyl phosphate
Guanidinoethyl methyl phosphate
Guanidinoethyl methyl phosphate
Guanidinoethyl methyl phosphate
(3S,4S)-7,12-Dimethyl-3,4-dihydro-3,4-tetraphenediol
(3S,4S)-7,12-Dimethyl-3,4-dihydro-3,4-tetraphenediol
(3S,4S)-7,12-Dimethyl-3,4-dihydro-3,4-tetraphenediol
(3S,4S)-7,12-Dimethyl-3,4-dihydro-3,4-tetraphenediol
(3S,4S)-7,12-Dimethyl-3,4-dihydro-3,4-tetraphenediol
Tetrahydro-2,5-furan-diacetic acid

Tetrahydro-2,5-furan-diacetic acid
Tetrahydro-2,5-furan-diacetic acid
Tetrahydro-2,5-furan-diacetic acid
Tetrahydro-2,5-furan-diacetic acid
5-Hydroxy-7-methoxy-2,2-dimethyl-6-(3-phenylpropanoyl)-3,4-dihydro-2H-chromen-4-yl hydrogen sulfate
5-Hydroxy-7-methoxy-2,2-dimethyl-6-(3-phenylpropanoyl)-3,4-dihydro-2H-chromen-4-yl hydrogen sulfate
5-Hydroxy-7-methoxy-2,2-dimethyl-6-(3-phenylpropanoyl)-3,4-dihydro-2H-chromen-4-yl hydrogen sulfate
5-Hydroxy-7-methoxy-2,2-dimethyl-6-(3-phenylpropanoyl)-3,4-dihydro-2H-chromen-4-yl hydrogen sulfate
5-Hydroxy-7-methoxy-2,2-dimethyl-6-(3-phenylpropanoyl)-3,4-dihydro-2H-chromen-4-yl hydrogen sulfate
1-(2-Carboxyethyl)-2,3,4,9-tetrahydro-1H-beta-carboline-3-carboxylic acid_b
1-(2-Carboxyethyl)-2,3,4,9-tetrahydro-1H-beta-carboline-3-carboxylic acid_b
1-(2-Carboxyethyl)-2,3,4,9-tetrahydro-1H-beta-carboline-3-carboxylic acid_b
1-(2-Carboxyethyl)-2,3,4,9-tetrahydro-1H-beta-carboline-3-carboxylic acid_b
1-(2-Carboxyethyl)-2,3,4,9-tetrahydro-1H-beta-carboline-3-carboxylic acid_b
Gly-Trp_b
Gly-Trp_b
Gly-Trp_b
Gly-Trp_b
Gly-Trp_b
Dihydrouridine_b
Dihydrouridine_b
Dihydrouridine_b
Dihydrouridine_b
Dihydrouridine_b
1ÃŽÂ²-Hydroxycholic acid_b
1ÃŽÂ²-Hydroxycholic acid_b
1ÃŽÂ²-Hydroxycholic acid_b
1ÃŽÂ²-Hydroxycholic acid_b
1ÃŽÂ²-Hydroxycholic acid_b
Methdilazine
Methdilazine
Methdilazine
Methdilazine
Methdilazine
2'-Deoxyadenosine
2'-Deoxyadenosine
2'-Deoxyadenosine
2'-Deoxyadenosine
2'-Deoxyadenosine
Hydroxyphenobarbital
Hydroxyphenobarbital
Hydroxyphenobarbital
Hydroxyphenobarbital
Hydroxyphenobarbital
4,4'-Thiobis(butan-2-one)
4,4'-Thiobis(butan-2-one)
4,4'-Thiobis(butan-2-one)
4,4'-Thiobis(butan-2-one)

4,4'-Thiobis(butan-2-one)
His-pro_b
His-pro_b
His-pro_b
His-pro_b
His-pro_b
L-alpha-Aspartyl-L-phenylalanine
L-alpha-Aspartyl-L-phenylalanine
L-alpha-Aspartyl-L-phenylalanine
L-alpha-Aspartyl-L-phenylalanine
L-alpha-Aspartyl-L-phenylalanine
2-Hydroxyhepta-2_4-dienedioate
2-Hydroxyhepta-2_4-dienedioate
2-Hydroxyhepta-2_4-dienedioate
2-Hydroxyhepta-2_4-dienedioate
2-Hydroxyhepta-2_4-dienedioate
Choline Alfoscerate
Choline Alfoscerate
Choline Alfoscerate
Choline Alfoscerate
Choline Alfoscerate
4-(9H-beta-Carbolin-1-yl)-1,2,4-butanetriol_a
4-(9H-beta-Carbolin-1-yl)-1,2,4-butanetriol_a
4-(9H-beta-Carbolin-1-yl)-1,2,4-butanetriol_a
4-(9H-beta-Carbolin-1-yl)-1,2,4-butanetriol_a
4-(9H-beta-Carbolin-1-yl)-1,2,4-butanetriol_a
Val-Trp_b
Val-Trp_b
Val-Trp_b
Val-Trp_b
Val-Trp_b
9-(alpha-D-glucosyl)dihydrozeatin
9-(alpha-D-glucosyl)dihydrozeatin
9-(alpha-D-glucosyl)dihydrozeatin
9-(alpha-D-glucosyl)dihydrozeatin
9-(alpha-D-glucosyl)dihydrozeatin
SECONAL_d
SECONAL_d
SECONAL_d
SECONAL_d
SECONAL_d
epsilon-(gamma-Glutamyl)-lysine_d
epsilon-(gamma-Glutamyl)-lysine_d
epsilon-(gamma-Glutamyl)-lysine_d
epsilon-(gamma-Glutamyl)-lysine_d
epsilon-(gamma-Glutamyl)-lysine_d
3,4-Dihydroxyphenylglycol
3,4-Dihydroxyphenylglycol

3,4-Dihydroxyphenylglycol
3,4-Dihydroxyphenylglycol
3,4-Dihydroxyphenylglycol
(1S,3R,4s)-1,3,4,5-Tetrahydroxycyclohexanecarboxylic acid
(1S,3R,4s)-1,3,4,5-Tetrahydroxycyclohexanecarboxylic acid
(1S,3R,4s)-1,3,4,5-Tetrahydroxycyclohexanecarboxylic acid
(1S,3R,4s)-1,3,4,5-Tetrahydroxycyclohexanecarboxylic acid
(1S,3R,4s)-1,3,4,5-Tetrahydroxycyclohexanecarboxylic acid
4-Hydroxyaminoquinoline N-oxide
4-Hydroxyaminoquinoline N-oxide
4-Hydroxyaminoquinoline N-oxide
4-Hydroxyaminoquinoline N-oxide
4-Hydroxyaminoquinoline N-oxide
6-imino-5-oxocyclohexa-1,3-dienecarboxylic acid
6-imino-5-oxocyclohexa-1,3-dienecarboxylic acid
6-imino-5-oxocyclohexa-1,3-dienecarboxylic acid
6-imino-5-oxocyclohexa-1,3-dienecarboxylic acid
6-imino-5-oxocyclohexa-1,3-dienecarboxylic acid
3-[(2Z)-1-Oxo-2-buten-2-yl]pentanedioic acid_b
3-[(2Z)-1-Oxo-2-buten-2-yl]pentanedioic acid_b
3-[(2Z)-1-Oxo-2-buten-2-yl]pentanedioic acid_b
3-[(2Z)-1-Oxo-2-buten-2-yl]pentanedioic acid_b
3-[(2Z)-1-Oxo-2-buten-2-yl]pentanedioic acid_b
2-glyceryl 14,15-epoxy-(5Z,8Z,11Z)-icosatrienoate
2-glyceryl 14,15-epoxy-(5Z,8Z,11Z)-icosatrienoate
2-glyceryl 14,15-epoxy-(5Z,8Z,11Z)-icosatrienoate
2-glyceryl 14,15-epoxy-(5Z,8Z,11Z)-icosatrienoate
2-glyceryl 14,15-epoxy-(5Z,8Z,11Z)-icosatrienoate
Methylol Dimethylhydantoin_b
Methylol Dimethylhydantoin_b
Methylol Dimethylhydantoin_b
Methylol Dimethylhydantoin_b
Methylol Dimethylhydantoin_b
o-Succinylbenzoate
o-Succinylbenzoate
o-Succinylbenzoate
o-Succinylbenzoate
o-Succinylbenzoate
Gly-Trp_a
Gly-Trp_a
Gly-Trp_a
Gly-Trp_a
Gly-Trp_a
Glycylglycylglycine
Glycylglycylglycine
Glycylglycylglycine
Glycylglycylglycine
Glycylglycylglycine

trp-ser
trp-ser
trp-ser
trp-ser
trp-ser
asp-gln_a
asp-gln_a
asp-gln_a
asp-gln_a
asp-gln_a
N,N-Dimethyl-L-histidine_b
N,N-Dimethyl-L-histidine_b
N,N-Dimethyl-L-histidine_b
N,N-Dimethyl-L-histidine_b
N,N-Dimethyl-L-histidine_b
9-[(5R)-5-Ethyl-alpha-D-xylopyranosyl]-N-(3-methyl-2-buten-1-yl)-9H-purin-6-amine
9-[(5R)-5-Ethyl-alpha-D-xylopyranosyl]-N-(3-methyl-2-buten-1-yl)-9H-purin-6-amine
9-[(5R)-5-Ethyl-alpha-D-xylopyranosyl]-N-(3-methyl-2-buten-1-yl)-9H-purin-6-amine
9-[(5R)-5-Ethyl-alpha-D-xylopyranosyl]-N-(3-methyl-2-buten-1-yl)-9H-purin-6-amine
9-[(5R)-5-Ethyl-alpha-D-xylopyranosyl]-N-(3-methyl-2-buten-1-yl)-9H-purin-6-amine
Guanfacine
Guanfacine
Guanfacine
Guanfacine
Guanfacine
Sinapinic acid_a
Sinapinic acid_a
Sinapinic acid_a
Sinapinic acid_a
Sinapinic acid_a
Propantheline
Propantheline
Propantheline
Propantheline
Propantheline
Losalen
Losalen
Losalen
Losalen
Losalen
2-Methoxy-2-methyldihydro-3(2H)-thiophenone_b
2-Methoxy-2-methyldihydro-3(2H)-thiophenone_b
2-Methoxy-2-methyldihydro-3(2H)-thiophenone_b
2-Methoxy-2-methyldihydro-3(2H)-thiophenone_b
2-Methoxy-2-methyldihydro-3(2H)-thiophenone_b
Desonide
Desonide
Desonide

Desonide
Desonide
2-Methoxy-8-(7-methoxy-2-oxo-2H-chromen-6-yl)-6-methylnaphthoquinone
2-Methoxy-8-(7-methoxy-2-oxo-2H-chromen-6-yl)-6-methylnaphthoquinone
2-Methoxy-8-(7-methoxy-2-oxo-2H-chromen-6-yl)-6-methylnaphthoquinone
2-Methoxy-8-(7-methoxy-2-oxo-2H-chromen-6-yl)-6-methylnaphthoquinone
2-Methoxy-8-(7-methoxy-2-oxo-2H-chromen-6-yl)-6-methylnaphthoquinone
2-{(2S,4S)-4-[(3-Amino-2,3,6-trideoxyhexopyranosyl)oxy]-2,5,12-trihydroxy-7-methoxy-6,11-dioxo-1,2,3,4,6,11-h
2-{(2S,4S)-4-[(3-Amino-2,3,6-trideoxyhexopyranosyl)oxy]-2,5,12-trihydroxy-7-methoxy-6,11-dioxo-1,2,3,4,6,11-h
2-{(2S,4S)-4-[(3-Amino-2,3,6-trideoxyhexopyranosyl)oxy]-2,5,12-trihydroxy-7-methoxy-6,11-dioxo-1,2,3,4,6,11-h
2-{(2S,4S)-4-[(3-Amino-2,3,6-trideoxyhexopyranosyl)oxy]-2,5,12-trihydroxy-7-methoxy-6,11-dioxo-1,2,3,4,6,11-h
2-{(2S,4S)-4-[(3-Amino-2,3,6-trideoxyhexopyranosyl)oxy]-2,5,12-trihydroxy-7-methoxy-6,11-dioxo-1,2,3,4,6,11-h
2-BUTYL PROPENYL DISULFIDE, E(R)-_b
2-BUTYL PROPENYL DISULFIDE, E(R)-_b
2-BUTYL PROPENYL DISULFIDE, E(R)-_b
2-BUTYL PROPENYL DISULFIDE, E(R)-_b
2-BUTYL PROPENYL DISULFIDE, E(R)-_b
shinorine
shinorine
shinorine
shinorine
shinorine
ferrileghemoglobin
ferrileghemoglobin
ferrileghemoglobin
ferrileghemoglobin
ferrileghemoglobin
2-(1-Ethoxyethoxy)propanoic acid_c
2-(1-Ethoxyethoxy)propanoic acid_c
2-(1-Ethoxyethoxy)propanoic acid_c
2-(1-Ethoxyethoxy)propanoic acid_c
2-(1-Ethoxyethoxy)propanoic acid_c
Uramustine
Uramustine
Uramustine
Uramustine
Uramustine
2-Aminomuconic acid
2-Aminomuconic acid
2-Aminomuconic acid
2-Aminomuconic acid
2-Aminomuconic acid
(3R)-2-(3,4-Dihydroxyphenyl)-6,8-dihydroxy-3,4-dihydro-2H-chromen-3-yl sulfate
(3R)-2-(3,4-Dihydroxyphenyl)-6,8-dihydroxy-3,4-dihydro-2H-chromen-3-yl sulfate
(3R)-2-(3,4-Dihydroxyphenyl)-6,8-dihydroxy-3,4-dihydro-2H-chromen-3-yl sulfate
(3R)-2-(3,4-Dihydroxyphenyl)-6,8-dihydroxy-3,4-dihydro-2H-chromen-3-yl sulfate
(3R)-2-(3,4-Dihydroxyphenyl)-6,8-dihydroxy-3,4-dihydro-2H-chromen-3-yl sulfate
Artesunate

Artesunate
Artesunate
Artesunate
Artesunate
Bentazone
Bentazone
Bentazone
Bentazone
Bentazone
3-(3,4-dihydroxypyridinium-1-yl)-L-alanine
3-(3,4-dihydroxypyridinium-1-yl)-L-alanine
3-(3,4-dihydroxypyridinium-1-yl)-L-alanine
3-(3,4-dihydroxypyridinium-1-yl)-L-alanine
3-(3,4-dihydroxypyridinium-1-yl)-L-alanine
2,3,4,5-tetrahydrodipicolinic acid
2,3,4,5-tetrahydrodipicolinic acid
2,3,4,5-tetrahydrodipicolinic acid
2,3,4,5-tetrahydrodipicolinic acid
2,3,4,5-tetrahydrodipicolinic acid
Tetraacetylethylenediamine_e
Tetraacetylethylenediamine_e
Tetraacetylethylenediamine_e
Tetraacetylethylenediamine_e
Tetraacetylethylenediamine_e
L-fucopyranose 1-phosphate
L-fucopyranose 1-phosphate
L-fucopyranose 1-phosphate
L-fucopyranose 1-phosphate
L-fucopyranose 1-phosphate
4-(METHYLNITROSAMINO)-1-(3-PYRIDYL-N-OXIDE)-1-BUTANOL_e
4-(METHYLNITROSAMINO)-1-(3-PYRIDYL-N-OXIDE)-1-BUTANOL_e
4-(METHYLNITROSAMINO)-1-(3-PYRIDYL-N-OXIDE)-1-BUTANOL_e
4-(METHYLNITROSAMINO)-1-(3-PYRIDYL-N-OXIDE)-1-BUTANOL_e
4-(METHYLNITROSAMINO)-1-(3-PYRIDYL-N-OXIDE)-1-BUTANOL_e
3-Methoxy-4-hydroxyhippuric acid_c
3-Methoxy-4-hydroxyhippuric acid_c
3-Methoxy-4-hydroxyhippuric acid_c
3-Methoxy-4-hydroxyhippuric acid_c
3-Methoxy-4-hydroxyhippuric acid_c
3-Methoxy-4-hydroxyhippuric acid_f
3-Methoxy-4-hydroxyhippuric acid_f
3-Methoxy-4-hydroxyhippuric acid_f
3-Methoxy-4-hydroxyhippuric acid_f
3-Methoxy-4-hydroxyhippuric acid_f
Spaglumic acid
Spaglumic acid
Spaglumic acid
Spaglumic acid

Spaglumic acid
gamma-L-glutamyl-L-tyrosine
gamma-L-glutamyl-L-tyrosine
gamma-L-glutamyl-L-tyrosine
gamma-L-glutamyl-L-tyrosine
gamma-L-glutamyl-L-tyrosine
3-Hydroxytetradecanedioic acid
3-Hydroxytetradecanedioic acid
3-Hydroxytetradecanedioic acid
3-Hydroxytetradecanedioic acid
3-Hydroxytetradecanedioic acid
6-[(Z)-2-(3,4-Dimethoxyphenyl)vinyl]-4-methoxy-5,6-dihydro-2H-pyran-2-one
6-[(Z)-2-(3,4-Dimethoxyphenyl)vinyl]-4-methoxy-5,6-dihydro-2H-pyran-2-one
6-[(Z)-2-(3,4-Dimethoxyphenyl)vinyl]-4-methoxy-5,6-dihydro-2H-pyran-2-one
6-[(Z)-2-(3,4-Dimethoxyphenyl)vinyl]-4-methoxy-5,6-dihydro-2H-pyran-2-one
6-[(Z)-2-(3,4-Dimethoxyphenyl)vinyl]-4-methoxy-5,6-dihydro-2H-pyran-2-one
Zinecard_b
Zinecard_b
Zinecard_b
Zinecard_b
Zinecard_b
Aminohippuric acid_a
Aminohippuric acid_a
Aminohippuric acid_a
Aminohippuric acid_a
Aminohippuric acid_a
Benzamide
Benzamide
Benzamide
Benzamide
Benzamide
3-Benzyl-6-isobutyl-2,5-piperazinedione_c
3-Benzyl-6-isobutyl-2,5-piperazinedione_c
3-Benzyl-6-isobutyl-2,5-piperazinedione_c
3-Benzyl-6-isobutyl-2,5-piperazinedione_c
3-Benzyl-6-isobutyl-2,5-piperazinedione_c
L-Glutamic acid, 5-[2-(4-carboxyphenyl)hydrazide]
L-Glutamic acid, 5-[2-(4-carboxyphenyl)hydrazide]
L-Glutamic acid, 5-[2-(4-carboxyphenyl)hydrazide]
L-Glutamic acid, 5-[2-(4-carboxyphenyl)hydrazide]
L-Glutamic acid, 5-[2-(4-carboxyphenyl)hydrazide]
Pyrimidine 5'-nucleotide
Pyrimidine 5'-nucleotide
Pyrimidine 5'-nucleotide
Pyrimidine 5'-nucleotide
Pyrimidine 5'-nucleotide
Dihydrouracil_a
Dihydrouracil_a

Dihydrouracil_a
Dihydrouracil_a
Dihydrouracil_a
9,11-Dihydroxy-2-methoxy-12H-benzo[a]xanthen-12-one
9,11-Dihydroxy-2-methoxy-12H-benzo[a]xanthen-12-one
9,11-Dihydroxy-2-methoxy-12H-benzo[a]xanthen-12-one
9,11-Dihydroxy-2-methoxy-12H-benzo[a]xanthen-12-one
9,11-Dihydroxy-2-methoxy-12H-benzo[a]xanthen-12-one
2-Amino-6-[(E)-(5-amino-5-carboxy-2-hydroxypentylidene)amino]-5-hydroxyhexanoic acid (non-preferred name)
2-Amino-6-[(E)-(5-amino-5-carboxy-2-hydroxypentylidene)amino]-5-hydroxyhexanoic acid (non-preferred name)
2-Amino-6-[(E)-(5-amino-5-carboxy-2-hydroxypentylidene)amino]-5-hydroxyhexanoic acid (non-preferred name)
2-Amino-6-[(E)-(5-amino-5-carboxy-2-hydroxypentylidene)amino]-5-hydroxyhexanoic acid (non-preferred name)
2-Amino-6-[(E)-(5-amino-5-carboxy-2-hydroxypentylidene)amino]-5-hydroxyhexanoic acid (non-preferred name)
Mono(3-carboxypropyl) phthalate
Mono(3-carboxypropyl) phthalate
Mono(3-carboxypropyl) phthalate
Mono(3-carboxypropyl) phthalate
Mono(3-carboxypropyl) phthalate
3-Methyladenine_b
3-Methyladenine_b
3-Methyladenine_b
3-Methyladenine_b
3-Methyladenine_b
(2E)-5-Hydroxyferulic acid
(2E)-5-Hydroxyferulic acid
(2E)-5-Hydroxyferulic acid
(2E)-5-Hydroxyferulic acid
(2E)-5-Hydroxyferulic acid
Tyrosol
Tyrosol
Tyrosol
Tyrosol
Tyrosol
N-Nonanoylglycine_b
N-Nonanoylglycine_b
N-Nonanoylglycine_b
N-Nonanoylglycine_b
N-Nonanoylglycine_b
Valylvaline_b
Valylvaline_b
Valylvaline_b
Valylvaline_b
Valylvaline_b
N-Acetyl-S-(allylcarbamothioyl)cysteine
N-Acetyl-S-(allylcarbamothioyl)cysteine
N-Acetyl-S-(allylcarbamothioyl)cysteine
N-Acetyl-S-(allylcarbamothioyl)cysteine
N-Acetyl-S-(allylcarbamothioyl)cysteine

L-gamma-Glutamyl-L-valine_d
L-gamma-Glutamyl-L-valine_d
L-gamma-Glutamyl-L-valine_d
L-gamma-Glutamyl-L-valine_d
L-gamma-Glutamyl-L-valine_d
N-(4-Amino-1-carboxybutyl)glutamic acid_b
N-(4-Amino-1-carboxybutyl)glutamic acid_b
N-(4-Amino-1-carboxybutyl)glutamic acid_b
N-(4-Amino-1-carboxybutyl)glutamic acid_b
N-(4-Amino-1-carboxybutyl)glutamic acid_b
1,4-Naphthoquinone
1,4-Naphthoquinone
1,4-Naphthoquinone
1,4-Naphthoquinone
1,4-Naphthoquinone
Lisdexamfetamine
Lisdexamfetamine
Lisdexamfetamine
Lisdexamfetamine
Lisdexamfetamine
asp-gln_b
asp-gln_b
asp-gln_b
asp-gln_b
asp-gln_b
pterin
pterin
pterin
pterin
pterin
13a-Hydroxy-2,3-dimethoxy-6a,13a-dihydrochromeno[3,4-b]furo[3,2-g]chromen-13(6H)-one
13a-Hydroxy-2,3-dimethoxy-6a,13a-dihydrochromeno[3,4-b]furo[3,2-g]chromen-13(6H)-one
13a-Hydroxy-2,3-dimethoxy-6a,13a-dihydrochromeno[3,4-b]furo[3,2-g]chromen-13(6H)-one
13a-Hydroxy-2,3-dimethoxy-6a,13a-dihydrochromeno[3,4-b]furo[3,2-g]chromen-13(6H)-one
13a-Hydroxy-2,3-dimethoxy-6a,13a-dihydrochromeno[3,4-b]furo[3,2-g]chromen-13(6H)-one
6-(alpha-D-glucosaminyl)-1D-myo-inositol_b
6-(alpha-D-glucosaminyl)-1D-myo-inositol_b
6-(alpha-D-glucosaminyl)-1D-myo-inositol_b
6-(alpha-D-glucosaminyl)-1D-myo-inositol_b
6-(alpha-D-glucosaminyl)-1D-myo-inositol_b
Dinoseb
Dinoseb
Dinoseb
Dinoseb
Dinoseb
Methional
Methional
Methional

Methional
Methional
epsilon-(gamma-Glutamyl)-lysine_c
epsilon-(gamma-Glutamyl)-lysine_c
epsilon-(gamma-Glutamyl)-lysine_c
epsilon-(gamma-Glutamyl)-lysine_c
epsilon-(gamma-Glutamyl)-lysine_c
hydroxyhexamide
hydroxyhexamide
hydroxyhexamide
hydroxyhexamide
hydroxyhexamide
1-Methylinosine
1-Methylinosine
1-Methylinosine
1-Methylinosine
1-Methylinosine
AAMU_b
AAMU_b
AAMU_b
AAMU_b
AAMU_b
Minoxidil
Minoxidil
Minoxidil
Minoxidil
Minoxidil
N-[(4-Methoxy-1-benzofuran-5-yl)carbonyl]glycine
N-[(4-Methoxy-1-benzofuran-5-yl)carbonyl]glycine
N-[(4-Methoxy-1-benzofuran-5-yl)carbonyl]glycine
N-[(4-Methoxy-1-benzofuran-5-yl)carbonyl]glycine
N-[(4-Methoxy-1-benzofuran-5-yl)carbonyl]glycine
Ethosuximide
Ethosuximide
Ethosuximide
Ethosuximide
Ethosuximide
5-Methoxy-3-indoleaceate
5-Methoxy-3-indoleaceate
5-Methoxy-3-indoleaceate
5-Methoxy-3-indoleaceate
5-Methoxy-3-indoleaceate
16alpha-hydroxydehydroepiandrosterone 3-sulfate
16alpha-hydroxydehydroepiandrosterone 3-sulfate
16alpha-hydroxydehydroepiandrosterone 3-sulfate
16alpha-hydroxydehydroepiandrosterone 3-sulfate
16alpha-hydroxydehydroepiandrosterone 3-sulfate
7-Aminomethyl-7-deazaguanine

7-Aminomethyl-7-deazaguanine
7-Aminomethyl-7-deazaguanine
7-Aminomethyl-7-deazaguanine
7-Aminomethyl-7-deazaguanine
2-(4-Isopropyl-4-methyl-5-oxo-4,5-dihydro-1H-imidazol-2-yl)-4-methylbenzoic acid_b
2-(4-Isopropyl-4-methyl-5-oxo-4,5-dihydro-1H-imidazol-2-yl)-4-methylbenzoic acid_b
2-(4-Isopropyl-4-methyl-5-oxo-4,5-dihydro-1H-imidazol-2-yl)-4-methylbenzoic acid_b
2-(4-Isopropyl-4-methyl-5-oxo-4,5-dihydro-1H-imidazol-2-yl)-4-methylbenzoic acid_b
2-(4-Isopropyl-4-methyl-5-oxo-4,5-dihydro-1H-imidazol-2-yl)-4-methylbenzoic acid_b
his-asn
his-asn
his-asn
his-asn
his-asn
meprobamate_d
meprobamate_d
meprobamate_d
meprobamate_d
meprobamate_d
6-Sulfatoxymelatonin
6-Sulfatoxymelatonin
6-Sulfatoxymelatonin
6-Sulfatoxymelatonin
6-Sulfatoxymelatonin
Toluene_b
Toluene_b
Toluene_b
Toluene_b
Toluene_b
Trifluoromethyl-bismethyl ketone
Trifluoromethyl-bismethyl ketone
Trifluoromethyl-bismethyl ketone
Trifluoromethyl-bismethyl ketone
Trifluoromethyl-bismethyl ketone
Taxifolin
Taxifolin
Taxifolin
Taxifolin
Taxifolin
17-Hydroxypregnenolone sulfate
17-Hydroxypregnenolone sulfate
17-Hydroxypregnenolone sulfate
17-Hydroxypregnenolone sulfate
17-Hydroxypregnenolone sulfate
5-O-alpha-L-Arabinofuranosyl-alpha-L-arabinofuranose
5-O-alpha-L-Arabinofuranosyl-alpha-L-arabinofuranose
5-O-alpha-L-Arabinofuranosyl-alpha-L-arabinofuranose
5-O-alpha-L-Arabinofuranosyl-alpha-L-arabinofuranose

5-O-alpha-L-Arabinofuranosyl-alpha-L-arabinofuranose
Sinapinic acid_b
Sinapinic acid_b
Sinapinic acid_b
Sinapinic acid_b
Sinapinic acid_b
N-Desalkylflurazepam
N-Desalkylflurazepam
N-Desalkylflurazepam
N-Desalkylflurazepam
N-Desalkylflurazepam
MFCD09953737_a
MFCD09953737_a
MFCD09953737_a
MFCD09953737_a
MFCD09953737_a
L-Proline, 4-hydroxy-5-oxo-4-(tetrahydro-2,3,4-trihydroxy-2-furanyl)-
L-Proline, 4-hydroxy-5-oxo-4-(tetrahydro-2,3,4-trihydroxy-2-furanyl)-
L-Proline, 4-hydroxy-5-oxo-4-(tetrahydro-2,3,4-trihydroxy-2-furanyl)-
L-Proline, 4-hydroxy-5-oxo-4-(tetrahydro-2,3,4-trihydroxy-2-furanyl)-
L-Proline, 4-hydroxy-5-oxo-4-(tetrahydro-2,3,4-trihydroxy-2-furanyl)-
Cilazapril
Cilazapril
Cilazapril
Cilazapril
Cilazapril
MFCD02728197_c
MFCD02728197_c
MFCD02728197_c
MFCD02728197_c
MFCD02728197_c
2-Hydroxyphenylacetic acid
2-Hydroxyphenylacetic acid
2-Hydroxyphenylacetic acid
2-Hydroxyphenylacetic acid
2-Hydroxyphenylacetic acid
N-(1-{[Methyl(2-methyl-2-propanyl)carbamoyl]amino}ethyl)-alpha-asparagine
N-(1-{[Methyl(2-methyl-2-propanyl)carbamoyl]amino}ethyl)-alpha-asparagine
N-(1-{[Methyl(2-methyl-2-propanyl)carbamoyl]amino}ethyl)-alpha-asparagine
N-(1-{[Methyl(2-methyl-2-propanyl)carbamoyl]amino}ethyl)-alpha-asparagine
N-(1-{[Methyl(2-methyl-2-propanyl)carbamoyl]amino}ethyl)-alpha-asparagine
Hypericin
Hypericin
Hypericin
Hypericin
Hypericin
(2E)-N-3,7-dimethyl-2,6-octadienyl cyclopropylcarboxamide
(2E)-N-3,7-dimethyl-2,6-octadienyl cyclopropylcarboxamide

(2E)-N-3,7-dimethyl-2,6-octadienyl cyclopropylcarboxamide
(2E)-N-3,7-dimethyl-2,6-octadienyl cyclopropylcarboxamide
(2E)-N-3,7-dimethyl-2,6-octadienyl cyclopropylcarboxamide
Casimiroin
Casimiroin
Casimiroin
Casimiroin
Casimiroin
pro-gln_c
pro-gln_c
pro-gln_c
pro-gln_c
pro-gln_c
Histidylphenylalanine
Histidylphenylalanine
Histidylphenylalanine
Histidylphenylalanine
Histidylphenylalanine
L-gamma-Glutamyl-L-leucine_d
L-gamma-Glutamyl-L-leucine_d
L-gamma-Glutamyl-L-leucine_d
L-gamma-Glutamyl-L-leucine_d
L-gamma-Glutamyl-L-leucine_d
ELK (Peptide Glu-Leu-Lys)_b
ELK (Peptide Glu-Leu-Lys)_b
ELK (Peptide Glu-Leu-Lys)_b
ELK (Peptide Glu-Leu-Lys)_b
ELK (Peptide Glu-Leu-Lys)_b
(3aS,5S,6R,7aR,7bS,9aS,10R,12aS,12bS)-10-[(2S,3R,4R)-3,4-Dihydroxy-6-methyl-2-heptanyl]-5,6-dihydroxy-7a,[illegible]
(3aS,5S,6R,7aR,7bS,9aS,10R,12aS,12bS)-10-[(2S,3R,4R)-3,4-Dihydroxy-6-methyl-2-heptanyl]-5,6-dihydroxy-7a,[illegible]
(3aS,5S,6R,7aR,7bS,9aS,10R,12aS,12bS)-10-[(2S,3R,4R)-3,4-Dihydroxy-6-methyl-2-heptanyl]-5,6-dihydroxy-7a,[illegible]
(3aS,5S,6R,7aR,7bS,9aS,10R,12aS,12bS)-10-[(2S,3R,4R)-3,4-Dihydroxy-6-methyl-2-heptanyl]-5,6-dihydroxy-7a,[illegible]
(3aS,5S,6R,7aR,7bS,9aS,10R,12aS,12bS)-10-[(2S,3R,4R)-3,4-Dihydroxy-6-methyl-2-heptanyl]-5,6-dihydroxy-7a,[illegible]
UQ3675000
UQ3675000
UQ3675000
UQ3675000
UQ3675000
S-Sulfocysteine
S-Sulfocysteine
S-Sulfocysteine
S-Sulfocysteine
S-Sulfocysteine
cis-3-Hexenyl Pyruvate
cis-3-Hexenyl Pyruvate
cis-3-Hexenyl Pyruvate
cis-3-Hexenyl Pyruvate
cis-3-Hexenyl Pyruvate

3,6-Dichloro-3,5-cyclohexadiene-1,2-diol
3,6-Dichloro-3,5-cyclohexadiene-1,2-diol
3,6-Dichloro-3,5-cyclohexadiene-1,2-diol
3,6-Dichloro-3,5-cyclohexadiene-1,2-diol
3,6-Dichloro-3,5-cyclohexadiene-1,2-diol
Lys-phe_a
Lys-phe_a
Lys-phe_a
Lys-phe_a
Lys-phe_a
felbamate
felbamate
felbamate
felbamate
felbamate
Urothion
Urothion
Urothion
Urothion
Urothion
[7-Hydroxy-1-(4-hydroxy-3-methoxyphenyl)-3-(hydroxymethyl)-6-methoxy-1,2,3,4-tetrahydro-2-naphthalenyl]met
[7-Hydroxy-1-(4-hydroxy-3-methoxyphenyl)-3-(hydroxymethyl)-6-methoxy-1,2,3,4-tetrahydro-2-naphthalenyl]met
[7-Hydroxy-1-(4-hydroxy-3-methoxyphenyl)-3-(hydroxymethyl)-6-methoxy-1,2,3,4-tetrahydro-2-naphthalenyl]met
[7-Hydroxy-1-(4-hydroxy-3-methoxyphenyl)-3-(hydroxymethyl)-6-methoxy-1,2,3,4-tetrahydro-2-naphthalenyl]met
[7-Hydroxy-1-(4-hydroxy-3-methoxyphenyl)-3-(hydroxymethyl)-6-methoxy-1,2,3,4-tetrahydro-2-naphthalenyl]met
4-(METHYLNITROSAMINO)-1-(3-PYRIDYL-N-OXIDE)-1-BUTANOL_b
4-(METHYLNITROSAMINO)-1-(3-PYRIDYL-N-OXIDE)-1-BUTANOL_b
4-(METHYLNITROSAMINO)-1-(3-PYRIDYL-N-OXIDE)-1-BUTANOL_b
4-(METHYLNITROSAMINO)-1-(3-PYRIDYL-N-OXIDE)-1-BUTANOL_b
4-(METHYLNITROSAMINO)-1-(3-PYRIDYL-N-OXIDE)-1-BUTANOL_b
3,4-dihydroxyphenylpyruvic acid
3,4-dihydroxyphenylpyruvic acid
3,4-dihydroxyphenylpyruvic acid
3,4-dihydroxyphenylpyruvic acid
3,4-dihydroxyphenylpyruvic acid
imazamethabenz-methyl_a
imazamethabenz-methyl_a
imazamethabenz-methyl_a
imazamethabenz-methyl_a
imazamethabenz-methyl_a
Dimeric mercapto propanone
Dimeric mercapto propanone
Dimeric mercapto propanone
Dimeric mercapto propanone
Dimeric mercapto propanone
N-(4-Hydroxy-3,5-dimethoxybenzoyl)glycine_b
N-(4-Hydroxy-3,5-dimethoxybenzoyl)glycine_b
N-(4-Hydroxy-3,5-dimethoxybenzoyl)glycine_b

N-(4-Hydroxy-3,5-dimethoxybenzoyl)glycine_b
N-(4-Hydroxy-3,5-dimethoxybenzoyl)glycine_b
H-DL-MET-DL-MET-OH
H-DL-MET-DL-MET-OH
H-DL-MET-DL-MET-OH
H-DL-MET-DL-MET-OH
H-DL-MET-DL-MET-OH
3-(4,7-Dimethoxy-1,3-benzodioxol-5-yl)propanoic acid
3-(4,7-Dimethoxy-1,3-benzodioxol-5-yl)propanoic acid
3-(4,7-Dimethoxy-1,3-benzodioxol-5-yl)propanoic acid
3-(4,7-Dimethoxy-1,3-benzodioxol-5-yl)propanoic acid
3-(4,7-Dimethoxy-1,3-benzodioxol-5-yl)propanoic acid
2-methoxyacetaminophen sulfate
2-methoxyacetaminophen sulfate
2-methoxyacetaminophen sulfate
2-methoxyacetaminophen sulfate
2-methoxyacetaminophen sulfate
NPYR
NPYR
NPYR
NPYR
NPYR
2-Acetamidohexanedioic acid_c
2-Acetamidohexanedioic acid_c
2-Acetamidohexanedioic acid_c
2-Acetamidohexanedioic acid_c
2-Acetamidohexanedioic acid_c
DIBOA
DIBOA
DIBOA
DIBOA
DIBOA
3-(Sulfooxy)benzenepropanoic acid_a
3-(Sulfooxy)benzenepropanoic acid_a
3-(Sulfooxy)benzenepropanoic acid_a
3-(Sulfooxy)benzenepropanoic acid_a
3-(Sulfooxy)benzenepropanoic acid_a
Redul
Redul
Redul
Redul
Redul
(S)-2-hydrazino-3-(4-hydroxy-3-methoxyphenyl)-2-methylpropionic acid
(S)-2-hydrazino-3-(4-hydroxy-3-methoxyphenyl)-2-methylpropionic acid
(S)-2-hydrazino-3-(4-hydroxy-3-methoxyphenyl)-2-methylpropionic acid
(S)-2-hydrazino-3-(4-hydroxy-3-methoxyphenyl)-2-methylpropionic acid
(S)-2-hydrazino-3-(4-hydroxy-3-methoxyphenyl)-2-methylpropionic acid
S-Allylcysteine_b

S-Allylcysteine_b
S-Allylcysteine_b
S-Allylcysteine_b
S-Allylcysteine_b
Scopoletin acetate
Scopoletin acetate
Scopoletin acetate
Scopoletin acetate
Scopoletin acetate
Azulfidine
Azulfidine
Azulfidine
Azulfidine
Azulfidine
S(8)-aminomethyldihydrolipoamide
S(8)-aminomethyldihydrolipoamide
S(8)-aminomethyldihydrolipoamide
S(8)-aminomethyldihydrolipoamide
S(8)-aminomethyldihydrolipoamide
GLY-MET
GLY-MET
GLY-MET
GLY-MET
GLY-MET
3-Benzyl-6-isobutyl-2,5-piperazinedione_b
3-Benzyl-6-isobutyl-2,5-piperazinedione_b
3-Benzyl-6-isobutyl-2,5-piperazinedione_b
3-Benzyl-6-isobutyl-2,5-piperazinedione_b
3-Benzyl-6-isobutyl-2,5-piperazinedione_b
(3aS,5S,6R,7aR,7bS,9aS,10R,12aS,12bS)-10-[(2S,3R,4R)-3,4-Dihydroxy-6-methyl-2-heptanyl]-5,6-dihydroxy-7a,[illegible]
(3aS,5S,6R,7aR,7bS,9aS,10R,12aS,12bS)-10-[(2S,3R,4R)-3,4-Dihydroxy-6-methyl-2-heptanyl]-5,6-dihydroxy-7a,[illegible]
(3aS,5S,6R,7aR,7bS,9aS,10R,12aS,12bS)-10-[(2S,3R,4R)-3,4-Dihydroxy-6-methyl-2-heptanyl]-5,6-dihydroxy-7a,[illegible]
(3aS,5S,6R,7aR,7bS,9aS,10R,12aS,12bS)-10-[(2S,3R,4R)-3,4-Dihydroxy-6-methyl-2-heptanyl]-5,6-dihydroxy-7a,[illegible]
(3aS,5S,6R,7aR,7bS,9aS,10R,12aS,12bS)-10-[(2S,3R,4R)-3,4-Dihydroxy-6-methyl-2-heptanyl]-5,6-dihydroxy-7a,[illegible]
Trolox
Trolox
Trolox
Trolox
Trolox
1ÃŽÂ²-Hydroxycholic acid_a
1ÃŽÂ²-Hydroxycholic acid_a
1ÃŽÂ²-Hydroxycholic acid_a
1ÃŽÂ²-Hydroxycholic acid_a
1ÃŽÂ²-Hydroxycholic acid_a
Xanthurenic acid
Xanthurenic acid
Xanthurenic acid
Xanthurenic acid

Xanthurenic acid
N-[(10Z)-7-Isobutyl-3-isopropyl-5,8-dioxo-2-oxa-6,9-diazabicyclo[10.2.2]hexadeca-1(14),10,12,15-tetraen-4-yl]-
N-[(10Z)-7-Isobutyl-3-isopropyl-5,8-dioxo-2-oxa-6,9-diazabicyclo[10.2.2]hexadeca-1(14),10,12,15-tetraen-4-yl]-
N-[(10Z)-7-Isobutyl-3-isopropyl-5,8-dioxo-2-oxa-6,9-diazabicyclo[10.2.2]hexadeca-1(14),10,12,15-tetraen-4-yl]-
N-[(10Z)-7-Isobutyl-3-isopropyl-5,8-dioxo-2-oxa-6,9-diazabicyclo[10.2.2]hexadeca-1(14),10,12,15-tetraen-4-yl]-
N-[(10Z)-7-Isobutyl-3-isopropyl-5,8-dioxo-2-oxa-6,9-diazabicyclo[10.2.2]hexadeca-1(14),10,12,15-tetraen-4-yl]-
Serotonin
Serotonin
Serotonin
Serotonin
Serotonin
mesifurane_c
mesifurane_c
mesifurane_c
mesifurane_c
mesifurane_c
2,3-Dihydroxypropyl 3,4,5-trihydroxybenzoate
2,3-Dihydroxypropyl 3,4,5-trihydroxybenzoate
2,3-Dihydroxypropyl 3,4,5-trihydroxybenzoate
2,3-Dihydroxypropyl 3,4,5-trihydroxybenzoate
2,3-Dihydroxypropyl 3,4,5-trihydroxybenzoate
methocarbamol
methocarbamol
methocarbamol
methocarbamol
methocarbamol
N-[(2S)-2-Hydroxypropanoyl]-L-tryptophan
N-[(2S)-2-Hydroxypropanoyl]-L-tryptophan
N-[(2S)-2-Hydroxypropanoyl]-L-tryptophan
N-[(2S)-2-Hydroxypropanoyl]-L-tryptophan
N-[(2S)-2-Hydroxypropanoyl]-L-tryptophan
g-Aminobutyryl-lysine_c
g-Aminobutyryl-lysine_c
g-Aminobutyryl-lysine_c
g-Aminobutyryl-lysine_c
g-Aminobutyryl-lysine_c
quinol sulfate_a
quinol sulfate_a
quinol sulfate_a
quinol sulfate_a
quinol sulfate_a
Nitrendipine
Nitrendipine
Nitrendipine
Nitrendipine
Nitrendipine
(2S)-3-(1H-Indol-3-yl)-2-({[(3S,4S,5R)-2,3,4-trihydroxy-5-(hydroxymethyl)tetrahydro-2-furanyl]methyl}amino)prop
(2S)-3-(1H-Indol-3-yl)-2-({[(3S,4S,5R)-2,3,4-trihydroxy-5-(hydroxymethyl)tetrahydro-2-furanyl]methyl}amino)prop

(2S)-3-(1H-Indol-3-yl)-2-({[(3S,4S,5R)-2,3,4-trihydroxy-5-(hydroxymethyl)tetrahydro-2-furanyl]methyl}amino)prop
(2S)-3-(1H-Indol-3-yl)-2-({[(3S,4S,5R)-2,3,4-trihydroxy-5-(hydroxymethyl)tetrahydro-2-furanyl]methyl}amino)prop
(2S)-3-(1H-Indol-3-yl)-2-({[(3S,4S,5R)-2,3,4-trihydroxy-5-(hydroxymethyl)tetrahydro-2-furanyl]methyl}amino)prop
N-D-Glucosylarylamine
N-D-Glucosylarylamine
N-D-Glucosylarylamine
N-D-Glucosylarylamine
N-D-Glucosylarylamine
3,7,12,17-Tetramethyl-21H,23H-porphine-2,18-dipropanoic Acid
3,7,12,17-Tetramethyl-21H,23H-porphine-2,18-dipropanoic Acid
3,7,12,17-Tetramethyl-21H,23H-porphine-2,18-dipropanoic Acid
3,7,12,17-Tetramethyl-21H,23H-porphine-2,18-dipropanoic Acid
3,7,12,17-Tetramethyl-21H,23H-porphine-2,18-dipropanoic Acid
1,2-dihydroxy-3-keto-5-methylthiopentene
1,2-dihydroxy-3-keto-5-methylthiopentene
1,2-dihydroxy-3-keto-5-methylthiopentene
1,2-dihydroxy-3-keto-5-methylthiopentene
1,2-dihydroxy-3-keto-5-methylthiopentene
(5Z)-2-Amino-4,5-dihydroxy-5-{[3-(hydroxymethyl)-2,5-dihydro-2-furanyl]imino}pentanoic acid (non-preferred nar
(5Z)-2-Amino-4,5-dihydroxy-5-{[3-(hydroxymethyl)-2,5-dihydro-2-furanyl]imino}pentanoic acid (non-preferred nar
(5Z)-2-Amino-4,5-dihydroxy-5-{[3-(hydroxymethyl)-2,5-dihydro-2-furanyl]imino}pentanoic acid (non-preferred nar
(5Z)-2-Amino-4,5-dihydroxy-5-{[3-(hydroxymethyl)-2,5-dihydro-2-furanyl]imino}pentanoic acid (non-preferred nar
(5Z)-2-Amino-4,5-dihydroxy-5-{[3-(hydroxymethyl)-2,5-dihydro-2-furanyl]imino}pentanoic acid (non-preferred nar
2-(2-Amino-2-carboxyethyl)-5-oxotetrahydro-2-furancarboxylic acid
2-(2-Amino-2-carboxyethyl)-5-oxotetrahydro-2-furancarboxylic acid
2-(2-Amino-2-carboxyethyl)-5-oxotetrahydro-2-furancarboxylic acid
2-(2-Amino-2-carboxyethyl)-5-oxotetrahydro-2-furancarboxylic acid
2-(2-Amino-2-carboxyethyl)-5-oxotetrahydro-2-furancarboxylic acid
Tetraacetylethylenediamine_a
Tetraacetylethylenediamine_a
Tetraacetylethylenediamine_a
Tetraacetylethylenediamine_a
Tetraacetylethylenediamine_a
4-(METHYLNITROSAMINO)-1-(3-PYRIDYL-N-OXIDE)-1-BUTANONE_a
4-(METHYLNITROSAMINO)-1-(3-PYRIDYL-N-OXIDE)-1-BUTANONE_a
4-(METHYLNITROSAMINO)-1-(3-PYRIDYL-N-OXIDE)-1-BUTANONE_a
4-(METHYLNITROSAMINO)-1-(3-PYRIDYL-N-OXIDE)-1-BUTANONE_a
4-(METHYLNITROSAMINO)-1-(3-PYRIDYL-N-OXIDE)-1-BUTANONE_a
L-gamma-Glutamyl-L-valine_a
L-gamma-Glutamyl-L-valine_a
L-gamma-Glutamyl-L-valine_a
L-gamma-Glutamyl-L-valine_a
L-gamma-Glutamyl-L-valine_a
(19R,25S)-22,25,28,28-Tetrahydroxy-22,28-dioxido-16-oxo-17,21,23,27-tetraoxa-22lambda~5~,28lambda~5~-d
(19R,25S)-22,25,28,28-Tetrahydroxy-22,28-dioxido-16-oxo-17,21,23,27-tetraoxa-22lambda~5~,28lambda~5~-d
(19R,25S)-22,25,28,28-Tetrahydroxy-22,28-dioxido-16-oxo-17,21,23,27-tetraoxa-22lambda~5~,28lambda~5~-d
(19R,25S)-22,25,28,28-Tetrahydroxy-22,28-dioxido-16-oxo-17,21,23,27-tetraoxa-22lambda~5~,28lambda~5~-d
(19R,25S)-22,25,28,28-Tetrahydroxy-22,28-dioxido-16-oxo-17,21,23,27-tetraoxa-22lambda~5~,28lambda~5~-d

1-(4-Methylphenyl)pyrrolidine-2,5-dione
1-(4-Methylphenyl)pyrrolidine-2,5-dione
1-(4-Methylphenyl)pyrrolidine-2,5-dione
1-(4-Methylphenyl)pyrrolidine-2,5-dione
1-(4-Methylphenyl)pyrrolidine-2,5-dione
Selsun
Selsun
Selsun
Selsun
Selsun
Diacetin_a
Diacetin_a
Diacetin_a
Diacetin_a
Diacetin_a
FB9500000_c
FB9500000_c
FB9500000_c
FB9500000_c
FB9500000_c
Coprine_a
Coprine_a
Coprine_a
Coprine_a
Coprine_a
N,N-Dimethyl-L-histidine_c
N,N-Dimethyl-L-histidine_c
N,N-Dimethyl-L-histidine_c
N,N-Dimethyl-L-histidine_c
N,N-Dimethyl-L-histidine_c
N-[(2S)-2-Hydroxypropanoyl]methionine_a
N-[(2S)-2-Hydroxypropanoyl]methionine_a
N-[(2S)-2-Hydroxypropanoyl]methionine_a
N-[(2S)-2-Hydroxypropanoyl]methionine_a
N-[(2S)-2-Hydroxypropanoyl]methionine_a
gamma-Glu-gln
gamma-Glu-gln
gamma-Glu-gln
gamma-Glu-gln
gamma-Glu-gln
(DL)-3-O-Methyldopa_b
(DL)-3-O-Methyldopa_b
(DL)-3-O-Methyldopa_b
(DL)-3-O-Methyldopa_b
(DL)-3-O-Methyldopa_b
vinyl sulfide
vinyl sulfide
vinyl sulfide

vinyl sulfide
vinyl sulfide
Valylvaline_a
Valylvaline_a
Valylvaline_a
Valylvaline_a
Valylvaline_a
1-(2,3-Dihydro-1H-pyrrolizin-5-yl)-1,4-pentanedione
1-(2,3-Dihydro-1H-pyrrolizin-5-yl)-1,4-pentanedione
1-(2,3-Dihydro-1H-pyrrolizin-5-yl)-1,4-pentanedione
1-(2,3-Dihydro-1H-pyrrolizin-5-yl)-1,4-pentanedione
1-(2,3-Dihydro-1H-pyrrolizin-5-yl)-1,4-pentanedione
Glycylleucine
Glycylleucine
Glycylleucine
Glycylleucine
Glycylleucine
O-heptanoylcarnitine
O-heptanoylcarnitine
O-heptanoylcarnitine
O-heptanoylcarnitine
O-heptanoylcarnitine
Tetraacetylethylenediamine_d
Tetraacetylethylenediamine_d
Tetraacetylethylenediamine_d
Tetraacetylethylenediamine_d
Tetraacetylethylenediamine_d
riboprine
riboprine
riboprine
riboprine
riboprine
Midodrine_c
Midodrine_c
Midodrine_c
Midodrine_c
Midodrine_c
Hydroxycarteolol_c
Hydroxycarteolol_c
Hydroxycarteolol_c
Hydroxycarteolol_c
Hydroxycarteolol_c
Val-Ser_a
Val-Ser_a
Val-Ser_a
Val-Ser_a
Val-Ser_a
MFCD09953737_b

MFCD09953737_b
MFCD09953737_b
MFCD09953737_b
MFCD09953737_b
2-Methoxy-3,4,13-trimethyl-12-oxo-11,14-dioxatetracyclo[8.3.1.0~1,10~.0~3,8~]tetradec-5-yl (2E)-3-(methylsul[illegible]
2-Methoxy-3,4,13-trimethyl-12-oxo-11,14-dioxatetracyclo[8.3.1.0~1,10~.0~3,8~]tetradec-5-yl (2E)-3-(methylsul[illegible]
2-Methoxy-3,4,13-trimethyl-12-oxo-11,14-dioxatetracyclo[8.3.1.0~1,10~.0~3,8~]tetradec-5-yl (2E)-3-(methylsul[illegible]
2-Methoxy-3,4,13-trimethyl-12-oxo-11,14-dioxatetracyclo[8.3.1.0~1,10~.0~3,8~]tetradec-5-yl (2E)-3-(methylsul[illegible]
2-Methoxy-3,4,13-trimethyl-12-oxo-11,14-dioxatetracyclo[8.3.1.0~1,10~.0~3,8~]tetradec-5-yl (2E)-3-(methylsul[illegible]
2-Methylbutyrylglycine
2-Methylbutyrylglycine
2-Methylbutyrylglycine
2-Methylbutyrylglycine
2-Methylbutyrylglycine
S-Methyl-1-thio-D-glycerate
S-Methyl-1-thio-D-glycerate
S-Methyl-1-thio-D-glycerate
S-Methyl-1-thio-D-glycerate
S-Methyl-1-thio-D-glycerate
Yangonin
Yangonin
Yangonin
Yangonin
Yangonin
Zalcitabine_c
Zalcitabine_c
Zalcitabine_c
Zalcitabine_c
Zalcitabine_c
alpha-ketoadipic acid
alpha-ketoadipic acid
alpha-ketoadipic acid
alpha-ketoadipic acid
alpha-ketoadipic acid
N-Acetylaspartic acid
N-Acetylaspartic acid
N-Acetylaspartic acid
N-Acetylaspartic acid
N-Acetylaspartic acid
leu-gln_b
leu-gln_b
leu-gln_b
leu-gln_b
leu-gln_b
Nisinic acid_b
Nisinic acid_b
Nisinic acid_b
Nisinic acid_b

Nisinic acid_b
delta-Guanidinovalericacid
delta-Guanidinovalericacid
delta-Guanidinovalericacid
delta-Guanidinovalericacid
delta-Guanidinovalericacid
4-(Nitrosoamino)-1-(3-pyridinyl)-1-butanol_a
4-(Nitrosoamino)-1-(3-pyridinyl)-1-butanol_a
4-(Nitrosoamino)-1-(3-pyridinyl)-1-butanol_a
4-(Nitrosoamino)-1-(3-pyridinyl)-1-butanol_a
4-(Nitrosoamino)-1-(3-pyridinyl)-1-butanol_a
Tetrahydropapaveroline_a
Tetrahydropapaveroline_a
Tetrahydropapaveroline_a
Tetrahydropapaveroline_a
Tetrahydropapaveroline_a
Leucylasparagine_b
Leucylasparagine_b
Leucylasparagine_b
Leucylasparagine_b
Leucylasparagine_b
lys-tyr_a
lys-tyr_a
lys-tyr_a
lys-tyr_a
lys-tyr_a
Glu-Gly
Glu-Gly
Glu-Gly
Glu-Gly
Glu-Gly
His-pro_a
His-pro_a
His-pro_a
His-pro_a
His-pro_a
[3-({3-[(Cyclopropylmethyl)amino]-3-oxetanyl}methyl)-1,2-oxazol-5-yl]methanol
[3-({3-[(Cyclopropylmethyl)amino]-3-oxetanyl}methyl)-1,2-oxazol-5-yl]methanol
[3-({3-[(Cyclopropylmethyl)amino]-3-oxetanyl}methyl)-1,2-oxazol-5-yl]methanol
[3-({3-[(Cyclopropylmethyl)amino]-3-oxetanyl}methyl)-1,2-oxazol-5-yl]methanol
[3-({3-[(Cyclopropylmethyl)amino]-3-oxetanyl}methyl)-1,2-oxazol-5-yl]methanol
N-(4-Amino-1-carboxybutyl)glutamic acid_a
N-(4-Amino-1-carboxybutyl)glutamic acid_a
N-(4-Amino-1-carboxybutyl)glutamic acid_a
N-(4-Amino-1-carboxybutyl)glutamic acid_a
N-(4-Amino-1-carboxybutyl)glutamic acid_a
D-Alanyl-D-alanine
D-Alanyl-D-alanine

D-Alanyl-D-alanine
D-Alanyl-D-alanine
D-Alanyl-D-alanine
(S)-?-glycerophosphorylethanolamine
(S)-?-glycerophosphorylethanolamine
(S)-?-glycerophosphorylethanolamine
(S)-?-glycerophosphorylethanolamine
(S)-?-glycerophosphorylethanolamine
mesifurane_b
mesifurane_b
mesifurane_b
mesifurane_b
mesifurane_b
9-Methyluric acid_c
9-Methyluric acid_c
9-Methyluric acid_c
9-Methyluric acid_c
9-Methyluric acid_c
asn-pro_b
asn-pro_b
asn-pro_b
asn-pro_b
asn-pro_b
3-Hydroxy-3-[(3-methylbutanoyl)oxy]-4-(trimethylammonio)butanoate
3-Hydroxy-3-[(3-methylbutanoyl)oxy]-4-(trimethylammonio)butanoate
3-Hydroxy-3-[(3-methylbutanoyl)oxy]-4-(trimethylammonio)butanoate
3-Hydroxy-3-[(3-methylbutanoyl)oxy]-4-(trimethylammonio)butanoate
3-Hydroxy-3-[(3-methylbutanoyl)oxy]-4-(trimethylammonio)butanoate
NicotinateD-ribonucleoside
NicotinateD-ribonucleoside
NicotinateD-ribonucleoside
NicotinateD-ribonucleoside
NicotinateD-ribonucleoside
Corticosterone
Corticosterone
Corticosterone
Corticosterone
Corticosterone
mesifurane_a
mesifurane_a
mesifurane_a
mesifurane_a
mesifurane_a
(4R)-4-{[3-(2-Aminoethyl)-1H-indol-5-yl]methyl}-1,3-oxazolidin-2-one
(4R)-4-{[3-(2-Aminoethyl)-1H-indol-5-yl]methyl}-1,3-oxazolidin-2-one
(4R)-4-{[3-(2-Aminoethyl)-1H-indol-5-yl]methyl}-1,3-oxazolidin-2-one
(4R)-4-{[3-(2-Aminoethyl)-1H-indol-5-yl]methyl}-1,3-oxazolidin-2-one
(4R)-4-{[3-(2-Aminoethyl)-1H-indol-5-yl]methyl}-1,3-oxazolidin-2-one

Isoquinoline_a
Isoquinoline_a
Isoquinoline_a
Isoquinoline_a
Isoquinoline_a
Propamocarb
Propamocarb
Propamocarb
Propamocarb
Propamocarb
7alpha-Hydroxy-3-oxochol-4-en-24-oic acid_b
7alpha-Hydroxy-3-oxochol-4-en-24-oic acid_b
7alpha-Hydroxy-3-oxochol-4-en-24-oic acid_b
7alpha-Hydroxy-3-oxochol-4-en-24-oic acid_b
7alpha-Hydroxy-3-oxochol-4-en-24-oic acid_b
Leu-Leu_d
Leu-Leu_d
Leu-Leu_d
Leu-Leu_d
Leu-Leu_d
Bis-D-fructose 2',1:2,1'-dianhydride_b
Bis-D-fructose 2',1:2,1'-dianhydride_b
Bis-D-fructose 2',1:2,1'-dianhydride_b
Bis-D-fructose 2',1:2,1'-dianhydride_b
Bis-D-fructose 2',1:2,1'-dianhydride_b
Zalcitabine_e
Zalcitabine_e
Zalcitabine_e
Zalcitabine_e
Zalcitabine_e
trans-Zeatin
trans-Zeatin
trans-Zeatin
trans-Zeatin
trans-Zeatin
Oxprenolol
Oxprenolol
Oxprenolol
Oxprenolol
Oxprenolol
TDP-2_b
TDP-2_b
TDP-2_b
TDP-2_b
TDP-2_b
L-gamma-Glutamyl-L-valine_e
L-gamma-Glutamyl-L-valine_e
L-gamma-Glutamyl-L-valine_e

L-gamma-Glutamyl-L-valine_e
L-gamma-Glutamyl-L-valine_e
N,N-dimethyl-9H-purin-6-amine
N,N-dimethyl-9H-purin-6-amine
N,N-dimethyl-9H-purin-6-amine
N,N-dimethyl-9H-purin-6-amine
N,N-dimethyl-9H-purin-6-amine
MFCD18695608_c
MFCD18695608_c
MFCD18695608_c
MFCD18695608_c
MFCD18695608_c
Caffeic acid
Caffeic acid
Caffeic acid
Caffeic acid
Caffeic acid
Phloionolic acid
Phloionolic acid
Phloionolic acid
Phloionolic acid
Phloionolic acid
Isophthalic acid
Isophthalic acid
Isophthalic acid
Isophthalic acid
Isophthalic acid
Flemichapparin B
Flemichapparin B
Flemichapparin B
Flemichapparin B
Flemichapparin B
Asparaginyl-4-hydroxyproline_b
Asparaginyl-4-hydroxyproline_b
Asparaginyl-4-hydroxyproline_b
Asparaginyl-4-hydroxyproline_b
Asparaginyl-4-hydroxyproline_b
Glycylproline
Glycylproline
Glycylproline
Glycylproline
Glycylproline
Butenylcarnitine
Butenylcarnitine
Butenylcarnitine
Butenylcarnitine
Butenylcarnitine
KYNURAMINE

KYNURAMINE
KYNURAMINE
KYNURAMINE
KYNURAMINE
Spermic acid_c
Spermic acid_c
Spermic acid_c
Spermic acid_c
Spermic acid_c
4-(2,5-Difluorophenyl)-5-(3-isopropyl-[1,2,4]triazolo[4,3-a]pyridin-6-yl)oxazole
4-(2,5-Difluorophenyl)-5-(3-isopropyl-[1,2,4]triazolo[4,3-a]pyridin-6-yl)oxazole
4-(2,5-Difluorophenyl)-5-(3-isopropyl-[1,2,4]triazolo[4,3-a]pyridin-6-yl)oxazole
4-(2,5-Difluorophenyl)-5-(3-isopropyl-[1,2,4]triazolo[4,3-a]pyridin-6-yl)oxazole
4-(2,5-Difluorophenyl)-5-(3-isopropyl-[1,2,4]triazolo[4,3-a]pyridin-6-yl)oxazole
butyrin
butyrin
butyrin
butyrin
butyrin
2-Acetamidohexanedioic acid_b
2-Acetamidohexanedioic acid_b
2-Acetamidohexanedioic acid_b
2-Acetamidohexanedioic acid_b
2-Acetamidohexanedioic acid_b
3,8,9-trihydroxy-10-propyl-3,4,5,8,9,10-hexahydro-2H-oxecin-2-one_b
3,8,9-trihydroxy-10-propyl-3,4,5,8,9,10-hexahydro-2H-oxecin-2-one_b
3,8,9-trihydroxy-10-propyl-3,4,5,8,9,10-hexahydro-2H-oxecin-2-one_b
3,8,9-trihydroxy-10-propyl-3,4,5,8,9,10-hexahydro-2H-oxecin-2-one_b
3,8,9-trihydroxy-10-propyl-3,4,5,8,9,10-hexahydro-2H-oxecin-2-one_b
8-Amino-7-oxononanoic acid_a
8-Amino-7-oxononanoic acid_a
8-Amino-7-oxononanoic acid_a
8-Amino-7-oxononanoic acid_a
8-Amino-7-oxononanoic acid_a
4-Hydroxyprolylleucine_c
4-Hydroxyprolylleucine_c
4-Hydroxyprolylleucine_c
4-Hydroxyprolylleucine_c
4-Hydroxyprolylleucine_c
DNOP_d
DNOP_d
DNOP_d
DNOP_d
DNOP_d
8-Amino-7-oxononanoic acid_b
8-Amino-7-oxononanoic acid_b
8-Amino-7-oxononanoic acid_b
8-Amino-7-oxononanoic acid_b

8-Amino-7-oxononanoic acid_b
N(alpha)-Benzyloxycarbonyl-L-leucine
N(alpha)-Benzyloxycarbonyl-L-leucine
N(alpha)-Benzyloxycarbonyl-L-leucine
N(alpha)-Benzyloxycarbonyl-L-leucine
N(alpha)-Benzyloxycarbonyl-L-leucine
MFCD00025555_c
MFCD00025555_c
MFCD00025555_c
MFCD00025555_c
MFCD00025555_c
APM_b
APM_b
APM_b
APM_b
APM_b
Caffeic acid 3-glucoside
Caffeic acid 3-glucoside
Caffeic acid 3-glucoside
Caffeic acid 3-glucoside
Caffeic acid 3-glucoside
glu-pro
glu-pro
glu-pro
glu-pro
glu-pro
Leu-Val_b
Leu-Val_b
Leu-Val_b
Leu-Val_b
Leu-Val_b
2-Acetamidohexanedioic acid_f
2-Acetamidohexanedioic acid_f
2-Acetamidohexanedioic acid_f
2-Acetamidohexanedioic acid_f
2-Acetamidohexanedioic acid_f
MFCD28369208
MFCD28369208
MFCD28369208
MFCD28369208
MFCD28369208
L-Homocysteic acid
L-Homocysteic acid
L-Homocysteic acid
L-Homocysteic acid
L-Homocysteic acid
Gly-Lys
Gly-Lys

Gly-Lys
Gly-Lys
Gly-Lys
Asparaginyl-4-hydroxyproline_a
Asparaginyl-4-hydroxyproline_a
Asparaginyl-4-hydroxyproline_a
Asparaginyl-4-hydroxyproline_a
Asparaginyl-4-hydroxyproline_a
N-{4-[(2R,3R)-3-(Hydroxymethyl)-4-methyl-5-oxo-2-morpholinyl]phenyl}acetamide
N-{4-[(2R,3R)-3-(Hydroxymethyl)-4-methyl-5-oxo-2-morpholinyl]phenyl}acetamide
N-{4-[(2R,3R)-3-(Hydroxymethyl)-4-methyl-5-oxo-2-morpholinyl]phenyl}acetamide
N-{4-[(2R,3R)-3-(Hydroxymethyl)-4-methyl-5-oxo-2-morpholinyl]phenyl}acetamide
N-{4-[(2R,3R)-3-(Hydroxymethyl)-4-methyl-5-oxo-2-morpholinyl]phenyl}acetamide
Methanesulfonic acid
Methanesulfonic acid
Methanesulfonic acid
Methanesulfonic acid
Methanesulfonic acid
3-(1-hydroxyethyl)-2,3,6,7,8,8a-hexahydropyrrolo[1,2-a]pyrazine-1,4-dione_b
3-(1-hydroxyethyl)-2,3,6,7,8,8a-hexahydropyrrolo[1,2-a]pyrazine-1,4-dione_b
3-(1-hydroxyethyl)-2,3,6,7,8,8a-hexahydropyrrolo[1,2-a]pyrazine-1,4-dione_b
3-(1-hydroxyethyl)-2,3,6,7,8,8a-hexahydropyrrolo[1,2-a]pyrazine-1,4-dione_b
3-(1-hydroxyethyl)-2,3,6,7,8,8a-hexahydropyrrolo[1,2-a]pyrazine-1,4-dione_b
Leupeptin
Leupeptin
Leupeptin
Leupeptin
Leupeptin
4-Acetamidobutanoic acid
4-Acetamidobutanoic acid
4-Acetamidobutanoic acid
4-Acetamidobutanoic acid
4-Acetamidobutanoic acid
Panthenol_a
Panthenol_a
Panthenol_a
Panthenol_a
Panthenol_a
Methyl 1-hydroxy-7-(hydroxymethyl)-1,4a,5,7a-tetrahydrocyclopenta[c]pyran-4-carboxylate_b
Methyl 1-hydroxy-7-(hydroxymethyl)-1,4a,5,7a-tetrahydrocyclopenta[c]pyran-4-carboxylate_b
Methyl 1-hydroxy-7-(hydroxymethyl)-1,4a,5,7a-tetrahydrocyclopenta[c]pyran-4-carboxylate_b
Methyl 1-hydroxy-7-(hydroxymethyl)-1,4a,5,7a-tetrahydrocyclopenta[c]pyran-4-carboxylate_b
Methyl 1-hydroxy-7-(hydroxymethyl)-1,4a,5,7a-tetrahydrocyclopenta[c]pyran-4-carboxylate_b
2,6-Dimethylpyrazine
2,6-Dimethylpyrazine
2,6-Dimethylpyrazine
2,6-Dimethylpyrazine
2,6-Dimethylpyrazine

3-hydroxy-3-methyloxindole_b
3-hydroxy-3-methyloxindole_b
3-hydroxy-3-methyloxindole_b
3-hydroxy-3-methyloxindole_b
3-hydroxy-3-methyloxindole_b
MFCD00142316
MFCD00142316
MFCD00142316
MFCD00142316
MFCD00142316
Leu-Val_d
Leu-Val_d
Leu-Val_d
Leu-Val_d
Leu-Val_d
Spermic acid_d
Spermic acid_d
Spermic acid_d
Spermic acid_d
Spermic acid_d
Lys-Pro_b
Lys-Pro_b
Lys-Pro_b
Lys-Pro_b
Lys-Pro_b
quinol sulfate_b
quinol sulfate_b
quinol sulfate_b
quinol sulfate_b
quinol sulfate_b
N-Butyryl-L-homoserinelactone
N-Butyryl-L-homoserinelactone
N-Butyryl-L-homoserinelactone
N-Butyryl-L-homoserinelactone
N-Butyryl-L-homoserinelactone
4-(METHYLNITROSAMINO)-1-(3-PYRIDYL-N-OXIDE)-1-BUTANOL_c
4-(METHYLNITROSAMINO)-1-(3-PYRIDYL-N-OXIDE)-1-BUTANOL_c
4-(METHYLNITROSAMINO)-1-(3-PYRIDYL-N-OXIDE)-1-BUTANOL_c
4-(METHYLNITROSAMINO)-1-(3-PYRIDYL-N-OXIDE)-1-BUTANOL_c
4-(METHYLNITROSAMINO)-1-(3-PYRIDYL-N-OXIDE)-1-BUTANOL_c
2-Isopropylmalic acid
2-Isopropylmalic acid
2-Isopropylmalic acid
2-Isopropylmalic acid
2-Isopropylmalic acid
Glycitein
Glycitein
Glycitein

Glycitein
Glycitein
Homocitrulline
Homocitrulline
Homocitrulline
Homocitrulline
Homocitrulline
(S)-3-sulfolactic acid
(S)-3-sulfolactic acid
(S)-3-sulfolactic acid
(S)-3-sulfolactic acid
(S)-3-sulfolactic acid
Hydroxycarteolol_a
Hydroxycarteolol_a
Hydroxycarteolol_a
Hydroxycarteolol_a
Hydroxycarteolol_a
Valylvaline_h
Valylvaline_h
Valylvaline_h
Valylvaline_h
Valylvaline_h
Leucyltyrosine_b
Leucyltyrosine_b
Leucyltyrosine_b
Leucyltyrosine_b
Leucyltyrosine_b
L-gamma-Glutamyl-L-valine_b
L-gamma-Glutamyl-L-valine_b
L-gamma-Glutamyl-L-valine_b
L-gamma-Glutamyl-L-valine_b
L-gamma-Glutamyl-L-valine_b
7-Hydroxy-2,11,11-trimethyl-3-oxotricyclo[4.3.2.01,5]undecane-9-carboxylic acid
7-Hydroxy-2,11,11-trimethyl-3-oxotricyclo[4.3.2.01,5]undecane-9-carboxylic acid
7-Hydroxy-2,11,11-trimethyl-3-oxotricyclo[4.3.2.01,5]undecane-9-carboxylic acid
7-Hydroxy-2,11,11-trimethyl-3-oxotricyclo[4.3.2.01,5]undecane-9-carboxylic acid
7-Hydroxy-2,11,11-trimethyl-3-oxotricyclo[4.3.2.01,5]undecane-9-carboxylic acid
Scymnol
Scymnol
Scymnol
Scymnol
Scymnol
Bromazine
Bromazine
Bromazine
Bromazine
Bromazine
4-Methylcatechol

4-Methylcatechol
4-Methylcatechol
4-Methylcatechol
4-Methylcatechol
entecavir
entecavir
entecavir
entecavir
entecavir
N6-METHYLLYSINE_a
N6-METHYLLYSINE_a
N6-METHYLLYSINE_a
N6-METHYLLYSINE_a
N6-METHYLLYSINE_a
feruloylagmatine
feruloylagmatine
feruloylagmatine
feruloylagmatine
feruloylagmatine
Tetraacetylethylenediamine_b
Tetraacetylethylenediamine_b
Tetraacetylethylenediamine_b
Tetraacetylethylenediamine_b
Tetraacetylethylenediamine_b
LW8000000_c
LW8000000_c
LW8000000_c
LW8000000_c
LW8000000_c
3-Hydroxy-3-(2,3,4-trimethoxyphenyl)propanoic acid
3-Hydroxy-3-(2,3,4-trimethoxyphenyl)propanoic acid
3-Hydroxy-3-(2,3,4-trimethoxyphenyl)propanoic acid
3-Hydroxy-3-(2,3,4-trimethoxyphenyl)propanoic acid
3-Hydroxy-3-(2,3,4-trimethoxyphenyl)propanoic acid
MFCD00870462
MFCD00870462
MFCD00870462
MFCD00870462
MFCD00870462
7-Methylxanthine
7-Methylxanthine
7-Methylxanthine
7-Methylxanthine
7-Methylxanthine
Diethylpyrocarbonate_b
Diethylpyrocarbonate_b
Diethylpyrocarbonate_b
Diethylpyrocarbonate_b

Diethylpyrocarbonate_b
1-PYRENYL B-D-GLUCURONIDE
1-PYRENYL B-D-GLUCURONIDE
1-PYRENYL B-D-GLUCURONIDE
1-PYRENYL B-D-GLUCURONIDE
1-PYRENYL B-D-GLUCURONIDE
N-(4-Hydroxy-3,5-dimethoxybenzoyl)glycine_d
N-(4-Hydroxy-3,5-dimethoxybenzoyl)glycine_d
N-(4-Hydroxy-3,5-dimethoxybenzoyl)glycine_d
N-(4-Hydroxy-3,5-dimethoxybenzoyl)glycine_d
N-(4-Hydroxy-3,5-dimethoxybenzoyl)glycine_d
2-Hydroxybutyric acid
2-Hydroxybutyric acid
2-Hydroxybutyric acid
2-Hydroxybutyric acid
2-Hydroxybutyric acid
Sinapyl alcohol
Sinapyl alcohol
Sinapyl alcohol
Sinapyl alcohol
Sinapyl alcohol
MFCD00153469
MFCD00153469
MFCD00153469
MFCD00153469
MFCD00153469
N~6~-Octanoyllysine_a
N~6~-Octanoyllysine_a
N~6~-Octanoyllysine_a
N~6~-Octanoyllysine_a
N~6~-Octanoyllysine_a
pro-gln_a
pro-gln_a
pro-gln_a
pro-gln_a
pro-gln_a
(9cis)-O~15~-[(2S,3R,4R,5S,6R)-6-Carboxy-3,4,5-trihydroxytetrahydro-2H-pyran-2-yl]-2-oxoretinoic acid
(9cis)-O~15~-[(2S,3R,4R,5S,6R)-6-Carboxy-3,4,5-trihydroxytetrahydro-2H-pyran-2-yl]-2-oxoretinoic acid
(9cis)-O~15~-[(2S,3R,4R,5S,6R)-6-Carboxy-3,4,5-trihydroxytetrahydro-2H-pyran-2-yl]-2-oxoretinoic acid
(9cis)-O~15~-[(2S,3R,4R,5S,6R)-6-Carboxy-3,4,5-trihydroxytetrahydro-2H-pyran-2-yl]-2-oxoretinoic acid
(9cis)-O~15~-[(2S,3R,4R,5S,6R)-6-Carboxy-3,4,5-trihydroxytetrahydro-2H-pyran-2-yl]-2-oxoretinoic acid
1,1'-[1,12-Dodecanediylbis(oxy)]dibenzene_d
1,1'-[1,12-Dodecanediylbis(oxy)]dibenzene_d
1,1'-[1,12-Dodecanediylbis(oxy)]dibenzene_d
1,1'-[1,12-Dodecanediylbis(oxy)]dibenzene_d
1,1'-[1,12-Dodecanediylbis(oxy)]dibenzene_d
2-Hydroxy-4-(methylthio)butanoic acid
2-Hydroxy-4-(methylthio)butanoic acid

2-Hydroxy-4-(methylthio)butanoic acid
2-Hydroxy-4-(methylthio)butanoic acid
2-Hydroxy-4-(methylthio)butanoic acid
N-Benzoylaspartic acid_b
N-Benzoylaspartic acid_b
N-Benzoylaspartic acid_b
N-Benzoylaspartic acid_b
N-Benzoylaspartic acid_b
Homoanserine_d
Homoanserine_d
Homoanserine_d
Homoanserine_d
Homoanserine_d
Primaquine
Primaquine
Primaquine
Primaquine
Primaquine
1-(3,4-dimethoxyphenyl)ethan-1-one oxime
1-(3,4-dimethoxyphenyl)ethan-1-one oxime
1-(3,4-dimethoxyphenyl)ethan-1-one oxime
1-(3,4-dimethoxyphenyl)ethan-1-one oxime
1-(3,4-dimethoxyphenyl)ethan-1-one oxime
asn-lys
asn-lys
asn-lys
asn-lys
asn-lys
Leu-Val_c
Leu-Val_c
Leu-Val_c
Leu-Val_c
Leu-Val_c
Tiglic acid_b
Tiglic acid_b
Tiglic acid_b
Tiglic acid_b
Tiglic acid_b
tert-Butyl 3-amino-1-methyl-2,3-dioxopropylcarbamate_d
tert-Butyl 3-amino-1-methyl-2,3-dioxopropylcarbamate_d
tert-Butyl 3-amino-1-methyl-2,3-dioxopropylcarbamate_d
tert-Butyl 3-amino-1-methyl-2,3-dioxopropylcarbamate_d
tert-Butyl 3-amino-1-methyl-2,3-dioxopropylcarbamate_d
Methylol Dimethylhydantoin_c
Methylol Dimethylhydantoin_c
Methylol Dimethylhydantoin_c
Methylol Dimethylhydantoin_c
Methylol Dimethylhydantoin_c

4-(4-Deoxy-alpha-D-gluc-4-enuronosyl)-D-galacturonate
4-(4-Deoxy-alpha-D-gluc-4-enuronosyl)-D-galacturonate
4-(4-Deoxy-alpha-D-gluc-4-enuronosyl)-D-galacturonate
4-(4-Deoxy-alpha-D-gluc-4-enuronosyl)-D-galacturonate
4-(4-Deoxy-alpha-D-gluc-4-enuronosyl)-D-galacturonate
tert-Butyl 3-amino-1-methyl-2,3-dioxopropylcarbamate_e
tert-Butyl 3-amino-1-methyl-2,3-dioxopropylcarbamate_e
tert-Butyl 3-amino-1-methyl-2,3-dioxopropylcarbamate_e
tert-Butyl 3-amino-1-methyl-2,3-dioxopropylcarbamate_e
tert-Butyl 3-amino-1-methyl-2,3-dioxopropylcarbamate_e
Menadiol
Menadiol
Menadiol
Menadiol
Menadiol
N-Acetyl-5-oxo-L-norvaline_b
N-Acetyl-5-oxo-L-norvaline_b
N-Acetyl-5-oxo-L-norvaline_b
N-Acetyl-5-oxo-L-norvaline_b
N-Acetyl-5-oxo-L-norvaline_b
Nicotine glucuronide
Nicotine glucuronide
Nicotine glucuronide
Nicotine glucuronide
Nicotine glucuronide
pentobarbital_f
pentobarbital_f
pentobarbital_f
pentobarbital_f
pentobarbital_f
Agomelatine
Agomelatine
Agomelatine
Agomelatine
Agomelatine
3,4-Dimethoxyphenylacetic acid
3,4-Dimethoxyphenylacetic acid
3,4-Dimethoxyphenylacetic acid
3,4-Dimethoxyphenylacetic acid
3,4-Dimethoxyphenylacetic acid
Pirbuterol
Pirbuterol
Pirbuterol
Pirbuterol
Pirbuterol
Homocysteine thiolactone
Homocysteine thiolactone
Homocysteine thiolactone

Homocysteine thiolactone
Homocysteine thiolactone
Esculin
Esculin
Esculin
Esculin
Esculin
Ectoine
Ectoine
Ectoine
Ectoine
Ectoine
Arg-pro
Arg-pro
Arg-pro
Arg-pro
Arg-pro
Alanyltryptophan
Alanyltryptophan
Alanyltryptophan
Alanyltryptophan
Alanyltryptophan
3-Methoxy-4-hydroxyhippuric acid_b
3-Methoxy-4-hydroxyhippuric acid_b
3-Methoxy-4-hydroxyhippuric acid_b
3-Methoxy-4-hydroxyhippuric acid_b
3-Methoxy-4-hydroxyhippuric acid_b
NSC 92778
NSC 92778
NSC 92778
NSC 92778
NSC 92778
SECONAL_a
SECONAL_a
SECONAL_a
SECONAL_a
SECONAL_a
(1R,3R,5R)-2-[(2S)-2-Amino-2-(3,5-dihydroxyadamantan-1-yl)acetyl]-2-azabicyclo[3.1.0]hexane-3-carbonitrile
(1R,3R,5R)-2-[(2S)-2-Amino-2-(3,5-dihydroxyadamantan-1-yl)acetyl]-2-azabicyclo[3.1.0]hexane-3-carbonitrile
(1R,3R,5R)-2-[(2S)-2-Amino-2-(3,5-dihydroxyadamantan-1-yl)acetyl]-2-azabicyclo[3.1.0]hexane-3-carbonitrile
(1R,3R,5R)-2-[(2S)-2-Amino-2-(3,5-dihydroxyadamantan-1-yl)acetyl]-2-azabicyclo[3.1.0]hexane-3-carbonitrile
(1R,3R,5R)-2-[(2S)-2-Amino-2-(3,5-dihydroxyadamantan-1-yl)acetyl]-2-azabicyclo[3.1.0]hexane-3-carbonitrile
2-[(carboxymethyl)methylamino]-5-methoxy-Benzoic acid_a
2-[(carboxymethyl)methylamino]-5-methoxy-Benzoic acid_a
2-[(carboxymethyl)methylamino]-5-methoxy-Benzoic acid_a
2-[(carboxymethyl)methylamino]-5-methoxy-Benzoic acid_a
2-[(carboxymethyl)methylamino]-5-methoxy-Benzoic acid_a
Zalcitabine_f

Zalcitabine_f
Zalcitabine_f
Zalcitabine_f
Zalcitabine_f
4-Hydroxyprolylleucine_a
4-Hydroxyprolylleucine_a
4-Hydroxyprolylleucine_a
4-Hydroxyprolylleucine_a
4-Hydroxyprolylleucine_a
N-Ethylpropanamide_b
N-Ethylpropanamide_b
N-Ethylpropanamide_b
N-Ethylpropanamide_b
N-Ethylpropanamide_b
Paraldehyde
Paraldehyde
Paraldehyde
Paraldehyde
Paraldehyde
pimethixene
pimethixene
pimethixene
pimethixene
pimethixene
Octyl benzoate
Octyl benzoate
Octyl benzoate
Octyl benzoate
Octyl benzoate
7Î±-Hydroxytestosterone
7Î±-Hydroxytestosterone
7Î±-Hydroxytestosterone
7Î±-Hydroxytestosterone
7Î±-Hydroxytestosterone
Allyl mercaptan
Allyl mercaptan
Allyl mercaptan
Allyl mercaptan
Allyl mercaptan
N-Pentanoylphenylalanine_a
N-Pentanoylphenylalanine_a
N-Pentanoylphenylalanine_a
N-Pentanoylphenylalanine_a
N-Pentanoylphenylalanine_a
Piperidine_c
Piperidine_c
Piperidine_c
Piperidine_c

Piperidine_c
6-Hydroxypentadecanedioic acid
6-Hydroxypentadecanedioic acid
6-Hydroxypentadecanedioic acid
6-Hydroxypentadecanedioic acid
6-Hydroxypentadecanedioic acid
2-Methylthiazolidine_a
2-Methylthiazolidine_a
2-Methylthiazolidine_a
2-Methylthiazolidine_a
2-Methylthiazolidine_a
O-succinylcarnitine
O-succinylcarnitine
O-succinylcarnitine
O-succinylcarnitine
O-succinylcarnitine
Valylproline
Valylproline
Valylproline
Valylproline
Valylproline
Leu-Leu_c
Leu-Leu_c
Leu-Leu_c
Leu-Leu_c
Leu-Leu_c
asn-val_a
asn-val_a
asn-val_a
asn-val_a
asn-val_a
Hyodeoxycholic acid
Hyodeoxycholic acid
Hyodeoxycholic acid
Hyodeoxycholic acid
Hyodeoxycholic acid
N-Pentanoylphenylalanine_b
N-Pentanoylphenylalanine_b
N-Pentanoylphenylalanine_b
N-Pentanoylphenylalanine_b
N-Pentanoylphenylalanine_b
N-LACTOYL ETHANOLAMINE PHOSPHATE
N-LACTOYL ETHANOLAMINE PHOSPHATE
N-LACTOYL ETHANOLAMINE PHOSPHATE
N-LACTOYL ETHANOLAMINE PHOSPHATE
N-LACTOYL ETHANOLAMINE PHOSPHATE
heptabarbital
heptabarbital

heptabarbital
heptabarbital
heptabarbital
Pregabalin
Pregabalin
Pregabalin
Pregabalin
Pregabalin
Dibutyl malate
Dibutyl malate
Dibutyl malate
Dibutyl malate
Dibutyl malate
Lysylvaline_b
Lysylvaline_b
Lysylvaline_b
Lysylvaline_b
Lysylvaline_b
MFCD02728197_b
MFCD02728197_b
MFCD02728197_b
MFCD02728197_b
MFCD02728197_b
Sulfoacetate
Sulfoacetate
Sulfoacetate
Sulfoacetate
Sulfoacetate
Zalcitabine_a
Zalcitabine_a
Zalcitabine_a
Zalcitabine_a
Zalcitabine_a
Leu-Val_f
Leu-Val_f
Leu-Val_f
Leu-Val_f
Leu-Val_f
N~5~-[P-Amino-P-(sulfoamino)phosphorimidoyl]-L-ornithyl-L-alanyl-L-arginine
N~5~-[P-Amino-P-(sulfoamino)phosphorimidoyl]-L-ornithyl-L-alanyl-L-arginine
N~5~-[P-Amino-P-(sulfoamino)phosphorimidoyl]-L-ornithyl-L-alanyl-L-arginine
N~5~-[P-Amino-P-(sulfoamino)phosphorimidoyl]-L-ornithyl-L-alanyl-L-arginine
N~5~-[P-Amino-P-(sulfoamino)phosphorimidoyl]-L-ornithyl-L-alanyl-L-arginine
4-Phenolsulfonic acid
4-Phenolsulfonic acid
4-Phenolsulfonic acid
4-Phenolsulfonic acid
4-Phenolsulfonic acid

hexobarbital_d
hexobarbital_d
hexobarbital_d
hexobarbital_d
hexobarbital_d
N,N-Diethyl-4-methyl-1-piperazinecarboxamide 4-oxide_c
N,N-Diethyl-4-methyl-1-piperazinecarboxamide 4-oxide_c
N,N-Diethyl-4-methyl-1-piperazinecarboxamide 4-oxide_c
N,N-Diethyl-4-methyl-1-piperazinecarboxamide 4-oxide_c
N,N-Diethyl-4-methyl-1-piperazinecarboxamide 4-oxide_c
meprobamate_e
meprobamate_e
meprobamate_e
meprobamate_e
meprobamate_e
Naphthalen-2-amine
Naphthalen-2-amine
Naphthalen-2-amine
Naphthalen-2-amine
Naphthalen-2-amine
N-Acetyl-5-oxo-L-norvaline_a
N-Acetyl-5-oxo-L-norvaline_a
N-Acetyl-5-oxo-L-norvaline_a
N-Acetyl-5-oxo-L-norvaline_a
N-Acetyl-5-oxo-L-norvaline_a
pro-gln_b
pro-gln_b
pro-gln_b
pro-gln_b
pro-gln_b
L-(+)-Erythrulose_b
L-(+)-Erythrulose_b
L-(+)-Erythrulose_b
L-(+)-Erythrulose_b
L-(+)-Erythrulose_b
N-(1-Methyl-4-oxo-4,5-dihydro-1H-imidazol-2-yl)alanine_a
N-(1-Methyl-4-oxo-4,5-dihydro-1H-imidazol-2-yl)alanine_a
N-(1-Methyl-4-oxo-4,5-dihydro-1H-imidazol-2-yl)alanine_a
N-(1-Methyl-4-oxo-4,5-dihydro-1H-imidazol-2-yl)alanine_a
N-(1-Methyl-4-oxo-4,5-dihydro-1H-imidazol-2-yl)alanine_a
2,4-Quinolinediol_a
2,4-Quinolinediol_a
2,4-Quinolinediol_a
2,4-Quinolinediol_a
2,4-Quinolinediol_a
pentobarbital_a
pentobarbital_a
pentobarbital_a

pentobarbital_a
pentobarbital_a
3,3-Dimethylglutaric acid_a
3,3-Dimethylglutaric acid_a
3,3-Dimethylglutaric acid_a
3,3-Dimethylglutaric acid_a
3,3-Dimethylglutaric acid_a
Glycylprolylhydroxyproline_b
Glycylprolylhydroxyproline_b
Glycylprolylhydroxyproline_b
Glycylprolylhydroxyproline_b
Glycylprolylhydroxyproline_b
threonylphenylalanine_a
threonylphenylalanine_a
threonylphenylalanine_a
threonylphenylalanine_a
threonylphenylalanine_a
2-methylcitric acid
2-methylcitric acid
2-methylcitric acid
2-methylcitric acid
2-methylcitric acid
Leucylasparagine_c
Leucylasparagine_c
Leucylasparagine_c
Leucylasparagine_c
Leucylasparagine_c
(7E,7'E)-5,5'-diferulic acid_a
(7E,7'E)-5,5'-diferulic acid_a
(7E,7'E)-5,5'-diferulic acid_a
(7E,7'E)-5,5'-diferulic acid_a
(7E,7'E)-5,5'-diferulic acid_a
3,3-Dimethylglutaric acid_b
3,3-Dimethylglutaric acid_b
3,3-Dimethylglutaric acid_b
3,3-Dimethylglutaric acid_b
3,3-Dimethylglutaric acid_b
(3aR,4R,5R,6aS)-5-Hydroxy-4-[(1E,3S)-3-hydroxy-1-octen-1-yl]hexahydro-2H-cyclopenta[b]furan-2-one
(3aR,4R,5R,6aS)-5-Hydroxy-4-[(1E,3S)-3-hydroxy-1-octen-1-yl]hexahydro-2H-cyclopenta[b]furan-2-one
(3aR,4R,5R,6aS)-5-Hydroxy-4-[(1E,3S)-3-hydroxy-1-octen-1-yl]hexahydro-2H-cyclopenta[b]furan-2-one
(3aR,4R,5R,6aS)-5-Hydroxy-4-[(1E,3S)-3-hydroxy-1-octen-1-yl]hexahydro-2H-cyclopenta[b]furan-2-one
(3aR,4R,5R,6aS)-5-Hydroxy-4-[(1E,3S)-3-hydroxy-1-octen-1-yl]hexahydro-2H-cyclopenta[b]furan-2-one
5-(2-Carboxyethyl)-4-hydroxy-6-oxo-5,6-dihydro-2-pyridinecarboxylic acid
5-(2-Carboxyethyl)-4-hydroxy-6-oxo-5,6-dihydro-2-pyridinecarboxylic acid
5-(2-Carboxyethyl)-4-hydroxy-6-oxo-5,6-dihydro-2-pyridinecarboxylic acid
5-(2-Carboxyethyl)-4-hydroxy-6-oxo-5,6-dihydro-2-pyridinecarboxylic acid
5-(2-Carboxyethyl)-4-hydroxy-6-oxo-5,6-dihydro-2-pyridinecarboxylic acid
5-Phospho-beta-D-ribosylamine

5-Phospho-beta-D-ribosylamine
5-Phospho-beta-D-ribosylamine
5-Phospho-beta-D-ribosylamine
5-Phospho-beta-D-ribosylamine
8-Hydroxyhexadecanedioic acid
8-Hydroxyhexadecanedioic acid
8-Hydroxyhexadecanedioic acid
8-Hydroxyhexadecanedioic acid
8-Hydroxyhexadecanedioic acid
2-(Carboxyacetamido)benzoic acid_b
2-(Carboxyacetamido)benzoic acid_b
2-(Carboxyacetamido)benzoic acid_b
2-(Carboxyacetamido)benzoic acid_b
2-(Carboxyacetamido)benzoic acid_b
6-Acetamido-2-oxohexanoic acid
6-Acetamido-2-oxohexanoic acid
6-Acetamido-2-oxohexanoic acid
6-Acetamido-2-oxohexanoic acid
6-Acetamido-2-oxohexanoic acid
Triacetin
Triacetin
Triacetin
Triacetin
Triacetin
Nicotinic acid ribonucleoside
Nicotinic acid ribonucleoside
Nicotinic acid ribonucleoside
Nicotinic acid ribonucleoside
Nicotinic acid ribonucleoside
3,4,15-Trihydroxy-12,13-epoxytrichothec-9-en-8-yl 3-methylbutanoate
3,4,15-Trihydroxy-12,13-epoxytrichothec-9-en-8-yl 3-methylbutanoate
3,4,15-Trihydroxy-12,13-epoxytrichothec-9-en-8-yl 3-methylbutanoate
3,4,15-Trihydroxy-12,13-epoxytrichothec-9-en-8-yl 3-methylbutanoate
3,4,15-Trihydroxy-12,13-epoxytrichothec-9-en-8-yl 3-methylbutanoate
Rivastigmine
Rivastigmine
Rivastigmine
Rivastigmine
Rivastigmine
asn-pro_d
asn-pro_d
asn-pro_d
asn-pro_d
asn-pro_d
Aspartyl-L-proline_a
Aspartyl-L-proline_a
Aspartyl-L-proline_a
Aspartyl-L-proline_a

Aspartyl-L-proline_a
2,2-Bis(hydroxymethyl)propionic acid_b
2,2-Bis(hydroxymethyl)propionic acid_b
2,2-Bis(hydroxymethyl)propionic acid_b
2,2-Bis(hydroxymethyl)propionic acid_b
2,2-Bis(hydroxymethyl)propionic acid_b
Threonylserine
Threonylserine
Threonylserine
Threonylserine
Threonylserine
(âˆ’)-nabilone_b
(âˆ’)-nabilone_b
(âˆ’)-nabilone_b
(âˆ’)-nabilone_b
(âˆ’)-nabilone_b
Phenyl D-glucopyranosiduronic acid_a
Phenyl D-glucopyranosiduronic acid_a
Phenyl D-glucopyranosiduronic acid_a
Phenyl D-glucopyranosiduronic acid_a
Phenyl D-glucopyranosiduronic acid_a
porphobilinogen_a
porphobilinogen_a
porphobilinogen_a
porphobilinogen_a
porphobilinogen_a
Bis-D-fructose 2',1:2,1'-dianhydride_a
Bis-D-fructose 2',1:2,1'-dianhydride_a
Bis-D-fructose 2',1:2,1'-dianhydride_a
Bis-D-fructose 2',1:2,1'-dianhydride_a
Bis-D-fructose 2',1:2,1'-dianhydride_a
Hydroxypropionylcarnitine_a
Hydroxypropionylcarnitine_a
Hydroxypropionylcarnitine_a
Hydroxypropionylcarnitine_a
Hydroxypropionylcarnitine_a
Homoanserine_a
Homoanserine_a
Homoanserine_a
Homoanserine_a
Homoanserine_a
Tranexamic acid
Tranexamic acid
Tranexamic acid
Tranexamic acid
Tranexamic acid
Chenodeoxycholic acid
Chenodeoxycholic acid

Chenodeoxycholic acid
Chenodeoxycholic acid
Chenodeoxycholic acid
2,2-Bis(hydroxymethyl)propionic acid_a
2,2-Bis(hydroxymethyl)propionic acid_a
2,2-Bis(hydroxymethyl)propionic acid_a
2,2-Bis(hydroxymethyl)propionic acid_a
2,2-Bis(hydroxymethyl)propionic acid_a
Î´-Valerolactam_c
Î´-Valerolactam_c
Î´-Valerolactam_c
Î´-Valerolactam_c
Î´-Valerolactam_c
DNOP_h
DNOP_h
DNOP_h
DNOP_h
DNOP_h
Rutinose (beta-anomer)
Rutinose (beta-anomer)
Rutinose (beta-anomer)
Rutinose (beta-anomer)
Rutinose (beta-anomer)
N-Acetylasparagine
N-Acetylasparagine
N-Acetylasparagine
N-Acetylasparagine
N-Acetylasparagine
N6-METHYLLYSINE_b
N6-METHYLLYSINE_b
N6-METHYLLYSINE_b
N6-METHYLLYSINE_b
N6-METHYLLYSINE_b
DNOP_c
DNOP_c
DNOP_c
DNOP_c
DNOP_c
N'-Hydroxy-4-pentylbenzenecarboximidamide
N'-Hydroxy-4-pentylbenzenecarboximidamide
N'-Hydroxy-4-pentylbenzenecarboximidamide
N'-Hydroxy-4-pentylbenzenecarboximidamide
N'-Hydroxy-4-pentylbenzenecarboximidamide
4-pyridoxic acid
4-pyridoxic acid
4-pyridoxic acid
4-pyridoxic acid
4-pyridoxic acid

coenzyme Q2
coenzyme Q2
coenzyme Q2
coenzyme Q2
coenzyme Q2
Butylparaben
Butylparaben
Butylparaben
Butylparaben
Butylparaben
3-(Sulfooxy)butanoic acid
3-(Sulfooxy)butanoic acid
3-(Sulfooxy)butanoic acid
3-(Sulfooxy)butanoic acid
3-(Sulfooxy)butanoic acid
Valylvaline_e
Valylvaline_e
Valylvaline_e
Valylvaline_e
Valylvaline_e
4-Amino-1-piperidinecarboxylic acid
4-Amino-1-piperidinecarboxylic acid
4-Amino-1-piperidinecarboxylic acid
4-Amino-1-piperidinecarboxylic acid
4-Amino-1-piperidinecarboxylic acid
2,4-Quinolinediol_b
2,4-Quinolinediol_b
2,4-Quinolinediol_b
2,4-Quinolinediol_b
2,4-Quinolinediol_b
Glucosamine
Glucosamine
Glucosamine
Glucosamine
Glucosamine
1-pyrroline
1-pyrroline
1-pyrroline
1-pyrroline
1-pyrroline
TO0127900_b
TO0127900_b
TO0127900_b
TO0127900_b
TO0127900_b
1,1'-[1,12-Dodecanediylbis(oxy)]dibenzene_g
1,1'-[1,12-Dodecanediylbis(oxy)]dibenzene_g
1,1'-[1,12-Dodecanediylbis(oxy)]dibenzene_g

1,1'-[1,12-Dodecanediylbis(oxy)]dibenzene_g
1,1'-[1,12-Dodecanediylbis(oxy)]dibenzene_g
2'-Deoxy-5-hydroxymethylcytidine-5'-diphosphate
2'-Deoxy-5-hydroxymethylcytidine-5'-diphosphate
2'-Deoxy-5-hydroxymethylcytidine-5'-diphosphate
2'-Deoxy-5-hydroxymethylcytidine-5'-diphosphate
2'-Deoxy-5-hydroxymethylcytidine-5'-diphosphate
4-Hydroxybenzaldehyde
4-Hydroxybenzaldehyde
4-Hydroxybenzaldehyde
4-Hydroxybenzaldehyde
4-Hydroxybenzaldehyde
Naringenin
Naringenin
Naringenin
Naringenin
Naringenin
MFCD00025555_b
MFCD00025555_b
MFCD00025555_b
MFCD00025555_b
MFCD00025555_b
Oxypeucedanin
Oxypeucedanin
Oxypeucedanin
Oxypeucedanin
Oxypeucedanin
1-Vinylimidazole_a
1-Vinylimidazole_a
1-Vinylimidazole_a
1-Vinylimidazole_a
1-Vinylimidazole_a
4-Acetamidobenzaldehyde
4-Acetamidobenzaldehyde
4-Acetamidobenzaldehyde
4-Acetamidobenzaldehyde
4-Acetamidobenzaldehyde
Procaine_a
Procaine_a
Procaine_a
Procaine_a
Procaine_a
Piperidine_b
Piperidine_b
Piperidine_b
Piperidine_b
Piperidine_b
4-{[(3-Hydroxy-3-methylbutanoyl)oxy]methyl}-1-[(3-methylbutanoyl)oxy]-6,7a-dihydro-1H-spiro[cyclopenta[c]pyr[illegible]

4-{[(3-Hydroxy-3-methylbutanoyl)oxy]methyl}-1-[(3-methylbutanoyl)oxy]-6,7a-dihydro-1H-spiro[cyclopenta[c]pyra
4-{[(3-Hydroxy-3-methylbutanoyl)oxy]methyl}-1-[(3-methylbutanoyl)oxy]-6,7a-dihydro-1H-spiro[cyclopenta[c]pyra
4-{[(3-Hydroxy-3-methylbutanoyl)oxy]methyl}-1-[(3-methylbutanoyl)oxy]-6,7a-dihydro-1H-spiro[cyclopenta[c]pyra
4-{[(3-Hydroxy-3-methylbutanoyl)oxy]methyl}-1-[(3-methylbutanoyl)oxy]-6,7a-dihydro-1H-spiro[cyclopenta[c]pyra
(âˆ’)-nabilone_e
(âˆ’)-nabilone_e
(âˆ’)-nabilone_e
(âˆ’)-nabilone_e
(âˆ’)-nabilone_e
L-(+)-Erythrulose_a
L-(+)-Erythrulose_a
L-(+)-Erythrulose_a
L-(+)-Erythrulose_a
L-(+)-Erythrulose_a
(+/-)-2-Hydroxyglutaric acid_a
(+/-)-2-Hydroxyglutaric acid_a
(+/-)-2-Hydroxyglutaric acid_a
(+/-)-2-Hydroxyglutaric acid_a
(+/-)-2-Hydroxyglutaric acid_a
Pyrrolidine
Pyrrolidine
Pyrrolidine
Pyrrolidine
Pyrrolidine
Ursodeoxycholic acid
Ursodeoxycholic acid
Ursodeoxycholic acid
Ursodeoxycholic acid
Ursodeoxycholic acid
Prolinamide
Prolinamide
Prolinamide
Prolinamide
Prolinamide
1,7-Dimethyluric acid
1,7-Dimethyluric acid
1,7-Dimethyluric acid
1,7-Dimethyluric acid
1,7-Dimethyluric acid
Alanine
Alanine
Alanine
Alanine
Alanine
alpha-Chaconine
alpha-Chaconine
alpha-Chaconine
alpha-Chaconine

alpha-Chaconine
Homoanserine_e
Homoanserine_e
Homoanserine_e
Homoanserine_e
Homoanserine_e
Saccharin
Saccharin
Saccharin
Saccharin
Saccharin
Ethyl sulfate
Ethyl sulfate
Ethyl sulfate
Ethyl sulfate
Ethyl sulfate
Leucylproline
Leucylproline
Leucylproline
Leucylproline
Leucylproline
Xanthine
Xanthine
Xanthine
Xanthine
Xanthine
5-Hydroxyindole
5-Hydroxyindole
5-Hydroxyindole
5-Hydroxyindole
5-Hydroxyindole
5-Allyl-5-sec-butyl-1,3-dimethyl-2,4,6(1H,3H,5H)-pyrimidinetrione_a
5-Allyl-5-sec-butyl-1,3-dimethyl-2,4,6(1H,3H,5H)-pyrimidinetrione_a
5-Allyl-5-sec-butyl-1,3-dimethyl-2,4,6(1H,3H,5H)-pyrimidinetrione_a
5-Allyl-5-sec-butyl-1,3-dimethyl-2,4,6(1H,3H,5H)-pyrimidinetrione_a
5-Allyl-5-sec-butyl-1,3-dimethyl-2,4,6(1H,3H,5H)-pyrimidinetrione_a
8-(3-Furyl)-5-hydroxy-1,1,5a,7a,11b-pentamethyldecahydrooxireno[4,4a]isochromeno[6,5-g][2]benzoxepine-3,1[illegible]
8-(3-Furyl)-5-hydroxy-1,1,5a,7a,11b-pentamethyldecahydrooxireno[4,4a]isochromeno[6,5-g][2]benzoxepine-3,1[illegible]
8-(3-Furyl)-5-hydroxy-1,1,5a,7a,11b-pentamethyldecahydrooxireno[4,4a]isochromeno[6,5-g][2]benzoxepine-3,1[illegible]
8-(3-Furyl)-5-hydroxy-1,1,5a,7a,11b-pentamethyldecahydrooxireno[4,4a]isochromeno[6,5-g][2]benzoxepine-3,1[illegible]
8-(3-Furyl)-5-hydroxy-1,1,5a,7a,11b-pentamethyldecahydrooxireno[4,4a]isochromeno[6,5-g][2]benzoxepine-3,1[illegible]
7-ketodeoxycholic acid_b
7-ketodeoxycholic acid_b
7-ketodeoxycholic acid_b
7-ketodeoxycholic acid_b
7-ketodeoxycholic acid_b
2-[(Sulfooxy)methyl]butanoic acid
2-[(Sulfooxy)methyl]butanoic acid

2-[(Sulfooxy)methyl]butanoic acid
2-[(Sulfooxy)methyl]butanoic acid
2-[(Sulfooxy)methyl]butanoic acid
5-Allyl-5-sec-butyl-1,3-dimethyl-2,4,6(1H,3H,5H)-pyrimidinetrione_b
5-Allyl-5-sec-butyl-1,3-dimethyl-2,4,6(1H,3H,5H)-pyrimidinetrione_b
5-Allyl-5-sec-butyl-1,3-dimethyl-2,4,6(1H,3H,5H)-pyrimidinetrione_b
5-Allyl-5-sec-butyl-1,3-dimethyl-2,4,6(1H,3H,5H)-pyrimidinetrione_b
5-Allyl-5-sec-butyl-1,3-dimethyl-2,4,6(1H,3H,5H)-pyrimidinetrione_b
N-Pentanoylphenylalanine_c
N-Pentanoylphenylalanine_c
N-Pentanoylphenylalanine_c
N-Pentanoylphenylalanine_c
N-Pentanoylphenylalanine_c
N-Acetylvaline_d
N-Acetylvaline_d
N-Acetylvaline_d
N-Acetylvaline_d
N-Acetylvaline_d
4-methylpyridine-3-sulfonic acid
4-methylpyridine-3-sulfonic acid
4-methylpyridine-3-sulfonic acid
4-methylpyridine-3-sulfonic acid
4-methylpyridine-3-sulfonic acid
5-amino-2-(dimethylamino)benzoic acid_a
5-amino-2-(dimethylamino)benzoic acid_a
5-amino-2-(dimethylamino)benzoic acid_a
5-amino-2-(dimethylamino)benzoic acid_a
5-amino-2-(dimethylamino)benzoic acid_a
p-Cresylsulfate
p-Cresylsulfate
p-Cresylsulfate
p-Cresylsulfate
p-Cresylsulfate
Asp-lys
Asp-lys
Asp-lys
Asp-lys
Asp-lys
1-(4-Aminobutyl)urea_a
1-(4-Aminobutyl)urea_a
1-(4-Aminobutyl)urea_a
1-(4-Aminobutyl)urea_a
1-(4-Aminobutyl)urea_a
N-(3-acetamidopropyl)pyrrolidin-2-one_d
N-(3-acetamidopropyl)pyrrolidin-2-one_d
N-(3-acetamidopropyl)pyrrolidin-2-one_d
N-(3-acetamidopropyl)pyrrolidin-2-one_d
N-(3-acetamidopropyl)pyrrolidin-2-one_d

Methionine sulfoxide
Methionine sulfoxide
Methionine sulfoxide
Methionine sulfoxide
Methionine sulfoxide
3,8,9-trihydroxy-10-propyl-3,4,5,8,9,10-hexahydro-2H-oxecin-2-one_a
3,8,9-trihydroxy-10-propyl-3,4,5,8,9,10-hexahydro-2H-oxecin-2-one_a
3,8,9-trihydroxy-10-propyl-3,4,5,8,9,10-hexahydro-2H-oxecin-2-one_a
3,8,9-trihydroxy-10-propyl-3,4,5,8,9,10-hexahydro-2H-oxecin-2-one_a
3,8,9-trihydroxy-10-propyl-3,4,5,8,9,10-hexahydro-2H-oxecin-2-one_a
7-ketodeoxycholic acid_d
7-ketodeoxycholic acid_d
7-ketodeoxycholic acid_d
7-ketodeoxycholic acid_d
7-ketodeoxycholic acid_d
O-propenoyl-D-carnitine
O-propenoyl-D-carnitine
O-propenoyl-D-carnitine
O-propenoyl-D-carnitine
O-propenoyl-D-carnitine
(2Z)-2-({[(6S,7S)-7-Hydroxy-2,3,10,11,12-pentamethoxy-6,7-dimethyl-5,6,7,8-tetrahydrodibenzo[a,c][8]annulen-
(2Z)-2-({[(6S,7S)-7-Hydroxy-2,3,10,11,12-pentamethoxy-6,7-dimethyl-5,6,7,8-tetrahydrodibenzo[a,c][8]annulen-
(2Z)-2-({[(6S,7S)-7-Hydroxy-2,3,10,11,12-pentamethoxy-6,7-dimethyl-5,6,7,8-tetrahydrodibenzo[a,c][8]annulen-
(2Z)-2-({[(6S,7S)-7-Hydroxy-2,3,10,11,12-pentamethoxy-6,7-dimethyl-5,6,7,8-tetrahydrodibenzo[a,c][8]annulen-
(2Z)-2-({[(6S,7S)-7-Hydroxy-2,3,10,11,12-pentamethoxy-6,7-dimethyl-5,6,7,8-tetrahydrodibenzo[a,c][8]annulen-
Genistein
Genistein
Genistein
Genistein
Genistein
NL8513000
NL8513000
NL8513000
NL8513000
NL8513000
Docosahexaenoic acid ethyl ester_a
Docosahexaenoic acid ethyl ester_a
Docosahexaenoic acid ethyl ester_a
Docosahexaenoic acid ethyl ester_a
Docosahexaenoic acid ethyl ester_a
Î´-Gluconolactone
Î´-Gluconolactone
Î´-Gluconolactone
Î´-Gluconolactone
Î´-Gluconolactone
Lysine
Lysine
Lysine

Lysine
Lysine
(2E)-3-(3,4-dimethoxyphenyl)prop-2-enoic acid
(2E)-3-(3,4-dimethoxyphenyl)prop-2-enoic acid
(2E)-3-(3,4-dimethoxyphenyl)prop-2-enoic acid
(2E)-3-(3,4-dimethoxyphenyl)prop-2-enoic acid
(2E)-3-(3,4-dimethoxyphenyl)prop-2-enoic acid
Indole-3-acetic acid
Indole-3-acetic acid
Indole-3-acetic acid
Indole-3-acetic acid
Indole-3-acetic acid
DNOP_a
DNOP_a
DNOP_a
DNOP_a
DNOP_a
N-(1-Methyl-4-oxo-4,5-dihydro-1H-imidazol-2-yl)alanine_b
N-(1-Methyl-4-oxo-4,5-dihydro-1H-imidazol-2-yl)alanine_b
N-(1-Methyl-4-oxo-4,5-dihydro-1H-imidazol-2-yl)alanine_b
N-(1-Methyl-4-oxo-4,5-dihydro-1H-imidazol-2-yl)alanine_b
N-(1-Methyl-4-oxo-4,5-dihydro-1H-imidazol-2-yl)alanine_b
3-Benzyl-6-isobutyl-2,5-piperazinedione_a
3-Benzyl-6-isobutyl-2,5-piperazinedione_a
3-Benzyl-6-isobutyl-2,5-piperazinedione_a
3-Benzyl-6-isobutyl-2,5-piperazinedione_a
3-Benzyl-6-isobutyl-2,5-piperazinedione_a
3-O-beta-D-galactosyl-sn-glycerol
3-O-beta-D-galactosyl-sn-glycerol
3-O-beta-D-galactosyl-sn-glycerol
3-O-beta-D-galactosyl-sn-glycerol
3-O-beta-D-galactosyl-sn-glycerol
paracetamol sulfate
paracetamol sulfate
paracetamol sulfate
paracetamol sulfate
paracetamol sulfate
3-[4-methyl-1-(2-methylpropanoyl)-3-oxocyclohexyl]butanoic acid
3-[4-methyl-1-(2-methylpropanoyl)-3-oxocyclohexyl]butanoic acid
3-[4-methyl-1-(2-methylpropanoyl)-3-oxocyclohexyl]butanoic acid
3-[4-methyl-1-(2-methylpropanoyl)-3-oxocyclohexyl]butanoic acid
3-[4-methyl-1-(2-methylpropanoyl)-3-oxocyclohexyl]butanoic acid
N-Acetylvaline_a
N-Acetylvaline_a
N-Acetylvaline_a
N-Acetylvaline_a
N-Acetylvaline_a
D-Alanine methyl ester

D-Alanine methyl ester
D-Alanine methyl ester
D-Alanine methyl ester
D-Alanine methyl ester
APM_c
APM_c
APM_c
APM_c
APM_c
3-(2-Oxo-2,3-dihydro-1,3-benzoxazol-3-yl)propanoic acid_a
3-(2-Oxo-2,3-dihydro-1,3-benzoxazol-3-yl)propanoic acid_a
3-(2-Oxo-2,3-dihydro-1,3-benzoxazol-3-yl)propanoic acid_a
3-(2-Oxo-2,3-dihydro-1,3-benzoxazol-3-yl)propanoic acid_a
3-(2-Oxo-2,3-dihydro-1,3-benzoxazol-3-yl)propanoic acid_a
Diethylpyrocarbonate_a
Diethylpyrocarbonate_a
Diethylpyrocarbonate_a
Diethylpyrocarbonate_a
Diethylpyrocarbonate_a
(DL)-3-O-Methyldopa_a
(DL)-3-O-Methyldopa_a
(DL)-3-O-Methyldopa_a
(DL)-3-O-Methyldopa_a
(DL)-3-O-Methyldopa_a
Lovastatin_a
Lovastatin_a
Lovastatin_a
Lovastatin_a
Lovastatin_a
hexobarbital_e
hexobarbital_e
hexobarbital_e
hexobarbital_e
hexobarbital_e
(S)-2-methylbutanal
(S)-2-methylbutanal
(S)-2-methylbutanal
(S)-2-methylbutanal
(S)-2-methylbutanal
Piperine
Piperine
Piperine
Piperine
Piperine
Caprolactam
Caprolactam
Caprolactam
Caprolactam

Caprolactam
7alpha-Hydroxy-3-oxochol-4-en-24-oic acid_c
7alpha-Hydroxy-3-oxochol-4-en-24-oic acid_c
7alpha-Hydroxy-3-oxochol-4-en-24-oic acid_c
7alpha-Hydroxy-3-oxochol-4-en-24-oic acid_c
7alpha-Hydroxy-3-oxochol-4-en-24-oic acid_c
Arginine
Arginine
Arginine
Arginine
Arginine
Cytosine
Cytosine
Cytosine
Cytosine
Cytosine
1,3-dimethyluracil
1,3-dimethyluracil
1,3-dimethyluracil
1,3-dimethyluracil
1,3-dimethyluracil
N-Acetylhistamine
N-Acetylhistamine
N-Acetylhistamine
N-Acetylhistamine
N-Acetylhistamine
Prolylleucine_a
Prolylleucine_a
Prolylleucine_a
Prolylleucine_a
Prolylleucine_a
N-lauroylglycine
N-lauroylglycine
N-lauroylglycine
N-lauroylglycine
N-lauroylglycine
Capryloylglycine_a
Capryloylglycine_a
Capryloylglycine_a
Capryloylglycine_a
Capryloylglycine_a
Metirosine
Metirosine
Metirosine
Metirosine
Metirosine
Triethyl citrate_b
Triethyl citrate_b

Triethyl citrate_b
Triethyl citrate_b
Triethyl citrate_b
3-[2-[(Z)-[3-(2-carboxyethyl)-4-methyl-5-[(3-methyl-5-oxo-4-vinyl-1,2-dihydropyrrol-2-yl)methyl]pyrrol-2-ylidene]
3-[2-[(Z)-[3-(2-carboxyethyl)-4-methyl-5-[(3-methyl-5-oxo-4-vinyl-1,2-dihydropyrrol-2-yl)methyl]pyrrol-2-ylidene]
3-[2-[(Z)-[3-(2-carboxyethyl)-4-methyl-5-[(3-methyl-5-oxo-4-vinyl-1,2-dihydropyrrol-2-yl)methyl]pyrrol-2-ylidene]
3-[2-[(Z)-[3-(2-carboxyethyl)-4-methyl-5-[(3-methyl-5-oxo-4-vinyl-1,2-dihydropyrrol-2-yl)methyl]pyrrol-2-ylidene]
3-[2-[(Z)-[3-(2-carboxyethyl)-4-methyl-5-[(3-methyl-5-oxo-4-vinyl-1,2-dihydropyrrol-2-yl)methyl]pyrrol-2-ylidene]
Creatinine
Creatinine
Creatinine
Creatinine
Creatinine
Maltotriose
Maltotriose
Maltotriose
Maltotriose
Maltotriose
1,5-Isoquinolinediol_c
1,5-Isoquinolinediol_c
1,5-Isoquinolinediol_c
1,5-Isoquinolinediol_c
1,5-Isoquinolinediol_c
Choline sulfate
Choline sulfate
Choline sulfate
Choline sulfate
Choline sulfate
7-ketodeoxycholic acid_c
7-ketodeoxycholic acid_c
7-ketodeoxycholic acid_c
7-ketodeoxycholic acid_c
7-ketodeoxycholic acid_c
2-(5-Benzyl-3,6-dioxopiperazin-2-yl)acetic acid
2-(5-Benzyl-3,6-dioxopiperazin-2-yl)acetic acid
2-(5-Benzyl-3,6-dioxopiperazin-2-yl)acetic acid
2-(5-Benzyl-3,6-dioxopiperazin-2-yl)acetic acid
2-(5-Benzyl-3,6-dioxopiperazin-2-yl)acetic acid
Piperidine_a
Piperidine_a
Piperidine_a
Piperidine_a
Piperidine_a
Carbofuran
Carbofuran
Carbofuran
Carbofuran
Carbofuran

(âˆ’)-nabilone_c
(âˆ’)-nabilone_c
(âˆ’)-nabilone_c
(âˆ’)-nabilone_c
(âˆ’)-nabilone_c
Crotamiton
Crotamiton
Crotamiton
Crotamiton
Crotamiton
N6,N6,N6-Trimethyl-L-lysine_c
N6,N6,N6-Trimethyl-L-lysine_c
N6,N6,N6-Trimethyl-L-lysine_c
N6,N6,N6-Trimethyl-L-lysine_c
N6,N6,N6-Trimethyl-L-lysine_c
TO0127900_a
TO0127900_a
TO0127900_a
TO0127900_a
TO0127900_a

(3R,4S,5S,6R,7R,9R,11R,12R,13S,14R)-6-{[(2S,3R,4S,6R)-4-(Dimethylamino)-3-hydroxy-6-methyltetrahydro-2H-pyran-2-yl]oxy}-12,13-dihydroxy-14-[(1S)-1-hydroxyethyl]-4-{[(2R,4R,5S,6S)-5-hydroxy-4-methoxy-4,6-dimethyltetrahydro-2H-pyran-2-yl]oxy}-7-methoxy-3,5,7,9,11,13-hexamethyloxacyclotetradecane-2,10-dione

(3R,4S,5S,6R,7R,9R,11R,12R,13S,14R)-6-{[(2S,3R,4S,6R)-4-(Dimethylamino)-3-hydroxy-6-methyltetrahydro-2H-pyran-2-yl]oxy}-12,13-dihydroxy-14-[(1S)-1-hydroxyethyl]-4-{[(2R,4R,5S,6S)-5-hydroxy-4-methoxy-4,6-dimethyltetrahydro-2H-pyran-2-yl]oxy}-7-methoxy-3,5,7,9,11,13-hexamethyloxacyclotetradecane-2,10-dione

(3R,4S,5S,6R,7R,9R,11R,12R,13S,14R)-6-{[(2S,3R,4S,6R)-4-(Dimethylamino)-3-hydroxy-6-methyltetrahydro-2H-pyran-2-yl]oxy}-12,13-dihydroxy-14-[(1S)-1-hydroxyethyl]-4-{[(2R,4R,5S,6S)-5-hydroxy-4-methoxy-4,6-dimethyltetrahydro-2H-pyran-2-yl]oxy}-7-methoxy-3,5,7,9,11,13-hexamethyloxacyclotetradecane-2,10-dione

(3R,4S,5S,6R,7R,9R,11R,12R,13S,14R)-6-{[(2S,3R,4S,6R)-4-(Dimethylamino)-3-hydroxy-6-methyltetrahydro-2H-pyran-2-yl]oxy}-12,13-dihydroxy-14-[(1S)-1-hydroxyethyl]-4-{[(2R,4R,5S,6S)-5-hydroxy-4-methoxy-4,6-dimethyltetrahydro-2H-pyran-2-yl]oxy}-7-methoxy-3,5,7,9,11,13-hexamethyloxacyclotetradecane-2,10-dione

(3R,4S,5S,6R,7R,9R,11R,12R,13S,14R)-6-{[(2S,3R,4S,6R)-4-(Dimethylamino)-3-hydroxy-6-methyltetrahydro-2H-pyran-2-yl]oxy}-12,13-dihydroxy-14-[(1S)-1-hydroxyethyl]-4-{[(2R,4R,5S,6S)-5-hydroxy-4-methoxy-4
,6-dimethyltetrahydro-2H-pyran-2-yl]oxy}-7-methoxy-3,5,7,9,11,13-hexamethyloxacyclotetradecane-2,10-dione
3,3,5,5-Tetramethylpyrroline-N-oxide
3,3,5,5-Tetramethylpyrroline-N-oxide
3,3,5,5-Tetramethylpyrroline-N-oxide
3,3,5,5-Tetramethylpyrroline-N-oxide
3,3,5,5-Tetramethylpyrroline-N-oxide
N-Acetylvaline_b
N-Acetylvaline_b
N-Acetylvaline_b
N-Acetylvaline_b
N-Acetylvaline_b
Biotin
Biotin
Biotin
Biotin
Biotin
Hexamethylene bisacetamide
Hexamethylene bisacetamide
Hexamethylene bisacetamide
Hexamethylene bisacetamide

Hexamethylene bisacetamide
(3Z,6Z,9Z,12Z,21R)-27-Amino-24-hydroxy-24-oxido-18-oxo-19,23,25-trioxa-24lambda~5~-phosphaheptacosa-3,
(3Z,6Z,9Z,12Z,21R)-27-Amino-24-hydroxy-24-oxido-18-oxo-19,23,25-trioxa-24lambda~5~-phosphaheptacosa-3,
(3Z,6Z,9Z,12Z,21R)-27-Amino-24-hydroxy-24-oxido-18-oxo-19,23,25-trioxa-24lambda~5~-phosphaheptacosa-3,
(3Z,6Z,9Z,12Z,21R)-27-Amino-24-hydroxy-24-oxido-18-oxo-19,23,25-trioxa-24lambda~5~-phosphaheptacosa-3,
(3Z,6Z,9Z,12Z,21R)-27-Amino-24-hydroxy-24-oxido-18-oxo-19,23,25-trioxa-24lambda~5~-phosphaheptacosa-3,
Î´-Valerolactam_a
Î´-Valerolactam_a
Î´-Valerolactam_a
Î´-Valerolactam_a
Î´-Valerolactam_a
L-Urobilin
L-Urobilin
L-Urobilin
L-Urobilin
L-Urobilin
UROBILIN, (-)-_a
UROBILIN, (-)-_a
UROBILIN, (-)-_a
UROBILIN, (-)-_a
UROBILIN, (-)-_a
N-(5-acetamidopentyl)acetamide
N-(5-acetamidopentyl)acetamide
N-(5-acetamidopentyl)acetamide
N-(5-acetamidopentyl)acetamide
N-(5-acetamidopentyl)acetamide
Cyclamic acid
Cyclamic acid
Cyclamic acid
Cyclamic acid
Cyclamic acid
urobilinogen
urobilinogen
urobilinogen
urobilinogen
urobilinogen
N-Acetylputrescine
N-Acetylputrescine
N-Acetylputrescine
N-Acetylputrescine
N-Acetylputrescine
presqualene diphosphate
presqualene diphosphate
presqualene diphosphate
presqualene diphosphate
presqualene diphosphate
Acetophenone
Acetophenone

Acetophenone
Acetophenone
Acetophenone
Prilocaine
Prilocaine
Prilocaine
Prilocaine
Prilocaine
4-Acetamidophenol
4-Acetamidophenol
4-Acetamidophenol
4-Acetamidophenol
4-Acetamidophenol
(Â±)-Albuterol
(Â±)-Albuterol
(Â±)-Albuterol
(Â±)-Albuterol
(Â±)-Albuterol
(-)-Erythromycin
(-)-Erythromycin
(-)-Erythromycin
(-)-Erythromycin
(-)-Erythromycin
N6,N6,N6-Trimethyl-L-lysine_b
N6,N6,N6-Trimethyl-L-lysine_b
N6,N6,N6-Trimethyl-L-lysine_b
N6,N6,N6-Trimethyl-L-lysine_b
N6,N6,N6-Trimethyl-L-lysine_b
Pipecolinic acid
Pipecolinic acid
Pipecolinic acid
Pipecolinic acid
Pipecolinic acid
DNOP_f
DNOP_f
DNOP_f
DNOP_f
DNOP_f
N-Methylcaprolactam
N-Methylcaprolactam
N-Methylcaprolactam
N-Methylcaprolactam
N-Methylcaprolactam
Limonin
Limonin
Limonin
Limonin
Limonin

Atenolol
Atenolol
Atenolol
Atenolol
Atenolol
Styrene
Styrene
Styrene
Styrene
Styrene
Valylvaline_g
Valylvaline_g
Valylvaline_g
Valylvaline_g
Valylvaline_g
2-(Hydroxymethyl)-1-methyl-3,4,5-piperidinetriol
2-(Hydroxymethyl)-1-methyl-3,4,5-piperidinetriol
2-(Hydroxymethyl)-1-methyl-3,4,5-piperidinetriol
2-(Hydroxymethyl)-1-methyl-3,4,5-piperidinetriol
2-(Hydroxymethyl)-1-methyl-3,4,5-piperidinetriol
Theobromine
Theobromine
Theobromine
Theobromine
Theobromine
6-hydroxypseudooxynicotine_b
6-hydroxypseudooxynicotine_b
6-hydroxypseudooxynicotine_b
6-hydroxypseudooxynicotine_b
6-hydroxypseudooxynicotine_b
Isoprene
Isoprene
Isoprene
Isoprene
Isoprene
Tyramine
Tyramine
Tyramine
Tyramine
Tyramine
1-[(4E)-4-(4-Methyl-5-oxo-2(5H)-furanylidene)butyl]-2-pyrrolidinone
1-[(4E)-4-(4-Methyl-5-oxo-2(5H)-furanylidene)butyl]-2-pyrrolidinone
1-[(4E)-4-(4-Methyl-5-oxo-2(5H)-furanylidene)butyl]-2-pyrrolidinone
1-[(4E)-4-(4-Methyl-5-oxo-2(5H)-furanylidene)butyl]-2-pyrrolidinone
1-[(4E)-4-(4-Methyl-5-oxo-2(5H)-furanylidene)butyl]-2-pyrrolidinone
Methylimidazoleacetic acid
Methylimidazoleacetic acid
Methylimidazoleacetic acid

Methylimidazoleacetic acid
Methylimidazoleacetic acid
butalbital_d
butalbital_d
butalbital_d
butalbital_d
butalbital_d
Piperidine_d
Piperidine_d
Piperidine_d
Piperidine_d
Piperidine_d
UROBILIN, (-)-_b
UROBILIN, (-)-_b
UROBILIN, (-)-_b
UROBILIN, (-)-_b
UROBILIN, (-)-_b
(2S)-6-Amino-2-[(E)-(hydroxymethylene)amino]hexanimidic acid_b
(2S)-6-Amino-2-[(E)-(hydroxymethylene)amino]hexanimidic acid_b
(2S)-6-Amino-2-[(E)-(hydroxymethylene)amino]hexanimidic acid_b
(2S)-6-Amino-2-[(E)-(hydroxymethylene)amino]hexanimidic acid_b
(2S)-6-Amino-2-[(E)-(hydroxymethylene)amino]hexanimidic acid_b
Î´-Valerolactam_b
Î´-Valerolactam_b
Î´-Valerolactam_b
Î´-Valerolactam_b
Î´-Valerolactam_b
MFCD00025555_d
MFCD00025555_d
MFCD00025555_d
MFCD00025555_d
MFCD00025555_d
N-Acetylneuraminic acid
N-Acetylneuraminic acid
N-Acetylneuraminic acid
N-Acetylneuraminic acid
N-Acetylneuraminic acid
Methylol Dimethylhydantoin_a
Methylol Dimethylhydantoin_a
Methylol Dimethylhydantoin_a
Methylol Dimethylhydantoin_a
Methylol Dimethylhydantoin_a
Deoxysugar II (possibly Fucose)
Deoxysugar II (possibly Fucose)
Deoxysugar II (possibly Fucose)
Deoxysugar II (possibly Fucose)
Deoxysugar II (possibly Fucose)
DNOP_e

DNOP_e
DNOP_e
DNOP_e
DNOP_e
Tropinone
Tropinone
Tropinone
Tropinone
Tropinone
Thymidine
Thymidine
Thymidine
Thymidine
Thymidine
8-Methyl-8-azabicyclo[3.2.1]octane-1,2,3,4,6-pentol_a
8-Methyl-8-azabicyclo[3.2.1]octane-1,2,3,4,6-pentol_a
8-Methyl-8-azabicyclo[3.2.1]octane-1,2,3,4,6-pentol_a
8-Methyl-8-azabicyclo[3.2.1]octane-1,2,3,4,6-pentol_a
8-Methyl-8-azabicyclo[3.2.1]octane-1,2,3,4,6-pentol_a
Solanidine
Solanidine
Solanidine
Solanidine
Solanidine
Tetrahydrofuran
Tetrahydrofuran
Tetrahydrofuran
Tetrahydrofuran
Tetrahydrofuran
Crotonic acid
Crotonic acid
Crotonic acid
Crotonic acid
Crotonic acid
N-{6-[(7-Chloro-4-quinazolinyl)oxy]-3-pyridinyl}-2-thiophenesulfonamide
N-{6-[(7-Chloro-4-quinazolinyl)oxy]-3-pyridinyl}-2-thiophenesulfonamide
N-{6-[(7-Chloro-4-quinazolinyl)oxy]-3-pyridinyl}-2-thiophenesulfonamide
N-{6-[(7-Chloro-4-quinazolinyl)oxy]-3-pyridinyl}-2-thiophenesulfonamide
N-{6-[(7-Chloro-4-quinazolinyl)oxy]-3-pyridinyl}-2-thiophenesulfonamide
Prolylleucine_b
Prolylleucine_b
Prolylleucine_b
Prolylleucine_b
Prolylleucine_b
3-[2-[(Z)-[3-(2-carboxyethyl)-4-methyl-5-[(3-methyl-5-oxo-4-vinyl-1,2-dihydropyrrol-2-yl)methyl]pyrrol-2-ylidene]r
3-[2-[(Z)-[3-(2-carboxyethyl)-4-methyl-5-[(3-methyl-5-oxo-4-vinyl-1,2-dihydropyrrol-2-yl)methyl]pyrrol-2-ylidene]r
3-[2-[(Z)-[3-(2-carboxyethyl)-4-methyl-5-[(3-methyl-5-oxo-4-vinyl-1,2-dihydropyrrol-2-yl)methyl]pyrrol-2-ylidene]r
3-[2-[(Z)-[3-(2-carboxyethyl)-4-methyl-5-[(3-methyl-5-oxo-4-vinyl-1,2-dihydropyrrol-2-yl)methyl]pyrrol-2-ylidene]r

3-[2-[(Z)-[3-(2-carboxyethyl)-4-methyl-5-[(3-methyl-5-oxo-4-vinyl-1,2-dihydropyrrol-2-yl)methyl]pyrrol-2-ylidene]r
3-Hydroxy-2-methyl-2-[(sulfooxy)methyl]propanoic acid
3-Hydroxy-2-methyl-2-[(sulfooxy)methyl]propanoic acid
3-Hydroxy-2-methyl-2-[(sulfooxy)methyl]propanoic acid
3-Hydroxy-2-methyl-2-[(sulfooxy)methyl]propanoic acid
3-Hydroxy-2-methyl-2-[(sulfooxy)methyl]propanoic acid
Docosahexaenoic acid ethyl ester_b
Docosahexaenoic acid ethyl ester_b
Docosahexaenoic acid ethyl ester_b
Docosahexaenoic acid ethyl ester_b
Docosahexaenoic acid ethyl ester_b
3-(2-Oxo-2,3-dihydro-1,3-benzoxazol-3-yl)propanoic acid_b
3-(2-Oxo-2,3-dihydro-1,3-benzoxazol-3-yl)propanoic acid_b
3-(2-Oxo-2,3-dihydro-1,3-benzoxazol-3-yl)propanoic acid_b
3-(2-Oxo-2,3-dihydro-1,3-benzoxazol-3-yl)propanoic acid_b
3-(2-Oxo-2,3-dihydro-1,3-benzoxazol-3-yl)propanoic acid_b
N-{3-Carboxy-3-[(3-carboxy-3-hydroxypropyl)amino]propyl}homoserine
N-{3-Carboxy-3-[(3-carboxy-3-hydroxypropyl)amino]propyl}homoserine
N-{3-Carboxy-3-[(3-carboxy-3-hydroxypropyl)amino]propyl}homoserine
N-{3-Carboxy-3-[(3-carboxy-3-hydroxypropyl)amino]propyl}homoserine
N-{3-Carboxy-3-[(3-carboxy-3-hydroxypropyl)amino]propyl}homoserine
2-Hydroxy-13,20-dimethoxy-4,7,17,22,22-pentamethyl-11-oxo-5,10,21,23-tetraoxahexacyclo[18.2.1.0~1,17~.0~
2-Hydroxy-13,20-dimethoxy-4,7,17,22,22-pentamethyl-11-oxo-5,10,21,23-tetraoxahexacyclo[18.2.1.0~1,17~.0~
2-Hydroxy-13,20-dimethoxy-4,7,17,22,22-pentamethyl-11-oxo-5,10,21,23-tetraoxahexacyclo[18.2.1.0~1,17~.0~
2-Hydroxy-13,20-dimethoxy-4,7,17,22,22-pentamethyl-11-oxo-5,10,21,23-tetraoxahexacyclo[18.2.1.0~1,17~.0~
2-Hydroxy-13,20-dimethoxy-4,7,17,22,22-pentamethyl-11-oxo-5,10,21,23-tetraoxahexacyclo[18.2.1.0~1,17~.0~
Cadaverine
Cadaverine
Cadaverine
Cadaverine
Cadaverine
Indole-3-carboxyaldehyde
Indole-3-carboxyaldehyde
Indole-3-carboxyaldehyde
Indole-3-carboxyaldehyde
Indole-3-carboxyaldehyde
4-Hydroxyprolylleucine_d
4-Hydroxyprolylleucine_d
4-Hydroxyprolylleucine_d
4-Hydroxyprolylleucine_d
4-Hydroxyprolylleucine_d
3',5,7-Trihydroxy-4'-methoxyflavanone
3',5,7-Trihydroxy-4'-methoxyflavanone
3',5,7-Trihydroxy-4'-methoxyflavanone
3',5,7-Trihydroxy-4'-methoxyflavanone
3',5,7-Trihydroxy-4'-methoxyflavanone
6-(alpha-D-glucosaminyl)-1D-myo-inositol_a
6-(alpha-D-glucosaminyl)-1D-myo-inositol_a

6-(alpha-D-glucosaminyl)-1D-myo-inositol_a
6-(alpha-D-glucosaminyl)-1D-myo-inositol_a
6-(alpha-D-glucosaminyl)-1D-myo-inositol_a
Ethyl malate_c
Ethyl malate_c
Ethyl malate_c
Ethyl malate_c
Ethyl malate_c
Safrole
Safrole
Safrole
Safrole
Safrole
1,1'-[1,12-Dodecanediylbis(oxy)]dibenzene_b
1,1'-[1,12-Dodecanediylbis(oxy)]dibenzene_b
1,1'-[1,12-Dodecanediylbis(oxy)]dibenzene_b
1,1'-[1,12-Dodecanediylbis(oxy)]dibenzene_b
1,1'-[1,12-Dodecanediylbis(oxy)]dibenzene_b
DNOP_g
DNOP_g
DNOP_g
DNOP_g
DNOP_g
(2E)-3-Methyl-4-(sulfooxy)-2-butenoic acid_a
(2E)-3-Methyl-4-(sulfooxy)-2-butenoic acid_a
(2E)-3-Methyl-4-(sulfooxy)-2-butenoic acid_a
(2E)-3-Methyl-4-(sulfooxy)-2-butenoic acid_a
(2E)-3-Methyl-4-(sulfooxy)-2-butenoic acid_a
Citrulline
Citrulline
Citrulline
Citrulline
Citrulline
g-Butyrobetaine
g-Butyrobetaine
g-Butyrobetaine
g-Butyrobetaine
g-Butyrobetaine
Piperidine_e
Piperidine_e
Piperidine_e
Piperidine_e
Piperidine_e
Isopelletierine
Isopelletierine
Isopelletierine
Isopelletierine
Isopelletierine

2-Acetamidohexanedioic acid_d
2-Acetamidohexanedioic acid_d
2-Acetamidohexanedioic acid_d
2-Acetamidohexanedioic acid_d
2-Acetamidohexanedioic acid_d
4-Vinylphenol
4-Vinylphenol
4-Vinylphenol
4-Vinylphenol
4-Vinylphenol
2-Hydroxycinnamic acid
2-Hydroxycinnamic acid
2-Hydroxycinnamic acid
2-Hydroxycinnamic acid
2-Hydroxycinnamic acid
Methyl (2Z,3E,5Z)-6-{4,4-dimethyl-3-[(3-methyl-2-buten-1-yl)oxy]-3,4-dihydro-2H-1,5-benzodioxepin-7-yl}-3-(hyd
Methyl (2Z,3E,5Z)-6-{4,4-dimethyl-3-[(3-methyl-2-buten-1-yl)oxy]-3,4-dihydro-2H-1,5-benzodioxepin-7-yl}-3-(hyd
Methyl (2Z,3E,5Z)-6-{4,4-dimethyl-3-[(3-methyl-2-buten-1-yl)oxy]-3,4-dihydro-2H-1,5-benzodioxepin-7-yl}-3-(hyd
Methyl (2Z,3E,5Z)-6-{4,4-dimethyl-3-[(3-methyl-2-buten-1-yl)oxy]-3,4-dihydro-2H-1,5-benzodioxepin-7-yl}-3-(hyd
Methyl (2Z,3E,5Z)-6-{4,4-dimethyl-3-[(3-methyl-2-buten-1-yl)oxy]-3,4-dihydro-2H-1,5-benzodioxepin-7-yl}-3-(hyd
Tiglic acid_a
Tiglic acid_a
Tiglic acid_a
Tiglic acid_a
Tiglic acid_a
O-Ethyl (4-hydroxyphenyl)carbamothioate
O-Ethyl (4-hydroxyphenyl)carbamothioate
O-Ethyl (4-hydroxyphenyl)carbamothioate
O-Ethyl (4-hydroxyphenyl)carbamothioate
O-Ethyl (4-hydroxyphenyl)carbamothioate
porphobilinogen_b
porphobilinogen_b
porphobilinogen_b
porphobilinogen_b
porphobilinogen_b
DL-4-Hydroxyphenyllactic acid
DL-4-Hydroxyphenyllactic acid
DL-4-Hydroxyphenyllactic acid
DL-4-Hydroxyphenyllactic acid
DL-4-Hydroxyphenyllactic acid
4-(METHYLNITROSAMINO)-1-(3-PYRIDYL-N-OXIDE)-1-BUTANONE_b
4-(METHYLNITROSAMINO)-1-(3-PYRIDYL-N-OXIDE)-1-BUTANONE_b
4-(METHYLNITROSAMINO)-1-(3-PYRIDYL-N-OXIDE)-1-BUTANONE_b
4-(METHYLNITROSAMINO)-1-(3-PYRIDYL-N-OXIDE)-1-BUTANONE_b
4-(METHYLNITROSAMINO)-1-(3-PYRIDYL-N-OXIDE)-1-BUTANONE_b
Leu-Val_e
Leu-Val_e
Leu-Val_e

Leu-Val_e
Leu-Val_e
Valylvaline_d
Valylvaline_d
Valylvaline_d
Valylvaline_d
Valylvaline_d
Phenyl D-glucopyranosiduronic acid_d
Phenyl D-glucopyranosiduronic acid_d
Phenyl D-glucopyranosiduronic acid_d
Phenyl D-glucopyranosiduronic acid_d
Phenyl D-glucopyranosiduronic acid_d
Lanthionine ketimine_a
Lanthionine ketimine_a
Lanthionine ketimine_a
Lanthionine ketimine_a
Lanthionine ketimine_a
9-Methyluric acid_b
9-Methyluric acid_b
9-Methyluric acid_b
9-Methyluric acid_b
9-Methyluric acid_b
2-Hydroxy-4-methylthiobutanoic acid
2-Hydroxy-4-methylthiobutanoic acid
2-Hydroxy-4-methylthiobutanoic acid
2-Hydroxy-4-methylthiobutanoic acid
2-Hydroxy-4-methylthiobutanoic acid
Seryltyrosine_b
Seryltyrosine_b
Seryltyrosine_b
Seryltyrosine_b
Seryltyrosine_b
2-Amino-4-methylpyrimidine
2-Amino-4-methylpyrimidine
2-Amino-4-methylpyrimidine
2-Amino-4-methylpyrimidine
2-Amino-4-methylpyrimidine
LU3453000
LU3453000
LU3453000
LU3453000
LU3453000
Brilliant blue FCF
Brilliant blue FCF
Brilliant blue FCF
Brilliant blue FCF
Brilliant blue FCF
Coumarone

Coumarone
Coumarone
Coumarone
Coumarone
1-Methylhistidine
1-Methylhistidine
1-Methylhistidine
1-Methylhistidine
1-Methylhistidine
Setoclavine
Setoclavine
Setoclavine
Setoclavine
Setoclavine
Butabarbital_a
Butabarbital_a
Butabarbital_a
Butabarbital_a
Butabarbital_a
Mevalonic acid
Mevalonic acid
Mevalonic acid
Mevalonic acid
Mevalonic acid
adrenaline
adrenaline
adrenaline
adrenaline
adrenaline
DNOP_b
DNOP_b
DNOP_b
DNOP_b
DNOP_b
Capryloylglycine_b
Capryloylglycine_b
Capryloylglycine_b
Capryloylglycine_b
Capryloylglycine_b
Lidocaine
Lidocaine
Lidocaine
Lidocaine
Lidocaine
N6,N6,N6-Trimethyl-L-lysine_a
N6,N6,N6-Trimethyl-L-lysine_a
N6,N6,N6-Trimethyl-L-lysine_a
N6,N6,N6-Trimethyl-L-lysine_a

N6,N6,N6-Trimethyl-L-lysine_a
Indole-3-acetaldehyde
Indole-3-acetaldehyde
Indole-3-acetaldehyde
Indole-3-acetaldehyde
Indole-3-acetaldehyde
Prolylhydroxyproline
Prolylhydroxyproline
Prolylhydroxyproline
Prolylhydroxyproline
Prolylhydroxyproline
1-Methylxanthine
1-Methylxanthine
1-Methylxanthine
1-Methylxanthine
1-Methylxanthine
Methyl 3-formyl-1H-indole-2-sulfinate
Methyl 3-formyl-1H-indole-2-sulfinate
Methyl 3-formyl-1H-indole-2-sulfinate
Methyl 3-formyl-1H-indole-2-sulfinate
Methyl 3-formyl-1H-indole-2-sulfinate
8-Hydroxyquinoline
8-Hydroxyquinoline
8-Hydroxyquinoline
8-Hydroxyquinoline
8-Hydroxyquinoline
Glycylprolylhydroxyproline_a
Glycylprolylhydroxyproline_a
Glycylprolylhydroxyproline_a
Glycylprolylhydroxyproline_a
Glycylprolylhydroxyproline_a
(15Z)-9,12,13-Trihydroxy-15-octadecenoic acid
(15Z)-9,12,13-Trihydroxy-15-octadecenoic acid
(15Z)-9,12,13-Trihydroxy-15-octadecenoic acid
(15Z)-9,12,13-Trihydroxy-15-octadecenoic acid
(15Z)-9,12,13-Trihydroxy-15-octadecenoic acid
2-(1,3-Benzodioxol-5-yl)-5,7-dimethoxy-3-chromanol
2-(1,3-Benzodioxol-5-yl)-5,7-dimethoxy-3-chromanol
2-(1,3-Benzodioxol-5-yl)-5,7-dimethoxy-3-chromanol
2-(1,3-Benzodioxol-5-yl)-5,7-dimethoxy-3-chromanol
2-(1,3-Benzodioxol-5-yl)-5,7-dimethoxy-3-chromanol
Methyl [9-(3-furyl)-4-hydroxy-3,3,5a,9a,11b-pentamethyl-5,7-dioxotetradecahydro[2]benzofuro[5,4-f]oxireno[d]is
Methyl [9-(3-furyl)-4-hydroxy-3,3,5a,9a,11b-pentamethyl-5,7-dioxotetradecahydro[2]benzofuro[5,4-f]oxireno[d]is
Methyl [9-(3-furyl)-4-hydroxy-3,3,5a,9a,11b-pentamethyl-5,7-dioxotetradecahydro[2]benzofuro[5,4-f]oxireno[d]is
Methyl [9-(3-furyl)-4-hydroxy-3,3,5a,9a,11b-pentamethyl-5,7-dioxotetradecahydro[2]benzofuro[5,4-f]oxireno[d]is
Methyl [9-(3-furyl)-4-hydroxy-3,3,5a,9a,11b-pentamethyl-5,7-dioxotetradecahydro[2]benzofuro[5,4-f]oxireno[d]is
Midodrine_b
Midodrine_b

Midodrine_b
Midodrine_b
Midodrine_b
7-[4-(tert-butyl)phenyl]-2-methyl-5-(methylthio)pyrazolo[1,5-a]pyrimidine-6-carbonitrile
7-[4-(tert-butyl)phenyl]-2-methyl-5-(methylthio)pyrazolo[1,5-a]pyrimidine-6-carbonitrile
7-[4-(tert-butyl)phenyl]-2-methyl-5-(methylthio)pyrazolo[1,5-a]pyrimidine-6-carbonitrile
7-[4-(tert-butyl)phenyl]-2-methyl-5-(methylthio)pyrazolo[1,5-a]pyrimidine-6-carbonitrile
7-[4-(tert-butyl)phenyl]-2-methyl-5-(methylthio)pyrazolo[1,5-a]pyrimidine-6-carbonitrile
Lysylvaline_a
Lysylvaline_a
Lysylvaline_a
Lysylvaline_a
Lysylvaline_a
11-dehydro Thromboxane B2
11-dehydro Thromboxane B2
11-dehydro Thromboxane B2
11-dehydro Thromboxane B2
11-dehydro Thromboxane B2
2-Hydroxy-13,20-dimethoxy-4,7,17,22,22-pentamethyl-5,10,21,23-tetraoxahexacyclo[18.2.1.0~1,17~.0~4,16~.0
2-Hydroxy-13,20-dimethoxy-4,7,17,22,22-pentamethyl-5,10,21,23-tetraoxahexacyclo[18.2.1.0~1,17~.0~4,16~.0
2-Hydroxy-13,20-dimethoxy-4,7,17,22,22-pentamethyl-5,10,21,23-tetraoxahexacyclo[18.2.1.0~1,17~.0~4,16~.0
2-Hydroxy-13,20-dimethoxy-4,7,17,22,22-pentamethyl-5,10,21,23-tetraoxahexacyclo[18.2.1.0~1,17~.0~4,16~.0
2-Hydroxy-13,20-dimethoxy-4,7,17,22,22-pentamethyl-5,10,21,23-tetraoxahexacyclo[18.2.1.0~1,17~.0~4,16~.0
3b-Hydroxy-5-cholenoic acid_a
3b-Hydroxy-5-cholenoic acid_a
3b-Hydroxy-5-cholenoic acid_a
3b-Hydroxy-5-cholenoic acid_a
3b-Hydroxy-5-cholenoic acid_a
4-[(2E,4Z)-6-Carboxy-2,4-heptadien-2-yl]-3-(carboxymethyl)proline
4-[(2E,4Z)-6-Carboxy-2,4-heptadien-2-yl]-3-(carboxymethyl)proline
4-[(2E,4Z)-6-Carboxy-2,4-heptadien-2-yl]-3-(carboxymethyl)proline
4-[(2E,4Z)-6-Carboxy-2,4-heptadien-2-yl]-3-(carboxymethyl)proline
4-[(2E,4Z)-6-Carboxy-2,4-heptadien-2-yl]-3-(carboxymethyl)proline
2-Hydroxyphenylalanine
2-Hydroxyphenylalanine
2-Hydroxyphenylalanine
2-Hydroxyphenylalanine
2-Hydroxyphenylalanine
asn-pro_c
asn-pro_c
asn-pro_c
asn-pro_c
asn-pro_c
Dodecanedioic acid
Dodecanedioic acid
Dodecanedioic acid
Dodecanedioic acid
Dodecanedioic acid

Cys-tyr
Cys-tyr
Cys-tyr
Cys-tyr
Cys-tyr
N-(3-acetamidopropyl)pyrrolidin-2-one_c
N-(3-acetamidopropyl)pyrrolidin-2-one_c
N-(3-acetamidopropyl)pyrrolidin-2-one_c
N-(3-acetamidopropyl)pyrrolidin-2-one_c
N-(3-acetamidopropyl)pyrrolidin-2-one_c
MFCD12974029
MFCD12974029
MFCD12974029
MFCD12974029
MFCD12974029
N,N-Dimethyl-L-histidine_a
N,N-Dimethyl-L-histidine_a
N,N-Dimethyl-L-histidine_a
N,N-Dimethyl-L-histidine_a
N,N-Dimethyl-L-histidine_a
Leucyltyrosine_a
Leucyltyrosine_a
Leucyltyrosine_a
Leucyltyrosine_a
Leucyltyrosine_a
L-gamma-Glutamyl-L-leucine_b
L-gamma-Glutamyl-L-leucine_b
L-gamma-Glutamyl-L-leucine_b
L-gamma-Glutamyl-L-leucine_b
L-gamma-Glutamyl-L-leucine_b
Î±-Muricholic acid
Î±-Muricholic acid
Î±-Muricholic acid
Î±-Muricholic acid
Î±-Muricholic acid
Dihydrouridine_a
Dihydrouridine_a
Dihydrouridine_a
Dihydrouridine_a
Dihydrouridine_a
1-(4-Amino-4-carboxybutanoyl)-2-piperidinecarboxylic acid_a
1-(4-Amino-4-carboxybutanoyl)-2-piperidinecarboxylic acid_a
1-(4-Amino-4-carboxybutanoyl)-2-piperidinecarboxylic acid_a
1-(4-Amino-4-carboxybutanoyl)-2-piperidinecarboxylic acid_a
1-(4-Amino-4-carboxybutanoyl)-2-piperidinecarboxylic acid_a
Coprine_c
Coprine_c
Coprine_c

Coprine_c
Coprine_c
1-(beta-D-Ribofuranosyl)-1,4-dihydronicotinamide
1-(beta-D-Ribofuranosyl)-1,4-dihydronicotinamide
1-(beta-D-Ribofuranosyl)-1,4-dihydronicotinamide
1-(beta-D-Ribofuranosyl)-1,4-dihydronicotinamide
1-(beta-D-Ribofuranosyl)-1,4-dihydronicotinamide
Isoprenaline
Isoprenaline
Isoprenaline
Isoprenaline
Isoprenaline
(2E,6E)-9-[(2R)-6-Hydroxy-2,5,7,8-tetramethyl-3,4-dihydro-2H-chromen-2-yl]-2,6-dimethyl-2,6-nonadienoic acid
(2E,6E)-9-[(2R)-6-Hydroxy-2,5,7,8-tetramethyl-3,4-dihydro-2H-chromen-2-yl]-2,6-dimethyl-2,6-nonadienoic acid
(2E,6E)-9-[(2R)-6-Hydroxy-2,5,7,8-tetramethyl-3,4-dihydro-2H-chromen-2-yl]-2,6-dimethyl-2,6-nonadienoic acid
(2E,6E)-9-[(2R)-6-Hydroxy-2,5,7,8-tetramethyl-3,4-dihydro-2H-chromen-2-yl]-2,6-dimethyl-2,6-nonadienoic acid
(2E,6E)-9-[(2R)-6-Hydroxy-2,5,7,8-tetramethyl-3,4-dihydro-2H-chromen-2-yl]-2,6-dimethyl-2,6-nonadienoic acid
Indole-3-carboxylate
Indole-3-carboxylate
Indole-3-carboxylate
Indole-3-carboxylate
Indole-3-carboxylate
Piceid
Piceid
Piceid
Piceid
Piceid
Bile acid I (similar to Deoxycholic acid)
Bile acid I (similar to Deoxycholic acid)
Bile acid I (similar to Deoxycholic acid)
Bile acid I (similar to Deoxycholic acid)
Bile acid I (similar to Deoxycholic acid)
N-Ethylpropanamide_a
N-Ethylpropanamide_a
N-Ethylpropanamide_a
N-Ethylpropanamide_a
N-Ethylpropanamide_a
MFCD18695608_b
MFCD18695608_b
MFCD18695608_b
MFCD18695608_b
MFCD18695608_b
7,8-Diaminononanoic acid
7,8-Diaminononanoic acid
7,8-Diaminononanoic acid
7,8-Diaminononanoic acid
7,8-Diaminononanoic acid
tyramine sulfate_a

tyramine sulfate_a
tyramine sulfate_a
tyramine sulfate_a
tyramine sulfate_a
Nitrosoheptamethyleneimine
Nitrosoheptamethyleneimine
Nitrosoheptamethyleneimine
Nitrosoheptamethyleneimine
Nitrosoheptamethyleneimine
1,3,7-Trimethyluric acid
1,3,7-Trimethyluric acid
1,3,7-Trimethyluric acid
1,3,7-Trimethyluric acid
1,3,7-Trimethyluric acid
N2-Acetyllysine
N2-Acetyllysine
N2-Acetyllysine
N2-Acetyllysine
N2-Acetyllysine
3-Methylsulfolene
3-Methylsulfolene
3-Methylsulfolene
3-Methylsulfolene
3-Methylsulfolene
N-(Carboxymethyl)norleucine_a
N-(Carboxymethyl)norleucine_a
N-(Carboxymethyl)norleucine_a
N-(Carboxymethyl)norleucine_a
N-(Carboxymethyl)norleucine_a
tert-Butyl 3-amino-1-methyl-2,3-dioxopropylcarbamate_a
tert-Butyl 3-amino-1-methyl-2,3-dioxopropylcarbamate_a
tert-Butyl 3-amino-1-methyl-2,3-dioxopropylcarbamate_a
tert-Butyl 3-amino-1-methyl-2,3-dioxopropylcarbamate_a
tert-Butyl 3-amino-1-methyl-2,3-dioxopropylcarbamate_a
meticillin
meticillin
meticillin
meticillin
meticillin
2-Acetamidohexanedioic acid_a
2-Acetamidohexanedioic acid_a
2-Acetamidohexanedioic acid_a
2-Acetamidohexanedioic acid_a
2-Acetamidohexanedioic acid_a
Uric acid
Uric acid
Uric acid
Uric acid

Uric acid
7-ketodeoxycholic acid_a
7-ketodeoxycholic acid_a
7-ketodeoxycholic acid_a
7-ketodeoxycholic acid_a
7-ketodeoxycholic acid_a
N-Propionylmethionine_c
N-Propionylmethionine_c
N-Propionylmethionine_c
N-Propionylmethionine_c
N-Propionylmethionine_c
Butabarbital_c
Butabarbital_c
Butabarbital_c
Butabarbital_c
Butabarbital_c
Leucyltyrosine_c
Leucyltyrosine_c
Leucyltyrosine_c
Leucyltyrosine_c
Leucyltyrosine_c
MFCD00025555_a
MFCD00025555_a
MFCD00025555_a
MFCD00025555_a
MFCD00025555_a
Hept-2-ulose
Hept-2-ulose
Hept-2-ulose
Hept-2-ulose
Hept-2-ulose
1,5-Isoquinolinediol_b
1,5-Isoquinolinediol_b
1,5-Isoquinolinediol_b
1,5-Isoquinolinediol_b
1,5-Isoquinolinediol_b
hypaphorine
hypaphorine
hypaphorine
hypaphorine
hypaphorine
meprobamate_c
meprobamate_c
meprobamate_c
meprobamate_c
meprobamate_c
2,5-Dimethylphenol
2,5-Dimethylphenol

2,5-Dimethylphenol
2,5-Dimethylphenol
2,5-Dimethylphenol
N-Stearoyltyrosine
N-Stearoyltyrosine
N-Stearoyltyrosine
N-Stearoyltyrosine
N-Stearoyltyrosine
1-(4-Aminobutyl)urea_b
1-(4-Aminobutyl)urea_b
1-(4-Aminobutyl)urea_b
1-(4-Aminobutyl)urea_b
1-(4-Aminobutyl)urea_b
3,3-Dimethylglutaric acid_c
3,3-Dimethylglutaric acid_c
3,3-Dimethylglutaric acid_c
3,3-Dimethylglutaric acid_c
3,3-Dimethylglutaric acid_c
Pilocarpine
Pilocarpine
Pilocarpine
Pilocarpine
Pilocarpine
Hydroxypropionylcarnitine_b
Hydroxypropionylcarnitine_b
Hydroxypropionylcarnitine_b
Hydroxypropionylcarnitine_b
Hydroxypropionylcarnitine_b
NPC
NPC
NPC
NPC
NPC
1-methylhypoxanthine
1-methylhypoxanthine
1-methylhypoxanthine
1-methylhypoxanthine
1-methylhypoxanthine
Ethanoic anhydride
Ethanoic anhydride
Ethanoic anhydride
Ethanoic anhydride
Ethanoic anhydride
7-Sulfocholic acid
7-Sulfocholic acid
7-Sulfocholic acid
7-Sulfocholic acid
7-Sulfocholic acid

3b-Hydroxy-5-cholenoic acid_b
3b-Hydroxy-5-cholenoic acid_b
3b-Hydroxy-5-cholenoic acid_b
3b-Hydroxy-5-cholenoic acid_b
3b-Hydroxy-5-cholenoic acid_b
Aurorix
Aurorix
Aurorix
Aurorix
Aurorix
2-(1-Ethoxyethoxy)propanoic acid_b
2-(1-Ethoxyethoxy)propanoic acid_b
2-(1-Ethoxyethoxy)propanoic acid_b
2-(1-Ethoxyethoxy)propanoic acid_b
2-(1-Ethoxyethoxy)propanoic acid_b
2-Acetamidohexanedioic acid_g
2-Acetamidohexanedioic acid_g
2-Acetamidohexanedioic acid_g
2-Acetamidohexanedioic acid_g
2-Acetamidohexanedioic acid_g
4-Iodoanisole
4-Iodoanisole
4-Iodoanisole
4-Iodoanisole
4-Iodoanisole
Phenethylamine
Phenethylamine
Phenethylamine
Phenethylamine
Phenethylamine
Arabic acid
Arabic acid
Arabic acid
Arabic acid
Arabic acid
6-Methylquinoline
6-Methylquinoline
6-Methylquinoline
6-Methylquinoline
6-Methylquinoline
S-Allylcysteine_c
S-Allylcysteine_c
S-Allylcysteine_c
S-Allylcysteine_c
S-Allylcysteine_c
YV8195000
YV8195000
YV8195000

YV8195000
YV8195000
N-Methyl-1H-indole-3-propanamide
N-Methyl-1H-indole-3-propanamide
N-Methyl-1H-indole-3-propanamide
N-Methyl-1H-indole-3-propanamide
N-Methyl-1H-indole-3-propanamide
1,1'-[1,12-Dodecanediylbis(oxy)]dibenzene_a
1,1'-[1,12-Dodecanediylbis(oxy)]dibenzene_a
1,1'-[1,12-Dodecanediylbis(oxy)]dibenzene_a
1,1'-[1,12-Dodecanediylbis(oxy)]dibenzene_a
1,1'-[1,12-Dodecanediylbis(oxy)]dibenzene_a
Methohexital_b
Methohexital_b
Methohexital_b
Methohexital_b
Methohexital_b
3-Aminosalicylic acid
3-Aminosalicylic acid
3-Aminosalicylic acid
3-Aminosalicylic acid
3-Aminosalicylic acid
3-(1-hydroxyethyl)-2,3,6,7,8,8a-hexahydropyrrolo[1,2-a]pyrazine-1,4-dione_a
3-(1-hydroxyethyl)-2,3,6,7,8,8a-hexahydropyrrolo[1,2-a]pyrazine-1,4-dione_a
3-(1-hydroxyethyl)-2,3,6,7,8,8a-hexahydropyrrolo[1,2-a]pyrazine-1,4-dione_a
3-(1-hydroxyethyl)-2,3,6,7,8,8a-hexahydropyrrolo[1,2-a]pyrazine-1,4-dione_a
3-(1-hydroxyethyl)-2,3,6,7,8,8a-hexahydropyrrolo[1,2-a]pyrazine-1,4-dione_a
Procaine_b
Procaine_b
Procaine_b
Procaine_b
Procaine_b
Acrylic acid
Acrylic acid
Acrylic acid
Acrylic acid
Acrylic acid
3,4-Methylenedioxyamphetamine
3,4-Methylenedioxyamphetamine
3,4-Methylenedioxyamphetamine
3,4-Methylenedioxyamphetamine
3,4-Methylenedioxyamphetamine
N-Acetylvaline_e
N-Acetylvaline_e
N-Acetylvaline_e
N-Acetylvaline_e
N-Acetylvaline_e
3,7-Dimethyluric acid

3,7-Dimethyluric acid
3,7-Dimethyluric acid
3,7-Dimethyluric acid
3,7-Dimethyluric acid
N~6~,N~6~-Dimethyllysine_b
N~6~,N~6~-Dimethyllysine_b
N~6~,N~6~-Dimethyllysine_b
N~6~,N~6~-Dimethyllysine_b
N~6~,N~6~-Dimethyllysine_b
CYS-ASP
CYS-ASP
CYS-ASP
CYS-ASP
CYS-ASP
Chenodeoxycholic acid 3-sulfate
Chenodeoxycholic acid 3-sulfate
Chenodeoxycholic acid 3-sulfate
Chenodeoxycholic acid 3-sulfate
Chenodeoxycholic acid 3-sulfate
Spermic acid_f
Spermic acid_f
Spermic acid_f
Spermic acid_f
Spermic acid_f
(2R,3S)-3-Hydroxy-8-methyl-8-azabicyclo[3.2.1]octane-2-carboxylic acid
(2R,3S)-3-Hydroxy-8-methyl-8-azabicyclo[3.2.1]octane-2-carboxylic acid
(2R,3S)-3-Hydroxy-8-methyl-8-azabicyclo[3.2.1]octane-2-carboxylic acid
(2R,3S)-3-Hydroxy-8-methyl-8-azabicyclo[3.2.1]octane-2-carboxylic acid
(2R,3S)-3-Hydroxy-8-methyl-8-azabicyclo[3.2.1]octane-2-carboxylic acid
4-Hydroxy-5-methylfuran-3(2H)-one
4-Hydroxy-5-methylfuran-3(2H)-one
4-Hydroxy-5-methylfuran-3(2H)-one
4-Hydroxy-5-methylfuran-3(2H)-one
4-Hydroxy-5-methylfuran-3(2H)-one
14-Hydroxy-3,9-dimethyl-12-methylene-5,8,10-trioxatetracyclo[9.3.1.0~1,9~.0~2,7~]pentadecane-6,13-dione
14-Hydroxy-3,9-dimethyl-12-methylene-5,8,10-trioxatetracyclo[9.3.1.0~1,9~.0~2,7~]pentadecane-6,13-dione
14-Hydroxy-3,9-dimethyl-12-methylene-5,8,10-trioxatetracyclo[9.3.1.0~1,9~.0~2,7~]pentadecane-6,13-dione
14-Hydroxy-3,9-dimethyl-12-methylene-5,8,10-trioxatetracyclo[9.3.1.0~1,9~.0~2,7~]pentadecane-6,13-dione
14-Hydroxy-3,9-dimethyl-12-methylene-5,8,10-trioxatetracyclo[9.3.1.0~1,9~.0~2,7~]pentadecane-6,13-dione
1-Vinylimidazole_b
1-Vinylimidazole_b
1-Vinylimidazole_b
1-Vinylimidazole_b
1-Vinylimidazole_b
Lanthionine ketimine_b
Lanthionine ketimine_b
Lanthionine ketimine_b
Lanthionine ketimine_b

Lanthionine ketimine_b
Tetraacetylethylenediamine_c
Tetraacetylethylenediamine_c
Tetraacetylethylenediamine_c
Tetraacetylethylenediamine_c
Tetraacetylethylenediamine_c
3-Phenylpropyl hydrogen sulfate
3-Phenylpropyl hydrogen sulfate
3-Phenylpropyl hydrogen sulfate
3-Phenylpropyl hydrogen sulfate
3-Phenylpropyl hydrogen sulfate
5beta-Cholestane-3alpha,7alpha,12alpha,25,26-pentol
5beta-Cholestane-3alpha,7alpha,12alpha,25,26-pentol
5beta-Cholestane-3alpha,7alpha,12alpha,25,26-pentol
5beta-Cholestane-3alpha,7alpha,12alpha,25,26-pentol
5beta-Cholestane-3alpha,7alpha,12alpha,25,26-pentol
pentoxyl
pentoxyl
pentoxyl
pentoxyl
pentoxyl
Vorinostat_a
Vorinostat_a
Vorinostat_a
Vorinostat_a
Vorinostat_a
GLK (Peptide Gly-Leu-Lys))
GLK (Peptide Gly-Leu-Lys))
GLK (Peptide Gly-Leu-Lys))
GLK (Peptide Gly-Leu-Lys))
GLK (Peptide Gly-Leu-Lys))
Semilicoisoflavone B
Semilicoisoflavone B
Semilicoisoflavone B
Semilicoisoflavone B
Semilicoisoflavone B
Nonivamide
Nonivamide
Nonivamide
Nonivamide
Nonivamide
Dipivefrin
Dipivefrin
Dipivefrin
Dipivefrin
Dipivefrin
1,9-Nonanedithiol
1,9-Nonanedithiol

1,9-Nonanedithiol
1,9-Nonanedithiol
1,9-Nonanedithiol
Aminohippuric acid_b
Aminohippuric acid_b
Aminohippuric acid_b
Aminohippuric acid_b
Aminohippuric acid_b
IN00150
IN00150
IN00150
IN00150
IN00150
feruloylserotonin
feruloylserotonin
feruloylserotonin
feruloylserotonin
feruloylserotonin
Procaine_c
Procaine_c
Procaine_c
Procaine_c
Procaine_c
N(2)-succinyl-L-ornithine_a
N(2)-succinyl-L-ornithine_a
N(2)-succinyl-L-ornithine_a
N(2)-succinyl-L-ornithine_a
N(2)-succinyl-L-ornithine_a
Varanic acid
Varanic acid
Varanic acid
Varanic acid
Varanic acid
Arenaine
Arenaine
Arenaine
Arenaine
Arenaine
5-guanidino-2-oxopentanoic acid
5-guanidino-2-oxopentanoic acid
5-guanidino-2-oxopentanoic acid
5-guanidino-2-oxopentanoic acid
5-guanidino-2-oxopentanoic acid
4-Hydroxyproline
4-Hydroxyproline
4-Hydroxyproline
4-Hydroxyproline
4-Hydroxyproline

N-[(1R,2S,6R)-4-Azatricyclo[5.2.1.0~2,6~]dec-4-yl]-4-chloro-3-sulfamoylbenzamide
N-[(1R,2S,6R)-4-Azatricyclo[5.2.1.0~2,6~]dec-4-yl]-4-chloro-3-sulfamoylbenzamide
N-[(1R,2S,6R)-4-Azatricyclo[5.2.1.0~2,6~]dec-4-yl]-4-chloro-3-sulfamoylbenzamide
N-[(1R,2S,6R)-4-Azatricyclo[5.2.1.0~2,6~]dec-4-yl]-4-chloro-3-sulfamoylbenzamide
N-[(1R,2S,6R)-4-Azatricyclo[5.2.1.0~2,6~]dec-4-yl]-4-chloro-3-sulfamoylbenzamide
Tetrahydropapaveroline_b
Tetrahydropapaveroline_b
Tetrahydropapaveroline_b
Tetrahydropapaveroline_b
Tetrahydropapaveroline_b
N,N-Diethyl-4-methyl-1-piperazinecarboxamide 4-oxide_d
N,N-Diethyl-4-methyl-1-piperazinecarboxamide 4-oxide_d
N,N-Diethyl-4-methyl-1-piperazinecarboxamide 4-oxide_d
N,N-Diethyl-4-methyl-1-piperazinecarboxamide 4-oxide_d
N,N-Diethyl-4-methyl-1-piperazinecarboxamide 4-oxide_d
1_2-Dihydroxydibenzothiophene
1_2-Dihydroxydibenzothiophene
1_2-Dihydroxydibenzothiophene
1_2-Dihydroxydibenzothiophene
1_2-Dihydroxydibenzothiophene
N-(3-acetamidopropyl)pyrrolidin-2-one_a
N-(3-acetamidopropyl)pyrrolidin-2-one_a
N-(3-acetamidopropyl)pyrrolidin-2-one_a
N-(3-acetamidopropyl)pyrrolidin-2-one_a
N-(3-acetamidopropyl)pyrrolidin-2-one_a
LysoSM(d18:1)
LysoSM(d18:1)
LysoSM(d18:1)
LysoSM(d18:1)
LysoSM(d18:1)
1-(2-Hydroxy-4,6-dimethoxyphenyl)-3-(4-methoxyphenyl)-1-propanone
1-(2-Hydroxy-4,6-dimethoxyphenyl)-3-(4-methoxyphenyl)-1-propanone
1-(2-Hydroxy-4,6-dimethoxyphenyl)-3-(4-methoxyphenyl)-1-propanone
1-(2-Hydroxy-4,6-dimethoxyphenyl)-3-(4-methoxyphenyl)-1-propanone
1-(2-Hydroxy-4,6-dimethoxyphenyl)-3-(4-methoxyphenyl)-1-propanone
Ethyl acetate
Ethyl acetate
Ethyl acetate
Ethyl acetate
Ethyl acetate
Valylvaline_i
Valylvaline_i
Valylvaline_i
Valylvaline_i
Valylvaline_i
Valyl-4-hydroxyproline_b
Valyl-4-hydroxyproline_b
Valyl-4-hydroxyproline_b

Valyl-4-hydroxyproline_b
Valyl-4-hydroxyproline_b
Coprine_d
Coprine_d
Coprine_d
Coprine_d
Coprine_d
Ferulic acid
Ferulic acid
Ferulic acid
Ferulic acid
Ferulic acid
Prenisteine
Prenisteine
Prenisteine
Prenisteine
Prenisteine
N-[1-Carboxy-3-(methylsulfinyl)propyl]glutamine
N-[1-Carboxy-3-(methylsulfinyl)propyl]glutamine
N-[1-Carboxy-3-(methylsulfinyl)propyl]glutamine
N-[1-Carboxy-3-(methylsulfinyl)propyl]glutamine
N-[1-Carboxy-3-(methylsulfinyl)propyl]glutamine
Salicylic acid
Salicylic acid
Salicylic acid
Salicylic acid
Salicylic acid
4-[(E)-2-(3,4,5-Trimethoxyphenyl)vinyl]phenol_b
4-[(E)-2-(3,4,5-Trimethoxyphenyl)vinyl]phenol_b
4-[(E)-2-(3,4,5-Trimethoxyphenyl)vinyl]phenol_b
4-[(E)-2-(3,4,5-Trimethoxyphenyl)vinyl]phenol_b
4-[(E)-2-(3,4,5-Trimethoxyphenyl)vinyl]phenol_b
AAMU_a
AAMU_a
AAMU_a
AAMU_a
AAMU_a
IN00260_b
IN00260_b
IN00260_b
IN00260_b
IN00260_b
L-gamma-Glutamyl-L-valine_k
L-gamma-Glutamyl-L-valine_k
L-gamma-Glutamyl-L-valine_k
L-gamma-Glutamyl-L-valine_k
L-gamma-Glutamyl-L-valine_k
2-Keto-glutaramic acid

2-Keto-glutaramic acid
2-Keto-glutaramic acid
2-Keto-glutaramic acid
2-Keto-glutaramic acid
gamma-Glu-His
gamma-Glu-His
gamma-Glu-His
gamma-Glu-His
gamma-Glu-His
L-gamma-Glutamyl-L-valine_f
L-gamma-Glutamyl-L-valine_f
L-gamma-Glutamyl-L-valine_f
L-gamma-Glutamyl-L-valine_f
L-gamma-Glutamyl-L-valine_f
Aspartyl-L-proline_b
Aspartyl-L-proline_b
Aspartyl-L-proline_b
Aspartyl-L-proline_b
Aspartyl-L-proline_b
(4S)-4-[(2E)-2-Octenoyloxy]-4-(trimethylammonio)butanoate_a
(4S)-4-[(2E)-2-Octenoyloxy]-4-(trimethylammonio)butanoate_a
(4S)-4-[(2E)-2-Octenoyloxy]-4-(trimethylammonio)butanoate_a
(4S)-4-[(2E)-2-Octenoyloxy]-4-(trimethylammonio)butanoate_a
(4S)-4-[(2E)-2-Octenoyloxy]-4-(trimethylammonio)butanoate_a
4-(Nitrosoamino)-1-(3-pyridinyl)-1-butanol_b
4-(Nitrosoamino)-1-(3-pyridinyl)-1-butanol_b
4-(Nitrosoamino)-1-(3-pyridinyl)-1-butanol_b
4-(Nitrosoamino)-1-(3-pyridinyl)-1-butanol_b
4-(Nitrosoamino)-1-(3-pyridinyl)-1-butanol_b
MFCD02728197_a
MFCD02728197_a
MFCD02728197_a
MFCD02728197_a
MFCD02728197_a
Sular
Sular
Sular
Sular
Sular
butalbital_c
butalbital_c
butalbital_c
butalbital_c
butalbital_c
1,1'-[1,12-Dodecanediylbis(oxy)]dibenzene_e
1,1'-[1,12-Dodecanediylbis(oxy)]dibenzene_e
1,1'-[1,12-Dodecanediylbis(oxy)]dibenzene_e
1,1'-[1,12-Dodecanediylbis(oxy)]dibenzene_e

1,1'-[1,12-Dodecanediylbis(oxy)]dibenzene_e
5-(5-Methyl-2-furyl)-2,3-dihydro-1H-pyrrolizine
5-(5-Methyl-2-furyl)-2,3-dihydro-1H-pyrrolizine
5-(5-Methyl-2-furyl)-2,3-dihydro-1H-pyrrolizine
5-(5-Methyl-2-furyl)-2,3-dihydro-1H-pyrrolizine
5-(5-Methyl-2-furyl)-2,3-dihydro-1H-pyrrolizine
3-Deoxy-D-manno-octulosonic acid
3-Deoxy-D-manno-octulosonic acid
3-Deoxy-D-manno-octulosonic acid
3-Deoxy-D-manno-octulosonic acid
3-Deoxy-D-manno-octulosonic acid
2-(1-Naphthyl)acetamide_b
2-(1-Naphthyl)acetamide_b
2-(1-Naphthyl)acetamide_b
2-(1-Naphthyl)acetamide_b
2-(1-Naphthyl)acetamide_b
asn-pro_a
asn-pro_a
asn-pro_a
asn-pro_a
asn-pro_a
ophthalmic acid_b
ophthalmic acid_b
ophthalmic acid_b
ophthalmic acid_b
ophthalmic acid_b
2-Isopropyl-4,6-dimethyl-1,3,5-dithiazinane
2-Isopropyl-4,6-dimethyl-1,3,5-dithiazinane
2-Isopropyl-4,6-dimethyl-1,3,5-dithiazinane
2-Isopropyl-4,6-dimethyl-1,3,5-dithiazinane
2-Isopropyl-4,6-dimethyl-1,3,5-dithiazinane
L-gamma-Glutamyl-L-valine_c
L-gamma-Glutamyl-L-valine_c
L-gamma-Glutamyl-L-valine_c
L-gamma-Glutamyl-L-valine_c
L-gamma-Glutamyl-L-valine_c
2-(2-Hydroxy-3,4,5-trimethoxyphenyl)-7-methoxy-8-chromanol
2-(2-Hydroxy-3,4,5-trimethoxyphenyl)-7-methoxy-8-chromanol
2-(2-Hydroxy-3,4,5-trimethoxyphenyl)-7-methoxy-8-chromanol
2-(2-Hydroxy-3,4,5-trimethoxyphenyl)-7-methoxy-8-chromanol
2-(2-Hydroxy-3,4,5-trimethoxyphenyl)-7-methoxy-8-chromanol
N(1),N(8)-bis(coumaroyl)spermidine
N(1),N(8)-bis(coumaroyl)spermidine
N(1),N(8)-bis(coumaroyl)spermidine
N(1),N(8)-bis(coumaroyl)spermidine
N(1),N(8)-bis(coumaroyl)spermidine
Spermic acid_g
Spermic acid_g

Spermic acid_g
Spermic acid_g
Spermic acid_g
2-Hydroxy-5-[(5-oxotetrahydro-2-furanyl)methyl]phenyl hydrogen sulfate
2-Hydroxy-5-[(5-oxotetrahydro-2-furanyl)methyl]phenyl hydrogen sulfate
2-Hydroxy-5-[(5-oxotetrahydro-2-furanyl)methyl]phenyl hydrogen sulfate
2-Hydroxy-5-[(5-oxotetrahydro-2-furanyl)methyl]phenyl hydrogen sulfate
2-Hydroxy-5-[(5-oxotetrahydro-2-furanyl)methyl]phenyl hydrogen sulfate
4-(METHYLNITROSAMINO)-1-(3-PYRIDYL-N-OXIDE)-1-BUTANOL_a
4-(METHYLNITROSAMINO)-1-(3-PYRIDYL-N-OXIDE)-1-BUTANOL_a
4-(METHYLNITROSAMINO)-1-(3-PYRIDYL-N-OXIDE)-1-BUTANOL_a
4-(METHYLNITROSAMINO)-1-(3-PYRIDYL-N-OXIDE)-1-BUTANOL_a
4-(METHYLNITROSAMINO)-1-(3-PYRIDYL-N-OXIDE)-1-BUTANOL_a
(-)-Physostigmine_a
(-)-Physostigmine_a
(-)-Physostigmine_a
(-)-Physostigmine_a
(-)-Physostigmine_a
N-(3,5-Dimethoxybenzoyl)glycine_a
N-(3,5-Dimethoxybenzoyl)glycine_a
N-(3,5-Dimethoxybenzoyl)glycine_a
N-(3,5-Dimethoxybenzoyl)glycine_a
N-(3,5-Dimethoxybenzoyl)glycine_a
Homoanserine_b
Homoanserine_b
Homoanserine_b
Homoanserine_b
Homoanserine_b
S(6)-acetyldihydrolipoamide
S(6)-acetyldihydrolipoamide
S(6)-acetyldihydrolipoamide
S(6)-acetyldihydrolipoamide
S(6)-acetyldihydrolipoamide
4-Methyleneglutamic acid
4-Methyleneglutamic acid
4-Methyleneglutamic acid
4-Methyleneglutamic acid
4-Methyleneglutamic acid
asn-phe
asn-phe
asn-phe
asn-phe
asn-phe
2-O-beta-D-Galactopyranosyl-D-xylopyranose
2-O-beta-D-Galactopyranosyl-D-xylopyranose
2-O-beta-D-Galactopyranosyl-D-xylopyranose
2-O-beta-D-Galactopyranosyl-D-xylopyranose
2-O-beta-D-Galactopyranosyl-D-xylopyranose

N-Propionylmethionine_a
N-Propionylmethionine_a
N-Propionylmethionine_a
N-Propionylmethionine_a
N-Propionylmethionine_a
N4-(beta-N-Acetyl-D-glucosaminyl)-L-asparagine
N4-(beta-N-Acetyl-D-glucosaminyl)-L-asparagine
N4-(beta-N-Acetyl-D-glucosaminyl)-L-asparagine
N4-(beta-N-Acetyl-D-glucosaminyl)-L-asparagine
N4-(beta-N-Acetyl-D-glucosaminyl)-L-asparagine
3-Methoxy-4-hydroxyhippuric acid_d
3-Methoxy-4-hydroxyhippuric acid_d
3-Methoxy-4-hydroxyhippuric acid_d
3-Methoxy-4-hydroxyhippuric acid_d
3-Methoxy-4-hydroxyhippuric acid_d
Spermic acid_e
Spermic acid_e
Spermic acid_e
Spermic acid_e
Spermic acid_e
L-gamma-Glutamyl-L-valine_l
L-gamma-Glutamyl-L-valine_l
L-gamma-Glutamyl-L-valine_l
L-gamma-Glutamyl-L-valine_l
L-gamma-Glutamyl-L-valine_l
N-(1H-Pyrrol-2-ylcarbonyl)glycine
N-(1H-Pyrrol-2-ylcarbonyl)glycine
N-(1H-Pyrrol-2-ylcarbonyl)glycine
N-(1H-Pyrrol-2-ylcarbonyl)glycine
N-(1H-Pyrrol-2-ylcarbonyl)glycine
N-(3,5-Dimethoxybenzoyl)glycine_e
N-(3,5-Dimethoxybenzoyl)glycine_e
N-(3,5-Dimethoxybenzoyl)glycine_e
N-(3,5-Dimethoxybenzoyl)glycine_e
N-(3,5-Dimethoxybenzoyl)glycine_e
N-{3-[(4-Acetamidobutyl)amino]propyl}acetamide_a
N-{3-[(4-Acetamidobutyl)amino]propyl}acetamide_a
N-{3-[(4-Acetamidobutyl)amino]propyl}acetamide_a
N-{3-[(4-Acetamidobutyl)amino]propyl}acetamide_a
N-{3-[(4-Acetamidobutyl)amino]propyl}acetamide_a
3-hydroxy-3-methyloxindole_a
3-hydroxy-3-methyloxindole_a
3-hydroxy-3-methyloxindole_a
3-hydroxy-3-methyloxindole_a
3-hydroxy-3-methyloxindole_a
tyramine sulfate_b
tyramine sulfate_b
tyramine sulfate_b

tyramine sulfate_b
tyramine sulfate_b
N-Hydroxy-2-acetamidofluorene
N-Hydroxy-2-acetamidofluorene
N-Hydroxy-2-acetamidofluorene
N-Hydroxy-2-acetamidofluorene
N-Hydroxy-2-acetamidofluorene
Endothal
Endothal
Endothal
Endothal
Endothal
2,4,6-Triisobutyl-1,3,5-dithiazinane
2,4,6-Triisobutyl-1,3,5-dithiazinane
2,4,6-Triisobutyl-1,3,5-dithiazinane
2,4,6-Triisobutyl-1,3,5-dithiazinane
2,4,6-Triisobutyl-1,3,5-dithiazinane
N-(3,5-Dimethoxybenzoyl)glycine_b
N-(3,5-Dimethoxybenzoyl)glycine_b
N-(3,5-Dimethoxybenzoyl)glycine_b
N-(3,5-Dimethoxybenzoyl)glycine_b
N-(3,5-Dimethoxybenzoyl)glycine_b
TOLMETIN
TOLMETIN
TOLMETIN
TOLMETIN
TOLMETIN
linatine
linatine
linatine
linatine
linatine
2,3,4,5,6-Pentahydroxy-N-(2-hydroxyethyl)hexanamide
2,3,4,5,6-Pentahydroxy-N-(2-hydroxyethyl)hexanamide
2,3,4,5,6-Pentahydroxy-N-(2-hydroxyethyl)hexanamide
2,3,4,5,6-Pentahydroxy-N-(2-hydroxyethyl)hexanamide
2,3,4,5,6-Pentahydroxy-N-(2-hydroxyethyl)hexanamide
UK3870000
UK3870000
UK3870000
UK3870000
UK3870000
(+)-Etomidate_b
(+)-Etomidate_b
(+)-Etomidate_b
(+)-Etomidate_b
(+)-Etomidate_b
7,8-Didehydro-4,5-epoxymorphinan-3,6-diol

7,8-Didehydro-4,5-epoxymorphinan-3,6-diol
7,8-Didehydro-4,5-epoxymorphinan-3,6-diol
7,8-Didehydro-4,5-epoxymorphinan-3,6-diol
7,8-Didehydro-4,5-epoxymorphinan-3,6-diol
LW8000000_b
LW8000000_b
LW8000000_b
LW8000000_b
LW8000000_b
N(2)-succinyl-L-ornithine_b
N(2)-succinyl-L-ornithine_b
N(2)-succinyl-L-ornithine_b
N(2)-succinyl-L-ornithine_b
N(2)-succinyl-L-ornithine_b
3-(2,3-Dihydroxy-3-methylbutyl)-4-methoxy-1-methyl-2(1H)-quinolinone_a
3-(2,3-Dihydroxy-3-methylbutyl)-4-methoxy-1-methyl-2(1H)-quinolinone_a
3-(2,3-Dihydroxy-3-methylbutyl)-4-methoxy-1-methyl-2(1H)-quinolinone_a
3-(2,3-Dihydroxy-3-methylbutyl)-4-methoxy-1-methyl-2(1H)-quinolinone_a
3-(2,3-Dihydroxy-3-methylbutyl)-4-methoxy-1-methyl-2(1H)-quinolinone_a
Formylkynurenine_a
Formylkynurenine_a
Formylkynurenine_a
Formylkynurenine_a
Formylkynurenine_a
indoline-2-carboxylic acid
indoline-2-carboxylic acid
indoline-2-carboxylic acid
indoline-2-carboxylic acid
indoline-2-carboxylic acid
{[(15-Hydroxy-3-methoxy-1,2,6,7-tetradehydroerythrinan-16-yl)oxy]sulfonyl}acetic acid
{[(15-Hydroxy-3-methoxy-1,2,6,7-tetradehydroerythrinan-16-yl)oxy]sulfonyl}acetic acid
{[(15-Hydroxy-3-methoxy-1,2,6,7-tetradehydroerythrinan-16-yl)oxy]sulfonyl}acetic acid
{[(15-Hydroxy-3-methoxy-1,2,6,7-tetradehydroerythrinan-16-yl)oxy]sulfonyl}acetic acid
{[(15-Hydroxy-3-methoxy-1,2,6,7-tetradehydroerythrinan-16-yl)oxy]sulfonyl}acetic acid
(7E,7'E)-5,5'-diferulic acid_b
(7E,7'E)-5,5'-diferulic acid_b
(7E,7'E)-5,5'-diferulic acid_b
(7E,7'E)-5,5'-diferulic acid_b
(7E,7'E)-5,5'-diferulic acid_b
MFCD01664827
MFCD01664827
MFCD01664827
MFCD01664827
MFCD01664827
Estrone glucuronide
Estrone glucuronide
Estrone glucuronide
Estrone glucuronide

Estrone glucuronide
ELK (Peptide Glu-Leu-Lys)_a
ELK (Peptide Glu-Leu-Lys)_a
ELK (Peptide Glu-Leu-Lys)_a
ELK (Peptide Glu-Leu-Lys)_a
ELK (Peptide Glu-Leu-Lys)_a
Tetrahydro-2-furanylmethyl hydrogen sulfate
Tetrahydro-2-furanylmethyl hydrogen sulfate
Tetrahydro-2-furanylmethyl hydrogen sulfate
Tetrahydro-2-furanylmethyl hydrogen sulfate
Tetrahydro-2-furanylmethyl hydrogen sulfate
2-(3-CARBOXYPROPIONYL)-6-HYDROXY-CYCLOHEXA-2,4-DIENE CARBOXYLIC ACID_a
2-(3-CARBOXYPROPIONYL)-6-HYDROXY-CYCLOHEXA-2,4-DIENE CARBOXYLIC ACID_a
2-(3-CARBOXYPROPIONYL)-6-HYDROXY-CYCLOHEXA-2,4-DIENE CARBOXYLIC ACID_a
2-(3-CARBOXYPROPIONYL)-6-HYDROXY-CYCLOHEXA-2,4-DIENE CARBOXYLIC ACID_a
2-(3-CARBOXYPROPIONYL)-6-HYDROXY-CYCLOHEXA-2,4-DIENE CARBOXYLIC ACID_a
Queuosine
Queuosine
Queuosine
Queuosine
Queuosine
Methyl alpha-aspartylphenylalaninate_b
Methyl alpha-aspartylphenylalaninate_b
Methyl alpha-aspartylphenylalaninate_b
Methyl alpha-aspartylphenylalaninate_b
Methyl alpha-aspartylphenylalaninate_b
Zalcitabine_g
Zalcitabine_g
Zalcitabine_g
Zalcitabine_g
Zalcitabine_g
(2S)-6-Amino-2-[(E)-(hydroxymethylene)amino]hexanimidic acid_a
(2S)-6-Amino-2-[(E)-(hydroxymethylene)amino]hexanimidic acid_a
(2S)-6-Amino-2-[(E)-(hydroxymethylene)amino]hexanimidic acid_a
(2S)-6-Amino-2-[(E)-(hydroxymethylene)amino]hexanimidic acid_a
(2S)-6-Amino-2-[(E)-(hydroxymethylene)amino]hexanimidic acid_a
3-Methyladenine_c
3-Methyladenine_c
3-Methyladenine_c
3-Methyladenine_c
3-Methyladenine_c
N-Benzyl-3-[(2S,5aS,8aR)-6-(1H-imidazol-2-ylmethyl)-1-methyl-5-oxodecahydropyrrolo[3,2-E][1,4]diazepin-2-yl]
N-Benzyl-3-[(2S,5aS,8aR)-6-(1H-imidazol-2-ylmethyl)-1-methyl-5-oxodecahydropyrrolo[3,2-E][1,4]diazepin-2-yl]
N-Benzyl-3-[(2S,5aS,8aR)-6-(1H-imidazol-2-ylmethyl)-1-methyl-5-oxodecahydropyrrolo[3,2-E][1,4]diazepin-2-yl]
N-Benzyl-3-[(2S,5aS,8aR)-6-(1H-imidazol-2-ylmethyl)-1-methyl-5-oxodecahydropyrrolo[3,2-E][1,4]diazepin-2-yl]
N-Benzyl-3-[(2S,5aS,8aR)-6-(1H-imidazol-2-ylmethyl)-1-methyl-5-oxodecahydropyrrolo[3,2-E][1,4]diazepin-2-yl]
4-[(E)-2-(3,4,5-Trimethoxyphenyl)vinyl]phenol_a
4-[(E)-2-(3,4,5-Trimethoxyphenyl)vinyl]phenol_a

4-[(E)-2-(3,4,5-Trimethoxyphenyl)vinyl]phenol_a
4-[(E)-2-(3,4,5-Trimethoxyphenyl)vinyl]phenol_a
4-[(E)-2-(3,4,5-Trimethoxyphenyl)vinyl]phenol_a
Harmane
Harmane
Harmane
Harmane
Harmane
1-(beta-D-ribofuranosyl)thymine_b
1-(beta-D-ribofuranosyl)thymine_b
1-(beta-D-ribofuranosyl)thymine_b
1-(beta-D-ribofuranosyl)thymine_b
1-(beta-D-ribofuranosyl)thymine_b
N(6),N(6)-Dimethyladenine
N(6),N(6)-Dimethyladenine
N(6),N(6)-Dimethyladenine
N(6),N(6)-Dimethyladenine
N(6),N(6)-Dimethyladenine
MC0555300
MC0555300
MC0555300
MC0555300
MC0555300
4-ethylphenylsulfonic acid
4-ethylphenylsulfonic acid
4-ethylphenylsulfonic acid
4-ethylphenylsulfonic acid
4-ethylphenylsulfonic acid
5-Hydroxy-N-[(6-oxo-2-piperidinyl)methyl]-2-(2,2,2-trifluoroethoxy)benzamide
5-Hydroxy-N-[(6-oxo-2-piperidinyl)methyl]-2-(2,2,2-trifluoroethoxy)benzamide
5-Hydroxy-N-[(6-oxo-2-piperidinyl)methyl]-2-(2,2,2-trifluoroethoxy)benzamide
5-Hydroxy-N-[(6-oxo-2-piperidinyl)methyl]-2-(2,2,2-trifluoroethoxy)benzamide
5-Hydroxy-N-[(6-oxo-2-piperidinyl)methyl]-2-(2,2,2-trifluoroethoxy)benzamide
g-Aminobutyryl-lysine_b
g-Aminobutyryl-lysine_b
g-Aminobutyryl-lysine_b
g-Aminobutyryl-lysine_b
g-Aminobutyryl-lysine_b
pentobarbital_e
pentobarbital_e
pentobarbital_e
pentobarbital_e
pentobarbital_e
Spermic acid_b
Spermic acid_b
Spermic acid_b
Spermic acid_b
Spermic acid_b

3-Hydroxyhexadecadienoylcarnitine
3-Hydroxyhexadecadienoylcarnitine
3-Hydroxyhexadecadienoylcarnitine
3-Hydroxyhexadecadienoylcarnitine
3-Hydroxyhexadecadienoylcarnitine
5beta-Cyprinolsulfate
5beta-Cyprinolsulfate
5beta-Cyprinolsulfate
5beta-Cyprinolsulfate
5beta-Cyprinolsulfate
Iminoglycine
Iminoglycine
Iminoglycine
Iminoglycine
Iminoglycine
Biocytin
Biocytin
Biocytin
Biocytin
Biocytin
3-Methyladenine_a
3-Methyladenine_a
3-Methyladenine_a
3-Methyladenine_a
3-Methyladenine_a
TLK (Peptide Thr-Leu-Lys)
TLK (Peptide Thr-Leu-Lys)
TLK (Peptide Thr-Leu-Lys)
TLK (Peptide Thr-Leu-Lys)
TLK (Peptide Thr-Leu-Lys)
N-(4-Heptanyl)-1,3-benzodioxole-5-carboxamide
N-(4-Heptanyl)-1,3-benzodioxole-5-carboxamide
N-(4-Heptanyl)-1,3-benzodioxole-5-carboxamide
N-(4-Heptanyl)-1,3-benzodioxole-5-carboxamide
N-(4-Heptanyl)-1,3-benzodioxole-5-carboxamide
N-Acetylvaline_c
N-Acetylvaline_c
N-Acetylvaline_c
N-Acetylvaline_c
N-Acetylvaline_c
N-[(10Z)-7-Isobutyl-3-isopropyl-5,8-dioxo-2-oxa-6,9-diazabicyclo[10.2.2]hexadeca-1(14),10,12,15-tetraen-4-yl]-
N-[(10Z)-7-Isobutyl-3-isopropyl-5,8-dioxo-2-oxa-6,9-diazabicyclo[10.2.2]hexadeca-1(14),10,12,15-tetraen-4-yl]-
N-[(10Z)-7-Isobutyl-3-isopropyl-5,8-dioxo-2-oxa-6,9-diazabicyclo[10.2.2]hexadeca-1(14),10,12,15-tetraen-4-yl]-
N-[(10Z)-7-Isobutyl-3-isopropyl-5,8-dioxo-2-oxa-6,9-diazabicyclo[10.2.2]hexadeca-1(14),10,12,15-tetraen-4-yl]-
N-[(10Z)-7-Isobutyl-3-isopropyl-5,8-dioxo-2-oxa-6,9-diazabicyclo[10.2.2]hexadeca-1(14),10,12,15-tetraen-4-yl]-
Midodrine_e
Midodrine_e
Midodrine_e

Midodrine_e
Midodrine_e
2-[4-(3-Hydroxypropyl)-2-methoxyphenoxy]-1,3-propanediol
2-[4-(3-Hydroxypropyl)-2-methoxyphenoxy]-1,3-propanediol
2-[4-(3-Hydroxypropyl)-2-methoxyphenoxy]-1,3-propanediol
2-[4-(3-Hydroxypropyl)-2-methoxyphenoxy]-1,3-propanediol
2-[4-(3-Hydroxypropyl)-2-methoxyphenoxy]-1,3-propanediol
4,9a-Dimethoxy-3,4a,5-trimethyl-2-oxo-2,4,4a,5,6,7,8,8a,9,9a-decahydronaphtho[2,3-b]furan-6-yl (2E)-3-(methy
4,9a-Dimethoxy-3,4a,5-trimethyl-2-oxo-2,4,4a,5,6,7,8,8a,9,9a-decahydronaphtho[2,3-b]furan-6-yl (2E)-3-(methy
4,9a-Dimethoxy-3,4a,5-trimethyl-2-oxo-2,4,4a,5,6,7,8,8a,9,9a-decahydronaphtho[2,3-b]furan-6-yl (2E)-3-(methy
4,9a-Dimethoxy-3,4a,5-trimethyl-2-oxo-2,4,4a,5,6,7,8,8a,9,9a-decahydronaphtho[2,3-b]furan-6-yl (2E)-3-(methy
4,9a-Dimethoxy-3,4a,5-trimethyl-2-oxo-2,4,4a,5,6,7,8,8a,9,9a-decahydronaphtho[2,3-b]furan-6-yl (2E)-3-(methy
epsilon-(gamma-Glutamyl)-lysine_b
epsilon-(gamma-Glutamyl)-lysine_b
epsilon-(gamma-Glutamyl)-lysine_b
epsilon-(gamma-Glutamyl)-lysine_b
epsilon-(gamma-Glutamyl)-lysine_b
Nimodipine
Nimodipine
Nimodipine
Nimodipine
Nimodipine
his-gln_a
his-gln_a
his-gln_a
his-gln_a
his-gln_a
Dehydroacetic acid
Dehydroacetic acid
Dehydroacetic acid
Dehydroacetic acid
Dehydroacetic acid
trimethadione
trimethadione
trimethadione
trimethadione
trimethadione
(2E,6E)-9-[(2R)-6-Hydroxy-2,5,7,8-tetramethyl-3,4-dihydro-2H-chromen-2-yl]-2,6-dimethyl-2,6-nonadienoic acid
(2E,6E)-9-[(2R)-6-Hydroxy-2,5,7,8-tetramethyl-3,4-dihydro-2H-chromen-2-yl]-2,6-dimethyl-2,6-nonadienoic acid
(2E,6E)-9-[(2R)-6-Hydroxy-2,5,7,8-tetramethyl-3,4-dihydro-2H-chromen-2-yl]-2,6-dimethyl-2,6-nonadienoic acid
(2E,6E)-9-[(2R)-6-Hydroxy-2,5,7,8-tetramethyl-3,4-dihydro-2H-chromen-2-yl]-2,6-dimethyl-2,6-nonadienoic acid
(2E,6E)-9-[(2R)-6-Hydroxy-2,5,7,8-tetramethyl-3,4-dihydro-2H-chromen-2-yl]-2,6-dimethyl-2,6-nonadienoic acid
Coixol
Coixol
Coixol
Coixol
Coixol
Methyl 2,3-dihydro-3-hydroxy-2-oxo-1H-indole-3-acetate_b

Methyl 2,3-dihydro-3-hydroxy-2-oxo-1H-indole-3-acetate_b
Methyl 2,3-dihydro-3-hydroxy-2-oxo-1H-indole-3-acetate_b
Methyl 2,3-dihydro-3-hydroxy-2-oxo-1H-indole-3-acetate_b
Methyl 2,3-dihydro-3-hydroxy-2-oxo-1H-indole-3-acetate_b
ala-met
ala-met
ala-met
ala-met
ala-met
Leu-pro_b
Leu-pro_b
Leu-pro_b
Leu-pro_b
Leu-pro_b
SECONAL_e
SECONAL_e
SECONAL_e
SECONAL_e
SECONAL_e
N-(4-Hydroxy-3,5-dimethoxybenzoyl)glycine_a
N-(4-Hydroxy-3,5-dimethoxybenzoyl)glycine_a
N-(4-Hydroxy-3,5-dimethoxybenzoyl)glycine_a
N-(4-Hydroxy-3,5-dimethoxybenzoyl)glycine_a
N-(4-Hydroxy-3,5-dimethoxybenzoyl)glycine_a
1-Methyl-1,2,3,4-tetrahydro-ÃŽÂ^2-carboline-3-carboxylic acid_a
1-Methyl-1,2,3,4-tetrahydro-ÃŽÂ^2-carboline-3-carboxylic acid_a
1-Methyl-1,2,3,4-tetrahydro-ÃŽÂ^2-carboline-3-carboxylic acid_a
1-Methyl-1,2,3,4-tetrahydro-ÃŽÂ^2-carboline-3-carboxylic acid_a
1-Methyl-1,2,3,4-tetrahydro-ÃŽÂ^2-carboline-3-carboxylic acid_a
imazamethabenz-methyl_b
imazamethabenz-methyl_b
imazamethabenz-methyl_b
imazamethabenz-methyl_b
imazamethabenz-methyl_b
cys-met
cys-met
cys-met
cys-met
cys-met
2-(3-CARBOXYPROPIONYL)-6-HYDROXY-CYCLOHEXA-2,4-DIENE CARBOXYLIC ACID_b
2-(3-CARBOXYPROPIONYL)-6-HYDROXY-CYCLOHEXA-2,4-DIENE CARBOXYLIC ACID_b
2-(3-CARBOXYPROPIONYL)-6-HYDROXY-CYCLOHEXA-2,4-DIENE CARBOXYLIC ACID_b
2-(3-CARBOXYPROPIONYL)-6-HYDROXY-CYCLOHEXA-2,4-DIENE CARBOXYLIC ACID_b
2-(3-CARBOXYPROPIONYL)-6-HYDROXY-CYCLOHEXA-2,4-DIENE CARBOXYLIC ACID_b
Quinaldic acid
Quinaldic acid
Quinaldic acid
Quinaldic acid

Quinaldic acid
Gly-DL-Phe
Gly-DL-Phe
Gly-DL-Phe
Gly-DL-Phe
Gly-DL-Phe
4-Formyl-2-methoxyphenyl hydrogen sulfate
4-Formyl-2-methoxyphenyl hydrogen sulfate
4-Formyl-2-methoxyphenyl hydrogen sulfate
4-Formyl-2-methoxyphenyl hydrogen sulfate
4-Formyl-2-methoxyphenyl hydrogen sulfate
L-gamma-Glutamyl-L-valine_h
L-gamma-Glutamyl-L-valine_h
L-gamma-Glutamyl-L-valine_h
L-gamma-Glutamyl-L-valine_h
L-gamma-Glutamyl-L-valine_h
Imidazolelactic acid
Imidazolelactic acid
Imidazolelactic acid
Imidazolelactic acid
Imidazolelactic acid
Ethynodiol diacetate
Ethynodiol diacetate
Ethynodiol diacetate
Ethynodiol diacetate
Ethynodiol diacetate
(+)-Etomidate_a
(+)-Etomidate_a
(+)-Etomidate_a
(+)-Etomidate_a
(+)-Etomidate_a
3-methyl-4-(methylamino)-1,2-diphenylbutan-2-yl propanoate
3-methyl-4-(methylamino)-1,2-diphenylbutan-2-yl propanoate
3-methyl-4-(methylamino)-1,2-diphenylbutan-2-yl propanoate
3-methyl-4-(methylamino)-1,2-diphenylbutan-2-yl propanoate
3-methyl-4-(methylamino)-1,2-diphenylbutan-2-yl propanoate
Maleamate
Maleamate
Maleamate
Maleamate
Maleamate
Lys-Pro_d
Lys-Pro_d
Lys-Pro_d
Lys-Pro_d
Lys-Pro_d
2,3,8,9-Tetrahydroxybenzo[b][1]benzofuro[2,3-f][1]benzofuran-6,12-dione
2,3,8,9-Tetrahydroxybenzo[b][1]benzofuro[2,3-f][1]benzofuran-6,12-dione

2,3,8,9-Tetrahydroxybenzo[b][1]benzofuro[2,3-f][1]benzofuran-6,12-dione
2,3,8,9-Tetrahydroxybenzo[b][1]benzofuro[2,3-f][1]benzofuran-6,12-dione
2,3,8,9-Tetrahydroxybenzo[b][1]benzofuro[2,3-f][1]benzofuran-6,12-dione
4-morpholinobenzoic acid
4-morpholinobenzoic acid
4-morpholinobenzoic acid
4-morpholinobenzoic acid
4-morpholinobenzoic acid
1-Methyl-1,2,3,4-tetrahydro-ÃŽÂ²-carboline-3-carboxylic acid_b
1-Methyl-1,2,3,4-tetrahydro-ÃŽÂ²-carboline-3-carboxylic acid_b
1-Methyl-1,2,3,4-tetrahydro-ÃŽÂ²-carboline-3-carboxylic acid_b
1-Methyl-1,2,3,4-tetrahydro-ÃŽÂ²-carboline-3-carboxylic acid_b
1-Methyl-1,2,3,4-tetrahydro-ÃŽÂ²-carboline-3-carboxylic acid_b
Lovastatin_b
Lovastatin_b
Lovastatin_b
Lovastatin_b
Lovastatin_b
Sulfurous acid
Sulfurous acid
Sulfurous acid
Sulfurous acid
Sulfurous acid
4H-1-Benzopyran-4-one, 6-Î²-D-glucopyranosyl-2,3-dihydro-5,7-dihydroxy-2-(4-hydroxyphenyl)-, (S)-
4H-1-Benzopyran-4-one, 6-Î²-D-glucopyranosyl-2,3-dihydro-5,7-dihydroxy-2-(4-hydroxyphenyl)-, (S)-
4H-1-Benzopyran-4-one, 6-Î²-D-glucopyranosyl-2,3-dihydro-5,7-dihydroxy-2-(4-hydroxyphenyl)-, (S)-
4H-1-Benzopyran-4-one, 6-Î²-D-glucopyranosyl-2,3-dihydro-5,7-dihydroxy-2-(4-hydroxyphenyl)-, (S)-
4H-1-Benzopyran-4-one, 6-Î²-D-glucopyranosyl-2,3-dihydro-5,7-dihydroxy-2-(4-hydroxyphenyl)-, (S)-
S-Allylcysteine_a
S-Allylcysteine_a
S-Allylcysteine_a
S-Allylcysteine_a
S-Allylcysteine_a
L-gamma-Glutamyl-L-leucine_c
L-gamma-Glutamyl-L-leucine_c
L-gamma-Glutamyl-L-leucine_c
L-gamma-Glutamyl-L-leucine_c
L-gamma-Glutamyl-L-leucine_c
2-(1-Naphthyl)acetamide_a
2-(1-Naphthyl)acetamide_a
2-(1-Naphthyl)acetamide_a
2-(1-Naphthyl)acetamide_a
2-(1-Naphthyl)acetamide_a
L-Arogenate
L-Arogenate
L-Arogenate
L-Arogenate
L-Arogenate

4-Indolecarbaldehyde
4-Indolecarbaldehyde
4-Indolecarbaldehyde
4-Indolecarbaldehyde
4-Indolecarbaldehyde
Phenyl D-glucopyranosiduronic acid_c
Phenyl D-glucopyranosiduronic acid_c
Phenyl D-glucopyranosiduronic acid_c
Phenyl D-glucopyranosiduronic acid_c
Phenyl D-glucopyranosiduronic acid_c
Zalcitabine_d
Zalcitabine_d
Zalcitabine_d
Zalcitabine_d
Zalcitabine_d
L-N2-(2-Carboxyethyl)arginine_b
L-N2-(2-Carboxyethyl)arginine_b
L-N2-(2-Carboxyethyl)arginine_b
L-N2-(2-Carboxyethyl)arginine_b
L-N2-(2-Carboxyethyl)arginine_b
Coenzyme M
Coenzyme M
Coenzyme M
Coenzyme M
Coenzyme M
Leucylasparagine_a
Leucylasparagine_a
Leucylasparagine_a
Leucylasparagine_a
Leucylasparagine_a
Ro 20-1724_d
Ro 20-1724_d
Ro 20-1724_d
Ro 20-1724_d
Ro 20-1724_d
3-Methylxanthine
3-Methylxanthine
3-Methylxanthine
3-Methylxanthine
3-Methylxanthine
beta-D-GlcpA-(1->6)-beta-D-Galp
beta-D-GlcpA-(1->6)-beta-D-Galp
beta-D-GlcpA-(1->6)-beta-D-Galp
beta-D-GlcpA-(1->6)-beta-D-Galp
beta-D-GlcpA-(1->6)-beta-D-Galp
L-Saccharopine
L-Saccharopine
L-Saccharopine

L-Saccharopine
L-Saccharopine
1,5-Isoquinolinediol_a
1,5-Isoquinolinediol_a
1,5-Isoquinolinediol_a
1,5-Isoquinolinediol_a
1,5-Isoquinolinediol_a
(6alpha,11beta)-6,9-Difluoro-11,21-dihydroxy-3,20-dioxopregna-1,4-dien-17-yl butyrate
(6alpha,11beta)-6,9-Difluoro-11,21-dihydroxy-3,20-dioxopregna-1,4-dien-17-yl butyrate
(6alpha,11beta)-6,9-Difluoro-11,21-dihydroxy-3,20-dioxopregna-1,4-dien-17-yl butyrate
(6alpha,11beta)-6,9-Difluoro-11,21-dihydroxy-3,20-dioxopregna-1,4-dien-17-yl butyrate
(6alpha,11beta)-6,9-Difluoro-11,21-dihydroxy-3,20-dioxopregna-1,4-dien-17-yl butyrate
2-(Carboxyacetamido)benzoic acid_a
2-(Carboxyacetamido)benzoic acid_a
2-(Carboxyacetamido)benzoic acid_a
2-(Carboxyacetamido)benzoic acid_a
2-(Carboxyacetamido)benzoic acid_a
Ro 20-1724_c
Ro 20-1724_c
Ro 20-1724_c
Ro 20-1724_c
Ro 20-1724_c
8-Amino-7-oxononanoic acid_d
8-Amino-7-oxononanoic acid_d
8-Amino-7-oxononanoic acid_d
8-Amino-7-oxononanoic acid_d
8-Amino-7-oxononanoic acid_d
glu-ser
glu-ser
glu-ser
glu-ser
glu-ser
GAMMA-HYDROXYHOMOARGININE
GAMMA-HYDROXYHOMOARGININE
GAMMA-HYDROXYHOMOARGININE
GAMMA-HYDROXYHOMOARGININE
GAMMA-HYDROXYHOMOARGININE
Coumarin
Coumarin
Coumarin
Coumarin
Coumarin
4-Guanidinobutyric acid
4-Guanidinobutyric acid
4-Guanidinobutyric acid
4-Guanidinobutyric acid
4-Guanidinobutyric acid
26Q0EO75R3

26Q0EO75R3
26Q0EO75R3
26Q0EO75R3
26Q0EO75R3
4-Guanidinobutanal
4-Guanidinobutanal
4-Guanidinobutanal
4-Guanidinobutanal
4-Guanidinobutanal
APM_a
APM_a
APM_a
APM_a
APM_a
dopaquinone
dopaquinone
dopaquinone
dopaquinone
dopaquinone
ophthalmic acid_c
ophthalmic acid_c
ophthalmic acid_c
ophthalmic acid_c
ophthalmic acid_c
MFCD00025555_e
MFCD00025555_e
MFCD00025555_e
MFCD00025555_e
MFCD00025555_e
L-N2-(2-Carboxyethyl)arginine_a
L-N2-(2-Carboxyethyl)arginine_a
L-N2-(2-Carboxyethyl)arginine_a
L-N2-(2-Carboxyethyl)arginine_a
L-N2-(2-Carboxyethyl)arginine_a
CMPF
CMPF
CMPF
CMPF
CMPF
Glaucine
Glaucine
Glaucine
Glaucine
Glaucine
pentobarbital_b
pentobarbital_b
pentobarbital_b
pentobarbital_b

pentobarbital_b
Glycine
Glycine
Glycine
Glycine
Glycine
2-(1-Ethoxyethoxy)propanoic acid_a
2-(1-Ethoxyethoxy)propanoic acid_a
2-(1-Ethoxyethoxy)propanoic acid_a
2-(1-Ethoxyethoxy)propanoic acid_a
2-(1-Ethoxyethoxy)propanoic acid_a
Leu-Leu_a
Leu-Leu_a
Leu-Leu_a
Leu-Leu_a
Leu-Leu_a
(8)-Gingerol
(8)-Gingerol
(8)-Gingerol
(8)-Gingerol
(8)-Gingerol
Vorinostat_c
Vorinostat_c
Vorinostat_c
Vorinostat_c
Vorinostat_c
Glycocyamine
Glycocyamine
Glycocyamine
Glycocyamine
Glycocyamine
2-Acrylamido-2-methyl-1-propane sulfonic acid
2-Acrylamido-2-methyl-1-propane sulfonic acid
2-Acrylamido-2-methyl-1-propane sulfonic acid
2-Acrylamido-2-methyl-1-propane sulfonic acid
2-Acrylamido-2-methyl-1-propane sulfonic acid
Monomethyl glutarate
Monomethyl glutarate
Monomethyl glutarate
Monomethyl glutarate
Monomethyl glutarate
2-methyl-1H-benzimidazole-5-carboxylic acid
2-methyl-1H-benzimidazole-5-carboxylic acid
2-methyl-1H-benzimidazole-5-carboxylic acid
2-methyl-1H-benzimidazole-5-carboxylic acid
2-methyl-1H-benzimidazole-5-carboxylic acid
N-Nonanoylglycine_c
N-Nonanoylglycine_c

N-Nonanoylglycine_c
N-Nonanoylglycine_c
N-Nonanoylglycine_c
8-hydroxy-11-(hydroxymethyl)-1,5,11-trimethyltricyclo[6.2.1.02,6]undec-2-en-9-one
8-hydroxy-11-(hydroxymethyl)-1,5,11-trimethyltricyclo[6.2.1.02,6]undec-2-en-9-one
8-hydroxy-11-(hydroxymethyl)-1,5,11-trimethyltricyclo[6.2.1.02,6]undec-2-en-9-one
8-hydroxy-11-(hydroxymethyl)-1,5,11-trimethyltricyclo[6.2.1.02,6]undec-2-en-9-one
8-hydroxy-11-(hydroxymethyl)-1,5,11-trimethyltricyclo[6.2.1.02,6]undec-2-en-9-one
4-Hydroxyprolylleucine_b
4-Hydroxyprolylleucine_b
4-Hydroxyprolylleucine_b
4-Hydroxyprolylleucine_b
4-Hydroxyprolylleucine_b
Methyl 1-hydroxy-7-(hydroxymethyl)-1,4a,5,7a-tetrahydrocyclopenta[c]pyran-4-carboxylate_c
Methyl 1-hydroxy-7-(hydroxymethyl)-1,4a,5,7a-tetrahydrocyclopenta[c]pyran-4-carboxylate_c
Methyl 1-hydroxy-7-(hydroxymethyl)-1,4a,5,7a-tetrahydrocyclopenta[c]pyran-4-carboxylate_c
Methyl 1-hydroxy-7-(hydroxymethyl)-1,4a,5,7a-tetrahydrocyclopenta[c]pyran-4-carboxylate_c
Methyl 1-hydroxy-7-(hydroxymethyl)-1,4a,5,7a-tetrahydrocyclopenta[c]pyran-4-carboxylate_c
2-Methylthiazolidine_c
2-Methylthiazolidine_c
2-Methylthiazolidine_c
2-Methylthiazolidine_c
2-Methylthiazolidine_c
N1-(5-methylisoxazol-3-yl)-2-morpholinoacetamide
N1-(5-methylisoxazol-3-yl)-2-morpholinoacetamide
N1-(5-methylisoxazol-3-yl)-2-morpholinoacetamide
N1-(5-methylisoxazol-3-yl)-2-morpholinoacetamide
N1-(5-methylisoxazol-3-yl)-2-morpholinoacetamide
Aprobarbital
Aprobarbital
Aprobarbital
Aprobarbital
Aprobarbital
N-Phenylacetylglutamic acid_b
N-Phenylacetylglutamic acid_b
N-Phenylacetylglutamic acid_b
N-Phenylacetylglutamic acid_b
N-Phenylacetylglutamic acid_b
6-(1-Hydroxyethyl)-3-(hydroxymethyl)-2,7-dioxabicyclo[4.1.0]hept-3-en-5-one_a
6-(1-Hydroxyethyl)-3-(hydroxymethyl)-2,7-dioxabicyclo[4.1.0]hept-3-en-5-one_a
6-(1-Hydroxyethyl)-3-(hydroxymethyl)-2,7-dioxabicyclo[4.1.0]hept-3-en-5-one_a
6-(1-Hydroxyethyl)-3-(hydroxymethyl)-2,7-dioxabicyclo[4.1.0]hept-3-en-5-one_a
6-(1-Hydroxyethyl)-3-(hydroxymethyl)-2,7-dioxabicyclo[4.1.0]hept-3-en-5-one_a
8-hydroxy-7-methylguanine
8-hydroxy-7-methylguanine
8-hydroxy-7-methylguanine
8-hydroxy-7-methylguanine
8-hydroxy-7-methylguanine

Glycylvaline
Glycylvaline
Glycylvaline
Glycylvaline
Glycylvaline
Gln-Gln
Gln-Gln
Gln-Gln
Gln-Gln
Gln-Gln
(âˆ’)-nabilone_d
(âˆ’)-nabilone_d
(âˆ’)-nabilone_d
(âˆ’)-nabilone_d
(âˆ’)-nabilone_d
Furaneol
Furaneol
Furaneol
Furaneol
Furaneol
norhaman
norhaman
norhaman
norhaman
norhaman
Acetanilide
Acetanilide
Acetanilide
Acetanilide
Acetanilide
Ethyl malate_b
Ethyl malate_b
Ethyl malate_b
Ethyl malate_b
Ethyl malate_b
Toxopyrimidine
Toxopyrimidine
Toxopyrimidine
Toxopyrimidine
Toxopyrimidine
5-Nitro-2-propoxyaniline
5-Nitro-2-propoxyaniline
5-Nitro-2-propoxyaniline
5-Nitro-2-propoxyaniline
5-Nitro-2-propoxyaniline
Glycyrin
Glycyrin
Glycyrin

Glycyrin
Glycyrin
Cystine
Cystine
Cystine
Cystine
Cystine
2-(4-Isopropyl-4-methyl-5-oxo-4,5-dihydro-1H-imidazol-2-yl)-4-methylbenzoic acid_a
2-(4-Isopropyl-4-methyl-5-oxo-4,5-dihydro-1H-imidazol-2-yl)-4-methylbenzoic acid_a
2-(4-Isopropyl-4-methyl-5-oxo-4,5-dihydro-1H-imidazol-2-yl)-4-methylbenzoic acid_a
2-(4-Isopropyl-4-methyl-5-oxo-4,5-dihydro-1H-imidazol-2-yl)-4-methylbenzoic acid_a
2-(4-Isopropyl-4-methyl-5-oxo-4,5-dihydro-1H-imidazol-2-yl)-4-methylbenzoic acid_a
Bicine_a
Bicine_a
Bicine_a
Bicine_a
Bicine_a
Histidylglycine_a
Histidylglycine_a
Histidylglycine_a
Histidylglycine_a
Histidylglycine_a
Zalcitabine_b
Zalcitabine_b
Zalcitabine_b
Zalcitabine_b
Zalcitabine_b
butalbital_b
butalbital_b
butalbital_b
butalbital_b
butalbital_b
Hyocholic acid
Hyocholic acid
Hyocholic acid
Hyocholic acid
Hyocholic acid
5-amino-2-(dimethylamino)benzoic acid_b
5-amino-2-(dimethylamino)benzoic acid_b
5-amino-2-(dimethylamino)benzoic acid_b
5-amino-2-(dimethylamino)benzoic acid_b
5-amino-2-(dimethylamino)benzoic acid_b
Melatonin
Melatonin
Melatonin
Melatonin
Melatonin
L-gamma-Glutamyl-L-leucine_e

L-gamma-Glutamyl-L-leucine_e
L-gamma-Glutamyl-L-leucine_e
L-gamma-Glutamyl-L-leucine_e
L-gamma-Glutamyl-L-leucine_e
L-gamma-Glutamyl-L-valine_g
L-gamma-Glutamyl-L-valine_g
L-gamma-Glutamyl-L-valine_g
L-gamma-Glutamyl-L-valine_g
L-gamma-Glutamyl-L-valine_g
N-acetyl-beta-D-glucosaminylamine
N-acetyl-beta-D-glucosaminylamine
N-acetyl-beta-D-glucosaminylamine
N-acetyl-beta-D-glucosaminylamine
N-acetyl-beta-D-glucosaminylamine
6-Hydroxynicotinic acid
6-Hydroxynicotinic acid
6-Hydroxynicotinic acid
6-Hydroxynicotinic acid
6-Hydroxynicotinic acid
bis-noryangonin
bis-noryangonin
bis-noryangonin
bis-noryangonin
bis-noryangonin
Isoquinoline_b
Isoquinoline_b
Isoquinoline_b
Isoquinoline_b
Isoquinoline_b
meprobamate_a
meprobamate_a
meprobamate_a
meprobamate_a
meprobamate_a
Temozolomide
Temozolomide
Temozolomide
Temozolomide
Temozolomide
MFCD09953737_c
MFCD09953737_c
MFCD09953737_c
MFCD09953737_c
MFCD09953737_c
clavulanic acid
clavulanic acid
clavulanic acid
clavulanic acid

clavulanic acid
1,1'-[1,12-Dodecanediylbis(oxy)]dibenzene_f
1,1'-[1,12-Dodecanediylbis(oxy)]dibenzene_f
1,1'-[1,12-Dodecanediylbis(oxy)]dibenzene_f
1,1'-[1,12-Dodecanediylbis(oxy)]dibenzene_f
1,1'-[1,12-Dodecanediylbis(oxy)]dibenzene_f
8-Methyl-8-azabicyclo[3.2.1]octane-1,2,3,4,6-pentol_b
8-Methyl-8-azabicyclo[3.2.1]octane-1,2,3,4,6-pentol_b
8-Methyl-8-azabicyclo[3.2.1]octane-1,2,3,4,6-pentol_b
8-Methyl-8-azabicyclo[3.2.1]octane-1,2,3,4,6-pentol_b
8-Methyl-8-azabicyclo[3.2.1]octane-1,2,3,4,6-pentol_b
Vorinostat_d
Vorinostat_d
Vorinostat_d
Vorinostat_d
Vorinostat_d
Threonylglutamine
Threonylglutamine
Threonylglutamine
Threonylglutamine
Threonylglutamine
pentobarbital_d
pentobarbital_d
pentobarbital_d
pentobarbital_d
pentobarbital_d
acetyltaurine
acetyltaurine
acetyltaurine
acetyltaurine
acetyltaurine
8-Methyl-8-azabicyclo[3.2.1]octane-1,2,3,4,6-pentol_c
8-Methyl-8-azabicyclo[3.2.1]octane-1,2,3,4,6-pentol_c
8-Methyl-8-azabicyclo[3.2.1]octane-1,2,3,4,6-pentol_c
8-Methyl-8-azabicyclo[3.2.1]octane-1,2,3,4,6-pentol_c
8-Methyl-8-azabicyclo[3.2.1]octane-1,2,3,4,6-pentol_c
2-BUTYL PROPENYL DISULFIDE, E(R)-_a
2-BUTYL PROPENYL DISULFIDE, E(R)-_a
2-BUTYL PROPENYL DISULFIDE, E(R)-_a
2-BUTYL PROPENYL DISULFIDE, E(R)-_a
2-BUTYL PROPENYL DISULFIDE, E(R)-_a
2-[(carboxymethyl)methylamino]-5-methoxy-Benzoic acid_b
2-[(carboxymethyl)methylamino]-5-methoxy-Benzoic acid_b
2-[(carboxymethyl)methylamino]-5-methoxy-Benzoic acid_b
2-[(carboxymethyl)methylamino]-5-methoxy-Benzoic acid_b
2-[(carboxymethyl)methylamino]-5-methoxy-Benzoic acid_b
Furan
Furan

Furan
Furan
Furan
butalbital_e
butalbital_e
butalbital_e
butalbital_e
butalbital_e
2,4-Bis(3-methyl-2-buten-1-yl)-1,3,5-benzenetriol
2,4-Bis(3-methyl-2-buten-1-yl)-1,3,5-benzenetriol
2,4-Bis(3-methyl-2-buten-1-yl)-1,3,5-benzenetriol
2,4-Bis(3-methyl-2-buten-1-yl)-1,3,5-benzenetriol
2,4-Bis(3-methyl-2-buten-1-yl)-1,3,5-benzenetriol
N-Acetyl-5-oxo-L-norvaline_c
N-Acetyl-5-oxo-L-norvaline_c
N-Acetyl-5-oxo-L-norvaline_c
N-Acetyl-5-oxo-L-norvaline_c
N-Acetyl-5-oxo-L-norvaline_c
1-(3-Amino-2,3-dideoxypentofuranosyl)-5-methyl-2,4(1H,3H)-pyrimidinedione
1-(3-Amino-2,3-dideoxypentofuranosyl)-5-methyl-2,4(1H,3H)-pyrimidinedione
1-(3-Amino-2,3-dideoxypentofuranosyl)-5-methyl-2,4(1H,3H)-pyrimidinedione
1-(3-Amino-2,3-dideoxypentofuranosyl)-5-methyl-2,4(1H,3H)-pyrimidinedione
1-(3-Amino-2,3-dideoxypentofuranosyl)-5-methyl-2,4(1H,3H)-pyrimidinedione
3-Hydroxypyridine
3-Hydroxypyridine
3-Hydroxypyridine
3-Hydroxypyridine
3-Hydroxypyridine
(2E)-3-(3,4-Dimethoxyphenyl)acrylic acid
(2E)-3-(3,4-Dimethoxyphenyl)acrylic acid
(2E)-3-(3,4-Dimethoxyphenyl)acrylic acid
(2E)-3-(3,4-Dimethoxyphenyl)acrylic acid
(2E)-3-(3,4-Dimethoxyphenyl)acrylic acid
Leu-arg
Leu-arg
Leu-arg
Leu-arg
Leu-arg
Dihydrocortisol
Dihydrocortisol
Dihydrocortisol
Dihydrocortisol
Dihydrocortisol
Resveratrol-4'-O-Glucuronide
Resveratrol-4'-O-Glucuronide
Resveratrol-4'-O-Glucuronide
Resveratrol-4'-O-Glucuronide
Resveratrol-4'-O-Glucuronide

N,N'-Bis[4-(4,5-dihydro-1H-imidazol-2-yl)phenyl]-2-methoxyterephthalamide
N,N'-Bis[4-(4,5-dihydro-1H-imidazol-2-yl)phenyl]-2-methoxyterephthalamide
N,N'-Bis[4-(4,5-dihydro-1H-imidazol-2-yl)phenyl]-2-methoxyterephthalamide
N,N'-Bis[4-(4,5-dihydro-1H-imidazol-2-yl)phenyl]-2-methoxyterephthalamide
N,N'-Bis[4-(4,5-dihydro-1H-imidazol-2-yl)phenyl]-2-methoxyterephthalamide
Tauropine
Tauropine
Tauropine
Tauropine
Tauropine
Hydroxycarteolol_b
Hydroxycarteolol_b
Hydroxycarteolol_b
Hydroxycarteolol_b
Hydroxycarteolol_b
Coprine_b
Coprine_b
Coprine_b
Coprine_b
Coprine_b
5,8,12-Trihydroxy-2-oxododecanoic acid
5,8,12-Trihydroxy-2-oxododecanoic acid
5,8,12-Trihydroxy-2-oxododecanoic acid
5,8,12-Trihydroxy-2-oxododecanoic acid
5,8,12-Trihydroxy-2-oxododecanoic acid
Leu-Leu_e
Leu-Leu_e
Leu-Leu_e
Leu-Leu_e
Leu-Leu_e
Lys-Pro_a
Lys-Pro_a
Lys-Pro_a
Lys-Pro_a
Lys-Pro_a
QJ9720000
QJ9720000
QJ9720000
QJ9720000
QJ9720000
Fluocinolone Acetonide
Fluocinolone Acetonide
Fluocinolone Acetonide
Fluocinolone Acetonide
Fluocinolone Acetonide
Leu-Leu_b
Leu-Leu_b
Leu-Leu_b

Leu-Leu_b
Leu-Leu_b
Ethyl malate_a
Ethyl malate_a
Ethyl malate_a
Ethyl malate_a
Ethyl malate_a
MFCD00037215
MFCD00037215
MFCD00037215
MFCD00037215
MFCD00037215
N-Nonanoylglycine_a
N-Nonanoylglycine_a
N-Nonanoylglycine_a
N-Nonanoylglycine_a
N-Nonanoylglycine_a
(4R,5S,9S,10R,12S,13S)-1,5,9-Trimethyl-11,14,15,16-tetraoxatetracyclo[10.3.1.0~4,13~.0~8,13~]hexadecan-10
(4R,5S,9S,10R,12S,13S)-1,5,9-Trimethyl-11,14,15,16-tetraoxatetracyclo[10.3.1.0~4,13~.0~8,13~]hexadecan-10
(4R,5S,9S,10R,12S,13S)-1,5,9-Trimethyl-11,14,15,16-tetraoxatetracyclo[10.3.1.0~4,13~.0~8,13~]hexadecan-10
(4R,5S,9S,10R,12S,13S)-1,5,9-Trimethyl-11,14,15,16-tetraoxatetracyclo[10.3.1.0~4,13~.0~8,13~]hexadecan-10
(4R,5S,9S,10R,12S,13S)-1,5,9-Trimethyl-11,14,15,16-tetraoxatetracyclo[10.3.1.0~4,13~.0~8,13~]hexadecan-10
1-Methylguanine
1-Methylguanine
1-Methylguanine
1-Methylguanine
1-Methylguanine
Valyl-4-hydroxyproline_a
Valyl-4-hydroxyproline_a
Valyl-4-hydroxyproline_a
Valyl-4-hydroxyproline_a
Valyl-4-hydroxyproline_a
Sulfurol
Sulfurol
Sulfurol
Sulfurol
Sulfurol
trp-pro
trp-pro
trp-pro
trp-pro
trp-pro
lys-leu
lys-leu
lys-leu
lys-leu
lys-leu
Penbutolol

Penbutolol
Penbutolol
Penbutolol
Penbutolol
(6S)-2-Amino-6-[(3-carboxypropanoyl)amino]heptanedioic acid
(6S)-2-Amino-6-[(3-carboxypropanoyl)amino]heptanedioic acid
(6S)-2-Amino-6-[(3-carboxypropanoyl)amino]heptanedioic acid
(6S)-2-Amino-6-[(3-carboxypropanoyl)amino]heptanedioic acid
(6S)-2-Amino-6-[(3-carboxypropanoyl)amino]heptanedioic acid
Leu-Val_a
Leu-Val_a
Leu-Val_a
Leu-Val_a
Leu-Val_a
Methohexital_a
Methohexital_a
Methohexital_a
Methohexital_a
Methohexital_a
dihydroxybenzylamine
dihydroxybenzylamine
dihydroxybenzylamine
dihydroxybenzylamine
dihydroxybenzylamine
N~6~-Octanoyllysine_b
N~6~-Octanoyllysine_b
N~6~-Octanoyllysine_b
N~6~-Octanoyllysine_b
N~6~-Octanoyllysine_b
L-gamma-Glutamyl-L-leucine_f
L-gamma-Glutamyl-L-leucine_f
L-gamma-Glutamyl-L-leucine_f
L-gamma-Glutamyl-L-leucine_f
L-gamma-Glutamyl-L-leucine_f
Voglibose
Voglibose
Voglibose
Voglibose
Voglibose
Butabarbital_d
Butabarbital_d
Butabarbital_d
Butabarbital_d
Butabarbital_d
3-Oxo-4,6-choladienoic acid
3-Oxo-4,6-choladienoic acid
3-Oxo-4,6-choladienoic acid
3-Oxo-4,6-choladienoic acid

3-Oxo-4,6-choladienoic acid
1-Methylhistamine
1-Methylhistamine
1-Methylhistamine
1-Methylhistamine
1-Methylhistamine
3-Sulfino-L-alanine
3-Sulfino-L-alanine
3-Sulfino-L-alanine
3-Sulfino-L-alanine
3-Sulfino-L-alanine
3-Succinoylpyridine
3-Succinoylpyridine
3-Succinoylpyridine
3-Succinoylpyridine
3-Succinoylpyridine
L-gamma-Glutamyl-L-valine_j
L-gamma-Glutamyl-L-valine_j
L-gamma-Glutamyl-L-valine_j
L-gamma-Glutamyl-L-valine_j
L-gamma-Glutamyl-L-valine_j
N-{3-[(4-Acetamidobutyl)amino]propyl}acetamide_b
N-{3-[(4-Acetamidobutyl)amino]propyl}acetamide_b
N-{3-[(4-Acetamidobutyl)amino]propyl}acetamide_b
N-{3-[(4-Acetamidobutyl)amino]propyl}acetamide_b
N-{3-[(4-Acetamidobutyl)amino]propyl}acetamide_b
5-Amino-6-(4-hydroxybutanoyl)-2,2-dimethyl-2,3-dihydro-4H-chromen-4-one
5-Amino-6-(4-hydroxybutanoyl)-2,2-dimethyl-2,3-dihydro-4H-chromen-4-one
5-Amino-6-(4-hydroxybutanoyl)-2,2-dimethyl-2,3-dihydro-4H-chromen-4-one
5-Amino-6-(4-hydroxybutanoyl)-2,2-dimethyl-2,3-dihydro-4H-chromen-4-one
5-Amino-6-(4-hydroxybutanoyl)-2,2-dimethyl-2,3-dihydro-4H-chromen-4-one
8-Amino-7-oxononanoic acid_c
8-Amino-7-oxononanoic acid_c
8-Amino-7-oxononanoic acid_c
8-Amino-7-oxononanoic acid_c
8-Amino-7-oxononanoic acid_c
pro-met
pro-met
pro-met
pro-met
pro-met
beta-D-Ethyl glucuronide_b
beta-D-Ethyl glucuronide_b
beta-D-Ethyl glucuronide_b
beta-D-Ethyl glucuronide_b
beta-D-Ethyl glucuronide_b
gamma-Glutamyl-S-[1-(6,7-dimethoxy-1,3-benzodioxol-5-yl)-1,3-dihydroxy-2-propanyl]cysteinylglycine
gamma-Glutamyl-S-[1-(6,7-dimethoxy-1,3-benzodioxol-5-yl)-1,3-dihydroxy-2-propanyl]cysteinylglycine

gamma-Glutamyl-S-[1-(6,7-dimethoxy-1,3-benzodioxol-5-yl)-1,3-dihydroxy-2-propanyl]cysteinylglycine
gamma-Glutamyl-S-[1-(6,7-dimethoxy-1,3-benzodioxol-5-yl)-1,3-dihydroxy-2-propanyl]cysteinylglycine
gamma-Glutamyl-S-[1-(6,7-dimethoxy-1,3-benzodioxol-5-yl)-1,3-dihydroxy-2-propanyl]cysteinylglycine
his-gln_b
his-gln_b
his-gln_b
his-gln_b
his-gln_b
3-Hydroxysebacic acid
3-Hydroxysebacic acid
3-Hydroxysebacic acid
3-Hydroxysebacic acid
3-Hydroxysebacic acid
Valylvaline_c
Valylvaline_c
Valylvaline_c
Valylvaline_c
Valylvaline_c
FB9500000_b
FB9500000_b
FB9500000_b
FB9500000_b
FB9500000_b
13(S)-HOTrE
13(S)-HOTrE
13(S)-HOTrE
13(S)-HOTrE
13(S)-HOTrE
thr-trp
thr-trp
thr-trp
thr-trp
thr-trp
6-hydroxypseudooxynicotine_c
6-hydroxypseudooxynicotine_c
6-hydroxypseudooxynicotine_c
6-hydroxypseudooxynicotine_c
6-hydroxypseudooxynicotine_c
2-Aminooctanedioic acid_b
2-Aminooctanedioic acid_b
2-Aminooctanedioic acid_b
2-Aminooctanedioic acid_b
2-Aminooctanedioic acid_b
1,1'-[1,12-Dodecanediylbis(oxy)]dibenzene_c
1,1'-[1,12-Dodecanediylbis(oxy)]dibenzene_c
1,1'-[1,12-Dodecanediylbis(oxy)]dibenzene_c
1,1'-[1,12-Dodecanediylbis(oxy)]dibenzene_c
1,1'-[1,12-Dodecanediylbis(oxy)]dibenzene_c

N-[(2E)-3-(4-Hydroxyphenyl)-2-propenoyl]tryptophan
N-[(2E)-3-(4-Hydroxyphenyl)-2-propenoyl]tryptophan
N-[(2E)-3-(4-Hydroxyphenyl)-2-propenoyl]tryptophan
N-[(2E)-3-(4-Hydroxyphenyl)-2-propenoyl]tryptophan
N-[(2E)-3-(4-Hydroxyphenyl)-2-propenoyl]tryptophan
ALA-PRO
ALA-PRO
ALA-PRO
ALA-PRO
ALA-PRO
threonylphenylalanine_b
threonylphenylalanine_b
threonylphenylalanine_b
threonylphenylalanine_b
threonylphenylalanine_b
Phenyl D-glucopyranosiduronic acid_b
Phenyl D-glucopyranosiduronic acid_b
Phenyl D-glucopyranosiduronic acid_b
Phenyl D-glucopyranosiduronic acid_b
Phenyl D-glucopyranosiduronic acid_b
3-Methyl-2-buten-1-yl hydrogen sulfate
3-Methyl-2-buten-1-yl hydrogen sulfate
3-Methyl-2-buten-1-yl hydrogen sulfate
3-Methyl-2-buten-1-yl hydrogen sulfate
3-Methyl-2-buten-1-yl hydrogen sulfate
2-Methylthiazolidine_b
2-Methylthiazolidine_b
2-Methylthiazolidine_b
2-Methylthiazolidine_b
2-Methylthiazolidine_b
N-(3-acetamidopropyl)pyrrolidin-2-one_b
N-(3-acetamidopropyl)pyrrolidin-2-one_b
N-(3-acetamidopropyl)pyrrolidin-2-one_b
N-(3-acetamidopropyl)pyrrolidin-2-one_b
N-(3-acetamidopropyl)pyrrolidin-2-one_b
Midodrine_d
Midodrine_d
Midodrine_d
Midodrine_d
Midodrine_d
glu-thr
glu-thr
glu-thr
glu-thr
glu-thr
N-(4-Hydroxy-3,5-dimethoxybenzoyl)glycine_c
N-(4-Hydroxy-3,5-dimethoxybenzoyl)glycine_c
N-(4-Hydroxy-3,5-dimethoxybenzoyl)glycine_c

N-(4-Hydroxy-3,5-dimethoxybenzoyl)glycine_c
N-(4-Hydroxy-3,5-dimethoxybenzoyl)glycine_c
Tetraacetylethylenediamine_f
Tetraacetylethylenediamine_f
Tetraacetylethylenediamine_f
Tetraacetylethylenediamine_f
Tetraacetylethylenediamine_f
2_7-Anhydro-alpha-N-acetylneuraminicacid
2_7-Anhydro-alpha-N-acetylneuraminicacid
2_7-Anhydro-alpha-N-acetylneuraminicacid
2_7-Anhydro-alpha-N-acetylneuraminicacid
2_7-Anhydro-alpha-N-acetylneuraminicacid
1-(beta-D-ribofuranosyl)thymine_a
1-(beta-D-ribofuranosyl)thymine_a
1-(beta-D-ribofuranosyl)thymine_a
1-(beta-D-ribofuranosyl)thymine_a
1-(beta-D-ribofuranosyl)thymine_a
{2-[2-(Isobutyryloxy)-4-methylphenyl]-2-oxiranyl}methyl 2-methylbutanoate
{2-[2-(Isobutyryloxy)-4-methylphenyl]-2-oxiranyl}methyl 2-methylbutanoate
{2-[2-(Isobutyryloxy)-4-methylphenyl]-2-oxiranyl}methyl 2-methylbutanoate
{2-[2-(Isobutyryloxy)-4-methylphenyl]-2-oxiranyl}methyl 2-methylbutanoate
{2-[2-(Isobutyryloxy)-4-methylphenyl]-2-oxiranyl}methyl 2-methylbutanoate
ala-ser_a
ala-ser_a
ala-ser_a
ala-ser_a
ala-ser_a
nicotianamine_a
nicotianamine_a
nicotianamine_a
nicotianamine_a
nicotianamine_a
1,3-Dihydroxy-2-propanyl (7Z,10Z,13Z,16Z,19Z)-7,10,13,16,19-docosapentaenoate
1,3-Dihydroxy-2-propanyl (7Z,10Z,13Z,16Z,19Z)-7,10,13,16,19-docosapentaenoate
1,3-Dihydroxy-2-propanyl (7Z,10Z,13Z,16Z,19Z)-7,10,13,16,19-docosapentaenoate
1,3-Dihydroxy-2-propanyl (7Z,10Z,13Z,16Z,19Z)-7,10,13,16,19-docosapentaenoate
1,3-Dihydroxy-2-propanyl (7Z,10Z,13Z,16Z,19Z)-7,10,13,16,19-docosapentaenoate
2-Despiperidyl-2-amino Repaglinide
2-Despiperidyl-2-amino Repaglinide
2-Despiperidyl-2-amino Repaglinide
2-Despiperidyl-2-amino Repaglinide
2-Despiperidyl-2-amino Repaglinide
Furfuranol
Furfuranol
Furfuranol
Furfuranol
Furfuranol
p-Cresol

p-Cresol
p-Cresol
p-Cresol
p-Cresol

nethyl]-5-[(3-ethyl-4-methyl-5-oxo-1,2-dihydropyrrol-2-yl)methyl]-4-methyl-1H-pyrrol-3-yl]propanoic acid_a
nethyl]-5-[(3-ethyl-4-methyl-5-oxo-1,2-dihydropyrrol-2-yl)methyl]-4-methyl-1H-pyrrol-3-yl]propanoic acid_a
nethyl]-5-[(3-ethyl-4-methyl-5-oxo-1,2-dihydropyrrol-2-yl)methyl]-4-methyl-1H-pyrrol-3-yl]propanoic acid_a
nethyl]-5-[(3-ethyl-4-methyl-5-oxo-1,2-dihydropyrrol-2-yl)methyl]-4-methyl-1H-pyrrol-3-yl]propanoic acid_a
nethyl]-5-[(3-ethyl-4-methyl-5-oxo-1,2-dihydropyrrol-2-yl)methyl]-4-methyl-1H-pyrrol-3-yl]propanoic acid_a

nethyl]-5-[(3-ethyl-4-methyl-5-oxo-1,2-dihydropyrrol-2-yl)methyl]-4-methyl-1H-pyrrol-3-yl]propanoic acid_b
nethyl]-5-[(3-ethyl-4-methyl-5-oxo-1,2-dihydropyrrol-2-yl)methyl]-4-methyl-1H-pyrrol-3-yl]propanoic acid_b
nethyl]-5-[(3-ethyl-4-methyl-5-oxo-1,2-dihydropyrrol-2-yl)methyl]-4-methyl-1H-pyrrol-3-yl]propanoic acid_b
nethyl]-5-[(3-ethyl-4-methyl-5-oxo-1,2-dihydropyrrol-2-yl)methyl]-4-methyl-1H-pyrrol-3-yl]propanoic acid_b

nethyl]-5-[(3-ethyl-4-methyl-5-oxo-1,2-dihydropyrrol-2-yl)methyl]-4-methyl-1H-pyrrol-3-yl]propanoic acid_b
