# Supplementary material for: A young child formula with Limosilactobacillus reuteri and GOS modulates gut microbiome and enhances bone and muscle development: a randomized trial
Source: Nat Commun. 2025 Dec 12;17:237. doi: 10.1038/s41467-025-66930-2 (PMC12783733; doi:10.1038/s41467-025-66930-2)
Supplement: Supplementary file 14 — Supplementary data 12 [file 41467_2025_66930_MOESM14_ESM.pdf]

Muscle progenitor	Fusion index		Myotube area	
	Mean (relative to veh.)	Std. Deviation	Mean (relative to veh.)	Std. Deviation
hydroxyphenyllatic 100µM	1.013	0.04951	0.9924	0.07978
hydroxyphenyllatic 10µM	1.029**	0.04647	1.024	0.07018
4-hydroxyproline 100µM	0.9847	0.05744	0.9707*	0.08451
4-hydroxyproline 10µM	1.033***	0.04226	1.043**	0.0592
pyruvic acid 100µM	1.009	0.06349	0.9716	0.09011
pyruvic acid 10µM	1.032**	0.04372	1.043**	0.06871
4-hydroxybenzoic acid 100µM	1.014	0.05719	0.9995	0.07729
4-hydroxybenzoic acid 10µM	1.011	0.05782	1.015	0.08135
indole 3 lactic acid 100µM	0.9484***	0.09969	0.8877***	0.1364
indole 3 lactic acid 10µM	1.002	0.05327	0.9921	0.07183
phenethylamine 100µM	0.9961	0.08381	0.9673	0.1132
phenethylamine 10µM	1.005	0.05463	1.002	0.07078
Indole−3−carboxyaldehyde 100µM	0.9762	0.09365	0.9651	0.1533
Indole−3−carboxyaldehyde 10µM	1.052***	0.03375	1.043	0.08285
pipecolinic acid 100µM	0.9759*	0.05328	0.9634*	0.08614
pipecolinic acid 10µM	0.9889	0.05691	0.9721	0.09071

Osteoblast	Proliferation (nuclei count)		Runx2 mRNA level		Atf4 mRNA level		Ctnnb1 mRNA level	
	Mean (relative to veh.)	Std. Deviation	Mean (relative to veh.)	Std. Deviation	Mean (relative to veh.)	Std. Deviation	Mean (relative to veh.)	Std. Deviation
4-hydroxybenzaldehyde 10µM	1.171**	0.2122	1.031	0.05334	1.103	0.1323	1.03	0.05937
4-hydroxybenzaldehyde 100µM	1.213***	0.2302	1.03	0.0719	1.096	0.12	1.023	0.04243
L-Citrulline 10µM	1.398****	0.2654	0.9838	0.07845	1.047	0.1094	0.9838	0.06174
L-Citrulline 100µM	1.299****	0.2199	0.9163*	0.07512	1.199**	0.1426	0.9896	0.06422
3-Hydroxypyridine 10µM	1.22****	0.1944	1.057*	0.04892	1.116	0.109	1.05*	0.02639
3-Hydroxypyridine 100µM	1.259****	0.1867	1.065*	0.05223	1.101	0.1281	1.062**	0.02543
Indole-3-lactic acid 10µM	1.107	0.2159	1.015	0.05633	1.15	0.1303	1.035	0.03778
Indole-3-lactic acid 100µM	1.245***	0.3329	0.9377*	0.06195	1.271***	0.169	1.005	0.04977
L-Histidine 10µM	1.335***	0.3898	1.074*	0.03883	1.147*	0.1277	1.08**	0.03416
L-Histidine 100µM	1.369***	0.4201	1.069*	0.06613	1.165*	0.1342	1.088***	0.03689
2-amino-4-methylpyrimidine 10µM	1.325***	0.3794	1.015	0.06658	1.262**	0.1367	1.073*	0.05903
2-amino-4-methylpyrimidine 100µM	1.399****	0.3419	0.9515	0.06746	1.376****	0.2036	1.025	0.04231
Indole−3−carboxyaldehyde 10µM	1.199****	0.2031	0.994	0.06083	1.291*	0.3315	1.031	0.03246
Indole−3−carboxyaldehyde 100µM	0.9861	0.1542	1.004	0.115	1.458**	0.2493	0.9013***	0.03892
Indole-3-propionic acid 10µM	1.284*	0.2139	1.192*	0.03669	1.081	0.1045	1.057	0.07092
Indole-3-propionic acid 100µM	1.125	0.2557	1.216*	0.04097	1.033	0.06624	0.9653	0.01388
Pyruvic acid 10µM	1.209	0.1557	1.163	0.06141	1.194	0.0437	1.126*	0.02714
Pyruvic acid 100µM	1.382**	0.2778	1.097	0.01956	1.405*	0.1691	1.05	0.05229
pipecolinic acid 10µM	1.319*	0.2517	1.143	0.0921	1.285*	0.1352	1.076	0.03338
pipecolinic acid 100µM	1.224	0.2663	1.11	0.08361	1.402**	0.1029	1.072	0.05199
4-hydroxyproline 10µM	1.214	0.2969	1.095	0.06465	1.529**	0.1446	1.041	0.07147
4-hydroxyproline 100µM	1.11	0.2115	1.148	0.06004	1.592***	0.05816	1.024	0.06255
