# Supplementary material for: A young child formula with Limosilactobacillus reuteri and GOS modulates gut microbiome and enhances bone and muscle development: a randomized trial
Source: Nat Commun. 2025 Dec 12;17:237. doi: 10.1038/s41467-025-66930-2 (PMC12783733; doi:10.1038/s41467-025-66930-2)
Supplement: Supplementary file 15 — Supplementary data 13 [file 41467_2025_66930_MOESM15_ESM.pdf]

Clinical variable	Grouping	Characteristic	Beta	95% CI low
Tibia SOS	Experimental + Control	Increase / Non-increase	55	9,6
Tibia SOS	Experimental + Control	Tibia SOS (V1)	0.34	0,2
Tibia SOS	Experimental + Control	Sex	62	21
Tibia SOS	Experimental + Control	Computed BMI	-16	-30
Tibia SOS	Experimental + Control	Vitamin D (V1)	-0.49	-3
Tibia SOS	Experimental + Control + Reference	Increase / Non-increase	46	4,7
Tibia SOS	Experimental + Control + Reference	Tibia SOS (V1)	0.33	0,21
Tibia SOS	Experimental + Control + Reference	Sex	32	-0,65
Tibia SOS	Experimental + Control + Reference	Computed BMI	-12	-24
Tibia SOS	Experimental + Control + Reference	Vitamin D (V1)	-0.49	-2,5
Vitamin D	Experimental + Control	Vitamin D (V1)	0.02	0,02
Vitamin D	Experimental + Control	Increase / Non-increase	0.10	0,03
Vitamin D	Experimental + Control	Sex	-0.03	-0,1
Vitamin D	Experimental + Control + Reference	Vitamin D (V1)	0.02	0,02
Vitamin D	Experimental + Control + Reference	Increase / Non-increase	0.07	0,01
Vitamin D	Experimental + Control + Reference	Sex	-0.04	-0,09
Tibia length	Experimental + Control	Increase / Non-increase	-0.29	-0,81
Tibia length	Experimental + Control	Tibia length (V1)	0.16	0,09
Tibia length	Experimental + Control	Sex	0.17	-0,3
Tibia length	Experimental + Control	Computed BMI	0.17	0,01
Tibia length	Experimental + Control	Vitamin D (V1)	-0.01	-0,04
Tibia length	Experimental + Control + Reference	Increase / Non-increase	0.02	-0,41
Tibia length	Experimental + Control + Reference	Tibia length (V1)	0.18	0,13
Tibia length	Experimental + Control + Reference	Sex	0.20	-0,14
Tibia length	Experimental + Control + Reference	Computed BMI	0.20	0,07
Tibia length	Experimental + Control + Reference	Vitamin D (V1)	-0.01	-0,03
Radius SOS	Experimental + Control	Increase / Non-increase	19	-26
Radius SOS	Experimental + Control	Radius SOS (V1)	0.34	0,21
Radius SOS	Experimental + Control	Sex	51	11
Radius SOS	Experimental + Control	Computed BMI	-3.2	-17
Radius SOS	Experimental + Control	Vitamin D (V1)	-0.99	-3,4
Radius SOS	Experimental + Control + Reference	Increase / Non-increase	15	-27
Radius SOS	Experimental + Control + Reference	Radius SOS (V1)	0.46	0,35
Radius SOS	Experimental + Control + Reference	Sex	32	-1,3
Radius SOS	Experimental + Control + Reference	Computed BMI	-0.84	-13
Radius SOS	Experimental + Control + Reference	Vitamin D (V1)	0.33	-1,7
Radius length	Experimental + Control	Increase / Non-increase	-0.02	-0,24
Radius length	Experimental + Control	Radius length (V1)	0.87	0,78
Radius length	Experimental + Control	Sex	0.27	0,07
Radius length	Experimental + Control	Computed BMI	-0.01	-0,08
Radius length	Experimental + Control	Vitamin D (V1)	-0.01	-0,02
Radius length	Experimental + Control + Reference	Increase / Non-increase	0.07	-0,12
Radius length	Experimental + Control + Reference	Radius length (V1)	0.83	0,76
Radius length	Experimental + Control + Reference	Sex	0.20	0,05
Radius length	Experimental + Control + Reference	Computed BMI	0.00	-0,05
Radius length	Experimental + Control + Reference	Vitamin D (V1)	-0.01	-0,02

95% CI high	P-value
101	0.018
0,49	<0.001
103	0.004
-1,1	0.035
2	0.7
87	0.029
0,46	<0.001
65	0.055
-0,59	0.040
1,5	0.6
0,03	<0.001
0,18	0.005
0,03	0.3
0,02	<0.001
0,13	0.023
0,01	0.086
0,22	0.3
0,24	<0.001
0,63	0.5
0,33	0.042
0,01	0.3
0,45	>0.9
0,24	<0.001
0,55	0.3
0,32	0.002
0,01	0.4
63	0.4
0,48	<0.001
91	0.014
11	0.7
1,4	0.4
58	0.5
0,57	<0.001
66	0.059
11	0.9
2,4	0.8
0,2	0.9
0,96	<0.001
0,47	0.009
0,06	0.8
0	0.046
0,25	0.5
0,91	<0.001
0,35	0.010
0,06	>0.9
0	0.13
