# Supplementary material for: A young child formula with Limosilactobacillus reuteri and GOS modulates gut microbiome and enhances bone and muscle development: a randomized trial
Source: Nat Commun. 2025 Dec 12;17:237. doi: 10.1038/s41467-025-66930-2 (PMC12783733; doi:10.1038/s41467-025-66930-2)
Supplement: Supplementary file 16 — Supplementary data 14 [file 41467_2025_66930_MOESM16_ESM.pdf]

Stool consistency and Descriptive statistics for the TCGQ

		Visit	Arm	n	min	Q1	Median
Average consistency of stools averaged over 3 days		V2	CM	72	3	3.65	4
		V2	EYCF	70	1.29	3	3.75
		V3	CM	69	2.5	4	4
		V3	EYCF	69	2.67	3.5	4
Gastro intestinal TOTAL SCORE		V1	REF	91	9	10	10
		V1	CM	91	9	10	11
		V1	EYCF	91	9	10	11
		V2	REF	87	9	9	10
		V2	CM	72	9	10	10
		V2	EYCF	70	9	9.25	10
		V3	REF	87	9	9	10
		V3	CM	69	9	10	10
		V3	EYCF	69	9	9	10
	Gastro intestinal symptoms	V1	REF	91	6	6	6
		V1	CM	91	6	6	6
		V1	EYCF	91	6	6	6
		V2	REF	87	6	6	6
		V2	CM	72	6	6	6
		V2	EYCF	70	6	6	6
		V3	REF	87	6	6	6
		V3	CM	69	6	6	6
		V3	EYCF	69	6	6	6
	Q1. Did your child have stooling issues	V1	REF	91	1	1	1
		V1	CM	91	1	1	1
		V1	EYCF	91	1	1	1
		V2	REF	87	1	1	1
		V2	CM	72	1	1	1
		V2	EYCF	70	1	1	1
		V3	REF	87	1	1	1
		V3	CM	69	1	1	1
		V3	EYCF	69	1	1	1
	Q1.a Did your child have constipation?	V1	REF	91	1	1	1
		V1	CM	91	1	1	1
		V1	EYCF	91	1	1	1
		V2	REF	87	1	1	1
		V2	CM	72	1	1	1
		V2	EYCF	70	1	1	1
		V3	REF	87	1	1	1
		V3	CM	69	1	1	1
		V3	EYCF	69	1	1	1
		V1	REF	91	1	1	1
		V1	CM	91	1	1	1

Gastro intestinal related Questions	Q1.b Did your child have diarrhea?	V1	EYCF	91	1	1	1
		V2	REF	87	1	1	1
		V2	CM	72	1	1	1
		V2	EYCF	70	1	1	1
		V3	REF	87	1	1	1
		V3	CM	69	1	1	1
		V3	EYCF	69	1	1	1
	Q2. Did your child experience gassiness?	V1	REF	91	1	1	1
		V1	CM	91	1	1	1
		V1	EYCF	91	1	1	1
		V2	REF	87	1	1	1
		V2	CM	72	1	1	1
		V2	EYCF	70	1	1	1
		V3	REF	87	1	1	1
		V3	CM	69	1	1	1
		V3	EYCF	69	1	1	1
	Q3. Did your child have abdominal pain?	V1	REF	91	1	1	1
		V1	CM	91	1	1	1
		V1	EYCF	91	1	1	1
		V2	REF	87	1	1	1
		V2	CM	72	1	1	1
		V2	EYCF	70	1	1	1
		V3	REF	87	1	1	1
		V3	CM	69	1	1	1
		V3	EYCF	69	1	1	1
	Q4. Did your child feel bloated?	V1	REF	91	1	1	1
		V1	CM	91	1	1	1
		V1	EYCF	91	1	1	1
		V2	REF	87	1	1	1
		V2	CM	72	1	1	1
		V2	EYCF	70	1	1	1
		V3	REF	87	1	1	1
		V3	CM	69	1	1	1
		V3	EYCF	69	1	1	1
	Gastro intestinal-RELATED BEHAVIORS domain	V1	REF	91	3	4	4
		V1	CM	91	3	4	5
		V1	EYCF	91	3	4	5
		V2	REF	87	3	3	4
		V2	CM	72	3	3	4
		V2	EYCF	70	3	3	4
		V3	REF	87	3	3	4
		V3	CM	69	3	4	4
		V3	EYCF	69	3	3	4
		V1	REF	91	1	1	1

Gastro intestinal related behavior questions	Q1. Did your child seem fussy and irritable?	V1	CM	91	1	1	1
		V1	EYCF	91	1	1	1
		V2	REF	87	1	1	1
		V2	CM	72	1	1	1
		V2	EYCF	70	1	1	1
		V3	REF	87	1	1	1
		V3	CM	69	1	1	1
		V3	EYCF	69	1	1	1
	Q2. Do you consider the sleep of your toddler a problem?	V1	REF	91	1	1	1
		V1	CM	91	1	1	1
		V1	EYCF	91	1	1	1
		V2	REF	87	1	1	1
		V2	CM	72	1	1	1
		V2	EYCF	70	1	1	1
		V3	REF	87	1	1	1
		V3	CM	69	1	1	1
		V3	EYCF	69	1	1	1
	Q3. Generally, how sleepy did your toddler get during the day?	V1	REF	91	1	1	1
		V1	CM	91	1	1	1
		V1	EYCF	91	1	1	1
		V2	REF	87	1	1	1
		V2	CM	72	1	1	1
		V2	EYCF	70	1	1	1
		V3	REF	87	1	1	1
		V3	CM	69	1	1	1
		V3	EYCF	69	1	1	1
	Q4. How many times did your toddler wake up during the night (between 7 in the evening and 7 in the morning)?	V1	REF	91	0	0	1
		V1	CM	91	0	1	1
		V1	EYCF	91	0	1	1
		V2	REF	87	0	0	1
		V2	CM	72	0	0	1
		V2	EYCF	70	0	0	1
		V3	REF	87	0	0	1
		V3	CM	69	0	0	1
		V3	EYCF	69	0	0	1

questionnaire

Q3	max	mean	sd
4	5	3.78	0.39
4	4	3.5	0.62
4	5	3.87	0.37
4	4	3.72	0.4
11.5	23	11.21	2.75
12	26	11.37	2.62
12	29	12.05	3.43
11	15	10.15	1.25
11	18	10.85	1.96
11	24	10.64	2.28
11	13	10.17	0.97
11	18	10.57	1.44
11	17	10.62	1.63
6	16	6.55	1.72
6	18	6.45	1.56
6	17	6.79	2.04
6	7	6.05	0.21
6	12	6.49	1.19
6	13	6.5	1.25
6	7	6.09	0.29
6	11	6.22	0.68
6	9	6.14	0.49
1	3	1.12	0.44
1	4	1.05	0.35
1	4	1.13	0.52
1	1	1	0
1	4	1.14	0.48
1	3	1.1	0.35
1	2	1.01	0.11
1	2	1.01	0.12
1	2	1.01	0.12
1	2	1.04	0.21
1	3	1.05	0.27
1	5	1.15	0.59
1	1	1	0
1	3	1.12	0.37
1	3	1.1	0.35
1	2	1.05	0.21
1	2	1.09	0.28
1	2	1.06	0.24
1	3	1.09	0.32
1	3	1.1	0.37

1	3	1.14	0.44
1	1	1	0
1	2	1.03	0.17
1	4	1.13	0.51
1	1	1	0
1	4	1.04	0.36
1	1	1	0
1	3	1.07	0.29
1	4	1.07	0.36
1	3	1.12	0.39
1	1	1	0
1	3	1.03	0.24
1	3	1.04	0.27
1	1	1	0
1	1	1	0
1	2	1.01	0.12
1	3	1.08	0.31
1	2	1.05	0.23
1	3	1.09	0.32
1	2	1.01	0.11
1	3	1.04	0.26
1	2	1.03	0.17
1	2	1.01	0.11
1	3	1.03	0.24
1	1	1	0
1	3	1.15	0.45
1	5	1.12	0.55
1	3	1.15	0.45
1	2	1.03	0.18
1	3	1.12	0.47
1	4	1.1	0.46
1	2	1.02	0.15
1	2	1.04	0.21
1	2	1.06	0.24
5	11	4.66	1.57
6	10	4.92	1.53
6	12	5.26	1.88
5	9	4.1	1.21
5	9	4.36	1.26
4	11	4.14	1.37
5	7	4.08	0.92
5	7	4.35	1.07
5	11	4.48	1.48
1	3	1.14	0.46

1	4	1.13	0.48
1	5	1.22	0.66
1	2	1.01	0.11
1	2	1.01	0.12
1	2	1.01	0.12
1	1	1	0
1	2	1.01	0.12
1	1	1	0
1	5	1.14	0.61
1	2	1.02	0.15
1	5	1.05	0.43
1	2	1.01	0.11
1	4	1.11	0.46
1	2	1.01	0.12
1	2	1.01	0.11
1	2	1.03	0.17
1	6	1.1	0.62
2	4	1.54	0.72
2	6	1.68	0.89
2	6	1.77	1.11
1	6	1.32	0.74
2	3	1.33	0.53
1	6	1.37	0.89
2	3	1.4	0.56
2	3	1.45	0.58
2	5	1.65	0.84
1	3	0.84	0.76
2	3	1.09	0.75
2	4	1.22	0.95
1	5	0.76	0.86
1	3	0.9	0.89
1	3	0.74	0.77
1	2	0.67	0.6
1	3	0.86	0.81
1	2	0.72	0.7
