# Supplementary material for: A young child formula with Limosilactobacillus reuteri and GOS modulates gut microbiome and enhances bone and muscle development: a randomized trial
Source: Nat Commun. 2025 Dec 12;17:237. doi: 10.1038/s41467-025-66930-2 (PMC12783733; doi:10.1038/s41467-025-66930-2)
Supplement: Supplementary file 17 — Supplementary data 15 [file 41467_2025_66930_MOESM17_ESM.pdf]

Comparison of stool consistency and Gastrointestinal total score

	Treatment	Visit	Estimate	CI95%	p-value
Stool consistency average	(EYCF/CM-1)%	V2	-8%	[-12%; -4%]	< 0.001
	(EYCF/CM-1)%	V3	-4%	[-8%; 0%]	0,079
Gastrointestinal total score	(EYCF/CM-1)%	V2	-3%	[-8%; 2%]	0,236
	(EYCF/CM-1)%	V3	0%	[-5%; 5%]	1
