# Supplementary material for: A young child formula with Limosilactobacillus reuteri and GOS modulates gut microbiome and enhances bone and muscle development: a randomized trial
Source: Nat Commun. 2025 Dec 12;17:237. doi: 10.1038/s41467-025-66930-2 (PMC12783733; doi:10.1038/s41467-025-66930-2)
Supplement: Supplementary file 18 — Supplementary data 16 [file 41467_2025_66930_MOESM18_ESM.pdf]

EYCF

Parameter	UoM	Oper.	/100g
ENERGY (KCAL) 449	kcal	=	480.2
Protein	g	=	10.6
Fat	g	=	22.2
Available Carbohydrates	g	=	59.5
Sum of Fibers (incl. GOS)	g	=	2.92
Na (Sodium)	mg	=	240
K (Potassium)	mg	=	715
Cl (Chloride)	mg	=	360
Ca (Calcium)	mg	=	865
P (Phosphorus)	mg	=	473
Mg (Magnesium)	mg	=	68
Fe (Iron)	mg	=	7
Cu (Copper)	mg	=	0.4
Zn (Zinc)	mg	=	4.4
I (Iodine)	µg	=	112
Mn (Manganese)	µg	=	80
Se (Selenium)	µg	=	15
Vit A	µgRE	=	620
Total Vitamin D [Sum of Vita	µgD	=	10
Vit E	mgTE	=	10
Vit K1 (Phytomenadione)	µg	=	40
Vit C	mg	=	110
Vit B1	mg	=	0.6
Vit B2	mg	=	1.2
Niacin	mg	=	5.8
Vit B6	mg	=	0.48
Total Folic Acid	µg	=	126
Pantothenic Acid	mg	=	3.5
Vit B12	µg	=	1.2
Biotin	µg	=	18
Choline	mg	=	64
Taurine	mg	=	1.461
L.reuteri	CFU	=	1.00E+07

CM

Parameter	UoM	Oper.	/100g
ENERGY (KCAL) 449	kcal	=	478
Protein	g	=	15.75
Fat	g	=	21
Available Carbohydrates	g	=	56.5
Sum of Fibers	g	=	0
Na (Sodium)	mg	=	310
K (Potassium)	mg	=	860
Cl (Chloride)	mg	=	480
Ca (Calcium)	mg	=	720
P (Phosphorus)	mg	=	542
Mg (Magnesium)	mg	=	58
Fe (Iron)	mg	=	7.5
Cu (Copper)	mg	=	0.5
Zn (Zinc)	mg	=	5.1
I (Iodine)	µg	=	112
Mn (Manganese)	µg	=	66
Se (Selenium)	µg	=	6.77
Vit A	µgRE	=	612
Total Vitamin D [Sum of Vitamin D2+D3]	µgD	=	3.05
Vit E	mgTE	=	7.37
Vit K1	µg	=	22
Vit C	mg	=	80
Vit B1	mg	=	1
Vit B2	mg	=	1.1
Niacin	mg	=	1.3
Vit B6	mg	=	1.5
Total Folic Acid	µg	=	30
Pantothenic Acid	mg	=	2
Vit B12	µg	=	3.2
Biotin	µg	=	9.4
