# Supplementary material for: A young child formula with Limosilactobacillus reuteri and GOS modulates gut microbiome and enhances bone and muscle development: a randomized trial
Source: Nat Commun. 2025 Dec 12;17:237. doi: 10.1038/s41467-025-66930-2 (PMC12783733; doi:10.1038/s41467-025-66930-2)
Supplement: Supplementary file 21 — Reporting summary [file 41467_2025_66930_MOESM21_ESM.pdf]

nature portfolio

Corresponding author(s): NCOMMS-25-26999

Last updated by author(s): Oct 30, 2025

Reporting Summary

Nature Portfolio wishes to improve the reproducibility of the work that we publish. This form provides structure for consistency and transparency in reporting. For further information on Nature Portfolio policies, see our Editorial Policies and the Editorial Policy Checklist.

Statistics

For all statistical analyses, confirm that the following items are present in the figure legend, table legend, main text, or Methods section.

n/a	Confirmed	
☐	☒	The exact sample size (*n*) for each experimental group/condition, given as a discrete number and unit of measurement
☐	☒	A statement on whether measurements were taken from distinct samples or whether the same sample was measured repeatedly
☐	☒	The statistical test(s) used AND whether they are one- or two-sided<br>*Only common tests should be described solely by name; describe more complex techniques in the Methods section.*
☐	☒	A description of all covariates tested
☐	☒	A description of any assumptions or corrections, such as tests of normality and adjustment for multiple comparisons
☐	☒	A full description of the statistical parameters including central tendency (e.g. means) or other basic estimates (e.g. regression coefficient) AND variation (e.g. standard deviation) or associated estimates of uncertainty (e.g. confidence intervals)
☐	☒	For null hypothesis testing, the test statistic (e.g. *F*, *t*, *r*) with confidence intervals, effect sizes, degrees of freedom and *P* value noted<br>*Give P values as exact values whenever suitable.*
☒	☐	For Bayesian analysis, information on the choice of priors and Markov chain Monte Carlo settings
☒	☐	For hierarchical and complex designs, identification of the appropriate level for tests and full reporting of outcomes
☐	☒	Estimates of effect sizes (e.g. Cohen's *d*, Pearson's *r*), indicating how they were calculated

Our web collection on statistics for biologists contains articles on many of the points above.

Software and code

Policy information about availability of computer code

Data collection	Tool or System Used (first and last version)<br>Rave Version 2021 Version 2022.3.0<br>Coder Version 2022.1.4 Version 2022.1.4<br>RTSM Version 2021 Version 2022.3.0<br>TSDV Version 2021 Version 2022.3.0<br>SAS Version 9.4 Version 9.4<br>LSAF Version 5.2.3 Version 5.2.3<br>Arisg Version 7.4.6.2.2.2 Version 7.4.6.2.2.2
Data analysis	The data analysis was performed in R version 3.6.1 (2019-07-05) and Python version 3.9 or higher (ref SAP https://nestle-clinical.veevavault.com/ui/#doc_info/49997/1/0)

For manuscripts utilizing custom algorithms or software that are central to the research but not yet described in published literature, software must be made available to editors and reviewers. We strongly encourage code deposition in a community repository (e.g. GitHub). See the Nature Portfolio guidelines for submitting code & software for further information.

Data

Policy information about availability of data

All manuscripts must include a data availability statement. This statement should provide the following information, where applicable:

- Accession codes, unique identifiers, or web links for publicly available datasets
- A description of any restrictions on data availability
- For clinical datasets or third party data, please ensure that the statement adheres to our policy

Following statement is included in page 40: All clinical outcomes necessary to interpret the results are included in supplementary data files or are available upon request in a deidentified format. Shotgun metagenomics data (depleted from reads representing the human genome) were deposited in the European Nucleotide Archive under BioProject: PRJEB83333. The metabolomics data have been deposited to MetaboLights repository with the study identifier MTBLS12557.

Research involving human participants, their data, or biological material

Policy information about studies with human participants or human data. See also policy information about sex, gender (identity/presentation), and sexual orientation and race, ethnicity and racism.

Reporting on sex and gender	Sex was collected at baseline and it is reported in supplementary table S1. Randomization was stratified by sex as well as statistical adjustments (page 29).
Reporting on race, ethnicity, or other socially relevant groupings	All kids were reported as Healthy Filipino toddlers (page 21, methods section) and Asian (see result section page 6).
Population characteristics	done on page 21 and 22 of the manuscript
Recruitment	Procedure on the parent/child recruitment is described in Appendix F of the study protocol.
Ethics oversight	The Asian Hospital and Medical Center Research Ethics Committee approved the study (REF:QF-REC-007). page 22

Note that full information on the approval of the study protocol must also be provided in the manuscript.

Field-specific reporting

Please select the one below that is the best fit for your research. If you are not sure, read the appropriate sections before making your selection.

☒ Life sciences ☐ Behavioural & social sciences ☐ Ecological, evolutionary & environmental sciences

For a reference copy of the document with all sections, see nature.com/documents/nr-reporting-summary-flat.pdf

Life sciences study design

All studies must disclose on these points even when the disclosure is negative.

Sample size

described page 22 and 23: The sample size calculation was based on the primary endpoint – tibia SoS. Assuming an expected difference of 70 m/s between the two study groups, with a standard deviation of 133 m/s , an overall type I error of 5% and 90% statistical power, the number of completed subjects per milk feeding group is equal to 77. Assuming a drop-out rate of approximately 15% over the 6 months, 273 toddlers (approx. 91 per feeding arm) were enrolled. The drop-out rate was to be monitored during the conduct of the trial and the number of enrolled subjects will be adjusted to ensure 231 completed subjects (77 subjects/arm).

Data exclusions

Exclusion criteria were pre defined as repoted page 21 and 22:
1. Chronic infectious, metabolic, genetic illness or other disease including any condition that impacts feeding or growth.
2. History of bone malabsorption, metabolic, congenital, or chromosomal abnormality known to affect feeding or growth.
3. Use of systemic antibiotics or anti-mycotic medication in the 4 weeks preceding enrollment.
4. Known or suspected cows' milk protein intolerance / allergy, or lactose intolerance, or severe food allergies that impact diet.
5. Current breast milk feeding in place of all other milk, and/or milk alternatives.
6. Clinical signs of severe micronutrient deficiencies.
7. Parent(s) not willing / not able to comply with the requirements of study protocol.
8. Child’s participation in another interventional clinical trial.
No changes in eligibility criteria were performed after trial commencement.

Replication

For Tibia SoS, handgrip measures, coefficient of variation was determined within the first 10 subjects included. Inter-obervatory CV was < 5% (not reported in the manuscript)
All anthropometric measures were repeated until reproduced within a pre-defined acceptable range (i.e. 10 grams for weight, 0.5 cm for height and 0.2 cm for head circumference).

Randomization

As per defined in the protocol, the toddlers who meet the eligibility criteria were randomized to receive either the EYCF or CM milk in a 1:1 allocation ratio. The randomization was stratified by sex (female/male). The toddlers receiving the habitual diet (REF) will not be randomized.

	This stratification ensured that an approximately equal proportion of males (and proportion of females) are assigned to the EYCF and CM groups. Subjects belonging to the reference group (REF) were not randomized. Confounding can occur if some covariates are related to both the treatment assignment (exposed to either EYCF or CM) and the outcome. In the presence of confounding, propensity score methods are used to remove the effects of confounding when estimating the effect of treatment. For this analysis gender, gestational age (GA), delivery mode, breastfeeding history, age at enrollment (months), BMI at baseline were used as variables to build the propensity scores. see page 29
Blinding	The study is a double-blinded study. The identity of specific product will be masked to subjects, support staff, investigators and sponsor's personnel except the manufacture and quality unit at Nestle Nutrition, (production factory). Products will be coded with group non-speaking codes. A total of 8 non-speaking codes will be used, 4 per formula group. The product cans will be labeled the same, but will only be distinguished by the non-speaking code printed on the can label. The non-speaking codes for this study are as follows: "7E2" "4G1" "7R5" "3G7" "2E7" "7B1" "4U7" "5T1". see page 30 of the protocol

Reporting for specific materials, systems and methods

We require information from authors about some types of materials, experimental systems and methods used in many studies. Here, indicate whether each material, system or method listed is relevant to your study. If you are not sure if a list item applies to your research, read the appropriate section before selecting a response.

Materials & experimental systems

n/a	Involved in the study
☐	☒ Antibodies
☒	☐ Eukaryotic cell lines
☒	☐ Palaeontology and archaeology
☒	☐ Animals and other organisms
☐	☒ Clinical data
☒	☐ Dual use research of concern
☒	☐ Plants

Methods

n/a	Involved in the study
☒	☐ ChIP-seq
☒	☐ Flow cytometry
☒	☐ MRI-based neuroimaging

Antibodies

Antibodies used	Human CTX-1 ELISA Kit (Colorimetric): Ref.NBP2-69073. Biotinylated Detection Ab (100x).- P1NP. Human Pro-Collagen I α1/COLIA1 (colorimetric): Ref. ab210966. Monoclonal antibody specific for human Pro-Collagen I α1 conjugated to horseradish.
Validation	Validation by manufacter website. P1NP: https://www.rndsystems.com/products/human-pro-collagen-i-alpha-1-quantikine-elisa-kit_dpca00?gad_source=1&gclid=EAIaIQobChMI74XTpcyEjQMVPQgGAB1hSC-nEAAYAiAAEgJyqPD_BwE&gclsrc=aw.ds.<br>CTX: https://www.novusbio.com/products/ctx-1-elisa-kit_nbp2-69073?srsltid=AfmBOop2FAWVy6RyKAqfKtc6dEef6WGtVzRf4xuMsH7gZNcZmegmlZYw

Clinical data

Policy information about clinical studies

All manuscripts should comply with the ICMJE guidelines for publication of clinical research and a completed CONSORT checklist must be included with all submissions.

Clinical trial registration	clinicaltrial.gov as NCT04799028.
Study protocol	full protocol was sent with manuscript submission - Trial N° 20.10.INF
Data collection	see Trial plan table in the protocol file (p. 18)
Outcomes	Primary endpoint<br>Speed of sound (SOS) using a quantitative ultrasound method as measured by Pediatric Sunlight 9000 device of the tibia at V3 (~6 months).<br>Secondary Endpoints<br><br>1. Additional bone mass index parameters<br>• Assessed at V1 (baseline), V2 (~3 months) and V3 (~6 months).<br>• Speed of sound (SOS) using quantitative ultrasound method as measured by Pediatric Sunlight 9000 device of the radius.<br>2. GI tolerance and stooling patterns<br>• Assessed at V1 (baseline) using 24-hour GI symptom and Behavior recall for the past 24 hours. At V2 and V3, stooling patterns will be collected using 3-day GI Symptom and Behavior diaries.<br>• Parent perceptions of GI symptoms and GI-related behaviors using parent-reported questionnaire including constipation, diarrhea, gassiness, abdominal pain, difficult to pass / hard stools, bloating, fussiness and sleep problems will be assessed using parent-reported Toddler Gut Comfort Questionnaire (GCQ). GCQ will be collected at V1 (baseline), V2 and V3 recalling the past week.<br>• Stool patterns including stool frequency and consistency will be recorded retrospectively via 24-hour GI Symptom and Behavior

Recall at V1 (baseline) and via 3-day GI Symptom and Behavior dairies prospectively for over three days prior to V2 and V3.
i. Stool frequency will be reported as daily mean stool frequency.
ii. Stool consistency will be reported as daily mean stool consistency score using a published, validated 5-point stool scale (1=watery, 2=runny, 3=mushy soft, 4=formed, and 5=hard).
3. Excretion of fecal calcium fatty acid soaps
• Assessed at V1 (baseline) and at home prior to V3.
• Stool parameters including individual soap fatty acids, total non-soap fatty acids, total fatty acids, and stool calcium content, requiring total of 27g of stool sample.
4. Serum vitamin D
• Assessed at V1 (baseline) and V3.
• Measured by serum 25[OH]D concentration requiring 1mL of whole blood sample.
5. Blood and urine bone turnover markers
• Assessed at V1 (baseline) and V3 in EYCF and CM groups only.
• Bone resorption markers in urinary [C-terminal cross-linked telopeptides of type I collagen (CTX) and bone accretion marker in serum procollagen type I N-terminal propeptide (PINP). This will lead to rate of bone turnover determination.
• CTX increases during periods of rapid growth in infancy and toddlerhood. PINP is released during processing of type I procollagen into collagen.
• CTX requires 5mL of urine and PINP requires 2 mL of whole
blood sample and both will be determined through ECLIA assay.
Other secondary endpoints:
6. Gut microbiota
• Assessed at V1 (baseline) and at home prior to V3.
• Gut microbiota analyzed for microbiome composition and diversity using next generation shotgun metagenomics sequencing, requiring 3g of stool sample.
7. Fecal markers of GI environment, metabolism and gut health
• Assessed at V1 (baseline) and at home prior to V3.
• Determination of fecal short chain fatty acids (SCFAs) and pH to describe gut environment and metabolism.
8. Physical strength
• Assessed at V1 (baseline) and V3.
• Hand grip test (Jamar hand dynamometer) tailored for toddlers will be used as previously described Bohannon et al Pediatric Physical Therapy 2017.
• Nicotinamide (vitamin B3) serum concentration requiring 2 mL of whole blood sample.
9. Safety endpoints will be
• Weight, height, head circumference, BMI, and corresponding z-scores at V1 (Baseline) and V3.
• Standard AEs reporting for safety assessment from V1 (baseline, after ICF signing) to V3+14 days
i. Type, incidence, severity, seriousness and relation to intervention
ii. AEs of interest, anemia and infections, will also be described.
iii. All concomitant medications used to treat illnesses and other conditions will be recorded (both dose and duration).

Plants

Seed stocks

Report on the source of all seed stocks or other plant material used. If applicable, state the seed stock centre and catalogue number. If plant specimens were collected from the field, describe the collection location, date and sampling procedures.

Novel plant genotypes

Describe the methods by which all novel plant genotypes were produced. This includes those generated by transgenic approaches, gene editing, chemical/radiation-based mutagenesis and hybridization. For transgenic lines, describe the transformation method, the number of independent lines analyzed and the generation upon which experiments were performed. For gene-edited lines, describe the editor used, the endogenous sequence targeted for editing, the targeting guide RNA sequence (if applicable) and how the editor was applied.

Authentication

Describe any authentication procedures for each seed stock used or novel genotype generated. Describe any experiments used to assess the effect of a mutation and, where applicable, how potential secondary effects (e.g. second site T-DNA insertions, mosiacism, off-target gene editing) were examined.
