# Supplementary material for: A young child formula with Limosilactobacillus reuteri and GOS modulates gut microbiome and enhances bone and muscle development: a randomized trial
Source: Nat Commun. 2025 Dec 12;17:237. doi: 10.1038/s41467-025-66930-2 (PMC12783733; doi:10.1038/s41467-025-66930-2)
Supplement: Supplementary file 19 — Supplementary Data 17 [file 41467_2025_66930_MOESM19_ESM.pdf]

MGS Group	Anotation	Level
Hgn3C.0001	Phocaeicola vulgatus	species
Hgn3C.0010	Anaerostipes hadrus	species
Hgn3C.0100	Bifidobacterium bifidum	species
Hgn3C.1000	Bacteroidales sp.	order
Hgn3C.1001	Archaea sp.	superkingdom
Hgn3C.1002	Eggerthellales sp.	order
Hgn3C.1003	Veillonellaceae sp.	family
Hgn3C.1004	Bacteroidales sp.	order
Hgn3C.1005	Barnesiella sp. An22	species
Hgn3C.1006	Eubacteriales sp.	order
Hgn3C.1007	Firmicutes sp.	phylum
Hgn3C.1008	Oscillospiraceae sp.	family
Hgn3C.1009	Prevotellaceae sp.	family
Hgn3C.0101	Bifidobacterium pseudocatenulatum	species
Hgn3C.1010	Parabacteroides massiliensis	species
Hgn3C.1011	Eubacteriales sp.	order
Hgn3C.1012	Erysipelotrichaceae sp.	family
Hgn3C.1013	Oscillospiraceae sp.	family
Hgn3C.1014	Burkholderiales sp.	order
Hgn3C.1015	Bacteria sp.	superkingdom
Hgn3C.1016	Eubacteriales sp.	order
Hgn3C.1017	Emergencia timonensis	species
Hgn3C.1018	Bacteroidia sp.	class
Hgn3C.1019	Prevotellaceae sp.	family
Hgn3C.0102	Eubacteriales sp.	order
Hgn3C.1020	Phocaeicola barnesiae	species
Hgn3C.1021	Bacteroides stercorirosoris	species
Hgn3C.1022	Eubacteriales sp.	order
Hgn3C.1023	Firmicutes sp.	phylum
Hgn3C.1024	Prevotellaceae sp.	family
Hgn3C.1025	Eubacteriales sp.	order
Hgn3C.1026	Bacteroidales sp.	order
Hgn3C.1027	Lachnospiraceae sp.	family
Hgn3C.1028	Bacteroidales sp.	order
Hgn3C.1029	Eubacteriales sp.	order
Hgn3C.0103	Holdemanella porci	species
Hgn3C.1030	Lachnospiraceae sp.	family
Hgn3C.1031	Eubacteriales sp.	order
Hgn3C.1032	Bacteroidales sp.	order
Hgn3C.1033	Hafnia paralvei	species
Hgn3C.1034	Odoribacter sp. AF15-53	species
Hgn3C.1035	Klebsiella sp.	genus
Hgn3C.1036	Escherichia coli	species
Hgn3C.1037	Rothia sp.	genus
Hgn3C.1038	Veillonella sp.	genus
Hgn3C.1039	Prevotellaceae sp.	family

Hgn3C.0104	Clostridium sp. AM32-2	species
Hgn3C.1040	Lachnospiraceae sp.	family
Hgn3C.1041	Bacteroides stercoris	species
Hgn3C.1042	Dorea formicigenerans	species
Hgn3C.1043	Clostridium phoceensis	species
Hgn3C.1044	Eubacteriales sp.	order
Hgn3C.1045	Lactococcus lactis subsp. lactis	subspecies
Hgn3C.1046	Bacteroidales sp.	order
Hgn3C.1047	Eubacteriales sp.	order
Hgn3C.1048	Akkermansia sp. BIOML-A59	species
Hgn3C.1049	Enterococcus faecium	species
Hgn3C.0105	Vescimonas coprocola	species
Hgn3C.1050	Staphylococcus epidermidis	species
Hgn3C.1051	Holdemania massiliensis	species
Hgn3C.1052	Enterococcus gallinarum	species
Hgn3C.1053	Eubacteriales sp.	order
Hgn3C.1054	Streptococcus lutetiensis	species
Hgn3C.1055	Veillonella seminalis	species
Hgn3C.1056	Muribaculum sp. NM65_B17	species
Hgn3C.1057	Eubacteriales sp.	order
Hgn3C.1058	Mediterraneibacter glycyrrhizinilyticus	species
Hgn3C.1059	Faecalicatena sp.	genus
Hgn3C.0106	Faecalibacterium sp. CLA-AA-H233	species
Hgn3C.1060	Eubacteriales sp.	order
Hgn3C.1061	Acidaminococcales sp.	order
Hgn3C.1062	Eubacteriales sp.	order
Hgn3C.1063	Enterococcus avium	species
Hgn3C.1064	Eubacteriales sp.	order
Hgn3C.1065	Klebsiella variicola subsp. variicola	subspecies
Hgn3C.1066	Lacrimispora saccharolytica	species
Hgn3C.1067	Lachnospiraceae sp.	family
Hgn3C.1068	Eubacteriales sp.	order
Hgn3C.1069	Bacteroidales sp.	order
Hgn3C.0107	Parasutterella excrementihominis	species
Hgn3C.1070	Raoultella planticola	species
Hgn3C.1071	Lactobacillus gasseri	species
Hgn3C.1072	Eubacteriales sp.	order
Hgn3C.1073	Streptococcus gallolyticus subsp. gallolyticus	subspecies
Hgn3C.1074	Eubacteriales sp.	order
Hgn3C.1075	Brachyspira aalborgi	species
Hgn3C.1076	Clostridia sp.	class
Hgn3C.1077	Eubacteriales sp.	order
Hgn3C.1078	Eubacteriales sp.	order
Hgn3C.1079	Eubacteriales sp.	order
Hgn3C.0108	Eubacteriales sp.	order
Hgn3C.1080	Bacteroidaceae sp.	family
Hgn3C.1081	Lacticaseibacillus rhamnosus	species
Hgn3C.1082	Lacticaseibacillus paracasei subsp. paracasei	subspecies

Hgn3C.1083	Firmicutes sp.	phylum
Hgn3C.1084	Massilimicrobiota timonensis	species
Hgn3C.1085	Eubacteriales sp.	order
Hgn3C.1086	Bacteroidales sp.	order
Hgn3C.1087	Ligilactobacillus salivarius	species
Hgn3C.1088	Enterococcus casseliflavus	species
Hgn3C.1089	Eubacteriales sp.	order
Hgn3C.0109	Bacteroides eggerthii	species
Hgn3C.1090	Turicibacter sanguinis	species
Hgn3C.1091	Eubacteriales sp.	order
Hgn3C.1092	Eubacteriales sp.	order
Hgn3C.1093	Enterobacter kobei	species
Hgn3C.1094	Eubacteriales sp.	order
Hgn3C.1095	Clostridia sp.	class
Hgn3C.1096	Clostridia sp.	class
Hgn3C.1097	Alistipes provencensis	species
Hgn3C.1098	Bacteroidales sp.	order
Hgn3C.1099	Eubacteriales sp.	order
Hgn3C.0011	Roseburia faecis	species
Hgn3C.0110	Eubacteriales sp.	order
Hgn3C.1100	Bacteria sp.	superkingdom
Hgn3C.1101	Firmicutes sp.	phylum
Hgn3C.1102	Eubacteriales sp.	order
Hgn3C.1103	Eubacteriales sp.	order
Hgn3C.1104	Bacteroidales sp.	order
Hgn3C.1105	Barnesiellaceae sp.	family
Hgn3C.1106	Clostridium butyricum	species
Hgn3C.1107	Eubacteriales sp.	order
Hgn3C.1108	Eubacteriales sp.	order
Hgn3C.1109	Eubacteriales sp.	order
Hgn3C.0111	Eubacteriales sp.	order
Hgn3C.1110	Porphyromonas sp.	genus
Hgn3C.1111	Eubacteriales sp.	order
Hgn3C.1112	Eubacteriales sp.	order
Hgn3C.1113	Firmicutes sp.	phylum
Hgn3C.1114	Bacteria sp.	superkingdom
Hgn3C.1115	Bacteria sp.	superkingdom
Hgn3C.1116	Bacteroidales sp.	order
Hgn3C.1117	Atopobiaceae sp.	family
Hgn3C.1118	Rhodospirillales sp.	order
Hgn3C.1119	Bacteroidales sp.	order
Hgn3C.0112	Clostridium sp.	genus
Hgn3C.1120	Actinomycetaceae sp.	family
Hgn3C.1121	Roseburia sp.	genus
Hgn3C.1122	Ruminococcus sp.	genus
Hgn3C.1123	Eubacteriales sp.	order
Hgn3C.1124	Clostridium porci	species
Hgn3C.1125	Bacteroidales sp.	order

Hgn3C.1126	Phocaeicola sartorii	species
Hgn3C.1127	Eubacteriales sp.	order
Hgn3C.1128	Actinobacteria sp.	phylum
Hgn3C.1129	Eubacteriales sp.	order
Hgn3C.0113	Clostridium sp. MCC345	species
Hgn3C.1130	Bacteroidales sp.	order
Hgn3C.1131	Lachnospiraceae sp.	family
Hgn3C.1132	Bacteroidales sp.	order
Hgn3C.1133	Lachnospiraceae sp.	family
Hgn3C.1134	Eubacteriales sp.	order
Hgn3C.1135	Eubacteriales sp.	order
Hgn3C.1136	Bacteria sp.	superkingdom
Hgn3C.1137	Eubacteriales sp.	order
Hgn3C.1138	Eubacteriales sp.	order
Hgn3C.1139	Firmicutes sp.	phylum
Hgn3C.0114	Eubacteriales sp.	order
Hgn3C.1140	Eubacteriales sp.	order
Hgn3C.1141	Archaea sp.	superkingdom
Hgn3C.1142	Prevotella sp.	genus
Hgn3C.1143	Gemmiger sp.	genus
Hgn3C.1144	Culturomica massiliensis	species
Hgn3C.1145	Lachnospiraceae sp.	family
Hgn3C.1146	Bacteria sp.	superkingdom
Hgn3C.1147	Methanomassiliicoccales sp.	order
Hgn3C.1148	Eggerthellales sp.	order
Hgn3C.1149	Bacteria sp.	superkingdom
Hgn3C.0115	Eubacteriales sp.	order
Hgn3C.1150	Oscillospiraceae sp.	family
Hgn3C.1151	Lachnospiraceae sp.	family
Hgn3C.1152	Acidaminococcus fermentans	species
Hgn3C.1153	Eubacterium sp. OM08-24	species
Hgn3C.1154	Lactococcus garvieae	species
Hgn3C.1155	Firmicutes sp.	phylum
Hgn3C.1156	Bacteria sp.	superkingdom
Hgn3C.1157	Eubacteriales sp.	order
Hgn3C.1158	Bacteroidales sp.	order
Hgn3C.1159	Firmicutes sp.	phylum
Hgn3C.0116	Anaerostipes hadrus	species
Hgn3C.1160	Deltaproteobacteria sp.	class
Hgn3C.1161	Eubacteriales sp.	order
Hgn3C.1162	Bacteria sp.	superkingdom
Hgn3C.1163	Bacteria sp.	superkingdom
Hgn3C.1164	Eubacteriales sp.	order
Hgn3C.1165	Desulfovibrio legallii	species
Hgn3C.1166	Eubacteriales sp.	order
Hgn3C.1167	Firmicutes sp.	phylum
Hgn3C.1168	Bacteria sp.	superkingdom
Hgn3C.1169	Oscillospiraceae sp.	family

Hgn3C.0117	Methanobrevibacter smithii	species
Hgn3C.1170	Bacteria sp.	superkingdom
Hgn3C.1171	Clostridia sp.	class
Hgn3C.1172	Treponemataceae sp.	family
Hgn3C.1173	Negativicutes sp.	class
Hgn3C.1174	Sutterella parvirubra	species
Hgn3C.1175	Firmicutes sp.	phylum
Hgn3C.1176	Eubacteriales sp.	order
Hgn3C.1177	Bacteroidaceae sp.	family
Hgn3C.1178	Clostridium sp. 12(A)	species
Hgn3C.1179	Lacrimispora celerecrescens	species
Hgn3C.0118	Eubacteriales sp.	order
Hgn3C.1180	Enterocloster citroniae	species
Hgn3C.1181	Acidovorax delafieldii	species
Hgn3C.1182	Comamonas kerstersii	species
Hgn3C.1183	Acinetobacter bereziniae	species
Hgn3C.1184	Acinetobacter johnsonii	species
Hgn3C.1185	Acinetobacter ursingii	species
Hgn3C.1186	Acinetobacter guillouiae	species
Hgn3C.1187	Actinomyces sp. HMSC065F12	species
Hgn3C.1188	Actinomyces oris	species
Hgn3C.1189	Aeromonas caviae	species
Hgn3C.0119	Bacteroides faecis	species
Hgn3C.1190	Fenollaria massiliensis	species
Hgn3C.1191	Aliarcobacter cryaerophilus	species
Hgn3C.1192	Methylobacterium sp. UNCCL125	species
Hgn3C.1193	Atopobium sp.	genus
Hgn3C.1194	Bacteroides gallinaceum	species
Hgn3C.1195	Bifidobacterium pullorum subsp. saeculare	subspecies
Hgn3C.1196	Blautia marasmi	species
Hgn3C.1197	Blautia hansenii	species
Hgn3C.1198	Turicimonas muris	species
Hgn3C.1199	Butyricimonas synergistica	species
Hgn3C.0012	Lachnospira sp.	genus
Hgn3C.0120	Flavonifractor plautii	species
Hgn3C.1200	Megasphaera sp. DJF_B143	species
Hgn3C.1201	Caulobacter sp. CCH5-E12	species
Hgn3C.1202	Chryseobacterium gambrini	species
Hgn3C.1203	Citrobacter sp. CRE-46	species
Hgn3C.1204	Citrobacter amalonaticus	species
Hgn3C.1205	Citrobacter braakii	species
Hgn3C.1206	Citrobacter pasteurii	species
Hgn3C.1207	Citrobacter sp.	genus
Hgn3C.1208	Citrobacter sp. S-77	species
Hgn3C.1209	Cloacibacillus evryensis	species
Hgn3C.0121	Oscillospiraceae sp.	family
Hgn3C.1210	Dielma sp.	genus
Hgn3C.1211	Lachnoclostridium pacaense	species

Hgn3C.1212	Clostridium jeddahense	species
Hgn3C.1213	Rubneribacter badeniensis	species
Hgn3C.1214	Corynebacterium tuberculostearicum	species
Hgn3C.1215	Cutibacterium acnes subsp. acnes	subspecies
Hgn3C.1216	Cutibacterium avidum	species
Hgn3C.1217	Cutibacterium granulosum	species
Hgn3C.1218	Enterobacter bugandensis	species
Hgn3C.1219	Enterobacter sp. NFIX58	species
Hgn3C.0122	Bacteroides finegoldii	species
Hgn3C.1220	Enterobacter ludwigii	species
Hgn3C.1221	Phytobacter diazotrophicus	species
Hgn3C.1222	Enterococcus durans	species
Hgn3C.1223	Enterococcus gilvus	species
Hgn3C.1224	Enterococcus raffinosus	species
Hgn3C.1225	Enterococcus faecium	species
Hgn3C.1226	Enterococcus thailandicus	species
Hgn3C.1227	Erysipelatoclostridium sp. An15	species
Hgn3C.1228	Escherichia marmotae	species
Hgn3C.1229	Escherichia sp. 93.0750	species
Hgn3C.0123	Eubacteriales sp.	order
Hgn3C.1230	Eubacterium limosum	species
Hgn3C.1231	Flavobacterium sp. Leaf359	species
Hgn3C.1232	Sanguibacteroides justesenii	species
Hgn3C.1233	Gardnerella vaginalis	species
Hgn3C.1234	Granulicatella adiacens	species
Hgn3C.1235	Haemophilus sp. CCUG 66565	species
Hgn3C.1236	Hafnia sp.	genus
Hgn3C.1237	Kosakonia cowanii	species
Hgn3C.1238	Klebsiella quasivariicola	species
Hgn3C.1239	Lactobacillus amylovorus	species
Hgn3C.0124	Ruthenibacterium lactatiformans	species
Hgn3C.1240	Lactobacillus helveticus	species
Hgn3C.1241	Limosilactobacillus reuteri subsp. reuteri	subspecies
Hgn3C.1242	Limosilactobacillus vaginalis	species
Hgn3C.1243	Lysinibacillus fusiformis	species
Hgn3C.1244	Megasphaera genomosp. type_1	species
Hgn3C.1245	Megasphaera massiliensis	species
Hgn3C.1246	Morganella morganii subsp. morganii	subspecies
Hgn3C.1247	Mycobacteroides chelonae	species
Hgn3C.1248	Neisseria sp. HMSC061E12	species
Hgn3C.1249	Pantoea sp. 3.5.1	species
Hgn3C.0125	Sutterella sp. KLE1602	species
Hgn3C.1250	Parabacteroides gordonii	species
Hgn3C.1251	Prevotella jejuni	species
Hgn3C.1252	Prevotella sp.	genus
Hgn3C.1253	Pseudoflavonifractor sp. An176	species
Hgn3C.1254	Pseudomonas extremaustralis	species
Hgn3C.1255	Pseudomonas fulva	species

Hgn3C.1256	Pseudomonas nitroreducens	species
Hgn3C.1257	Robinsoniella peoriensis	species
Hgn3C.1258	Rothia sp. HMSC072E10	species
Hgn3C.1259	Selenomonas artemidis	species
Hgn3C.0126	Ruminococcus sp. BSD2780120874_150323_B10	species
Hgn3C.1260	Serratia marcescens	species
Hgn3C.1261	Shewanella xiamenensis	species
Hgn3C.1262	Sphingomonas sp. CCH9-F2	species
Hgn3C.1263	Staphylococcus borealis	species
Hgn3C.1264	Staphylococcus hominis subsp. hominis	subspecies
Hgn3C.1265	Staphylococcus lugdunensis	species
Hgn3C.1266	Stenotrophomonas bentonitica	species
Hgn3C.1267	Stenotrophomonas maltophilia	species
Hgn3C.1268	Streptococcus sp.	genus
Hgn3C.1269	Streptococcus constellatus subsp. constellatus	subspecies
Hgn3C.0127	Faecalibacterium sp.	genus
Hgn3C.1270	Streptococcus sp.	genus
Hgn3C.1271	Streptococcus agalactiae	species
Hgn3C.1272	Veillonella sp. CNR 79/14	species
Hgn3C.1273	Citrobacter koseri	species
Hgn3C.1274	Kluyvera cryocrescens	species
Hgn3C.1275	Lachnospiraceae sp.	family
Hgn3C.1276	Citrobacter sp. MGH106	species
Hgn3C.1277	Lachnospiraceae sp.	family
Hgn3C.1278	Clostridium sp. NSJ-6	species
Hgn3C.1279	Enterococcus malodoratus	species
Hgn3C.0128	Eubacteriales sp.	order
Hgn3C.1280	Clostridium sp.	genus
Hgn3C.1281	Atlantibacter sp. RC6	species
Hgn3C.1282	Terrisporobacter othiniensis	species
Hgn3C.1283	Clostridium baratii	species
Hgn3C.1284	Enterobacteriaceae sp.	family
Hgn3C.1285	Enterobacteriaceae sp.	family
Hgn3C.1286	Corynebacterium variabile	species
Hgn3C.1287	Macrococcus caseolyticus subsp. caseolyticus	subspecies
Hgn3C.1288	Enterobacter hormaechei	species
Hgn3C.1289	Anaerosporobacter mobilis	species
Hgn3C.0129	Haemophilus parainfluenzae	species
Hgn3C.1290	Veillonella ratti	species
Hgn3C.1291	Lachnoclostridium edouardi	species
Hgn3C.1292	Limosilactobacillus portuensis	species
Hgn3C.1293	Peptostreptococcaceae sp.	family
Hgn3C.1294	Eubacteriales sp.	order
Hgn3C.1295	Butyricicoccus pullicaecorum	species
Hgn3C.1296	Raoultella planticola	species
Hgn3C.1297	Clostridium sp.	genus
Hgn3C.1298	Firmicutes sp.	phylum
Hgn3C.1299	Erysipelotrichaceae sp.	family

Hgn3C.0013	Ruminococcus bromii	species
Hgn3C.0130	Phocaeicola plebeius	species
Hgn3C.1300	Rothia sp.	genus
Hgn3C.1301	Enterobacteriaceae sp.	family
Hgn3C.1302	Schaalia sp.	genus
Hgn3C.1303	Clostridium paraputrificum	species
Hgn3C.1304	Citrobacter sp. RHBSTW-00671	species
Hgn3C.1305	Isoptericola variabilis	species
Hgn3C.1306	Peptostreptococcaceae sp.	family
Hgn3C.1307	Bifidobacterium longum spp.	subspecies
Hgn3C.1308	Eisenbergiella sp.	genus
Hgn3C.1309	Enterobacter cloacae subsp. cloacae	subspecies
Hgn3C.0131	Streptococcus thermophilus	species
Hgn3C.1310	Erysipelotrichaceae sp.	family
Hgn3C.1311	Lachnospiraceae sp.	family
Hgn3C.1312	Candida tropicalis	species
Hgn3C.1313	Klebsiella aerogenes	species
Hgn3C.1314	Eubacteriales sp.	order
Hgn3C.1315	Eubacteriales sp.	order
Hgn3C.1316	Eubacteriales sp.	order
Hgn3C.1317	Lachnospiraceae sp.	family
Hgn3C.1318	Eubacterium maltosivorans	species
Hgn3C.1319	Atlantibacter hermannii	species
Hgn3C.0132	Eubacteriales sp.	order
Hgn3C.1320	Proteus mirabilis	species
Hgn3C.1321	Veillonellaceae sp.	family
Hgn3C.1322	Enterococcus hirae	species
Hgn3C.1323	Lachnospiraceae sp.	family
Hgn3C.1324	Eubacterium sp. c-25	species
Hgn3C.1325	Luxibacter massiliensis	species
Hgn3C.1326	Eggerthella guodeyinii	species
Hgn3C.1327	Erysipelotrichaceae sp.	family
Hgn3C.1328	Lacrimispora saccharolytica	species
Hgn3C.1329	Eubacteriales sp.	order
Hgn3C.0133	Dialister hominis	species
Hgn3C.1330	Lachnospiraceae sp.	family
Hgn3C.1331	Lachnospiraceae sp.	family
Hgn3C.1332	Erysipelotrichales sp.	order
Hgn3C.1333	Tannerellaceae sp.	family
Hgn3C.1334	Eubacteriales sp.	order
Hgn3C.1335	Eubacteriales sp.	order
Hgn3C.1336	Eubacteriales sp.	order
Hgn3C.1337	Lachnospiraceae sp.	family
Hgn3C.1338	Erysipelatoclostridium sp.	genus
Hgn3C.1339	Eubacteriales sp.	order
Hgn3C.0134	Eubacteriales sp.	order
Hgn3C.1340	Clostridiaceae sp.	family
Hgn3C.1341	Lachnospiraceae sp.	family

Hgn3C.1342	Eubacteriales sp.	order
Hgn3C.1343	Enterococcus italicus	species
Hgn3C.1344	Eubacteriaceae sp.	family
Hgn3C.1345	Limosilactobacillus mucosae	species
Hgn3C.1346	Erysipelotrichales sp.	order
Hgn3C.1347	[Clostridium] hylemonae	species
Hgn3C.1348	Eubacteriales sp.	order
Hgn3C.1349	Corynebacterium falsenii	species
Hgn3C.0135	Firmicutes sp.	phylum
Hgn3C.1350	Enterococcus asini	species
Hgn3C.1351	Actinomyces sp. ICM47	species
Hgn3C.1352	Akkermansia sp.	genus
Hgn3C.1353	Beduini sp.	genus
Hgn3C.1354	Actinomyces sp.	genus
Hgn3C.1355	Collinsella stercoris	species
Hgn3C.1356	Eubacteriales sp.	order
Hgn3C.1357	Bacteroidales sp.	order
Hgn3C.1358	Lachnospiraceae sp.	family
Hgn3C.1359	Extibacter muris	species
Hgn3C.0136	Alistipes ihumii	species
Hgn3C.1360	Bifidobacterium crudilactis	species
Hgn3C.1361	Senegalimassilia anaerobia	species
Hgn3C.1362	Christensenella intestinihominis	species
Hgn3C.1363	Oscillospiraceae sp.	family
Hgn3C.1364	Clostridia sp.	class
Hgn3C.1365	Erysipelotrichales sp.	order
Hgn3C.1366	Lachnospiraceae sp.	family
Hgn3C.1367	Oscillospiraceae sp.	family
Hgn3C.1368	Lachnospiraceae sp.	family
Hgn3C.1369	Lachnospiraceae sp.	family
Hgn3C.0137	Clostridium sp. OF03-18AA	species
Hgn3C.1370	Kluyvera genomosp. 3	species
Hgn3C.1371	Lachnospiraceae sp.	family
Hgn3C.1372	Eubacteriales sp.	order
Hgn3C.1373	Eubacteriales sp.	order
Hgn3C.1374	Eubacterium sp. An3	species
Hgn3C.1375	Atopobiaceae sp.	family
Hgn3C.1376	Kluyvera sp. CRP	species
Hgn3C.1377	Collinsella tanakaei	species
Hgn3C.1378	Collinsella sp. AF08-23	species
Hgn3C.1379	Gordonibacter urolithinfaciens	species
Hgn3C.0138	Anaerobutyricum soehngenii	species
Hgn3C.1380	Kluyvera georgiana	species
Hgn3C.1381	Eubacteriales sp.	order
Hgn3C.1382	Oscillospiraceae sp.	family
Hgn3C.1383	Sellimonas monacensis	species
Hgn3C.1384	Anaerococcus sp.	genus
Hgn3C.1385	Eubacteriales sp.	order

Hgn3C.1386	Veillonella sp.	genus
Hgn3C.1387	Alistipes sp.	genus
Hgn3C.1388	Streptococcus infantarius subsp. infantarius	subspecies
Hgn3C.1389	Eubacteriales sp.	order
Hgn3C.0139	Ruminococcus callidus	species
Hgn3C.1390	Erysipelatoclostridium sp.	genus
Hgn3C.1391	Clostridium sp.	genus
Hgn3C.1392	Enterobacter sp. JBIWA005	species
Hgn3C.1393	Anaerofustis stercorihominis	species
Hgn3C.1394	Eubacteriales sp.	order
Hgn3C.1395	Bilophila sp.	genus
Hgn3C.1396	Anaerostipes sp.	genus
Hgn3C.1397	Erysipelotrichales sp.	order
Hgn3C.1398	Actinomyces sp.	genus
Hgn3C.1399	Clostridium sp.	genus
Hgn3C.0014	Lacrimispora saccharolytica	species
Hgn3C.0140	Veillonella parvula	species
Hgn3C.1400	Eubacterium sp.	genus
Hgn3C.1401	Eubacteriales sp.	order
Hgn3C.1402	Collinsella phocaeensis	species
Hgn3C.1403	Eubacteriaceae sp.	family
Hgn3C.1404	Clostridium sp.	genus
Hgn3C.1405	Eubacteriales sp.	order
Hgn3C.1406	Slackia piriformis	species
Hgn3C.1407	Christensenella minuta	species
Hgn3C.1408	Eubacterium callanderi	species
Hgn3C.1409	Eggerthella timonensis	species
Hgn3C.0141	Adlercreutzia equolifaciens subsp. celatus	subspecies
Hgn3C.1410	Erysipelatoclostridium sp.	genus
Hgn3C.1411	Eubacteriales sp.	order
Hgn3C.1412	Christensenella hongkongensis	species
Hgn3C.1413	Streptococcus sp.	genus
Hgn3C.1414	Collinsella sp.	genus
Hgn3C.1415	Klebsiella quasipneumoniae subsp. quasipneumoniae	subspecies
Hgn3C.1416	Limosilactobacillus fermentum	species
Hgn3C.1417	Oscillospiraceae sp.	family
Hgn3C.1418	Carnobacteriaceae sp.	family
Hgn3C.1419	Agathobaculum sp. NSJ-28	species
Hgn3C.0142	Eubacterium ramulus	species
Hgn3C.1420	Eubacteriales sp.	order
Hgn3C.1421	Bilophila sp.	genus
Hgn3C.1422	Eubacteriales sp.	order
Hgn3C.1423	Lachnospiraceae sp.	family
Hgn3C.1424	Mogibacterium sp. NSJ-24	species
Hgn3C.1425	Eubacteriales sp.	order
Hgn3C.1426	Limosilactobacillus oris	species
Hgn3C.1427	Erysipelotrichales sp.	order
Hgn3C.1428	Mediterraneibacter glycyrrhizinilyticus	species

Hgn3C.1429	Lachnospiraceae sp.	family
Hgn3C.0143	Streptococcus salivarius	species
Hgn3C.1430	Corynebacterium provencense	species
Hgn3C.1431	Bacteroidales sp.	order
Hgn3C.1432	Eggerthella timonensis	species
Hgn3C.1433	Robinsoniella sp.	genus
Hgn3C.1434	Blautia sp.	genus
Hgn3C.1435	Acinetobacter baumannii	species
Hgn3C.1436	Bacteria sp.	superkingdom
Hgn3C.1437	Candidatus Stoquefichus sp. SB1	species
Hgn3C.1438	Dielma fastidiosa	species
Hgn3C.1439	Streptococcus sp. IMAU 99161	species
Hgn3C.0144	Eubacteriales sp.	order
Hgn3C.1440	Eubacteriales sp.	order
Hgn3C.1441	Lancefieldella sp.	genus
Hgn3C.1442	Oscillospiraceae sp.	family
Hgn3C.1443	Streptococcus sp.	genus
Hgn3C.1444	Ruminococcus sp. 1001175B_160314_E1	species
Hgn3C.1445	Oscillospiraceae sp.	family
Hgn3C.1446	Eubacteriales sp.	order
Hgn3C.1447	Bacteria sp.	superkingdom
Hgn3C.1448	Leuconostoc falkenbergense	species
Hgn3C.1449	Bifidobacterium scardovii	species
Hgn3C.0145	Faecalibacterium sp.	genus
Hgn3C.1450	Lactococcus sp. NH2-7C	species
Hgn3C.1451	Faecalicatena orotica	species
Hgn3C.1452	Raoultibacter timonensis	species
Hgn3C.1453	Collinsella sp.	genus
Hgn3C.1454	Escherichia fergusonii	species
Hgn3C.1455	Enterocloster sp.	genus
Hgn3C.1456	Eubacteriales sp.	order
Hgn3C.1457	Eisenbergiella sp. OF01-20	species
Hgn3C.1458	Intestinimonas timonensis	species
Hgn3C.1459	Eggerthellaceae sp.	family
Hgn3C.0146	Phascolarctobacterium succinatutens	species
Hgn3C.1460	Blautia sp.	genus
Hgn3C.1461	Eubacteriales sp.	order
Hgn3C.1462	Eubacteriales sp.	order
Hgn3C.1463	Enterobacter sp.	genus
Hgn3C.1464	Oscillospiraceae sp.	family
Hgn3C.1465	Clostridia sp.	class
Hgn3C.1466	Lachnospiraceae sp.	family
Hgn3C.1467	Eubacteriales sp.	order
Hgn3C.1468	Staphylococcus aureus	species
Hgn3C.1469	Lachnospiraceae sp.	family
Hgn3C.0147	Oscillospiraceae sp.	family
Hgn3C.1470	Erysipelotrichales sp.	order
Hgn3C.1471	Eggerthella sp.	genus

Hgn3C.1472	Amedibacillus dolichus	species
Hgn3C.0148	Agathobaculum sp. M2	species
Hgn3C.0149	Eggerthellales sp.	order
Hgn3C.0015	Faecalibacterium sp. Marseille-Q4896	species
Hgn3C.0150	Eubacteriales sp.	order
Hgn3C.0151	Clostridium sp. AF34-13	species
Hgn3C.0152	Bacteroides salyersiae	species
Hgn3C.0153	Lachnospiraceae sp.	family
Hgn3C.0154	Prevotella copri	species
Hgn3C.0155	Blautia sp.	genus
Hgn3C.0156	Eubacteriales sp.	order
Hgn3C.0157	Eubacteriales sp.	order
Hgn3C.0158	Eubacteriales sp.	order
Hgn3C.0159	Firmicutes sp.	phylum
Hgn3C.0016	Parabacteroides merdae	species
Hgn3C.0160	Eubacteriales sp.	order
Hgn3C.0161	Blautia sp.	genus
Hgn3C.0162	Prevotella sp.	genus
Hgn3C.0163	Bacteria sp.	superkingdom
Hgn3C.0164	Kineothrix sp.	genus
Hgn3C.0165	Butyrivibrio crossotus	species
Hgn3C.0166	Eubacteriales sp.	order
Hgn3C.0167	Lachnospiraceae sp.	family
Hgn3C.0168	Evtepia gabavorous	species
Hgn3C.0169	Bacteroides intestinalis	species
Hgn3C.0017	Subdoligranulum sp. APC924/74	species
Hgn3C.0170	Oscillospiraceae sp.	family
Hgn3C.0171	Catenibacterium mitsuokai	species
Hgn3C.0172	Eubacteriales sp.	order
Hgn3C.0173	Proteobacteria sp.	phylum
Hgn3C.0174	Lachnospiraceae sp.	family
Hgn3C.0175	Oscillospiraceae sp.	family
Hgn3C.0176	Alistipes indistinctus	species
Hgn3C.0177	Clostridia sp.	class
Hgn3C.0178	Clostridia sp.	class
Hgn3C.0179	Bacteroidales sp.	order
Hgn3C.0018	Faecalibacterium prausnitzii	species
Hgn3C.0180	Bacteria sp.	superkingdom
Hgn3C.0181	Alistipes onderdonkii	species
Hgn3C.0182	Butyricimonas faecihominis	species
Hgn3C.0183	Intestinibacter bartlettii	species
Hgn3C.0184	Eubacteriales sp.	order
Hgn3C.0185	Bifidobacterium catenulatum subsp. kashiwanohense	subspecies
Hgn3C.0186	Butyricimonas virosa	species
Hgn3C.0187	Bacteria sp.	superkingdom
Hgn3C.0188	Eubacteriales sp.	order
Hgn3C.0189	Eubacteriales sp.	order
Hgn3C.0019	Phocaeicola dorei	species

Hgn3C.0190	Eubacteriales sp.	order
Hgn3C.0191	Prevotella sp.	genus
Hgn3C.0192	Erysipelatoclostridium ramosum	species
Hgn3C.0193	Eubacteriales sp.	order
Hgn3C.0194	Faecalibacillus faecis	species
Hgn3C.0195	Eubacteriales sp.	order
Hgn3C.0196	Phocaeicola coprocola	species
Hgn3C.0197	Bacteroides clarus	species
Hgn3C.0198	Oscillospiraceae sp.	family
Hgn3C.0199	Bacteroidales sp.	order
Hgn3C.0002	Bacteroides uniformis	species
Hgn3C.0020	Collinsella aerofaciens	species
Hgn3C.0200	Bacteroidales sp.	order
Hgn3C.0201	Veillonella dispar	species
Hgn3C.0202	Eggerthella lenta	species
Hgn3C.0203	Faecalibacterium sp. Marseille-Q3530	species
Hgn3C.0204	Coprobacter fastidiosus	species
Hgn3C.0205	Proteobacteria sp.	phylum
Hgn3C.0206	Gemmiger formicilis	species
Hgn3C.0207	Eubacteriales sp.	order
Hgn3C.0208	Romboutsia timonensis	species
Hgn3C.0209	Bifidobacterium breve	species
Hgn3C.0021	Bifidobacterium longum subsp. longum	subspecies
Hgn3C.0210	Firmicutes sp.	phylum
Hgn3C.0211	Eubacteriales sp.	order
Hgn3C.0212	Holdemanella biformis	species
Hgn3C.0213	Akkermansia sp. GGCC_0220	species
Hgn3C.0214	Duodenibacillus massiliensis	species
Hgn3C.0215	Alistipes sp. cv1	species
Hgn3C.0216	Eggerthellales sp.	order
Hgn3C.0217	Eubacteriales sp.	order
Hgn3C.0218	Eubacteriales sp.	order
Hgn3C.0219	Tyzzerella nexilis	species
Hgn3C.0022	Alistipes shahii	species
Hgn3C.0220	Clostridium sp. MCC334	species
Hgn3C.0221	Clostridium sp.	genus
Hgn3C.0222	Parabacteroides merdae	species
Hgn3C.0223	Firmicutes sp.	phylum
Hgn3C.0224	Veillonellales sp.	order
Hgn3C.0225	Eubacteriales sp.	order
Hgn3C.0226	Eubacteriales sp.	order
Hgn3C.0227	Lachnospiraceae sp.	family
Hgn3C.0228	Coprococcus eutactus	species
Hgn3C.0229	Eubacteriales sp.	order
Hgn3C.0023	Bacteroides xylanisolvens	species
Hgn3C.0230	Gemmiger sp.	genus
Hgn3C.0231	Firmicutes sp.	phylum
Hgn3C.0232	Megamonas funiformis	species

Hgn3C.0233	Eubacteriales sp.	order
Hgn3C.0234	Eubacteriales sp.	order
Hgn3C.0235	Parasutterella sp.	genus
Hgn3C.0236	Oscillospiraceae sp.	family
Hgn3C.0237	Pseudoruminococcus massiliensis	species
Hgn3C.0238	Oscillospiraceae sp.	family
Hgn3C.0239	Blautia sp. BIOML-A1	species
Hgn3C.0024	Bacteroides caccae	species
Hgn3C.0240	Lachnospiraceae sp.	family
Hgn3C.0241	Eubacteriales sp.	order
Hgn3C.0242	Enterocloster bolteae	species
Hgn3C.0243	Acidaminococcales sp.	order
Hgn3C.0244	Desulfovibrio piger	species
Hgn3C.0245	Blautia stercoris	species
Hgn3C.0246	Roseburia sp. AM59-24XD	species
Hgn3C.0247	Eubacteriales sp.	order
Hgn3C.0248	Coprococcus catus	species
Hgn3C.0249	Clostridium sp. AM33-3	species
Hgn3C.0025	Ruminococcus bicirculans	species
Hgn3C.0250	Eubacteriales sp.	order
Hgn3C.0251	Alistipes senegalensis	species
Hgn3C.0252	Firmicutes sp.	phylum
Hgn3C.0253	Waltera sp.	genus
Hgn3C.0254	Acidaminococcus intestini	species
Hgn3C.0255	Firmicutes sp.	phylum
Hgn3C.0256	Bacteria sp.	superkingdom
Hgn3C.0257	Eubacteriales sp.	order
Hgn3C.0258	Eubacteriales sp.	order
Hgn3C.0259	Clostridiaceae sp.	family
Hgn3C.0026	Dorea longicatena	species
Hgn3C.0260	Hydrogenoanaerobacterium saccharovorans	species
Hgn3C.0261	Firmicutes sp.	phylum
Hgn3C.0262	Oscillospiraceae sp.	family
Hgn3C.0263	Eubacterium sp. AF22-9	species
Hgn3C.0264	Clostridium sp. AF27-2AA	species
Hgn3C.0265	Eubacteriales sp.	order
Hgn3C.0266	Dorea sp. AF36-15AT	species
Hgn3C.0267	Oscillospiraceae sp.	family
Hgn3C.0268	Eubacteriales sp.	order
Hgn3C.0269	Flintibacter sp. NSJ-23	species
Hgn3C.0027	Barnesiella intestinihominis	species
Hgn3C.0270	Eubacteriales sp.	order
Hgn3C.0271	Oscillibacter sp.	genus
Hgn3C.0272	Blautia faecicola	species
Hgn3C.0273	Slackia isoflavoniconvertens	species
Hgn3C.0274	Eubacteriales sp.	order
Hgn3C.0275	Lachnospiraceae sp.	family
Hgn3C.0276	Eubacteriales sp.	order

Hgn3C.0277	Faecalibacterium sp.	genus
Hgn3C.0278	Firmicutes sp.	phylum
Hgn3C.0279	Oscillospiraceae sp.	family
Hgn3C.0028	Oscillibacter sp. ER4	species
Hgn3C.0280	Clostridia sp.	class
Hgn3C.0281	Eubacteriales sp.	order
Hgn3C.0282	Eubacteriales sp.	order
Hgn3C.0283	Eubacteriales sp.	order
Hgn3C.0284	Erysipelotrichaceae sp.	family
Hgn3C.0285	Colidextribacter sp.	genus
Hgn3C.0286	Clostridia sp.	class
Hgn3C.0287	Ruminococcus sp. 1001095IJ_161003_G2	species
Hgn3C.0288	Butyricimonas paravirosa	species
Hgn3C.0289	Bacteroides cellulosilyticus	species
Hgn3C.0029	Anaerobutyricum hallii	species
Hgn3C.0290	Sellimonas intestinalis	species
Hgn3C.0291	Parabacteroides johnsonii	species
Hgn3C.0292	Clostridium sp. OM07-9AC	species
Hgn3C.0293	Eubacteriales sp.	order
Hgn3C.0294	Firmicutes sp.	phylum
Hgn3C.0295	Eubacteriales sp.	order
Hgn3C.0296	Firmicutes sp.	phylum
Hgn3C.0297	Eubacteriales sp.	order
Hgn3C.0298	Monoglobus pectinilyticus	species
Hgn3C.0299	Escherichia coli	species
Hgn3C.0003	Lachnospiraceae sp.	family
Hgn3C.0030	Faecalibacterium prausnitzii	species
Hgn3C.0300	Clostridia sp.	class
Hgn3C.0301	Prevotella hominis	species
Hgn3C.0302	Lachnospiraceae sp.	family
Hgn3C.0303	Firmicutes sp.	phylum
Hgn3C.0304	Eubacteriales sp.	order
Hgn3C.0305	Candidatus Borkfalkia ceftriaxoniphila	species
Hgn3C.0306	Bacteroides nordii	species
Hgn3C.0307	Veillonella atypica	species
Hgn3C.0308	[Clostridium] symbiosum	species
Hgn3C.0309	Eubacteriales sp.	order
Hgn3C.0031	Blautia massiliensis	species
Hgn3C.0310	Ligilactobacillus ruminis	species
Hgn3C.0311	Mesosutterella multiformis	species
Hgn3C.0312	Bifidobacterium angulatum	species
Hgn3C.0313	Ruminococcus bromii	species
Hgn3C.0314	Ruminococcus sp.	genus
Hgn3C.0315	Bacteroidia sp.	class
Hgn3C.0316	Firmicutes sp.	phylum
Hgn3C.0317	Intestinimonas butyriciproducens	species
Hgn3C.0318	Eubacterium sp. AF34-35BH	species
Hgn3C.0319	Clostridiaceae sp.	family

Hgn3C.0032	Alistipes onderdonkii subsp. vulgaris	subspecies
Hgn3C.0320	Eubacteriales sp.	order
Hgn3C.0321	Blautia luti	species
Hgn3C.0322	Eubacteriales sp.	order
Hgn3C.0323	Bacteroides sp.	genus
Hgn3C.0324	Prevotella stercorea	species
Hgn3C.0325	Roseburia sp. BX0805	species
Hgn3C.0326	Eubacteriales sp.	order
Hgn3C.0327	Ruminococcus sp. 1001270H_150608_F2	species
Hgn3C.0328	Eubacteriales sp.	order
Hgn3C.0329	Eubacteriales sp.	order
Hgn3C.0033	Gemmiger formicilis	species
Hgn3C.0330	Faecalibacillus intestinalis	species
Hgn3C.0331	Clostridia sp.	class
Hgn3C.0332	Klebsiella pneumoniae subsp. pneumoniae	subspecies
Hgn3C.0333	Eubacteriales sp.	order
Hgn3C.0334	Bacteroides cellulosilyticus	species
Hgn3C.0335	Alistipes sp. Marseille-P2263	species
Hgn3C.0336	Parabacteroides goldsteinii	species
Hgn3C.0337	Eubacteriales sp.	order
Hgn3C.0338	Eubacteriales sp.	order
Hgn3C.0339	Eubacteriales sp.	order
Hgn3C.0034	Roseburia inulinivorans	species
Hgn3C.0340	Eubacterium sp. AF19-12LB	species
Hgn3C.0341	Eubacteriales sp.	order
Hgn3C.0342	Eubacteriales sp.	order
Hgn3C.0343	Bacteroidales sp.	order
Hgn3C.0344	Akkermansia sp. BIOML-A8	species
Hgn3C.0345	Oscillospiraceae sp.	family
Hgn3C.0346	Eubacteriales sp.	order
Hgn3C.0347	unclassified sp.	unclassified
Hgn3C.0348	Streptococcus sp.	genus
Hgn3C.0349	Eubacteriales sp.	order
Hgn3C.0035	Odoribacter splanchnicus	species
Hgn3C.0350	[Clostridium] symbiosum	species
Hgn3C.0351	Prevotella sp.	genus
Hgn3C.0352	Eubacteriales sp.	order
Hgn3C.0353	Prevotella sp. P4-67	species
Hgn3C.0354	Sutterella seckii	species
Hgn3C.0355	Oscillospiraceae sp.	family
Hgn3C.0356	Blautia sp. M29	species
Hgn3C.0357	Eubacteriales sp.	order
Hgn3C.0358	Firmicutes sp.	phylum
Hgn3C.0359	Eubacterium sp. BX4	species
Hgn3C.0036	Bacteroides thetaiotaomicron	species
Hgn3C.0360	Eubacteriales sp.	order
Hgn3C.0361	Dorea sp. AF24-7LB	species
Hgn3C.0362	Dysosmobacter sp. NSJ-60	species

Hgn3C.0363	Anaerostipes sp.	genus
Hgn3C.0364	Eubacteriales sp.	order
Hgn3C.0365	Eubacteriales sp.	order
Hgn3C.0366	Eubacteriales sp.	order
Hgn3C.0367	Lachnospiraceae sp.	family
Hgn3C.0368	Eubacteriales sp.	order
Hgn3C.0369	Prevotella sp.	genus
Hgn3C.0037	Roseburia intestinalis	species
Hgn3C.0370	Ruminococcus sp. AF41-9	species
Hgn3C.0371	Lachnospiraceae sp.	family
Hgn3C.0372	Eubacteriales sp.	order
Hgn3C.0373	Enterocloster clostridioformis	species
Hgn3C.0374	Bacteroidia sp.	class
Hgn3C.0375	Ellagibacter isourolithinifaciens	species
Hgn3C.0376	Eubacteriales sp.	order
Hgn3C.0377	Butyricicoccus sp. MSJd-7	species
Hgn3C.0378	Firmicutes sp.	phylum
Hgn3C.0379	Eubacteriales sp.	order
Hgn3C.0038	Bifidobacterium adolescentis	species
Hgn3C.0380	Victivallis lenta	species
Hgn3C.0381	Clostridium sp. AM30-24	species
Hgn3C.0382	Eubacteriales sp.	order
Hgn3C.0383	Eubacteriales sp.	order
Hgn3C.0384	Eggerthellales sp.	order
Hgn3C.0385	Veillonella rogosae	species
Hgn3C.0386	Clostridium sp. AT4	species
Hgn3C.0387	Eubacteriales sp.	order
Hgn3C.0388	Clostridium sp.	genus
Hgn3C.0389	Vescimonas fastidiosa	species
Hgn3C.0039	Coprococcus comes	species
Hgn3C.0390	Bacteria sp.	superkingdom
Hgn3C.0391	Firmicutes sp.	phylum
Hgn3C.0392	Clostridia sp.	class
Hgn3C.0393	Rikenellaceae sp.	family
Hgn3C.0394	Hungatella hathewayi	species
Hgn3C.0395	Bacteria sp.	superkingdom
Hgn3C.0396	Bacteroidales sp.	order
Hgn3C.0397	Parabacteroides sp.	genus
Hgn3C.0398	Eubacteriales sp.	order
Hgn3C.0399	Butyricimonas faecalis	species
Hgn3C.0004	Blautia wexlerae	species
Hgn3C.0040	Eubacteriales sp.	order
Hgn3C.0400	Prevotellamassilia timonensis	species
Hgn3C.0401	Eubacteriales sp.	order
Hgn3C.0402	Firmicutes sp.	phylum
Hgn3C.0403	Desulfovibrionales sp.	order
Hgn3C.0404	Eubacteriales sp.	order
Hgn3C.0405	Bacteroides ovatus	species

Hgn3C.0406	Phocaeicola coprophilus	species
Hgn3C.0407	Eubacteriales sp.	order
Hgn3C.0408	Eubacteriales sp.	order
Hgn3C.0409	Eubacteriales sp.	order
Hgn3C.0041	Ruminococcus sp.	genus
Hgn3C.0410	Eubacteriales sp.	order
Hgn3C.0411	Eubacteriales sp.	order
Hgn3C.0412	Eubacteriales sp.	order
Hgn3C.0413	Blautia caecimuris	species
Hgn3C.0414	Eubacteriales sp.	order
Hgn3C.0415	Clostridium sp. C5-48	species
Hgn3C.0416	Firmicutes sp.	phylum
Hgn3C.0417	Eubacteriales sp.	order
Hgn3C.0418	Blautia sp.	genus
Hgn3C.0419	Prevotella sp. 885	species
Hgn3C.0042	Blautia faecis	species
Hgn3C.0420	Clostridium sp. 1001270J_160509_D11	species
Hgn3C.0421	Bacteroidales sp.	order
Hgn3C.0422	Bacteroidia sp.	class
Hgn3C.0423	Eubacteriales sp.	order
Hgn3C.0424	Eubacteriales sp.	order
Hgn3C.0425	Eubacteriales sp.	order
Hgn3C.0426	Bifidobacterium animalis subsp. lactis	subspecies
Hgn3C.0427	Bacteria sp.	superkingdom
Hgn3C.0428	Eubacteriales sp.	order
Hgn3C.0429	Firmicutes sp.	phylum
Hgn3C.0043	Lachnoclostridium sp. 210928-DFI.6.3	species
Hgn3C.0430	Enterocloster aldenensis	species
Hgn3C.0431	Bacteria sp.	superkingdom
Hgn3C.0432	Eubacteriales sp.	order
Hgn3C.0433	Firmicutes sp.	phylum
Hgn3C.0434	Firmicutes sp.	phylum
Hgn3C.0435	Eubacteriales sp.	order
Hgn3C.0436	Eubacteriales sp.	order
Hgn3C.0437	Prevotella sp.	genus
Hgn3C.0438	Coprobacillus cateniformis	species
Hgn3C.0439	Enterococcus faecalis	species
Hgn3C.0044	Lachnospira pectinoschiza	species
Hgn3C.0440	Bacteria sp.	superkingdom
Hgn3C.0441	Firmicutes sp.	phylum
Hgn3C.0442	Holdemanella sp.	genus
Hgn3C.0443	Prevotella stercorea	species
Hgn3C.0444	Eubacteriales sp.	order
Hgn3C.0445	Aeromonadales sp.	order
Hgn3C.0446	Akkermansia sp. KLE1798	species
Hgn3C.0447	Bacteria sp.	superkingdom
Hgn3C.0448	Firmicutes sp.	phylum
Hgn3C.0449	Coprococcus sp. OM04-5BH	species

Hgn3C.0045	Faecalibacterium sp.	genus
Hgn3C.0450	Eubacteriales sp.	order
Hgn3C.0451	Roseburia sp. AM16-25	species
Hgn3C.0452	Oscillospiraceae sp.	family
Hgn3C.0453	Blautia sp. Marseille-P3087	species
Hgn3C.0454	Eubacteriales sp.	order
Hgn3C.0455	Peptostreptococcaceae sp.	family
Hgn3C.0456	Alistipes sp.	genus
Hgn3C.0457	Coprobacter secundus subsp. similis	subspecies
Hgn3C.0458	Eubacteriales sp.	order
Hgn3C.0459	Eubacteriales sp.	order
Hgn3C.0046	Clostridium sp. AF37-5	species
Hgn3C.0460	Sutterella sp.	genus
Hgn3C.0461	Dialister succinatiphilus	species
Hgn3C.0462	Eubacteriales sp.	order
Hgn3C.0463	Eubacteriales sp.	order
Hgn3C.0464	Bifidobacterium longum subsp. infantis	subspecies
Hgn3C.0465	Eisenbergiella massiliensis	species
Hgn3C.0466	Prevotella pectinovora	species
Hgn3C.0467	Eubacteriales sp.	order
Hgn3C.0468	Clostridium sp. NSJ-42	species
Hgn3C.0469	Firmicutes sp.	phylum
Hgn3C.0047	Dysosmobacter sp. BX15	species
Hgn3C.0470	Eubacteriales sp.	order
Hgn3C.0471	Mitsuokella jalaludinii	species
Hgn3C.0472	Parabacteroides goldsteinii	species
Hgn3C.0473	Bacteroidales sp.	order
Hgn3C.0474	Oscillospiraceae sp.	family
Hgn3C.0475	Coriobacteriia sp.	class
Hgn3C.0476	Streptococcus sp.	genus
Hgn3C.0477	Eubacteriales sp.	order
Hgn3C.0478	Burkholderiales sp.	order
Hgn3C.0479	Eubacteriales sp.	order
Hgn3C.0048	Akkermansia muciniphila	species
Hgn3C.0480	Eubacteriales sp.	order
Hgn3C.0481	Firmicutes sp.	phylum
Hgn3C.0482	Lachnospiraceae sp.	family
Hgn3C.0483	Bacteroidia sp.	class
Hgn3C.0484	Bacteroidales sp.	order
Hgn3C.0485	Prevotella sp.	genus
Hgn3C.0486	Rikenellaceae sp.	family
Hgn3C.0487	Bacteria sp.	superkingdom
Hgn3C.0488	Clostridia sp.	class
Hgn3C.0489	Eubacteriales sp.	order
Hgn3C.0049	Eubacteriales sp.	order
Hgn3C.0490	Mogibacterium kristiansenii	species
Hgn3C.0491	Eubacteriales sp.	order
Hgn3C.0492	Prevotellaceae sp.	family

Hgn3C.0493	Eisenbergiella tayi	species
Hgn3C.0494	Prevotellaceae sp.	family
Hgn3C.0495	Ruminococcus sp.	genus
Hgn3C.0496	Sutterella sp. AM11-39	species
Hgn3C.0497	Odoribacter laneus	species
Hgn3C.0498	Prevotella sp.	genus
Hgn3C.0499	Bacteroides sp.	genus
Hgn3C.0005	Fusicatenibacter saccharivorans	species
Hgn3C.0050	Escherichia coli	species
Hgn3C.0500	Anaerotignum sp.	genus
Hgn3C.0501	Oscillospiraceae sp.	family
Hgn3C.0502	Lachnospiraceae sp.	family
Hgn3C.0503	Eubacteriales sp.	order
Hgn3C.0504	Megasphaera sp. BL7	species
Hgn3C.0505	Bacteroides sp.	genus
Hgn3C.0506	Lachnospiraceae sp.	family
Hgn3C.0507	[Clostridium] spiroforme	species
Hgn3C.0508	Erysipelotrichales sp.	order
Hgn3C.0509	Bacteroidales sp.	order
Hgn3C.0051	Alistipes communis	species
Hgn3C.0510	Methanosphaera stadtmanae	species
Hgn3C.0511	Clostridia sp.	class
Hgn3C.0512	Eubacteriales sp.	order
Hgn3C.0513	Eubacteriales sp.	order
Hgn3C.0514	Blautia glucerasea	species
Hgn3C.0515	Eubacteriales sp.	order
Hgn3C.0516	Eubacteriales sp.	order
Hgn3C.0517	Ruminococcus callidus	species
Hgn3C.0518	Eubacteriales sp.	order
Hgn3C.0519	Bacteroidales sp.	order
Hgn3C.0052	Phocaeicola massiliensis	species
Hgn3C.0520	Bacteroidales sp.	order
Hgn3C.0521	Firmicutes sp.	phylum
Hgn3C.0522	Roseburia sp. BX1005	species
Hgn3C.0523	Phascolarctobacterium succinatutens	species
Hgn3C.0524	Butyricicoccus sp.	genus
Hgn3C.0525	Firmicutes sp.	phylum
Hgn3C.0526	Anaerostipes sp.	genus
Hgn3C.0527	Sutterella sp.	genus
Hgn3C.0528	Coprococcus sp. AM27-12LB	species
Hgn3C.0529	Bacteria sp.	superkingdom
Hgn3C.0053	Eubacteriales sp.	order
Hgn3C.0530	Eubacteriales sp.	order
Hgn3C.0531	Clostridia sp.	class
Hgn3C.0532	Paraprevotella xylaniphila	species
Hgn3C.0533	Flintibacter sp. KGMB00164	species
Hgn3C.0534	Eubacteriales sp.	order
Hgn3C.0535	Victivallis vadensis	species

Hgn3C.0536	Bacteroidaceae sp.	family
Hgn3C.0537	Bifidobacterium dentium	species
Hgn3C.0538	Eubacteriales sp.	order
Hgn3C.0539	Eubacteriales sp.	order
Hgn3C.0054	Prevotella copri	species
Hgn3C.0540	Firmicutes sp.	phylum
Hgn3C.0541	Phocaeicola plebeius	species
Hgn3C.0542	[Clostridium] innocuum	species
Hgn3C.0543	Oscillospiraceae sp.	family
Hgn3C.0544	Eubacteriales sp.	order
Hgn3C.0545	Eubacteriales sp.	order
Hgn3C.0546	Bacteroidia sp.	class
Hgn3C.0547	Eubacteriales sp.	order
Hgn3C.0548	Bacteria sp.	superkingdom
Hgn3C.0549	Longicatena caecimuris	species
Hgn3C.0055	Alistipes finegoldii	species
Hgn3C.0550	Eubacteriales sp.	order
Hgn3C.0551	Eubacteriales sp.	order
Hgn3C.0552	Firmicutes sp.	phylum
Hgn3C.0553	Firmicutes sp.	phylum
Hgn3C.0554	Eubacteriales sp.	order
Hgn3C.0555	Bacteria sp.	superkingdom
Hgn3C.0556	Firmicutes sp.	phylum
Hgn3C.0557	Enterocloster bolteae	species
Hgn3C.0558	Olsenella sp.	genus
Hgn3C.0559	Oscillospiraceae sp.	family
Hgn3C.0056	Bacteroides fragilis	species
Hgn3C.0560	Oscillospiraceae sp.	family
Hgn3C.0561	Enterocloster clostridioformis	species
Hgn3C.0562	Eubacteriales sp.	order
Hgn3C.0563	Parasutterella sp.	genus
Hgn3C.0564	Neglecta timonensis	species
Hgn3C.0565	Ruminococcus champanellensis	species
Hgn3C.0566	Eubacteriales sp.	order
Hgn3C.0567	Prevotella rara	species
Hgn3C.0568	Eubacteriales sp.	order
Hgn3C.0569	Bacteria sp.	superkingdom
Hgn3C.0057	Oscillibacter sp. MSJ-31	species
Hgn3C.0570	Eubacteriales sp.	order
Hgn3C.0571	Eubacterium sp.	genus
Hgn3C.0572	Eubacteriales sp.	order
Hgn3C.0573	Prevotella sp. Marseille-P4119	species
Hgn3C.0574	Eubacteriales sp.	order
Hgn3C.0575	Bacteria sp.	superkingdom
Hgn3C.0576	Eubacteriales sp.	order
Hgn3C.0577	Eubacteriales sp.	order
Hgn3C.0578	Firmicutes sp.	phylum
Hgn3C.0579	Eubacteriales sp.	order

Hgn3C.0058	Lachnospiraceae sp.	family
Hgn3C.0580	Methanobrevibacter smithii	species
Hgn3C.0581	Negativibacillus massiliensis	species
Hgn3C.0582	Bacteroides sp.	genus
Hgn3C.0583	Clostridia sp.	class
Hgn3C.0584	Bacteroides fragilis	species
Hgn3C.0585	Bacteria sp.	superkingdom
Hgn3C.0586	Lawsonibacter sp. NSJ-51	species
Hgn3C.0587	Eubacteriales sp.	order
Hgn3C.0588	Eubacteriales sp.	order
Hgn3C.0589	Erysipelotrichales sp.	order
Hgn3C.0059	Bacteroides cellulosilyticus	species
Hgn3C.0590	Clostridia sp.	class
Hgn3C.0591	Eubacteriales sp.	order
Hgn3C.0592	Clostridia sp.	class
Hgn3C.0593	Eubacteriales sp.	order
Hgn3C.0594	Eubacteriales sp.	order
Hgn3C.0595	Eubacteriales sp.	order
Hgn3C.0596	Butyricimonas sp.	genus
Hgn3C.0597	Butyricimonas sp.	genus
Hgn3C.0598	Eubacteriales sp.	order
Hgn3C.0599	Rhodospirillales sp.	order
Hgn3C.0006	Bacteroides ovatus	species
Hgn3C.0060	Eubacteriales sp.	order
Hgn3C.0600	Eubacteriales sp.	order
Hgn3C.0601	Bacteroidales sp.	order
Hgn3C.0602	Eubacteriales sp.	order
Hgn3C.0603	Clostridia sp.	class
Hgn3C.0604	Megasphaera elsdenii	species
Hgn3C.0605	Oscillospiraceae sp.	family
Hgn3C.0606	Oscillospiraceae sp.	family
Hgn3C.0607	Eubacteriales sp.	order
Hgn3C.0608	Lachnospiraceae sp.	family
Hgn3C.0609	Eubacteriaceae sp.	family
Hgn3C.0061	Faecalibacillus sp. TM498	species
Hgn3C.0610	Eubacteriales sp.	order
Hgn3C.0611	Clostridia sp.	class
Hgn3C.0612	Bacteroidales sp.	order
Hgn3C.0613	Clostridiaceae sp.	family
Hgn3C.0614	Eubacteriales sp.	order
Hgn3C.0615	Firmicutes sp.	phylum
Hgn3C.0616	Lachnospiraceae sp.	family
Hgn3C.0617	Prevotella sp.	genus
Hgn3C.0618	Eubacteriales sp.	order
Hgn3C.0619	Erysipelotrichales sp.	order
Hgn3C.0062	Waltera intestinalis	species
Hgn3C.0620	Alistipes sp. An31A	species
Hgn3C.0621	Eubacteriales sp.	order

Hgn3C.0622	Lachnospiraceae sp.	family
Hgn3C.0623	Bacteroidales sp.	order
Hgn3C.0624	Eubacteriales sp.	order
Hgn3C.0625	Eubacteriales sp.	order
Hgn3C.0626	Oscillospiraceae sp.	family
Hgn3C.0627	Clostridia sp.	class
Hgn3C.0628	Eubacteriales sp.	order
Hgn3C.0629	Collinsella intestinalis	species
Hgn3C.0063	Eubacteriales sp.	order
Hgn3C.0630	Allisonella histaminiformans	species
Hgn3C.0631	Firmicutes sp.	phylum
Hgn3C.0632	Eubacteriales sp.	order
Hgn3C.0633	Oscillospiraceae sp.	family
Hgn3C.0634	Blautia schinkii	species
Hgn3C.0635	Eubacteriales sp.	order
Hgn3C.0636	Anaerostipes caccae	species
Hgn3C.0637	Veillonellales sp.	order
Hgn3C.0638	Blautia sp.	genus
Hgn3C.0639	Veillonellaceae sp.	family
Hgn3C.0064	Blautia sp. DFI.9.9	species
Hgn3C.0640	Eubacterium sp. AF22-8LB	species
Hgn3C.0641	Eubacteriales sp.	order
Hgn3C.0642	Eubacteriales sp.	order
Hgn3C.0643	Eubacteriales sp.	order
Hgn3C.0644	Bacteroides cellulosilyticus	species
Hgn3C.0645	Eubacteriales sp.	order
Hgn3C.0646	Lachnospiraceae sp.	family
Hgn3C.0647	Mitsuokella multacida	species
Hgn3C.0648	Eubacteriales sp.	order
Hgn3C.0649	Lachnospiraceae sp.	family
Hgn3C.0065	Blautia obeum	species
Hgn3C.0650	Eubacteriales sp.	order
Hgn3C.0651	Eubacteriales sp.	order
Hgn3C.0652	Firmicutes sp.	phylum
Hgn3C.0653	Clostridia sp.	class
Hgn3C.0654	Firmicutes sp.	phylum
Hgn3C.0655	Eubacteriales sp.	order
Hgn3C.0656	Eubacteriales sp.	order
Hgn3C.0657	Parolsenella catena	species
Hgn3C.0658	Eubacteriales sp.	order
Hgn3C.0659	Oscillospiraceae sp.	family
Hgn3C.0066	Oscillospiraceae sp.	family
Hgn3C.0660	Eubacteriales sp.	order
Hgn3C.0661	Alistipes sp.	genus
Hgn3C.0662	Eubacteriales sp.	order
Hgn3C.0663	Prevotella lascolaii	species
Hgn3C.0664	Blautia hansenii	species
Hgn3C.0665	Desulfovibrionales sp.	order

Hgn3C.0666	Burkholderiales sp.	order
Hgn3C.0667	Oscillospiraceae sp.	family
Hgn3C.0668	Oscillospiraceae sp.	family
Hgn3C.0669	Burkholderiales sp.	order
Hgn3C.0067	Roseburia hominis	species
Hgn3C.0670	Eubacteriales sp.	order
Hgn3C.0671	Coriobacteriia sp.	class
Hgn3C.0672	Clostridium sp.	genus
Hgn3C.0673	Eubacteriales sp.	order
Hgn3C.0674	Eubacteriales sp.	order
Hgn3C.0675	Eubacteriales sp.	order
Hgn3C.0676	Bacteroides sp.	genus
Hgn3C.0677	Bacteroides sp.	genus
Hgn3C.0678	Bacteroidales sp.	order
Hgn3C.0679	Holdemania filiformis	species
Hgn3C.0068	[Ruminococcus] lactaris	species
Hgn3C.0680	Eubacteriales sp.	order
Hgn3C.0681	Pseudoflavonifractor sp.	genus
Hgn3C.0682	[Clostridium] scindens	species
Hgn3C.0683	Clostridium disporicum	species
Hgn3C.0684	Prevotella buccae	species
Hgn3C.0685	Lachnospiraceae sp.	family
Hgn3C.0686	Eubacteriales sp.	order
Hgn3C.0687	Bacteroidales sp.	order
Hgn3C.0688	Firmicutes sp.	phylum
Hgn3C.0689	Eubacteriales sp.	order
Hgn3C.0069	Oscillibacter sp. KLE 1728	species
Hgn3C.0690	Eubacteriales sp.	order
Hgn3C.0691	Firmicutes sp.	phylum
Hgn3C.0692	Clostridia sp.	class
Hgn3C.0693	Hungatella hathewayi	species
Hgn3C.0694	Anaeromassilibacillus sp. An250	species
Hgn3C.0695	Eubacteriales sp.	order
Hgn3C.0696	Eubacteriales sp.	order
Hgn3C.0697	Eubacteriales sp.	order
Hgn3C.0698	Clostridia sp.	class
Hgn3C.0699	Firmicutes sp.	phylum
Hgn3C.0007	Parabacteroides distasonis	species
Hgn3C.0070	Faecalibacterium sp. OF04-11AC	species
Hgn3C.0700	Eubacteriales sp.	order
Hgn3C.0701	Bacteroides sp.	genus
Hgn3C.0702	Eubacteriales sp.	order
Hgn3C.0703	Firmicutes sp.	phylum
Hgn3C.0704	[Clostridium] scindens	species
Hgn3C.0705	Bacteria sp.	superkingdom
Hgn3C.0706	Eubacteriales sp.	order
Hgn3C.0707	Bacteroidales sp.	order
Hgn3C.0708	Eubacteriales sp.	order

Hgn3C.0709	Firmicutes sp.	phylum
Hgn3C.0071	Faecalicatena fissicatena	species
Hgn3C.0710	Oscillospiraceae sp.	family
Hgn3C.0711	Enterocloster asparagiformis	species
Hgn3C.0712	Megasphaera micronuciformis	species
Hgn3C.0713	Veillonellales sp.	order
Hgn3C.0714	Intestinimonas sp. MSJ-38	species
Hgn3C.0715	Desulfovibrio fairfieldensis	species
Hgn3C.0716	Eubacteriales sp.	order
Hgn3C.0717	Eubacteriales sp.	order
Hgn3C.0718	Eubacteriales sp.	order
Hgn3C.0719	Eubacteriales sp.	order
Hgn3C.0072	Clostridium fessum	species
Hgn3C.0720	Eubacteriales sp.	order
Hgn3C.0721	Desulfovibrionales sp.	order
Hgn3C.0722	Eubacteriales sp.	order
Hgn3C.0723	Bacteroidales sp.	order
Hgn3C.0724	Eubacteriales sp.	order
Hgn3C.0725	Sutterellaceae sp.	family
Hgn3C.0726	Oscillospiraceae sp.	family
Hgn3C.0727	Firmicutes sp.	phylum
Hgn3C.0728	Firmicutes sp.	phylum
Hgn3C.0729	Eubacteriales sp.	order
Hgn3C.0073	Blautia sp.	genus
Hgn3C.0730	Eubacteriales sp.	order
Hgn3C.0731	Eubacteriales sp.	order
Hgn3C.0732	Eubacterium ramulus	species
Hgn3C.0733	Desulfovibrio piger	species
Hgn3C.0734	Bacteria sp.	superkingdom
Hgn3C.0735	Bacteroidales sp.	order
Hgn3C.0736	Coprococcus sp.	genus
Hgn3C.0737	Alistipes timonensis	species
Hgn3C.0738	Sutterella seckii	species
Hgn3C.0739	Clostridia sp.	class
Hgn3C.0074	Clostridium sp. D43t1_170807_D5	species
Hgn3C.0740	Anaerostipes sp. NSJ-7	species
Hgn3C.0741	Blautia sp. OF01-4LB	species
Hgn3C.0742	Eubacteriales sp.	order
Hgn3C.0743	Enterobacter sp.	genus
Hgn3C.0744	Oscillospiraceae sp.	family
Hgn3C.0745	Eubacteriales sp.	order
Hgn3C.0746	Bacteroidia sp.	class
Hgn3C.0747	Eubacteriales sp.	order
Hgn3C.0748	Eubacteriales sp.	order
Hgn3C.0749	Eubacteriales sp.	order
Hgn3C.0075	Agathobaculum butyriciproducens	species
Hgn3C.0750	Merdimonas faecis	species
Hgn3C.0751	Eubacteriales sp.	order

Hgn3C.0752	Oscillospiraceae sp.	family
Hgn3C.0753	Firmicutes sp.	phylum
Hgn3C.0754	Collinsella bouchesdurhonensis	species
Hgn3C.0755	Oscillibacter sp.	genus
Hgn3C.0756	Oscillibacter valericigenes	species
Hgn3C.0757	Oxalobacter formigenes	species
Hgn3C.0758	Bacteroides sp.	genus
Hgn3C.0759	Thermoanaerobacterales sp.	order
Hgn3C.0076	Lachnospira pectinoschiza	species
Hgn3C.0760	Klebsiella oxytoca	species
Hgn3C.0761	Bacteroides sp.	genus
Hgn3C.0762	Enterocloster lavalensis	species
Hgn3C.0763	Prevotellaceae sp.	family
Hgn3C.0764	Bacteroides sp.	genus
Hgn3C.0765	Marseilla massiliensis	species
Hgn3C.0766	Eubacteriales sp.	order
Hgn3C.0767	Citrobacter freundii	species
Hgn3C.0768	Eubacteriales sp.	order
Hgn3C.0769	Rikenellaceae sp.	family
Hgn3C.0077	Sutterella wadsworthensis	species
Hgn3C.0770	Eubacteriales sp.	order
Hgn3C.0771	Eubacteriales sp.	order
Hgn3C.0772	Eubacteriales sp.	order
Hgn3C.0773	Eubacteriales sp.	order
Hgn3C.0774	Eubacteriales sp.	order
Hgn3C.0775	Clostridiaceae sp.	family
Hgn3C.0776	Oscillospiraceae sp.	family
Hgn3C.0777	Holdemanella sp.	genus
Hgn3C.0778	Oscillibacter sp.	genus
Hgn3C.0779	Clostridium neonatale	species
Hgn3C.0078	Clostridium sp. MCC328	species
Hgn3C.0780	Eubacteriales sp.	order
Hgn3C.0781	Collinsella sp.	genus
Hgn3C.0782	Streptococcus sp.	genus
Hgn3C.0783	Clostridia sp.	class
Hgn3C.0784	Eubacteriales sp.	order
Hgn3C.0785	Eubacteriales sp.	order
Hgn3C.0786	Hallella sp. CLA-AA-H145	species
Hgn3C.0787	Bacteroides mediterraneensis	species
Hgn3C.0788	Eubacteriales sp.	order
Hgn3C.0789	Eubacteriales sp.	order
Hgn3C.0079	[Ruminococcus] torques	species
Hgn3C.0790	Oscillospiraceae sp.	family
Hgn3C.0791	Eubacteriales sp.	order
Hgn3C.0792	Clostridia sp.	class
Hgn3C.0793	Prevotellaceae sp.	family
Hgn3C.0794	Bacteroides sp.	genus
Hgn3C.0795	Oscillospiraceae sp.	family

Hgn3C.0796	Eubacteriales sp.	order
Hgn3C.0797	Senegalimassilia sp.	genus
Hgn3C.0798	Streptococcus vestibularis	species
Hgn3C.0799	Bacteroidales sp.	order
Hgn3C.0008	Alistipes putredinis	species
Hgn3C.0080	Phascolarctobacterium faecium	species
Hgn3C.0800	Archaea sp.	superkingdom
Hgn3C.0801	Marseilla massiliensis	species
Hgn3C.0802	Prevotellaceae sp.	family
Hgn3C.0803	Bacteroidales sp.	order
Hgn3C.0804	Lachnospiraceae sp.	family
Hgn3C.0805	Eubacteriales sp.	order
Hgn3C.0806	Bacteroidia sp.	class
Hgn3C.0807	Enorma massiliensis	species
Hgn3C.0808	Bacteria sp.	superkingdom
Hgn3C.0809	Frisingicoccus sp.	genus
Hgn3C.0081	Coprococcus eutactus	species
Hgn3C.0810	Eubacteriales sp.	order
Hgn3C.0811	Eubacteriales sp.	order
Hgn3C.0812	Clostridia sp.	class
Hgn3C.0813	Clostridia sp.	class
Hgn3C.0814	Massilimicrobiota timonensis	species
Hgn3C.0815	Eubacteriales sp.	order
Hgn3C.0816	Lachnospiraceae sp.	family
Hgn3C.0817	Prevotella sp.	genus
Hgn3C.0818	Rothia sp.	genus
Hgn3C.0819	Archaea sp.	superkingdom
Hgn3C.0082	Roseburia sp. CLA-AA-H204	species
Hgn3C.0820	Eubacteriales sp.	order
Hgn3C.0821	Bacteroidales sp.	order
Hgn3C.0822	Bacteroidales sp.	order
Hgn3C.0823	Anaerotignum lactatifermentans	species
Hgn3C.0824	Eubacteriales sp.	order
Hgn3C.0825	Bacteroidales sp.	order
Hgn3C.0826	Bacteria sp.	superkingdom
Hgn3C.0827	Eubacteriales sp.	order
Hgn3C.0828	Bacteroides congonensis	species
Hgn3C.0829	Dorea phocaeensis	species
Hgn3C.0083	Eubacteriales sp.	order
Hgn3C.0830	Dakarella massiliensis	species
Hgn3C.0831	Eubacteriales sp.	order
Hgn3C.0832	Veillonellales sp.	order
Hgn3C.0833	Eisenbergiella tayi	species
Hgn3C.0834	Olsenella sp. AF21-51	species
Hgn3C.0835	Eubacteriales sp.	order
Hgn3C.0836	Lachnospiraceae sp.	family
Hgn3C.0837	Anaerotruncus colihominis	species
Hgn3C.0838	Eubacteriales sp.	order

Hgn3C.0839	Eubacteriales sp.	order
Hgn3C.0084	Flavonifractor plautii	species
Hgn3C.0840	Eubacteriales sp.	order
Hgn3C.0841	Barnesiella sp. GGCC_0306	species
Hgn3C.0842	Ruminococcus sp. 210702-SL.1.03	species
Hgn3C.0843	Eubacteriales sp.	order
Hgn3C.0844	Bacteroidales sp.	order
Hgn3C.0845	Eubacteriales sp.	order
Hgn3C.0846	Eubacteriales sp.	order
Hgn3C.0847	Clostridia sp.	class
Hgn3C.0848	Eubacteriales sp.	order
Hgn3C.0849	Clostridia sp.	class
Hgn3C.0085	Faecalibacterium sp.	genus
Hgn3C.0850	Clostridia sp.	class
Hgn3C.0851	Oscillospiraceae sp.	family
Hgn3C.0852	Bacteria sp.	superkingdom
Hgn3C.0853	Eubacteriales sp.	order
Hgn3C.0854	Eubacteriales sp.	order
Hgn3C.0855	Eubacteriales sp.	order
Hgn3C.0856	Bilophila sp.	genus
Hgn3C.0857	Blautia glucerasea	species
Hgn3C.0858	Turicibacter sanguinis	species
Hgn3C.0859	Hydrogeniiclostidium mannosilyticum	species
Hgn3C.0086	Dialister invisus	species
Hgn3C.0860	Bacteria sp.	superkingdom
Hgn3C.0861	Firmicutes sp.	phylum
Hgn3C.0862	Eubacteriales sp.	order
Hgn3C.0863	Eubacteriales sp.	order
Hgn3C.0864	Eubacteriales sp.	order
Hgn3C.0865	Eubacteriales sp.	order
Hgn3C.0866	Victivallales sp.	order
Hgn3C.0867	Bacteria sp.	superkingdom
Hgn3C.0868	Blastocystis sp. subtype 4	species
Hgn3C.0869	Bacteroidales sp.	order
Hgn3C.0087	Eubacteriales sp.	order
Hgn3C.0870	Eubacteriales sp.	order
Hgn3C.0871	Anaerotruncus massiliensis	species
Hgn3C.0872	Eubacteriales sp.	order
Hgn3C.0873	Clostridium perfringens	species
Hgn3C.0874	Methanomassiliicoccales sp.	order
Hgn3C.0875	Lachnospiraceae sp.	family
Hgn3C.0876	Clostridiaceae sp.	family
Hgn3C.0877	Bacteroidales sp.	order
Hgn3C.0878	Eubacterium sp.	genus
Hgn3C.0879	Massilistercora sp.	genus
Hgn3C.0088	[Ruminococcus] gnavus	species
Hgn3C.0880	Firmicutes sp.	phylum
Hgn3C.0881	Oscillospiraceae sp.	family

Hgn3C.0882	Eubacteriales sp.	order
Hgn3C.0883	Oscillospiraceae sp.	family
Hgn3C.0884	Eggerthellales sp.	order
Hgn3C.0885	Eubacteriales sp.	order
Hgn3C.0886	Bacteroides sp.	genus
Hgn3C.0887	Eubacteriales sp.	order
Hgn3C.0888	Eubacteriales sp.	order
Hgn3C.0889	Clostridium paraputrificum	species
Hgn3C.0089	Paraprevotella clara	species
Hgn3C.0890	Enterobacter roggenkampii	species
Hgn3C.0891	Eubacteriales sp.	order
Hgn3C.0892	Eggerthellales sp.	order
Hgn3C.0893	Phocea massiliensis	species
Hgn3C.0894	Eubacteriales sp.	order
Hgn3C.0895	Eubacteriales sp.	order
Hgn3C.0896	Lachnospiraceae sp.	family
Hgn3C.0897	Prevotella sp.	genus
Hgn3C.0898	Bacteria sp.	superkingdom
Hgn3C.0899	Eubacteriales sp.	order
Hgn3C.0009	Faecalibacterium longum CLA-AA-H243	subspecies
Hgn3C.0090	Clostridium sp. AM49-4BH	species
Hgn3C.0900	Bacteria sp.	superkingdom
Hgn3C.0901	Firmicutes sp.	phylum
Hgn3C.0902	Candidatus Methanomassiliicoccus intestinalis	species
Hgn3C.0903	Archaea sp.	superkingdom
Hgn3C.0904	Bacteroidia sp.	class
Hgn3C.0905	Eubacteriales sp.	order
Hgn3C.0906	Bacteria sp.	superkingdom
Hgn3C.0907	Klebsiella sp.	genus
Hgn3C.0908	Eubacteriales sp.	order
Hgn3C.0909	Eubacteriales sp.	order
Hgn3C.0091	Lachnospira sp. NSJ-43	species
Hgn3C.0910	Firmicutes sp.	phylum
Hgn3C.0911	Bacteroides sp.	genus
Hgn3C.0912	Clostridia sp.	class
Hgn3C.0913	Erysipelotrichaceae sp.	family
Hgn3C.0914	Eubacteriales sp.	order
Hgn3C.0915	Firmicutes sp.	phylum
Hgn3C.0916	Firmicutes sp.	phylum
Hgn3C.0917	Firmicutes sp.	phylum
Hgn3C.0918	Eubacteriales sp.	order
Hgn3C.0919	Eubacteriales sp.	order
Hgn3C.0092	Lachnospiraceae sp.	family
Hgn3C.0920	Eubacteriales sp.	order
Hgn3C.0921	Bacteroides sp.	genus
Hgn3C.0922	Eubacteriales sp.	order
Hgn3C.0923	Eubacteriales sp.	order
Hgn3C.0924	Clostridioides difficile	species

Hgn3C.0925	Bacteroides sp. Marseille-P3684	species
Hgn3C.0926	Victivallales sp.	order
Hgn3C.0927	Eubacteriales sp.	order
Hgn3C.0928	Eubacteriales sp.	order
Hgn3C.0929	Lachnospiraceae sp.	family
Hgn3C.0093	Bilophila wadsworthia	species
Hgn3C.0930	Eubacteriales sp.	order
Hgn3C.0931	Bacteroides sp.	genus
Hgn3C.0932	Eubacteriales sp.	order
Hgn3C.0933	Oscillospiraceae sp.	family
Hgn3C.0934	Phocaeicola coprophilus	species
Hgn3C.0935	Bacteria sp.	superkingdom
Hgn3C.0936	Lactobacillus acidophilus	species
Hgn3C.0937	Elusimicrobiaceae sp.	family
Hgn3C.0938	Firmicutes sp.	phylum
Hgn3C.0939	Eubacteriales sp.	order
Hgn3C.0094	Eubacteriales sp.	order
Hgn3C.0940	Eubacteriales sp.	order
Hgn3C.0941	Bacteria sp.	superkingdom
Hgn3C.0942	Eubacteriales sp.	order
Hgn3C.0943	Lactiplantibacillus plantarum subsp. plantarum	subspecies
Hgn3C.0944	Bacteria sp.	superkingdom
Hgn3C.0945	Eubacteriales sp.	order
Hgn3C.0946	Eubacteriales sp.	order
Hgn3C.0947	Eubacteriales sp.	order
Hgn3C.0948	Enterobacter sp.	genus
Hgn3C.0949	Eubacteriales sp.	order
Hgn3C.0095	Eubacteriales sp.	order
Hgn3C.0950	Clostridia sp.	class
Hgn3C.0951	Eubacteriales sp.	order
Hgn3C.0952	Bacteroidales sp.	order
Hgn3C.0953	Lachnospiraceae sp.	family
Hgn3C.0954	Bacteria sp.	superkingdom
Hgn3C.0955	Eubacteriales sp.	order
Hgn3C.0956	Erysipelotrichales sp.	order
Hgn3C.0957	Alistipes sp.	genus
Hgn3C.0958	Firmicutes sp.	phylum
Hgn3C.0959	Eubacteriales sp.	order
Hgn3C.0096	Eubacteriales sp.	order
Hgn3C.0960	Clostridia sp.	class
Hgn3C.0961	Eubacteriales sp.	order
Hgn3C.0962	Clostridia sp.	class
Hgn3C.0963	Eubacteriales sp.	order
Hgn3C.0964	Bacteria sp.	superkingdom
Hgn3C.0965	Lactobacillus delbrueckii subsp. bulgaricus	subspecies
Hgn3C.0966	Lachnospiraceae sp.	family
Hgn3C.0967	Firmicutes sp.	phylum
Hgn3C.0968	Bacteria sp.	superkingdom

Hgn3C.0969	Bacteroides ndongoniae	species
Hgn3C.0097	Eubacterium ventriosum	species
Hgn3C.0970	Eubacteriales sp.	order
Hgn3C.0971	Bacteroides cutis	species
Hgn3C.0972	Eubacteriales sp.	order
Hgn3C.0973	Desulfovibrio piger	species
Hgn3C.0974	Bacteroides finegoldii	species
Hgn3C.0975	Eubacteriales sp.	order
Hgn3C.0976	Eubacteriales sp.	order
Hgn3C.0977	Eubacteriales sp.	order
Hgn3C.0978	Veillonella sp.	genus
Hgn3C.0979	Eubacteriales sp.	order
Hgn3C.0098	Coprococcus catus	species
Hgn3C.0980	Firmicutes sp.	phylum
Hgn3C.0981	Rikenellaceae sp.	family
Hgn3C.0982	Acidaminococcales sp.	order
Hgn3C.0983	Bacteroidales sp.	order
Hgn3C.0984	Firmicutes sp.	phylum
Hgn3C.0985	Firmicutes sp.	phylum
Hgn3C.0986	Desulfovibrio sp.	genus
Hgn3C.0987	Fusobacterium mortiferum	species
Hgn3C.0988	Eubacteriales sp.	order
Hgn3C.0989	Bacteria sp.	superkingdom
Hgn3C.0099	Dorea longicatena	species
Hgn3C.0990	Raoultella ornithinolytica	species
Hgn3C.0991	Eubacteriales sp.	order
Hgn3C.0992	Eubacteriales sp.	order
Hgn3C.0993	Faecalitalea cylindroides	species
Hgn3C.0994	Bacteroides sp.	genus
Hgn3C.0995	Bacteria sp.	superkingdom
Hgn3C.0996	Oscillospiraceae sp.	family
Hgn3C.0997	Mediterranea massiliensis	species
Hgn3C.0998	Eubacteriales sp.	order
Hgn3C.0999	Burkholderiales sp.	order

subspecies	species
unclassified	Phocaeicola vulgatus
unclassified	Anaerostipes hadrus
unclassified	Bifidobacterium bifidum
unclassified	unclassified
unclassified	unclassified
unclassified	unclassified
unclassified	unclassified
unclassified	unclassified
unclassified	Barnesiella sp. An22
unclassified	unclassified
unclassified	unclassified
unclassified	unclassified
unclassified	unclassified
unclassified	Bifidobacterium pseudocatenulatum
unclassified	Parabacteroides massiliensis
unclassified	unclassified
unclassified	unclassified
unclassified	unclassified
unclassified	unclassified
unclassified	unclassified
unclassified	unclassified
unclassified	Emergencia timonensis
unclassified	unclassified
unclassified	unclassified
unclassified	unclassified
unclassified	Phocaeicola barnesiae
unclassified	Bacteroides stercorirosoris
unclassified	unclassified
unclassified	unclassified
unclassified	unclassified
unclassified	unclassified
unclassified	unclassified
unclassified	unclassified
unclassified	unclassified
unclassified	unclassified
unclassified	Holdemanella porci
unclassified	unclassified
unclassified	unclassified
unclassified	unclassified
unclassified	Hafnia paralvei
unclassified	Odoribacter sp. AF15-53
unclassified	unclassified
unclassified	Escherichia coli
unclassified	unclassified
unclassified	unclassified
unclassified	unclassified

unclassified	Clostridium sp. AM32-2
unclassified	unclassified
unclassified	Bacteroides stercoris
unclassified	Dorea formicigenerans
unclassified	Clostridium phoceensis
unclassified	unclassified
Lactococcus lactis subsp. lactis	Lactococcus lactis
unclassified	unclassified
unclassified	unclassified
unclassified	Akkermansia sp. BIOML-A59
unclassified	Enterococcus faecium
unclassified	Vescimonas coprocola
unclassified	Staphylococcus epidermidis
unclassified	Holdemania massiliensis
unclassified	Enterococcus gallinarum
unclassified	unclassified
unclassified	Streptococcus lutetiensis
unclassified	Veillonella seminalis
unclassified	Muribaculum sp. NM65_B17
unclassified	unclassified
unclassified	Mediterraneibacter glycyrrhizinilyticus
unclassified	unclassified
unclassified	Faecalibacterium sp. CLA-AA-H233
unclassified	unclassified
unclassified	unclassified
unclassified	unclassified
unclassified	Enterococcus avium
unclassified	unclassified
Klebsiella variicola subsp. variicola	Klebsiella variicola
unclassified	Lacrimispora saccharolytica
unclassified	unclassified
unclassified	unclassified
unclassified	unclassified
unclassified	Parasutterella excrementihominis
unclassified	Raoultella planticola
unclassified	Lactobacillus gasseri
unclassified	unclassified
Streptococcus gallolyticus subsp. gallolyticus	Streptococcus gallolyticus
unclassified	unclassified
unclassified	Brachyspira aalborgi
unclassified	unclassified
unclassified	unclassified
unclassified	unclassified
unclassified	unclassified
unclassified	unclassified
unclassified	unclassified
unclassified	Lacticaseibacillus rhamnosus
Lacticaseibacillus paracasei subsp. paracasei	Lacticaseibacillus paracasei

unclassified	unclassified
unclassified	Massilimicrobiota timonensis
unclassified	unclassified
unclassified	unclassified
unclassified	Ligilactobacillus salivarius
unclassified	Enterococcus casseliflavus
unclassified	unclassified
unclassified	Bacteroides eggerthii
unclassified	Turicibacter sanguinis
unclassified	unclassified
unclassified	unclassified
unclassified	Enterobacter kobei
unclassified	unclassified
unclassified	unclassified
unclassified	unclassified
unclassified	Alistipes provencensis
unclassified	unclassified
unclassified	unclassified
unclassified	Roseburia faecis
unclassified	unclassified
unclassified	unclassified
unclassified	unclassified
unclassified	unclassified
unclassified	unclassified
unclassified	unclassified
unclassified	unclassified
unclassified	Clostridium butyricum
unclassified	unclassified
unclassified	unclassified
unclassified	unclassified
unclassified	unclassified
unclassified	unclassified
unclassified	unclassified
unclassified	unclassified
unclassified	unclassified
unclassified	unclassified
unclassified	unclassified
unclassified	unclassified
unclassified	unclassified
unclassified	unclassified
unclassified	unclassified
unclassified	unclassified
unclassified	unclassified
unclassified	unclassified
unclassified	unclassified
unclassified	unclassified
unclassified	Clostridium porci
unclassified	unclassified

unclassified	Phocaeicola sartorii
unclassified	unclassified
unclassified	unclassified
unclassified	unclassified
unclassified	Clostridium sp. MCC345
unclassified	unclassified
unclassified	unclassified
unclassified	unclassified
unclassified	unclassified
unclassified	unclassified
unclassified	unclassified
unclassified	unclassified
unclassified	unclassified
unclassified	unclassified
unclassified	unclassified
unclassified	unclassified
unclassified	unclassified
unclassified	unclassified
unclassified	unclassified
unclassified	unclassified
unclassified	Culturomica massiliensis
unclassified	unclassified
unclassified	unclassified
unclassified	unclassified
unclassified	unclassified
unclassified	unclassified
unclassified	unclassified
unclassified	unclassified
unclassified	unclassified
unclassified	Acidaminococcus fermentans
unclassified	Eubacterium sp. OM08-24
unclassified	Lactococcus garvieae
unclassified	unclassified
unclassified	unclassified
unclassified	unclassified
unclassified	unclassified
unclassified	unclassified
unclassified	Anaerostipes hadrus
unclassified	unclassified
unclassified	unclassified
unclassified	unclassified
unclassified	unclassified
unclassified	unclassified
unclassified	Desulfovibrio legallii
unclassified	unclassified
unclassified	unclassified
unclassified	unclassified
unclassified	unclassified

unclassified	Methanobrevibacter smithii
unclassified	unclassified
unclassified	unclassified
unclassified	unclassified
unclassified	unclassified
unclassified	Sutterella parvirubra
unclassified	unclassified
unclassified	unclassified
unclassified	unclassified
unclassified	Clostridium sp. 12(A)
unclassified	Lacrimispora celerecrescens
unclassified	unclassified
unclassified	Enterocloster citroniae
unclassified	Acidovorax delafieldii
unclassified	Comamonas kerstersii
unclassified	Acinetobacter bereziniae
unclassified	Acinetobacter johnsonii
unclassified	Acinetobacter ursingii
unclassified	Acinetobacter guillouiae
unclassified	Actinomyces sp. HMSC065F12
unclassified	Actinomyces oris
unclassified	Aeromonas caviae
unclassified	Bacteroides faecis
unclassified	Fenollaria massiliensis
unclassified	Aliarcobacter cryaerophilus
unclassified	Methylobacterium sp. UNCCL125
unclassified	unclassified
unclassified	Bacteroides gallinaceum
Bifidobacterium pullorum subsp. saeculare	Bifidobacterium pullorum
unclassified	Blautia marasmi
unclassified	Blautia hansenii
unclassified	Turicimonas muris
unclassified	Butyricimonas synergistica
unclassified	unclassified
unclassified	Flavonifractor plautii
unclassified	Megasphaera sp. DJF_B143
unclassified	Caulobacter sp. CCH5-E12
unclassified	Chryseobacterium gambrini
unclassified	Citrobacter sp. CRE-46
unclassified	Citrobacter amalonaticus
unclassified	Citrobacter braakii
unclassified	Citrobacter pasteurii
unclassified	unclassified
unclassified	Citrobacter sp. S-77
unclassified	Cloacibacillus evryensis
unclassified	unclassified
unclassified	unclassified
unclassified	Lachnoclostridium pacaense

unclassified	Clostridium jeddahense
unclassified	Rubneribacter badeniensis
unclassified	Corynebacterium tuberculostearicum
Cutibacterium acnes subsp. acnes	Cutibacterium acnes
unclassified	Cutibacterium avidum
unclassified	Cutibacterium granulosum
unclassified	Enterobacter bugandensis
unclassified	Enterobacter sp. NFIX58
unclassified	Bacteroides finegoldii
unclassified	Enterobacter ludwigii
unclassified	Phytobacter diazotrophicus
unclassified	Enterococcus durans
unclassified	Enterococcus gilvus
unclassified	Enterococcus raffinosus
unclassified	Enterococcus faecium
unclassified	Enterococcus thailandicus
unclassified	Erysipelatoclostridium sp. An15
unclassified	Escherichia marmotae
unclassified	Escherichia sp. 93.0750
unclassified	unclassified
unclassified	Eubacterium limosum
unclassified	Flavobacterium sp. Leaf359
unclassified	Sanguibacteroides justesenii
unclassified	Gardnerella vaginalis
unclassified	Granulicatella adiacens
unclassified	Haemophilus sp. CCUG 66565
unclassified	unclassified
unclassified	Kosakonia cowanii
unclassified	Klebsiella quasivariicola
unclassified	Lactobacillus amylovorus
unclassified	Ruthenibacterium lactatiformans
unclassified	Lactobacillus helveticus
Limosilactobacillus reuteri subsp. reuteri	Limosilactobacillus reuteri
unclassified	Limosilactobacillus vaginalis
unclassified	Lysinibacillus fusiformis
unclassified	Megasphaera genomosp. type_1
unclassified	Megasphaera massiliensis
Morganella morganii subsp. morganii	Morganella morganii
unclassified	Mycobacteroides chelonae
unclassified	Neisseria sp. HMSC061E12
unclassified	Pantoea sp. 3.5.1
unclassified	Sutterella sp. KLE1602
unclassified	Parabacteroides gordonii
unclassified	Prevotella jejuni
unclassified	unclassified
unclassified	Pseudoflavonifractor sp. An176
unclassified	Pseudomonas extremaustralis
unclassified	Pseudomonas fulva

unclassified	Pseudomonas nitroreducens
unclassified	Robinsoniella peoriensis
unclassified	Rothia sp. HMSC072E10
unclassified	Selenomonas artemidis
unclassified	Ruminococcus sp. BSD2780120874_150323_B10
unclassified	Serratia marcescens
unclassified	Shewanella xiamenensis
unclassified	Sphingomonas sp. CCH9-F2
unclassified	Staphylococcus borealis
Staphylococcus hominis subsp. hominis	Staphylococcus hominis
unclassified	Staphylococcus lugdunensis
unclassified	Stenotrophomonas bentonitica
unclassified	Stenotrophomonas maltophilia
unclassified	unclassified
Streptococcus constellatus subsp. constellatus	Streptococcus constellatus
unclassified	unclassified
unclassified	unclassified
unclassified	Streptococcus agalactiae
unclassified	Veillonella sp. CNR 79/14
unclassified	Citrobacter koseri
unclassified	Kluyvera cryocrescens
unclassified	unclassified
unclassified	Citrobacter sp. MGH106
unclassified	unclassified
unclassified	Clostridium sp. NSJ-6
unclassified	Enterococcus malodoratus
unclassified	unclassified
unclassified	unclassified
unclassified	Atlantibacter sp. RC6
unclassified	Terrisporobacter othiniensis
unclassified	Clostridium baratii
unclassified	unclassified
unclassified	unclassified
unclassified	Corynebacterium variabile
Macrococcus caseolyticus subsp. caseolyticus	Macrococcus caseolyticus
unclassified	Enterobacter hormaechei
unclassified	Anaerosporobacter mobilis
unclassified	Haemophilus parainfluenzae
unclassified	Veillonella ratti
unclassified	Lachnoclostridium edouardi
unclassified	Limosilactobacillus portuensis
unclassified	unclassified
unclassified	unclassified
unclassified	Butyricicoccus pullicaecorum
unclassified	Raoultella planticola
unclassified	unclassified
unclassified	unclassified
unclassified	unclassified

unclassified	Ruminococcus bromii
unclassified	Phocaeicola plebeius
unclassified	unclassified
unclassified	unclassified
unclassified	unclassified
unclassified	Clostridium paraputrificum
unclassified	Citrobacter sp. RHBSTW-00671
unclassified	Isoptericola variabilis
unclassified	unclassified
Bifidobacterium longum spp.	Bifidobacterium longum
unclassified	unclassified
Enterobacter cloacae subsp. cloacae	Enterobacter cloacae
unclassified	Streptococcus thermophilus
unclassified	unclassified
unclassified	unclassified
unclassified	Candida tropicalis
unclassified	Klebsiella aerogenes
unclassified	unclassified
unclassified	unclassified
unclassified	unclassified
unclassified	unclassified
unclassified	Eubacterium maltosivorans
unclassified	Atlantibacter hermannii
unclassified	unclassified
unclassified	Proteus mirabilis
unclassified	unclassified
unclassified	Enterococcus hirae
unclassified	unclassified
unclassified	Eubacterium sp. c-25
unclassified	Luxibacter massiliensis
unclassified	Eggerthella guodeyinii
unclassified	unclassified
unclassified	Lacrimispora saccharolytica
unclassified	unclassified
unclassified	Dialister hominis
unclassified	unclassified
unclassified	unclassified
unclassified	unclassified
unclassified	unclassified
unclassified	unclassified
unclassified	unclassified
unclassified	unclassified
unclassified	unclassified
unclassified	unclassified
unclassified	unclassified
unclassified	unclassified
unclassified	unclassified
unclassified	unclassified

unclassified	unclassified
unclassified	Enterococcus italicus
unclassified	unclassified
unclassified	Limosilactobacillus mucosae
unclassified	unclassified
unclassified	[Clostridium] hylemonae
unclassified	unclassified
unclassified	Corynebacterium falsenii
unclassified	unclassified
unclassified	Enterococcus asini
unclassified	Actinomyces sp. ICM47
unclassified	unclassified
unclassified	unclassified
unclassified	unclassified
unclassified	Collinsella stercoris
unclassified	unclassified
unclassified	unclassified
unclassified	unclassified
unclassified	Extibacter muris
unclassified	Alistipes ihumii
unclassified	Bifidobacterium crudilactis
unclassified	Senegalimassilia anaerobia
unclassified	Christensenella intestinihominis
unclassified	unclassified
unclassified	unclassified
unclassified	unclassified
unclassified	unclassified
unclassified	unclassified
unclassified	unclassified
unclassified	unclassified
unclassified	Clostridium sp. OF03-18AA
unclassified	Kluyvera genomosp. 3
unclassified	unclassified
unclassified	unclassified
unclassified	unclassified
unclassified	Eubacterium sp. An3
unclassified	unclassified
unclassified	Kluyvera sp. CRP
unclassified	Collinsella tanakaei
unclassified	Collinsella sp. AF08-23
unclassified	Gordonibacter urolithinfaciens
unclassified	Anaerobutyricum soehngenii
unclassified	Kluyvera georgiana
unclassified	unclassified
unclassified	unclassified
unclassified	Sellimonas monacensis
unclassified	unclassified
unclassified	unclassified

unclassified	unclassified
unclassified	unclassified
Streptococcus infantarius subsp. infantarius	Streptococcus infantarius
unclassified	unclassified
unclassified	Ruminococcus callidus
unclassified	unclassified
unclassified	unclassified
unclassified	Enterobacter sp. JBIWA005
unclassified	Anaerofustis stercorihominis
unclassified	unclassified
unclassified	unclassified
unclassified	unclassified
unclassified	unclassified
unclassified	unclassified
unclassified	unclassified
unclassified	Lacrimispora saccharolytica
unclassified	Veillonella parvula
unclassified	unclassified
unclassified	unclassified
unclassified	Collinsella phocaeensis
unclassified	unclassified
unclassified	unclassified
unclassified	unclassified
unclassified	Slackia piriformis
unclassified	Christensenella minuta
unclassified	Eubacterium callanderi
unclassified	Eggerthella timonensis
Adlercreutzia equolifaciens subsp. celatus	Adlercreutzia equolifaciens
unclassified	unclassified
unclassified	unclassified
unclassified	Christensenella hongkongensis
unclassified	unclassified
unclassified	unclassified
Klebsiella quasipneumoniae subsp. quasipneumoniae	Klebsiella quasipneumoniae
unclassified	Limosilactobacillus fermentum
unclassified	unclassified
unclassified	unclassified
unclassified	Agathobaculum sp. NSJ-28
unclassified	Eubacterium ramulus
unclassified	unclassified
unclassified	unclassified
unclassified	unclassified
unclassified	unclassified
unclassified	Mogibacterium sp. NSJ-24
unclassified	unclassified
unclassified	Limosilactobacillus oris
unclassified	unclassified
unclassified	Mediterraneibacter glycyrrhizinilyticus

unclassified	unclassified
unclassified	Streptococcus salivarius
unclassified	Corynebacterium provencense
unclassified	unclassified
unclassified	Eggerthella timonensis
unclassified	unclassified
unclassified	unclassified
unclassified	Acinetobacter baumannii
unclassified	unclassified
unclassified	Candidatus Stoquefichus sp. SB1
unclassified	Dielma fastidiosa
unclassified	Streptococcus sp. IMAU 99161
unclassified	unclassified
unclassified	unclassified
unclassified	unclassified
unclassified	unclassified
unclassified	unclassified
unclassified	Ruminococcus sp. 1001175B_160314_E1
unclassified	unclassified
unclassified	unclassified
unclassified	unclassified
unclassified	Leuconostoc falkenbergense
unclassified	Bifidobacterium scardovii
unclassified	unclassified
unclassified	Lactococcus sp. NH2-7C
unclassified	Faecalicatena orotica
unclassified	Raoultibacter timonensis
unclassified	unclassified
unclassified	Escherichia fergusonii
unclassified	unclassified
unclassified	unclassified
unclassified	Eisenbergiella sp. OF01-20
unclassified	Intestinimonas timonensis
unclassified	unclassified
unclassified	Phascolarctobacterium succinatutens
unclassified	unclassified
unclassified	unclassified
unclassified	unclassified
unclassified	unclassified
unclassified	unclassified
unclassified	unclassified
unclassified	unclassified
unclassified	unclassified
unclassified	Staphylococcus aureus
unclassified	unclassified
unclassified	unclassified
unclassified	unclassified
unclassified	unclassified

unclassified	Amedibacillus dolichus
unclassified	Agathobaculum sp. M2
unclassified	unclassified
unclassified	Faecalibacterium sp. Marseille-Q4896
unclassified	unclassified
unclassified	Clostridium sp. AF34-13
unclassified	Bacteroides salyersiae
unclassified	unclassified
unclassified	Prevotella copri
unclassified	unclassified
unclassified	unclassified
unclassified	unclassified
unclassified	unclassified
unclassified	unclassified
unclassified	Parabacteroides merdae
unclassified	unclassified
unclassified	unclassified
unclassified	unclassified
unclassified	unclassified
unclassified	unclassified
unclassified	Butyrivibrio crossotus
unclassified	unclassified
unclassified	unclassified
unclassified	Evtepia gabavorous
unclassified	Bacteroides intestinalis
unclassified	Subdoligranulum sp. APC924/74
unclassified	unclassified
unclassified	Catenibacterium mitsuokai
unclassified	unclassified
unclassified	unclassified
unclassified	unclassified
unclassified	unclassified
unclassified	Alistipes indistinctus
unclassified	unclassified
unclassified	unclassified
unclassified	unclassified
unclassified	Faecalibacterium prausnitzii
unclassified	unclassified
unclassified	Alistipes onderdonkii
unclassified	Butyricimonas faecihominis
unclassified	Intestinibacter bartlettii
unclassified	unclassified
Bifidobacterium catenulatum subsp. kashiwanohense	Bifidobacterium catenulatum
unclassified	Butyricimonas virosa
unclassified	unclassified
unclassified	unclassified
unclassified	unclassified
unclassified	Phocaeicola dorei

unclassified	unclassified
unclassified	unclassified
unclassified	Erysipelatoclostridium ramosum
unclassified	unclassified
unclassified	Faecalibacillus faecis
unclassified	unclassified
unclassified	Phocaeicola coprocola
unclassified	Bacteroides clarus
unclassified	unclassified
unclassified	unclassified
unclassified	Bacteroides uniformis
unclassified	Collinsella aerofaciens
unclassified	unclassified
unclassified	Veillonella dispar
unclassified	Eggerthella lenta
unclassified	Faecalibacterium sp. Marseille-Q3530
unclassified	Coprobacter fastidiosus
unclassified	unclassified
unclassified	Gemmiger formicilis
unclassified	unclassified
unclassified	Romboutsia timonensis
unclassified	Bifidobacterium breve
Bifidobacterium longum subsp. longum	Bifidobacterium longum
unclassified	unclassified
unclassified	unclassified
unclassified	Holdemanella biformis
unclassified	Akkermansia sp. GGCC_0220
unclassified	Duodenibacillus massiliensis
unclassified	Alistipes sp. cv1
unclassified	unclassified
unclassified	unclassified
unclassified	unclassified
unclassified	Tyzzerella nexilis
unclassified	Alistipes shahii
unclassified	Clostridium sp. MCC334
unclassified	unclassified
unclassified	Parabacteroides merdae
unclassified	unclassified
unclassified	unclassified
unclassified	unclassified
unclassified	unclassified
unclassified	unclassified
unclassified	Coprococcus eutactus
unclassified	unclassified
unclassified	Bacteroides xylanisolvens
unclassified	unclassified
unclassified	unclassified
unclassified	Megamonas funiformis

unclassified	unclassified
unclassified	unclassified
unclassified	unclassified
unclassified	unclassified
unclassified	Pseudoruminococcus massiliensis
unclassified	unclassified
unclassified	Blautia sp. BIOML-A1
unclassified	Bacteroides caccae
unclassified	unclassified
unclassified	unclassified
unclassified	Enterocloster bolteae
unclassified	unclassified
unclassified	Desulfovibrio piger
unclassified	Blautia stercoris
unclassified	Roseburia sp. AM59-24XD
unclassified	unclassified
unclassified	Coprococcus catus
unclassified	Clostridium sp. AM33-3
unclassified	Ruminococcus bicirculans
unclassified	unclassified
unclassified	Alistipes senegalensis
unclassified	unclassified
unclassified	unclassified
unclassified	Acidaminococcus intestini
unclassified	unclassified
unclassified	unclassified
unclassified	unclassified
unclassified	unclassified
unclassified	unclassified
unclassified	Dorea longicatena
unclassified	Hydrogenoanaerobacterium saccharovorans
unclassified	unclassified
unclassified	unclassified
unclassified	Eubacterium sp. AF22-9
unclassified	Clostridium sp. AF27-2AA
unclassified	unclassified
unclassified	Dorea sp. AF36-15AT
unclassified	unclassified
unclassified	unclassified
unclassified	Flintibacter sp. NSJ-23
unclassified	Barnesiella intestinihominis
unclassified	unclassified
unclassified	unclassified
unclassified	Blautia faecicola
unclassified	Slackia isoflavoniconvertens
unclassified	unclassified
unclassified	unclassified
unclassified	unclassified

unclassified	unclassified
unclassified	unclassified
unclassified	unclassified
unclassified	Oscillibacter sp. ER4
unclassified	unclassified
unclassified	unclassified
unclassified	unclassified
unclassified	unclassified
unclassified	unclassified
unclassified	unclassified
unclassified	unclassified
unclassified	Ruminococcus sp. 1001095IJ_161003_G2
unclassified	Butyricimonas paravirosa
unclassified	Bacteroides cellulosilyticus
unclassified	Anaerobutyricum hallii
unclassified	Sellimonas intestinalis
unclassified	Parabacteroides johnsonii
unclassified	Clostridium sp. OM07-9AC
unclassified	unclassified
unclassified	unclassified
unclassified	unclassified
unclassified	unclassified
unclassified	unclassified
unclassified	Monoglobus pectinilyticus
unclassified	Escherichia coli
unclassified	unclassified
unclassified	Faecalibacterium prausnitzii
unclassified	unclassified
unclassified	Prevotella hominis
unclassified	unclassified
unclassified	unclassified
unclassified	unclassified
unclassified	Candidatus Borkfalkia ceftriaxoniphila
unclassified	Bacteroides nordii
unclassified	Veillonella atypica
unclassified	[Clostridium] symbiosum
unclassified	unclassified
unclassified	Blautia massiliensis
unclassified	Ligilactobacillus ruminis
unclassified	Mesosutterella multiformis
unclassified	Bifidobacterium angulatum
unclassified	Ruminococcus bromii
unclassified	unclassified
unclassified	unclassified
unclassified	unclassified
unclassified	Intestinimonas butyriciproducens
unclassified	Eubacterium sp. AF34-35BH
unclassified	unclassified

Alistipes onderdonkii subsp. vulgaris	Alistipes onderdonkii
unclassified	unclassified
unclassified	Blautia luti
unclassified	unclassified
unclassified	unclassified
unclassified	Prevotella stercorea
unclassified	Roseburia sp. BX0805
unclassified	unclassified
unclassified	Ruminococcus sp. 1001270H_150608_F2
unclassified	unclassified
unclassified	unclassified
unclassified	Gemmiger formicilis
unclassified	Faecalibacillus intestinalis
unclassified	unclassified
Klebsiella pneumoniae subsp. pneumoniae	Klebsiella pneumoniae
unclassified	unclassified
unclassified	Bacteroides cellulosilyticus
unclassified	Alistipes sp. Marseille-P2263
unclassified	Parabacteroides goldsteinii
unclassified	unclassified
unclassified	unclassified
unclassified	unclassified
unclassified	Roseburia inulinivorans
unclassified	Eubacterium sp. AF19-12LB
unclassified	unclassified
unclassified	unclassified
unclassified	unclassified
unclassified	Akkermansia sp. BIOML-A8
unclassified	unclassified
unclassified	unclassified
unclassified	unclassified
unclassified	unclassified
unclassified	unclassified
unclassified	Odoribacter splanchnicus
unclassified	[Clostridium] symbiosum
unclassified	unclassified
unclassified	unclassified
unclassified	Prevotella sp. P4-67
unclassified	Sutterella seckii
unclassified	unclassified
unclassified	Blautia sp. M29
unclassified	unclassified
unclassified	unclassified
unclassified	Eubacterium sp. BX4
unclassified	Bacteroides thetaiotaomicron
unclassified	unclassified
unclassified	Dorea sp. AF24-7LB
unclassified	Dysosmobacter sp. NSJ-60

unclassified	unclassified
unclassified	unclassified
unclassified	unclassified
unclassified	unclassified
unclassified	unclassified
unclassified	unclassified
unclassified	unclassified
unclassified	Roseburia intestinalis
unclassified	Ruminococcus sp. AF41-9
unclassified	unclassified
unclassified	unclassified
unclassified	Enterocloster clostridioformis
unclassified	unclassified
unclassified	Ellagibacter isourolithinifaciens
unclassified	unclassified
unclassified	Butyricicoccus sp. MSJd-7
unclassified	unclassified
unclassified	unclassified
unclassified	Bifidobacterium adolescentis
unclassified	Victivallis lenta
unclassified	Clostridium sp. AM30-24
unclassified	unclassified
unclassified	unclassified
unclassified	unclassified
unclassified	Veillonella rogosae
unclassified	Clostridium sp. AT4
unclassified	unclassified
unclassified	unclassified
unclassified	Vescimonas fastidiosa
unclassified	Coprococcus comes
unclassified	unclassified
unclassified	unclassified
unclassified	unclassified
unclassified	unclassified
unclassified	Hungatella hathewayi
unclassified	unclassified
unclassified	unclassified
unclassified	unclassified
unclassified	unclassified
unclassified	Butyricimonas faecalis
unclassified	Blautia wexlerae
unclassified	unclassified
unclassified	Prevotellamassilia timonensis
unclassified	unclassified
unclassified	unclassified
unclassified	unclassified
unclassified	unclassified
unclassified	Bacteroides ovatus

unclassified	Phocaeicola coprophilus
unclassified	unclassified
unclassified	unclassified
unclassified	unclassified
unclassified	unclassified
unclassified	unclassified
unclassified	unclassified
unclassified	unclassified
unclassified	Blautia caecimuris
unclassified	unclassified
unclassified	Clostridium sp. C5-48
unclassified	unclassified
unclassified	unclassified
unclassified	unclassified
unclassified	Prevotella sp. 885
unclassified	Blautia faecis
unclassified	Clostridium sp. 1001270J_160509_D11
unclassified	unclassified
unclassified	unclassified
unclassified	unclassified
unclassified	unclassified
unclassified	unclassified
Bifidobacterium animalis subsp. lactis	Bifidobacterium animalis
unclassified	unclassified
unclassified	unclassified
unclassified	unclassified
unclassified	Lachnoclostridium sp. 210928-DFI.6.3
unclassified	Enterocloster aldenensis
unclassified	unclassified
unclassified	unclassified
unclassified	unclassified
unclassified	unclassified
unclassified	unclassified
unclassified	unclassified
unclassified	unclassified
unclassified	Coprobacillus cateniformis
unclassified	Enterococcus faecalis
unclassified	Lachnospira pectinoschiza
unclassified	unclassified
unclassified	unclassified
unclassified	unclassified
unclassified	Prevotella stercorea
unclassified	unclassified
unclassified	unclassified
unclassified	Akkermansia sp. KLE1798
unclassified	unclassified
unclassified	unclassified
unclassified	Coprococcus sp. OM04-5BH

unclassified	unclassified
unclassified	unclassified
unclassified	Roseburia sp. AM16-25
unclassified	unclassified
unclassified	Blautia sp. Marseille-P3087
unclassified	unclassified
unclassified	unclassified
unclassified	unclassified
Coprobacter secundus subsp. similis	Coprobacter secundus
unclassified	unclassified
unclassified	unclassified
unclassified	Clostridium sp. AF37-5
unclassified	unclassified
unclassified	Dialister succinatiphilus
unclassified	unclassified
unclassified	unclassified
Bifidobacterium longum subsp. infantis	Bifidobacterium longum
unclassified	Eisenbergiella massiliensis
unclassified	Prevotella pectinovora
unclassified	unclassified
unclassified	Clostridium sp. NSJ-42
unclassified	unclassified
unclassified	Dysosmobacter sp. BX15
unclassified	unclassified
unclassified	Mitsuokella jalaludinii
unclassified	Parabacteroides goldsteinii
unclassified	unclassified
unclassified	unclassified
unclassified	unclassified
unclassified	unclassified
unclassified	unclassified
unclassified	unclassified
unclassified	unclassified
unclassified	Akkermansia muciniphila
unclassified	unclassified
unclassified	unclassified
unclassified	unclassified
unclassified	unclassified
unclassified	unclassified
unclassified	unclassified
unclassified	unclassified
unclassified	unclassified
unclassified	unclassified
unclassified	unclassified
unclassified	unclassified
unclassified	Mogibacterium kristiansenii
unclassified	unclassified
unclassified	unclassified

unclassified	Eisenbergiella tayi
unclassified	unclassified
unclassified	unclassified
unclassified	Sutterella sp. AM11-39
unclassified	Odoribacter laneus
unclassified	unclassified
unclassified	unclassified
unclassified	Fusicatenibacter saccharivorans
unclassified	Escherichia coli
unclassified	unclassified
unclassified	unclassified
unclassified	unclassified
unclassified	unclassified
unclassified	Megasphaera sp. BL7
unclassified	unclassified
unclassified	unclassified
unclassified	[Clostridium] spiroforme
unclassified	unclassified
unclassified	unclassified
unclassified	Alistipes communis
unclassified	Methanosphaera stadtmanae
unclassified	unclassified
unclassified	unclassified
unclassified	unclassified
unclassified	Blautia glucerasea
unclassified	unclassified
unclassified	unclassified
unclassified	Ruminococcus callidus
unclassified	unclassified
unclassified	unclassified
unclassified	Phocaeicola massiliensis
unclassified	unclassified
unclassified	unclassified
unclassified	Roseburia sp. BX1005
unclassified	Phascolarctobacterium succinatutens
unclassified	unclassified
unclassified	unclassified
unclassified	unclassified
unclassified	unclassified
unclassified	Coprococcus sp. AM27-12LB
unclassified	unclassified
unclassified	unclassified
unclassified	unclassified
unclassified	unclassified
unclassified	Paraprevotella xylaniphila
unclassified	Flintibacter sp. KGMB00164
unclassified	unclassified
unclassified	Victivallis vadensis

unclassified	unclassified
unclassified	Bifidobacterium dentium
unclassified	unclassified
unclassified	unclassified
unclassified	Prevotella copri
unclassified	unclassified
unclassified	Phocaeicola plebeius
unclassified	[Clostridium] innocuum
unclassified	unclassified
unclassified	unclassified
unclassified	unclassified
unclassified	unclassified
unclassified	unclassified
unclassified	unclassified
unclassified	Longicatena caecimuris
unclassified	Alistipes finegoldii
unclassified	unclassified
unclassified	unclassified
unclassified	unclassified
unclassified	unclassified
unclassified	unclassified
unclassified	unclassified
unclassified	unclassified
unclassified	Enterocloster bolteae
unclassified	unclassified
unclassified	unclassified
unclassified	Bacteroides fragilis
unclassified	unclassified
unclassified	Enterocloster clostridioformis
unclassified	unclassified
unclassified	unclassified
unclassified	Neglecta timonensis
unclassified	Ruminococcus champanellensis
unclassified	unclassified
unclassified	Prevotella rara
unclassified	unclassified
unclassified	unclassified
unclassified	Oscillibacter sp. MSJ-31
unclassified	unclassified
unclassified	unclassified
unclassified	unclassified
unclassified	Prevotella sp. Marseille-P4119
unclassified	unclassified
unclassified	unclassified
unclassified	unclassified
unclassified	unclassified
unclassified	unclassified
unclassified	unclassified

unclassified	unclassified
unclassified	Methanobrevibacter smithii
unclassified	Negativibacillus massiliensis
unclassified	unclassified
unclassified	unclassified
unclassified	Bacteroides fragilis
unclassified	unclassified
unclassified	Lawsonibacter sp. NSJ-51
unclassified	unclassified
unclassified	unclassified
unclassified	unclassified
unclassified	Bacteroides cellulosilyticus
unclassified	unclassified
unclassified	unclassified
unclassified	unclassified
unclassified	unclassified
unclassified	unclassified
unclassified	unclassified
unclassified	unclassified
unclassified	unclassified
unclassified	unclassified
unclassified	unclassified
unclassified	Bacteroides ovatus
unclassified	unclassified
unclassified	unclassified
unclassified	unclassified
unclassified	unclassified
unclassified	unclassified
unclassified	Megasphaera elsdenii
unclassified	unclassified
unclassified	unclassified
unclassified	unclassified
unclassified	unclassified
unclassified	unclassified
unclassified	Faecalibacillus sp. TM498
unclassified	unclassified
unclassified	unclassified
unclassified	unclassified
unclassified	unclassified
unclassified	unclassified
unclassified	unclassified
unclassified	unclassified
unclassified	unclassified
unclassified	unclassified
unclassified	unclassified
unclassified	Waltera intestinalis
unclassified	Alistipes sp. An31A
unclassified	unclassified

unclassified	unclassified
unclassified	unclassified
unclassified	unclassified
unclassified	unclassified
unclassified	unclassified
unclassified	unclassified
unclassified	unclassified
unclassified	Collinsella intestinalis
unclassified	unclassified
unclassified	Allisonella histaminiformans
unclassified	unclassified
unclassified	unclassified
unclassified	unclassified
unclassified	Blautia schinkii
unclassified	unclassified
unclassified	Anaerostipes caccae
unclassified	unclassified
unclassified	unclassified
unclassified	unclassified
unclassified	Blautia sp. DFI.9.9
unclassified	Eubacterium sp. AF22-8LB
unclassified	unclassified
unclassified	unclassified
unclassified	unclassified
unclassified	Bacteroides cellulosilyticus
unclassified	unclassified
unclassified	unclassified
unclassified	Mitsuokella multacida
unclassified	unclassified
unclassified	unclassified
unclassified	Blautia obeum
unclassified	unclassified
unclassified	unclassified
unclassified	unclassified
unclassified	unclassified
unclassified	unclassified
unclassified	unclassified
unclassified	unclassified
unclassified	Parolsenella catena
unclassified	unclassified
unclassified	unclassified
unclassified	unclassified
unclassified	unclassified
unclassified	unclassified
unclassified	unclassified
unclassified	Prevotella lascolaii
unclassified	Blautia hansenii
unclassified	unclassified

unclassified	unclassified
unclassified	unclassified
unclassified	unclassified
unclassified	unclassified
unclassified	Roseburia hominis
unclassified	unclassified
unclassified	unclassified
unclassified	unclassified
unclassified	unclassified
unclassified	unclassified
unclassified	unclassified
unclassified	unclassified
unclassified	unclassified
unclassified	unclassified
unclassified	Holdemania filiformis
unclassified	[Ruminococcus] lactaris
unclassified	unclassified
unclassified	unclassified
unclassified	[Clostridium] scindens
unclassified	Clostridium disporicum
unclassified	Prevotella buccae
unclassified	unclassified
unclassified	unclassified
unclassified	unclassified
unclassified	unclassified
unclassified	unclassified
unclassified	Oscillibacter sp. KLE 1728
unclassified	unclassified
unclassified	unclassified
unclassified	unclassified
unclassified	Hungatella hathewayi
unclassified	Anaeromassilibacillus sp. An250
unclassified	unclassified
unclassified	unclassified
unclassified	unclassified
unclassified	unclassified
unclassified	unclassified
unclassified	Parabacteroides distasonis
unclassified	Faecalibacterium sp. OF04-11AC
unclassified	unclassified
unclassified	unclassified
unclassified	unclassified
unclassified	unclassified
unclassified	[Clostridium] scindens
unclassified	unclassified
unclassified	unclassified
unclassified	unclassified
unclassified	unclassified

unclassified	unclassified
unclassified	Faecalicatena fissicatena
unclassified	unclassified
unclassified	Enterocloster asparagiformis
unclassified	Megasphaera micronuciformis
unclassified	unclassified
unclassified	Intestinimonas sp. MSJ-38
unclassified	Desulfovibrio fairfieldensis
unclassified	unclassified
unclassified	unclassified
unclassified	unclassified
unclassified	unclassified
unclassified	Clostridium fessum
unclassified	unclassified
unclassified	unclassified
unclassified	unclassified
unclassified	unclassified
unclassified	unclassified
unclassified	unclassified
unclassified	unclassified
unclassified	unclassified
unclassified	unclassified
unclassified	unclassified
unclassified	unclassified
unclassified	unclassified
unclassified	unclassified
unclassified	Eubacterium ramulus
unclassified	Desulfovibrio piger
unclassified	unclassified
unclassified	unclassified
unclassified	unclassified
unclassified	Alistipes timonensis
unclassified	Sutterella seckii
unclassified	unclassified
unclassified	Clostridium sp. D43t1_170807_D5
unclassified	Anaerostipes sp. NSJ-7
unclassified	Blautia sp. OF01-4LB
unclassified	unclassified
unclassified	unclassified
unclassified	unclassified
unclassified	unclassified
unclassified	unclassified
unclassified	unclassified
unclassified	unclassified
unclassified	unclassified
unclassified	Agathobaculum butyriciproducens
unclassified	Merdimonas faecis
unclassified	unclassified

unclassified	unclassified
unclassified	unclassified
unclassified	Collinsella bouchesdurhonensis
unclassified	unclassified
unclassified	Oscillibacter valericigenes
unclassified	Oxalobacter formigenes
unclassified	unclassified
unclassified	unclassified
unclassified	Lachnospira pectinoschiza
unclassified	Klebsiella oxytoca
unclassified	unclassified
unclassified	Enterocloster lavalensis
unclassified	unclassified
unclassified	unclassified
unclassified	Marseilla massiliensis
unclassified	unclassified
unclassified	Citrobacter freundii
unclassified	unclassified
unclassified	unclassified
unclassified	Sutterella wadsworthensis
unclassified	unclassified
unclassified	unclassified
unclassified	unclassified
unclassified	unclassified
unclassified	unclassified
unclassified	unclassified
unclassified	unclassified
unclassified	unclassified
unclassified	unclassified
unclassified	Clostridium neonatale
unclassified	Clostridium sp. MCC328
unclassified	unclassified
unclassified	unclassified
unclassified	unclassified
unclassified	unclassified
unclassified	unclassified
unclassified	unclassified
unclassified	Hallella sp. CLA-AA-H145
unclassified	Bacteroides mediterraneensis
unclassified	unclassified
unclassified	unclassified
unclassified	[Ruminococcus] torques
unclassified	unclassified
unclassified	unclassified
unclassified	unclassified
unclassified	unclassified
unclassified	unclassified
unclassified	unclassified

unclassified	unclassified
unclassified	unclassified
unclassified	Streptococcus vestibularis
unclassified	unclassified
unclassified	Alistipes putredinis
unclassified	Phascolarctobacterium faecium
unclassified	unclassified
unclassified	Marseilla massiliensis
unclassified	unclassified
unclassified	unclassified
unclassified	unclassified
unclassified	unclassified
unclassified	unclassified
unclassified	Enorma massiliensis
unclassified	unclassified
unclassified	unclassified
unclassified	Coprococcus eutactus
unclassified	unclassified
unclassified	unclassified
unclassified	unclassified
unclassified	unclassified
unclassified	Massilimicrobiota timonensis
unclassified	unclassified
unclassified	unclassified
unclassified	unclassified
unclassified	unclassified
unclassified	unclassified
unclassified	Roseburia sp. CLA-AA-H204
unclassified	unclassified
unclassified	unclassified
unclassified	unclassified
unclassified	Anaerotignum lactatifermentans
unclassified	unclassified
unclassified	unclassified
unclassified	unclassified
unclassified	unclassified
unclassified	Bacteroides congonensis
unclassified	Dorea phocaeensis
unclassified	unclassified
unclassified	Dakarella massiliensis
unclassified	unclassified
unclassified	unclassified
unclassified	Eisenbergiella tayi
unclassified	Olsenella sp. AF21-51
unclassified	unclassified
unclassified	unclassified
unclassified	Anaerotruncus colihominis
unclassified	unclassified

unclassified	unclassified
unclassified	Flavonifractor plautii
unclassified	unclassified
unclassified	Barnesiella sp. GGCC_0306
unclassified	Ruminococcus sp. 210702-SL.1.03
unclassified	unclassified
unclassified	unclassified
unclassified	unclassified
unclassified	unclassified
unclassified	unclassified
unclassified	unclassified
unclassified	unclassified
unclassified	unclassified
unclassified	unclassified
unclassified	unclassified
unclassified	unclassified
unclassified	unclassified
unclassified	unclassified
unclassified	unclassified
unclassified	unclassified
unclassified	Blautia glucerasea
unclassified	Turicibacter sanguinis
unclassified	Hydrogeniiclostidium mannosilyticum
unclassified	Dialister invisus
unclassified	unclassified
unclassified	unclassified
unclassified	unclassified
unclassified	unclassified
unclassified	unclassified
unclassified	unclassified
unclassified	unclassified
unclassified	unclassified
unclassified	Blastocystis sp. subtype 4
unclassified	unclassified
unclassified	unclassified
unclassified	unclassified
unclassified	Anaerotruncus massiliensis
unclassified	unclassified
unclassified	Clostridium perfringens
unclassified	unclassified
unclassified	unclassified
unclassified	unclassified
unclassified	unclassified
unclassified	unclassified
unclassified	unclassified
unclassified	[Ruminococcus] gnavus
unclassified	unclassified
unclassified	unclassified

unclassified	unclassified
unclassified	unclassified
unclassified	unclassified
unclassified	unclassified
unclassified	unclassified
unclassified	unclassified
unclassified	unclassified
unclassified	Clostridium paraputrificum
unclassified	Paraprevotella clara
unclassified	Enterobacter roggenkampii
unclassified	unclassified
unclassified	unclassified
unclassified	Phocea massiliensis
unclassified	unclassified
unclassified	unclassified
unclassified	unclassified
unclassified	unclassified
unclassified	unclassified
unclassified	unclassified
Faecalibacterium longum CLA-AA-H243	Faecalibacterium longum
unclassified	Clostridium sp. AM49-4BH
unclassified	unclassified
unclassified	unclassified
unclassified	Candidatus Methanomassiliicoccus intestinalis
unclassified	unclassified
unclassified	unclassified
unclassified	unclassified
unclassified	unclassified
unclassified	unclassified
unclassified	unclassified
unclassified	unclassified
unclassified	Lachnospira sp. NSJ-43
unclassified	unclassified
unclassified	unclassified
unclassified	unclassified
unclassified	unclassified
unclassified	unclassified
unclassified	unclassified
unclassified	unclassified
unclassified	unclassified
unclassified	unclassified
unclassified	unclassified
unclassified	unclassified
unclassified	unclassified
unclassified	unclassified
unclassified	unclassified
unclassified	unclassified
unclassified	Clostridioides difficile

unclassified	Bacteroides sp. Marseille-P3684
unclassified	unclassified
unclassified	unclassified
unclassified	unclassified
unclassified	unclassified
unclassified	Bilophila wadsworthia
unclassified	unclassified
unclassified	unclassified
unclassified	unclassified
unclassified	unclassified
unclassified	Phocaeicola coprophilus
unclassified	unclassified
unclassified	Lactobacillus acidophilus
unclassified	unclassified
unclassified	unclassified
unclassified	unclassified
unclassified	unclassified
unclassified	unclassified
unclassified	unclassified
unclassified	unclassified
Lactiplantibacillus plantarum subsp. plantarum	Lactiplantibacillus plantarum
unclassified	unclassified
unclassified	unclassified
unclassified	unclassified
unclassified	unclassified
unclassified	unclassified
unclassified	unclassified
unclassified	unclassified
unclassified	unclassified
unclassified	unclassified
unclassified	unclassified
unclassified	unclassified
unclassified	unclassified
unclassified	unclassified
unclassified	unclassified
unclassified	unclassified
unclassified	unclassified
unclassified	unclassified
unclassified	unclassified
unclassified	unclassified
unclassified	unclassified
unclassified	unclassified
unclassified	unclassified
unclassified	unclassified
Lactobacillus delbrueckii subsp. bulgaricus	Lactobacillus delbrueckii
unclassified	unclassified
unclassified	unclassified
unclassified	unclassified

unclassified	Bacteroides ndongoniae
unclassified	Eubacterium ventriosum
unclassified	unclassified
unclassified	Bacteroides cutis
unclassified	unclassified
unclassified	Desulfovibrio piger
unclassified	Bacteroides finegoldii
unclassified	unclassified
unclassified	unclassified
unclassified	unclassified
unclassified	unclassified
unclassified	unclassified
unclassified	Coprococcus catus
unclassified	unclassified
unclassified	unclassified
unclassified	unclassified
unclassified	unclassified
unclassified	unclassified
unclassified	unclassified
unclassified	unclassified
unclassified	Fusobacterium mortiferum
unclassified	unclassified
unclassified	unclassified
unclassified	Dorea longicatena
unclassified	Raoultella ornithinolytica
unclassified	unclassified
unclassified	unclassified
unclassified	Faecalitalea cylindroides
unclassified	unclassified
unclassified	unclassified
unclassified	unclassified
unclassified	Mediterranea massiliensis
unclassified	unclassified
unclassified	unclassified

genus	family	order
Phocaeicola	Bacteroidaceae	Bacteroidales
Anaerostipes	Lachnospiraceae	Eubacteriales
Bifidobacterium	Bifidobacteriaceae	Bifidobacteriales
unclassified	unclassified	Bacteroidales
unclassified	unclassified	unclassified
unclassified	unclassified	Eggerthellales
unclassified	Veillonellaceae	Veillonellales
unclassified	unclassified	Bacteroidales
Barnesiella	Barnesiellaceae	Bacteroidales
unclassified	unclassified	Eubacteriales
unclassified	unclassified	unclassified
unclassified	Oscillospiraceae	Eubacteriales
unclassified	Prevotellaceae	Bacteroidales
Bifidobacterium	Bifidobacteriaceae	Bifidobacteriales
Parabacteroides	Tannerellaceae	Bacteroidales
unclassified	unclassified	Eubacteriales
unclassified	Erysipelotrichaceae	Erysipelotrichales
unclassified	Oscillospiraceae	Eubacteriales
unclassified	unclassified	Burkholderiales
unclassified	unclassified	unclassified
unclassified	unclassified	Eubacteriales
Emergencia	Eubacteriales Family XIII. Incertae Sedis	Eubacteriales
unclassified	unclassified	unclassified
unclassified	Prevotellaceae	Bacteroidales
unclassified	unclassified	Eubacteriales
Phocaeicola	Bacteroidaceae	Bacteroidales
Bacteroides	Bacteroidaceae	Bacteroidales
unclassified	unclassified	Eubacteriales
unclassified	unclassified	unclassified
unclassified	Prevotellaceae	Bacteroidales
unclassified	unclassified	Eubacteriales
unclassified	unclassified	Bacteroidales
unclassified	unclassified	unclassified
unclassified	unclassified	Bacteroidales
unclassified	unclassified	unclassified
Holdemanella	Erysipelotrichaceae	Erysipelotrichales
unclassified	Lachnospiraceae	Eubacteriales
unclassified	unclassified	Eubacteriales
unclassified	unclassified	Bacteroidales
Hafnia	Hafniaceae	Enterobacterales
Odoribacter	Odoribacteraceae	Bacteroidales
Klebsiella	Enterobacteriaceae	Enterobacterales
Escherichia	Enterobacteriaceae	Enterobacterales
Rothia	Micrococcaceae	Micrococcales
Veillonella	Veillonellaceae	Veillonellales
unclassified	Prevotellaceae	Bacteroidales

Clostridium	Clostridiaceae	Eubacteriales
unclassified	Lachnospiraceae	Eubacteriales
Bacteroides	Bacteroidaceae	Bacteroidales
Dorea	Lachnospiraceae	Eubacteriales
Clostridium	Clostridiaceae	Eubacteriales
unclassified	unclassified	Eubacteriales
Lactococcus	Streptococcaceae	Lactobacillales
unclassified	unclassified	Bacteroidales
unclassified	unclassified	Eubacteriales
Akkermansia	Akkermansiaceae	Verrucomicrobiales
Enterococcus	Enterococcaceae	Lactobacillales
Vescimonas	Oscillospiraceae	Eubacteriales
Staphylococcus	Staphylococcaceae	Bacillales
Holdemania	Erysipelotrichaceae	Erysipelotrichales
Enterococcus	Enterococcaceae	Lactobacillales
unclassified	unclassified	Eubacteriales
Streptococcus	Streptococcaceae	Lactobacillales
Veillonella	Veillonellaceae	Veillonellales
Muribaculum	Muribaculaceae	Bacteroidales
unclassified	unclassified	Eubacteriales
Mediterraneibacter	Lachnospiraceae	Eubacteriales
Faecalicatena	Lachnospiraceae	Eubacteriales
Faecalibacterium	Oscillospiraceae	Eubacteriales
unclassified	unclassified	unclassified
unclassified	unclassified	Acidaminococcales
unclassified	unclassified	Eubacteriales
Enterococcus	Enterococcaceae	Lactobacillales
unclassified	unclassified	Eubacteriales
Klebsiella	Enterobacteriaceae	Enterobacterales
Lacrimispora	Lachnospiraceae	Eubacteriales
unclassified	Lachnospiraceae	Eubacteriales
unclassified	unclassified	Eubacteriales
unclassified	unclassified	Bacteroidales
Parasutterella	Sutterellaceae	Burkholderiales
Raoultella	Enterobacteriaceae	Enterobacterales
Lactobacillus	Lactobacillaceae	Lactobacillales
unclassified	unclassified	unclassified
Streptococcus	Streptococcaceae	Lactobacillales
unclassified	unclassified	Eubacteriales
Brachyspira	Brachyspiraceae	Brachyspirales
unclassified	unclassified	unclassified
unclassified	unclassified	Eubacteriales
unclassified	unclassified	unclassified
unclassified	unclassified	Eubacteriales
unclassified	unclassified	unclassified
unclassified	Bacteroidaceae	Bacteroidales
Lacticaseibacillus	Lactobacillaceae	Lactobacillales
Lacticaseibacillus	Lactobacillaceae	Lactobacillales

unclassified	unclassified	unclassified
Massilimicrobiota	Erysipelotrichaceae	Erysipelotrichales
unclassified	unclassified	unclassified
unclassified	unclassified	Bacteroidales
Ligilactobacillus	Lactobacillaceae	Lactobacillales
Enterococcus	Enterococcaceae	Lactobacillales
unclassified	unclassified	Eubacteriales
Bacteroides	Bacteroidaceae	Bacteroidales
Turicibacter	Turicibacteraceae	Erysipelotrichales
unclassified	unclassified	unclassified
unclassified	unclassified	Eubacteriales
Enterobacter	Enterobacteriaceae	Enterobacterales
unclassified	unclassified	unclassified
unclassified	unclassified	unclassified
unclassified	unclassified	unclassified
Alistipes	Rikenellaceae	Bacteroidales
unclassified	unclassified	Bacteroidales
unclassified	unclassified	unclassified
Roseburia	Lachnospiraceae	Eubacteriales
unclassified	unclassified	Eubacteriales
unclassified	unclassified	unclassified
unclassified	unclassified	unclassified
unclassified	unclassified	Eubacteriales
unclassified	unclassified	Eubacteriales
unclassified	unclassified	Bacteroidales
unclassified	Barnesiellaceae	Bacteroidales
Clostridium	Clostridiaceae	Eubacteriales
unclassified	unclassified	Eubacteriales
unclassified	unclassified	Eubacteriales
unclassified	unclassified	Eubacteriales
unclassified	unclassified	Eubacteriales
Porphyromonas	Porphyromonadaceae	Bacteroidales
unclassified	unclassified	unclassified
unclassified	unclassified	unclassified
unclassified	unclassified	unclassified
unclassified	unclassified	unclassified
unclassified	unclassified	unclassified
unclassified	unclassified	Bacteroidales
unclassified	Atopobiaceae	Coriobacteriales
unclassified	unclassified	unclassified
unclassified	unclassified	Bacteroidales
Clostridium	Clostridiaceae	Eubacteriales
unclassified	Actinomycetaceae	Actinomycetales
Roseburia	Lachnospiraceae	Eubacteriales
Ruminococcus	Oscillospiraceae	Eubacteriales
unclassified	unclassified	Eubacteriales
Clostridium	Clostridiaceae	Eubacteriales
unclassified	unclassified	Bacteroidales

Phocaeicola	Bacteroidaceae	Bacteroidales
unclassified	unclassified	Eubacteriales
unclassified	unclassified	unclassified
unclassified	unclassified	Eubacteriales
Clostridium	Clostridiaceae	Eubacteriales
unclassified	unclassified	Bacteroidales
unclassified	Lachnospiraceae	Eubacteriales
unclassified	unclassified	Bacteroidales
unclassified	Lachnospiraceae	Eubacteriales
unclassified	unclassified	unclassified
unclassified	unclassified	Eubacteriales
unclassified	unclassified	unclassified
unclassified	unclassified	Eubacteriales
unclassified	unclassified	Eubacteriales
unclassified	unclassified	unclassified
unclassified	unclassified	Eubacteriales
unclassified	unclassified	unclassified
unclassified	unclassified	unclassified
unclassified	unclassified	unclassified
Gemmiger	unclassified	Eubacteriales
Culturomica	Odoribacteraceae	Bacteroidales
unclassified	Lachnospiraceae	Eubacteriales
unclassified	unclassified	unclassified
unclassified	unclassified	Methanomassiliicoccales
unclassified	unclassified	Eggerthellales
unclassified	unclassified	unclassified
unclassified	unclassified	Eubacteriales
unclassified	Oscillospiraceae	Eubacteriales
unclassified	Lachnospiraceae	Eubacteriales
Acidaminococcus	Acidaminococcaceae	Acidaminococcales
Eubacterium	Eubacteriaceae	Eubacteriales
Lactococcus	Streptococcaceae	Lactobacillales
unclassified	unclassified	unclassified
unclassified	unclassified	unclassified
unclassified	unclassified	Eubacteriales
unclassified	unclassified	Bacteroidales
unclassified	unclassified	unclassified
Anaerostipes	Lachnospiraceae	Eubacteriales
unclassified	unclassified	unclassified
unclassified	unclassified	Eubacteriales
unclassified	unclassified	unclassified
unclassified	unclassified	unclassified
unclassified	unclassified	Eubacteriales
Desulfovibrio	Desulfovibrionaceae	Desulfovibrionales
unclassified	unclassified	Eubacteriales
unclassified	unclassified	unclassified
unclassified	unclassified	unclassified
unclassified	Oscillospiraceae	Eubacteriales

Methanobrevibacter	Methanobacteriaceae	Methanobacteriales
unclassified	unclassified	unclassified
unclassified	unclassified	unclassified
unclassified	Treponemataceae	Spirochaetales
unclassified	unclassified	unclassified
Sutterella	Sutterellaceae	Burkholderiales
unclassified	unclassified	unclassified
unclassified	unclassified	unclassified
unclassified	Bacteroidaceae	Bacteroidales
Clostridium	Clostridiaceae	Eubacteriales
Lacrimispora	Lachnospiraceae	Eubacteriales
unclassified	unclassified	Eubacteriales
Enterocloster	Lachnospiraceae	Eubacteriales
Acidovorax	Comamonadaceae	Burkholderiales
Comamonas	Comamonadaceae	Burkholderiales
Acinetobacter	Moraxellaceae	Moraxellales
Acinetobacter	Moraxellaceae	Moraxellales
Acinetobacter	Moraxellaceae	Moraxellales
Acinetobacter	Moraxellaceae	Moraxellales
Actinomyces	Actinomycetaceae	Actinomycetales
Actinomyces	Actinomycetaceae	Actinomycetales
Aeromonas	Aeromonadaceae	Aeromonadales
Bacteroides	Bacteroidaceae	Bacteroidales
Fenollaria	unclassified	Eubacteriales
Aliarcobacter	Arcobacteraceae	Campylobacterales
Methylobacterium	Methylobacteriaceae	Hyphomicrobiales
Atopobium	Atopobiaceae	Coriobacteriales
Bacteroides	Bacteroidaceae	Bacteroidales
Bifidobacterium	Bifidobacteriaceae	Bifidobacteriales
Blautia	Lachnospiraceae	Eubacteriales
Blautia	Lachnospiraceae	Eubacteriales
Turicimonas	Sutterellaceae	Burkholderiales
Butyricimonas	Odoribacteraceae	Bacteroidales
Lachnospira	Lachnospiraceae	Eubacteriales
Flavonifractor	Oscillospiraceae	Eubacteriales
Megasphaera	Veillonellaceae	Veillonellales
Caulobacter	Caulobacteraceae	Caulobacterales
Chryseobacterium	Weeksellaceae	Flavobacteriales
Citrobacter	Enterobacteriaceae	Enterobacterales
Citrobacter	Enterobacteriaceae	Enterobacterales
Citrobacter	Enterobacteriaceae	Enterobacterales
Citrobacter	Enterobacteriaceae	Enterobacterales
Citrobacter	Enterobacteriaceae	Enterobacterales
Citrobacter	Enterobacteriaceae	Enterobacterales
Cloacibacillus	Synergistaceae	Synergistales
unclassified	Oscillospiraceae	Eubacteriales
Dielma	Erysipelotrichaceae	Erysipelotrichales
Lachnoclostridium	Lachnospiraceae	Eubacteriales

Clostridium	Clostridiaceae	Eubacteriales
Rubneribacter	Eggerthellaceae	Eggerthellales
Corynebacterium	Corynebacteriaceae	Corynebacteriales
Cutibacterium	Propionibacteriaceae	Propionibacteriales
Cutibacterium	Propionibacteriaceae	Propionibacteriales
Cutibacterium	Propionibacteriaceae	Propionibacteriales
Enterobacter	Enterobacteriaceae	Enterobacterales
Enterobacter	Enterobacteriaceae	Enterobacterales
Bacteroides	Bacteroidaceae	Bacteroidales
Enterobacter	Enterobacteriaceae	Enterobacterales
Phytobacter	Enterobacteriaceae	Enterobacterales
Enterococcus	Enterococcaceae	Lactobacillales
Enterococcus	Enterococcaceae	Lactobacillales
Enterococcus	Enterococcaceae	Lactobacillales
Enterococcus	Enterococcaceae	Lactobacillales
Enterococcus	Enterococcaceae	Lactobacillales
Erysipelatoclostridium	Erysipelotrichaceae	Erysipelotrichales
Escherichia	Enterobacteriaceae	Enterobacterales
Escherichia	Enterobacteriaceae	Enterobacterales
unclassified	unclassified	Eubacteriales
Eubacterium	Eubacteriaceae	Eubacteriales
Flavobacterium	Flavobacteriaceae	Flavobacteriales
Sanguibacteroides	Porphyromonadaceae	Bacteroidales
Gardnerella	Bifidobacteriaceae	Bifidobacteriales
Granulicatella	Carnobacteriaceae	Lactobacillales
Haemophilus	Pasteurellaceae	Pasteurellales
Hafnia	Hafniaceae	Enterobacterales
Kosakonia	Enterobacteriaceae	Enterobacterales
Klebsiella	Enterobacteriaceae	Enterobacterales
Lactobacillus	Lactobacillaceae	Lactobacillales
Ruthenibacterium	Oscillospiraceae	Eubacteriales
Lactobacillus	Lactobacillaceae	Lactobacillales
Limosilactobacillus	Lactobacillaceae	Lactobacillales
Limosilactobacillus	Lactobacillaceae	Lactobacillales
Lysinibacillus	Bacillaceae	Bacillales
Megasphaera	Veillonellaceae	Veillonellales
Megasphaera	Veillonellaceae	Veillonellales
Morganella	Morganellaceae	Enterobacterales
Mycobacteroides	Mycobacteriaceae	Corynebacteriales
Neisseria	Neisseriaceae	Neisseriales
Pantoea	Erwiniaceae	Enterobacterales
Sutterella	Sutterellaceae	Burkholderiales
Parabacteroides	Tannerellaceae	Bacteroidales
Prevotella	Prevotellaceae	Bacteroidales
Prevotella	Prevotellaceae	Bacteroidales
Pseudoflavonifractor	Oscillospiraceae	Eubacteriales
Pseudomonas	Pseudomonadaceae	Pseudomonadales
Pseudomonas	Pseudomonadaceae	Pseudomonadales

Pseudomonas	Pseudomonadaceae	Pseudomonadales
Robinsoniella	Lachnospiraceae	Eubacteriales
Rothia	Micrococcaceae	Micrococcales
Selenomonas	Selenomonadaceae	Selenomonadales
Ruminococcus	Oscillospiraceae	Eubacteriales
Serratia	Yersiniaceae	Enterobacterales
Shewanella	Shewanellaceae	Alteromonadales
Sphingomonas	Sphingomonadaceae	Sphingomonadales
Staphylococcus	Staphylococcaceae	Bacillales
Staphylococcus	Staphylococcaceae	Bacillales
Staphylococcus	Staphylococcaceae	Bacillales
Stenotrophomonas	Xanthomonadaceae	Xanthomonadales
Stenotrophomonas	Xanthomonadaceae	Xanthomonadales
Streptococcus	Streptococcaceae	Lactobacillales
Streptococcus	Streptococcaceae	Lactobacillales
Faecalibacterium	Oscillospiraceae	Eubacteriales
Streptococcus	Streptococcaceae	Lactobacillales
Streptococcus	Streptococcaceae	Lactobacillales
Veillonella	Veillonellaceae	Veillonellales
Citrobacter	Enterobacteriaceae	Enterobacterales
Kluyvera	Enterobacteriaceae	Enterobacterales
unclassified	Lachnospiraceae	Eubacteriales
Citrobacter	Enterobacteriaceae	Enterobacterales
unclassified	Lachnospiraceae	Eubacteriales
Clostridium	Clostridiaceae	Eubacteriales
Enterococcus	Enterococcaceae	Lactobacillales
unclassified	unclassified	Eubacteriales
Clostridium	Clostridiaceae	Eubacteriales
Atlantibacter	Enterobacteriaceae	Enterobacterales
Terrisporobacter	Peptostreptococcaceae	Eubacteriales
Clostridium	Clostridiaceae	Eubacteriales
unclassified	Enterobacteriaceae	Enterobacterales
unclassified	Enterobacteriaceae	Enterobacterales
Corynebacterium	Corynebacteriaceae	Corynebacteriales
Macrococcus	Staphylococcaceae	Bacillales
Enterobacter	Enterobacteriaceae	Enterobacterales
Anaerosporobacter	Lachnospiraceae	Eubacteriales
Haemophilus	Pasteurellaceae	Pasteurellales
Veillonella	Veillonellaceae	Veillonellales
Lachnoclostridium	Lachnospiraceae	Eubacteriales
Limosilactobacillus	Lactobacillaceae	Lactobacillales
unclassified	Peptostreptococcaceae	Eubacteriales
unclassified	unclassified	Eubacteriales
Butyricicoccus	Clostridiaceae	Eubacteriales
Raoultella	Enterobacteriaceae	Enterobacterales
Clostridium	Clostridiaceae	Eubacteriales
unclassified	unclassified	unclassified
unclassified	Erysipelotrichaceae	Erysipelotrichales

Ruminococcus	Oscillospiraceae	Eubacteriales
Phocaeicola	Bacteroidaceae	Bacteroidales
Rothia	Micrococcaceae	Micrococcales
unclassified	Enterobacteriaceae	Enterobacterales
Schaalia	Actinomycetaceae	Actinomycetales
Clostridium	Clostridiaceae	Eubacteriales
Citrobacter	Enterobacteriaceae	Enterobacterales
Isoptericola	Promicromonosporaceae	Micrococcales
unclassified	Peptostreptococcaceae	Eubacteriales
Bifidobacterium	Bifidobacteriaceae	Bifidobacteriales
Eisenbergiella	Lachnospiraceae	Eubacteriales
Enterobacter	Enterobacteriaceae	Enterobacterales
Streptococcus	Streptococcaceae	Lactobacillales
unclassified	Erysipelotrichaceae	Erysipelotrichales
unclassified	Lachnospiraceae	Eubacteriales
Candida	Debaryomycetaceae	Saccharomycetales
Klebsiella	Enterobacteriaceae	Enterobacterales
unclassified	unclassified	Eubacteriales
unclassified	unclassified	Eubacteriales
unclassified	unclassified	Eubacteriales
unclassified	Lachnospiraceae	Eubacteriales
Eubacterium	Eubacteriaceae	Eubacteriales
Atlantibacter	Enterobacteriaceae	Enterobacterales
unclassified	unclassified	Eubacteriales
Proteus	Morganellaceae	Enterobacterales
unclassified	Veillonellaceae	Veillonellales
Enterococcus	Enterococcaceae	Lactobacillales
unclassified	unclassified	unclassified
Eubacterium	Eubacteriaceae	Eubacteriales
Luxibacter	Lachnospiraceae	Eubacteriales
Eggerthella	Eggerthellaceae	Eggerthellales
unclassified	Erysipelotrichaceae	Erysipelotrichales
Lacrimispora	Lachnospiraceae	Eubacteriales
unclassified	unclassified	Eubacteriales
Dialister	Veillonellaceae	Veillonellales
unclassified	unclassified	unclassified
unclassified	Lachnospiraceae	Eubacteriales
unclassified	unclassified	Erysipelotrichales
unclassified	Tannerellaceae	Bacteroidales
unclassified	unclassified	Eubacteriales
unclassified	unclassified	Eubacteriales
unclassified	unclassified	Eubacteriales
unclassified	unclassified	unclassified
Erysipelatoclostridium	Erysipelotrichaceae	Erysipelotrichales
unclassified	unclassified	Eubacteriales
unclassified	unclassified	Eubacteriales
unclassified	Clostridiaceae	Eubacteriales
unclassified	Lachnospiraceae	Eubacteriales

unclassified	unclassified	Eubacteriales
Enterococcus	Enterococcaceae	Lactobacillales
unclassified	Eubacteriaceae	Eubacteriales
Limosilactobacillus	Lactobacillaceae	Lactobacillales
unclassified	unclassified	Erysipelotrichales
Lachnoclostridium	Lachnospiraceae	Eubacteriales
unclassified	unclassified	Eubacteriales
Corynebacterium	Corynebacteriaceae	Corynebacteriales
unclassified	unclassified	unclassified
Enterococcus	Enterococcaceae	Lactobacillales
Actinomyces	Actinomycetaceae	Actinomycetales
Akkermansia	Akkermansiaceae	Verrucomicrobiales
Beduini	Clostridiaceae	Eubacteriales
Actinomyces	Actinomycetaceae	Actinomycetales
Collinsella	Coriobacteriaceae	Coriobacteriales
unclassified	unclassified	Eubacteriales
unclassified	unclassified	Bacteroidales
unclassified	Lachnospiraceae	Eubacteriales
Extibacter	Lachnospiraceae	Eubacteriales
Alistipes	Rikenellaceae	Bacteroidales
Bifidobacterium	Bifidobacteriaceae	Bifidobacteriales
Senegalimassilia	Coriobacteriaceae	Coriobacteriales
Christensenella	Christensenellaceae	Eubacteriales
unclassified	Oscillospiraceae	Eubacteriales
unclassified	unclassified	unclassified
unclassified	unclassified	Erysipelotrichales
unclassified	Lachnospiraceae	Eubacteriales
unclassified	Oscillospiraceae	Eubacteriales
unclassified	Lachnospiraceae	Eubacteriales
unclassified	Lachnospiraceae	Eubacteriales
Clostridium	Clostridiaceae	Eubacteriales
Kluyvera	Enterobacteriaceae	Enterobacterales
unclassified	Lachnospiraceae	Eubacteriales
unclassified	unclassified	Eubacteriales
unclassified	unclassified	Eubacteriales
Eubacterium	Eubacteriaceae	Eubacteriales
unclassified	Atopobiaceae	Coriobacteriales
Kluyvera	Enterobacteriaceae	Enterobacterales
Collinsella	Coriobacteriaceae	Coriobacteriales
Collinsella	Coriobacteriaceae	Coriobacteriales
Gordonibacter	Eggerthellaceae	Eggerthellales
Anaerobutyricum	Lachnospiraceae	Eubacteriales
Kluyvera	Enterobacteriaceae	Enterobacterales
unclassified	unclassified	Eubacteriales
unclassified	Oscillospiraceae	Eubacteriales
Sellimonas	Lachnospiraceae	Eubacteriales
Anaerococcus	Peptoniphilaceae	Tissierellales
unclassified	unclassified	Eubacteriales

Veillonella	Veillonellaceae	Veillonellales
Alistipes	Rikenellaceae	Bacteroidales
Streptococcus	Streptococcaceae	Lactobacillales
unclassified	unclassified	Eubacteriales
Ruminococcus	Oscillospiraceae	Eubacteriales
Erysipelatoclostridium	Erysipelotrichaceae	Erysipelotrichales
Clostridium	Clostridiaceae	Eubacteriales
Enterobacter	Enterobacteriaceae	Enterobacterales
Anaerofustis	Eubacteriaceae	Eubacteriales
unclassified	unclassified	Eubacteriales
Bilophila	Desulfovibrionaceae	Desulfovibrionales
Anaerostipes	Lachnospiraceae	Eubacteriales
unclassified	unclassified	Erysipelotrichales
Actinomyces	Actinomycetaceae	Actinomycetales
Clostridium	Clostridiaceae	Eubacteriales
Lacrimispora	Lachnospiraceae	Eubacteriales
Veillonella	Veillonellaceae	Veillonellales
Eubacterium	Eubacteriaceae	Eubacteriales
unclassified	unclassified	Eubacteriales
Collinsella	Coriobacteriaceae	Coriobacteriales
unclassified	Eubacteriaceae	Eubacteriales
Clostridium	Clostridiaceae	Eubacteriales
unclassified	unclassified	Eubacteriales
Slackia	Eggerthellaceae	Eggerthellales
Christensenella	Christensenellaceae	Eubacteriales
Eubacterium	Eubacteriaceae	Eubacteriales
Eggerthella	Eggerthellaceae	Eggerthellales
Adlercreutzia	Eggerthellaceae	Eggerthellales
Erysipelatoclostridium	Erysipelotrichaceae	Erysipelotrichales
unclassified	unclassified	Eubacteriales
Christensenella	Christensenellaceae	Eubacteriales
Streptococcus	Streptococcaceae	Lactobacillales
Collinsella	Coriobacteriaceae	Coriobacteriales
Klebsiella	Enterobacteriaceae	Enterobacterales
Limosilactobacillus	Lactobacillaceae	Lactobacillales
unclassified	Oscillospiraceae	Eubacteriales
unclassified	Carnobacteriaceae	Lactobacillales
Agathobaculum	Oscillospiraceae	Eubacteriales
Eubacterium	Eubacteriaceae	Eubacteriales
unclassified	unclassified	Eubacteriales
Bilophila	Desulfovibrionaceae	Desulfovibrionales
unclassified	unclassified	Eubacteriales
unclassified	Lachnospiraceae	Eubacteriales
Mogibacterium	Eubacteriales Family XIII. Incertae Sedis	Eubacteriales
unclassified	unclassified	Eubacteriales
Limosilactobacillus	Lactobacillaceae	Lactobacillales
unclassified	unclassified	Erysipelotrichales
Mediterraneibacter	Lachnospiraceae	Eubacteriales

unclassified	Lachnospiraceae	Eubacteriales
Streptococcus	Streptococcaceae	Lactobacillales
Corynebacterium	Corynebacteriaceae	Corynebacteriales
unclassified	unclassified	Bacteroidales
Eggerthella	Eggerthellaceae	Eggerthellales
Robinsoniella	Lachnospiraceae	Eubacteriales
Blautia	Lachnospiraceae	Eubacteriales
Acinetobacter	Moraxellaceae	Moraxellales
unclassified	unclassified	unclassified
Candidatus Stoquefichus	Erysipelotrichaceae	Erysipelotrichales
Dielma	Erysipelotrichaceae	Erysipelotrichales
Streptococcus	Streptococcaceae	Lactobacillales
unclassified	unclassified	Eubacteriales
unclassified	unclassified	Eubacteriales
Lancefieldella	Atopobiaceae	Coriobacteriales
unclassified	Oscillospiraceae	Eubacteriales
Streptococcus	Streptococcaceae	Lactobacillales
Ruminococcus	Oscillospiraceae	Eubacteriales
unclassified	Oscillospiraceae	Eubacteriales
unclassified	unclassified	Eubacteriales
unclassified	unclassified	unclassified
Leuconostoc	Lactobacillaceae	Lactobacillales
Bifidobacterium	Bifidobacteriaceae	Bifidobacteriales
Faecalibacterium	Oscillospiraceae	Eubacteriales
Lactococcus	Streptococcaceae	Lactobacillales
Faecalicatena	Lachnospiraceae	Eubacteriales
Raoultibacter	Eggerthellaceae	Eggerthellales
Collinsella	Coriobacteriaceae	Coriobacteriales
Escherichia	Enterobacteriaceae	Enterobacterales
Enterocloster	Lachnospiraceae	Eubacteriales
unclassified	unclassified	Eubacteriales
Eisenbergiella	Lachnospiraceae	Eubacteriales
Intestinimonas	unclassified	Eubacteriales
unclassified	Eggerthellaceae	Eggerthellales
Phascolarctobacterium	Acidaminococcaceae	Acidaminococcales
Blautia	Lachnospiraceae	Eubacteriales
unclassified	unclassified	Eubacteriales
unclassified	unclassified	Eubacteriales
Enterobacter	Enterobacteriaceae	Enterobacterales
unclassified	Oscillospiraceae	Eubacteriales
unclassified	unclassified	unclassified
unclassified	Lachnospiraceae	Eubacteriales
unclassified	unclassified	Eubacteriales
Staphylococcus	Staphylococcaceae	Bacillales
unclassified	Lachnospiraceae	Eubacteriales
unclassified	Oscillospiraceae	Eubacteriales
unclassified	unclassified	Erysipelotrichales
Eggerthella	Eggerthellaceae	Eggerthellales

Amedibacillus	Erysipelotrichaceae	Erysipelotrichales
Agathobaculum	Oscillospiraceae	Eubacteriales
unclassified	unclassified	Eggerthellales
Faecalibacterium	Oscillospiraceae	Eubacteriales
unclassified	unclassified	Eubacteriales
Clostridium	Clostridiaceae	Eubacteriales
Bacteroides	Bacteroidaceae	Bacteroidales
unclassified	unclassified	unclassified
Prevotella	Prevotellaceae	Bacteroidales
Blautia	Lachnospiraceae	Eubacteriales
unclassified	unclassified	Eubacteriales
unclassified	unclassified	Eubacteriales
unclassified	unclassified	Eubacteriales
unclassified	unclassified	unclassified
Parabacteroides	Tannerellaceae	Bacteroidales
unclassified	unclassified	Eubacteriales
Blautia	Lachnospiraceae	Eubacteriales
unclassified	unclassified	unclassified
unclassified	unclassified	unclassified
Kineothrix	Lachnospiraceae	Eubacteriales
Butyrivibrio	Lachnospiraceae	Eubacteriales
unclassified	unclassified	Eubacteriales
unclassified	unclassified	unclassified
Evtepia	unclassified	Eubacteriales
Bacteroides	Bacteroidaceae	Bacteroidales
Subdoligranulum	Oscillospiraceae	Eubacteriales
unclassified	Oscillospiraceae	Eubacteriales
Catenibacterium	Coprobacillaceae	Erysipelotrichales
unclassified	unclassified	Eubacteriales
unclassified	unclassified	unclassified
unclassified	Lachnospiraceae	Eubacteriales
unclassified	Oscillospiraceae	Eubacteriales
Alistipes	Rikenellaceae	Bacteroidales
unclassified	unclassified	unclassified
unclassified	unclassified	unclassified
unclassified	unclassified	Bacteroidales
Faecalibacterium	Oscillospiraceae	Eubacteriales
unclassified	unclassified	unclassified
Alistipes	Rikenellaceae	Bacteroidales
Butyricimonas	Odoribacteraceae	Bacteroidales
Intestinibacter	Peptostreptococcaceae	Eubacteriales
unclassified	unclassified	Eubacteriales
Bifidobacterium	Bifidobacteriaceae	Bifidobacteriales
Butyricimonas	Odoribacteraceae	Bacteroidales
unclassified	unclassified	unclassified
unclassified	unclassified	Eubacteriales
unclassified	unclassified	Eubacteriales
Phocaeicola	Bacteroidaceae	Bacteroidales

unclassified	unclassified	Eubacteriales
unclassified	unclassified	unclassified
Erysipelatoclostridium	Erysipelotrichaceae	Erysipelotrichales
unclassified	unclassified	Eubacteriales
Faecalibacillus	Erysipelotrichaceae	Erysipelotrichales
unclassified	unclassified	Eubacteriales
Phocaeicola	Bacteroidaceae	Bacteroidales
Bacteroides	Bacteroidaceae	Bacteroidales
unclassified	Oscillospiraceae	Eubacteriales
unclassified	unclassified	Bacteroidales
Bacteroides	Bacteroidaceae	Bacteroidales
Collinsella	Coriobacteriaceae	Coriobacteriales
unclassified	unclassified	Bacteroidales
Veillonella	Veillonellaceae	Veillonellales
Eggerthella	Eggerthellaceae	Eggerthellales
Faecalibacterium	Oscillospiraceae	Eubacteriales
Coprobacter	Barnesiellaceae	Bacteroidales
unclassified	unclassified	unclassified
Gemmiger	unclassified	Eubacteriales
unclassified	unclassified	Eubacteriales
Romboutsia	Peptostreptococcaceae	Eubacteriales
Bifidobacterium	Bifidobacteriaceae	Bifidobacteriales
Bifidobacterium	Bifidobacteriaceae	Bifidobacteriales
unclassified	unclassified	unclassified
unclassified	unclassified	Eubacteriales
Holdemanella	Erysipelotrichaceae	Erysipelotrichales
Akkermansia	Akkermansiaceae	Verrucomicrobiales
Duodenibacillus	Sutterellaceae	Burkholderiales
Alistipes	Rikenellaceae	Bacteroidales
unclassified	unclassified	Eggerthellales
unclassified	unclassified	Eubacteriales
unclassified	unclassified	Eubacteriales
Tyzzerella	Lachnospiraceae	Eubacteriales
Alistipes	Rikenellaceae	Bacteroidales
Clostridium	Clostridiaceae	Eubacteriales
Clostridium	Clostridiaceae	Eubacteriales
Parabacteroides	Tannerellaceae	Bacteroidales
unclassified	unclassified	unclassified
unclassified	unclassified	Veillonellales
unclassified	unclassified	Eubacteriales
unclassified	unclassified	Eubacteriales
unclassified	Lachnospiraceae	Eubacteriales
Coprococcus	Lachnospiraceae	Eubacteriales
unclassified	unclassified	Eubacteriales
Bacteroides	Bacteroidaceae	Bacteroidales
Gemmiger	unclassified	Eubacteriales
unclassified	unclassified	unclassified
Megamonas	Selenomonadaceae	Selenomonadales

unclassified	unclassified	Eubacteriales
unclassified	unclassified	unclassified
Parasutterella	Sutterellaceae	Burkholderiales
unclassified	Oscillospiraceae	Eubacteriales
Pseudoruminococcus	Oscillospiraceae	Eubacteriales
unclassified	Oscillospiraceae	Eubacteriales
Blautia	Lachnospiraceae	Eubacteriales
Bacteroides	Bacteroidaceae	Bacteroidales
unclassified	Lachnospiraceae	Eubacteriales
unclassified	unclassified	Eubacteriales
Enterocloster	Lachnospiraceae	Eubacteriales
unclassified	unclassified	Acidaminococcales
Desulfovibrio	Desulfovibrionaceae	Desulfovibrionales
Blautia	Lachnospiraceae	Eubacteriales
Roseburia	Lachnospiraceae	Eubacteriales
unclassified	unclassified	Eubacteriales
Coprococcus	Lachnospiraceae	Eubacteriales
Clostridium	Clostridiaceae	Eubacteriales
Ruminococcus	Oscillospiraceae	Eubacteriales
unclassified	unclassified	Eubacteriales
Alistipes	Rikenellaceae	Bacteroidales
unclassified	unclassified	unclassified
Waltera	Lachnospiraceae	Eubacteriales
Acidaminococcus	Acidaminococcaceae	Acidaminococcales
unclassified	unclassified	unclassified
unclassified	unclassified	unclassified
unclassified	unclassified	Eubacteriales
unclassified	unclassified	Eubacteriales
unclassified	Clostridiaceae	Eubacteriales
Dorea	Lachnospiraceae	Eubacteriales
Hydrogenoanaerobacterium	Oscillospiraceae	Eubacteriales
unclassified	unclassified	unclassified
unclassified	Oscillospiraceae	Eubacteriales
Eubacterium	Eubacteriaceae	Eubacteriales
Clostridium	Clostridiaceae	Eubacteriales
unclassified	unclassified	Eubacteriales
Dorea	Lachnospiraceae	Eubacteriales
unclassified	Oscillospiraceae	Eubacteriales
unclassified	unclassified	unclassified
Flintibacter	unclassified	Eubacteriales
Barnesiella	Barnesiellaceae	Bacteroidales
unclassified	unclassified	Eubacteriales
Oscillibacter	Oscillospiraceae	Eubacteriales
Blautia	Lachnospiraceae	Eubacteriales
Slackia	Eggerthellaceae	Eggerthellales
unclassified	unclassified	Eubacteriales
unclassified	unclassified	unclassified
unclassified	unclassified	Eubacteriales

Faecalibacterium	Oscillospiraceae	Eubacteriales
unclassified	unclassified	unclassified
unclassified	Oscillospiraceae	Eubacteriales
Oscillibacter	Oscillospiraceae	Eubacteriales
unclassified	unclassified	unclassified
unclassified	unclassified	Eubacteriales
unclassified	unclassified	unclassified
unclassified	unclassified	Eubacteriales
unclassified	Erysipelotrichaceae	Erysipelotrichales
Colidextribacter	unclassified	Eubacteriales
unclassified	unclassified	unclassified
Ruminococcus	Oscillospiraceae	Eubacteriales
Butyricimonas	Odoribacteraceae	Bacteroidales
Bacteroides	Bacteroidaceae	Bacteroidales
Anaerobutyricum	Lachnospiraceae	Eubacteriales
Sellimonas	Lachnospiraceae	Eubacteriales
Parabacteroides	Tannerellaceae	Bacteroidales
Clostridium	Clostridiaceae	Eubacteriales
unclassified	unclassified	Eubacteriales
unclassified	unclassified	unclassified
unclassified	unclassified	unclassified
unclassified	unclassified	unclassified
unclassified	unclassified	Eubacteriales
Monoglobus	Oscillospiraceae	Eubacteriales
Escherichia	Enterobacteriaceae	Enterobacterales
unclassified	unclassified	unclassified
Faecalibacterium	Oscillospiraceae	Eubacteriales
unclassified	unclassified	unclassified
Prevotella	Prevotellaceae	Bacteroidales
unclassified	unclassified	unclassified
unclassified	unclassified	unclassified
unclassified	unclassified	Eubacteriales
Candidatus Borkfalkia	Candidatus Borkfalkiaceae	Candidatus Borkfalkiales
Bacteroides	Bacteroidaceae	Bacteroidales
Veillonella	Veillonellaceae	Veillonellales
Lachnoclostridium	Lachnospiraceae	Eubacteriales
unclassified	unclassified	Eubacteriales
Blautia	Lachnospiraceae	Eubacteriales
Ligilactobacillus	Lactobacillaceae	Lactobacillales
Mesosutterella	Sutterellaceae	Burkholderiales
Bifidobacterium	Bifidobacteriaceae	Bifidobacteriales
Ruminococcus	Oscillospiraceae	Eubacteriales
Ruminococcus	Oscillospiraceae	Eubacteriales
unclassified	unclassified	unclassified
unclassified	unclassified	unclassified
Intestinimonas	unclassified	Eubacteriales
Eubacterium	Eubacteriaceae	Eubacteriales
unclassified	Clostridiaceae	Eubacteriales

Alistipes	Rikenellaceae	Bacteroidales
unclassified	unclassified	Eubacteriales
Blautia	Lachnospiraceae	Eubacteriales
unclassified	unclassified	Eubacteriales
Bacteroides	Bacteroidaceae	Bacteroidales
Prevotella	Prevotellaceae	Bacteroidales
Roseburia	Lachnospiraceae	Eubacteriales
unclassified	unclassified	Eubacteriales
Ruminococcus	Oscillospiraceae	Eubacteriales
unclassified	unclassified	Eubacteriales
unclassified	unclassified	Eubacteriales
Gemmiger	unclassified	Eubacteriales
Faecalibacillus	Erysipelotrichaceae	Erysipelotrichales
unclassified	unclassified	unclassified
Klebsiella	Enterobacteriaceae	Enterobacterales
unclassified	unclassified	unclassified
Bacteroides	Bacteroidaceae	Bacteroidales
Alistipes	Rikenellaceae	Bacteroidales
Parabacteroides	Tannerellaceae	Bacteroidales
unclassified	unclassified	Eubacteriales
unclassified	unclassified	Eubacteriales
unclassified	unclassified	Eubacteriales
Roseburia	Lachnospiraceae	Eubacteriales
Eubacterium	Eubacteriaceae	Eubacteriales
unclassified	unclassified	Eubacteriales
unclassified	unclassified	Eubacteriales
unclassified	unclassified	Bacteroidales
Akkermansia	Akkermansiaceae	Verrucomicrobiales
unclassified	Oscillospiraceae	Eubacteriales
unclassified	unclassified	Eubacteriales
unclassified	unclassified	unclassified
Streptococcus	Streptococcaceae	Lactobacillales
unclassified	unclassified	Eubacteriales
Odoribacter	Odoribacteraceae	Bacteroidales
Lachnoclostridium	Lachnospiraceae	Eubacteriales
Prevotella	Prevotellaceae	Bacteroidales
unclassified	unclassified	Eubacteriales
Prevotella	Prevotellaceae	Bacteroidales
Sutterella	Sutterellaceae	Burkholderiales
unclassified	Oscillospiraceae	Eubacteriales
Blautia	Lachnospiraceae	Eubacteriales
unclassified	unclassified	Eubacteriales
unclassified	unclassified	unclassified
Eubacterium	Eubacteriaceae	Eubacteriales
Bacteroides	Bacteroidaceae	Bacteroidales
unclassified	unclassified	unclassified
Dorea	Lachnospiraceae	Eubacteriales
Dysosmobacter	Oscillospiraceae	Eubacteriales

Anaerostipes	Lachnospiraceae	Eubacteriales
unclassified	unclassified	Eubacteriales
unclassified	unclassified	unclassified
unclassified	unclassified	Eubacteriales
unclassified	Lachnospiraceae	Eubacteriales
unclassified	unclassified	Eubacteriales
unclassified	unclassified	unclassified
Roseburia	Lachnospiraceae	Eubacteriales
Ruminococcus	Oscillospiraceae	Eubacteriales
unclassified	Lachnospiraceae	Eubacteriales
unclassified	unclassified	Eubacteriales
Enterocloster	Lachnospiraceae	Eubacteriales
unclassified	unclassified	unclassified
Ellagibacter	Eggerthellaceae	Eggerthellales
unclassified	unclassified	Eubacteriales
Butyricicoccus	Clostridiaceae	Eubacteriales
unclassified	unclassified	unclassified
unclassified	unclassified	unclassified
Bifidobacterium	Bifidobacteriaceae	Bifidobacteriales
Victivallis	Victivallaceae	Victivallales
Clostridium	Clostridiaceae	Eubacteriales
unclassified	unclassified	Eubacteriales
unclassified	unclassified	unclassified
unclassified	unclassified	Eggerthellales
Veillonella	Veillonellaceae	Veillonellales
Clostridium	Clostridiaceae	Eubacteriales
unclassified	unclassified	Eubacteriales
Clostridium	Clostridiaceae	Eubacteriales
Vescimonas	Oscillospiraceae	Eubacteriales
Coprococcus	Lachnospiraceae	Eubacteriales
unclassified	unclassified	unclassified
unclassified	unclassified	unclassified
unclassified	unclassified	unclassified
unclassified	Rikenellaceae	Bacteroidales
Hungatella	Clostridiaceae	Eubacteriales
unclassified	unclassified	unclassified
unclassified	unclassified	Bacteroidales
Parabacteroides	Tannerellaceae	Bacteroidales
unclassified	unclassified	unclassified
Butyricimonas	Odoribacteraceae	Bacteroidales
Blautia	Lachnospiraceae	Eubacteriales
unclassified	unclassified	Eubacteriales
Prevotellamassilia	Prevotellaceae	Bacteroidales
unclassified	unclassified	Eubacteriales
unclassified	unclassified	unclassified
unclassified	unclassified	Desulfovibrionales
unclassified	unclassified	Eubacteriales
Bacteroides	Bacteroidaceae	Bacteroidales

Phocaeicola	Bacteroidaceae	Bacteroidales
unclassified	unclassified	Eubacteriales
unclassified	unclassified	unclassified
unclassified	unclassified	Eubacteriales
Ruminococcus	Oscillospiraceae	Eubacteriales
unclassified	unclassified	Eubacteriales
unclassified	unclassified	Eubacteriales
unclassified	unclassified	Eubacteriales
Blautia	Lachnospiraceae	Eubacteriales
unclassified	unclassified	Eubacteriales
Clostridium	Clostridiaceae	Eubacteriales
unclassified	unclassified	unclassified
unclassified	unclassified	Eubacteriales
Blautia	Lachnospiraceae	Eubacteriales
Prevotella	Prevotellaceae	Bacteroidales
Blautia	Lachnospiraceae	Eubacteriales
Clostridium	Clostridiaceae	Eubacteriales
unclassified	unclassified	Bacteroidales
unclassified	unclassified	unclassified
unclassified	unclassified	Eubacteriales
unclassified	unclassified	unclassified
unclassified	unclassified	Eubacteriales
Bifidobacterium	Bifidobacteriaceae	Bifidobacteriales
unclassified	unclassified	unclassified
unclassified	unclassified	unclassified
unclassified	unclassified	unclassified
Lachnoclostridium	Lachnospiraceae	Eubacteriales
Enterocloster	Lachnospiraceae	Eubacteriales
unclassified	unclassified	unclassified
unclassified	unclassified	Eubacteriales
unclassified	unclassified	unclassified
unclassified	unclassified	unclassified
unclassified	unclassified	Eubacteriales
unclassified	unclassified	Eubacteriales
Prevotella	Prevotellaceae	Bacteroidales
Coprobacillus	Coprobacillaceae	Erysipelotrichales
Enterococcus	Enterococcaceae	Lactobacillales
Lachnospira	Lachnospiraceae	Eubacteriales
unclassified	unclassified	unclassified
unclassified	unclassified	unclassified
Holdemanella	Erysipelotrichaceae	Erysipelotrichales
Prevotella	Prevotellaceae	Bacteroidales
unclassified	unclassified	Eubacteriales
unclassified	unclassified	Aeromonadales
Akkermansia	Akkermansiaceae	Verrucomicrobiales
unclassified	unclassified	unclassified
unclassified	unclassified	unclassified
Coprococcus	Lachnospiraceae	Eubacteriales

Faecalibacterium	Oscillospiraceae	Eubacteriales
unclassified	unclassified	Eubacteriales
Roseburia	Lachnospiraceae	Eubacteriales
unclassified	Oscillospiraceae	Eubacteriales
Blautia	Lachnospiraceae	Eubacteriales
unclassified	unclassified	Eubacteriales
unclassified	Peptostreptococcaceae	Eubacteriales
Alistipes	Rikenellaceae	Bacteroidales
Coprobacter	Barnesiellaceae	Bacteroidales
unclassified	unclassified	Eubacteriales
unclassified	unclassified	unclassified
Clostridium	Clostridiaceae	Eubacteriales
Sutterella	Sutterellaceae	Burkholderiales
Dialister	Veillonellaceae	Veillonellales
unclassified	unclassified	Eubacteriales
unclassified	unclassified	Eubacteriales
Bifidobacterium	Bifidobacteriaceae	Bifidobacteriales
Eisenbergiella	Lachnospiraceae	Eubacteriales
Prevotella	Prevotellaceae	Bacteroidales
unclassified	unclassified	unclassified
Clostridium	Clostridiaceae	Eubacteriales
unclassified	unclassified	unclassified
Dysosmobacter	Oscillospiraceae	Eubacteriales
unclassified	unclassified	Eubacteriales
Mitsuokella	Selenomonadaceae	Selenomonadales
Parabacteroides	Tannerellaceae	Bacteroidales
unclassified	unclassified	Bacteroidales
unclassified	Oscillospiraceae	Eubacteriales
unclassified	unclassified	unclassified
Streptococcus	Streptococcaceae	Lactobacillales
unclassified	unclassified	unclassified
unclassified	unclassified	Burkholderiales
unclassified	unclassified	Eubacteriales
Akkermansia	Akkermansiaceae	Verrucomicrobiales
unclassified	unclassified	Eubacteriales
unclassified	unclassified	unclassified
unclassified	Lachnospiraceae	Eubacteriales
unclassified	unclassified	unclassified
unclassified	unclassified	Bacteroidales
unclassified	unclassified	unclassified
unclassified	Rikenellaceae	Bacteroidales
unclassified	unclassified	unclassified
unclassified	unclassified	unclassified
unclassified	unclassified	Eubacteriales
unclassified	unclassified	unclassified
Mogibacterium	Eubacteriales Family XIII. Incertae Sedis	Eubacteriales
unclassified	unclassified	unclassified
unclassified	Prevotellaceae	Bacteroidales

Eisenbergiella	Lachnospiraceae	Eubacteriales
unclassified	Prevotellaceae	Bacteroidales
Ruminococcus	Oscillospiraceae	Eubacteriales
Sutterella	Sutterellaceae	Burkholderiales
Odoribacter	Odoribacteraceae	Bacteroidales
unclassified	unclassified	unclassified
Bacteroides	Bacteroidaceae	Bacteroidales
Fusicatenibacter	Lachnospiraceae	Eubacteriales
Escherichia	Enterobacteriaceae	Enterobacterales
Anaerotignum	Lachnospiraceae	Eubacteriales
unclassified	Oscillospiraceae	Eubacteriales
unclassified	unclassified	unclassified
unclassified	unclassified	Eubacteriales
Megasphaera	Veillonellaceae	Veillonellales
Bacteroides	Bacteroidaceae	Bacteroidales
unclassified	unclassified	unclassified
Erysipelatoclostridium	Erysipelotrichaceae	Erysipelotrichales
unclassified	unclassified	Erysipelotrichales
unclassified	unclassified	Bacteroidales
Alistipes	Rikenellaceae	Bacteroidales
Methanosphaera	Methanobacteriaceae	Methanobacteriales
unclassified	unclassified	unclassified
unclassified	unclassified	Eubacteriales
unclassified	unclassified	Eubacteriales
Blautia	Lachnospiraceae	Eubacteriales
unclassified	unclassified	unclassified
unclassified	unclassified	Eubacteriales
Ruminococcus	Oscillospiraceae	Eubacteriales
unclassified	unclassified	Eubacteriales
unclassified	unclassified	Bacteroidales
Phocaeicola	Bacteroidaceae	Bacteroidales
unclassified	unclassified	Bacteroidales
unclassified	unclassified	unclassified
Roseburia	Lachnospiraceae	Eubacteriales
Phascolarctobacterium	Acidaminococcaceae	Acidaminococcales
Butyricicoccus	Clostridiaceae	Eubacteriales
unclassified	unclassified	unclassified
Anaerostipes	Lachnospiraceae	Eubacteriales
Sutterella	Sutterellaceae	Burkholderiales
Coprococcus	Lachnospiraceae	Eubacteriales
unclassified	unclassified	unclassified
unclassified	unclassified	Eubacteriales
unclassified	unclassified	Eubacteriales
unclassified	unclassified	unclassified
Paraprevotella	Prevotellaceae	Bacteroidales
Flintibacter	unclassified	Eubacteriales
unclassified	unclassified	Eubacteriales
Victivallis	Victivallaceae	Victivallales

unclassified	Bacteroidaceae	Bacteroidales
Bifidobacterium	Bifidobacteriaceae	Bifidobacteriales
unclassified	unclassified	Eubacteriales
unclassified	unclassified	Eubacteriales
Prevotella	Prevotellaceae	Bacteroidales
unclassified	unclassified	unclassified
Phocaeicola	Bacteroidaceae	Bacteroidales
Erysipelatoclostridium	Erysipelotrichaceae	Erysipelotrichales
unclassified	Oscillospiraceae	Eubacteriales
unclassified	unclassified	Eubacteriales
unclassified	unclassified	Eubacteriales
unclassified	unclassified	unclassified
unclassified	unclassified	unclassified
unclassified	unclassified	unclassified
Longicatena	Erysipelotrichaceae	Erysipelotrichales
Alistipes	Rikenellaceae	Bacteroidales
unclassified	unclassified	Eubacteriales
unclassified	unclassified	unclassified
unclassified	unclassified	unclassified
unclassified	unclassified	unclassified
unclassified	unclassified	Eubacteriales
unclassified	unclassified	unclassified
unclassified	unclassified	unclassified
Enterocloster	Lachnospiraceae	Eubacteriales
Olsenella	Atopobiaceae	Coriobacteriales
unclassified	Oscillospiraceae	Eubacteriales
Bacteroides	Bacteroidaceae	Bacteroidales
unclassified	Oscillospiraceae	Eubacteriales
Enterocloster	Lachnospiraceae	Eubacteriales
unclassified	unclassified	unclassified
Parasutterella	Sutterellaceae	Burkholderiales
Neglecta	unclassified	Eubacteriales
Ruminococcus	Oscillospiraceae	Eubacteriales
unclassified	unclassified	unclassified
Prevotella	Prevotellaceae	Bacteroidales
unclassified	unclassified	Eubacteriales
unclassified	unclassified	unclassified
Oscillibacter	Oscillospiraceae	Eubacteriales
unclassified	unclassified	Eubacteriales
Eubacterium	Eubacteriaceae	Eubacteriales
unclassified	unclassified	unclassified
Prevotella	Prevotellaceae	Bacteroidales
unclassified	unclassified	Eubacteriales
unclassified	unclassified	unclassified
unclassified	unclassified	Eubacteriales
unclassified	unclassified	Eubacteriales
unclassified	unclassified	unclassified
unclassified	unclassified	Eubacteriales

unclassified	unclassified	unclassified
Methanobrevibacter	Methanobacteriaceae	Methanobacteriales
Negativibacillus	unclassified	unclassified
Bacteroides	Bacteroidaceae	Bacteroidales
unclassified	unclassified	unclassified
Bacteroides	Bacteroidaceae	Bacteroidales
unclassified	unclassified	unclassified
Lawsonibacter	unclassified	Eubacteriales
unclassified	unclassified	Eubacteriales
unclassified	unclassified	unclassified
unclassified	unclassified	Erysipelotrichales
Bacteroides	Bacteroidaceae	Bacteroidales
unclassified	unclassified	unclassified
unclassified	unclassified	unclassified
unclassified	unclassified	unclassified
unclassified	unclassified	unclassified
unclassified	unclassified	Eubacteriales
unclassified	unclassified	Eubacteriales
Butyricimonas	Odoribacteraceae	Bacteroidales
Butyricimonas	Odoribacteraceae	Bacteroidales
unclassified	unclassified	Eubacteriales
unclassified	unclassified	unclassified
Bacteroides	Bacteroidaceae	Bacteroidales
unclassified	unclassified	Eubacteriales
unclassified	unclassified	Eubacteriales
unclassified	unclassified	Bacteroidales
unclassified	unclassified	unclassified
unclassified	unclassified	unclassified
Megasphaera	Veillonellaceae	Veillonellales
unclassified	Oscillospiraceae	Eubacteriales
unclassified	Oscillospiraceae	Eubacteriales
unclassified	unclassified	Eubacteriales
unclassified	Lachnospiraceae	Eubacteriales
unclassified	Eubacteriaceae	Eubacteriales
Faecalibacillus	Erysipelotrichaceae	Erysipelotrichales
unclassified	unclassified	Eubacteriales
unclassified	unclassified	unclassified
unclassified	unclassified	Bacteroidales
unclassified	Clostridiaceae	Eubacteriales
unclassified	unclassified	Eubacteriales
unclassified	unclassified	unclassified
unclassified	Lachnospiraceae	Eubacteriales
Prevotella	Prevotellaceae	Bacteroidales
unclassified	unclassified	Eubacteriales
unclassified	unclassified	Erysipelotrichales
Waltera	Lachnospiraceae	Eubacteriales
Alistipes	Rikenellaceae	Bacteroidales
unclassified	unclassified	Eubacteriales

unclassified	unclassified	unclassified
unclassified	unclassified	Bacteroidales
unclassified	unclassified	Eubacteriales
unclassified	unclassified	unclassified
unclassified	Oscillospiraceae	Eubacteriales
unclassified	unclassified	unclassified
unclassified	unclassified	Eubacteriales
Collinsella	Coriobacteriaceae	Coriobacteriales
unclassified	unclassified	unclassified
Allisonella	Veillonellaceae	Veillonellales
unclassified	unclassified	unclassified
unclassified	unclassified	Eubacteriales
unclassified	Oscillospiraceae	Eubacteriales
Blautia	Lachnospiraceae	Eubacteriales
unclassified	unclassified	Eubacteriales
Anaerostipes	Lachnospiraceae	Eubacteriales
unclassified	unclassified	Veillonellales
Blautia	Lachnospiraceae	Eubacteriales
unclassified	Veillonellaceae	Veillonellales
Blautia	Lachnospiraceae	Eubacteriales
Eubacterium	Eubacteriaceae	Eubacteriales
unclassified	unclassified	Eubacteriales
unclassified	unclassified	Eubacteriales
unclassified	unclassified	Eubacteriales
Bacteroides	Bacteroidaceae	Bacteroidales
unclassified	unclassified	Eubacteriales
unclassified	unclassified	unclassified
Mitsuokella	Selenomonadaceae	Selenomonadales
unclassified	unclassified	Eubacteriales
unclassified	Lachnospiraceae	Eubacteriales
Blautia	Lachnospiraceae	Eubacteriales
unclassified	unclassified	Eubacteriales
unclassified	unclassified	Eubacteriales
unclassified	unclassified	unclassified
unclassified	unclassified	unclassified
unclassified	unclassified	unclassified
unclassified	unclassified	unclassified
unclassified	unclassified	Eubacteriales
Parolsenella	Atopobiaceae	Coriobacteriales
unclassified	unclassified	Eubacteriales
unclassified	Oscillospiraceae	Eubacteriales
unclassified	Oscillospiraceae	Eubacteriales
unclassified	unclassified	Eubacteriales
Alistipes	Rikenellaceae	Bacteroidales
unclassified	unclassified	Eubacteriales
Prevotella	Prevotellaceae	Bacteroidales
Blautia	Lachnospiraceae	Eubacteriales
unclassified	unclassified	Desulfovibrionales

unclassified	unclassified	Burkholderiales
unclassified	Oscillospiraceae	Eubacteriales
unclassified	Oscillospiraceae	Eubacteriales
unclassified	unclassified	Burkholderiales
Roseburia	Lachnospiraceae	Eubacteriales
unclassified	unclassified	unclassified
unclassified	unclassified	unclassified
Clostridium	Clostridiaceae	Eubacteriales
unclassified	unclassified	Eubacteriales
unclassified	unclassified	unclassified
unclassified	unclassified	Eubacteriales
Bacteroides	Bacteroidaceae	Bacteroidales
Bacteroides	Bacteroidaceae	Bacteroidales
unclassified	unclassified	Bacteroidales
Holdemania	Erysipelotrichaceae	Erysipelotrichales
Mediterraneibacter	Lachnospiraceae	Eubacteriales
unclassified	unclassified	Eubacteriales
Pseudoflavonifractor	Oscillospiraceae	Eubacteriales
Lachnoclostridium	Lachnospiraceae	Eubacteriales
Clostridium	Clostridiaceae	Eubacteriales
Prevotella	Prevotellaceae	Bacteroidales
unclassified	Lachnospiraceae	Eubacteriales
unclassified	unclassified	Eubacteriales
unclassified	unclassified	Bacteroidales
unclassified	unclassified	unclassified
unclassified	unclassified	unclassified
Oscillibacter	Oscillospiraceae	Eubacteriales
unclassified	unclassified	Eubacteriales
unclassified	unclassified	unclassified
unclassified	unclassified	unclassified
Hungatella	Clostridiaceae	Eubacteriales
Anaeromassilibacillus	Oscillospiraceae	Eubacteriales
unclassified	unclassified	Eubacteriales
unclassified	unclassified	Eubacteriales
unclassified	unclassified	Eubacteriales
unclassified	unclassified	unclassified
unclassified	unclassified	unclassified
Parabacteroides	Tannerellaceae	Bacteroidales
Faecalibacterium	Oscillospiraceae	Eubacteriales
unclassified	unclassified	Eubacteriales
Bacteroides	Bacteroidaceae	Bacteroidales
unclassified	unclassified	unclassified
unclassified	unclassified	unclassified
Lachnoclostridium	Lachnospiraceae	Eubacteriales
unclassified	unclassified	unclassified
unclassified	unclassified	Eubacteriales
unclassified	unclassified	Bacteroidales
unclassified	unclassified	unclassified

unclassified	unclassified	unclassified
Faecalicatena	Lachnospiraceae	Eubacteriales
unclassified	Oscillospiraceae	Eubacteriales
Enterocloster	Lachnospiraceae	Eubacteriales
Megasphaera	Veillonellaceae	Veillonellales
unclassified	unclassified	Veillonellales
Intestinimonas	unclassified	Eubacteriales
Desulfovibrio	Desulfovibrionaceae	Desulfovibrionales
unclassified	unclassified	Eubacteriales
unclassified	unclassified	Eubacteriales
unclassified	unclassified	Eubacteriales
unclassified	unclassified	Eubacteriales
Clostridium	Clostridiaceae	Eubacteriales
unclassified	unclassified	Eubacteriales
unclassified	unclassified	Desulfovibrionales
unclassified	unclassified	Eubacteriales
unclassified	unclassified	Bacteroidales
unclassified	unclassified	Eubacteriales
unclassified	Sutterellaceae	Burkholderiales
unclassified	Oscillospiraceae	Eubacteriales
unclassified	unclassified	unclassified
unclassified	unclassified	unclassified
unclassified	unclassified	Eubacteriales
Blautia	Lachnospiraceae	Eubacteriales
unclassified	unclassified	unclassified
unclassified	unclassified	Eubacteriales
Eubacterium	Eubacteriaceae	Eubacteriales
Desulfovibrio	Desulfovibrionaceae	Desulfovibrionales
unclassified	unclassified	unclassified
unclassified	unclassified	Bacteroidales
Coprococcus	Lachnospiraceae	Eubacteriales
Alistipes	Rikenellaceae	Bacteroidales
Sutterella	Sutterellaceae	Burkholderiales
unclassified	unclassified	unclassified
Clostridium	Clostridiaceae	Eubacteriales
Anaerostipes	Lachnospiraceae	Eubacteriales
Blautia	Lachnospiraceae	Eubacteriales
unclassified	unclassified	Eubacteriales
Enterobacter	Enterobacteriaceae	Enterobacterales
unclassified	Oscillospiraceae	Eubacteriales
unclassified	unclassified	Eubacteriales
unclassified	unclassified	unclassified
unclassified	unclassified	Eubacteriales
unclassified	unclassified	unclassified
unclassified	unclassified	Eubacteriales
Agathobaculum	Oscillospiraceae	Eubacteriales
Merdimonas	Lachnospiraceae	Eubacteriales
unclassified	unclassified	Eubacteriales

unclassified	Oscillospiraceae	Eubacteriales
unclassified	unclassified	unclassified
Collinsella	Coriobacteriaceae	Coriobacteriales
Oscillibacter	Oscillospiraceae	Eubacteriales
Oscillibacter	Oscillospiraceae	Eubacteriales
Oxalobacter	Oxalobacteraceae	Burkholderiales
Bacteroides	Bacteroidaceae	Bacteroidales
unclassified	unclassified	unclassified
Lachnospira	Lachnospiraceae	Eubacteriales
Klebsiella	Enterobacteriaceae	Enterobacterales
Bacteroides	Bacteroidaceae	Bacteroidales
Enterocloster	Lachnospiraceae	Eubacteriales
unclassified	Prevotellaceae	Bacteroidales
Bacteroides	Bacteroidaceae	Bacteroidales
Marseilla	Prevotellaceae	Bacteroidales
unclassified	unclassified	Eubacteriales
Citrobacter	Enterobacteriaceae	Enterobacterales
unclassified	unclassified	Eubacteriales
unclassified	Rikenellaceae	Bacteroidales
Sutterella	Sutterellaceae	Burkholderiales
unclassified	unclassified	Eubacteriales
unclassified	unclassified	unclassified
unclassified	unclassified	unclassified
unclassified	unclassified	Eubacteriales
unclassified	unclassified	Eubacteriales
unclassified	Clostridiaceae	Eubacteriales
unclassified	Oscillospiraceae	Eubacteriales
Holdemanella	Erysipelotrichaceae	Erysipelotrichales
Oscillibacter	Oscillospiraceae	Eubacteriales
Clostridium	Clostridiaceae	Eubacteriales
Clostridium	Clostridiaceae	Eubacteriales
unclassified	unclassified	Eubacteriales
Collinsella	Coriobacteriaceae	Coriobacteriales
Streptococcus	Streptococcaceae	Lactobacillales
unclassified	unclassified	unclassified
unclassified	unclassified	Eubacteriales
unclassified	unclassified	Eubacteriales
Hallella	Prevotellaceae	Bacteroidales
Bacteroides	Bacteroidaceae	Bacteroidales
unclassified	unclassified	Eubacteriales
unclassified	unclassified	Eubacteriales
Mediterraneibacter	Lachnospiraceae	Eubacteriales
unclassified	Oscillospiraceae	Eubacteriales
unclassified	unclassified	unclassified
unclassified	unclassified	unclassified
unclassified	Prevotellaceae	Bacteroidales
Bacteroides	Bacteroidaceae	Bacteroidales
unclassified	Oscillospiraceae	Eubacteriales

unclassified	unclassified	Eubacteriales
Senegalimassilia	Coriobacteriaceae	Coriobacteriales
Streptococcus	Streptococcaceae	Lactobacillales
unclassified	unclassified	Bacteroidales
Alistipes	Rikenellaceae	Bacteroidales
Phascolarctobacterium	Acidaminococcaceae	Acidaminococcales
unclassified	unclassified	unclassified
Marseilla	Prevotellaceae	Bacteroidales
unclassified	Prevotellaceae	Bacteroidales
unclassified	unclassified	Bacteroidales
unclassified	Lachnospiraceae	Eubacteriales
unclassified	unclassified	unclassified
unclassified	unclassified	unclassified
Enorma	Coriobacteriaceae	Coriobacteriales
unclassified	unclassified	unclassified
Frisingicoccus	Lachnospiraceae	Eubacteriales
Coprococcus	Lachnospiraceae	Eubacteriales
unclassified	unclassified	Eubacteriales
unclassified	unclassified	Eubacteriales
unclassified	unclassified	unclassified
unclassified	unclassified	unclassified
Massilimicrobiota	Erysipelotrichaceae	Erysipelotrichales
unclassified	unclassified	Eubacteriales
unclassified	Lachnospiraceae	Eubacteriales
unclassified	unclassified	unclassified
Rothia	Micrococcaceae	Micrococcales
unclassified	unclassified	unclassified
Roseburia	Lachnospiraceae	Eubacteriales
unclassified	unclassified	Eubacteriales
unclassified	unclassified	Bacteroidales
unclassified	unclassified	Bacteroidales
Anaerotignum	Lachnospiraceae	Eubacteriales
unclassified	unclassified	unclassified
unclassified	unclassified	Bacteroidales
unclassified	unclassified	unclassified
unclassified	unclassified	unclassified
Bacteroides	Bacteroidaceae	Bacteroidales
Dorea	Lachnospiraceae	Eubacteriales
unclassified	unclassified	Eubacteriales
Dakarella	Sutterellaceae	Burkholderiales
unclassified	unclassified	Eubacteriales
unclassified	unclassified	Veillonellales
Eisenbergiella	Lachnospiraceae	Eubacteriales
Olsenella	Atopobiaceae	Coriobacteriales
unclassified	unclassified	Eubacteriales
unclassified	Lachnospiraceae	Eubacteriales
Anaerotruncus	Oscillospiraceae	Eubacteriales
unclassified	unclassified	unclassified

unclassified	unclassified	Eubacteriales
Flavonifractor	Oscillospiraceae	Eubacteriales
unclassified	unclassified	Eubacteriales
Barnesiella	Barnesiellaceae	Bacteroidales
Ruminococcus	Oscillospiraceae	Eubacteriales
unclassified	unclassified	Eubacteriales
unclassified	unclassified	Bacteroidales
unclassified	unclassified	Eubacteriales
unclassified	unclassified	Eubacteriales
unclassified	unclassified	unclassified
unclassified	unclassified	Eubacteriales
unclassified	unclassified	unclassified
Faecalibacterium	Oscillospiraceae	Eubacteriales
unclassified	unclassified	unclassified
unclassified	Oscillospiraceae	Eubacteriales
unclassified	unclassified	unclassified
unclassified	unclassified	Eubacteriales
unclassified	unclassified	unclassified
unclassified	unclassified	unclassified
Bilophila	Desulfovibrionaceae	Desulfovibrionales
Blautia	Lachnospiraceae	Eubacteriales
Turicibacter	Turicibacteraceae	Erysipelotrichales
Hydrogeniiclostidium	Oscillospiraceae	Eubacteriales
Dialister	Veillonellaceae	Veillonellales
unclassified	unclassified	unclassified
unclassified	unclassified	unclassified
unclassified	unclassified	Eubacteriales
unclassified	unclassified	Eubacteriales
unclassified	unclassified	Eubacteriales
unclassified	unclassified	unclassified
unclassified	unclassified	Victivallales
unclassified	unclassified	unclassified
Blastocystis	Blastocystidae	Opalinata
unclassified	unclassified	Bacteroidales
unclassified	unclassified	Eubacteriales
unclassified	unclassified	unclassified
Anaerotruncus	Oscillospiraceae	Eubacteriales
unclassified	unclassified	Eubacteriales
Clostridium	Clostridiaceae	Eubacteriales
unclassified	unclassified	Methanomassiliicoccales
unclassified	Lachnospiraceae	Eubacteriales
unclassified	Clostridiaceae	Eubacteriales
unclassified	unclassified	Bacteroidales
Eubacterium	Eubacteriaceae	Eubacteriales
Massilistercora	unclassified	Eubacteriales
Mediterraneibacter	Lachnospiraceae	Eubacteriales
unclassified	unclassified	unclassified
unclassified	Oscillospiraceae	Eubacteriales

unclassified	unclassified	Eubacteriales
unclassified	Oscillospiraceae	Eubacteriales
unclassified	unclassified	Eggerthellales
unclassified	unclassified	Eubacteriales
Bacteroides	Bacteroidaceae	Bacteroidales
unclassified	unclassified	Eubacteriales
unclassified	unclassified	Eubacteriales
Clostridium	Clostridiaceae	Eubacteriales
Paraprevotella	Prevotellaceae	Bacteroidales
Enterobacter	Enterobacteriaceae	Enterobacterales
unclassified	unclassified	unclassified
unclassified	unclassified	Eggerthellales
Phocea	Oscillospiraceae	Eubacteriales
unclassified	unclassified	unclassified
unclassified	unclassified	Eubacteriales
unclassified	unclassified	unclassified
unclassified	unclassified	unclassified
unclassified	unclassified	unclassified
unclassified	unclassified	Eubacteriales
Faecalibacterium	Oscillospiraceae	Eubacteriales
Clostridium	Clostridiaceae	Eubacteriales
unclassified	unclassified	unclassified
unclassified	unclassified	unclassified
Methanomassiliicoccus	Methanomassiliicoccaceae	Methanomassiliicoccales
unclassified	unclassified	unclassified
unclassified	unclassified	unclassified
unclassified	unclassified	Eubacteriales
unclassified	unclassified	unclassified
Klebsiella	Enterobacteriaceae	Enterobacterales
unclassified	unclassified	Eubacteriales
unclassified	unclassified	Eubacteriales
Lachnospira	Lachnospiraceae	Eubacteriales
unclassified	unclassified	unclassified
Bacteroides	Bacteroidaceae	Bacteroidales
unclassified	unclassified	unclassified
unclassified	Erysipelotrichaceae	Erysipelotrichales
unclassified	unclassified	Eubacteriales
unclassified	unclassified	unclassified
unclassified	unclassified	unclassified
unclassified	unclassified	unclassified
unclassified	unclassified	unclassified
unclassified	unclassified	Eubacteriales
unclassified	unclassified	unclassified
unclassified	unclassified	Eubacteriales
Bacteroides	Bacteroidaceae	Bacteroidales
unclassified	unclassified	Eubacteriales
unclassified	unclassified	Eubacteriales
Clostridioides	Peptostreptococcaceae	Eubacteriales

Bacteroides	Bacteroidaceae	Bacteroidales
unclassified	unclassified	Victivallales
unclassified	unclassified	unclassified
unclassified	unclassified	unclassified
unclassified	unclassified	unclassified
Bilophila	Desulfovibrionaceae	Desulfovibrionales
unclassified	unclassified	unclassified
Bacteroides	Bacteroidaceae	Bacteroidales
unclassified	unclassified	Eubacteriales
unclassified	Oscillospiraceae	Eubacteriales
Phocaeicola	Bacteroidaceae	Bacteroidales
unclassified	unclassified	unclassified
Lactobacillus	Lactobacillaceae	Lactobacillales
unclassified	Elusimicrobiaceae	Elusimicrobiales
unclassified	unclassified	unclassified
unclassified	unclassified	unclassified
unclassified	unclassified	Eubacteriales
unclassified	unclassified	Eubacteriales
unclassified	unclassified	unclassified
unclassified	unclassified	Eubacteriales
Lactiplantibacillus	Lactobacillaceae	Lactobacillales
unclassified	unclassified	unclassified
unclassified	unclassified	Eubacteriales
unclassified	unclassified	Eubacteriales
unclassified	unclassified	unclassified
Enterobacter	Enterobacteriaceae	Enterobacterales
unclassified	unclassified	Eubacteriales
unclassified	unclassified	Eubacteriales
unclassified	unclassified	unclassified
unclassified	unclassified	Eubacteriales
unclassified	unclassified	Bacteroidales
unclassified	Lachnospiraceae	Eubacteriales
unclassified	unclassified	unclassified
unclassified	unclassified	Eubacteriales
unclassified	unclassified	Erysipelotrichales
Alistipes	Rikenellaceae	Bacteroidales
unclassified	unclassified	unclassified
unclassified	unclassified	Eubacteriales
unclassified	unclassified	Eubacteriales
unclassified	unclassified	unclassified
unclassified	unclassified	unclassified
unclassified	unclassified	unclassified
unclassified	unclassified	Eubacteriales
unclassified	unclassified	unclassified
Lactobacillus	Lactobacillaceae	Lactobacillales
unclassified	Lachnospiraceae	Eubacteriales
unclassified	unclassified	unclassified
unclassified	unclassified	unclassified

Bacteroides	Bacteroidaceae	Bacteroidales
Eubacterium	Eubacteriaceae	Eubacteriales
unclassified	unclassified	Eubacteriales
Bacteroides	Bacteroidaceae	Bacteroidales
unclassified	unclassified	Eubacteriales
Desulfovibrio	Desulfovibrionaceae	Desulfovibrionales
Bacteroides	Bacteroidaceae	Bacteroidales
unclassified	unclassified	Eubacteriales
unclassified	unclassified	Eubacteriales
unclassified	unclassified	Eubacteriales
Veillonella	Veillonellaceae	Veillonellales
unclassified	unclassified	Eubacteriales
Coprococcus	Lachnospiraceae	Eubacteriales
unclassified	unclassified	unclassified
unclassified	Rikenellaceae	Bacteroidales
unclassified	unclassified	Acidaminococcales
unclassified	unclassified	Bacteroidales
unclassified	unclassified	unclassified
unclassified	unclassified	unclassified
Desulfovibrio	Desulfovibrionaceae	Desulfovibrionales
Fusobacterium	Fusobacteriaceae	Fusobacteriales
unclassified	unclassified	unclassified
unclassified	unclassified	unclassified
Dorea	Lachnospiraceae	Eubacteriales
Raoultella	Enterobacteriaceae	Enterobacterales
unclassified	unclassified	Eubacteriales
unclassified	unclassified	Eubacteriales
Faecalitalea	Erysipelotrichaceae	Erysipelotrichales
Bacteroides	Bacteroidaceae	Bacteroidales
unclassified	unclassified	unclassified
unclassified	Oscillospiraceae	Eubacteriales
Mediterranea	Bacteroidaceae	Bacteroidales
unclassified	unclassified	Eubacteriales
unclassified	unclassified	Burkholderiales

class	phylum	superkingdom	PHL018-0047_V3 EYCF
Bacteroidia	Bacteroidetes	Bacteria	0.121883321
Clostridia	Firmicutes	Bacteria	2.030872148
Actinomycetia	Actinobacteria	Bacteria	7.616228544
Bacteroidia	Bacteroidetes	Bacteria	0
unclassified	unclassified	unclassified	0
Coriobacteriia	Actinobacteria	Bacteria	0
Negativicutes	Firmicutes	Bacteria	0
Bacteroidia	Bacteroidetes	Bacteria	0
Bacteroidia	Bacteroidetes	Bacteria	0
Clostridia	Firmicutes	Bacteria	0
unclassified	Firmicutes	Bacteria	0
Clostridia	Firmicutes	Bacteria	0
Bacteroidia	Bacteroidetes	Bacteria	0
Actinomycetia	Actinobacteria	Bacteria	0.632164511
Bacteroidia	Bacteroidetes	Bacteria	0
Clostridia	Firmicutes	Bacteria	0
Erysipelotrichia	Firmicutes	Bacteria	0
Clostridia	Firmicutes	Bacteria	0
Betaproteobacteria	Proteobacteria	Bacteria	0
unclassified	unclassified	unclassified	0
Clostridia	Firmicutes	Bacteria	0
Clostridia	Firmicutes	Bacteria	0
Bacteroidia	Bacteroidetes	Bacteria	0
Bacteroidia	Bacteroidetes	Bacteria	0
Clostridia	Firmicutes	Bacteria	0
Bacteroidia	Bacteroidetes	Bacteria	0
Bacteroidia	Bacteroidetes	Bacteria	0
Clostridia	Firmicutes	Bacteria	0
unclassified	Firmicutes	Bacteria	0
Bacteroidia	Bacteroidetes	Bacteria	0
Clostridia	Firmicutes	Bacteria	0
Bacteroidia	Bacteroidetes	Bacteria	0
unclassified	unclassified	unclassified	0
Bacteroidia	Bacteroidetes	Bacteria	0
unclassified	unclassified	unclassified	0
Erysipelotrichia	Firmicutes	Bacteria	5.910912985
Clostridia	Firmicutes	Bacteria	0
Clostridia	Firmicutes	Bacteria	0
Bacteroidia	Bacteroidetes	Bacteria	0
Gammaproteobacteria	Proteobacteria	Bacteria	0
Bacteroidia	Bacteroidetes	Bacteria	0
Gammaproteobacteria	Proteobacteria	Bacteria	0.001688946
Gammaproteobacteria	Proteobacteria	Bacteria	0.240978237
Actinomycetia	Actinobacteria	Bacteria	0.011869077
Negativicutes	Firmicutes	Bacteria	0.001140958
Bacteroidia	Bacteroidetes	Bacteria	0

Clostridia	Firmicutes	Bacteria	0
Clostridia	Firmicutes	Bacteria	0.008138359
Bacteroidia	Bacteroidetes	Bacteria	0.005257099
Clostridia	Firmicutes	Bacteria	0.56691284
Clostridia	Firmicutes	Bacteria	0
Clostridia	Firmicutes	Bacteria	0.00454935
Bacilli	Firmicutes	Bacteria	0
Bacteroidia	Bacteroidetes	Bacteria	0
Clostridia	Firmicutes	Bacteria	0
Verrucomicrobiae	Verrucomicrobia	Bacteria	0
Bacilli	Firmicutes	Bacteria	0
Clostridia	Firmicutes	Bacteria	1.743315985
Bacilli	Firmicutes	Bacteria	0
Erysipelotrichia	Firmicutes	Bacteria	0.014641743
Bacilli	Firmicutes	Bacteria	0
Clostridia	Firmicutes	Bacteria	0
Bacilli	Firmicutes	Bacteria	0
Negativicutes	Firmicutes	Bacteria	0
Bacteroidia	Bacteroidetes	Bacteria	0
Clostridia	Firmicutes	Bacteria	0
Clostridia	Firmicutes	Bacteria	0.005982435
Clostridia	Firmicutes	Bacteria	0.010071724
Clostridia	Firmicutes	Bacteria	0
unclassified	unclassified	unclassified	0
Negativicutes	Firmicutes	Bacteria	0
Clostridia	Firmicutes	Bacteria	0
Bacilli	Firmicutes	Bacteria	0
Clostridia	Firmicutes	Bacteria	0
Gammaproteobacteria	Proteobacteria	Bacteria	0
Clostridia	Firmicutes	Bacteria	0
Clostridia	Firmicutes	Bacteria	0
Clostridia	Firmicutes	Bacteria	0
Bacteroidia	Bacteroidetes	Bacteria	0
Betaproteobacteria	Proteobacteria	Bacteria	0
Gammaproteobacteria	Proteobacteria	Bacteria	0
Bacilli	Firmicutes	Bacteria	0
unclassified	unclassified	unclassified	0
Bacilli	Firmicutes	Bacteria	0
Clostridia	Firmicutes	Bacteria	0
Spirochaetia	Spirochaetes	Bacteria	0
Clostridia	Firmicutes	Bacteria	0
Clostridia	Firmicutes	Bacteria	0
unclassified	unclassified	unclassified	0
Clostridia	Firmicutes	Bacteria	0
unclassified	unclassified	unclassified	0.028731044
Bacteroidia	Bacteroidetes	Bacteria	0
Bacilli	Firmicutes	Bacteria	0
Bacilli	Firmicutes	Bacteria	1.462561322

unclassified	Firmicutes	Bacteria	0
Erysipelotrichia	Firmicutes	Bacteria	0
unclassified	unclassified	unclassified	0
Bacteroidia	Bacteroidetes	Bacteria	0
Bacilli	Firmicutes	Bacteria	0
Bacilli	Firmicutes	Bacteria	0
Clostridia	Firmicutes	Bacteria	0
Bacteroidia	Bacteroidetes	Bacteria	0
Erysipelotrichia	Firmicutes	Bacteria	0
unclassified	unclassified	unclassified	0
Clostridia	Firmicutes	Bacteria	0
Gammaproteobacteria	Proteobacteria	Bacteria	0
unclassified	unclassified	unclassified	0
Clostridia	Firmicutes	Bacteria	0
unclassified	unclassified	unclassified	0
Bacteroidia	Bacteroidetes	Bacteria	0
Bacteroidia	Bacteroidetes	Bacteria	0
unclassified	unclassified	unclassified	0
Clostridia	Firmicutes	Bacteria	0.028241153
Clostridia	Firmicutes	Bacteria	0
unclassified	unclassified	Bacteria	0
unclassified	Firmicutes	Bacteria	0
Clostridia	Firmicutes	Bacteria	0
Clostridia	Firmicutes	Bacteria	0
Bacteroidia	Bacteroidetes	Bacteria	0
Bacteroidia	Bacteroidetes	Bacteria	0
Clostridia	Firmicutes	Bacteria	0.004389511
Clostridia	Firmicutes	Bacteria	0
Clostridia	Firmicutes	Bacteria	0
Clostridia	Firmicutes	Bacteria	0
Clostridia	Firmicutes	Bacteria	0.015591786
Bacteroidia	Bacteroidetes	Bacteria	0.003089682
unclassified	unclassified	unclassified	0
unclassified	unclassified	unclassified	0
unclassified	Firmicutes	Bacteria	0
unclassified	unclassified	Bacteria	0
unclassified	unclassified	Bacteria	0
Bacteroidia	Bacteroidetes	Bacteria	0
Coriobacteriia	Actinobacteria	Bacteria	0
unclassified	unclassified	unclassified	0
Bacteroidia	Bacteroidetes	Bacteria	0
Clostridia	Firmicutes	Bacteria	0
Actinomycetia	Actinobacteria	Bacteria	0
Clostridia	Firmicutes	Bacteria	0
Clostridia	Firmicutes	Bacteria	0
Clostridia	Firmicutes	Bacteria	0
Clostridia	Firmicutes	Bacteria	0
Bacteroidia	Bacteroidetes	Bacteria	0

Bacteroidia	Bacteroidetes	Bacteria	0
Clostridia	Firmicutes	Bacteria	0
unclassified	Actinobacteria	Bacteria	0
Clostridia	Firmicutes	Bacteria	0
Clostridia	Firmicutes	Bacteria	0
Bacteroidia	Bacteroidetes	Bacteria	0
Clostridia	Firmicutes	Bacteria	0
Bacteroidia	Bacteroidetes	Bacteria	0
Clostridia	Firmicutes	Bacteria	0
unclassified	unclassified	unclassified	0
Clostridia	Firmicutes	Bacteria	0
unclassified	unclassified	Bacteria	0
Clostridia	Firmicutes	Bacteria	0
Clostridia	Firmicutes	Bacteria	0
unclassified	Firmicutes	Bacteria	0
Clostridia	Firmicutes	Bacteria	0
unclassified	unclassified	unclassified	0
unclassified	unclassified	unclassified	0
unclassified	unclassified	unclassified	0
Clostridia	Firmicutes	Bacteria	0
Bacteroidia	Bacteroidetes	Bacteria	0
Clostridia	Firmicutes	Bacteria	0
unclassified	unclassified	Bacteria	0
Thermoplasmata	Candidatus Thermoplasmatota	Archaea	0
Coriobacteriia	Actinobacteria	Bacteria	0
unclassified	unclassified	Bacteria	0
Clostridia	Firmicutes	Bacteria	0
Clostridia	Firmicutes	Bacteria	0
Clostridia	Firmicutes	Bacteria	0
Negativicutes	Firmicutes	Bacteria	0
Clostridia	Firmicutes	Bacteria	0
Bacilli	Firmicutes	Bacteria	0
unclassified	Firmicutes	Bacteria	0
unclassified	unclassified	Bacteria	0
Clostridia	Firmicutes	Bacteria	0
Bacteroidia	Bacteroidetes	Bacteria	0
unclassified	Firmicutes	Bacteria	0
Clostridia	Firmicutes	Bacteria	0
Deltaproteobacteria	Proteobacteria	Bacteria	0
Clostridia	Firmicutes	Bacteria	0
unclassified	unclassified	Bacteria	0
unclassified	unclassified	Bacteria	0
Clostridia	Firmicutes	Bacteria	0
Deltaproteobacteria	Proteobacteria	Bacteria	0
Clostridia	Firmicutes	Bacteria	0
unclassified	Firmicutes	Bacteria	0
unclassified	unclassified	Bacteria	0
Clostridia	Firmicutes	Bacteria	0

Methanobacteria	Euryarchaeota	Archaea	0
unclassified	unclassified	Bacteria	0
Clostridia	Firmicutes	Bacteria	0
Spirochaetia	Spirochaetes	Bacteria	0
Negativicutes	Firmicutes	Bacteria	0
Betaproteobacteria	Proteobacteria	Bacteria	0
unclassified	Firmicutes	Bacteria	0
unclassified	unclassified	unclassified	0
Bacteroidia	Bacteroidetes	Bacteria	0.005535507
Clostridia	Firmicutes	Bacteria	0
Clostridia	Firmicutes	Bacteria	0
Clostridia	Firmicutes	Bacteria	0
Clostridia	Firmicutes	Bacteria	0
Betaproteobacteria	Proteobacteria	Bacteria	0
Betaproteobacteria	Proteobacteria	Bacteria	0
Gammaproteobacteria	Proteobacteria	Bacteria	0
Gammaproteobacteria	Proteobacteria	Bacteria	0
Gammaproteobacteria	Proteobacteria	Bacteria	0
Gammaproteobacteria	Proteobacteria	Bacteria	0
Actinomycetia	Actinobacteria	Bacteria	0
Actinomycetia	Actinobacteria	Bacteria	0
Gammaproteobacteria	Proteobacteria	Bacteria	0
Bacteroidia	Bacteroidetes	Bacteria	0
Clostridia	Firmicutes	Bacteria	0
Epsilonproteobacteria	Proteobacteria	Bacteria	0
Alphaproteobacteria	Proteobacteria	Bacteria	0
Coriobacteriia	Actinobacteria	Bacteria	0.001310985
Bacteroidia	Bacteroidetes	Bacteria	0
Actinomycetia	Actinobacteria	Bacteria	0
Clostridia	Firmicutes	Bacteria	0
Clostridia	Firmicutes	Bacteria	0
Betaproteobacteria	Proteobacteria	Bacteria	0
Bacteroidia	Bacteroidetes	Bacteria	0
Clostridia	Firmicutes	Bacteria	0
Clostridia	Firmicutes	Bacteria	0.001220569
Negativicutes	Firmicutes	Bacteria	0
Alphaproteobacteria	Proteobacteria	Bacteria	0
Flavobacteriia	Bacteroidetes	Bacteria	0
Gammaproteobacteria	Proteobacteria	Bacteria	0
Gammaproteobacteria	Proteobacteria	Bacteria	0
Gammaproteobacteria	Proteobacteria	Bacteria	0
Gammaproteobacteria	Proteobacteria	Bacteria	0
Gammaproteobacteria	Proteobacteria	Bacteria	0
Gammaproteobacteria	Proteobacteria	Bacteria	0
Synergistia	Synergistetes	Bacteria	0
Clostridia	Firmicutes	Bacteria	0.141777998
Erysipelotrichia	Firmicutes	Bacteria	0
Clostridia	Firmicutes	Bacteria	0.009567916

Clostridia	Firmicutes	Bacteria	0
Coriobacteriia	Actinobacteria	Bacteria	0
Actinomycetia	Actinobacteria	Bacteria	0
Actinomycetia	Actinobacteria	Bacteria	0
Actinomycetia	Actinobacteria	Bacteria	0
Actinomycetia	Actinobacteria	Bacteria	0
Gammaproteobacteria	Proteobacteria	Bacteria	0
Gammaproteobacteria	Proteobacteria	Bacteria	0
Bacteroidia	Bacteroidetes	Bacteria	0
Gammaproteobacteria	Proteobacteria	Bacteria	0
Gammaproteobacteria	Proteobacteria	Bacteria	0
Bacilli	Firmicutes	Bacteria	0
Bacilli	Firmicutes	Bacteria	0
Bacilli	Firmicutes	Bacteria	0
Bacilli	Firmicutes	Bacteria	0
Bacilli	Firmicutes	Bacteria	0
Erysipelotrichia	Firmicutes	Bacteria	0
Gammaproteobacteria	Proteobacteria	Bacteria	0
Gammaproteobacteria	Proteobacteria	Bacteria	0.001946101
Clostridia	Firmicutes	Bacteria	0.015611
Clostridia	Firmicutes	Bacteria	0.005741396
Flavobacteriia	Bacteroidetes	Bacteria	0
Bacteroidia	Bacteroidetes	Bacteria	0
Actinomycetia	Actinobacteria	Bacteria	0
Bacilli	Firmicutes	Bacteria	0.002073908
Gammaproteobacteria	Proteobacteria	Bacteria	0
Gammaproteobacteria	Proteobacteria	Bacteria	0
Gammaproteobacteria	Proteobacteria	Bacteria	0
Gammaproteobacteria	Proteobacteria	Bacteria	0
Bacilli	Firmicutes	Bacteria	0
Clostridia	Firmicutes	Bacteria	0.010170329
Bacilli	Firmicutes	Bacteria	0
Bacilli	Firmicutes	Bacteria	0.00429487
Bacilli	Firmicutes	Bacteria	0
Bacilli	Firmicutes	Bacteria	0
Negativicutes	Firmicutes	Bacteria	0
Negativicutes	Firmicutes	Bacteria	0
Gammaproteobacteria	Proteobacteria	Bacteria	0
Actinomycetia	Actinobacteria	Bacteria	0
Betaproteobacteria	Proteobacteria	Bacteria	0
Gammaproteobacteria	Proteobacteria	Bacteria	0
Betaproteobacteria	Proteobacteria	Bacteria	0.007237311
Bacteroidia	Bacteroidetes	Bacteria	0
Bacteroidia	Bacteroidetes	Bacteria	0
Bacteroidia	Bacteroidetes	Bacteria	0
Clostridia	Firmicutes	Bacteria	0
Gammaproteobacteria	Proteobacteria	Bacteria	0
Gammaproteobacteria	Proteobacteria	Bacteria	0

Gammaproteobacteria	Proteobacteria	Bacteria	0
Clostridia	Firmicutes	Bacteria	0
Actinomycetia	Actinobacteria	Bacteria	0
Negativicutes	Firmicutes	Bacteria	0
Clostridia	Firmicutes	Bacteria	0
Gammaproteobacteria	Proteobacteria	Bacteria	0
Gammaproteobacteria	Proteobacteria	Bacteria	0
Alphaproteobacteria	Proteobacteria	Bacteria	0
Bacilli	Firmicutes	Bacteria	0
Bacilli	Firmicutes	Bacteria	0
Bacilli	Firmicutes	Bacteria	0
Gammaproteobacteria	Proteobacteria	Bacteria	0
Gammaproteobacteria	Proteobacteria	Bacteria	0
Bacilli	Firmicutes	Bacteria	0.000814336
Bacilli	Firmicutes	Bacteria	0.001568298
Clostridia	Firmicutes	Bacteria	0
Bacilli	Firmicutes	Bacteria	0.002042211
Bacilli	Firmicutes	Bacteria	0
Negativicutes	Firmicutes	Bacteria	0
Gammaproteobacteria	Proteobacteria	Bacteria	0
Gammaproteobacteria	Proteobacteria	Bacteria	0
Clostridia	Firmicutes	Bacteria	0.005300161
Gammaproteobacteria	Proteobacteria	Bacteria	0
Clostridia	Firmicutes	Bacteria	0
Clostridia	Firmicutes	Bacteria	0
Bacilli	Firmicutes	Bacteria	0
Clostridia	Firmicutes	Bacteria	0
Clostridia	Firmicutes	Bacteria	0
Gammaproteobacteria	Proteobacteria	Bacteria	0
Clostridia	Firmicutes	Bacteria	0
Clostridia	Firmicutes	Bacteria	0
Gammaproteobacteria	Proteobacteria	Bacteria	0
Gammaproteobacteria	Proteobacteria	Bacteria	0
Actinomycetia	Actinobacteria	Bacteria	0
Bacilli	Firmicutes	Bacteria	0
Gammaproteobacteria	Proteobacteria	Bacteria	0
Clostridia	Firmicutes	Bacteria	0
Gammaproteobacteria	Proteobacteria	Bacteria	0.042849882
Negativicutes	Firmicutes	Bacteria	0
Clostridia	Firmicutes	Bacteria	0
Bacilli	Firmicutes	Bacteria	0
Clostridia	Firmicutes	Bacteria	0
Clostridia	Firmicutes	Bacteria	0
Clostridia	Firmicutes	Bacteria	0
Gammaproteobacteria	Proteobacteria	Bacteria	0
Clostridia	Firmicutes	Bacteria	0
unclassified	Firmicutes	Bacteria	0
Erysipelotrichia	Firmicutes	Bacteria	0

Clostridia	Firmicutes	Bacteria	0.212464784
Bacteroidia	Bacteroidetes	Bacteria	0
Actinomycetia	Actinobacteria	Bacteria	0
Gammaproteobacteria	Proteobacteria	Bacteria	0
Actinomycetia	Actinobacteria	Bacteria	0.002671254
Clostridia	Firmicutes	Bacteria	0
Gammaproteobacteria	Proteobacteria	Bacteria	0
Actinomycetia	Actinobacteria	Bacteria	0.007488701
Clostridia	Firmicutes	Bacteria	0
Actinomycetia	Actinobacteria	Bacteria	0.792226137
Clostridia	Firmicutes	Bacteria	0
Gammaproteobacteria	Proteobacteria	Bacteria	0
Bacilli	Firmicutes	Bacteria	0.427182811
Erysipelotrichia	Firmicutes	Bacteria	0
Clostridia	Firmicutes	Bacteria	0
Saccharomycetes	Ascomycota	Eukaryota	0
Gammaproteobacteria	Proteobacteria	Bacteria	0
Clostridia	Firmicutes	Bacteria	0
Clostridia	Firmicutes	Bacteria	0
Clostridia	Firmicutes	Bacteria	0
Clostridia	Firmicutes	Bacteria	0
Clostridia	Firmicutes	Bacteria	0
Gammaproteobacteria	Proteobacteria	Bacteria	0
Clostridia	Firmicutes	Bacteria	0
Gammaproteobacteria	Proteobacteria	Bacteria	0
Negativicutes	Firmicutes	Bacteria	0
Bacilli	Firmicutes	Bacteria	0
unclassified	unclassified	unclassified	0
Clostridia	Firmicutes	Bacteria	0
Clostridia	Firmicutes	Bacteria	0
Coriobacteriia	Actinobacteria	Bacteria	0
Erysipelotrichia	Firmicutes	Bacteria	0
Clostridia	Firmicutes	Bacteria	0
Clostridia	Firmicutes	Bacteria	0.002179431
Negativicutes	Firmicutes	Bacteria	0
unclassified	unclassified	unclassified	0
Clostridia	Firmicutes	Bacteria	0
Erysipelotrichia	Firmicutes	Bacteria	0
Bacteroidia	Bacteroidetes	Bacteria	0
Clostridia	Firmicutes	Bacteria	0
Clostridia	Firmicutes	Bacteria	0
Clostridia	Firmicutes	Bacteria	0
unclassified	unclassified	unclassified	0
Erysipelotrichia	Firmicutes	Bacteria	0.004603075
Clostridia	Firmicutes	Bacteria	0
Clostridia	Firmicutes	Bacteria	0.000522537
Clostridia	Firmicutes	Bacteria	0
Clostridia	Firmicutes	Bacteria	0

Clostridia	Firmicutes	Bacteria	0
Bacilli	Firmicutes	Bacteria	0
Clostridia	Firmicutes	Bacteria	0
Bacilli	Firmicutes	Bacteria	0
Erysipelotrichia	Firmicutes	Bacteria	0
Clostridia	Firmicutes	Bacteria	0.005596292
Clostridia	Firmicutes	Bacteria	0
Actinomycetia	Actinobacteria	Bacteria	0
unclassified	Firmicutes	Bacteria	0
Bacilli	Firmicutes	Bacteria	0
Actinomycetia	Actinobacteria	Bacteria	0.00594986
Verrucomicrobiae	Verrucomicrobia	Bacteria	0
Clostridia	Firmicutes	Bacteria	0
Actinomycetia	Actinobacteria	Bacteria	0
Coriobacteriia	Actinobacteria	Bacteria	0.002068208
Clostridia	Firmicutes	Bacteria	0
Bacteroidia	Bacteroidetes	Bacteria	0
Clostridia	Firmicutes	Bacteria	0
Clostridia	Firmicutes	Bacteria	0
Bacteroidia	Bacteroidetes	Bacteria	0
Actinomycetia	Actinobacteria	Bacteria	0
Coriobacteriia	Actinobacteria	Bacteria	0
Clostridia	Firmicutes	Bacteria	0
Clostridia	Firmicutes	Bacteria	0
Clostridia	Firmicutes	Bacteria	0.001898916
Erysipelotrichia	Firmicutes	Bacteria	0
Clostridia	Firmicutes	Bacteria	0
Clostridia	Firmicutes	Bacteria	0
Clostridia	Firmicutes	Bacteria	0
Clostridia	Firmicutes	Bacteria	0.064417103
Clostridia	Firmicutes	Bacteria	0
Gammaproteobacteria	Proteobacteria	Bacteria	0
Clostridia	Firmicutes	Bacteria	0
Clostridia	Firmicutes	Bacteria	0
Clostridia	Firmicutes	Bacteria	0
Clostridia	Firmicutes	Bacteria	0
Coriobacteriia	Actinobacteria	Bacteria	0
Gammaproteobacteria	Proteobacteria	Bacteria	0
Coriobacteriia	Actinobacteria	Bacteria	0.002999228
Coriobacteriia	Actinobacteria	Bacteria	0
Coriobacteriia	Actinobacteria	Bacteria	0.012183109
Clostridia	Firmicutes	Bacteria	0.199262422
Gammaproteobacteria	Proteobacteria	Bacteria	0
Clostridia	Firmicutes	Bacteria	0
Clostridia	Firmicutes	Bacteria	0
Clostridia	Firmicutes	Bacteria	0
Tissierellia	Firmicutes	Bacteria	0
Clostridia	Firmicutes	Bacteria	0

Negativicutes	Firmicutes	Bacteria	0
Bacteroidia	Bacteroidetes	Bacteria	0
Bacilli	Firmicutes	Bacteria	0.009266149
Clostridia	Firmicutes	Bacteria	0
Clostridia	Firmicutes	Bacteria	0
Erysipelotrichia	Firmicutes	Bacteria	0
Clostridia	Firmicutes	Bacteria	0
Gammaproteobacteria	Proteobacteria	Bacteria	0
Clostridia	Firmicutes	Bacteria	0.043482114
Clostridia	Firmicutes	Bacteria	0
Deltaproteobacteria	Proteobacteria	Bacteria	0
Clostridia	Firmicutes	Bacteria	0
Erysipelotrichia	Firmicutes	Bacteria	0
Actinomycetia	Actinobacteria	Bacteria	0
Clostridia	Firmicutes	Bacteria	0
Clostridia	Firmicutes	Bacteria	0.020577566
Negativicutes	Firmicutes	Bacteria	0.003197395
Clostridia	Firmicutes	Bacteria	0
Clostridia	Firmicutes	Bacteria	0
Coriobacteriia	Actinobacteria	Bacteria	0
Clostridia	Firmicutes	Bacteria	0
Clostridia	Firmicutes	Bacteria	0
Clostridia	Firmicutes	Bacteria	0
Coriobacteriia	Actinobacteria	Bacteria	0
Clostridia	Firmicutes	Bacteria	0.011156227
Clostridia	Firmicutes	Bacteria	0
Coriobacteriia	Actinobacteria	Bacteria	0.001623648
Coriobacteriia	Actinobacteria	Bacteria	0
Erysipelotrichia	Firmicutes	Bacteria	0
Clostridia	Firmicutes	Bacteria	0
Clostridia	Firmicutes	Bacteria	0.01502315
Bacilli	Firmicutes	Bacteria	0.003681809
Coriobacteriia	Actinobacteria	Bacteria	0.016651506
Gammaproteobacteria	Proteobacteria	Bacteria	0
Bacilli	Firmicutes	Bacteria	0
Clostridia	Firmicutes	Bacteria	0
Bacilli	Firmicutes	Bacteria	0
Clostridia	Firmicutes	Bacteria	0.047505132
Clostridia	Firmicutes	Bacteria	0
Clostridia	Firmicutes	Bacteria	0.000506613
Deltaproteobacteria	Proteobacteria	Bacteria	0
Clostridia	Firmicutes	Bacteria	0
Clostridia	Firmicutes	Bacteria	0.001853318
Clostridia	Firmicutes	Bacteria	0
Clostridia	Firmicutes	Bacteria	0
Bacilli	Firmicutes	Bacteria	0
Erysipelotrichia	Firmicutes	Bacteria	0
Clostridia	Firmicutes	Bacteria	0

Clostridia	Firmicutes	Bacteria	0
Bacilli	Firmicutes	Bacteria	0.13762537
Actinomycetia	Actinobacteria	Bacteria	0
Bacteroidia	Bacteroidetes	Bacteria	0
Coriobacteriia	Actinobacteria	Bacteria	0
Clostridia	Firmicutes	Bacteria	0
Clostridia	Firmicutes	Bacteria	0
Gammaproteobacteria	Proteobacteria	Bacteria	0
unclassified	unclassified	Bacteria	0
Erysipelotrichia	Firmicutes	Bacteria	0
Erysipelotrichia	Firmicutes	Bacteria	0
Bacilli	Firmicutes	Bacteria	0.002803714
Clostridia	Firmicutes	Bacteria	0
Clostridia	Firmicutes	Bacteria	0
Coriobacteriia	Actinobacteria	Bacteria	0
Clostridia	Firmicutes	Bacteria	0
Bacilli	Firmicutes	Bacteria	0.028032309
Clostridia	Firmicutes	Bacteria	0.00369584
Clostridia	Firmicutes	Bacteria	0
Clostridia	Firmicutes	Bacteria	0.001846066
unclassified	unclassified	Bacteria	0.02145544
Bacilli	Firmicutes	Bacteria	0
Actinomycetia	Actinobacteria	Bacteria	0
Clostridia	Firmicutes	Bacteria	0
Bacilli	Firmicutes	Bacteria	0
Clostridia	Firmicutes	Bacteria	0
Coriobacteriia	Actinobacteria	Bacteria	0.033661225
Coriobacteriia	Actinobacteria	Bacteria	0.028324926
Gammaproteobacteria	Proteobacteria	Bacteria	0
Clostridia	Firmicutes	Bacteria	0
Clostridia	Firmicutes	Bacteria	0
Clostridia	Firmicutes	Bacteria	0
Clostridia	Firmicutes	Bacteria	0
Coriobacteriia	Actinobacteria	Bacteria	0
Negativicutes	Firmicutes	Bacteria	0
Clostridia	Firmicutes	Bacteria	0.643916289
Clostridia	Firmicutes	Bacteria	0
Clostridia	Firmicutes	Bacteria	0
Gammaproteobacteria	Proteobacteria	Bacteria	0
Clostridia	Firmicutes	Bacteria	0
Clostridia	Firmicutes	Bacteria	0
Clostridia	Firmicutes	Bacteria	0
Clostridia	Firmicutes	Bacteria	0.001079201
Bacilli	Firmicutes	Bacteria	0.001389264
Clostridia	Firmicutes	Bacteria	0
Clostridia	Firmicutes	Bacteria	0
Erysipelotrichia	Firmicutes	Bacteria	0
Coriobacteriia	Actinobacteria	Bacteria	0

Erysipelotrichia	Firmicutes	Bacteria	0
Clostridia	Firmicutes	Bacteria	0.043391514
Coriobacteriia	Actinobacteria	Bacteria	0
Clostridia	Firmicutes	Bacteria	0.103068052
Clostridia	Firmicutes	Bacteria	0
Clostridia	Firmicutes	Bacteria	0
Bacteroidia	Bacteroidetes	Bacteria	0
unclassified	unclassified	unclassified	0
Bacteroidia	Bacteroidetes	Bacteria	0
Clostridia	Firmicutes	Bacteria	0
Clostridia	Firmicutes	Bacteria	0
Clostridia	Firmicutes	Bacteria	0.002207831
Clostridia	Firmicutes	Bacteria	0
unclassified	Firmicutes	Bacteria	0
Bacteroidia	Bacteroidetes	Bacteria	0.024347316
Clostridia	Firmicutes	Bacteria	0.442468306
Clostridia	Firmicutes	Bacteria	0
unclassified	unclassified	unclassified	0
unclassified	unclassified	unclassified	0
Clostridia	Firmicutes	Bacteria	0.001792412
Clostridia	Firmicutes	Bacteria	0.000634721
Clostridia	Firmicutes	Bacteria	0
unclassified	unclassified	unclassified	0
Clostridia	Firmicutes	Bacteria	0.247609519
Bacteroidia	Bacteroidetes	Bacteria	0
Clostridia	Firmicutes	Bacteria	0
Clostridia	Firmicutes	Bacteria	0.002683116
Erysipelotrichia	Firmicutes	Bacteria	0
Clostridia	Firmicutes	Bacteria	0
unclassified	Proteobacteria	Bacteria	0
Clostridia	Firmicutes	Bacteria	0
Clostridia	Firmicutes	Bacteria	0
Bacteroidia	Bacteroidetes	Bacteria	0
unclassified	unclassified	unclassified	0
Clostridia	Firmicutes	Bacteria	0
Bacteroidia	Bacteroidetes	Bacteria	0
Clostridia	Firmicutes	Bacteria	0.002991428
unclassified	unclassified	Bacteria	0
Bacteroidia	Bacteroidetes	Bacteria	0.003551999
Bacteroidia	Bacteroidetes	Bacteria	0
Clostridia	Firmicutes	Bacteria	0.16231731
Clostridia	Firmicutes	Bacteria	0
Actinomycetia	Actinobacteria	Bacteria	8.093218493
Bacteroidia	Bacteroidetes	Bacteria	0
unclassified	unclassified	Bacteria	0
Clostridia	Firmicutes	Bacteria	0
Clostridia	Firmicutes	Bacteria	0
Bacteroidia	Bacteroidetes	Bacteria	0.000925855

Clostridia	Firmicutes	Bacteria	0
unclassified	unclassified	unclassified	0
Erysipelotrichia	Firmicutes	Bacteria	0.013756822
Clostridia	Firmicutes	Bacteria	0
Erysipelotrichia	Firmicutes	Bacteria	0
Clostridia	Firmicutes	Bacteria	0.003670629
Bacteroidia	Bacteroidetes	Bacteria	0
Bacteroidia	Bacteroidetes	Bacteria	0
Clostridia	Firmicutes	Bacteria	0
Bacteroidia	Bacteroidetes	Bacteria	0
Bacteroidia	Bacteroidetes	Bacteria	0.001307106
Coriobacteriia	Actinobacteria	Bacteria	1.468938081
Bacteroidia	Bacteroidetes	Bacteria	0
Negativicutes	Firmicutes	Bacteria	0.02774341
Coriobacteriia	Actinobacteria	Bacteria	0.134141685
Clostridia	Firmicutes	Bacteria	0
Bacteroidia	Bacteroidetes	Bacteria	0
unclassified	Proteobacteria	Bacteria	0
Clostridia	Firmicutes	Bacteria	0
Clostridia	Firmicutes	Bacteria	0
Clostridia	Firmicutes	Bacteria	0.00310405
Actinomycetia	Actinobacteria	Bacteria	0.030398326
Actinomycetia	Actinobacteria	Bacteria	13.58102237
unclassified	Firmicutes	Bacteria	0
Clostridia	Firmicutes	Bacteria	0
Erysipelotrichia	Firmicutes	Bacteria	0.011377549
Verrucomicrobiae	Verrucomicrobia	Bacteria	0
Betaproteobacteria	Proteobacteria	Bacteria	0
Bacteroidia	Bacteroidetes	Bacteria	0.001249608
Coriobacteriia	Actinobacteria	Bacteria	0
Clostridia	Firmicutes	Bacteria	0.003423687
Clostridia	Firmicutes	Bacteria	0.162554418
Clostridia	Firmicutes	Bacteria	0
Bacteroidia	Bacteroidetes	Bacteria	0.001876798
Clostridia	Firmicutes	Bacteria	0.001772043
Clostridia	Firmicutes	Bacteria	0.612118888
Bacteroidia	Bacteroidetes	Bacteria	0
unclassified	Firmicutes	Bacteria	0
Negativicutes	Firmicutes	Bacteria	0.017094397
Clostridia	Firmicutes	Bacteria	0
Clostridia	Firmicutes	Bacteria	0.007324846
Clostridia	Firmicutes	Bacteria	0
Clostridia	Firmicutes	Bacteria	0
Clostridia	Firmicutes	Bacteria	0
Bacteroidia	Bacteroidetes	Bacteria	0.004164713
Clostridia	Firmicutes	Bacteria	0
unclassified	Firmicutes	Bacteria	0
Negativicutes	Firmicutes	Bacteria	0.374829919

Clostridia	Firmicutes	Bacteria	0
unclassified	unclassified	unclassified	0
Betaproteobacteria	Proteobacteria	Bacteria	0
Clostridia	Firmicutes	Bacteria	0.005324351
Clostridia	Firmicutes	Bacteria	0
Clostridia	Firmicutes	Bacteria	0
Clostridia	Firmicutes	Bacteria	0
Bacteroidia	Bacteroidetes	Bacteria	0.010524566
Clostridia	Firmicutes	Bacteria	0
Clostridia	Firmicutes	Bacteria	0
Clostridia	Firmicutes	Bacteria	0.003294399
Negativicutes	Firmicutes	Bacteria	0
Deltaproteobacteria	Proteobacteria	Bacteria	0
Clostridia	Firmicutes	Bacteria	0.373328016
Clostridia	Firmicutes	Bacteria	0
Clostridia	Firmicutes	Bacteria	0
Clostridia	Firmicutes	Bacteria	0
Clostridia	Firmicutes	Bacteria	0
Clostridia	Firmicutes	Bacteria	0
Clostridia	Firmicutes	Bacteria	0
Bacteroidia	Bacteroidetes	Bacteria	0
unclassified	Firmicutes	Bacteria	0
Clostridia	Firmicutes	Bacteria	0
Negativicutes	Firmicutes	Bacteria	0
unclassified	Firmicutes	Bacteria	0
unclassified	unclassified	Bacteria	0
Clostridia	Firmicutes	Bacteria	0
Clostridia	Firmicutes	Bacteria	0
Clostridia	Firmicutes	Bacteria	0
Clostridia	Firmicutes	Bacteria	0
Clostridia	Firmicutes	Bacteria	0
unclassified	Firmicutes	Bacteria	0
Clostridia	Firmicutes	Bacteria	0
Clostridia	Firmicutes	Bacteria	0
Clostridia	Firmicutes	Bacteria	0.001921
Clostridia	Firmicutes	Bacteria	0
Clostridia	Firmicutes	Bacteria	0
Clostridia	Firmicutes	Bacteria	0.003532437
unclassified	unclassified	unclassified	0
Clostridia	Firmicutes	Bacteria	0
Bacteroidia	Bacteroidetes	Bacteria	0
Clostridia	Firmicutes	Bacteria	0
Clostridia	Firmicutes	Bacteria	0
Clostridia	Firmicutes	Bacteria	0.001805482
Coriobacteriia	Actinobacteria	Bacteria	0
Clostridia	Firmicutes	Bacteria	0
unclassified	unclassified	unclassified	0
Clostridia	Firmicutes	Bacteria	0

Clostridia	Firmicutes	Bacteria	0
unclassified	Firmicutes	Bacteria	0
Clostridia	Firmicutes	Bacteria	0
Clostridia	Firmicutes	Bacteria	0
Clostridia	Firmicutes	Bacteria	0
Clostridia	Firmicutes	Bacteria	0
unclassified	unclassified	unclassified	0
Clostridia	Firmicutes	Bacteria	0
Erysipelotrichia	Firmicutes	Bacteria	0.011282111
Clostridia	Firmicutes	Bacteria	0
unclassified	unclassified	unclassified	0
Clostridia	Firmicutes	Bacteria	0
Bacteroidia	Bacteroidetes	Bacteria	0
Bacteroidia	Bacteroidetes	Bacteria	0
Clostridia	Firmicutes	Bacteria	2.899949319
Clostridia	Firmicutes	Bacteria	0
Bacteroidia	Bacteroidetes	Bacteria	0
Clostridia	Firmicutes	Bacteria	0
Clostridia	Firmicutes	Bacteria	0
unclassified	Firmicutes	Bacteria	0
unclassified	unclassified	unclassified	0.027644947
unclassified	Firmicutes	Bacteria	0
Clostridia	Firmicutes	Bacteria	0
Clostridia	Firmicutes	Bacteria	0
Gammaproteobacteria	Proteobacteria	Bacteria	0.012965909
unclassified	unclassified	unclassified	3.727093195
Clostridia	Firmicutes	Bacteria	0
Clostridia	Firmicutes	Bacteria	0
Bacteroidia	Bacteroidetes	Bacteria	0
unclassified	unclassified	unclassified	0
unclassified	Firmicutes	Bacteria	0
Clostridia	Firmicutes	Bacteria	0.001359321
Clostridia	Firmicutes	Bacteria	0
Bacteroidia	Bacteroidetes	Bacteria	0.001127265
Negativicutes	Firmicutes	Bacteria	0.006368148
Clostridia	Firmicutes	Bacteria	0
Clostridia	Firmicutes	Bacteria	0
Clostridia	Firmicutes	Bacteria	2.862777612
Bacilli	Firmicutes	Bacteria	0
Betaproteobacteria	Proteobacteria	Bacteria	0
Actinomycetia	Actinobacteria	Bacteria	0
Clostridia	Firmicutes	Bacteria	0.001276187
Clostridia	Firmicutes	Bacteria	5.460129436
Bacteroidia	Bacteroidetes	Bacteria	0
unclassified	Firmicutes	Bacteria	0
Clostridia	Firmicutes	Bacteria	0
Clostridia	Firmicutes	Bacteria	0
Clostridia	Firmicutes	Bacteria	0.124840528

Bacteroidia	Bacteroidetes	Bacteria	0.001899872
Clostridia	Firmicutes	Bacteria	0
Clostridia	Firmicutes	Bacteria	0
Clostridia	Firmicutes	Bacteria	0
Bacteroidia	Bacteroidetes	Bacteria	0
Bacteroidia	Bacteroidetes	Bacteria	0
Clostridia	Firmicutes	Bacteria	0
Clostridia	Firmicutes	Bacteria	0
Clostridia	Firmicutes	Bacteria	0
Clostridia	Firmicutes	Bacteria	0
Clostridia	Firmicutes	Bacteria	0
Clostridia	Firmicutes	Bacteria	2.327145982
Erysipelotrichia	Firmicutes	Bacteria	0.240993222
unclassified	unclassified	unclassified	0
Gammaproteobacteria	Proteobacteria	Bacteria	0.001458379
unclassified	unclassified	unclassified	0
Bacteroidia	Bacteroidetes	Bacteria	0
Bacteroidia	Bacteroidetes	Bacteria	0
Bacteroidia	Bacteroidetes	Bacteria	0
Clostridia	Firmicutes	Bacteria	0.003071014
Clostridia	Firmicutes	Bacteria	0.131756546
Clostridia	Firmicutes	Bacteria	0
Clostridia	Firmicutes	Bacteria	0.390489754
Clostridia	Firmicutes	Bacteria	0
Clostridia	Firmicutes	Bacteria	0
Clostridia	Firmicutes	Bacteria	0
Bacteroidia	Bacteroidetes	Bacteria	0
Verrucomicrobiae	Verrucomicrobia	Bacteria	0
Clostridia	Firmicutes	Bacteria	0
Clostridia	Firmicutes	Bacteria	0
unclassified	unclassified	unclassified	0
Bacilli	Firmicutes	Bacteria	0.015770425
Clostridia	Firmicutes	Bacteria	0
Bacteroidia	Bacteroidetes	Bacteria	0.187511449
Clostridia	Firmicutes	Bacteria	0
Bacteroidia	Bacteroidetes	Bacteria	0.002368166
Clostridia	Firmicutes	Bacteria	0
Bacteroidia	Bacteroidetes	Bacteria	0
Betaproteobacteria	Proteobacteria	Bacteria	0
Clostridia	Firmicutes	Bacteria	0
Clostridia	Firmicutes	Bacteria	0
Clostridia	Firmicutes	Bacteria	0
unclassified	Firmicutes	Bacteria	0
Clostridia	Firmicutes	Bacteria	0.029910371
Bacteroidia	Bacteroidetes	Bacteria	0.004423631
unclassified	unclassified	unclassified	0
Clostridia	Firmicutes	Bacteria	0
Clostridia	Firmicutes	Bacteria	0

Clostridia	Firmicutes	Bacteria	0
Clostridia	Firmicutes	Bacteria	0.003052252
unclassified	unclassified	unclassified	0
Clostridia	Firmicutes	Bacteria	0
Clostridia	Firmicutes	Bacteria	0
Clostridia	Firmicutes	Bacteria	0
unclassified	unclassified	unclassified	0
Clostridia	Firmicutes	Bacteria	0
Clostridia	Firmicutes	Bacteria	0
Clostridia	Firmicutes	Bacteria	0
Clostridia	Firmicutes	Bacteria	0
Clostridia	Firmicutes	Bacteria	0
Bacteroidia	Bacteroidetes	Bacteria	0
Coriobacteriia	Actinobacteria	Bacteria	0
Clostridia	Firmicutes	Bacteria	0
Clostridia	Firmicutes	Bacteria	0
unclassified	Firmicutes	Bacteria	0
unclassified	unclassified	unclassified	0
Actinomycetia	Actinobacteria	Bacteria	0.012888361
Lentisphaeria	Lentisphaerae	Bacteria	0
Clostridia	Firmicutes	Bacteria	0
Clostridia	Firmicutes	Bacteria	0
unclassified	unclassified	unclassified	0
Coriobacteriia	Actinobacteria	Bacteria	0
Negativicutes	Firmicutes	Bacteria	0
Clostridia	Firmicutes	Bacteria	0.043054256
Clostridia	Firmicutes	Bacteria	0
Clostridia	Firmicutes	Bacteria	0.017955966
Clostridia	Firmicutes	Bacteria	0.178211852
Clostridia	Firmicutes	Bacteria	0
unclassified	unclassified	unclassified	0
unclassified	Firmicutes	Bacteria	0
Clostridia	Firmicutes	Bacteria	0
Bacteroidia	Bacteroidetes	Bacteria	0
Clostridia	Firmicutes	Bacteria	0.00098083
unclassified	unclassified	Bacteria	0
Bacteroidia	Bacteroidetes	Bacteria	0
Bacteroidia	Bacteroidetes	Bacteria	0
unclassified	unclassified	unclassified	0
Bacteroidia	Bacteroidetes	Bacteria	0
Clostridia	Firmicutes	Bacteria	1.786907598
Clostridia	Firmicutes	Bacteria	1.392953949
Bacteroidia	Bacteroidetes	Bacteria	0
Clostridia	Firmicutes	Bacteria	0
unclassified	Firmicutes	Bacteria	0
Deltaproteobacteria	Proteobacteria	Bacteria	0
Clostridia	Firmicutes	Bacteria	0
Bacteroidia	Bacteroidetes	Bacteria	0

Bacteroidia	Bacteroidetes	Bacteria	0
Clostridia	Firmicutes	Bacteria	0
unclassified	unclassified	unclassified	0
Clostridia	Firmicutes	Bacteria	0
Clostridia	Firmicutes	Bacteria	3.59705331
Clostridia	Firmicutes	Bacteria	0
Clostridia	Firmicutes	Bacteria	0
Clostridia	Firmicutes	Bacteria	0
Clostridia	Firmicutes	Bacteria	0.038647161
Clostridia	Firmicutes	Bacteria	0
Clostridia	Firmicutes	Bacteria	0
unclassified	Firmicutes	Bacteria	0
Clostridia	Firmicutes	Bacteria	0.021893714
Clostridia	Firmicutes	Bacteria	0.002387718
Bacteroidia	Bacteroidetes	Bacteria	0
Clostridia	Firmicutes	Bacteria	0.365328625
Clostridia	Firmicutes	Bacteria	0.000751345
Bacteroidia	Bacteroidetes	Bacteria	0
Bacteroidia	Bacteroidetes	Bacteria	0
Clostridia	Firmicutes	Bacteria	0
unclassified	unclassified	unclassified	0
Clostridia	Firmicutes	Bacteria	0
Actinomycetia	Actinobacteria	Bacteria	0
unclassified	unclassified	Bacteria	0
unclassified	unclassified	unclassified	0.014796694
unclassified	Firmicutes	Bacteria	0
Clostridia	Firmicutes	Bacteria	0.040669521
Clostridia	Firmicutes	Bacteria	0
unclassified	unclassified	unclassified	0
Clostridia	Firmicutes	Bacteria	0
unclassified	Firmicutes	Bacteria	0
unclassified	Firmicutes	Bacteria	0
Clostridia	Firmicutes	Bacteria	0
Clostridia	Firmicutes	Bacteria	0.012171403
Bacteroidia	Bacteroidetes	Bacteria	0
Erysipelotrichia	Firmicutes	Bacteria	0
Bacilli	Firmicutes	Bacteria	0
Clostridia	Firmicutes	Bacteria	0
unclassified	unclassified	Bacteria	0
unclassified	Firmicutes	Bacteria	0
Erysipelotrichia	Firmicutes	Bacteria	6.084977804
Bacteroidia	Bacteroidetes	Bacteria	0
Clostridia	Firmicutes	Bacteria	0
Gammaproteobacteria	Proteobacteria	Bacteria	0
Verrucomicrobiae	Verrucomicrobia	Bacteria	0
unclassified	unclassified	Bacteria	0
unclassified	Firmicutes	Bacteria	0
Clostridia	Firmicutes	Bacteria	0

Clostridia	Firmicutes	Bacteria	0.07970807
Clostridia	Firmicutes	Bacteria	0
Clostridia	Firmicutes	Bacteria	0
Clostridia	Firmicutes	Bacteria	0
Clostridia	Firmicutes	Bacteria	0
Clostridia	Firmicutes	Bacteria	0
Clostridia	Firmicutes	Bacteria	0.000749518
Bacteroidia	Bacteroidetes	Bacteria	0
Bacteroidia	Bacteroidetes	Bacteria	0
Clostridia	Firmicutes	Bacteria	0
unclassified	unclassified	unclassified	0
Clostridia	Firmicutes	Bacteria	0.000819335
Betaproteobacteria	Proteobacteria	Bacteria	0
Negativicutes	Firmicutes	Bacteria	0
Clostridia	Firmicutes	Bacteria	0.004050553
Clostridia	Firmicutes	Bacteria	0
Actinomycetia	Actinobacteria	Bacteria	0
Clostridia	Firmicutes	Bacteria	0
Bacteroidia	Bacteroidetes	Bacteria	0
unclassified	unclassified	unclassified	0
Clostridia	Firmicutes	Bacteria	0.58121356
unclassified	Firmicutes	Bacteria	0
Clostridia	Firmicutes	Bacteria	0
Clostridia	Firmicutes	Bacteria	0.000874849
Negativicutes	Firmicutes	Bacteria	0
Bacteroidia	Bacteroidetes	Bacteria	0
Bacteroidia	Bacteroidetes	Bacteria	0
Clostridia	Firmicutes	Bacteria	0
Coriobacteriia	Actinobacteria	Bacteria	0
Bacilli	Firmicutes	Bacteria	0
unclassified	unclassified	unclassified	0
Betaproteobacteria	Proteobacteria	Bacteria	0
Clostridia	Firmicutes	Bacteria	0
Verrucomicrobiae	Verrucomicrobia	Bacteria	1.96237527
Clostridia	Firmicutes	Bacteria	0
unclassified	Firmicutes	Bacteria	0
Clostridia	Firmicutes	Bacteria	0.000656798
Bacteroidia	Bacteroidetes	Bacteria	0
Bacteroidia	Bacteroidetes	Bacteria	0
unclassified	unclassified	unclassified	0
Bacteroidia	Bacteroidetes	Bacteria	0
unclassified	unclassified	Bacteria	0
Clostridia	Firmicutes	Bacteria	0
Clostridia	Firmicutes	Bacteria	0
unclassified	unclassified	unclassified	0.016783461
Clostridia	Firmicutes	Bacteria	0
unclassified	unclassified	unclassified	0
Bacteroidia	Bacteroidetes	Bacteria	0

Clostridia	Firmicutes	Bacteria	0.000941369
Bacteroidia	Bacteroidetes	Bacteria	0
Clostridia	Firmicutes	Bacteria	0
Betaproteobacteria	Proteobacteria	Bacteria	0
Bacteroidia	Bacteroidetes	Bacteria	0
unclassified	unclassified	unclassified	0
Bacteroidia	Bacteroidetes	Bacteria	0
Clostridia	Firmicutes	Bacteria	1.960597123
Gammaproteobacteria	Proteobacteria	Bacteria	0.338670518
Clostridia	Firmicutes	Bacteria	0.004937639
Clostridia	Firmicutes	Bacteria	0
unclassified	unclassified	unclassified	0
Clostridia	Firmicutes	Bacteria	0.008908612
Negativicutes	Firmicutes	Bacteria	0
Bacteroidia	Bacteroidetes	Bacteria	0
unclassified	unclassified	unclassified	0
Erysipelotrichia	Firmicutes	Bacteria	0
Erysipelotrichia	Firmicutes	Bacteria	0
Bacteroidia	Bacteroidetes	Bacteria	0
Bacteroidia	Bacteroidetes	Bacteria	0
Methanobacteria	Euryarchaeota	Archaea	0
unclassified	unclassified	unclassified	0
Clostridia	Firmicutes	Bacteria	0
Clostridia	Firmicutes	Bacteria	0
Clostridia	Firmicutes	Bacteria	0
unclassified	unclassified	unclassified	0
Clostridia	Firmicutes	Bacteria	0
Clostridia	Firmicutes	Bacteria	0
Clostridia	Firmicutes	Bacteria	0
Bacteroidia	Bacteroidetes	Bacteria	0
Bacteroidia	Bacteroidetes	Bacteria	0
Bacteroidia	Bacteroidetes	Bacteria	0
unclassified	Firmicutes	Bacteria	0
Clostridia	Firmicutes	Bacteria	0
Negativicutes	Firmicutes	Bacteria	0
Clostridia	Firmicutes	Bacteria	0
unclassified	Firmicutes	Bacteria	0
Clostridia	Firmicutes	Bacteria	0
Betaproteobacteria	Proteobacteria	Bacteria	0
Clostridia	Firmicutes	Bacteria	0.033644163
unclassified	unclassified	unclassified	0
Clostridia	Firmicutes	Bacteria	0.17820347
Clostridia	Firmicutes	Bacteria	0
Clostridia	Firmicutes	Bacteria	0
Bacteroidia	Bacteroidetes	Bacteria	0
Clostridia	Firmicutes	Bacteria	0
Clostridia	Firmicutes	Bacteria	0
Lentisphaeria	Lentisphaerae	Bacteria	0

Bacteroidia	Bacteroidetes	Bacteria	0
Actinomycetia	Actinobacteria	Bacteria	0
Clostridia	Firmicutes	Bacteria	0
Clostridia	Firmicutes	Bacteria	0
Bacteroidia	Bacteroidetes	Bacteria	0.139696757
unclassified	Firmicutes	Bacteria	0
Bacteroidia	Bacteroidetes	Bacteria	0
Erysipelotrichia	Firmicutes	Bacteria	0.008142929
Clostridia	Firmicutes	Bacteria	0
Clostridia	Firmicutes	Bacteria	0
Clostridia	Firmicutes	Bacteria	0
Bacteroidia	Bacteroidetes	Bacteria	0
unclassified	unclassified	unclassified	0
unclassified	unclassified	Bacteria	0
Erysipelotrichia	Firmicutes	Bacteria	0
Bacteroidia	Bacteroidetes	Bacteria	0
Clostridia	Firmicutes	Bacteria	0
unclassified	unclassified	unclassified	0
unclassified	Firmicutes	Bacteria	0
unclassified	Firmicutes	Bacteria	0
Clostridia	Firmicutes	Bacteria	0
unclassified	unclassified	Bacteria	0
unclassified	Firmicutes	Bacteria	0
Clostridia	Firmicutes	Bacteria	0.003523517
Coriobacteriia	Actinobacteria	Bacteria	0
Clostridia	Firmicutes	Bacteria	0
Bacteroidia	Bacteroidetes	Bacteria	0.000758272
Clostridia	Firmicutes	Bacteria	0
Clostridia	Firmicutes	Bacteria	0
unclassified	unclassified	unclassified	0
Betaproteobacteria	Proteobacteria	Bacteria	0
Clostridia	Firmicutes	Bacteria	0.001457631
Clostridia	Firmicutes	Bacteria	0
unclassified	unclassified	unclassified	0
Bacteroidia	Bacteroidetes	Bacteria	0
Clostridia	Firmicutes	Bacteria	0
unclassified	unclassified	unclassified	0
Clostridia	Firmicutes	Bacteria	0.000583239
Clostridia	Firmicutes	Bacteria	0
Clostridia	Firmicutes	Bacteria	0
unclassified	unclassified	unclassified	0
Bacteroidia	Bacteroidetes	Bacteria	0
Clostridia	Firmicutes	Bacteria	0
unclassified	unclassified	Bacteria	0
Clostridia	Firmicutes	Bacteria	0
Clostridia	Firmicutes	Bacteria	0
unclassified	Firmicutes	Bacteria	0
Clostridia	Firmicutes	Bacteria	0

unclassified	unclassified	unclassified	0.025136407
Methanobacteria	Euryarchaeota	Archaea	0
unclassified	Firmicutes	Bacteria	0
Bacteroidia	Bacteroidetes	Bacteria	0
Clostridia	Firmicutes	Bacteria	0
Bacteroidia	Bacteroidetes	Bacteria	0
unclassified	unclassified	Bacteria	0
Clostridia	Firmicutes	Bacteria	0
Clostridia	Firmicutes	Bacteria	0
unclassified	unclassified	unclassified	0.012088521
Erysipelotrichia	Firmicutes	Bacteria	0
Bacteroidia	Bacteroidetes	Bacteria	0
unclassified	unclassified	unclassified	0
unclassified	unclassified	unclassified	0
unclassified	unclassified	unclassified	0
unclassified	unclassified	unclassified	0
Clostridia	Firmicutes	Bacteria	0
Clostridia	Firmicutes	Bacteria	0
Bacteroidia	Bacteroidetes	Bacteria	0
Bacteroidia	Bacteroidetes	Bacteria	0
Clostridia	Firmicutes	Bacteria	0.002476791
unclassified	unclassified	unclassified	0
Bacteroidia	Bacteroidetes	Bacteria	0
Clostridia	Firmicutes	Bacteria	0.100679186
Clostridia	Firmicutes	Bacteria	0
Bacteroidia	Bacteroidetes	Bacteria	0
unclassified	unclassified	unclassified	0
Clostridia	Firmicutes	Bacteria	0
Negativicutes	Firmicutes	Bacteria	0
Clostridia	Firmicutes	Bacteria	0
Clostridia	Firmicutes	Bacteria	0
Clostridia	Firmicutes	Bacteria	0
Clostridia	Firmicutes	Bacteria	0
Clostridia	Firmicutes	Bacteria	0
Erysipelotrichia	Firmicutes	Bacteria	1.57654949
Clostridia	Firmicutes	Bacteria	0
Clostridia	Firmicutes	Bacteria	0
Bacteroidia	Bacteroidetes	Bacteria	0
Clostridia	Firmicutes	Bacteria	0.005664476
Clostridia	Firmicutes	Bacteria	0
unclassified	Firmicutes	Bacteria	0
Clostridia	Firmicutes	Bacteria	0
Bacteroidia	Bacteroidetes	Bacteria	0
Clostridia	Firmicutes	Bacteria	0
Erysipelotrichia	Firmicutes	Bacteria	0
Clostridia	Firmicutes	Bacteria	0.01514933
Bacteroidia	Bacteroidetes	Bacteria	0
Clostridia	Firmicutes	Bacteria	0

unclassified	unclassified	unclassified	0
Bacteroidia	Bacteroidetes	Bacteria	0
Clostridia	Firmicutes	Bacteria	0
unclassified	unclassified	unclassified	0
Clostridia	Firmicutes	Bacteria	0
unclassified	unclassified	unclassified	0
Clostridia	Firmicutes	Bacteria	0
Coriobacteriia	Actinobacteria	Bacteria	0.098406962
unclassified	unclassified	unclassified	0.012025064
Negativicutes	Firmicutes	Bacteria	0
unclassified	Firmicutes	Bacteria	0
Clostridia	Firmicutes	Bacteria	0
Clostridia	Firmicutes	Bacteria	0
Clostridia	Firmicutes	Bacteria	0.000559191
Clostridia	Firmicutes	Bacteria	0
Clostridia	Firmicutes	Bacteria	0
Negativicutes	Firmicutes	Bacteria	0
Clostridia	Firmicutes	Bacteria	0
Negativicutes	Firmicutes	Bacteria	0
Clostridia	Firmicutes	Bacteria	1.186618511
Clostridia	Firmicutes	Bacteria	0.00601288
Clostridia	Firmicutes	Bacteria	0
Clostridia	Firmicutes	Bacteria	0
Clostridia	Firmicutes	Bacteria	0.008520636
Bacteroidia	Bacteroidetes	Bacteria	0
Clostridia	Firmicutes	Bacteria	0
unclassified	unclassified	unclassified	0
Negativicutes	Firmicutes	Bacteria	0
Clostridia	Firmicutes	Bacteria	0
Clostridia	Firmicutes	Bacteria	0
Clostridia	Firmicutes	Bacteria	0
Clostridia	Firmicutes	Bacteria	0
Clostridia	Firmicutes	Bacteria	0
unclassified	Firmicutes	Bacteria	0
unclassified	unclassified	unclassified	0
unclassified	Firmicutes	Bacteria	0
unclassified	unclassified	unclassified	0
Clostridia	Firmicutes	Bacteria	0
Coriobacteriia	Actinobacteria	Bacteria	0
Clostridia	Firmicutes	Bacteria	0
Clostridia	Firmicutes	Bacteria	0
Clostridia	Firmicutes	Bacteria	0
Clostridia	Firmicutes	Bacteria	0
Bacteroidia	Bacteroidetes	Bacteria	0
Clostridia	Firmicutes	Bacteria	0
Bacteroidia	Bacteroidetes	Bacteria	0
Clostridia	Firmicutes	Bacteria	0
Deltaproteobacteria	Proteobacteria	Bacteria	0

Betaproteobacteria	Proteobacteria	Bacteria	0
Clostridia	Firmicutes	Bacteria	0
Clostridia	Firmicutes	Bacteria	0
Betaproteobacteria	Proteobacteria	Bacteria	0
Clostridia	Firmicutes	Bacteria	0.078273487
unclassified	unclassified	unclassified	0
Coriobacteriia	Actinobacteria	Bacteria	0
Clostridia	Firmicutes	Bacteria	0
Clostridia	Firmicutes	Bacteria	0
unclassified	unclassified	unclassified	0
Clostridia	Firmicutes	Bacteria	0
Bacteroidia	Bacteroidetes	Bacteria	0.001358392
Bacteroidia	Bacteroidetes	Bacteria	0
Bacteroidia	Bacteroidetes	Bacteria	0
Erysipelotrichia	Firmicutes	Bacteria	0
Clostridia	Firmicutes	Bacteria	0
Clostridia	Firmicutes	Bacteria	0.001915402
Clostridia	Firmicutes	Bacteria	0
Clostridia	Firmicutes	Bacteria	0.049029079
Clostridia	Firmicutes	Bacteria	0.000895343
Bacteroidia	Bacteroidetes	Bacteria	0
Clostridia	Firmicutes	Bacteria	0
Clostridia	Firmicutes	Bacteria	0
Bacteroidia	Bacteroidetes	Bacteria	0
unclassified	Firmicutes	Bacteria	0
unclassified	unclassified	unclassified	0
Clostridia	Firmicutes	Bacteria	0.002511178
Clostridia	Firmicutes	Bacteria	0
unclassified	Firmicutes	Bacteria	0
Clostridia	Firmicutes	Bacteria	0
Clostridia	Firmicutes	Bacteria	0
Clostridia	Firmicutes	Bacteria	0
Clostridia	Firmicutes	Bacteria	0
Clostridia	Firmicutes	Bacteria	0
Clostridia	Firmicutes	Bacteria	0
Clostridia	Firmicutes	Bacteria	0
unclassified	Firmicutes	Bacteria	0
Bacteroidia	Bacteroidetes	Bacteria	0.004714062
Clostridia	Firmicutes	Bacteria	0
Clostridia	Firmicutes	Bacteria	0
Bacteroidia	Bacteroidetes	Bacteria	0
unclassified	unclassified	unclassified	0
unclassified	Firmicutes	Bacteria	0
Clostridia	Firmicutes	Bacteria	0
unclassified	unclassified	unclassified	0
Clostridia	Firmicutes	Bacteria	0
Bacteroidia	Bacteroidetes	Bacteria	0
unclassified	unclassified	unclassified	0

unclassified	Firmicutes	Bacteria	0
Clostridia	Firmicutes	Bacteria	0.037477689
Clostridia	Firmicutes	Bacteria	0
Clostridia	Firmicutes	Bacteria	0
Negativicutes	Firmicutes	Bacteria	0
Negativicutes	Firmicutes	Bacteria	0
Clostridia	Firmicutes	Bacteria	0
Deltaproteobacteria	Proteobacteria	Bacteria	0
Clostridia	Firmicutes	Bacteria	0
Clostridia	Firmicutes	Bacteria	0
Clostridia	Firmicutes	Bacteria	0.026415386
Clostridia	Firmicutes	Bacteria	0
Clostridia	Firmicutes	Bacteria	0.04862583
Clostridia	Firmicutes	Bacteria	0
Deltaproteobacteria	Proteobacteria	Bacteria	0
Clostridia	Firmicutes	Bacteria	0
Bacteroidia	Bacteroidetes	Bacteria	0
Clostridia	Firmicutes	Bacteria	0
Betaproteobacteria	Proteobacteria	Bacteria	0
Clostridia	Firmicutes	Bacteria	0
unclassified	Firmicutes	Bacteria	0
unclassified	Firmicutes	Bacteria	0
Clostridia	Firmicutes	Bacteria	0.013054459
Clostridia	Firmicutes	Bacteria	0.066998265
unclassified	unclassified	unclassified	0
Clostridia	Firmicutes	Bacteria	0
Clostridia	Firmicutes	Bacteria	0
Deltaproteobacteria	Proteobacteria	Bacteria	0
unclassified	unclassified	Bacteria	0
Bacteroidia	Bacteroidetes	Bacteria	0
Clostridia	Firmicutes	Bacteria	0
Bacteroidia	Bacteroidetes	Bacteria	0
Betaproteobacteria	Proteobacteria	Bacteria	0
Clostridia	Firmicutes	Bacteria	0
Clostridia	Firmicutes	Bacteria	0
Clostridia	Firmicutes	Bacteria	0
Clostridia	Firmicutes	Bacteria	0
Clostridia	Firmicutes	Bacteria	0
Gammaproteobacteria	Proteobacteria	Bacteria	0
Clostridia	Firmicutes	Bacteria	0
Clostridia	Firmicutes	Bacteria	0
Bacteroidia	Bacteroidetes	Bacteria	0
Clostridia	Firmicutes	Bacteria	0
unclassified	unclassified	unclassified	0
Clostridia	Firmicutes	Bacteria	1.916164768
Clostridia	Firmicutes	Bacteria	0.101988982
Clostridia	Firmicutes	Bacteria	0
Clostridia	Firmicutes	Bacteria	0

Clostridia	Firmicutes	Bacteria	0
unclassified	Firmicutes	Bacteria	0
Coriobacteriia	Actinobacteria	Bacteria	0
Clostridia	Firmicutes	Bacteria	0
Clostridia	Firmicutes	Bacteria	0
Betaproteobacteria	Proteobacteria	Bacteria	0
Bacteroidia	Bacteroidetes	Bacteria	0
unclassified	unclassified	unclassified	0
Clostridia	Firmicutes	Bacteria	0
Gammaproteobacteria	Proteobacteria	Bacteria	0
Bacteroidia	Bacteroidetes	Bacteria	0
Clostridia	Firmicutes	Bacteria	0
Bacteroidia	Bacteroidetes	Bacteria	0
Bacteroidia	Bacteroidetes	Bacteria	0
Bacteroidia	Bacteroidetes	Bacteria	0
Clostridia	Firmicutes	Bacteria	0
Gammaproteobacteria	Proteobacteria	Bacteria	0
Clostridia	Firmicutes	Bacteria	0
Bacteroidia	Bacteroidetes	Bacteria	0
Betaproteobacteria	Proteobacteria	Bacteria	0.000630932
Clostridia	Firmicutes	Bacteria	0
unclassified	unclassified	unclassified	0
unclassified	unclassified	unclassified	0
Clostridia	Firmicutes	Bacteria	0
Clostridia	Firmicutes	Bacteria	0
Clostridia	Firmicutes	Bacteria	0
Clostridia	Firmicutes	Bacteria	0
Erysipelotrichia	Firmicutes	Bacteria	0
Clostridia	Firmicutes	Bacteria	0
Clostridia	Firmicutes	Bacteria	0
Clostridia	Firmicutes	Bacteria	0.000722715
Clostridia	Firmicutes	Bacteria	0
Coriobacteriia	Actinobacteria	Bacteria	0.322651459
Bacilli	Firmicutes	Bacteria	0.020580378
unclassified	unclassified	unclassified	0
Clostridia	Firmicutes	Bacteria	0
Clostridia	Firmicutes	Bacteria	0
Bacteroidia	Bacteroidetes	Bacteria	0
Bacteroidia	Bacteroidetes	Bacteria	0
Clostridia	Firmicutes	Bacteria	0
Clostridia	Firmicutes	Bacteria	0
Clostridia	Firmicutes	Bacteria	0.00276143
Clostridia	Firmicutes	Bacteria	0.107444137
unclassified	unclassified	unclassified	0
Clostridia	Firmicutes	Bacteria	0
Bacteroidia	Bacteroidetes	Bacteria	0
Bacteroidia	Bacteroidetes	Bacteria	0
Clostridia	Firmicutes	Bacteria	0

Clostridia	Firmicutes	Bacteria	0
Coriobacteriia	Actinobacteria	Bacteria	0
Bacilli	Firmicutes	Bacteria	0.006690003
Bacteroidia	Bacteroidetes	Bacteria	0
Bacteroidia	Bacteroidetes	Bacteria	0.068825042
Negativicutes	Firmicutes	Bacteria	0.002999726
unclassified	unclassified	unclassified	0
Bacteroidia	Bacteroidetes	Bacteria	0
Bacteroidia	Bacteroidetes	Bacteria	0
Bacteroidia	Bacteroidetes	Bacteria	0
Clostridia	Firmicutes	Bacteria	0
unclassified	unclassified	unclassified	0
Bacteroidia	Bacteroidetes	Bacteria	0
Coriobacteriia	Actinobacteria	Bacteria	0
unclassified	unclassified	unclassified	0
Clostridia	Firmicutes	Bacteria	0
Clostridia	Firmicutes	Bacteria	0
Clostridia	Firmicutes	Bacteria	0
Clostridia	Firmicutes	Bacteria	0
Clostridia	Firmicutes	Bacteria	0
Clostridia	Firmicutes	Bacteria	0
Erysipelotrichia	Firmicutes	Bacteria	0
Clostridia	Firmicutes	Bacteria	0
Clostridia	Firmicutes	Bacteria	0
unclassified	unclassified	unclassified	0
Actinomycetia	Actinobacteria	Bacteria	0
unclassified	unclassified	unclassified	0
Clostridia	Firmicutes	Bacteria	0
Clostridia	Firmicutes	Bacteria	0
Bacteroidia	Bacteroidetes	Bacteria	0
Bacteroidia	Bacteroidetes	Bacteria	0
Clostridia	Firmicutes	Bacteria	0
unclassified	unclassified	unclassified	0
Bacteroidia	Bacteroidetes	Bacteria	0
unclassified	unclassified	Bacteria	0
unclassified	unclassified	unclassified	0
Bacteroidia	Bacteroidetes	Bacteria	0
Clostridia	Firmicutes	Bacteria	0.002548167
Clostridia	Firmicutes	Bacteria	0
Betaproteobacteria	Proteobacteria	Bacteria	0
Clostridia	Firmicutes	Bacteria	0
Negativicutes	Firmicutes	Bacteria	1.780351553
Clostridia	Firmicutes	Bacteria	0.000642285
Coriobacteriia	Actinobacteria	Bacteria	0
Clostridia	Firmicutes	Bacteria	0
Clostridia	Firmicutes	Bacteria	0
Clostridia	Firmicutes	Bacteria	0
unclassified	unclassified	unclassified	0

Clostridia	Firmicutes	Bacteria	0
Clostridia	Firmicutes	Bacteria	0.001239823
Clostridia	Firmicutes	Bacteria	0
Bacteroidia	Bacteroidetes	Bacteria	0
Clostridia	Firmicutes	Bacteria	0
Clostridia	Firmicutes	Bacteria	0
Bacteroidia	Bacteroidetes	Bacteria	0
Clostridia	Firmicutes	Bacteria	0
Clostridia	Firmicutes	Bacteria	0
Clostridia	Firmicutes	Bacteria	0
Clostridia	Firmicutes	Bacteria	0
unclassified	unclassified	unclassified	0
Clostridia	Firmicutes	Bacteria	0.031454475
Clostridia	Firmicutes	Bacteria	0
Clostridia	Firmicutes	Bacteria	0
unclassified	unclassified	unclassified	0
Clostridia	Firmicutes	Bacteria	0
unclassified	unclassified	unclassified	0
unclassified	unclassified	unclassified	0.009120723
Deltaproteobacteria	Proteobacteria	Bacteria	0
Clostridia	Firmicutes	Bacteria	0
Erysipelotrichia	Firmicutes	Bacteria	0
Clostridia	Firmicutes	Bacteria	0
Negativicutes	Firmicutes	Bacteria	0
unclassified	unclassified	unclassified	0
unclassified	Firmicutes	Bacteria	0
Clostridia	Firmicutes	Bacteria	0
Clostridia	Firmicutes	Bacteria	0
Clostridia	Firmicutes	Bacteria	0
unclassified	unclassified	unclassified	0
Lentisphaeria	Lentisphaerae	Bacteria	0
unclassified	unclassified	Bacteria	0
Bigyra	unclassified	Eukaryota	0
Bacteroidia	Bacteroidetes	Bacteria	0
Clostridia	Firmicutes	Bacteria	0
unclassified	unclassified	unclassified	0
Clostridia	Firmicutes	Bacteria	0.001112835
Clostridia	Firmicutes	Bacteria	0
Clostridia	Firmicutes	Bacteria	0
Thermoplasmata	Candidatus Thermoplasmatota	Archaea	0
Clostridia	Firmicutes	Bacteria	0.026054444
Clostridia	Firmicutes	Bacteria	0
Bacteroidia	Bacteroidetes	Bacteria	0
Clostridia	Firmicutes	Bacteria	0
Clostridia	Firmicutes	Bacteria	0
Clostridia	Firmicutes	Bacteria	0.167310559
unclassified	Firmicutes	Bacteria	0
Clostridia	Firmicutes	Bacteria	0

Clostridia	Firmicutes	Bacteria	0
Clostridia	Firmicutes	Bacteria	0
Coriobacteriia	Actinobacteria	Bacteria	0
Clostridia	Firmicutes	Bacteria	0
Bacteroidia	Bacteroidetes	Bacteria	0
Clostridia	Firmicutes	Bacteria	0
Clostridia	Firmicutes	Bacteria	0
Clostridia	Firmicutes	Bacteria	0
Bacteroidia	Bacteroidetes	Bacteria	0
Gammaproteobacteria	Proteobacteria	Bacteria	0.00076107
unclassified	unclassified	unclassified	0
Coriobacteriia	Actinobacteria	Bacteria	0
Clostridia	Firmicutes	Bacteria	0
unclassified	unclassified	unclassified	0
Clostridia	Firmicutes	Bacteria	0
unclassified	unclassified	unclassified	0
unclassified	unclassified	unclassified	0
unclassified	unclassified	unclassified	0
Clostridia	Firmicutes	Bacteria	0
Clostridia	Firmicutes	Bacteria	0.889209833
Clostridia	Firmicutes	Bacteria	0
unclassified	unclassified	Bacteria	0
unclassified	Firmicutes	Bacteria	0
Thermoplasmata	Candidatus Thermoplasmatota	Archaea	0
unclassified	unclassified	unclassified	0
Bacteroidia	Bacteroidetes	Bacteria	0
Clostridia	Firmicutes	Bacteria	0
unclassified	unclassified	Bacteria	0
Gammaproteobacteria	Proteobacteria	Bacteria	0
Clostridia	Firmicutes	Bacteria	0
Clostridia	Firmicutes	Bacteria	0
Clostridia	Firmicutes	Bacteria	0
unclassified	Firmicutes	Bacteria	0
Bacteroidia	Bacteroidetes	Bacteria	0
unclassified	unclassified	unclassified	0
Erysipelotrichia	Firmicutes	Bacteria	0
Clostridia	Firmicutes	Bacteria	0
unclassified	Firmicutes	Bacteria	0
unclassified	Firmicutes	Bacteria	0
unclassified	Firmicutes	Bacteria	0
unclassified	unclassified	unclassified	0
Clostridia	Firmicutes	Bacteria	0
unclassified	unclassified	unclassified	0.007521717
Clostridia	Firmicutes	Bacteria	0
Bacteroidia	Bacteroidetes	Bacteria	0.002072918
Clostridia	Firmicutes	Bacteria	0
Clostridia	Firmicutes	Bacteria	0
Clostridia	Firmicutes	Bacteria	0

Bacteroidia	Bacteroidetes	Bacteria	0
Lentisphaeria	Lentisphaerae	Bacteria	0
unclassified	unclassified	unclassified	0
unclassified	unclassified	unclassified	0
unclassified	unclassified	unclassified	0
Deltaproteobacteria	Proteobacteria	Bacteria	0.027236241
unclassified	unclassified	unclassified	0
Bacteroidia	Bacteroidetes	Bacteria	0
Clostridia	Firmicutes	Bacteria	0
Clostridia	Firmicutes	Bacteria	0.009000854
Bacteroidia	Bacteroidetes	Bacteria	0
unclassified	unclassified	Bacteria	0
Bacilli	Firmicutes	Bacteria	0
Elusimicrobia	Elusimicrobia	Bacteria	0
unclassified	Firmicutes	Bacteria	0
unclassified	unclassified	unclassified	0
Clostridia	Firmicutes	Bacteria	0.006382219
Clostridia	Firmicutes	Bacteria	0
unclassified	unclassified	Bacteria	0
Clostridia	Firmicutes	Bacteria	0
Bacilli	Firmicutes	Bacteria	0
unclassified	unclassified	unclassified	0
Clostridia	Firmicutes	Bacteria	0
Clostridia	Firmicutes	Bacteria	0
unclassified	unclassified	unclassified	0
Gammaproteobacteria	Proteobacteria	Bacteria	0
Clostridia	Firmicutes	Bacteria	0
Clostridia	Firmicutes	Bacteria	0
Clostridia	Firmicutes	Bacteria	0
Clostridia	Firmicutes	Bacteria	0
Bacteroidia	Bacteroidetes	Bacteria	0
Clostridia	Firmicutes	Bacteria	0
unclassified	unclassified	unclassified	0
Clostridia	Firmicutes	Bacteria	0
Erysipelotrichia	Firmicutes	Bacteria	0
Bacteroidia	Bacteroidetes	Bacteria	0
unclassified	Firmicutes	Bacteria	0
Clostridia	Firmicutes	Bacteria	0
Clostridia	Firmicutes	Bacteria	1.992222309
unclassified	unclassified	unclassified	0
unclassified	unclassified	unclassified	0
unclassified	unclassified	unclassified	0
Clostridia	Firmicutes	Bacteria	0
unclassified	unclassified	unclassified	0
Bacilli	Firmicutes	Bacteria	0
Clostridia	Firmicutes	Bacteria	0
unclassified	Firmicutes	Bacteria	0
unclassified	unclassified	Bacteria	0

Bacteroidia	Bacteroidetes	Bacteria	0
Clostridia	Firmicutes	Bacteria	0.010741911
Clostridia	Firmicutes	Bacteria	0
Bacteroidia	Bacteroidetes	Bacteria	0
Clostridia	Firmicutes	Bacteria	0
Deltaproteobacteria	Proteobacteria	Bacteria	0
Bacteroidia	Bacteroidetes	Bacteria	0
Clostridia	Firmicutes	Bacteria	0
Clostridia	Firmicutes	Bacteria	0
Clostridia	Firmicutes	Bacteria	0
Negativicutes	Firmicutes	Bacteria	0.000680597
Clostridia	Firmicutes	Bacteria	0
Clostridia	Firmicutes	Bacteria	0.684935321
unclassified	Firmicutes	Bacteria	0
Bacteroidia	Bacteroidetes	Bacteria	0
Negativicutes	Firmicutes	Bacteria	0
Bacteroidia	Bacteroidetes	Bacteria	0
unclassified	Firmicutes	Bacteria	0
unclassified	Firmicutes	Bacteria	0
Deltaproteobacteria	Proteobacteria	Bacteria	0
Fusobacteriia	Fusobacteria	Bacteria	0
unclassified	unclassified	unclassified	0
unclassified	unclassified	Bacteria	0
Clostridia	Firmicutes	Bacteria	1.45804161
Gammaproteobacteria	Proteobacteria	Bacteria	0
Clostridia	Firmicutes	Bacteria	0
Clostridia	Firmicutes	Bacteria	0
Erysipelotrichia	Firmicutes	Bacteria	0
Bacteroidia	Bacteroidetes	Bacteria	0
unclassified	unclassified	Bacteria	0
Clostridia	Firmicutes	Bacteria	0
Bacteroidia	Bacteroidetes	Bacteria	0
Clostridia	Firmicutes	Bacteria	0
Betaproteobacteria	Proteobacteria	Bacteria	0

PHL018-0056_V2	PHL018-0247_V1	PHL018-0046_V3	PHL018-0097_V2	PHL018-0234_V1
REF	REF	CTRL	CTRL	CTRL
0.546842458	0.977717031	0.000538193	0.387206409	0.01245673
3.025926573	1.945555306	0.039779184	3.555190428	0.043354332
0.008250213	2.097812698	0.004147746	0	0
0	0	0	0	0
0	0	0	0	0
0	0	0	0	0
0.010423819	0	0	0	0
0	0	0	0	0
0	0	0	0	0
0	0	0	0	0
0	0	0	0	0
0	0	0	0	0
0	0	0	0	0
0.219898814	11.15617075	0.649029382	2.411935164	0.34758004
0	0	0	0	0
0	0	0	0	0
0	0.005728779	0	0	0
0	0	0	0	0
0	0	0	0	0
0	0	0	0	0
0	0	0	0	0
0	0	0	0	0
0	0	0	0	0
0	0	0	0	0
0	0	0	0	0
0	0	0	0	0
0	0	0	0	0
0	0	0	0	0
0	0	0	0	0
0	0	0	0	0
0	0	0	0	0
0	0	0	0	0
0	0	0	0	0
0	0	0	0	0
0	0	0	0	0
0	0	2.345745919	0	0.336720511
0	0	0	0	0
0	0	0	0	0
0	0	0	0	0
0	0	0	0	0
0	0	0	0	0
0.013961058	0.854668847	0.189313681	0	0.553782791
0.10385799	0.101038512	6.361958862	0.141734765	0.122987807
0.049151898	0.012660339	0.003037137	0.009407475	0.054976502
0.006472472	0.003439407	0.001843931	0	0.004251192
0	0	0	0	0

0	0	0	0.007768095	0
0.030586036	0.423902007	0	0.043034544	0.003567459
0	0	0	0.006976961	0
0.001137067	0	0.22392157	0.842565043	0.957564254
0.004654329	0	0	0.003128942	0.013393822
0.007831312	0	0.009127019	0	0
0.002164935	0.001517044	0.035463962	1.288214204	0
0	0	0	0	0
0	0	0	0	0
0	0	0	0	0
0.013355761	0	0	0	0.001537059
0.003492227	0	0.207684269	0.364884225	0.021925401
0	0	0	0	0
0.099672306	0.137980282	0	0.026988606	0.009071091
0.003342806	0	0	0	0
0	0	0	0	0
0.003036448	0.08688284	1.275081648	0.045582177	0.000938529
0	0	0	0	0
0	0	0	0	0
0	0	0	0	0
0.094556589	0.55584875	0.000500088	0.113390132	0.002351124
0.012965312	0.026768084	0.000782556	0.006212085	0
0	0	0	0	0.002666399
0	0	0	0	0
0	0	0	0	0
0	0	0	0	0
0	0.017804354	0	0	0
0	0	0	0	0
0	0.018845758	0.006807587	0	0.004127385
0	0	0	0	0
0.129232132	0.602959682	0	0.349764376	0
0	0	0	0	0
0	0	0	0	0
0.001828622	0	0	0	0
0	0	0	0	0
0	0	0	0	0
0	0	0	0	0
0	5.879021295	54.52732704	0.004675535	0
0	0	0	0	0
0	0	0	0	0
0	0	0	0	0
0	0	0	0	0
0	0	0	0	0
0	0	0	0	0
0.030182702	0	0	0	0
0	0	0	0	0
0	0	0	0	0
0	0	0	0	0

0	0	0	0	0
0.003230882	0	0	0	0
0	0	0	0	0
0	0	0	0	0
0	0	0	0	0
0.013505132	0	0	0	0
0	0	0	0	0
0	0	0	0	0
0.023364727	0.007987777	0.002117925	0.004141012	0.010504361
0	0	0	0	0
0	0	0	0	0
0.009124195	0.011692864	0.004917739	0	0.008003202
0	0	0	0	0
0	0	0	0	0
0	0	0	0	0
0	0	0	0	0
0	0	0	0	0
0	0	0	0	0
5.57983038	0	0.00719006	0	0.165682357
0	0	0	0	0
0	0	0	0	0
0	0	0	0	0
0	0	0	0	0
0	0	0	0	0
0	0	0	0	0
0	0	0	0	0
0.00124505	0.002290178	0	0	0.007937107
0.001143748	0	0	0	0.000662847
0	0	0	0	0
0	0	0	0	0
0.001572439	0	0.000559962	0.121665093	0.082471771
0.00061861	0	0	0	0.028999361
0	0	0	0	0
0	0	0	0	0
0	0	0	0	0
0	0	0	0	0
0	0	0	0	0
0	0	0	0	0
0	0	0	0	0
0	0	0	0	0
0	0	0	0	0
0	0	0	0	0
0	0	0	0.011616418	0.003130865
0	0	0	0	0
0	0	0	0	0.004319684
0	0	0	0	0
0	0	0	0	0
0	0	0	0	0

0.003703399	0	0	0	0
0	0	0	0	0
0	0	0	0	0
0	0	0	0	0
0.001435812	0	0	0	0.002080274
0	0	0	0	0
0	0	0	0	0
0	0	0	0	0
0	0	0	0	0
0	0	0	0	0
0	0	0	0	0
0	0	0	0	0
0	0	0	0	0
0	0	0	0	0
0	0	0	0	0
0	0	0.224444004	0	3.581799711
0	0	0	0	0
0	0	0	0	0
0	0	0	0	0
0	0	0	0	0
0	0	0	0	0
0	0	0	0	0
0	0	0	0	0
0	0	0	0	0
0	0	0	0	0
0	0	0	0	0
0	0	0	0	0
0	0	0	0	0
0	0	0	0	0
0	0	0	0	0
0	0	0	0	0.007518461
0	0	0.010844626	0	0
0	0	0	0	0
0	0	0	0	0
0	0	0	0	0
0	0	0	0	0
0	0	0	0	0
0	0	0	0	0
0	0	0	0	0
0	0	0	0	0
0	0	0	0	0
0	0	0	0	0
0	0	0	0	0
0	0	0	0	0
0	0	0	0	0
0	0	0	0	0
0	0	0	0	0
0	0.087753719	0	0	0

0	0	0	0	0
0	0	0	0	0
0	0	0	0	0
0	0	0	0	0
0	0	0	0	0
0	0	0	0	0
0	0	0	0	0
0	0	0	0	0
0	0	0	0	0
0	0	0	0	0
0	0	0	0	0
0	0	0	0	0
0.028685204	0	0	0	0.001055505
0	0	0	0	0
0	0	0	0.001204066	0
0	0	0	0	0
0	0	0	0	0
0	0	0	0	0
0	0	0	0	0
0	0	0	0	0
0.011067424	0.045239378	0.052145432	0.106223746	0.012828016
0	0	0	0	0
0	0	0	0	0
0	0	0	0.002596172	0.009604029
0	0	0	0	0
0	0	0	0	0
0.002726903	0.009322554	0.000706239	0.00314144	0.000957786
0	0	0	0	0
0	0	0	0	0
0	0	0	0	0
0.002965374	0.061818672	0	0.064556714	0.001336651
0	0	0	0	0
0	0	0	0	0
0	0	0.000741117	0	0.609686374
0.012186402	0.040982786	0	0.242171886	0.001926136
0	0	0	0	0
0	0	0	0	0
0	0	0	0	0
0	0	0.004206331	0	0.001037189
0.003894809	0	0	0	0
0	0	0	0	0
0	0	0	0	0
0	0	0	0	0
0	0	0	0	0.002619384
0	0	0	0	0
0.005010292	0	0.001502499	0	0
0.001799268	0	0	0.008184857	0
0.210398352	0.001290848	0	0	0.001372617

0	0	0	0	0
0	0	0	0	0
0	0.00117955	0	0	0.002229811
0	0	0	0	0
0	0	0	0	0
0	0	0	0	0
0.00224765	0.002067194	0	0	0.043637159
0	0	0.002020753	0	0.10041201
0	0	0	0	0
0	0	0	0	0
0	0	0	0	0
0	0	0	0	0
0	0	0	0	0
0	0	0	0	0
0.012136407	0	0	0	0
0	0	0	0	0
0	0	0	0	0
0	0	0.043628843	0	0
0	0	0.032738092	0.001760979	0.001085733
0	0	0.019823049	0	0.046924112
0.321258774	0.259973815	0	0.05368422	0
0	0	0	0	0
0	0	0	0	0
0	0	0	0	0
0.005882474	0.006251777	0.002513772	0.006880971	0
0	0	0	0	0
0	0	0	0	0
0	0	0	0	0
0	0.103719419	0.037783136	0	0.011061484
0	0	0	0	0
0.301753847	1.096759349	0.006512586	1.461174115	0.053161635
0	0	0	0	0
0	0	0	0	0
0.000845026	0	0	0	0
0	0	0	0	0
0	0	0	0	0
0	0	0	0	0
0	0	0	0	0
0	0	0	0	0
0	0	0	0	0
0	0	0	0	0
0.053927744	0.029572882	0	0.034179956	0.002400798
0	0.00563234	0	0.000726032	0
0	0	0	0	0
0	0	0	0	0
0	0	0	0	0
0	0	0	0	0
0	0	0	0	0

0	0	0	0	0
0	0	0	0	0
0.061305903	0	0.001984697	0.004346183	0.075149605
0	0	0	0	0
0.369276883	0	0.096969711	0	0.830814765
0	0	0	0	0
0	0	0	0	0
0	0	0	0	0
0	0	0	0	0
0	0	0	0	0
0	0	0	0	0
0	0	0	0	0
0	0	0	0	0
0	0.001510651	0	0.006604561	0
0.005338022	0.003324919	0.002896648	0.005698982	0.001571349
0.009525095	0	0	0	0.010304313
0.004923683	0.006866561	0	0.00762278	0
0	0	0	0	0
0	0	0	0	0
0	0	0	0	0
0	0	0	0	0
0.193474352	0.002351172	0	0	0.000808115
0	0	0	0	0.001007632
0	0.005632955	0	0	0
0	0.002741794	0	0.002043258	0.001619707
0	0	0	0	0
0	0	0	0	0.00638916
0	0	0	0	0
0	0	0	0	0
0.012858544	0.008966252	0	0.081472978	0
0	0	0.001141574	0	0.001548179
0	0	0	0	0
0	0	0	0	0.01212522
0	0	0.003005816	0.000881555	0
0	0	0	0	0
0.013577534	0.011953791	0	0	0.016544478
0	0	0	0	0
0.090662453	0.051560804	0.006316795	0	0.138844082
0	0	0	0	0
0	0	0	0	0
0	0	0	0	0
0	0	0	0	0
0	0	0	0	0
0.030194398	0.026686075	0	0.001193231	0
0	0	0	0	0
0	0.025185986	0.001798513	0	0.011707711
0	0.004948596	0.006086378	0	0
0	0.013011055	0	0.009308338	0.001537249

0	0.001501067	1.346764187	0.02173348	0
0	0	0	0	0
0.000821551	0.015671533	0.000780168	0	0
0	0	0	0	0
0.002755199	0.009685488	0.009615319	0	0.002128995
0.004043342	0.015058506	0	0	0.023606333
0	0	0	0	0
0.004634422	0.010419054	0.002475548	0.220984003	0
0	0.005474245	0	0	0
0.027466593	0.005206858	0.003685646	0.584759641	0.006459467
0.001799518	0	0	0	0
0.059615152	0.005748439	0.060531503	0.001490049	0.089769189
0.004869869	0.006066637	0	0.003616816	0.002508694
0.033810626	0	0	0	0
0.000954009	0.006239398	0	0	0
0	0	0.013636533	0	0
0	0	0.202658004	0	0
0	0	0	0	0
0.318012798	0	0	0.068278969	0
0	0.280065551	0	0	0
0	0	0	0	0.023292153
0	0	0	0	0
0.129216222	0	0	0	0
0	0	0	0	0
0	0	0	0	0
0.005402053	0	0	0	0
0	0	3.368060375	0	0
0.005879325	1.566913926	0	0.00222875	0
0.041205375	0	0	0	0
0.001389508	0.003635056	0	0.028714773	0
0.006666201	0.00284336	0	0	0
0	0	0	0	0
0	0	0	0	0
0	0	0	0.095381415	0
0	0	0	0	0
0	0	0	0	0
0	0	0	0.005372427	0
0	0	0	0	0
0	0	0	0.000703129	0
0.344587633	0	0	0	0
0	0	0	0.002962382	0
0	0	0	0.036796727	0
0	0	0	0	0
0	0.027929747	0	0.054301983	0
0	0	0	0	0
0	0	0	0	0.047796056
0.004030728	0.003569814	0	0	0
0	0.036390199	0	0	0

0	0	0	0	0
0.00600616	0	0.002138856	0.004756951	0
0	0	0	0	0
0	0	0	0	0
0	0	0	0	0
0.050265866	0.085214962	0	0.002578861	0
0	0	0	0	0
0	0	0	0	0
0	0	0	0	0
0	0	0	0	0
0.005829994	0.031857985	0.006556173	0	0
0	0	0	0	0
0	0.061383328	0	0	0
0	0.02275111	0.003303436	0.001978052	0.013440153
0	0.023259827	0	0	0
0	0	0	0	0
0	0	0	0	0
0	0	0	0.006874873	0
0	0	0	0	0
0	0	0	0.006947207	0.091203057
0	0	0	0	0
0	0	0.150095995	0	0.463211018
0	0	0	0	0
0	0	0	0	0
0	0	0	0	0
0	0	0	0	0
0	0.032120087	0	0.031864596	0
0	0	0	0	0
0	0	0	0	0
0.266847324	0.088581587	0.191264737	0.103136169	0.151365116
0.004719995	0	0	0.008243415	0
0	0.004288855	0.018485457	0	0
0.014233885	0	0	0	0
0	0	0	0.001086093	0
0	0	0	0.00178636	0
0	0	0	0	0
0	0	0	0	0.307469809
0	0.00362335	0.029251519	0	0.008904413
0	0	0	0	0.010039642
0	0	0	0	0
0	0.120406279	0.005261831	0.016029432	0
0	0	0.682751012	0	0.493672378
0	0.001106408	0.032579654	0	0.007320401
0.004025139	0	0	0	0.00599844
0.004032562	0	0	0.149897443	0
0	0	0	0	0
0.001750938	0.006543691	0	0.018043172	0.003092533
0	0	0	0	0

0.001543117	0.005298442	0	0	0.005485021
0	0	0	0	0
0.001682092	0.005844384	0.003893568	0.114367441	0
0.019821102	0	0	0	0
0.065357569	0	0	0	0.078151863
0.012786126	0	0.000929239	0	0
0	0	0	0	0.002202922
0	0	0.00073908	0	0.064148834
0.118038572	0	0	0	0
0	0	0	0.008560317	0
0	0	0	0	0
0	0	0	0	0
0	0	0	0	0
0.006421687	0.002282556	0.000795419	0	0
0	0	0	0	0
0.6705069	0	0	0.03998591	0.13707331
0.073520613	0.053975696	0.010334784	0.003771933	0.048961926
0.325858776	0.019457317	0	0.101966219	0
0.004996134	0	0	0	0
0	0	0	0	0
0	0	0.001085397	0.026739612	0.016957363
0.005891059	0.005733425	0	0.00191417	0
0	0	0	0	0.001711359
0	0	0.128533549	0	0
0	0	0.000482943	0	0.020783965
0.009239023	0.749842042	0	0	0
0	0.01305192	0	0.002693531	0
0.021372712	0	0	0	0
0	0	0	0	0
0	0	0	0	0.006901965
0.009806587	0.030159612	0	0.019573816	0.039873333
0	0.0196363	0	0	0
0.028172673	0.583924183	0	0.026816102	0.00922005
0	0.00543642	0.004397875	0.002315066	0.031931975
0	0	0	0.002974262	0
0	0	0	0	0
0	0.000734906	0	0.035343087	0.000926173
0.034682059	0.141958512	0	0.05093971	0.000543233
0	0	0.002355723	0	0.023944049
0	0.131728958	0	0.002054406	0
0.038371335	0	0	0	0
0	0	0	0	0
0.018924433	0.001719018	0	0	0
0.036585976	0	0	0.018366665	0.043819559
0.270629533	0	0	0	0
0	0	0	0	0
0	0	0	0	0
0	0	0	0	0

0.003127013	0	0	0.004064216	0
0.441294131	2.83419686	0.083464307	0	0.267714263
0	0	0	0	0
0	0	0	0	0
0	0	0	0.001221457	0
0	0	0	0	0
0	0.001207516	0	0.066518648	0
0	0	0.00174685	0	0
0	0	0.000832749	0.0031343	0.005872654
0	0	0	0.004976164	0
0.019457484	0.01144524	0	0.024055512	0
0.012370568	0.014158517	0.00679673	0.200862378	0.023214653
0	0	0.001118972	0	0.744888775
0	0	0	0	0
0	0	0	0.001592348	0.006942467
0	0	0	0	0
0.022611052	0.023969637	0.012564706	0.010657136	0.004991998
0.039351394	0.084118406	0	0.032385153	0
0	0	0	0	0
0.019897624	0	0	0.039812612	0
0.019217884	0.034983305	0.009307415	0	0.085932153
0.004463866	0	0	0.00124323	0
0	0	0	0	0
0	0	0	0	0
0	0	0	0.017206562	0
0.031017077	0	0	0	0
0.025292015	0	0	0.005423936	0.007622013
0.029957801	0.116667153	0.006788908	0.489007265	0.03724879
0	0	0	0	0
0.01445445	0	0	0	0
0	0.001911205	0	0	0
0	0	0	0	0
0	0	0	0	0
0	0	0	0	0
0	0	0.004901879	0	0.012103627
0.469222028	0.088552034	0.163812025	0.090309848	0.047726224
0	0	0	0	0
0	0	0	0	0
0.030908904	0.032959233	0.015314061	0.001309979	0.047508195
0	0	0	0	0
0.113421496	0	0	0	0
0	0	0	0	0
0.007754701	0.318317066	0.008284589	0.014919394	0
0.077431531	0	0	0.052047878	0.000608985
0	0	0	0	0
0.001424985	0	0.186307367	0	0.387749162
0	0	0	0	0
0.001910871	0.00364509	0	0	0

0.001425459	0	0	0	0
0	0	0	0	0
0	0	0	0	0
5.579734491	0.55319337	0.041304168	0	0.343257787
0	0	0	0	0
0	0	0	0	0
0	0	0	0	0
0	0	0	0.006839813	0.004271858
0	0	0.057765991	0.014261126	6.156415091
0.070525398	0	0	0	0.064345058
0	0	0	0	0
0	0	0.00373032	0	0.020059966
0	0	0	0	0
0	0	0.039622179	0	0
0.145447141	0	0.00307827	0	0.062003152
0	0	0	0	0
0	0	0.001396638	0	0.243580082
0	0	0.093677813	0	1.793025482
0	0	0	0	0
0	0	0	0	0.068984977
0	0	0.056760276	0	0
0	0	0	0	0.000816851
0	0	0	0	0.000810168
0.156031884	0.357078314	0.020498299	0.42488462	0.012028908
2.263814298	0	0	0.001039146	0
0	0	0	0.287014199	1.742213984
0	0	0	0	0.266397296
0	0	0.647922215	0	1.229876579
0	0	0	0	0
0	0	0	0	0
0.005755982	0	0	0	0.732169353
0.387133271	0	0	0	0
0.018533838	0	0	0.023650678	0
0	0	0	0	0
0	0	0.000582315	0	0
0	0	0.001203211	0	0.703329022
0.006303101	0.000594569	0.457380351	0	1.101863023
0	0	0	0	0
0	0	0	0.091045628	0
0.001303975	0	0	0	0
0.122554875	0.084832332	0.084641091	0.129899059	0.154795642
0	0	0.2193202	0	0.326356538
0.070496037	0.23074282	0.170411078	20.06577776	0.018427885
0	0	0	0.007420266	0.002941052
0	0	0	0	0
0	0	0	0	0
0	0	0	0	0
0.452742128	0.010734547	0	0.013311452	0.0008117

0	0	0	0	0
0	0	0	0	0.020335735
0.175716415	2.065052398	0	0.497906046	0.000583579
0.001904853	0	0	0	0.002943832
0	0	0	0	0
0.028953728	0	0	0	0.027736514
0	0	0	0	0
0	0	0	0	0
0	0	0	0	0
0	0	0	0	0
0.101832682	0.117803607	0.001056224	0.035658258	0.005729713
0.267527159	7.628904493	0.356904005	6.490554891	0.728845522
0	0	0	0	0
0.077091439	0.082214747	0.011969155	0.001820182	0.129240167
0.078408743	0.618496964	0.016319122	0.100406119	0.017542888
0	0	0	0	6.204996625
0	0	0	0	0
0	0	0	0	0
0	0	0	0	0.006494114
0	0	0	0.002381596	0.004877102
0.51894105	1.050200192	0.0258204	0.030896563	0.190492956
0	0.586574023	0.002953869	1.681177457	0
0.492696103	0.691686266	0.442328522	12.07623605	0.188089887
0	0	0	0	0
0	0	0	0	0.036085208
0	0	0.00424328	0	1.869301467
0	0.011951171	0	0	0.003637757
0	0	0	0	0
0	0	0	0	0.024588652
0	0	0	0	0
0	0	0.001301908	0	0
0.000782308	0	0.082462003	0	0.213087675
3.825468917	3.344688659	0.001265564	0.888082524	0.024126715
0.002710088	0	0.002068051	0	0.008226966
0.008544639	0.02794176	0.000715961	0.029723734	0.000647314
1.191475748	0	0	0.002243879	0.159019404
0.016899565	0	0	0	0.003264654
0	0	0	0	0
5.379888952	0	0.00383704	0.017067643	0.477076507
0	0	0	0	0
0	0	0	0	0.002506033
0	0	0	0	0
0	0	0	0	0
0	0	0	0	0
6.448515823	0.110951611	0	0.009212	0.055376731
0.312057433	0	0.018641572	0	0.274527595
0	0	0	0	0
0	0.356987524	0	0.002160704	0.137269241

0	0	0	0	0
0	0	0	0	0
0	0	0	0	0
0	0	0	0.003053303	0.004322103
0	0	0	0	0
0.000988006	0	0	0	0
0.02155533	0	0.312276757	0	0.000757101
0.54083746	0.69090752	0.001173036	0.095526108	0
0	0	0	0	0
0	0	0	0	0
1.714616225	0.015278359	0	0.003400571	0.000770188
0	0	0	0	0
0.302612432	0	0	0.129307086	0.011295534
0	0	0.00075721	0	0.086424948
0	0	0	0	0
0	0	0	0	0
0	0	0.023494663	0	0.071888591
0.00539338	0	0	0	0.007052808
0	0	0	0	0.913509865
0.010289095	0	0	0	0
0	0	0	0	0
0	0	0	0	0
0.004783217	0	0.002214359	0	0.013490703
0	0	0	0	0
0	0	0	0	0
0	0	0	0	0
0	0	0	0	0
0	0	0	0	0
0	0	0	0	0
0	0	0.010172809	0	0.017620348
0	0.035297999	0	0	0
0	0	0	0	0
0	0	0	0	0.512311807
0	0	0	0	0
0.205071586	0	0	0	0
0	0	0	0	0
0	0	0.014742506	0	0
0.004640598	0.01431296	0	0.042974026	0
0	0	0	0	0
0	0	0	0	0
0	0	0	0	0.002599796
0	0	0	0	0
0	0	0	0	0
0	0	0.008899549	0	0.365326349
0	0	0.015182354	0.323131297	0.175454493
0	0	0	0	0
0	0	0	0	0.004181546
0	0	0	0	0

0	0	0	0	0
0	0	0	0	0
0	0	0	0	0
0	0	0.001195504	0	1.778585443
0	0	0	0	0
0	0	0	0	0
0	0	0	0	0
0	0	0	0	0
7.051813024	0.535119476	0	0.597504457	0
0	0	0	0	0
0	0	0	0	0.002732522
0	0	0	0	0
0	0	0	0	0.000655711
0.01919581	0	0	0	0
0.001254361	0.001196381	0.0248658	1.968189803	0.621624415
0	1.32179701	0	0.309267357	0
0	0	0	0	0
0	0	0	0	0
0	0	0	0	0
0	0	0	0	0
0	0	0.000633726	0	0.033862188
0	0	0	0	0
0	0	0	0	0
0	0	0	0	0
0.003421094	0.013984117	0.190052769	0.010671449	0.010309824
0.008061309	0.002636123	4.667553975	0.351725673	2.402200698
0.330326528	0	0	0	0.123691845
0	0	0	0	0
0.491423641	0	0.005751708	0	0.062171655
0	0	0	0	0.006044154
0	0	0	0	0
0.000881281	0	0	0	0.032687181
0	0	0	0.000807447	0
0.005115834	0	0	0.035442142	0.001129458
0.008128233	0.013290038	0.003644987	0.003228	0.013492178
0.087887558	0.06123787	0	0.014935449	0
0	0	0	0.061437811	0.002715108
2.228940664	0.000589331	0.283408401	0.084557748	0.427986787
0	0	0	0	0.107991601
0	0	0	0	0
0	0	0	0	0
0	0	0.00378121	0	0
1.602592296	0	0.02381799	0.153922075	0.293016378
0	0	0	0	0
0	0	0	0	0
0	0	0	0.015918146	0
0	0	0	0	0
0.9889109	1.28843822	0.102022733	0	0.592657058

0	0	0	0.067004287	0
0	0	0	0	0.55455488
0	0	0	0	0
0	0	0	0	0
0.003099898	0.003119063	0	0	0.001842577
0.440654893	0	0	0	0.053408672
0	0	0	0	0
0	0	0	0	0
0	0	0	0	0
0	0	0	0	0
0	0	0	0	0.000563958
0.004839753	0.365087176	0.082570651	0.630243682	1.981057822
0.009816671	0	0.003290182	0.73654194	0.024764523
0	0	0	0	0
0.222547754	3.980315237	1.772210309	0.013440893	0.139363515
0	0	0	0	0
0.027241144	0	0	0.000748007	0
0	0	0	0	0
0.019337032	0	0	0.005890447	0.005416509
0.000804063	0	0	0	0.053640187
0.710011985	0	0.002565483	0	0.113501027
0	0	0	0	0
0.019977801	0	0.003013116	0.020848681	0.244634676
0	0	0	0	0
0	0	0	0	0
0	0	0	0	0
0	0	0	0	0
0	0	0	0	0
0	0	0	0	0
0	0	0	0	0
0	0	0	0	0
0.04294228	0.047393516	0.029310033	0.018487925	0.010784254
0	0	0.118384318	0	0
0.007687405	0	0	0	0.001519587
0.082906633	0.053200123	0	0.017156484	0
0	0.016840275	0.002976752	0	0.010842253
0	0	0	0	0
0	0	0	0	0.134497332
0	0	0	0	0
0	0	0	0	0
0.005327353	0	0.002670029	0	0.014484149
0.008923141	0	0	0	0.678993145
0	0	0	0	0
0	0	0	0	0
0.023041357	0.148643205	0	0.006671391	0.006875004
0	0	0	0	0
0	0	0.154342699	0	0
0.001941951	0	0	0	0.00116712

0	0	0	0	0
0	0	0	0	0
0.003539187	0	0	0.001379977	0
0	0	0	0	0
0	0	0	0	0
0	0	0	0	0
0	0	0.057529095	0.019241307	0
0.084229279	0	0.000634813	0.0458313	0.026344161
0	0	0	0	0
0	0	0.004060422	0	0
0	0	0	0	0
0.143081218	0	0	0	0
0	0	0	0	0
0	0	0	0	0.504230047
0	0	0	0	0
0	0	0	0	0
0.003117011	0	0.001009091	0	0.44317764
0	0	0	0	0
0.001764811	0.042664884	0	0	0.278585084
0.000669568	0	0	0	0
0	0	0	0	0
0	0	0	0	0
0	0	0	0	0
0	0	0	0	0
0	0.020784658	0	0	0
0.536478184	0.070666437	0	0.006831894	0
0	0	0	0	0
0.598279274	10.2330124	0.002665019	0	2.341386942
0	0	0	0	0.789354134
0	0	0.001653551	0	0.715361212
0	0	0	0	0
0	0	0	0	0
0	0	0	0	0.000678458
0	0	0	0	0
0.018027661	0.005640481	0	0	0
0	0	0	0.005669064	0.508503433
0	0	0	0	0
0.00707125	0.003096916	0	0.004677353	0
0	0	0	0	0
0	0	0	0	0
3.18232544	2.418633568	1.958620622	2.5890164	1.605750603
0.090213542	0.138737754	0.019222921	0.032977023	0.026569692
0	0	0.002472584	0	1.291846044
0	0	0	0	0
0	0	0	0	0
0	0	0	0	0
0	0	0	0	0
0.206646828	0.003441038	0	0.025203928	0.002942696

0	0	0	0	0
0.003092434	0.005898986	0	0.019493481	0.006531527
0.000963144	0	0	0	0
0	0	0	0	0
1.828831545	0.001687139	0.056655054	2.33556269	1.753274077
0	0	0	0	0
0	0	0	0	0
0	0	0	0	0
2.061877121	1.044095729	0.001026316	0.240800925	0.001590707
0	0	0	0	0
0	0	0	0	0
0	0	0	0	0
0.291868867	0	0.012697928	0	0.127239426
0.00837336	0.008657437	0.00964713	0.010322805	0.00589935
0	0	0	0	2.193022946
0.336482706	0	0.028786783	0	0.423350125
0.064147031	0.025262605	0.011231982	0.006232177	0.078056679
0	0	0	0	0
0	0	0	0	0
0	0	0	0	0
0	0	0	0	0
0	0	0	0	0
0	0	0	0	0
0	0	0	0	0
0	0	0	0	0
0	0	0	0	0
0	0	0.00778694	0	0.083624722
0.054575686	0.0230777	0	0.007337864	0
0	0	0	0	0
0	0	0	0	0
0	0	0	0	0
0	0	0	0	0
0.018707786	0	0	0	0.108157729
0	0	0.005151802	0	0.07113798
0	0	0	0	2.24185768
0.005805309	0.034408319	0	0	0.00934557
0	0.001414863	0	0	0
0.001142054	0	0	0	0.003000455
0	0	0	0	0
0	0	0	0	0
0	0	2.52350864	0	0.372221282
0	0	0.002511859	0	0
0	0	0.009339135	0	0.143443444
0	0	0	0	0.003559619
0	0	0	0	0
0	0	0	0	0
0	0	0	0	0
0	0	0	0	0

0	0	0.004728771	0	0.427773254
0	0	0	0	0
0	0	0	0	0
0	0	0	0	0
0.548695448	0	0.003712607	0	0.069876497
0.007975983	0	0	0	0
0.021684675	0.086379206	0.034825328	0	0.659568594
0	0	0	0	0
0	0	0	0	0
0	0	0	0	0
0	0	0	0	0.003785022
0	0	0.001721393	0	0.02241136
0	0	0	0	0
0	0	0	0	0
0	0	0	0	0
0	0	0	0	0
0	0	0	0	0
0	0	0	0	0
0.002128076	0	0	0	0
0	0	0	0	0
1.404063734	0.289073379	0.651868966	0.000986492	0.870678885
0	0	0	0	0
0.004464977	0	0	0	0.029671491
0.003374758	0	0	0.003096151	0.00306793
0	0	0	0	0
0.006791896	0	0	0.006164037	0.001990707
0	0	0	0	0
0	0	0	0	0
0	0	0.021111361	0	0.04444182
0.020856824	0.00677366	0	0.365956206	0.339991215
0	0	0	0	0.117657867
0	0	0	0	0
0.011548293	0	0	0	0
0	0	0	6.29041558	0.013269275
0	0	0	0	0
0	0	0	0	0
0.101096329	0.002639881	0.00406896	1.082081003	0.018809997
0	0	0	0	0
0	0	0	0	0
0	0	0	0	0
0	0	0	0	0
0	0	0	0	0
0	0	0	0	0
0	0	0	0	0
0	0	0.050899694	0	0
0	0	0	0	0
0	0	0	0	0
0	0	0	0	0

0.851660686	0.048023424	0	0.016241398	0.001815661
0	0	0	0	0.181745568
0.002030828	0	0	0	0
0	0	0	0	0
0	0	0	0	0
0	0	0	0	0
0.002463192	0	0	0	0
0.376367841	0.339785236	0.005154942	0.266606122	0.211679756
0.162281347	0.11348113	8.206667713	0.215712652	0.169442619
0.061622949	0.751283808	0	0.333337837	0.000541105
0	0	0	0	0.676462207
0	0	0	0	0
0	0	0	0	0
0	0	0	0	0
0	0	0	0	0
0	0	0	0	0
0	0	0	0	0.000963773
0	0	0	0	0
0	0	0	0	0
0	0	0	0	0.016452231
0	0	0	0	0
0	0	0	0	0
0	0	0	0	0
0	0	0	0	0
0	0	0.003714699	0	0.040302383
0	0	0	0	0
0	0	0	0	0
0	0	0	0	0
0	0	0	0	0
0.0005994	0	0	0	0
0	0	0	0	0
0	0	0	0	0
0	0	0	0	0
0	0	0	0	0
0	0	0	0	0
0	0	0	0	0
0	0	0	0	0
0	0	0	0	0
0	0	0	0	0
0.007890216	1.330532827	0	0	0.007415174
0	0	0	0	0
0.899597466	0	0.070041148	0.230540918	0.206721835
0	0	0	0	0
0	0	0	0	0
0.001662451	0	0	0	0
0	0	0	0	0.005870878
0	0	0	0	0
0	0	0	0	0

0	0	0	0	0
0.004434212	0	0	0.005637912	0
0	0	0	0	0
0	0	0	0	0
0.005930102	0.919576614	0.00230375	0.009459113	0.224116278
0	0	0	0	0.383749093
0	0	0	0	0
0.637470672	0.054202846	0	0.375327592	0
0	0	0	0.001057871	0.006918306
0	0	0.005473283	0	0
0	0	0	0	0
0	0	0	0	0
0	0	0	0	0
0	0	0	0	0
0	0	0	0.018086864	0
0	0	0	0.019756258	0.026040871
0	0	0	0	0
0	0	0	0	0
0	0	0	0	0
0	0	0	0	0
0	0	0	0	0
0	0	0	0	0
0	0	0	0	0
0.708763259	0.012111518	0	0	0.000726841
0.000627671	0	0	0	0.10670303
0	0	0	0	0
0	0.001406649	0	10.04055913	0.122651702
0	0	0	0.000662278	0
0.117644662	0	0	0	0
0	0	0	0	0
0	0	0	0	0
0	0	0	0	0
0	0	0	0	0
0	0	0	0	0
0	0	0	0	0
0	0	0	0.031160058	0
0	0	0	0	0
0	0	0.000628392	0	0
0	0	0.002040399	0	0
0	0	0	0	0
0	0	0	0	0
0	0	0	0	0.080324295
0	0	0	0	0
0	0	0	0	0
0	0	0	0	0
0	0	0	0	0
0	0	0	0	0
0	0	0	0	0.002354933

0.011528941	0	0.005474101	0	0.143163297
0	0	0	0	0
0	0	0	0	0
0.062384293	0	0	0	0
0	0	0	0	0
1.237977908	0.619735873	0	0.019872842	0
0	0	0	0	0
0	0	0	0	0
0	0	0	0	0
0	0	0	0	0
0	0	0	0	0
0.019509954	0	0	0.001056555	0
0	0	0	0	0
0	0	0	0	0
0	0	0	0	0
0	0	0	0	0
0	0	0	0	0
0	0	0	0	0
0	0	0	0	0.000397523
0	0	0	0	0
0.003980958	0.262467893	0	0.004565382	0
0	0	0	0	0
0.064472345	0.00265956	0	0.061405181	0.003427914
0.049805297	0	0	0	0.156661108
0	0	0	0	0.006568457
0	0	0	0	0
0	0	0	0	0
0	0	0	0	0
0	0	0	0	0
0	0	0	0	0
0	0	0	0	0
0	0	0.046382849	0	0
0.056777557	0.149892843	0	0.004859637	0.002996212
0	0	0	0	0
0.011643907	0.004455779	0.002470265	0.713136668	0.033118347
0.168244579	0.009266494	0	0.012348906	0.000686953
0	0	0	0	0
0	0	0	0	0
0.170694022	0.211841397	0	0.002756458	0.057010361
0	0	0	0	0
0	0	0	0	0
0	0	0.007225969	0	0.038373595
0	0	0.005138092	0.001465057	0.812489056
0	0	0	0	0
0	0	0	0	0
0.017187943	0	0.004250559	0	0.016048422
0	0	0	0	0
0	0	0	0	0

0	0	0	0	0
0	0	0	0	0
0	0	0	0	0
0	0	0	0	0
0	0	0	0	0.007929069
0	0	0	0	0
0	0	0	0	0
0.002537486	1.301813809	0	0	0
0.034667276	0	0	0	0.001901089
0	0.000804052	0	0	0.000759986
0	0	0	0	0
0	0	0	0	0
0	0	0	0	0
0.006132917	0	0.003313648	0	0
0	0	0	0	0
0.240467656	0.009295403	0	0.001179095	0
0	0	0	0	0
0	0	0	0	0
0	0	0	0	0
0.001215254	0	0.529126919	0	0.52383352
0	0	0.004858784	0	0.003514337
0	0	0	0	0
0	0	0	0	0
0.008700515	0	0	0	0
0.004250483	0	0	0	0
0	0	0	0	0.102612539
0	0	0	0	0
0	0	0	0	0
0	0	0	0	0
0	0	0.072144531	0	0.538415609
0.058201032	0	0.017224979	0.086058497	0.666391221
0	0	0	0	0
0	0	0	0	0
0	0	0	0	0
0	0	0	0	0
0	0	0	0	0
0	0	0	0	0
0	0	0	0	0
0	0	0	0	0
0	0	0	0	0
0	0	0	0	0
0	0	0	0	0
0	0	0	0	0
0	0	0	0.002015107	0
0	0	0	0	0
0	0	0	0	0
0.335319307	0.153667398	0	0.071214815	0.002870469
0	0	0	0	0

0	0	0	0	0
0	0	0	0	0
0	0	0	0	0
0	0	0	0	0
0	0	0.000790624	0.04238188	0.153257016
0	0	0	0	0
0	0	0	0	0
0	0	0	0	0
0	0	0	0	0
0	0	0	0	0
0	0	0	0	0.089667982
0.019264846	0.023729183	0	0.29495653	0.006351495
0.002216094	0	0	0	0
0	0	0	0	0
0	0	0	0.00133569	0
0	0	0.017214081	3.577050698	0.004321789
0	0	0	0.005199599	0.076786928
0	0	0	0	0
0.00914147	0.005247255	0	0.347906611	0.011873177
0.058918001	1.87767592	0.268054339	0.00396085	0.257659646
0	0	0	0	0
0	0	0.000475759	0	0.259806095
0	0	0	0	0
0	0	0	0	0
0	0	0	0	0
0	0	0	0	0
0.005087683	0	0	0.08133403	0.001100778
0	0	0	0	0
0	0	0	0	0
0	0	0	0	0
0.109806638	0.001320139	0	0.000899477	0
0	0	0	0	0
0	0	0	0	0
0	0	0	0	0
0	0	0	0	0.115949042
0	0	0	0	0
0	0	0	0	0
0.046839224	0.034714683	0	0.094633981	0.0063871
0.016959878	0	0.053592672	0	0.100473373
0	0	0	0	0
0.08235698	0.011111226	0	0.161506881	0
0	0	0	0	0
0	0	0	0	0
0.78709502	0.251885955	0	0.006793949	0.002042821
0	0	0	0	0
0	0	0	0	0.047815709
0	0	0	0	0
0	0	0	0	0.002518057

0	0	0	0	0
0	0	0.000731505	0	0.024761627
0	0	0	0	0.014980173
0.095711168	0.004324126	0	0.006874569	0
0	0.003437621	0	0	0.000541537
0.001205968	0.002711243	0	0	0.000931874
0	0	0	0	0.095001446
0	0	0	0	0
0	0	0	0	0
0	0	0	0.001134852	0.017992125
0	0	0	0	0.036185046
0	0	0	0	0
0.686848892	0	0.013606218	0.005569259	0.623865885
0.001260172	0	0	0	0
0	0	0	0	0
0	0	0	0	0
0	0	0	0	0
0	0	0	0	0
0	0	0	0	0
0	0	0	0	0
0	0	0	0	0
0	0	0	0	0
0.062771146	0.027099918	0	0.051104848	0
0.018864807	0	0.001383108	0	0.046304039
0	0	0	0	0
0	0	0	0	0.002533035
0	0	0	0	0
0.001540969	0	0	0.001502112	0
0	0	0	0	0
0	0	0	0	0
0	0	0	0	0.014989092
0	0	0	0	0
0	0	0	0	0
0	0	0	0	0
0	0	0	0	0.056209693
0	0	0	0	0
0	0.10245509	0	0.064339509	0
0	0	0	0	0
0.022400065	0.021757511	0	0	0.029476642
0	0	0	0	0
0	0	0	0	0
0	0	0	0	0
0	0	0.144272858	0	0
0	0	0	0	0
0	0	0	0	0
0.687646019	0	0.030019082	0	0.087906148
0.054577322	0	0	0	0
0	0	0	0	0

0	0	0	0	0
0	0	0	0	0
0	0	0	0	0.076247389
0.000883825	0	0.009232355	0	0
0	0	0	0	0
0.006108061	0	0	0	0
0.056810492	0	0	0	0
0	0	0	0	0
0	0	0	0	0
0	0	0	0	0
0.056496538	0.000735465	0	0.025782081	0.000556127
0.014505967	0	0	0	0
0	0	0	0	0
0	0	0	0	0
0	0	0	0	0
0	0	0	0	0
0	0	0	0	0.096462926
0	0	0	0	0
0	0	0	0	0
0.000715835	0	0	0.00279114	0
0	0	0	0	0
0	0	0	0	0
0	0	0	0	0
0	0	0	0	0
0	0	0	0	0
0	0	0	0	0
0	0	0	0	0
0	0	0	0	0.002652618
0	0	0	0	0
0	0	0	0	0
0.001229954	0.002513789	0	0.000999116	0
0	0	0	0	0
0.004760338	0.097097574	0.017630139	0.138048971	0.011219132
0.00097291	0.035593088	0.000866158	0	0
0	0	0	0	0
0	0	0	0	0
0	0	0	0	0
0	0	0	0	0
0	0	0	0	0
0	0	0	0	0
0	0	0	0	0
0.324855976	1.228955286	0	0.115098649	0
0.011689296	0.033934512	0.010704034	0.025812122	0.100095322
0	0	0	0	0
0	0	0	0	0
0.001378926	0	0	0	0.130526525
0	0	0	0.003745382	0
0	0	0	0	0

0	0	0	0	0
0	0	0.001601985	0	0.021049872
0.005254797	0.029832764	0	0	0.008797711
0	0	0	0	0
0	0	0	0	0.026837378
0	0	0	0.030300502	0
0	0	0	0	0
0	0	0	0	0
0	0	0	0	0
0	0	0	0	0
0.006997798	0	0	0	0
0	0	0	0	0
0	0	0	0	0
0.001472264	0.00240722	0	0	0
0	0	0	0	0
0	0	0	0	0
2.345514433	0	0.020837774	0	0
0	0	0	0	0
0	0	0	0	0
0	0	0	0	0
0	0	0	0	0
0.004696529	0.066807674	0	0	0
0	0	0	0	0
0	0	0	0	0
0	0	0.003084408	0	0.013757455
0.004356573	0	0	0	0.002805339
0	0	0	0	0
0.492567009	0	0	0	0.057509655
0	0	0	0	0
0	0	0	0	0
0	0	0	0	0
0	0	0	0.001034478	0
0	0	0	0	0
0	0	0	0	0
0	0	0	0	0
0	0	0	0	0
0	0	0	0.004313944	0
0.005541214	0.196959598	0	0.040628578	0
0.016920433	0	0.023632644	3.225067384	0.138388505
0	0	0	0	0
0	0	0	0	0
0.081395838	0	0	0	0.004260705
0.734788704	0.035148794	0	0.013496476	0.001126186
0	0	0	0	0
0	0	0	0	0
0	0	0	0	0.010365093
0.016721895	0.007898532	0	0.065925404	0.000861422
0	0	0	0	0

0	0	0	0	0
0.047826565	0.040632632	0	0.094612345	0.001449275
0	0	0.021184514	0	0.343685935
0	0	0	0	0
0	0	0	0	0
0	0	0.00061125	0	0
0	0	0	0	0
0	0	0	0	0
0	0	0	0	0
0	0	0	0	0
0	0	0	0	0
0	0	0	0	0
0.039404656	0	0.04571564	0	0.492733249
0	0	0	0	0
0.002923163	0	0	0.006268796	0.003388176
0	0	0	0	0
0	0	0	0	0
0	0	0	0	0
0.000970132	0	0	0	0
0	0	0	0	0
0	0	0	0	0
0.022104979	0.304161643	0	0.002798387	0.036158353
0	0.016771813	0	0	0
0	0	0	0	0
0	0	0	0	0
0	0	0	0	0
0	0	0	0	0
0	0	0	0	0
0	0	0	0	0
0	0	0	0	0
0	0	0	0	0
0	0	0	0	0
0	0	0	0	0
0	0	0	0	0
6.147182042	0.001917791	0.00519794	0	15.80683065
0	0	0	0	0
0	0	0	0	0
0	0	0	0	0
0.008027089	0.216088204	0.079834654	0.006427415	0.174118245
0	0	0	0	0
0.076343292	0.014499853	0.064076251	0.01328002	0.039526574
0	0	0	0	0
0	0	0	0	0
0	0	0	0	0
0	0	0	0	0
4.370982272	9.591968128	0.081118459	0.166965034	0.234690476
0	0	0	0	0
0	0	0	0	0

0	0	0	0	0
0	0	0	0	0
0	0	0	0	0
0	0	0	0	0.011804643
0.209590674	0	0	0	0
0	0	0	0	0
0	0	0	0	0
0.004453393	0.012482579	0.0015859	0	0.026546572
0.048446858	0	0	0	0.00246491
0.023026273	0.020001043	0.021524723	0	2.067305779
0	0	0.009728955	0	0.04620904
0	0	0	0	0
0.005345233	0.008279719	0	0	0.000652163
0	0	0	0	0
0	0	0	0	0
0	0	0	0	0
0	0	0	0	0
0	0	0	0	0
0	0	0	0	0
1.531958254	2.011268647	0.05243236	0.295102032	0.372855743
0	0	0	0	0
0	0	0	0	0
0	0	0	0	0
0	0	0	0	0
0	0	0	0	0
0	0	0	0	0
0	0	0	0	0
0	0	0	0	0
0	0	0	0	0
0	0	0.004766245	0.036791652	0
0	0	0	0	0
0	0	0	0	0.160957151
0	0	0	0	0
0	0	0	0	0
0	0	0	0	0
0	0	0	0.003791591	0
0	0	0	0	0
0	0	0	0	0
0	0	0	0	0
0	0	0	0	0.030954475
0	0	0	0	0
0	0	0	0	0
0.000624432	0	0	0	0
0	0	0	0	0
0.235656953	0.011151688	0	0.009170246	0
0	0	0	0	0
0	0	0	0	0.006142554
0	0	0	0	0

0	0	0	0	0
0	0	0	0	0
0	0	0	0	0
0	0	0	0	0
0	0	0	0	0
0.012114739	0.008612243	0	0.013349592	0.003256101
0	0	0	0	0
0	0	0	0	0
0	0	0	0	0
0	0	0	0	0.579319951
0	0	0	0	0
0	0	0	0	0
0	0	0	0	0
0	0	0	0	0.012489174
0	0	0	0	0
0	0	0	0	0
0	0	0	0	0.068131077
0.005405778	0.001237418	0	0	0
0	0	0	0	0
0	0	0	0	0
0	0	0	0	0
0	0	0	0	0
0	0	0	0	0
0	0	0.021145954	0	0
0	0	0	0	0
0	0	0.001564779	0	0.016764749
0	0	0	0	0
0	0	0	0	0
0	0	0	0	0
0	0	0	0	0
0	0	0	0	0
0.006450193	0	0	0	0
0	0	0	0	0
0	0	0	0	0
0	0	0	0	0
0	0	0	0	0
0	0	0	0	0
0.119863938	0	0.00059491	0	0
0.003965459	0	0.073784363	0	0.002106628
0	0	0	0	0
0	0	0	0	0
0	0	0	0	0
0	0	0	0	0
0	0	0	0	0
0	0	0	0	0
0	0	0	0	0
0	0	0	0	0
0	0	0	0	0

0	0	0	0	0
0.001179428	0	0	0	0.001518945
0.000721986	0	0	0	0
0	0	0	0	0
0	0	0	0	0
0.005391157	0	0	0	0
0	0	0	0	0
0	0	0	0	0
0	0	0	0	0
0	0	0	0	0
0.001286973	0.002104261	0.001283253	0	0.010740264
0	0	0	0	0
0	0	0	0	0.014796028
0	0	0	0	0
0	0	0	0	0
0	0	0	0	0
0	0	0	0	0
0	0	0	0	0
0	0	0.004632331	0	0
0.002570179	0	0	0.001461466	0
0.170067303	0.190504289	0	0.633321254	0
0	0	0.001151062	0	0
0	0	0	0	0
0.005193869	1.476230149	0.428026058	1.125651541	1.162715875
0	0	0	0	0
0	0	0	0	0
0	0	0	0	0
0	0	0	0	0.002782174
0.00879039	0	0	0	0
0	0	0	0	0
0	0	0	0	0
0	0	0	0	0
0	0	0	0	0.028878637
0	0	0	0	0

PHL018-0099_V2	PHL018-0038_V3	PHL018-0229_V1	PHL018-0219_V1	PHL018-0237_V1
EYCF	CTRL	REF	REF	CTRL
1.832785982	0.072620604	0.015211216	0.098557984	0.017623724
2.3462854	1.944871547	0.061926585	0.195969569	0.169582546
1.79906268	14.35775995	0	18.1583808	2.163872044
0	0	0	0	0
0	0	0	0	0
0	0	0	0	0
0	0	0	0.105821604	0
0	0	0	0	0
0	0	0	0.01901673	0
0	0	0	0	0
0	0	0	0	0
0	0	0	0	0
0	0	0	0	0
0.519463505	6.747604331	0.042753723	1.492602487	0.517928358
0	0	0	0	0
0	0	0	0	0
0.001667377	0	0	0.001483711	0
0	0	0	0	0
0	0	0	0	0
0	0	0	0	0
0	0	0	0	0
0	0	0	0	0
0	0	0	0	0
0	0	0	0	0
0	0	0	0	0
0	0	0	0	0
0	0	0	0	0
0	0	0	0	0
0	0	0	0	0
0	0	0.012661191	0	0
0	0	0	0	0
0	0	0	0	0
0	0	0	0	0
0	0	0	0	0
0	0	0	0	0
0	0	1.935908988	0.447082565	0.967629013
0	0	0	0	0
0	0	0	0	0
0	0	0	0	0
0	0	0	0	0
0	0	0	0	0
0.023883141	0	0.344404196	0.001092978	0.0049111
0.001585701	0	1.529552373	0.597816781	2.035132237
0.285885636	0	0.000531517	0.00433118	0.008321417
0	0	0.012454173	0	0.01006359
0	0	0	0	0

0.003659281	0	0	0	0
0.0196361	0.013401541	0	0	0
0	0.004599608	0.001553784	0.005874223	0
0.538182061	1.212990065	0.490878864	0.411116215	0.341576187
0.023664052	0	0	0.0013884	0
0.100157815	0	0	0	0
0.011332855	0	0.004943925	0.000823226	0
0	0	0	0	0.038527746
0	0	0	0	0.013232083
0	0	0	0	0
0	0	0	0	0
0.715239363	0	0.360147864	0	0.348891907
0	0	0	0.001782919	0
0.084833974	0.027173876	0	0.04878677	0
0	0	0	0	0
0	0	0	0	0
0	0	43.39945796	0.002222648	0
0	0	0	0	0
0	0	0	0	0
0	0	0	0	0
0.035122238	0.349700593	0.01856849	0.033485823	0
0.007477198	0.020797745	0	0.007604075	0
0	0	0	0	0
0	0	0	0	0
0	0	0	0	0
0	0	0	0	0
0	0	0	0	0
0	0	0	0	0
0	0	0	0	0
0	0	0	0	0
0.848986937	0	0	0.020380759	0
0	0	0	0	0
0	0	0	0	0
0	0	0	0	0
0	0	0	0	0
0	0	0	0	0
0	0	0	0	0
0	0	3.176132635	0	0
0	0	0	0	0
0	0	0	0	0
0	0	0	0	0
0	0	0	0	0
0	0	0	0	0
0	0	0	0	0
0	0	0	0	0
0	0	0	0	0
0	0	0	0	0
0	0	0	0	0

0	0	0	0	0
0	0.048536206	0	0.009060603	0
0	0	0	0	0
0	0	0	0	0
0.049756621	0	0	0	0
0	0	0	0.000539215	0
0	0	0	0	0
0.07525352	0	0	0.001359423	0
0.068751983	0.002047492	0	0	0
0	0	0	0	0
0	0	0	0	0
0	0	0	0	0
0	0	0	0	0
0	0	0	0	0
0	0	0	0	0
0	0	0	0	0
0	0	0	0	0
0	0	0	0	0
0.012254153	0	0	0.105440951	1.455462416
0	0	0	0	0
0	0	0	0	0
0	0	0	0	0
0	0	0	0	0
0	0.005085254	0	0	0
0	0	0	0	0
0	0	0	0	0
0	0	0	0	0
0.011475495	0	0	0.002283303	0
0	0	0	0	0
0	0	0	0	0
0.51942925	0	0	0.222615534	0.005994644
0	0	0	0.003018771	0
0	0	0	0	0
0	0	0	0	0
0	0	0	0	0
0	0	0	0	0
0	0	0	0	0
0	0	0	0	0
0	0	0	0.235990693	0
0	0	0	0	0
0	0	0	0	0
0	0	0	0	0
0	0.009816422	0	0	0
0	0	0	0	0
0	0	0	0.000507272	0
0	0	0	0	0
0	0	0	0	0
0	0	0	0	0

0	0	0	0	0
0	0	0	0	0
0	0	0	0	0
0	0	0	0	0
0.014719018	0	0	0	0.03123274
0	0	0	0	0
0	0	0	0	0
0	0	0	0	0
0	0	0	0	0
0	0	0	0	0
0	0	0	0	0
0	0	0	0	0
0	0	0	0	0
0	0	0	0	0
0	0	0	0	0
0	0	9.155734585	0	0.571902171
0	0	0	0	0
0	0	0	0	0
0	0	0	0	0
0	0	0	0	0
0	0	0	0	0
0	0	0	0	0
0	0	0	0	0
0	0	0	0	0
0.128889938	0	0	0	0.013302453
0	0	0	0	0
0	0	0	0	0
0	0	0	0	0
0	0	0	0	0
0	0	0	0	0
0	0	0	0.022841287	0
0	0	0.174804697	0	0
0	0	0	0	0
0	0	0	0	0
0	0	0	0	0
0	0	0	0	0
0	0	0	0	0
0	0	0	0	0
0	0	0	0	0
0	0	0	0	0
0	0	0	0	0
0	0	0	0	0
0	0	0	0	0
0	0	0	0	0
0	0	0	0	0
0	0	0	0	0
0	0	0	0	0
0.019340805	0	0	0	0

0	0	0	0	0
0	0	0	0	0
0	0	0	0	0
0	0	0	0	0
0	0	0	0	0
0	0	0	0	0
0	0	0	0	0
0	0	0	0	0
0	0	0	0	0
0	0	0	0	0
0	0	0	0	0
0	0	0	0.04810168	0
0.000595871	0.000798011	0	0.000757477	0
0	0	0	0	0
0	0	0	0.006904518	0
0	0	0	0	0
0	0	0	0	0
0	0	0	0	0
0	0	0	0	0
0	0	0	0	0
0.116612781	0.032452937	0.002520197	0.047021164	0.011964112
0	0	0	0	0
0.001955168	0	0	0	0
0	0.003111898	0.001051224	0.002363066	0
0	0	0	0	0
0	0	0	0	0
0.004974485	0.001303436	0.001320933	0.002309495	0.001185977
0	0	0	0	0
0	0	0	0	0
0	0.029285059	0	0	0
0	0.08683686	0	0	0
0	0	0	0	0
0	0	0	0	0
0	0	0.017866182	0.021638867	0.01291219
0.153198813	0.056793712	0	0.000737216	0.006293842
0	0	0	0	0
0	0	0	0	0
0	0	0	0	0
0	0	0	0	0
0	0	0	0	0
0	0	0	0	0
0	0	0	0	0
0	0	0	0.001915634	0
0	0	0	0	0
0	0	0	0	0
0	0	0	0	0
0.001046521	0	0	0.001795971	0
0	0	0	0	0.002242581

0	0	0	0	0
0	0	0	0	0
0	0	0	0	0
0	0	0	0.000834752	0
0	0	0	0	0
0	0	0	0	0
0	0	0	0	0
0	0	0	0	0
0.015778735	0	0	0	0.00034888
0	0	0	0	0
0	0	0	0	0
0	0	0	0	0
0	0	0	0	0
0	0	0	0	0
0	0	0	0	0
0	0	0	0	0
0	0	0	0	0
0	0	0	0	0
0	0	0.008199997	0.002537868	0.003361017
0	0	0	0	0
0	0.001556819	0	0.007487226	0
0	0	0	0	0
0	0	0	0	0
0	0	0	0	0
0.005132197	0.00309295	0	0.007828925	0.002814229
0	0	0	0	0
0	0	0	0	0
0	0	0	0	0
0	0	0.00415454	0	0
0	0	0	0	0
0.285490635	1.196622569	0	0.379579683	0.050646402
0	0	0	0	0
0.021965153	0	0	0	0
0	0	0	0	0
0	0	0	0	0
0	0	0	0	0
0	0	0	0	0
0	0	0	0	0
0	0	0	0	0
0	0	0	0	0
0	0	0	0	0
0.037949438	0	0.000788349	0.00118143	0.031585834
0	0	0	0	0
0	0	0	0	0
0	0	0	0	0
0	0	0	0	0
0	0	0	0	0
0	0	0	0	0

0	0	0	0	0
0	0	0	0	0
0.295149191	0	0	0.00482131	0.010331912
0	0	0	0	0
0	0	0	2.922891868	0
0	0	0	0	0
0	0	0	0	0
0	0	0	0	0
0	0	0	0	0
0	0	0	0	0
0	0	0	0	0
0	0	0	0	0
0	0	0	0	0
0.025794516	0.000809647	0.00109402	0.001639513	0.001657543
0.008649061	0.001559268	0.002257427	0.002706407	0.000456028
0	0	0.016434739	0.001992077	0.020506014
0.003284943	0.002791872	0	0.002312781	0.00155881
0	0	0	0	0
0	0	0	0	0
0.001544483	0	0	0	0
0	0	0	0	0
0	0	0	0	0.002032565
0	0	0	0	0
0	0	0	0	0
0	0	0	0	0
0	0	0	0	0
0	0	0	0	0
0	0	0	0	0
0	0	0	0	0
0	0.003314167	0	0	0
0	0	0	0	0
0	0	0	0	0
0	0	0	0.001440264	0
0	0	0	0	0
0	0	0	0	0
0	0	0	0	0
0	0	0	0	0
0	0	0	0.011148094	0.012768537
0	0	0	0	0
0	0	0	0	0
0	0	0	0	0
0	0	0	0	0
0	0	0	0	0
0	0	0	0	0
0	0	0	0	0
0	0	0	0	0
0	0	0	0	0
0.030200562	0.003486696	0	0	0

0.006407716	0	0	0	0
0	0	0	0	0
0.001911378	0	0	0	0
0	0	0	0	0
0	0.007001846	0	0.005683643	0
0	0	0	0.008071863	0
0	0	0	0	0
0.048182977	0.025721094	0.00297248	0.002312964	0.001385718
0	0	0	0	0
0.03229847	0.191858013	0	0.034170984	0.0635589
0	0	0	0	0
0	0	0.003077034	0	0
0.012139268	0.002845035	0	0.006481258	0.014145149
0.017514596	0	0	0	0
0	0	0	0	0
0	0	0.000824419	0	0
0	0	0	0	0
0	0	0	0	0
0.143791391	0.123091972	0	0	0
0	0	0	0	0
0	0	0	0	0
0	0	0	0	0
0	0	0	0	0
0	0	0	0	0
0	0	0	0	0
0	0	0.023187727	0	0.013406528
0	0	0.818790503	0	0
0	0.001144924	0	0.006955319	0
0	0.22295488	0	0.046926684	0
0	0.002435301	0	0	0
0	0.021588926	0	0.000771476	0
0	0	0	0	0
0	0	0	0	0
0	0.00278599	0	0	0
0	0	0	0	0
0.064998282	0	0	0	0
0.045413171	0.245780052	0	0	0
0	0	0	0	0
0	0	0	0	0
0	0	0	0	0
0	0	0	0	0
0	0	0	0	0
0	0	0	0	0
0.056385448	0.019069041	0	0	0
0	0	0	0	0
0	0	0	0	0
0	0	0	0	0
0.003723941	0.011755625	0	0	0

0.014956998	0	0	0	0
0	0	0.001333489	0	0
0	0.052967637	0	0	0
0.002207767	0	0	0	0
0	0	0	0.0008837	0
0.02054248	0.011823643	0	0.003697013	0
0	0	0	0	0
0.002388238	0	0	0	0
0	0	0	0	0
0	0	0	0	0
0	0.007260054	0	0.014293041	0
0	0	0	0	0
0	0	0	0	0
0.003793725	0.001016138	0.003947485	0.003279384	0.041432258
0.0056299	0.52281396	0	0.060897764	0
0	0	0	0	0
0.1976777	0.001242064	0	0	0
0.046764158	0.005438767	0	0	0
0	0	0	0	0
0.224906708	0	0	0	0
0	0	0	0	0
0.314542618	0	0.434275115	0	0.098391287
0	0	0	0	0
0	0	0	0	0
0	0	0	0	0
0	0	0	0	0
0.021389692	0.002754407	0	0	0
0	0	0	0	0
0	0	0	0	0
0.319931723	1.302520505	0.346919591	0.115648126	2.632270129
0.001201079	0	0.002328743	0	0.036458716
0	0	0	0	0
0	0	0	0	0
0	0	0	0	0
0.009593015	0	0	0	0
0	0	0	0	0
0	0	0.384361006	8.733967976	0.386370921
0	0	0	0	0
0	0	0	0.195973025	0
0	2.341279374	0	0	0
0.014138808	0.036756556	0	0	0
2.063261134	11.03822241	0.698372357	1.74637743	2.952994448
0	0	0.000641012	0	0
0	0	0	0	0
0.016887507	0.00706761	0	0	0
0	0	0	0	0
0	0.005041527	0.001184743	0	0.005684153
0	0	0	0	0

0	0	0.072357803	0	0.004798567
0	0.002419353	0	0	0
0.001100663	0	2.362000644	0	0.000754433
0	0	0	0	0
0	0	0	0.064136729	0
0	0.003430012	0	0	0
0	0	0	0	0
0	0	0	0	0
0.073462998	0.009959143	0	0	0
0	0	0	0.088261158	0
0	0	0	0	0
0	0	0	0	0
0	0	0	0	0
0	0	0	0.058339543	0
0	0	0	0	0
1.240409453	0.030784218	0.004004642	0.012631997	0.005088792
0.011182907	0	2.716931399	0	0.159666581
0	0	0	0	0
0	0	0	0	0
0	0	0	0	0
0	0.036859105	0	0	0
0.00244747	0.001229152	0	0	0
0	0	0	0	0
0	0	0	0.273049342	0
0	0	0	0.004361841	0
0	0	0	0.021412211	0
0	0.003497646	0	0	0
0	0	0	0	0
0	0	0	0	0
0.004584009	0	0	0	0
0.072316975	0.014732032	0	0.020354104	0
0.102956436	0	0	0	0.000832683
0.02139854	0.062156199	0.966444028	0.020732375	1.09826384
0	0	0.00253098	0	0
0.002662037	0	0	0	0
0	0	0	0	0
0.002091433	0.001312932	0	0.001196393	0.008780438
0.033938649	0.002053547	0	0.01528204	0
0	0	0.061530665	0	0.153032148
0.018888912	0	0	0.001529955	0
0	0	0	0	0
0	0	0	0	0
0.025377598	0	0	0.00069962	0
0.049635035	0.08287752	0	0.118929965	0
4.144144471	0.048309848	0	0	0
0	0	0	0	0
0.003180671	0	0	0	0
0	0	0	0	0

0.000909393	0.003349202	0	0	0
0.407853774	0.102414806	0.017959948	0.394163227	0.030361739
0	0	0	0	0
0	0	0	0	0.001850387
0	0.001098074	0	0	0
0	0	0	0	0
0	0.090605089	0	0	0
0	0	0	0	0
0.0040804	0	0.001730616	0	0
0	0	0	0	0
0.003312349	0.001663506	0	0.008210853	0.001844699
0.032069947	0.002168109	0.005649978	0.007055941	0.052841
0	0	0.27480111	0	0.158781813
0	0	0	0	0
0	0	0.000805953	0	0.012373746
0	0	0	0	0
0.018209665	0.02395155	0	0.018022621	0.003417564
0.316307798	0.057379648	0	0.011375965	0
0	0	0	0	0
0.101052265	0.057632681	0	0.024251519	0
0.011401389	0.004490925	0.216789317	0.011253829	0.105424646
0	0	0	0	0
0	0	0	0	0
0	0	0	0	0
0.010892889	0	0	0	0
0	0	0	0	0
0.082968757	0	0	0	0.028132336
0.016394933	0.034605633	0.032087189	0.340893031	0.013192031
0	0	0	0	0
0	0	0	0	0.002946792
0	0	0	0	0
0	0	0	0	0
0.000865362	0.001738386	0	0	0
0	0.001084532	0	0	0
0	0	0.005620453	0	0.004541605
0.545699759	0.830049505	0.236597996	0.097843815	0.082967217
0	0	0	0	0
0	0	0	0	0
0.002638041	0.005299438	0.001392372	0.011401921	0
0	0	0	0	0
2.450704912	0.015888142	0	0	0
0	0	0	0	0
0.001246301	0.001430649	0	0.001493779	0
0.008766753	0.001553923	0	0.009439954	0
0	0	0	0.008956936	0
0	0	0.203444556	0	0.407452893
0	0	0	0	0
0.001389292	0.015070772	0	0	0

0	0.000999324	0	0	0
0	0	0	0	0
0.002610643	0	0	0	0
0.394613121	0	0.004502988	3.548833784	0.421143884
0	0	0	0	0
0	0	0	0	0
0.003858347	0	0	0	0
0	0	0	0	0
0.371949055	0.018668532	0.185544267	0.118362967	0.098694037
0	0	0.014085973	0.144184879	0.005716489
0	0	0	0	0
0	0.002394674	0.001483057	0.000606144	0.041398697
0	0	0	0	0
0	0	0	0	0
0.569721144	0	0.012349477	0.020632899	0.002921776
0	0	0	0	0
0.027587488	0	0.001741493	0.015332715	0.107153394
0	0	0.01452843	0	0.405019404
0	0	0	0	0
0	0	0	0	0
0	0	0	0	0
0	0	0	0	0
0	0	0	0	0
1.121421559	0.318674696	0	0.168393507	0.115282957
0.200893152	0	0	0	0
0	0	0	2.834006326	0
0	0	0	0	0.015322142
0	0	0.398548507	0	1.497884271
0	0	0	0	0
0	0	0	0	0
0.004828691	0	0.007733204	0.004124927	0.014430521
0	0	0	0	0.044406029
0.666201907	0	0	0	0
0	0	0	0	0
0	0	0	0	0
0	0	0.005667822	0	0.004826836
0.188664018	0	0.219170103	0.676099583	0.086549521
0	0	0	0	0
0.176626395	0	0	0.001735239	0
0.063993373	0	0	0	0
0.154953567	0.02680139	0.000905372	0.002471319	0.000653201
0	0	0	0	0
0.01851999	30.43627196	0	0.050747832	0.861014558
0	0	0	0	0
0	0	0	0	0
0	0	0	0	0
0	0	0	0	0
0.459379196	0	0	0.001048521	0

0	0	0	0	0
0.163854542	0	0.007648929	0	0.031046645
0.029211365	0.471436268	0	0.005025627	0
0	0	0.000644443	0	0.001301856
0	0	0	0	0
0.002422272	0	0.000587061	0	0.000711561
0	0	0	0	0
0.448516656	0	0	0	0
0	0	0	0	0.131282089
0	0	0	0	0
1.799747782	0.015594951	0	0.012993589	0.001625893
1.380692531	1.658955603	1.046480756	0.861485428	0.421451107
0	0	0	0	0
0.000698189	0	0.015003524	0	0.097148257
0.103070712	1.052033028	0	0.028396432	0.001005669
0	0	2.059691716	0	8.812288763
0.002532655	0	0	0	0
0	0	0	0	0
0.002122514	0	0	0	0
0.039079088	0	0	0.319202149	0
0.292075381	0.206955371	0	0.270609275	0
0	3.428047655	0	0	0.105734688
0.257776694	1.868666327	0.014365027	3.604139309	2.311935938
0	0	0	0	0
0	0	0	0	0
0	0	5.67300601	0	3.180578243
0.031345102	0	0	0	0
0	0	0.007291607	0	0.001292101
0	0	0	0	0
0	0	0	0.085531736	0
0	0	0	0	0
0	0	0	0	0.151927405
0.019710822	0.178182684	0	0.014020439	0
0	0	0.001146085	0.00437972	0.001041855
0.015786672	0.004551419	0	0.019176267	0
0.014459948	0	0.003543445	0.002144525	0.280683949
0.0372483	0	0	0	0
0	0	0	0	0
0	0.017940184	0.00255172	0	8.968371558
0	0	0	0	0
0	0	0	0	0.018545817
0	0	0	0	0
0	0	0	0	0
0	0	0	0	0
0.372626601	0	0.000466257	0	0.008634061
0	0	0	0	0
0	0	0	0	0
0.39865603	0.01193215	0.000562434	0.001896458	1.888151491

0	0	0	0	0
0	0	0	0	0
0	0	0	0	0
0.000585596	0	0	0.003870961	0.002006939
0	0	0	0	0
0	0	0	0	0.513793159
0	0	0.423753766	0	0
0.429107537	0	0.00036567	0.003150987	0.037427403
0	0	0	0	0
0	0	0	0	0
0.006304587	0.001528534	0	0	0.000745066
0	0	0	0	0
0	0	0	0	0.025572653
0	0	0	0	0
0	0	0	0	0
0	0	0	0	0
0	0	0.006473631	0	0
0.003619604	0.003272064	0	0	0.105070435
0	0	0	0.121431478	0
0.044394249	0	0	0.06834377	0
0	0	0	0	0
0	0	0	0	0
0.006885687	0	0.004672673	0.00190978	0.007580107
0	0	0	0	0
0	0	0	0	0
0	0	0	0	0
0	0	0	0	0
0	0	0	0	0
0	0	0	0	0
0	0	0.002670455	1.397024013	0.203797924
0.111125913	0.001719843	0	0	0.006220319
0	0	0	0	0
0	0	0	0	0
0	0	0	0	0
0	0	0	0	0.001895809
0	0	0	0	0
0	0	0	0	0.115994574
0.018771319	0.004066638	0	0	0
0	0	0	0	0
0.007079261	0	0	0	0
0	0	0	0	0.001893643
0	0	0	0	0
0	0	0	0	0
0	0	0.04438804	0	0.006124956
0	0	0.173003813	0	0.030508686
0	0	0	0	0
0.009617395	0	0	0	0
0	0	0	0	0

0	0	0	0	0
0	0	0	0	0
0	0	0	0	0
0	0	0.024472257	0.002094353	0.797767573
0	0	0	0	0
0	0	0	0	0
0	0	0	0	0
0	0	0	0	0
0.184013413	0.321983101	0.006717291	0.078403469	0.006262966
0	0	0	0	0
0	0	0	0	0
0	0	0	0	0
0	0	0	0	0
0	0	0	0	0
1.66691446	0	0.000866425	0	0.059759733
0.195424042	0.429124581	0	0.080719516	0
0	0	0	0	0
0	0	0	0	0
0	0	0	0	0
0	0	0	0	0
0	0	0	0	0.006917352
0	0	0	0	0
0	0	0	0	0
0	0	0	0.022364061	0
0	0	0.086082577	0.015480535	2.413901773
3.672983555	0	2.912892808	0.363917946	0.728705165
0	0	0.04188658	0.001524135	1.271980754
0.002038052	0	0	0	0
0.249731727	0.013367501	0.034531421	0	0.065286105
0	0	0	0	0
0	0	0	0	0
0.049016021	0	0	0	0
0	0	0	0	0
0	0	0	0	0
0.001838543	0	0.037964122	0	0.003600575
0.003898881	0.000932414	0	0	0.002799687
2.007936531	0	0	0	0
2.529137241	1.87366405	0.841214979	0.892724549	0.392579734
0	0	0	16.18852221	7.200008522
0	0.021550653	0	0.001893449	0
0	0	0	0.252191256	0
0	0	0	0	0
5.188096137	0.375877401	0.72190301	0.042378818	0.00347802
0	0	0	0	0
0	0	0	0	0
0	0	0	0	0
0	0	0	0	0
0.036708114	0.272167024	0	0.05492786	0.008835166

0.147314221	0	0	0	0
0	0	0	0	0
0	0	0	0	0
0	0	0	0	0
3.386063075	0	0	0.015709101	0
0	0	0.001223566	0	0
0	0	0	0	0
0	0	0	0	0
0	0	0	0	0
0.01983714	0	0	0.002866796	0
0	0	0	0	0.000982013
0.854282222	0	0.005079564	0.103058832	0.79512305
0.999792139	0	0.034188234	0.095296967	0.001899273
0	0	0	0	0
0.028631226	0.010874862	0.056328752	0.005688837	0.936385074
0	0	0	0	0
0.002343195	0	0	0	0
0	0	0	0.001715285	0
0.000632651	0	0	0	0.002601847
0	0	0.001904195	0	0
0	0	0	0	0
0	0	0	0	0
0.553855715	0	0.026160615	0.039100368	0.141957893
0	0	0	0	0
0	0	0	0	0
0	0	0	0	0
0	0	0	0	0
0	0	0	0	0
0	0	0	0	0
0	0	0	0	0
0	0	0	0	0
0.111771268	0.009721362	0.003178019	0.020717397	0.010913987
0	0	0	0	0
0.092961015	0	0	0.05829341	0.00363496
0.005455233	0	0	0	0.002631293
0.10444501	0	0.003358265	0	0.010176169
0	0	0	0	0.00049173
0	0	0	0	0.006488958
0	0	0	0	0.000636414
0	0	0	0	0
0	0	0.059109812	0	0
0	0	0	0	0.012806734
0	0	0	0	0
0	0	0	0.055200693	0
0.617805379	0.010795481	0	0.004706092	0.012960438
0	0	0	0	0
3.18934723	0	0.00738673	0	0.538114313
0	0.004348956	0	0	0

0	0	0	0	0
0	0	0	0	0
0.0366417	0.00413527	0	0.003140179	0
0	0	0	0	0
0	0	0	0	0
0	0	0	0	0
0	0	0	0	0.017861576
0.003499348	0.005662795	0	0.021500639	0.010660331
0	0	0	0	0
0	0	0	0	0
0	0	0	0	0
0	0	0	0	0.000861384
0	0	0	0	0
0	0	0.16116171	0.188586802	0.061596759
0	0	0	0	0
0	0	0	0	0
0	0	0	0	0
0	0	0	0	0
1.281046298	0	0	17.11380086	0.002261522
0	0	0	0	0
0.000935176	0	0.000507693	0	0
0	0	0	0	0
0	0	0	0	0
0	0	0	0	0
0	0	0	0	0
0.010191186	0.005583436	0	0.001130631	0
0	0	0	0	0
0.620635301	0.011112315	0	0.002213325	0.000372944
0	0	0	0	0
0	0	0.621416636	0	1.351369269
0	0	0	0	0
0	0	0	0	0
0	0	0	0	0
0	0	0	0	0
0.004077716	0.001365256	0	0.010219168	0.001996434
0	0	0	0	0
0	0	0	0	0
0.022474064	0.000885237	0	0	0.000906147
0	0	0	0	0
0	0	0	0	0.003358528
6.714392543	6.612878616	1.425437627	2.097059348	1.897992196
0.911907978	0.15713944	0.212112877	0.08032989	0.003758722
0	0	0.129876016	0	0.004281932
0	0	0	0	0
0	0	0	0	0
0	0	0	0	0
0	0	0	0	0
0.015388492	0	0	0.000800264	0

0	0	0	0	0
0.015828334	0.007226546	0	0.000548759	0
0.060711678	0	0	0	0
0	0	0	0	0
2.86296556	0.252857636	3.893985078	0.090762129	0.003365804
0	0	0	0	0
0	0	0	0	0
0	0	0	0.001454566	0
0.457446922	0.738907317	0	0.012876813	0
0.001526432	0	0	0	0
0	0.000816449	0	0	0
0	0	0	0	0
0.561542002	0	0.09962512	0.089579694	0.007307477
0.024064794	0.018991753	0.019684117	0.002622122	0.148011787
0	0	0.027810125	0	0
0.934651302	0.095632129	0.056833378	0.327192019	0.063810456
0.004648292	0.002427812	0	0	0
0	0	0	0	0
0	0	0	0	0
0	0	0	0	0
0	0	0	0	0
0	0	0	0	0
0	0	0	0	0
0	0	0	0	0
0	0	0	0	0
0	0	0	0	0
0	0	0.11146929	0.028855468	0.122896959
0	0	0	0	0
0	0	0	0	0
0	0	0	0	0
0	0	0	0	0
0	0	0	0	0
0	0	0	0	0.241676263
0	0	0	0	0
0.63693253	0	0.003683749	0	0.050541077
0	0	0	0	0
0.001941342	0	0.102757769	0	0.000887109
0	0	0	0	0
0	0	0	0	0
0	0	0	0	0
0	0	0.294841125	0.489691617	0.277257148
0	0	0.072733932	0	0
0	0	0	0	0
0	0	0	0	0
0	0	0	0	0
0	0	0	0	0
0	0	0	0	0
0	0	0	0	0

0.079299322	0	0.422642463	0	0.213036821
0	0	0	0	0
0	0	0	0	0
0.001653281	0	0	0	0
0	0	0.000536733	0.019304503	0
0	0	0	0	0
0.030047689	0.005589019	0	0	0
0	0	0	0	0
0.000654593	0	0	0	0
0	0	0	0.085641351	0
0	0	0	0	0
0	0	0	0.024125074	0
0	0	0	0	0
0	0	0	0	0
0	0	0	0	0
0	0	0	0	0
0	0	0	0	0
0.027996103	0	0	0	0
0	0	0	0	0
0	0	0	0	0
0.046089175	0.014366855	0.001677662	0.518681464	0.123822673
0	0	0	0	0
0	0	0.002643508	0.012033345	0.002803612
0.004849479	0.003827172	0.00070519	0.002377817	0.011515291
0	0	0	0	0
0	0	0	0	0.000840337
0	0	0	0	0
0	0	0	0	0
0	0	0	0	0
0.091980333	0	0.009811031	0	0.710939167
0	0	0	0	0
0	0	0	0	0
0	0	0	0	0
0	1.600506175	0	2.471225232	0
0	0	0	0	0
0	0	0	0	0
0.025788806	0	0	0.014380445	0
0	0	0	0	0
0	0	0	0	0
0	0	0	0	0.028991156
0	0	0	0	0
0	0	0	0	0
0	0	0	0	0
0.003722333	0	0	0	0
0	0	0	0	0
0	0	0	0	0
0	0	0	0	0
0	0	0	0	0

0.077154988	0.001310328	0	0.011371622	0
0	0	0.00659681	0	0
0.039422713	0	0	0	0
0	0	0	0	0
0	0	0	0	0
0	0	0	0	0
0	0	0	0	0
3.908796973	0	0.132451459	0.529802412	0.61055434
0.004972966	0	2.031958674	0.820629359	2.625658013
0.125854956	0.000613651	0.079601707	0	0
0	0	0.007459922	0	0
0	0	0	0	0
0	0	0	0	0
0.016675363	0	0	0	1.182027002
0.007833719	0	0	0	0
0	0	0	0	0
0.025027893	0	0	0.00269742	0
0	0	0	0	0
0	0	0	0	0
0.074640752	0	0	0.002060832	0.002662243
0	0	0	0	0
0	0	0	0	0
0	0	0	0.263005407	0
0	0	0	0	0
0	0	0	0	0.004024545
0	0	0	0	0
0	0	0	0	0
0	0	0	0	0
0	0	0	0	0
0	0	0	0	0
0	0	0	0.015041072	0
0	0	0	0	0
0	0	0	0	0
0	0	0	0	0
0	0	0	0	0
0	0	0.008337331	0	0
0	0	0	0	0
0.028349763	0	0	0	0
0	0	0.000751904	0	0.00197462
0	0	0	0	0
0	0	0	0	0
0.768735494	0	0.211793684	0	0.107357079
0	0	0	0	0
0	0	0	0	0
0	0	0	0	0
0.002320025	0	0	0.010490871	0
0	0	0	0	0
0	0	0	0	0

0	0	0	0	0
0.072326988	0	0.000570716	0	0
0	0	0	0	0
0	0	0	0	0
0.277030801	0.005196653	0.117297338	0	0.135241769
0	0	0	0	0
0	0	0	0	0
0.281164309	0.021430696	0	0.057500471	0.0147871
0	0	0	0	0.002433688
0	0	0	0	0
0	0	0	0	0
0	0	0	0	0
0	0	0	0	0
0	0	0	0	0
0.027870379	0	0	0	0
0	0.019619276	0	0	0.082162903
0	0	0	0	0
0	0	0	0	0
0	0	0	0	0
0	0	0	0	0
0	0	0	0	0
0	0	0	0	0
0	0	0	0	0
0.006360085	0	0	0	0
0	0	0.051778419	0	0.200189156
0	0	0	0	0
0.582077278	0.008104488	0.000382013	0.008873589	0.038328448
0	0	0	0	0
0	0	0	0	0
0	0	0	0	0
0	0	0	0	0
0	0	0	0	0
0	0	0	0	0
0	0	0	0	0
0	0	0	0	0
0	0	0	0.008476687	0
0	0	0	0	0
0	0	0	0	0
0	0	0.028834392	0	0.014795882
0	0	0	0	0
0	0	0	0	0
0	0	0.014824498	0	0.083943261
0	0	0	0	0
0	0	0	0	0.055550586
0	0	0	0	0
0	0	0	0	0
0	0	0	0	0
0	0	0	0	0

0	0	0.14717288	0	0.145992733
0	0	0	0	0
0.004020356	0	0	0	0
0.004389316	0	0	0	0
0	0	0	0	0
0	0	0.049529261	0	0
0	0	0	0	0
0	0	0	0	0
0	0	0	0	0
0	0	0	0	0
0	0	0	0	0
0.000756514	0	0	0	0
0	0	0	0	0
0	0	0	0	0
0	0	0	0	0
0	0	0	0	0
0	0	0	0	0
0	0	0	0	0
0	0	0	0	0
0	0	0	0	0
0.019000495	0.001846897	0	0.005454046	0
0	0	0	0	0
0.010578988	0.004146661	0	0.015153738	0.000884293
0	0	0.002403866	0	0.001294962
0	0	0	0	0
0	0	0	0	0
0	0	0	0	0
0	0	0	0	0
0	0	0	0.045147494	0.002143449
0	0	0	0	0
0	0	0	0	0
0	0	0	0	0
0.010873733	0.065531206	0.026892306	0	0.56578329
0	0	0	0	0
0.857012926	0	0.042389674	0.082099839	0.0025186
0	0.025319207	0	0.033572458	0
0	0	0	0	0
0	0	0	0	0.076216123
0.031175134	0.000675823	0	0.000855327	0
0	0	0	0	0
0	0	0	0	0
0	0	0	0	0
0.05297497	0.002107306	0.054813574	0.011601553	0.020492282
0	0	0	0	0
0	0	0	0	0
0.869751522	0	0.001696032	0.021286697	0.001284823
0	0	0	0	0
0	0	0	0	0

0	0	0	0	0
0	0	0	0	0
0	0	0	0	0
0	0	0	0	0
0	0	0	0	0
0	0	0	0	0
0	0	0	0	0.152860521
0	0	0	0	0
1.338028836	0	0	0	0.010566339
0.003432316	0	0.00160132	0.003599637	0.331867673
0	0	0	0	0
0	0	0	0	0
0	0	0	0	0
0.221039148	0	0.001126868	0	0
0.177001813	0	0	0	0
0.010131058	0.005511949	0	0.010624936	0
0	0	0	0	0
0	0	0	0	0
0	0	0	0	0
0	0	1.542360041	0	0.274100531
0	0	0.00703086	0	0
0	0	0	0	0
0	0	0	0	0
0	0	0	0	0
0	0	0	0	0
0	0	0	0	0
0	0	0	0	0
0	0	0	0	0.000570261
0	0	0	0	0
0	0	0	0.005005423	0.02653934
0.007891147	0	0.064841869	0.718852377	0.055499668
0	0	0	0	0
0	0	0	0	0
0	0	0	0	0
0	0	0	0	0
0	0	0	0	0
0	0	0	0	0
0	0	0	0	0
0	0	0	0.102930832	0.004903307
0	0	0	0	0
0	0	0	0	0
0	0	0	0	0
0	0	0	0	0
0.007665171	0	0	0	0
0	0	0	0	0
0	0	0	0	0
0.20180414	0.08485515	0	0.006591921	0
0	0	0	0	0

0	0	0	0	0
0	0	0	0	0
0	0	0	0	0
0	0	0	0	0.000796376
0.081838032	0	0	0.083472936	0.186207618
0	0	0	0	0
0	0	0	0	0
0	0	0	0	0
0	0	0	0	0
0	0	0	0	0
0	0.050639038	0	0	0
0.117654052	0.003826615	0	0.001880222	0.003379375
0.023781359	0	0	0.001032282	0
0	0	0	0	0
0	0	0	0	0
0.312120824	0	0.012535772	0	0
0.005343215	0	0	0	0
0	0	0	0	0
0.090106474	1.735793743	0	0.143018604	0
0.055169959	0.018693941	0	0.007604744	0
0	0	0	0	0
0	0	0	0	0.038215719
0	0	0	0	0
0	0	0	0	0
0	0	0	0	0
0	0	0	0	0
0.070310224	0.005171774	0	0.010969254	0
0.013019778	0	0	0	0
0	0	0	0	0
0	0	0	0	0
0.006641694	0.001212926	0	0.000614036	0
0.008912907	0	0	0.221353088	0
0	0	0	0	0
0	0	0	0	0
0	0	0	0	0
0	0	0	0	0
0	0	0	0	0
0.131645139	0.007385446	0	0.002157019	0.002326122
0	0	0	0	0.259034107
0.0024673	0	0	0.008382898	0
0	0	0.003161243	0	0
0	0	0	0	0
0	0	0	0	0
0.001467767	0.026115545	0	0.002718798	0
0	0	0	0	0
0	0	0	0	0.108701673
0	0	0	0	0
0	0	0	0	0

0	0	0	0	0
0	0	0.005715999	0	0.011301346
0	0	0	0	0
0.002197467	0	0	0	0.004970526
0.000815246	0	0.002212925	0	0.007124675
0	0	0	0	0
0	0	0.004630756	0	0.015493725
0.003609079	0	0	0	0
0	0	0	0	0
0	0	0	0	0
0	0	0	0	0
0	0	0	0	0
0.056855853	0	0.066172067	0.001663333	0.471281897
0	0	0	0	0
0	0	0	0	0
0	0	0	0	0
0	0	0	0	0
0	0	0	0	0
0	0	0	0	0
0	0	0	0	0
0	0	0	0	0
0	0	0	0	0
0.128195303	0.004120409	0	0.000938668	0
0.029770894	0.044003132	0.011168985	0.026214639	0.00331805
0	0	0	0	0
0	0	0	0	0
0	0	0	0	0
0	0	0	0	0
0	0	0	0	0
0	0	0	0	0
0	0	0.020175608	0	0.036838269
0	0	0	0	0
0	0	0	0	0
0	0	0	0	0
0	0	0	0	0
0	0	0	0	0
0	0	0	0	0
0.041015296	0	0	0	0
0	0.001166113	0.001444382	0	0
0	0	0	0	0
0	0	0	0	0
0	0	0	0	0
0	0	0	0	0
0	0	0	0	0
0	0	0	0	0
0.107764581	0.101401711	0.297486743	0.116085676	0.293128525
0	0	0	0	0
0	0	0	0	0

0	0	0	0	0
0	0	0	0	0
0	0	0	0.342961769	0.057691635
0	0	0.000872121	0	0
0	0	0	0	0
0	0	0	0.009085528	0.009561453
0	0	0	0	0
0	0	0	0	0
0	0	0	0.001502101	0.001227168
0	0	0	0	0
0.003610467	0	0	0	0
0	0	0	0	0.00048193
0	0	0	0	0
0	0	0	0	0
0	0	0	0	0
0	0	0	0	0
0	0	0	0	0
0	0	0	0	0
0	0	0	0	0
0.003955386	0	0	0.001429047	0.000856155
0	0	0	0	0
0	0	0	0	0
0	0	0	0	0
0	0	0	0	0
0	0	0	0	0
0	0	0	0	0
0	0	0	0	0
0	0	0.034707558	0	0.007106106
0	0	0	0	0
0	0	0	0	0
0.005007706	0.006646618	0	0	0.069874985
0	0	0	0	0
0.024919098	0.038587003	0.07158678	0.027717745	0.058073412
0	0.008952069	0	0.007930868	0
0	0	0	0	0
0	0	0	0	0
0	0	0	0	0
0	0	0	0	0
0	0	0	0	0
0	0	0	0	0
0	0	0	0	0
0.223799479	0	0	0.038309271	0.003630075
0.041417956	0	0.005225831	0.008096075	0.036235597
0	0	0	0	0
0	0	0	0	0
0.063761745	0	0.030410856	0	0.01525535
0	0	0	0	0
0	0	0	0	0

0	0	0	0	0
0.007413504	0	0.014648658	0	0.003885832
0.026488664	0.001023305	0.058074307	0.006022228	0.002967849
0	0	0	0	0
1.722701887	0	0	0.061269075	0.017702935
3.742121038	0.001789472	0	0	0
0	0	0	0	0
0	0	0	0	0
0	0	0	0	0
0	0	0	0	0
0	0	0	0	0
0	0	0	0	0
0	0	0	0	0
0	0	0	0	0
0	0	0	0	0
0	0	0	0	0
0	0	0	0	0.005440025
0	0	0	0	0
0	0	0	0	0
0	0	0	0	0
0	0	0	0	0
0.017209554	0	0	0.011407277	0
0.005183929	0	0	0	0
0	0	0	0	0
0.00167926	0	0	0	0.163157552
0.013725531	0	0	0	0.0130264
0	0	0	0	0
0	0	0	0.004218283	0.057002681
0	0	0	0	0
0	0	0	0	0
0	0	0	0	0
0	0.014135719	0	0	0
0	0	0	0	0
0	0	0	0	0
0	0	0	0	0
0	0	0	0	0
0	0	0	0	0
0	0	0	0.003206411	0
0	0	0.019385114	0	2.528338559
0	0	0	0	0
0	0	0	0	0
0	0	0	0	0.112110975
0.061881844	0	0	0.005980685	0
0	0	0	0	0
0	0	0	0	0
0	0	0	0	0
0.039552697	0.003256376	0	0.004451	0.002222196
0	0	0	0	0

0.002361298	0	0	0	0
0.103702753	0.041294921	0	0.005460333	0.003505003
0	0	0	0	0
0	0	0	0	0
0	0	0	0	0
0	0	0	0	0
0	0	0	0	0
0	0	0	0	0
0	0	0	0	0
0	0	0	0	0
0	0	0	0	0
0	0	0	0	0
0.149191064	0	0.003081279	0.016986337	0.167618688
0	0	0	0	0
0	0	0	0.007975335	0
0	0	0	0	0
0	0	0	0	0
0	0	0	0	0
0	0	0	0	0
0	0	0	0	0
0	0	0	0	0
0.044582322	0.001257856	0	0.000955172	0
0.004234118	0	0	0.004991005	0
0	0	0	0	0
0	0	0	0	0
0	0	0	0	0
0	0	0	0	0
0	0	0	0	0
0	0	0	0	0
0	0	0	0	0
0	0	0	0	0
0	0	0	0	0
0	0	0	0	0
0	0	0	0	0
0	0	0	0.126964344	17.05613435
0	0	0	0	0
0	0	0	0	0
0	0	0	0	0
0.014506791	0.002763467	0.091485062	0.001907709	0.015943822
0	0	0	0	0
0.04260998	0.052259346	0.39461928	0.039512863	0.148798723
0	0	0	0	0
0	0	0	0	0
0	0	0	0	0
0	0	0	0	0
0.621630328	0.832251574	0.014984902	0.00724616	0.047518999
0	0	0	0	0
0	0	0	0	0

0	0	0	0.003314175	0
0	0	0	0.003030326	0
0	0	0	0.009255861	0
0	0	0	0	0
0.017721115	0	0	0	0
0	0	0	0	0
0	0	0	0	0
0	0	0	0.015240794	0
0	0	0	0	0
0	0	0	0	0
0	0	0	0	0
0	0	0	0	0
0.009817848	0	0	0.0022465	0
0	0	0	0	0
0	0	0	0	0
0	0	0	0	0
0	0	0	0	0
0	0	0	0	0
0	0	0	0	0
0.997945568	0.127928577	0.033653068	0.07731567	0.248573576
0	0	0	0	0
0	0	0	0	0
0	0	0	0	0
0	0	0	0	0
0	0	0	0	0
0	0	0	0	0
0	0	0	0	0
0	0	0	0	0
0	0	0	0	0
0	0	0	0	0
0	0	0	0	0
0	0	0	0	0
0	0	0	0	0
0	0	0	0	0
0	0	0	0	0
0.00706993	0	0	0	0
0	0	0	0	0
0	0	0	0	0
0	0	0	0	0
0	0	0	0	0
0	0	0	0	0
0	0	0	0	0
0.007990235	0	0	0	0.000746834
0	0	0	0	0
0.038165309	0	0	0	0.000843865
0.153332067	0	0	0	0
0	0	0	0	0
0	0.002888351	0	0	0

0	0	0	0	0
0	0	0	0	0
0	0	0	0	0
0	0	0	0	0
0	0	0	0	0
0.012407757	0	0	0.047669128	0.034858802
0	0	0	0	0
0	0	0	0	0
0	0	0	0	0
0	0	0	0	0
0	0	0	0	0
0	0	0	0	0
0	0	0	0	0
0	0	0	0	0
0	0	0	0	0
0	0	0	0	0
0	0	0	0	0
0	0	0	0	0
0	0	0	0	0
0	0	0	0	0
0	0	0	0	0
0	0	0	0	0
0	0	0	0	0
0	0	0	0	0
0	0	0	0	0
0	0	0	0	0
0	0	0	0	0
1.449312912	0	0	0.506484092	0
0	0	0	0	0
0	0	0	0.00971603	0
0	0	0	0	0
0	0	0	0	0
0	0	0	0	0
0	0	0	0	0
0	0	0	0	0
0	0	0	0	0
0	0	0	0	0
0.237391063	0	0	0	0
0	0	0	0	0.479168818
0	0	0	0	0
0	0	0	0	0
0	0	0	0	0
0	0	0	0	0
0	0	0	0	0
0	0	0	0	0
0	0	0	0	0
0	0	0	0	0
0	0	0	0	0

0	0	0	0	0
0	0	0	0.024722604	0.008463739
0	0	0	0	0
0	0	0	0	0
0	0	0	0	0
0	0	0	0	0
0	0	0	0	0
0	0	0	0	0
0	0	0	0	0
0	0	0	0	0
0	0	0.011429366	0	0.018624916
0	0	0	0	0
0	0	0.00549996	0	0.192281444
0	0	0	0	0
0	0	0	0	0
0	0	0	0	0
0	0	0	0	0
0	0	0	0	0
0	0	0	0	0
0	0	0	0	0
0	0	0.000773341	0	0.000781123
0	0	0	0	0
0	0	0	0	0
2.112396636	0	0.519171085	0.051267459	0.661188095
0	0	0	0	0
0	0	0	0	0
0	0	0	0	0
0	0	0	0.189478383	0
0	0	0	0	0
0	0	0	0	0
0.002460741	0	0	0	0
0	0	0	0	0
0.001918004	0	0	0	0
0	0	0	0	0

PHL018-0029_V3	PHL018-0058_V2	PHL018-0102_V2	PHL018-0243_V1	PHL018-0217_V1
EYCF	EYCF	CTRL	REF	REF
1.061200361	0.013717676	0.03865884	0.909487982	0.036246467
1.99655061	0.20075359	0.652832967	22.83567174	1.676590255
5.082994643	2.749759714	0	0.033717616	0.103008143
0	0	0	0	0
0	0	0	0	0
0	0	0	0	0
0	0	0	0	0
0	0	0	0	0
0	0	0	0	0
0	0	0	0	0
0	0	0	0	0
0	0	0	0	0
0	0	0	0	0
5.052572284	0.682259871	37.62770525	9.741686287	9.264426392
0	0	0	0	0
0	0	0	0	0
0	0	0	0	0
0	0	0	0	0
0	0	0	0	0
0	0	0	0	0
0	0	0	0	0
0	0	0	0	0
0	0	0	0	0
0	0	0	0	0
0.014851107	0	0	0	0.051546281
0	0	0	0	0
0	0	0	0	0
0	0	0	0	0
0	0	0	0	0
0	0	0	0	0
0	0	0	0	0
0	0	0	0	0
0	0	0	0	0
0	0	0	0	0
0	0	0	0	0
0.67367999	0.662976547	1.440814567	0.046621529	2.061771808
0	0	0	0	0
0	0	0	0	0
0	0	0	0	0
0	0	0	0	0
0	0	0	0	0
0.001441628	0	0	0.002270931	0.007878462
0.595488088	24.40301521	0.149123531	0.132467951	0.666931786
0	0	0.000866574	0.037935829	0.04106307
0	0	0	0	0.002993766
0	0	0	0	0

0.001104403	0	0	0	0
0.002026101	0.009742876	0.004456425	0.024236402	0
0	0.001039453	0.016350997	0.009896061	0.000735687
0.383905627	0.167196845	2.352841766	1.492352137	0.819488147
0	0	0.001549203	0	0
0	0	0	0	0
0	0	0.063886606	0.006093491	0
0.025048147	0	0	0	0
0	0	0	0	0
0	0	0	0	0
0	0	0	0	0
0.625871832	0	0.159066515	0	0.58374753
0	0	0	0	0
0.017691193	0.010132565	0.033780679	0.050759818	0.023904878
0.000800191	0	0	0	0
0	0	0	0	0
0	0	0	0.289680674	0.273716346
0	0	0	0	0
0	0	0	0	0
0	0	0	0	0
0.026089629	0.113953085	0.284888428	0.32595094	0.001684006
0.012344249	0.007180593	0.008060534	0.07200851	0
0	0	0	0	0
0	0	0	0	0
0	0	0	0	0
0	0	0	0	0
0	0.125178662	0	0	0
0	0	0	0	0
0	0	0	0.010203132	0
0	0	0	0	0
0	0	0	0	0
0	0	0	0	0
0	0	0	0	0
0	0	0	0	0
0	0	0	0	0
0	0	0	0.024105783	0
0	0	0	0	0
0	0	0	0.001381612	0
0	0	0	0	0
0	0	0	0	0
0	0	0	0	0
0	0	0	0	0
0	0	0	0	0
0	0	0	0	0
0	0	0	0	0
0	0	0	0	0
0	0	0	0.001972118	0
0	0	0	0	0

0	0	0	0	0
0	0	0	0	0
0	0	0	0	0
0	0	0	0	0
0	0	0.031629572	0.145253481	0.110040293
0.00071122	0	0	0	0
0	0	0	0	0
0	0	0	0	0
0	0	0	0.00704829	0.01026127
0	0	0	0	0
0	0	0	0	0
0	0	0	0.000767149	0
0	0	0	0	0
0	0	0	0	0
0	0	0	0	0
0	0	0	0	0
0	0	0	0	0
0	0	0	0	0
0.158145537	0.01984021	0.001482885	0.02730908	0.008346358
0	0	0	0	0
0	0	0	0	0
0	0	0	0	0
0	0	0	0	0
0	0	0	0	0
0	0	0	0	0
0	0	0	0	0
0	0	0	0	0
0	0	0	0	0
0	0	0	0	0
0	0	0	0	0
0.233418553	0	0.059764553	0.002445856	0.037577857
0.000904939	0	0.00099521	0.028652727	0.402914347
0	0	0	0	0
0	0	0	0	0
0	0	0	0	0
0	0	0	0	0
0	0	0	0	0
0	0	0	0	0
0.163156122	0	0.0473394	0	0
0	0	0	0	0
0	0	0	0	0
0	0	0	0	0
0.010878046	0	0.024846592	0.028175033	0
0	0	0	0	0
0	0	0	0	0.001305909
0	0	0	0	0
1.439382593	0	0	0	0.007448234
0	0	0	0	0

0	0	0	0	0
0	0	0	0	0
0	0	0	0	0
0	0	0	0	0
0.000630117	0	0.007449471	0.001675005	0.000737909
0	0	0	0	0
0	0	0	0	0
0	0	0	0	0
0	0	0	0	0
0	0	0	0	0
0	0	0	0	0
0	0	0	0	0
0	0	0	0	0
0	0	0	0	0
0	0	0	0	0
2.283204056	0	0	0	0.689815117
0	0	0	0	0
0	0	0	0	0
0	0	0	0	0
0	0	0	0	0
0	0	0	0	0
0	0	0	0	0
0.001613442	0	0	0	0
0	0	0	0	0
0	0	0	0	0
0	0	0	0	0
0	0	0	0	0.017958171
0	0	0	0	0
0	0	0	0	0
0	0	0	0	0
0	0	0	0	0
0	0	0	0	0
0	0	0	0	0
0	0	0	0	0
0	0	0	0	0
0	0	0	0	0
0	0	0	0	0
0	0	0	0	0
0	0	0	0	0
0	0	0	0	0
0	0	0	0	0
0	0	0	0	0
0	0	0	0	0
0	0	0	0	0
0	0	0	0	0
0	0	0	0	0
0	0	0	0	0
0	0	0	0	0

0	0	0	0	0
0	0	0	0	0
0	0	0	0	0
0	0	0	0	0
0	0	0	0	0
0	0	0	0	0
0	0	0	0	0
0	0	0	0	0
0	0	0	0	0
0	0	0	0	0
0	0	0	0	0
0	0	0	0	0.001186886
0.000999105	0.001487808	0	0.00141646	0
0	0	0	0	0
0	0	0	0	0.01218845
0	0	0	0	0
0	0	0	0	0
0	0	0	0	0
0	0	0	0	0
0	0	0	0	0
0.013450222	0.021327462	0.024653225	0.112117322	0.053816265
0	0	0	0	0
0	0	0	0	0
0.001558431	0	0	0	0
0	0	0	0	0
0	0	0	0	0
0	0	0	0	0.022423061
0	0	0	0	0
0	0	0	0	0
0	0.001444416	0	0.039879326	0
0	0	0	0	0
0	0	0	0	0
0	0	0	0	0
0	0	0	0	0.008556529
0.027955961	0	0.006148936	0.011200908	0.001138723
0	0	0	0	0
0	0	0	0	0
0	0	0	0	0
0	0	0	0	0
0	0	0	0	0
0	0	0	0	0
0	0	0	0	0
0	0	0	0	0
0	0	0	0	0
0	0	0	0	0
0	0	0	0.015586555	0
0.005264152	0	0	0	0
0	0.002794709	0.001333621	0	0

0	0	0	0	0
0	0	0	0	0
0	0	0	0	0
0	0	0	0	0
0	0	0	0	0
0	0	0	0	0
0	0	0	0.001165372	0
0	0	0	0	0
0	0.005083529	0	0	0
0	0	0	0	0
0	0	0	0	0
0	0	0	0	0
0	0	0	0	0
0	0.01704348	0	0	0
0	0	0	0	0
0.011921315	0	0	0	0
0	0	0	0	0
0	0.181518031	0	0	0
0.002995063	0.124619571	0	0	0.00309478
0	0	0	0	0.00141859
0	0.057405486	0.026294346	0	0.001521705
0	0	0	0	0
0	0	0	0	0
0	0	0	0	0
0	0.00192216	0	0	0.006046378
0	0	0	0	0.001760196
0	0	0	0	0
0	0	0	0	0
0	0	0	0	0
0	0	0	0	0
0.850743205	0.184966178	0.008616338	0.098207496	0.070491277
0	0	0	0	0
0.038017338	0.026537415	0	0	0
0	0	0	0.011975633	0
0	0	0	0	0
0	0	0	0	0
0	0	0	0	0
0	0	0	0	0
0	0	0	0	0
0	0	0	0	0.003129377
0	0	0	0	0
0	0.00290065	0.008782924	0.004372455	0.005930818
0	0	0	0	0
0	0	0	0	0
0	0	0	0	0
0	0	0	0	0
0	0	0	0	0
0	0	0	0	0

0	0	0	0	0
0	0	0	0	0
0	0	0	0.035253743	0.038954152
0	0	0	0	0
0	0	0	0	0
0	0	0	0	0
0	0	0	0	0
0	0	0	0	0
0	0	0	0	0
0	0	0	0	0
0	0	0	0	0
0	0	0	0	0
0	0	0	0	0
0.002432815	0	0	0.004151662	0.002532434
0.001115539	0	0	0.012915848	0.017505362
0	0	0.002101554	0	0.002517555
0	0.001892782	0	0	0.009228649
0	0	0	0	0
0	0	0	0	0
0	0	0	0	0
0	0	0	0	0
0	0	0.001009488	0	0
0	0	0	0	0
0	0.008387819	0	0	0
0	0	0	0.001738884	0.004739939
0	0	0	0	0
0	0	0.006207645	0	0
0	0	0	0	0
0	0	0	0	0
0	0	0.001825291	0.003921739	0
0	0	0	0	0.002883121
0	0	0	0	0
0	0	0	0	0
0	0	0	0	0
0	0	0	0	0
0	0	0	0.075812598	0
0	0	0	0	0
0.002306546	0	0	0.001021893	0.432178932
0	0	0	0	0
0	0	0	0	0
0	0	0	0.002458134	0
0	0	0	0	0
0	0	0	0	0
0	0	0	0	0
0	0	0	0	0
0	0	0.001462513	0.004189718	0.090845276
0	0	0	0	0.032573038
0	0	0	0.009489561	0

0.001611587	0	0.012912828	0	5.969990132
0	0	0.007076897	0.013370006	0
0	0	0	0	0.006755537
0	0	0	0	0
0.003627421	0	0.001329757	0	0.006796721
0	0	0	0	0.007388447
0	0	0	0	0
0.022372369	0	0	0.001201438	0.012305839
0	0	0	0	0
0.009433508	0.356831896	0.02151756	0.071824191	0.04091579
0	0	0	0	0
0	0	0	0.009510626	0.010684057
0	0	0	0.019718669	0.008819316
0	0	0	0	0
0	0	0	0	0
0	0	0	0	0
0	0	0	0	0
0	0	0	0	0
0	0	0	0	0
0	0.008829699	0	0	0
0	0	0	0	0
0	0	0	0	0
0	0	0	0	0
0.067823616	0	0	0	0
0	0	0	0	0
0	0	0	0.001120352	0
0	2.345450033	0	0	0
0	0	0	0	0
0	0	0.286161811	0	0
0.015366869	0	0	0	0
0	0	0	0	0
0	0	0	0	0
0	0	0	0	0
0	0	0	0	0
0	0	0	0	0
0	0	0	0	0
0	0	0.214573218	0	0
0	0	0.008143163	0	0
0	0	0	0	0
0	0	0.006855327	0.006775362	0
0	0	0	0	0
0	0	0	0	0
0	0	0	0	0
0	0	0	0	0
0	0	0	0	0
0	0	0	0	0
0.025649282	0	0.003566516	0	0.00345253
0	0	0	0	0

0	0	0	0	0
0.001317925	0	0	0	0
0	0	0	0	0
0	0.030318713	0	0	0
0	0	0.001281865	0	0
0	0	0.20480626	0.171735349	0
0	0	0	0.001220193	0
0	0	0	0	0
0	0	0.004658212	0	0
0.000908733	0	0	0	0
0.001346599	0	0.005035154	0	0.029331394
0.01893105	0	0	0	0
0	0	0	0	0
0	0	0.001119285	0	0.005661352
0.025744735	0.031947936	0.007078219	0	0
0	0	0.001426783	0	0
0	0	0	0	0
0	0	0	0	0
0	0	0	0	0
0	0	0	0.002163042	0
0	0	0	0	0
0.008917825	0.094958377	0.261003752	0.002039203	0
0	0	0	0	0.016682567
0	0	0	0	0
0	0	0	0	0
0	0	0	0	0
0.00082764	0	0.016383613	0	0
0	0	0	0	0
0	0	0	0	0
0.516170342	1.401879277	0.799810518	0.613349815	0.527600724
0.006904687	0	0	0	0
0	0	0	0	0
0	0	0	0	0
0	0	0	0.000866534	0
0	0	0	0	0
0	0	0	0	0
2.595508059	0	0.211982686	0.032532606	0
0	0	0	0	0
0.146077487	0.028303144	0.237390691	0.192149776	0
0	0.160278551	0	0	0
0	0.008046934	0.000920174	0.015816354	0
0.146439594	0	6.729570711	7.014546141	0.113554505
0	0	0	0	0
0	0.001252625	0	0	0
0	0	0	0	0
0	0	0	0	0.007988351
0.012294603	0	0.021971687	0.032941414	0.001028414
0	0	0.003389942	0	0

0	0	0	0	0
0	0	0.001903526	0	0
0	0	0	0.024855953	0.010806001
0	0	0	0	0
0	0	0	0.005790797	0.044006253
0	0	0	0	0
0	0	0	0.001591835	0
0	0	0	0	0
0	0	0	0	0
0	0	0	0	0
0	0	0	0	0
0	0	0	0	0
0	0	0	0	0
0	0	0.006468176	0	0
0	0	0	0	0
0.368850794	0	0.017481073	0.003167631	0.003364087
0	0.002107335	0	0.002257064	0.015909288
0	0.006400826	0	0	0
0	0	0	0	0
1.429319961	0.237830277	0	0	0.002652135
0.004012815	0	0.014710365	0.016751196	0
0	0	0	0	0
0	0	0	0	0
0.002108878	0	0	0.453732546	0.187966734
0.025591941	0	0.039708223	0	0.000929298
0	0.270664185	0	0	0
0	0	0.001778163	0.001273494	0
0	0	0	0	0
0	0	0	0	0
0	0	0	0	0
0.026232055	0	0.043949355	0	0.017519701
0	0	0	0.007580461	0.010376329
0.034618626	0.43718112	0.020475682	0.021309173	0.084147519
0	0	0	0	0.001464675
0	0	0	0.011249781	0.001393874
0	0	0	0	0
0	0	0	0	0
0.010284115	0	0.016964994	0.048843302	0.010357307
0.035448483	0	0	0.682540456	0.004473332
0	0	0	0	0
0	0	0	0	0
0	0	0.003982351	0	0
0.00246078	0	0	0.001453633	0.001080651
0.051700443	0.081292961	0.214894112	0	0.001128161
0	0	0.006219782	0	0
0	0	0	0.010595659	0
0	0	0	0	0
0	0	0	0	0

0	0.002270631	0	0	0
0.020179312	0.002782391	0.01972647	0.139401677	1.244910675
0	0	0	0	0
0	0	0.019414036	0	0
0	0	0	0	0
0	0	0	0	0
0	0	0	0	0
0	0	0	0	0
0	0	0	0	0
0	0	0	0	0
0.038321667	0	0	0.005905407	0
0.005894255	0.001924863	0	0.030053929	0.027246963
0	0	0	0	0.45862552
0	0	0	0	0
0	0	0	0	0.001679054
0	0	0	0	0
0	0.001082549	0.008154692	0	0.025284235
0	0	0.322870476	0.06823328	0
0.0023116	0	0	0	0
0	0	0.034976232	0.010208014	0
0.004947898	0.009489238	0.006925508	0.003719959	0.097186491
0	0	0	0	0
0	0	0	0	0
0	0	0	0	0
0	0	0	0	0
0	0	0	0	0
0	0.008539909	0.052733486	0	0.008318953
0.101353056	0.027883882	0.042587442	0.112399948	0.036951019
0	0	0	0	0
0	0	0	0	0
0.001025961	0	0	0	0.015318729
0	0	0.001088581	0	0
0	0	0	0	0
0	0	0	0	0
0.06415853	0	0.002978123	0	0.007074295
0.008900349	0.043677523	0.195763888	0.447375562	0.506458144
0	0	0.000824237	0.001549554	0
0	0	0	0	0
0	0	0.001297194	0.019509656	0.021755625
0	0	0.00240055	0	0
0	0	0.002034991	0	0
0	0	0	0	0
0	0	0	0	0
0	0	0.003613501	0	0.001620132
0.018629947	0	0.015741056	0	0
0.121499626	0	0.041657906	0.012032419	0
0	0	0	0	0
0	0	0	0	0

0	0	0	0	0
0	0	0	0	0.446710732
0	0	0	0	0
0	0	0.006653294	0.00640809	1.796831489
0	0	0	0	0
0	0	0	0	0.006143531
0	0	0	0	0
0.004105805	0	0	0	0
0.549234711	0	0.054505772	0.005056965	2.814682944
0.013051465	0	0	0	0.158081581
0	0	0	0	0
0.01858833	0.002728378	0	0	0
0	0	0	0	0
0	0	0	0	0
0.698178137	0.037456177	0	0	0.001158913
0	0	0	0	0
0.0718587	0	0	0.034568763	0
0	0	0.007106044	0.002290159	0
0	0	0	0	0
0	0	0	0	0
0	0	0	0	0
0	0	0	0	0.003259701
0	0	0	0	0
0.244352154	0.01419537	0.906823439	0.200091706	0.046141638
0	0	0.001234806	0	0
0	0	0.113897376	0	1.971694511
0	0	0	0	0
1.053769504	0.834505727	0.095795719	2.346569335	0.679087582
0	0	0	0	0
0	0	0	0	0
0	0	0	0	0.007054123
0.005492341	0	0	0.001297775	0
0.000887315	0	0.036756183	0	0.000692736
0	0	0	0	0
0	0	0	0	0
0.292440014	0	0	0	0.014470422
0	0	0.006786224	0.007290289	5.943233005
0	0	0	0	0
0.005409804	0	0	0	0.001949304
0.006008724	0.012605082	0	0	0
0.087243459	0.001189723	0.013495749	0.199349981	0.041989875
0	0	0	0	0
30.84549284	10.04019897	0.876273344	0.200011992	0.257516795
0.002004408	0	0	0	0.000782431
0	0	0	0	0
0	0	0	0	0
0	0	0	0	0
0.031808741	0.022882937	0.00202793	0.007080318	0

0	0	0	0	0
0.083996128	0.165473094	0.000692813	0	0.041559228
0.01303655	0.013161527	0.007289999	0.112251244	0.016042936
0	0	0	0	0.004195559
0	0	0	0	0.498188439
0.003307189	0	0	0.006991918	0.098250306
0	0	0	0	0
0	0	0	0	0
0	0	0	0	0.006824473
0	0	0	0	0.036820906
0.033626016	0.0169605	0.012644892	0.204530769	0.006097284
1.447622389	1.718384917	1.538282803	3.13532251	0.791654212
0	0	0	0	0
0.001873062	0	0	0	0.027418491
0.001476044	0.023583107	0.016029945	0.094386384	0.040836894
0	0	0.020280876	0	0
0	0	0.000659315	0	0
0	0	0	0	0
0	0	0	0	0.006137813
0.002323141	0	0.052276845	0.001689018	0.005231831
0.006909516	0.004964107	0.020596661	0.196990565	0.638163967
11.09316232	8.221359123	0.038137386	0.0857883	0.041956563
0.139798192	0.387884945	0.095570357	9.455701445	3.114166322
0	0	0	0	0
0	0	0	0	0
0.072120309	0.007570804	0.002635833	0	0.054093731
0.6791788	0	0	0	0.001018717
0.015740513	0	0	0	0
0.059454385	0.006176925	0	0	0.01101047
0	0	0	0	0
0.004612721	0	0	0	0
0.171832528	0	0.512739306	0.159203645	0.129661938
0	0.005806292	0.91249478	6.602889237	0.000782759
0.057428281	0.013283241	0	0	0.019698166
0	0	0.001132064	0.002258565	0.004305254
0.033135119	0.011533388	0.003555169	0.02410894	0.076423813
0.036106667	0.000726593	0	0	0
0	0	0	0	0
0.049414202	0	0.008580547	0.00251397	0.000830631
0	0	0	0	0
0	0	0	0.001050947	0.066044952
0	0	0	0	0
0	0	0	0	0
0	0	0	0	0
0.217965564	0.031165754	0.057266512	0.051992675	0.005396451
0	0	1.324741329	0	0.057143126
0	0	0.00456579	0	0
0.558370404	0.053115046	0.011615064	0.029881053	0

0	0	0	0	0
0	0	0	0	0
0	0	0	0	0
0.006087638	0	0.005183152	0	0
0	0	0	0	0
0	0	0.002098125	0.00163925	0.117598876
0.012039648	0	0.015841493	0	0
0.016624488	0.018163513	0	0	0.001058064
0	0	0	0	0
0	0	0	0	0
0.003280654	0.000814226	0	0.001808753	0.001792869
0	0	0	0	0
0.036193106	0	0	0	0
0	0	1.452957531	0	0.003715488
0	0	0	0	0
0	0	0	0	0
0	0	0	0.00057653	0
0.004551782	0	0	0	0
0	0	0	0	0.135738654
0	0	0	0	0
0	0	0	0	0.002651978
0	0	0	0	0
0.043032608	0	0	0	0
0	0	0	0	0
0	0	0	0	0
0	0	0	0	0
0	0	0	0	0
0	0	0	0	0
0	0	0	0	0
0.028592263	0	0.602282259	0.005196934	2.747187185
0	0	0	0	0.002622442
0	0	0	0	0
0	0	0	0	0
0	0	0	0	0
0	0	0	0.001849156	0
0	0	0	0	0
0.125454135	0	0	0	0.04842146
0.006479972	0.00551405	0.001425275	0.010280517	0
0	0	0	0	0
0	0	0	0	0
0.011812216	0.0030179	0	0	0.001423974
0	0	0	0	0
0	0	0	0	0
0.038116511	0	1.6099186	0	0.040004825
0.260050648	0	0	0.000745112	0.410398092
0	0	0	0	0
0	0	0	0	0.107124807
0	0	0	0	0

0	0	0	0	0
0	0	0	0	0
0.164966364	0	0	0	0
0.159852678	0	0.069873875	0	0.066209462
0	0	0	0	0
0	0	0	0	0
0	0	0	0	0
0	0	0	0	0
0.02834294	0.026616785	0.03903303	0.292922814	0
0	0	0	0	0
0	0	0	0	0.004276203
0	0	0	0	0
0	0.001293978	0	0.000879947	0
0	0	0	0	0
1.209234416	0	0.208862743	0	1.349049507
0	0.039319068	0	0.003022584	0.020280895
0	0	0	0.026870212	0
0	0	0	0	0
0	0	0	0	0
0	0	0	0	0
0	0	0	0.000922684	0.005030198
0	0	0	0	0
0	0	1.138104953	0	0
0	0	0	0	0
0.011410438	0.715441851	0.010237077	0.006030866	0.097053075
0	0	0.518860289	5.298141831	0.872010331
0	0	0.068290902	0	0.009416868
0	0	0	0	0
0.043054255	0	0	0	0
0	0	0	0	0.016714468
0	0	0	0	0
0	0	0	0	0
0	0	0	0	0
0.00080183	0.001393043	0.002116357	0.02652481	0
0.001321162	0	0	0	0.002166036
0.065746669	0.006489982	0.002875773	0.007502777	0
0	0	0.309066699	0	0
0.57894041	0	9.618108578	0.006976885	0.072120023
0	0	0	0.041525707	0
0	0	0	0	0
0	0	0	0	0
0	0	0	0	0.015874816
0.004083818	0	1.520269514	0	0.066953903
0	0	0.009491415	0	0
0	0	0.007588799	0	0
0.006901085	0	0.027049741	0	0.000828885
0	0	0	0	0.002018661
0	0.004547775	0.003410275	0.033305294	1.586926589

0.002522593	0.032869336	0	0	0.001723239
0	0	0	0	0
0	0	0	0	0
0	0	0	0	0
0	0	0.021636211	0.153554114	0
0	0	0	0	0
0	0	0	0	0
0	0	0	0	0
0	0	0	0	0
0	0	0	0	0
0	0	0	0	0
0.150994447	0	0.025694202	0.437993331	0.077784696
0.003801284	0	1.149073859	0	0.742816271
0	0	0	0	0
0.001936393	0	0.002661944	0.06691621	0.030896652
0	0	0	0	0
0	0	0	0	0
0	0	0	0	0
0	0	0.000933274	0	0
0	0	0	0	0.15427378
0	0	0	0	0.107165509
0	0	0	0	0
0	0	0.056490757	0.01023632	0.053389551
0	0	0	0	0
0	0	0	0	0
0	0	0	0	0
0	0	0.000921687	0	0
0	0	0	0	0
0	0	0	0	0
0	0	0	0	0
0	0	0	0	0
0	0.002338639	0.005526792	0.096481266	0.129841582
0	0	0.004643131	0	0
0.043779344	0.01518847	0	0.010752392	0.160788355
0.063621564	0.006053766	0.002235402	0.011046632	0
0.057384934	0.004552356	0.001440852	0.022289397	0.49158404
0.001082586	0	0	0	0
0.014577548	0	0	0	0.048710038
0.007706163	0	0	0	0
0	0	0	0	0
0.003117267	0	0	0.000818415	0.023119999
0	0	0	0	0.006420236
0	0	0	0	0
0	0	0	0	0.001356479
0.241884119	0.003727229	0.017176338	0.175058897	0.006565683
0	0	0	0	0
0	0	0	0	0
0.008901173	0	0.001457951	0	0

0	0	0	0	0
0	0	0	0	0
0	0	0.006377047	0.001957348	0
0	0	0	0	0
0	0	0	0	0
0	0	0	0.000631458	0.003755476
0	0	0	0.00269326	0
0	0	0	0	0
0	0	0	0	0
0	0	0	0	0
0	0	0	0	0
0	0	0.002780782	0	0
0	0	0	0	0
0.291533529	0	0	0.001967046	0.524878653
0	0	0	0	0
0	0	0	0	0
0	0	0	0	2.136392412
0	0	0	0	0
0	0.00109842	0.018738731	0.045751359	0.784159345
0	0	0	0	0
0	0	0.001655462	0	0
0	0	0	0	0
0	0	0	0	0
0	0	0	0	0
0	0	0	0	0.002305793
0	0	0.003485114	0	0.0008732
0	0	0	0	0
0	0.003849198	0	0.59258916	0.14657926
0	0	0	0	0.295797517
0.131266838	0	2.566006819	0.197616775	0.070795723
0	0	0	0	0
0	0	0	0	0
0	0	0	0	0
0	0	0	0	0
0.070715837	0.0014545	0.01890542	0.002538709	0.000686295
0	0	0.004102348	0	0
0	0	0.00066556	0	0
0.001108312	0	0	0	0.001297906
0	0	0	0	0
0	0	0	0	0
0.550744465	1.602452238	3.810315418	7.782380811	6.106830986
0	0.011477172	0	0.512217202	0.849935656
0	0	0.015289335	0.001124999	0
0	0	0	0	0
0	0	0.002446375	0	0
0	0	0	0	0
0	0	0	0	0
0.014601649	0.002183123	0.004062924	0.001870589	0

0	0	0	0	0
0.001809518	0	0.006169077	0	0.00932389
0	0	0	0	0.00148497
0	0	0	0	0
0.044789977	0	4.677513688	0	1.854333645
0	0	0	0	0
0	0	0	0	0
0	0	0	0	0.016850696
0.023669741	0	0.372778271	0.418398703	0.002750724
0	0	0	0	0
0	0	0	0	0
0	0	0	0	0
0	0	0.213474785	0.00166182	0.043239708
0.001080798	0.081545978	0.011648398	0.015067411	0.02025098
0.064689694	0	0.003256534	0.001345547	5.376269773
0.05268157	0	1.756244803	0.004191257	0.131488406
0.018705263	0	0	0.061877661	0.001533357
0	0	0	0	0
0	0	0	0	0
0	0	0	0	0.00604713
0	0	0	0	0
0	0	0	0	0
0	0	0	0	0
0	0	0	0	0
0	0	0	0	0
0	0	0	0	0
0.009271047	0	0.009480371	0	0.003047582
0	0	0	0.001517828	0
0	0	0	0	0
0	0	0	0	0
0	0	0	0	0
0	0	0	0	0
0	0	0.008702691	0	0
0.002501081	0	0	0	0.012616934
0.005461129	0	0.017767451	0.001075334	0.059156939
0	0	0	0	0
0.4548427	0	0.007636871	0.005896711	0
0	0	0	0	0
0	0	0	0	0
0	0	0	0	0
0.17109552	0.665035948	1.542192928	0.035942696	2.207355767
0.011178288	0	0	0	0
0	0	0	0	0
0.265509801	0	0	0	0
0	0	0	0	0
0	0	0	0	0
0	0	0	0	0
0	0	0	0	0

0	0	0	0.003323759	5.369680956
0	0	0	0	0
0	0	0	0	0
0	0	0	0	0
0.002387105	0	0	0	0.008153421
0	0	0	0	0.166508639
0.002798967	0	0.00123127	1.415757434	0.266373919
0	0	0	0	0
0	0	0	0	0
0	0	0.004090708	0.005319726	0
0	0	0	0	0.028088869
0	0	0	0	0
0	0	0	0	0
0	0	0	0	0
0	0	0	0	0
0	0	0	0	0
0	0	0	0	0
0	0	0	0	0
0	0	0	0	0
0	0	0	0	0
0.00124356	0.014991054	0	1.015632393	0.045145046
0	0	0	0	0
0.026061215	0	0	0	0.001835754
0.015681587	0.004756906	0.001341347	0	0
0	0	0	0	0
0	0	0.000904279	0	0.001203645
0	0	0	0	0
0	0	0	0	0
0	0	0	0	0.128918464
0	0.014314998	0	0.272866824	0.005286072
0	0	0	0	0
0	0	0	0	0
0	0	0	0	0
0	1.40649195	0.050955276	0	0
0	0	0	0	0
0	0	0	0	0
0	0	0.528687282	0.013393994	0.002042519
0	0	0	0	0
0	0	0	0	0
0	0	0	0	0
0	0	0	0	0
0	0	0	0	0
0	0	0	0	0
0	0	0	0	0
0	0	0.004765358	0	0
0	0	0	0	0
0	0	0	0	0
0.02659918	0	0	0	0

0.006937066	0	0.000824764	0	0.001097806
0	0	0	0	0
0	0	0	0	0
0	0	0	0	0
0	0	0	0	0
0.007333814	0	0	0	0
0	0	0	0	0
0.012705534	0.635694176	0.254053839	1.254997078	2.435876329
0.910830667	32.90967888	0.2116656	0.172105297	0.814715456
0.301988065	0.035339588	0.07187511	0.012344529	0.000959696
0	0	0	0	0.0042232
0	0	0	0	0
0	0	0	0	0.017245819
0	0.644007847	0	0	0
0	0	0	0	0
0	0	0.002434773	0	0
0	0	0.023175717	0	0
0	0	0	0	0
0	0	0	0	0
0	0	0	0	0
0	0	0	0	0
0	0	0	0	0
0	0	0	0	0
0	0	0	0	0
0.022446297	0	0	0	0.042369047
0	0	0	0	0
0	0	0	0	0
0	0	0	0	0
0	0	0	0	0
0	0	0	0	0
0	0.005736524	0	0	0
0	0	0	0	0
0	0	0.008287341	0	0
0	0	0	0	0
0.003631695	0	0	0	0
0	0	0	0	0.003735332
0	0	0	0	0
0.019088645	0	0	0	0
0.021402068	0	0	0	0
0	0	0	0	0
0	0	0	0	0
0.420574306	0	0.000637091	0	0.155806501
0	0	0	0	0
0	0	0	0	0
0	0	0	0	0
0	0	0.00128342	0	0.001366642
0	0	0	0	0
0	0	0	0	0

0	0	0	0	0
0	0	0	0	0
0	0	0	0	0
0	0	0	0	0
0.27278628	0.164706395	0.045012679	1.336920684	0.377356482
0	0	0	0	0
0	0	0	0	0
0.037205792	0.040843168	0.007868696	0.022259996	0.000837893
0.003095717	0	0	0	0
0	0	0	0	0
0	0	0	0	0
0.263872587	0	0	0.000841998	0
0	0	0	0	0
0	0	0	0	0
0	0	0	0.002380291	0
0.046333976	0.001540129	0.032984895	0	0.021558724
0	0	0	0	0
0	0	0	0	0
0	0	0	0	0.02196894
0	0	0	0	0
0	0	0	0	0
0	0	0	0	0
0	0	0	0	0
0.001238406	0.0007684	0	0	0.001691964
0.234139716	0	0	0.003688297	1.203223995
0	0	0	0	0
11.37079959	0.002108367	0	0.113298735	0
0.001391433	0	0	0	0
0	0	0.001851505	0	0
0	0	0	0	0
0	0	0	0	0
0	0	0	0	0
0	0	0	0	0
0	0	0.13323294	0	0
0	0	0	0	0
0	0	0	0	0
0	0	0	0	0
0.070858323	0	0	0	0.008388668
0	0	0	0	0
0	0	0	0	0
0	0	0	0	0
0	0	0	0	0
0	0	0	0	0
0	0	0	0	0
0	0	0	0	0
0	0	0	0	0
0	0	0	0	0
0	0	0	0	0

0.003373043	0	0.002538091	0.325515794	0.092306602
0	0	0	0	0
0	0	0.000580181	0	0.000617803
0	0	0	0	0
0	0	0	0	0
0	0.027310157	0.071477385	0	0.150538485
0	0	0	0	0
0	0	0	0	0
0	0	0	0	0
0	0	0	0	0
0	0	0	0	0
0	0.000944456	0	0	0
0	0	0	0	0
0	0	0	0	0
0	0	0	0	0
0	0	0	0	0
0	0	0	0	0
0	0	0	0	0
0	0.00130745	0	0.000533465	0
0.098795774	0	0	0	0
0	0	0.001356248	0.000971325	0.002647687
0	0	0	0	0
0.045426447	0	0.017413902	0.000920034	0
0	0	0	0.007017195	0.011407141
0	0	0	0	0
0.019099155	0	0	0	0
0	0	0	0	0
0	0	0	0	0
0	0.006692603	0	0	0
0	0	0.005438334	0	0
0	0	0	0	0
0	0	0	0	0
0.10015527	0.335456153	0.006950971	0.084150414	0
0	0	0	0	0
0.006088533	0	0.934076423	0	0.743819701
0	0	0	0.004839832	0
0	0	0	0	0
0	0	0	0	0
0	0	0.009181238	0	0
0	0	0	0	0
0	0	0	0	0
0	0	0	0	0
0.28045492	0	0.005512885	0	0.219057197
0	0	0	0	0
0	0	0	0	0
0.049658489	0	0.019356195	0	0
0	0	0	0	0
0	0	0	0	0

0	0	0	0	0
0	0	0	0	0
0	0	0	0	0
0	0	0	0	0
0	0	0	0	0
0	0	0	0	0
0	0	0	0	0
0	0	0	0	0
0	0	0	0	0.030575594
0.001294878	0	0	0	0
0	0	0	0	0
0	0	0	0	0
0	0	0	0	0
0	0	0	0	0
0	0	0	0	0
0	0	0	0.020069015	0
0	0	0	0	0
0	0	0	0	0
0	0	0	0	0
0	0	0.032063361	0	3.181703915
0	0.002476851	0.004633958	0	0.003635905
0	0	0	0	0
0	0	0	0	0
0	0	0	0.00233908	0
0	0	0	0	0
0	0	0	0	0
0	0	0	0	0
0	0	0	0	0
0	0	0	0	0
0	0	0.038481664	0	0.048450752
0.121669533	0	0.024483752	1.183809449	0.345395185
0	0	0	0	0
0	0	0	0	0
0	0	0	0	0
0	0	0	0	0
0	0	0	0	0
0	0	0	0	0
0	0	0	0	0
0.031704385	0	0.003636438	0	0.005238911
0	0	0	0	0
0	0	0	0	0
0.026435344	0	0.000726809	0	0
0	0	0	0	0.001092965
0	0	0	0	0
0	0	0	0	0
0	0	0	0	0
0	0.005664575	0	0	0.00203641
0	0	0	0	0

0	0	0	0	0
0	0	0	0	0
0	0	0	0	0
0	0	0	0	0
0.172457537	0.009068273	0.00446471	0.114344682	0.00456405
0	0	0	0	0
0	0	0	0	0
0	0	0	0	0
0	0	0	0	0
0	0	0	0	0
0.019793097	0	0	0	0
0.399730023	0.001678664	0.003967104	0.201635018	0.001320108
0	0	0	0	0
0	0	0	0	0
0.002920799	0	0	0	0.002414435
0	0	0	0	0
0.002427613	0	0	0	0.00101532
0	0	0	0	0
0	0.294681883	0.088052219	0.001411829	0.021591219
0	0	0.001470823	0.260975336	1.023771053
0	0	0	0	0
0	0	0	0	0
0	0	0	0	0
0	0	0	0	0
0	0	0	0	0
0	0	0	0	0
0.037867759	0.001773283	0.008054028	0	0.000627533
0	0	0	0	0
0	0	0	0	0
0	0	0	0	0
0.011136227	0	0	0	0.015886627
0	0	0	0	0
0	0	0	0	0
0	0	0	0	0
0	0	0	0	0
0	0	0	0	0
0	0	0	0	0
0.043387581	0.004766326	0.007196461	0.021176208	0.019490947
0	0	0	0	0.00360671
0	0	0.009926481	0	0
0	0.002467282	0.007809117	0	0.005987153
0	0	0	0	0
0	0	0	0	0
0	0.012303307	0	0.114391654	0
0	0	0	0	0
0	0	0	0	0
0	0	0	0	0
0	0	0	0	0

0	0	0	0	0
0	0	0	0	0
0	0	0	0	0
0	0	0	0.001305915	0
0	0	0	0	0
0.00317549	0	0	0.010317049	0
0.016948115	0	0	0	0
0	0.000858227	0	0	0.018222652
0	0	0	0	0
0	0	0	0	0
0.033064143	0	0	0	0
0	0	0	0	0
0.004231132	0	0.009651089	0.006479968	0.147179941
0	0	0	0	0
0	0	0	0	0
0	0	0	0	0
0	0	0	0	0
0	0	0	0	0
0	0	0	0	0
0	0	0	0	0
0	0	0	0	0
0	0	0	0	0
0.093476135	0	0	0.000731368	0
0.007061471	0	0.084353539	0	0.00085546
0	0	0	0	1.098617488
0	0	0	0	0
0	0	0	0.001397517	0.000692623
0	0	0	0	0
0	0	0	0	0
0	0	0	0	0
0.002300783	0	0.05516044	0.01440667	0.001616622
0	0	0.001565354	0	0
0	0	0	0	0
0	0	0	0	0
0	0	0	0	0.02100776
0	0	0	0	0
0	0	0	0	0
0	0	0	0	0
0	0	0	0.165127017	0.001823699
0	0	0	0	0
0	0	0	0	0
0	0	0	0	0
0	0	0	0	0
0	0	0	0	0
0	0	0.042882535	0	0
0.016270706	0.08544039	0.023167727	0.061715559	0.300452683
0	0	0	0	0
0	0	0	0	0

0	0	0	0	0
0	0	0	0	0
0.102769918	0.085457581	0.578245689	0.001245298	0
0	0	0	0	0
0	0	0	0	0
0	0	0	0.000931452	0
0	0	0	0	0
0	0	0	0	0
0	0	0	0	0
0	0	0	0	0
0.001895078	0	0.001215737	0	0
0	0	0	0.00194992	0
0	0	0	0	0
0	0	0	0	0
0	0	0	0	0
0	0	0	0	0
0	0	0	0	0
0	0	0	0	0
0	0	0	0	0
0.000837733	0.004678134	0	0	0
0	0	0	0	0
0	0	0	0	0
0	0	0	0	0
0	0	0	0	0
0	0	0	0	0
0	0	0	0	0
0	0	0	0	0
0.005099014	0.289632532	0	0	0
0	0	0	0	0
0	0	0	0	0
0.001439399	0.008261279	0	0.003613701	0.00210704
0	0	0	0	0
0.064762441	0.166955899	0.046179913	0.229785829	0.018593305
0	0	0.000939122	0	0.013000234
0	0	0	0	0
0	0	0	0	0
0	0	0	0	0
0	0	0	0	0
0	0	0	0	0
0	0	0	0	0
0	0	0	0	0
0	0.002985937	0.229273873	0.041422894	0.002817784
0.009841106	0	0.002302722	0.022263867	0.043891514
0	0	0	0	0
0	0	0	0	0
0.091680592	0.012015425	0	0.034675167	0.086103845
0	0.00221559	0	0	0
0	0	0	0	0

0	0	0	0	0
0	0.004627627	0.395393518	0	0
0	0	0	0.024823502	0.007501682
0	0	0	0	0
0.037440099	0.040114129	0	0	0.003593839
0	0.08847325	0.007446446	0	0
0	0	0	0	0
0	0	0	0	0
0	0	0	0	0.077973518
0	0	0	0	0
0	0	0	0	0
0	0	0	0	0
0	0	0	0	0
0	0	0	0	0.003531001
0	0	0	0	0
0	0	0	0	0
0	0	0	0	0
0	0	0	0	0
0	0	0	0	0
0	0	0.104093534	0	0
0	0	0	0	0
0.000824443	0	0	0	0.000965478
0	0	0	0	0
0	0	0	0	0
0	0	0	0	0.003956762
0	0	0	0.007278406	0.00447796
0	0	0	0	0
0	0	0.000906503	0.006086472	0.174233746
0	0	0	0	0
0	0	0	0	0
0	0	0	0	0
0.001304051	0	0	0	0
0	0	0	0	0
0	0	0	0	0
0	0	0	0	0
0	0	0	0	0
0	0	0.014061485	0	0
0	0	0.013023101	0	0
0	0.749875692	0.156328271	0.197107273	0.59426046
0	0	0	0	0
0	0	0	0	0
0.002074134	0	0.010644839	0.051187527	0
0.005330045	0	0	0	0.001997387
0	0	0	0	0
0	0	0	0	0
0	0	0	0	0
0.020874049	0.003237957	0	0	0
0	0	0	0	0

0	0	0	0	0
0.162356536	0.011235692	0.006562757	0.007779556	0.002409763
0	0	0.00107332	0	0
0	0	0	0	0
0	0	0	0	0
0	0	0	0	0
0	0	0	0	0
0	0	0	0	0
0	0	0	0	0
0	0	0	0	0
0	0	0	0	0
0	0	0	0	0
0	0	0.001435327	0	0.204805544
0	0	0	0	0
0.019242788	0	0.001692987	0.003940598	0
0	0	0	0	0
0	0	0	0	0
0	0	0	0	0
0	0	0	0	0
0	0	0	0	0
0	0	0	0	0
0	0	0	0.022624488	0.233995661
0.09003341	0	0.004471627	0	0.003627874
0	0	0	0	0
0	0	0	0	0
0	0	0	0	0
0	0	0	0	0
0	0	0	0	0
0	0	0	0	0
0	0	0	0	0
0	0	0	0	0
0	0	0	0	0
0	0	0	0	0
0	0	0	0	0
0	0	0.018115086	0.584629765	0.007501565
0	0	0	0	0
0	0	0	0	0
0	0	0	0	0
0.000754875	0	0.001106902	0.010404804	0.480900763
0	0	0	0	0
0.004512302	0.0097992	0.02009781	0.070102899	0.184418759
0	0	0	0	0
0	0	0	0	0
0	0	0	0	0
0	0	0	0	0
0.001808198	1.351138339	0.333796103	2.721314062	0.008470077
0	0	0	0	0
0	0	0	0	0

0	0	0	0	0
0	0	0	0	0
0	0	0	0	0
0	0	0	0	0
0	0	0	0	0
0	0	0	0	0
0	0	0	0	0
0	0	0	0	0.009481919
0	0	0	0	0
0	0	0	0.003880099	0.004437723
0.047692033	0	0	0	0
0	0	0	0	0
0	0	0	0.002333828	0
0	0	0	0	0
0	0	0	0	0
0	0	0	0	0
0	0	0	0	0
0	0	0	0	0
0	0	0	0	0
0.529673505	0.180552531	0.038672899	0.646291061	0.114790925
0	0	0	0	0
0	0	0	0	0
0	0	0	0	0
0	0	0	0	0
0	0	0	0	0
0	0	0	0	0
0	0	0	0	0
0	0	0	0	0
0	0	0	0	0
0	0	0.012919361	0.000832739	0
0	0	0	0	0
0	0	0	0	0
0	0	0	0	0
0	0	0	0	0
0	0	0	0	0
0	0	0.005006118	0	0.002998538
0	0	0	0	0
0	0	0	0	0
0	0	0	0	0
0	0	0	0	0
0	0	0	0	0
0	0	0	0	0
0	0	0	0	0.002353377
0	0	0	0	0
0.017546286	0.003073986	0	0.016096159	0
0.012929012	0	0	0	0
0	0	0	0	0
0	0	0	0	0

0	0	0	0	0
0	0	0	0	0
0	0	0	0	0
0	0	0	0	0
0	0	0	0	0
0.006318334	0.001147425	0.000508435	0.007282672	0.00812106
0	0	0	0	0
0	0	0	0	0
0	0	0	0	0
0	0	0	0	0
0	0	0	0	0
0	0	0	0	0
0	0	0	0	0
0.015368373	0	0	0	0.004153242
0	0	0	0	0
0	0	0	0	0
0	0	0	0	0
0	0	0	0	0
0	0	0	0	0
0	0	0	0	0
0	0	0	0.001266625	0
0	0	0	0	0
0	0	0	0	0
0	0	0	0.021674601	0.002685533
0	0	0	0	0
0	0	0	0	0
0	0	0	0	0
0	0	0	0	0.083873692
0	0	0	0	0
0	0	0	0	0
0	0	0	0	0
0	0	0	0	0
0	0	0	0	0
0	0	0	0	0
0	0	0	0	0
0	0	0	0	0
0	0	0	0	0
0	0	0	0	0
0	0	0.413156946	0	0.006878163
0	0	0	0	0
0	0	0	0	0
0	0	0	0	0
0	0	0	0	0
0	0	0	0	0
0	0	0	0	0
0	0	0	0	0
0	0	0	0	0
0	0	0	0	0

0	0	0	0	0
0	0	0	0.113945697	0
0	0	0	0	0
0	0	0	0	0
0	0	0	0	0
0	0	0	0	0
0	0	0	0	0
0	0	0	0	0
0	0	0	0	0
0	0	0	0	0.001097334
0	0	0	0	0.001851965
0	0	0	0	0
0.289204891	0	0	0.098689654	0.086715626
0	0	0	0	0
0	0	0	0	0
0	0	0	0	0
0	0	0	0	0
0	0	0	0	0
0	0	0	0	0
0	0	0	0	0
0.134137266	0	0	0	0
0	0	0.003788647	0	0
0	0	0	0	0
1.368571345	0	0.9569501	0.091111152	0.945596931
0	0	0	0	0
0	0	0	0	0
0	0	0	0	0
0	0	0	0	0.04255942
0	0	0	0	0
0	0	0	0	0
0.001575367	0	0	0	0
0	0	0	0	0
0.000723588	0	0	0	0
0	0	0	0	0

PHL018-0262_V1	PHL018-0244_V1	PHL018-0258_V1	PHL018-0264_V1	PHL018-0030_V3
REF	REF	REF	REF	CTRL
0.028126862	0.10197194	0.11110906	0.012985722	0.027372903
0.509422584	3.599791855	0	4.671229371	0.875846781
0.008210908	8.067993652	0	0	9.886192779
0	0	0	0	0
0	0	0	0	0
0	0	0	0	0
0	0	0	0	0
0	0	0	0	0
0	0	0	0	0
0	0	0	0	0
0	0	0	0	0
0	0	0	0	0
0	0	0	0	0
1.353662591	17.32448435	0	4.450002489	3.185537692
0	0	0	0	0
0	0	0	0	0
0	0	0	0	0
0	0	0	0	0
0	0	0	0	0
0	0	0	0	0
0	0	0	0	0
0	0	0	0	0
0	0	0	0	0
0	0	0	0	0
0.005978387	0	0	0	0
0	0	0	0	0
0	0	0	0	0
0	0	0	0	0
0	0	0	0	0
0	0	0	0	0
0	0	0	0	0
0	0	0	0	0
0	0	0	0	0
0	0	0	0	0
0	0	0	0	0
0.005201155	2.161816994	0	0.413692816	3.941821847
0	0	0	0	0
0	0	0	0	0
0	0	0	0	0
0	0	0	0	0
0	0	0	0	0
0.004631515	0.001288629	0.021195873	0.019093729	0
0.513094906	0.213282478	0.130371575	0.001837264	0.008030762
0.003037813	0.000704344	0.108129664	0.026544562	0.0011257
0.002190156	0	0.000759328	0	0.00081159
0	0	0	0	0

0	0	0	0.001059951	0.000876532
0	0.050030884	0.052582498	0.004375242	0.000723625
0	0.00074873	0	0	0.004188234
1.863526058	0.192842821	0	0.296167863	0.568886245
0	0	0.001506289	0	0
0	0	0	0.050197206	0
0	0	0.000967553	0.127555865	0.061014612
0	0	0	0.001966908	0
0	0	0	0	0
0	0	0	0	0
0	0	0	0	0
0.022156886	1.962950698	0	0.152238723	0.629472443
0	0	0	0.001354201	0
0	0.021026933	0.001732327	0.019927165	0.068322455
0	0.016927989	0	0	0
0	0	0	0	0
0	0	0	0	0
0	0	0	0	0
0	0	0	0	0
0	0	0	0	0
0.005807869	0.055272041	0.092625147	0.159937625	0.103500204
0	0.005689492	0.002062434	0.093298305	0.107096334
0	0	0	0.043602055	0
0	0	0	0	0
0	0	0	0	0
0	0	0	0	0
0	0	0.001436528	0	0
0	0	0	0	0
0	0	0	0	0
0	0	0	0	0
0	0	0	0.00453074	0
0	0	0	0	0
0	0	0	0	0
0	0	0	0	0
0	0	0	0	0
0	0.007816408	0	0	0
0	0	0	0	0
0	0	0	0	0
0	0	0	0	0
0	0	0	0	0
0	0	0	0	0
0	0	0	0	0
0	0	0	0	0
0	0	0	0	0
0	0	0	0	0
0	0	0	0	0
0	0	0	0.005492786	0
0	0.001916076	0	0	0

0	0	0	0	0
0	0	0	0	0
0	0	0	0	0
0	0	0	0	0
0	0.288089453	0	0.059925904	0
0	0	0	0	0
0	0	0	0	0
0	0	0	0	0
0	0	0.001495128	0.000858858	0
0	0	0	0	0
0	0	0	0	0
0	0	0.000771473	0	0
0	0	0	0	0
0	0	0	0	0
0	0	0	0	0
0	0.00107669	0	0	0
0	0	0	0	0
0	0	0	0	0
0.016832655	0	0	0.02440317	0
0	0	0	0	0
0	0	0	0	0
0	0	0	0	0
0	0	0	0	0
0	0	0	0	0
0	0	0	0	0
0	0	0	0	0
0	0	0.00316474	0	0
0	0	0	0	0
0	0	0	0	0
0	0	0	0	0
0	0.009098387	0	0	0.341103583
0	0.004368051	0	0	0.005688325
0	0	0	0	0
0	0	0	0	0
0	0	0	0	0
0	0	0	0	0
0	0	0	0	0
0	0	0	0	0
0.002974022	0	0	0	0
0	0	0	0	0
0	0	0	0	0
0	0	0	0	0
0	0	0	0	0
0	0	0	0	0
0	0	0	0	0
0	0	0	0	0
0	0	0	0	0
0	0	0	0	0

0	0	0	0	0
0	0	0	0	0
0	0	0	0	0
0	0	0	0	0
0.263371432	0	0	0.021952631	0.087768496
0	0	0	0	0
0	0	0	0	0
0	0	0	0	0
0	0	0	0	0
0	0	0	0	0
0	0	0	0	0
0	0	0	0	0
0	0	0	0	0
0	0	0	0	0
0	0	0	0	0
0.014536101	0	0	0	0.326037724
0	0	0	0	0
0	0	0	0	0
0	0	0	0	0
0	0	0	0	0
0	0	0	0	0
0	0	0	0	0
0	0	0	0	0
0	0	0	0	0
0	0	0	0	0
0	0	0	0	0
0.003284424	0	0	0	0
0	0	0	0	0
0	0	0	0	0
0	0	0	0	0
0	0	0	0	0
0	0	0.014699922	0.001202496	0
0	0	0	0	0
0	0	0	0	0
0	0	0	0	0
0	0	0	0	0
0	0	0	0	0
0	0	0	0	0
0	0	0	0	0
0	0	0	0	0
0	0	0	0	0
0	0	0	0	0
0	0	0	0	0
0	0	0	0	0
0	0	0	0	0
0	0	0	0	0
0	0	0	0	0
0	0	0	0	0

0.027789603	0	0	0	0
0	0	0	0	0
0	0	0	0	0
0	0	0	0	0
0	0	0	0	0
0	0	0	0	0
0	0	0	0	0
0	0	0	0	0
0	0	0	0	0
0	0	0	0	0
0	0	0	0	0
0	0	0	0	0
0	0	0	0.000690402	0
0	0	0	0	0
0	0	0	0	0
0	0	0	0	0
0	0	0	0	0
0	0	0	0	0
0	0	0	0	0
0	0	0	0	0
0.001920511	0.035400357	0.042613886	0.146730738	0.017791736
0	0	0	0	0
0	0	0	0	0
0	0	0	0	0
0	0	0	0	0
0	0	0	0	0
0	0.002139431	0.005622675	0.001754157	0.006424122
0	0	0	0	0
0	0	0	0	0
0	0	0.000622305	0	0
0	0	0.016621285	0	0
0	0	0	0	0
0	0	0	0	0
0	0	0	0.006311276	0.004349281
0.004998348	0.069969261	0.008231413	0.003639651	0.006251176
0	0	0	0	0
0	0	0	0	0
0	0	0	0	0
0	0	0	0	0
0	0	0	0	0
0	0	0	0	0
0	0	0	0	0
0	0	0	0	0
0	0	0	0	0
0	0	0	0	0
0	0	0	0	0
0.001014728	0	0	0	0.0025068
0	0.002322763	0.003859163	0	0

0	0	0	0	0
0	0	0	0	0
0	0	0	0	0
0	0	0	0	0
0	0	0	0	0
0	0	0	0	0
0	0	0	0	0
0	0	0	0	0
0	0	0	0	0
0	0	0	0	0
0	0	0	0	0
0	0	0	0	0
0	0	0	0	0
0	0	0	0	0
0	0	0	0	0
0	0	0	0	0
0	0	0	0	0
0	0	0	0	0
0.001584838	0	0.000470969	0	0
0.001556702	0	0	0	0
0	0.007898284	0.063220201	0	0
0	0	0	0	0
0	0	0	0	0
0	0	0	0	0
0	0	0.007361189	0.010703506	0.002458698
0.00676048	0	0	0	0
0	0	0	0	0
0	0	0	0	0
0	0	0	0	0
0	0	0	0	0
0	0.082331343	0.107785728	0.04291585	0.189459077
0	0	0	0	0
0.029007832	0	0	0	0
0	0.320881002	0	0.004840511	0
0	0	0	0	0
0	0	0	0	0
0	0	0	0	0
0	0	0	0	0
0	0	0	0	0
0.000981157	0	0	0	0
0	0	0	0	0
0.044556355	0.018804349	0.008678486	0.004935398	0.012800595
0	0	0	0	0
0	0	0	0	0
0	0	0	0	0
0	0	0	0	0
0	0	0	0	0
0	0	0	0	0

0	0	0	0	0
0	0	0	0	0
0.003143137	0	0.125318567	0.027887419	0
0	0	0	0	0
0	0	0	0	0
0	0	0	0	0
0	0	0	0	0
0	0	0	0	0
0	0	0	0	0
0	0	0	0	0
0	0	0	0	0
0	0	0	0	0
0	0	0	0	0
0	0	0.001445214	0	0
0	0	0.00516895	0.020556281	0.002337377
0	0	0	0	0
0.000980045	0	0.009287376	0.004684408	0.003389568
0	0	0	0	0
0	0	0	0	0
0	0	0	0	0
0	0	0	0	0
0	0.00389739	0.003067262	0	0
0	0	0	0	0
0	0.002225942	0.176567378	0	0
0	0	0	0	0
0	0	0	0	0
0	0	0	0	0
0	0	0	0	0
0	0	0	0	0
0	0	0.001183153	0	0
0	0	0	0	0
0	0	0	0	0
0	0	0	0	0
0	0	0	0	0
0	0	0	0	0
0	0	0	0.016364501	0
0	0	0	0	0
0.017414055	0	0.007707395	0.003320564	0
0	0	0	0	0
0	0	0	0	0
0	0.00409158	0	0	0
0	0	0	0	0
0	0	0	0	0
0	0	0	0	0
0	0	0	0	0
0	0	0	0	0
0	0	0	0	0
0	0.001872976	0.004356608	0	0

0	0	1.264950112	0	0
0	0	0	0	0
0	0	0.001070906	0	0
0	0	0	0	0
0	0	0	0.001114055	0.013358451
0	0	0	0	0
0	0	0	0	0
0.006534152	0	0	0.044114997	0.022276066
0	0	0	0	0
0.087104604	0.105560387	0.430260726	0.023741121	0.035272535
0	0	0	0	0
0	0	0.002391057	0.021323802	0
0	0.095700074	0.017955649	0.019691135	0
0	0.001934232	0.005509091	0.006052355	0
0	0	0	0	0
0	0	0	0	0
0	0	0	0	0
0	0	0	0	0
0	0	0.368540748	0	0
0	0	0.03099673	0	0
0	0	0	0	0
0	0	0	0	0
0	0.001689641	0.001497205	0	0
0.000869414	0	0	0	0
0	0	0	0	0
0.001827948	0.00148339	0	0	0
0	0	0	0	0
0	0.008712895	1.065523722	0	0
0	0	0	0	0
0	0	0.003042896	0	0
0.004903718	0	0	0	0
0	0	0	0	0
0	0	0	0	0
0	0	0.005525525	0	0
0	0	0	0	0
0	0	0	0	0
0	0.107620794	0	0	0.002047645
0	0	0	0	0
0	0	0	0	0
0	0	0.266172797	0	0
0	0	0	0	0
0	0	0	0	0
0	0	0	0	0
0	0.164066249	0	0	0
0	0	0	0	0
0.004903796	0	0	0	0
0	0.016338887	0.001050824	0	0.001123149
0	0	0	0	0

0	0	0	0	0
0	0	0	0	0.004079391
0	0	0	0	0
0	0	0	0	0
0	0.068504204	0.004414177	0	0
0	0	0.026691762	0	0
0	0	0	0	0
0	0	0	0	0
0	0	0	0	0
0	0	0	0	0
0	0	0.029038077	0	0.005899531
0	0	0	0	0
0	0	0	0	0
0	0	0.003174149	0.001758232	0.011631819
0.003639243	0	0.475097489	0	0
0	0	0	0	0
0	0	0	0	0
0	0	0	0	0
0	0	0	0	0
0.00137246	0	0	0	0
0	0	0	0.002841634	0
0.353045405	0	0	0.479739624	0.369598811
0	0.026546625	0	0.025583324	0
0	0	0	0	0
0	0	0	0.003266788	0
0	0	0	0	0
0	0.00197281	0	0	0
0	0	0	0	0
0	0	0	0	0
0.062785331	2.683994436	0.177132553	0.405908863	0.11188938
0	0	0	0.00119282	0
0	0	0	0	0
0	0	0.019659464	0	0
0	0	0	0	0
0	0	0	0	0
0	0	0	0	0
1.25429125	0	0	0	0
0	0	0	0	0
0.056176777	0	0	0	0
0	0	2.676297479	0	0
0	0.006918165	0.008946843	0.025054599	0.004980532
0.001155937	0.310199552	0	4.655406664	0.136728282
0	0	0	0	0
0	0	0	0	0
0	0.001476442	0	0	0
0	0	0	0	0
0	0.000981231	0.001956324	0	0
0	0	0	0	0

0.001392427	0.001937079	0	0	0
0.0022243	0	0.001233865	0	0
0	0.002969346	0.002631163	0.002975646	0.002460724
0	0	0	0	0
0	0	0	0.004390577	0.046163122
0	0	0	0.018464368	0
0	0	0	0	0
0	0	0	0	0
0	0.046000461	0.001212068	0	0
0	0	0	0	0
0	0	0	0	0
0	0	0.019335749	0	0
0	0	0	0	0
0	0	0	0	0.003500964
0	0	0	0	0
0.025102937	0	0.021331366	0.120207264	0.013862111
0.014184779	0.000758969	0	0.006845215	0.000808669
0	0.008068531	0.159946625	0	0
0	0	0.003907528	0	0.001461764
0	0	0	0.378526315	0
0	0	0	0.004929668	0
0	0	0	0	0.000938014
0.361505144	0	0	0	0
0.001806717	0	0	0	0.089490031
0	0	0	0	0
0.006468212	0	0.002740872	0	0
0	0.001445276	0.005522895	0.004034669	0
0	0	0	0	0
0	0	0	0	0
0	0	0.00678038	0	0
0	0.064482199	0	0.014633689	0
0	0.004915997	0.012796062	0.005278315	0
0.015701534	0.016902504	0.008295204	0.003015402	1.675144855
0	0	0.004829433	0	0
0	0.22129938	0	0.004797885	0
0	0	0	0	0
0	0.000940371	0.001171787	0.001514517	0
0.024317262	0.064348701	0	0.009001612	0.009011064
0.021206225	0	0	0.447326874	0.003201502
0	0	0	0	0
0	0	0	0	0
0	0	0	0	0
0	0	0	0	0
0	0	0.0204592	0.093013535	0.389789006
0	0	15.23771318	0	0
0	0.00420872	0	0.004389799	0
0	0	0	0	0
0	0	0	0	0

0	0	0	0	0
0	0.003256807	0.776191858	0.743374336	0.464455875
0	0	0	0	0
0	0.001820719	0	0	0
0	0.000983102	0	0	0
0	0	0	0	0
0	0	0.012014185	0	0
0	0	0	0	0.002050294
0	0	0	0	0
0	0	0	0.006098604	0
0	0	0.002969346	0	0.017852146
0.004385286	0.009150907	0.652381403	0.096467025	0.009750135
0	0	0	0.009075282	0
0	0	0	0	0
0	0.01495219	0	0.006880369	0.00091036
0	0	0	0	0
0	0	0.036729003	0.074347198	0.017032105
0.002182907	0.021257302	0.033804386	0.008477487	0.012133551
0	0	0	0	0
0	0.006408713	0.039642492	0	0.005602769
0.032659266	0.008041422	0.007815862	0.426869399	0.019063797
0	0	0.000997496	0	0
0	0	0	0	0
0	0	0	0	0
0	0	0	0.164072829	0
0	0	0	0	0
0	0.111518862	0.138270196	0.064939759	0
0.034097607	0.189953623	0.003834049	0.011562721	0.021172654
0.011679666	0	0	0	0
0	0	0.001284656	0	0
0	0	0	0	0.020764015
0	0	0	0	0
0	0	0	0	0
0	0	0	0	0
0	0	0	0.005997682	0
0.026514563	0.116805075	0.152128656	0.197089959	0.062419564
0	0	0	0	0
0	0	0	0	0
0	0	0.006831831	0.010867741	0
0	0	0	0	0
0	0	14.6938887	0	0
0	0	0	0	0
0.003913038	0	0	0	0.118390283
0	0.000772901	0.01433099	0.009958383	0
0	0.07250047	0	0	0
0.215835966	0	0	0	0
0	0	0	0	0
0.003233005	0	0	0	0

0	0	0	0	0
0	0	0	0	0
0	0	0	0	0
0.041553232	0	0	0.047331381	0.007748705
0	0	0	0	0
0	0	0	0	0
0	0	0	0	0
0.018227165	0	0	0	0
4.772179967	0.035240102	0	0.07705849	0.367753189
0	0.000999971	0	0.051750914	0.047945332
0	0	0	0	0
0.162588963	0	0.004096373	0.004373718	0.016561437
0	0	0	0	0
0	0	0	0	0
0.151125392	0	0	0.001519665	0.18441975
0	0	0	0	0
0	0	0	0	0
0	0	0	0	0
0	0	0	0	0
0	0	0	0	0
0	0	0	0	0
0	0	0	0	0
0	0	0	0	0
0	0.494780768	0.032419741	0.574185928	0.069302144
0	0	0	0	0
0.018092421	0	0	6.021839806	0
0	0	0	0	0
0.656144179	0	0	2.134495296	1.886440695
0	0	0	0	0
0	0	0	0	0
0	0	0	0	0
0.000705809	0	0	0	0
0.003990944	0.004362297	0	0	0
0	0	0	0	0
0.000922204	0	0	0	0
0.481670193	0	0	0	0
0.118126776	0	0	1.342689964	4.155428388
0	0	0	0	0
0	0	0	0	0
0	0	0	0	0
0	0.082269251	0.004954886	0.285535278	0.008948267
0	0	0	0	0
10.53143498	9.328661903	0	2.316678441	23.37501775
0	0	0	0.00057712	0
0	0	0	0	0
0	0	0	0	0
0	0	0	0	0
0.0035545	0	0.19327302	0	0

0	0	0	0	0
0.069892242	0	0	0	0
0.011074017	0.14832878	1.684398674	0.004580606	0.086070698
0	0	0	0	0
0	0	0	0	0
0.0085	0	0	0.003007021	0.004641782
0	0	0	0	0
0	0	0	0	0
0	0	0	0	0
0	0	0	0	0
0.011709084	0.019779655	0.58592748	0.001998819	0.075828268
0.849962564	1.477599843	1.779544653	0.289718749	0.617318751
0	0	0	0	0
0.02286681	0	0.011057378	0	0.001783912
0	0.290266491	0.108180633	0.066935996	0.288890045
0	0	0	0	0
0	0	0	0	0
0	0	0	0	0
0	0	0	0	0
0	0.00063895	0	0.005762753	0.004935732
0.002803982	0.0081266	0.263450363	0.043557922	0.020088309
0.20312374	2.781577327	0	0	0.977506842
0.755243658	10.54128915	9.831594068	1.11438116	5.025465101
0	0	0	0	0
0	0	0	0	0
0.899643085	0.005842863	0	11.60796818	0.004150314
0	0	0	0	0
0	0	0	0	0.001083711
0	0	0	0	0
0	0	0	0	0
0	0	0	0	0
0.063090979	0	0	0.341431361	0.250631015
0	0	0.16458639	0.077238074	0
0.017904117	0	0	0.000782724	0.038674826
0.002078739	0.004732108	0.005896632	0.002032349	0.005462145
0.262238223	0.011377846	0.284096512	0.003955791	0.037330841
0.007775124	0	0	0	0.006691747
0	0	0	0	0
0.136725005	0	0	0.012253433	0.096826741
0	0	0	0	0
0	0	0	0	0
0	0	0	0	0
0.001779743	0	0	0	0
0	0	0	0	0
0.093861166	0.022243101	0.051738223	0	0
0.001373135	0	0	0	0.006105986
0	0	0	0	0
4.276013771	0.068071896	0.686762448	0.01632497	0.095558871

0	0	0	0	0
0.009477179	0	0	0	0
0	0	0	0	0
0.194568024	0	0	0	0.001309202
0	0	0	0	0
0	0	0	0.005193453	0
0	0	0	0	0
0	0	0.083729404	0	0.036313177
0	0	0	0	0
0	0	0	0	0
0	0.000586496	0.007795488	0	0.003957711
0	0	0	0	0
0	0	0	0.000882193	0.002735752
0	0	0	0	0.021685361
0	0	0	0	0
0	0	0	0	0
0	0	0	0	0
0.014272539	0	0	0	0.001213838
0	0	0	0	0
0.003376122	0	0	0	0
0	0	0	0	0.007394741
0	0	0	0	0
0.002427819	0	0	0	0.056378598
0	0	0	0	0
0	0	0	0	0
0	0	0	0	0
0	0	0	0	0
0	0	0	0	0
0	0	0	0	0
0.295077172	0.012778897	0	0.015451647	0.12924439
0	0	0.005065348	0	0.012632612
0	0	0	0	0
0	0	0	0	0
0	0	0	0	0
0	0	0	0.002002897	0
0	0	0	0	0
0.283792889	0	0	0.170640993	0.085472901
0.003330911	0.01870072	0.003299509	0.007036534	0.004760912
0	0	0	0	0
0	0	0	0.000841266	0
0.009822116	0.015837895	0	0	0
0	0	0	0	0
0	0	0	0	0
0	0	0	0.002898979	0
0	0	0	0.091278682	0
0	0	0	0	0
0.000678198	0	0	0	0
0.002542914	0	0	0	0

0	0	0	0	0
0	0	0	0	0
0	0	0	0	0
0.027926218	0	0	0	0.246432436
0	0	0	0	0
1.162448109	0	0	0	0
0	0	0	0	0
0	0	0	0	0
0	0.103165665	0.335832324	0.002646697	0
0.002378207	0	0	0.001184096	0
0	0	0	0	0
0	0	0	0	0
0	0	0	0	0
0	0	0	0	0
0.002593867	2.759984269	0	8.126975727	1.589108794
0	0.001583216	0.089610328	0.012692604	0.001499458
0	0	0.003609057	0	0
0	0	0	0	0
0	0	0	0	0
0	0	0	0	0
0	0	0	0	0
0	0	0	0	0
0	0	0	0	0
0	0	0	0	0
0.026625499	0.003220882	0.004013509	0	0
0.035643086	0	0	4.081621782	2.322719355
0	0	0	0	0
0	0	0	0	0
0.239530329	0	0	0.018141085	0.006250771
0	0	0	0	0
0	0	0	0	0
0	0	0	0	0
0	0	0	0	0
0	0	0.006144618	0	0.014509657
0.040068039	0	0.002354509	0.001217267	0.005033123
0	0.020368814	0.001664352	0	0.001956794
0	0.003962797	0	0	0
0.042010045	0.629292674	0	1.238072048	0.008034759
0.00242515	19.38032423	0	0	0
0	0	0	0	0
0.036893565	0	0	0	0
0	0	0.006039645	0	0
0.125624717	0.107230683	0	0.45269623	1.241705578
0.156997332	0	0	0	0
0	0	0	0	0
0	0.011704674	0	0.000611384	0
0	0	0	0	0
0.00724538	0.000755957	0.025370955	0.244475852	0.045642718

0	0	0	0	0
0	0	0	0	0
0	0	0	0	0
0	0	0	0	0
0	0	0.009511351	0	0
0.139603424	0	0	0	0
0	0	0	0	0
0	0	0	0	0
0	0	0	0	0
0	0	0	0	0
0	0	0	0	0
0.57939006	0.238354156	0	2.482640405	3.597584744
0	0	0	0.168988597	0.035962236
0	0	0	0	0
0.001866314	0.01016895	0.186566538	0.025089136	0.003688457
0.001891689	0	0	0	0
0	0	0	0	0.001122562
0	0	0	0	0
0.000817909	0.000568918	0.001323323	0.000977357	0.001414402
0	0	0	0	0
0	0	0	0	0.756797639
0	0	0	0	0
0.380420874	0.02065263	0	0.249703707	0.148533925
0	0	0	0	0
0	0	0	0	0
0	0	0	0	0
0	0	0	0.017567069	0
0	0	0	0	0
0	0	0	0	0
0	0	0	0	0
0	0	0	0	0
0	0	0.114470907	0.070539436	0.02722202
0	0	0	0	0
0.122803932	0.015148291	0.007969923	0	0.01733582
0.00130605	0.021439608	0.003984712	0	0.002129482
0.045122017	0.003513337	0	0	0.001497361
0	0	0	0	0
0	0	0	0	0
0.62749205	0	0	0	0
0	0	0	0	0
0	0	0	0.12445886	0
0	0	0	0	0
0	0	0	0	0
0	0	0	0	0
0.011064679	0.008770237	0.035863337	0.002536722	0.008200309
0	0	0	0	0
0	0	0	0.108851371	0
0	0	0	0	0.008477516

0	0	0	0	0
0	0	0	0	0
0	0	0.000738143	0.007393797	0
0	0	0	0	0
0	0.004881059	0	0	0
0	0	0	0	0
0.02079958	0	0	0	0
0	0	0	0.016218	0.002980344
0	0	0	0	0
0	0	0	0	0
0	0	0	0	0
0	0	0	0	0.001404784
0	0	0	0	0
0	0	0	0	0
0	0	0	0	0
0	0	0	0	0
0	0	0	0	0
0	0	0	0	0
0.012227912	1.37689356	0	0	0
0.004342565	0	0	0	0
0	0	0	0	0
0	0	0	0	0
0	0	0	0	0
0	0	0	0	0
0	0	0	0	0.000833447
0	0.073316204	0.026577043	0	0.001597851
0	0	0	0	0
0.000703425	0.001467859	0.047149721	0.038875717	0.022590801
0	0	0	0	0
0.029242215	0	1.334319525	0.965558134	0.306644528
0	0	0	0	0
0	0	0	0	0
0	0	0	0	0
0	0	0	0	0
0	0.000873078	0.000696277	0	0.002976799
0	0	0	0	0
0	0	0	0	0.044958044
0	0.001386963	0.003357802	0	0.000844449
0	0	0	0	0
0	0	0	0	0
0.173833412	2.857503171	19.41476039	9.154359352	0.907002223
0	0.031038038	0	0.147629286	0.460359933
0	0	0	0	0
0	0	0	0	0
0	0	0	0	0
0	0	0	0	0
0	0	0	0	0
0.002938983	0	0.000783805	0	0.001172852

0	0	0	0	0
0	0.010675341	0	0	0.011719073
0	0	0	0	0
0	0	0	0	0
2.347420417	1.756010883	0	3.092614389	2.884915384
0	0	0	0	0
0	0	0	0	0
0	0	0	0	0
0.002321946	0.026810513	2.459334754	0.136510426	0.015659755
0	0	0	0	0
0	0	0	0	0
0	0	0	0	0
0.011975338	0	0	0.02105984	0.04420865
0	0.140856512	0.070111648	0.023152468	0.002264585
0.004098026	0	0	0	0.086558757
0.006154545	0	0	0.94115302	0.204244286
0	0.000668804	0	0.002585145	0
0	0	0	0	0
0	0	0	0	0
0	0	0	0	0
0	0	0	0	0
0	0	0	0	0
0	0	0	0	0
0	0	0	0	0
0	0	0	0	0
0	0	0	0	0
0	0	0	0.003933806	0.005576706
0	0.001148379	0	0	0
0	0	0	0	0
0	0	0	0	0
0	0	0	0	0
0	0	0	0	0
0	0	0	0	0
0	0	0	0	0
1.110011208	0	0	0	0.033807821
0	0	0	0	0.01556969
0	0	0	0	0
0	0	0	0	0
0	0	0	0	0
0	0	0	0	0
0.00367566	2.242460834	0	0.542339159	1.338782852
7.948699354	0	0	0	0
0	0	0	0	0
0.109981082	0	0	0	0
0	0	0	0	0
0.001042118	0	0	0	0
0	0	0	0	0
0	0	0	0	0

0	0	0	0.002545786	0.134161773
0	0	0	0	0
0	0	0	0	0
0	0.000743365	0	0	0
0	0.017603553	0	0.037114627	0.005115351
0.002481761	0	0	3.061026389	0
0	0.001000766	0.050546874	0.018696724	0
0	0	0	0	0
0	0	0	0	0
0	0	0	0	0
0.003780247	0	0	0	0
0	0	0	0.007893412	0
0	0	0	0	0
0	0	0	0	0
0	0	0	0	0
0	0	0	0	0
0	0	0	0	0
0	0	0	0	0
0	0	0	0	0
0	0	0	0	0
0.007077184	0.003493545	0.034034671	0.052582565	0.134849255
0	0	0	0	0
0.085363585	0	0	0.009026986	0.005225434
0.027989005	0.005606926	0.000931565	0.001204033	0
0.000803073	0	0	0	0
0	0.002021223	0	0	0
0.001297368	0	0	0	0
0	0	0	0	0
0	0	0	0	0
0.023847382	0.277507377	0.010279006	0.364772575	0
0	0	0	0	0
0	0	0	0	0
0	0	0	0	0
0.001434747	0	0	0	1.085125278
0	0	0	0	0
0	0	0	0	0
0	0	0	0.065786349	0
0	0	0	0	0
0	0	0	0	0
0	0	0	0	0
0	0	0.000726634	0	0
0	0	0	0	0
0	0	0	0	0
0	0	0	0	0
0	0	0	0	0
0.002245511	0	0	0	0
0	0	0	0	0
0	0	0	0	0

0	0.002513855	0.006682644	0.002375235	0
0.139387382	0	0	0	0.072989825
0	0	0	0	0
0	0	0	0	0
0	0	0	0	0
0	0	0	0	0
0	0	0	0	0
0.059867934	0.175518934	0	3.959328718	0.199846628
0.614554339	0.283576723	0.18094238	0.001694676	0.009249373
0.02790796	0.196318808	0.01478739	0	0.162734526
0	0	0	0	0
0	0	0	0	0
0	0	0	0	0
0.382730845	0.012463781	0	0	0
0	0	0	0	0
0	0	0	0	0
0	0.002446368	0	0	0
0	0	0	0	0
0	0	0	0	0
0	0	0	0	0
0	0	0	0	0
0	0	0	0	0
0	0	0	0.004076806	0
0	0	0	0	0
0	0	0	0	0
0	0	0	0	0
0	0	0	0	0
0	0	0	0	0
0	0	0	0	0
0	0	0	0	0
0	0.004304256	0	0	0
0	0	0	0	0
0	0	0	0	0
0	0	0	0	0
0	0	0	0.002091314	0
0.256955901	0	0	0	0
0	0	0	0	0
0	0	0	0	0
0.022059983	0	0	0	0
0	0.192738387	0	0	0
0	0	0	0	0
0.014640867	0.010183843	0	0.36828579	0.165151512
0	0	0	0	0
0	0	0	0	0
0	0	0	0	0
0	0	0	0	0
0	0	0	0	0
0	0	0	0	0

0	0	0	0	0
0	0	0	0	0
0	0	0	0	0
0	0	0	0	0
0.463044843	0.048851738	0	0.0051771	0.039432346
0	0	0	0	0
0	0	0	0	0
0	0.022171451	0.346407886	0	0.00318006
0	0	0	0	0
0	0	0	0	0
0	0	0	0	0
1.562457333	0	0	0	0
0	0	0	0	0
0	0	0	0	0
0	0	0.017503985	0	0
0.00212653	0.00166406	0	0.001429363	0.005319082
0	0	0	0	0
0	0	0	0	0
0	0	0	0	0
0	0	0	0	0
0	0	0	0	0
0	0	0	0	0
0	0	0	0	0
0	0	0.000735675	0	0
0.207199809	0	0	0.149105543	0
0.003384405	0	0	0	0
0.007035213	0.006580952	0	0	0
0	0	0	0	0
0	0	0	0	0.001870672
0	0	0	0	0
0	0	0	0	0
0	0	0	0	0
0	0	0	0	0
0	0	0	0	0
0	0	0	0	0
0	0	0	0	0
0	0	0	0	0
0.011693313	0	0	0	0.071911069
0.000881278	0	0	0	0
0	0	0	0	0
0	0	0	0	0
9.592447078	0	0	0	0.05222224
0	0	0	0	0
0	0	0	0	0
0	0	0	0	0
0	0	0	0	0
0	0	0	0	0
0	0	0	0	0

0.015285267	0	0	1.092224602	0
0	0	0	0	0
0	0	0	0	0
0	0	0	0	0
0	0	0	0	0
0	0	0	0.018002773	0
0	0	0	0	0
0	0	0	0	0
0	0	0	0	0
0	0	0	0	0
0	0	0	0.187973053	0
0	0	0.000508631	0	0
0	0	0	0	0
0	0	0	0	0
0	0	0	0	0
0	0	0	0	0
0	0	0	0	0
0	0	0	0	0
0	0	0	0	0
0	0	0	0	0
0	0.012493271	0.245420834	0.001893748	0
0	0	0	0	0
0.004002966	0.001160154	0.002775659	0	0.01384459
0	0	0	0	0
0	0	0	0	0
5.905371669	0	0	0	0
0	0	0	0	0
0	0	0	0	0
12.2821722	0	0	0	0
0	0	0	0	0
0	0	0	0	0
0	0	0	0	0
0.001561984	0.536720731	0.744076428	0.108349603	0.00138915
0	0	0	0	0
0	0	0	0.122212104	0.053638392
0.138628975	0	0.002085904	0	0.004087361
0	0	0	0	0
0	0	0	0	0
0	0.000605062	0.050867304	0	0.001719156
0	0	0	0	0
0	0	0	0	0
0	0	0	0	0
0.510943624	0.030422461	0	0.012154289	0.0346761
0	0	0	0	0
0	0	0	0	0
0	0	0	0.036921247	0.002394681
0	0	0	0	0
0	0	0	0	0

0	0	0	0	0
0	0	0	0	0
0	0	0	0	0
0	0	0	0	0
0	0	0	0	0
0	0	0	0	0
0	0	0	0	0
0	0	0	0.184217779	0.003663866
0	0	0	0	0.000934831
0	0	0	0	0
0	0	0	0	0
0	0	0	0	0
0	0	0	0	0
0	0	0	0	0
0	0	0	0	0
0	0.081234896	6.080181639	0	0
0	0	0	0	0
0	0	0	0	0
0	0	0	0	0
0.003655271	0	0	0.409486343	0.355388567
0	0.002775274	0	0	0.002323363
0	0	0	0	0
0	0	0	0	0
0	0	0	0	0
0	0	0	0	0
0.001190328	0	0	0	0
0	0	0	0	0
0	0	0	0	0
0	0	0	0	0
0	0	0	0.988728151	0
0.005988064	0.070653394	0	0.905557869	0.466472957
0	0	0	0	0
0	0	0	0	0
0	0	0	0	0
0	0	0	0	0
0	0	0	0	0
0	0	0	0	0
0	0	0	0	0
0.018746618	0	0	0	0
0	0	0	0	0
0	0	0	0	0
0.023213867	0	0	0	0
0	0	0	0	0
0	0	0	0	0
0	0	0	0	0
0	0	0	0	0
0	0.011657884	1.628935377	0	0
0	0	0	0	0

0	0	0	0	0
0	0	0	0	0
0	0	0	0	0
0	0	0	0	0
0.003964982	0	0	0.003553457	0.02582828
0	0	0	0	0
0	0	0	0	0
0	0	0	0	0
0	0	0	0	0
0	0	0	0	0
0	0	0	0	0
0.002317811	0.001612215	0.006629583	0	0.001717787
0	0	0	0	0.002408279
0	0	0	0	0
0	0	0.014850421	0	0
0	0.044977398	0	2.21545623	0
0	0.002634967	0	0	0
0	0	0	0	0
0	0.305652321	0	0.003735642	0.013982722
0	0	0.017876006	0	0
0	0	0	0	0
0	0	0	0	0
0	0	0	0	0
0	0	0	0	0
0	0	0	0	0
0	0	0	0	0
0	0.003193292	0	0.002674342	0.007145035
0	0	0	0	0
0	0	0	0	0
0	0	0	0	0
0	0	0.058095899	0	0.001157039
0	0	0	0	0
0	0	0	0	0
0	0	0	0	0
0	0	0	0	0
0	0	0	0	0
0	0	0	0	0
0.006215477	0.010426878	0.063886684	0	0.00406451
0.000659642	0	0	0	0
0	0	0	0	0
0	0	0	0.001635596	0
0	0	0	0	0
0	0	0	0	0
0	0.008107984	0	0	0
0	0	0	0	0
0	0	0	0	0
0	0	0	0	0
0	0	0	0	0

0	0	0	0	0
0	0	0	0	0.018373496
0	0	0	0	0
0	0.001185656	0.001181948	0	0
0	0	0	0	0.001757531
0	0	0.0014148	0	0
0.008546454	0	0	0	0
0.005776847	0.000772738	0	0	0
0	0	0	0	0
0	0	0	0	0
0	0	0	0	0
0	0	0	0	0
0.084379456	0.015128342	0	0.014979959	0.011044494
0	0	0	0	0
0	0	0	0	0
0	0	0	0	0
0	0	0	0	0
0	0	0	0	0
0	0	0	0	0
0	0	0	0	0
0	0	0	0	0
0	0	0	0	0
0.044814564	0	0.00441294	0	0.039502079
0.003129155	0.013494715	0	0.020191542	0
0	0	0	0	0
0	0	0	0	0
0	0	0	0	0
0	0	0	0	0
0	0	0	0	0
0	0	0	0	0
0.003252358	0	0.02603716	0.031002828	0.014024165
0	0	0	0	0
0.006520084	0	0	0	0
0	0	0	0	0
0	0	0	0	0
0	0.00128995	0.064510087	0	0
0	0	0.008059176	0	0
0	0	0	0	0
0	0	0	0.029817625	0.00129778
0	0	0	0	0
0	0	0	0	0
0	0	0	0	0
0	0	0	0	0
0	0	0	0	0
0	0	0	0	0
0.54165696	0.077771652	0	0.293511743	0.040616592
0	0	0	0	0
0	0	0	0	0

0	0	0	0	0
0	0	0	0	0
0.327121028	0	0	0	0
0	0	0	0	0
0	0	0	0	0
0	0	0	0	0
0	0	0	0	0
0	0	0	0	0
0	0	0	0	0
0	0	0	0	0
0	0	0	0	0.004060982
0	0	0	0	0
0	0	0	0	0
0	0	0	0	0
0	0	0	0	0
0	0	0	0	0
0	0	0	0	0
0	0	0	0	0
0	0	0	0	0
0.399401374	0	0	0	0.004986629
0	0	0	0	0
0	0	0	0	0
0	0	0	0	0
0	0	0	0	0
0	0	0	0	0
0	0	0	0	0
0	0	0	0	0
0.005361248	0	0	0.025358693	0.009491903
0	0	0	0	0
0	0	0	0	0
0.282085634	0	0	0.001864977	0.005312198
0	0	0	0	0
0.022014321	0.037161173	0.193601657	0.008180507	0.065062481
0	0	0.004375305	0.002704573	0.010979472
0.001331933	0	0	0	0
0	0	0	0	0
0	0	0	0	0
0	0	0	0	0
0	0	0	0	0
0	0	0	0	0
0	0	0	0	0
0	0.009064108	0.037827653	0.042359592	0.068258326
0.196156859	0.010106822	0	0.117198506	0.18765365
0	0	0	0	0
0	0	0	0	0
0.110428978	0.002434177	0	0	0.025071215
0	0	0	0	0
0	0	0	0	0

0	0	0	0	0
0.012121418	0	0	0.011873881	0.012535096
0	0.095738863	0.011872843	0.014460157	0.004148656
0	0	0	0	0
0.014986171	0.001828777	0	0.025683379	0.002727943
0.025080176	0.00178012	0.020584806	0	0
0	0	0	0	0
0	0	0	0	0
0	0	0	0	0
0	0	0	0	0
0	0	0	0	0
0	0	0	0	0
0.161770999	0	0	0	0
0	0	0	0.004960872	0
0	0	0	0	0
0	0	0	0	0
0	0	0	0.00298239	0
0	0	0	0	0
0	0	0	0	0
0	0	0	0	0
0	0	0	0	0
0	0	0	0	0
0	0	0	0	0
0	0	0	0	0
0.014925575	0	0	0	0
0	0.014051827	0.004353798	0.00146797	0
0	0	0	0	0
0	0	0	0.143584886	0.017271005
0	0	0	0	0
0	0	0	0	0
0	0	0	0	0
0	0.001332175	0	0	0
0	0	0	0	0
0	0	0	0	0
0	0	0	0	0
0	0	0	0	0
0	0	0	0	0
0	0	0	0	0
0.010410072	0	0	1.546272022	1.429325908
0	0	0	0	0
0	0	0	0	0
0.001480792	0	0	0	0.001975414
0	0.001905749	0.00455949	0.00466535	0
0	0	0	0	0
0	0	0	0	0
0	0	0	0	0
0.003353101	0.001166169	0	0	0.013978501
0	0	0	0	0

0	0	0	0	0
0.003173248	0.080564128	0.007701162	0.002606905	0.031552953
0	0	0	0	0
0	0	0	0	0
0	0	0	0	0
0	0	0	0	0
0	0	0	0	0
0	0	0	0	0
0	0	0	0	0
0	0	0	0	0
0	0	0	0	0
0	0	0	0	0
0.003633937	0	0	0.027056256	0.003521879
0	0	0	0	0
0.002637708	0.002752091	0	0.004136895	0
0	0	0	0	0
0	0	0	0	0
0	0	0	0	0
0	0	0	0	0
0	0	0	0	0
0	0	0	0	0
0	0	0.000898105	0	0
0	0	0	0	0
0	0	0	0	0
0	0	0	0	0
0	0	0	0	0
0	0	0	0	0
0	0	0	0	0
0	0	0	0	0
0	0	0	0	0
0	0	0	0	0
0	0	0	0	0
0	0	0	0	0
0	0	0	0	0
1.808373009	0	0	0	14.36558695
0	0	0	0	0
0	0	0	0	0
0	0	0	0	0
0.004527013	0.000674761	0.001121084	0.001738781	0
0	0	0	0	0
0.0034792	0.023797154	0.158620531	0.061582355	0.009454579
0	0	0	0	0
0	0	0	0	0
0	0	0	0	0
0	0	0	0	0
0.068382505	0.024475329	9.477541105	0.251982878	0.022387758
0	0	0	0	0
0	0	0	0	0

0	0	0	0	0
0	0	0	0	0
0	0	0	0	0
0	0	0	0	0
0	0	0	0	0
0	0	0	0	0
0	0	0	0	0
0	0	0	0	0
0	0	0	0	0
0	0	0.007203633	0	0
0.008559783	0	0	0	0
0	0	0	0	0
0	0.003752239	0.002200295	0	0
0	0	0	0	0
0	0	0	0	0
0	0	0	0	0
0	0	0	0	0
0	0	0	0	0
0	0	0	0	0
12.64892792	0.087423068	0.038384995	0.010884273	0.080797862
0	0	0	0	0
0	0	0	0	0
0	0	0	0	0
0	0	0	0	0
0	0	0	0	0
0	0	0	0	0
0	0	0	0	0
0	0	0	0	0
0	0	0	0	0
0.004528941	0.006090435	0.237412046	0.018670861	0
0	0	0	0	0
0	0	0	0	0
0	0	0	0	0
0	0	0	0	0
0	0	0	0	0
0	0	0	0	0
0	0	0	0	0
0	0	0	0	0
0	0	0	0	0
0	0	0	0	0
0	0	0	0	0
0	0	0	0	0
0	0	0	0.001052028	0
0	0	0	0	0
0	0.002952304	0.003310953	0	0
0	0	0	0	0
0	0	0	0	0
0	0	0	0	0

0	0	0	0	0
0	0	0	0	0
0	0	0	0	0
0	0	0	0	0
0.001889541	0	0	0	0
0.108128777	0.003168264	0	0.001952321	0.00308219
0	0	0	0	0
0	0	0	0	0
0	0	0	0	0
0	0	0	0	0
0	0	0	0	0
0	0	0	0	0
0	0	0	0	0
0	0	0	0	0.083693071
0	0	0	0	0
0	0	0	0	0
0	0	0	0	0
0	0	0	0	0
0	0	0	0	0
0	0	0	0	0
0	0.004472165	0	0	0
0	0	0	0	0
0	0	0	0	0
0	0	0	0	0
0	0	0	0	0
0	0	0	0	0
0	0	0	0	0
0	0	0	0	0
0	0	0	0	0
0	0	0	0	0
0	0	0	0	0
0	0	0	0	0
0	0	0	0	0
0	0	0	0	0
0	0	0	0	0
0	0	0	0	0
0	0	0	0	0
0	0	0	0	0
0	0	0	0.13553898	0
0	0	0	0	0
0	0	0	0	0
0	0	0	0	0
0	0	0	0	0
0	0	0	0	0
0	0	0	0	0
0	0	0	0	0
0	0	0	0	0
0	0	0	0	0

0	0	0	0	0
0	0	0	0	0
0	0	0	0	0
0	0	0	0	0
0	0	0	0	0
0	0	0	0	0
0	0	0	0	0
0	0	0	0	0
0	0	0	0	0
0	0	0	0	0
0.002032269	0	0.001409177	0	0
0	0	0	0	0
0	0	0	0	0.645493135
0	0	0	0	0
0	0	0	0	0
0	0	0	0	0
0	0	0	0	0
0	0	0	0	0
0	0	0	0	0
0	0	0	0	0
0.007955863	0.003484314	0	0	0.000655143
0	0	0	0	0
0	0	0	0	0
2.087278004	0.597825327	0.00389292	3.82244023	0.796351589
0	0	0	0	0
0	0	0	0	0
0	0	0	0	0
0	0	0	0	0
0	0	0	0	0
0	0	0	0	0
0	0	0	0	0
0	0	0	0	0
0	0	0	0	0
0	0	0	0	0

PHL018-0260_V1	PHL018-0041_V3	PHL018-0122_V2	PHL018-0105_V2	PHL018-0218_V1
REF	EYCF	CTRL	CTRL	REF
8.097828946	0.212515807	0.025619503	0.008729296	1.104637958
0.347423552	2.346958249	0.630984331	2.645176338	0.519886983
0	9.099618512	26.02033471	10.89512463	9.39126418
0	0	0	0	0
0	0	0	0	0
0	0	0	0	0
0	0	0	0	1.401167234
0	0	0	0	0
0	0	0	0	0
0	0	0	0	0
0	0	0	0	0
0	0	0	0	0
0	0	0	0	0
0.477090289	7.934515795	15.97029689	4.086615263	2.176589041
0	0	0	0	0
0	0	0	0	0
0	0	0.001705116	0	0
0	0	0	0	0
0	0	0	0	0
0	0	0	0	0
0	0	0	0	0
0	0	0	0	0
0	0	0	0	0
0	0	0	0	0
0	0	0	0	0.145719652
0	0	0	0	0
0	0	0	0	0
0	0	0	0	0
0	0	0	0	0
0	0	0	0	0
0	0	0	0	0
0	0	0	0	0
0	0	0	0	0
0	0	0	0	0
0	0	0	0	0
0.86976966	3.010199748	0	4.785323972	0
0	0	0	0	0
0	0	0	0	0
0	0	0	0	0
0	0	0	0	0
0	0	0	0	0
0.00415473	0.208046371	0	0.053588243	0.009128462
0.377870408	0.014818232	0.003772682	0.041343185	7.57817601
0	0.00512357	0	0.062855379	0.121858201
0.00463886	0	0	0	0
0	0	0	0	0

0.023517778	0	0	0	0
0	0	0	0.235402227	0.009868751
0	0.003857898	0	0	0
0.405873607	0.311670095	0.094637826	0.221703447	0.308198341
0.000811956	0.031753212	0	0.001052282	0.007273105
0	0.001692961	0	0	0.07533068
0	0.032065533	0.011116326	0.024333306	0
0	0	0	0	0
0	0.037933006	0	0	0
0	0	0	0	0
0.001198017	0.002702937	0	0	0.00145783
0	0.017646829	0	0	0.392470173
0	0	0	0	0
0.023345016	0.088697076	0.019479441	0.009128297	0.121206755
0	0	0	0	0
0	0	0	0	0
0	0	0	0.10636839	0.854434237
0	0	0	0	0
0	0	0	0	0
0	0	0	0	0
0.006512482	0	0.274032624	0.688140042	0.058435685
0.001482324	0.005852672	0.005377712	0.056602906	0.00751581
0.020782489	0.204904756	0	0	0
0	0	0	0	0
0	0	0	0	0
0	0	0	0	0
0	0	0	0	0
0	0	0	0	0
0.001422241	0.209108484	0.000644967	0	0
0	0	0	0	0
0.001049773	0	0.107017824	0.079063846	0
0	0	0	0	0
0	0	0	0	0
0.038375287	0	0	0	0
0	0	0	0	0
0	0	0	0	0
0	0	0	0	0
0	0	0	0	0.002050585
0	0	0	0	0
0	0	0	0	0
0	0	0	0	0
0	0	0	0	0
0	0	0	0	0
0	0	0	0	0
0	0	0	0	0
0	0	0	0	0
0	0	0	0.001017898	0
0	0.002956546	0	0	0

0	0	0	0	0
0	0	0.06953539	0	0.001775908
0	0	0	0	0
0	0	0	0	0
0	0.005710172	0	0	0
0	0	0	0	0
0	0	0	0	0
0	0	0	0	0
0.682720719	0.125869725	0.000649748	0	0.018706395
0	0	0	0	0
0	0	0	0	0
0	0.191637334	0	0	0
0	0	0	0	0
0	0	0	0	0
0	0	0	0	0
0	0	0.002273897	0	0
0	0	0	0	0
0	0	0	0	0
0	0.101034713	0	0	0.119156742
0	0	0	0	0
0	0	0	0	0
0	0	0	0	0
0	0	0	0	0
0	0	0	0	0
0	0	0	0	0
0	0	0	0	0
0	0	0	0	0
0	0	0	0	0
0	0	0	0	0
0	0	0	0	0
0.001657319	0.16396405	0.032768531	0	0.149238768
0.006780819	0.001176825	0	0	0
0	0	0	0	0
0	0	0	0	0
0	0	0	0	0
0	0	0	0	0
0	0	0	0	0
0	0	0	0	0
0	0.248792565	0	0	0.155784203
0	0	0	0	0
0	0	0	0	0
0	0	0	0	0
0	0	0.000729072	0.001562669	0
0	0	0	0	0
0	0	0	0	0
0	0	0	0	0
0	0	0	0	0
0	0	0	0	0

0.015613228	0	0	0	0.003166543
0	0	0	0	0
0	0	0	0	0
0	0	0	0	0
0.002118644	0.004950749	0	0	0.129519451
0	0	0	0	0
0	0	0	0	0
0	0	0	0	0
0	0	0	0	0
0	0	0	0	0
0	0	0	0	0
0	0	0	0	0
0	0	0	0	0
0	0	0	0	0
0	0	0	0	0
0	0	0	0	0
0	0	0	0	0
0	0	0	0	0
0	0	0	0	0
0	0	0	0	0
0	0	0	0	0
0	0	0	0	0
0	0	0	0	0
0	0	0	0	0
0	0	0	0	0.281369805
0	0	0	0	0
0.018697761	0	0	0	0
0	0	0	0	0
0	0	0	0	0
0	0	0	0	0
0	0	0	0	0
0.001404236	0.001357802	0	0	0.000813701
0	0	0	0	0
0	0	0	0	0
0	0	0	0	0
0	0	0	0	0
0	0	0	0	0
0	0	0	0	0
0	0	0	0	0
0	0	0	0	0
0	0	0	0	0
0	0	0	0	0
0	0	0	0	0
0	0	0	0	0
0	0	0	0	0
0	0	0	0	0
0	0	0	0	0
0	0	0	0	0

0	0	0	0	0
0	0	0	0	0
0	0	0	0	0
0	0	0	0	0
0	0	0	0	0
0	0	0	0	0
0	0	0	0	0
0	0	0	0	0
0	0	0	0	0
0	0	0	0	0
0	0	0	0	0
0	0	0	0	0
0	0	0	0	0
0	0	0	0	0
0	0	0	0	0.008660455
0	0.005244197	0	0	0
0	0	0	0	0
0	0	0	0	0
0	0	0	0	0
0	0	0	0	0
0.011126462	0.021459238	0.017253044	0.092498837	0.102637762
0	0	0	0	0
0	0	0	0	0.000958067
0	0	0	0.005404956	0.005693114
0	0	0	0	0
0	0	0	0	0
0.001254153	0.028295896	0.000758322	0.010680951	0.001695711
0	0	0	0	0
0	0	0	0	0
0	0	0.010547123	0	0.005442646
0	0	0.091655086	0.623780845	0
0	0	0	0	0
0	0	0	0	0
2.735494617	0	0	0	0.084079298
0.015413063	0.010010866	0.004871552	0.007004251	0.006393983
0	0	0	0	0
0	0	0	0	0
0	0	0	0	0
0	0.0048516	0	0	0
0	0	0	0	0
0	0	0	0	0
0	0	0	0	0
0	0	0	0	0
0	0	0	0	0
0	0	0	0	0
0	0.0027591	0	0	0.870550833
0.001264261	0	0	0	0
0	0	0	0	0.029533032

0	0	0	0	0
0	0	0	0	0
0	0	0	0	0.001850514
0	0	0	0	0
0	0	0	0	0
0	0	0	0	0
0	0.001336208	0	0	0
0	0	0	0.003454753	0
0	0	0	0	0
0	0	0	0	0
0	0	0	0	0
0	0	0	0	0
0	0	0	0	0
0	0	0	0	0
0	0.002959993	0	0	0
0	0	0	0	0
0	0	0	0	0
0	0	0	0	0.056951845
0.002030985	0	0	0	0.032266063
0	0.000656382	0	0	0
0	0	0.001358605	0.001941326	0.004557038
0	0	0	0	0
0	0	0	0	0
0	0	0	0	0
0	0.003916727	0	0.002754895	0
0	0	0	0	0
0	0	0	0	0
0	0	0	0	0
0	0.064610359	0	0	0
0	0	0	0	0
0.061313798	0.193420133	0.366126752	0.356863982	0.747039567
0	0	0	0	0
0	0.022659789	0	0	0
0	0	0	0.005012767	0
0	0	0	0	0
0	0	0	0	0
0	0	0	0	0
0	0	0	0	0
0	0	0	0	0
0	0	0	0	0
0	0	0	0	0
0	0.004221834	0.00084858	0	0.005882373
0	0	0	0	0
0	0	0	0	0
0	0	0	0	0
0	0	0	0	0
0	0	0	0	0
0	0	0	0	0

0	0	0	0	0
0	0	0	0	0
0	0	0	0.077258524	0.156779893
0	0	0	0	0
0	0	0	0	0
0	0	0	0	0
0	0	0	0	0
0	0	0	0	0
0	0	0	0	0
0	0	0	0	0
0	0	0	0	0
0	0	0	0	0
0	0	0	0	0
0	0.002636456	0	0.004038462	0.010006479
0.036221791	0.033849659	0	0.001190436	0.007607009
0	0.012684185	0	0	0.022338673
0	0.000826471	0	0.002712791	0.001238215
0.001136627	0	0	0	0
0	0	0	0	0
0	0	0	0	0
0	0	0	0	0
0	0	0	0	0.03263844
0	0	0	0	0
0	0	0	0	0
0.00212089	0	0	0	0
0	0	0	0	0
0	0	0	0	0
0	0	0	0	0
0	0	0	0	0
0	0.00287785	0.008098182	0.001062696	0
0	0.001524596	0	0	0
0	0	0	0	0
0	0	0	0	0
0	0	0	0	0
0	0	0	0	0
0.001761285	0	0	0.003423897	0
0	0	0	0	0
0.054841048	0.001249809	0	0	0.074149319
0	0	0	0	0
0	0	0	0	0
0	0	0	0	0
0	0	0	0	0
0	0	0	0	0
0	0	0	0	0
0	0	0	0	0
0	0.003362732	0	0	0
0	0	0	0	0
0	0.006055328	0.436333211	0	0

18.50542413	0	0	0.95816787	0
0	0	0	0	0
0	0	0	0	0
0	0	0	0	0
0.007666358	0.011531104	0.00337124	0	0
0.001515235	0	0	0	0
0	0	0	0	0
0	0.002571447	0.003248805	0.031470861	0
0	0	0	0	0
0.054038557	0.878812155	0.309898964	0.021625132	0.064668704
0	0	0	0	0
0.001031108	0.100615145	0	0.016035598	0
0.000586599	0.001764628	0.00053203	0.003258099	0.024388659
0	0	0	0	0
0	0	0	0	0
0	0	0	0	0
0	0	0	0	0
0	0	0	0	0
0	0	1.324845677	0.012279482	0
0	0	0	0.032340513	0
0	0	0	0	0
0	0	0	0	0
0	0.005917821	0	0	0
0	0	0	0	0
0	0	0	0	0
0	0	0	0.001517942	0.002309477
0	0	0	0	0
0	0	0	0	0
0	0	0.041662994	0	0.007330172
0	0	0	0.002602952	0
0	0	0	0.002443251	0
0	0	0	0	0
0	0	0	0.002249957	0
0	0	0.002701421	0	0
2.60959172	0	0	0	0
0	0	0	0.056696602	0
0	0	0.008195478	0.739058991	0
0	0	0	0	0
0	0	0	0	0
0	0	0	0.018093512	0
0	0	0	0	0
0	0	0.555285373	0	0
0	0	0	0	0
0	0	0	0.031591778	0
0	0	0	0	0
0	0	0	0	0
0	0.00127799	0	0.001573067	0.004595228
0	0	0.021683586	0	0

0	0	0	0	0
0	0.035706077	0	0	0
0.001029594	0	0	0	0
0	0	0	0	0
0	0	0	0	0
0.000892281	0	0.007485795	0.015363365	0.044562614
0	0	0	0	0
0	0	0	0	0
0	0	0	0	0
0	0	0	0	0
0.009314063	0.020138579	0.001173279	0.036500038	0
0	0	0.002419185	0	0
0	0	0	0	0
0.001222147	0.003584593	0	0.001357614	0.01611126
0	0	0.008523833	0.062913702	0
0	0	0.021929408	0	0
0	0	0	0	0
0	0	0.003020375	0	0
0	0	0	0	0
0	0	0	0.002605032	0
0	0.002970963	0	0	0
0	0	0	0	0
0	0	0	0	0
0	0	0	0	0
0	0	0	0	0
0	0	0	0	0
0	0	0.023796795	1.873872872	0
0	0	0	0	0
0	0	0	0	0
0.094944815	0.574735497	0.090661742	0.205763735	0.828367241
0.0044773	0.001309464	0	0	0.001345257
0	0.041758996	0	0	0
0	0	0	0	0
0	0	0.001065087	0	0
0	0.001743114	0.007407657	0	0
0	0	0	0	0
0	0	0	0	0
0	0.05508588	0	0.031273215	0
0.001304186	0	0	0.005215487	0
0	0.005872834	0	0	0
0.02150139	0.057351705	0.022781759	0.05067124	0.030565867
0.74573295	1.769067294	0	0.067384896	0.003855564
0	0.012615557	0	0.030676485	0
0	0	0	0	0
0	0	0	0	0
0	0	0	0	0
0	0.019747678	0.002104176	0.004920023	0.009624321
0	0	0	0	0

0.002342036	0	0	0	0.012138682
0.000831384	0	0	0	0
0	0	0	0.247357161	0.058249156
0	0	0	0	0
0	0	0	0	0.078342495
0	0.003723054	0.045099111	0	0
0	0	0	0	0
0	0	0	0	0
0.000653358	0	0	0.0573365	0
0.02195857	0	0	0.072043073	0
0	0	0	0	0
0	0	0	0	0
0	0	0	0	0.003244967
0.00094168	0.011685282	0	0	0
0	0	0	0	0
0.041026808	0.013697769	0	0	0.039364796
0.027529066	0.002300386	0	0	0.016749638
0	0	0.001572941	0	0
0	0	0	0	0
0	0.019327519	3.594725675	0.003323059	0
0.028783549	0.008987338	0.029369147	0	0
0.000709606	0.001334164	0	0.001051016	0.000959442
0	0	0	0	0
0	0.525642831	0	0.566617747	0
0	0	0	0	0.042903982
0	0	0	0.004774447	0.024788732
0.001294384	0.006132753	0.002347944	0.003738432	0
0.040137951	0	0	0	0.491749032
0	0	0	0	0
0	0	0	0	0
0.001771876	0.00266511	0	0.062110071	0.064284953
0.006750882	0	0	0.002282357	0.325025922
0	0.017970046	0.002027756	0.037563805	0.043926407
0	0.038855145	0	0.004004077	0
0	0	0	0.012701744	0.002608889
0	0	0	0	0
0	0	0	0.018523783	0
0.003161443	0.031428778	0	0.001975426	0.039672833
0.197864549	0.035269617	0	0.230558118	0
0	0	0	0	0
0	0	0	0	0
0	0	0	0	0
0	0	0	0	0.005693346
0.006323983	0.006438009	0.223271958	0	0.009384708
0	0	0.344937639	0.947546722	0
0	0	0	0	0
0	0	0.001393998	0	0
0	0	0	0	0

0	0	0.005314149	0	0
0.507520551	0.591662817	0.005128095	0.080154883	0.067437571
0	0	0	0	0
0.000978378	0	0	0	0
0	0	0	0	0
0	0	0	0	0
0	0	0.16807866	0.527930128	0
0	0	0	0	0
0	0	0	0	0
0	0	0	0	0
0.003201216	0	0.001088781	0	0
0.006258396	0.004706682	0	0.051644168	0.052886462
0	0.030742155	0	0	0
0	0	0.029727219	0	0
0	0	0	0.001275038	0.026072404
0	0	0	0	0
0.002304593	0.005435912	0.000570056	0.017687565	0
0.004351535	0.001840843	0.298224443	0.14380776	0
0	0	0	0	0
0.007770563	0	0.060065561	0.041197608	0.003581727
0.020525326	0.03265986	0	0.018240342	0.00197184
0	0	0	0	0
0	0	0	0	0
0	0	0	0	0
0	0	0	0	0
0	0	0	0	0.006429272
0	0	0	0	0.033952202
0.037889891	0.105174025	0.067688868	0.04719153	0.060311742
0	0	0	0	0
0	0	0	0	0.011797311
0	0	0	0	0
0	0	0	0	0
0	0	0	0	0.00169617
0	0	0	0	0
0	0	0	0	0
0.070344696	0.429657014	3.295785003	0.246945847	0.212030213
0	0	0	0	0
0	0	0	0	0
0.046458138	0.041224055	0.001284643	0.008811075	0.010628754
0	0	0	0	0
0	0	0.188632103	0.908334923	0
0	0	0	0	0
0.004990013	0	0	0.017967366	0
0.012127473	0.002998544	0	0.011995372	0.00488549
0	0	0.172812408	0	0
0	0.078421026	0	0	0.363261682
0	0	0	0	0
0	0	0	0	0

0	0	0	0	0
0	0	0	0	0
0	0	0	0	0.001918891
0.4847831	0.289829558	0	0	1.74691068
0	0	0	0	0
0.012859933	0	0	0	0
0	0.000876359	0	0	0
0	0	0	0	0
0	0.258542356	0	0	0
0.023105711	0	0	0	0.071199737
0	0	0	0	0
0.009408533	0.000649816	0	0	0.00077884
0	0	0	0	0
0.06807658	0	0	0	0
0.004278092	0.088120381	0.00143708	0.005632346	1.192049966
0	0	0	0	0
0.014467695	0.009093037	0	0	0.069582829
0	0	0	0	0
0	0	0	0	0
0	0	0	0	0
0	0	0	0	0
0	0	0	0	0
0	0	0	0	0
0	0	0.440615099	0.693602502	0.330901434
0.261567175	0	0.000489143	0	0
0	0	0	0	0
0	0.167702182	0	0	0.008025559
0	2.025397542	0	0	0.819739312
0	0	0	0	0
0	0	0	0	0
0.159227221	0	0	0	0.027068676
0	0	0	0	0
0.006393039	0	0	0	0.014838662
0	0	0	0	0
0.009996162	0	0	0	0
0	0	0	0	0
0.390832781	8.116182223	0	0	2.109529289
0	0	0	0	0.022487555
0	0	0	0	0.006080798
0.016629788	0.060310649	0	0	0.002369261
0.185305318	0.225456461	0.153198219	0.060157551	0.03791828
0	0.014618262	0	0	0
0.017235711	4.781581129	0.241299022	13.13550603	1.046215524
0	0	0	0	0.110485602
0	0	0	0	0
0	0	0	0	0
0	0	0	0	0
0.063771745	0.001998338	0	0.014020503	1.104748979

0	0	0	0	0
0	0	0	0	0
0.004882103	0	0.592188395	0.578165885	0.463862268
0	0	0	0	0.001628719
0	0	0	0	0
0.071734999	0.006602143	0	0	0.025773897
0	0	0	0	0
0.246558454	0	0	0	0
0.185827397	0	0	0	0
0.017000729	0	0	0	0
1.049537252	0.060891258	0.006993722	0.01389045	0.068895749
1.251882491	2.24906625	2.213847648	3.001326203	1.199871168
0	0	0	0	0
0.053306335	0	0	0.001249262	0.001140416
0.218013276	0.10557347	0.162847747	0.094705635	0.630521796
0	0.051045846	0	0	0
0.006479158	0.007633909	0	0	0
0	0	0	0	0
0	0	0	0	0
0.003605121	0.007165481	0.038614176	0.042717078	0.019149436
2.21576112	0.182073811	0.040557101	0.032598278	0.018421649
0	0.006228874	16.21811555	1.089337252	0.291533821
2.160649084	7.000646326	8.599659321	0.86065	0.93590275
0	0	0	0	0
0.003595184	0	0	0	0
0	0.006139237	0	0.010230659	0
0	0	0.098342761	0.025760628	0
0	0	0	0	0.000738979
0	0	0	0	0
0	0	0	0	0
0	0	0	0	0
0	0.293927254	0	0	0.299500809
0.004066747	0	0.029313321	0.069427154	0.147158712
0.157182368	0	0	0	0.068854695
0	0	0.001793776	0.002040055	0.0047274
0.030861017	0.022333476	0.002112462	0.008193126	0.296612228
0	0.003172622	0	0	1.199329371
0	0	0	0	0
0	0	0	0.005298417	0
0	0	0	0	0
0	0.005591243	0	0	0
0	0	0	0	0
0	0	0	0	0
0	0	0	0	0
0.195666933	0.069664644	0.004617294	0.008113944	0.089557252
0	0	0	0	0
0	0	0	0	0
0.286223746	0	0.008717819	0.023431003	0.031678122

0	0	0	0	0
0	0	0	0	0
0	0	0	0	0
0.003207041	0.002766572	0	0	0.031182137
0.064716588	0	0	0	0
0	0	0	0	0.173349099
0	0.051024803	0	0	0.057176757
0	0.188385287	0	0.003278164	0.048408792
0	0	0	0	0
0	0	0	0	0
0	0.000711053	0	0	0.047725254
0	0	0	0	0
0	0	0	0	0.004290649
0	0	0	0	0
0	0	0	0	0
0	0	0	0	0
0.170326906	0.058348245	0	0	0
0.008395588	0.00118387	0	0	0.000709468
0.005337441	0	0	0	0
0.002050596	0	0	0	0.029111914
0	0	0	0	0
0	0	0	0	0
0	0.000909945	0	0	0.003271861
0	0	0	0	0
0	0	0	0	0
0	0	0	0	0
0	0	0	0	0
0	0	0	0	0
0	0	0	0	0
0.139449685	0	0	0.002816487	0.054421438
0	0	0	0	0
0	0	0	0	0
0.057931484	0	0	0	0
0	0	0	0	0
0	0	0	0	0
0	0	0	0	0
0	0	0	0.316725488	0.068201056
0	0	0.001129187	0.001975725	0.007935766
0	0	0	0	0
0	0.009697088	0	0.002728244	0
0.314725971	0.247359905	0.004843233	0	0
0	0	0	0	0
0	0	0	0	0
0	0.00818348	0	0.002110529	0
0	0	0	0	0
0	0	0	0	0
0.00067598	0	0	0	0
0	0	0	0	0

0	0	0	0	0
0.183323967	0	0	0	0
0	0	0	0	0
0	0.137908078	0	0	0
0	0	0	0	0
0	0.429492138	0	0	0
0	0	0	0	0
0.001642811	0	0	0	0
0.003434143	0.002213724	1.283246456	0.174935635	0.049002615
0	0	0	0	0
0	0	0	0	0
0	0	0	0	0
0	0	0	0	0
0.013289224	0	0	0	0
0.027499076	0.675311637	2.507202627	2.741217105	0.505159301
0.001512451	0	0.141633471	0	0.321696266
0.223875311	0	0	0	0
0	0	0	0	0
0	0	0	0	0
0	0	0	0	0
0	0.005712893	0	0	0.00874923
0	0	0	0	0
0	0	0	0	0
0	0	0	0	0
0.01673074	0.002277866	0.001831385	0.007690442	0.242495883
0.142740435	3.458791147	0	0	0.958087582
0	1.134079686	0	0	0.274172672
0	0	0	0	0
0	0.016785555	0	0	0
0.368296667	0	0	0	0
0	0	0	0	0
0	0.006286965	0	0	0.077236625
0	0	0	0	0
0.006470382	0.001390316	0	0.001369063	0.021558691
0.008630451	0	0	0.004793541	0
0.006638978	0	0	0	0.018559334
0	0.012115424	0	0	0
0.64754016	1.130007812	0	1.735270681	1.177478636
0	0	0	0	0
0	0	0	0	0
0	0	0	0	0
0.120254995	0	0	0.007233001	0
0.329054115	0.175919901	0.145218675	0.074630832	0.655424854
0	0	0	0	0
0	0	0	0	0
0	0	0	0	0
0	0	0	0	0
2.148631019	0.025967588	0	5.319594217	0.091723116

0	0	0	0.016151759	0.003686121
0	0	0	0	0
0	0	0	0	0
0	0	0	0	0
0.02312193	0.025856762	0	0	0
0	0	0	0	0
0	0	0	0	0
0.004507894	0	0	0	0
0	0	0	0	0
0	0	0	0	0
0	0	0	0	0
4.605182956	0.809379417	0	0	1.218125892
0.181856191	0.207896134	0	0	0.057767828
0	0	0	0	0
0.502489068	2.441319449	0.010755646	0.486895902	0.005659079
0	0	0	0	0
0.095921037	0	0	0	0
0	0.018563815	0	0	0
0.033220763	0	0	0	0
0	0	0	0	0.045604405
0	0.006471817	0	0	0
0	0	0	0	0
0.53012064	0.028168552	0.001617662	0	0.170616609
0	0	0	0	0
0	0	0	0	0
0	0	0	0	0
0	0	0	0	0
0	0	0	0	0
0	0	0	0	0
0	0	0	0	0
0	0	0	0	0
0.042544668	0.01463651	0	0.013742407	0.012545055
0	0	0	0	0
0.225660356	0.117164417	0.003691405	0.001004703	0.265519228
0.006639071	0	0	0	0.019801182
0	0.002271722	0	0	0.104912372
0.003466646	0	0	0	0
0	0	0	0	0
0	0	0	0	0
0	0	0	0	0
0.116623991	0	0	0	0
0	0	0	0	0
0	0	0	0	0
0.025752355	0.003227881	0	0	0.032723569
0.065593865	0.041229255	0	0.001495753	0.319315655
0	0	0	0	0
0.053247055	0.338860319	0	0.231922199	0
0.000909678	0.001847155	0.000990065	0	0.00055348

0	0	0	0	0
0	0	0	0	0
0	0.002244283	0.001263069	0.001988978	0.006455757
0	0	0	0	0
0	0	0.006587662	0	0.030484797
0.00171151	0	0	0	0
0	0	0	0	0
0.455058879	0.002119515	0.003748958	0	0.022291639
0	0	0	0	0
0	0.004588504	0	0	0
0.040153202	0	0	0	0
0	0	0	0	0
0	0	0	0	0
0	0	0	0	0
0	0	0	0	0
0	0	0	0	0
0.275962343	0	0	0	0.926748663
0	0	0	0	0
7.527456528	0	0	0.341462909	2.219276273
0	0	0	0	0
0.007230398	0	0	0	0
0	0	0	0	0
0	0	0	0	0
0	0	0	0	0
0.004203343	0	0	0	0
0.001969852	0	0.003573206	0.004774249	0
0	0	0	0	0
1.42363391	0.580345325	0	0.305889407	0.10238127
0	0	0	0	0
0.186625067	0.536997527	0	0.831600848	0
0	0	0	0	0
0	0	0	0	0
0	0	0	0	0
0	0	0	0	0
0.001501297	0	0	0	0.150146852
0	0	0	0	0
0	0	0	0	0
0.006814128	0	0	0	0
0	0	0	0	0
0	0	0	0	0
1.873039493	11.42927763	2.475409544	9.31804723	1.674646053
1.352208198	0.127835519	0.15565242	1.01466951	0.150952442
0	0	0	0	0
0	0	0	0	0
0	0	0	0	0
0	0	0	0	0
0	0	0	0	0
0.033124423	0.001715844	0.001686085	0	0.003941693

0	0	0	0	0
0.005910311	0.003529774	0.001261293	0	0.004759462
0.021926889	0	0	0	0
0	0	0	0	0
3.138341005	1.895525786	0.45725335	2.529383991	1.134931076
0	0	0	0	0
0	0	0	0	0
0	0	0	0	0
0.001735764	0.001370666	1.171745061	1.332360027	0.014433351
0	0	0	0	0
0	0	0	0	0
0	0	0	0	0
0.021451324	0.097177028	0	0	0
0.00353014	0.086673786	0.004331769	0.030448776	0.022531467
0	0	0	0	0
0.512734888	0.135712055	0	0.010032276	0.209428601
1.204844542	0.095476433	0	0.021558022	0.00400883
0	0	0	0	0
0	0	0	0	0
0	0	0	0	0
0	0	0	0	0
0	0	0	0	0
0	0	0	0	0
0	0	0	0	0
0	0	0	0	0
0	0	0	0	0
1.377351114	0.002115177	0	0	0.001742921
0	0	0	0	0.009833457
0	0	0	0	0
0	0	0	0	0
0	0	0	0	0
0	0	0	0	0
0.183017556	0	0	0	2.0727149
0	0.012926694	0	0	0
0	0	0	0	0
0	0	0	0.009742214	0.019234546
0.003543948	0	0	0	0.001902583
0	0	0	0	0
0	0	0	0	0
0	0	0	0	0
0.928232717	3.159701339	0	4.985788119	0
0	0	0	0	0
0	0	0	0	0
0	0	0	0	0
0	0	0	0	0
0	0	0	0	0
0	0	0	0	0
0	0	0	0	0

0	0.339941187	0	0	0
0.000736938	0	0	0	0
0	0	0	0	0
0	0	0	0.008874594	0
0.01337694	0	0	0	0.012402276
0.270168264	0	0	0	0.820958013
0.879432415	1.357281466	0	0.019514342	0.052896947
0	0	0	0	0
0	0	0	0	0
0.005265939	0	0.107461478	0	0
0	0.019862933	0	0	0
0.124156455	0	0	0	0.017486363
0	0	0	0	0
0	0	0	0	0
0	0	0	0	0
0	0	0	0	0
0	0	0	0	0.011841373
0	0	0	0	0
0	0	0	0	0.228539737
0	0	0	0	0
0.008703771	0.030418475	0	0.346739977	0.098471886
0.004139383	0	0	0	0
0.002823597	0.006582884	0	0	0
0	0.008497115	0.001669949	0	0.00152764
0	0	0	0	0
0.006714197	0	0	0	0
0	0	0	0	0
0	0	0	0	0
0	0	0	0	0
0	0	0	0.464301273	8.408548575
0	0	0	0	0
0	0	0	0	0
0	0	0	0	0
0	0	0	0	0
0	0	0	0	0
0	0	0	0	0
0.006911576	0	0.024314626	0.078870712	0.143785306
0	0	0	0	0
0	0	0	0	0
0	0	0	0	0
0	0	0	0	0
0	0	0	0	0
0	0	0	0	0
0	0	0	0	0
0.530835196	0	0	0	0
0	0	0	0	0
0	0	0	0	0
0	0	0	0	0

0.001621008	0	0.002450351	0	0.005296674
0	0	0	0	0
0	0.013521861	0	0	0.00998449
0	0	0	0	0
0.000458552	0	0	0	0
0	0	0	0	0
0	0	0	0	0
1.377945236	0.609802577	0	0.080118888	1.384563736
0.472130312	0.012438072	0.005641084	0.054330591	11.17506191
0	0	0.022491879	0	0.001197498
0	0	0	0	0.00398152
0	0	0	0	0
0.022158278	0	0	0	0
0	0	0	0	0
0	0	0	0	0
0	0	0	0	0
0.001840405	0.035294363	0.030760935	0.002920574	0
0	0	0	0	0
0	0	0	0	0
0.00522255	0.042161023	0	0	0
0	0	0	0	0
0	0	0	0	0
0	0	0	0	0
0	0	0	0	0
0.055326621	0	0	0	0
0	0	0	0	0
0	0	0	0	0
0	0	0	0	0
0	0	0.004786157	0	0
0	0	0	0	0.116339481
0	0.003131021	0	0	0
0	0	0	0	0
0	0	0	0	0
0	0	0	0	0
0	0	0	0	0
0	0	0	0	0
0.004553834	0	0	0	0
0	0	0	0.023725354	0
0	0	0	0.002616969	0
0	0	0	0.69429177	0
0	0	0	0	0
0.37453258	0.164691453	0	0	0
0	0	0	0	0
0	0	0	0	0
0	0	0	0	0
0.016656237	0.003794075	0	0	0
0	0	0	0	0
0	0	0	0	0

0	0	0	0	0
0	0.004951281	0	0.022030459	0
0	0	0	0	0
0	0	0	0	0
0	0.179218096	0	0	1.084616607
0.39901085	0	0	0	0
0	0	0	0	0
0.004353194	0.003618474	0.169773893	0.058283117	0.012081487
0	0.000774195	0	0	0
0	0	0	0	0
0	0	0	0	0
0	0	0	0	0
0	0	0	0	0
0	0	0	0	0
0.019354703	0	0.000438855	0.001791671	0
0.175063612	0.019950465	0	0.002207359	0
0	0	0	0	0
0	0	0	0	0
0	0	0	0	0
0	0	0	0	0
0	0	0	0	0
0	0	0	0	0
0	0	0	0	0
0	0	0	0	0.053886205
0	0.437027439	0	0	0.418433296
0	0	0	0	0
0.316455869	0	0.003454073	0	0.006068682
0.000572867	0	0	0	0
0	0	0	0	0
0	0	0	0	0
0	0	0	0	0
0	0	0	0	0
0	0	0	0	0
0	0	0	0	0
0	0	0	0	0
0	0	0	0	0
0	0	0	0	0
0	0.002307884	0	0	0
0	0	0	0	0
0	0	0	0	0
0	0	0	0	0
0	0	0	0	0
0	0	0	0	0
0	0	0	0	0
0	0	0	0	0
0	0	0	0	0
0	0	0	0	0
0	0	0	0	0

0.222389519	0.108892057	0	0	1.147121478
0	0	0	0	0
0	0	0	0	0
0.009332509	0	0	0	0
0	0	0	0	0
0	0	0	0.003731267	0.637955232
0	0	0	0	0
0	0	0	0	0
0	0	0	0	0
0	0	0	0	0
0	0	0	0	0
0.075854961	0	0	0	0
0	0	0	0	0
0	0	0	0	0
0	0	0	0	0
0	0	0	0	0
0	0	0	0	0
0	0	0	0	0
0.00144591	0.001631113	0	0	0
0	0	0	0	0
0	0	0.001969917	0.011844248	0
0	0	0	0	0
0.328915196	0.001687848	0.024652452	0	0
0	0	0	0	0.005091729
0	0	0	0	0
0	0	0	0	0
0	0	0	0	0
0	0	0	0	0
0	0	0	0	0.001532352
0	0	0	0	0
0	0	0	0	0
0	0	0	0	0.00076279
0	0.002897882	0.001694453	0.046176172	0.354510825
0	0	0	0	0
0.201790249	0.179564743	0	0	0.333630924
0	0	0	0	0
0	0	0	0	0
0	0	0	0	0
0.004551889	0	0	0.001926262	0.00527529
0	0	0	0	0
0	0	0	0	0
0	0	0	0	0
0	0.022873428	0	0	0
0	0	0	0	0
0	0	0	0	0
0	0.002043615	0	0	0.037965508
0	0	0	0	0
0	0	0	0	0

0	0	0	0	0
0	0	0	0	0
0	0	0.087426221	0	0
0	0	0	0	0
0	0	0	0	0
0	0	0	0	0
0	0	0	0	0
0	0	0.392692623	1.944592145	0
0.478820675	0	0	0	0
0	0	0	0	0.0039945
0	0	0	0	0
0	0	0	0	0
0	0	0	0	0
0	0.087905436	0	0	0.0790197
0	0	0	0	0
0	0	0.001110039	0.014275322	0
0	0	0	0	0
0	0	0	0	0
0	0	0	0	0
0	2.035211359	0	1.887617841	1.01778597
0.00127827	0.008411675	0	0.00757311	0
0	0	0	0	0
0	0	0	0	0
0	0	0	0	1.109276593
0.039974689	0	0	0	0
0	0	0	0	0
0	0	0	0	0
0	0	0	0	0
0	0	0	0	0
0	0.022179696	0	0	0
1.61696035	0.415012803	0	0	0.240918431
0	0	0	0	0
0	0	0	0	0
0	0	0	0	0
0	0	0	0	0
0	0	0	0	0
0	0	0	0	0
0	0	0	0	0
0	0	0	0	0
0	0	0	0	0
0	0	0	0	0
0	0	0	0	0
0.00376567	0	0	0	0.026002896
0	0	0	0	0
0	0	0	0	0
0	0	0	0	0
0	0	0.265145695	0.941297752	0
0	0	0	0	0

0	0	0	0	0
0	0	0	0	0
0	0	0	0	0
0	0	0	0	0
0.002652009	0.013198677	0.003395719	0	0.060746324
0	0	0	0	0
0	0	0	0	0
0	0	0	0	0
0	0	0	0	0
0	0	0	0	0
0	0	0	0	0
0.035736367	0.00537517	0	0	0.059519385
0.205356346	0	0	0	0
0	0	0	0	0
0	0.00055858	0	0	0.076321827
0	0	0	0	0.09683933
0	0.001315409	0.001510825	0.004996162	0
0	0	0	0	0
0.010823913	0	0.050423678	0.034977961	0
2.116055414	1.451534748	0.003690033	0.006898157	0.041908507
0	0	0	0	0
0	0	0	0	0
0	0	0	0	0
0	0	0	0	0
0	0	0	0	0
0	0	0	0	0
0.002230722	0.001742159	0.004824567	0.001143685	0
0	0	0	0	0
0	0	0	0	0
0	0	0	0	0
0.000583533	0	0.001234913	0	0.00453664
0	0	0	0	0
0	0	0	0	0
0	0	0	0	0
0	0	0	0	0
0	0	0	0	0
0	0	0	0	0
0.170685432	0.005241505	0.000495779	0.002277079	0.006513202
0.020875139	0.113109357	0	0	0
0	0	0	0	0
0.021964973	0	0	0	0.039290344
0	0	0	0	0
0	0	0	0	0
0	0	0.364648286	0	0
0	0	0	0	0
0	0	0	0	0
0	0	0	0	0
0	0	0	0	0

0	0	0	0	0
0.509216449	0.002540045	0	0	0.001405102
0	0	0	0	0.001657601
0	0	0	0	0.043287244
0	0.001777627	0	0.00196926	0
0	0	0	0	0.002320081
0.007625432	0	0	0	0
0.004650654	0	0	0	0
0	0.014597057	0	0	0
0	0	0	0	0
0.018777203	0.0075227	0	0	0
0	0	0	0	0
0.321409764	0.005094781	0	0.009532108	0.024883501
0	0	0	0	0
0	0	0	0	0
0	0	0	0	0
0	0	0	0	0
0	0	0	0	0
0	0	0	0	0
0	0	0	0	0
0	0	0	0	0
0	0	0	0	0
0	0	0.005933069	0	0.004422383
0.064561664	0.007124796	0	0.04677255	0.010437128
0	0	0	0	0
0	0	0	0	0
0	0	0	0	0
0	0	0	0	0
0	0	0	0	0
0	0	0	0	0
0.006409765	0.014710897	0	0.018659956	0
0	0.001349698	0	0	0
0	0	0	0	0
0	0	0	0	0
0	0	0	0	0
0	0	0	0	0
0	0	0	0.004434503	0
0	0	0	0	0
0.004301084	0	0	0.005608759	0
0	0	0	0	0
0	0	0	0	0
0	0	0	0	0
0	0	0	0	0
0	0	0	0	0
0.119954623	0	0	0	0
0.184276191	0.184864529	0	0	0.013347621
0	0	0	0	0
0	0	0	0	0

0	0	0	0	0
0	0	0	0	0
0	0	0	0	0
0	0.001120909	0	0	0.088417286
0	0	0	0	0
0	0	0	0	0.005376224
0.301856603	0	0	0	0
0	0	0	0	0
0	0	0	0	0.019125194
0	0	0	0	0
0.002427361	0	0	0	0
0	0	0	0	0.004364067
0	0	0	0	0
0	0	0	0	0
0	0	0	0	0
0	0	0	0	0
0	0	0	0	0
0	0	0	0	0
0	0	0	0	0
0.026758748	0.001134821	0	0	0
0	0	0	0	0
0	0	0	0	0
0	0	0	0	0
0	0	0	0	0
0	0	0	0	0
0	0	0	0	0
0	0	0	0	0
0	0	0	0	0
0	0	0	0	0
0	0	0	0	0
0.065681524	0.002534814	0.000627067	0.002880075	0
0	0	0	0	0
0.012242208	0.041883668	1.483117076	0.034332353	0.016077544
0.002255939	0.001850835	0	0.002278178	0
0	0	0	0	0
0	0	0	0	0
0	0	0	0	0
0	0	0	0	0
0	0	0	0	0
0	0	0	0	0
0	0	0	0	0
0.052504166	0.017694296	0.011530598	0.004218396	0.070319962
0.20557266	0.112321451	0	0	0.077918487
0	0	0	0	0
0	0	0	0	0
0	0.005246458	0	0	0
0.180091312	0	0	0	0
0	0	0	0	0

0	0	0	0	0
0	0	0	0	0
0.009353824	0.002221461	0	0	0.051170791
0	0	0	0	0
0.891905432	0	0	0.015282882	0.05859551
0	0	0.013187154	0	0
0	0	0	0	0
0	0	0	0	0
0	0	0	0	0
0	0	0	0	0
0	0	0	0	0
0	0	0	0	0
0	0	0	0	0
0	0	0	0	0
0	0	0	0	0
0	0	0	0	0
0	0	0	0	0.30818292
0	0	0	0	0
0	0	0	0	0
0	0	0	0	0
0	0	0	0	0
0	0	0	0	0
0	0	0	0	0
0	0	0	0	0
0	0	0	0	0
0	0	0	0.002493677	0.006001438
0	0	0	0	0
2.064744489	0.139127538	0	0	0.029710257
0	0	0	0	0
0	0	0	0	0
0	0	0	0	0
0	0	0	0	0
0	0	0	0	0
0	0	0	0	0
0	0	0	0	0
0	0	0	0	0
0	0	0	0	0
0	0	0.00442186	0	0
1.627368104	1.225896992	0	0.123288524	3.82282702
0	0	0	0	0
0	0	0	0	0
0	0.023976012	0	0	0
0.000614441	0.001617336	0.002414889	0	0.003115396
0.02823106	0	0	0	0
0	0	0	0	0
0.021442424	0	0	0	0
0.002819926	0.001767292	0	0	0.018428323
0	0	0	0	0

0	0	0	0	0
0.004744303	0.010480985	0.005378695	0.005270179	0.005412372
0	0	0	0	0
0	0	0	0	0
0	0	0	0	0
0	0	0	0	0
0	0	0	0	0
0	0	0	0	0
0	0	0	0	0
0	0	0	0	0
0	0	0	0	0
0	0	0	0	0
0.023195064	0.263075152	0	0	0.479958277
0	0	0	0	0
0.002218287	0.001112188	0.002682569	0	0.000999765
0	0	0	0	0
0	0	0	0	0
0	0	0	0	0
0.001908666	0	0	0	0.145162333
0	0	0	0	0
0	0	0	0	0
0.57779525	0.179130044	0.013392013	0	0.027982666
0	0.005875167	0.003205289	0.005578736	0
0.156572836	0	0	0	0.06649289
0	0	0	0	0
0	0	0	0	0
0	0	0	0	0
0	0	0	0	0
0	0	0	0	0
0	0	0	0	0
0	0	0	0	0
0	0	0	0	0
0	0	0	0	0
0	0	0	0	0
0.042197042	0	4.324888803	0	0
0	0	0	0	0
0	0	0	0	0
0	0	0	0	0
0.139233816	0.139616019	0.001753908	0.00590742	0.000980494
0	0	0	0	0
0.021023661	0.267972484	0.019854203	0	0.538255006
0	0	0	0	0
0	0	0	0	0
0	0	0	0	0
0	0	0	0	0
0.039952334	0.001399682	0.055366346	3.040222192	0.113237814
0	0	0	0	0
0	0	0	0	0

0	0	0	0	0
0	0	0	0	0
0	0	0	0	0
0	0	0	0	0
0.058343197	0	0	0	0
0	0	0	0	0
0	0	0	0	0
0.004559677	0	0	0	0
0	0	0	0	0
0.006309997	0.031758272	0	0.069824608	0
0	0	0	0	0
0	0	0	0	0
0	0.000802785	0	0	0.002646004
0	0	0	0	0
0	0	0	0	0
0	0	0	0	0
0	0	0	0	0
0	0	0	0	0
0	0	0	0	0
0.071363531	0.056210141	0.017617405	0.024860989	0.044961585
0	0	0	0	0
0	0	0	0	0
0	0	0	0	0
0	0	0	0	0
0	0	0	0	0
0	0	0	0	0
0	0	0	0	0
0	0	0	0	0
0	0	0	0	0
0	0.001018467	0	0.025072452	0
0	0	0	0	0
0	0	0	0	0
0	0	0	0	0
0	0	0	0	0
0	0	0	0	0
0.000819927	0.0018499	0	0	0
0	0	0	0	0
0	0	0	0	0
0	0	0	0	0
0	0	0	0	0
0	0	0	0	0
0	0	0	0	0
0	0	0	0	0.09966358
0	0	0	0	0
0.091220519	0.004474121	0	0	0.017294016
0	0	0	0	0
0	0	0	0	0
0	0	0	0	0

0	0	0	0	0
0	0	0	0	0
0	0	0	0	0
0	0	0	0	0
0	0	0	0	0
0.011103206	0.001503047	0.000537083	0.001480071	0.196962551
0	0	0	0	0
0.004055892	0	0	0	0
0	0	0	0	0
0	0	0	0	0
0	0	0	0	0
0	0	0	0	0
0	0	0	0	0
0	0	0	0	0
0	0	0	0	0
0	0	0	0	0
0	0	0	0	0
0	0	0	0	0
0	0	0	0	0
0	0	0	0	0
0	0	0	0	0
0	0	0	0	0
0	0	0	0	0
0	0	0	0	0
0	0	0	0	0
0	0.001044897	0	0.000771693	0
0	0	0	0	0
0	0	0	0	0
0	0	0	0	0
0	0	0.002184631	0	0
0	0	0	0	0
0	0	0	0	0
0	0	0	0	0
0	0	0	0	0
0	0	0	0	0
0	0	0	0	0
0	0	0	0	0
0	0	0	0	0
0.796823998	1.242365153	0	0	0.441203567
0	0	0	0	0
0	0	0	0	0
0	0	0	0	0
0	0	0	0	0
0	0	0	0	0
0	0	0	0	0
0	0	0	0	0
0	0	0	0	0
0	0	0	0	0

0	0	0	0	0
0.14320436	0.045809086	0	0.097214117	0.086222886
0	0	0	0	0
0	0	0	0	0
0	0	0	0	0
0	0	0	0	0
0	0	0	0	0
0	0	0	0	0
0	0	0	0	0
0	0	0	0	0
0.001953279	0	0	0	0
0	0	0	0	0
0.056588417	0.128935756	0	0	0
0	0	0	0	0
0	0	0	0	0
0	0	0	0	0
0	0	0	0	0
0	0	0	0	0
0	0	0	0	0
0	0	0	0	0
0	0	0	0.006117232	0.038196257
0	0	0	0	0
0	0	0	0	0
0.794675893	1.176004949	0	4.096171445	2.50506546
0	0	0	0	0
0	0	0	0	0
0	0	0	0	0
0	0	0	0	0
0	0	0	0	0
0	0	0	0	0
0	0	0.002549118	0	0
0	0	0	0	0
0	0	0	0	0
0	0	0	0	0

PHL018-0022_V3	PHL018-0001_V3	PHL018-0235_V1	PHL018-0240_V1	PHL018-0067_V2
CTRL	EYCF	EYCF	REF	EYCF
0.082035874	0.213691486	0	0.547707929	0.023498432
0.966535588	1.148171572	0	5.326191263	2.164278086
0.006841335	4.057316733	0	0	6.833401224
0	0	0	0	0
0	0	0	0	0
0	0	0	0	0
0	0	0	0	0
0	0	0	0	0
0	0	0	0	0
0	0	0	0	0
0	0	0	0	0
0	0	0	0	0
0	0	0	0	0
1.420409414	0.652376645	0	0.179728143	5.340543495
0	0	0	0	0
0	0	0	0	0
0.001548339	0	0	0	0
0	0	0	0	0
0	0	0	0	0
0	0	0	0	0
0	0	0	0	0
0.001973144	0	0	0	0.002195935
0	0	0	0	0
0	0	0	0	0
0	0.061677738	0	0	0
0	0	0	0	0
0	0	0	0	0
0	0	0	0	0
0	0	0	0	0
0	0	0	0	0
0	0	0	0	0
0	0	0	0	0
0	0	0	0	0
0	0	0	0	0
0	0	0	0	0
0	0	0.001440632	6.988329182	0.238374238
0	0	0	0	0
0	0	0	0	0
0	0	0	0	0
0	0	0	0	0
0	0	0	0	0
0	0.819396169	0.240890946	0.00543003	0.000789831
0.177708901	0.142171171	0.598920626	0.398528028	0.001748005
0.016146727	0.000782759	0.118967841	0.208320554	0.007123196
0	0	0	0.000885434	0.003734962
0	0	0	0	0

0	0.002194197	0	0.00731558	0
0.002244212	0	0.038462979	0.004736796	0
0.027138076	0.103802811	0	0	0
0.236936786	1.219282698	0	0.967526017	0.16677109
0	0.011614732	0	0.015478694	0.001080493
0	0	0	0	0
0.001503396	0.002157291	0	0.017135148	0
0	0	0	0	0
0	0	0	0	0
0	0	0	0	0
0	0	6.139919013	0.001166214	0
0	0.00913473	0	0.070018284	1.503844217
0	0	0	0.00054979	0
0.134585605	0.072227997	0	0.271339909	0.025136728
0	0	0	0	0
0	0	0	0	0
0	0	0	0.004361554	0.420818312
0	0	0	0	0
0	0	0	0	0
0	0	0	0	0
0.112179616	0.016937936	0	0.481713386	0.015531889
0.024996167	0.08602987	0	0.149858784	0.003804243
0	0	0	0	0.00071115
0	0	0	0	0
0	0	0	0	0.000700247
0	0	0	0	0
0	0	0	0	0
0	0	0	0	0
0	0.007108364	0.070262977	0.001538317	0
0	0	0	0	0
0.056404749	0	0	0.010219049	0
0	0	0	0	0
0	0	0	0	0
0	0	0	0	0
0	0	0	0	0
0	0	0	0	0
0	0	0	0	0
0	0	0	0	0
0	0	0	0	0
0	0	0	0	0
0	0	0	0	0
0	0	0	0	0
0	0	0	0	0
0	0	0	0	0
0	0.017895301	0	0	0
0	0	0	0	0
0	0	0	0	0
0	0	0.001156051	0.00106303	0

0	0	0	0	0
0.01346173	0	0	0	0
0	0	0	0	0
0	0	0	0	0
0.001459085	0	0.003646837	0	0
0	0	0	0	0
0	0	0	0	0
0	0	0	0	0
0.001652017	0.001666796	0	0	0
0	0	0	0	0
0	0	0	0	0
0	0	0	0.00118072	0
0	0	0	0	0
0	0	0	0	0
0	0	0	0	0
0	0.01116788	0	0	0
0	0	0	0	0
0	0	0	0	0
0.034777971	0.013394609	0	0	0
0	0	0	0	0
0	0	0	0	0
0	0	0	0	0
0	0.003443628	0	0	0
0	0	0	0	0
0	0	0	0	0
0	0	0	0	0
0	0	0.008751974	0	0
0	0.003988984	0	0	0
0	0	0	0	0
0	0.001715672	0	0	0
0	0.039245628	0	0.010083265	0.01776955
0.001403298	0.09061454	0	0	0.000594951
0	0	0	0	0
0	0	0	0	0
0	0	0	0	0
0	0	0	0	0
0	0	0	0	0
0	0	0	0	0
0	0	0	0	0
0	0	0	0	0
0	0	0	0	0
0	0	0	0	0
0	0.013299819	0	0	0
0	0	0	0	0
0	0	0	0	0
0	0	0	0	0
0	0	0	0	0
0	0	0	0	0

0	0	0	0	0
0	0	0	0	0
0	0	0	0	0
0	0	0	0	0
0.047111621	0.026915851	0	0.016081816	0
0	0	0	0	0
0	0	0	0	0
0	0	0	0	0
0	0	0	0	0
0	0	0	0	0
0	0	0	0	0
0	0	0	0	0
0	0	0	0	0
0	0	0	0	0
0	0	0	0	0
0	4.154374364	0	11.05196415	0
0	0	0	0	0
0	0	0	0	0
0	0	0	0	0
0	0	0	0	0
0	0	0	0.002089151	0
0	0	0	0	0
0	0	0	0	0
0	0	0	0	0
0	0	0	0	0
0	0	0	0	0
0	0	0	0	0
0	0	0	0	0
0	0	0	0	0
0	0	0	0	0
0	0.020030679	0	0	0
0.000925202	0.015972859	0	0.039055959	0
0	0	0	0	0
0	0	0	0	0
0	0	0	0	0
0	0	0	0	0
0	0	0	0	0
0	0	0	0	0
0	0	0	0	0
0	0	0	0	0
0	0	0	0	0
0	0	0	0	0
0	0	0	0	0
0	0	0	0	0
0	0	0	0	0
0	0	0	0	0
0	0	0	0	0
0	0	0	0	0

0	0	0	0	0
0	0	0	0	0
0	0	0	0	0
0	0	0	0	0
0	0	0	0	0
0	0	0	0	0
0	0	0	0	0
0	0	0	0	0
0	0	0	0	0
0	0	0	0	0
0	0	0	0	0
0	0	0	0	0
0	0	0.000711253	0.001245757	0.001313722
0	0	0	0	0
0	0.001507794	0	0	0
0	0	0	0	0
0	0	0	0	0
0	0	0	0	0
0	0	0	0	0
0	0	0	0	0
0.050902008	0.048249024	0.024381	0.021836879	0.003275125
0	0	0	0	0
0	0	0	0	0
0	0.01199796	0	0	0
0	0	0	0	0
0	0	0	0	0
0.013014496	0	0	0.017125942	0
0	0	0	0	0
0	0	0	0	0
0	0	0	0	0
0	0	0	0	0.001188319
0	0	0	0	0
0	0	0	0	0
0	0.002721859	0	0	0
0.005385294	0.018111569	0	0.003978306	0.016515027
0	0	0	0	0
0	0	0	0	0
0	0	0	0	0
0	0	0	0	0
0	0	0	0	0
0	0	0	0	0
0	0	0	0	0
0	0	0	0	0
0	0	0	0	0
0	0	0	0	0
0	0.72172669	0	0	0
0.002623879	0.001568801	0	0.001640933	0.004037731
0.000767541	0	0.00102771	0.000540009	0.009680996

0	0	0	0	0
0	0	0	0	0
0	0.001887033	0	0	0
0	0	0	0	0
0	0	0	0	0
0	0	0	0	0
0	0	0	0	0
0	0.02647002	0	0	0
0	0	0	0	0.001496238
0	0	0	0	0
0	0	0	0	0
0	0	0	0	0
0	0	0	0	0
0	0	0.008147714	0	0
0	0	0.027546096	0.001064269	0
0	0	0	0	0
0	0	0	0	0
0	0	0	0	0
0	0	0	0.002745929	0
0	0	0	0	0
0.025043851	0.009552102	0.015725739	0	0.006549646
0	0	0	0	0
0	0	0	0	0
0	0	0	0	0
0.009722223	0.004103194	0.018990542	0.010059031	0
0	0	0	0	0
0	0	0	0	0
0	0	0	0	0
0	0.082475214	1.448374438	0	0
0	0	0	0	0
1.090490885	0.265190503	0	0.036484783	0.101972554
0	0	0	0	0
0	0.029504616	0	0.02851575	0.013278313
0	0	0	0	0
0	0	0	0	0
0	0	0	0	0
0	0	0	0	0
0	0	0	0	0
0	0	0	0	0
0	0	0	0	0.008366029
0	0	0	0	0
0.009277501	0.219427475	0	0.125991186	0.029240937
0	0	0	0	0
0	0	0	0	0
0	0	0	0	0
0	0	0	0	0
0	0	0	0	0
0	0	0	0	0

0	0	0	0	0
0	0	0	0	0
0.010836679	0.000728908	0.12565597	0.188699566	0.007504165
0	0	0	0	0
0	1.759800515	0	0	0
0	0	0	0	0
0	0	0	0	0
0	0	0	0	0
0	0	0	0	0
0	0	0	0	0
0	0	0	0	0
0	0	0	0	0
0	0	0	0	0
0.005688831	0	0	0.003581112	0
0.02273555	0.005097543	0	0.006085345	0
0	0.004983019	0	0	0
0.002815774	0.00075759	0.012969561	0.003764011	0
0	0	0	0	0
0	0	0	0	0
0	0.001029012	0	0	0
0	0	0	0	0
0.000762552	0	0.001633648	0	0.007543583
0	0	0	0	0
0.002758303	0	0	0	0
0	0	0.003056036	0	0
0	0	0	0	0
0	0	0	0	0
0	0	0	0	0
0	0	0	0	0
0	0.015168502	0	0	0
0	0	0	0.015074687	0
0	0	0	0	0
0	0	0	0	0
0	0.001051361	0	0	0
0	0	0	0	0
0.002030786	0.03414922	0.00598213	0	0
0	0	0	0	0
0	0	0	0.002022167	0.00505479
0	0	0	0	0
0	0	0	0	0
0	0	0	0	0
0	0.005382625	0	0	0
0	0	0	0	0
0	0	0	0	0
0	0	0	0	0
0	0	0	0	0
0	0	0	0	0
0	0	0	0	0

2.048246464	0.037507467	0.001638962	0.256563013	0.282543087
0	0	0	0	0
0.022896465	0	0	0.002247764	0
0	0	0	0	0
0.019018136	0.006966634	0	0.035618112	0
0	0.002350289	0	0	0
0	0	0	0	0
0.014267717	0.002020401	0	0.028793661	0
0	0	0	0	0
0.026703082	0.087116696	0	0.000816833	0.563927397
0	0	0	0	0
0	0.060242427	0	0.003764009	0.00176416
0.002479975	0	0.019078343	0.003489609	0.003791505
0.143124789	0	0	0.157067261	0.00812939
0	0	0	0	0.001376286
0	0	0	0	0
0	0	0	0	0
0	0.001316662	0	0	0.00203327
0.016751409	0	0	0	0
0	0	0	0	0
0	0	0	0.000702882	0
0	0	0	0	0
0	0.046526472	0	0	0
0	0	0	0	0
0	0	0	0	0
0	0	0	0	0.020781819
0	0	0.083501576	0.005682115	0
0.001270198	0	0	0.010500474	0
0	0	0	0	0
0	0.001453832	0	0.003991779	0.00080182
0.003381337	0	0	0	0
0	0	0	0	0
0	0	0	0.034504405	0
0	0	0	0	0
0	0	0	0	0
0	0	0	0	0
0.001488388	0	0	0	0
0	0	0	0	0
0	0	0	0	0
0	0	0	0.004080508	0
0	0	0	0.577458653	0
0.123975665	0	0	0	0
0	0	0	0	0
0	0	0	0.00119073	0.000837128
0.004456385	0	0	0	0
0	0	0	0	0
0	0.003807304	0	0.001148757	0.001292192
0	0	0	0	0

0	0	0	0	0
0	0	0	0	0
0	0	0	0	0
0	0	0.013552831	0	0
0	0	0	0	0
0.031121573	0	0	0.129384338	0.002798833
0	0.002308433	0	0	0
0	0.00179006	0	0	0
0	0	0	0	0
0	0	0	0	0
0.01312574	0.002140309	0	0.020778099	0
0	0.002005957	0	0	0
0	0	0	0	0
0.007327591	0	0	0.005551948	0
0.065302041	0	0	0	0.007898733
0	0	0	0	0
0	0	0	0	0
0	0	0	0	0
0	0.002421189	0	0	0
0	0.13428381	0	0	0
0	0	0	0	0
0	0.254048859	0	0.471453518	0
0	0	0	0	0
0	0	0	0	0
0	0	0	0	0
0	0	0	0	0
0.049809346	0	0	0.008384696	0.002116067
0	0	0	0	0
0	0	0.155758528	0	0
1.599127091	0.624644263	0	2.264687325	0.139763595
0	0.000857378	0	0.004842714	0
0	0	0	0	0
0	0	0	0	0
0	0	0	0	0
0	0	0	0	0
0	0	0	0	0
0	0	0	0	0
0	0	0	0	0
0.242403512	0	0	0.74671525	0
0.02434386	0	0	0	0.049154241
0.096153543	0.045299047	0	0.048088221	0.002750468
0	0.191399945	0	1.960785032	0
0	0	0	0	0
0	0	0	0.028711894	0
0	0	0	0	0
0	0	0	0	0
0	0.006106646	0	0.007185849	0.000962271
0	0	0	0	0

0	0	0.011891761	0	0.005936406
0	0	0	0	0
0.002044167	0.001833292	0.002627584	0.002013464	0.017795377
0	0	0	0	0
0	0	0	0	0
0.004312692	0.389282495	0	0.05068942	0
0	0	0	0	0
0	0	0	0	0
0.009039967	0	0	0	0
0	0	0	0	0
0.104128729	0	0	0	0
0	0	0	0	0
0	0	0	0	0
0.064060437	0.001947526	0	0.003246582	0.001074103
0	0	0	0	0
0.094608934	0.079712548	0	0.017610788	0.004826511
0	0.004849929	0.006044528	0.000992528	0.003954114
0.00085699	0	0.007037871	0	0.001695563
0	0	0	0	0
0	0	0	0.163693088	0
0	0.002923269	0	0.194718456	0.007328396
0.001363643	0	0	0.000959401	0
0	0	0	0.20623879	0
0	0	0	0	0
0	0.103464425	0	0	0
0	0.042778027	0	0	0
0.008059189	0.004550843	0	0.005460098	0
0.008311439	0.00067764	0	0	0
0	0	0	0	0
0	0	0	0	0
0.057884913	0.004682399	0.002674711	0.073784761	0.044238417
0.002030572	0.001214066	0.012325514	0.008333638	0
0.012889193	0.006068766	0	0.022897422	0
0	0.009525432	0	0	0
0	0	0	0	0
0	0	0	0	0
0	0	0.027772584	0.004509096	0
0.044881358	0.00408644	0	0.063313505	0.052512638
0	0.112927792	0	0	0
0	0.003341082	0	0	0.001474144
0	0	0	0	0
0	0	0	0	0
0.135262132	0.001833379	0.002919676	0	0.012807886
0	0.010526901	0	0.397268813	0
0	0.053685106	0	0	0.051642755
0	0	0	0	0
0	0	0	0	0
0	0	0	0	0

0.00135115	0	0	0	0
0.509741346	0.16760562	0	1.16872183	0.365146138
0	0	0	0	0
0.000940069	0.023775206	0	0	0
0	0	0	0	0
0	0	0.156365503	0	0
0	0	0	0	0.001420554
0	0	0	0	0
0	0	0	0	0
0	0	0	0	0
0.00645933	0.006827453	0	0.010549753	0.016659441
0.013057553	0.001232689	0	0.033120606	0.002039565
0	0.275733521	0	0	0
0	0	0	0	0
0	0	0.005670532	0.002420904	0
0.004185157	0	0	0	0
0.042274066	0.002166463	0.003363863	0.06254366	0.006213231
0.115399707	0	0	0.185546335	0.006514549
0	0	0	0	0
0.027285946	0.004930754	0	0.007736196	0.012690622
0.004484081	0	0	0.058889745	0.00394302
0	0	0	0	0
0	0	0	0	0
0	0	0	0	0
0	0.001126147	0	0	0
0.001827568	0	0	0	0
0.04180122	0.064613465	0	0	0
0.132916092	0.176907194	0	0.292278107	0.017025323
0	0	0	0	0
0	0	0	0	0
0	0	0	0	0
0	0	0	0	0
0	0	0	0	0
0	0	0	0	0
0.069450126	0	0	0	0.086507528
0.197169571	0.296591672	0	0.091429156	0.168896379
0.00539601	0.004094845	0	0	0
0.00708992	0	0	0	0
0	0.04891975	0.001399495	0.006204626	0
0	0	0	0	0
0	0.008823204	0	0	0.047850871
0	0	0	0	0
0.056146737	0.059251274	0	0.00753757	0.019822919
0	0	0	0	0
0.035482952	0	0	0.067987857	0.002240533
0	0.090167072	0	0	0
0.106104479	0	0	0	0
0	0	0	0	0

0.002217334	0	0	0.001560022	0
0	0	0	0	0
0	0	0	0	0
13.9474645	0.014713628	0	0.005202301	0.101950425
0	0	0	0	0
0	0	0	0	0
0	0	0	0	0
0	0.230072973	0	0.00145438	0
0.81971428	0.053709826	0.003207505	0.239991101	1.48481008
0	0.025930296	0	0	0
0	0.163611163	0	0	0
0.005091997	0.026010395	0	0.002647943	0
0	0	0	0	0
0	5.675702765	0	0	0
0	0.296397661	0.028180088	0.076349601	0.104823093
0	0	0	0	0
0	0	0	0.001173636	0
0	0	0	0	0
0	0	0	0	0
0	0	0	0	0
0	0	0	0	0
0	0	0	0	0
0	0	0	0	0
0.253410835	0.610628111	0	0.620792576	0.12360305
0	0.016916536	0	0.011666596	0
0	0	0	0	0
0	0.014156003	0	0	0
0	0	0.00227501	0	0
0	0	0	0	0
0	0	0	0	0
0	0.024321404	0	0	0
0	0	0	0	0
0.047503681	0.078595635	0	0	0
0	0	0	0	0
0	0.350735862	0	0	0
0	0	0	0	0
3.63973024	0.543866974	0.001262312	0	0
0	0	0	0	0
0	0.0031004	0	0	0
0	0.001705424	0.003225139	0	0
0.064777498	0.168571045	0.034125153	0.538827249	0.069334026
0	0.398854669	0	0	0
6.407007147	7.445870106	0	0.093929024	0.09527281
0	0	0	0	0
0	0	0	0	0
0	0	0	0	0
0	0	0	0	0
0	0.00183179	0	0.003233272	0.026014508

0	0	0	0	0
0	0.029204215	0	0	0
0.379843965	0.002194972	0	0.176324624	0.004115955
0	0.001423596	0	0	0
0	0	0	0	0
0	0.001556203	0	0.00718925	0.045774923
0	0	0	0.013719835	0
0	0	0	0	0
0	0	0	0	0
0	0	0	0	0
0.055988971	0.120468569	0.001801788	0.075232601	0.137398678
1.067319943	3.521332904	0	2.879111283	1.074199125
0	0	0	0	0
0.001556024	0	0	0.007115886	0.076195532
0.096665923	0.098974062	0.041155618	0.469599836	0.11746177
0	0	0.002372561	0	0
0	0.004019941	0	0	0
0	0	0	0	0
0	0	0	0	0
0	0.010651277	0	0.030637774	0.000783255
0.059212613	0.087965269	0	0.163520498	0
0.011035271	1.514032564	0	0.202459818	13.60723956
2.936777461	6.984626959	0	0.058621052	30.58329507
0	0	0	0	0
0	0	0	0	0
0	0	0	0.014602607	0
0	0.714654062	0.022964871	0	0.005957488
0	0	0	0	0
0	0	0	0	0
0	0.032651489	0	0	0
0	0.038450757	0	0	0
0	0.204971745	0	0.4149688	0.033105105
0.004195435	0.502201375	0	0.013022333	0
0	0.04438979	0	0	0
0.002606154	0.01460816	0	0.01650221	0.106832111
0.068257029	0.007024751	0	0.0198389	0.802755867
0	0.015704312	0	0.003346114	0.138580358
0	0	0	0	0
0	0.19681933	0	0.04956329	0
0	0	0	0	0
0	0.016082683	0	0	0
0	0	0	0	0
0	0	0	0	0
0	0	0	0	0
0.000765633	0.029754889	0	0.026215126	0.351689067
0	0	0	0	0
0	0	0	0	0
0.01200632	0.002760964	16.07438625	0.008880321	0.87222086

0	0	0	0	0
0	0	0	0	0
0	0	0	0	0
0	0.000975383	0	0	0
0	0	0	0	0
0	0	0	0	0
0	1.067645617	0.00076526	0	0
0.028021497	0.02566934	0	0.041682571	0
0	0	0	0	0
0	0	0	0	0
0.00581411	0.015860257	0.011417832	0	0.004313727
0	0	0	0	0
0	0	0	0	0
0	0.741589692	0	0	0
0	0	0	0	0
0	0	0	0	0
0	0.486861797	0	0	0
0	0.00253214	0	0.003121523	0.004189592
0	0	0	0	0
0.001636868	0	0	0.005758152	0
0	0	0	0	0
0	0	0	0	0
0	0.573448911	0	0	0
0	0	0	0	0
0	0	0	0	0
0	0	0	0	0
0	0	0	0	0
0	0	0	0	0
0	0	0	0	0
0	0.394367044	0	0	0
0	0.036961796	0	0	0
0	0	0	0	0
0	0	0	0	0
0	0	0	0	0
0	0	0	0	0.02572549
0	0	0	0	0
0	0.117467478	0	0	0
0.001435509	0.001655258	0	0.020199259	0.001014346
0	0	0	0	0
0	0	0	0	0
0	0.263499464	0	0.09448878	0
0	0	0	0	0
0	0	0	0	0
0	0.008573077	0	0.022558255	0
0	0	0	0	0
0	0	0	0	0
0	0	0	0	0
0	0	0	0	0

0	0	0	0	0
0	0	0	0	0
0	0	0	0	0
0	1.034183022	0	0	0
0	0	0	0	0
0	0	0	0	0
0	0	0	0	0
0	0	0	0	0
0.010747523	0.062398659	0.030295895	0.060690974	0.002518114
0	0	0	0	0
0	0	0	0	0
0	0	0	0	0
0	0.007398822	0.001590665	0	0
0	0	0	0	0
3.157876217	0.093692846	0	2.497445519	0.990043164
0.704744197	0	0	0	0.081728431
0.019670432	0.000773739	0	0	0
0	0	0	0	0
0	0	0	0	0
0	0	0	0	0
0	0.205979317	0	0	0
0	0	0	0	0
0	0	0	0	0
0	0	0	0	0.012613612
0.033592549	0.203134677	0	0.2080285	0
6.630289849	0.436625378	0	6.642527298	0
0	0.039718585	0	0	0
0	0	0	0	0
0	0	0	0.025368586	0
0	0	0	0	0
0	0	0	0	0
0	0.022091459	0	0	0
0	0	0	0	0
0	0.005257077	0	0	0
0	0	0	0.001647322	0.040824067
0.001655099	0.002783175	0.017507336	0.003638928	0.020875759
0	0.019530688	0	1.107955717	0.016263008
1.263137817	0.605909878	0	2.517488972	0.123639232
0	0	0	0	0
0	0	0	0	0
0	0	0	0	0
0.007507581	0	0	0.000825315	0.001856728
0.716965882	0.107758998	0	0.007359536	0.040553224
0	0	0.000910217	0	0
0	0	0	0	0
0	0.007558567	0	0	0.000969469
0	0	0	0	0
0.024667842	0.052087366	0	0.553390551	0

0	0.180425524	0	0	0
0	0	0	0	0
0	0	0	0	0
0	0	0	0	0
0.001159126	0.001871193	0	0.017615051	0.002064008
0	0	0	0	0
0	0	0	0	0
0	0	0	0	0
0	0	0	0	0
0	0	0	0	0
0	0	0	0	0
3.599705692	0.973630262	0	3.490957454	1.351120668
0	0.136696415	0	0.47140462	0
0	0	0	0	0
0.00321727	3.347761267	66.2664697	0.041875379	0.0053045
0	0	0	0	0
0	0.001003599	0	0	0
0	0	0	0	0
0	0.00210752	0	0	0
0	0.001168452	0	0	0
0	0.042573943	0	0.005201418	0
0	0	0	0	0
0.357601803	1.249444381	0	0.291623244	0.1598133
0	0	0	0.050917638	0
0	0	0	0	0
0	0	0	0	0
0	0	0	0	0
0	0	0	0	0
0	0	0	0	0
0	0	0	0	0
0	0	0	0	0
0.075495347	0.002808137	0	0.113573741	0.012389994
0	0	0	0	0
0.104979582	0.13109293	0	0	0.021492457
0.001125726	0.003028792	0.019534707	0.004435273	0.022718056
0.002031677	0.00390448	0	0.001429401	0
0	0	0	0	0
0	0	0	0	0
0	0	0	0	0
0	0	0	0	0
0	0.378135434	0	0	0
0	0	0	0	0
0	0	0	0	0
0	0	0	0	0
0.064762876	0.125512797	0.001425455	0.014199863	0.116066555
0	0	0	0	0
0	0.660733988	0	0	0.249216071
0	0	0	0.000885529	0

0	0	0	0	0
0	0	0	0	0
0	0.011520526	0	0.009199056	0.007488434
0	0	0	0	0
0.002326316	0	0	0.004255412	0
0	0.036104334	0	0	0
0	0.26880369	0	0	0
0.082537794	0	0	0.049230008	0
0	0	0	0	0
0	0.006691497	0	0	0
0	0	0	0	0
0.001867166	0	0	0	0
0	0	0	0	0
0	0	0	0	0
0	0	0	0	0
0	0	0	0	0
0	0	0	0	0
0	0	0	0	0
0	0	0	0.44474144	0
0	0	0	0	0
0	0	0	0	0
0	0	0	0	0
0	0	0	0	0
0	0	0	0	0
0	0	0	0	0
0.04872898	0.000740714	0	0.005084437	0.019608966
0	0	0	0	0
0.04021822	0.048032039	0.065378617	0.06114173	0
0	0	0	0	0
0.066209613	0.61015794	0	0.095017221	0
0	0	0	0	0
0	0	0	0	0
0	0	0	0	0
0	0	0	0	0
0.008438701	0.001552445	0.883764989	0.003653606	0.002140519
0	0	0	0	0
0	0	0	0	0
0	0.000880785	0.145175335	0	0
0	0	0	0	0
0	0	0	0	0
9.555838485	10.92824414	0	1.715830231	5.384096411
1.437081163	0.386682159	0	0.093444221	0.139212561
0	0	0.000903841	0	0
0	0	0	0	0
0	0	0	0	0
0	0	0	0	0
0	0.00074963	0	0	0
0.004676683	0.01188368	0	0	0.116817377

0	0	0	0	0
0.031668166	0.006291428	0	0.008601915	0.001387944
0.002809111	0	0	0	0
0	0	0	0	0
2.90483518	1.120359307	0	0.040166003	0.381080606
0	0	0	0	0
0	0	0	0	0
0	0	0	0	0
0.206740416	0	0	0.36736573	0.007523446
0	0.002397181	0	0	0
0	0	0	0	0
0	0	0	0	0
0	0.128027767	0	0.126880623	0
0.007901169	0.025552875	0	0.1066303	0
0	0	0	0	0
0.278993096	0.478046652	0	0.722670266	0
0	0.220005603	0	0.036150794	0
0	0	0	0	0
0	0	0	0	0
0	0	0	0	0
0	0	0	0	0
0	0	0	0	0
0	0	0	0	0
0	0	0	0	0
0	0	0	0	0
0	0	0	0	0
0	0.005170376	0	0	0
0	0	0	0	0.002011053
0	0	0	0	0
0	0	0	0	0
0	0	0	0	0
0	0	0	0	0
0	0.020045479	0.001173627	0.288298245	0
0	0.017200658	0	0	0
0	0	0	0	0
0.008230738	0.370559433	0	0.180341937	0
0	0	0	0	0
0	0	0	0	0
0	0	0	0	0
0	0	0	0	0
0	0	0	7.340868828	0.256143557
0	0	0	0	0
0	0	0	0	0
0	0	0	0	0
0	0	0	0	0
0	0	0	0	0
0	0	0	0	0
0	0	0	0	0

0	0	0	0	0
0	0	0	0	0
0	0	0	0	0
0.00598747	0	0	0	0
0	0.028060596	0	0	0
0	0	0	0	0
0.053324779	0.076555215	0.001549767	0.323111865	0
0	0	0	0	0
0	0	0	0	0
0.610422612	0.008168022	0	0	0
0	0.015060519	0	0	0
0	0.024801923	0	0	0
0	0	0	0	0
0	0	0	0	0
0	0	0	0	0
0	0.008643293	0	0	0
0.166882624	0	0	0	0
0.110878013	0.000712831	0	0	0
0	0	0	0	0
0	0	0	0	0
0.091697532	0.052942977	0	0.540481749	0.012069008
0	0	0	0	0
0	0.02413698	0	0.005955387	0.026838858
0	0.00207705	0	0.000950491	0.031502314
0	0	0	0	0
0	0.002042044	0	0	0
0	0	0	0	0
0	0	0	0	0
0	0	0	0	0
0	0	2.515079974	0.040843946	0
0	0	0	0	0
0	0	0	0	0
0	0	0	0	0
0	0	0	8.772062897	0
0	0	0	0	0
0	0	0	0	0
0.004829783	0.668357577	0	0.00934458	0
0	0	0	0	0
0	0	0	0	0
0	0	0	0	0
0	0	0	0	0
0	0	0	0	0
0	0	0	0	0
0	0.007026682	0	0	0
0	0	0.001224088	0	0
0	0	0	0	0
0	0	0	0	0
0	0	0	0	0

0.119826474	0.005773698	0.009342977	0.004675483	0.034924809
0	0	0	0	0
0	0.171757974	0	0	0
0	0	0	0	0
0	0	0	0	0
0	0	0	0	0
0	0	0	0	0.001579326
1.017755033	1.311297852	0	1.195965387	0.384333593
0.227202247	0.172775716	0.002095029	0.46349564	0.002257283
0.105750168	0.089753029	0	0.220809027	0
0	0.017434518	0	0	0
0	0.001425195	0	0	0
0	0.018154308	0	0	0
0	0	0	0	0
0	0	0	0	0
0	0	0	0	0
0.117316942	0	0	0.434450493	0
0	0	0	0	0
0	0	0	0	0
0.079975317	0.016538971	0	0	0.007073898
0	0	0	0	0
0	0	0	0	0
0	0	0	0	0
0	0	0	0	0
0	0.071147737	0	0.005869129	0
0	0	0	0	0
0	0	0	0	0
0	0	0	0	0
0.001170102	0	0	0	0
0	0	0	0	0
0	0.120160324	0	0	0
0	0	0	0	0
0	0	0	0	0
0	0	0	0	0
0.003016987	0	0	0	0.004725568
0	0	0	0	0
0	0	0	0	0
0	0	0	0	0
0	0	0	0	0
0	0	0	0.001021037	0
0	0	0	0	0
0	0.267978218	0	0.626008947	0.202280377
0	0	0	0	0
0	0	0	0	0
0	0	0	0	0
0.049304806	0	0	0.004936977	0
0	0	0	0	0
0	0.000520323	0	0	0

0	0	0	0	0
0.000749731	0.001176681	0	0.001714305	0
0	0	0	0	0
0	0	0	0	0
0.235851464	0.107170754	0	0.033002633	0
0	0	0	0	0
0	0	0	0	0
0.020605512	0.0388551	0.007640321	0.021188155	0.001829342
0	0.002602129	0	0	0
0	0	0	0	0
0	0	0	0	0
0	0	0	0	0
0	0	0	0	0
0	0	0	0	0
0	0.005670665	0	0	0
0	0.058767311	0	0	0
0	0	0	0	0
0	0	0	0	0
0	0	0	0	0
0	0	0	0	0
0	0	0	0	0
0	0	0	0	0
0	0	0	0	0
0.001600341	0.004100718	0	0	0.003844781
0	0	0	0	0.061372772
0	0	0	0	0
0	0.352928839	0	0	0.033303216
0.001321047	0	0	0	0
0	0	0	0	0
0	0	0	0	0
0	0	0	0	0
0	0.000901218	0	0	0
0	0	0	0	0
0	0	0.010787796	0	0
0	0	0	0	0
0	0	0	0	0
0	0	0	0	0
0	0.554847749	0	0	0
0	1.276873584	0	0	0
0	0	0	0	0
0	0	0	0	0
0	0	0	0	0
0	0	0	0	0
0	0	0	0	0
0	0	0	0	0
0	0	0	0	0
0	0	0	0	0
0	0	0	0	0

0	0.13579296	0	0.12907802	0.005252222
0	0	0	0	0
0	0	0	0	0
0	0	0	0	0
0	0	0	0	0
0.018681113	0	0	0.014746067	0
0	0	0	0	0
0	0.000992256	0	0	0
0	0	0	0	0
0	0	0	0	0
0	0	0	0	0
0	0	0	0	0
0	0	0	0	0
0	0	0	0	0
0	0	0	0	0
0	0	0	0	0
0	0	0	0	0
0	0	0	0	0
0	0.001495171	0	0	0
0	0	0	0	0
0.055550131	0.000612536	0	0.006246825	0.015765271
0	0	0	0	0
0.037090551	0.084579048	0	0.000809157	0.065135418
0	0.03020923	0	0	0
0	0	0	0	0
0	0	0	0	0
0	0	0	0	0
0	0	0	0	0
0	0	0	0	0
0	0	0	0	0
0	0	0	0	0
0	0	0	0	0
0.016694412	0	0.095758326	0.444812548	0.019711483
0	0	0	0	0
0	0.16049506	0	2.495409206	0.009054182
0.001512516	0	0.00601775	0	0.000855008
0	0	0	0	0
0	0	0	0	0
0.010496781	0	0	0.001934189	0.009889515
0	0	0	0	0
0	0	0	0	0
0	0.019089023	0	0	0
0.101405644	0.011269807	0	0.028990203	0.146571239
0	0	0	0	0
0	0	0	0	0
0	0.031429707	0	0.005551706	0.008724484
0	0	0	0	0
0	0	0	0	0

0	0	0	0	0
0	0	0	0	0
0	0	0	0	0
0	0	0	0	0
0	0	0	0	0
0	0	0	0	0
0	0	0	0	0
0	0	0	0.04465329	0.002440441
0.079801433	0.007410433	0	0.002447733	0
0.01171323	0	0	0.003700009	0
0	0	0	0	0
0	0	0	0	0
0	0	0	0	0
0	1.253862656	0	0	0
0	0.002598794	0	0	0
0.010113357	0	0	0.001985673	0
0	0	0	0	0
0	0	0	0	0
0	0	0	0	0
0.221723079	1.617822119	0	0.917959324	0
0	0	0	0.009159699	0.000972017
0	0	0	0	0
0	0	0	0	0
0	0.043549672	0	0	0
0	0	0	0	0
0	0	0	0	0
0	0	0	0	0
0	0	0	0	0
0	0	0	0	0
0	0.014428572	0	0	0
0.186571539	0.176068391	0	0.5027295	0.07015807
0	0	0	0	0
0	0.460017668	0	0	0
0	0	0	0	0
0	0	0	0	0
0	0	0	0	0
0	0	0	0	0
0	0	0	0	0
0	0	0	0	0
0	0	0	0	0
0	0	0	0	0
0	0	0	0	0
0	0	0	0	0
0	0	0	0	0
0	0	0	0	0
0	0	0	0	0
0.030818143	0	0.110640163	1.066330243	0.04692139
0	0	0	0	0

0	0	0	0	0
0	0	0	0	0
0	0	0	0	0
0	0	0	0	0
0.142097883	0.845454132	0	0.126549377	0.356053905
0	0	0	0	0
0	0	0	0	0
0	0	0	0	0
0	0	0	0	0
0	0	0	0	0
0	0.041372535	0	0.074713285	0
0.012236493	0.034714264	0.002407478	0.002986822	0.044220339
0.079500751	0.004637367	0	0	0
0	0	0	0	0
0.00361584	0	0	0	0
0	1.257630842	0	0.761018387	0
0	0.003445082	0	0.018648024	0.01235025
0	0	0	0	0
0.075069804	0.032560523	0	0.290420993	0.027685007
0.044935337	0.056464069	0	0.025882271	0
0	0	0	0	0
0	0	0	0	0
0	0	0	0	0
0	0	0	0	0
0	0	0	0	0
0	0	0	0	0
0.022950629	0.004613445	0	0.009047935	0.079268341
0	0	0	0	0
0	0	0	0	0
0	0	0	0	0
0.016147703	0.000804551	0.000720707	0	0
0.108766506	0	0	0	0
0	0	0	0	0
0	0	0	0	0
0	0	0	0	0
0	0	0	0	0
0	0	0	0	0
0.021901973	0.013566101	2.547265781	0.004212613	0.004052751
0	0.026005601	0	0	0
0.010497646	0	0	0	0
0.001573038	0.007335977	0	0.000774706	0.001400522
0	0	0	0	0
0	0	0	0	0
0.001635575	0.002514601	0	0.005917993	0.001155715
0	0	0	0	0
0	0	0	0	0
0	0	0	0	0
0	0	0	0	0

0	0	0	0	0
0	0	0	0.728373292	0
0	0	0	0	0
0.00073461	0	0.002885276	0	0
0	0	0	0.003195741	0.002696072
0	0.020504177	0	0	0
0	0.006606543	0	0	0
0.000574529	0	0	0	0
0	0.028009828	0	0	0
0	0	0	0	0
0	0	0	0	0
0	0	0	0	0
0.010414702	0.009807347	0	0.00781583	0.466544854
0	0	0	0	0
0	0	0	0	0
0	0	0	0	0
0	0	0	0	0
0	0	0	0	0
0	0	0	0	0
0	0	0	0	0
0	0	0	0	0
0	0	0	0	0
0.030170279	0.032592517	0	0.004663402	0.037533722
0	0.035799516	0	0.069071827	0.005069461
0	0.081362349	0	0	0
0	0	0	0	0
0	0	0	0	0
0	0	0	0	0
0	0	0	0	0
0	0	0	0	0
0	0.027655358	0	0	0
0	0	0	0	0
0	0	0	0	0
0	0	0	0	0
0	0	0	0	0
0	0	0	0	0
0	0	0	0	0
0	0	0	0	0
0.005174826	0.065554057	0.004850235	0.002578891	0.000853204
0	0	0	0	0
0	0	0	0	0
0	0	0	0	0
0	0	0	0	0
0	0	0	0	0
0	0	0.005615908	0	0
0.187810915	0.037940747	0	0.039774195	0.228956835
0	0.006074878	0	0	0
0	0	0	0	0

0	0	0	0	0
0	0	0	0	0
0	0	0	0	0
0	0.020036035	0	0	0
0	0	0	0	0
0	0	0	0	0
0	0.017297473	0	0	0
0	0	0	0	0
0	0	0	0.01684348	0
0	0	0	0	0
0	0.022590542	0	0.000615344	0.007613943
0.002350455	0	0	0	0.000775066
0	0	0	0	0
0	0	0	0	0
0	0	0	0	0
0	0	0	0	0
0	0	0	0	0
0	0	0	0	0
0	0	0	0	0
0	0.002704627	0	0	0.001606404
0	0	0	0	0
0	0	0	0	0
0	0	0	0	0
0	0	0	0	0
0	0	0	0	0
0	0	0	0	0
0	0	0	0	0
0	0	0	0	0
0	0	0	0	0
0	0	0	0	0
0.008968227	0.018230963	0	0.002103221	0.003943049
0	0	0	0	0
0.02568546	0.026356241	0	0.128940409	0.019915525
0.00567519	0.004453524	0	0.0123112	0.000935701
0	0	0	0	0
0	0	0	0	0
0	0	0	0	0
0	0	0	0	0
0	0	0	0	0
0	0	0	0	0
0	0	0	0	0
1.366674303	0.186439998	0	0.010581023	0.008662975
0.181597829	0.0468001	0	0.14685573	0.084202135
0	0	0	0	0
0	0	0	0	0
0.005697529	0.013225305	0	0.001886371	0
0	0.00177359	0	0	0
0	0	0	0	0

0	0	0	0	0
0	0.006319333	0	0.01743643	0
0.009933632	0.001272698	0	0.044529146	0.007299977
0	0	0	0	0
1.403653616	0.202831156	0	0.016421978	0.023090542
0	0.007517551	0	0	0
0	0	0	0	0
0	0	0	0	0
0	0	0	0	0
0	0	0	0	0
0	0	0	0	0
0	0	0	0	0
0	0	0	0	0
0	0	0	0	0
0	0	0	0	0
0	0	0	0	0
0	0	0	0	0
0	0	0	0	0
0	0	0	0	0
0	0	0	0	0
0	0	0	0	0
0.00182639	0	0	0	0
0	0	0	0	0
0	0	0	0	0
0.00233906	0.011118151	0	0	0.00092557
0	0	0.009829982	0.005628692	0
0	0	0	0	0
0	0.27512565	0.000733494	0.020876577	0
0	0	0	0	0
0	0	0	0	0
0	0	0	0	0
0	0	0	0	0
0	0	0	0	0
0	0	0	0	0
0	0	0	0	0
0	0	0	0	0
0	0	0	0	0
0	0	0	0.122769207	0
0	0.002873982	0	0	0
0	0	0	0	0
0	0	0	0	0
0	0.002060416	0	0	0
0.106741211	0.005506586	0.006576973	0.002658356	0.031070875
0	0	0	0	0
0	0	0	0	0
0	0	0	0	0
0.002528876	0.003887999	0	0.004320938	0.006432951
0	0	0	0	0

0	0	0	0	0
0.008433296	0.021872316	0	0.002084676	0.016459879
0	0	0	0	0
0	0	0	0	0
0	0	0	0	0
0	0	0	0	0
0	0	0	0	0
0	0	0	0	0
0	0	0	0	0
0	0	0	0	0
0	0	0	0	0
0	0	0	0	0
0.09444807	0.061367826	0	0	0
0	0	0	0	0
0.084972965	0.017586283	0	0.073379567	0.004498189
0	0	0	0	0
0	0	0	0	0
0	0	0	0	0
0.00188633	0	0	0.012386651	0
0	0	0	0	0
0	0	0	0	0
0.003070073	0.017521424	0	0.002749056	0
0	0.005385512	0	0	0.002758069
0	0	0	0	0
0	0	0	0	0
0	0	0	0	0
0	0	0	0	0
0	0	0	0	0
0	0	0	0	0
0	0	0	0	0
0	0	0	0	0
0	0	0	0	0
0	0	0	0	0
0	0	0	0	0
17.70920961	1.469261552	0.001085761	0	12.99912129
0	0	0	0	0
0	0	0	0	0
0	0	0	0	0
0	0.126480206	1.101645988	0.145499022	0.001378589
0	0	0	0	0
0.28638855	0.288222166	0	0.040790452	0.030902185
0	0	0	0	0
0	0	0	0	0
0	0	0	0	0
0	0	0	0	0
0.423460027	0.006158534	0.446549158	0.025968084	0.014718454
0	0	0	0	0
0	0	0	0	0

0	0	0	0	0
0	0.010239806	0	0	0
0	0.002385768	0	0	0
0	0	0	0	0
0	0	0	0.000610692	0
0	0	0	0	0
0	0	0	0	0
0	0.001386772	0	0	0
0	0	0	0	0.002109195
0	0.495898252	0	0.002362496	0
0	0.050552705	0	0	0
0	0	0	0	0
0.006564186	0.003434101	0	0	0
0	0	0	0	0
0	0	0	0	0
0	0	0	0	0
0	0	0	0	0
0	0	0	0	0
0	0	0	0	0
0.253422277	0.529882054	0	0.26821639	5.592687197
0	0	0	0	0
0	0	0	0	0
0	0	0	0	0
0	0	0	0	0
0	0	0	0	0
0	0	0	0	0
0	0	0	0	0
0	0	0	0	0
0	0	0	0	0
0.038255657	0	0	0.030393864	0
0	0	0	0	0
0	0	0	0	0
0	0	0	0	0
0	0	0	0	0
0	0	0	0	0
0	0.001695723	0	0	0
0	0	0	0	0
0	0	0	0	0
0	0	0	0	0
0	0	0	0	0
0	0	0	0	0
0	0	0	0	0
0.102595463	0.170956837	0	0	0
0	0	0	0	0
0	0.003486049	0	0.004826029	0
0	0.021479655	0	0	0
0	0	0	0	0
0	0	0.017407419	0	0

0	0	0	0	0
0	0	0	0	0
0	0	0	0	0
0	0	0	0	0
0	0	0	0	0
0	0.030004957	0	0.088148946	0.005572418
0	0	0	0	0
0	0	0	0	0
0	0	0	0	0
0	0	0	0	0
0	0	0	0	0
0	0	0	0	0
0	0	0	0	0
0	0	0	0	0
0	0	0	0	0
0	0	0	0	0
0	0.194606126	0	0	0
0	0	0	0	0
0	0	0	0	0
0	0	0	0	0
0	0	0	0	0
0	0	0	0	0
0	0	0	0	0
0	0	0	0	0
0	0	0	0	0
0	0.003831248	0	0	0
0	0	0	0	0
0	0.167744326	0	0	0
0	0	0	0	0
0	0	0	0	0
0	0	0	0	0
0	0	0	0	0
0	0	0	0	0
0	0	0	0	0
0	0	0	0	0
0	0	0	0	0
0	0	0	0	0
0	0.094132408	0	0	0
0	0	0.000774302	1.566226233	0.006197429
0	0	0	0	0
0	0	0	0	0
0	0	0	0	0
0	0	0	0	0
0	0	0	0	0
0	0	0	0	0
0	0	0	0	0
0	0	0	0	0
0	0	0	0	0

0	0	0	0	0
0	0.016967877	0	0	0.00415918
0	0	0	0	0
0	0	0	0	0
0	0	0	0	0
0	0	0	0	0
0	0	0	0	0
0	0	0	0	0
0	0	0	0	0
0	0	0	0	0
0	0	0	0.001056348	0.005198566
0	0	0	0	0
0	0	0	0.197625589	0
0	0	0	0	0
0	0	0	0	0
0	0	0	0	0
0	0	0	0	0
0	0	0	0	0
0	2.88147991	0	0	0
0	0	0	0	0
0	0	0	0.001250818	0.006532478
0.007407241	0	0	0	0
0	0	0	0	0
0.771520055	1.270457964	0	2.87346617	0
0	0	0	0	0
0	0	0	0	0
0	0	0	0	0
0	0	0	0	0
0	0	0	0	0
0	0	0	0	0
0.001246397	0	0	0	0
0	0	0	0	0
0	0	0	0	0.00132145
0	0	0	0	0

PHL018-0021_V3	PHL018-0228_V1	PHL018-0037_V3	PHL018-0256_V1	PHL018-0088_V2
EYCF	REF	CTRL	REF	EYCF
2.188907648	0.035634512	0.040458178	0.10884487	2.118541257
0.042070814	0.230860609	0.393533851	1.134292678	0.353996039
0.007506118	0.088483055	2.447429105	3.392689832	0.179856078
0	0	0	0	0
0	0	0	0	0
0	0	0	0	0
0	0	0	0	0
0	0	0	0	0
0	0	0	0	0
0	0	0	0	0
0	0	0	0	0
0	0	0	0	0
0	0	0	0	0
6.440923356	0.07295886	0.373799759	1.326369622	0.037486673
0	0	0	0	0
0	0	0	0	0
0	0	0	0	0
0	0	0	0	0
0	0	0	0	0
0	0	0	0	0
0	0	0	0	0
0	0	0	0	0
0	0	0	0	0
0	0	0	0	0
0	0	0.111862065	0	0
0	0	0	0	0
0	0	0	0	0
0	0	0	0	0
0	0	0	0	0
0	0	0	0	0
0	0	0	0	0
0	0	0	0	0
0	0	0	0	0
0	0	0	0	0
0	0	0	0	0
0	0.11599374	1.322737139	0	0
0	0	0	0	0
0	0	0	0	0
0	0	0	0	0
0	0	0	0	0
0	0	0	0	0
0.008064696	0.023816218	0	0.051167971	0
0.456661162	3.635320868	0.002394522	21.80191622	0.000737358
0.067773493	0.09185869	0	0.004510901	0.027619551
0.002621879	0.058514685	0	0.009431381	0.006126991
0	0	0	0	0

0	0.125970767	0	0	0.153851332
0	0.001091476	0	0	0.00191202
0.29678782	0	0.000916778	0	0.126022439
0.290599915	0.638861785	0.423387067	0.140168312	0.708971783
0	0.008256478	0.024668727	0	0.00470975
0	0	0	0	0
0.000683357	0.109969288	0	0.008702457	0
0	0.023225393	0	0	0
0	0	0	0	0
0	0	0	0	0
0	0	0	0	0
0.000643017	0.390352351	0.128825363	0.150248519	0.228721629
0	0	0	0	0
0	0.098026763	0	0	0.049482438
0.001566738	0	0	0	0
0	0	0	0	0
0	0.889463338	1.063414456	0.00671314	0
0	0	0	0	0
0	0	0	0	0
0	0	0	0	0
0	0.053474185	0.022484233	0	0.017281527
0	0.01895233	0.005910941	0	0.006032621
0	0	0	0	0.0506305
0	0	0	0	0
0	0	0	0	0
0	0	0	0	0
0.110154629	0	0	0.001845794	0
0	0	0	0	0
0	0.163763538	0	0.003107631	0
0	0	0	0	0
0	0	0	0	0
0	0	0.002628185	0	0
0	0	0	0	0
0	0	0	0	0.003077362
0	0	0	0	0
0	0	0	0	0
0	0	0	0	0
0	18.25875325	0.00552932	0	0
0	0	0	0	0
0	0	0	0	0
0	0	0	0	0
0	0	0	0	0
0	0	0	0	0
0	0	0	0	0
0	0	0.153558456	0	0
0	0	0	0	0
0	0	0	0	0
0	0	0	0.005354757	0

0	0	0	0	0
0	0	0	0	0
0	0	0	0	0
0	0	0	0	0
0	0	0.073817883	0	0
0	0	0	0.034472252	0
0	0	0	0	0
0	0	0.002442225	0	0
0	0	0	0	0.00402138
0	0	0	0	0
0	0	0	0	0
0	0.002052165	0	0	0
0	0	0	0	0
0	0	0	0	0
0	0	0	0	0
0	0	0	0	0
0	0	0.000876732	0	0
0	0	0	0	0
0.009051653	0	0.36717753	0	0.005816948
0	0	0	0	0
0	0	0	0	0.056884285
0	0	0	0	0
0	0	0	0	0
0	0	0	0	0
0	0	0	0	0
0	0	0	0	0
0	0	0	0	0
0	0	0.001977746	0	0
0	0	0	0	0
0	0	0	0	0
0	0.008475893	0.098942473	0	0.01265231
0.00273368	0.013103927	0	0	0.04430965
0	0	0	0	0
0	0	0	0	0
0	0	0	0	0
0	0	0	0	0
0	0	0	0	0
0	0	0	0	0
0	0.607399752	0.465253902	0	0
0	0	0	0	0
0	0	0	0	0
0	0	0	0	0
0	0	0	0	0
0	0	0	0	0
0	0	0	0	0
0	0	0	0	0
0	0.004536889	0	0	0.029093924
0	0	0	0	0

0	0	0	0	0
0	0	0	0	0
0	0	0	0	0
0	0	0	0	0
0	0	0	0	0.0202177
0	0	0	0	0
0	0	0	0	0
0	0	0	0	0
0	0	0	0	0
0	0	0	0	0
0	0	0	0	0
0	0	0	0	0
0	0	0	0	0
0	0	0	0	0
0	0	0	0	0
0	0	0	0	1.457492679
0	0	0	0	0
0	0	0	0	0
0	0	0	0	0
0	0	0	0	0
0	0	0	0	0
0	0	0	0	0
0	0	0.020989033	0	0
0	0	0	0	0
0	0.002743801	0	0	0
0	0	0	0	0
0	0	0	0	0.001484251
0	0	0	0	0
0	0	0	0	0
0	0	0	0	0
0	0	0	0	0
0	0	0	0	0
0	0	0	0	0
0	0	0	0	0
0	0	0	0	0
0	0	0	0	0
0	0	0	0	0
0	0	0	0	0
0	0	0	0	0
0	0	0	0	0
0	0	0	0	0
0	0	0	0	0
0	0	0	0	0
0	0	0	0	0
0	0	0	0	0
0	0	0	0	0
0	0	0	0	0
0	0	0	0	0
0	0	0	0	0

0	0	0	0	0
0	0	0	0	0
0	0	0	0	0
0	0	0	0	0
0	0	0	0	0
0	0	0	0	0
0	0	0	0	0
0	0	0	0	0
0	0	0	0	0
0	0	0	0	0
0	0	0	0	0
0	0	0	0	0
0	0.004994732	0	0	0.006249748
0	0	0	0	0
0	0	0	0	0
0	0	0	0	0
0.004499748	0	0	0	0
0.003024792	0	0	0	0
0	0	0	0	0
0	0	0	0	0
0.031978141	0.013203342	0.007851309	0.0042777	0.008864882
0.032406646	0	0	0	0
0	0	0	0	0
0	0	0.008869614	0	0
0	0	0	0	0
0	0	0	0	0
0.014241359	0.006564049	0	0.000747369	0.003989358
0	0	0	0	0
0	0	0	0	0
0	0	0	0	0
0	0	0	0	0
0	0	0	0	0
0	0	0	0	0
0	0	0.017393593	0	0.009357786
0.022030605	0.012362368	0.001489973	0.019483097	0.041162295
0	0	0	0	0
0	0	0	0	0
0.000647036	0	0	0	0
0.000790872	0	0	0	0
0	0	0	0	0
0	0	0	0	0
0.000611165	0	0	0	0
0	0	0	0	0
0	0	0	0	0
0	0	0	0	0
0	0.159597566	0.664520712	0	0.031702265
0	0.001588069	0	0	0
0.000581467	0.000895908	0	0	0.001307857

0	0	0	0	0
0	0	0	0	0
0	0	0	0	0
0	0	0	0	0
0	0	0	0	0
0	0	0	0	0
0	0	0	0	0
0	0.001931825	0	0	0
0	0	0	0	0
0	0	0	0	0
0	0	0	0	0
0	0	0	0	0
0	0	0	0	0
0	0	0	0	0
0	0	0	0	0
0	0	0	0	0
0	0	0	0	0
0	0	0	0	0
0.002217555	0.016248521	0	0.097832265	0
0	0	0	0	0.41240843
0.009377208	0.007840073	0	0.004165723	0
0	0	0	0	0
0	0	0	0	0
0	0	0.008454543	0	0
0.004765764	0	0	0.006502642	0.005011637
0	0	0	0.00200775	0
0	0	0	0	0
0	0	0	0	0
0	0.005521051	0	0.006769708	0
0	0	0	0	0
0	0.564621228	0.103068968	0.02209775	0.097894949
0	0	0	0	0
0.163493942	0	0	0	0.032801601
0	0	0.002597692	0	0
0	0	0	0	0
0	0	0	0	0
0	0	0	0	0
0	0	0	0	0
0	0	0	0	0
0	0	0	0.000655621	0.001235159
0	0	0	0	0
0	0.015949842	0	0	0.354403619
0	0	0	0	0
0	0	0	0	0
0	0	0	0	0
0	0	0	0	0
0	0	0	0	0
0.000663314	0	0	0	0

0	0	0	0	0
0	0	0	0	0
0.05603019	0.075050988	0	0.001400189	0.022158277
0	0	0	0	0
0	0	0	0	0
0	0	0	0	0
0	0	0	0	0
0	0	0	0	0
0	0	0	0	0
0	0	0	0	0
0	0	0	0	0
0	0	0	0	0
0.002501174	0	0	0	0
0.00113413	0	0.000946739	0	0
0.005990883	0	0.000976762	0.002043563	0
0	0	0.006065365	0	0.028338121
0.002773093	0.1220459	0	0.003201629	0.001370845
0	0	0	0	0
0	0	0	0	0
0	0	0	0	0
0	0	0	0	0
0.001347937	0.001186779	0	0	0.001484978
0	0	0	0	0
0	0	0	0	0
0	0	0	0	0
0	0	0	0	0
0	0	0	0	0
0	0	0	0	0
0	0	0	0	0
0	0	0	0	0
0	0	0	0	0
0	0	0	0	0
0.018331906	0	0	0	0
0.000666072	0	0	0	0
0	0	0	0	0
0	0	0	0.002099156	0
0	0	0	0	0
0.001935478	0.011182984	0	0.305679612	0.009950513
0	0	0	0	0
0	0	0	0	0
0.001396721	0	0	0	0
0	0	0	0	0
0	0	0	0	0
0	0.004630338	0	0	0
0	0	0	0	0
0	0	0.001940692	0	0
0	0	0	0	0
0	0	0	0	0

0	0	1.670136846	0	0
0	0	0.001942913	0.481693797	0
0	0.000621529	0	0.004403221	0
0	0	0	0	0
0	0.002084394	0.004234875	0.001938153	0
0	0	0	0	0
0	0	0	0	0
0.011946575	0.006135647	0	0	0.003655875
0	0	0	0	0
2.86056511	0.001204599	0.028450918	0.055204171	0
0	0	0	0	0
0.031973503	0.001387714	0	0	0
0.003757513	0.009298237	0	0.00279651	0.001975684
0	0	0	0	0
0	0	0	0	0
0	0	0	0	0
0	0	0	0	0
0	0	0	0	0
0	0.011618433	0	0	0
0	0	0	0	0
0	0	0	0	0
0	0	0	0	0
0	0	0	0	0
0	0	0	0	0
0	0	0	0	0
0.016710442	0.095018591	0	0.001357175	0.002556854
0	3.866695869	0	1.857285214	0
0	0.013096572	0	0	0
0	0	0	0	0
0	0	0	0	0
0	0	0	0	0
0	0	0	0	0
0	0	0	0	0
0	0	0.000814431	0	0
0	0	0	0	0
0	0	0	0	0
0	0	0	0	0
0	0	0	0	0
0	0	0	0	0
0	0	0	0	0
0	0	0	0	0.0014384
0	0	0	0	0
0	0	0	0	0
0	0	0	0	0
0	0.009587421	0	0	0
0	0	0.017161772	0	0
0	0.003049371	0	0	0.00381558
0	0	0.001640164	0	0

0	0	0	0	0
0.007741316	0	0	0	0
0	0	0	0	0
0.010117549	0	0	0	0
0	0.028884014	0	0	0
0	0.01601166	0.001829868	0	0
0	0.012685411	0	0	0
0	0.001294319	0	0	0
0	0	0	0	0
0	0	0	0	0
0.016723457	0	0.005659569	0.002466844	0.005518807
0	0	0	0	0
0	0	0	0	0
0	0	0.001336719	0.006700331	0
0.022899203	0	0.015779395	0	0
0	0	0	0	0
0	0	0	0	0
0	0	0	0	0
0	0	0	0	0
0	0.002761621	0.302983263	0	0
0	0	0	0	0
0	0.108851605	0.275309568	0.030301801	0.043435881
0	0	0.012473412	0	0
0	0	0	0	0
0	0	0	0	0
0	0	0	0	0
0	0.003091269	0.008213026	0	0
0	0	0	0	0
0	0	0	0	0
0.064781856	0.118941041	0.155441791	2.825966935	0.098547675
0.001303625	0.032137406	0	0	0.002420197
0	0	0	0	0
0	0	0	0	0.002293468
0	0	0	0	0
0	0	0.001408375	0	0
0	0	0	0	0
0	0	0	0.146400187	0
0	0	0	0	0
0	0	0	0	0
0	0.002075997	0.044550678	0	0
0	0	0	0	0
0	0.424406438	0.169177372	0.006385268	0.030073858
0	0	0	0	0
0	0	0.003977251	0	0
0	0	0	0	0
0	0	0	0	0
0.003131854	0	0.010188406	0	0
0	0	0	0	0

0.001591134	0.048097539	0	0.004652187	0.0029215
0	0	0.000909324	0	0
0	0.926101888	0.063020523	0.00169039	0
0	0	0	0	0
0	0.019089539	0	0	0
0	0	0	0	0.010868576
0.002261216	0	0	0	0
0	0	0	0	0
0	0	0	0	0
0	0	0.067257219	0	0
0	0	0	0	0
0	0	0	0	0
0	0	0	0	0
0	0	0	0	0
0	0	0	0	0
0	0.307194806	0.032531198	0.000657808	0.124547523
0.030993072	0.25962394	0	0.157731103	0.005723381
0	0	0	0	0
0	0.007181489	0	0	0
0	0.124442097	0	0	0
0	0.000691755	0	0	0
0	0.004668997	0	0	0.003186636
0	0	0	0	0
0	0	0	0	0
0	0	0	0	0
0	0	0	0	0
0	0	0	0	0
0	0	0.000895934	0	0.001103558
0	0	0	0	0
0	0	0	0	0.004476327
0	0.012188338	0.009689916	0	0
0.006665975	0.001316762	0	0	0
0.018796616	0.002758218	0.051453879	1.329981304	0.012550072
0.002099017	0	0	0.013842673	0
0.000998776	0.001795367	0	0	0
0	0	0	0	0
0.000662082	0.000906771	0	0.004215759	0
0	0.020033112	0.084824577	0	0.00776405
0	0	0	0	0
0	0.029134533	0	0	0.002176424
0	0	0	0	0
0	0	0	0	0
0	0.081924614	0	0	0
0	0	0.113734127	0	0.00277065
0	0	0	0	0
0	0.002125796	0.002159497	0	0
0	0	0.00180114	0	0
0	0	0.001097726	0	0

0	0	0	0	0
0.284567509	0.037443687	0.003190223	0.037223328	0.136928989
0	0.005753624	0	0	0
0	0	0	0	0
0	0	0	0	0
0	0	0	0	0
0	0	0	0	0
0.001083857	0	0	0	0
0	0	0	0	0
0	0	0	0	0
0	0.005744465	0	0	0
0.006247592	0.025669632	0	0.013141969	0.007695317
0	0	0	0	0
0	0	0	0	0
0.001604161	0.001235823	0	0	0
0	0	0	0	0
0.009333174	0.007331128	0	0.011735844	0.020463302
0.000712691	0.003416293	0.008924018	0	0.001221342
0	0	0	0	0
0.001234092	0.000633818	0.008209287	0	0
0.004340622	0.02209917	0.005120063	0.00206002	0.005336347
0	0	0	0	0
0	0	0	0	0
0	0	0	0	0
0	0.001954249	0	0	0
0	0	0	0	0
0	0	0.182518404	0	0
0.011633893	0.005562984	0.174349167	0.02846346	0.006960783
0	0	0	0	0
0	0	0	0	0
0.015926812	0.005305855	0	0	0.002489642
0	0	0	0	0
0	0	0	0	0
0	0	0	0	0
0	0	0	0	0
1.981867147	0.155870863	0.12398133	0.168141278	0.071556906
0	0	0.007710779	0	0
0	0	0	0	0
0.017815914	0	0.00154919	0.002700989	0.010177072
0	0	0	0	0
0	0	0	0	0
0	0	0	0	0
0.000601203	0	0.048147916	0	0
0.001451126	0.001788683	0.001968459	0	0.00074604
0	0	0.008355087	0	0
0	0.675164547	0.254227939	0	0
0	0	0	0	0
0	0	0	0	0

0	0	0.0015337	0	0
0	0	0	0	0
0	0	0	0	0.012038441
0	0.611896805	1.046467841	0	0.958239708
0	0	0	0	0
0	0	0	0	0
0	0	0	0	0
0	0	0	0	0.071655402
0.012854621	0.67204589	0	0	8.670040925
0	0	0.247983548	0	0
0	0	0	0	0
0	0	0.001750094	0	0.002155662
0	0	0	0	0
0	0	0	0	0
0	0.031276181	0.003754873	0.179589876	0.537215162
0	0	0	0	0
0	0.283168527	0	0	0
0	0.769500086	0	0	0
0	0	0	0	0
0	0	0	0	0
0	0	0	0	0
0	0	0	0	0
0	0	0	0	0
0	0.1251639	0.134381921	0	0.080705677
0	0	0.003113216	0	0
0	0	0.064001033	0	0
0	0.004797948	0.021835561	0	0.000960563
0	1.460752409	0	0	2.8399175
0	0	0	0	0
0	0	0	0	0
0	0.639955764	0.023309036	0	0
0	0	0	0	0
0.000372283	0.00305922	0.007769294	0	0.005741856
0	0	0	0	0
0	0	0.188347525	0	0
0	0.005396288	0	0.132798893	0
0	0	0.91385558	0	0
0	0	0	0	0
0	0	0	0	0
0	0	0	0	0
0	0.002479053	0.14623237	0.003732104	0.077755777
0	0	0	0	0
0.184522849	0.272411829	2.64243244	0.07690107	0.11461237
0	0.002591495	0.002632578	0	0.007025751
0	0	0	0	0
0	0	0	0	0
0	0	0	0	0
0.018181149	0.002384085	0.004238289	0.016362228	0.014418455

0	0	0	0	0
0	0.016470763	0	0	0
0.001668704	0	0	0.075136414	0
0	0	0.014011861	0	0
0	0	0	0	0
0	0.001800361	0	0	0.070022491
1.042534454	0	0	0	0.532373625
0	0	0	0	0
0	0	0.1804016	0	0
0	0	0.187750178	0	0
0.036954373	0.003552797	0.01253694	0.063338424	0.178755912
1.562735142	0.197861446	1.315779076	0.374735975	0.312254445
0	0	0	0	0
0.046955454	0.506332462	0	0.162449372	0.043684916
0.004335044	0.000636125	0.001130866	0.036334669	0.025271817
0	0	0	0	6.713879562
0	0	0	0	0
0	0	0	0	0
0	0	0.066798215	0	0
0	0.018329567	0.014551877	0	0
0.000457732	0	0.137556675	0.158428043	0.233756471
1.138889115	0	0	0.384540491	0
3.684970057	0.073126898	0.224963793	0.058597187	0.047047246
0	0	0.012494304	0	0
0	0	0.029647696	0	0
0	0.496175661	0	0	0
0	0	0	0	0
0	0.000653844	0	0	0
0	0	0	0	0.029525956
0	0	0.210602607	0	0
0	0.017286315	0.007199745	0	0.00454226
0	0.066138125	0	0	0.021846238
0	0.005377201	0.047016046	0	0.081460714
0	0.002196714	0.004909383	0	0.077329448
0	0.004182773	0.00090132	0	0.079299532
4.62571439	0.075580102	0.002830535	0.12537973	0.151444266
0	0.000672897	0	0.009608763	0.025048745
0	0	0	0	0
0	4.990767554	0	2.424663266	0.816743681
0	0	0	0	0
0	0	0.007633063	0	0
0	0	0	0	0
0	0	0	0	0
0	0	0.004050367	0	0
0.007346939	0.01012838	0.000806976	0.01556439	0.060881617
0	0	0.001715254	0	0
0	0	0	0	0
0.74560906	0.031622008	0.002676942	0.27207706	0.01378871

0	0	0	0	0
0	0	0	0	0
0	0	0	0	0
0	0	0.020805416	0	0.010166038
0	0	0	0	0
0	0	0.073652711	0	0
0	0	0.006965164	0	0.677531218
0	0.009968125	0.0030062	0	0.83879541
0	0	0	0	0
0	0	0	0	0
0	0.008671615	0	0	0.001179404
0	0	0	0	0
0	0.003961397	0.001676749	0.010962697	0
0	0	0	0	0
0	0	0	0	0
0	0	0	0	0
0	0.687982596	0	0	0
0	0.004551059	0	0.000833844	0.055375021
0	0	0.234183327	0	0
0	0	0.000575085	0	0
0	0.000836536	0	0	0.008722756
0	0	0	0	0
0	0.19703562	0	0	0.024224283
0	0	0	0	0
0	0	0	0	0
0	0	0	0	0
0	0	0	0	0
0	0	0	0	0
0	0	0	0	0
0	0.032796261	0	0	0
0	0	0	0	0
0	0	0	0	0
0	0	0	0	0
0	0	0	0	0
0	0	0	0	0
0	0	0	0	0
0	0.034694341	0.036341447	0	0.041047062
0	0.005425716	0	0	0
0	0	0	0	0
0	0	0.000959376	0	0.001378654
0	0	0.033613879	0	0.049084809
0	0	0	0	0
0	0	0	0	0
0	0	0	0	0.002714743
0	0.056534804	0	0.00385116	0
0	0	0	0	0
0	0	0	0	0.005502076
0	0	0	0	0

0	0	0	0	0
0	0	0	0	0
0	0	0	0	0
0	0	0.111263614	0	0.348975786
0	0	0	0	0
0	0	0	0	0
0	0	0	0	0
0	0	0.03570602	0	0
0.001928371	0.001760698	0.104857294	0.007308787	0.000826164
0	0	0	0	0
0	0	0.015666675	0	0.002953667
0	0	0	0	0
0	0	0	0	0
0	0	0	0	0
0	0	2.874508407	0	0.208587864
0	0	0.317062338	0	0
0	0.002819672	0	0	0
0	0	0	0	0
0	0	0	0	0
0	0	0	0	0
0	0	0.286351567	0	0.048642534
0	0	0	0	0
0	0	0	0	0
0	0	0.022836018	0	0
0.034015635	0.172630535	0	0.820987238	0.000971546
0	6.689954224	1.746086732	0.095592686	4.323736423
0	0	0.295913868	0	1.165739392
0	0	0	0	0
0	0.375850315	0	0	0
0	0	0	0	0
0	0	0	0	0
0	0.058671074	0	0	0.011470817
0	0	0.000636595	0	0
0	0	0	0.002386926	0.005707547
0.045675095	0.406990021	0	0.028135359	0.003989675
0	0.00579571	0	0	0.000805776
0	0	0	0	0
0	0.592628006	0.330189238	0	0.116007224
0	0	11.40004599	0.125319905	0.03689012
0	0	0	0	0
0	0	0	0	0
0	0	0.009273018	0	0
0.130433686	0.844353706	0.030841064	0	0.156907819
0	0	0	0	0
0	0	0	0	0
0	0.000610079	0	0	0.005725288
0	0	0	0	0
3.082987075	0.001700875	0.608692819	0.029048726	0.588613093

0	0.002174304	0.000552193	0	0
0	0	0	0	0
0	0	0	0	0
0	0	0	0	0
0.000702496	0	0	0.00311519	0.003160161
0	0.002953244	0.009176659	0	0
0	0	0	0.03128576	0
0	0	0.069114954	0	0
0	0	0	0	0
0	0	0.002759059	0	0
0	0	0	0	0.001036319
0	0	1.429120913	0.019233434	0.532277874
0.026793886	0.108122809	0.233652978	0	0.780859052
0	0	0	0	0
0.067229133	0.010014234	0	0.011639547	0
0	0	0	0	0
0	0	0	0	0
0	0	0	0	0
0	0.000914317	0.001857622	0	0
0	0	0.00617942	0	0
0	0	0.023069116	0	0
0	0	0	0	0
0	2.435637897	0.143960952	0	4.446192568
0	0	0	0	0
0	0	0	0	0
0	0	0	0	0
0	0	0	0	0
0	0	0	0	0
0	0	0	0	0
0	0	0	0	0
0	0	0	0	0
0.028728317	0.014889954	0	0.012226999	0.039972651
0	0	0	0	0
0.000526685	0.023533545	0	0.016348947	0.042477809
0	0.006131985	0	0	0
0.05474938	0.002484389	0	0.876032016	0.365405969
0	0	0	0	0.005254097
0	0	0	0.042282452	0
0	0.003421734	0	0	0
0	0	0	0	0
0	0	0.0896175	0	0
0	0	0	0	0
0	0	0	0	0
0	0	0.015213415	0	0.013920392
0.007392974	0.012944186	0.001051951	0.04332969	0.1872332
0	0	0	0	0
0	0	2.277773736	0	0.092121834
0	0.001305908	0	0	0.000612765

0	0	0	0	0
0	0	0	0	0.015922412
0	0	0.013055768	0	0
0	0	0	0	0
0	0	0	0	0
0	0	0.009203793	0	0
0.127207878	0.738014539	0	0	0
0	0	0.118847044	0	0.004218697
0	0	0	0	0
0	0	0	0	0.089254225
0	0	0	0	0
0	0.002179437	0	0.001206267	0
0	0	0	0	0
0	0.161015719	0	0	0
0	0	0	0	0
0	0	0	0	0
0	0	0	0	0
0	0	0	0	0
0	0	7.499371399	0	0.000715976
0	0	0	0	0
0	0	0	0	0
0	0	0	0	0
0	0	0	0	0
0	0	0	0	0
0	0.003394235	0	0	0.00070785
0.0014078	0.01092583	0	0.017928135	0.066948376
0	0	0	0	0
0	0	0.163755036	0.003133585	0.172776541
0	0	0	0	0
0	0.584597087	0.648879652	0.118212245	0.75145052
0	0	0	0	0
0	0	0	0	0
0	0	0	0	0.01371395
0	0	0	0	0
0	0.002020517	0	0.000670985	0.000632052
0	0	0	0	0
0	0	0	0	0
0	0	0	0.002791695	0.001195323
0	0	0	0	0
0	0	0	0	0
1.375618154	1.411794284	3.675944527	1.720931387	1.85228682
0	0	0.449232748	0.076911159	0.316743401
0	0	0	0	0
0	0	0.135416978	0	0
0	0	0	0	0
0	0	0	0	0
0	0	0.006827678	0	0
0.003838162	0.001819609	0.000616152	0.001208533	0.06849429

0	0	0	0	0
0	0.022771356	0.009664885	0	0.00097579
0	0	0	0	0.015955346
0	0	0	0	0
0.087881029	5.134303316	0.485610309	0.008107067	0.63046884
0	0	0	0	0
0	0	0	0	0
0	0	0	0	0
0.001971023	0.023204998	0.013131193	0	0.007015329
0	0	0.001536681	0	0
0.000857744	0	0	0	0
0	0	0	0	0
0	0	0.081902561	0	0.017825149
0.002358001	0.009129431	0.002460491	0.120280216	0.004196338
0	0	0	17.46417153	8.296783179
0	0.096625642	0.350229471	0	0.202974268
0	0	0.097286849	0	0.002017377
0	0	0	0	0
0	0	0	0	0
0	0	0	0	0
0	0	0	0	0
0	0	0	0	0
0	0	0	0	0
0	0	0	0	0
0	0	0	0	0
0	0	0	0	0
0	0	0.003133145	0	0.154368946
0	0.017084775	0	0.000840535	0.005146466
0	0	0	0	0
0	0	0	0	0
0	0	0	0	0
0	0	0.002818914	0	0
0	0	0	0	0
0	0	0	0	0.032576717
0.004200684	0.518568024	0	0	0
0	0	0.004833315	0	0
0	0	0	0	0
0	0	0.006319412	0	0.002594627
0	0	0	0	0
0	0	0	0	0
0	0.147024476	1.409995437	0	0
0	0.093532887	0	0	0
0	0	0	0	0
0	0	0.00255688	0	0
0	0	0	0	0
0	0	0	0	0
0	0	0	0	0
0	0	0	0	0

0	0	0.003167129	0	0
0	0	0	0	0
0	0	0	0	0
0	0	0	0	0
0	0	0.037448348	0	0
0	0	0	0	0.042177258
0	0	0.064046655	0.010254892	0.050915541
0	0	0	0	0
0	0	0	0	0
0	0.009280793	0.077308952	0	0
0	0	0.009708382	0	0.046856675
0	0	0.041293094	0	0.273143431
0	0	0	0	0
0	0	0.837221041	0	0
0	0	0	0	0.001977421
0	0	0	0	0
57.98604524	0	0	0.010621081	0
0	0	0	0	0
0	0	0	0	0
0	0	0	0	0
0	0	0.001399959	0.69379945	0.196963309
0	0	0	0	0
0	0	0	0	0.058750444
0	0.003003659	0.000762819	0.010573207	0.022926127
0	0	0	0.001192496	0
0	0	0.000539973	0	0
0	0	0	0	0
0	0	0	0	0
0	0	0.194312476	0	0
0.329321047	0.314712136	0	0.152747478	0
0	0	0	0	0
0	0	0	0	0
0	0	0	0	0
0	0	0	0.006178394	0.056794207
0	0	0	0	0
0	0	0	0	0
0	0.007328792	0.056505448	0	0.069600086
0	0	0	0	0
0	0	0	0	0
0	0	0	0	0
0	0	0	0	0
0	0	0	0	0
0	0	0	0	0
0	0	0	0	0
0	0	0.146342213	0	0.30955027
0	0	0	0	0
0	0	0	0	0
0	0	0	0	0

0	0.059954402	0	0.006010568	0.003437529
0	0	0	0	0
0	0	0	0	0
0	0	0	0	0
0	0	0	0	0
0	0	0	0	0
0	0	0	0	0.005440668
0.105161324	0.191316019	0.614567401	0	0.104225991
0.681078764	4.372064217	0.003092161	28.74250746	0.001269581
0	0.317155089	0	0	0
0	0.010982919	0	0	0
0	0	0	0	0.025724152
0	0	0.023303405	0	0
1.042616213	0	0.028042468	0.143182607	0
0	0	0	0	0
0	0	0	0	0
0	0	0	0	0.122494998
0	0	0	0	0
0	0.00579865	0	0	0
0	0	0.014131611	0.071580605	0.003114845
0	0	0	0	0
0	0	0	0	0
0	0	0	0	0
0	0	0	0	0
0	0.01641965	0.095036918	0	0.002070465
0	0	0	0	0
0	0	0	0	0
0	0	0	0	0
0	0	0.004254832	0	0
0	0	0	0	0
0	0	0	0	0
0	0	0	0	0
0	0	0	0	0
0	0	0	0	0
0	0	0	0	0
0	0	0	0	0
0	0	0	0	0
0	0	0	0	0
0	0.003074522	0	0	0
0	0	0	0	0.003027998
0	0	0	0	0
0	0	0.214560793	0	0.339882151
0	0	0	0	0
0	0	0	0	0
0	0	0	0	0.008458714
0	0	0.004963156	0	0
0	0	0	0	0
0	0	0	0	0

0	0	0	0	0
0.001135948	0	0.084009677	0	0
0	0	0	0	0
0	0	0	0	0
1.781977208	0.077898597	0.109378263	1.810560381	19.81488581
0	0	0	0	0
0	0	0	0.042286215	0
0.001801169	0.002877975	0.012320883	0.001365335	0
0	0	0	0	0.004879719
0	0	0	0	0
0	0	0	0	0
0	0	0	0	0
0	0	0	0	0
0	0	0	0	0
0	0	0	0	0.000724303
0	0.002317757	0.000905577	0	0.039263369
0	0	0	0	0
0	0	0	0	0
0	0	0	0	0
0	0	0	0	0
0	0	0	0	0
0	0	0	0	0
0	0	0	0	0
0	0.00195694	0	0	0
0	0.137549094	0	0.171358585	0
0	0	0	0	0.00101442
0.00966285	0.00162713	0.000826463	0.123631371	0.275874741
0	0	0.000522143	0	0
0	0.003627793	0	0	0
0	0	0	0	0
0	0	0	0	0
0	0	0	0	0
0	0	0	0	0
0	0	0	0	0
0	0	0	0	0
0	0	0	0	0
0	0	0	0	0
0	0	0	0	0.040298644
0	0	0	0	0
0	0	0	0	0.000716523
0	0	0	0	0
0	0.113274446	0	0	0
0	0	0	0	0
0	0	0	0	0
0	0	0	0	0
0	0	0	0	0
0	0	0	0	0
0	0	0	0	0

0	0	0.030777786	0	0.098030587
0	0	0	0	0
0	0	0	0	0
0	0	0	0	0
0	0	0	0	0
0	0.048929206	0	0	0
0	0	0	0	0
0	0	0	0	0
0	0	0	0	0
0	0	0	0	0
0	0	0	0	0
0	0	0	0	0
0	0	0	0	0
0	0	0	0	0
0	0	0	0	0.009018618
0	0	0	0	0
0	0	0	0	0
0	0	0	0	0
0	0	0	0	0
0	0	0	0	0
0	0.04163258	0.000539905	0	0
0	0	0	0	0
0.060118189	0.005146022	0.002045583	0.028531546	0.558237769
0	0	0.799231705	0	0
0	0	0.025314599	0	0
0	0	0	0	0
0	0	0	0	0
0	0	0	0	0
0	0	0	0	0
0	0	0	0	0
0	0	0	0	0
0	0	0	0	0
0	0.701302452	0.00830128	0.392166903	0.008651947
0	0	0	0	0
0.029924339	0.131384975	0.193061851	0	0.743659641
0.013258978	0	0.001195641	0	0
0	0	0	0	0
0	0	0	0	0
0	0.011085413	0	0	0.00973391
0	0	0	0	0
0	0	0	0	0
0	0	0	0	0.023757614
0.065309732	0.084140401	0	0.115392217	0.897743986
0	0	0	0	0
0	0	0	0	0
0	0.071156476	0.004036191	0	0.2652996
0	0	0	0	0
0	0	0	0	0

0	0	0	0	0
0	0	0	0	0
0	0	0	0	0
0	0	0.10493215	0	0
0	0	0	0	0
0	0	0	0	0
0	0	0	0	0
0	0	0	0	0
0	0.008798693	0	0	0.380040225
0	0.221359285	0.001133791	0	0
0	0	0	0	0
0	0	0.012907652	0	0
0	0	0	0	0
0	0	0	0	0
0	0	0.003206901	0	0
0	0	0.001115525	0	0
0	0	0	0	0
0	0	0	0	0
0	0	0	0	0
0	0	0	0	0.134020173
0	0	0.002524354	0	0
0	0	0	0	0
0	0	0	0	0
0	0.009263852	0.031946888	0	0.001525205
0	0	0	0	0
0	0	1.724535139	0	0
0	0	0	0	0
0	0	0.015756528	0.36535192	0
0	0	0	0	0
0	0	0	0	0
0.044024293	0.007288898	0.14808896	0.008298992	0.329449864
0	0	0	0	0
0	0	0	0	0
0	0	0	0	0
0	0	0	0	0
0	0	0	0	0
0	0	0.000762026	0	0
0	0	0	0	0
0	0.002850058	0	0.001781579	0
0	0	0	0	0
0	0	0	0	0
0	0	0	0	0
0	0	0.008008562	0	0.001811842
0	0	0	0	0
0	0	0	0	0
0	0	0	0	0
0.05362497	0	0	0	0.000703296
0	0	0	0	0

0	0	0	0	0
0	0	0	0	0
0	0	0	0	0
0	0	0	0	0
0	0	0.20361256	0.00873854	0.006129839
0	0	0	0	0
0	0	0	0	0
0	0	0	0	0
0	0	0	0	0
0	0	0	0	0
0	0	0	0	0
0.003594494	0.002914891	0.00118444	0.014197245	0.079268196
0.021055124	0	0	0	0.007342322
0	0	0.066586302	0	0.063145901
0	0	0.000451313	0	0
0	0	0	0	0.00226904
0	0.003736495	0.002732925	0	0.004114314
0	0	0	0	0
0	0.002207172	0.001494775	0	0
0	0	0.017760658	0.001786468	0.008894854
0	0	0	0.000764198	0
0	0	0	0	0
0	0	0	0	0
0	0	0	0	0
0	0	0	0	0
0	0	0	0	0
0	0	0.031749264	0	0.000770579
0	0	0	0	0
0	0	0	0	0
0	0	0.000447892	0	0
0	0.00261782	0	0	0
0	0	0.013978891	0	0
0	0	0	0	0
0	0	0	0	0
0	0	0	0	0
0	0	0	0	0
0	0	0	0	0
0	0.005885453	0.000747344	0.118245758	0.036514545
0	0	0	0	1.046871454
0	0	0	0	0
0	0.002570566	0	0.002601595	0.005207612
0	0	0	0	0
0	0	0	0	0
0	0	0	0	0
0	0	0	0	0
0	0	0	0	0
0	0	0	0	0
0	0	0	0	0

0	0	0	0	0
0	0	0.002683734	0	1.254597368
0	0	0	0	0
0	0.001143292	0	0	0.005006975
0.001204379	0	0	0.000704277	0.002211376
0.002442577	0	0.001853631	0	0
0	0	0	0	0.047200633
0	0	0	0.001583656	0
0	0	0.107874938	0	0
0	0	0.001118403	0	0
0	0	0	0	0.0157613
0	0	0	0	0
0.003418946	0.428854083	0.002607055	0.001794222	0.179659162
0	0	0	0	0
0	0	0	0	0
0	0	0	0	0
0	0	0	0	0
0	0	0	0	0
0	0	0	0	0
0	0	0	0	0
0	0	0	0	0
0	0	0	0	0
0	0.029702436	0	0	0
0	0	0.006822618	0	0.074582836
0	0	0	0	0
0	0	0.001889463	0	0.015515533
0	0	0	0	0.032744469
0	0	0	0	0
0	0	0	0	0
0	0	0	0	0
0	0.008329099	0.022160127	0.003687967	0.054839224
0	0	0	0	0
0	0	0	0	0
0	0	0	0	0
0	0	0	0	0
0	0	0	0	0
0	0	0	0	0
0	0.004899334	0	0	0
0	0	0	0.006463429	0
0	0	0	0	0
0	0	0	0	0
0	0	0.001242875	0	0
0	0	0	0	0
0	0	0	0	0
0	0	0	0	6.693649983
0.001293455	0.418070079	0.212348852	0.048827612	0.10639681
0	0	0	0	0
0	0	0	0	0

0	0	0	0	0
0	0	0	0	0
0.322834801	0	0.279645545	0	0
0	0	0	0	0
0	0	0	0	0
0	0	0	0	0
0	0	0	0	0
0	0	0	0	0
0	0	0	0	0.013792085
0	0	0	0.003052324	0
0.00185524	0	0.000829662	0	0.011581857
0	0	0	0	0
0	0	0	0	0
0	0	0	0	0
0	0	0	0	0
0	0	0	0	0
0.107721337	0	0	0	0
0	0	0	0	0
0	0	0	0	0
0	0	0	0	0.001355253
0	0	0	0	0
0	0	0	0	0
0	0	0	0	0
0	0	0	0	0
0	0	0	0	0
0	0	0	0	0
0	0	0	0	0
0	0	0	0	0
0	0	0	0	0
0	0	0	0	0
0	0.002636411	0	0.001236021	0
0	0	0	0	0
0.009466324	0.003781411	0.013719133	0.085869576	0.01487061
0	0.001840091	0	0.002199846	0.00391416
0	0	0	0	0
0	0	0	0	0
0	0	0	0	0
0	0	0	0	0
0	0	0	0	0
0	0	0	0	0
0	0	0	0	0
0.001442196	0	0.004966107	0	0.025765369
0	0.008482365	0.071868071	0.011507226	0.032066955
0	0	0	0	0
0	0	0	0	0
0.085310039	0.044075225	0	0.066514965	0.610891337
0	0	0	0	0
0	0	0	0	0

0	0	0	0	0
0	0.003025158	0.00691451	0	0
0.005590319	0	0	0	0.003039857
0	0.897845008	0	0	0
0	0.002645149	0	0.01218071	0.034421821
0.001503984	0.009612484	0	0	0
0	0	0	0	0
0	0	0	0	0
0	0	0	0.107301218	0
0	0	0	0	0
0	0	0	0	0
0	0	0	0	0
0	0	0	0	0
0	0	0.049407568	0	0
0	0	0	0	0
0	0	0	0	0
0	0	0	0	0
0	0	0	0	0
0	0	0	0	0
0	0	0	0	0
0	0	0	0	0
0	0	0.001082819	0	0
0	0	0	0	0
0	0	0	0	0
2.404608589	0.027848573	0	0	0.040312067
0.001426075	0.002197255	0	0.009242579	0
0	0	0	0	0
0	0	0.023095504	0	0.031781396
0	0	0	0	0
0	0	0	0	0
0	0	0	0	0
0	0	0	0	0
0	0	0	0	0
0	0	0	0	0
0	0	0	0	0
0	0	0	0	0
0	0	0	0	0
0	0	0	0	0
0	0	0	0.801391662	0.092567354
0	0	0	0	0
0	0	0	0	0
0	0.061578485	0	0.025594659	0.007208014
0	0.055680936	0	0.00610259	0.003908977
0	0	0	0	0
0	0	0	0	0
0	0	0	0	0
0	0	0.000856746	0.013443507	0
0	0	0	0	0

0	0	0.030784124	0	0
0.020202482	0.011351305	0.001261231	0.022853244	0.029294767
0	0	0	0	0
0	0	0	0	0
0	0	0	0	0
0	0	0	0	0
0	0	0	0	0
0	0	0	0	0
0	0	0	0	0
0	0	0	0	0
0	0	0	0	0
0	0	0	0	0
0	0.001499917	0.007999399	0	0
0	0	0	0	0
0	0.002653756	0.001347913	0	0.110685331
0	0	0	0	0
0	0	0	0	0
0	0	0	0	0
0	0	0.014911408	0	0
0	0	0	0	0
0	0	0	0	0
0	0	0.03618269	0	0.01005491
0	0	0.005764127	0	0.017540961
0	0	0	0.015451657	0
0	0	0	0	0
0	0	0	0	0
0	0	0	0	0
0	0	0	0	0
0	0	0	0	0
0	0	0	0	0
0	0	0	0	0
0	0	0	0	0
0	0	0	0	0
0	0	0	0	0
0	19.72710864	31.07266023	0.001571618	0
0	0	0	0	0
0	0	0	0	0
0	0	0	0	0
0.041595479	0	0.054199192	0.014404798	0.015740016
0	0	0	0	0
0	0.007195201	0.055313367	0.038747421	0.046475588
0	0	0	0	0
0	0	0	0	0
0	0	0	0	0
0	0	0	0	0
0.000650272	0.018257225	0.017868119	0.094937161	0.102243865
0	0	0	0	0
0	0	0	0	0

0	0	0	0	0
0	0	0	0	0
0	0	0.155087149	0	0
0	0	0	0	0
0	0	0	0	0
0	0	0	0	0
0	0	0	0	0
0	0	0.000977869	0	0
0	0.002262442	0	0	0.23232422
0.001271934	0.114537107	0	0.010702186	0
0	0	0.099848249	0	0
0	0	0	0	0
0	0.002554001	0.000648622	0	0
0	0	0	0	0
0	0	0	0	0
0	0	0	0	0
0	0	0	0	0
0	0	0	0	0
0	0	0	0	0
0.742121928	0.198150853	0.070240558	3.242343222	1.783696881
0	0	0.01501501	0	0
0	0	0	0	0
0	0	0	0	0
0	0	0	0	0
0	0	0	0	0
0	0	0	0	0
0	0	0	0	0
0	0	0	0	0
0	0	0	0	0
0	0	0.00761169	0	0
0	0	0	0	0
0	0	0	0	0
0	0	0	0	0
0	0	0	0	0
0	0	0	0	0
0	0	0.001494654	0	0.002454703
0	0	0	0	0
0	0	0	0	0
0	0	0	0	0
0	0	0	0	0
0	0	0	0	0
0	0	0	0	0
0	0	0.949544054	0	0
0	0	0	0	0
0.036722161	0.001245482	0.001265226	0.008272136	0.106195962
0	0	0	0	0
0	0	0	0	0
0	0	0	0	0

0	0	0	0	0
0	0	0	0	0
0	0	0	0	0
0	0	0	0	0
0	0	0	0	0
0	0.002258087	0.001889081	0.014997573	0.073628471
0	0	0	0	0
0	0	0	0	0
0	0	0	0	0
0	0	0	0	0
0	0	0	0	0
0	0	0	0	0
0	0	0	0	0
0	0.000679307	0	0	0
0	0	0	0	0
0	0	0	0	0
0	0	0.272517952	0	0.000806418
0	0	0	0	0
0	0	0	0	0
0	0	0	0	0
0	0	0	0	0
0	0	0	0	0
0	0	0	0	0
0	0	0	0	0
0	0	0	0	0
0	0	0	0	0
0	0	0	0	0
0	0	0.328722723	0	0
0	0	0	0	0
0	0	0.095135189	0	0
0	0	0	0	0
0	0	0	0	0
0	0	0	0	0
0	0	0	0	0
0	0	0	0	0
0	0	0	0	0
0	0	0	0	0
0	0	0.047343057	0	0
0	1.862808046	0	0	0.928829546
0	0	0	0	0
0	0	0	0	0
0	0	0	0	0
0	0	0	0	0
0	0	0	0	0
0	0.000553207	0	0	0
0	0	0	0	0
0	0	0	0	0
0	0	0	0	0

0	0	0	0	0
0	0.006692057	0.062996151	0	0.132302186
0	0.001638615	0	0	0
0	0	0	0	0
0	0	0	0	0
0	0	0	0	0
0	0	0	0	0
0	0	0	0	0
0	0	0.08798649	0	0
0	0	0	0	0.004042413
0.001516594	0.051797403	0	0.098551134	0.006335049
0	0	0	0	0
0	0.013482076	0	0	0
0	0	0	0	0
0	0	0	0	0
0	0	0	0	0
0	0	0	0	0
0	0	0	0	0
0	0	0	0	0
0	0	0	0	0
0.011159575	0.007905451	0	0	0.571995432
0	0	0	0	0
0	0	0	0	0
0	1.357090007	1.081727506	0.028272237	1.4594214
0	0	0	0	0
0	0	0	0	0
0	0	0	0	0
0	0.001134954	0.059953198	0	0
0	0	0	0	0
0	0	0	0	0
0	0.001357859	0	0	0
0	0	0	0	0
0	0	0	0	0.001300658
0	0	0	0	0

PHL018-0251_V1	PHL018-0042_V3	PHL018-0248_V1	PHL018-0261_V1	PHL018-0055_V3
REF	CTRL	REF	REF	EYCF
0.077231429	0.045404955	0.083258236	0.363388413	0.015676306
26.48355036	0.100679742	0.311379608	2.917898435	0.193658013
0	0	2.830300359	19.16276109	0
0	0	0	0	0
0	0	0	0	0
0	0	0	0	0.303410181
0	0	0	0	0
0	0	0	0	0
0	0	0	0	0
0	0	0	0	0
0	0	0	0	0
0	0	0	0	0
0	0	0	0	0
0	0.012162785	0.081086937	0.173031293	0.025860353
0	0	0	0	0
0	0	0	0	0
0	0	0	0	0
0	0	0	0	0
0	0	0	0	0
0	0	0	0	0
0	0	0	0	0
0	0	0	0.048377836	0
0	0	0	0	0
0	0	0	0	0
0	0.037381081	0	0	0.020745408
0	0	0	0	0
0	0	0	0	0
0	0	0	0	0.096316218
0	0	0	0	0
0	0	0	0	0
0	0	0	0	0
0	0	0	0	0
0	0	0	0	0
0	0	0	0	0
0	0	0	0	0
0	3.566371639	3.976728655	0	1.878580689
0	0	0	0	0
0	0	0	0	0
0	0	0	0	0
0	0	0	0	0
0	0	0	0	0
0.001951962	0	0.001822943	0.002733059	0.246573977
0.319060042	2.791925128	0.091796248	0.495901835	0.268811344
0.14216587	0.006556875	0.006114217	0.032939298	0.043980658
0	0.001609285	0	0	0
0	0	0	0	0

0	0.017467474	0.006601733	0	0.000529119
0.114191433	0	0.009956905	0.002016583	0
0.001984754	0	0.001733205	0	0
1.925405466	0.291903347	0.179204172	1.069962011	1.051774584
0	0	0	0.003204719	0.004184373
0	0.051444343	0	0	0.069605635
0	0	0.005804176	0	0.002861206
0	0	0	0	0.012909692
0	0.009275796	0	0	0.003462182
0	0	0	0	0
0	0	0.012730184	0	0.004301827
0.014525882	0.023878005	0.111653256	0	0.025148536
0	0	0.000486601	0	0.000901341
0.162412374	0.027810975	0.024940612	0.045996645	0
0	0	0	0	0
0	0	0	0	0
0.001804296	0	0.041543827	0	0
0	0	0	0	0
0	0	0	0	0
0	0	0	0	0
0.637802833	0	0.200067843	0.054212476	0
0.021153777	0.007102924	0.038047764	0.07525336	0
0	0.006741102	0.005670106	0	0
0	0	0	0	0
0	0	0	0	0
0	0	0	0.001016816	0
0	0	0	0	0
0	0	0	0	0
0	0	0	0	0.00709301
0	0	0	0	0
1.368632499	0	0	0	0
0	0	0	0	0
0	0	0	0	0
0	0	0	0	0
0	0	0	0	0
0	0	0	0	0
0	0	0	0	0
0	0	0.002581086	0	0
0	0	0	0	0
0	0	0	0	0
0	0	0	0	0
0	0	0	0	0
0	0	0	0	0
0	0	0	0	0
0	0	0	0	0
0	0	0	0	0
0	0	0	0	0
0	0	0	0	0

0	0	0	0	0
0	0	0.000558837	0	0
0	0	0	0	0
0	0	0	0	0
0	0.005130852	0	0	0
0	0	0	0	0
0	0	0	0	0
0	0.001410921	0	0	0
0	0	0.011572913	0	0.005537826
0	0	0	0	0
0	0	0	0	0
0	0.001226269	0	0	0.00207397
0	0	0	0	0
0	0	0	0	0
0	0	0	0	0
0	0	0	0	0
0	0	0	0	0
0	0	0	0	0
0	0.035740792	0.349951587	0	1.781135535
0	0.053284383	0	0	0
0	0	0	0	0
0	0	0	0	0
0	0.000722052	0	0	0
0	0	0	0	0
0	0	0	0	0
0	0	0	0	0
0	0	0	0	0
0	0	0	0	0
0	0	0	0	0
0	0	0	0	0
0	0.011851093	0.257616627	0.045331139	0.002149002
0.031612369	0.001281735	0.007489948	0.096553727	0.004682403
0	0	0	0	0
0	0	0	0	0
0	0	0	0	0
0	0	0	0	0
0	0	0	0	0
0	0	0	0	0
0	0	0.078055144	0	0
0	0	0	0	0
0	0	0	0	0
0	0	0	0	0.001657821
0	0	0	0	0
0	0	0	0	0
0	0	0	0	0
0	0	0	0	0
0	0	0	0.775606858	0
0	0	0	0	0

0	0	0	0	0
0	0	0	0	0
0	0	0	0	0
0	0	0	0	0
0	0.089359978	0	0	0.000754723
0	0	0	0	0
0	0	0	0	0
0	0	0	0	0
0	0	0	0	0
0	0	0	0	0
0	0	0	0	0
0	0	0	0	0
0	0	0	0	0
0	0	0	0	0
0	0	0	0	0
0	0.008766358	0	0	6.872092249
0	0	0	0	0
0	0	0	0	0
0	0	0	0	0
0	0	0	0	0
0	0	0	0	0
0	0	0	0	0
0	0	0	0	0
0	0	0	0	0
0	0	0	0	0.301974713
0	0	0	0	0
0	0.043300757	0	0	0.01902669
0	0	0	0	0
0	0	0	0	0
0	0	0	0	0
0	0	0	0	0
0	0.03149941	0.000432089	0	0.001400644
0	0	0	0	0
0	0	0	0	0
0	0	0	0	0
0	0	0	0	0
0	0	0	0	0
0	0	0	0	0.099221413
0	0	0	0	0
0	0	0	0	0
0	0	0	0	0
0	0	0	0	0
0	0	0	0	0
0	0	0	0	0
0	0	0	0	0
0	0	0	0	0
0	0	0	0	0
0.046450929	0	0	0	0

0	0	0	0	0.071883561
0	0	0	0	0
0	0	0	0	0
0	0	0	0	0
0	0	0	0	0
0	0	0	0	0
0	0	0	0	0
0	0	0	0	0
0	0	0	0	0
0	0	0	0	0
0	0	0	0	0
0	0.000430653	0	0	0.000776915
0.003517246	0.002122665	0.001240401	0	0
0	0	0	0	0
0	0	0	0	0
0	0	0	0	0
0	0	0	0	0
0	0	0	0	0
0	0	0	0	0
0	0	0	0	0
0.158339585	0.011377439	0.002576946	0.194643322	0.013961994
0	0	0	0	0
0	0	0	0	0
0.002110113	0	0	0	0.001119965
0	0	0	0	0
0	0	0	0	0
0.025041935	0	0.001350677	0.006930042	0.005629248
0	0	0	0	0
0	0	0	0	0
0	0	0	0.015887885	0
0	0	0	0.00814166	0
0	0	0	0	0
0	0	0	0	0
0	0.0413957	0	0	0.003719383
0.020736495	0.001549417	0.025653489	0.029863962	0.003028139
0	0	0	0	0
0	0	0	0	0
0	0	0	0	0
0	0	0	0	0.00225775
0	0	0	0	0
0	0	0	0	0
0	0	0	0	0.006513661
0	0	0	0	0.002905302
0	0	0	0	0
0	0	0	0	0
0	0	2.110440393	0	0
0	0	0.001815419	0	0
0.010789838	0.000490736	0.006930192	0.010246825	0

0	0	0	0	0
0	0	0	0	0
0	0	0	0	0
0	0	0	0	0
0	0	0	0	0
0	0	0	0	0
0	0	0	0	0.021738831
0	0	0	0	0.002386207
0	0	0	0	0
0	0	0	0	0
0	0	0	0	0
0	0	0	0	0
0	0	0	0	0
0	0	0	0	0
0	0	0.010361446	0	0.002878908
0	0	0	0	0
0	0	0	0	0
0	0.018789053	0	0	0
0	0.007236592	0	0.002404835	0.006752629
0	0.025736283	0	0	0.087635141
0.058764317	0	0.021330664	0.007685983	0
0	0	0	0	0
0	0	0	0.000984844	0
0	0	0	0	0
0.003495441	0	0.001780583	0.001761903	0.00296839
0	0	0	0	0
0	0	0	0	0
0	0	0	0	0
0	0	0	0	0.014399951
0	0	0	0	0
0.668063557	0.053184488	0.467106015	1.031398811	0.004577614
0	0	0	0	0
0	0	0	0	0
0	0.00087543	0	0	0.015398265
0	0	0	0	0
0	0	0	0	0
0	0	0	0	0
0	0	0	0	0
0	0	0	0	0
0	0	0	0	0
0	0	0	0	0
0.016088212	0	0.015450267	0.08042914	0
0	0	0.007327107	0.002788554	0
0	0	0	0	0
0	0	0	0	0
0	0	0	0	0
0	0	0	0	0
0	0	0	0	0

0	0	0	0	0
0	0	0	0	0
0.136773393	0.011258896	0.006073154	0.033385792	0.014296149
0	0	0	0	0
0	0.035625771	0	0	0
0	0	0	0	0
0	0	0	0	0
0	0	0	0	0
0	0	0	0	0
0	0	0	0	0
0	0	0	0	0
0	0	0	0	0
0	0	0	0	0
0	0	0	0.001537389	0.073818859
0.017521085	0.000474007	0.001538839	0.002030261	0.069906638
0	0.035524128	0	0	0.008926383
0.002409413	0	0	0	0.010717747
0	0	0	0	0
0	0	0	0	0
0	0	0	0	0
0	0	0	0	0
0.010253629	0	0.005539772	0.009397125	0
0	0	0	0	0
0.002408404	0	0	0	0
0	0	0	0	0
0	0	0	0	0
0	1.047753957	0	0	0.039019398
0	0	0	0	0
0	0	0	0	0
0	0	0	0	0.002862629
0	0	0	0	0.009773208
0	0	0	0	0
0	0	0	0	0.016837643
0	0	0	0	0
0	0	0	0	0
0	0	0	0	0.006587922
0	0	0	0	0
0	0.026543891	0	0.04427443	0.002762664
0	0	0	0	0
0	0	0	0	0
0	0	0	0	0
0	0	0	0	0
0	0	0	0	0
0	0	0	0	0
0	0	0	0	0
0	0	0	0	0
0	0	0	0	0
0.010402725	0	0.003372195	0.196633946	0

0	4.70544919	0	0	0
0	0	0	0	0
0	0	0	0	0.026716588
0	0	0	0	0
0.006221263	0.00137008	0.002501937	0.006601841	0.014830049
0	0	0	0	0
0	0	0	0	0
0.014228134	0.004080995	0	0	0.001299224
0	0	0	0	0
0.016557536	0	0.00506066	0.022255869	0.027787112
0.037211522	0	0	0	0
0	0.284666725	0	0	0.008999113
0.007165447	0	0.006879014	0.001852204	0.015992687
0.084129827	0	0.003837838	0.105488243	0
0	0	0	0.022859393	0
0	0	0	0	0
0	0	0	0	0.002341991
0	0	0	0.008744421	0
0.018251255	0	0	0	0
0	0	0	0	0
0	0	0	0	0
0	0	0	0	0
0	0	0	0	0
0	0.04791215	0.072444694	0	0.037599358
0	0	0	0	0
0	0.001511034	0	0	0
0	0	0.008527522	0	0.011543016
0.188652775	0	0.00118642	0	0
0	0	0	0	0
0	0	0	0	0
0	0	0	0	0
0	0	0	0	0
0	0	0	0	0
0	0	0.000855397	0	0
0	0	0	0	0
0	0	0	0	0
0.536727171	0	0	0	0
0	0	0	0	0
0	0	0	0	0
0.409854334	0	0	0	0
0	0	0	0	0
0	0	0	0	0
0	0	0	0	0
0.108614682	0	0	0	0
0	0	0	0	0
0	0	0	0	0.001163412
0.002794295	0	0.00264349	0.002012122	0
0	0	0	0	0

0	0	0	0	0
0	0	0	0	0
0	0	0	0	0
0	0	0	0	0
0	0	0	0.08187111	0
0	0	0.088087527	0.088484092	0
0	0	0.126620678	0	0
0	0	0	0	0
0	0.441950685	0.00233718	0	0.123111807
0	0	0	0	0
0.077307626	0	0	0.007658705	0.018580467
0	0	0	0	0
0.089232349	0	0	0	0
0.004478645	0	0.000877472	0.379143323	0.004876081
0	0	0	0	0
0	0	0	0	0
0	0	0	0.009501299	0
0.011063744	0	0	0	0
0.023467464	0	0	0	0
0.017146205	0	0.291704645	0.060915231	0
0	0	0	0	0
0	0.136496389	0.168057958	0	0.177516611
0	0.010772562	0.006161827	0.212577555	0
0	0	0	0	0
0	0	0	0	0
0	0	0	0	0
0.006723743	0	0	0	0
0	0	0	0	0
0	0	0	0	0
1.659873042	0.252501492	0.313830538	0.367385427	0.143660999
0	0.001589192	0.005476716	0.0007854	0
0	0	0	0	0.002089969
0	0	0	0	0
0	0	0	0	0
0	0	0	0	0
0	0	0	0	0
0	0	2.27366286	0	1.596182388
0	0	0	0	0.008697545
0	0	0	0	0
0	0	0	0	0
0.04729868	0.002814602	0.001587031	0.00214143	0
1.975726034	0.576248487	1.55699434	0	0.288566183
0	0	0	0	0.005691081
0	0	0	0	0
0	0	0	0.00375767	0
0	0	0	0	0
0.002378125	0	0	0.00149839	0
0	0	0.003401693	0	0

0	0.00057551	0	0	0
0	0	0	0	0
0	0	0.005855308	0	0
0	0	0	0	0
0	0.03522591	0.740244855	0	0.02243924
0	0	0	0	0
0	0	0	0	0
0	0	0	0	0.000872744
0	0	0	0.06163203	0
0	0	0	0.041117165	0
0	0	0	0	0
0	0	0	0	0
0.143424876	0	0	0	0
0.003649942	0	0.005577857	0	0.002582995
0	0	0	0	0
0.001815142	0.144713137	0	0	0.014313469
0.011496549	0.025856327	0	0.018350587	0.001423781
0.185772518	0	0.001067287	0	0
0.002078137	0	0.008015159	0.000997619	0
0	0	0	0.375787232	0
0.09924912	0	0.009594882	0	0
0.00466739	0	0.006113765	0.002520678	0
0	0	0	0	0
0	0.029205907	0.067071102	0.02105635	0
0.036531763	0	0	0	0
0.003786169	0	0	0	0
0.002554118	0	0	0.002758763	0
0	0	0	0.000581954	0
0	0	0	0	0
0	0	0	0	0
0.110716782	0.000435404	0.024877884	0.541058926	0
0.004550668	0	0.012644274	0	0.03161869
0.012604658	0	0.004414322	0.027796443	0.004738792
0	0	0	0	0.052731292
0	0	0	0.001692385	0.009979435
0	0	0	0	0
0	0	0	0	0.002268103
0.055698707	0.013546558	0.149242001	0.002105652	0
0	0.024779598	0.070082524	0	0.024303769
0	0.00512102	0	0.005738617	0
0	0	0	0	0
0	0	0	0	0
0	0	0.138327612	0.03848776	0
0.486755311	0	0	0.052964328	0
0.014889955	0	0	0	0
0	0	0	0	0
0	0	0	0	0
0	0	0	0	0

0	0	0	0	0
0.491809676	0.105592869	1.750027166	0	0.444454928
0	0	0	0	0
0	0	0	0	0
0	0	0	0.002502077	0
0	0	0	0	0
0	0	0	0	0
0	0	0	0	0
0	0	0	0	0
0.043018983	0	0	0	0
0.005357947	0.00132745	0.000718249	0.000947619	0
0.022262269	0.001318183	0.004065438	0.00564602	0.023780513
0	0.028041775	0	0	0.011514526
0	0	0	0	0
0.00097067	0	0	0.043490961	0
0	0	0	0	0
0.011221087	0.002007824	0.008874902	0.001736514	0.009194787
0.336179974	0	0	0.039932159	0
0	0	0	0	0
0.061979623	0	0.000887581	0	0
0.007308487	0.051764319	0.065306823	0.001790784	0.065728505
0	0	0	0	0
0	0	0	0	0
0	0	0	0	0.014254485
0	0	0	0	0
0	0	0	0	0
0.021942103	0.015878434	0.02740745	0.559316966	0
0.072176209	0.011511423	0.019290135	0.020936727	0.050161554
0	0	0	0	0
0.00156164	0.000612594	0.001392128	0	0
0.032992321	0	0	0	0.003686533
0	0	0	0	0
0	0	0	0	0
0	0	0	0	0
0	0.190596668	0.021263944	0	0
0.578998662	0.077929585	0.131984799	0.53427353	0.12676168
0	0	0	0	0
0	0	0	0.008154617	0
0	0.110681363	0.009152515	0.005366815	0.066118236
0	0	0	0	0
0.016535944	0	0	0	0
0	0	0	0	0
0.006790652	0.004820232	0.027920429	0.001833685	0
0.021776105	0	0.00083494	0	0.001657047
0	0	0	0	0
0	0.101229039	0	0	0.606073313
0	0	0	0	0
0	0	0	0	0

0	0	0	0	0
0	0	0	0	0
0	0	0	0	0.002831173
0	0.21362918	0	0	1.014660294
0	0	0	0	0
0	0	0	0	0
0	0	0	0	0
0	0.00581537	0	0	0
0.175392222	4.567350945	0.587035874	0	0.053318399
0	0.023239297	0.002314793	0	0.064673394
0	0	0	0	0.008142243
0	0.004529565	0.008547272	0	0.001340637
0	0	0	0	0
0	0	0	0	0.001199689
0.054491155	0.034925149	0.030146872	0	0
0	0	0	0	0
0	0.049975349	0.483016866	0	0
0	0.637277548	0.164005559	0	0.138068012
0	0	0	0	0
0	0.002907361	0	0	0
0	0.041927692	0	0	0
0	0.03968817	0	0	0.023648987
0	0.022809955	0	0	0
0.314718573	0.079772862	0.13555263	0.744386871	0
0	0	0.003614001	0	0
0	0.078944309	0	0	0.741229039
0	0	0.045765385	0	0.012801167
0	3.947440669	0.708300084	0	3.628382266
0	0	0	0	0
0	0	0	0	0
0	0.007430922	0	0	0.017874215
0	0.06521588	0.156453756	0	0.001403402
0	0	0.001326014	0.038690232	0
0	0	0	0	0
0	0	0	0	0
0.019407782	0.394020301	0.060227207	0	0.006946195
0	1.541556358	0.803961246	0	0.738684316
0	0	0	0.099590362	0
0	0.001178819	0.000861069	0.071949676	0
0.00968548	0	0.00049338	0.009601344	0
0.023106262	0.001357909	0.103023749	0.117995602	0.042441364
0	0	0.001033761	0	0.035265284
0	0	0.074407623	0	0.002865269
0	0.007807245	0.007258114	0	0
0	0	0	0	0
0	0.000926923	0	0	0
0	0	0	0	0
0	0.031341352	0.00397453	0.00524378	0

0	0	0	0	0
0	0.279877505	0.044754444	0	0.052516485
0.974720167	0.013457309	0.011430108	1.181036127	0.013550225
0	0	0	0	0.002059755
0	0.042301641	0	0	0
0	0.01343634	0.015967445	0	0.005337172
0	0	0	0	0
0	0	0	0	0
0	0	0	0	0
0	0	0	7.051597763	0
0.027611486	0.016439045	0.078556374	0.325488453	0.000623621
4.780113838	0.220343474	1.116644639	1.219653514	0.610627269
0	0	0	0	0
0.015216736	0.008124698	0.002422321	0.001331616	0.001794766
0.616306528	0.007186562	0.028760489	0.158034338	0
0	4.820209132	0	0	0.06791405
0	0	0	0	0
0	0	0	0	0
0	0.023920308	0	0	0.048733274
0	0.002657667	0.379877205	0.085171302	0.009760294
0.056379048	0.043009026	0.168680446	0.106930516	2.631900068
0.932077945	0	0.002378635	6.315711184	0
1.4715053	0.007297557	0.190950412	15.00598842	0.570705567
0	0	0	0	0
0	0	0	0	0.023735814
0	0.123906541	0.006011294	0	0.798785963
0	0	0	0	0
0	0	0	0	0
0	0	0	0	0
0	0	0	0	0.002132469
0	0.016475418	0	0	0.002498208
0	0.055556721	0.178783182	0	0.258570941
0.376192244	0	0.002458802	0.001013753	0.001067459
0.016793847	0.015040706	0.028008215	0	0
0.033649777	0.021244991	0.207216369	0.237359573	0
0.012584059	0.042210044	0.077063412	0.483086027	0.029814071
0.112575396	0.001935053	0.001480767	0	0
0	0	0	0	0
0.029131651	0.030808633	0.231564649	0.081757229	0.209330902
0	0	0	0	0
0	0.019402992	0.189953914	0	0.014149231
0	0	0	0	0
0	0	0	0	0.367589515
0	0	0	0	0
0.010919003	0.025291672	0.005562157	0.101951455	0
0	0.025097711	0	0	0.052728607
0	0.283234451	0	0	0
0.016558208	0	0.088948545	0	0

0	0	0	0	0
0	0	0	0	0
0	0	0	0	0
0	0.001251638	0.001760796	0	0.003010669
0	0	0	0	0
0	0.079591598	0.219033163	0	0.027587165
0	1.526672461	0	0	0.026986895
0.009052747	0	0.053343637	0.208217373	0
0	0	0	0	0
0	0	0	0	0
0.015695068	0.002633099	0.019007197	0.009453656	0
0	0	0	0	0
0.009075077	0.007458918	0.067097475	0	0.024037606
0	0.48480143	0	0	0
0	0	0	0	0
0	0	0	0	0
0	0.026149494	0.055535662	0	0.006078673
0	0.00606022	0.005274353	0.004556288	0
0	0	0	0	0.001101473
0	0.002441949	0.045300882	0	0
0.001533122	0.008362427	0	0	0
0	0	0	0	0.689797564
0	0.03909747	0	0	0.006235358
0	0	0	0	0
0	0	0	0	0
0	0	0	0	0
0	0	0	0	0
0	0	0	0	0
0	0	0	0	0
0	0	0.637440484	0	1.808207806
0	0	0	0.003722903	0
0	0	0	0	0
0	0.057660691	0	0	0.004010195
0	0	0	0	0
0	0	0	0	0
0	0	0	0	0
0	0.024734687	0.005293305	0	0.080440197
0.012032739	0	0.083897466	0.034537905	0
0	0.009928821	0	0	0.001235308
0	0	0	0	0
0	0.010948675	0.006589669	0.186329338	0.000665792
0	0	0	0	0
0	0.026901306	0	0	0.007799618
0	0.347225422	0	0	0.205873787
0	0	0.050314844	0	0.535224108
0	0	0	0	0.011732952
0	0.0333879	0	0	0.001685625
0	0	0	0	2.504016605

0	0	0	0	0
0	0	0	0	0
0	0.145806594	0	0	0
0	0.586766463	0.118384411	0	0.227814994
0	0	0	0	0
0	0	0	0	0.65163484
0	0	0	0	0
0	0	0	0.033367818	0
0.036301906	0	0.038041189	0.065060443	0
0	0	0	0	0
0	0	0	0	0
0	0	0	0	0.001342455
0	0	0.00493167	0.001863864	0
0	0	0	0	0
0	0.125271194	0	3.786877542	2.119044507
0	0	0.324820973	3.28621583	0
0	0	0	0.027288143	0
0	0	0	0	0
0	0	0	0	0
0	0	0	0	0.011586059
0	0.078122911	0.130626418	0	0.064731078
0	0.004744754	0	0	0
0	0	0	0	0
0	0	0	0	0
0.010570859	2.078665429	0.008077217	0.062163748	0.012084367
0	0.143309319	0	0	11.73687228
0	0.102030807	0	0	0.306921654
0	0	0	0.005724583	0
0	0	0.000724287	0	0.285260183
0	0	0	0	0
0	0	0	0	0
0	0.000821691	0	0	0
0	0.001235719	0	0	0
0	0.000794986	0	0.003101046	0
0.014668585	0.008607818	0	0	0
0.002276102	0	0.026925005	0.054814805	0
0	0	0	0	0
0	0.040925235	0.496339629	0	0.717827916
0	0	3.38049247	0	4.973209459
0	0	0	0	0
0	0	0	0	0
0	0.013800161	0	0	0
0	0.247998811	0.000528136	0	0.24408034
0	1.48168863	0	0	0.078654314
0	1.04903802	0	0	0.005142673
0	0.000501259	0	0.002817915	0
0	0	0	0	0
0	0.211020822	0.042776211	0.262622663	5.642541266

0	0	0.001449921	0.042084847	0
0	0	0	0	0.016625467
0	0	0	0	0
0	0	0	0	0
0	0	0	0.004523332	0
0	0	0	0	0
0	0.014631548	0	0	0
0	0	0	0	0
0	0	0	0	0
0	0	0	0	0.01006449
0	0.001360972	0.013917711	0	0.000767264
0	0.026254548	2.187582569	0	0.422762345
0	0.101758624	0.003670598	0	0.015055203
0	0	0	0	0
0.014748035	0.002571247	0.007512671	0.002753281	0.515351275
0	0	0	0	0
0	0.002027163	0	0.003447548	0
0	0	0	0.018382179	0.000619392
0	0	0	0	0
0	0.002998777	0.001947073	0	0.00247954
0	0.124265452	0.002665244	0	0.110406725
0	0	0	0	0
0.636009711	0.019430467	0.016078272	0	0.621471066
0	0	0	0	0
0	0	0	0	0.006787318
0	0	0	0	0
0	0.210937929	0	0	0.004015263
0	0	0	0	0
0	0.000463209	0	0	0.002089114
0	0	0	0	0
0	0	0	0	0
0.135664586	0.006005794	0.017764372	0.001429107	0.112259293
0	0.804131549	0	0	0.003371243
0	0.032337552	0.009740606	0.102631332	0.001202848
0.003027408	0.000719746	0.025095245	0.047398101	0
0	0.023752692	0.070304283	0.022652411	0.142110469
0	0	0	0	0
0	0	0.018232261	0	0.008567161
0	0	0	0	0
0	0	0	0	0
0	0.004415227	0.122076135	0	0.341841316
0	0.032863131	0	0	0.019699566
0	0	0	0	0
0	0	0	0	0
0.033889772	0.057005344	0.0034527	0.061952153	0
0	0	0	0	0
0	0.161904986	0	0	0.125270516
0	0.000536486	0	0	0

0	0	0	0	0
0	0.022140539	0	0	0
0	0.001173289	0	0.005418025	0
0	0	0	0	0
0	0	0	0	0
0	0	0.027648519	0	0.002902122
0	0.252978944	0.277516878	0	0.138924635
0	0	0.012545454	0	0.060906587
0	0	0	0	0
0	0.004770393	0	0	0.002028549
0	0	0	0	0
0	0	0	0.004074611	0
0	0	0	0	0
0	0	0.321083467	0	0
0	0	0	0	0
0	0	0	0	0
0	0.145488466	0	0	0
0	0.039664264	0	0	0.000662554
0	0.029775332	3.180753658	0	5.034584819
0	0	0	0	0
0	0	0	0	0
0	0	0	0	0
0	0	0	0	0
0	0	0	0	0.006529859
0	0	0	0	0.002934812
0.012872439	0	0.010543013	0.14906472	0
0	0	0	0	0
0.000988201	0.012792376	0.049206456	0.001037729	0.496699842
0	0.058942922	0	0	0.025165182
0	0.495715636	2.170230812	0	0.214785685
0	0	0	0	0.036321189
0	0	0	0	0
0	0.000818645	0	0	0.081227642
0	0	0	0	0
0.002909504	0	0.192808495	0.004221908	0
0	0.853552107	0	0	0
0	0.100075423	0	0	0.080315079
0	0	0	0.085474912	0
0	0	0	0	0
0	0	0	0	0.000372895
27.37884794	0.959111571	1.229977488	17.336107	1.406875536
0.214204448	0.099970994	0	0	0
0	2.634439629	0.129253917	0	0
0	0.020544055	0	0	0.031889443
0	0.241378318	0	0	0
0	0	0	0	0
0	0.000712552	0	0	0
0.003334802	0.004609721	0.002305451	0.002401332	0

0	0	0	0	0
0.00171506	0.004613331	0.05653789	0.006792412	0
0	0	0	0	0
0	0	0	0	0.00487813
0	0.41165722	0	0	1.000203925
0	0.001576871	0	0	0
0	0	0	0	0
0	0	0	0	0.005331249
3.49356341	0	0.006106561	0.277872763	0
0	0	0	0	0
0	0	0	0	0
0	0	0	0	0
0	0.027767446	0.137522398	0	0.066716465
0.022243667	0.007807174	0.00775276	0.052126373	0.013877309
0	1.716517762	0.057972434	0	0.214330569
0	0.071602175	0.012234344	0	0.199198352
0	0	0	0.001489392	0.006810881
0	0	0	0	0
0	0	0	0	0.007198717
0	0	0	0	0.000927737
0	0.002537318	0	0	0
0	0	0	0	0
0	0	0	0	0
0	0	0	0	0
0	0	0	0	0.005936409
0	0	0	0	0
0	0.204456717	0.014696152	0	0.076522877
0	0	0.001772225	0.008559401	0
0	0	0	0	0
0	0.003709346	0	0	0
0	0	0	0	0
0	0	0	0	0
0	1.361379935	0	0	0
0	0.013216156	0.022027785	0	0.038897586
0	0.270081192	0.227100543	0	0.426259293
0.003162123	0	0	0.060133199	0
0	0.354848508	0	0	0
0	0	0	0	0.058590336
0	0	0	0	0
0	0	0	0	0.003961428
0	0.413407346	3.262120358	0	0.214023656
0	0	0.002253689	0	0
0	0.137762799	0.007315632	0	0
0	0	0.049578282	0	0.259577031
0	0	0	0	0
0	0	0	0	0
0	0	0	0	0
0	0	0	0	0

0	0.110148725	0.296745742	0	0.134015344
0	0	0	0	0
0	0	0	0	0
0	0	0.002150982	0.001655436	0
0	0.003521919	0.002058069	0	0.01753614
0	0	0	0	1.097338298
0	0.000792879	0.005276777	0.060279816	0.032720013
0	0	0	0	0
0	0	0	0	0
0	0	0.432768666	0	0
0	0.071310914	0.006867173	0	0.003252326
0	0.014445574	0	0	0
0	0	0	0	0
0	0	0	0	0
0	0	0	0	0
0	0	0	0	0
0	0	0	0.03674987	0
0	0	0	0	0
0	0	0	0	0.005548282
0	0	0	0	0
0.001202699	0.025917028	0.015806547	0.001212459	3.455919121
0	0	0.003090857	0	0.002477275
0	0.016374729	0.053384911	0	0.003755164
0.00471841	0.004318816	0.004086061	0.001387371	0
0	0	0	0	0
0	0	0	0	0
0	0	0	0	0.157921928
0	0.024292995	0	0	0
0	0	0	0	0
0.592845475	0	0.005361906	0.163733682	0
0	0.005458112	0	0	0.10920339
0	0	0	0	0.001550276
0	0	0	0	0.002466646
0	0	0	0	0
0	0.254266172	0	0	0
0	0	0	0	0
0.010922314	0	0.004962346	0.000743983	0.002715776
0	0	0	0	0
0	0.001565038	0	0	0
0	0	0.041197498	0	0.06837599
0	0	0	0	0
0	0	0	0	0
0	0	0	0	0
0	0	0	0	0.000797387
0	0.602919096	0	0	0.005140554
0	0	0	0	1.531860138
0	0	0	0	0
0	0	0	0	0

0	0	0.13552325	0.185327969	0
0	0	0.009934068	0	0.186225748
0	0	0	0	0.029035662
0	0	0	0	0
0	0	0	0	0
0	0	0	0	0
0	0	0	0	0
0	0.076345967	0.002257233	0	0.505976854
0.407634408	3.200191092	0.110421668	0.687916766	0.281707462
0.110961035	0	0.026142218	0.128640716	0
0	0	0	0	0.191667311
0	0	0	0	0
0	0	0	0	0.031962295
0	0	0	0	0.027139025
0	0	0	0	0
0	0.567999035	0	0	0
0	0	0	0	0
0	0	0	0	0
0	0	0	0	0
0	0	0	0	0
0	0	0	0	0.197251242
0	0	0	0	0
0	0	0	0	0
0	0	0	0	0
0	0.012758807	0.001731524	0	0.01542352
0	0	0	0	0
0	0	0	0	0
0	0	0	0	0
0	0	0	0	0
0	0	0	0	0.006385404
0	0	0	0	0
0	0	0	0	0
0	1.010151722	0	0	0
0	0.007179252	0	0	0
0	0.011412922	0.001722108	0	0
0	0	0	0	0.007831906
0	0	0.003405775	0	0.003595895
0	0	0	0.031675606	0.002797405
0	0	0	0	0
0.497991213	0	0	0	0
0	0	0	0	0
0	0.08259826	0	0.43402913	0.067165671
0	0	0	0	0.002991725
0	0	0	0	0
0	0	0.000377409	0	0
0	0	0	0	0
0	0	0	0	0
0	0	0	0	0

0	0	0	0	0
0	0.001198371	0	0	0.01718716
0	0	0	0	0
0	0	0	0	0.012271242
0.178109144	0.28114753	0.42786259	0.854983053	0.943293414
0	0	0	0	0
0	0	0	0	0
0.007750244	0	0.082085209	0.101733572	0
0	0.001180497	0	0	0.002738136
0	0	0	0	0.014395153
0	0	0	0	0
0	0	0.26421416	0	0.007170416
0	0	0	0	0
0	0	0	0	0
0	0	0.002663441	0	0
0.037529654	0.0011719	0.005706765	0.291285775	0
0	0	0	0	0
0	0	0	0	0
0	0	0	0	0
0	0	0	0	0
0	0	0	0	0.020558085
0	0.036869389	0	0	0
0	0	0	0	0
0.013414397	0	0.018649252	0.00187823	0
0	0.00598235	0.307786515	0	0.455039705
0	0	0	0	0
0	0.015909091	0.274081558	0.183595694	0
0	0	0	0	0
0	0	0	0.006064274	0
0	0	0	0	0
0	0	0	0	0
0	0	0	0	0
0	0	0	0	0
0	19.14269311	0	0	0
0	0	0	0	0
0	0	0	0	0
0	0	0	0	0
0	0.042228884	0.018828135	0	0.002040602
0	0	0	0	0
0	0	0.001260138	0	0
0	0	0	0	0
0	0.354041908	0.001990485	0	0
0	0	0	0	0
0	0	0.060058991	0	0
0	0	0	0	0
0	0	0	0	0
0	0	0	0	0
0	0	0	0	0.002929264

0	0.907348462	0	0	0.024665628
0	0	0	0	0
0	0	0	0	0
0	0	0	0	0
0	0	0	0	0.003466351
0.007797624	0	0	0	0
0	0	0	0	0
0	0	0	0	0
0	0	0	0	0
0	0	0	0	0
0	0	0	0	0
0	0.000404239	0	0.004545	0
0	0	0	0	0.011251074
0	0	0	0	0
0	0	0	0	0.000890287
0	0	0	0	0
0	0	0	0	0
0	0	0	0	0
0	0	0.005502081	0	0
0	0	0	0	0
0.37653766	0	0	0.23543388	0
0	0	0	0	0
0.023899997	0.024633415	0.020231476	0.031593779	0
0	0	0.46013681	0	0.144557642
0	0	0	0	0.02077707
0	0	0	0	0.283662371
0	0	0	0	0
0	0	0	0	0
0	0	0	0	0
0	0.63620149	0	0	0.000592292
0	0	0	0	0
0	0.021626558	0	0	0
0.536186511	0	0.01257524	0.006359918	0
0	0	0	0	0
0	0.145524306	0.012448331	0	0.017502085
0	0	0.000403644	0.04082854	0
0	0	0	0	0
0	0	0	0	0
0.103872625	0.001438122	0.014628852	0.056977584	0
0	0	0	0	0.010461326
0	0	0	0	0
0	0	0	0	0.000449094
0.015003663	0.442633174	0.066784284	0	0.028314161
0	0	0	0	0
0	0	0	0	0
0	0.023890219	0	0	0.001405397
0	0	0	0	0
0	0	0	0	0

0	0	0	0	0
0	0	0	0	0
0	0	0	0	0
0	0	0	0	0
0	0	0	0	0.001473412
0	0	0	0	0.000606383
0	0	0	0	0
0	0	0.000977555	0.001151547	0
0	0.005560974	0.019768469	0.008262102	0
0	0	0.02024394	0	0.0384633
0	0	0	0	0
0	0	0	0	0
0	0	0	0	0
0	0	0	0	0.17981663
0.07533058	0	0	0	0
0	0	0	0.013042621	0
0	0	0	0	0
0	0	0	0	0
0	0	0	0	0
0	0.1183441	0	0	0.058087128
0	0.940603055	0.004588837	0	0.0019125
0	0	0	0	0
0	0	0	0	0
0	0	0.093248376	0	0
0	0.000609704	0	0.00228504	0
0	0.020225832	0	0	0.590072635
0	0	0	0	0
0	0	0	0	0.030275478
0	0	0	0	0
0	0.006701604	0	0	0.058200422
0	0.097531458	0.913229079	0	0.227379493
0	0	0	0	0
0	0	0	0	0
0	0	0	0	0
0	0	0	0	0
0	0	0	0	0
0	0	0	0	0
0	0	0	0	0.000635592
0	0	0.04306408	0	0.013046248
0	0	0	0	0
0	0	0	0	0
0	0.1493017	0	0	0.022797296
0	0.001189724	0	0	0.040958677
0	0	0	0.001031954	0
0	0	0	0.000905443	0
0	0	0	0	0
1.248188356	0	0	0.343186493	0
0	0.018515446	0	0	0

0	0	0	0	0
0	0	0	0	0
0	0	0	0	0
0	0	0	0	0.019786682
0	0.018170373	0	0	0.005912854
0	0	0	0	0
0	0	0	0	0
0	0	0	0	0
0	0	0	0	0
0	0	0	0	0
0	0.004526404	0	0	0.010273105
0.00824214	0.012294132	0.012284671	0.016925794	0
0	0.007713991	0	0	0
0	0	0	0	0.005373747
0	0	0	0	0
0	0	0	0	0
0	0.006754033	0.07271657	0	0
0	0	0	0	0
0.046229599	0.00084629	0.082658486	0.569743143	0
0	0.001104997	0.073027391	0	0.016018834
0	0	0	0	0
0	0.027542709	0	0	0
0	0	0	0	0.027437156
0	0	0	0	0
0	0	0	0	0
0	0	0	0	0
0.008706718	0.001897466	0	0.037791468	0
0	0	0	0	0
0	0	0	0	0
0	0	0	0	0
0.003015687	0	0.017317113	0.001381891	0
0	0	0	0.00348274	0
0	0	0	0	0
0	0	0	0	0
0	0	0	0	0.026134781
0	0.005782925	0	0	0
0	0	0	0	0
0.043530359	0.00544012	0.00932111	0.168608859	0
0	0.042047077	0	0	0.218218295
0	0	0	0	0
0	0	0	0	0
0	0	0	0	0.001397916
0	0	0	0	0
0	0	0.001600445	0.220991879	0
0	0	0	0	0
0	0	0	0	0.009396571
0	0	0	0	0
0	0	0	0	0

0	0	0	0	0
0	0.071758291	0	0	0.005528312
0	0	0	0	0.007919279
0.006884602	0	0.005794217	0.002263197	0
0.007495855	0.001887694	0	0.001399386	0
0	0	0	0	0.000760688
0	0	0	0	0.002055655
0	0	0	0	0
0	0	0	0	0
0	0	0	0	0.000734348
0	0.009185148	0	0	0.021004667
0	0	0	0	0
0	0.02885461	0.317017503	0.043137523	0.031383034
0	0	0.008544368	0	0
0	0	0.003607684	0	0
0	0	0	0	0
0	0	0	0	0
0	0	0	0	0
0	0	0	0	0
0	0.026211663	0	0	0
0	0	0	0	0
0	0	0	0	0
0.008661287	0	0	0.005867994	0
0	0	0.011252536	0	0.499772722
0	0	0	0	0
0	0.020248814	0	0	0.000861548
0	0.000837711	0.020260893	0	0.007178494
0	0	0	0	0
0	0	0	0	0.035151834
0	0	0	0	0
0	0.035031864	0.040625064	0	0.007936592
0	0	0	0.002023831	0
0	0	0	0	0
0	0	0	0	0
0	0.014616661	0	0	0.000739318
0.005210568	0	0	0	0
0.01522477	0	0	0	0
0	0	0	0	0
0.000790717	0	0	0	0.015668117
0	0	0	0	0
0	0	0	0	0
0	0	0	0	0
0	0	0	0	0
0	0	0	0	0
0	8.830829025	0	0	0.001727765
0	0.153798158	0.494138918	0.080654796	0.129155643
0	0	0	0	0
0	0	0	0	0

0	0.011387473	0	0	0
0	0	0	0	0
0	0	0.149036852	0	0.077432601
0	0	0	0	0.000991094
0	0	0	0	0
0	0	0.059054973	0	0
0	0	0.000545909	0	0
0	0	0	0	0
0	0	0.012795695	0	0
0	0	0	0	0
0	0.000671037	0	0	0
0.001276982	0	0.000975745	0	0
0	0	0	0	0
0	0	0	0	0
0	0	0	0	0
0	0	0	0	0
0	0	0	0	0.053384801
0	0	0	0	0
0	0	0	0	0
0.002552151	0	0	0	0
0	0	0	0	0
0	0	0	0	0
0	0	0	0	0
0	0	0	0	0
0	0	0	0	0
0	0.010363193	0	0	0
0	0	0	0	0
0	0.014772537	0.004316241	0	0.014657614
0	0	0	0	0
0	0	0	0	0
0.005846833	0.00152905	0.013154534	0	0.00706856
0	0	0	0	0
0.3394384	0.006361786	0.127809621	0.12095571	0.014412851
0.003179635	0	0.008540292	0	0.049503688
0	0	0	0	0.007945081
0	0	0	0	0
0	0	0	0	0
0	0	0	0	0
0	0	0	0	0
0	0	0	0	0
0	0	0	0	0
1.719499684	0	0.045757674	0.373403743	0.003129015
0	0.033808818	0.098493785	0	0.027486692
0	0	0	0	0
0	0	0	0	0
0.006145316	0.479561116	0.034643462	0	0.038270539
0.000805809	0.003090753	0	0.006543914	0
0	0	0	0	0

0	0	0	0	0
0	0.005281814	0.003025962	0	0.006095494
0.00451023	0.003084858	0.056377557	0	0.033391193
0	0	0	0	0
0.165655283	0.011880887	0	0	0
0	0	0	0	0
0	0	0	0	0
0	0	0	0	0
0	0	0	0	0
0	0	0	0	0
0	0	0	0	0
0	0	0	0	0
0	0	0	0	0.00074458
0	0	0	0	0
0	0	0	0	0
0	0	0	0	0
0	0.185528772	0	0	0
0	0	0	0	0
0	0	0	0	0
0	0	0	0	0
0	0	0	0	0
0	0	0	0	0
0	0.001025926	0	0	0.005552427
0	0	0	0	0
0	0.014356838	0.006699999	0	0.005058647
0.005177462	0	0	0.002658067	0.017098633
0	0	0	0	0
0	1.216523187	0.24418285	0	0.010267691
0	0	0	0	0
0	0	0	0	0
0	0	0	0	0
0	0	0	0	0
0	0	0	0	0
0	0	0	0	0
0	0	0	0	0
0	0	0	0	0
0	0	0	0	0
0	0	0	0.030307384	0
0	0.085369672	0	0	4.648763179
0	0	0	0	0
0	0.001094351	0	0	0
0	0	0.002384317	0	0.003533215
0	0	0.103671259	0.142113565	0
0	0	0	0	0.09954742
0	0	0	0	0
0	0	0	0	0
0.012806868	0	0.006973757	0.015210996	0
0	0	0	0	0

0	0	0	0	0
0.010308909	0.002914552	0.015612192	0.020831947	0.002563256
0	0.31396258	0.014136164	0	0.001636544
0	0	0	0	0
0	0	0	0	0
0	0	0	0	0
0	0	0	0	0
0	0	0	0	0
0	0	0	0	0
0	0	0	0	0
0	0	0	0	0
0	0	0	0	0
0	0.615725891	0	0	0.039184823
0	0	0	0	0
0.009032299	0.024711252	0.121751294	0	0
0	0	0	0	0
0	0	0	0	0
0	0	0	0	0
0	0	0	0	0
0	0	0	0	0
0	0	0	0	0
0.021834905	0	0.063086751	0.001433079	0.005070237
0	0	0.00707791	0	0
0	0	0	0.000885639	0
0	0	0	0	0
0	0	0	0	0
0	0	0	0	0
0	0	0	0	0.003358993
0	0	0	0	0
0	0	0	0	0
0	0	0	0	0
0	0	0	0	0
0	0	0	0	0
0	0	0	0	0
0	1.294680441	42.94035156	1.022448546	0.060064628
0	0	0	0	0
0	0	0	0	0
0	0	0	0	0
0.0020442	0	0.019785047	0.003148427	4.493268728
0	0	0	0	0
0.369939354	0.183425271	0.752701326	0.102910486	0.294442949
0	0	0	0	0
0	0	0	0	0
0	0	0	0	0
0	0	0	0	0
9.038063318	0	0.641577888	0.83917615	0
0	0	0	0	0
0	0	0	0	0

0	0	0	0	0.000661364
0	0	0	0	0
0	0	0	0	0
0	0.006281873	0	0	0.002174303
0	0	0.000405378	0	0
0	0	0	0	0
0	0	0	0	0
0	0	0	0	0
0	0	0.00772452	0	0
0	0	0	0	0.027354055
0	0.033020219	0	0	0.016339119
0	0	0	0	0
0.007355423	0.002972794	0.032188963	0.009549767	0
0	0	0	0	0
0	0	0	0	0
0	0	0	0	0
0	0	0	0	0
0	0	0	0	0
0	0	0	0	0
0.124092482	0.042491587	1.078105764	1.365994572	0.013899239
0	0	0	0	0
0	0	0	0	0
0	0	0	0	0
0	0	0	0	0
0	0	0	0	0
0	0.023488841	0	0	0
0	0	0	0	0
0	0	0	0	0
0	0	0	0	0
0	0	0	0.04302773	0
0	0	0	0	0
0	0	0	0	0
0	0	0	0	0
0	0	0	0	0
0	0	0	0	0
0	0.002417775	0	0	0
0	0	0	0	0
0	0	0	0	0
0	0	0	0	0
0	0.019458515	0	0	0
0	0	0	0	0
0	0	0	0	0
0	0.008150927	0.03893626	0	0
0	0	0	0	0
0.00167701	0.002485217	0.003417086	0.010331563	0
0	0.016575848	0	0	0.013456551
0	0	0	0	0
0	0	0	0	0

0	0	0	0	0
0	0	0	0	0
0	0	0	0	0.009703867
0	0	0	0	0
0	0	0.008789351	0	0.000939271
0.009180992	0.015169896	0.136690426	0.17424173	0
0	0	0	0	0
0	0	0	0	0
0	0	0	0	0
0	0	0	0	0
0	0	0	0	0
0	0	0	0	0
0	0	0	0	0
0	0	0	0	0.001887944
0	0	0	0	0
0	0	0	0	0
0	0.026740989	0	0	0.031345191
0	0	0	0	0
0	0	0	0	0
0	0.102670478	0	0	0
0	0	0	0	0
0	0	0	0	0
0	0	0	0	0
0	0	0	0	0
0	0	0	0	0
0	0	0	0	0.002540684
0	0	0	0	0
0.002686981	0	0	0.001436478	0.036128998
0	0	0	0	0
0	0	0	0	0
0	0	0	0	0
0	0	0	0	0
0	0	0	0	0
0	0	0	0	0
0	0	0	0	0.555586197
0	0	0	0	0
0	0	0	0	0
0	0	0	0	0
0	0.456311414	0	0	0.001667528
0	0	0	0	0
0	0	0	0	0
0	0	0	0	0
0	0	0	0	0
0	0	0	0	0
0	0	0	0	0
0	0	0	0	0
0	0	0	0	0
0	0	0	0	0

0	0	0	0	0
0	0.004276527	0	0	0
0.001144036	0	0	0	0
0	0	0	0	0
0	0	0	0	0
0	0	0	0	0
0	0	0	0	0
0	0	0	0	0
0	0	0	0	0
0	0	0	0	0.002244674
0	0.001759929	0	0	0
0	0	0	0	0
0	0	0	0	0.040020771
0	0	0	0	0
0	0	0	0	0
0	0	0	0	0
0	0	0	0	0
0	0	0	0	0
0	0	0	0	0
0	0	0.000388987	0	0
0.012108111	0	0.001265208	0	0
0	0.00330078	0	0	0
0	0	0	0	0
0.817345283	0.194648512	1.736043186	0	0.583516104
0	0	0	0	0
0	0	0	0	0
0	0	0	0	0
0	0	0	0	0
0	0	0	0	0
0	0	0	0	0
0	0	0.007295581	0	0.002587741
0	0	0	0	0
0	0.000854061	0	0.003566648	0
0	0	0	0	0

PHL018-0059_V2	PHL018-0245_V1	PHL018-0027_V3	PHL018-0048_V3	PHL018-0254_V1
CTRL	REF	EYCF	CTRL	REF
0.12365609	0.004603559	0.163829071	0.003883267	0.397303528
5.877302843	0.12192661	1.526201917	5.511339193	1.517436271
0	0	2.969561322	7.986912789	0
0	0	0	0	0
0	0	0	0	0
0	0	0	0	0
0	0	0	0	0
0	0	0	0	0
0	0	0	0	0
0	0	0	0	0
0	0	0	0	0
0	0	0	0	0
0	0	0	0	0
0.088461536	2.517529401	5.226607122	6.330177372	2.737212599
0	0	0	0	0
0	0	0	0	0
0	0	0	0	0
0	0	0.001574787	0	0
0	0	0	0	0
0	0	0	0	0
0	0	0	0	0
0	0	0	0	0
0	0	0	0	0
0	0	0	0	0
0	0	0	0	0
0	0	0	0	0
0	0	0	0	0
0	0	0	0	0
0	0	0	0	0
0	0	0	0	0
0	0	0	0	0
0	0	0	0	0
0	0	0	0	0
0	0	0	0	0
0	0	0	0	0
0	1.806564193	0	0	1.133318274
0	0	0	0	0
0	0	0	0	0
0	0	0	0	0
0	0	0	0	0
0	0	0	0	0
0.39361315	0.004447203	0.001094556	0	0.036924075
0.087224401	0.023726912	0.002941491	0.073653356	0.277555595
0.130943155	0.253635557	0.0008974	0.002306746	0.03377413
0.003939029	0	0	0.004324014	0
0	0	0	0	0

0	0	0	0	0
0.030672301	0.001464894	0.002115187	0	0.005030545
0	0	0	0	0
0.898214089	3.540364174	0.345533185	0.407279446	0.28639977
0.005079038	0	0.006738115	0	0
0	0	0	0	0
23.83793625	0.673585672	0	0.001907223	0.005391933
0	0.006585488	0	0	0
0	0	0	0	0
0	0	0	0	0
0	0	0	0	0
0	0.008421412	0.002472693	0.181482428	0.05767919
0	0	0	0	0.000983385
0.09687428	0	0	0	0.082921337
0	0.004936933	0.00364527	0	0
0	0	0	0	0
0.002463903	0.004008003	0.001011753	0	0.018388825
0	0	0	0	0
0	0	0	0	0
0	0	0	0	0
0.343373353	0.01568459	0.084225372	0.082510285	0.075595725
0.042795679	0.004685641	0.000732217	0.006775725	0.002016393
0	0	0	0	0.049755628
0	0	0	0	0
0	0	0	0	0
0	0	0	0	0
0	0	0	0	0
0	0	0	0	0
0.005474805	0	0.001311404	0	0.005417053
0	0	0	0	0
0	0.035648483	0.178381727	0	0.002513275
0	0	0	0	0
0	0	0	0	0
0	0	0	0	0
0	0	0	0	0
0	0	0	0	0
0	0	0	0	0
0	0	0	0	0.002689597
0	0	0	0	0
0	0	0	0	0
0	0	0	0	0
0	0	0	0	0
0	0	0	0	0
0	0	0	0	0
0	0	0	0	0
0	0	0	0	0
0	0	0	0	0
0.007746716	0.326421544	0	0	0

0	0	0	0	0
0	0	0	0	0
0	0	0	0	0
0	0	0	0	0
0.457086975	0	0.014835476	0	0
0	0	0	0	0
0	0	0	0	0
0	0	0	0.000933176	0
0	0.611636001	0	0	0
0	0	0	0	0
0	0	0	0	0
0.123862896	0.002754256	0	0	0
0	0	0	0	0
0	0	0	0	0
0	0	0	0	0
0	0	0	0	0
0	0	0	0	0
0	0	0	0	0
0	0.001114163	0	0	0.013089334
0	0	0	0	0
0	0	0	0	0
0	0	0	0	0
0	0	0	0	0
0	0	0	0	0
0	0	0	0	0
0	0	0	0	0
0	0	0	0	0
0	0	0	0	0
0	0	0	0	0
0	0	0	0	0
0.244821717	0	0	0	0.032103174
0	0	0	0	0.010028024
0	0	0	0	0
0	0	0	0	0
0	0	0	0	0
0	0	0	0	0
0	0	0	0	0
0	0	0	0	0
0	0	0	0	0
0	0	0	0	0
0	0	0	0	0
0	0	0	0	0
0	0	0.00095298	0.001224806	0.001312167
0	0	0	0	0
0	0	0	0	0
0	0	0	0	0
0	0	0	0	0
0	0	0	0	0

0	0	0	0	0.001061927
0	0	0	0	0
0	0	0	0	0
0	0	0	0	0
0	0	0	0.002951421	0.010869155
0	0	0	0	0
0	0	0	0	0
0	0	0	0	0
0	0	0	0	0
0	0	0	0	0
0	0	0	0	0
0	0	0	0	0
0	0	0	0	0
0	0	0	0	0
0	0	0	0	0
0	0	0	0	0
0	0	0	0	0
0	0	0	0	0
0	0	0	0	0
0	0	0	0	0
0	0	0	0	0
0	0	0	0	0
0	0	0	0	0
0	0	0	0	0
0	0	0	0	0
0	0	0	0	0
0	0	0	0	0
0	0	0	0	0
0	0	0	0	0
0	0	0	0	0
0.011404227	0	0	0	0
0	0	0	0	0.003056273
0	0	0	0	0
0	0	0	0	0
0	0	0	0	0
0	0	0	0	0
0	0	0	0	0
0	0	0	0	0
0	0	0	0	0
0	0	0	0	0
0	0	0	0	0
0	0	0	0	0
0	0	0	0	0
0	0	0	0	0
0	0	0	0	0
0	0	0	0	0
0	0	0	0	0
0	0	0	0	0

0	0	0	0	0
0	0	0	0	0
0	0	0	0	0
0	0	0	0	0
0	0	0	0	0
0	0	0	0	0
0	0	0	0	0
0	0	0	0	0
0	0	0	0	0
0	0	0	0	0
0	0	0	0	0
0	0	0	0	0
0.002586266	0	0	0	0.00376014
0	0	0	0	0
0	0	0	0	0
0	0	0	0	0
0	0	0	0	0
0	0	0	0	0
0	0	0	0	0
0	0	0	0	0
0.054919688	0.092492341	0.039997336	0.004812485	0.033590448
0	0	0	0	0
0	0	0	0	0
0	0.002704231	0	0	0
0	0	0	0	0
0	0	0	0	0
0.011063624	0.010823413	0	0.019873535	0.004776837
0	0	0	0	0
0	0	0	0	0
0.004304292	0	0	0	0
0.002606749	0.011040015	0	0	0
0	0	0	0	0
0	0	0	0	0
0	0	0	0	0.021483205
0.054836585	0.001687302	0.008121089	0.00227728	0.044473881
0	0	0	0	0
0	0	0	0	0
0	0	0	0	0
0.067528269	0.001362872	0	0	0
0.0474062	0	0	0	0
0.000593237	0	0	0	0
0	0	0	0	0
0	0	0	0	0
0	0	0	0	0
0	0	0	0	0
0	0	0	0	0
0.001460003	0	0.000799362	0	0.001650973
0.000800779	0.001202417	0	0	0.001267732

0	0	0	0	0
0	0	0	0	0
0	0.006592462	0	0	0
0	0.001273691	0	0	0
0	0	0	0	0
0	0	0	0	0
0	0	0	0	0
0	0	0	0.009914732	0
0	0	0	0	0
0	0	0	0	0
0	0	0	0	0
0	0	0	0	0
0	0	0	0	0
0	0	0	0	0
0	0	0	0	0
0	0	0	0	0
0	0	0	0	0
0	0	0	0	0
0	0	0	0	0.002762731
0	0	0	0	0
0.175630674	0	0.001775851	0.030127562	0.003531935
0	0	0	0	0
0	0	0	0	0
0	0	0	0	0
0	0.012542905	0	0	0.011874815
0	0	0	0	0
0	0	0	0	0
0	0	0	0	0
0.035986233	0	0	0	0.002833077
0.00218282	0	0	0	0
1.258375521	0.32805169	0.076605906	0.174537087	0.436101381
0	0	0	0	0
0.041736816	0	0.05285844	0	0
0.021942079	0	0.001501681	0	0
0	0	0	0	0
0	0	0	0	0
0	0	0	0	0
0	0	0	0	0
0	0	0	0	0
0	0	0	0.001788085	0
0	0	0	0	0
0.006843012	0.004957326	0	0	0.007534455
0	0	0	0	0
0	0	0	0	0
0	0	0	0	0
0	0	0	0	0
0	0	0	0	0
0	0	0	0	0

0	0	0	0	0
0	0	0	0	0
0.142455064	0.28379147	0.000557108	0.00085922	0.021989847
0	0	0	0	0
1.356751128	0	0	0	0
0	0	0	0	0
0	0	0	0	0
0	0	0	0	0
0	0	0	0	0
0	0	0	0	0
0	0	0	0	0
0	0	0	0	0
0	0	0	0	0
0.029488494	0	0.001641881	0	0.003391082
0.006806639	0.002580951	0.003218499	0.00104502	0.007930199
0	0.006631798	0	0	0
0.002115151	0	0	0.003572119	0.010896053
0.088418655	0.00068436	0.007186616	0	0
0	0	0	0	0
0	0	0	0	0
0	0	0	0	0
0.001272919	0	0	0	0.001679322
0	0	0	0	0
0	0	0	0	0
0	0	0	0	0
0	0	0	0	0
0	0	0	0	0
0	0	0	0	0
0	0	0	0	0
0	0	0	0	0
0	0.001627452	0	0	0
0	0	0	0	0
0	0	0	0	0
0	0.00122433	0	0	0
0	0	0	0	0.00304447
0	0	0	0.004293793	0
0	0	0	0	0
0	0.003335317	0	0.002025691	0
0	0	0	0	0
0	0	0	0	0
0	0	0	0	0
0	0	0	0	0
0	0	0	0	0
0	0	0	0	0
0	0	0	0	0
0	0.006153598	0	0	0
0	0.001446145	0	0	0
0	0.006059859	0	0	0

0	0	0	0	0
0	0	0	0	0
0	0	0	0	0.004774316
0	0	0	0	0
0	0.008112782	0	0	0.000758435
0	0	0	0	0
0	0	0	0	0
0.054528214	0.216065132	0.002316304	0.001984668	0.044650773
0	0	0	0	0
0.102393572	0.01374211	1.06109541	0.075688686	0.016801872
0	0	0	0	0
0.054079948	0.004035378	0	0	0
0.010913993	0.01106661	0	0.002383425	0.005106853
0	0	0	0	0.004201162
0.00212884	0.00096866	0	0	0
0	0.004948499	0	0	0
0	0	0	0	0
0	0	0	0	0
0.108204329	0.006465537	0.086118161	0	0
0	0	0	0.074060862	0
0	0	0	0	0
0	0	0	0	0
0.000582509	0	0	0	0
0	0	0	0	0
0	0.053121214	0	0	0
0.005917664	0	0	0.000832825	0.000991364
0	0	0	0	0
0.003710571	0.001326581	0	0	0
0	0	0.204564055	0	0
0	0.001693016	0	0	0
0	0	0	0	0
0	0	0	0	0
0	0	0.009604818	0	0
0.019204725	0	0	0	0
0	0	0	0	0
0	0	0	0	0
0	0	0.027546227	0	0
0	0	0	0	0
0	0	0	0	0
0	0	0	0	0
0	0	0	0	0
0	0.016426414	0.00105697	0	0
0	0	0	0	0
0.008475518	0	0	0	0
0	0	0	0	0
0	0	0	0	0
0	0	0.0026861	0.001726139	0
0	0	0	0	0.001119014

0	0	0	0	0
0.090771795	0.092619406	0	0	0
0	0	0	0	0
0	0	0.001927261	0	0
0	0	0	0	0
0.319003986	0	0.003173444	0	0.02136222
0	0	0	0	0
0	0	0	0	0
0	0	0	0	3.202082161
0	0	0	0	0
0	0.086300454	0.001226887	0.075373026	0.123319788
0	0	0	0	0
0	0	0	0	0
0.00352842	0.025313287	0	0.001191777	0.00771389
0	0	0	0	0
0.001439287	0	0	0	0
0	0	0	0	0
0.072566004	0.012030517	0.001315991	0	0
0	0	0	0	0
0	0	0	0	0
0.125203573	2.406457277	0	0	0
0	1.407343106	0	2.673643067	0
0	0	0	0	0
0	0	0	0	0
0	0	0	0	0
0	0	0	0	0
0	0.011489151	0.400491467	0	0
0	0	0	0	0
0	0	0	0	0
0.322728106	0.455170872	0.352542359	1.027174106	1.412978451
0	0	0	0.001617051	0.001624116
0	0	0	0	0
0	0	0	0	0
0	0	0	0	0.001022357
0	0	0.003663706	0.021256568	0.019697926
0	0	0	0	0
0	0.005992445	0	0.937327314	0
0.0011988	0.000900034	0	0	0.000813363
0	0	0	0	0
0	0	0	0	0
0.063042863	0	0.009529072	0	0.005248268
3.806842539	5.482111965	0	0	3.106763946
0	0	0	0	0
0	0	0.002490778	0	0
0	0	0.001433239	0	0
0	0	0.000647397	0	0
0	0.006857334	0.00233367	0	0.001606626
0	0	0	0	0

0.005008602	0	0	0.002220396	0
0.016481798	0	0	0	0
0.011943058	0.003415848	0	0	0.030869094
0	0	0	0	0
0	0	0	0	0
0	0	0.033561313	0	0.010535125
0	0	0	0	0
0.014876113	0	0	0	0
0	0	0	0	0.023463191
0	0	0	0	0
0	0	0	0	0
0	0	0	0	0
0	0.003853401	0	0	0
0	0.005102848	0	0.200586642	0
0	0	0	0	0
0.007082041	0.005981681	0	0.003363841	0.028629968
0.111662034	0	0.004190324	0.054435622	0.000887646
0.006199104	0	0	0.000905988	0
0	0	0	0	0
0	0	0	0	0
0.033883046	0.00495157	0.001421808	0	0
0	0.002563512	0	0.002883219	0.002574057
0	0	0	0	0
0	0	0	0	0
0	0	0	0	0
0.00155109	0	0.010403103	0.116568602	0
0.002491315	0	0	0	0.001408592
0	0	0	0	0.063290633
0	0	0	0	0
0	0	0	0	0
0.208220759	0.004741518	0.012447936	0	0.004927658
0.088412276	0.040646872	0	0.005008897	0.008304777
0.08418044	0.104325292	5.420951415	0.120931937	0.023417686
0.009250258	0.003375994	0	0	0.003050893
0.056670004	0	0	0	0
0	0	0	0	0
0.002431467	0.001216997	0	0	0.004674162
0.008746992	0	0.031389035	0	0.004730532
0.008192975	0.224303651	0	0	0.597089404
0.013370307	0	0	0	0
0	0	0	0	0.001228213
0	0	0	0	0
0	0	0	0.015128015	0
0.253827393	0	0.017920112	0	0
0.820596343	0	0.25883433	0	0
0.030055775	0	0.000851159	0	0
0	0	0	0	0.001911532
0	0	0	0	0

0	0	0	0	0
0.060112643	0.68053637	0	0.369540494	0.517433984
0	0	0	0	0
0.000470774	0	0	0	0
0	0	0	0	0
0	0	0	0	0
0.001917656	0	0	0	0
0	0	0	0	0.011252657
0	0.003561553	0	0	0.001341077
0.010920671	0	0	0	0.017459089
0.011745199	0	0	0	0.006314151
0.974260027	0.025121088	0.00306199	0.002179598	0.010702363
0	0	0	0	0
0	0	0	0	0
0.090135545	0.001382188	0	0	0
0	0	0	0	0
0.000604865	0.047682605	0	0.008682841	0.13451649
0.005234654	0.017357811	0.24396914	0.001326062	0
0	0	0	0	0
0.007817955	0.021479185	0.02163456	0	0
0	0.042408284	0.000853794	0.006847329	0.049139952
0	0	0	0	0
0	0	0.212360652	0	0
0	0	0	0	0
0.179216048	0	0	0	0
0	0	0	0	0
0	0	0	0	0.030169696
0.034253636	0.057240891	0.007078164	0.207134529	0.030737355
0	0	0	0	0
0	0	0	0	0
0.001659866	0	0	0.003003453	0.028690969
0	0	0	0	0
0	0	0	0	0
0	0	0	0	0
0	0.033013328	0	0	0
0.01608262	0.141805161	3.863807467	0.623907096	0.233883663
0	0	0	0	0
0	0	0	0	0
0.022082006	0.043322977	0	0	0
0	0	0	0	0
0.807786911	0	0.233778822	0	0
0	0	0	0	0
0.0534862	0	0	0.002726644	0.009362582
0.000479628	0.001000263	0	0	0
0	0.185815628	0.299110645	0	0
0	0	0	0	0
0.061851036	0	0	0	0
0.002067416	0	0	0	0

0	0.002605229	0	0	0
0	0	0	0	0
0	0	0	0	0
0	0.000965313	0	0	0.286568822
0	0	0	0	0
0	0	0	0	0
0	0	0	0	0
0	0	0	0	0.002341247
0	0.937670988	0.019841169	0.511771142	0.325714316
0	0.012078536	0	0.000654986	0.073678946
0	0	0	0	0
0	0	0.000455264	0.029724252	0
0	0	0	0	0
0	0	0	0	0
0	0	0.026423201	0.020665768	0
0	0	0	0	0
0	0	0	0	0.165764211
0	0	0	0	0
0	0	0	0	0
0	0	0	0	0
0	0	0	0	0
0	0	0	0	0
0	0	0	0	0
0.019681972	0.151995381	0.154402669	0.024929549	0.119918842
0	0	0	0	0
0	0	0	0	0
0	0	0	0	0
0	4.556984706	0	7.055323613	1.939809244
0	0	0	0	0
0	0	0	0	0
0	0.018657051	0	0	0.004265889
0	0	0	0	0
0.006972694	0	0	0	0.039269062
0	0	0	0	0
0	0	0	0	0
0	0.022299203	0	0	0
0	0.003446099	0	0	0
0.006344627	0	0	0	0
0	0	0	0	0
0	0	0.005105236	0	0
0.086683078	0.069427394	0.004658611	0.002694341	0.017238931
0	0	0	0	0
0.831895939	9.07984481	24.07802983	17.85059073	0.065166719
0.002161906	0	0	0	0
0	0	0	0	0
0	0	0	0	0
0	0	0	0	0
0.008949951	0.002133155	0	0	0.218075244

0	0	0	0	0
0	0	0	0.026310727	0
1.46750966	0.056745141	0.136391154	0.017335455	0.023099419
0	0	0	0	0
0	0	0	0	0
0.000643679	0	0	0	0.144118906
0	0	0	0	0
0.046313842	0	0	0	0
0	0	0	0	0
0	0	0	0	0
0.05395106	0	0.016471332	0.006096839	0.160797889
3.823251035	0.812879639	0.340154276	1.296375939	1.595197858
0	0	0	0	0
0.009091077	0.005958676	0	0.021933191	0.017133674
1.438964112	0.016861707	0.101982531	1.058440954	0.2825774
0	0.447740386	0	0	0
0	0	0	0	0
0	0	0	0	0
0	0	0	0	0
0	0	0.000814083	0	0.001494557
0.109265079	2.406119259	0	0.002129187	0.000760352
2.132766666	0.069815588	27.22179905	1.311102965	0
2.194265508	1.527012439	12.51560773	1.72469264	0.355494374
0	0	0	0	0
0	0	0	0	0
0	0.417150368	0	0	0.002050041
0	0	0	0.016298285	0
0	0.022377214	0	0	0
0	0	0	0	0
0	0	0	0	0
0	0	0	0	0
0.200754742	0	0	0	0
1.603981416	0	0	0	0.018168128
0	0.005699965	0	0	0.084370985
0.003126865	0	0.001116509	0.001721976	0.018140518
0.002988606	0.005375731	0.016260562	0.171290407	0.422651261
0	0	0.001926389	0	0
0	0	0	0	0
0.011147556	0.231388042	0.010411624	0.088594196	0.042760424
0	0	0	0	0
0	0	0	0	0
0	0	0	0	0
0	0	0	0	0
0	0	0	0	0
0.160183793	0.017858155	0.001399496	0.001079211	0.045284022
0	0	0	0	0
0	0	0	0	0
0	0.140182561	0.001899197	0.200480878	0.09908028

0	0	0	0	0
0	0	0	0	0
0	0	0	0	0
0	0.004089072	0	0	0.004105892
0	0	0	0	0
0	0	0	0	0
0	1.311937863	0	0	0
0	0.001045191	0.081769567	0.064537192	0.323410856
0	0	0	0	0
0	0	0	0	0
0	0	0	0	0.007773891
0	0	0	0	0
0	0.004209037	0	0	0
0	0.188695674	0	0	0.208375719
0	0	0	0	0
0	0	0	0	0
0	0	0	0	0
0	0	0	0.013005321	0.007994313
0	0	0	0	0
0	0	0.001745345	0	0
0	0	0	0	0
0	0	0	0	0
0	0.011898849	0	0	0.038623077
0	0	0	0	0
0	0	0	0	0
0	0	0	0	0
0	0	0	0	0
0	0	0	0	0
0	0	0	0	0
0	0	0	0	0.737155469
0	0	0.005100711	0	0.003421567
0	0	0	0	0
0	0	0	0	0
0	0	0	0	0
0	0	0	0	0
0	0	0	0	0
0	0.225572868	0	0	0
0.007017669	0.044976888	0.001967968	0.008671912	0.00387102
0	0	0	0	0
0.000506476	0	0	0	0
0	0	0	0	0
0	0	0	0	0
0	0	0	0	0
1.248638841	0	0	0	0.022179394
0	0.002918331	0	0	0
0	0	0	0	0
0	0	0	0	0
0	0	0	0	0

0	0	0	0	0
0	0	0	0	0
0	0	0	0	0
0	0	0	0	0
0	0	0	0	0
0	0	0	0	0
0	0	0	0	0
0	0	0	0	0
0.069638262	0.037809152	0.055446349	0.013455016	0.041108633
0	0	0	0	0
0	0	0	0	0
0	0	0	0	0
0	0	0	0	0
0	0	0	0	0
0	0	0.911842297	0	0.533477941
0	0	0.165407642	0	0
0	0	0	0	0
0	0	0	0	0
0	0	0	0	0
0	0	0	0	0
0	0	0	0	0
0	0	0	0	0
0	0	0	0	0
0	0	0	0	0
0.003331225	0	0	0.088958825	0.524862317
0.109370338	1.16850583	0	0	14.09405145
0	0.137984025	0	0	0
0	0	0	0	0
0	0.154884319	0	0.004918606	0
0	0	0	0	0
0	0	0	0	0
0.003217991	0	0	0	0
0	0	0	0	0
0	0	0.003165706	0	0
0.007572719	0	0.000802474	0.021349368	0.004419738
0.005698349	0.009291046	0	0.003062026	0
0	0	0.033220794	0.008125525	0.195058507
0.619264228	0.803674439	0	0	4.934707278
0	0	0	0	0
0	0	0	0	0
0	0	0	0	0
0	0	0	0	0
1.138066485	1.259303669	0	0.653948419	0.082717705
0	0	0	0	0
0	0	0	0	0
0.02470209	0	0	0	0.00258983
0	0	0	0	0
0	7.263484019	0	0	0.003536493

0	0	0	0	0
0	0	0	0	0
0	0	0	0	0
0	0	0	0	0
0.00135444	0	0.020816806	0	0.001312803
0	0.017719685	0	0	0
0	0	0	0	0
0	0	0	0	0
0	0	0	0	0
0	0	0	0	0
0	0	0	0	0
0	2.17514913	0.002638832	1.077827258	1.367785554
0	0.16651465	0	0	0.438457454
0	0	0	0	0
1.487418202	0.018200438	0.000918879	0.035429354	0.142969128
0	0	0	0	0
0	0	0	0	0.003344527
0	0	0.049201606	0	0
0.006472492	0	0	0	0
0	0	0	0	0
0	0	0.004267473	0	0
0	0	0	0	0
0.035663839	0.014795827	0	0.599078832	3.496522126
0	0	0	0	0
0	0	0	0	0
0	0	0	0	0
0	0	0	0	0
0	0	0	0	0
0	0	0	0	0
0	0	0	0	0
0	0	0	0	0
0.045298746	0.113727891	0	0.015079649	0.041044787
0	0	0	0	0
0	0.004538059	0.007624827	0	0.140584118
0.006201231	0.009170429	0	0	0
0	0.099424327	0	0.015341703	0.027119355
0	0	0	0	0
0	0	0	0	0
0	0	0	0.007929979	0
0	0	0	0	0
0	1.18620996	0	0	0
0	0	0	0	0
0	0	0	0	0
0	0	0	0	0
0.015919992	0	0.014138664	0.001641301	0.002511956
0	0	0	0	0
0	0	0	0	0
0	0	0	0	0.004949711

0	0	0	0	0
0	0	0	0	0
0	0	0.001257885	0	0.004329986
0	0	0	0	0
0	0.007774652	0.011481098	0	0
0	0	0	0	0
0	0	0	0.150821937	0
0.014645832	0	0	0	0.178700948
0	0	0	0	0
0	0	0	0	0
0	0	0	0	0
0	0	0	0	0
0	0	0	0	0
0	0	0	0	0
0	0	0	0	0
0	0	0	0	0
0	0	0	0	0
0	0	0	0	0
0	0	0	0	0
0	0	0	0	0
0	0.002829712	0	0	0
0	0	0	0	0
0	0	0	0	0
0	0	0	0	0
0.000606767	0	0	0.008453959	0
0.002585043	0.003665949	0.000707665	0.00785823	0
0	0	0	0	0
0.018555112	1.341577761	0	0	0.004577924
0	0	0	0	0
0.181483585	0.074059696	0	0.543456704	1.213046206
0	0	0	0	0
0	0	0	0	0
0	0	0	0	0
0	0	0	0	0
0.001986574	0	0	0	0.002042201
0	0	0	0.139777308	0
0	0	0	0	0
0	0	0	0	0.00208557
0	0	0	0	0
0	0	0	0	0
1.84254071	4.284898297	8.851973413	16.90794091	23.46637711
0.255160801	0	0	0.000570002	0.989945612
0	0.607616429	0	0	0
0	0	0	0	0
0	0	0	0	0
0	0	0	0	0
0	0	0	0	0
0.008619929	0	0	0	0.008276119

0	0	0	0	0
0.017398039	0.00251194	0.000549551	0	0.000756682
0	0	0	0	0
0	0	0	0	0
15.79184432	16.60748255	0	3.601571916	1.982285786
0	0	0	0	0
0	0	0	0	0
0	0	0	0	0
0.163868396	0.039922766	0.236918813	0.014598932	0.01076682
0	0	0	0	0
0	0	0.038804884	0	0
0	0	0	0	0
0	0.014644667	0.001868938	0	0.168555022
0.00219822	0.010002294	0.008041844	0.068088951	0.042935702
0	0.382866544	0	0.153367872	0
0.157590354	0.012927301	0.002828177	0.295359976	0.659222856
0.011586242	0.609126165	0	0.001095175	0.001759935
0	0	0	0	0
0	0	0	0	0
0	0	0	0	0
0	0	0	0	0
0	0	0	0	0
0	0	0	0	0
0	0	0	0	0
0	0	0	0	0
0	0	0	0	0
0	0.037443122	0	0	0.120046367
0.000509023	0	0	0.002578953	0.016116923
0	0	0	0	0
0	0	0	0	0
0	0	0	0	0
0	0	0	0	0
0	0	0	0	0
0	0	0	0	0
0	0.467760668	0	0	0
0	0	0	0	0
0.001003099	0	0	0	0.001134306
0	0	0	0	0
0	0	0	0	0
0	0	0	0	0
0	0.178675183	0	0	1.249890893
0	0	0	0	0
0	0	0	0	0
0	0	0	0	0
0	0	0	0	0
0	0	0	0	0
0	0	0	0	0
0	0	0	0	0

0	0.01743475	0	0	0.107349669
0	0	0	0	0
0	0	0	0	0
0	0	0	0	0
0	0.088826524	0	0.013044521	0.040968205
0	0	0	0	0
0.003105153	4.224589987	0	0.042389473	0.002340875
0	0	0	0	0
0	0	0	0	0.005782979
0	0	0.004459186	0	0
0.00522987	0	0	0	0
0	0	0	0	0
0	0	0	0	0
0	0	0	0	0
0	0	0	0	0
0	0	0	0	0
0	0	0	0	0
0	0	0	0	0
0	0	0	0	0
0	0	0	0	0
0.001313902	0.389236802	0	0.005200663	0.005757328
0	0	0	0	0
0	0	0	0	0.007374556
0.004510343	0.000604691	0	0.001224189	0.004189534
0	0	0	0	0
0.001900425	0	0	0	0
0	0	0	0	0
0	0	0	0	0
0	0	0	0	0
7.132238316	0.056569028	0.041697323	0	0.000786485
0	0	0	0	0
0	0	0	0	0
0	0	0	0	0
0.30122041	0	0	0	0.007392107
0	0	0	0	0
0	0	0	0	0
0.045148854	0	0	0	0
0	0	0	0	0
0	0	0	0	0
0	0	0	0	0
0	0	0	0	0
0	0	0	0	0
0	0	0	0	0
0	0	0	0	0
0	0	0	0	0
0	0	0	0	0
0	0	0	0	0
0	0.043751678	0	0	0

0.03189304	0	0	0.017783159	0
0	0.074873843	0	0	0
0	0.162383745	0	0	0
0	0	0	0	0
0	0	0	0	0
0	0	0	0	0
0.000799496	0	0	0	0
0.326757551	0.406867092	0	0.284258753	1.13324782
0.097673202	0.039491172	0.006926657	0.092699856	0.418106424
0.144707033	0.052852095	0.00062221	0	0
0	0	0	0	0
0	0	0	0	0
0	0	0	0	0
0	0.01108952	0	0.01403161	0
0	0	0	0	0
0	0	0	0	0
0	0	0	0	0.090983875
0	0	0	0	0
0	0	0	0	0
0	0	0	0.007161706	0
0	0	0	0	0
0	0	0	0	0
0	0	0	0	0
0	0	0	0	0
0	0	0	0	0
0	0	0	0	0
0	0	0	0	0
0	0	0	0	0
0	0	0	0	0
0	0	0	0	0
0	0	0	0	0
0	0	0	0	0
0	0	0	0	0
0	0	0	0	0
0	0.002100605	0	0	0
0	0	0	0	0
0	0	0	0	0
0.067475096	0	0	0	0.015494961
0	0.001031596	0	0.014880213	0
0	0.003464383	0	0	0.032481748
0	0	0	0	0
0.027492205	0	0.004105139	0.004748473	0
0	0	0	0	0
0	0	0	0	0
0	0	0	0	0
0	0	0	0	0
0	0	0	0	0
0	0	0	0	0

0	0	0	0	0
0.07164928	0	0.024410741	0	0
0	0	0	0	0
0	0	0	0	0
0	0.819700287	0.001437038	0.186171105	0.312630023
0	0	0	0	0
0	0	0	0	0
0.046743992	0.001379498	0.008148609	0.020387248	0.054104835
0	0	0	0	0.003983165
0	0	0	0	0
0	0	0	0	0
0	0	0	0	0
0	0	0	0	0
0	0	0	0	0
0	0	0	0	0
0	0	0.003298039	0	0
0	0	0	0	0
0	0	0	0	0
0	0	0	0	0
0	0	0	0	0
0	0	0	0	0
0	0	0	0	0
0	0	0	0	0
0	0	0	0	0.001510428
0	0.274680906	0	0.301267876	0
0	0	0	0	0
0.033508463	0.004149234	0	0	0.013025177
0	0	0	0	0
0	0	0	0	0
0	0	0	0	0
0	0	0	0	0
0	0	0	0	0
0	0	0	0	0
0	0	0	0	0
0	0	0	0	0
0	0	0	0	0
0	0	0	0	0
0	0	0	0	0.02185868
0	0	0	0	0
0	0	0	0	0
0	0	0	0	0
0	0.174113291	0	0.145571046	0
0	0	0	0	0
0	0	0	0	0
0	0	0	0	0
0	0	0	0	0
0	0	0	0	0
0	0	0	0	0

0	0	0	0	1.898974908
0	0	0	0	0
0	0	0	0	0
0	0	0	0	0
0	0	0	0	0
0	0	0.005309455	0	0.026662331
0	0	0	0	0
0	0	0	0	0
0	0	0	0	0
0	0	0	0	0
0	0	0	0	0
0	0	0	0	0.001392374
0	0	0	0	0
0	0	0	0	0
0	0	0	0	0
0	0	0	0	0
0	0	0	0	0
0	0	0	0	0
0	0	0	0	0
0	0	0	0	0
0.007790768	0	0	0	0
0	0	0	0	0
0.002879758	0	0.01320476	0	0.078968539
0	0	0	0	0
0	0	0	0	0
0	0	0	0	0
0	0	0	0	0
0	0	0	0	0
0	0	0	0.427331844	0
0	0	0	0	0
0	0	0	0	0
0	0	0	0	0
0.143384185	0.014904503	0.021594714	0.003700579	0.206612945
0	0	0	0	0.088775718
0.001206174	0.15973254	0	0	0.518753048
0.001442756	0	0.018809951	0	0.268988762
0	0	0	0	0
0	0	0	0	0
0	0.002088137	0	0	0
0	0	0	0	0
0	0	0	0	0
0	0	0	0	0
0	0.086068444	0.001402209	0.056845673	0.034477058
0	0	0	0	0
0	0	0	0	0
0	0	0	0	0.271617178
0	0	0	0	0
0	0	0	0	0

0	0	0	0	0
0	0	0	0	0
0	0	0	0	0
0	0	0	0	0
0	0	0	0	0
0	0	0	0	0
0	0	0	0	0
0	0	0	0	0
0	0	0	0	0
0	0.009736601	0	0.007581389	0
0	0	0	0	0
0	0	0	0	0
0	0	0	0	0
0	0.92998524	0	0	0
0.00612703	0	0	0	0
0.027286185	0.001228172	0.002257029	0	0
0	0	0	0	0
0	0	0	0	0
0	0	0	0	0
0.030569377	0.001028264	0	0	1.237010436
0	0	0	0	0
0	0	0	0	0
0	0	0	0	0
0	0	0	0	0
0	0	0	0	0.002700111
0	0	0	0	0
0	0	0	0	0
0	0	0	0	0
0	0	0	0	0
0	0.012474476	0	0	2.690616486
0	0.000598934	0	0.152779227	2.478420629
0	0	0	0	0
0	0	0	0	0
0	0	0	0	0
0	0	0	0	0
0	0	0	0	0
0	0	0	0	0
0	0	0	0	0
0	0	0	0	0
0	0	0	0	0
0	0	0	0	0
0	0	0	0	0
0	0	0	0	0
0	0	0	0	0
0	0	0	0	0
0	0	0	0	0
0.048831931	0.005280536	0.082022883	0.005599717	0
0	0	0	0	0

0	0	0	0	0
0	0	0	0	0
0	0	0	0	0
0	0	0	0	0
0	0.003193535	0.01787766	0	0
0	0	0	0	0
0	0	0	0	0
0	0	0	0	0
0	0	0	0	0
0	0	0	0	0
0	0	0	0	0.059489731
0.00479391	0	0.004108225	0.001584014	0.01013484
0	0	0	0	0
0	0	0	0	0
0.028273319	0	0	0.017704565	0
1.096015653	0	0	0	1.608513142
0	0	0	0	0
0	0	0	0	0
0.373571867	0	0.027737716	0	0.055860736
0.041214283	0.763772231	0	0	0.001864207
0	0	0	0	0
0	0	0	0	0
0	0	0	0	0
0	0	0	0	0
0	0	0	0	0
0	0	0	0	0
0.006935648	0	0.005695974	0	0
0	0	0	0	0
0	0	0	0	0
0	0	0	0	0
0.002059075	0	0	0	0
0	0	0	0	0
0	0	0	0	0
0	0	0	0	0
0	0	0	0	0
0	0	0	0	0
0	0	0	0	0
0.027486274	0.00065825	0.002700167	0.002665234	0.044912055
0	0.025929466	0	0	0
0	0	0	0	0
0	0	0	0	0.001484659
0	0	0	0	0
0	0	0	0	0
0.014626313	0	0.006566568	0	0.010364727
0	0	0	0	0
0	0	0	0	0
0	0	0	0	0
0	0	0	0	0

0	0	0	0	0
0	0	0	0	0
0	0	0	0	0
0.000817517	0.001790176	0	0	0
0	0	0	0.003841572	0.007502355
0	0	0	0	0.003043074
0	0	0	0	0
0	0	0	0	0
0	0	0	0	0
0	0	0	0	0
0.011821721	0	0	0	0
0	0	0	0	0
0.004346283	0.044051843	0	0.082025725	0.084206628
0	0	0	0	0
0	0	0	0	0
0	0	0	0	0
0	0	0	0	0
0	0	0	0	0
0	0	0	0	0
0	0	0	0	0
0	0	0	0	0
0	0	0	0	0
0.029950645	0.00143225	0.003760095	0.003866093	0
0.018684406	0.039999135	0	0	0.028001435
0	0	0	0	0
0	0	0	0	0
0	0	0	0	0
0	0	0	0	0
0	0	0	0	0
0	0	0	0	0
0.004679531	0	0	0.058194136	0.048586705
0.00361871	0	0	0	0.005952062
0	0	0	0	0
0	0	0	0	0
0	0	0	0	0
0	0	0	0	0
0	0.002262193	0	0	0
0	0	0	0	0
0	0.001351133	0.000443392	0.001823566	0
0	0	0	0	0
0	0	0	0	0
0	0	0	0	0
0	0	0	0	0
0	0	0	0	0
0	0	0	0	0
0.078535939	0.011491476	0.004421267	0.438612852	0.292925024
0	0	0.165757268	0	0
0	0	0	0	0

0	0	0	0	0
0	0	0	0	0
0	0.001219345	0	0	0
0	0	0	0	0
0	0	0.001645742	0	0
0	0	0	0	0
0	0	0	0	0
0	0	0	0	0
0	0	0	0	0.002447834
0	0	0	0	0
0.00437997	0	0	0	0
0.000871908	0	0	0	0
0	0	0	0	0
0	0	0	0	0
0	0	0	0	0
0	0	0	0	0
0	0	0	0	0
0	0	0	0	0
0	0	0	0	0
0.000968099	0.002665038	0.002544197	0.000980969	0.002846292
0	0	0	0	0
0	0	0	0	0
0	0	0	0	0
0	0	0	0	0
0	0	0	0	0
0	0	0	0	0
0	0	0	0	0
0	0.004826152	0	0	0
0	0	0	0	0
0	0	0	0	0
0.001330715	0.00603607	0	0.034342238	0.004326228
0	0	0	0	0
0.049443074	0.011116918	0.34641696	0.005626511	0.054163109
0	0.005680142	0	0.002249876	0.007141784
0	0	0	0	0
0	0	0	0	0
0	0	0	0	0
0	0	0	0	0
0	0	0	0	0
0	0	0	0	0
0	0	0	0	0
0.092951858	0	0.037973114	0.025760719	0.452422652
0	0.058375166	0	0.042907566	0.061071788
0	0	0	0	0
0	0	0	0	0
0	0.17956314	0	0.031179363	0
0	0	0	0	0.006636405
0	0	0	0	0

0	0	0	0	0
0	0.037556214	0	0.054455368	0
0.009949252	0.019859898	0	0.005100771	0.006964676
0	0	0	0	0
0	0	0	0	0.861093
0.068489105	0	0	0	0
0	0	0	0	0
0	0	0	0	0
0	0	0	0	0
0	0	0	0	0
0	0	0	0	0
0	0	0	0	0
0	0	0	0	0
0	0	0	0	0
0	0	0	0	0
0	0	0	0	0
0	0	0	0	0
0	0	0	0	0
0	0	0	0	0
0	0	0	0	0
0	0	0	0	0
0	0	0	0	0
0	0	0	0	0.003331809
0	0	0	0	0
0	0.001221444	0	0.00568743	0.000883057
0.084646204	0.00393198	0	0	0.001110418
0	0	0	0	0
0	0	0	0	0.055365229
0	0	0	0	0
0	0	0	0	0
0	0	0	0	0
0	0	0	0	0
0	0	0	0	0
0	0	0	0	0
0	0	0	0	0
0	0	0	0	0
0	0	0	0	0
0	0	0	0	0
1.516357943	0	0	11.37165717	1.734460149
0	0	0	0	0
0	0	0	0	0
0	0.002132796	0	0	0
0.029762662	0	0	0.01248276	0
0	0	0	0	0
0	0	0	0	0
0	0	0	0	0
0.005126012	0	0	0	0.00477359
0	0	0	0	0

0	0	0	0	0
0.049081346	0.006903285	0.009999027	0.012047934	0.024738935
0	0	0	0	0
0	0	0	0	0
0	0	0	0	0
0	0	0	0	0
0	0	0	0	0
0	0	0	0	0
0	0	0	0	0
0	0	0	0	0
0	0	0	0	0
0	0	0	0	0
0	0.011323527	0	0	0
0.001190826	0	0	0	0
0.004269561	0	0	0	0
0	0	0	0	0
0	0	0	0	0
0	0	0	0	0
0.001312011	0	0	0	0
0	0	0	0	0
0	0	0	0	0
0	1.176438771	0	0	0.00079025
0.009129036	0	0.002940125	0.063256438	0
0	0	0	0	0
0	0	0	0	0
0	0	0	0	0
0	0	0	0	0
0	0	0	0	0
0	0	0	0	0
0	0	0	0	0
0	0	0	0	0
0	0	0	0	0
0	0	0	0	0
0	0	0	0	0
0.596015191	0	0	0	0
0	0	0	0	0
0	0	0	0	0
0	0	0	0	0
0.00162838	0.036676629	0	0	0
0	0	0	0	0
0.006048813	0.046457221	0.038542129	0.127868429	0
0	0	0	0	0
0	0	0	0	0
0	0	0	0	0
0	0	0	0	0
2.122301187	0.03884689	0.299875052	0.040834892	0.038886668
0	0	0	0	0
0	0	0	0	0

0	0	0	0	0
0	0	0	0	0
0	0	0	0	0
0	0	0	0	0
0	0	0	0	0
0	0	0	0	0
0	0	0	0	0
0	0.001291938	0	0	0
0	0	0	0	0
0.018217383	0.003214735	0.002877158	0.314463658	0.001584634
0	0	0	0	0
0	0	0	0	0
0	0	0	0	0
0	0	0	0	0
0	0	0	0	0
0	0	0	0	0
0	0	0	0	0
0	0	0	0	0
0	0	0	0	0
0.082290597	0.024661983	0.022694212	0.133055104	0.136479695
0	0	0	0	0
0	0	0	0	0
0	0	0	0	0
0	0	0	0	0
0	0	0	0	0
0	0	0	0	0
0	0	0	0	0
0	0	0	0	0
0	0	0	0	0
0	0	0.000713544	0	0
0	0	0	0	0
0	0	0	0	0
0	0	0	0	0
0	0	0	0	0
0	0	0	0	0
0	0	0	0	0.00148712
0	0	0	0	0
0	0	0	0	0
0	0	0	0	0
0	0	0	0	0
0	0	0	0	0
0	0	0	0	0
0	0	0	0	0
0	0	0	0	0
0	0.005969959	0	0	0.00496346
0	0	0	0	0.103702147
0	0	0	0	0
0	0	0.000941348	0	0

0	0	0	0	0
0	0	0	0	0
0	0	0	0	0
0	0	0	0	0
0	0	0	0	0
0.001852107	0.003743717	0	0.004691825	0
0	0	0	0	0
0	0	0	0	0
0	0	0	0	0
0	0	0	0	0
0	0	0	0	0
0	0	0	0	0
0	0	0	0	0
0	0	0	0	0
0	0	0	0	0
0	0	0	0	0
0	0	0	0	0
0	0	0	0	0
0	0	0	0	0
0	0	0	0	0
0.000495576	0	0	0	0
0	0	0	0	0
0	0	0	0	0
0	0	0	0	0
0	0	0	0	0
0.002897006	0.001394239	0	0.002258093	0
0	0	0	0	0
0	0	0	0	0
0	0	0	0	0
0	0	0	0	0.009611187
0	0	0	0	0
0	0	0	0	0
0	0	0	0	0
0	0	0	0	0
0	0	0	0	0
0	0	0	0	0
0	0	0	0	0
0	0	0	0	0
0	0	0	0.001273641	0
0	0	0	0	0
0	0	0	0	0
0	0	0	0	0
0	0	0	0	0
0	0	0	0	0
0	0.003464865	0	0	0
0	0	0	0	0
0	0	0	0	0
0	0	0	0	0

0	0	0	0	0
0.132390296	0	0	0	0
0	0	0	0	0
0	0	0	0	0
0	0	0	0	0
0	0	0	0	0
0	0	0	0	0
0	0	0	0	0
0	0	0	0	0
0	0	0	0	0
0.000626583	0	0	0.005026396	0
0	0	0	0	0
0.577656663	0	0	0	0
0	0	0	0	0
0	0	0	0	0
0	0	0	0	0
0	0	0	0	0
0	0	0	0	0
0	0	0	0	0
0	0	0	0	0
0.01950228	0.006896551	0.002089103	0.005369989	0.001198544
0	0	0	0	0
0	0	0	0	0
1.075230811	4.372719094	0	1.819879868	0.638417565
0	0	0	0	0
0	0	0	0	0
0	0	0	0	0
0	0	0	0	0
0	0	0	0	0
0	0	0	0	0
0	0	0	0	0
0	0	0	0	0
0	0	0	0	0
0	0	0	0	0

PHL018-0062_V2	PHL018-0131_V2	PHL018-0252_V1	PHL018-0044_V3	PHL018-0031_V3
EYCF	EYCF	REF	CTRL	CTRL
0.008854071	0	0.216805811	0.016968487	0.180267464
2.24963331	0.12446868	7.320441836	2.573517137	2.636154334
0	0.019356662	0	0	14.49174452
0	0	0	0	0
0	0	0	0	0
0	0	0	0	0
0	0	0	0	0
0	0	0	0	0
0	0	0	0	0
0	0	0	0	0
0	0	0	0	0
0	0	0	0	0
0	0	0	0	0
2.986305611	3.36210968	3.605195637	1.973765349	18.69039985
0	0	0	0	0
0	0	0	0	0
0	0	0	0	0
0	0	0	0	0
0	0	0	0	0
0	0	0	0	0
0	0	0	0	0
0	0	0.215814156	0	0
0	0	0	0	0
0	0	0	0	0
0	0.004019795	0	0	0
0	0	0	0	0
0	0	0	0	0
0	0	0	0	0
0	0	0	0	0
0	0.008139659	0	0	0
0	0	0	0	0
0	0	0	0	0
0	0	0	0	0
0	0	0	0	0
0	0	0	0	0
0	2.152490621	0	0.750219275	0
0	0	0	0	0
0	0	0	0	0
0	0	0	0	0
0	0	0	0	0
0	0	0	0	0
0	0.020855532	0.003340769	0.008781517	0.002487783
0.026306779	3.188965131	0.695058945	0.401187995	0.098711278
0.002784448	0.003017461	0.001933422	0.190793453	0.005779074
0	0	0	0.007613126	0.00196071
0	0	0	0	0

0	0	0	0	0.009529209
0.07994914	0	0.008492793	0.007096518	0.016607875
0	0	0.253653571	0.001785808	0.00090342
0	0.071680589	0.040813657	0.791935189	0.326081671
0	0.00129466	0	0.003260715	0.001458557
0	0	0	0	0.004987314
0	0	0	0	0.269122442
0	0.006200862	0	0	0
0	0	0	0	0
0	0	0	0	0
0	0	0	0	0
0	0.079621641	0.836706443	0.031040902	0.051738043
0	0	0	0.001145987	0
0.006182832	0.003771984	0.057718887	0.083369043	0.012245259
0	0	0	0	0
0	0	0	0	0
0	0.084913098	0.010463006	0	2.325108084
0	0	0	0	0
0	0	0	0	0
0	0	0	0	0
0.067995226	0	0.088378272	0.246516971	0.085081435
0.000908767	0	0.190882296	0.010809085	0.019637947
0	0	0	0	0
0	0	0	0	0
0	0	0	0	0
0	0	0	0	0
0	0	0	0	0
0	0	0	0	0
0	0	0	0	0
0	0	0	0	0
2.964602578	0	0.001430021	0	0.029983575
0	0	0	0	0
0	0	0	0	0
0	0	0	0	0
0	0	0	0	0
0	0	0	0	0
0	0	0	0	0
0	4.244878764	0	0	0.05105685
0	0	0	0	0
0	0	0	0	0
0	0	0	0	0
0	0	0	0	0
0	0	0	0	0
0	0	0	0	0
0	0	0	0	0
0	0	0	0	0.021840755
0	0	0	0	0
0.005967968	0	0.071403386	0	0.045566313

0	0	0	0	0
0	0	0	0	0
0	0	0	0	0
0	0	0	0	0
0	0	0	0	0.024058004
0	0	0	0	0
0	0	0	0	0
0	0	0	0	0
0	0	0	0	0
0	0	0	0	0
0	0	0	0	0
0	0.005083641	0	0	0
0	0	0	0	0
0	0	0	0	0
0	0	0	0	0
0	0	0	0	0
0	0	0	0	0
0	0	0	0	0
0.012252239	0.012392382	0	0	0.007645414
0	0	0	0	0
0	0	0	0	0
0	0	0	0	0
0	0	0	0	0
0	0	0	0	0
0	0	0	0	0
0	0	0	0	0
0	0	0	0	0
0	0	0	0	0
0	0	0	0	0
0	0	0	0	0
0.01056693	0.044219288	0	0	0.020988726
0	0	0	0	0.016709455
0	0	0	0	0
0	0	0	0	0
0	0	0	0	0
0	0	0	0	0
0	0	0	0	0
0	0	0	0	0
0	1.530694771	0	0	0
0	0	0	0	0
0	0	0	0	0
0	0	0	0	0
0	0	0.002395362	0	0.005414996
0	0	0	0	0
0	0	0.000729654	0	0
0	0	0	0	0
0	0	0	0	0.051472129
0	0	0	0	0

0	0	0	0	0
0	0	0	0	0
0	0	0	0	0
0	0	0	0	0
0	0.002573842	0	0.010555299	0.045262137
0	0	0	0	0
0	0	0	0	0
0	0	0	0	0
0	0	0	0	0
0	0	0	0	0
0	0	0	0	0
0	0	0	0	0
0	0	0	0	0
0	0	0	0	0
0	0	0	0	0
0	0	0	0	1.568036522
0	0	0	0	0
0	0	0	0	0
0	0	0	0	0
0	0	0	0	0
0	0	0	0	0
0	0	0	0	0
0	0	0	0	0
0	0	0	0	0
0	0	0	0	0
0	0	0	0	0
0	0	0	0	0.003901604
0	0	0	0	0
0	0	0	0	0
0	0	0	0	0
0	0	0.039580773	0	0.002456231
0	0.000852971	0	0.091584637	0.000900893
0	0	0	0	0
0	0	0	0	0
0	0	0	0	0
0	0	0	0	0
0	0	0	0	0
0	0	0	0	0
0	0	0	0	0
0	0	0	0	0
0	0	0	0	0
0	0	0	0	0
0	0	0	0	0
0	0	0	0	0
0	0	0	0	0
0	0	0	0	0
0	0	0	0	0
0	0	0	0	0

0	0	0	0	0
0	0	0	0	0
0	0	0	0	0
0	0	0	0	0
0	0	0	0	0
0	0	0	0	0
0	0	0	0	0
0	0	0	0	0
0	0	0	0	0
0	0	0	0	0
0	0	0	0	0
0	0	0	0	0
0	0	0.002288046	0	0
0	0	0	0	0
0	0	0	0	0
0	0	0	0	0
0	0	0	0	0
0	0	0	0	0
0	0	0	0	0
0	0	0	0	0
0.015402915	0	0.010084085	0.136550917	0.011282995
0	0	0	0	0
0	0	0	0	0
0	0	0	0	0.001680842
0	0	0	0	0
0	0	0	0	0
0	0.003110708	0.001245731	0.003446042	0.003379346
0	0	0	0	0
0	0	0	0	0
0	0	0	0	0
0.052884719	0	0	0	0
0	0	0	0	0
0	0	0	0	0
0	0	0.007096519	0	0
0.00515415	0.001489458	0	0.005923154	0.003565783
0	0	0	0	0
0	0	0	0	0
0	0	0	0	0
0	0	0	0	0
0	0	0	0	0
0	0	0	0	0
0	0	0	0	0
0	0	0	0	0
0	0	0	0	0
0	0	0	0	0
0	0	0	0	0.003994134
0	0	0.001937477	0	0
0.000612164	0	0.005242473	0	0

0	0	0	0	0
0	0	0	0	0
0	0	0	0	0
0	0	0	0	0
0	0	0	0	0
0	0	0	0	0
0	0.000755465	0	0.001802562	0
0	0	0	0	0
0	0	0	0	0
0	0	0	0	0
0	0	0	0	0
0	0	0	0	0
0	0	0	0	0
0	0	0	0	0
0	0	0	0	0
0	0	0	0	0
0	0	0	0	0
0	0	0	0	0
0.00083009	0.015652248	0.007781196	0.00300491	0
0	0.007628276	0	0	0
0.008877382	0	0.02061799	0	0.015248155
0	0	0	0	0
0	0	0	0	0
0	0	0	0	0
0	0	0.002111443	0.045288996	0
0	0	0	0	0
0	0	0	0	0
0	0	0	0	0
0	0	0	0	0
0	0	0	0	0
0.010128935	0.021074998	2.127613621	0.064253802	0.144601339
0	0	0	0	0
0.036384215	0.221772844	0	0	0
0	0	0	0	0
0	0	0	0	0
0	0	0	0	0
0	0	0	0	0
0	0	0	0	0
0	0	0	0	0
0	0.001364416	0	0	0
0	0	0	0	0
0	0.003182586	0	0.045799786	0.009075757
0	0	0	0	0
0	0	0	0	0
0	0	0	0	0
0	0	0	0	0
0	0	0	0	0
0	0	0	0	0

0	0	0	0	0
0	0	0	0	0
0	0.002747429	0	0.198749299	0.002954546
0	0	0	0	0
0	0	2.401652605	0	0.124722076
0	0	0	0	0
0	0	0	0	0
0	0	0	0	0
0	0	0	0	0
0	0	0	0	0
0	0	0	0	0
0	0	0	0	0
0	0	0	0	0
0	0	0	0.100441422	0.009329449
0.006832758	0	0.00091239	0.04321737	0.005390167
0	0	0	0.002328045	0
0	0	0.001247504	0.023537208	0
0	0	0	0	0
0	0	0	0	0
0	0	0	0	0
0	0	0	0	0
0	0	0.003378419	0	0
0	0	0	0	0
0	0	0.052955129	0	0
0	0	0	0	0
0	0	0	0	0
0.256451358	0	0	0	0.13254327
0	0	0	0	0
0	0	0	0	0
0	0	0	0	0
0	0	0	0	0.001517339
0	0	0	0	0
0	0	0	0	0
0	0	0	0	0.000684895
0	0	0	0	0
0	0	0	0.003350418	0
0	0	0	0	0
0.004754535	0.000883271	0	0.059712804	0.001492632
0	0	0	0	0
0	0	0	0	0
0	0	0	0	0
0	0	0	0	0
0	0	0	0	0
0	0	0.0017575	0	0
0	0	0	0	0
0	0	0	0	0
0	0	0	0	0
0	0	0.004855687	0	0

0.205557247	0	0	0	0
0	0	0	0	0
0.003397475	0	0	0.001171315	0
0	0	0	0	0
0.001424245	0	0.000988945	0.015614534	0.002086585
0	0	0	0	0
0	0	0	0	0
0	0.001384613	0	0.015279748	0.011991715
0	0	0	0	0
0.026338883	0.364280256	0.177172666	0.009790023	0.327693916
0	0	0	0	0
0	0.006850405	0	0.015691463	0.001620702
0	0	0.007158396	0.007935021	0.055496829
0	0	0.003539642	0.003012813	0.001867081
0	0	0.002568226	0	0
0	0	0	0	0
0	0	0	0	0
0	0	0	0	0
1.813134368	0	0.016402728	0	0.093896204
0	0	0	0	0
0	0	0	0	0
0	0	0	0	0
0	0	0	0	0
0	0	0	0	0
0	0	0	0	0
0	0.002420934	0.000969501	0	0.001022779
0	0.317253446	0	0	0
0	0	0	0	0
0	0	0	0	0.0058642
0	0	0.000997494	0	0
0	0	0	0	0.000877998
0	0	0	0	0
0	0	0	0	0
0	0	0.014707975	0	0.046281219
0	0	0	0	0
0.00424759	0	0.014746884	0	0.003733751
0.03830334	0	0	0.122450727	0.12962374
0	0	0	0	0
0	0	0	0	0
0.039061795	0	0.0024472	0.004556489	0.003657387
0	0	0	0	0.081704137
0	0	0.070365796	0	0.008408238
0	0	0	0	0
0	0	0.000937277	0	0
0	0	0.219502527	0	0
0	0	0	0	0.004789158
0.002430871	0.006322309	0	0.002873375	0.006613916
0	0	0	0	0

0	0	0	0	0
0	0.001009375	0	0.007626595	0.003127179
0	0	0	0	0
0	0	0	0	0
0	0	0	0	0
0.034189382	0	0.085653565	0	0.00681211
0	0	0	0	0
0	0	0	0	0
0	0	0	0	0.066515747
0	0	0	0	0
0.004124008	0.007425616	0	0.012796149	0.009062797
0	0	0	0	0
0	0	0	0	0
0	0	0	0.003099784	0.001975863
0	0	0.024846545	0	0
0	0	0	0	0
0	0	0	0	0
0	0	0	0	0
0	0	2.037309296	0	0
0	0	0	0	0
0	0	0	0	0.386279234
0	0.827237768	0	0.359225815	0.352215969
0	0	0	0	0
0	0	0	0	0
0	0	0	0	0
0	0	0.001289089	0	0
0.04874382	0	0	0.003360987	0.001904319
0	0	0	0	0
0	0	0	0	0
0.761752653	0.077313163	0.198274059	1.662970157	0.688330683
0	0	0	0.003364731	0
0	0	0	0	0
0	0	0	0	0
0	0	0.002199584	0	0
0	0	0	0	0.001982648
0	0	0	0	0
0	3.697293713	0	0.087354965	0
0	0.000794501	0	0	0
0	0	0	0	0.337125183
0	0	0.134201019	0	0
0	0	0	0.015799831	0.015160831
8.03926728	0.087262603	2.963539528	4.491225604	7.347761559
0	0	0	0	0
0	0	0	0	0
0	0	0	0	0.003868357
0	0	0	0	0
0	0	0.001436521	0	0.002652063
0	0	0	0	0

0	0.001106479	0	0.008580297	0
0.003262055	0	0.00353915	0	0
0	0	0.001509417	0.002248329	5.350031346
0	0	0	0	0
0	0	0	0	0
0	0	0	0	0.081023291
0	0	0	0	0
0	0	0	0.015164912	0
0	0	0	0	0
0	0	0	0	0.000917332
0	0	0	0	0
0	0	0	0	0
0	0	0	0	0
0.161936903	0	0.009219948	0.002686977	0
0	0	0	0	0
0.005639523	0.0023468	0	0	0.013384706
0	0.01105503	0.000520842	0.323513948	0.004395719
0.010024749	0	0	0	0.001557683
0	0	0.133242342	0	0
0	0	0	0	0
0.011028942	0	0.138502364	0	0.096716663
0.004350378	0	0	0.004499513	0.001912052
0	0	0	0	0
0	0	0	0	0.025676868
0	0	0	0	0
0.001317497	0	0	0	0.062248788
0	0	0	0	0.003952784
0.004017525	0	0.001255333	0	0
0	0	0	0	0
0	0	0	0	0
0.06396997	0	0.24891117	0	0.011847495
0	0	0	0.075562567	0.006590732
0.033238585	0.015447801	0.006424247	0.028353016	0.004267182
0	0.009791898	0	0	0
0	0	0	0	0.009756522
0	0	0	0	0
0	0	0	0.002563308	0
0	0.002940528	0	0	0.008518586
0	0.045560971	0	0.468345902	0.333417117
0	0.000858637	0	0.002253606	0.000580401
0	0	0	0	0
0	0	0	0	0
0	0	0.091324032	0	0
0	0	0.588580448	0	0.020919184
0.171724811	0	0.15958583	0.560395489	0
0	0	0	0.001365754	0
0	0	0	0	0
0	0	0	0	0

0	0	0	0	0
0.017166633	0.021751434	0.04538733	1.595912139	0.273747191
0	0	0	0	0
0	0	0	0	0
0	0	0	0	0.0016607
0	0	0	0	0
0.01221644	0	0	0	0
0.001775009	0.003077678	0	0	0
0	0	0.001888562	0.02250464	0
0	0	0	0	0
0	0	0.006813687	0	0
0.00146165	0	0.002029836	0.07143048	0.015257367
0	0	0	0.02711972	0
0	0	0	0	0
0	0	0	0.000873373	0.001030935
0	0	0	0	0
0.017211386	0	0.009810506	0.059515153	0.000564526
0.00833685	0	0.004862604	0.063807566	0.081588677
0	0	0	0	0
0.002598487	0	0.049618187	0.00358342	0.006027599
0.001059659	0.015158018	0	0.233992712	0.004463292
0	0	0	0	0
0	0	0	0	0
0	0	0	0	0
0	0	0	0	0.038147914
0	0	0	0	0
0	0	0	0.070316452	0.168749495
0.187803963	0.018307654	0.132359539	0.054166447	0.108076599
0	0	0	0	0
0	0	0	0	0
0	0	0	0	0
0	0	0	0	0
0	0	0	0	0
0	0	0	0	0
0.0110414	0.14613723	0	0	0
4.792961257	0.006610051	0.062537678	1.272092757	0.052709719
0	0	0	0	0
0	0	0	0	0
0	0.001806763	0	0.002155496	0
0	0	0	0	0
0.161725564	0	0.174649859	0.585218365	0
0	0	0	0	0
0.005063542	0.002012002	0.002050968	0	0.000618194
0	0	0.004526105	0	0.008505176
0	0	0.041623767	0	0
0	0.38407724	0	0	0
0	0	0	0	0
0	0	0.002514909	0	0.000723577

0.007663367	0	0.002455926	0	0
0	0	0	0	0
0	0	0	0	0
0	0	0	0.074465861	0.011160002
0	0	0	0	0
0	0	0	0	0
0	0	0	0	0
0	0	0	0.013187121	0.002093381
0	5.106909126	0	0.689155777	0
0	0	0	0	0
0	0	0	0	0
0	0.014491596	0	0.009740109	0.003966566
0	0	0	0	0
0	0	0	0	0.025494956
0.001554232	0.07613018	0	0.030743678	0.01963932
0	0	0	0	0
0	0	0	0	0
0	7.292673258	0	0.023878052	0
0	0	0	0	0
0	0	0	0	0
0	0	0	0	0
0	0	0	0	0
0	0	0	0	0
0.137173979	0.020117869	0.821894265	0	0.536001471
0.014768438	0	0	0	0.002906389
0	0	0	0.25023031	0
0	0	0	0	0
0	0	0	1.884129401	6.801636772
0	0	0	0	0
0	0	0	0	0
0	0	0	0.004024345	0.00050298
0	0	0	0	0
0.00661939	0	0	0	0.000893211
0	0	0	0	0
0	0	0	0	0
0	0.035162939	0	0	0
0.068674129	0.093433705	0	3.577858078	0
0	0	0	0	0
0	0	0.41217145	0	0.001077181
0	0	0	0	0.004320464
0.025521597	0	0.109777947	0	0.08437641
0	0	0	0	0
28.83675068	10.53134384	0.136771676	5.977277835	0.719256427
0	0	0	0	0
0	0	0	0	0
0	0	0	0	0
0	0	0	0	0
0	0	0.001131134	0	0.001988826

0	0	0	0	0
0	1.673466335	0	0.011768023	0
0.291758958	0	0.093612966	0.006191344	0.141654007
0	0	0	0	0
0	0	0	0	0
0	0.010842656	0.003274378	0.005725528	0
0	0	0	0	0
0	0	0	0	0.000913207
0	0	0	0	0
0	0	0	0	0
0.007410536	0	0	0	0.015162057
4.079293454	0.768862951	0.366585449	1.660423119	2.528031545
0	0	0	0	0
0	0.030364506	0.004404387	0.077014453	0.013535249
1.792528152	0.005087139	0.215794827	0.051474401	0.062724184
0	0	0	0	0
0	0	0	0	0.001939806
0	0	0	0	0
0	0	0	0	0
0	0	0	0	0.002775454
0	0	0	0.0035443	0.002510229
21.02304216	0.464665964	2.881422104	0	5.592197664
2.301307527	7.852770176	5.97628733	0.698827569	6.17134509
0	0	0	0	0
0	0	0	0	0
0	0.152857506	0	5.18495452	0
0	0	0	0	0
0.004020881	0	0	0	0
0	0	0	0	0
0	0	0	0	0
0	0	0	0	0
0	0.091504457	0	0.272288124	0
0.327789877	0	0.007106984	0.054016279	0.002114693
0.008805818	0.000694016	0	0.005381816	0.010115506
0.003637513	0	0.066872515	0	0.003172099
0.004521747	0.394280972	0.023961184	0.021414619	0
0	0.002989561	0	0	0.001010407
0	0	0	0	0
0	2.987073147	0.014696526	0.152948928	0
0	0	0	0	0
0	0	0	0	0
0	0	0	0	0
0	0	0	0	0
0	0	0	0	0
0.01004168	0.419751294	0.319796846	0.005333309	0.011729464
0	0	0	0	0
0	0	0	0	0
0	0.014190997	0	0.339278695	0.098083318

0	0	0	0	0
0	0	0	0	0
0	0	0	0	0
0	0	0	0	0.002202723
0	0	0	0	0
0	1.718145999	0	0.00739533	0
0	0.007903668	0	0	0
0	0	0	0	0.070629809
0	0	0	0	0
0	0	0	0	0
0	0.000670025	0.035239597	0	0
0	0	0	0	0
0	0.010950968	0.005481857	0	0
0	0	0	0	0
0	0	0	0	0
0	0	0	0	0
0	0	0	0	1.543167115
0	0.014316361	0	0.002439948	0.003770354
0	0	0.140714416	0	0
0.002127497	0	0.010072218	0	0
0	0	0	0	0.000418708
0	0	0	0	0
0	0	0	0.052937152	0.006701545
0	0	0	0	0
0	0	0	0	0
0	0	0	0	0
0	0	0	0	0
0	0	0	0	0
0	0	0	0	0
0	0	0.011512794	0.043943581	0
0	0	0	0.010912666	0
0	0	0	0	0
0	0.021368798	0	0	0
0	0	0	0	0
0	0	0	0.001906812	0
0	0	0	0	0
0	0.137707014	0	0	0
0.005451964	0.003025001	0.022865336	0	0.012300568
0	0	0	0	0
0	0	0	0	0.000630265
0	0	0	0.0057139	0.014388751
0	0	0	0	0
0	0	0	0	0
0	0.007344085	0	0	0
0	0.265986743	0	0.047381236	0
0	0	0	0	0
0	0	0	0	0
0	0	0	0	0

0	0	0	0	0
0	0	0	0	0
0	0	0	0	0
0	0.019558429	0	0.019968094	0
0	0	0	0	0
0	0	0	0.901636296	0
0	0	0	0	0
0	0	0	0	0
0.185272328	0	0.551760135	0.023952953	0.218996762
0	0	0	0	0
0	0	0	0	0
0	0	0	0	0
0	0	0	0	0
0	0	0	0	0
0	0.035602388	0	0.723402509	0.234165358
0	0	0	0	1.088794336
0	0	0	0	0
0	0	0	0	0
0	0	0	0	0
0	0	0	0	0
0	0	0	0	0
0	0	0	0	0
0	0	0	0	0
0	0	0.017064834	0	0
0	0.091989959	0.021857693	0.036947624	0
0.003667076	0.012138612	0.452776341	0.881650446	0.393653136
0	0	0	0.037553291	0
0	0	0	0	0
0	0.217417015	0	0.012153524	0
0	0	0	0	0
0	0	0	0	0
0	0	0	0	0.008009012
0	0	0	0	0
0	0	0	0	0
0	0.037236228	0.004322268	0.009979166	0.027130794
0	0.005149859	0.026428516	0	0.00128929
0	0	0	0.087589973	0.017795587
2.527996912	0.116732355	0	4.354424438	2.463265642
0	0	0	0	0
0	0	0	0	0
0	0.000661288	0	0	0
0	0	0	0	0
2.995929685	0.017202468	11.19294791	0.07556124	0.582947866
0	0	0	0	0
0	0	0	0	0
0	0.000542094	0	0.000862302	0
0	0	0	0	0
0.028224618	0	0	0	0.468475511

0	0	0.24724099	0	0
0	0	0	0	0
0	0	0	0	0
0	0	0	0	0
0	0	0	0	0
0	0.112375195	0	0	0
0	0	0	0	0
0	0	0	0	0
0	0	0	0	0
0	0	0	0	0
0	0	0	0	0
0.219704663	5.664677317	0	0.138788233	0.094630677
0	0.015527115	0	0.17381765	0.137536858
0	0	0	0	0
0.009408606	0.031391177	0.022370591	0.360347114	0.011695555
0	0	0	0	0
0	0	0	0	0
0	0	0	0	0
0	0	0	0	0
0	0	0	0	0
0	0	0	0	0
0	0	0	0	0
0	0.000842642	0.083181167	0.15355718	0.233175646
0	0	0	0	0
0	0	0	0	0
0	0	0	0	0
0	0.008900654	0	0	0
0	0	0	0	0
0	0.008015105	0	0	0
0	0	0	0	0
0	0	0	0	0
0.006289476	0.001282975	0.003853403	0.340560301	0.00514921
0	0	0	0	0
0.005175246	0	0.152082408	0	0.004197166
0	0.008302721	0.029093344	0	0.000701535
0	0.336616078	0	0.014046006	0.003391364
0	0	0	0	0
0	0.414234342	0	0	0
0	0	0	0.00113797	0
0	0	0	0	0
0	0	0	0	0
0	0	0	0	0
0	0	0	0	0
0	0	0	0	0.001856121
0.001650997	0	0.107924841	0	0.001727706
0	0	0	0	0
0	0	0	0.177744258	0
0	0	0	0	0.002614561

0	0	0	0	0
0	0	0	0	0
0.002683284	0	0	0	0
0	0	0	0	0
0	0	0	0	0
0	0	0	0	0.003535641
0	1.796915949	0	0	0
0	0	0.031992679	0	0.008606461
0	0	0	0	0
0	0	0	0.003048083	0
0	0	0	0	0
0	0	0.003619137	0	0
0	0	0	0	0
0	0.036724556	0	0	0
0	0	0	0	0
0	0	0	0	0
0	0	0	0	0
0	0	0	0	0
0	0.044516455	0	0	0.318668864
0	0	0	0	0
0	0.013642457	0	0	0
0	0	0	0	0
0	0	0	0	0
0	0	0	0	0
0.00128847	0	0	0	0
0.004391475	0	0	0.002271009	0.001608429
0	0	0	0	0
0	0	0	0	0.031486639
0	0.048708268	0	0	0
0	0.055144613	2.242240867	0.216605074	0.031795426
0	0	0	0	0
0	0	0	0	0
0	0	0	0	0
0	0	0	0	0
0	0	0.011343891	0	0
0	0	0	0	0
0	0	0	0.00239619	0
0	0	0.003263322	0	0
0	0	0	0	0
0	0	0	0	0.000447712
3.974365545	0.130118246	1.049895439	30.47594938	3.855643281
0.010894027	0	0.044192185	0.436071376	0.040320651
0	0	0	0	0
0	0	0	0	0
0	0	0	0	0
0	0	0	0	0
0	0	0	0	0
0	0.010060332	0.011798676	0	0

0	0	0	0	0
0.001918286	0	0.000739995	0.000881799	0.006245286
0	0	0	0	0
0	0	0	0	0
1.984633822	0.104088566	11.34852135	0.76433425	0.394990014
0	0	0	0	0
0	0	0	0	0
0	0	0	0	0
0.579958134	0	0.010344651	0.091351719	0.219509972
0	0	0	0	0
0	0	0	0	0
0	0	0	0	0
0	0.001795488	0	0.162366933	0.084501915
0.002800755	0.001765896	0.002651926	0.108233776	0.003170683
0.039440464	0.709674843	0	0.122932552	0
0.087334626	0.005434054	0.0474401	0.190597525	0.330433666
0	0	0	0	0.000968377
0	0	0	0	0
0	0.004441593	0	0	0
0	0	0	0	0
0	0	0	0	0
0	0	0	0	0
0	0	0	0	0
0	0	0	0	0
0	0	0	0	0
0	0	0	0	0
0.004023288	0.006643767	0	0.004755669	0.009964182
0	0	0.030772437	0	0.000475076
0	0	0	0	0
0	0	0	0	0
0	0	0	0	0
0	0	0	0	0
0	0	0	1.399816357	0.207738987
0	0	0	0	0
0	2.562759241	0	0	0
0	0	0	0.024571853	0
0	0	0	0	0
0	0	0	0	0.001383852
0	0	0	0	0
0	0	0	0	0
0	0.198881783	0	0.745385748	0
0	0	0	0	0
0	0	0	0	0
0	0	0	0	0
0	0	0	0	0
0	0	0	0	0
0	0	0	0	0
0	0	0	0	0

0	0.001846853	0	0.07222006	0
0	0	0	0	0
0	0	0	0	0
0	0	0	0	0
0	0.001218827	0	0	0
0	0	0	0	0.003957519
0.003516693	0	0	0.020686847	0.004508104
0	0	0	0	0
0	0	0.007863762	0	0
0	0	0	0	0.003040544
0	0.12085066	0	0	0
0	0	0	0	0
0	0	0	0	0
0	0	0	0	0
0	0	0	0	0
0	0	0	0	0
0	0	0	0	0
0	0	0	0	0
0	0	0	0	0
0	0	0	0	0
0	0.001269894	0.014820561	0	0.045985352
0	0	0	0	0
0	0.002801377	0	0.00668417	0.009298975
0.004104738	0	0.000427528	0.002971816	0.001503408
0	0	0	0	0
0	0	0	0	0
0	0	0	0	0
0	0	0	0	0
0	0.085651458	0	0	0
0	0.001792576	0	0.032689573	0.644799529
0	0	0	0	0
0	0	0	0	0
0	0	0	0	0
0	0	0	0	0
0	0	0	0	0
0	0	0	0	0
0	0	0	0.052590383	0
0	0	0	0	0
0	0	0	0	0
0	0.031576959	0	0	0
0	0	0	0	0
0	0	0	0	0
0	0	0	0	0
0	0.003611624	0	0	0
0	0	0	0	0
0	0	0	0	0
0	0	0	0	0
0	0.116289173	0	0	0

0	0	0.328618393	0	0
0	3.476136519	0	0	0
0	0	0	0	0
0	0	0.012476785	0	0
0	0	0	0	0
0	0.014253471	0	0.020751386	0
0	0	0.002004035	0	0
0.556910312	0.02024821	1.437052409	0.591949606	0.617240004
0.038130948	4.350067618	0.915374692	0.528829197	0.146007184
0.014238106	0.010251533	0.067529405	0.016722974	0.004596159
0	0	0	0	0
0	0	0	0	0
0	0	0	0	0
0	0	0	0	0
0	0	0	0	0
0	0	0	0	0
0.073500398	0	0	0	0
0	0	0	0	0
0	0	0	0	0
0	0	0	0	0
0	0	0	0	0
0	0	0	0	0
0	0	0	0	0
0	0	0	0	0
0	0.001658801	0	0	0
0	0	0	0	0
0	0	0	0	0
0	0	0	0	0.00152478
0	0	0	0	0
0	0	0	0	0
0	0	0	0	0.01745026
0	0	0	0	0
0	0	0	0	0
0	0	0	0	0
0	0.005331111	0	0	0
0	0	0	0	0
0	0	0	0	0
0	0	0.303615965	0	0
0	0	0	0	0
0.315933028	0	0.000612345	0	0.043120279
0	0	0	0	0
0	0.054621683	0	0.115272936	0.223264178
0	0	0	0	0
0	0	0	0	0
0	0	0	0	0
0	0.004766873	0	0	0
0	0	0	0	0
0	0	0	0	0

0	0	0	0	0
0	0	0	0.126577687	0.027595201
0	0	0	0	0
0	0	0	0	0
0	1.527142099	0	0.065716508	0.255988314
0	0	0	0	0
0	0	0	0	0
0.017136563	0	0.052862881	0.056658748	0.051034871
0	0.03331493	0	0	0
0	0	0	0	0
0	0	0	0	0
0	0.185401756	0	0	0
0	0	0	0	0
0	0	0	0	0
0	0	0	0	0.006229235
0	0	0	0	0
0	0	0	0	0
0	0	0	0	0
0	0	0	0	0
0	0	0	0	0
0	0	0	0	0
0	0	0	0	0
0	0	0	0	0
0	0	0.006414915	0	0
0	1.479030692	0	0.036243183	0
0	0	0	0	0
0.008672051	0	0.188521353	0.002759796	0.001140189
0	0	0	0	0
0	0	0	0	0
0	0	0	0	0
0	0	0	0	0
0	0	0	0	0
0	0	0	0	0
0	0	0	0	0
0	0	0	0	0
0	0	0	0	0
0	0	0	0	0
0	0	0	0	0.052619864
0	0	0	0	0
0	0	0	0	0
0	0	0	0	0
0	0.369282489	0	0.083568398	0
0	0	0	0	0
0	0	0	0.025606256	0
0	0	0	0	0
0	0	0	0	0
0	0	0	0	0
0	0	0	0.043528201	0

0	0.011602424	0	0.035686065	0.002144498
0	0	0	0	0
0	0	0	0	0.00257944
0	0	0	0	0
0	0	0	0	0
0	0.000630097	0	0	0
0	0	0	0	0
0	0	0	0	0
0	0	0	0	0
0	0	0	0	0
0	0	0	0	0
0	0	0	0	0
0	0	0	0	0
0	0	0	0	0
0	0	0	0	0
0	0	0	0	0
0	0	0	0	0
0	0	0	0	0.025175698
0	0	0	0	0
0	0	0	0	0
0.000726309	0	0.003530265	0.019531402	0.001064077
0	0	0	0	0
0	0.006096759	0.007643087	0.001897441	0.000895901
0	0	0	0	0.102291935
0	0	0	0	0
0	0.344360296	0	0.079741654	0
0	0	0	0	0
0	0	0	0	0
0	0	0	0	0
0	0	0	0	0
0	0	0	0	0
0	0	0	0	0
0.030924898	0.003723645	0.006561243	0.021323353	0.007341318
0	0	0	0	0
0	0.015099549	0	1.225746159	0.147283356
0	0	0.054884912	0	0.013802005
0	0	0	0	0
0	0.021476547	0	0.010381265	0
0.006378567	0	0.001291807	0.020616344	0
0	0	0	0	0
0	0	0	0	0
0	0	0	0	0
0	0.503815877	0	0.080998458	0
0	0	0	0	0
0	0	0	0	0
0.001234023	0	0	0.003828978	0.00072316
0	0	0	0	0
0	0	0	0	0

0	0	0	0	0
0	0	0	0	0
0	0	0	0	0.001806606
0	0	0	0	0
0	0	0	0	0
0	0	0	0	0
0	0	0	0	0
0	0	0	0	0
0	0	0	0	0
0	0.094323941	0	0	0
0	0	0	0	0
0	0	0	0	0
0	0	0	0	0
0	0	0	0	0
0	0	0	0	0
0	0	0.00086834	0	0
0	0	0	0	0
0	0	0	0	0
0	0	0	0	0
0	0	0	1.791107203	0
0	0	0	0.018912434	0
0	0	0	0	0
0	0	0	0	0
0	0	0	0	0
0	0	0	0	0
0	0	0	0	0.284389026
0	0	0	0	0
0	0	0	0	0
0	0	0	0	0
0	0.001797842	0	0	0.011393098
0	0.01304142	0	0	0.05450084
0	0	0	0	0
0	0	0	0	0
0	0	0	0	0
0	0	0	0	0
0	0	0	0	0
0	0	0	0	0
0	0	0	0	0
0	0.049656051	0	0	0
0	0	0	0	0
0	0	0	0	0
0	0	0	0	0
0	0	0	0	0
0	0	0.336686423	0	0
0	0	0	0	0
0	0	0	0	0
0.221215377	0	0.073601992	0	0.001875526
0	0	0	0	0

0	0	0	0	0
0	0	0	0	0
0	0	0	0	0
0	0	0	0.009967975	0
0.055853249	0.008788889	0.10359716	0.023146746	0.008126715
0	0	0	0	0
0	0	0	0	0
0	0	0	0	0
0	0	0	0	0
0	0	0	0	0
0	0	0	0	0
0	0	0.028765916	0	0
0	0.010130074	0.007954403	0	0
0	0	0	0	0
0	0	0	0	0.00103772
0.298616516	0	12.71696163	0	0.001966574
0	0	0	0.003168758	0.001496169
0	0	0	0	0
0.006233637	0	0.031834817	0.004783504	0.06643208
0	0	0	0	0
0	0	0	0	0
0	0	0	0	0
0	0	0	0	0
0	0	0	0	0
0	0	0	0	0
0	0	0	0	0
0	0.000729616	0	0.002176107	0.000924729
0	0	0	0	0
0	0	0	0	0
0	0	0	0	0
0	0	0.010267452	0	0.001921753
0	0	0	0	0
0	0	0	0	0
0	0	0	0	0
0	0	0	0	0
0	0	0	0	0
0	0	0	0	0
0	0	0.042583589	0	0.00196388
0	0	0	0.28349314	0
0	0	0	0	0
0	0	0	0	0
0	0	0	0	0
0	0	0	0	0
0.227848865	0	1.783272423	0	0.001820116
0	0	0	0	0
0	0	0	0	0
0	0	0	0	0
0	0	0	0	0

0	0	0	0	0
0	0.000552342	0	0.001098255	0
0	0	0	0	0
0.001301999	0	0.001808125	0	0
0	0.028057207	0	0.002747757	0
0	0	0	0	0.002739934
0	0	0	0.002874971	0
0.004073104	0	0	0	0
0	0	0	0	0.002169452
0	0	0	0	0
0	0	0	0	0.001891426
0	0	0	0	0
0	0.032805531	0	0.027491705	0.007571993
0	0	0	0	0
0	0	0	0	0
0	0	0	0	0
0	0	0	0	0
0	0	0	0	0
0	0	0	0	0
0	0	0	0	0
0	0	0	0	0
0	0	0	0	0
0.013611265	0	0.050462564	0.005530592	0.001068275
0	0	0	0.072085829	0.016808021
0	0	0	0	0
0	0.009365349	0	0	0
0	0	0	0	0
0	0	0	0	0
0	0	0	0	0
0	0	0	0	0
0	0.001879603	0.069626248	0.00448479	0.000595561
0	0	0	0	0
0	0	0	0	0
0	0	0	0	0
0	0	0	0	0
0	0	0	0	0
0	0	0	0	0
0	0	0	0	0
0	0.000993922	0	0.004031596	0
0	0	0	0	0
0	0	0	0	0
0	0	0	0	0
0	0	0	0	0
0	0	0	0	0
0	0	0	0	0
0.002017394	0.044948619	0	0.064891765	0.007536719
0	0	0	0	0
0	0	0	0	0

0	0	0	0	0
0	0	0	0	0
0	0.122706252	0	0.176139351	0
0	0	0	0	0
0	0	0	0	0
0	0	0	0	0
0	0	0	0	0
0	0	0	0	0
0	0	0	0	0
0	0	0	0	0
0	0.002257743	0.003616592	0	0
0	0	0	0	0
0	0	0	0	0
0	0	0	0	0
0	0	0	0	0
0	0	0	0	0
0	0	0	0	0
0	0	0	0	0
0	0	0	0	0
0	0.195261644	0	0.005613256	0
0	0	0	0	0
0	0	0	0	0
0	0	0	0	0
0	0	0	0	0
0	0	0	0	0
0	0	0	0	0
0	0	0	0	0
0	0.01278079	0	0.006776745	0
0	0	0	0	0
0	0	0	0	0
0	0.008819275	0	0.007671948	0.001707707
0	0	0	0	0
0.042817242	0.022186832	0	0.016288755	0.02667796
0.001508779	0	0.002095285	0.002340751	0
0	0	0	0	0
0	0	0	0	0
0	0	0	0	0
0	0	0	0	0
0	0	0	0	0
0	0	0	0	0
0	0	0	0	0
0.101627178	0	0.539649674	0	0.115077212
0.008138941	0.263211303	0	0.03520234	0.007587954
0	0	0	0	0
0	0	0	0	0
0	0.628163362	0	0.005897951	0
0	0	0	0	0
0	0	0	0	0

0	0	0	0	0
0	0.015232262	0	0	0.009652829
0	0	0	0.034650264	0.002210887
0	0	0	0	0
0	0	0	0	0.035482265
0	0	0	0	0.094507581
0	0	0	0	0
0	0	0	0	0
0	0.186075691	0	0	0
0	0	0	0	0
0	0	0	0	0
0	0	0	0	0
0	0	0	0	0
0	0	0.255594378	0	0.011149848
0	0	0	0	0
0	0	0	0	0
0	0	0	0	0
0	0	0	0	0
0	0	0	0	0
0	0	0	0	0
0	0	0	0	0
0	0	0	0	0
0	0	0	0	0
0	0	0	0	0
0	0.084964034	0	0	0
0	0	0	0.010611005	0
0	0	0	0	0
0.000728187	0	0.796531374	0	0.004800724
0	0	0	0	0
0	0	0	0	0
0	0.009078509	0	0	0
0	0	0	0	0
0	0	0	0	0
0	0	0	0	0
0	0	0	0	0
0	0	0	0	0
0	0	0.047919547	0	0
0.095646698	0	0	0	0.050905466
0	0.001772627	0	5.18917894	0
0	0	0	0	0
0	0	0	0	0
0	0.034359557	0	0.001965345	0
0	0	0.255635047	0	0
0	0	0	0	0
0	0	0	0	0
0	0	0	0	0
0.034192012	0	0.051484869	0	0.001688524
0	0	0	0	0

0	0	0	0	0
0.023753454	0	0.003702636	0.035598158	0.013671397
0	0	0	0	0
0	0	0	0	0
0	0	0	0	0
0	0	0	0	0
0	0	0	0	0
0	0	0	0	0
0	0	0	0	0
0	0	0	0	0
0	0	0	0	0
0	0	0	0	0
0	0.005108959	0	0.016430178	0
0	0	0	0	0
0.004230997	0	0.009233259	0.011252672	0.003099304
0	0	0	0	0
0	0	0	0	0
0	0	0	0	0
0	0	0	0	0
0	0	0	0	0
0	0	0	0	0
0	0	0	0	0
0	0.039544053	0.028979936	0.00212295	0.012529721
0	0	0	0	0
0	0	0	0	0
0	0	0	0	0
0	0	0	0	0
0	0	0	0	0
0	0	0	0	0
0	0	0	0	0
0	0	0	0	0
0	0	0	0	0
0	0	0	0	0
0	0	0	0	0
0	9.540634653	13.09222945	0	0
0	0	0	0	0
0	0	0	0	0
0	0	0	0	0
0	0	0	0	0.006296239
0	0	0	0	0
0.060325643	0	0.01623852	0.169040088	0.022192305
0	0	0	0	0
0	0	0	0	0
0	0	0	0	0
0	0	0	0	0
0.547986035	0.001582703	0.081973818	0.228156199	0.269911497
0	0	0	0	0
0	0	0	0	0

0	0	0	0	0
0	0	0	0	0
0	0	0	0	0
0	0	0	0	0
0.00115382	0	0	0	0
0	0	0	0	0
0	0	0	0	0
0	0	0	0	0
0	0	0	0	0.00226482
0	0.003095773	0	0.007078833	0
0	0.00897857	0	0	0
0	0	0	0	0
0.000872561	0	0.001615669	0	0
0	0	0	0	0
0	0	0	0	0
0	0	0	0	0
0	0	0	0	0
0	0	0	0	0
0	0	0	0	0
0	0.496451438	0.170769535	0.106834252	0.141278758
0	0	0	0	0
0	0	0	0	0
0	0	0	0	0
0	0	0	0	0
0	0	0	0	0
0	0	0	0	0
0	0	0	0	0
0	0	0	0	0
0	0	0	0	0
0	0	0.023443956	0.00772833	0.004459925
0	0	0	0	0
0	0	0	0	0
0	0	0	0	0
0	0	0	0	0
0	0	0	0	0
0	0	0	0	0
0	0	0	0	0
0	0	0	0	0
0	0	0	0	0
0	0.415167424	0	0	0
0	0	0	0	0
0	0	0	0	0
0	0	0	0	0
0	0	0	0	0
0	0	0.010467755	0	0.004809052
0	0	0	0	0
0	0	0	0	0
0	0	0	0	0

0	0	0	0	0
0	0	0	0	0
0	0	0	0	0
0	0	0	0	0
0	0	0	0	0
0.001089125	0.022818504	0	0.004130359	0
0	0	0	0	0
0	0	0	0	0
0	0	0	0	0
0	0	0	0	0
0	0	0	0	0
0	0	0	0	0
0	0	0	0	0
0	0	0	0	0
0	0	0	0	0
0	0	0	0	0
0	0	0	0	0
0	0	0	0	0
0	0	0	0	0
0	0	0	0	0
0	0	0	0	0
0	0	0	0	0
0	0	0	0	0
0	0	0	0	0
0	0	0	0	0
0	0	0	0.0023493	0
0	0	0	0	0
0	0	0	0	0
0	0	0	0	0
0	0	0	0	0
0	0	0	0	0
0	0	0	0	0
0	0	0	0	0
0	0	0	0	0
0	0	0	0	0
0	0	0	0	0
0	0	0	0	0
0	0	0	0	0
0	0.001999264	0	0	0
0	0	0	0	0
0	0	0	0	0
0	0	0	0	0
0	0	0	0	0
0	0	0	0	0
0	0	0	0	0
0	0	0	0	0
0	0	0	0	0
0	0	0	0	0

0	0	0	0	0
0	0	0.084386086	0	0.018459722
0	0	0	0.005404146	0
0	0	0	0	0
0	0	0	0	0
0	0	0	0	0
0	0	0	0	0
0	0	0	0	0
0	0	0	0	0
0	0	0	0	0
0	0.001845626	0.00073911	0.014862555	0
0	0	0	0	0
0	0.155141178	0.199111238	0.21954863	0.013783404
0	0	0	0	0
0	0	0	0	0
0	0	0	0	0
0	0	0	0	0
0	0	0	0	0
0	0	0	0	0
0	0	0	0	0
0	0	0	0	0.004352569
0	0	0	0	0
0	0	0	0	0
0	0.166038066	0	3.075422955	1.687615703
0	0	0	0	0
0	0	0	0	0
0	0	0	0	0
0	0	0	0	0
0.002707073	0	0	0	0
0	0	0	0	0
0	0	0	0	0
0	0	0	0	0
0	0.00147782	0	0	0
0	0	0	0	0

PHL018-0241_V1	PHL018-0208_V1	PHL018-0231_V1	PHL018-0084_V2	PHL018-0053_V2
REF	REF	EYCF	EYCF	CTRL
1.280773725	0.00676566	0	0.054501573	0
0.566350267	0.139109401	4.8764886	0.031031746	7.057208353
0	0.096275662	8.745479499	0	0
0	0	0	0	0
0	0	0	0	0
0	0	0	0	0
0	0	0	0	0
0	0	0	0	0
0	0	0	0	0
0	0	0	0	0
0	0	0	0	0
0	0	0	0	0
0	0	0	0	0
1.352454179	4.555911	1.602791265	6.13556448	9.585200833
0	0	0	0	0
0	0	0	0	0
0	0	0	0.006771099	0
0	0	0	0	0
0	0	0.017092708	0	0
0	0	0	0	0
0	0	0	0	0
0	0	0	0	0
0	0	0	0	0
0	0	0	0	0
0	0	0	0.009131472	0
0	0	0	0	0
0	0	0	0	0
0	0	0	0	0
0	0	0	0	0
0	0	0	0	0
0	0	0	0	0
0	0	0	0	0
0	0	0	0	0
0	0	0	0	0
0	0	0	0	0
0.083137303	1.265651188	12.04963559	4.823869794	10.0781726
0	0	0	0	0
0	0	0	0	0
0	0	0	0	0
0	0	0	0	0
0	0	0	0	0
0.001579348	0.320164237	0.162106541	0.001039154	0.008520886
0.172768505	0.257842141	0.256305537	0.348714118	0.15399405
0.183008217	0.009301	0.050855552	0	0.054566855
0.001555925	0	0.000848728	0	0.001089018
0	0	0	0	0

0	0	0	0	0
0.05410414	0.003764488	0.138785826	0.113696335	0.291295301
0.044735239	0	0.077211182	0.270250926	0.006021334
0.724900272	0.319961193	0.001093418	0.364301033	0
0	0.000615841	0	0.036189063	0
0	0	0	0.000945892	0
0.160887601	0.952716793	1.177881595	0.001408834	0.451680535
0	0	0	0	0
0	0	0	0	0
0	0	0	0	0
0	0.00239885	0	0	0
0.066110682	0.001005017	0.000572415	0.719008377	0
0	0.004112342	0	0	0.000901601
0.020446161	0.004929464	0.019517749	0.269897465	0.00708077
0	0	0.002231541	0	0.00230089
0	0	0	0	0
0.444968662	0.288486999	0	0	0.017114949
0	0	0	0	0
0	0	0	0	0
0	0	0	0	0
1.472535964	0.016524867	0.11220574	0.315406446	0.561164506
0	0.021923708	0.005186829	0.01051074	0.001663828
0	0	0	0.558013629	0
0	0	0	0	0
0	0	0	0	0
0	0	0	0.007345593	0
0	0	0	0	0
0	0	0	0	0
0	0.00798254	0.003244	0.000640299	0.016082127
0	0	0	0	0
0.643113639	0.004968396	0.0494995	0	0.209268347
0	0	0	0	0
0	0	0	0	0
0	0	0	0	0
0	0	0	0	0
0	0	0	0	0
0	0	0	0	0
31.31456062	0.015541884	0	0.006069227	0.009415308
0	0	0	0	0
0	0	0	0	0
0	0	0	0	0
0	0	0	0	0
0	0	0	0	0
0	0	0	0	0
0	0	0	0.000611067	0
0	0	0	0	0
0.001044979	0	0	0	0
0	0	0	0	0

0	0	0	0	0
0	0	0	0	0
0	0	0	0	0
0	0	0	0	0
0	0	0	0	0
0	0.00310928	0	0	0
0	0	0	0	0
0	0	0	0.075997672	0
0	0.134481229	0.001114106	0	0.149743142
0	0	0	0	0
0	0	0	0	0
0	0.045798148	0	0	0
0	0	0	0	0
0	0	0	0	0
0	0	0	0	0
0	0	0	0	0
0	0	0	0	0
0	0	0	0	0
0	0	0	0	0
0	0	0	0	0
0	0	0	0	0
0	0	0	0	0
0	0	0	0.000705533	0
0	0	0	0	0
0	0	0	0	0
0	0	0	0	0
0	0	0	0	0
0	0.001316622	0	0.006946756	0
0	0	0	0	0
0	0	0	0	0
0	0	0	0.079089982	0.0014882
0	0	0	0.001408963	0
0	0	0	0	0
0	0	0	0	0
0	0	0	0	0
0	0	0	0	0
0	0	0	0	0
0	0	0	0	0
0	0	0	0	0
0	0	0	0	0
0	0	0	0	0
0	0	0	0	0
0	0	0	0.001085692	0
0	0	0	0	0
0	0	0	0	0
0	0	0	0	0
0	0	0	0	0
0.010848517	0	0	0	0

0	0	0	0	0
0	0	0	0	0
0	0	0	0.985554739	0
0	0	0	0	0
0	0	0	0.001362603	0
0	0	0	0	0
0	0	0	0	0
0	0	0	0	0
0	0	0	0	0
0	0	0	0	0
0	0	0	0	0
0	0	0	0	0
0	0	0	0	0
0	0	0	0	0
0	0	0	0	0
0	0	0	0	0
0	0	0	0	0
0	0	0	0	0
0	0	0	0	0
0	0	0	0	0
0	0	0	0.001464546	0
0	0	0	0	0
0	0	0	0	0
0	0	0	0	0
0	0	0	0	0
0	0	0	0	0
0	0	0	0.015645226	0
0	0	0	0	0
0	0	0	0	0
0	0	0	0	0
0	0	0	0	0
0.543556834	0.003286489	0.008360904	0	0.001401048
0	0	0	0	0
0	0	0	0	0
0	0	0	0	0
0	0	0	0	0
0	0	0	0	0
0	0	0	0	0
0	0	0	0	0
0	0	0	0	0
0	0	0	0	0
0	0	0	0	0
0	0	0	0	0
0	0	0	0	0
0	0	0	0	0
0	0	0	0	0
0	0	0	0	0
0	0	0	0	0

0	0	0	2.326824227	0
0	0	0	0	0
0	0	0	0	0
0	0	0	0	0
0	0	0	0	0
0	0	0	0	0
0	0	0	0	0
0	0	0	0	0
0	0	0	0	0
0	0	0	0	0
0	0	0	0	0
0	0	0	0	0
0	0.000524141	0	0.00086421	0
0	0	0	0	0
0	0	0	0	0
0	0	0	0	0
0	0	0	0	0
0	0	0	0	0
0	0	0	0	0
0	0	0	0	0
0.040521635	0.068601277	0.052245334	0.009372015	0.116383515
0	0	0	0	0
0.001077431	0.000859905	0	0	0
0	0	0	0	0.002987436
0	0	0	0	0
0	0	0	0	0
0.03075001	0.002473205	0.007671627	0	0.014390006
0	0	0	0	0
0	0	0	0	0
0	0	0	0	0.000892501
0.003089016	0.360512228	0.308355055	0	0.00852348
0	0	0	0	0
0	0	0	0	0
0	0	0	0	0
0.003195822	0.016578955	0.002179077	0.046890998	0
0	0	0	0	0
0	0	0	0	0
0	0	0	0	0
0	0.013391192	0	0	0
0	0	0	0	0
0	0	0	0	0
0	0	0	0	0
0	0	0	0	0
0	0	0	0	0
0	0	0	0	0
0	0	0	0	0
0.001441761	0	0	0	0
0.002277429	0.003483796	0	0.004045858	0

0	0	0	0	0
0	0	0	0	0
0	0.00304501	0.00208117	0	0.003944887
0	0.001732836	0	0	0.000633186
0	0	0	0	0
0	0	0	0	0
0	0.001078074	0	0	0
0	0.00696768	0	0	0
0.001308948	0	0	0.005610368	0
0	0	0	0	0
0	0	0	0	0
0	0	0	0	0
0	0	0	0	0
0	0	0	0	0
0	0	0	0	0
0	0	0	0	0
0	0	0	0	0
0	0	0	0	0
0	0	0	0.001066751	0
0	0	0	0	0
0	0.015906074	0	0	0.057789395
0	0	0	0	0
0	0	0	0	0
0	0	0	0	0
0.009615846	0.004965837	0.00555381	0	0.002474372
0	0	0	0	0
0	0	0	0	0
0	0	0	0	0
0	0	0	0	0
0	0	0	0	0
0.155335976	6.844842329	1.263570423	2.139867902	0.004029463
0	0	0	0	0
0	0	0	0.001644219	0
0	0	0	0	0
0	0	0	0	0
0	0	0	0	0
0	0	0	0	0
0	0	0	0	0
0	0	0	0	0
0	0	0	0	0
0	0	0	0	0
0.012803709	0	0	0.006907989	0.010641816
0	0	0	0	0
0	0	0	0	0
0	0	0	0	0
0	0	0	0	0
0	0	0	0	0
0	0	0	0	0

0	0	0	0	0
0	0	0	0	0
0.143086752	0	0.057295977	0	0.053919065
0	0	0	0	0
0	0.002598249	0	0	0
0	0	0	0	0
0	0	0	0	0
0	0	0	0	0
0	0	0	0	0
0	0	0	0	0.006003439
0	0	0	0	0
0	0	0	0	0
0	0	0	0	0
0.012141582	0.030488936	0	0	0.00155453
0.003666324	0.00760791	0.001599929	0	0.002993808
0	0	0	0	0
0.010582867	0.00155589	0.001671061	0	0.00268021
0	0	0	0	0
0	0	0	0	0
0	0.00679281	0	0	0
0	0	0	0	0
0.002514029	0.00220711	0	0.002778955	0
0	0	0	0	0
0	0	0	0	0
0	0.012225542	0.000879556	0	0.032093798
0	0	0	0	0
0	0	0	0	0
0	0	0	0	0
0	0	0	0	0
0	0	0.003173886	0.002584147	0.005408747
0	0.000922551	0	0	0
0	0	0	0	0
0	0	0	0	0
0	0	0	0	0
0	0	0	0	0
0	0.310991825	0	0	0
0	0	0	0	0
0.006001372	0	0.00051689	0	0
0	0	0	0	0
0	0	0	0	0
0	0	0	0	0
0	0	0	0	0.001298366
0	0	0	0	0
0.003923496	0	0	0	0
0	0	0	0	0
0	0.036820728	0	0	0.006373166
0	0	0	0	0.004493236
0	0.022290255	0.000834778	0	0.001338898

0	0.072668939	0	4.534870243	0
0	0	0.063516942	0.007101648	0
0.009743022	0	0	0	0
0	0.001962537	0	0	0
0.033646142	0.031504962	0.001011602	0	0
0	0.025858237	0	0	0.011338461
0	0	0	0	0
0.163768905	0.001778301	0.012559271	0.007916621	0.038338155
0	0	0	0	0.00262885
0.006124269	1.379282273	0.149077588	0.023270633	0.064966928
0	0	0.0007551	0	0
0	0.105108965	0.007376296	0.006731035	0
0.039022453	0.00854238	0.001824516	0	0.003316514
0.007111805	0.41813064	0	0	0
0	0	0.003752946	0.001448586	0
0	0.001335784	0.002008528	0	0.004100054
0	0.009908153	0	0	0.000638909
0	0.002317778	0	0	0
0	0.248077516	0.368969904	1.417348726	1.030432537
0	0.140093267	0.2003178	0	0
0	0	0	0	0
0	0	0	0	0
0	0.002387296	0.006526571	0	0
0	0	0	0.209209131	0
0	0	0	0	0
0.001558327	0	0	0	0
3.790778242	0.001385912	0	0	0
0.556541105	0	0.001884543	0.04786015	0.062925309
0	0	1.85377449	0	0
0	0	0	0	0
0	0	0	0.001760363	0
0	0	0	0	0
0	0	0	0	0
0	0	0.003335082	0	0.034472156
0	0	0	0	0
0	0.302055079	0.003878929	5.31219926	0
0.001177682	0.000939917	2.148198015	0.063229578	3.410198729
0	0	0.001106222	0	0.013011285
0	0	0	0	0
0	0	0.394860061	0	0
0	0	0	0	0
0	0	0	0.715273308	3.129237933
0	0	0	0.011288609	0
0	0	0	0	0.095925369
0	0	0	0	0.05716268
0	0	0	0.003150701	0
0.002583871	0	0	0.004590252	0.009325048
0	0	0	0.096985611	0

0	0	0	0	0.001068322
0.002165742	0.033417551	0.004331697	0	0.002842198
0	0	0	0	0
0	0	0	0	0
0	0	0	0.005293147	0
0	0.00913633	0	0.049008473	0.002670765
0	0	0	0.025460651	0
0	0	0	0	0
0	0	0	0.001709773	0
0	0	0	0	0
0.005310869	0.028728573	0.013197353	0	0.021941603
0	0	0	0	0
0	0	0	0	0
0.013101098	0.005339274	0.001824614	0	0.00390199
0	0	0	0	0.043429241
0	0	0	0	0
0	0	0	0	0
0	0	0.071617643	0.000642539	0.491508711
0	0	0	0	0.0007787
0	0	0	0.021247391	0
0	0	0	0	0
0.095176343	0	0	0.297104763	0
0	0	0	0.184206938	0
0	0	0.055745266	0	0
0	0	0	0	0
0	0	0	0	0
0	0	0.059515801	1.46877904	0.05764452
0	0	0	0	0
0	0	0	0	0
3.850499273	0.003260941	0.031648272	0.230659331	1.305118498
0	0	0	0.0013438	0.003308988
0	0	0.001448259	0	0
0	0	0	0	0
0	0.001496361	0	0.001903281	0
0.003020802	0	0	0	0.006166737
0	0	0	0	0
0	0	0	0	0
0	0	0.005889264	0	0
0	0	0.020606747	0	0.609753719
0	0	0	0	0.092629627
0	0.102969521	0	0.005970852	0.012831442
0	0.368389255	1.210104554	3.399025515	0
0	0	0.003265363	0	0
0	0	0	0	0
0	0	0	0.228392385	0
0	0	0.001358811	0	0
0.0012026	0.031673523	0.001180793	0	0.033668798
0.000675385	0	0	0	0

0.001483807	0	0	0	0
0	0	0	0	0
0.418108367	0.596714201	0	0	0.050235764
0	0	0	0	0
0	0	0	0.116513029	0
0	0.152393782	0	0.154921402	0
0	0	0	0	0
0	0	0	0	0
0	0.235088908	0	0	0.085612834
0	0	0	0	0
0	0	0	0	0
0	0	0	0	0
0	0	0	0	0
0.001610835	0.013927538	0.000732234	0.002331717	0.011744275
0	0	0	0	0
0.242326681	0	0.007438965	0.037437851	0.000734236
0.006511366	0.013363116	0.022960036	0	0.006510591
0	0	0	0	0.12928093
0	0	0	0.01631039	0
0	0	0.011334532	0	0
0.036634707	0	0.024619732	0.068032002	0
0	0	0.001029984	0.002129785	0.016991881
0	0	0	0	0
0	0.972350003	0.153050162	0	0
0	0	0	0	0
0	0.055255587	0	0.000725623	0.002412156
0	0.011545341	0	0.006293562	0.004907491
0	0	0	0	0
0	0	0	0	0
0	0	0	0.011767547	0
0	0.019710652	0	0.028894686	0
0.0833467	0.00614438	0	0	0.006442703
0.12525072	0.103524522	0.002260013	0.079768268	0.022306632
0	0.18700014	0	0	0.002797163
0.152564225	0	0.000889113	0	0
0	0	0	0	0
0.003169435	0.001609713	0.002357547	0	0.001260421
0	0	0.000737484	0.3850013	0.004534255
0	0.017100374	0	0.038195794	0
0.002947703	0	0	0	0
0	0	0	0	0
0	0	0	0	0
0	0	0	0.011440878	0
0	0.025409898	0.205236159	0	0
0.004474817	0	0.481614119	1.657033789	0.221649171
0	0	0	0	0
0	0	0	0.000593103	0
0	0	0	0	0

0.003675009	0	0.002915852	0	0.02104527
7.469732939	0.308766628	0	0.00193942	0.033847419
0	0	0	0	0
0	0	0	0	0
0	0	0	0.00428099	0
0	0	0	0	0
0	0.413284734	0.284791451	0	0
0	0.015999248	0.001157822	0.004096617	0
0	0	0	0.006213828	0
0	0.004629954	0	0	0
0.002966161	0	0	0	0
0.819062811	0.011663523	0.091210567	0	0.192677727
0	0	0	0	0
0	0	0	0	0
0.001570753	0	0.000714014	0	0
0	0	0.000790281	0	0
0.062358899	0.028221545	0.028541838	0	0.001672262
0.009614806	1.285706779	0.770124586	0.447649656	0.040431649
0	0	0	0	0
0	0.046613887	0.003515491	0	0.005462283
0.09781678	0.011012148	0.035347483	0.011477785	0.036861696
0	0	0	0	0
0	0	0	0	0
0	0	0	0	0
0.123780304	0.003304011	0.00203237	0	0.025353314
0	0	0.112157101	0	0
0	0	0	0.01036905	0
0.030115747	0.590021483	0.107279226	0.439791176	0.041927912
0	0	0	0	0
0	0	0	0.004738755	0
0	0	0	0	0.006637675
0	0	0	0	0
0	0	0	0.032129586	0
0	0	0	0	0
0.052487554	0	0	0.989380083	0
0.123212273	0.003891016	0.041583162	0.027645184	1.09155071
0	0	0.000895759	0.023824396	0
0	0	0	0	0
0	0.151862581	0.002995733	0.001785482	0.06641995
0	0	0	0	0
0.006892402	0	0.442314867	0.772755554	0.164941883
0	0	0	0	0
0.001177365	0.000939664	0.065079396	0.015028478	0
0	0	0.035756899	0	0
0	0	0.082412136	0	0
0	0	0	0	0
0	0	0	0	0
0	0	0	0	0

0	0.060823179	0	0	0.001439033
0	0	0	0	0
0	0	0	0	0
0	0	0	0	0
0	0	0	0	0
0	0	0	0	0
0	0	0	0	0
0	0	0	0	0
0	0.134533652	0	0.108149692	0
0	0.004238581	0	0	0
0	0	0	0	0
0.004817314	0	0	0.002420435	0
0	0	0	0	0
0	0	0	0	0
0.043005079	0	0	0.034810946	0
0	0	0	0	0
0.001414194	0.010158103	0	0	0
0	0	0	0	0
0	0	0	0	0
0	0	0	0	0
0	0	0	0	0
0	0	0	0	0
0	0	0	0	0
0.00121838	0.022781884	0.139186946	0.216995134	0.502826387
0	0.004744995	0	0.002266146	0
0	0	0	1.037629488	0
0	0	0	0.032163769	0
0	0	0	0.15448242	0.502774651
0	0	0	0	0
0	0	0	0	0
0	0	0	0	0
0	0	0	0	0
0	0.000387912	0	0.000767513	0
0	0	0	0	0
0	0	0	0.004965864	0
0	0	0	0	0
0	0	0	0.000736212	0
0	0	0	0.021226489	0
0	0	0	0.011698484	0
0	0	0	0	0
0.038651301	0.071251886	0.079177945	0.030351531	0.767289252
0	0	0	0.015125041	0
0.307181241	0.067173595	0.020206366	0.225426056	0.20591795
0	0	0	0.010547164	0
0	0	0	0.001263924	0
0	0	0	0	0
0	0	0	0	0
0.011110789	0	0	0	0

0	0	0	0	0
0	0	0	0	0
0.303067803	0.746120348	0.505724665	0.321090881	0.599982234
0	0	0	0	0
0	0	0	0	0
0	0	0	0.001806736	0
0.011963007	0	0	0.066311054	0
0	0	0	0.00411965	0
0	0	0	0	0
0	0	0	0	0
0.141887082	0.006449241	0.026317489	0.1983475	0.004574541
1.834841557	6.132196137	2.87346317	2.618525929	0.940932166
0	0	0	0	0
0.03437096	0	0.003357979	0	0.005385852
0.015968397	1.064245556	0.357900762	0.401539314	0.196827478
0	0	0	0	0
0	0	0	0.001555704	0
0.027609947	0	0	0.000531698	0
0	0	0	0	0
0	0.038281084	0.268473954	0.080224602	0
0.000597599	2.559940196	0.073671318	0.006920297	3.129180453
0.232983194	0.084931876	1.797092759	0	0.464966497
0.273192377	36.07577362	31.19020764	1.793941522	6.448133949
0	0	0	0	0
0	0	0	0	0
0	0	0.017577943	0.007350252	0.015871733
0	0	0	0.001504909	0
0	0	0	0	0
0	0	0	0.10979085	0
0	0	0	0.092697047	0
0	0	0	0	0
0	0	0	0.107799303	0
3.51001418	0.194030707	0.004660129	1.50362365	0.006833706
0	0	0	0.06731047	0
0	0.001928655	0.010984796	0.138774624	0.006201705
0.092079474	0.015545808	0.011953211	0.015554022	0.002130191
0.004703955	0	0	0	0
0	0	0	0	0
0	0.007442074	0	0	0
0	0	0	0	0
0	0	0	0.014003877	0
0	0	0	0	0
0	0	0	0	0
0	0	0	0	0
0.11636503	0.060638605	0.00468145	0.01554531	0.004195962
0	0	0	0	0
0	0	0	0	0
0.005176261	0.001215062	0	0.966203239	0.00612705

0	0	0	0	0
0	0	0	0	0
0	0	0	0	0
0	0	0	0	0
0	0	0	0	0
0	0	0	0	0
0	0	0	0	0
0.103930868	0.050084767	0.01274228	0.021413568	0
0	0	0	0	0
0	0	0	0	0
0.003114851	0	0.004182383	0.043700774	0
0	0	0	0	0
0	0	0	0.077686387	0
0	0	0.071378947	0	0
0	0	0	0	0
0	0	0	0	0
0	0	0	0	0
0.001595718	0	0	0.000944932	0
0	0	0	0	0
0	0	0	0.06959409	0.00141642
0	0	0	0	0
0	0	0	0	0
0	0	0	0	0
0	0.003580984	0	0	0
0	0	0	0	0
0	0	0	0	0
0	0	0	0	0
0	0	0	0	0
0	0	0	0	0
1.531248045	0	0	0	0
0	0	0	0.000596004	0
0	0	0	0	0
0	0	0	0	0
0	0	0	0	0
0	0	0	0	0
0	0	0	0	0
0	0	0	0	0
0.021499865	0	0.007744757	0.06613984	0.005146171
0	0	0	0	0
0	0	0	0.008529736	0
0	0	0	0.132375133	0
0	0	0	0	0
0	0	0	0	0
0.022257713	0	0	0	0
0.062874667	0	0	0	0
0	0	0	0	0
0	0	0	0.023433328	0
0	0	0	0	0

0	0	0	0	0
0	0	0	0	0
0	0	0	0	0
0	0	0	0	0
0	0	0	0	0
0	0	0	0	0
0	0	0	0	0
0	0	0	0	0
0.011469133	0.076800901	0.287785104	0.015460659	0.001713173
0	0	0	0	0
0	0	0	0	0
0	0	0	0	0
0	0	0	0.003994331	0
0	0	0	0	0
0	0.14247019	0	0.028569696	0
0	0.810800834	0.426319956	0.367862694	0.046779523
0	0	0.002792751	0	0
0	0	0	0	0
0	0	0	0	0
0	0	0	0	0
0	0.000512139	0	0.013510732	0
0	0	0	0	0
0	0	0	0	0
0	0	0	0.020847852	0
0.187178432	0.019690917	0.010587062	0.053504952	0.005180508
0.002170412	0	0	2.432177072	0
0	0	0	0	0
0	0	0	0.001451054	0
0	0	0	0	0
0	0	0	0	0
0	0	0	0	0
0	0	0	0.009199814	0
0	0	0	0	0
0.001229802	0	0	0.002635566	0
0.011000048	0	0.004421289	0	0.003039125
0.002046237	0	0	0.036674566	0
0	0	0	0	0.04397008
4.63774617	0.2755041	0	0.015689099	0
0	0	0	0	0
0	0	0	0	0
0	0	0	0	0
0	0	0	0.019786957	0
0	0.012051084	0	0.033778927	0
0	0	0	0	0
0	0	0	0	0
0	0	0	0	0
0	0	0	0	0
0	1.056426637	0.316206065	0.005608376	1.359907311

0	0	0	0.006000507	0
0	0	0	0	0
0	0	0	0	0
0	0	0	0	0
0.004127196	0	0.057033149	0.004163837	0
0	0	0	0	0
0	0	0	0	0
0	0	0	0	0
0	0	0	0	0
0	0	0	0.079951499	0
0	0	0	0	0
9.731172877	0.036635313	0	0.151819003	0.19487842
0	0.271875193	0	0	0
0	0	0	0	0
0.025191373	1.280185686	0.135533833	0.184663066	0.026911789
0	0	0	0	0
0	0	0	0	0
0	0	0	0	0
0	0.001298487	0	0.006606388	0
0	0	0	0	0
0	0	0	0.288059484	0
0	0	0	0	0
0	0	0	0	0.369549792
0	0	0	0	0
0	0	0	0	0
0	0	0	0	0
0	0	0	0	0
0	0	0	0	0
0	0	0	0	0
0	0	0	0	0
0	0	0	0	0
1.413211345	0.023617939	0.013514339	0.000815055	0.039738611
0	0	0	0	0
0.033349674	0	0	0.005564894	0.001654394
0.003340228	0	0	0.030065203	0
0.00143532	0	0	0.002266532	0.001004604
0	0	0	0	0
0	0	0	0	0
0	0	0	0	0
0	0	0	0	0
0	0	0	0	0
0	0	0	0	0
0	0	0	0	0
0	0	0	0	0
0.558061855	0.168948246	0.009572715	0.023728431	0
0	0	0	0	0
0	0	0	0	0
0	0	0	0.005242125	0

0	0.001287303	0	0	0
0	0	0	0.001502362	0
0	0	0.005940349	0.018988032	0
0	0	0	0	0
0	0	0.003639725	0	0
0	0	0	0	0
0	0	0	0	0
0	0	0	0	0
0	0	0	0	0
0	0	0	0	0
0	0	0	0	0
0	0	0	0.001640366	0
0	0	0	0	0
0	0	0	0	0
0	0	0	0	0
0	0	0	0	0
0	0	0	0	0
0	0	0	0	0
0	13.40866773	0	0.104317804	0
0	0	0	0	0
0	0	0	0	0
0	0	0	0	0
0	0	0	0	0
0	0	0	0	0
0	0.002869284	0	0	0
0.016337563	0.007497496	0.021722599	0.118997232	0.014114995
0	0	0	0	0
0	0.414947567	0.039907333	0	0.145502816
0	0	0	0	0
0	0	0	0	0
0	0	0	0	0
0	0	0	0	0
0	0	0	0	0
0	0	0	0	0
0.000642028	0.000854012	0.00186781	0.137036734	0
0	0	0	0.002886957	0
0	0	0	0.010862195	0
0.000971352	0.00251954	0	0.000766937	0
0	0	0	0	0
0	0	0	0	0
2.874329655	0.07937734	1.403092357	0.007487047	18.78105263
0	0.000993092	0.07999527	0.387928535	1.123633466
0	0	0	0	0
0	0	0	0.16487202	0
0	0	0	0	0
0	0	0	0	0
0	0	0	0	0
0.034498616	0.003999292	0.000630783	0.00152171	0

0	0	0	0	0
0	0	0.003568466	0.011425991	0.002601569
0	0	0	0	0
0	0	0	0	0
0	0.149737723	0	0.138447853	0
0	0	0	0	0
0	0	0	0	0
0	0	0	0.018254746	0
0.224272969	1.32310095	1.968077091	9.704024859	0.59453465
0	0	0	0.013282992	0
0	0	0.000366511	0	0
0	0	0	0	0
0	0	0	0	0
0.196078385	0	0.006717112	0	0.017196253
0	0	0	0	0
0	0.006203663	0.000954002	0	0
0	0.011448479	0.012072362	0.021357272	0.079602725
0	0	0	0	0
0	0	0	0	0
0	0	0	0	0
0	0	0	0	0
0	0	0	0	0
0	0	0	0	0
0	0	0	0	0
0	0	0	0	0
0	0	0	0	0
0	0	0	0	0
0	0.000641887	0.000987097	0.003016297	0
0	0	0	0	0
0	0	0	0.000937367	0
0	0	0	0	0
0	0	0	0	0
0	0	0	0	0
0	0	0	0.004493204	0
0	0	0	0	0
0.001162954	0	0.002156858	0	0.001627942
0	0.270946853	0	0	0
0	0	0	0	0
0	0	0	0	0
0	0	0	0	0
0.086313789	0.114358035	12.70809585	5.254942522	10.95673657
0	0	0	0	0
0	0	0	0	0
0	0	0	0	0
0	0	0	0	0
0	0	0	0	0
0	0	0	0	0
0	0	0	0	0

0	0	0	0	0
0	0	0	0.000497659	0
0	0	0	0	0
0	0	0	0	0
0	0.038786548	0	0	0
0	0	0	0	0
0	0.263327361	0.006021504	0.121537279	0.000715398
0	0	0	0	0
0.001587192	0	0	0.002278505	0
0	0	0	0.05012439	0.000562927
0	0	0	0.000587187	0
0	0	0	0.030347449	0
0	0	0	0	0
0	0	0	0	0.009887969
0	0	0	0	0
0	0	0	0	0
0	0	0	0	0
0	0.05176167	0	0.065638218	0
0	0	0	0	0
0	0	0	0	0
0	0.149271954	0.148416008	0	0.073047831
0	0	0	0	0
0	0	0	0	0
0.003626819	0.001371122	0.00312373	0.044912973	0.000835023
0	0	0	0	0
0	0	0	0.002311523	0
0	0	0	0	0
0	0	0	0	0
0	0	0	0	0
6.315735754	0	0.117489707	0	1.553920314
0	0	0	0	0
0	0	0	0	0
0	0	0	0	0
0	2.356018914	0	2.098520764	0
0	0	0	0	0
0	0	0	0	0
0.09052296	0.111134586	0.008989769	0.010749236	0.007313814
0	0	0	0	0
0	0	0	0	0
0	0	0	0	0
0	0	0	0	0
0	0	0	0	0
0	0	0	0	0
0	0	0	0.013676494	0
0	0	0	0	0
0	0	0	0	0
0	0	0	0	0
0	0	0	0	0

0	0.001475376	0.00605024	0.134766877	0.001976733
0	0	0	0	0
0	0	0	0	0
0	0	0	0	0
0	0	0	0	0
0	0	0	0	0
0	0.000378066	0	0	0
0.966759375	0.002356476	0.263904676	0.1635191	0.148534045
0.216011367	0.332192014	0.353314636	0.493840552	0.169806628
0.101450815	0.000537402	0.003917843	0.745011594	0.002945539
0	0	0	0.003535296	0
0	0	0	0	0
0	0	0	0.041898156	0
0	0.009524693	0.533024971	0	0
0	0	0	0.001859153	0
0	0	0	0	0
0	0.012204024	0	0	0.004197088
0	0	0	0	0
0	0	0	0	0
0	0	0	0.013078629	0
0	0	0	0	0
0	0	0	0	0
0	0	0	0	0
0	0	0	0	0
0	0	0	0	0
0	0	0	0.000538512	0
0	0	0	0	0
0	0	0	0	0
0	0	0	0	0
0	0	0	0	0
0	0	0	0.013661711	0
0	0	0	0	0
0	0	0	0	0
0	0	0	0	0
0.002735312	0	0	0.042211997	0
0	0	0	0	0
0	0	0	0	0
0	0	0	0	0
0	0	0	0	0
0.198081015	0.000654616	0.016330462	0	1.279120913
0	0	0	0	0
0	0	0	0.186237768	0
0	0	0	0	0
0	0	0	0	0
0	0	0	0	0
0	0	0	0	0
0	0	0	0	0
0	0	0	0	0

0	0	0	0	0
0	0.010208864	0	0	0
0	0	0	0	0
0	0	0	0	0
0.108600556	0	0	0.059756355	0.048527525
0	0	0	0	0
0	0	0.005640343	0.001064884	0
0.029786235	0.147848914	0.029360123	0.001031488	0.001828762
0	0	0	0	0
0	0	0	0	0
0	0	0	0	0
0	0	0	0	0
0	0	0	0	0
0	0	0	0	0
0	0.005871956	0	0	0.033954744
0	0.0034363	0	0	0
0	0	0	0	0
0	0	0	0	0
0	0	0	0	0
0	0	0	0	0
0	0	0	0	0
0	0	0	0	0
0	0	0	0	0
0	0	0.000616718	0.013390013	0
0.192732619	0	0	0.065761736	0
0	0	0	0	0
0.076933777	0.524222609	0	0.041312155	0.027502618
0	0	0	0	0
0	0	0	0.001213546	0
0	0	0	0	0
0	0	0	0	0
0	0	0	0	0
0	0	0	0	0
0	0	0	0	0
0	0	0	0	0
0	0	0	0.018794777	0
0	0	0	0	0
0	0	0	0.001004776	0
0	0	0	0	0
0	0	0	0	0
0	0	0	0	0
0	0	0	0	0
0	0	0	0	0
0	0	0	0	0
0	0	0	0	0
0	0	0	0	0
0	0	0	0	0
0	0	0	0	0

0	0	0.009335889	0	0
0	0	0	0.014827158	0
0	0	0	0	0
0	0	0	0	0
0	0	0	0	0
0.015096826	0	0.019665759	0	0
0	0	0	0	0
0	0	0	0	0
0	0	0	0	0
0	0	0	0	0
0	0	0	0	0
0	0	0	0	0
0	0	0	0	0
0	0	0	0	0
0	0	0	0	0
0	0	0	0	0
0	0	0	0	0
0	0	0	0	0
0	0	0	0.003905728	0
0	0	0	0	0
0.010808327	0	0.008475127	0.550606896	0
0	0	0	0	0
0.165507819	0.000907855	0	0.017738049	0
0	0	0	0	0
0	0	0	0	0
0	0	0	0	0
0	0	0	0	0
0	0	0	0	0
0	0	0.001074296	0	0
0	0	0	0	0
0	0	0	0	0
0	0	0	0	0
0.82612011	0.004463565	0.006682502	0.008831489	0.030290127
0	0	0	0	0
0	0.319410996	0	0	0
0	0.022173194	0.001632043	0	0.001308815
0	0	0	0	0
0	0	0	0	0
0.009393174	0.071613774	0.008899234	0.230690381	0.012975867
0	0	0	0	0
0	0	0	0	0
0	0	0	0	0
0	0.024913773	0	0.011410583	0
0	0	0	0	0
0	0	0	0	0
0	0	0	0	0
0	0	0	0	0
0	0	0	0	0

0	0	0	0	0
0	0	0	0	0
0	0	0	0	0
0	0	0	0	0
0	0	0	0	0
0	0	0	0	0
0	0	0	0	0
0	0	0	0	1.320996261
0	0	0	0.024451882	0
0.011585069	0	0	0	0.001654813
0	0	0	0	0
0	0	0	0	0
0	0	0	0	0
0	0	0	0	0
0	0	0	0.025495125	0
0	0.100440127	0.074357769	0.012489383	0.025439888
0	0	0	0	0
0	0	0	0	0
0	0	0	0	0
0.930162415	0	0	0	0
0	0	0.022794817	0	0.012966237
0	0	0	0	0
0	0	0	0	0
0	0	0	0	0
0	0	0	0	0
0	0	0	0	0
0	0	0	0	0
0	0	0	0	0
0	0	0	0	0
0	0	0	0	0
0	0.497806993	0	0.003284153	0
0	0	0	0	0
0	0	0	0	0
0	0	0	0	0
0	0	0	0	0
0	0	0	0	0
0	0	0	0.001656139	0
0	0	0	0	0
0	0	0	0	0
0	0	0	0	0
0	0	0	0	0
0	0	0	0.121762232	0
0	0	0	0.002098969	0
0	0	0	0.000980915	0
0	0	0	0	0
0	0	0	0	0
0.356484175	0.177321436	0.005845355	0.649981583	0
0	0	0	0.003667268	0

0	0	0	0	0
0	0	0	0	0
0	0	0	0	0
0.009603921	0	0	0	0
0	0	0	0	0
0	0	0	0	0
0	0	0	0	0
0	0	0	0	0
0	0	0	0	0
0	0	0	0	0
0	0	0	0	0
0.10250167	0.046127492	0.000943107	0.007020508	0
0.005327296	0	0.00197604	0	0
0	0	0	0	0
0	0.007811638	0	0	0
0	0	0	0	0
0	0	0	0.038997141	0
0	0	0	0	0
0	0.06015378	0	0.38053479	0.003599778
0	0.26408213	0.059275944	0.010989974	1.325246608
0	0	0	0	0
0	0	0	0	0
0	0	0	0	0
0	0	0	0	0
0	0	0	0	0
0	0	0	0	0
0	0.000624712	0.014090045	0.577229661	0.000856022
0	0	0	0	0
0	0	0	0	0
0	0	0	0	0
0.001330918	0	0	0.011909467	0
0	0	0	0	0
0	0	0	0	0
0	0	0	0	0
0	0	0	0	0
0	0	0	0	0
0	0	0	0	0
0.011064932	0.011567367	0	0.018334112	0
0	0	0	0	0
0	0	0	0.002537413	0
0	0	0.001951951	0.001351254	0
0	0	0	0	0
0	0	0	0	0
0	0	0	0.007481032	0.142136654
0	0	0	0	0
0	0	0	0	0
0	0	0	0.079229757	0
0	0	0	0	0

0	0	0	0	0
0	0	0	0	0
0	0	0	0	0
0	0	0.002113769	0.043407688	0
0.004717189	0	0.002695669	0	0.001768735
0	0	0	0	0
0	0	0	0	0
0	0	0	0	0
0	0	0	0.001398116	0
0	0	0	0	0
0	0	0	0.00805855	0
0	0	0	0	0
0.019743103	0.000959128	0	0.000677752	0.003154229
0	0	0	0	0
0	0	0	0	0
0	0	0	0	0
0	0	0	0	0
0	0	0	0	0
0	0	0	0	0
0	0	0	0	0
0	0	0	0	0
0	0	0	0	0
0	0	0.002589563	0.071395455	0.003955612
0.040547643	0	0	0	0
0	0	0	0	0
0	0	0	0.000622188	0
0	0	0	0	0
0	0	0	0	0
0	0	0	0	0
0	0	0	0	0
0	0	0	0	0
0	0	0	0.002770178	0.000852665
0	0	0	0	0
0	0	0	0	0
0	0	0	0	0
0	0	0.001293582	0	0
0	0.032914054	0	0	0
0	0	0	0.203040708	0
0	0.773573327	0.001046957	0	0.002798685
0	0	0	0	0
0	0	0	0	0
0	0	0	0	0
0	0	0	0	0
0	0	0	0	0
0	0	0	0.421894719	0
0.119146508	0.000599003	0	0.110368994	0
0	0	0.105614102	0	0
0	0	0	0	0

0	0	0	0	0
0	0	0	0	0
0.004811439	0	0	0	0
0	0	0	0	0
0	0	0	0.001499943	0
0	0	0	0	0
0	0	0	0	0
0	0	0	0	0
0	0	0.003659585	0	0
0	0	0	0	0
0.004325245	0.001380804	0	0	0
0	0	0	0.006118373	0
0	0	0	0	0
0	0	0	0	0
0	0	0	0	0
0	0	0	0	0
0	0.003044582	0	0	0
0	0	0	0	0
0	0	0	0	0
0.000688322	0.000732473	0	0.000724626	0
0	0	0	0	0
0	0	0	0	0
0	0	0	0	0
0	0	0	0	0
0	0	0	0	0
0	0	0	0	0
0	0	0	0	0
0	0.008308704	0	0	0
0	0	0	0.000884927	0
0	0	0	0	0
0.0088701	0.00062927	0	0	0.002586805
0	0	0	0	0
0.047604681	0.049866641	0.040074789	0.073366124	0.026631419
0.021049105	0.067757738	0	0	0
0	0	0	0	0
0	0	0	0	0
0	0	0	0	0
0	0	0	0	0
0	0	0	0	0
0	0	0	0	0
0	0	0	0	0
0	0.01532796	0.083809468	0.361699876	0.24182736
0.433772862	0.001098457	0	0.009599087	0.010435915
0	0	0	0	0
0	0	0	0	0
0	0	0	0	0
0	0	0	0.00102956	0
0	0	0	0	0

0	0	0	0.02898363	0
0	0	0	0.013851153	0
0.113127193	0.004032697	0	0	0
0	0	0	0	0
0	0	0	0.388797963	0
0	0	0	0	0.012788822
0	0	0	0	0
0	0	0	0	0
0	0	0	0	0
0	0	0	0	0
0	0	0	0	0
0	0	0	0	0
0	0	0	0	0
0	0	0	0.47746873	0
0	0	0	0	0
0	0	0	0	0
0	0	0	0	0
0	0	0	0	0
0	0	0	0	0
0	0	0	0	0
0	0	0	0	0
0	0	0	0.095737725	0
0	0	0	0	0
0	0	0	0	0
0	0	0	0	0
0.009309172	0	0.001904242	0	0
0	0	0	0	0
0	0	0	0.398203234	0
0	0	0	0	0
0	0	0	0	0
0	0	0	0	0
0	0	0	0.043991337	0
0	0	0	0	0
0	0	0	0	0
0	0	0	0	0
0	0	0	0	0
0	0	0	0.000979974	0
0	0.000924457	0.085045354	0	1.191964071
0	0	1.429225908	1.734064454	0
0	0	0	0	0
0	0	0	0	0
0	0	0	0	0
0	0.001864133	0.00382223	0.105117186	0.000817395
0	0	0	0	0
0	0	0	0	0
0	0	0	0	0
0.003215837	0	0.006626899	0.008745738	0.002188295
0	0	0	0	0

0	0	0	0	0
0.016682046	0.011514861	0.045375752	0.408847924	0.005719683
0	0	0	0	0
0	0	0	0	0
0	0	0	0	0
0	0	0	0	0
0	0	0	0	0
0	0	0	0	0
0	0	0	0	0
0	0	0	0	0
0	0	0	0	0
0	0	0	0	0
0	0	0	0	0
0	0	0	0	0
0.014054052	0	0	0.025299947	0.005164249
0	0	0	0	0
0	0	0	0	0
0	0	0	0	0
0	0	0	0.002455113	0
0	0	0	0	0
0	0	0	0	0
0	0.087023426	0.008131143	0	0.573585145
0	0.013035492	0	0	0
0	0	0	0	0
0	0	0	0	0
0	0	0	0	0
0	0	0	0	0
0	0	0	0	0
0	0	0	0.000881894	0
0	0	0	0	0
0	0	0	0	0
0	0	0	0	0
0	0	0	0	0
0	0	0	0	0
0.078197301	0	0	9.659725423	0
0	0	0	0	0
0	0	0	0	0
0	0	0	0	0
0	0.114404503	0.003909596	0	0.012058825
0	0	0	0	0
0.011369748	0	0.095052162	0	0.341020926
0	0	0	0	0
0	0	0	0	0
0	0	0	0	0
0	0	0	0	0
1.173280795	0.434097405	0.4464274	0.473016989	1.569705811
0	0	0	0	0
0	0	0	0	0

0	0	0	0	0
0	0	0	0	0
0	0	0	0.006751516	0
0	0	0	0	0
0	0	0	0	0
0	0	0	0	0
0	0	0	0	0
0	0.032712032	0	0	0.004549324
0	0	0	0	0
0	0.058535487	0.002264558	0	0.000726423
0	0	0	0.00161324	0
0	0	0	0	0
0.000811545	0	0.003541463	0.020290764	0.00923023
0	0	0	0	0
0	0	0	0	0
0	0	0	0	0
0	0	0	0	0
0	0	0	0	0
0	0	0	0	0
0.177693843	0.356852763	0.083794707	0.001901075	0.13987511
0	0	0	0	0
0	0	0	0	0
0	0	0	0	0
0	0	0	0	0
0	0	0	0	0
0	0	0	0	0
0	0	0	0	0
0	0	0	0	0
0	0	0	0	0
0	0	0	0.468846958	0.002477133
0	0	0	0	0
0	0	0	0	0
0	0	0	0	0
0	0	0	0	0
0	0	0	0	0
0	0	0	0	0
0	0	0	0.524963069	0
0	0	0	0	0
0	0	0	0	0
0	0	0	0	0
0	0	0	0	0
0	0	0	0	0
0	0	0	0	0
0	0	0	0	0
0.013342681	0	0	0.001428448	0
0	0	0	0	0
0	0	0	0.043414243	0
0	0	0	0	0

0	0	0	0	0
0	0	0	0	0
0	0	0	0	0
0	0	0	0	0
0	0	0	0	0
0.001350621	0.005524443	0.001749754	0.029858966	0.00088624
0	0	0	0	0
0	0	0	0	0
0	0	0	0	0
0	0	0	0	0
0	0	0	0	0
0	0	0	0	0
0	0	0	0	0
0	0	0	0	0
0	0	0	0	0
0	0	0	0	0
0	0	0	0	0
0	0	0	0	0
0	0	0	0	0
0	0	0	0	0
0	0	0	0	0
0	0	0	0	0
0	0	0	0	0
0	0	0	0	0
0	0	0	0	0
0	0	0	0	0.000924152
0	0	0	0	0
0	0	0	0	0
0	0	0	0	0
0	0	0	0	0
0	0	0	0	0
0	0	0	0	0
0	0	0	0	0
0	0	0	0	0
0	0	0	0	0
0	0	0	0	0
0	0	0	0	0
0	0	0	0	0
0	0	0	0	0
0	0	0	0	0
0	0	0	0	0
0	0	0	0	0
0	0	0	0	0
0	0	0	0	0
0	0	0	0	0
0	0	0	0	0
0	0	0	0	0
0	0	0	0	0

0	0	0	0	0
0	0	0	0.178340512	0
0	0	0	0	0
0	0	0	0	0
0	0	0	0	0
0	0	0	0	0
0	0	0	0	0
0	0	0	0	0
0	0	0	0	0
0	0	0	0	0
0.003712525	0	0	0	0
0	0	0	0	0
0	0	0	0	0
0	0	0	0	0
0	0	0	0	0
0	0	0	0	0
0	0	0	0	0
0	0	0	0	0
0	0	0	0	0
0	0	0	0	0
0.035921498	0	0	0	0.001318639
0	0	0	0	0
0	0	0	0	0
0.01415035	0.00830404	0	0.631739205	0
0	0	0	0	0
0	0	0	0	0
0	0	0	0	0
0	0	0	1.134644191	0
0	0	0	0	0
0	0	0	0	0
0	0	0.004303664	0	0
0	0	0	0	0
0	0	0	0	0
0	0	0	0	0

PHL018-0056_V3	PHL018-0255_V1	PHL018-0061_V2	PHL018-0013_V3	PHL018-0249_V1
REF	REF	REF	EYCF	REF
0.962587992	0.069117394	0.23969291	0.136067405	0.020030577
6.054554103	0.099828388	0.070050171	0.025367294	0.331169733
0.001312353	2.20906641	0	0.961812368	0
0	0	0	0	0
0	0	0	0	0
0	0	0	0.03169018	0
0	0	0	0	0
0	0	0	0	0
0	0	0	0	0
0	0	0	0	0
0	0	0	0	0
0	0	0	0	0
0	0	0	0	0
2.409821407	0.174785658	0.013175468	0.082338493	3.700442572
0	0	0	0	0
0	0	0	0	0
0	0	0	0	0
0	0	0	0	0
0	0	0	0	0
0	0	0	0	0
0	0	0	0	0
0	0	0	0	0
0	0	0	0	0
0	0	0	0	0
0.328554678	0.028504621	0.001869785	0.194275088	0
0	0	0	0	0
0	0	0	0	0
0	0	0	0	0
0	0	0	0	0
0	0	0	0	0
0	0	0	0	0
0	0	0	0	0
0	0	0	0	0
0	0	0	0	0
0	0	0	0	0
0.000748173	0.348720317	0.466998421	2.978648184	1.81195333
0	0	0	0	0
0	0	0	0	0
0	0	0	0	0
0	0	0	0	0
0	0	0	0	0
0.005922056	0.00197674	0.023852639	0	0.016376706
2.167038057	0.194479682	12.63685868	0.005188783	4.22689936
0.038357268	0.001620681	0	0.000981137	0
0.004200643	0.00382403	0	0	0.000944419
0	0	0	0	0

0.017920246	0.001652009	0.000480869	0	0.020195795
0.023096355	0	0.006836951	0	0.003368231
0.016000099	0.007517726	0.00091178	0.034119893	0.000522183
0	0.165460845	0.503498436	0.700162234	0.384227132
0.014235324	0.001791087	0.01140842	0	0.000585455
0	0.015773362	0	0	0
0	0.000609083	0.004964202	0	0
0	0	0	0	0
0	0	0	0	0
0	0	0	0	0
0	0	0	0	0
0	0.372102977	0.449765196	0.53323293	0
0	0	0	0	0
0.249645711	0.010468951	0.016421664	0.003181452	0.006140591
0	0	0.00394867	0	0
0	0	0	0	0
0.006568864	0	0.113954328	0.005309572	0.285993854
0	0	0	0	0
0	0	0	0	0
0	0	0	0	0
0.052911627	0	0.017173748	0.046919249	0.013660449
0.015688061	0.005193285	0.031577099	0.005901127	0.000801614
0	0.560967762	0	0.011544423	0.145834677
0	0	0	0	0
0	0	0	0	0
0	0	0	0	0
0	0	0	0	0
0	0	0	0	0
0.020981821	0.000922738	0	0	0
0	0	0	0	0
1.065231594	0	0.003616098	0	0
0	0	0	0	0
0	0	0	0	0
0	0	0	0	0
0.004653684	0	0	0	0
0	0	0	0	0
0	0	0	0	0
0	0	5.05211262	0	15.12652496
0	0	0	0	0
0	0	0	0	0
0	0	0	0	0
0	0	0	0	0
0	0	0	0	0
0	0	0	0	0
0	0	0	0	0
0	0	0	0	0
0	0	0	0	0
0	0	0	0	0

0	0	0	0	0
0.006989489	0	0	0	0
0	0	0	0	0
0	0	0	0	0
0.083062596	0	0	0.057016583	0
0.002921617	0	0	0	0
0	0	0	0	0
0	0	0	0.004110868	0
0	0.000976056	0.00519519	0.007030391	0
0	0	0	0	0
0	0	0	0	0
0	0	0	0	0
0	0	0	0	0
0	0	0	0	0
0	0	0	0	0
0	0	0	0.005285037	0
0	0	0	0	0
0	0	0	0	0
0	0.432349243	0.031032388	0.019461843	0.533013721
0	0	0	0	0
0	0	0	0	0
0	0	0	0	0
0	0	0	0	0
0	0	0	0	0
0	0	0	0	0
0	0	0	0	0
0	0	0	0	0
0	0	0	0	0
0	0	0	0	0
0	0	0	0	0
0	0.031999573	0.168261705	0.065670935	0.010754981
0.002676523	0.011235234	2.501084528	0	0.003610532
0	0	0	0	0
0	0	0	0	0
0	0	0	0	0
0	0	0	0	0
0	0	0	0	0
0	0	0	0	0
0	0	0	0	0.518999588
0	0	0	0	0
0	0	0	0	0
0	0	0	0	0
0	0	0.025868162	0.00044653	0.000695534
0	0	0	0	0
0	0	0	0	0
0	0	0	0	0
0	0	0	0	0
0	0	0	0	0

0.004256218	0	0	0	0
0	0	0	0	0
0	0	0	0	0
0	0	0	0	0
0	0.05714235	0.249119065	0.132034848	0.007987338
0	0	0	0	0
0	0	0	0	0
0	0	0	0	0
0	0	0	0	0
0	0	0	0	0
0	0	0	0	0
0	0	0	0	0
0	0	0	0	0
0	0	0	0	0
0	0	0	0	0
0	0	8.447248341	0	2.678735856
0	0	0	0	0
0	0	0	0	0
0	0	0	0	0
0	0	0	0	0
0	0	0	0	0
0	0	0	0	0
0	0	0.004817216	0	0
0	0	0	0	0
0	0.006825141	0.001478454	0	0
0	0	0	0	0
0	0.007646176	0.012326738	0	0
0	0	0	0	0
0	0	0	0	0
0	0	0	0	0
0	0	0	0	0
0	0	1.515867022	0	0
0	0	0	0	0
0	0	0	0	0
0	0	0	0	0
0	0	0	0	0
0	0	0	0	0
0	0	0	0	0
0	0	0	0	0
0	0	0	0	0
0	0	0	0	0
0	0	0	0	0
0	0	0	0	0
0	0	0	0	0
0	0	0	0	0
0	0	0	0	0
0	0	0	0	0
0	0	0	0	0

0	0	0	0	0.048820349
0	0	0	0	0
0	0	0	0	0
0	0	0	0	0
0	0	0	0	0
0	0	0	0	0
0	0	0	0	0
0	0	0	0	0
0	0	0	0	0
0	0	0	0	0
0	0	0	0	0
0	0	0	0.048603699	0
0.001477519	0.0005978	0.001044052	0	0
0	0	0	0	0
0	0.005351735	0	0	0
0	0	0	0	0
0	0	0	0	0
0	0	0	0	0
0	0	0	0	0
0	0	0	0	0
0.029928225	0.002794357	0.013339529	0.026051628	0.013353842
0	0	0	0	0
0.000606004	0	0	0	0
0	0	0.001357117	0	0
0	0	0	0	0
0	0	0	0	0
0.002413314	0.001952841	0	0.000464445	0.004702349
0	0	0	0	0
0	0	0	0	0
0.0117623	0	0	0	0
0.025660423	0	0	0	0
0	0	0	0	0
0	0	0	0	0
0	0.266577806	0.061639278	0.022582021	0.00835083
0.03145622	0.028545073	0.007832637	0.006918603	0.00181857
0	0	0	0	0
0	0	0	0	0
0	0	0	0	0
0	0	0	0	0
0	0	0	0	0
0	0	0	0	0
0	0	0	0	0
0.001556915	0	0	0	0
0	0	0	0	0
0	0	0	0	0
0	0.072878637	0.04897779	0	0.00153909
0.001946213	0	0	0	0
0.005764256	0	0.000905148	0.001602375	0

0	0	0	0	0
0	0	0	0	0
0	0	0	0	0
0	0	0	0	0
0	0	0	0	0
0	0	0	0	0
0.004558526	0	0	0	0
0	0	0	0	0
0	0	0.019620195	0	0
0	0	0	0	0
0	0.000801535	0	0	0
0	0	0	0	0
0	0	0	0	0
0	0	0	0	0
0	0	0	0	0
0	0	0	0	0
0	0	0	0	0
0	0	0	0	0
0.011724421	0.002240066	0.069193172	0	0.008933007
0	0.060551306	0.194977927	0.074698074	0
0.489696661	0	0	0.001109461	0
0	0	0	0	0
0	0	0	0	0
0	0	0	0	0
0.015270937	0.001544645	0.002248089	0.00110209	0
0	0	0	0	0
0	0	0	0	0
0	0	0	0	0
0	0	0	0	0
0	0	0	0	0
0.40999896	0.025440067	0.261259849	0.079267239	0.013818898
0	0	0	0	0
0	0	0	0	0
0	0	0	0	0
0	0	0	0	0
0	0	0	0	0
0	0	0	0	0
0	0	0	0	0
0	0	0	0	0
0.001411368	0.000713795	0	0	0
0	0	0	0	0
0.066253596	0.053466371	0.00491912	0.001940303	0.001133362
0	0	0	0	0
0	0	0	0	0
0	0	0	0	0
0	0	0	0	0
0	0	0	0	0
0	0	0	0	0

0	0	0	0	0
0	0	0	0	0
0.041897568	0.001097588	0	0	0
0	0	0	0	0
0	0	0	0	0.049930289
0	0	0	0	0
0	0	0	0	0
0	0	0	0	0
0	0	0	0	0
0	0.001505769	0	0	0
0	0	0	0	0
0	0	0	0	0
0	0	0	0	0
0.0013325	0	0.013417486	0	0
0.00494912	0.003671068	0.001165724	0.001904925	0
0	0.060563317	0.012272604	0	0.002581126
0.001879693	0	0	0	0
0	0	0.001202059	0	0
0	0	0	0	0
0	0	0	0	0
0	0	0	0	0
0.002828043	0.000858164	0	0.002449168	0
0	0	0	0	0
0	0	0	0	0
0	0	0	0	0
0	0	0	0	0
0	0.012212744	0	0	0
0	0	0	0	0
0	0	0	0	0
0.004908949	0	0.001156262	0	0
0	0	0	0	0
0	0	0	0	0
0	0	0	0	0
0	0	0	0	0
0	0	0	0	0
0.002824307	0	0	0	0
0	0	0	0	0
0.00568503	0.025229748	0.001757518	0	0.001677571
0	0	0	0	0
0	0	0	0	0
0	0	0	0	0
0	0	0	0	0
0	0	0	0	0
0.018536953	0	0	0	0
0	0	0	0.003013151	0
0	0	0	0	0
0	0	0	0	0
0.027113615	0	0	0	0

0	0	0	0.047340773	0.001339567
0	0	0	0	0
0	0	0	0	0
0	0	0	0	0
0.00655647	0	0.001052947	0.004817776	0.002814139
0	0	0	0	0
0	0	0	0	0
0.00167097	0.001267631	0.001771124	0.009406206	0.043109232
0	0	0	0	0
0	0.044162238	0.007302144	0.01591004	0
0	0	0	0	0
0.003637551	0	0.017525352	0	0.004460839
0.01956532	0	0	0.001158573	0
0.010666806	0	0	0	0
0	0	0	0	0
0	0	0	0	0
0	0	0	0	0
0	0	0	0	0
2.34833301	0	0	0	0
0	0	0	0	0
0	0	0.005759853	0	0
0	0	0	0	0
0	0	0	0	0
0	0.052877118	0.264086709	0	0.006467035
0	0	0	0	0
0.001460808	0	0	0	0.004138211
0	0	17.05163477	0	3.652984767
0.498159573	0	0.002496539	0	0
0.008375679	0	0	0	0
0.045690832	0	0	0.000867754	0
0.004389075	0	0	0	0
0	0	0	0	0
0	0	0	0	0
0.004967219	0	0	0	0
0	0	0	0	0
0	0	0	0	0
2.126935414	0	0	0	0
0	0	0	0	0
0	0	0	0	0
2.544887043	0	0	0	0
0	0	0	0	0
0	0	0	0	0
0.00214443	0	0	0	0
0	0	0	0	0
0	0	0	0	0
0	0.000518913	0	0	0
0.003633261	0	0	0	0
0	0	0	0	0

0	0	0	0	0
0	0	0.001434598	0	0
0	0	0	0	0
0	0	0	0	0
0	0	0	0	0
0.017169799	0	0.151051	0.006278252	0.001158072
0	0	0	0.001285987	0
0	0	0	0	0
0	0	0	0	0
0	0	0	0	0
0.013607952	0.001007148	0	0.001916241	0.00335792
0	0	0	0.000897977	0
0	0	0	0	0
0	0.001141803	0.000664716	0	0.002326422
0	0	0	0	0
0	0	0	0	0
0	0	0	0	0
0	0	0	0	0
0	0	0	0	0
0	0.000570554	0	0.068715903	0
0	0	0	0	0
0	0.088644011	0.32842003	0.15514876	0.238159225
0	0	0	0.102561779	0
0	0	0	0	0
0	0	0	0	0
0	0	0	0	0
0.151973741	0	0	0	0
0	0	0	0	0
0	0	0	0	0
0.184766682	0.103208058	0.116595313	0.110390609	0.149112085
0.0053182	0	0.001653508	0	0.002726142
0	0	0	0	0.004431747
0.075016274	0	0	0	0
0	0	0	0	0
0	0	0	0	0
0	0	0	0	0
0	0.200240465	0.159782857	0	1.087317773
0	0	0.000903363	0	0.002155676
0	0	0	0	0
0	0	0	0	0
0.002577834	0	0	0.003125471	0
0	0.418186534	0.980234427	0	0.378474885
0	0	0.000620654	0	0
0	0	0	0	0
0.022391072	0	0	0	0
0	0	0	0	0
0	0	0.00191186	0	0
0	0	0	0	0

0	0.001823392	0	0	0
0.037968551	0	0.00195946	0	0.000431614
0.0027292	0	0	0.015231874	0
0	0	0	0	0
0	0	0.014543873	0	0
0.098223755	0	0	0	0.000761496
0	0	0	0	0
0	0	0	0	0
0.041069506	0	0.00276388	0	0
0	0	0	0	0
0	0	0	0	0
0	0	0	0	0
0	0	0	0	0
0	0.000733145	0	0.002266732	0
0	0	0	0	0
1.787323011	0.037097883	0.009339253	0.049872067	0.002228606
0.027729102	0.009843191	0.001109099	0.006645441	0.076046174
0.091216661	0	0	0	0
0.002431874	0	0	0	0
0	0	0	0	0
0	0	0	0	0
0.002124059	0	0	0	0
0	0	0	0	0
0	0	0	0.056018317	0
0	0.001928909	0	0	0
0.007925931	0	0	0.005769812	0
0	0	0	0.000958686	0
0.02080638	0.001020389	0	0	0
0	0	0	0	0
0.004546598	0	0	0	0
0.024245672	0.002452434	0.042653035	0.022601429	0
0.005271485	0	0	0	0
0.191803842	0.006527382	0.015200008	0.018007937	0.051505461
0	0.001870875	0.001815258	0	0.001039611
0	0	0	0	0
0	0	0	0	0
0.001620597	0	0	0	0
0.066157345	0.000769169	0	0.00248787	0
0	0.036112012	0.019426395	0.348976795	0.173361692
0	0	0	0	0
0.004102247	0	0	0	0
0	0	0	0	0
0	0	0	0	0
0.058173658	0	0.077320243	0.38247732	0
0.609857472	0	0	0	0
0	0	0	0	0
0	0	0	0	0
0	0	0	0	0

0.002254928	0	0	0	0
0.479751954	0.053033379	0.016108217	0.169701336	0.008386624
0	0	0	0	0
0	0	0	0	0
0	0	0	0	0
0	0	0	0	0
0.020130657	0	0	0	0
0	0	0	0	0
0	0	0	0.000912736	0
0.018071345	0	0	0	0
0.032853164	0	0	0	0.001558045
0.011469309	0.002784271	0	0.002648735	0
0	0.113597728	0.824982638	0	0
0	0	0	0	0
0	0	0	0	0
0	0	0	0	0
0.0266078	0.000815564	0.001709249	0.00062069	0
0.041867394	0	0	0	0.00141194
0	0	0	0	0
0.421164163	0	0	0.004839661	0
0.013581095	0.003364209	0.002546074	0.129937923	0.129177508
0	0	0	0	0
0	0	0	0	0
0	0	0	0	0
0	0	0	0	0
0	0	0.001796017	0	0
0.049790814	0.00863367	0.002126471	0.045173638	0
0.28692646	0.025387168	0.014363152	0.03197501	0.029406512
0	0	0	0	0
0.002842715	0	0	0	0
0	0.001342839	0	0	0
0	0	0	0	0
0.003576249	0	0	0	0
0	0	0	0	0
0	1.052719175	0	0	0.19773366
0.098716603	0.035099205	0.066232614	0.101611043	0.057503103
0	0	0	0	0
0	0	0	0	0
0.010175323	0.005513717	0.005392604	0.006923813	0.002451096
0	0	0	0	0
0.612966528	0	0	0	0
0	0	0	0	0
0.001765899	0.000937752	0.002651636	0	0
0	0	0.005421397	0	0
0	0	0	0	0
0	0.219028135	0.108916519	0.330410737	0.106486853
0	0	0	0	0
0.003100395	0	0	0	0

0	0	0	0	0
0	0.013509967	0.008481869	0	0
0	0	0	0	0
0.960228159	3.074188275	0.152054166	0.314525587	0.061731096
0	0	0	0	0
0.002675226	0	0	0	0
0	0	0	0	0
0	0	0.001950233	0.003983627	0
0	4.775904779	0.183231795	0.088640307	0.594162389
0.001148873	0.092733741	0.110407814	0.319845516	0.021387061
0	0	0	0	0
0	0.02152657	0.000696221	0.001137704	0
0	0	0	0	0
0	0	0	0	0
1.110426526	0.10707612	0.303491938	0.143993996	0.017000061
0	0	0	0	0
0	0.012229295	0.020234214	0.030921895	0.027361199
0	2.928921009	0.451832606	0	0
0	0	0	0	0
0	0.000667493	0.013989245	0.017399008	0
0	0	0.005687703	0	0
0	0	0	0	0
0	0.001651861	0	0	0
0.511806821	0	0.424606277	0.465395893	0.06048339
0.01937188	0	0.002933283	0	0
0	0.089287254	0.123273475	0.316435382	0
0	0.001498787	0.332146353	0	0.101422383
0	1.357468606	0.564852198	0.511214983	7.036146793
0	0	0	0	0
0	0	0	0	0
0	0.008429131	0.007276089	0.059726278	0.004199369
0	0	0	0	0
0.029342218	0	0.041467894	0.09459495	0.001966786
0	0	0	0	0
0	0.0021469	0	0	0
0	0.129877516	0.339312039	0	0.033140924
0	0.914280863	0.249592743	0.105372129	3.090918253
0	0	0	0	0
0	0	0	0.003405006	0
0.011460061	0	0	0.002035845	0
0.001181496	0.036282477	0.141427732	0.304234307	0
0	0.026405234	0.018272563	0	0
0.032882499	0.568409249	0	0.007855779	0.049328362
0	0.003622911	0.005672821	0	0
0	0	0	0	0
0	0	0	0	0
0	0	0	0	0
0.720088721	0.000689576	0.001405061	0.001476018	0

0	0	0	0	0
0	0.086223678	0.032915577	0	0.076625727
0.464820028	0.002313628	0.010198028	0	0
0	0.002322284	0.00998362	0	0.021044267
0	0	0	0	0.00134644
0	0.065482958	0	0.00396312	0.042102626
0	0	0	0	0
0	0	0	0	0
0	0	0	0	0
0	0	0	0	0
0.811147701	0.001135786	0.060453768	0.159759211	0.005770381
3.033647436	0.713260252	0.6119147	0.857109439	1.009232517
0	0	0	0	0
0.092043556	0.064616487	0.001223328	0.006996699	0.019266732
0.291727059	0.005795803	0.009318952	0.008139223	0
0	10.23244621	1.297496838	0.126943498	0
0	0	0	0.001812877	0
0	0	0	0.001457866	0
0	0.197161957	0.027554067	0.135493754	0.004195199
0	0.00334139	0.059135118	0	0.017676243
0.243799937	0.068948834	0.115747363	0.211008451	0
0.000944447	0	0	0	0.465178004
0.001693198	0.365823885	0.158231339	0.369522408	0.977007641
0	0	0	0	0
0	0.004931626	0.013967081	0	0
0	0	0.010815189	2.145593879	2.833633797
0	0	0	0.709778305	0
0	0	0	0	0
0	0	0	0.14343489	0
0	0	0	0	0
0	0.011549814	0.110376374	0	0
0	0.057774838	0.210327028	0.216063287	0.06463687
3.092572631	0.024496127	0.003880475	0	0.005555936
0.055278175	0.02605061	0.017015184	0.367264313	0.000988594
0.111997004	0	0.000768348	0.00146483	0.004278151
0.115781265	0.143556617	0.004222655	0.009919177	0.280628611
0.032470583	0.003503336	0.009007852	0.005693527	0
0	0	0	0	0
0.136652945	4.527778842	0.115916052	0.056018513	0.552822925
0	0	0	0	0
0	0.052692522	0.031915069	0	0.049983468
0	0	0	0	0
0	0	0	0.027027913	0
0	0	0	0	0
2.37294571	0.034809921	0.024879871	0.033935085	0.016661988
0	0.004661814	0	0	0.061727517
0	0	0	0	0
0	0.155696624	0	0.003757318	0.032343074

0	0	0	0	0
0	0	0	0	0
0	0	0	0	0
0.002662076	0.003818699	0.009576457	0	0
0	0	0	0	0
0	0.277335676	0.151788498	0	0.389240203
0	0	0	0	0.053075368
1.520083254	0.434371414	0.003147169	0.170741998	0.000600802
0	0.27483781	0	0	0
0	0	0	0	0
0.110507976	0.00212651	0.007618313	0.003890371	0
0	0	0	0	0
0.027139544	0	0	0	0.01050555
0	0	0.015927266	0	0.295678109
0	0	0	0	0
0	0	0	0	0
0	0.020438666	0.047594558	0.099764826	0.07503988
0.001346274	0.011847198	0.004598002	0	0.014679931
0	0	0	0	0
0.010319985	0	0	0.001168288	0
0	0	0.028594736	0	0
0	0	0	0.009549669	0
0.003363007	0.026061945	0.031075807	0.00298714	0
0	0	0	0	0
0	0	0	0	0
0	0	0	0	0
0	0	0	0	0
0	0	0	0	0
0	0	0	0	0
0	0.065634624	0.038305829	1.050361929	0.101828931
0	0	0.004500202	0	0
0	0	0	0	0
0	0	0	0	0
0	0	0	0	0
0.056151431	0.033167596	0	0	0.001590092
0	0	0	0	0
0	0.040413502	0.040644185	0.021953336	0.023433426
0.034452652	0	0	0.000395192	0
0	0	0	0	0
0	0.002458449	0	0	0
0	0.001818886	0	1.040183695	0
0	0	0	0	0
0	0	0	0	0.018736456
0	0.041955296	0.074840186	0	0.00224171
0	0.059905699	0.002196844	0.102760645	0.018697469
0	0.007276315	0.015045113	0	0
0	0.005920695	0.00382979	0.002252992	0
0	0	0	0	0

0	0	0	0	0
0	0	0	0	0
0	0.032047163	0.213059285	0	0.035259868
0	1.008933966	0.1918055	0.836255205	0.03964704
0	0	0	0	0
0	0.37191677	0	0	0
0	0	0	0	0
0	0	0	0	0
5.794115191	0.002673652	0.092500683	0.002906858	0.02292221
0	0.002460663	0.000895317	0	0
0	0	0.026710735	0	0
0	0	0	0	0
0	0.000334234	0	0.010916751	0
0.034751565	0	0	0	0
0	0.278047791	0.25183204	0.502130045	0.433623444
0	0	0	0	0
0	0	0	0	0
0	0	0	0	0
0	0	0	0	0
0	0	0	0	0
0	0.037821222	0.005440772	0.035285512	0.031484254
0	0	0	0	0
0	0	0	0	0
0	0	0	0	0
0.20204682	0.062658191	0.397458694	0	4.452460166
0	7.900007173	0.409618908	1.091844139	1.534772655
0	1.592601148	0.007586069	0.017482238	0.052357925
0	0	0	0	0
1.327114935	0.223837066	0.000914454	0.007845188	0.119799605
0	0	0	0	0
0	0	0	0	0
0	0.014607793	0	0	0
0	0	0	0	0
0.055929287	0.004437811	0.019690693	0.005362804	0
0.005535741	0.001580997	0	0.000564013	0.007467511
0.010588381	0	0.000975915	0	0.002018298
0	0	0	0	0
0.051983098	0.452718598	0.087423493	1.195401128	0.387431207
0	0.662320064	0.001437959	2.071337727	0
0	0	0	0	0
0	0	0	0	0
0	0	0	0	0
0.528126226	0.357361001	0.205597489	1.552542682	0.560306421
0	0	0.000835069	0	0
0	0	0	0	0
0	0	0	0.001007221	0
0	0	0	0	0.002149235
0	0.179636978	0.112188182	1.063263415	0.033507966

0	0.000471674	0	0.000897427	0
0	0	0	0	0
0	0	0	0	0
0	0	0	0	0
0.021923878	0	0.00109355	0.006552278	0
0.954527414	0	0.03931471	0	0
0	0	0	0	0
0	0	0	0.049843136	0
0	0	0	0	0
0	0.033937088	0	0.004782971	0
0	0.001916434	0.001255137	0	0
0	0.87572619	0.237420713	1.025708729	0.066766462
1.454406997	0.019332773	0.026481952	0.015146078	0.006319331
0	0	0	0	0
0.015511272	0.015930926	0.038362288	0.000861103	0.031587401
0	0	0	0	0
0.001452544	0	0	0.000359413	0
0	0	0	0.025526115	0
0.027452603	0.0019041	0.025495431	0	0
0	0.002287288	0.001024288	0.007532115	0.009385872
0	0.038359558	0.063935459	0.059112428	0
0	0	0	0	0
0	4.32582524	0.02658728	1.383686985	0.203708784
0	0	0	0.002446776	0
0	0	0	0	0
0	0	0	0	0
0	0	0	0	0
0	0	0	0	0
0	0.001956782	0.003797222	0.000930764	0
0	0	0	0	0
0	0	0	0	0
0.053416735	0.011510882	0.001641113	0.013855785	0
0	0	0	0	0.00370431
0.143679402	0.013965836	0.063266779	0.132189997	0
0.008516861	0	0.00106204	0	0.001182684
0	0.229294985	0.013234619	0.005593155	0.070350486
0	0.005397914	0	0	0
0	0.047536356	0	0	0
0	0.002969122	0	0.000996913	0
0	0	0	0	0
0.007683244	0.069037267	0.033329087	0.117429228	0.038146803
0	0.002782403	0	0	0
0	0	0	0	0
0	0	0	0	0.002200718
0.047872135	0.059604413	0.111945112	0.045875217	0.006324626
0	0	0	0	0
0	0.074216675	0	0.027450395	0
0.011436316	0.003257858	0	0	0

0	0	0	0	0
0	0	0	0	0
0.032922774	0	0	0.002357588	0
0	0	0	0	0
0	0	0	0	0
0	0	0	0	0
0	0.432384833	0	0.261464758	0.313788787
0.260791206	0.181823948	0.007323576	0.00055663	0.112659826
0	0	0	0	0
0.001660258	0	0.000670389	0	0
0	0	0	0	0
0.003895131	0	0	0	0
0	0	0	0	0
0	0.117178224	0.00072494	0	0.117218614
0	0	0	0	0
0	0.002832589	0	0	0
0	0	0	0	0
0	0	0	0	0
0	1.561034936	0	0.904220954	0.302757247
0	0	0	0.000418148	0
0	0	0	0	0.000938417
0	0	0	0	0
0	0	0	0	0
0	0	0	0	0
0	0.001309005	0.000571541	0.000466981	0
0.18722797	0	0	0	0
0	0	0	0	0
0.002023718	0.582842767	0.196546947	1.117508972	0.076286153
0	0.090991487	0	0	0
0	0.252237526	0.347218862	0.202382141	0.14877397
0	0	0	0	0
0	0	0	0	0
0	0.052018452	0.005536546	0.002193295	0
0	0	0	0	0
0.003611104	0	0	0	0.104894193
0	0.007635572	0	0	0
0	0	0	0	0
0.033326704	0	0	0.001734868	0
0	0	0	0	0
0	0	0.000451855	0	0
3.223176861	0.319021482	0.538674347	1.171286122	1.696613087
3.078606712	0.002063049	0.097354172	0.451402812	0.005259943
0	0	0.029022708	0	0.250905555
0	0.005856755	0.004804419	0	0
0	0	0	0	0
0	0	0	0	0
0	0.003762637	0	0.002326659	0
0.076748119	0.048420199	0.012102654	0.024408454	0.001316064

0	0	0	0	0
0.033449905	0.001218037	0.011030386	0.06476089	0
0.003125408	0	0.005889311	0	0
0	0	0	0	0
1.791289206	0.255432178	0.768610667	1.942928756	1.906799521
0	0	0	0	0.005552379
0	0	0	0	0
0	0	0	0.000819046	0
2.808544169	0	0.000944068	0	0
0	0	0	0	0
0	0	0	0	0
0	0	6.562143555	0	0
0	0.035505911	0.218338931	0.106586796	0.022506787
0.012520252	0.00080835	0	0.005229201	0.005030874
0	10.66276149	0.012298103	0	0.188197545
0.199141276	0.094590324	0.294257082	1.495176598	0.230742403
0	0.058898038	0.016126076	0.011179566	0
0	0	0	0	0
0	0	0	0	0
0	0.000724142	0	0	0
0	0	0	0.0031034	0
0	0	0	0	0
0	0	0	0	0
0	0	0	0	0
0	0.000751402	0.001603942	0	0
0	0	0	2.02088011	0
0	0.053039001	0.004815734	0.046522369	0.121271062
0.005202127	0.000686337	0	0.003264632	0
0	0	0	0	0
0	0.000945591	0	0	0
0	0	0	0	0
0	0	0	0	0
0	0	0	0	0.004275454
0	0.014389286	0.013235523	0.018890578	0.001439255
0	1.954766838	0.002774144	0	0.007754716
0	0.000952739	0.011092995	0.007401944	0
0	0	0	0	0
0	0.08126892	0	0.000570574	0
0	0	0	0	0
0	0	0	0	0
0	0.342084587	0.259946706	0.314146535	0.211379277
0.001907415	0	0	0	0
0	0.023460935	0	0.00433532	0.007690778
0	0	0	0	0.015683868
0	0	0	0	0
0	0	0	0	0
0	0	0	0	0
0	0	0	0	0

0	1.535112443	0.032023603	0.110093577	0.083349072
0	0	0	0	0
0	0	0	0.024648586	0
0.001024868	0	0	0	0
0.007354484	0.013687782	0.046598537	0.09199951	0.03108558
0	0	0	0	0.004815729
0.048750942	0.144305195	0.00909964	0.216409315	0
0	0	0	0.000770018	0
0	0	0	0.004404449	0
0	0	0	0	0
0	0.010492851	0	0.002898019	0
0	0	0.001705244	0	0.000406919
0	0	0	0	0
0	0	0	0	0
0	0	0	0	0.001676406
0	0	0	0	0
0	0	0	0	0
0	0	0	0	0
0	0	0	0	0
0	0	0	0	0
0.023206807	0.108554006	0.048731425	0.131709905	0.008416946
0	0	0	0	0
0	0.017586491	0.055115686	0.056186231	0.001954497
0.016534036	0.029712369	0.001213856	0.002603446	0.001158639
0	0	0	0	0
0.012919854	0.000922471	0.006623357	0	0
0	0	0	0	0
0	0	0	0	0.002274042
0	0	0	0	0
0	0	0	0.00124899	0.001042221
0	0	0	0	0
0	0	0	0	0
0	0	0	0	0
0	0	0	0	0
0	0.093353763	0	0	0.022232123
0	0	0	0	0
0.080604007	0.022991577	0.003797121	0	0.006705131
0	0	0	0	0
0	0.143947317	0	0	0
0	0	0	0	0
0	0	0	0	0
0	0	0	0	0
0	0	0	0	0
0	0.030497494	0.030436319	0	0
0	0.009259486	0	0	0
0	0	0	0	0
0	0	0	0	0
0	0	0	0	0.00984564

0.051756248	0	0.002285766	0.0004002	0
0	0.223290021	0	0	0
0	0	0.01358203	0	0.00129642
0	0	0	0	0
0	0	0	0	0
0	0	0	0	0
0	0	0	0	0
0	0.28940095	0.320025501	0.245727213	0.225940705
2.75778084	0.223407607	18.09636469	0.007119308	5.612996748
0.690037317	0	0.031578773	0	0
0	0.016722713	0.001465536	0	0
0	0	0	0.000637996	0
0	0.01950727	0.007570943	0	0
0	0	0	0	0
0	0	0	0	0
0	0	0	0	0
0	0	0	0	0
0	0	0	0.119268453	0.397018299
0	0	0	0	0
0	0.020711269	0.010651883	0.100207577	0.001553344
0	0	0	0	0
0	0	0	0	0
0	0	0	0.197081771	0
0	0	0	0	0
0.014013044	0.013143257	0.026876771	0	0.008469563
0	0	0	0	0
0	0	0	0	0
0	0	0	0	0
0	0	0	0	0
0	0	0	0	0
0.00451001	0	0	0	0
0	0	0	0	0
0	0	0	0	0
0	0	0	0	0.000619587
0	0.037857261	0	0	0.007735423
0	0	0.001215222	0	0
0	0	0	0	0
0	0	0	0	0
0	0.007003074	0.001358977	0.000634492	0
0	0	0.028523883	0	0
0	0	0	0	0
2.568191761	0.206237669	0.907878799	0.335927508	0.115886506
0	0	0	0	0
0	0	0	0	0
0.009440663	0	0	0	0
0.051569095	0	0	0	0
0	0	0	0	0
0	0	0	0	0

0	0	0	0	0
0.000625611	0	0	0.002769186	0.006610755
0	0	0	0	0
0	0	0	0	0
0	1.334903336	0.359310064	0.149313363	0.194294707
0	0	0.025042071	0	0
0	0	0	0	0
0.473502352	0.005529713	0.114021823	0.00526053	0.023590857
0	0.013892085	0.001658966	0	0
0	0	0	0.018978656	0.12691253
0	0	0	0	0
0	0	1.929211156	0	0
0	0	0	0	0
0	0	0	0	0
0	0	0	0	0
0	0.009591757	0	0.124215515	0
0	0	0	0	0
0	0	0	0	0
0	0	0	0	0
0	0	0	0	0
0	0	0	0	0
0	0	0	0	0
0	0	0	0	0
0.031286534	0	0	0.00088114	0
0	0.080210768	0.031503646	0.191418703	0.080137357
0	0	0	0	0
0.005816074	0.001976661	0.017096701	0.001208853	0
0	0	0	0	0
0.004149541	0	0	0	0
0	0	0	0	0
0	0	0	0	0
0	0	0	0	0
0	0	0	0	0
0	0	0	0	0
0	0	0	0	0
0	0	0	0	0
0	0	0	0	0
0	0.037358068	0.008935428	0.006061	0.002574783
0	0	0	0.064733115	0
0	0	0	0	0
0	0	0	0	0
0	1.412933329	0.014112038	0	0.02288072
0	0	0	0	0
0	0	0	0	0
0	0	0	0	0
0	0	0	0	0
0	0	0	0	0
0	0.006144776	0	0	0

0	0.048729201	0.038030593	0.148671118	0.044564194
0	0	0	0	0
0	0	0	0	0
0.000870699	0	0	0	0
0	0	0	0	0
0.522401812	0.128448025	0.290692775	0	0.001196726
0	0	0	0	0
0	0	0	0	0
0	0	0	0	0
0	0	0	0	0
0	0	0	0	0
0.039158369	0	0	0.000541514	0
0	0	0	0	0
0	0	0	0	0
0	0	0	0	0
0	0	0	0	0
0	0	0	0	0
1.654987494	0	0	0	0
0	0	0	0.00235601	0
0	0	0	0	0
0.006839067	0	0	0	0
0	0	0	0	0
0.042866317	0.090859741	0.104660161	0.031028576	0
0	0.03198445	0.003310253	0.921952269	1.112600147
0	0.001569433	0	0	0
0	0	0	0	0
0	0	0	0	0
0	0	0	0	0
0	0	0	0	0
0	0	0	0	0
0	0	0	0	0
0	0	0.012728292	0	0.026251167
0.087178668	0.00127271	0.014818502	0	0
0	0	0	0	0
1.732998312	0.042122032	0.04090139	0.021969735	0.006145318
0.075726895	0	0	0	0.001297179
0	0	0	0	0
0	0	0	0	0
0.101215265	0.001518803	0	0.002087032	0
0	0	0	0	0
0	0	0	0	0
0	0	0.004489562	0.597809766	0.002597175
0	0.458586057	0.048477693	0.011263228	0.068860514
0	0	0	0	0
0	0	0	0	0
0	0.033379489	0.11933081	0.00059633	0.002089956
0	0	0	0	0
0	0	0	0	0

0	0	0	0	0
0	0	0	0	0
0	0	0	0	0
0	0	0	0	0
0	0	0	0	0
0	0	0	0	0
0	0	0	2.106562357	0
0	0	0	0	0
0.069190816	0.017325218	0.145137336	0	0.000815873
0	0.180134477	0.001691414	0	0
0	0.480146839	0	0	0
0	0	0	0	0
0	0	0	0	0
0	0	0.111370901	0	0.013423102
0	0	0	0	0
0.182126828	0	0	0	0
0	0	0	0	0
0	0	0	0	0
0	0	0	0	0
0	0.145326476	0.4176777	0.585136419	0.764847295
0	0	0.014870425	0.138699115	0.01198194
0	0	0	0	0
0	0	0	0	0
0	0.003807697	0.002216701	0	0
0.006719592	0	0	0	0
0	0	0	0	0
0	0	0	0	0
0	0	0	0	0
0	0	0	0	0
0	2.052003337	0.033920647	0.013622671	0.342617678
0.077416718	0.269382945	0.389244222	0.299738374	0.211016399
0	0	0	0	0
0	0	0	0.001555651	0
0	0	0	0	0
0	0	0	0	0
0	0	0	0	0
0	0	0	0	0
0	0	0	0	0
0	0.00261854	0.000846899	0	0.038640295
0	0	0	0	0
0	0	0	0	0
0	0.009720993	0.001342862	0	0.00531022
0	0.000837646	0	0	0
0	0	0	0	0
0	0	0	0	0
0	0	0	0	0
0	0	0	0	0
0	0.005284918	0.002461348	0	0

0	0	0	0	0
0	0	0	0	0
0	0	0	0	0
0	0	0.002544674	0	0.000647712
2.316430707	0.012752692	0.086261535	0.516241211	0.000944858
0	0	0.08581964	0	0
0	0	0	0	0
0	0	0	0	0
0	0	0	0	0
0	0	0	0	0
0	0.013107395	0.064995125	0	0
0.012502913	0.011972127	0.02081103	0.010426853	0.001124398
0.01126628	0.001309301	0	0.010794896	0
0	0	0	0	0
0	0	0	0.000733476	0
0	0.036846721	0	0	0.016758929
0	0	0.032012305	0	0
0	0	0	0	0
0	0	0.033895064	0.001821986	0.001986602
0.033513072	0.03084185	0.231376744	0.192378445	0
0	0	0	0	0
0	0.160641255	0	0	0.023717458
0	0	0	0	0
0	0	0	0	0
0	0	0	0	0
0	0	0	0	0
0.008364847	0.001202351	0.000777738	0.001525095	0
0	0	0	0	0
0	0	0	0	0
0	0	0	0	0
0.002744787	0	0	0	0
0	0	0	0	0
0	0	0	0	0
0	0	0	0	0
0	0	0	0	0
0	0	0	0	0
0	0	0	0	0
0.186179029	0.007660428	0.013626614	0.016396927	0.001537162
0	0.207697216	0.001788003	0.006817531	0.216462512
0.004449389	0	0	0	0
0.031502828	0.007010278	0.013727424	0.000303137	0
0	0	0	0	0
0	0	0	0	0
0.094106328	0	0.001285871	0	0
0	0	0	0	0
0	0.061073529	0.002956054	0.003622895	0
0	0	0	0	0
0	0	0	0	0

0	0	0	0	0
0	0.080099174	0.00989138	0.059010099	0.020381512
0	0	0	0	0
0.005721274	0	0	0	0
0.001010741	0.001380183	0	0	0.000681726
0.000978344	0	0.001382644	0	0
0	0.018612608	0	0	0.008381138
0	0.00375008	0	0	0
0	0	0.030653376	0	0
0	0.006878314	0.084535248	0	0
0	0.005055165	0	0	0
0	0	0	0	0
0.346655318	0.096928881	0.019514548	0.005574997	0.147842547
0.00245356	0	0.005393879	0	0
0	0	0	0	0
0	0	0	0	0
0	0	0	0	0
0	0	0	0	0
0	0	0	0	0
0	0	0	0	0
0	0	0	0	0
0	0	0	0	0
0.099939171	0	0	0	0.002401272
0	0.003642354	0.014631067	0.012993924	0.004452774
0	0	0	0	0
0	0.018425906	0	0	0
0	0.001916868	0.004292038	0	0
0	0	0	0	0
0	0	0	0	0
0	0	0	0	0
0	0.006022803	0.009617155	0.01522441	0.005928546
0	0	0.031607268	0	0
0	0	0	0	0
0	0	0	0	0
0	0	0	0.562156315	0.008177043
0	0	0	0	0
0	0	0	0	0
0	0	0	0	0
0.004318121	0	0	0	0
0	0	0	0	0
0	0	0	0	0
0	0	0	0	0
0	0.580023158	0.021785042	0	0
0	0	0	0	0
0	0	0	0	0
0.046012934	0.431685644	0.017151824	0.118205139	0.451998841
0	0	0	0	0
0	0	0	0	0

0	0	0	0	0
0	0	0	0	0
0	0	0	0	0.053444379
0	0	0.03692942	0	0.000644809
0	0	0	0	0
0	0	0	0	0.000655328
0	0	0	0	0
0	0	0	0	0
0	0	0	0.006663976	0
0	0	0	0	0
0.024327451	0.000944911	0	0.000561821	0
0	0	0	0.00050328	0
0	0	0	0	0
0	0	0	0	0
0	0	0	0	0
0	0	0	0	0
0	0	0	0	0
0	0	0	0	0
0	0	0	0	0
0.000774297	0.000469918	0	0	0
0	0	0	0	0
0	0	0	0	0
0	0	0	0	0
0	0	0	0	0
0	0	0	0	0
0	0	0	0	0
0	0	0	0	0
0	0	0	0.017645302	0.020228496
0	0	0	0	0
0	0	0	0	0
0.006652013	0.004709927	0.001253461	0	0.020489086
0	0	0	0	0
0.023943361	0.015312373	0.004548113	0.015756118	0.012850027
0.00157855	0.000958015	0	0	0
0	0	0	0	0
0	0	0	0	0
0	0	0	0	0
0	0	0	0	0
0	0	0	0	0
0	0	0	0	0
0	0	0	0	0
0.749158883	0.121217365	0.001197341	0.030571734	0
0	0.067182824	0.01531634	0.053483809	0.044380994
0	0	0	0	0
0	0	0	0	0
0	0.149586074	0.016599883	0.008826743	0.025653449
0.001222371	0	0	0.000423444	0
0	0	0	0	0

0	0	0	0	0
0	0.00180469	0.011843401	0.00795989	0.005652331
0.006315501	0.000574928	0	0.004922466	0
0	0	0	0	0
0	0.016066827	0.005701191	1.023929612	0.001190405
0	0	0.002081064	0.000850174	0
0	0	0	0	0
0	0	0	0	0
0	0	0	0	0
0	0	0	0	0
0	0	0	0	0
0	0	0	0	0
0	0	0	0	0
0.010882102	0	0	0	0
0	0	0	0	0
0	0	0	0	0
0	0	0.133235331	0	0.02905824
0	0	0	0	0
0	0	0	0	0
0	0	0	0	0
0	0	0	0	0
0	0	0	0	0
0	0	0	0	0
0	0	0	0	0
0	0.019900497	0	0.006160333	0.011233866
0.000785395	0	0	0	0
0	0	0	0	0
0	0.075674577	0.005742397	0	0.047446498
0	0	0	0	0
0	0	0	0	0
0	0	0	0	0
0.002525398	0	0.000648913	0	0
0	0	0	0	0
0	0	0	0	0
0	0	0	0	0
0	0	0	0	0
0	0	0	0	0
0	0	0	0.043030729	0
0	0.654338485	0.006046537	0	0.371780026
0	0	0	0	0
0	0	0	0	0
0.001136043	0.056191002	0.002207581	0	0.004789003
0.045454574	0	0.001670948	0.001213564	0
0	0	0	0	0
0	0	0	0	0
0	0.000733802	0	0.000523561	0
0.008440859	0	0	0.002552709	0
0	0	0	0	0

0	0	0	0	0
0.078613135	0.020161321	0.01200597	0.006442097	0
0	0.054015761	0	0.010068939	0.013588123
0	0	0	0	0
0	0	0	0	0
0	0	0	0	0
0	0	0	0	0
0	0	0	0	0
0	0	0	0	0
0	0	0	0	0
0	0	0	0	0
0	0	0	0	0
0	0.822882669	0.038263547	0.012226808	0.019527144
0	0	0	0	0
0.038891303	0.031278741	0.002681129	0	0
0	0	0	0	0
0	0	0	0	0
0	0	0	0	0
0	0	0.011122585	0	0
0	0	0	0	0
0	0	0	0	0
0.007763075	0.019410882	0.03423004	0.035856258	0
0	0	0.062553892	0	0.086263355
0	0	0	0	0
0	0	0	0	0
0	0	0	0	0
0	0	0	0	0
0	0	0	0	0
0	0	0	0	0
0	0	0	0	0
0	0	0	0	0
0	0	0	0	0
0	0	0	0	0
0	0	0	0	0
14.45747847	7.572331619	0	45.37632605	17.86037193
0	0	0	0	0
0	0	0	0	0
0	0	0	0	0
0.006822085	0.009033374	0.024760645	0.001790341	0
0	0	0	0	0
0.051715625	0.0566973	0.323390827	0.072719055	0.032068238
0	0	0	0	0
0	0	0	0	0
0	0	0	0	0
0	0	0	0	0
1.50223709	0.073801661	0.827574931	0.004411039	0.002147132
0	0	0	0	0
0	0	0	0	0

0	0	0	0	0
0	0	0	0	0
0	0	0	0	0
0	0	0	0	0
0.003138659	0	0	0	0
0	0	0	0	0
0	0	0	0	0
0	0	0	0	0
0.196243434	0	0	0	0
0	0.003401064	0.008139895	0	0.003149847
0	0.001992727	0.076102046	0.009668182	0.010851748
0	0	0	0	0
0.009433419	0.004617019	0	0.001581216	0.000615742
0	0	0	0	0
0	0	0	0	0
0	0	0	0	0
0	0	0	0	0
0	0	0	0	0
0	0	0	0	0
0.865642613	0.159940692	0.013780132	0.001876525	0.147974553
0	0	0	0	0
0	0	0	0	0
0	0	0	0	0
0	0	0	0	0
0	0	0	0	0
0	0	0	0	0
0	0	0	0	0
0	0	0	0	0
0	0	0	0	0
0.189072911	0	0.001636798	0	0
0	0	0	0	0
0	0	0	0.10862241	0
0	0	0	0	0
0	0	0	0	0
0	0	0	0	0
0.019283635	0	0	0	0
0	0	0	0	0
0	0	0	0	0
0	0	0	0	0
0	0	0	0.352724878	0.014322575
0	0	0	0	0
0	0	0	0	0
0	0.00778838	0	0.764842215	0.012148374
0	0	0	0	0
0.075809424	0.006947583	0.003235702	0.001468751	0
0	0.005168374	0	0	0
0	0	0	0	0
0	0	0	0	0

0	0	0	0	0
0	0	0	0	0
0	0	0	0	0
0	0	0	0	0
0	0.006781593	0	0	0
0.005697451	0.24250426	0.014090866	0.012170924	0.002689979
0	0	0	0	0
0	0	0	0	0
0	0	0	0	0
0	0	0	0	0
0	0	0	0	0
0	0	0	0	0
0	0	0	0	0
0	0	0	0	0
0	0	0	0	0
0	0	0	0	0
0	0.008108862	0	0.298190943	0.014760852
0.048181443	0	0	0	0
0	0	0	0	0
0	0	0	0	0
0.000880817	0	0	0	0
0	0	0	0	0
0	0	0	0	0
0	0	0	0	0
0	0	0	0	0
0	0	0	0	0
0	0	0	0	0
0	0	0	0.588795316	0
0	0	0	0	0
0	0	0	0	0
0	0	0	0	0
0	0	0	0	0
0	0	0	0	0
0	0	0	0	0
0	0	0	0	0
0	0	0	0	0
0	0	0	0	0
0.025749921	0	0	0	0
0	0.098594962	0.010987145	0.831312238	0.325288831
0	0	0	0	0
0	0	0	0	0
0	0	0	0	0
0	0	0	0	0
0	0	0	0	0
0	0	0	0	0
0	0	0	0	0
0	0	0	0	0
0	0	0	0	0

0	0	0	0	0
0	0.000774248	0.00285468	0.005646948	0.003585295
0.003644433	0	0	0	0
0	0	0	0	0
0	0	0	0	0
0	0	0	0	0
0	0	0	0	0
0	0	0	0	0
0	0	0	0	0
0	0.053963865	0	0	0
0.00334099	0.005238053	0	0	0.001502291
0	0	0	0	0
0	0.020286092	0.093319288	0.20044474	0
0	0	0	0.002591371	0
0	0	0	0	0
0	0	0	0	0
0	0	0	0	0
0	0	0	0	0
0	0	0	0	0
0	0	0	0	0
0.083642296	0	0.016772684	0	0
0	0	0.004941142	0	0
0	0	0	0	0
0	0.983376839	0.593162147	0.446805129	1.124781535
0.004343745	0	0	0	0
0	0	0	0	0
0	0	0	0	0
0	0	0	0	0
0.126642371	0	0	0	0
0	0	0	0	0
0	0.002693138	0	0	0
0	0	0	0	0
0	0.025796503	0	0	0.011628098
0	0	0	0	0

PHL018-0011_V3	PHL018-0063_V2	PHL018-0078_V2	PHL018-0098_V2	PHL018-0239_V1
CTRL	EYCF	CTRL	CTRL	REF
0.075641342	0.141060743	0.008398554	0.062140208	0.109443957
1.312394481	0.070392687	1.13040426	2.766522256	1.069292312
11.97875047	8.548873543	0	0	0.001250191
0	0	0	0	0
0	0	0	0	0
0	0	0	0	0
0	0	0	0	0
0	0	0	0	0
0	0	0	0	0
0	0	0	0	0
0	0	0	0	0
0	0	0	0	0
0	0	0	0	0
26.02259811	1.750423831	0.599108149	3.144708307	0.31584346
0	0	0	0	0
0	0	0	0	0
0	0	0	0	0
0	0	0	0	0
0	0	0	0	0
0	0	0	0	0
0	0	0	0	0
0	0	0	0	0
0	0	0	0	0
0	0	0	0	0
0.017491045	0	0.001469662	0	0.084549878
0	0	0	0	0
0	0	0	0	0
0	0	0	0	0
0	0	0	0	0
0	0.001959418	0	0	0
0	0	0	0	0
0	0	0	0	0
0	0	0	0	0
0	0	0	0	0
0	0	0	0	0
0	0.549841896	1.633293625	2.761103036	2.216998681
0	0	0	0	0
0	0	0	0	0
0	0	0	0	0
0	0	0	0	0
0	0	0	0	0
0.001806731	0.077400762	0.032316165	0	0
0.279327022	0.310863528	13.71642452	0.008804754	0.019620111
0	0.026065957	0	0.018266053	0.081329387
0	0.017513555	0.011082153	0	0.006947348
0	0	0	0	0

0.00259519	0.013771789	0.001700847	0	0.013325793
0.001666368	0	0	0.009050996	0.009910971
0.499358558	0.002176066	0.003654987	0.003842974	0.008194745
2.176197959	0.090458361	0.691485484	1.547493044	1.015124155
0.008871343	0	0.00390502	0.017824104	0.003583202
0	0	0	0	0
0.019391695	0.294686256	0.013935326	0.065548697	0
0	0	0	0	0
0	0	0	0	0
0	0	0	0	0
0	0	0.002816851	0.000722719	0
0.701367157	0.039815109	0.369581029	0	0.099146426
0	0	0	0	0
0.116218308	0.000538724	0.056287436	0.130128533	0.188293815
0	0	0	0	0
0	0	0	0	0
0.002004063	4.051777791	0.055273585	0.003346467	0
0	0	0	0	0
0	0	0	0	0
0	0	0	0	0
0.071637394	0.019924278	0.060322152	1.57050259	0.04783285
0.05819577	0	0.004752724	0.014531246	0.007925362
0	0	0.106392563	0	3.49515579
0	0	0	0	0
0	0	0	0	0
0	0	0	0	0
0	0	0.006384284	0	0
0	0	0	0	0
0.001159645	0.003076921	0.000760013	0	0
0	0	0	0	0
0.000616283	0	0	0.195686197	0
0	0	0	0	0
0	0	0	0	0
0.001005746	0	0	0	0
0	0	0	0	0
0	0	0.005294711	0	0
0	0	0	0	0
0	0.088468189	3.167826348	0	0.001372905
0	0	0	0	0
0	0	0	0	0
0	0	0	0	0
0	0	0	0	0
0	0	0	0	0
0	0	0	0	0
0	0	0	0	0
0	0	0	0.016173981	0
0	0	0.021300449	0	0
0	0.002835012	0.003601342	0	0

0	0	0	0	0
0	0	0	0	0
0	0	0	0	0
0	0	0	0	0
0	0	0	0.013147456	0
0.012478785	0	0	0	0
0	0	0	0	0
0	0	0	0	0
0.000700944	0	0	0.021068382	0.021157526
0	0	0	0	0
0	0	0	0	0
0	0	0.020222489	0	0
0	0	0	0	0
0	0	0	0	0
0	0	0	0	0
0	0	0	0.001939046	0
0	0	0	0	0
0	0	0	0	0
0	0.14558912	0.225260024	0.119632262	0
0	0	0	0	0
0	0	0	0	0
0	0	0	0	0
0	0	0	0	0
0	0	0	0	0
0	0	0	0	0
0	0	0	0	0
0	0	0	0	0
0	0	0	0	0
0	0	0	0	0
0	0	0	0	0
0.356424013	0.013385851	0.002834028	0	0.101943522
0.000510355	0.005767629	0.006875392	0.003321439	0.007082625
0	0	0	0	0
0	0	0	0	0
0	0	0	0	0
0	0	0	0	0
0	0	0	0	0
0	0	0	0	0
0	0	0.027324908	0	0
0	0	0	0	0
0	0	0	0	0
0	0	0	0	0
0	0	0	0	0.001227959
0	0	0	0	0
0	0	0	0	0
0	0	0	0	0
0	0	0	0	0
0	0	0	0.001594108	0

0.038764307	0	0	0	0
0	0	0	0	0.114519186
0	0	0	0	0
0	0	0	0	0
0.049602999	0	0.01342413	0	0.010171629
0	0	0	0	0
0	0	0	0	0
0	0	0	0	0
0	0	0	0	0
0	0	0	0	0
0	0	0	0	0
0	0	0	0	0
0	0	0	0	0
0	0	0	0	0
0	0	0	0	0
0	0	0	0	5.332712196
0	0	0	0	0
0	0	0	0	0
0	0	0	0	0
0	0	0	0	0
0	0	0	0	0
0	0	0	0	0
0	0	0	0	0
0	0	0	0	0
0	0	0	0	0
0	0	0	0	0
0	0	0	0	0
0	0	0	0	0
0	0	0	0	0
0	0	0	0	0
0	0	0	0.003666081	0
0.001373958	0.004339956	0.015222244	0.000806784	0
0	0	0	0	0
0	0	0	0	0
0	0	0	0	0
0	0	0	0	0
0	0	0	0	0
0	0	0	0	0
0	0	0	0	0
0	0	0	0	0
0	0	0	0	0
0	0	0	0	0
0	0	0	0	0
0	0	0	0	0
0	0	0	0	0
0	0	0	0	0
0	0	0	0	0
0	0	0.004468553	0	0

0	0	0	0	0
0	0	0	0	0
0	0	0	0	0
0	0	0	0	0
0	0	0	0	0
0	0	0	0	0
0	0	0	0	0
0	0	0	0	0
0	0	0	0	0
0	0	0	0	0
0	0	0	0	0
0	0	0	0	0.000991546
0	0.003737621	0	0	0.000586472
0	0	0	0	0
0	0	0	0	0
0	0	0	0	0
0	0	0	0	0
0	0	0	0	0
0	0	0	0	0
0	0	0	0	0
0.094115995	0.004141306	0.014960227	0.04871742	0.04678666
0	0	0	0	0
0	0	0	0	0
0.001464837	0	0	0	0
0	0	0	0	0
0	0	0	0	0
0.001227111	0	0.000670191	0.003152431	0.00319306
0	0	0	0	0
0	0	0	0	0
0	0	0	0	0
0	0	0	0	0
0	0	0	0	0
0	0	0	0	0
0	0.035432726	0	0.017863837	0
0.004569917	0.055406775	0.011730623	0.010096448	0.025685349
0	0	0	0	0
0	0	0	0	0
0	0	0	0	0
0	0	0	0	0
0	0	0	0	0
0	0	0	0	0
0	0	0	0	0
0	0	0	0	0
0	0	0	0	0
0	0	0	0	0
0	0	0	0.041134347	0.010642562
0.006679806	0	0.001080949	0.003304953	0
0.000651329	0.000840093	0	0.000836628	0.001016893

0	0	0	0	0
0	0	0	0	0
0	0	0	0	0
0	0	0	0	0
0	0	0	0	0
0	0	0	0	0
0	0	0.001519114	0	0
0	0.006900851	0	0	0
0	0	0.000700981	0	0.010854209
0	0	0	0	0
0	0	0	0	0
0	0	0	0	0
0	0	0	0	0
0	0	0	0	0
0.000770198	0	0	0	0
0	0	0	0	0
0	0	0	0	0
0	0	0.078585344	0	0
0	0.001220527	0.059511312	0	0
0	0	0	0	0
0	0	0	0	0.025425185
0	0	0	0	0
0	0	0	0	0
0	0	0	0	0
0	0.003934566	0	0.002493488	0.027276753
0	0	0	0	0
0	0	0	0	0
0	0	0	0	0
0	0	0	0	0
0	0	0	0	0
0.696012006	0.046863744	0.100648502	0.07585928	0.175921158
0	0	0	0	0
0	0.128559196	0	0	0
0	0	0	0	0
0	0	0	0	0
0	0	0	0	0
0	0	0	0	0
0	0	0	0	0
0	0	0	0	0
0	0.004892609	0	0	0
0	0	0	0	0
0.01629489	0	0.010799412	0.091624632	0.004192446
0	0	0	0	0
0	0	0	0	0
0	0	0	0	0
0	0	0	0	0
0	0	0	0	0
0	0	0	0	0

0	0	0	0	0
0	0	0	0	0
0	0.018808178	0	0.019608077	0.055035931
0	0	0	0	0
0	0	0	0.665280957	0
0	0	0	0	0
0	0	0	0	0
0	0	0	0	0
0	0	0	0	0
0	0	0	0	0
0	0	0	0	0
0	0	0	0	0
0	0	0	0	0
0.020580376	0	0	0.006266159	0.017982937
0.00650097	0	0.001374398	0.002370451	0.007639553
0	0	0	0.004152896	0
0.001194721	0	0	0.002946455	0.004725347
0.000556059	0	0	0.014666027	0
0	0	0	0	0
0	0	0	0	0
0	0	0	0	0
0.001078492	0.000794888	0	0.001329904	0
0	0	0	0	0
0	0	0	0	0
0.003112739	0	0	0.024167462	0
0	0	0	0	0
0	0.000837944	0	0	0
0	0	0	0	0
0	0	0	0	0
0.001560049	0	0	0.022122758	0.00162376
0	0	0.008305346	0.001019126	0.001032261
0	0	0	0	0
0	0	0	0	0
0	0	0	0	0
0	0	0	0	0
0.015078948	0.001587675	0.012706083	0	0
0	0	0	0	0
0.011924124	0.020373361	0.000888055	0.003898735	0.128624024
0	0	0	0	0
0	0	0	0	0
0	0	0	0	0
0	0	0	0	0
0	0	0	0	0
0	0	0	0	0
0	0	0	0	0
0	0	0	0.003853442	0.003469428
0	0	0	0.004226086	0.001223015
0.001641262	0	0	0	0.005124867

0.616740137	0	0	0	0
0	0	0	0.015860053	0
0	0.0009991	0	0	0.000705462
0	0	0	0	0
0.009319493	0.001507786	0.000993147	0.004437965	0.006151276
0.002594487	0	0	0.00571303	0.005400884
0	0	0	0	0
0.095903892	0	0	0.024555731	0.011275403
0	0	0	0	0
0.122495453	0.128284948	0	0.17852074	0.021876334
0	0	0	0	0
0.23229353	0.018217646	0.020111537	0	0
0.023723299	0.075862013	0	0.004128522	0.043808613
0	0	0.032372968	0	0.003326699
0.001770886	0	0	0.007481207	0
0	0	0	0	0
0	0	0	0	0
0	0	0	0	0
0	0	0	0.560796008	0
0	0	0	0	0
0	0	0	0	0
0	0	0	0	0
0	0	0.000862549	0	0
0	0	0	0	0
0	0	0	0	0
0	0.001149664	0.004381287	0.004121721	0.000927743
0	0	0.18682644	0.005205378	0
0	0	0.00647557	0.032121089	0
0	0	0	0	0
0	0	0	0	0
0	0	0	0	0
0	0	0	0	0
0	0	0	0	0
0.106662579	0	0	0	0
0	0	0	0	0
0	0	0	0	0
0	0	0.000827773	0	0.009728149
0	0	0	0	0
0	0	0	0	0
0.245615408	0	0	0	0
0	0	0	0	0
0	0	0	0	0
0	0	0	0	0
0	0	0	0	0
0	0	0	0	0
0.000815176	0	0	0	0
0.002216908	0.003267882	0	0.004271405	0.001442152
0	0	0	0	0

0	0	0	0	0
0.001858162	0.16228959	0.001014842	0.003818875	0
0	0	0	0	0
0	0	0	0	0
0	0	0	0	0
0.138595373	0	0	0	0.023398912
0	0	0	0	0
0	0.0020806	0	0	0
0	0	0	0	0
0	0	0.004354014	0	0
0.01417614	0	0.002488613	0.013266672	0.038205104
0.001898039	0	0	0	0
0	0	0	0	0
0.006218134	0	0	0.000983034	0.003236037
0.09510047	0	0.003171877	0.010195202	0.003274288
0	0	0	0	0
0	0	0	0	0
0	0	0	0	0
0	0	0	0	0
0.070556739	0	0	0	0
0	0	0.105269851	0	0
0	0.006576602	0.304806044	0.145319036	0.162952468
0	0	0	0	0
0	0	0	0	0
0	0	0	0	0
0	0	0	0	0
0	0	0	0	0
0	0	0	0	0
0	0	0	0	0
0.211385873	2.98085078	0.071222386	0.185158492	0.334953683
0.003082757	0.001674181	0.001417818	0.005502012	0
0	0	0	0	0
0	0	0	0	0
0	0	0	0	0.004544551
0.020734291	0	0	0	0
0	0	0	0	0
0	1.988671022	0.138279882	0	0
0	0.003772962	0.002662682	0	0
0	0	0	0	0
0.448816281	0	0	0	0.027392508
0.068618538	0	0.052402353	0.07172429	0.015962248
0.075248147	0.017448276	0.272071766	0	1.543719324
0	0.002962523	0.003170946	0	0
0	0	0	0	0
0	0	0	0.014358452	0
0	0	0	0	0
0.009492618	0	0.002930329	0.000848223	0
0	0	0	0	0

0	0.012948577	0.007509188	0	0.014840746
0	0	0	0	0
0.003816259	9.334652271	0.04016946	0.003565062	0.003611009
0	0	0	0	0
0	0	0.022738877	0	0
0.212694017	0	0.001175745	0	0
0	0	0	0	0
0	0	0	0	0
0.115920992	0	0.006284517	0	0
0	0	0	0	0
0	0	0	0	0
0	0	0	0	0
0	0	0	0	0
0	0	0	0.007574406	0.018940317
0	0	0	0	0
0.288555697	0	0.006881912	0	0.013490003
0.001197133	0.152643047	0.454839688	0.039365346	0.14204649
0	0	0	0	0.001261557
0.033592873	0	0	0	0
0.502374711	0	0	0.464285727	0
0	0	0	0.034883112	0.031406841
0	0	0	0.00285386	0.001445321
0	0	0	0	0
0	0	0	0	0
0	0	0.013239558	0	0
0	0	0.001722581	0	0
0.007852142	0	0.003596787	0.005465974	0.002109113
0.059149183	0	0	0	0
0	0	0	0	0
0	0	0	0	0
0.014061994	0.001277778	0.007364394	0	0
0.007179694	0.003598351	0	0.006197365	0.004184826
0.019687794	0.229348296	0.1012533	0.030627597	0.029030075
0	0	0.003424327	0	0
0.000839083	0.000824579	0.003360635	0	0.009897969
0	0	0	0	0
0	0	0	0	0.002058446
0.028419468	0.001282419	0.037378063	0.068141955	0.073447141
0	0	0.103694985	0.549595168	0.58552894
0	0	0	0.002274	0
0	0	0	0	0
0	0	0	0	0
0	0	0.000947438	0	0.002106522
0.619215191	0.00255833	0	0.067305675	0
0	0	0	0.002427843	0
0	0	0	0	0
0	0	0	0	0
0	0	0	0	0

0	0	0	0	0
0.459696269	0.246197419	0.011222383	0.222249387	0.537147318
0	0.00047839	0.005435567	0	0
0	0	0	0	0
0	0	0	0	0
0	0	0	0	0
0	0	0	0	0
0.002832855	0.010588719	0	0	0
0.002652696	0	0	0	0
0	0	0	0	0
0.01213724	0.001009985	0.002779819	0.014483804	0.007742751
0.002041151	0.002149145	0	0.229224138	0.061914253
0.328670249	0.022245304	0	0	0
0	0	0	0	0
0	0	0	0	0
0	0	0	0	0
0.001844919	0.00710103	0	0.024013836	0.047579854
0.226190652	0	0.003197373	0.012305241	0.01218686
0	0	0	0	0
0	0	0	0.024503832	0
0.003170962	0.013711062	0.069504218	0.017812934	0.011661624
0	0.025010191	0	0	0
0	0	0	0	0
0	0	0	0	0.004093297
0.013141924	0.000785357	0	0	0
0	0	0	0	0
0.174439629	0	0.036923156	0	0.056684303
0.214348801	0.0415657	0.037304498	0.070419736	0.062440644
0	0	0	0	0
0	0	0	0	0
0	0	0	0.016103044	0
0	0	0	0	0
0.004091477	0	0	0	0
0	0	0	0	0
0.145868546	0	0.002994183	0	0
0.146401923	0.100896767	0.044444166	0.203975696	0.279826535
0.007572978	0	0	0	0
0	0	0	0	0
0.105880158	0.006332885	0.014229643	0.047850047	0.006923822
0	0	0	0	0
0	0	0	0	0
0	0	0	0	0
0.003198825	0	0	0.003806111	0.013668314
0	0.001557445	0	0.014364849	0.013704058
0.148203664	0	0	0	0.017114871
0	0	0.480083046	0.679791247	0.070970388
0	0	0	0	0
0.007094134	0	0	0.001619977	0

0	0	0	0	0
0	0	0	0	0
0	0	0	0	0
0.067845488	0	0.328131984	0	0.956584311
0	0	0	0	0
0	0	0	0	0
0	0	0	0	0
0.006490513	0.001034322	0.016287002	0.026858727	0.10060332
0.046585273	3.858944442	0.151962441	0	0.289824534
0	0.002195839	0.19832049	0.03241584	0.118018642
0	0	0	0	0
0.014278175	0.013154411	0.00082085	0.009073583	0.001564345
0	0	0	0	0
2.556079688	0	0	0	0.58280388
0.15286096	0.095981526	0.038101942	0.0202323	0.071483667
0	0	0	0	0
0	0	0	0	0
0	5.752689212	0	0	0
0.01078756	0	0	0	0
0	0	0	0	0
0	0	0	0	0
0	0	0	0	0
0	0	0	0	0
1.384633868	0.017500375	0.586904735	0.130590912	0.825507943
0	0	0	0	0
0.027010452	0	0	0	0
0	0	0	0.001075316	0.256827405
0	0.243277448	1.667726197	4.32525411	0
0	0	0	0	0
0	0	0	0	0
0	0	0.029725745	0.02083274	0.014637797
0.438639563	0	0.001127805	0	0.024538158
0.03850418	0	0.005162404	0	0
0	0	0	0	0
0	0	0	0	0
0.184748352	0.050791242	0	0	0
0	0	0.08673049	0.776667485	1.462607977
0	0	0	0	0
0.123993561	0	0	0.012862095	0
0	0	0	0	0
0.001982512	0.002258192	0.137608504	0.011297306	0.919747237
0	0	0	0	0.168300219
1.220267011	3.189582597	2.921342601	12.45097494	1.239946343
0	0.002430046	0.000514485	0.012422788	0
0	0	0	0	0
0	0	0	0	0
0	0	0	0	0
0.028381829	0.000958096	0	0	0.054346247

0	0	0	0	0
0	0.200174197	0.021344259	0	0
0.306340469	0	0.002722316	0.06295877	0.091007645
0	0	0	0	0
0	0	0	0	0
0.000818045	0.055348831	0.002859381	0.002858103	0
0	0	0	0.021740243	0
0	0	0	0	0
0	0	0	0	0
0	0	0	0	0
0.188007999	0.002855536	0.004009247	0.047565617	0.014007891
8.106267121	2.320297372	1.555051254	1.778865461	1.182336734
0.020695094	0	0	0	0
0.000660214	0.200317734	0.200481694	0.006558184	0.251735733
1.601404221	0	0.026710077	0.125460099	0.094405265
0	0	0	0	0
0	0	0	0	0
0.002118508	0	0	0	0
0	0	0	0	0
0.007894629	0	0	0	0.01276263
0.722433677	0.002015461	0.011574503	1.24904864	0.185894035
0.669917673	3.408756916	0	0.001480438	0.585187205
4.657520381	4.404098487	0.209831521	3.249307431	0.566901161
0	0	0	0	0
0	0	0	0	0
0	0.263852615	1.239327169	0.005471753	0
0.296402478	0	0.002232846	0	0
0	0	0.001362967	0	0
0	0	0.018880509	0	0
0.0357222	0	0	0	0
0.003393154	0	0	0	0
1.106871255	0.01213003	0	0.378032996	0.144429156
0.000837697	0	0	0	0.022669588
0	0.002118712	0.000697776	0.00919011	0.02775944
0.004976013	0	0.00286865	0.011078869	0
0.010255106	1.751305089	0.004356228	0.015221689	0.202606639
0.006237295	0.002839392	0.002003837	0.001131073	0.003818834
0	0	0	0	0
0	4.081890534	0.069910854	0.104362689	0.504251403
0	0	0	0	0
0	0	0	0	0
0	0	0	0	0
0	0	0	0	0
0	0	0	0	0
0.044613435	0.003671259	0.003075295	0.018026247	0.045308317
0	0	0	0	0
0	0	0	0	0
0.00679231	0.205060706	0	0.032482645	0.439408982

0	0	0	0	0
0	0	0	0	0
0	0	0	0	0
0.007752435	0.003401086	0.002016201	0.005500583	0.003073918
0	0	0	0	0
0	0.011654811	0	0	0
0	0	0.973443981	0	0
0	0.000500738	0.000556581	0.006108746	0.006010692
0	0	0	0	0
0	0	0	0	0
0.001027876	0.003636392	0	0.001689984	0.007916915
0	0	0	0	0
0.001439978	0	0.002162735	0	0
0	0	0	0	0
0	0	0	0	0
0	0	0	0	0
0	0	0	0	0.174892485
0	0.032794815	0.002990942	0.005803353	0.002315634
0	0	0	0	0
0.00354975	0	0.023433096	0	0.084496577
0	0	0	0	0
0	0	0	0	0
0	0	0.035776555	0.001216518	0.019099055
0	0	0	0	0
0	0	0	0	0
0	0	0	0	0
0	0	0	0	0
0	0	0	0	0
0	0	0	0	0
0	0	0	0	0.006885576
0	0	0	0	0
0	0	0	0	0
0	0	0	0	0
0	0	0	0	0
0	0.000602392	0	0.008062759	0
0	0	0	0	0
0.754627725	0.014973452	0.15356861	0	0
0	0.00820872	0.001900867	0.040950961	0.001086779
0	0	0	0	0
0.004119521	0	0.001687419	0	0
0.040166063	0.002767838	0.00214018	0	0.358753233
0	0	0	0	0
0	0	0	0	0
0.002196961	0	0	0	0
0	0.445947583	0.071443522	0.287930739	0.272514264
0	0	0	0	0
0	0	0	0	0
0	0	0	0	0

0	0	0	0	0
0	0	0	0	0
0	0.005194732	0	0	0
0	0	0.002268966	0	0.149001561
0	0	0	0	0
0	0	0	0	0
0	0	0	0	0
0.031653193	0	0	0	0
0.230166556	0.004245448	0.002883774	0.068316444	0.063701142
0	0	0	0	0
0	0	0	0	0.000714483
0	0	0	0	0
0	0	0	0	0
0	0	0	0	0
1.859800588	0	1.365682682	6.749450118	0
0.002219759	0	0	0	0.854082076
0	0	0	0	0
0	0	0	0	0
0	0	0	0	0
0	0	0	0	0
0	0	0.001202759	0	0.001719129
0	0	0	0	0
0	0	0	0	0
0	0	0	0	0
0.00705603	0.010401096	0.376735504	0	0.024676476
0.076880965	0.007058496	5.269089431	0.780183602	6.946570543
0	0	0	0.147355643	0
0	0	0	0	0
0	0.670738954	0.061454501	0	0
0	0	0	0	0
0	0	0	0	0
0.003271768	0	0	0	0
0	0	0	0	0
0	0	0.000823244	0.04352533	0.001568907
0.00347709	0.118252153	0.100105776	0.007656512	0.004394609
0.003511258	0.00168215	0	0.006494671	0.002192792
1.232359635	0	0	0	0
3.002358058	0.013880513	3.963764712	3.772325458	0.698243641
0	6.03123268	0.867300624	0	0
0	0	0	0	0
0	0.117538998	0	0	0
0.002189987	0	0	0	0
0.112757629	0.02015773	0.194445059	1.162389408	5.971753768
0	0.870005431	0	0	0
0	0	0	0	0
0	0.00257432	0.000726707	0	0.002423628
0	0	0	0	0
0.31690865	0.004296495	0.077278574	3.106234897	7.248719211

0.097214892	0	0	0.009949142	0
0	0	0	0	0
0	0	0	0	0
0	0	0	0	0
0.00196725	0	0.001719073	0	0
0	0.954905426	0.016968281	0	0
0	0	0	0	0
0	0	0	0	0
0	0	0	0	0
0.020140385	0	0	0	0
0	0	0	0.000618732	0
0	0.544559967	0.048966357	0.047184385	2.607619932
0.337052224	0.006495778	0.050216102	0	0.418005269
0	0	0	0	0
0.002730149	0.123080677	0.047217572	0.034834795	0
0	0	0	0	0
0.00142441	0	0	0	0
0	0	0	0	0
0	0	0	0	0
0	0	0.003139877	0	0.000920593
0	0	0	0	0.063408474
0	0	0	0	0
0	0	0.17134745	0.050610401	0.811524615
0	0	0	0	0
0	0	0	0	0
0	0	0	0	0
0	0.02879786	0	0	0
0	0	0	0	0
0	0	0	0	0.000853201
0	0	0	0	0
0	0	0	0	0
0.006199813	0.006527836	0	0.047326649	0.200043161
0	0	0	0	0
0.051474503	0.007283334	0.011277419	0.014246772	0.063555115
0.00324796	0.002112232	0	0.005497201	0.000795436
0	0.851004311	0.011568281	0	0.012561311
0	0	0	0	0.004413023
0	0.008348439	0	0	0
0	0.026045969	0	0	0
0	0	0	0	0
0	0	0.070944866	0	0
0	0	0	0	0
0	0	0	0	0
0	0	0	0	0
0.085447225	0.001387174	0.00143908	0.00870315	0.029384398
0	0	0	0	0
0	0.153478453	0.416691549	0	0
0.003026217	0	0	0	0.002779238

0	0	0	0	0
0	0	0	0	0
0	0	0	0	0
0	0	0	0	0
0.066329525	0	0	0	0
0	0	0	0	0
0	0.115286784	0.033001373	0	0
0.00882405	0	0.006626521	0.373280696	0.776656895
0	0	0	0	0
0	0.003866565	0	0	0
0	0	0	0	0
0	0	0	0	0
0	0	0	0	0
0	0.147870239	0	0	0
0	0	0	0	0
0	0	0	0	0
0	0	0	0	0
0	0	0	0	0
0	0.595573433	0.582530335	0	0
0	0	0	0	0
0	0	0	0	0
0	0	0	0	0
0	0	0	0	0
0	0	0	0	0
0.003290168	0.001060925	0.002021555	0	0
0.00455562	0	0	0.007742202	0.007659614
0	0	0	0	0
0.011833622	0.007836985	0.151177469	3.043742861	0.235270484
0	0	0	0	0
0	0.059997906	1.798421065	0.108909852	0.204345821
0	0.313266244	0	0	0
0	0	0	0	0
0	0	0	0	0
0	0	0	0	0
0.013771177	0.000405994	0.001203389	0	0.000573343
0	0	0	0	0
0	0	0	0	0
0.0012501	0	0	0.001712795	0.002602305
0	0	0	0	0
0	0	0	0	0
3.844673306	1.926249087	2.127063441	6.199902903	3.867573153
0.122620589	0	0.005997286	1.985977767	0.922130007
0	0	0.055139894	0	1.36068194
0.064403857	0	0	0	0
0	0	0	0	0
0	0	0	0	0
0	0	0	0	0
0.017362602	0	0	0	0.002237445

0	0	0	0.192545293	0
0.002211098	0	0.001114707	0	0.001947336
0	0	0	0	0
0	0	0	0	0
0.984398036	0.246750952	2.445383418	5.637232739	5.926889613
0	0	0	0	0
0	0	0	0	0
0	0	0	0	0.014077368
0.496933443	0	0.030980277	0.476789262	0.050025768
0	0	0	0	0
0	0	0	0	0
0	0	0	0	0
0.095689046	0	0.093510062	0	0.346438822
0.012190668	0.136421662	0.005770249	0.021713582	0.005921309
0	0.173895659	0	0	0
0.40547821	0	0.225277893	0.051398101	1.029410326
0	0	0.003456869	0.002348717	0.636653833
0	0	0	0	0
0	0	0	0	0
0	0	0	0	0
0	0	0	0	0
0	0	0	0	0
0	0	0	0	0
0	0	0	0	0
0	0	0	0	0
0	0	0	0	0
0	0	0.032396812	0.006126918	0.035803177
0.00086255	0.004958695	0	0.000531811	0.00071822
0	0	0	0	0
0	0	0	0	0
0	0	0	0	0
0	0	0	0	0
0	0	0	0	2.008615586
0	0	0.010073981	0	0
0	0.349484007	0.071255384	0	0.083480995
0	0	0	0.000615195	0
0.34858902	0	0.002228007	0	0
0	0	0	0	0
0	0	0	0	0
0	0	0	0	0
0	0.326228261	0.216511125	2.94349915	2.37522755
0	0.003455018	0	0	0
0	0.017822591	0	0	0
0	0.071272	0	0	0
0	0	0	0	0
0	0	0	0	0
0	0	0	0	0
0	0	0	0	0

0	0	0.036311877	0	0.408919545
0	0	0	0	0
0.394362715	0	0.040152074	0	0
0	0	0	0.0016065	0
0	0	0.1235641	0.002882074	0.006616897
0	0	0	0	0
0.001929305	0.005946408	0.003065301	0.081284288	0.814739006
0	0	0	0	0
0.005075718	0	0	0	0
0.229097528	0	0	0	0
0	0	0	0.002404159	0.009740577
0.006748855	0	0	0	0
0	0	0	0	0
0	0	0	0	0
0	0	0	0	0
0.001784355	0	0	0	0
0	0.013739453	0	0	0.006258964
0.001854824	0	0	0	0
0	0	0	0	0
0	0	0	0	0
0.052933405	0.0092304	0.279248394	0.473588902	0.313190645
0	0	0	0	0
0.002762714	0.001085984	0.006639014	0	0.00364235
0.00409439	0.003500547	0.00107336	0.007909862	0.008352735
0	0	0	0	0
0	0	0	0	0
0	0	0	0	0
0	0	0	0	0
0	0	0	0	0
0	0.293773795	0	1.085369373	1.653575676
0	0	0	0	0
0	0	0	0	0
0	0	0	0	0
0	0	0	1.184053743	0
0	0	0	0	0
0	0	0	0	0
0.003073885	0	0	0	0.029647948
0	0	0	0	0
0	0.00389701	0	0	0
0	0	0	0	0
0	0	0	0	0
0	0	0	0	0
0	0	0	0	0
0.000977743	0	0	0	0
0	0	0	0	0
0	0	0	0	0
0	0	0	0	0
0	0	0	0	0

0.000528685	0	0.00173246	0	0.002384529
0	0.39985506	0.015700329	0	0
0	0	0	0	0.001307896
0	0	0	0	0
0	0	0	0	0
0	0.219779587	0.002428929	0	0
0.003928805	0	0	0	0
0.098435004	0.003600834	0.137366361	1.17625564	3.403425725
0.361538265	0.394093464	17.70905271	0.011085752	0.022169344
0.007125178	0.035483193	0.055742288	0.072030634	0
0	0	0	0	0
0	0	0	0	0
0.011173796	0	0	0	0.096859798
0	8.906128112	0	0	0
0	0	0	0	0
0	0	0	0	0
0	0	0	0	0
0	0	0	0	0
0	0	0	0	0
0.023633476	0	0.006104904	0	0
0	0	0	0	0
0	0	0	0	0
0	0	0	0	0
0	0	0	0	0
0	0	0	0	0.019648308
0	0	0	0	0
0	0	0	0	0
0	0	0	0	0
0	0	0	0	0
0	0	0	0	0
0	0	0	0	0
0	0	0	0	0
0	0	0	0	0
0	0	0	0	0
0.196921348	0	0	0	0
0	0	0	0.020988325	0
0	0	0	0	0
0	0	0	0	0
0	0.061314812	0	0.001076659	0
0	0	0.000576513	0.000542358	0
0	0	0	0	0
0.728194638	0.158657255	0.143340784	0	0.775502392
0	0	0	0	0
0.00824353	0	0	0	0
0.001885849	0	0	0	0
0.002820651	0.030259853	0.010954731	0	0
0	0	0	0	0
0	0	0	0	0

0	0	0	0	0
0.010974713	0.000820599	0	0.0045764	0.000827747
0	0	0	0	0
0	0	0	0	0
0.02134844	0.895231381	0.040565451	0.079066565	0.515695713
0	0	0	0	0
0	0	0	0.04934758	0
0.013898865	0	0.000734605	0.004146503	0.007699899
0.005371936	0.062029193	0.001711443	0	0
0	0	0	0	0
0	0	0	0	0
0	0.322158882	0	0	0
0	0	0	0	0
0	0	0	0	0
0.079063541	0	0	0	0
0.082436416	0	0.001274235	0	0
0	0	0	0	0
0	0	0	0	0
0	0	0	0	0
0	0	0	0	0
0	0	0	0	0
0	0	0	0	0
0	0	0	0	0
0.000582016	0.003145754	0	0	0.008279053
0	0.12429391	0.086164371	0	0.105460086
0	0	0	0	0
0.031761721	0	0	0	0.005725272
0.003923605	0	0	0	0
0	0	0	0	0
0	0	0	0	0
0	0	0	0	0
0	0	0	0	0
0	0	0	0	0
0	0	0	0	0
0	0	0	0	0
0	0	0	0	0
0	0	0	0	0
0.176151697	0.002011831	0	0	0.016667773
0	0	0	0	0
0	0	0	0	0
0	0	0	0	0
0	3.121010492	0	0	0
0	0	0	0	0
0	0.167851082	0	0	0
0	0	0	0	0
0	0	0	0	0
0	0	0	0	0
0	0	0	0	0

0.009344542	0	0.004556747	0.017147147	0.032131067
0	0	0	0	0
0	0	0	0	0
0	0	0	0	0
0	0	0	0	0
0	0.001282386	0.024706914	0.018078025	0
0	0	0	0	0
0	0	0	0	0
0	0	0	0	0
0	0	0	0	0
0	0	0	0	0
0.001073051	0	0	0	0
0.00139902	0	0	0	0
0	0	0	0	0
0	0	0	0	0
0	0	0	0	0
0	0	0	0	0
0	0	0	0	0
0	0	0	0	0
0	0	0	0	0
0.005795826	0	0.010340347	0.034741936	0.048260162
0	0	0	0	0
0.160789552	0.000539489	0.002132099	0	0.000761863
0	0	0.310517787	0	0.218951277
0	0	0	0	0
0	0.14141018	0	0	0
0	0	0	0	0
0	0	0	0	0
0	0.005604312	0	0	0
0	0	0	0	0
0	0	0	0	0
0	0	0	0	0
0.005026908	0.44359102	0	0.050247438	0.016885738
0	0	0	0	0
0.301064165	0.005121816	0.058716072	0	3.042116102
0.055190857	0	0	0.092890099	0
0	0	0	0	0
0	0.090684613	0	0	0
0	0	0	0.017216887	0.009050508
0	0	0	0	0
0	0	0	0	0
0	0	0.782917119	0	0
0.002975862	0.40576367	0.020045096	0	0.022197976
0	0	0	0	0
0	0	0	0	0
0	0	0.001505878	0	0.006149666
0	0	0	0	0
0	0	0	0	0

0	0	0	0	0
0	0	0	0	0
0	0	0	0	0
0	0	0	0	0
0	0	0	0	0
0	0	0	0	0
0	0	0	0	0
0.038823666	0	0.177321065	0.497122921	0
0.00147616	0	0.001410868	0	0.022470501
0	0.072362485	0.004653113	0	0.054473158
0	0	0	0	0
0	0	0	0	0
0	0	0	0	0
0	0	0	0	0.022699587
0.005081896	0	0	0	0
0	0	0	0.017432775	0.001246409
0	0	0	0	0
0	0	0	0	0
0	0	0	0	0
0.315648192	0	1.096594844	0	0
0	0.016439066	0	0.001499424	0.001952678
0	0	0	0	0
0	0	0	0	0
0	0	0	0.001912302	0
0	0	0	0	0
0	0	0	0	0
0	0	0	0	0
0	0	0	0	0
0	0	0	0	0
0	0	0.043607893	2.14878486	0
0	0.004543251	0.083633706	0	0.373307389
0	0	0	0	0
0	0	0	0	0
0	0	0	0	0
0	0	0	0	0
0	0	0	0	0
0	0	0	0	0
0	0	0	0	0
0	0.062388293	0	0	0
0	0	0	0	0
0	0	0.002222159	0	0
0	0	0	0	0.529749692
0	0	0	0	0
0.003410948	0	0	0.013801221	0
0	0	0	0	0
0	0	0	0	0
0.001225876	0	0	0.078311589	0
0.000498134	0	0	0	0

0	0	0	0	0
0	0	0	0	0
0	0	0	0	0
0	0	0	0	0
0.248034858	0.002699972	0.010170308	0.067288139	0.016045903
0	0	0	0	0
0	0	0	0	0
0	0	0	0	0
0	0	0	0	0
0	0	0	0	0
0	0	0.001058423	0	0.005042753
0.018648435	0	0	0.002177617	0.003529093
0.019928298	0.005390045	0.001397934	0	0.004376783
0	0	0	0	0
0.023901008	0	0	0	0.005210743
0	0	0.025740695	0.8148671	0
0	0.010330949	0.01620091	0	0.050892967
0	0	0	0	0
0.010269601	0.001655733	0.001226921	0.190118679	0
0.129061339	0.000617681	0.142118948	1.109423769	0.073708162
0	0	0	0	0
0	0	0	0	0
0	0	0	0	0
0	0	0	0	0
0	0	0	0	0
0	0	0	0	0
0.055572947	0	0.002384096	0.002242852	0.015902311
0	0	0	0	0
0	0	0	0	0
0	0	0	0	0
0.024550865	0	0	0	0
0	0	0	0	0
0	0	0	0	0
0	0	0	0	0
0	0	0	0	0
0	0	0	0	0
0	0	0	0	0
0.03195694	0.000689849	0	0.016350691	0.051911018
0	0	0	0.114859149	0.921174408
0.008484016	0	0	0	0
0.001334867	0	0.001603894	0.001646043	0
0	0	0	0	0
0	0	0	0	0
0	0	0	0.002852471	0.021875631
0	0	0	0	0
0	0	0	0	0.001844996
0	0	0	0	0
0	0	0	0	0

0	0	0	0	0
0	0	0.000740446	0	0
0	0	0	0	0
0	0	0	0	0.002162808
0	0	0.008210137	0.000594133	0
0	0	0	0	0
0	0	0.025330209	0.013468872	0.002728493
0	0	0	0	0
0.019750592	0	0	0	0.018448751
0	0	0	0	0
0	0	0	0	0.039138838
0	0	0	0	0
0.007954048	0.034523138	0.013675975	0.019828398	0.028669458
0	0	0	0	0
0	0	0	0	0
0	0	0	0	0
0	0	0	0.053542202	0
0	0	0	0	0
0	0	0	0	0
0	0	0	0	0
0	0	0	0	0
0	0	0	0	0
0.004073086	0	0.007626963	0.0392638	0.005046935
0.003433129	0	0.008250073	0	0
0	0	0	0	0
0	0	0	0	0
0	0	0	0.028563322	0
0	0	0	0	0
0	0	0	0	0
0	0	0	0	0
0	0.00175331	0.037323244	0	0.006077494
0	0	0	0	0
0	0.000984173	0	0	0
0	0	0	0	0
0	0	0	0	0
0	0	0	0	0
0	0	0	0	0
0	0	0	0	0
0.026530861	0.005394262	0.020985415	0	0
0	0	0	0	0
0	0	0	0	0
0	0	0	0	0
0	0	0	0	0
0	0	0	0	0
0	0	0	0	0.000882031
0.072604078	0.361293764	0.444359676	0.035732228	0.508709289
0	0	0	0	0
0	0	0	0	0

0	0	0	0	0
0	0	0	0	0
0	0.039310001	0	0	0
0	0	0.192213609	0	0
0	0	0	0	0
0	0	0	0	0
0	0	0	0	0
0	0	0	0	0
0	0	0	0	0
0	0	0	0	0
0.002375022	0	0	0	0
0	0.000816708	0	0.000683206	0
0	0	0	0	0
0	0	0	0	0
0	0	0	0	0
0	0	0	0	0
0	0	0	0	0
0	0	0	0	0
0	0	0	0	0
0	0.006093761	0	0	0.001024474
0	0	0	0	0
0	0	0	0	0
0	0	0	0	0
0	0	0	0	0
0	0	0	0	0
0	0	0	0	0
0	0	0	0	0
0	0	0.018085216	0	0
0	0	0	0	0
0	0	0	0	0
0.003551503	0.064068523	0.004987715	0.00260679	0.006336929
0	0	0	0	0
0.197542791	0.034734667	0.013941885	0.05246364	0.009606042
0	0	0	0	0.009398617
0	0	0	0	0
0	0	0	0	0
0	0	0	0	0
0	0	0	0	0
0	0	0	0	0
0	0	0	0	0
0	0	0	0	0
0.458794554	0	0.000705838	0.006142194	0.078691748
0	0.039745411	0.007739184	0.013550159	0.127005593
0	0	0	0	0
0	0	0	0	0
0.002844045	0.293462898	0.013668916	0	0.021017364
0.001118782	0	0	0	0
0	0	0	0	0

0	0	0	0	0
0	0	0.013738253	0.61189344	0.005365129
0.004094376	0.004970334	0	0.035143828	0.010277941
0	0	0	0	0
0.418260496	0.006094593	0.007561971	0.191089106	0
0	0	0	0	0
0	0	0	0	0
0	0	0	0	0
0	0	0	0	0
0	0	0	0	0
0	0	0	0	0
0	0	0	0	0
0	0	0	0	0
0	0	0	0	0
0	0	0	0	0
0	0	0	0	0
0	0	0	0	0
0	0	0	0	0
0	0	0	0	0
0	0	0	0	0
0	0	0	0	0
0	0	0	0	0
0	0	0	0	0
0	0	0	0	0
0	0.009655456	1.314753305	0	0
0	0.001030184	0	0.000615561	0.001870483
0	0.000932249	0	0	0
0	0.000856555	0	0	0.511872037
0	0	0	0	0
0	0	0	0	0
0	0.004773162	0	0	0
0	0	0	0	0
0	0	0	0	0
0	0	0	0	0
0	0	0	0	0
0	0	0	0	0
0	0	0	0	0
0	0	0	0.001225478	0.04923715
0	0.930114715	0.549916806	0	1.238969149
0	0	0	0	0
0	0	0	0	0
0	0.057955047	0.001183071	0.000890385	0.006313023
0.001001988	0	0	0	0.002085816
0	0	0	0	0
0	0	0	0	0
0	0.001189467	0	0	0
0	0.015816673	0.011720372	0	0
0	0	0	0	0

0	0	0	0	0
0.02262978	0.065575406	0.007605747	0.052471111	0.021540792
0	0.03820082	0	0	0
0	0	0	0	0
0	0	0	0	0
0	0	0	0	0
0	0	0	0	0
0	0	0	0	0
0	0	0	0	0
0	0	0	0	0
0	0	0	0	0
0	0	0	0	0
0	0.000753468	0.15990565	0	0.115767908
0	0	0	0	0
0.007717176	0.012975377	0.00658557	0.013877726	0.006024252
0	0	0	0	0
0	0	0	0	0
0	0	0	0	0
0	0	0	0	0
0	0	0	0	0
0	0	0	0	0
0.007815716	0	0.00258702	0.054759466	0.168121254
0	0.001475482	0.005963739	0	0
0	0	0	0	0.008125001
0	0	0	0	0
0	0	0	0	0
0	0	0	0	0
0	0	0	0	0
0	0	0	0	0
0	0	0	0	0
0	0	0	0	0
0	0	0	0	0
0	0	0	0	0
0	0	0	0	0
0	0	15.30829022	2.501616346	0.034915806
0	0	0	0	0
0	0	0	0	0
0	0	0	0	0
0.00141908	0.008890258	1.284234112	0.192001433	0.115454744
0	0	0	0	0
0.057893862	0.03157257	0.02802852	0.040750538	1.028361976
0	0	0	0	0
0	0	0	0	0
0	0	0	0	0
0	0	0	0	0
0.063856527	0.001610574	0.096007031	0.178392854	0.05231224
0	0	0	0	0
0	0	0	0	0

0	0	0	0	0
0	0	0	0	0
0.020316744	0	0	0	0
0	0	0	0	0
0	0	0	0	0
0	0	0	0	0
0	0	0	0	0
0.002361897	0	0	0.007011544	0.005463009
0.10822826	0.001212282	0	0	0
0	0.149113646	0.018675254	0	0
0.312542497	0	0	0	0
0	0	0	0	0
0.001624675	0	0	0.008586038	0.005073074
0	0	0	0	0
0	0	0	0	0
0	0	0	0	0
0	0	0	0	0
0	0	0	0	0
0	0	0	0	0
0.107835908	3.030096729	0.130426447	0.147748815	0.3979997
0	0	0	0	0
0	0	0	0	0
0	0	0	0	0
0	0	0	0	0
0	0	0	0	0
0	0.008217121	0	0	0
0	0	0	0	0
0	0	0	0	0
0	0	0	0	0
0	0	0.001929799	0	0
0	0	0	0	0
0	0	0	0	0
0	0	0	0	0
0	0	0	0	0
0	0	0	0	0
0.003743823	0	0	0	0
0	0	0	0	0
0	0	0	0	0
0	0	0	0	0
0	0	0	0	0
0	0	0	0	0
0	0	0	0	0
0	0	0.021570579	0	0.01036646
0	0	0	0	0
0	0.00357517	0	0.001395687	0.002625396
0	0	0	0	0
0	0	0	0	0
0	0	0	0	0

0	0	0	0	0
0	0	0	0	0
0	0	0	0	0
0	0	0	0	0
0	0	0	0	0
0.004345519	0.000747321	0.000791107	0.00133963	0.002412258
0	0	0	0	0
0	0	0	0	0
0	0	0	0	0
0	0	0	0	0
0	0	0	0	0
0	0	0	0	0
0	0	0	0	0
0	0	0	0	0
0	0	0	0	0
0	0	0	0	0
0.102083328	0	0	0	0
0	0	0	0	0.00055703
0	0	0	0	0
0	0	0	0	0
0	0	0	0	0
0	0	0	0	0
0	0	0	0	0
0	0	0	0	0
0	0	0	0	0
0	0.086668204	0.00098994	0	0
0	0	0	0	0
2.051488252	0	0	0	0
0	0	0	0	0
0	0	0	0	0
0	0	0	0	0
0	0	0	0	0
0	0	0	0	0
0	0	0	0	0
0	0	0	0	0
0	0	0	0	0
0	0	0	0	0
0	0	0	0	0
0	0	1.003930138	0	0.00127692
0	0	0	0	0
0	0	0	0	0
0	0	0	0	0
0	0	0	0	0
0	0	0	0	0
0	0.01141233	0.001757235	0	0
0	0	0	0	0
0	0	0	0	0
0	0	0	0	0

0	0	0	0	0
0.005351675	0	0.001239992	0	0
0	0	0.00151812	0	0
0	0	0	0	0
0	0	0	0	0
0	0	0	0	0
0	0	0	0	0
0	0	0	0	0
0	0	0	0	0
0	0	0	0	0
0.000637054	0.041788323	0.045694732	0.001745689	0.01458755
0	0	0	0	0
0.542733998	0	0.087301837	0	0.631644482
0	0	0	0	0
0	0	0	0	0
0	0	0	0	0
0	0	0	0	0
0	0	0	0	0
0	0	0	0	0
0	0	0	0	0
0	0	0	0.004429423	0
0	0	0	0	0
0	0.001112812	0	0	0
1.135156321	0.051583394	0.611974061	5.953674685	3.045772982
0	0	0	0	0
0	0	0	0	0
0	0	0	0	0
0	0	0	0	0
0	0	0	0	0
0	0	0	0	0
0	0	0	0	0.006605261
0	0	0	0	0
0	0.043110193	0	0	0
0	0	0	0	0

PHL018-0020_V3	PHL018-0023_V3	PHL018-0074_V2	PHL018-0250_V1	PHL018-0032_V3
EYCF	EYCF	CTRL	REF	EYCF
0.141235152	0.024076939	0.010742551	0.199310178	0.02189519
2.103285564	4.414497564	5.169660037	0.298156914	6.456326992
3.005420248	13.32070614	5.776239803	0	0
0	0	0	0	0
0	0	0	0	0
0	0	0	0	0
0	0	0	0	0
0	0	0	0	0
0	0	0	0	0
0	0	0	0	0
0	0	0	0	0
0	0	0	0	0
0	0	0	0	0
6.539803596	13.8274268	4.902405444	0	4.750044162
0	0	0	0	0
0	0	0	0	0
0	0	0	0	0
0	0	0	0	0
0	0	0	0	0
0	0	0	0	0
0	0	0	0	0
0	0	0	0	0
0	0	0	0	0
0	0	0	0	0
0	0	0	0	0
0	0	0	0	0
0	0	0	0	0
0	0	0	0	0
0	0	0	0	0
0	0	0	0	0
0	0	0	0	0
0	0	0	0	0
0	0	0	0	0
0	0	0	0	0
0	0	0	0	0
0	1.169581554	0	0	6.254472152
0	0	0	0	0
0	0	0	0	0
0	0	0	0	0
0	0	0	0	0
0	0	0	0	0
0.001094424	0	0	0.009726346	0.545017749
0	0.03884664	0	0.977385406	0.272277304
0.079858971	0.004290669	0.015953279	0.223714119	0.012660314
0	0	0.0010617	0.003418477	0
0	0	0	0	0

0	0	0	0	0
0	0.002398382	0.003470967	0.203659433	0.001600984
0	0	0.003913526	0.018786692	0.011472509
0.200968432	0.459171072	0.419455459	0.17761703	0.735691647
0.00336865	0	0	0	0.000742075
0.034511282	0	0	0	0
0	0	0	0.324019661	0.018589965
0	0	0	0	0
0	0	0	0	0
0	0	0	0	0
0	0	0	0	0
0.254802041	0	0.008831301	0	0.652539333
0	0.003563203	0	0	0
0.10626268	0	0.011626357	0.08401541	0.048338421
0	0	0	0.006420088	0
0	0	0	0	0
0.038846635	0	0.412740843	0.011663416	0
0	0	0	0	0
0	0	0	0	0
0	0	0	0	0
0.01918235	0.021399526	0.118759628	0.043602563	0.100859036
0	0.001826552	0.006007743	0	0.00304818
0	0	0	0	0
0	0	0	0	0
0	0	0	0	0
0	0	0	0	0
0	0	0	0.01411029	0
0	0	0	0	0
0	0	0	0	0.007279075
0	0	0	0	0
0	0	0.228457559	0.045192339	0
0	0	0	0	0
0	0	0	0	0
0	0	0.005110339	0	0
0	0	0	0	0
0	0	0	0.023549687	0
0	0	0	0	0
2.677197271	0	0.011073231	0	0
0	0	0	0	0
0	0	0	0	0
0	0	0	0	0
0	0	0	0	0
0	0	0	0	0
0	0	0	0	0
0	0	0	0	0
0	0	0	0	0
0	0	0	0.002608969	0
0	0	0	0	0

0	0	0	0	0
0	0	0	0	0
0	0	0	0	0
0	0	0	0	0
0	0	0.026259375	0	0
0	0	0	0	0
0	0	0	0	0
0	0	0.00124111	0	0
0	0	0	0.065882551	0
0	0	0	0	0
0	0	0	0	0
0	0.001639774	0	0	0.023488135
0	0	0	0	0
0	0	0	0	0
0	0	0	0	0
0	0	0	0	0
0	0	0	0	0
0	0	0	0	0
0	0	0	0.014330746	0
0	0	0	0	0
0	0	0	0	0
0	0	0	0	0
0	0	0	0	0
0	0	0	0	0
0	0	0	0	0
0	0	0	0	0
0	0	0	0	0
0	0	0	0	0
0	0	0	0	0
0	0	0	0	0
0.006875898	0	0	0	0.053256168
0.008656099	0	0	0	0.020593852
0	0	0	0	0
0	0	0	0	0
0	0	0	0	0
0	0	0	0	0
0	0	0	0	0
0	0	0	0	0
0	0	0	0	0
0	0	0	0	0
0	0	0	0	0
0	0	0	0	0
0.002382162	0	0	0	0.003526405
0	0	0	0	0
0	0	0	0	0
0	0	0	0	0
0	0	0	0.002639445	0
0	0	0	0	0

0	0	0	0	0
0	0	0	0	0
0	0	0	0	0
0	0	0	0	0
0	0	0	0	0
0.045577233	0	0	0	0
0	0	0	0	0
0	0	0	0	0
0	0	0	0	0
0	0	0	0	0
0	0	0	0	0
0	0	0	0	0
0	0	0	0	0
0	0	0	0	0
0	0	0	0	0
0	0	0	0.268992844	0.066108025
0	0	0	0	0
0	0	0	0	0
0	0	0	0	0
0	0	0	0	0
0	0	0	0	0
0	0	0	0	0
0	0	0	0	0
0	0	0	0	0
0	0	0	0	0
0	0	0	0	0
0	0	0	0	0
0	0	0	0	0
0	0	0	0	0
0	0	0	0	0
0	0	0	0	0
0	0	0	0	0
0	0	0	0	0
0	0	0	0	0
0	0	0	0	0
0	0	0	0	0
0	0	0	0	0
0	0	0	0	0
0	0	0	0	0
0	0	0	0	0
0	0	0	0	0
0	0	0	0	0
0	0	0	0	0
0	0	0	0	0
0	0	0	0	0
0	0	0	0	0
0	0	0	0	0
0	0	0	0	0

0	0	0	0	0
0	0	0	0	0
0	0	0	0	0
0	0	0	0	0
0	0	0	0	0
0.000758597	0	0	0	0
0	0	0	0	0
0	0	0	0	0
0	0	0	0	0
0	0	0	0	0
0	0	0	0	0
0	0	0	0	0
0	0	0	0	0
0	0	0	0	0
0	0	0	0	0
0	0	0	0	0
0	0	0	0	0
0	0	0	0	0
0	0	0	0	0
0	0	0	0	0
0.032334365	0.118173666	0.105201532	0.179856337	0.040150582
0	0	0	0	0
0	0	0	0.001075996	0
0	0	0	0	0.009851525
0	0	0	0	0
0	0	0	0	0
0.004955466	0.006799735	0	0.015473559	0.010545163
0	0	0	0	0
0	0	0	0	0
0	0	0	0	0
0	0	0.433453949	0.196009938	0
0	0	0	0	0
0	0	0	0	0
0	0	0	0	0
0.017993421	0.016575108	0.024532866	0.030053921	0.002305068
0	0	0	0	0
0	0	0	0	0
0	0	0	0	0
0	0	0	0	0
0	0	0	0	0
0	0	0	0	0
0	0	0	0	0
0	0	0	0	0
0	0	0	0	0
0	0	0	0	0
0	0	0	0	0
0.001498622	0	0	0	0
0	0	0	0.002274397	0

0	0	0	0	0
0	0	0	0	0
0	0.003897631	0	0.002424679	0
0	0	0	0	0
0	0	0	0	0
0	0	0	0	0
0	0	0	0	0
0	0	0	0	0
0	0.000646557	0	0	0
0	0	0	0	0
0	0	0	0	0
0	0	0	0	0
0	0	0	0	0
0	0	0	0	0
0	0	0	0	0
0	0	0	0	0
0	0	0	0	0
0	0	0	0	0
0	0	0	0.003084062	0.002041806
0	0	0	0	0
0	0	0.00194275	0	0.016154582
0	0	0	0	0
0	0	0	0	0
0	0	0	0	0
0.010583031	0	0	0.027114471	0
0	0	0	0	0
0	0	0	0	0
0	0	0	0	0
0	0	0	0	0
0	0	0	0	0
0.120117476	0.001447707	0.029284318	0.005435792	0.391183792
0	0	0	0	0
0.034362855	0.038477347	0	0	0.027286821
0	0	0.007084628	0	0
0	0	0	0	0
0	0	0	0	0
0	0	0	0	0
0	0	0	0	0
0	0	0	0	0
0	0	0	0.001252985	0
0	0	0	0	0
0.050572958	0.014203577	0	0.003622887	0.009645446
0	0	0	0	0
0	0	0	0	0
0	0	0	0	0
0	0	0	0	0
0	0	0	0	0
0	0	0	0	0

0	0	0	0	0
0	0	0	0	0
0.088290994	0.006022184	0.019198203	0.181162432	0.010822993
0	0	0	0	0
0	0	0	0	0
0	0	0	0	0
0	0	0	0	0
0	0	0	0	0
0	0	0	0	0
0	0	0	0	0
0	0	0	0	0
0	0	0	0	0
0	0	0	0	0
0.006464124	0	0.003367854	0.201695908	0
0.006097472	0.002957945	0.002779723	0.046866458	0.000940242
0	0	0	0	0
0	0.004092523	0.002217071	0.007509393	0
0	0	0	0	0
0	0	0	0	0
0	0	0	0	0
0	0	0	0	0
0	0	0	0.001757477	0
0	0	0	0	0
0	0	0	0	0
0	0	0	0.005233449	0
0	0	0	0	0
0	0	0	0	0
0	0	0	0	0
0	0	0	0	0
0	0	0	0.023969373	0
0	0	0	0.232416159	0
0	0	0	0	0
0	0	0	0	0
0	0	0	0	0
0	0	0	0	0
0	0	0	0	0
0	0	0	0	0
0.001641595	0	0	0.132800693	0.002126351
0	0	0	0	0
0	0	0	0	0
0	0	0	0	0
0	0	0	0	0
0	0	0	0	0
0	0	0	0	0
0	0	0	0	0
0	0	0	0.035403955	0
0	0	0	0.00205156	0
0.00119303	0	0	0	0

0.016778743	0	0	0	0
0	0	0.001935237	0	0
0	0	0	0	0
0	0	0	0	0
0.001652273	0	0	0.013493451	0.002547836
0	0	0	0.034081987	0
0	0	0	0	0
0.060216628	0.135144205	0.016048643	0.003708636	0.090712363
0	0	0	0	0
0.638967509	15.1892261	0.121015476	0.906713841	1.553043749
0	0	0	0	0
0	0	0	0	0
0.000695339	0.002313025	0.007607809	0.050995669	0.002358898
0	0	0	0	0.00325687
0	0	0	0	0.004411041
0	0	0	0	0.011589044
0	0	0	0	0
0	0.001004138	0	0	0
0	0	0.218735152	0.084528786	0
0	0.023209082	0	0	0
0	0	0	0	0
0	0	0	0	0
0	0	0	0	0
0	0	0	0	0
0	0	0	0	0
0.001619786	0.000673521	0	0.002334378	0.000999096
0	0	0	0	0
0.039175428	0	0.017502153	0	0
0	0	0.033568427	0	0
0	0	0	0	0
0	0.008431787	0	0.004174858	0.00241219
0	0	0	0	0
0	0	0	0	0
0	0	0.038242931	0.00644509	0
0	0	0	0	0
0	0	0	0	0
0	0.014506595	0.043863301	0	0
0	0	0	0	0
0	0	0	0	0
0	0.000944496	0	0.865855999	0
0	0	0	0	0
0	0	0	0	0
0	0	0	0	0
0	0	0	0	0
0	0	0	0	0
0	0	0	0	0
0.002350054	0.000837577	0	0	0.00248491
0	0	0	0	0

0	0	0	0	0
0.001500771	0	0	0.002162858	0.197171313
0	0.154526219	0	0	0
0	0	0	4.602804519	0
0	0	0	0	0
0	0	0.051207462	0	0.045259501
0	0	0	0	0.004896591
0	0	0	0	0
0	0	0	0	0
0	0	0	0	0
0.002760162	0.017598054	0	0.028581572	0.013336161
0.002874334	0	0	0	0
0	0	0	0	0
0.001158961	0	0.0069742	0.009186395	0.003752993
0	0.009264443	0.021169911	3.99120821	0
0	0	0	0	0
0	0	0	0	0
0	0	0	0	0
0	0	0	0	0
0	0	0.003421613	0	0
0	0	0	0	0
0.867111585	0	0	0	0.324602192
0	0	0	0	0.002543865
0	0	0	0	0
0	0	0	0	0
0	0	0	0	0
0	0.027431968	0.272915093	0	0
0	0	0	0	0
0	0	0	0	0
0.921595782	0.377203312	1.439079653	1.026221384	0.609443533
0.006093538	0.005721361	0.003656113	0	0
0	0	0	0	0
0	0	0	0	0
0	0	0	0	0
0	0	0	0	0.005810215
0	0	0	0	0
0	0	0	0	0
0	0	0	0	0
0	0.001799892	0	0	0.117000856
0	0.040485486	0.104278601	0.003623285	0
0.002143782	0	0.096688522	0.020596952	0.003746519
0.629535838	0	3.677925229	5.279042892	5.649213063
0	0	0	0	0
0	0	0	0	0
0	0	0	0	0
0	0	0	0	0
0.013000307	0.004781912	0	0.004563796	0.006245324
0	0	0	0	0

0	0	0	0	0
0.001576802	0	0	0	0
0	0	0.848450577	0.02180637	0
0	0	0	0	0
0	0	0	0	0
0.251940247	0	0	0	0.030886687
0	0	0	0	0
0	0	0	0	0
0	0	0	0	0
0	0	0.007947583	0	0
0	0	0	0	0
0	0	0	0	0
0	0	0	0	0
0.002232486	0.001856574	0	0.015818787	0.021687977
0	0	0	0	0
0.01264871	0.003264482	0.004772117	0.000838109	0.015334611
0.001450322	0.013066219	0.010314319	0.103114175	0.004025562
0	0	0	0	0.00081515
0	0	0	0	0.00900922
0	0.360841463	0	0	0
0.003655636	0	0	0	0.014092648
0	0.00186537	0	0.001616312	0.00077824
0.000948681	0	0	0	0
0.001867802	0.003550391	0	0	0
0	0	0.012358626	0	0
0	0	0.006619453	0	0
0	0	0.003357464	0.002653465	0
0	0	0.000849899	0	0
0	0	0	0	0
0	0	0	0	0
0.002100335	0	0.001532005	0	0.001943254
0	0	0.002284032	0.039779317	0.003219059
0.442250083	0.00303042	0.019572365	0	2.500410353
0	0	0	0	0
0	0	0.010380702	0	0
0	0	0	0	0
0	0.003735966	0	0.001726481	0
0.001639528	0.00077912	0.005637756	0.012826787	0.02232024
0	0	0	0	0.090954217
0	0	0	0	0.006378318
0.003344624	0	0	0	0
0	0	0	0	0
0.001050819	0	0.00344915	0	0.001296306
0.002011198	0	0.002600569	0	0.055654337
0	0	0	0	0.785211305
0	0	0.001955423	0	0
0	0	0	0	0
0	0	0	0	0

0	0	0.001899755	0	0
0.090000879	0.011412731	0.04085511	5.524313847	0.511430309
0	0	0	0	0
0	0	0	0	0
0	0	0	0	0
0	0	0	0	0
0	0	0.441279864	0.183254806	0
0	0	0	0	0
0	0	0	0	0
0	0	0	0	0
0.008063606	0	0	0	0.006143973
0.002119583	0.148359042	1.008407584	0.053286982	0.012093216
0	0	0	0	0
0	0	0	0	0.004814258
0	0.047748715	0.002084091	0.000784331	0
0	0	0	0	0
0.011175563	0	0.007608128	0.064900515	0.001148862
0.014829812	0.008847392	0.220455391	0.014557876	0.017300026
0	0	0.023616374	0	0
0	0.000522276	0.010765046	0.05490857	0
0.005890466	0.00532458	0	0.05807058	0.04810152
0	0	0	0	0
0	0	0	0	0
0	0	0	0	0
0	0	0	0.004134297	0.012341898
0	0	0	0	0
0.006066975	0	0.053380319	0	0.057067879
0.114053549	0.127672506	0.146230901	0.111672177	0.010325702
0	0	0	0	0
0	0	0	0.00094639	0
0	0.00995869	0	0	0.010809255
0	0	0	0	0
0	0	0	0	0
0	0	0	0	0
0.070450475	0	0	0	0.030286389
0.515511355	0.969286515	0.039315666	0.173506817	0.294102131
0	0	0	0	0
0	0	0	0	0
0	0	0.006613155	0.093560738	0.001242723
0	0	0	0	0
0	0.001401858	0	0	0.719769195
0	0	0	0	0
0.006118998	0.013400149	0.00312428	0.004899157	0
0.002756963	0.021453474	0.022623251	0	0.005101546
0	0	0	0	0.003111758
0	0	0	0.025412974	0
0	0	0	0	0
0	0.002117733	0	0.00122332	0

0	0	0	0.061871824	0
0	0	0	0	0
0	0	0	0	0
0	0	0	0.102479179	0
0	0	0	0	0
0	0	0	0	0
0	0	0	0	0
0.004250396	0	0	0	0.013960426
0.715478481	0	0.314361222	0.337175869	0.065271213
0.007855733	0.005120431	0	0	0
0	0	0	0	0
0.017525561	0	0	0	0.016846595
0	0	0	0	0
0	0	0	0	0
0	0.001405749	0.015823238	0.053414086	0.024675788
0	0	0	0	0
0.047038981	0	0	0	0
0	0	0	0	0
0	0	0	0	0
0	0	0	0	0
0	0	0	0	0
0	0	0	0	0
0	0	0	0	0
0.010855126	0.006620028	0	0	0.19366802
0.004475019	0	0	0	0
0	0	0	0.007385128	0
0	0	0	0	0
0	0.170166696	0	0	0
0	0	0	0	0
0	0	0	0	0
0	0	0	0	0
0	0	0	0	0
0.002189241	0	0	0	0.005920723
0	0	0	0	0
0	0	0	0	0
0	0	0	0	0.030203161
0.147562415	0	0	0	0
0	0	0	0	0
0	0	0.002333122	0	0
0	0	0	0	0.001959481
0	0.063553111	0	0.363761462	0.027676219
0	0	0	0	0
31.90080132	0.487934448	23.97566738	0	7.8790631
0	0	0	0	0
0	0	0	0	0
0	0	0	0	0
0	0	0	0	0
0	0	0	0.00151309	0

0	0	0	0	0
0	0	0	0.193215243	0
0.046296938	0.066749594	0.033310619	0.144321524	0.888582056
0	0	0	0	0
0	0	0	0	0
0.018631895	0	0.001951797	0	0.010819872
0	0	0	0	0
0	0	0.00131864	0	0
0	0	0	0	0
0	0	0	0	0
0.095357489	0	0.012973932	0.290991497	0.011428216
3.061240362	0.757446635	2.436507165	1.940399412	0.224417313
0	0	0	0	0
0.0050657	0.001330334	0.011376622	0.055330159	0.009127009
0.006933414	0.340889767	0.160914107	0.621332595	0.108664062
0	0	0	0	0
0	0	0.001493875	0	0
0	0	0	0	0
0	0	0	0	0
0	0	0.001781184	0	0.000941384
0.013044528	0.011881203	0.231526982	2.914788637	0.086973033
0	7.654251615	1.559466797	4.587010965	0
7.750807061	12.29094719	3.753128282	9.181095455	7.700935514
0	0	0	0	0
0	0	0	0	0
0	0.001238021	0	0	0.012166626
0.260278707	0	0	0	0
0	0	0	0	0
0	0	0	0	0
0	0	0	0	0
0	0	0	0	0
0.034403906	0	0	0	0.172718713
0	0	0	0.571872609	0.495774067
0.024958738	0	0	0	0.006802326
0.001172193	0	0.001099297	0.005309301	0.029592071
0.170255125	0.00880136	0.009062205	0	0.417007477
0	0	0.001215829	0.002775908	0.000616881
0	0	0	0	0
0	0	0	0	0
0	0	0	0	0
0	0	0	0	0
0	0	0	0	0
0	0	0	0	0
0	0	0	0	0
0.009970206	0	0.002870671	0.21653895	0
0	0	0	0	0
0	0	0	0	0
0.239902872	0.006580058	0.031165323	0.288572393	0.041873881

0	0	0	0	0
0	0	0	0	0
0	0	0	0	0
0.008944806	0	0	0	0
0	0	0	0	0
0.001974999	0	0	0	0
0	0	0	0	0
0	0.003080209	0	0.507495766	0.009138321
0	0	0	0	0
0	0	0	0	0
0	0	0	0	0.001152136
0	0	0	0	0
0	0	0	0	0
1.094076755	0	0	0	0
0	0	0	0	0
0	0	0	0	0
0	0	0	0	0
0.013683878	0	0	0	0.010934049
0	0	0	0	0
0	0	0	0	0
0	0	0	0	0
0	0	0	0	0
0	0	0	0	0
0	0	0	0	0
0	0	0	0	0
0	0	0	0	0
0	0	0	0	0
0	0	0	0	0
0	0	0	0	0
0.013023638	5.516774597	0.120571462	0	0.045881234
0	0	0	0	0
0	0	0	0	0
0	0	0	0	0
0	0	0	0	0
0	0	0	0	0
0	0	0	0	0
0.026752693	0	0	0	0
0.002108283	0.006311824	0	0	0.003120971
0	0	0	0	0
0	0	0.001137602	0	0
0	0	0	0.007221912	0
0	0	0	0	0
0	0	0	0	0
0.365664264	0	0	0.005114424	0
0.100066408	0	0	0	0
0	0	0	0	0
0	0	0	0	0
0	0	0	0	0

0	0	0	0	0
0	0	0	0	0
0	0	0	0	0
0	0	0	0	0
0	0	0	0	0
0	0	0.362843401	0	0
0	0	0	0	0
0	0	0	0	0
0.048558155	0.079554565	0.063626581	0.441672038	0.016141277
0	0	0	0	0
0	0	0	0	0
0	0	0	0	0
0	0	0	0	0
0	0	0	0	0
0.154834266	0	0	0	0
0	0	0	0	0
0	0	0	0	0
0	0	0	0	0
0	0	0	0	0
0	0	0	0	0
0	0	0	0	0
0	0	0	0	0
0	0	0	0	0
0	0	0	0	0
0	0.000853075	0	0.057162878	0.066435887
0.474084494	0.052531844	0	0.139031032	0.271645419
0	0	0	0.010508412	0
0	0	0	0	0
0	0	0	0	0
0	0	0	0	0
0	0	0	0	0
0.005505188	0	0	0	0
0	0	0	0	0
0	0	0	0	0.001351658
0	0.004754305	0	0.045676136	0.002227106
0	0	0	0.004087024	0.001967867
0.106454659	0.001095211	0	0	0.112302389
1.348783991	0.828072002	0	4.688103708	1.343458875
0	0.004021041	0	0	0
0	0	0	0	0
0	0	0	0	0
0	0	0	0	0
0.619374934	0	0	0	0.1860875
0	0	0	0	0
0	0	0	0	0
0.019344067	0	0	0	0
0	0	0	0	0
0.321977811	0	0.115553683	9.105584384	0.028512623

0	0	0.003404835	0	0
0	0	0	0	0
0	0	0	0	0
0	0	0	0	0
0.001668321	0	0	0.005724584	0.022050708
0	0	0	0	0
0	0	0	0	0
0	0	0	0	0
0	0	0	0	0
0	0	0	0	0
0	0	0	0	0
0.496700093	0.026330797	0	4.315450958	0.04963737
1.318800352	0	0.000947212	0	0
0	0	0	0	0
0.051818527	0.002292191	0.002111	0.676613171	0.062479023
0	0	0	0	0
0.000958704	0	0	0	0
0	0	0.002617459	0	0
0.001932713	0	0	0	0
0	0	0	0	0
0	0	0	0	0
0	0	0	0	0
0.728229028	0	1.817654884	0.107658171	0
0	0	0	0	0
0	0	0	0	0
0	0	0	0	0
0	0	0	0	0
0	0	0	0	0
0	0	0	0	0
0	0	0	0	0
0	0	0	0	0
0.004649698	0.003271882	0.028567164	0.256011803	0.040372175
0	0	0	0	0
0	0	0.012121138	0	0.016366845
0	0.000769958	0	0.004447706	0.005996292
0.01491927	0.006699846	0.002611743	0	0.001932487
0	0	0	0	0
0	0	0	0	0
0.001861427	0	0	0	0
0	0	0	0	0
0	0	0	0	0
0	0	0	0	0
0	0	0	0	0
0	0	0	0	0
0.057231869	0.001706594	0	0.169342524	0.004430211
0	0	0	0	0
0.05928073	0	0	3.007952206	0
0.000862647	0	0	0	0

0	0	0	0	0.001163378
0.003213984	0	0	0	0
0	0	0.004644353	0	0.010909398
0	0	0	0	0
0	0	0	0	0
0.006897848	0	0	0	0
0	0	0	0	0
0	0	0.037282337	0	0
0	0	0	0	0
0	0	0.00119893	0	0
0	0	0	0	0
0	0	0	0	0
0	0	0	0	0
0	0	0	0	0
0	0	0	0	0
0	0	0	0	0
0	0	0	0	0
0	0	0	0	0
0	0	0	0	0
0	0	0	0	0
0.008142189	0	0	0	0
0	0	0	0	0
0	0	0	0	0
0	0	0	0	0
0	0	0	0	0
0.032690155	0.00335408	0.01021908	0.003875004	0.009034311
0	0	0	0	0
0.039477015	0	0.396687959	0.013574441	0.015378771
0	0	0	0	0
0.32506262	0	0.204380439	0	0.308265928
0	0	0	0	0
0	0	0	0	0
0	0	0	0	0
0	0	0	0	0
0	0	0	0	0.003087195
0	0	0	0	0
0	0	0	0	0
0	0	0	0.004122749	0
0	0	0	0	0
0	0	0	0	0
15.22085314	16.7207288	19.61952537	6.415354302	14.43073999
0.042127365	0.014136463	3.229686598	4.937414735	0.230441275
0	0	0	0	0
0	0	0	0	0
0	0	0	0	0
0	0	0	0	0
0	0	0	0	0
0.046076072	0	0	0.005389246	0

0	0	0	0	0
0	0	0	0	0.004003566
0	0	0	0	0
0	0	0	0	0
3.397206949	0	1.181737686	0	1.659440267
0	0	0	0	0
0	0	0	0	0
0	0	0	0	0
0.075099814	0.091029285	0.699439469	0.292570168	0.192268573
0	0	0	0	0
0	0.012777698	0	0	0
0	0	0	0	0
0.028431129	0	0	0	0
0.029045601	0.067961029	0.021006604	0.035474146	0.022318519
0.698776253	0	0	0	0
0.062212372	0	0	0	0.010278498
0	0	0	0.371642838	0.000985368
0	0	0	0	0
0	0	0	0	0
0	0	0	0	0
0	0	0	0	0
0	0	0	0	0
0	0	0	0	0
0	0	0	0	0
0	0	0	0	0
0	0	0	0	0
0.006482548	0	0	0.024023363	0
0	0	0	0	0
0	0	0	0	0
0	0	0	0	0
0	0	0	0	0
0	0	0	0	0
0	0	0	0	0
0	0	0	0	0
0.224913094	0	0	0	0.004075537
0	0	0	0.000696843	0.056368067
0	0	0	0	0
0	0	0	0.016450445	0
0	0	0	0	0
0	0	0	0	0
0	1.222093142	0	0	6.703651544
0	0	0	0	0
0	0	0	0	0
0	0	0	0	0
0	0	0	0	0
0	0	0	0	0
0	0	0	0	0
0	0	0	0	0

0	0	0.009513924	0	0
0	0	0	0	0
0	0	0	0	0
0	0	0	0	0
0.055724889	0	0	0	0
0	0	0	0	0
0.000849944	0	0	0.379721827	0.001376161
0	0	0	0	0
0	0	0	0.011960136	0
0	0	0	0	0
0.003092089	0	0	0	0
0	0	0	0	0
0	0	0	0	0
0	0	0	0	0
0	0	0	0	0
0	0	0	0	0
0	0	0	0	0
0	0	0	0	0
0	0	0	0	0
0	0	0	0	0
0.010114925	0	0.268704063	0.186200352	0.006738079
0	0	0	0	0
0.009148516	0	0.001464809	0	0.00061934
0.001388895	0	0.00151961	0	0.001835744
0	0	0	0	0
0.0014045	0	0	0	0
0	0	0	0	0
0	0	0	0	0
0	0	0	0	0.007773183
0	0.540028658	0.79203376	0.016187403	0.006076753
0	0	0	0	0
0	0	0	0	0
0	0	0	0	0
0	0.027489456	0	0	7.019639206
0	0	0	0	0
0	0	0	0	0
0	0	0	0.050329579	0.002067293
0	0	0	0	0
0	0	0	0	0
0	0	0	0	0
0	0	0	0	0
0	0	0.008448867	0	0
0	0	0	0	0
0	0	0	0	0
0	0	0	0	0
0	0	0	0	0
0	0	0	0	0
0	0	0	0	0

0.0040565	0	0.003503903	0.004717893	0.006518577
0	0	0	0	0
0	0	0	0	0
0	0	0	0	0
0	0	0	0	0
0	0	0	0	0
0	0	0	0	0
0.151478434	0.658482142	0	0	1.553003153
0	0.060197441	0	1.322032252	0.368657916
0.15491168	0	0.00918928	0.012328241	0.137066316
0	0	0	0	0
0	0	0	0	0
0	0	0	0	0
0	0	0	0	0
0	0	0	0	0
0	0	0	0	0
0	0	0.021140988	0	0
0	0	0	0	0
0	0	0	0	0
0	0	0.000634998	0	0
0	0	0	0	0
0	0	0	0	0
0	0	0	0	0
0	0	0	0	0
0	0	0	0	0
0	0	0	0	0
0	0	0	0	0
0	0	0	0	0
0	0	0	0	0
0	0	0	0	0
0	0	0	0	0
0	0	0	0	0
0	0	0	0	0
0	0	0	0	0
0.003360134	0	0	0	0.000956564
0	0	0	0	0
0	0	0	0	0
0	0	0.050363435	0	0
0	0	0	0	0
0	0	0	0.696660741	0
0	0	0	0	0
0.004617724	0	2.17418159	2.508580372	0.083006049
0	0	0	0	0
0	0	0	0	0
0	0	0	0	0
0	0	0	0	0
0	0	0	0	0
0	0	0	0	0

0	0	0	0	0
0	0	0.032467568	1.138024727	0
0	0	0	0	0
0	0	0	0	0
0.475781665	0.151008154	0.060427378	0	0.023929254
0	0	0	0	0
0	0	0.013293305	0	0
0.003802225	0.007453276	0.019314611	0.036009007	0.001256379
0.011117971	0	0	0	0
0	0	0	0	0
0	0	0	0	0
0	0	0	0	0
0	0	0	0	0
0	0	0	0	0
0	0	0	0	0.001592004
0.000942183	0	0	0.004752444	0.020049526
0	0	0	0	0
0	0	0	0	0
0	0	0	0	0
0	0	0	0	0
0	0	0	0	0
0	0	0	0	0
0	0	0	0	0
0.00141022	0	0	0	0.000869834
0	0	0	0	0
0	0	0	0	0
0.007523867	0	0	0.022718942	0.012728991
0	0	0	0	0
0	0	0	0	0
0	0	0	0	0
0	0	0	0	0
0	0	0	0	0
0	0	0	0	0
0	0	0	0	0
0	0	0	0	0
0	0	0	0	0
0	0	0	0	0
0	0	0	0	0
0	0	0	0	0
0	0	0	0	0
0	0	0	0	0
0	0	0	0	0.027364516
0	0	0	0	0
0	0	0	0	0
0	0	0	0	0
0	0	0	0	0
0	0	0	0	0
0	0	0	0	0

0.072777074	0.003530489	0.106610998	0	0.10305869
0	0	0	0	0
0	0	0	0	0
0	0	0	0	0
0	0	0	0	0
0	0.017334979	0	0.323586975	0
0	0	0	0	0
0	0	0	0	0
0	0	0	0	0
0	0	0	0	0
0	0	0	0	0
0.001083331	0	0	0	0
0	0	0	0	0
0	0	0	0	0
0	0	0	0	0
0	0	0	0	0
0	0	0	0	0
0	0	0	0	0
0	0	0	0	0
0	0	0	0	0
0.203158934	0	0.008706789	0.103441869	0.095065219
0	0	0	0	0
0.029263731	0.002458204	0.004527781	0.003691987	0.009298531
0	0	0	0	0
0	0	0	0	0
0	0	0	0	0
0	0	0	0	0
0	0	0	0	0
0	0	0.035556568	0	0
0	0	0	0	0
0	0	0	0	0
0	0	0	0	0
0	0	0	0.147875932	0.034576026
0	0	0	0	0
1.203660578	0	0.034677779	0	0
0	0	0.081905963	0.121225336	0.012742521
0	0	0	0	0
0	0	0	0	0
0.019527262	0	0.001967835	0.001728017	0.003565824
0	0	0	0	0
0	0	0	0	0
0	0	0	0	0
0.075710154	0.000749546	0.032871269	0.034927	0.006671228
0	0	0	0	0
0	0	0	0	0
0.001670194	0	0.025061269	0	0.014349053
0	0	0	0	0
0	0	0	0	0

0	0	0	0	0
0	0	0	0	0
0	0	0	0	0
0	0	0	0	0
0	0	0	0	0
0	0	0	0	0
0	0	0	0	0
0	0.003643054	0.091066481	0	0
0.005141542	0	0	0	0
0	0	0	0.003069497	0
0	0	0	0	0
0	0	0	0	0
0.002993287	0	0	0	0
0	0	0	0	0
0	0	0	0	0
0	0.009651894	0.002645518	0	0
0	0	0	0	0
0	0	0	0	0
0	0	0	0	0
0.00303658	0	0	0.421088782	0
0	0.001050075	0	0	0.006542229
0	0	0	0	0
0	0	0	0	0
0	0	0	0	0
0	0	0	0	0
0	0	0	0	0
0	0	0	0	0
0	0	0	0	0
0	0	0	0	0
0.080693925	0	0	0	0
0	0	0.858575922	0	0.637120442
0	0	0	0	0
0	0	0	0	0
0	0	0	0	0
0	0	0	0	0
0	0	0	0	0
0	0	0	0	0
0	0	0	0	0
0	0	0	0	0
0	0	0	0	0
0	0	0	0	0
0	0	0	0	0
0	0	0	0	0
0	0	0	0	0
0	0	0	0	0
0	0	0	0	0
0	0.378755331	0	0.297744732	0.003206169
0	0	0	0	0

0	0	0	0	0
0	0	0	0	0
0	0	0	0	0
0	0	0	0	0
0.049743201	0.003844545	0.44470501	0	0
0	0	0	0	0
0	0	0	0	0
0	0	0	0	0
0	0	0	0	0
0	0	0	0	0
0	0	0	0	0
0.010526056	0	0	0.02885958	0.002612856
0	0	0	0.005107394	0.001844375
0	0	0	0	0
0	0	0.011987469	0	0
0.051109468	0	0.005897773	0	0
0	0	0.000864168	0	0
0	0	0	0	0
0.01535758	0	0	0	0.069066566
0	0	0	2.304579463	0
0	0	0	0	0
0	0	0	0	0
0	0	0	0	0
0	0	0	0	0
0	0	0	0	0
0	0	0	0	0
0.009559945	0	0	0.000977124	0.000564572
0.001542983	0	0	0	0
0	0	0	0	0
0	0	0	0	0
0	0	0	0	0
0	0	0	0	0
0	0	0	0	0
0	0	0	0	0
0	0	0	0	0
0	0	0	0	0
0	0	0	0	0
0.010367388	0	0.001240653	0.171355886	0.003447134
0	0	0	0	0
0	0	0	0	0
0	0.00067244	0.002654082	0.012274663	0
0	0	0	0	0
0	0	0	0	0
0.000960845	0.007391262	0	0	0
0	0	0	0	0
0	0	0	0	0
0	0	0	0	0
0	0	0	0	0

0	0	0	0	0
0	0	0	0	0
0	0	0	0	0
0.00201394	0	0	0.012577149	0
0	0.000776688	0	0.020189605	0.0017282
0.00120535	0	0	0.00144759	0.001951607
0.001710079	0	0	0	0
0	0	0	0	0
0	0	0	0	0
0	0	0	0	0
0	0	0	0	0
0	0	0	0	0
0.01909415	0	0	0.008058176	0.049200979
0	0	0	0	0
0	0	0	0	0
0	0	0	0	0
0	0	0	0	0
0	0	0	0	0
0	0	0	0	0
0	0	0	0	0
0	0	0	0	0
0	0	0	0	0
0.00093991	0.00136788	0	0.00270913	0
0.016082331	0.016833226	0	0.028238992	0
0	0	0	0	0
0	0	0	0	0
0	0	0	0	0
0	0	0	0	0
0	0	0	0	0
0	0	0	0	0
0.010741955	0	0.001719941	0	0.007999343
0	0	0	0	0
0	0	0	0	0
0	0	0	0	0
0	0	0	0	0
0	0	0	0	0
0	0	0	0.007220788	0.002575369
0	0	0	0	0
0	0	0	0	0
0	0	0	0	0
0	0	0	0	0
0	0	0	0	0
0	0	0	0	0
0	0	0	0	0
0.496642133	0	0	0	0
0.109608159	0.012164418	0	0.22017437	0.114041935
0	0	0	0	0
0	0	0	0	0

0	0	0	0	0
0	0	0	0	0
0	0	0	0	0
0	0	0	0	0
0	0	0	0	0
0	0	0	0	0
0	0	0	0	0
0	0	0	0	0
0	0	0	0	0
0	0	0	0	0
0	0	0	0.001555015	0
0	0	0	0.001289801	0.001242057
0	0	0	0	0
0	0	0	0	0
0	0	0	0	0
0	0	0	0	0
0	0	0	0	0
0	0	0	0	0
0	0	0	0	0
0	0	0	0	0
0	0	0	0	0
0	0	0	0	0
0	0	0	0	0
0	0	0	0	0
0	0	0	0	0
0	0	0	0	0
0	0	0	0	0
0	0	0	0	0
0	0	0	0	0
0	0	0	0	0
0.004917295	0	0.002017532	0.003740166	0.007772125
0	0	0	0	0
0.071368498	0.039171842	0.02290503	0.121595992	0.434483593
0	0	0	0.003970648	0
0	0	0	0	0
0	0	0	0	0
0	0	0	0	0
0	0	0	0	0
0	0	0	0	0
0	0	0	0	0
0	0	0	0	0
0.079068477	0.003580702	0	0.012410471	0.037482909
0.020743836	0.001586293	0	0.186015092	0
0	0	0	0	0
0	0	0	0	0
0.088550637	0.002005765	0.009801551	0	0.001275145
0.004969801	0	0	0	0
0	0	0	0	0

0	0	0	0	0
0.025728594	0	0	0	0.007857759
0.001167135	0	0	0.078775214	0.005669192
0	0	0	0	0
0	0.001162477	0.062450893	0	0.001077756
0	0.005846333	0.059548727	0.004793397	0
0	0	0	0	0
0	0	0	0	0
0	0	0	0	0
0	0	0	0	0
0	0	0	0	0
0	0	0	0	0
0	0	0	0	0
0	0	0	0	0
0	0	0	0	0
0	0	0	0	0
0	0	0	0	0
0	0	0	0	0
0	0	0	0	0
0	0	0	0	0
0	0	0	0	0
0	0	0	0	0
0	0	0	0	0
0	0	0	0	0.337922626
0.001923766	0	0	0	0
0.001935263	0.010461088	0	0	0
0	0	0	0	0
0.004458523	0	0	0	0
0	0	0	0	0
0	0	0	0	0
0	0	0	0	0
0.001484972	0	0	0	0
0	0	0	0	0
0	0	0	0	0
0	0	0	0	0
0	0	0	0	0
0.001474424	0	0	0	0
0	0	0	0	0.002450689
0	0	0	0	0
0	0	0	0	0
0	0	0	0	0
0	0	0	0	0
0.004612838	0	0.003453193	0.005364939	0.003593979
0	0	0	0	0
0	0	0	0	0
0	0	0	0	0
0	0	0	0	0
0	0	0	0	0

0	0	0	0	0
0.042412623	0.00974339	0.028457872	0.022963552	0.013007946
0	0	0	0	0
0	0	0	0	0
0	0	0	0	0
0	0	0	0	0
0	0	0	0	0
0	0	0	0	0
0	0	0	0	0
0	0	0	0	0
0	0	0	0	0
0	0	0	0	0
0.032696547	0	0	0	0
0	0	0	0	0
0.029508824	0	0.006712939	0	0.008650106
0	0	0	0	0
0	0	0	0	0
0	0	0	0	0
0	0	0	0	0
0	0	0	0	0
0	0	0	0	0
0	0	0	0.096210986	0
0	0	0	0	0.008159724
0	0	0.000970055	0	0
0	0	0	0	0
0	0	0	0	0
0	0	0	0	0
0	0	0	0	0
0	0	0	0	0
0	0	0	0	0
0	0	0	0	0
0	0	0	0	0
0	0	0	0	0
0	0	0	0	0
0	0	0	0	6.333873464
0	0	0	0	0
0	0	0	0	0
0	0	0	0	0
0.00171921	0	0	0.36807078	0.009543794
0	0	0	0	0
0.137450318	0.009187216	0.003935346	0.156002433	0.01640144
0	0	0	0	0
0	0	0	0	0
0	0	0	0	0
0	0	0	0	0
0.045593543	0.087819223	0.083355505	1.308786322	0.501305185
0	0	0	0	0
0	0	0	0	0

0	0	0	0	0
0	0	0	0	0
0	0	0	0	0
0	0	0	0	0
0	0	0	0	0
0	0	0	0	0
0	0	0	0	0
0	0	0	0.038488716	0
0	0.00149143	0	0	0.001290553
0.004315215	0	0	0.054450234	0.003193989
0	0	0.024564685	0	0
0	0	0	0	0
0	0	0.001538238	0	0.001300773
0	0	0	0	0
0	0	0	0	0
0	0	0	0	0
0	0	0	0	0
0	0	0	0	0
0	0	0	0	0
0.567119917	0.098238222	0.379525628	0.097144605	0.177603405
0	0	0	0	0
0	0	0	0	0
0	0	0	0	0
0	0	0	0	0
0	0	0	0	0
0	0	0	0	0
0	0	0	0	0
0	0	0	0	0
0	0	0	0	0
0	0	0.037469001	0.058093833	0.156443411
0	0	0	0	0
0	0	0	0	0
0	0	0	0	0
0	0	0	0	0
0	0	0	0	0
0	0	0	0	0
0	0	0	0	0
0	0	0	0	0
0	0	0	0	0
0	0	0	0	0
0	0	0	0	0
0	0	0	0	0
0	0	0	0	0
0	0	0	0	0
0.003996124	0	0	0	0
0	0	0	0	0
0	0	0	0	0
0	0	0	0	0

0	0	0	0	0
0	0	0	0	0
0	0	0	0	0
0	0	0	0	0
0	0	0	0	0
0	0	0	0.035743797	0
0	0	0	0	0
0	0	0	0	0
0	0	0	0	0
0	0	0	0	0
0	0	0	0	0
0	0	0	0	0
0	0	0	0	0
0	0	0	0	0
0	0	0	0	0
0	0	0	0	0
0	0	0	0	0
0	0	0	0	0
0	0	0	0	0
0	0	0	0	0
0.001220843	0	0	0	0
0	0	0	0	0
0	0	0	0	0
0	0	0	0	0
0	0	0	0	0
0	0	0	0	0
0	0	0	0	0
0	0	0	0	0
0	0	0	0	0
0	0	0	0	0
0	0	0	0	0
0	0	0	0	0
0	0	0	0	0
0	0	0	0	0
0	0	0	0	0
0	0	0	0	0
0	0	0	0	0
0	0	0	0	0
1.33864773	0	0	0	0
0	0	0	0	0
0	0	0	0	0
0	0	0	0	0
0	0	0	0	0
0	0	0	0	0
0	0	0	0	0
0	0	0	0	0
0	0	0	0	0
0	0	0	0	0

0	0	0	0	0
0.023380017	0	0.000859846	0	0
0	0	0	0	0
0	0	0	0	0
0	0	0	0	0
0	0	0	0	0
0	0	0	0	0
0	0	0	0	0
0	0	0	0	0
0	0	0	0	0
0	0	0	0.002471721	0
0	0	0	0	0
0.125061486	0	0	0	0
0	0	0	0	0
0	0	0	0	0
0	0	0	0	0
0	0	0	0	0
0	0	0	0	0
0	0	0	0	0
0	0	0	0	0
0.010705359	0.002388546	0	0	0.019084703
0	0	0	0	0
0	0	0	0	0
1.177106566	0	6.6458137	1.185799706	2.452313852
0	0	0	0	0
0	0	0	0	0
0	0	0	0	0
0	0	0	0	0
0	0	0	0	0
0	0	0	0	0
0	0	0	0	0
0	0	0	0	0
0.001647953	0	0	0	0
0	0	0	0	0

PHL018-0004_V3	PHL018-0115_V2	PHL018-0052_V2	PHL018-0052_V3	PHL018-0060_V3
CTRL	EYCF	EYCF	EYCF	CTRL
0.012506613	0.672846464	0.087158427	0.263343243	0.005899917
1.862318548	8.489266613	1.846378318	1.826745136	0.020557931
6.618097711	2.38920984	6.747872454	5.827401616	0.965089237
0	0	0	0	0
0	0	0	0	0
0	0	0	0	0
0	0	0	0	0
0	0	0	0	0
0	0	0	0	0
0	0	0	0	0
0	0	0	0	0
0	0	0	0	0
0	0	0	0	0
5.890400462	0.385875418	28.88625569	7.378073355	0.168832023
0	0	0	0	0
0	0	0	0	0
0	0	0	0	0
0	0	0	0	0
0	0	0	0	0
0	0	0	0	0
0	0	0	0	0
0	0	0	0	0
0	0	0	0	0
0	0	0	0	0
0	0	0	0	0.098500778
0	0	0	0	0
0	0	0	0	0
0	0	0	0	0
0	0	0	0	0
0	0	0	0	0.001960495
0	0	0	0	0
0	0	0	0	0
0	0	0	0	0
0	0	0	0	0
0	0	0	0	0
4.981411513	0	5.5638658	0.89358681	0.419537501
0	0	0	0	0
0	0	0	0	0
0	0	0	0	0
0	0	0	0	0
0	0	0	0	0
0	0.209604904	0.009841344	0.284959921	0.004579981
0.095915802	0.011581031	0.019602229	0.719182545	2.160559637
0.046581103	0.257496885	0.010690999	0	0.009462637
0	0.002004818	0	0	0
0	0	0	0	0

0.010204362	0	0	0	0.003508633
0.029513578	0	0.002334028	0	0
0.000947685	0	0.031735442	0.008998355	0
1.902814004	1.038054735	0	0	0.160308841
0	0	0	0	0
0	0	0	0	0.043714284
0.057739352	0.102694305	0.001945768	0.34877431	0.001655817
0	0	0	0	0
0	0	0	0	0.004476815
0	0	0	0	0
0	0	0	0	0.010079109
0.024925717	0.213906421	0	0.010043126	0.029944141
0	0	0	0	0
0.029033756	0.004116405	0.014134049	0.023557958	0
0	0	0	0.002521551	0
0	0	0	0	0
0	0.002257262	0.022378244	0	0.472052807
0	0	0	0	0
0	0	0	0	0
0	0	0	0	0
0.150842052	0.181530489	0.085194529	0.009004035	0
0.003273328	0.054181227	0.000987523	0.000994578	0
0.018846618	0	0	0	0.293774966
0	0	0	0	0
0	0	0	0	0
0	0.002839153	0	0	0
0	0	0	0	0
0	0	0	0	0
0.001395844	0.002786471	0	0.002968823	0
0	0	0	0	0
0.532865657	0	0.001342767	0	0
0	0	0	0	0
0	0	0	0	0
0	0	0	0	0
0	0	0	0	0
0	0	0	0	0
0	0	0	0	0
0	0	0	0	26.53683926
0	0	0	0	0
0	0	0	0	0
0	0	0	0	0
0	0	0	0	0
0	0	0	0	0
0	0	0	0	0
0	0	0	0	0
0	0	0	0	0
0	0	0.004395294	0	0
0	0	0	0	0

0	0	0	0	0
0	0	0	0	0
0	0	0	0	0
0	0	0	0	0
0	0	0.333620617	0.000754726	0
0	0	0	0	0
0	0	0	0	0
0	0	0	0	0
0	0	0	0	0
0	0	0	0	0
0	0	0	0	0
0	0.001833196	0.088432519	0.083893041	0
0	0	0	0	0
0	0	0	0	0
0	0	0	0	0
0	0	0	0	0
0	0	0	0	0
0	0	0	0	0
0.015691333	2.031981886	0.04576087	0	0.295944696
0	0	0	0	0
0	0	0	0	0
0	0	0	0	0
0	0	0	0	0
0	0.00128389	0	0	0
0	0	0	0	0
0	0	0	0	0
0	0	0	0	0
0	0	0	0	0
0	0	0	0	0
0	0	0	0	0
0.017371653	0.140272044	0.006182984	0.015271356	0.015127151
0	0.006387048	0.001297298	0.000466631	0
0	0	0	0	0
0	0	0	0	0
0	0	0	0	0
0	0	0	0	0
0	0	0	0	0
0	0	0	0	0
0	0.30022084	0	0	0.278976649
0	0	0	0	0
0	0	0	0	0
0	0	0	0	0.012202401
0	0	0	0.002157404	0
0	0	0	0	0
0	0	0	0	0
0	0	0	0	0
0	0	0	0	0
0	0	0	0	0

0	0	0	0	0
0	0	0	0	0
0	0	0	0	0
0	0	0	0	0
0	0.00466973	0.006452291	0	0
0	0	0	0	0
0	0	0	0	0
0	0	0	0	0
0	0	0	0	0
0	0	0	0	0
0	0	0	0	0
0	0	0	0	0
0	0	0	0	0
0	0	0	0	0
0	0	0	0	0
0	0	0	0	0.589240223
0	0	0	0	0
0	0	0	0	0
0	0	0	0	0
0	0	0	0	0
0	0	0	0	0
0	0	0	0	0
0	0	0	0	0
0	0	0	0	0
0	0.020071236	0	0	0
0	0	0	0	0
0	0	0	0	0.021585903
0	0	0	0	0
0	0	0	0	0
0	0	0	0	0.251891083
0	0	0	0	0
0	0	0	0	0.000477658
0	0	0	0	0
0	0	0	0	0
0	0	0	0	0
0	0	0	0	0
0	0	0	0	0
0	0	0	0	0
0	0	0	0	0
0	0	0	0	0
0	0	0	0	0
0	0	0	0	0
0	0	0	0	0
0	0	0	0	0
0	0	0	0	0
0	0	0	0	0
0	0	0	0	0
0.008689728	0	0	0	0

0	0	0	0	0.886293453
0	0	0	0	0
0	0	0	0	0
0	0	0	0	0
0	0	0	0	0
0	0	0	0	0
0	0	0	0	0
0	0	0	0	0
0	0	0	0	0
0	0	0	0	0
0	0	0	0	0
0	0	0	0	0.028901535
0	0.001692402	0	0	0
0	0	0	0	0
0	0	0	0	0
0	0	0	0.001386274	0
0	0	0	0	0
0	0	0	0	0
0	0	0	0	0
0	0	0	0	0
0.049879549	0.040609603	0.022572074	0.048484922	0.007691525
0	0	0	0	0
0	0	0	0	0
0	0	0	0	0
0	0	0	0	0
0	0	0	0	0
0.009600833	0.008907171	0.001114021	0	0.001659023
0	0	0	0	0
0	0	0	0	0
0	0	0	0	0
0	0	0.011771188	0	0
0	0	0	0	0
0	0	0	0	0
0	0.003223126	0	0	0.004352391
0.006600873	0.134859084	0.03036889	0.13078373	0.002965639
0	0	0	0	0
0	0	0	0	0
0	0	0	0	0
0	0	0	0	0
0	0	0	0	0
0	0	0	0	0
0	0	0	0	0
0	0	0	0	0
0	0	0	0	0
0	0	0	0	0
0.026710188	0	0	0.004575029	0
0	0	0	0	0
0	0.00293448	0.002306082	0.027840901	0

0	0	0	0	0
0	0	0	0	0
0	0	0	0	0
0.000996558	0	0	0	0
0	0	0	0	0
0	0	0	0	0
0	0	0	0	0
0	0	0.015300162	0.003852366	0
0	0	0	0	0
0	0	0	0	0
0	0	0	0	0
0	0	0	0	0
0	0	0	0	0
0	0	0	0	0
0	0	0	0	0.000624773
0	0	0	0	0
0	0	0	0	0
0	0	0	0	0
0.000597987	0	0	0.006510709	0.01289585
0	0	0	0	0.141469056
0	0.050992332	0.033796773	0.042212746	0
0	0	0	0	0
0	0	0	0	0
0	0	0	0	0
0.005257386	0.007288277	0	0	0
0	0	0	0	0
0	0	0	0	0
0	0	0	0	0
0	0	0	0.00508147	0
0	0	0	0	0
0.052969146	0.796019321	0.108795424	0.342742989	0.06935408
0	0	0	0	0
0	0.04058431	0.01362518	0.000816817	0
0	0	0.016455399	0	0.00301609
0	0	0	0	0
0	0	0	0	0
0	0	0	0	0
0	0	0	0	0
0	0	0	0	0
0	0	0	0	0
0	0	0	0	0
0.049365245	0	0.002792417	0	0.001336669
0	0	0	0	0
0	0	0	0	0
0	0	0	0	0
0	0	0	0	0
0	0	0	0	0
0	0	0	0	0

0	0	0	0	0
0	0	0	0	0
0.036804299	0.257562868	0.015528073	0	0.012308327
0	0	0	0	0
0	0	0	0	0.008253891
0	0	0	0	0
0	0	0	0	0
0	0	0	0	0
0	0	0	0	0
0.001771628	0	0	0	0
0	0	0	0	0
0	0	0	0	0
0	0	0	0	0
0.00152915	0	0.00996464	0.003484662	0.002061049
0.004291185	0	0.005711462	0	0.00068045
0	0	0	0	0.016391521
0.005177034	0.001435377	0	0	0
0	0	0	0	0
0	0	0	0	0
0	0	0	0	0
0	0	0	0	0
0	0.001943604	0.003524757	0.02701897	0
0	0	0	0	0
0	0	0	0.003974543	0
0	0	0	0	0
0	0	0	0	0
0	0	0	0	0.123119705
0	0	0	0	0
0	0	0	0	0
0	0	0	0	0
0	0	0	0	0
0	0	0	0	0
0	0	0	0	0
0	0	0	0	0
0	0	0	0	0
0	0	0	0	0.042636628
0	0	0	0	0
0	0.021570443	0.004723725	0.001238925	0
0	0	0	0	0
0	0	0	0	0
0	0	0	0	0
0	0	0	0	0
0	0	0	0	0
0	0	0	0	0
0	0	0	0	0
0	0	0	0	0
0	0	0	0	0
0	0	0	0	0

0	0.00129995	0	0	0
0	0	0	0	0
0.004351116	0	0.001476763	0	0.002199227
0	0	0	0	0
0.013132927	0.004779097	0	0	0.004056497
0	0	0	0	0
0	0	0	0	0
0.019942745	0.058855116	0.002429733	0.004078485	0
0	0	0	0	0
0	0.006904759	0.365639281	0.295766154	0.04129171
0	0	0	0	0
0	0.016666313	0.001648616	0.001614272	0.002864347
0.005987405	0.012067354	0.002501068	0	0
0	0.001309086	0	0	0
0	0	0	0	0
0	0	0	0	0
0	0	0	0	0
0	0	0	0	0
0.217972561	0	0.163276957	0	0
0	0	0	0	0
0	0	0	0	0
0	0	0	0	0
0	0	0.012903894	0	0
0	0	0	0	0.060095813
0	0	0	0	0
0	0	0.001213794	0.002648675	0
0	0	0	0	1.904946933
0.001297425	0	0	0	0
0	0	0	0.008135584	0
0	0.034775811	0	0	0
0	0	0	0	0
0	0	0	0	0
0	0	0	0	0
0.004560224	0	0	0.014655303	0
0	0	0	0	0
0.011967671	0	0	0	0
0.118278691	0	0	0	0
0	0	0.006318425	0	0
0	0	0.002593182	0	0
0	0	0	0	0
0.016296473	0	0	0	0
0	0	0	0	0
0	0	0	0	0
0	0	0	0	0
0	0	0	0	0
0	0	0	0	0.00224828
0	0.014981991	0.023547406	0.010388255	0.001123952
0	0	0	0	0

0	0	0	0	0
0.022366369	0	0	0.046721569	0
0	0	0	0	0
0	0	0	0	0
0	0	0	0	0
0	0.00229447	0	0	0
0	0.042643569	0	0	0
0	0	0	0	0
0	0	0	0	0.233390646
0	0	0	0	0
0.006094131	0	0	0	0
0	0	2.408400148	1.161434171	0
0	0	0	0	0
0.0060453	0.003950834	0.008076798	0	0.000776009
0.079226575	0	0	0	0
0	0	0	0	0
0	0	0	0	0
0.030255705	0	0	0	0
0.001531975	0.047402443	0.002218454	0.001861919	0
0.001726177	0.048888716	0	0	0
0	0	0	0	0
0	0.185577161	0.902565075	0.922165538	0.029391638
0	0.002385824	0.010136913	0.068062204	0
0	0	0	0	0
0	0	0	0	0
0	0	0	0	0
0.247206063	0	0	0	0
0	0	0	0	0
0	0	0	0	0
0.770919552	0.874455383	0.30666556	6.0764828	0.021896233
0	0.000730998	0	0	0.003685223
0	0	0	0	0
0	0	0	0	0
0	0.001725573	0.004506271	0	0
0.005719427	0	0	0	0
0	0	0	0	0
0	0	0	0.789854293	3.263050672
0	0.00219652	0	0	0
0.136394714	0.402440221	0.27358269	0.638906424	0
0.009537517	0	0	0	0
0.081884933	0.019488093	0.003641294	0.00251678	0
2.224681423	5.152609426	0	0	0.057336624
0	0	0	0	0
0	0	0	0	0
0.004271467	0.009992538	0	0.002433479	0
0	0	0.000873129	0	0
0.00149036	0	0.000899247	0	0
0	0	0	0	0

0	0.003059029	0.00305118	0	0
0	0	0	0	0
0.004175967	0	0.044220319	0	0.00168856
0.06265348	0	0	0	0
0	0	0	0	0.001384154
0	0	0	0	0
0	0	0	0	0
0	0	0.001554433	0	0
0.001282464	0.098885142	0	0	0
0	0	0	0	0
0	0	0	0	0
0	0	0	0	0
0	0	0	0	0
0	0.001383713	0	0	0.004484438
0	0	0	0	0
0.02123411	0.269525999	0.00372598	0	0.005986873
0.000720484	0.049740305	0.066077769	0.006384989	0.001456644
0	0.014926107	0.001056341	0.001773146	0
0	0	0	0.001695911	0
0	0	0	0	0
0	0	0.006574483	0.003908495	0
0	0.004518375	0.027229776	0	0
0.11624931	0	0	0	0
0.534192196	0	0	0	0
0	0.09717489	0	0	0
0.00094911	0	0.108234598	0.188408574	0
0.005487958	0.001901979	0	0	0
0	0.027684048	0	0	0
0	0	0	0	0
0	0	0	0	0
0.003709846	0.041368469	0.011961612	0.04508843	0
0.007259339	0	0.005318702	0	0
0.004607915	0.035726723	2.284813399	5.445821725	0.008428847
0	0.017655131	0	0	0
0	0	0	0	0.020192671
0	0	0	0	0
0	0.021532947	0	0	0
0.003025198	0.078972681	0.008003372	0.019267537	0.001725088
0	0	0	0	0.011300642
0	0	0	0	0
0	0	0.000601514	0	0
0	0	0	0	0
0.002436096	0.014328736	0	0	0
0.018710663	0	0.038636587	0	0.006121109
1.738319316	0	0	0	0
0	0	0.008265154	0.000578069	0.01572771
0	0	0	0	0
0	0	0	0	0

0	0	0	0	0
1.431994486	0.018990052	0.361607064	0.000963467	0.039533686
0	0	0	0	0
0	0.002635668	0	0	0
0	0	0	0	0
0	0	0	0	0
0	0	0.012213133	0	0
0	0	0	0.001480088	0.001313126
0	0.031870355	0	0	0
0	0	0	0	0
0.000706906	0.004997888	0.001279589	0.001073941	0
0.015092367	0.057804327	0.003494303	0	0.005913398
0	0	0	0	0.028839206
0	0	0	0	0
0	0.001349283	0.010521869	0.003080534	0
0	0	0	0	0
0.019739524	0.032016771	0	0	0.002494285
0.189622251	0.075531291	0.057399978	0	0
0	0	0	0	0
0.111067292	0.009342109	0	0	0
0.061832652	0.007936867	0.019805645	0	0.00617337
0	0	0	0	0
0	0	0	0	0
0	0	0	0	0
0.075673247	0	0	0.010067463	0
0	0	0	0	0
0	0.150101062	0	0	0.017772487
0.107368048	0.066136031	0.082488498	0.035745637	0.01931962
0	0	0	0	0
0	0	0	0.001486814	0
0.012188041	0	0.004202266	0.009919422	0
0	0	0	0	0
0.000984968	0	0	0.002743359	0
0	0	0	0	0
0.054441393	0.048517532	0	0	0.001429447
1.185489419	0.63047011	0.200503624	0.233297282	0.046578236
0	0.027771345	0	0	0
0	0	0	0	0
0	0.007908866	0.007246917	0.004054827	0.011466779
0	0	0	0	0
1.648031417	0	0	0	0
0	0	0	0	0
0.032626814	0.0169403	0.006052596	0	0
0	0	0	0	0.001977844
0	0	0	0	0
0	0	0	0	0.066073832
0	0	0	0	0
0	0.006708013	0	0	0

0.029726325	0	0	0	0
0	0	0	0	0
0	0	0	0	0
0.009913021	0.374282738	0	0	0.821711395
0	0	0	0	0
0	0	0	0	0
0	0	0	0	0
0	0.00263443	0	0	0
0.115970953	0	0.172551356	0.329523679	9.520151665
0	0	0	0	0.021608595
0	0	0	0	0
0.019900026	0	0	0	0.000914388
0	0	0	0	0
0	0	0	0	0
0.005038431	0.797191756	0	0.49980786	0.032068591
0	0	0	0	0
0	0	0	0	0
0	0.035267944	0	0	0.728819706
0	0	0	0	0
0	0	0	0	0.001360955
0	0	0	0	0
0	0	0	0	0.031976814
0	0	0	0	0
0.193233249	0.205431187	0.072232943	0.00163849	0
0.000846887	0.014528661	0	0	0
0	0	0	0	0.094259178
0.001007665	0	0	0	0.013411929
3.682982358	0	1.028272932	1.535348719	0.698829295
0	0	0	0	0
0	0	0	0	0
0	0	0	0	0.006518899
0	0.063057561	0	0	0.0047461
0.000669268	0.093939879	0	0.001398047	0
0	0	0	0	0
0	0	0	0	0.002188661
0	0.298384432	0	0	0.034294369
0	0	0.585021015	0	1.90306846
0	0	0	0	0
0	0	0	0	0
0	0	0.014796085	0.009959115	0
0.048810983	0.362691472	0.005497586	0.0026366	0.025584755
0	0	0	0	0.007110645
0.087702027	1.30073308	1.899603887	7.006670575	0.005787644
0.00170083	0.01367552	0	0	0
0	0	0	0	0
0	0	0	0	0
0	0	0	0	0
0	0	0.002832312	0.018818881	0

0	0	0	0	0.009377681
0	0.003260641	0.007525924	0.004511719	0.03410363
0.042748446	0.226442712	0.006108955	0.009114958	0
0	0	0	0	0.00946983
0	0	0	0	0
0	0	0	0	0.016324212
0.004790564	0	0	0	0
0	0	0	0	0
0	0	0	0	0
0	0	0	0	0
0.01202689	0.065840518	0.095744423	0.315088981	0.001488703
1.965836687	2.914439897	6.978262056	2.311179544	0.362231214
0	0	0	0	0
0.005033026	0.051227696	0.005753946	0.00140852	0.00142815
0.092058004	0.095844742	0.00453433	0.003329902	0
0	0	0	0	1.587186784
0	0	0	0	0
0	0	0	0	0
0	0	0	0	0.006216407
0.011120093	0.027748272	0	0	0.019484531
0.105740344	0.025667	0.047664661	0	0.091085897
0	0	6.704087984	35.81959916	0.001899074
0.11425311	0.779173346	5.343010313	5.875574064	1.296385218
0	0	0	0	0
0	0	0	0	0.032747054
0.008381506	0.120874086	0.059124566	0	0.069775122
0	0.055872048	0.204876652	0.095880944	0
0	0	0	0	0
0	0	0	0	0
0	0	0	0	0
0	0	0	0	0.007034106
0	0.19724535	0.030409544	0	0.049998433
0	0.955170787	0.684217445	0	0
0.009995995	0.032138454	0	0.019128601	0
0.001996513	0.001453069	0	0	0
0	0.033939287	0.014689112	0.000520917	0.017869994
0	0.024795293	0	0.017611963	0.001190494
0	0	0	0	0
0.001069988	0.097120198	0.014041904	0.009143676	4.717526581
0	0	0	0	0
0	0	0	0	0.040966376
0	0	0	0	0
0	0	0	0	0
0	0	0	0	0
0.000782045	0.085538881	0.014155998	0.113859007	0.008608244
0	0	0	0	0.009912348
0	0	0	0	0
0.085845722	0.555541841	0.010814802	0.00477722	0

0	0	0	0	0
0	0	0	0	0
0	0	0	0	0
0.001777423	0.007207283	0	0	0.004042711
0	0	0	0	0
0	0	0	0	0.009521324
0	0	0	0	0.009835552
0.010426635	0.272422365	0.142106572	0.109950319	0.004408919
0	0	0	0	0
0	0	0	0	0
0	0.00957066	0.001119777	0.002819442	0
0	0	0	0	0
0	0.076770339	0	0	0.06964728
0	0	0	0	0.017787594
0	0	0	0	0
0	0	0	0	0
0	0	0	0	0.000620126
0	0.000771034	0	0	0.000971765
0	0	0	0	0
0.008173997	0	0	0	0.000500783
0.001281067	0.019865097	0	0	0.025283428
0	0	0	0	0.176994661
0	0	0	0	0
0	0	0	0	0
0	0	0	0	0
0	0	0	0	0
0	0	0	0	0
0	0	0	0	0
0	0	0	0	0
0.35807956	0.253962475	0	0	0.578389103
0	0	0	0	0
0	0	0	0	0
0	0	0	0	0.002175705
0	0	0	0	0
0	0	0	0	0
0	0	0	0	0
0	0	0	0	0.029854802
0.011101828	0.003136154	0.001327075	0	0
0	0	0	0	0.006512188
0	0	0	0	0
0.00845093	0.010053503	0.025969754	0.012390915	0.004503219
0	0	0	0	0
0	0	0	0	0.003501042
0.062449535	0	0	0	0.042771413
0	0	0.712108625	0.123941045	0.026448066
0	0	0	0	0
0	0.004966452	0	0	0.000536521
0	0	0	0	0.212736818

0	0	0	0	0
0	0	0	0	0
0	0	0.020182967	0	0.011598044
0	0	0.018292136	0	0.908502648
0	0	0	0	0
0	0	0	0	0.091783027
0	0	0	0	0
0	0	0	0	0
0.010400111	0.266004724	0.082361639	0.034672232	0.00116814
0	0	0	0	0
0	0	0	0	0
0	0	0	0	0
0	0	0	0	0
0	0	0	0	0
4.691259165	0.651439577	0	0	0.031479609
0	0.004444818	0.571708686	0.204311439	0
0	0	0	0	0
0	0	0	0	0
0	0	0	0	0
0	0	0	0	0
0	0	0	0	0.015928922
0	0	0	0	0
0	0	0	0	0
0	0	0	0	0
0.181754874	0.001695469	0	0.028386654	0.079903514
2.849855584	3.342321454	0	0	1.383460595
0	0	0	0	1.418184712
0	0	0	0.001121331	0
0.052572551	0.050769614	0.0330651	0.023915579	0.073861874
0	0	0	0	0.030994757
0	0	0	0	0
0	0.100181021	0	0.001661924	0.026392655
0	0	0	0	0
0	0.007243913	0	0	0
0.00388636	0.00186495	0.002976263	0.001816682	0
0.00105661	0.011337325	0	0.000802609	0
0.305091717	0.005713859	0.349155979	0	0
0	1.741342514	0	0	0.063519978
0	0	0	0	2.41357389
0	0	0	0	0
0	0	0	0	0
0	0	0	0	0
0.248057926	0.043595617	0	0	0.082320591
0	0	0	0	1.079051427
0	0	0	0	0
0	0.025977489	0.005617033	0.00288941	0
0	0	0	0	0
2.60928292	1.172316916	0.033773774	0.041186315	0.015690826

0	0.007418517	0	0	0
0	0	0	0	0
0	0	0	0	0.027502751
0	0	0	0	0
0	0.005317915	0.001500197	0.002877932	0
0.001140147	0	0.019606202	0.105833066	0
0	0	0	0	0.037095743
0	0	0	0	0
0	0	0	0	0
0	0.005041107	0	0	0.295998561
0	0	0	0	0
0.402181016	0	2.227295923	0.095748688	0.169159338
0	0	0	0	0
0	0	0	0	0
0.001369263	0.734603436	1.361462024	0.033283287	0.044108578
0	0	0	0	0
0	0.002139165	0	0	0
0	0	0	0	0
0	0.002096345	0	0	0
0	0	0	0	0.004484193
0	0	0	0	0.028257907
0	0	0	0	0
0.193953261	0.002329612	0.084707016	0	0.005977063
0	0	0	0	0
0	0	0	0	0
0	0	0	0	0
0	0	0	0	0
0	0	0	0	0
0	0	0	0	0.014462629
0	0	0	0	0
0	0	0	0	0
0.092394156	0.050544455	0.004502753	0	0.015566561
0	0	0	0	0
0.009764304	0.2188432	0.003374247	0.005259355	0.002033929
0.001379828	0.012337851	0	0.000698752	0
0.002964608	0.054002976	0.011269264	0.004729071	0.179212352
0	0	0	0	0
0	0	0	0	0.021933435
0	0.003626002	0	0	0.000534155
0	0	0	0	0
0	0	0	0	0.074737681
0	0	0	0	0.303359027
0	0	0	0	0
0.018721777	0	0	0	0
0.002491992	0.191637802	0.003485634	0.023919826	0.000916038
0	0	0	0	0
0	0	0	0	0.087221344
0.000857083	0	0.000581786	0.00195314	0

0	0	0	0	0
0	0	0	0	0
0	0.002923325	0	0	0
0	0	0	0	0
0	0	0	0	0
0	0	0	0	0.000679208
0.09130259	0	0	0	0.513450399
0	0	0	0	0.001491237
0	0	0	0	0
0	0	0	0	0.001614178
0	0	0	0	0
0	0	0	0.002173072	0
0	0	0	0	0
0	0	0	0	0.112475469
0	0	0	0	0
0	0	0	0	0
0	0	0	0	0.179395804
0	0	0	0	0.001845249
0.208300729	0	0	0	6.168700706
0	0	0	0	0
0.015540687	0	0	0	0
0	0	0	0	0
0	0	0	0	0
0	0	0	0	0
0	0	0	0	0
0.008225279	0.012104347	0	0.002883685	0
0	0	0	0	0
0.037364489	0.001802918	0.002428862	0	0.007373344
0	0	0	0	0.015165762
1.073607369	0.268475475	0.366902347	0	0.399181846
0	0	0	0	0
0	0	0	0	0
0	0	0	0	0.002937968
0	0	0	0	0
0.001105075	0.001103009	0	0.000503654	0.000446839
0	0	0	0	0
0	0	0	0	0.270748634
0.000752362	0.002816083	0	0.002857498	0
0	0	0	0	0
0	0	0	0	0
10.48626066	20.77749447	7.709826407	4.852719547	0.286089344
0.65665636	0.494503497	0	0	0.21390571
0	0	0	0	0
0	0	0	0	0.007463351
0	0	0	0	0
0	0	0	0	0
0	0	0	0	0.00134254
0	0.001738333	0	0.004233355	0.00067068

0	0	0	0	0
0.001228366	0.045466764	0.006670494	0	0
0	0.001789976	0	0	0
0	0	0	0	0
5.32423627	0.046256852	0	0	0.281870745
0	0	0	0	0
0	0	0	0	0
0	0	0	0	0.010678732
0.546225816	0.348914787	0.094174321	0.002795101	0
0	0	0	0	0.00133814
0	0	0.00774561	0	0
0	0	0	0	0
0	0	0	0	0.023728865
0.036439667	0.080249325	0.01150987	0.074679848	0
0	0.241794081	0.708593284	0.491744137	0.195384905
2.847324694	0.097651278	0.07501443	0	0.069651619
0	0.071291646	0.007278467	0	0
0	0	0	0	0
0	0	0	0	0
0	0	0	0	0
0	0	0	0	0.000831415
0	0	0	0	0
0	0	0	0	0
0	0	0	0	0
0	0	0	0	0.010085909
0	0	0	0	0
0	0	0	0	0.011037371
0.00290706	0.017357945	0	0	0
0	0	0	0	0
0	0	0	0	0
0	0	0	0	0
0	0	0	0	0
0	0	0	0	0
0	0	0	0	0.00469414
0	0	0.13514243	0.190668364	0.082584759
0	0.031467935	0	0	0.007770176
0	0	0	0	0
0	0	0	0	0.001375734
0	0	0	0	0
0	0	0	0	0
5.262451257	0	3.266206307	0.079778113	0.040841501
1.046396497	0	0	0	0
0	0	0	0	0
0.021856222	0	0	0	0
0	0	0	0	0
0	0	0	0	0
0	0	0	0	0
0	0	0	0	0

0	0	0	0	0.534579267
0	0	0	0	0
0	0	0	0	0
0	0	0	0	0
0	0	0	0	0.014560734
0	0	0	0	0
0.039056362	0.013433456	0.003821457	0	0.009674692
0	0	0	0	0
0	0	0	0	0
0	0	0.00521213	0.000504747	0.008657631
0	0	0	0	0.050551755
0	0	0	0	0
0	0	0	0	0
0	0	0	0	0
0	0	0	0	0
0	0	0	0	0
0	0	0	0	0
0	0	0	0	0
0	0	0	0	0
0	0	0	0	0
0.035575884	0	0.003820152	0	0
0	0	0	0	0
0	0.012170453	0.010433712	0.009430481	0.008217268
0.00571688	0.023611839	0.001605767	0	0.005579799
0	0	0	0	0
0	0	0	0	0
0	0	0	0	0
0	0	0	0	0
0	0	0	0	0.012731241
0	1.196837798	0.38492241	0.021120682	0.000573617
0	0	0	0	0.003750925
0	0	0	0	0
0	0	0	0	0
0	0	0	0	0
0	0	0	0	0.026199988
0	0	0	0	0
0	0	0	0	0
0	0	0	0	0
0	0	0	0	0
0	0	0	0	0.09954803
0	0	0	0	0
0	0	0	0	0
0	0	0	0	0
0	0	0	0	0.001744888
0	0.000873802	0	0	0.016833957
0	1.268273981	0	0	0
0	0	0	0	0
0	0	0	0	0.057198838

0.004878828	0.024613203	0.009215266	0.003705994	0
0	0	0	0	0.078363398
0	0	0	0	0
0	0	0	0	0
0	0	0	0	0
0	0	0	0	0
0	0	0	0	0
3.312555688	0.065218571	0.041001978	0.000992666	0.127112597
0.145170053	0.014539722	0.019889022	0.98536864	2.797439335
0.036160169	0	0	0	0
0	0	0.002215664	0	0.003849549
0	0	0	0	0
0	0	0	0	0.019616159
0	0	0.003569501	0.003557553	0.273762495
0	0	0	0	0
0	0	0	0	0
0	0	0	0	0
0	0	0	0	0
0	0	0	0	0
0	0.01798608	0	0	0
0	0	0	0	0
0	0	0	0	0
0	0	0	0	0
0	0	0	0	0
0	0	0	0	0.009120274
0	0	0	0	0
0	0	0	0	0
0	0	0	0	0.023205378
0	0	0	0	0
0	0	0	0	0
0	0.178904053	0	0	0
0	0	0	0	0
0	0	0	0	0
0	0	0	0	0
0.004108875	0.005767306	0	0	0
0	0	0	0	0
0	0	0	0	0
0	0	0	0	0
0	0.005664585	0	0	0
0.100800207	0	0	0	0
0	0	0	0	0
0	0.302837733	0	0.170434732	0.100707703
0	0	0	0	0
0	0	0	0	0
0	0	0	0	0
0	0	0	0.000716387	0
0	0	0	0	0
0	0	0	0	0

0	0	0	0	0
0	0.001433193	0.277239341	0.176403001	0.251980156
0	0	0	0	0
0	0	0	0	0
0.119382764	1.544599957	0.352976076	0.117319938	2.633167886
0	0	0.351586281	0	0
0	0	0	0	0
0.013761624	0.085512722	0.008303427	0.003689447	0.002545867
0	0.001680734	0	0	0.001452545
0	0	0	0	0.069078605
0	0	0	0	0
0	0.138077408	0	0	0.074717724
0	0	0	0	0
0	0	0	0	0
0.002849323	0	0	0	0
0.004446504	0	0	0	0
0	0	0	0	0
0	0	0	0	0
0	0	0	0	0
0	0	0	0	0
0	0	0	0	0
0	0	0	0	0
0	0	0	0	0
0	0.007575235	0	0.001596456	0
0	0	0.288834204	0.66301046	0
0	0	0	0	0
0	0	0.004252697	0.003082519	0.012378542
0	0.001964144	0	0.001024987	0
0	0	0	0	0
0	0	0	0	0
0	0	0	0	0
0	0.001024502	0	0.000623741	0
0	0	0	0	0
0	0	0	0	0
0	0	0	0	0
0	0	0	0	0
0	0	0	0	0
0	0	0	0	0.018451918
0	0	0	0	0
0	0	0	0	0
0	0	0	0	0
0	0	0	0	0.168246213
0	0	0	0	0
0	0	0	0	0
0	0	0	0	0
0	0	0	0	0
0	0	0	0	0
0	0	0	0	0.000437045

0	0.222281331	0	0	0.025718381
0	0	0	0	0.005550223
0	0	0	0	0
0	0	0	0	0
0	0	0	0	0
0	0.054582692	0.000631829	0.001944382	0
0	0	0	0	0
0	0	0	0	0
0	0	0	0	0
0	0	0	0	0
0	0	0	0	0
0.003229032	0	0	0	0
0	0	0	0	0.00479165
0	0	0	0	0
0	0	0	0	0
0	0	0	0	0
0	0	0	0	0
0	0	0	0	0
0	0	0	0	0
0	0	0	0	0.001399294
0.008604144	0	0	0	0
0	0	0	0	0
0	0.002931378	0	0.004015559	0.001187528
0	0	0	0	0.446167157
0	0	0	0	0.01439966
0	0	0	0	0.013045571
0	0	0	0	0
0	0	0	0	0
0	0	0	0	0
0	0	0	0	0
0	0	0	0	0
0	0	0	0	0
0.062708295	0.36511467	0.005476357	0.139140518	0
0	0	0	0	0
0	0	0	0	0
0	0	0.074907054	0.005197128	0
0	0	0	0	0
0	0	0	0	0
0.007147852	0.002866537	0.004158809	0	0
0	0	0	0	0
0	0	0	0	0
0	0	0	0	0
0.014029468	0.021973178	0.019181415	0.030610214	0.937829295
0	0	0	0	0
0	0	0	0	0
0	0.048506515	0	0	0.016455236
0	0	0	0	0
0	0	0	0	0

0	0	0	0	0
0	0	0	0	0
0	0	0	0	0
0	0	0	0	0
0	0	0	0	0
0	0	0	0	0
0	0	0	0	0
0.029321637	0	0	0	0
0	0.012747079	0	0	0.006136876
0.003418372	0	0.003093843	0	0.04722602
0	0	0	0	0
0	0	0	0	0
0	0	0	0	0
0	0	0	0	0
0	0.011571012	0	0	0
0.001201179	0.02397867	0	0	0
0	0	0	0	0
0	0	0	0	0
0	0	0	0	0
8.547949045	0	0	0	0.060454167
0.007025457	0	0.005677217	0	0
0	0	0	0	0
0	0	0	0	0
0	0	0	0	0
0	0.001620391	0	0	0
0	0	0	0	0.28497193
0	0	0	0	0
0	0	0	0	0.091578139
0	0	0	0	0
0	0	0	0	0.012584738
0	0.009744595	0.242106135	0.009789025	0.19922339
0	0	0	0	0
0	0	0	0	0.000500119
0	0	0	0	0
0	0	0	0	0
0	0	0	0	0
0	0	0	0	0
0	0	0	0	0
0	0	0	0.007689398	0.025656627
0	0	0	0	0
0	0	0	0	0
0	0	0	0	0.010078054
0	0	0	0	0.00042697
0	0	0	0	0
0	0	0	0	0
0	0	0	0	0
0.169690016	0	0.125312543	0	0
0	0	0	0	0

0	0	0	0	0
0	0	0	0	0
0	0	0	0	0
0	0	0	0	0
0.077716205	0.04149037	0	0	0.011267324
0	0	0	0	0
0	0	0	0	0
0.005629064	0	0	0	0
0	0	0	0	0
0	0	0	0	0
0	0	0	0	0.002515268
0	0.043600319	0.003232054	0.006975307	0
0	0.00439312	0.01473894	0.028919509	0.004745847
0	0	0	0	0
0	0.028133376	0	0	0.001048008
0	0	0.050985707	0	0
0.002354215	0.024966786	0	0	0.004627444
0	0	0	0	0
0.056978038	0.325083507	0	0.003374447	0
0.00807007	0.001887887	0	0	0.018695117
0	0	0	0	0
0	0	0	0	0.008940822
0	0	0	0	0
0	0	0	0	0
0	0	0	0	0
0	0	0	0	0
0.018390295	0.059757601	0.028350319	0.082895375	0.000953353
0	0	0	0	0
0	0	0	0	0
0	0	0	0	0
0	0.002858159	0	0	0
0	0	0	0	0
0	0	0	0	0
0	0	0	0	0
0	0	0	0	0
0	0	0	0	0
0	0	0	0	0
0.003540804	0.1006038	0.001893658	0.002567365	0
0	0	0	0	0.091583254
0	0	0	0	0
0	0.005212197	0	0	0
0	0	0	0	0.00375423
0	0	0	0	0
0.001670634	0.005062087	0.101089946	0.157721776	0
0	0	0	0	0
0	0	0	0	0
0	0	0	0	0
0	0	0	0	0

0	0	0	0	0
0	0	0	0	0.000824821
0	0	0	0	0.02020222
0.001500713	0.003120641	0.00067912	0	0
0	0	0	0	0
0	0	0	0.001364531	0
0.007038917	0	0	0	0.000981448
0	0	0.00548835	0.003566167	0
0	0	0	0	0
0	0	0	0	0.052396051
0	0	0	0	0.012953388
0	0	0	0	0
0.042729062	0	0	0	0.081488977
0	0	0	0	0.001518021
0	0	0	0	0
0	0	0	0	0
0	0	0	0	0
0	0	0	0	0
0	0	0	0	0
0	0	0	0	0
0	0	0	0	0
0	0	0	0	0
0.008638092	0	0.016903827	0.001064037	0
0	0.037809502	0	0	0.459137805
0	0	0	0	0
0	0	0	0	0.001919566
0	0	0	0	0.002254794
0	0	0	0	0
0	0	0	0	0
0	0	0	0	0
0.080175182	0.008119453	0.00918825	0	0.007894233
0	0	0.016579102	0	0
0	0	0	0	0
0	0	0	0	0
0	0	0	0	0.013972066
0.00190484	0	0	0	0
0	0.003105477	0	0	0
0	0	0	0	0
0	0.003297418	0.000996653	0.000836478	0.074657141
0	0	0	0	0
0	0	0	0	0
0	0	0	0	0
0	0	0	0	0
0	0	0	0	0
0	0	0	0	0
0.033135418	0.280447671	0.098211719	0.002502272	0.085274207
0	0	0.000834931	0	0
0	0	0	0	0.017627455

0	0	0	0	0
0	0	0	0	0
0	0	0	0	0.081037726
0	0	0	0	0.011435329
0	0	0	0	0
0	0	0	0	0
0	0	0	0	0
0	0	0	0	0
0	0	0	0	0
0	0	0	0	0
0	0	0	0	0
0	0	0	0	0
0	0	0	0	0
0	0	0	0	0
0	0	0	0	0
0	0	0	0	0
0	0	0.005942689	0	0
0	0	0	0	0
0	0	0	0	0
0.00165866	0.001478178	0	0.002879841	0
0	0	0	0	0
0	0	0	0	0
0	0	0	0	0
0	0	0	0	0
0	0	0	0	0
0	0	0	0	0
0	0	0	0	0.003058952
0	0	0.017325224	0.007768388	0.000706879
0	0	0	0	0
0	0	0	0	0
0.004682013	0.005333614	0.007001117	0.001700931	0.00137187
0	0	0	0	0
0.04372671	0.027130663	0.193170929	0.361596133	0.005415794
0.010144465	0	0	0	0.002441627
0	0	0	0	0
0	0	0	0	0
0	0	0	0	0
0	0	0	0	0
0	0	0	0	0
0	0	0	0	0
0	0	0	0	0
0.090808951	0.251101692	0.174055206	0.002806433	0
0.016108997	0.004137949	0.105059329	0.011156823	0.035122906
0	0	0	0	0
0	0	0	0	0
0.001711663	0	0.025406283	0.032764845	2.29781915
0	0.003640382	0	0	0
0	0	0	0	0

0	0	0	0	0
0	0.004025404	0.021563477	0.034499193	0.013547582
0.015364788	0	0.003673311	0	0.000781482
0	0	0	0	0
0.025461978	0.004620798	0.007960897	0.009670555	0.007643701
0	0	0	0	0
0	0	0	0	0
0	0	0	0	0
0	0	0	0	0
0	0	0	0	0
0	0	0	0	0
0	0	0	0	0
0	0	0	0	0
0	0	0	0	0
0	0	0	0	0
0	0	0	0	0
0	0	0	0	0.109088442
0	0	0	0	0
0	0	0	0	0
0	0	0	0	0
0	0	0	0	0
0	0.001163783	0	0	0
0	0	0	0	0
0	0	0	0	0
0.003822716	0	0	0	0.0367109
0.003124519	0.327161394	0	0	0.0006479
0	0	0	0	0
0	0	0	0	0.009741526
0	0	0	0	0
0	0	0	0	0
0	0	0	0	0.00152517
0	0	0	0	0
0	0	0	0	0
0	0	0	0	0
0	0	0	0	0
0	0	0	0	0
0	0	0	0	0
0.004067179	0	0	0	0
3.400253144	0.010618182	0.0017775	0	1.075012709
0	0	0	0	0
0	0	0	0	0
0	0	0	0	0.040590833
0.001688505	0.01835827	0.007641021	0.002198745	0
0	0	0	0	0
0	0	0	0	0
0	0	0	0	0
0.001107035	0.02992617	0	0	0
0	0	0	0	0

0	0	0	0	0
0.016762501	0.140878779	0.046777634	0.030771227	0.001412073
0	0	0	0	0.000744142
0	0	0	0	0
0	0	0	0	0
0	0	0	0	0
0	0	0	0	0
0	0	0	0	0
0	0	0	0	0
0	0.008541406	0	0	0
0	0	0	0	0
0	0	0	0	0
0	0.012896282	0	0	0.079941618
0	0	0	0	0
0.012482131	0.023541339	0.005254474	0	0
0	0	0	0	0
0	0	0	0	0
0	0	0	0	0
0	0	0	0	0
0	0	0	0	0
0	0	0	0	0
0	0	0	0	0
0	0	0.035687469	0.000998401	0
0	0	0.002126037	0	0
0	0	0	0	0
0	0	0	0	0
0.004715857	0	0	0	0
0	0	0	0	0
0	0	0	0	0
0	0	0	0	0
0	0	0	0	0
0	0	0	0	0
0	0	0	0	0
0	0	0	0	0
0	10.33250293	1.780637818	0.470498076	2.976736383
0	0	0	0	0
0	0.001877186	0	0	0
0	0	0	0	0
0	0.001420774	0	0	0.011127652
0	0	0	0	0
0.196549734	0.467525499	0.032805679	0.088223112	0.030964382
0	0	0	0	0
0	0	0	0	0
0	0	0	0	0
0	0	0	0	0
0.037700848	3.636874232	0.032005582	0.012209935	0.001772605
0	0	0	0	0
0	0	0	0	0

0	0	0	0	0
0	0	0	0	0
0	0	0	0	0
0	0	0	0	0.010066379
0	0.00165929	0	0	0
0	0	0	0	0
0	0	0	0	0
0	0	0	0	0
0	0	0	0	0
0	0.006062439	0.147970773	0.033869969	0.004430339
0	0	0	0	0.009751148
0	0	0	0	0
0.001955592	0.004182723	0	0	0.003012371
0	0	0	0	0
0	0	0	0	0
0	0	0	0	0
0	0	0	0	0
0	0	0	0	0
0	0	0	0	0
0.11537793	1.176442218	0.579960117	1.158611341	0.262089536
0	0	0	0	0
0	0	0	0	0
0	0	0	0	0
0	0	0	0	0
0	0	0	0	0
0	0	0	0	0.008708846
0	0	0	0	0
0	0	0	0	0
0	0	0	0	0
0	0	0.035847119	0	0
0	0	0	0	0
0	0	0	0	0
0	0	0	0	0
0	0	0	0	0
0	0	0	0	0
0	0	0	0	0
0	0	0	0	0
0	0	0	0	0
0	0	0	0	0
0	0	0	0	0
0	0	0	0	0
0	0	0	0	0
0	0	0	0	0.001253665
0	0	0	0	0
0	0.022145778	0.004650305	0.005854408	0
0	0	0	0	0.005948769
0	0	0	0	0
0	0	0	0	0

0	0	0	0	0
0	0	0	0	0
0	0	0	0	0
0	0	0	0	0
0	0	0	0	0
0.002440958	0.054601365	0	0	0.00340752
0	0	0	0	0.005772144
0	0	0	0	0
0	0	0	0	0
0	0	0	0	0.004833968
0	0	0	0	0
0	0	0	0	0
0	0	0	0	0
0	0	0	0	0
0	0	0	0	0
0	0	0	0	0
0	0	0	0	0.017103302
0	0	0	0	0
0	0	0	0	0
0	0	0	0	0
0	0	0	0	0
0	0	0	0	0
0	0	0	0	0
0	0	0	0	0
0	0	0	0	0
0	0	0.000987312	0	0
0	0	0	0	0
0	1.054811665	0	0	0
0	0	0	0	0
0.008510401	0	0	0	0
0	0	0	0	0
0	0	0	0	0
0	0	0	0	0
0	0	0	0	0
0	0	0	0	0
0	0	0	0	0
0	0	0	0	0
0	0	0	0	0
0	0	0	0	0.089565894
0	0	0	0	0
0	0	0	0	0
0	0	0	0	0
0	0	0	0	0
0	0	0	0	0
0	0	0	0	0
0	0	0	0	0
0	0	0	0	0
0	0	0	0	0

0	0	0	0	0
0	0	0	0	0.008419296
0	0	0	0	0
0	0	0	0	0
0	0	0	0	0
0	0.00233784	0	0	0
0	0	0	0	0
0	0	0	0	0
0	0	0	0	0
0	0	0	0	0
0	0.004464701	0	0	0.000689025
0	0	0	0	0
0	0.421864771	0	0	0.00697815
0	0	0	0	0
0	0	0	0	0
0	0	0	0	0
0	0	0	0	0
0	0	0	0	0
0	0	0	0	0
0	0	0	0	0
0.001037689	0.009386478	0.003756696	0.005517653	0
0	0.012014343	0	0	0
0	0	0	0	0
2.401351197	6.594107656	0	0.080642355	0.27894043
0	0	0	0	0
0	0	0	0	0
0	0	0	0	0
0	0	0	0	0
0	0	0	0	0
0	0	0	0	0
0	0	0	0	0.000857976
0	0	0	0	0
0.001637324	0	0	0	0
0	0	0	0	0

PHL018-0263_V1	PHL018-0233_V1	PHL018-0080_V2	PHL018-0024_V3	PHL018-0253_V1
REF	REF	CTRL	EYCF	REF
0.062998175	0.073284629	0	0.120919577	0.66265759
0.204649623	0.12193644	1.868074331	0.420934241	1.10608701
7.20893639	0	0	1.663642814	0
0	0	0	0	0
0	0	0	0	0
0	0	0	0	0
0	0	0	0	0
0	0	0	0	0
0	0	0	0	0
0	0	0	0	0
0	0	0	0	0
0	0	0	0	0
0	0	0	0	0
4.321911908	0.038619667	2.380214082	3.747853502	1.025350406
0	0	0	0	0
0	0	0	0	0
0	0	0	0	0
0	0	0	0	0
0	0	0	0	0
0	0	0	0	0
0	0	0	0	0
0	0	0	0	0
0	0	0	0	0
0	0	0	0	0
0	0	0	0	0
0	0	0	0	0
0	0	0	0	0
0	0.021686591	0	0	0
0	0	0	0	0
0	0	0	0	0
0	0	0	0	0
0	0	0	0	0
0	0	0	0	0
0	0	0	0	0
0	0	0	0	0
2.166828924	0.074013453	18.33607364	0.071017163	2.365185956
0	0	0	0	0
0	0	0	0	0
0	0	0	0	0
0	0	0	0	0
0	0	0	0	0
0.19928894	0	0.006898281	0.001645248	0.023096542
4.213550263	5.967211539	0.031360968	0	1.863475386
0.044030328	0	0.049146371	0.046705637	0.078364025
0.022266152	0.002630072	0.002249701	0	0
0	0	0	0	0

0	0	0.002368975	0	0
0.002146251	0	0	0	0.021755425
0.007763864	0	0	0.02437636	0.020129612
0.521026168	0.209152066	0.686862573	0.768957715	0.636686595
0	0	0	0.03026399	0.013541224
0	0	0	0	0
0.128536823	0	0.001074983	0.00162644	0.017488729
0	0	0	0	0
0	0	0	0	0
0	0	0	0	0
0.007926205	0	0	0	0
0.093958657	0.343766666	0.18340186	0.013937819	0.600459827
0	0	0	0	0
0.013797807	0	0.006736352	0.003328016	0
0.001412738	0	0	0	0
0	0	0	0	0
0.859659532	0.310437378	0	0	0.005450883
0	0	0	0	0
0	0	0	0	0
0	0	0	0	0
0.042216813	0.101763416	0.037828412	0.016704266	0.020797761
0.003882021	0.001607315	0.000763811	0.004457466	0.04339349
0	0.010015505	0	0	0.030529904
0	0	0	0	0
0.005415638	0	0	0	0
0	0	0	0	0
0	0	0.000684013	0	0
0	0	0	0	0
0.04208224	0	0	0	0.00504662
0	0	0	0	0
0.013427787	0	0	0.000935334	0
0	0	0	0	0
0	0	0	0	0
0	0	0	0	0.000547109
0	0	0	0	0
0	0	0	0	0
0	0	0	0	0
0	34.9308777	0.010188207	0	0
0	0	0	0	0
0	0	0	0	0
0	0	0	0	0
0	0	0	0	0
0	0	0	0	0
0	0	0	0	0
0	0	0	0	0
0	0	0	0	0
0	0	0	0	0
0.007637768	0	0	0.001834753	0

0	0	0	0	0
0	0	0	0	0
0	0	0	0	0
0	0	0	0	0
0	0	0	0	0
0.000753396	0	0	0	0
0	0	0	0	0
0	0	0	0	0
0.020143804	0	0	0	0
0	0	0	0	0
0	0	0	0	0
0.016966718	0	0	0.003519977	0
0	0	0	0	0
0	0	0	0	0
0	0	0	0	0
0	0	0	0	0
0	0	0	0	0
0	0	0	0	0
0	0.10201958	0.014302634	0.377067895	0.405393107
0	0	0	0	0
0	0	0	0	0
0	0	0	0	0
0	0	0	0	0
0	0	0	0	0
0	0	0	0	0
0	0	0	0	0
0	0	0	0	0
0	0	0	0	0
0	0	0	0	0
0	0	0	0	0
0.011330493	0	0	0	1.18979556
0.128836208	0.002010963	0.000537541	0	0
0	0	0	0	0
0	0	0	0	0
0	0	0	0	0
0	0	0	0	0
0	0	0	0	0
0	0	0	0	0
0.310606408	0	0	0	0
0	0	0	0	0
0	0	0	0	0
0	0	0	0	0
0.004210374	0	0.00103552	0	0.004064605
0	0	0	0	0
0	0	0	0	0
0	0	0	0	0
0	0	0	0	0
0	0	0	0	0

0	0	0	0	0
0	0	0	0	0
0	0	0	0	0
0	0	0	0	0
0	0	0.006706113	0.000404504	0.02158656
0	0	0	0	0
0	0	0	0	0
0	0	0	0	0
0	0	0	0	0
0	0	0	0	0
0	0	0	0	0
0	0	0	0	0
0	0	0	0	0
0	0	0	0	0
0	0	0	0	0
0	0	0	2.608980126	0
0	0	0	0	0
0	0	0	0	0
0	0	0	0	0
0	0	0	0	0
0	0	0	0	0
0	0	0	0	0
0	0	0	0	0
0	0	0	0	0
0	0	0	0.008900695	0
0	0	0	0	0
0	0	0	0	0
0	0	0	0	0
0	0	0	0	0
0	0	0	0	0
0	0	0	0	0
0.005530104	0	0	0	0.001281274
0	0	0	0	0
0	0	0	0	0
0	0	0	0	0
0	0	0	0	0
0	0	0	0	0
0	0	0	0	0
0	0	0	0	0
0	0	0	0	0
0	0	0	0	0
0	0	0	0	0
0	0	0	0	0
0	0	0	0	0
0	0	0	0	0
0	0	0	0	0
0	0	0	0	0
0	0	0	0	0

0	0	0	0.003427664	0
0	0	0	0	0
0	0	0	0	0
0	0	0	0	0
0	0	0	0	0
0	0	0	0	0
0	0	0	0	0
0	0	0	0	0
0	0	0	0	0
0	0	0	0	0
0	0	0	0	0
0	0	0	0	0
0	0	0	0	0
0	0	0	0	0
0.01492996	0.001003852	0.004293354	0	0
0	0	0	0	0
0	0	0	0	0
0	0	0	0	0
0	0	0	0	0
0	0	0	0	0
0.047097023	0.006572858	0.042167009	0.014070815	0.017957341
0	0	0	0	0
0	0	0	0	0
0.019397461	0	0	0	0
0	0	0	0	0
0	0	0	0	0
0.016134233	0.002820541	0.001292477	0.006145883	0.00556273
0	0	0	0	0
0	0	0	0	0
0	0	0	0	0
0	0	0	0	0
0	0	0	0	0
0	0	0	0	0
0.004595569	0	0	0	0.028953766
0.002575112	0.003376304	0.018290736	0.012692467	0.073087167
0	0	0	0	0
0	0	0	0	0
0	0	0	0	0
0.003244762	0	0	0	0
0	0	0	0	0
0	0	0	0	0
0.0108014	0	0	0	0
0.005888388	0	0	0	0
0	0	0	0	0
0.009397763	0	0	0	0
0	0	0	0	0
0	0	0	0	0
0.001284566	0	0	0	0.001594405

0	0	0	0	0
0	0	0	0	0
0	0	0	0	0
0	0	0	0	0
0	0	0	0	0
0	0	0	0	0
0.00156734	0	0	0	0
0.004484569	0	0	0	0
0	0	0	0	0
0.001886095	0	0	0	0
0.000681143	0	0	0	0
0	0	0	0	0
0	0	0	0	0
0	0	0	0	0
0.004340003	0	0	0	0
0	0	0	0	0.00070333
0	0	0	0	0
0	0.028044052	0	0	0
0.016609882	0.030505092	0	0	0.007206663
0	0	0	0	0.589657228
0	0.026950743	0	0.002446872	0
0	0	0	0	0
0	0	0	0	0
0	0	0	0	0
0.026252735	0	0.003066945	0	0
0.005041984	0	0	0	0
0	0	0	0	0
0	0	0	0	0
0.027924065	0	0	0	0
0	0	0	0	0
0.121454643	0.07891361	0.093835247	0.038273555	0.10357058
0	0	0	0	0
0	0	0	0.025218719	0
0	0	0	0	0
0	0	0	0	0
0	0	0	0	0
0	0	0	0	0
0	0	0	0	0
0	0	0	0	0
0.012738212	0	0.002078653	0	0
0	0	0	0	0
0.003507746	0.031562507	0.002121258	0	0.005577441
0	0.000756632	0	0	0
0	0	0	0	0
0	0	0	0	0
0	0	0	0	0
0	0	0	0	0
0	0	0	0	0

0	0	0	0	0
0	0	0	0	0
0.022800038	0.001132341	0.047944582	0.0385204	0.070284067
0	0	0	0	0
0	0	0	0	0
0	0	0	0	0
0	0	0	0	0
0	0	0	0	0
0	0	0	0	0
0	0.001864134	0	0	0
0	0	0	0	0
0	0	0	0	0
0	0	0	0	0
0.004295141	0	0	0	0.000829287
0.018670735	0	0.001546158	0.000954826	0
0	0.021011272	0	0	0.021495864
0.022620061	0	0.002768399	0	0
0	0	0	0	0
0	0	0	0	0
0	0	0	0	0
0	0	0	0	0
0.002187799	0	0	0	0.001173364
0	0	0	0	0
0.001507372	0	0	0	0
0.005067123	0	0	0	0
0	0	0	0	0
0	0	0	0	0
0	0	0	0	0
0	0	0	0	0
0.05415105	0	0	0	0
0.024961898	0	0	0	0
0	0	0	0	0
0.006037046	0	0	0	0
0.002242245	0	0	0	0
0	0	0	0	0
0.013109454	0	0	0	0.022967545
0	0	0	0	0
5.202454749	0.001067839	0.009704922	0	0.005601998
0	0	0	0	0
0	0	0	0	0
0	0	0	0	0
0	0	0	0	0
0	0	0	0	0
0	0	0	0	0
0	0	0	0	0
0.374718588	0	0	0	0
0.023836298	0	0	0	0
0	0	0	0	0

0.268023511	0	0	0.007882342	0.02236642
0	0	0	0	0
0.003564632	0	0	0	0
0	0	0	0	0
0.069677921	0.001791309	0.002633543	0.002690851	0.011623095
0.131995255	0	0	0	0
0	0	0	0	0
0.012567695	0.006591148	0.017115091	0.011605601	0.005199679
0.00830111	0	0	0	0
0.049964639	0.00097052	0.24143813	0.086725548	0.071458953
0	0	0	0	0
0.346496646	0	0.014610984	0	0.000823217
0.032557285	0.000753851	0.004836196	0.006097606	0.003746635
0.005894239	0	0	0.003704276	0.055637243
0	0	0	0	0.001712591
0	0	0	0	0
0.131102602	0	0	0	0
0	0	0	0.005672383	0
0.613643443	0	0	0.131134263	0
0	0	0	0	0
0	0	0	0	0
0	0	0	0	0
0.000889931	0	0	0	0
0	0	0	0	0
0	0	0	0	0
0.00251132	0	0.001173528	0	0
0.022835382	5.477576579	0	0	0.00842889
0	0	0	0	0
0.004628214	0	0	0	0
0	0	0	0	0
0.001347392	0	0	0	0
0	0	0	0	0
0	0	0	0	0
0.024632591	0	0	0	0
0	0	0	0	0
0	0	0	0.002675016	0
0.007686465	0	0.00186244	0.001150146	0
0	0	0	0	0
0	0	0	0	0
0	0	0	0	0
0	0	0	0	0
0	0	0	0	0
0	0	0	0	0
0	0	0	0	0
0	0	0	0	0
0	0	0	0	0
0	0	0.002626875	0	0.007537254
0	0	0	0	0

0	0	0	0	0
0.009772558	0	0	0.001692082	0
0.014475291	0	0	0	0
0	0	0	0	0
0	0	0	0	0
0.003935617	0	0	0	0.097833533
0	0	0	0.003757034	0.071027431
0	0	0	0	0
0	0	0	0	0
0.000962622	0	0	0	0
0.060196364	0	0	0.016366892	0.002478723
0	0	0	0	0
0	0	0	0	0
0.006199154	0	0	0	0.002341773
0	0	0.006881659	0.008373589	0
0	0	0	0	0
0	0	0	0	0
0	0	0	0.005086521	0
0	0.004513508	0	0	0
0.009535495	0	0	0	0
0	0	0	0	0
0.450697316	0.061529439	0.188821645	0.342010244	0.239463249
0	0	0	0	0
0	0	0	0	0
0	0	0	0	0
0	0	0	0	0
0.003799122	0	0	0.036364759	0
0	0	0	0	0
0	0	0	0	0
0.134719628	0.282132249	0.162830792	1.341063205	0.873011482
0	0	0.005810372	0.001329738	0
0.021821265	0	0	0	0
0	0	0	0	0
0	0	0	0	0
0.000973634	0	0	0.001180068	0
0	0	0	0	0
0.008086545	0.060838177	0	0.987343035	0.963554698
0.02802157	0	0	0	0
0	0	0	0	0
0	0	0	0.031696291	0
0.033901999	0	0	0.011279621	0
0.857182113	7.039427175	2.707189696	1.32826024	5.478313007
0.016609722	0	0	0	0
0	0	0	0	0
0	0	0	0	0
0	0	0	0	0
0.006976977	0	0	0	0
0	0	0	0	0

0.01692989	0	0	0	0
0	0	0	0	0
1.961585073	0.021188925	0	0	0.010568631
0	0	0	0	0
0	0	0	0	0
0.001455685	0	0	0	0.006558007
0.002446745	0	0	0	0
0	0	0	0	0
0.015129396	0	0	0.001358311	0
0.034630745	0	0	0	0
0	0	0	0	0
0	0	0	0	0
0	0	0	0	0
0	0	0	0.020765856	0
0	0	0	0	0
0.001014341	0.005142404	0.00151679	0.041144119	0.136112011
0.036651895	0.019064909	0.013239477	0.001090135	0.006946347
0	0	0	0	0
0	0	0	0	0
0	0	0	0	0
0	0	0.006886083	0	0
0	0	0	0.000843	0
0	0	0	0	0
0	0	0	0	0
0	0	0	0.011207198	0
0	0	0	0	0
0.002569134	0	0	0.000691967	0
0	0	0	0.058825462	0
0	0	0	0	0
0	0	0	0	0
0.009986188	0	0	0.006665697	0
0.019419366	0	0.007864635	0	0.008436168
0.165176096	0.00754214	0.122691212	0.422058042	0.030642014
0.0940671	0	0	0	0
0	0	0	0	0
0	0	0	0	0
0.001671611	0	0	0	0
0.007190025	0	0.010385038	0.02640753	0.005468742
0.043053737	0.049799623	0	0.137452296	0.084220079
0	0	0.000832434	0	0
0	0	0	0	0
0	0	0	0	0
0.050830807	0	0	0.001316414	0.001572788
0.018879084	0	0.026863716	0.027485776	0
0	0	0	0	0
0	0	0.001775771	0	0
0	0	0	0	0
0	0	0	0	0

0	0	0	0	0
4.202160017	0.010899256	0.4167588	0.397498939	0.65948919
0	0	0	0	0
0	0	0	0	0
0	0	0	0	0
0	0	0	0	0
0	0	0	0	0
0	0	0	0	0
0.002989547	0	0	0	0
0	0	0	0.001743239	0
0.000661862	0	0.00061857	0.000891325	0.005324561
0.123232756	0.003734156	0.011670782	0.093466854	0.010786275
0	0.438498918	0	0	0
0	0	0	0	0
0.000759404	0	0	0	0
0	0	0	0	0
0.050824761	0	0.01597739	0.001306685	0.0066907
0.012595716	0	0.012052121	0.143939533	0
0	0	0	0	0
0.018889595	0	0	0.002360273	0
0.099346744	0.058099978	0.144059969	0.146109828	0.068998001
0	0	0	0	0
0	0	0	0	0
0	0	0	0	0
0.004403193	0	0	0	0
0	0	0	0	0
0	0.0065775	0	0.010070538	0.198184021
0.185100635	0.005975971	0.031474847	0.094730885	0.070156852
0	0.003668247	0	0	0
0	0	0	0	0.001179451
0.026083257	0	0.001269644	0	0
0.00052427	0	0	0	0
0	0	0	0	0
0	0	0	0	0
0	0	0.002268493	0	0
0.027427352	0.432653343	0.471413282	0.181449283	0.167554691
0	0	0	0	0
0	0	0	0	0
0.183361084	0	0.003795193	0.002019199	0.007237323
0	0	0	0	0
0	0	0	0	0
0	0	0	0	0
0	0	0	0.005519215	0.002014861
0	0	0	0	0.00442116
0	0	0	0.008361917	0
0	0.541153273	0.206786189	0	0
0	0	0	0	0
0	0	0	0	0

0	0	0	0	0
0	0	0	0	0
0	0.010943197	0	0.000562002	0
0	0.048096807	0.128668494	0.278374151	0.423681627
0	0	0	0	0
0	0	0	0	0
0	0	0	0	0.001323146
0	0	0	0	0.008969963
0	0.662280124	0.38160607	0.139219824	0.495313244
0	0.038325507	0	0.030162173	0.007054726
0	0	0	0	0
0.001058637	0	0.006529987	0.001368633	0.001226382
0	0	0	0	0
0	0	0	0	0.076053031
0.022538615	0	0.002286057	0.022729164	0.256039491
0	0	0	0	0
0	0	0	0	0.642220538
0	1.024657916	0.00341171	0.057702553	0
0	0	0	0	0
0	0	0	0	0
0	0	0	0	0
0	0	0	0	0
0	0	0	0	0
0.110740191	0	0.003303088	0.065138081	0.574499029
0	0	0	0	0
0.314005531	0.092144053	0	1.808075611	0.062322976
0	0	0	0	0
7.038499208	0.295476592	0	6.985878267	2.08771767
0	0	0	0	0
0	0	0	0	0
0	0.000539753	0	0	0.059413927
0.000775739	0	0	0	0
0.008302747	0	0	0	0.000907392
0	0	0	0	0
0	0	0	0	0
0	0.022424847	0.006361273	0.00973032	0
0	0.190714515	0.088054579	0.026226285	3.505697
0	0	0	0	0
0	0	0	0.002137121	0
0	0	0	0.011755572	0
0.418415957	0.006391425	0.032081094	0.02084322	0.149022649
0	0	0	0	0
4.242865968	0.009584124	19.06703082	18.69314848	0.408585734
0	0	0	0.001286728	0
0	0	0	0	0
0	0	0	0	0
0	0	0	0	0
0.022707529	0.002561075	0	0	0.421220164

0	0	0	0	0
0.076742847	0.027415243	0.098333479	0	0.029849103
0.021533669	0.00777442	0	0	0.004519162
0	0.001548064	0	0	0
0	0	0	0	0
0	0	0.002929512	0	0.018156108
0	0	0	0	0
0	0.019762759	0	0	0
0.004580083	0	0	0	0
0	0	0	0	0
0.013558613	0.009039199	0.000644326	0.004642194	0.292177451
2.051774525	0.211108116	3.278064412	2.49263346	0.67617515
0	0	0	0	0
0.180804453	0.014091237	0.022947651	0	0.007661772
0.039480288	0	0.000547989	0.021793556	0.019811434
0	3.016132276	0	0	0
0	0	0	0	0
0	0	0	0	0
0	0	0	0	0
0.003596709	0	0	0.00642423	0.132856805
2.716023322	0	0.005220391	0.081552061	0.102826814
0.493379728	0.000532196	1.023504449	0.923425663	0
2.11690028	0.015901968	9.415080456	10.6927658	0.921697017
0	0	0	0	0
0	0	0	0	0
0.00161565	1.441268022	0.012511189	2.925560562	1.731504813
0.05736289	0	0	0	0
0	0	0.007322281	0.002759493	0
0	0	0	0	0
0	0	0	0	0
0	0	0	0	0.047581108
0	0.209488617	0.098229092	0.075375165	0.266652882
0.145622967	0	0	0	0.023923883
0.004139582	0.009753815	0	0.043337613	0.229177047
0.089414656	0	0	0.000629342	0
0.049653768	0.206344984	0.460058981	0.032857756	0.00206615
0.00082698	0	0	0	0.011176888
0	0	0	0	0
0.030805634	0.012259344	0.065539711	0.010118476	0.220262764
0	0	0	0	0
0	0	0	0	0
0	0	0	0	0
0	0	0	0	0
0	0	0	0	0
0.00219664	0.008960242	0	0.029187579	0.538865179
0	0	0	0	0
0	0	0	0	0
0.132192962	0.007205667	0.32056003	0.003092612	0.009379369

0	0	0	0	0
0	0	0	0	0
0	0	0	0	0
0	0.00090914	0	0	0.002409826
0	0	0	0	0
0	0	0	0	0.156610554
0	1.011061764	0	0	0
0.006891004	0	0	0	0.200866351
0	0	0	0	0
0	0	0	0	0
0.004865281	0.017415714	0	0.002059214	0.004920497
0	0	0	0	0
0	0.051952373	0	0	0
0	0.099363533	0	0	0
0	0	0	0	0
0	0	0	0	0
0.0249848	0	0.321915673	0	0
0	0.000842918	0	0.000779204	0
0.003247179	0	0	0	0
0.001565418	0	0	0	0
0	0	0	0	0
0	0	0	0	0
0	0.190088736	0	0	0.002146649
0	0	0	0	0
0	0	0	0	0
0	0	0	0	0
0	0	0	0	0
0	0	0	0	0
0	0	0	0	0
0.003727732	0	0.713764789	6.999293012	0.00607276
0	0	0	0	0.011274006
0	0	0	0	0
0	0	0	0	0
0	0	0	0	0
0	0	0	0	0
0	0	0	0	0
0	0.03785007	0	0.104699138	0.059101164
0	0	0	0	0
0	0	0	0	0
0	0	0	0	0
0.003312184	0	0	0	0
0	0	0	0	0
0	0	0	0	0.003841869
0	0	0	0	0.008273838
0.134952335	0.053918987	0.286591412	0	0.122325701
0	0	0	0	0
0	0	0	0	0.030042695
0	0	0	0	0

0	0	0	0	0
0	0	0	0	0
0	0	0	0	0
0	0	0	0.064313177	0.664425012
0	0	0	0	0
0	0.235449345	0	0	0
0	0	0	0	0
0	0	0	0	0
0.241271661	0.016313409	0.021487299	0.007649439	0.003394552
0	0	0	0	0
0	0	0	0	0
0	0	0	0	0
0	0	0	0	0
0	0	0	0	0
2.178381774	0	0	0.880947714	0.087255989
0.047322573	0	0	0.00050534	0.006641315
0	0	0	0	0
0	0	0	0	0
0	0	0	0	0
0	0	0	0	0
0	0	0	0	0.855157581
0	0	0	0	0
0	0	0	0	0
0	0	0	0	0
0.587814415	0.611111044	0.00148638	0	0.060799656
0.177484542	0.460693281	1.407138963	4.548677065	0.448319794
0	0.634942797	0	0	0.068526882
0	0	0	0	0.003431016
0	0.002430782	0.048342173	0	0
0	0	0	0	0
0	0	0	0	0
0	0	0	0	0
0	0	0	0	0
0.000849379	0	0	0.001372625	0.06133396
0.052061735	0	0	0.001809322	0.004593591
0.000989283	0.000518831	0	0	0.000764026
0	0	0	0	0
1.579092588	0.123223798	0	0.702954267	1.014340347
0.61671344	0.00065526	0	0	0
0	0	0	0	0
0	0	0	0	0
0.001121855	0	0	0	0
0.572112702	0.326136339	0.004548413	0.144843672	0.072300843
0	1.384777042	0	0.037099748	0
0	0	0	0	0
0	0	0	0.003332077	0.019905022
0	0	0	0	0
4.510077282	0	0.001395443	0.47244746	0.963728729

0	0	0	0.001439447	0
0	0	0	0	0
0	0	0	0	0
0	0	0	0	0
0	0	0	0	0.003210453
0	0	0.071632945	0	0
0	0	0	0	0
0	0	0	0	0
0	0	0	0	0
0	0	0	0	0
0	0	0	0	0.001310165
0	0.186471407	0.206834258	0.599929829	1.019665352
0	0	0.0842984	0	0.135796182
0	0	0	0	0
1.134070117	0	0.052718992	0.057181049	0.442576296
0	0	0	0	0
0	0	0	0	0.032888292
0	0	0	0	0
0	0.002357266	0	0	0
0	0	0	0	0.002886829
0.095077172	0	0	0	0
0	0	0	0	0
0.0120189	0.47924422	0.065354196	0.347140774	0.879702379
0	0	0	0	0
0	0	0	0	0
0	0	0	0	0
0	0	0	0	0
0	0	0	0	0
0	0	0	0	0
0	0	0	0	0
0	0	0	0	0
0.439854049	0	0.034205132	0.021238522	0.050107116
0	0	0	0	0
0	0	0	0.00349203	0.286270834
0.00086127	0	0	0	0
0.002775705	0.00485241	0.020753185	0.008074133	0.019025204
0	0	0	0	0
0	0.009178403	0.016163801	0	0.019478992
0	0	0	0.000599632	0
0	0	0	0	0
0	0.53333173	0	0	0
0	0	0	0	0.102517654
0	0	0	0	0
0	0	0	0	0.024875285
0.008908596	0.009270094	0	0.011311598	0.115692731
0	0	0	0	0
0	0	0	0.387037811	0.872625562
0	0	0	0	0

0	0	0	0	0
0	0	0	0	0
0.004606859	0	0	0	0
0	0	0	0	0
0	0	0	0	0
0.01453186	0	0	0	0
0	0.050447296	0.074251147	0	0
0	0.003440744	0	0.003348071	0.00460013
0	0	0	0	0
0	0	0.00743092	0	0.009840659
0	0	0	0	0
0	0	0.004797986	0	0
0	0	0	0	0
0	0.047481759	0	0.167875478	0.469414322
0	0	0	0	0
0	0	0	0	0
0	0	0	0	0
0	0	0	0	0
0	0.025611655	0	1.929477959	0.604654887
0	0	0	0	0
0	0.002439133	0	0	0
0	0	0	0	0
0	0	0	0	0
0	0	0	0	0
0	0	0	0	0
0.002567056	0	0	0.006382224	0
0	0	0	0	0
0.930062371	0	0.013367175	0.005466062	0.22222652
0	0	0	0	0
0.23834769	0.063837413	1.884276547	1.134893069	1.119915134
0	0	0	0	0
0	0	0	0	0
0	0	0	0	0.018716994
0	0	0	0	0
0.00413864	0.000904382	0	0	0.005793264
0	0	0	0	0
0	0.494935736	0	0	0
0.000939229	0	0	0	0.002040101
0	0	0	0	0
0	0	0	0	0
1.902887651	3.868392232	19.59053188	7.824105931	3.369355768
0.190099215	0.096302465	0	0.76398866	1.196349659
0	0	0.318826784	0	0
0	0	0	0	0
0	0	0	0	0
0	0	0	0	0
0	0	0	0	0
0	0.000814456	0	0.003764467	0.028244942

0	0	0	0	0
0.001150095	0	0	0.002942769	0.007031756
0	0	0	0	0.025934823
0	0	0	0	0
0.315028044	1.338537979	0.033093977	0.997894888	1.334025024
0	0	0	0	0
0	0	0	0	0
0	0	0	0	0
0.115605551	0	0.014489327	0.145220328	0
0	0	0	0	0
0	0	0	0	0
0	0	0	0	0
0	0	0	0.151096808	0.159126476
0.001144891	0.005403959	0.015408064	0.020166971	0.010610431
1.264592078	0.800602016	0.001804047	0	0
0.018601887	0.144530215	0.029502073	0.210580585	0.172685715
0.00079258	0	0.003148138	0	0.069436471
0	0	0	0	0
0	0	0	0	0
0	0	0	0	0
0	0	0	0	0
0	0	0	0	0
0	0	0	0	0
0	0	0	0	0
0	0	0	0	0
0	0	0	0	0
0.101137349	0.007981849	0	0.002645131	0.067197404
0	0	0	0.000785456	0
0	0	0	0	0
0	0	0	0	0
0	0	0	0	0
0	0	0	0	0
0	0	0	0.905923307	0
0	0	0	0.009551068	0.023412538
0	0.322145768	0.034917562	0	0
0.002248989	0	0	0	0
0.002298738	0	0.007519327	0	0.078779915
0	0	0	0	0
0	0	0	0	0
0	0	0	0	0
2.239078945	0.010834253	1.776244821	0.014466575	0.356315528
0	0.007324761	0	0	0
0	0	0	0	0
0	0	0.008450602	0	0
0	0	0	0	0
0	0	0	0	0
0	0	0	0	0
0	0	0	0	0

0	0.09786984	0.036406715	0	0.716569912
0	0	0	0	0
0	0	0	0	0
0.001541646	0	0	0	0
0	0.026891615	0	0.243651763	0.032751666
0	0	0	0	0
0.073728411	0	0.012192462	0.015492369	0.049422359
0	0	0	0	0
0	0	0	0	0
0.001659048	0	0	0.003351344	0
0	0	0	0	0.012727022
0	0	0	0	0.083270937
0	0	0	0	0
0	0	0	0	0
0	0	0	0	0
0	0	0	0	0
0	0	0	1.652632156	0
0.002090358	0	0	0	0
0	0	0	0	0
0	0	0	0	0
0.074521845	0	0.056808378	0.024785409	0.186757992
0	0	0	0	0
0.002283259	0.001088599	0	0.00201263	0.004608802
0	0	0	0.002833614	0.014789136
0	0	0	0	0
0	0	0	0	0
0	0	0	0	0
0	0	0	0	0
0	0	0.145892625	0.075780074	0
0.031080247	0	0.477170331	0.070617862	0
0	0	0	0	0
0	0	0	0	0
0	0	0	0	0
0	0	0	0.560311723	0
0	0	0	0	0
0	0	0	0	0
0.176444296	0	0	0.001679488	0.031882177
0	0	0	0	0
0	0.002418245	0	0	0
0	0	0	0	0
0	0	0	0	0
0	0	0	0	0
0	0	0	0	0
0	0	0	0	0
0	0	0	0	0
0	0	0	0	0
0	0	0	0	0
0	0.002693767	0	0	0

0.052630746	0	0	0.000641909	0.001342114
0	0.007079553	0.068126433	0	0
0	0.031977343	0	0	0
0	0	0	0	0
0	0	0	0	0
0	0	0	0	0
0	0	0	0	0
0.281007382	0.162917931	0.103487952	0.35195718	1.671374873
5.608565979	7.945265973	0.035836294	0	2.383824126
0.031685801	0	0	0	0.009637565
0.002546762	0	0	0	0.326254854
0	0	0	0	0
0	0	0	0	0
0.028387011	0	0	0	0
0	0	0	0	0
0	0	0	0	0
0.028411393	0	0	0	0.081443628
0	0.082130849	0	0	0
0	0	0	0	0
0.001079779	0	0	0.002617437	0
0	0	0	0	0
0	0	0	0	0
0	0	0	0	0
0	0	0	0	0
0	0	0	0.051064854	0
0	0	0	0	0
0	0	0	0	0
0	0	0	0	0
0	0	0	0	0
0	0	0	0	0
0.018363069	0	0	0	0.001772731
0	0	0	0	0
0	0	0	0	0
0	0	0	0	0
0	0	0	0	0
0	0	0	0	0
0	0	0	0	0
0	0	0	0	0
0	0	0	0	0.001595878
0	0	0	0	0
0	0	0	0	0
0.469038459	0.018237225	0.001338209	0.504108835	0.429300711
0	0	0	0	0
0	0	0	0	0
0	0	0	0	0
0	0	0	0	0
0	0	0	0	0
0	0	0	0	0

0	0	0	0	0
0	0	0.001675264	0	0
0	0	0	0	0
0	0	0	0	0
0.255380032	0.091567892	0.168173921	0.185898337	0.868328149
0	0	0	0	0
0	0	0	0	0
0.020211357	0.000662492	0.015111477	0.003266221	0.003414532
0	0	0.00424357	0	0.001217601
0	0	0	0	0
0	0	0	0	0
0	0	0.016580898	0	0
0	0	0	0	0
0	0	0	0	0
0.03112393	0	0	0	0
0.007230778	0	0	0	0.922687044
0	0	0	0	0
0	0	0	0	0
0	0	0	0	0
0	0	0	0	0
0	0	0	0	0
0	0	0	0	0
0	0	0	0	0
0.000655922	0	0	0.001766653	0.00189964
0	0.050160359	0	0.136748875	0.067793696
0	0	0	0.002576226	0
1.420199996	0	0.000747567	0	0.455980636
0	0	0	0.000850695	0
0	0	0.00861154	0	0
0	0	0	0	0
0	0	0	0	0
0	0	0	0	0
0	0	0	0	0
0	0	0	0	0
0	0	0	0	0
0	0	0	0	0
0	0	0	0	0
0	0	0	0	0.716103008
0	0	0	0	0
0	0.000768937	0	0	0
0	0	0	0	0
0	0.349635254	0.016263346	0	0
0	0	0	0	0
0	0	0	0	0
0	0	0	0	0
0	0	0	0	0
0	0	0	0	0
0	0	0	0	0

0	0.403710813	0	0.065947394	0.040666194
0	0	0	0	0
0	0	0	0	0
0	0	0	0	0
0	0	0	0	0
0	0.014663933	0	0.007569969	0
0	0	0	0	0
0	0	0	0	0
0	0	0	0	0
0	0	0	0	0
0	0	0	0	0
0	0	0	0	0.024321743
0	0	0	0	0
0	0	0	0	0
0	0	0	0	0
0	0	0	0	0
0	0	0	0	0
0	0	0	0	0
0	0	0	0	0
0	0	0	0	0
0.028957668	0	0	0.029027923	0
0	0	0	0	0
0.001924812	0.006008754	0.001027949	0.034438326	0.046719759
0	0.002419791	0	0	0.318759034
0	0	0	0	0
0	0.014184282	0	0	0
0	0	0	0	0
0	0	0	0	0
0	0	0	0.10117071	0
0	0	0	0	0
0	0	0	0	0
0	0	0	0	0
0.001287556	0	0.04067282	0	0.08949449
0	0	0	0	0
0	0	0.5534746	0	0.830543609
0.018804417	0	0	0	0
0	0	0	0	0
0	0	0	0	0
0.009560564	0	0	0	0.002076652
0	0	0	0	0
0	0	0	0	0
0	0	0	0	0
0	0.087455382	0.031343828	0.013323595	0.475393139
0	0	0	0	0
0	0	0	0	0
0	0.006014237	0	0.010581245	0.013499041
0	0	0	0	0
0	0	0	0	0

0	0	0	0	0
0	0	0	0	0
0	0	0	0	0
0	0	0	0	0
0	0	0	0	0.003369604
0	0	0	0	0
0	0	0	0	0
0.009651543	0	0	0	0
0.001247707	0	0	0	0.52516614
0	0.004396166	0.009400942	0	0
0	0	0	0	0
0	0	0	0	0
0	0	0	0	0
0	0.226694686	0	0	0
0	0	0	0	0
0	0.010026929	0	0	0
0	0	0	0	0
0	0	0	0	0
0	0	0	0	0
0	0.108145573	0.038191822	0	0.872807701
0.003053978	1.123833923	0	0	0
0	0	0	0	0
0	0	0	0	0
0	0	0	0	0
0	0	0	0	0.00997803
0	0	0	0	0
0	0	0	0	0
0	0	0	0	0
0	0	0	0	0
0	0.017727772	0	0	0.063238958
0.213345319	0.088207501	0.138565326	0.187158238	0.298084628
0	0	0	0	0
0	0	0	0	0
0	0	0	0	0
0	0	0	0	0
0	0	0	0	0
0	0	0	0	0
0	0	0	0	0
0	0	0	0.033629678	0.011536576
0	0	0	0	0
0	0	0	0	0
0	0	0	0	5.570927114
0	0	0	0	0
0	0	0	0.002523692	0
0	0	0	0	0
0	0	0	0	0
0.0016118	0	0	0.023442493	0
0	0	0	0	0

0	0	0	0	0
0	0	0	0	0
0	0	0	0	0
0	0	0	0	0
0.038876364	0.217269731	0	0.160816509	0.10113283
0	0	0	0	0
0	0	0	0	0.031916112
0	0	0	0	0
0	0	0	0	0
0	0	0	0	0
0	0	0	0.002470644	0
0.050152994	0.002713783	0	0.004631364	0.039194413
0	0	0	0.010322225	0.154355085
0	0	0	0	0
0	0	0	0.005735312	0.00245979
0	0	0.00329786	0	0.839074776
0	0	0	0.002523132	0
0	0	0	0	0
0.068357042	0	0.001014061	0.00467586	0.001920356
1.570672152	0	0	0	0.009345764
0	0	0	0	0
0	0	0	0	0
0	0	0	0	0
0	0	0	0	0
0	0	0	0	0
0	0	0	0	0
0.002081354	0	0	0.023544749	0.001461305
0.000956898	0	0	0	0
0	0	0	0	0
0	0	0	0	0
0	0	0	0.000866533	0
0.004170019	0	0	0	0
0	0	0	0	0
0	0	0	0	0
0	0	0	0	0
0	0	0	0	0
0	0	0	0	0
0.013562122	0.017386543	0	0.002435203	0.041896263
0	0.003960691	0	0	0
0	0	0	0	0
0	0.000657476	0	0.000364668	0
0	0	0	0	0
0	0	0	0	0
0	0	0	0	0
0	0	0	0	0
0	0	0	0	0.004821336
0	0	0	0	0
0	0	0	0	0

0	0	0	0	0
0	0	0.000535487	0.227932773	0.379998489
0	0.079676061	0	0	0
0	0	0	0	0
0.003475191	0	0.000608978	0.000702003	0
0.008970136	0	0	0	0.001082447
0	0.000993203	0	0	0
0.009890105	0	0	0	0
0	0	0	0	0
0	0	0	0	0
0	0	0	0	0
0	0	0	0	0
0.054448528	0.078208732	0.131406596	0.00737726	0.013140861
0	0	0	0	0
0	0	0	0	0
0	0	0	0	0
0	0	0	0	0
0	0	0	0	0
0	0	0	0	0
0	0	0	0	0
0	0	0	0	0
0	0	0	0	0
0.003934546	0	0	0.000706484	0.001266109
0.010059615	0.005861979	0	0.008336751	0.018426639
0	0	0	0	0
0	0	0	0	0
0	0.012595537	0	0	0
0	0	0	0	0
0	0	0	0	0
0	0	0	0	0
0.004630723	0.00404765	0.052617311	0.017329976	0.027057691
0	0	0	0	0.098433194
0	0	0	0	0
0	0	0	0	0
0	0	0	0	0.826506991
0	0	0	0	0
0	0	0	0	0
0	0	0	0	0
0.031343304	0	0.000770873	0	0.035035758
0	0	0	0	0
0	0	0	0	0
0	0	0	0	0
0	0	0	0	0
0	0	0	0	0
0	0	0	0	0
0.053883869	0.060895854	0.251796811	0.353003387	0.187230973
0	0	0	0	0
0	0	0	0	0

0	0	0	0	0
0	0	0	0	0
0	0.039689323	0.181399231	0.13051772	0.214861463
0	0	0	0	0
0	0	0	0	0
0	0	0	0	0
0	0	0	0	0
0	0	0	0	0
0	0	0	0	0
0.002353345	0	0	0	0
0	0	0	0	0.004198909
0	0	0	0	0
0	0	0	0	0
0	0	0	0	0
0	0	0	0	0
0	0	0	0	0
0.012295326	0	0	0	0
0	0	0	0	0
0	0	0	0	0
0	0	0	0	0
0	0	0	0	0
0	0	0	0	0
0	0	0	0	0
0	0	0	0	0
0	0	0	0	0
0	0	0	0	0
0	0	0	0	0
0	0.003004452	0	0.014878678	0.01516913
0	0	0	0	0
0	0	0	0	0
0	0.001166173	0	0.000616014	0.001287972
0	0	0	0	0
0.032309773	0.004062137	0.043846847	0.265002107	0.024995659
0.020126853	0.000988348	0.00253623	0.001827283	0.021394868
0	0	0	0	0
0	0	0	0	0
0	0	0	0	0
0	0	0	0	0
0	0	0	0	0
0	0	0	0	0
0	0	0	0	0
0.036048148	0	0	0.004266147	0.15712777
0	0.017060919	0.018449179	0.03136344	0.072373561
0	0	0	0	0
0	0	0	0	0
0.006730907	0.208720338	0.009286188	0.005179697	0.040224934
0	0	0	0	0.053556682
0	0	0	0	0

0	0	0	0	0
0.013942024	0.031278757	0.004994866	0.007322498	0.00919721
0.038814387	0	0.006595557	0.007895493	0.007336897
0	0	0	0	0
0	0.009092867	0	0.109272302	6.393343347
0	0	0	0	0
0	0	0	0	0
0	0	0	0	0
0	0.057592368	0	0	0
0	0	0	0	0
0	0	0	0	0
0	0	0	0	0
0	0	0	0	0
0.005931727	0	0	0.003318184	0
0	0	0	0	0
0	0	0	0	0
0	0	0	0	0
0	0	0	0	0
0	0	0	0	0
0	0	0	0	0
0	0	0	0	0
0	0	0	0	0
0	0	0	0	0
0	0	0	0	0
0	0.296618175	0.00459941	0	0
0.002025295	0	0.002523762	0.000909151	0.002824144
0	0	0	0	0
0	0.016599941	0	0.019754735	0.037510322
0	0	0	0	0
0	0	0	0	0
0	0	0	0	0
0	0	0	0	0
0	0	0	0	0
0	0	0	0	0
0	0	0	0	0
0	0	0	0	0
0	0	0	0	0
0.001120006	0	0	0	0
0	4.122308252	1.307613021	0	14.11793768
0	0	0	0	0
0	0	0	0	0
0	0	0	0	0.001885391
0.045394834	0	0.001055363	0	0.001962233
0	0	0	0.007262629	0
0	0	0	0	0
0	0	0	0	0
0.008637464	0.027783584	0	0.002791684	0.049363465
0	0	0	0	0

0	0	0	0	0
0.003269667	0.003429565	0.017112484	0.047202736	0.070704858
0	0	0	0	0
0	0	0	0	0
0	0	0	0	0
0	0	0	0	0
0	0	0	0	0
0	0	0	0	0
0	0	0	0	0
0.002060261	0	0	0	0
0	0	0	0	0
0	0	0	0	0
0	0	0.001292101	0	0.162606818
0	0	0	0	0
0	0	0.004064131	0.009662712	0
0	0	0	0	0
0	0	0	0	0
0	0	0	0	0
0	0	0	0.001943538	0
0	0	0	0	0
0	0	0	0	0
0.803947394	0	0.00124728	0	0.000773022
0.00184592	0	0	0.001822985	0
0	0	0	0	0
0	0	0	0	0
0	0	0	0	0
0	0	0	0	0
0	0	0	0	0
0	0	0	0	0.000625571
0	0	0	0	0
0	0	0	0	0
0	0	0	0	0
0	0	0	0	0
0	0	0	0	0
3.36688227	4.410921947	0	0	0.080480007
0	0	0	0	0
0	0	0	0	0
0	0	0	0	0
0.745264657	0	0.002491121	0	0.00180123
0	0	0	0	0
0.004301899	0.072279975	0.270491308	0.144833509	0.278940572
0	0	0	0	0
0	0	0	0	0
0	0	0	0	0
0	0	0	0	0
0.052811038	0.001674247	0.792264609	0.009286173	0.008453081
0	0	0	0	0
0	0	0	0	0

0	0	0	0	0
0	0	0	0	0
0	0	0	0	0
0.003714839	0	0	0	0
0	0	0	0	0
0	0	0	0	0
0	0	0	0	0
0.130133462	0	0	0	0
0	0	0	0.00389427	0
0.036662959	0	0.001500651	0	0
0	0	0.002373976	0	0.041414612
0	0	0	0	0
0	0	0	0.000634056	0
0	0	0	0	0
0	0	0	0	0
0	0	0	0	0
0	0.000529306	0	0	0
0	0	0	0	0
0	0	0	0	0
0.964552072	0.007529509	1.369055046	0.997402697	0.080462114
0	0	0	0	0
0	0	0	0	0
0	0	0	0	0
0	0	0	0	0
0	0	0	0	0
0	0.016473819	0	0	0
0	0	0	0	0
0	0	0	0	0
0	0	0	0	0
0	0	0	0.009451766	0
0	0	0	0	0
0	0	0	0	0
0	0	0	0	0
0	0	0	0	0
0	0	0	0	0
0	0	0	0	0
0	0	0	0	0
0	0	0	0	0
0	0	0	0	0
0	0	0	0	0
0	0	0	0	0
0	0	0	0	0
0.002902874	0	0	0	0.028584196
0	0	0	0	0
0	0.002293619	0	0.001766875	0.026176114
0	0	0	0	0
0	0	0	0	0
0	0	0	0	0

0	0	0	0	0
0	0	0	0	0
0	0	0	0	0
0	0	0	0	0
0	0	0	0	0
0.014039017	0.027111031	0	0.001055233	0.017492783
0	0	0	0	0
0	0	0	0	0.000938593
0	0	0	0	0
0	0	0	0	0
0	0	0	0	0
0	0	0	0	0
0	0	0	0	0
0	0	0	0	0
0	0	0	0	0
0	0	0	0	0
0	0	0	0	0.266092992
0.008242811	0	0	0	0
0	0	0	0	0
0	0	0	0	0
0	0	0	0	0
0	0	0	0	0
0	0	0	0	0
0	0	0	0	0
0	0	0	0	0
0.00153205	0	0	0	0
0	0	0	0	0
0	0	0	0.002316246	0
0	0	0	0	0
0	0	0	0	0
0	0	0	0	0
0	0	0	0	0
0	0	0	0	0
0	0	0	0	0
0	0	0	0	0
0	0	0	0	0
0	0	0	0	0
0	0	0	0	0
0	0	0.00689157	1.346186044	0
0	0	0	0	0
0	0	0	0	0
0	0	0	0	0
0	0	0	0	0
0	0	0	0	0
0	0	0	0	0
0	0	0	0	0
0	0	0	0	0
0	0	0	0	0

0	0	0	0	0
0	0	0	0.0097467	0
0	0	0	0	0
0	0	0	0	0
0	0	0	0	0
0	0	0	0	0
0	0	0	0	0
0	0	0	0	0
0	0	0	0	0
0	0	0	0	0
0.434838269	0	0.002012968	0	0.000693093
0	0	0	0	0
0	0.016485926	0	0	0.247591688
0	0	0	0	0
0	0	0	0	0
0	0	0	0	0
0	0	0	0	0
0	0	0	0	0
0	0	0	0	0
0	0	0	0	0
0	0	0	0	0.004455171
0	0	0	0	0
0	0	0	0	0
4.492652409	0.650390339	3.088353039	0.05040049	3.063832134
0	0	0	0	0
0	0	0	0	0
0	0	0	0	0
0	0	0.003389374	0	0
0	0	0	0	0
0	0	0	0	0
0	0	0	0	0
0	0	0	0	0
0	0	0	0	0
0	0	0	0	0

PHL018-0045_V3	PHL018-0059_V3	PHL018-0118_V2	PHL018-0225_V1	PHL018-0012_V3
EYCF	CTRL	EYCF	CTRL	EYCF
0.407326007	0.062026929	0.251855657	0.03119785	0.019439522
0.317317095	15.47103419	1.557366013	0.780260091	3.001134954
1.486131544	0.001978596	8.797902518	0	2.148194174
0	0	0	0	0
0	0	0	0	0
0	0	0	0	0
0	0	0	0	0
0	0	0	0	0
0	0	0	0	0
0	0	0	0	0
0	0	0	0	0
0	0	0	0	0
0	0	0	0	0
0.047388155	2.018707367	8.36742032	0.626047408	8.771779084
0	0	0	0	0
0	0	0	0	0
0	0	0	0	0
0	0	0	0	0
0.015630999	0	0	0	0
0	0	0	0	0
0	0	0	0	0
0	0	0	0	0
0	0	0	0	0
0	0	0	0	0
0	0	0	0.046937791	0
0	0	0	0	0
0	0	0	0	0
0	0	0	0	0
0	0	0	0	0
0	0	0	0	0
0	0	0	0	0
0	0	0	0	0
0	0	0	0	0
0	0	0	0	0
0	0	0	0	0
0	0	0.005059736	0.867868083	1.647619811
0	0	0	0	0
0	0	0	0	0
0	0	0	0	0
0	0	0	0	0
0	0	0	0	0
0.003067598	1.219411483	0.007509308	0	0.016412465
0.061101162	0	0.06677332	17.46871311	0.182001687
0	0.081254997	0.016623088	0	0.00171781
0	0.002638828	0	0.002569436	0
0	0	0	0	0

0	0	0	0.007992108	0
0.007959288	0.001176409	0	0	0.042145442
0.11989803	0	0.097842954	0	0.004260812
0.326063634	0.393272557	0.450329031	0.284828338	0.433816617
0.002075187	0	0	0.046765204	0.005425413
0	0	0	0	0
0.044254938	0.41332972	0	0.004419945	0
0	0	0	0	0
0	0	0	0	0
0	0	0	0	0
0	0	0.001559023	0	0
0	0	2.230136399	0.069663651	0.019072204
0	0	0.00055123	0	0
0.08228983	0.028355191	0.156151762	0.063484375	0.063149122
0	0	0	0	0.002325935
0	0	0	0	0
0	0.07799152	0.001665895	0.595711937	0
0	0	0	0	0
0	0	0	0	0
0	0	0	0	0
0.055481734	0.033588764	0.149274341	0	0.122859309
0.01591172	0.001075111	0.035113866	0.006281033	0.001401615
0	0	0	0	0
0	0	0	0	0
0	0	0	0	0
0	0	0	0	0
0	0	0	0	0.000669431
0	0	0	0	0
0	0.01031533	0.001156759	0	0.000896535
0	0	0	0	0
0.162911533	0	0.009733517	0	0.032716601
0	0	0	0	0
0	0	0	0	0
0	0	0	0	0
0	0	0	0	0
0	0.004790853	0	0	0
0	0	0	0	0
0	5.700578237	0	25.29601344	0
0	0	0	0	0
0	0	0	0	0
0	0	0	0	0
0	0	0	0	0
0	0	0	0	0
0	0	0	0	0
0	0	0	0	0
0	0	0	0	0
0	0.000664602	0	0.002372791	0
0.926474446	0	0	0.000528823	0

0	0	0	0	0
0.015914387	0	0.007754984	0	0
0	0	0	0	0
0	0	0	0	0
0	0.052009639	0.563507738	0	0
0	0	0	0	0
0	0	0	0	0
0	0	0	0	0
0	0	0	0	0.000812862
0	0	0	0	0
0	0	0	0	0
0	0.011461425	0	0	0
0	0	0	0	0
0	0	0	0	0
0	0	0	0	0
0.002563073	0	0	0	0
0	0	0	0	0
0	0	0	0	0
0	0.004473742	0	0	0.287513523
0	0	0	0	0
0	0	0	0	0
0	0	0	0	0
0	0	0	0	0
0	0	0	0	0
0	0	0	0	0
0	0	0	0	0
0	0	0	0	0
0.002218297	0	0.002549209	0	0
0	0	0	0	0
0	0	0	0	0
0.036596915	0.000961626	0.739084094	0.050094093	0.007521992
0.002843952	0.001008829	0.000848474	0.004256635	0
0	0	0	0	0
0	0	0	0	0
0	0	0	0	0
0	0	0	0	0
0	0	0	0	0
0	0	0	0	0
0	0	0	0.004814886	0
0	0	0	0	0
0	0	0	0	0
0	0	0	0	0
0	0.001166047	0	0	0.000608067
0	0	0	0	0
0	0	0	0	0
0	0	0	0	0
0	0	0	0	0
0	0	0	0	0

0	0	0	0	0
0	0	0	0	0
0	0	0	0	0
0	0	0	0	0
0	0	0	0.038189163	0
0	0	0	0	0
0	0	0	0	0
0	0	0	0	0
0	0	0	0	0
0	0	0	0	0
0	0	0	0	0
0	0	0	0	0
0	0	0	0	0
0	0	0	0	0
0	0	0	0	0
0	0	0	0	3.850905894
0	0	0	0	0
0	0	0	0	0
0	0	0	0	0
0	0	0	0	0
0	0	0	0	0
0	0	0	0	0
0	0	0	0	0
0	0	0	0	0
0	0	0	0.009598473	0
0	0	0	0	0
0	0	0	0.00978121	0
0	0	0	0	0
0	0	0	0	0
0	0	0	0	0
0	0	0	0	0
0	0	0.218470259	0	0
0	0	0	0	0
0	0	0	0	0
0	0	0	0	0
0	0	0	0	0
0	0	0	0	0
0	0	0	0	0
0	0	0	0	0
0	0	0	0	0
0	0	0	0	0
0	0	0	0	0
0	0	0	0	0
0	0	0	0	0
0	0	0	0	0
0	0	0	0	0
0	0	0	0	0
0	0	0	0	0

0	0	0	0	0
0	0	0	0	0
0	0	0	0	0
0	0	0	0	0
0	0	0	0	0
0	0	0	0	0
0	0	0	0	0
0	0	0	0	0
0	0	0	0	0.007714811
0	0	0	0	0
0	0	0	0	0
0	0	0	0	0
0.000981215	0	0.002341911	0	0
0	0	0	0	0
0	0	0	0	0
0	0	0	0	0
0	0	0	0	0
0	0	0	0	0
0	0	0	0	0
0	0	0	0	0
0.092465697	0.020131314	0.046123481	0.015546359	0.008416523
0	0	0	0	0
0.487763311	0	0	0	0
0.006887366	0	0	0	0
0	0	0	0	0
0	0	0	0	0
0	0.004244898	0.007990365	0.001377757	0
0	0	0	0	0
0	0	0	0	0
0	0	0	0	0
0	0.047351853	0	0	0
0	0	0	0	0
0	0	0	0	0
0	0	0	0	0.057575761
0.000716228	0.006323417	0.152331245	0.001319386	0
0	0	0	0	0
0	0	0	0	0
0	0	0	0	0
0	0.00853693	0	0	0
0	0	0	0	0
0	0	0	0	0
0	0	0	0	0
0	0	0	0	0
0	0	0	0	0
0	0	0	0	0
0	0	0	0	0
0	0	0.005141341	0.001904725	0
0.000850672	0	0	0	0

0	0	0	0	0
0	0	0	0	0
0	0	0	0	0
0	0	0.001032329	0	0
0	0	0	0	0
0	0	0	0	0
0	0	0	0	0
0	0	0	0	0
0	0	0	0	0
0	0	0	0	0
0	0	0	0	0
0	0	0	0	0
0	0	0	0	0
0	0	0	0	0
0	0	0	0	0
0	0	0	0	0
0	0	0	0	0
0	0	0	0.104492796	0
0	0	0	0.083030689	0.001152242
0	0	0	0	0
0.214393418	0.021728948	0.022945392	0	0
0	0	0	0	0
0	0	0	0	0
0	0	0	0	0
0	0	0.005647807	0	0.003001566
0	0	0	0	0
0	0	0	0	0
0	0	0	0	0
0.003387322	0.04417228	0	0	0
0	0	0	0	0
1.655067956	0.074915721	0.752061433	0.128951402	0.002499539
0	0	0	0	0
0.028702518	0	0.126986006	0.013755792	0.016921227
0	0.002296782	0	0	0
0	0	0	0	0
0	0	0	0	0
0	0	0	0	0
0	0	0	0	0
0	0	0	0	0
0	0	0	0	0
0	0	0	0	0
0	0.001809576	0.004261434	0.011629139	0.000566192
0	0	0	0	0
0	0	0	0	0
0	0	0	0	0
0	0	0	0	0
0	0	0	0	0
0	0	0	0	0

0	0	0	0	0
0	0	0	0	0
0	0.092706611	0.019301576	0.000884988	0
0	0	0	0	0
0	0	0	0	0
0	0	0	0.000775958	0
0	0	0	0	0
0	0	0	0	0
0	0	0	0	0
0	0.001039078	0	0	0
0	0	0	0	0
0	0	0	0	0
0	0	0	0	0
0	0.018331865	0.027034267	0	0
0.000876452	0.003109018	0.007147212	0	0.000648513
0	0	0.001306445	0	0
0	0.001416979	0.005958737	0	0.001108384
0	0.01373968	0	0	0
0	0	0	0	0
0	0	0	0	0
0	0	0	0	0
0	0	0	0	0
0	0	0	0	0
0.018516977	0	0	0	0
0	0	0	0	0
0	0	0	0	0
0	0	0	0	0
0	0	0	0	0
0	0	0	0	0
0	0	0	0	0
0	0	0	0	0
0	0	0	0	0
0	0	0	0	0
0	0.003687075	0	0.005744188	0
0	0	0	0	0
0	0	0.001074387	0	0.001665388
0	0	0	0	0
0	0	0.025906456	0	0.002304671
0	0	0	0	0
0	0	0	0	0
0	0	0	0	0
0	0	0	0	0
0	0	0	0	0
0	0	0	0	0.002342259
0	0	0	0	0
0	0	0	0	0
0	0	0	0	0
0.007545396	0	0.006002981	0	0

0	0	0	0	0
0	0	0	0	0
0	0	0.011268249	0	0
0	0	0	0	0
0.002849976	0	0.011714843	0	0.001054392
0	0	0	0	0
0	0	0	0	0
0.011318793	0.016375274	0.030458178	0.007359074	0
0	0	0	0	0
12.61391551	0.08374258	0.242961515	0.060049163	0.479353043
0	0	0	0	0
0.001317647	0.206157189	0.005451138	0.003883646	0.028469032
0.002998445	0.002269082	0.004293916	0	0.002662369
0.353683505	0	0	0	0
0	0	0	0	0
0.000900233	0.001135423	0	0	0
0	0	0.00069444	0	0
0.002082711	0	0	0	0
0	0.003970555	0	0	0.003105833
0	0	0.011324746	0	0
0	0	0	0	0
0	0	0	0	0
0	0.038633086	0	0	0
0	0	0	0	0
0	0	0	0	0
0	0.080608501	0	0	0
0	0	0	3.478726933	0
0	0	0	0	0.015416454
0.373545491	0	0	0	0
0	0	0	0	0.000886255
0.013741057	0	0	0	0
0	0	0	0	0
0.02514357	0	0	0	0.049247187
0	0.004032501	0	0	0
0	0	0	0	0
0	0	0	0	0
0.00448689	0	0	0	0.01347524
0	0	0	0	0
0	0	0	0	0
0.263976064	0	0	0	0
0	0	0	0	0
0.002734392	0	0	0	0
0	0	0	0	0
0.001500597	0.019695236	0	0	0
0	0	0	0	0
0	0	0	0.008943316	0
0	0	0.006219533	0.004000287	0
0.018133745	0	0	0	0

0	0	0.001088602	0	0
0.003559398	0.101621579	0	0.00089412	0
0	0	0	0	0
0	0	0	0	0
0.015167558	0	0	0	0
0.007981684	0.004745841	0	0.002310521	0.080320115
0	0	0	0	0
0	0.002232057	0	0.007606766	0
0	0	0	0	2.517183383
0	0	0	0	0
0	0	0.003787728	0	0.000587129
0	0	0	0	0
0	0	0	0	0
0	0	0.003578455	0	0.00110938
0.093802731	0	0.047874407	0	0
0.003312751	0	0.014394237	0	0
0	0	0	0	0.001291463
0	0.00161022	0	0	0
0	0	0.051629253	0	0
0	0	0	0	0
0.000807719	0	0	0.005207736	0
0	0	0	0.14884601	0
0	0	0	0	0
0	0	0	0	0
0	0	0	0	0
0	0	0	0	0
0.089681224	0	0	0	0
0	0	0	0	0
0	0	0	0	0
0.00842438	0.464625367	6.445557389	0.151477178	0.575917061
0	0.000962171	0	0.003747479	0
0	0	0	0	0
0	0	0	0	0
0.002641181	0	0	0	0
0.002708013	0	0	0	0
0	0	0	0	0
0	0	0	0.61704473	0
0	0	0.001621066	0	0.001696131
0	0	0.42026529	0	0
0.595492573	0	0	0	0
0.005135793	0.004469504	0.010623454	0	0
0	4.847069432	1.839974444	0.056305928	1.20024015
0	0	0	0	0
0.277821219	0	0	0	0
0	0	0	0	0
0.044661938	0	0	0	0
0.001724923	0.002447512	0	0	0.000797702
0	0	0	0	0

0	0.009814414	0	0.000980136	0
0	0.004663141	0	0	0
0	0.005829209	0.005767818	0.2500744	0
0	0	0	0	0
0	0	0	0	0
0	0	0	0	0
0	0	0	0	0
0	0.027076843	0	0	0
0.049872676	0	0.02054743	0.008407933	0
0	0	0.132493902	0	0
0	0	0	0	0
0	0	0	0	0
0	0	0	0	0
0	0	0	0	0.003027388
0	0	0	0	0
0	0.005515353	0	0.004677374	0
0.002293164	0.131729466	0.004312218	0.236369397	0.000462759
0.067373082	0.00119795	0.020755198	0	0
0	0	0	0	0
0	0	0	0	0
0.000788193	0.031811566	0	0	0.112753476
0	0	0.011158192	0.006904519	0.001431403
0	0	0	0	0
0	0	0	0.009326217	0
0.001948349	0	0	0.054016187	0
0.737634067	0	0	0	0
0	0	0.001052768	0	0
0	0	0.000799484	0	0
0	0	0	0	0
0.000679361	0	0.038554735	0	0.001172919
0.064003338	0	0.067412818	0.01668433	0
0	0.009366889	0.014323645	0	0.004884622
0.102567666	0.028377188	0.460284055	0.187627421	0.00443942
0.001228324	0.006584226	0.001302977	0	0.002726624
0	0.076297148	0.00302248	0.001345848	0
0	0	0	0	0
0.002324663	0.024188937	0.001438469	0	0
0.013735959	0.051591465	0.041944542	0.021396299	0.038412634
0	0.942529465	0	0.048098643	0
0.004294034	0	0.010905738	0	0
0	0	0	0	0
0	0	0	0	0
0	0	0	0	0
0.00551899	0.006563088	0.033495594	0	0.000583381
0.757242279	0	0	0	0
0	0.016246796	0.032584189	0.001014074	0
0	0	0	0	0
0	0	0	0	0

0	0	0	0	0
0	0.049470311	0.698996133	0	0.192464093
0	0	0	0	0
0	0	0	0	0.010607901
0	0	0	0	0
0	0	0	0	0
0	0.04648911	0	0	0
0	0	0	0	0.00046931
0.010834647	0	0	0	0.001118635
0	0.005676175	0	0	0
0.002045407	0.002708768	0.000813644	0.006405446	0
0.063066518	0.195398952	0.173308327	0	0.002930642
0	0	0	0	0
0	0	0.04212934	0	0
0	0.065045423	0	0.003674724	0
0	0	0	0	0
0	0.005065127	0.024026487	0	0.011727593
1.704662885	0.005786206	0.01061777	0	0.03723717
0	0	0	0	0
0.063010068	0.024592974	0	0	0
0	0	0.086808655	0.007934262	0.015853087
0	0	0.001093314	0.016454859	0
0	0	0	0	0
0	0	0	0	0
0	0.41564679	0	0	0
0	0	0	0	0
0.153483961	0	0.09973396	0.006024241	0.040651987
0.175835259	0.012435871	0.247097796	0.03719147	0.013722781
0	0	0	0	0
0	0	0.001351736	0	0
0	0.000714841	0	0	0
0	0	0	0	0
0	0	0	0	0
0	0	0	0	0
0	0	0	0.004145437	0
0.023441723	0.195436145	0.10557689	0.128816334	18.41271201
0	0	0	0	0
0	0	0	0	0
0	0.062185992	0.00345604	0.002667432	0.008142864
0	0	0	0	0
0.367452453	0	0	0	0
0	0	0	0	0
0.022868187	0	0.063817254	0.001944287	0.010673163
0.003566576	0.000642623	0.014052397	0	0
0	0	0	0	0.001816078
0	0	0	0.005656893	0
0	0	0.01861251	0	0
0.004117924	0	0	0	0

0	0	0.015445554	0	0
0	0	0	0	0
0	0	0	0	0
0	0	0	0.003585425	0.002122342
0	0	0	0	0
0	0	0	0	0
0	0	0	0	0
0	0	0	0	0
0	0	0.137647733	0.248004044	0
0	0	0.001019758	0	0
0	0	0	0	0
0	0.000742736	0	0.001265608	0.042266322
0	0	0	0	0
0	0	0	0	0
0.391352454	0	0	0.102200367	0.033320141
0	0	0	0	0
0	0	0	0.006227687	0
0	0	0	0.708205682	0
0	0	0	0	0
0	0	0	0	0
0	0	0	0	0
0	0	0	0.06294775	0
0	0	0	0	0
0.089562227	0.016973635	0.548308867	0	0.071003377
0	0	0	0.012864986	0.00040796
0	0	0.062535989	0.086530553	0
0	0	0	0.013293223	0
0	0	0	0.636052871	0
0	0	0	0	0
0	0	0	0	0
0	0	0	0	0
0	0	0	0.108141743	0
0	0	0.021838674	0	0.018913989
0	0	0	0	0
0	0	0	0.001785145	0
0	0	0	0	0
0	0	0	0.157446092	0
0	0	0	0	0
0	0	0	0	0.002570403
0	0	0	0	0
0.008097353	0.013181668	0.012434766	0	0.023965913
0	0	0.218728306	0	0.715084368
0.798336294	19.26069709	3.243644804	2.796048627	0.115835976
0	0	0	0.002719701	0.019299496
0	0	0	0	0
0	0	0	0	0
0	0	0	0	0
0.002942821	0.010278407	0.001080579	0	0

0	0	0	0	0
0	0	0	0.020514784	0
1.757499517	0.201576236	0.002589643	0.002998098	0.031310451
0	0	0	0	0
0	0	0	0	0
0	0	0.018224189	0.001102167	0.009992555
0	0	0	0	0
0	0.004260681	0	0	0
0	0	0	0	0
0	0	0	0	0
0.014914026	0.002418478	0.038308063	0.105380901	0.005832967
0.389892336	1.240115996	4.343438498	0.847331953	0.482048243
0	0	0	0	0
0	0.048287221	0.004207537	0.010165932	0
0.462608254	0.377436507	0.104084159	0.011182222	0.425827744
0	0	0	0	0
0	0	0	0	0
0	0	0	0	0
0	0	0	0	0
0.0528265	0	0.274507196	0.002909703	0
0.001428589	0	0.034665056	0	0.01109909
5.51462698	12.55750373	8.775572715	0.030329047	0.287502028
53.40134409	2.38270611	3.763181299	1.349089845	8.035245619
0	0	0	0	0
0	0	0	0	0
0	0	1.676376483	0.071742231	0.003641677
0	0	0.027697322	0	0
0	0	0	0.002401667	0
0	0	0	0.029516941	0.005023767
0	0	0	0	0
0	0	0	0	0
0.068614968	0.107963549	0	0.122205273	0.167280044
2.618128938	0.006623573	0.046829065	0	0.00097145
0	0	0	0.023514972	0.088526303
0.011120419	0	0	0	0
0	0.013138927	0.112556933	0.052270256	0.188224766
0.018400942	0	0	0.005649494	0.035598643
0	0	0	0	0
0	0.007248308	0.04692208	0.057744839	0
0	0	0	0	0
0	0	0	0.004827964	0
0	0	0	0	0
0	0	0	0	0
0	0	0	0	0
0.059964716	0.010274369	0.057068183	0.13714076	0.005860152
0	0	0	0	0
0	0	0	0	0
0.019925924	0	0.486439134	0.058327632	0

0	0	0	0	0
0	0	0	0	0
0	0	0	0	0
0	0	0.002301529	0.002664541	0
0	0	0	0	0
0	0.000966744	0.038892248	0.047379881	0.002772744
0	0	0	0.002917165	0
0	0	0	0.001471116	0
0	0	0	0	0
0	0	0	0	0
0.004081087	0	0.004784815	0.001978395	0.001907192
0	0	0	0	0
0	0	0.006882717	0.016109828	0
0	0	0	0	0
0	0	0	0	0
0	0	0	0	0
0	0	0	0.00294281	0
0	0	0	0.008070147	0
0.001881568	0	0.000748471	0	0
0	0	0	0	0
0	0	0	0	0
0	0	0	0	0
0	0	0	0.004367325	0
0	0	0.380163947	0	0
0	0	0	0	0
0	0	0	0	0
0	0	0	0	0
0	0	0	0	0
0	0	0	0	0
0	0	1.059783524	0	0
0	0	0.050606578	0.013244755	0
0	0	0	0	0
0	0	0	0.000688321	0
0	0	0	0	0
0	0	0	0	0
0	0	0	0	0
0	0	0	0.030035318	0
0.000909135	0	0.003037822	0.006029086	0.001749011
0	0	0	0	0
0	0	0	0	0
0	0	0	0.03645477	0
0	0	0	0	0
0	0	0	0	0
0	0.181895661	0	0.051230871	0
0	0	0	0.010649278	0
0	0	0	0	0
0	0	0	0	0
0	0	0	0	0

0	0	0	0	0
0	0	0	0	0
0	0	0	0.013759636	0
0	0	0	0	0
0	0	0	0	0
0	0	0	0.333581009	0
0	0	0	0	0
0	0	0	0	0
1.203193063	0.013046734	0.05985235	0	0.013359754
0	0	0	0	0
0	0	0	0	0
0	0	0	0	0
0	0	0	0	0
0	0	0	0	0
0.003964614	0	0.171759171	0.293129612	0
0.208157378	0	0.000768831	0	0.010868775
0	0	0	0	0
0	0	0	0	0
0	0	0	0	0
0	0	0	0	0
0	0	0	0.059342256	0
0	0	0	0	0
0	0	0	0	0
0	0	0	0	0
0.094072474	0	0.002815386	0.568773447	0.379238615
0	0	0.069215229	0.487194063	0.61617653
0	0	0.022862927	0.007880087	0
0	0	0	0	0
0	0	0	0.008549065	0
0	0	0	0	0
0	0	0	0	0
0	0	0	0.026763546	0
0	0	0	0	0
0.019529316	0	0.000375899	0.027706998	0.013634591
0	0.021356132	0.001651635	0.004541336	0
0.028249123	0.000520558	0.016199145	0	0.000407189
0.014954758	0.11084267	0	0	0
0	0.78412587	0.424497765	0.750914542	0
0	0	7.588548564	0	0
0	0	0	0	0
0	0	0	0	0
0	0	0	0	0
0	0.342871876	0	0.165311596	0.865250958
0	0	0	0.138095613	0
0	0	0	0	0
0.001042695	0	0	0.001280521	0
0	0	0	0	0
0	0	0.01563859	0.001020012	0.00512136

0	0	0	0.036700098	0.000916561
0	0	0	0	0
0	0	0	0	0
0	0	0	0	0
0	0	0.001144706	0.003029156	0
0	0	0	0	0
0	0	0	0	0
0	0	0	0	0
0	0	0	0	0
0	0	0	0	0
0	0	0	0	0
0	0	0.816337581	1.14525795	7.209147681
0	0	0.044551222	0	0.098522835
0	0	0	0	0
0.04136241	1.336143978	0.185717552	0.02364673	0.311681625
0	0	0	0	0
0	0	0	0	0
0	0	0	0	0
0.019793853	0	0	0.153527958	0
0	0	0.003308515	0	0
0	0	0	0	0
0	0	0	0	0
0	0.203528018	0	0.008770498	0
0	0	0	0	0
0	0	0	0	0
0	0	0	0	0
0	0	0	0	0
0	0	0	0	0
0	0	0	0	0
0	0	0	0	0
0	0	0	0	0
0.002776216	0.048145835	0.168843394	0	0.012325241
0	0	0	0	0
0.02203709	0	0	0.019162083	0
0.02814701	0	0.010481947	0.001654803	0.001772496
0	0	0.023339775	0.002844323	0.006093233
0	0	0	0	0
0	0	0	0	0
0	0	0	0.000633708	0
0	0	0	0	0
0	0	0	0	0
0	0	0	0.005136336	0
0	0	0	0	0
0	0	0	0	0.343187563
0.025367828	0.009859014	0.031446659	0.047093143	0.013532169
0	0	0	0	0
0	0	0	0.09677182	0
0.001301969	0	0	0	0.000825743

0	0	0	0	0
0	0	0	0	0
0.002440622	0	0.022329715	0	0
0	0	0	0	0
0	0	0	0	0.001526195
0	0	0	0	0
0	0	0	0.348907694	0
0	0.01399049	0	0.001769167	0.014354579
0	0	0	0	0
0	0	0	0	0
0	0	0	0	0
0.003931849	0	0	0	0
0	0	0	0	0
0	0	0	0.110244607	0
0	0	0	0	0
0	0	0	0	0
0	0	0	0	0
0	0	0	0	0
0	0	7.952479614	0	0
0	0	0	0	0
0	0	0	0	0
0	0	0	0	0
0	0	0	0	0
0	0	0	0	0
0	0	0	0	0
0.003478398	0.000692707	0.01441935	0	0.020861102
0	0	0	0	0
0	0	0.002138436	0	0.00079554
0	0	0	0.170490041	0
0	0.415535836	1.206614429	0.066513122	0.288758941
0	0	0	0	0
0	0	0	0	0
0	0	0	0	0
0	0	0	0	0
0.003645146	0	0.001068425	0.002120476	0
0	0	0	0	0
0	0	0	0	0
0.00130616	0	0.004503014	0	0.010792219
0	0	0	0	0
0	0	0	0.000938736	0
0.749842488	6.628174868	4.693377147	2.266795927	15.29625829
0	0.050497241	0.068713644	0	1.427061681
0	0	0	0	0
0	0	0	0	0.020951182
0	0	0	0	0
0	0	0	0	0
0	0	0	0.000910147	0
0.001382188	0	0.001099643	0.008911602	0.001406244

0	0	0	0	0
0.002310253	0	0.010745203	0.000818421	0
0	0	0	0	0
0	0	0	0	0
0	4.897592194	0	0.784696304	3.080217607
0	0	0	0	0
0	0	0	0	0
0	0	0	0	0
1.510097285	0.029374971	0.01411758	0.000980658	0.714537132
0	0	0.00891315	0	0
0	0	0	0	0
0	0	0	0	0
0	0	0.077481694	0.007952357	0
0	0.007630893	0.352649362	0.004106178	0.018535343
0	0	0	0	0
0	0.534564712	0.076503159	0.021340167	0
0	0	0.000584604	0	0.000951494
0	0	0	0	0
0	0	0	0	0
0	0	0	0	0
0	0	0	0	0
0	0	0	0	0
0	0	0	0	0
0	0	0	0	0
0	0	0	0	0
0	0	0	0	0
0	0	0.049562884	0	0.001536531
0.005948117	0	0.002581208	0	0.002133912
0	0	0	0	0
0	0	0	0	0
0	0	0	0	0
0	0	0	0	0
0	0	0	0	0
0	0	0	0.007309329	0
0	0	0	0	0
0.039407859	0	0	0.071442186	0.003394178
0	0	0.001865094	0	0
0	0	0	0	0
0	0	0	0	0
0	0	0	0	0
0	0	0.006704474	0.213571108	1.759353557
0	0	0	0	0
0	0	0	0	0
0	0	0	0	0
0	0	0	0	0
0	0	0	0	0
0	0	0	0	0
0	0	0	0	0

0	0	0	0	0
0	0	0	0	0
0	0	0	0	0
0	0	0	0	0
0	0	0	0.001259599	0
0	0	0	0	0
0	0	0.010643082	0	0.055594831
0	0	0	0	0
0	0	0	0	0
0	0	0	0	0
0	0.001051076	0	0	0
0	0	0	0	0
0	0	0	0	0
0	0	0	0	0
0	0	0	0	0
0	0	0	0	0
0	0	0.062733442	0	1.234779692
0	0	0	0	0
0	0	0	0	0
0	0	0	0	0
0	0	0.036367061	0.014462897	0
0	0	0	0	0
0	0	0	0.010812267	0
0.002909039	0.000647478	0.003403498	0.000630451	0.002785571
0	0	0	0	0
0.006460259	0	0	0.072147686	0
0	0	0	0	0
0	0	0	0	0
0	0	0	0	0
0.04383815	3.294167295	0.025863722	0.005897885	0.002186776
0	0	0	0	0
0	0	0	0	0
0	0	0	0	0
0	0	0	0.07978413	0.007572274
0	0	0	0	0
0	0	0	0	0
0.53807197	0	0.043438312	0	0.000792152
0	0	0	0	0
0	0	0	0	0
0	0	0	0	0
0	0	0	0	0
0	0	0	0.000791506	0
0	0	0	0	0
0	0	0	0.005833897	0
0	0	0	0	0
0	0	0	0	0
0	0	0	0	0
0	0	0	0	0

0.009390689	0	0.026807927	0.005087908	0.012670715
0	0	0	0	0
0	0	0	0	0
0	0	0	0	0
0	0	0	0	0
0	0	0	0	0
0	0	0	0	0
0	2.99289769	0.093858065	0.119632621	0.01916521
0.077457761	0	0.089216822	23.72524453	0.268389036
0.089738811	0.012371514	0.102129454	0	0.117764077
0	0	0	0.001087384	0
0	0	0	0	0
0	0	0	0	0
0	0	0.549668812	0	0
0	0	0	0	0
0	0	0	0	0
0	0	0.035283275	0	0
0	0	0	0	0
0	0	0	0	0
0.003336932	0	0	0	0
0	0	0	0	0
0	0	0	0	0
0	0	0	0	0
0	0	0	0	0
0	0	0	0	0
0	0	0	0	0
0	0	0	0	0
0	0	0	0	0
0	0	0	0	0
0	0	0	0	0
0	0	0	0	0
0	0	0	0	0
0	0	0	0	0
0	0	0	0	0
0	0	0	0.001231926	0
0	0	0	0.001081989	0
0	0	0	0	0
0	0.159809326	0	0	0.034760004
0	0	0	0.001411648	0
0	0	0	0	0
0	0	0	0	0
0	0.232132698	0	0.06690022	0.422091635
0	0	0	0	0
0	0	0	0	0
0	0	0	0	0
0	0	0.051192427	0	0
0	0	0	0	0
0	0	0	0	0

0	0	0	0	0
0	0.037099799	0.013089275	0	0
0	0	0	0	0
0	0	0	0	0
0	0	0.870667145	0.037666025	0.274563109
0	0	0	0	0
0	0	0	0	0
0.082916768	0.005317584	0.052550045	0	0.006065939
0	0	0	0.002369488	0
0	0	0	0	0
0	0	0	0	0
0	0	0	0	0
0	0	0	0	0
0	0	0	0	0
0	0	0	0	0
0	0	0.03070741	0.004116405	0.001202501
0	0	0	0	0
0	0	0	0	0
0	0	0	0	0
0	0	0	0	0
0	0	0	0	0
0	0	0	0	0
0	0	0	0	0
0.001418936	0	0	0.000746819	0.000899926
0	0	0	0.21628171	0
0	0	0	0	0
0	0.008593351	0.023894742	0	0
0	0	0	0	0.001011129
0.00716481	0	0	0	0
0	0	0	0	0
0	0	0	0	0
0	0	0	0	0
0	0	0	0	0
0	0	0	0	0
0	0	0	0	0
0	0	0	0	0
0	0	0	0	0
0	0	0	0.004903563	0
0	0	0	0	0
0	0	0	0	0
0	0	0	0	0
0	0	0	0.190366021	0
0	0	0	0	0
0	0	0	0	0
0	0	0	0	0
0	0	0	0	0
0	0	0	0	0
0	0	0	0	0

0	0	0	0.007547621	0
0	0	0	0	0
0	0	0	0	0
0	0	0	0	0
0	0	0	0	0
0.265622549	0	0	0.293773131	0.343911504
0	0	0	0	0
0	0	0	0	0
0	0	0	0	0
0	0	0	0	0
0	0	0	0	0
0	0	0	0	0
0	0	0	0	0
0	0	0	0	0
0	0	0	0	0
0	0	0	0	0
0	0	0	0	0
0	0	0	0	0
0	0	0	0	0
0	0	0	0	0
0.004037156	0	0	0	0.030618952
0	0	0	0	0
0	0	0.001216914	0.066685739	0.026785707
0	0	0.651934556	0	0
0	0	0	0	0
0	0	0	0.024295207	0
0	0	0	0	0
0	0	0	0	0
0	0	0	0	0
0	0	0	0	0
0	0	0	0	0
0	0	0	0	0
0.005491078	0.00609758	1.308301266	0	0.007242767
0	0	0.216726707	0	0
0	0	0.328076359	0	0.74282914
0	0	0.003048365	0	0
0	0	0	0	0
0	0	0	0	0
0.001772748	0	0	0	0.004918915
0	0	0	0	0
0	0	0	0	0
0	0.069520528	0	0	0
0	0	0.012986986	0.024581644	0
0	0	0	0	0
0	0	0	0	0
0	0	0	0	0
0	0	0	0	0
0	0	0	0	0

0	0	0	0	0
0	0	0	0	0
0	0	0	0	0
0	0	0	0	0
0	0	0	0	0
0	0	0	0	0
0	0	0	0	0
0	0	0	0	0
0	0	0	0	0
0	0	0	0.004099595	0
0	0	0	0	0
0.006392706	0	0	0	0
0	0	0	0	0
0	0	0	0	0
0.016614501	0	0	0	0
0.779292121	0.008284962	0.001161342	0	0.031323056
0	0	0	0	0
0	0	0	0	0
0	0	0	0	0
0	0.004624288	0.281276027	0	0
0	0	0	0.027483209	0.004028905
0	0	0	0	0
0	0	0	0	0
0	0	0	0	0
0	0	0	0	0
0	0	0	0	0
0	0	0	0	0
0	0	0	0	0
0	0	0	0	0
0	0	0	0.007763717	0
0	0	0.045712025	0.036530237	0.098698717
0	0	0	0	0
0	0	0	0	0
0	0	0	0	0
0	0	0	0	0
0	0	0	0	0
0	0	0	0	0
0	0	0	0	0
0	0	0	0	0
0	0	0	0	0
0	0	0	0	0
0	0	0	0.12255266	0
0	0	0	0	0
0	0	0	0.00184646	0.001648153
0	0	0	0	0
0	0	0	0	0
0.011100694	0	0	0	0.066815629
0	0	0	0	0

0	0	0	0	0
0	0	0	0	0
0	0	0	0	0
0	0	0	0	0
0.004784451	0.06426647	0	0.024678051	1.61195315
0	0	0	0.001456631	0
0	0	0	0.000684404	0
0	0	0	0	0
0	0	0	0	0
0	0	0	0	0
0	0	0	0	0
0.009299519	0.00167558	0.005108508	0.011216688	0.003276665
0	0	0.002431644	0.002815178	0.009752931
0	0	0	0	0
0	0.002713432	0	0	0.00049941
0	0.978065185	0	0	0
0	0	0	0.001254831	0.001638094
0	0	0	0	0
0.169502548	0.081676352	0.034813722	0.002316366	0.244141196
0	0	0.002264098	0	0
0	0	0	0	0
0	0	0	0	0
0	0	0	0	0
0	0	0	0	0
0	0	0	0	0
0	0	0	0	0
0.020524508	0.00132752	0.020794958	0	0.002596019
0	0	0	0	0
0	0	0	0	0
0	0	0	0	0
0.002187365	0	0.003480456	0	0.000735681
0	0	0.007005964	0	0
0	0	0	0	0
0	0	0	0	0
0	0	0	0	0
0	0	0	0	0
0	0	0	0	0
0.024588465	0.000925083	0.015116197	0.001286795	0.080528036
0	0	0	0	0
0	0	0	0	0
0.020641975	0	0	0.02248114	0.022291238
0	0	0	0	0
0	0	0	0	0
0.002693185	0.003331469	0.001813011	0	0.006591468
0	0	0	0	0
0	0	0	0	0
0	0	0	0	0
0	0	0	0	0

0	0	0	0	0
0	0	0.043811195	0	0
0	0	0	0	0
0.001302678	0	0.002245506	0	0
0	0.000952415	0.00176226	0	0
0.001299429	0	0.001033802	0.00119686	0
0	0	0	0.001552488	0
0.010867276	0	0	0	0
0	0	0	0	0
0	0	0	0	0
0	0.003224404	0	0.007270684	0
0	0	0	0	0
0	0.001455828	0	0.01913685	0.013551388
0	0	0	0.006603457	0
0	0	0	0	0
0	0	0	0	0
0	0	0	0	0
0	0	0	0	0
0	0	0	0	0
0	0	0	0	0
0	0	0	0	0
0	0	0	0	0
0.094842185	0.003067183	0.001773508	0	0.087870725
0	0.024883249	0	0	0
0	0	0	0	0
0	0	0	0.003253324	0
0	0	0	0	0
0	0	0	0	0
0	0	0	0	0
0	0	0	0	0
0	0.013038352	0.035054867	0	0.007858101
0	0.002479337	0.010704241	0	0
0	0	0	0	0
0	0	0	0	0
0	0	0	0	0
0.005905274	0	0	0	0.004194713
0.023046188	0	0.000763964	0	0.121321973
0	0	0	0	0
0	0	0.002585643	0	0.002970606
0	0	0	0	0
0	0	0	0	0
0	0	0	0	0
0	0	0	0	0
0	0	0	0	0
0	0	0	0	0
0.000672815	0.567389511	0.231776128	0.100547526	0.08948613
0.024623874	0	0	0	0
0	0	0	0	0

0	0	0	0	0
0	0	0	0	0
0	0	0	0.042270335	0
0	0	0	0	0
0	0	0	0	0
0	0	0	0	0
0	0	0	0	0
0	0	0	0	0
0	0	0	0	0
0	0	0	0	0
0.005428341	0	0.000987129	0.003999888	0
0	0	0	0	0
0	0	0	0	0
0	0	0	0	0
0	0	0	0	0
0	0	0	0	0
0	0	0	0	0
0	0	0	0	0
0	0	0	0	0
0	0.002140206	0.000654551	0.000757791	0
0	0	0	0	0
0	0	0	0	0
0	0	0	0	0
0	0	0	0	0
0	0	0	0	0
0	0	0	0	0
0	0	0	0	0
0	0	0.001629836	0.006499337	0
0	0	0	0	0
0	0	0	0	0
0	0.000835754	0.006185601	0.001790308	0.015297523
0	0	0	0	0
0.022363572	0.016884879	0.063333177	0.02248808	0.159706027
0	0	0.012843846	0	0.001396217
0	0	0	0	0
0	0	0	0	0
0	0	0	0	0
0	0	0	0	0
0	0	0	0	0
0	0	0	0	0
0	0	0	0	0
0.127269027	0.082707552	0.06969532	0	0.231304061
0	0	0.037300866	0.0609882	0.31876756
0	0	0	0	0
0	0	0	0	0
0	0	0.02245931	0.01231661	0
0	0	0	0	0
0	0	0	0	0

0	0	0	0	0
0	0	0	0	0
0	0.010473866	0.08008205	0	0.001117204
0	0	0	0	0
0	0.001330458	0	0.013324849	0.010407058
0.039507459	0	0.000466803	0	0
0	0	0	0	0
0	0	0	0	0
0	0	0	0	0
0	0	0	0	0
0	0	0	0	0
0	0	0	0	0
0	0	0	0	0
0.685309045	0	0	0	0
0	0	0	0	0
0	0	0	0	0
0	0	0	0	0
0	0	0	0	0
0	0	0	0	0
0	0	0	0	0
0	0	0	0	0
0	0	0	0	0
0	0	0	0	0
0	0	0	0	0
0	0	0.000824986	0.015663767	0
0.00166905	0.022695578	0.00049795	0	0
0	0	0	0	0
0	0	0	0	0.001796962
0	0	0	0	0
0	0	0	0	0
0	0	0	0	0
0	0	0.052255135	0	0
0	0	0	0	0
0	0	0	0	0
0	0	0	0	0.013016162
0	0	0	0	0
0	0	0	0	0
0.006437922	0	0	0	0
0.610882056	0.715648454	4.575696479	0	0
0	0	0	0	0
0	0	0	0	0
0	0	0.001260464	0	0
0.010887981	0	0.017824307	0.002507137	0.008985927
0	0	0	0	0
0	0	0	0	0
0	0	0	0	0.000534724
0.026906621	0	0.028287055	0	0.002607135
0	0	0	0	0

0	0	0	0	0
0.009296164	0.004205645	0.122192457	0.006887132	0.006429916
0	0	0	0	0
0	0	0	0	0
0	0	0	0	0
0	0	0	0	0
0	0	0	0	0
0	0	0	0	0
0	0	0	0	0
0	0	0	0	0
0	0	0	0	0
0	0	0	0	0
0	0	0	0.046436564	0
0	0	0	0	0
0.009575104	0	0	0	0.001678005
0	0	0	0	0
0	0	0	0	0
0	0	0	0	0
0	0	0.002661232	0.002567482	0
0	0	0	0	0
0	0	0	0	0
0	0	0.000984376	0	0
0.004753833	0	0.006051301	0	0
0	0	0	0	0
0	0	0	0	0
0	0	0	0	0
0	0	0	0	0
0	0	0	0	0
0	0	0	0	0
0	0	0	0	0
0	0	0	0	0
0	0	0	0	0
0	0	0	0	0
0	0	0	0	0
0	0	0.002681277	4.485546588	0
0	0	0	0	0
0	0	0	0	0
0	0	0	0	0
0	0	0.039713963	0.077616152	0.006765495
0	0	0	0	0
0.018390749	0.011318246	0.045833135	0.090818035	0.124274332
0	0	0	0	0
0	0	0	0	0
0	0	0	0	0
0	0	0	0	0
0.215635338	0.155983403	0.306781718	0.011916863	0.306533603
0	0	0	0	0
0	0	0	0	0

0	0	0.00359527	0	0
0	0	0	0	0
0	0	0	0	0
0	0	0	0.033588299	0
0	0	0	0.002658247	0
0	0	0	0	0
0	0	0	0	0
0	0	0	0	0
0	0	0	0	0
0	0.028163449	0.01440948	0	0.002570156
0	0	0	0.004820218	0
0	0	0	0	0
0	0	0	0	0
0	0	0	0	0
0	0	0	0	0
0	0	0	0	0
0	0	0	0	0
0	0	0	0	0
0	0	0	0	0
0.115046012	0.10739749	1.608360483	0.355072117	0.346892636
0	0	0	0	0
0	0	0	0	0
0	0	0	0	0
0	0	0	0	0
0	0	0	0	0
0	0	0	0.002384299	0
0	0	0	0	0
0	0	0	0	0
0	0	0	0	0
0.014998298	0	0.001652175	0	0.030219884
0	0	0	0	0
0	0	0	0	0
0	0	0	0	0
0	0	0	0	0
0	0	0	0	0
0	0	0	0	0
0	0	0	0	0
0	0	0	0	0
0	0	0	0	0
0	0	0	0	0
0	0	0	0	0
0	0	0	0	0
0	0	0	0	0
0	0	0	0	0
0.00304096	0	0.007903153	0.005041665	0
0	0	0	0	0
0	0	0	0	0
0.003652917	0	0	0	0

0	0	0	0	0
0	0	0	0	0
0	0	0	0	0
0	0	0	0	0
0	0	0	0.002438438	0
0.009080779	0	0.009873488	0.004878998	0.001455815
0	0	0	0	0
0	0	0	0	0
0	0	0	0	0
0	0	0	0	0
0	0	0	0	0
0	0	0	0	0
0	0	0	0	0
0	0	0	0	0
0	0	0	0	0
0	0	0	0	0
0	0	0	0	0
0	0	0	0	0
0	0	0	0	0
0	0	0	0	0
0	0	0	0	0
0	0	0	0	0
0	0	0	0	0
0	0	0	0	0
0	0	0	0	0
0	0.002687203	0	0	0
0	0	0	0	0
0	0	0.016069289	0	0
0	0	0	0	0
0	0	0.012407537	0	0
0	0	0	0	0
0	0	0	0	0
0	0	0	0	0
0	0	0	0	0
0	0	0	0	0
0	0	0	0	0
0	0	0	0	0
0	0	0	0	0
0	0.071539784	0	0.003148408	3.855046394
0	0	0	0	0
0	0	0	0	0
0	0	0	0	0
0	0	0	0	0
0	0	0	0	0
0	0	0	0	0
0	0	0	0	0
0	0	0	0	0
0	0	0	0	0

0	0	0	0	0
0	0.026607211	0.107980324	0.002497112	0
0	0	0	0	0
0	0	0	0	0
0	0	0	0	0
0	0	0.000554581	0	0
0	0	0	0	0
0	0	0	0	0
0	0	0	0	0
0	0	0	0.000635715	0
0	0.001049399	0	0	0
0	0	0	0	0
0	1.364694271	0.101671832	0.177916918	0.341479476
0	0	0	0	0
0	0	0	0	0
0	0	0	0	0
0	0	0	0	0
0	0	0	0	0
0	0	0	0	0
0	0	0	0	0
0.053144551	0	0	0.000622241	0.005498593
0	0	0	0	0
0	0	0	0	0
0	2.585870827	0.740876924	0.141409049	0
0	0	0	0	0
0	0	0	0	0
0	0	0	0	0
0	0	0	0	0
0	0	0	0	0
0	0	0	0	0
0	0	0.013539804	0	0
0	0	0	0	0
0	0	0	0	0
0	0	0	0	0

PHL018-0230_V1 REF	PHL018-0014_V3 CTRL	PHL018-0246_V1 REF	PHL018-0100_V2 EYCF	PHL018-0173_V2 CTRL
0.232357286	0.081658939	0.123619404	0.156896512	0
0	9.761997892	0.00913389	0.094307924	8.926578693
0	3.137903172	0	0.675975296	14.50660328
0	0	0	0	0
0	0	0	0	0
0	0	0	0	0
0	0	0	0	0
0	0	0	0	0
0	0	0	0	0
0	0	0	0	0
0	0	0	0	0
0	0	0	0	0
0	0	0	0	0
40.17244681	0.252539702	0.681212743	0.522242462	21.22202704
0	0	0	0	0
0	0	0	0	0
0	0	0	0	0
0	0	0	0	0
0	0	0	0	0
0	0	0	0	0
0	0	0	0	0
0	0	0	0	0
0	0	0	0	0
0	0	0	0	0
0	0	0.042689931	0.089298483	0
0	0	0	0	0
0	0	0	0	0
0	0	0	0	0
0	0	0	0	0
0	0	0	0	0
0	0	0	0	0
0	0	0	0	0
0	0	0	0	0
0	0	0	0	0
0	0	0	0	0
0	3.846065555	0.912039169	1.072172219	0
0	0	0	0	0
0	0	0	0	0
0	0	0	0	0
0	0	0	0	0
0	0	0	0	0
0.002252664	0.110376026	0.073740081	0.017083901	0.002481971
0.3089559	0.127016209	3.168688489	0.136729821	0.005778292
0.006279476	0.018776983	0.014079191	0.000933778	0
0.001864174	0.005264599	0.015225899	0	0
0	0	0	0	0

0	0.004142557	0	0.000747865	0
0.018758266	0.002346997	0	0.004287528	0.013318679
0	0.011920711	0.006162646	0	0
0.189946148	1.710131234	0.727977999	0.364608868	0.460019271
0	0	0	0	0
0	0	0	0.008357156	0
0	0.001078115	0.002282492	0	0.010451687
0	0	0	0	0
0	0	0	0.007340256	0
0	0	0	0	0
0	0	0	0.004897996	0
0	0	0.125844764	0.048907276	0
0	0	0	0	0
0.019684937	0.044010391	0.006742928	0.018693901	0.043085367
0	0	0	0	0
0	0	0	0	0
0	0.053629854	0.017087099	0.16982637	0
0	0	0	0	0
0	0	0	0	0
0	0	0	0	0
1.572587788	0.047321315	0.004534217	0.001112884	0.062311579
0	0.003064145	0.00060817	0.152031483	0.006701754
0	0	0.041382754	0.079254195	0
0	0	0	0	0
0	0	0	0	0
0	0	0	0	0
0.281777851	0	0	0	0
0	0	0	0	0
0.002313385	0.012086444	0.020747333	0.002005075	0
0.001699623	0	0	0	0
0	0	0	0	0
0	0	0	0	0
0	0	0	0	0
0	0	0	0	0
0	0	0	0	0
0	0.00385882	0.001885283	0	0
0	0	0	0	0
0	3.744084354	0	8.37813635	0
0	0	0	0	0
0	0	0	0	0
0	0	0	0	0
0	0	0	0	0
0	0	0	0	0
0	0	0	0	0
0	0	0	0.011959595	0
0	0.277364303	0	0	0
0	0	0.00250635	0	0
0.001827005	0	0	0	0

0	0	0	0	0
0.002088979	0	0	0	0.002092385
0	0	0	0	0
0	0	0	0	0
0	0.483931725	0.496578147	0.006838804	0
0	0	0	0	0.001113152
0	0	0	0	0
0	0	0	0	0
0.00052437	0	0.105028078	0	0
0	0	0	0	0
0	0	0	0	0
0	0.013180983	0.002502408	0.002638244	0
0	0	0	0	0
0	0	0	0	0
0	0	0	0	0
0	0	0	0.001223497	0
0	0	0	0	0
0	0	0	0	0
0	0.050491975	0.207113438	0.350100032	0.006331169
0	0	0	0	0
0	0	0	0	0
0	0	0	0	0
0	0	0	0	0
0	0	0	0	0
0	0	0	0	0
0	0	0	0	0
0	0	0	0	0
0	0	0	0	0
0	0	0	0	0
0	0	0	0	0
0	0	0	0.014486221	0
0	0.004073253	0.001712027	0.008211449	0
0	0	0	0	0
0	0	0	0	0
0	0	0	0	0
0	0	0	0	0
0	0	0	0	0
0	0	0	0	0
0	0.017769926	0	0.00517159	0
0	0	0	0	0
0	0	0	0	0
0	0	0	0	0
0	0.001384717	0	0	0.004911222
0	0	0	0	0
0	0	0	0	0
0	0	0	0	0
0	0	0	0	0
0	0	0	0	0

0	0	0	0	0
0	0	0	0	0
0	0	0	0	0
0	0	0	0	0
0	0	0.001821264	0.083738841	0
0	0	0	0	0
0	0	0	0	0
0	0	0	0	0
0	0	0	0	0
0	0	0	0	0
0	0	0	0	0
0	0	0	0	0
0	0	0	0	0
0	0	0	0	0
0	0	0	0	0
0	0	8.899976832	1.11340781	0
0	0	0	0	0
0	0	0	0	0
0	0	0	0	0
0	0	0	0	0
0	0.001120749	0	0	0
0	0	0	0	0
0	0	0	0.002497302	0
0	0	0	0	0
0	0	0	0	0
0	0	0	0	0
0	0	0	0.025295045	0
0	0	0	0	0
0	0	0	0	0
0	0	0	0	0
0	0	0	0	0
0	0	0	0	0
0	0	0	0	0
0	0	0	0	0
0	0	0	0	0
0	0	0	0	0
0	0	0	0	0
0	0	0	0.268257843	0
0	0	0	0	0
0	0	0	0	0
0	0	0	0	0
0	0	0	0	0
0	0	0	0	0
0	0	0	0	0
0	0	0	0	0
0	0	0	0	0
0	0	0	0	0
0	0	0	0	0

0	0	0	0	0
0	0	0	0	0
0	0	0	0	0
0	0	0	0	0
0	0	0	0	0
0	0	0	0	0
0	0	0	0	0
0	0	0	0	0
0	0	0	0	0
0	0	0	0	0
0	0	0	0	0
0	0	0	0.008784831	0
0.001124053	0	0.001260118	0	0
0	0	0	0	0
0	0	0	0	0
0	0	0	0	0
0	0	0	0	0
0	0	0	0	0
0	0	0	0	0
0	0	0	0	0
0.112091186	0.036107149	0.024346554	0.003542019	0.050523276
0	0	0	0	0
0	0	0	0	0
0	0	0	0	0.007683319
0	0	0	0	0
0	0	0	0	0
0	0.002880541	0.004345134	0.000442026	0.000612991
0	0	0	0	0
0	0	0	0	0
0.186607203	0	0	0	0.006339687
0.001220106	0.019238451	0	0	0.015887245
0	0	0	0	0
0	0	0	0	0
0	0	0	0	0.001929793
0	0.02317141	0.001533016	0.004444636	0.002054571
0	0	0	0	0
0	0	0	0	0
0	0	0	0	0
0	0	0	0	0
0	0	0	0	0
0	0	0	0	0
0	0	0	0	0
0	0	0	0	0
0	0	0	0	0
0	0	0	0	0
0	0	0	0	0
0	0	0	0	0
0.125873733	0	0	0	0

0	0	0	0	0
0.019972733	0	0	0	0
0	0	0	0	0
0	0	0	0	0
0	0	0	0	0
0	0	0	0	0
0	0.348337551	0	0	0
0.01050653	0.019121574	0.001046962	0	0
0	0	0	0	0
0	0	0	0	0
0	0	0	0	0
0	0	0	0	0
0	0	0	0	0
0	0	0	0	0
0	0	0	0.006658519	0
0	0	0	0	0
0	0	0	0	0
0	0	0.018261141	0	0
0.002808022	0	0.031664453	0	0
0	0	0	0.041425018	0
0.018761358	0.012041773	0	0	0.014643076
0	0	0	0	0
0	0	0	0	0
0	0	0	0	0
0.003388491	0.012303528	0	0.007342246	0.003878875
0	0	0.001316489	0	0
0	0	0	0	0
0	0	0	0	0
0	0.060011782	0	0	0
0	0	0	0.000599572	0
0.568828822	0.019479484	0.071300167	0.06366552	0.129025122
0	0	0	0	0
0	0	0	0.000965403	0
0.002086122	0.0016365	0	0.006529246	0
0	0	0	0	0
0	0	0	0	0
0	0	0	0	0
0	0	0	0	0
0	0	0	0	0
0	0	0.003811719	0	0
0	0	0	0	0
0	0.056731698	0.007779688	0.017543136	0
0	0	0	0	0.004757782
0	0	0	0	0
0	0	0	0	0
0	0	0	0	0
0	0	0	0	0
0	0	0	0	0

0	0	0	0	0
0	0	0	0	0
0.003898314	0.015542396	0.004884339	0	0
0	0	0	0	0
0	0	0	0	0
0	0	0	0	0
0	0	0	0	0
0	0	0	0	0
0	0	0	0	0
0	0	0	0	0
0	0	0	0	0
0	0	0	0	0
0	0	0	0	0
0.002280886	0.003936431	0.005398077	0.002013516	0
0.003555971	0.006793382	0.00140697	0	0.003352253
0	0	0.0143103	0.004529069	0
0.002621695	0.014134733	0.002939047	0.000860718	0.004535766
0	0	0	0	0
0	0	0	0	0
0	0	0	0	0
0	0	0	0	0
0.114889732	0	0	0	0
0	0	0	0	0
0.163763488	0	0	0	0
0	0	0.001160216	0	0
0	0	0	0	0
0	0	0	0.99748939	0
0	0	0	0	0
0	0	0	0	0
0.004979447	0	0.002093325	0	0
0	0	0	0	0.001651412
0	0	0	0	0
0	0	0	0	0
0	0	0	0	0
0	0	0	0	0
0	0.002427195	0.004817483	0.009621856	0
0	0	0	0	0
0	0.007633883	3.861255961	0.008981023	0
0	0	0	0	0
0	0	0	0	0
0	0.003856248	0	0	0
0	0	0	0	0
0	0	0	0	0
0	0	0	0	0.002690546
0	0	0	0	0
0.003740415	0	0	0	0
0.002051065	0	0	0	0
0	0	0	0.003783747	0.00688696

0	0	0	0.182249367	0.06853572
0	0	0	0	0
0	0.012728303	0.002021045	0.001302126	0
0	0	0	0	0
0	0	0.0330422	0.001146306	0.003860626
0	0	0.019479079	0	0
0	0	0	0	0
0.037183343	0	0.002493934	0	0.005093192
0	0	0	0	0
0	0.010175963	0	0.191641835	0.379616306
0	0	0	0	0
0.005534686	0.057903761	0.011563213	0.01090242	0
0.001144979	0.004580828	0.018184003	0	0.001529128
0	0.009821783	0	0	0
0	0	0	0	0.000589752
0	0.008899108	0	0	0
0	0	0	0	0.001043299
0	0	0	0	0
0	0	0	0	0.02284675
0	0	0	0	0.075232132
0	0	0	0	0
0.649338664	0.256510664	0	0	0
0	0	0	0	0
0	0	0	0.25369164	0
0	0	0	0	0
0	0.069989166	0	0	0
0	0	0	0	0
0	0	0	0	0.009153553
0	0	0	0	0.084338355
0	0.00904162	0	0	0
0	0	0	0	0.002388946
0	0	0	0	0
0	0	0	0	0
0	0	0	0	0
0	0	0	0	0
0	0	0	0.000651177	0
0	0.028818525	0	0	0
0	0	0	0	0
0	0	0	0	0
0	0	0	0	1.699462756
0	0	0	0	0
0	0	0	0	0.290223499
0	0	0	0	0
0	0	0	0	0.018294614
0	0.031644311	0	0	0
0	0	0	0.014329647	0
0.004975343	0.002732105	0	0	0.002768586
0	0	0	0	0.001256485

0	0	0	0	0.005233511
0	0	0	0	0.003712906
0	0.001773581	0	0	0
0	0	0	0	0
0	0	0	0	0
0	0.010451909	0	0	0
0	0	0	0	0
0	0	0	0	0
0	0	0	0.456245646	0
0	0	0	0	0
0	0	0.007642772	0	0.015680576
0.084720984	0	0	0	0.013511089
0	0	0	0	0
0.002862601	0.009768467	0	0.000516895	0.000955756
0	0	0	0	0.052946175
0	0	0	0	0.009015629
0	0	0	0.000601733	0
0	0.018684789	0	0	0.008603472
0	0	0	0	0.037892765
0	0	0	0.046492198	0
0	0	0	0	0
0	0.362938918	0	0.335448815	0
0	0	0	0	0
0.00840939	0	0	0	0
0	0	0	0	0
0	0	0	0	0
0	0	0	0	0
0	0	0	0	0
0	0	0	0	0
0.40176446	0.599896896	0.252442042	0.785945444	0.796083177
0	0	0	0.002337814	0
0	0	0	0	0
0	0.005982348	0	0	0
0	0	0	0	0
0	0	0	0	0
0	0	0	0	0
0	0	0.006590709	0.071448358	0
0	0.001716667	0	0	0
0	0	0	0	0
0	0	0	0	0
0.001176685	0	0	0	0
0	0	0.200050038	0.005482044	0
0	0.000943547	0	0	0
0	0	0	0	0
0	0.003123826	0	0	0.001994289
0	0	0	0	0
0.019142734	0.002906497	0	0	0.007009829
0	0	0	0	0

0.001523801	0.961441302	0.017367259	0	0
0	0	0	0	0
0	0.006840927	0.003103504	0	0.001386454
0	0	0	0	0
0	0	1.361367558	0.08949315	0
0	0	0	0	0
0	0	0	0	0
0	0.00160773	0	0	0
0	0	0	0.000921104	0
0	0	0	0	0
0	0	0	0	0
0.045399762	0	0	0	0
0	0	0	0	0
0	0	0	0	0
0	0	0	0	0
0	0.012169677	0.001006429	0.03566342	0.003596884
0.179311692	2.230151286	0.093257642	0.002012395	0.002591401
0	0	0	0	0.001210943
0	0.002612424	0	0	0
0	0.022486337	0	0	0
0	0.006374906	0	0	0
0.009002994	0.004889478	0	0	0.00393078
0	0	0	0	0
0	0.001861245	0.126587526	0	0
0	0	0.005838354	0	0
0	0	0	0	0.067486512
0	0	0	0	0
0	0	0	0	0
0	0	0	0	0
0	0	0	0	0
0	0.006782749	0.002800171	0.005689866	0.008852832
0.001145826	0.004449388	0.013487529	0	0
0	0.070478611	0.162358956	0.024821571	0.012293554
0.001563483	0.008278912	0.013583734	0.000846946	0
0	0.023242211	0.014073866	0.001813512	0
0	0	0	0	0
0.006904257	0.029595571	0	0	0
0.528181851	0.026957244	0.015132609	0.008774721	0.013713784
0	0	0.01557759	0.044387718	0
0	0	0.000883747	0	0
0	0	0	0	0
0	0	0	0	0.004080631
0	0.003257739	0.001293188	0	0
0	0.025507214	0.001687552	0	0
0	0.409303652	0	0	0
0	0	0	0	0
0	0	0	0	0
0	0	0	0	0

0	0	0	0	0
0.006306373	0.09572755	1.280213609	0.033972127	0.263193955
0	0	0	0	0
0	0	0	0.001724146	0
0	0	0	0	0
0	0	0	0	0
0	0.007208409	0	0	0.012174435
0	0	0	0	0
0.003127023	0	0	0	0
0	0.002696253	0	0	0
0	0.00248149	0.001094499	0.001128268	0
0.047117789	0.965542429	0.068472078	0.002941029	0.00670047
0	0	0.119261891	0.176888551	0
0	0	0	0	0
0.005825052	0.043657052	0	0	0
0	0	0	0	0
0.002044691	0.002021038	0.009168789	0.010337774	0.016179395
0.008493732	0.002623587	0	0	0.136121288
0	0	0	0	0
0	0.019469946	0	0	0.004488557
0	0.027987833	0.122446582	0.007919491	0.020909098
0	0	0	0	0
0	0	0	0	0
0	0	0	0	0
0	0	0	0	0
0	0	0	0	0
0	0.016457679	0	0.016836471	0.28706033
0	0.015822838	0.008542181	0.034316478	0.057690748
0	0	0.008817739	0	0
0.005136304	0	0	0	0
0	0	0.001078326	0	0
0	0	0	0	0
0	0	0	0	0.002180114
0	0	0	0	0
0	0	0.010727997	0	0
0.785902452	1.132224961	0.076537757	0.114239494	0.926992546
0	0	0	0	0
0	0	0	0	0
0.015205712	0.032792324	0.061986681	0.005191809	0.003323019
0	0	0	0	0
0	0.372352105	0	0	0
0	0	0	0	0
0.145427875	0.035331728	0	0	0.000841022
0.013781373	0	0.006906873	0	0
0	0	0	0	0
0	0	0	0.188265818	0
0	0	0	0	0
0.011007223	0	0	0	0

0	0	0	0	0
0	0	0.035737098	0.035256759	0.03475742
0	0	0	0	0
0	0.025132052	2.490808697	0.448520984	0
0	0	0	0	0
0	0	0	0	0
0	0	0	0	0
0	0	0	0.002780316	0
0	0	7.231466665	1.6289586	0
0	0	0	0.093430525	0
0	0	0	0	0
0	0.001852247	0.003361215	0.008662301	0
0	0	0	0	0
0	0	0	0	0
0	0.029696158	0.013348349	0.037192731	0
0	0	0	0	0
0	0	0	0.042978451	0
0	0	0	0.321599624	0
0	0	0	0	0
0	0	0	0	0
0	0	0	0	0
0	0	0	0	0
0	0	0	0.003988257	0
0.919497693	0.249242195	0.023544952	0.04819852	0
0	0.005945747	0	0.023316615	0
0	0	0	0.156718763	0
0	0	0	0.01914882	0
0	0	4.008725466	3.848164853	0
0	0	0	0	0
0	0	0	0	0
0	0.06688313	0	0.027105844	0.003284539
0	0	0	0.020083858	0
0	0	0	0.00060086	0
0	0	0	0	0
0	0	0	0	0
0	0	0.471871563	0.000557833	0
0	0.907900716	5.056323917	1.013953596	0
0	0.00137658	0	0	0.000976473
0	0	0.001968201	0.026066083	0.000781574
0	0	0	0.001743819	0
0.368347705	0.143167343	0.059451437	0.004154964	0.021787575
0	0	0	0.089652463	0
1.44704896	0.788086388	0.015230901	0.008099634	0.422726091
0	0	0.001580033	0.138220309	0
0	0	0	0	0
0	0	0	0.087391084	0
0	0	0	0	0
0.407139111	0.142809345	0	0.027471092	0

0	0	0	0	0
0	0	0.171931556	0.116460603	0
0.892029501	0.187212143	0.029261735	0	0.024899691
0	0.002529592	0	0.016173834	0
0	0	0	0	0
0	0	0.0557987	0.026756364	0
0	0	0	0	0
0	0	0	0.00955596	0
0	0	0	0	0
0	0	0	0	0
0.4102458	0.016083298	0.052899377	0.258113864	0.013853346
0	0.813752396	0.911676767	1.017199421	1.381555452
0	0	0	0	0
0	0.015188032	0.185300202	0.001268376	0.001978821
1.263988277	0.070835471	0.11111761	0.001749175	0.843801751
0	0	0	0.083653424	0
0	0	0	0	0
0	0	0	0	0
0	0	0	0.051528838	0
0	0	0	0.004235416	0.004195408
0.004773277	0.051032082	2.307459443	0.059594374	0.101426758
0	0.380208496	0	0.028802198	19.78631551
0	0.169613086	0.211916175	0.266555482	0.757046033
0	0	0	0	0
0	0	0.045328118	0.022808206	0
0	0.004615226	2.909302859	0.001180363	0
10.52587771	0	0	0.017377608	0.00081691
0	0	0	0	0
0	0	0.001743876	0	0.000779056
0	0	0	0	0
0	0	0	0.011407868	0
0	0	0.034460655	0.071838232	0
2.995914553	0	0.000936708	0	0.057329452
0	0.007422777	0.021965095	0.080883567	0
0	0	0.006336946	0	0
0	0.009094252	0.151834195	0.019291218	0.02466551
0	0.001550281	0	0.00264327	0
0	0	0	0	0
0	0.055088576	0	1.207021765	0
0	0	0	0	0
0	0	0.05366605	0.011686016	0
0	0	0	0	0
0	0	0.008733686	1.060415848	0
0	0	0	0	0
0	0.001525141	0.046980468	0.084253192	0.006058385
0	0	0	0	0
0	0	0	0	0
0	0.081684341	0.047031323	0.013927399	0.047241435

0	0	0	0	0
0	0	0	0	0
0	0	0	0	0
0	0	0	0.001196811	0
0	0	0	0	0
0	0	0.001822896	0.048387835	0
0	0	0	0	0
0	0.086120456	0.004938018	0.098993076	0
0	0	0	0	0
0	0	0	0	0
1.115892061	0	0.002758476	0.00088862	0
0	0	0	0	0
0	0	0	0.002852874	0
0	0	0	0.12574896	0
0	0	0	0	0
0	0	0	0	0
0	0	0	0.016302188	0
0	0	0.017414127	0.001602805	0
0	0	0	0.004411045	0
0	0.00108688	0	0	0
0	0	0.000850059	0.025850457	0
0	0	0	0	0
0	0	0.05493663	0.013219731	0
0	0	0	0	0
0	0	0	0	0
0	0	0	0	0
0	0	0	0	0
0	0	0	0	0
0	0	0	0	0
0	0	0.469035904	0.1633833	0
0	0	0	0	0.001617645
0	0	0	0	0
0	0	0	0.010047949	0
0	0	0	0	0
0	0	0.008956407	0	0
0	0	0	0	0
0	0	0.079180165	0.069044535	0
0.011282698	0.00759819	0	0	0.010084053
0	0	0	0.079224923	0
0	0.005076875	0	0	0
0	0	0.004929525	0	0
0	0	0	0	0
0	0	0	0.001272012	0
0	0	0	0.007670324	0
0	0	0	0.134813715	0
0	0	0	0	0
0	0	0.058611568	0.004169357	0
0	0	0	0	0

0	0	0	0	0
0	0	0	0.042396667	0
0	0	0	0.026113978	0
0	0.012921694	0	0.419145259	0
0	0	0	0.013904644	0
0	0	0	0	0
0	0	0	0	0
0	0	0	0	0
2.85287926	0.025014799	0	0.003976909	0.733745417
0	0	0	0	0
0	0	0.001151375	0.001359989	0
0	0	0	0	0
0	0	0	0	0
0	0	0	0	0
0	0	1.209237164	0.170480634	0
1.475518226	0	0	0.002265519	0.045832831
0	0	0	0	0
0	0	0	0	0
0	0	0	0	0
0	0	0	0	0
0	0	0	0.028954818	0
0	0	0	0	0
0	0	0	0	0
0	0	0	0	0
0.080796985	0.155033915	0.44657461	0.006506757	0
0	0	0.713919744	1.2158453	0
0	0	0.035919708	0.059898169	0
0	0	0	0	0
0	0	0	0	0
0	0	0	3.308160184	0
0	0	0	0	0
0	0	0	0.007202228	0
0	0	0	0	0
0	0.010190531	0	0.037465122	0
0	0.137824287	0.012497338	0	0.00099254
0.140968314	0.000989087	0	0	0.002806423
0	0	0	0	0
0	2.937036549	0.471052498	0.283496618	0
0	0	0.001735544	17.87047012	0
0	0	0	0	0
0	0	0	0	0
0	0	0	0.001290879	0
0.028955181	2.542706729	4.722013948	0.093333167	0.221472808
0	0	0	0	0
0	0	0	0	0
0	0.012415723	0	0.000838777	0
0	0	0	0	0
0.006343179	0.416867249	3.506024514	0.000572688	0.00132365

0	0	0	0.015658658	0.001184457
0	0	0	0	0
0	0	0	0	0
0	0	0	0	0
0.006867843	0	0	0.005243912	0
0	0	0	0.001228332	0
0	0	0	0	0
0	0	0	0	0
0	0	0	0	0
0	0	0	0.00867744	0
0	0	0	0	0
0	2.884178869	0.282901895	1.288435363	0.321125532
0	0	0	0.22480513	0.297475452
0	0	0	0	0
0.080334339	2.457134408	0.059021504	0.068513407	0.011137855
0	0	0	0	0
0	0	0	0.006955309	0
0	0	0	0	0
0	0	0.00334475	0.003878941	0
0	0.085644342	0	0.000477903	0
0	0	0.120734789	0.003925054	0
0	0	0	0	0
0	3.913560715	0.651113498	0.971852518	0.08000822
0	0	0	0	0
0	0	0	0	0
0	0	0	0	0
0	0	0	0	0
0	0	0	0	0
0	0	0	0.00679141	0
0	0	0	0	0
0	0	0	0	0
0.012662533	0.029522921	0.301732894	0.011485429	0.029495764
0	0	0	0.113406249	0
0	0.031783364	0.011544681	0.068111263	0
0.13625755	0.003229123	0	0	0.003244972
0	0.044749363	0.638389982	0.034955527	0
0	0	0	0.00256582	0
0	0	0.09826235	0.129118381	0
0	0	0	0.02229663	0
0	0	0	0	0
0	0	0.488543042	0	0
0	0	0	0	0
0	0	0	0	0
0	0	0	0	0
0.06476704	0.019616229	0.025465057	0.059389598	0
0	0	0	0	0
0	0	0	0.061833868	0
0	0	0	0	0

0	0	0	0	0
0	0	0	0	0
0	0	0	0	0.001944769
0	0	0	0	0
0	0	0	0	0
0	0	0	0	0
0	0	0.608821253	0	0
0	0.697276077	0.021995188	0.005562502	0.014509558
0	0	0	0	0
0	0	0	0.007167959	0
0	0	0	0	0.380540397
0.068155945	0	0	0	0
0	0	0	0	0
0	0	0.578002608	0.296632729	0
0	0	0	0	0
0	0	0	0	0
0	0	0.003136892	0.006568404	0
0	0	0	0	0
0	0	0.07535599	1.725345153	0.023897573
0	0	0	0	0
0	0	0	0.000382253	0.017139872
0	0	0	0	0
0	0	0	0	0
0	0	0	0	0
0	0.008688824	0.069671935	0	0
0.151700399	0.001974267	0.012539243	0	0.003676155
0	0	0	0	0
0.004960888	1.415252057	1.472810927	0	0.022617412
0	0	0.212708787	0	0
0.007164445	0	0.054080042	0.379821863	0
0	0	0	0	0
0	0	0	0	0
0	0	0	0.000913249	0
0	0	0	0	0
0.176005417	0	0	0	0.002201362
0	0	0	0	0
0	0	0	0.582448837	0
0.01433955	0.004401762	0	0	0
0	0	0	0	0
0	0	0	0	0
0.473132643	22.13288265	2.400043573	3.392772974	15.83448266
0	0.296648883	0.224600281	0.231398149	0.33765833
0	0	0	0	0
0	0	0	0.18776419	0
0	0	0	0	0
0	0	0	0	0
0	0	0	0.007835399	0
0	0	0.001109415	0.003335629	0

0	0	0	0	0
0	0.001197778	0	0.020463283	0.001359424
0	0	0	0.004293406	0
0	0	0	0	0
0	3.370488829	3.578296395	1.074718462	2.375845984
0	0	0	0	0
0	0	0	0	0
0	0	0.006452737	0.089903592	0
0.697425159	0.110511524	0	0	0.49749444
0	0	0	0	0
0	0	0	0	0.0011519
0	0	0	0	0
0	0.050743666	0	0.053340347	0.174525548
0	0.077837138	0.009542073	0.013466609	0.033696494
0	0	2.16327555	0	0
0	0.926387415	0.121926714	0.139333679	0.001665729
0	0.049774081	0.016907567	0.000506664	0
0	0	0	0	0
0	0	0	0	0
0	0	0.010854672	0	0
0	0	0	0	0
0	0	0	0	0
0	0	0	0	0
0	0	0	0	0
0	0	0	0	0
0	0	0	0	0
0	0	0.035728853	0	0
0.030112376	0	0	0	0
0	0	0	0	0
0	0	0	0	0
0	0	0	0	0
0	0	0	0	0
0	0	0	0	0
0	0	0.045092887	0.005471791	0
0	0	1.169496557	0.004468534	0
1.15807352	0	0	0.00287537	0
0	0	0	0	0
0	0	0.074170601	0	0
0	0	0	0	0
0	0	0	0	0
0	4.040640658	0.952706192	1.122506305	0
0	0	0	0.061371734	0
0	0	0	0.008710565	0
0	0	0	0	0
0	0	0	0	0
0	0	0	0	0
0	0	0	0.02839565	0
0	0	0	0	0

0	0.019686319	0.983590927	0.046538392	0
0	0	0	0	0
0	0	0	0	0
0	0	0	0	0
0	0	0	0.018589395	0
0	0	0	0	0
0.041461917	0.171685923	0.42440708	0.008592336	0.00070092
0	0	0	0	0
0	0	0	0	0
0	0	0	0.00503766	0.021877534
0	0	0	0.006435644	0
0	0	0	0	0
0	0	0	0	0
0	0	0	0	0
0	0	0	0	0
0	0	0	0	0
0	0	0	0	0.149895971
0	0	0	0	0
0	0	0	0	0
0	0	0	0	0
0	0.041392811	0.018485148	0	0
0	0	0	0	0
0	0.003631737	0.003501151	0.086115944	0
0	0.00069201	0	0.000589948	0
0	0	0	0	0
0	0	0.000864223	0.000974408	0
0	0	0	0	0
0	0	0	0	0
0	0	0.115296966	0	0
0.207133097	4.277997897	0.008170707	0	0
0	0	0	0.155349198	0
0	0	0	0	0
0	0	0	0	0
0	0	0	0	0
0	0	0	0	0
0	0	0	0	0
0.396339234	0	0.005728667	0	0.082713833
0	0	0	0	0
0	0	0	0	0
0	0	0	0	0
0	0	0	0	0
0	0	0	0	0
0	0	0	0	0
0	0	0	0.004930796	0
0	0	0	0	0
0	0	0	0	0
0	0	0	0	0
0	0	0	0	0

0	0.003474926	0.00078823	0	0
0	0	0	0	0
0	0	0	0	0
0	0	0	0	0
0	0	0	0	0
0	0	0	0	0
0	0	0	0	0
0	3.851616466	0.181290832	0.609564028	0.64104683
0.434010508	0.137918852	3.816902021	0.193106381	0.008567236
0	0.024139356	0	0	0
0	0	0	0.298907894	0
0	0	0	0.001943041	0
0	0	0	0.012495494	0
0	0	0	0.05650536	0
0	0	0	0	0
0	0	0	0	0
0.000769773	0	0	0	0.103574779
0	0	0	0	0
0	0	0	0	0
0	0	0	0	0
0	0	0	0	0
0	0	0	0	0
0	0	0	0.024719523	0
0	0	0	0	0
0	0	0	0.016499885	0
0	0	0	0	0
0	0	0	0	0
0	0	0	0.274169882	0
0	0	0	0	0
0	0	0	0	0
0	0	0	0.064823946	0
0	0	0	0	0
0	0	0	0	0
0	0	0	0.003735242	0
0	0	0	0	0
0	0	0	0	0
0	0	0	0	0
0	0	0	0	0
0	0	0	0.04347833	0
0.002807731	0.000743371	0.003344327	0	0
0	0	0	0	0
0	0	0.061495927	0.066950015	0
0	0	0	0	0
0	0	0	0	0
0	0	0	0	0
0	0	0	0.000338682	0
0	0	0	0	0
0	0	0	0	0

0	0	0	0	0
0	0.013889207	0	0.005385625	0
0	0	0	0	0
0	0	0	0	0
0	0.514605186	1.240084972	1.397670272	0
0	0	0	0.005417345	0
0	0	0	0	0
0.180448807	0.014839771	0	0	0.125422667
0	0	0	0.001935063	0
0	0	0	0	0
0	0	0	0	0
0	0	0	1.17865087	0
0	0	0	0	0
0	0	0	0	0
0	0	0	0	0
0	0	0.001304433	0	0
0	0	0	0	0
0	0	0	0	0
0	0	0	0	0
0	0	0	0	0
0	0	0	0	0
0	0	0	0	0
0	0	0	0	0
0.121525068	0	0.000867742	0	0
0	0	0.358422831	0.119131406	0
0	0	0	0	0
0.155041492	0.199932165	0	0.112877104	0.100161265
0	0	0	0	0
0.010522833	0	0	0	0
0	0	0	0	0
0	0	0	0	0
0	0	0	0	0
0	0	0	0	0
0	0	0	0	0
0	0	0	0	0
0	0	0	0	0
0	0	0	0	0
0	0	0.257406298	0.073285397	0
0	0	0	0	0
0	0	0	0	0
0	0	0	0	0
0	0	0	0	0
0	0	0	0	0
0	0	0	0.023148126	0
0	0	0	0	0
0	0	0	0	0
0	0	0	0	0
0	0	0	0.061715881	0

0	0	0	0.081987668	0
0	0	0	0	0
0	0	0.000554482	0	0
0	0	0	0	0
0	0	0	0	0
0	0	0.003891145	0	0
0	0	0	0	0
0	0	0	0	0
0	0	0	0	0
0	0	0	0	0
0	0	0	0	0
0	0	0	0.004123	0
0	0	0	0.006831333	0
0	0	0	0	0
0	0	0	0	0
0	0	0	0	0
0	0	0	0	0
0	0.176820269	0	0	0
0	0	0	0	0
0	0	0	0	0
0	0.04734619	0	0	0
0	0	0	0	0
0	0	0.007366367	0.019863008	0
0	0.005661446	0	0.014157611	0
0	0	0	0	0
0	0	0	0	0
0	0	0	0	0
0	0	0	0	0
0	0	0	0	0
0	0	0	0	0
0	0	0	0	0
0	0	0	0	0
0.085011945	0.004183727	0.002299524	0.010864666	0.228285666
0	0	0	0	0
0	0	0.006856378	0.209534425	0.26219337
0.004023613	0	0	0	0.017769399
0	0	0	0	0
0	0	0	0	0
0	0.002090958	0	0.000611167	0
0	0	0	0	0
0	0	0	0	0
0	0	0	0	0
0	0	0.613108592	0.20992469	0
0	0	0	0	0
0	0	0	0	0
0	0	0.001761789	0.016175059	0
0	0	0	0	0
0	0	0	0	0

0	0	0	0	0
0	0	0	0	0
0	0	0	0	0
0	0	0	0	0
0	0	0	0.002380051	0
0	0	0	0	0
0	0	0	0	0
0	0.003752633	0	0	0
0	0	0	0.098238578	0
0	0	0.001360968	0.039458207	0.000810662
0	0	0	0	0
0	0	0	0	0
0	0	0	0	0
0	0	0	0	0
0	0	0	0	0
5.632054683	0	0	0	0.160916146
0	0	0	0	0
0	0	0	0	0
0	0	0	0	0
0	0	1.108008805	0.274353079	0
0	0.008807803	0.00489487	0	0
0	0	0	0	0
0	0	0	0	0
0	0	0	0	0
0	0	0	0.003497978	0
0	0	0	0	0
0	0	0	0	0
0	0	0	0	0
0	0	0	0	0
0	0	0.132073641	0.0104317	0
0	0	0.219118234	0.187102638	0
0	0	0	0	0
0	0	0	0	0
0	0	0	0	0
0	0	0	0	0
0	0	0	0	0
0	0	0	0	0
0	0	0	0	0
0	0	0.1289971	0	0
0	0	0	0	0
0	0	0	0.002748056	0
0	0	0	0	0
0	0	0	0	0
0	0	0	0	0
0	0	0	0	0
0	0	0	0	0
0	0.002302115	0.002741535	0.000441581	0.300675778
0	0	0	0.001196242	0

0	0	0	0	0
0	0	0	0	0
0	0	0	0	0
0	0	0	0	0
0	0.052885443	0.017409115	0.031999744	0
0	0	0	0	0
0	0	0	0	0
0	0	0	0	0
0	0	0	0	0
0	0	0	0	0
0	0	0	0.005584681	0
0.015218963	0.012834231	0.007345791	0.068396189	0
0	0	0	0.001712305	0
0	0	0	0	0
0	0	0	0	0.001290758
0	0	0	0.004036569	0
0	0	0.002733767	0.023366858	0
0	0	0	0	0
0	0	0.000717713	0.000809219	0.124725017
0.094041773	0.009738029	0.75133148	0.007094266	0
0	0	0	0	0
0	0	0	0.020513134	0
0	0	0	0	0
0	0	0	0	0
0	0	0	0	0
0	0	0	0	0
0.143351433	0	0	0	0
0	0	0	0	0
0	0	0	0	0
0	0	0	0	0
0	0	0	0.002742213	0
0	0	0	0.002073337	0
0	0	0	0	0
0	0	0	0	0
0	0	0	0.003542126	0
0	0	0	0	0
0	0	0	0	0
0.094159648	0.007156366	0.040069991	0.002504579	0.005744287
0	0	0.240800104	0.047968201	0
0	0	0	0	0
0	0	0	0	0
0	0	0	0	0
0	0	0	0	0
0	0	0	0	0.002179036
0	0	0	0	0
0	0	0	0.001021636	0
0	0	0	0	0
0	0	0	0	0

0	0	0	0	0
0	0	0	0.06067907	0
0	0	0	0.336455492	0
0.06549594	0.000878006	0	0	0
0.000768942	0	0	0	0
0.018607383	0	0	0	0.001491018
0	0	0	0.004939342	0
0	0.001373353	0	0	0.001136548
0	0	0	0	0
0	0	0	0.015742068	0
0	0	0	0.01249384	0
0	0	0	0	0
0	0.001244765	0.019105993	0.109619784	0
0	0	0	0.001198393	0
0	0	0	0	0
0	0	1.784629328	0	0
0	0	0	0	0
0	0	0	0	0
0	0	0	0	0
0	0	0	0	0
0	0	0	0	0
0	0	0	0	0
0	0.012019838	0	0	0
0	0.003223596	0.006654101	0.003132901	0
0	0	0	0	0
0	0	0	0	0
0	0	0.001036055	0.005740612	0
0	0	0	0	0
0	0	0	0	0
0	0	0	0	0
0	0	0.001450928	0.007322669	0
0	0	0	0	0
0	0	0	0	0
0	0	0	0	0
0	0	0.005879389	0.000914343	0
0.142739823	0	0	0	0
0	0	0	0	0
0	0	0	0	0
0	0.00489642	0.00593333	0.017531906	0
0	0	0	0	0
0	0	0	0	0
0	0	0	0	0
0	0	0	0	0
0	0	0	0	0
0	0	0	0	0
0	0.221977448	0.467851388	0.046739459	0.050181014
0.00095557	0	0	0	0.021248249
0	0	0	0	0

0	0	0	0	0
0	0	0	0	0
0	0	0	0.034379839	0
0	0	0	0.038522768	0
0	0	0	0	0.001628429
0	0	0	0.035449668	0
0	0	0	0	0.000557449
0	0	0	0	0
0	0	0	0.000365902	0
0	0	0	0	0
0	0	0	0.002245748	0
0.005747441	0	0	0	0.001107079
0	0	0	0	0
0	0	0	0	0
0	0	0	0	0
0	0	0	0	0
0	0	0	0	0
0	0	0	0	0
0	0	0	0	0
0	0.003049873	0	0	0
0	0	0	0	0
0	0	0	0	0
0	0	0	0	0
0	0	0	0	0
0	0	0	0	0.000680537
0	0	0	0	0
0	0	0	0	0
0	0	0.007551688	0	0
0	0	0	0	0
0	0	0	0	0
0	0.00547851	0.00397127	0.00146207	0.002703418
0	0	0	0	0
0	0.0486763	0.001976164	0.016410256	0.037471143
0	0	0.019745487	0.004481505	0.002205266
0	0	0	0	0
0	0	0	0	0
0	0	0	0	0
0	0	0	0	0
0	0	0	0	0
0	0	0	0	0
0	0	0	0	0
0.386887692	0.402730823	0	0.017690401	0.000484196
0	0.13111445	0.033671024	0.072454071	0.01789326
0	0	0	0	0
0	0	0	0	0
0	0.010414814	0.291941827	0.039544669	0
0	0	0	0.009537768	0
0	0	0	0	0

0	0	0	0	0
0	0.004646419	0	0.398985317	0
0	0.112960022	0.021275663	0	0.003609362
0	0	0	0	0
0	0	0.009676477	0.02382924	0
0	0	0	0.00067428	0.010098814
0	0	0	0	0
0	0	0	0	0
0	0	0.157294294	0.021483963	0
0	0	0	0	0
0	0	0	0	0
0	0	0	0	0
0	0	0	0	0
0	0	0	0	0
0	0	0	0	0
0	0	0	0	0
0	0	0.592201108	0	0
0	0	0	0	0
0	0	0	0	0
0	0	0	0	0
0	0	0	0	0
0	0	0	0	0
0	0	0	0.027075216	0
0	0	0	0	0
0	0	0.029075595	0.003145988	0
0.054373014	0.341138247	0	0	0
0	0	0	0	0
0	0.011457932	0.156159249	0.000697717	0
0	0	0	0	0
0	0	0	0	0
0	0	0	0	0
0	0	0	0	0.040936848
0	0	0	0	0
0	0	0	0	0
0	0	0	0	0
0	0	0	0	0
0	0	0	0	0
0	0.001119784	0	0	0
0	0.668733953	0.177378676	0.222033892	0
0	0	0	0	0
0	0	0	0	0
0	0	0	0.012172638	0
0	0.004233753	0.001120417	0	0
0	0	0	0	0
0	0	0	0	0
0	0	0	0	0
0.003363674	0.002158938	0	0.001987765	0
0	0	0	0	0

0	0	0	0	0
0.346299414	0.01225882	0.003892998	0.002647538	0.020483336
0	0	0	0.01652227	0
0	0	0	0	0
0	0	0	0	0
0	0	0	0	0
0	0	0	0	0
0	0	0	0	0
0	0	0	0	0
0	0	0	0	0
0	0	0	0	0
0	0	0	0	0
0	0	0.367628416	0.081898286	0
0	0	0	0	0
0	0.002377651	0.009438297	0.01250936	0
0	0	0	0	0
0	0	0	0	0
0	0	0	0	0
0	0	0	0.003267445	0
0	0	0	0	0
0	0	0	0	0
0.012993027	0.005670814	1.727240375	0.00341255	0
0.000544586	0	0	0	0
0.015329339	0	0	0	0
0	0	0	0	0
0	0	0	0	0
0	0	0	0	0.00090329
0	0	0	0	0
0	0	0	0	0
0	0	0	0	0
0	0	0	0	0
0	0	0	0	0
0	0	0	0	0
0	0	0	0	0
0	0	0	20.51361107	0.297767737
0	0	0	0	0
0	0	0	0	0
0	0	0	0	0
0.009200532	0.025650033	0.157622765	0.140062377	0.023629573
0	0	0	0	0
9.992041133	0.266724567	0.202035011	0.078528473	0.317593691
0	0	0	0	0
0	0	0	0	0
0	0	0	0	0
0	0	0	0	0
4.912667778	0.085835107	0.114029181	0.00104953	0.574422714
0	0	0	0	0
0	0	0	0	0

0	0	0	0	0
0	0	0	0	0
0	0	0	0	0
0	0	0	0.004470102	0
0	0	0	0.001591979	0
0	0	0	0	0
0	0	0	0	0
0.001308824	0	0.012618394	0	0
0	0	0.002574898	0	0
0.498105357	0.120067436	0.0509809	0.002566106	0
0	0	0	0	0
0	0	0	0	0
0	0	0	0.001839312	0
0	0	0	0	0
0	0	0	0	0
0	0	0	0	0
0	0	0	0	0
0	0	0	0	0
0	0	0	0	0
0.663088929	0.138206413	0.380686324	0.185291891	0.152730221
0	0	0	0.000852278	0
0	0	0	0	0
0	0	0	0	0
0	0	0	0	0
0	0	0	0	0
0	0	0	0	0
0	0	0	0	0
0	0	0	0	0
0	0	0	0	0
0	0.014618968	0	0	0
0	0	0	0	0
0	0	0	0	0
0	0	0	0	0
0	0	0	0	0
0	0	0	0	0
0	0	0	0	0.002938869
0	0	0	0	0
0	0	0	0	0
0	0	0	0	0
0	0	0	0	0
0	0	0	0	0
0	0	0	0	0
0	0	0	0.00618562	0
0	0	0	0	0
0.005612532	0.008471715	0.008027621	0.0096452	0
0	0	0	0.003471851	0
0	0	0	0	0
0.000968676	0	0	0	0.043661508

0	0	0	0	0
0	0	0	0.000372846	0
0	0	0	0	0
0	0	0	0	0
0	0	0	0	0
0.002600673	0.003060225	0.008098556	0.010331176	0
0	0	0	0	0
0	0	0	0	0
0	0	0	0	0
0	0	0	0	0
0	0	0	0	0
0	0	0	0	0
0	0	0	0	0
0	0	0.093396089	0	0
0	0	0	0	0
0	0	0	0	0
0	0	0	0.01620253	0
0	0	0	0	0
0	0	0	0	0
0	0	0	0	0
0	0	0	0	0
0	0	0	0	0
0	0	0	0	0
0	0	0	0	0
0	0	0	0	0
0.003841895	0.007020501	0	0	0
0	0	0	0	0
0	0	0	0.657129801	0
0	0	0	0	0
0	0	0	0	0
0	0	0	0	0
0	0	0	0	0
0	0	0	0	0
0	0	0	0	0
0	0	0	0	0
0	0	0	0	0
0	0	0	0	0
0	0	0	0	0
0	0	0	1.486326428	0
0	0	0	0	0
0	0	0	0	0
0	0	0	0	0
0	0	0	0	0
0	0	0	0	0
0	0	0	0	0
0	0	0	0	0
0	0	0	0	0
0	0	0	0	0

0	0	0	0	0
0	0	0	0.051874378	0.001296182
0	0	0	0	0
0	0	0	0	0
0	0	0	0	0
0	0	0	0	0
0	0	0	0	0
0	0	0	0	0
0	0	0	0	0
0	0	0.002708376	0	0
0	0	0.017096399	0	0
0	0	0	0	0
0	0	0.157229638	0.183445101	0
0	0	0	0	0
0	0	0	0	0
0	0	0	0	0
0	0	0	0	0
0	0	0	0	0
0	0	0	0	0
0	0	0	0	0
0	0.208542081	0.001204987	0	0
0	0	0	0	0
0	0	0	0	0
0	1.615911451	2.902797395	0.582805142	0
0	0	0	0	0
0	0	0	0	0
0	0	0	0	0
0	0	0.050652989	0	0
0	0	0	0	0
0	0	0	0	0
0	0	0	0	0.004226839
0	0	0	0	0
0	0	0	0	0
0	0	0	0	0

PHL018-0090_V2	PHL018-0043_V3	PHL018-0259_V1	PHL018-0107_V2	PHL018-0232_V1
CTRL	EYCF	REF	EYCF	REF
0.995340362	1.015118403	0.022544457	0.038155764	0.087047144
0.409503878	0.393038628	1.157779547	3.089843312	0.876555166
0.031280271	0.019574364	3.108616478	4.770358185	0
0	0	0	0	0
0	0	0	0	0
0	0	0	0	0
0	0	0	0	0
0	0	0	0	0
0	0	0	0	0
0	0	0	0	0
0	0	0	0	0
0	0	0	0	0
0	0	0	0	0
2.796317424	2.734491688	1.527864592	19.91818168	5.532108869
0	0	0	0	0
0	0	0	0	0
0	0	0	0	0
0	0	0	0	0
0	0	0	0	0
0	0	0	0	0
0	0	0	0	0
0	0	0	0	0
0	0	0	0	0
0	0	0	0	0
0	0	0	0	0
0	0	0	0	0
0	0	0	0	0
0	0	0	0	0
0	0	0	0	0
0	0	0	0	0
0	0	0	0	0
0	0	0	0	0
0	0	0	0	0
0	0	0	0	0
0	0	0	0	0
1.614485706	1.623965122	1.668184252	0	1.059980114
0	0	0	0	0
0	0	0	0	0
0	0	0	0	0
0	0	0	0	0
0	0	0	0	0
0	0	0.027954272	0.130325842	0.008238612
0.036903538	0.03672556	0.00951395	1.163549266	0.00404184
0.206605087	0.213787337	0.004718624	0.008094772	0.314672952
0	0	0.000583193	0.003209825	0.015785048
0	0	0	0	0

0	0	0.002393466	0	0
0	0	0	0.031221017	0.073964303
0.000788599	0	0	0	0.002971158
3.386027086	3.450366607	0.600607595	0.014011907	0.546158493
0	0	0.001735333	0	0.008494487
0	0	0	0	0
0	0	1.509830237	0	0.001283851
0	0	0.018700858	0	0
0	0	0	0	0
0	0	0	0	0
0.002113357	0	0	0	0
0.055911849	0.051132487	0.020584166	0	0
0	0	0.000804712	0	0
0.193279392	0.198745157	0.001729643	0.006790306	0.066430531
0	0	0	0	0
0	0	0	0	0
0.00401463	0.004362021	0	0	0.016386158
0	0	0	0	0
0	0	0	0	0
0	0	0	0	0
0.275797037	0.267170982	0.018417591	0.024480309	0.37114334
0.024151395	0.025740403	0.008712163	0	0
0	0	0	0	0
0	0	0	0	0
0	0	0	0	0
0	0	0	0	0
0	0	0	0	0
0	0	0	0	0
0	0	0	0.002281343	0.011086416
0	0	0	0	0
0	0	0	0.002918737	0
0	0	0	0	0
0	0	0	0	0
0	0	0	0	0
0	0	0	0	0
0	0	0.001227595	0	0
0	0	0	0	0
0	0	0	0	0
0	0	0	0	0
0	0	0	0	0
0	0	0	0	0
0	0	0	0	0
0	0	0	0	0
0	0	0	0	0
0	0	0	0	0
0	0	0	0	0
0	0	0	0	0
0	0	0.002533939	0	0

0	0	0	0	0
0	0	0	0	0
0	0	0	0	0
0	0	0	0	0
0	0	0.021035453	0	0
0	0	0	0	0
0	0	0	0	0
0	0	0	0	0
0.008600501	0.008449851	0	0	0
0	0	0	0	0
0	0	0	0	0
0	0	0.013331729	0	0.006142031
0	0	0	0	0
0	0	0	0	0
0	0	0	0	0
0	0	0	0	0.000949464
0	0	0	0	0
0	0	0	0	0
0	0	0.146198424	0	0
0	0	0.001471229	0	0
0	0	0	0	0
0	0	0	0	0
0	0	0	0	0
0	0	0	0	0
0	0	0	0	0
0	0	0	0	0
0	0	0	0	0
0	0	0	0	0
0	0	0	0	0
0	0	0	0	0
0.026149859	0.027801763	0.005195025	0	0
0.00238552	0	0.003592067	0	0.013909663
0	0	0	0	0
0	0	0	0	0
0	0	0	0	0
0	0	0	0	0
0	0	0	0	0
0	0	0	0	0
0	0	0.096455925	0	0
0	0	0	0	0
0	0	0	0	0
0	0	0	0	0
0.001181693	0.002370363	0	0	0
0	0	0	0	0
0	0	0.001526366	0	0
0	0	0	0	0
0	0	0	0	0
0	0	0	0	0

0	0	0	0	0
0	0	0	0	0
0	0	0	0	0
0	0	0	0	0
0.000741545	0.001586632	0.004959257	0	0
0	0	0	0	0
0	0	0	0	0
0	0	0	0	0
0	0	0	0	0
0	0	0	0	0
0	0	0	0	0
0	0	0	0	0
0	0	0	0	0
0	0	0	0	0
0	0	0	0	0
0	0	0	0	0
0	0	0	0	0
0	0	0	0	0
0	0	0	0	0
0	0	0	0	0
0	0	0	0	0
0	0	0	0	0
0	0	0	0	0
0	0	0	0	0
0	0	0	0.009592585	0
0	0	0	0	0
0	0	0	0	0
0	0	0	0	0
0	0	0	0	0
0	0	0	0	0
0	0	0	0	0
0	0	0	0	0
0	0	0	0	0
0	0	0	0	0
0	0	0	0	0
0	0	0	0	0
0	0	0	0	0
0	0	0	0	0
0	0	0	0	0
0	0	0	0	0
0	0	0	0	0
0	0	0	0	0
0	0	0	0	0
0	0	0	0	0
0	0	0	0	0
0	0	0	0	0
0	0	0	0	0
0.002892401	0.003545593	0	0	0

0	0	0	0	0
0	0	0	0	0
0	0	0	0	0
0	0	0	0	0
0	0	0	0	0
0	0	0	0	0
0	0	0	0	0
0	0	0	0	0
0	0	0	0	0
0	0	0	0	0
0	0	0	0	0
0	0	0	0	0
0	0.001509445	0	0	0
0	0	0	0	0
0	0	0	0	0
0	0	0	0	0
0	0	0	0	0
0	0	0	0	0
0	0	0	0	0
0	0	0	0	0
0.0517071	0.055975593	0.015853166	0.003070518	0.046825989
0	0	0	0	0
0.002006146	0	0	0	0
0	0	0	0	0.009673885
0	0	0	0	0
0	0	0	0	0
0.002048503	0.0027394	0.000893469	0.022352467	0.095188965
0	0	0	0	0
0	0	0	0	0
0	0	0	0	0
0	0	0	0	0
0	0	0	0	0
0	0	0	0	0
0.012898028	0.011498758	0.01328258	0	0
0.010527865	0.01254831	0.00831848	0	0.023856615
0	0	0	0	0
0	0	0	0	0
0	0	0	0	0
0	0	0	0	0
0	0	0	0	0
0	0	0	0	0
0	0	0	0	0
0	0	0	0	0
0	0	0	0	0
0	0	0	0	0
0	0	0	0.000713311	0
0.001982414	0.001656888	0	0	0
0	0	0	0	0

0	0	0	0	0
0	0	0	0	0
0	0	0	0	0.004492059
0	0	0	0	0
0	0	0	0	0
0	0	0	0	0
0	0	0	0	0
0	0	0.002726902	0	0.001177786
0	0	0	0	0
0	0	0	0	0
0	0	0	0	0
0	0	0	0	0
0	0	0	0	0
0	0	0	0	0
0	0.001375511	0	0	0
0	0	0	0	0
0	0	0	0	0
0	0	0	0	0
0	0	0	0.006696586	0
0	0	0.03385226	0	0
0.001223362	0.002617543	0.002667888	0.049924685	0
0	0	0	0	0
0	0	0	0	0
0	0	0	0	0
0.003402653	0.008450491	0	0.010077716	0.023808527
0	0	0	0	0.001184793
0	0	0	0	0
0	0	0	0	0
0	0	0	0.007339526	0.003495511
0	0	0	0	0
0.060808729	0.052206894	0.010593164	0.121907283	0.117038092
0	0	0	0	0
0.003579213	0.003589779	0	0.078109962	0
0	0	0.005752794	0	0
0	0	0	0	0
0	0	0	0	0
0	0	0	0	0
0	0	0	0	0
0	0	0	0	0
0	0	0	0.000980422	0.020537326
0	0	0	0	0
0.205758826	0.209431682	0	0	0.011976149
0	0	0	0	0
0	0	0	0	0.001247784
0	0	0	0	0
0	0	0	0	0
0	0	0	0	0
0	0	0	0	0

0	0	0	0	0
0	0	0	0	0
0.200566043	0.195230381	0.0070304	0.003517678	0.361201825
0	0	0	0	0
0	0	0	0	0
0	0	0	0	0
0	0	0	0	0
0	0	0	0	0
0	0	0	0	0
0	0	0	0	0
0	0	0	0	0
0	0	0	0	0
0	0	0	0	0
0	0	0	0	0
0.002310539	0.001404461	0	0.001833578	0.012926026
0	0	0	0	0
0.002153988	0.002560411	0.001391817	0.006789894	0.020739495
0	0	0	0	0
0	0	0	0	0
0	0	0	0	0
0	0	0	0	0
0	0	0	0.001178719	0
0	0	0	0	0
0	0	0	0	0
0	0	0	0	0
0	0	0	0	0
0	0	0	0	0
0	0	0	0	0
0	0	0	0	0
0.001562581	0	0	0	0
0	0	0	0	0
0	0	0	0	0
0	0	0	0	0
0	0	0	0	0
0	0	0	0	0
0	0	0	0	0.005961398
0	0	0	0	0
0	0	0	0.074935459	0.172836009
0	0	0	0	0
0	0	0	0	0
0	0	0	0	0
0	0	0	0	0
0	0	0	0	0
0	0	0	0	0
0	0	0	0	0
0	0	0	0	0
0	0	0	0	0
0	0	0	0.006098877	0.001651659

0	0	0	0	0
0	0	0	0	0
0.003620704	0.001815696	0	0.000987693	0
0	0	0	0	0
0.002276736	0.003044609	0	0.007949713	0.042317753
0	0	0	0	0
0	0	0	0	0
0.065741734	0.068709803	0	0.024375908	0.009218283
0	0	0	0	0
0.046709335	0.046627282	0.002152035	0.879008249	0.033874854
0	0	0	0	0
0	0	0.00073457	0.017090792	0.001903624
0.003832548	0.005637664	0.017969615	0.001881867	0.083028068
0	0	0	0	0
0	0	0	0	0
0	0	0	0	0
0	0	0	0	0
0	0	0	0	0
0.173953377	0.175087769	0	0.007092587	0.035374164
0	0	0	0	0
0	0	0	0	0
0	0	0	0	0
0	0	0.051027318	0	0
0	0	0	0	0
0	0	0	0	0
0	0	0.028717935	0	0.350666296
0	0	0	0	0
0.02888008	0.031401671	0	0.080401858	0.033219182
0	0	0	0	0
0	0	0.002503995	0	0
0	0	0	0	0.011129103
0	0	0	0	0
0	0	0	0	0
0	0	0	0	0
0	0	0	0	0
0	0	0	0	0
0.050856363	0.048722619	0	0	0
0	0	0	0	0
0	0	0	0	0
0.023474618	0.017579457	0	0.108093556	0
0	0	0	0	0.035323072
0.047969426	0.056085721	0	0	0
0	0	0	0	0
0	0	0	0	0
0	0	0	0	0
0	0	0	0	0
0	0	0	0	0.002091517
0	0	0	0	0

0	0	0	0	0
0	0	0.285470832	0	0
0	0	0	0	0
0	0	0	0	0
0	0	0	0	0
0.051811666	0.045313729	0	0	0.005491076
0	0	0.00123695	0	0
0	0	0	0	0
0	0	0	0	0
0	0	0	0	0
0.005324679	0.00712053	0	0.001383353	0.146480642
0	0	0	0	0
0	0	0	0	0
0.002874565	0.003523729	0.013234148	0.001829691	0.005716007
0	0	0	0.006611852	0.014002333
0	0	0	0	0
0	0	0	0	0
0	0	0	0	0
0	0	0	0	0
0	0	0.000417666	0	0
0	0	0.009381087	0	0
0	0	0.433129038	0	0
0	0	0	0	0
0	0	0	0	0
0	0	0	0	0
0	0	0	0	0
0	0	0	0	0
0	0	0	0	0
0	0	0	0	0
0.98771712	1.025966509	10.05122513	0.250187068	1.140353806
0	0	0.002717122	0	0.001428684
0	0	0	0	0
0	0	0	0	0
0.0046035	0.004309284	0	0	0
0.000865331	0	0	0.007081614	0
0	0	0	0	0
0	0	0.52151236	0	0
0	0	0	0	0.005826134
0	0	0.002044048	0.448241032	0
0	0	0	0	0
0	0	0.019039842	0	0
10.35384127	10.41084932	0	0	3.06047389
0	0	0	0	0.004775314
0	0	0	0.098770292	0
0	0	0	0	0
0	0	0	0	0
0.001033481	0	0	0.001127694	0.008047154
0	0	0	0	0

0	0	0.015943306	0.004452432	0.01889682
0	0	0	0	0.004707012
0	0	0.019955746	0	0.024875539
0	0	0	0	0
0	0	0	0	0
0.014015735	0.014417549	0	0	0
0	0	0	0	0
0	0	0.022648188	0	0
0	0	0	0	0
0	0	0	0	0
0	0	0	0	0
0	0	0	0	0
0	0	0	0	0
0.008997991	0.00894742	0	0	0.495479047
0	0	0	0	0
0.435609419	0.446780947	0.118090943	0	0.000679313
0.000999229	0	0.028764169	0.011339329	0.01104323
0.005098904	0.006493912	0	0.123196763	0
0	0	0	0	0
0	0	0	0	0
0.010074506	0.004715315	0.012999109	0	0.016131777
0	0	0	0	0.002329013
0	0	0	0	0
0.190381884	0.180249857	0	0.064030263	1.36282438
0.095815722	0.090166561	0	0.001426613	0.00539505
0	0	0.002679213	0.002585348	0
0	0	0.000553278	0	0
0	0	0	0	0
0	0	0	0	0
0	0	0	0	0
0.087210078	0.077662905	0	0.002105315	0.006057822
0.039120919	0.041159758	0.079041179	0.007846916	0.09031469
0.084082794	0.096692231	0.43661521	0.017333473	0.138618774
0	0	0	0	0.002464693
0	0	0.000814591	0	0
0	0	0	0	0
0.001485667	0	0.005399867	0.001080735	0.00248776
0.058480321	0.057228712	0.012246543	0	0
0	0	0.209976525	0	0.009213698
0.001741556	0.001058604	0	0	0
0	0	0	0	0
0	0	0	0	0
0.054733165	0.045697208	0	0	0
0.060465031	0.056600623	0	0	0
0	0	0	0	0
0	0	0.006905034	0	0
0	0	0	0	0
0	0	0	0	0

0	0	0	0	0.024519065
2.490873906	2.529631281	0.040471907	0.481110711	0.042085425
0	0	0	0	0
0	0	0	0	0
0	0	0	0	0
0	0	0	0	0
0	0	0	0	0
0	0	0	0.00176921	0
0	0	0.010535175	0	0.001516762
0	0	0	0.003347588	0
0.013333414	0.009177395	0	0	0.01354389
0.021028764	0.020309699	0.206366396	0.029000899	0.361298138
0	0	0	0	0
0	0	0	0	0
0	0	0.010466688	0	0.00282544
0	0	0	0	0
0.020943014	0.02608444	0	0.04772077	0.111912086
0.011461155	0.009267278	0	0.026175276	0.031800341
0	0	0	0	0
0	0	0	0.001321978	0
0.19818911	0.198774168	0.060182769	0.005545031	0.689532438
0	0	0	0	0.002316269
0	0	0	0	0
0	0	0	0	0
0	0	0.000775844	0	0
0	0	0	0	0
0	0	0.001671195	0	0.039896511
0.103747541	0.10924521	0.040407809	0.130885917	0.286026087
0	0	0	0	0
0	0	0	0	0
0	0	0	0	0.242310353
0	0	0	0	0
0	0	0	0	0
0	0	0	0	0
0.063148851	0.053762857	0	0	0.00197037
0.245287898	0.251105409	0.226482696	12.96308533	0.061227774
0	0	0	0	0
0	0	0	0	0
0.001943361	0	0.002623555	0.00878501	0.003835274
0	0	0	0	0
0	0	0	0	0
0	0	0	0	0
0	0	0	0.00680819	0.022237127
0	0	0.00106517	0	0.002862604
0	0	0	0	0
0	0	0.089877447	0	0
0	0	0	0	0
0	0	0	0	0.008593329

0	0	0	0	0
0	0	0	0	0
0	0	0.001011062	0	0
0.010016575	0.011556349	0.009137355	0	0
0	0	0	0	0
0	0	0	0	0
0	0	0	0	0
0	0	0.001021793	0	0
2.281292436	2.291430391	0.069996687	0	0.253313847
0.639303448	0.649993412	0	0	0
0	0	0	0	0
0.00639797	0.007045961	0.003009383	0	0.001890608
0	0	0	0	0
0	0	0.61351228	0	0
0.220501838	0.221360419	0.010610679	0	0.009555825
0	0	0	0	0
0	0	0.2132057	0	0
0	0	0	0	0
0	0	0	0	0
0	0	0	0	0
0	0	0	0	0
0	0	0.000507998	0	0
0	0	0	0	0
0	0	0.447323262	0.000979278	0.006950494
0	0	0.001024564	0	0
0	0	0.086849465	0	0
0	0	0	0	0
2.901743733	2.972385309	1.175407183	0	1.907948295
0	0	0	0	0
0	0	0	0	0
0	0	0.012101484	0	0
0	0	0.001879425	0	0
0.303242582	0.300693286	0	0	0.001923416
0	0	0	0	0
0	0	0	0	0
0	0	0.00045102	0	0
0.491804006	0.488098903	0.51492537	0	0
0	0	0	0	0
0	0	0	0	0
0	0	0	0	0
0.240092732	0.243094838	0.00091858	0	0.006347952
0	0	0	0	0
5.221187347	5.170717547	5.311867415	0.585783885	4.107284434
0	0	0	0	0
0	0	0	0	0
0	0	0	0	0
0	0	0	0	0
0.314008799	0.314282817	0	0.001894315	0

0	0	0	0	0
0.069985558	0.063714914	0.034634879	0	0
0.00416051	0.002225489	0	0.494609678	0.013585266
0	0	0	0	0
0	0	0	0	0
0.003605238	0.003725453	0.002739874	0	0
0	0	0	0	0
0	0	0	0	0
0	0	0	0	0
0	0	0	0	0
0.223851578	0.213040956	0.001187766	0.007800213	0.014620853
2.672623203	2.622906939	0.519946841	1.20710851	4.359990083
0	0	0	0	0
0	0	0	0.084664128	0.071699472
0.272023897	0.248320114	0.002020351	0.00530716	0.008071705
0	0	0	0	0
0	0	0	0	0
0	0	0	0	0
0	0	0	0	0
0	0	0.000978407	0.006058161	0
0.036291573	0.029988872	0	0	0.015694267
0.086400889	0.081771373	0.377892385	3.163110939	0.075888462
2.305375386	2.234881168	0.075458738	14.15954764	0.71559482
0	0	0	0	0
0	0	0	0	0
2.832476129	2.879516498	1.362630488	0	17.57706536
0	0	0	0	0
0	0	0.001038313	0	0
0	0	0.001608651	0	0
0	0	0	0	0
0.015293971	0.014897252	0.000411764	0	0
0.575828316	0.58417388	0.113134471	0	0
0.001678112	0.001683066	0	0	0
0.070702908	0.078505479	0.004651216	0.000698174	0
0.010245069	0.011293587	0	0	0
0.010461965	0.008139907	0.002350666	0.097344686	0
0.012678581	0.009583079	0	0	0
0	0	0	0	0
0.166054337	0.161037698	0.046601012	0.009472505	0.018450306
0	0	0	0	0
0	0	0.001339334	0	0
0	0	0	0	0
0	0	0	0	0
0	0	0	0	0
0.313885442	0.302846134	0.000788432	0.000710089	0.01607322
0	0	0	0	0
0	0	0	0	0
0.048408327	0.044789603	0.000760851	4.313356981	0.118303909

0	0	0	0	0
0	0	0	0	0
0	0	0	0	0
0	0	0.000806373	0	0
0	0	0	0	0
0.005878297	0.005677292	0	0	0
0	0	0	0	0
0	0	0.006678079	0	0
0	0	0	0	0
0	0	0	0	0
0	0.001101422	0	0	0
0	0	0	0	0
0.008293308	0.011572577	0.00445595	0	0
0	0	0	0	0
0	0	0	0	0
0	0	0	0	0
0	0	0.013136138	0	0
0.000685658	0.000916909	0.000996848	0	0
0	0	0.236406687	0	0
0	0	0	0	0
0.019188314	0.023012489	0	0	0.000956279
0	0	0	0	0
0.003162057	0.003964239	0	0	0
0	0	0	0	0
0	0	0	0	0
0	0	0	0	0
0	0	0	0	0
0	0	0	0	0
0	0	0	0	0
0.065019065	0.067841595	1.702865427	0.004066983	0
0	0	0	0	0
0	0	0	0	0
0	0	0	0	0
0	0	0	0	0
0	0	0.000357057	0	0
0	0	0	0	0
0.042909292	0.038845747	0.093254345	0	0
0	0	0	0	0
0	0	0	0	0
0	0	0.000499909	0	0
0	0	0	0	0
0	0	0.01024681	0	0
0	0	0	0	0
0	0	0	0	0
0	0	0.094669808	0.152039215	0
0	0	0	0	0
0	0	0.017697826	0	0
0	0	0	0	0

0	0	0	0	0
0	0	0	0	0
0	0	0.006304788	0	0
0.210660136	0.195689455	0.089612202	0	0
0	0	0	0	0
1.272269007	1.240090999	0	0	0
0	0	0	0	0
0	0	0	0	0
0	0	0.017300334	0.864052707	0.013887814
0.001742311	0.002071057	0	0	0
0	0	0.000624761	0	0
0	0	0	0	0
0	0	0	0	0
0	0	0	0	0
0	0	17.54900538	0	0
0	0	0.001077483	0.004852091	0
0	0	0	0	0
0	0	0	0	0
0	0	0	0	0
0	0	0	0	0
0	0	0	0	0
0	0	0	0	0
0	0	0	0	0
0	0	0	0	0
0	0	0	1.10987637	0
2.309905354	2.242341776	1.816658045	0.0037797	2.163090934
0	0	0.004586075	0	0
0	0	0	0	0
0.094662266	0.099486353	0.038448851	0	0
0	0	0	0	0
0	0	0	0	0
0	0	0	0	0
0	0	0.001492718	0	0
0.007850936	0.008479813	0.000548756	0	0
0	0	0.001265848	0.01112922	0.175580632
0	0	0	0.002110661	0.015459067
0.818067135	0.830917084	0	0	0
1.462611967	1.484056418	0.012846324	0.517826338	2.137606775
0	0	0.134965372	0	0
0	0	0	0	0
0	0	0	0	0
0	0	0	0	0
0.303738158	0.323594137	0.160705809	0	0
0	0	0	0	0
0	0	0	0	0
0	0	0.002300932	0	0
0	0	0	0	0
0.35325244	0.32013455	0	0	0.979613921

0	0	0.000431603	0	0
0	0	0	0	0
0	0	0	0	0
0	0	0	0	0
0.004334973	0.003425516	0	0	0
0.041365641	0.039330897	0	0	0
0	0	0	0	0
0	0	0	0	0
0	0	0	0	0
0	0	0.002444062	0	0
0.00090464	0	0	0	0
0.47409754	0.460181995	0.073604909	7.688937357	0.258437058
0	0	0.176383266	0	0
0	0	0	0	0
0	0	0.441963714	0.307338253	0.084426655
0	0	0	0	0
0.001743771	0.002119901	0	0	0
0	0	0	0	0
0	0	0.001451948	0	0
0	0	0	0	0
0	0	0.016107865	0	0
0	0	0	0	0
0.25267699	0.252708664	0.141040992	0	0
0	0	0	0	0
0	0	0	0	0
0	0	0	0	0
0	0	0	0	0
0	0	0	0	0
0	0	0	0	0
0	0	0	0	0
0	0	0	0	0
0.08279832	0.095696874	0.009888099	0.120354173	0.453819284
0	0	2.405132787	0	0
0.027625653	0.032399155	0.00902073	0	0
0	0	0	0.002296928	0.012737671
0.001233473	0	0.061330603	0.001615101	0.005886559
0	0	0	0	0
0	0	0.002660422	0	0
0	0	0	0	0
0	0	0	0	0
0.712017297	0.693351351	0	0	0
0	0	0	0	0
0	0	0	0	0
0	0	0	0	0
0.420200322	0.416209779	0.000685185	0	0
0	0	0	0	0
0	0	4.145605085	0.044825564	0
0	0	0.000388837	0	0

0	0	0	0	0
0	0	0	0	0
0.024956399	0.022944232	0	0	0.000734584
0	0	0	0	0
0	0	0	0	0
0	0	0	0	0
0	0	0.063809736	0	0
0	0	0	0	0
0	0	0	0	0
0	0	0	0	0
0	0	0	0	0
0	0	0	0	0
0	0	0	0	0
0.547842141	0.536620321	0	0.06448199	0
0	0	0	0	0
0	0	0	0	0
0	0	5.069160637	0	0
0	0	0	0	0
0.086666874	0.072714195	1.605606314	0	0.36028152
0	0	0	0	0
0	0	0	0	0
0	0	0	0	0
0	0	0	0	0
0	0	0	0	0
0	0	0	0	0
0.001404004	0.000938766	0	0.038874358	0.016310164
0	0	0	0	0
0.051018685	0.053064452	0	0	0.012081172
0	0	0	0	0
0	0	0.223719389	0.131011732	0
0	0	0	0	0
0	0	0	0	0
0	0	0.035336819	0	0
0	0	0	0	0
0.001287396	0.002705363	0	0.001404756	0
0	0	0	0	0
0	0	0	0	0
0.003756387	0.003348867	0	0	0.001572524
0	0	0	0	0
0	0	0	0	0
24.78930124	24.66360018	6.963625143	9.562986673	15.89333695
0.053771755	0.043307818	0.03464636	0	0.034532841
0.041955795	0.044357465	0	0	0.220223264
0	0	0	0	0
0	0	0.062586977	0	0
0	0	0	0	0
0	0	0	0	0
0.013415743	0.013510719	0	0	0

0	0	0	0	0
0.056559681	0.05535974	0	0	0
0	0	0.001301741	0	0
0	0	0	0	0
1.410934778	1.466151908	1.629812518	0	0
0	0	0	0	0
0	0	0	0	0
0	0	0	0	0
0.037934378	0.040946756	0.010510866	0.189328983	1.438203888
0	0	0	0	0
0	0	0	0.066365625	0
0	0	0	0	0
0.591453869	0.609857484	0	0	0
0.028287546	0.034290191	0.500909061	0.009770617	0.053416447
0.065835635	0.068539265	0.052690318	0	0
0.3027652	0.313540865	0.42864458	0	0
0.024126275	0.024962867	0	0	0
0	0	0	0	0
0	0	0	0	0
0	0	0	0	0
0	0	0	0	0
0	0	0	0	0
0	0	0	0	0
0	0	0	0	0
0	0	0	0	0
0	0	0	0	0
0	0	0.000556572	0	0
0	0	0	0.001319791	0
0	0	0	0	0
0	0	0	0	0
0	0	0	0	0
0	0	0	0	0
0	0	0	0	0
0	0	0.001185016	0	0
0.138177342	0.142094638	0.01759734	0	0
0	0	0	0	0
0	0	0	0	0
0	0	0	0	0
0	0	0	0	0
0	0	0	0	0
0.924380173	0.91794263	1.674912111	0	1.099421924
0	0	0	0	0
0	0	0	0	0
0	0	0.099771187	0	0
0	0	0	0	0
0	0	0	0	0
0	0	0	0	0
0	0	0	0	0

0	0	0.002888059	0	0
0	0	0	0	0
0	0	0	0	0
0	0	0	0	0
0.07041806	0.087906749	0	0	0
0	0	0	0	0
0.151432698	0.157400494	0	0	0.041919321
0	0	0	0	0
0	0	0	0	0
0	0	0	0	0
0	0	0.000619447	0	0
0	0	0	0	0
0	0	0	0	0
0	0	0	0	0
0	0	0	0	0
0	0	0	0	0
0	0	0.007443268	0	0
0	0	0	0	0
0	0	0	0	0
0	0	0	0	0
0.032614399	0.03086546	0	0	3.794556123
0	0	0	0	0
0.002951677	0.002713692	0.004291315	0	0
0	0	0.001192463	0	0.001442114
0	0	0	0	0
0	0	0.000562735	0	0
0	0	0	0	0
0	0	0	0	0
0	0	0	0	0
0.004013598	0.003472934	4.758601117	0.005409948	12.32693229
0	0	0	0	0
0	0	0	0	0
0	0	0	0	0
0	0	0	0.007263946	0
0	0	0	0	0
0	0	0	0	0
0.001026291	0.001372428	0	0	0
0	0	0	0	0
0	0	0	0	0
0	0	0	0	0
0	0	0	0	0
0	0	0	0	0
0	0	0	0	0
0	0	0	0	0
0	0	0.007766776	0	0
0	0	0	0	0
0	0	0	0	0
0.033454942	0.035319686	0	0	0

0.042892986	0.048389681	0	0	0
0	0	0.018618834	0	0
0	0	0	0	0
0	0	0	0	0
0	0	0	0	0
0	0	0	0	0
0	0	0	0	0
0.635791107	0.629669859	0.794181152	0	1.281934269
0.041005735	0.039644738	0.014702704	1.435129005	0.003479614
0.039541401	0.041786125	0	0	0.030764521
0	0	0	0	0
0	0	0	0	0
0	0	0	0	0
0	0	0.006560623	0.460164219	0
0	0	0	0	0
0	0	0	0	0
0	0	0	0.003936132	0
0	0	0	0	0
0	0	0	0	0
0	0	0.001162684	0	0
0	0	0	0	0
0	0	0	0	0
0	0	0	0	0
0	0	0	0	0
0	0	0.063771677	0	0.102492801
0	0	0	0	0
0	0	0	0	0
0	0	0	0	0
0	0	0	0	0
0	0	0	0	0
0	0	0	0	0
0	0	0	0	0
0	0	0	0	0
0	0	0	0	0
0.003846518	0.002857684	0	0	0
0	0	0	0	0
0	0	0	0	0
0	0	0	0	0
0.001259336	0	0	0	0
0	0	0	0.001730526	0.350107595
0	0	0	0	0
0	0	0.189906133	0	0.95961801
0	0	0	0	0
0	0	0	0	0
0	0	0	0	0
0	0	0	0	0
0	0	0	0	0
0	0	0	0	0

0	0	0	0	0
0.000477936	0	0	0	0
0	0	0	0	0
0	0	0	0	0
0.030738183	0.032765039	0.052460882	0.094787821	0.188541627
0	0	1.643698465	0	0
0	0	0	0	0
0.001347234	0	0.011588885	0.077177592	0.007049854
0.00403549	0.001499038	0	0	0
0	0	0	0	0
0	0	0	0	0
0	0	0.031703698	0	0
0	0	0	0	0
0	0	0	0	0
0	0	0	0	0
0	0	0.000424688	0	0
0	0	0	0	0
0	0	0	0	0
0	0	0	0	0
0	0	0	0	0
0	0	0	0	0
0	0	0	0	0
0	0	0	0	0
0	0	0	0	0
0.279217941	0.256864299	0.06447326	0.558407614	0
0	0	0	0	0
0.001421822	0.004040387	0	0	0.001785638
0	0	0	0	0
0	0	0	0	0
0	0	0	0	0
0	0	0	0	0
0	0	0	0	0
0	0	0	0	0
0	0	0	0	0
0	0	0	0	0
0	0	0	0	0
0	0	0	0	0
0	0	0.001059979	0	0
0	0	0	0	0
0	0	0	0	0
0	0	0	0	0
0	0	0	0	0
0	0	0	0	0
0	0	0	0	0
0	0	0	0	0
0	0	0	0	0
0	0	0	0	0
0	0	0	0	0

0.016880899	0.020339229	0.003766408	0	0
0	0	0	0	0
0	0	0	0	0
0	0	0	0	0
0	0	0	0	0
0.07590633	0.071210414	0	0.003591945	0
0	0	0	0	0
0	0	0	0	0
0	0	0	0	0
0	0	0	0	0
0	0	0	0	0
0.001074792	0.001676835	0	0	0
0	0	0	0	0
0	0	0	0	0
0	0	0.003815248	0	0
0	0	0	0	0
0	0	0	0	0
0	0	0	0	0
0	0	0	0	0
0	0	0	0	0
0.00174157	0.002328948	0	0	0.09963924
0	0	0	0	0
0.065495492	0.065035215	0.001954163	0	0
0	0	0.043575624	0	0
0	0	0	0	0
0	0	0.021921293	0	0
0	0	0	0	0
0	0	0	0	0
0	0	0	0	0
0	0	0	0	0
0	0	0	0	0
0	0	0	0	0
0	0	1.526773738	0.134604653	0.027593193
0	0	0	0	0
0	0	0.174115667	0	0
0	0	0	0	0.000691949
0	0	0	0	0
0	0	0	0	0
0.002973979	0.001704433	0	0.007417348	0.003201396
0	0	0	0	0
0	0	0.170598377	0	0
0	0	0	0	0
0.231251933	0.250452823	0.009930928	0	0.025891787
0	0	0	0	0
0	0	0	0	0
0.200816237	0.188614894	0.06940454	0	0
0	0	0	0	0
0	0	0	0	0

0	0	0	0	0
0	0	0	0	0
0	0	0	0	0
0	0	0	0	0
0	0	0	0	0
0	0	0	0	0
0	0	0	0	0
0	0	0	0	0.733033813
0	0	0.031169305	0	0
0.003860444	0.004890745	0.001476982	0	0
0	0	0	0	0
0	0	0	0	0
0	0	0	0	0
0	0	0	0	0
0	0	0	0	0
0	0	0	0	0
0	0	0	0	0
0	0	0	0	0
0	0	0	0	0
0	0	0	0	0
0.004593371	0.006980198	0.008044077	0	0.023074898
0	0	0	0	0
0	0	0	0	0
0	0	0	0	0
0.002161447	0	0	0	0
0	0	0	0	0
0	0	0	0	0
0	0	0.000859225	0	0
0	0	0	0	0
0	0	0	0	0
0.105774165	0.106901208	0.018897749	0.025352657	0.019589263
0	0	0	0	0
0	0	0	0	0
0	0	0	0	0
0	0	0	0	0
0	0	0	0	0
0	0	0	0	0
0	0	0	0	0
0	0	0.006788898	0	0
0	0	0	0	0
0	0	0	0	0
0	0	0.01567958	0	0
0.001230151	0.000940025	0	0	0
0	0	0	0	0
0	0	0	0	0
0	0	0	0	0
0.043998513	0.036397376	0	0.271309275	0
0	0	0	0	0

0	0	0	0	0
0	0	0	0	0
0	0	0	0	0
0	0	0	0	0
0.060083622	0.061129893	0.090687031	0	0
0	0	0	0	0
0	0	0	0	0
0	0	0	0	0
0	0	0	0	0
0	0	0	0	0
0	0	0	0	0
0.08002124	0.084869962	0	0	0
0	0	0	0	0
0	0	0	0	0
0	0	0	0	0
0	0	0	0	0
0	0	0.002610787	0	0
0	0	0	0	0
0	0	0.013786413	0	0
0.015109572	0.016276707	0	0	0
0	0	0	0	0
0	0	0	0	0
0	0	0	0	0
0	0	0	0	0
0	0	0	0	0
0	0	0	0	0
0.00538133	0.00449768	0.001833675	0	0.001267182
0	0	0	0	0
0	0	0	0	0
0	0	0	0	0
0	0	0	0	0
0	0	0	0	0
0	0	0	0	0
0	0	0	0	0
0	0	0	0	0
0	0	0	0	0
0	0	0	0	0
0.061205111	0.075042001	0.001168273	0.001753645	0.002691161
0	0	0.036769205	0	0
0	0	0	0	0
0.00441221	0.002503163	0	0	0
0	0	0	0	0
0	0	0	0	0
0	0	0	0	0
0	0	0	0	0
0	0	0	0	0
0	0	0	0	0
0	0	0	0	0

0	0	0	0	0
0	0	0	0	0
0	0	0	0	0
0	0	0	0	0
0	0	0	0.00252764	0.022546352
0	0	0	0	0.001251537
0	0	0.001027755	0	0
0	0	0	0	0
0	0	0	0	0
0	0	0	0	0
0	0	0.001750266	0	0
0	0	0	0	0
0.024343477	0.027029507	0.007614624	0	0.011487846
0	0	0	0	0
0	0	0	0	0
0	0	0	0	0
0	0	0	0	0
0	0	0	0	0
0	0	0	0	0
0	0	0	0	0
0	0	0	0	0
0	0	0	0	0
0.016901595	0.022601985	0	0.000635943	0.006831479
0.017193503	0.019313569	0.188976424	0.004002321	0.04261015
0	0	0	0	0
0	0	0	0	0
0	0	0	0	0.008158608
0	0	0	0	0
0	0	0	0	0
0	0	0	0	0
0	0	0.008187973	0.005672593	0
0	0	0.000608828	0	0
0	0	0.001250038	0	0
0	0	0	0	0
0	0	0	0	0
0	0	0	0	0
0	0	0	0	0
0	0	0	0	0
0	0	0	0.000799901	0.020714683
0	0	0	0	0
0	0	0	0	0
0	0	0	0	0
0	0	0	0	0
0	0	0	0	0
0	0	0	0	0
0.015963344	0.017034707	0.741848525	0.010204823	0.020048049
0	0	0	0	0
0	0	0	0	0

0	0	0	0	0
0	0	0	0	0
0.053256412	0.057284179	0.104238128	0	0.413972585
0	0	0.003008467	0	0
0	0	0	0	0
0	0	0	0	0
0	0	0	0	0
0	0	0	0	0
0	0	0	0	0
0	0	0	0	0
0.002824915	0.002385899	0	0	0
0	0	0	0	0
0	0	0	0	0
0	0	0	0	0
0	0	0	0	0
0	0	0	0	0
0	0	0	0	0
0	0	0	0	0
0	0	0	0	0
0	0	0	0	0.001733396
0	0	0	0	0
0	0	0	0	0
0	0	0	0	0
0	0	0	0	0
0	0	0	0	0
0	0	0	0	0
0	0	0	0	0
0.016147344	0.013422892	0.002855179	0	0.080842482
0	0	0	0	0
0	0	0	0	0
0.006098171	0.007475322	0.000369411	0	0
0	0	0	0	0
0.053694531	0.060757274	0.039460988	0.02146131	0.054836361
0.004019779	0.002418987	0	0.004386226	0.005048362
0	0	0	0	0
0	0	0	0	0
0	0	0	0	0
0	0	0	0	0
0	0	0	0	0
0	0	0	0	0
0	0	0	0	0
0	0	0	0.001235922	0.00060964
0.030555094	0.026097927	0.006735057	0.312540549	0.010893134
0	0	0	0	0
0	0	0	0	0
0.009115713	0.011809221	0.002070771	0	0.006439638
0.001680896	0.001998054	0	0	0
0	0	0	0	0

0	0	0	0	0
0	0	0.009457862	0	0
0.080814233	0.079681756	0.019991223	0.006580697	0.038173488
0	0	0	0	0
0	0	0.003360427	0	0
0	0	0	0	0
0	0	0	0	0
0	0	0	0	0
0	0	0	0	0
0	0	0	0	0
0	0	0	0	0
0	0	0	0	0
0	0	0	0	0
0	0	0	0	0
0	0	0	0	0
0	0	0	0	0
0	0	0	0	0
0	0	0	0	0
0	0	0	0	0
0	0	0	0	0
0	0	0	0	0
0	0	0	0	0
0	0	0	0	0
0	0	0	0	0
0.002186941	0	0.001734271	0	0
0.008000039	0.0077562	0.130847703	0	0.010047092
0	0	0	0	0
1.810675992	1.864860884	0.008743849	0	0
0	0	0	0	0
0	0	0	0	0
0	0	0	0	0
0	0	0	0	0
0	0	0	0	0
0	0	0	0	0
0	0	0	0	0
0	0	0	0	0
0	0	0	0	0.018562413
0	0	0	0	0
0	0	1.056654949	0.086784732	0.705356307
0	0	0	0	0
0	0	0	0	0
0.002169702	0	0.000630887	0	0
0.041951068	0.041336752	0	0	0.000756251
0	0	0	0	0
0	0	0	0	0
0	0	0.000671462	0	0
0.007369599	0.004516939	0	0	0
0	0	0	0	0

0	0	0	0	0
0.012979963	0.007253967	0.006196448	0	0.041118155
0	0	0	0	0
0	0	0	0	0
0	0	0	0	0
0	0	0	0	0
0	0	0	0	0
0	0	0	0	0
0	0	0	0	0
0	0	0	0	0
0	0	0	0	0
0	0	0	0	0
0.062461139	0.069012776	0.004763778	0	0.00102877
0	0	0	0	0
0.017150271	0.014212949	0.001931509	0	0.003033618
0	0	0	0	0
0	0	0	0	0
0	0	0	0	0
0	0	0	0	0
0	0	0	0	0
0	0	0	0	0
0.048868188	0.054881253	0	0	0
0.007291496	0.009263159	0	0	0.001144656
0	0	0	0	0
0	0	0	0	0
0	0	0	0	0
0	0	0	0	0
0	0	0	0	0
0	0	0	0	0
0	0	0	0	0
0	0	0	0	0
0	0	0	0	0
0	0	0	0	0
0	0	0	0	0
3.188580055	3.196840687	2.767069034	0	0
0	0	0	0	0
0	0	0	0	0
0	0	0	0	0
0.002132074	0.001900771	0	0	0.001785086
0	0	0	0	0
0.549715958	0.568949756	0.462910646	0.083438291	0
0	0	0	0	0
0	0	0	0	0
0	0	0	0	0
0	0	0	0	0
0.008998196	0.009106063	0.002474989	0.825813846	2.163923216
0	0	0	0	0
0	0	0	0	0

0.316893792	0.316598215	0	0	0
0	0	0	0	0
0	0	0	0	0
0	0	0.002400037	0	0
0	0	0	0	0
0	0	0	0	0
0	0	0	0	0
0	0	0	0	0
0	0	0.003952068	0	0
0	0	0.03993896	0.013754918	0.031065252
0	0	0	0	0
0	0	0	0	0
0.019295265	0.016165714	0	0	0.004087417
0	0	0	0	0
0	0	0	0	0
0	0	0	0	0
0	0	0	0	0
0	0	0	0	0
0	0	0	0	0
0.403695927	0.423634366	0.01098023	1.445896641	0.067044793
0	0	0	0	0
0	0	0	0	0
0	0	0	0	0
0	0	0	0	0
0	0	0	0	0
0	0	0	0	0
0	0	0	0	0
0	0	0	0	0
0	0	0	0	0
0	0	0	0	0
0	0	0	0	0
0	0	0	0	0
0	0	0	0	0
0	0	0	0	0
0	0	0	0	0
0	0	0	0	0
0	0	0	0	0
0	0	0	0	0
0	0	0	0	0
0	0	0.018804334	0	0
0	0	0	0	0
0	0	0	0	0
0	0	0	0	0
0	0	0	0	0
0.015936246	0.021830835	0	0	0.001708514
0	0	0	0	0
0	0	0	0	0
0	0	0	0	0

0	0	0	0	0
0	0	0	0	0
0	0	0	0	0
0	0	0	0	0
0	0	0	0	0
0.002466457	0.000776075	0.001371069	0.005699227	0.00674178
0	0	0	0	0
0	0	0	0	0
0	0	0	0	0
0	0	0	0	0
0	0	0	0	0
0	0	0	0	0
0	0	0	0	0
0	0	0	0	0
0	0	0	0	0
0	0	0	0	0
0	0	0.007036182	0	0
0	0	0	0	0
0	0	0	0	0
0	0	0	0	0
0	0	0	0	0
0	0	0	0	0
0	0	0	0	0
0	0	0	0	0
0	0	0	0	0
0	0	0.003299358	0	0
0	0	0	0	0
0	0	0	0	0
0	0	0	0	0
0	0	0	0	0
0	0	0	0	0
0	0	0	0	0
0	0	0	0	0
0	0	0	0	0
0	0	0	0	0
0	0	0	0	0
0	0	0	0	0
0	0	0	0	0
0	0	1.248920323	0	0
0	0	0	0	0
0	0	0	0	0
0	0	0	0	0
0	0	0	0	0
0	0	0	0	0
0	0	0	0	0
0	0	0	0	0
0	0	0	0	0
0	0	0	0	0

0	0	0	0	0
0	0	0	0	0
0	0	0	0	0
0	0	0	0	0
0	0	0	0	0
0	0	0	0	0
0	0	0	0	0
0	0	0	0	0
0	0	0	0	0
0	0	0	0	0
0	0	0.000773073	0.093066286	0.037396888
0	0	0	0	0
0	0	0.915265707	0	0
0	0	0	0	0
0	0	0	0	0
0	0	0	0	0
0	0	0	0	0
0	0	0	0	0
0	0	0	0	0
0	0	0	0	0
0	0	0	0	0.023044466
0	0	0	0	0
0	0	0	0	0
3.410101205	3.420705963	0	3.456760836	2.251050959
0	0	0	0	0
0	0	0	0	0
0	0	0	0	0
0	0	0	0	0
0	0	0	0	0
0	0	0	0	0
0	0	0	0	0
0	0	0	0	0
0	0.00121466	0	0	0
0	0	0	0	0

PHL018-0081_V2	PHL018-0226_V1	PHL018-0227_V1	PHL018-0057_V3	PHL018-0035_V3
EYCF	REF	REF	REF	CTRL
0.472677985	0.56019522	0.039768227	2.4557838	0.005306179
1.169777072	0	0.909507407	2.522438291	2.342994599
1.917713938	0	0.047350304	6.060829594	0
0	0	0	0	0
0	0	0	0	0
0	0	0	0	0
0	0	0	0	0
0	0	0	0	0
0	0	0	0	0
0	0	0	0	0
0	0	0	0	0
0	0	0	0	0
0	0	0	0	0
4.815010719	0	1.513464863	1.17030236	0.3962533
0.005138662	0	0	0	0
0	0	0	0	0
0	0	0	0.0007443	0
0	0	0	0	0
0	0	0	0	0
0	0.060077806	0	0	0
0	0	0	0	0
0	0	0	0	0
0	0	0	0	0
0	0	0	0	0
0.084003833	0	0	0	0
0	0	0	0	0
0	0.000875351	0	0	0
0	0	0	0	0
0	0	0	0	0
0	0	0	0	0
0	0	0	0	0
0	0	0	0	0
0	0	0	0	0
0	0	0	0	0
0	0	0	0	0
1.483143766	0	32.13807342	0	0.016513704
0	0	0	0	0
0	0	0	0	0
0	0	0	0	0
0	0	0	0	0
0	0	0	0	0
0	0.002155096	0	0	0.612423906
0.116110831	0.042414716	0.032011686	5.986308665	0.04321022
0.005254793	0.00905542	0.07166589	0.009964569	0.082503254
0	0.008598694	0	0	0
0	0	0	0	0

0	0	0	0	0
0	0.097963655	0.08113167	0.022700017	0.026137573
0	0.01502606	0	0	0
0.399556396	0	0.364424884	0.005756878	0.49164181
0	0.001105569	0	0	0
0	0	0	0	0
0.001086169	0	0	0	0.022151602
0	0	0	0	0
0	0	0	0	0
0	0	0	0	0
0	0	0	0	0
0.118046727	0	0	0	0.011264919
0	0	0	0	0.001975455
0.0941235	0.179604726	0.019253154	0	0.003266175
0	0	0	0.001183521	0
0	0	0	0	0
0	0	0.000807981	0.000946048	0
0	0	0	0	0
0	0	0	0	0
0.000811423	0	0	0	0
0.145764414	0.0260266	0.121860331	0.049769188	0.230253786
0.010804625	0.020327659	0	0	0.002546086
0	0	0	0	0
0	0	0	0	0
0	0	0	0	0
0	0	0	0	0
0.008600735	0.035633272	0	0.023299167	0
0	0	0	0	0
0	0	0	0	0.019246976
0	0	0	0	0
0	0	0.081718182	0.246316978	0
0	0	0	0	0
0	0	0	0	0
0	0	0	0	0
0	0	0	0	0
0	0	0	0	0
0	0	0	0	0
0.001559731	0	0	0	0
0	0	0	0	0
0	0	0	0	0
0	0	0	0	0
0	0	0	0	0
0	0	0	0	0
0	0	0	0	0
0	0	0	0	0
0	0	0	0	0
0	0	0	0	0
0	0	0	0.001556401	0

0	0	0	0	0
0	0	0	0	0
0	0	0	0	0
0	0	0	0	0
0.008784629	0.081968223	0	0	0
0	0	0	0	0
0	0	0	0	0
0	0	0.001610659	0	0
0	0.001045121	0	0	0.080541552
0	0	0	0	0
0	0	0	0	0
0	0	0	0	0.016883235
0	0	0	0	0
0	0	0	0	0
0	0	0	0	0
0	0	0	0	0
0	0	0	0	0
0	0	0	0	0
0	0	0	0.775390643	0.002542741
0	0	0	0	0
0	0	0	0	0
0	0	0	0	0
0	0	0	0	0
0	0	0	0	0
0	0	0	0	0
0	0	0	0	0
0	0	0	0	0.029178713
0	0	0	0	0
0	0	0	0	0
0	0	0	0	0
0.372759132	0	0	0	0
0.006034827	0.034090488	0	0	0
0	0	0	0	0
0.001837089	0	0	0	0
0	0	0	0	0
0	0	0	0	0
0	0	0	0	0
0	0	0	0	0
0	0	0	0	0
0	0	0	0	0
0	0	0	0	0
0	0	0	0	0
0.001953086	0	0	0.009096563	0
0	0	0	0	0
0	0	0	0	0
0	0	0	0	0
0	0.001229472	0	0	0
0	0	0	0	0

0	0.00933884	0	0	0
0	0	0	0	0
0	0	0	0	0
0	0	0	0	0
0	0	0	0	0.001512332
0	0	0	0	0
0	0	0	0	0
0	0	0	0	0
0	0	0	0	0
0	0	0	0	0
0	0	0	0	0
0	0	0	0	0
0	0	0	0	0
0	0	0	0	0
0	0	0	0	0
0	0	0	0	0
0	0	0	0	0
0	0	0	0	0
0	0	0	0	0
0	0	0	0	0
0	0	0	0	0
0	0	0	0	0
0	0	0	0	0
0	0	0	0	0
0.030569456	0	0	0	0.164773838
0	0	0	0	0
0	0	0	0	0.001179646
0	0	0	0	0
0	0	0	0	0
0	0	0	0	0
0	0	0	0	0
0	0	0	15.93697497	0
0	0	0	0	0
0	0	0	0	0
0	0	0	0	0
0	0	0	0	0
0	0	0	0	0
0	0	0	0	0
0	0	0	0	0
0	0	0	0	0
0	0	0	0	0
0	0	0	0	0
0	0	0	0	0
0	0	0	0	0
0	0	0	0	0
0	0	0	0	0
0	0	0	0	0
0	0	0	0.206523274	0

0.021720655	0	0	0	0
0	0	0	0	0
0	0	0	0	0
0	0	0	0	0
0	0	0	0	0
0	0	0	0	0
0	0	0	0	0
0	0	0	0	0
0	0	0	0	0
0	0	0	0	0
0	0	0	0	0
0	0	0	0	0
0	0.064522092	0	0	0
0	0	0	0	0
0	0	0	0	0
0	0	0	0	0
0	0	0	0	0
0	0	0	0	0
0	0	0	0	0
0	0	0	0	0
0.110625597	0.011728968	0.025296581	0.033487448	0.138990262
0	0	0	0	0
0	0.001653982	0	0	0
0	0.012667774	0	0	0
0	0	0	0	0
0	0	0	0	0
0	0.000975811	0.006596477	0.015447367	0.026633402
0	0	0	0	0
0	0	0	0	0
0.002173409	0.001827009	0	0.000619759	0
0	0	0.005260454	0.28179075	0.0278504
0	0	0	0	0
0	0	0	0	0
0	0	0	0.059370063	0
0	0.009811918	0.003684919	0	0.004084137
0	0	0	0	0
0	0	0	0	0
0	0	0	0	0
0	0	0	0	0
0	0	0	0	0
0	0	0	0	0
0	0	0	0	0
0	0	0	0	0
0	0	0	0	0
0	0	0	0	0
0	0	0	0	0
0.001755271	0.002655924	0	0	0
0	0.064289742	0	0.001537349	0.002286795

0	0	0	0	0
0	0	0	0	0
0	0	0	0	0
0	0	0	0	0
0	0	0	0	0
0	0	0	0	0
0	0	0	0	0.091146278
0	0	0	0	0.002390771
0	0	0	0	0.000819323
0	0	0	0	0.001869596
0	0	0	0	0
0	0	0	0	0
0	0	0	0	0
0	0	0	0	0
0	0	0	0	0
0	0	0	0	0
0	0	0	0	0
0	0	0	0.024586294	0
0	0	0	0.027204452	0
0	0	0.002448305	0	0
0.003119588	0.038170259	0.001313134	0.000922513	0.006861158
0	0	0	0	0
0	0	0	0	0
0	0	0	0	0
0	0.0028944	0.003652346	0.004123728	0.004956778
0	0	0	0	0
0	0	0	0	0
0	0	0	0	0
0	0	0	0	0.117666848
0	0	0	0	0
0.734026133	0.06941657	0	0.715970763	0.018574797
0	0	0	0	0
0.012993354	0	0	0	0
0	0	0	0	0
0	0	0	0	0
0	0	0	0	0
0	0	0	0	0
0	0	0	0	0
0	0	0	0	0
0	0	0	0	0
0	0	0	0	0
0.016886855	0.005459772	0.013385319	0.031287584	0
0	0.011727234	0	0	0
0	0	0	0	0
0	0	0	0	0
0	0	0	0	0
0	0	0	0	0
0	0	0	0	0

0	0	0	0	0
0	0	0	0	0
0.006850596	0.006032966	0.066982711	0.002821697	0.059583378
0	0	0	0	0
0	0	0	0	0
0	0	0	0	0
0	0	0	0	0
0	0	0	0	0
0	0	0	0	0
0	0	0.000678176	0	0
0	0	0	0	0
0	0	0	0	0
0	0	0	0	0
0	0	0.000819498	0.002878602	0
0	0.000750431	0.004283793	0.006929734	0.016332037
0	0	0	0	0
0	0.001425082	0.002312052	0.002030351	0.00884685
0	0	0	0	0
0	0	0	0	0
0	0	0	0	0
0	0	0	0	0
0	0.049656665	0	0.000610943	0.000826157
0	0	0	0	0
0	0.00279199	0	0	0
0	0	0	0	0.010155987
0	0	0	0	0
0	0	0	0	0
0	0	0	0	0
0	0	0	0	0
0	0.010420762	0	0	0.004381825
0	0	0	0	0.027012138
0	0	0	0	0
0	0	0	0	0
0	0	0	0	0
0	0	0	0	0
0	0	0	0	0
0	0	0	0	0
0.021150007	0.221430535	0	0.004605517	0.003459934
0	0	0	0	0
0	0	0	0	0
0	0	0	0	0
0	0	0	0	0
0	0	0	0	0
0	0	0	0	0
0	0	0	0	0
0	0	0	0	0.012235067
0	0	0	0	0.002250265
0	0.011746332	0.004587855	0	0

0	0	0	0	0
0	0	0	0.411556416	0
0	0	0.003157698	0.010451941	0.002307559
0	0	0	0	0
0	0.010031902	0.0075758	0.004077501	0.001160814
0	0.004863167	0	0	0.026026161
0	0	0	0	0
0	0.001900259	0.183951106	0.008422864	0.039051256
0	0	0	0	0
0.285657494	0	0.004236925	0.013332501	0.010901306
0	0	0	0.001121331	0
0	0	0	0	0.073096633
0	0.002510263	0.002262583	0.007225126	0.010258804
0	0.042630031	0	0	0.053418777
0.00195443	0	0	0.000557316	0
0	0	0	0	0
0	0	0	0	0
0	0	0	0	0
0.006577451	0	0	0.027133594	0.015386895
0	0	0	0	0
0	0	0	0	0
0	0	0	0	0
0	0	0	0	0
0	0	0	0	0
0	0	0	0	0
0.000948592	0	0.002395756	0	0.001706984
0	0.002132583	0	3.188575923	0
0.002294223	0	0.01477539	0.016028135	0
0	0	0	0	0
0.004229255	0	0	0	0
0	0	0	0.005869639	0.003052812
0	0	0	0	0
0	0	0	0	0
0.013232899	0	0.119688587	0	0
0	0	0	0.286251087	0
0	0	0	0	0
0	0	0.092609983	0.007393308	0.009997715
0	0	0	0	0
0	0	0	0	0
0	0	0	0.023454827	0
0	0	0	0	0
0	0	0	0	0.016037789
0	0	0	0	0
0	0.847990581	0	0.008474708	0.005042425
0	0	0	0.255596669	0
0	0	0	0	0
0.049938487	0.000991635	0	0	0
0	0	0	0	0

0	0	0	0	0
0	0	0	0	0.020164923
0	0	0	0	0
0	0	0	0	0
0	0	0	0	0
0	0.00598792	0	0	0.012818195
0.048212578	0.02634346	0	0	0
0	0	0	0	0.009370596
0.027116937	0	0	0	0
0	0	0	0	0
0	0	0	0.04995147	0
0	0.807685363	0	0	0
0	0	0	0	0
0.012556323	0.001996907	0.006942388	0.000677392	0.010076152
0	0.014432223	0.003902472	0	0
0	0	0	0	0
0	0	0	0	0
0	0	0.001501348	0.009228965	0
0	0	0.001642023	0.03388604	0
0	0	0	0	0
0	0	0	0	0
1.451474138	0	0.270649366	0	0.443259455
0	0	0	0	0
0	0	0	0	0
0	0	0	0	0
0	0	0	0	0
0	0	0	0.015913564	0.019201895
0	0	0	0	0
0	0	0	0	0
0.141270343	1.856166414	0.923762529	0.493147631	1.386097841
0.00391389	0.011612081	0	0	0
0	0	0	0	0
0	0	0	0	0
0	0	0	0	0
0	0.007986431	0	0	0
0	0	0	0	0
0.543628638	0	0.406109352	0	0
0	0	0	0	0
0	0	0	0	0
0	0.078311677	0	0	0
0	0.0009381	0	0.0018563	0.002008168
0	0	5.295837722	1.07156812	0
0	0	0	0	0
0	0	0	1.269541284	0
0	0	0.000953814	0	0
0	0	0	0	0
0	0.00295381	0	0.00097416	0
0	0	0	0	0

0	0.00242967	0	0	0
0	0	0	0	0
0	0	0	0.001310199	0.005315211
0	0	0.006190465	0.001522141	0
0	0	0	0.363603323	0
0	0	0.445309313	0.255078638	0
0	0	0	0	0
0	0	0	0	0
0.013908972	0	0	0	0
0	0	0	0.191761514	0
0	0.032740199	0	0	0
0	0	0	0	0
0	0	0	0	0.010792912
0	0	0.002228834	0.016963028	0
0	0	0	0	0
0	0	0.000774131	0	0.004366604
0.078423236	0.20967179	0	0.000941874	0.023180692
0	0.002313217	0	0	0
0	0.009587321	0	0	0
0	0	0	0	0
0	0	0.01703172	0.031337587	0.059391644
0	0	0	0.000655515	0
0	0	0	0	0
0	0	0	0	0
0.065620706	0	0	0	0
0.010815722	0.165535267	0	0	0
0	0	0	0.001913148	0
0	0	0	0	0
0	0	0	0	0
0	0	0	0	0
0.009020113	0.024585607	0	0	0.001721536
0	0.001027686	0.003705148	0	0.020166135
0.038024599	0	0.552688963	0.011101075	0.015709816
0	0	0	0	0.643391482
0.002778151	0.002668989	0	0	0
0	0	0	0	0
0	0.002359007	0.005847189	0	0.049236224
0	0.185638307	0	0.025919044	0.002961924
0	0	0.14082008	0	0
0	0	0	0	0
0	0	0	0	0
0	0	0	0	0
0	0.643875642	0	0	0
0	0	0	0	0
0	0	0.063820951	0	0
0	0	0	0	0
0	0	0	0	0
0	0	0	0.000741707	0

0	0	0.00616355	0	0
0.00971903	0.054361918	0.331658266	0.312887508	0.020516318
0	0	0	0	0
0	0	0	0	0
0	0	0	0.000732013	0
0	0	0	0	0
0	0	0	0.267372413	0.023647545
0.002584121	0	0	0	0
0	0	0	0	0
0	0	0	0	0
0.011944576	0.027086904	0	0	0.001249661
0.023584459	0.003478165	0.369928196	0.005781338	1.328671521
0	0	0	0	0
0	0	0	0	0
0.002868469	0	0.017145508	0.00063619	0.197868543
0	0	0	0.002347149	0
0.001454383	0.006357426	0.008815627	0.018192597	0.002355436
0.020767892	0.002221912	0.016879754	0.033414841	0.050961361
0	0	0	0	0
0	0	0.05777658	0.056773326	0.010589326
0.134385299	0.001765106	0.171822453	0.005987603	0.003778521
0	0	0	0	0
0	0	0	0	0
0	0	0	0	0
0	0	0	0	0
0	0	0	0	0.001485004
0.005921652	0.160286838	0.06842221	0	0.015717553
0.145382301	0	0.207986055	0.023970835	0.244402876
0	0	0	0	0
0.002307436	0.002909511	0	0.022517441	0
0	0	0	0	0.003385786
0	0	0	0	0
0	0	0	0	0
0	0	0	0	0
0.542602307	0	0	0	0
0.51260598	0.274872818	0.327474096	0.31306504	1.656976599
0	0	0	0	0
0	0	0	0	0
0	0.008926642	0	0	0.274250201
0	0	0	0	0
0	0	0.072553546	0	0
0	0	0	0	0
0	0.33697835	0.001086042	0.009060326	0.016980796
0	0	0	0.015960486	0.028431209
0	0	0	0	0
0	0	0	0	0.151808123
0	0	0	0	0
0	0	0	0	0

0	0	0.025792807	0.050185763	0
0	0	0	0	0
0	0	0	0	0.004727646
0	0.005379548	0	0.632219105	0
0	0	0	0	0
0	0	0	0	0
0	0.042069106	0	0	0
0	0	0.003017558	0.00248428	0
2.657049229	0	0	0	0.259261845
0	0	0.017145994	0.00231645	0.002684962
0	0	0	0	0
0.002399251	0	0	0	0.001199287
0	0	0	0	0
0	0	0	0	0
1.962488937	0.046046024	0	0	0
0	0	0	0	0
0	0	0	0	0.279354631
0	0	0	0	0
0	0	0	0	0
0	0	0	0	0
0	0	0	0	0
0.032422913	0	0	0	0
0	0	0	0	0
0	0	0.051216582	0	0.028217418
0	0.001258861	0	0	0
0	0	0	0.001711503	1.142159546
0	0	0	0	0
1.007246824	0	3.225956648	0	0
0	0	0	0	0
0	0.012595392	0	0	0
0.052092071	0	0	0	0
1.164499476	0	0	0	0.010985049
0	0	0	0.001968572	0
0	0	0	0	0
0.004067818	0	0	0	0
0.328295354	0	0	0	0.041519628
0	0	0.006665864	0	0
0	0	0	0	0
0	0.10054495	0	0	0
0.178792059	0.011067686	0	0	0
0.001278696	0.025797495	0.058517868	0.481209963	0.958827993
0	0	0	0	0.326348147
16.79769902	0	0.0197581	0.131457621	3.741911047
0.000891129	0	0	0	0.000601342
0	0.00982967	0	0	0
0	0	0	0	0
0	0	0	0	0
0.0055337	0.679669765	0.001164655	0.017795917	0

0	0	0	0	0
0.023526334	0	0	0	0
0.024165746	1.896650868	0.018086551	0.00941208	0.007689611
0	0	0	0	0
0	0	0	0	0
0.003018036	0	0.001055399	0	0
0	0	0	0	0
0	0.001186628	0	0	0
0	0	0	0	0
0	0	0	0	0
0.105033065	0.042078924	0.00460387	0.002310248	0
2.932903697	0	2.124012355	0.822978759	1.978100293
0	0	0	0	0
0.008431435	0.052500913	0	0.006856614	0.004776466
0.008120804	0.42158815	0.083158058	0.172413594	0.084137331
0	0	0	0	0
0	0.001176279	0	0	0
0	0	0	0	0
0	0	0	0	0
0	0	0	0.143996442	0
0	0.423831608	0.005879956	0.004034018	7.632506067
0.047018669	0	0.003678667	0.015132146	0
7.059687625	0	0.678600195	1.085152735	0.417141485
0	0	0	0	0
0	0	0	0	0
3.619503667	0	0.016734134	0	0
0	42.14526527	0	0	0
0	0	0	0	0
0	0	0	0	0
0	0	0	0	0
0.006687212	0	0	0	0.000962687
0.263296353	0	0	0	0.062005972
0	0	0.003827667	0.019179206	0.003743251
0.541378455	0	0	0	0.001019471
0	0.901365979	0.003715159	0.007308008	0.000882354
0.02398338	0.387360744	0.03858927	0.024922592	0.010425787
0.089026127	0.001094109	0	0	0
0	0	0	0	0
0.029326765	0	0.019830957	0	0.002269819
0.340510513	0	0	0	0
0	0	0	0	0
0	0	0	0	0
0	0	0	0	0
0	0	0	0	0
0.028272244	0.913447968	0.010477781	0.068702059	0.00165899
0	0	0	0	0
0	0	0	0	0
0.003706962	3.354215424	0.003370418	0.059935268	0

0	0	0	0	0
0	0	0	0	0
0	0	0	0	0
0	0.00110086	0	0	0.001178292
0	0	0	0	0
0	0	0	0	0
0	0	0	0	0
0	0.460302757	0	0	0.000650545
0	0	0	0	0
0	0	0	0	0
0	0.823426083	0	0.023484873	0
0	0	0	0	0
0.008939388	0	0	0	0
0	0	0	0	0
0	0	0	0	0
0	0	0	0	0
0.360140636	0	0	0	0
0.002185545	0	0	0	0
0	0	0	0	0
0	0	0	0.119485672	0
0	0	0	0	0
0	0	0	0	0
0	0	0.003181963	0	0
0	0	0	0	0
0	0	0	0	0
0	0	0	0	0
0	0	0	0	0
0	0	0	0	0
0	0	0	0	0
0.064536226	0	0.036450971	0.014833803	0.109797951
0	0	0	0	0
0	0	0	0	0
0	0	0	0	0
0	0	0	0	0
0	0	0	0	0
0	0	0	0	0
0.129723831	0	0	0	0
0.012099762	0.006850051	0.310766908	0.131933272	0.005554447
0	0	0	0	0
0	0	0	0	0.00219144
0	0.009348426	0	0	0.001459205
0	0	0	0	0
0	0	0	0	0
0	0	0	0.00366977	0
0.393668849	0	0	0	0
0	0	0	0	0
0.004223314	0	0	0	0
0	0	0	0	0

0	0	0	0	0
0	0	0	0	0
0.08372362	0	0	0	0
0	0	0	0	0
0	0	0	0	0
0.581046176	0	0	0	0
0	0	0	0	0
0	0	0	0	0
0.035759128	0.014314189	0.121019356	0.058915422	0
0	0	0	0	0
0.003896212	0	0	0	0
0	0	0	0	0.015131507
0	0.010354779	0	0	0
0	0.016504011	0	0	0
0.804207918	0	3.223924282	0	3.290475077
0	0	0.001193258	0	0
0	0.00733551	0	0	0
0	0	0	0	0
0	0	0	0	0
0	0	0	0	0
0	0	0	0	0.001171512
0	0	0	0	0
0	0	0	0	0
0	0	0	0	0
0.010813299	0.033666156	0	3.37220993	0.012972299
0	0	1.054228359	9.670218923	0.478208917
0	0	0.011512052	0	0
0	0	0	0	0
0	0	0	0	0
0	0	0	0	0
0	0	0	0	0
0	0	0	0	0
0	0	0	0	0
0	0	0.000486176	0	0
0.002114526	0.004443777	0.000801064	0.003048339	0.036148204
0.004982379	0.03685688	0.00924884	0.001823297	0
0.117744417	0	0	0	0
1.232649461	0	12.84832301	0.231336261	3.065512581
0	0	0	0	0
0	0	0	0	0
0	0	0	0	0
0	0	0	0	0
0.218553174	0	1.031730936	1.565837433	0.042268925
1.385662435	0	0	0	0
0.308487828	0	0	0	0
0.06513847	0	0	0	0
0	0	0	0	0
0	5.933167058	1.754858117	0.100067936	1.394118089

0	0.069764185	0	0	0
0	0	0	0	0
0	0	0	0	0
0	0	0	0	0
0.002512326	0.012906108	0	0	0
0	0	0	0	0
0.522231499	0	0	0	0
0	0	0	0	0
0	0	0	0	0
0	0	0	0	0
0	0	0	0	0
0.49111208	0	0.117465623	0	0.006951383
0	0.253442083	0	0.694689447	1.326540314
0	0	0	0	0
0.07037779	0.01058383	0.002201436	0	5.251671833
0	0	0	0	0
0	0.087784636	0	0	0
0	0	0	0	0
0	0.01308251	0.000857578	0	0
0	0	0	0	0
0	0	0	0	0
0	0	0	0	0
0	0	0.037732984	0.195701402	0.082437054
0	0	0	0	0
0	0	0	0	0
0	0	0	0	0
0.882390812	0	0	0	0
0	0	0	0	0
0.00523425	0	0	0	0
0	0	0	0	0
0	0	0	0	0
0	0.021129229	0.080304188	0.028988428	0.07236933
0	0	0	0	0
0.192334086	0.007213129	0	0	0
0.002711039	0.039653731	0.006778506	0.002344969	0
0.189421276	0	0.005825545	0.012091797	0
0	0	0	0	0
0	0	0	0	0
0	0	0.00094394	0	0
0.003054727	0	0	0	0
0	0	0	0.34994373	0
0	0	0	0	0
0	0	0	0	0
0	0	0.025417755	0	0
0.054481153	0.583025001	0.005463416	0.063970044	0
0	0	0	0	0
0.057523717	0	0	0	0.014152235
0.003788928	0	0	0	0

0	0	0	0	0
0	0	0	0	0
0	0	0	0.006616103	0
0	0	0	0	0
0	0	0	0	0
0	0	0	0	0
0	0	0.197888026	0	0
0	0	0	0.001388509	0.010326982
0	0	0	0	0
0	0	0	0	0
0	0	0	0	0
0	0.050556698	0	0	0
0	0	0	0	0
0.419922933	0	0	0	0
0	0	0	0	0
0	0	0	0	0
0	0	0	0	0
0	0	0	0	0
0.127500931	0	1.698630575	5.410541883	0
0	0	0	0	0
0.00175674	0	0	0	0
0	0	0	0	0
0.001601567	0	0	0	0
0	0	0	0	0
0	0	0	0	0
0	0.067716154	0	0.008436773	0
0	0	0	0	0
0	0.74974867	0	0.001781121	7.052009924
0	0	0	0	0
0.839376192	0	0.020936083	0.799757542	1.519621391
0	0	0	0	0
0	0	0	0	0
0	0	0	0	0
0	0	0	0	0
0.00182382	0.241140548	0	0.005374073	0
0	0	0	0	0.001413612
0	0	0	0	0
0	0.006958646	0	0.004327612	0
0	0	0	0	0
0	0	0	0	0
14.67047865	15.43707299	5.085505519	1.062881235	20.45673241
0	0	0.166430062	0.20994083	0.00331017
0	0	0	0	0
0	0	0	0	0
0	0	0	0	0
0	0	0	0	0
0	0	0	0	0
0.00164247	0.048126963	0	0.001405077	0

0	0	0	0	0
0.011343225	0	0	0	0
0	0	0	0	0
0	0	0	0	0
2.418103869	0	2.866529716	1.125217254	1.796467292
0	0	0	0	0
0	0	0	0	0
0	0	0	0	0.009209389
0.154233206	0.02917155	0.040718067	0.100536435	0.36687515
0	0	0	0	0
0	0	0	0	0
0	0	0	0	0
0	0	0	0	0.036291602
0.047281813	0.032717729	0.030800216	0.005179312	0.020233215
1.647525193	0	0	0	0
0	0	0.579160975	1.594261938	0.025543431
0	0.000629158	0	0	0.035915302
0	0	0	0	0
0	0	0	0	0
0	0	0	0	0
0	0	0	0	0
0.001198538	0	0	0	0
0	0	0	0	0
0	0	0	0	0
0	0	0	0	0
0	0	0	0	0
0.002820155	0	0.000986201	0.001732084	0
0.000979148	0.00082309	0	0.007329236	0
0	0	0	0	0
0	0	0	0	0
0	0	0	0	0
0.00184025	0	0	0	0
0	0	0	0	0
0	0	0	0	0.00138526
0.116237351	0	0	0	0
0	0	0	0.023927941	0
0	0	0	0	0
0	0	0	0.007472283	0
0	0	0	0	0
0	0	0	0	0
1.593580332	0	2.796116491	0	0.016226013
0	0	0	0	0
0	0	0	0	0
0	0	0	0	0
0	0	0	0	0
0	0	0	0	0
0	0	0	0	0
0	0	0	0	0

0	0	0	0	0
0	0	0	0	0
0	0	0	0	0
0	0	0.011555531	0.005350556	0
0	0	0.132073522	0.021713466	0.019813634
0	0	0	0	0
0	0.053348411	0.002074241	0	0.574367156
0	0	0	0	0
0	0.000590672	0	0	0
0	0	0	0.073836656	0
0	0	0	0	0
0	0	0	0	0
0	0	0	0	0
0	0	0	0	0
0	0	0	0	0
0	0	0	0	0
0	0	0	0	0
0	0	0	0	0.003875044
0	0	0	0	0
0	0	0	0	0
0	0.095805129	0.216329624	0.002049491	0.088047018
0	0	0	0	0
0.02639612	0	0	0	0
0.000697179	0.016019029	0.001408632	0.031698236	0
0	0	0	0	0
0	0.002304738	0	0	0
0	0	0	0	0
0	0	0	0	0
0	0	0	0	0
0.409389009	0.001405801	0.766085567	0	5.279929904
0	0	0	0	0
0	0	0	0	0
0	0	0	0	0
0	0	0	0	0
0	0	0	0	0
0	0	0	0	0
0	0	0	0.01992809	0
0	0	0	0	0
0.003483391	0	0	0	0
0	0	0	0	0
0	0	0	0	0
0	0	0	0	0
0	0	0	0	0
0	0	0	0	0
0	0	0	0	0
0	0	0	0	0
0	0	0	0	0
0	0	0	0	0

0.011002782	2.621081625	0	0.016471875	0
0	0	0	0	0
0	0	0	0	0
0	0	0	0	0
0	0	0	0	0
0	0	0	0	0
0	0.001777577	0	0	0
0.141987399	0	2.273200603	0	0.890572088
0.171527647	0.054112257	0.03895905	8.076448456	0.031784488
0	0	0	0	0
0.798442968	0	0	0	0
0	0	0	0	0
0	0	0	0	0
0	0	0	0	0
0	0	0	0	0
0	0	0	0	0
0	0	0	0	0
0	0	0	0	0
0	0	0	0	0
0	0	0	0	0
0	0	0	0	0
0	0	0	0	0
0	0	0	0	0
0	0	0	0	0
0	0	0	0	0.006911327
0	0	0	0	0
0	0	0	0	0
0	0	0	0	0
0	0	0	0.00206242	0
0	0	0	0	0
0	0	0	0	0
0	0	0	0	0
0	0	0	0	0
0	0	0	0	0
0.026035358	0	0	0	0
0	0	0	0	0
0	0	0	0	0
0	0	0	0	0
0	0	0	0	0
0	0	0.307932401	0.090525544	0.154309753
0	0	0	0	0
0.694095665	0.006495026	0	0	0
0	0	0	0	0
0	0	0	0	0
0	0	0	0	0
0	0	0.001516274	0	0
0	0	0	0	0
0	0	0	0	0

0	0	0	0	0
0.004739553	0.000758887	0	0	0.000812266
0	0	0	0	0
0	0	0	0	0
1.519691361	0	0.043513566	0.163555216	0.007665716
0	0	0	0	0
0	0	0	0.026948736	0
0.003817182	0.023531165	0.010604708	0.079157356	0.001717248
0.002223267	0	0	0	0
0	0	0	0	0
0	0	0	0	0
0.585776359	0	0	0	0
0.10703705	0	0	0	0
0	0	0	0	0
0	0	0.011537344	0.017730359	0
0	0	0.002090314	0	0
0	0	0	0	0
0	0	0	0	0
0	0	0	0	0
0	0	0	0	0
0	0	0	0	0
0	0	0	0	0
0	0	0	0	0
0.001101152	0.083077028	0	0.012455304	0
0.975553091	0	0	0	0
0	0	0	0	0
0.002517822	0.020530326	0.008012327	0.170039206	0
0	0	0	0	0
0	0.013213025	0	0	0
0	0	0	0	0
0	0	0	0	0
0	0	0	0	0
0	0	0	0	0
0	0	0	0	0
0	0	0	0	0
0	0	0	0	0
0	0	0	0	0
0.228005954	0	0	0	0
0	0	0	0	0
0	0	0	0	0
0	0	0	0	0
0.001480413	0	0	0	0
0	0	0	0	0
0	0	0	0	0
0	0	0	0	0
0	0	0	0	0
0	0	0	0	0
0	0	0	0	0

0	0	0	0	0.001704331
0	0	0	0	0
0	0	0	0	0
0	0	0	0	0
0	0	0	0	0
0	1.925978019	0	0.013324245	0
0	0	0	0	0
0	0	0	0	0
0	0	0	0	0
0	0	0	0	0
0	0	0	0	0
0	0.079996972	0	0	0
0	0	0	0	0
0	0	0	0	0
0.17820043	0	0	0	0
0	0	0	0	0
0	0	0	0	0
0	0	0	0	0
0	0.009112578	0	0	0
0	0	0	0	0
0.012610315	0	0.009969967	0.013862442	0
0	0	0	0	0
0.001038646	0.032886978	0	0.008523269	0
0	0	0	0	0
0	0	0	0	0
0	0	0	0	0
0	0	0	0	0
0	0	0	0	0
0	0	0	0	0
0	0	0	0	0
0	0	0	0	0
0	0	0	0	0
0.003242293	0.035704478	0.028251044	0.002588759	0.07818206
0	0	0	0	0
0	0.245747987	0.002856657	0.599748768	1.477475262
0	0.010060779	0.027007202	0	0
0	0	0	0	0
0	0	0	0	0
0	0	0.009804659	0.004805615	0
0	0	0	0	0
0	0	0	0	0
0	0	0	0	0
0.156238647	0	0.00453251	0.001638934	0.035460393
0	0	0	0	0
0	0	0	0	0
0.039962891	0	0.003176117	0	0
0	0	0	0	0
0	0	0	0	0

0	0	0	0	0
0	0	0	0	0
0	0	0	0	0
0	0	0	0	0
0.005566724	0	0	0	0
0	0	0	0	0
0	0	0	0	0
0	0.054027696	0.174736095	0	0
0	0	0	0	0
0	0	0	0	0
0	0	0	0	0
0	0	0	0	0
0	0	0	0	0
0	0	0	0	0
0	0	0	0	0
0	0.104749394	0.004720704	0.014886234	0
0	0	0	0	0
0	0	0	0	0
0	0	0	0	0
0	0	2.176113025	2.280928145	0
0.014197763	0	0	0	0
0	0	0	0	0
0	0	0	0	0
0	0	0	0	0
0	0.041827974	0	0	0
0	0	1.104689523	0	0
0	0	0	0	0
0	0	0	0.00129984	0
0	0	0	0	0
0	0	0	0.073431559	0.002905023
0	0	1.203551907	0.978802946	0.09257548
0	0	0	0	0
0	0	0	0	0
0	0	0	0	0
0	0	0	0	0
0	0	0	0	0
0	0	0	0	0
0	0	0	0	0
0	0	0.00255526	0	0
0	0	0	0	0
0	0	0	0	0
0.509775426	0	0	0	0
0	0	0	0	0
0	0.00406863	0	0	0
0	0	0	0	0
0	0	0	0	0
0.002609227	0	0.006589842	0.150074521	0
0	0	0	0	0

0	0	0	0	0
0	0	0	0	0
0	0	0	0	0
0.001039314	0	0	0	0
0.888008701	0	0.006564138	0.021328146	0.001948738
0	0	0	0	0
0	0	0	0	0
0	0	0	0	0
0	0	0	0	0
0	0	0	0	0
0	0	0	0	0
0.021349715	0.101615251	0.001594836	0.012604694	0
0	0.003271207	0	0	0
0	0	0	0	0
0	0	0	0	0
0	0	0	0	0.027140825
0.020583399	0	0	0	0
0	0	0	0	0
0.006375374	0.190445103	0	0.15043708	0.00122919
0	0.101714527	0.003604075	0	1.190603306
0	0	0	0	0
0	0	0	0	0
0	0	0	0	0
0	0	0	0	0
0	0	0	0	0
0	0	0	0	0
0.014770702	0.032242925	0.003429631	0.228418592	0
0	0	0	0	0
0	0	0	0	0
0	0	0	0	0
0	0.093529242	0	0	0
0	0	0	0.030664564	0
0	0	0	0	0
0	0	0	0	0
0	0	0	0	0
0	0	0	0	0
0	0	0	0	0
0.02219868	0.159811535	0.004312677	0.120559848	0.002902086
0	0	0	0	0
0.00842701	0	0	0	0
0	0.091235911	0	0.003780867	0
0.005837438	0	0	0	0
0	0	0	0	0
0	0.003311101	0	0.002807984	0.11720505
0	0	0	0	0
0	0	0	0	0
0	0	0	0	0
0	0	0	0	0

0	0	0	0	0
0	0	0.248702068	0	0.825856433
0	0	0	0	0
0.00176913	0.024538204	0.001117025	0.027073573	0
0.002461259	0.394715603	0	0	0
0.001764718	0.044800335	0	0	0
0	0	0	0	0
0	0	0	0	0
0	0	0	0	0
0	0	0	0	0
0	0	0	0	0
0	0	0	0	0
0	0.005095251	0.011085387	0.00625805	0.001880566
0	0	0	0	0
0	0	0	0	0
0	0	0	0	0
0	0	0	0	0.004251436
0	0	0	0	0
0	0	0	0	0
0	0	0	0	0
0	0	0	0	0
0	0	0	0	0
0	0	0.004796125	0.125620462	0
0.011691639	0	0.011811322	0.002593058	0.029513065
0	0	0	0	0
0	0	0	0	0
0	0	0	0	0
0	0	0	0	0
0	0	0	0	0
0	0	0	0	0
0.023321956	0	0	0.015313366	0.040311064
0	0	0.000539397	0	0
0	0	0	0	0
0	0	0	0	0
0	0	0	0	0
0	0	0	0.001067212	0
0	0	0	0	0.024640511
0	0	0	0	0
0	0	0	0	0.003737639
0	0	0	0	0
0	0	0	0	0
0	0	0	0	0
0	0	0	0	0
0	0	0	0	0
0.26845158	0	0.021182725	0	0
0.238712287	0.003648473	0.230021185	0	0.055287988
0	0	0	0	0
0	0	0	0	0

0.000784624	0	0	0	0
0	0	0	0	0
0.318657947	0	0.073852219	0	0
0	0	0	0	0
0	0	0	0	0
0	0	0	0	0
0	0.00155294	0	0	0
0	0	0	0	0
0	0	0	0	0
0	0	0	0	0.010130838
0	0.041963247	0	0	0
0.000943417	0.007666188	0.000953074	0.00397552	0
0	0	0	0	0
0	0	0	0	0
0	0	0	0	0
0	0	0	0	0
0	0	0	0	0
0	0	0	0	0
0	0	0	0	0
0.004189987	0.001643685	0	0	0
0	0	0	0	0
0	0	0	0	0
0	0	0	0	0
0	0	0	0	0
0	0	0	0	0
0	0	0	0.001731478	0
0	0	0	0	0
0.010201244	0	0	0	0
0	0	0	0	0
0	0	0	0	0
0	0.002219007	0	0.027942353	0.00215917
0	0	0	0	0
0.022569515	0	0.077268534	0.00630339	0.0298335
0	0	0.00302033	0.000568357	0
0	0	0	0	0
0	0	0	0	0
0	0	0	0	0
0	0	0	0	0
0	0	0	0	0
0	0	0	0	0
0	0	0	0	0
0.018109273	0	0.002431568	0.031114449	0.00515625
0.024296348	0	0.005501479	0	0.001758895
0	0	0	0	0
0	0	0	0	0
0.060130768	0	0.00886733	0	0.002541725
0	0.188831756	0	0	0
0	0	0	0	0

0	0	0	0	0
0.055003281	0	0.004433484	0	0.017110556
0	0.007469393	0.003366205	0.003638236	0.001537457
0	0	0	0	0
0	0.18281803	0	0	0
0	1.076431776	0	0.001590563	0
0	0	0	0	0
0	0	0	0	0
0	0	0	0	0
0	0	0	0	0
0	0	0	0	0
0	0	0	0	0
0	0	0	0	0
0	0	0	0	0
0	0	0	0	0
0	0	0	0	0
0	0	0	0	0
0	0	0	0	0
0	0	0	0	0
0	0	0	0	0
0	0	0	0	0
0	0	0	0.008596712	0
0	0	0	0	0
0	0	0	0	0
0.001126611	0	0.007042267	0	0
0.001700017	0	0.039285962	0	1.501796128
0	0	0	0	0
0.034080893	0	0.002082446	0	0
0	0	0	0	0
0	0	0	0	0
0	0	0	0	0
0	0	0	0	0
0	0	0	0	0
0	0	0	0	0
0	0	0	0	0
0	0	0	0	0
0	0	0	0	0
0	0	0.001495853	0	0
0	0	0	0	0.124523837
0	0	0	0	0
0	0	0	0	0
0	0	0	0	0
0.006824609	2.071019043	0	0.013811709	0
0	0	0	0	0
0	0	0	0	0
0	0	0	0.000870678	0
0	0	0	0.037337923	0
0	0	0	0	0

0	0	0	0	0
0.08370826	0.006690608	0.025577492	0.195393574	0.004691828
0	0	0	0	0
0	0	0	0	0
0	0	0	0	0
0	0	0	0	0
0	0	0	0	0
0	0	0	0	0
0	0	0	0	0
0	0	0	0	0
0	0	0	0	0
0	0	0	0	0
0	0	0	0.001930358	0.002088286
0	0	0	0	0
0.013688077	0.03567001	0.001296393	0	0
0	0	0	0	0
0	0	0	0	0
0	0	0	0	0
0	0	0	0	0
0	0	0	0	0
0	0	0	0	0
0	0.020905456	0	0	0.850284313
0.088318676	0	0	0.018728844	0
0	0.107371625	0	0	0
0	0	0	0	0
0.888318597	0	0	0	0
0	0	0	0	0
0	0	0	0	0
0	0	0	0	0
0	0	0	0	0
0	0	0	0	0
0	0	0	0	0
0	0	0	0	0
0	0	0	0	0
0	0	0	2.190013346	0
0	0	0	0	0
0	0	0	0	0
0	0	0	0	0
0	0.123002834	0	0.000893198	3.413054889
0	0	0	0	0
0.005717386	0.079680774	0.978789141	5.080717786	0.005414943
0	0	0	0	0
0	0	0	0	0
0	0	0	0	0
0	0	0	0	0
0.248748661	3.624255542	0.103633087	0.043783951	0.013019457
0	0	0	0	0
0	0	0	0	0

0	0	0	0	0
0	0	0	0	0
0	0	0	0	0
0	0	0	0	0
0	0.01054329	0	0	0
0	0	0	0	0
0	0	0	0	0
0	0.001878204	0	0	0.022783551
0	0	0	0	0
0	0	0	0	0.01546156
0	0	0	0	0
0	0	0	0	0
0	0.001937937	0.006238308	0.008984302	0.001481605
0	0	0	0	0
0	0	0	0	0
0	0	0	0	0
0	0	0	0	0
0	0	0	0	0
0	0	0	0	0
0.428562612	1.192645507	0.013770286	0.783477925	0.004219946
0	0	0	0	0
0	0	0	0	0
0	0	0	0	0
0	0	0	0	0
0	0	0	0	0
0.019042528	0	0	0	0
0	0	0	0	0
0	0	0	0	0
0	0	0	0	0
0	0	0	0	0
0	0	0	0	0
0	0	0	0	0
0	0	0	0	0
0	0	0	0	0
0	0	0	0	0
0	0	0	0	0.008535349
0	0	0	0	0
0	0	0	0	0
0	0	0	0	0
0	0	0	0	0
0	0	0	0	0
0	0	0	0	0
0	0	0	0.028696196	0
0	0	0	0	0
0.017345329	0.031938916	0	0.021799522	0
0	0	0	0	0
0	0	0	0	0
0	0	0	0	0

0	0	0	0	0
0	0	0	0	0
0	0	0	0	0
0	0	0	0	0
0	0	0	0	0
0.003288625	0.013476842	0	0.011487664	0
0	0	0	0	0
0	0.005145232	0	0	0
0	0	0	0	0
0	0	0	0	0
0	0	0	0	0
0	0	0	0	0
0	0	0	0	0
0	0	0	0	0
0	0	0	0	0
0	0	0	0	0
0.001495905	0	0	0	0
0	0	0	0	0
0	0	0	0	0
0	0	0	0	0
0	0.043072313	0	0	0
0	0	0	0	0
0	0	0	0	0
0	0	0	0	0
0	0	0	0	0
0	0	0	0	0
0	0	0	0	0
0	0	0	0	0
0	0	0	0	0
0	0	0	0	0
0	0	0	0	0
0	0	0	0	0
0	0	0	0	0
0	0	0	0	0
0	0	0	0	0
0	0	0	0	0
0	0	0	0	0
0	0	0	0	0
0	0	0.093207983	0	0
0	0	0	0	0
0	0	0	0	0
0	0	0	0	0
0	0	0	0	0
0	0	0	0	0
0	0	0	0	0
0	0	0	0	0
0	0	0	0	0
0	0	0	0	0

0	0	0	0	0
0	0	0	0	0
0.017467419	0	0	0	0
0	0	0	0	0
0	0	0	0	0
0	0	0	0	0
0	0	0	0	0
0	0	0	0	0
0	0	0	0	0
0	0	0	0	0
0.001205283	0.014437864	0	0	0.000813337
0	0	0	0	0
0	0	0	0	1.005428199
0	0	0	0	0
0	0	0	0	0
0	0	0	0	0
0	0	0	0	0
0	0	0	0	0
0	0	0	0	0
0	0	0	0	0
0	0.04267529	0.003707436	0.002848757	0
0	0	0	0	0
0	0	0	0	0
3.300047813	0	3.008661809	1.069038685	6.080648383
0	0	0	0	0
0	0	0	0	0
0	0	0	0	0
0	0	0	0	0
0	0	0	0	0
0	0	0	0	0
0	0	0	0	0
0	0	0	0	0
0	0	0	0	0
0	0	0	0	0

PHL018-0094_V2	PHL018-0053_V3	PHL018-0017_V3	PHL018-0257_V1	PHL018-0242_V1
REF	CTRL	EYCF	REF	REF
0.152808747	0	0.03740675	0.014982231	3.731851996
1.253173324	5.697791029	0.280354957	0.022371179	0.541932916
0	0	0	0	0
0	0	0	0	0
0	0	0	0	0
0	0	0	0	0
0	0	0	0	0
0	0	0	0	0
0	0	0	0	0
0	0	0	0	0
0	0	0	0	0
0	0	0	0	0
0	0	0	0	0
0.076108881	15.58751381	4.540237188	0.123502899	0.48461873
0	0	0	0	0
0	0	0	0	0
0	0	0	0	0
0	0	0	0	0
0	0	0	0	0
0	0	0	0	0
0	0	0	0	0
0	0	0	0	0
0	0	0	0	0
0	0	0	0	0
0	0	0	0	0.000937048
0	0	0	0	0
0	0	0	0	0.000490647
0	0	0	0	0
0	0	0	0	0
0	0	0	0	0
0	0	0	0	0
0	0	0	0	0
0	0	0	0	0
0	0	0	0	0
0	0	0	0	0
0	1.66706427	2.222215426	0.410400407	0.716631715
0	0	0	0	0
0	0	0	0	0
0	0	0	0	0
0	0	0	0	0
0	0	0	0	0
0	0.003220576	0	0.40746561	0.008304755
0.052221264	0.001527339	0.093707711	3.411362513	0.510092798
0.00350067	0.002200394	0.032167951	0	0
0.003453702	0	0.001955948	0.009030521	0.002380098
0	0	0	0	0

0	0	0	0	0.049161757
0.006632492	0.010184133	0.00124568	0	0
0	0.003742491	0.007209809	0	0
2.859756517	0.230574311	0.279637954	0.098109961	0.335200565
0.025164821	0	0.004295759	0	0.000885269
0	0	0	0	0
0.000761674	0.250152538	0.016823116	0	0
0	0	0	0	0
0	0	0	0	0
0	0	0	0	0
0	0	0	0	0.001306188
0	0	0.823725402	0.441242969	0
0	0	0	0	0
0.094551127	0.007817515	0	0	0.044186182
0	0	0	0	0
0	0	0	0	0
0	0.001786164	0.000786514	0.539597996	0
0	0	0	0	0
0	0	0	0	0
0	0	0	0	0
0.187526056	3.774913003	0.012327057	0	0.005809503
0.004509956	0.003016221	0.003984459	0	0.001212124
0.414589903	0	0.672270641	0	0.056647422
0	0	0	0	0
0	0	0	0	0
0	0	0	0	0
0	0	0	0	0
0	0	0	0	0
0	0.001378078	0	0.038603826	0
0	0	0	0	0
0.265121227	0.02929467	0	0	0.001831293
0	0	0	0	0
0	0	0	0	0
0.00100077	0	0	0	0.011767569
0	0	0	0	0
0	0	0	0	0
0	0	0	0	0
0	0	0	17.80228731	0
0	0	0	0	0
0	0	0	0	0
0	0	0	0	0
0	0	0	0	0
0	0	0	0	0
0	0	0	0	0
0.449961476	0	0	0	0
0	0	0	0	0
0.04750618	0	0	0	0
0	0	0	0.029020208	0

0	0	0	0	0
0.010419745	0	0	0	0
0	0	0	0	0
0	0	0	0	0
0.045174856	0	0.003887446	0	0
0	0	0	0	0
0	0	0	0	0
0	0	0	0	0
0.065562761	0.006663804	0.000733579	0	0.694180444
0	0	0	0	0
0	0	0	0	0
0	0.022242566	0	0.061387735	0
0	0	0	0	0
0	0	0	0	0
0	0	0	0	0
0	0	0.001579865	0	0
0	0	0	0	0
0	0	0	0	0
0.487336205	0	0.007958449	1.198202085	0
0	0	0	0	0
0	0	0	0	0
0	0	0	0	0
0.006675207	0	0	0	0
0	0	0	0	0
0	0	0	0	0
0	0	0	0	0
0	0	0	0	0
0.00422517	0	0	0	0
0	0	0	0	0
0	0	0	0	0
0.503914882	0	0.034790191	0.003897056	0.009576889
0	0	0.024391307	0.002574147	0.019146152
0	0	0	0	0
0	0	0	0	0
0	0	0	0	0
0	0	0	0	0
0	0	0	0	0
0	0	0	0	0
0	0	0.347631142	0.024589704	0
0	0	0	0	0
0	0	0	0	0
0	0	0	0	0
0.001173941	0	0	0	0
0	0	0	0	0
0.004171949	0	0	0	0
0	0	0	0	0
0	0	0	0.010091875	0
0	0	0	0	0

0	0	0	0	0.005958039
0	0	0	0	0
0	0	0	0	0
0	0	0	0	0
0	0	0.083369864	0	0.000989974
0	0	0	0	0
0	0	0	0	0
0	0	0	0	0
0	0	0	0	0
0	0	0	0	0
0	0	0	0	0
0	0	0	0	0
0	0	0	0	0
0	0	0	0	0
0	0	0	0	0
0	0	0	0	0
0	0	0	0	0
0	0	0	0	0
0	0	0	0	0
0	0	0	0	0
0	0	0	0	0
0	0	0	0	0
0	0	0	0	0
0	0	0	0	0
0	0	0.018579911	0	0
0	0	0	0	0
0	0	0	0	0.078249324
0	0	0	0	0
0	0	0	0	0
0	0	0	0	0
0.00687809	0	0	0	0
0	0	0.133316465	0	0.003280771
0	0	0	0	0
0	0	0	0	0
0	0	0	0	0
0	0	0	0	0
0	0	0	0	0
0	0	0	0	0
0	0	0	0	0
0	0	0	0	0
0	0	0	0	0
0	0	0	0	0
0	0	0	0	0
0	0	0	0	0
0	0	0	0	0
0	0	0	0	0
0	0	0	0	0
0.051961149	0	0	0	0

0	0	0	0	0
0	0	0	0	0
0	0	0	0	0
0	0	0	0	0
0	0	0	0	0
0	0	0	0	0
0	0	0	0	0
0	0	0	0	0
0	0	0	0	0
0	0	0	0	0
0	0	0	0	0
0.334808054	0	0	0	0
0.002055803	0	0	0.00167177	0
0	0	0	0	0
0	0	0	0.11410685	0
0	0	0	0	0
0	0	0	0	0
0	0	0	0	0
0	0	0	0	0
0	0	0	0	0
0.058007302	0.063851169	0.033812724	0.006251616	0.013696386
0	0	0	0.153133438	0
0	0	0	0	0.000515046
0	0	0	0	0
0	0	0	0	0
0	0	0	0	0
0.003866624	0.003645629	0.003424649	0.002548555	0.002278986
0	0	0	0	0
0	0	0	0	0
0	0	0	0	0
0	0.070985561	0	0	0
0	0	0	0	0
0	0	0	0	0
0	0	0.031670189	0.00095515	1.049645734
0.032285875	0.00190075	0.026783048	0.004677779	0.004073874
0	0	0	0	0
0	0	0	0	0
0	0	0	0	0
0	0	0.001158925	0.016756148	0
0	0	0	0	0
0	0	0	0.003758005	0
0	0	0	0.002234288	0
0	0	0.002485533	0.18306589	0
0	0	0	0	0
0	0	0	0	0
0	0	0	0.056156048	0
0	0	0	0	0
0.027058454	0	0	0.003188577	0.001088682

0	0	0	0	0
0	0	0	0	0
0	0.004597312	0	0	0
0	0	0	0	0
0	0	0	0	0
0	0	0	0	0
0	0	0	0.030328096	0
0	0	0	0.04250282	0
0	0	0	0	0
0	0	0	0	0
0	0	0	0.006276257	0
0	0	0	0	0
0	0	0	0	0
0	0	0	0	0
0	0	0	0	0.001144327
0	0	0	0	0
0	0	0	0	0
0	0	0	0.023964727	0
0	0	0	0.017392993	0.003506078
0	0	0	0.045943961	0
0.002916808	0.002612594	0	0	0
0	0	0	0	0
0	0	0	0	0
0	0	0	0	0
0.002897428	0	0.004063209	0	0.002163141
0	0	0	0	0
0	0	0	0	0
0	0	0	0	0
0	0	0	0.048437141	0
0	0	0	0	0
0.652533466	0.005763119	0.427463635	0.010551704	0.136702053
0	0	0	0	0
0	0	0.024308628	0	0
0.00508713	0	0.00194562	0	0
0	0	0	0	0
0	0	0	0	0
0	0	0	0	0
0	0	0	0	0
0	0	0	0	0
0	0	0	0	0
0	0	0	0	0
0.04572788	0.0089206	0.008047748	0.029496712	0
0.001987155	0	0	0	0
0	0	0	0	0
0	0	0	0	0
0	0	0	0	0
0	0	0	0	0
0	0	0	0	0

0	0	0	0	0
0	0	0	0	0
0	0	0.037774471	0	0
0	0	0	0	0
1.13079645	0	0	0	0
0	0	0	0	0
0	0	0	0	0
0	0	0	0	0
0	0	0	0	0
0	0.000999474	0	0	0
0	0	0	0	0
0	0	0	0	0
0	0	0	0	0
0	0	0.001063633	0	0.001132498
0.012937612	0.009469976	0.00263367	0.000746638	0.03061238
0	0	0.016213228	0.001598749	0
0.001426572	0	0	0	0.00159756
0	0	0	0	0.002271966
0	0	0	0	0
0	0	0	0	0
0	0	0	0	0
0.03305322	0	0	0.002687873	0.001442141
0	0	0	0	0
0	0	0	0	0
0.001376595	0.012741212	0	0	0
0	0	0	0	0
0	0	1.045476089	1.372206829	0
0	0	0	0	0
0	0	0	0.016083289	0
0.017075638	0.054504759	0	0	0
0	0	0	0	0
0	0	0	0	0
0	0	0.001868741	0.176417631	0
0	0	0.001561673	0	0
0	0	0	0	0
0	0	0.003156201	0.105838992	0.007201178
0	0	0	0	0
0.004314592	0	0.000850861	0.018814906	0.056772887
0	0	0	0	0
0	0	0	0	0
0	0	0	0	0
0	0	0	0	0
0	0	0	0	0
0	0	0	0	0
0	0	0	0	0
0	0	0	0	0
0.000876911	0	0	0	0
0	0	0	0	0

0.025408867	0	0	0.005393231	13.45649619
0	0	0	0	0
0.000899238	0	0	0	0
0	0	0	0.002816816	0
0.15742134	0.03700648	0	0.000809286	0.018490157
0.00258165	0	0	0	0.00206506
0	0	0	0	0
0.008243071	0.032183808	0.002334165	0	0
0	0	0	0	0
0.005882062	0.140105234	0.097953439	0	0.06989624
0	0	0	0	0
0	0.001498636	0	0.718840328	0.001967365
0.011612592	0.00363766	0.001401571	0	0
0.001734741	0	0	0	0
0.01488016	0	0	0	0
0	0.001023887	0	0	0
0	0	0	0.000929484	0
0	0	0	0	0
0	0.312882099	0	0	0
0	0	0	0	0
0	0	0	0	0
0	0	0	0	0
0	0	0	0.050606329	0
0	0	0	0	0
0	0	0	0	0
0	0	0.003498156	0.003371839	0
0	0	0	3.609452711	0
0	0.004162963	0.066837846	0	0
0	0	0	0	0
0	0	0	0	0
0.004044835	0	0	0	0
0	0	0	0	0
0	0	0	0	0
0	0.066147454	0	0	0
0	0	0	0	3.512962256
0	0	0	0	0
0.011562422	0.727656532	0.012425292	0	0
0	0.03204939	0	0	0
0	0	0	0	0
0.00186565	0	0	0	0
0	0	0	0	0
0.107809121	0.37568594	0	0	0
0	0	0	0	0
0	0.016000471	0	0	0
0	0.122394624	0	0	0
0	0	0	0	0
0.004136136	0	0.013630733	0	0
0	0	0	0	0

0.009903583	0.004357522	0	0	0
0	0	0	0	0
0	0	0	0	0.001964476
0	0	0	0	0
0	0	0	0	0
0.014116736	0.025418497	0	0	0.000972846
0	0	0	0	0
0	0	0	0	0
0	0	0.020277793	0	0
0	0	0	0	0
0.011083287	0.039408511	0.00688822	0.002703864	0.016642987
0.003541215	0	0	0	0
0	0	0	0	0
0.008567126	0.00227366	0	0.000851492	0.001865495
0	0.066140386	0	0	0
0	0	0	0	0
0	0	0	0	0
0.001621122	0.078825238	0	0	0
0	0.012099807	0	0	0
0.049801767	0	0	0	0
0	0	0	0	0
1.568335376	0	0.418534068	0.212998157	0
0	0	0	0	0
0	0	0	0	0
0	0	0	0	0
0	0	0	0	0
0	0.060398606	0.035008659	0	0
0	0	0	0	0
0	0	0	0	0
0.234408769	0.69237032	0.504458026	0.028082436	0.211722613
0.00403619	0.001349685	0.006112965	0.000722087	0.00253118
0	0.000812107	0	0.187347862	0
0	0	0	0	0
0	0.001092352	0	0	0
0.001934222	0.001283325	0	0	0
0	0	0	0	0
0	0	0	0.068429285	0
0	0.003476198	0	0.87180227	0
0	0.787935425	0	0	0
0	0.077683033	0	0	0
0.063583631	0.0221967	0	0	0.039217362
3.092793112	0	1.440181331	0.371913695	2.242668368
0	0.002122948	0	0.675792124	0
0	0.021422527	0	0	0
0.007503606	0.003689963	0	0	0
0	0	0	0	0
0	0.012016363	0.001079846	0	0.001149761
0	0	0	0	0

0	0	0	0.007478799	0.002269779
0.003399619	0	0	0	0.002900643
0	0.002885971	0.002541598	0.16737019	0
0.003437653	0	0	0	0
0.568114821	0	0.030536837	0	0
0.00771164	0	0	0	0
0	0	0	0	0
0	0	0	0.013716354	0
0	0.107875187	0	0	0.001662151
0	0	0	0	0.053728596
0	0	0	0	0
0	0	0	0	0
0	0	0	0	0
0.014669072	0.010128083	0	0.00184524	0.002310088
0	0	0	0	0
0.155833676	0.002994835	0.045213806	0.004646519	0.004412941
0.022632981	0.000948418	0.035706787	0.062512557	0.02734673
0.000964849	0.006625672	0	0	0
0.001230429	0	0	0	0
0	0	0	0	0
0.028774207	0	0	0	0.029140851
0.007138996	0.001375144	0	0	0
0	0	0	0	0
0	0	0	0	0
0	0	0	0	0
0	0	0	0	0
0.004284709	0.009030181	0	0	0.003387014
0.005263592	0.002857343	0	0	0.04608413
0	0	0	0	0
0.00215662	0	0	0	0
0.002300124	0	0.005846362	0	0.003005117
0.009716073	0.005801812	0.001803354	0	0.004800279
0.108563388	0.05686583	0.032337519	0.044783291	0
0	0	0	0.101151406	0
0	0	0.0017563	0.089125173	0
0	0	0	0	0
0	0	0	0	0
0.079642499	0.006662625	0.015984144	0.013078135	0.004739479
0.247517087	0	0.027956835	0	0.217170346
0	0	0	0	0
0.007508682	0	0	0	0
0	0	0	0	0
0	0.003779428	0	0	0.002577412
0	0	0	0	0.008913028
0	1.960111535	0	0	0
0	0	0	0	0
0	0	0	0	0
0	0	0	0	0

0	0.006472096	0	0	0
0.916416509	0.23854993	0.178378915	0.020868882	0.507257639
0	0	0	0	0
0	0	0	0.001420111	0.000711145
0	0	0	0	0
0	0	0	0	0
0	0.062847346	0	0	0
0	0	0	0	0
0	0	0.003533349	0.010088494	0
0	0	0	0	0
0.006622975	0	0	0	0.002399553
0.020020369	0.037423946	0.014037784	0	0.009097968
0	0	0.514311985	0	0
0	0	0	0	0
0	0	0.002585771	0	0
0	0	0	0	0
0.004691466	0.012180177	0.007508731	0	0.003197954
0.041572038	0.103407551	0.216670114	0	0.002965275
0	0	0	0	0
0.087345937	0.00369619	0	0	0.012708266
0.033866596	0.013314485	0.101991588	0.024837466	0.019551818
0	0	0	0	0
0	0	0	0	0
0	0	0	0	0
0	0.002110455	0	0	0
0.002700576	0	0	0	0
0	0	0	0	0
0.075567546	0.111875627	0.051261684	0.022198789	0.045817679
0	0	0	0	0
0.001348405	0	0	0	0
0	0	0.000756933	0.001931296	0
0	0	0	0	0
0	0	0	0	0
0	0	0	0	0
0	0	0.049588896	0.960089628	0
0.233084126	0.009941628	0.372897382	0.019631237	0.063984136
0	0	0	0	0
0	0	0	0	0
0.045507303	0.018445398	0.027847495	0.318501208	0.043858005
0	0	0	0	0
0	1.642763972	0	0	0
0	0	0	0	0
0.084265702	0	0.062726436	0	0.008442261
0.023452798	0	0	0	0.010686662
0.003187401	0	0	0	0
0	0	1.571687049	0.036773626	0
0	0	0	0	0
0.001307229	0	0	0	0

0.002574416	0	0	0	0
0	0	0.11236325	0	0
0	0	0	0.003433312	0
6.651667713	0	0.043095096	0.087441223	1.821427495
0	0	0	0	0
0	0	0	0	0.001705266
0	0	0.001272787	0	0
0	0	0	0	0
2.132530854	0	1.380631259	3.026006795	0
0.094516607	0	0.004585288	0.09406192	0.037299721
0	0	0	0	0
0.050474126	0	0.009241036	0.001337774	0.002512174
0	0	0	0	0
0	0	0	0	0.30397461
0.137758073	0	0.181719436	0.006761598	0
0	0	0	0	0
0.067007705	0	0.001904762	0.093239076	0.011717831
0	0	0	9.810619275	0
0	0	0	0	0
0	0	0	0.006272009	0
0	0	0	0	0
0	0	0	0	0
0	0	0	0	0
0.334192666	0.331857708	0	0.021932043	0
0.008576212	0	0	0	0.312624863
0	0	0.159173671	0	0
0	0	0	0.003166844	0
0	0.608567406	3.78936812	0.367589517	0
0	0	0	0	0
0	0.006629328	0	0	0
0	0	0	0.010729491	0.084665055
3.378570566	0	0	0	0
0.03693051	0	0	0	0.011617126
0	0	0	0	0
0.009564435	0	0	0	0.084934921
0	0	0.185732728	0.011716359	0
0	0	3.235170419	1.074887707	0.982174659
0	0	0	0	0
0	0	0.00716377	0	0
0	0	0.002392474	0	0.014385176
0.170728511	0.363029275	0.018673342	0	0.215693353
0	0	0	0	0
0.003227045	0.391562964	15.02810674	0.004426183	0.015659956
0	0	0.004765058	0.001397462	0
0	0	0	0	0
0	0	0	0	0
0	0	0	0	0
0.002586999	0	0.012924318	0	0.030902126

0	0	0	0	0
0	0	0.069613674	0.266865458	0
0.006199827	0.643431263	0.002173582	0	0.004860052
0	0	0	0	0
0	0	0	0	0
0	0	0.001369811	0.055920613	0.095167111
0	0	0	0	0
0	0	0	0	0.127851427
0	0	0	0	0.165195765
0	0	0	0	0.096457873
0.060668398	0.00266555	0.038199891	0	1.443328559
4.55863522	2.338156876	1.11490355	0.653537567	1.446081615
0	0	0	0	0
0.015547767	0.006015065	0.012437159	0.127520016	0.038255832
0.310620087	0.65557675	0	0	0.323694425
0	0	3.997096811	0.079373634	0
0	0	0	0	0.002825668
0	0	0	0	0
0	0	0	0	0
0.060170319	0	0.026368706	0	0.008797131
1.821212349	0.79352008	0.031480534	0	1.904854882
0	1.821196597	0.083869141	0	0
1.795835601	12.34342468	5.765041839	0.016380129	2.71046474
0	0	0	0	0
0	0	0	0	0.007092969
0	0.005110923	1.853148404	0.68176783	0
0.69340467	0	0	0	0
0	0	0	0.013962359	0
0.001551831	0	0	0	0
0	0	0	0	0
0	0	0	0	0
0.008348766	0	0.146911548	0.024845785	0
0	1.277458805	0.001972573	0	0.006767592
0.213895575	0	0.054654038	0	0.211237412
0.007289551	0.004763495	0	0	0.001386216
0.038171892	0.001354086	0.316213384	0.191349102	0.066660143
0.00492864	0	0.004799777	0	0
0	0	0	0	0
4.501821139	0	0.342356591	0.485219633	0
0	0	0	0	0
0	0	0	0	0
0	0	0	0	0
0	0	0	0	0
0	0	0	0	0
0.070683528	0.003088364	0.110606832	0.006168535	0.094841912
0	0	0	0	0
0	0	0	0	0
0.007018654	0.031976442	6.968842229	0	0.223279484

0	0	0	0	0
0	0	0	0	0
0	0	0	0	0
0	0	0.003477163	0	0.000617049
0.003618695	0	0	0	0.038378886
0	0	0.570866337	0.057751265	0
0	0	0	0	0
0.001183058	0	0	0.004988956	0
0	0	0	0	0
0	0	0	0	0
0.009409674	0.000977191	0.007960424	0.005855371	0
0	0	0	0	0
0	0	0.11509876	0.251695559	0
0	0	0	0	0
0	0	0	0	0
0	0	0	0	0
0.288148217	0	0.012160945	0	0.256921637
0.010898558	0	0.005731348	0.012490811	0.015256062
1.054176044	0	0	0.019234603	0.004615359
0.08692252	0	0	0	0.002235747
0	0	0	0	0
0	0	0	0	0
0	0	0.05554711	0.002985575	0
0	0	0	0	0
0	0	0	0	0
0	0	0	0	0
0	0	0	0	0
0	0	0	0	0
0	0	0	0	0
1.056931654	0	0.105149408	0.011592605	0.113339498
0	0	0	0	0
0	0	0	0	0
0	0	0	0	0.430180397
0	0	0	0	0
0	0	0	0	0
0	0	0	0	0
0	0	0.101686144	0.004455195	0
0.035325055	0.005997269	0.018030411	0	0.000581751
0	0	0	0	0.03139448
0.012980607	0	0	0	0
0	0	0.008373055	0	0.344598551
0	0	0	0	0
0	0	0	0	0
0.011603099	0	0.004775363	0.027376882	0
0	0	0.141659007	0.04713661	0
0	0	0	0	0
0.000493599	0	0	0	0
0	0	0	0	0

0	0	0	0	0
0	0	0	0	1.350609331
0	0	0	0	0
0	0	0.101631523	0.050382911	0
0	0	0	0	0
0	0	0.351328602	0	0
0	0	0	0	0
0	0	0	0	0.026040461
0.01886673	0.518235685	0	0	0.004546549
0	0	0.000809101	0	0
0	0	0.001077615	0	0
0	0	0	0.016019238	0
0	0	0	0	0
0	0	0	0	0.007801837
0.064358213	14.50133993	0.00884477	0.037612005	0.085525593
0.009571366	0.013629035	0	0	0
0	0	0	0	0.200035768
0	0	0	0	0
0	0	0	0	0
0	0	0	0	0
0.246708346	0	0.010947655	0	0.001840494
0	0	0	0	0
0	0	0	0	0
0.029271525	0	0	0	0
0.002808448	0	0.003544585	0.479578793	0.018555888
2.570843193	0	3.089793047	0.087847319	0.112349075
0	0	0.623811363	0.013642083	0
0	0	0	0	0
1.655667081	0	0	3.842128103	0
0	0	0	0	0.215535451
0	0	0	0	0
0	0	0.147616677	0	0
0	0	0	0	0
0.016798784	0	0.006783378	0	0.003695267
0	0.001180583	0.00441876	0.144355937	0.011346999
0.012577989	0.000625897	0	0.008282134	0.014085575
0	0.055548114	0.086462815	0	0
0.922585256	0	1.459971734	0.158983342	0.891787265
0	0	0	0.422345438	0
0	0	0	0	0
0	0	0	0	0
0	0	0	0	0.090514548
0.244929656	0	0.184041491	0.175744417	0.333902621
0	0	0.074899479	0	0
0	0	0	0	0
0.008440547	0	0.005744234	0	0
0	0	0	0	0
0.569511631	0.510743136	0.078201613	0.004717007	0.922703665

0	0	0.004342609	0	0
0	0	0	0	0
0	0	0	0	0
0	0	0	0	0
0.076147392	0	0.001235306	0	0.023675141
0.006786213	0	0.038066591	0.0023607	0
0	0	0	0	0
0	0	0	0	0
0	0	0	0	0
0	0	0	0	0
0.001048491	0	0	0	0.001006424
1.714011848	0.392101558	0.602494077	0.198052353	6.505022143
0.746852938	0	0	0.006148545	0.120239807
0	0	0	0	0
0	0.038121766	0.001666736	1.702066254	0.934225941
0	0	0	0	0
0.00267724	0	0	0	0.071249713
0	0	0	0	0
0.000595282	0	0	0.004259907	0.007555157
0	0	0	0.004920372	0
0.092653653	0	0.054945018	0.016458458	0
0	0	0	0	0
1.20531798	0	0.315760266	0.184672232	0.112788289
0	0	0	0	0
0	0	0	0	0
0	0	0	0	0
0	0	0	0	0
0	0	0	0	0
0	0	0.005361813	0	0
0	0	0	0	0
0	0	0	0	0
0.090774466	0.040697343	0.013594889	0	0.046386084
0	0	0	0	0
0.043588219	0.002103278	0.049240348	0	0.110937869
0.012737448	0	0.001999518	0.006632235	0.011709374
0.486966697	0.003804938	0.011599297	0.111804494	0
0	0	0	0	0
0	0	0.007074411	0.200015491	0
0.010702074	0	0.002067436	0.001758337	0
0	0	0	0	0
0	0	0	0.094467924	0.090694816
0	0	0	0	0
0	0	0	0	0
0	0	0	0	0.017678471
0.011049435	0	0.092577119	0.001005141	0.051592495
0	0	0	0	0
0	0	0.220750086	0	0.311449032
0.044460132	0	0.010246545	0	0.001190177

0	0	0	0	0
0	0	0	0	0.000909594
0.058107868	0	0	0	0.001301452
0	0	0	0	0
0	0	0	0	0
0	0	0	0	0.004105297
0	0	0	4.08603566	0
0	0.099181655	0	0	0.059816209
0	0	0	0	0
0	0	0.008330193	0	0
0	0	0	0	0.121363319
0	0	0	0.002820625	0
0	0	0	0	0
0	0	0.036523518	0.067279818	0
0	0	0	0	0
0	0	0	0	0
0	0	2.503126987	0	0.429827663
0	0	0	0	0
1.37943641	0	0	0.245489401	8.654676768
0	0	0	0	0
0	0	0	0	0.003744539
0	0	0	0	0
0	0	0	0	0
0	0	0	0	0
0.003273889	0	0	0.000732136	0
0.015691433	0.010202792	0.043826395	0	0.002538206
0	0	0	0	0
2.188286509	0.027513836	0.010589716	0	1.656711629
0	0	0	0	0
0	0	0.535555433	0.008524366	0.144780788
0	0	0	0	0
0	0	0	0	0
0	0	0	0.001611872	0
0	0	0	0	0
0.242635206	0	0	0	0.006956616
0	0	0	0	0
0	0	0	0.550625131	0
0	0	0	0	0.003250356
0	0	0	0	0
0	0	0	0	0
1.059020089	14.47331529	8.945157177	0.392565053	1.99008229
0.206239924	0.041141676	0	0	0.619801215
0.42856179	0	0.172542153	0	0
0	0	0	0.004764691	0
0	0	0	0	0
0	0	0	0	0
0	0	0	0	0
0.005594359	0	0.008652851	0	0.018978944

0	0	0	0	0
0.043833926	0.001819097	0.004094079	0	0.018384241
0	0	0	0	0.016823232
0	0	0	0	0
0.879264621	0	1.240164334	0.17813357	2.997075849
0	0	0	0	0
0	0	0	0	0
0.006639333	0	0	0	0
0.047994136	3.599529954	0.251858982	0	0.001892488
0	0	0	0	0
0	0	0	0	0
0	0	0	0	0
0.135012118	0	0.030614038	0.021918133	0.015101039
0.001213035	0.010140849	0.029131344	0.000904232	0.003848881
0	0	0.239529961	0.44650298	0
0.316871551	0	0.032629412	0.022111787	0.590799855
0.013296123	0.00710384	0.003496092	0	1.637288508
0	0	0	0	0
0	0	0	0	0
0	0	0	0	0
0	0	0	0	0
0	0	0	0	0
0	0	0	0	0
0	0	0	0	0
0	0	0	0	0
0	0	0	0	0
0.072563785	0	0.066879896	0.084640774	1.287402503
0.006007975	0.000615013	0.00198596	0	0
0	0	0	0	0
0	0	0	0	0
0	0	0	0	0
0	0	0	0	0
0	0	0	0	0.217776874
0	0	0.008516521	0.002092488	0
1.730464971	0	0.581087183	1.15118868	0
0.14396387	0	0	0	0
0	0	0	0	0.005454968
0.095441154	0	0	0	0
0	0	0	0	0
0	0	0	0	0
0	1.777811003	2.078011945	0.050470318	0.767748225
0.168083792	0	0	0.071651713	0
0	0	0	0	0
0	0	0	2.570707976	0
0	0	0	0	0
0	0	0	0	0
0	0	0	0	0
0	0	0	0	0

0	0	0.307584459	0.034969391	0
0	0	0	0	0.001807824
0	0	0	0.002061024	0
0.002916796	0	0	0	0
0.094701252	0	0.001369789	0.052591156	0.017293361
0	0	0.000712404	0	0.253095873
0.539975974	0.474591548	0.017805067	0	0.880660853
0	0	0	0	0
0	0	0	0	0
0.155088786	0	0	0	0.024811084
0	0	0.004229275	0	0
0	0	0	0	0.045976826
0	0	0	0	0
0	0	0	0	0
0	0	0	0	0
0	0	0	0	0
0	0	0	0	0
0	0	0	0	0
0	0	0	0	0
0	0	0	0	0
0.158845308	0.016073274	0.067805665	0.053803777	0.01116429
0	0	0	0	0.005235235
0.072758066	0	0.015613254	0.001639375	0.003078543
0.048237728	0.001751622	0.011826657	0	0
0	0	0	0.000697106	0
0	0	0	0.000859908	0.001076534
0	0	0	0.103678855	0
0	0	0	0	0
0	0	0	0	0
0	1.646770149	0.067098582	0	0.003151892
0	0	0	0	0
0	0	0	0	0
0	0	0	0	0
0	0	0	0	0
0	0	0	0	0
0	0	0	0	0
0	0.000974104	0.004932737	0	0.007763984
0	0	0	0	0
0	0	0	0	0
0	0	0	0.352458204	0
0	0	0	0	0
0	0	0	0	0
0	0	0	0	0
0	0	0	0	0
0	0	0	0	1.142834028
0	0	0	2.259049542	0
0	0	0	0	0
0	0	0	0	0

0.006312832	0.004607308	0	0.004235193	0.003141992
0	0	0	0.752004032	0
0	0	0	0.002982581	0
0	0	0	0	0
0	0	0	0	0.002666428
0	0	0	0.081545635	0
0	0	0	0	0
1.89972273	0.004945694	0.598602945	0.028829514	1.781824098
0.066059587	0.001972326	0.112613893	4.478381929	0.625725754
0.127235412	0.002288457	0.103792245	0	0
0.008319902	0	0	0	0
0	0	0	0	0
0	0	0	0	0.041627781
0	0	0	0.640154552	0
0	0	0	0	0
0	0	0	0	0
0	0	0.008076693	0	0.002006578
0	0	0	0.218022509	0
0	0	0	0	0
0	0	0	0	0.015658778
0	0	0	0	0
0	0	0	0	0
0	0	0	0	0
0	0	0	0	0
0	0	0.005374873	0.000741289	0.103011652
0	0	0	0	0
0	0	0	0	0
0	0	0	0	0
0	0	0	0	0
0	0	0	0	0
0	0	0	0	0
0	0	0	0	0
0	0	0	0	0
0	0	0	0	0
0	0	0.004019086	0.050703272	0
0	0	0.000588321	0	0
0	0	0	0	0.004965006
0	0	0	0	0
0	0	0.002193057	0.003357317	0
0	0	0	0	0
0	0	0	0	0
0.100958826	0	0.278663672	0.790649755	0.571770403
0	0	0	0	0
0	0	0	0	0
0	0	0.001076538	0	0
0	0	0	0	0.026716377
0	0	0	0	0
0	0	0	0	0

0	0	0	0	0
0.039250182	0	0.009654632	0	0
0	0	0	0	0
0	0	0	0	0
0.872283102	0.066331131	0.551810741	1.916630794	0
0	0	0	0	0.763864383
0	0	0	0	0
0.014722363	0.080186805	0.001407676	0	0.00574546
0.040758352	0	0.003045276	0	0
0	0	0.001055596	0	0
0	0	0	0	0
0	0	0	0.0602221	0
0	0	0	0	0
0	0	0	0	0
0	0.027755498	0	0	0.024267606
0.013929376	0	0	0	0.206252635
0	0	0	0	0
0	0	0	0	0
0	0	0	0	0
0	0	0	0	0
0	0	0	0	0
0	0	0	0	0
0	0	0	0	0
0.004246997	0.001613839	0.004872909	0.00345364	0.001080918
1.078457434	0	0.086710466	0.049010699	0
0	0	0	0	0
0.001235933	0.038798737	0	0	0.153236422
0.008180212	0	0.000586612	0	0
0	0	0	0.002347543	0
0	0	0	0	0
0	0	0	0	0
0	0	0	0	0
0	0	0	0	0
0	0	0	0	0
0	0	0	0	0
0	0	0	0	0.001767636
0	0	0	0	0
2.543192009	0	0.269292695	0	0
0	0	0.15943116	0	0
0	0	0	0	0
0	0	0	0	0
0	0	0.300920507	2.232982645	0
0	0	0	0	0
0	0	0	0.02144947	0
0	0	0	0	0
0	0	0	0	0
0	0	0	0	0
0	0	0	0	0

0.02573639	0	0.458594038	0.018565798	0.298995979
0	0	0	0	0
0	0	0	0	0
0	0	0	0	0.012580191
0	0	0	0	0
0.056941036	0	0	0	0
0	0	0	0	0
0	0	0	0	0
0	0	0	0	0
0	0	0	0	0
0	0	0	0	0
0.003203226	0	0	0	0.050220267
0	0	0	0	0
0	0	0	0	0
0	0	0	0.002953882	0
0	0	0	0	0
0	0	0	0	0
0	0	0	0	0
0	0	0	0	0
0	0	0	0	0
0.024798748	0	0	0	0
0	0	0	0	0
0.059724628	0	0.101884706	0	0.147632356
0	0	0	0.525542963	0
0	0	0	0	0
0	0	0	1.231880683	0
0	0	0	0	0
0	0	0	0	0
0	0	0	0	0
0	0	0	0	0
0	0	0	0	0
0	0	0	0	0
0.003183114	0.007603002	0.001434806	0	0
0.111929489	0	0	0.013029656	0
0.670737988	0	0	0.008368503	0.116154437
0.002006962	0.013291661	0.001918953	0	0
0	0	0	0	0
0	0	0	0.086005137	0
0.022791629	0.001008125	0	0	0.019851543
0	0	0	0	0
0	0	0	0	0
0	0	0	0	0
0.232202583	0	0.966766197	2.793803734	0
0	0	0	0	0
0	0	0	0	0
0.010386486	0	0.228582028	0.001051796	0
0	0	0	0	0
0	0	0	0	0

0	0	0	0	0
0	0	0	0	0
0	0	0	0	0
0	0	0	0	0
0	0	0	0	0
0	0	0	0	0
0	0	0	0	0
0	2.438453488	0	0	0
0.01505578	0	0.003282888	0.00476294	0.403003524
0.022405174	0	0.003609043	0.005416694	0
0	0	0	0	0
0	0	0	0	0
0	0	0	0	0
0	0	0	0.020033086	0
0	0	0	0	0
0	0.012807615	0	0	0
0	0	0	0	0
0	0	0	0	0
0	0	0	0	0
1.501097259	0	0.254797164	0.000892393	0
0	0.002477156	0.004799445	0.002597564	0.000929125
0	0	0	0	0
0	0	0	0	0
0	0	0	0.004732605	0
0	0	0	0	0.029456812
0	0	0	0	0
0	0	0	0	0
0	0	0	0.157206263	0
0	0	0	0	0
0	0	0.682142273	0	0
3.442308213	0	0.05042136	0.09471786	1.693907392
0	0	0	0	0
0	0	0	0	0
0	0	0	0	0
0	0	0	0	0
0	0	0	0	0
0	0	0	0	0
0	0	0	0	0
0	0	0	0	0
0	0	0	0	0
0	0	0	0	0
0	0	0	0	0
0	0	0	0.000937003	0.008993389
0	0	0	0	0
0	0	0	0	0
0	0	0	0	0
0.067699506	0.001699582	0	0	0
0	0	0	0.076262035	0

0	0	0	0	0
0	0	0	0	0
0	0	0	0	0
0	0	0	0	0
0.037818529	0	0.326355857	0	0.000510258
0	0	0	0	0
0	0	0.004279589	0	0
0	0	0	0	0
0	0	0	0	0
0	0	0	0	0
0	0	0	0	0
0.005060773	0	0.018851345	0	0.018891177
0	0	0	0	0.212285697
0	0	0	0	0
0	0	0.016394188	0	0
0	0	0	0.001466807	0
0.035193199	0	0	0	0
0.003312033	0	0	0	0
0.169727733	0.001334813	0.001679335	0	0.014483314
1.679920199	0.029130644	0.004823939	0.033008238	2.232407667
0	0	0	0	0
0	0	0.114966428	0	0
0	0	0	0	0
0	0	0	0	0
0	0	0	0	0
0	0	0	0	0
0.004176585	0.000598557	0.002459955	0	0.002806312
0	0	0	0	0
0	0	0	0	0
0	0	0	0	0
0.00549188	0	0	0	0.00063622
0	0	0	0	0
0	0	0	0	0
0	0	0	0	0
0	0	0	0	0
0	0	0	0	0
0	0	0	0	0
0.010910058	0	0.007836443	0.005593695	0.082097171
0.164992241	0	0.742268794	0.282865178	0.043190206
0.026592426	0	0	0	0
0.004516093	0	0	0	0.006098615
0	0	0	0	0
0	0	0	0	0
0.001972955	0.085767245	0	0	0
0	0	0	0	0
0	0	0	0	0
0	0	0	0	0
0	0	0	0	0

0	0	0	0	0
0	0	0.00124151	0	0.068360659
0	0	0	0	0
0.011785696	0	0.006306612	0	0.001620846
0	0	0.001008497	0	0
0	0	0	0	0
0.030498995	0	0.050649007	0	0.005392828
0.003072478	0	0	0	0.011952053
0	0	0	0	0
0	0	0	0	0
0.068512638	0	0.036838161	0.001375487	0.036353254
0	0	0	0	0
0.172657591	0	0.100817493	0.241500316	0.62798422
0	0	0	0	0
0	0	0	0	0
0	0	0	0	0
0	0	0	0	0
0	0	0	0	0
0	0	0	0	0
0	0	0	0	0
0	0	0	0	0
0	0	0	0	0
0.04805621	0.001843924	0.066782677	0	0
0.00706003	0	0.034732064	0.005296706	0.082632943
0	0	0	0	0
0	0	0	0	0
0	0.005725557	0	0	0
0	0	0.001330494	0	0
0	0	0	0	0
0	0	0	0	0
0	0	0.012674449	0.000577474	0.006024579
0	0.002186101	0	0	0
0	0	0	0	0
0	0	0	0	0
0	0	0	0	0
0	0	0	0	0
0	0	0	0	0.000768081
0	0	0	0	0
0	0	0.004978751	0.214976452	0.012437196
0	0	0	0	0
0	0	0	0	0
0	0	0	0	0
0	0	0	0	0
0	0	0	0	0
0	0	0	0	0.136451289
0.293785081	0	0.072951808	0.201036895	0.226029086
0	0	0	0	0
0	0	0	0	0

0	0	0	0	0
0	0	0	0	0
0.210806707	0	0.055127235	0.029248159	0
0	0	0.023401956	0	0
0	0	0	0	0
0	0	0	0	0
0.009623493	0	0	0	0.435223218
0	0	0	0	0
0	0	0	0	0
0	0	0	0.01208133	0
0.002954089	0	0.00170885	0	0.000992449
0.000882093	0	0	0	0
0	0	0	0	0
0	0	0	0	0
0	0	0	0	0
0	0	0	0	0
0	0	0.003653725	0.220629796	0
0	0	0	0	0
0	0	0	0	0
0.00803114	0	0	0	0.003948473
0	0	0	0	0
0	0	0	0	0
0	0	0	0	0
0	0	0	0	0
0	0	0	0	0
0	0	0	0	0
0	0	0	0	0
0	0	0.0072959	0.002908641	0
0.007272109	0	0	0	0
0	0	0	0	0
0	0.004421446	0.000884966	0.026945085	0.030340874
0	0	0	0	0
0.08616834	0.075372775	0.020345206	0.004719115	0.015535701
0.181899842	0.002146146	0	0	0.002236028
0	0	0	0.003988831	0
0	0	0	0	0
0	0	0	0	0
0	0	0	0	0
0	0	0	0	0
0	0	0	0	0
0	0	0	0	0
0.085035745	0.013630351	0	0	0.013861172
0.085580555	0.017307256	0.081359546	0.026977523	0.304181992
0	0	0	0	0
0	0	0	0	0
0.115179931	0	0.052088063	0.349593841	0
0.005937316	0	0	0.000829847	0.140874772
0	0	0	0	0

0	0	0	0	0
0.028928225	0	0.012083937	0.012663083	0
0.009346508	0.001144847	0.004032947	0	0.009930014
0	0	0	0	0
0	0	0.030792341	0.001027005	1.231510428
0.002048872	0.0235793	0	0	0
0	0	0	0	0
0	0	0	0	0
0	0	0	0	0
0	0	0	0	0
0	0	0	0	0
0	0	0	0	0
0	0	0	0	0
0	0	0	0	0
0	0	0	0	0
0	0	0	0	0
0.02260524	0	0	0	0
0	0	0	0	0
0	0	0	0	0
0	0	0	0	0
0	0	0	0	0
0	0	0	0	0
0	0	0	0.000727199	0
0	0	0	0	0
0.012245535	0	0.007893824	5.201826153	0
0	0	0.001044868	0	0
0	0	0	0	0
0	0	0.114126727	0.033101687	0.433399389
0	0	0	0	0
0	0	0	0	0
0	0	0	0.021012023	0
0	0	0	0	0
0	0	0	0	0
0	0	0	0	0
0	0	0	0	0
0	0	0	0	0
0	0	0	0	0
0.024524499	0.315328019	0	0	0
0	0	0.555534389	0.480222756	2.944987406
0	0	0	0	0
0	0	0	0	0
0.054964472	0	0.00430738	0.0057843	0
0.002991092	0.005715478	0	0.003210692	0.00133984
0	0	0	0	0.086270932
0	0	0	0	0
0	0	0	0	0.010790072
0.017387983	0	0.017967189	0.001637244	0.001366462
0	0	0	0	0

0	0	0	0	0
0.076214145	0.012411864	0.021254332	0.00103295	0.002801864
0	0	0	0	0
0	0	0	0	0
0	0	0	0	0
0	0	0	0	0
0	0	0	0	0
0	0	0	0	0
0	0	0	0	0
0	0	0	0	0
0	0	0	0	0
0	0	0	0	0
0.020751618	0	0	0	0.04898393
0	0	0	0	0
0.074630224	0	0.038363277	0	0
0	0	0	0	0
0	0	0	0	0
0	0	0	0	0
0.001061869	0	0	0	0.011594156
0	0	0	0	0
0	0	0	0	0
0.551698958	0.006754836	0.000991468	0	0.738962089
0.013581872	0	0.053330316	0	0
0	0	0	0	0.197077643
0	0	0	0	0
0	0	0	0	0
0	0	0	0	0
0	0	0	0	0
0	0	0	0	0
0	0	0	0	0
0	0	0	0	0
0	0	0	0	0
0	0	0	0	0
0	0	0	0	0
11.36627206	0	0.759091413	0.153504828	0.108308313
0	0	0	0	0
0	0	0	0	0
0	0	0	0	0
0.13038007	0.040192065	0.001980201	0	0.091188678
0	0	0	0	0
0.013505037	0	0.069910775	0.043414153	0.022685608
0	0	0	0	0
0	0	0	0	0
0	0	0	0	0
0	0	0	0	0
0.12853077	0.814245427	0.10240432	0	0.019750665
0	0	0	0	0
0	0	0	0	0

0	0	0	0	0
0	0	0	0	0
0	0	0	0	0
0	0	0	0	0
0.001099408	0	0	0	0.073050149
0	0	0	0	0
0	0	0	0	0
0.001827942	0.001247462	0.001098606	0	0.003216773
0	0	0.012910401	0	0
0	0.004515001	0	1.227367302	0.01825776
0	0	0.062116436	0.005126913	0
0	0	0	0	0
0.022863862	0	0	0	0
0	0	0	0	0
0	0	0	0	0
0	0	0	0	0
0	0	0	0	0
0	0	0	0	0
0	0	0	0	0
0.181304012	0.145578207	1.56248063	0.077412059	0.24147807
0	0	0	0	0
0	0	0	0	0
0	0	0	0	0
0	0	0	0	0
0	0	0	0	0
0	0	0.001296439	0	0
0	0	0	0	0
0	0	0	0	0
0	0	0	0	0
0.098666437	0.001574625	0	0	0
0	0	0	0	0
0	0	0	0	0
0	0	0	0	0
0	0	0	0	0
0	0	0	0	0
0.002660928	0	0	0	0.001489932
0	0	0	0	0
0	0	0	0	0
0	0	0	0	0
0	0	0	0	0
0	0	0	0	0
0	0	0	0	0
0.022725665	0	0.000898572	0.004126829	0
0	0	0	0	0
0.003282183	0	0.0083256	0	0.051458159
0	0	0	0.039537438	0
0	0	0	0	0
0	0	0	0	0

0	0	0	0	0
0	0	0	0	0
0	0	0	0.00317728	0
0	0	0	0	0
0	0	0	0	0
0.01816088	0	0.050784233	0.007993661	0.007424848
0	0	0	0	0
0	0	0	0	0.004614369
0	0	0	0	0
0	0	0	0	0
0	0	0	0	0
0	0	0	0	0
0	0	0	0	0
0	0	0	0	0
0	0	0	0	0
0	0	0	0	0
0	0	0	0	0
0	0	0	0	0
0	0	0	0	0
0	0	0	0	0
0	0	0	0	0
0	0	0	0	0
0	0	0	0	0
0	0	0	0	0
0	0	0	0	0
0.000676349	0	0	0.010083409	0
0	0	0	0	0
0	0	0	0	0
0	0	0	0	0
0.142269878	0.001867129	0	0	0
0	0	0	0	0
0	0	0	0	0
0	0	0	0	0
0	0	0	0	0
0	0	0	0	0
0	0	0	0	0
0	0	0	0	0
0	0	0	0	0
0	0	0.274976568	0.147780973	1.015046382
0	0	0	0	0
0	0	0	0	0
0	0	0	0	0
0	0	0	0	0
0	0	0	0	0
0	0	0	0	0
0	0	0	0	0
0	0	0	0	0
0	0	0	0	0

0	0	0	0	0
0	0	0.004243063	0	0.041382866
0	0	0	0	0
0	0	0	0	0
0	0	0	0	0
0	0	0	0.004156812	0
0	0	0	0	0
0.425937829	0	0	0	0
0	0	0	0	0
0	0	0	0	0
0.001056503	0	0.000888953	0.011151688	0.001183135
0	0	0	0	0
0	0	0	0.003898066	0.092895371
0	0	0	0	0
0	0	0	0	0
0	0	0	0	0
0	0	0	0	0
0	0	0	0	0
0	0	0	0	0
0	0	0.001664347	0.002673746	0
0.027450558	0.006659129	0.005413401	0.003261203	0
0	0	0	0	0
0	0	0	0	0
0.977643673	0	0.434245135	0.087245114	0.702493283
0	0	0	0	0
0	0	0	0	0
0	0	0	0	0
0	0	0	0	0
0	0	0	0	0
0	0	0	0	0
0.023153889	0	0	0	0
0	0	0	0	0
0	0	0.001898127	0	0
0	0	0	0	0

PHL018-0034_V3	PHL018-0054_V2	PHL018-0236_V1	PHL018-0009_V3	PHL018-0111_V2
CTRL	CTRL	EYCF	EYCF	EYCF
0.029490996	0.006836642	0.00716298	0.151417376	2.119648988
0.09565281	5.252191898	2.094012294	3.41340299	0.859236901
0.104882531	6.271163174	20.05370321	4.06753337	1.932943991
0	0	0	0	0
0	0	0	0	0
0	0	0	0	0
0	0	0	0	0
0	0	0	0	0
0	0	0	0	0
0	0	0	0	0
0	0	0	0	0
0	0	0	0	0
0	0	0	0	0
0.280320465	32.28802011	14.24105721	22.30738169	3.346899062
0	0	0	0	0
0	0	0	0	0
0	0	0	0	0
0	0	0	0	0
0	0	0	0	0
0	0	0	0	0
0	0	0	0	0
0	0	0	0	0
0	0	0	0	0
0	0	0	0	0
0.128850714	0.135663066	0	0	0
0	0	0	0	0
0	0	0	0	0
0	0	0	0	0
0	0	0	0	0
0.002320963	0	0	0	0
0	0	0	0	0
0	0	0	0	0
0	0	0	0	0
0	0	0	0	0
0	0	0	0	0
0.061498444	1.829152144	0	0	0
0	0	0	0	0
0	0	0	0	0
0	0	0	0	0
0	0	0	0	0
0	0	0	0	0
0.001927851	1.108720471	0	2.3208484	0.207619731
0.291652695	0.001566058	0.010020672	0.044851192	3.902584647
0.023906552	0.020756812	0	0	0.001825985
0	0.001301298	0	0	0.001462745
0	0	0	0	0

0.082336561	0	0	0	0
0	0	0	0	0.008086071
0	0.002158518	0	0	0
0.290114813	0.555328718	0.383148204	0.193798629	0.242409636
0.025572494	0.009357584	0	0.007762672	0
0	0	0	0	0
0	0.004974431	1.610540417	0.148520231	0
0	0	0	0	0
0	0	0	0	0
0	0	0	0	0
0	0	0	0	0
0.665188888	0.234111956	0	0.105945597	0.261825512
0	0	0	0	0
0.036266892	0.012468858	0.003449307	0.081878492	0.041847237
0	0.058348335	0	0	0
0	0	0	0	0
0.051321876	0	0.144737353	0	0
0	0	0	0	0
0	0	0	0	0
0	0	0	0	0
0.001442261	0.035527449	0	0.037195665	0.156144002
0.003482081	0.049482954	0.001934267	0.004744987	0.030393512
0	0.000991088	0	0	0
0	0	0	0	0
0	0	0	0	0
0	0	0	0	0
0	0	0	0	0
0	0	0	0	0
0	0.013847533	0	0.034235988	0.001778919
0	0	0	0	0
0	0	0	0	0.271461956
0	0	0	0	0
0	0	0	0	0
0	0	0	0	0
0	0	0	0	0
0	0	0	0	0
0	0	0	0	0
0.602394191	0	0.006314813	0	0.004576806
0	0	0	0	0
0	0	0	0	0
0	0	0	0	0
0	0	0	0	0
0	0	0	0	0
0	0	0	0	0
0	0	0	0	0
0	0	0	0	0
0	0	0.000551864	0	0
0	0	0.012326716	0.003068142	0

0	0	0	0	0
0	0	0	0	0
0	0	0	0	0
0	0	0	0	0
0	0.026653561	0	0	0
0	0.001900656	0	0	0
0	0	0	0	0
0	0	0	0	0
0	0	0	0.082371777	0
0	0	0	0	0
0	0	0	0	0
0	0.015121655	0	0.000851956	0.002006291
0	0	0	0	0
0	0	0	0	0
0	0	0	0	0
0	0	0	0	0
0	0	0	0	0
0	0	0	0	0
0.045980363	0.007280323	0	0.227649718	0.397770826
0	0	0	0	0
0	0	0	0	0
0	0	0	0	0
0	0	0	0	0
0	0	0	0	0
0	0	0	0	0
0	0	0	0	0
0	0	0	0	0
0	0	0	0	0
0	0	0	0	0
0	0	0	0	0
0.010381748	0	0	0.010525424	0.224589483
0.031403415	0.015753811	0	0	0
0	0	0	0	0
0	0	0	0	0
0	0	0	0	0
0	0	0	0	0
0	0	0	0	0
0	0	0	0	0
0.030870776	0	0	0	0
0	0	0	0	0
0	0	0	0	0
0	0	0	0	0
0	0.000958364	0	0	0
0	0	0	0	0
0	0	0	0	0
0	0	0	0	0
0	0	0	0	0
0	0	0	0	0

0	0	0	0	0
0	0	0	0	0
0	0	0	0	0
0	0	0	0	0
0.1519911	0.195755546	0	0.013951287	0
0	0	0	0	0
0	0	0	0	0
0	0	0	0	0
0	0	0	0	0
0	0	0	0	0
0	0	0	0	0
0	0	0	0	0
0	0	0	0	0
0	0	0	0	0
0	0	0	0	0
0	0	0	0	0
0	0	0	0	0
0	0	0	0	0
0	0	0	0	0
0	0	0	0	0
0	0	0	0.04109754	0
0	0	0	0	0
0	0	0	0	0
0	0	0	0	0
0	0	0	0	0
0	0	0	0	0
0.080253144	0	0	0	0
0	0	0	0	0
0	0	0	0	0
0	0	0	0	0
0	0	0	0	0.06303518
0	0	0	0	0
0	0	0	0	0
0	0	0	0	0
0	0	0	0	0
0	0	0	0	0
0	0	0	0	0
0	0	0	0	0
0	0	0	0	0
0	0	0	0	0
0	0	0	0	0
0	0	0	0	0
0	0	0	0	0
0	0	0	0	0
0	0	0	0	0
0	0	0	0	0
0	0	0	0	0
0	0	0	0	0

0	0	0	0	0
0	0	0	0	0
0	0	0	0	0
0	0	0	0	0
0	0	0	0	0
0	0	0	0	0
0	0	0	0	0
0	0	0	0	0
0	0	0	0	0
0	0	0	0	0
0	0	0	0	0
0	0	0	0	0
0.00260535	0.000915428	0	0.000983154	0.001234802
0	0	0	0	0
0	0	0	0	0
0	0	0	0	0
0	0	0	0	0
0	0	0	0	0
0	0	0	0	0
0	0	0	0	0
0.005246101	0.0975629	0.023921773	0.01286783	0.009619926
0	0	0	0	0
0	0	0	0	0
0	0	0	0	0
0	0	0	0	0
0	0	0	0	0
0.001309374	0.016696615	0.006210417	0.00214112	0
0	0	0	0	0
0	0	0.00727411	0	0
0	0	0	0	0
0	0	0.335470723	0	0.004691114
0	0	0	0	0
0	0	0	0	0
0.07923625	0	0	0	0
0.017798406	0.033688747	0.013314451	0.004305855	0.061841257
0	0	0	0	0
0	0	0	0	0
0	0	0	0	0
0	0	0	0	0
0	0	0	0	0
0	0	0	0	0
0	0	0	0	0
0	0	0	0	0
0	0	0	0	0
0	0	0	0	0
0.086819339	0	0	0	0
0	0	0	0	0
0.002258728	0.001388864	0	0	0.002497881

0	0	0	0	0
0	0	0	0	0
0	0	0	0	0
0	0	0	0	0
0	0	0	0	0
0	0	0	0	0
0	0	0	0.007765203	0
0	0.002281735	0	0.000980218	0.000769446
0	0	0	0	0
0	0	0	0	0
0	0	0	0.004429217	0
0	0	0	0	0
0	0	0	0	0
0	0	0	0	0
0	0	0	0	0
0	0	0	0	0
0	0	0	0	0
0	0	0	0	0
0	0	0	0	0.017056489
0	0	0	0	0
0	0	0.001603826	0.069304051	0.005353204
0	0	0	0	0
0	0	0	0	0
0	0	0	0	0
0	0.004730714	0.002788047	0.002032283	0
0	0	0	0	0
0	0	0	0	0
0	0	0	0	0
0	0.020033488	0	0.064486274	0
0	0	0	0	0
0.086054052	0.100894423	0.070462743	0.094960947	0.088367881
0	0	0	0	0
0	0	0	0.013795075	0.446855663
0	0	0	0	0
0	0	0	0	0
0	0	0	0	0
0	0	0	0	0
0	0	0	0	0
0	0	0	0	0
0	0	0	0	0
0	0	0	0	0
0.029304386	0	0.003305755	0.002491801	0.112545297
0	0	0	0	0
0	0	0	0	0
0	0	0	0	0
0	0	0	0	0
0	0	0	0	0
0	0	0	0	0

0	0	0	0	0
0	0	0	0	0
0.016435761	0.01680767	0	0	0
0	0	0	0	0
0	0	0	0	5.257370942
0	0	0	0	0
0	0	0	0	0
0	0	0	0	0
0	0	0	0	0
0	0.00640507	0	0	0
0	0	0	0	0
0	0	0	0	0
0	0	0	0	0
0	0.002476735	0.007298321	0.000797991	0
0.001342603	0.00102211	0.001721088	0.001317275	0.002527623
0	0	0	0	0
0	0.004658411	0.00078441	0	0.000785454
0.002966683	0	0	0	0
0	0	0	0	0
0	0	0	0	0
0	0	0	0	0
0.003682537	0.000788478	0	0	0.000945388
0	0	0	0	0
0	0	0	0.007001343	0.001831959
0	0	0	0	0
0	0	0	0	0
3.103582911	0	0	0	0
0	0	0	0	0
0	0	0	0	0
0	0	0	0	0
0.004585728	0	0	0	0.016300486
0	0	0	0	0
0	0	0	0	0
0	0	0.00346985	0.000781097	0
0	0	0	0	0
0	0	0	0	0
0	0	0	0	0
0.008385941	0	0	0.00113486	0.002078619
0	0	0	0	0
0	0	0	0	0
0	0	0	0	0
0	0	0	0	0
0	0	0	0	0
0	0	0	0	0
0	0	0	0	0
0	0	0	0	0
0	0	0	0	0
0	0	0.002693981	0	0

0	5.47295938	0	0	0
0	0.019568746	0	0	0
0	0	0	0	0
0	0	0	0	0
0.001212714	0.006370265	0.005596497	0.019275342	0.001494386
0	0	0	0	0.002436732
0	0	0	0	0
0	0.056681822	0.005752797	0	0
0	0	0	0	0
0.004905905	0.056822546	0.074658203	0.508496474	0
0	0	0	0	0
0	0.003687907	0	0.021388047	0
0.002857994	0.002098056	0.014916386	0.001001456	0.002725209
0	0	0	0	0
0	0	0	0	0
0	0	0.025456136	0	0.009440749
0	0	0	0	0
0	0	0	0	0
0	0	0.536400712	0	0.233299949
0	0	0.00081316	0	0
0	0	0	0	0
0	0	0	0	0
0	0	0	0.005580217	0
0.213062912	0	0	0	0
0	0	0	0	0
0	0.001357611	0.000731528	0	0.002441672
0.002225664	0	0	0	0
0	0	0	0	0.462091335
0	0	0	0	0
0.002446394	0	0	0	0
0	0	0	0	0
0	0	0	0.001944761	0
0	0	0	0	0
0	0.002130608	0.008849512	0	0
0	0	0	0	0
0	0	0.03542135	0	0
0	0	0	0	0.234993477
0	0	0	0	0
0	0	0	0	0
0	0	0	0	0
0	0	0	0	0
0	0	0	0	0
0	0	0	0	0
0	0	0	0	0
0	0	0	0	0
0.001565683	0	0	0	0
0.00887072	0	0.003638851	0.000870337	0.000910924
0	0	0	0	0

0	0	0	0	0
0	0.013584888	0.001270835	0.034043194	0
0	0	0	0	0
0	0	0	0	0
0	0	0	0	0
0	0	0.003403418	0.001827989	0
0.00543823	0	0	0	0
0	0	0	0	0
0	0	0	0	0
0	0	0	0	0
0.00189081	0.001850727	0.013296476	0.007685579	0.024409321
0	0	0	0	0
0	0	0	0	0
0	0	0.001766509	0.002503775	0.00078616
0	0	0.083014321	0	0
0	0	0	0	0
0	0	0	0	0
0	0	0	0	0.005355426
0	0	0	0	0
0	0.01904676	0	0	0
0	0	0	0.004855888	0
0	0	0.33930719	0.110117714	0
0	0	0	0	0
0	0	0	0	0
0	0	0	0	0
0	0	0	0	0
0	0	0	0	0.015059169
0	0	0	0	0
0	0	0	0	0
0.081170522	0.294733125	8.790495366	0.415078164	0.120612237
0.01956339	0.009489605	0.001198436	0.001019168	0
0	0	0	0.093961081	0
0	0	0	0	0
0.000735623	0	0	0	0
0	0	0	0	0
0	0	0	0	0
0.088869031	0	0	0	0
0	0	0	0.097231738	0
0	0.301644665	0.722407966	0	0
0	0	0	0	0
0.008182021	0.100620515	0.001129538	0.021407108	0.010556384
0.533900989	1.893531452	2.59335526	0	2.665501508
0	0	0	0.012156612	0
0	0	0	0	0
0	0	0	0	0.003159305
0	0	0	0	0
0	0.003017384	0.008976162	0.000864165	0
0	0	0	0	0

0	0	0	0	0.006416751
0	0	0	0	0.005704507
0.003331714	0	0.357050268	0	0
0	0	0	0	0
0	0	0	0	0
0	0	0.067138023	0	0
0	0	0	0	0
0	0	0	0.007170198	0.001563451
0	0	0	0.037924577	0.051834539
0	0	0	0	0
0	0.007334399	0	0	0
0	0	0	0	0
0	0	0	0	0
0.002703643	0.001122685	0.000756177	0.020015363	0.0045431
0	0	0	0	0
0.20360196	0.040797193	0	0.067089532	0
0.004045056	0.029903175	0	0	0.070614937
0	0	0	0.003299175	0
0	0	0	0.005609724	0
0	0	6.025534641	0	0
0	0.006127888	0.055513485	0	0
0	0	0	0	0.006339757
0	0	0	0	0
0.914902862	0.064676852	0	0	0.130077585
0.003780496	0	0	0	0
0.001682731	0	0.008844107	0.223158269	0.010655855
0	0.004012278	0	0.001591059	0
0	0	0	0	0
0	0	0	0.00182857	0
0	0	0	0	0.001662374
0.003699785	0.125104364	0	0	0.023850943
0.003064403	0.00944824	0.002121266	0	0.020139514
0	0	0.019075244	7.008419484	0
0	0.03772147	0	0.138254361	0
0	0.002385629	0	0	0.010726421
0	0	0	0	0
0	0.000903667	0	0	0
0.017122085	0.024028131	0.005711994	0.012548705	0.380247292
0.076470137	0	0	0.284322696	0
0	0.00077041	0.005967399	0	0
0	0	0	0	0
0	0	0	0	0
0	0	0	0	0
0.019163155	0.009930133	0	0	0.479723385
0	0	0.077561364	0	0
0	0	0	0	0.005772989
0	0	0	0	0
0	0	0	0	0

0	0	0	0	0
0.093324053	0.344426438	0.056429043	0.007998014	1.02414685
0	0	0	0	0
0	0	0	0	0
0	0	0	0	0
0	0	0	0	0
0	0	0.008600694	0	0
0	0	0	0.001906547	0
0	0.001763061	0	0.007321524	0
0	0	0	0.001202483	0
0.002506626	0	0.000481988	0.001434614	0
0.004355972	0.007461352	0.00311105	0	0.017253368
0	0	0	0	0.521432667
0	0	0	0	0
0	0.002189499	0	0	0
0	0	0	0	0
0.00809313	0.017734313	0.005383583	0.001716854	0.002246146
0	0.002269734	0.103300755	0	0.36156009
0	0	0	0	0
0	0	0	0	0
0.231659513	0.019061307	0.002949411	0.002434451	0.028722649
0	0	0.0047495	0	0
0	0	0	0	0
0	0	0	0	0
0	0	0.006704582	0	0
0	0	0	0	0
0.025381874	0.077037595	0.001027488	0	0
0.012946322	0.102939425	0.001290795	0.085386251	0.052192923
0	0	0	0	0
0	0	0	0	0
0	0.002937609	0.000593582	0	0
0	0	0	0	0
0	0	0	0	0
0	0	0	0	0
0	0	0	0	0
0.066949904	0.307663051	0.111102205	0.226122107	0.205416842
0	0	0	0	0
0	0	0	0	0
0.001478772	0.005403715	0.002957203	0.010736472	0.003340773
0	0	0	0	0
0	0	0.065661832	0	0
0	0	0	0	0
0.04706718	0.004513161	0.011330196	0.00370139	0.003505045
0	0.003168998	0	0	0.001424865
0	0	0	0	0
0	0	0	0.569491573	0
0	0.197945823	0	0	0
0	0	0.002156365	0	0

0	0	0	0	0.021132797
0	0	0	0	0
0	0	0	0	0.002874033
0.926053848	0.211928665	0	0.00061585	0
0	0	0	0	0
0	0	0	0	0
0	0	0	0	0
0.040056317	0	0	0	0
17.17239801	0.396829099	0	0	0
0.033847098	0	0	0	0
0	0	0	0	0
0.056531196	0.003891609	0	0.002163518	0
0	0	0	0	0
0	0	0	6.26416102	0
0.02431739	0.099552345	0	0.063136122	0.026330217
0	0	0	0	0
0.096670186	0.084271727	0	0	0
8.098998917	0	0	0	0
0	0	0	0	0
0	0	0	0	0
0	0	0	0	0
0	0	0	0	0
0	0	0	0	0
0.112657705	0.23982704	0.055029364	0.108030953	0.199302107
0.0028153	0	0	0	0
0.005416061	0	0	0.931068773	0
0.004913	0	0	0	0
0.536821188	1.070736553	0.233493092	0	0
0	0	0	0	0
0	0	0	0	0
0.265825036	0.020251825	0	0.001912098	0
0	0	0	0	0
0	0.00643625	0	0.006985186	0
0	0	0	0	0
0	0.003904032	0	0	0
0	0	0	0	0
2.586965297	0.970905372	0	0	0
0	0	0	0.002251081	0
0	0.153705992	0	0	0
0.006600433	0	0	0	0.026816932
0.067693317	0.021741003	0	0.044151724	0.180892982
0.168026922	0	0	0	0
0.003691202	0.612771822	4.688529114	0.46808749	0.130059861
0	0	0	0	0
0	0	0	0	0
0	0	0	0	0
0	0	0	0	0
0.028897419	0	0	0.000907272	0.04011981

0	0	0	0	0
0.241360137	0	0	0	0
0.003324173	0.014930886	0.047044414	0.019786199	0.110371556
0	0	0	0	0
0	0	0	0	0
0.082749499	0.004983888	0	0.000685134	0
0	0	0	0	0
0	0	0	0	0
0.067120873	0	0	0	0
0	0.037585312	0	0	0
0.145563162	0.009193235	0.001673524	0.014089578	0.536240637
0.518466253	0.208724326	3.003949533	0.529185966	0.237627903
0	0	0	0	0
0.005166143	0.004022316	0	0	0.092114963
0.102148063	0.354799505	0.263738265	0.139619498	0.006650928
0	0	0	0	0
0	0	0	0	0
0	0	0	0	0
0	0	0	0	0
0.003046932	0.001228023	0	0	0.01527619
0.015683542	0.057685507	0.094819805	0.136699752	0.143449018
0	0.002413751	3.638314588	1.348952842	6.44107462
0.192919808	5.495743469	5.320847346	16.06009848	0.409303323
0	0	0	0	0
0.00589833	0	0	0	0
4.634149017	0.003992753	0	0	0
0	0	0	0	0
0	0	0	0	0
0.040076975	0.138087645	0	0	0
0	0	0	0	0
0.005430937	0	0	0	0
0.037704837	0.098892234	0	0.189528482	0.065415067
0	0	0	0.003727215	0.032126098
0.039023503	0.029773058	0	0.016050578	0.167465681
0	0	0.002155357	0	0.006663996
0.061197444	0.027074064	0.001091016	0.011929061	0.032669998
0.00097874	0.003576503	0	0.004417269	0.026935512
0	0	0	0	0
6.936300027	0	0	0.011167528	0
0	0	0	0	0
0.030690854	0	0	0	0
0	0	0	0	0
0	0	0	0	0
0	0	0	0	0
0.008781364	0.065972001	0	0.001133645	0.818217517
0	0	0	0	0
0	0	0	0	0
3.694909762	0.311317985	0.022726714	0	0.547312174

0	0	0	0	0
0	0	0	0	0
0	0	0	0	0
0.001969558	0.019117407	0	0.004444525	0
0	0	0	0	0
0.337463259	0	0	0	0
0.280533483	0	0	0	0
0.176341662	0	0.002230328	0.078416843	0.452242611
0	0	0	0	0
0	0	0	0	0
0.003948419	0.001502946	0	0	0.00810917
0	0	0	0	0
0.206852801	0	0	0	0.063310224
0.099768702	1.08051183	0	0	0
0	0	0	0	0
0	0	0	0	0
0.06150513	0	0	0.231775339	0
0.038347972	0.001251168	0.001123621	0.00609159	0
0	0	0	0	0.009274047
0	0.002632808	0	0	0
0	0.004816778	0	0	0
0	0	0	0	0
0.033054083	0.098330901	0	0	0
0	0	0	0	0
0	0	0	0	0
0	0	0	0	0
0	0	0	0	0
0	0	0	0	0
0	0	0	0	0
0.003308858	0.025441895	0	0	0
0	0	0	0	0
0	0	0	0	0
0.029000949	0	0	0	0
0	0	0	0	0
0	0.004182742	0	0	0
0	0	0	0	0
0.084197443	0	0	0	0
0	0	0.002713607	0	0.01201297
0	0	0	0	0
0	0	0	0	0
0.012544017	0.506926268	0	0	0
0	0	0	0	0
0.09398983	0	0	0	0
0	0	0	0	0
0	0	0	0	0
0	0	0	0	0
0.051872144	0	0	0	0
0	0	0	0	0

0	0	0	0	0
0	0	0	0	0
0	0	0	0	0
0.967676599	0.452218029	0	0.06034687	0
0	0	0	0	0
0	0	0	0	0
0	0	0	0	0
0	0	0	0	0
0.001792675	0.008773363	0.088835547	0.001256326	0.158578388
0	0	0	0	0
0	0	0	0	0
0	0	0	0	0
0	0	0	0	0
0	0	0	0	0
0.160296814	0.011979063	0	0	0
0	0	0.011993291	0	1.340374757
0	0	0	0	0
0	0	0	0	0
0	0	0	0	0
0	0	0	0	0
0.32134467	0	0	0	0
0	0	0	0	0
0	0	0	0	0
0	0	0	0	0
0.039452241	0	0	0	0.128033564
5.335108676	1.261580661	0	0.002506525	3.178118369
0	0	0	0.066380386	0
0	0.000853918	0	0	0
0	0	0	0	0
0	0	0	0	0
0	0	0	0	0
0.029286463	0	0	0	0
0	0	0	0	0
0.002090919	0.001285681	0	0.005523197	0.00396395
0.00079504	0.004842047	0	0	0.130082482
0.002622653	0	0	0	0.001923693
0	0	0	0	0
0.251086802	0.219089571	0	0.950325723	0.37057431
0.164904743	0	0	0	0
0	0	0	0	0
0	0	0	0	0
0	0.026442141	0	0	0
0.497138347	0.312205495	0	0.281805568	0
0	0	0	0	0
0	0	0	0	0
0.000709895	0.010133178	0.000682513	0.001567132	0
0	0.0248757	0	0	0
0.661604927	0.358382754	0	2.378732888	0.041518536

0	0.114602966	0	0	0
0.027237942	0	0	0	0
0	0	0	0	0
0	0	0	0	0
0.00272887	0	0	0	0.009484518
0.006872867	0	0	0	0
0	0	0	0	0
0	0	0	0	0
0	0	0	0	0
0	0	0	0	0
0.000963722	0	0	0	0
0.677029038	1.059284548	0	1.621500871	9.224764219
0.137504839	0	0	0.136407019	0
0	0	0	0	0
0.000728293	0.567748875	0.044626173	1.480317548	0.142594976
0	0	0	0	0
0	0	0	0	0
0	0	0	0	0
0	0	0	0	0
0	0	0	0	0
0.033118855	0	0	0.337903666	0
0	0	0	0	0
0.888022293	0.010395804	0	0.053185607	1.318557406
0	0	0	0	0
0	0	0	0	0
0	0	0	0	0
0	0	0	0	0
0	0	0	0	0
0.00349871	0	0	0.003432706	0
0	0	0	0	0
0	0	0	0	0
0.028666894	0.049283446	0.004361325	0.004018012	0.070197574
0	0	0	0	0
0.045954659	0.028574923	0	0.038448085	0.015514617
0.003057968	0	0	0	0.00209346
0.49302422	0.002400859	0	0	0
0.002352535	0	0	0	0
0.124177894	0	0	0	0
0	0	0	0	0
0	0	0	0	0
0.166398198	0.305453483	0	0	0
0.024743646	0	0	0	0
0	0	0	0	0
0	0	0	0	0
0.041571186	0.013301215	0	0.001970383	0.034439882
0	0	0	0	0
0.032780431	0	1.406589113	0.435347768	2.07749039
0	0.003687408	0	0	0.004096127

0	0	0	0	0.07922931
0	0	0	0	0
0	0	0	0.002445443	0
0	0	0	0	0
0	0	0	0	0
0	0	0	0	0
0	0	0	0	0
0.050608841	0	0	0	3.352845931
0	0	0	0	0
0	0	0	0	0
0	0	0	0	0
0	0	0	0	0
0	0	0	0	0
0	0	0	0	0
0	0	0	0	0
0	0	0	0	0
0	0	0	0	0
0	0	0	0	0
0.00599238	0.307423887	0	0	0
0	0	0	0	0
0.02623193	0.001293028	0	0	0
0	0	0	0	0
0	0	0	0	0
0	0	0	0	0
0.001535946	0	0	0	0.06489237
0.002991397	0.002134988	0.004745421	0	0.004991724
0	0	0	0	0
0.06368363	0.092157017	0	0.005027305	0.040590597
0	0	0	0	0
0.479782085	0.178542295	0	0.025733957	0
0	0	0	0	0
0	0	0	0	0
0	0	0	0	0
0	0	0	0	0
0.006269599	0	0	0.001730059	0
0	0	0	0	0
0	0	0	0	0
0.00088927	0	0	0	0.001826361
0.087462499	0	0	0	0
0	0	0	0	0
1.531338787	10.20648686	5.533489994	9.51782609	3.473627513
0	0.852241367	0.044184372	0.423916684	1.650241595
0	0.146357716	0	0	0
0	0	0	0	0
0	0	0	0	0
0	0	0	0	0
0.004541106	0	0	0	0
0.010057275	0.001611895	0	0.001731148	0.099834395

0	0	0	0	0
0.007259465	0.023211537	0	0.001780628	0
0	0	0	0	0
0	0	0	0	0
2.014238986	1.10800751	0	1.273148347	0
0	0	0	0	0
0	0	0	0	0
0	0	0	0	0
0	0.008484553	0.150812657	0.007112013	1.106873368
0	0	0	0	0
0	0	0	0	0
0	0	0	0	0
0.029978682	0.05074642	0	0	0
0.002601582	0.023023981	0.410202213	0.025099606	0.004897811
0	0	0	0	0
0.120440508	0.156834809	0	0.151506864	0.121852055
0.006378585	0	0	0.027978068	0
0	0	0	0	0
0	0	0	0	0
0	0	0	0	0
0	0	0	0	0
0	0	0	0	0
0	0	0	0	0
0	0	0	0	0
0.014946445	0	0	0	0
0	0	0	0	0
0.028384734	0	0	0.000960319	0
0	0	0	0.001625419	0.002646338
0	0	0	0	0
0	0	0	0	0
0	0	0	0	0
0	0	0	0	0
0	0	0	0	0
0.029136872	0.029746851	0	0	0
4.065275152	0	0	0	0
0	0	0	0.005431897	0.006778512
0	0	0	0	0
0.041219791	0	0	0	0
0	0	0	0	0
0	0	0	0	0
0.073804158	1.901203144	0	0	0
0.400330532	0	0	0	0
0	0	0	0	0
0	0	0	0	0
0	0	0	0	0
0	0	0	0	0
0	0	0	0	0
0	0	0	0	0

0.310580156	0.343384857	0	0	0
0	0	0	0	0
0	0	0	0	0
0	0	0	0	0.001284763
0.000798053	0	0	0	0
0	0	0	0	0
0.039114199	0.006197655	0	0.712073188	0.01556669
0	0	0	0	0
0.00092468	0	0	0	0
0.00098175	0	0	0	0
0.014297796	0	0	0	0
0	0	0	0	0
0	0	0	0	0
0	0	0	0	0
0	0	0	0	0
0	0	0	0	0
0	0	0	0	0
0	0	0	0	0
0.361746965	0	0	0	0
0	0	0	0	0
0.066103497	0.002848524	0	0.093001688	0
0	0	0	0	0
0.076842488	0.011894379	0	0.000771282	0
0.060814677	0.004589847	0.000940879	0	0.024585149
0	0	0	0	0
0	0	0	0	0
0	0	0	0	0
0	0	0	0	0
0	0	0	0	0
0	0.124937042	0.027469814	0.056016406	0
0.099831084	0	0	0	0
0	0	0	0	0
0	0	0	0	0
0.063266763	0	0.003445036	2.317766504	0
0	0	0	0	0
0	0	0	0	0
0	0	0	0.007079767	0.043336796
0	0	0	0	0
0	0	0	0	0
0.849294185	0	0	0	0
0	0	0	0	0
0	0	0	0	0
0	0	0	0	0
0	0	0	0	0
0.044286987	0	0	0	0
0	0	0	0	0
0	0	0	0	0
1.06356858	0	0	0	0

0.006581484	0.013098659	0	0.001291465	0.033985334
0.460470256	0	0	0	0
0	0	0	0	0
0	0	0	0	0
0	0	0	0	0
0	0	0	0	0
0	0	0	0	0.003711125
0.386347148	0.433466348	0	1.056287311	0
0.390792866	0.001348217	0.01089699	0.053574613	5.137495564
0	0.025811181	0.001738494	0	0.049586668
0	0	0	0	0
0	0	0	0	0
0.030430003	0	0	0	0
0	0.02420414	0.031932795	0	0
0	0	0	0	0
0	0	0	0	0
0	0	0	0	0.085407166
0	0	0	0	0
0	0	0	0	0
0.014483208	0.009534176	0	0.023739024	0
0	0	0	0	0
0	0	0	0	0
0	0	0	0	0
0	0	0	0	0
0.01081199	0.008625697	0	0	0
0	0	0	0	0
0	0	0	0	0
0	0	0	0	0
0	0	0	0	0
0	0	0	0	0
0	0	0	0	0
0	0	0	0	0
0	0	0	0	0
0	0	0	0	0
0	0	0	0	0
0	0	0	0	0
0	0	0	0	0
0	0.056070646	0.024253394	0	0
0	0	0	0	0
0	0	0	0	0.017156776
0	0	0	0	0
0.031762184	0.137340734	0.189776576	0.266405508	2.465506935
0	0	0	0	0
0	0	0	0	0
0	0	0	0	0
0	0	0	0	0
0	0	0	0	0
0	0.009598365	0	0	0

0	0	0	0	0
0	0.058722955	0	0	0.005054115
0	0	0	0	0
0	0	0	0	0
2.814941642	0.108386728	0.027193669	0.015365528	0
0	0	0	0	0
0	0.017624918	0	0	0
0.001913628	0.015023515	0.00588741	0.002816291	0.052811582
0.011702958	0.001909145	0	0	0
0	0	0	0	0
0	0	0	0	0
0	0	0	0	0
0	0	0	0	0
0	0	0	0	0
0	0	0	0	0.003112534
0.015559458	0.020373841	0	0.020558113	0
0	0	0	0	0
0	0	0	0	0
0	0	0	0	0
0	0	0	0	0
0	0	0	0	0
0	0	0	0	0
0	0	0	0	0
0.006831362	0	0	0	0.007015044
0	0	0	0	0
0	0	0	0	0
0.177785584	0	0.006261899	0	0.003888518
0.000996818	0	0	0	0
0	0	0	0	0
0	0	0	0	0
0	0	0	0	0
0	0	0	0	0
0	0	0	0	0
0	0	0	0	0
0	0	0	0	0
0	0	0	0	0
0	0	0	0	0
0.031067865	0.190469678	0	0.015812415	0
0.001146339	0	0	0	0
0	0	0	0	0
0	0	0	0	0
1.986688277	0	0	0	0
0	0	0	0	0
0	0	0	0	0
0	0	0	0	0
0	0	0	0	0
0	0	0	0	0
0	0	0	0	0

0	0	0	0.106039414	0
0	0	0	0	0
0	0	0	0	0
0	0	0	0	0
0	0	0	0	0
0	0	0	0.025906356	0
0	0	0	0	0
0	0	0	0	0
0	0	0	0	0
0	0	0	0	0
0	0	0	0	0
0	0	0	0	0.009602146
0	0	0	0	0
0	0	0	0	0
0.019196543	0	0	0	0
0	0	0	0	0
0	0	0	0	0
0	0	0	0	0
0	0	0	0	0
0	0	0	0	0
0	0.010122406	0	0	0
0	0	0	0	0
0.017182894	0.001189196	0	0.015581558	1.040514935
0.014296982	0	0	0	0
0	0	0	0	0
0	0	0	0	0
0	0	0	0	0
0	0	0	0	0
0	0	0	0	0
0	0	0	0	0
0	0	0	0	0
0	0	0	0	0
0	0	1.717751676	0	0.328234364
0	0	0	0	0
0.109699403	0	0	0.129455447	0
0.001173902	0	0.000902898	0	0
0	0	0	0	0
0	0	0	0	0
0.005883815	0.002325784	0.015142987	0	0.031372022
0	0	0	0	0
0	0	0	0	0
0	0	0	0	0
1.787197323	0.039886565	0	0	0
0	0	0	0	0
0	0	0	0	0
0.024377255	0.002639737	0	0	0
0	0	0	0	0
0	0	0	0	0

0	0	0	0	0
0	0	0	0	0
0	0	0	0	0
0	0	0	0	0
0	0	0	0	0
0	0	0	0	0
0	0	0	0	0
0	0.04452345	0.353613977	0	0
0.008269375	0	0	0	0
0.113853168	0.003954764	0	0	0
0	0	0	0	0
0	0	0	0	0
0	0	0	0	0
0	0	0	0	0
0	0	0	0	0
0	0.004377432	0.023587116	0	0.4520321
0	0	0	0	0
0	0	0	0	0
0	0	0	0	0
0.320048474	0	0	0	0
0.007784893	0.003047945	0	0	0
0	0	0	0	0
0	0	0	0	0
0	0	0	0	0
0	0	0	0	0
0	0	0	0	0
0	0	0	0	0
0	0	0	0	0
0	0	0	0	0
0.031342322	0	0	0.072763031	0
0.102989118	0.085586156	0	0.164900617	0
0	0	0	0	0
0	0	0	0	0
0	0	0	0	0
0	0	0	0	0
0	0	0	0	0
0	0	0	0	0
0	0	0	0	0
0	0	0	0	0
0	0	0	0	0
0	0	0	0	0
0.002872293	0	0	0	0
0.017598031	0	0	0	0
0	0.00545501	0	0	0
0	0	0	0	0
0	0	0	0	0
0	0	0.236093819	0	0.120890499
0.00088588	0	0	0	0

0	0	0	0	0
0	0	0	0	0
0	0	0	0	0
0	0	0	0	0
0.007980597	0.000929931	0	0	0.13513685
0	0	0	0	0
0	0	0	0	0
0	0	0	0	0
0	0	0	0	0
0	0	0	0	0
0	0.082648214	0	0	0
0.014019463	0.003615006	0	0	0.057353477
0.022239679	0	0	0	0
0	0	0	0	0
0	0	0	0	0.018226144
0.492881315	0.332120752	0	0	0
0.017739167	0	0	0.00136506	0
0	0	0	0	0
0	0.03226107	0.058076285	0.123136334	0.442195864
0.001341364	0.001787042	0	0.014476648	0.009642015
0	0	0	0	0
0	0	0	0	0
0	0	0	0	0
0	0	0	0	0
0	0	0	0	0
0	0	0	0	0
0.004478735	0	0	0.004921552	0.045255734
0	0	0	0	0
0	0	0	0	0
0	0	0	0	0
0.002030753	0	0	0	0.000834142
0	0	0	0	0
0	0	0	0	0
0	0	0	0	0
0	0	0	0	0
0	0	0	0	0
0	0	0	0	0
0.025253438	0.015477902	0	0.001539815	0.004981375
0.409457963	0.072507321	0	0.016668739	0
0	0	0	0	0
0	0	0	0.001816729	0
0	0	0	0	0
0	0	0	0	0
0	0.001449585	0	0.001868196	0.012818181
0	0	0	0	0
0	0	0	0	0
0	0	0	0	0
0	0	0	0	0

0	0	0	0	0
0.057503654	0.009911761	0	0	0
0	0	0	0	0
0.002438974	0	0	0.000652626	0.00068306
0	0.001409008	0.000474514	0	0.027452842
0	0.00181846	0	0	0
0.010758357	0	0	0	0
0	0	0	0	0
0	0	0	0	0
0	0	0	0	0
0.018687901	0	0	0	0
0	0	0	0	0
0.206837987	0.007538153	0	0.000771033	0
0.001996814	0	0	0	0
0	0	0	0	0
0	0	0	0	0
0	0	0	0	0
0	0	0	0	0
0	0	0	0	0
0	0	0	0	0
0	0	0	0	0
0	0	0	0	0
0	0.006144673	0	0.016853533	0.022314985
0.003907499	0.003067699	0	0.001916891	0
0	0	0	0	0
0.001983936	0	0	0	0
0	0	0	0	0
0.001865228	0	0	0	0
0	0	0	0	0
0	0	0	0	0
0.017306873	0.012648523	0	0	0
0	0	0	0	0
0	0	0	0	0
0	0	0	0	0
0.001934626	0	0	0	0
0	0.008264014	0	0	0
0	0	0	0	0
0	0	0	0	0
0	0	0	0	0
0	0	0	0	0
0	0	0	0	0
0.000889785	0	0	0	0
0	0	0	0	0
0	0	0	0	0
0	0	0	0	0
0.412607801	0.71931082	0.164358239	0.111065333	0.325626128
0	0	0	0	0
0	0	0	0	0

0	0	0	0	0
0	0	0	0	0
0	0	0	0	0
0	0	0	0	0
0	0	0	0	0
0	0	0	0	0
0	0	0	0	0
0	0	0	0	0
0	0	0	0	0.015025814
0	0	0	0	0
0	0.001266097	0	0	0.028463529
0	0	0	0	0
0	0	0	0	0
0	0	0	0	0
0	0	0	0	0
0	0	0	0	0
0	0	0	0	0
0	0	0	0	0
0	0	0	0	0
0	0.013912226	0	0	0
0	0	0	0	0
0	0	0	0	0
0	0	0	0	0
0	0	0	0	0
0	0	0	0	0
0	0	0	0	0
0	0	0	0	0
0.013250125	0	0	0	0
0	0	0	0	0
0	0	0	0	0
0.009383745	0.001030348	0	0.002124627	0.028908168
0	0	0	0	0
0.005866799	0	11.26489138	0.395322128	0.001721299
0.000856462	0	0	0	0.007915413
0	0	0	0	0
0	0	0	0	0
0	0	0	0	0
0	0	0	0	0
0	0	0	0	0
0	0	0	0	0
0	0	0	0	0
0.414567215	0	0	0	0.139308534
0.049980966	0.062111026	0	0.091482776	0.383858186
0	0	0	0	0
0	0	0	0	0
0.342011214	0	0	0	0
0	0	0	0	0
0	0	0	0	0

0	0	0	0	0
0	0	0.007445537	0	0
0.001541949	0.002641208	0.000988315	0	0.026917957
0	0	0	0	0
0.026470202	0.867451518	0	0	0
0.01917465	0.061811141	0	0	1.142032761
0	0	0	0	0
0	0	0	0	0
0	0	0	0	0
0	0	0	0	0
0	0	0	0	0
0	0	0	0	0
0	0	0	0	0
0	0	0.029240802	0	0
0	0	0	0	0
0	0	0	0	0
0.000865735	0	0	0	0
0	0	0	0	0
0	0	0	0	0
0	0	0	0	0
0	0	0	0	0
0	0	0	0	0
0	0	0	0	0
0	0	0	0	0
0.027321832	0	0	0	0
0.000639189	0.001459824	0	0	0
0	0	0	0	0
0.018394384	0	0	0	0
0	0	0	0	0
0	0	0	0	0
0	0	0	0	0
0	0	0	0	0
0	0	0	0	0
0	0	0	0	0
0	0	0	0	0
0	0	0	0	0
0	0	0	0	0
0	0	0	0	0
2.425405793	0	0	1.874225855	0
0	0	0	0	0
0	0	0	0	0
0.08228599	0	0.020089125	0	0
0.002565983	0.012209103	0	0.000629394	0.01998191
0	0	0	0	0
0	0	0	0	0
0.001968048	0	0	0	0
0	0	0.001258013	0.002246648	0
0	0	0	0	0

0	0	0	0	0
0.018161725	0.05726931	0.010159236	0.004859755	0.061036557
0	0	0	0	0
0	0	0	0	0
0	0	0	0	0
0	0	0	0	0
0	0	0	0	0
0	0	0	0	0
0	0	0	0	0
0	0	0	0	0
0	0	0	0	0
0	0	0	0	0
0.873534147	0	0	0.022689261	0
0	0	0	0	0
0.046319183	0.001763116	0.003760526	0.00176732	0.013476642
0	0	0	0	0
0	0	0	0	0
0	0	0	0	0
0	0	0	0.006982547	0
0	0	0	0	0
0	0	0	0	0
0	0.003174452	0	0.123726862	0
0	0	0	0.015623377	0
0	0.000891728	0	0	0
0	0	0	0	0
0	0	0	0	0
0	0	0	0	0
0	0	0	0	0
0	0	0	0	0
0	0	0	0	0
0	0	0	0	0
0	0	0	0	0
0	0	0	0	0
0	0	0	0	0
0	0	0.006000392	0	18.78501951
0	0	0	0	0
0	0	0	0	0
0	0	0	0	0
0.021451339	0	0.001358752	0.007428231	0.394951305
0	0	0	0	0
0.168806303	0.21602169	0.072575921	0.197369603	0.039502253
0	0	0	0	0
0	0	0	0	0
0	0	0	0	0
0	0	0	0	0
0.073059822	0.001183398	1.206763143	0.001779329	0.428596045
0	0	0	0	0
0	0	0	0	0

0	0	0	0	0
0	0	0	0	0
0	0	0	0	0
0	0	0	0	0
0	0	0	0	0
0	0	0	0	0
0	0	0	0	0
0	0	0.001076899	0	0.002300443
0	0	0	0	0
0	0.064812434	0	0.006960746	0.007285346
0	0.042110926	0	0	0
0	0	0	0	0
0.005200771	0	0	0	0
0	0	0	0	0
0	0	0	0	0
0	0	0	0	0
0	0	0	0	0
0	0	0	0	0
0	0	0	0	0
1.735674436	1.091197116	0.057814118	1.174360301	0.870120401
0	0	0	0	0
0	0	0	0	0
0	0	0	0	0
0	0	0	0	0
0	0	0	0	0
0	0	0	0	0
0	0	0	0	0
0	0	0	0	0
0	0	0	0	0
0	0	0.002537417	0	0
0	0	0	0	0
0	0	0	0	0
0	0	0	0	0
0	0	0	0	0
0	0	0	0	0
0.001141372	0	0	0.002799599	0
0	0	0	0	0
0	0	0	0	0
0	0	0	0	0
0	0	0	0	0
0	0	0	0	0
0	0	0	0	0
0	0	0	0	0
0	0	0	0	0
0	0	0	0	0.040394725
0	0	0	0	0
0	0	0	0	0
0	0	0	0	0

0	0	0	0	0
0	0	0	0	0
0	0	0	0	0
0	0	0	0	0
0.009585644	0	0	0	0
0.235705766	0	0	0.000454936	0.056661973
0	0	0	0	0
0	0	0	0	0
0	0	0	0	0
0	0	0	0	0
0	0	0	0	0
0	0	0	0	0
0	0	0	0	0
0	0	0	0	0
0	0	0	0	0
0	0	0	0	0
0.024185126	0	0	0.056838918	0
0	0	0	0	0
0	0	0	0	0
0	0	0	0	0
0	0	0	0	0
0	0	0	0	0
0	0	0	0	0
0	0	0	0	0
0	0	0	0	0
0	0	0	0.000711596	0
0	0	0	0	0
0	0	0	0	0
0	0	0	0	0
0	0	0	0	0
0	0	0	0	0
0	0	0	0	0
0	0	0	0	0
0	0	0	0	0
0	0	0	0	0
0	0	0	0	0
0	0	0	0	0
0	0	0	0	0
0	0.612395454	0	0.466438869	0
0	0	0	0	0
0	0	0	0	0
0	0	0	0	0
0	0	0	0	0
0	0	0	0	0
0	0	0	0	0
0	0	0	0	0
0	0	0	0	0
0	0	0	0	0

0	0	0	0	0
0.057969631	0	0	0	0
0	0	0	0	0
0	0	0	0	0
0	0	0	0	0
0.002372943	0	0	0	0
0	0	0	0	0
0	0	0	0	0
0	0	0	0	0
0.035007841	0	0	0	0
0.002945631	0	0	0	0.002094115
0	0	0	0	0
0.033879231	0.273102046	0	0.012772055	0
0	0	0	0	0
0	0	0	0	0
0	0	0	0	0
0	0	0	0	0
0	0	0	0	0
0	0	0	0	0
0.002639645	0	0	0	0.000774463
0.041624958	0	0	0.000676901	0.006848514
0	0	0	0	0
0	0	0	0	0
1.200967657	2.836616625	0	0.357568088	0
0	0	0	0	0
0	0	0	0	0
0	0	0	0	0
0	0	0	0	0
0	0	0	0	0
0	0	0	0	0
0	0	0	0	0
0	0	0	0	0
0	0	0	0	0
0	0	0	0	0

PHL018-0024_V1	PHL018-0019_V3	PHL018-0014_V1	PHL018-0093_V2	PHL018-0008_V3
EYCF	CTRL	CTRL	CTRL	CTRL
0.064626105	0.029245444	0.068813854	0.225546222	0.420643212
0.644104153	0.087268701	1.314577306	1.99580469	0.78476302
0	0	0	0	2.694661336
0	0	0	0	0
0	0	0	0	0
0	0	0	0	0
0	0	0	0	0
0	0	0	0	0
0	0	0	0	0
0	0	0	0	0
0	0	0	0	0
0	0	0	0	0
0	0	0	0	0
0.522289059	4.819552729	0.203684563	14.15305378	2.270273182
0	0	0	0	0
0	0	0	0	0
0	0	0	0	0
0	0	0	0	0
0	0	0	0	0
0	0	0	0	0
0	0	0	0	0
0.027362899	0	0	0	0
0	0	0	0	0
0	0	0	0	0
0	0	0.041082168	0	0
0	0	0	0	0
0	0	0	0	0
0	0	0	0	0
0	0	0	0	0
0	0	0	0	0
0	0	0	0	0
0	0	0	0	0
0	0	0	0	0
0	0	0	0	0
0	0	0	0	0
0.144308132	10.11511569	5.182144894	0	2.891828439
0	0	0	0	0
0	0	0	0	0
0	0	0	0	0
0	0	0	0	0
0	0	0	0	0
0.048985573	0.01726829	0.020773952	0	0.106424175
0.043704189	0	0.089105148	0	1.007177209
0.045113572	0.011080058	0.11316857	0	0.01299124
0	0	0.003547415	0	0
0	0	0	0	0

0	0.008244096	0	0	0
0.002357726	0.031391812	0	0.007684015	0.024429976
0.006628136	0.001744933	0.001609367	0	0.011542616
1.08642671	0.284832038	0.833491115	0.311211946	1.568320162
0.003278499	0.02875857	0.002571232	0	0.001525216
0	0	0	0	0
0.003032522	0	2.366062046	0	1.554800729
0	0	0	0	0
0	0	0	0	0
0	0	0	0	0
0	0	0	0	0.051800371
0.094165621	0.740500878	0.65149975	0	0.001885655
0	0	0	0	0
0.002714746	0.009112271	0.134842767	0	0.050326574
0	0	0	0	0
0	0	0	0	0
0	0	0.00128016	0	5.059005725
0	0	0	0	0
0	0	0	0	0
0	0	0	0	0
0.113981041	1.28170876	0.267212988	0.768968335	0.394095883
0.029986332	0.02933161	0.014638161	0.042670587	0.00550565
0	0	0	0	0
0	0	0	0	0
0	0	0	0	0
0	0	0	0	0
0	0.001919067	0	0	0
0	0	0	0	0
0	0	0.003792697	0	0.002185857
0	0	0	0	0
0.007324561	0	0	0	0.002581452
0	0	0	0	0
0	0	0	0	0
0	0	0	0	0
0	0	0	0	0
0	0	0	0	0
0	0	0	0	0
0	0	0.003819687	0	0.11303462
0	0	0	0	0
0	0	0	0	0
0	0	0	0	0
0	0	0	0	0
0	0	0	0	0
0	0	0	0	0
0	0	0	0	0
0	0	0	0	0
0	0	0	0	0.006337421
0.561653334	0	0	0	0.052555921

0	0	0	0	0
0	0	0	0	0
0	0	0	0	0
0	0	0	0	0
0	0	0	0	0.03241482
0	0	0	0	0
0	0	0	0	0
0	0	0	2.001784853	0.015884172
0.003124039	0	0.005193911	0	0.001835049
0	0	0	0	0
0	0	0	0	0
0.051784752	0	0.015594899	0	0
0	0	0	0	0
0	0	0	0	0
0	0	0	0	0
0	0	0	0	0
0	0	0	0	0
0	0	0	0	0
0.010580056	0	0.001110299	0	0.002085606
0	0	0	0	0
0	0	0	0	0
0	0	0	0	0
0	0	0	0	0
0	0	0.005766808	0	0
0	0	0	0	0
0	0	0	0	0
0	0	0	0	0
0	0	0.001928807	0	0
0	0	0	0	0
0	0	0	0	0
0.022324131	0	0.025191608	0	0.003905632
0	0.000754063	0.043293549	0	0.015320518
0	0	0	0	0
0	0	0	0	0
0	0	0	0	0
0	0	0	0	0
0	0	0	0	0
0	0	0	0	0
0	0	0	0	0
0	0	0	0	0
0	0	0	0	0
0	0	0	0	0
0	0	0.000803863	0	0
0	0	0	0	0
0	0	0	0	0
0	0	0	0	0
0	0	0.00653146	0	0
0	0	0	0	0

0	0	0	0	0
0	0	0	0	0
0	0	0	0	0
0	0	0	0	0
0.007479177	0	0.196885372	0	0
0	0	0	0	0
0	0	0	0	0
0	0	0	0	0
0	0	0	0	0
0	0	0	0	0
0	0	0	0	0
0	0	0	0	0
0	0	0	0	0
0	0	0.001780136	0	0
0	0	0	0	0
0	0	0	0	0
0	0	0	0	0
0	0	0	0	0
0	0	0	0	0
0	0	0	0	0
0	0	0	0	0
0	0	0	0	0
0	0	0.00265712	0	0
0	0	0	0	0
0	0	0	0	0
0	0	0	0	0
0	0	0	0	0
0	0	0	0	0
0	0	0	0	0
0	0	0	0	0
0	0	0	0	0
0.088840725	0	0.001203649	0	0.111403511
0	0	0	0	0
0	0	0	0	0
0	0	0	0	0
0	0	0	0	0
0	0	0	0	0
0	0	0	0	0
0	0	0	0	0
0	0	0	0	0
0	0	0	0	0
0	0	0	0	0
0	0	0	0	0
0	0	0	0	0
0	0	0	0	0
0	0	0	0	0
0	0	0	0	0
0.005243439	0	0	0.359576517	0

1.700981429	0	0	0	0
0	0	0	0	0
0	0	0	0	0
0	0	0	0	0
0	0	0	0	0
0	0	0	0	0
0	0	0	0	0
0	0	0	0	0
0	0	0	0	0
0	0	0	0	0
0	0	0	0	0
0	0	0	0	0
0.001674194	0	0	0.001768256	0
0	0	0	0	0
0	0	0	0	0
0	0	0	0	0
0	0	0	0	0
0	0.000750927	0	0	0
0	0	0	0	0
0	0	0	0	0
0.11790951	0.179012409	0.109830535	0.015114118	0.147100051
0	0	0	0	0
0	0	0	0	0
0	0	0	0	0
0	0	0	0	0
0	0	0	0	0
0.038486332	0.001586453	0.050166835	0	0.002570028
0	0	0	0	0
0	0	0	0	0
0	0	0.002683637	0	0
0	0.022139936	0.015002345	0.212500278	0.002561882
0	0	0	0	0
0	0	0	0	0
0	0	0.003509626	0	0.006967127
0.004752456	0.008862229	0.006071901	0.106023358	0.028952618
0	0	0	0	0
0	0	0	0	0
0	0	0	0	0
0	0	0.003169003	0	0
0	0	0	0	0
0	0	0	0	0
0	0	0	0	0
0	0	0	0	0
0	0	0	0	0
0	0	0	0	0
0	0	0	0	0
0	0	0	0	0
0.000645091	0	0	0	0

0	0	0	0	0
0	0	0	0	0
0	0	0	0	0
0	0	0	0	0
0	0	0	0	0
0	0	0	0	0
0	0	0.001658308	0.010287558	0
0.002781991	0	0.005023961	0	0
0	0	0	0	0.01092041
0	0	0	0	0
0	0	0	0	0
0	0	0	0	0
0	0	0	0	0
0	0	0	0	0.18099822
0	0	0	0	0.033068177
0	0	0	0	0
0	0	0	0	0
0	0	0	0	0
0	0	0	0	0.005375818
0	0	0	0	0
0.010161328	0	0	0	0
0	0	0	0	0
0	0	0	0	0
0	0	0	0	0
0.001922631	0.009680207	0.009920156	0	0.003049235
0	0	0	0	0
0	0	0	0	0
0	0	0	0	0
0.012155515	0	0.029584215	0	0
0	0	0	0	0
0.568910532	0.02965705	0.500247077	1.572401594	0.068841496
0	0	0	0	0
0	0	0	0	0
0	0	0	0	0.004866967
0	0	0	0	0
0	0	0	0	0
0	0	0	0	0
0	0	0	0	0
0	0	0	0	0
0	0	0	0	0
0	0	0	0	0
0.002176021	0.019274436	0.021893758	0.011080978	0.006327025
0	0	0	0	0
0	0	0	0	0
0	0	0	0	0
0	0	0	0	0
0	0	0	0	0
0	0	0	0	0

0	0	0	0	0
0	0	0	0	0
0.041668381	0.010445171	0.126882532	0	0.010352082
0	0	0	0	0
0	0	0	0	0
0	0	0	0	0
0	0	0	0	0
0	0	0	0	0
0	0	0	0	0
0	0	0	0	0
0	0	0	0	0
0	0	0	0	0
0	0	0	0	0
0.000754936	0	0.005453315	0	0.000798204
0.005192506	0.004182979	0.004072331	0	0.002196042
0	0	0	0	0
0.002603214	0.006619624	0.003174775	0	0.001251097
0	0	0	0	0
0	0	0	0	0
0	0	0	0	0
0	0	0	0	0
0	0	0	0	0
0	0	0	0	0
0	0	0	0	0
0	0	0.019795265	0	0.00833014
0	0	0	0	0
0	0	0	0	0.01388984
0	0	0	0	0
0	0	0	0	0
0	0	0.003826684	0	0.00751485
0	0	0	0	0
0	0	0	0	0
0	0	0	0	0
0	0	0	0	0.00104174
0	0	0	0	0
0.029442357	0	0.206513696	0.008691574	0.007218503
0	0	0	0	0
0.001610444	0	0.012464055	0.002915869	0.003405487
0	0	0	0	0
0	0	0	0	0
0	0	0	0	0
0	0	0	0	0
0	0	0	0	0
0	0	0	0	0
0	0	0	0	0
0	0	0	0	0.004363236
0	0	0	0	0.009228559
0	0.043286406	0.00335491	0	0

0.094304088	0	0	0	0.033085248
0	0	0.004476549	0	0.002402525
0	0	0	0	0
0	0	0	0	0
0.000675383	0	0.005807931	0.00152856	0
0.004772185	0	0	0	0.005433825
0	0	0	0	0
0.083309428	0	0.078480147	0	0.072068864
0	0	0	0	0.00655634
0.054643802	0.060410981	0	0	0.008941465
0	0	0	0.001198028	0
0.100920487	0	0.062382946	0	0.002112962
0.077688679	0.002332076	0.004497325	0.002058484	0.097968496
0.002302226	0	0	0	0
0	0	0.001809938	0	0
0	0	0	0	0.01353377
0	0	0	0	0
0	0	0	0	0
0.02846399	0.003081992	0	0.056524331	0.102906701
0.011285161	0	0	0	0
0	0	0	0	0
0	0	0	0	0
0	0	0.011702529	0	0
0	0	0	0	0.005327365
0	0	0	0	0
0	0.004691746	0.000910998	0	0.001166752
0	0	0	0	0
0	0	0	0.979626745	0
0	0	0	0	0
0	0	0.020854969	0	0
0	0	0	0	0
0	0	0	0	0
0	0	0	0	0
0	0	0	0	0
0	0	0	0	0
0.021485073	0	0	0	0.005915743
0	0.069981252	0	0.129781439	0.028304338
0	0	0	0	0
0	0	0	0	0
0	0	0	0	0
0	0	0	0	0
0	0	0	0.343880837	0.003288627
0	0	0	0	0
0	0	0	0.011348142	0
0	0	0	0	0
0	0	0	0	0
0	0.00307082	0.009912854	0	0
0.015694518	0	0.019537706	0	0

0	0	0	0	0
0	0	0.077231634	0	0.01264798
0	0	0	0	0
0	0	0	0	0
0	0	0.010077757	0	0
0.008214461	0.103034168	0.03479947	0.010861278	0.009599497
0	0	0	0	0
0	0	0	0	0
0	0	0	0	0
0	0	0	0	0
0.005265138	0	0.02742514	0	0
0	0	0	0	0
0	0	0	0	0
0	0	0.001466598	0	0
0.022289152	0	0.001978578	0	0
0	0	0	0	0
0	0	0	0	0
0.013369653	0	0	0	0.001949781
0	0	0	0	0.001332797
0	0	0	0	0.005706649
0	0	0.002106932	0	0.399907797
0	0.22352929	0	0	0.288534217
0	0	0	0	0
0	0	0	0	0
0	0	0	0	0
0	0	0	0	0
0.017207484	0.171550925	0	0.002790077	0.847229081
0	0	0	0	0
0	0	0	0	0
0.153809395	0.102944034	0.206373609	1.110334256	0.093610166
0	0.004494924	0	0	0.000849532
0	0	0	0	0
0	0	0	0	0
0	0	0.057543078	0	0
0.002139136	0	0	0	0.000904695
0	0	0	0	0
9.691202358	0.164706894	0	0	0
0	0	0	0	0
0	0	0	0	0
0.09249108	0	0	0	0
0.038556865	0.021134133	0.104494253	0.092552522	0
2.183235797	0.632608321	0.032491269	4.862366404	4.782294694
0	0	0	0	0
0.028660823	0	0	0	2.086220007
0.003954014	0	0	0	0
0	0	0	0	0
0.008992933	0.002972817	0.001898208	0	0.00172879
0	0	0	0	0.004854478

0	0.022572046	0.0026023	0	0.001066519
0	0	0	0	0
0.012713567	0	0	0	11.43533587
0	0	0	0	0
0.083485622	0	0	0	0
0	0	0	0	0.194776366
0	0	0	0	0
0	0	0	0	0
0.007386721	0	0	0	0
0	0	0	0	0
0	0	0	0	0
0	0	0	0	0
0	0	0	0	0
0	0	0.16809236	0.002788197	0
0	0	0	0	0
0.004278858	0	0.124002104	0.03776808	0.037135267
0.000790445	0.095293857	0.089725664	0.001073384	0
0.00072027	0	0	0	0
0.001224704	0	0	0	0
0	0	0	0	0.00334373
0	0.043747768	0.003854995	0.009807578	0.00105328
0.001375312	0.005642205	0	0.001556337	0
0	0	0	0	0
0	0	0.233883293	0	0
0.050788852	0	0	0	0
0	0	0	0	0
0.001756083	0	0.008025298	0.006132014	0
0.386582186	0	0	0	0
0	0	0	0	0
0	0	0	0	0
0.055327689	0	0.031894227	0.014508291	0.001815477
0.003413247	0.024186676	0.000880563	0.001158754	0.114882398
0.532979058	0.006363272	0.04359756	0.022433579	0.016607977
0.007762307	0	0.007609689	0	0.005745033
0	0.001239768	0	0	0.0038076
0	0	0	0	0
0.000734527	0.04273536	0.001515969	0	0.025110885
0.017233028	0.00492746	0.021537641	0.061364159	0.024699185
0.11080754	0.023076823	1.118508697	0	0.582933914
0	0	0	0	0.001158678
0	0	0	0	0
0	0	0	0	0
0	0	0.001773	0.010110255	0
0.195360232	0	0.09362715	0.78998182	0.002212546
0	0.802464739	0	0.006752459	0
0.001878541	0	0	0	0
0	0	0	0	0
0	0	0	0	0

0	0.185821122	0	0.018890472	0
2.605218222	0.004962736	0.005384912	0.040390997	0.028690167
0	0	0	0	0
0	0	0	0	0
0	0	0	0	0
0	0	0	0	0
0	0	0.001743611	0.005900042	0
0	0.004260363	0.004963416	0	0
0.001671912	0	0	0	0
0.015168041	0.017819478	0.001956555	0	0
0	0.003037057	0.004201657	0.005265777	0
0.424536338	0.855318068	0.132024277	0	0.199700422
0	0	0	0	0.022105028
0	0	0	0	0
0.120796389	0.009458318	0.002754786	0	0.013642244
0	0	0	0	0
0.010760468	0.002498758	0.00984704	0.001378507	0.001931975
0.026092243	0.185482539	0.06554594	0.1453068	0.020899754
0	0	0	0	0
0.008385899	0	0	0.115967898	0.001266646
0.081864758	0.043806444	0.015556262	0	0.013946476
0	0	0	0	0
0	0	0	0	0
0	0	0	0	0
0	0	1.192519914	0	0.004463368
0	0	0	0	0
0.043398894	0	0	0	0.052441404
0.107038065	0.064486545	0.016074732	0.155626694	0.045880853
0	0	0	0	0.002752599
0	0	0	0.001093528	0.001135242
0	0.000801476	0.001478417	0	0
0	0	0	0	0
0	0	0	0	0
0	0	0	0	0
0	0	0	0	0.012097109
0.107714105	0.33331833	0.186552176	0.049097736	0.041015449
0	0	0	0	0.009405732
0	0	0	0	0
0.047217192	0.003071481	0.035410679	0	0.009288068
0	0	0	0	0
0	0.762853225	0	0	0
0	0	0	0	0
0.001333974	0	0.011012602	0.005887277	0
0.00563468	0.002701896	0	0	0.000851089
0.008460187	0	0	0.097077895	0
0	0	0	0	0
0	0	0	0	0
0	0	0	0	0

0	0	0	0	0
0	0	0	0	0
0	0	0	0	0
1.923428267	0	0.056114608	0	1.622579879
0	0	0	0	0
0	0	0	0	0
0	0	0	0	0
0	0	0	0	0
0.012163945	0.225499724	0.296929335	0	0
0.080885246	0	0.945198506	0	0.171225616
0	0	0	0	0
0.000558214	0	0.004032287	0	0.000786943
0	0	0	0	0
0	0	0	0	0.201368163
0.028406118	0.185712879	0.008873209	0.025229533	0.056172009
0	0	0	0	0
0	0	0	0	0
0	0	0	0	0
0	0	0	0	0
0	0	0	0	0
0	0	0	0	0
0	0	0	0	0
0	0	0	0	0
0.507461075	0.007612302	0.153543889	0.418163798	0
0	0	0.016898743	0.17387816	0
0.509719755	0	0	0	0.161926826
0	0	0	0	0
3.933424273	19.68746339	1.042324633	0	1.115141168
0	0	0	0	0
0	0	0	0	0
0.001989824	0	0	0	0
0	0	0	0	0.013695432
0	0	0.010797289	0	0.004512467
0	0	0	0	0
0	0	0	0	0
0.036043627	0.033120288	0	0	0
0.090327855	0	0.617124098	0	1.757624218
0	0	0.00439527	0	0
0	0	0	0	0
0.01185346	0	0	0	0.000985727
0.471245903	0	0.125442099	0.000969588	0.421062854
0.007286505	0	0	0	0
2.33685901	2.628756289	1.194584664	0.438503825	0.181579335
0.001088491	0	0	0	0
0	0	0	0	0
0	0	0	0	0
0	0	0	0	0
0.001502061	0	0	0	0.063299092

0	0	0	0	0
0	0.000656174	0	0.157685439	0
0.00287979	2.956033213	0.016344668	0.362848793	0.000652466
0	0	0	0	0
0	0	0	0	0.024514164
0	0	0.001170518	0	0.001713292
0	0	0	0	0
0	0	0	0	0.000520878
0	0	0	0	0
0	0	0	0	0
0.004847046	0	0.052102486	0.686726663	0.078580975
3.629685733	2.423283531	0.351085837	5.598428424	1.139528268
0	0	0	0	0
0.000653891	0	0.02744079	0.002071887	0
0.624189576	0.084366936	0.033677336	0.18053285	0.002542514
0	0	0	0	0
0	0	0	0	0
0	0	0	0	0
0	0	0	0	0
0.120778877	0	0.009098772	0	0.000879484
0.512218369	0	0.133454162	0.007125767	0.193649859
0.899472631	2.058269708	0.004785473	0	0
4.98287964	0.899079272	0.019138586	0.055146345	0.139293304
0	0	0	0	0
0	0	0	0	0
0.584582413	0.017248111	0.014024233	0	0.00471822
0	0	0	0	0
0.009003952	0	0	0	0
0	0	0	0	0
0	0	0	0	0
0	0	0	0	0
0	0	0.086907376	0	0.11084115
0.016178638	0	0.07106209	0.602760546	0.060089919
0.009490357	0	0	0.00923599	0.012208375
0.010130507	0.001378536	0.014550879	0.003346229	0.007671478
0.007711711	0.00294626	0.007569793	0.038823452	0.024063342
0	0.006708539	0	0	0.030160685
0	0	0	0	0
0.047542436	0.035462255	0.049969258	0.026900099	0
0	0	0	0	0
0	0	0	0	0.000525347
0	0	0	0	0
0	0	0	0	0
0	0	0	0	0
0.009652314	0	0.001328075	0.206804969	0
0	0	0	0	0
0	0	0	0	0
0.00413985	0.56451491	0.04699264	0.11419095	0.022706303

0	0	0	0	0
0	0	0	0	0
0	0	0	0	0
0.041864395	0	0	0	0.002706083
0	0	0	0	0
0	0	0	0	0.003243565
0	0	0	0	0.083588923
0	0	0.010762839	0	0.455863611
0	0	0	0	0
0	0	0	0	0
0.002646882	0	0.001260652	0.018248154	0
0	0	0	0	0
0	0.004388187	0	0	0
0	0	0	0	0
0	0	0	0	0
0	0	0	0	0
0	0	0	0	0.066925327
0.001016986	0	0	0	0.000896061
0	0	0	0	0
0	0	0.059152655	0	0.00468697
0	0	0.003574069	0	0.002706669
0	0	0	0	0
0	0	0.011091269	0	0.033885472
0	0	0	0	0
0	0	0	0	0
0	0	0	0	0.003821055
0	0	0	0	0
0	0	0	0	0
0	0	0	0	0
3.260874418	0	0.002112905	0	0.06018328
0	0.000897118	0	0.090807771	0
0	0	0	0	0
0	0	0	0	0
0	0	0	0	0
0	0	0	0	0
0	0	0	0	0
0.122476828	0	0.067106107	0	0.050719416
0.031885921	0.004242541	0.001067164	0.073960189	0
0	0	0	0	0
0.001020017	0	0	0	0
0	0	0	0	0.013336533
0	0	0	0	0
0	0	0	0	0
0	0	0	0	0
0	0.390425285	0.068262103	0	0.029176503
0	0	0	0	0
0	0	0	0	0
0	0	0	0	0

0	0	0	0	0
0	0	0	0	0
0	0	0	0	0
0	0	0.716699585	0	0.03262861
0	0	0	0	0
0	0	0	0	1.874302131
0	0	0	0	0
0	0	0	0	0
0.082009318	2.406427959	0.010793177	0.058748824	0.004021312
0	0	0	0	0
0	0	0	0	0
0	0	0	0	0
0	0	0	0	0.004838538
0	0	0	0	0
0.969764228	0	1.84422761	0	0.00379289
0	2.972687135	0.462816767	0.172161055	0
0	0	0	0	0
0	0	0	0	0
0	0	0	0	0
0	0	0	0	0
0	0	0.017443697	0	0
0	0	0	0	0
0	0	0	0	0
0	0	0	0	0
0.01705182	0.001251057	0.003173117	0	0.044333893
0.547240169	0	13.5648882	0.021109462	1.096757429
0	0	0	0	0
0	0	0.003044084	0	0.001100804
0	0.015312941	0.061032662	0	0
0	0	0	0	0
0	0	0	0	0
0	0	0.030095257	0	0
0	0	0	0	0
0	0	0.002310884	0.0040546	0.001262781
0.001475909	0.006054903	0.002030724	0	0.001820581
0.000521644	0.013618444	0.021891002	0.006611424	0
0	0.151380119	0.148692619	0	0.024664276
1.359473531	0	2.463565388	0	1.171459951
0	0	0	0	0
0	0	0	0	0
0	0	0	0	0
0	0	0	0	0
0.122370236	0	4.166689756	0.042914484	0.321323739
0.084808366	0	0	0	0
0	0	0	0	0
0	0.005529328	0.002379883	0	0
0	0	0	0	0
0.101300295	0	0.155178148	0.017105995	0.088237709

0	0	0	0	0.004138299
0	0	0	0	0
0	0	0	0	0
0	0	0	0	0
0	0	0.001206379	0.007672938	0.00185407
0	0	0.001161725	0	0.001190295
0	0	0	0	0
0	0	0	0	0
0	0	0	0	0
0	0	0	0	0
0	0	0	0	0
2.204492093	0	0.499348785	0	1.846577792
0.008728969	0.020060928	0	0	0.578200387
0	0	0	0	0
0.196716077	0.060508031	1.262867979	0.026621257	0.048840845
0	0	0	0	0
0	0	0	0	0
0	0	0	0	0
0	0	0.010190566	0	0
0	0	0.002259942	0	0
0	0	0	0	0.071575112
0	0	0	0	0
0.061070547	0	2.848424092	0	0.458086486
0	0	0	0	0
0	0	0	0	0
0	0	0	0	0
0	0	0	0	0
0	0	0	0	0
0	0	0	0	0
0	0	0	0	0
0	0	0	0	0
0.096492536	0.008833234	0.031984398	0.003970667	0.010511444
0	0	0	0	0
0.004090207	0	0.023214562	0	0.017761873
0.000756906	0.013973406	0.015816841	0.004111363	0
0.012440738	0	0.127121752	0	0.053130863
0	0	0	0	0
0	0	0	0	0
0	0	0	0	0
0	0	0	0	0
0	0	0	0	0.225521101
0	0	0	0	0
0	0	0	0	0
0.037125537	0	0	0	0.027037559
0.002982515	0.001459946	0.007886767	0.029110129	0.067405046
0	0	0	0	0
0.297366328	0	0	0	0
0.002820925	0	0.000545815	0	0.000932063

0	0	0	0	0
0	0	0	0	0
0.00115675	0	0	0	0
0	0	0	0	0
0	0	0	0.003717062	0
0	0	0	0	0
0	0	0	0	0
0	0.000611146	0.16421438	0	0.106264937
0	0	0	0	0
0	0	0	0	0
0	0	0.447737953	0	0
0	0	0.000607276	0.00079913	0
0	0	0	0	0
0	0	0	0	0.088823428
0	0	0	0	0
0	0	0	0	0
0	0	0	0	0
0	0	0	0	0
25.34119546	0	0	0	0
0	0	0	0	0
0	0	0	0	0
0	0	0	0	0
0	0	0	0	0
0	0	0	0	0
0	0.000683622	0	0	0
0.006767983	0.01689235	0.0008954	0.065041032	0.002018325
0	0	0	0	0
0.619816255	0	0.018229509	0.128939088	1.383056493
0	0	0	0	0
4.019218268	0	0.55988332	0	0.331788952
0	0	0	0	0
0	0	0	0	0
0	0	0	0	0
0	0	0	0	0
0.008910999	0.002238201	0	0.003210387	0
0	0	0	0	0
0	0	0	0	0
0	0.003001467	0	0.001120876	0.00305454
0	0	0	0	0
0	0	0	0	0
2.153721957	4.979617827	8.589304306	17.0974614	1.449749549
2.005070529	0	0.792319762	2.46884434	0.804617922
0	0.454457883	0.218631546	0	0
0	0	0	0	0
0	0	0	0	0
0	0	0	0	0
0	0	0	0	0
0.003111714	0.001282686	0	0.012899049	0.001904769

0	0	0	0	0
0.002863743	0	0.005562726	0	0.027072755
0	0	0.005278772	0	0.001664182
0	0	0	0	0
1.206601957	0	3.370771703	0	2.182935578
0	0	0	0	0
0	0	0	0	0
0	0	0	0	0
0.036500947	5.012906102	0.109354707	0.596624736	0.185139387
0	0	0	0	0
0	0	0	0	0
0	0	0	0	0
0.160736918	0	0.130398918	0	0.170295412
0.004829565	0.005178462	0.026164946	0.015302714	0.004042537
0	0.05765293	0	0	0
0.486636384	0	0.720974665	0.154051122	0.546652333
0.005572319	0	0.128123283	0	0.036823044
0	0	0	0	0
0	0	0	0	0
0	0	0	0	0
0	0	0	0	0
0	0	0	0	0
0	0	0	0	0
0	0	0	0	0
0	0	0	0	0
0	0	0	0	0
0.014990334	0	0.346413125	0	0.011366798
0	0	0.006170995	0.004176292	0
0	0	0	0	0
0	0	0	0	0
0	0	0	0	0
0	0	0	0	0
0	0	0	0	0
0.007522389	0	0	0	0
0	0	0	0	0
0.000592941	0	0	0	0.117234821
0.006060569	0	0	0	2.986517938
0	0	0	0	0
0	0	0	0	0
0	0	0	0	0
0.013313665	10.94676803	5.415352222	0	3.008065994
0	0	0.120272966	0	0.067032125
0	0	0	0	0
0	0	0	0	0
0	0	0	0	0
0	0	0	0	0
0	0	0	0	0.617756817
0	0	0	0	0

0	0	0	0	0.044899464
0	0	0	0	0
0	0	0	0	0
0.000774193	0	0	0	0
0.007407514	0	0.125553957	0	1.301884718
0	0	0	0	0
0.366705073	0	0.019359983	0	0.054181921
0	0	0	0	0
0	0	0	0	0
0.153091412	0	0.024449357	0	0.017342741
0	0	0.001739048	0	0.001559089
0	0	0	0	0.017507659
0	0	0	0	0
0	0	0	0	0
0	0	0	0	0
0	0	0	0	0
0.092910358	0	0	0	0
0.001002033	0.001121135	0.014304122	0	0
0	0	0	0	0
0	0	0	0	0
0.014222944	0	0.082601695	0.011229095	0.207386004
0	0	0	0	0
0.022072451	0	0.019953707	0	0.031245243
0.00162207	0.004174204	0.001004323	0.027093087	0.003601577
0	0	0	0	0
0	0	0.006121857	0	0
0	0	0	0	0
0	0	0	0	0
0.170445071	0	0	0	0
0.781370396	2.794867276	0.368350501	0	0.72445772
0	0	0	0	0
0	0	0	0	0
0	0	0	0	0
0.389068615	0.004070164	0	18.35013567	2.276990123
0	0	0	0	0
0	0	0	0	0
0.016642967	0	0.073724546	0.015709973	0.022103336
0	0	0	0	0
0	0	0	0	0
0	0	0	0	0
0	0	0	0	0
0	0	0	0	0
0	0	0	0	0.157389533
0	0	0	0	0
0	0	0	0	0
0	0	0	0	0
0	0	0	0	0
0	0	0	0	0

0.00314173	0	0.017110884	0.067549921	0.00092272
0	0	0	0	0
0	0	0	0	0.091233885
0	0	0	0	0
0	0	0	0	0
0	0	0	0	0
0	0	0	0	0
0.619362472	0	0.768228092	0	6.310040215
0.05205384	0.002145722	0.127505304	0	1.38693789
0.002670192	0.028168556	0.068296192	0.571092899	0.032467141
0	0	0	0	0
0	0	0	0	0
0	0	0	0	0.00611249
0	0	0	0	0
0	0	0	0.002264288	0.002820796
0	0	0	0	0
0	0	0	0	0
0	0	0	0	0
0	0	0	0	0
0	0	0.011750907	0	0
0	0	0	0	0
0	0	0	0	0
0	0	0	0	0
0	0	0	0	0
0.225046354	0	0.078451449	0	0.029361509
0	0	0	0	0
0	0	0	0	0
0	0	0	0	0.03246911
0	0	0	0	0
0	0	0	0	0
0	0	0	0	0.082470449
0	0	0	0	0
0	0	0	0	0
0	0	0	0	0
0	0	0	0	0
0	0	0	0	0.012362154
0	0	0	0	0
0	0	0	0	0.009062873
0.002075423	0	0	0	0
0.001742461	0	0	0	0.051769398
0	0	0	0	0
0	0.03821526	0.378062638	0	0.62532622
0	0	0	0	0
0	0	0	0	0
0	0	0	0	0
0	0	0	0	0
0	0	0	0	0
0	0	0	0	0

0	0	0	0	0
0	0.014276631	0.016418689	0	0.006828921
0	0	0	0	0
0	0	0	0	0
0.096249135	0.011335728	0.713670233	0	1.838765026
0	0	0	0	0
0	0	0	0	0
0.016208058	0.249163966	0.005957088	0.029547306	0.001173767
0	0	0	0	0.003281496
0	0	0	0	0
0	0	0	0	0
0	0.022269928	0	0	0
0	0	0	0	0
0	0	0	0	0
0	0	0	0	0.008262904
0	0	0	0	0.002646798
0	0	0	0	0
0	0	0	0	0
0	0	0	0	0
0	0	0	0	0
0	0	0	0	0
0	0	0	0	0
0	0	0	0	0
0.000960736	0	0	0.004174818	0
0.276950464	0.307573771	0.241338005	0	0.146592461
0	0	0	0	0
0	0	0.072541079	0.041524501	0.000557438
0	0	0	0	0.000782619
0	0	0	0	0
0	0	0	0	0
0	0	0	0	0
0	0	0	0	0
0	0	0	0	0
0	0	0	0	0
0	0	0	0	0
0	0	0	0	0
0	0	0	0	0
0	0	0.01022933	0	0.032967177
0	0	0	0	0
0	0	0	0	0
0	0	0	0	0
0	0	0	0	0
0	0	0	0	0
0	0	0	0	0
0	0	0	0	0
0	0	0	0	0
0	0	0	0	0
0	0	0	0	0

0.153534577	0	0	0	0.787381795
0	0	0	0	0
0.008676511	0	0	0	0
0	0	0.000678738	0.005359008	0
0	0	0	0	0
0.002489153	0	0	0	0
0	0	0	0	0
0	0	0	0	0
0	0	0	0	0
0	0	0	0	0
0	0	0	0	0
0	0	0	0	0
0	0	0	0	0
0	0	0	0	0
0	0	0	0	0
0	0	0	0	0
0	0	0	0	0
0	0.002357952	0	0	0
0	0	0	0	0.000592596
0	0	0	0	0
0.089548939	0.001070436	0.020930182	2.990965042	0.008901659
0	0	0	0	0
0.052438787	0.008111297	0.002244336	0.134542796	0.014052684
0	0	0.247408982	0	0
0	0	0	0	0
0	0	0	0	0
0	0	0	0	0
0	0	0	0	0
0	0	0	0	0
0	0	0	0	0
0	0	0	0	0
0	0	0	0	0
0.009052296	1.906392135	0.006071901	0.0218193	0.002392778
0	0	0	0	0
0.012955578	0.017892873	0	0	0.667140506
0	0	0	0.107273901	0
0	0	0	0	0
0	0	0	0	0
0.001050258	0	0	0.046779298	0.000888362
0	0	0	0	0
0	0	0	0	0
0	0	0	0	0
0.002456134	0.221769433	0.025852628	0	0
0	0	0	0	0
0	0	0	0	0
0	0	0.126409443	0	0.015055455
0	0	0	0	0
0	0	0	0	0

0	0	0	0	0
0	0	0	0	0
0	0	0	0	0
0	0	0	0	0
0	0	0	0	0
0	0	0	0	0
0	0	0	0	0
0	0	0.110481036	0.04760839	0
0.000548258	0	0.150682657	0	0.046181236
0.011050008	0.01910711	0	0	0.000849696
0	0	0	0	0
0	0	0	0	0
0	0	0	0	0
0	0	0	0	0.549939864
0.009417001	0	0	0	0
0	0.080284001	0	0	0
0	0	0	0	0
0	0	0	0	0
0	0	0	0	0
0	0	0	0	0
0.004542007	0.017324575	0.007030588	0	0.006548625
0	0	0	0	0
0	0	0	0	0
0	0	0	0	0
0	0	0	0	0
0	0	0	0	0
0	0	0	0	0
0	0	0	0	0
0	0	0	0	0
0	0	0	0	0
0.709164904	3.847745224	0.070296435	6.837067555	2.584583459
0	0	0	0	0
0	0	0	0	0
0	0	0	0	0
0	0	0	0	0
0	0	0	0	0
0	0	0	0	0
0	0	0	0	0
0.384237092	0.003646721	0	0	0
0	0	0	0	0
0	0	0	0	0
0	0	0	0	0
0	0	0	0	0
0	0	0	0	0
0	0	0	0	0
0	0	0	0	0
0.005261253	0.001584857	0	0.575408392	0.001497675
0	0	0	0	0

0	0	0	0	0
0	0	0	0	0
0	0	0	0	0
0	0	0	0	0
0.018292169	0	0.299681371	0	0.019180715
0	0	0	0	0
0	0	0	0	0
0	0	0	0	0
0	0	0	0	0
0	0	0	0	0
0.014203534	0	0	0	0.049720386
0.001679075	0	0.002382459	0.0114005	0.011761421
0.004526934	0	0	0	0.005169299
0	0	0	0	0
0.005438184	0	0	0	0
0.070998479	0	0	1.62837159	1.170537423
0.003389947	0	0	0	0.004437625
0	0	0	0	0
0.04720093	0.001600342	0	0.067117872	0.001008205
0.384305791	0	0.001498948	0	0.110578369
0	0	0	0	0
0	0	0	0	0
0	0	0	0	0
0	0	0	0	0
0	0	0	0	0
0	0	0	0	0
0.033090932	0	0.002402351	0.021451774	0.000879082
0	0	0	0	0
0	0	0	0	0
0	0	0	0	0
0.00094247	0	0.000583541	0	0
0.016084044	0	0	0.002488252	0
0	0	0	0	0
0	0	0	0	0
0	0	0	0	0
0	0	0	0	0
0	0	0	0	0
0.022248312	0.041932464	0.003689811	0.024637239	0.075050962
0	0	0	0	0
0	0	0	0	0
0	0	0	0.001615789	0
0	0	0	0	0
0	0	0	0	0
0	0.084591882	0	0	0
0	0	0	0	0
0	0	0	0	0.001299374
0	0	0	0	0
0	0	0	0	0

0	0	0	0	0
0	0	0	0	0
0	0	0	0	0
0.000617414	0.001842134	0.000637131	0.004471555	0
0.000763522	0.012600552	0.000590929	0	0
0	0	0	0.001003591	0
0	0	0	0	0
0	0	0.132877758	0	0
0	0	0	0	0
0	0	0	0	0
0	0	0	0	0
0	0	0	0	0
0.022758286	0.00587616	0.033421078	0	0.005861412
0	0	0	0	0
0	0	0	0	0
0	0	0	0	0
0	0	0	0	0
0	0	0	0	0
0	0	0	0	0
0	0	0	0	0
0	0	0	0	0
0	0	0	0	0
0.004802471	0.009457004	0.019625126	0.104605082	0.008530563
0.016094509	0	0.071580274	0	0.016058238
0	0	0	0	0
0	0	0	0	0
0	0	0	0	0
0	0	0	0	0
0	0	0	0	0
0	0	0	0	0
0.064899372	0	0.0156931	0	0.011096797
0	0	0	0	0
0	0	0	0	0
0	0	0	0	0
0	0	0	0	0
0	0	0	0	0
0	0	0	0	0
0	0	0	0	0
0.066869826	0	0.464523785	0.019194735	0.022226215
0	0	0	0	0
0	0	0	0	0
0	0	0	0	0
0	0	0	0	0
0	0	0	0	0
0	0	0	0	0
0.550715495	0.007135775	0.024680205	0.678126015	0.389927885
0	0	0	0	0
0	0	0	0	0

0	0	0	0	0
0	0	0	0	0
0.413600594	0.093320847	0	0	0.070722781
0	0	0	0	0
0	0	0	0	0
0	0	0	0	0
0	0	0	0	0
0	0	0	0	0
0	0	0	0	0
0	0	0	0	0
0	0	0	0	0.001088103
0	0	0.002038562	0.000894198	0
0	0	0	0	0
0	0	0	0	0
0	0	0	0	0
0	0	0	0	0
0	0	0	0	0
0	0	0	0	0
0	0	0	0	0
0	0.001090722	0.000804786	0	0
0	0	0	0	0
0	0	0	0	0
0	0	0	0	0
0	0	0	0	0
0	0	0	0	0
0	0	0	0	0
0	0	0	0	0
0.002373441	0	0	0	0
0	0	0	0	0
0	0	0	0	0
0.000669998	0	0.002074183	0.002956926	0.00301069
0	0	0	0	0
0.313313592	0.045478502	0.011640301	0.170080002	0.020768674
0.002981122	0	0	0	0
0	0	0	0	0
0	0	0	0	0
0	0	0	0	0
0	0	0	0	0
0	0	0	0	0
0	0	0	0	0
0	0	0	0	0
0.116480113	0	0.056302454	0	0.154790819
0.104285037	0	0.025344942	0	0.086354692
0	0	0	0	0
0	0	0	0	0
0	0.001890969	0.004941496	0	0
0	0	0	0	0
0	0	0	0	0

0	0	0	0	0
0	0.005255103	0	0	0.004534195
0.0324413	0.004270272	0.00073847	0	0
0	0	0	0	0
0.045139467	0	0	0	0.043900379
0.000926972	0.001244584	0	0	0
0	0	0	0	0
0	0	0	0	0
0	0	0	0	0
0	0	0	0	0
0	0	0	0	0
0	0	0	0	0
0	0	0	0	0
0.036891669	0	0	0	0
0	0	0	0	0
0	0	0	0	0
0	0	0	0	0
0	0	0	0	0
0	0	0	0	0
0	0	0	0	0
0	0	0	0	0
0	0	0	0	0
0	0	0	0	0
0	0	0	0	0
0	0	0	0	0
0.003361997	0.263755084	0.004897926	0	0.145951155
0	0	0	0	0
0	0	0	0	0.001217
0	0	0	0	0
0	0	0	0	0
0	0	0	0	0
0	0	0	0.029909288	0
0	0	0	0	0
0	0	0	0	0
0	0	0	0	0
0	0	0	0	0
0	0	0	0	0
0	0	0.062568575	0	0.007701201
0	1.031202864	0.137299015	0	0
0	0	0	0	0
0	0	0	0	0
0.00107272	0	0	1.564787166	0
0.002183261	0	0.012084191	0.051209474	0.000629561
0.07070639	0	0	0	0
0	0	0	0	0
0	0	0	0	0
0.005769026	0	0.02412638	0	0.001284141
0	0	0	0	0

0	0	0	0	0
0.003831289	0.026791753	0.00454669	0.034337809	0.027545938
0	0	0	0	0
0	0	0	0	0
0	0	0	0	0
0	0	0	0	0
0	0	0	0	0
0	0	0	0	0
0	0	0	0	0
0	0	0	0	0
0	0	0	0	0
0	0	0	0	0
0	0	0.020687789	0	0.028262071
0	0	0	0	0
0.030095354	0.002672422	0.009119755	0	0.005808453
0	0	0	0	0
0	0	0	0	0
0.001474033	0	0	0	0
0.016118163	0	0	0	0
0.001908848	0	0	0	0
0	0	0	0	0
0.017592865	0	0.144027297	0	0.024305529
0.003965479	0	0.006510188	0	0
0	0	0.008975643	0	0
0	0	0	0	0
0	0	0	0	0
0	0	0	0	0
0	0	0	0	0
0	0	0	0	0
0	0	0	0	0
0	0	0	0	0
0	0	0	0	0
0	0	0	0	0
0	0	0	0	0
0	0	19.27399815	0	0
0	0	0	0	0
0	0	0	0	0
0	0	0	0	0
0.003747969	0	0.03239167	0	0.018823193
0	0	0	0	0
0.022473677	0.19904439	0.03966377	0.253842337	0.036641902
0	0	0	0	0
0	0	0	0	0
0	0	0	0	0
0	0	0	0	0
0.229894153	3.966993843	0.188349446	2.892606777	0.096109326
0	0	0	0	0
0	0	0	0	0

0	0	0	0	0
0	0	0.00121256	0	0
0	0	0	0	0
0	0	0	0	0
0	0	0.001693859	0.012135623	0
0	0	0	0	0
0	0	0	0	0
0.004418632	0	0	0	0.00384743
0.008145266	0	0	0	0.012573663
0.025870348	0.001052557	0.047082971	0.002554955	0
0	0	0	0	0
0	0	0	0	0
0.002758482	0.001800376	0.000948859	0.005306664	0
0	0	0	0	0
0	0	0	0	0
0	0	0	0	0
0	0	0	0	0
0	0	0	0	0
0	0	0	0	0
0.405933652	0.289129835	0.135761585	0.040009173	0.535849824
0	0	0	0	0
0	0	0	0	0
0	0	0	0	0
0	0	0	0	0
0	0	0	0	0
0	0	0	0	0
0	0	0	0	0
0	0	0	0	0
0	0	0	0	0
0	0	0.030922227	0	0.00531899
0	0	0	0	0
0	0	0	0	0
0	0	0	0	0
0	0	0	0	0
0	0	0	0	0
0	0	0	0	0
0	0	0	0	0
0	0	0	0	0
0	0	0	0	0
0	0	0	0	0.005959061
0	0	0	0	0
0	0	0	0	0
0.033164552	0	0	0	0.017622572
0	0	0	0	0
0	0	0.0023797	0.013569864	0.012191115
0	0	0	0	0
0	0	0	0	0
0	0	0	0	0

0	0	0	0	0
0	0	0	0	0
0	0	0	0	0
0	0	0	0	0
0	0	0	0	0
0.06125884	0.004494435	0.0072542	0.044028412	0.001365171
0	0	0	0	0
0	0	0	0	0
0	0	0	0	0
0	0	0	0	0
0	0	0	0	0
0	0	0	0	0
0	0	0	0	0
0	0	0	0	0.005042345
0	0	0	0	0
0	0	0	0	0
0	0	0.008978871	0	0
0	0	0	0.00191941	0
0	0	0	0	0
0	0	0	0	0
0	0	0	0	0
0	0	0	0	0
0	0	0	0	0
0	0	0.018625763	0	0
0	0	0	0	0
0	0	0.000926268	0	0
0	0	0	0	0
0.015619204	0	0.055286509	0	0
0	0	0	0	0
0	0	0.037736926	0	0
0	0	0	0	0
0	0	0	0	0
0	0	0	0	0
0	0	0	0	0
0	0	0	0	0
0	0	0	0	0
0	0	0	0	0
0	0	0	0	0
0	0	0	0	0
0	0	0	0	0
0	0	0	0	0
0	0	0	0	0
0	0	0	0	0
0	0	0	0	0
0	0	0	0	0.088022838
0	0	0	0	0
0	0	0	0	0
0	0	0	0	0

0	0	0	0	0
0.000642475	0	0.040111062	0	0
0	0	0	0	0
0	0	0	0	0
0	0	0	0	0
0	0	0	0	0
0	0	0	0	0.000905558
0	0	0	0	0
0	0	0	0	0
0	0	0	0	0
0	0	0.003255512	0	0
0	0	0	0	0
0	0	0	0	0.522974677
0	0	0	0	0
0	0	0	0	0
0	0	0	0	0
0	0	0	0	0
0	0	0	0	0
0	0	0	0	0
0	0	0	0	0
0.003415354	0.072843663	0.010793552	0.035363829	0.02369784
0	0	0	0	0
0	0	0	0	0
0.578410519	1.885689914	1.92184796	1.134835845	3.462023117
0	0	0	0	0
0	0	0	0	0
0	0	0	0	0
0	0	0	0	0
0	0	0	0	0
0	0	0	0	0
0.006494826	0	0	0	0
0	0	0	0	0
0	0	0	0	0
0	0	0	0	0

PHL018-0016_V1	PHL018-0013_V1	PHL018-0111_V3	PHL018-0019_V1	PHL018-0054_V3
CTRL	EYCF	EYCF	CTRL	CTRL
0.108495653	1.865747242	0.626841976	0.004827902	0.059529279
0.207110349	0.101030447	0	3.18585813	3.423068236
0.016731769	0	12.10059255	0	0
0	0	0	0	0
0	0	0	0	0
0	0	0	0	0
4.603652268	0	0	0	0
0	0	0	0	0
0	0	0	0	0
0	0	0	0	0
0	0	0	0	0
0	0	0	0	0
0	0	0	0	0
0.567185289	0.045556846	18.29107201	0.533230741	13.44070461
0	0	0	0	0
0	0	0	0	0
0	0	0	0	0
0	0	0	0	0
0	0	0	0	0
0	0	0	0	0
0	0.008455999	0	0	0
0	0	0	0	0
0	0	0	0	0
0	0	0	0	0
0	0.073485816	0	0	0.10819444
0	0	0	0	0
0	0	0	0	0
0	0	0	0	0
0	0	0	0	0
0	0	0	0	0
0	0	0	0	0
0	0	0	0	0
0	0	0	0	0
0	0	0	0	0
0	0	0	0	0
0	5.208077002	0	8.919387798	2.162442061
0	0	0	0	0
0	0	0	0	0
0	0	0	0	0
0	0	0	0	0
0	0	0	0	0
0.143406513	0.000746155	0.430626496	0.007995302	0.006971535
1.375771628	0.097134414	0	0.015640859	0.007141409
0.070947279	0.030128899	0	0.02294304	0.056472025
0	0	0	0	0
0	0	0	0	0

0.160002556	0	0	0.330178144	0
0.001033575	0	0.00310125	0.026336259	0.001175757
0	0.573406895	0	0.00087103	0.008749421
0.332795448	0.279972299	0.176278195	0.774907195	1.375112093
0	0	0	0	0.02027314
0	0	0	0	0
0.39909436	0.00105375	0	0.006210209	0.774091216
0	0	0	0	0
0	0	0	0	0
0	0	0	0	0
0	0.004647323	0	0	0
2.836837775	0.241837656	0.02687075	0.70880247	0.335070357
0.002639229	0	0	0	0.002456418
0	0.015696983	0.027268375	0.053774996	0.034747451
0	0	0	0	0
0	0	0	0	0
0.77848833	0.005172813	0.001483415	0.028268504	0
0	0	0	0	0
0	0	0	0	0
0	0	0	0	0
0.20833473	0.019138705	0.03681824	0.185851657	0.069858344
0.005706815	0.003893365	0.004723679	0.000802283	0.001119286
0	0.013436503	0	0	0.00125541
0	0	0	0	0
0	0	0	0	0
0	0	0	0	0
0	0	0	0	0
0	0	0	0	0
0.006797173	0	0.004669553	0	0
0	0	0	0	0
0	0.002545161	0.015327468	0	0
0	0	0	0	0
0	0	0	0	0
0	0	0	0	0
0	0	0	0	0
0	0	0	0	0
0	0	0	0	0
0.014794725	0.000726325	0	0	0
0	0	0	0	0
0	0	0	0	0
0	0	0	0	0
0	0	0	0	0
0	0	0	0	0
0	0	0	0	0
0	0	0	0	0
0.032778567	0	0	0	0
0	0	0	0	0
0	0	0	0	0

0	0	0	0	0
0	0	0.010823332	0	0
0	0	0	0	0
0	0	0	0	0
0	1.234730085	0	0	0.00738943
0	0	0	0	0
0	0	0	0	0
0	0	0	0	0
0	0	0	0	0
0	0	0	0	0
0	0	0	0	0
0.006404065	0	0	0.001125381	0.000753623
0	0	0	0	0
0	0	0	0	0
0	0	0	0	0
0	0	0	0	0.001164987
0	0	0	0	0
0	0	0	0	0
0.593908245	1.880118698	0	0	0.017437928
0	0	0	0	0
0	0	0	0	0
0	0	0	0	0
0	0	0	0	0
0	0	0	0	0
0	0	0	0	0
0	0	0	0	0
0	0	0	0	0
0	0	0	0	0
0	0	0	0	0
0	0	0	0	0
0.05896489	0	0	0	0
0	0.070115943	0	0	0.005461462
0	0	0	0	0
0	0	0	0	0
0	0	0	0	0
0	0	0	0	0
0	0	0	0	0
0	0	0	0	0
0	0	0	0	0
0	0	0	0	0
0	0	0	0	0
0	0	0	0	0
0	0	0	0	0.030106184
0	0	0	0	0
0	0	0	0	0
0	0	0	0	0
0	0	0	0	0
0	0	0	0	0

0	0	0	0	0
0	0	0	0	0
0	0	0	0	0
0	0	0	0	0
0	0	0	0	0.050278303
0	0	0	0	0
0	0	0	0	0
0	0	0	0	0
0	0	0	0	0
0	0	0	0	0
0	0	0	0	0
0	0	0	0	0
0	0	0	0	0
0	0	0	0	0
0	0	0	0	0
0.006096931	0	0	0	0
0	0	0	0	0
0	0	0	0	0
0	0	0	0	0
0	0	0	0	0
0	0	0	0	0
0	0	0	0	0
0	0	0.001539499	0	0
0	0	0	0	0
0	0	0	0.001765474	0
0	0	0	0	0
0	0	0	0.000613324	0
0	0	0	0	0
0	0	0	0	0
0	0	0	0	0
0	0	0	0	0
0.007456806	0	0	0.006731602	0
0	0	0	0	0
0	0	0	0	0
0	0	0	0	0
0	0	0	0	0
0	0	0	0	0
0	0	0.658416037	0	0
0.000517497	0	0	0	0
0	0	0	0	0
0	0	0	0	0
0	0	0	0	0
0	0	0	0	0
0	0	0	0	0
0	0	0	0	0
0	0	0	0	0
0	0	0	0	0
0	0	0	0	0

0	0	0	0	0
0	0	0	0	0
0	0	0	0	0
0	0	0	0	0
0	0	0	0	0
0	0	0	0	0
0	0	0	0	0
0	0	0	0	0
0	0	0	0	0
0	0	0	0	0
0	0	0	0	0
0	0	0	0	0
0.006523824	0.005274571	0	0	0.000927657
0	0	0	0	0
0.025883484	0.018517637	0	0	0
0	0	0	0	0
0.00172771	0	0	0	0
0	0	0	0	0
0	0	0	0	0
0	0	0	0	0
0.09110369	0.018564106	0.014140958	0.071875634	0.146564955
0	0	0	0	0
0	0	0	0	0
0	0	0	0	0.008139289
0	0	0	0	0
0	0	0	0	0
0.002885926	0	0	0.001583842	0.004545584
0	0	0	0	0
0	0	0	0	0
0	0	0	0	0
0	0	0.001690141	0	0
0	0	0	0	0
0	0	0	0	0
0.001397747	0	0	0	0
0.031498612	0.053033467	0.00378859	0	0.010721281
0	0	0	0	0
0	0	0	0	0
0	0	0	0	0
0	0	0	0	0
0	0	0	0	0
0	0	0	0	0
0	0	0	0	0
0	0	0	0	0
0	0	0	0	0
0	0	0	0	0
0	0	0	0	0
0	0	0.009376081	0	0
0.003888416	0.004438333	0	0	0

0	0	0	0	0
0	0	0	0	0
0	0	0	0	0.014697944
0	0	0	0	0
0	0	0	0	0
0	0	0	0	0
0	0.001531615	0	0	0
0.002540758	0	0	0	0
0	0	0	0	0
0	0	0	0	0
0	0	0	0	0
0	0	0	0	0
0	0	0	0	0
0	0	0	0	0
0	0.003604917	0	0	0
0	0	0	0	0
0	0	0	0	0
0	0	0	0	0
0.009167234	0	0	0	0
0.047170536	0	0	0	0
0.003712087	0.000807061	0	0	0.002111366
0	0	0	0	0
0	0	0	0	0
0	0	0	0	0
0.013169367	0.001603396	0	0.00644285	0
0	0	0	0	0
0	0	0	0	0
0	0	0	0	0
0.049014101	0	0.015906561	0	0
0	0	0	0	0
0.601853892	0.054892394	0.007232613	0.039146466	0.092705628
0	0	0	0	0
0	0	0.064274836	0	0
0	0	0	0	0
0	0	0	0	0
0	0	0	0	0
0	0	0	0	0
0	0	0	0	0
0	0	0	0	0
0	0	0	0	0
0	0	0	0	0
0.009340469	0.006200271	0.0065051	0.003038323	0
0	0	0	0	0
0	0	0	0	0
0	0	0	0	0
0	0	0	0	0
0	0	0	0	0
0	0	0	0	0

0	0	0	0	0
0	0	0	0	0
0.064881042	0.017279925	0	0.02289069	0.045419236
0	0	0	0	0
0	0	0	0	0
0	0	0	0	0
0	0	0	0	0
0	0	0	0	0
0	0	0	0	0
0.001141148	0	0	0	0.007529141
0	0	0	0	0
0	0	0	0	0
0	0	0	0	0
0.005791579	0	0	0.000843278	0
0.008649868	0.005542834	0	0.008584194	0.002589413
0	0.001483584	0	0	0
0.003890427	0.004341949	0	0.014274855	0
0	0	0	0	0
0	0	0	0	0
0	0	0	0	0
0	0	0	0	0
0.003043686	0.004275863	0	0	0
0	0	0	0	0
0	0	0	0	0
0	0	0	0	0
0	0	0	0	0
0	0	0	0	0
0	0	0	0	0
0	0	0	0	0
0	0	0	0	0
0	0	0	0	0.002449182
0	0	0	0	0
0	0	0	0	0
0	0	0	0	0
0	0	0	0	0
0.009352816	0	0	0.002383166	0
0	0	0	0	0
0	0.031785393	0.001925734	0.000899449	0.001673126
0	0	0	0	0
0	0.001683874	0	0	0
0	0	0	0	0.001931835
0	0	0	0	0
0	0	0	0	0
0	0	0	0	0
0	0	0	0	0
0	0	0	0	0
0	0	0	0	0
0	0	0	0	0

0	0.02984965	0.392633545	0	10.254736
0	0	0	0	0.036312401
0	0.002425926	0	0	0
0	0	0	0	0
0.000986911	0.001126484	0.002422826	0	0.020488779
0	0	0	0	0
0	0	0	0	0
0	0	0	0	0.080650746
0	0	0	0	0
0	0.057777156	0.050562178	0.003269904	0.019058723
0	0	0	0	0
0.068442665	0.005624795	0.014338047	0.000837105	0
0.02616576	0.003160449	0	0.004444818	0
0	0	0	0	0
0	0	0	0	0
0	0	0	0	0
0.001360188	0	0	0	0
0	0	0	0	0
0	0.002041962	0.049773999	0	0
0	0.016367593	0	0	0
0	0	0	0	0
0	0	0	0	0
0	0	0	0	0
0	0	0	0	0
0	0	0	0	0
0	0	0	0	0
0	0	0	0	0
0	0.003116046	0.012446473	0.031600764	0
0	0	0.044854127	0	0
0	0	0	0	0
0	0	0	0	0
0	0	0	0	0
0	0	0	0	0
0	0	0.062471344	0	0
0	0	0	0	0
0	0	0	0	0.01841509
0	0	0.002991688	0	0
0	0	0	0	0
0	0	0	0	0
0	0	0	0	0
0	0	0.005938671	0	0
0	0	0	0	0
0	0	0	0	0
0	0	0	0	0
0	0	0	0	0
0	0	0	0	0
0.003308718	0	0	0	0
0	0	0	0	0

0	0	0	0	0
0.021850186	0	0	0	0
0	0	0	0	0
0	0	0	0	0
0	0	0	0	0
0	0.011177177	0	0.003621993	0
0	0	0	0	0
0	0	0	0	0
0	0	0	0	0
0	0	0	0	0
0	0	0.005696326	0	0.010627562
0	0.002155625	0	0	0
0	0	0	0	0
0.1118854	0.002765542	0.001982696	0	0.00324836
0	0	0	0	0
0	0	0.09936277	0	0
0	0	0	0	0
0.002021022	0	0	0	0
0	0	0	0	0
0	0	0	0	0.006020563
0	0	0	0	0
0.203102124	0.131769951	0	0.274315029	0
0	0.02399421	0	0	0
0	0	0	0	0
0	0	0	0	0
0	0	0	0	0
0	0.001070921	0.001842657	0	0
0	0	0	0	0
0	0	0	0	0
2.110698656	0.105396321	1.326480268	0.614708516	0.449304527
0.001232802	0.000536057	0	0.011308567	0.003205461
0.002225333	0	0	0	0
0	0	0	0	0
0	0	0	0	0
0	0	0.002046347	0	0
0	0	0	0	0
0.345754366	0	0	0.059537108	0
0.010319258	0	0.001732196	0.00188779	0
0.152638371	0	0.928226162	0	0.521852575
0	0	0	0	0
0.03414644	0	0.007917102	0	0.246657619
0	0	0	3.092920683	2.670491127
0.00557491	0	0.002115738	0	0
0	0	0	0	0
0	0	0.001050695	0	0
0	0	0	0	0
0	0.000511346	0	0	0.001019232
0	0	0	0	0

0	0	0	0.017464545	0
0	0	0	0	0
1.753722433	0.009742929	0.003697933	0.003454371	0.017135232
0	0	0	0	0
0.019447464	0.035375566	0	0	0
0	0	0	0	0
0	0	0	0	0
0.052039127	0	0	0.002209992	0
0	0	0	0	0
0	0	0	0	0
0	0	0	0	0.014864768
0	0	0	0	0
0	0	0	0	0
0	0	0.011184894	0	0
0	0	0	0	0
0.27667429	0.008920985	0.028567498	0.003385528	0.048232598
0.019274823	0.01071198	0.00590748	0.045471524	0.008622711
0	0	0	0	0
0	0	0	0	0
0	0	0	0	0
0	0.004361585	0.011614365	0.001390948	0.006830729
0.006280279	0	0	0.001536251	0
0	0	0	0	0
0	0.049845575	0.274408555	0	0.666831155
0	0	0	0	0
0.018829416	0.003256418	0	0	0.008438041
0.003849142	0	0	0	0.019703881
0	0	0	0	0
0	0	0	0	0
0	0	0	0	0
0.013172676	0.011773923	0.095133041	0	0.048997741
0.055175798	0.001660464	0	0.015568377	0.01702127
1.450495277	0.003613682	0	0.022688571	0
0.085495997	0	0.0027846	0	0.002903196
0	0.03049461	0.006956236	0	0
0	0	0	0	0
0.019588374	0	0	0.021606007	0
0.010072778	0.005429289	0.096165474	0.011977549	0.022678117
0	0.092883495	0	0.395950339	0
0	0	0	0	0.002146931
0	0	0	0	0
0	0	0	0	0
0	0.020298732	0.003081757	0	0.002856007
0.017528037	0	0.051833361	0	0.007826619
0	0	0.005946057	0	0
0	0.002262905	0.001247955	0	0
0	0	0	0	0
0	0	0	0	0

0	0	0	0	0
0.025163303	0.073980947	0.056470991	0.002040116	0.760509858
0	0	0	0	0
0	0	0	0	0
0	0	0	0	0
0	0	0	0	0
0	0	0.002020448	0	0
0	0	0	0	0
0.001832324	0	0.033415811	0	0
0	0	0	0	0
0	0	0.069321945	0.00346521	0.001450322
0.353540879	0.083814992	0	0.837135429	0.011881623
0	0	0	0	0
0	0	0	0	0
0.011215041	0	0	0.004721376	0
0	0	0	0	0
0.038938775	0.007619259	0.002427762	0.001814288	0.008099712
0.002021916	0	0.028048976	0.004121593	0.011828863
0	0	0	0	0
0.001125366	0.005708979	0.058938195	0	0.007040959
0.002294611	0.325819294	0	0.741612933	0.02871291
0	0	0	0.001164072	0
0	0	0	0	0
0	0	0	0	0
0.005397525	0	0	0	0
0	0	0.002295904	0	0
0.102146817	0.009134403	0	0	0.029371662
0.021705616	0.033776099	0	0.042008841	0.13816143
0	0	0	0	0
0.001176722	0.002462422	0	0	0
0	0	0	0	0.000893056
0	0	0	0	0
0	0	0	0	0
0	0	0	0	0
0	0	0.045217992	0.045272344	0
0.129606004	0.05433724	0.664727574	0.329824894	0.678952105
0	0	0	0	0
0	0	0	0	0
0.029784879	0.025640983	0.003282634	0.001226571	0.007529371
0	0	0	0	0
0	0	0.004943688	0	0
0	0	0	0	0
0.014619628	0.00125154	0.00717812	0	0.03617187
0.001058623	0.005236134	0	0.002517619	0.020672964
0	0.002469408	0.034143224	0	0
0	0.175200964	0	0.000929007	0
0	0	0	0	0.300591328
0.000855591	0	0	0	0

0	0	0.001114221	0	0
0	0	0	0	0
0	0	0	0	0
0.034693003	4.182965245	2.242401694	0	0.035591558
0	0	0	0	0
0	0	0	0	0
0	0	0	0	0
0	0	0	0	0.026208819
3.08291329	2.57383775	0	0.352568722	0.62829634
0	0	0	0.129709068	0
0	0	0	0	0
0	0.014121015	0	0.006235362	0.031084845
0	0	0	0	0
0	0	0	0	0
0.020529999	0.12779552	0.977343924	0.003258777	0.024694155
0	0	0	0	0
0.168140668	0.034742682	0	0	0.315122003
11.77182615	0	0	0.031580078	0
0	0	0	0	0
0	0.001247187	0	0	0
0	0	0	0	0
0	0	0	0.000771877	0
0	0	0	0	0
0.1439408	0.147152414	0.093029043	0	0.331908845
0.001718326	0	0	0	0
0	0	0	0	0
0	0	0	0	0
3.461789751	0	0	1.17526464	3.966933362
0	0	0	0	0
0	0	0	0	0
0.00198249	0.214821114	0	0	0
0	0	0	0.088145526	0
0.002866759	0	0	0	0.015104125
0	0	0	0	0
0	0	0	0	0.001457543
0	0	0.346452238	0.001541928	0
0.035820342	0.512110263	0	3.723489925	0.169165474
0	0	0	0	0
0.019530219	0	0.004864077	0	0.023665776
0	0	0.047937171	0	0
0.00762952	0.046147724	0.083885521	0	0.008679058
0	0	0	0	0
2.545815706	0.116791446	0.675994365	0.775225789	0.278977353
0.029823181	0	0	0	0
0	0	0	0	0
0	0	0	0	0
0	0	0	0	0
0	0.014852946	0.003592265	0	0

0	0	0	0	0
0.024638157	0	0	0.040397455	0
0.007664798	0.002916263	0.111424793	0.001608391	0.216184583
0	0.030039954	0	0	0
0	0	0	0	0
0.147043839	0.148244682	0	0.001810041	0.01212113
0	0	0	0	0
0	0	0	0	0
0	0	0	0	0
0	0	0	0	0
0.112218067	0.102066554	0.108191889	0	0.065464152
0.3665066	0.815819062	0.291304076	0.909083271	0.299191181
0	0	0	0	0
0.001433258	0.001999502	0.003648909	0	0.003260842
0.063249893	0.003437857	0.203132154	0.00383727	0.371102991
0	0.063133704	0	0	0
0	0	0	0	0
0	0	0	0	0
0.003168834	0.036019148	0	0	0
0.005469713	0	0.020688952	0	0.002696262
0.053427843	0.696373557	0.013966453	0.020042547	0.192884231
0.02169756	0	9.519745051	0	0
0.038899251	1.12508098	4.389187923	0.044290628	2.248794286
0	0	0	0	0
0	0	0	0	0
0	5.669722976	0	13.52065541	0.004046095
0	0.854675767	0	0	0
0	0	0	0	0
0	0	0	0	0.008505068
0	0	0	0	0
0	0	0	0	0
0.185296869	0.081695642	0.102234637	0.105318902	0.28749091
0	0.011589564	0	0	0
0	0	0.022696732	0	0.06566589
0	0.0028542	0.001309605	0	0.005461523
0.830937954	0.155134393	0.150242958	0.173333155	0.037753485
0	0.005909454	0.060834043	0	0
0	0	0	0	0
0	0.840143607	0	0.411324378	0
0	0	0	0	0
0	0	0	0.000925022	0
0	0	0	0	0
0	0	0	0	0
0	0	0	0	0
0.030324774	0.022538988	0.009746604	0.00551071	0.043588391
0	0	0	0	0
0	0	0	0	0
0.467316131	0.011652076	0.027536291	2.106076928	0.742242054

0	0	0	0	0
0	0	0	0	0
0	0	0	0	0
0.013824434	0.00792789	0	0	0.010256192
0	0	0	0	0
0	0	0	0	0
0	0	0	0	0
0	0	0.120090249	0	0.000838894
0	0	0	0	0
0	0	0	0	0
0.007140526	0.004414785	0.007060575	0	0.000761512
0	0	0	0	0
0	0	0.247070255	0.01174895	0
0	0	0	0.91641978	9.261020812
0	0	0	0	0
0	0	0	0	0
0	0	0	0.023680964	0
0.003715203	0.006784999	0	0.007005291	0.005705474
0	0.022311309	0	0	0
0	0	0.002010638	0	0.001905701
0	0	0.009363467	0	0.010137728
0	0	0	0	0
0.014991772	0.002444568	0	0.027940668	1.396972907
0.007312496	0.17329262	0	0	0
0	0	0	0	0
0	0	0	0	0
0	0	0	0	0
0	0	0	0	0
0	0	0	0	0
0.281393349	0.310090269	2.424382149	0	0.02782391
0	0	0	0	0
0	0	0	0	0
0	0	0	0	0
0	0	0	0	0
0	0	0	0.000723372	0.007669887
0	0	0	0	0
0	0	0	0.178666076	0
0	0	0.0026793	0.000770102	0.001289267
0	0	0	0	0
0	0	0	0	0
0	0	0	0	0.207051986
0	0	0	0	0
0	0	0	0	0
0.010639381	0	0	0.037019067	0
0	0.053370726	0	0.059024866	0
0	0	0	0	0
0	0.004780137	0	0.007317255	0.002245869
0.175391179	0	0	0	0

0	0	0	0	0
0	0	0	0	0
0	0	0	0	0
1.83797229	0.653263601	0	0.101306863	0.186809134
0	0	0	0	0
0	0	0	1.852980102	0
0	0	0	0	0.13035586
0	0	0	0	0
0.015630911	0.002378868	0.171969042	0.006638118	0.028449828
0	0	0	0.003421214	0
0	0	0	0	0
0	0	0	0	0
0	0	0	0	0
0	0	0	0	0
1.38331747	1.716212683	1.864344258	0.157420773	0.016185464
0	0	0.174384947	0	0
0	0	0	0	0
0	0	0	0	0
0	0	0	0	0
0	0	0	0	0
0	0	0	0	0
0	0	0	0	0
0	0	0	0	0
0	0	0	0	0
0.068930647	0.036600371	0	0	0
9.211880844	3.674739534	0	11.28612915	0.032190806
0	0.121911576	0	0	0
0	0	0	0	0.006489941
0.141154249	0	0	0.064426901	0
0	0	0	0	0
0	0	0	0	0
0	0	0	0	0
0	0	0	0	0
0	0.003859588	0.008390407	0	0.001488979
0.00134793	0.002256555	0	0.00137385	0.003066709
0	0.009280645	0.004574322	0.007963389	0.001300675
0	0	0	0	0
0.195814126	0.518241685	0.299816402	5.103752064	0.632759315
0	5.237103061	0	0	0
0	0	0	0	0
0	0	0	0	0
0	0	0.002357872	0	0.054819942
0.530454976	1.887463165	0.444665776	0	0.59809365
0	0	0	0	0
0	0	0	0	0
0.093878734	0.00123641	0	0	0.001026855
0	0	0	0	0.042813636
0.001725693	0.01335052	0.012552614	0.120189931	0.004253349

0.013511939	0	0.003042162	0	0.015370678
0	0	0	0	0
0	0	0	0	0
0	0	0	0	0
0.002775954	0.043222169	0	0	0
0	0	0	0	0
0	0	0	0	0
0	0.001691444	0	0	0
0	0	0	0	0
0.00514385	0	0	0	0
0	0	0	0	0
2.035399815	0.719702549	0.170666271	0.650150758	0.936287321
0.010859241	0.063942489	0	1.183754985	0
0	0	0	0	0
1.534072861	0.027624083	0.326572058	0.039265491	0.046633499
0	0	0	0	0
0	0.001437972	0	0	0
0	0	0	0	0
0	0	0.001180855	0	0
0	0.008218683	0	0	0
0	0.112364763	0.133154517	0.34100688	0
0.002575957	0	0	0	0
0.767379654	1.527590454	0.088413017	0.066500851	0.05171574
0	0	0	0	0
0	0	0	0	0
0	0	0	0	0
0	0	0	0	0
0	0	0	0	0
0	0	0	0.001813758	0
0	0	0	0	0
0	0	0	0	0
0.053836646	0.074130733	0.011888019	0.015351063	0.077282284
0	0	0	0	0
0.006243682	0.083692954	0	0.000979037	0.052267705
0	0.004892027	0.004299189	0.005072876	0.002123191
0.631993124	0.07750788	0.002625241	0.024523321	0.003345284
0	0	0	0	0
0	0	0	0.000808474	0
0	0.002900757	0	0	0
0	0	0	0	0
0.120679437	0	0	0	1.438128134
0	0	0	0.000814431	0
0	0	0	0	0
0	0	0	0	0
0.021654961	0.138977678	0.018055038	0.001041102	0.033232495
0	0	0	0	0
0.319881231	0	0	0.096881178	0
0	0.000735168	0	0	0.007473341

0	0	0	0	0
0	0	0	0	0
0	0.00385873	0	0	0
0	0	0	0	0
0	0.001467491	0.001168985	0	0
0	0	0	0	0
0	0	0	0.037217649	0
0.003192685	0	0.017853027	0.001220279	0.000680978
0	0	0	0	0
0	0	0	0	0
0	0	0	0	0
0	0	0	0	0
0	0	0	0	0
0	0	0	0.215033917	0
0	0	0	0	0
0	0	0	0	0
0	0	0	0	0
0	0	0	0	0
1.34400505	2.444014758	0	0	0.126615627
0	0	0	0	0
0	0	0	0	0.005022826
0	0	0	0	0
0	0	0	0	0
0	0	0	0	0
0	0	0	0	0
0.011411292	0	0.041087587	0.015507631	0.00216351
0	0	0	0	0
0.000744626	0.543107922	0.091198193	0	0.001270588
0	0	0	0.039760651	0
0.939043969	0.511797516	0	2.093967723	0.471042307
0	0	0	0	0
0	0	0	0	0
0.04009928	0	0	0	0
0	0	0	0	0
0.005181942	0.002426583	0.000869843	0.000812552	0.00272067
0	0	0	0	0
0	0	0	0.002202635	0
0	0.027878796	0.001480526	0	0.0028299
0	0	0	0	0
0	0	0	0	0
1.319321654	1.692347382	31.48643771	4.820709843	14.30302985
0.020196523	1.163977922	0.097176423	0	2.473747015
0	0	0	0.062184601	0.229653058
0	0	0	0	0
0	0	0	0	0
0.038747943	0	0	0	0
0	0	0	0	0
0.003410268	0.055451969	0.002741731	0	0

0	0	0	0	0
0.004430832	0.00084291	0	0	0.013020904
0.001293741	0	0	0	0
0	0	0	0	0
2.421460724	2.042685288	1.094724167	0	3.72772933
0	0	0	0	0
0	0	0	0	0
0	0	0	0	0
0.007005142	0.001262501	0.091719152	0.142233238	0.029568399
0	0	0	0	0
0	0	0	0	0
0	0	0	0	0
0	0.069481128	0	0.053754188	0.064892659
0.089759432	0.007551865	0.059916563	0.018207176	0.030105255
0	0	0	0.114168634	0
0.066236055	0.161397567	0.033172982	0.089459611	0.321977476
0.002671794	0.000726108	0	0	0.016499238
0	0	0	0	0
0	0	0	0	0
0	0	0	0	0
0	0	0	0	0
0	0	0	0	0
0	0	0	0	0
0	0	0	0	0
0	0	0	0	0
0	0	0	0	0
0.002157289	0.083342203	0	0.008118544	0
0.011984048	0.007836863	0.002247388	0	0.000852038
0	0	0	0	0
0	0	0	0	0
0	0	0	0	0
0	0	0	0	0.00320271
0	0	0	0	0
0	0.018521371	0	0	0.092665677
0	0	0	0.241117326	0
0	0	0	0.025830734	0
0	0.000673902	0	0	0
0	0.007470998	0	0.003544069	0
0	0	0	0	0
0	0	0	0	0
0	0.53900292	0	1.241375139	2.110691963
0	0	0	0	0
0	0	0	0.002112035	0
0	0	0	0	0
0	0	0	0	0
0	0	0	0	0
0	0	0	0	0
0	0	0	0	0

0	0.231208998	0	0.008149614	0.079529871
0	0	0	0	0
0	0.004064187	0	0	0
0	0	0	0	0.002654278
0	0	0	0.016548678	0
0	0	0	0	0
0.000951892	0.053601302	0.001246323	0	0.083161863
0	0	0	0	0
0	0	0	0	0
0	0.004255734	0	0	0.00204493
0	0	0	0	0
0	0	0	0	0.024620991
0	0	0	0	0
0	0	0	0	0
0	0	0	0	0
0	0	0	0	0
0	0	0	0	0
0	0	0	0	0
0	0	0	0	0
0	0	0	0	0
0.001993759	0.039859682	0.409959687	0.006465765	0
0	0	0	0	0
0.070771335	0.021602224	0	0	0.014327488
0	0.001758563	0.000581891	0.001630696	0.013549049
0	0	0	0	0
0	0	0	0	0
0	0	0	0.014435076	0
0	0	0	0	0
0	0	0	0	0
2.830755354	0.035239095	0	0.826910122	0.09284451
0	0	0	0	0
0	0	0	0	0
0	0	0	0	0
0	0	1.469047935	0	0
0	0	0	0	0
0	0	0	0	0
0	0.020650077	0	0	0
0	0	0	0	0
0	0	0	0	0
0	0	0	0	0
0	0	0	0	0
0.026558783	0	0	0	0
0	0	0	0	0
0	0	0	0	0
0	0	0	0	0
0	0	0	0.576502307	0
0	0	0	0	0
0	0	0	0	0

0	0.015574891	0.008139762	0	0.004569618
0	0	0	0	0
0	0	0	0	0
0	0	0	0	0
0	0	0	0	0
0	0	0	0.001476055	0
0	0.000783296	0	0	0
0.131200819	0.360639595	0	0.737282113	1.329166618
1.682320451	0.141427789	0	0.020197794	0.004440244
0	0.0497856	0.031245382	0.318718223	0.065390021
0	0	0	0	0
0	0	0	0	0
0	0	0	0	0
0.098693624	0	0	0	0
0	0	0	0	0
0	0	0	0	0
0.010052186	0.023585061	0.013405177	0	0
0	0.145044704	0	0	0
0	0	0	0	0
0.015946417	0	0	0	0.007886979
0	0	0	0	0
0	0	0	0	0
0	0	0	0	0
0	0	0	0	0
0.001958644	0	0	0.077548876	0.011140405
0	0	0	0	0
0	0	0	0	0
0	0.235826527	0	0	0
0	0	0	0	0
0	0	0	0	0
0	0	0	0	0
0	0	0	0	0
0	0	0	0	0
0	0	0	0	0
0	0	0.011370378	0	0
0	0	0	0	0
0	0	0	0	0
0	0	0	0	0.032058837
0	0.005884784	0	0	0
0	0	0.006875873	0.095566441	0
0	0	0	0	0
0.873951857	0.458147503	0	0.284937929	0.484088869
0	0	0	0	0
0	0	0	0	0
0	0	0	0	0
0	0	0	0	0
0	0	0	0	0
0	0	0	0	0.003444836

0	0	0	0	0
0	0.001182366	0	0	0.022781738
0	0	0	0	0
0	0	0	0	0
2.526300781	2.797069102	0.177774703	0.044284192	0.127133701
0	0	0	0	0
0	0	0	0	0.027385916
0.01532984	0.004629063	0.022301712	0.002976118	0.020206695
0.012147839	0	0	0	0.007462222
0	0	0	0	0
0	0	0	0	0
0	0	0	0.002223328	0
0	0	0	0	0
0	0	0	0	0
0	0	0.001495202	0	0
0.144983775	0	0.016809213	0	0.107551396
0	0	0	0	0
0	0	0	0	0
0.004593566	0	0	0	0
0	0	0	0	0
0	0	0	0	0
0	0	0	0	0
0	0	0	0	0
0.002948159	0.000961457	0.007812024	0	0.001437307
0	0.247361151	0.327900622	0.087842713	0
0	0	0	0	0
0.003274231	1.202234329	0	0	0
0	0	0	0	0
0	0	0	0	0
0	0	0	0	0
0	0	0	0	0
0	0	0	0	0.000842342
0	0	0	0	0
0	0	0	0	0
0	0	0	0	0
0	0	0	0	0
0	0	0	0	0
0	0.012024496	0	0.020333595	0.122684278
0	0.169130372	0	0	0
0	0	0	0	0
0	0	0	0	0
0	0	0	0.057942058	0
0	0	0	0	0
0	0	0	0	0
0	0	0	0	0
0	0	0	0	0
0	0	0	0	0
0	0	0	0	0

0	0.113569739	0	1.432006865	0
0	0	0	0	0
0	0	0	0	0
0	0	0	0	0
0	0	0	0	0
0	0	0	0	0
0	0	0	0	0
0	0	0	0	0
0	0	0	0	0
0	0	0	0	0
0	0	0	0	0
0	0	0	0	0
0	0	0	0	0
0	0	0	0	0
0	0	0	0	0
0	0	0	0	0
0	0	0	0	0
0	0	0	0	0
0	0	0	0	0
0	0	0	0	0
0	0	0.01670276	0.080578053	0.00811069
0	0	0	0	0
0.009269363	0.349653043	0.068195497	0	0
0.117318887	1.305905739	0	0	0
0	0	0	0	0
0.120164378	0	0	0	0
0	0	0	0	0
0	0	0	0	0
0	0	0	0	0
0	0	0	0	0
0	0	0	0	0
0	0	0	0.433179931	0
0.237603705	0	0.245717118	0.034884996	0
0	0	0	0	0
0.051691801	0.310964897	0	1.092608464	0
0.000597078	0.022565883	0	0	0.001358428
0	0	0	0	0
0	0	0	0.002828006	0
0.012200875	0.137336873	0.001004701	0.100657069	0.001571237
0	0	0	0	0
0	0	0	0	0
0	0.086722845	0	0	0
0.52920559	0.281028925	0	0.330872074	0.062772604
0	0	0	0	0
0	0	0	0	0
0.022873891	0.019195272	0	0.011112156	0.036477318
0	0	0	0	0
0	0	0	0	0

0	0	0	0	0
0	0	0	0	0
0	0	0	0	0
0	0	0	0	0
0	0	0	0	0
0	0	0	0	0
0	3.946242644	0	0	0
0	0	0.023666114	0	0.047703166
0.036652531	0	0	0	0.022100393
0.000880742	0.016755016	0	0.049372306	0.011020896
0	0	0	0	0
0	0	0	0	0
0	0	0	0	0
0	0	0	0	0
0	0	0	0	0.001821761
0	0	0.036637743	0	0
0	0	0	0	0
0	0	0	0	0
0	0	0	0	0
0.567889496	0.209373807	0	6.688939013	0
0	0	0	0.040818707	0.004118219
0	0	0	0	0
0	0	0	0	0
0	0	0	0	0
0	0	0	0	0
0	0	0	0	0
0	0	0	0	0
0	0	0	0	0
0	0	0	0	0
0.010103801	0.025799141	0	0	0
0.439137256	0.795743614	0	0.062632703	0.578664381
0	0	0	0	0
0	0	0	0	0
0	0	0	0	0
0	0	0	0	0
0	0	0	0	0
0	0	0	0	0
0	0	0	0	0
0	0	0	0.001618098	0
0	0	0	0	0
0	0	0	0	0
0	0	0	0.031842087	0
0	0	0	0	0
0	0	0	0	0
0	0	0	0	0
0	0	0	0	0
0	0.025144733	0.010888778	0	0
0	0	0	0	0

0	0	0	0	0
0	0	0	0	0
0	0	0	0	0
0	0	0	0	0
0.152922178	0.299929524	0.066704787	0.001688654	0.010554367
0	0	0	0	0
0	0	0	0	0
0	0	0	0	0
0	0	0	0	0
0	0	0	0	0
0	0	0	0	0.064130379
0.003910372	0.07036411	0.006525358	0	0.005233287
0	0.026577969	0	0	0
0	0	0	0.026826385	0
0	0	0	0.038591313	0.005583382
0	0	0	0	0.040696221
0	0	0	0	0
0	0	0	0	0
0.057303207	0.000662688	0.013682876	0	0.021398442
0	0.01124849	0.00521082	0	0.00336313
0	0	0	0	0
0	0	0	0	0
0	0	0	0	0
0	0	0	0	0
0	0	0	0	0
0	0	0	0	0
0	0.009429958	0.00298262	0	0.00352427
0	0	0	0	0
0	0	0	0	0
0	0	0	0	0
0	0	0.000901588	0	0
0	0.00424476	0	0	0
0	0	0	0	0
0	0	0	0	0
0	0	0	0	0
0	0	0	0	0
0	0	0	0	0
0.012336604	0.064608339	0.00190029	0	0.035992215
0	0.086876455	0	0	0.058582207
0	0	0	0	0
0	0.025028185	0	0	0
0	0	0	0	0
0	0	0	0	0
0.001936971	0	0	0	0.000979301
0	0	0	0	0
0	0.002928244	0	0	0
0	0	0	0	0
0	0	0	0	0

0	0	0	0	0
0.000919746	0.374435977	0	0	0.006696119
0	0	0	0	0
0.000902202	0.008581632	0	0	0
0	0	0	0	0
0	0	0	0.001375886	0
0	0	0	0.008031189	0
0.009525603	0	0	0	0
0	0	0	0	0
0	0	0	0.00185954	0
0	0	0	0	0
0	0	0	0	0
0.064592968	0.278244149	0	0.80224239	0.024007845
0	0	0	0	0
0	0	0	0	0
0	0	0	0	0
0	0	0	0	0
0	0	0	0	0
0	0	0	0	0
0	0	0	0	0
0	0	0	0	0
0	0	0	0	0
0	0.047580183	0.025956968	0.004506142	0.006705745
0.004471784	0	0.009758267	0.110146189	0.007347795
0.005702493	0	0	0	0
0	0	0	0	0
0.034194316	0	0	0	0
0	0	0.000941024	0	0
0.301156024	0	0	0	0
0	0	0	0	0
0.022300284	0.036439546	0	0.051439549	0.031509693
0	0	0	0	0
0	0	0	0	0
0	0	0	0	0
0	0	0	0	0
0	0	0	0	0.001954031
0	0.000569329	0	0	0
0	0	0	0	0
0.011320552	0.003173716	0	0.003643654	0
0	0	0	0	0
0	0	0	0	0
0	0	0	0	0
0	0	0.010940479	0	0
0	0	0	0	0
0	0	0	0	0
0.721329313	0.555543832	0.051096429	0.230284291	0.350048823
0	0	0	0	0
0	0	0	0	0

0	0	0	0	0
0	0	0	0	0
0	0	0	0.130982208	0
0	0	0	0	0
0	0	0	0	0
0	0	0	0	0
0	0	0	0	0
0	0	0	0	0
0	0.003076366	0	0	0
0.00181329	0	0	0	0
0	0.001716489	0	0	0
0	0.003294931	0	0	0
0	0	0	0	0
0	0	0	0	0
0	0	0	0	0
0	0	0	0	0
0	0	0	0	0
0	0	0	0	0
0	0	0	0	0
0.001923089	0	0	0	0.102089598
0	0	0	0	0
0	0	0	0	0
0	0	0	0	0
0	0	0	0	0
0	0	0	0	0
0	0	0	0	0
0	0	0	0	0
0	0.034548709	0	0.109180403	0
0	0	0	0	0
0	0	0	0	0
0.002753554	0.001815941	0	0.004116204	0.015035234
0	0	0	0	0
0.108703318	0.010055885	0.007209351	0.007820725	0.004121895
0.003920577	0.007789889	0	0	0
0	0	0	0	0
0	0	0	0	0
0	0	0	0	0
0	0	0	0	0
0	0	0	0	0
0	0	0	0	0
0	0	0	0	0
0	0.020548781	0.011862136	0.000893617	0
0.086732763	0.051531456	0.007924976	0.053998379	0.041312497
0	0	0	0	0
0	0	0	0	0
0.033031604	0.067889051	0.007747036	0.01132714	0.004565251
0	0.002464221	0	0	0
0	0	0	0	0

0	0	0	0	0
0	0.005960603	0	0.007755628	0
0.024835441	0.002983977	0	0.002664527	0.005947763
0	0	0	0	0
0.412670739	0	0.066958575	0	0.288976787
0.044496904	0.00108228	0	0	0.253321242
0	0	0	0	0
0	0	0	0	0
0	0	0	0	0
0	0	0	0	0
0	0	0	0	0
0	0	0	0	0
0	0	0	0	0
0	0	0	0	0
0	0	0	0	0
0	0	0	0	0
0	0	0	0	0
0	0	0	0	0
0	0	0	0	0
0	0	0	0	0
0	0	0	0	0
0	0	0	0	0
0	0.007872853	0	0	0
0	0	0	0	0
0	0.003115008	0	0.00241554	0
0.147165875	0.000824638	0	0.462579004	0.001725881
0	0	0	0	0
0.004415135	0.002239797	0	0	0
0	0	0	0	0
0	0	0	0	0
0	0	0	0	0
0	0	0	0.001356056	0
0	0	0	0	0
0	0	0	0	0
0	0	0	0	0
0	0	0	0	0
0	0	0	0	0
0	0.003940118	0.001883188	0	0
0	0.331377503	0	0.052548315	0
0	0	0	0	0
0	0	0	0	0
0	0.009303893	0	0.006710197	0
0	0.012910782	0.004984058	0	0.004453996
0	0	0	0	0
0	0	0	0	0
0	0	0	0	0
0.009982954	0.005570786	0	0.004409132	0.004921036
0	0	0	0	0

0	0	0	0	0
0.053745715	0.048566127	0.004352305	0.00663342	0.018389503
0	0	0	0.003298376	0
0	0	0	0	0
0	0	0	0	0
0	0	0	0	0
0	0	0	0	0
0	0	0	0	0
0	0	0	0	0
0	0	0	0	0
0	0	0	0	0
0	0	0	0	0
4.133661185	0.028540264	0.008959399	0	0
0	0	0	0	0
0	0.009162873	0.002856141	0	0.005062233
0	0	0	0	0
0	0	0	0	0
0	0	0	0	0
0	0.000661077	0.021169534	0	0
0	0	0	0	0
0	0	0	0	0
0	0.003521217	0.003646443	0	0.002047093
0	0	0	0.006644276	0
0	0	0	0	0
0	0	0	0	0
0	0	0	0	0
0	0	0	0	0
0	0	0	0	0
0	0	0	0	0
0	0	0	0	0
0	0	0	0	0
0	0	0	0	0
0	0	0	0	0
0	0	0	0	0
5.292153778	21.80827157	0	2.977086914	0
0	0	0	0	0
0	0	0	0	0
0	0	0	0	0
0.000770169	0.228563456	0	0.002616597	0.054611224
0	0	0	0	0
0.113942171	0.056401566	0.195147132	0.148509726	0.303223757
0	0	0	0	0
0	0	0	0	0
0	0	0	0	0
0	0	0	0	0
0.001581285	0.052543168	0.081090492	1.050554713	0.00479683
0	0	0	0	0
0	0	0	0	0

0	0.009466872	0	0	0
0	0	0	0	0
0	0	0	0	0
0	0	0	0	0
0	0	0	0	0
0	0	0	0	0
0	0	0	0	0
0	0	0	0	0
0	0	0	0.005459054	0
0.024743991	0.001765213	0.012936542	0.003940594	0.004984514
0	0.010549457	0	0	0.11163143
0	0	0	0	0
0	0	0.001099511	0	0
0	0	0	0	0
0	0	0	0	0
0	0	0	0	0
0	0	0	0	0
0	0	0	0	0
0	0	0	0	0
0.418421466	0.737809095	0.152178944	0.221461374	0.35226652
0	0	0	0	0
0	0	0	0	0
0	0	0	0	0
0	0	0	0	0
0	0	0	0	0
0	0	0	0	0
0	0	0	0	0
0	0	0	0	0
0.00165834	0	0	0	0
0	0	0.013600404	0	0.00299954
0	0	0	0	0
0.001465459	0	0	0	0
0	0	0	0	0
0	0	0	0	0
0	0	0	0	0
0	0	0	0.006508655	0
0	0	0	0	0
0	0	0	0	0
0	0	0	0	0
0	0	0	0	0
0	0	0	0	0
0	0	0	0	0
0	0.003262213	0	0	0
0	0	0	0	0
0.004422794	0.025481825	0.006664035	0	0.003114556
0	0	0	0	0
0	0	0	0	0
0	0	0	0	0

0	0	0	0	0
0	0	0	0	0
0	0	0	0	0
0	0	0	0	0
0	0	0	0	0
0.028615492	0.012682115	0.011836976	0.018108404	0
0	0	0	0	0
0	0	0	0	0
0	0	0	0	0
0	0	0	0	0
0	0	0	0	0
0	0	0	0	0
0	0	0	0	0
0	0	0	0	0
0	0	0	0	0
0	0	0	0	0
0	0	0	0	0
0	0	0	0	0
0	0	0	0	0
0	0	0	0	0
0	0	0	0	0
0	0	0	0	0
0	0	0	0	0
0	0	0	0	0
0	0	0	0	0
0.004918619	0	0	0	0
0	0	0	0	0
0.523473216	0	0	0	0
0	0	0	0	0
0	0	0	0	0
0	0	0	0	0
0	0	0	0	0
0	0	0	0	0
0	0	0	0	0
0	0	0	0	0
0	0	0	0	0
0	0	0	0	0
0	0	0	0	0
2.687266271	0.262111459	0	0	0.535746129
0	0	0	0	0
0	0	0	0	0
0	0	0	0	0
0	0	0	0	0
0	0	0	0	0
0	0	0	0	0
0	0	0	0	0
0	0	0	0	0
0	0	0	0	0

0	0	0	0	0
0	0.030406543	0.003265096	0.039829977	0
0	0	0	0	0
0	0	0	0	0
0	0	0	0	0
0	0	0.00526755	0	0
0	0	0	0	0
0	0	0	0	0
0	0	0	0	0
0	0	0	0	0
0	0	0	0	0.002359839
0	0	0	0	0
0.159416241	0	0	0	1.001555127
0	0	0	0	0
0	0	0	0	0
0	0	0	0	0
0	0	0	0	0
0	0	0	0	0
0	0	0	0	0
0	0	0.002929793	0	0
0	0.00373835	0	0.009299106	0
0	0	0	0	0
0	0	0	0	0
2.919535121	1.32220655	0	2.85321179	3.268465742
0	0	0	0	0
0	0	0	0	0
0	0	0	0	0
0	0	0	0	0
0	0	0	0	0
0	0	0	0	0
0	0	0	0	0
0	0	0	0	0
0	0	0	0	0.002799345
0	0	0	0	0

PHL018-0068_V2	PHL018-0274_V2	PHL018-0186_V2	PHL018-0242_V3	PHL018-0026_V3
REF	REF	REF	REF	EYCF
0.064461069	0.326506078	0.437960208	0.001218356	0.130330436
2.503328993	13.76688767	0	1.362809562	1.262472822
23.49127568	0.860238762	0	0	0
0	0	0	0	0
0	0	0	0	0
0	0	0	0	0
0	0	0	0	0
0	0	0	0	0
0	0	0	0	0
0	0	0	0	0
0	0	0	0	0
0	0	0	0	0
0	0	0	0	0
21.03401696	1.913462613	0	0.709844673	0
0	0	0	0	0
0	0	0	0	0
0	0	0.011616649	0	0.046102173
0	0	0	0	0
0	0	0	0	0
0	0	0	0	0
0	0	0	0	0
0	0	0	0	0
0	0	0	0	0
0	0	0	0	0
0	0	0	0	0
0	0	0	0	0
0	0	0	0	0
0	0	0	0	0
0	0	0	0	0
0	0	0	0	0
0	0	0	0	0
0	0	0	0	0
0	0	0	0	0
0	0	0	0	0
0	28.33361207	0	6.307201068	0
0	0	0	0	0
0	0	0	0	0
0	0	0	0	0
0	0	0	0	0
0	0	0	0	0
0	0	0	0.615970256	0
0.020481854	0.008214465	0.335452728	10.1174546	0.313351975
0	0.011439868	0.056274342	0.001736953	0
0	0	0.00265561	0	0
0	0	0	0	0

0	0.012716524	0	0	0
0.01482164	0.001521477	0.098920598	0	0.00363163
0	0.003983704	0	0	0.039536358
0	0.11408337	0	1.142704488	0
0.005888572	0.001551489	0	0	0
0	0	0	0	0
0.000851058	0	0	0.034706165	0
0	0	0	0.003137192	0
0	0.003165521	0	0	0
0	0	0	0	0
0	0	0	0	0
0	0.087275183	0	0.076223977	0
0	0	0	0.001036768	0
0.23567357	0.018686591	0.136316153	0.003642611	0.142327633
0	0	0	0.010583342	0
0	0	0	0	0
0	0	0	0.027905601	0
0	0	0	0	0
0	0	0	0	0
0	0	0	0	0
0.128166835	0.142335546	0.042466363	0.389893019	0.24809691
0.022777198	0	0.009216591	0.00085034	0.005685182
0	0.281805545	0	0.090606816	0
0	0	0	0	0
0	0	0	0	0
0	0	0	0	0
0	0	0	0	0.127326633
0	0	0	0	0
0	0	0	0.610817284	0
0	0	0	0	0
0.003882782	0	0	0.015898237	0.353262
0	0	0	0	0
0	0	0	0	0
0	0	0	0	0
0	0	0	0	0
0	0	0	0	0
0	0	0	0	0
0	0	0	0.001288909	0
0	0	0	0	0
0	0	0	0	0
0	0	0	0	0
0	0	0	0	0
0	0	0	0	0
0	0	0	0	0
0	0	0	0	0
0	0	0	0	0
0.215813161	0	0	0.004993725	0
0	0	0	0.019974517	0

0	0	0	0	0
0	0	0	0	0
0	0	0	0	0
0	0	0	0	0
0	0	0	0.010647001	0
0	0	0	0.002351653	0
0	0	0	0	0
0	0.53959018	0	0	0
0	0	0.001936643	0.003082202	0
0	0	0	0	0
0	0	0	0	0
0	0	0	0.017891881	0
0	0	0	0	0
0	0	0	0	0
0	0	0	0	0
0	0	0	0	0
0	0	0	0	0
0	0	0	0	0
0.001006517	0	0.381185676	0.033332015	0
0	0	0	0	0
0	0	0	0	0
0	0	0	0	0
0	0	0	0	0
0	0	0	0	0
0	0	0	0	0
0	0	0	0	0
0	0	0	0	0
0	0	0	0	0
0	0	0	0	0
0	0	0	0	0
0	0.001209146	0.018996398	0	0
0	0.000543643	0	0	0
0	0	0	0	0
0	0	0	0	0
0	0	0	0	0
0	0	0	0	0
0	0	0	0	0
0	0	0	0	0
0	0	0	0	0
0	0	0	0	0
0	0	0	0	0
0	0	0	0	0
0	0	0.002390386	0	0.015331807
0	0	0	0	0
0	0	0	0	0
0	0	0	0	0
0	0	0	0	0
0	0	0	0	0

0	0	0	0	0
0	0	0	0	0
0	0	0	0	0
0	0	0	0	0
0	0	0	0.011111931	0
0	0	0	0	0
0	0	0	0	0
0	0	0	0	0
0	0	0	0	0
0	0	0	0	0
0	0	0	0	0
0	0	0	0	0
0	0	0	0	0
0	0	0	0	0
0	0	0	0	0
0	0	0	0	0
0	0	0	0	0
0	0	0	0	0
0	0	0	0	0
0	0	0	0	0
0	0	0	0	0
0	0	0	0	0
0	0	0	0	0
0	0	0	0	0
0	0.02124865	0	0.008888326	0
0	0	0	0	0
0	0	0	0	0
0	0	0	0	0
0	0	0	0	0
0	0	0	0	0
0	0	0.875797611	0	0
0	0	0	0	0
0	0	0	0	0
0	0	0	0	0
0	0	0	0	0
0	0	0	0	0
0	0	0	0	0
0	0	0	0	0
0	0	0	0	0
0	0	0	0	0
0	0	0	0	0
0	0	0	0	0
0	0	0	0	0
0	0	0	0	0
0	0	0	0	0
0	0	0	0	0
0	0	0	0	0
0	0	0	0	0

0	0	0	0	0
0	0	0	0	0
0	0	0	0	0
0	0	0	0	0
0	0	0	0	0
0	0	0	0	0
0	0	0	0	0
0	0	0	0	0
0	0	0	0	0
0	0	0	0	0
0	0	0	0	0
0	0	0	0	0
0.000835292	0.001000355	0	0	0
0	0	0	0	0
0	0	0	0	0
0	0	0	0	0
0	0	0	0	0
0	0	0	0	0
0	0	0	0	0
0	0	0	0	0
0.024988724	0.012344798	0.056664051	0.058474085	0.02113213
0	0	0	0	0
0	0	0	0	0
0.002442957	0	0	0	0
0	0	0	0	0
0	0	0	0	0
0.000909553	0.005010738	0.002486287	0.023502007	0.002773376
0	0	0	0	0
0	0	0	0	0
0.001702954	0	0.028776802	0	0.042838841
0.252960829	0	0.034697682	0	0
0	0	0	0	0
0	0	0	0	0
0	0	0.015417265	0	0
0.003048562	0.056712084	0.020959576	0.009913487	0.003292179
0	0	0	0	0
0	0	0	0	0
0	0	0	0	0
0	0	0	0.000779093	0
0	0	0	0.029114831	0
0	0	0	0.001414497	0
0	0	0	0	0
0	0	0	0.067950426	0
0	0	0	0	0
0	0	0	0	0
0	0	0	0.027295756	0
0	0.000790611	0.001913922	0	0
0.00289665	0.001907984	0.034191668	0	0.002346096

0	0	0	0	0
0	0	0	0	0
0	0	0.003617684	0	0.002522134
0	0	0	0	0
0	0	0	0	0
0	0	0	0	0
0	0	0	0.001358972	0
0	0	0	0.00548947	0
0	0.037768963	0	0	0
0	0	0	0	0
0	0	0	0	0
0	0	0	0	0
0	0	0	0	0
0	0	0	0	0
0	0	0	0	0
0	0	0	0	0
0	0	0	0	0
0	0	0	0	0
0	0	0.001464114	0.055341383	0.001403508
0	0	0	0	0
0	0	0.086658636	0	0.21241459
0	0	0	0	0
0	0	0	0	0
0	0	0	0	0
0.004856165	0	0.03378953	0	0
0	0	0	0	0
0	0	0	0	0
0	0	0	0	0
0	0	0	0.055392839	0
0	0	0	0	0
0.930806886	0.182721564	0.286641016	0.047177996	0.987213964
0	0	0	0	0
0	0	0	0	0.060003829
0	0	0	0	0
0	0	0	0	0
0	0	0	0	0
0	0	0	0	0
0	0	0	0	0
0	0	0	0	0
0	0	0	0	0
0	0	0	0	0
0	0.012091899	0	0.018033796	0
0	0	0	0	0
0	0	0	0	0
0	0	0	0	0
0	0	0	0	0
0	0	0	0	0
0	0	0	0	0

0	0	0	0	0
0	0	0	0	0
0	0.013714011	0.068346054	0.004852371	0
0	0	0	0	0
0	0	14.25354405	0	0
0	0	0	0	0
0	0	0	0	0
0	0	0	0	0
0	0	0	0	0
0	0	0	0	0
0	0	0	0	0
0	0	0	0	0
0	0	0	0	0
0	0	0.002246386	0.002085512	0
0.003963701	0.001340322	0.014369256	0.00786888	0.00071094
0	0	0	0.006845048	0
0.001859645	0	0.0047533	0.001681103	0.001822617
0.003915515	0.004343741	0	0	0
0	0	0	0	0
0	0	0	0	0
0	0	0	0	0
0.002638003	0.001378605	0.032181554	0	0.002010926
0	0	0	0	0
0	0	0.030302797	0	0
0	0	0	0.001216656	0
0	0	0	0	0
0.032865109	0	0	0	0
0	0	0	0	0
0	0	0	0	0
0	0	0	0.002926879	0
0	0	0	0	0
0	0	0	0	0
0	0	0	0.051821148	0
0	0	0	0	0
0	0	0	0	0
0	0	0	0	0
0	0	0	0	0
0	0	0.138070489	0.007944385	0
0	0	0	0	0
0	0	0	0	0
0	0	0	0	0
0	0	0	0	0
0	0	0	0	0
0.026198932	0	0	0	0.005991364
0	0	0	0	0
0	0	0	0	0
0	0	0	0	0
0.006569251	0	0.011608711	0	0.004451272

0	0	0	0	0
0	0	0	0	0
0	0	0	0	0
0	0	0	0	0
0.020975967	0	0.015072518	0.01039488	0.006164739
0	0	0	0	0
0	0	0	0	0
0.003542462	0.003054595	0	0.035119153	0
0	0	0	0	0
0.03286163	0.10704687	0.02867242	0.177139499	2.333280162
0	0	0	0	0
0	0	0	0.041109172	0
0.003403367	0.003056934	0.014589103	0.042399588	0.001297178
0	0.004023668	0.00224782	0	0
0.004375357	0	0.001739659	0	0.006170297
0	0	0	0.003030896	0
0	0	0	0	0
0	0	0	0	0.001267054
0.008589499	0	0.19637505	0	0.20900252
0.202694841	0	0.429788896	0	0
0	0	0	0	0
0.030424327	0	0	0	0
0	0	0	0.007176073	0
0	0	0	0	0
0	0	0	0	0
0	0.026348061	0	0	0
0	0	0	0	0
0	0	0.006849587	0	0
0	0	0	0	0
0.001019629	0	0	0	0.002331761
0.126811848	0	0.400618787	0	0.27966709
0	0	0	0	0
0.108595318	0	0.268454045	0	0
0.069339424	0	0	0	0
0	0	0	0	0
0	0	0	0	0
0.003370247	0	0	0	0
0	0	0	0	0
0	0	0	0	0
0	0	0	0.003022967	0
0	0	0	0	0
0	0	0	0	0
0	0	0	0	0
0	0	0	0	0.031495562
0	0	0	0	0
0	0	0	0	0
0	0.001771129	0	0.00454917	0
0	0	0	0	0

0	0	0	0	0
0	0	0	0.003631439	0
0	0	0	0	0
0	0	0	0	0
0	0	0	0	0
0.176176139	0.089976145	0.059338455	0	0
0	0	0	0	0
0	0	0	0	0
0	0	0	0	0
0	0	0	0	0
0.0208275	0	0	0.049868428	0.052779023
0	0	0.391472313	0	0
0	0	0	0	0
0.001595414	0.012483151	0.025638026	0.003926099	0
0	0	0	0	0
0	0	0	0	0
0	0	0	0	0
0	0	0.025207156	0	0
0	0	0.014065844	0	0
0	0.007332567	0	0	0
0	0	0	0.027798525	0
0	0.476980572	0	0.199115485	0
0	0	0	0	0
0	0	0	0	0
0	0	0	0	0
0	0	0	0	0
0.037191804	0	0	0	0
0	0	0	0	0
0	0	0	0	0
0.422496767	0.784186479	0.489314534	0.372831311	0
0	0.005703495	0	0	0
0	0	0	0.012019935	0
0	0	0	0	0
0	0	0	0	0
0.009605355	0	0	0	0
0	0	0	0	0
0	0	0	0	0
0	0	0	0.04087492	0
0	0	0	0	0
0	0	0	0	0
0	0	0.008691648	0	0.012831075
0	0.366557089	0	2.824322	0
0	0	0	0.025137279	0
0	0	0	0	0
0.001479558	0.002126322	0	0	0
0	0	0	0	0.173214339
0.004359305	0	0.005701568	0	0.041100993
0	0	0	0	0

0	0.006780562	0.003376693	0.002089916	0
0	0	0	0	0
0	0	0	0.080141574	0
0	0	0.126504915	0	0
0	0	0	0	0
0.001196751	0	0.005234162	0	0
0	0	0	0	0
0	0	0	0.020412095	0
0.240472097	0	0	0	0.008668834
0	0.02049137	0	0	0
0	0	0	0	0
0.010025583	0	0	0	0
0	0	0	0	0
0.016134397	0	0	0.062933096	0.230364221
0	0	0	0	0
0.01501042	0.274395618	0	0.002110778	0
0	0.008713974	0.043218846	0.038603027	0.075081032
0	0	0.005358083	0	0.016025227
0.009165475	0	0.041680868	0	0.002445366
0	0	0	0	0
0	0.06321396	0	0.007371333	0
0	0.004437552	0	0.004342061	0
0	0	0	0.027546457	0
0	0	0	0	0
0	0	0	0	0
0	0	0.324170736	0	0.720410104
0	0	0.0069983	0	0.002790783
0	0	0	0	0
0	0	0	0	0
0	0	0	0	0
0	0.000820777	0.069614212	0	0.044406774
0.006386036	0.006118393	0.003491281	0.001010261	0
0.0082085	0.056798986	0	0.046492083	0
0	0	0	0.27156527	0
0	0	0	0	0
0	0	0	0	0
0	0.001316672	0.00601056	0	0
0.018915452	0.048395822	0.275408688	0.018589123	0.008356512
0.306423311	0	0	0.053889951	0
0	0	0	0.002224172	0.004019002
0	0	0	0	0
0	0	0	0	0
0	0.00246386	0	0	0.010536814
0	0.004662078	0	0	0.057430515
0.043671281	2.664295383	0.975249065	0	0
0	0	0	0	0
0	0	0	0	0
0	0	0	0	0

0	0	0	0	0
0.059457555	0.00252557	0.731343322	0.518339072	0
0	0	0	0	0
0	0	0	0	0
0	0	0.007140547	0	0
0	0	0	0	0
0.012735375	0	0.005117504	0	0
0	0	0	0	0
0	0	0.005063777	0.001979426	0
0.001277044	0	0	0	0
0.001958873	0.00375355	0.01038474	0	0.002820548
0.005187194	0.202208515	0.171441041	0.007180303	0.03978865
0	0	0	0	0
0	0	0	0	0
0	0.059576418	0.00471643	0.001843648	0
0	0	0	0	0
0.007749063	0	0.035568093	0.002644076	0.010076718
0.089054872	0.004251952	0.098226419	0.00374441	3.590501185
0	0	0	0	0
0.018827584	0.101236508	0	0.001823568	0.039980847
0.002115328	0.081742472	0.022428338	0.174509405	0.000537499
0	0	0	0	0
0	0	0	0	0
0	0	0	0	0
0	0	0	0	0
0	0	0	0	0
0	0.030228681	0	0	0
0.016987014	0.063664265	0	0.319053321	0
0	0	0	0	0
0	0	0	0	0
0	0	0	0.002826957	0
0	0	0	0	0
0	0	0	0	0
0	0	0	0	0
0	0.053184731	0	0.030076211	0
1.033713949	0.21387567	0.324856578	0.221163866	0
0	0	0	0	0
0	0	0	0	0
0	0	0.019910891	0.026325867	0
0	0	0	0	0
0.032100521	2.686444951	0.965320129	0	0
0	0	0.036236296	0	0
0.080115427	0	0.008931086	0.00276383	0.022545003
0.004156655	0.001212049	0.010059928	0	0
0.064343432	0	0	0	0
0	0	0	0.59020231	0
0	0	0	0	0
0.043818988	0	0.183518063	0	0.075490379

0	0	0	0	0
0	0	0	0	0
0	0	0	0	0
0.018531017	0.008146122	12.85781869	0.022302988	0
0	0	0	0	0
0	0	0	0	0
0	0.062021514	0	0	0
0	0	0	0	0
0	0	0	0.159407017	0
0	0	0	0	0
0	0	0	0	0
0	0.004802991	0	0	0
0	0	0	0	0
0	0	0	0	0
0.004309185	0.489154586	0.174076238	0.004544709	0
0	0	0	0	0
0	0	0	0.007350937	0
0	0.046796558	0	0.031097861	0
0	0	0	0	0
0	0	0	0	0
0	0	0	0	0
0	0	0	0	0
0	0	0	0	0
0.668618851	0	0.974557676	0.019612185	0.385520708
0	0.053399819	0	0	0
0	0	0	0.687435635	0
0	0	0	0.000736227	0
0	0.179367719	0	2.239839731	0
0	0	0	0	0
0	0	0	0	0
0	0	0	0	0
0	0	0	0	0
0	0.000444212	0	0	0
0	0	0	0	0
0	0	0	0	0
0	0.036291441	0	0.013074319	0
0	0	0	0.738742969	0
0	0	0	0	0
0	0.011666466	0	0	0.117188656
0	0.001145951	0	0	0
0.019837815	0	0.435411049	0.382092083	0
0	0	0	0	0
0.524515192	0.121142077	0	2.406930416	0
0	0	0	0	0
0	0	0	0	0
0	0	0	0	0
0	0	0	0	0
0.014452927	0.003000225	0.002154953	0	0.000918111

0	0	0	0	0
0	0	0	0.004397451	0
0.216596109	0.354860878	0.894705906	0.002922403	2.600911168
0	0	0.002232993	0	0
0	0.439327218	0	0	0
0	0.004357009	0	0.00364508	0
0	0	0	0	0
0	0	0	0	0
0	0	0	0	0
0	0	0	0	0
0.067561165	0.026282833	1.027403676	0.004782135	0.014862783
2.590849635	1.545737825	0	2.801985322	0
0	0	0	0	0
0.000734041	0.001172128	0.039157571	0.044901354	0
0.931501581	0.042674111	0.009774789	0	3.568602528
0	0	0	0	0
0	0	0	0	0
0	0	0	0	0
0	0	0	0	0
0	0	0	0	0
0.001900208	0	0.070830959	0.222651813	0
2.581875531	0.074238787	0.782958905	0	31.34433749
19.44083584	1.21367359	2.843762186	2.938143222	40.94995703
0	0	0	0	0
0	0	0	0	0
0	1.785193255	0	2.570776924	0
0	0	0.038856922	0	0
0	0	0	0	0
0	0.026857171	0	0	0
0	0	0	0	0
0	0	0	0	0
0	0	0	0	0
0	0.020969889	2.182089483	0.025539162	0.004259851
0	0.026028171	0	0	0
0.004303011	0.000883429	0.235858963	0.004214041	0.00866896
0	0.018004364	0.586616546	0.015588017	0
0	0.656986553	0.006082257	0	0
0	0	0	0	0
0	0	0	0.179284223	0
0	0	0	0	0
0	0	0	0.000784345	0
0	0	0	0	0
0	0	0	0	0
0	0	0	0	0
0.038766784	0.20854908	0.43704417	0.015998727	0
0	0	0.475991874	0	0
0	0	0	0	0
0	0.078753885	0.441446165	0.037372351	0

0	0	0	0	0
0	0	0	0	0
0	0	0	0	0
0	0.002556075	0	0	0
0	0	0	0	0
0	0.009203692	0	0.024276997	0
0	0	0	0	0
0	0.234843331	0	0	0.039155008
0	0	0	0	0
0	0	0	0	0
0.001828505	0.002408823	0.573210555	0	0.020385105
0	0	0	0	0
0	0	0	0	0
0	0	0	0	0
0	0	0	0	0
0	0	0	0	0
0	0.041042327	0	0.07691194	0
0	0.007656563	0	0.001605386	0
0	0	0	0	0
0	0	0.032748943	0.008686756	0
0	0.000485875	0	0	0
0	0	0	0	0
0.003071206	0	0.001308347	0.022673472	0
0	0	0	0	0
0	0	0	0	0
0	0	0	0	0
0	0	0	0	0
0	0	0	0	0
0	0	0	0	0
0	0	0	0.20289847	0
0	0	0	0	0
0	0	0	0	0
0	0	0	0	0
0	0	0	0	0
0	0	0	0.002108433	0
0	0	0	0	0
0	0	0	0.010184802	0
0.001547862	0	0.021732581	0	0.003687254
0	0	0	0	0
0	0	0	0	0
0	0.015653378	0	0.001914567	0
0	0	0	0	0
0	0	0	0	0
0	0.536279108	0	0	0
0	0.007156658	0	0.041011877	0
0	0	0	0	0
0	0	0	0	0
0	0	0	0	0

0	0	0	0	0
0	0	0	0	0
0	0	0	0.010153628	0
0	0	0	0.121786619	0
0	0	0	0	0
0	0.61650432	0	0	0
0	0	0	0	0
0	0	0	0	0
0.644697525	0.025054809	2.468384316	0.003095728	0.644617567
0	0	0	0	0
0	0	0	0	0
0	0	0	0	0
0	0.005369328	0	0	0
0	0	0	0	0
3.211244123	1.078649293	0	0.137525151	0
0.292044107	0	0	0	0.341772741
0	0	0	0	0
0	0	0	0	0
0	0	0	0	0
0	0	0	0	0
0	0	0	0	0
0	0	0	0	0
0	0	0	0	0
0	0	0.112292503	0	0
0	0.002705854	0.07080638	0.518934947	0.027508984
0.333334571	11.67110574	1.995082937	0.153161255	0
0	0	0	0.161746917	0
0	0	0	0	0
0	0.391127417	0	0.222797898	0
0	0	0	0	0
0	0	0	0	0
0	0	0	0	0
0	0	0	0	0
0	0.002247933	0.012493993	0	0
0.002485226	0	0	0.002912288	0
0.001171169	0.001309098	0.069073092	0	0.00282711
0	0	0	0	0
0	2.036323536	0	1.133452556	0
0	0	0	0	0
0	0	0	0	0
0	0	0	0	0
0	0	0	0	0
0	0.093278671	0	2.016473496	0.309266998
0	0	0	0	0
0	0	0	0	0
0	0.007441237	0	0	0
0.016032787	0	0	0	0
0.032995628	0	0.012299241	0.005281999	0

0	0.007366771	0	0.004170486	0.084904741
0	0	0	0	0
0	0	0	0	0
0	0	0	0	0
0.00131234	0.011316045	0.016305979	0	0
0	0	0	0	0
0	0	0	0	0
0	0	0	0	0
0	0	0	0	0
0	0	0	0	0
0	0	0	0	0
0	0	0	0.309433096	0
0	0.409022504	0	0.108372459	0
0	0	0	0	0
0	0	0	1.130875978	0
0	0	0	0	0
0	0	0	0	0
0	0	0	0	0
0	0	0	0	0
0	0	0.002688086	0.012186208	0
0	0	0	0.178877584	0
0	0	0	0	0
0.001293513	0.211920497	0.084217601	0.406307829	0
0	0	0	0	0
0	0	0	0	0
0	0	0	0	0
0	0	0	0	0
0	0	0	0	0
0	0	0	0	0
0	0	0	0	0
0	0	0	0	0
0.011488486	0.007547653	0.037195488	0.015924418	0.005254542
0	0	0	0	0
0	0.012568925	0	0	0
0.000637263	0.001424627	0.068834381	0	0.002590824
0	0.008133146	0	0.00548726	0
0	0	0	0	0
0	0	0	0.008997475	0
0	0	0	0.001544271	0
0	0	0	0	0
0	0	0	0.018133148	0
0	0	0	0	0
0	0	0	0	0
0	0	0.430251286	0	0
0.024901444	0.126706997	0.324067652	0.001324158	0
0	0	0	0	0
0	0	0	0.057005634	0
0	0	0	0	0

0	0	0	0	0
0	0	0	0	0
0.00519415	0	0	0	0.002309681
0	0	0	0	0
0.002414325	0	0	0	0
0	0	0	0	0
0	0	0	0.080150289	0
0	0	0.1507498	0.022202935	0
0	0	0	0	0
0	0	0	0	0
0	0	0	0	0
0.013212303	0	0.001969974	0.000928963	0.016786034
0	0	0	0	0
0	0	0	0.410319631	0
0	0	0	0	0
0	0	0	0	0
0	0	0	0	0
0	0	0	0	0
0	0	0	4.004991898	0
0	0	0	0	0
0	0	0	0	0
0	0	0	0	0
0	0	0	0	0
0	0	0	0	0
0	0	0	0.001446758	0
0.018506881	0.003546249	0.056543557	0	0
0	0	0	0	0
0	0	0.681386649	0.002212115	0
0	0	0	0	0
0	0.087904108	0	0	0
0	0	0	0	0
0	0	0	0	0
0	0	0	0	0
0	0	0	0	0
0	0	0.268064154	0	0.007625392
0	0	0	0	0
0	0	0	0	0
0	0	0.002072346	0	0
0	0	0	0	0
0	0	0	0	0
7.844048836	8.490586264	7.407500939	5.879776671	0
1.037753561	0.002274452	0.975240878	0.113739393	0
0	0	0	0.35872397	0
0	0	0	0	0
0	0	0	0	0
0	0	0	0	0
0	0	0	0	0
0.000735395	0.012506197	0.01809201	0	0

0	0	0	0	0
0	0.021741391	0	0	0.000432456
0	0	0	0	0
0	0	0	0	0
0	0.357778933	0.1264108	1.671551245	0.997760321
0	0	0	0	0
0	0	0	0	0
0	0	0	0	0
0.357823268	0.056987009	1.481655177	0.003584618	3.399132845
0	0	0	0	0
0	0	0	0	0.04576418
0	0	0	0	0
0	0.006865777	0.015890188	0.167240284	0
0.007454623	0.007142194	0.023128081	0.037404371	0
0	0	0	0.080587916	0
0	0.000532803	8.060250893	0.129422082	0
0	0	0	0	0
0	0	0	0	0
0	0	0	0	0
0	0	0	0	0
0	0	0	0	0
0	0	0	0	0
0	0	0	0	0
0	0	0	0	0
0	0	0	0	0
0	0	0	0	0
0.007648981	0.008305527	0	0.012907275	0
0	0	0.010676485	0.001820552	0.000877247
0	0	0	0	0
0	0	0	0	0
0	0	0	0	0
0	0	0	0	0
0	0	0	0	0
0	0.002118947	0	0	0
0	0	0	0	0
0.002218733	0	0	0.004446002	0
0	0	0	0.001435058	0
0	0	0.004581638	0	0
0	0	0	0	0
0	0	0	0	0
0	2.717262066	0	0.824805726	0
0	0	0	0.006444496	0
0	0	0	0	0
0	0	0	0	0
0	0	0	0	0
0	0	0	0	0
0	0	0	0	0
0	0	0	0	0

0.002310039	0	0	0.140862264	0
0	0	0	0	0
0	0	0	0	0
0	0.004787823	0	0	0
0	0	0	0	0
0	0	0	0	0
0	0	0.109323472	0.254198572	0
0	0.000902986	0	0	0
0	0	0	0	0
0	0	0	0	0
0	0	0	0	0
0	0	0.82502072	0	0
0	0	0	0	0
0	0	0	0	0
0	0	0	0	0
0	0	0	0	0
0	0	0	0	0
0	0	0	0	0
0	0	0	0	0
0	0	0	0	0
0.000928274	0	0.086919574	0.072642523	0
0	0	0	0	0
0	0.003727689	0	0.006911004	0
0	0.003489158	0	0.005377169	0.003608933
0	0	0	0	0
0	0	0	0	0
0	0	0	0	0
0	0	0	0	0
0	0	0	0	0
0.006289664	0.545107279	0.002083996	0	0.016980715
0	0	0	0	0
0	0	0	0	0
0	0	0	0	0
0	0.001117863	0	0	0
0	0	0	0	0
0	0	0	0	0
0.001139205	0	0	0.030997921	0.003473623
0	0	0	0	0
0	0	0	0	0
0	0	0	0.040198626	0
0	0	0	0	0
0	0	0	0	0
0	0	0	0	0
0	0	0	0	0
0	0	0	0	0
0	0	0	0	0
0	0	0	0	0
0	0	0	0.049701117	0

0.004898366	0.016144168	0.096990961	0	0.048691112
0	0	0	0	0
0	0	0	0	0
0	0	0	0	0
0	0	0	0	0
0	0	0	0	0
0	0	0	0	0
1.560759182	1.804515876	1.952560735	0.398467306	0
0.029832241	0.006482504	0.443274555	12.51698994	0.502174243
0.06251904	0.091284156	0	0.019871155	0
0	0	0	0	0
0	0	0	0	0
0	0	0	0	0
0	0	0	0	0
0	0	0	0	0
0	0	0	0	0
0.070644934	0	0.377548545	0	0
0	0	0	0	0
0	0	0	0	0
0	0	0	0	0
0	0	0	0	0
0	0	0	0	0
0	0	0	0	0
0	0	0	0	0
0	0	0	0	0
0	0	0	0	0
0	0	0	0	0
0	0	0	0	0
0	0	0	0	0
0	0	0	0	0
0	0	0	0	0
0	0	0	0	0
0	0	0	0	0
0	0	0	0	0
0	0.00363624	0	0	0
0	0	0	0	0
0	0	0	0	0
0	0	0	0	0
0	0	0	0.004177164	0
0	0.002811093	0.12676995	0	0
0	0	0	0	0
0	0.10498513	0	0	0
0	0	0	0	0
0	0	0	0	0
0	0	0	0	0
0.002090709	0	0	0	0
0	0	0	0	0
0	0	0	0	0

0	0	0	0	0
0	0	0.031288892	0.013614866	0.012267095
0	0	0	0	0
0	0	0	0	0
0	0.357458438	0	0.165061223	0
0	0	0	0	0
0	0	0	0	0
0.056827421	0.035103174	0.195722543	0.002628658	0.034769225
0	0	0	0	0
0	0	0	0	0
0	0	0	0	0
0	0	0	0	0
0	0	0	0	0
0	0	0	0	0
0	0	0	0	0
0	0.029202079	0	0	0
0	0	0	0	0
0	0	0	0	0
0	0	0	0	0
0	0	0	0	0
0	0	0	0	0
0	0	0	0	0
0	0	0	0	0
0.002372693	0	0.037092458	0	0.012331927
0	0	0	0.062744365	0
0	0.006215576	0	0	0
0	0.020980478	0.763220349	0.004577413	0.087825397
0	0	0	0	0
0.010556448	0	0	0	0.006258845
0	0	0	0	0
0	0	0	0	0
0	0	0	0	0
0	0	0	0	0
0	0	0	0	0
0	0	0	0	0
0	0	0	0	0
0	0	0	0	0
0	0.000581533	0	0	0
0	0	0	0	0
0	0.001829291	0	0	0
0	0	0	0	0
0	0	0	0	0
0	0	0	0	0
0	0	0	0	0
0	0	0	0	0
0	0	0	0	0
0	0	0	0	0
0	0	0	0	0

0.009276314	0	3.743807775	0.117791222	0
0	0	0	0	0
0	0	0	0	0
0	0.001414817	0	0	0
0	0	0	0	0
0	0	0	0	0
0	0	0	0	0
0	0	0	0	0
0	0	0	0	0
0	0	0	0	0
0	0	0	0	0
0.000795362	0	0	0	0
0	0	0	0	0
0	0	0	0	0
0	0	0	0	0
0	0	0	0	0
0	0	0	0	0
0	0	2.481466358	0	0
0	0.002863317	0	0	0
0	0	0	0	0
0.003221965	0	0.389017012	0.00996766	0
0	0	0	0	0
0	0.068094982	0.005662625	0.001430503	0
0	0	0.1475844	0	0
0	0	0	0	0
0	0	0	0	0
0	0	0	0	0
0	0	0	0	0
0	0	0	0	0
0	0	0	0	0
0	0	0	0	0
0	0	0	0	0
0.052841736	0.009492581	0.063131253	0.028668733	0.012394087
0	0	0	0	0
0	0.364680497	0	0.056800363	0
0.003669514	0	0.116315783	0	0
0	0	0	0	0
0	0	0	0.07113841	0
0.002357986	0.005196069	0.061409149	0.00422767	0
0	0	0	0	0
0	0	0	0	0
0	0	0	0	0
0	0.008717397	0	0.018901283	0
0	0	0	0	0
0	0	0	0	0
0	0	0.117534868	0.048958618	0
0	0	0	0	0
0	0	0	0	0

0	0	0	0	0
0	0	0	0	0
0	0	0	0	0
0	0	0	0	0
0	0	0	0	0
0	0	0	0	0
0	0	0	0	0
0	0.017231644	0	0	0
0	0	0	0	0
0	0	0	0.020694032	0
0	0	0	0	0
0	0	0	0	0
0	0	0	0	0
0	0	0	0	0
0	0	0	0	0
0.076556184	0	0.001544009	0	0.007950233
0	0	0	0	0
0	0	0	0	0
0	0	0	0	0
0	0.007831869	0	0	0
0	0	0	0.039108494	0
0	0	0	0	0
0	0	0	0	0
0	0	0	0.002805599	0
0	0	0	0	0
0	0	0	0	0
0	0	0	0	0
0	0	0	0	0
0	0	0	0	0
0	0	0	0	0
0	0.237422873	0	0.170811535	0
0	0	0	0	0
0	0	0	0.013253823	0
0	0	0	0	0
0	0	0	0	0
0	0	0	0	0
0	0	0	0	0
0	0	0	0	0
0	0	0	0	0
0	0	0	0	0
0	0	0	0	0
0	0	0	0	0
0	0	0	0	0
0	0	0	0	0.00325225
0	0	0	0	0
0	0	0	0	0
0.19149541	0	0.937516235	0	0.001558455
0	0.005836875	0	0	0

0	0	0	0	0
0	0	0	0	0
0	0	0	0	0
0	0	0	0	0
0	0.42111482	0	0.016108248	0
0	0	0	0	0
0	0	0	0	0
0	0	0	0	0
0	0	0	0	0
0	0	0	0	0
0	0	0	0.019997391	0
0.005183437	0.022573616	0.064404844	0	0
0	0.003700315	0.002222606	0	0.002014389
0	0	0	0	0
0	0	0	0	0
0	0.158865387	0	0	0
0	0.001041707	0	0	0
0	0	0	0	0
0.056376246	0	0.291896952	0.00301051	0
0	0	0.021533991	0.155758285	0
0	0	0	0	0
0	0	0	0	0
0	0	0	0	0
0	0	0	0	0
0	0	0	0	0
0	0	0	0	0
0.006160058	0.00464997	0.034697897	0.001181226	0.00426887
0	0	0	0	0
0	0	0	0	0
0	0	0	0	0
0	0.001013651	0	0	0.00419379
0	0	0	0	0
0	0	0	0	0
0	0	0	0	0
0	0	0	0	0
0	0	0	0	0
0	0	0	0	0
0.002675933	0.026065123	0.043445093	0.003919708	0
0	0	0	0	0
0	0	0	0	0
0	0.001279741	0.014752451	0	0
0	0	0	0	0
0	0	0	0	0
2.840476383	0	0.161935421	0	0
0	0	0	0	0
0	0	0	0	0
0	0	0	0	0
0	0	0	0	0

0	0	0	0	0
0	0	0	0	0
0	0	0	0	0
0	0	0.008956229	0	0
0	0	0	0.000903956	0
0	0	0.002474001	0	0
0	0	0	0	0
0	0	0	0	0
0	0	0	0	0
0	0	0	0.005631222	0
0	0	0	0	0
0	0	0	0	0
0	0.008472852	0.093602709	0.020208159	0
0	0	0	0	0
0	0	0	0	0
0	0	0	0	0
0	0	0	0	0
0	0	0	0	0
0	0	0	0	0
0	0	0	0	0
0	0	0	0	0
0	0	0	0	0
0.018976773	0.001239644	0.05251658	0.005458357	0.013972671
0	0	0	0.019054769	0
0	0	0	0	0
0	0	0	0	0
0	0.074812851	0	0	0
0	0	0	0	0
0	0	0	0	0
0	0	0	0	0
0	0.003225127	0	0	0
0	0	0	0	0
0	0	0	0	0
0	0	0	0	0
0	0	0.003005622	0	0
0.346113074	0	0	0	0
0.01966992	0	0	0	0
0	0	0	0	0
0	0	0	0.003647361	0
0	0	0	0	0
0	0	0	0	0
0	0	0	0	0
0	0	0	15.19739582	0
0	0	0	0	0
0	0	0	0	0
0	0.39595863	0	0.060028575	0
0.18064715	0	0	0	0.665631714
0	0	0	0	0

0	0	0	0	0
0	0	0	0	0
0	0	0	0	0
0	0	0	0	0
0	0	0	0	0
0	0	0	0	0
0	0	0	0	0
0	0	0	0	0
0.000941143	0	0	0	0
0	0	0	0	0
0.001155264	0.002213691	0.002788834	0	0
0	0	0.000734777	0	0
0	0	0	0	0
0	0	0	0	0
0	0	0	0	0
0	0	0	0	0
0	0	0	0.005884729	0
0	0	0	0	0
0	0	0	0	0
0	0	0.003698473	0	0
0	0	0	0	0
0	0	0	0	0
0	0	0	0	0
0	0	0	0	0
0	0	0	0	0
0	0	0	0	0
0	0	0	0	0
0	0.160355785	0	0.029121683	0
0	0	0	0	0
0	0	0	0	0
0	0.0014412	0.004859509	0.016261222	0
0	0	0	0	0
0.053925679	0.052293128	0	0.086575995	0
0.004015836	0	0.010201216	0	0.00510206
0	0	0	0	0
0	0	0	0	0
0	0	0	0	0
0	0	0	0	0
0	0	0	0	0
0	0	0	0	0
0	0	0	0	0
0.010417497	0.156940908	0.407597905	0	0.184152866
0	0	0	0.022616232	0
0	0	0	0	0
0	0	0	0	0
0	0.006665006	0	0.04602163	0
0	0	0	0	0
0	0	0	0	0

0	0	0	0	0
0	0.007906798	0	0.189548017	0
0	0	0.008251381	0.005365854	0
0	0	0	0	0
0	0	0	0	0
0.011654664	0	0	0	0
0	0	0	0	0
0	0	0	0	0
0	0	0	0	0
0	0	0	0	0
0	0	0	0	0
0	0	0	0	0
0	0	0	0	0
0	0	0	0	0
0	0	0	0	0
0	0	0	0	0
0	0	0	0	0
0	0	0	0	0
0	0	0	0	0
0	0	0	0	0
0	0	0	0	0
0	0	0.138740611	0	0.627223219
0	0	0	0	0
0	0	0	0	0
0	0	0	0.003025728	0
0	0.008082634	0.003089456	0	0
0	0	0	0	0
0	0	0	0.00340685	0
0	0	0	0	0
0	0	0	0	0
0	0	0	0	0
0	0	0	0	0.002374512
0	0	0	0	0
0	0	0	0	0
0	0	0	0	0
0	0	0	0	0
0	0	0	0	0
0	0	0	0	0
0	1.380391057	0	0	0
0	0	0	0	0
0	0	0	0	0
0	0	0	0	0
0.005347358	0.0074714	0.070649539	0	0.038214777
0	0	0	0	0
0	0	0	0	0
0	0	0	0	0
0.003749355	0.000979695	0.009825452	0	0.001818791
0	0	0	0	0

0	0	0	0	0
0.022149664	0.049653848	0.014321727	0.007937961	0.012950241
0	0	0	0	0
0	0	0	0	0
0	0	0	0	0
0	0	0	0	0
0	0	0	0	0
0	0	0	0	0
0	0	0	0	0
0	0	0	0	0
0	0	0	0	0
0	0	0	0	0
0	0	0.045418003	0.029728568	0
0	0	0	0	0
0.003485678	0.001541349	0	0.002262272	0
0	0	0	0	0
0	0	0	0	0
0	0	0	0	0
0	0	0	0	0
0	0	0	0	0
0	0	0	0	0
0.012639547	0	0.019369243	0.008053771	0
0	0	0	0	0
0	0	0.048797181	0.035469753	0
0	0	0	0	0
0	0	0	0	0
0	0	0	0	0
0	0	0	0	0
0	0.005308291	0	0	0
0	0	0	0	0
0	0	0	0	0
0	0	0	0	0
0	0	0	0	0
0	0	0	0	0
0	0	3.34803129	0	0
0	0	0	0	0
0	0	0	0	0
0	0	0	0	0
0	0	0.001045542	0.029951992	0
0	0	0	0	0
0.041497124	0	0.13827638	0.076091779	0
0	0	0	0	0
0	0	0	0	0
0	0	0	0	0
0	0	0	0	0
0.60253072	0.173545546	2.837900101	0.025623503	0.473708468
0	0	0	0	0
0	0	0	0	0

0	0	0	0	0
0	0	0	0	0
0	0	0	0	0
0	0	0	0	0
0	0.004119291	0	0	0
0	0	0	0	0
0	0	0	0	0
0	0	0	0	0
0	0	0	0	0
0	0	0	0.125020688	0
0	0	0	0	0
0	0	0	0	0
0	0	0.001539026	0.000816462	0
0	0	0	0	0
0	0	0	0	0
0	0	0	0	0
0	0	0	0	0
0	0	0	0	0
0	0	0	0	0
0.098763578	0.018704807	0	0.046993855	0.103300208
0	0	0	0	0
0	0	0	0	0
0	0	0	0	0
0	0	0	0	0
0	0	0	0	0
0	0	0	0	0
0	0	0	0	0
0	0	0	0	0
0	0	0	0	0
0.010557986	0	0	0.000776864	0
0	0	0	0	0
0	0	0	0	0
0	0	0	0	0
0	0.003747561	0	0	0
0	0	0	0	0
0	0.002278865	0	0	0
0	0	0	0	0
0	0	0	0	0
0	0	0	0	0
0	0	0	0	0
0	0	0	0	0
0	0	0	0	0
0	0	0	0	0
0	0	0	0	0
0.002372991	0.012607797	0.011150579	0	0
0	0	0	0	0
0	0	0	0	0
0	0	0	0	0.007407722

0	0	0	0	0
0	0	0	0	0
0	0	0	0	0
0	0	0	0	0
0	0	0	0	0
0	0.008177823	0.006723506	0.020721762	0.000982125
0	0	0	0	0
0	0	0	0	0
0	0	0	0	0
0	0	0	0	0
0	0	0	0	0
0	0	0	0	0
0	0	0	0	0
0	0	0	0	0
0	0	0	0	0
0	0	0	0	0
0	0	0	0	0
0	0	0	0	0
0	0	0	0	0
0	0	0	0	0
0	0	0	0	0
0	0	0	0	0
0	0	0	0	0
0	0.029849395	0	0	0
0	0	0	0	0
0	0	0	0.002125398	0
0	0	0	0	0
0	0	0	0	0
0	0	0	0	0
0	0	0	0	0
0	0	0	0	0
0	0	0	0	0
0	0	0	0	0
0	0	0	0	0
0	0	0	0	0
0	0	0	0	0
0	0	0	0	0
0	0	0	0	0
0	0	4.333468609	0	0
0	0	0	0	0
0	0	0	0	0
0	0	0	0	0
0	0	0	0	0
0	0	0	0	0
0	0	0	0.004244376	0
0	0	0	0	0
0	0	0	0	0
0	0	0	0	0

0	0	0	0	0
0	0.001036498	0	0	0
0	0	0	0	0
0	0	0	0	0
0	0	0	0	0
0	0	0	0	0
0	0	0	0	0
0	0	0	0	0
0	0	0	0	0
0	0	0	0	0
0	0	0.004928337	0.00423302	0
0	0	0	0	0
0	0.271739593	0	0	0
0	0	0	0	0
0	0	0	0	0
0	0	0	0	0
0	0	0	0	0
0	0	0	0	0
0	0	0	0	0
0	0	0	0	0
0	0	0	0.017943207	0
0	0	0	0	0
0	0	0	0	0
0	0.499235964	0	4.154214555	0
0	0	0	0	0
0	0	0	0	0
0	0	0	0	0
0	0	0	0	0
0	0	0	0	0
0	0	0	0	0
0	0	0	0	0
0	0	0	0	0
0	0	0	0	0
0	0	0	0	0

PHL018-0018_V1	PHL018-0010_V1	PHL018-0235_V2	PHL018-0139_V2	PHL018-0017_V1
CTRL	CTRL	EYCF	CTRL	EYCF
0.05027702	0.067402363	0.410235362	0.019312761	0.298077447
0.085267051	2.35944805	0.396750839	0.058210441	1.335366247
0	13.35917996	14.4474223	0	0
0	0	0	0	0
0	0	0	0	0
0	0	0	0	0
0	0	0	0	0
0	0	0	0	0
0	0	0	0	0
0	0	0	0	0
0	0	0	0	0
0	0	0	0	0
0	0	0	0	0
0.840878845	20.49290142	1.457807747	0.303968175	3.262085247
0	0	0	0	0
0	0	0	0	0
0	0	0	0	0
0	0	0	0	0
0	0	0	0	0
0	0.027316721	0	0	0
0	0	0	0.003624572	0
0	0	0	0	0
0	0	0	0	0
0	0	0	0	0
0	0	0	0.015034917	0
0	0	0	0	0
0	0	0	0	0
0	0	0	0	0
0	0	0	0	0
0	0	0	0	0
0	0	0	0	0
0	0	0	0	0
0	0	0	0	0
0	0	0	0	0
0	0	0	0	0
0.625270185	0	0.281256514	0.097817128	0.549626544
0	0	0	0	0
0	0	0	0	0
0	0	0	0	0
0	0	0	0	0
0	0	0	0	0
0.007460153	0.002401713	0.047981315	0	0.003742896
0.012621811	0.017540563	0.477393639	7.175885957	0.444116464
0.00595109	0	0.008184565	0	0.033448937
0.004052163	0	0	0	0
0	0	0	0	0

0.057768608	0	0	0	0
0	0.042277245	0.005940091	0	0
0	0.002511833	0.00210492	0	0.013863892
0.332347298	0	1.423978943	0.431898172	1.462710953
0	0	0	0.000937566	0.038950866
0	0	0	0.02417394	0
0	1.441106024	0	0	0.000845739
0.078663398	0	0.066886951	0	0
0	0	0	0	0
0	0	0	0	0
0	0	0	0.011205138	0
0.621180013	0	0.821875297	0.083515384	0.427351453
0	0	0	0	0
0.053672982	0.03750526	0	0	0.023167708
0	0	0	0	0
0	0	0	0	0
0	0	0.005715097	0.674936974	0.000622754
0	0	0	0	0
0	0	0	0	0
0	0	0	0	0
0.04568295	0.216433221	0.084938254	0	0.007200333
0.004208256	0.1629148	0.021843659	0.002727926	0.023886786
0	0	0	0.008279153	0.414346397
0	0	0	0	0
0	0.039818016	0	0	0
0	0	0	0	0
0	0	0	0	0
0	0	0	0	0
0	0	0.001488161	0	0
0	0	0	0	0
0	0	0	0	0
0	0	0	0	0
0	0	0	0	0
0	0.004456504	0	0	0
0	0	0	0	0
0	0	0	0	0
0	0	0	0	0
0	0	0	32.70673229	0
0	0	0	0	0
0	0	0	0	0
0	0	0	0	0
0	0	0	0	0
0	0	0	0	0
0	0	0	0	0
0.191629874	0	0	0	0
0	0	0	0	0
0	0	0	0	0
0.238585796	0	0	0	0.000910694

0	0	0	0	0
0	0	0	0	0
0	0	0	0	0
0	0	0	0	0
0	0	0.000882738	0	0.000513007
0	0.001184873	0	0	0.000553962
0	0	0	0	0
0	0	0	0	0
0	0	0	0.005428611	0
0	0	0	0	0
0	0	0	0	0
0	0	0	0	0
0	0	0	0	0
0	0	0	0	0
0	0	0	0	0
0	0	0	0	0.004378228
0	0	0	0	0
0	0	0	0	0
0.94981638	0	0	0.078364897	0
0.00045099	0	0	0	0
0	0	0	0.005485016	0
0	0	0	0	0
0	0	0	0	0
0	0	0	0	0
0	0	0	0	0
0	0	0	0	0
0	0	0	0	0
0	0	0	0	0
0	0	0	0	0
0	0	0	0	0
0.053565281	0	0.011676507	0.014783379	0.13894215
0	0.002713686	0	0.001505733	0.002255511
0	0	0	0	0
0	0	0	0	0
0	0	0	0	0
0	0	0	0	0
0	0	0	0	0
0	0	0	0	0
0	0	0	0	0.053175572
0	0	0	0	0
0	0	0	0	0
0	0	0	0.004318193	0
0	0.001254637	0	0	0
0	0	0	0	0
0	0	0	0	0
0	0	0	0	0
0	0	0	0	0
0	0	0	0	0

0	0	0	0	0
0	0	0	0	0
0	0	0	0	0
0	0	0	0	0
0.115668092	0	0	0.004980168	0.452755464
0	0	0	0	0
0	0	0	0	0
0	0	0	0	0
0	0	0	0	0
0	0	0	0	0
0	0	0	0	0
0	0	0	0	0
0	0	0	0	0
0	0	0	0	0
0	0	0	0	0
0	0	0	0	0
0	0	0	0	0
0	0	0	0	0
0	0	0	0	0
0	0	0	0	0
0	0	0	0	0
0	0	0	0.004460639	0
0	0	0	0	0.001795275
0	0	0	0	0
0	0	0.027589509	0.06673889	0.041324172
0	0	0	0	0
0	0	0	0.004252637	0
0	0	0	0	0
0	0	0	0	0
0	0	0	0	0
0	0	0	0.010854758	0.001662941
0	0	0	0	0.009921548
0	0	0	0	0
0	0	0	0	0
0	0	0	0	0
0	0	0	0	0
0	0	0	0	0
0.002945585	0	0	0.008135102	0
0	0	0	0	0
0	0	0	0	0
0	0	0	0	0
0	0	0	0	0
0	0	0	0	0
0	0	0	0	0
0	0	0	0	0
0	0	0	0	0
0	0	0	0	0
0	0	0	0	0

0.161079534	0	0	0	0
0	0	0	0	0
0	0.010422047	0	0	0
0	0	0	0	0
0	0	0	0	0
0	0	0	0	0
0	0	0	0	0
0	0	0	0	0
0	0	0	0	0
0	0	0	0	0
0	0	0	0	0
0	0	0	0.001180469	0
0.002347541	0.00179764	0	0	0
0	0	0	0	0
0	0.004118115	0	0	0
0	0	0	0	0
0	0	0	0	0
0	0	0	0	0
0	0	0	0	0
0	0	0	0	0
0.006897532	0.02390155	0.09639215	0.025799065	0.145503305
0	0	0	0	0
0	0	0.002086993	0	0
0	0	0	0	0.001517307
0	0	0	0	0
0	0	0	0	0
0.001278124	0	0.0016039	0.002353277	0.005253737
0	0	0	0	0
0	0	0	0	0
0	0.561958219	0	0	0
0	0.005203341	0	0	0
0	0	0	0	0
0	0	0	0	0
0.059589623	0	0	0	0
0.01876759	0.085533922	0.013032285	0.001213464	0.02404674
0	0	0	0	0
0	0	0	0	0
0	0	0	0	0
0	0	0	0	0
0	0	0	0	0
0	0	0	0	0
0.001629841	0	0	0	0
0	0	0	0	0
0	0	0	0	0
0	0	0	0	0
0	0	0	0.004813956	0
0	0.00263098	0.000705525	0	0
0.019479947	0	0.006036653	0	0.003373299

0	0	0	0	0
0	0	0	0	0
0	0	0.014214739	0	0
0	0	0.000590259	0	0
0	0	0	0	0
0	0	0	0	0
0	0	0.00082626	0	0
0	0	0	0	0
0	0	0	0	0
0	0	0	0	0
0	0	0	0	0
0	0	0	0	0
0	0	0	0	0
0	0	0	0	0
0	0	0	0.001590656	0
0	0	0	0	0
0	0	0	0	0
0	0	0	0	0
0	0	0.003541867	0.03444481	0.003018948
0	0	0	0.029226203	0
0.006978677	0.177166871	0.039881224	0	0
0	0	0	0	0
0	0	0	0	0
0	0	0	0	0
0	0	0	0	0.010455939
0	0	0.000839368	0	0
0	0	0	0	0
0	0	0	0	0
0	0	0	0	0
0	0	0	0	0
0.140793282	1.738598489	0.034446857	0.094434069	0.118625363
0	0	0	0	0
0	0	0	0	0
0	0	0	0.00370232	0.002695917
0	0	0	0	0
0	0	0	0	0
0	0	0	0	0
0	0	0	0	0
0	0	0	0	0
0.001281393	0	0.000383728	0	0
0	0	0	0	0
0.004413342	0	0.002349558	0	0.030950366
0	0	0	0	0
0	0	0	0	0
0	0	0	0	0
0	0	0	0	0
0	0	0	0	0
0	0	0	0	0

0	0	0	0	0
0	0	0	0	0
0.00759415	0	0.00983419	0	0.032195558
0	0	0	0	0
0	0	0	0.487564597	0.05736833
0	0	0	0	0
0	0	0	0	0
0	0	0	0	0
0	0	0	0	0
0	0	0	0	0
0	0	0	0	0
0	0	0	0.004627824	0
0	0	0	0.002125544	0
0	0.001351001	0	0.002023988	0.044424746
0.001497783	0.001561104	0.000897057	0.002041768	0.009905252
0.220491474	0	0	0.022853451	0.006697819
0	0	0.001192469	0	0.003960052
0	0	0	0	0.038244891
0	0	0	0	0
0	0	0	0	0
0	0	0	0	0
0.016368483	0	0.008611671	0	0.002144878
0	0	0	0	0
0	0	0	0	0
0	0	0	0	0
0	0	0	0	0
3.713738189	0	0	0	2.123510355
0	0	0	0	0
0	0	0	0	0
0	0	0	0	0
0	0	0	0	0
0	0	0	0	0
0	0	0	0	0
0	0	0	0	0
0	0	0	0	0
0	0	0.010443215	0	0.002142044
0	0	0	0	0
0.111052643	0	0.071873543	0.000719602	0
0	0	0	0	0
0	0	0	0	0
0	0	0	0	0
0	0	0	0	0
0	0	0	0	0
0	0	0	0	0
0	0	0	0	0
0	0	0	0	0
0	0	0	0	0
0	0.016057694	0	0	0

0	0	0.128978085	0	0
0	0	0	0	0
0	0	0	0	0
0	0	0	0	0
0.007914353	0.002900729	0	0.02977607	0
0	0	0	0	0
0	0	0	0	0
0	0	0	0.001974081	0.00184817
0	0	0	0	0
0	0.195227228	0.005385069	0.010754992	0.030207051
0	0	0	0	0
0.001351049	0	0.032007354	0	0.002717034
0.002562047	0.004476035	0.002864343	0	0.510961179
0	0	0	0	0
0.00122967	0	0	0	0
0	0	0	0	0
0	0	0	0	0
0	0.001589851	0	0	0
0	0	0	0	0
0	0.705699122	0	0	0
0	0	0	0	0
0	0	0	0	0
0	0	0	0	0
0	0	0	0.037711194	0
0	0	0	0	0
0	0	0.002383021	0	0.001661885
0	0	0	1.436073394	0
0	0.16477666	0	0	0
0	0	0	0	0
0.000818745	0	0.003922925	0	0
0	0	0	0	0
0	0	0	0	0
0	0	0	0	0
0	0.067872297	0	0	0
0	0	0	0	0
0	0.210266753	0	0	0
0	0.911478321	0	0	0
0	0	0	0	0
0	0	0	0	0
0	0	0	0	0
0	0	0	0	0
0	1.577453318	0	0	0
0	0	0	0	0
0	0	0	0	0
0	0	0	0	0
0	0	0	0.008513943	0
0.002474002	0	0	0	0.016533483
0	0	0	0	0

0	0	0	0	0
0	0	0	0	0
0	0	0	0	0
0	0	0	0.008183258	0
0	0.035244483	0	0	0
0.006235444	0.11141244	0	0	0
0	0.003355205	0	0	0.004625518
0	0	0	0	0
0	0	0	0.702899042	0.00637832
0	0	0	0	0
0.000678006	0.003499699	0	0.000672184	0
0	0.020941639	0	0	0
0	1.884869564	0	0	0
0	0	0.225579105	0.00211682	0.00396361
0	0.076430025	0	0	0
0	0	0	0	0
0	0	0	0	0
0	0.039601267	0	0	0.002121478
0	0	0.001451817	0	0
0.04084195	0.001830088	0	0	0
0	0	0	0	0
0.175016643	0	0.424876622	0.214917866	0.357607028
0.057542907	0	0	0	0
0	0	0	0	0
0	0	0	0	0
0	0	0	0	0
0	0.008548722	0	0	0
0	0	0	0	0
0	0	0	0	0
0.077616262	3.927449688	3.776127622	0.099910643	0.565295713
0.001883092	0	0.01283988	0	0.034822478
0	0	0	0	0
0	0	0	0	0
0.000820647	0.00073315	0	0	0
0	0.041343563	0	0	0
0	0	0	0	0
0	0	17.12487076	1.01389243	0
0	0	0	0	0
0	0	0.01473805	0	0
0	0	0	0	0
0.048096003	0.012127226	0	0	0.005865334
0.415608486	0	0	1.026133447	0.499679288
0	0	0	0	0
0	0	0	0	0
0	0.001651052	0	0	0
0	0	0	0	0
0	0.00087782	0.000735615	0	0
0	0	0	0	0

0.004773599	0	0.058451066	0	0
0	0	0	0	0
0	0	0.001731393	0.002149515	0.019836658
0	0	0	0	0
0.088965063	0	0	0	0.095301197
0	0.02060432	0	0	0
0	0	0	0	0
0	0	0	0	0
0	0.008157989	0	0	0
0	0	0	0	0
0	0	0	0	0
0	0	0	0	0
0	0	0	0	0
0.032080692	0	0	0.001427158	0
0	0	0	0	0
0.183850387	0.053600841	0	0	0.022076593
0.007303268	0.003182729	0.067282855	0.000529797	0.025296277
0	0.021139008	0.001036948	0	0
0	0.034409364	0	0	0
0	0	0	0	0
0.006061299	0	0.053064441	0	0.011460313
0.003719156	0.00073836	0	0	0
0	0	0	0	0
0	0	0	0	0
0	0.136631826	0	0	0
0	0	0	0	0
0.003392033	0.001346833	0	0	0
0	0	0	0	0
0	0	0	0	0
0	0	0	0	0
0.007050993	0.092593376	0.12057877	0.015515389	0
0.002820337	0	0.012284839	0	0.009519193
0.025876934	0	1.484522426	0.017667889	0.017447351
0.002798805	0.007084459	0.005866943	0	0
0	0	0	0.001980482	0.001854163
0	0	0	0	0
0	0	0.002291193	0	0.001433964
0.031240615	0.06455735	0.009372594	0	0
0	0	0	0.005084738	0.072834475
0	0.022861093	0	0	0
0	0	0.001049727	0	0
0	0	0	0	0
0.003355601	0.001537347	0	0	0.004791691
0.000808414	0	0.125133069	0	0
0	0.782348045	0	0	0
0	0	0	0.000746131	0.001397082
0	0	0	0	0
0	0	0	0	0

0	0.109434546	0	0	0
0.16674936	0.031937274	0.010705395	0.165084401	1.436182538
0	0	0	0	0
0	0	0	0	0
0	0	0	0	0
0	0	0	0	0
0	0	0	0	0
0	0	0.013993309	0	0.004191897
0	0	0	0	0.006994185
0	0	0	0	0
0.001747715	0.003955481	0.011863135	0	0
0.006508178	0	0.151758211	0	0.135312924
0	0	0	0.065229244	0.795121787
0	0	0	0	0
0	0	0.010889172	0	0.00409478
0	0	0	0	0
0.00549033	0.015913845	0	0.000907198	0.056565712
0	7.11836454	0	0	0.008599923
0	0	0	0	0
0	0.137819848	0	0	0
0.005473185	0.002248108	0.001205708	0.100852283	0.432508886
0	0	0	0	0
0	0	0	0	0
0	0	0	0	0
0	0.004154965	0	0	0
0.001730712	0	0	0	0
0.005402307	0.187948875	0	0.006464042	0
0.017050378	0	0.542870881	0.096849834	0.038354305
0	0	0.001405505	0	0
0.001693732	0	0.000772888	0	0
0	0	0	0	0
0	0	0	0	0
0	0	0	0	0
0	0	0	0	0
0	0	0	0	0.141155837
0.087588714	0.048527145	0.129046224	0.091548702	0.480855729
0	0	0	0	0
0	0.003071135	0	0	0
0	0	0.011066022	0.004415918	0.004823305
0	0	0	0	0
0	0.298900977	0	0	0
0	0	0.006531526	0	0
0	0.00608742	0.001440358	0	0.085381259
0	0.004148675	0	0	0.00444497
0	0	0	0	0
0	0	0	0.084433208	2.024071236
0	0	0	0	0
0	0.023471041	0	0	0

0	0.005002505	0	0	0
0	0	0	0	0.094071803
0	0	0	0.022806225	0
1.034955533	0.339064917	0.233880012	0.186400205	0.055245638
0	0	0	0	0
0	0	0	0	0
0	0	0	0	0.003401263
0.00109627	0	0.034017194	0	0
0.857514366	0	7.833156829	0.311872097	0.562553319
0.011891014	0	0.000874608	0.18769189	0.076532822
0	0	0	0	0
0.001677264	0	0	0	0.020394103
0	0	0	0	0
0	0	0	0.034790308	0
0.124152224	0.007913677	0.113291109	0.041028731	0.302798318
0	0	0	0	0
0.042607787	0	0	0.000894956	0.016589908
4.003218452	0	0	0	0
0	0.208281268	0	0	0
0	0	0	0.001039487	0
0	0	0	0	0
0	0	0	0.002007	0
0	0	0	0	0
0.162653627	0.855194063	0.068032226	0	0
0.012719819	0	0	0	0
0.082053278	0	0	0.65399886	0.12317814
0	0	0	0	0
0	0	1.180472664	0.396525948	1.44866882
0	0	0	0	0
0	0	0	0	0
0.275080008	0	0.382955994	0	0.009988947
0	0	0	0.026502354	0
0.004095288	0.008573804	0.133093368	0	0.015435017
0	0	0	0	0
0	0	0	0.003283662	0
1.022399671	0	0.197295408	0.024398176	0.174908209
0.023391068	0	0	0.528418401	1.70788478
0	0.001870897	0	0	0
0.0162381	0.013726862	0	0	0.023499146
0	0	0.040074047	0	0.008691674
0	0.045424375	0.449721354	0.126500893	0.022252748
0	0	0	0.056250217	0
0.098055954	0.249927964	0.525469074	0.820960743	10.3156987
0.005046048	0	0.001678997	0	0.007415758
0	0.007546445	0	0	0
0	0	0	0	0
0	0	0	0	0
0	0.047693245	0.004942781	0	0.487251446

0	0	0	0.000660147	0
0.484591791	0	0.163374955	0.006988085	0.112365221
0.049506931	0.880373758	0.004442075	0	0.007400397
0.017036491	0	0	0	0
0	0	0	0.065192253	0
0.061347767	0	0	0	0.006507625
0	0	0	0	0
0	0	0	0	0
0	0	0	0	0
0	0.013396015	0	0	0
0.008192196	0.031009794	0.010031059	0.001985342	0.090907955
0.854352401	0	0.490848078	1.314665544	1.387015819
0	0	0	0	0
0.053048109	0	0.002667258	0	0.002735453
0.347746881	0.218732456	0.110782562	0	0.053603622
1.868206501	0	0	0.507034843	1.144944588
0	0	0	0	0
0	0.001024059	0	0	0
0	0	0	0.131782429	0
0.0101973	0.005716119	0.025148117	0.007433633	0
0.00701885	0.005270856	0.007798265	0.151273539	0.032715377
0.009807329	0.512869072	0.380419212	0	0.066854511
0.042659223	3.992718104	0.484291522	0.578384409	2.339606708
0	0	0	0	0
0	0	0	0.005633989	0
0	0	0.000876062	2.932240921	9.128659982
0	3.63121323	0	0.092817192	0
0	0	0.005083406	0.002682184	0
0	0.003455211	0	0	0
0	0	0	0	0
0	0	0	0.003503867	0.016089498
0.091760299	0	0.505228242	0.172376277	0.413816549
0	0.014476303	0	0.002965815	0.0295019
0.105222729	0	0.042924422	0.006643637	0.05187655
0.01048915	0.029986555	0.000492721	0	0
0.069683602	0.011107761	0.220556447	0.010589712	0.109529177
0.004585828	0	0.005885484	0.002435599	0.038612266
0	0	0	0	0
5.603116562	0	0.131503581	0.009835002	0.002394003
0	0	0	0	0
0	0	0	0.002812234	0
0	0	0	0	0
0	0	0	0.001810594	0
0	0	0	0	0
0.00773394	0	0.101750406	0.022810911	0.003589241
0.238959605	0	0	0.002963199	1.16086418
0	0	0	1.016812153	0
0.755902131	0.133353011	0.419621403	0	0.429713368

0	0	0	0	0
0	0	0	0.055033337	0
0	0	0	0	0
0.017467756	0	0.005263821	0	0.003976824
0	0.021612162	0	0	0
0	0	0	0.03737161	1.142174941
0	0	0	0.506366015	0
0	0	0	0.003006695	0
0	0	0	0	0
0	0	0	0	0
0.013397879	0.00787029	0.013190632	0	0.010221068
0	0	0	0	0
0.295361336	0	0	0.004938837	0.027444655
0	0	0	0.031674849	0
0	0	0	0	0
0	0	0	0	0
0	0	0	0.017592624	0.081212385
0	0	0.0012201	0	0.003261709
0	0	0	0.02980624	0
0	0	0	0	0
0.012487983	0	0.028941805	0	0
0	0	0	0.034006153	0
0.017967517	0	0	0.000523919	0.010954549
0	0	0	0	0
0	0	0	0	0
0	0	0	0	0
0	0	0	0	0
0	0	0	0	0
0	0	0	0	0
0.05130906	0	0.110540247	1.546548676	0.057731253
0	0	0	0.002579601	0
0	0	0	0	0
0	0	0	0.038458485	0
0	0	0	0	0
0.000729681	0	0	0	0
0	0	0	0	0
0	0	0	0.025408908	0
0	0.006662339	0	0	0
0	0	0	0.014077242	0
0	0.000730151	0	0	0
0.021136572	0.029344608	0	0.002890356	0.002164803
0	0	0	0.004003821	0
0	0	0	0.006251069	0
0.003922787	0	0.001686783	0.020492586	0.007282098
0	0	0.372480617	0.039702058	0.224819862
0	0	0	0.006140167	0
0.037643455	0	0	0	0
0.597374535	0	0	0.002057836	0

0	0	0	0	0
0	0	0	0	0
1.0515511	0	0	0.389096511	0
0.433642602	0	0	0.036618202	0.105860137
0	0	0	0	0
0	0	0	0.065970097	1.535130254
0	0.297036056	0	0	0
0	0	0	0	0
0.009642253	0.0474738	0	0.00870973	0.025854799
0	0	0	0	0.001601597
0	0	0.019820558	0.000759479	0.01592725
0	0	0	0	0
0	0	0	0	0
0	0	0	0	0
0	0	0.017952789	0.248224446	0.103047212
0	0.548840673	0	0	0
0	0	0	0	0
0	0	0	0	0
0	0	0	0	0
0	0	0	0	0
2.005916279	0	0	0.012345065	0.096719465
0	0	0	0.012386568	0
0	0	0	0	0
0	0	0	0	0
0.005795496	0	0.057951569	0.33550088	0.164652104
0.83481204	0	0.756739096	0.726395898	0.980968812
0.003936279	0	0	0.138259304	0.314903133
0	0	0	0	0
0.606047836	0	0	0.01288615	0
0	0	0	0.019389768	0.037150427
0	0	0	0	0
0	0	0	0.055343381	0
0	0	0	0	0
0.00053829	0	0.003116479	0.001734421	0.001498889
0.001552131	0	0.000708272	0	0.00493939
0.002351075	0.012135665	0.025033125	0	0.007419543
0	0.013679702	0.003100019	0	0.108846145
0.034158037	1.287895941	0.013992029	0.415676527	1.042643675
0	0	0	0.205477525	0
0	0	0	0	0
0	0	0	0	0
0	0	0.000567755	0	0
0.153785333	0	0.016590983	0.032276683	1.312893057
4.054589371	0	0	1.984597241	0.324009958
0.0293818	0	0	0.035082992	0
0.005494126	0.005660071	0	0	0.004410407
0	0	0	0	0
0.643212558	0.004226799	0.002833652	0.066372256	0

0.008643829	0.009876044	0	0	0.013590017
2.610517163	0	0	0.008002013	0
0	0	0	0.042717071	0
0	0	0	0	0
0	0	0.000561012	0	0.001630173
0.006934343	0	0.269987807	0.027834561	0.145837424
0	0	0	0.018297525	0
0	0	0	0	0
0	0	0	0	0
0	0	0	0	0
0	0	0	0	0
0.130435013	0	0.664429117	0.02377469	0.191000209
0	0	0.07421206	0.015812453	0.467605948
0	0	0	0	0
0.020717993	0.009435983	0.401613271	0	0.116134175
0	0	0	0	0
0	0	0	0	0
0	0	0	0	0
0.00872356	0	0.010236231	0.002824051	0.027595988
0.449500021	0	0	0.001174283	0
0.049082213	0	0	0.02528022	0.080306337
0	0	0	0	0
0.577500855	0	0	0.013558402	0.058808417
0	0	0	0	0
0	0	0	0	0
0	0	0	0	0
0.163443767	0	0	0	0
0	0	0	0	0
0.022503779	0	0	0	0.001018904
0	0	0	0	0
0	0	0	0	0
0.020031583	0.003139617	0.005472489	0.003658344	0.165868224
0	0	0	0	0
0.025281938	0.02556653	0	0.058484662	0.174529624
0.001705706	0.013003465	0.026833679	0	0.009024242
1.255549857	0.019120347	0.823883414	0.045997598	0.013470186
0	0	0	0	0
0.115478345	0	0	0	0
0	0	0	0	0
0	0	0	0	0
0	0	0	0.131544036	0
0	0	0	0.001791856	0
0	0	0	0	0
0	0	0	0.008397737	0
0.002184378	0.00220167	0.481579359	0.004331244	0.099035305
0	0	0	0	0
0	0	0	0.345779585	0.077552295
0.007310571	0.003028925	0	0	0

0	0	0	0	0
0	0	0	0.030344837	0
0	0.16291457	0	0	0
0	0	0	0	0
0	0	0	0	0
0	0	0	0	0
0.242183231	0	0.001069346	0.297190005	0
0	0	0	0.001301709	0
0	0	0	0	0
0.016957834	0	0	0	0.004347213
0	0	0	0	0
0.001414569	0	0.008895793	0	0
0	0	0	0	0
0	0	1.402352119	0.284374229	0
0	0	0	0	0
0	0	0	0	0
0	0	0	0.455012754	0
0	0	0	0.00632786	0
0	0	4.231416447	1.295324745	0
0	0	0	0	0
0	0	0	0.001095799	0
0	0	0	0	0
0	0	0	0	0
0	0	0	0	0
0.013401806	0.00065605	0	0	0
0.002659289	0.010062024	0.024484009	0	0
0	0	0	0	0
0.022048218	0.003282907	0.000978162	0.508826651	0.003552899
0	0	0	0.029561198	0
0.503600508	0	0	1.022428689	0.767545271
0	0	0	0	0
0	0	0	0	0
0	0	0	0	0
0	0	0	0	0
0.003114625	0.008005917	0.054719029	0	0.008520637
0	0	0	0	0
1.29768394	0	0	0	0
0	0	0.009506614	0	0.008632524
0	0	0	0	0
0.013933612	0	0	0	0
1.391891374	2.630037833	2.771098093	0.545779642	10.72532575
0	0.062605444	0.000326198	0.162529077	0.006571809
3.468279142	0	0	0	0.141512393
0.017174788	0	0	0.062630914	0
0	0	0	0	0
0	0	0	0	0
0	0	0	0.003348315	0
0.002066788	0	0.009549109	0.001317241	0.006029099

0	0	0	0	0
0.006073892	0.009586454	0	0.000752717	0
0.010286246	0	0.000566499	0.000879131	0.000658447
0	0	0	0	0
1.793068383	0	0.477918785	0.207413362	8.105198896
0	0	0	0.014665211	0
0	0	0	0	0
0	0	0	0	0
0	8.599916908	0.00339027	0	0.003377609
0	0	0	0	0
0	0	0	0	0
0	0	0	0	0
0.076281038	0	0	0.04812567	0.048342886
0.003809237	0.186035943	0.19946307	0.001258843	0.035019812
0.213125645	0	0	0	0
0.218659393	0	0	0.030399637	0.14796794
0	0	0	0.043106474	0
0	0	0	0	0
0	0	0	0	0
0	0	0	0.020137615	0
0	0	0	0	0
0	0	0	0	0
0	0	0	0	0
0	0	0	0	0
0	0	0	0.009333402	0.019954166
0	0	0	0	0
0.013239444	0	0	0.003518246	0.039146082
0.002926241	0	0.001844836	0	0.001715417
0	0.006969453	0	0	0
0	0	0	0	0
0	0	0	0	0
0	0	0	0	0
0	0	0	0.000987525	0
0.027284444	0	0	0.005442045	0.029820389
0.000763792	0	0.612682034	0.007950958	0.244760203
0.011402286	0	0.003841366	0	0.033734475
0.001638915	0	0	0	0
0	0	0	0.003836163	0.015927454
0	0	0	0	0
0	0	0	0.041817027	0
0.668349804	0	0.304933247	0.117249488	0.1085559
0.139550848	0	0.00069146	0	0.001071588
0	0	0	0.026847914	0.009140182
0.87180409	0	0.149022643	0.011212779	0
0	0	0	0	0
0	0	0	0	0
0	0	0	0	0
0	0	0	0	0

0.02276498	0	0	0.157652199	0.071372376
0	0	0	0	0
0	0	0	0	0
0	0	0	0	0
0.00317167	0	0.001599652	0.058420322	0.010226103
1.869759595	0	0	0	0
0.002818533	0	0.006877382	0.184581122	0.007993649
0	0	0	0	0
0	0.001184878	0	0	0
0	0.523666273	0	0	0
0.018418859	0	0	0.000753019	0
0	0	0	0	0
0	0	0	0	0
0	0	0	0	0
0	0	0	0	0
0	0	0	0	0
0	0	0	0	0.001494908
0	0	0	0	0
0	0	0	0	0
0	0	0	0	0
0.066637774	0.006392791	0.003214302	0.058823501	0
0	0	0	0	0
0	0	0	0.00407551	0.042276466
0.005409951	0.003657514	0	0.000869757	0.00339284
0	0	0	0	0
0.002760011	0	0.001928541	0	0.006244351
0	0	0	0	0
0	0	0	0	0
0.100363401	0	0	0	0
0.002104375	0	1.359339324	0	0.2718906
0	0	0	0.02633005	0
0	0	0	0	0
0	0	0	0.037880557	0
0	0.002232002	0	0	0
0	0	0	0.015452778	0
0	0	0	0.393842736	0
0	0.018741844	0	0.002539345	0.029887067
0	0	0	0	0
0.058668701	0	0	0.006685626	0
1.28279235	0	0	0	0
0	0	0	0	0
0	0	0	0	0
0	0	0	0	0
0	0	0	0.065425023	0.053149955
0	0	0	0.067292352	0
0	0	0	0	0.012372815
0	0	0	0.001268305	0
0.086493614	0	0	0	0

0.007079958	0.006371947	0	0	0.010076254
0.277089253	0	0.335902129	0.018787385	0
0	0	0	0.000556105	0
0	0	0	0	0
0	0	0	0	0
0	0	0	0	0
0	0	0	0	0
0.124712607	0	0.00361211	0.536047912	1.184845972
0.016052195	0.018238445	0.600310065	9.633821121	0.593909542
0	0.049354695	0.01825988	0	0
0	0	0	0.023802071	0
0	0	0	0	0
0	0	0	0.045759878	0
0	0.080360109	0.0112419	0	0
0	0	0	0	0
0	0	0	0	0
0	0.227061835	0	0	0
0	0	0	0	0
0	0	0	0	0
0.166799265	0	0	0.009611076	0
0	0	0	0	0
0	0	0	0.001605907	0
0	0	0	0.071415246	0
0	0	0	0	0
0	0	0	0.038699967	0.025419634
0	0	0	0.008546144	0
0	0	0	0	0
0	0	0	0	0
0	0.025342596	0	0	0
0	0	0	0	0
0	0.034655715	0	0	0
0	0	0	0	0
0	0	0	0.009135256	0
0	0	0	0	0
0	0	0	0	0.013789862
0	0	0	0	0.013043163
0	0	0	0	0
0	0	0	0	0
0	0	0.001493958	0	0.000868221
0	0	0	0	0
0	0	0	0	0
0.231720781	0	0	0.343150814	0.110396885
0	0	0	0	0
0	0	0	0	0
0	0	0	0	0
0	0	0	0	0
0	0	0	0	0
0	0	0	0	0

0	0	0	0	0
0	0	0	0.105585081	0.023724156
0	0	0	0	0
0	0	0	0	0
2.44615097	0.185171486	0.728663845	0.266311509	0.695089612
0.086728313	0	0	0	0
0	0	0	0	0
0.004403041	0.012396767	0.001118763	0.00753995	0.02879344
0	0	0	0	0.002967658
0	0	0	0	0.143878963
0	0	0	0	0
0	0	0	0	0
0	0	0	0	0
0	0	0	0	0
0	0.024166762	0	0	0
0	0.005996098	0	0	0
0	0	0	0	0
0	0	0	0	0
0	0	0	0	0
0	0	0	0	0
0	0	0	0.004341767	0
0	0	0	0	0
0	0	0	0	0
0.012817033	0.00247578	0	0	0.001286108
0.152253025	0	12.80334373	0.163473591	0.070229939
0	0	0	0	0
0.005069246	0.116426971	0.001518045	0.218932884	0
0	0	0	0	0
0.000941851	0	0.001128193	0	0
0	0	0	0	0
0	0	0	0	0
0	0	0	0	0
0	0	0	0	0
0	0	0	0	0
0	0	0	0	0
0	0	0	0	0
0	0	0	0	0
0.117135661	0	0	0.039139474	0
0	0	0	0	0
0	0	0	0	0
0	0	0	0	0
0	0	0.715097556	0.002031556	0.199189098
0	0	0	0	0
0	0	0.057268259	0	0
0	0	0	0	0
0	0	0	0	0
0	0	0	0	0
0	0	0	0.001271662	0

0	0	0	0.002363128	0.151687861
0	0	0	0	0
0.006197841	0	0	0	0
0	0	0	0	0
0	0	0	0	0
0	0	0.053201922	0.418373164	0
0	0	0	0	0
0	0	0	0	0
0	0	0	0	0
0	0	0	0	0
0	0	0	0	0
0	0	0	0	0
0	0	0	0.003577273	0
0	0	0	0	0
0	0	0	0	0
0	0	0	0.08977468	0
0	0	0	0	0
0	0.760317638	0	0	0
0	0	0	0	0
0	0	0	0	0
0	0.043761394	0.025343362	0	0
0	0	0	0	0
0.009148799	0	0.094107009	0.006262787	0.171047599
0.042465393	0	0	0.016800775	0.00259069
0.049500557	0	0	0.010862566	0
0	0	0.002133128	0.007944802	0
0	0	0	0	0
0	0	0	0	0
0	0	0	0	0
0	0	0	0	0
0	0	0	0	0
0	0	0	0	0.009961493
0.00183596	0.586587749	0.76923061	0.002224683	0
0	0.267431972	0	0	0
0	0	0.099524343	0.015738843	0.435274926
0	0.16944855	0	0.000649169	0.001367469
0	0	0	0	0
0	0	0	0	0
0	0.063827752	0	0	0
0	0	0	0	0
0	0	0	0	0
0	0	0	0.000909824	0
0.133281654	0	0.781105141	0.031388081	0.39825338
0	0	0	0	0
0	0	0	0	0
0.015824564	0	0	0.001045912	0.06283213
0	0	0	0	0
0	0	0	0	0

0	0	0	0	0
0	0	0	0	0
0	0	0	0	0
0	0	0	0	0
0	0	0	0	0
0	0	0	0	0
0	0	0	0	0
0	3.762991914	0.002035213	0	0
0.220580153	0	0.00355189	0.026947882	0.024923284
0.070448445	0	0.000867727	0	0.039166029
0	0	0	0	0
0	0	0	0	0
0	0	0	0	0
0	0	0	0.003860662	0
0	0	0	0	0
0	0.017828814	0	0	0
0	0	0	0	0
0	0	0	0	0
0	0	0	0	0
0.202438723	0	0.001191307	0.201735789	0.39504569
0	0	0	0	0
0	0	0	0	0
0	0	0	0	0
0	0	0	0	0
0	0	0	0	0
0	0	0	0	0
0	0	0	0	0
0	0	0.001261986	0.005918832	0
0	0	0	0	0
0	0	0	5.594989071	0.076106202
0.263287771	0	0.003006907	0.49075384	0.238866943
0	0	0	0	0
0	0	0	0	0
0	0	0	0	0
0	0	0	0	0
0	0	0	0	0
0	0	0	0	0
0	0	0	0.002069438	0
0	0	0.601399831	0.009870985	0
0	0	0	0	0
0	0	0	0	0.001264345
0	0	0	0.038670973	0
0.014724024	0	0	0.004969394	0
0	0	0	0	0
0	0	0	0	0
0	0	0	0	0
0	0.075177672	0	0	0
0	0	0	0	0

0	0	0	0	0
0	0	0	0	0
0	0	0	0	0
0	0	0	0	0
0.041289952	0	0	0.001891405	0
0.035539017	0	0	0.006028608	0
0	0	0	0	0
0	0	0	0	0
0	0	0	0	0
0	0	0	0	0
0.009342871	0	0	0.00308755	0.022322006
0.017784021	0.004281843	0.084907946	0.009378361	0.018262796
0.003101271	0	0.020464241	0	0
0	0	0	0	0
0	0.035721959	0	0.006146391	0
0	0	0	0.083431982	0.043015409
0	0.004853204	0	0	0
0	0	0	0	0
0.021488558	1.577091964	0.005834398	0	0.010770425
0.00093525	0	0	0.276496942	0.001041696
0	0	0	0	0
0	0	0	0	0.032727952
0	0	0	0	0
0	0	0	0	0
0	0	0	0	0
0	0	0	0	0
0	0.042494217	0	0	0
0	0	0	0	0
0	0	0	0	0
0	0	0	0	0
0.002888474	0.111113511	0	0	0
0	0	0	0	0
0	0	0	0	0
0	0	0	0	0
0	0	0	0.005128885	0
0	0	0	0	0
0	0	0	0	0
0.0527633	0.027017075	0.022592689	0.000710094	0.174178295
0	0	0	0.024057077	0.317449676
0	0	0	0	0
0	0	0.001427518	0.029773908	0
0	0	0	0.001972321	0
0	0	0	0	0
0	0.031206888	0.314099792	0	0
0	0	0	0	0
0	0	0	0	0
0	0	0	0	0
0.023487359	0	0	0	0

0	0	0	0	0
0.001664269	0	0	0.005699926	0.110799952
0	0	0	0.001213392	0
0.006492992	0.001325877	0.002518464	0	0.002238474
0	0	0	0	0
0	0.002380626	0	0	0
0	0	0	0.000535445	0.022892371
0.004642818	0	0.059321419	0	0
0	0	0	0	0.055534001
0	0	0	0.001062658	0
0	0	0	0.024164329	0.083520425
0	0	0	0	0
0.06693521	0	0.00430557	0.002868233	0.112416043
0	0	0	0.00110424	0
0	0.001355512	0	0	0
0	0	0	0	0
0	0	0	0	0
0	0	0	0	0
0	0	0	0	0
0	0	0	0	0
0	0	0	0	0
0	0	0	0	0
0	0.001031316	0	0	0.004500248
0.000613003	0.028234125	0	0.374367468	0.003793177
0	0	0	0	0
0.033047724	0	0	0.008677213	0
0	0	0	0.00328037	0.00261047
0	0	0	0	0
1.014714041	0	0	0	0
0	0	0	0	0
0.005985238	0	0	0.021629827	0.018458177
0	0	0	0.00074011	0
0	0	0	0	0
0	0	0	0	0
0.626750566	0	0	0.012677725	0
0	0	0	0	0
0	0	0	0	0
0	0	0	0	0
0.00065341	0	0.027785309	0	0.002729167
0	0	0	0	0
0	0	0	0	0
0	0	0	0	0
0	0	0	0	0
0	0	0	0	0
0	0	0	5.289256477	0
0.388052629	0	0.28693054	0.03889953	0.087377574
0	0	0	0	0
0	0	0	0	0

0	0	0	0	0
0	0	0	0	0
0	0	0.403321606	0	0.080730577
0	0	0	0.003872312	0.643294384
0	0	0	0	0
0	0	0	0	0
0	0	0	0	0
0	0	0	0	0
0	0	0.02667439	0	0
0	0	0	0	0
0.005963518	0	0.002010729	0.003021849	0.002460095
0.001384997	0.000942727	0	0	0
0.08084518	0	0	0	0
0	0	0	0	0
0	0	0	0	0
0	0	0	0	0
0.00077921	0	0	0	0
0	0	0	0	0
0	0	0	0	0
0	0	0	0	0.002283756
0	0	0	0	0
0	0	0	0	0
0	0	0	0	0
0	0	0	0	0
0	0	0	0	0
0	0	0	0	0
0	0	0	0	0
0	0	0	0.010991001	0.041159892
0	0	0	0	0
0	0	0	0	0
0.001207884	0.004316399	0.017482889	0.001047824	0.003923965
0	0	0	0	0
0.014200074	0	0.113116735	0.040496395	0.028638488
0.003762095	0.026247658	0	0.000888046	0.006152391
0	0	0	0.003966516	0
0	0	0	0	0
0	0	0	0	0
0	0	0	0	0
0	0	0	0	0
0	0	0	0	0
0	0	0	0	0
0	0.005841122	0	0	0
0.015110839	0	0.028483678	0.007838942	0.054145207
0	0	0	0	0
0	0	0	0	0
0.246292166	0	0.074414031	0.008558795	0.029694856
0	0	0	0	0
0	0	0	0	0

0	0	0	0	0
0.004233767	0	0.01308275	0	0.007175964
0.001290127	0	0	0.0008527	0.030934618
0	0	0	0	0
0.109019379	0.036197477	0.048533671	0.01736141	0.04732801
0	0	0	0	0
0	0	0	0	0
0	0	0	0	0
0	0	0	0	0
0	0	0	0	0
0	0	0	0.002705251	0
0	0	0	0	0
0	0	0	0	0
0	0.188586908	0	0	0
0	0	0	0	0
0	0	0	0	0
0.024808936	0	0	0.007540353	0
0	0	0	0	0
0	0	0	0	0
0	0	0	0	0
0	0	0	0	0
0	0	0	0	0
0	0	0	0.003133569	0
0	0	0	0	0
0.009037592	0	0.007641634	0.005973327	0.002302727
0	0	0.220369342	0	0.003474733
0	0	0	0	0
0.671813447	0	0	0.001714401	0.016050534
0	0	0	0	0
0	0	0	0	0
0.007087316	0	0.005957555	0	0
0	0	0	0	0
0	0	0	0	0
0	0	0	0	0
0	0	0	0	0
0	0	0	0	0
0	0	0	0	0
0	0	0	0	0
0	0	0.888072515	0.068196541	6.760252348
0	0	0	0	0
0	0	0	0	0
0.064206119	0	0.002316538	0	0
0.005725124	0.005754051	0	0	0.00763879
0	0	0	0	0
0	0	0	0.26609182	0
0	0	0	0	0
0.024085379	0.000978064	0.019452289	0	0.051824221
0	0	0	0	0

0	0	0	0	0
0.013468879	0.07857335	0.084529292	0	0.013463189
0	0	0	0.045266576	0.021474754
0	0	0	0	0
0	0	0	0	0
0	0	0	0	0
0	0	0	0	0
0	0	0	0	0
0	0	0	0	0
0	0	0	0	0
0	0	0	0	0
0	0	0	0	0
0	0	0.001894966	0.063700927	0.020500546
0	0	0	0	0
0.018946696	0.003334028	0.00240707	0	0
0	0	0	0	0
0	0	0	0	0
0	0	0	0	0
0.000952725	0	0	0.001180681	0
0	0	0	0	0
0	0	0	0	0
0	0	0	0.051568816	0
0.000649912	0.009096362	0	0	0
0.004355745	0	0	0	0
0	0	0	0	0
0	0	0	0	0
0	0	0	0	0
0	0	0	0	0
0	0	0	0	0
0	0	0	0	0
0	0	0	0	0
0	0	0	0	0
0	0	0	0	0
0	0	0	0	0
26.17688429	0	0	0.001522654	0
0	0	0	0	0
0.000929949	0.004430922	0	0	0
0	0	0	0	0
0.006334599	0	0.002023434	0.019049963	0.000783952
0	0	0	0	0
0.113974778	0.001127606	0.041123595	0.012388281	0.10684339
0	0	0	0	0
0	0	0	0	0
0	0	0	0	0
0	0	0	0	0
0.005635926	3.209505431	0.051238163	0.265192927	0.006438337
0	0	0	0.193188835	0
0	0	0	0	0

0	0	0	0	0
0	0	0	0	0
0	0	0	0	0
0	0	0	0.020422903	0
0.00180841	0	0	0	0
0	0	0	0	0
0	0	0	0	0
0	0	0	0.003948795	0
0	0	0	0	0.005451914
0.001059989	0	0.001015764	0	0.000787088
0	0	0	0.064060388	0
0	0	0	0	0
0.003522571	0.000740469	0	0	0
0	0	0	0	0
0	0	0	0	0
0	0	0	0	0
0	0	0	0.049778361	0
0	0	0	0	0
0	0	0	0	0
0.073403769	0.070081118	0.575130276	0.013713597	0.14739076
0	0	0	0	0
0	0	0	0	0
0	0	0	0	0
0	0	0	0	0
0	0	0	0	0
0.093267459	0	0	0.024121727	0.004106035
0	0	0	0	0
0	0	0	0	0
0	0	0	0	0
0	0	0	0	0
0	0	0	0	0
0	0	0	0	0
0	0	0	0	0
0	0	0	0	0
0	0	0	0	0
0	0	0	0	0.004431915
0	0	0	0.138531857	0
0	0	0	0.032750141	0
0	0	0	0	0
0	0	0	0.033901238	0
0	0	0	0	0
0	0	0	0	0
0.003679363	0	0	0.001215924	0
0	0	0	0	0
0.012125744	0.000825364	0.005671581	0.003949963	0.005948999
0	0	0	0.011127242	0.010591149
0	0	0	0	0
0	0	0	0	0

0	0	0	0	0
0	0	0	0	0
0	0	0	0.002106337	0
0	0	0	0	0
0	0	0	0	0
0.086255864	0.011861198	0.001755584	0.003589845	0.007802036
0	0	0	0.000921683	0
0	0	0	0	0
0	0	0	0	0
0	0	0	0	0
0	0	0	0	0
0	0	0	0	0
0	0	0	0	0
0.044145723	0	0	0	0
0	0	0	0	0
0	0	0	0	0
0.111529738	0	0	0.009175462	0.035525706
0	0	0	0	0
0	0	0	0	0
0	0	0	0.003254933	0
0	0	0	0	0
0	0	0	0	0
0	0	0	0	0
0	0	0	0	0
0	0	0	0	0
0	0.001927575	0	0	0
0	0	0	0	0
0.004390317	0	0	0.057184412	0
0	0	0	0	0
0	0	0	0	0
0	0	0	0	0
0	0	0	0	0
0	0	0	0	0
0	0	0	0	0
0	0	0	0	0
0	0	0	0	0
0	0	0	0	0
0	0	0	0	0
3.804614457	0	1.0992845	0.445387184	0.006777399
0	0	0	0	0
0	0	0	0	0
0	0	0	0	0
0	0	0	0	0
0	0	0	0	0
0	0	0	0	0
0	0	0	0	0
0	0	0	0	0
0	0	0	0	0

0	0	0	0	0
0	0	0	0.00043062	0
0	0	0	0	0
0	0	0	0	0
0	0	0	0	0
0.007743092	0	0	0	0
0	0	0	0	0
0	0	0	0	0
0	0	0	0	0
0	0	0	0.005067219	0
0.007583285	0	0.012717055	0	0
0	0	0	0.000807758	0
0.114921102	0	0	0.082093634	0
0	0	0	0	0
0	0	0	0	0
0	0	0	0	0
0	0	0	0	0
0	0	0	0	0
0	0	0	0	0
0.001577539	0	0	0	0
0.000769658	0.010313955	0	0	0
0	0	0	0	0
0	0	0	0	0
1.062693432	0	2.623532848	0.435681528	3.289181887
0	0	0	0	0
0	0	0	0	0
0	0	0	0	0
0	0	0	0	0
0	0	0	0	0
0	0	0	0	0
0	1.879304851	0	0	0
0	0	0	0	0
0	0	0	0	0.005448084
0	0	0	0	0

PHL018-0011_V1	PHL018-0003_V3	PHL018-0005_V3	PHL018-0050_V2	PHL018-0253_V2
CTRL	CTRL	EYCF	EYCF	REF
0.107748806	0.1236639	1.062242076	0.13062384	0.003877013
1.156941535	3.462859792	0.722364721	0.891117908	0.084231129
5.713159401	0	9.985277611	0.037532889	0
0	0	0	0	0
0	0	0	0	0
0	0	0	0	0
0	0	0	0	0
0	0	0	0	0
0	0	0	0	0
0	0	0	0	0
0	0	0	0	0
0	0	0	0	0
0	0	0	0	0
1.456137144	1.06115304	6.328559663	7.251654923	0.236375229
0	0	0	0	0
0	0	0	0	0
0	0	0	0	0
0	0	0	0	0
0	0	0	0	0
0	0	0	0	0
0	0	0	0	0
0	0	0	0	0
0	0	0	0	0
0	0	0	0	0
0.095384081	0.041633578	0	0	0.147010577
0	0	0	0	0
0	0	0	0	0
0	0	0	0	0
0	0	0	0	0
0	0	0	0	0
0	0	0	0	0
0	0	0	0	0
0	0	0	0	0
0	0	0	0	0
0	0	0	0	0
0	0.658524913	0	0	0.577664997
0	0	0	0	0
0	0	0	0	0
0	0	0	0	0
0	0	0	0	0
0	0	0	0	0
0.0098074	0.029192731	0.003094998	0.115958014	0.003433222
0.001674396	0.295174663	0.37536133	0.309290496	0.06275912
0.037791991	0.124413781	0.008289212	0.004465464	0.007037039
0	0.027023337	0	0	0.001475915
0	0	0	0	0

0.003305833	0	0	0	0
0.001654029	0	0.004784769	0.013704328	0
0	0	0	0	0
3.684142816	0.607627891	0.682070563	0.700471848	0.336638236
0.045539686	0	0	0	0
0	0	0	0	0.034091976
0.007977834	0.031737129	0	0.003771456	0
0	0.08526184	0	0	0
0	0	0	0	0
0	0	0	0	0
0	0	0.014778858	0	0
3.575933723	0.596393638	0.001316114	0.022693748	0.011944963
0	0.002156405	0	0	0.001832871
0.276151224	0	0	0.021181511	0.009217559
0	0	0	0.003705789	0
0	0	0	0	0
0	0.635709487	0.324765061	0.18474935	0.006058499
0	0	0	0	0
0	0	0	0	0
0	0	0	0	0
0.091399257	0.101251065	0.021698842	0.314554419	0
0.05967685	0.009285407	0.001159445	0.001269352	0.00551207
0	0.94475909	0	0	0.004215292
0	0	0	0	0
0	0	0	0	0
0	0	0	0	0
0	0	0	0.009935935	0
0	0	0	0	0
0	0.001131307	0.012501804	0	0
0	0	0	0	0
0.003033108	0	0.108668262	0	0
0	0	0	0	0
0	0	0	0	0
0.006696918	0	0	0	0
0	0	0	0	0
0	0.00796703	0	0	0
0	0	0	0	0
0	0.017425467	0.008435691	0.003249471	0
0	0	0	0	0
0	0	0	0	0
0	0	0	0	0
0	0	0	0	0
0	0	0	0	0
0	0	0	0	0
0	0	0	0	0.989824949
0	0	0	0	0
0	0	0	0	0
0	0	0	0.001852437	0

0	0	0	0	0
0	0	0.001913404	0.001036811	0
0	0	0	0	0
0	0	0	0	0
0	0	0.016214075	0	0
0	0	0	0	0
0	0	0	0	0
0	0	0	0	0
0.030743628	0	0	0.002181206	0.001210874
0	0	0	0	0
0	0	0	0	0
0	0.002232832	0	0	0.00042174
0	0	0	0	0
0	0	0	0	0
0	0	0	0	0
0	0	0	0	0
0	0	0	0	0
0	0	0	0	0
0.069977074	0.218582779	0	0	0.006505706
0.051478538	0	0	0	0.001512593
0	0	0	0	0
0	0	0	0	0
0	0	0	0	0.003443497
0	0	0	0	0
0	0	0	0	0
0	0	0	0	0
0	0	0	0.000998862	0
0	0	0	0	0
0	0	0	0	0
0	0	0	0	0
0.202947423	0	0	0	0.048293888
0	0.017218455	0	0.001058752	0
0	0	0	0	0
0	0	0	0	0
0	0	0	0	0
0	0	0	0	0
0	0	0	0	0
0	0	0	0	0
0	0	0	0	0
0	0	0	0	0
0	0	0	0	0
0	0	0	0	0
0	0	0.014910523	0	0
0	0	0	0	0
0	0	0	0	0
0	0	0	0	0
0	0	0	0	0
0	0	0	0	0

0	0	0	0	0
0	0	0	0	0
0	0	0	0	0
0	0	0	0	0
0	0.245565475	0	0	0.001534723
0	0	0	0	0
0	0	0	0	0
0	0	0	0	0
0	0	0	0	0
0	0	0	0	0
0	0	0	0	0
0	0	0	0	0
0	0	0	0	0
0	0	0	0	0
0	0	0	0	0
0	0.003669445	0	0	0
0	0	0	0	0
0	0	0	0	0
0	0	0	0	0
0	0	0	0	0
0	0	0	0	0
0	0	0	0	0.016854694
0.001505314	0	0	0	0.00568874
0	0	0	0	0
0	0.007054288	0	0	0
0	0	0	0	0
0.011876807	0	0	0	0.23840076
0	0	0	0	0
0	0	0	0	0
0	0	0	0	0
0.02246459	0	0	0	0
0	0	0	0.003053927	0
0	0	0	0	0
0	0	0	0	0
0	0	0	0	0
0	0	0	0	0
0	0	0	0	0
0	0	0	0	0
0	0	0	0	0.011420529
0	0	0	0	0
0	0	0	0	0
0	0	0	0	0
0	0	0	0	0
0	0	0	0	0
0	0	0	0	0
0	0	0	0	0
0	0	0	0	0
0	0	0	0	0

0	0	0	0	0
0	0	0	0	0
0	0	0	0	0
0	0	0	0	0
0	0	0	0	0
0	0	0	0	0
0	0	0	0	0
0	0	0	0	0
0	0	0	0	0
0	0	0	0	0
0	0	0	0	0
0	0	0	0	0.104533477
0	0.004580766	0	0	0
0	0	0	0	0
0	0	0	0	0
0	0	0	0	0
0	0	0	0	0
0	0	0	0	0
0	0	0	0	0
0	0	0	0	0
0.085096783	0.035972714	0.092403134	0.01830445	0.033487549
0	0	0	0	0
0	0	0	0	0
0	0	0	0.022506581	0
0	0	0	0	0
0	0	0	0	0
0.000532886	0	0	0.000795528	0.004522286
0	0	0	0	0
0	0	0	0	0
0	0	0	0	0
0.003895441	0	0	0.122123035	0
0	0	0	0	0
0	0	0	0	0
0.038957801	0.032452997	0	0	0.033960882
0.019249981	0.023405765	0.021710868	0.010132261	0.000473669
0	0	0	0	0
0	0	0	0	0
0	0	0	0	0
0	0	0	0	0
0	0	0	0	0
0	0	0	0	0
0	0	0	0	0
0	0	0	0	0
0	0	0	0	0
0	0	0	0	0
0	0	0	0	0
0.018049271	0.002111852	0	0.001154794	0
0.002121349	0.001389965	0	0	0

0	0	0	0	0
0	0	0	0	0
0	0	0.00081564	0.00092603	0
0	0	0	0	0
0	0	0	0	0
0	0	0	0	0
0	0	0	0	0
0	0	0	0	0
0	0	0	0	0
0	0	0	0	0
0	0	0	0	0
0	0	0	0	0
0	0	0	0	0
0	0	0	0	0
0	0	0.010555019	0	0
0	0	0	0	0
0	0	0	0	0
0	0	0.004234127	0	0
0	0.001817467	0.001059064	0.002447741	0.000801
0	0	0	0	0.083923099
0	0	0.003347631	0.055870335	0
0	0	0	0	0
0	0	0	0	0
0	0	0	0	0
0	0.007101695	0.00177354	0	0
0	0	0	0	0
0	0	0	0	0
0	0	0	0	0
0	0	0.010789784	0	0
0	0	0	0	0
1.251122069	0.141276528	0.247792147	1.805487117	0.08824503
0	0	0	0	0
0	0	0.057132998	0.042567951	0
0	0	0.003821574	0	0
0	0	0	0	0
0	0	0	0	0
0	0	0	0	0
0	0	0	0	0
0	0	0	0	0
0	0.024722579	0	0	0
0	0	0	0	0
0	0.027685245	0.056036601	0	0.00215073
0	0	0	0	0
0	0	0	0	0
0	0	0	0	0
0	0	0	0	0
0	0	0	0	0
0	0	0	0	0

0	0	0	0	0
0	0	0	0	0
0.041132259	0.079058657	0.009031691	0.003219295	0.006513202
0	0	0	0	0
1.78981067	0	0	0.003569847	0.001102895
0	0	0	0	0
0	0	0	0	0
0	0	0	0	0
0	0	0	0	0
0	0.001794841	0	0	0.000726454
0	0	0	0	0
0	0	0	0	0
0	0	0	0	0
0.005075472	0	0.00510689	0.009092417	0
0.002003501	0.003324491	0.002107535	0	0.001449079
0	0.088708861	0	0	0.001861717
0.001660225	0.004953485	0.004366085	0	0
0	0	0.020998444	0	0
0	0	0	0	0
0	0	0	0	0
0	0	0	0	0
0.001873387	0.002630343	0	0	0
0	0	0	0	0
0	0	0	0	0
0	0	0	0.005112191	0
0	0	0	0	0
0	0	0	0	0
0	0	0	0	0
0	0	0	0	0
0.009213561	0	0	0.000970916	0
0	0	0	0	0
0	0	0	0	0
0	0	0	0	0
0	0	0	0.001934766	0
0	0	0	0	0
0.005238553	0	0	0	0
0	0	0	0	0
0.001647601	0.025777079	0	0.00084331	0.0117975
0	0	0	0	0
0	0	0	0	0
0	0	0	0	0
0	0	0	0	0
0	0	0	0	0
0	0	0	0.006547015	0
0	0	0	0	0
0	0	0	0.003241438	0
0	0	0	0	0
0	0	0	0	0

1.933420792	0	0	0	1.142720493
0	0	0	0	0
0.003139562	0.012948873	0.000825647	0	0
0	0	0	0	0
0.007896756	0.004157813	0	0	0.019162135
0	0	0	0.009803599	0
0	0	0	0	0
0.035974385	0.008159326	0.02707187	0	0.02685995
0	0	0	0	0
0.042213673	0	0.311439761	0.625425802	0.032450606
0	0	0	0	0
0.001971516	0.018146579	0.004839075	0.007063716	0
0.013957671	0.016331148	0.008564757	0.007739448	0.001454192
0	0	0	0	0
0	0	0	0	0.001631567
0	0	0	0	0
0	0	0	0	0
0	0	0	0.002132228	0
0	0	0.027103735	0.226462602	0
0	0	0	0	0
0	0	0	0	0
0	0	0	0	0
0	0	0	0	0
0	0.004071204	0	0	0.026254986
0	0	0	0	0
0	0.052988808	0	0	0
0	0	0	0	0
0	0	0.013786817	0.086788862	0
0	0	0.105264028	0	0
0	0	0	0	0
0	0	0	0.019532082	0
0	0	0	0	0
0	0	0	0	0
0.155157138	0	0.001597526	0.00385419	0
0	0	0	0	0
0	0	0	0.003515834	0
0.045634014	0.014477987	0.018232142	0	0
0	0	0	0	0
0	0	0	0	0
0.065340078	0	0	0	0
0	0	0	0	0
0.105692088	0	0	0	0
0	0	0	0	0
0	0	0	0	0
0	0	0	0	0
0.042055059	0	0	0	0.010139105
0.062817182	0.001689635	0.010127056	0.014228359	0
0	0	0	0	0.001042831

0	0	0	0	0
0	0.00642017	0.00848827	0	0
0	0	0	0	0
0	0	0.010899157	0	0
0.001427315	0	0	0	0
0.43125754	0	0.007976283	0.005093889	0
0	0	0	0	0.004527909
0	0	0	0	0
0	0	0	0	1.568107652
0	0	0	0	0
0.009893792	0.004013087	0	0.019496578	0.01206965
0.069854617	0	0	0.023440782	0
0	0	0	0	0
0.005608294	0.002333146	0.001311003	0	0.003305159
0	0	0	0	0
0	0	0	0	0
0	0	0	0	0
0.019607566	0	0	0	0
0	0	0	0	0
0.051440329	0	0	0.074636756	0
0	0.007855842	0	0.001202796	0
0	0.256863078	0	0	0.146670995
0	0	0	0	0.013800327
0	0	0	0	0
0	0	0	0	0
0	0	0	0	0
0.002026959	0	0.013030177	0.014121257	0
0.007016161	0	0	0	0
0	0	0	0	0
0.551388102	0.601475606	0.018896276	1.570030614	0.169079012
0	0.015234974	0	0.001514678	0.001569601
0	0	0	0	0
0	0	0	0	0
0	0	0	0	0
0.015381975	0	0.00453849	0.006048862	0
0	0	0	0	0
0	1.326257256	0	0	1.002611356
0	0.002378098	0	0	0
0	0	0	0	0
0	0	0	0	0
0.037738845	0	0.010957499	0.014989783	0
0.332060071	4.264131685	0	0.138784672	1.130829292
0	0	0	0	0
0	0	0.002817197	0	0.199796406
0	0	0.001013166	0	0
0	0	0	0.007482072	0
0	0.001006592	0.023755506	0.018462021	0
0	0	0	0	0

0	0.017077026	0	0	0.001231567
0	0	0	0	0
0.007231639	1.380471823	0.759447191	0.4302162	0
0	0	0	0	0
0	0	0	0	0
0.801926687	0	0.010030765	0	0
0	0	0	0	0
0	0	0	0	0
0.014805854	0	0	0	0.008098443
0	0	0	0	0
0	0	0	0	0
0	0	0	0	0
0	0	0	0	0
0	0	0	0.001971164	0
0	0	0	0	0
0.010943959	0.032927038	0.001973526	0.009802738	0.013558082
0.005025384	0.058880517	0.011484103	0.004501344	0.023024122
0	0	0	0.01074933	0
0.027565163	0	0	0	0
0	0	0	0	0
0	0.009812391	0.002756807	0.138890063	0
0	0.012135655	0.003805989	0.024306169	0
0	0	0	0	0
0	0	0	0	0
0	0	0	0	0.044196203
0	0	0.017049334	0	0
0.002419911	0	0.001851322	0.002758721	0
0.204375451	0	0	0	0.000664583
0	0	0	0	0
0	0	0	0	0
0.004349884	0	0.023406757	0	0
0.016961144	0	0.001574283	0.002457604	0.003770467
0.005224774	0.013671422	5.136083949	0.010636232	0.017761026
0	0.003345058	0	0.000914565	0
0	0.003751817	0	0.009356328	0.001932677
0	0	0	0	0
0	0.004220456	0.001806848	0.000769271	0
0.412892908	0	0.002826078	0.022560208	0.002003851
0	0.866575347	0	0	0.09001073
0	0.015805919	0	0	0
0	0	0	0	0
0	0	0	0	0
0	0	0	0.006747751	0
0.033948465	0	0.031023313	0	0
0.32421731	0	0	0	0
0	0	0.00577623	0	0
0	0	0	0	0
0	0	0	0	0

0.000746868	0	0	0	0
1.113340338	0.869297865	0.018773088	0.296959468	0.36133638
0	0	0	0	0
0	0	0	0	0
0	0	0	0	0
0	0	0	0	0
0	0	0	0.121721334	0
0	0.014558401	0	0	0
0	0	0	0	0
0.001995173	0	0	0	0
0.080591066	0.009787627	0	0.015077061	0
0.012915984	0.011734275	0.05381384	0.002268456	0.008261074
0.655988211	0	0	0	0.007549004
0	0	0	0	0
0	0	0	0	0
0	0	0	0	0
0.003204702	0.040746254	0.003183826	0.021528899	0
0.204540489	0	0.020665229	0.111582923	0
0	0	0	0	0
0	0	0.003946741	0	0
0.011383393	0.05748703	0.000965685	0.005756003	0.054047684
0	0.001603887	0	0.001841766	0
0	0	0.61062369	0	0
0	0	0	0	0
0	0.002598805	0	0	0
0	0	0	0	0
0.099311543	0	0	0	0.079156549
0.06049433	0.012603634	0.008416317	0.096835744	0.073591331
0	0	0	0	0
0	0	0	0	0
0	0.00235195	0	0	0
0	0	0	0	0
0.019425981	0	0	0	0
0	0	0	0	0
0.17634552	0.262880259	0	0	0.006900033
0.148620548	0.115928799	0.091370788	0.070998723	0.06437656
0	0	0	0	0
0	0	0	0	0
0.009388498	0.003717998	0.001519386	0.00733134	0.009384755
0	0	0	0	0
0.080665919	0	0	0	0
0	0	0	0	0
0.00541027	0.006364494	0.000922898	0.001309755	0.002326712
0.000564704	0.001387535	0.000594027	0.005311085	0
0.42361942	0	0.102624435	0	0
0	0.376834638	0	0	0.496575897
0	0	0	0	0
0.002966602	0	0	0.013490648	0

0.03472716	0	0	0	0
0	0.613017825	0	0	0
0	0	0	0	0
0.314722021	0.162121211	0	0	0.036583417
0	0	0	0	0
0	0	0	0	0.02432127
0	0	0	0	0
0.01234078	0.036579576	0	0	0.002181853
0.231727087	1.991915328	0.118397615	0	0.228550737
0	0.133997198	0	0	0.150888651
0	0	0	0	0
0.005384599	0.004811092	0.003432849	0	0.012722159
0	0	0	0	0
2.512739158	0	0	0	0.707147043
0.069613024	0	0.145039132	0	0.020568211
0	0	0	0	0
0	2.640387427	0	0	0.004052362
0	0	0	0	0.164930968
0.089319914	0	0	0	0
0	0.00184133	0	0	0
0.137427708	0	0	0	0
0.009543937	0	0	0	0
0	0	0	0	0.004662074
1.77871335	0	0.117743757	0.063780382	0.122486402
0	0	0	0.019430998	0
0	0.498270032	0	0	0.84978107
0	0	0	0	0
0	7.300782248	0	0	0.651684932
0	0	0	0	0
0	0	0	0	0
0	0.053453736	0	0	0.003786155
3.476236344	0	0	0	0
0.042013382	0	0	0.005406938	0
0	0	0	0	0
0	0	0	0	0
0.767588208	0.067228806	0	0	0.01209189
0	1.887254036	0	0	0.125486742
0	0	0	0	0
0.097159707	0	0	0	0
0	0	0.028134271	0	0.000650442
0.269235244	0.001318681	0.004939807	0.178205853	0.173480201
0	0	0	0	0.012655008
0.070792868	1.31431148	30.76413841	0.975044437	0.003286746
0	0	0	0	0
0	0	0	0	0
0	0	0	0	0
0	0	0	0	0
0.112525399	0	0.005740274	0.211695667	0.002994158

0	0	0	0	0.130259833
0	0	0	0	0.000409164
0.01118367	0.002026131	0.377599139	0.378724327	0.002296191
0	0	0	0	0.040799064
0	0.006962646	0	0	0
0.001420978	0.137464821	0	0	0.001695797
0	0	0	0	0
0	0	0	0.050691596	0
0	0	0	0	0
0	0	0	0	0
0.062340224	0.005719243	0.025895588	0.052666776	0
3.360170267	0.414208736	0.327214395	1.590739449	1.052017014
0.015129298	0	0	0	0
0.017011165	0.408186121	0	0.00359531	0.03953782
0.391017396	0.010151205	0.148462325	0.58567155	0.002037209
0.820211048	3.222865542	0	0	0.325270222
0	0	0	0	0
0	0	0	0	0
0	0	0	0	0
0.027280717	0	0.000460385	0.032537671	0.006731898
0.261548318	0	0.000468438	0.147318992	0.4224354
0	0	26.36981831	23.77363912	0.021527829
1.038067377	0.0149609	4.184706191	8.140313118	0.524712951
0	0	0	0	0.000759671
0	0	0	0	0.105098528
0	1.704258899	0	0	1.184775674
14.47973747	0	0	11.48196713	0
0	0	0	0	0
0	0	0	0	0
0.077284188	0	0	0	0
0.004747985	0.003678039	0	0	0.005991899
0.334647518	0.321900437	0	0.014058671	0.151073371
0.016551927	0.001787682	0.008227367	0.118770241	0.004630762
0	0.056282571	0	0	0.000993182
0.009243788	0	0.00088398	0.008172328	0
0.009566535	0.523846748	0.013012874	0.102827402	0.005905199
0.003505261	0	0.005698517	0	0.000887342
0	0	0	0	0
0	0	0.010151793	0	0.846585481
0	0	0	0	0
0.006680957	0	0	0	0.01247958
0	0	0	0	0
0	0	0	0	0
0	0	0	0	0
0.012038121	0.026409715	0.002967941	0.20605262	0.010026489
0	0	0	0	0
0	0	0	0	0
0.024504632	0.007008613	0.00620559	0	0.002707765

0	0	0	0	0
0	0	0	0	0
0	0	0	0	0
0.051781147	0.020708181	0	0	0.002423361
0	0	0	0	0
0	0	0	0	0.066123912
0	0.011828617	0	0	0.297149293
0	0	0.300112557	0	0.000352094
0	0	0	0	0
0	0	0	0	0
0.001606918	0	0.001878176	0.024468959	0
0	0	0	0	0
0.005002603	0.003292473	0	0	0
0.182678161	0.088777013	0	0	0.193583217
0	0	0	0	0
0	0	0	0	0
0	0.187938534	0	0	0.079236799
0	0.027756203	0.001563539	0.005857996	0
0	0	0	0	0.311586273
0.009919321	0.010539579	0.001410053	0	0
0	0.00222489	0	0	0
0	0	0	0	0
0.069403411	0	0	0	0.013906611
0	0	0	0	0
0	0	0	0	0
0	0	0	0	0
0	0	0	0	0
0	0	0	0	0
0	0	0	0	0
0	0	0	0	1.586786048
0	0.014610994	0.003254401	0	0
0	0	0	0	0
0	0	0	0	0
0	0	0	0	0
0	0	0	0	0
0	0	0	0	0
0.534965929	0.112381225	0	0	0.038960862
0.004383137	0	0.008267528	0.055100356	0
0	0	0	0	0
0.005664993	0	0	0	0
0.102953491	0	0	0	0
0	0	0	0	0
0	0	0	0	0.012064802
0.007045316	0.009479863	0	0	0.016465454
0	0.457834807	0	0	0.214370596
0	0	0	0	0
0	0.010686809	0	0	0.002856423
0	0	0	0	0

0	0	0	0	0
0	0	0	0	0
0	0	0	0	1.617196783
0.012328097	0.566564901	0	0	0.155018137
0	0	0	0	0
0	0.672183019	0	0	0
0	0	0	0	0
0.704468678	0	0	0	0
0.100890266	0.001463386	0.082666686	0.077112877	0.001824282
0	0	0	0	0
0	0	0	0	0.006166341
0	0	0	0	0
0	0	0	0	0
0.000723917	0	0	0	0
2.908934413	0.002103256	0.640234635	0	0.403065855
0.001285272	0	0.180662458	0	0
0	0	0	0	0
0	0	0	0	0
0	0	0	0	0
0	0	0	0	6.525278608
0	0.028869386	0	0	0.068858573
0	0	0	0	0
0	0	0	0	0
0	0	0	0	0
0	0.020994983	0.003610057	0.011198087	0.007996131
6.489431131	6.787830142	0	0	1.074437424
0	1.102205638	0	0	0.013637278
0.000760825	0	0	0	0
0	0	0	0	0.018074047
0	0	0	0	0.011617067
0	0	0	0	0
0.034572789	0	0	0	0.041573217
0	0	0	0	0
0	0	0.00123943	0	0
0.00194138	0.373133142	0.001361459	0.002318581	0.002745892
0.002287201	0.003853635	0.015237789	0.06432902	0
0.187252701	0.00265123	0	0	0.434040554
1.159091194	0.187594543	0	0.14651094	0.120682624
0	0	0	0	0
0	0	0	0	0
0	0	0	0	0
0.011239385	0	0	0	0.004952529
0.275252447	0.498812968	0.038357624	0.891199016	0.127482347
0	0.009452753	0	0	0.842939498
0	0	0	0	0
0.011556458	0	0.000911741	0	0.006780818
0	0	0	0	0
1.256078372	0.001615616	0	0.115436775	0.221362905

0.08288866	0	0	0	0
0	0	0	0	2.139444012
0	0	0	0	0.079726279
0	0	0	0	0
0	0	0.035407176	0.000765214	0
0	0	0	0	0.005890704
0	0	0	0	2.232307114
0	0	0	0	0
0	0	0	0	0
0	0	0	0	1.874555707
0.00169959	0	0	0	0.001456211
0	0.733234047	0.223019976	2.559381883	0.447336827
0.323769096	0	0	0	0.003291898
0	0	0	0	0
0.048609429	0.252193725	0.398259543	0.065133765	0.010690294
0	0	0	0	0
0.006460566	0	0	0	0
0	0	0.02053898	0.005553959	0
0	0	0	0	0
0	0	0	0	0.336905822
0	0	0.044496422	0	0.024185162
0	0	0	0	0.060169969
0.009936113	1.349215236	0	0	0.120453526
0	0	0	0	0
0	0	0	0	0
0	0	0	0	0
0	0	0	0	0
0	0	0	0	0
0	0.057228018	0	0	0.00042482
0	0	0	0	11.49322303
0	0	0	0	0
0.051282546	0.063003201	0.016183636	0.00734958	0.029784326
0	1.194231267	0	0	0
0.104400995	0.023021943	0.00121276	0	0.000407664
0.001825302	0.003960736	0.015884321	0.061979917	0
0.004492158	0.659858464	0	0	0.163211103
0	0	0	0	0.001406273
0	0	0	0	0.002524821
0	0	0	0	0
0.006170105	0	0	0	0
0	0	0	0	0.176370051
0	0	0	0	0.009029156
0	0	0	0	0
0	0	0	0	0.018055941
0.0127502	0.008032957	0.041440475	0.042314888	0.000650261
0	0	0	0	0
0	0	0	0	0.195210622
0.012368624	0	0	0	0.000615031

0	0	0	0	0
0	0	0	0	0
0	0	0.00444604	0.004997294	0
0	0	0	0	0
0.100815017	0	0.004869641	0	0
0	0	0	0	0
0	0	0	0	0
0.014050432	0	0	0	0.058814356
0	0	0	0	0
0	0	0	0	0.001500015
0	0	0	0	0
0	0	0	0	0
0	0	0	0	0
0	0	0	0	0.703548017
0	0	0	0	0
0	0	0	0	0
0	0	0	0	4.325523309
0	0	0	0	0.007522417
0	0	0	7.203353551	1.472746382
0	0	0	0	0
0	0	0	0	0.000855479
0	0	0	0	0
0	0	0	0	0
0	0	0	0	0
0	0	0	0	0
0.00136961	0	0.001920972	0.029170319	0
0	0	0	0	0
0.078051308	0.009202092	0	0.313003979	3.187365987
0	0	0	0	0.032735812
0	0.110168878	0	0	2.105848017
0	0	0	0	0
0	0	0	0	0
0	0	0	0	0.002127265
0	0	0	0	0
0.005262645	0.001791287	0	0.138987547	0.000507511
0	0	0	0	0
0	0	0	0	0.985129127
0.000723825	0	0.00418776	0.001296692	0
0	0	0	0	0
0	0	0	0	0
3.854681494	5.405965884	1.716699526	1.492837679	1.456976249
0.607862987	0.042962011	0	0.043306537	0.235537742
0	0.549876719	0	0	0.021522701
0.282731553	0.004283864	0	0	0
0	0	0	0	0
0	0	0	0	1.387773935
0	0	0	0	0.003920993
0.001005319	0	0.000906447	0.00475971	0

0	0	0	0	0
0.028214888	0	0	0.002381718	0.000587639
0	0	0	0	0.002882581
0	0	0	0	0
3.414638476	8.909589562	0.599859932	0.646126472	0.408770866
0	0	0	0	0
0	0	0	0	0
0	0	0	0	0.004610565
0.230401459	0.013668886	0.029791385	0.182857693	0
0	0	0	0	0
0	0	0.015976073	0	0
0	0	0	0	0
0.283916243	0.216649353	0.022044593	0	0.047506455
0.004058676	0.011892763	0.001670646	0.082824876	0.003088692
0	2.477968015	0	0	0.009024516
1.433137343	0.345025398	0.026809097	0	0.273271452
0.006718905	0	0	0.00341947	0.722623517
0	0	0	0	0
0	0	0	0	0
0.232643296	0	0	0	0
0	0	0	0	0
0	0	0	0	0
0	0	0	0	0
0	0	0	0	0
0	0	0	0	0
0	0	0	0	0
0.031734657	0.147840723	0	0	0.00612716
0	0	0	0.005636593	0
0	0	0	0	0
0	0	0	0	0
0	0	0	0	0
0.00087607	0	0	0	0.000497859
0	0	0	0	0
0	0.07100798	0	0	0.017369065
0	0.762372123	0	0	0.016552613
0	0	0.018049714	0	0
0	0.00099494	0	0	0.001973219
0	0	0	0	0
0	0	0	0	0
0	0	0	0	0
0	0.652715346	0	0	0.078193573
0	0	0	0	0
0	0	0	0	0.075778055
0	0	0	0	0.011817495
0	0	0	0.017612846	0
0	0	0	0	0
0	0	0	0	0
0	0	0	0	0

0	0	0	0	0.011224453
0	0	0	0	0
0.027149597	0	0	0	0
0.00458261	0	0	0	0
0	0.011628601	0	0	0.312538487
0	0	0	0	0
0.363004935	0.001711053	0	0.00395694	0.147615211
0	0	0	0	0
0.003010592	0	0	0	0
0.094293847	0	0.002353665	0	0
0	0	0	0	0.008083267
0.097780836	0	0	0	0.013460362
0	0	0	0	0
0	0	0	0	0
0	0	0	0	0
0	0	0	0	0
0	0	0	15.22240122	0
0	0	0	0	0
0	0	0	0	0
0	0	0	0	0
0.017113265	0	0.001373026	0.036373228	0.775381562
0	0	0	0	0
0.032312932	0.050310426	0	0	0.011199613
0.009103523	0.016177056	0.000748143	0.010829823	0.001584356
0	0	0	0	0
0	0	0	0	0
0	0	0	0	1.320760496
0	0	0	0	0
0	0	0	0	0
0	0	0.135024898	0	0
0	0	0	0	0.121842497
0	0	0	0	0
0	0	0	0	0.011285793
0	0	0	0.011973796	0
0	0	0	0	0.024368922
0	0	0	0	0.750175068
0.020111995	0.004248255	0.00711452	0.130726464	0.003681675
0	0	0	0	0
0	0.305116705	0	0	0.053759804
0	1.410470245	0	0	0.041712631
0	0	0	0	0
0	0	0	0	0
0	0	0	0	0
0.034477136	0	0	0	0.027565168
0	0	0	0	0
0	0	0	0	0
0	0	0	0	0
0	0	0	0	0

0.013469106	0	0	0.138605417	0
0	1.575266242	0	0	0.002194293
0	0	0	0	0
0	0	0	0	0
0	0	0	0	0
0	0	0	0	0.012599676
0	0	0	0	0
2.704026068	0.381955708	0	0.190177553	0.04751272
0.002162228	0.384883772	0.537793901	0.46826405	0.089645224
0.153036623	0.000704499	0.091839615	0.155055644	0
0	0	0	0	0.513113844
0	0	0	0	0
0	0	0	0	0.0617684
0	0	0	0.141150907	0
0	0	0	0	0
0	0	0	0	0.041700204
0	0	0	0	0
0	0	0	0	0
0	0	0	0	0
0.054089227	0	0	0	0
0	0	0	0	0
0	0	0	0	0
0	0.002535872	0	0	0.013076123
0	0	0	0	0
0	0	0	0	0.019662209
0	0	0	0	0
0	0	0	0	0
0	0	0	0	0
0	0	0	0	0
0	0	0	0	0
0	0	0	0	0
0	0	0	0	0
0	0	0	0	0
0	0	0	0	0.00455089
0.206422285	0.013268644	0	0	0
0	0.077691519	0	0	0
0	0	0	0	0
0	0	0	0	0
0	0	0	0	0
0	0	0	0.00068433	0
0	0	0	0	0
0.75746093	0	0.015753452	0	0.032358016
0	0	0	0	0
0.003049493	0	0	0	0
0.004615898	0	0	0	0
0.000408299	0	0	0	0.000974529
0	0	0	0	0
0	0	0	0	0

0	0	0	0	0
0.002762831	0	0.032986443	0.014105944	0
0	0	0	0	0
0	0	0	0	0
0.182518549	5.17902573	0.005485601	0	0.486959425
0	0	0	0	0.047044737
0	0	0	0	0
0.01304496	0.003007084	0.011674225	0.020055742	0.002168659
0.006415253	0	0	0	0.01159996
0	0	0	0	0
0	0	0	0	0
0	0	0	0	0
0	0	0	0	0
0	0	0	0	0
0.007264316	0	0	0	0
0.04694381	0	0	0.012100309	0.000671736
0	0	0	0	0
0	0	0	0	0
0	0	0	0	0
0	0	0	0	0
0	0	0	0	0
0	0	0	0	0
0	0	0	0	0
0.001179483	0	0	0.003924099	0
0	0.131556371	0	0	0.161495672
0	0	0	0	0
0.052089236	0.001298277	0	0.451528096	0.010176647
0.003407733	0	0	0.002761677	0.000645523
0	0	0	0	0
0	0	0	0	0
0	0	0	0	0
0	0	0	0	0
0	0	0	0	0
0	0	0	0	0
0	0	0	0	0
0	0	0	0	0
0	0	0	0	0
0.374260044	0.012426956	0	0	0.64217436
0	0	0	0	0
0	0	0	0	0
0	0	0	0	0
0	1.527214861	0	0	0.004613861
0	0	0	0	0
0	0	0	0	0
0	0	0	0	0
0	0	0	0	0
0	0	0	0	0
0	0	0	0	0

0.230434452	0.289764552	0	0	0.036551473
0	0	0	0	0
0	0	0	0	0
0	0	0	0	0
0	0	0	0	0
0	0	0.916332853	0.07083728	0
0	0	0	0	0
0.002772467	0	0	0	0
0	0	0	0	0
0	0	0	0	0
0	0	0	0	0
0.006523772	0	0	0	0
0.008640557	0	0	0	0.030720136
0	0	0	0	0
0	0	0	0	0
0	0	0	0	0
0	0	0	0	0
0.077002956	0.2320903	0	0	0
0	0	0	0	0
0	0	0	0	0
0.003355864	0	0.000706024	0.076049763	0
0	0	0	0	0
0.014198011	0.001487674	0.010476973	0.004365696	0
0	0	0	0	2.247960698
0.003045422	0	0	0	0.047247344
0	0	0	0	0
0	0	0	0	0
0	0	0	0	0
0	0	0	0.001150224	0
0	0	0	0	0
0	0	0	0	0
0.025356037	0	0	0	0.014748539
0.002182988	0	0.055738478	0.332409273	0.001578897
0	0	0	0	0
0.28917297	0	0	0	0.004660644
0.122941422	0	0.031326207	0	0
0	0	0	0	0
0	0.365335484	0	0	0.011186828
0.012893891	0	0	0.015839087	0
0	0	0	0	0
0	0	0	0	0
0	0	0	0	0.00821907
0.018738337	0.226807821	0.011101793	0	0.032558225
0	0	0	0	0
0	0	0	0	0
0.001197361	0	0	0	0.013744972
0	0	0	0	0
0	0	0	0	0

0	0	0	0	0
0	0	0	0	0
0	0	0	0	0
0	0	0	0	0
0	0	0	0	0.003281524
0	0	0	0	0
0	0	0	0	0
0	0	0	0.034940701	0
0	0	0	0	0.063751308
0	0	0	0	0
0	0	0	0	0
0	0	0	0	0
0	0	0	0	0
0	0	0	0	0
0	0	0	0	0
0	0	0.01440517	0	0
0	0	0	0	0
0	0	0	0	0
0	0	0	0	0
0.773823263	1.194693542	0	0	1.650097468
0	0.195223078	0	0	0
0	0	0	0	0
0	0	0	0	0
0	0	0	0	0
0.001639943	0	0	0	0
0	0	0	0	0
0	0	0	0	0
0	0	0	0	0
0	0	0	0	0
0	0	0	0	0.013150017
0.945219123	0.351319819	0	0	0.062546677
0	0	0	0	0
0	0	0	0	0.049702209
0	0	0	0	0
0	0	0	0	0
0	0	0	0	0
0	0	0	0	0
0	0	0	0	0
0	0.019619203	0	0	0.01023279
0	0	0	0	0
0	0	0	0	0
0	0	0	0	0.639999942
0	0	0	0	0.005334388
0.003147634	0	0	0	0
0	0	0	0	0
0	0	0	0	0
0	0	0.230343553	0.399430355	0
0.002018986	0	0	0	0

0	0	0	0.026319434	0
0	0	0	0	0
0	0	0	0	0
0	0	0	0	0
0.003579344	0.167706316	0.063171781	0	0.10283468
0	0	0	0	0
0	0	0	0	0.040172966
0	0	0	0	0
0	0	0	0	0
0	0	0	0	0
0	0	0	0	0.032674555
0.006441812	0.003359451	0.0087124	0.024728867	0.001171454
0.007621935	0	0	0	0
0	0	0	0	0
0.005189631	0	0	0	0
0	0	0	0	0
0	0	0.001042357	0	0
0	0	0	0	0
0.009616189	0	0.085322223	0.00112349	0.011309656
0.048221601	0.003321422	0	0.355487525	0.021716126
0	0	0	0	0
0	0.644258739	0	0	0
0	0	0	0	0
0	0	0	0	0
0	0	0	0	0
0	0	0	0	0
0.048703727	0	0.013651834	0.018285916	0.000464057
0	0	0	0	0
0	0	0	0	0
0	0	0	0	0
0.001322353	0	0	0	0
0	0	0	0	0
0	0	0	0	0
0	0	0	0	0
0	0	0	0	0.067341136
0	0	0	0	0
0	0	0	0	0
0.02345843	0.010761591	0.33020139	0.035887204	0.002771815
0	0.873327009	0	0	0.001555832
0.011789673	0	0	0	0.015535419
0	0	0.033294024	0.009865405	0
0.005210572	0	0	0	0.000829107
0	0	0	0	0
0	0	0.14147767	0.070029398	0
0	0	0	0	0
0	0	0	0.069363839	0.013595979
0	0	0	0	0
0	0	0	0	0.010880797

0	0	0	0	0
0	0	0	0	0.002342038
0	0	0	0	0
0.00090237	0	0	0.000646618	0
0	0.004700414	0	0	0
0	0.003033168	0.006362896	0.007159557	0
0	0	0	0	0.007524288
0	0	0.004602741	0	0
0.20584563	0	0	0	0
0	0	0	0	0.017255804
0	0	0	0	0
0	0	0	0	0
0	0.535273807	0	0	0.006208689
0	0	0	0	0
0.000790748	0	0	0	0
0	0	0	0	0
0	0	0	0	0
0	0	0	0	0
0	0	0	0	0
0	0	0	0	0
0	0	0	0	0
0	0	0	0	0
0.014487151	0	0.009037327	0.009506005	0
0	0.00502385	0	0	0.133163936
0	0	0	0	0
0	0	0	0	0.013782014
0	0	0	0	0.002304856
0	0	0	0	0
0	0	0	0	0
0	0	0	0	0
0	0.006856713	0	0	0.039450901
0	0	0	0	0.000462237
0	0	0	0	0
0	0	0	0	0
0	0	0	0	0.050238538
0	0	0	0.01248706	0
0	0	0	0	0
0	0	0	0	0
0.004608522	0.002008127	0.001170165	0	0.00050573
0	0	0	0	0
0	0	0	0	0
0	0	0	0	0
0	0	0	0	13.38447884
0	0	0	0	0
0	0	0	0	0.000585566
0.164706241	0.25736675	0.021620582	0	0.431054129
0	0	0.136540023	0	0
0	0	0	0	0

0	0	0	0	0
0	0	0	0	0
0	0.011880347	0	0	0.052714421
0	0	0	0	0
0	0	0	0	0
0	0	0	0	0
0	0	0	0.022282166	0
0	0	0	0	0
0	0	0	0	0.006083155
0	0	0	0	0
0	0	0	0.000808349	0
0	0	0	0.001206868	0
0	0	0	0	0
0	0	0	0	0
0	0	0	0	0
0	0	0	0	0
0	0	0	0	0
0	0	0	0	0
0	0	0	0	0
0	0	0.008452999	0	0
0	0	0	0	0
0	0	0	0	0
0	0	0	0	0
0	0	0	0	0
0	0	0	0	0
0	0	0	0	0
0	0	0	0	0
0	0.006907228	0	0	0.005268765
0	0	0	0	0
0	0	0	0	0
0.009547431	0.023098097	0.003244724	0.047890296	0.003505821
0	0	0	0	0
0.075381672	0.007183726	0.355934791	0.036662974	0.01736796
0.007842614	0.001957597	0	0.004683198	0.001525233
0	0	0	0	0
0	0	0	0	0
0	0	0	0	0
0	0	0	0	0
0	0	0	0	0
0	0	0	0	0
0	0	0	0	0
0.236137153	0	0.326475506	0.089481478	0
0	0.078000316	0.010788586	0.114678708	0.023799148
0	0	0	0	0
0	0	0	0	0
0.009880451	0.343696729	0	0	0.020438332
0.010364646	0	0	0	0
0	0	0	0	0

0	0	0	0	0
0	0.004274358	0	0	0.006126336
0.006693744	0.008811004	0	0.003559965	0.007156208
0	0	0	0	0
0.527114391	0.022043508	0	0.040392859	0.003189146
0.002763793	0	0.000684072	0.218725509	0
0	0	0	0	0
0	0	0	0	0
0	0	0	0	0
0	0	0	0	0
0	0	0	0	0.000703987
0	0	0	0	0
0	0	0	0	0.006964856
0	0	0	0	0
0	0	0	0	0
0	0	0	0	0
0	0	0	0	0.555590038
0	0	0	0	0
0	0	0	0	0
0	0	0	0	0
0	0	0	0	0
0	0	0	0	0
0	0	0	0	0
0	0	0	0	0
0	0.007261506	0	0	0
0.002601354	0.001947974	0	0	0
0	0	0	0	0
0.011944113	0.385478982	0	0	0.019540864
0	0	0	0	0
0	0	0	0	0
0	0	0	0	0
0	0	0	0	0
0	0	0	0	0
0	0	0	0	0
0	0	0	0	0
0	0	0	0	0
0	0	0	0	0
0.067325164	0	0	0	0
2.789945286	0	0	0	0.706029424
0	0	0	0	0
0	0	0	0	0
0	0	0	0	0.013321759
0.010442971	0	0	0.103673419	0
0	0	0	0	0
0	0	0	0	1.53035479
0	0	0	0	0.001115166
0.008520374	0	0.002800877	0.00190797	0
0	0	0	0	0

0.049839802	0	0	0	0
0.0540917	0.020991803	0.012546378	0.061541916	0
0	0	0	0	0.204745185
0	0	0	0	0
0	0	0	0	0
0	0	0	0	0
0	0	0	0	0
0	0	0	0	0
0	0	0	0	0
0	0	0	0	0
0	0	0	0	0
0	0	0	0	0
0	0.726043267	0	0	0.01610595
0	0	0	0	0
0.016965776	0	0	0.019699308	0
0	0	0	0	0
0	0	0	0	0
0	0	0	0	0
0	0	0	0	0
0	0	0	0	0
0	0	0	0	0
0.071583735	0	0	0.010686514	0.002618487
0	0.011318474	0.01163901	0.002973206	0.001383314
0	0	0	0.002087546	0.002359901
0	0	0	0	0
0	0	0	0	0
0	0	0	0	0
0	0	0	0	0
0	0	0	0	0
0	0	0	0	0.009612351
0	0	0	0	0
0	0	0	0	0
0	0	0	0	0
0	0	0	0	0
0.020544095	0	0	0	0
0	0	0	0	0
0	0	0	0	0
0	0	0	0	0
0.0012325	0.050761384	0.005402081	0.040847147	0.004249174
0	0	0	0	0
0.002026016	0.024825979	0.027318355	0.022106903	0.054804612
0	0	0	0	0
0	0	0	0	0
0	0	0	0	0
0	0	0	0	0
0.039644933	0.001184336	0.931231257	0.726082829	0
0	0	0	0	0
0	0	0	0	0

0	0	0	0	0
0	0	0	0	0
0.053753474	0	0	0	0
0	0	0	0	0
0.001919214	0	0	0.001575824	0
0	0	0	0	0
0	0	0	0	0
0	0	0	0.01674116	0
0.15314043	0	0	0	0
0	0.02142822	0.003037265	0.009421337	0
0.07330012	0.072072862	0	0	0.015920546
0	0	0	0	0
0.010885353	0	0	0.002527841	0
0	0	0	0	0
0	0	0	0	0
0	0	0	0	0
0	0	0	0	0
0	0	0	0	0
0	0	0	0	0
0.266260713	0.210854866	0.199118184	0.546418076	0.001998465
0	0	0	0	0.035354822
0	0	0	0	0
0	0	0	0	0
0	0	0	0	0
0	0	0	0	0
0	0	0	0	0.014979667
0	0	0	0	0
0	0	0	0	0
0	0	0	0	0
0	0	0	0	0
0	0	0	0	0.001531005
0	0	0	0	0
0	0	0	0	0
0	0	0	0	0
0	0	0	0	0
0.002787076	0	0	0	0
0	0	0	0	0
0	0	0	0	0
0	0	0	0	0
0	0	0	0	0
0	0	0	0	0
0	0	0	0	0
0.010738208	0	0	0	0.069414553
0	0	0	0	0
0.002359263	0.032652192	0	0.009660535	0
0	0	0	0	0
0	0	0	0	0
0	0	0.021116813	0	0

0	0	0	0	0
0	0	0	0	0
0	0	0	0	0
0	0	0	0	0
0	0	0	0	0
0.024657939	0.10280368	0.010587074	0.022875452	0.01461332
0	0	0	0	0
0	0	0	0	0
0	0	0	0	0
0	0	0	0	0
0	0	0	0	0
0	0	0	0	0
0	0	0	0	0
0	0	0	0	0
0	0	0	0	0
0	0	0	0	0
0.068670703	0	0	0	0.086929532
0.001549366	0	0	0	0
0	0	0	0	0
0	0	0	0	0
0	0	0	0	0.000696327
0	0	0	0	0
0	0	0	0	0
0	0	0	0	0
0	0	0	0	0
0	0	0	0	0
0	0	0	0	0
0.004418318	0	0	0	0.003398045
0	0	0	0	0
0	0	0	0	0
0	0	0	0	0
0	0	0	0	0
0	0	0	0	0
0	0	0	0	0
0	0	0	0	0
0	0	0	0	0
0	0	0	0	0
0	0	0	0	0
0	0	0	0	0.458902602
0	0	0	0	0
0	0	0	0	0.006736855
0	0	0	0	0
0	0	0	0	0
0	0	0	0	0
0	0.035557251	0	0	0
0	0	0	0	0
0	0	0	0	0
0	0	0	0	0

0	0	0	0	0
0.070284022	0.002175373	0	0	0.134024001
0	0	0	0	0
0	0	0	0	0
0	0	0	0	0
0	0	0	0	0
0	0	0	0	0
0	0	0	0	0
0	0	0	0	0
0	0	0	0	0
0.000553295	0.043245082	0	0	0.005135688
0	0	0	0	0
0.646206016	0.168988265	0	0.041370399	0.20296856
0	0	0	0	0
0	0	0	0	0
0	0	0	0	0
0	0	0	0	0
0	0	0	0	0
0	0	0	0	0
0	0	0	0	0
0	0	0.094712199	0.023641097	0
0	0	0	0	0
0	0	0	0	0
0.705858573	1.521434703	0	0.215843714	0.494221601
0	0	0	0	0
0	0	0	0	0
0	0	0	0	0
0	0	0	0	0
0	0	0	0	0
0	0	0	0	0
0.007900127	0	0	0	0
0	0	0	0	0
0	0	0	0	0
0	0	0	0	0

PHL018-0138_V2	PHL018-0012_V1	PHL018-0006_V3	PHL018-0109_V2	PHL018-0199_V2
EYCF	EYCF	EYCF	REF	CTRL
0	0	0	0.010559662	0.063380655
0.108605697	36.12438425	3.268846967	1.074779333	1.816154914
0	0	4.098461158	0	6.052957816
0	0	0	0	0
0	0	0	0	0
0	0	0	0	0
0	0	0	0	0
0	0	0	0	0
0	0	0	0	0
0	0	0	0	0
0	0	0	0	0
0	0	0	0	0
0	0	0	0	0
0.61497973	9.262498767	3.06285306	0.583610301	2.213670556
0	0	0	0	0
0	0	0	0	0
0	0	0	0	0
0	0	0	0	0
0	0	0	0	0
0	0	0	0	0
0	0	0	0	0
0	0	0	0	0
0	0	0	0	0
0	0	0	0	0
0	0	0	0.23196912	0
0	0	0	0	0
0	0	0	0	0
0	0	0	0	0
0	0	0	0	0
0	0	0	0	0
0	0	0	0	0
0	0	0	0	0
0	0	0	0	0
0	0	0	0	0
0	0	0	0	0
1.7832696	0	0	0	4.154910719
0	0	0	0	0
0	0	0	0	0
0	0	0	0	0
0	0	0	0	0
0	0	0	0	0
0	0.006274566	0.009572725	0	0.022224917
11.34172875	0.053333251	0.075045782	0.145133037	0.80836811
0	0.01167375	0.029151366	0.038980173	0.043163388
0	0	0.006235845	0	0
0	0	0	0	0

0	0	0	0	0.000966018
0	0.113706964	0.000617774	0	0
0	0	0.016686068	0	0
0.074855708	0.006682779	0.569658566	1.542424685	0.546259058
0	0	0	0.002669969	0.009241277
0	0	0	0.002250311	0
0.004964187	0	0	0	0.001602737
0	0	0	0	0
0	0	0	0	0
0	0	0	0	0
0	0	0	0.057295497	0
0.205479283	0.299016911	0	0.013362183	0.034812497
0	0	0	0	0
0.009428995	0.167927822	0	0.006190395	0.003698562
0	0	0	0	0
0	0	0	0	0
0.716024133	0.016596626	0.643379832	0	0.001223874
0	0	0	0	0
0	0	0	0	0
0	0	0	0	0
0	0.266584661	0.001670328	0.002180447	0.036252302
0.00230036	0	0	0.130583854	0.012653301
0	0	0	0.075938752	0.530786508
0	0	0	0	0
0	0	0	0	0
0	0	0	0	0
0	0	0	0	0
0	0	0	0	0
0	0.016604917	0	0.000561213	0.000971234
0	0	0	0	0
0	2.884434826	0	0	0
0	0	0	0	0
0	0	0	0	0
0	0	0	0	0
0	0	0	0	0
0	0	0	0	0
0	0	0	0	0
37.30153548	0	0	0	0
0	0	0	0	0
0	0	0	0	0
0	0	0	0	0
0	0	0	0	0
0	0	0	0	0
0	0	0	0	0
0	0	0	1.029943046	0
0	0	0	0	0
0.012324124	0	0	0.003254234	0
0	0.123113114	0	0.001181921	0

0	0	0	0	0
0	0	0	0	0
0	0	0	0	0
0	0	0	0	0
0	0.000882054	0.009282553	0.030959415	0.074174194
0	0	0	0	0
0	0	0	0	0
0	0	0	0	0
0	0.001310775	0	0.017667907	0
0	0	0	0	0
0	0	0	0	0
0.000860471	0	0.008623435	0	0.001135941
0	0	0	0	0
0	0	0	0	0
0	0	0	0	0
0	0	0	0	0
0	0	0	0	0
0	0	0	0	0
0	0	0	0.593062005	0.010109342
0	0	0	0	0
0	0	0	0	0
0	0	0	0	0
0	0	0	0	0
0	0	0	0	0
0	0	0	0	0
0	0	0	0	0
0.003909964	0	0	0	0
0	0	0	0.001902731	0
0	0	0	0	0
0	0	0	0	0
0.003977914	0	0	0.002877488	0.281583424
0	0	0	0.009605069	0.008192517
0	0	0	0	0
0	0	0	0	0
0	0	0	0	0
0	0	0	0	0
0	0	0	0	0
0	0	0	0	0
0.015824592	0	0	0	0
0	0	0	0	0
0	0	0	0	0
0	0	0	0	0
0	0	0	0.015542717	0
0	0	0	0	0
0	0	0	0.001014533	0
0	0	0	0	0
0	0	0	0	0
0	0	0	0	0

0	0	0	0	0
0	0	0	0	0
0	0	0	0	0
0	0	0	0	0
0.00250502	0	0	0	0.060765615
0	0	0	0	0
0	0	0	0	0
0	0	0	0	0
0	0	0	0	0
0	0	0	0	0
0	0	0	0	0
0	0	0	0	0
0	0	0	0	0
0	0	0	0	0
0	0	0	0	0
0	0	0	0	0
0	0	0	0	0
0	0	0	0	0
0	0	0	0	0
0	0	0	0	0
0	0	0	0	0
0	0	0	0	0
0	0	0	0	0
0	0	0	0	0
0.01653614	0	0	0	0
0	0	0	0	0
0	0	0	0.001043368	0
0	0	0	0	0
0	0	0	0	0
0	0	0	0	0
0	0	0	0	0
0	0	0	0	0
0	0	0	0	0
0	0	0	0	0
0	0	0	0	0
0	0	0	0	0
0	0	0	0	0
0	0	0	0	0
0	0	0	0	0
0	0	0	0	0
0	0	0	0	0
0	0	0	0	0
0	0	0	0	0
0	0	0	0	0
0	0	0	0	0
0	0	0	0	0
0	0	0	0	0
0	0	0	0	0

0	0	0	9.379638901	0
0	0	0	0	0
0	0	0	0	0
0	0	0	0	0
0	0	0	0	0
0	0	0	0	0
0	0	0	0	0
0	0	0	0	0
0	0	0	0	0
0	0	0	0	0
0	0	0	0	0
0	0	0	0.075609437	0
0	0	0	0	0
0	0	0	0	0
0	0	0	0.001808305	0
0	0	0	0	0
0	0	0	0	0
0	0	0	0	0
0	0	0	0	0
0	0	0	0	0
0.001188247	0.055914145	0.018227025	0.126615771	0.02352975
0	0	0	0	0
0	0	0	0	0
0	0	0.000950356	0.002953798	0.000766775
0	0	0	0	0
0	0	0	0	0
0	0.001092721	0.001592248	0	0.011562051
0	0	0	0	0
0	0	0	0	0
0	0	0	0	0
0	0.266867989	0.005643383	0	0
0	0	0	0	0
0	0	0	0	0
0	0	0	0.044142779	0.006441007
0	0.008789973	0	0	0
0	0	0	0	0
0	0	0	0	0
0	0	0	0	0
0	0	0	0	0
0	0	0	0	0
0	0	0	0	0
0	0	0	0	0
0	0	0.009039502	0	0
0	0	0	0	0
0	0	0	0	0
0	0	0	0	0
0	0	0	0	0
0	0	0.000845138	0	0

0	0	0	0	0
0	0	0	0	0
0	0.001907962	0	0.000960115	0
0.00105051	0	0	0	0
0	0	0	0	0
0	0	0	0	0
0	0	0	0	0
0	0	0.001822352	0	0
0	0	0	0	0
0	0	0	0	0
0	0	0	0	0
0	0	0	0	0
0	0	0	0	0
0	0	0	0	0
0	0	0	0.067300138	0
0	0	0	0	0
0	0	0	0	0
0.066831343	0	0	0	0
0.067938909	0	0	0	0.004045239
0	0	0	0.101941677	0
0	0.00287131	0	0.027584199	0
0	0	0	0	0
0	0	0	0	0
0	0	0	0	0
0	0.009853167	0.002938661	0	0.00440328
0	0	0	0	0
0	0	0	0	0
0	0	0	0	0
0	0	0.004807554	0	0.004202114
0	0	0	0	0
0.062294114	0.044336742	0.007302621	0.091706553	0.289834645
0	0	0	0	0
0	0	0.026467925	0	0
0	0	0.001407143	0.000937186	0
0	0	0	0	0
0	0	0	0	0
0	0	0	0	0
0	0	0	0	0
0	0	0	0	0
0	0	0	0	0
0	0	0	0	0
0.001238995	0	0.001267031	0	0.018145427
0	0	0	0	0
0	0	0	0	0
0	0	0	0	0
0	0	0	0	0
0	0	0	0	0
0	0	0	0	0

0	0	0	0	0
0	0	0	0	0
0	0.014985515	0.029234003	0.04153696	0.032572308
0	0	0	0	0
0	0.022598581	0	0	0
0	0	0	0	0
0	0	0	0	0
0	0	0	0	0
0	0	0	0	0
0	0	0	0	0
0	0	0	0	0
0	0	0	0	0
0	0	0	0	0
0	0.005430065	0.002857245	0.012296207	0
0	0.00537817	0.003265314	0.003044673	0.018002758
0	0	0	0.004889586	0.00423095
0	0.007404597	0.005580798	0	0.001834454
0	0	0	0	0
0	0	0	0	0
0	0	0	0	0
0	0	0	0	0
0	0	0	0	0
0	0	0	0	0
0	0	0	0	0
0	0	0	0.001115866	0
0	0	0	0	0
0	0	0	2.551755657	7.272108997
0	0	0	0	0
0	0	0	0	0
0	0	0	0	0
0	0	0	0.006132879	0.001153646
0	0	0	0	0
0	0	0.004633856	0	0
0	0	0	0	0
0	0	0	0	0
0	0	0.034286782	0	0
0	0	0	0	0
0	0.003185468	0.001875425	0	0.007944024
0	0	0	0	0
0	0	0	0	0
0	0	0	0	0
0	0	0	0	0
0	0	0	0	0
0	0.021582817	0	0	0
0	0	0	0	0
0	0	0	0	0
0	0	0	0	0
0	0	0	0	0

0	0	0	1.63584168	0
0	0	0	0	0
0	0	0	0	0
0	0	0	0	0
0	0.002186038	0	0	0.001586448
0	0	0	0.001195818	0
0	0	0	0	0
0.002156144	0.018385281	0.00771727	0	0.019346679
0	0	0	0	0
0.216404718	0	0.070168792	0.050328777	0.026817262
0	0	0	0	0
0	0.003773221	0.054108447	0	0.0110901
0	0.002044373	0.001654967	0.010339031	0
0	0	0	0	0
0	0	0	0	0
0	0.002455155	0	0	0
0	0	0	0	0
0	0	0	0	0
0	6.796191268	0	0	0
0	0	0	0.00179814	0
0	0	0	0	0
0	0	0	0	0
0	0	0	0	0
0	0	0	0.080015567	0
0	0	0	0	0
0	0	0.004626275	0	0.000933153
3.094258039	0	0	0	0.002703599
0	5.206137185	0	0	0.001128442
0	0	0	0	0
0	0	0	0	0
0	0	0	0	0
0	0	0	0	0
0	0	0	0	0
0	0.005294034	0	0	0
0	0	0	0	0
0	0.00386342	0	0	0
0	1.211717613	0.00677396	0	0
0	0	0	0	0
0	0	0	0	0
0	0	0	0	0.001472158
0	0	0	0	0
0	0	0	0	0
0	0	0	0	0
0	0	0	0	0
0	0	0	0	0
0	0	0	0.077060311	0
0	0	0	0	0.003094533
0	0	0	0	0

0	0	0	0	0
0	0.00430212	0	0	0.001513031
0	0	0	0	0
0	0	0	0	0
0	0	0	0.013255351	0
0	0	0	0.016372296	0
0	0	0	0.000783015	0
0.001910328	0	0	0	0
0	0	0	0	0
0	0	0	0	0
0	0.001082024	0	0	0.00406364
0	0	0	0	0
0	0	0	0	0
0	0	0	0.003086444	0.001836102
0	0	0.043330766	0	0
0	0	0	0	0
0	0	0	0.401655154	0
0	0.023875345	0	0	0
0	0	0	0	0
0	0	0	0.152917253	0
0.239335956	0	0	0	0
0.16148113	0	0	0.448408357	1.248189669
0	0	0	0.234860161	0
0	0	0	0	0
0	0	0	0	0
0	0	0	0	0
0	0.008589948	0.046657484	0	0
0	0	0	0	0
0	0	0	0	0
0.096967127	0.13485527	0.083695794	0.153535962	0.447007323
0	0	0	0.000392608	0.001245649
0	0	0	0	0
0	0.008009568	0	0	0
0	0	0	0	0
0	0.140553616	0	0	0
0	0	0	0	0
1.779273052	0	0	0	0.308706204
0	0	0	0	0
0	0	0	0	0
0	0	0.205872018	0	0
0	0	0	0.035364908	0
1.165337299	0	0	1.779500445	0.673418451
0	0	0	0	0
0	0	0	0	0
0	0	0	0	0
0	0	0	0	0
0	0.002205134	0.004284255	0.002996072	0.000864165
0	0	0	0	0

0	0	0.001982269	0	0.001599353
0	0	0	0.025063982	0
0.002054288	0.052642962	0.035413032	0.019868047	0
0	1.37945419	0	0	0
0.009670684	0	0	0	0.244614463
0	0.002760491	0	0	0
0	0	0	0	0
0	0	0.009133746	0	0
0.004145808	0	0	0.007046909	0.00386682
0	0	0	0	0
0	0	0	0	0
0	0	0	0	0
0	0	0	0	0
0	0	0	0	0
0	0	0	0	0
0	0	0	0.002903874	0.320622682
0.005400812	0.005543335	0.05902731	0.001126525	0.019913356
0	0.007253007	0	0.001759736	0
0	0	0	0.004654459	0
0	0	0	0	0
0	0.178416262	0.004785399	0	0
0	0	0	0	0
0	0	0	0	0
0	0	0	0	0
0.007255898	0	0	0	0
0	0	0	0	0
0	0	0	0.002655959	0
0	0	0	0	0
0	0.026663169	0	0	0
0	0	0	0	0
0	0	0	0.03775565	0
0	0	0.010434007	0.023627499	0.001803956
0.118658828	0.004967298	0	0.535065555	0.05171914
0	0.00167497	0	0	0.007384507
0	0	0.002661413	0.023687797	0.001952096
0	0	0	0	0
0	0	0.001069232	0.00059819	0.000862688
0.000981224	0.00661082	0	0.003274685	0.032653626
0	0	0	0.022589032	0.221724626
0	0	0	0	0
0	0	0	0.004750458	0
0	0	0	0	0.022013378
0	0	0	0	0
0	0	0	0.541325957	0
0	0.542489682	0	0	0.005771442
0	0	0.009115144	0	0
0	0	0	0	0
0	0	0	0	0

0	0	0	0	0
0.000668521	0.329357974	0.105965479	0.066295153	0.275058192
0	0	0	0	0
0	0	0	0	0
0	0	0	0	0
0	0	0	0	0
0	0.01197962	0	0	0
0	0	0	0	0
0	0	0	0	0
0	0	0	0	0
0.00281511	0	0	0	0
0	0.006854985	0.002270155	0.089844613	0.003663254
0	0	0	0	0.09421217
0	0	0	0	0
0	0	0	0	0
0	0	0	0	0
0	0.016647736	0.011171494	0.004133581	0.018742329
0	0.156395837	0.007596361	0	0
0	0	0	0	0
0	0	0	0	0
0.079754046	0.008358113	0.003657343	0.003239701	0.041164361
0	0	0	0	0
0	0	0	0	0
0	0	0	0	0
0.002403617	0	0	0	0
0	0	0	0	0
0	0	0	0.16089717	0.128884173
0.020336424	0.020888089	0.106315336	0.343969971	0.095971338
0	0	0	0	0
0	0	0	0	0.002648194
0	0.00231858	0	0	0
0	0	0	0	0
0	0	0	0	0
0	0	0	0	0
0	0	0	0	0.248847207
0.039243164	3.81068504	0.102630526	0.166240554	0.260889748
0	0	0	0	0
0	0	0	0	0
0	0	0.022298214	0.010507532	0.007351099
0	0	0	0	0
0	0.389553031	0	0	0.007283451
0	0	0	0	0
0	0.002338768	0.032622771	0.015209207	0
0	0	0.002390372	0	0.002495864
0	0	0	0	0
0.022020012	0	0	0.786631227	1.015789798
0	0	0	0	0
0	0	0	0.001483492	0

0	0	0	0	0
0	0	0	0	0.053673548
0	0	0	0	0
0	0	0	0.200230492	0.604627681
0	0	0	0	0
0	0	0	0.003428719	0
0	0	0	0	0
0	0	0	0	0.035137966
0.00823952	0	0	0.222888732	0.345679504
0	0	0	0.530085358	0.017858016
0	0	0	0.056464941	0
0	0.008030191	0	0.003182219	0.004195914
0	0	0	0	0
0	0	0	4.257055241	0
0.002229393	0.006792197	0	0	0.326338601
0	0	0	0.324600517	0
0.004447603	0	0	0.014190754	0.027522421
0.005137504	0	0	0	0
0	0	0	0	0
0	0	0	0.001217918	0
0	0	0	0.009903542	0
0	0	0	0.002110326	0
0	0	0	0	0
0	0.056489998	0	0.297274953	0
0	0	0	0.001276873	0
0.131261534	0	0	0.076178943	0
0	0	0	0	0
0.700203435	0	0	1.569373048	0.445290957
0	0.001132496	0	0	0
0	0	0	0	0
0.000463491	0	0	0	0.013384689
0.003639118	0	0	0.446103339	2.115862111
0	0	0	0	0.179383487
0	0	0	0	0
0	0	0	0	0
0	0	0	0	0.70772778
0.018779217	0	0	0.379101815	1.022495571
0	0	0	0	0
0	0	0	0	0
0	0	0	0	0
0	0.016947772	0.007327519	0.174153267	0.101385455
0	0	0	0.100247389	0
2.185881322	0.108816699	0.061718834	0.545140535	6.048017244
0	0	0	0	0
0	0	0	0	0
0	0	0	0	0
0	0	0	0	0
0	0	0	0.004019283	0

0	0	0	0	0
0.003172289	0	0	0	0.104007668
0.001229783	0.297388331	0.041141843	0.007873391	0.076100778
0	0	0	0	0.00172357
0	0.004880966	0	0	0
0	0	0	0.00290323	0.002283781
0	0	0	0	0
0	0	0	0	0
0	0	0	0.015392283	0
0	0	0	0	0
0.00051747	0	0.007584898	0.014473705	0.068740071
0.952225628	1.170825145	1.289920473	5.528735999	2.24447435
0	0	0	0	0
0.00390932	0.003292294	0.077100053	0	0.030258501
0.003374093	0.228126599	0.053106816	0.094280664	0.015251027
0	0	0	0.008590859	0
0	0	0	0	0
0	0	0	0	0
0	0	0	0	0
0	0	0	0.102831765	0
0	0.010409919	0.005470202	0.092223901	0.017415503
0.564854184	1.395927361	29.54058938	0	0.001885329
9.534309689	0	41.22973434	2.186937358	2.550778252
0	0	0	0	0
0	0	0	0	0
1.365308316	0	0	0	0.812318562
0	0	0	0.004781492	0
0	0	0	0.002731793	0.002488227
0	0	0	0	0
0	0	0	0	0
0	0	0	0.027520813	0.034470675
0.073566465	0	0	0.093833928	0.361827159
0	2.591669989	0	0.002027028	0.004384959
0	0	0	0	0.214007739
0	0.00413565	0	0.000445955	0
0.004176744	0.015625858	0.076554055	0.003523107	0.000662726
0	0.007057106	0	0	0.012547606
0	0	0	0	0
0.018046648	0	0	0.37750021	0.044825225
0	0	0	0	0
0	0	0	0.015241603	0.264470914
0	0	0	0	0
0	0	0	0.090598228	0
0	0	0	0	0
0.000915981	0.010414004	0	0.006113897	0.030683996
0	0	0	0	0
0	0	0	0	0
0.005082649	0	0	0.030132268	0

0	0	0	0	0
0	0	0	0	0
0	0	0	0	0
0	0	0	0.000595526	0.015974526
0	0	0	0	0
0.093358014	0	0	0	0.079759104
0.001367526	0	0	0.484100503	0
0.001005723	0	0	0.013699786	0.049669936
0	0	0	0	0
0	0	0	0	0
0.000521687	0.007029551	0	0.008290724	0
0	0	0	0	0
0	0	0	0	0
0	0	0	0	0.010162082
0	0	0	0	0
0	0	0	0	0
0	0	0	0.067210661	0.2290009
0	0	0	0	0.000358329
0	0	0	0.134647946	0
0	0	0	0	0
0	0	0	0	0
0	0	0	0	0
0.00116832	0	0	0.027214054	0.000688546
0	0	0	0	0
0	0	0	0	0
0	0	0	0	0
0	0	0	0	0
0	0	0	0	0
0	0	0	0	0
0	0	0.052755142	1.372162579	0.502548278
0	0	0	0	0
0	0	0	0	0
0.006050159	0	0	0.015143616	0
0	0	0	0	0
0	0	0	0	0.001254962
0	0	0	0	0
0.090782014	0	0.009551055	0.185238131	0.514765371
0	0.001301702	0.002709662	0	0
0	0	0	0	0
0	0	0	0	0
0.001795501	0	0	0	0
0	0	0	3.758893524	0
0	0	0	0	0
0.006147033	0	0	0	1.526975304
0.006184574	0	0	0	0.199009453
0	0	0	0	0
0	0	0	0.001867182	0
0	0	0	0	0

0	0	0	0	0
0	0	0	0	0
0	0	0	0	1.560079711
0.021671138	0	0	0.093686289	0.134708249
0	0	0	0.118747023	0
0.036461217	0	0	0	0.811631709
0	0	0	0	0
0	0	0	0	0
0	0.732467887	0.01930823	0.009872916	0
0	0	0	0	0
0	0	0	0.010519953	0.060366576
0	0	0	0	0
0	0	0	0	0
0	0	0	0	0
0.009045584	0	0	4.961865574	0.255560784
0	0	0	0.282738854	0
0	0	0	0	0
0	0	0	0	0
0	0	0	0.000839407	0
0	0	0	0	0
0.008693381	0	0	0.065574943	0.69409538
0	0	0	0	0
0	0	0	0	0
0	0	0	0.00177274	0
0.304164115	0.003920763	0.002441498	0.004097742	0.127450675
0.064358386	0	0.002761456	4.02214815	4.757386113
0	0	0	0.023369796	0.172032198
0	0	0	0	0
0.001669868	0	0	0	0.07595038
0	0	0	0.030682118	0
0	0	0	0	0
0	0	0	0.018912605	0.026006465
0	0	0	0	0
0	0	0	0	0
0.005882528	0.001592377	0.082715039	0	0.014560778
0	0.001125617	0	0	0
0	0	0	0	0.13327813
0.145556318	0	0.264088709	3.126818438	0.782149253
0	0	5.288787139	0	0
0	0	0	0	0
0	0	0	0.374927649	0
0	0	0	0.004817501	0
0.088684484	0	0.096502139	0.054569355	0.105153847
0.056878948	0	0	0	0
0	0	0	0	0
0	0	0	0	0.000464336
0	0	0	0	0
0	0	0.008480873	0.427235513	0.415550136

0.001838563	0	0	0.046218658	0
0	0	0	1.707356355	0
0	0	0	0	0
0	0	0	0	0
0	0	0	0.000634704	0.00137302
0	0	0	0	0
0	0	0	0.585494158	0
0	0	0	0.002795267	0
0	0	0	0	0
0	0	0	0	0
0.000763994	0	0	0.001092735	0
0.454582225	6.894492318	0	1.631355659	5.224625833
0	0.241044658	0.204403504	0.025553126	0.092600514
0	0	0	0	0
0.001347167	0.096273571	0.117100296	0	0.023659678
0	0	0	0	0
0	0.002705946	0	0.000893598	0
0	0	0	0	0
0	0	0	0.008524772	0
0	0	0	0.004458779	0
0	0	0	0.008256212	0.109465825
0	0	0	0	0
0.010497413	0	0	0.034095128	0.060493563
0	0	0	0.043635418	0
0	0	0	0	0
0	0	0	0	0
0	0	0	0	0
0	0	0	0	0
0	0	0	0.001818244	0
0	0	0	2.743876769	0
0	0	0	0	0
0	0.020505928	0.024772334	0.011430156	0.015247844
0	0	0	0	0.091246612
0.001871441	0	0	0.011896477	0.085028443
0	0.001224954	0	0	0
0	0	0	0.065755955	0.108468158
0	0	0	0	0
0	0	0	0.018419869	0
0	0	0	0	0.006127783
0	0	0	0	0
0	0	0	0.819949737	0
0	0	0	0.003711119	0
0	0	0	0	0
0	0.075622909	0	0.028603666	0
0.000636825	0.019709471	0.003093368	0.001821692	0.061870049
0	0	0	0.006273453	0
0.027594232	0	0	0	0.230218213
0	0	0	0	0

0	0	0	0	0
0	0	0	0	0
0	0	0	0	0
0	0	0	0	0
0	0.120872918	0	0.001592279	0
0	0	0	0	0
0.006343868	0	0	0	0
0	0	0	0.020017606	0
0	0	0	0	0
0	0	0	0	0.01035309
0	0	0	0	0
0	0	0	0	0
0	0	0	0	0
0.233650596	0	0	0	0.347771954
0	0	0	0	0
0	0	0	0	0
0	0	0	0	0
0	0	0	0.060154941	0
0.01900192	0	0	6.079885835	0
0	0	0	0	0
0	0	0	0.000713275	0
0	0	0	0	0
0	0	0	0	0
0	0	0	0	0
0	0	0	0	0
0	0.00786376	0.030616915	0	0
0	0	0	0	0
0	0	0.00741779	0.394382342	0.431271056
0	0	0	0	0
0.01107157	0	0	0.675265173	1.271658738
0	0	0	0	0
0	0	0	0	0
0	0	0	0.002483119	0
0	0	0	0	0
0	0	0	0	0.000640763
0	0	0	0	0
0	0	0	0	0
0	0	0	0.001008316	0.00232665
0	0	0	0	0
0	0	0	0	0
0.505115694	3.033336713	3.336923648	1.664839294	4.248325086
0	2.319749475	0.010422463	0.0451466	0
0	0	0	0	0
0	0	0	0	0
0	0	0	0	0
0	0	0	0	0
0	0	0	0.001334931	0.01518149
0	0	0	0.002000638	0.001615738

0	0	0	0	0
0	0	0	0.007270967	0.057811079
0	0	0	0	0
0	0	0	0	0
0.223886477	0	0.487227266	0.401995445	0.98142247
0	0	0	0	0
0	0	0	0	0
0	0	0	0.001454549	0
0	1.333883854	0.070811998	0	0
0	0	0	0.000997917	0
0	0	0.002825842	0	0
0	0	0	0	0
0.006989913	0	0	0.161028308	0.165405617
0.002234281	0.004342232	0.000703027	0.006063587	0.016307656
0	0	0	0.045590464	0.449483469
0.007051672	0	0	0.517342042	0.178154412
0	0.004509034	0.001977173	0.170317842	0.000613554
0	0	0	0	0
0	0	0	0	0
0	0	0	0	0
0	0	0	0.011573837	0
0	0	0	0	0
0	0	0	0	0
0	0	0	0	0
0	0	0	0	0
0	0	0	0	0
0.000775934	0	0	0.004439252	0.002774255
0	0.000921703	0	0	0
0	0	0	0	0
0	0	0	0.000821589	0
0	0	0	0	0
0	0	0	0.001278512	0
0	0.003974079	0	0.497954263	0
0	0	0	0.019253584	0.004796087
0	0	0	0	0.156441382
0	0.002772169	0	0	0
0	0	0	0	0
0	0	0	0.009271573	0
0	0	0	0	0
0	0	0	0	0
0.30308646	0	0	0	0.3896081
0	0	0	0	0
0	0	0	0	0
0.00285761	0	0	0	0
0	0	0	0	0
0	0	0	0.000983694	0
0	0	0	0.456083923	0.004081008
0	0	0	0	0

0	0	0	0.07587035	0.053785934
0	0	0	0	0
0	0	0	0	0
0	0	0	0	0
0	0	0	0.087321705	0.007307982
0	0	0	0	0.165966487
0	0.001499357	0.014868603	0.539843899	0.012608484
0	0	0	0	0
0	0	0	0	0
0	0	0	0	0
0	0	0	0.006175935	0.030876592
0	0	0	0.004577525	0
0	0	0	0	0
0	0	0	0	0
0	0	0	0	0
0	0	0	0	0
0	0	0	0	0
0	0.016036399	0	0	0
0	0	0	0	0
0	0	0	0	0
0	0.005353017	0.191824709	0.091721113	0.002563957
0	0	0	0	0
0	0	0	0.00237693	0.120577142
0	0.001050043	0	0	0.001143054
0	0	0	0	0
0	0	0	0.002181866	0
0	0	0	0	0
0	0	0	0	0
0	0	0	0	0
0.003588983	0	0.006116992	2.570069275	0.005264391
0	0	0	0.15590007	0
0	0	0	0	0
0	0	0	0.024517737	0
0	0	0.005446711	0	0
0	0	0	0.001408189	0
0	0	0	0	0
0	0.010073051	0	0.00297523	0.00686523
0	0	0	0	0
0	0	0	0	0
0	0	0	0	0.097093723
0	0	0	0	0
0	0	0	0	0
0	0	0	0	0
0.005458671	0	0	0	0
0	0	0	0.004536616	0
0	0	0	0	0
0	0	0	0	0
0	0	0	0	0

0	0.002448082	0	0	0
0	0	0	0	0
0	0	0	0	0
0	0	0	0	0
0	0	0	0.007479007	0
0	0	0	0	0
0	0	0	0	0
0.185970933	0	0.296616826	0.366624881	0.565020344
14.97955506	0.084833494	0.096670943	0.193664511	0.975540768
0	0.02716281	0	0	0.00107523
0	0	0	0	0
0	0	0	0	0
0	0	0	0	0.008031422
0	0	0	0	0
0	0	0	0	0
0	0	0	0	0
0	0.414530722	0.000890112	0	0
0	0	0	0	0
0	0	0	0	0
0	0	0	0	0.117246277
0	0	0	0	0
0	0	0	0	0
0	0	0	0	0.017126216
0	0	0	0	0
0.000587071	0	0	0.082177043	0.080795339
0	0	0	0.072097779	0
0	0	0	0	0
0	0	0	0	0
0	0	0	0	0
0	0	0	0	0
0	0	0	0	0
0	0	0	0	0
0	0	0	0	0
0	0	0	0	0
0	0	0	0	0.013103618
0	0	0	0.006196763	0
0	0	0	0	0
0	0	0	0	0
0.001418059	0	0	0	0.008628904
0	0.008835828	0	0	0.000491157
0	0	0	0	0
0.003531283	0.116564901	0	0	0.280973613
0	0	0	0	0
0	0	0	0	0
0	0	0	0	0
0	0	0	0	0
0	0	0	0	0
0	0	0	0.000794214	0

0	0	0	0	0
0.00080726	0	0.006879391	0.054908486	0.000777069
0	0	0	0	0
0	0	0	0	0
0.003197878	0	0	0.071926829	0.129753364
0	0	0	0	0.086807945
0	0	0	0	0
0	0.080009376	0.001163523	0.01084902	0
0	0	0	0	0.027182305
0	0	0	0	0.070999974
0	0	0	0	0
0	0	0	0	0
0	0	0	0	0
0	0	0	0	0
0	0.008600337	0	0	0
0	0	0	0	0.102994128
0	0	0	0	0
0	0	0	0	0
0	0	0	0	0
0	0	0	0	0
0	0	0	0	0
0	0	0	0.034118375	0
0	0	0	0.002149775	0
0	0.004353509	0	0	0
0.163093966	0	0	0	0.098907037
0	0	0	0	0
0	0	0.200614577	0.02461677	0.006068265
0	0	0	0	0.00078241
0	0	0	0	0
0	0	0	0	0
0	0	0	0	0
0	0	0	0.006327803	0
0	0	0	0	0
0	0	0	0	0
0	0	0	0	0
0	0	0	0	0
0	0	0	0	0
0.003694374	0	0	0	0.079760627
0	0	0	0.01195815	0
0	0	0	0	0
0	0	0	0	0
0.003353595	0	0	0	0
0	0	0	0	0
0	0	0	0	0
0	0	0	0	0
0	0	0	0	0
0	0	0	0	0
0	0	0	0	0

0.000423457	0	0	0.018035427	0.080359371
0	0	0	0.015666177	0
0	0	0	0	0
0	0	0	0	0
0	0	0	0	0
0	0.157829382	0	0	0
0	0	0	0	0
0	0	0	0	0
0	0	0	0	0
0	0	0	0.042636372	0
0	0	0	0	0
0	0.001911067	0	0.00086551	0
0	0	0	0	0
0	0	0	0	0
0	0	0	0	0
0	0	0	0	0
0	0	0	0	0
0	0	0	0	0.477377243
0	0	0	0	0
0	0	0	0	0
0	0.19591473	0	0	0.00121354
0	0	0	0	0
0.001031954	0	0	0.019089554	0.003065225
0	0	0	0.39158345	0
0	0	0	0.008838302	0
0.003005497	0	0	0	0
0	0	0	0	0
0	0	0	0	0
0	0	0	0	0
0	0	0	0	0
0	0	0	0	0
0	0	0	0	0.352727068
0	0.067145629	0	0	0
0	0	0	0	0
0	1.938565085	0.199965388	0.030663442	0.090868466
0	0	0.000793061	0	0
0	0	0	0	0
0	0	0	0	0
0	0.037620151	0	0	0
0	0	0	0	0
0	0	0	0	0
0	0	0	0	0
0.001258638	0	0	0.023002814	0.034962325
0	0	0	0	0
0	0	0	0	0
0	0	0	0.023192702	0.062130196
0	0	0	0	0
0	0	0	0	0

0	0	0	0	0
0	0	0	0	0
0	0	0	0	0
0	0	0	0	0
0	0	0	0	0
0	0	0	0	0
0	0	0	0	0
0	0	0	0	0
0	0	0	0	0
0	0	0	0.006708338	0.00084947
0	0	0	0	0
0	0	0	0	0
0	0	0	0	0
0	0	0	0	0
0	0	0	0.049320172	0.021281121
0	0.203247587	0.002071776	0	0
0	0	0	0	0
0	0	0	0	0
0	0	0	0	0
0	0	0	0.09690627	0.013645035
0.033326108	0	0	0	0.001745836
0	0	0	0	0
0	0	0	0	0
0	0	0	0	0
0	0	0	0	0
0	0	0	0	0
0	0	0	0	0
0	0	0	0	0
0	0	0	0	0
0	0	0	0.049052578	0.093263741
0.051594141	0	0	0.116318238	0.192808646
0	0	0	0	0
0	0	0	0	0
0	0	0	0	0
0	0	0	0	0
0	0	0	0	0
0	0	0	0	0
0	0	0	0	0
0.003711614	0	0	0	0
0	0	0	0	0
0	0	0	0	0.00159735
0	0	0	0.105887892	0
0	0	0	0.002122751	0
0	0	0	0	0
0	0	0	0	0
0	0	0	0	0
0	0	0.220923164	0	0
0	0	0	0	0

0	0	0	0	0
0	0	0	0	0
0	0	0	0	0
0	0	0	0	0
0	0	0	0.126931571	0
0	0	0	0.013795497	0
0	0	0	0	0
0	0	0	0	0
0	0	0	0	0
0	0	0	0	0
0	0	0	0.036108366	0
0	0.004076045	0.005866048	0.002905764	0.010353214
0	0	0.000474461	0	0.003445283
0	0	0	0	0
0	0.0032788	0	0	0
0	0	0	0.139300353	0
0.002481668	0	0	0	0.016608231
0	0	0	0	0
0	0.003086405	0	0.006859637	0
0	0.001119418	0	0.178962918	0.002047203
0	0	0	0	0
0	0	0	0	0
0	0	0	0	0
0	0	0	0	0
0	0	0	0	0
0	0	0	0	0
0	0.00197349	0	0.001083371	0
0	0	0	0	0
0	0	0	0	0
0	0	0	0	0
0	0	0	0	0.001992442
0	0	0	0	0
0	0	0	0	0
0	0	0	0	0
0	0	0	0	0
0	0	0	0	0
0	0	0	0	0
0.00056553	0	0.005089274	0.010137871	0.01735792
0	0	0	0.00843187	0.0347967
0	0	0	0	0
0	0.00984224	0	0.000753681	0
0	0	0	0	0
0	0	0	0	0
0	0	0	0.011034992	0
0	0	0	0	0
0	0	0	0	0
0	0	0	0	0
0	0	0	0.011771626	0

0	0	0	0	0
0	0	0	0.014489249	0
0	0	0	0	0
0	0	0	0	0.000580112
0	0	0.002167306	0	0.000807067
0	0	0	0.001203744	0
0	0	0	0	0.003799944
0	0	0	0.573349836	0
0	0	0	0	0.41801831
0	0	0	0.000774708	0
0.023384301	0	0	0.027837362	0.037869094
0	0	0	0	0
0.014079623	0	0	0.004989941	0.03906569
0	0	0	0	0
0	0	0	0	0
0	0	0	0	0
0	0	0	0	0
0	0	0	0	0
0	0	0	0	0
0	0	0	0	0
0	0	0	0	0
0	0	0	0	0
0	0.002694352	0	0.00484973	0
0	0	0.002771806	0.010153441	0.041692396
0	0	0	0	0
0	0	0	0.002317691	0.02866677
0	0	0	0.019281328	0
0	0	0	0	0
0	0	0	0	0
0	0	0	0	0
0	0	0	0.014826817	0.042715083
0	0	0	0	0.02241029
0	0	0	0	0
0	0	0	0	0
0	0	0	0.007458282	0
0	0.004348384	0	0	0
0	0.003682769	0	0	0
0	0	0	0	0
0	0	0.064577229	0.00059033	0.000383109
0	0	0	0	0
0	0	0	0	0
0	0	0	0	0
0	0	0	0	0
0	0	0	0	0
0.016128766	0	0	0	0
0.030503566	0.072078422	0.054449906	0.094139983	0.047527206
0	0	0	0	0
0	0	0	0	0

0	0	0	0	0
0	0	0	0	0
0.146243525	0	0	0	0
0	0	0	0	0.076336211
0	0	0	0	0
0	0	0	0	0
0	0	0	0	0
0	0	0	0	0
0	0.003075428	0	0.000819319	0
0	0	0	0	0
0	0	0	0	0
0	0	0	0	0
0	0	0	0	0
0	0	0	0	0
0	0	0	0	0
0	0	0	0	0
0.002768609	0	0	0	0
0	0	0	0	0
0	0	0	0	0
0	0	0	0.004128311	0
0	0	0	0	0
0	0	0	0	0
0	0	0	0	0
0	0	0	0	0
0	0	0	0	0
0	0	0	0	0
0	0	0	0	0
0.016585395	0	0	0	0.008210628
0	0	0	0	0
0	0	0	0	0
0	0.000903586	0	0.000954866	0.005193534
0	0	0	0	0
0.024417358	0.14667198	0.038389066	0.076024939	0.06222093
0	0	0	0	0.000420152
0.000824086	0	0	0	0
0	0	0	0	0
0	0	0	0	0
0	0	0	0	0
0	0	0	0	0
0	0	0	0	0
0	0	0	0	0
0	0	0	0.074141918	0.372526691
0.018146478	0.311254915	0	0.076038734	0.248487038
0	0	0	0	0
0	0	0	0	0
0	0	0	0.002064575	0.011314328
0	0.003785773	0	0.000751997	0
0	0	0	0.000961442	0

0	0	0	0	0
0	0	0	0.011641432	0.051373884
0	0.004117803	0.003125115	0.025642739	0.002857625
0	0	0	0	0
0	0	0	0	0
0	0.031003786	0	0.001962778	0
0	0	0	0	0
0	0	0	0	0
0	0	0	0	0
0	0	0	0	0
0	0	0	0	0
0	0	0	0	0
0	0	0	0	0
0	0	0	0.053883471	0
0	0	0	0	0
0	0	0	0	0
0	0	0	0	0
0	0	0	0	0
0	0	0	0	0
0	0	0	0	0
0	0	0	0.000545578	0
0	0.000621056	0	0	0
0	0	0	0	0
0	0	0	0	0
0	0	0	0	0.001108275
0	0	0.001381827	0.001771627	0.001254261
0	0	0	0	0
0	0	0	0.037651645	0.179392235
0	0	0	0	0
0	0	0	0	0
0	0	0	0	0
0	0	0.000454418	0	0
0	0	0	0	0
0	0	0	0	0
0	0	0	0	0
0	0	0	0	0
0	0	0	0	0
0	0	0	0	0
0	0	0	0.376680971	14.25702477
0	0	0	0	0
0	0	0	0	0
0	0	0	0.004193328	0
0	0	0	0	0
0	0	0	0	0
0	0	0	0	0
0	0	0	0	0
0	0	0	0	0.011340226
0	0	0	0	0

0	0	0	0	0
0	0.014674374	0.012213868	0	0.001079946
0	0	0	0	0
0	0	0	0	0
0	0	0	0	0
0	0	0	0	0
0	0	0	0	0
0	0	0	0	0
0	0	0	0	0
0	0	0	0	0
0	0	0	0	0
0	0	0	0	0
0	0	0	0.004287764	0.28411526
0	0	0	0	0
0	0.00566941	0	0.001750663	0
0	0	0	0	0
0	0	0	0	0
0	0	0	0	0
0	0	0	0.004518936	0
0	0	0	0	0
0	0	0	0	0
0	0.002024658	0	0.104303552	0.001983597
0	0	0	0.030386062	0.043186578
0	0	0	0	0
0	0	0	0	0
0	0	0	0	0
0	0	0	0	0
0	0	0	0	0
0	0	0	0	0
0	0	0	0	0
0	0	0	0	0
0	0	0	0	0
0	0	0	0	0
0	0	0	0	0
6.176588973	0	0	7.769885439	0.074291083
0	0	0	0.300094709	0
0	0	0	0	0
0	0	0	0	0
0.001800595	0	0.00122756	0.108738166	0.08864357
0	0	0	0	0
0.008610517	0.026739831	0.023847844	0.104682222	0.281660601
0	0	0	0	0
0	0	0	0	0
0	0	0	0	0
0	0	0	0	0
0.000821538	4.614346324	0.021633295	0.00058752	0.003728118
0	0	0	0.027010961	0
0	0	0	0.008320342	0

0	0	0	0	0
0	0	0	0	0
0	0	0	0	0
0.002296251	0	0	0	0
0	0	0	0	0
0	0	0	0	0
0	0	0	0	0
0	0	0	0.000846701	0
0	0	0	0	0
0	0	0.019719515	0	0.001325857
0.00656711	0	0	0	0
0	0	0	0	0
0	0.001488081	0	0	0.002429837
0	0	0	0	0
0	0	0	0	0
0	0	0	0	0
0	0	0	0	0
0	0	0	0	0
0	0	0	0	0
0.006465658	0.085650795	0.03877828	0.009164467	0.023213264
0	0	0	0	0
0	0	0	0	0
0	0	0	0	0
0	0	0	0	0
0	0	0	0	0
0.000523934	0	0	0	0
0	0	0	0	0
0	0	0	0	0
0	0	0	0	0
0	0.01439507	0	0	0
0	0	0	0	0
0	0	0	0.00108897	0
0	0	0	0	0
0	0	0	0	0
0	0	0	0	0
0	0	0.002313253	0	0
0	0	0	0	0
0	0	0	0	0
0	0	0	0	0
0	0	0	0	0.328424978
0	0	0	0	0
0	0	0	0	0
0	0	0	0.064682612	0
0	0	0	0	0
0	0	0	0	0.001760471
0	0	0	0	0
0	0	0	0	0
0	0	0	0	0

0	0	0	0	0
0	0	0	0	0
0.001677519	0	0	0	0
0	0	0	0	0
0	0	0	0	0
0	0	0	0.003154538	0
0	0	0	0	0
0	0	0	0	0
0	0	0	0	0
0	0	0	0	0
0	0	0	0	0
0	0	0	0	0
0	0	0	0	0
0	0	0	0	0
0	0	0	0	0
0	0	0	0	0
0	0	0	0.292934687	0
0	0.00800626	0	0	0
0	0	0	0	0
0	0	0	0	0
0	0	0	0	0
0	0	0	0	0
0	0	0	0	0
0	0	0	0	0
0	0	0	0	0
0	0	0.002743896	0	0
0	0	0	0	0
0	0	0	0.037202831	0
0	0	0	0	0
0	0	0	0.031048695	0
0	0	0	0	0
0	0	0	0	0
0	0	0	0	0
0	0	0	0	0
0	0	0	0	0
0	0	0	0	0
0	0	0	0	0
0	0	0	0	0
0.192505066	0	0	0.125176349	0
0	0	0	0	0
0	0	0	0	0
0	0	0	0	0
0	0	0	0	0
0	0	0	0	0
0	0	0	0	0
0	0	0	0	0
0	0	0	0	0
0	0	0	0	0

0	0	0	0	0
0	0	0	0.010202862	0
0	0	0	0	0
0	0	0	0	0
0	0	0	0	0
0	0	0	0	0
0	0	0	0	0
0	0	0	0	0
0	0	0	0	0
0	0	0	0	0.003534633
0	0	0.009184614	0	0.001926707
0	0	0	0	0
0.000396914	0	0	0.331412788	0
0	0	0	0	0
0	0	0	0	0
0	0	0	0	0
0	0	0	0	0
0	0	0	0	0
0	0	0	0	0
0	0	0	0	0
0	0	0.002237239	0	0.00180507
0	0	0	0	0
0	0	0	0	0
0.001208212	0	2.900303965	0.158984893	0.761241955
0	0	0	0	0
0	0	0	0	0
0	0	0	0	0
0	0	0	0	0
0	0	0	0	0
0	0	0	0	0
0	0	0	0	0
0	0	0	0	0
0	0	0	0	0.00063292
0	0	0	0	0

PHL018-0162_V3 REF	PHL018-0007_V3 EYCF	PHL018-0254_V2 REF	PHL018-0020_V1 EYCF	PHL018-0022_V1 CTRL
0.063505085	0.044720885	1.955664155	0.372176419	0.436803633
0.174987194	5.319147292	0	6.955510862	0.890818016
0	4.670267392	0	0	0
0	0	0	0	0
0	0	0	0	0
0	0	0	0	0
0	0	0	0	0
0	0	0	0	0
0	0	0	0	0
0	0	0	0	0
0	0	0	0	0
0	0	0	0	0
0	0	0	0	0
0.62431038	3.167454081	0	0	0.341779741
0	0	0	0	0
0	0	0	0	0
0	0	0	0	0
0	0	0	0	0
0	0	0	0	0
0	0	0	0	0
0	0	0	0	0
0	0	0	0	0
0	0	0	0	0
0	0	0	0	0
0	0.022209872	0	0	0
0	0	0	0	0
0	0	0	0	0
0	0	0	0	0
0	0	0	0	0
0	0	0	0	0
0	0	0	0	0
0	0	0	0	0
0	0	0	0	0
0	0	0	0	0
0	0	0	0	0
10.34108483	0	0	0	0
0	0	0	0	0
0	0	0	0	0
0	0	0	0	0
0	0	0	0	0
0	0	0	0	0
0.026678728	0	0.050581962	0	0.045156823
7.259076374	0.004609862	1.167047993	0.036216223	5.908268838
0.056585125	0.002535768	0.338027147	0.13752791	0.134696683
0.007084998	0	0.012975069	0.003424428	0
0	0	0	0	0

0	0	0	0	0
0.001018885	0	0.044263083	1.047271701	0
0	0.006931455	0	0	0.060121849
1.643505549	0.473348367	0	0.435700501	0.291407682
0	0	0	0.010189627	0
0	0	0	0	0
0.789574176	0.000998371	0.002426057	0	0
0	0	0	0	0
0	0	0	0	0
0	0	0	0	0
0	0	0	0	0
2.167541897	1.348382241	0	0.340346095	0
0	0	0	0	0
0.101674778	0	0.006563758	0.049431662	0.028825961
0.014593694	0	0	0	0
0	0	0	0	0
0.007076487	0	0.001058614	0.083830688	0
0	0	0	0	0
0	0	0	0	0
0	0	0	0	0
0.10776951	0.642017551	0.182576606	0.219968366	0.097976961
0.003569412	0.005142973	0	0.005284771	0.015665763
0	0	0	0	0
0	0	0	0	0
0	0	0	0	0
0	0	0	0	0
0	0	0.005488719	0	0
0	0	0	0	0
0	0	0.008085843	0	0.003961601
0	0	0	0	0
0	0.525685741	0.016711119	0	0
0	0	0	0	0
0	0	0	0	0
0	0	0	0	0
0	0	0	0	0
0	0	0	0	0
0	0	0	0	0
0	0	0	5.878242858	0
0	0	0	0	0
0	0	0	0	0
0	0	0	0	0
0	0	0	0	0
0	0	0	0	0
0	0	0	0	0
0	0	0	0	0
0	0	0	0	0
0	0.078274924	0	0.010454045	0.002026907
0.004233437	0	0	0	0

0	0	0	0	0
0	0	0	0	0.045720722
0	0	0	0	0
0	0	0	0	0
0	0	0	0	0.021149254
0	0	0	0	0
0	0	0	0	0
0	0	0	0	0
0	0	0.003517493	0.102367237	0.051236938
0	0	0	0	0
0	0	0	0	0
0	0	0.114129794	0	0.016966533
0	0	0	0	0
0	0	0	0	0
0	0	0	0	0
0	0	0	0	0
0	0	0	0	0
0	0	0	0	0
0.300521625	0	0.771627316	0	0.230857408
0	0.05517952	0	0	0
0	0	0	0	0
0	0	0	0	0
0	0	0	0	0
0	0	0	0	0
0	0	0	0	0
0	0	0	0	0
0	0	0	0	0.003096207
0	0	0	0	0
0	0	0	0	0
0	0	0	0	0
0	0.109291995	0	0	0
0.007426829	0	0.013838779	0.00119015	0
0	0	0	0	0
0	0	0	0	0
0	0	0	0	0
0	0	0	0	0
0	0	0	0	0
0	0	0	0	0
0	0	0	0	0
0	0	0	0	0
0	0	0	0	0
0	0	0	0	0
0.001178229	0.049817147	0.003739194	0.030034494	0.000592703
0	0	0	0	0
0	0	0	0	0
0	0	0	0	0
0	0	0.250237151	0.00793212	0
0	0	0	0	0

0	0	0.012709626	0	0
0	0	0	0	0
0	0	0	0	0
0	0	0	0	0
0.011153938	0.026505993	0	0	0
0	0	0	0.03190237	0
0	0	0	0	0
0	0	0	0	0
0	0	0	0	0
0	0	0	0	0
0	0	0	0	0
0	0	0	0	0
0	0	0	0	0
0	0	0	0	0
0	0	0	0	0
0	0	0	0	0
0	0	0	0	0
0	0	0	0	0
0	0	0	0	0
0	0	0	0	0
0	0	0	0	0
0	0	0	0	0
0	0	0	0	0
0	0	0	0	0
0	0	0	0	0
0	0	0	0	0
0	0	0	0	0
0	0	0	0	0
0	0	0	0	0
0	0	0	0	0
0.003626572	0	0	0	0
0	0	0	0	0
0	0	0	0	0
0	0	0	0	0
0	0	0	0	0
0	0	0	0	0
0	0	0	0	0
0	0	0	0	0
0	0	0	0	0
0	0	0	0	0
0	0	0	0	0
0	0	0	0	0
0	0	0	0	0
0	0	0	0	0
0	0	0	0	0
0	0	0	0	0
0	0	0	0	0
0	0	0	0.082774181	0

0	0	0	0	0
0	0	0	0	0
0	0	0	0	0
0	0	0	0	0
0	0	0	0	0
0	0	0	0	0
0	0	0	0	0
0	0	0	0	0
0	0	0	0	0
0	0	0	0	0
0	0	0	0	0
0	0	0	0	0
0	0.001286088	0.002381115	0	0.000943582
0	0	0	0	0
0	0	0	0	0
0	0	0	0	0
0	0	0	0	0
0	0	0	0	0
0	0	0	0	0
0	0	0	0	0
0.031564607	0.067330925	0.035987908	0.065925634	0.012702753
0	0	0	0	0
0	0	0	0	0.001238431
0	0.007881007	0	0	0
0	0	0	0	0
0	0	0	0	0
0.045868672	0.001200366	0.048399032	0.008107952	0.004315374
0	0	0	0	0
0	0	0	0	0
0	0	0	0	0.000659565
0	0	0	0	0
0	0	0	0	0
0	0	0	0	0
0.061361613	0.00335906	0	0	0
0	0	0.02679523	0.13161489	0.02181065
0	0	0	0	0
0	0	0	0	0
0	0	0	0	0
0	0	0	0	0
0	0	0	0	0
0	0	0	0	0
0	0	0	0	0
0	0	0.02082525	0	0
0	0	0	0	0
0	0	0	0	0
0	0	0	0	0
0.001058894	0	0	0	0.005468768
0	0.001274267	0.011009733	0	0.007526016

0	0	0	0	0
0	0	0	0	0
0	0	0	0	0
0	0.00121482	0	0	0
0	0	0	0	0
0	0	0	0	0
0	0	0.005387324	0	0
0	0	0.003956673	0	0.004939011
0	0	0	0	0
0	0	0	0	0
0	0	0.001915569	0	0
0	0	0	0	0
0	0	0	0	0
0	0	0	0	0
0	0	0	0	0
0	0	0	0	0
0	0	0	0	0
0	0	0	0	0
0.033454393	0	0.00454871	0	0.045257892
0	0.12214668	0	0	0
0	0.006451702	0	0	0
0	0	0	0	0
0	0	0	0	0
0	0	0	0	0
0.011632042	0	0.007690648	0.004526945	0.004388587
0	0	0	0	0
0	0	0	0	0
0	0	0	0	0
0	0	0	0	0
0	0	0	0	0
0.238626782	0.107529175	0.011195752	0.333626542	0.624435869
0	0	0	0	0
0	0	0	0	0
0	0	0	0	0
0	0	0	0	0
0	0	0	0	0
0	0	0	0	0
0	0	0	0	0
0	0	0	0	0
0	0	0.00322222	0	0
0	0	0	0	0
0.009560348	0.032058582	0.00560939	0.150931755	0.014900922
0	0	0	0	0
0	0	0	0	0
0	0	0	0	0
0	0	0	0	0
0	0	0	0	0
0	0	0	0	0

0	0	0	0	0
0	0	0	0	0
0.063762084	0.001574211	0.312343335	0.166734407	0.157538296
0	0	0	0	0
0.731645034	0	0	0	0
0	0	0	0	0
0	0	0	0	0
0	0	0	0	0
0	0	0	0	0
0	0	0	0.000735502	0
0	0	0	0	0
0	0	0	0	0
0	0	0.004228416	0	0
0.001957462	0.001491246	0.001073704	0.003258823	0.003829361
0.008616684	0.002461657	0.010191322	0.004890421	0.010114035
0	0.005490686	0	0	0
0.00531805	0	0.018175458	0.003343318	0.002881009
0	0	0	0	0
0	0	0	0	0
0	0	0	0	0
0	0	0	0	0
0	0.001054987	0.010482411	0	0.006935277
0	0	0	0	0
0	0	0.016603392	0	0
0	0	0	0.008468751	0.002780078
0	0	0	0	0
0	0	0	0	0
0	0	0	0	0
0	0	0	0	0
0	0	0	0.094589179	0.005015971
0	0	0	0.001541859	0
0	0	0	0	0
0	0	0.015091472	0	0
0	0	0	0	0
0	0	0	0	0
0	0	0.008647943	0	0
0	0	0	0	0
0.045236696	0	0.058978981	0.086897305	0.022328235
0	0	0	0	0
0	0	0	0	0
0	0	0	0	0
0	0	0	0	0
0	0	0	0	0
0	0	0	0	0
0	0	0	0	0
0	0	0.003521524	0	0.002093251
0	0	0.002172415	0	0.014167604
0	0	0	0	0

0	0	9.564074914	0	0
0	0.042845007	0	0	0
0.001547181	0	0.015275837	0	0.101244259
0	0	0	0	0
0.027629894	0.013563386	0.009605603	0.010336484	0.002512279
0	0	0	0.008211203	0
0	0	0	0	0
0.199101732	0.002181713	0.059916434	0.118882932	0.019208246
0	0	0	0	0
0.006184666	0.064892181	0.177291642	0.029102113	0.01022915
0	0	0	0	0
0	0	0.052492823	0	0.021034021
0.00294787	0.009357358	0.006467845	0.00758459	0.004613509
0	0	0	0	0.155607794
0	0	0	0	0.000988518
0.015980041	0	0	0	0
0	0	0	0	0
0	0.001706144	0	0	0
0.021162393	0	0.010447167	0	0.013351422
0	0.031014819	0	0	0
0	0	0	0	0
0	0	0	0	0
0	0	0	0	0
0	0	0	0	0
0	0	0	0	0
0.0101098	0	0.004943787	0.003637576	0
0	0	0	0	0
0.01245623	0	0	0.084834786	0.003790576
0	0.033476817	0	0	0
0	0	0	0	0
0	0	0.00075785	0	0.000720765
0	0	0	0	0
0	0	0	0	0
0	0	0.003694609	0	0
0	0	0	0	0
0	0	0	0	0
0.006703308	0	0	0	0.001522871
0	0	0	0	0
0	0	0	0	0
0	0	0	0	0
0	0	0	0	0
0	0	0	0	0
0	0	0	0	0
0	0	0.004931196	0.003139885	0
0	0	0	0	0
0	0	0	0	0
0.001897711	0.001897516	0	0.001292461	0
0	0	0	0	0

0	0	0	0	0
0	0	0	0	0
0	0	0	0	0
0	0	0	0	0
0	0	0	0	0
0.001121008	0.022631364	0	0	0
0	0	0	0	0
0	0	0	0	0
0	0	0	0	0
0	0	0	0	0
0.068259477	0.013371914	0.037174144	0.001180673	0.011700233
0	0	0	0	0
0	0	0	0	0
0.002047242	0.002573412	0.003284629	0	0.004085097
0.262865855	0.003550678	0	0	0.032823932
0	0	0	0	0
0	0	0	0	0
0	0	0	0	0
0	0	0	0.004748861	0
0	0.005891862	0	0	0
0.017425972	0	0	0	0
0	0.007406054	0	0	0
0	0	0	0	0
0	0	0	0	0
0	0	0	0	0
0	0	0	0	0
0	0	0	0	0.109951521
0	0	0	0	0
0	0	0	0	0
0.376003739	0.589130726	0.098513672	0.839982559	0.811183867
0.004305562	0	0	0.004918796	0
0	0	0.022140489	0	0
0	0	0	0	0
0	0	0	0	0
0.074508436	0	0	0	0
0	0	0	0	0
0	0	0	0	0
0	0	0.01982994	0	0
0	0.289844318	0	0	0.599790586
0.992461864	0	0	0	0.009383094
0	0.270992264	0	0	0.040498283
0.745606689	0	0	2.410768501	0
0	0	0.002123241	0	0
0	0	0	0	0
0	0	0	0	0
0	0	0	0	0
0.001059893	0.003835409	0.003052196	0.003368651	0
0	0	0	0	0

0.003487279	0	0.002689927	0.001484411	0
0	0.004137788	0	0.003035183	0
0.023461444	0.001018114	0.002199139	0	0
0	0	0	0	0
0	0	0	0	0
0	0	0	0	0
0	0	0	0	0
0	0	0.015677286	0	0
0	0	0	0.007702375	0
0	0	0	0	0
0	0	0	0	0.081284799
0	0	0	0	0
0	0	0	0	0
0.000591535	0	0.000730053	0.001074327	0.009720593
0	0	0	0	0
0.004930927	0.0364499	0	0.093192056	0.129682688
0.016567049	0.001171041	0.013490473	0.033965939	0.003007108
0	0.021104409	0	0	0
0.001058506	0.000604798	0.00348366	0	0
0	0	0	0	0
0	0	0	0	0
0	0	0	0	0
0	0	0	0	0
0	0	0	0.635602536	0
0	0	0	0	0
0	0	0	0	0
0	0.02006972	0	0	0.004326553
0	0	0	0	0
0	0	0	0	0
0	0	0	0	0
0	0.014132376	0	0	0.051677562
0.010571019	0.029778421	0.02700301	0.005692652	0
0.070221202	0.03209783	0.043621212	0.296515434	0.009890854
0	0	0.00455396	0	0.020474361
0.003591348	0	0	0	0.0039344
0	0	0	0	0
0	0	0	0	0.001987114
0.011915535	0.013616358	0	0.02772704	0.0101011
0	0	0	0	0
0.000541232	0.006030247	0	0	0
0	0	0	0.01416373	0
0	0	0	0	0
0	0.028847926	0	0	0.015396682
0.002971326	0.116774075	0	0	0
0	0	0	0	0
0	0	0	0	0
0	0	0	0	0
0	0	0	0	0

0	0	0	0	0
0.375241889	0.736491329	1.477279552	0.777133917	0.485976135
0	0	0	0	0
0	0	0	0	0
0	0	0	0	0
0	0	0	0	0
0	0	0	0	0
0	0	0	0	0.001219872
0	0	0	0	0.001211523
0	0	0	0.003124977	0
0.012903319	0	0	0.005478218	0.017702618
0.080873176	0.00370809	0.020947982	0.009519954	0.039259843
0	0	0	0	0
0	0	0	0	0
0.002114992	0	0.001423774	0	0.000677051
0	0	0	0	0
0.025619468	0.009224068	0.048510869	0.010755909	0.008032785
0.006378172	0.020043627	0	0	0.037432642
0	0	0	0	0
0.001035413	0.018086174	0	0	0
0.087765518	0.013441352	0.063650354	0.01947272	0.01890412
0	0	0	0	0
0	0	0	0	0
0	0	0	0	0
0.023411563	0	0	0	0
0	0	0	0	0
0	0.337193183	0	0.629898715	0.067089657
0.013271017	0.026814048	0.048898567	0.105840978	0.069307108
0	0	0	0	0
0	0	0	0	0
0.006810345	0	0	0	0
0	0	0	0	0
0	0	0	0	0
0	0	0	0	0
0.027319669	0.001532234	0	0.384684849	0.041706905
0.166662717	0.683693461	0.340673326	0.358259082	0.100486685
0	0	0	0	0
0	0	0	0	0
0.029658124	0	0.035431767	0.016159243	0.02812803
0	0	0	0	0
0	0	0	0	0
0	0	0	0	0
0.005908854	0.130928418	0.006581045	0.005496608	0.016070421
0	0.005088158	0	0	0.000489969
0	0	0	0	0
0	0	0	0	0
0	0	0	0	0
0	0	0.001110329	0.00520816	0.001055996

0	0	0	0	0
0	0	0	0	0
0	0	0	0	0
0	0.0199484	0.122637363	0.000685908	6.415581111
0	0	0	0	0
0	0	0	0	0
0	0	0	0	0
0	0	0	0.012681446	0
0.340592586	0	0	1.943123896	11.16060801
0	0	0	0.026564769	0
0	0	0	0	0
0.002251488	0.009556356	0	0.0041621	0.007361909
0	0	0	0	0
0	0.00138142	0	0	0
0.005971929	0.061874214	0	0	0
0	0	0	0	0
0	0	0	0.369254785	0
0	0	0	0	0
0	0	0	0	0
0	0	0	0	0
0	0	0	0	0
0	0	0	0	0
0	0	0	0	0
0.434761024	0.232559699	0.081723604	0.364269661	0.384121142
0	0.00378535	0	0.001845848	0
0	0	0	0	0
0	0	0	0	0
9.249220882	0	0	0	0
0	0	0	0	0
0	0	0	0	0
0.723095625	0	0	0	0
0	0	0	0	0
0	0	0	0.00275535	0
0	0	0	0	0
0	0	0	0	0
0.006628154	0.033326728	0	0	0
0	0.16003668	0	0.318090376	0
0	0	0	0	0
0	0	0	0	0
0	0	0	0	0
0.022216115	0.014809225	0.030655279	0.119993874	0.088370988
0	0.222789025	0	0	0
0.013140402	26.83095507	0	0	1.510451599
0	0	0	0	0
0	0	0	0	0
0	0	0	0	0
0	0	0	0	0
0	0	3.552818233	0.001010483	0.004571462

0	0	0	0	0
0	0	0	0	0
0.005866901	1.297188589	0.147447648	0.227635504	0.135015414
0	0	0	0	0
0	0	0	0	0
0.002801041	0	0	0.019649165	0
0	0	0	0	0
0	0	0	0	0
0	0	0	0	0
0	0	0	0	0
0.007680341	0.004188848	0	0.114839774	0.286225637
3.380255399	3.272339151	2.965520751	2.413237182	0.856897044
0	0	0	0	0
0.067253425	0	0.097881827	0.044905712	0.001326727
0.515434233	0.054795322	0.71839643	0.360345077	0.092527822
0	0	0	0	0
0	0	0	0	0
0	0	0	0	0
0	0	0	0	0
0	0	0	0	0
0.955201175	0.09663255	0.70328187	0.235350664	0.458504619
0.052721413	3.186742174	0.351590139	0	0.015018388
0.63758615	1.959027279	6.704404426	0.314713163	1.682317905
0	0	0	0	0
0	0	0	0	0
0.005785324	0	0	0	0
0	0.001199762	0	0.001906793	0
0	0	0	0	0
0	0.033816498	0	0	0
0	0	0	0	0
0	0.008298	0	0	0
0.10358973	0.230123189	0	0.105920783	0
0.072604633	0	0.376567335	0.001220947	0.010100313
0.00615185	0.008748401	0	0	0
0.006862611	0.021363181	0.009053716	0.01595563	0.033331659
0.153461987	0.01318969	2.871862323	0.031000893	0.076135288
0	0.002512322	0	0	0
0	0	0	0	0
0	0.002391276	0	0	0
0	0	0	0	0
0	0	0	0	0
0	0	0	0	0
0	0	0	0	0
0	0	0	0	0
0.002966203	0.002118499	0.067724589	0.01944415	0.005440079
0	0	0	0	0
0	0	0	0	0
0.034215116	1.295888459	20.20445069	0.03929467	0.004724799

0	0	0	0	0
0	0	0	0	0
0	0	0	0	0
0	0.006139	0	0.013774271	0
0	0	0	0	0
0	0	0	0	0
0	0	0	0	0.042233542
0	0.02807881	0.15360032	0	0.105637837
0	0	0	0	0
0	0	0	0	0
0.000703904	0.001809853	0.093606133	0	0.008881902
0	0	0	0	0
0.007704034	0.001760742	0	0	0
0	0	0	0.485792231	0
0	0	0	0	0
0	0	0	0	0
0	0	0	0	0
0	0	0	0.005587287	0
0	0	0	0	0
0	0	0	0	0
0	0	0	0	0
0	0	0	0	0
0	0	0	0	0
0	0	0	0	0
0	0	0	0	0
0	0	0	0	0
0	0	0	0	0
0	0	0	0	0
0	0	0	0	0
0	0.002224485	0	0.077859066	0
0	0	0	0	0
0	0	0	0	0
0	0	0	0	0
0	0	0	0	0
0	0	0	0	0
0	0	0	0	0
0	0	0	0.288722043	0
0.005213834	0	0	0.010348484	0.001049119
0	0	0	0	0
0	0	0	0	0
0	0	0	0	0
0	0	0	0	0
0	0	0	0	0
0	0	0	0.954712357	0
1.176946715	0.003672598	0	0	0
0	0	0	0	0
0	0	0	0	0
0	0	0	0	0

0	0	0	0	0
0	0	0	0	0
0	0	0	0	0
0	0	0	0	0
0	0	0	0	0
0	0	0	0	0
0	0	0	0	0
0	0	0	0	0
0.036570093	0.020895053	0.084435304	0.087872515	0.079580062
0	0	0	0	0
0	0	0	0	0
0	0	0	0	0
0	0	0	0	0
0	0	0	0	0
0	3.051400184	0	0.051684426	3.541344168
0	0	0	0	0.451087858
0	0	0	0	0.199790285
0	0	0	0	0
0	0	0	0	0
0	0	0	0	0
0	0.049008913	0	0	0
0	0	0	0	0
0	0	0	0	0
0	0	0	0	0
0.257307515	0	0.054864884	0.001645471	0.588727722
2.138529601	1.499682995	0	6.403458899	12.06641108
0	0.243989145	0	0	0
0	0	0	0	0
0	0	0	0	0
0	0	0	0	0
0	0	0	0	0
0	0	0	0	0
0	0	0	0	0
0	0.001179597	0	0.003515149	0
0.04634755	0.000971802	0.044868358	0.0159276	0
0	0.001545629	0.088657791	0	0.010584022
0	0	0	0	0
0	0.012798734	0	3.776752055	0.610506658
0	0	0	0	0
0	0	0	0	0
0	0	0	0	0
0	0	0.034915095	0	0
7.75978967	0.534133975	0	1.397279754	0.581663912
0	0	0	0	0
0	0	0	0	0
0	0.01480559	0	0.001357539	0
0	0	0	0	0
3.311148949	0.085276365	0.005318789	0.808858122	0.001064951

0	0	0	0	0
0	0	0	0	0
0	0	0	0	0
0	0	0	0	0
0	0	0	0.002064438	0.005929901
0.003405499	0.001853143	0	0	0
0	0	0	0	0
0	0	0	0	0
0	0	0	0	0
0	0	0	0	0
0	0	0	0	0
0	2.790329128	0	2.134817634	6.109509288
0	0	0	0	0
0	0	0	0	0
0.391067093	0	0.831163252	0.001856962	0.585667283
0	0	0	0	0
0	0	0	0.007012548	0
0	0	0	0	0
0	0	0	0.014416066	0
0	0	0	0	0
0	0.108526712	0	0	0
0	0	0	0	0
0	0.059368818	1.496854296	1.167309758	0.651698974
0	0	0	0	0
0	0.003177021	0	0	0
0	0	0	0	0
0	0	0	0	0
0	0	0	0	0
0	0	0	0	0
0	0	0	0	0
0	0	0	0	0
0.046247986	0.01732768	0.107605535	0.049226065	0.088991241
0	0	0	0	0
0	0.018755988	0	0	0.241261407
0	0.000934462	0.091233924	0	0.011134133
0	0.014937469	0	0.042502744	0.024499509
0	0	0	0	0
0	0	0	0	0
0	0	0	0.001023731	0
0	0	0	0	0
0	0.0149679	0	0	0
0	0	0	0	0
0	0	0	0	0
0	0	0	0	0
0	0.006075554	0.063031494	0.114554567	0.146936553
0	0	0	0	0
0.243185597	0	0	0.848249772	0
0	0	0	0	0

0	0	0	0	0
0	0	0	0.027363769	0
0	0.032941526	0	0	0
0	0	0	0	0
0	0	0	0	0
0	0	0	0	0
0	0	0	0	0
0	0	0	0	0.082381235
0	0	0	0	0
0	0	0	0	0
0	0	0	0	0
0	0	0.033897139	0	0.007562095
0	0	0	0	0
0.615964191	0	0	0	0
0	0	0	0	0
0	0	0	0	0
0	0	0	0	0
0	0	0	0	0
0	0.63887385	9.186727351	0	0.03134827
0	0.005133384	0	0	0
0	0	0	0.004949331	0
0	0	0	0	0
0	0	0	0	0
0	0	0	0	0
0	0.001005768	0	0	0
0.013348935	0.005656112	0.024804769	0.013279671	0.023062807
0	0	0	0	0
2.613777635	0.315060575	0.001087116	1.092045111	0.008788313
0	0	0	0	0
0	0.044499174	0	1.077967257	0
0	0	0	0	0
0	0	0	0	0
0	0.00212573	0	0	0
0	0	0	0	0
0.002986385	0	0	0	0.017342208
0	0	0	0	0
0	0	0	0	0
0	0.001426663	0.01342702	0	0.016956855
0	0	0	0	0
0	0	0	0	0
4.79591257	7.463270988	1.135377541	11.80026859	5.258982602
0.095695996	1.351206059	2.157950326	3.416130946	0.916072781
0	0	0	0	0
0	0	0	0	0
0	0	0	0	0
0	0	0	0	0
0	0	0	0	0
0	0.000647017	0.058872387	0.053600656	0.10068502

0	0	0	0	0
0	0	0	0	0.011279123
0	0	0	0	0
0	0	0	0	0
6.203632003	3.406940974	0	7.469797762	2.471645747
0	0	0	0	0
0	0	0	0	0
0	0	0	0	0
0.509122733	0.258169411	0	0.139608807	0.212963515
0	0	0	0	0
0	0	0	0	0
0	0	0	0	0
0.021927938	0.185752024	0	0.371334387	0
0.006261252	0.02583744	0.005580921	0.02132154	0.003062205
1.715811328	0	0	1.327597963	0
0	1.510725223	0.243426703	0.187988039	0.552486054
0.001655544	0	0.007429879	0.00533014	0
0	0	0	0	0
0	0	0	0	0
0	0	0	0	0
0	0	0	0	0
0	0	0	0	0
0	0	0	0	0
0	0	0	0	0
0	0	0	0	0
0	0	0	0	0
0.345102568	0.013160372	0.001938175	0.019608668	0
0.000738357	0.005062503	0.051030331	0	0
0	0	0	0	0
0	0	0	0	0
0	0	0	0	0
0	0	0	0	0
0	0	0	0	0
0.257430919	0.002299637	0	0	0
0.00091543	0	0	0	0
0	0	0	0.001396113	0
0	0	0	0	0
0	0	0	0	0
0	0	0	0	0
0	0	0	0	0
0.861973859	0	0	0	0
0.118584214	0.155750594	0	0	0
0	0	0	0	0
0.005783586	0	0	0	0
0	0	0	0	0
0	0	0	0	0
0	0	0	0	0
0	0	0	0	0

0	0.011270698	0	0	0
0	0	0	0	0
0	0	0	0	0
0	0	0	0	0.023169549
0	0	0	0.198396129	0
0	0	0	0	0
0.003453174	0.002916668	0.063000404	0.14765399	0
0	0	0	0	0
0	0	0	0	0
0	0	0	0	0
0	0.000832221	0	0	0
0	0	0	0	0
0	0	0	0	0
0	0	0	0	0
0	0	0	0	0
0	0	0	0	0
0	0	0	0	0.156791721
0	0	0.000712568	0	0
0	0	0	0	0
0	0	0	0	0
2.275600782	0.00277683	0.025050427	1.718050245	0
0	0	0	0	0
0.005518171	0.008287669	0	0	0
0.008692072	0.00656845	0	0.004010221	0
0	0	0	0	0
0	0	0	0.003154106	0
0	0	0	0	0
0	0	0	0	0
0	0	0	0	0
0.341803575	0	0.005976518	0.001374202	0.34649087
0	0	0	0	0
0	0	0	0	0
0	0	0	0	0
0	0	0	0	0
0	0	0	0	0
0	0	0	0	0
0	0	0	0.000716832	0.009471548
0	0	0	0	0
0	0	0	0	0
0	0	0	0	0
0	0	0	0	0
0	0	0	0	0
0	0	0	0	0
0	0.012242347	0	0	0
0	0.114211218	0	0	0
0	0	0	0	0
0	0	0	0	0
0	0	0	0	0

0	0.026202894	0	0.00143838	0.093492429
0	0	0	0	0
0	0	0	0	0
0	0	0	0	0
0	0	0	0	0
0	0	0	0	0
0	0	0	0	0
0.483112731	2.264575872	9.100378533	0.318586527	0.944138493
9.381571607	0.007305876	1.37994218	0.040639562	7.442858392
0	0.013186268	0	0.108677376	0.010642003
0	0	0	0	0
0	0	0	0	0
0	0.075859286	0	0	0
0	0.003004816	2.622138046	0	0
0	0	0	0	0
0	0	0	0	0
0.006826416	0	0	0	0.074181775
0	0	0	0	0
0	0	0	0	0
0	0.000937232	0	0	0
0	0	0	0	0
0	0	0	0	0
0	0	0	0	0
0	0	0	0	0
0	0.008010962	0	0	0
0	0	0	0	0
0	0	0	0	0
0	0	0	0	0
0	0	0	0	0
0	0	0	0	0
0	0.011688498	0	0	0
0	0	0	0	0
0	0	0	0	0
0	0	0	0	0
0.001461045	0	0	0.014179658	0.001714936
0	0	0	0	0
0	0	0	0	0
0	0	0	0	0
0	0	0	0	0
0.003012001	0	0.165606178	4.405916253	0
0	0	0	0	0
0.21829577	0.998353861	0	0.148144109	0
0	0	0	0	0
0	0	0	0	0
0	0	0	0	0
0	0.006438074	0	0	0
0	0	0	0	0
0	0.01589278	0	0	0

0	0	0	0	0
0	0	0.003024634	0.002781859	0.00447475
0	0	0	0	0
0	0	0	0	0
0	0.671088723	0	0.777626705	1.107593871
0.207023676	0	0	0	0
0	0.003773154	0	0	0
0.020916864	0.005701523	0.005920847	0.006796116	0.034011932
0	0	0	0	0
0	0	0	0	0
0	0	0	0	0
0	0	0	0	0
0	0	0	0	0
0	0	0	0	0
0	0	0	0	0
0	0.021301056	0	0.015642355	0
0	0	0	0	0
0	0	0	0	0
0	0	0	0	0
0	0	0	0	0
0	0	0	0	0
0	0	0	0	0
0	0	0	0	0
0	0	0.114982692	0	0.002144237
0	0	0	0	0
0	0	0	0	0
0.033568019	0	4.138916032	0.020068852	0.001248003
0	0	0	0	0
0	0	0.015603574	0	0.008480013
0	0	0	0	0
0	0	0	0	0
0	0	0	0	0
0	0	0	0	0
0	0	0	0	0
0	0	0	0	0
0	0	0	0	0
0	0	0	0	0
0	0.090250434	0	0	0
0	0	0	0	0
0	0	0	0	0
0	0	0	0	0
0.481549803	0	0	0	0
0	0	0	0	0
0	0	0	0	0
0	0	0	0	0
0	0	0	0	0
0	0	0	0	0
0	0	0	0	0

0.099417234	0	0	0.093197723	0
0	0	0	0	0
0	0	0	0	0
0	0	0	0	0
0	0	0	0	0
0	0	0.005923003	0	0.078670057
0	0	0	0	0
0	0	0	0	0
0	0	0	0	0
0	0	0	0	0
0	0	0	0	0
0	0	0	0.005213255	0
0	0.030107627	0	0	0
0	0	0	0	0
0	0	0	0	0
0	0	0	0	0
0	0	0	0	0
0	0	0	0	0
0	0	0	0	0
0	0	0	0	0
0.039194479	0.108665533	0.317584955	0.225039828	0.06425232
0	0	0	0	0
0.003759476	0.009546898	0.779489775	0.042674011	0.382440369
0	0	0	0	0
0	0	0	0	0
0	0.08396285	0	0	0
0	0	0	0	0
0	0	0	0	0
0	0	0.004686047	0	0
0	0	0	0	0
0	0	0	0	0
0	0	0	0	0
0.007823854	0	0.064694789	0.107370041	0
0	0	0	0	0
0	0	0	0.018112137	0
0.112067916	0.003407879	0.192937302	0.16077491	0.000921165
0	0	0	0	0
0	0	0	0	0
0.002360106	0.006431277	0.003808999	0.015331942	0.031964186
0	0	0	0	0
0	0	0	0	0
0	0	0	0	0
0.037786262	0	0	0.192288331	1.353249924
0	0	0	0	0
0	0	0	0	0
0	0.060107866	0	0.01928972	0.015039284
0	0	0	0	0
0	0	0	0	0

0	0	0	0	0
0	0	0	0	0
0	0	0	0	0
0	0	0	0	0
0	0	0	0	0
0	0	0	0	0
0	0	0	0	0
0.026880216	0.204780654	0	0	0
0	0	0	0	0.018354619
0	0	0.001071533	0	0.002038197
0	0	0	0	0
0	0	0	0	0
0	0	0	0	0
0	0	0	0	0
0	0	0	0	0
0	0.034946897	0	0	0
0	0	0	0	0
0	0	0	0	0
0	0	0	0	0
0	0	0	0.010131517	0
0	0	0	0	0
0	0	0	0	0
0	0	0	0	0
0	0	0	0	0
0	0	0	0.001572607	0
0	0	0	0	0
0	0	0	0	0
0	0	0	0	0
0	0	0	0	0
0	3.090619378	0	0	0
0	0.05395146	0	0	0.113604076
0	0	0	0	0
0	0	0	0	0
0	0	0	0	0
0	0	0	0	0
0	0	0	0	0
0	0	0	0	0
0	0	0	0	0
0	0	0	0	0
0	0	0	0	0
0	0	0	0	0
0	0	0	0	0
0	0	0	0	0
0	0	0	0	0
0	0	0	0	0
0	0	0	0	0
0	0.194263615	0	0	0
0	0	0	0	0

0	0	0	0	0
0	0	0	0	0
0	0	0	0	0
0	0	0	0	0
0.417588538	0.015229631	0	0.204137394	0.017023508
0	0	0	0	0
0	0	0	0	0
0	0	0	0	0
0	0	0	0	0
0	0	0	0	0
0	0	0	0	0
0	0.000621886	0.179328148	0.021497045	0.041520355
0	0	0	0	0.103056001
0	0	0	0	0
0	0	0.095201747	0	0.001298109
0	0	0	0.634093106	0
0	0.004942477	0	0	0
0	0	0	0	0
0	0.046304745	0.001356188	0.051078255	0.060944165
0.005918503	0	0.006861726	0.125323529	0.148623292
0	0	0	0	0
0	0	0	0	0
0	0	0	0	0
0	0	0	0	0
0	0	0	0	0
0	0	0	0	0
0.003880446	0.006240924	0	0	0.097337002
0	0	0	0	0
0	0	0	0	0
0	0	0	0	0
0	0.021781752	0.016688273	0	0.006692862
0	0	0	0	0
0	0	0	0	0
0	0	0	0	0
0	0	0	0	0
0	0	0	0	0
0	0	0	0	0
0	0.007847809	0.486080482	0.005768578	0.101433394
0	0.211440442	0	0	0
0	0	0	0	0
0	0	0.073614743	0	0.00308484
0	0	0	0	0
0	0	0	0	0
0.001527553	0.003297236	0.014453619	0	0
0	0	0	0	0
0	0	0	0	0
0	0.01456199	0	0	0
0	0	0	0	0

0	0	0	0	0
0	0	0	0	1.337863588
0	0	0	0	0
0.001600882	0.001219595	0	0	0.00104393
0.004949309	0	0.002443309	0.008764059	0
0.001703349	0.013625395	0.008671642	0.00145011	0
0	0	0	0.001880987	0
0	0	0	0	0
0	0	0	0	0
0	0	0	0	0
0	0.016628208	0	0	0
0	0	0	0	0
0	0.025647458	0.321500014	0.039158775	0
0	0	0	0	0
0	0	0	0	0
0	0	0	0	0
0	0	0	0	0
0	0	0	0	0
0	0	0	0	0
0	0	0	0	0
0	0	0	0	0
0	0	0	0	0
0	0.002845938	0	0.026007695	0
0	0	0	0.048036483	0
0	0	0	0	0
0	0	0	0	0
0	0	0	0	0
0	0	0	0	0
0	0	0	0	0
0	0	0	0	0
0	0	0	0.035302479	0
0	0	0	0	0
0	0	0	0	0
0	0	0	0	0
0	0	0	0	0
0	0	0	0	0
0	0	0	0	0
0	0	0	0	0
0	0	0.010631689	0.001280072	0
0	0	0	0	0
0	0	0	0	0
0	0	0	0	0
0	0	0	0	0
0	0	0	0	0
0	0	0	4.866879619	0
0.501750248	0.062360491	0	0.192401358	0.178413085
0	0	0	0	0
0	0	0	0	0

0	0	0	0	0
0	0	0	0	0
0.125377007	0	0	0	0
0	0	0	0	0
0	0	0	0	0
0	0	0	0	0
0	0	0	0	0
0	0	0	0	0
0	0	0.015381709	0	0
0	0	0	0	0
0	0	0	0	0
0	0	0	0	0
0	0	0	0	0
0	0	0	0	0
0	0	0	0	0
0	0	0	0	0
0	0	0	0	0
0	0	0	0	0
0	0	0	0	0
0	0	0.002287686	0	0
0	0	0	0	0
0	0	0	0	0
0	0	0	0	0
0	0	0	0	0
0	0	0	0	0
0	0	0	0	0
0	0	0	0	0
0	0	0	0	0
0	0	0	0	0
0	0	0	0	0
0.014042585	0	0	0.005521419	0.029737035
0	0	0	0	0
0.067068493	0.619089413	0.092938882	0.045334433	0.019532828
0.001030627	0.001962899	0.01717154	0.002339741	0.002217826
0	0	0	0	0
0	0	0	0	0
0	0	0	0	0
0	0	0	0	0
0	0	0	0	0
0	0	0	0	0
0	0	0	0	0
0.146031409	0.024652126	0	0.239600003	0.231140805
0	0.11474238	0	0.101660268	0.263599509
0	0	0	0	0
0	0	0	0	0
0.02093714	0	0	0.232131622	0.066296587
0	0	0	0.010001194	0
0	0	0	0	0

0	0	0	0	0
0	0	0	0	0
0.004535696	0.014135786	0.012722284	0.034260877	0.003387924
0	0	0	0	0
0	0.049473564	0	0	1.941484517
0	0	0	0	0
0	0	0	0	0
0	0	0	0	0
0	0	0	0	0
0	0	0	0	0
0	0	0	0	0
0	0	0	0	0
0	0	0	0	0
0	0	0	0	0
0	0	0	0	0
0	0	0	0	0
0	0	0	0	0
0	0	0	0	0
0	0	0	0	0
0	0	0	0	0
0	0	0	0	0
0	0	0	0	0
0	0	0	0	0
0	0	0	0	0
0	0	0	0	0.025926878
0.020511226	0	0.00653952	0.00465648	0.019461055
0	0	0	0	0
0.110757765	0	0	0	0
0	0	0	0	0
0	0	0	0	0
0.001072975	0	0	0	0
0.046166967	0	0	0	0
0	0	0	0	0
0	0	0	0	0
0	0	0	0	0
0	0	0	0	0
0	0	0	0.007450052	0
0	0	0	0	0
0	0	0	0	0
0	0	0	0	0
0	0	0	0	0
0	0.101710795	0	0	0
0	0.023327562	0	0.002336643	0.065842605
0	0	0	0	0
0	0	0	0	0
0.001381485	0	0	0	0
0	0.003298762	0	0.001429842	0.013553386
0	0	0	0	0

0	0	0	0	0
0.003145796	0.019298542	0.033920229	0.124264151	0.027207536
0	0	0	0	0
0	0	0	0	0
0	0	0	0	0
0	0	0	0	0
0	0	0	0	0
0	0	0	0	0
0	0	0	0	0
0	0	0	0	0
0	0	0	0	0
0	0	0	0	0
0	0	0	0.2176589	0.681424978
0	0	0	0	0
0.034681682	0	0	0.09363807	0.041919997
0	0	0	0	0
0	0	0	0	0
0	0	0	0	0
0	0	0	0	0
0	0	0	0	0
0	0	0	0	0
0	0	1.443232477	0.127860266	0.175145648
0	0	0	0	0
0	0	0.394567634	0	0
0	0	0	0	0
0	0	0	0	0
0	0	0	0	0
0	0	0	0	0
0	0	0	0	0
0	0	0	0	0
0	0	0	0	0
0	0	0	0	0
0	0	0	0	0
0	0	0	0	0
3.011110517	2.912725822	0	0	7.117337253
0	0	0	0	0
0	0	0	0	0
0	0	0	0	0
0.063572253	0.001850868	0.243871783	0.039987413	0.011406791
0	0	0	0	0
0.033038757	0.121976752	0.038534927	0.289551995	0.152989292
0	0	0	0	0
0	0	0	0	0
0	0	0	0	0
0	0	0	0	0
0.725574209	0.012587958	3.934876698	0.186566968	0.34568944
0	0	0	0	0
0	0	0	0	0

0	0	0	0	0
0	0	0	0	0
0	0	0	0	0
0	0	0	0	0
0	0	0	0.000858858	0
0	0	0	0	0
0	0	0	0	0
0	0	0	0.001835987	0
0	0	0	0	0
0.006504614	0	0.053936627	0.101337452	0.099015667
0	0	0	0	0
0	0	0	0	0
0.005165641	0	0.002452024	0	0
0	0	0	0	0
0	0	0	0	0
0	0	0	0	0
0	0	0	0	0
0	0	0	0	0
0	0	0	0	0
0.883658972	0.013943502	0.457228349	0.575210777	1.195381952
0	0	0	0	0
0	0	0	0	0
0	0	0	0	0
0	0	0	0	0
0	0	0	0	0
0	0	0	0	0
0	0	0	0	0
0	0	0	0	0
0	0	0	0	0
0	0	0	0	0.098964343
0	0	0	0	0
0	0	0	0	0
0	0	0	0	0
0	0	0	0	0
0	0	0	0	0
0	0	0	0	0
0	0	0	0	0
0	0	0	0	0
0	0	0	0	0
0	0	0	0	0
0	0	0	0	0
0	0	0	0	0
0	0.008062879	0	0	0
0	0	0	0	0
0.000996556	0.003416413	0.043662018	0.004524783	0
0	0	0	0	0
0	0	0	0	0
0	0	0	0	0

0	0	0	0	0
0	0	0	0	0
0	0	0	0	0
0	0	0	0	0
0	0	0	0	0
0	0.000708468	0	0	0
0	0	0	0	0
0	0	0	0	0
0	0	0	0	0
0	0	0	0	0
0	0	0	0	0
0	0	0	0	0
0	0	0	0	0
0	0	0	0	0
0	0	0	0	0
0	0	0	0	0
0	0.030077837	0	0	0
0	0.003141058	0	0.000624014	0
0	0	0	0	0
0	0	0	0	0
0	0	0	0	0
0	0	0	0	0
0	0	0	0	0
0	0	0	0.011424255	0
0	0	0	0	0
0	0	0.004547928	0.001585093	0.001138257
0	0	0	0	0
0	0.121421726	0	0	0
0	0	0	0	0
0	0	0	0	0
0	0	0	0	0
0	0	0	0	0
0	0	0	0	0
0	0	0	0	0
0	0	0	0	0
0	0	0	0	0
0	0	0	0	0
0	0	0	0	0.063008652
0	0	0	0.59722282	0
0	0	0	0	0
0	0	0	0	0
0	0	0	0	0
0	0	0	0	0
0	0	0	0	0
0	0	0	0	0
0	0	0	0	0
0	0	0	0	0
0	0	0	0	0

0	0	0	0	0
0	0	0	0.343960254	0
0	0	0	0	0
0	0	0	0	0
0	0	0	0	0
0	0.001305234	0	0	0
0	0	0	0	0
0	0	0	0	0
0	0	0	0	0
0	0	0	0	0
0.015632768	0.002284954	0.004262504	0.011637311	0
0	0	0	0	0
0	0.551865902	0	0.377326961	0
0	0	0	0	0
0	0	0	0	0
0	0	0	0	0
0	0	0	0	0
0	0	0	0	0
0	0	0	0	0
0	0	0	0	0
0.000553476	0	0.026184805	0.039956938	0
0	0	0	0	0.007655403
0	0	0	0	0
0.624017792	2.783548783	0	4.28055713	0.948442873
0	0	0	0	0
0	0	0	0	0
0	0	0	0	0
0	0	0	0	0
0	0	0	0	0
0	0	0	0	0
0	0	0	0	0
0	0	0	0	0
0	0	0	0	0
0	0	0	0	0

PHL018-0008_V1	PHL018-0114_V2	PHL018-0060_V2	PHL018-0026_V1	PHL018-0138_V3
CTRL	EYCF	CTRL	EYCF	EYCF
0.221190043	0.04895508	0.047537996	0.03693267	0.275994922
0.981826418	0.288155405	13.53364556	0.00233776	0.03424169
0	0	6.050857771	14.81137721	0.073713645
0	0	0	0	0
0	0	0	0	0
0	0	0	0	0
0	0	0	0	0
0	0	0	0	0
0	0	0	0	0
0	0	0	0	0
0	0	0	0	0
0	0	0	0	0
0	0	0	0	0
1.758704888	3.029174536	3.178077059	10.76620672	6.540699844
0	0	0	0	0
0	0	0	0	0
0	0	0	0	0
0	0	0	0	0
0	0	0	0	0
0	0	0	0	0
0	0	0	0	0
0	0	0	0	0
0	0	0	0	0
0	0	0	0	0
0	0.012520147	0	0	0
0	0	0	0	0
0	0	0	0	0
0	0	0	0	0
0	0	0	0	0
0	0	0	0	0
0	0	0	0	0
0	0	0	0	0
0	0	0	0	0
0	0	0	0	0
0	0	0	0	0
0	0	0	0	0
5.91631577	5.27798025	2.353930685	0	1.627901015
0	0	0	0	0
0	0	0	0	0
0	0	0	0	0
0	0	0	0	0
0	0	0	0	0
0.386692156	0.032347974	0.014728724	0	0.001524301
1.798786465	0.788236076	1.052694426	0.059404645	0.44787087
0.036485367	0.001221377	0.068367172	0.013036109	0
0.007206757	0	0.002143057	0	0
0	0	0	0	0

0	0	0.001909496	0	0
0.089316523	0	0	0.01492032	0.107828706
0.008146533	0.003153123	0.007307048	0	0.090485125
0.32439388	1.315506796	1.209934548	0	0.111153563
0.001548748	0	0.002922708	0	0.013206596
0	0.011761321	0	0	0
0	0.561661865	0.052225251	0	0
0	0	0	0	0
0	0	0	0	0
0	0	0	0	0
0	0	0	0	0.006680893
0.008910942	0.007296361	0.059801784	0	0.106951734
0	0	0	0	0
0.028443728	0.010331624	0.039418861	0.079700981	0.005207457
0	0	0	0	0.14514961
0	0	0	0	0
2.431786915	0.190263854	0.001508067	0	0
0	0	0	0	0
0	0	0	0	0
0	0	0	0	0
0.078528498	0	0.018986291	0	0.147413866
0.000489362	0.001195871	0.010186444	0.004669713	0.004214759
0	0.113053172	0	0	0
0	0	0	0	0
0	0	0	0	0
0	0	0	0	0
0	0	0	0	0.465431616
0	0	0	0	0
0.006886397	0.000874208	0.001163519	0	0
0	0	0	0	0
0.129568549	0	0	0.205126056	0.110613395
0	0	0	0	0
0	0	0	0	0
0	0	0	0	0
0	0	0	0	0
0.001327365	0	0	0	0
0	0	0	0	0
0.045098659	0.646338909	0	0	0
0	0	0	0	0
0	0	0	0	0
0	0	0	0	0
0	0	0	0	0
0	0	0	0	0
0	0	0	0	0
0	0	0	0	0
0	0	0	0	0
0.002621748	0	0	0	0
0	0	0	0	0

0	0	0	0	0
0	0	0	0	0
0	0	0	0	0
0	0	0	0	0
0.056816252	0	0	0	0
0	0	0	0	0
0	0	0	0	0
0	0	0	0	0
0	0	0	0	0.013141597
0	0	0	0	0
0	0	0	0	0
0.003294912	0.001533692	0.001020626	0	0
0	0	0	0	0
0	0	0	0	0
0	0	0	0	0
0	0	0	0	0
0	0	0	0	0
0	0	0	0	0
0.419321376	0.075582127	0	0	0
0	0	0	0	0
0	0	0	0	0
0	0	0	0	0
0	0	0	0	0
0	0	0	0	0
0	0	0	0	0
0	0	0	0	0
0	0	0	0	0
0	0	0	0	0
0	0	0	0	0
0	0	0	0	0
0.004231168	0.021851196	0.015619196	0	0
0.003214349	0.025649016	0.024578846	0	0.001020623
0	0	0	0	0
0	0	0	0	0
0	0	0	0	0
0	0	0	0	0
0	0	0	0	0
0	0	0	0	0
0	0	0.054781996	0	0
0	0	0	0	0
0	0	0	0	0
0	0	0	0	0
0	0	0	0	0
0	0	0	0	0
0	0	0	0	0
0	0	0	0	0
0	0	0	0	0
0	0	0	0	0

0	0	0	0	0
0	0	0	0	0
0	0	0	0	0
0	0	0	0	0
0	0.120692183	0.048283047	0	0
0	0	0	0	0
0	0	0	0	0
0	0	0	0	0
0	0	0	0	0
0	0	0	0	0
0	0	0	0	0
0	0	0	0	0
0	0	0	0	0
0	0	0	0	0
0	0	0	0	0
0	19.95309181	0	0	0
0	0	0	0	0
0	0	0	0	0
0	0	0	0	0
0	0	0	0	0
0	0	0	0	0
0	0	0	0	0
0	0	0	0	0
0	0	0	0	0
0	0.032894849	0	0	0
0	0	0	0	0
0	0	0	0	0
0	0	0	0	0
0	0	0	0	0
0	0	0	0	0
0	0	0	0	0
0	0.010912577	0	0	0
0	0	0	0	0
0	0	0	0	0
0	0	0	0	0
0	0	0	0	0
0	0	0	0	0
0	0	0	0	0
0	0	0	0	0
0	0	0	0	0
0	0	0	0	0
0	0	0	0	0
0	0	0	0	0
0	0	0	0	0
0	0	0	0	0
0	0	0	0	0
0	0	0	0	0
0	0	0	0.011570274	0

0	0	0	0	0
0	0	0	0	0
0	0	0	0	0
0	0	0	0	0
0	0	0	0	0
0	0	0	0	0
0	0	0	0	0
0	0	0	0	0
0	0	0	0	0
0	0	0	0	0
0	0	0	0	0
0	0	0	0	0
0.000844959	0	0	0	0
0	0	0	0	0
0	0	0	0	0
0	0	0	0	0
0	0	0	0	0
0	0	0.002266355	0	0
0	0	0	0	0
0	0	0	0	0
0.048976002	0.022502847	0.072232174	0.027739523	0.032656905
0	0	0	0	0
0	0	0	0	0.001386504
0	0	0	0	0.003570238
0	0	0	0	0
0	0	0	0	0
0	0.006745286	0.008823666	0.007199442	0
0	0	0	0	0
0	0	0	0	0
0	0	0	0.019726102	0.002625513
0	0	0	0	0.633269254
0	0	0	0	0
0	0	0	0	0
0	0.000808961	0	0	0
0.029399321	0.00495229	0.002521849	0.012359513	0.036499135
0	0	0	0	0
0	0	0	0	0
0	0	0	0	0
0	0	0	0	0
0	0	0	0	0
0	0	0	0	0
0.002874511	0	0	0	0
0	0	0	0	0
0	0	0	0	0
0	0	0	0	0
0	0.03526089	0	0	0
0	0	0	0.009133791	0
0	0	0	0	0.00061057

0	0	0	0	0
0	0	0	0	0
0	0	0	0	0
0	0	0.001246035	0	0
0	0	0	0	0
0	0	0	0	0
0	0	0	0	0
0.002527308	0	0.006575975	0	0
0.005485396	0	0	0	0
0	0	0	0	0
0	0	0.00343634	0	0
0	0	0	0	0
0	0	0	0	0
0	0	0	0	0
0	0	0	0	0.008086395
0	0	0	0	0
0	0	0	0	0
0	0	0	0	0
0.013558857	0.003745155	0.004984575	0	0.002980538
0	0.003218832	0	0	0
0.024836027	0	0	0.002936238	0.17256642
0	0	0	0	0
0	0	0	0	0
0	0	0	0	0
0.003929895	0	0.007303902	0.008750186	0
0	0	0	0	0
0	0	0	0	0
0	0	0	0	0
0.166057433	0.005891701	0	0	0
0	0	0	0	0
0.081774618	0.033004985	0.043792474	3.864481954	0.340523259
0	0	0	0	0
0	0.00210457	0	0	0.015409052
0.002090871	0.002846738	0.003031069	0	0
0	0	0	0	0
0	0	0	0	0
0	0	0	0	0
0.060804937	0	0	0	0
0	0	0	0	0
0	0	0.001080064	0	0
0	0	0	0	0
0.034758967	0.013802295	0.004592505	0	0.01647659
0	0	0	0	0
0	0	0	0	0
0	0	0	0	0
0	0	0	0	0
0	0	0	0	0
0	0	0	0	0

0	0	0	0	0
0	0	0	0	0
0.02771806	0.001083191	0.065509091	0.015593538	0
0	0	0	0	0
0	0	0	0	0
0	0	0	0	0
0	0	0	0	0
0	0	0	0	0
0	0	0	0	0
0	0	0	0	0
0	0	0	0	0
0	0	0	0	0
0	0	0	0	0
0.00114304	0.004549061	0	0.008944059	0
0.003773718	0	0.011362095	0	0
0	0	0	0	0
0.001074953	0	0.00503459	0.002051536	0
0	0	0	0	0
0	0	0	0	0
0	0	0	0	0
0	0	0	0	0
0	0	0	0	0.000485281
0	0	0	0	0
0	0	0	0	0
0	0	0	0	0
0	0	0	0	0
1.05652431	0	0	0	0
0	0	0	0	0
0	0	0	0	0
0	0	0	0	0.008657544
0	0	0	0	0
0	0	0	0	0
0	0	0	0	0
0	0	0	0	0
0	0	0	0	0
0	0	0	0	0
0	0	0	0	0
0	0.001787606	0.020121193	0	0
0	0	0	0	0
0	0	0	0	0
0	0	0	0	0
0	0	0	0	0
0	0	0	0	0
0	0	0	0	0
0	0	0	0	0
0	0	0	0	0.002499765
0	0	0	0	0.000660898
0	0	0	0.001972825	0

0	0.007952833	0	0	0
0.005254508	0	0	0.095088523	0
0.001219672	0	0.00566705	0	0
0	0	0	0	0
0	0.001927751	0.002280639	0	0.003409283
0	0	0	0	0.00305753
0	0	0	0	0
0	0.042634187	0.013106954	0.015592908	0
0	0	0	0	0.00082858
0.007978071	0.028780212	0.111042497	0.472289698	10.62394377
0	0	0	0	0.003340086
0.281398743	0	0.022522452	0	0.001513185
0.048715012	0.010636661	0.008254106	0	0
0.000784299	0	0	0	0
0	0	0	0.024158314	0
0	0	0	0	0
0	0	0	0	0
0	0	0	0	0
0.074238056	0	0	0.041390385	1.920036774
0	0	0	0	0
0	0	0	0	0
0	0	0	0.116880695	0
0	0	0	0	0
0	0.001198473	0	0	0
0	0	0	0	0
0	0.003149745	0.001341478	0.001147937	0
0	2.526547207	0	0	0.003351949
0	0	0	0	0
0	0	0	0	0.035417514
0	0	0.005750885	0.002165318	0
0	0	0	0	0.004124379
0	0	0	0	0
0	0	0	0	0.01571133
0	0	0	0	0.038246679
0	0	0	0	0
0	0	0	0.021535285	0.001016766
0	0	0	0	0.117262832
0	0	0	0	0
0	0	0	0	0
0.008223978	0	0	0	3.80185553
0	0	0	0	0
0	0	0	0	0
0	0	0	0	0
0	0	0	0	0
0	0	0.003347896	0	0
0	0	0	0	0
0.000748001	0.001566783	0	0.005710201	0.001039088
0	0	0	0	0

0	0	0	0	0
0.018669391	0.008171284	0	0	0
0	0	0	0	0
0	0	0	0	0
0	0	0	0.009171396	0
0.003534846	0	0.004379789	0	0.005565172
0	0	0	0	0
0	0	0	0	0
0	0	0	0	0
0	0	0	0	0
0	0.008587611	0.001015964	0.001086732	0
0	0	0.011188224	0.011611077	0
0	0	0	0	0
0	0.001802921	0	0.124229469	0
0	0	0	0	0.08111895
0	0	0	0	0
0	0	0	0	0
0.002303495	0	0	0	0.026530164
0	0	0	0	0
0.000903222	0	0	0	0
0	0	0	0	0
0.150838993	0.397742284	1.985903833	0	0.422794858
0	0	0.014801053	0	0
0	0	0	0.001206432	0
0	0	0	0	0
0	0	0	0	0
0	0	0	0	2.708152708
0	0	0	0	0
0	0	0	0	0
0.163069289	0.234209654	0.431218417	0.121295927	0.027078248
0.005401451	0.001528923	0	0	0
0	0.00214656	0	0	0
0	0	0	0	0
0	0	0	0	0
0.01574077	0	0	0	0.017655058
0	0	0	0	0
0	0	0.216494764	0	0.051120306
0	0.003215899	0.033018428	0	0
0	0	0.010242616	0	5.044752285
0	0	0	0	0
0	0.015007245	0.004537243	0.002869773	0.020200461
0	1.288614916	3.344203076	0	0.005960166
0	0.001893839	0.037640731	0	0
0	0	4.541064285	0	0
0	0	0	0	0
0	0	0	0.001376258	0
0	0.001555672	0	0.003720746	0.007737895
0	0	0	0	0

0.049712659	0	0	0	0
0	0	0	0	0
5.261034799	0.41878801	0.003480915	0	0
0.010361387	0	0	0	0
0	0.036017385	0	0	0
0.081933533	0	0.033479442	0	0
0	0	0	0	0
0	0	0	0	0
0.003067652	0	0	0	0
0	0	0	0	0.248993803
0	0	0	0	0
0	0	0	0	0
0	0	0	0	0
0	0	0	0	0
0	0	0	0	0
0.025752313	0.100928311	0.051293538	0	0
0.327804367	0.002068157	0.019818663	0.017987168	0
0.083100048	0	0	0	0.025087483
0.003523191	0	0	0.061223502	0
0.00335179	0	0	0	0
0.006561159	0	0.004289006	0.026986845	0.041109365
0	0.001090434	0	0.009935324	0
0	0	0	0	0
0	0	0.177228342	0	0
0	0.001581458	0	0.002561638	0
0.001418919	0	0	0	0.712157649
0	0.001193428	0	0.000652424	0.001329686
0	0	0	0	0
0	0	0	0	0
0	0	0	0	0
0	0.015066141	0.007344391	0.029770016	0
0.054780348	0	0.030255436	0	0
0.021896644	0.020613629	0.094652445	0.043902443	0.055162194
0.007404276	0.020309672	0.002752241	0	0
0.021894043	0.001844938	0	0	0
0	0	0	0	0
0.002446706	0	0.001488215	0.017829038	0
0.0534023	0.007287163	0.015518037	0.012117248	0.023342597
0.606435903	0.066991002	0.069023195	0	0
0.002133314	0	0	0	0.006401166
0	0	0	0	0
0	0	0	0	0
0	0	0	0	0
0	0	0.076165141	0	0.104463957
0	0	0	0.001774963	0
0.00246504	0	0	0	0
0	0	0	0	0
0	0	0	0	0

0	0	0	0	0
0.008058931	0.105006413	0.150924532	0.010178191	0.003951211
0	0	0	0	0
0	0.000451035	0	0	0
0	0	0	0	0
0	0	0	0	0
0	0	0	0	0.626715744
0.002366806	0	0.041416628	0	0
0	0	0.265187831	0	0
0	0	0	0	0
0	0	0	0.04050679	0
0.114914317	0.002472974	0.022527653	0.08562206	0
0.013494912	0.142032049	0	0	0
0	0	0	0	0
0.001010477	0	0	0.00250703	0
0	0	0	0	0.023038351
0.000737765	0.000772671	0.011312164	0.002464026	0
0.008380053	0	0.01121381	0.37744032	10.87379849
0	0	0	0	0
0.024720268	0	0.005201187	0.001483596	0.058184069
0.031193677	0.039641741	0.022482975	0	0.008877945
0	0	0	0	0
0	0	0	0	0
0	0	0	0	0
0	0	0	0	0
0	0	0	0	0
0.003567101	0.446928677	0.075159089	0	0
0.04973141	0.031335333	0.013526085	0.219724726	0.234719131
0	0	0	0	0
0.007315577	0	0	0.002326949	0
0	0	0.012094584	0	0
0	0	0	0	0
0	0	0	0	0
0	0	0	0	0
0.1500956	0.104798153	0	0	0
0.140963364	0.413157147	0.151469343	0.111659244	0.625387753
0.003010751	0	0	0	0
0	0	0	0	0
0.086038593	0	0.008899167	0	0.01309533
0	0	0	0	0
0	0	0	0.001194651	0
0	0	0	0	0
0	0.002062435	0	0.095403297	0.02701938
0	0	0	0.000697804	0
0.014410655	0	0	0	0.612890118
0	0.093214382	0	0	0
0	0	0	0	0
0	0	0	0	0.00295564

0	0	0	0	0
0	0	0	0	0
0	0.000436175	0	0	0
0.194070955	0.135440565	0.329736534	0	0
0	0	0	0	0
0.00091794	0	0	0	0
0	0	0	0	0
0	0.001322413	0.001232036	0	0
0	0.358570001	0.049290376	0	0.014133205
0.005834285	0.051855218	0	0	0
0	0	0	0	0
0.002197483	0.003363664	0.001130989	0	0
0	0	0	0	0
0.002329487	0	0	0	0
0.137676496	0.011833265	0.012443938	0	0.081965949
0	0	0	0	0
0	0.032967088	0.129044613	0	0
0	0.636412535	0	0	0
0	0	0	0	0
0	0	0	0	0
0	0	0	0	0
0	0	0	0	0
0	0	0	0	0
0	0	0.378030604	0.079878904	0.731306436
0	0	0	0	0
0.049584068	0.105397584	0	0	0
0	0	0	0	0
0.762851222	2.823116189	0.943159505	0	0
0	0	0	0	0
0	0	0	0	0
0.002464994	0.022029845	0	0	0
0.016412201	0	0	0	0
0.000875486	0.003536648	0	0	0
0	0	0	0	0
0	0	0	0	0
0	0	0	0	0
0.511966589	0.194498372	0.374652698	0	0
0	0	0	0	0
0	0	0	0	0.052067043
0.01923777	0	0	0.007389195	0
0.026756549	0.003057001	0.002893286	0	0.042980848
0	0	0	0	0
0.37077782	8.907134172	3.351324033	0.128456618	15.86548609
0.001130107	0	0.005355914	0	0
0	0	0	0	0
0	0	0	0	0
0	0	0	0	0
0.008382248	0	0.00195641	0	0.00211221

0	0	0	0	0
0	0.065098191	0.009505838	0	0
0.056994792	0	0.005209554	0.765161993	0.066973885
0	0	0	0	0
0	0	0	0	0
0.001472075	0.002620933	0.001969865	0	0
0	0	0	0	0
0.002983618	0	0	0	0
0	0	0	0	0
0	0	0	0	0
0.041281067	0.001345065	0.002455132	0.046745433	0.031654863
1.268737095	3.163170545	1.359535655	2.001362147	2.143924707
0	0	0	0	0
0	0.016590277	0.009715488	0	0
0.008894242	0.049680344	0.03549682	1.061952169	0.254522753
0	7.729331341	0	0	0
0	0	0	0	0
0	0	0	0	0
0	0	0	0	0
0.215514447	0	0.000842659	0	0.00151161
0.005382162	0.004348399	0.007716608	0.012326153	0.400278632
0	0	0.208693375	51.7312779	9.746751368
0.447936235	5.09653968	2.816609274	4.308501082	0.618136428
0	0	0	0	0
0	0	0	0	0
0.010503381	0	0.002465816	0	0.001382288
0	0	0.91391792	1.128690478	0
0	0	0	0	0
0	0.00342906	0	0	0
0	0	0	0	0
0	0.001421088	0	0	0
0.100607235	0.117378945	0.069774775	0	0
0	0.010656646	0	0	0.467552503
0.012070161	0.002708839	0	0	0
0.004352813	0	0.001386843	0	0.004457303
0.02323851	0.007874064	0.09717728	0	0.0014242
0.022283023	0	0.001472501	0	0.002338789
0	0	0	0	0
0	0	0.014716323	0	0.004833146
0	0	0	0	0
0.000752304	0.000630319	0.005872416	0	0
0	0	0	0	0
0	0	0	0	0
0	0	0	0	0
0.000779438	0	0	0	0.161276978
0	0.004574373	0	0	0
0	0	0	0	0
0.130219761	0.004677326	0.004193844	0	0.002546902

0	0	0	0	0
0	0	0	0	0
0	0	0	0.00544677	0
0.005480571	0.002087229	0.001481587	0	0
0	0	0	0	0
0.052364385	0.082646981	0	0	0
0	0.799410395	0	0	0
0.082523735	0.001920628	0.032890284	0.009916389	0
0	0	0	0	0
0	0	0	0	0
0.000924834	0	0.001443833	0.012884756	0.001079179
0	0	0	0	0
0	0.005597313	0	0.001081798	0
0	0.613944291	0	0	0
0	0	0	0	0
0	0	0	0	0
0.307323792	0.012966789	0	0	0
0.001693787	0.002096459	0.002232208	0	0
0	0	0	0	0
0.020829756	0	0	0	0
0.008618379	0.003581805	0	0	0
0	0	0	0	0
0.003550573	0.009296413	0	0	0
0	0	0	0	0
0	0	0	0	0
0.003283084	0	0	0	0
0	0	0	0	0
0	0	0	0	0
0	0	0	0	0
0.039061521	0.124871926	1.447184084	0	0
0	0.003966944	0	0	0
0	0	0	0	0
0	0.058804966	0	0	0
0	0	0	0	0
0	0.000770177	0	0	0
0	0	0	0	0
0.085567661	0.057986961	0.050220526	0	0
0.00532364	0	0.006285755	0.004781226	0.005089745
0	0	0	0	0
0	0	0.004994381	0	0.000772345
0.006757794	0.001077016	0	0	0
0	0	0	0	0
0	0	0	0	0
0	0.295089867	0.004747721	0	0
0.109697841	0	0	0	0
0	0	0	0	0
0	0.002337212	0	0	0
0	0	0	0	0

0	0	0	0	0
0	0	0	0	0
0	0	0	0	0
0.042363689	0.062311062	0.008162965	0	0
0	0	0	0	0
1.423506985	0.979332498	0	0	0
0	0	0	0	0
0	0	0	0	0
0.002375414	0	0	0.121372817	0.05435697
0	0	0	0	0
0	0.000808572	0	0	0
0	0	0	0	0
0.013731739	0	0	0	0
0	0	0	0	0
0	2.028829987	1.032251222	0	0
0	0	0	0	0.006381271
0	0	0	0	0
0	0	0	0	0
0	0	0	0	0
0	0	0	0	0
0	0.105663181	0	0	0
0	0	0	0	0
0	0	0	0	0
0	0	0	0	0
0.054598645	0.045213617	0.054654498	0	0.018414688
6.818050135	3.877027635	2.196958056	0	0
0	0.078939979	0	0	0
0	0	0	0	0
0	0.281357957	0	0	0
0	0.009921499	0	0	0
0	0	0	0	0
0	0	0	0	0
0.001967587	0	0	0	0
0.00298373	0	0	0	0
0.001787725	0.006319046	0.01520078	0.001279445	0
0	0	0	0.000904413	0.003686511
0.142799908	0	0.499898362	0	0
3.037780158	0.488873885	8.147465732	0	0
0	0.958822856	0	0	0
0	0	0	0	0
0	0	0	0	0
0	0	0	0	0
0.380991928	0.592988511	0.202197391	0	0
0	0	0.010128866	0	0
0	0	0	0	0
0	0	0	0.009996202	0
0	0	0	0	0
0.181669019	0.165545108	0.028181036	0	0

0.004266796	0	0	0.001357189	0.042083391
0	0	0	0	0
0	0	0	0	0
0	0	0	0	0
0	0	0	0.001520143	0
0.005113559	0.123533591	0	0	0
0	0	0	0	0
0	0	0	0	0
0	0	0	0	0
0	0	0	0	0
0	0	0	0	0
1.883307304	0.018470539	11.6032557	0	0.071320091
0.274330259	0.049605855	0.618519238	0	0.000714538
0	0	0	0	0
6.729466821	0.069891221	0.521602311	0.072470549	0.015867655
0	0	0	0	0
0.000854411	0	0	0	0
0	0	0	0	0
0.004844423	0	0	0.000684854	0.003439599
0	0	0	0	0
0.082442941	0.009178874	0.297503461	0	0
0	0	0	0	0
1.114479512	0.292533386	0.045330269	0	0
0	0	0	0	0
0	0	0	0	0
0	0	0	0	0
0	0	0	0	0
0	0	0	0	0
0.002581422	0	0	0	0
0	0	0	0	0
0	0	0	0	0
0.005312663	0.009180644	0.041766356	0.002027831	0
0	0	0	0	0
0.022427271	0.010840798	0.014946389	0	0
0	0	0	0.000984227	0.002435765
0.056509131	0.014389545	0	0	0.009419987
0	0	0	0	0
0	0	0	0	0
0.002369901	0.001241015	0.001541602	0	0
0	0	0	0	0
0.002929248	0.130536697	0	0	0
0	0	0	0	0
0	0	0	0	0
0	0	0	0	0
0.022522507	0	0.009253081	0.003231876	0.222445186
0	0	0	0	0
0	0.312769326	0	0.894353804	0
0.000961004	0	0	0	0

0	0	0	0	0
0	0	0	0	0
0	0	0	0	0.007298985
0	0	0	0	0
0.006039062	0	0	0	0.026055833
0	0	0	0	0
0	0.03977719	0	0	0
0	0.006929256	0	0	0
0	0	0	0	0
0	0	0	0	0
0	0	0	0	0
0	0	0	0	0
0	0	0	0	0
0.133204279	0	0	0	0
0	0	0	0	0
0	0	0	0	0
0.052164587	0	0	0	0
0	0	0	0	0
0	2.56563667	0.817860143	0	0.152864402
0	0	0	0	0
0	0	0	0	0
0	0	0	0	0
0	0	0	0	0
0	0	0	0	0
0	0	0	0	0
0.003941279	0	0	0	0.029565237
0	0	0	0	0
0.068821867	0.001454491	0	0	0.028938486
0	0	0	0	0
0.862408878	0.111754738	0.659495363	0	0
0	0	0	0	0
0	0	0	0	0
0	0.00324228	0	0	0
0	0	0	0	0
0	0	0	0.003625936	0.010465213
0	0	0	0	0
0	0	0	0	0
0.001499707	0	0	0	0
0	0	0	0	0
0	0	0	0	0
4.432849896	2.441017082	4.50505358	1.514841179	0.686477473
1.945014144	0.227359831	0.932978697	0	0.00457501
0	0.276303063	0	0	0
0.007676165	0.007724087	0	0	0
0	0	0	0	0
0	0	0	0	0
0	0	0	0	0
0.002380502	0	0	0	0.00731648

0	0	0	0	0
0.054174002	0.003846589	0	0	0
0	0.004305408	0	0	0
0	0	0	0	0
2.65282615	2.231687328	0.561862915	0	0
0	0	0	0	0
0	0	0	0	0
0	0	0	0	0
0.068763726	0	0.03646581	0.594056088	10.03543557
0	0	0	0	0
0	0	0	0.028377494	0.082573822
0	0	0	0	0
0.019479506	0.105743418	0.009119994	0	0
0.010054534	0.005935234	0.016308516	0.00750114	0
0	0	0	0	0
0.195616069	0.152553821	0.440958317	0	0
0.00047458	0	0	0	0.020041664
0	0	0	0	0
0	0	0	0	0
0	0	0	0	0
0	0	0	0	0
0	0	0	0	0
0	0	0	0	0
0	0	0	0	0
0	0.012013038	0	0	0
0	0	0	0	0
0.173732853	0.009652289	0.00322124	0	0
0	0	0.001557775	0.00281417	0
0	0	0	0	0
0	0	0	0	0
0	0	0	0	0
0	0	0	0	0
0	0	0	0	0
0	0.047713794	0	0	0
0	0.34484413	0	0	0
0.003411506	0.004325112	0	0	0
0	0.001345456	0.099461749	0	0
0.021871807	0	0	0	0
0	0	0	0	0
0	0	0	0	0
6.136481755	0.846852891	2.430658304	0	1.715045917
0.251321902	0	0	0	0
0	0.001599069	0	0	0
0	0.041582536	0	0	0
0	0	0	0	0
0	0	0	0	0
0	0.051630542	0	0	0
0	0	0	0	0

0.061520897	0.093265775	0	0	0
0	0	0	0	0
0	0	0	0	0
0	0	0	0	0
0.04323626	0.204385417	0	0	0
0	0	0	0	0
0	0.001322203	0	0	0.022228983
0	0	0	0	0
0	0	0	0	0
0.00115897	0	0	0	0
0.000956845	0.025052929	0	0	0
0.039745948	0	0.000615582	0	0
0	0	0	0	0
0	0	0	0	0
0	0	0	0	0
0	0	0	0	0
0	0	0	0	0
0	0	0	0	0
0	0	0	0	0
0	0	0	0	0
0.002554127	0.03147027	0.042220103	0	0.000375679
0	0	0	0	0
0.017731745	0.007115878	0	0	0
0.007662589	0.001234636	0.002136194	0.001265535	0
0	0	0	0	0
0.000869244	0	0	0	0.001159213
0	0	0	0	0
0	0	0	0	0
0	0	0.020690876	0	0
0.222898144	0.002665397	0.949306165	0.014773593	0
0	0.036456147	0	0	0
0	0	0	0	0
0	0	0	0	0
0.391533853	0.011701842	0	0	0
0	0.120426014	0	0	0
0	0	0	0	0
0	0.010427737	0	0	0.056862091
0	0	0	0	0
0	0	0	0	0
0	0	0	0	0
0	0	0	0	0
0	0	0	0	0
0	0	0	0	0
0	0.004422481	0	0	0
0	0	0.003502701	0	0
0	0	0	0	0
0	0	0	0	0
0	0	0	0	0

0	0	0	0.007111418	0.000660801
0	0.037326188	0	0	0
0	0.243925402	0	0	0
0	0	0	0	0
0	0	0	0	0
0	0.004400369	0	0	0
0	0	0	0	0
1.527131681	0.306577487	0.545924109	0.041635902	0
2.309917347	1.019711783	1.421008991	0.087637014	0.593085799
0.002252477	0.001814654	0	0	0
0	0	0	0	0
0	0.000635371	0	0	0
0.010053649	0.026716205	0	0	0
0	0	0	0	0.011825299
0.002272175	0	0	0	0
0	0	0	0	0
0	0	0.027359153	0	0
0	0.672769883	0	0	0
0	0	0	0	0
0	0.004062838	0	0	0
0	0	0	0	0
0	0	0	0	0
0	0	0	0	0
0	0	0	0	0
0.016485409	0.007586312	0	0	0
0	0	0	0	0
0	0	0	0	0
0	0	0	0	0
0	0	0	0	0
0	0	0	0	0
0.040235078	0	0	0	0.118257244
0	0	0	0	0
0	0	0	0	0
0	0	0	0	0
0.008446305	0.005026099	0	0	0
0	0	0	0	0
0	0	0	0	0
0	0	0.151006475	0	0
0.009238581	0.001382242	0.002312741	0	0
0.330835326	0	0	0	0
0	0	0	0	0
0.007022278	0.034549187	0.181564063	0	0.060708024
0	0	0	0	0
0	0	0	0	0
0.002313814	0	0	0	0
0	0	0	0	0
0	0	0	0	0
0	0	0	0	0

0	0	0	0	0
0.007012343	0	0.00063834	0	0
0	0	0	0	0
0	0	0	0	0
1.313368101	0.013201867	0.052879999	0	0.00533666
0	0.079476478	0	0	0
0	0	0	0.006789082	0
0.002420426	0	0.000899696	0.009431185	0.012776912
0.005034804	0.001265525	0	0	0
0	0	0	0	0
0	0	0	0	0
0	0	0	0	0
0	0	0	0	0
0	0	0	0	0
0.000851947	0	0	0	0
0.002624022	0	0.013265094	0.001836236	0
0	0	0	0	0
0	0	0	0	0
0	0	0	0	0
0	0	0	0	0
0	0	0	0	0
0	0	0	0	0
0	0	0	0	0
0.000698227	0	0	0.009327901	0
0.096445324	0	0.19829888	0	0.117537684
0	0	0	0	0
0.013570408	0	0.009613752	0.045704059	0.004923539
0	0	0	0	0
0	0	0	0	0
0	0	0	0	0
0	0	0	0	0
0	0	0	0	0
0	0	0	0	0
0	0	0	0	0
0	0	0	0	0
0	0	0	0	0
0	0	0	0	0
0.014899643	0.001371833	0	0	0
0	0	0.059284909	0	0
0	0	0	0	0
0	0	0	0	0
0	0	0	0	0
0	0	0	0	0
0	0	0	0	0
0	0	0	0	0
0	0	0	0	0
0	0	0	0	0
0	0	0	0	0

0.114105498	0.00251588	0.078019667	0	0
0	0	0	0	0
0	0	0	0	0
0	0	0	0	0
0	0	0	0	0
0	0.001821745	0.016681471	0.003983653	0.104966563
0	0	0	0	0
0	0	0	0	0
0	0	0	0	0
0	0	0	0	0
0	0.046911202	0	0	0
0.001287307	0	0	0	0
0	0	0	0	0
0	0	0	0	0
0	0.019750905	0	0	0
0	0	0	0	0
0	0	0	0	0
0	0	0	0	0
0.005346218	0	0	0.000850266	0
0	0	0	0	0
0.010429616	0	0	0.014099235	0.004346504
0	0	0	0	0
0.021074954	0.000919671	0.002937662	0.009426868	0.021225361
0	0.002735624	0	0	0
0	0	0	0	0
0	0	0	0	0
0	0	0	0	0
0	0	0	0	0
0	0	0	0	0
0	0	0	0	0
0	0	0	0	0
0	0	0	0	0
0.0059621	0	0.022696645	0.001177096	0.003255791
0	0	0	0	0
0.308950275	0.052194594	0.517394434	0	0.025750774
0	0	0	0.002518855	0.000687536
0	0	0	0	0
0	0	0	0	0
0.001144933	0	0	0	0.023698269
0	0	0	0	0
0	0	0	0	0
0	0.018404059	0	0	0
0	0.031547516	0.003981069	0	0.00130183
0	0	0	0	0
0	0	0	0	0
0.003012442	0.004639667	0.012251428	0	0
0	0	0	0	0
0	0	0	0	0

0	0	0	0	0
0	0	0	0	0
0	0	0	0	0
0	0	0	0	0
0	0	0	0	0
0	0	0	0	0
0	0	0	0	0
0	0	0	0	0
0.004482602	0.131625263	0	0	0
0.075744395	0.003058435	0	0	0
0	0	0	0	0
0	0	0	0	0
0	0	0	0	0
0	1.520614778	0	0	0
0	0	0	0	0
0	0	0	0.134345867	0.011525005
0	0	0	0	0
0	0	0	0	0
0	0	0	0	0
0	0.129432421	0.869967847	0	0
0.006001746	0	0	0	0.003439157
0	0	0	0	0
0	0	0	0	0
0	0.004008266	0	0	0
0	0	0	0	0
0	0	0	0	0
0	0	0	0	0
0	0	0	0	0
0	0	0	0	0
0	0.032552118	0.03569764	0	0
2.033583632	0.074901481	1.108382626	0	0
0	0	0	0	0
0	0	0	0	0
0	0	0	0	0
0	0	0	0	0
0	0	0	0	0
0	0	0	0	0
0	0	0	0	0
0	0	0	0	0.57817819
0	0	0	0	0
0	0.000958518	0	0	0
0	0	0	0	0
0	0.004463956	0	0	0
0	0	0	0	0.002078371
0	0	0	0	0
0	0	0	0	0
0	0	0	2.250456709	0.04949058
0	0	0	0	0

0	0	0	0	0
0	0	0	0	0
0	0	0	0	0
0.000659016	0	0	0	0
0.201196288	0.008917661	0.024197173	0	0
0	0	0	0	0
0	0	0	0	0
0	0	0	0	0
0	0	0	0	0
0	0	0	0	0
0.088055925	0.008583057	0	0	0
0.004957424	0	0.001913597	0.003275026	0.046564188
0.010199019	0	0	0	0.002742195
0	0	0	0	0
0	0	0	0	0.003633293
2.765885866	0.15109568	0	0	0
0.048100536	0	0.002943566	0	0.00195568
0	0	0	0	0
0.011405746	0.001058453	0.008049924	0	0.073646095
0	0.00394862	0	0.003058066	0.068505688
0	0	0	0	0
0	0	0	0	0
0	0	0	0	0
0	0	0	0	0
0	0	0	0	0
0	0	0	0	0
0.001510631	0	0.00067382	0	0.034751152
0	0	0	0	0
0	0	0	0	0
0	0	0	0	0
0	0	0	0	0.004567218
0	0	0	0	0
0	0	0	0	0
0	0	0	0	0
0	0	0	0	0
0	0	0	0	0
0	0	0	0	0
0.023580138	0.000755994	0.001207419	0.002525646	0.001704672
0	0.017579917	0	0	0
0	0	0	0	0
0	0	0.001339326	0	0.004905224
0	0	0	0	0
0	0	0	0	0
0	0	0	0	0.001784338
0	0	0	0	0
0	0	0	0	0
0	0	0	0	0
0	0	0	0	0

0	0	0	0	0
0	0	0	0	0
0	0	0	0	0
0.007852478	0	0	0.003746591	0.000779164
0	0	0.00077348	0	0
0	0	0	0	0.002984529
0	0.010831055	0	0	0
0	0	0	0	0
0	0	0	0	0
0	0	0	0	0
0	0.002912412	0.048754533	0	0
0	0	0	0	0
0.013253099	0.026094694	0.010049664	0	0
0	0	0	0	0
0	0	0	0	0
0	0	0	0	0
0	0	0	0	0
0	0	0	0	0
0	0	0	0	0
0	0	0	0	0
0	0	0	0	0
0	0	0	0	0
0.022512148	0	0.00408669	0.026144865	0.007563665
0.004324553	0.036448991	0	0	0
0	0	0	0	0
0	0	0	0	0
0	0.004889377	0	0	0
0	0	0	0	0
0	0	0	0	0
0	0	0	0	0
0.028603402	0.004279535	0.019745446	0	0
0	0	0	0	0
0	0	0	0	0
0	0	0	0	0
0	0	0	0	0
0	0	0	0	0
0	0	0	0	0
0	0	0	0	0
0	0.002069019	0.00073433	0	0.00137217
0	0	0	0	0
0	0	0	0	0
0	0	0	0	0
0	0	0	0	0
0	0	0	0	0
0	0	0	0	0
0.594449287	0.334345877	0.138424664	0	0
0	0	0	0.336579728	0.076730097
0	0	0	0	0

0	0	0	0	0
0	0	0	0	0
0.058537078	0.060061898	0.463396216	0	2.413753546
0	0.013513024	0	0	0
0	0	0	0	0
0	0	0	0	0
0	0	0	0	0
0	0	0	0	0
0	0	0	0	0
0	0	0	0	0
0.000534233	0	0	0	0
0.00139582	0	0	0.000570837	0
0	0	0	0	0
0	0	0	0	0
0	0	0	0	0
0	0	0	0	0
0.000981624	0	0	0	0
0	0	0	0	0
0	0	0	0	0
0	0	0	0	0
0	0	0	0	0
0	0	0	0	0
0	0	0	0	0
0	0	0	0	0
0	0	0	0	0
0	0	0	0	0
0	0	0	0	0
0	0	0	0	0
0	0	0	0	0
0	0	0	0	0
0.003804135	0.003665394	0.003223997	0.003339668	0
0	0	0	0	0
0.018374669	0.111763515	0.055362757	0.036753798	0.027096033
0	0.000567269	0	0	0
0	0.001468839	0	0	0
0	0	0	0	0
0	0	0	0	0
0	0	0	0	0
0	0	0	0	0
0	0	0	0	0
0	0	0	0	0
0.192446884	0	0.014749914	0.012483172	0.117152369
0.078269576	0.006491061	0.47471218	0	0.002509119
0	0	0	0	0
0	0	0	0	0
0	0.041808153	0	0	0
0	0	0	0	0
0	0	0	0	0

0	0	0	0	0
0.004266852	0.013017616	0.035995994	0	0.009277537
0.006501044	0.001588681	0.003866402	0	0
0	0	0	0	0
0.139285675	0.003443032	0	0.009576308	0.002019019
0	0	0	0.010285816	0
0	0	0	0	0
0	0	0	0	0
0	0	0	0	0
0	0	0	0	0
0	0	0	0	0
0	0	0	0	0
0	0	0	0	0
0	0	0.018889529	0	0
0	0	0	0	0
0	0	0	0	0
0	0.02542124	0	0	0
0	0	0	0	0
0	0	0	0	0
0	0	0	0	0
0	0	0	0	0
0	0	0	0.003659381	0
0	0.004875871	0	0	0
0	0	0	0	0
0	0.552149158	0	0	0
0.493884787	0	0.002604471	0.001200075	0
0	0	0	0	0
0	0.002555308	0	0	0
0	0	0	0	0
0	0	0	0	0
0	0	0	0	0
0	0	0	0.009772239	0.003676892
0	0	0	0	0
0	0	0	0	0
0	0	0	0	0
0	0	0	0	0
0	0	0	0	0
0.011087846	0	0	0	0
0.43258623	0	0	0	0
0	0	0	0	0
0	0	0	0	0
0	0	0	0	0
0.000540925	0	0	0.003957336	0.000601142
0	0	0	0	0
0	0	0	0	0
0	0	0	0	0
0	0	0.003691117	0.001316076	0.009426207
0	0	0	0	0

0	0	0	0	0
0.055688745	0.003827487	0.012420029	0.014613682	0.03669777
0	0.002809434	0	0	0
0	0	0	0	0
0	0	0	0	0
0	0	0	0	0
0	0	0	0	0
0	0	0	0	0
0	0	0	0	0
0	0	0	0	0
0	0	0	0	0
0	0	0	0	0
1.093104019	0.055102563	0	0	0
0	0	0	0	0
0.010198335	0	0.005807214	0	0.00090428
0	0	0	0	0
0	0	0	0	0
0	0	0	0	0
0	0	0	0	0
0	0	0	0	0
0	0	0	0	0
0	0	0	0	0.020248104
0	0.042530846	0.0010956	0	0.004640407
0	0	0.000979035	0	0
0	0	0	0	0
0	0	0	0	0
0	0	0	0	0
0	0	0	0	0
0	0	0	0	0
0	0	0	0	0
0	0	0	0	0
0	0	0	0	0
0	0	0	0	0
0	0	0	0	0
11.50241127	0	0	0	0
0	0	0	0	0
0	0	0	0	0
0	0	0	0	0
0	0.275023991	0	0	0.081412836
0	0	0	0	0
0.061058037	0.06354727	0.156814256	0	0.004930106
0	0	0	0	0
0	0	0	0	0
0	0	0	0	0
0	0	0	0	0
0.457892792	0	0.005603064	0.057953125	0.152587251
0	0	0	0	0
0	0	0	0	0

0	0	0	0	0
0	0	0	0	0
0	0	0	0	0
0	0	0	0	0
0	0	0	0	0
0	0	0	0	0
0	0	0	0	0
0.001652873	0	0	0	0.005313821
0.073551944	0	0	0	0
0.057045757	0.001118817	0.084103097	0	0
0	0.055693743	0.005527966	0	0
0	0	0	0	0
0.000626488	0	0	0	0
0	0	0	0	0
0	0	0	0	0
0	0	0	0	0
0	0	0	0	0
0	0	0	0	0
0	0	0	0	0
0.471745212	0.010122695	0.703689156	0	0.035632428
0	0	0	0	0
0	0	0	0	0
0	0	0	0	0
0	0	0	0	0
0	0	0	0	0
0	0	0	0	0
0	0	0	0	0
0	0	0	0	0
0	0	0	0	0
0	0	0.001551041	0	0
0	0	0	0	0
0	0	0	0	0
0	0	0	0	0
0	0	0	0	0
0	0	0	0	0
0	0	0.002683091	0	0
0	0	0	0	0
0	0	0	0	0
0	0	0	0	0
0	0.404030939	0	0	0
0	0	0	0	0
0	0	0	0	0
0.002781087	0	0	0	0
0	0	0	0	0
0.007681458	0	0.001168066	0	0.005820398
0	0.024877658	0	0	0
0	0	0	0	0
0	0	0	0.013674524	0

0	0	0	0	0
0	0	0	0	0
0	0.001868742	0	0	0
0	0	0	0	0
0	0	0	0	0
0.008080441	0.003821891	0.020637493	0.001243664	0.001738061
0	0	0	0	0
0	0	0	0	0
0	0	0	0	0
0	0	0	0	0
0	0	0	0	0
0	0	0	0	0
0	0.009575271	0	0	0
0	0	0	0	0
0	0	0	0	0
0	0	0	0	0
0	0	0	0	0
0	0	0	0	0
0	0	0	0	0
0	0	0	0	0
0	0.007280218	0	0	0
0	0	0	0	0
0	0	0	0	0
0	0.007201494	0	0	0
0	0	0	0	0
0	0	0.00181862	0	0
0	0	0	0	0
0	0	0	0	0
0	0	0	0	0
0	0	0	0	0
0	0	0	0	0
0	0	0	0	0
0	0	0	0	0
0	0	0	0	0
0	0	0	0	0
0	0	0	0	0
0	0	0	0	0
0	0	0	0	0
0.252409656	0	0.653000765	0	0
0	0	0	0	0
0	0	0	0	0
0	0	0	0	0
0	0	0	0	0
0	0	0	0	0
0.001266531	0	0	0	0
0	0	0	0	0
0	0	0	0	0
0	0	0	0	0

0	0	0	0	0
0.273735792	0.011308562	0.000976282	0	0
0	0	0	0	0
0	0	0	0	0
0	0	0	0	0
0	0	0	0	0
0	0	0	0	0
0	0	0	0	0
0	0	0	0	0
0	0	0	0	0
0	0	0.002343667	0	0
0	0	0	0	0
0.068112356	0.087547532	0.478950096	0	0
0	0	0	0	0
0	0	0	0	0
0	0	0	0	0
0	0	0	0	0
0	0	0	0	0
0	0	0	0	0
0	0	0	0	0
0	0	0.001081218	0	0
0	0	0	0	0
0	0	0	0	0
2.204967172	1.070188927	1.301782686	0	0
0	0	0	0	0
0	0	0	0	0
0	0	0	0	0
0	0	0.000993448	0	0
0	0	0	0	0
0	0	0	0	0
0	0	0	0	0
0	0	0	0	0
0	0	0	0	0
0	0	0	0	0

PHL018-0187_V2	PHL018-0264_V2	PHL018-0112_V2	PHL018-0025_V1	PHL018-0015_V1
CTRL	REF	CTRL	CTRL	CTRL
3.991336379	0.00229368	0.053442633	0.019514605	0.040120809
0.753458119	0.174855614	0.121052758	0.22926343	0
0.162775075	8.355559309	0	10.71114525	0
0	0	0	0	0
0	0	0	0	0
0	0	0	0	0
0	0	0	0	0
0	0	0	0	0
0	0	0	0	0
0	0	0	0	0
0	0	0	0	0
0	0	0	0	0
0	0	0	0	0
5.401837052	0.392807354	0.628014371	13.96058661	24.67702285
0	0	0	0	0
0	0	0	0	0
0	0	0	0	0
0	0	0	0	0
0	0	0	0	0
0	0	0	0	0
0	0	0	0	0
0.002466407	0	0	0	0
0	0	0	0	0
0	0	0	0	0
0	0	0.313466876	0	0
0	0	0	0	0
0	0	0	0	0
0	0	0	0	0
0	0	0	0	0
0	0	0.178558241	0	0
0	0	0	0	0
0	0	0	0	0
0	0	0	0	0
0	0	0	0	0
0	0	0	0	0
0	6.378289809	0.22782455	0.17081064	0
0	0	0	0	0
0	0	0	0	0
0	0	0	0	0
0	0	0	0	0
0	0	0	0	0
0.033387746	0.03784446	0	0.418863596	0.032014746
0.012621253	0.362174205	2.154702498	0.00984836	1.9976092
0	0.021552212	0.006225825	1.561311571	0.010467039
0	0	0	0.006268124	0
0	0	0	0	0

0	0	0.000786125	0	0
0.001275109	0.004060728	0	0	0.044925339
0.211071953	0.000790013	0.001430954	0	0
0.137176174	0.384046691	1.348910573	0.215951266	0
0.022837305	0	0.010107352	0	0
0	0	0.016279485	0	0
0.001640053	0.02841891	0.000695612	0.005241459	0
0	0	0	0	0
0	0	0.029898614	0	0
0	0	0	0	0
0	0.014114312	0	0.049993485	0
0.000964523	0.001626158	0.132055066	0.006693484	0
0	0	0	0	0
0.058140438	0.003667158	0.027233587	0	0
0	0.027924096	0	0	0
0	0	0	0	0
0.03810782	0.002765007	0	0.000980194	0
0	0	0	0	0
0	0	0	0	0
0	0	0	0	0
0	0.076338626	0.010352886	0	0.056591947
0.007186088	0.004365961	0.010379358	0	0
0	0	0.014043908	0	0
0	0	0	0	0
0	0	0	0	0
0	0	0	0	0
0	0	0	0	0
0	0	0	0	0
0	0.000698166	0	0.017923091	0.00322726
0	0	0	0	0
0	0.033599162	0.001493455	0	1.697946034
0	0	0	0	0
0	0	0	0	0
0.000538719	0	0	0	0
0	0	0	0	0
0	0	0	0.00103079	0
0	0	1.131974246	0	0
0.001413063	0	0.003596018	0	0
0	0	0	0	0
0	0	0	0	0
0	0	0	0	0
0	0	0.042756493	0	0
0	0	0	0	0
0	0	0	0	0
0	0	0	0	0
0	0	0	0	0
0	0	0	0	0
0	0	0	0	0

0	0	0	0	0
0	0	0	0	0
0	0	0	0	0
0	0	0	0	0
0	0	0.003188017	0.000807456	0
0	0.001117986	0	0	0
0	0	0	0	0
0	0	0.00136141	0	0
0	0	0.003025664	0	0
0	0	0	0	0
0	0	0	0	0
0	0.006124234	0	0.003980237	0
0	0	0	0	0
0	0.00057238	0	0	0
0	0	0	0	0
0	0	0.00120035	0	0
0	0	0	0	0
0	0	0	0	0
0	0.09937681	0.010530258	0.109277647	0
0	0	0	0	0
0	0	0	0	0
0	0	0	0	0
0	0	0	0	0
0	0	0	0	0
0	0	0	0	0
0	0	0	0	0
0	0	0.001400158	0	0
0	0.001183527	0	0	0
0	0	0	0	0
0	0	0	0	0
0	0.009274625	0.039787495	0	0
0	0.006315893	0.001236757	0.00832054	0
0	0	0	0	0
0	0	0	0	0
0	0	0	0	0
0	0	0	0	0
0	0	0	0	0
0	0	0	0	0
0	0.617928275	0	0	0
0	0	0	0	0
0	0	0	0	0
0	0	0	0	0
0	0.000591906	0.001072122	0.001923446	0
0	0	0	0	0
0	0	0	0	0
0	0	0	0	0
0	0	0	0	0
0	0	0	0	0

0	0	0	0	0
0	0	0	0	0
0	0	0	0	0
0	0	0	0	0
0	0	0.107511142	0	0
0	0	0	0	0
0	0	0	0	0
0	0	0	0	0
0	0	0	0	0
0	0	0	0	0
0	0	0	0	0
0	0	0	0	0
0	0	0	0	0
0	0	0	0	0
0	0	0	0	0
0	0	2.962562237	0	0
0	0	0	0	0
0	0	0	0	0
0	0	0	0	0
0	0	0	0	0
0	0	0	0	0
0	0	0	0	0
0.018567409	0	0.001575036	0	0
0	0	0	0	0
0	0	0	0	0
0	0	0	0	0
0	0	0.017968633	0	0
0	0	0	0	0
0	0	0	0	0
0	0	0	0	0
0	0	0.007092051	0	0
0.003574611	0.080355909	0	0.001344015	0
0	0	0	0	0
0	0	0	0	0
0	0	0	0	0
0	0	0	0	0
0	0	0	0	0
0	0	0.002569958	0	0
0	0	0	0	0
0	0	0	0	0
0	0	0	0	0
0	0	0	0	0
0	0	0	0	0
0	0	0	0	0
0	0	0	0	0
0	0	0	0	0
0	0	0	0	0
0	0	0	0	0.272275926

0	0	1.376910644	0	0
0	0	0	0	0
0	0	0	0	0
0	0	0	0	0
0	0	0	0	0
0	0	0	0	0
0	0	0	0	0
0	0	0	0	0
0	0	0	0	0
0	0	0	0	0
0	0	0	0	0
0	0	0.112715547	0	0
0.003018134	0	0	0	0
0	0	0	0	0
0	0	0	0	0
0	0	0	0	0
0	0	0	0	0
0	0	0	0	0
0	0	0	0	0
0	0	0	0	0
0.005266973	0.082456852	0.014041868	0.201649635	0.040009102
0	0	0	0	0
0.000660206	0	0	0	0
0	0	0	0.001791141	0
0	0	0	0	0
0	0	0	0	0
0.004820139	0.003283482	0.001672702	0.009002759	0.012272708
0	0	0	0	0
0	0	0	0	0
0.01734625	0	0	0	0
0	0.001636537	0	0	0.020744279
0	0	0	0	0
0	0	0	0	0
0	0	0.044662899	0	0
0.005385244	0.000917108	0.006229355	0	0.011923078
0	0	0	0	0
0	0	0	0	0
0	0	0	0	0
0	0.037112512	0	0	0.001733497
0	0	0	0	0
0	0.000605212	0	0.000786674	0
0	0	0	0	0
0	0.001429846	0	0.00092928	0
0	0	0	0	0
0	0	0	0	0
0	0	0.18228016	0	0
0	0	0	0	0
0	0	0	0	0.004389966

0	0	0	0	0
0	0	0	0	0
0	0	0	0	0.002588038
0	0	0	0	0
0	0	0	0	0
0	0	0	0	0
0	0	0	0.005668457	0
0	0.003288246	0	0.001373839	0
0.341682066	0	0	0	0
0	0	0	0	0
0	0	0	0	0
0	0	0	0	0
0	0	0	0	0
0	0	0	0	0
0	0.004379375	0	0.052487747	0
0	0	0	0	0
0	0	0	0	0
0	0	0	0	0.010364101
0	0.000830828	0.009932217	0	0.010513333
0	0	0.139858971	0	0
0.023290302	0	0.001331912	0	0.000637325
0	0	0	0	0
0	0	0	0	0
0	0	0	0	0
0	0.00194786	0	0.018989178	0.017726526
0	0	0	0	0
0	0	0	0	0
0	0	0	0	0
0	0	0	0.039641105	0
0	0	0	0	0
0.302868546	0.221322538	0.280095335	0	0.008903869
0	0	0	0	0
0	0	0	0	0
0	0	0.005701795	0	0
0	0	0	0	0
0	0	0	0	0
0	0	0	0	0
0	0	0	0	0
0	0	0	0	0
0.000768802	0	0	0	0
0	0	0	0	0
0	0.00440915	0.003993156	0.001790986	0
0	0	0	0	0
0	0	0	0	0
0	0	0	0	0
0	0	0	0	0
0	0	0	0	0
0	0	0	0	0

0	0	0	0	0
0	0	0	0	0
0	0.019146764	0.001044597	1.701275614	0.010396742
0	0	0	0	0
0	0	1.18646623	0	0
0	0	0	0	0
0	0	0	0	0
0	0	0	0	0
0	0	0	0	0
0	0	0	0	0
0	0	0	0	0
0	0	0	0	0
0	0	0	0	0
0	0.007138526	0	0.006959163	0.001767735
0	0.016623629	0.000952862	0.045540747	0.005288997
0	0	0	0	0
0	0.001678328	0.000868561	0.02127006	0.007896581
0	0	0	0	0
0	0	0	0	0
0	0	0	0	0
0	0	0	0	0
0.001848594	0	0.000588047	0	0.004126945
0	0	0	0	0
0	0.004361826	0	0	0
0	0	0	0	0
0	0	0	0	0
0	0	3.985090937	0	0
0	0	0	0	0
0	0	0	0	0
0	0	0	0	0
0	0	0	0	0
0	0	0	0	0
0	0	0	0	0
0	0	0	0	0
0	0	0	0.003123924	0
0	0.008646	0	0.003792942	0.017235333
0	0	0	0	0
0.086515616	0.00842981	0.002462733	0.520119958	0
0	0	0	0	0
0	0	0	0	0
0	0	0	0	0
0	0	0	0	0
0	0	0	0	0
0	0	0	0	0.001463791
0	0	0	0	0
0	0	0	0	0
0	0	0	0	0
0	0	0	0	0.007993276

2.90779105	0.183917482	0	0	0
0	0	0	0.044069234	0
0	0	0.000821244	0.154923586	0
0	0	0	0	0
0	0.014369155	0.003718129	0.003779967	0.003953642
0	0	0.003031374	0	0
0	0	0	0	0
0	0.04411955	0.01100264	0.013090304	0.062623457
0	0	0	0	0
0.102730265	0.036577698	0	0.060394819	1.107015673
0	0	0	0	0
0.001350989	0.033406707	0	0.096715091	0.001316096
0.002049547	0.014781767	0.007475933	0.015533245	0.154570983
0	0.01049596	0	0	0
0	0	0.00107286	0	0
0	0	0	0.001685637	0
0	0	0	0.001430102	0.002179608
0	0	0	0	0
0	0.183877796	0	0	0.08573179
0	0	0	0	0
0	0.003969822	0	0	0.01499165
0	0	0	0	0
0	0.002971339	0	0.003089793	0
0	0	0	0	0
0	0	0	0	0
0	0	0	0.003269687	0.006201448
0	0.003587939	0	0	0
0	0	0	0	0.604863358
0	0	0	0	0
0	0	0	0	0
0	0.007437893	0	0	0.45687239
0.000852875	0	0	0	0
0	0	0	0	0.00221594
0	0	0	0	0
0	0	0	0	0
0	0	0	0	0
0	0.036499741	0	0	0.004613422
0	0	0	0	0
0	0	0	0	0
0	0	0	0	0
0	0	0	0	0
0	0	0	0	0
0	0	0	0	0
0.027501931	0	0	0	0.045745587
0	0	0	0	0
0	0	0	0	0
0	0	0.002014611	0	0
0	0	0	0	0

0	0	0	0	0
0	0.018645149	0	0.016661947	0
0	0.015162569	0	0	0
0	0	0.042822103	0	0
0	0	0	0	0
0.005611651	0.025842782	0.000991719	0	0
0	0	0	0	0
0	0	0	0	0
0	0	0.549936498	0	0
0	0	0	0	0
0.001627143	0.001524065	0.002070408	0.026248622	0.01673179
0	0	0	0	1.202317056
0	0	0	0	0.037263376
0	0.006959323	0.000652006	0.098725622	0.001871924
0	0	0	0.004970958	0
0	0	0	0	0
0	0	0	0	0
0	0	0	0	0
0	0	0.0023132	0	0
0	0.004460847	0	0	0
0	0	0	0	0
0	0.331552576	0.339095171	0	0
0	0	0.025573862	0	0
0	0	0	0	0
0	0	0	0	0
0	0	0	0	0
0	0.071294312	0	0	0.002931732
0	0	0	0	0
0	0	0	0	0
0.543414972	0.54029645	0.225227611	0.064468445	0.441454627
0	0	0	0.002698145	0
0	0	0	0	0
0	0	0	0	0.015862176
0	0	0	0	0
0	0.002600647	0.001962734	0	0
0	0	0	0	0
0	0	0.163481195	0	0
0	0.000978401	0	0.001430726	0
0	0	0	0	0
0	0	0	0	0
0.045285474	0.147176039	0.001429386	0	0
0.04383849	0.055314075	1.627175204	0	2.953847864
0	0.002688835	0	0.000655319	0
0	0	0	0	0.003919411
0	0	0	0	0
0	0	0	0	0
0.000884286	0	0	0	0.007717124
0	0	0	0	0

0	0	0	0.003237862	0
0	0	0	0	0
0.08808419	0.010443563	0.001891647	0.183600204	0.007844719
0	0	0	0	0
0	0.001883385	0	0	0
0	0.007344454	0	0	0
0	0	0	0	0
0	0	0	0	0
0.019403789	0.023092406	0.001161871	0	0.001853199
0.049512519	0.161187772	0	0	0
0	0	0	0	0
0	0	0	0	0
0	0	0	0	0.016337702
0	0.002542446	0	0.015998003	0
0	0	0	0	0
0.001157049	0	0.009978598	0	0
0.001923702	0	0.002538415	0.035131296	0.023772257
0.002856607	0	0	0	0
0.003311732	0	0	0	0
0	0	0	0	0
0	0	0.001371054	0	0
0	0.002322265	0	0	0.000805097
0	0	0	0	0
0	0	0	0.134870091	0
0	0	0.015420561	0	0
0.071237462	0	0.005254722	0	0
0.005969714	0.007116504	0.001150906	0	0.004625989
0	0	0	0	0
0	0	0	0	0
0	0	0	0	0
0.051590349	0.003479195	0.002800835	0	0
0	0	0.002870795	0.230642742	0.001998089
0.930063735	0.168192755	0.055661659	0.010164332	0
0	0.002359249	0	0.127648358	0.001363199
0	0	0	0.39918087	0
0	0	0	0	0
0	0	0	0.002901871	0.001074967
0.008698659	0.001745916	0.014757794	0	0
0	0.125375555	0.204905958	0	0
0	0	0	0	0.002749347
0	0	0.000669017	0	0
0	0	0	0	0
0.13845113	0	0	0	0
0.017784317	0.070719506	0.002605777	0	0
0	0	0	0	0.00693476
0	0	0.001915143	0	0
0	0	0	0	0
0	0	0	0	0

0	0.053210401	0.005991202	0	0
0.134052086	2.647598422	0.542234719	0.522090371	0.303564016
0	0	0	0	0
0	0	0	0	0
0	0	0	0	0.001123813
0	0	0	0	0
0	0	0	0	0
0	0	0	0.001781433	0
0	0	0	0	0
0.008613363	0.003457592	0.001739653	0	0
0.001887449	0	0.00213478	0	0
0.001874272	0.040377403	0.010334399	1.620723647	0.006339805
0	0	0	0	0
0.003987703	0	0	0	0
0	0	0	0.041966011	0
0	0	0	0	0
0	0.012547202	0.002794278	0.050932996	0.053482898
0.001425378	0.072628467	0	0	0.023605585
0	0	0	0	0
0	0.04820145	0	0	0
0.002264666	0.156544693	0.027855522	0.03846302	0.013972438
0	0	0	0	0
0	0	0	0	0
0	0	0	0	0
0	0.001069204	0	0	0
0	0	0	0	0
0	0	0.056127301	0	0
0.004573311	0.122420673	0.030627215	0.251437126	0
0	0	0	0	0
0	0	0	0	0
0	0	0.001533608	0.001414994	0.005451361
0	0	0	0	0
0	0	0	0	0
0	0	0	0	0.000443982
0	0	0.061385818	0	0
0.550358683	0.277016045	0.288201125	0.094110324	15.3600714
0	0	0	0	0
0	0	0	0	0
0.004157035	0.014184161	0.008312061	0.062360523	0.003133671
0	0	0	0	0
0	0	0	0	0
0	0	0	0	0
0	0.001182544	0	0	0
0.021766829	0	0	0	0.003251378
0	0	0	0	0
0	0.002696311	0.684805684	0	0
0	0	0	0	0
0	0.001581864	0	0	0.1048835

0	0	0	0	0.002045497
0	0	0	0	0
0	0	0	0	0
0	0	0.032609154	0.010166031	0
0	0	0	0	0
0	0	0.004481071	0	0
0	0	0	0	0
0.018228605	0	0.059620066	0	0
0	0.236697356	0.646559967	0	0
0	0	0.063066925	0	0
0	0	0	0	0
0.001207577	0.002073643	0.001707272	0	0
0	0	0	0	0
0	0	0	0	0
0.656026199	0.000622246	0.063820682	0	0
0	0	0	0	0
0.01191521	0	0.002021486	0	0
0	0	0.269337868	0	0
0	0	0	0	0
0	0	0.005793633	0	0
0	0	0	0	0
0	0	0.061025454	0	0
0	0	0.047163257	0	0
0.178537785	0.052296088	0	0	0
3.415167359	0	0	0	0
0	1.158690018	1.22036607	0	0
0	0	0.00399399	0	0
0	6.71036613	2.569041018	1.745124384	0
0	0	0	0	0
0	0	0	0	0
0	0	0.042987767	0	0
0	0.014677062	0	0	0
0.009828256	0.000836877	0	0	0
0	0	0	0	0
0	0	0	0	0
0	0	0.132566747	0	0
0	0	1.495821486	0	0
0.000628225	0	0	0	0
0	0	0	0	0
0	0	0	0	0
0.001737681	0.026945838	0.076486294	0	0.012696004
0	0	0.319345533	0	0
0.158195086	0.739132415	0.038705944	12.31679052	0.457476551
0.007400543	0	0.00499365	0	0
0	0	0	0	0
0	0	2.803929857	0	0
0	0	0	0	0
0.029012391	0.001956563	0.000984425	0.007417675	0

0	0	0	0	0
0	0.213700068	0.017757414	0.00617848	0
0.277004053	0.013129106	0	0.001422136	1.610608483
0	0	0.000612045	0	0
0	0	0	0	0
0.015073356	0	0.001932831	0	0
0	0	0	0	0
0	0	0	0	0
0	0	0	0	0
0	0	0	0	0
0.697725729	0	0.008894678	0	0
0.151747446	2.749584583	0.501592362	2.850748294	0
0	0	0	0	0
0.003536384	0.002649886	0.001399927	0.079436629	0.003062264
0.23706432	0.111719344	0.075332532	0	0.009803549
0	0	0.157140695	0	0
0	0	0	0	0
0	0	0	0	0
0	0	0.061018497	0	0
0.118583349	0	0.009616521	0	0
0.076898779	0.133765029	0.170844888	0.805328514	0
0.000964613	0.022045532	0	1.12478649	13.3169396
0.600430822	1.502525428	0.05750538	1.154637659	7.225617975
0	0	0	0	0
0	0	0.014308911	0	0
0	0.009453037	0.000558337	0	0
0	0.042048445	0	0	0.103998855
0	0	0	0	0
0	0	0	0	0
0	0	0	0	0
0	0	0.00376875	0	0
0	0	0.3523468	0	0
0	0.001681121	0.058997299	0	0.038247673
0.182035821	0.000961489	0.002757452	0	0
0.020878654	0.002219122	0	0	0.005770043
0.032958377	0.008194021	0.00845641	0.52090087	0
0.027120699	0	0.003500904	0	0
0	0	0	0	0
0	0.029880939	0.034533999	0	0
0	0	0	0	0
0	0	0.015956346	0	0
0	0	0	0	0
0	0	0.127760467	0	0
0	0	0.723238952	0	0
0.170757849	0	0.04132961	0.001059252	0.005462045
0	0	0.164894619	0	0
0	0	0	0	0
0	0.010485433	0.005222884	0.720393716	0

0	0	0	0	0
0	0	0	0	0
0	0	0	0	0
0	0	0.00100643	0	0
0	0	0	0	0
0	0	0.127405104	0	0
0	0	0.357550152	0	0
0	0.001193005	0.002160895	0	0
0	0	0	0	0
0	0	0	0	0
0.0314928	0	0.003549507	0	0.031466058
0	0	0	0	0
0	0	0.005725025	0	0
0	0	0	0	0
0	0	0	0	0
0	0	0	0	0
0	0.206958958	0.010003825	0	0
0.004400062	0	0.001555202	0	0
0	0	0.037639146	0	0
0	0.001858375	0.005750391	0	0
0	0	0.004421357	0	0
0	0	0	0	0
0	0	0	0	0
0	0	0	0	0
0	0	0	0	0
0	0	0	0	0
0	0	0	0	0
0	0	0	0	0
0	0	0	0	0
0.063339893	0.003526453	2.330114076	0	0
0	0	0	0	0
0	0	0	0	0
0	0	0	0	0
0	0	0	0	0
0.008055321	0	0	0	0
0	0	0	0	0
0	0	0.093914972	0.008784452	0
0.009880685	0	0	0	0.000908065
0	0	0.047489074	0	0
0	0	0.000935902	0	0
0.665903749	0.001966019	0.00623185	0	0
0	0	0	0	0
0	0	0	0	0
0	0	3.412298731	0	0
0	0.053931325	0.191960205	0	0
0	0	0	0	0
0	0	0	0	0
0	0	0	0	0

0	0	0	0	0
0	0	0.414994978	0	0
0	0	0.314393164	0	0
0	0	0.378163764	0	0
0	0	0.017743123	0	0
0	0	0.609451368	0	0
0	0	0	0	0
0	0	0	0	0
0.007713461	0.003130756	0.002181058	0.001408658	1.396187264
0	0	0	0	0
0	0	0.007641669	0	0
0	0	0	0	0
0	0	0	0	0
0	0	0	0	0
1.010876058	4.331641678	0.260496182	0.439000245	0
0	0.047702376	0	0	0.052605513
0	0	0	0	0
0	0	0	0	0
0	0	0	0	0
0	0	0.098994937	0	0
0	0	0.034355269	0	0
0	0	0	0	0
0	0	0	0	0
0.027710606	0	0	0	0
0	0.038232829	0.083358012	0.003865264	1.248405397
0	0.46659992	1.202700895	0.092380441	0
0	0	0.006487042	0	0
0	0	0.001910555	0	0
0	0	0	0	0
0	0	0	0	0
0	0	0	0	0
0	0	0.227285649	0	0
0	0	0	0	0
0.047798032	0	0	0	0
0.001064271	0.000996851	0.0006771	0.017978368	0.020087674
0.003761553	0	0.000797712	0	0
0	0	0.240050121	0	0
0.015503169	22.87086827	0.326073383	0	0
0	0.536636198	0	0	0
0	0	0	0	0
0	0	0	0	0
0.018555497	0.001398393	0	0	0
0	0.523347156	0.340543564	0	0
0	0	0.646271751	0	0
0	0	0.052610939	0	0
0.005701759	0	0.001058025	0	0
0	0	0	0	0
0.192742332	0.032437322	0.834111992	0.904864307	0

0	0	0	0	0
0	0	0.101327203	0	0
0	0	0	0	0
0	0	0	0	0
0.013909383	0	0	0	0
0	0	0.007919185	0	0
0	0	0	0	0
0	0	0	0	0
0	0	0	0	0
0	0	0	0	0
0	0	0	0	0
10.57789249	0	0.528435504	5.183875784	0
0.004848404	0	0.189621873	0	0
0	0	0	0	0
0.018523504	0.187198008	0.001654012	1.459733066	0.279975754
0	0	0	0	0
0.039505065	0	0	0	0
0	0	0	0	0
0	0	0	0	0
0	0	0.007434805	0	0
0	0.100390446	0	0	0
0	0	0	0	0
0	0.015565262	0.13867638	0.00587999	0
0	0	0	0	0
0	0	0	0	0
0	0	0	0	0
0	0	0	0	0
0	0	0	0	0
0	0	0	0	0
0	0	0	0	0
0	0	0	0	0
0.005060387	0.161746237	0.045609129	0.477378483	0.030040283
0	0	2.139732241	0	0
0.137622459	0	0.012867126	0	0
0.004912215	0	0	0	0
0	0.6371191	0.049688056	0.474145082	0
0	0	0	0	0
0	0	0	0	0
0	0	0	0	0
0	0	0	0	0
0	0	0.009911092	0	0
0	0	0.002341449	0	0
0	0	0	0	0
0.001666248	0.002497109	0.062191561	0	0
0.681495876	0	0.05712578	0	0.022915483
0	0	0	0	0
0.748806689	0	0.137429481	0	0
0	0.000714485	0	0	0

0	0	0	0	0
0	0	0	0	0
0	0.002734501	0	0	0
0	0	0	0	0
0	0	0	0	0
0	0.037303352	0	0	0
0	0	0.418648941	0	0
0	0	0.000501177	0	0
0	0	0	0	0
0	0	0	0	0
0	0	0	0	0
0	0	0	0	0
0	0	0	0	0
0	0	0.390666599	0	0
0	0	0	0	0
0	0	0	0	0
0	0	0.161245072	0	0
0	0	0.058324046	0	0
0.175584525	2.054724355	0.073716341	2.704638949	0
0	0	0	0	0
0	0	0	0	0
0	0	0	0	0
0	0	0	0	0
0	0	0	0	0
0	0	0	0	0
0.035288763	0	0	0.007541496	0.017981049
0	0	0	0	0
0.197506545	0.017552987	0.288014361	0.050832554	0
0	0	0	0	0
0.003419886	2.407394957	1.555817248	0.004371857	0
0	0	0	0	0
0	0	0	0	0
0	0	0.005759816	0	0
0	0	0	0	0
0.014162722	0	0	0	0
0	0	0	0	0
0	0	0	0	0
0.008258472	0	0	0	0
0	0	0	0	0
0	0	0.0171746	0	0
15.21479401	9.156811105	5.196227265	2.982982047	13.9372796
0.585571285	0.378301503	0.386821633	0	0
0	0	0	0	0
0	0	0.301755524	0	0
0	0	0	0	0
0	0	0	0	0
0	0	0	0	0
0.042337756	0	0.005559949	0	0

0	0	0	0	0
0.00273313	0	0.098148115	0	0
0	0	0	0	0
0	0	0	0	0
0	0.294931281	7.335614259	0	0
0	0	0.107458488	0	0
0	0	0	0	0
0	0	0.002622046	0	0
0.004730441	0.511583868	0.002469377	0.044357377	1.983030572
0	0	0	0	0
0	0	0	0	0
0	0	0	0	0
0	0	0.055268165	0	0
0.042661562	0.012843964	0.006646949	0.011129995	0.017316543
0	0.03308426	0	0	0
0	0.284069028	0.269843174	0.021202057	0
0.001130111	0.00141136	0.012941782	0	0
0	0	0	0	0
0	0	0	0	0
0	0	0.050034521	0	0
0	0	0	0	0
0	0	0	0	0
0	0	0	0	0
0	0	0	0	0
0	0	0	0	0
0	0	0	0	0
0	0.002147662	0.050015163	0.027517193	0
0	0	0	0	0
0	0	0	0	0
0	0	0	0	0
0	0	1.869361006	0	0
0	0	0.000785697	0	0
0	0	0	0	0
0	0	0.015776142	0	0
0	0	0.183479894	0	0
0	0	0	0	0
0.001092562	0.001023349	0	0	0
0	0	0.002739905	0	0
0	0	0	0	0
0	0	0	0	0
0	6.749333615	0.337155132	0.176202724	0
0	0	0	0	0
0	0	0.016963034	0	0
0	0	0	0	0
0	0	0	0	0
0	0	0.000831215	0	0
0	0	0	0	0
0	0	0	0	0

0	0	0.017164639	0	0
0	0	0	0	0
0	0	0.004208457	0	0
0	0.003921761	0	0	0
0	0	0.003228414	0	0
0	0	0	0	0
0.006200498	0.017071126	0.067739298	0	0
0	0	0	0	0
0	0	0	0	0
0.031146737	0.033605061	0	0	0
0	0	0.01410961	0	0
0	0	0.003205891	0	0
0	0	0	0	0
0	0	0	0	0
0	0	0	0	0
0	0	0	0	0
0	0	0	0	0.106163187
0	0.001691969	0	0	0
0	0	0	0	0
0	0	0	0	0
0.021824023	0.308633058	0.182549128	0	0
0	0	0	0	0
0	0.002217728	0.008201331	0	0
0.001052701	0	0.000744154	0	0
0	0	0	0	0
0	0	0	0	0
0	0	0	0	0
0	0	0	0	0
0	0	0	0	0
0	0.033585463	0	22.96615148	0.003689879
0	0	0.000840411	0	0
0	0	0	0	0
0	0	0	0	0
36.24431743	0	0.020503558	0	0
0	0	0	0	0
0	0	0	0	0
0	0.005346284	0.083242959	0	0
0	0	0	0	0
0	0	0	0	0
0	0	0	0	0
0	0	0	0	0
0	0	0	0	0
0	0	0	0	0
0	0	0.030565174	0	0
0	0	0.323945149	0	0
0	0	0	0	0
0	0	0.002474137	0	0
0	0	0.005502566	0	0

0.15782889	0.001591478	0	0	0.001226097
0	0	0	0	0
0	0	0	0	0
0	0	0	0	0
0	0	0	0	0
0	0	0	0	0
0.0008708	0	0	0	0
0	0.060279688	0.424406704	0.285152313	0
0.019854452	0.461031463	2.848582592	0.005141182	2.546169884
0.016916568	0.004057846	0	0	0.027466081
0	0	0.003491106	0	0
0	0	0	0	0
0	0	0.114878486	0	0
0	0.011507129	0	0.005809321	0
0	0	0	0	0
0	0	0	0	0
0	0.050334466	0	0	0.030649828
0	0	0	0	0
0	0	0	0	0
0	0	0	0	0
0	0	0	0	0
0	0	0	0	0
0	0	0	0	0
0	0	0	0	0
0	0	0.119452807	0	0
0	0	0	0	0
0	0	0	0	0
0	0	0	0	0
0	0	0	0	0
0	0	0	0	0
0	0.00163497	0.000987142	0	0
0	0	0	0	0
0	0	0	0	0
0	0	0.003692873	0	0
0	0	0.003489853	0	0
0	0	0.001021702	0	0
0	0	0	0	0
0	0	0	0	0
0	0	0	0	0
0	0.008650096	0	0	0
0	0	0	0	0
0	0.279197135	0.920538894	0	0
0	0	0	0	0
0	0	0	0	0
0	0	0	0	0
0	0	0.007547381	0	0
0	0	0	0	0
0	0	0	0	0

0	0	0	0	0
0	0	0.000722699	0.003422925	0
0	0	0	0	0
0	0	0	0	0
0	0.336941189	0.195822347	1.013052235	0
0	0	2.42806797	0	0
0	0	0	0.004905109	0
0.01561009	0.002474358	0.006519003	0	0.089486844
0	0	0	0	0
0	0	0.107241656	0	0
0	0	0	0	0
0	0	0.170756534	0	0
0	0	0	0	0
0	0	0	0	0
0	0	0	0	0
0.041032114	0.001365632	0.001060103	0	0
0	0	0	0	0
0	0	0	0	0
0	0	0	0	0
0	0	0	0	0
0	0	0	0	0
0	0	0.00150744	0	0
0	0	0	0	0
0.011430902	0	0	0	0.004049333
0	0	0.103056533	0.479698678	0
0	0	0	0	0
0.47274963	0.00249265	0	0.004860039	0.183481258
0	0	0	0	0
0	0	0	0	0
0	0	0	0	0
0	0	0	0	0
0	0	0	0	0
0	0	0	0	0
0	0	0	0	0
0	0	0	0	0
0	0	0	0	0
0	0	0	0	0
0	0	0.114435596	0	0
0	0	1.649141523	0	0
0	0	0	0	0
0	0	0	0	0
0	0	0	0	0
0	0	0	0	0
0	0	0.06767023	0	0
0	0	0	0	0
0	0	0	0	0
0	0	0	0	0
0	0	0	0	0

0	0	0.043975578	0	0
0	0	0.002406875	0	0
0	0	0.000751039	0	0
0.067052469	0	0	0	0
0	0	0	0	0
0	0	0.070976186	0	0
0	0	0.000709024	0	0
0	0	0	0	0
0	0	0	0	0
0	0	0	0	0
0	0	0	0	0
0.00555612	0	0	0	0
0	0	0	0	0
0	0	0	0	0
0	0	0	0	0
0	0	0	0	0
0	0	0	0	0
0	0	0	0	0
0	0	0	0	0
0	0	0	0	0
0.00393235	0.071726242	0.000526695	0.000566952	0.583522693
0	0	0	0	0
0.012807752	0	0.033480576	0.001432037	0
0	0	0	0	0
0	0	0	0	0
0	0	0.024640879	0	0
0	0	0	0	0
0	0	0	0	0
0	0	0	0.364055615	0
0	0	0	0	0
0	0	0	0	0
0	0	0.203102874	0	0
0.026681436	0	0.002907032	0.002011646	0.015301284
0	0	0	0	0
0.040069307	0	0.505392077	0	0
0	0.008463319	0	0	0.003189257
0	0	0	0	0
0	0	0.011459957	0	0
0.002272009	0.001489656	0	0	0.164155161
0	0	0	0	0
0	0	0	0	0
0	0	0.128108178	0	0
0	0.019409232	0.063776619	0.011158865	0
0	0	0	0	0
0	0	0	0	0
0	0	0.011275374	0	0
0	0	0	0	0
0	0	0	0	0

0	0	0	0	0
0	0	0	0	0
0	0	0	0	0
0	0	0	0	0
0	0	0	0	0
0	0	0	0	0
0	0	0	0	0
0	0.019860124	0	0.019733446	0
0.006523217	0.002777263	0.041585192	0	0
0	0.010380824	0	0.017461952	0
0	0	0	0	0
0	0	0	0	0
0	0	0	0	0
0	0	0	0	0
0	0	0	0	0
0.001496677	0.003204262	0	0	0.030549018
0	0	0	0	0
0	0	0	0	0
0	0	0	0	0
0	0.00251504	0.645666378	0.000490369	0
0	0.011085691	4.702404596	0	0
0	0	0	0	0
0	0	0	0	0
0	0	0.009180447	0	0
0	0	0	0	0
0	0	0.634272529	0	0
0	0	0	0	0
0	0	0	0	0
0	0	0	0	0
0	0	0.346630964	0	0
0.416897301	0.526563741	0.467006748	0	0
0	0	0	0	0
0	0	0.000560269	0	0
0	0	0	0	0
0	0	0	0	0
0	0	0	0	0
0	0	0	0	0
0	0	0	0	0
0	0	0.003488962	0	0
0	0	0	0	0
0	0	0	0	0
0	0	0	0	0
0	0	0.003188819	0	0
0	0	0	0	0
0	0	0	0	0
0	0	0	0	0
0	0.016605907	0	0.002997901	1.53769814
0	0	0	0	0

0	0	0	0	0.001950675
0	0	0	0	0
0	0	0	0	0
0	0	0.00133121	0	0
0	0.037523876	0.025383647	0.000895862	0
0	0	0.014029785	0	0
0	0	0	0	0
0	0	0	0	0
0	0	0	0	0
0	0	0	0	0
0	0	0.032052298	0	0
0.143703668	0	0.010399131	0	0.008477668
0.021152996	0	0.001163015	0	0
0	0	0	0	0
0	0	0.039917834	0	0
0	0	0.14571213	0	0
0.005587371	0.001962531	0.008886851	0	0
0	0	0	0	0
0.298935584	0.279837321	0.012394702	0	0.011443169
0.010324706	0.082410732	0.015041326	0	0
0	0	0	0	0
0	0	0	0	0
0	0	0	0	0
0	0	0	0	0
0	0	0	0	0
0	0	0	0	0
0.035252987	0.001347745	0	0	0
0	0	0	0	0
0	0	0	0	0
0	0	0	0	0
0.013660176	0	0	0	0
0	0.000825068	0	0	0
0	0	0	0	0
0	0	0	0	0
0	0	0	0	0
0	0	0	0	0
0	0	0	0	0
0.156563985	0.002012523	0.005346426	0.000653985	0
0	0	0.001607664	1.250238392	0
0	0.001064128	0.001734713	0	0
0	0	0.002668728	0	0
0	0	0.001246151	0	0
0	0	0	0	0
0.004035797	0.006565495	0	0	0
0	0	0	0	0
0	0	0	0	0
0	0	0	0	0
0	0	0	0	0

0	0	0	0	0
0	0	0.010010258	0	0
0	0	0	0	0
0	0	0	0	0
0	0	0	0	0
0	0	0	0.005109533	0
0	0	0.007513174	0	0
0	0.001956829	0	0	0
0	0	0	0	0
0	0	0.01789302	0	0
0	0	0	0	0
0	0	0	0	0
0.050968447	0.003990621	0.005354242	0	0
0	0	0	0	0
0	0	0	0	0
0	0	0.158599354	0	0
0	0	0	0	0
0	0	0	0	0
0	0	0	0	0
0	0	0	0	0
0	0	0	0	0
0	0	0	0	0
0.019739323	0.00447625	0.001233803	0	0.006241153
0	0.126771091	0.050749562	0	0
0	0	0	0	0
0	0	0.007219323	0	0
0	0	0	0	0
0	0	0	0	0
0	0	0	0	0
0	0	0	0	0
0	0.051645987	0.038519178	0	0
0	0	0	0	0
0	0	0	0	0
0	0	0	0	0
0	0.001455423	0.089466488	0	0
0	0	0	0	0.001769498
0	0	0	0	0
0	0	0	0	0
0	0.018726894	0	0.00501155	0.031029645
0	0	0	0	0
0	0	0	0	0
0	0	0	0	0
0	0	0	0	0
0	0	0	0	0
0	0	0	0	0
0.205572562	0.258940657	0.696508181	0.183459875	0
0	0.001345799	0	0	0
0	0	0	0	0

0	0	0	0	0
0	0	0	0	0
0	0	0	0	0
0	0	0	0	0
0	0	0	0	0
0	0	0	0	0
0	0	0	0	0
0	0	0	0	0
0	0.000509628	0.002461577	0	0
0	0	0	0	0
0.008428066	0	0.003777019	0	0
0.00379868	0	0	0	0
0	0	0	0	0
0	0	0	0	0
0	0	0	0	0
0	0	0	0	0
0	0.001970502	0	0.001920991	0
0	0	0	0	0
0	0	0	0	0
0	0	0	0	0
0	0	0	0	0
0	0	0	0	0
0	0	0	0	0
0	0	0	0	0
0	0	0	0	0
0	0	0	0	0
0	0	0	0	0
0	0	0	0	0
0	0	0	0	0
0	0	0	0	0
0	0.00067879	0	0	0
0	0	0	0	0
0.056377059	0.038224973	0.006424055	0.033806995	0
0	0.003825638	0.003464696	0.009421932	0.001396101
0	0	0	0	0
0	0	0	0	0
0	0	0	0	0
0	0	0	0	0
0	0	0	0	0
0	0	0	0	0
0	0	0	0	0
0.336434555	0.01556157	0	0	0
0.457100217	0	0.033802833	0.271647328	0
0	0	0	0	0
0	0	0	0	0
0	0.009132059	0.038509361	0	0
0	0	0	0	0
0	0	0	0	0

0	0	0	0	0
0	0.00889626	0.004309512	0	0
0.004128224	0.045675457	0.015320765	0.028036029	0.011310752
0	0	0	0	0
1.713193776	0	0.002271823	0.001128677	0
0.165838817	0	0.003572228	0.001831078	0
0	0	0	0	0
0	0	0	0	0
0	0	0	0	0
0	0	0	0	0
0	0	0	0	0
0	0	0	0	0
0	0	0	0	0
0	0.01198462	0	0	0
0	0	0	0	0.005799216
0	0	0	0	0
0	0	0	0	0
0	0	0	0	0
0	0	0	0	0
0	0	0	0	0
0	0	0	0	0
0	0	0	0	0
0	0	0	0	0
0	0	0	0	0
0	0	0.525982432	0	0
0	0	0	0.047268736	0
0	0	0	0	0
0	0.000971779	0.018129928	0	0
0	0	0	0	0
0	0	0	0	0
0	0	0	0	0
0	0	0	0	0
0	0	0	0	0
0	0	0	0	0
0	0	0	0	0
0	0	0	0	0
0	0	0	0	0
0	0.031711143	0	0	0
0.533894376	0	0.041516458	0	0
0	0	0	0	0
0	0	0	0	0
0	0	0	0	0
0.136108802	0.000804331	0	0	0
0	0	0	0	0
0	0	0	0	0
0	0	0	0	0
0.00262738	0	0.000835782	0	0
0	0	0	0	0

0	0	0	0	0
0.017197962	0.000970391	0.006151855	0	0.011606531
0	0	0.033762464	0	0
0	0	0	0	0
0	0	0	0	0
0	0	0	0	0
0	0	0	0	0
0	0	0	0	0
0	0	0	0	0
0	0.000815275	0	0	0
0	0	0	0	0
0	0	0	0	0
0	0	0.017651151	0	0
0	0	0	0	0
0.001808468	0.007501574	0.001095777	0	0.001048666
0	0	0	0	0
0	0	0	0	0
0	0	0	0	0
0.012003805	0	0.005333735	0	0
0	0	0	0	0
0	0	0	0	0
0.004059562	0.024240258	0.011407074	0	0
0.012282801	0	0	0	0
0	0	0	0	0
0	0	0	0	0
0	0	0.006191421	0	0
0	0	0	0	0
0	0	0	0	0
0	0	0	0	0
0	0	0	0	0
0	0	0	0	0
0	0	0	0	0
0	0	0	0	0
0	0	0	0	0
0	1.78659714	6.800242176	0	0
0	0	0	0	0
0	0.001881358	0	0	0
0	0	0	0	0
0.001520237	0.001186609	0.024502135	0.002313589	0.000617072
0	0	0	0	0
0.282614651	0.062773181	0.382537443	0.034850567	0.147543038
0	0	0	0	0
0	0	0	0	0
0	0	0.576266244	0	0
0	0	0	0	0
0.207046089	0.027042994	0.174529802	0.081465618	0.353056881
0	0	0	0	0
0	0	0	0	0

0	0	0	0	0
0	0	0.002515653	0	0
0	0	0	0	0
0	0	0.244219357	0	0
0.327274831	0	0	0	0
0	0	0	0	0
0	0	0	0	0
0	0	0.005246682	0	0.002282324
0	0.002310602	0.004334673	0	0
0	0.032404868	0	0.028803249	0
0	0	0	0	0
0	0	0	0	0
0.001740488	0.011877386	0.000632752	0	0
0	0	0	0	0
0	0	0	0	0
0	0	0	0	0
0	0	0	0	0
0	0	0	0	0
0	0	0	0	0
1.701195398	0.108826447	0.000563194	1.341812001	0
0	0	0	0	0
0	0	0	0	0
0	0	0	0	0
0	0	0	0	0
0	0	0	0	0
0	0	0.009756278	0	0
0	0	0	0	0
0	0	0	0	0
0	0	0	0	0
0	0	0	0	0
0	0	0	0	0
0	0	0.020331429	0	0
0	0	0	0	0
0	0	0	0	0
0	0	0	0	0
0	0	0.001458082	0	0
0	0	0	0	0
0	0	0	0	0
0	0	0	0	0
0	0	0	0	0
0	0	0	0	0
0	0	0	0	0
0	0.022744438	0.335194496	0	0
0	0	0	0	0
0.150075429	0	0.003173833	0.00360622	0.001012461
0	0	0	0	0
0	0	0	0	0
0	0	0	0	0

0	0	0	0	0
0	0	0	0	0
0	0	0	0	0
0	0	0	0	0
0	0	0	0	0
0.015983138	0.007412639	0.001711226	0	0.00642465
0	0	0	0	0
0	0	0	0	0
0	0	0	0	0
0	0	0	0	0
0	0	0	0	0
0	0	0	0	0
0	0	0	0	0
0	0	0.013127232	0	0
0	0	0	0	0
0	0	0	0	0
0	0	0.163182338	0	0
0	0.019332127	0	0	0
0	0	0.002653131	0	0
0	0	0	0	0
0	0	0	0	0
0	0	0	0	0
0	0	0	0	0
0	0	0	0	0
0	0	0	0	0
0	0	0	0.003546128	0
0	0	0	0	0
0	0	0.020957425	0	0
0	0	0	0	0
0	0	0	0	0
0	0	0	0	0
0	0	0	0	0
0	0	0	0	0
0	0	0	0	0
0	0	0	0	0
0	0	0	0	0
0	0	0	0	0
0	0	0	0	0
0	0	0	0	0
0	0	0	0	0
0	0	0	0	0
0	0	0	0	0
0	0	0	0	0
0	0	0	0	0
0	0	0	0	0
0	0	0	0	0
0	0	0	0	0
0	0	0	0	0

0	0	0	0	0
0	0	0.004863325	0.001903653	0
0	0	0.00396944	0	0
0	0	0	0	0
0	0	0	0	0
0	0	0	0	0
0.002593908	0	0	0	0
0	0	0	0	0
0	0	0	0	0
0	0	0	0	0
0	0	0	0.002077235	0
0	0	0	0	0
0	0	0.04775737	0	0
0	0	0	0	0
0	0	0	0	0
0	0	0	0	0
0	0	0	0	0
0	0	0	0	0
0	0	0	0	0
0	0	0	0	0
0.001385323	0	0.001370998	0	0.023804434
0	0	0	0	0
0	0	0	0	0
0.013197408	5.133012724	0.740174292	0.435748994	0
0	0	0	0	0
0	0	0.010404772	0	0
0	0	0	0	0
0	0	0.05106299	0	0
0	0	0	0	0
0	0	0	0	0
0	0	0.004229135	0	0
0	0	0	0	0
0	0	0	0.001108849	0.000788661
0	0	0	0	0

PHL018-0009_V1	PHL018-0055_V2	PHL018-0003_V1	PHL018-0130_V2	PHL018-0189_V2
EYCF	EYCF	CTRL	REF	EYCF
0.099228151	1.566319314	0.417643892	0.202415032	0.115491228
2.231969728	0.110882609	0.365190601	0.723025603	0.791950631
4.667916553	0	0	0	0
0	0	0	0	0
0	0	0	0	0
0	0	0	0	0
0	0	0	0.110106838	0
0	0	0	0	0
0	0	0	0	0
0	0	0	0	0
0	0	0	0	0
0	0	0	0	0
0	0	0	0	0
1.212360122	0.681418937	0.68544311	0.179650378	0
0	0	0	0	0
0	0	0	0	0
0	0	0	0	0
0	0	0	0	0
0	0.015758634	0	0	0
0	0	0	0	0
0	0	0	0	0
0	0	0	0	0
0	0	0	0	0
0	0	0	0	0
0	0	0.362347697	0.026926962	0
0	0	0	0	0
0	0	0	0	0
0	0	0	0	0
0	0	0	0	0
0	0	0	0	0
0	0	0	0	0
0	0	0	0	0
0	0	0	0	0
0	0	0	0	0
0	0	0	0	0
0	3.594759573	2.199595994	0	0
0	0	0	0	0
0	0	0	0	0
0	0	0	0	0
0	0	0	0	0
0	0	0	0	0
0.089428451	0.00488008	0.014927981	0	0.036993819
0.054053107	1.768793253	0.342112268	0.848321931	13.97282659
0.132263919	0.041283655	0.117925281	0.013770742	0.163386623
0.002072992	0.001311192	0	0.001378919	0.005727098
0	0	0	0	0

0	0	0.002163574	0	0
0	0.001402893	0	0	0.013230124
0.003056497	2.230221215	0	0.056114402	0
0.732806818	0.340913346	0.780670818	0.37321882	0.167534539
0	0	0.005085677	0	0
0	0	0.051152592	0	0
0.008914903	0.00100245	1.210442781	0	0
0	0	0.076951354	0	0
0	0	0	0.032230448	0
0	0	0	0	0
0	0	0	0	0
0.01817537	1.696299444	0.402591486	0	0.059869915
0	0	0	0	0
0.043036754	0	0.003264474	0.00943758	0
0	0	0	0	0
0	0	0	0	0
0	0.00639728	2.467875517	0.000776275	0.01563211
0	0	0	0	0
0	0	0	0	0
0	0	0	0	0
0.008245756	0.086862438	0.031045892	0.000598357	0.0165678
0.001173025	0.055023161	0.003562664	0.010674179	0
0.022366625	0	0	0	0
0	0	0	0	0
0	0	0	0	0
0	0	0	0	0
0	0.00233882	0	0	0
0	0	0	0	0
0.00480204	0	0.002486006	0	0.00293978
0	0	0	0	0
0	0.007263765	0.002018446	0	0
0	0	0	0	0
0	0	0	0	0
0	0	0	0	0
0	0	0	0	0
0	0	0	0	0
0	0	0	0	0
0	0	0.055375885	0.047686815	0
0	0	0	0	0
0	0	0	0	0
0	0	0	0	0
0	0	0	0	0
0	0	0	0	0
0	0	0	0	0
0	0	0	0	0
0	0	0	0	0
0	0.0033023	0.035237332	0	0
0.002133242	0	0.009653053	0	0

0	0	0	0	0
0	0	0	0	0
0	0	0	0	0
0	0	0	0	0
0	0	0	0	0
0	0	0	0	0
0	0	0	0	0
0	0	0.021745254	0	0
0	0	0	0	0
0	0	0	0	0
0	0	0	0	0
0.004738834	0	0	0	0
0	0	0	0	0
0	0	0	0	0
0	0	0	0	0
0	0	0	0	0
0	0	0	0	0
0	0	0	0	0
0.017103659	2.220442193	0.401566956	0.912284922	0
0	0	0.118220166	0	0
0	0	0	0	0
0	0	0	0	0
0	0	0	0	0
0	0	0	0	0
0	0	0	0	0
0	0	0	0	0
0	0	0	0	0
0	0	0	0	0
0	0	0	0	0
0	0	0	0	0
0.003986975	0.095244765	0.027230956	0.105524437	0
0	0.001002542	0.028415689	0	0
0	0	0	0	0
0	0	0	0	0
0	0	0	0	0
0	0	0	0	0
0	0	0	0	0
0	0	0	0	0
0	0	0	0	0
0	0	0	0	0
0	0	0	0	0
0	0	0	0	0
0	0	0.000878183	0.075961591	0
0	0	0	0	0
0	0	0	0	0
0	0	0	0	0
0	0	0	0	0
0	0	0	0	0

0	0	0	0	0
0	0	0.077566414	0	0
0	0	0	0	0
0	0	0	0	0
0.006514675	0	0.156860498	0.012388575	0.003753641
0	0	0	0	0
0	0	0	0	0
0	0	0	0	0
0	0	0	0	0
0	0	0	0	0
0	0	0	0	0
0	0	0	0	0
0	0	0	0	0
0	0	0	0.00209894	0
0	0	0	0	0
0.029593707	4.326308574	0	0	0
0	0	0	0	0
0	0	0	0	0
0	0	0	0	0
0	0	0	0	0
0	0	0	0	0
0	0	0	0	0
0	0	0.005999076	0.001790276	0
0	0	0	0	0
0	0	0.007661683	0	0
0	0	0	0	0
0	0	0.010233994	0.004390245	0
0	0	0	0	0
0	0	0	0	0
0	0.011956345	0	0	0
0	0	0	0.021189527	0
0	0	0	0	0
0	0	0	0	0
0	0	0	0	0
0	0	0	0	0
0	0	0	0	0
0	0	0	0	0
0	0	0	0	0
0	0	0	0	0
0	0	0	0	0
0	0	0	0	0
0	0	0	0	0
0	0	0	0	0
0	0	0	0	0
0	0	0	0	0
0	0	0	0	0
0	0	0	0	0
0	0	0	0	0

0	0	0	0	0
0	0	0	0	0
0	0	0	0	0
0	0	0	0	0
0	0	0	0	0
0	0	0	0	0
0	0	0	0	0
0	0	0	0	0
0	0	0	0	0
0	0	0	0	0
0	0	0	0	0
0	0	0.003403729	0	0
0	0.011806563	0.000671071	0	0
0	0	0	0	0
0	0	0	0	0
0	0	0	0	0
0	0	0	0	0
0.002046108	0	0	0	0
0	0	0	0	0
0	0	0	0	0
0.034537661	0.020695721	0.019134814	0.015960796	0.044513147
0	0	0	0	0
0	0	0	0.00047743	0
0.002369463	0.00287753	0	0.007565409	0
0	0	0	0	0
0	0	0	0	0
0.026994998	0.003816688	0	0.004013833	0.025125764
0	0	0	0	0
0	0	0	0	0
0	0	0	0	0
0.376995826	0	0	0	0
0	0	0	0	0
0	0	0	0	0
0	0	0.030097698	0.001330125	0
0.008870543	0.021320754	0.003061507	0.024782254	0.032750046
0	0	0	0	0
0	0	0	0	0
0	0	0	0	0
0	0	0	0	0
0	0	0	0	0
0	0	0	0	0
0	0	0	0	0
0	0	0	0	0
0	0	0	0	0
0	0	0	0	0
0	0	0.005829777	0.321346946	0
0.002241036	0.000728991	0.00066296	0	0
0	0	0	0	0

0	0	0	0	0
0	0	0	0	0
0.002310541	0	0	0	0
0	0	0	0	0
0	0	0	0	0
0	0	0	0	0
0	0	0	0	0
0	0	0	0	0
0	0	0	0.033806856	0
0	0	0	0	0
0	0	0	0	0
0	0	0	0	0
0	0	0	0	0
0	0	0	0	0
0	0	0	0	0
0	0	0	0	0
0	0	0	0	0
0	0	0	0	0.095968828
0	0.007807295	0.004585494	0	0.057158163
0	0	0.109333033	0.025090488	0
0.042358072	0	0	0	0.006668913
0	0	0	0	0
0	0	0	0	0
0	0	0	0	0
0.013188198	0.004766682	0.002167461	0.003007738	0.010883275
0	0	0	0	0
0	0	0	0	0
0	0	0	0	0
0.051241734	0	0	0	0
0	0	0	0	0
0.074145773	0.365893287	0.031285666	0.076253551	0.179508571
0	0	0	0	0
0	0	0	0	0.374113067
0	0.080342914	0	0	0
0	0	0	0	0
0	0	0	0	0
0	0	0	0	0
0	0	0	0.001297498	0
0	0	0	0	0
0	0	0	0	0
0	0	0	0	0
0.006396993	0	0.010957261	0.000756475	0.008925812
0	0	0	0	0
0	0	0	0	0
0	0	0	0	0
0	0	0	0	0
0	0	0	0	0
0	0	0	0	0

0	0	0	0	0
0	0	0	0	0
0.134767119	0.041096715	0.106783504	0.001187349	0.131804026
0	0	0	0	0
0	0	0	0.772604422	0
0	0	0	0	0
0	0	0	0	0
0	0	0	0	0
0	0	0	0	0
0	0	0	0	0
0	0	0	0	0
0	0	0	0	0
0	0	0	0	0
0.003616693	0.003244235	0	0.01627171	0.003220534
0.004613347	0.001853786	0.00917864	0.014296629	0.007360974
0	0	0.014038494	0	0
0.01607869	0.000704074	0.005122402	0.005183095	0.004892516
0	0	0	0	0
0	0	0	0	0
0	0	0	0	0
0	0	0	0	0
0	0	0	0	0
0	0	0	0	0
0.019471793	0.002955874	0	0	0
0	0	0	0	0
0	0	0	0	0
0	0	0	0.870197232	6.132134193
0	0	0	0	0
0	0	0	0	0
0	0	0	0	0
0	0	0	0	0
0	0	0	0	0
0	0	0	0	0
0	0	0	0	0
0	0	0	0	0
0	0	0.001924149	0	0.003780609
0	0	0	0	0
0.012624873	0.090234826	0.006535869	0	0.042541813
0	0	0	0	0
0	0	0	0	0
0	0	0	0	0
0	0	0	0	0
0	0	0	0	0
0	0	0	0	0
0	0	0	0	0
0	0	0	0	0
0	0	0	0	0
0	0.00161205	0	0	0

0	0	0.05373632	0.332832155	0
0	0	0	0	0
0	0.001109531	0	0	0.003744847
0	0	0	0	0
0.066476411	0	0	0.02324432	0.011081403
0	0	0	0	0
0	0	0	0	0
0.032984539	0.00281652	0.00711501	0	0.065549365
0	0	0	0	0
0	0.154184139	0	0.021710226	0.796348603
0	0.003208005	0	0	0
0.004569369	0	0.011039244	0	0
0.00643691	0.00413406	0.04836215	0.01324041	0.002051929
0	0	0	0.005102225	0.030596903
0	0	0	0	0
0	0	0	0	0
0	0	0	0	0
0	0	0	0	0
0	0	0	0	0
0	0	0	0	0
0	0	0	0	0
0	0	0	0	0
0.003831957	0	0	0	0
0	0	0.119142304	0	0
0	0	0	0	0
0	0	0	0	0.021292499
0	0	0	0	0
0	0.005028804	0	0	0
0	0	0	0	0
0	0.000900756	0	0	0
0	0	0	0	0
0	0	0	0	0
0	0	0	0	0
0	0	0	0	0
0	0	0	0	0
0	0	0	0	0
0	0	0	0	0
0	0	0	0	0
0	0	0	0	0
0	0.004143501	0	0	0
0	0	0	0	0
0	0	0	0	0
0	0	0	0	0
0	0	0	0.013166014	0
0	0	0	0	0
0	0	0.11548364	0	0
0.003585984	0.012248159	0.010148627	0.002289923	0.002431736
0	0.00790565	0	0	0

0	0.044247824	0	0	0
0.002003794	0	0.016044498	0	0
0	0	0	0	0
0	0	0	0	0
0	0	0	0	0
0	0	0	0	0
0.003768689	0	0	0	0
0	0	0	0	0.0019267
0.001373867	0	0	0	0
0	0	0	0	0
0.018344618	0.005967355	0	0.005491139	0.035789387
0	0	0	0	0.001850638
0	0	0	0	0
0.004951735	0.000939609	0	0	0.000932745
0	0.004278222	0	0	0
0	0	0	0	0
0	0	0	0.000517648	0
0	0	0	0	0
0	0.019251344	0	0	0
0	0	0	0.033922051	0
0	0	0.050177584	0	0
0	0	0.09153853	0	0.214077626
0	0	0	0	0
0	0	0	0	0
0	0	0	0	0
0	0	0	0	0
0	0	0	0	0
0	0	0	0	0
0	0	0	0	0
1.047903569	0.460352728	0.307180349	0.852752077	0.683262746
0	0.018167335	0.007246386	0.000837971	0.005062348
0	0	0	0	0
0	0	0	0	0
0	0	0	0	0
0	0	0	0	0
0	0	0	0	0
0	0	0.213567194	0	0
0	0	0	0	0
0	0	0	0	0
0	0	0	0	0
0	0.000579346	0	0.019496672	0
0	0.685608441	0.748117654	0.289072665	0.154322038
0	0	0	0	0
0	0	0	0	0
0	0	0	0	0
0	0	0	0	0
0.010948704	0.019660772	0	0.026431577	0
0	0	0	0	0

0.002306391	0	0	0	0.000744769
0	0	0	0.023526988	0
0	0.006474399	5.608743166	0	0.033488577
0	0	0	0	0
0	0.399382433	0	0	0
0	0	0	0	0
0	0	0	0	0
0.001846426	0	0	0	0
0.003584736	0	0	0	0
0	0	0	0	0
0	0.097904882	0	0	0
0	0	0	0	0
0	0	0	0	0
0.036365307	0.004072395	0	0.028075789	0
0	0	0	0	0
0	0.026520888	0.023475495	0.068425373	0
0.00774574	0.068589824	0.010336767	0.002679215	0.008754267
0.00125477	0	0	0	0.001654503
0	0	0	0.001064395	0
0	0	0	0	0
0	0	0	0	0
0	0.002045845	0	0.002390577	0.006092697
0	0	0	0	0
0	0	0	0	0
0	0	0	0	0
0.161217628	0	0	0	0
0	0	0	0.001831461	0
0	0	0	0.004139383	0
0	0	0	0	0
0	0	0	0	0
0	0.030272249	0	0.005173335	0
0.011520686	0.005077373	0.014365467	0.035597575	0.013720765
1.880341807	0.005371496	0.0153876	0.04236713	0.075184786
0.002028388	0	0.007350644	0	0
0	0.014834679	0.038224423	0	0
0	0	0	0	0
0.002559215	0	0	0	0.002169326
0.036025647	0.027533871	0.001899578	0.033549118	0
1.399634712	0	0.371642724	0	0
0	0	0	0	0
0	0	0	0	0
0	0	0	0	0
0	0	0	0	0.00902092
0.002538833	0.379917551	0	0.036477845	0.004708758
0.009936487	0	0	0	0
0	0	0	0	0
0	0.008862429	0	0	0
0	0	0	0	0

0	0	0	0.001480423	0
0.548507138	0.341805488	0.794643585	3.261814651	3.919641805
0	0	0.011788749	0	0
0	0	0	0	0
0	0	0	0	0
0	0	0	0	0
0.028383872	0	0	0	0
0	0	0	0	0
0	0	0	0	0
0	0	0	0	0
0.001773279	0.006345164	0.007868765	0.001011047	0.001526984
0.112068662	0.054416588	0.011981209	0.087347055	0.328663275
0	0	0.217497146	0.690848796	0
0	0	0	0	0
0	0	0	0	0.002628035
0	0.00261621	0	0	0
0.043769285	0.00402685	0.01666257	0.012281065	0.037375991
0.002410485	0.006011519	0	0.005222549	0
0	0.003627972	0	0	0
0	0.003903566	0	0	0
0.012583697	0.021178762	0.066467321	0.076644338	0.084920639
0	0	0	0	0
0	0	0	0	0
0	0	0	0	0
0	0	1.386133489	0	0
0	0	0	0	0
0	0.012706655	0	0.012500866	0
0.02849374	0.043695684	0.027696031	0.15526445	0.075361134
0	0	0	0	0
0	0	0	0	0
0.007487463	0	0	0	0
0	0.00502609	0	0	0
0	0	0	0	0
0	0	0	0	0
0	0	0.560530424	0	0
0.770599109	0.061626948	0.153868609	0.41399957	0.32664669
0	0	0	0	0
0	0	0	0	0
0.013988351	0.010889599	0.007675005	0.008589061	0
0	0.008396656	0	0	0
0.011703684	0	0	0	0
0	0.000947195	0	0	0
0.01546842	0	0	0	0.023802426
0	0.000798273	0.00043558	0.001846914	0
0	0	0	0	0
4.270087364	0.001154693	0.836481342	0.215457959	0
0	0	0	0	0
0	0	0	0	0

0	0.01178178	0	0.005830751	0
0	0	0	0	0
0	0	0	0	0
0	2.014931348	0.12085364	0.485699076	0
0	0	0	0	0
0	0	0	0.001405088	0
0	0	0	0.001099192	0
0	0	0.005484172	0	0
0	0	0.599017904	0	0
0	0.017901809	0.415929987	0.019188543	0
0	0	0	0	0
0.00437605	0	0.012250329	0	0
0	0	0	0	0
0	0	0.266581245	0.188659862	0
0.006018546	0	0.025618705	0.11465841	0.003023194
0	0	0	0	0
0	0.049855028	0.15263603	0.057234563	0
0	0	0	0	0
0	0	0	0	0
0	0.00675629	0.005844591	0	0
0	0	0	0	0
0	0	0.002804433	0.011891151	0
0	0	0.027196775	0.000536085	0
0.026088312	0	0	0.378383597	0.009901175
0	0	0	0	0
0.030964937	0	0.379711896	0.636645577	0
0	0	0.272563331	0.008214875	0
0	0	1.009154179	1.279214676	0
0	0	0	0	0
0	0	0	0	0
0.001890785	0	0.00570999	0.004150472	0.036506627
0	0	0.038581354	0	0
0	0.22814199	0.000744981	0.001005077	0
0	0	0	0	0
0	0	0.016267772	0	0
0	0	0.243593255	0	0
0.463276304	0.41017343	0.963814791	0.160933719	0
0	0	0	0	0
0	0	0	0	0
0	0	0.000600579	0.007639599	0
0.007696403	0.104619181	0.001448877	0.005398765	0
0.00202559	0	0.179527809	0.168154114	0
0.030496153	1.033546159	0.513127385	0.004689865	0
0	0	0.002243842	0	0
0	0	0	0	0
0	0	0	3.492602908	0
0	0	0	0	0
0.001682174	0.012980744	0.030576794	0.266087312	0

0	0	0	0	0
0	0	1.270812617	0	0
0.443452378	0.058953672	0.000556544	0.041189527	0.01437762
0	0.03109128	0	0.000927581	0
0	0	0.017680189	0	0
0.000635154	0	0.017975375	0	0
0	0	0	0	0
0	0	0	0.027230808	0
0	0	0	0	0
0	0	0	0	0
0.023484759	0.337077828	0.041528479	0.477707084	0.051096932
0.253165432	0.754244505	0.492048915	0.935549324	1.865590606
0	0	0	0	0
0.03246529	0.014914665	0.003734936	0.002955155	0.009655896
0.490582383	0.89648935	0.001858909	0.254015698	0.028576833
0	0	0	0	0
0	0	0	0	0
0	0	0	0	0
0	0	0.097625375	0	0
0	0.017158048	0	0.005552097	0
0.422425176	0.14671612	0.049615214	0.237620563	0.052658577
0.014215398	1.338629169	0	0	4.79952198
0.198033138	3.967042119	0.002595477	2.087310289	8.781152139
0	0	0	0	0
0	0	0	0	0
0	0	2.33059323	0	0
0	0	0	0	0.282622256
0	0	0.00079612	0	0
0	0	0	0	0
0	0	0	0	0
0	0	0.012797066	0.002531323	0
0.201071248	0.079556813	0.118902974	0.108166037	0
0	0	0	0.109458781	0
0	0	0.023822814	0	0
0	0	0	0.0011422	0
0.038091561	0.29826034	0.01645447	0.005884924	0.024441993
0	0	0.000819319	0.004358313	0
0	0	0	0	0
0.020705722	0	0.635019713	0	0
0	0	0	0	0
0	0	0.019716974	0.019000563	0
0	0	0	0	0
0	0	0.037707067	0	0
0	0	0	0	0
0.001121009	0.657928995	0.027082718	0.012079958	0.024917057
0	0	0	0	0
0	0	0.306352782	0	0
0.029749352	1.598831007	0	0	0.01502839

0	0	0	0	0
0	0	0	0	0
0	0	0	0	0
0.002627436	0	0.006594997	0	0
0	0	0	0	0
0	0	0.276816962	0.260668763	0
0	0	6.471885657	0	0
0.156272451	0	0.229544469	0.052983702	0.021197683
0	0	0	0	0
0	0	0	0	0
0	0.023422296	0.002387151	0.005945687	0
0	0	0	0	0
0	0	0.061096329	0	0
0	0	0.796006551	0	0
0	0	0	0	0
0	0	0	0	0
0.465150348	0	0.097511091	0.172774871	0
0.007086696	0.006891721	0.003821633	0.004242556	0
0	0	0.003056242	0.056361717	0
0	0	0.029915444	0.002072462	0
0	0	0.003259411	0	0
0	0	0	0	0
0	0.057751389	0.305076211	0	0
0	0	0	0	0
0	0	0	0	0
0	0	0	0	0
0	0	0	0	0
0	0	0	0	0
0	0	0	0	0
0	0	0.442814491	2.127076151	0.012497262
0	0.055024936	0.018222968	0	0
0	0	0	0	0
0	0	0.043443353	0	0
0	0	0	0	0
0	0	0	0	0
0	0	0	0	0
0	0	0.045892885	0.016879142	0
0	0.03435607	0	0	0.140153226
0	0	0	0	0
0.000666358	0	0	0	0
0	0	0.008458979	0.071678073	0
0	0	0	0	0
0	0	0.001226644	0	0
0	0	0.015095404	0.001396504	0
0	0.315193043	0.095312413	0	0
0	0	0	0	0
0	0	0.011224998	0	0
0	0	0	0	0

0	0	0	0	0
0	0	0.058242913	0	0
0	0	0	0	0
0	0	0.753930568	0.085646697	0
0	0	0	0	0
0	0	0.99569422	0	0
0	0	0	0	0
0	0	0	0	0
0.012733804	0.022159101	0.002786973	0.005454055	0.011466635
0	0	0	0	0
0	0	0.000919741	0	0
0	0	0.001437678	0	0
0	0	0	0	0
0	0	0	0	0
0	0.193799863	1.745356412	1.172087105	0.342557992
0	0.003088689	0	0	0
0	0	0	0	0
0	0	0	0	0
0	0	0	0	0
0	0	0	0	0
0	0	0.099503195	0.036585845	0
0	0	0	0	0
0	0	0	0	0
0	0	0	0	0
0.001460936	0.054334685	0.047900472	0.054808951	0.495346642
0	0.189696041	9.607059853	18.81952647	3.166257458
0.016029756	0	0.051948109	0.088265673	0
0	0	0	0	0
0	0	0.17060064	0	0
0	0	0.431855962	0	0
0	0	0	0	0
0	0	0.103260186	0	0
0	0	0	0	0
0.028868638	0.070176692	0	0.001556995	0.002498499
0.00353534	0.004878862	0.00221847	0.006413588	0.029543638
0.001363122	0.071216971	0.002665922	0.086320304	0.000855892
0	0	0	0	0
0.810902043	0.660659573	0.73433295	1.921357365	6.027750324
0	10.21526119	0	0	0
0	0	0	0	0
0	0	0	0	0
0	0	0	0	0
7.468307407	0.03228266	0.220510476	0.07359999	2.226903138
0	0	0.030916591	0	0
0	0	0	0	0
0.008178784	0	0.001337096	0	0
0.021595908	0	0.002026177	0	0
0	0.035395047	0.067454907	0.85695735	0.34748935

0	0	0	0	0
0	0	0	0	0
0	0	0.005871505	0	0
0	0	0	0	0
0	0.004057696	0	0.007315352	0
0	0	0	0	0
0	0	0.025320107	0.086060933	0
0	0	0	0.003742398	0
0	0	0	0	0
0	0	0	0	0
0	0	0	0	0
0.444352612	3.445787039	0.31288086	0.448987921	0
2.314895478	0	0	0.000738134	0.590149064
0	0	0	0	0
1.953424989	0.000670389	0.072753505	0	0.315664919
0	0	0	0	0
0	0	0	0	0
0	0	0	0	0
0	0.201544667	0	0.000617945	0
0	0.011727859	0	0.003426011	0
0.460827454	0.061295695	0.182272753	0	0
0	0	0	0	0
0.482016886	0.088750494	0.045725019	0.531967981	0.115137763
0	0	0	0	0
0	0	0	0	0
0	0	0	0	0
0	0	0	0	0
0	0	0	0	0
0.000795573	0	0.00109831	0	0
0	0	0	0	0
0	0	0	0	0
0.183379684	0.035086927	0.053532941	0.346121343	0.05382911
0	0	0.663651812	0	0
0.030919499	0.469077117	0.094065577	0.005941616	0.004170432
0	0.065116618	0.00307185	0.080321041	0.002794276
0.003505894	0.159661448	0.092399692	0	0
0.000658392	0	0.00075744	0	0
0	0	0.033780107	0	0
0	0	0.000588184	0	0
0	0	0	0	0
0	0	0.22682636	0.140398624	0
0	0	0.249052893	0	0
0	0	0	0	0
0	0.122979226	0	0.098645393	0
0.003653289	0.020334592	0.017315902	0.072514385	0.005872302
0	0	0	0	0
0	0	0.356026197	0.243393583	0
0	0	0	0	0

0	0	0	0	0
0	0	0.017316801	0	0
0	0.000955957	0	0.002211738	0.000759179
0	0	0	0	0
0	0	0	0	0
0	0	0	0	0
0	0	1.22436271	0	0
0.014511331	0.00668081	0.009524024	0.551817614	0
0	0	0	0	0
0	0	0.000969518	0	0
0	0.421043062	0	0	0
0	0	0	0	0
0	0	0	0	0
0	0.210891381	0.160197025	0	0
0	0	0	0	0
0	0	0	0	0
0	0	0	0	0
0	0	0.062263001	0	0
0	7.361336756	0.011333165	3.690208085	0
0	0	0	0	0
0	0	0.012006456	0	0
0	0	0	0	0
0	0	0	0	0
0	0	0	0	0
0	0.000807902	0	0	0
0	0.038033571	0	0	0.008542018
0	0	0	0	0
0.002441248	0.017013382	0.011795738	0.315327492	0.002006625
0	0	0.105711041	0	0
1.02738612	0.30430103	0.924170331	0.661142111	1.039453258
0	0	0	0	0
0	0	0	0	0
0	0	0.089289798	0.000935277	0
0	0	0	0	0
0	0.021822065	0.001968144	0.110573636	0
0	0	0	0	0
0	0	0.457554289	0	0
0	0.014938848	0	0	0
0	0	0	0	0
0	0	0	0	0
30.20839674	0.824566778	3.837782852	7.610159499	5.244615113
0.542146553	0.140061775	0.004631824	1.148912064	0
0	0	0.146144966	0	0
0	0	0.063504698	0.02108066	0
0	0	0.093797398	0	0
0	0	0	0	0
0	0	0	0	0
0	0.020464292	0.002215555	0.000683217	0

0	0	0	0	0
0	0	0.00167118	0.001581177	0
0	0	0	0	0
0	0	0.017874344	0	0
14.81762643	0.15278487	2.495356164	0.815374312	1.901819946
0	0	0	0	0
0	0	0	0	0
0	0	0.030605266	0	0
0.013625934	0.008840981	0	0.008069655	0
0	0	0	0	0
0	0	0	0	0
0	0	0	0	0
0	0.000973948	0.092853885	0.067430128	0
0.080717059	0.009778517	0.010344676	0.013641523	0.029715553
0	0	1.077842261	0	0
0.946935117	0.120690227	0.067314428	0.933074191	0
0.002957737	0	0	0	0
0	0	0	0	0
0	0	0.090962762	0	0
0	0	0	0	0
0	0	0	0	0
0	0	0	0	0
0	0	0	0	0
0	0	0	0	0
0	0	0	0	0
0	0	0	0	0
0.036995416	0.054208013	0.003823661	0.007895849	0
0	0.022366021	0	0	0
0	0	0	0	0
0	0	0	0	0
0	0	0	0	0
0	0	0	0	0
0	0	0	0	0
0	0	0.014699182	0.021481128	0
0	0	0.621282655	0	0
0	0.00215607	0	0	0.001556596
0	0	0	0.010323832	0
0	0.014251591	0	0.005450084	0
0	0	0	0	0
0	0	0	0	0
0	0.374009432	0.27594802	0	0
0	0	0	0	0
0	0	0.079577877	0	0
0	0	0	0	0
0	0	0	0	0
0	0	0	0	0
0	0	0	0	0
0	0	0	0	0

0	0	0.138009882	0.145351864	0
0	0	0	0	0
0	0	0.021846498	0.01225791	0
0	0.001535534	0	0	0
0.003145474	0.138839956	0.114572898	0.053112645	0
0	0	0	0	0
0.077848994	0.014298384	0.002663317	0.001630512	0
0	0	0	0	0
0	0	0	0.00166238	0
0	0	0	0.003611419	0
0	0	0.005319519	0.00790905	0
0	0	0	0.161128206	0
0	0	0	0	0
0	0	0	0	0
0	0	0	0	0
0	0	0	0	0
0	0.010336125	0	0	0.173550975
0	0	0	0.000696696	0
0	0	0	0	0
0	0	0	0	0
0	0	0.268627242	0.004484532	0.522301444
0	0	0	0	0
0	0.119034065	0.041458255	0.021878411	0
0.000847735	0.058714144	0	0.002706714	0.006706791
0	0	0	0	0
0	0.087488315	0	0	0
0	0	0	0	0
0	0	0	0	0
0	0	0	0	0
2.502707112	0.048618405	0.141276213	0.016556285	0.88413633
0	0	0.030426723	0	0
0	0	0	0	0
0	0	0	0	0
0	0	0	1.946643669	0
0	0	0.60600735	0	0
0	0	0.645298964	0.199405742	0
0	0	0	0.153041051	0
0	0	0	0	0
0	0	1.015929265	0	0
0	0	0.151999454	0	0
0	0	0	0	0
0	0	0	0	0
0	0	0	0	0
0	0	0	0	0
0	0	0.04192408	0	0
0	0	0	0	0
0	0	0	0	0
0	0	0	0	0

0	0	0	0	0.007732227
0	0	0.070763532	0	0
0	0	0	0	0
0	0	0	0	0
0	0	0	0	0
0	0	0	0	0
0	0	0	0	0
2.349031705	0.981403791	0.218520974	2.656498931	8.156785434
0.064074084	2.314829151	0.462788154	1.263486619	17.29803824
0	0.0501234	0.001720124	0	0
0	0	0.005651898	0.008169807	0
0	0	0	0	0
0	0	0.022345134	0	0
0	0.171773136	0	0	0
0	0	0	0.004347511	0
0	0	0	0	0
0	0.084896259	0	0.000797156	0
0	0	0	0	0
0	0	0	0	0
0.00392618	0.046582521	0.049780251	0	0
0	0	0	0	0
0	0	0	0	0
0	0	0.260235107	0	0
0	0	0	0	0
0	0.000818003	0.033847861	0.050755055	0
0	0	0.065337846	0	0
0	0	0	0	0
0	0	0	0	0
0	0	0	0	0
0	0	0	0	0
0	0	0	0	0
0	0	0	0	0
0	0	0	0	0
0	0	0.0033795	0.007381868	0
0	0	0.038876489	0	0
0	0	0	0	0
0	0	0	0	0
0	0	0	0	0
0	0	0.002058945	0	0
0	0	0	0	0.058322727
0	0	0	0	0
0.231352115	0	0.549930959	0.443770099	0
0	0	0	0	0
0	0	0	0	0
0	0	0	0	0
0	0	0	0	0
0	0	0	0	0
0	0	0	0	0

0	0	0	0	0
0	0	0	0	0.028690102
0	0	0	0	0
0	0	0	0	0
0.103884989	6.198804835	0.438392397	0	0
0	0	0.042115733	0	0
0	0	0	0	0
0.03191031	0.023559826	0	0.005788964	0.004808688
0	0	0.003798735	0.000924811	0
0	0	0	0	0
0	0	0	0	0
0	0	0.19716149	0	0
0	0	0	0	0
0	0	0	0	0
0	0	0	0	0.000769352
0.004277134	0	0	0.11909161	0.003601829
0	0	0	0	0
0	0	0	0	0
0	0	0.005250175	0	0
0	0	0	0	0
0	0	0	0	0
0	0	0	0	0
0	0	0	0	0
0	0.008955955	0	0	0
0	0	0.003391994	0.048852904	0
0	0	0	0	0
0	5.011120218	0.775676672	5.174836448	0
0	0	0	0	0
0	0	0	0	0
0	0	0	0	0
0	0	0	0	0
0	0	0	0	0
0	0	0	0	0
0	0	0	0	0
0	0	0	0	0
0	0	0	0	0
0	0	0	0	0
0.001883875	0	0.722843661	0.009586385	0
0	0	0.190580431	0	0
0	0	0	0	0
0	0	0	0	0
0	0	0.592695692	0	0
0	0	0	0.004754167	0
0	0	0.032966638	0	0
0	0	0	0	0
0	0	0.00487846	0	0
0	0	0	0	0
0	0	0.024222954	0	0

0.197795387	0.55184076	0.115514757	0.12444585	0
0	0	0	0	0
0	0	0	0.002902491	0
0	0	0	0	0
0	0	0	0	0
0.232159463	0	0	0	0.008482357
0	0	0	0	0
0	0	0	0	0
0	0	0	0	0
0	0	0	0	0
0	0	0	0	0
0	0	0	0	0
0	0	0	0	0
0	0	0	0	0
0	0	0.000936101	0	0
0	0	0	0	0
0	0	0	0	0
0	0	0	0	0
0	0	0	0.000438398	0
0	0	0	0	0
0	0	0	0	0
0	0	0	0	0
0.013260881	0.124377102	0.001307644	0.004284445	0
0	1.66280114	0.000797882	0.044980159	0
0	0	0	0	0
0	0	0	0	0
0	0	0	0	0
0	0	0	0	0
0	0	0	0	0
0	0	0	0.001200287	0
0	0	0	0	0
0.053017749	0	0.088532201	0	0
0.207866401	0.433821229	0	0.038707521	0.10938961
0	0	0	0	0
2.080467583	0	0	0.045179698	2.30352475
0	0.021611383	0	0.007765291	0
0	0	0	0	0
0	0	0.024355299	0	0
0	0	0	0	0
0	0	0	0	0
0	0	0	0	0
0	0	0.465515159	1.006867979	0
0	0.031421135	0.097465281	0	0
0	0	0	0	0
0	0	0	0	0
0	0.025147271	0.00844411	0.001627459	0
0	0	0	0	0
0	0	0	0	0

0	0	0	0	0
0	0	0	0	0
0	0	0	0	0
0	0	0	0	0
0	0	0	0	0
0	0	0	0	0
0	0	0	0.70977995	0
0	0	0	0.005700824	0
0	0	0.011867022	0.01257942	0
0	0.064554288	0.126473813	0	0
0	0	0	0	0
0	0	0	0	0
0	0	0	0	0
0	0	0	0	0
0	0	0	0	0
0	0	0	0	0
0	0	0	0	0
0	0	0	0	0
0	0	0	0	0
0	0.19596402	2.268719011	0.692822436	0
0	0	0.253412808	0	0
0	0	0	0	0
0	0	0	0	0
0	0	0	0.013181084	0
0	0	0	0	0
0	0	0	0	0
0	0	0	0	0
0	0	0	0	0
0	0	0	0	0
0.376088355	0	0.01699873	0.389727392	0
0.560265243	0.822138114	0.404118473	1.054113382	0.018030824
0	0	0	0	0
0	0	0.002937094	0	0
0	0	0	0	0
0	0	0	0	0
0	0	0	0	0
0	0	0	0	0
0	0	0	0	0
0	0	0.002612878	0	0
0	0	0	0	0
0	0	0.004315782	0	0
0	0	0	0	0
0	0	0	0.001087378	0
0	0	0	0	0
0	0	0	0	0
0	0	0	0	0
0	0.062209641	0	0	0.077293569
0	0	0	0	0

0	0	0	0	0
0	0	0	0	0
0	0	0	0	0
0	0	0.004743257	0	0
0	0.020539061	0.067079564	0.005991233	0.115488137
0	0	0	0	0
0	0	0	0	0
0	0	0	0	0
0	0	0	0	0
0	0	0	0	0
0	0	0.019214672	0.017615709	0
0.00191959	0.181083818	0.027257624	0.179492705	0.002066217
0	0.221235634	0	0.00245505	0
0	0	0	0	0
0	0	0.010674609	0.007339735	0.005038726
0.047981355	0.756534384	1.24780653	0.137197932	0.968725833
0	0.018409907	0.041055181	0.012626633	0
0	0	0	0	0
0.016404062	0.011662885	0	0.008287372	0.03301204
0	0.010083521	0	0.001731325	0
0	0	0	0	0
0	0	0.031340455	0.00721046	0
0	0	0	0	0
0	0	0	0	0
0	0	0	0	0
0	0	0	0	0
0	0	0.000899813	0	0
0	0	0	0	0
0	0	0	0	0
0	0	0	0	0
0	0	0	0	0.000556692
0	0.074906974	0	0	0
0	0	0	0	0
0	0	0	0	0
0	0	0	0	0
0	0	0	0	0
0	0	0	0	0
0.003460573	0.549883783	0.005852334	0.010911077	0.045890866
0.000832472	0	0.347073637	0	0
0	0	0	0	0
0.012955376	0.087188736	0	0	0
0	0	0.01209567	0	0
0	0	0	0	0
0	0	0	0	0
0	0	0	0	0
0	0	0	0	0
0	0	0	0	0
0	0	0	0	0

0	0	0	0	0
0	0.352711796	0.137751244	1.421054177	0
0	0	0.002244972	0	0
0	0	0	0	0.002026053
0.001995178	0	0	0	0.002067045
0.001609355	0.001465828	0	0.019526213	0
0	0.022578835	0.008285517	0	0
0	0	0	0	0
0	0	0.007086926	0	0
0	0	0.013941367	0	0
0	0	0.062575787	0	0
0	0	0	0	0
0.01601122	0.312933199	0.036445171	0.000456446	0
0	0	0.001671566	0	0
0	0	0	0	0
0	0	0	0	0
0	0	0	0	0
0	0	0	0	0
0	0.024335373	0	0	0
0	0	0	0	0
0	0	0	0	0
0	0	0	0	0
0	0	0	0	0.016641911
0.002073231	0.012364093	0.045998873	0.054138673	0.005802145
0	0	0	0	0
0	0	0.016758834	0	0
0	0	0.0048002	0	0
0	0	0.000567787	0	0
0	0	0	0	0
0	0	0	0	0
0.033582213	0.011045398	0.048099714	0.015860211	0.029309504
0	0	0	0	0
0	0	0	0	0
0	0	0	0	0
0	0	0.067695383	0.087834081	0
0	0	0	0	0
0	0	0	0.003559925	0
0	0	0	0	0
0	0	0.004739659	0	0.002497628
0	0	0	0	0
0	0	0	0	0
0	0	0	0	0
0	0	0	0	0
0	0	0	0	0
0	0	7.714060872	0	0
0.244361722	0.449533434	0.055074304	0.088131988	0.054309633
0	0	0	0	0
0	0	0	0	0

0	0	0	0	0
0	0	0	0	0
0	0	0.008407221	0	0
0	0	0.351707337	0.043684072	0
0	0	0	0	0
0	0	0	0	0
0	0	0	0	0
0	0	0	0	0
0	0	0.003175667	0.016787823	0
0	0	0	0	0
0	0.005102892	0	0.000919967	0
0	0.001741402	0	0	0.00086434
0	0	0	0	0
0	0	0	0	0
0	0	0	0	0
0	0	0	0	0
0	0	0.000974514	0	0
0	0	0	0	0
0	0	0	0	0
0	0	0	0.000813356	0
0	0	0	0	0
0	0	0	0	0
0	0	0	0	0
0	0	0	0	0
0	0	0	0	0
0	0	0	0	0
0	0	0	0	0
0	0.016262205	0.017513521	0	0
0	0	0	0	0
0	0	0	0	0
0.002626182	0.034550704	0.007704223	0	0.001648956
0	0	0	0	0
0.101896266	0.024108705	0.015435178	0.013589743	0.018188761
0.004414359	0.001182551	0	0.010363614	0
0	0	0	0	0
0	0	0	0	0
0	0	0	0	0
0	0	0	0	0
0	0	0	0	0
0	0	0	0	0
0	0	0	0	0
0.053725904	1.211937528	0	0.010679554	0.029139892
0.070801624	0.166244332	0.039906107	0.023378616	0
0	0	0	0	0
0	0	0	0	0
0	0.158387242	0.167158374	0	0
0	0	0	0	0
0	0	0	0	0

0	0	0	0	0
0	0	0.051383859	0	0.003819677
0.005609989	0.00733333	0.012262513	0.126628031	0.0220741
0	0	0	0	0
0	0	0.005153172	0.612796084	0
0.00896249	0.015811584	0	0.1104042	0.0014601
0	0	0	0	0
0	0	0	0	0
0	0	0	0	0
0	0	0	0	0
0	0	0	0	0
0	0	0	0	0
0	0	0	0	0
0	0	0	0	0
0	0	0	0	0
0	0	0	0	0
0	0	0	0.188983957	0
0	0	0	0	0
0	0	0	0	0
0	0	0	0	0
0	0	0	0	0
0	0	0	0	0
0	0.003477304	0	0	0
0	0	0	0	0
0	0	0.040069541	0	0
0.006459793	0.000980615	0.003210452	0	0.004088495
0	0	0	0	0
0	0.029107665	0.027163186	0.312914185	0
0	0	0	0	0
0	0	0	0	0
0	0	0	0	0
0	0	0	0	0
0	0	0	0	0
0	0	0	0	0
0	0	0	0	0
0	0	0	0	0
0	0	0	0	0
0	0	0	0	0
0	0	0	0	0
0	0	0	0	0
0	0	0	0	0
0	0	0.008707114	0	0
0	0	0	0	0.007229538
0	0	0	0	0
0	0	0	0	0
0	0	0.001029678	0	0
0	0	0.002464545	0	0
0	0	0	0	0

0	0	0	0	0
0.00775902	0.051293094	0.002245968	0.03156638	0.019990177
0	0	0.178221699	0	0
0	0	0	0	0
0	0	0	0	0
0	0	0	0	0
0	0	0	0	0
0	0	0	0	0
0	0	0	0	0
0	0	0	0	0
0	0	0	0	0
0	0	0	0	0
0	0	0.45474819	0	0
0	0	0	0	0
0.002184526	0	0	0.001992836	0.005878476
0	0	0	0	0
0	0	0	0	0
0	0	0	0	0
0	0	0	0	0
0	0	0	0	0
0	0	0	0	0
0.003677789	0.003256743	0	0.000978561	0
0	0.00554134	0	0.000751944	0
0	9.00592596	0	0	0
0	0	0	0	0
0	0	0	0	0
0	0	0	0	0
0	0	0	0	0
0	0	0	0	0
0	0	0	0	0
0	0	0	0	0
0	0	0	0	0
0	0	0	0	0
0	0	0	0	0
0	0	0	9.46452468	0.001887004
0	0	0	0	0
0	0	0	0	0
0	0	0	0	0
0	0.070852637	0.001901358	0.0329809	0.008763052
0	0	0	0	0
0.41136013	0.039575293	0.029739612	0.101858213	0.128197
0	0	0	0	0
0	0	0	0	0
0	0	0	0	0
0	0	0	0	0
0.411910561	0.213677217	0	0.456701792	0.106294841
0	0	0	0	0
0	0	0	0	0

0	0	0	0	0
0	0	0	0	0
0	0	0	0	0
0	0	0.014894897	0	0
0	0	0	0	0
0	0	0	0	0
0	0	0	0	0
0	0	0	0	0.003326428
0	0	0	0	0
0.008911233	0	0.001272642	0	0.000694587
0	0	0.038475893	0	0
0	0	0	0	0
0	0.008890654	0	0.001198703	0
0	0	0	0	0
0	0	0	0	0
0	0	0	0	0
0	0	0.233738201	0	0
0	0	0	0	0
0	0	0	0	0
0.049990237	1.581038724	0.454673219	0.08151349	0.340606401
0	0	0	0.174436055	0
0	0	0	0	0
0	0	0	0	0
0	0	0	0	0
0	0	0	0	0
0	0	0.000737671	0	0
0	0	0	0	0
0	0	0	0	0
0	0	0	0	0
0	0	0	0.001596792	0
0	0	0	0	0
0	0.021217052	0	0.055384007	0
0	0	0	0	0
0	0	0	0	0
0	0	0	0	0
0.017994562	0	0	0	0
0	0	0	0	0
0	0	0	0	0
0	0	0	0	0
0	0	0	0	0
0	0	0	0	0
0	0	0	0	0
0	0	0.002760942	0.105005964	0
0	0	0	0	0
0.001757586	0.057935162	0.00727919	0.004609662	0
0	0.086640669	0.022458775	0	0
0	0	0	0	0
0	0	0	0	0

0	0	0	0	0
0	0	0	0	0
0	0	0	0	0
0	0	0	0	0
0	0	0.000617254	0	0
0	0	0.016561366	0.00658334	0.0004237
0	0	0	0	0
0	0	0	0	0
0	0	0	0	0
0	0	0	0	0
0	0	0	0	0
0	0	0	0	0
0	0	0	0	0
0	0	0	0	0
0	0	0	0	0
0	0	0	0	0
0	0	0.121160696	0.013611725	0
0	0	0	0	0
0	0	0	0	0
0	0	0	0	0
0	0	0	0	0
0	0	0	0	0
0	0	0	0	0
0	0	0.040082919	0	0
0	0	0	0	0
0	0	0	0	0
0	0	0	0	0
0	0	0	0.001050945	0
0	0	0	0	0
0	0	0	0	0
0	0	0	0	0
0	0	0	0	0
0	0	0	0	0
0	0	0	0	0
0	0	0	0	0
0	0	0	0	0
0	0	0	0	0
0	0	0	0	0
0	0	1.436094168	0	0
0	0	0	0	0
0	0	0.033866702	0	0
0	0	0	0	0
0	0	0	0	0
0	0	0	0	0
0	0	0.008262634	0	0
0	0	0	0	0
0	0	0	0	0
0	0	0	0	0

0	0	0	0	0
0	0	0	0.240046393	0
0	0	0	0	0
0	0	0	0	0
0	0	0	0	0
0	0	0.002781002	0	0
0	0	0	0.001250528	0
0	0	0	0	0
0	0	0	0	0
0	0	0	0	0
0.003022723	0.001042859	0.001517437	0	0
0	0	0	0	0
0.080959578	0	0.004330968	0.213257695	0
0	0	0	0	0
0	0	0	0	0
0	0	0	0	0
0	0	0	0	0
0	0	0	0	0
0	0	0	0	0
0	0	0.002367525	0	0
0.011713731	0	0.03137969	0	0.006934665
0	0	0	0	0
0	0	0	0	0
3.067739684	1.074619138	1.782326708	0.249889305	0.689534961
0	0	0	0	0
0	0	0	0	0
0	0	0	0	0
0	0	0	0	0
0	0	0	0	0
0	0	0	0	0
0	0	0	0	0
0	0	0	0	0
0.00264037	0	0.002025057	0	0
0	0	0	0	0

PHL018-0023_V1	PHL018-0004_V1	PHL018-0057_V2	PHL018-0029_V1	PHL018-0188_V2
EYCF	CTRL	REF	EYCF	CTRL
0.103991558	0.35467679	2.481226671	0.04922078	0.004712992
5.556569401	1.396059576	0.912387912	0.271814669	0.112025801
12.98403287	1.796963099	3.487829338	0.138103721	11.04361099
0	0	0	0	0
0	0	0	0	0
0	0	0	0	0
0	0	0	0	0
0	0	0	0	0
0	0	0	0	0
0	0	0	0	0
0	0	0	0	0
0	0	0	0	0
0	0	0	0	0
2.220159364	0.940620489	1.488540988	1.095409067	15.00895778
0	0	0	0	0
0	0	0	0	0
0	0	0.003288645	0	0
0	0	0	0	0
0	0	0	0	0
0	0	0	0	0
0	0	0	0	0
0	0	0	0	0
0	0	0	0	0
0	0	0	0	0
0	0	0	0	0
0	0	0	0	0
0	0	0	0	0
0	0	0	0	0
0	0	0	0	0
0	0	0	0	0
0	0	0	0	0
0	0	0	0	0
0	0	0	0	0
0	0	0	0	0
0	0	0	0	0
0	0.151314742	2.260857211	0.657478293	0
0	0	0	0	0
0	0	0	0	0
0	0	0	0	0
0	0	0	0	0
0	0	0	0	0
0	0	0	0.037627921	0.461196472
0.02098128	13.07971512	0.771388182	0.128482652	0.0463552
0.004121882	0.007852963	0.002703465	0.1344069	0.022047014
0	0.000918115	0.001670659	0.038953414	0
0	0	0	0	0

0	0.068815712	0	0.013400199	0
0.111920751	0.078586054	0.003475697	0	0.029919341
0	0.006768517	0.870975832	0.003899865	0.000930014
0	1.242307611	0.325455746	0.425238141	0.960944969
0.001650618	0	0.654260424	0.023730262	0
0	0	0	0.016001384	0
0	0.006141885	0.001064396	0.002068138	7.832721439
0	0	0	0	0
0	0	0	0	0
0	0	0	0	0
0	0	0	0	0.068899209
0	0.435512522	0.011830948	0.104113704	0.011571096
0.000984122	0	0	0	0.005848718
0.007972921	0.047128127	0.053169512	0.01300108	0.001036087
0	0	0	0	0
0	0	0	0	0
0	0.004020033	0	0.011280445	4.652644944
0	0	0	0	0
0	0	0	0	0
0	0	0	0	0
0.515295723	0.311415479	0.025977456	0	0.187580032
0.018726142	0.002285911	0.049347816	0.009469985	0.001370579
0	0	0.218428396	0.172877204	0
0	0	0	0	0
0.000713156	0	0.024222535	0	0
0	0	0	0	0
0	0	0.000677276	0	0
0	0	0	0	0
0	0	0	0.027153973	0.008986004
0	0	0.001066233	0	0
0	0.03626265	0	0	0
0	0	0.00183897	0	0
0	0	0	0	0
0	0	0	0	0
0	0	0	0.006957379	0
0	0	0	0	0
0	0	0	0	0
0	0	0	0	0.099510505
0	0	0	0	0
0	0	0	0	0
0	0	0	0	0
0	0	0	0	0
0	0	0	0	0
0	0	0	0	0
0	0	0	1.635141774	0
0	0	0	0	0.009685282
0	0	0	0	0
0	0	0	0	0

0	0	0	0	0
0	0	0.002779826	0	0
0	0	0	0	0
0	0	0	0	0
0	0	0	0	0
0	0	0	0.001605496	0
0	0	0	0	0
0	0	0	0	0
0	0	0	0.00236728	0
0	0	0	0	0
0	0	0	0	0
0	0	0	0.013741826	0.048255812
0	0	0	0	0
0	0	0	0	0
0	0	0	0	0
0	0	0	0	0
0	0	0	0	0
0	0	0	0	0
0	0	2.383521643	0.489183313	0
0	0	0	0	0
0	0	0	0	0
0	0	0	0	0
0	0	0	0	0
0	0	0	0	0
0	0	0	0	0
0	0	0	0	0
0	0	0	0	0
0	0	0.001968144	0	0
0	0	0	0	0
0	0	0	0	0
0	0.00594798	0.018771686	0.03694832	0
0.000757399	0.006824944	0.002128987	0.006894428	0.00048228
0	0	0	0	0
0	0	0	0	0
0	0	0	0	0
0	0	0	0	0
0	0	0	0	0
0	0	0	0	0
0	0	0	0	0
0	0	0	0	0
0	0	0	0	0
0	0	0	0	0
0	0.001126937	0.028503951	0	0
0	0	0	0	0
0	0	0	0	0
0	0	0	0	0
0	0	0	0	0.009155899
0	0	0	0	0

0	0	0	0	0
0	0	0	0	0
0	0	0	0	0
0	0	0	0	0
0	0	0.018376006	0.027070362	0
0	0	0	0	0
0	0	0	0	0
0	0	0	0	0
0	0	0	0	0
0	0	0	0	0
0	0	0	0	0
0	0	0	0	0
0	0	0	0	0
0	0	0	0	0
0	0	0	0	0
0	0	0	3.919027628	0
0	0	0	0	0
0	0	0	0	0
0	0	0	0	0
0	0	0	0	0
0	0	0	0	0
0	0	0	0	0
0	0	0.00090377	0.003512079	0
0	0	0	0	0
0	0	0	0	0
0	0	0	0	0
0	0	0	0.001997119	0
0	0	0	0	0
0	0	0	0	0
0	0	0	0	0
0	0	0	0	0
0	0	14.7467453	0.003005102	0
0	0	0	0	0
0	0	0	0	0
0	0	0	0	0
0	0	0	0	0
0	0	0	0	0
0	0	0	0	0
0	0	0	0	0
0	0	0	0	0
0	0	0	0	0
0	0	0	0	0
0	0	0	0	0
0	0	0	0	0
0	0	0	0	0
0	0	0	0	0
0	0	0	0	0
0	0	0	0	0

0	0	0	1.601645575	0
0	0	0	0	0
0	0	0	0	0
0	0	0	0	0
0	0	0	0	0
0	0	0	0	0
0	0	0	0	0
0	0	0	0	0
0	0	0	0	0
0	0	0	0	0
0	0	0	0	0
0	0	0	0.010981816	0
0	0.001937606	0.000783509	0.001353218	0
0	0	0	0	0
0	0	0	0	0
0	0	0	0	0
0	0	0	0	0
0	0	0	0	0
0	0	0	0	0
0	0	0	0	0
0.072859887	0.012478735	0.019044652	0.078119688	0.031194937
0	0	0	0	0
0	0.001236213	0	0	0
0.003260867	0	0	0	0
0	0	0	0	0
0	0	0	0	0
0.00855922	0.001406578	0.008318367	0.00663086	0.001352876
0	0	0	0	0
0	0	0	0	0
0	0	0	0	0
0.125984471	0	0.157760607	0	0.002889831
0	0	0	0	0
0	0	0	0	0
0	0	0	0.012950232	0
0.063276521	0.080931388	0.169667756	0.003086771	0.028934148
0	0	0	0	0
0	0	0	0	0
0	0	0	0	0
0	0	0	0	0
0	0	0	0	0
0	0	0	0	0
0	0	0	0.006371028	0
0	0	0	0.002352792	0
0	0	0	0	0
0	0	0	0	0
0	0.01870279	0.058309468	0.052117246	0
0	0.003402997	0	0	0
0	0.001306528	0	0.002346364	0.0006155

0	0	0	0	0
0	0	0	0	0
0.002649816	0	0	0	0.042182342
0	0	0	0.001677676	0
0	0	0	0	0
0	0	0	0	0
0	0	0	0	0
0	0	0	0.001686471	0.002654378
0.007142912	0	0	0	0
0	0	0	0.008101382	0
0	0	0	0	0.001332676
0	0	0	0.00885982	0
0	0	0	0	0
0	0	0	0	0
0	0	0	0	0.067930781
0	0	0	0	0
0	0	0	0	0
0	0.074118544	0.008976038	0	0
0	0.023347697	0.003626762	0	0
0	0	0	0	0
0.048722845	0.002800019	0.001019018	0	0.112880046
0	0	0	0	0
0	0	0	0	0
0	0	0	0	0
0.004753485	0	0	0.001748278	0.005044705
0	0	0	0	0
0	0	0	0	0
0	0	0	0	0
0	0	0	0.008967686	0.162152199
0	0	0	0	0
0.749624725	0.467153652	0.204179702	0.058234779	0.037511542
0	0	0	0	0
0	0	0	0	0
0	0	0	0	0
0	0	0	0	0
0	0	0	0	0
0	0	0	0	0
0	0	0	0	0
0	0	0	0	0
0	0	0	0	0
0	0	0	0	0
0	0.030010807	0.010310892	0	0
0	0	0	0	0
0	0	0	0	0
0	0	0	0	0
0	0	0	0	0
0	0	0	0	0
0	0	0	0	0

0	0	0	0	0
0	0	0	0	0
0	0.006588026	0.000959039	0.156527897	0.023463156
0	0	0	0	0
0	0	0.066166275	0	0
0	0	0	0	0
0	0	0	0	0
0	0	0	0	0
0	0	0	0	0
0.002621166	0	0	0	0.16432202
0	0	0	0	0
0	0	0	0	0
0	0	0	0	0
0.001809932	0	0.001324889	0.006635921	0.004321837
0	0	0.039585497	0.002644109	0.00277442
0	0	0	0.023657971	0
0.004468069	0	0.002242747	0.002367142	0.008806206
0	0	0	0	0
0	0	0	0	0
0	0	0	0	0
0	0	0	0	0
0	0.00098898	0	0.001748335	0
0	0	0	0	0
0	0	0	0	0
0	0	0	0	0
0	0	0	0	0
2.496452232	0.18874481	0	0	0
0	0	0	0	0
0	0	0	0	0
0	0	0	0	0
0	0	0	0	0
0	0	0	0	0
0	0	0	0.003216254	0
0	0	0	0	0
0	0	0	0	0
0.00230175	0	0	0.017072242	0.004071277
0	0	0	0	0
0	0	0	0.204772054	0
0	0	0	0	0
0	0	0	0	0
0	0	0	0	0
0	0	0	0	0
0	0	0	0	0
0	0	0	0	0
0	0	0	0.004301866	0
0	0	0	0	0
0	0	0	0	0
0	0	0	0	0

0	0	0.002031145	0.166826878	0
0	0	0	0	0
0	0	0.006126088	0.001627773	0
0	0	0	0	0
0.004452819	0.00182384	0	0.001228272	0.003222013
0	0	0	0	0
0	0	0	0	0
0.085396516	0.032504237	0.010633121	0.021234222	0.243582384
0	0	0	0	0
0.199434109	0.006775851	0.05068902	0.049392671	0.211652369
0	0	0	0	0
0	0	0	0.034299622	0.074125134
0.036796886	0	0.012969039	0.006547446	0.071413137
0	0.002997507	0.00545445	0	0.050247693
0	0	0	0	0
0	0	0	0.001293263	0.001899799
0	0	0	0.000752373	0
0.001553065	0	0	0	0
0.071818574	0.003896415	0	0	0.10191671
0.274821932	0	0	0	0
0	0	0	0	0
0	0	0	0	0
0	0	0	0	0
0	0	0	0.029923987	0
0	0	0	0	0
0	0	0.002788731	0.018262514	0
0	5.829675503	1.505312597	0	0
0	0.131892017	0	0	0
0	0	0.036166599	0	0
0	0.004730415	0	0	0.001516604
0.064300872	0	0	0	0
0	0	0	0	0
0	0	0	0	0
0	0.002672398	0	0	0
0	0	0	0	0
0	0.032894937	0.029928839	0	0
0.192540782	0.074993744	0.002897869	0	0
0	0	0	0	0
0	0	0	0	0
2.96003586	0	0	0	0
0	0.448037472	0	0	0
0	0	0.016648692	0	0
0	0	0	0	0
0	0	0	0	0
0	0	0	0	0
0	0	0	0	0
0	0.001270568	0.000867002	0	0
0	0	0	0	0

0	0	0.00327782	0	0
0	0.001064961	0	0	0
1.119035233	0	0	0	0
0	0	0	0	0
0	0	0	0	0
0.095618964	0.037026866	0.024128033	0	0
0	0	0	0	0
0	0	0	0	0
0	0	0	0	0
0	0	0	0	0
0.001127052	0.002031179	0.031152507	0.011399213	0.00215298
0.027675173	0	0	0	0
0	0.016839018	0	0	0
0.001064782	0	0.002244762	0.082278241	0.004068055
0.044387558	0	0	0	0
0	0	0	0	0
0	0	0	0	0
0	0	0	0	0
0	0.043474657	0	0	0
0	0.065752704	0	0	0
0	0	0	0	0
0	0	0	0.208452746	0
0	0	0.025562061	0	0
0	0	0	0	0
0	0	0	0	0
0	0	0	0	0
0.002770813	0.001486183	0.038672224	0	0
0	0	0	0	0
0	0	0	0	0
0.127356975	0.206538964	0.599209437	0.172695443	10.97739917
0.01199134	0	0.00575315	0	0
0	0	0	0.002051531	0
0	0	0	0	0
0	0	0	0	0
0	0.006436822	0	0	0
0	0	0	0	0
0	0	0	0	0
0	0	0	0.009440126	0.001382144
1.422366716	0	0	0	0
0.09020952	0	0	0	0
0.019258065	0.014874425	0.019889726	0	0.028241466
0	1.769453837	1.228634355	0.039138469	0.19223349
0	0	0	0.005027771	0.000844089
0	0	0	0	0
0.000592475	0	0	0	0
0	0	0	0	0
0.009555104	0.002365421	0.026471242	0	0.006435314
0	0	0.002618735	0	0

0	0	0	0.012611939	0
0.015645619	0.026294469	0	0	0
0.001853533	0	0.002532703	0.017497183	9.551201983
0	0	0	0	0
0	0	0.072426867	0.65726182	0
0.028945376	0	0.000898048	0	0
0	0	0	0	0
0	0	0	0.001285039	0
0.194297436	0	0	0	0.056383086
0	0.006586382	0	0	0
0	0	0	0	0
0	0	0	0	0
0	0	0	0	0
0.00348682	0	0.002642472	0	0.000870692
0	0	0	0	0
0.042636514	0.266999615	0.008072455	0.087543679	0
0.003553243	0.00434535	0.010820202	0.122549137	0.001319826
0	0	0.006573062	0	0.004352457
0.000550535	0	0	0	0.000779017
0	0	0	0	0
0	0	0	0.001585231	0
0	0.001061133	0.001930903	0.000833728	0.001312225
0	0.354548272	0	0	0
0	0	0	0	0.012904166
0	0	0	0	0
0	0.008435712	0	0	0.002483769
0.002255435	0.001451699	0.003434083	0	0.001436168
0	0.009921935	0.000501516	0	0
0	0	0	0	0
0	0	0	0	0
0.09674022	0.004416053	0.008236618	0.003469676	0
0.003068677	0	0	0.009828437	0.003799458
0.044070407	0.007835826	0.010266162	0.032999265	0.065116751
0	0	0	0.010234655	0.029995282
0	0	0	0	0
0	0	0	0	0
0.005062883	0	0	0.001335833	0
0.013427561	0.011301836	0.012298991	0.004526962	0.002740422
0	0	0.173894012	0.020695039	0.000542874
0	0	0	0	0
0	0	0	0	0
0	0	0	0	0
0	0.025187112	0.001206106	0	0
0	0.063833209	0.048476566	0.002990182	0.027524853
0	0.026743371	0	0	0
0	0	0	0	0
0	0	0	0	0
0	0	0	0	0

0	0	0	0	0
0.034247772	0.077604856	0.330508184	0.900847027	0.0460548
0	0	0	0	0
0	0.000731524	0	0	0
0	0	0.004312473	0	0
0	0	0	0	0
0.114147946	0	0.141571121	0	0
0	0	0	0	0.001720943
0	0.001105694	0	0.007167104	0.019598414
0	0.003072043	0	0	0.016281303
0.002789023	0.01480991	0	0	0.000739973
0.074518288	0.001002717	0.010947642	0.190392552	0.053732776
0	0	0	1.051100515	0
0	0	0	0	0
0.015400268	0	0	0.028522504	0
0	0	0	0	0
0.001460255	0.005404343	0.014109765	0.004615404	0.009298303
0.015401854	0.06314025	0.03496772	0	0.090277141
0	0	0	0	0
0.032157574	0.033077278	0.001982288	0	0
0.004517674	0.002423148	0.014991659	0.071013918	0.001398381
0	0	0	0	0.00447444
0	0	0	0	0
0	0	0	0	0
0	0	0	0	0.021481511
0	0	0	0	0
0	0	0	0.010523539	0.04653487
1.583734562	0.136756329	0.036554288	0.042089224	0.763519245
0	0	0	0	0
0	0	0.000847943	0	0
0.008801578	0	0	0.001736991	0.000911298
0	0	0	0	0
0	0	0	0	0
0	0	0	0	0
0	0.535495776	0	0.007229764	0
0.379911845	0.319063674	0.214547946	0.23424863	0.582860365
0	0	0.001836704	0	0
0	0	0	0	0
0.005676531	0	0	0.030454113	0.021739869
0	0	0	0	0
0	0.020335223	0	0	0
0	0	0	0	0
0.080203742	0.025473645	0.002809299	0.010007273	0.041524561
0.02113185	0	0.008136976	0	0
0.048489039	0.012483888	0.016925991	0	0
0	0	0	0.437119788	0
0	0	0	0	0
0.014037533	0	0	0	0

0	0	0	0	0
0	0	0	0	0
0	0	0	0	0
0	0	3.151086826	0.184718954	0
0	0	0	0	0
0	0	0	0	0
0	0	0	0	0
0	0	0	0	0
0	1.973073744	0	1.6215601	0.033016994
0.245780479	0	0.077494308	0.506486094	0
0	0	0	0	0
0	0.004306945	0.003526734	0.007783086	0
0	0	0	0	0
0	0	0	0	0
0.032440792	0.378511866	0	0.387870557	0
0	0	0	0	0
0	0	0.151636766	0.060465489	0
0	0	0	2.339602244	0
0	0	0	0	0
0	0	0	0.026895442	0
0	0	0	0	0
0	0	0	0.024662737	0
0	0	0	0	0
0	0.399694931	0.261488905	0.176453496	0.03711461
0	0.006250222	0	0.005386012	0
0	0	0	0.472311936	0
0	0	0.002400921	0.003958205	0
0	0	0	5.467225644	0
0	0	0	0	0
0	0	0	0	0
0	0.042498913	0	0	0
0	0	0	0.318636835	0
0.001361522	0.004780019	0.170046291	0.206309972	0
0	0	0	0	0
0	0	0	0	0
0	0	0	0.31631376	0
0	0	2.385138352	1.233128013	0
0	0	0	0	0
0	0	0	0.015323714	0
0	0	0	0.002179927	0
0.161284695	0	0.045486192	0.168645059	0
0	0	0	0.133219453	0
0.078162389	0.022304403	0.67486301	2.212174235	0.174483095
0.004613434	0.010257974	0	0.00381773	0
0	0	0	0	0
0	0	0	0	0
0	0	0	0	0
0.000578754	0.001986732	0.022142987	0.020877946	0

0	0	0	0	0
0	0	0	0.127632218	0
0.127974185	0.094749222	0.012562654	0.008230002	0.512051983
0	0	0.025286203	0.01536623	0
0	0	0.028780401	0.11261288	0
0	0	0	0.046414378	0
0	0.028332401	0	0	0
0	0	0	0	0
0	0	0	0	0
0	0	0	0	0
0.00581031	0.393845184	1.06117496	0.122675199	0
3.894571339	1.59576764	1.339652918	0.717463119	5.100615439
0	0	0	0	0
0	0.010594812	0.02455775	0.208900395	0.001247793
0.777216396	0.20992069	0.199312133	0.062787336	0.087186722
0	0	0	0.695154969	0
0	0	0	0	0
0	0	0	0	0
0	0	0	0.083970342	0
0	0.17655639	0.041867094	0.366389417	0
0.16177184	0.016064226	0.007129626	0.414973656	0.04086146
5.508513865	0.993102511	0.006385552	0	4.002100912
21.49800808	0.270524399	1.508102782	0.703944679	5.829468509
0	0	0	0	0
0	0	0	0.018404749	0
0	0	0.005126059	1.494186526	0
3.68716762	0	0	0	0
0	0	0	0	0
0	0	0	0	0
0	0	0	0	0
0	0	0.001572759	0.034973023	0
0	0	0.103824345	0.11000281	0
0.026107112	0.041049059	0.197877359	0.001131652	0.17890518
0	0	0	0.06227785	0
0.018461805	0.030103209	0.00648683	0	0
0.025027186	0	0.155671807	0.130498591	0.024586379
0.001143456	0.022079381	0	0.014043365	0
0	0	0	0	0
0	0	0	0.111880373	0.007770278
0	0	0	0	0
0	0	0	0.150603641	0
0	0	0	0	0
0	0	0	0.140851678	0
0	0	0	0	0
0	0.031526981	0.265385597	0.043884976	0
0	0	0	0	0
0	0	0	0	0
0.07676478	0.003892851	0.402952043	0.15881182	0.060977374

0	0	0	0	0
0	0	0	0	0
0	0	0	0	0
0	0.043373347	0	0.016789785	0
0	0	0	0	0
0	0	0	0.2760181	0
0	0	0	0	0
0.010133066	0.059574964	0	0.000764834	0.004654658
0	0	0	0.006988511	0
0	0	0	0	0
0.009701762	0.009661285	0.001500755	0.011849109	0.000971334
0	0	0	0	0
0	0	0	0.258722105	0.000680383
0	0	0	0.17631378	0
0	0	0	0	0
0	0	0	0	0
0	0	0.4542799	0.013218926	0
0	0	0.002855645	0.000770634	0
0	0	0	0.022705512	0
0	0.006191818	0.160152627	0	0
0.000406151	0	0	0	0
0	0	0	0	0
0	0	0.003703898	0.114970041	0
0	0	0	0	0
0	0	0	0	0
0	0	0	0	0
0	0	0	0	0
0	0	0	0	0
0	0	0	0	0
0	0.573222156	0	0.824607835	0
0.007497044	0	0.016548167	0	0
0	0	0	0	0
0	0	0.003170141	0	0
0	0	0	0	0
0	0	0	0	0
0	0	0	0	0
0	0	0	0.020285256	0
0.010382106	0.006981601	0.002722319	0	0.006084657
0	0	0	0	0
0	0	0	0	0
0	0	0	0.007352399	0.00154295
0	0	0	0	0
0	0	0	0	0
0	0	0.014452197	0.006544588	0
0	0	0	0.068514985	0
0	0	0	0	0
0	0	0	0.006701227	0
0	0	0	0	0

0	0	0	0	0
0	0	0	0	0
0	0	0	0.644553091	0
0	0	1.003370819	0.273598623	0
0	0	0	0	0
0	0	0	0	0
0	0	0	0	0
0	0	0	0	0
0.134210712	0.011004336	0.034792005	0.004971488	0.031072185
0	0	0	0.012532749	0
0	0	0	0.017928397	0
0	0	0	0	0
0	0	0	0	0
0	0	0	0	0
0	0	0.894152471	2.005805561	0
0	0.086085678	0.548649866	0	0.105931443
0	0	0	0	0
0	0	0	0	0
0	0	0	0	0
0	0	0	0	0
0	0.008204033	0	0.038509988	0
0	0	0	0	0
0	0	0	0	0
0	0	0	0	0
0	13.73987898	0.019721333	0.055921396	0.005601023
2.053969982	6.700243178	5.467143867	10.70504483	0
0	0	0	0.790946245	0
0	0	0	0	0
0	0	0.545827776	0.191342735	0
0	0	0	0	0
0	0	0	0	0
0	0	0	0.055657518	0
0	0	0	0	0
0.003892385	0.009157354	0	0	0
0.002874982	0.000854062	0.009583643	0.419395746	0
0.001406952	0.072043705	0.014830604	0	0
0	0	0	0	0
0.986577579	0	1.51998656	0.795025039	0
0	0.90987905	0	0.08666526	0
0	0	0	0	0
0	0	0	0.02686105	0
0	0	0	0.00125511	0
0	0.012374875	1.158931247	0.117322906	0
0	0	0	0	0
0	0	0	0	0
0	0	0	0.003445222	0
0	0	0	0	0
0.040110286	0	0	4.594239926	0

0	0	0	0.008897594	0
0	0	0	0.692558226	0
0	0	0	0	0
0	0	0	0	0
0	0.008794362	0.025235136	0.002657576	0
0	0.003040096	0	0.001194296	0
0	0	0	0	0
0	0	0	0	0
0	0	0	0	0
0	0	0	0.01209237	0
0	0	0	0	0
0	0	0.536031368	2.178327709	0
0.068084729	0	0.203453752	0.06950574	0
0	0	0	0	0
0.002633616	0	0	0.203259895	0.777429763
0	0	0	0	0
0	0	0	0	0
0	0	0.559200233	0	0
0.000532697	0	0	0.001975526	0
0	0	0.010838351	0.022502856	0
0	0	0	0.150173667	0
0	0	0	0	0
0.02641676	0.047563981	0.031344182	1.003599181	0
0	0	0	0	0
0	0	0	0	0
0	0	0	0	0
0	0	0	0	0
0	0	0	0	0
0	0	0	0	0
0	0	0	0	0
0	0	0	0	0
0.054153987	0.001692036	0.052957659	0.021802588	0.025945967
0	0	0	0	0
0	0.112088355	0.119516757	0.015275642	0
0.001190868	0.069860647	0.011158113	0	0
0.003070347	0.01242191	0.597713256	0.108689214	0.011404576
0	0	0	0.001527382	0
0	0	0	0	0
0	0	0	0	0
0	0	0	0	0
0	0	0	0.146161575	0
0	0	0	0	0
0	0	0	0	0
0	0.237654251	0.118088507	0	0
0.027652219	0.363730659	0.198834893	0.059156496	0
0	0	0	0	0
0	0	0	0.395331205	0
0	0	0.005198166	0.001282555	0

0	0	0	0	0
0	0	0	0	0
0	0	0.029029853	0	0
0	0	0	0	0
0.001230466	0	0	0	0
0	0	0.006636472	0	0
0	0.011789338	0	0.343689919	0
0	0	0.003450963	0.010264867	0
0	0	0	0	0
0	0	0.012451557	0.009286422	0
0	0	0	0	0
0	0.012713352	0	0	0
0	0	0	0	0
0	0	0	0.317259367	0
0	0	0	0	0
0	0	0	0	0
0	0	0	0	0
0	0	0	0	0
0	3.241507139	1.137303822	5.547000994	0
0	0	0	0	0
0	0	0	0	0
0	0	0	0	0
0	0	0	0	0
0	0	0	0	0
0	0	0	0	0
0.006708826	0.084755295	0.03819104	0	0.026989161
0	0	0	0	0
0.13316467	0.003538517	0.004113746	0.446067622	0
0	0	0	0.139931551	0
0	0.251753712	0.937354333	0.179833019	0
0	0	0	0	0
0	0	0	0	0
0	0	0	0	0
0	0	0	0	0
0.002452477	0.003367519	0.001531937	0.004299501	0
0	0	0	0	0
0	0	0	0	0
0	0.012896372	0.009126072	0.000750565	0
0	0	0	0	0
0	0	0	0	0
5.218900528	7.806098253	5.032909681	2.365498535	7.034282304
0.078630283	1.469175312	0.171779822	0.03420122	0.013115244
0	0	0	0	0
0	0	0.007327258	0.547031461	0
0	0	0	0	0
0	0	0	0	0
0	0	0	0.011924277	0
0.004269996	0.004549011	0.00776031	0.005510134	0

0	0	0	0	0
0	0.000974799	0.001951185	0.016390166	0
0	0	0	0	0
0	0	0	0	0
0	0.208996562	4.016911935	1.917523498	0
0	0	0	0	0
0	0	0	0	0
0	0	0	0.159323814	0
0.276290769	0.540799614	0.032589881	0	0.883504666
0	0	0.00129028	0	0
0	0	0	0	0.034865181
0	0	0	0	0
0	0	0.065150312	0.103741218	0
0.020262742	0.012343331	0.006144907	0.002561765	0.517635167
0	0	0	0	0
0	0	0.532762629	0.353313947	0
0	0	0	0.069512823	0
0	0	0	0	0
0	0	0	0	0
0	0	0	0	0
0	0	0	0	0
0	0	0	0	0
0	0	0	0	0
0	0	0	0	0
0	0	0	0.019418846	0
0	0	0	0	0
0	0	0.172354392	0.034834617	0
0.002611361	0.00079096	0.002158919	0	0.000489061
0	0	0	0	0
0	0	0	0	0
0	0	0	0	0
0	0	0	0	0
0	0	0	0	0
0	0	0.104858067	0	0
0	0	0	0.137725344	0
0.176805829	0.000686232	0.007908502	0	0
0	0	0	0.001102193	0
0	0	0	0	0
0	0	0	0	0
0	0	0	0	0
0	0.184060261	2.354433462	0.088266054	0
0	1.153593921	0	0.078030306	0
0	0	0	0	0
0	0.012584859	0	0	0
0	0	0	0	0
0	0	0	0	0
0	0	0	0	0
0	0	0	0	0

0	0	0	0.594188985	0
0	0	0	0	0
0	0	0	0	0
0	0	0	0	0
0.03013523	0	0.007409973	0.033173641	0
0	0	0	0	0
0.020458953	0	0	0.167278089	0
0	0	0	0	0
0	0	0	0.000557466	0
0	0	0.037230068	0	0
0	0	0	0.06187084	0
0	0	0	0	0
0.00500993	0	0	0	0
0	0	0	0	0
0	0	0	0.008536407	0
0	0	0	0	0
0	0	0	0	0.130131414
0	0	0	0	0
0	0	0	0	0
0	0	0	0	0
0.002676375	0	0.001567307	0.169333881	0
0	0	0	0	0
0	0	0.003841632	0.006966729	0
0	0.005256389	0.029036159	0.000589989	0.003250095
0	0	0	0	0
0.001032295	0	0	0	0
0	0	0	0	0
0	0	0	0	0
0	0	0	0	0
1.57330982	0	0	0.911968689	0.533029285
0	0	0	0	0
0	0	0	0	0
0	0	0	0	0
0	0	0	0	0
0	0	0	0	0
0	0	0	1.474507717	0
0.024633953	0.005167724	0.18037648	0.001476459	0.145046033
0	0	0	0	0
0	0	0	0	0
0	0	0	0	0
0	0	0	0	0
0	0	0	0	0
0	0	0	0	0
0	0	0.000611811	0	0
0	0	0	0	0
0	0	0	0	0
0	0	0	0	0
0	0	0	0	0

0.013024403	0.06241867	0.039881968	0.001110986	0
0	0	0	0	0
0	0	0	0.001508911	0
0	0	0	0	0
0	0	0	0	0
0	0	0	0	0
0	0	0	0	0
1.514384785	0	1.666485697	0.363241647	0
0.032512962	16.26551467	0.981129552	0.163175476	0.053848489
0.021434232	0.241706503	0.249032894	0	0
0	0	0	0.005087981	0
0	0	0	0	0
0	0	0	0.043557088	0
0	0	0	0	0
0	0	0	0	0
0	0	0	0	0
0.262733802	0	0.076191757	0	0
0	0	0	0	0
0	0	0	0	0
0	0	0.122570155	0	0
0	0	0	0	0
0	0	0	0	0
0	0	0	0	0
0	0	0	0	0
0	0	0.022148039	0.022126411	0
0	0	0	0	0
0	0	0	0	0
0	0	0	0	0
0	0	0	0	0
0	0	0	0	0
0	0	0	0	0
0	0	0	0	0
0	0	0	0	0
0	0	0	0	0
0	0.022254238	0	0	0
0	0	0	0	0
0	0	0	0	0
0	0	0	0	0
0	0	0	0.000566164	0
0.396025909	0.263369822	0	0	0
0	0	0	0	0
0	0	0	0	0
0	0	0	0	0
0	0	0	0	0
0	0	0	0	0
0	0	0	0	0
0	0	0	0	0
0	0	0	0	0

0	0	0	0	0
0	0	0	0.02549758	0
0	0	0	0	0
0	0	0	0	0
0.072671629	0.340550555	0.400291843	0.504095739	0.009246454
0	0	0	0	0
0	0	0	0	0
0.035930567	0.006167073	0.064058866	0.013728765	0.009321117
0	0	0.002100889	0.027415325	0
0	0	0	0.031793404	0
0	0	0	0	0
0	0	0	0	0
0	0	0	0	0
0	0	0	0	0
0	0.008682804	0.037524458	0	0
0	0.19968318	0	0.042199295	0
0	0	0	0	0
0	0	0	0	0
0	0	0	0	0
0	0	0	0	0
0	0	0	0	0
0	0	0	0	0
0	0	0	0	0
0.001209248	0.005114715	0.000809309	0.008910836	0
0	0	0	0.263960646	0
0	0	0	0	0
0	0.13362773	0.119543111	0.567301915	0.000503037
0	0	0.000584559	0	0
0	0.003325468	0	0	0
0	0	0	0	0
0	0	0	0	0
0	0	0	0	0
0	0	0	0	0
0	0	0	0	0
0	0	0	0	0
0	0	0	0	0
0	0	0	0	0
0	0	0	0.312369969	0
0	0	0	0	0
0	0	0	0	0
0	0	0	0	0
0	0	0	0	0
0	0	0	0	0
0	0	0	0	0
0	0	0	0	0
0	0	0	0	0
0	0	0	0	0
0	0	0	0	0

0	0	0.090840923	0.184094573	0
0	0	0	0.004174293	0
0	0	0	0	0
0	0	0	0	0
0	0	0	0	0
0.040625961	0	0.030645898	0	0
0	0	0	0	0
0	0	0	0	0
0	0	0	0	0
0	0	0	0	0
0	0	0	0	0
0	0.007584922	0	0	0
0	0	0	0	0
0	0	0	0	0
0	0	0	0	0
0	0	0	0	0
0	0	0	0	0
0	0	0	0	0
0	0	0	0	0
0	0	0	0	0
0	0.830434913	0.103762966	0	0.012049407
0	0	0	0	0
0.011297431	0.092292536	0.029008035	0.143709363	0
0	0	1.336100245	0.662076057	0
0	0	0	0	0
0	0	0	0.089933352	0
0	0	0	0.038129885	0
0	0	0	0	0
0	0	0	0	0
0	0	0	0	0
0	0	0	0	0
0	0	0	0	0
0.345477598	0.532994739	0.021446767	0.001234708	6.25409451
0	0	0	0	0
0.072070151	0	0.222961073	0.0999519	0
0.000816347	0	0.057175882	0	0.00086636
0	0	0	0	0
0	0	0	0	0
0	0.152667131	0.124082226	0	0.07495574
0	0	0	0	0
0	0	0	0	0
0	0	0	0	0
0	0.184483027	0	1.475160302	0.002577815
0	0	0	0	0
0	0	0	0	0
0	0	0	0.088330582	0
0	0	0	0	0
0	0	0	0	0

0	0	0	0	0
0	0	0	0	0
0	0	0.00410494	0	0
0	0	0	0	0
0	0	0	0	0
0	0	0	0	0
0	0	0	0	0
1.595646736	0.002617748	0	0	0
0	0.012267376	0.001924352	0.021271021	0
0	0.003952834	0	0.006211454	0.00230032
0	0	0	0	0
0	0	0.001126021	0	0
0	0	0	0	0
0	0	0	0.01398458	0
0	0	0	0	0
0.022392377	0.01075236	0	0	0
0	0	0	0	0
0	0	0	0	0
0	0	0	0	0
0	0	0.937899025	1.235669438	0
0	0	0.006956578	0	0
0	0	0	0	0
0	0	0	0	0
0	0	0.004436063	0.004309671	0
0	0	0	0	0
0	0	0	0	0
0	0	0	0	0
0	0	0	0	0
0	0	0	0	0
0	0	0	0.045083035	0
0	0	0.28336765	0.351061408	0
0	0	0	0	0
0	0	0	0	0
0	0	0	0	0
0	0	0	0	0
0	0	0	0	0
0	0	0	0	0
0	0	0	0	0
0	0	0	0	0
0	0	0	0	0
0	0	0	0	0
0	0	0.001511626	0.998991879	0
0	0	0	0	0
0	0	0	0	0
0	0	0	0	0
0	0	0	0	0
0.07295308	0.008899368	0.015980768	0	0.355641621
0	0	0	0	0

0	0	0	0	0
0	0	0	0	0
0	0	0	0	0
0	0	0	0	0
0.055724623	0.393135855	0.005253084	0.047975519	0
0	0	0	0.040212789	0
0	0	0	0	0
0	0	0	0	0
0	0	0	0	0
0	0	0	0	0
0	0	0	0.0586433	0
0.006981767	0.080888016	0.038012743	0.067561283	0
0	0	0.014109657	0	0
0	0	0	0	0
0	0	0	0	0.002289145
0	0	0	0	0
0	0.00261554	0	0	0
0	0	0	0	0
0.167171946	0.014530512	0.088860587	0	0.004397433
0	0.001200786	0.006336574	0.081703048	0
0	0	0	0	0
0	0	0	0.045330617	0
0	0	0	0	0
0	0	0	0	0
0	0	0	0	0
0	0	0	0	0
0.003587985	0.028482452	0.046750659	0.015423059	0.002221218
0	0	0.01129013	0	0
0	0	0	0	0
0	0	0	0	0
0.001016792	0.002399662	0.000595442	0.001028403	0
0	0	0.129272465	0	0
0	0	0	0	0
0	0	0	0	0
0	0	0	0	0
0	0	0	0	0
0	0	0	0	0
0.00488152	0.091500158	0.370788849	0.014932203	0
0	0	0	0.519112909	0
0	0	0	0	0
0.001664064	0.002601161	0.004733235	0	0
0	0	0	0	0
0	0	0	0	0
8.883266709	0	0.009305182	0.002499967	0.216973892
0	0	0	0	0
0	0	0	0	0
0	0	0	0	0
0	0	0	0.004496551	0

0	0	0	0	0
0	0	0	0.018314898	0
0	0	0	0	0
0.001480227	0.012861998	0.001733666	0	0.00333819
0.001029665	0	0	0.001909275	0
0.008859212	0	0	0	0
0	0	0.003364783	0	0
0	0	0	0	0
0	0	0	0	0
0	0	0	0.017732705	0
0	0	0	0	0
0	0	0	0	0
0	0	0.450093891	0.064736495	0
0	0	0	0.002059884	0
0	0	0	0	0
0	0	0	0	0
0	0	0	0	0
0	0	0	0	0
0	0	0	0	0
0	0	0	0	0
0	0	0	0	0
0	0	0	0	0
0.019688517	0.018230489	0.014361626	0	0.010997225
0.016507749	0	0.010741191	0.045553954	0
0	0	0	0	0
0	0	0	0.043536748	0
0	0	0	0.005006702	0
0	0	0	0	0
0	0	0	0	0
0	0	0	0	0
0	0.022805806	0.025711255	0.013828358	0
0.003127174	0.015718926	0	0	0
0	0	0	0	0
0	0	0	0	0
0	0	0	3.236840347	0
0	0.00077741	0	0	0
0	0.019225582	0	0	0.051023692
0	0	0	0	0
0.001140474	0	0	0.028178272	0.006570442
0	0	0	0	0
0	0	0	0	0
0	0	0	0	0
0	0	0	0	0
0	0	0	0	0
0	0	0	0	0
0	0.487341171	0.346861288	0.303596007	0
0	0	0	0	0
0	0	0	0	0

0	0	0	0	0
0	0	0	0	0
0	0	0	0.12566185	0
0	0	0	0	0
0	0	0	0	0
0	0	0.003864212	0	0
0	0	0	0	0
0	0	0	0	0
0	0	0.015890351	0	0
0	0	0	0.004701567	0.000789324
0	0.001701484	0.004024964	0	0
0	0.002794353	0	0	0
0	0	0	0	0
0	0	0	0	0
0	0	0	0	0
0	0	0	0	0
0	0	0	0.002161622	0
0	0	0	0	0
0	0	0	0	0
0	0.000902584	0	0	0.001302186
0	0	0	0	0
0	0	0	0	0
0	0	0	0	0
0	0	0	0	0
0	0	0	0	0
0	0	0	0	0
0	0	0	0	0
0	0	0	0.007455619	0
0	0	0	0	0
0	0	0	0	0
0	0.001744679	0.001940111	0.001827717	0.008789885
0	0	0	0	0
0.172402693	0.047042427	0.001638303	0.055353164	0.027834414
0	0	0	0.002168624	0.0007585
0	0	0	0.033516021	0
0	0	0	0	0
0	0	0	0	0
0	0	0	0	0
0	0	0	0	0
0	0	0	0	0
0	0	0	0	0
0.003740048	0.086291111	0.349758523	0	0.027326283
0	0	0.040229519	0.105285625	0
0	0	0	0	0
0	0	0	0	0
0	0.006519986	0.012753987	0.033297738	0
0	0	0	0	0
0	0	0	0	0

0	0	0	0	0
0	0	0	0.002599679	0
0.002144583	0	0.006781779	0.019738545	0.000682791
0	0	0	0	0
0.076713099	0.001983857	6.542030822	0.059404126	0
0.038669466	0	0	0.001517238	0
0	0	0	0	0
0	0	0	0	0
0	0	0	0	0
0	0	0	0	0
0	0	0	0	0
0	0	0	0	0
0	0	0	0	0
0	0	0	0	0
0	0	0	0	0
0	0	0	0	0
0	0	0	0.034342976	0
0	0	0	0	0
0	0	0	0	0
0	0	0	0	0
0	0	0	0	0
0	0	0.006869457	0	0
0	0	0	0	0
0	0	0	0	0
0	0.003185286	0	0.010368192	0
0.016002003	0	0	0.002157963	0.00132085
0	0	0	0	0
0	0	0.020806237	0.11321263	0
0	0	0	0	0
0	0	0	0	0
0	0	0	0	0
0	0	0.001095697	0	0
0	0	0	0	0
0	0	0	0	0
0	0.001084179	0	0	0
0	0	0	0	0
0	0	0	0	0
0.355385868	0.010935886	0.016583017	0	0
0	0	1.105856691	0	0
0	0	0	0	0
0	0	0.006248479	0	0
0	0	0	0.000975441	0
0.010528063	0.056967143	0.030513107	0.000902397	0
0	0	0	0	0
0	0	0	0	0
0	0	0	0	0
0.009008371	0.073092358	0.02461842	0.000828294	0
0	0	0	0	0

0	0	0	0	0
0.040128628	0.078261722	0.127079663	0.004180619	0.016267173
0	0	0	0	0
0	0	0	0	0
0	0	0	0	0
0	0	0	0	0
0	0	0	0	0
0	0	0	0	0
0	0	0	0	0
0	0	0	0	0
0	0	0	0	0
0	0	0	0	0
0	0	0.010661465	0.004603422	0
0	0	0	0	0
0	0.069660918	0	0	0.004330017
0	0	0	0	0
0	0	0	0	0
0	0	0	0	0
0	0	0.001391157	0	0
0	0	0	0	0
0	0	0	0	0
0	0.001085913	0.000740998	0.015997458	0
0.108899437	0	0.006073562	0.007703449	0
0	0	3.277286281	0	0
0	0	0	0	0
0	0	0	0	0
0	0	0	0	0
0	0	0	0	0
0	0	0	0	0
0	0	0	0	0
0	0	0	0	0
0	0	0	0	0
0	0	0	0	0
0	0	0	0	0
0	0.018733023	0.092620078	0.647418833	0
0	0	0	0	0
0	0	0	0	0
0	0	0	0	0
0	0	0	0.00681617	0
0	0	0	0	0
0	0.185956573	0.133139648	0.198053961	0.103406346
0	0	0	0	0
0	0	0	0	0
0	0	0	0	0
0	0	0	0	0
3.135532565	0.809883892	0.173199522	0.000656003	1.431961519
0	0	0	0	0
0	0	0	0	0

0.004621472	0	0.038791332	0	0
0	0	0	0	0
0	0	0	0	0
0	0	0	0	0
0	0.00168862	0	0	0
0	0	0	0	0
0	0	0	0	0
0	0	0	0	0
0	0	0	0.003703307	0
0.000845771	0	0	0.064584893	0.070685002
0	0	0.009402385	0.045559691	0
0	0	0	0	0
0.001033338	0.00744915	0.002178472	0	0
0	0	0	0	0
0	0	0	0	0
0	0	0	0	0
0	0	0	0	0
0	0	0	0	0
0	0	0	0	0
0.071004214	1.016562334	0.136375906	0.02790738	0.020693139
0	0	0	0	0
0	0	0	0	0
0	0	0	0	0
0	0	0	0	0
0	0	0	0	0
0	0	0	0	0
0	0	0	0	0
0	0	0	0	0
0	0	0	0	0
0	0	0	0	0
0	0	0	0	0
0	0	0	0	0
0	0	0	0	0
0	0	0	0	0
0	0	0	0	0
0	0	0	0	0
0	0	0	0	0
0	0	0	0	0
0	0	0	0	0
0	0	0	0	0
0	0	0	0	0
0	0	0	0	0
0	0	0.001253597	0.124803704	0
0	0	0	0	0
0.002246028	0.005782579	0.018414096	0.004193862	0
0	0	0	0	0
0	0	0	0	0
0	0	0	0	0.026063445

0	0	0	0	0
0	0	0	0	0
0	0	0	0.03196961	0
0	0	0	0	0
0	0	0	0	0
0.000515922	0.031214651	0.056044804	0.029221555	0
0	0	0	0	0
0	0	0	0	0
0	0	0	0	0
0	0	0	0	0
0	0	0	0	0
0	0	0	0	0
0	0	0	0	0
0	0	0	0	0
0	0	0	0	0
0	0	0	0	0
0	0	0	0.49386399	0
0	0.001226889	0	0	0
0	0	0	0	0
0	0	0	0	0
0	0	0	0.000453777	0
0	0	0	0	0
0	0	0	0	0
0	0	0	0	0
0	0	0	0	0
0	0	0	0.003264815	0.004496252
0	0	0	0	0
0	0	0	1.244804793	0
0	0	0	0	0
0	0	0.001638576	0	0
0	0	0	0	0
0	0	0	0	0
0	0	0	0	0
0	0	0	0	0
0	0	0	0	0
0	0	0	0	0
0	0	0	0	0
0	0	0.008444443	0	0
0	0	0.232432263	0	0
0	0	0	0	0
0	0	0	0	0
0	0	0	0	0
0	0	0	0	0
0	0	0	0	0
0	0	0	0	0
0	0	0	0	0
0	0	0	0	0
0	0	0	0	0

0	0	0	0	0
0	0	0.229845638	0.001168425	0
0	0	0	0.003755063	0
0	0	0	0	0
0	0	0	0	0
0	0	0	0.004406208	0
0	0	0	0	0
0	0	0	0	0
0	0	0	0	0
0	0	0	0	0
0	0	0	0.048193718	0
0	0	0	0	0
0	0	0.000652467	0.066768374	0
0	0	0	0	0
0	0	0	0	0
0	0	0	0	0
0	0	0	0	0
0	0	0	0	0
0	0	0	0	0
0	0	0	0.001750512	0
0.00825216	0.003458626	0.016857671	0	0.005906373
0	0	0	0	0
0	0	0	0	0
0	0.285489135	0.457675927	0.846580827	0
0	0	0	0.002424285	0
0	0	0	0	0
0	0	0	0	0
0	0	0	0	0
0	0	0	0	0
0	0	0	0	0
0	0	0.002867938	0	0
0	0	0	0	0
0	0	0	0	0
0	0	0	0	0

PHL018-0030_V1	PHL018-0028_V1	PHL018-0248_V2	PHL018-0034_V1	PHL018-0021_V1
CTRL	CTRL	REF	CTRL	EYCF
0.672799023	0.024413801	0.506863697	0.044836225	0.309738372
0.103082275	0.877863146	0.012545431	0.092225097	2.591521221
0	3.320101994	0.111456787	6.93784612	0.077748542
0	0	0	0	0
0	0	0	0	0
0	0	0	0	0
0	0	0	0	0
0	0	0	0	0
0	0	0	0	0
0	0	0	0	0
0	0	0	0	0
0	0	0	0	0
0	0	0	0	0
0.383628685	0.578454619	0.12209345	2.424410033	0.127981959
0	0	0	0	0
0	0	0	0	0
0	0	0	0	0
0	0	0	0	0
0	0	0	0	0.011884758
0	0	0	0	0
0	0	0	0	0
0	0	0	0	0
0	0	0	0	0
0	0	0	0	0
0	0	0	0.187952956	0
0	0	0	0	0
0	0	0	0	0
0	0	0	0	0
0	0	0	0	0
0	0	0	0	0
0	0	0	0	0
0	0	0	0	0
0	0	0	0	0
0	0	0	0	0
0	0	0	0	0
0.971793224	0.235884931	1.497577643	0.112568253	0
0	0	0	0	0
0	0	0	0	0
0	0	0	0	0
0	0	0	0	0
0	0	0	0	0
0	0.002211057	0.715401266	0.030230984	0.470689442
4.281727666	0.308282596	0.306672361	0.022825791	0.66054609
0.002380263	0.668718757	0.000909083	0.013371206	0.101929509
0.00343217	0.013360024	0.002883833	0	0.01276691
0	0	0	0	0

0.089281078	0	0.006115925	0	0
0.000655751	0	0	0	0.141595438
0.044098829	0	0	0.025307544	0.023185674
0.082978084	0.302055765	0.078603535	0.299057911	0.563996747
0.002066849	0	0	0.055037875	0
0	0.073316843	0	0	0
0	0	0	0.033255924	0.01279608
0	0	0	0	0
0	0	0	0	0
0	0	0	0	0
0	0	0.001151007	0.001032289	0
0.261571031	2.820486972	0.065421642	0.579444196	0
0	0	0	0.001362631	0
0.013423094	0.001987793	0.007715563	0	0.110182923
0	0	0	0	0
0	0	0	0	0
0.072456457	0.000817516	0.026812023	0	0.621726355
0	0	0	0	0
0	0	0	0	0
0	0	0	0	0
0.024822134	0	0.008152974	0	0.630115112
0.004494645	0.001577721	0.002492276	0.011016423	0.003383083
1.455619491	0.001327199	0.141166613	0.034919704	0
0	0	0	0	0
0	0	0	0	0
0	0	0	0	0
0	0	0	0	0
0	0	0	0	0
0	0	0	0.002042491	0.020287228
0	0	0	0	0
0	0	0	0	0.022760852
0	0	0	0	0
0	0	0	0	0
0	0	0.000658383	0	0
0	0	0	0	0
0	0	0	0	0.002212169
0	0	0	0	0
0	0	0	0.002904037	0.024101272
0	0	0	0	0
0	0	0	0	0
0	0	0	0	0
0	0	0	0	0
0	0	0	0	0
0	0	0	0	0
0	0	0	0.161104994	0
0	0	0	0	0
0	0	0	0	0
0	0	0	0	0.001495379

0	0	0	0	0
0	0	0	0	0
0	0	0	0	0
0	0	0	0	0
0	0.498350553	0	0.001272129	1.615904979
0	0	0.000875238	0.000588722	0
0	0	0	0	0
0.002235342	0	0	0	0
0	0	0	0	0.008992542
0	0	0	0	0
0	0	0	0	0
0	0	0	0	0.172974519
0	0	0	0	0
0	0	0	0	0
0	0	0	0	0
0	0	0	0	0
0	0	0	0	0
0	0	0	0	0
4.184503083	0	0.261878578	0.066330599	0
0	0	0	0	0
0	0	0	0	0
0	0	0	0	0
0	0	0	0.000506381	0
0	0	0	0	0
0	0	0	0	0
0	0	0	0	0
0	0	0	0	0
0	0	0	0	0
0	0	0	0	0
0	0	0	0	0
0.027843644	0.007937899	0.028661144	0.025704957	0
0.109968595	0	0.018040822	0.008239837	0.008531504
0	0	0	0	0
0	0	0	0	0
0	0	0	0	0
0	0	0	0	0
0	0	0	0	0
0	0	0	0	0
0	0	0.038234088	0	0
0	0	0	0	0
0	0	0	0	0
0	0	0	0	0
0	0	0	0.003290091	0
0	0	0	0	0
0	0	0	0	0
0	0	0	0	0
0	0	0	0	0
0	0	0	0	0

0.004383502	0	0	0	0
0	0	0	0	0
0	0	0	0	0
0	0	0	0	0
0.053295973	0	0.006688099	0.030252148	0
0	0	0	0	0
0	0	0	0	0
0	0	0	0	0
0	0	0	0	0
0	0	0	0	0
0	0	0	0	0
0	0	0	0	0
0	0	0	0	0
0	0	0	0	0
0	0	0	0	0
0	0	0	0	0
0	0	0	0	0
0	0	0	0	0
0	0	0	0	0
0	0	0	0	0
0	0	0	0	0
0	0	0	0	0
0	0	0	0	0
0	0	0	0	0
0	0	0	0.095036913	0
0	0	0	0	0
0	0	0	0.049961006	0
0	0	0	0	0
0	0	0	0	0
0	0	0	0	0
0	0	0	0	0
0	0	0.177314799	0.11079969	0.036169192
0	0	0	0	0
0	0	0	0	0
0	0	0	0	0
0	0	0	0	0
0	0	0	0	0
0	0	0	0	0
0	0	0	0	0
0	0	0	0	0
0	0	0	0	0
0	0	0	0	0
0	0	0	0	0
0	0	0	0	0
0	0	0	0	0
0	0	0	0	0
0	0	0	0	0
0	0	0	0	0

0	0	0	0	0
0	0	0	0	0
0	0	0	0	0
0	0	0	0	0
0	0	0	0	0
0	0	0	0	0
0	0	0	0	0
0	0	0	0	0
0	0	0	0	0
0	0	0	0	0
0	0	0	0	0
0	0	0	0	0
0.001379679	0	0	0.000661619	0.000876212
0	0	0	0	0
0.017217119	0.000665124	0	0	0
0	0	0	0	0
0	0	0	0	0
0	0	0	0	0
0	0	0	0	0
0	0	0	0	0
0	0.03208941	0	0.017009692	0.048330019
0	0	0	0	0
0	0	0	0	0
0	0	0	0	0
0	0	0	0	0
0	0	0	0	0
0.002253507	0	0.002008233	0.000900549	0.016696936
0	0	0	0	0
0	0	0	0	0
0	0	0	0	0
0	0	0	0	0.038518995
0	0	0	0	0
0	0	0	0	0
0.145435162	0.001634255	0.006111497	0.003024078	0
0.008182539	0.002485605	0	0.003219611	0.020253412
0	0.005982797	0	0	0
0	0	0	0	0
0	0	0	0	0
0	0	0	0	0
0	0	0	0	0
0	0	0	0	0
0	0	0	0	0
0	0	0	0	0
0	0	0	0	0
0	0	0	0	0
0	0	0.545377831	1.377139998	0
0	0	0	0	0
0.001046607	0.000708524	0.000639562	0	0.024498336

0	0	0	0	0
0	0	0	0	0
0	0	0	0	0.002082425
0	0	0	0	0
0	0	0	0	0
0	0	0	0	0
0	0	0.003186427	0	0.002433005
0	0	0.005286443	0	0.015014918
0	0	0	0	0
0	0	0	0	0
0	0	0	0	0.001174831
0	0	0	0	0
0	0	0	0	0
0	0	0	0	0
0	0	0.001764659	0	0
0	0	0	0	0
0	0	0	0	0
0	0	0	0	0
0.021439913	0.001981551	0	0	0.001158821
0	0	0.036150091	0	0
0	0	0.001439175	0	0.187176957
0	0	0	0	0
0	0	0	0	0
0	0	0	0	0
0.001336848	0.0147818	0	0.002136931	0.033960419
0	0.001280719	0	0	0
0	0	0	0	0
0	0	0	0	0
0	0	0.010232253	0	0.137467841
0	0	0	0	0
0.018471759	0.003516992	0.043563597	0.03021228	0.732356876
0	0	0	0	0
0	0	0	0	0
0	0	0	0	0.001626155
0	0	0	0	0
0	0	0	0	0
0	0	0	0	0
0	0	0	0	0
0	0	0	0	0
0	0.004098483	0	0	0
0	0	0	0	0
0.037321543	0.008564138	0.022832154	0	0
0.000705274	0	0	0	0
0	0	0	0	0
0	0	0	0	0
0	0	0	0	0
0	0	0	0	0
0	0	0	0	0

0	0	0	0	0
0	0	0	0	0
0.000844384	0.698239264	0	0.009920561	0.110200404
0	0	0	0	0
0	0	0	0	0
0	0	0	0	0.001567296
0	0	0	0	0
0	0	0	0	0
0	0	0	0	0
0	0	0	0.001851688	0
0	0	0	0	0
0	0	0	0	0
0	0	0	0	0
0	0.017136171	0	0.00671266	0.003259622
0.00096279	0.00250936	0.000823679	0.001846807	0.017854416
3.697458469	0.006350148	0.183867311	0.001384076	0
0.000658208	0.003119117	0	0	0.010311092
0	0	0	0	0
0	0	0	0	0
0	0	0	0	0
0	0	0	0	0
0	0	0.000635404	0.00132969	0.028427056
0	0	0	0	0
0	0	0	0	0
0	0	0	0	0
0	0	0	0	0
0	0	0	0	0
0	0	0	0	0
0	0	0	0	0
0	0	0	0	0
0	0	0	0	0
0	0	0	0	0
0	0	0	0	0
0	0	0	0	0
0	0	0	0	0
0	0	0.005076519	0	0.037685178
0	0	0	0	0
0.083858854	0.035376012	0.00691877	0.012648959	0
0	0	0	0	0
0	0	0	0	0
0	0	0	0	0.009579676
0	0	0	0	0
0	0	0	0	0
0	0	0	0	0
0	0	0	0	0
0	0	0	0	0
0	0	0	0	0
0	0	0.010958941	0	0.009188095

0	0	0.027836229	0	0
0	0.135450056	0	0	0
0	0.007372994	0	0	0
0	0	0	0	0
0.00250458	0.002225383	0	0.023821028	0
0	0	0	0	0
0	0	0	0	0
0	0.107444758	0	0.001402959	0.039389631
0	0	0	0	0
0	0	0.006449453	0.145473616	0.012256527
0	0	0	0	0
0.000926366	0	0.010897124	0	0.134137016
0	0.020187332	0	0.006570874	0.016734817
0	0	0	0	0
0	0	0	0.020273981	0
0	0	0	0	0.007369043
0	0	0.002050784	0	0
0	0	0	0	0
0	0	0	0	0
0	0	0	0	0
0	0	0.001109948	0	0
0	0	0	0	0
0	0	0	0.000521561	0
0	0	0.172497296	0.543219182	0
0	0	0	0	0
0.005115281	0.021089065	0	0	0.107334821
0	0	0	0.001574483	0
0	0	0	0	2.958631777
0	0	0	0	0
0	0	0	0	0
0	0	0	0	0
0	0	0	0	0
0	0	0	0	0
0	0	0	0	0
0	0	0	0	0
0	0	0	0	0.01159589
0	0	0	0	0.265445977
0	0	0	0	0
0	0	0	0	0
0	0	0	0	0
0	0	0	0	0
0	0	0	0	0
0	0	0	0	0
0	0	0	0	0.035384719
0	0	0	0	0
0	0	0	0	0
0.003307853	0	0	0	0.002908751
0	0	0	0	0

0	0	0	0	0
0	0	0	0	0.092104078
0	0	0	0	0
0	0.010381763	0.124755209	0.016284114	0
0	0	0	0.006030224	0
0.004609439	0	0	0.026909657	0
0	0	0	0	0
0	0	0	0	0.00131694
0	0	2.055809098	0.544855719	0
0	0	0	0	0
0	0	0.000745715	0.005350401	0
0	0	0	0	0
0	0	0	0	0
0	0.006504	0.000704514	0.00168493	0
0	0	0	0	0
0	0	0	0	0
0	0.16863259	0	0	0
0	0	0	0	0.038273403
0	0	0	0	0
0	0	0.145463981	0	0
0	0	0	0	0.012922967
0.193659477	0.114420109	0.082308066	0.297179363	0
0	0	0.003788452	0.320583035	0.008734767
0	0	0	0	0
0	0	0	0	0
0	0	0	0	0
0	0	0	0	0.007863244
0	0	0	0	0
0	0	0	0	0
0.117166979	0.578874239	0.052666181	0.18121822	0.076455119
0	0.039535526	0.010515051	0	0
0	0	0	0	0
0	0	0	0	0
0	0	0	0	0
0	0	0	0	0
0	0	0	0	0
0	0	1.744576736	1.401921455	0
0	0	0	0	0
0	0	0	0	0
0	0	0	0	0
0	0.006202515	0	0.025279821	0.021325798
0.147349008	0.747003427	0.111580121	0.616958349	0
0	0	0	0	0
0	0	0	0.002924432	0
0	0	0	0	0.00957091
0	0	0	0	0
0	0	0.001823696	0.001453862	0.002888123
0	0	0	0	0

0	0.071182166	0.001750105	0	0.136005373
0	0	0	0	0
0.003504153	0.00301918	0.006131962	0.00366633	1.448553506
0	0	0	0	0
0.014884459	0.713141013	0.166880403	0	0
0	0	0	0	0
0	0	0	0	0
0	0	0.00140108	0	0.001664131
0	0	0	0.005817414	0
0	0	0	0	0
0	0	0	0	0
0	0	0	0	0
0	0	0	0	0.00219098
0	0	0	0.004259917	0
0	0	0	0	0
0.153717843	0.11298629	0.016438449	0.005865472	0
0.006595363	0.137170859	0.002742842	0.001581389	0.437709616
0	0	0	0	0.008764383
0	0	0	0	0
0	0	0	0	0
0	0	0	0	0.014076904
0.00467517	0	0.001818031	0	0
0	0	0	0	0
0	0	0	0	0
0	0.001014236	0	0	0
0	0	0	0	0.003678503
0	0	0	0.002007586	0.001181661
0	0	0	0.031903324	0
0	0	0	0	0
0	0	0	0	0.017188692
0	0.000838181	0.002837246	0.023749649	0
0	0.058732379	0	0.011633995	0.043207781
0.069548782	0.011600667	0.003490516	0.005297761	0.015945601
0	0	0.028474347	0.043827746	0.170015908
0	0.181031647	0	0	0.325552167
0	0	0	0	0
0	0.011025639	0	0	0.012109374
0.002307741	0	0.024299108	0.008002056	0.030214104
0.074112742	0.015310566	0.000846144	0.002150133	0
0	0	0	0	0
0	0	0	0	0
0	0	0	0	0
0	0.001258051	0.000851702	0.002291565	0.00134881
0	0	0	0.067383436	0.034146652
0	0	0	0	0.292775056
0	0	0	0	0.009831594
0	0	0	0	0
0	0	0	0	0

0	0	0	0	0
0.124332272	0.008597073	0.122871429	1.587781214	0.514631866
0	0	0	0	0
0	0	0	0	0
0	0	0	0	0
0	0	0	0	0
0	0	0	0	0.006821753
0	0	0	0	0.00188796
0	0	0	0	0.003937578
0	0	0	0	0.018754463
0.001977271	0	0	0	0.006849452
0.00722913	0.225210791	0	0.011298865	0.145555
0	0	0	0.04892022	0
0	0	0	0	0
0	0.000733008	0	0	0.025672374
0	0	0	0	0
0.001882262	0.014271466	0.001006437	0.009387356	0.017213676
0	0	0	0	0.355669528
0	0	0	0	0
0	0	0	0	0.004836716
0.005629151	0.00344941	0.01079406	0.115237943	0.004931012
0	0.000858447	0	0	0
0	0	0	0	0
0	0	0	0	0
0	0	0	0	0
0	0	0	0	0
0	0.001134913	0.012498286	0.039875278	0
0.007427119	0.01735341	0.002647498	0.082115642	0.028301063
0	0	0	0	0
0.001244284	0	0	0	0.014856226
0	0	0	0	0
0	0	0	0	0
0	0	0	0	0
0	0	0	0	0
0	0	0.009228442	0	0
0.084148872	0.275908888	0.020163298	0.231068239	0.019070445
0	0	0	0	0
0	0	0	0	0
0.013488768	0	0.015241264	0.003173216	0.117668148
0	0	0	0	0
0	0	0	0	0.132258151
0	0	0	0	0
0.042048551	0.001282002	0.008100555	0.013788753	0.001963556
0	0	0.000798054	0.034355564	0
0	0	0	0	0
0	0.161317776	0.083444937	0.011684225	0
0	0	0	0	0
0	0	0	0	0

0	0	0	0	0
0	0	0	0	0
0	0	0	0.001358766	0
0.680584218	3.927137014	2.161867281	0.053531779	0
0	0	0	0	0
0	0	0	0	0
0	0	0	0	0
0.028670977	0	0	0	0
0.032859267	4.747881848	14.88975173	0.614822884	0
0.001931031	0.010103982	0.017724822	0.110041764	0
0	0	0	0	0
0.030016051	0.005722304	0.003874005	0	0
0	0	0	0	0
0	0	0	0	0
0.261643757	0.385522471	0.064850155	0.07741192	0
0	0	0	0	0
0.106026858	0.01341894	0.01926141	0.008904503	0
0	6.383000787	0.637917099	0	0
0	0	0	0	0
0	0	0	0	0
0	0	0	0	0
0	0	0	0.13102702	0
0	0	0	0	0
0.032909249	0.065304241	0.032553392	0.21936265	0.357218138
0	0	0.002418039	0	0
0	0.605985101	0.211289568	0.005587515	0
0	0	0.013152426	0.061559589	0
0.735951937	1.987993357	0.184303852	1.030765022	0
0	0	0	0	0
0	0	0	0	0
0	0.004370972	0	0.020909807	0
0	0.018345005	0.101891283	0	0
0	0.401615054	0.000409479	0	0.00405298
0	0	0	0	0
0	0	0	0	0
0	0	0.147500542	0	0
0	1.502725387	2.58448494	0.020079196	0
0	0.000850557	0	0	0
0	0.102969603	0	0	0
0	0.002194218	0.000660217	0.080528322	0
0.004468195	0	0.018759048	0.015554439	0.005885556
0	0	0.015370312	0.244131941	0
1.290207624	0.290931917	0.135659582	0.03674771	0.001260794
0	0.138851584	0.002004156	0	0
0	0	0	0	0
0	0	0	0	0
0	0	0	0	0
0.004376601	0.008248803	0.024890676	0.003243569	0

0	0	0	0	0
0	0.011164984	1.378785094	0	0
0.001144218	0	0.000611808	0	0.005086722
0	0.001953721	0	0	0
0	0	0	0	0
0.097197827	0.101268213	0.030202763	0	0
0	0	0	0	0
0	0	0	0	0
0	0	0	0	0
0	0	0	0	0
0.099984312	0.033494705	0.022625886	0.050461114	0.146736071
0.045539735	0.654777408	0.439376298	1.370096435	2.151720906
0	0	0	0	0
0.034958682	0.260942037	0.023986588	0.002519488	0.032596711
0.004777245	0.011507864	0.027246632	0.021381799	0.224107904
0	14.34545782	0	1.798148232	0
0	0	0	0	0
0.001414727	0	0	0	0
0	0	0	0	0
0.001388103	0	0.012700077	0.029436899	0
0.02008727	0	0.103378018	0.005869964	0.011162464
0	0.00803186	0	0	1.017183436
0.0755952	0.137672978	0.274541802	2.113666692	2.442637664
0	0	0	0	0
0	0	0	0	0
0	0	0.001407703	2.087287839	0
0	0	0	0	0
0	0	0	0.000784906	0
0	0	0	0.241607534	0
0	0	0	0	0
0	0.011688525	0	0	0
0	0.150596225	0.048164641	0.079288884	0
0	0.009568194	0.001233843	0	0.693179277
0.015543967	0	0.044222335	0.125030993	0
0.014849439	0	0.002443055	0	0.031757881
0.112648159	0.102879661	0.067691596	0.004850087	0.050056575
0.011454377	0.0223506	0.003962979	0.002584892	0
0	0	0	0	0
2.744572322	0.410002821	0.278663343	0.412360293	0
0	0	0	0	0
0	0	0.023645299	0.054783354	0
0	0	0	0	0
0	0	0	0	0
0	0	0	0	0
0.008153189	0.00848115	0.016587265	0.003623741	0.010861085
0	0	0	0	0
0	0	0	0.15579189	0
13.59769074	0.789460109	0.036939277	0.050614149	1.407642712

0	0	0	0	0
0	0	0	0	0
0	0	0	0	0
0.024744969	0	0.001087481	0.005039131	0
0	0	0	0	0
0	0	0.121657854	0.319145982	0
0	0	0	0	0
0.447443168	0.390589818	0.004669839	0.14090232	0
0.012096212	0	0	0	0
0	0	0	0	0
0.004719066	0.003578035	0.011506081	0	0.052027967
0	0	0	0	0
0	0	0.124251838	0	0.058150282
0.119196658	0	0	0	0
0	0	0	0	0
0	0	0	0	0
0	0	0.005554865	0.074594173	0
0.076998884	0.001116987	0.006721789	0	0
0	0	0	0	0
0	0	0	0	0
0.007538779	0.000826963	0	0	0
0	0	0	0	0
0.002174062	0.006224397	0.000581231	0	0
0	0	0	0	0
0	0	0	0	0
0	0	0	0	0
0	0	0	0	0
0	0	0	0	0
0	0	0	0	0
0.049923822	0.058470125	0.134992696	0.023485602	0
0.03464048	0	0	0	0
0	0	0	0	0
0	0	0	0.104086699	0
0	0	0	0	0
0.022063942	0.001955992	0	0	0
0	0	0	0	0
0.082727667	0.083754789	0.01170903	0.032800449	0
0.000798954	0.000757217	0.001367031	0	0.157700869
0	0	0	0	0
0	0	0	0	0
0.152022307	0.338355271	0.01907888	0.062261155	0
0	0	0	0	0
0	0	0	0	0
0	0	0	0.132903231	0.067210892
0	0	0.018238938	0	0
0	0	0	0	0
0	0.00449677	0.003409636	0.001310553	0
0	0	0	0	0

0	0	0	0	0
0	0	0	0	0
0	0.910626313	0	0	0
1.05269325	0	0.124591269	1.580984552	0
0	0	0	0	0
0	0	0	0	0
0	0	0	0	0
0	0	0	0	0
0.001101891	0.001044329	0.001178353	0.004861346	1.002384284
0.001064818	0	0	0	0
0	0.004107003	0.001348094	0	0
0	0	0	0	0
0	0	0.001787315	0.000616526	0
0	0	0	0	0
0.03230741	0.068669149	0.064187098	0.092197804	0
0	0	0	0	0
0	0	0	0	0
0	0	0	0	0
0	0	0	0	0
0	0	0	0.006043114	0
0	0	0.589807537	0	0
0	0	0	0	0
0	0	0	0	0
0	0	0	0	0
0.08863224	0.085967095	0.007759973	0.008202372	0.013825346
12.18993923	0.168132208	1.593167239	0.408810012	18.34077334
0.063618488	0.173119986	0.003092411	0.073958706	0
0	0	0	0.017126271	0
4.771974467	0	0.700250643	0	0
0	0	0	0	0
0	0	0	0	0
0	0	0	0	0
0	0	0	0	0
0.030726427	0.030006738	0	0.002256669	0.006328819
0.025541775	0.122928631	0.002194887	0	0.499090653
0.002095662	0	0.001896301	0	0.029689788
0	0	0	0	0
0.110855106	0.06900761	0.211789222	0.446230414	0
0	0	6.54472103	22.34013078	0
0	0	0.013994895	0	0
0	0	0	0	0
0	0	0	0	0
0.135257592	1.370650093	0.042639137	0.105004903	0
0	0	0	0	0
0	0	0	0	0
0	0.003075794	0	0.002197105	0
0	0	0	0	0
0	0	0.429307633	0.062304482	0.019778311

0	0.078238926	0	0	0
0	0	0	0	0
0	0	0	0	0
0	0	0	0	0
0.001806362	0	0	0	0.002523821
0	0	0.005022554	0.001001003	0
0	0.00528125	0	0	0
0	0	0	0	0
0	0	0	0	0
0	0	0.01434766	0.02521394	0
0	0	0.014041901	0	0
0.391290413	2.495692927	0.125283244	0.680720241	0
0.038931933	0.263521173	0.017776084	0.004824738	1.220694888
0	0	0	0	0
0.004178114	0.001237454	0.037978324	0.040673006	6.510512819
0	0	0	0	0
0.005812964	0.007230953	0	0	0
0	0	0	0.012708091	0
0.002746577	0.003036946	0	0	0
0	0.038004514	0	0.009541694	0
0.068369004	0	0.008429904	0	0
0	0	0	0	0
0.351460519	0.006538825	0.264084195	0	0.000678441
0	0	0	0	0
0	0	0	0	0
0	0	0	0	0
0	0	0	0	0
0	0	0	0	0
0	0	0	0.00162426	0
0	0	0	0	0
0	0	0	0	0
0.041746994	0.059413457	0.004348428	0.05147894	0.053370013
0	0	0	0	0
0.084914035	0.158517599	0.020710211	0.198989707	0
0.002456037	0	0.001313232	0	0.030972967
0.071916374	0.101542646	1.195681958	0.30235931	0.007181279
0	0	0	0	0
0	0	0.046104124	0	0
0	0	0	0	0
0	0	0	0	0
0	0.002479048	0.027971963	0.136185813	0
0	0	0	0.130957283	0
0	0	0	0	0
0	0	0	0	0
0.101616595	0.244865215	0.002956949	0.006298408	0.106680415
0	0	0	0	0
0	0.062875237	0.127816387	1.697601299	0
0	0	0	0.001097373	0.000622843

0	0	0	0	0
0	0	0	0	0
0	0	0	0	0
0	0	0	0	0
0	0	0	0	0
0	0	0	0	0
0	0	7.162502546	0	0
0.061443127	0.000999886	0.027257462	0.00145705	0
0	0	0	0	0
0	0	0	0	0
0	0	0	0	0
0	0	0	0	0
0	0	0	0	0
0	0	0.072839029	0	0
0	0	0	0	0
0	0	0	0	0
0	0	0	0	0
0	0	0	0	0
0	2.248354001	4.170751898	17.56351281	0
0	0	0	0	0
0	0	0.005036342	0	0
0	0	0	0	0
0	0	0	0	0
0	0	0	0	0
0.001699362	0	0.003028803	0.000543281	0
0.035234124	0.001906022	0	0	0.173904186
0	0	0	0	0
0.003149516	0	0.050184156	0.008608918	0
0	0	0	0	0
1.430741672	0.096539671	0.319890968	0.638172646	0
0	0	0	0	0
0	0	0	0	0
0	0	0.001066915	0	0
0	0	0	0	0
0.001180193	0.001597915	0	0.160569911	0.014562134
0	0	0	0	0
0.119089422	0	0	0	0
0.003443589	0.028557352	0.000613757	0	0.001214982
0	0	0	0	0
0	0	0	0	0
1.052818638	5.696094315	0.384209435	4.403072591	0.828392813
0.605393547	0.340770187	0	0.140623901	0
0	0	0	0	0
0	0	0	0	0
0	0	0	0	0
0	0.004165916	0	0	0
0	0.005915491	0	0.002706809	0
0.031581595	0.051984894	0	0.054899954	0.005399952

0	0	0	0	0
0	0	0.001336092	0	0
0	0	0	0.003148938	0
0	0	0	0	0
1.349156424	0.909877167	0.135416966	0.585984568	0
0	0	0	0	0
0	0	0	0	0
0	0	0	0	0
0	0	0	0	3.877124995
0	0	0	0	0
0	0	0	0	0
0	0	0	0	0
0.021093258	0.064180882	0.014767494	0.064329628	0
0.001865613	0.020996827	0	0.011451473	0
0	0	2.562315759	0	0
0.052663242	0.046611264	0.061229019	0.251516467	0
0	0	0.013811385	0	0
0	0	0	0	0
0	0	0	0	0
0	0	0	0	0
0	0	0	0	0
0	0	0	0	0
0	0	0	0	0
0	0	0	0	0
0	0	0.001699974	0.101041684	0
0	0	0	0	0
0.074260264	0.052053958	0.003302631	0.000673179	0
0	0	0	0	0.001207179
0	0	0	0	0
0.001714707	0	0	0	0
0	0	0	0	0
0	0	0	0.001269011	0
0	0	0	0	0
0.02324659	0.038753064	0.00941117	0	0
9.307680873	0	2.825990187	0.028064952	0
0	0	0	0	0
0	0	0.000801149	0.009520344	0
0.032010238	0	0.002775524	0.014271694	0.084833149
0	0	0	0	0
0	0	0	0	0
0.106599028	0.263476504	0.349907871	0.127421071	0
0	0	0.187454238	0.053952048	0
0	0	0.002499426	0	0
0	0	0	0	0
0	0	0	0	0
0	0	0	0	0
0	0	0	0	0
0	0	0	0	0

0	2.25460408	0.305839457	0.096809773	0
0	0	0	0	0
0	0	0	0	0
0	0	0	0	0.006381653
0	0.020543157	0.045532914	0.007903844	0
0	0	0	0	0
0.000805236	0	0.009816684	0.000463377	0.025978749
0	0	0	0	0
0	0.001750696	0	0.004797019	0
0	0	0.007950336	0	0
0	0	0.024644065	0	0
0	0	0	0	0
0	0	0	0	0
0	0	0	0	0
0	0	0	0	0
0	0	0	0	0
0	0	0	0	0
0	0	0	0	0
0	0	0	0.332110075	0
0	0	0	0	0
0.522994941	0	0.007378445	0.052939298	0.003310746
0	0	0.001908938	0.02011653	0
0.121088508	0.040471554	0.018808744	0.066664041	0
0.073686934	0.008373381	0.00048245	0.001298066	0.012606643
0	0.00255588	0	0	0
0.001064498	0	0	0	0
0	0	0	0	0
0	0	0	0	0
0	0	0	0	0
0	1.036921074	0	0.001245481	3.759914011
0	0.002515024	0	0	0
0	0	0	0	0
0	0	0	0	0
0.377245593	0.026636539	0	0.621967056	0
0	0	0	0	0
0	0	0	0	0
0	0	0.001609786	0	0.640766128
0	0	0	0	0
0	0	0	0	0
0	0	0.21319591	0	0
0	0	0	0	0
0	0	0	0	0
0	0	0	0	0
0	0	0.02688219	0	0
0	0	0	0.110336711	0
0	0	0	0	0
0	0	0	0	0
0	0	0	0	0

0	0.004409176	0.000865222	0.036781485	0.004727265
1.462525638	0	3.628409019	0.084632728	0
0	0	0	0	0
0	0	0	0	0
0	0	0	0	0
0	0	0	0	0
0	0.002357959	0	0	0
0.483989957	0.385798503	0.062542477	0.205403903	0
5.13017585	0.37071759	0.439479834	0.02533478	0.827830932
0.001060939	0	0.003592769	0	1.133082345
0	0	0.000665692	0	0
0	0	0	0	0
0	0	0	0	0
0	1.507463091	0.027548568	0.215150452	0
0	0	0	0	0
0	0	0	0	0
0	0	0	0	0
0	0	0	0	0
0	0	0	0	0
0.010996952	0.020671245	0	0.433414686	0
0	0	0	0	0
0	0	0	0	0
0	0	0	0	0
0	0	0	0	0
0	0.001358936	0.02998522	0.014546379	0
0	0	0	0	0
0	0	0	0	0
0	0	0	0.754479018	0
0	0	0	0	0
0	0	0	0	0
0	0	0	0	0
0	0	0	0	0
0	0	0	0	0
0	0	0	0	0
0	0	0	0	0
0	0	0	0	0
0	0	0.001093786	0.021908324	0
0	0	0	0	0.024126274
0	0.001139751	0	0	0
0.002907772	0	0	0	0.142194289
0	0	0	0	0
0.060330972	0.038501262	0.021208759	0.21882408	0
0	0	0	0	0
0	0	0	0	0
0	0	0	0	0
0	0	0	0	0
0	0	0	0	0
0	0	0	0	0

0	0	0	0	0
0	0.001903227	0	0	0.035431027
0	0	0	0	0
0	0	0	0	0
1.984548377	4.241358108	12.66018024	0.579880393	0.336907262
0	0	0	0	0
0	0.005664508	0	0	0
0.002058415	0.004145628	0.001761	0.003356152	0.138570194
0	0.005113199	0	0.002759642	0
0	0	0	0.024871228	0
0	0	0	0	0
0	0	1.273303458	0	0
0	0	0	0	0
0	0	0	0	0
0	0	0	0	0
0.001428198	0.266487961	0.003818256	0.098452376	0
0	0	0	0	0
0	0	0	0	0
0	0	0	0	0
0	0	0	0	0
0	0	0	0	0
0	0	0	0	0
0	0	0	0	0
0.002493944	0	0.0013335	0	0.00837185
0	0.03736573	0.154237415	0.484455362	0
0	0	0	0	0
0	0.020653258	0.893297383	0.223456062	0.535990474
0.00085779	0	0	0	0
0	0	0	0	0
0	0	0	0	0
0	0	0	0	0
0	0	0	0	0
0	0	0	0	0
0	0	0	0	0
0	0	0	0	0
0	0	0	0	0
0	0	0	0	0
0.110949348	0.086308142	0.001429497	0.42243704	0
0	0	0	0	0
0	0	0	0	0
0	0	0	0	0
5.453175731	0	0.185425393	0	0
0	0	0	0	0
0	0	0	0	0
0	0	0	0	0
0	0	0	0	0
0	0	0	0	0
0	0	0	0	0

0.053473748	0.070611168	0.126329892	0.00867016	0
0	0	0	0	0
0	0	0	0	0
0	0.000722408	0	0	0
0	0	0	0	0
1.84792208	3.251885778	0	0	0
0	0	0	0	0
0	0	0	0	0
0	0	0	0	0
0	0	0	0	0
0	0	0	0	0
0.002299018	0.004669111	0	0	0
0	0	0	0	0
0	0	0	0	0
0	0	0	0	0
0	0	0	0	0
0	0	0	0	0
0	0	0	0	0
0	0	0.001944841	0	0
0	0	0	0	0
0	0	0	0	0.183185445
0	0	0	0	0
0.204992847	1.284876238	0.031145658	0.080576573	0.078824005
0	0.003886753	0.069730379	0.686740242	0
0	0	0	0	0
0	0	0.345210677	0	0
0	0	0	0	0
0	0	0	0	0
0	0.003216718	0.984537175	0	0
0	0	0	0	0
0	0	0	0	0
0.040548224	0	0	0	0
0.005455026	0	0	0	6.580226817
0	0	0	0	0
0.047856585	0.24968352	0.015252873	0.004499885	1.347539859
0.002862166	0	0	0	0.053676066
0	0	0	0	0
0	0	0	0	0
0.029600187	0	0.000833005	0	0.847668592
0	0	0	0	0
0	0	0	0	0
0	0	0	0	0
0.129109961	0.457521114	1.550658975	0.057655471	0
0	0	0.004470978	0	0
0	0	0	0	0
0.383197798	0.024637605	0.023399898	0	0
0	0	0	0	0
0	0	0	0	0

0	0	0	0	0
0	0	0	0	0
0	0	0	0	0
0	0	0	0	0
0	0	0	0	0
0	0	0	0	0
0	0	0	0	0
0	0	0	0	0
0.005421739	0.007025307	0.017031507	0.060774329	0
0	0	0.00139431	0.003215563	0
0	0	0	0	0
0	0	0	0	0
0	0	0	0	0
0	0	0	0	0
0	0	0	0	0
0	0	0	0	0.015130225
0	0	0	0	0
0	0	0	0	0
0	0	0	0	0
0.358417651	0.282654267	0.174744175	0.003090265	5.817502094
0	0	0	0.005507202	0
0	0	0	0	0
0	0	0	0	0
0	0	0.025843635	0.059466925	0
0.001238413	0	0	0	0
0	0	0	0	0
0	0	0	0	0
0	0.349381474	0	0	0
0	0	0	0	0
0	0	0.015233717	0.010019145	0
0.056898783	0.172578652	0.228893224	0.428283314	0
0	0	0	0	0
0	0	0	0	0
0	0	0	0	0
0	0	0	0	0
0	0	0	0	0
0	0	0	0	0
0	0	0	0	0
0	0	0.031236648	0	0
0	0	0	0	0
0	0	0	0	0
0	0	0	0	0
0	0	0.000516844	0	0
0	0.002087139	0	0	0
0	0	0	0	0
0	0	0	0	0
0	0	0	0	4.360426868
0	0	0	0	0

0	0	0	0	0
0	0	0	0	0
0	0	0	0	0
0	0	0	0	0
0.990045711	0.012453012	0.004346494	0.027556136	0
0	0	0	0	0
0	0	0	0	0
0	0	0	0	0
0	0	0	0	0
0	0	0	0	0
0	0	0.002093238	0	0
0.020431216	0.092900612	0.036206848	0.011197371	0.033118514
0.034246727	0	0	0.02044808	0.011301235
0	0	0	0	0
0.002224288	0	0	0	0.011112527
0.864316289	0.001971904	0	0	0
0	0	0.01104292	0	0.003041441
0	0	0	0	0
0.005009539	0	0.001418071	0	0
0.000961902	0	0.025304766	0	0.010995989
0	0	0	0	0
0	0	0	0	0
0	0	0	0	0
0	0	0	0	0
0	0	0	0	0
0	0	0	0	0
0.005087385	0	0	0	0.034463106
0	0	0	0	0
0	0	0	0	0
0	0	0	0	0
0	0	0.048775232	0	0.000665893
0	0	0	0	0.001678232
0	0	0	0	0
0	0	0	0	0
0	0	0	0	0
0	0	0	0	0
0	0	0	0	0
0.043953708	0.053817412	0.008534192	0.003532593	0.022612137
0	1.101832004	0.086540865	0.064584585	0
0	0	0	0	0
0.059446325	0.148112614	0	0.003644222	0
0	0	0	0	0
0	0	0	0	0
0	0	0	0	0
0	0	0	0	0
0	0	0	0	0
0	0	0	0	0
0	0	0	0	0

0	0	0	0	0
0	0	0.001497658	0	0
0	0	0	0	0
0.003816011	0	0.001836364	0	0.011148042
0	0.001257899	0	0	0.069455346
0	0	0	0	0.004351398
0	0	0	0.000887914	0
0	0.002121409	0	0	0
0	0	0	0.028840388	0
0	0	0	0	0
0	0	0	0	0
0	0	0	0	0
0.554798544	0.184426609	0.108621587	0.003372663	0
0	0	0.011025307	0	0
0	0	0.003933621	0	0
0	0	0	0	0
0	0	0	0	0
0	0	0	0	0
0	0	0	0	0
0	0	0	0	0
0	0	0	0	0
0	0	0	0	0
0.013357055	0	0	0	0.03483624
0	0	0.006067984	0.006046795	0
0	0	0	0	0
0	0.006067682	0	0	0
0	0.00886956	0.002183527	0	0
0	0	0.001092293	0	0
0	0	0	0	0
0	0	0	0	0
0.107428581	0.003058261	0.007644721	0.01733101	0
0	0	0	0	0
0	0	0	0	0
0	0	0	0	0
0.018312805	0	0	0	0
0	0	0	0	0
0	0	0	0	0
0	0	0	0	0
0	0.000796154	0.008983283	0.002094748	0.081517906
0	0	0	0	0
0	0	0	0	0
0	0	0	0	0
0	0	0	0	0
0	0	0	0	0
0	0	0	0	0
0.283812097	0.465821911	0.085677249	0.262702982	0.975385601
0	0	0	0	0.432624859
0	0	0	0	0

0	0	0	0	0
0	0	0	0	0
0	0	0.073774281	0	0
0	0	0	0	0
0	0	0	0	0
0	0	0.019646964	0	0
0	0	0	0	0
0	0	0	0	0
0	0	0	0	0
0	0	0	0	0.001461253
0.095136737	0	0.00145757	0.00039217	0.001558104
0	0	0	0	0.002067782
0	0	0	0	0
0	0	0	0	0
0	0	0	0	0
0	0	0	0	0
0	0	0	0	0
0	0	0	0	0
0	0	0	0	0
0.004699655	0	0	0	0
0	0	0	0	0
0	0	0	0	0
0	0	0	0	0
0	0	0	0	0
0	0	0	0	0
0	0	0	0	0
0	0.011850515	0	0	0
0	0	0.005134027	0.006331174	0
0	0	0	0	0
0	0	0	0	0
0.009317284	0.032378697	0.005812229	0.001191483	0.006903467
0	0	0	0	0
0.006671295	0.013243681	0.017739149	0.018157565	0.022214741
0.003316544	0.001964556	0	0.004594587	0.000702095
0	0.001271713	0	0	0
0	0	0	0	0
0	0	0	0	0
0	0	0	0	0
0	0	0	0	0
0	0	0	0	0
0	0	0	0	0
0.136617001	0.015113052	0	0.031583336	1.353732956
0.018613591	0.122460932	0.015267101	0.034837654	0
0	0	0	0	0
0	0	0	0	0
0.519417354	0.133651557	0.688390941	0.037318815	0
0.00462278	0.011358894	0	0	0
0	0	0	0	0

0	0	0	0	0
0.007572895	0.002018613	0.001822137	0.246218987	0
0.002874931	0.004453911	0.00331092	0.014210808	0.013763892
0	0	0	0	0
0	0	0	0.770386448	0
0	0.001018123	0	0	0.107629047
0	0	0	0	0
0	0	0	0	0
0	0	0	0	0
0	0	0	0	0
0	0	0	0	0
0	0	0	0	0
0	0	0	0	0
0	0	0	0	0
0	0	0	0	0
0	0	0	0	0
0	0	0.155353674	0.322602725	0
0	0	0	0	0
0	0	0	0	0
0	0	0	0	0
0	0	0	0	0
0	0	0	0	0
0	0	0	0	0
0	0	0	0	0
0	0.008420901	0.263510907	0.002622023	0
0	0.068421451	0	0	0.01071253
0	0	0	0	0
0.009248341	0.000842809	0.004374464	0.002217501	0
0	0	0	0	0
0	0	0	0	0
0	0	0.003897545	0	0
0	0	0	0	0.005514019
0	0	0	0	0
0	0	0	0	0
0	0	0	0	0
0	0	0	0	0
0	0	0	0	0
0	0	0	0	0.235290763
0.002164037	0.046738884	0	0.797155162	0
0	0	0	0	0
0	0	0	0	0
0.020487	0.009896897	0.00212705	0.003433789	0
0	0.002615939	0.001377439	0.031943038	0.004908154
0	0	0	0.01815863	0
0	0	0	0	0
0.003175428	0	0	0	0
0	0	0.001505151	0	0.00214529
0	0	0	0	0

0	0	0	0	0
0.008347137	0.001893611	0	0.012604665	0.017820828
0	0	0.004729079	0	0
0	0	0	0	0
0	0	0	0	0
0	0	0	0	0
0	0	0	0	0
0	0	0	0	0
0	0	0	0	0
0	0	0	0	0
0	0	0	0	0
0	0	0	0	0
1.196263433	0.233978203	0.011644359	0.016565281	0
0	0	0	0	0
0	0.001049354	0.048544948	0	0.109974374
0	0	0	0	0
0	0	0	0	0
0	0	0	0	0
0	0	0	0	0
0	0	0	0	0
0	0	0	0	0
0	0	0.00837219	0.001042869	0
0	0	0	0	0
0	0	0	0	0
0	0	0	0	0
0	0	0	0	0
0	0	0	0	0
0	0	0	0	0
0	0	0	0	0
0	0	0	0	0
0	0	0	0	0
0	0	0	0	0
0	0	0	0	0
0	0	0	0	0
0	6.995619505	6.972670988	0.181419793	0.043889275
0	0	0	0	0
0	0	0	0	0
0	0	0	0	0
0.001520193	0	0.010683046	0	0.038066322
0	0	0	0	0
0.042449401	1.104992139	0.243425536	0.089642966	0.013603131
0	0	0	0	0
0	0	0	0	0
0	0	0	0	0
0	0	0	0	0
0.049716393	0.01479079	0.042676061	0.013684679	4.755934527
0	0	0	0	0
0	0	0	0	0

0	0	0	0	0
0	0	0	0	0
0	0	0	0	0
0	0	0	0	0
0	0	0	0	0
0	0	0	0	0
0	0	0	0	0
0	0	0	0	0
0	0	0.006460357	0	0
0	0.004907909	0.017021339	0	0.374431266
0	0	0	0.056239284	0
0	0	0	0	0
0.001278689	0	0.007292899	0.004087931	0.004060374
0	0	0	0	0
0	0	0	0	0
0	0	0	0	0
0	0	0	0	0
0	0	0	0	0
0	0	0	0	0
1.457746378	1.764296929	0.053755205	0.051849297	4.130824101
0	0	0	0	0
0	0	0	0	0
0	0	0	0	0
0	0	0	0	0
0	0	0	0	0
0	0	0	0	0
0	0	0	0	0
0	0	0	0	0
0	0	0	0	0
0	0	0	0	0
0	0	0	0	0
0	0	0	0	0
0	0	0	0	0
0	0	0	0	0
0	0	0	0	0
0	0.00319988	0	0	0.006861455
0	0	0	0	0
0	0	0	0	0
0	0	0	0	0
0	0	0	0	0
0	0	0	0	0
0	0	0	0	0
0.000788378	0.000933991	0.001854785	0.010283313	0
0	0	0	0	0
0.012293124	0	0.067445502	0	0.00633625
0	0	0	0	0
0	0	0	0	0
0	0	0	0	0

0	0	0	0	0
0	0	0	0	0
0	0.001725811	0	0	0
0	0	0	0	0
0	0	0.004297462	0	0
0.021280684	0.049319486	0.175942945	0.175654571	0
0	0	0	0	0
0	0	0	0	0
0	0	0	0	0
0	0	0	0	0
0	0	0	0	0
0	0	0	0	0
0	0	0	0	0
0.066490093	0	0	0	0
0	0	0	0	0
0	0	0	0	0
0	0	0	0.061738588	0
0	0	0	0	0.005409452
0	0	0	0	0
0	0	0	0	0
0	0	0	0	0
0	0	0	0	0
0	0	0	0	0
0	0	0	0	0
0	0	0	0	0
0	0	0.006006884	0	0.047300213
0	0	0	0	0
0	0	1.316585129	3.645258609	0
0	0	0	0	0
0	0	0	0.009224429	0
0	0	0	0	0
0	0	0	0	0
0	0	0	0	0
0	0	0	0	0
0	0	0	0	0
0	0	0	0	0
0	0	0	0	0
0	0	0	0	0
0.020088978	0	0	0	0
0	0	0	0	0
0	0	0	0	0
0	0	0	0	0
0	0	0	0	0
0	0	0	0	0
0	0	0	0	0
0	0	0	0	0
0	0	0	0	0
0	0	0	0	0

0	0	0	0	0
0.023229805	0	0.007643614	0.024136127	0
0	0	0	0	0
0	0	0	0	0
0	0	0	0	0
0	0	0.000873472	0	0
0	0	0	0	0
0	0	0	0	0
0	0	0	0	0
0	0	0	0	0
0.001559876	0.008546937	0.003336236	0	0.005696251
0	0	0	0	0
0.145770216	0.195833362	0	0.042837395	0
0	0	0	0	0
0	0	0	0	0
0	0	0	0	0
0	0	0	0	0
0	0	0	0	0
0	0	0	0	0
0	0	0.001214556	0	0
0	0	0.003386078	0	0.070381679
0	0	0	0	0
0	0	0	0	0
0.929857872	0.939819764	0.226155217	1.777190515	0
0	0	0	0	0
0	0	0	0	0
0	0	0	0	0
0	0	0	0	0
0	0	0	0	0
0	0	0	0	0
0	0	0.002908005	0	0.015542905
0	0	0	0	0
0.089096477	0.001972947	0	0	0
0	0	0	0	0

PHL018-0236_V2	PHL018-0270_V2	PHL018-0031_V1	PHL018-0002_V1	PHL018-0176_V2
EYCF	REF	CTRL	CTRL	REF
0.019621469	0.063556803	0.768218383	0.659727742	0.135828505
0.259375352	2.764144366	0.492458397	5.555062784	1.195227277
12.94911487	0	6.791173506	3.855698681	6.699702213
0	0	0	0	0
0	0	0	0	0
0	0	0	0	0
0	0	0.039003004	0	0
0	0	0	0	0
0	0	0	0	0
0	0	0	0	0
0	0	0	0	0
0	0	0	0	0
0	0	0	0	0
3.574056606	4.602091562	16.93022118	1.569982994	0.969878494
0	0	0	0	0
0	0	0	0	0
0	0	0	0	0
0	0	0	0	0
0	0	0	0	0
0	0	0	0	0
0	0	0	0	0
0	0	0	0	0
0	0	0	0	0
0	0	0	0	0
0	0	0	0.544462652	0
0	0	0	0	0
0	0	0	0	0
0	0	0	0	0
0	0	0	0	0
0	0	0	0	0
0	0	0	0	0
0	0	0	0	0
0	0	0	0	0
0	0	0	0	0
0	0	0	0	0
0	4.353688365	0	0	2.473588122
0	0	0	0	0
0	0	0	0	0
0	0	0	0	0
0	0	0	0	0
0	0	0	0	0
0.009021871	0	0.059433009	0	0
0.008245966	0.005813602	0.145626342	0.010725725	0.025295539
0	0.022613827	0.075121843	0	0.009206543
0	0	0.012546764	0	0
0	0	0	0	0

0	0	0.002845654	0	0
0.009509677	0	0.005406991	0	0.147461169
0	0	0.104521727	0.076001884	0
0.174539829	0.564354421	0.32279041	0.290409319	0.346907388
0	0	0.00674082	0.032639423	0.003428927
0	0	0.047612662	0	0
0.002223892	0	1.038779377	0	0.927711681
0	0	0	0	0
0	0	0	0	0
0	0	0	0	0
0	0	0	0	0
0	0.105447771	0.081517463	0.771415438	0.078900917
0	0	0	0	0
0.041011554	0.165185492	0.014025823	0.016363957	0.051675394
0	0	0	0	0
0	0	0	0	0
2.272189478	0.065111435	0	0	4.172641779
0	0	0	0	0
0	0	0	0	0
0	0	0	0	0
0	0.199140408	0.030398124	0.065448017	0.211019237
0.004082051	0.008137508	0.009371631	0.008379451	0.095025601
0	0	0	0	0
0	0	0	0	0
0	0	0	0	0
0	0	0	0	0
0	0	0	0	0
0	0	0	0	0
0	0	0	0	0
0	0	0	0	0
0	0	0	0.108597266	0.0046815
0	0	0	0	0
0	0	0	0	0
0	0	0	0	0
0	0	0	0	0
0	0	0.004621813	0	0
0	0	0	0	0
0.125504315	0	0	0	0.088357057
0	0	0	0	0
0	0	0	0	0
0	0	0	0	0
0	0	0	0	0
0	0	0	0	0
0	0	0	0	0
0	0	0	0.588884164	0
0	0	0	0	0
0	0	0.001228874	0.12043334	0.003947092
0	0	0.00473419	0.048679257	0.088417155

0	0	0	0	0
0	0	0	0	0
0	0	0	0	0
0	0	0	0	0
0	0.005385236	5.067319311	0	0
0	0	0	0	0
0	0	0	0	0
0	0	0.003666741	0	0
0	0	0	0	0
0	0	0	0	0
0	0	0	0	0
0	0	0	0	0
0	0	0	0	0
0	0	0	0	0
0	0	0	0	0
0	0	0	0	0
0	0	0	0	0
0	0	0	0	0
0	0.014930665	0.24816377	0.833531805	0.012378361
0	0	0	0	0
0	0	0	0	0
0	0	0	0	0
0	0	0	0.003506649	0
0	0	0	0.000823202	0
0	0	0	0	0
0	0	0	0	0
0	0	0	0	0
0	0	0	0.003861559	0
0	0	0	0	0
0	0	0	0	0
0	0.081756629	0.087634116	0.008275669	0.031915128
0.000617805	0	0.032643879	0	0
0	0	0	0	0
0	0	0	0	0
0	0	0	0	0
0	0	0	0	0
0	0	0	0	0
0	0	0	0	0
0	0	0	0	0.178010967
0	0	0	0	0
0	0	0	0	0
0	0	0	0	0
0	0	0.001540047	0	0.007739921
0	0	0	0	0
0	0	0	0	0
0	0	0	0	0
0	0	0	0	0
0	0	0	0	0

0	0	0	0	0
0	0	0	0	0
0	0	0	0	0
0	0	0	0	0
0	0	0	0	0.074082218
0	0	0	0	0
0	0	0	0	0
0	0	0	0	0
0	0	0	0	0
0	0	0	0	0
0	0	0	0	0
0	0	0	0	0
0	0	0	0	0
0	0	0	0.00111809	0
0	0	0	0	0
0	0	3.001129829	0	2.675437322
0	0	0	0	0
0	0	0	0	0
0	0	0	0	0
0	0	0	0	0
0	0	0	0	0
0	0	0	0	0
0	0	0	0	0
0	0	0	0	0
0	0	0	0	0
0	0	0	0	0
0	0	0.079748797	0.15391883	0
0	0	0	0	0
0	0	0	0	0
0	0	0	0	0
0	0.012801459	0	0.007300071	0
0	0	0	0	0
0	0	0	0	0
0	0	0	0	0
0	0	0	0	0
0	0	0	0	0
0	0	0	0	0
0	0	0	0	0
0	0	0	0	0
0	0	0	0	0
0	0	0	0	0
0	0	0	0	0
0	0	0	0	0
0	0	0	0	0
0	0	0	0	0
0	0	0	0	0
0	0	0	0	0
0	0	0	0	0

0	0	0	0	0
0	0	0	0	0
0	0	0	0	0
0	0	0	0	0
0	0	0	0	0
0	0	0	0	0
0	0	0	0	0
0	0	0	0	0
0	0	0	0	0
0	0	0	0	0
0	0	0	0	0
0	0	0	0	0
0	0	0	0.001085131	0.000972784
0	0	0	0	0
0	0	0	0	0
0	0	0	0	0
0	0	0	0	0
0	0	0	0	0
0	0	0	0	0
0	0	0	0	0
0.038260463	0.015029657	0.008251535	0.015555158	0.018916279
0	0	0	0	0
0	0	0	0	0
0	0	0	0.001763141	0.001517375
0	0	0	0	0
0	0	0	0	0
0.004159305	0.002775316	0.00048055	0	0
0	0	0	0	0
0	0	0	0	0
0.002383916	0	0.002399288	0	0
0	0	0.004949943	0	0
0	0	0	0	0
0	0	0	0	0
0	0.006272815	0.088249451	0.001653278	0
0.001991544	0.008825038	0.02362311	0.015181523	0.000946765
0	0	0	0	0
0	0	0	0	0
0	0	0	0	0
0	0	0	0	0
0	0	0	0	0
0	0	0	0	0
0	0	0	0	0
0	0	0	0	0
0	0	0	0	0
0	0	0	0	0
0	0	0.172777713	0	0.047550062
0	0.004131965	0.000775077	0	0
0	0	0.001785474	0.002195111	0

0	0	0	0	0
0	0	0	0	0
0	0	0	0	0
0	0	0	0.001395136	0.001072024
0	0	0	0	0
0	0	0	0	0
0	0	0	0	0
0	0	0.000549997	0	0
0	0	0	0	0
0	0	0	0	0
0	0	0	0	0
0	0	0	0	0
0	0	0	0	0
0	0	0	0	0
0	0	0	0	0
0	0	0	0	0
0	0	0	0	0
0	0	0	0	0
0	0	0	0	0
0	0.057446091	0	0.045317459	0
0.050033412	0.007904583	0.057396685	0.012701717	0.070597386
0	0	0	0	0
0	0	0	0	0
0	0	0	0	0
0	0	0	0.002336541	0
0	0	0	0	0
0	0	0	0	0
0	0	0	0	0
0	0	0	0	0
0	0	0.001303652	0	0
0.582501171	0.111182465	0.197935629	0.09408749	0.110518552
0	0	0	0	0
0.092300914	0	0	0	0
0	0.002183149	0.051690109	0	0
0	0	0	0	0
0	0	0	0	0
0	0	0	0	0
0	0	0	0	0
0	0	0	0	0
0	0	0	0	0
0	0	0	0	0
0.00239367	0	0.110417267	0.008991239	0.003413797
0	0	0	0	0
0	0	0	0	0
0	0	0	0	0
0	0	0	0	0
0	0	0	0	0
0	0	0	0	0

0	0	0	0	0
0	0	0	0	0
0	0.006239431	0.089490368	0	0.005715444
0	0	0	0	0
0	2.297728549	0.001250096	1.191868301	0.005786691
0	0	0	0	0
0	0	0	0	0
0	0	0	0	0
0	0	0	0	0
0	0	0	0	0
0	0	0	0	0
0	0	0	0	0
0	0	0	0	0
0.008673542	0.001060877	0.034626005	0.016636622	0.00500064
0.004874126	0	0.013304126	0.002019317	0
0	0	0	0.017295562	0
0.001041306	0.001496527	0	0	0.002227629
0	0	0	0	0
0	0	0	0	0
0	0	0	0	0
0	0	0	0	0
0	0	0.000675759	0.001869294	0
0	0	0	0	0
0	0	0	0	0
0	0	0	0	0
0	0	0	0	0
0	0	0	0	0
0	0	0	0	0
0	0	0	0	0
0	0	0	0	0
0	0	0	0	0
0	0	0	0	0
0	0	0	0	0
0	0	0	0	0
0	0	0	0	0
0	0	0	0	0
0	0	0	0	0
0	0.001131541	0.003396086	0.000782858	0
0	0	0	0	0
0	0	0	0	0
0	0	0.004288823	0	0
0	0	0	0	0
0	0	0	0	0
0	0	0.002372889	0.009724321	0
0	0	0	0	0
0	0	0	0	0
0	0	0	0	0
0	0	0.001799661	0	0

0	0	0	0.933310974	0
0	0	0	0.310289798	0
0	0.003537502	0	0	0
0	0	0	0	0
0.003797227	0.002609983	0	0.002626501	0
0	0	0	0	0
0	0	0	0	0
0.004396972	0	0.007985545	0	0
0	0	0	0	0
0.615405196	0.019539911	0.12372033	0.151472784	0
0	0	0	0	0
0	0	0.00059263	0	0
0.043910743	0.012381739	0.001198749	0	0.089973158
0	0	0	0.004196784	0
0	0	0	0	0.001019112
0	0	0	0	0.010709972
0	0	0	0	0
0	0	0	0.002879101	0
0.009651418	0	0.007261034	0	0.021447213
0.041559634	0	0	0.005962898	0
0	0	0	0	0
0	0	0	0	0
0	0	0	0	0
0	0	0	0	0
0	0	0	0	0
0.000809254	0	0	0	0
0	0	0	0	0
0	0	0	0	0.003349619
0.020216836	0	0	0.13532926	0
0.00166524	0	0.001256983	0	0
0	0	0	0	0
0	0	0	0	0
0	0	0	0	0
0	0	0	0.004209303	0
0	0	0	0	0
0	0	0	0	0
0	0	0.001780625	0	0
0	0	0	0	0
0	0	0	0	0
0	0.000978568	0	0	0.006312064
0	0	0	0	0
0	0	0	0	0
0	0	0	0	0
0.002868638	0.109251613	0	0	0.018596296
0	0	0	0	0
0	0	0.000893849	0	0
0.001006371	0	0.01758035	0	0
0	0	0	0.007023812	0

0	0	0	0	0
0.019344661	0	0.057001342	0	0.475106687
0	0	0	0	0
0	0	0	0	0
0.014522504	0.005146328	0	0	0.00624187
0.027266775	0.017998598	0.004786498	0.021016625	0
0	0.002280013	0	0	0
0.000820144	0	0	0	0
0	0	0	0	0
0	0	0	0	0
0.00165479	0.012155263	0	0.002437575	0
0	0	0	0	0
0	0	0	0	0
0.000868534	0.004243969	0	0.001842318	0.080018973
0.018630576	0	0	0	0.002255986
0.003188576	0	0	0	0
0.000849314	0	0	0	0.001211271
0	0	0	0	0
0	0	0	0	0.013447562
0.00479139	0	0	0.09417719	0
0	0	0.00746748	0	1.264963246
0.399269968	0	0.218556387	0.006769557	0.341993879
0	0	0	0	0
0	0	0	0	0
0	0	0	0	0
0	0	0	0	0
0	0	0	0	0
0	0	0	0	0
0	0	0	0	0
0.988681377	0.878592288	0.075779648	0.059409962	0.11075471
0	0	0.000762468	0	0
0	0	0	0	0
0	0	0	0	0
0.001001464	0	0.00089993	0	0
0.005490556	0	0	0	0.002013558
0	0	0	0	0
0	0	0	0	0
0	0	0	0	0
0.416520223	0	0.051771092	0.46692192	0
0	0	0	0	0
0.003284537	0.026475387	0.010779508	0.321092262	0.010590662
0	3.15778619	1.402941713	0	6.490622306
0	0	0	0	0
0	0	0	0	0
0.003382944	0	0.009206741	0	0
0	0	0	0	0
0.012140694	0	0.003717413	0	0.001710101
0	0	0	0	0

0	0	0.005783186	0	0.001318736
0	0	0	0.045744183	0
5.397276978	0.158619249	0	0	9.410616641
0	0	0	0	0
0	0.044885014	0	0	0
0.010007109	0	0.00067444	0	0
0	0	0	0	0
0	0	0	0	0
0	0.003558932	0	0	0
0.02739972	0	0.00802246	0	0
0	0	0	0	0
0	0	0	0	0
0	0	0	0	0
0.006190258	0.251263616	0	0	0
0	0	0	0	0
0	0.001127415	0.098127385	0.01300006	0.014730821
0.000579671	0	0.183461072	0.001536982	0
0	0	0.004366827	0.036880632	0.003515484
0.004340981	0.008820232	0.000807076	0.001786035	0
1.448917325	0.013887765	0	0	0
0	0.343848757	0.017725029	0.000726388	0.129194784
0	0	0.006706811	0.005125607	0
0	0	0	0	0
0	0.683045846	0.014835218	0	0
0	0.000583946	0	0	0
0.207130259	0.031276988	0.219444931	0	0
0	0.002115208	0.001388703	0.022926737	0
0	0	0	0	0
0	0	0	0	0
0	0	0	0	0
0.162577516	0.076006795	0.028816568	0.033016573	0
0	0	0.009896998	0.006083823	0.000892464
0.012710857	0.008848361	0.003640813	0.019747575	0.026908648
0	0	0	0	0
0	0.002043706	0.05914695	0	0.00144863
0	0	0	0.003719289	0
0	0	0.005227749	0	0.03200957
0.00365092	0.010897552	0.019078929	0.039093617	0.026835341
0	0	0.038132556	0	3.452503174
0.002985	0	0.000619007	0.093606004	0
0	0	0	0.003721698	0
0	0	0	0	0
0	0	0	0.003897629	0
0	0.031822041	0.051417884	0.420994612	0.018759664
0.008580234	0	0	0	1.83921086
0	0	0.120989159	0	0
0	0	0	0.002896135	0
0	0	0	0	0

0	0	0.011449792	0	0
0	0.096245442	0.199313873	0.148902369	0.112701402
0	0	0	0	0
0	0	0	0	0.008924546
0	0	0	0	0
0	0	0	0	0
0	0	0.001717931	0	0
0	0	0	0.000779371	0
0	0	0	0.000928844	0
0.009176842	0	0	0	0
0.002843731	0.032695262	0.002913176	0.00263903	0.004055664
0.018002229	0.0140014	0.09866103	0	0.577220106
0	0	0	0	0
0	0	0	0	0
0	0	0	0	0.004188031
0	0	0	0	0
0.002233348	0.007917227	0.002408302	0.002960834	0.001486403
0.01488253	0.049443853	0.002501047	0.024598856	0.004686066
0	0	0	0.004602831	0
0	0.010461588	0	0.015806824	0.005011812
0.000767715	0.031775969	0.002317996	0.003867598	0.012481831
0	0	0	0	0.001907394
0	0	0	0	0
0	0	0	0	0
0	0	0.005841998	0	0.286614669
0	0	0	0	0
0.105027355	0	0.004085674	0.252558571	0.151516739
0.000652774	0.043389135	0.07443855	0.01968802	0.049806976
0	0	0	0	0
0	0	0	0	0
0	0.008304687	0	0	0
0	0	0	0	0
0	0	0	0	0
0	0	0	0	0
0	0	0	0.201167472	0
0.012704986	1.097930394	0.027996179	0.097399258	0.319849592
0	0	0	0	0
0.005593459	0	0	0	0
0	0.002893262	0	0	0
0	0	0	0	0
0.006316389	0	0	0	1.763102115
0	0	0	0	0
0.0022011	0.006326682	0.000659313	0.146390412	0.023020491
0	0	0.000509244	0.001408676	0
0.013959578	0	0	0	0
0	0	0	0	0.327757272
0	0	0	0	0
0.000954192	0	0.001852094	0.001518011	0.001360847

0	0	0	0	0
0	0	0	0	0
0	0	0	0	0
0	0.529309845	0.013975621	0.142554122	0.051388875
0	0	0	0	0
0	0	0	0	0
0	0	0	0	0
0	0.013000864	0	0	0.001090265
0	0	0	0	0
0	0.116347377	0	0	0
0	0	0	0	0
0	0.005098812	0.009564391	0	0.001751481
0	0	0	0	0
0	0	0	4.165377809	0
0.097288997	0.027834581	0.371800276	2.153393743	0.178256906
0	0	0	0	0
0	0	0	0	0
0	0	0	0	0
0	0	0	0	0
0	0	0	0	0
0	0	0	0	0
0	0	0	0	0
0	0	0	0	0
0.438200842	1.54869529	0.342582957	0.64622321	2.41086034
0	0	0.082521135	0.156160933	0
0	0	0	0	3.347366027
0	0	0	0	0
0.021692691	0	0.581776001	0	0.877988193
0	0	0	0	0
0	0	0	0	0
0	0.005338216	0.000429149	0.001758691	0
0	0	0	0	0
0	0	0.057592859	0	0.002447827
0	0	0	0	0
0	0	0	0.124543729	0
0	0	0	0.512217991	0
0	0.278084747	0	0.045835427	0
0.002413621	0	0	0	0.025310665
0.012926519	0	0.037375289	0	0.002431055
0	0	0.020432486	0	0.016436917
0.011519567	0	0.105727958	0.116795522	0.004107235
0	0.016339453	0	0.557536393	0
11.21108717	0.721743537	0.437398335	12.07553216	5.646494151
0	0	0	0	0
0	0	0	0	0
0	0	0	0	0
0	0	0	0	0
0	0.107423494	0.006278497	0.004172411	0

0	0	0	0	0
0	0	0	0	0
0.056870103	0.004119102	0.014151956	0.043197047	0.008605499
0	0	0	0	0
0	0	0	0	0
0	0.001537044	0	0	0.001355816
0	0	0	0	0
0	0	0.00038958	0	0
0	0	0	0	0
0.46837446	0	0	0	0
0.032435443	0	0.36381783	0.037699413	0.086391782
1.795948251	1.239625104	1.712743881	2.414262751	2.087972625
0	0	0	0	0
0.000799216	0	0.021717966	0	0
0.098880595	0.063903167	0.030425938	0.31795525	0.37078701
0	0	0	0	0
0	0	0.006178915	0	0
0	0	0	0	0
0	0	0	0	0
0.002318023	0.032612448	0.003946753	0.003073091	0.018791496
0	0.090807161	0.001204739	0.007405702	0.000885196
24.03024261	0	29.09048684	0.816226425	0.031899092
18.00733627	0.373072327	2.475576675	4.646766266	0.163204593
0	0	0	0	0
0	0	0	0	0
0	0.008551215	0	0	0.002970045
0	0	0	0	0
0	0	0	0	0
0	0	0	0.397452817	0
0	0	0	0	0
0	0	0.001624084	0	0
0	0.62839804	0	0.072712119	0.286809806
0.005932282	0.00131164	0.257356216	0	0.641261273
0	0	0.120204036	0.278341101	0.018197311
0.004216563	0.001009982	0	0.027950284	0.006299925
0.001793164	0.030726611	0.001933635	0.014080726	0.015540937
0.002131904	0.018191879	0.016236061	0.100511924	0.00817127
0	0	0	0	0
0	0.023893567	0	0.004707798	0.021178673
0	0	0	0	0
0	0	0	0	0
0	0	0	0	0
0	0	0	0	0
0	0	0	0	0
0.012426701	0	0.481558095	0.037328405	0.035221011
0	0	0	0	2.187035081
0	0	0	0	0
0.000948734	0	0.060360248	0.071693034	0.067653127

0	0	0	0	0
0	0	0	0	0
0	0	0	0	0
0	0.004046176	0.001301117	0.006576253	0
0	0	0	0	0
0	0	0	0	0.0567398
0	0	0	0	0
0.01751785	0	0.847396016	0.192334596	0.01724217
0	0	0	0	0
0	0	0	0	0
0	0.001072945	0.004991342	0.001583612	0
0	0	0	0	0
0	0	0.014379731	0.014905692	0
0	0.082951059	0	0	0
0	0	0	0	0
0	0	0	0	0
0	0	0.065143904	0	0
0.000497203	0	0.004289217	0	0.004254594
0	0.018609733	0	0	0
0	0	0	0	0.006235026
0	0	0.007621262	0	0.001417452
0	0	0	0	0
0	0.014623133	0	0.014629321	0
0	0	0	0	0
0	0	0	0	0
0	0	0	0	0
0	0	0	0	0
0	0	0	0	0
0	0	0	0	0
0	0	0	0.001791585	0.07131072
0	0.004901369	0	0.009666348	0.003186692
0	0	0	0	0
0	0	0	0	0.004074882
0	0	0	0	0
0	0	0	0	0
0	0	0	0	0
0	0.22727879	0	0	0
0.002401545	0	0.003816363	0.004021666	0.022172547
0	0	0	0	0
0.000623356	0.001791728	0.007797373	0.005619561	0
0	0.000681743	0.052815217	0.016351043	0
0	0	0	0	0
0	0	0	0	0
0	0	0	0	2.377116547
0	0	0	0	0
0	0	0	0	0
0	0.003624622	0	0	0
0	0	0	0	0

0	0	0	0	0
0	0	0	0	0
0	0	0	0	0
0	0.038668047	0	0	0
0	0	0	0	0
0	0	0	0	0
0	0	0	0	0
0	0	0	0	0
0.015166074	0.009019089	0.062972839	0.023572831	0.092982011
0	0	0	0	0
0	0.002507917	0	0	0
0	0	0	0	0
0	0	0	0	0
0	0	0	0	0
0	0	0	1.92502174	0.014828888
0.08115063	0	0.250643011	0	0
0	0	0.005848748	0	0
0	0	0	0	0
0	0	0	0	0
0	0	0	0	0
0	0.004597591	0	0.106205006	0
0	0	0	0	0
0.063002007	0	0	0	0
0	0.127415995	0	0	0
0	0.004419247	0.019895176	0	0.012864023
0	0.243254939	0.812796427	2.800581958	3.188842908
0	0	0	1.235245745	0
0	0	0	0	0
0	0	0	0	0.054444905
0	0	0	0	0
0	0	0	0	0
0	0.381746064	0.003416694	0.151570911	0.004707103
0	0.002853366	0	0	0
0.001532753	0	0.014403186	0.017127101	0.011554451
0	0	0.132859741	0	0
0.002550311	0	0.01251293	0.003719148	0.001273018
0.238971988	0	0	0	0.30797335
0	0.164925613	0	0	0.209252719
0	0	0	0	0
0	0	0	0	0
0	0	0	0	0
0	0	0	0.00440925	0
0	0.007512871	0.586820014	0.392952766	0
0	0	0	0	0
0	0	0	0	0
0.001932879	0.001215316	0.000521074	0.293725566	0
0	0	0	0	0
0	0	0.002905466	0.094659625	0.058463692

0.010189582	0	0.0267731	0	0.001228069
0	0	0	0	0
0	0	0	0	0
0	0	0	0	0
0.000571544	0	0.003697904	0	0
0	0	0	0.001276924	0
0	0	0	0	0
0	0	0	0	0
0	0	0	0	0
0	0	0	0.002091454	0
0	0	0.001768466	0	0
0	7.176520464	1.453179051	1.315948359	0
0	0.338155336	0.014178791	0	0.152980532
0	0	0	0	0
0.029248844	0.005462224	0.164115502	0.000657225	0.000707017
0	0	0	0	0
0	0	0	0.004114757	0.002787052
0	0	0	0	0
0	0.00083263	0.002030409	0.023042228	0.000619698
0	0	0.002597768	0	0
0	0	0	0.111440611	0.028902002
0	0	0	0	0
0	2.421368443	0.054369092	0.029127067	0.130356476
0	0	0	0	0.018679703
0	0	0	0	0
0	0	0	0	0
0	0	0	0	0
0	0	0	0	0
0	0	0	0	0
0	0	0	0	0
0	0	0	0	0
0.00107216	0.069030956	0.00624321	0.00909698	0.029664344
0	3.734387883	0	0	0.022837488
0.003535176	0.020168547	0.044590146	0.125405158	0.038042566
0.001942764	0	0.0151136	0.00331148	0.002176998
0	0.015168549	0.074460787	0.03083094	0
0	0	0.001062643	0	0
0	0	0	0	0
0	0.006873593	0	0	0.007730518
0	0	0	0	0.004459971
0	0	0	0	0
0	0	0	0	0
0	0	0	0	0
0	0	0.000609102	0	0
0.002460622	0	0.134880156	0.075572906	0.018716174
0	0	0	0	0
0	0.081822877	0	0	0
0.000905064	0.000557454	0.005716363	0.003599634	0.002397166

0	0	0	0	0
0	0	0	0	0
0.001413833	0	0	0.013682913	0
0	0	0	0	0
0.846390363	0	0	0	0
0	0	0.000393476	0	0
0	0	0	0	0
0	0.03012749	0.013822379	0.003186304	0.012758665
0	0	0	0	0
0	0	0	0	0
0	0	0	0	0
0	0	0	0	0
0	0	0	0	0
0	0	0	0	0
0	0	0	0	0
0	0	0	0	0
0	0.038800276	0	0	0
0	0	0	0	0
0	0	0	1.346100393	0
0	0	0	0.000797865	0
0	0.002400023	0	0	0
0	0	0	0	0
0	0	0	0	0
0	0	0	0	0
0	0	0	0.003762181	0
0.003054328	0	0.005901025	0.005905139	0.001633505
0	0	0	0	0
0	0	0.00644755	0.006605665	0
0	0	0	0	0
0	0.62847135	0.209775146	0.236385871	0.206345749
0	0	0	0	0
0	0	0	0	0
0	0.001890312	0	0.001918128	0
0	0	0	0	0
0	0	0.010210641	0.003005707	0.002092215
0	0	0	0	0
0	0	0	0	0
0	0.003914738	0.00489553	0.054168312	0.016186709
0	0	0	0	0
0	0	0.035620913	0	0
0.599036	27.61912682	1.580600708	0.759730831	1.482879272
0	2.130100246	0.649458698	0.857939888	0.566054017
0	0	0	0	0
0	0	0	0	0
0	0	0	0	0
0	0	0	0	0
0	0	0	0	0
0.001201035	0	0.027715591	0.004936	0.006166401

0	0	0	0	0
0	0.006391514	0.03077241	0.045202422	0.000528554
0	0	0	0.01759796	0
0	0	0	0	0
0	0.060102584	0.383440223	2.384633072	0.000480993
0	0	0	0	0.090512573
0	0	0	0	0
0	0	0	0	0
0.016652828	0.011345939	0.053340015	0.490279223	0.104499465
0	0	0	0	0
0	0	0	0	0
0	0	0	0	0
0	0.099367124	0.044397921	0.335938555	0.055466708
0.046780606	0.088440034	0	0.001565144	0.003367441
0	0	0	0	0
0	0.540035494	0.300603563	1.070502038	0.741254567
0	0	0.002203283	0	0.002913998
0	0	0	0	0
0	0	0	0	0
0	0	0	0	0
0	0	0	0	0
0	0	0	0.007970616	0
0	0	0	0	0
0	0	0	0	0
0	0	0	0	0
0	0	0	0	0
0	0.007340912	0.009220006	0.003533091	0.022646202
0.001127683	0	0.007701474	0.001162787	0
0	0	0	0	0
0	0	0	0	0
0	0	0	0	0
0	0	0	0	0
0	0	0	2.415996003	0
0	0	0	0.005572165	0.000936612
0	0	0	0	0
0	0.022080658	0	0.014027504	0.001653725
0	0	0	0	0
0	0	0.023171624	0	0.001171189
0	0	0	0	0
0	0	0	0	0
0	4.635344741	0	0	2.630472111
0	0	0	0.068797688	0
0	0	0	0	0
0	0	0	0	0
0	0	0	0	0
0	0	0	0	0
0	0	0	0	0
0	0	0	0	0

0	0.005126265	0	0.058018934	0
0	0	0	0.00295081	0
0	0	0	0	0
0	0	0	0	0.001012145
0	0.010149203	0	0	0
0	1.936981005	0	0	0
0.001656082	0	0.002884778	0	0.00090841
0	0	0	0.001795768	0
0	0	0	0	0
0	0	0.000720619	0.010985775	0
0	0	0	0.001228819	0
0	0	0	0	0
0	0	0	0	0
0	0	0	0	0
0	0	0	0	0
0	0	0	0	0
0	0	0	0	0
0.009063003	0	0	0.006332317	0
0	0	0	0	0
0	0	0	0	0
0	0	0	0.228482321	0
0	0	0	0	0
0	0	0.023802003	0	0.005151245
0.001070587	0	0.009235631	0.001892424	0.001017898
0	0	0	0	0
0	0	0	0.012279491	0
0	0	0	0	0
0	0	0	0	0
0	0	0	0	0
0.145180042	0	0.625289018	0	0.351110127
0	0	0	0	0
0	0	0	0	0
0	0	0	0	0
0.001778502	0	0	0	0.024821055
0	0	0	0	0
0	0	0	0	0
0	0.002994753	0.321325613	0	0.002971859
0	0	0	0	0
0	0	0	0	0
0	0	0	0	0
0	0	0	0	0
0	0	0	0	0
0	0	0	0	0
0	0	0	0	0
0	0	0	0.039778643	0
0	0	0	0	0
0	0	0	0	0
0	0	0	0	0

0.002815975	0	0.003588674	0.003393859	0
0	0	0	0	0
0	0	0	0	0
0	0	0	0	0
0	0	0	0	0
0	0	0	0	0
0	0	0.000530539	0	0.00084201
0	0.394760353	0.413259682	0.698861768	3.779658902
0.013863028	0.006929893	0.176355067	0.015981513	0.025501892
0.036506047	0.05990276	0.126242126	0.037271597	0.028525531
0	0	0.005132789	0	0.002106768
0	0	0	0	0
0	0	0	0	0
0.010560522	0	0.03255949	0.014789242	0
0	0	0	0	0
0	0	0	0	0
0	0	0	0.005944945	0
0	0	0	0	0
0	0	0	0	0
0	0	0	0.095641025	0
0	0	0	0	0
0	0	0	0	0
0	0	0	0	0
0	0	0	0	0
0	0	0	0	0
0	0	0	0	0
0	0	0	0	0
0	0	0	0	0
0	0	0	0	0
0	0	0	0	0
0	0	0	0.045848868	0
0	0	0	0	0
0	0	0	0	0
0	0	0	0	0
0	0	0	0.046428804	0
0	0	0	0	0
0	0	0	0	0
0	0.022915418	0	0	0
0	0.015749083	0	0	0.013458034
0	0	0	0	0.537975722
0	0	0	0	0
0.037703607	0	0.188785868	0.897809249	0.027074021
0	0	0	0	0
0	0	0	0	0
0	0	0.021215642	0	0
0	0	0	0.047832607	0
0	0	0	0	0
0	0	0	0	0

0	0	0	0	0
0	0	0.412964243	0	0
0	0	0	0	0
0	0	0	0	0
0.003391912	0.118386358	4.127889913	1.63190482	0
0	0	0	0.174755167	0
0	0	0	0.013928164	0
0.000814119	0.005148099	0.02563453	0.00885033	0.008359761
0	0	0	0	0
0	0	0	0	0
0	0	0	0	0
0	0	0	0	0
0	0	0	0	0
0	0	0	0	0
0	0	0	0	0
0.020052656	0	0	0.107648764	0.003222381
0	0	0	0	0
0	0	0	0	0
0	0	0	0	0
0	0	0	0	0
0	0	0	0	0
0	0	0	0	0
0	0	0	0	0
0	0	0	0.002988968	0
0	0	0	0	0
0	0	0	0	0
0.007861604	0	1.56471651	0	0.0141529
0	0	0	0.001799094	0
0	0	0	0	0
0	0	0	0	0
0	0	0	0	0
0	0	0	0	0
0	0	0	0	0
0	0	0	0.006449957	0
0	0	0	0	0
0	0	0	0	0
0	0	0	0	0
0	0	0.648211578	1.174367042	0.009990588
0	0	0	0	0.225686175
0	0.006518463	0	0	0
0	0	0	0	0
0	0	0	0	0
0	0	0	0	0
0	0	0	0	0
0	0	0	0	0
0	0	0	0	0
0	0	0	0	0
0	0.012747643	0	0	0

0	0.956092261	0.005489138	0	0.015902358
0	0	0	0	0
0	0	0	0	0
0	0	0.001300331	0.003197326	0
0	0	0	0	0
0	0	0	0	0
0	0	0	0	0
0	0	0	0	0
0	0	0	0	0
0	0	0	0	0
0	0	0	0	0
0	0	0	0.003271981	0.003334613
0	0	0	0.00464014	0
0	0	0	0	0
0	0	0	0	0
0	0	0	0	0
0	0	0	0	0
0	0	0	0	4.113442405
0	0	0.00110805	0	0
0	0	0	0	0
0	0	0.124380064	0.010417658	0.00120074
0	0	0	0	0
0.003367102	0	0.085421027	0.057795693	0.019461076
0	0	0.3921332	0	0
0	0	0	0	0
0	0	0	0.056360769	0
0	0	0	0	0
0	0	0	0	0
0	0	0	0	0
0	0	0	0	0
0	0	0	0	0
0	0	0	0	0
0.213095176	0	0.013422221	0.015621567	0.0186986
0	0.063695316	0	0	0
0	2.361572515	0.017929129	0	0.943420307
0	0.164219417	0.011919652	0.00353117	0.000759739
0	0	0	0	0
0	0	0	0	0
0	0.005534549	0.008139292	0.012457287	0.00505286
0	0	0	0	0
0	0	0	0	0
0	0	0	0	0
0	0	0	0	0
0	0	0	0	0
0	0	0	0	0
0	0	0	0.824184657	0.001020048
0	0	0	0	0
0	0	0	0	0

0	0	0	0	0
0	0	0	0	0
0	0	0	0	0
0	0	0	0	0
0	0	0	0	0
0	0	0	0	0
0	0	0	0	0
0	0	0	0.103355462	0.10527163
0	0.009245306	0	0.40226092	0
0	0	0	0	0
0	0	0	0	0
0	0	0	0	0
0	0	0	0.001081413	0
0	0	0	0	0
0	0	0	0	0
0.00275429	0	0	0	0.002274166
0	0	0	0	0
0	0	0	0	0
0	0	0	0	0
0	1.617963304	0	0	0.04777318
0	0	0	0	0.004534482
0	0	0	0	0
0	0	0	0	0
0	0	0.009786319	0	0.00220308
0	0	0	0	0.000838253
0	0	0	0	0
0	0	0	0	0
0	0	0	0	0
0	0	0	0	0
0	0.853760612	0	0.412904989	0
0	0.055539867	0	1.886745634	0.46629539
0	0	0	0	0
0	0	0	0	0
0	0	0	0	0
0	0	0	0	0
0	0	0	0	0
0	0	0	0	0
0	0	0	0	0
0	0	0	0	0
0	0	0	0	0
0	0	0	0	0
0	0	0	0	0
0	0	0	0	0
0	0	0.002170617	0	0
0	0	0	0	0
0	0	0	0	0
0.096161373	0	0	0.964792133	0.015238126
0	0	0.00039015	0	0

0	0	0	0	0
0	0	0	0	0
0	0	0	0	0
0	0	0	0	0
0	1.049343459	0.21183998	0.038801743	0.135185212
0	0	0	0	0
0	0	0	0	0
0	0	0	0	0
0	0	0	0	0
0	0	0	0	0
0	0	0	0	0
0.000923509	0	0.070207732	0.012855478	0.00570738
0	0	0.003150295	0.009682653	0
0	0	0	0	0
0	0	0.00050594	0	0
0	1.35516399	0	0	1.075774919
0	0.002381868	0.001659515	0.043316177	0.009116971
0	0	0	0	0
0.384724153	0.002009979	0.039965531	0.080500633	0.096240062
0	0	0	0	0
0	0	0.033576966	0	0
0	0	0	0	0
0	0	0	0	0
0	0	0	0	0
0	0	0	0	0
0	0	0	0	0
0.020608788	0.001051534	0.005785927	0.051087009	0.004521812
0	0	0	0	0
0	0	0	0	0
0	0	0	0	0
0	0	0.001937784	0.016859817	0
0	0	0	0.028701437	0
0	0	0	0.01840158	0
0	0	0	0	0
0	0	0	0	0
0	0	0	0	0
0	0	0	0	0
0.00038847	0.016609281	0.090901671	0.105576946	0.03601176
0	0	0	0.080373751	0.036484362
0	0	0	0	0
0	0	0.025091936	0	0.040376125
0	0	0	0	0
0	0	0	0	0
0.014401297	0	0	0.002291081	0.00102694
0	0	0	0	0
0	0	0	0	0
0	0	0	0.598792731	0
0	0	0	0	0

0	0	0	0	0
0	0.421726792	0	0	0
0	0	0	0	0
0	0	0.001139246	0.001000443	0.000645743
0	0	0.001358527	0	0
0	0	1.060167744	0	0
0	0	0	0	0
0.00271486	0	0	0	0
0	0.001233471	0.002115432	0	0
0	0	0	0	0
0	0	0	0.040342069	0
0	0	0	0	0
0	0.006150225	0.02111205	0.007800907	0.040433752
0	0	0	0	0
0	0	0	0	0
0	0	0	0	0
0	0	0	0	0
0	0	0	0	0
0	0	0	0	0
0	0	0	0	0
0	0	0	0	0
0	0	0	0	0
0.003944506	0	0.007443637	0.065367253	0.024109555
0	0	0	0	0.005215852
0	0	0	0	0
0	0	0	0	0
0	0.449153078	0	0	0
0	0	0	0.001071138	0
0	0	0	0	0
0	0	0	0	0
0	0.018510912	0.004234501	0.004789533	0.007840597
0	0	0	0	0
0	0	0	0	0
0	0	0	0	0
0	0	0	0	0
0	0	0	0	0
0	0	0	0	0
0	0.139012405	0	0	0
0.000797379	0	0	0	0
0	0	0	0	0
0	0	0	0	0
0	0	0	0	0
0	0	0	0	0
0	0	0	0	0
0	0	0	0	0
0.010757284	0.093936172	0	0.198418609	0.02351296
0	0	0	0	0
0	0	0	0	0

0	0	0	0	0
0	0	0	0	0
0	0	0	0	0
0	0	0	0	0
0	0	0	0	0
0	0	0	0	0
0	0	0	0.004119071	0
0	0	0	0	0
0	0	0	0	0
0	0	0	0	0
0	0	0.004185381	0	0.002613968
0	0	0	0	0
0	0	0	0	0
0	0	0	0	0
0	0	0	0	0
0	0	0	0	0
0	0	0	0	0
0	0	0	0	0
0	0	0	0	0
0	0	0.002312722	0	0
0	0	0	0	0
0	0	0	0	0
0	0	0	0	0
0	0	0	0	0
0	0	0	0	0
0	0	0	0	0
0	0	0	0	0
0	0	0	0	0
0	0	0	0	0
0	0	0	0	0
0	0.002824551	0.011656292	0	0.009284799
0	0	0	0	0
6.548452036	0.005739285	0.023377155	1.013481796	0.051665501
0	0.021784015	0	0.003284769	0.000831442
0	0	0	0	0
0	0	0	0	0
0	0	0	0	0
0	0	0	0	0
0	0	0	0	0
0	0	0	0	0
0	0	0	0	0
0.001056021	0.028329948	0.266087235	0.019600106	0.063589766
0	0.292751529	0.071215505	0.053821281	0
0	0	0	0	0
0	0	0	0	0
0	0	0.094669537	0	0
0	0	0	0.004309168	0.00540824
0	0	0	0	0

0	0	0	0	0
0.008685904	0	0.004521678	0	0.009611097
0	0.001508435	0.001509085	0	0
0	0	0	0	0
0.001396747	0	0.356057315	1.148437343	0.008366438
0	0	0.137519395	0	0.002132911
0	0	0	0	0
0	0	0	0	0
0	0	0	0	0
0	0	0	0	0
0	0	0	0	0
0	0	0	0	0
0	0	0	0	0
0.018673427	0	0.001109872	0	0
0	0	0	0	0
0	0	0	0	0
0	0	0	0	0
0	0	0	0	0
0	0	0	0	0
0.014176617	0	0	0	0
0	0	0	0	0
0	0	0	0	0
0	0	0	0	0
0	0	0	0	0
0	0	0	0	0
0	0.001250592	0.01595192	0	0.027302699
0	0	0	0	0
0	0	0	0	0
0	0	0	0	0
0	0	0	0	0
0	0	0	0	0
0	0	0.001920044	0	0
0	0	0	0	0
0	0	0	0	0
0	0	0	0	0
0	0	0	0	0
0	0	0.05778789	0	0.056257726
0	0.022407472	0.000934047	0	0.02182439
0	5.396933393	0	0	0
0	0	0	0	0
0	0	0	0	0
0.010226797	0	0	0.251345404	0
0.001309982	0	0.002354337	0.003087454	0.000622755
0	0	0	0	0
0	0	0	0	0
0	0	0.001805721	0	0
0.006234717	0	0.007443462	0.0023616	0
0	0	0	0	0

0	0	0	0	0
0.001826283	0.007268312	0.024238141	0.082506117	0.028249925
0	0	0	0	0
0	0	0	0	0
0	0	0	0	0
0	0	0	0	0
0	0	0	0	0
0	0	0	0	0
0	0	0	0	0
0	0	0	0	0
0	0	0	0	0
0	0	0	0	0
0	0.005122166	0	0	0.028168338
0	0	0	0	0
0.001926777	0	0.014355121	0	0
0	0	0	0	0
0	0	0	0	0
0	0	0	0	0
0	0.027013044	0	0	0.000829068
0	0	0	0	0
0	0	0	0	0
0	0	0	0.00136834	0
0.008459851	0	0.008552411	0	0.024319008
0	0	0.078717649	0	0
0	0	0	0	0
0	0	0	0	0
0	0	0	0	0
0	0	0	0	0
0	0	0	0.004244786	0
0	0	0	0	0
0	0	0	0	0
0	0	0	0	0
0	0	0	0	0
0	0	0	0	0
3.069227061	0.083276897	0	12.54531162	0.265963533
0	0	0	0	0
0	0	0	0	0
0	0	0	0	0
0	0	0.00463106	0	0
0	0	0	0	0
0.005699043	0.125955958	0.010796129	0.01449827	0.018672072
0	0	0	0	0
0	0	0	0	0
0	0	0	0	0.001078968
0	0	0	0	0
0.452342328	0.025851533	0.299702669	0.073645812	1.201206243
0	0	0	0	0
0	0	0	0	0

0.009738391	0	0	0	0
0.013212873	0	0	0	0
0	0	0	0	0
0	0	0	0	0.005824719
0	0	0.005913308	0.010816322	0
0	0	0	0	0
0	0	0	0.00577924	0
0	0	0	0	0
0	0	0.47806901	0	0
0	0	0.001859833	0	0
0	0	0	0	0
0	0	0	0	0
0	0	0.001090698	0.004693272	0
0	0	0	0	0
0	0	0	0	0
0	0	0	0	0
0	0	0	0	0
0	0	0	0	0
0	0	0	0	0
0.075923065	0.007978683	1.345688228	0.271129461	0.05264192
0	0	0	0	0
0	0	0	0	0
0	0	0	0	0
0	0	0	0	0
0	0	0	0	0
0	0	0	0	0
0	0	0	0	0
0	0	0	0	0
0	0	0	0	0
0.004812035	0	0.019545216	0	0
0	0	0	0	0
0	0	0	0	0
0	0	0	0	0
0	0	0	0	0
0	0	0	0	0
0	0.004466258	0	0	0
0	0	0	0	0
0	0	0	0	0
0	0	0	0	0
0	0	0	0	0
0	0	0	0	0
0	0	0	0	0
0	0	0	0	0
0	0	0	0	0
0.000563713	0.004050742	0.04999755	0.019430737	0.002813842
0	0	0	0	0
0	0	0	0	0
0	0	0	0	0

0	0	0	0	0
0	0	0	0	0
0	0	0	0	0
0	0	0	0	0
0	0	0	0	0
0	0.002116825	0.038913418	0.001813225	0.007652341
0	0	0	0	0
0	0	0	0.001162995	0
0	0	0	0	0
0	0	0	0	0
0	0	0	0	0
0	0	0	0	0
0	0	0	0	0
0	0	0	0	0
0	0	0	0	0
0	0	0	0	0
0	0	0	0	0.009828249
0	0	0	0.001374205	0
0	0	0	0	0
0	0	0	0	0
0	0	0	0	0
0	0	0	0	0
0	0	0	0	0
0	0	0	0	0
0	0	0	0	0
0	0	0	0	0
0	0	0	0	0
0	0	0	0.394493945	0
0	0	0	0	0
0	0	0	0.0451352	0
0	0	0	0	0
0	0	0	0	0
0	0	0	0	0
0	0	0	0	0
0	0	0	0	0
0	0	0	0	0
0	0	0	0	0
0	0	0	0.071498563	0
0	0	0	0	0.630114623
0	0	0	0	0
0	0	0	0	0
0	0	0	0	0
0	0	0	0	0
0	0	0	0	0
0	0	0	0	0
0	0	0	0	0
0	0	0	0	0
0	0	0	0	0

0	0	0	0	0
0	0	0.011304481	0	0
0	0	0	0	0
0	0	0	0	0
0	0	0	0	0
0	0	0	0.021842158	0
0	0	0	0	0
0	0	0	0	0
0	0	0	0	0
0	0	0	0	0
0	0	0.001663182	0	0
0	0	0	0	0
0	0.255673602	0.000612509	0.25121268	0.470174076
0	0	0	0	0
0	0	0	0	0
0	0	0	0	0
0	0	0	0.007828867	0
0	0	0	0	0
0	0	0	0	0
0	0	0	0	0
0	0	0.03443581	0	0.03750665
0	0	0	0	0
0	0	0	0	0
0	1.520577852	0.748823521	1.906922164	4.474890812
0	0	0	0	0
0	0	0	0	0
0	0	0	0	0
0	0	0	0	0
0	0	0	0	0
0	0	0	0	0
0	0	0	0	0
0	0	0	0	0
0	0	0	0	0
0	0	0	0	0

PHL018-0199_V3	PHL018-0243_V3	PHL018-0269_V3	PHL018-0073_V2	PHL018-0241_V2
CTRL	REF	REF	CTRL	REF
0.104952947	0.077899896	0.045371866	0.27372041	0.061560867
5.600896097	9.14940569	1.089803372	0.018433556	9.440831662
2.333841528	0	0.107001813	1.295082277	2.869186889
0	0	0	0	0
0	0	0	0	0
0	0	0	0	0
0	0	0	0	0
0	0	0	0	0
0	0	0	0	0
0	0	0	0	0
0	0	0	0	0
0	0	0	0	0
0	0	0	0	0
6.709097343	2.085869423	3.237456616	0.152034249	3.441999872
0	0	0	0	0
0	0	0	0	0
0	0	0	0	0
0	0	0	0	0
0	0	0	0	0
0	0	0	0	0
0	0	0	0	0
0	0	0	0	0
0	0	0	0	0
0	0	0	0	0
0	0	0.006199603	0	0
0	0	0	0	0
0	0	0	0	0
0	0	0	0	0
0	0	0	0	0
0	0	0	0	0
0	0	0	0	0
0	0	0	0	0
0	0	0	0	0
0	0	0	0	0
0	0	0	0	0
7.481678571	2.905377651	6.490671426	0.110414156	0.00302362
0	0	0	0	0
0	0	0	0	0
0	0	0	0	0
0	0	0	0	0
0	0	0	0	0
0.001235327	0.003104921	0.002881736	0.075039163	0
0.185908539	0.04122972	0.040695673	8.351686939	0.077696617
0.006414492	0.004751879	0.049353453	0.130003511	0.104621611
0	0	0.001514132	0.083431069	0.004244058
0	0	0	0	0

0.002050446	0.000951448	0.002698228	0	0
0	0.022691429	0	0	0.20261943
0	0	0	0	0
1.273538122	0.226557249	0.573892425	0.301024213	0.092181454
0.001448514	0.004854341	0.021776126	0	0
0	0	0	0	0
0.013026185	0.098455508	0	0.003784762	0
0	0	0	0	0
0	0	0	0	0
0	0	0	0	0
0	0	0.012962803	0.005795773	0
0.051217643	0.467398099	0.339034673	0.581210199	0
0	0	0	0	0
0.006330355	0.088766487	0.020985075	0.001807018	0.001848453
0	0	0	0	0
0	0	0	0	0
1.642015523	0.000688808	0	1.128874028	0.134303792
0	0	0	0	0
0	0	0	0	0
0	0	0	0	0
0	0.047253378	0.343024404	0.00801974	0.182768016
0.002809715	0.069623546	0.010795525	0.000717119	0
0.001112267	0	0	0	0
0	0	0	0	0
0	0	0	0	0
0	0	0	0	0
0	0	0	0	0.002815393
0	0	0	0	0
0	0.005952082	0	0.00298156	0
0	0	0	0	0
0	0.000753138	0	0	0.111322483
0	0	0	0	0
0	0	0	0	0
0	0	0	0	0
0	0	0	0	0
0	0	0	0	0
0	0	0	0	0
0.038880743	0	0	0.028478834	9.578567075
0	0	0	0	0
0	0	0	0	0
0	0	0	0	0
0	0	0	0	0
0	0	0	0	0
0	0	0	0	0
0	0	0	0	0
0	0	0	0	0
0	0.001232627	0	0	0.00194343
0	0	0	0	0

0	0	0	0	0
0	0	0	0	0
0	0	0	0	0
0	0	0	0	0
0.002257545	0.002458823	0	0	0
0	0	0	0	0
0	0	0	0	0
0	0	0	0	0
0	0	0	0.00086644	0
0	0	0	0	0
0	0	0	0	0
0	0	0	0	0
0	0	0	0	0
0	0	0	0	0
0	0	0	0	0
0	0	0	0	0
0	0	0	0	0
0	0	0	0	0
0.022283134	0.190673893	0.046719151	0.046909616	0
0	0	0	0	0
0	0	0	0	0
0	0	0	0	0
0	0	0	0	0
0	0	0	0	0
0	0	0	0	0
0	0	0	0	0
0	0	0	0	0
0	0	0	0	0
0	0	0	0	0
0	0	0	0	0
0.024244328	0.503693754	0.411988744	0.039768212	0
0.026209851	0.001403295	0	0.002018725	0
0	0	0	0	0
0	0	0	0	0
0	0	0	0	0
0	0	0	0	0
0	0	0	0	0
0	0	0	0	0
0	0	0	0	0
0	0	0	0	0
0	0	0	0	0
0	0	0	0	0
0	0	0	0	0
0	0	0	0	0
0	0	0	0	0
0	0	0	0	0
0	0	0	0	0
0	0	0	0	0

0	0	0	0	0
0	0	0	0	0
0	0	0	0	0
0	0	0	0	0
0.011743821	0.028906696	0	0	0
0	0	0.002308989	0	0
0	0	0	0	0
0	0	0	0	0
0	0	0	0	0
0	0	0	0	0
0	0	0	0	0
0	0	0	0	0
0	0	0	0	0
0	0	0	0	0
0	0	0	0	0
11.35386036	0	2.91894715	0	0
0	0	0	0	0
0	0	0	0	0
0	0	0	0	0
0	0	0	0	0
0	0	0	0	0
0	0	0	0	0
0	0	0	0	0
0	0	0	0	0
0	0	0.203624729	0	0
0	0	0	0	0
0.003537806	0	0	0	0
0	0	0	0	0
0	0	0	0	0
0	0	0	0.009192412	0
0	0.122212922	0	0	0
0	0	0	0	0
0	0	0	0	0
0	0	0	0	0
0	0	0	0	0
0	0	0	0	0
0	0	0	0	0
0	0	0	0	0
0	0	0	0.009429174	0
0	0	0	0	0
0	0	0	0	0
0	0	0	0	0
0	0	0	0	0
0	0	0	0	0
0	0	0	0	0
0	0	0	0	0
0	0	0	0	0
0	0	0	0	0

0	0	0	0	0
0	0	0	0	0
0	0	0	0	0
0	0	0	0	0
0	0	0	0	0
0	0	0	0	0
0	0	0	0	0
0	0	0	0	0
0	0	0	0	0
0	0	0	0	0
0	0	0	0	0
0	0	0	0	0
0	0	0	0.001671593	0
0	0	0	0	0
0	0	0	0.000604636	0
0	0	0	0	0
0	0	0	0	0
0	0	0	0	0
0	0	0	0	0
0	0	0	0	0
0.029774099	0.089158785	0.126465027	0.023961988	0.03806124
0	0	0	0	0
0	0	0	0	0
0	0.001678243	0	0	0
0	0	0	0	0
0	0	0	0	0
0.007271501	0	0.020297313	0.005258367	0.021574877
0	0	0	0	0
0	0	0	0	0
0	0	0.001551128	0	0
0	0	0	0	0.004124532
0	0	0	0	0
0	0	0	0	0
0.002543538	0.005114426	0.011258425	0	0.006202844
0.005207692	0.010471382	0.008746819	0.002485513	0.016179585
0	0	0	0	0
0	0	0	0	0
0	0	0	0	0
0	0	0	0	0
0	0	0	0	0
0	0	0	0	0
0	0	0	0	0
0	0	0	0	0
0	0	0	0	0
0	0	0	0	0
0	0	0	0	0
0.000902167	0	0	0	0
0	0.000447731	0	0.017712439	0

0	0	0	0	0
0	0	0	0	0
0	0	0	0	0
0	0	0	0	0
0	0	0	0	0
0	0	0	0	0
0	0	0	0	0
0	0	0	0	0
0	0.007940555	0	0	0
0	0	0	0	0
0	0	0	0	0
0	0	0	0	0
0	0	0	0	0
0	0	0	0	0
0	0	0	0.001523272	0
0	0	0	0.002770199	0
0	0	0	0	0
0	0	0	0.053904013	0
0	0	0	0.048800029	0
0	0.043831081	0	0	0
0	0	0	0	0
0	0	0	0	0
0	0	0	0	0
0	0	0	0	0
0.001769712	0.005782486	0.013073057	0.017756702	0.004909153
0	0	0	0.002095645	0
0	0	0	0	0
0	0	0	0	0
0	0	0	0.039386789	0
0	0	0	0	0
0.061404941	0.44992527	0.185389222	0.060035321	0.006229444
0	0	0	0	0
0	0	0	0	0
0	0.002768875	0	0	0
0	0	0	0	0
0	0	0	0	0
0	0	0	0	0
0	0	0	0	0
0	0	0	0	0
0	0	0	0.004790258	0
0	0	0	0	0
0.014688286	0.003020575	0.005451158	0.059566547	0.003174941
0	0.000844791	0	0	0
0	0	0	0	0
0	0	0	0	0
0	0	0	0	0
0	0	0	0	0
0	0	0	0	0

0	0	0	0	0
0	0	0	0	0
0.003353375	0.001053564	0.040743013	0.143452963	0.082723219
0	0	0	0	0
0	12.00418062	0.072695868	0	0
0	0	0	0	0
0	0	0	0	0
0	0	0	0	0
0	0	0	0	0
0	0	0	0	0
0	0	0	0	0
0	0	0	0	0
0	0	0	0	0
0	0	0	0.001256271	0
0.001338263	0.005189625	0.021109741	0.005391815	0
0.032749429	0	0	0	0
0	0.001971037	0.006097857	0.007088641	0.003107651
0	0	0	0.001466341	0
0	0	0	0	0
0	0	0	0	0
0	0	0	0	0
0	0.000790793	0.001834875	0.012371439	0
0	0	0	0	0
0	0	0	0	0
0	0	0	0.004446197	0
0	0	0	0	0
0.963391226	0	0	0	0
0	0	0	0	0
0	0	0	0	0
0	0	0	0	0
0	0	0	0	0
0	0	0	0	0
0	0	0	0	0
0.000906906	0	0	0	0
0	0	0	0	0
0	0	0	0	0
0	0	0	0	0
0.000741177	0.000745162	0.008837099	3.078939564	0.002349731
0	0	0	0	0
0	0	0	0	0
0	0	0	0	0
0	0	0	0	0
0	0	0	0	0
0	0	0	0	0.003891644
0	0	0	0	0
0	0	0	0	0
0	0	0	0	0
0	0.003911173	0	0	0

0	0	0.001534036	0	0
0	0.003249793	0.017446614	0	0
0	0	0	0.004468317	0
0	0	0	0	0
0.005802211	0.003541711	0.011279363	0.004046002	0.00525559
0	0	0	0.002199124	0
0	0	0	0	0
0.029918099	0.009052888	0.09666997	0.003150757	0.038215615
0	0	0	0	0
0.017364655	0	0.091724519	0.008768393	0.005220317
0	0	0	0	0
0	0	0	0.040405233	0
0	0.044714595	0.014104731	0.055432712	0.006358787
0	0	0.038311437	0	0
0	0	0	0	0
0	0	0	0	0
0	0	0	0	0
0	0	0	0	0
0	0	0	0	0
0	0	0	0	0
0	0.004662179	0	0	0
0	0	0	0.012506804	0
0	0	0	0	0
0	0	0	0	0
0	0	0	0	0
0	0	0.001579655	0.014543647	0.025117239
0	0	0.653974107	0.046851528	0
0	0.000740947	0	0	0
0	0	0	0.002256151	0
0	0	0	0	0
0	0	0	0	0
0	0	0	0	0
0	0	0	0	0
0	0	0	0	0
0	0	0	0	0
0	0	0	0	0
0	0.053019576	0	0	0.196755479
0	0	0	0	0
0	0	0	0	0
0	0	0.001661393	0	0
0	0	0.227497195	0	0
0	0.106019861	0	0	0
0	0	0	0	0
0	0.044426512	0	0	0
0	0.027320781	0	0	0
0	0	0	0	0
0.002273669	0.003301845	0.001178656	0	0
0	0	0	0	0

0	0	0	0	0
0.004234973	0	0	0	0
0	0	0	0	0
0	0	0	0.00265433	0
0	0	0	0	0
0.022086404	0.325275444	0.032491696	0	0
0	0	0	0	0
0.002316164	0	0	0	0
0	0	0	0	0
0	0	0	0	0
0.011538969	0	0.019739708	0.007009275	0
0	0	0	0	0
0	0	0	0	0
0.001526201	0	0.0566254	0.020339033	0.003110443
0	0	0.013380206	0.02034015	0
0	0	0	0	0
0	0	0	0	0
0	0.000639954	0.01319896	0	0
0	0	0	0	0
0	0.100683405	0	0	0
0	0	0	0	0
0	0	0.218515317	0.256693752	0
0	0	0	0.004488551	0
0	0	0	0	0
0	0	0	0	0
0	0	0	0	0
0	0.04800972	0	0	0.00262309
0	0	0	0	0
0	0	0	0	0
1.225926056	0.916997912	0.858836442	1.233763023	3.750243131
0.000443746	0	0	0.012354397	0.001641258
0	0	0	0	0
0	0	0	0	0
0	0	0	0	0
0	0.003365291	0	0	0
0	0	0	0	0
0	0	0	1.397583468	0
0	0	0	0	0
0	0	0.028584979	0.013628297	0
0	0	0.001604846	0	0
0	0.066856813	0.03902583	0	0
9.175724092	7.059597046	6.951075638	0	3.30447048
0	0	0	0	0
0	0	0	0	0
0	0	0	0	0
0	0	0	0	0
0.00150503	0.001134841	0.001755446	0	0
0	0	0	0	0

0	0	0.001082963	0.069492585	0.002943522
0	0	0	0	0
3.716294266	0.003179808	0	2.51886385	0
0.001679739	0.001477674	0	0	0
0	0.014857426	0.222566684	0	0
0	0	0.092175043	0	0
0	0	0	0	0
0	0	0	0	0
0	0.024608745	0	0	0
0	0	0	0	0
0	0	0	0	0
0	0	0	0	0
0	0	0	0	0
0	0	0.038536017	0	0
0	0	0	0	0
0.091242663	0.001154915	0.010208553	0	0
0	0.000548615	0.037905642	0.984350111	0.004324892
0	0	0	0	0
0	0	0	0	0
0	0	0	0	0
0	0.043789392	0.005704107	0	0
0	0	0.001640618	0.00091545	0.00200666
0	0	0	0	0
0	0.815544604	0.034973309	0	0
0.018529715	0	0	0	0
0	0	0	0	0
0	0.006036087	0	0	0
0	0.005436005	0	0	0
0	0	0	0	0
0	0	0	0	0
0.013170805	0.018538264	0.026764442	0.001523909	0
0.001832698	0.005264434	0.010179209	0.031807466	0.017430436
0.075781845	0.01428549	0.176006413	0.055973185	0.463111109
0	0.003591667	0	0.005425171	0
0.001529896	0	0	0	0
0	0	0	0	0
0	0	0.004205866	0.001711233	0
0.014276135	0.020554756	0.206944378	0.012809097	0.005028797
0.04503984	0.155064501	0.225203207	0	0
0	0	0	0	0
0	0	0	0	0
0.001034312	0	0.002573668	0	0
0	0	0	0	0
0	0.045370092	0.346413663	0.074172273	0
0	0	0.647749705	0	0
0	0.002897374	0	0	0
0	0	0	0	0
0	0	0	0	0

0	0	0	0	0.013255178
0.072288477	0.246039854	0.689469918	2.726995211	1.759918141
0.008481401	0	0	0	0
0	0	0	0	0
0	0	0	0	0
0	0	0	0	0
0	0	0	0	0
0	0	0	0	0
0	0	0	0	0
0	0.002105504	0	0	0
0.003747784	0.005203337	0	0	0.00113157
0.005582431	0.008819556	0.241433744	0.071222811	0.018119179
0.076975746	0	0	0	0
0	0	0.004465559	0	0
0	0	0.012419563	0	0
0	0	0	0	0
0.012147173	0.011460944	0.038654008	0.053718738	0.020439814
0	0.009512092	0.037728002	0	0.00423001
0	0	0	0	0
0	0.012986815	0	0.002904873	0
0.265182707	0.018019321	0.027873452	0.063968516	0.061402891
0	0	0	0	0
0	0	0	0	0
0	0	0	0	0
0	0.005859836	0	0	0
0	0.003198237	0	0	0
0	0.876293705	0	0.008666291	0
0.173698299	0.061574317	0.235728711	0.100650515	0.031494698
0	0	0	0.001299673	0
0	0	0	0	0
0.000879143	0	0	0	0.00209034
0	0	0	0	0
0	0	0	0	0
0	0	0	0	0
0.065151629	0	0.01304702	0	0.006507681
0.206960475	0.380247645	0.251234946	0.045500539	1.380380181
0	0	0	0	0
0	0	0	0	0
0	0.0053349	0.00157188	0.02494237	0
0	0	0	0	0
0	0	0.635217527	0	0
0	0	0	0	0
0	0.000925857	0.009547837	0	0.024815905
0	0	0	0.017359995	0.000704685
0	0.020355817	0	0	0
0.229813449	0.011390825	0.188344213	0	0
0	0	0	0	0
0	0	0	0	0

0	0	0	0	0
0	0	0	0	0
0	0	0.003281248	0	0
0.068107804	0.99458008	1.33998516	0	0
0	0	0	0	0
0	0	0	0	0
0	0.020482136	0	0	0
0.008315869	0	0	0	0
0.020076423	1.330615274	0.397786392	17.49514449	0
0.066783045	0	0.440285821	0	0
0	0	0	0	0
0	0.003616048	0	0.007617072	0
0	0	0	0	0
0.01180142	0	0	0	0
0.065578983	0.130158001	0.045718406	0	0
0	0	0	0	0
0.146380124	0.17719367	0.097481359	0.005599351	0.198717685
0	0	0	0	0
0	0	0	0	0
0	0	0	0	0
0	0	0	0	0
0	0	0	0	0
0	0	0	0	0
0	1.105385001	0.167261773	0.010780267	0
0	0	0	0	0
0	0	0.852766327	0	0
0	0	0	0	0
0.025115159	0.389844044	0.401902333	1.644506252	0
0	0	0	0	0
0	0	0	0	0
0.002830593	0.002845812	0.01035785	0.012101006	0
0	0	0.028545122	0	0
0.008363712	0.000509617	0.03547388	0.083849998	0
0	0	0	0	0
0	0	0	0	0
0.092612796	0	0.030518488	0	0
0.234843885	0	0.801056031	0	0
0	0	0	0	0
0	0.002509537	0	0	0
0	0	0	0	0
0	0.032046502	0.171456427	0.028159528	0.004167122
0	0.08220747	0	0	0
18.69627652	0.04399229	0.036477318	0.006200275	0.259321774
0	0.057560189	0	0	0
0	0	0	0	0
0	0	0	0	0
0	0	0	0	0
0	0.047856602	0	0.00064274	0

0	0	0	0	0
0.014395209	0.094795557	0	0.125894221	0
0.00870958	0.00913712	0.010600417	0.002259176	0.482004308
0	0	0	0	0
0	0	0	0	0
0	0.001949423	0	0	0
0	0	0.024946436	0	0
0	0	0	0	0
0	0	0	0	0
0	0	0	0	0
0.056884489	0.140172364	0.00722759	0.083279972	0.016796287
1.847931998	1.611292262	2.256854136	2.22727234	1.669306277
0	0	0	0	0
0.004012553	0	0.029641176	2.128155246	0.075371146
0.14782563	0.303916806	0.101793376	0.003258441	0.376671799
0.025131345	0.628216112	0	0	0
0	0	0	0	0
0	0.004059952	0	0	0
0	0	0.007501985	0	0
0	0.033100006	0.344358056	0.002657657	0
0	0.315643256	0.095955307	0.097349412	0.011114023
0	0	0	0.007479506	0.005985485
0.899180913	0.904607126	4.441889005	0.474111406	0.504811365
0	0	0	0	0
0	0	0	0	0
5.559171576	0.003941906	0.013356639	1.048061449	1.639585506
0	0	0	0	0
0	0	0	0	0
0	0	0	0	0
0	0	0	0	0
0	0	0	0	0
0.252563343	0.564926681	0.564123438	0	0
0	0	0.017518098	0.009733518	0.046303234
0.04484237	0.018735984	0.018566615	0	0
0	0.009501583	0.000979844	0	0
0.003289478	0.006440266	0.006192732	0.099157465	0.005214264
0.003177591	0.004371661	0	0	0
0	0	0	0	0
0.025324696	0.001222121	0	1.968846435	0
0	0	0	0	0
0.010366604	0	0	0	0
0	0	0	0	0
0	0.040054814	0	0	0
0	0	0	0	0
0.019546125	0.002580462	0.007983253	0.007067365	0.001564809
0	0	0	0	0
0	0	0	0	0
0	0.024183617	0.072224881	2.386273414	0.007550351

0	0	0	0	0
0	0	0	0	0
0	0	0	0	0
0.000673094	0.002368496	0	0	0
0	0	0	0	0
0.019023123	0	0	0	0
0	0	0	0	0
0.126196343	0.020237703	0.003130499	0	0.005399802
0	0	0	0	0
0	0	0	0	0
0	0.007159952	0	0.00223619	0
0	0	0	0	0
0	0	0	0	0
0.123941255	0.042929334	0	0	0
0	0	0	0	0
0	0	0	0	0
0.536633509	0	0	0	0
0	0.000627421	0	0	0
0	0.239987432	0	0	0
0	0.005092466	0.070896212	0	0
0	0	0	0	0
0	0	0	0	0
0	0.008318769	0	0	0
0	0	0	0	0
0	0	0	0	0
0	0	0	0	0
0	0	0	0	0
0	0	0	0	0
0	0	0	0	0
0.012250315	0	1.317188766	0	0.063333831
0	0	0	0	0
0	0	0	0	0
0	0	0	0	0
0	0	0	0	0
0.000745105	0	0	0	0
0	0	0	0	0
1.639092796	0.246113093	0.122935561	0	0
0	0.000957004	0.008635408	0.004302213	0.015591628
0	0	0	0	0
0	0.003303775	0	0	0
0	0.024393128	0.03148264	0.066037798	0
0	0	0	0	0
0	0	0	0	0
0.783270083	0.003252775	0.054149629	0	0
0	0.069184535	0	0	0
0	0	0	0	0
0	0.00712296	0	0	0
0	0	0	0	0

0	0	0	0	0
0	0	0	0	0
0	0	0	0	0
0.011993405	0.12137215	0	0	0
0	0	0	0	0
0.070676874	0	0	0	0
0	0	0	0	0
0	0	0	0	0
0	0.013198683	0.023138788	0.002373388	0.00589611
0	0	0	0	0
0.0009387	0	0	0	0
0	0	0	0	0
0	0	0	0	0
0	0	0	0	0
0.190570829	0.245343311	0.201249444	0.593882675	0
0	0	0	0.04207243	0
0	0.008589019	0	0.016506608	0
0	0	0	0	0
0	0	0	0	0
0	0	0	0	0
0.080139218	0	0	0	0
0	0	0	0	0
0	0	0	0	0
0	0	0	0	0
0.014666359	0.004397695	0.002400931	0.372045179	0.038339046
0.9244872	1.357612843	3.978315122	0.08970579	0.942615277
0.140299203	0	0	0	0
0	0	0	0	0
0.009448225	0	0	0.393598471	0.07666646
0	0	0	0	0
0	0	0	0	0
0.026347182	0	0	0	0
0	0.008393083	0	0	0
0	0.000552624	0	0	0
0	0	0.024296573	0.252479472	0.020816555
0.001120361	0.000482736	0.00298691	0.001041668	0
0	0	0	0	0
0.889894003	9.015102845	12.82061812	0	0
0	0	0	0.55473561	0
0	0	0	0	0
0	0	0	0	0
0	0	0	0	0
0.082673611	0.767852927	0.629937032	0	0
0	0	0	0	0
0	0	0	0	0
0	0	0	0	0
0	0	0	0	0
0.062806312	1.912534184	0.438035736	0.076774262	0.152015001

0	0.001267715	0	0	0
0	0	0	0	0
0	0	0	0	0
0	0	0	0	0
0.001076063	0.020014199	0	0	0
0	0	0	0	0
0	0	0	0	0
0	0.00173255	0	0	0
0	0	0	0	0
0	0	0	0	0
0.000548918	0	0	0	0
0.259766172	0.429338315	0.003087772	1.461096101	0
0	0.085363703	0.313060713	0	0
0	0	0	0	0
0.00705198	0.006255789	0.001290248	1.567008509	0
0	0	0	0	0
0	0	0	0	0.001600971
0	0	0	0	0
0	0.010235263	0	0.002169167	0
0	0	0.001567481	0	0
0	0	0	0	0
0	0	0	0	0
0.034823904	1.624729788	0.050308731	0.054072745	0.317753452
0	0	0	0	0
0	0	0	0	0
0	0	0	0	0
0	0	0	0	0
0	0	0	0	0
0	0	0	0	0
0	0	0	0	0
0	0	0	0	0
0.01399554	0.031929861	0.087062414	0.09488286	0.058448435
0.117980366	0	0	0	0
0.033883971	0.107740947	0	0.060236801	0
0.000522528	0.001050676	0.002979631	0.001133595	0
0.015268325	0.158018972	0.018856336	0.015587647	0
0	0	0	0	0
0	0	0	0	0
0.002801438	0	0	0	0
0	0	0	0	0
0	0.403875154	0	0	0
0	0	0	0	0
0	0	0	0	0
0	0	0.015215715	0	0
0.018873908	0.075211514	0.00613731	0.027173156	0.003535725
0	0	0	0	0
0.232570987	0	0	0	0
0	0.001142102	0.001261912	0.000880169	0

0	0	0	0	0
0	0	0	0	0
0	0.022479882	0	0	0
0	0	0	0	0
0	0	0	0	0
0	0	0	0	0
0	0	0	0	0
0.001005553	0.000673973	0.041701798	0	0
0	0	0	0	0
0	0	0	0	0
0	0	0	0	0
0	0	0	0.001175136	0
0	0	0	0	0
0.349825569	0	0	0	0
0	0	0	0	0
0	0	0	0	0
0	0	0	0	0
0	0	0	0	0
0	0	0	1.844574972	0.063721279
0	0	0	0	0
0	0	0	0	0.002044648
0	0	0	0	0
0	0	0	0	0
0	0	0	0	0
0	0	0	0.003050243	0
0	0.000802971	0.00099367	0.03915865	0.00126601
0	0	0	0	0
0.069731739	0.089598181	1.011023315	0.345126363	0
0	0	0	0	0
1.754896704	0.854662215	0.485188033	0.163997747	0.156848064
0	0	0	0	0
0	0	0	0	0
0	0.000663915	0	0	0
0	0	0	0	0
0.001673928	0.001009757	0	0.001089447	0
0	0	0	0	0
0	0	0	0	0
0.003418953	0	0	0.002266373	0.0018065
0	0	0	0	0
0	0	0	0	0
3.923102132	7.054947139	6.898381906	0.853564085	35.79820902
0	1.073694788	0.727396849	0	0.02248022
0	0	0	0	0
0	0	0	0	0
0	0	0	0	0
0	0	0	0.862772783	0
0	0	0	0	0
0	0	0	0.016024951	0.000955825

0	0	0	0	0
0.015195597	0.011224135	0.014468516	0	0
0	0	0	0	0
0	0	0	0	0
0.639061968	2.655676662	1.680055489	0.026479199	0
0.011288932	0	0	0	0
0	0	0	0	0
0	0	0	0	0
0	0.086236158	0.326747166	0.002855078	0.053256996
0	0	0	0	0
0	0	0	0	0
0	0	0	0	0
0.184865237	0.382018567	0.244620155	0	0
0.016485165	0.027188475	0.02189263	0.058065641	0.049326304
0.068900153	0	0	0	0
0.186650581	0.852389693	0.806058212	0.056221631	0
0	0	0	0	0
0	0	0	0	0
0	0	0	0	0
0	0	0	0	0
0	0	0	0	0
0	0	0	0	0
0	0	0	0	0
0	0	0	0	0
0	0	0	0	0
0	0	0	0	0
0.001114994	0.03601177	0.008670092	0.068183183	0
0	0	0	0.002047107	0
0	0	0	0	0
0	0	0	0	0
0	0	0	0	0
0.001182306	0	0	0	0
0	0	0	0	0
0	0.004143637	0	0	0
0.020278381	0	0	0	0
0	0.01646144	0	0	0
0	0	0.081840101	0.007664792	0
0	0.016753264	0	0	0
0	0	0	0	0
0	0	0	0	0
0.752886753	3.125855398	6.714775002	0.120603551	0.004238271
0	0	0	0	0
0	0	0	0	0
0	0.040058146	0	0	0
0	0	0	0	0
0	0	0	0	0
0	0	0	0	0
0	0	0	0	0

0.003099493	0.453054615	0	0.079195787	0
0	0	0	0	0
0	0	0.012516049	0	0
0	0	0	0	0
0.016704947	0	0.053814105	0	0
0	0	0	0	0
0	0.069285315	0.006962646	0.019772344	0
0	0.006992586	0	0	0
0	0.001361603	0	0	0
0	0	0	0	0
0	0.007602719	0	0	0
0	0	0.039360812	0	0
0	0	0	0	0
0	0	0	0	0
0	0.019586415	0	0	0
0	0	0	0	0
0	0	0	0.047869627	0
0	0	0	0	0
0	0	0	0	0
0	0	0	0	0
0.08022472	0.17156627	0.22703732	0.007760926	0.009169532
0	0	0	0	0
0.034756984	0.003038596	0	0	0
0.00104514	0.001050759	0.000928789	0.001133685	0
0	0	0	0	0
0	0.003364789	0	0	0
0	0	0	0	0
0	0	0	0	0
0	0	0	0.15971314	0
0.005801917	0.002376453	2.11339507	1.837224782	0.521834205
0	0	0	0	0
0	0	0	0	0
0	0	0	0	0
0	0.084321327	0	0	0
0	0	0	0	0
0	0	0	0	0
0	0	0.020047194	0.013172096	0
0	0	0	0	0
0	0	0	0	0
0.050050104	0	0	0	0
0	0	0	0	0
0	0	0	0	0
0	0	0	0	0
0	0	0	0	0
0	0	0	0	0
0	0	0	0	0
0	0	0	0	0
0	0	0	0	0

0	0.000484566	0.001249263	0.002265501	0
0	0	0	0	0
0	0.020922852	0.005938499	0.004556238	0
0	0	0	0	0
0	0	0	0	0
0	0	0	0	0
0	0	0	0	0
0.289999042	1.294929718	1.607749696	1.39945218	3.284489711
0.233263853	0.053495453	0.053336642	10.28790936	0.112725149
0	0.052244602	0	0	0
0	0	0	0	0
0	0	0	0	0
0	0	0	0	0
0	0	0	0.132642629	0
0	0.001697367	0	0	0
0	0	0	0	0
0	0	0	0	0
0	0	0	0	0
0	0	0	0	0
0.007715584	0.001463597	0.016300707	0	0
0	0	0	0	0
0	0	0	0	0
0	0	0	0	0
0	0	0	0	0
0	0.098977878	0.020860118	0	0
0	0	0	0	0
0	0	0	0	0
0	0	0	0	0
0	0	0	0	0
0	0	0	0	0
0	0	0.001796764	0	0
0	0	0	0	0
0	0	0	0	0
0	0.002440238	0	0	0
0.003500988	0	0	0	0
0	0	0	0	0
0	0	0	0	0
0	0	0	0	0
0.005540019	0	0	0	0
0	0	0	0	0.569486785
0	0	0	0	0
0.117813098	0.075526287	0.312401649	0	0.008393238
0	0	0	0	0
0	0	0	0	0
0	0	0	0	0
0	0	0	0	0
0	0	0	0	0
0	0	0	0	0

0	0	0	0	0
0	0.027260976	0	0	0.002068615
0	0	0	0	0
0	0	0	0	0
0.017637669	0.109452307	0.669664839	0.947141556	0
0.028717679	0.01397036	0	0	0
0	0	0.001093737	0	0
0.001226213	0.00431482	0.020023328	0.003325248	0.015549696
0.002244602	0	0	0	0
0.007224799	0	0	0	0
0	0	0	0	0
0	0.447900889	0	0	0
0	0	0	0	0
0	0	0	0	0
0	0.010269578	0	0	0
0.008685131	0	0	0	0
0	0	0	0	0
0	0	0	0	0
0	0	0	0	0
0	0	0	0	0
0	0	0	0	0
0	0	0	0	0
0	0	0	0	0
0	0.006934797	0	0.003836971	0
0	0	0	0	0
0	0	0	0	0
0.001617626	0	0.001006281	0.387080785	0
0	0	0	0	0
0	0	0	0	0
0	0	0	0	0
0	0	0	0	0
0	0	0	0	0
0	0	0	0	0
0	0	0	0	0
0	0	0	0	0
0	0	0.001124506	0	0
0	0	0	0	0
0	0	0	0	0
0	0	0	0	0
0	0	0	0	0
0	0	0	0	0
0	0	0	0	0
0	0	0	0	0
0	0	0	0	0
0	0	0	0	0
0	0	0	0	0
0	0	0	0	0
0	0.032756176	0	0	0

0.003042475	0.03181186	0	0	0
0	0	0	0	0
0	0.087868353	0	0	0
0	0	0	0	0
0	0	0	0	0
0	0	0	0	0.002793706
0	0	0	0	0
0	0	0	0	0
0	0	0	0	0
0	0	0	0	0
0	0	0	0	0
0	0	0	0	0
0	0	0	0	0
0	0	0	0	0
0	0	0	0	0
0	0	0	0	0
0	0	0	0	0
0	0	0	0	0
0	0	0	0	0
0	0	0.019157705	0	0
0	0.00088536	0.00684766	0	0.000837545
0	0	0	0	0
0	0	0	0.206051318	0.001410345
0	0	0.265317104	0	0
0	0	0	0	0
0	0	0	0	0
0	0	0	0	0
0	0	0	0	0
0	0	0	4.607674132	0
0	0	0	0	0
0	0	0	0	0
0.001565594	0	0.461913099	0	0
0.001041538	0	0	0.234768022	0.058444625
0	0	0	0	0
0.004099265	0.087858724	0.352659003	0	0
0	0.022519626	0	0	0.002384713
0	0	0	0	0
0	0	0	0	0
0	0	0	0.002306978	0
0	0	0	0	0
0	0	0	0	0
0	0	0.261076433	0	0
0.005199797	0.144558734	0.041136319	1.538093205	0
0	0	0	0	0
0	0	0	0	0
0	0.014621354	0.025967962	0	0
0	0	0	0	0
0	0	0	0	0

0	0	0	0	0
0	0	0	0	0
0	0	0	0	0
0	0	0	0	0
0	0	0	0	0
0	0	0	0	0
0	0	0	0	0
0	0.017268607	0.008364426	0	0
0	0.001860338	0.01857419	0	0
0.000924648	0	0.010353567	0.008425076	0
0	0	0	0	0
0	0	0.004337219	0	0
0	0	0	0	0
0	0.112577839	0.033146478	0.036576782	0
0	0	0	0	0
0	0	0.001131863	0	0
0	0	0	0	0
0	0	0	0	0
0	0	0	0	0
0.010968087	1.837842644	1.968704121	0	0
0.01596287	0.003439006	0.013003654	0.002473609	0
0	0	0	0	0
0	0	0	0	0
0	0	0	0	0
0	0	0	0	0
0	0	0	0	0
0	0	0	0	0
0	0	0	0.000777974	0
0	0	0	0	0
0.082031217	0.473788873	0	0	0.331204065
0.506059346	1.287412984	0.423175306	0.04186779	0.044773476
0	0	0	0	0
0	0	0	0	0
0	0	0	0	0
0	0	0	0	0
0	0	0	0	0
0	0	0	0	0
0	0	0	0	0
0	0	0	0.014463337	0
0	0	0	0	0
0	0	0	0	0
0	0	0	0	0
0	0	0	0	0
0	0	0	0	0
0	0	0	0	0
0	0	0	0	0
0	0	0.248247129	0.007475533	0.890766082
0	0.00243501	0	0	0

0	0	0	0	0
0	0	0	0	0
0	0	0	0	0
0	0	0	0	0
0.232521349	0.010072739	0.057780154	0	0.000882292
0	0	0	0	0
0	0	0	0	0
0	0	0	0	0
0	0	0	0	0
0	0	0	0	0
0	0	0	0	0
0.003477437	0.102553238	0	0.026404344	0
0	0.00134057	0	0	0
0	0	0	0	0
0	0	0	0	0.002800456
0.19380101	2.061861521	0.201164122	0	0
0.00505195	0.010158223	0	0	0
0	0	0	0	0
0.134143606	0	0.067340045	0.013487692	0
0	0.051272258	0.032080955	0.497039402	0
0	0	0	0	0
0	0	0	0	0
0	0	0	0	0
0	0	0	0	0
0	0	0	0	0
0	0	0	0	0
0	0.011387356	0.021423283	0.014610437	0
0	0	0	0	0
0	0	0	0	0
0	0	0	0	0
0	0	0	0	0
0	0	0	0	0
0	0	0	0	0
0	0	0	0	0
0	0	0	0	0
0	0	0	0	0
0	0	0	0	0
0.01572476	0.003431477	0.004360168	0.061881132	0.014298551
0.066417749	0.168779672	0	0	0
0	0	0.009622757	0	0
0	0	0	0	0
0	0	0	0	0
0	0	0	0	0
0.001446067	0.025260493	0.003092232	0	0
0	0	0	0	0
0	0	0	0	0.005379281
0	0.018707963	0	0	0
0	0	0	0	0

0	0	0	0	0
0	0.036501604	0.366889664	0	2.747241415
0	0	0	0	0
0.001136613	0.00095227	0	0	0
0	0	0.002732429	0.026872317	0.006405628
0	0	0	0	0
0.005147319	0	0	0	0
0	0	0.001382447	0	0
0.044475324	0	0.002871994	0	0
0	0	0	0	0
0	0	0	0	0
0	0	0	0	0
0.005908458	0.004860184	0.005429701	0	0.003192852
0	0	0	0	0
0	0	0	0	0
0	0	0	0	0
0	0	0.035269924	0	0
0	0	0	0	0
0	0	0	0	0
0	0	0	0	0
0	0	0	0	0
0	0	0	0	0
0	0.012088402	0	0	0.0016817
0.015023061	0.028319684	0.018496171	0	0.027121005
0	0	0	0	0
0.000924556	0	0	0	0
0	0	0	0	0
0	0	0	0	0
0	0	0	0	0
0	0	0	0	0
0.0626948	0.053319416	0.005825578	0.006201839	0.003437661
0.006708239	0	0	0	0
0	0	0	0	0
0	0	0	0	0
0	0	0.046269795	0	0
0	0	0	0	0
0	0	0	0	0
0	0	0	0	0
0	0.001509321	0	0	0
0	0	0	0	0
0	0	0	0	0
0	0	0	0	0
0	0	0	0	0
0	0	0	0	0
0	0.013980694	0	0	0
0.013355277	0.040281242	0.305842158	0	1.506785377
0	0	0	0	0
0	0	0	0	0

0	0	0	0	0
0	0	0	0	0
0	0	0	0	0
0.006204199	0	0	0	0
0	0	0.002888594	0	0
0	0	0	0.008940357	0
0	0	0	0	0
0	0	0	0	0
0	0	0	0	0
0	0	0	0	0
0	0.002312876	0	0.003669718	0
0	0	0	0	0
0	0	0	0	0
0	0	0	0	0
0	0	0	0	0
0	0	0	0	0
0	0	0	0	0
0	0	0	0	0
0	0	0	0	0
0	0	0	0	0
0	0	0	0	0
0	0	0	0	0
0	0	0	0	0
0	0	0	0	0
0	0	0	0	0
0	0	0	0	0
0	0	0	0	0
0.029195071	0	0	0.003446913	0.003462987
0	0	0	0	0
0	0	0	0	0
0.005242022	0.002790109	0	0.177106284	0.00171074
0	0	0	0	0
0.049766263	0.091608699	0.054566658	0.02951562	0.051361158
0	0.003126602	0.004551935	0.01646991	0
0	0	0	0	0
0	0	0	0	0
0	0	0	0	0
0	0	0	0	0
0	0	0	0	0
0	0	0	0	0
0	0	0	0	0
0.001767292	0.250527845	0.25720994	0.100643464	0.001167247
0.014891994	0.023991373	0	0.067144731	0
0	0	0	0	0
0	0	0	0	0
0.001814937	0.017204266	0.77821905	0.103216148	0
0	0	0.001057456	0	0.002155645
0	0	0	0	0

0	0	0	0	0
0	0	0.004969512	0.00876739	0
0	0.004194191	0.03858561	0.033581731	0.029235543
0	0	0	0	0
0	1.1063574	0.213752758	0.906520965	0
0	0	0	0	0
0	0	0	0	0
0	0	0	0	0
0	0	0	0	0
0	0	0	0	0
0	0	0	0	0
0	0	0	0	0
0	0	0	0	0
0	0	0	0	0
0	0	0	0	0
0	0	0	0	0
0	0	0	0	0
0	0	0	0	0
0	0	0	0	0
0	0	0	0	0
0	0	0	0	0
0	0	0	0	0
0	0.004243296	0	0	0
0	0	0	0	0
0	0.001819265	0	0.021394974	0
0	0	0.003680267	0.004541516	0.002885499
0	0	0	0	0
0.195121256	0	0.001098458	0.001532323	0
0	0	0	0	0
0	0	0	0	0
0	0	0	0	0
0	0	0.013902544	0	0
0	0	0	0	0
0	0	0	0	0
0	0	0	0	0
0	0	0	0	0
0	0	0	0	0
0	0.011294836	0	0	0
0	2.864468069	0	0	0.237878429
0	0	0	0	0
0	0	0	0	0
0	0.153869795	0	0.021849486	0
0	0	0	0.001783529	0
0	0	0	0.38568398	0
0	0	0	0	0
0	0	0	0	0
0.019563812	0.003371828	0.00130394	0.00272845	0
0	0	0	0	0

0	0	0	0	0
0.007934764	0.006027387	0.006032887	0.001147601	0.015372701
0	0	0	0	0
0	0	0	0	0
0	0	0	0	0
0	0	0	0	0
0	0	0	0	0
0	0	0	0	0
0	0	0	0	0
0	0	0	0	0
0	0	0	0	0
0	0	0	0	0
0.025536034	0.475237648	0.009565945	0	0
0	0	0	0	0
0	0.015030488	0.002051482	0	0
0	0	0	0	0
0	0	0	0	0
0	0	0	0	0
0	0.00195619	0	0	0
0	0	0	0	0
0	0	0	0	0
0	0	0	0.159729196	0
0.011779797	0	0.019093794	0	0
0	0	0	0	0
0	0	0	0	0
0	0	0	0	0
0	0	0	0	0
0	0	0	0	0
0	0.027931409	0	0	0
0	0	0	0	0
0	0	0	0	0
0	0	0	0	0
0	0	0	0	0
0	0	0	0	0
0	0.016161573	2.958814004	5.690007132	0
0	0	0	0	0
0	0	0	0	0
0	0	0	0	0
0	0	0.007712433	0.375617801	0
0	0	0	0	0
0.215948441	0.050341409	0.422429534	0.342453154	4.095735114
0	0	0	0	0
0	0	0	0	0
0	0	0	0	0
0	0	0	0	0
0	0.033380784	0.061618433	0.069869502	1.117160649
0	0	0	0	0
0	0	0	0	0

0	0	0	0	0
0	0	0	0	0
0	0	0	0	0
0	0	0	0	0
0	0	0	0	0
0	0	0	0	0
0	0	0	0	0
0	0	0	0.002076125	0
0	0	0.009794272	0	0
0	0	0	0.012445435	0
0	0	0	0	0
0	0	0	0	0
0	0.001063639	0.002632489	0	0
0	0	0	0	0
0	0	0	0	0
0	0	0	0	0
0	0	0	0	0
0	0	0	0	0
0	0	0	0	0
0.011864801	0.109061411	0.032510523	1.057587177	0.100007076
0	0	0	0	0
0	0	0	0	0
0	0	0	0	0
0	0	0	0	0
0	0	0	0	0
0	0	0	0	0
0	0	0	0	0
0	0	0	0	0
0	0	0	0	0
0	0.00465544	0	0	0
0	0	0	0	0
0	0	0.00246103	0	0
0	0	0	0	0
0	0	0	0	0
0	0	0	0	0
0	0	0	0	0
0	0	0	0	0
0	0	0	0	0
0	0	0	0	0
0	0	0	0	0
0	0	0	0	0
0	0	0	0	0
0	0.161796037	0.04260545	0	0
0	0	0	0	0
0.00159198	0	0	0	0
0	0.114264607	0	0	0
0	0	0	0	0
0	0	0	0	0

0	0	0	0	0
0	0	0	0	0
0	0	0	0	0
0	0	0	0	0
0	0	0	0	0
0.007394954	0.012877984	0.005955613	0.197528509	0
0	0	0	0	0
0	0	0	0	0
0	0	0	0	0
0	0	0	0	0
0	0	0	0	0
0	0	0	0	0
0	0	0	0	0
0	0	0	0	0
0	0	0	0	0
0	0	0	0	0
0	0	0	0	0
0	0	0	0	0
0	0	0	0	0
0	0	0	0	0
0	0	0	0	0
0	0	0	0	0
0	0	0	0	0
0	0.003771665	0	0	0
0	0	0	0	0
0	0	0	0	0
0	0	0	0	0
0	0	0	0	0
0	0	0	0	0
0	0	0	0	0
0	0	0	0	0
0	0	0	0	0
0	0	0	0	0
0	0	0	0	0
0	0	0	0	0
0	0	0	0	0
0	0	0	0	0
0	0	0	0	0
0	0	0.075082152	0	0
0	0	0	0	0
0	0	0	0	0
0	0	0	0	0
0	0	0	0	0
0	0	0	0	0
0	0	0	0	0
0	0	0	0	0
0	0	0	0	0
0	0	0	0	0

0	0	0	0	0
0	0.066590076	0.003218933	0	0
0	0	0.005629905	0	0
0	0	0	0	0
0	0	0	0	0
0	0	0	0	0
0	0	0	0	0
0	0	0	0	0
0	0	0	0	0
0.0022081	0	0	0	0
0.000774358	0	0.002107469	0.044518032	0.003682386
0	0	0	0	0
0	0.270654513	0	0	0
0	0	0	0	0
0	0	0	0	0
0	0	0	0	0
0	0	0	0	0
0	0	0	0	0
0	0	0	0	0
0	0	0	0	0
0	0.001580305	0	0	0.008720599
0	0	0.01161229	0	0
0	0	0	0	0
0.549007805	1.158084033	1.856960001	0.979351128	3.330844955
0	0	0	0	0
0	0	0	0	0
0	0	0	0	0
0	0	0	0	0
0	0	0	0	0
0	0	0	0	0
0	0	0	0	0
0	0	0	0	0
0	0	0	0	0
0	0	0	0	0

PHL018-0005_V1	PHL018-0064_V2	PHL018-0263_V3	PHL018-0237_V2	PHL018-0185_V2
EYCF	CTRL	REF	CTRL	REF
0.048470004	0.067828844	0.012354479	0.034029708	0
0.384757696	2.163026346	0.103594078	0.047461628	2.625351398
5.528516598	17.22541266	0.00876096	1.695829952	0
0	0	0	0	0
0	0	0	0	0
0	0	0	0	0
0	0	0	0	0
0	0	0	0	0
0	0	0	0	0
0	0	0	0	0
0	0	0	0	0
0	0	0	0	0
0	0	0	0	0
23.16253484	11.06576545	0.158845351	0.539935535	0.636324341
0	0	0	0	0
0	0	0	0	0
0	0	0	0	0
0	0	0	0	0
0	0	0	0	0
0	0	0	0	0
0	0	0	0	0
0	0	0	0	0
0	0	0	0	0
0	0	0	0	0
0	0	0	0	0
0	0	0	0	0
0	0	0	0	0
0	0	0	0	0
0	0	0	0	0
0	0	0	0	0
0	0	0	0	0
0	0	0	0	0
0	0	0	0	0
0	0	0	0	0
0	0	0	0	0
0	0.18714897	1.52650147	0.563709885	0
0	0	0	0	0
0	0	0	0	0
0	0	0	0	0
0	0	0	0	0
0	0	0	0	0
0.001089863	0.001231528	0.095665436	0	0
0.026015341	0.051395918	0.98962248	6.085382278	0.039435275
0.003276359	0.056687423	0.003822308	0	0.013585785
0	0	0	0	0.001780886
0	0	0	0	0

0	0	0	0	0
0.007467145	0	0.001769088	0	0.022230091
0	0.011039949	0.000487582	0	0.057505128
0.098713655	0.516536701	0.394011587	0.168564575	0.423405826
0.001810426	0	0	0	0
0	0	0	0	0
0.222253115	0.008944524	0.005477844	0	0
0	0	0	0.052890397	0
0	0	0	0	0
0	0	0	0	0
0	0	0.002322959	0	0
0	0.192878229	0	0	0
0	0	0	0	0
0.017930586	0.002704666	0	0	0.057774501
0.005444461	0	0	0	0
0	0	0	0	0
0.006044492	0	0.047989191	0.358780238	0.007352152
0	0	0	0	0
0	0	0	0	0
0	0	0	0	0
0.038981343	0.575059412	0.221144647	0	0.144768013
0.009186375	0.010168606	0.002245493	0	0.002902272
0	0	0	0.229133132	0
0	0	0	0	0
0	0	0	0	0
0	0	0	0	0
0	0	0	0	0
0	0	0	0	0
0	0	0.008234888	0	0
0	0	0.001829099	0	0
0	0.128504011	0.042745779	0	0
0	0	0	0	0
0	0	0	0	0
0	0	0	0	0
0	0	0.002068828	0	0
0	0.00738796	0	0	0
0	0	0	0	0
0	0	0	46.12183967	4.273321654
0	0	0	0	0
0	0	0	0	0
0	0	0	0	0
0	0	0	0	0
0	0	0	0	0
0	0	0	0	0
0	0	0	0	0
0	0	0	0	0
0.001983054	0.009079678	0	0	0
0	0.481431525	0	0	0

0	0	0	0	0
0	0	0	0	0
0	0	0	0	0
0	0	0	0	0
0.108382349	3.392943666	0	0	0.021473023
0	0	0	0	0
0	0	0	0	0
0	0	0	0	0
0	0.002184168	0.007668906	0	0
0	0	0	0	0
0	0	0	0	0
0	0.000950916	0.019318804	0	0
0	0	0	0	0
0	0	0	0	0
0	0	0	0	0
0	0	0	0	0
0	0	0	0	0
0	0	0	0	0
0	0	0.313208546	0.423265171	0
0	0	0	0	0
0	0	0	0	0
0	0	0	0	0
0	0	0	0	0
0	0	0	0	0
0	0	0	0	0
0	0	0	0	0
0	0	0	0	0
0	0	0	0	0
0	0	0	0	0
0	0	0.002680908	0	0
0	0.078572795	0	0.104208836	0
0	0.000397571	0.025003737	0	0.004085016
0	0	0	0	0
0	0	0	0	0
0	0	0	0	0
0	0	0	0	0
0	0	0	0	0
0	0	0	0	0
0	0	0.784751392	0	0
0	0	0	0	0
0	0	0	0	0
0	0	0	0	0
0	0.004901644	0.013963098	0	0.004459319
0	0	0	0	0
0	0	0	0	0
0	0	0	0	0
0	0	0	0	0
0	0	0	0	0

0	0	0	0	0
0	0	0	0	0
0	0	0	0	0
0	0	0	0	0
0	0	0	0.00340773	0
0	0	0	0	0
0	0	0	0	0
0	0	0	0	0
0	0	0	0	0
0	0	0	0	0
0	0	0	0	0
0	0	0	0	0
0	0	0	0	0
0	0	0	0	0
0	0	0	0	0
0	0	0	0	0
0	0	0	0	0
0	0	0	0	0
0	0	0	0	0
0	0	0	0	0
0	0	0	0	0
0	0	0	0	0
0	0	0	0	0
0	0	0	0	0
0	0	0	0.017833639	0
0	0	0	0	0
0	0	0	0	0
0	0	0	0	0
0	0	0	0	0
0	0	0	0	0
0	0	0	0	0
0	0	0.011507152	0	0
0	0	0	0	0
0	0	0	0	0
0	0	0	0	0
0	0	0	0	0
0	0	0	0	0
0	0	0	0	0
0	0	0	0	0
0	0	0	0	0
0	0	0	0	0
0	0	0	0	0
0	0	0	0	0
0	0	0	0	0
0	0	0	0	0
0	0	0	0	0
0	0	0	0	0
0	0	0	0	0

0	0	0	0	0
0	0	0	0	0
0	0	0	0	0
0	0	0	0	0
0	0	0	0	0
0	0	0	0	0
0	0	0	0	0
0	0	0	0	0
0	0	0	0	0
0	0	0	0	0
0	0	0	0	0
0	0	0	0	0
0.000453191	0	0	0	0
0	0	0	0	0
0	0	0	0	0
0	0	0	0	0
0	0	0	0	0
0	0	0	0	0
0	0	0	0	0
0	0	0	0	0
0.058467726	0.124748702	0.174275853	0.001554273	0.026703853
0	0	0	0	0
0	0	0	0	0.002260883
0	0	0.01360742	0.003889906	0
0	0	0	0	0
0	0	0	0	0
0	0.001911861	0.005404015	0	0.009276449
0	0	0	0	0
0	0	0	0	0
0.005455673	0.012784191	0	0	0
0.588824995	1.191760521	0.051848768	0	0.007615213
0	0	0	0	0
0	0	0	0	0
0	0	0.009924074	0.049583482	0
0.038777334	0.001246002	0.000754697	0.003438396	0.032616134
0	0	0	0	0
0	0	0	0	0
0	0	0	0	0
0	0	0	0	0
0	0	0	0	0
0	0	0	0	0
0	0	0	0	0
0	0	0	0	0
0	0	0	0	0
0	0	0	0	0
0	0	0	0.035048139	0
0	0	0.001225704	0	0.001650215
0.000785795	0	0.002151269	0	0

0	0	0	0	0
0	0	0	0	0
0	0	0	0	0
0	0	0	0	0
0	0	0	0	0
0	0	0	0	0
0	0	0.00287091	0	0
0	0	0.006571537	0	0
0	0	0	0	0
0	0	0	0	0
0	0	0	0	0
0	0	0	0	0
0	0	0	0	0
0	0	0	0	0
0	0	0.004451787	0	0
0	0	0	0	0
0	0	0	0	0
0	0	0.007835768	0	0
0	0	0.004922609	0.032101955	0
0	0	0.089317152	0.175033946	0
0.024165903	0.005518503	0.002622154	0	0.005539876
0	0	0	0	0
0	0	0	0	0
0	0	0	0	0
0.004293637	0	0.004007279	0	0.005179358
0	0	0	0	0
0	0	0	0	0
0	0	0	0	0
0	0.012266721	0.00860446	0	0
0	0	0	0	0
0.449430194	2.107232929	0.154542112	0.015104185	0.497921633
0	0	0	0	0
0	0	0	0	0
0.01570004	0.078204284	0	0	0
0	0	0	0	0
0	0	0	0	0
0	0	0	0	0
0	0	0	0	0
0	0	0	0	0
0	0	0	0	0
0	0	0	0	0
0.007804669	0.008985547	0.004233051	0.007292939	0
0	0	0	0	0
0	0	0	0	0
0	0	0	0	0
0	0	0	0	0
0	0	0	0	0
0	0	0	0	0

0	0	0	0	0
0	0	0	0	0
0.00425285	0.056414163	0.002657646	0	0.010120907
0	0	0	0	0
0	0.049890299	0	0	0
0	0	0	0	0
0	0	0	0	0
0	0	0	0	0
0	0	0	0	0
0	0	0	0	0
0	0	0	0	0
0	0	0	0	0
0	0	0	0	0
0.015530988	0.004552382	0.038602925	0	0.002033714
0.003879373	0.00098019	0.016103992	0	0.006434499
0	0	0.003522447	0.023848805	0
0.00230619	0.001675258	0.001578413	0	0.004143911
0	0	0	0	0
0	0	0	0	0
0	0	0	0	0
0	0	0	0	0
0.000693944	0	0.001424857	0	0
0	0	0	0	0
0	0	0	0	0
0	0	0	0	0
0	0	0	0	0
0	0	0	0	0
0	0	0	0	0
0	0	0	0	0
0	0.000972234	0	0	0
0	0	0	0	0
0	0	0	0	0
0	0	0	0	0
0	0	0	0	0
0	0	0	0	0
0	0	0.277835748	0	0
0	0	0	0	0
0	0	0.004251689	0	0.015907317
0	0	0	0	0
0	0	0	0	0
0	0	0	0	0
0	0	0	0	0
0	0	0	0	0
0	0	0	0	0
0	0	0	0	0
0	0	0	0	0
0	0	0	0	0
0	0	0	0	0

0	0	0	0	0
0	0	0	0	0.175570216
0	0	0	0	0.001506986
0	0	0	0	0
0	0	0.010698364	0	0.000909704
0	0.025697208	0	0	0
0	0	0	0	0
0.007687908	0.005212326	0.16311561	0	0.024227858
0	0	0	0	0
0.011093731	0	0.046641626	0.006686019	0
0	0	0	0	0
0	0.001768327	0.064977976	0	0
0.052317806	0.000596151	0.057186835	0	0.002297028
0	0	0.003118996	0	0
0	0	0	0	0
0.00138596	0	0	0	0
0	0	0.00189699	0	0
0	0	0	0	0
0	2.256868065	0.001275841	0	0
0	0.008490331	0	0	0
0	0	0	0	0
0	0	0	0	0
0	0	0	0	0
0	0	0	0	0
0	0	0	0	0
0	0.004860544	0	0	0.005053632
0	0	0.000738137	4.166364875	0
0.29432516	0.171714219	0	0	0
0	0.034657737	0	0	0
0	0	0	0	0
0	0	0.001579538	0	0
0	0	0	0	0
0	0	0	0	0
0	0.183209115	0	0	0
0	0	0	0	0
0	0	0.005597582	0	0
0.007923679	0.0497861	0.018563897	0	0.038416723
0	0	0	0	0
0	0	0	0	0
0	0.2399011	0.011611269	0	0
0.018350445	0	0	0	0
0	0.489286262	0	0	0
0	0	0	0	0
0	0	0	0	0
0	0	0	0	0
0	0	0	0	0
0.003566111	0.005396848	0	0	0.001848409
0.003843773	0	0	0	0

0	0	0.002595244	0	0
0.001743603	0.001447525	0	0	0
0	0	0	0	0
0	0.043678502	0	0	0
0	0.012162021	0	0	0
0.014394814	0	0	0	0.007861568
0	0	0	0	0.032376414
0	0.000879635	0	0	0.001129774
0	0	0	0.481398513	0
0	0	0	0	0
0	0	0.008779168	0	0
0.071558892	0	0	0	0
0	0.000572202	0	0	0
0	0	0.006516806	0	0
0.002335547	0.182476951	0	0	0.014204083
0	0	0	0	0
0	0	0	0	0
0	0.12473911	0.004420115	0	0
0	0.028356215	0	0	0
0	0	0	0	0
0	0.002408858	0	0	0
0	0	0.701518668	0.102201079	0
0	0	0	0	0
0	0	0	0	0
0.009768804	0	0	0	0
0	0	0	0	0
0.404196972	0.827821257	0.052673391	0	0.007610535
0	0	0	0	0
0	0	0	0	0
0.098965307	0.322515873	0.183519411	0.080989331	1.859878899
0.001043982	0.001263945	0	0.004164975	0
0	0	0.000940475	0	0
0	0	0	0	0
0.008871814	0	0	0	0
0	0	0	0	0
0	0	0	0	0
0	0	0	0.409215726	0
0	0	0.002012837	0	0
0	0.003777451	0	0	0
0	0.776641531	0	0	0
0.029887876	0.047174716	0.000974095	0	0
2.301194732	2.024265349	0.045937335	0.010178256	1.017379006
0	0	0	0	0
0	0	0	0	0
0	0.011057774	0	0	0
0	0	0	0	0
0.001991712	0.004340447	0.009542239	0.001461325	0.002477657
0	0	0	0	0

0	0.001190085	0	0	0.002377676
0	0.012293608	0	0	0
0.017579356	0	0.008021163	0.03715859	0.003239766
0	0	0	0	0
0	0	0	0	0
0.004501828	0	0.002310871	0	0
0	0	0	0	0
0	0	0	0	0
0	0	0	0	0
0	0	0	0	0
0	0	0	0	0
0	0.001505255	0	0	0
0	0	0	0	0
0	0	0.001141201	0	0
0	0	0	0	0
0	0	0	0.029159218	0
0.004974734	0.017874396	0.008072744	0.000529835	0.047644682
0.000974856	0.001321886	0.003602964	0	0
0.012597674	0.00899063	0	0	0
0	0.093692917	0	0	0
0.03478606	0.078158286	0	0	0
0.004839718	0.001983192	0	0	0.004013685
0	0	0	0	0
0	0	0	0	0
0	0	0	0	0
0.101682398	0	0	0	0
0.005296842	0.008682038	0.002509815	0	0
0	0	0	0	0
0	0	0	0	0
0	0	0	0	0
0.022929943	0.054772108	0.014792483	0	0.003083726
0.006005601	0	0.025136564	0	0
4.399012481	0.041963658	0.156921867	0.007503345	0
0	0	0.049183337	0	0
0.014172338	0.115478771	0	0	0
0	0	0	0	0
0.008549718	0	0	0	0.002638314
0.068884187	0.016265406	0.00143673	0	0
0	0	0.031307572	0.011822872	0
0	0.006033638	0	0.000699583	0.079286438
0	0	0	0	0
0	0	0	0	0
0.00209288	0	0	0	0.002314229
0.059234826	0.450748969	0.095954404	0	0
0	0	0	0	0
0.036103927	0.048923379	0	0	0
0	0	0	0	0
0	0	0	0	0

0	0.004912559	0.012544476	0	0
0	0.015186224	1.60772902	0.00303143	1.208939084
0	0	0	0	0
0	0	0	0	0
0	0	0	0	0
0	0	0	0	0
0.566883651	1.09128388	0	0	0
0	0.00063052	0	0	0
0	0	0	0.001494244	0
0	0	0	0	0
0.005353335	0	0.00161645	0	0.003917328
0.270175979	0.087681283	0.058749067	0	0.022950888
0	0	0	0.175377741	0
0	0	0	0	0
0.00234852	0.020997009	0	0	0
0	0	0	0	0
0.005111092	0	0.015910974	0	0.030628326
1.416873006	1.177253914	0.026807089	0	0.008874955
0	0	0	0	0
0.007921823	0.074977474	0.007419429	0	0
0	0.000823403	0.065982025	0.020583588	0.011844574
0	0	0	0	0
0.119324	0	0	0	0
0	0	0	0.005028279	0
0.060563409	0	0	0	0
0	0.001510167	0	0	0
0	0.288197468	0	0	0
0.005240683	0.085940272	0.111872694	0.004375463	0.01648564
0	0	0	0	0
0	0	0	0	0
0	0	0	0	0
0	0	0	0	0
0	0.003612271	0	0	0
0	0	0.000562055	0	0
0	0	0	0.001733142	0
0.061169265	0.113455976	0.167358341	0.051163271	1.431065683
0	0	0	0	0
0	0	0	0	0
0	0.0095005	0.052181933	0	0
0	0	0	0	0
0	0	0	0	0
0	0	0	0	0
0.002166577	0.030952197	0	0	0.011005339
0	0	0	0	0.008890681
0	0.235062948	0.009345648	0	0
0	0	0.753011572	0.363624242	0
0	0	0.000786747	0	0
0	0.003479582	0	0	0

0	0.008061861	0.004272645	0	0
0	0	0	0	0
0	0	0	0	0
0	0	0	0.325095761	0
0	0	0	0	0
0	0	0	0	0
0	0	0	0	0
0	0	0	0	0
0	0	1.880137937	1.631449012	0
0	0	0	0.063757809	0
0	0	0	0	0
0.001813249	0	0.000930774	0.003658562	0
0	0	0	0	0
0	0	0	0	0
0.009476555	0.013043255	0.019713988	0.122683411	0
0	0	0	0	0
0	0	0	0.382173981	0.002967548
0	0	0	0	0
0	0	0	0	0
0	0	0	0	0
0	0	0	0	0
0	0	0	0	0
0	0	0	0	0
0.457138208	1.10760942	0.097414301	0	0
0	0.001096191	0	0	0
0	0	0.316285426	0.107349582	0
0	0	0	0.001667306	0
0	0	3.817410189	0.258444231	0
0	0	0	0	0
0	0	0	0	0
0	0	0	0.009538547	0
0	0	0	0	0
0	0.014293698	0	0	0
0	0	0	0	0
0	0	0	0	0
0	0	0	0.054557173	0
0	0	0.000825735	0.24572791	0
0	0	0	0	0
0	0	0	0	0
0	0	0	0	0
0.002609233	0.557947815	0.162956388	0	0.005770386
0	0	0	0	0
0.462384656	0.309736326	0.579139	0.600741782	6.876425802
0	0.007216075	0	0.05642365	0
0	0	0	0	0
0	0	0	0	0
0	0	0	0	0
0	0	0.004114632	0	0

0	0	0	0	0
0	0	0.169336514	0.020441628	0
0.026392872	0.209843188	0.068425716	0	0.872756986
0	0	0	0	0
0	0	0	0	0
0	0	0	0.007385942	0
0	0	0	0	0.468065239
0	0	0	0	0
0	0	0	0	0
0	0	0	0	0
0.004428191	0.01779123	0.004882896	0.012815407	0.032371498
0.302338643	3.845598243	2.560658622	0.489390339	0.080567591
0	0	0	0	0
0	0	0.013447129	0.001558412	0.026129084
0.177564733	1.254128385	0.070168353	0	0.095550195
0	0	0	5.366306782	0
0	0	0	0	0
0	0	0	0	0
0	0	0	0	0
0.006349638	0.09172642	0	0	0
0.003848939	0.476108345	0.466837073	0	0.004788015
43.41332222	0.859966469	0.009665536	0	0.22294457
0.787067507	1.500998294	1.929574344	0.236628925	0.620644977
0	0	0	0	0
0	0	0	0	0
0	0	0.003043953	0.492005298	0
0.004439099	0	0	0	0
0	0	0	0	0
0	0	0	0	0
0	0	0	0	0
0	0	0	0.001835492	0
0.02868022	0	0	0.085837315	0
0.061480436	0.022350641	0.022307488	0	0
0	0	0	0	0
0.008449087	0.006783629	0.046224061	0	0.000553185
0.013899686	0.006213527	0.026187676	0.102878935	0.370394202
0.000885291	0.000571636	0	0.007307327	0
0	0	0	0	0
0.016980404	0	0.100192206	0.44988552	0.031425593
0	0	0	0	0
0	0	0	0	0
0	0	0	0	0
0	0	0	0	0
0	0	0	0	0
0.001916057	0.003374201	0	0.041982782	0.21321867
0	0	0	0	0
0	0	0	0	0
0.007774332	0.032765181	0.377499426	0.005549886	0.069701892

0	0	0	0	0
0	0	0	0	0
0	0	0	0	0
0	0	0.00548688	0.001797257	0
0	0	0	0	0
0	0	0	0	0
0.003575752	0	0	0.001967654	0
0.00409827	0	0.06409327	0.137415899	0
0	0	0	0	0
0	0	0	0	0
0.002976189	0.008167395	0.006959672	0.000909849	0.004936439
0	0	0	0	0
0	0.004346803	0	0.014817582	0
0	0	0	0	0
0	0	0	0	0
0	0	0	0	0
0	0	0.196946709	0	0
0	0	0	0.002272286	0
0	0	0	0	0
0	0	0	0	0
0	0	0	0	0
0	0	0	0	0
0	0	0	0	0
0	0	0	0	0
0	0	0	0	0
0	0	0	0	0
0	0	0	0	0
0	0	0	0	0
0	0	0	0	0
0	0.033336482	0	0.479808808	0
0.006641561	0	0	0.002723109	0
0	0	0	0	0
0	0	0	0	0
0	0	0	0	0
0	0	0	0.003617339	0
0	0	0	0	0
0	0	0	0.012249283	0
0.006158524	0.018572479	0.001149558	0	0.002089391
0	0	0	0	0
0	0	0	0	0
0	0	0	0	0
0	0	0	0	0
0	0	0	0	0
0	0	0	0.017651826	0
0	0	0.095287708	0.024662534	0
0	0	0	0	0
0	0	0	0	0
0	0	0	0	0

0	0	0	0	0
0	0	0	0	0
0	0	0	0	0
0	0	0	0.445272782	0
0	0	0	0	0
0	0	0	0.232766761	0
0	0	0	0	0
0	0	0	0	0
0.011582227	0.24324474	0.007927161	0	0.064035976
0	0	0	0	0
0	0	0	0	0
0	0	0	0	0
0	0	0	0	0
0	0	0	0	0
0	0.146452174	1.802425582	0.440607372	0
0.919008344	0.776272437	0	0	0
0	0	0	0	0
0	0	0	0	0
0	0	0	0	0
0	0	0	0	0
0	0	0	0	0
0	0	0	0	0
0	0	0	0	0
0	0	0	0	0
0	0.001319211	0.032161301	0.692286083	0.001882612
0.004707181	0	0.297861774	2.467334507	26.63576977
0	0	0.280329189	0.182872312	0
0	0	0	0	0
0	0	0	0.06530911	0.272567886
0	0	0	0	0
0	0	0	0	0
0	0	0	0	0
0.00054964	0	0	0	0
0	0	0	0	0
0	0.000967387	0	0.003297674	0.118946789
0	0.000957356	0.003769122	0.000932422	0.001171044
0	0.145089672	0.005535243	0	0
0.658826001	0	4.89831775	0.572408166	3.161381347
0	0	8.485291552	0	0
0	0	0	0	0
0	0	0	0	0
0	0	0	0	0
0.029136826	0	0.053090519	0.156723131	0
0	0	0	0	0
0	0	0	0	0
0	0.00116611	0	0	0
0	0	0	0	0
0.000639354	0.042315485	0.035225964	0.008209177	0.030753362

0	0	0	0	0
0	0	0	0	0
0	0	0	0	0
0	0	0	0	0
0.005696121	0	0	0	0
0	0	0	0	0
0	0	0	0	0
0	0	0	0	0
0	0	0	0	0
0	0	0	0	0
0	0	0	0	0
0	0	0	1.821465808	0
0	0	0	0.004638655	0
0	0	0	0	0
0.008051467	0.178652131	0.225246683	0	0.003642141
0	0	0	0	0
0	0	0	0	0
0.014171858	0	0	0	0
0	0	0	0.0243592	0
0	0	0	0.004110288	0
0	0	0	0.024843632	0
0	0	0	0	0
0	0.026434116	0.348879561	0.170951215	0
0	0.018288666	0	0	0
0	0	0	0	0
0	0	0	0	0
0	0	0	0	0
0	0	0	0	0
0	0	0	0	0
0	0	0	0	0
0	0	0	0	0
0.016146761	0	0.238658048	0	0.06537893
0	0	0	0	0
0.016133287	0.004710783	0	0	0
0	0.000893008	0.002681913	0	0.001274389
0	0.002110526	0.536493363	0.010900693	0.080827844
0	0	0	0	0
0	0	0	0.034445553	0
0	0	0	0	0
0	0	0	0	0
0	0	0	0.021901577	0
0	0	0	0.000814539	0
0	0	0	0	0
0	0	0	0.002340522	0
0.000756884	0.004838365	0.006527179	0.054144473	0.429849375
0	0	0	0	0
0	0	0	0	0
0	0.003466839	0	0	0

0	0	0	0	0
0	0	0	0	0
0.020666117	0	0.003857564	0	0
0	0	0	0	0
0	0.010457288	0	0	0
0	0	0.032633131	0	0
0	0	0	0.0128975	0
0	0.220541331	0.002428739	0.010577152	0
0	0	0	0	0
0	0	0	0.001281024	0.037164631
0	0	0	0	0
0	0	0	0	0
0	0	0	0	0
0	0	0	0.072587861	0
0	0	0	0	0
0	0	0	0	0
0	0	0	0	0
0	0	0	0	0
0	8.740925014	38.47996317	0.540923746	0
0	0	0	0	0
0	0	0	0.001095878	0
0	0	0	0	0
0	0	0	0	0
0	0	0	0	0
0	0	0	0	0
0.021420764	0.006551759	0.001302119	0	0.019634682
0	0	0	0	0
0	0.010688116	0.080152882	0	0
0	0	0	0	0
0	0	0.203101888	0.119247237	0
0	0	0	0	0
0	0	0	0	0
0	0	0.001046717	0.007373666	0
0	0	0	0	0
0.000886091	0.00171646	0.010309861	0	0.010287966
0	0	0	0	0
0	0	0	0	0
0	0	0	0.011987692	0.008338449
0	0	0	0	0
0	0	0	0	0
1.934035	2.894513254	3.494908613	0.739761619	28.15227166
0.193076443	0.057860057	0.376541319	0.003240017	0
0	0	0.064445483	0	0
0	0	0	0	0
0	0	0	0	0
0	0	0	0	0
0	0	0	0	0
0	0	0	0.003805638	0.009264996

0	0	0	0	0.766406115
0.000957591	0	0	0	0
0	0	0	0	0
0	0	0	0	0
0.188476283	0	0.031951241	0.351422569	0
0	0	0	0	0
0	0	0	0	0
0	0	0	0	0
1.584115236	1.412059382	0.348345012	0	0.130275328
0	0	0	0	0
0	0	0	0	0
0	0	0	0	0
0.00824124	0	0	0.022967407	0
0.008170779	0.008546978	0.008052887	0.001798477	0.251261777
0	0	0.056925706	0	0
0.010459617	0.061693599	0.01362929	0.050915698	0
0	0	0.168696367	0	0
0	0	0	0	0
0	0	0	0	0
0	0	0	0	0
0	0	0	0	0
0	0	0	0	0
0	0	0	0	0
0	0	0	0	0
0	0	0	0.007383673	0
0	0	0	0	0
0	0	0.00416585	0.002571212	0
0	0.000806321	0	0	0
0	0	0	0	0
0	0	0	0	0
0	0	0	0	0
0	0	0	0	0
0	0	0	0.209864304	0
0	0	0	0	0
0	0	0	0.072510408	0
0	0	0.008074205	0	0
0	0	0	0	0
0	0	0.009024727	0.001000811	0
0	0	0	0	0
0	0	0	0	0
0	0.194393941	1.592907801	0.157065106	0
0	0	0	0	0
0	0	0	0	0
0	0	0	0	0
0	0	0	0	0
0	0	0	0	0
0	0	0	0	0
0	0	0	0	0

0	0	0	0.540202664	0
0	0	0	0	0
0	0	0	0	0
0	0	0	0	0
0	0	0	0.002648138	0
0	0	0	0	0
0	0.050963538	0.466479991	0	0
0	0	0	0	0
0	0	0	0	0
0	0	0	0	0
0	0	0	0	0
0	0	0	0	0
0	0	0	0	0
0	0	0	0	0
0	0	0	0	0
0	0	0	0	0
0.013784186	0.039115426	0	0	0
0.00366177	0	0	0	0
0	0	0	0	0
0	0	0	0	0
0	0	0.012409297	0.018771671	0
0	0	0	0	0
0	0	0	0.009618899	0
0.000790347	0.003189567	0.002163731	0.001739639	0
0	0	0	0	0
0	0	0	0.007525404	0
0	0	0	0	0
0	0	0	0	0
0	0	0	0	0
0.311673426	0.460706097	0.01459742	0.003129685	0
0	0	0	0	0
0	0	0	0	0
0	0	0	0	0
0	0	0	0	0
0	0	0	0	0
0	0	0	0	0
0	0.041186859	0.007614529	0	0
0	0	0	0	0
0	0	0	0	0
0	0	0	0.011654408	0
0	0	0	0	0
0	0	0	0	0
0	0	0	0	0
0	0	0	0	0
0	0	0	0	0
0	0	0	0	0
0	0	0	0	0
0	0	0	0	0

0.002409583	0.001235552	0.000582063	0	0.010109151
0	0	0	0	0
0	0	0	0	0
0	0	0	0	0
0	0	0	0	0
0	0	0	0	0
0	0	0	0	0
0	0	0.076050583	0.206458248	5.223942842
0.035819686	0.081023141	1.37913218	7.935475778	0.048341532
0.658264035	0.021752268	0.077120498	0	0
0	0	0	0	0
0	0	0	0	0
0	0	0	0	0
0	0	0.078460088	0	0
0	0	0	0	0
0	0	0	0	0
0.060208639	0.055309564	0	0	0.09917835
0	0	0	0	0
0	0	0	0	0
0	0	0	0	0
0	0	0	0	0
0	0	0	0	0
0	0	0	0	0
0	0	0	0	0
0	0	0	0	0
0	0	0	0	0
0	0	0	0	0
0	0	0	0	0
0	0	0	0	0
0	0	0	0	0
0	0	0.00254137	0	0
0	0	0	0	0
0	0	0	0	0
0	0	0	0	0
0	0	0	0	0
0	0	0	0	0
0	0	0	0	0
0	0	0	0	0
0	0	0	0	0
0	0	0.004793918	0	0
0	0	0	0	0
0.438388442	0	0	0	0
0	0	0	0	0
0	0	0	0	0
0	0	0	0	0
0	0	0	0	0
0	0	0	0	0
0	0	0	0	0

0	0	0	0	0
0.018805228	16.7342759	0	0	0.003819328
0	0	0	0	0
0	0	0	0	0
0.047755498	0.078822254	0.280944438	0.449593127	0.577902119
0	0	0	0	0
0	0	0	0	0.01775269
0.020915239	0.053612809	0.006108516	0	0.011364273
0	0	0	0	0
0	0	0	0	0
0	0	0	0	0
0	0	0	0	0
0	0	0	0	0
0	0	0	0	0
0.002411623	0	0	0	0
0.004534908	0	0	0	0
0	0	0	0	0
0	0	0	0	0
0	0	0	0	0
0	0	0	0	0
0	0	0	0	0
0	0	0	0	0
0	0	0	0	0
0	0	0.003364084	0	0.004140986
0	0	0	0	0
0	0	0	0	0
0	0	0.029889267	0.012879337	0.270799242
0	0	0	0	0
0	0	0	0	0
0	0	0	0	0
0	0	0	0	0
0	0	0	0	0
0	0	0	0	0
0	0	0	0	0
0	0	0	0	0
0	0	0.002619677	0	0
0	0	0	0	0
0	0	0	0	0
0	0	0	0	0
0	0	0	0	0
0	0	0	0	0
0	0	0	0.052270177	0
0	0	0	0	0
0	0	0	0	0
0	0	0	0	0
0	0	0	0	0
0	0	0	0	0
0	0	0	0	0

0	0	0	0.212106067	0
0	0	0	0	0
0	0	0	0	0
0	0	0	0	0
0	0	0	0	0
0.041205084	0.003162639	0	0	0
0	0	0	0	0
0	0	0	0	0
0	0	0	0	0
0	0	0	0	0
0	0	0	0	0
0	0	0	0	0
0	0	0	0	0
0	0	0	0	0
0	0	0	0	0
0	0	0	0	0
0	0	0	0	0
0	0	0	0	0
0	0	0	0	0
0	0	0	0	0
0.001087701	0.012642004	0.082314796	0	0
0	0	0	0	0
0.005887228	0	0.003223491	0.023109197	0.009764817
0	0	0	0.466699994	0
0	0	0	0	0
0	0	0	0	0
0	0	0	0	0
0	0	0	0	0
0	0	0	0	0
0	0	0	0	0
0	0	0	0	0
0	0	0	0	0
0.024993921	0.00551801	0.001698067	0.000606776	0.011888124
0	0	0	0	0
0	0	0	0.004342064	0
0.060907097	0.001571216	0	0	0
0	0	0	0	0
0	0	0	0	0
0.000682312	0.004791221	0.010857541	0	0
0	0	0	0	0
0	0	0	0	0
0	0	0	0	0
0	0	0.187364231	0.206342173	0
0	0	0	0	0
0	0	0	0	0
0	0.015188839	0.000487868	0.015689822	0
0	0	0.028060463	0	0
0	0	0	0	0

0	0	0	0	0
0	0	0	0	0
0	0	0	0	0
0	0	0	0	0
0	0	0	0	0
0	0	0	0	0
0	0	0	0	0
0.148628746	0.046552024	0.004714349	0	0.714939763
0	0	0	0.002776649	0
0	0.001264189	0	0.003950306	0
0	0	0	0	0
0	0	0	0	0
0	0	0	0	0
0	0	0	0	0
0	0	0	0	0
0.03306817	0.03467154	0.005273632	0	0
0	0	0	0	0
0	0	0	0	0
0	0	0	0	0
0	0	0.059743869	0.308985746	0
0	0	0.002237598	0	0
0	0	0	0	0
0	0	0	0	0
0	0	0	0.000941294	0
0	0	0	0	0
0	0	0	0	0
0	0	0	0	0
0	0	0.004709686	0	0
0	0	0	0	0
0	0	0	0.037535775	0
0	0	0.11988133	0.047958936	0
0	0	0	0	0
0	0	0	0	0
0	0	0	0	0
0	0	0	0	0
0	0	0	0	0
0	0	0	0	0
0	0	0	0	0
0	0	0	0.003883947	0
0	0	0	0	0
0	0	0	0	0
0	0	0	0	0
0	0	0	0	0
0	0	0	0	0
0	0	0	0	0
0	0	0	0	0
0	0.325166839	0.031547943	0	0.050696585
0	0	0	0	0

0	0	0	0	0
0	0	0	0	0
0	0	0	0	0
0	0	0	0	0
0	0.088108967	0.011091138	0.048234327	0.315821419
0	0	0	0	0
0	0	0	0	0
0	0	0	0	0
0	0	0	0	0
0	0	0	0	0
0	0	0	0.008405605	0
0	0	0.001924804	0.008816298	0.083680037
0	0	0	0	0.010975559
0	0	0	0	0
0	0.004654776	0	0	0
0	0	0	0.087084785	0.257434424
0.015075375	0	0	0	0
0	0	0	0	0
0.211657992	0.370255241	0	0	0
0	0.782612203	0.071795571	0	0
0	0	0	0	0
0	0	0	0.029106357	0
0	0	0	0	0
0	0	0	0	0
0	0	0	0	0
0	0	0	0	0
0.00175548	0.008632208	0.079437242	0	0
0	0	0	0	0
0	0	0	0	0
0	0	0	0	0
0	0	0	0	0
0	0.004804094	0.011881734	0	0
0	0	0	0	0
0	0	0	0	0
0	0	0	0	0
0	0	0	0	0
0	0	0	0	0
0.000967892	0.001562432	0.007949391	0.046514515	0.173025235
0	0	0	0.210977326	0
0	0	0	0	0
0.002898805	0	0	0	0
0	0	0	0	0
0	0	0	0	0
0.033967854	0.007414043	0	0	0
0	0	0	0	0
0	0	0	0	0
0	0	0	0	0
0	0	0	0	0

0	0	0	0	0
0	0	0	0.009900587	0
0	0	0	0	0
0.001002774	0.001456866	0.001029484	0	0
0.000775047	0.000750678	0.003501045	0	0.007198953
0	0	0.000821534	0	0
0	0	0	0	0
0.001699221	0	0	0	0
0	0	0	0	0
0	0	0	0	0
0	0	0	0	0
0	0	0	0	0
0	0.002294917	0.010824767	0.08840011	0
0	0	0	0	0
0	0	0	0	0
0	0	0	0	0
0	0	0	0	0
0	0	0	0	0
0	0	0	0	0
0	0	0	0	0
0	0	0	0	0
0	0	0	0	0
0.005147963	0.002417502	0.014413878	0	0
0.016015311	0	0.02986002	0.026742468	0.032670299
0	0	0	0	0
0	0	0	0	0
0	0	0	0.003608668	0
0	0	0	0	0
0	0	0	0	0
0	0	0	0	0
0	0	0.004821423	0.003062852	0
0	0	0	0	0
0	0	0	0	0
0	0	0	0	0
0	0	0	0.002888805	0
0	0.056532239	0	0	0
0	0	0	0	0
0	0.075996114	0	0	0
0	0	0.585146935	0	0.002196829
0	0	0	0	0
0	0	0	0	0
0	0.00233857	0	0	0
0	0	0	0	0
0	0	0	0	0
0	0	0	0	0
0.045447375	0	0.079092543	0.151049397	0.360798051
0	0	0.047462997	0	0
0	0	0	0	0

0	0	0	0.002936752	0
0	0	0	0	0
0	0	0	0.061242726	0
0	0	0	0	0
0	0	0	0	0
0	0	0	0	0
0	0	0	0	0
0	0	0	0	0
0	0	0.004053984	0	0
0	0	0	0	0
0	0	0	0	0.001980241
0	0	0	0	0
0	0	0	0	0
0	0	0	0	0
0	0	0	0	0
0	0	0	0	0
0	0	0	0	0
0	0	0	0	0
0	0	0	0	0
0.010133153	0	0	0.001219759	0.005777508
0	0	0	0	0
0	0	0	0	0
0	0	0	0	0
0	0	0	0	0
0	0	0	0	0
0	0	0	0	0
0	0	0	0	0
0	0	0	0.001542708	0
0	0	0	0	0
0	0	0	0	0
0.000952156	0.004742834	0.00404972	0.002993998	0.021207583
0	0	0	0	0
0.284916073	0.039772153	0.02496997	0.00608359	0.003929388
0	0	0.036452288	0	0
0	0	0	0	0
0	0	0	0	0
0	0	0	0	0
0	0	0	0	0
0	0	0	0	0
0	0	0	0	0
0	0	0	0	0
0.143445259	0.013465616	0.014806656	0.005290909	0.037067969
0	0	0.009425618	0.093377271	0
0	0	0	0	0
0	0	0	0	0
0	0	0.013855512	0.026433485	0.006449942
0	0	0	0	0
0	0	0	0	0

0	0	0	0	0
0	0	0.021451684	0.004015208	0
0.001355983	0.001125726	0.027643164	0	0.021848327
0	0	0	0	0
0	0.071757227	0	0.050045332	0
0	0.064015233	0	0	0
0	0	0	0	0
0	0	0	0	0
0	0	0	0	0
0	0	0	0	0
0	0	0	0	0
0	0	0	0	0
0	0	0	0	0
0	0	0	0	0
0	0	0	0	0
0	0	0	0	0
0	0	0	0	0
0	0	0	0	0
0	0	0	0	0
0	0	0	0	0
0	0	0	0	0
0	0	0	0	0
0	0	0	0	0
0	0	0	0	0
0	0	0.003605761	0.129665718	0
0	0.002022152	0	0	0
0	0	0	0	0
0	0	0.000799686	0.005143576	0
0	0	0	0	0
0	0	0	0	0
0	0	0	0	0
0.001267529	0	0	0	0.019155013
0	0	0	0	0
0	0	0	0	0
0	0	0	0	0
0	0	0	0	0
0	0	0	0	0
0.017265274	0	0.009519072	0	0
0	0	0.083680082	1.44073612	0
0	0	0	0	0
0	0	0	0	0
0	0	0.001430935	0.004601886	0
0.001934154	0.002185562	0.001654728	0	0.006950824
0	0	0	0	0
0	0	0	0	0
0	0	0	0.001836694	0
0	0.006686964	0.018479294	0	0.010633401
0	0	0	0	0

0	0	0	0	0
0.026134907	0.012204557	0.006228639	0.002396907	0.196616456
0	0	0	0	0
0	0	0	0	0
0	0	0	0	0
0	0	0	0	0
0	0	0	0	0
0	0	0	0	0
0	0	0	0	0
0	0	0	0	0
0	0	0	0	0
0	0	0	0	0
0	0	0.002870046	0.163245438	0
0	0	0	0	0
0	0.005448149	0.003982653	0	0.001608603
0	0	0	0	0
0	0	0	0	0
0	0	0	0	0
0	0	0	0	0
0	0	0	0	0
0	0	0	0	0
0	0.076383175	0.065318377	0	0
0.009514435	0.012901386	0.015478318	0	0
0	0	0	0	0
0	0	0	0	0
0	0	0	0	0
0	0	0	0	0
0	0	0	0	0
0	0	0	0	0
0	0	0	0	0
0	0	0	0	0
0	0	0	0	0
0	0	0	0	0
0	0	0	0	0
0	0.20487221	7.028761354	0	0
0	0	0	0	0
0	0	0	0	0
0	0	0	0	0
0	0.0184246	0.009764716	0.001046777	1.120724579
0	0	0	0	0
0.014327366	0.042787034	0.045702976	0.089727677	0.361552321
0	0	0	0	0
0	0	0	0	0
0	0	0	0	0
0	0	0	0	0
2.924183239	0.374882996	0.072174893	0	0.813005893
0	0	0	0	0
0	0	0	0	0

0	0	0	0	0
0	0	0	0	0
0	0	0	0	0
0	0	0	0	0
0	0	0	0	0
0	0	0	0	0
0	0	0	0	0
0	0.02412365	0	0	0
0.000661458	0	0.00203723	0	0
0.000763952	0.002219795	0.220780947	0	0
0	0	0	0.007093705	0
0	0	0	0	0
0	0.00379691	0.001916472	0	0
0	0	0	0	0
0	0	0	0	0
0	0	0	0	0
0	0	0	0	0
0	0	0	0	0
0	0	0	0	0
0.078258409	0.074510321	0.790124538	0.023406233	1.453185638
0	0	0	0	0
0	0	0	0	0
0	0	0	0	0
0	0	0	0	0
0	0	0	0	0
0	0	0	0	0
0	0	0	0	0
0	0	0	0	0
0	0	0	0	0
0.001953831	0	0	0	0
0	0	0	0	0
0	0	0	0	0
0	0	0	0	0
0	0	0	0	0
0	0	0	0	0
0	0	0	0	0
0	0	0	0	0
0	0	0	0	0
0	0	0	0	0
0	0	0	0	0
0	0	0	0	0
0	0	0	0	0
0	0	0.008932925	0	0
0	0	0	0	0
0.001560577	0.001209207	0	0.002919749	0
0	0	0	0	0
0	0	0	0	0
0	0	0	0	0

0	0	0	0	0
0	0	0	0	0
0	0	0	0	0
0	0	0	0	0
0	0	0	0	0
0.002912578	0.002256799	0.04915824	0.012565366	0
0	0	0	0	0
0	0	0	0	0
0	0	0	0	0
0	0	0	0	0
0	0	0	0	0
0	0	0	0	0
0	0	0	0	0
0	0	0	0	0
0	0	0	0	0
0	0	0	0	0
0	0	0	0.010731401	0
0	0	0	0	0
0	0	0	0	0
0	0	0	0	0
0	0	0	0	0
0	0	0	0	0
0	0	0	0	0
0	0	0	0	0
0	0	0	0	0
0	0	0.00374169	0	0
0	0	0	0	0
0	0	0	0	0
0	0	0	0	0
0	0	0.092761076	0	0
0	0	0	0	0
0	0	0	0	0
0	0	0	0	0
0	0	0	0	0
0	0	0	0	0
0	0	0	0	0
0	0	0	0	0
0	0	0	0	0
0	0	0	1.45191163	0
0	0	0	0	0
0	0	0	0	0
0	0	0	0	0
0	0	0	0	0
0	0	0	0	0
0	0	0	0	0
0	0	0	0	0
0	0	0	0	0
0	0	0	0	0

0	0	0	0	0
0	0	0	0	0
0	0	0	0	0
0	0	0	0	0
0	0	0	0	0
0	0	0	0	0
0	0	0	0	0
0	0	0	0	0
0	0	0	0	0
0	0	0	0	0
0	0	0.003682209	0	0.004532579
0	0	0	0	0
0	0	0	0.169737062	0
0	0	0	0	0
0	0	0	0	0
0	0	0	0	0
0	0	0	0	0
0	0	0	0	0
0	0	0	0	0
0	0	0	0	0
0.005547055	0.016789502	0.00373722	0	0
0	0	0	0	0
0	0	0	0	0
1.154538468	1.810597697	1.573017915	0.417064472	2.306536636
0	0	0	0	0
0	0	0	0	0
0	0	0	0	0
0	0	0	0	0
0	0	0	0	0
0	0	0	0	0
0	0	0	0	0
0	0	0	0	0
0	0	0	0	0
0	0	0	0	0

PHL018-0095_V2	PHL018-0027_V1	PHL018-0006_V1	PHL018-0045_V2	PHL018-0140_V2
CTRL	EYCF	EYCF	EYCF	CTRL
0.068307994	0.383877232	0.008804776	0.097384282	0.01874823
3.889068111	4.411332954	6.243075024	1.321128018	1.470644254
10.73897932	15.34303176	0	4.048177918	7.577410985
0	0	0	0	0
0	0	0	0	0
0	0	0	0	0
0	0	0	0	0
0	0	0	0	0
0	0	0	0	0
0	0	0	0	0
0	0	0	0	0
0	0	0	0	0
0	0	0	0	0
3.522492914	3.615227506	2.133175699	46.25759854	6.859366816
0	0	0	0	0
0	0	0	0	0
0	0	0	0.004698401	0
0	0	0	0	0
0	0	0	0	0
0	0	0	0	0
0	0	0	0	0
0	0	0	0	0
0	0	0	0	0
0	0	0	0	0
0	0	0.014251903	0	0
0	0	0	0	0
0	0	0	0	0
0	0	0	0	0
0	0	0	0	0
0	0	0	0	0
0	0	0	0	0
0	0	0	0	0
0	0	0	0	0
0	0	0	0	0
0	0	0	0	0
1.421869832	0	8.0888034	0	3.362507458
0	0	0	0	0
0	0	0	0	0
0	0	0	0	0
0	0	0	0	0
0	0	0	0	0
0.00449918	0.044064349	0.989668086	0.001062613	0
0.018017984	0.008065605	0.023824572	0.003023628	0.107842815
0	0.134368168	0.112776839	0.015681796	0.02474858
0.001181987	0.005788111	0.032240437	0	0
0	0	0	0	0

0	0	0.005497935	0	0.001734354
0	0.005160764	0	0.005152314	0.015908947
0.061650373	0	0	0.081497894	0.003069287
0.218550072	0.328421678	0.974293193	0.19229484	4.496216368
0	0.049100462	0	0.034887854	0
0	0	0.001399846	0	0
0.129620029	0.106845422	0.685682575	0	0.064796934
0	0	0.003400763	0	0
0	0	0	0	0
0	0	0	0	0
0	0	0	0	0
1.441696046	0.682535516	0.246815651	0	0.034096069
0	0	0	0	0
0.056021462	0.074434335	0.016065564	0.176771491	0.029308753
0.001664878	0	0	0	0.000837746
0	0	0	0	0
1.062161929	1.041853213	0.919178121	0	0.001116089
0	0	0	0	0
0	0	0	0	0
0	0	0	0	0
0.091894933	0.136598278	0.031702829	0.06813948	0.32174728
0.124404279	0.07219373	0.001344265	0.043162698	0.008481118
0	0	0	0	0.004529792
0	0	0	0	0
0.033019012	0	0	0	0
0	0	0	0	0
0	0	0	0	0
0	0	0	0	0
0	0	0.004422095	0	0
0	0	0	0	0
0	0	0	0.052967666	0.508417004
0	0	0	0	0
0	0	0	0	0
0	0	0	0	0
0	0	0	0	0
0	0	0	0	0
0	0	0	0	0
1.269600579	0.022073751	0	0	0
0	0	0	0	0
0	0	0	0	0
0	0	0	0	0
0	0	0	0	0
0	0	0	0	0
0	0	0	0	0
0	0	0	0	0
0	0	0	0	0
0	0	0.000949697	0	0
0	1.422206608	0	0.315255218	0

0	0	0	0	0
0	0	0	0.131506192	0
0	0	0	0	0
0	0	0	0	0
0	0.006121942	0.001967302	0	0
0	0	0	0	0
0	0	0	0	0
0	0.002136697	0	0	0
0	0	0	0	0
0	0	0	0	0
0	0	0	0	0
0	0	0.008404528	0	0
0	0	0	0	0
0	0	0	0	0
0	0	0	0	0
0	0	0	0	0
0	0	0	0	0
0	0	0	0	0
0	0.025616797	0.329871183	0	0.078246446
0	0	0	0	0
0	0	0	0	0
0	0	0	0	0
0	0	0	0	0
0	0	0	0	0
0	0	0	0	0
0	0	0	0	0
0	0	0	0	0
0	0	0	0.000665961	0
0	0	0	0	0
0	0	0	0	0
0	0.065498325	0.002576505	0	0.019867781
0.036150082	0.000776423	0.01729906	0	0
0	0	0	0	0
0	0	0	0	0
0	0	0	0	0
0	0	0	0	0
0	0	0	0	0
0	0	0	0	0
0	0	0	0	0
0	0	0	0	0
0	0	0	0	0
0	0	0	0	0
0.000652872	0	0	0	0
0	0	0	0	0
0	0	0	0	0
0	0	0	0	0
0	0	0	0	0
0	0	0	0	0

0	0	0	0	0
0	0	0.001503812	0	0
0	0	0	0	0
0	0	0	0	0
0	0	0.215344666	0	0
0	0	0	0	0
0	0	0	0	0
0	0	0	0	0
0	0	0	0	0
0	0	0	0	0
0	0	0	0	0
0	0	0	0	0
0	0	0	0	0
0	0	0	0	0
0	0	0	0	0
0	0	0	0	0
0	0	0	0	0
0	0	0	0	0
0	0	0	0	0
0	0	0	0	0
0	0	0	0	0
0	0	0	0	0
0	0	0	0	0
0	0	0	0	0
0.018103437	0	0.007395345	0	0
0	0	0	0	0
0	0	0.000782975	0	0
0	0	0	0	0
0	0	0	0	0
0	0	0	0	0
0	0	0	0	0
0	0	0	0	0
0	0	0	0	0
0	0	0	0	0
0	0	0	0	0
0	0	0	0	0
0	0	0	0	0
0	0	0	0	0
0	0	0	0	0
0	0	0	0	0
0	0	0	0	0
0	0	0	0	0
0	0	0	0	0
0	0	0	0	0
0	0	0	0.003059222	0
0	0	0	0	0
0	0	0	0	0
0	0	0	0	0.005628722

0	0	0	0	0
0	0	0	0	0
0	0	0	0	0
0	0	0	0	0
0	0	0	0	0
0	0	0	0	0
0	0	0	0	0
0	0	0	0	0
0	0	0	0	0
0	0	0	0	0
0	0	0	0	0
0	0	0	0	0
0.001455118	0	0	0.001590695	0
0	0	0	0	0
0.001127859	0	0	0	0
0	0	0	0	0
0	0	0	0	0
0	0	0	0	0
0	0	0	0	0
0	0	0	0	0
0.002332046	0.050888544	0.038686767	0.049570304	0.056325954
0	0	0	0	0
0.001193632	0	0	0	0
0.007295565	0	0	0.002067674	0
0	0	0	0	0
0	0	0	0	0
0.000905419	0.008167481	0.009315396	0	0.008200727
0	0	0	0	0
0	0	0	0	0
0	0	0	0.112013413	0
0	0	0	0	0
0	0	0	0	0
0	0	0	0	0
0.007601076	0	0.048195243	0	0
0.008092552	0.003910724	0.003146459	0	0.00381757
0	0	0	0	0
0	0	0	0	0
0	0	0	0	0
0	0	0	0	0
0	0	0	0	0
0	0	0	0	0
0	0	0	0	0
0	0	0	0	0
0	0	0	0	0
0	0	0	0	0
0	0	0	0	0.023262349
0	0	0	0	0
0.000901089	0	0	0.001225836	0

0	0	0	0	0
0	0	0	0.002964042	0
0	0	0.003467408	0	0
0	0	0	0.000779098	0
0	0	0	0	0
0	0	0	0	0
0	0	0.000736573	0	0
0	0	0.007438334	0	0
0	0	0	0	0.001072186
0	0	0	0	0
0	0	0	0	0
0	0	0	0	0
0	0	0	0	0
0	0	0	0.254659524	0
0	0	0	0	0
0	0	0	0	0
0	0	0	0	0
0	0	0	0	0
0	0	0	0	0.001106694
0	0	0	0	0
0.040012865	0.008640325	0	0.018619473	0
0	0	0	0	0
0	0	0	0	0
0	0	0	0	0
0	0.014950885	0.008225	0	0.003243278
0	0	0	0	0
0	0	0	0	0
0	0	0	0	0
0	0	0.044151959	0	0
0	0	0	0	0
0.325425023	0.323809641	0.19634741	1.490785941	0.098229953
0	0	0	0	0
0	0	0	0.008616858	0
0	0	0	0	0
0	0	0	0	0
0	0	0	0	0
0	0	0	0	0
0	0	0	0	0
0	0	0	0	0
0	0	0.002660594	0	0
0	0	0	0	0
0.036069378	0.013996643	0.013575627	0	0.009992517
0	0	0	0	0
0	0	0.021434702	0	0
0	0	0	0	0
0	0	0	0	0
0	0	0	0	0
0	0	0	0	0

0	0	0	0	0
0	0	0	0	0
0	0.1209271	0.112749986	0.019254256	0.024326301
0	0	0	0	0
0	0	0	0	0
0	0	0	0	0
0	0	0	0	0
0	0	0	0	0
0	0	0	0	0
0	0.000959644	0.001336332	0	0
0	0	0	0	0
0	0	0	0	0
0	0	0	0	0
0	0	0	0.001912758	0
0.003249389	0.003349903	0.005331239	0.004538852	0.003270107
0	0	0	0	0
0	0.002999004	0.010124115	0	0.001862997
0	0	0	0	0
0	0	0	0	0
0	0	0	0	0
0	0	0	0	0
0	0	0	0.001826801	0
0	0	0	0	0
0	0	0	0	0
0	0	0	0	0
0	0	0	0	0
0	0	4.476100271	0.000713242	0
0	0	0	0	0
0	0	0	0	0
0	0	0	0	0
0	0	0	0	0
0	0	0	0	0
0	0	0	0	0
0.006055571	0	0.001844054	0	0
0	0	0	0	0
0	0.00245788	0	0.001216259	0
0	0	0	0	0
0.004199128	0.005565882	0.129751597	0	0
0	0	0	0	0
0	0	0	0	0
0	0	0	0	0
0	0	0	0	0
0	0	0	0	0
0	0	0	0.002850981	0
0	0	0	0	0
0	0	0	0	0
0	0	0	0	0
0.020344788	0.002247226	0	0.002162259	0

0	0	0	0	0
0	0	0	0	0
0	0	0	0	0.00201315
0	0	0	0	0
0	0.010114879	0.001926189	0.00641699	0.016456566
0	0	0	0	0
0	0	0	0	0
0.003878999	0.144327106	0.00607495	0.004197585	0.009936769
0	0	0	0	0
0.010177155	0.025480486	0	0.097965256	0
0	0	0	0.001397054	0
0.001395741	0.008057924	0.104995452	0	0
0	0.028814759	0.023913099	0	0.007458283
0	0	0	0.024334874	0
0	0	0	0.006295477	0
0.002225038	0	0	0	0
0	0	0	0	0
0	0	0	0.001875769	0
0	0	0	0	0.267098525
0	0.316262115	0	0	0
0	0	0	0	0
0	0	0	0	0
0	0.012839297	0.008364484	0	0
0	0	0.001010392	0	0
0	0	0	0	0
0	0	0.413305201	0	0
0	0	0	0	0
0	0.060570938	0	0	0.003001427
0	0	0	0.82507818	0
0	0.002092783	0	0	0
0	0	0	0	0
0	0	0	0	0
0	0	0	0	0
0	0	0	0	0.007465798
0	0	0	0	0
0	0	0	0	0.007047264
0	0	0	1.873864774	0.091442023
0	0.009264751	0	0	0
0	0	0	0	0
0	0	0	0.005293065	0
0	0	0	0	0.030718365
0	0	0	0.037679258	0
0	0	0	0.028089072	0
0	0	0	0	0
0	0	0	0.025538424	0
0	0	0.002417742	0	0
0.057353047	0.002845692	0.002054737	0.015646261	0
0	0.021237644	0	0	0

0	0	0	0.001232351	0
0.058954648	0.023321902	0.017058316	0	0.010348348
0	0	0	0.006872883	0
0	0	0	0	0
0	0	0	0	0
0	0.022164232	0	0.007188607	0
0	0	0	0	0
0	0	0.00089706	0	0
0	0	0.011245909	0	0
0	0	0	0	0
0.001961215	0.029172859	0.010189527	0.025965586	0.00169176
0.002888451	0	0	0	0
0	0	0	0	0
0.003970409	0.002728817	0.001773312	0.001575384	0.003196579
0	0	0	0.030741536	0.088944986
0	0	0	0	0
0	0	0	0	0
0	0	0	0.001095074	0.026700925
0	0	0	0	0.005953966
0	0.035016669	0	0	0.008625506
0	0	0.001637718	0	0
0.137661983	0.385652923	0.148650634	0	0
0	0.035217428	0	0	0
0	0	0	0	0
0	0	0	0	0
0	0	0	0	0
0	0	0	0.046363621	0.322621024
0	0	0	0	0
0	0	0	0	0
0.192828968	1.269959997	0.57667425	0.380355442	0.691503894
0	0	0.002234238	0	0
0	0	0	0	0
0	0	0	0	0
0	0	0	0.001946248	0.003327475
0	0	0	0	0.011547198
0	0	0	0	0
0	0	0.325466852	0	0
0	0.001946974	0.03072712	0	0
0	0	0	0	0.13900454
0	0	0	0	0.005673556
0.054184194	0.314522494	0	0.003515372	0.270239367
3.753133841	7.721865856	4.039620594	4.912496411	4.426663638
0	0.001783554	0.033351867	0	0
0	0	0.006030038	1.766245957	0
0	0	0	0.002921964	0.001235187
0	0	0	0.054051314	0
0.001141972	0.001412756	0	0.003495444	0
0	0	0	0	0

0.002818003	0.003776724	0.039646245	0	0
0	0	0	0	0.00054363
2.335717185	2.336714462	2.121887895	0	0
0	0	0	0	0.048741166
0	0.002725712	0	0	0
0	0	0	0.007292938	0
0	0	0	0	0
0	0	0	0	0
0	0	0	0	0.005696287
0	0	0	0.001410561	0
0	0	0	0	0
0	0	0	0	0
0	0	0	0	0
0.026768453	0.002102589	0.000975972	0.018207809	0
0	0	0	0	0
0.023309837	0.029371074	0.008199078	0	0.046315412
0.00485815	0.076492335	0.281933876	0	0.002222329
0.00214635	0.015212608	0	0.001140578	0
0	0.022574516	0	0	0
0	0	0	0	0
0	0	0.001331773	0.115709794	0
0	0.007393928	0	0	0
0	0	0	0	0.070066635
0	0	0	0	0.665300316
0.002063823	0	0	0	0
0.150103368	0	0	0	0
0.004205086	0.049709861	0	0	0.016363336
0	0.452124989	0	0.008899714	0.000714167
0	0	0	0	0
0	0	0	0.001223716	0
0.174821617	0.015385434	0	0.045498747	0.008796812
0	0.001638414	0.103125507	0	0.002878895
0.396756903	0.155063154	0.023750834	0	0.003654797
0	0.001788496	0.003320708	0.001475034	0
0	0.004148726	0.014220846	0	0
0	0	0	0	0
0	0	0	0	0
0.012410344	0.077427169	0.009829701	0.148817356	0.003660707
0	0	2.393292387	0	0
0.014695256	0	0	0	0
0	0	0	0	0
0	0.00647265	0	0	0
0	0.025071817	0	0	0.001932205
0.580098989	0.097636186	0	0.097566	0.058833617
0	0	0	0.087542927	1.244078479
0	0	0	0	0
0	0	0	0	0
0	0	0	0	0

0.007931216	0	0	0	0
0.017785358	0.622205086	7.253246055	0.042390968	0.725926522
0	0	0.001145889	0	0
0	0	0	0	0
0	0	0	0	0
0	0	0	0	0
0	0	0	0	0
0.001567659	0	0.003215053	0	0
0	0.003026741	0	0	0
0	0	0	0	0
0	0.004243943	0	0	0.000654134
0	0.03027653	0.054361342	0.003567156	0.008769165
0	0	0.059626055	0	0
0	0	0	0	0
0	0	0	0	0
0	0	0	0.004511761	0
0.0020419	0.026196333	0.01715388	0.001543215	0.013471137
0	0.073781871	0	0.364601776	0.238302828
0	0	0	0	0
0	0	0	0.099677059	0.094307572
0.008890795	0.022432122	0.114201122	0	0.038615442
0	0	0.018947328	0	0
0	0	0	0	0
0	0	0	0	0
0.004324214	0.011752842	0.669319753	0	0.088222533
0	0	0	0	0
0.035333572	0	0	0	0
0.275530503	0.269953349	0.036175682	0	0.109863802
0	0	0	0	0
0.001799753	0	0	0	0
0.002401444	0	0.016343805	0.010889694	0.000805585
0	0	0	0	0
0	0	0	0.021817723	0
0	0	0	0	0
0	0	0.28911366	0	0.010904175
0.071967995	0.08287186	0.085393019	0.001789195	0.805166303
0	0	0	0	0
0	0	0	0	0
0.007975939	0.004743841	0.037286824	0	0.003704673
0	0	0	0	0
0	0	0	0.083471918	1.169712325
0	0	0	0.006049914	0
0.053291777	0.003649877	0	0.006337217	0.018564769
0	0	0	0	0
0	0	0	0.270687838	0
0	0	0.331421768	0	0
0	0	0	0	0
0	0	0	0.01310428	0

0	0	0	0	0.019386044
0	0	0.0008816	0	0
0	0	0	0	0
0	0	0.076642859	0	0.003057668
0	0	0	0	0
0	0	0	0	0
0	0	0	0.008726371	0
0	0	0.003902088	0	0
0.13710051	0	0.453531622	0	0.052359605
0.017844647	0	0.165216481	0	0
0	0	0	0	0
0.004158592	0.002786699	0.003980059	0	0.004394362
0	0	0	0	0
0	0	0.006980766	0	0
0.134178725	0	0.005419186	0.017068966	0.007770511
0	0	0	0	0
0.01410043	0	0.027632791	0	0
0	0	0	0	0
0	0	0	0	0
0	0	0	0	0
0	0	0	0	0
0	0	0	0	0
0	0	0.00091624	0	0
1.167203229	0.709682664	0	0.324338878	0.581168208
0	0	0	0	0.002938747
0	0	0.045508695	0	0
0	0	0.023277369	0	0
0	0	3.117251316	0	3.825016075
0	0	0	0	0
0	0	0	0	0
0	0	0.012768678	0	0
0	0	0.129600901	0	0
0.005692291	0.015701855	0	0	0.001238613
0	0	0	0	0
0	0	0.00875256	0	0
0.440141571	0	0.005249169	0	0
0	0	0.446588388	0	0
0	0	0	0	0
0	0	0	0	0
0	0	0	0	0
0.03391012	0.048531402	0	0.001187197	0.041553818
0	0	0.030010965	0	0
24.15348492	4.375827361	3.304032824	0.872010493	0.091269253
0	0	0.001330445	0	0.002273355
0	0	0	0	0
0	0	0	0	0
0	0	0	0	0
0	0.002224842	0	0	0

0	0	0	0	0
0	0	0.07338521	0	0
0.198831734	0.209010739	0.01891957	0.396548287	0.013417073
0	0	0	0	0
0	0	0.007459161	0	0
0.001629697	0.002613502	0.001213125	0	0
0	0	0	0	0.004666256
0	0	0	0	0
0	0	0	0	0
0	0	0	0	0
0.562858326	0.055839859	0.00107998	0.07349298	0.01748854
6.054233465	3.162099965	0.687796682	0	1.930178825
0	0	0	0	0
0.009986305	0.13685098	0.371113938	0	0.000980485
0.070250501	0.848107549	0.002204412	1.193787723	0.136760846
0	0	0	0	0
0	0	0	0	0
0	0	0	0	0
0	0	0.006764545	0	0
0.04889302	0.229411768	0	0.174977891	0.022076657
0.316081753	0.0323714	0	0.001125811	0.158572604
0.148288909	0.411162263	0	16.03376734	0
2.468416296	1.749806838	0.132006889	1.637871579	0.12945796
0	0	0	0	0
0	0	0	0	0
0.00158667	0	0.018439565	0	0.006387144
0.291172004	0	0	0	0
0	0	0	0	0
0	0	0	0	0
0	0	0	0	0
0	0	0.003394536	0	0
0	0	0.16134126	0.066959663	0
0	0	0.018190355	0.043356201	0
0	0.043915266	0.000845826	0	0.016542882
0.011932483	0.001261702	0	0.005072771	0.00076978
0.010929668	0.034886702	0.002615111	0	0.000846104
0.004669845	0	0	0	0.001021656
0	0	0	0	0
1.551269537	0	0.262236319	0	0
0	0	0	0	0
0	0	0.004782608	0	0
0	0	0	0	0
0	0	0	0	0
0	0	0	0	0
0.206855216	0.02372233	0.001376417	0.009170914	0.00844275
0	0	0	0	0
0	0	0	0	0
0.051755251	0.049183084	0	0	0.022405379

0	0	0	0	0
0	0	0	0	0
0	0	0	0.003160471	0
0	0	0.000782077	0	0.001233552
0	0	0	0	0
0	0	0.275599326	0	0
0	0	0.777880396	0	0
0.023121951	0	0	0.01102841	0.012486005
0	0	0	0	0
0	0	0	0	0
0.00819088	0	0	0.020118974	0
0	0	0	0	0
0	0	0.014681043	0	0
0	0	1.203542717	0	0
0	0	0	0	0
0	0	0	0	0
0	0	0.143814757	0	0
0.009470445	0	0.007432372	0	0
0	0	0	0	0
0	0	0.01128027	0	0.002234759
0	0	0.001449456	0	0.00152413
0	0	0	0	0
0.023147741	0	0.003552997	0	0
0	0	0	0	0
0	0	0	0	0
0	0	0	0	0
0	0	0	0	0
0	0	0	0	0
0	0	0	0	0
0.014185864	0	0.216791636	0	0.34263145
0	0	0.001886583	0.004113848	0
0	0	0	0	0
0	0	0.004924504	0	0
0	0	0	0	0
0	0.002424664	0	0	0
0	0	0	0	0
0.008146694	0	0.131456537	0	0
0.008089344	0.012707918	0	0.001473842	0.004651954
0	0	0	0	0
0.011398402	0.004112846	0	0.008560774	0
0	0.108053744	0.000518855	0	0.01582198
0	0	0	0	0
0	0	0	0	0
0.005237141	0	0.010382631	0	0.080940306
0.134281302	0	0.120353364	0	0
0	0	0	0	0
0	0	0.002452084	0	0
0	0	0	0	0

0	0	0	0	0
0	0	0	0	0
0	0	0	0	0
0	0	0.005317412	0	0
0	0	0	0	0
0	0	0.7783994	0	0
0	0	0	0	0
0	0	0	0	0
0.016203569	0.147604651	0	0.331325914	0.028336545
0	0	0	0	0
0	0	0.001999593	0	0
0	0	0	0	0
0	0	0	0	0
0	0	0	0	0.001114056
0.02061616	0	0.245542402	0	8.322478741
0	0	0	0.17250432	0
0	0.027566364	0	0	0
0	0	0	0	0
0	0	0	0	0
0	0	0	0	0
0	0	0.000777576	0	0
0	0	0	0	0
0	0	0	0	0
0	0.136960769	0	0	0
0	0	0.002989654	0.001062385	0.212512527
0	2.465614062	2.71924758	0	2.65370405
0	0	0.001334369	0	0
0	0	0	0	0
0	0	0.015160111	0	0.034355902
0	0	0.552055858	0	0
0	0	0	0	0
0	0	0.35368743	0	0
0	0	0	0	0
0.011344361	0.002407851	0	0	0.000503678
0.002473931	0.011335365	1.066790547	0.004674332	0.001659803
0.008743847	0	0	0.004130232	0
0	0	0	0.482149723	0.19291951
0.408535803	2.342237736	0.013649194	0	0
0	0	0	0	0
0	0	0	0	0
0	0	0	0	0
0	0	0	0	0
0.722227836	0.177108752	0.407109322	0	0.269355614
0	0	0.001336667	0	0
0	0	0	0	0
0.013806162	0	0.004756842	0	0
0	0	0	0	0
3.264332107	0.143609836	0	0	0.55957547

0	0	0	0	0
0	0	0	0	0
0	0	0	0	0
0	0	0	0	0
0.012628295	0	0	0.002591727	0
0	0	0	0	0
0	0	0	0	0
0	0	0	0	0
0	0	0	0	0
0	0	0	0	0
0	0	0	0	0
1.604140705	2.858153834	0.028067855	0	0.416832905
0	0	0	0	0
0	0	0	0	0
0.071260489	0.178859497	1.406920854	0.001070476	0
0	0	0	0	0
0	0	0	0	0
0	0	0	0	0
0.001544936	0	0	0	0
0	0	0	0	0
0	0	0.006295648	0	0
0	0	0	0	0
0.006223568	0.46129412	0.56174858	0	0.187465495
0	0	0	0	0
0	0	0	0	0
0	0	0	0	0
0	0	0	0	0
0	0	0	0	0
0	0	0.001302446	0	0
0	0	0	0	0
0	0	0	0	0
0.000980252	0.075793066	0.212651603	0.006945459	0.066424468
0	0	0	0	0
0.140059079	0.083639377	0.007342857	0	0.008706829
0.00655512	0.000871986	0	0.003775571	0
0	0.003372286	0.026610687	0	0.001920308
0	0	0	0	0
0.207053271	0	0.004644475	0	0
0.006073279	0	0	0	0
0	0	0	0	0
0	0	0	0	0
0	0	0.013256307	0	0
0	0	0	0	0
0	0	0	0	0.044349895
0.22101143	0.008589933	0.000797448	0.007969963	0.00356376
0	0	0	0	0
0	0	0.204305807	0	0
0	0.001218681	0	0	0.001189652

0	0	0	0	0
0	0	0.013834349	0	0
0	0.028207145	0	0.010624229	0
0	0	0	0	0
0	0	0	0.010031435	0
0	0	0	0	0.001119132
0	0	0.556530399	0	0.067780338
0	0	0.00077891	0	0
0	0	0	0	0
0	0	0.001532954	0	0
0	0	0	0	0
0	0	0	0.011555405	0
0	0	0	0	0
0	0	0.078243183	0	0
0	0	0	0	0
0	0	0	0	0
0	0	0	0	0
0	0	0	0	0
0	0	0.100907055	0	0.234220107
0	0	0	0	0
0	0	0.002060766	0	0.003348897
0	0	0	0	0
0	0	0	0	0
0	0	0	0	0
0.019345245	0	0.002831665	0	0
0.001163544	0.003998451	0	0.037263554	0.002732245
0	0	0	0	0
0.047453238	0.075925559	0	0	0.104298646
0	0	0.015573723	0	0
1.67966872	2.471773866	1.873343587	0	0.624735809
0	0	0	0	0
0	0	0	0	0
0	0	0	0	0
0	0	0	0	0
0.001016101	0	0	0.008639358	0
0	0	0	0	0
0	0	0.158621472	0	0
0.001383573	0	0	0	0
0	0	0	0	0
0	0	0	0	0
2.81234756	10.56327578	5.190218622	0	6.674020093
0.014516538	2.230915079	0	0	0.644838523
0	0	0.073760177	0	0
0	0	0.040223278	0	0
0	0	0.081700576	0	0
0	0	0	0	0
0	0	0	0	0
0.019948454	0	0	0	0

0	0	0	0	0
0.034825201	0.001552538	0	0.002880971	0.001894445
0	0	0	0	0
0	0	0.004147147	0	0
0.561196244	5.919952915	4.028276153	0	4.939189288
0	0	0	0	0
0	0	0	0	0
0	0	0.000764077	0	0
0.10093817	0.338535699	0	0.346005883	0.661510827
0	0	0	0	0
0	0	0	0.004159035	0
0	0	0	0	0
0.111027252	0.213083473	0.023632947	0	0
0.011243551	0.00834577	0.011406505	0	0.022856781
0.029620044	0	0.16691552	0	0
0	0.299240476	0.270029922	0	2.180536933
0	0.002607934	0	0	0.000783327
0	0	0	0	0
0	0	0.00295263	0	0
0	0	0	0	0
0	0	0	0	0
0	0	0	0	0
0	0	0	0	0
0	0	0	0	0
0	0	0	0	0
0	0	0	0	0
0	0	0	0	0
0.000954644	0.000984174	0	0.001623364	0
0	0	0	0	0
0	0	0	0	0
0	0	0	0	0
0	0	0	0	0
0	0	0	0	0
0	0	0.005746557	0	0
0	0	0.184642207	0	0
0	0	0	0	0
0	0	0	0	0
0	0	0	0	0
0	0	0	0	0
0	0	0	0	0
1.467477387	0	0.972907759	0	3.540317508
0	0	0	0	0.235318799
0	0	0.021120568	0	0
0	0	0.004472962	0	0.005173743
0	0	0	0	0
0	0	0	0	0
0	0	0	0	0
0	0	0	0	0

0	0	0.020807739	0	0
0	0	0	0	0
0	0	0.037710727	0	0
0	0	0	0	0
0.019038691	0	0.344620979	0	0
0	0	0	0	0
0	0.001200735	0	0	0.060755557
0	0	0	0	0
0	0	0	0	0
0	0	0	0.154460553	0
0.008238988	0	0.03109053	0	0
0	0	0	0	0
0	0	0	0	0
0	0	0	0	0
0	0	0	0	0
0	0	0	0	0
0	0	0	6.565850912	0
0	0	0	0	0
0	0	0	0	0
0	0	0	0	0
0.000924055	0.00762111	0.026885283	0	0.02715444
0	0	0.000858024	0	0
0	0	0.023801536	0	0
0.005981642	0.000934344	0.001387841	0.001695288	0.002918681
0	0	0	0	0
0.000641545	0	0	0	0
0	0	0	0	0
0	0	0	0	0
0	0	0	0	0
0	0	0.021472354	0.047023862	0.010501665
0	0	0.0009796	0	0
0	0	0	0	0
0	0	0	0	0
0	0	0	0	0
0	0	0.056403963	0	0
0	0	0.353674781	0	0
0	0	0.015411866	0	0
0	0	0	0	0
0	0	0.048806214	0	0
0	0	0.126043848	0	0
0	0	0	0	0
0	0	0	0	0
0	0	0	0	0
0	0	0	0	0
0	0	0.011093705	0	0
0	0	0	0	0
0	0	0.001669628	0	0
0	0	0	0	0

0.008581945	0.022319602	0	0.002321697	0.004514619
0	0	0.051695018	0	0
0	0	0	0	0
0	0	0	0	0
0	0	0	0	0
0	0	0	0	0
0.001049585	0	0	0	0
0.615218115	1.967574691	0.488755097	0	2.506390857
0.025410547	0.008206155	0.025491676	0.002863341	0.155592078
1.080798759	0.237743642	0.004079664	0.005074038	0.056411436
0	0	0.00670238	0	0
0	0	0	0	0
0	0	0	0	0
0	0	0	0	0
0	0	0	0	0
0	0	0	0	0
0	0.019078737	0	0	0
0	0	0	0	0
0	0	0	0	0
0	0	0.000507443	0	0
0	0	0	0	0
0	0	0	0	0
0	0	0	0	0
0	0	0	0	0
0	0	0.011909356	0	0
0	0	0.005423723	0	0
0	0	0	0	0
0	0	0	0	0
0	0	0	0.002803144	0
0	0	0	0	0
0	0	0	0	0
0	0	0	0	0
0	0	0	0	0
0	0	0.004156055	0	0
0	0	0.018757271	0	0.001901072
0	0	0.240565031	0	0
0	0	0	0	0
0	0	0	0	0
0.00997268	0.000956387	0	0	0
0	0	0	0	0.055651691
0	0	0	0	0
0.33185918	0	0.04056668	0	0
0	0	0	0	0
0	0	0	0	0
0	0	0	0	0
0.075617194	0	0	0	0
0	0	0	0	0
0	0	0	0	0

0	0	0	0	0
0	0	0	0	0
0	0	0	0	0
0	0	0	0	0
0.03396497	0.141584845	0.212000982	0	0.035915369
0	0	0.021957922	0	0
0	0	0	0	0
0.026299697	0.023020662	0	0.028900801	0.04219799
0	0	0.005690249	0	0
0	0	0	0	0
0	0	0	0	0
0	0	0.015078575	0	0
0	0	0	0	0
0	0	0	0	0
0	0	0	0.001039533	0.002988167
0.182261062	0.093394866	0	0	0.006713243
0	0	0	0	0
0	0	0	0	0
0	0	0	0	0
0	0	0	0	0
0	0	0	0	0
0	0	0	0	0
0	0	0	0	0
0.001503034	0	0	0.02391588	0
0.057120897	0	0.056131342	0	0
0	0	0	0	0
0.13098877	0.009920527	0.003195217	0.008515749	0.002569282
0	0	0	0	0
0	0	0	0	0
0	0	0	0	0
0	0	0	0	0
0	0	0	0	0
0	0	0	0	0
0	0	0	0	0
0	0	0	0	0
0	0	0	0	0
0	0	0	0	0
0.008660384	0	0.043948775	0	0
0	0	0.194821778	0	0
0	0	0	0	0
0	0	0	0	0
0.080135618	0	0.041773526	0	0
0	0	0	0	0
0	0	0.032424966	0	0
0	0	0	0	0
0	0	0.003018358	0	0
0	0	0	0	0
0	0	0.000570698	0	0

0.00646391	0.061878677	0.014317114	0	0
0	0	0	0	0
0	0	0	0	0
0	0	0	0	0
0	0	0	0	0
0	0	0	0	0
0	0	0	0	0
0	0	0	0	0
0	0	0	0	0
0	0	0	0	0
0	0	0	0	0
0	0	0	0	0.00637436
0	0	0	0	0
0	0	0	0	0
0	0	0	0	0
0	0	0	0	0
0	0	0	0	0
0	0	0	0	0
0	0	0	0	0
0	0	0	0	0
0	0.032844907	0	0.001090805	0.004303695
0	0	0	0	0
0.12583909	0.000835182	0	0	0
0	0	0	0	0
0	0	0	0	0
0	0	0	0	0
0	0	0	0	0
0	0	0	0	0
0	0	0	0	0
0	0	0	0	0
0	0	0	0	0
0	0	0.006756596	0	0
0.051590019	0.044842972	0	0.054400198	0.039957228
0	0	0	0	0
0	0	0	0	0
0	0	0	0	0.001021147
0	0	0	0	0
0	0	0.009025643	0	0
0.001408362	0	0	0.002594482	0.002126011
0	0	0	0	0
0	0	0	0	0
0	0	0.848199493	0	0
0.011253111	0.002546605	0.05805294	0	0.008838914
0	0	0	0	0
0	0	0	0	0
0.009808816	0.000674149	0.002086159	0	0
0	0	0	0	0
0	0	0	0	0

0	0	0	0	0
0	0	0	0	0
0	0.146997407	0	0.539070116	0
0	0	0	0	0
0	0	0	0	0
0	0	0	0	0
0	0	0	0	0
0	0.029532006	0	2.118618679	0.034975807
0	0	0.002149991	0	0.00061657
0.002469615	0.004629105	0.14761708	0	0.00112971
0	0	0	0	0
0	0.009034304	0	0	0
0	0	0	0	0
0	0	0	0	0
0	0.001683427	0	0	0
0	0.009336783	0	0.015588542	0.002445321
0	0	0	0	0
0	0	0	0	0
0	0	0	0	0
0.534103935	0	1.646802457	0	11.6783567
0	0	0.495482102	0	0.007894071
0	0	0	0	0
0	0	0	0	0
0	0	0	0	0.001184443
0	0	0	0	0
0	0	0	0	0
0	0	0	0	0
0	0	0	0	0
0	0	0	0	0
0	0	0.02300661	0	0
0	1.640819538	0.302761589	0	0
0	0	0	0	0
0	0	0	0	0
0	0	0	0	0
0	0	0	0	0
0	0	0	0	0
0	0	0	0	0
0	0	0	0	0
0	0	0.005422404	0	0
0	0	0	0	0
0	0	0	0	0
0	0	0	0	0
0	0	0.001115085	0	0
0	0	0	0	0
0	0	0	0	0
0	0	0	0	0
0	0	0	0.107283075	0.084655601
0	0.001705121	0	0	0

0	0	0	0	0
0	0	0	0	0
0	0	0	0	0
0	0	0.002133566	0	0
0.048652939	0.054860223	0.031689536	0	0.08092519
0	0	0	0	0
0	0	0	0	0
0	0	0	0	0.001132358
0	0	0	0	0
0	0	0	0	0
0	0	0	0	0
0.052771505	0.00145077	0	0.001395917	0.000944142
0.001821141	0	0	0	0
0	0	0	0	0
0.00303851	0	0.009066315	0	0
0	0	0.137028029	0	0
0	0.000743876	0	0.003374251	0.003449245
0	0	0	0.004176275	0
0.197663288	0.103628141	0	0.244770067	0.033243288
0.018782687	0.016973112	0	0	0.003500452
0	0	0	0	0
0	0	0.037943836	0	0
0	0	0	0	0
0	0	0	0	0
0	0	0	0	0
0	0	0	0	0
0.014679787	0.013601332	0	0.002685876	0.007854203
0	0.026157675	0	0	0
0	0	0	0	0
0	0	0	0	0
0.002948916	0	0	0	0
0	0	0	0	0
0	0	0	0	0
0	0	0	0	0
0	0	0	0	0
0	0	0	0	0
0	0	0	0	0
0.010359113	0.021206537	0.000566536	0.0054105	0.004170064
0	0	0.922311515	0	0
0	0	0	0	0
0.001772869	0	0	0	0
0	0	0.002541941	0	0
0	0	0	0	0
0	0	0	0.004291418	0
0	0	0	0	0
0	0	0	0	0
0	0	0	0	0
0	0	0	0	0

0	0	0	0	0
0	0	0	0	0
0	0	0	0	0
0.001609866	0	0	0.000977701	0
0	0.001539312	0.079208732	0.001632244	0
0.002752887	0.001135217	0	0	0
0	0	0.001922379	0	0.002246019
0	0	0	0	0
0	0	0	0	0
0	0	0.010173882	0	0
0	0	0.021825394	0	0
0	0	0	0	0
0.061134021	0.002016802	0.167259077	0	0.013453204
0	0	0	0	0
0	0	0	0	0
0	0	0	0	0
0	0	0	0	0
0	0	0	0	0
0	0	0	0	0
0	0	0	0	0
0	0	0	0	0
0	0	0	0	0
0.037137065	0	0	0.009490941	0.005184811
0.008865928	0.003394923	0.267408292	0	0
0	0	0	0	0
0.001122441	0	0.002148507	0	0
0	0	0.007262694	0	0
0	0	0	0	0
0	0	0	0	0
0	0	0	0	0
0.12494104	0.082415995	0.12828204	0	0.043116929
0	0	0	0	0
0	0	0	0	0
0	0	0	0	0
0	0	0.067986191	0	0
0	0	0	0	0.000755418
0	0	0	0	0
0	0	0	0.04375941	0
0	0.003340342	0.000775254	0.002582714	0
0	0	0	0	0
0	0	0	0	0
0	0	0	0	0
0	0	0	0	0
0	0	0	0	0
0	0	6.206713848	0	0
0.011759413	0.55803303	0.22833133	0	0.119957667
0	0	0	0	0
0	0	0	0	0

0	0	0	0	0
0	0	0	0	0
0	0	0.016091633	0	0
0	0	0.00901092	0	0
0	0	0	0	0
0	0	0	0	0
0	0	0	0	0
0	0	0	0	0
0	0.003283874	0	0	0
0	0	0	0	0
0.008542957	0	0	0	0
0	0	0	0	0
0	0	0	0	0
0	0	0	0	0
0	0	0	0	0
0	0	0	0	0
0	0	0	0	0
0	0	0	0	0
0	0	0	0	0
0	0	0	0	0
0	0	0	0	0
0	0	0	0	0
0	0	0	0	0
0	0	0	0	0
0	0	0	0	0
0	0	0	0	0
0	0	0	0	0
0	0	0	0	0
0	0	0	0	0
0	0	0	0	0
0.001310232	0.004438216	0.002507965	0	0.000565108
0	0	0	0	0
0.029991593	0.015459661	0.016016012	0	0.042649627
0.002442969	0.002289579	0.001912982	0	0.008046146
0	0	0	0	0
0	0	0	0	0
0	0	0	0	0
0	0	0	0	0
0	0	0	0	0
0	0	0	0	0
0	0	0	0	0
0.116396023	0.058431474	0	0.170414759	0.018173506
0.066436204	0.131817628	0.013550694	0	0.014906444
0	0	0	0	0
0	0	0	0	0
0.002203392	0	0.043079729	0	0
0	0	0	0	0
0	0	0	0	0

0	0	0	0	0
0.003651186	0.013880222	0.078406424	0	0
0	0.010992248	0.080361837	0	0.010462148
0	0	0	0	0
0	0.44739639	0.000814794	0	0.053548217
0	0	0	0	0
0	0	0	0	0
0	0	0	0	0
0	0	0	0	0
0	0	0	0	0
0	0	0	0	0
0	0	0	0	0
0	0	0	0	0
0	0	0	0	0
0	0	0	0	0
0	0	0	0	0
0	0	0	0	0
0	0	0	0	0
0	0	0	0	0
0	0	0	0	0
0	0	0	0	0
0	0	0	0	0
0	0	0	0	0
0	0	0	0	0
0	0	0.018081692	0	0.002874094
0	0.001594827	0.003595649	0	0
0	0	0	0	0
0	0.002210054	0	0	0
0	0	0	0	0
0	0	0	0	0
0	0	0	0	0
0.00271321	0	0	0	0
0	0	0	0	0
0	0	0	0	0
0	0	0	0	0
0	0	0	0	0
0	0	0	0	0
0	0	0	0.04488957	0.003099402
5.646015751	1.348067289	0.41018322	0	4.393923567
0	0	0	0	0
0	0	0	0	0
0.023255459	0	0.002294544	0	0
0.004879468	0.016005831	0	0.002451532	0.002901705
0	0	0	0	0
0	0	0	0	0
0	0	0	0	0
0.093986488	0.003847767	0	0.004615833	0.001707324
0	0	0	0	0

0	0	0	0	0
0.017339506	0.005296554	0.001639022	0.029667691	0.004739522
0	0	0.039597121	0	0
0	0	0	0	0
0	0	0	0	0
0	0	0	0	0
0	0	0	0	0
0	0	0	0	0
0	0	0	0	0
0	0	0	0	0
0	0	0	0	0
0	0	0	0	0
0	0.091682421	0.56114323	0	0
0	0	0	0	0
0.019751377	0.019536845	0	0	0.00349196
0	0	0	0	0
0	0	0	0	0
0	0	0	0	0
0	0	0	0	0
0	0	0	0	0
0	0	0	0	0
0	0	0	0	0
0.068886764	0	0	0.052747858	0
0	0	0.003359184	0	0
0	0	0	0	0
0	0	0	0	0
0	0	0	0	0
0	0	0	0	0
0	0	0	0	0
0	0	0	0	0
0	0	0	0	0
0	0	0	0	0
0	0	0	0	0
0	0	0	0	0
0.174213726	0	0	0	0
0	0	0	0	0
0	0	0	0	0
0	0	0	0	0
0.011779457	0.001079452	0.001753695	0.00734465	0
0	0	0	0	0
0	0	0.019542926	0	0.127786377
0	0	0	0	0
0	0	0	0	0
0	0	0	0	0
0	0	0	0	0
3.06049995	0.428298642	0	0.533047562	0.027584628
0	0	0	0	0
0	0	0	0	0

0	0	0	0.071632407	0
0	0	0	0	0
0	0	0	0	0
0	0	0.009447819	0	0
0	0	0	0	0
0	0	0	0	0
0	0	0	0	0
0	0	0	0	0
0	0	0	0	0
0.00394219	0.003793192	0.09331758	0	0
0	0	0.018038643	0	0
0	0	0	0	0
0.003853159	0.00079447	0.000983397	0	0
0	0	0	0	0
0	0	0	0	0
0	0	0	0	0
0	0	0.027509466	0	0
0	0	0	0	0
0	0	0	0	0
0.443102204	0.4388946	0.954287951	0.216366361	0.048780492
0	0	0	0	0
0	0	0	0	0
0	0	0	0	0
0	0	0	0	0
0	0	0	0	0
0	0	0	0	0
0	0	0	0	0
0	0	0	0	0
0	0	0	0	0
0	0.011087088	0	0	0
0	0	0	0	0
0	0	0	0	0
0	0	0	0	0
0	0	0	0	0
0	0	0	0	0
0	0	0	0	0
0	0	0	0	0
0	0	0	0	0
0	0	0	0	0
0	0	0	0	0
0	0	0	0	0
0	0	0	0	0
0	0	0	0	0
0	0	0	0	0
0.008804589	0.002878054	0	0	0.000864461
0	0	0	0	0
0	0	0	0	0
0	0	0	0	0

0	0	0	0	0
0	0	0	0	0
0	0	0	0	0
0	0	0	0	0
0	0	0	0	0
0.012664987	0	0.001994639	0.00381663	0.003549445
0	0	0	0	0
0	0	0	0	0
0	0	0	0	0
0	0	0	0	0
0	0	0	0	0
0	0	0	0	0
0	0	0	0	0
0	0	0	0	0
0	0	0	0	0
0	0	0	0	0
0	0	0	0	0
0	0	0	0	0
0	0	0	0	0
0	0	0	0	0
0	0	0	0	0
0	0	0	0	0
0	0	0	0	0
0	0	0.01079349	0	0
0	0	0	0	0
0	0.01421851	0.005039918	0	0
0	0	0	0	0
0	0	0	0	0
0	0	0	0	0
0	0.015835724	0	0	0
0	0	0	0	0
0	0	0	0	0
0	0	0	0	0
0	0	0	0	0
0	0	0	0	0
0	0	0	0	0
0	0	0	0	0
0	0	0	0	0
0	0.720433376	0.122154207	0	0
0	0	0	0	0
0	0	0.002065437	0	0
0	0	0	0	0
0	0	0	0	0
0	0	0	0	0
0	0	0.003621131	0	0
0	0	0	0	0
0	0	0	0	0
0	0	0	0	0

0	0	0	0	0
0	0.00795668	0	0	0
0	0	0	0	0
0	0	0	0	0
0	0	0	0	0
0	0	0	0	0
0	0	0	0	0
0	0	0	0	0
0	0	0	0	0
0	0	0	0	0
0	0.00242294	0.444694859	0	0
0	0	0	0	0
0	0.51182838	0.007621068	0	0
0	0	0	0	0
0	0	0	0	0
0	0	0	0	0
0	0	0	0	0
0	0	0	0	0
0	0	0	0	0
0	0	0.001871708	0	0
0.001192676	0	0	0	0
0	0	0	0	0
0	0	0	0	0
0.921222756	3.110319214	1.297735897	0	2.33205879
0	0	0	0	0
0	0	0	0	0
0	0	0	0	0
0	0	0	0	0
0	0.01073948	0	0	0
0	0	0	0	0
0	0	0	0	0
0	0	0	0	0
0	0	0.00864519	0	0
0	0	0	0	0

PHL018-0243_V2	PHL018-0115_V3	PHL018-0216_V3	PHL018-0230_V3	PHL018-0277_V2
REF	EYCF	CTRL	REF	REF
0.002375916	0.541304829	0.00984782	0.121060391	0.009536649
2.913791971	10.5460983	0.481737296	2.561845506	0.292290984
0	4.915466678	0	0.919632055	3.061609822
0	0	0	0	0
0	0	0	0	0
0	0	0	0	0
0	0	0	0	0
0	0	0	0	0
0	0	0	0	0
0	0	0	0	0
0	0	0	0	0
0	0	0	0	0
0	0	0	0	0
1.165519394	0	0.737477737	0.844369646	2.531599393
0	0	0	0	0
0	0	0	0	0
0	0	0	0	0
0	0	0	0	0
0	0	0	0	0
0	0	0	0	0
0	0	0.001692406	0	0
0	0	0	0	0
0	0	0	0	0
0	0	0	0	0
0	0	0.000556055	0.151875249	0
0	0	0	0	0
0	0	0	0	0
0	0	0	0	0
0	0	0	0	0
0	0	0	0	0
0	0	0	0	0
0	0	0	0	0
0	0	0	0	0
0	0	0	0	0
0	0	0	0	0
0	3.964157141	5.940940544	0	2.380321955
0	0	0	0	0
0	0	0	0	0
0	0	0	0	0
0	0	0	0	0
0	0	0	0	0
0.006311855	0.058293331	0.003733436	0	0.013426529
0.024491119	0.001994084	1.742938427	0.002041553	0.171586012
0.009879425	0.004700979	0.184269384	0.001544134	0
0	0	0	0	0.023391614
0	0	0	0	0

0	0	0.002097415	0	0.005413579
0.1418327	0.079375127	0	0.003403215	0
0.001250238	0.013659076	0.002820001	0.009379666	0
0.561297722	0.414142827	0.880491323	0.141932125	0.684570372
0	0.009188484	0.000729623	0.000946458	0.05910764
0	0	0.047253495	0	0
0	0.010364751	0.016872027	0	0.001862883
0	0	0	0	0
0	0	0	0	0
0	0	0	0	0
0	0	0	0	0.001222592
0	0.013715003	0.004167456	0.00707925	0.968358508
0.001404028	0	0	0.001580017	0.000691642
0	0.057527541	0	0.020463535	0.246243767
0	0	0	0	0
0	0	0	0	0
0.008592427	0	2.860190178	0	0.002090242
0	0	0	0	0
0	0	0	0	0
0	0	0	0	0
1.25617864	0.074352009	0.002340843	0.082353918	0.010875392
0	0.040811585	0	0.006047554	0.017018223
0	0	0.033615216	0	0
0	0	0	0	0
0	0	0	0	0
0	0	0	0	0
0	0	0	0	0
0	0	0	0	0
0.005303449	0	0	0	0.000725708
0	0	0	0	0
0	0	0	0.029123323	0
0	0	0	0.023318041	0
0	0	0	0	0
0	0	0	0	0
0	0	0	0	0
0.048994952	0	0.001161324	0	0
0	0	0	0	0
4.572058349	0	0.561123127	0	0
0	0	0	0	0
0	0	0	0	0
0	0	0	0	0
0	0	0	0	0
0	0	0	0	0
0	0	0	0	0
0	0	0	0.035202837	0
0	0	0	0	0
0	0	0	0	0
0	0.289560431	0	0	0

0	0	0	0	0
0	0	0	0	0
0	0	0	0	0
0	0	0	0	0
2.074436242	0	0	0	0
0	0	0	0	0
0	0	0	0	0
0	0	0	0	0
0	0.003114284	0	0	0.000913858
0	0	0	0	0
0	0	0	0	0
0	0.001205207	0	0	0.004668273
0	0	0	0	0
0	0	0	0	0
0	0	0	0	0
0	0.003672903	0	0	0
0	0	0	0	0
0	0	0	0	0
0	0	0.08360713	0	0
0	0	0	0	0
0	0	0	0	0
0	0	0	0	0
0	0.001784263	0	0.001141711	0
0	0	0	0	0
0	0	0	0	0
0	0	0	0	0
0	0	0	0	0
0	0.003194193	0	0.003372425	0
0	0	0	0	0
0	0	0	0.164147009	0
0	0.126527705	0.000714847	0.002511412	0.030274543
[truncated: 377,350 more chars]
